# Outcomes from integrating anti-cervical cancer teachings into the curriculum of high schools in a South-Eastern Nigerian State

**DOI:** 10.1186/s12889-022-14231-4

**Published:** 2022-10-14

**Authors:** Christian Ifediora, Lennert Veerman, Emmanuel Azuike, Uchenna Ekwochi, Williams Obiozor

**Affiliations:** 1 Griffith University School of Medicine, Gold Coast, Australia; 2 OCI (Onyebuchi Chris Ifediora) Foundation, Awka, Nigeria; 3 School of Medicine, Chukwuemeka Odumegwu Ojukwu University, Uli, Nigeria; 4 School of Medicine, Enugu State University of Science and Technology, Enugu, Nigeria; 5 School of Adult Education, Nnamdi Azikiwe University, Awka, Nigeria

**Keywords:** Cervical cancer, High school, Intervention, Knowledge, Secondary school, Teenage women

## Abstract

**Background:**

Quests for the global elimination of cervical cancer and its related SDG goals by 2030 are achievable if realistic approaches for improving outcomes in LMICs are entrenched. Targeting teenage high schoolers in these countries, which largely lack universally-affordable anti-cervical cancer measures, can be a game-changer. This paper evaluates a 2019 Harvard-endorsed measure that integrated relevant teachings into the curricula of some Nigerian high schools, in what was a global-first.

**Method:**

A 12-month, quasi-experimental (pre-and-post-tests) research that evaluated the impact of the above initiative on three public schools randomly selected from a pool of 261 in South-east Nigeria. The intervention was “exposure” to anti-cervical teachings, which included “repetitions” and “examination/assessments” designed to enhance “engagement”. Both genders were among the 2,498 recruited participants. Data collections with questionnaires were at three different intervals over 12 months.

**Results:**

At Phase-1 (baseline), there were 1,699 (68.0%) responses, while Phases 2 (one-month post-intervention) and 4 (12-month post-intervention) had 1,797 (71.9%) and 500 (20.0%) responses, respectively. COVID-19 lockdowns washed out Phase-3 (six-month post-intervention).

The majority in all groups were aged 15–19 years. Males dominated in phases 1 (55.9%) and 2 (67.3%), and females (65.6%) in Phase 4.

Overall, there were increased knowledge on “General Awareness”, “HPV Vaccinations”, “Risk Factors” and “Symptoms”, particularly between Phases 2 and 1. Levels at Phase-4 were higher than at Phase-2, with the exception of “Pap Smears”, as knowledge gained in half of its assessing items became negative (reversed) at Phase-4. These observed changes were non-different between gender, age groups, and classes of high schools.

Relative to Phase 2, knowledge changes at Phase-4 for questions associated with established myths (“spiritual attacks”; OR 0.39; CI 0.29–0.52 and “enemy poisons”; OR 0.49; CI 0.37–0.65) were reversed, even though they were originally increased significantly between Phases 2 and 1.

**Conclusion:**

Anti-cervical cancer enlightenment interventions to teenage high school students were largely effective, but appears guaranteed if engagement-enhancing measures are maintained over time. Extra efforts should be put into debunking prevailing myths.

**Supplementary Information:**

The online version contains supplementary material available at 10.1186/s12889-022-14231-4.

## Introduction and background

Cervical cancer disproportionately affects women in lower middle-income countries (LMICs) [1–3]. Despite its incidence ranking fourth worldwide[4], the cancer remains the second commonest among 15–44 -year-old women in these countries [5]. Around 84% or 445,000 of all global occurrences, as well as 85% of the 270,000 ensuing deaths, occur in LMICs [4]. The cancer also has direct economic impacts, with an associated global cost of US$2.7 billion in 2010, a figure expected to reach US$4.7 billion by 2030 [6].

In Nigeria, at least 53.1 million women aged = 15 years are at risk [7]. In 2018, 10,403 of the 14,943 women diagnosed with the cancer, died, though figures are likely to be underestimated, given that most affected patients prefer traditional or religious healing centres than hospitals [7]. Numbers are also likely to worsen, with projections revealing that cervical cancer deaths by 2025 in Nigeria will rise by 63% for those = 65 years, and by 50% for those > 65 years [8].

Unfortunately, these grim statistics are unlikely to change, as most of the affected LMICs (including Nigeria) lack universal, government-funded vaccination and screening programmes [4, 9]. In fact, only nine of 55 African countries have national anti-cervical cancer programs in place [10]. In Nigeria, with a Human Papilloma Virus (HPV) prevalence was 16.0% in 2017, the coverage of cervical cancer screening were 1.8% for those aged 25 - 34 years, 6.6% for those 35 - 44, 12.7% for those 45 - 54, and 2.8% for those 55 - 64 [5]. The proportion of Nigerian women (over the age of 18 years) who have ever been screened is only 8.7% [7].

Vaccinations against the HPV, the sexually-transmissible causative virus of cervical cancer, are neither universally available nor affordable to women in LMICs [9]. In 2014, for instance, only 3% of adolescent females in these countries received HPV vaccinations, contrasting with the coverage of over 30% reported in the high and upper middle income countries (HIC/UMICs) [11].

The foregoing leaves health education campaigns as an important and realistic anti-cervical cancer measure in LMICs. Research has shown that such anti-cervical cancer empowerments can enhance knowledge and preventive practices [12–14], perceptions [15, 16], screening uptake [15, 17–19], and the adoption of positive behaviours [20]. They can also improve participation rates in vaccination programs, since awareness of the cancer is linked to increased vaccination uptakes [21].

Recent publications argue that targeting young, mostly teenage adults in high schools of LMICs, through inclusions into their regular academic curricula can make an important contribution to tackling cervical cancer in these countries [2, 22, 23]. The authors argued that such an initiative would only be effective if measures that will improve engagement are built into the program (e.g., repetition of the teachings and having in-built assessment systems like examinations and quizzes). These arguments were based on the fact that cervical cancer has its roots in the teenage years [23], and that, in a country like Nigeria, 81.5% [24] of girls in tertiary institutions are already sexually active, while 51.7% do so before turning 20 year [5]. As such, it becomes obvious that, for a good proportion of women in Nigerian tertiary institutions (usually in their late teens to mid-twenties), exposures to the HPV would have already occurred. Therefore, delaying any awareness programs until women get into this demography, would come too late to many. Unfortunately, existing enlightenment campaigns generally target older, working, or post-high school women (those in tertiary institutions). They also usually have no in-built measures that will ensure engagement with the campaigns.

If proven to be effective, the measures above could be world-leading and timely, while offering potentially inexpensive and adaptable contributions to the fight against cervical cancer. In addition to the benefits to LMICs, high-income and upper-middle income countries (HIC/UMICs) with functional universal healthcare systems may also find any positive outcome from this work useful, as most of them are stuck below their screening targets, possibly due to vaccine hesitancy. For instance, screening uptakes have remained below expectations for women aged 20–69 years in countries like Australia (56.0%) [25], the USA (64.6%) [26] Canada (65.0%) [27] and the UK (71.4%) [28].

Given the foregoing, both LMICs and HIC/UMICs have key roles to play in the global quest to eradicate cervical cancer, and findings from this work may be vital in this. Globally, such an achievement could save up to 13.4 million lives over the next half century [11], and will make indirect contributions to the attainment of five of 17 items in the Sustainable Development Goals (SDGs): goals 1 (poverty eradication), 3 (good health), 4 (quality education), 5 (gender equality), and 10 (reducing inequalities) [6, 29].

In late 2019, a new program that inculcated the principles enunciated above was implemented in public high schools in Anambra State, South-east Nigeria, in what was a global first. A study was put in place to evaluate that initiative, and ascertain if such measures and theories are actually effective. This paper reports on the outcomes of the study. Therefore, the major aim of this work is to assess the short-to-medium term impacts (changes in the knowledge levels of various aspects of cervical cancer) of integrating anti-cervical cancer teachings and their engagement-improvement measures into the curriculum of high schools.

## Methods

### Study design, setting and participants

This is a 12-month, quasi-experimental (pre-test and post-test) research study that evaluated impacts of the above-mentioned initiative on both breast and cervical cancers. This paper is focused on just the quantitative outcomes from the anti-cervical cancer component. Findings on breast cancer, as well as the qualitative aspects, will be published separately.

The pre-test and post-test quasi-experimental design is a widely established statistical method for the immediate evaluation of the efficacy of new concepts, as is the case with this work [30, 31]. It obviates the need for randomisation, and yet allows a dependent variable (like “knowledge”, in this work) to be tested before and after an intervention (“anti-cervical cancer teachings” in this work) [30, 32].

The study for this work participants were derived from three school clusters randomly selected from the 261 (30 girls-only; 25 boys-only, and 206 co-educational/mixed) schools in Anambra State, where the proposed initiative is functional. Anambra State, with a 2016 population of 5,527,800 people [33], is one of Nigeria’s 36 states. As at 2020, Nigeria is estimated to have a population of 206,139,589, which is 2.61% of the global total [34].

#### Recruitment

Recruitment of the participant schools was through the government ministry in charge of high school education in Anambra State. The pre-test and post-test study method allows for Purposive (Judgement, Selective or Subjective) sampling, wherein a researcher relies on his/her own judgment in selecting participants, usually based on defined characteristics relevant to a study [30]. This principle justified this study’s selection process, which first allowed large schools from the 261 to be grouped into three different pools of male-only, female-only and co-educational groups. For the sake of this study, large senior secondary schools (SSS) were arbitrarily defined as those with at least 500 students across the relevant classes, and the focus on them ensures that numbers needed for a robust study, are realized [35]. As advocated in the literature regarding quasi-experimental methods, study validity can be improved through random sampling techniques [30]. A stratified random sampling approach was, therefore, used to select one participating school from the three pools.

#### Inclusion criteria

All male and female students in any of the three levels of Senior Secondary Schools (SSS I, II and III) were included. Senior secondary schools in Nigeria are equivalent to the high schools of many other countries, and these expressions are used interchangeably in this paper. The inclusion of males was necessary, given that sexual pressures from them significantly contributes to the problem of cervical cancer. In addition, the required behaviours needed by women to tackle the cancer (vaccinations and screenings) will only succeed with financial and moral support from males [23]. Students in private high schools were excluded, given that their set up is not entirely under the control of the board in charge of the targeted schools.

The schools selected, and their eligible populations, were: (i) Okongwu Memorial Grammar School, Nnewi (boys-only; 699 students) (ii) New Era Girls Secondary School, Onitsha (girls-only; 1,128) and (iii) Kenneth Dike Memorial Secondary School, Awka (co-educational; 671). This gives a total population of 2,498 students.

#### Sample size estimation

**Fig. 1:**
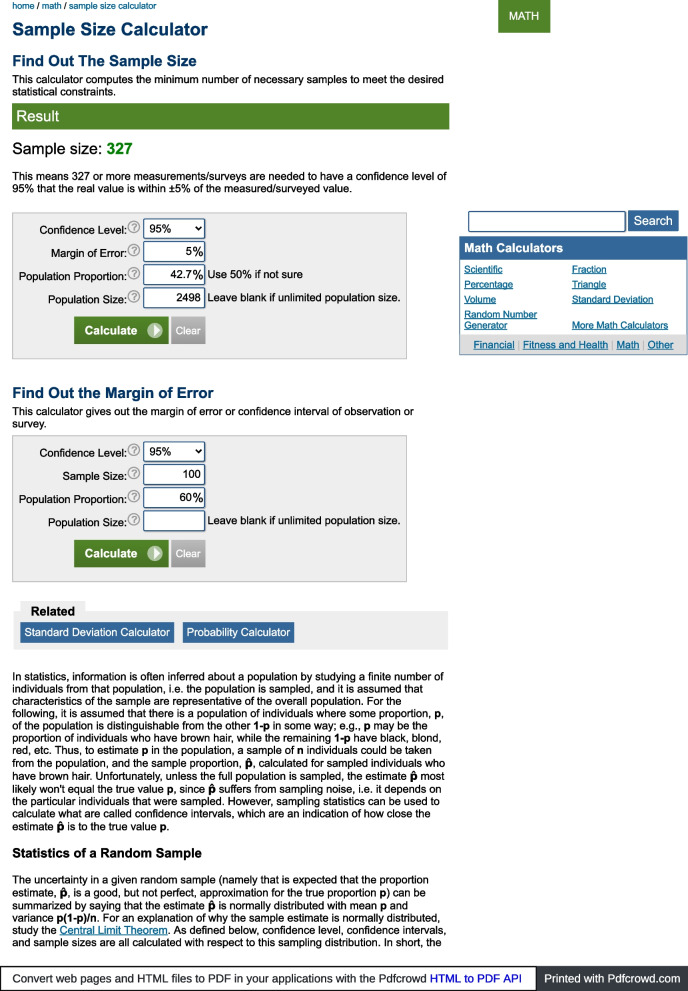
Sample Size Estimation for the Study

The sample size was estimated using data from a previous study of a similar demographic, which found that 42.7% of the students had heard of cervical cancer before an intervention was made [2]. With this population proportion, and allowing for a Confidence Interval of 95% and an error margin 5%, the estimated minimum sample size needed for this study was 327 (Fig. 1).

#### Details of the campaign/the intervention (Table 1)

**Table 1:** The intervention, engagement-ensuring measures, and commencement

a) **The Teachings**: A curriculum was developed for the Campaign, using the validated content that was piloted in a past intervention^a^. The content, developed with the input of the Harvard Medical School, USA (Appendix 1 in Additional File 1), was also vetted by a 28-man multi-stakeholder implementation committee (Appendix 2 in Additional File 1). This measure ensured that the teachings were religiously, culturally, and professionally appropriate to the targeted audience. Given the need to reach every student in the eligible schools, Civic Education, a subject that was compulsory to all the students, was used. The teachings were integrated into the subject. A 45-min teaching period was allocated to cervical cancer. Each teaching covered the various aspects of the cancer, including General Awareness, Risk Factors, Symptoms, Vaccination and Screening. In addition to the teachings, specially made books were donated to all the schools, with each receiving an average of 10, which were kept in the respective school libraries. This provided extra sources of knowledge to the participants. The teachers were trained to draw the attention of the participants to these books on a regular basis. The teachings, which commenced on September 16^th^, 2019, were held in week 3 of every school term
b) **The repetition component**: This was one of the two recommended measures put in place to enhance engagement. Teachings were repeated across all the three classes that make up the senior secondary school (SSS 1, 2 and 3) cadre. Each class is made of three terms. A 45-min teaching session was allocated to the anti-cervical cancer teaching per term. This means that the same teaching is delivered three times to each class, making a total of three teachings for each class over the 12 months of this study. This measure is expected to maximize the chances that the participants will gain the required knowledge, sustain them, and make them part of their habits, given that repetitions help transform preventive practices into habits^b,c^. It should be noted that, beyond the time-limitations of this study, this measure will amount to nine teachings for each cohort starting from the first year of senior secondary school
c) **The examination and assessment component**: This was the second measure to ensure engagement. The Campaign ensured that questions on the anti-cancer teachings were included in all Civic Education examinations across the high school classes, including the mid and end-of-term exams. The scores contributed to the final “pass or fail” outcomes for the Civic Education subject
d) **The Campaign Flag-off and Workshops**: The Campaign flag-off ceremony was officially held on September the 10^th^, 2019. Thereafter, all the teachers for Civic Education (and the Guidance Counsellors for those with no Civic Education teachers) were hosted to a 2-day workshop held on September the 11^th^ and 12^th^, 2019, where they were all trained. This training ensured that their approach was standardized. There were 321 teachers from the 261 schools (some schools have more than one Civic Education teacher). They were split into two groups of roughly equal parts, with each group attending on each of the two days

^a^Ifediora CO, Azuike EC. Knowledge and attitudes about cervical cancer and its prevention among female secondary school students in Nigeria. Journal of Tropical Medicine International Health. 2018;23(7):714–723 [2]

^b^Lally P, Gardner B. Promoting habit formation. Health Psychology Review. 2013;7(sup1):S137-S158 [39]

^c^Lally P, Van Jaarsveld CH, Potts HW, Wardle J. How are habits formed: Modelling habit formation in the real world. European journal of social psychology. 2010;40(6):998–1009 [38]

The Intervention was tagged the “Arm our Youths (ArOY) Health Campaign”, and was developed with the help of the Harvard Medical School (Appendix 1 in Additional File 1). It included both anti-cervical and anti-breast cancer teachings. Additional components include “repetition of teachings” and “examination/assessment” measures, with both designed to enhance “engagement” with the program. “Engagement” was proposed in the previous papers as the key to long term knowledge retention [2, 22].

#### Data collection

Using questionnaires, data was collected concurrently from all three schools at periods corresponding to the different phases of the Study. These were Phase 1 (0-Month or Pre-intervention), Phase 2 (One-month Post-intervention) and Phase 4 (12-month post-intervention). Unfortunately, no data was collected at Phase 3 (6-month post-intervention) given the hard COVID-19 lockdowns at the time. Phase 4 was also affected partially, as schools were not fully open at the time. The phases of the study were retained as such in this study, in keeping with the broad principles of “intention to treat” [36]. Three of the 5-person research team members were resident in Nigeria, and they oversaw the data collection.

#### The questionnaire (design, despatch, and return)

The Questionnaires were products of modified questions already utilized in a similar work [22]. Given the slight modification, piloting became necessary. Twenty high school students in the same class as the participants, who were not part of the selected intervention clusters, were engaged. Their feedback, along with inputs from professional associates, helped design the final documents that served as the pre-intervention questionnaire (Additional File 2) and post-intervention questionnaire (Additional File 3). Table 2 shows the structure of these questionnaires.

- Phase 1 (baseline) questionnaires were administered to students in the participating schools in the week commencing September 16^th^, 2019. Research team members in Nigeria delivered the questionnaires on days arranged with the management of the various schools. Students in each school completed the questionnaires on the same day and time, with the team members providing guidance as needed. Completed questionnaires were collected back on the same day for each school.
- Phase 2 questionnaires were despatched one-month post-intervention, which was in the first week of November 2019, while Phase 4 (12-month questionnaire) was dispatched in the week starting September 14, 2020. Both phases followed similar patterns as explained for Phase 1.
- Phase 3, which was due to commence in the first week of April 2020, did not eventuate, as activities in Nigerian secondary schools were suspended on March 31, 2020, due to the COVID-19 pandemic. They reopened on September 7, 2020.

**Table 2:** Structure of the questionnaires (Additional files 2 and 3)

The questionnaire is a 10-page document that was divided into 4 parts. The pre-intervention or Phase 1 questionnaire (Additional File 2) and post-intervention questionnaires for phases 2 and 4 (Additional File 3) are identical
Part 1 (Page 1 on both documents) contained the Introduction, Participant’s Information, and Consent, while Part 2 (Page 2) contained the seven major questions designed for collecting the basic demographics of the participants
Part 3 (Pages 3 to 6) explored knowledge on breast cancer and breast self-examination (BSE), which were not covered in this paper
Part 4 (pages 7 to 10), which is the main focus of this paper (along with Parts 1 and 2), had 10 main questions (some with sub-components) that were designed to collect information on the General Knowledge on Cervical Cancers, Pap Smear and HPV Vaccinations, as well as Risk Factors and Symptoms. Questions 1 to 5 (Page 7) explored the General Knowledge. Question 6 (Page 8; 10 sub-questions) explored Pap Smears, Question Numbers 7 (Page 8; 14 sub-questions) looked at Risk Factors and Question 8 (Page 9; 7 sub-questions) looked at Symptoms. Knowledge questions for Pap smear (Question 9, with 10 sub-questions) and HPV (Question 10, with 9 sub-questions) were explored on pages 9 and 10, respectively

#### Data analysis

The Statistical Package for the Social Sciences, SPSS (IBM SPSS Statistics for Windows, Version 28.0. Armonk, NY: IBM Corp, released 2021) was used for the quantitative data analyses, which included various Descriptive and Inferential components.

The Descriptive Component, presented either as actual numbers, percentages, or proportions, summarised the key demographics of the participants. The Inferential Analysis adopted Binary Logistics Regression (BLR) and Chi-square (?^2^) analysis, with Odds Ratios (OR) and ?^2^ statistics generated respectively from them, along with 95% Confidence Intervals (CIs) and Probability (*p*) values. Only *p*-values of < 0.05 were deemed significant.

The variables (dependent/outcome and independent/predictor) needed for the BLR analysis are shown as Additional File 4. The dependent variables included five key aspects of cervical cancers, including General Awareness (explored with four questions), Pap Smears (eight questions), HPV Vaccinations (two questions), Risk Factors (14 questions) and Symptoms (seven questions). The exploring questions made up the independent variables, and are also shown in Additional File 4.

Alongside the foregoing, four additional dependent (respondent) variables (Age, Gender of Individuals, Gender of Schools and Class of Study) were also explored, so as to enrich the study by showing how they impacted the knowledge changes. Also shown in Additional File 4, they were analysed using one representative question (as the independent variable) to assess knowledge across the five key aspects of cervical cancer explored in this study.

## Results

### Basic summary (Table 3)

**Table 3:** Demographics and characteristics of 3 senior secondary schools in South-east Nigeria (Population 2,498)

**S/N**	Variables		**Phase 1** *Pre-Intervention* *N*^a^ = 1699 (68.0%)	**Phase 2** *1-Month Post-Intervention* *N*^a^ = 1797 (71.9%)	**Phase 4** *12-Month Post-Intervention* *N*^a^ = 500 (20.0)
1	Age (Years)	Mean (SE^a^)	15.96 (0.038)	15.99 (0.038)	16.93 (SE: 0.130)
		SD^a^	1.538	2.458	2.904
2	**Age**	< 15	240 (15.0%)	332 (21.7%)	34 (7.1%)
	**Ranges**	= 15 to = 19	1343 (83.9%)	1162 (75.8%)	411 (85.8%)
	**(Years)**	> 19	18 (1.1%)	38 (2.5%)	34 (7.1%)
			*(N* = *1601; Missing* = *98)*	*(N* = 1532*; Missing* = *265)*	*(N* = 479*; Missing* = *21)*
3	**Gender**	Male	908 (55.9%)	1039 (67.3%)	161 (34.3%)
		Female	716 (44.1%)	505 (32.7%)	308 (65.7%)
			*(N* = *1624; Missing* = *75)*	*(N* = 1544*; Missing* = *253)*	*(N* = 479*; Missing* = *21)*
4	**Gender of School**	Boys- only	497 (30.2%)	362 (23.0%)	127 (26.5)
		Girls-only	576 (35.0%)	773 (49.1%)	211 (44.1%)
		Mixed	572 (34.8%)	438 (27.8%)	141 (29.4%)
			*(N* = *1645; Missing* = *54)*		*(N* = 469*; Missing* = *31)*
5	**Class of Study**	SSS^a^ 1	304 (19.8%)	573 (36.3%)	8 (1.7%)
		SSS^a^ 2	664(43.3%)	535 (33.9%)	3 (0.6%)
		SSS^a^ 3	564 (36.8%)	470 (29.8%)	468 (97.7%)
			*(N* = 1532*; Missing* = *167)*	*(N* = 1532*; Missing* = *167)*	*(N* = 479*; Missing* = *21)*

^a^*SE* Standard Error, *SD* Standard Deviation, *SSS* Senior Secondary school, *N* Number, *df* Degree of Freedom

A total of 1,699 (68.0%), 1,797 (71.9%) and 500 (20.0%) responses were received for Phases 1, 2 and 4, respectively. Across all phases, the age group of “ = 15 to = 19” years form an overwhelming majority, while the “ > 19-year” group was the least. With respect to the gender of individuals, males were more than the females in all the groups at phases 1 (55.9%) and 2 (67.3%), while females, with 65.6%, were more in Phase 4. In phases 1 and 2, participants from all three Senior Secondary School (SSS) classes (I, II and III) accounted for approximately 20% or more in each, except in Phase 4, where 97.7% of participants were in SSS 3.

### Impact of the interventions at the various phases

Table 4 reveals the findings. As shown in Section A of the Table, statistically significant improvements on knowledge at Phase 2 (relative to Phase 1) were observed on all four questions that defined “knowledge on General Awareness”. The trend was also maintained in Phase 4 (12 months), with the ORs being generally larger when compared to those observed at Phase 2. Analysis of the impacts at Phase 4 relative to Phase 2 (i.e., post-intervention impact at 12 months relative to the impact at one month) found that, with the exception of “knowledge on awareness that pap smears exist” (OR 1.17; CI 0.96–1.43), there were also significant improvements on all the other parameters, though the ORs were smaller than those between phases 2 and 1, as well as between phases 4 and 1.

**Table 4:** Knowledge changes on 3 aspects of cervical cancer (with respect to the study phases)

**A. KNOWLEDGE CHANGES ON THE GENERAL AWARENESS ON ASPECTS OF CERVICAL CANCER**													
S/N	Variables	Phase 1 Vs 2 (5,499)				Phase 1 Vs 4 (3,185)				Phase 2 Vs 4 (3314)			
	(Questions)	OR	Lower	Upper	p	OR	Lower	Upper	p	OR	Lower	Upper	p
1	Ever heard of cervical cancer?	Number (*N*) = 4758				*N* = 2897				*N* = 2791			
		2.50	2.22	2.81	< 0.01*	4.617	3.682	5.789	< 0.01*	1.850	1.474	2.322	< 0.01*
2	Ever heard of cervical cancer screening?	*N* = 4726				*N* = 2880				*N* = 2764			
		2.81	2.49	3.16	< 0.01*	3.78	3.07	4.65	< 0.01*	1.35	1.10	1.65	< 0.01*
3	Ever heard of Pap Smear?	*N* = 4727				*N* = 2879				*N* = 2776			
		3.25	2.86	3.68	< 0.01*	3.81	3.10	4.68	< 0.01*	1.17	0.96	1.43	0.12
4	Ever heard of HPV and Vaccinations?	*N* = 4721				*N* = 2875				*N* = 2770			
		3.66	3.23	4.14	< 0.01*	5.26	4.26	6.48	< 0.01*	1.44	1.17	1.76	< 0.01*
**B. CHANGES ON THE KNOWLEGDE OF PAP SMEARS**													
S/N	Variables	Phase 1 Vs 2 (5,499)				Phase 1 Vs 4 (3,185)				Phase 2 Vs 4 (3314)			
	(Statements)	OR	Lower	Upper	p	OR	Lower	Upper	p	OR	Lower	Upper	p
1	Pap smear detects cervical cancer (Ca)	*N* = 4554				*N* = 2779				*N* = 2649			
		0.95	0.77	1.17	0.63	13.90	10.96	17.64	< 0.01*	14.64	11.48	18.65	< 0.01*
2	Pap smear is for married women only	*N* = 4280				2593				*N* = 2419			
		3.20	2.75	3.71	< 0.01*	1.09	0.80	1.49	0.57	0.34	0.25	0.46	< 0.01*
3	Pap smear is for women > 18 years	*N* = 4311				*N* = 2618				*N* = 2447			
		1.64	1.40	1.91	< 0.01*	2.95	2.32	3.76	< 0.01*	1.80	1.42	2.28	< 0.01*
4	Pap smear should start 2 years after sexual debut	*N* = 4280				*N* = 2597				*N* = 2431			
		1.73	1.48	2.02	< 0.01*	2.26	1.76	2.91	< 0.01*	1.31	1.02	1.67	0.03*
5	Pap smear starts from menopause	*N* = 4314				*N* = 2613				*N* = 2441			
		2.66	2.29	3.08	< 0.01*	1.16	0.86	1.55	0.33	0.44	0.33	0.58	< 0.01*
6	Pap smear is for those with Cervix cancer history	*N* = 4282				*N* = 2600				*N* = 2430			
		2.03	1.77	2.34	< 0.01*	1.24	0.95	1.61	0.12	0.61	0.47	0.79	< 0.01*
7	Pap smear is to be done 2 yearly	*N* = 4272				*N* = 2594				*N* = 2418			
		1.12	0.94	1.33	0.20	3.26	2.55	4.17	< 0.01*	2.92	2.28	3.73	< 0.01*
8	Pap smear is to be done once only	*N* = 4246				*N* = 2575				*N* = 2409			
		2.28	1.96	2.65	< 0.01*	1.17	0.87	1.57	0.32	0.51	0.38	0.68	< 0.01*
**C. CHANGES ON THE KNOWLEGDE OF HUMAN PAPILLOMA VIRUS (HPV) VACCINATIONS**													
S/N	Variables	Phase 1 Vs 2 (5,499)				Phase 1 Vs 4 (3,185)				Phase 2 Vs 4 (3314)			
	(Statements)	OR	Lower	Upper	p	OR	Lower	Upper	p	OR	Lower	Upper	p
1	HPV vaccine is preventive	*N* = 4284				*N* = 2589				*N* = 2445			
		1.12	0.94	1.33	0.22	5.27	4.15	6.69	< 0.01*	4.72	3.72	5.99	< 0.01*
2	HPV Vaccine is best given before sexual debut	*N* = 4303				*N* = 2610				*N* = 2445			
		1.00	0.83	1.19	0.97	3.71	2.91	4.74	< 0.01*	3.73	2.91	4.77	< 0.01*

1. Binary Logistics Regression was used for all inferential analysis

2. Phase 1: Pre-intervention (0 Month); Phase 2 = One-month post-intervention; Phase 4 = Twelve-month post

3. Phase 3 data was not collected due toCOVID-19 pandemic lockdowns

4. * = Statistically significant

Findings from the analysis of “Knowledge on Pap Smears” are shown in Section B of Table 4. Compared to Phase 1, the knowledge in Phase 2 improved significantly in six out of the eight questions asked, while only four of the eight, was similarly improved at Phase 4 (a decrease). The findings between Phases 4 and 2 were mixed, with statistically significant increases in 4 of the 8 questions, while the other 4 showed negative increases (decreases) that were equally significant.

Section C of Table 4 revealed the findings regarding changes in “Knowledge on HPV vaccinations”. There were no statistically significant improvements at Phase 2 relative to Phase 1 on any of the two questions. However, at 12-month post intervention (Phase 4), the knowledge changes on both questions were statistically improved relative to Phases 1 and 2.

The changes in aspects of “Knowledge on Risk Factors” and “Symptoms” are captured in sections A and B of Table 5, respectively. After one month of intervention (Phase 2), knowledge on only five of the 14 items explored for Risk Factors were improved relative to Phase 1 (pre-intervention). In contrast, at the 12-month mark (Phase 4), there were positively significant improvements in 10 of the 14 items. The post-intervention changes in knowledge at 12 months relative to one month (i.e., Phases 4 vs 2) revealed that there were 12 (out of 14) statistically significant findings, with 10 of these being positive. Interestingly, knowledge on “spiritual attacks” (OR 0.39; CI 0.29–0.52) and “poisons” (OR 0.49; CI 0.37–0.65) were the 2 items whose knowledge levels changed negatively, and were significantly so. They were both significantly positive after one month (Phase 1), and statistically unchanged (Phase 4 relative to Phase 1), and then negatively so thereafter (Phase 4 relative to Phase 2).

**Table 5:** Knowledge changes on 2 aspects of cervical cancer (with respect to the study phases)

**A. CHANGES ON THE KNOWLEGDE OF RISK FACTORS OF CERVICAL CANCER**													
S/N	Variables	Phase 1 Vs 2 (5,499)				Phase 1 Vs 4 (3,185)				Phase 2 Vs 4 (3314)			
	(Risk Factors)	OR	Lower	Upper	p	OR	Lower	Upper	p	OR	Lower	Upper	p
1	Early sex	Number included (*n*) = 4444				*n* = 2707				*n* = 2575			
		1.10	0.90	1.36	0.35	13.21	10.35	16.84	< 0.01*	11.96	9.38	15.24	< 0.01*
2	Multiple partners	*n* = 4340				*n* = 2611				*n* = 2487			
		0.79	0.66	0.96	0.01*	8.00	6.31	10.13	< 0.01*	10.07	7.88	12.87	< 0.01*
3	Smoking	4297				*n* = 2611				*n* = 2450			
		0.84	0.71	0.99	0.04*	4.30	3.41	5.41	< 0.01*	5.14	4.05	6.51	< 0.01*
4	Alcohol	4259				2588				*n* = 2419			
		0.34	0.29	0.39	< 0.01*	1.40	1.12	1.75	< 0.01*	4.17	3.28	5.29	< 0.01*
5	Having more than 4 babies	4260				2574				*n* = 2430			
		0.48	0.41	0.55	< 0.01*	1.61	1.28	2.02	< 0.01*	3.38	2.66	4.30	< 0.01*
6	High fat diet	4247				2571				2420			
		1.03	0.88	1.20	0.73	1.52	1.18	1.96	< 0.01*	1.48	1.15	1.91	< 0.01*
7	Use of the oral birth pill	4238				2571				2405			
		1.19	1.02	1.40	0.03*	1.99	1.55	2.57	< 0.01*	1.68	1.30	2.16	< 0.01*
8	Spiritual attack	4206				2555				2373			
		2.02	1.76	2.32	< 0.01*	0.79	0.59	1.05	0.11	0.39	0.29	0.52	< 0.01*
9	Having a family history	4234				2568				2400			
		1.36	1.181	1.55	< 0.01*	1.25	0.97	1.60	0.08	0.92	0.72	1.18	0.51
10	Vaginal wart	4259				2587				2412			
		0.32	0.27	0.37	< 0.01*	1.69	1.36	2.12	< 0.01*	5.33	4.18	6.79	< 0.01*
11	Urinary Tract Infection	4233				2566				2413			
		0.35	0.30	0.41	< 0.01*	1.49	1.19	1.87	< 0.01*	4.28	3.36	5.45	< 0.01*
12	STDs	4228				2567				2397			
		0.33	0.29	0.39	< 0.01*	1.67	1.34	2.09	< 0.01*	5.02	3.95	6.38	< 0.01*
13	Poison	4218				2555				2387			
		1.85	1.61	2.12	< 0.01*	0.91	0.69	1.20	0.49	0.49	0.37	0.65	< 0.01*
14	Hereditary	4261				2579				2426			
		1.36	1.19	1.56	< 0.01*	1.16	0.90	1.49	0.26	0.85	0.66	1.09	0.20
**B. CHANGES ON THE KNOWLEGDE OF SYMPTOMS OF CERVICAL CANCER**													
S/N	Variables	Phase 1 Vs 2 (5,499)				Phase 1 Vs 4 (3,185)				Phase 2 Vs 4 (3,314)			
	(Symptoms)	OR	Lower	Upper	p	OR	Lower	Upper	p	OR	Lower	Upper	p
1	Offensive discharge	4459				2707				2612			
		0.10	0.09	0.12	< 0.01*	2.06	1.67	2.55	< 0.01*	20.22	15.73	26.01	< 0.01*
2	Bleeds after sexual intercourse	4260				*n* = 2578				2420			
		0.96	0.80	1.16	0.69	5.56	4.35	7.11	< 0.01*	5.78	4.51	7.42	< 0.01*
3	Pain with menstruation	4240				2584				2408			
		1.22	1.06	1.40	0.01*	1.66	1.32	2.10	< 0.01*	1.36	1.08	1.72	0.01*
4	Heavy period (menstruation)	4227				2563				2398			
		1.15	0.99	1.32	0.07	1.32	1.02	1.70	0.03*	1.15	0.89	1.48	0.28
5	No symptoms	4231				2560				2401			
		1.36	1.17	1.57	< 0.01*	1.41	1.09	1.83	0.01	1.04	0.80	1.35	0.77
6	Vaginal rash	4233				2558				2415			
		0.34	0.29	0.40	< 0.01*	1.50	1.19	1.88	< 0.01*	4.38	3.44	5.59	< 0.01*
7	Vaginal swelling	4282				2604				2438			
		0.24	0.21	0.29	< 0.01*	1.48	1.19	1.84	< 0.01*	6.07	4.78	7.72	< 0.01*

(1) Binary Logistics Regression was used for all inferential analysis

(2) Phase 1: Pre-intervention (0 Month); Phase 2 = 1 month post-intervention

(3) Phase 4 = 12 months post-intervention; Phase 3 data was not collected due to COVID-19 lockdowns

(4) * = Statistically significant

Section B of Table 5 showed that, compared to the baseline, only two of the seven items that explored changes in “Knowledge on Symptoms” were significantly improved after one month, while all seven were positively improved after 12 months (phases 4 vs 1). Five of these improvements remained when knowledge levels at 12 months were compared to that at one month post intervention (phases 4 vs 2).

### Impacts on various respondent variables at various phases

Section A of Table 6 reveals the findings for phases 1 (pre-intervention) Vs 2 (1-month post intervention). It shows that, relative to Phase 1, the “Knowledge on General Awareness” at Phase 2 improved significantly among both genders, the age ranges of “ < 15 and = 15- = 19 years”, class of SSS 3, and all school genders (male-only, female-only, and co-educational). Apart from General Awareness, though, these statistically-significant improvements were not observed on other knowledge aspects like Screening, HPV Vaccinations and Symptoms. “Knowledge on Risk Factors”, represented by “early onset of sexual activities can be associated with cervical cancers”, was only significantly improved among females (X^2^ = 5.96; p 0.02) and male-only schools (X^2^ = 5.87; p 0.02), with other areas also not being increased.

**Table 6:** Chi-square analysis exploring various knowledge and respondent variables (phases 1 vs 2; 1 vs 4)

	**A. PHASES 1 AND 2**										
**S/N**	**KNOWLEDGE VARIABLES**		**Phase of Study**	**KNOWLEDGE LEVELS BASED ON DIFFERENT RESPONDENT VARIABLES**							
				**Gender**		**Age (In years)**^b^		Class of Study^b^	**Gender of School**		
	Cervical Cancer Aspects	Sample Question^a^	Chi Square (X^2^)	Males (%)	Females (%)	< 15 (%)	= 15 to = 19 (%)	SSS^c^ (%)	Male-only (%)	Female-only (%)	Mixed (%)
1	**Awareness**	Having ever heard of cervical cancer	Phase 1	43.2	35.4	32.9	40.9	45.6	34.3	47.6	38.1
			Phase 2	72.6	45.5	57.9	64.4	67.7	50.3	84.7	47.4
			X^2^	244.3	19.76	50.36	201.87	69.18	29.10	251.17	17.15
			*(p)*	* < 0.01*	< 0.01*	< 0.01*	< 0.01*	< 0.01*	< 0.01*	< 0.01*	< 0.01*
2	**Screening**	Pap smear is to be done 2-yearly	Phase 1	11.0	16.8	14.5	13.2	11.9	16.2	9.8	14.3
			Phase 2	13.4	17.0	14.5	14.8	15.4	18.3	13.2	14.6
			X^2^	3.25	0.02	0.00	1.84	3.33	0.81	4.00	0.03
			*(p)*	*0.07*	*0.88*	*1.00*	*0.18*	*0.07*	*0.37*	*0.05*	*0.86*
3	**Vaccine**	HPV Vaccine is best given before sexual debut	Phase 1	9.3	16.5	13.0	12.3	11.2	16.1	5.8	14.9
			Phase 2	8.9	18.8	15.3	11.5	12.0	20.0	6.7	14.1
			X^2^	0.13	1.59	0.85	0.53	0.19	2.61	0.56	0.22
			(p)	*0.72*	*0.21*	*0.36*	*0.47*	*0.67*	*0.11*	*0.46*	*0.64*
4	**Risk Factors**	Early onset of sexual activities	Phase 1	6.5	10.3	7.7	8.8	7.7	10.5	5.5	8.5
			Phase 2	5.7	14.2	9.7	8.0	8.3	15.5	4.0	10.1
			X^2^	0.77	5.96	0.92	0.04	0.19	5.87	1.84	1.31
			*(p)*	*0.38*	0.02*	*0.34*	*0.83*	*0.67*	0.02*	*0.18*	*0.25*
5	**Symptoms**	Bleeding after intercourse	Phase 1	10.0	12.6	13.7	10.7	10.1	12.1	8.3	12.6
			Phase 2	8.9	13.8	12.3	10.7	9.4	15.6	5.8	13.5
			X^2^	0.92	0.60	0.33	0.00	0.60	2.62	3.48	0.27
			*(p)*	*0.34*	*0.44*	*0.57*	*0.99*	*0.68*	*0.11*	*0.06*	*0.60*
	**B. PHASES 1 AND 4**										
1	**Awareness**	Having ever heard of cervical cancer	Phase 1	46.4	36.7	36.4	42.8	47.1	34.2	49.8	40.5
			Phase 4	85.4	61.1	70.0	78.1	77.5	61.5	89.6	72.1
			X^2^	131.34	29.71	12.35	146.26	91.84	28.67	93.79	42.60
			*(p)*	< 0.01*	< 0.01*	< 0.01*	< 0.01*	< 0.01*	< 0.01*	< 0.01*	< 0.01*
2	**Screening**	Pap smear is to be done 2-yearly	Phase 1	11.4	17.3	17.3	13.3	11.8	14.7	9.2	18.7
			Phase 4	40.8	24.6	33.3	35.0	34.5	23.1	43.7	33.0
			X^2^	97.00	3.67	3.57	77.37	61.52	4.43	97.74	8.91
			*(p)*	< 0.01*	0*.06*	0*.06*	< 0.01*	< 0.01*	0.04*	< 0.01*	< 0.01*
3	**Vaccine**	HPV Vaccine is best given before sexual debut	Phase 1	8.2	17.0	14.8	11.7	10.2	15.5	4.7	17.3
			Phase 4	42.0	25.4	36.0	36.5	35.6	26.9	41.8	37.6
			X^2^	142.99	4.95	7.07	105.02	79.04	7.66	139.27	19.11
			*(p)*	< 0.01*	0.03*	*0.01*	< 0.01*	< 0.01*	*0.01*	< 0.01*	< 0.01*
4	**Risk Factors**	Early onset of sexual activities	Phase 1	7.0	10.1	8.0	8.0	7.9	9.5	5.7	10.1
			Phase 4	57.9	49.6	64.0	55.8	54.4	44.4	65.3	49.6
			X^2^	308.38	121.58	56.80	390.55	229.85	76.93	278.71	97.00
			*(p)*	< 0.01*	< 0.01*	< 0.01*	< 0.01*	< 0.01*	< 0.01*	< 0.01*	< 0.01*
5	**Symptoms**	Bleeding after intercourse	Phase 1	9.7	11.2	15.6	9.8	10.0	9.7	8.6	13.0
			Phase 4	48.6	33.6	44.0	43.5	43.1	31.4	53.1	37.9
			X^2^	167.26	40.13	11.78	191.29	120.10	32.98	150.80	31.27
			*(p)*	< 0.01*	< 0.01*	< 0.01*	< 0.01*	< 0.01*	< 0.01*	< 0.01*	< 0.01*

^a^Sample Questions: Each of the five Aspects of Knowledge has one representative question

^b^As is the standard practice for Chi Square analysis, the columns for Age > 19 years, SS 1 and SS 2 were all excluded, as the numbers in some of the cells included in the analyses were too small (< 5). Such low numbers introduce significant errors

^c^*SSS* Senior Secondary School, (4) *p* Probability Value (5) * = Statistically significant

The findings at Phase 4 relative to Phase 1 (Section B of Table 6) stand in contrast to those just described for Phase 2 Vs 1, as there were general increases for four of the five aspects (Awareness, Vaccines, Risk Factors and Symptoms) across all the eight respondent variables. Screening was the only aspect without 100% increase for all eight variables, with “being female” (X^2^ = 3.67; p 0.06) and being “aged < 15 years” (X^2^ = 5.57; p 0.06) just missing statistical significance.

Table 7 compares Phases 4 (12 months pot intervention) to 2 (1-month post intervention). While there were no increases for “General Awareness” on any of the eight variables, statistically significant improvements were noted on all eight with respect to Risk Factors and Symptoms. Knowledge on Screening and Vaccines were also improved, but this applies to six of the eight variables for each.

**Table 7:** Chi-square analysis exploring various knowledge variables and the respondent variables (Phase 2 vs 4)

S/N	**Knowledge Variables**		**Phase of Study**	**KNOWLEDGE LEVELS BASED ON DIFFERENT RESPONDENT VARIABLES**							
				Gender		**Age (In years)**^**2**^		**Class of Study**^**2**^	Gender of School		
	Cervical Cancer Aspects	Sample Questions^**1**^	Chi Square (X^2^)	Males (%)	Females (%)	< 15 (%)	= 15 to = 19 (%)	SSS 3 (%)	Male-only (%)	Female-only (%)	Mixed (%)
1	**Awareness**	Ever heard of cervical cancer	Phase 2	86.3	53.7	66.9	79.3	77.6	54.5	94.8	58.8
			Phase 4	85.4	61.1	70.0	78.1	77.5	61.5	89.6	72.1
			X^2^	0.16	2.41	0.12	0.21	0.004	1.74	6.77	7.44
			(p)	0*.69*	*0.12*	*0.73*	*0.64*	*0.95*	*0.19*	*0.01*	*0.01*
2	**Screening**	Pap smear is to be done 2-yearly	Phase 2	12.1	17.0	12.9	14.3	15.9	18.4	12.1	12.9
			Phase 4	40.8	24.6	33.3	35.0	34.5	23.1	43.7	33.0
			X^2^	97.54	3.64	7.42	64.00	34.67	1.18	84.23	19.85
			(p)	< 0.01*	*0.06*	0.01	< 0.01*	< 0.01*	*0.28*	< 0.01*	< 0.01*
3	**Vaccine**	HPV Vaccine is best given before sexual debut	Phase 2	6.0	18.9	13.9	8.8	10.4	19.4	5.1	11.9
			4	42.0	25.4	36.0	36.5	35.6	26.9	41.8	37.6
			X^2^	195.76	2.52	8.46	137.37	68.40	2.69	157.77	33.43
			(p)	< 0.01*	*0.11*	< 0.01*	< 0.01*	< 0.01*	0.10	< 0.01*	< 0.01*
4	**Risk Factors**	Early onset of sexual activities	2	4.1	14.4	9.4	6.3	7.4	15.0	2.9	10.2
			4	57.9	49.6	64.0	55.8	54.4	44.4	65.3	49.6
			X^2^	417.94	72.30	57.44	403.30	207.96	40.46	391.82	86.23
			(p)	< 0.01*	< 0.01*	< 0.01*	< 0.01*	< 0.01*	< 0.01*	< 0.01*	< 0.01*
5	**Symptoms**	Bleeding after intercourse	2	7.0	15.2	11.0	9.7	10.0	16.7	5.0	13.3
			4	48.6	33.6	44.0	43.5	43.1	31.4	53.1	37.9
			X^2^	234.32	20.45	21.18	182.00	108.26	241.41	241.71	27.67
			(p)	< 0.01*	< 0.01*	< 0.01*	< 0.01*	< 0.01*	< 0.01*	< 0.01*	< 0.01*

1. Sample Questions: Each of the five Aspects of Knowledge has one representative question

2. As is the standard practice for Chi Square analysis, the columns for Age > 19 years, SS 1 and SS 2 were all excluded, as the numbers in some of the cells included in the analyses were too small (< 5). Such low numbers introduce significant errors

3. *SSS* Senior Secondary School

4. *p* Probability Value

5. * = Statistically significant

Overall, it appears that improvements for the various respondent variables were sustained across all knowledge aspects of cervical cancer after 12 months (Phase 4) post intervention, but poorly so after the first month. Levels at 12 months relative to the first month (Phases 4 Vs 2) were largely positive as well.

## Discussion

One key finding of this study is that there were some inconsistent benefits if the interventions were delivered as one-offs (within one month). Knowledge improvements for various aspects of cervical of cancer were significantly raised and sustained only after measures that ensured engagement (repetitions and assessments) were continued for relatively prolonged periods (over 12 months). The two exceptions to these observations were with knowledge on cervical cancer screenings (pap smears) and knowledge that were related to myths (for instance, that cervical cancers are caused from “spiritual attacks” or by “poisons from enemies”). With these later aspects, observed positive changes became negative (reversed) over time, even when they were initially raised. It was also observed that benefits from such interventions were similarly applicable to all age groups, classes, and genders in high schools. A detailed discussion of the specifics of these findings, and how they relate to existing studies, will help reveal their implications for practice and policy, and will now be undertaken.

A notable fact was that, even though there were increases on the “Knowledge regarding General Awareness” at both the 12-month post-intervention (Phase 4) and the one-month post-intervention (Phase 2) marks on nearly all the questions asked, the levels at Phase 4 were much higher than that at Phase 2. These were particularly so with observations regarding “HPV Vaccinations”, “Risk Factors” and “Symptoms”, and might imply that interventions that were repeated and continued for longer periods (12 months as against one month) led to higher improvements in knowledge when compared to the baseline. Interestingly, a 2019 Nigerian publication, which had no engagement-enhancing (repetition or assessment) component, revealed an inconsistent improvement in knowledge after the six-month duration of that study [22]. Unfortunately, that 2019 study relied on a one-off intervention, and participants were only monitored for six months, not 12, as is the case with this current study. These observations appear to make the case that long-term interventions, with in-built engagement measures, would hold the key to sustained anti-cervical cancer knowledge among young adults. It also agrees with the recommendations of past publications, which recommended that engagement measures need to be put in place for awareness interventions on breast and cervical cancers [22, 37]. This is not entirely a surprise, as various publications have previously argued that repeated teachings, one of the engagement measures employed in this paper, is one way of sustaining knowledge over time [38, 39].

“Knowledge on Pap Smears (Section B of Table 4) is the only exception to the above, as improvements on them after 12 months (Phase 4), dropped relative to the level at one month. Previous studies found a 3-month post-intervention increase in knowledge regarding pap smears [40, 41], and these would appear to disagree with the findings of this work. This comparison should be treated with caution, though, as those studies had much shorter follow-ups, while the participants involved in them were working-class, older, non-high school adults, groups different to the much younger and inexperienced teenage high schoolers of this work. When compared to another study with similar participants (high school students) from 2019, the non-improvement from this work, as disappointing as it is, is actually a relatively better outcome, as there was no improvement at all from that study after six months of intervention with respect to “pap smears” [22]. As already stated, that 2019 study lacked engagement-improvement measures, and so differs from the current study in that regard. Overall, it would appear that, even though the engagement-enhancing measures included in this work improved knowledge of “pap smears” relative to situations where it was absent, that increase was not significantly sustained. The reasons behind this, which appear peculiar to “knowledge on pap smears” and not to other aspects of cervical cancer, are not clear. One possible explanation is that the teachings on “pap smears” might have been poorly structured or delivered. Future studies may be helpful in resolving this observation, even though the qualitative aspect of this work, which will use interviews to explore some of these findings, and is expected in a later publication, may also help understand these.

Of interest are observations that knowledge changes over a 12-month period regarding “risk factors” that bother on myths, including the fact that cervical cancers arise from “spiritual attacks” (OR 0.39; CI 0.29–0.52) and can be due to “poisons from enemies” (OR 0.49; CI 0.37–0.65), appear to have reversed over time. For instance, knowledge of both were significantly increased at Phase 1 (one month post intervention) but became statistically unchanged when Phase 4 (12-month post intervention) is compared to Phase 1. Surprisingly, however, they became statistically negative (reversed) when the knowledge levels at Phase 4 are compared to Phase 2. These suggest that the knowledge peaked after one month of intervention, but dropped back to the pre-intervention levels at the 12-month mark. As indicated, the two concerned knowledge aspects represent myths, with reports showing that these are held strongly among Nigerian communities, who generally attribute health mishaps to black magic and supernatural occurrences [42]. Such myths may, therefore, be hard to tackle, and this reversal of knowledge after an initial increase, may be a reflection of that. The real reasons for these reversals need to be unravelled. The concept of knowledge or attitude “decay”, wherein gained knowledge declines over time due to on-going external stimulations that lead to potential loss of positive results, might also have been a factor [30]. The repetition component and the use of pre-test, immediate post-test, and later post-test are all measures advocated by scholars for circumventing these reverasals [30]. Thought they were all adopted in this work, it appears that they might not have been effective with these very variables.

The forgoing observation with myths might have important practical implications, given that policy makers need to make extra efforts in ensuring that entrenched myths are debunked during health enlightenment campaigns. The qualitative component of this study may help unravel how the teachers and students approached these aspects, and whether the contents and repetition provisions were adhered to, given the potential for knowledge-decay explained above. There is also a chance that the disruptions from the COVID-19 lockdowns might have had an impact, even though one would expect such an impact to affect knowledge on other aspects of the risk factors, which was not the case in this study.

A final point for discussion is that improvements in knowledge regarding the various respondent variables like gender (for individuals and for schools), age, and class of study, were consistently raised at the 12-month post-intervention mark (Phase 4), but poorly so when the intervention ceased at one month-month mark. Improvements between Phases 4 (12 months) and 2 (one month) were varied and inconsistent (but not reversed as in the case with the myths), and might indicate that some aspects ok knowledge (like General Awareness) peaked at one month and remained sustained (with no further increases) after the initial enlightenment. Others, however, needed the repetitions and time to ensure that their knowledge peaked. The implication is that repetitions might be necessary, if benefits of anti-cervical cancer enlightenments in high schools are to be sustained for all gender, ages, and classes of study.

A key strength of this work is the inclusion of pre-tests and post-tests, which not only allowed baseline knowledge to be documented, but also for any observed changes to be quantified. The inclusion of males is also another strength, as it allowed insights to be gained on how a gender that is not directly affected by the cancer can key into the intervention. Alongside these strengths, a number of weaknesses are also acknowledged. One key limitation is based on the very nature of quasi-experimental designs, which are inherently unable to establish outcome causalities [30]. They also lack the ability to randomize, which can potentially limit widespread application of findings [30]. One measure advocated for mitigating these inherent limitations (and increase validity) is the need to include a measure of randomness in choosing study participants [30]. As explained in the Methods, this was adopted in this work, adding to its overall strength.

Another limitation arises from the potential impact of the lockdowns and school closures orchestrated by the COVID-19 pandemic. An obvious effect is that no data was collected at 6 months (Phase 3), while the 12-month (Phase 4) data was limited. In addition, teachings within the period of the lockdown were not possible, and this might have affected the levels of knowledge garnered.

The inability to do the study longer than 12 months is also another limitation. As such, the impact after 12 months, particularly when the engagement measures might no longer be in place (when students in SSS III might have left school), were not assessed in this study. A final limitation is that this study only assessed “Knowledge”, and not the actual “Preventive Practices” (vaccinations, screenings, and preventive behaviours) against cervical cancer. With this, it is not clear if these knowledge changes would actually translate to increased uptakes of cervical cancer vaccinations, screenings, and positive behaviours.

In summary, findings from this work appear to suggest that anti-cervical cancer enlightenment interventions to teenage high school students are largely effective, but can only be guaranteed and sustained if engagement-enhancing measures (like repetitions and in-built assessments/examinations) are put in place over time. An additional measure to improve engagement, which was not included in this work, is the introduction of inter-class or inter-school quiz competitions among the participating students and their schools, as the prizes from such contests can help incentivize both the students and their teachers. The impacts observed in this work appear to be similar for all gender, age groups, and classes of study in high schools, and targeting boys and girls in all three classes of high schools, is, therefore, recommended. Special effort should also be put into debunking myths associated with cervical cancer risk factors, and on ensuring that knowledge on cervical cancer screenings is well structured, delivered, and understood. One way of achieving these is by ensuring that teachers of these initiatives undergo a standardized training that lays emphasis on the need to debunk the myths, among other things. As such, we recommend that policy makers include teachers’ training exercises in any plan to roll out this initiative.

## Supplementary Information

**Additional file 1.**

**Additional file 2.**

**Additional file 3.**

**Additional file 4.**

**Additional file 5.**

**Additional file 6.**

**Additional file 7.**

**Additional file 8.**

**Additional file 9.**

## Data Availability

All data generated or analysed during this study are included in this published article as Additional files 5, 6, 7, 8 and 9.
