# Supplementary material for: Outcomes from integrating anti-cervical cancer teachings into the curriculum of high schools in a South-Eastern Nigerian State
Source: BMC Public Health. 2022 Oct 14;22:1914. doi: 10.1186/s12889-022-14231-4 (PMC9562070; doi:10.1186/s12889-022-14231-4)

**APPENDIX 1**

**ENDORSEMENT LETTER FROM THE HARVARD MEDICAL**

*
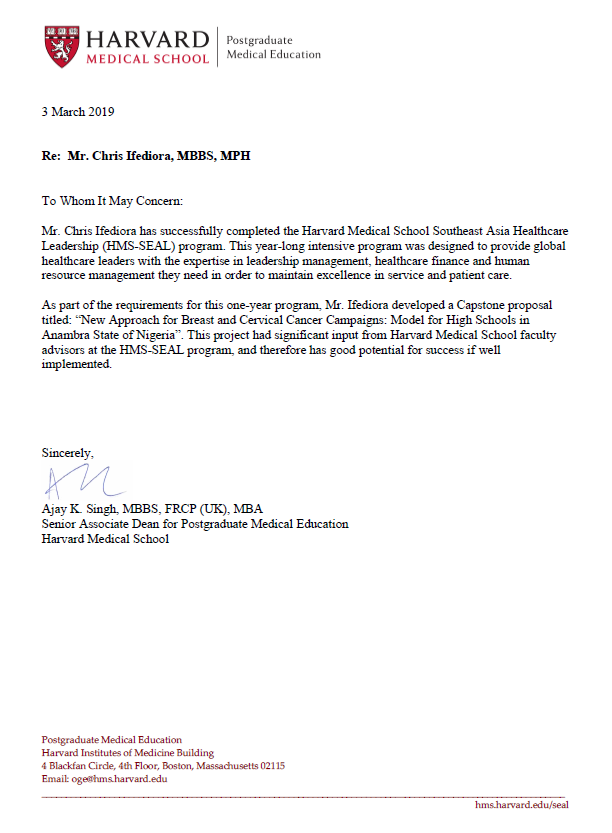
*

**APPENDIX 2: IMPLEMENTATION COMMITTEE**

**
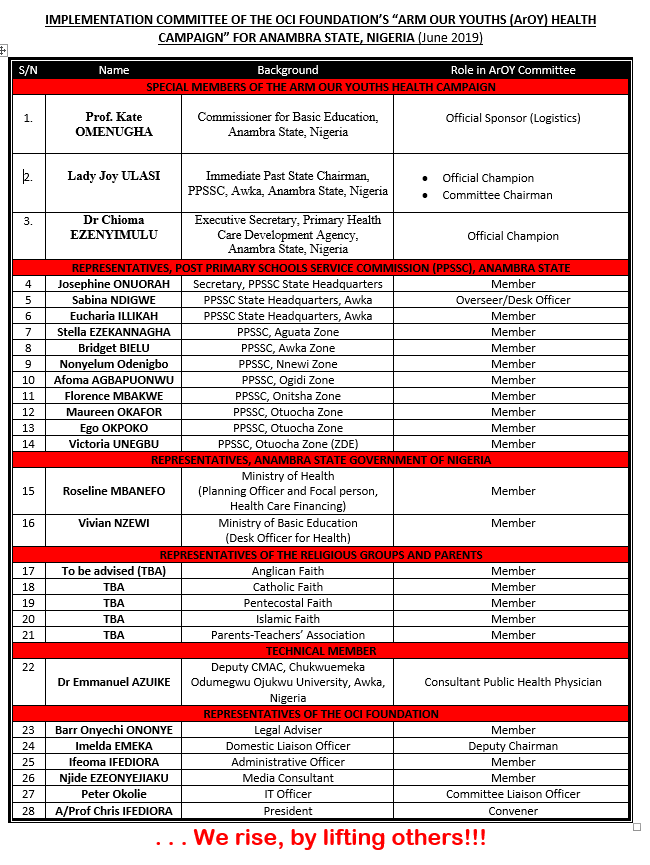
**

**APPENDIX 3: ETHICAL APPROVAL**


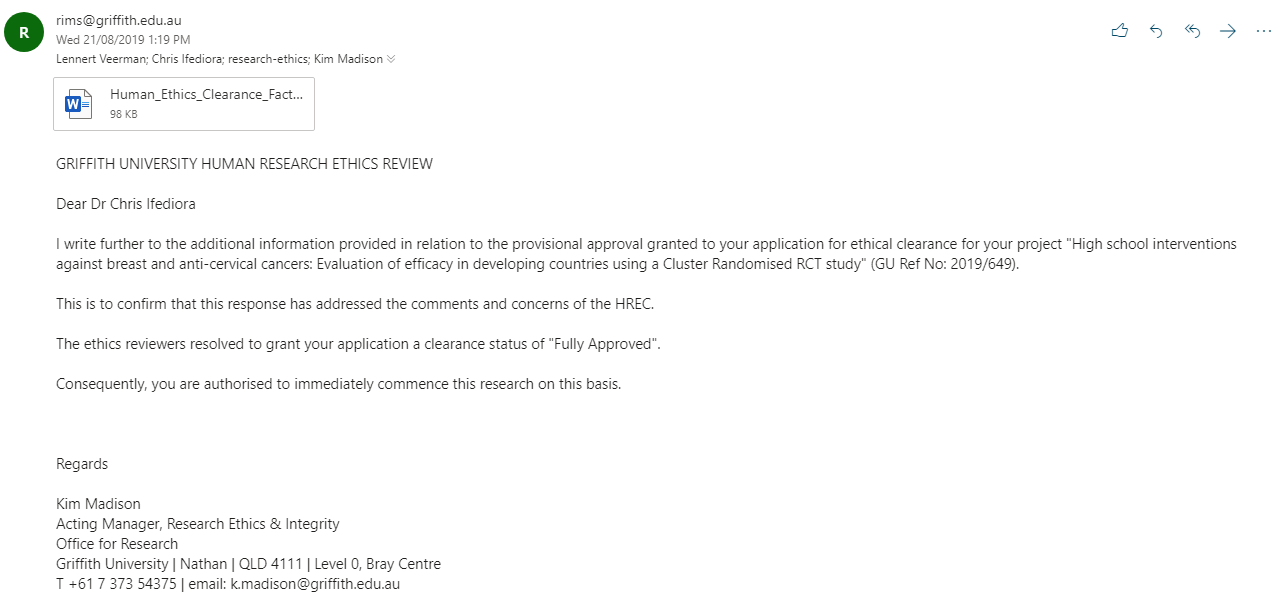

Supplement: Supplementary file 1 — Additional file 1. [file 12889_2022_14231_MOESM1_ESM.docx]
