# Supplementary material for: Outcomes from integrating anti-cervical cancer teachings into the curriculum of high schools in a South-Eastern Nigerian State
Source: BMC Public Health. 2022 Oct 14;22:1914. doi: 10.1186/s12889-022-14231-4 (PMC9562070; doi:10.1186/s12889-022-14231-4)
Supplement: Supplementary file 3 — Additional file 3. [file 12889_2022_14231_MOESM3_ESM.pdf]

Breast and Cervical Cancers

Introduction and Explanation of Survey

1. **Information Sheet and Introductory Letter to Participants**

Dear,

Thank you very much for your time in looking at this questionnaire. This is a follow-up study being conducted by Assoc. Prof. Chris Ifediora and Prof. Lennert Veerman (both of Griffith University, Gold Coast, Australia), Dr. Emmanuel Azuike (of Chukwuemeka Odumegwu Ojukwu University, Anambra State, Nigeria), and Dr Uche Ekwochi of the Enugu State University of Science and Technology, Enugu State, Nigeria). We also have Assoc. Prof. William Obiozor of the Nnamdi Azikiwe University, Awka, Nigeria.

You might remember completing a similar document recently. It is expected that there will be a total of 3 follow-up questionnaires like this, which will come about a week ater the original one, and then 6 months later, and then 12 months later.

Please, kindly answer all the questions contained in this document. The entire survey is expected to take about 30 minutes. Apart from this time consideration, you are not expected to face any risk or discomfort.

Please note that your participation in this study is voluntary, and strict confidentiality will be maintained throughout. As such, you will not be required to identify yourself at any point. However, it is very important that the answers you provide to the questions are as accurate as possible, and should be as it concerns you alone. It will also be appreciated if you can complete all parts of the questionnaire.

Ethical clearance has been obtained from the Griffith University Human Research Ethics Committee, with reference number GU Ref No: 2019/649.

The collated, anonymized data and computed results will be stored in the Griffith University Research Storage Service, based in Australia, during the active phase of this research. After the study, all the data will be moved to the Griffith Archive Storage System, and will be kept there for a duration of up to five years, in line with the Griffith University's ethical regulations.

If you have any concerns with this study, please contact Assoc. Prof. Chris O. Ifediora (MBBS, MPH, FRACGP, AFANZAHPE) through c.ifediora@griffith.edu.au or +61755808331. If, however, you have additional questions about the ethics of this research, please contact the Manager, Research Ethics of Griffith University, Australia, on +6173735 4375 or researchethics@griffith.edu.au.

If you wish to proceed with this study, please turn over to the next page to continue. Otherwise, kindly return this questionnaire to your school principal or teacher. Completion of this survey will be taken as your consent to participate in the research.

◯ Thank you.

2. **What is your gender? (*please write*):**

3. **What is your age (*in years*)?**

4. **Which State is your school located in? Please chose one:**

- ☐ Anambra
- ☐ Enugu

5. **What is your current level (class) of study? Please choose one:**

- ☐ SSS 1
- ☐ SSS 2
- ☐ SSS 3

6. **Which of these best describe the school you currently attend? *Please choose one:***

- ☐ Girls-only school
- ☐ Boys-only school
- ☐ Mixed boys and girls school

7. **Have you ever heard of breast cancer? *(Please tick only one answer):***

- ○ Yes
- ○ No
- ○ Not sure

8. **Have you ever heard of Breast Self-Examination? *(Please tick only one answer):***

- ○ Yes
- ○ No
- ○ Not Sure

[illegible] Breast Cancers and Breast Self-Examinations

Knowledge (Questions 1 to 8), Attitudes (Questions 9 to 10) and Practices (Questions 11 to 12) towards Breast Cancers and Breast Self-Examinations

[illegible]

4. **Who is advised to do breast self-examination? (*please tick only one box for each question*):**

	Agree	Unsure	Disagree
All women aged 20 years or older	☐	☐	☐
Only married women	☐	☐	☐
Only women who have started having sexual intercourse	☐	☐	☐
Only women that have ever been pregnant	☐	☐	☐
Only women whose periods (menstruation) have stopped (usually above 50 years of age)	☐	☐	☐
Only women that have had breast cancers or with breast cancers in their families	☐	☐	☐

5. **How often should breasts be examined? (*please tick only one box for each question*):**

	Agree	Unsure	Disagree
Once a Day	☐	☐	☐
Once a Week	☐	☐	☐
Once a Month	☐	☐	☐
Once every 6 months	☐	☐	☐
Once every year	☐	☐	☐

6. **At what stage of your monthly cylce should you examine your breasts (*please tick only one box for each question*)?**

	Agree	Unsure	Disagree
Anytime	☐	☐	☐
During your period (menstrual flow)	☐	☐	☐
7 to 10 days after your period (menstrual flow) stops	☐	☐	☐
Midway through your cycle	☐	☐	☐
7 to 10 days before your period (menstrual flow) starts	☐	☐	☐

7. **Which of the following may increase the risks of developing breast cancer (*please tick one box for each question*):**

	Agree	Unsure	Disagree
Starting your periods (menstruation) at an early age (before 11 years)	☐	☐	☐
Starting your periods (menstruation) at a late age (over 16 years)	☐	☐	☐
Having no babies at all	☐	☐	☐
Having many babies (more than 4)	☐	☐	☐
Eating a high fatty diet	☐	☐	☐
Using the birth control pill	☐	☐	☐
Having someone in the family with breast cancer	☐	☐	☐
Starting sexual intercourse at an early age	☐	☐	☐
Having a urinary tract infection (infection of urine)	☐	☐	☐

8. **Which of the following may indicate early breast cancer (please tick one box for each question):**

	Agree	Unsure	Disagree
Pain in the breasts	☐	☐	☐
Chest pain	☐	☐	☐
Headache	☐	☐	☐
Breast lump or swelling	☐	☐	☐
Lump or swelling in the armpit	☐	☐	☐
Nipple discharge from one breast only	☐	☐	☐
Nipple discharge in a pregnant person	☐	☐	☐
Nipple discharge when it is being queezed	☐	☐	☐

9. **Please answer the following about yourself (*tick only one box for each question*):**

	Yes	Unsure	No
I am healthy and do not need to examine my breasts	☐	☐	☐
Examining my breasts by myself is important and necessary	☐	☐	☐
I feel shy and embarrassed about examining my breasts by myself	☐	☐	☐
Examining my breasts by myself will be painful	☐	☐	☐
I am afraid that examining my breasts might reveal breast cancer	☐	☐	☐
Examining my breasts by myself will waste a lot of my time	☐	☐	☐
Examining my breasts by myself is a dirty practice and against my values or beliefs	☐	☐	☐
I will not like to touch my breasts in the way required for me to examine it	☐	☐	☐
If I find a suspicious lump while examining my breasts, I will see a medical doctor rather than a traditional healer	☐	☐	☐

10. **How do you respond to the following questions (*please tick only one box for each question*):**

	Agree	Unsure	Disagree
Cancer of the breast is a common cause of deaths arising from cancers among females in Nigeria	☐	☐	☐
Any young woman aged 20 years or more can develop breast cancer	☐	☐	☐
Cancer of the breast can be passed on from one person to another	☐	☐	☐
Self breast examination can help detect and prevent breast cancers	☐	☐	☐

11. **What is the best position to adopt while examining your breasts (*please tick only one box for each row*)?**

	Agree	Unsure	Disagree
Standing in front of a mirror	☐	☐	☐
Lying down	☐	☐	☐
Use right hand to examine the left breast (and left hand to examine right breast)	☐	☐	☐
Use the finger pads (finger pulps) of the fingers to examine	☐	☐	☐
Use my fingernails to examine	☐	☐	☐
Use my palms to examine	☐	☐	☐
Use one finger only	☐	☐	☐
Use the 3 middle fingers only	☐	☐	☐
Examine the breast by moving fingers in circles around the breast till all parts are covered	☐	☐	☐
Examine the breasts by moving fingers from top of breast to bottom (vertically) till all parts covered	☐	☐	☐
Examine breasts by checking small sections of it (in wedges) till all parts examined	☐	☐	☐
Press the nipple to check for any discharge	☐	☐	☐
Examine the armpit for lumps	☐	☐	☐
Look for any changes in breast size or shape	☐	☐	☐
Look at the nipple to see if it is drawn inwards	☐	☐	☐
Raise one hand above the head while examining breast on that side	☐	☐	☐
Undress to the waist	☐	☐	☐

12. **In the last 6 months, how often have you examined your breasts? (*please tick only one answer*):**

- ○ Not at all
- ○ Once every day
- ○ At least once every week
- ○ At least once every month
- ○ Once in the last 6 months

Griffith UNIVERSITY

[illegible] Cervical Cancers

Knowledge (Questions 1 to 7) and Attitude (Question 8) towards Cervical Cancers, Screening and Vaccination

1. **Have you ever heard about "cancer of the cervix", which is also called "cervical cancer"?** ***(please tick only one answer)***
Note: Cervical Cancer is the cancer of the lower part (or entrance) to the womb. It is the part of the womb that connects it to the vagina.

- ○ Yes
- ○ No
- ○ Not sure

2. **Have you ever heard of "screening for cervical cancer"?** ***(please tick only one answer)***

- ○ Yes
- ○ No
- ○ Not sure

3. **Have you ever heard about "Pap Test", which is also called "Pap Smear"?** ***(please tick only one answer):***
Note: Pap Test is a test done to look for Cervical Cancer.

- ○ Yes
- ○ No
- ○ Not sure

4. **Have you ever heard about "Human Papilloma Virus (HPV)" and Vaccinations against it to prevent Cervical Cancer?** ***(please tick only one answer)***

- ○ Yes
- ○ No
- ○ Not sure

5. **If you have ever heard about cervical cancer, screening for cervical cancer, pap test or HPV vaccination, how did you hear about it?** ***(kindly tick all the sources of your information, which may be more than one):***

- ○ Home/Family
- ○ Friends/Peers
- ○ TV, Radio
- ○ Newspaper/Magazines
- ○ School Teachers
- ○ Health Workers (doctors, nurses, etc.)
- ○ In the church
- ○ Internet: Facebook, Google, Twitter, etc.
- ○ During the health symposium at Umueri
- ○ From the information handout/fact-sheet that was given out after the health symposium at Umueri
- ○ I have never heard about any of those terms

6. **Regarding cervical cancers, what do you think concerning the following? (*please tick only one box for each question*):**

	Agree	Unsure	Disagree
Pap smear (test) can help detect early cervical cancer	☐	☐	☐
Pap smear is only for married women	☐	☐	☐
Pap smear is advisable for women 18 years or over who have started having sexual intercourse	☐	☐	☐
Pap smear is advised for all women after 2 years of starting sexual activity	☐	☐	☐
Only women whose periods (menstruation) have stopped (usually above 50 years of age) should have pap smears	☐	☐	☐
Only women that have had cervical cancers or those whose family members have had it should have pap smears	☐	☐	☐
Pap smears should be done at least every 2 years for those who are eligible	☐	☐	☐
Pap smears should be done only once in a lifetime	☐	☐	☐
Human Papilloma Virus (HPV) Vaccine can help protect against cervical cancers	☐	☐	☐
To be effective, HPV Vaccine is recommended for adolescent girls before the first exposure to sexual activity	☐	☐	☐

7. **Which of the following may increase the risks of developing cervical cancer? (*please tick one box for each question*):**

	Agree	Unsure	Disagree
Early onset of unprotected sexual activities	☐	☐	☐
Having multiple sexual partners	☐	☐	☐
Smoking cigarretes	☐	☐	☐
Drinking lots of alcohol	☐	☐	☐
Having many babies (more than 4)	☐	☐	☐
Eating a high fatty diet	☐	☐	☐
Using the birth control pill	☐	☐	☐
Spiritual attack	☐	☐	☐
Having someone in the family with cervical cancer	☐	☐	☐
Having vaginal warts (caused by the Human Papilloma Virus, a type of viral infection)	☐	☐	☐
Having a urinary tract infection (infection of urine)	☐	☐	☐
Having sexually transmitted diseases (infections)	☐	☐	☐
Poison from enemies	☐	☐	☐
Cervical cancer can be inherited from one's parents	☐	☐	☐

8. **Which of the following may indicate early cervical cancer (please tick one box for each question):**

	Agree	Unsure	Disagree
Vaginal discharge with an offensive (foul) smell	☐	☐	☐
Bleeding after sexual intercourse	☐	☐	☐
Having pain during menstruation	☐	☐	☐
Having heavy bleeds during menstruation	☐	☐	☐
There may be no symptoms	☐	☐	☐
Rash in vaginal area (private part)	☐	☐	☐
Swelling in vaginal area (private part)	☐	☐	☐

9. **How do you respond to the following questions regarding cervical cancer screening through pap smears (*please tick only one box for each question*):**

	Agree	Unsure	Disagree
If I am eligible, I will like to do regular pap smears to detect early cervical cancer	☐	☐	☐
I am afraid that pap smear will be painful or dangerous	☐	☐	☐
I will like to know more about pap smears	☐	☐	☐
I can only undertake pap smear if it is free	☐	☐	☐
No matter the financial costs, I will like to undertake regular pap smears	☐	☐	☐
I will be too embarrased to discuss pap smears with my doctor	☐	☐	☐
I am worried about what the doctor will find from the pap smear test	☐	☐	☐
I will be too busy to find time for the pap smear	☐	☐	☐
I do not think that pap smear for early detection is necessary	☐	☐	☐
It is against my religious and cultural beliefs to undertake pap smear	☐	☐	☐

10. **How do you respond to the following questions regarding cervical cancer prevention through HPV Vaccinations (*please tick only one box for each question*):**

	Agree	Unsure	Disagree
If available, I will like to have the HPV Vaccine that prevents cervical cancer	☐	☐	☐
I am afraid that HPV vaccination will be painful or dangerous	☐	☐	☐
I will like to know more about HPV Vaccines	☐	☐	☐
I can undertake HPV vaccination only if it is free	☐	☐	☐
No matter the financial costs, I will like to have HPV vaccines if I am eligible	☐	☐	☐
I will be too embarrased to ask for HPV vaccination from my doctor	☐	☐	☐
I will be too busy to find time for HPV vaccination	☐	☐	☐
I do not think that HPV vaccination for preventing cervical cancer is necessary	☐	☐	☐
It is against my religious and/or cultural beliefs to go for HPV vaccination	☐	☐	☐

OCI Foundation . . . *we rise, by lifting others!*

(Website: www.ocifoundationng.org)
