# Supplementary material for: Outcomes from integrating anti-cervical cancer teachings into the curriculum of high schools in a South-Eastern Nigerian State
Source: BMC Public Health. 2022 Oct 14;22:1914. doi: 10.1186/s12889-022-14231-4 (PMC9562070; doi:10.1186/s12889-022-14231-4)
Supplement: Supplementary file 4 — Additional file 4. [file 12889_2022_14231_MOESM4_ESM.docx]

**Table 1: Variables used for the study**

| S/N | 1. **DEPENDENT (OUTCOME) VARIABLES** | | | | |
| --- | --- | --- | --- | --- | --- |
| 1 | Knowledge on cervical cancer “General Awareness” | | | | |
| 2 | Knowledge on “Pap Smears” for cervical cancer | | | | |
| 3 | Knowledge on HPV Vaccinations | | | | |
| 4 | Knowledge on cervical cancer “Risk Factors” | | | | |
| 5 | Knowledge on cervical cancer “Symptoms” | | | | |
| S/N | 1. **INDEPENDENT/PREDICTOR VARIABLES (QUESTIONS ON THE 5 ASPECTS OF CERVICAL CANCER)** | | | | |
|  | **General Awareness**  *(4 Questions)* | **Pap Smears**  *(8 Questions)* | **HPV Vaccine**  *(2 Questions)* | **Risk Factors**  *(14 Questions)* | **Symptoms**  *(7 Questions)* |
| 1 | Ever heard of cervical cancer? | Pap smear detects cervical cancer (Ca) | HPV vaccine is preventive | Early sex | Offensive discharge |
| 2 | Ever heard of cervical cancer screening? | Pap smear is for married women only | HPV Vaccine is best first sex | Multiple partners | Bleeds after sexual intercourse |
| 3 | Ever heard of Pap Smear? | Pap smear is for women > 18 years |  | Smoking | Pain with menstruation |
| 4 | Ever heard of HPV and Vaccinations? | Pap smear should start 2 years after sexual debut |  | Alcohol | Heavy period (menstruation) |
| 5 |  | Pap smear starts from menopause |  | Having more than 4 babies | No symptoms |
| 6 |  | Pap smear is for those with a past history of Ca Cervix |  | High fat diet | Vaginal rash |
| 7 |  | Pap smear is to be done 2 yearly |  | Use of the oral birth pill | Vaginal swelling |
| 8 |  | Pap smear is to be done once only |  | Spiritual attack |  |
| 9 |  |  |  | Having a family history |  |
| 10 |  |  |  | Vaginal wart |  |
| 11 |  |  |  | Urinary Tract Infection |  |
| 12 |  |  |  | STDs |  |
| 13 |  |  |  | Poison |  |
| 14 |  |  |  | Hereditary |  |
| ***NOTE****: With respect to Supplementary Data S1 and S2, all questions with options of “Yes/Agree”, “Not Sure/Unsure” and “No/Disagree” were dichotomized into “correct” for the right ones, or “incorrect” for the “wrong and not sure/unsure” responses. These were used to derive the answers for the dependemt sand independent variables used in thr analysis.* | | | | | |
| S/N | 1. **RESPONDENT VARIABLES FOR LOGISTICS REGRESSION ANALYSIS** | | | | |
|  | Variables | | | Categories | |
| 1 | Age Ranges (Years) | | | <15 | |
|  |  |  |  | ≥15 to ≤19 | |
|  |  |  |  | >19 | |
| 2 | Gender | | | Male | |
|  |  |  |  | Female | |
| 3 | Gender of School | | | Boys- only school | |
|  |  |  |  | Girls-only school | |
|  |  |  |  | Mixed school | |
| 4 | Class of Study | | | Senior Secondary School (SSS) 1 | |
|  |  |  |  | Senior Secondary School (SSS) 2 | |
|  |  |  |  | Senior Secondary School (SSS) 3 | |
