# Supplementary material for: Outcomes from integrating anti-cervical cancer teachings into the curriculum of high schools in a South-Eastern Nigerian State
Source: BMC Public Health. 2022 Oct 14;22:1914. doi: 10.1186/s12889-022-14231-4 (PMC9562070; doi:10.1186/s12889-022-14231-4)
Supplement: Supplementary file 5 — Additional file 5. [file 12889_2022_14231_MOESM5_ESM.pdf]

Table: Phase 1 Data (Pre-Intervention) for Anti-cervical cancer teachings in High Schools in Southeast Nigeria

ID	Gender	Age	Age2	StateofSch	AnambraL	ClassofStu	SchoolGen	HeardofBr	HeardofBS
1	2	16	2	1	#NULL!	3	1	2	2
2	2	15	2	1	#NULL!	1	1	1	2
3	2	16	2	1	#NULL!	3	1	1	1
4	2	17	2	1	#NULL!	1	1	1	2
5	2	15	2	1	#NULL!	1	1	1	2
6	2	0	#NULL!	1	#NULL!	1	1	1	2
7	2	16	2	1	#NULL!	3	1	1	1
8	2	18	2	1	#NULL!	3	1	1	1
9	2	18	2	1	#NULL!	3	1	1	1
10	2	15	2	1	#NULL!	3	1	1	2
11	2	17	2	1	#NULL!	3	1	1	2
12	2	17	2	1	#NULL!	3	1	1	2
13	2	16	2	1	#NULL!	3	1	1	1
14	2	15	2	1	#NULL!	3	1	1	1
15	2	16	2	1	#NULL!	3	1	1	2
16	2	16	2	1	#NULL!	3	1	1	1
17	2	15	2	1	#NULL!	3	1	1	2
18	2	16	2	1	#NULL!	3	1	1	1
19	2	16	2	1	#NULL!	3	1	1	1
20	2	17	2	9	#NULL!	9	1	1	9
21	2	17	2	1	#NULL!	3	1	1	1
22	2	18	2	1	#NULL!	3	1	1	1
23	2	17	2	1	#NULL!	3	1	1	2
24	2	16	2	1	#NULL!	3	1	1	1
25	2	18	2	1	#NULL!	3	1	1	1
26	2	17	2	1	#NULL!	3	1	1	1
27	2	18	2	1	#NULL!	3	1	1	1
28	2	16	2	1	#NULL!	3	1	1	2
29	2	17	2	1	#NULL!	3	1	1	2
30	2	18	2	1	#NULL!	3	1	1	2
31	2	16	2	1	#NULL!	3	1	1	2
32	2	18	2	1	#NULL!	3	1	1	2
33	2	16	2	1	#NULL!	3	1	2	1
34	2	14	1	1	#NULL!	3	1	1	1
35	2	16	2	1	#NULL!	3	1	1	2
36	2	15	2	1	#NULL!	3	1	1	1
37	2	17	2	1	#NULL!	3	1	1	2
38	2	16	2	1	#NULL!	3	1	1	1
39	2	17	2	1	#NULL!	3	1	1	2
40	2	16	2	1	#NULL!	3	1	1	2
41	2	17	2	1	#NULL!	3	1	1	2
42	2	0	#NULL!	1	#NULL!	3	1	2	1
43	2	17	2	1	#NULL!	3	1	1	1
44	2	16	2	1	#NULL!	3	1	1	1
45	2	19	2	1	#NULL!	3	1	1	1
46	2	18	2	1	#NULL!	3	1	1	1

47	2	17	2	1	#NULL!	3	1	1	2
48	2	18	2	1	#NULL!	3	1	1	1
49	2	17	2	1	#NULL!	3	1	1	1
50	2	15	2	1	#NULL!	3	1	1	1
51	2	17	2	1	#NULL!	3	1	1	1
52	2	16	2	1	#NULL!	3	1	1	1
53	2	17	2	1	#NULL!	3	1	2	1
54	2	15	2	1	#NULL!	3	1	1	1
55	2	17	2	1	#NULL!	3	1	1	1
56	2	15	2	1	#NULL!	3	1	1	1
57	2	17	2	1	#NULL!	3	1	1	1
58	2	19	2	1	#NULL!	3	1	1	1
59	2	15	2	1	#NULL!	3	1	1	1
60	2	16	2	1	#NULL!	3	1	1	9
61	2	15	2	1	#NULL!	3	1	1	2
62	2	16	2	1	#NULL!	3	1	1	1
63	2	16	2	1	#NULL!	3	1	1	1
64	2	0	#NULL!	1	#NULL!	3	1	1	1
65	2	16	2	1	#NULL!	3	1	1	2
66	2	17	2	1	#NULL!	3	1	1	1
67	2	17	2	1	#NULL!	3	1	1	2
68	2	17	2	1	#NULL!	3	1	1	2
69	2	18	2	1	#NULL!	3	1	1	1
70	2	17	2	1	#NULL!	3	1	1	1
71	2	17	2	1	#NULL!	3	1	1	1
72	2	15	2	1	#NULL!	3	1	1	1
73	2	17	2	1	#NULL!	3	1	1	2
74	2	16	2	1	#NULL!	3	1	1	2
75	2	18	2	1	#NULL!	3	1	1	2
76	2	17	2	1	#NULL!	3	1	1	1
77	2	17	2	1	#NULL!	3	1	1	1
78	2	17	2	1	#NULL!	3	1	1	1
79	2	19	2	1	#NULL!	3	1	1	1
80	2	0	#NULL!	1	#NULL!	3	1	1	1
81	2	18	2	1	#NULL!	3	1	1	1
82	9	19	2	1	#NULL!	3	1	1	1
83	9	16	2	1	#NULL!	3	1	1	1
84	2	15	2	1	#NULL!	3	1	1	2
85	2	17	2	1	#NULL!	3	1	1	1
86	2	17	2	1	#NULL!	3	1	1	1
87	2	17	2	1	#NULL!	3	1	1	1
88	2	17	2	1	#NULL!	3	1	1	1
89	2	17	2	1	#NULL!	3	1	1	2
90	2	16	2	1	#NULL!	3	1	1	1
91	2	16	2	1	#NULL!	3	1	1	2
92	2	16	2	1	#NULL!	3	1	2	2
93	2	16	2	1	#NULL!	3	1	2	2

94	2	16	2	1	#NULL!	3	1	1	1
95	2	17	2	1	#NULL!	3	1	1	1
96	2	15	2	1	#NULL!	3	1	1	1
97	2	16	2	1	#NULL!	3	1	1	1
98	2	16	2	1	#NULL!	3	1	2	1
99	2	16	2	1	#NULL!	3	1	1	2
100	2	17	2	1	#NULL!	3	1	1	1
101	2	17	2	1	#NULL!	3	1	1	1
102	2	17	2	1	#NULL!	3	1	1	1
103	2	16	2	1	#NULL!	3	1	1	1
104	2	15	2	1	#NULL!	3	1	1	3
105	2	17	2	1	#NULL!	3	1	1	9
106	2	17	2	1	#NULL!	3	1	1	1
107	2	16	2	1	#NULL!	3	1	1	1
108	2	17	2	1	#NULL!	3	1	1	3
109	2	16	2	1	#NULL!	3	1	1	2
110	2	16	2	1	#NULL!	3	1	1	1
111	2	15	2	1	#NULL!	3	1	1	1
112	2	17	2	1	#NULL!	3	1	1	1
113	2	16	2	1	#NULL!	3	1	1	2
114	2	20	3	1	#NULL!	3	1	1	2
115	2	16	2	1	#NULL!	3	1	1	1
116	2	18	2	1	#NULL!	3	1	2	1
117	2	15	2	1	#NULL!	3	1	2	2
118	2	17	2	1	#NULL!	3	1	1	1
119	2	16	2	1	#NULL!	3	1	1	1
120	2	16	2	1	#NULL!	3	1	1	2
121	2	16	2	1	#NULL!	3	1	2	2
122	2	18	2	1	#NULL!	3	1	1	2
123	2	18	2	1	#NULL!	3	1	1	2
124	2	17	2	1	#NULL!	3	1	1	2
125	2	16	2	1	#NULL!	3	1	2	3
126	2	19	2	1	#NULL!	3	1	2	2
127	2	17	2	1	#NULL!	3	1	1	2
128	2	17	2	1	#NULL!	3	1	1	2
129	2	16	2	1	#NULL!	3	1	1	2
130	2	17	2	1	#NULL!	3	1	1	1
131	2	15	2	1	#NULL!	3	1	1	1
132	2	18	2	1	#NULL!	3	1	1	1
133	2	17	2	1	#NULL!	3	1	1	1
134	2	19	2	1	#NULL!	3	1	1	1
135	2	18	2	1	#NULL!	3	1	1	1
136	2	18	2	1	#NULL!	3	1	2	3
137	2	15	2	1	#NULL!	3	1	1	2
138	2	17	2	1	#NULL!	3	1	2	3
139	2	17	2	1	#NULL!	3	1	1	2
140	2	16	2	1	#NULL!	3	1	2	2

141	2	19	2	1	#NULL!	3	1	1	2
142	2	17	2	1	#NULL!	3	1	1	1
143	2	18	2	1	#NULL!	3	1	1	2
144	2	18	2	1	#NULL!	3	1	1	1
145	2	18	2	1	#NULL!	3	1	1	3
146	2	17	2	1	#NULL!	3	1	1	1
147	2	21	3	1	#NULL!	9	1	1	9
148	2	15	2	1	#NULL!	3	1	1	1
149	2	15	2	1	#NULL!	3	1	1	2
150	2	18	2	1	#NULL!	3	1	1	2
151	2	18	2	1	#NULL!	3	1	1	2
152	2	19	2	1	#NULL!	3	1	1	2
153	2	20	3	1	#NULL!	3	1	1	9
154	2	16	2	1	#NULL!	3	1	1	2
155	2	16	2	1	#NULL!	3	1	1	2
156	2	17	2	1	#NULL!	3	1	1	2
157	2	18	2	1	#NULL!	3	1	1	2
158	2	16	2	1	#NULL!	3	1	1	2
159	2	19	2	1	#NULL!	3	1	1	1
160	2	15	2	1	#NULL!	2	1	2	2
161	2	16	2	1	#NULL!	2	1	1	1
162	2	17	2	1	#NULL!	2	1	1	2
163	2	17	2	1	#NULL!	2	1	1	2
164	2	16	2	1	#NULL!	2	1	2	2
165	2	16	2	1	#NULL!	2	1	2	2
166	2	17	2	1	#NULL!	2	1	1	1
167	2	15	2	1	#NULL!	2	1	2	2
168	2	15	2	1	#NULL!	2	1	1	1
169	2	15	2	1	#NULL!	2	1	1	2
170	2	16	2	1	#NULL!	2	1	1	2
171	2	17	2	1	#NULL!	3	1	1	1
172	2	18	2	1	#NULL!	3	1	1	3
173	2	16	2	1	#NULL!	2	1	2	2
174	2	17	2	1	#NULL!	2	1	2	2
175	2	16	2	1	#NULL!	2	1	2	2
176	2	16	2	1	#NULL!	2	1	1	3
177	2	16	2	1	#NULL!	2	1	1	1
178	2	16	2	1	#NULL!	2	1	1	1
179	2	16	2	1	#NULL!	2	1	1	2
180	2	17	2	1	#NULL!	2	1	1	2
181	2	16	2	1	#NULL!	2	1	1	1
182	2	16	2	1	#NULL!	2	1	2	2
183	2	15	2	1	#NULL!	2	1	1	9
184	2	20	3	1	#NULL!	2	1	1	3
185	2	15	2	1	#NULL!	2	1	1	1
186	2	20	3	1	#NULL!	2	1	1	3
187	2	16	2	1	#NULL!	2	1	1	3

188	2	17	2	1	#NULL!	2	1	2	1
189	2	15	2	1	#NULL!	2	1	1	1
190	9	15	2	1	#NULL!	2	1	2	2
191	2	16	2	1	#NULL!	2	1	1	1
192	2	15	2	1	#NULL!	2	1	1	3
193	2	15	2	1	#NULL!	2	1	1	1
194	2	16	2	1	#NULL!	2	1	1	1
195	2	15	2	1	#NULL!	3	1	1	1
196	2	16	2	1	#NULL!	3	1	1	1
197	2	16	2	1	#NULL!	3	1	1	1
198	9	0	#NULL!	1	#NULL!	3	1	1	1
199	2	17	2	1	#NULL!	3	1	1	1
200	2	18	2	1	#NULL!	3	1	1	1
201	2	17	2	1	#NULL!	2	1	1	1
202	2	17	2	1	#NULL!	2	1	1	1
203	2	15	2	1	#NULL!	2	1	1	1
204	2	16	2	1	#NULL!	2	1	1	2
205	2	17	2	1	#NULL!	2	1	1	2
206	2	15	2	1	#NULL!	2	1	1	1
207	2	16	2	1	#NULL!	2	1	1	2
208	2	16	2	1	#NULL!	2	1	1	1
209	2	15	2	1	#NULL!	2	1	1	2
210	2	16	2	1	#NULL!	2	1	1	2
211	2	15	2	1	#NULL!	2	1	1	3
212	2	16	2	1	#NULL!	2	1	1	2
213	2	16	2	1	#NULL!	2	1	1	2
214	2	16	2	1	#NULL!	2	1	2	2
215	2	16	2	1	#NULL!	2	1	2	2
216	2	16	2	1	#NULL!	2	1	1	2
217	2	16	2	1	#NULL!	2	1	1	1
218	2	15	2	1	#NULL!	2	1	1	3
219	2	19	2	1	#NULL!	2	1	1	3
220	2	16	2	1	#NULL!	2	1	1	1
221	2	16	2	1	#NULL!	2	1	2	2
222	2	16	2	1	#NULL!	2	1	1	3
223	2	16	2	1	#NULL!	2	1	1	2
224	2	15	2	1	#NULL!	2	1	1	3
225	2	17	2	1	#NULL!	2	1	3	1
226	2	15	2	1	#NULL!	2	1	1	1
227	2	17	2	1	#NULL!	2	1	1	2
228	2	17	2	1	#NULL!	2	1	1	1
229	2	15	2	1	#NULL!	2	1	1	2
230	2	16	2	1	#NULL!	2	1	1	2
231	2	15	2	1	#NULL!	2	1	1	2
232	2	15	2	1	#NULL!	2	1	1	2
233	2	15	2	1	#NULL!	2	1	1	2
234	2	16	2	1	#NULL!	2	1	1	2

235	2	15	2	1	#NULL!	2	1	1	2
236	2	16	2	1	#NULL!	2	1	1	3
237	2	15	2	1	#NULL!	2	1	1	1
238	2	18	2	1	#NULL!	2	1	1	2
239	2	16	2	1	#NULL!	2	1	1	2
240	2	16	2	1	#NULL!	3	9	1	1
241	2	17	2	1	#NULL!	3	1	1	1
242	2	17	2	1	#NULL!	3	1	1	1
243	2	16	2	1	#NULL!	3	1	1	1
244	2	17	2	1	#NULL!	3	1	1	2
245	2	16	2	1	#NULL!	3	1	1	2
246	2	17	2	1	#NULL!	3	1	2	2
247	2	17	2	1	#NULL!	3	1	1	2
248	2	16	2	1	#NULL!	3	1	1	1
249	2	16	2	1	#NULL!	3	1	1	2
250	2	16	2	1	#NULL!	3	1	2	2
251	2	17	2	1	#NULL!	3	1	1	2
252	2	17	2	1	#NULL!	3	1	1	2
253	2	17	2	1	#NULL!	3	1	2	2
254	2	16	2	1	#NULL!	3	1	1	2
255	2	17	2	1	#NULL!	3	1	1	1
256	2	17	2	1	#NULL!	3	1	1	2
257	2	16	2	1	#NULL!	3	1	1	2
258	2	17	2	1	#NULL!	3	1	1	1
259	2	15	2	1	#NULL!	3	1	1	2
260	2	18	2	1	#NULL!	3	1	1	2
261	2	16	2	1	#NULL!	3	1	1	2
262	2	15	2	1	#NULL!	3	1	1	2
263	2	0	#NULL!	1	#NULL!	3	1	1	2
264	2	16	2	1	#NULL!	3	1	1	3
265	2	18	2	1	#NULL!	3	1	1	3
266	2	17	2	1	#NULL!	3	1	1	2
267	2	18	2	1	#NULL!	3	1	1	2
268	2	17	2	1	#NULL!	3	1	1	1
269	9	17	2	1	#NULL!	3	1	1	3
270	2	17	2	1	#NULL!	3	1	2	1
271	2	16	2	1	#NULL!	3	1	1	2
272	2	16	2	1	#NULL!	3	1	1	1
273	2	18	2	1	#NULL!	3	1	1	3
274	2	17	2	1	#NULL!	3	1	1	9
275	2	15	2	1	#NULL!	2	1	1	2
276	2	14	1	1	#NULL!	2	1	1	1
277	2	0	#NULL!	1	#NULL!	2	1	1	2
278	2	15	2	1	#NULL!	2	1	1	2
279	2	16	2	1	#NULL!	2	1	1	2
280	2	16	2	1	#NULL!	2	1	1	2
281	2	17	2	1	#NULL!	2	1	1	2

282	2	15	2	1	#NULL!	2	1	1	1
283	2	16	2	1	#NULL!	2	1	1	1
284	2	16	2	1	#NULL!	2	1	1	3
285	2	13	1	1	#NULL!	2	1	1	2
286	2	15	2	1	#NULL!	2	1	1	2
287	2	15	2	1	#NULL!	2	1	1	2
288	2	16	2	1	#NULL!	2	1	1	2
289	2	16	2	1	#NULL!	2	1	1	1
290	2	16	2	1	#NULL!	2	1	1	1
291	2	16	2	1	#NULL!	2	1	1	2
292	2	17	2	1	#NULL!	2	1	1	2
293	2	14	1	1	#NULL!	2	1	1	1
294	2	15	2	1	#NULL!	2	1	2	2
295	2	16	2	1	#NULL!	2	1	2	2
296	2	17	2	1	#NULL!	2	1	1	2
297	2	20	3	1	#NULL!	2	1	1	2
298	2	15	2	1	#NULL!	2	1	1	1
299	2	15	2	1	#NULL!	2	1	1	3
300	2	16	2	1	#NULL!	2	1	1	2
301	2	14	1	1	#NULL!	2	1	1	2
302	2	16	2	1	#NULL!	2	1	1	2
303	2	0	#NULL!	1	#NULL!	2	1	1	2
304	2	17	2	1	#NULL!	2	1	1	1
305	2	17	2	1	#NULL!	2	1	1	2
306	2	16	2	1	#NULL!	2	1	1	2
307	2	18	2	1	#NULL!	3	1	1	1
308	2	18	2	1	#NULL!	3	1	1	1
309	2	16	2	1	#NULL!	3	1	1	1
310	2	17	2	1	#NULL!	3	1	1	1
311	2	18	2	1	#NULL!	3	1	1	1
312	2	17	2	1	#NULL!	3	1	1	1
313	2	17	2	1	#NULL!	3	1	1	1
314	2	17	2	9	#NULL!	9	9	9	9
315	2	17	2	9	#NULL!	3	1	1	1
316	2	18	2	1	#NULL!	3	1	1	1
317	2	17	2	1	#NULL!	3	1	1	3
318	2	17	2	1	#NULL!	3	1	1	1
319	2	16	2	1	#NULL!	3	1	1	1
320	2	17	2	1	#NULL!	3	1	1	1
321	2	17	2	1	#NULL!	3	1	1	2
322	2	19	2	1	#NULL!	3	1	1	1
323	2	17	2	1	#NULL!	3	1	1	1
324	9	17	2	1	#NULL!	3	1	1	1
325	2	16	2	1	#NULL!	3	1	1	1
326	2	17	2	1	#NULL!	3	1	1	1
327	2	17	2	1	#NULL!	3	1	1	2
328	2	16	2	1	#NULL!	3	1	1	1

329	2	15	2	1	#NULL!	3	1	1	1
330	2	16	2	1	#NULL!	3	1	1	1
331	2	16	2	1	#NULL!	3	1	1	1
332	2	15	2	1	#NULL!	3	1	1	1
333	2	17	2	1	#NULL!	3	1	1	3
334	2	17	2	1	#NULL!	3	1	1	1
335	2	17	2	1	#NULL!	3	1	1	1
336	2	18	2	1	#NULL!	3	1	1	1
337	2	16	2	1	#NULL!	2	1	1	3
338	2	16	2	1	#NULL!	2	1	1	1
339	2	16	2	1	#NULL!	2	1	1	1
340	2	15	2	1	#NULL!	2	1	1	1
341	2	15	2	1	#NULL!	2	1	1	1
342	2	16	2	1	#NULL!	1	1	1	1
343	2	16	2	1	#NULL!	2	1	1	1
344	2	16	2	1	#NULL!	2	1	1	2
345	2	17	2	1	#NULL!	2	1	1	1
346	2	17	2	1	#NULL!	2	1	1	1
347	2	16	2	1	#NULL!	2	1	1	1
348	2	14	1	1	#NULL!	2	1	1	2
349	2	16	2	1	#NULL!	2	1	1	2
350	2	16	2	1	#NULL!	2	1	1	2
351	2	16	2	1	#NULL!	2	1	1	2
352	2	14	1	1	#NULL!	2	1	1	1
353	2	14	1	1	#NULL!	2	1	1	2
354	2	16	2	1	#NULL!	2	1	1	2
355	2	15	2	1	#NULL!	2	1	1	2
356	2	15	2	1	#NULL!	2	1	1	1
357	2	16	2	1	#NULL!	2	1	1	2
358	2	16	2	1	#NULL!	2	1	1	2
359	2	15	2	1	#NULL!	2	1	1	2
360	2	15	2	1	#NULL!	2	1	1	2
361	2	16	2	1	#NULL!	2	1	1	2
362	2	15	2	1	#NULL!	2	1	1	2
363	2	15	2	1	#NULL!	2	1	1	2
364	2	15	2	1	#NULL!	2	1	1	2
365	2	15	2	1	#NULL!	2	1	1	2
366	2	15	2	1	#NULL!	2	1	1	2
367	2	15	2	1	#NULL!	2	1	1	2
368	2	14	1	1	#NULL!	2	1	3	2
369	2	16	2	1	#NULL!	2	1	1	1
370	2	15	2	1	#NULL!	2	1	1	2
371	2	15	2	1	#NULL!	2	1	1	2
372	2	13	1	1	#NULL!	2	1	1	2
373	2	14	1	1	#NULL!	2	1	1	1
374	2	15	2	1	#NULL!	2	1	1	3
375	2	16	2	1	#NULL!	2	1	1	2

376	2	16	2	1	#NULL!	2	1	1	1
377	2	14	1	1	#NULL!	2	1	1	1
378	2	16	2	1	#NULL!	2	1	2	2
379	2	16	2	1	#NULL!	2	1	1	2
380	2	14	1	1	#NULL!	2	1	1	2
381	2	15	2	1	#NULL!	2	1	1	1
382	2	0	#NULL!	1	#NULL!	2	1	1	2
383	2	15	2	1	#NULL!	2	1	1	1
384	2	15	2	1	#NULL!	2	1	1	1
385	2	14	1	1	#NULL!	2	1	1	2
386	2	14	1	1	#NULL!	2	1	1	3
387	2	16	2	1	#NULL!	2	1	1	1
388	2	17	2	1	#NULL!	2	1	1	1
389	2	16	2	1	#NULL!	2	1	1	2
390	2	17	2	1	#NULL!	2	1	1	2
391	2	16	2	1	#NULL!	2	1	1	1
392	2	16	2	1	#NULL!	2	1	1	2
393	2	14	1	1	#NULL!	2	1	1	1
394	2	16	2	1	#NULL!	2	1	2	1
395	2	16	2	1	#NULL!	2	1	1	1
396	2	15	2	1	#NULL!	2	1	1	3
397	2	15	2	1	#NULL!	2	1	1	1
398	2	16	2	1	#NULL!	2	1	1	1
399	2	15	2	1	#NULL!	2	1	1	2
400	2	16	2	1	#NULL!	2	1	1	1
401	2	19	2	1	#NULL!	2	1	1	1
402	2	15	2	1	#NULL!	2	1	1	2
403	2	15	2	1	#NULL!	2	1	1	2
404	2	12	1	1	#NULL!	2	1	1	2
405	2	17	2	1	#NULL!	2	1	1	2
406	2	16	2	1	#NULL!	2	1	1	1
407	2	17	2	1	#NULL!	2	1	1	2
408	2	16	2	1	#NULL!	2	1	1	1
409	2	15	2	1	#NULL!	2	1	2	2
410	2	16	2	1	#NULL!	2	1	2	2
411	2	0	#NULL!	1	#NULL!	2	1	1	2
412	2	14	1	1	#NULL!	2	1	1	2
413	2	16	2	1	#NULL!	2	1	1	2
414	2	18	2	1	#NULL!	2	1	1	2
415	2	15	2	9	#NULL!	2	1	1	1
416	2	0	#NULL!	1	#NULL!	2	1	1	2
417	2	15	2	1	#NULL!	1	1	1	2
418	2	15	2	1	#NULL!	2	1	1	2
419	2	17	2	1	#NULL!	2	1	1	2
420	2	16	2	1	#NULL!	2	1	1	3
421	2	16	2	1	#NULL!	2	1	1	2
422	2	17	2	1	#NULL!	2	1	1	2

423	2	16	2	1	#NULL!	2	1	1	2
424	2	15	2	1	#NULL!	2	1	1	1
425	2	16	2	1	#NULL!	2	1	1	2
426	2	18	2	1	#NULL!	2	1	2	2
427	2	16	2	9	#NULL!	9	9	9	9
428	2	15	2	1	#NULL!	2	1	2	1
429	2	17	2	1	#NULL!	2	1	1	2
430	2	16	2	1	#NULL!	2	1	1	2
431	2	19	2	1	#NULL!	2	1	1	2
432	2	16	2	1	#NULL!	2	1	2	2
433	2	16	2	1	#NULL!	2	1	1	2
434	2	14	1	1	#NULL!	2	1	1	1
435	2	15	2	1	#NULL!	2	1	1	2
436	2	15	2	1	#NULL!	2	1	1	1
437	2	15	2	1	#NULL!	2	1	1	2
438	2	15	2	1	#NULL!	2	1	1	1
439	2	16	2	1	#NULL!	2	1	1	9
440	2	15	2	1	#NULL!	2	1	2	1
441	2	16	2	1	#NULL!	2	1	1	1
442	2	14	1	1	#NULL!	2	1	1	1
443	2	15	2	1	#NULL!	2	1	1	2
444	2	16	2	1	#NULL!	2	1	2	3
445	2	15	2	1	#NULL!	2	1	1	1
446	2	15	2	1	#NULL!	2	1	1	1
447	2	14	1	1	#NULL!	2	1	1	1
448	2	15	2	1	#NULL!	2	1	1	1
449	2	17	2	1	#NULL!	2	1	1	2
450	2	16	2	1	#NULL!	2	1	1	3
451	2	16	2	9	#NULL!	2	1	1	2
452	2	15	2	1	#NULL!	2	1	1	3
453	2	15	2	1	#NULL!	2	1	1	1
454	2	15	2	1	#NULL!	2	1	1	2
455	2	16	2	1	#NULL!	2	1	1	1
456	2	16	2	1	#NULL!	2	1	1	1
457	2	14	1	9	#NULL!	2	1	1	1
458	2	16	2	1	#NULL!	2	1	1	1
459	2	15	2	1	#NULL!	2	1	1	3
460	2	16	2	1	#NULL!	2	1	1	3
461	2	15	2	1	#NULL!	2	1	1	3
462	2	15	2	1	#NULL!	2	1	1	1
463	2	16	2	1	#NULL!	2	1	1	2
464	2	19	2	1	#NULL!	3	1	1	2
465	2	16	2	1	#NULL!	3	1	1	2
466	2	19	2	1	#NULL!	3	1	1	2
467	2	16	2	1	#NULL!	3	1	1	2
468	2	17	2	1	#NULL!	3	1	1	2
469	9	17	2	1	#NULL!	3	1	1	2

470	2	16	2	1	#NULL!	2	1	2	2
471	2	19	2	1	#NULL!	3	1	1	1
472	2	19	2	1	#NULL!	3	1	1	1
473	2	17	2	1	#NULL!	3	1	2	1
474	2	18	2	1	#NULL!	3	1	1	2
475	2	0	#NULL!	1	#NULL!	3	1	1	1
476	2	19	2	1	#NULL!	3	1	1	2
477	2	18	2	1	#NULL!	3	1	1	9
478	2	15	2	1	#NULL!	3	1	1	1
479	2	17	2	1	#NULL!	3	1	1	1
480	2	0	#NULL!	1	#NULL!	3	1	1	1
481	2	17	2	1	#NULL!	2	1	1	2
482	2	16	2	1	#NULL!	3	1	2	2
483	2	17	2	1	#NULL!	3	1	1	2
484	2	17	2	1	#NULL!	2	1	2	2
485	2	16	2	1	#NULL!	2	1	2	2
486	2	17	2	1	#NULL!	2	1	1	2
487	2	18	2	1	#NULL!	2	1	1	2
488	2	15	2	1	#NULL!	2	1	1	1
489	2	14	1	1	#NULL!	2	1	1	1
490	9	0	#NULL!	9	#NULL!	9	9	9	9
491	2	17	2	1	#NULL!	2	1	1	1
492	2	15	2	1	#NULL!	2	1	1	1
493	2	17	2	1	#NULL!	2	1	1	1
494	2	16	2	1	#NULL!	2	1	1	2
495	2	16	2	1	#NULL!	2	1	1	2
496	2	16	2	1	#NULL!	2	1	1	1
497	2	16	2	1	#NULL!	2	1	1	2
498	2	16	2	1	#NULL!	2	1	1	2
499	2	16	2	1	#NULL!	2	1	1	1
500	2	16	2	1	#NULL!	2	1	1	2
501	2	15	2	1	#NULL!	2	1	1	2
502	2	16	2	1	#NULL!	2	1	1	2
503	2	16	2	1	#NULL!	2	1	2	2
504	2	14	1	1	#NULL!	2	1	1	2
505	2	15	2	1	#NULL!	2	1	1	1
506	2	15	2	1	#NULL!	2	1	1	1
507	2	14	1	1	#NULL!	2	1	1	1
508	2	17	2	1	#NULL!	2	1	2	2
509	2	15	2	1	#NULL!	2	1	1	2
510	2	14	1	1	#NULL!	2	1	1	2
511	2	15	2	1	#NULL!	2	1	1	1
512	2	17	2	1	#NULL!	2	1	1	2
513	2	16	2	1	#NULL!	2	1	1	2
514	2	15	2	1	#NULL!	2	1	1	2
515	2	16	2	1	#NULL!	2	1	1	1
516	2	16	2	1	#NULL!	2	1	1	3

517	2	16	2	1	#NULL!	2	1	1	3
518	2	15	2	1	#NULL!	2	1	1	2
519	2	15	2	1	#NULL!	2	1	1	2
520	2	15	2	1	#NULL!	2	1	1	2
521	2	15	2	1	#NULL!	2	1	1	3
522	2	17	2	1	#NULL!	2	1	1	2
523	2	14	1	1	#NULL!	2	1	1	2
524	2	16	2	1	#NULL!	2	1	1	1
525	2	15	2	1	#NULL!	2	1	1	1
526	2	16	2	1	#NULL!	2	1	1	1
527	2	15	2	1	#NULL!	2	1	1	9
528	2	15	2	1	#NULL!	2	1	1	1
529	2	16	2	1	#NULL!	2	1	1	2
530	2	16	2	1	#NULL!	2	1	1	2
531	2	17	2	1	#NULL!	2	1	1	2
532	2	14	1	1	#NULL!	2	1	1	2
533	2	16	2	1	#NULL!	2	1	1	3
534	2	17	2	1	#NULL!	2	1	1	1
535	2	14	1	1	#NULL!	2	1	2	2
536	2	16	2	1	#NULL!	2	1	1	1
537	2	15	2	1	#NULL!	2	1	1	2
538	2	15	2	1	#NULL!	2	1	1	2
539	2	17	2	1	#NULL!	2	1	1	2
540	2	17	2	1	#NULL!	2	1	1	1
541	2	15	2	1	#NULL!	2	1	1	1
542	2	14	1	1	#NULL!	2	1	1	2
543	2	15	2	1	#NULL!	2	1	1	1
544	2	15	2	1	#NULL!	2	1	1	1
545	2	15	2	1	#NULL!	2	3	1	1
546	2	16	2	1	#NULL!	3	1	1	2
547	2	16	2	1	#NULL!	3	1	1	1
548	2	17	2	1	#NULL!	3	1	1	2
549	2	16	2	1	#NULL!	3	1	1	2
550	2	18	2	1	#NULL!	3	1	1	2
551	2	17	2	1	#NULL!	3	1	2	3
552	2	16	2	1	#NULL!	3	1	1	2
553	2	17	2	1	#NULL!	3	1	1	2
554	2	15	2	1	#NULL!	3	1	1	1
555	2	17	2	1	#NULL!	3	1	1	3
556	2	18	2	1	#NULL!	3	1	1	2
557	2	0	#NULL!	1	#NULL!	3	1	1	1
558	2	15	2	1	#NULL!	3	1	1	1
559	2	17	2	1	#NULL!	3	1	1	1
560	2	18	2	1	#NULL!	3	1	1	1
561	2	16	2	1	#NULL!	2	1	1	3
562	2	16	2	1	#NULL!	2	1	1	1
563	2	15	2	1	#NULL!	2	1	1	2

564	2	16	2	1	#NULL!	2	1	1	2
565	2	17	2	1	#NULL!	2	1	1	2
566	2	15	2	1	#NULL!	2	1	1	2
567	2	17	2	1	#NULL!	2	1	1	2
568	2	18	2	1	#NULL!	2	1	1	2
569	2	15	2	1	#NULL!	2	1	1	2
570	2	17	2	1	#NULL!	2	1	1	2
571	2	16	2	1	#NULL!	2	1	1	3
572	2	16	2	1	#NULL!	2	1	1	2
573	2	16	2	1	#NULL!	2	1	1	3
574	2	16	2	1	#NULL!	2	1	1	3
575	2	16	2	1	#NULL!	2	1	1	3
576	2	16	2	1	#NULL!	2	1	1	2
577	2	19	2	1	#NULL!	2	1	1	2
578	2	15	2	1	#NULL!	2	1	1	2
579	2	16	2	1	#NULL!	2	1	1	2
580	2	17	2	1	#NULL!	2	1	1	2
581	2	15	2	1	#NULL!	2	1	1	2
582	1	0	#NULL!	1	#NULL!	2	2	3	3
583	1	15	2	1	#NULL!	2	2	3	1
584	1	15	2	1	#NULL!	2	2	2	2
585	1	17	2	1	#NULL!	2	2	1	3
586	1	15	2	1	#NULL!	2	2	1	2
587	1	16	2	1	#NULL!	2	2	1	1
588	2	18	2	1	#NULL!	3	3	1	3
589	1	0	#NULL!	1	#NULL!	3	3	1	1
590	2	18	2	1	#NULL!	3	3	1	1
591	1	16	2	1	#NULL!	3	3	1	1
592	1	0	#NULL!	1	#NULL!	3	3	1	1
593	1	17	2	1	#NULL!	3	3	1	2
594	1	19	2	1	#NULL!	3	3	1	2
595	2	16	2	1	#NULL!	3	3	1	2
596	2	17	2	1	#NULL!	3	3	1	1
597	1	17	2	1	#NULL!	3	3	1	1
598	1	18	2	1	#NULL!	3	3	1	1
599	1	15	2	1	#NULL!	3	3	1	3
600	2	13	1	1	#NULL!	9	3	1	2
601	2	13	1	1	#NULL!	9	3	1	2
602	2	13	1	1	#NULL!	9	3	1	2
603	2	15	2	1	#NULL!	9	3	1	2
604	1	18	2	9	#NULL!	9	9	9	9
605	1	14	1	1	#NULL!	9	3	1	3
606	2	15	2	1	#NULL!	9	3	1	1
607	2	15	2	1	#NULL!	9	3	1	1
608	1	14	1	1	#NULL!	9	3	2	2
609	2	13	1	1	#NULL!	9	3	1	1
610	1	14	1	1	#NULL!	9	3	1	3

611	2	14	1	1	#NULL!	3	3	1	2
612	2	14	1	1	#NULL!	9	3	3	1
613	9	14	1	1	#NULL!	9	3	9	2
614	2	17	2	1	#NULL!	9	2	1	1
615	1	15	2	1	#NULL!	9	3	1	1
616	1	0	#NULL!	1	#NULL!	9	3	1	1
617	1	17	2	1	#NULL!	9	3	1	3
618	9	13	1	1	#NULL!	9	3	1	2
619	2	14	1	1	#NULL!	9	3	3	1
620	9	0	#NULL!	1	#NULL!	9	9	9	9
621	2	15	2	9	#NULL!	9	9	9	9
622	2	14	1	1	#NULL!	9	3	1	2
623	1	0	#NULL!	1	#NULL!	9	3	2	2
624	2	13	1	1	#NULL!	9	3	3	3
625	2	14	1	1	#NULL!	9	3	1	2
626	2	18	2	1	#NULL!	3	3	1	1
627	2	13	1	1	#NULL!	9	3	1	1
628	2	13	1	1	#NULL!	9	3	1	2
629	2	13	1	1	#NULL!	9	3	1	2
630	1	12	1	1	#NULL!	9	3	1	2
631	1	12	1	1	#NULL!	9	3	1	3
632	9	0	#NULL!	1	#NULL!	9	3	2	2
633	9	0	#NULL!	1	#NULL!	1	3	2	2
634	2	14	1	1	#NULL!	9	3	1	2
635	2	13	1	1	#NULL!	9	3	1	2
636	2	14	1	1	#NULL!	9	3	2	2
637	2	14	1	1	#NULL!	9	3	1	2
638	2	14	1	1	#NULL!	9	3	2	1
639	1	12	1	1	#NULL!	9	3	1	3
640	2	15	2	1	#NULL!	9	3	1	1
641	2	15	2	1	#NULL!	9	3	2	2
642	2	15	2	1	#NULL!	9	3	1	2
643	2	15	2	1	#NULL!	9	3	1	3
644	2	16	2	1	#NULL!	9	3	1	2
645	2	16	2	1	#NULL!	9	3	1	2
646	2	13	1	1	#NULL!	9	3	1	1
647	2	15	2	1	#NULL!	9	3	1	1
648	2	13	1	1	#NULL!	3	3	2	2
649	2	12	1	1	#NULL!	9	3	2	2
650	2	18	2	1	#NULL!	9	1	1	1
651	2	12	1	1	#NULL!	9	3	1	1
652	2	13	1	1	#NULL!	9	3	1	3
653	1	14	1	1	#NULL!	9	3	1	2
654	2	13	1	1	#NULL!	9	3	1	2
655	1	14	1	1	#NULL!	9	3	1	2
656	1	14	1	1	#NULL!	9	3	1	1
657	1	16	2	1	#NULL!	3	3	1	2

658	2	15	2	1	#NULL!	3	3	1	2
659	2	15	2	1	#NULL!	3	3	1	1
660	1	19	2	1	#NULL!	3	3	1	2
661	2	18	2	1	#NULL!	3	3	1	2
662	2	16	2	1	#NULL!	3	3	1	2
663	1	16	2	1	#NULL!	3	3	1	1
664	1	20	3	1	#NULL!	3	3	3	2
665	2	0	#NULL!	1	#NULL!	3	3	1	1
666	2	14	1	1	#NULL!	9	3	2	2
667	2	15	2	1	#NULL!	3	3	1	1
668	1	14	1	1	#NULL!	9	3	3	3
669	2	15	2	1	#NULL!	9	3	1	9
670	2	13	1	1	#NULL!	9	3	1	3
671	2	12	1	1	#NULL!	9	3	1	1
672	1	13	1	1	#NULL!	9	3	1	3
673	2	13	1	1	#NULL!	9	3	1	3
674	2	13	1	1	#NULL!	3	3	1	2
675	2	13	1	1	#NULL!	9	3	1	1
676	2	14	1	1	#NULL!	9	3	1	1
677	1	13	1	1	#NULL!	9	3	1	2
678	2	14	1	1	#NULL!	9	3	1	1
679	1	14	1	1	#NULL!	9	3	1	2
680	2	15	2	1	#NULL!	9	3	1	2
681	1	13	1	1	#NULL!	9	3	1	2
682	1	18	2	1	#NULL!	3	3	2	2
683	1	13	1	1	#NULL!	9	3	2	2
684	2	14	1	1	#NULL!	9	3	1	2
685	2	13	1	1	#NULL!	9	3	1	2
686	2	14	1	1	#NULL!	9	3	1	2
687	1	15	2	1	#NULL!	9	3	3	3
688	1	15	2	1	#NULL!	9	3	1	2
689	2	16	2	1	#NULL!	9	3	2	3
690	1	14	1	1	#NULL!	9	3	1	1
691	2	14	1	1	#NULL!	9	3	1	2
692	2	14	1	1	#NULL!	9	3	2	2
693	2	13	1	1	#NULL!	9	3	2	2
694	2	14	1	1	#NULL!	9	3	1	3
695	2	13	1	1	#NULL!	9	3	1	3
696	2	14	1	1	#NULL!	9	3	2	2
697	2	13	1	1	#NULL!	9	3	1	2
698	1	14	1	1	#NULL!	9	2	2	3
699	2	15	2	1	#NULL!	9	3	1	2
700	1	14	1	1	#NULL!	9	3	1	2
701	2	13	1	1	#NULL!	9	3	1	2
702	2	13	1	9	#NULL!	9	9	9	9
703	1	14	1	1	#NULL!	9	3	2	2
704	2	14	1	1	#NULL!	9	3	1	2

705	1	12	1	1	#NULL!	9	3	1	2
706	2	13	1	1	#NULL!	9	3	1	1
707	1	14	1	1	#NULL!	3	3	1	1
708	2	14	1	1	#NULL!	9	3	2	2
709	1	16	2	1	#NULL!	3	3	2	2
710	1	15	2	1	#NULL!	3	3	2	2
711	1	14	1	1	#NULL!	3	3	3	3
712	1	16	2	1	#NULL!	3	3	2	2
713	1	16	2	1	#NULL!	3	3	2	2
714	9	0	#NULL!	9	#NULL!	9	9	9	9
715	2	14	1	1	#NULL!	9	3	1	2
716	2	15	2	1	#NULL!	9	3	1	1
717	2	11	1	1	#NULL!	9	3	1	2
718	2	15	2	1	#NULL!	9	3	3	1
719	2	13	1	1	#NULL!	9	3	1	1
720	1	13	1	1	#NULL!	9	3	1	3
721	1	13	1	1	#NULL!	9	3	1	2
722	1	15	2	1	#NULL!	9	3	1	2
723	1	15	2	1	#NULL!	3	3	1	2
724	1	14	1	1	#NULL!	9	3	1	3
725	2	16	2	9	#NULL!	9	3	1	2
726	1	11	1	1	#NULL!	9	3	1	2
727	1	15	2	2	#NULL!	9	3	2	2
728	1	14	1	1	#NULL!	9	3	1	1
729	1	14	1	1	#NULL!	9	3	1	1
730	1	13	1	1	#NULL!	9	3	1	1
731	1	12	1	1	#NULL!	9	3	1	1
732	1	11	1	1	#NULL!	9	3	1	2
733	1	14	1	2	#NULL!	9	3	1	3
734	1	15	2	1	#NULL!	9	3	3	3
735	1	15	2	2	#NULL!	9	3	1	3
736	1	14	1	1	#NULL!	9	3	1	2
737	2	14	1	1	#NULL!	3	3	1	2
738	1	13	1	1	#NULL!	9	3	1	1
739	1	14	1	1	#NULL!	9	3	1	2
740	1	15	2	1	#NULL!	9	3	2	2
741	1	13	1	1	#NULL!	1	2	1	2
742	2	15	2	1	#NULL!	9	3	1	1
743	1	14	1	1	#NULL!	9	3	1	1
744	1	15	2	1	#NULL!	2	2	1	2
745	1	16	2	1	#NULL!	2	2	1	2
746	1	17	2	2	#NULL!	2	2	1	2
747	1	17	2	1	#NULL!	2	2	1	1
748	1	17	2	1	#NULL!	2	2	2	2
749	1	17	2	1	#NULL!	2	2	1	2
750	1	14	1	1	#NULL!	2	2	1	1
751	1	15	2	1	#NULL!	2	2	1	2

752	1	16	2	1	#NULL!	2	2	1	2
753	1	15	2	1	#NULL!	1	2	1	1
754	1	16	2	1	#NULL!	2	2	2	1
755	1	16	2	1	#NULL!	2	2	1	1
756	1	15	2	1	#NULL!	2	2	2	2
757	1	16	2	1	#NULL!	2	2	1	2
758	1	16	2	1	#NULL!	2	2	2	1
759	1	15	2	1	#NULL!	2	2	1	2
760	9	0	#NULL!	9	#NULL!	3	2	1	3
761	1	15	2	1	#NULL!	2	2	1	2
762	1	17	2	1	#NULL!	2	2	2	2
763	1	16	2	1	#NULL!	2	2	1	1
764	1	15	2	1	#NULL!	2	2	1	1
765	1	16	2	1	#NULL!	2	2	1	2
766	1	17	2	1	#NULL!	2	2	1	1
767	1	17	2	1	#NULL!	2	2	1	2
768	1	13	1	1	#NULL!	2	2	1	3
769	1	21	3	1	#NULL!	2	2	1	3
770	1	16	2	1	#NULL!	2	2	1	2
771	1	15	2	1	#NULL!	2	2	1	2
772	1	17	2	1	#NULL!	2	2	2	2
773	1	15	2	1	#NULL!	2	2	1	1
774	1	16	2	1	#NULL!	2	2	1	2
775	1	16	2	1	#NULL!	2	2	1	2
776	1	17	2	1	#NULL!	2	2	2	2
777	1	18	2	1	#NULL!	2	3	1	2
778	1	15	2	1	#NULL!	2	2	1	2
779	1	17	2	1	#NULL!	2	2	2	2
780	1	16	2	1	#NULL!	2	2	1	2
781	1	15	2	1	#NULL!	2	2	1	1
782	1	16	2	1	#NULL!	2	2	1	2
783	1	15	2	1	#NULL!	2	2	1	2
784	1	18	2	1	#NULL!	2	2	1	1
785	1	14	1	1	#NULL!	2	2	2	2
786	1	16	2	1	#NULL!	2	2	2	2
787	1	18	2	1	#NULL!	2	2	1	3
788	1	15	2	1	#NULL!	2	2	1	2
789	1	15	2	1	#NULL!	2	2	1	2
790	1	15	2	1	#NULL!	2	2	1	2
791	1	16	2	1	#NULL!	2	2	1	2
792	1	15	2	1	#NULL!	2	2	1	3
793	1	16	2	1	#NULL!	2	2	1	1
794	1	15	2	1	#NULL!	2	2	2	2
795	1	16	2	1	#NULL!	2	2	2	1
796	1	16	2	1	#NULL!	2	2	1	2
797	1	15	2	1	#NULL!	2	2	2	2
798	1	16	2	1	#NULL!	2	2	2	2

799	1	15	2	1	#NULL!	2	2	1	2
800	1	16	2	1	#NULL!	2	2	2	2
801	1	18	2	1	#NULL!	2	2	1	2
802	1	16	2	1	#NULL!	2	2	2	2
803	1	13	1	1	#NULL!	2	2	1	1
804	1	15	2	1	#NULL!	2	2	2	3
805	1	15	2	1	#NULL!	2	2	2	2
806	1	16	2	1	#NULL!	2	2	1	1
807	1	20	3	1	#NULL!	2	2	1	2
808	1	15	2	1	#NULL!	2	2	2	2
809	1	18	2	1	#NULL!	2	2	1	1
810	9	0	#NULL!	1	#NULL!	2	2	2	2
811	1	16	2	1	#NULL!	2	2	1	2
812	1	15	2	1	#NULL!	2	2	1	2
813	1	16	2	1	#NULL!	2	2	1	1
814	1	15	2	1	#NULL!	2	2	2	2
815	1	16	2	1	#NULL!	2	2	2	2
816	1	16	2	1	#NULL!	2	2	1	2
817	1	16	2	1	#NULL!	2	2	1	2
818	1	0	#NULL!	1	#NULL!	2	2	1	1
819	1	17	2	1	#NULL!	2	2	1	2
820	1	16	2	1	#NULL!	2	2	1	2
821	1	16	2	1	#NULL!	2	2	1	2
822	1	17	2	1	#NULL!	2	2	1	2
823	1	15	2	1	#NULL!	2	2	1	2
824	1	16	2	1	#NULL!	2	2	1	1
825	1	15	2	1	#NULL!	2	2	1	3
826	1	14	1	1	#NULL!	2	2	1	1
827	1	16	2	1	#NULL!	2	2	1	2
828	1	17	2	1	#NULL!	2	2	1	2
829	9	15	2	1	#NULL!	2	9	1	1
830	1	16	2	1	#NULL!	2	2	1	2
831	1	17	2	1	#NULL!	2	2	1	1
832	1	16	2	1	#NULL!	2	2	1	1
833	1	0	#NULL!	1	#NULL!	2	2	1	2
834	1	16	2	1	#NULL!	2	2	1	1
835	9	15	2	1	#NULL!	2	2	1	1
836	1	15	2	1	#NULL!	2	2	1	2
837	1	16	2	9	#NULL!	9	2	2	3
838	1	15	2	9	#NULL!	9	2	2	3
839	1	16	2	1	#NULL!	2	2	1	2
840	1	17	2	1	#NULL!	2	2	1	3
841	1	18	2	1	#NULL!	2	2	1	2
842	1	17	2	1	#NULL!	2	2	1	2
843	1	16	2	1	#NULL!	2	2	2	3
844	1	15	2	1	#NULL!	2	2	1	1
845	1	19	2	1	#NULL!	2	2	1	2

846	1	18	2	1	#NULL!	2	2	1	2
847	1	16	2	1	#NULL!	2	2	2	2
848	1	17	2	1	#NULL!	2	2	2	2
849	1	17	2	1	#NULL!	2	2	1	2
850	1	18	2	1	#NULL!	2	2	1	2
851	1	14	1	1	#NULL!	2	2	2	2
852	1	17	2	1	#NULL!	2	2	2	1
853	1	16	2	1	#NULL!	9	9	9	9
854	1	17	2	1	#NULL!	2	2	1	2
855	1	16	2	1	#NULL!	2	2	1	3
856	1	16	2	1	#NULL!	2	2	2	2
857	1	17	2	1	#NULL!	2	2	1	1
858	1	18	2	1	#NULL!	2	2	1	2
859	1	18	2	1	#NULL!	2	2	1	2
860	1	17	2	1	#NULL!	2	2	2	2
861	1	16	2	1	#NULL!	2	2	2	2
862	1	19	2	1	#NULL!	2	2	2	2
863	1	16	2	1	#NULL!	2	2	1	1
864	1	16	2	1	#NULL!	2	2	1	1
865	1	17	2	1	#NULL!	2	2	2	2
866	1	16	2	1	#NULL!	2	2	1	2
867	1	15	2	1	#NULL!	2	2	1	2
868	1	0	#NULL!	1	#NULL!	2	2	1	2
869	1	17	2	1	#NULL!	2	2	1	2
870	1	17	2	1	#NULL!	2	2	2	2
871	1	16	2	1	#NULL!	2	2	2	2
872	1	17	2	1	#NULL!	2	2	1	1
873	1	16	2	1	#NULL!	2	2	1	1
874	1	15	2	1	#NULL!	2	2	1	1
875	1	16	2	1	#NULL!	2	2	1	2
876	1	16	2	1	#NULL!	2	2	1	2
877	1	0	#NULL!	1	#NULL!	2	2	1	9
878	1	16	2	1	#NULL!	2	2	1	1
879	1	17	2	1	#NULL!	2	2	2	2
880	1	19	2	1	#NULL!	2	2	1	1
881	1	15	2	1	#NULL!	2	2	1	3
882	1	17	2	1	#NULL!	2	2	2	2
883	1	18	2	1	#NULL!	2	2	2	2
884	1	18	2	1	#NULL!	2	2	1	2
885	1	16	2	1	#NULL!	2	2	2	2
886	1	16	2	1	#NULL!	2	2	1	1
887	1	16	2	1	#NULL!	2	2	2	2
888	1	18	2	1	#NULL!	2	2	1	2
889	1	17	2	1	#NULL!	2	2	1	9
890	1	16	2	1	#NULL!	2	2	1	1
891	1	16	2	1	#NULL!	2	2	2	1
892	1	15	2	1	#NULL!	2	2	1	2

893	1	15	2	1	#NULL!	2	2	2	2
894	1	15	2	1	#NULL!	2	2	1	2
895	1	18	2	1	#NULL!	2	2	2	2
896	1	18	2	1	#NULL!	2	2	2	2
897	1	15	2	1	#NULL!	2	2	1	2
898	1	16	2	1	#NULL!	2	2	1	2
899	1	15	2	1	#NULL!	2	2	2	2
900	1	15	2	1	#NULL!	2	2	1	2
901	1	15	2	1	#NULL!	2	2	1	2
902	1	16	2	1	#NULL!	2	2	1	2
903	1	0	#NULL!	1	#NULL!	2	2	1	2
904	1	18	2	1	#NULL!	2	2	1	3
905	1	14	1	1	#NULL!	2	2	1	2
906	1	16	2	1	#NULL!	2	2	2	2
907	1	16	2	1	#NULL!	2	2	1	1
908	1	15	2	1	#NULL!	2	2	2	2
909	1	16	2	1	#NULL!	2	2	1	1
910	1	15	2	1	#NULL!	2	2	1	1
911	1	15	2	1	#NULL!	2	2	1	2
912	1	18	2	1	#NULL!	2	2	1	1
913	1	16	2	1	#NULL!	2	2	2	2
914	1	17	2	1	#NULL!	2	2	2	2
915	1	19	2	1	#NULL!	2	2	1	1
916	1	17	2	1	#NULL!	2	2	1	3
917	1	16	2	1	#NULL!	2	2	2	2
918	1	15	2	1	#NULL!	2	2	2	2
919	1	16	2	1	#NULL!	2	2	1	1
920	1	16	2	1	#NULL!	2	2	1	2
921	1	16	2	1	#NULL!	2	2	1	2
922	2	14	1	1	#NULL!	9	3	3	2
923	2	17	2	1	#NULL!	3	3	1	2
924	2	15	2	1	#NULL!	9	3	1	1
925	1	15	2	1	#NULL!	9	3	2	2
926	2	14	1	1	#NULL!	9	3	1	2
927	2	0	#NULL!	1	#NULL!	3	3	3	2
928	1	18	2	1	#NULL!	3	3	1	2
929	2	19	2	1	#NULL!	3	3	1	2
930	1	17	2	1	#NULL!	3	3	2	2
931	2	17	2	1	#NULL!	3	3	1	1
932	2	16	2	1	#NULL!	3	3	2	1
933	1	0	#NULL!	1	#NULL!	3	3	1	2
934	1	17	2	1	#NULL!	3	3	1	1
935	1	17	2	1	#NULL!	3	3	2	2
936	2	17	2	1	#NULL!	3	3	3	2
937	1	18	2	1	#NULL!	3	3	2	2
938	2	19	2	1	#NULL!	3	3	2	2
939	2	19	2	1	#NULL!	3	3	1	2

940	2	17	2	1	#NULL!	3	3	1	1
941	2	19	2	1	#NULL!	3	3	3	2
942	1	20	3	1	#NULL!	3	3	1	1
943	2	17	2	1	#NULL!	3	3	1	1
944	1	18	2	1	#NULL!	3	3	1	2
945	9	0	#NULL!	9	#NULL!	9	9	9	9
946	2	16	2	1	#NULL!	3	3	1	2
947	2	18	2	1	#NULL!	3	3	1	2
948	2	16	2	1	#NULL!	3	3	1	2
949	2	17	2	1	#NULL!	3	3	1	2
950	2	17	2	1	#NULL!	3	3	1	2
951	2	17	2	1	#NULL!	3	3	1	2
952	2	17	2	1	#NULL!	3	3	9	9
953	2	15	2	1	#NULL!	3	3	2	2
954	2	17	2	1	#NULL!	3	3	1	2
955	9	16	2	1	#NULL!	3	3	1	2
956	1	19	2	1	#NULL!	3	3	2	2
957	9	18	2	1	#NULL!	3	2	2	1
958	2	16	2	1	#NULL!	3	3	1	2
959	2	15	2	1	#NULL!	3	3	1	1
960	2	18	2	1	#NULL!	3	3	1	2
961	2	17	2	1	#NULL!	3	3	1	2
962	2	17	2	2	#NULL!	3	3	1	2
963	2	15	2	1	#NULL!	3	3	2	2
964	2	17	2	1	#NULL!	3	3	1	3
965	2	17	2	1	#NULL!	3	3	1	1
966	2	17	2	1	#NULL!	3	3	1	1
967	2	17	2	1	#NULL!	3	3	1	3
968	2	17	2	1	#NULL!	3	3	1	1
969	9	16	2	1	#NULL!	3	3	1	2
970	2	15	2	1	#NULL!	3	3	1	1
971	2	17	2	1	#NULL!	3	3	9	9
972	2	16	2	1	#NULL!	3	3	1	1
973	2	16	2	1	#NULL!	3	3	1	2
974	1	17	2	1	#NULL!	3	3	1	2
975	2	14	1	1	#NULL!	3	3	1	1
976	2	14	1	1	#NULL!	3	3	1	1
977	2	15	2	1	#NULL!	3	3	1	1
978	2	15	2	1	#NULL!	3	3	1	2
979	2	18	2	1	#NULL!	3	3	1	2
980	2	19	2	1	#NULL!	3	3	1	1
981	1	17	2	1	#NULL!	3	3	2	2
982	2	16	2	1	#NULL!	3	3	1	2
983	1	23	3	1	#NULL!	3	3	2	2
984	1	18	2	1	#NULL!	3	3	1	1
985	1	18	2	1	#NULL!	3	3	1	3
986	2	16	2	1	#NULL!	3	3	1	3

987	2	17	2	1	#NULL!	3	3	1	1
988	2	16	2	1	#NULL!	3	3	2	2
989	1	16	2	1	#NULL!	3	3	1	1
990	2	16	2	1	#NULL!	3	3	1	2
991	2	16	2	1	#NULL!	3	3	1	1
992	1	16	2	1	#NULL!	3	3	1	1
993	1	16	2	1	#NULL!	3	3	2	2
994	1	15	2	1	#NULL!	3	3	1	1
995	1	17	2	1	#NULL!	3	3	1	3
996	1	17	2	1	#NULL!	3	3	2	2
997	2	0	#NULL!	1	#NULL!	3	3	1	2
998	1	19	2	1	#NULL!	3	3	1	2
999	2	17	2	1	#NULL!	3	3	1	1
1000	2	18	2	1	#NULL!	3	3	1	2
1001	2	16	2	1	#NULL!	2	3	1	2
1002	2	16	2	1	#NULL!	3	3	1	2
1003	1	18	2	1	#NULL!	3	3	1	1
1004	2	16	2	1	#NULL!	3	3	1	1
1005	2	17	2	1	#NULL!	3	3	1	2
1006	2	15	2	1	#NULL!	3	3	1	1
1007	2	16	2	1	#NULL!	3	3	1	2
1008	1	17	2	1	#NULL!	3	3	1	2
1009	2	17	2	1	#NULL!	3	3	1	2
1010	2	16	2	1	#NULL!	3	3	1	3
1011	2	15	2	1	#NULL!	3	3	1	2
1012	2	15	2	1	#NULL!	3	3	1	2
1013	2	18	2	1	#NULL!	3	3	1	1
1014	2	17	2	2	#NULL!	3	3	2	2
1015	2	17	2	1	#NULL!	3	3	1	2
1016	1	19	2	1	#NULL!	3	3	1	2
1017	2	18	2	1	#NULL!	3	2	1	2
1018	1	17	2	1	#NULL!	3	3	3	3
1019	1	17	2	1	#NULL!	3	3	1	1
1020	2	17	2	1	#NULL!	3	3	1	3
1021	1	19	2	1	#NULL!	3	3	2	2
1022	2	17	2	1	#NULL!	3	3	1	1
1023	1	19	2	1	#NULL!	3	3	1	2
1024	1	18	2	1	#NULL!	3	3	1	2
1025	2	14	1	1	#NULL!	3	3	1	2
1026	1	17	2	1	#NULL!	3	3	1	2
1027	1	17	2	1	#NULL!	3	3	1	1
1028	1	18	2	1	#NULL!	3	3	1	2
1029	1	18	2	1	#NULL!	3	3	2	2
1030	1	19	2	1	#NULL!	3	3	2	2
1031	1	20	3	1	#NULL!	3	3	1	9
1032	1	0	#NULL!	1	#NULL!	3	3	1	2
1033	1	17	2	1	#NULL!	3	3	1	1

1034	1	18	2	1	#NULL!	3	3	2	2
1035	1	21	3	1	#NULL!	3	3	1	2
1036	1	17	2	1	#NULL!	3	3	2	2
1037	2	17	2	1	#NULL!	3	3	1	2
1038	1	17	2	1	#NULL!	3	3	1	1
1039	1	18	2	1	#NULL!	3	3	2	2
1040	2	18	2	1	#NULL!	3	3	1	1
1041	2	16	2	1	#NULL!	3	3	1	2
1042	2	16	2	1	#NULL!	3	3	1	1
1043	1	14	1	1	#NULL!	9	3	1	1
1044	1	17	2	1	#NULL!	2	3	1	2
1045	1	15	2	1	#NULL!	2	3	3	3
1046	1	17	2	1	#NULL!	2	3	1	3
1047	2	19	2	1	#NULL!	2	3	1	2
1048	2	16	2	1	#NULL!	2	3	3	2
1049	2	15	2	1	#NULL!	2	3	1	1
1050	2	16	2	1	#NULL!	2	3	1	1
1051	2	16	2	1	#NULL!	2	3	1	1
1052	2	14	1	1	#NULL!	2	3	1	2
1053	2	17	2	1	#NULL!	2	3	1	2
1054	2	16	2	1	#NULL!	2	3	1	2
1055	1	16	2	1	#NULL!	2	3	1	2
1056	1	16	2	1	#NULL!	2	3	1	2
1057	1	19	2	1	#NULL!	2	3	2	2
1058	2	16	2	1	#NULL!	2	3	1	2
1059	2	15	2	2	#NULL!	2	3	1	2
1060	2	14	1	1	#NULL!	2	3	1	2
1061	2	17	2	1	#NULL!	2	3	1	2
1062	2	13	1	1	#NULL!	2	3	1	2
1063	2	17	2	1	#NULL!	2	3	1	1
1064	2	16	2	1	#NULL!	2	3	1	2
1065	1	19	2	1	#NULL!	2	3	2	3
1066	1	18	2	1	#NULL!	2	3	2	1
1067	1	16	2	1	#NULL!	2	3	2	2
1068	2	15	2	2	#NULL!	2	3	1	2
1069	2	16	2	2	#NULL!	2	3	1	1
1070	2	13	1	1	#NULL!	2	3	1	2
1071	2	18	2	1	#NULL!	2	3	1	2
1072	2	18	2	9	#NULL!	2	3	1	2
1073	1	16	2	1	#NULL!	2	3	1	1
1074	1	16	2	1	#NULL!	2	3	1	2
1075	2	16	2	1	#NULL!	2	3	2	2
1076	2	16	2	1	#NULL!	2	3	1	1
1077	2	16	2	1	#NULL!	2	3	1	2
1078	2	16	2	1	#NULL!	2	2	2	2
1079	2	14	1	1	#NULL!	2	3	1	1
1080	2	16	2	1	#NULL!	2	3	1	2

1081	2	17	2	1	#NULL!	3	3	2	2
1082	2	17	2	1	#NULL!	3	3	1	3
1083	2	17	2	1	#NULL!	3	3	1	1
1084	1	15	2	1	#NULL!	9	3	1	2
1085	2	14	1	1	#NULL!	9	3	1	1
1086	2	15	2	1	#NULL!	2	3	2	2
1087	2	15	2	1	#NULL!	2	3	2	2
1088	2	0	#NULL!	1	#NULL!	2	3	1	9
1089	2	16	2	1	#NULL!	2	3	1	2
1090	2	14	1	1	#NULL!	2	3	1	1
1091	2	15	2	1	#NULL!	2	3	2	1
1092	1	14	1	1	#NULL!	2	3	1	9
1093	1	18	2	1	#NULL!	2	3	1	1
1094	2	16	2	1	#NULL!	2	3	2	2
1095	2	16	2	1	#NULL!	2	3	1	2
1096	2	15	2	1	#NULL!	2	3	1	1
1097	2	15	2	1	#NULL!	2	3	1	2
1098	2	15	2	1	#NULL!	2	3	1	3
1099	2	14	1	2	#NULL!	2	3	1	1
1100	1	17	2	1	#NULL!	2	3	1	2
1101	1	18	2	1	#NULL!	2	3	2	1
1102	1	0	#NULL!	1	#NULL!	2	3	2	2
1103	1	15	2	1	#NULL!	2	3	1	2
1104	2	18	2	1	#NULL!	2	3	1	2
1105	2	17	2	1	#NULL!	2	3	1	1
1106	1	16	2	1	#NULL!	2	3	3	1
1107	1	16	2	1	#NULL!	2	3	1	2
1108	1	16	2	1	#NULL!	2	3	1	1
1109	1	17	2	1	#NULL!	2	3	1	2
1110	2	17	2	1	#NULL!	2	3	1	2
1111	2	17	2	1	#NULL!	2	3	1	1
1112	2	16	2	1	#NULL!	2	3	1	2
1113	2	16	2	1	#NULL!	2	3	1	2
1114	2	15	2	1	#NULL!	2	3	1	1
1115	2	15	2	1	#NULL!	2	3	1	2
1116	1	17	2	1	#NULL!	2	3	1	2
1117	1	15	2	1	#NULL!	2	3	2	3
1118	2	17	2	1	#NULL!	2	3	1	1
1119	2	16	2	1	#NULL!	2	3	1	2
1120	2	15	2	1	#NULL!	2	3	1	2
1121	1	18	2	2	#NULL!	2	3	1	2
1122	2	0	#NULL!	1	#NULL!	2	3	1	9
1123	2	15	2	1	#NULL!	2	3	2	2
1124	2	16	2	1	#NULL!	2	3	2	2
1125	2	14	1	1	#NULL!	2	3	1	1
1126	2	15	2	1	#NULL!	2	3	1	1
1127	2	16	2	1	#NULL!	2	3	1	2

1128	2	17	2	1	#NULL!	2	3	1	2
1129	1	17	2	1	#NULL!	2	3	1	2
1130	2	17	2	1	#NULL!	2	3	1	2
1131	2	15	2	1	#NULL!	2	3	1	2
1132	2	15	2	1	#NULL!	2	3	1	2
1133	1	16	2	1	#NULL!	2	3	1	2
1134	2	16	2	1	#NULL!	2	3	1	2
1135	1	0	#NULL!	1	#NULL!	2	2	2	2
1136	1	17	2	1	#NULL!	2	3	1	2
1137	1	16	2	1	#NULL!	2	2	2	2
1138	1	16	2	1	#NULL!	2	3	1	2
1139	2	17	2	2	#NULL!	2	3	1	3
1140	1	17	2	1	#NULL!	2	3	1	1
1141	1	17	2	1	#NULL!	2	3	1	3
1142	2	16	2	1	#NULL!	2	3	1	2
1143	2	15	2	1	#NULL!	2	3	1	1
1144	2	16	2	1	#NULL!	2	3	1	3
1145	1	17	2	1	#NULL!	2	3	1	1
1146	1	16	2	1	#NULL!	2	3	1	1
1147	1	16	2	1	#NULL!	2	3	1	1
1148	1	17	2	1	#NULL!	2	3	1	1
1149	2	15	2	1	#NULL!	2	3	1	2
1150	2	16	2	1	#NULL!	2	3	1	3
1151	2	17	2	1	#NULL!	2	3	1	3
1152	2	16	2	1	#NULL!	2	3	1	1
1153	2	17	2	1	#NULL!	2	3	9	2
1154	2	15	2	1	#NULL!	2	3	1	2
1155	1	18	2	1	#NULL!	2	3	2	2
1156	2	17	2	1	#NULL!	2	3	1	1
1157	2	17	2	1	#NULL!	2	3	1	2
1158	2	16	2	1	#NULL!	2	3	1	1
1159	2	16	2	1	#NULL!	2	1	1	1
1160	2	16	2	1	#NULL!	2	3	1	2
1161	2	17	2	1	#NULL!	2	3	1	2
1162	2	15	2	1	#NULL!	2	3	1	2
1163	2	15	2	1	#NULL!	2	3	1	1
1164	2	16	2	1	#NULL!	2	3	1	3
1165	1	17	2	1	#NULL!	2	3	1	2
1166	1	16	2	1	#NULL!	2	3	1	2
1167	1	16	2	1	#NULL!	2	3	1	9
1168	1	15	2	1	#NULL!	2	3	1	1
1169	2	15	2	1	#NULL!	2	3	1	2
1170	2	15	2	1	#NULL!	2	3	1	2
1171	2	15	2	1	#NULL!	2	3	1	3
1172	2	17	2	1	#NULL!	2	3	1	2
1173	2	14	1	1	#NULL!	2	3	1	2
1174	2	16	2	1	#NULL!	2	3	1	1

1175	1	15	2	1	#NULL!	2	3	1	1
1176	2	16	2	1	#NULL!	2	3	1	2
1177	1	17	2	1	#NULL!	2	3	2	2
1178	1	18	2	1	#NULL!	2	3	1	3
1179	1	19	2	1	#NULL!	2	3	1	1
1180	9	9	1	1	#NULL!	2	3	1	2
1181	1	15	2	1	#NULL!	2	3	1	2
1182	9	9	1	1	#NULL!	2	3	1	2
1183	1	17	2	1	#NULL!	2	3	1	1
1184	1	16	2	1	#NULL!	2	3	1	2
1185	1	16	2	1	#NULL!	2	3	1	2
1186	1	16	2	1	#NULL!	2	3	1	2
1187	1	16	2	1	#NULL!	2	3	2	2
1188	1	18	2	1	#NULL!	2	3	1	1
1189	1	16	2	1	#NULL!	2	3	1	1
1190	9	0	#NULL!	9	#NULL!	9	9	9	9
1191	1	19	2	2	#NULL!	2	3	1	3
1192	1	17	2	1	#NULL!	2	3	1	2
1193	1	16	2	1	#NULL!	2	3	2	2
1194	2	17	2	1	#NULL!	2	1	1	1
1195	2	14	1	1	#NULL!	2	3	1	2
1196	1	16	2	1	#NULL!	2	3	1	1
1197	2	19	2	1	#NULL!	2	3	1	2
1198	2	17	2	1	#NULL!	2	3	1	3
1199	2	15	2	1	#NULL!	2	3	2	2
1200	1	18	2	1	#NULL!	2	3	1	2
1201	2	16	2	1	#NULL!	2	3	1	1
1202	1	17	2	1	#NULL!	2	2	2	2
1203	1	15	2	1	#NULL!	2	3	2	2
1204	2	15	2	1	#NULL!	2	3	1	2
1205	9	0	#NULL!	9	#NULL!	9	9	9	9
1206	1	17	2	1	#NULL!	2	3	3	3
1207	1	16	2	1	#NULL!	2	2	2	3
1208	1	15	2	1	#NULL!	2	3	1	1
1209	1	15	2	1	#NULL!	2	3	1	2
1210	1	16	2	1	#NULL!	2	3	1	3
1211	2	15	2	1	#NULL!	2	3	1	1
1212	2	15	2	1	#NULL!	2	3	1	2
1213	2	14	1	1	#NULL!	2	3	1	2
1214	2	14	1	1	#NULL!	2	3	1	2
1215	2	16	2	1	#NULL!	2	3	1	2
1216	2	16	2	1	#NULL!	2	3	1	2
1217	1	16	2	1	#NULL!	2	3	1	2
1218	1	16	2	1	#NULL!	2	3	1	1
1219	2	14	1	1	#NULL!	2	3	1	1
1220	1	17	2	1	#NULL!	2	3	1	2
1221	2	15	2	1	#NULL!	2	3	1	2

1222	2	15	2	1	#NULL!	2	3	1	1
1223	2	17	2	1	#NULL!	2	3	1	2
1224	2	18	2	1	#NULL!	2	3	1	2
1225	1	16	2	1	#NULL!	2	3	2	2
1226	2	16	2	2	#NULL!	2	3	9	2
1227	2	16	2	1	#NULL!	2	3	1	3
1228	2	16	2	1	#NULL!	2	3	1	3
1229	2	14	1	1	#NULL!	2	3	1	2
1230	2	16	2	1	#NULL!	2	3	1	2
1231	1	15	2	1	#NULL!	2	3	1	3
1232	2	15	2	1	#NULL!	2	3	1	2
1233	2	16	2	1	#NULL!	2	3	1	2
1234	1	17	2	1	#NULL!	2	3	1	1
1235	2	16	2	1	#NULL!	2	3	1	1
1236	1	15	2	2	#NULL!	1	2	1	2
1237	1	16	2	1	#NULL!	1	2	1	2
1238	1	15	2	1	#NULL!	1	2	1	3
1239	1	15	2	1	#NULL!	1	2	1	2
1240	1	16	2	1	#NULL!	1	2	2	2
1241	1	14	1	1	#NULL!	1	2	1	2
1242	1	13	1	1	#NULL!	1	2	2	2
1243	1	15	2	1	#NULL!	1	2	1	2
1244	1	14	1	1	#NULL!	1	2	1	1
1245	1	14	1	1	#NULL!	1	2	1	1
1246	1	15	2	1	#NULL!	1	2	1	9
1247	1	16	2	1	#NULL!	1	2	2	2
1248	1	17	2	1	#NULL!	1	2	1	1
1249	1	16	2	1	#NULL!	1	2	1	2
1250	1	15	2	1	#NULL!	1	9	9	9
1251	1	16	2	1	#NULL!	1	2	1	1
1252	1	16	2	1	#NULL!	1	2	1	2
1253	1	16	2	1	#NULL!	1	2	1	1
1254	1	15	2	1	#NULL!	1	2	1	2
1255	1	14	1	1	#NULL!	1	2	2	2
1256	1	14	1	1	#NULL!	1	2	1	2
1257	1	14	1	1	#NULL!	1	2	1	3
1258	1	15	2	1	#NULL!	1	2	1	2
1259	1	14	1	1	#NULL!	1	2	1	2
1260	1	15	2	1	#NULL!	1	2	3	2
1261	1	14	1	1	#NULL!	1	2	9	9
1262	1	15	2	2	#NULL!	1	2	1	2
1263	1	15	2	1	#NULL!	1	2	1	2
1264	1	16	2	1	#NULL!	1	2	1	2
1265	1	15	2	1	#NULL!	1	2	1	2
1266	1	14	1	1	#NULL!	1	2	3	3
1267	1	16	2	2	#NULL!	1	2	1	2
1268	1	14	1	1	#NULL!	1	2	3	3

1269	1	16	2	1	#NULL!	1	2	3	3
1270	1	16	2	2	#NULL!	1	2	2	2
1271	1	15	2	1	#NULL!	1	2	1	2
1272	1	15	2	1	#NULL!	1	2	1	1
1273	1	14	1	1	#NULL!	1	2	1	2
1274	1	16	2	1	#NULL!	1	2	1	1
1275	1	14	1	1	#NULL!	1	2	1	2
1276	1	16	2	1	#NULL!	1	2	1	9
1277	1	18	2	1	#NULL!	1	2	1	2
1278	1	16	2	9	#NULL!	9	9	9	9
1279	1	16	2	9	#NULL!	9	9	1	2
1280	1	15	2	1	#NULL!	1	2	2	2
1281	1	15	2	1	#NULL!	1	2	1	1
1282	1	15	2	1	#NULL!	1	2	1	2
1283	1	15	2	1	#NULL!	1	2	1	1
1284	1	14	1	1	#NULL!	1	2	1	1
1285	1	15	2	1	#NULL!	1	2	1	2
1286	1	17	2	1	#NULL!	1	2	1	1
1287	1	15	2	1	#NULL!	1	3	1	2
1288	1	14	1	1	#NULL!	1	2	1	2
1289	1	14	1	1	#NULL!	1	2	1	1
1290	1	14	1	1	#NULL!	1	2	1	1
1291	1	17	2	1	#NULL!	1	2	1	3
1292	1	0	#NULL!	1	#NULL!	1	2	1	3
1293	1	14	1	1	#NULL!	1	2	1	3
1294	1	15	2	1	#NULL!	1	2	1	1
1295	1	15	2	1	#NULL!	1	2	1	1
1296	2	17	2	1	#NULL!	1	9	9	9
1297	1	15	2	1	#NULL!	1	2	2	2
1298	1	14	1	1	#NULL!	1	2	1	2
1299	1	0	#NULL!	1	#NULL!	1	2	1	2
1300	1	15	2	1	#NULL!	1	2	1	1
1301	1	16	2	2	#NULL!	1	2	1	3
1302	1	15	2	1	#NULL!	1	2	1	1
1303	1	18	2	2	#NULL!	1	2	1	2
1304	9	19	2	1	#NULL!	1	2	2	2
1305	1	16	2	1	#NULL!	1	2	1	2
1306	9	18	2	1	#NULL!	1	2	1	1
1307	1	16	2	1	#NULL!	1	2	1	1
1308	1	16	2	1	#NULL!	1	2	1	1
1309	1	15	2	1	#NULL!	1	2	1	1
1310	1	17	2	1	#NULL!	1	2	1	1
1311	1	15	2	1	#NULL!	1	2	1	1
1312	1	17	2	1	#NULL!	1	2	1	1
1313	1	15	2	1	#NULL!	1	2	1	3
1314	1	18	2	1	#NULL!	1	2	1	2
1315	9	0	#NULL!	9	#NULL!	9	9	9	9

1316	1	15	2	1	#NULL!	1	2	2	2
1317	1	15	2	1	#NULL!	1	2	1	1
1318	1	14	1	1	#NULL!	1	2	1	2
1319	1	14	1	1	#NULL!	1	2	1	1
1320	1	16	2	1	#NULL!	1	2	2	2
1321	1	15	2	1	#NULL!	1	2	1	1
1322	1	18	2	1	#NULL!	1	2	1	2
1323	1	15	2	1	#NULL!	1	2	2	2
1324	1	16	2	1	#NULL!	1	2	1	1
1325	1	15	2	1	#NULL!	1	2	1	2
1326	1	15	2	1	#NULL!	1	2	1	2
1327	1	15	2	1	#NULL!	1	2	1	2
1328	1	16	2	1	#NULL!	1	2	1	2
1329	1	14	1	1	#NULL!	1	2	2	2
1330	1	16	2	1	#NULL!	1	2	1	2
1331	1	15	2	1	#NULL!	1	2	1	1
1332	1	18	2	1	#NULL!	1	2	1	1
1333	1	16	2	1	#NULL!	1	2	1	2
1334	1	14	1	1	#NULL!	1	2	1	1
1335	1	15	2	1	#NULL!	1	2	1	1
1336	1	13	1	1	#NULL!	1	2	1	2
1337	1	15	2	1	#NULL!	1	2	1	2
1338	1	13	1	1	#NULL!	1	2	1	2
1339	1	13	1	1	#NULL!	1	2	1	2
1340	1	15	2	1	#NULL!	1	2	1	2
1341	1	15	2	1	#NULL!	1	2	1	1
1342	1	15	2	1	#NULL!	1	2	1	2
1343	1	15	2	1	#NULL!	1	2	2	2
1344	1	16	2	9	#NULL!	9	9	9	9
1345	1	15	2	1	#NULL!	1	2	1	1
1346	1	0	#NULL!	1	#NULL!	1	2	1	2
1347	1	15	2	1	#NULL!	1	2	1	2
1348	1	13	1	1	#NULL!	1	2	1	2
1349	1	14	1	1	#NULL!	1	2	1	2
1350	1	14	1	1	#NULL!	1	2	1	2
1351	1	16	2	1	#NULL!	1	2	1	2
1352	1	15	2	1	#NULL!	1	2	1	2
1353	1	16	2	1	#NULL!	1	2	1	1
1354	1	18	2	1	#NULL!	1	2	2	9
1355	1	13	1	1	#NULL!	1	2	1	2
1356	1	15	2	1	#NULL!	1	2	2	2
1357	1	15	2	1	#NULL!	1	2	1	3
1358	1	15	2	1	#NULL!	1	2	1	1
1359	1	15	2	1	#NULL!	1	2	1	3
1360	1	15	2	1	#NULL!	1	2	1	2
1361	1	15	2	1	#NULL!	1	2	1	2
1362	1	15	2	1	#NULL!	1	2	1	2

1363	1	15	2	1	#NULL!	1	2	1	2
1364	1	14	1	1	#NULL!	1	2	1	2
1365	1	15	2	1	#NULL!	1	2	3	2
1366	1	13	1	1	#NULL!	1	2	1	2
1367	1	14	1	1	#NULL!	1	2	1	3
1368	1	15	2	1	#NULL!	1	2	1	1
1369	1	19	2	1	#NULL!	1	2	2	2
1370	1	16	2	1	#NULL!	1	2	1	1
1371	1	14	1	1	#NULL!	1	2	1	2
1372	1	15	2	1	#NULL!	1	2	1	2
1373	1	14	1	1	#NULL!	1	2	1	2
1374	1	16	2	1	#NULL!	1	2	1	2
1375	1	14	1	1	#NULL!	1	2	2	1
1376	1	15	2	1	#NULL!	1	2	2	2
1377	1	15	2	1	#NULL!	1	2	1	1
1378	1	14	1	1	#NULL!	1	2	1	3
1379	1	16	2	1	#NULL!	1	2	1	2
1380	1	14	1	1	#NULL!	1	2	1	2
1381	1	16	2	1	#NULL!	1	2	2	2
1382	1	14	1	1	#NULL!	1	2	2	2
1383	1	14	1	1	#NULL!	1	2	1	2
1384	1	15	2	1	#NULL!	1	2	1	2
1385	1	16	2	1	#NULL!	1	2	2	2
1386	1	14	1	1	#NULL!	1	2	2	2
1387	1	14	1	1	#NULL!	1	2	1	1
1388	1	14	1	1	#NULL!	1	2	1	2
1389	1	14	1	1	#NULL!	1	2	1	2
1390	1	14	1	1	#NULL!	1	2	2	2
1391	1	15	2	1	#NULL!	1	2	1	1
1392	1	15	2	1	#NULL!	1	2	1	2
1393	1	15	2	1	#NULL!	1	2	1	2
1394	1	15	2	1	#NULL!	1	2	2	2
1395	1	15	2	1	#NULL!	3	2	1	2
1396	1	14	1	1	#NULL!	1	2	1	2
1397	1	15	2	1	#NULL!	1	2	2	2
1398	1	15	2	1	#NULL!	1	2	3	3
1399	1	13	1	1	#NULL!	1	2	3	3
1400	1	14	1	1	#NULL!	1	2	1	3
1401	1	13	1	1	#NULL!	1	2	3	3
1402	1	16	2	1	#NULL!	1	2	1	3
1403	1	15	2	1	#NULL!	1	2	1	2
1404	1	15	2	1	#NULL!	1	2	1	1
1405	1	14	1	1	#NULL!	1	2	1	1
1406	1	17	2	1	#NULL!	3	2	1	2
1407	1	16	2	1	#NULL!	3	2	2	2
1408	1	18	2	1	#NULL!	3	2	1	1
1409	1	16	2	1	#NULL!	3	2	1	2

1410	1	16	2	1	#NULL!	3	2	1	2
1411	1	18	2	1	#NULL!	3	2	2	3
1412	1	17	2	1	#NULL!	3	2	1	2
1413	1	18	2	1	#NULL!	3	2	1	2
1414	1	0	#NULL!	1	#NULL!	3	2	1	2
1415	1	16	2	1	#NULL!	3	2	1	2
1416	1	17	2	1	#NULL!	3	2	1	2
1417	1	19	2	1	#NULL!	3	2	2	2
1418	1	16	2	1	#NULL!	3	2	3	2
1419	1	19	2	1	#NULL!	3	2	1	2
1420	1	17	2	1	#NULL!	3	2	1	2
1421	1	17	2	1	#NULL!	3	2	1	2
1422	1	17	2	1	#NULL!	3	2	2	2
1423	1	17	2	1	#NULL!	3	2	1	2
1424	1	0	#NULL!	1	#NULL!	3	2	1	2
1425	1	16	2	1	#NULL!	3	2	2	2
1426	1	17	2	1	#NULL!	3	2	1	2
1427	1	17	2	1	#NULL!	3	2	1	2
1428	1	16	2	1	#NULL!	3	2	1	2
1429	1	19	2	1	#NULL!	3	2	1	3
1430	1	19	2	1	#NULL!	3	2	2	2
1431	1	17	2	1	#NULL!	3	2	3	3
1432	1	17	2	1	#NULL!	3	2	1	3
1433	1	18	2	1	#NULL!	3	2	1	2
1434	1	17	2	1	#NULL!	3	2	1	2
1435	1	18	2	1	#NULL!	3	2	3	2
1436	1	21	3	1	#NULL!	3	2	1	2
1437	1	17	2	1	#NULL!	3	1	1	2
1438	1	19	2	1	#NULL!	3	2	1	2
1439	1	0	#NULL!	1	#NULL!	3	2	1	3
1440	1	18	2	1	#NULL!	3	2	1	2
1441	1	18	2	1	#NULL!	3	2	1	3
1442	1	18	2	1	#NULL!	3	2	2	2
1443	1	18	2	1	#NULL!	3	2	1	2
1444	1	17	2	1	#NULL!	3	2	1	2
1445	1	15	2	1	#NULL!	3	2	1	2
1446	1	17	2	1	#NULL!	3	2	3	2
1447	1	17	2	1	#NULL!	3	2	1	2
1448	1	15	2	1	#NULL!	3	2	1	2
1449	1	16	2	1	#NULL!	3	2	1	2
1450	1	15	2	1	#NULL!	3	2	1	2
1451	1	17	2	1	#NULL!	3	2	2	2
1452	1	19	2	1	#NULL!	3	2	1	2
1453	1	18	2	1	#NULL!	3	2	1	2
1454	1	18	2	1	#NULL!	3	2	1	2
1455	1	17	2	1	#NULL!	3	2	3	3
1456	1	16	2	1	#NULL!	3	2	1	2

1457	1	17	2	1	#NULL!	3	2	1	2
1458	1	16	2	1	#NULL!	3	2	1	2
1459	1	17	2	1	#NULL!	3	2	1	1
1460	1	18	2	1	#NULL!	3	2	2	9
1461	1	18	2	1	#NULL!	3	2	2	2
1462	1	17	2	1	#NULL!	3	2	1	2
1463	1	18	2	1	#NULL!	3	2	1	3
1464	1	17	2	1	#NULL!	3	2	2	2
1465	1	17	2	1	#NULL!	3	2	1	1
1466	1	18	2	1	#NULL!	3	2	1	1
1467	1	18	2	1	#NULL!	3	2	1	1
1468	1	18	2	1	#NULL!	3	2	2	2
1469	1	16	2	1	#NULL!	3	2	1	2
1470	1	17	2	1	#NULL!	3	2	1	1
1471	1	17	2	1	#NULL!	3	2	1	2
1472	1	18	2	1	#NULL!	3	2	1	2
1473	1	18	2	1	#NULL!	3	2	1	2
1474	1	18	2	1	#NULL!	3	2	1	3
1475	1	17	2	1	#NULL!	3	2	1	2
1476	1	0	#NULL!	1	#NULL!	3	2	1	1
1477	1	17	2	1	#NULL!	3	2	1	1
1478	1	16	2	1	#NULL!	3	2	2	2
1479	1	17	2	1	#NULL!	3	2	2	2
1480	1	17	2	1	#NULL!	3	2	2	2
1481	1	20	3	1	#NULL!	3	2	2	2
1482	1	18	2	1	#NULL!	3	2	1	2
1483	1	17	2	1	#NULL!	3	2	1	2
1484	1	19	2	1	#NULL!	3	2	1	2
1485	1	15	2	1	#NULL!	3	2	1	2
1486	1	18	2	1	#NULL!	3	2	3	2
1487	1	17	2	1	#NULL!	3	2	2	2
1488	1	17	2	1	#NULL!	3	2	2	2
1489	1	17	2	1	#NULL!	3	2	1	2
1490	1	15	2	1	#NULL!	3	2	1	1
1491	1	0	#NULL!	1	#NULL!	3	2	1	2
1492	1	16	2	1	#NULL!	3	2	1	1
1493	1	17	2	1	#NULL!	3	2	1	1
1494	1	16	2	1	#NULL!	3	2	1	1
1495	1	18	2	1	#NULL!	3	3	1	1
1496	1	17	2	1	#NULL!	3	2	1	1
1497	1	0	#NULL!	1	#NULL!	3	2	1	1
1498	1	17	2	1	#NULL!	3	2	1	2
1499	1	17	2	1	#NULL!	3	2	1	1
1500	1	18	2	1	#NULL!	3	2	3	3
1501	1	17	2	1	#NULL!	3	2	1	2
1502	1	0	#NULL!	1	#NULL!	3	2	1	1
1503	1	18	2	1	#NULL!	3	2	1	2

1504	1	17	2	1	#NULL!	3	2	1	2
1505	1	15	2	1	#NULL!	3	2	1	1
1506	1	17	2	1	#NULL!	3	2	2	1
1507	1	17	2	1	#NULL!	3	2	1	1
1508	1	18	2	1	#NULL!	3	2	1	1
1509	1	17	2	1	#NULL!	3	2	1	1
1510	1	17	2	1	#NULL!	3	2	1	1
1511	1	18	2	1	#NULL!	3	2	1	3
1512	1	17	2	1	#NULL!	3	2	1	2
1513	1	0	#NULL!	1	#NULL!	3	2	1	2
1514	1	19	2	1	#NULL!	3	2	1	1
1515	1	19	2	1	#NULL!	3	2	1	1
1516	1	17	2	1	#NULL!	3	3	1	1
1517	1	18	2	1	#NULL!	3	2	1	2
1518	1	16	2	1	#NULL!	3	2	1	1
1519	1	17	2	1	#NULL!	3	2	1	1
1520	1	17	2	1	#NULL!	3	2	1	2
1521	1	15	2	1	#NULL!	3	2	1	2
1522	9	0	#NULL!	9	#NULL!	9	9	9	9
1523	1	15	2	1	#NULL!	3	2	1	2
1524	1	18	2	1	#NULL!	3	2	1	2
1525	1	18	2	1	#NULL!	3	2	1	2
1526	1	16	2	1	#NULL!	3	2	1	2
1527	1	18	2	1	#NULL!	3	2	1	2
1528	1	16	2	1	#NULL!	3	2	1	2
1529	1	17	2	1	#NULL!	3	2	1	2
1530	1	20	3	1	#NULL!	3	2	1	3
1531	1	17	2	1	#NULL!	3	2	1	2
1532	1	0	#NULL!	1	#NULL!	3	2	1	1
1533	1	17	2	1	#NULL!	3	2	1	1
1534	1	17	2	1	#NULL!	3	2	1	1
1535	1	17	2	1	#NULL!	3	2	1	2
1536	1	17	2	1	#NULL!	3	2	1	2
1537	1	17	2	1	#NULL!	3	2	1	2
1538	1	17	2	1	#NULL!	3	9	1	2
1539	1	16	2	1	#NULL!	3	2	1	2
1540	1	17	2	1	#NULL!	3	2	1	1
1541	1	16	2	1	#NULL!	3	2	1	2
1542	1	17	2	1	#NULL!	3	2	1	2
1543	1	16	2	1	#NULL!	3	2	1	2
1544	1	14	1	1	#NULL!	3	2	1	3
1545	1	17	2	1	#NULL!	3	2	1	2
1546	1	16	2	1	#NULL!	3	2	1	2
1547	1	18	2	1	#NULL!	3	2	1	2
1548	1	19	2	1	#NULL!	3	2	1	2
1549	1	17	2	1	#NULL!	3	2	1	2
1550	1	18	2	1	#NULL!	3	2	2	2

1551	1	16	2	1	#NULL!	3	2	1	1
1552	1	17	2	1	#NULL!	3	2	1	2
1553	1	16	2	1	#NULL!	3	2	1	2
1554	1	18	2	1	#NULL!	3	2	1	2
1555	1	17	2	1	#NULL!	3	2	1	2
1556	1	17	2	1	#NULL!	3	2	1	2
1557	1	18	2	1	#NULL!	3	2	2	2
1558	1	30	3	1	#NULL!	3	2	3	2
1559	1	19	2	1	#NULL!	3	2	2	2
1560	1	18	2	1	#NULL!	3	2	1	3
1561	2	14	1	1	#NULL!	1	3	1	2
1562	1	17	2	1	#NULL!	1	3	1	2
1563	1	16	2	1	#NULL!	1	3	3	2
1564	2	15	2	1	#NULL!	1	3	2	2
1565	1	17	2	1	#NULL!	1	3	1	2
1566	1	12	1	1	#NULL!	1	3	1	2
1567	1	15	2	1	#NULL!	1	3	1	2
1568	9	0	#NULL!	2	#NULL!	1	3	2	3
1569	1	16	2	1	#NULL!	1	3	1	1
1570	1	17	2	1	#NULL!	1	2	1	2
1571	1	18	2	1	#NULL!	1	3	1	2
1572	1	17	2	1	#NULL!	1	3	2	2
1573	2	14	1	1	#NULL!	1	3	1	2
1574	2	16	2	1	#NULL!	1	3	1	2
1575	9	15	2	1	#NULL!	1	3	1	9
1576	2	15	2	1	#NULL!	1	3	1	2
1577	1	14	1	1	#NULL!	1	3	1	2
1578	2	14	1	1	#NULL!	1	3	1	2
1579	2	15	2	1	#NULL!	1	3	1	1
1580	2	19	2	1	#NULL!	1	3	3	1
1581	1	15	2	1	#NULL!	1	3	1	2
1582	1	15	2	1	#NULL!	1	3	2	2
1583	1	16	2	1	#NULL!	1	2	1	3
1584	1	16	2	1	#NULL!	1	3	2	2
1585	2	18	2	1	#NULL!	1	3	1	3
1586	2	14	1	1	#NULL!	1	3	2	2
1587	1	16	2	1	#NULL!	1	3	1	2
1588	9	17	2	1	#NULL!	1	3	1	2
1589	9	0	#NULL!	1	#NULL!	1	3	1	2
1590	2	17	2	1	#NULL!	1	3	1	1
1591	9	14	1	1	#NULL!	1	3	1	1
1592	1	14	1	1	#NULL!	1	3	2	1
1593	2	16	2	1	#NULL!	1	3	1	2
1594	2	15	2	1	#NULL!	1	3	1	2
1595	2	14	1	1	#NULL!	1	3	1	2
1596	1	14	1	1	#NULL!	1	3	1	2
1597	1	16	2	1	#NULL!	1	3	2	2

1598	1	16	2	1	#NULL!	1	3	1	2
1599	2	14	1	1	#NULL!	1	3	1	1
1600	2	15	2	1	#NULL!	1	3	1	2
1601	2	15	2	1	#NULL!	1	3	1	2
1602	2	13	1	1	#NULL!	1	3	1	2
1603	2	16	2	1	#NULL!	1	3	1	2
1604	2	15	2	1	#NULL!	1	3	1	2
1605	2	14	1	1	#NULL!	1	3	1	2
1606	1	14	1	1	#NULL!	1	3	1	2
1607	2	15	2	1	#NULL!	1	3	1	2
1608	1	16	2	2	#NULL!	1	3	1	2
1609	2	14	1	1	#NULL!	1	3	1	2
1610	2	14	1	1	#NULL!	1	3	1	2
1611	2	15	2	1	#NULL!	1	3	1	2
1612	2	14	1	1	#NULL!	1	3	2	2
1613	2	17	2	1	#NULL!	1	3	1	2
1614	1	15	2	1	#NULL!	1	3	1	2
1615	1	15	2	1	#NULL!	1	3	2	2
1616	2	15	2	1	#NULL!	1	3	1	2
1617	1	14	1	1	#NULL!	1	3	1	2
1618	2	15	2	1	#NULL!	1	3	1	2
1619	2	14	1	1	#NULL!	1	3	1	2
1620	2	15	2	1	#NULL!	1	3	1	2
1621	2	14	1	1	#NULL!	1	3	1	2
1622	2	16	2	1	#NULL!	1	3	1	2
1623	2	17	2	1	#NULL!	1	3	1	2
1624	1	16	2	1	#NULL!	1	3	2	2
1625	1	17	2	1	#NULL!	1	3	1	2
1626	2	16	2	1	#NULL!	1	1	1	2
1627	2	15	2	1	#NULL!	1	3	1	1
1628	2	16	2	1	#NULL!	1	3	1	1
1629	2	13	1	1	#NULL!	1	3	1	2
1630	2	16	2	1	#NULL!	1	3	3	3
1631	2	14	1	1	#NULL!	1	3	1	1
1632	2	14	1	1	#NULL!	1	3	1	1
1633	2	15	2	1	#NULL!	1	3	1	2
1634	2	14	1	1	#NULL!	1	3	1	1
1635	1	15	2	1	#NULL!	1	3	1	2
1636	2	15	2	1	#NULL!	1	3	2	2
1637	2	14	1	1	#NULL!	1	3	1	2
1638	1	18	2	1	#NULL!	1	3	2	2
1639	1	14	1	1	#NULL!	1	3	2	2
1640	2	15	2	1	#NULL!	1	3	3	3
1641	2	16	2	1	#NULL!	1	3	1	2
1642	2	15	2	1	#NULL!	1	3	1	1
1643	2	14	1	1	#NULL!	1	3	1	2
1644	2	16	2	1	#NULL!	1	3	2	2

1645	1	20	3	1	#NULL!	1	2	2	2
1646	2	15	2	1	#NULL!	1	3	1	2
1647	1	16	2	1	#NULL!	1	3	1	1
1648	2	16	2	1	#NULL!	1	3	1	1
1649	2	18	2	1	#NULL!	1	3	1	2
1650	9	16	2	1	#NULL!	1	3	1	2
1651	2	14	1	1	#NULL!	1	3	2	2
1652	2	16	2	1	#NULL!	1	3	1	2
1653	1	17	2	1	#NULL!	1	3	1	2
1654	2	16	2	1	#NULL!	1	3	1	1
1655	2	15	2	1	#NULL!	1	3	1	3
1656	2	15	2	1	#NULL!	1	3	1	2
1657	2	14	1	1	#NULL!	1	3	1	3
1658	2	15	2	1	#NULL!	1	3	1	1
1659	2	14	1	1	#NULL!	1	3	2	2
1660	2	14	1	1	#NULL!	1	3	1	1
1661	2	16	2	1	#NULL!	1	3	1	1
1662	2	14	1	1	#NULL!	1	3	2	2
1663	2	16	2	1	#NULL!	1	3	1	2
1664	2	15	2	1	#NULL!	1	3	1	1
1665	2	15	2	1	#NULL!	1	3	2	2
1666	2	16	2	1	#NULL!	1	3	1	1
1667	9	16	2	1	#NULL!	1	3	1	1
1668	2	14	1	1	#NULL!	1	3	2	1
1669	2	13	1	1	#NULL!	1	3	1	1
1670	2	15	2	1	#NULL!	1	3	1	1
1671	2	15	2	1	#NULL!	1	3	1	2
1672	2	15	2	1	#NULL!	1	3	1	2
1673	1	13	1	1	#NULL!	1	3	1	2
1674	2	15	2	1	#NULL!	1	3	1	2
1675	2	14	1	1	#NULL!	1	3	1	3
1676	2	15	2	1	#NULL!	1	3	1	1
1677	2	14	1	1	#NULL!	1	3	1	3
1678	2	15	2	1	#NULL!	1	3	1	3
1679	1	14	1	1	#NULL!	1	3	1	3
1680	2	16	2	1	#NULL!	1	3	1	1
1681	2	13	1	1	#NULL!	1	3	1	2
1682	1	15	2	1	#NULL!	1	3	2	2
1683	1	15	2	1	#NULL!	1	3	1	2
1684	1	15	2	1	#NULL!	1	3	1	1
1685	1	14	1	1	#NULL!	1	3	3	1
1686	1	15	2	1	#NULL!	1	3	1	2
1687	1	18	2	1	#NULL!	1	3	1	1
1688	1	16	2	1	#NULL!	3	3	2	1
1689	1	16	2	1	#NULL!	1	3	2	2
1690	1	16	2	1	#NULL!	1	3	1	1
1691	1	15	2	1	#NULL!	1	3	1	1

1692	1	14	1	1	#NULL!	1	3	1	2
1693	1	15	2	1	#NULL!	1	3	3	1
1694	1	0	#NULL!	1	#NULL!	1	3	1	2
1695	1	15	2	1	#NULL!	1	3	1	1
1696	1	15	2	1	#NULL!	1	3	1	2
1697	1	16	2	1	#NULL!	1	3	1	3
1698	1	15	2	1	#NULL!	1	3	1	3
1699	1	14	1	1	#NULL!	1	3	1	2

PainBreast	ChestPain	Headache	BreastLum	ArmpitLum	DischargeC	DischargeP	DischargeS	Women20	WomenMa
1	3	2	3	2	2	2	2	9	9
3	3	1	2	1	2	3	2	3	3
1	3	3	1	1	2	2	2	1	3
1	3	1	1	2	2	2	2	3	9
3	3	1	9	9	9	9	9	9	9
1	3	1	1	2	2	2	2	3	3
2	2	2	1	2	2	2	2	1	2
1	2	1	2	1	1	3	3	1	1
1	9	9	9	9	9	9	9	2	9
2	2	9	9	9	9	9	9	2	9
1	2	2	2	2	2	2	2	1	2
1	2	1	2	2	2	2	2	1	2
1	2	2	2	2	2	2	2	2	2
1	2	3	2	2	3	3	3	2	1
1	2	2	1	2	2	2	2	2	2
1	2	2	1	1	2	2	2	2	2
1	2	3	2	2	1	2	3	1	2
1	2	2	1	2	2	2	2	1	3
1	2	2	1	3	3	1	2	3	3
2	1	1	2	2	2	2	2	2	2
2	2	2	1	2	1	2	2	1	3
1	1	1	1	1	2	2	2	2	2
2	3	2	2	2	2	2	1	1	1
2	2	2	1	2	1	2	2	1	3
1	2	2	1	1	2	2	2	1	2
1	2	2	1	2	2	2	2	2	2
2	2	2	1	2	2	2	2	1	2
1	2	1	2	2	2	1	2	1	3
1	2	1	1	1	2	3	3	1	3
1	1	9	9	9	2	2	2	2	2
1	2	1	2	2	2	2	2	2	2
2	1	1	1	2	2	2	2	2	2
1	1	1	1	1	1	2	2	1	3
2	1	1	1	2	2	2	2	2	2
2	1	1	2	2	2	2	2	2	2
2	1	1	1	2	2	2	2	2	2
2	1	1	2	2	2	2	2	2	2
1	1	3	1	1	1	3	2	3	3
1	2	1	1	1	2	3	3	1	3
1	2	1	1	1	2	3	3	1	3
1	9	1	1	1	2	3	3	1	3
1	9	9	9	9	9	9	9	2	9
1	3	1	1	1	1	3	2	9	9
1	3	9	1	3	9	9	9	1	3
2	2	2	1	2	2	2	2	1	2
1	1	3	1	1	1	3	2	1	1

1	1	9	1	1	1	3	1	1	1
1	9	9	1	9	9	9	9	1	9
1	1	3	1	2	1	9	2	3	9
1	1	1	1	2	1	2	1	3	3
9	9	9	1	9	9	9	9	1	9
1	1	3	1	1	1	3	3	3	3
1	1	1	1	3	3	3	2	1	3
1	9	9	9	9	9	9	9	1	9
1	3	3	1	9	9	9	9	1	9
1	1	1	1	1	1	2	1	3	3
9	9	9	1	9	9	9	2	1	9
9	9	9	9	1	9	9	9	1	9
1	2	2	1	1	2	3	3	1	3
2	2	2	1	1	1	1	1	1	3
9	9	9	9	1	9	9	9	9	9
1	2	2	1	3	2	2	2	1	3
1	2	3	1	1	1	2	2	1	3
3	9	1	1	1	1	2	1	1	9
1	3	2	3	2	1	3	3	1	3
1	1	3	1	1	2	2	1	1	3
1	1	3	1	3	1	1	3	1	3
1	1	3	1	3	9	1	3	9	9
1	1	3	1	9	1	1	3	1	3
1	3	3	1	3	1	3	9	1	3
1	1	3	1	1	1	9	2	1	1
1	9	9	1	1	1	9	9	9	9
1	3	3	1	1	3	3	1	9	9
1	3	3	1	2	1	3	1	2	3
1	1	3	1	2	2	3	2	2	3
9	9	9	1	9	9	9	9	1	9
1	9	9	9	1	1	9	9	9	9
1	1	2	2	1	2	2	2	3	3
1	1	1	1	9	9	2	1	9	9
9	9	9	9	1	1	3	2	1	3
1	2	2	1	1	2	2	2	2	1
1	1	1	1	1	2	2	2	1	9
1	1	1	1	2	2	2	2	1	2
1	2	2	1	1	2	3	2	2	2
1	9	9	9	1	9	9	9	1	9
1	1	1	1	1	1	1	1	1	3
1	1	1	1	1	1	2	1	1	3
1	2	1	1	9	9	2	1	3	3
1	3	3	1	3	3	3	3	1	3
1	1	2	1	2	1	2	1	1	3
1	3	1	1	3	1	3	2	1	3
3	3	3	3	3	3	3	3	9	9
1	1	1	9	1	3	3	3	1	9

1	2	3	1	2	2	3	2	2	2
1	2	2	1	2	2	3	2	3	2
1	1	2	1	3	1	2	1	1	2
1	2	2	1	2	2	3	2	3	2
1	2	2	1	1	2	2	2	1	3
1	1	2	1	1	2	2	2	9	9
1	1	1	1	1	2	2	2	3	3
1	1	2	1	3	1	2	1	1	3
1	1	2	1	3	1	2	1	1	2
1	2	9	1	2	1	3	1	3	2
2	1	1	2	1	1	2	1	3	1
1	2	3	1	2	2	2	2	3	2
1	2	3	1	2	2	2	1	1	2
1	2	2	1	2	2	2	2	1	1
2	3	2	9	1	3	3	1	1	3
1	3	2	1	2	1	2	3	1	2
1	3	3	1	9	2	2	2	1	2
1	2	3	1	2	2	2	1	2	2
1	1	9	1	1	9	9	9	1	3
1	1	9	1	9	9	9	9	1	3
1	1	9	1	2	2	2	2	2	3
1	3	3	1	3	2	3	2	1	9
3	3	1	2	1	1	1	2	2	2
1	3	3	1	3	1	3	3	3	3
2	2	1	2	9	1	3	1	2	1
2	2	2	1	2	2	2	2	2	2
1	9	9	9	9	9	9	9	1	9
1	2	2	1	2	2	2	2	1	2
2	2	3	2	2	2	2	2	2	2
9	9	9	1	9	9	9	9	9	9
1	2	2	1	2	2	3	2	2	2
2	2	9	9	9	9	9	9	1	2
1	3	1	2	3	3	3	3	2	2
2	2	3	2	2	2	3	2	3	3
9	9	9	1	9	9	9	9	9	9
9	9	9	9	9	9	9	9	1	1
2	2	1	1	2	2	2	3	1	3
1	3	3	2	1	2	3	2	1	2
1	9	9	9	9	9	9	9	1	9
1	2	2	3	3	1	2	3	1	3
3	3	3	1	3	3	3	3	2	2
3	1	3	3	3	3	3	3	2	2
9	9	9	9	9	9	9	9	1	2
1	2	2	1	2	2	2	2	2	1
1	3	2	1	3	2	3	2	2	3
1	1	3	1	1	2	1	1	1	3
1	3	2	1	3	2	3	2	1	3

2	2	2	2	2	2	2	2	9	2
1	9	9	9	9	9	9	9	1	9
2	2	3	2	3	2	2	2	1	3
3	1	1	3	3	3	3	3	2	9
3	1	1	3	3	3	3	3	2	9
3	3	9	1	9	9	9	9	1	9
1	2	9	3	2	2	9	9	2	1
3	3	3	1	3	3	3	3	2	3
2	3	2	2	2	2	2	2	3	1
2	2	2	2	2	2	2	2	2	2
1	2	3	2	2	3	2	1	1	3
1	2	2	2	2	2	2	1	1	3
2	2	2	9	9	9	9	9	2	9
2	2	2	2	2	2	2	2	2	3
2	2	3	2	3	2	2	2	1	3
2	2	3	2	2	2	3	2	2	2
1	1	1	2	2	2	2	2	2	2
1	3	9	3	3	9	9	9	3	3
9	9	9	1	9	9	9	9	9	9
1	1	1	1	1	2	2	2	1	3
1	9	9	9	9	9	9	9	1	3
1	1	1	1	1	1	2	2	1	3
1	1	1	1	1	1	1	1	2	3
1	3	1	3	3	3	3	3	1	3
1	3	1	3	3	3	3	3	1	3
1	1	1	1	1	1	1	1	1	3
1	1	1	1	1	2	2	2	1	3
1	2	9	9	9	9	9	9	1	3
3	3	3	1	3	3	3	3	1	3
2	2	2	2	1	1	3	2	1	3
1	3	1	3	3	1	3	3	1	2
1	2	2	1	2	1	2	1	1	3
1	1	1	1	9	2	2	2	1	3
3	3	1	3	3	3	3	3	1	3
1	1	2	2	2	2	2	2	2	3
1	2	2	1	1	1	3	1	1	3
2	3	3	3	3	3	3	3	1	3
1	3	3	3	1	1	1	1	1	3
1	2	2	1	1	2	2	2	1	3
2	3	3	1	2	3	1	1	3	3
1	3	3	1	1	3	3	1	1	3
1	3	1	3	3	3	3	3	1	3
1	1	1	1	1	1	1	1	1	3
1	1	1	1	1	1	1	1	1	3
3	3	3	3	3	3	3	3	1	3
1	1	1	1	1	1	1	1	1	3
1	1	1	1	1	1	1	1	1	3

1	1	1	1	1	1	1	1	1	3
1	3	1	1	1	1	1	3	1	3
3	3	3	3	3	3	3	3	1	3
1	1	1	1	1	1	1	1	1	3
3	3	1	3	3	3	3	3	1	2
1	1	1	1	1	1	1	1	1	3
1	1	1	1	1	1	1	1	1	3
1	1	2	1	2	2	2	2	1	3
1	1	1	1	1	1	1	1	1	3
1	2	2	1	9	2	3	1	1	3
1	1	3	2	2	2	2	2	1	3
9	9	9	1	1	9	9	1	9	9
1	1	1	1	1	1	1	1	1	3
1	2	2	1	1	1	3	9	1	3
1	3	3	1	3	2	2	1	2	1
1	2	2	1	1	1	2	2	3	1
1	2	2	2	2	1	2	2	3	3
1	1	1	1	1	1	1	1	1	1
1	3	3	1	3	1	1	1	2	2
1	2	2	1	2	2	2	2	2	2
2	2	3	1	1	3	3	2	1	3
1	3	1	3	1	3	3	3	1	2
9	9	9	9	9	9	9	1	1	9
1	2	2	1	2	1	2	2	2	3
1	3	1	3	1	3	3	3	1	2
1	3	3	1	3	1	1	1	9	2
1	2	2	1	2	2	2	2	2	2
1	2	3	1	3	1	1	1	1	2
2	2	2	2	2	2	2	2	1	2
1	2	3	3	3	1	1	1	3	2
1	3	3	1	3	1	1	1	3	3
1	3	3	1	3	1	1	1	3	3
1	3	3	1	3	1	1	1	3	3
1	1	2	1	3	1	2	1	1	2
1	2	2	1	1	1	2	2	2	1
2	2	1	1	1	1	2	1	2	3
1	1	3	1	1	1	2	2	2	1
1	2	2	1	1	1	2	2	1	1
1	2	1	1	2	2	2	2	3	3
2	2	1	1	3	1	3	1	1	3
2	2	2	2	2	2	2	2	3	2
1	3	1	9	3	2	2	2	2	2
1	1	1	1	2	3	2	2	9	1
1	1	1	1	2	2	2	3	2	2
1	1	1	2	2	1	2	2	1	3
1	1	2	1	1	2	2	2	1	2
1	2	2	1	2	1	2	1	9	3

1	2	2	2	2	1	2	2	2	3
1	2	2	2	2	1	2	2	2	3
3	2	3	1	2	2	2	2	2	2
1	1	1	2	2	2	2	2	2	2
2	1	3	2	3	1	3	2	2	3
1	2	2	1	1	2	3	2	1	3
1	1	3	1	1	3	2	2	9	9
2	2	3	1	1	2	3	2	1	2
1	2	3	2	2	1	3	1	1	1
2	3	3	1	3	3	3	3	1	3
1	3	3	1	3	2	2	1	2	2
1	2	1	3	2	2	2	2	1	1
1	2	3	2	2	2	2	2	1	1
1	1	2	2	3	2	2	2	1	2
1	1	2	1	2	2	3	3	2	1
1	3	2	2	1	1	2	2	1	2
1	1	2	2	3	2	2	2	1	2
1	1	3	2	3	2	3	3	2	2
1	3	2	2	2	1	3	2	1	3
1	3	3	1	3	1	2	2	1	2
1	3	1	1	1	1	2	1	1	2
1	1	2	1	2	2	2	2	2	1
1	1	2	2	3	2	2	2	1	2
2	3	9	1	2	1	3	2	2	3
2	9	9	9	9	9	9	9	9	9
3	1	2	2	2	2	2	2	2	2
3	1	2	2	2	2	2	2	2	2
3	2	9	1	3	1	3	1	1	3
2	2	1	2	2	2	2	2	2	2
1	2	3	1	3	2	2	2	1	9
1	2	2	2	2	2	2	2	1	2
1	1	2	1	1	2	2	1	2	3
1	1	9	1	1	9	9	9	1	3
2	3	1	2	2	2	3	2	2	3
2	2	2	2	2	2	2	2	2	2
9	9	9	9	9	9	9	9	1	2
1	1	9	1	1	9	9	9	3	3
1	9	3	1	1	1	3	2	3	3
1	1	2	1	1	1	1	2	1	3
1	2	3	1	1	2	3	3	1	3
3	3	2	3	2	3	3	3	1	2
1	2	2	1	3	2	2	2	2	3
1	1	3	1	2	2	3	2	2	3
1	2	2	1	2	1	1	2	1	3
1	2	3	1	2	2	2	2	1	3
1	1	3	2	3	3	3	3	2	1
1	2	2	1	1	2	2	3	1	3

1	2	1	1	2	2	2	2	2	2
1	1	2	1	2	3	3	2	1	3
1	1	1	1	2	1	1	1	2	2
1	2	3	1	2	1	2	1	1	3
1	3	3	1	2	2	3	2	3	3
1	1	2	1	2	1	3	3	1	3
1	2	1	1	2	2	2	2	1	2
9	9	9	1	9	9	9	9	2	9
1	2	1	1	1	1	2	2	1	3
1	2	1	2	2	2	2	2	2	2
1	2	2	2	2	1	2	2	1	1
1	2	2	1	1	1	2	2	1	2
2	2	2	1	1	1	2	3	1	2
1	3	3	1	3	3	3	3	1	9
2	1	3	1	3	1	3	3	3	3
1	1	1	1	2	2	1	2	1	3
3	1	3	2	2	1	1	2	2	3
1	1	1	1	1	1	2	3	1	2
1	1	1	1	1	1	2	3	3	2
1	2	2	2	2	2	2	2	2	1
1	2	3	1	2	1	2	2	3	3
1	1	2	1	2	1	3	3	1	3
1	2	3	1	2	2	3	2	1	2
1	2	2	1	2	1	2	1	2	2
1	2	2	1	2	1	1	2	1	3
1	9	9	9	9	9	9	9	9	9
1	3	3	1	3	9	9	1	1	3
1	3	2	1	1	2	3	3	1	3
1	1	2	1	1	2	2	2	1	3
3	2	2	1	2	2	2	2	1	2
1	2	3	1	2	2	3	1	1	3
3	3	3	2	1	2	3	3	9	9
1	9	9	9	9	9	9	9	1	9
3	3	3	2	1	3	3	3	1	3
2	9	2	1	1	2	2	2	1	3
1	3	3	1	1	9	1	1	1	2
1	2	2	1	2	2	3	2	1	3
1	3	3	1	1	1	1	1	1	1
1	3	3	1	1	1	2	1	1	3
3	3	3	3	2	1	3	2	9	9
9	9	9	9	9	1	9	9	9	9
1	3	3	1	1	1	1	1	1	3
2	2	2	2	2	2	2	1	1	2
1	3	3	1	2	2	3	3	3	3
1	1	2	1	3	2	2	2	1	3
1	3	1	1	3	2	2	2	1	3
1	1	2	1	1	1	2	2	1	3

1	2	2	1	1	2	3	3	1	3
1	2	2	1	1	2	3	3	1	3
9	9	9	9	1	9	9	9	1	9
1	1	9	1	1	1	9	2	3	3
9	9	9	1	9	9	9	9	9	9
1	2	1	1	1	2	2	2	2	3
1	2	1	1	1	2	2	2	1	3
2	9	1	9	2	9	9	9	2	2
1	2	2	1	9	1	9	1	3	2
1	2	2	1	3	2	1	1	1	1
1	3	3	1	9	2	2	1	2	3
1	1	1	3	3	2	2	3	3	2
2	3	3	1	2	2	3	3	1	2
3	2	3	3	3	3	3	3	1	3
1	3	3	1	2	1	2	1	2	3
2	3	9	1	3	1	2	2	2	2
1	1	1	1	2	1	2	1	3	3
1	1	1	1	1	1	2	1	2	3
1	1	1	1	1	1	9	1	3	3
3	2	3	1	2	3	2	2	2	2
1	1	3	1	1	3	2	2	3	3
1	3	3	1	3	1	3	2	3	3
1	3	3	1	3	1	3	2	3	3
2	3	3	1	2	3	2	1	2	3
1	2	2	1	2	2	2	2	2	2
2	3	2	1	1	2	2	2	1	3
2	2	1	1	2	2	2	2	2	2
2	3	3	1	3	2	3	3	1	2
2	3	3	1	2	1	2	2	2	1
2	3	2	1	1	1	3	3	1	3
2	2	1	2	2	3	2	3	2	3
2	1	3	1	1	1	2	3	2	3
1	1	2	2	1	2	2	1	1	1
3	1	2	1	3	9	3	3	2	2
3	1	9	2	2	2	2	2	3	3
3	2	3	1	1	9	2	1	1	3
1	1	3	1	2	3	2	1	1	3
1	2	2	1	2	2	2	2	2	1
2	2	1	1	2	2	2	2	2	3
2	3	3	3	3	2	2	3	3	3
1	2	1	2	1	2	2	2	2	3
2	3	1	2	2	1	2	2	1	3
1	2	3	1	2	2	2	1	1	3
2	3	2	1	2	2	2	2	1	3
2	3	3	1	3	2	3	3	1	2
1	3	1	1	1	1	3	3	1	3
3	2	3	1	1	1	2	1	1	3

1	1	9	1	3	9	2	3	2	2
1	2	2	1	3	1	2	2	1	2
9	9	1	9	9	9	9	9	2	9
3	9	3	9	1	9	1	9	2	3
1	2	1	1	2	3	3	3	1	3
1	3	3	1	2	2	2	2	1	3
2	2	2	1	3	2	2	2	2	2
1	2	2	1	1	1	2	2	1	2
1	2	2	1	1	2	9	2	1	2
2	2	2	1	1	1	1	2	3	3
2	3	2	1	2	2	1	2	1	3
2	2	2	1	2	1	1	1	2	3
1	2	2	2	1	2	2	2	1	1
1	1	1	1	3	1	2	2	2	3
1	1	1	1	3	1	2	2	2	3
1	1	2	1	1	1	2	1	1	3
1	3	3	2	3	2	3	3	1	3
1	2	3	1	2	1	2	2	1	3
1	2	3	1	2	1	2	2	1	3
9	9	9	9	9	9	9	9	9	9
1	3	1	1	3	3	3	3	1	2
1	2	2	1	2	1	3	2	2	3
1	2	3	1	2	1	3	2	2	3
1	1	1	1	1	1	2	1	1	2
1	2	2	2	2	3	1	1	1	3
3	9	3	1	3	9	2	2	2	3
1	3	3	1	3	3	3	3	9	9
1	1	3	1	1	2	2	2	2	2
1	2	3	1	2	1	3	2	3	3
9	9	9	9	1	9	9	9	9	9
1	2	2	1	2	2	2	1	2	1
1	2	1	2	2	3	2	2	2	1
1	2	2	2	2	2	2	2	2	1
1	3	3	9	3	3	3	3	2	9
1	9	9	1	2	1	3	1	3	1
1	2	3	1	1	1	2	2	2	3
1	2	2	1	2	2	2	1	9	9
2	1	1	3	3	2	1	2	1	3
1	3	3	1	2	2	2	2	2	2
1	2	3	1	2	1	2	1	1	2
3	3	2	1	1	2	3	2	1	2
3	3	1	2	9	2	3	2	3	3
1	2	3	1	1	1	2	2	3	2
1	2	9	1	2	1	3	2	2	9
2	3	3	1	2	1	2	2	2	3
1	1	1	1	1	1	1	1	1	3
1	1	2	1	1	1	1	1	1	3

1	1	1	1	1	1	1	1	1	3
1	1	3	1	1	1	3	1	1	3
1	1	2	1	1	1	1	1	1	3
1	1	1	1	1	2	2	2	1	3
1	1	3	1	1	1	1	1	2	3
1	1	2	1	1	1	1	1	1	3
1	1	1	1	1	1	1	1	3	3
1	1	1	1	1	1	1	1	1	3
1	2	2	1	2	1	1	1	3	2
1	1	1	1	1	1	1	1	1	1
1	1	3	1	1	1	1	1	2	3
1	1	1	1	1	1	1	1	1	3
1	1	2	1	1	2	2	1	1	3
1	1	2	1	2	1	1	2	1	3
1	1	2	1	1	1	1	1	1	3
1	1	2	1	2	1	1	1	1	3
1	1	2	1	1	1	1	1	1	3
1	1	2	1	2	3	2	2	1	3
1	1	2	1	2	3	2	2	1	3
1	1	2	1	2	3	2	2	1	3
1	1	2	1	1	1	1	1	1	3
1	1	2	1	1	1	1	1	1	3
1	1	2	1	1	1	1	1	1	3
1	1	1	1	1	1	1	1	1	3
1	3	1	1	1	1	1	1	1	3
1	1	1	1	1	1	1	1	1	3
1	1	1	1	1	1	1	1	1	3
1	1	3	1	1	1	1	1	1	3
1	1	9	1	1	1	1	1	1	3
1	1	1	1	1	1	1	1	1	3
1	1	1	1	1	1	1	1	2	3
1	1	2	1	2	1	2	2	1	3
1	1	1	1	1	1	1	1	1	3
1	1	1	1	1	1	1	1	1	3
1	1	1	1	1	1	1	1	2	1
1	1	1	1	1	1	1	1	1	3
1	1	2	1	1	3	1	1	3	3
1	1	1	1	1	1	1	1	1	3
1	1	1	1	1	1	1	1	1	3
1	1	1	1	1	1	1	1	1	3
1	3	3	1	1	1	3	1	1	3
1	2	2	1	1	2	2	2	1	2
1	9	9	9	9	9	9	9	1	9
1	2	2	1	2	2	2	2	2	2
2	9	9	9	9	9	9	9	2	9
1	2	2	2	2	3	2	2	2	2
9	3	3	1	1	2	2	2	9	9

1	2	2	1	2	2	2	2	2	2
1	1	3	1	1	2	3	3	1	3
1	1	3	1	1	2	3	3	1	3
1	9	3	1	2	1	2	2	3	2
1	3	9	1	2	2	3	1	1	3
1	2	2	1	1	1	2	1	3	3
1	2	2	1	2	1	2	2	2	3
1	2	2	1	2	3	2	2	1	1
1	3	1	1	2	2	2	2	2	1
2	2	2	2	2	2	2	2	2	2
1	1	2	2	9	9	9	9	9	9
9	9	9	1	9	9	9	9	9	9
1	3	3	3	1	3	3	3	1	3
1	2	2	1	1	2	3	2	2	2
9	9	9	9	1	9	9	9	9	9
9	9	9	9	1	9	9	9	9	9
1	9	9	9	9	9	9	9	1	9
9	9	9	1	9	9	9	9	9	9
9	9	9	1	9	9	9	9	9	9
3	3	2	3	3	3	3	3	1	3
9	9	9	9	9	9	9	9	9	9
1	3	3	1	1	3	3	1	3	3
2	3	9	1	2	2	3	2	2	1
1	9	9	9	9	9	9	9	1	9
9	9	9	9	9	9	9	9	9	9
9	9	9	9	1	9	9	9	9	9
9	9	9	1	9	9	9	9	2	9
1	3	3	1	2	2	2	3	2	1
1	3	3	1	2	1	2	3	1	3
9	9	9	1	9	9	9	9	9	9
9	9	9	1	9	9	9	9	9	9
1	2	3	1	2	1	3	2	1	2
3	9	3	2	2	2	9	9	1	2
1	3	3	1	3	3	3	1	1	3
2	2	2	2	2	2	2	2	2	2
2	3	3	1	2	3	3	3	1	3
1	2	1	1	2	1	2	2	1	3
2	3	3	2	3	3	3	3	1	3
3	3	2	2	3	2	2	2	2	2
2	3	2	3	2	2	3	2	1	2
2	3	3	1	2	1	2	1	3	2
1	3	3	1	3	3	3	3	1	3
3	2	1	1	2	2	2	2	1	3
2	2	3	1	2	1	3	2	1	3
3	3	3	1	2	3	2	3	1	1
3	3	3	1	2	1	2	2	3	3
1	2	1	1	2	2	3	3	1	3

9	3	3	1	3	2	2	2	2	1
1	2	3	1	1	2	2	2	1	3
3	3	3	1	2	3	2	3	1	2
1	2	3	1	3	2	2	2	2	1
2	3	3	1	2	3	3	2	2	1
2	3	3	1	3	1	3	2	3	3
2	2	2	2	3	3	3	3	1	2
1	3	3	1	1	2	1	2	1	2
1	1	2	1	3	2	1	2	1	3
3	3	3	2	2	2	2	2	1	2
3	3	3	3	2	2	2	2	1	2
1	2	2	1	2	3	3	1	2	3
2	2	3	2	2	3	2	3	2	2
2	2	3	2	2	3	2	3	2	2
2	3	3	1	2	1	2	1	1	3
2	3	3	3	1	3	3	3	2	2
9	1	3	1	2	2	3	3	1	3
3	3	3	3	3	3	3	3	1	3
3	3	3	1	1	2	3	3	1	3
1	3	3	1	2	1	2	2	1	2
1	3	3	1	1	2	2	2	1	3
1	3	3	1	3	1	3	2	3	3
1	2	3	1	1	2	2	2	1	2
1	2	3	1	1	2	2	2	1	2
2	3	3	1	2	2	3	3	1	3
1	2	3	1	3	2	2	2	2	3
1	3	3	9	3	3	3	3	1	3
1	2	3	2	1	9	2	2	1	3
1	2	2	3	3	2	2	2	2	3
1	3	3	1	3	3	2	3	3	2
1	9	9	1	2	1	3	2	9	9
1	1	2	1	2	2	2	2	2	1
1	3	3	2	2	2	2	2	2	2
1	3	1	2	1	1	2	2	2	2
1	3	2	1	1	3	1	2	3	3
9	9	9	1	9	9	9	9	1	9
1	2	2	1	2	2	2	2	1	2
1	1	1	1	3	1	3	1	1	3
1	3	3	2	2	2	2	2	2	2
1	1	3	1	3	1	3	2	1	3
1	9	9	1	1	9	9	9	1	3
1	1	3	1	2	2	2	2	1	2
9	9	9	1	9	9	9	9	1	9
1	2	3	1	2	2	2	2	3	2
1	3	1	1	3	1	2	1	1	3
1	2	2	2	2	2	2	2	2	2
1	9	9	9	9	9	9	9	9	9

9	9	9	1	9	9	9	9	9	9
9	9	9	1	9	9	9	9	9	9
9	9	9	9	1	9	9	9	9	9
9	9	9	9	9	9	9	9	9	9
9	9	9	1	9	9	9	9	1	9
9	9	9	1	9	9	9	9	1	9
2	9	1	2	2	2	2	2	9	9
3	3	3	1	1	1	9	9	9	9
9	9	9	9	1	9	9	9	1	9
2	9	9	9	9	9	9	9	2	9
1	3	3	1	2	2	9	2	9	3
2	9	9	9	9	9	9	9	2	9
1	3	3	1	1	9	9	9	9	3
9	9	9	1	9	9	9	9	9	9
9	9	9	1	9	9	9	9	9	9
9	9	9	1	9	9	9	9	9	9
9	9	9	9	1	9	9	9	9	9
9	9	9	9	9	1	9	9	1	9
1	1	2	2	2	2	2	2	2	2
3	1	1	2	1	1	2	1	1	1
1	1	1	1	1	1	1	1	1	1
1	1	2	1	1	1	1	2	3	3
2	2	9	2	2	3	2	1	3	1
1	1	1	1	3	1	3	2	1	3
1	1	2	1	2	2	9	3	2	1
1	9	9	9	9	9	9	9	1	9
1	2	1	1	2	2	2	2	1	3
1	2	1	1	3	2	3	1	2	3
1	9	9	9	9	9	9	9	1	3
1	3	3	1	2	2	2	2	2	1
1	1	1	1	1	1	1	2	1	1
1	2	2	1	3	2	2	2	3	3
1	2	2	1	1	2	1	2	1	3
1	2	3	1	3	2	3	1	1	3
1	1	2	1	3	2	2	2	1	2
1	3	3	2	3	3	3	3	3	3
1	2	3	1	2	1	1	2	2	3
1	3	3	1	3	1	1	1	2	3
1	2	3	1	2	3	1	1	9	3
1	2	2	1	2	1	2	1	2	3
1	1	2	1	1	1	9	1	1	1
3	1	3	9	1	1	9	3	1	3
1	2	3	1	2	1	2	2	3	9
1	1	1	1	1	1	1	1	1	1
1	3	3	1	3	1	3	3	2	3
1	2	3	1	2	1	2	2	9	9
1	1	1	1	2	2	2	2	2	2

1	2	3	2	3	3	3	3	1	2
1	1	1	1	3	1	1	3	1	3
1	3	3	1	3	1	1	1	3	3
1	2	1	1	2	1	2	1	1	2
1	1	2	1	2	2	2	2	2	2
1	1	2	3	2	3	1	3	2	2
1	1	1	2	1	2	1	2	1	2
1	2	3	2	3	3	3	3	1	2
1	1	1	9	9	1	1	1	1	1
9	9	9	9	9	9	9	9	9	9
9	1	9	9	9	9	9	9	9	9
1	1	2	1	1	2	2	1	1	3
1	3	3	3	3	3	3	3	3	3
2	2	1	1	2	2	2	2	1	1
2	1	1	3	9	1	1	2	1	1
1	9	9	9	9	9	9	9	1	9
9	9	9	1	9	9	9	9	1	9
1	3	3	1	3	1	1	1	2	3
1	2	2	2	1	1	2	1	1	3
1	2	2	1	2	1	2	2	1	2
1	3	2	1	1	2	1	2	2	1
1	3	3	3	3	3	3	3	3	3
1	2	3	1	2	3	3	3	3	3
9	9	9	9	9	9	9	9	9	9
1	2	2	2	2	2	9	9	9	9
3	3	3	3	3	3	3	2	3	3
1	2	2	1	2	1	2	2	1	2
3	3	1	3	9	9	1	3	3	3
1	1	1	1	2	2	2	2	2	2
1	9	9	9	1	9	9	9	9	9
1	1	3	1	3	2	1	2	1	3
1	1	3	1	1	1	1	1	9	3
1	1	2	2	2	2	2	2	1	1
1	2	2	1	2	1	1	2	2	1
9	9	9	9	9	1	9	9	9	9
9	9	9	1	9	9	9	9	9	9
9	9	9	1	9	9	9	9	9	9
1	1	2	1	2	1	2	2	1	2
1	1	2	1	2	3	3	2	1	2
1	1	2	1	2	1	9	2	1	2
1	1	2	1	2	1	2	2	1	2
1	1	1	1	1	1	1	1	1	2
2	2	3	2	3	1	1	2	1	2
1	1	2	1	2	1	2	2	1	2
1	1	3	2	3	1	1	2	1	2
2	2	1	3	3	3	3	3	3	2
1	3	3	1	3	1	1	1	3	1

3	3	3	1	3	3	3	3	3	3
1	1	1	1	1	1	1	2	1	3
1	2	2	1	3	1	1	1	1	3
1	2	2	1	2	1	2	1	1	2
2	2	1	1	2	2	2	2	2	2
1	1	1	1	1	1	1	1	1	1
1	1	3	2	3	2	3	2	1	3
9	9	9	1	9	1	9	9	1	9
1	1	1	2	3	2	9	3	3	3
3	3	1	1	2	2	2	2	1	3
2	2	2	2	2	2	2	2	2	2
9	9	9	9	9	9	9	9	9	1
1	2	1	3	3	3	3	3	1	2
3	2	1	1	2	2	3	9	9	1
1	2	1	1	2	1	3	3	2	3
3	2	3	1	2	2	3	2	3	1
2	2	1	3	3	3	3	3	1	2
2	2	1	3	3	3	3	3	1	2
2	2	1	3	3	3	3	3	1	2
1	3	3	1	3	3	3	2	1	1
1	2	3	1	2	3	2	1	9	9
1	3	3	1	3	1	1	1	2	3
9	9	9	1	9	9	9	9	9	9
1	3	3	1	3	1	3	2	1	2
3	2	1	9	1	3	1	3	1	9
2	2	1	3	3	3	3	3	3	2
1	2	2	1	2	1	2	2	1	2
1	1	3	1	3	1	3	1	1	3
9	1	9	9	9	9	9	9	1	9
2	2	2	2	2	2	2	2	2	2
1	1	1	1	1	2	3	3	1	3
2	3	2	2	3	2	3	2	2	9
1	2	1	2	3	1	2	1	3	2
3	3	1	3	2	2	2	3	2	2
3	3	3	3	3	3	3	3	3	3
1	2	3	1	3	2	3	3	2	3
1	2	3	3	3	3	3	3	1	2
1	2	1	3	3	3	3	3	1	2
2	2	1	3	3	3	3	3	1	2
1	1	3	1	1	1	3	2	1	3
1	1	2	1	2	2	1	3	2	1
1	3	1	1	1	2	3	1	2	2
1	3	3	3	3	3	3	3	1	2
3	3	1	3	9	1	3	1	1	3
9	9	9	9	9	9	9	9	9	9
3	3	3	3	3	3	3	3	3	3
2	2	1	2	2	2	2	2	2	2

1	2	1	1	1	1	3	3	2	2
1	3	3	3	3	3	3	3	9	9
1	1	3	1	3	1	1	2	2	1
2	1	2	1	3	1	1	1	3	3
1	1	3	1	2	2	2	2	2	2
1	1	2	1	2	2	2	2	2	2
1	1	1	3	3	1	1	9	2	1
1	1	3	1	2	2	2	2	2	2
1	1	2	1	2	2	2	2	2	2
9	9	9	9	9	9	9	9	9	9
3	2	1	9	2	2	2	2	1	2
1	3	3	1	2	3	3	2	3	3
2	2	1	1	2	2	2	2	1	1
1	1	1	1	3	1	1	3	1	3
1	3	1	1	2	1	2	2	1	3
3	3	3	1	1	2	1	1	1	3
1	1	1	2	2	2	2	2	1	2
1	1	9	2	2	2	2	2	1	2
1	1	1	2	2	2	2	2	1	2
1	1	3	2	2	2	2	2	1	2
1	1	1	1	1	1	3	9	1	2
1	2	1	3	2	1	2	2	1	3
3	3	1	3	3	3	3	3	1	3
1	2	2	1	2	2	2	2	2	2
2	2	2	2	1	2	2	2	2	2
9	9	9	9	9	9	9	1	9	9
9	9	9	9	9	9	9	1	9	9
1	3	1	1	3	1	2	1	1	3
3	3	1	3	3	3	3	3	1	3
2	3	3	2	2	2	2	3	2	2
2	2	1	1	1	9	3	1	1	3
1	1	2	1	2	1	9	2	2	1
1	1	3	2	1	2	2	1	1	2
9	9	9	9	9	9	9	1	9	9
1	3	1	1	2	2	3	1	2	1
3	3	3	3	3	3	3	3	3	3
1	2	2	1	2	2	2	2	1	3
1	1	1	3	3	3	3	3	9	3
1	9	9	9	9	9	9	9	9	9
1	2	1	1	1	2	3	2	1	3
9	9	9	1	9	9	9	9	1	3
3	3	1	3	3	3	3	3	2	2
3	3	1	3	3	3	3	3	2	2
2	2	2	2	2	2	3	3	2	2
3	3	1	3	3	3	3	3	2	2
1	1	2	1	2	1	2	1	3	3
1	1	2	1	2	2	2	2	1	3

2	2	2	2	2	2	2	2	2	2
2	2	3	1	2	2	2	2	1	3
3	1	1	1	1	1	1	1	1	3
1	2	3	9	9	9	9	9	1	3
1	9	9	9	9	9	9	9	1	9
2	1	3	1	1	1	1	2	1	1
1	1	2	1	3	2	3	2	1	2
1	9	9	9	9	9	9	9	9	9
1	2	1	3	2	3	1	2	1	2
2	9	9	9	9	9	9	9	1	9
2	2	2	2	2	2	2	2	2	2
1	3	3	2	3	3	2	2	1	3
1	3	3	1	2	1	2	2	1	3
2	2	2	2	2	2	2	2	1	2
1	1	3	1	3	1	2	1	1	3
2	2	2	2	2	2	2	2	2	2
2	2	2	2	2	2	2	2	2	2
1	3	3	1	2	1	3	1	9	9
1	1	2	2	2	3	1	3	1	2
1	3	2	2	3	1	2	1	9	9
1	1	3	2	2	3	1	1	1	2
1	3	3	2	3	3	2	2	1	3
1	2	2	1	2	2	2	2	2	2
1	2	3	2	2	2	2	2	1	1
1	1	2	1	1	2	2	2	1	3
2	3	2	3	2	3	2	3	1	2
1	1	2	3	1	2	3	1	2	1
3	3	1	3	1	3	3	3	1	3
1	1	2	1	2	1	1	1	2	2
1	3	3	2	3	3	2	2	1	3
1	9	9	9	9	9	9	9	3	3
1	1	1	1	1	2	2	2	1	3
3	3	3	1	3	1	1	1	3	3
1	2	2	1	2	1	1	2	2	2
1	2	3	9	9	9	9	9	1	1
1	1	1	1	1	1	1	1	1	3
2	2	2	2	2	2	2	2	2	2
1	2	2	9	1	1	2	3	1	1
3	1	1	3	1	3	3	2	1	2
1	2	1	1	2	1	2	1	1	3
1	1	2	2	2	2	1	2	1	3
2	2	2	2	2	2	2	2	2	2
2	1	3	1	3	1	1	1	1	3
1	1	3	1	3	3	3	3	1	1
1	2	2	1	3	3	3	3	2	2
3	3	3	3	3	3	3	3	3	3
1	2	3	1	2	2	2	2	1	2

1	2	3	9	2	9	9	9	1	3
3	3	3	3	3	3	3	3	3	1
3	3	3	3	3	3	3	3	3	3
3	2	2	2	2	2	2	2	2	2
1	2	2	1	2	2	2	1	1	3
1	3	3	1	3	2	2	1	1	2
3	3	3	3	3	3	3	3	2	1
1	1	3	3	3	2	2	3	1	3
2	3	9	1	2	3	1	2	2	1
3	3	3	3	3	3	3	3	2	2
1	3	2	1	3	1	2	1	1	3
3	3	1	3	3	3	3	3	2	2
9	1	9	9	9	9	9	9	1	9
1	3	2	1	2	1	2	2	2	2
1	2	2	2	2	2	2	2	1	2
3	1	3	1	3	3	9	1	9	9
3	1	3	3	3	3	9	1	1	9
3	3	1	1	1	1	1	1	1	3
1	1	1	2	1	2	2	2	1	3
1	3	2	1	2	1	2	2	2	2
1	1	1	3	3	1	3	3	1	3
1	3	2	1	2	2	2	2	1	1
1	1	2	1	2	2	2	2	2	3
1	1	1	1	1	1	1	1	1	2
1	3	2	1	3	3	3	3	1	3
1	1	9	1	9	1	9	1	1	9
2	2	2	2	2	2	2	2	2	2
1	2	2	1	1	9	3	1	1	3
1	1	3	1	3	1	1	2	1	3
3	2	3	3	3	2	2	9	3	2
1	1	1	1	1	1	1	1	1	1
1	1	1	1	1	1	3	3	1	1
1	2	1	1	1	1	1	1	1	1
1	1	1	2	1	1	1	1	2	1
1	1	3	2	9	3	1	1	3	1
3	3	9	2	3	3	1	1	3	9
9	9	9	1	9	9	9	9	9	9
1	1	1	1	1	1	1	1	1	1
1	2	2	2	2	2	2	2	2	2
2	2	2	2	2	2	2	2	2	2
1	1	1	2	1	1	9	2	3	3
1	1	2	1	2	1	1	1	1	2
1	2	3	2	9	9	9	9	3	3
1	2	2	1	1	1	3	2	1	1
2	2	2	2	2	2	2	2	2	2
1	1	1	3	3	3	3	3	3	3
1	2	2	2	2	2	2	2	3	3

1	1	2	2	3	3	9	1	1	3
3	3	1	3	3	3	3	3	1	3
1	2	3	1	1	2	3	2	3	3
1	9	9	9	9	9	9	9	1	9
1	1	3	2	3	2	3	3	2	3
1	2	3	1	1	2	3	2	3	2
3	3	1	3	3	3	3	3	9	9
9	9	9	9	9	9	9	9	9	9
3	3	3	1	1	1	3	3	1	3
2	2	2	2	2	2	2	2	2	2
1	1	1	1	1	1	1	1	1	9
3	1	3	3	3	3	9	9	1	3
1	9	2	2	9	9	9	9	3	3
1	3	3	2	2	2	2	2	2	1
1	1	1	1	1	1	1	1	1	1
1	1	9	2	3	3	2	3	2	1
1	1	2	2	2	2	2	2	2	1
1	1	1	1	1	1	1	1	1	9
1	2	2	2	1	2	1	2	3	2
1	3	3	2	2	1	2	2	1	2
3	3	9	1	2	2	3	2	1	3
1	3	3	1	3	2	2	2	1	2
3	2	1	3	3	3	2	2	2	2
1	2	9	1	1	1	2	9	1	1
3	2	1	2	3	2	2	9	2	3
1	2	2	9	1	1	3	3	1	3
3	3	3	3	2	3	3	3	2	3
1	3	3	1	2	1	3	3	1	3
1	2	3	2	2	1	2	2	1	2
1	2	3	1	1	2	9	9	1	2
1	2	1	1	2	2	1	1	1	2
1	3	3	3	9	1	3	2	1	1
1	2	1	1	3	3	2	1	1	3
3	3	3	1	3	3	3	3	1	3
1	9	9	1	9	1	9	9	1	9
1	2	1	3	1	1	3	2	1	3
3	3	3	9	3	3	3	3	2	1
3	3	3	3	3	3	3	3	1	3
2	3	1	9	2	1	1	1	9	2
3	3	3	3	3	3	3	3	3	1
1	1	3	3	3	9	9	9	3	1
9	9	3	9	9	9	9	9	1	9
3	3	3	3	3	3	3	3	1	1
3	3	3	3	3	3	1	1	1	3
1	2	2	1	2	1	2	2	1	3
1	1	1	3	3	3	3	3	2	1
1	1	2	1	1	1	3	1	1	3

3	1	3	3	3	3	3	3	3	1
3	3	3	1	3	3	3	3	1	3
1	2	3	1	3	1	2	1	1	2
1	1	1	1	1	1	1	1	3	3
3	3	3	3	3	3	3	3	2	2
1	3	2	9	9	9	3	9	1	3
2	2	2	2	2	2	2	2	2	2
3	3	3	3	3	3	3	3	2	2
3	3	3	3	3	3	3	3	2	9
2	2	2	2	1	2	1	2	2	1
1	1	2	1	2	3	3	2	1	3
1	1	2	2	1	1	2	2	3	3
1	1	3	1	1	3	1	3	3	1
3	3	1	3	3	3	3	3	2	2
1	1	2	1	1	1	2	2	1	1
2	2	2	2	2	2	2	2	2	3
1	1	3	1	3	2	2	1	1	3
1	2	2	1	3	1	2	2	1	2
1	3	3	1	2	2	2	1	1	2
9	9	9	9	1	9	9	9	9	9
3	3	3	3	3	3	3	3	1	9
3	3	3	3	3	3	3	3	3	3
1	1	3	3	3	3	3	3	3	3
1	3	3	1	1	1	2	1	1	3
3	3	3	3	3	3	3	3	1	3
3	1	3	3	3	3	3	3	3	1
3	3	3	3	3	3	3	3	2	1
1	2	3	1	3	1	2	2	1	3
1	1	3	1	2	3	3	2	3	1
2	2	3	1	2	1	1	2	2	3
1	1	1	1	2	3	3	3	1	1
1	1	1	1	1	1	3	3	1	1
2	2	3	2	2	1	2	2	1	1
1	2	1	1	1	1	2	2	1	3
1	9	9	1	9	1	2	1	1	1
1	1	3	1	2	9	9	9	1	3
1	9	9	9	9	9	9	9	1	9
1	2	2	2	2	2	2	2	9	9
1	1	2	1	1	1	1	2	3	3
3	3	3	3	3	3	3	3	2	2
1	1	2	1	1	2	1	1	1	3
1	3	3	3	3	1	3	3	1	3
2	2	2	1	2	2	2	2	2	2
2	9	9	9	9	9	9	9	9	9
9	9	9	9	9	9	9	9	9	9
1	1	1	1	1	1	1	1	1	3
1	3	2	1	1	1	2	1	1	2

1	2	2	3	9	1	1	2	9	9
1	1	1	9	1	2	2	2	1	2
1	3	3	1	3	1	3	3	1	3
9	9	9	1	9	9	9	9	9	9
1	2	3	2	2	2	2	3	3	3
9	9	9	9	9	9	9	9	9	9
2	2	1	2	2	2	2	2	3	2
1	2	1	1	1	2	2	3	1	2
3	3	1	3	3	3	3	3	3	3
1	2	2	2	3	1	2	2	1	3
1	3	3	2	1	1	2	2	1	3
1	1	1	1	1	2	3	2	1	3
1	9	9	9	9	9	9	9	9	9
1	9	9	9	9	9	9	9	9	9
1	2	1	2	1	3	2	1	9	9
3	3	9	1	2	2	3	3	1	3
1	1	2	3	1	2	3	1	1	3
3	3	2	1	3	3	3	3	3	3
1	3	3	2	2	1	2	1	3	1
1	2	3	1	1	1	2	2	3	3
1	2	1	1	2	2	2	2	1	3
1	3	3	1	3	1	2	2	1	2
1	1	2	2	2	2	2	2	2	2
1	2	1	2	2	2	3	3	2	3
3	3	3	3	3	3	3	2	1	3
1	2	2	1	1	1	1	2	3	3
1	9	9	9	9	9	9	9	1	1
1	1	1	2	2	1	1	1	2	2
1	2	2	1	1	1	1	2	2	3
3	3	9	1	2	2	3	3	1	3
1	2	3	1	1	1	2	2	3	3
1	9	9	9	9	9	9	9	9	9
1	2	1	3	2	1	1	2	2	3
1	1	2	1	2	2	2	2	1	3
1	2	2	2	2	2	2	2	2	1
1	3	3	1	1	1	2	2	9	9
1	9	9	9	9	9	9	9	2	9
1	9	9	9	9	9	9	9	2	9
1	2	3	1	2	1	2	2	1	3
1	1	2	2	2	2	2	2	1	1
9	9	9	1	9	9	9	9	1	9
1	2	1	1	1	1	1	2	1	2
1	1	1	1	2	3	3	3	1	1
1	1	1	1	2	3	3	3	1	3
1	1	1	1	1	1	1	1	1	1
1	2	1	1	1	2	1	2	1	3
1	9	9	9	9	9	9	9	1	9

9	9	9	9	9	9	9	9	1	1
1	3	2	1	2	1	2	2	1	3
1	1	1	9	1	2	9	2	3	3
9	9	9	9	9	9	1	9	1	9
1	9	9	9	9	9	9	9	1	9
1	1	2	1	2	1	3	1	3	3
1	1	3	1	1	2	2	2	2	3
1	3	3	1	3	1	3	2	1	3
3	2	1	3	3	3	3	3	1	1
1	1	3	1	1	2	2	2	2	3
1	2	3	1	1	1	3	2	1	3
9	9	9	1	9	9	9	9	2	9
1	2	2	1	1	1	2	1	1	3
1	2	2	2	3	1	2	3	2	3
1	2	2	1	1	2	2	1	2	3
3	3	1	3	1	3	3	3	3	3
1	1	3	1	3	2	2	2	1	3
1	1	2	1	2	1	2	1	1	3
1	2	1	2	2	2	2	1	2	2
1	3	1	1	3	3	3	2	1	3
1	1	3	1	2	1	1	2	3	2
9	9	1	9	9	9	9	9	9	1
1	1	3	1	2	1	1	2	2	2
1	2	2	2	2	2	2	2	3	2
1	1	2	1	2	1	2	2	2	2
1	1	2	2	2	2	2	2	1	1
2	2	2	1	2	2	2	2	3	3
1	2	1	1	3	1	1	2	1	3
1	2	2	1	3	2	3	2	1	3
1	3	3	3	3	3	3	3	1	3
9	9	1	9	9	9	9	9	1	9
2	1	2	2	1	1	9	3	2	3
1	1	1	1	2	1	2	2	1	9
2	3	2	1	2	3	2	2	1	3
1	1	3	9	2	3	1	3	1	1
1	9	9	9	9	9	9	9	9	9
9	9	9	9	9	9	9	9	9	9
2	2	2	2	2	2	2	2	2	2
1	3	1	1	3	2	2	2	3	3
1	2	1	3	2	1	1	2	1	2
3	1	2	3	2	1	2	1	1	2
1	1	1	1	9	1	1	1	1	1
1	1	9	2	1	3	1	1	1	1
1	2	2	3	1	2	1	3	1	2
3	2	3	1	9	3	1	2	3	9
1	9	9	9	9	9	9	9	1	9
1	3	3	2	2	9	3	3	2	3

2	1	2	1	2	1	2	2	1	2
1	2	2	1	3	1	2	2	1	3
3	3	3	3	3	3	3	3	3	3
1	3	2	1	1	9	3	9	1	2
1	2	3	1	2	1	1	2	1	3
2	1	2	1	2	1	2	2	1	2
1	2	1	1	3	1	3	2	2	1
1	1	3	1	2	1	1	2	2	2
1	2	1	1	2	2	2	2	3	1
3	3	1	3	3	3	1	1	1	3
1	3	3	2	2	2	2	2	1	2
1	3	2	2	2	2	2	2	2	2
9	9	9	9	1	9	9	9	1	9
1	3	3	1	1	2	1	2	2	2
1	3	3	2	3	1	2	2	2	3
1	3	3	1	2	1	2	1	1	1
1	3	2	1	2	1	2	1	1	3
1	3	3	1	2	1	2	1	1	3
1	2	3	1	2	1	1	3	1	3
2	2	3	1	3	2	3	2	3	3
1	1	1	1	1	1	3	1	1	3
1	3	3	1	1	3	3	1	1	1
3	1	2	3	2	3	3	2	2	3
3	3	1	2	2	2	2	9	1	3
1	2	3	1	2	2	2	1	2	2
2	3	3	2	3	3	2	2	2	1
2	2	1	2	2	2	2	2	2	2
2	2	2	1	2	2	2	2	2	2
1	3	1	1	3	1	3	1	3	1
1	3	2	3	1	1	3	1	3	1
1	2	1	2	3	3	3	3	1	3
3	3	1	2	2	2	2	9	1	3
3	3	2	2	2	2	2	9	1	3
1	2	2	9	9	9	9	9	1	3
1	1	1	2	1	1	1	2	2	1
1	1	1	1	2	1	2	3	1	3
1	3	3	1	9	9	9	9	1	3
1	3	3	1	2	1	3	3	1	3
1	3	3	1	2	3	2	3	1	3
3	1	1	3	3	3	3	3	1	1
3	3	2	2	2	3	1	2	3	1
1	3	1	3	3	3	3	3	3	3
1	3	3	3	3	3	3	3	1	3
1	1	3	1	3	1	1	3	1	3
1	9	9	9	9	9	9	9	1	9
1	9	9	9	9	9	9	9	1	9
1	9	9	9	9	9	9	9	1	9

3	3	3	2	2	3	3	2	3	3
2	1	2	3	1	1	3	2	2	3
1	1	9	1	1	1	1	1	1	3
1	3	3	1	2	1	2	2	2	3
1	1	1	1	1	1	3	1	1	1
1	9	9	9	9	9	9	9	1	9
1	9	9	9	9	9	9	9	1	9
9	9	9	1	9	9	9	9	9	9
1	9	9	9	9	9	9	9	1	9
1	9	9	9	9	9	9	9	9	1
1	9	9	9	9	9	9	9	9	1
1	1	1	3	1	1	1	1	1	1
1	9	9	9	9	9	9	9	1	9
1	3	3	1	1	1	3	1	1	3
1	3	3	1	3	2	1	2	1	3
1	2	2	1	2	2	2	2	2	2
2	2	2	1	2	2	2	2	1	1
9	9	9	1	9	9	9	9	9	9
9	9	9	1	9	9	9	9	1	9
1	9	9	9	9	9	9	9	1	9
1	3	2	2	3	3	9	3	1	3
1	9	9	9	9	9	9	9	1	9
1	1	1	2	2	2	2	2	1	1
1	1	2	2	2	3	2	2	1	9
1	1	1	1	2	2	2	2	1	3
1	1	1	1	1	1	1	1	1	2
1	2	3	1	3	1	2	2	1	2
1	1	1	2	2	2	2	2	1	2
1	1	1	1	2	2	2	2	1	2
1	1	3	2	2	2	2	2	1	3
1	1	1	1	3	3	3	3	1	1
1	2	2	1	2	2	2	2	1	2
2	2	2	2	1	1	1	1	1	1
1	9	9	9	9	9	9	9	1	9
1	1	1	2	1	1	1	1	1	2
1	2	2	1	1	2	2	2	1	2
1	1	2	2	2	2	2	2	1	2
1	9	9	9	9	9	9	9	1	9
1	2	2	1	2	1	3	3	1	1
3	3	3	3	3	1	3	3	1	3
2	2	1	3	9	3	3	3	2	1
3	3	3	3	3	3	2	1	3	3
3	3	3	1	3	1	3	3	3	3
1	1	2	1	1	1	1	1	1	1
1	3	3	1	3	3	1	1	1	2
1	9	9	1	3	2	3	2	3	3
1	2	1	1	2	2	2	2	1	3

1	3	3	1	3	3	2	1	1	3
1	1	1	2	3	2	1	3	2	1
9	3	3	9	2	3	3	2	1	9
1	2	2	1	1	2	2	2	1	3
1	3	2	1	1	1	3	1	2	3
3	1	1	1	9	2	2	9	9	9
2	3	3	3	3	2	1	1	1	3
3	3	3	3	3	3	3	3	1	3
3	1	1	1	2	2	9	3	9	9
3	1	1	1	1	2	2	3	1	3
9	9	9	2	9	9	9	9	9	9
2	2	2	2	3	1	3	2	1	3
1	1	1	2	2	2	2	2	1	1
3	1	1	2	2	1	2	2	2	1
1	1	1	2	2	2	2	2	1	3
1	1	3	1	2	2	3	2	1	2
2	1	1	1	1	2	2	2	1	3
1	2	3	1	2	2	2	2	2	3
1	1	1	1	1	1	1	1	1	3
9	9	9	9	9	1	9	9	9	9
1	1	1	1	1	1	1	1	1	3
1	3	2	1	2	1	1	3	1	3
1	2	2	1	2	1	1	2	2	3
1	1	2	1	2	1	2	3	1	3
1	1	2	1	1	1	2	2	1	3
3	3	1	3	3	3	3	3	3	3
1	1	3	1	3	1	1	1	3	1
1	1	1	1	2	9	2	3	3	3
1	2	2	1	2	2	2	2	1	2
1	2	2	1	1	2	9	1	3	3
1	2	3	1	2	3	2	3	1	3
1	1	1	2	2	2	2	2	2	2
1	1	2	1	1	2	2	2	1	2
1	1	2	1	3	2	2	2	1	1
1	1	1	1	2	2	2	2	1	2
1	2	1	2	1	1	1	2	3	1
3	2	2	1	2	1	2	2	2	2
1	2	2	2	1	1	1	1	1	2
1	2	2	1	1	1	1	2	1	2
2	2	2	2	2	2	1	2	2	2
1	3	3	1	1	1	3	3	1	3
3	3	3	3	3	3	3	3	2	3
1	3	2	1	3	1	3	3	1	2
1	2	1	2	2	2	2	2	1	1
1	1	1	1	2	1	1	1	3	1
3	3	3	3	3	9	3	3	3	3
1	2	2	1	2	2	2	2	1	3

2	2	1	3	2	1	3	2	1	2
1	1	1	1	1	1	1	3	1	3
9	9	9	9	9	1	9	9	9	9
3	1	3	3	3	3	3	3	3	3
9	9	9	9	9	1	9	9	9	9
3	3	1	1	2	1	9	3	3	3
1	9	9	9	9	9	9	9	1	9
1	2	1	3	3	2	3	2	1	3
2	3	9	2	3	2	3	3	2	2
1	1	1	2	3	1	2	2	3	3
1	2	2	1	1	2	9	2	3	3
1	1	3	1	2	1	1	2	1	3
1	1	1	2	2	1	1	2	2	1
1	1	1	2	1	1	1	2	1	1
1	1	2	2	3	3	2	2	1	1
9	9	9	9	9	9	9	9	9	9
2	1	1	1	3	2	2	2	3	3
3	3	3	3	3	3	3	3	3	3
3	1	1	3	2	3	1	3	3	2
3	9	1	3	9	2	2	2	1	1
1	2	2	1	1	1	1	2	2	2
3	1	1	3	3	3	3	3	1	1
1	1	1	1	1	1	1	1	1	2
9	3	3	3	1	2	2	2	1	2
2	2	1	2	2	1	2	2	2	2
1	1	2	1	2	1	2	2	1	3
3	9	9	9	9	9	9	9	9	9
1	1	1	1	1	1	2	2	2	2
1	2	3	1	2	2	3	1	1	3
2	2	2	1	2	2	2	2	1	3
9	9	9	9	9	9	9	9	9	9
2	2	2	2	2	2	1	1	1	2
1	3	1	2	3	1	1	2	1	3
2	2	2	2	2	2	2	2	2	2
2	2	2	2	2	2	3	3	2	2
1	2	3	1	2	1	3	2	1	3
3	3	3	3	3	3	3	3	3	1
1	2	3	1	3	1	1	2	1	3
1	2	3	1	3	1	1	2	1	3
1	2	3	1	2	2	2	2	3	3
1	1	2	2	2	2	2	2	1	1
1	1	1	1	1	2	2	2	1	3
1	3	2	1	9	2	3	2	3	3
1	9	9	9	9	9	9	9	1	9
9	9	9	1	9	9	9	9	1	9
1	3	2	1	9	2	3	2	3	3
3	1	3	3	3	3	3	1	3	3

3	3	1	3	3	3	3	3	3	3
1	2	2	1	2	2	2	1	2	2
1	9	9	9	9	9	9	9	1	9
3	3	1	3	3	3	3	3	3	3
1	3	1	3	2	1	1	3	3	3
1	1	1	1	2	1	2	3	2	3
1	2	1	2	1	2	2	2	2	3
2	3	3	3	3	1	3	2	1	3
1	2	2	1	1	2	2	1	3	3
1	2	2	1	2	1	3	3	1	3
9	9	9	9	9	9	9	9	1	9
9	9	9	9	9	9	9	9	1	9
2	2	2	2	2	2	2	2	2	2
1	1	1	1	2	1	2	2	1	2
9	9	9	9	1	9	9	9	9	1
1	9	1	1	1	1	2	2	2	3
1	2	3	1	1	3	1	3	3	3
1	1	3	1	1	3	3	1	2	3
1	3	1	1	2	1	3	2	1	1
1	3	1	1	3	3	2	1	3	3
1	2	3	1	3	2	2	1	2	1
1	3	1	1	3	1	1	1	1	3
3	1	3	1	3	1	2	3	1	1
1	1	3	1	3	1	3	3	1	9
1	1	3	2	3	1	2	2	1	3
1	3	3	3	3	3	3	3	1	1
9	9	9	1	9	9	9	9	1	9
1	3	1	1	3	2	2	1	3	3
1	1	1	1	2	9	9	1	1	3
1	1	2	1	2	3	1	2	1	3
1	1	1	1	9	3	3	1	1	3
1	2	3	1	2	1	1	2	1	1
1	1	1	2	3	2	1	3	1	2
3	3	1	3	3	3	3	3	1	3
1	2	2	1	3	2	1	1	1	3
2	2	2	1	2	1	2	2	2	2
1	1	3	1	2	2	9	9	9	9
2	3	3	1	3	2	3	1	1	3
1	9	9	9	9	9	9	9	9	9
3	3	1	3	3	3	3	3	1	3
1	1	3	1	1	2	1	2	1	3
1	1	3	1	2	1	3	3	9	1
1	1	3	9	1	9	3	3	1	3
1	1	3	1	2	3	3	1	1	3
1	1	2	1	3	1	3	3	1	3
1	1	1	1	2	3	3	1	1	3
3	1	1	3	3	3	3	3	3	3

3	3	1	3	3	3	3	3	3	3
1	1	1	9	9	9	9	9	9	9
3	3	3	3	1	3	3	3	1	1
3	1	1	3	1	3	1	1	3	3
1	3	3	1	3	1	3	1	1	3
1	3	3	1	3	1	1	3	1	3
1	1	3	1	1	2	1	2	3	3
1	1	1	3	2	1	3	3	2	2
3	3	3	3	3	3	3	3	3	3
1	1	1	1	2	3	3	1	1	3
1	1	1	1	2	3	3	1	1	3
1	3	2	2	1	3	2	2	1	3
1	1	1	1	3	3	2	2	2	3
1	1	1	3	2	1	3	3	2	2
2	2	2	2	2	2	3	2	3	1
1	3	1	1	3	1	2	3	1	1
3	3	2	1	3	1	3	2	2	3
3	3	2	2	3	1	2	1	2	1
1	1	3	1	3	3	3	3	1	3
2	1	1	2	2	2	2	2	2	2
1	1	1	1	2	2	2	1	1	3
1	1	2	3	2	2	3	2	1	3
1	1	1	2	1	2	2	2	2	3
1	1	1	2	2	1	1	1	3	3
1	1	2	2	1	2	2	2	2	1
1	2	1	2	2	1	2	3	2	1
1	2	2	3	1	2	2	1	1	3
1	3	2	1	3	3	3	3	3	3
2	2	2	3	3	3	3	3	3	3
1	9	9	9	9	9	9	9	1	9
2	1	2	1	1	2	3	2	1	3
1	1	1	1	1	1	2	3	1	1
1	1	2	2	3	2	3	2	2	3
1	1	2	1	3	1	2	2	2	3
1	2	1	3	1	1	2	1	2	1
2	2	2	2	2	2	2	2	2	2
1	1	3	1	1	1	3	3	3	1
1	1	2	1	3	1	1	3	1	3
1	2	3	1	1	3	3	2	1	3
1	2	3	1	3	2	1	2	1	2
1	1	2	1	2	1	2	1	2	2
1	1	2	2	3	2	3	3	1	2
1	1	1	2	2	2	3	1	1	3
1	1	2	1	2	1	2	1	1	3
3	1	1	1	3	2	2	2	2	2
1	1	3	1	3	2	2	2	2	2
1	1	3	1	2	1	2	3	1	3

2	1	1	1	2	2	3	2	3	3
1	3	1	1	2	1	2	2	2	2
1	1	1	2	1	1	2	1	1	2
1	2	1	3	9	1	1	2	2	1
2	3	2	2	3	3	2	3	3	1
1	1	1	1	1	2	2	1	3	3
2	1	3	2	3	2	3	2	2	3
3	3	2	3	3	3	3	3	3	3
1	1	1	1	1	1	2	1	3	3
1	2	3	2	2	2	2	2	2	2
1	9	9	9	9	9	9	9	1	9
1	1	2	1	3	3	3	3	2	2
1	1	2	9	9	9	9	9	2	9
2	3	2	2	3	3	2	3	3	1
1	2	1	3	2	2	3	3	2	2
1	3	1	3	3	3	3	3	3	3
2	1	1	2	2	2	2	3	2	2
1	1	1	2	2	9	1	2	2	3
1	1	1	2	2	2	3	3	3	3
1	1	2	2	2	2	2	2	3	3
3	3	3	3	3	3	3	3	3	3
9	9	9	9	9	9	9	9	2	1
1	9	9	9	9	9	9	9	1	9
1	1	3	2	2	3	1	2	2	2
1	1	3	1	2	2	1	2	2	2
1	1	1	3	1	2	2	2	2	3
9	9	9	1	9	9	9	9	9	9
2	1	1	2	2	2	3	2	3	3
2	3	2	1	2	2	2	2	3	2
1	9	9	9	9	9	9	9	1	9
1	1	2	1	1	1	1	3	1	3
1	1	9	1	2	1	2	2	1	2
1	3	9	2	1	3	1	1	2	3
1	1	1	1	1	2	9	3	2	1
1	1	1	1	1	1	1	1	3	3
1	2	1	2	1	2	2	3	3	3
1	1	2	1	1	2	1	1	1	3
1	3	3	1	3	2	2	3	1	3
1	2	3	1	1	1	3	2	1	1
1	2	2	1	1	2	2	3	3	3
1	3	9	1	2	2	2	1	1	2
1	3	3	1	2	1	2	2	3	1
1	1	1	1	3	3	3	3	1	3
1	9	3	9	2	1	2	2	1	2
1	3	3	1	3	2	2	2	1	3
2	2	2	1	2	3	3	1	1	2
3	2	3	1	1	2	2	2	3	3

1	3	3	1	1	3	3	2	2	3
1	1	2	1	2	2	2	2	1	2
1	1	1	3	3	3	3	3	9	9
1	9	9	9	9	9	9	9	9	9
1	2	1	3	1	1	2	1	1	3
1	3	1	3	3	2	3	1	1	3
3	3	2	3	1	3	3	3	3	3
1	3	2	3	2	3	3	3	1	2
1	2	1	1	3	2	2	2	1	3
1	2	2	1	2	2	2	2	2	3
1	3	3	3	2	2	1	2	2	3
1	1	2	1	2	1	1	2	1	1
2	2	2	2	2	2	2	2	2	2
3	3	3	3	3	3	3	3	3	3
1	3	3	2	1	3	3	3	1	2
2	3	3	1	2	1	1	1	2	1
2	2	1	3	3	2	3	3	2	2
1	3	3	1	3	1	3	3	1	3
1	1	1	3	3	1	3	1	1	1
3	3	3	3	1	3	1	1	3	1
1	2	3	2	2	1	1	2	2	3
1	2	2	3	2	2	2	2	2	1
1	3	2	9	2	2	2	2	2	1
1	1	2	1	2	1	1	1	1	3
1	1	1	1	2	1	2	2	1	3
1	2	2	1	2	2	2	3	3	3
1	1	3	1	3	2	1	3	1	3
3	3	1	3	3	3	3	3	1	3
1	1	3	3	3	3	3	3	1	3
1	1	1	1	1	1	1	1	2	2
1	1	3	2	3	3	3	3	2	2
3	3	3	3	3	3	3	2	2	1
1	3	3	2	3	1	3	3	3	3
9	9	9	9	9	1	9	9	9	9
3	3	3	3	1	3	2	3	1	3
3	1	3	3	3	3	3	3	3	3
3	3	1	3	3	3	3	3	3	3
1	3	2	1	3	2	1	1	1	2
1	1	2	1	2	1	2	2	2	1
2	1	1	2	2	2	2	2	2	2
3	1	1	3	3	3	3	3	2	1
1	3	9	1	3	3	3	3	3	3
1	1	1	1	1	1	1	1	2	1
1	2	2	2	2	2	2	2	2	2
1	1	1	1	2	2	2	2	3	2
1	3	3	3	2	1	3	1	1	3
1	3	3	1	3	1	3	2	1	3

1	2	3	9	2	2	2	2	2	3
2	3	3	2	2	2	2	2	2	2
2	2	2	2	2	2	2	2	2	2
2	2	2	2	2	2	2	2	2	2
2	2	2	2	2	2	2	2	2	2
2	2	2	3	2	2	2	2	2	2
1	2	3	1	2	1	2	2	3	2
2	2	2	2	2	2	2	2	1	3
2	3	3	2	3	1	2	2	2	1
1	2	2	3	3	2	1	3	2	2
2	2	2	2	2	2	2	2	2	2
3	2	2	2	2	2	2	2	2	2
2	3	3	1	2	1	2	3	2	2
2	2	2	2	2	2	2	2	2	2
2	2	2	2	2	2	2	2	3	1
2	2	3	1	2	2	2	2	2	2
2	2	2	2	2	2	2	2	1	3
2	1	2	2	2	2	2	2	2	2
2	3	3	1	1	1	3	3	1	3
1	2	3	1	2	1	1	2	1	2
2	2	2	2	2	2	2	2	2	2
2	3	3	2	2	2	2	2	2	2
2	2	3	2	2	2	2	2	3	2
2	2	2	2	2	2	2	2	2	2
2	2	2	1	2	2	2	2	2	2
1	1	2	9	1	1	1	2	1	2
2	2	2	1	1	2	2	2	2	2
2	2	2	2	2	2	2	2	2	2
2	2	2	1	2	2	2	2	2	2
2	3	3	3	2	1	2	2	3	3
3	3	3	3	3	3	3	3	3	3
3	2	2	2	2	2	2	2	2	2
2	2	2	2	2	2	2	2	2	2
3	2	2	2	9	3	2	2	3	3
2	2	2	2	2	2	2	2	2	2
1	2	3	1	2	2	3	2	1	3
1	2	3	1	2	2	2	2	1	3
2	2	2	2	2	2	2	2	2	2
1	3	2	2	2	2	2	2	2	2
2	2	2	2	2	2	2	2	2	2
1	3	3	1	2	1	2	2	1	3
2	2	2	2	2	2	2	2	2	2
2	2	2	2	2	2	2	2	2	2
2	2	2	2	2	2	2	2	2	2
2	2	2	2	2	2	2	2	2	2
2	3	3	2	2	2	2	2	2	2
2	2	2	2	2	2	2	2	2	2

2	2	2	2	2	2	2	2	2	2
2	2	2	2	2	2	2	2	2	2
2	1	3	3	1	1	3	2	3	3
2	2	2	1	2	2	2	2	2	2
2	2	2	2	2	2	2	2	2	2
2	2	2	2	2	2	2	2	2	2
1	1	1	1	2	2	2	2	1	3
9	9	9	1	9	9	9	9	1	9
1	1	2	1	2	1	1	2	1	3
1	2	2	1	2	1	2	2	1	3
1	2	2	1	1	2	2	3	2	3
2	2	2	2	2	2	2	2	2	2
2	2	3	1	2	2	2	2	2	2
1	1	1	1	1	1	1	1	9	9
1	3	3	2	3	1	3	3	3	3
2	3	2	2	2	2	2	2	1	2
2	2	2	2	2	2	2	2	1	2
2	3	3	2	2	2	2	2	2	2
1	1	1	1	1	1	1	1	1	3
1	1	3	1	9	2	2	2	2	2
1	9	9	9	9	9	9	9	1	9
2	1	2	2	2	2	2	3	2	2
2	2	2	2	2	2	2	2	2	2
2	2	2	2	2	2	2	2	2	2
2	2	2	2	2	2	2	2	2	2
1	1	3	1	3	2	2	2	2	2
9	9	9	1	9	9	9	9	2	9
2	2	3	2	2	2	2	2	1	2
2	2	2	2	2	2	2	2	2	2
1	1	9	9	1	1	1	2	2	2
2	2	2	2	2	2	2	2	2	2
3	3	3	3	3	3	3	3	1	3
1	1	2	1	3	2	2	1	3	3
3	3	3	3	3	3	3	3	1	3
3	3	2	1	1	2	1	1	3	3
1	2	3	1	1	3	9	9	1	3
3	2	2	1	1	1	2	1	1	3
1	2	3	1	1	1	2	1	1	3
1	1	2	1	2	2	2	2	3	3
1	2	2	1	2	3	3	2	1	3
2	3	2	1	2	1	2	3	2	3
1	2	3	1	2	2	2	2	1	1
1	1	2	1	1	1	2	1	9	1
2	2	2	2	2	2	2	2	2	2
1	1	2	1	1	1	2	1	2	2
1	1	1	1	1	2	2	2	1	2
1	2	3	1	2	1	2	1	1	2

1	2	3	1	2	1	1	2	2	3
1	2	9	2	3	9	1	1	1	1
1	1	2	1	1	1	2	1	3	1
2	1	2	1	2	1	2	2	1	2
1	3	3	1	3	2	3	2	1	2
1	3	2	3	2	2	3	1	1	3
1	2	1	1	3	1	2	2	1	3
9	9	9	1	9	9	9	9	9	9
1	1	2	1	2	2	2	2	2	2
1	1	3	3	1	2	2	2	2	1
1	1	2	1	1	1	1	1	2	2
1	1	2	1	1	1	1	1	2	2
3	3	3	3	3	3	3	3	1	3
1	3	3	1	2	2	1	2	2	2
1	1	2	1	2	1	1	1	3	3
1	1	2	1	2	2	2	2	2	2
1	1	2	1	2	2	2	2	2	2
3	3	3	3	3	3	3	3	2	3
1	1	2	1	2	1	2	2	1	3
1	3	1	1	3	3	2	2	1	3
1	2	3	2	2	3	2	2	2	3
2	2	3	1	2	1	2	2	1	9
1	2	3	1	3	2	2	2	3	3
2	2	3	9	9	9	9	9	9	9
1	2	3	1	2	1	2	1	2	2
1	2	1	1	2	1	1	3	1	3
1	3	9	1	1	1	2	2	3	3
2	9	9	9	9	9	9	9	2	9
1	2	2	1	2	2	2	2	2	2
1	2	2	3	3	3	3	3	1	1
1	2	2	1	2	1	2	1	1	1
1	2	3	3	3	2	2	2	3	2
2	2	2	1	2	2	2	2	1	2
2	2	2	1	2	2	2	2	1	2
2	2	2	1	2	2	2	2	1	2
2	2	2	2	2	2	2	2	2	2
1	1	9	1	2	9	1	1	3	3
2	2	2	2	2	2	2	2	1	2
2	2	2	1	2	2	2	2	1	2
2	2	2	1	2	2	2	2	1	2
1	1	1	1	1	3	3	3	2	2
2	2	2	2	2	2	2	2	3	3
1	2	2	1	2	2	2	2	2	2
1	1	2	1	2	3	3	1	3	2
9	9	9	9	9	9	9	9	9	9
1	1	1	2	2	2	1	1	1	1
2	3	3	3	3	3	3	3	3	3

1	2	2	1	1	2	3	2	1	3
2	1	2	1	3	3	1	2	1	3
2	2	2	2	2	2	2	2	1	3
1	1	2	1	2	1	2	2	1	3
2	2	2	2	2	2	2	2	1	2
1	9	2	2	2	2	2	2	2	2
1	2	2	2	2	2	2	2	2	2
3	3	3	3	3	3	3	3	3	3
1	3	3	1	1	1	3	3	1	3
2	2	2	2	2	2	2	2	2	2
1	2	1	1	1	1	1	1	1	1
1	9	1	2	1	2	1	2	1	1
3	1	1	3	1	3	3	2	2	3
9	2	2	2	2	2	1	2	1	2
3	3	3	3	3	3	3	3	1	3
3	2	3	3	1	1	2	1	2	3
1	1	1	3	3	2	2	2	3	3
2	2	1	2	3	1	3	1	3	1
9	9	9	1	9	9	9	9	1	2
1	3	1	3	1	9	2	1	1	3
1	3	2	1	3	2	2	1	1	3
1	1	3	1	2	3	1	1	3	1
1	1	1	1	1	3	2	1	9	9
1	3	3	1	3	1	2	2	1	3
1	3	2	2	3	1	2	1	1	1
3	3	1	3	3	3	3	3	2	2
1	1	2	1	3	1	3	3	2	3
3	3	3	1	2	1	2	1	1	3
2	3	1	2	2	3	2	3	2	9
3	3	2	1	3	3	3	2	9	3
1	1	1	3	3	2	2	2	1	3
2	2	2	1	2	2	2	2	2	2
1	1	2	1	3	1	3	1	1	3
3	3	3	3	3	3	3	2	3	3
2	2	2	1	2	2	2	2	1	2
1	2	9	1	3	2	3	3	3	3
1	1	3	3	3	3	3	3	3	3
2	2	2	2	2	2	2	2	2	2
1	2	1	1	2	1	3	1	1	1
1	9	9	9	9	9	9	9	9	9
1	1	2	2	2	1	2	1	2	1
3	3	1	3	2	2	1	3	1	3
1	1	3	2	3	2	2	2	1	3
1	2	1	1	3	2	3	2	1	3
1	1	2	1	1	2	1	1	1	3
1	3	3	3	3	3	3	3	1	3
3	3	3	3	3	3	3	3	3	3

3	3	3	2	2	3	1	2	2	3
1	1	3	1	3	1	1	1	3	2
1	3	3	2	2	2	2	3	1	3
1	1	1	1	3	3	3	3	1	3
1	3	3	1	1	1	1	1	1	3
1	2	2	1	2	1	3	1	2	2
1	1	1	1	1	1	1	1	1	3
9	9	9	9	9	1	9	9	2	2
1	3	3	1	2	1	3	2	1	3
3	1	3	2	3	3	3	3	3	3
1	1	2	1	2	1	2	2	1	3
1	1	2	1	2	1	2	2	2	2
1	3	2	2	3	2	2	2	2	2
1	1	3	1	1	1	1	3	1	3
1	2	1	1	1	1	2	2	3	2
1	2	2	2	2	2	2	2	2	2
3	3	3	3	3	2	1	2	2	1
2	3	3	3	3	2	2	2	2	1
1	1	3	2	3	2	2	2	2	3
1	1	1	3	2	1	1	1	1	1
1	1	2	1	9	2	2	2	2	2
9	9	9	9	1	9	9	9	9	9
1	2	2	1	3	2	1	1	3	3
2	3	3	2	2	2	3	2	3	3
1	2	3	2	3	2	1	3	2	3
1	9	1	1	2	2	2	2	2	2
2	2	2	2	2	2	2	2	9	9
2	2	2	2	3	1	1	2	1	3
2	1	3	1	2	3	3	3	3	3
1	1	1	1	1	1	1	1	1	2
1	1	1	3	1	3	3	3	3	1
1	2	3	1	1	2	2	3	9	9
1	3	2	2	3	2	2	3	2	2
3	3	3	1	3	1	3	3	1	3
1	2	3	1	2	1	3	2	1	2
2	2	2	1	2	1	3	2	1	3
1	1	3	1	2	1	3	3	3	2
1	1	9	1	2	2	2	2	1	3
1	2	3	2	3	2	2	2	2	2
1	1	9	9	9	9	9	9	2	9
1	1	2	1	2	1	2	1	1	2
1	1	2	1	2	1	2	2	1	3
1	1	1	2	1	1	2	2	2	2
1	2	3	1	2	1	2	3	1	3
2	2	3	1	2	1	2	1	1	2
1	3	3	1	3	2	3	3	3	3
1	2	3	1	2	3	2	1	1	3

1	2	3	1	2	1	2	1	1	3
2	2	2	2	2	2	2	2	1	3
1	3	2	9	3	2	9	1	2	2
3	1	2	1	2	1	3	2	3	2
1	2	2	1	2	1	1	1	1	3
9	9	9	2	2	9	9	9	2	1
1	3	1	1	3	2	2	2	1	2
1	9	9	9	9	9	9	9	1	9
1	2	3	2	2	2	3	2	1	3
3	1	2	1	2	1	1	2	3	3
2	3	3	1	2	1	3	2	2	2
2	2	3	1	3	3	2	2	2	3
1	2	3	1	3	2	3	2	1	3
1	1	3	1	2	1	2	1	1	3
9	2	2	2	2	9	9	9	9	9
1	2	1	2	3	2	2	2	3	3
1	1	1	1	1	1	1	1	1	1
1	2	2	2	1	2	2	3	2	2
3	1	2	1	2	1	1	2	3	3
1	2	2	3	3	1	2	2	1	3
1	2	1	1	3	1	1	3	1	3
2	1	1	3	3	3	3	3	2	3
1	1	1	1	2	1	1	1	2	2
3	1	1	3	3	3	3	3	1	3
1	2	2	1	1	1	2	1	1	3
1	1	1	1	1	1	1	2	2	1
1	2	3	1	2	2	3	2	1	2
1	2	2	1	2	1	2	2	1	3
1	1	2	1	2	2	3	2	3	3
1	1	2	1	1	2	2	1	1	3
1	1	2	1	1	1	2	2	1	1
1	1	2	2	2	2	1	2	1	2
1	1	2	2	1	2	1	1	1	1
1	1	2	1	2	2	2	2	1	1
1	2	3	1	2	3	1	3	1	3
1	3	3	1	3	2	3	1	1	3
1	1	1	2	2	1	1	1	1	2
1	3	3	1	3	1	1	3	1	3
1	1	2	2	2	1	2	2	1	2
1	1	2	2	2	1	1	1	1	2
3	3	3	3	3	3	3	3	3	3
1	1	3	1	2	1	1	2	2	3
3	1	1	2	2	1	3	2	1	3
2	2	2	2	2	2	2	2	3	3
1	2	3	2	2	2	3	2	3	2
2	2	2	2	2	2	2	2	2	1
1	2	2	3	2	3	2	2	2	3

1	2	2	1	3	2	3	2	1	3
3	1	1	2	1	3	2	3	2	1
2	1	1	2	2	2	2	2	2	2
1	2	3	2	1	3	3	1	2	2
2	2	2	2	2	2	2	2	1	3
2	1	2	3	1	1	2	1	3	1
2	1	2	1	3	2	2	1	3	3
3	3	3	3	3	3	3	3	1	1

WomenInt	WomenPre	WomenMe	CABreastH	Daily	Weekly	Monthly	SixMonthly	Yearly	Anytime
9	9	9	1	9	9	9	9	9	1
1	2	3	3	2	3	1	3	3	2
3	3	2	2	1	1	2	2	2	2
3	1	9	3	2	9	1	1	2	2
9	9	9	9	9	9	9	9	9	9
1	2	3	3	2	3	1	1	9	2
2	2	2	2	2	2	1	2	2	1
3	3	3	2	3	2	1	1	2	3
9	9	9	9	2	9	9	9	9	2
9	9	9	9	2	9	9	9	9	9
2	2	2	2	2	9	9	1	9	2
3	3	3	3	1	2	1	3	2	1
2	2	2	2	2	2	2	2	2	2
2	1	2	1	2	2	1	1	3	3
3	3	2	2	2	2	2	2	2	2
2	2	9	1	1	2	2	2	3	2
2	1	3	1	2	1	1	1	2	1
3	3	3	3	1	3	3	3	3	1
3	3	3	3	2	2	2	2	2	1
2	2	2	2	2	2	2	2	2	2
3	3	3	2	3	3	3	2	3	2
2	3	3	3	2	1	2	2	2	2
3	2	2	2	9	1	9	9	9	1
3	3	3	2	2	1	2	2	2	2
2	2	2	2	1	2	3	3	3	1
2	2	2	2	1	2	2	2	2	2
2	2	2	2	2	2	1	2	2	1
3	3	3	1	1	2	1	2	2	2
3	2	2	2	2	2	1	3	3	1
2	2	2	2	2	2	2	2	2	2
2	2	2	2	2	2	2	2	2	2
2	2	2	2	2	2	2	2	2	2
3	3	3	2	1	2	2	3	3	2
2	2	2	2	2	2	2	2	2	2
2	2	2	2	2	2	2	2	2	2
2	2	2	2	2	2	2	2	2	2
2	2	2	2	2	2	2	2	2	2
3	3	3	3	1	3	3	3	3	9
3	2	2	2	2	2	1	3	3	1
3	2	2	2	2	2	1	3	3	1
3	2	2	2	2	2	1	3	3	9
9	9	9	9	2	9	9	9	9	2
9	9	9	9	2	1	1	3	3	1
3	3	3	3	1	1	1	3	3	1
2	2	2	2	2	2	2	1	2	1
1	3	1	3	3	3	3	3	3	9

1	3	3	1	3	3	9	1	1	1
9	9	9	9	9	1	9	9	9	9
9	9	3	9	9	9	9	9	9	9
1	2	3	2	1	3	3	3	3	1
9	9	9	9	9	1	9	9	9	1
3	3	3	3	3	3	1	3	3	3
3	3	3	1	1	3	3	3	3	1
9	9	9	9	9	9	1	9	9	9
9	9	9	9	9	9	9	1	9	1
1	3	2	2	1	3	3	3	3	1
2	9	9	9	9	9	1	2	9	9
9	9	9	9	1	9	9	9	9	9
2	2	2	3	1	9	9	1	9	9
3	3	3	2	2	1	9	1	9	2
9	9	9	9	9	9	1	9	9	9
3	3	3	3	3	3	3	1	3	3
2	3	2	3	9	9	1	9	9	1
3	3	2	3	1	1	1	3	3	1
3	2	2	1	1	1	3	2	3	1
3	1	2	3	1	2	2	1	1	1
3	3	3	3	3	3	1	3	3	3
9	9	9	9	3	3	1	3	3	3
3	3	3	3	3	3	1	3	3	3
3	1	9	1	1	1	1	2	3	2
1	2	1	2	3	1	3	1	3	3
9	9	9	9	1	9	9	9	9	9
9	9	9	9	9	9	9	9	9	9
3	3	3	1	3	1	3	2	2	2
3	3	3	3	1	3	3	3	3	2
9	9	9	9	9	9	1	9	9	9
9	9	9	9	1	9	9	9	9	9
3	1	3	3	1	3	3	3	3	9
1	9	9	9	9	9	9	9	9	9
3	3	3	9	3	1	9	9	9	9
2	2	2	1	1	1	3	3	3	1
2	1	2	2	1	2	1	2	1	9
1	2	2	1	1	2	1	2	2	9
2	2	2	2	1	9	9	9	9	1
9	9	9	9	9	9	1	9	9	9
3	3	3	3	1	3	3	3	3	1
3	3	2	3	1	1	9	9	3	1
3	3	3	1	2	1	9	3	3	3
3	3	3	3	3	1	3	3	3	9
2	2	3	3	1	2	1	3	3	1
9	9	9	9	9	9	1	9	9	9
9	9	9	9	9	9	1	9	9	9
9	9	9	9	9	9	1	9	9	9

2	3	2	1	3	1	2	2	2	1
2	3	3	1	2	2	2	2	2	1
3	3	3	3	1	2	2	3	3	1
2	3	2	1	2	2	2	1	2	2
2	3	3	2	2	2	2	1	2	1
3	9	9	1	2	3	3	3	3	2
3	3	3	3	1	3	3	3	3	3
3	3	3	2	3	1	1	1	3	1
3	3	3	3	1	2	2	3	3	1
2	2	2	1	1	1	1	2	2	1
3	2	2	1	3	2	1	1	2	1
2	2	2	1	1	2	2	2	2	1
3	3	3	3	1	2	1	3	3	1
9	9	9	9	1	2	3	3	3	9
2	1	2	3	2	1	1	2	2	1
3	1	2	2	2	1	3	2	1	2
3	3	3	1	3	2	1	1	1	1
2	2	2	2	2	2	2	2	2	2
3	3	3	3	1	2	2	2	2	2
3	3	3	3	1	3	9	9	9	9
3	3	3	3	1	3	9	9	9	2
9	9	9	9	9	9	9	1	9	9
2	2	1	2	3	2	2	1	3	1
3	3	1	1	9	9	1	9	9	1
1	2	1	2	3	2	3	3	3	1
2	2	2	2	2	2	2	2	2	2
9	9	9	9	9	9	9	1	9	9
2	2	2	2	9	9	9	1	9	9
2	3	3	2	2	2	2	2	2	2
9	9	9	1	9	9	1	9	9	9
2	2	2	2	2	2	2	2	2	2
2	2	2	2	2	2	2	2	2	1
2	2	2	1	2	2	2	2	3	2
3	3	2	2	2	2	2	2	2	2
9	9	9	1	9	9	1	9	9	9
1	3	3	3	3	1	3	3	3	3
3	3	3	2	1	3	2	3	3	1
3	3	3	2	3	3	2	1	3	2
9	9	9	9	9	1	9	9	9	1
3	3	3	3	2	2	2	1	2	1
2	2	2	2	3	3	1	3	3	3
2	2	2	2	3	3	1	3	3	3
2	2	2	2	2	2	2	2	2	1
1	2	2	2	3	2	1	1	2	3
2	1	3	1	2	1	2	2	3	1
3	3	3	3	3	1	1	2	3	2
1	2	3	3	1	2	3	3	3	3

2	2	2	2	3	3	3	3	3	9
9	9	9	9	9	9	9	1	9	9
2	3	2	1	2	2	1	3	3	1
9	9	9	9	2	2	1	2	2	3
9	9	9	9	2	2	1	2	2	3
9	9	9	9	9	1	9	9	9	1
2	3	2	1	1	2	2	2	3	2
3	3	3	2	2	2	2	3	3	2
3	3	3	2	2	2	2	2	2	2
2	2	2	2	2	2	2	2	2	1
3	2	3	2	2	2	2	2	3	2
3	2	3	2	2	2	2	2	2	2
9	9	9	9	1	9	9	9	9	9
3	3	3	3	2	2	2	2	2	1
2	3	2	1	2	1	2	3	3	1
2	2	2	2	2	2	2	2	2	2
2	2	2	2	2	2	2	2	2	2
3	3	2	2	3	1	9	9	9	9
9	1	9	9	9	9	1	9	9	9
3	3	3	1	3	3	1	3	3	3
3	3	3	3	3	3	1	3	3	3
3	3	3	3	3	3	1	3	3	3
1	3	3	3	2	3	3	2	3	2
3	3	3	3	3	3	1	3	3	1
3	3	3	3	3	3	1	3	3	1
3	3	3	1	9	9	1	9	9	9
3	3	3	1	3	3	1	3	3	3
3	3	3	3	9	9	1	9	9	9
3	3	3	3	3	3	1	3	3	3
3	3	3	3	3	3	1	3	3	3
2	1	1	1	1	1	1	2	3	1
2	2	3	1	1	2	1	2	3	2
1	3	3	1	3	3	1	3	3	3
3	3	3	3	3	3	1	3	3	3
3	3	3	3	3	3	1	3	3	3
3	3	3	2	3	2	1	3	3	2
3	3	3	2	3	3	1	3	3	1
3	3	3	1	9	9	1	9	9	1
3	3	3	3	3	3	1	3	3	3
3	3	3	3	3	3	1	3	3	3
3	3	3	3	3	3	1	3	3	3
3	3	3	3	3	3	1	3	3	1
3	3	3	1	9	9	1	9	9	9
3	3	3	1	3	3	1	3	3	3
3	3	3	3	3	3	1	3	3	3
3	3	3	1	3	3	1	3	3	3
3	3	3	1	3	3	1	3	3	3

3	3	3	3	3	3	1	3	3	3
3	3	3	3	3	3	1	3	3	3
3	3	3	3	3	3	1	3	3	3
3	3	3	3	3	3	3	1	3	3
3	3	3	3	3	3	1	3	3	3
3	3	3	3	3	3	1	3	3	1
3	3	3	3	3	3	1	3	3	3
3	3	3	3	1	2	3	3	3	2
3	3	3	3	1	3	3	3	3	1
3	2	3	1	3	1	2	3	3	9
3	3	3	3	1	3	3	3	3	9
9	9	9	9	9	9	9	9	9	1
3	3	3	3	1	9	9	9	9	9
3	3	3	3	3	3	1	3	3	3
3	2	2	3	1	2	1	9	3	1
2	1	2	1	2	1	1	1	2	1
2	1	1	2	9	1	9	1	1	3
1	1	1	1	1	1	1	1	1	1
2	2	2	2	2	2	1	2	2	1
2	2	2	1	2	2	2	2	2	2
1	2	1	1	1	1	1	1	2	1
2	2	2	2	2	2	2	2	2	1
9	9	9	9	2	9	1	2	3	2
1	1	1	1	1	2	2	3	3	1
2	2	2	2	2	2	2	2	2	1
2	9	9	9	2	2	2	2	2	9
2	2	2	1	2	2	2	2	2	2
2	2	2	2	2	2	2	2	2	1
3	2	2	2	2	2	2	2	2	2
3	2	2	2	1	2	2	2	3	1
3	3	3	3	2	2	1	2	2	2
3	3	3	3	2	2	1	2	2	2
3	3	3	3	1	2	1	2	2	1
1	1	2	2	1	2	2	2	2	1
2	1	2	1	2	2	2	2	2	2
1	2	2	3	1	2	2	2	2	2
2	2	2	1	3	3	2	1	1	2
2	1	2	1	2	2	2	2	2	2
3	3	3	3	2	2	2	1	2	2
3	3	3	3	2	3	3	1	3	2
2	3	3	3	2	2	2	2	2	2
2	2	2	1	2	2	2	2	2	1
9	9	9	9	1	9	9	9	9	9
3	2	2	3	1	9	9	9	9	9
3	3	2	2	2	3	1	1	1	3
3	3	3	2	2	2	1	1	3	3
2	2	3	1	3	2	3	2	1	2

3	1	1	2	1	2	2	2	2	1
3	1	1	2	1	2	2	2	2	1
3	1	2	2	2	2	2	1	2	2
2	1	1	2	3	2	2	1	2	2
3	3	3	2	2	2	1	1	2	1
3	2	3	1	2	1	3	3	3	1
9	9	9	9	9	9	9	9	9	1
3	2	3	1	2	3	3	1	2	3
3	3	3	1	2	3	1	1	2	1
3	3	3	3	3	9	9	9	9	1
2	2	2	2	2	2	2	2	2	2
3	3	1	2	2	2	1	3	1	2
3	3	3	1	2	3	1	1	2	1
2	2	2	3	2	1	2	3	3	3
2	3	2	3	1	2	3	3	3	3
3	3	3	1	1	1	1	2	3	1
2	2	2	3	2	1	3	3	3	3
2	2	3	3	2	2	1	2	2	2
2	2	3	1	2	1	2	3	3	1
3	3	3	2	3	3	2	1	2	2
3	2	2	1	2	1	2	1	3	2
2	1	2	2	2	1	1	2	2	2
2	2	2	3	2	1	2	3	3	3
2	3	2	2	3	3	1	2	2	2
9	9	9	9	2	9	9	9	9	9
2	2	2	2	2	2	2	2	2	2
2	2	2	2	2	2	2	2	2	2
3	2	3	2	3	9	1	1	3	3
1	1	1	2	2	2	1	1	3	3
9	9	9	9	2	2	2	2	2	1
2	2	2	2	2	2	1	2	2	2
3	2	2	2	2	2	2	2	2	2
3	3	3	3	1	9	9	9	9	9
3	3	3	3	2	2	3	3	3	1
2	2	2	2	2	2	2	2	2	2
2	2	2	2	2	2	2	2	2	1
3	3	2	2	3	2	1	1	2	3
1	9	9	9	9	9	9	9	9	1
2	3	3	3	3	3	2	1	3	3
9	3	2	2	3	9	2	2	1	2
2	2	2	2	1	3	3	3	3	1
3	3	3	2	1	1	1	2	2	1
2	3	2	3	3	3	1	2	3	1
3	3	3	3	2	2	2	2	2	2
1	3	3	2	2	1	2	1	1	9
3	3	3	1	1	2	3	3	3	1
2	3	2	1	3	1	2	2	3	1

2	2	2	2	2	1	2	2	2	1
2	2	3	1	3	2	1	3	3	2
2	2	2	1	2	2	1	1	2	2
2	3	3	1	2	3	3	1	3	3
3	3	3	2	1	2	3	3	3	2
3	2	3	3	3	3	1	1	3	1
2	1	2	1	2	1	1	1	2	1
9	9	9	9	9	9	9	1	9	9
9	2	3	2	2	2	1	2	2	2
2	2	2	2	2	2	2	2	2	2
1	1	1	3	2	2	2	2	2	1
2	2	2	2	1	3	3	3	3	1
3	3	3	3	3	1	1	1	1	1
9	9	9	9	9	1	9	9	9	9
1	3	1	3	1	3	3	1	3	3
3	3	3	3	2	2	2	2	2	2
3	3	3	3	1	3	3	3	3	1
3	3	2	2	2	1	1	1	1	1
2	2	1	1	1	2	3	1	1	2
2	2	2	1	2	1	2	1	2	1
2	3	2	1	2	2	2	2	2	2
3	2	3	3	2	1	1	3	3	2
2	3	3	3	3	1	2	3	3	3
2	2	2	2	2	2	2	2	2	1
3	3	3	3	2	2	2	2	2	2
9	9	9	1	9	9	9	9	9	9
3	3	3	1	2	2	2	2	2	9
3	3	3	2	2	2	1	2	3	2
3	3	3	3	2	2	2	2	2	2
2	2	2	2	2	1	1	3	2	2
3	3	3	2	2	1	2	2	2	1
9	9	9	9	9	9	1	3	2	2
9	9	9	9	9	1	9	9	9	1
3	3	3	3	3	3	1	2	3	3
2	2	2	2	2	2	1	2	2	2
1	9	3	1	3	1	9	1	2	1
3	3	3	3	1	1	2	2	3	1
1	3	3	1	3	1	1	1	2	1
1	3	3	1	3	1	1	3	3	3
9	9	9	9	3	3	2	2	1	3
9	9	9	9	1	9	9	9	9	9
1	3	3	1	3	1	3	3	3	1
2	2	2	2	2	2	1	2	2	1
3	3	3	2	3	3	1	2	3	2
3	3	3	3	2	3	1	1	3	2
2	3	3	3	3	2	2	1	2	2
3	3	3	1	2	1	2	2	2	1

3	2	2	1	2	1	1	2	3	1
3	2	2	1	2	1	1	2	3	1
9	9	9	9	9	9	1	9	9	9
3	3	2	1	3	1	1	3	3	3
9	9	9	9	9	9	9	1	9	9
3	1	2	3	3	1	3	2	2	2
3	3	3	1	1	2	2	2	2	1
2	1	2	9	2	9	9	9	9	2
3	3	3	1	3	3	1	2	3	3
3	1	2	1	3	3	1	2	2	3
3	3	3	3	3	3	3	1	3	2
2	3	3	3	2	2	2	2	2	2
3	3	3	1	2	1	3	2	3	3
3	3	3	3	3	1	3	3	3	1
3	3	3	3	3	3	3	1	3	2
3	2	2	2	3	3	2	3	3	3
3	3	3	3	3	3	3	1	3	2
2	2	2	2	2	2	2	1	2	2
3	3	3	3	2	3	3	1	3	9
1	2	3	2	2	1	2	2	3	1
3	9	2	1	3	3	3	1	1	3
3	3	3	2	3	2	1	3	3	1
3	3	2	3	3	2	1	3	3	1
2	2	3	1	3	1	1	3	2	3
2	2	2	2	2	2	2	2	1	2
2	3	3	3	3	2	2	3	2	2
3	2	3	3	3	2	1	2	3	2
3	3	3	3	3	3	2	1	3	3
1	2	3	2	2	1	1	2	3	2
2	3	3	3	3	1	1	3	3	1
3	2	3	3	1	3	2	3	3	2
3	2	3	3	1	3	2	3	3	2
1	1	2	2	1	1	1	2	2	1
2	2	2	2	2	2	2	2	2	1
2	2	2	2	2	2	2	2	2	1
3	3	3	2	3	2	1	2	3	2
3	3	3	3	2	2	2	2	2	3
2	2	2	1	3	2	1	2	2	1
1	2	2	2	3	1	3	2	2	2
3	3	2	3	3	2	1	3	3	1
2	1	2	2	2	2	2	2	2	3
3	2	2	1	1	3	2	1	2	1
1	2	3	1	2	1	2	1	1	2
3	2	2	3	3	2	2	1	3	3
3	3	3	3	3	3	2	1	2	2
2	3	3	3	2	1	1	3	3	3
3	3	3	3	2	2	1	2	2	2

3	3	9	1	2	2	1	2	3	1
3	1	3	1	2	2	1	1	2	1
9	9	9	9	2	9	9	9	9	9
3	2	2	1	3	2	2	2	3	1
2	1	3	1	3	3	1	1	2	1
2	2	2	3	3	1	3	2	3	1
2	2	2	2	3	1	1	2	1	2
2	2	2	2	1	3	3	2	3	1
2	2	2	2	1	3	3	3	3	1
3	3	3	3	1	2	2	2	2	1
2	1	2	1	3	2	1	1	3	1
3	3	3	1	3	1	1	1	3	1
1	3	3	3	3	2	1	2	3	2
2	2	2	2	3	2	2	2	2	2
2	2	2	2	3	2	2	2	2	2
3	3	3	3	2	2	1	2	3	1
3	3	3	3	2	3	2	2	2	2
3	3	3	3	3	2	1	3	3	1
3	3	3	3	2	1	1	3	3	1
9	9	9	9	9	9	9	9	9	9
3	2	3	1	1	2	2	3	3	3
3	3	3	3	3	3	3	2	3	1
3	3	3	3	3	3	3	2	3	1
2	2	2	1	2	2	2	2	2	1
3	3	3	1	3	2	1	2	3	2
2	3	2	1	2	1	2	2	2	1
9	9	9	9	9	1	9	9	9	9
2	2	2	2	3	2	1	2	3	1
2	3	2	1	2	2	2	2	2	2
9	9	9	1	9	1	9	9	9	9
2	2	2	1	2	2	2	1	2	3
1	2	2	2	1	2	3	3	2	1
2	2	2	1	2	2	2	1	2	3
9	9	9	9	9	9	9	1	9	9
3	2	3	2	1	2	3	2	2	2
2	3	2	3	3	3	1	2	3	1
9	9	9	9	9	9	9	9	9	9
3	2	3	1	3	2	1	1	3	2
3	2	2	2	3	2	1	2	2	2
2	2	1	1	3	1	2	3	3	1
3	3	2	3	3	2	2	1	3	1
1	2	3	3	2	3	1	3	3	2
3	2	1	1	2	3	3	1	2	3
3	3	3	3	3	2	1	3	3	1
3	3	3	3	2	2	2	2	2	2
3	3	3	2	9	9	1	9	9	9
3	3	3	3	3	3	3	3	3	3

3	3	3	2	9	9	1	9	9	9
3	3	3	1	3	3	1	3	3	3
3	3	3	2	3	3	1	3	3	3
3	3	3	1	3	3	1	3	3	3
3	3	3	3	3	1	9	9	9	1
3	3	3	3	3	3	1	3	3	3
3	3	3	3	2	2	1	2	2	2
9	9	3	3	3	3	1	1	1	3
3	3	3	3	2	2	2	9	2	2
3	3	3	3	2	2	2	2	2	3
3	3	3	1	3	1	9	9	9	9
3	3	3	9	3	3	1	3	3	9
3	3	3	1	3	3	1	3	3	3
3	3	3	3	3	3	1	3	3	3
3	3	3	2	3	3	1	3	3	3
3	3	3	1	3	3	1	3	3	3
9	3	3	1	3	3	1	3	3	2
3	3	3	3	3	3	1	3	3	2
3	3	3	1	3	3	1	3	3	3
3	3	3	1	3	3	1	3	3	3
3	3	3	1	9	9	1	9	9	3
3	3	3	1	3	3	1	3	3	3
3	3	3	1	3	3	1	3	3	3
3	3	3	3	3	3	1	3	3	1
3	3	3	3	3	3	1	3	3	3
3	3	3	3	3	3	1	3	3	3
3	3	3	3	3	3	1	3	3	3
3	3	3	1	3	3	1	3	3	3
3	3	3	9	3	3	1	3	3	3
3	3	3	3	3	3	1	3	3	3
3	3	3	3	3	3	1	3	3	9
3	3	2	2	2	2	1	2	2	2
3	3	3	1	3	3	1	3	3	3
3	3	3	1	3	3	1	3	3	3
2	2	2	1	9	9	1	9	9	9
3	3	3	3	3	9	1	2	3	3
2	2	3	2	2	1	1	2	2	1
3	3	3	3	2	2	1	3	3	1
3	3	3	3	3	3	1	3	3	3
3	3	3	3	3	3	1	3	3	3
3	3	3	3	3	3	1	3	3	3
2	2	2	2	2	2	1	1	2	2
9	9	9	9	9	1	9	9	9	9
2	2	2	2	2	3	3	3	3	2
9	9	9	9	2	9	9	9	9	2
2	2	2	2	2	2	1	2	2	1
9	9	9	9	9	1	1	2	9	3

2	2	2	2	2	2	2	2	2	2
1	3	2	1	3	3	1	3	3	1
1	3	2	1	3	3	1	3	3	1
2	9	2	9	3	1	2	2	3	1
2	3	1	3	3	2	1	1	3	3
2	2	2	1	9	9	9	9	1	9
3	2	1	2	1	2	3	3	3	1
2	3	2	2	2	3	1	2	1	2
2	1	2	1	1	2	2	3	3	2
2	2	2	2	2	2	2	2	2	2
9	9	9	9	9	9	9	9	9	9
9	9	1	9	9	9	9	9	1	9
3	3	3	3	9	1	2	3	9	3
2	2	2	2	2	2	1	1	2	1
1	9	9	9	9	9	1	9	9	9
1	9	9	9	9	9	1	9	9	9
9	9	9	9	9	9	1	9	9	9
1	9	9	9	9	1	9	9	9	9
1	9	9	9	9	1	9	9	9	9
3	9	9	9	9	9	9	9	9	9
9	9	9	9	9	9	9	9	9	9
3	3	3	1	1	3	3	3	3	1
3	2	3	3	3	2	1	1	3	9
9	9	9	9	9	1	9	9	9	1
9	9	9	9	1	9	9	9	9	1
1	9	9	9	9	9	1	9	9	9
9	9	9	9	9	1	9	9	9	9
2	2	2	1	2	2	3	3	2	1
3	2	1	2	2	1	1	3	3	9
9	9	1	9	9	1	9	9	9	9
9	9	9	1	9	1	9	9	9	9
2	3	2	2	3	2	2	1	2	1
3	2	3	3	3	2	3	3	3	3
3	3	3	3	3	3	3	1	1	1
2	2	2	2	2	2	2	2	2	1
3	3	2	3	3	3	2	1	3	1
3	2	2	3	3	3	1	1	2	3
3	3	3	3	3	3	1	2	3	3
2	2	3	2	2	2	3	3	2	3
3	2	3	2	1	2	2	3	3	2
3	2	3	3	2	1	2	2	2	2
3	3	3	1	3	1	3	1	3	1
3	1	1	1	3	2	1	1	3	1
3	2	3	1	2	1	3	3	3	2
3	2	2	1	2	1	3	3	2	1
3	3	3	3	3	3	2	1	2	3
3	3	2	1	2	2	1	1	3	3

2	2	3	3	1	2	2	3	3	2
3	3	2	1	9	9	9	9	9	1
3	2	3	3	2	1	2	3	3	1
3	1	1	2	3	1	2	3	3	2
2	3	3	3	3	3	2	1	2	3
3	3	3	3	3	1	3	3	3	2
2	2	3	3	2	2	1	9	9	1
3	1	2	1	3	1	2	1	2	1
3	2	3	2	2	1	1	2	3	1
3	3	3	1	2	2	1	2	2	2
3	2	3	2	2	2	2	3	3	2
2	2	2	1	3	2	2	1	1	2
3	3	3	3	3	2	2	3	3	3
3	3	3	3	3	2	2	3	3	3
3	3	3	3	3	2	1	1	2	2
2	2	1	1	2	2	2	2	2	2
3	3	3	3	2	2	1	2	2	1
3	3	2	3	3	3	2	2	2	2
3	3	3	1	3	3	2	1	3	3
3	2	3	1	3	2	1	3	3	3
3	1	3	1	3	3	1	2	2	1
3	3	3	3	3	2	1	3	3	1
3	3	3	2	3	2	1	2	1	1
2	3	3	2	3	2	1	2	1	1
3	3	2	2	2	2	3	1	2	1
2	3	3	2	1	2	3	3	3	2
3	3	3	1	3	3	1	1	3	1
3	3	3	2	3	3	1	1	3	1
2	2	2	2	3	2	1	1	3	2
3	2	1	1	1	2	3	3	1	2
3	3	2	3	2	3	1	2	2	3
2	2	2	3	1	2	3	3	1	2
2	2	2	2	2	2	2	2	2	3
2	2	2	1	2	2	1	1	2	2
2	3	3	3	3	2	2	2	3	1
9	9	9	9	9	9	9	9	2	9
2	2	3	2	3	2	2	1	1	2
3	3	2	1	3	1	1	2	1	1
2	2	2	2	2	2	2	2	2	3
2	3	2	2	3	2	1	2	2	1
2	3	2	1	1	1	1	3	3	1
1	2	1	2	3	2	2	1	3	1
9	9	9	9	1	9	9	9	9	9
2	2	2	1	1	2	2	2	2	1
1	2	3	3	3	3	3	3	3	1
2	2	2	2	1	2	1	2	2	1
1	9	9	9	9	2	9	9	9	9

9	9	9	1	9	9	9	1	9	1
9	9	9	1	9	9	1	9	9	9
9	9	1	9	9	9	1	9	9	9
9	9	1	9	9	9	9	1	9	9
9	9	9	9	9	9	9	1	9	9
9	9	9	9	1	9	9	9	9	9
9	9	9	2	9	9	1	9	9	9
9	9	9	9	9	9	9	1	9	1
9	9	9	9	9	9	9	1	9	1
9	9	9	9	2	9	9	9	9	2
9	9	9	9	9	9	9	9	9	9
9	9	9	9	2	9	9	9	9	2
9	9	9	9	1	9	9	9	9	9
1	9	9	9	9	9	1	9	9	9
9	9	9	1	9	9	1	9	9	1
9	9	9	1	1	9	9	9	9	1
9	9	9	9	9	9	1	9	9	1
9	9	9	9	9	9	1	9	9	9
2	2	2	2	2	2	2	2	2	2
1	1	1	1	1	1	2	1	3	1
1	1	1	1	1	1	1	1	1	1
3	3	3	3	3	3	1	1	2	3
2	2	2	1	3	3	1	3	3	2
1	1	1	3	3	3	1	1	1	1
2	3	2	3	2	2	1	3	3	2
3	3	3	3	3	3	1	3	3	2
3	3	3	3	3	1	1	3	3	2
1	2	2	2	3	2	1	2	1	3
3	3	3	3	3	3	1	3	3	3
9	1	2	3	2	2	2	2	2	3
1	2	2	3	1	1	2	2	1	1
3	3	3	3	3	1	3	3	3	3
2	2	2	2	2	2	2	1	2	1
3	2	3	3	3	1	2	3	3	1
1	2	2	1	1	1	3	3	3	1
3	3	3	3	2	1	3	3	3	2
2	2	2	1	3	1	2	3	3	2
3	2	2	2	2	9	9	9	9	2
9	9	9	9	2	9	3	9	1	9
3	3	3	2	2	3	3	3	3	1
9	1	9	9	9	9	9	9	9	9
1	3	3	3	1	3	3	3	3	1
9	9	9	9	9	9	9	9	9	9
1	3	1	1	1	1	1	3	1	1
3	1	2	2	3	2	2	1	2	1
9	9	9	9	2	2	1	1	1	9
2	2	1	1	2	1	1	2	2	1

1	2	1	1	1	2	3	1	3	2
3	1	1	3	3	3	2	2	3	1
1	3	1	1	3	3	3	1	3	3
1	2	1	1	1	2	1	1	2	2
2	2	2	2	2	1	1	2	2	2
2	2	2	2	2	2	2	1	3	2
1	2	2	2	2	2	2	2	2	2
1	2	1	1	1	2	3	1	3	2
2	1	1	1	1	1	1	1	3	1
9	9	9	9	9	9	9	9	9	9
9	9	9	9	9	9	9	9	1	1
3	2	3	2	3	2	1	3	2	1
3	3	3	3	3	3	3	3	3	3
2	3	2	1	3	2	1	2	2	2
9	3	3	2	9	9	1	3	3	1
9	9	9	9	1	9	9	9	9	9
9	9	9	9	9	1	9	9	9	9
2	2	2	9	2	2	2	2	2	2
3	2	3	1	3	3	1	3	3	1
3	2	2	3	3	3	3	1	2	3
2	1	2	1	3	1	1	3	3	3
3	3	3	3	3	3	3	3	3	3
3	3	3	3	3	3	3	3	3	3
9	9	9	9	9	9	9	9	9	9
9	9	9	9	9	9	9	9	9	9
3	9	2	1	3	3	3	1	3	3
1	2	2	3	3	3	3	2	3	3
1	9	3	1	9	9	9	9	9	9
2	9	1	1	2	1	1	2	2	1
9	9	9	1	9	9	9	9	9	1
3	3	3	3	3	1	3	3	3	1
3	3	1	1	3	3	1	3	3	3
3	1	3	1	3	1	2	1	1	2
1	1	1	1	3	1	2	2	3	1
1	9	9	1	9	1	9	9	9	9
9	9	9	1	9	9	9	1	9	9
9	9	9	1	9	9	9	1	9	1
2	2	2	1	1	3	3	2	2	1
2	2	2	1	1	3	3	2	2	1
2	2	2	1	1	3	3	2	2	1
2	2	2	1	1	3	3	2	2	1
3	2	3	2	2	3	1	2	3	2
3	2	3	3	1	2	3	1	2	1
1	2	2	1	1	3	3	2	2	1
3	2	1	1	1	2	3	1	2	1
3	2	2	3	3	2	3	2	2	3
1	1	3	3	3	3	3	3	1	1

3	3	3	1	3	2	1	3	1	3
2	2	2	3	1	3	3	3	3	1
3	3	3	3	1	3	3	3	3	1
3	3	3	3	2	2	1	1	2	1
2	2	2	2	3	3	1	2	3	1
1	1	1	1	1	1	1	1	1	1
2	2	3	2	2	9	1	2	2	1
9	9	9	9	9	1	9	9	9	9
3	3	1	1	2	2	2	1	3	3
3	3	2	2	2	2	1	2	2	2
2	2	2	2	2	2	2	2	2	2
1	1	3	1	9	1	9	9	9	1
3	2	2	1	2	2	1	2	2	1
2	2	2	2	2	2	1	2	2	1
1	3	3	3	3	2	3	2	2	3
2	2	2	2	2	2	3	1	3	2
2	3	3	1	2	3	2	2	1	1
3	2	3	1	2	3	2	2	1	1
3	2	3	1	2	3	2	2	1	1
3	3	1	3	3	3	1	3	3	3
9	9	9	1	2	1	3	1	2	1
3	2	2	2	2	9	9	9	9	2
9	9	9	1	3	9	9	9	1	1
1	2	3	3	3	3	3	1	3	3
2	1	3	9	1	2	3	2	3	1
3	2	2	3	1	2	1	3	2	3
2	2	1	2	2	3	2	3	1	3
1	3	3	3	1	1	1	1	3	1
9	9	9	9	9	9	9	9	1	1
2	2	2	2	9	1	1	2	3	3
2	3	3	1	3	1	3	3	3	1
9	9	9	9	3	9	9	9	9	3
1	2	3	1	1	2	3	2	1	3
1	1	1	3	2	1	1	2	2	3
3	3	3	3	3	3	3	3	3	3
3	3	3	1	3	3	2	1	2	1
3	2	3	1	2	3	2	2	1	1
3	2	3	1	2	3	2	2	1	1
3	2	3	1	2	3	2	2	1	1
1	1	3	1	1	3	3	3	3	3
3	2	1	1	2	1	3	1	1	1
9	1	2	3	1	2	2	1	3	1
1	3	3	3	3	1	3	3	3	1
3	9	3	1	1	3	3	3	3	3
9	9	9	9	9	9	9	9	9	9
3	3	3	3	3	3	3	3	3	3
2	1	2	2	2	1	2	2	2	2

2	2	2	2	1	1	1	1	1	1
9	1	1	9	1	9	9	9	9	1
3	1	1	1	9	9	9	9	9	9
1	3	3	1	3	3	3	1	3	3
2	2	2	2	9	9	1	1	2	3
2	2	2	2	2	1	1	2	2	2
1	1	2	3	1	2	1	2	2	1
2	2	2	2	2	1	1	2	2	2
2	2	2	2	2	1	1	2	2	2
9	9	9	9	9	9	9	9	9	9
3	3	2	3	2	3	1	2	2	2
3	1	3	1	1	2	3	3	3	3
3	3	2	3	3	3	1	2	3	3
3	1	1	3	3	3	2	2	3	1
3	3	3	3	3	3	1	1	3	1
3	3	1	2	1	1	1	1	1	1
3	3	3	2	3	3	3	1	3	3
3	3	3	2	1	9	9	9	9	9
3	3	3	2	1	9	9	9	9	3
1	3	3	1	1	3	3	3	3	1
2	1	3	3	1	2	1	1	1	1
3	2	2	3	9	1	1	2	3	3
3	3	3	3	9	9	9	1	9	1
2	2	2	2	2	1	2	2	2	2
2	2	2	2	2	3	3	3	3	3
9	9	9	1	9	9	1	9	9	2
9	9	9	1	9	9	1	9	9	2
1	1	3	3	3	3	3	1	3	1
3	3	3	3	9	9	9	1	9	1
2	2	2	2	2	2	2	2	2	3
2	3	3	1	9	9	1	9	9	9
1	2	3	1	3	2	1	1	3	3
1	2	2	1	2	2	1	1	3	3
9	9	9	1	9	9	1	9	9	2
2	3	2	3	3	1	3	2	3	1
3	3	3	3	3	3	3	3	3	3
2	2	2	2	1	2	2	2	2	1
9	9	9	9	9	9	9	9	9	9
9	9	9	1	9	9	9	9	1	9
3	2	1	3	2	2	2	2	2	2
3	3	3	3	3	1	3	3	3	9
2	2	2	2	2	2	1	2	2	2
2	2	2	2	3	1	3	3	3	2
2	2	2	2	2	2	2	2	2	2
2	2	2	2	2	2	2	2	2	2
3	3	3	3	3	3	1	3	3	2
3	3	3	3	3	1	3	3	3	1

2	2	2	2	3	3	2	1	2	2
3	3	3	3	2	2	2	2	2	1
3	3	3	3	3	3	1	3	3	9
3	2	3	3	3	2	1	1	2	9
9	9	9	9	1	9	9	9	9	1
2	1	2	2	3	1	2	2	1	1
2	2	2	3	2	2	2	2	2	2
9	9	9	1	1	9	9	9	9	9
2	1	2	3	1	2	1	2	3	1
9	9	9	9	9	9	2	9	9	9
2	2	2	2	2	2	2	2	2	2
2	3	3	9	9	1	9	9	9	9
2	2	2	2	9	9	1	9	9	9
2	2	2	2	2	2	2	2	2	2
3	3	1	1	1	2	1	2	3	3
2	2	2	2	2	2	2	2	2	2
2	2	2	2	2	2	2	2	2	2
9	9	9	9	1	3	2	2	3	3
1	3	2	1	9	2	1	1	2	1
9	9	9	9	9	9	9	9	9	9
2	9	9	9	9	9	1	9	9	1
3	3	3	2	3	3	1	2	2	3
2	2	2	2	2	2	2	2	2	2
2	1	1	1	1	1	1	1	1	2
3	2	3	1	2	2	2	2	2	2
1	3	3	3	1	2	2	2	2	1
3	2	9	2	3	3	1	3	3	3
3	3	3	1	3	3	1	3	3	3
2	2	2	2	2	2	2	2	2	2
3	3	3	3	3	3	1	2	2	1
3	3	3	3	9	9	1	9	9	1
3	2	2	2	3	2	1	2	3	3
1	3	3	1	3	3	1	3	3	1
2	2	2	2	2	1	2	2	2	2
2	2	3	1	3	2	2	1	2	1
3	3	3	2	3	3	3	3	1	3
2	2	2	2	2	2	1	2	2	2
3	2	1	2	2	2	1	1	1	1
1	3	2	3	1	9	9	9	9	1
1	3	1	1	2	1	2	2	3	1
3	2	3	3	3	3	3	1	2	3
2	2	2	2	2	2	2	1	2	2
1	3	3	3	3	3	3	1	3	1
9	3	3	1	3	3	3	1	1	1
2	2	2	2	2	2	2	2	2	2
3	3	3	3	3	3	3	3	3	3
2	2	3	2	3	2	2	1	3	3

3	2	1	1	3	3	2	1	2	9
3	3	3	3	9	9	9	9	9	3
3	3	3	3	3	3	3	3	3	3
2	2	2	2	2	2	2	2	2	2
3	3	3	2	2	2	2	1	2	2
1	1	3	1	1	1	1	2	3	1
3	1	3	1	2	2	2	2	2	2
3	3	2	3	3	1	1	2	3	2
2	3	1	2	1	2	3	1	2	9
2	2	2	2	2	2	2	2	2	2
3	3	3	1	3	3	1	2	3	1
2	2	2	2	2	2	2	2	2	2
9	9	9	9	9	9	9	1	9	9
1	1	1	2	1	2	2	2	2	9
2	3	2	3	1	2	2	2	2	1
9	9	9	9	3	3	3	1	3	3
9	9	9	9	3	3	3	9	1	3
3	3	1	3	3	3	1	2	3	3
2	2	2	3	1	1	1	1	2	2
1	1	1	3	1	2	2	2	2	1
3	3	3	1	1	1	1	3	3	1
1	3	1	2	1	1	1	1	1	2
3	3	3	1	3	3	1	2	2	2
1	2	3	1	3	2	1	2	3	2
3	3	3	3	3	3	3	3	1	3
1	2	3	9	9	2	1	2	3	2
2	2	2	2	2	2	2	2	2	2
2	3	3	3	1	1	2	2	2	1
2	2	2	2	1	9	9	9	9	1
3	2	2	2	3	2	2	2	1	2
1	1	1	1	2	1	2	1	2	1
1	1	1	1	1	1	1	1	1	1
1	1	1	1	1	1	1	1	1	1
1	2	2	2	1	1	1	1	1	1
1	2	3	1	3	9	2	1	2	2
1	9	1	1	3	3	1	1	1	3
1	9	9	9	9	9	9	1	9	9
1	1	1	1	1	1	1	1	1	1
2	2	2	2	2	2	2	2	2	2
2	2	2	2	2	2	2	2	2	2
1	1	3	1	3	1	2	2	3	2
1	3	1	1	1	1	1	2	3	2
9	9	9	1	9	9	1	9	9	2
3	2	1	9	2	2	2	2	1	2
2	2	2	2	2	2	2	2	2	2
3	3	1	3	3	3	3	1	1	3
3	2	3	3	2	2	2	2	2	1

2	9	3	2	3	3	1	3	3	3
3	3	3	3	3	1	2	2	3	2
1	3	3	2	1	2	3	3	3	1
9	9	9	9	9	9	9	1	9	9
1	2	3	1	2	9	1	2	3	9
1	3	2	1	2	2	1	2	3	2
9	9	9	9	9	9	9	3	9	9
9	9	9	9	9	9	9	9	9	9
3	3	3	3	3	3	3	1	1	3
2	2	2	2	2	2	1	2	2	2
1	9	9	1	9	9	1	9	9	9
3	3	3	3	1	3	3	2	2	2
9	9	9	1	2	2	1	2	2	1
2	3	3	1	2	1	2	2	3	3
1	2	2	2	2	2	2	2	2	2
2	9	3	2	2	1	2	3	3	2
1	2	2	2	3	2	1	1	2	1
1	9	9	1	9	9	1	9	9	9
1	2	3	3	3	2	1	2	3	2
1	2	1	1	2	2	2	2	2	3
3	9	2	2	2	1	2	3	2	9
2	2	3	2	3	3	1	2	2	1
1	2	1	2	2	3	1	2	3	1
1	2	2	2	2	2	2	2	2	2
1	1	3	9	2	3	2	1	3	1
1	3	9	9	3	3	1	1	3	9
3	9	1	3	3	3	2	2	1	3
3	3	3	1	3	3	1	1	3	1
2	2	2	2	3	9	1	2	2	2
3	2	1	1	2	2	1	3	2	3
3	2	1	3	3	1	2	2	3	1
3	2	2	9	3	2	1	1	2	1
3	3	2	2	2	2	2	3	3	1
1	3	1	3	1	1	1	3	3	3
9	9	9	9	9	9	9	1	9	9
2	3	3	3	3	1	2	3	3	1
1	1	9	1	1	2	1	1	1	1
1	3	1	3	1	1	1	3	3	3
9	9	3	9	1	1	9	1	2	2
3	3	3	3	1	3	3	3	3	1
3	3	3	1	3	9	1	1	3	3
9	9	9	9	1	9	9	9	9	1
1	1	1	1	3	3	1	1	3	1
3	1	3	3	3	3	1	1	3	1
2	2	2	2	3	2	1	1	2	1
2	1	9	9	9	9	9	9	9	9
3	1	2	1	1	2	2	1	2	2

1	1	3	2	9	9	9	9	1	9
3	3	2	2	2	9	1	3	2	2
1	2	1	3	1	2	1	2	1	1
3	3	3	3	3	1	3	3	2	3
2	2	2	2	2	2	2	2	2	2
2	3	3	2	3	3	2	1	2	2
2	2	2	1	3	3	2	1	3	2
1	2	2	2	2	2	2	2	2	2
1	2	2	2	2	2	2	2	2	2
2	1	3	2	2	2	2	2	2	2
3	3	2	2	3	3	2	1	2	2
3	2	2	3	3	3	3	1	2	3
1	1	1	3	3	1	1	1	3	3
3	3	2	3	2	3	2	3	2	3
9	9	9	9	9	9	9	9	9	9
2	2	2	1	3	3	2	1	3	2
3	3	3	3	3	1	3	3	3	1
3	3	2	1	2	2	1	2	2	2
3	2	2	1	2	2	1	1	3	9
1	9	9	9	9	9	9	1	9	1
9	9	9	9	9	9	1	9	9	9
3	3	3	3	3	3	3	3	3	3
3	3	3	1	1	1	3	3	3	1
3	3	1	1	3	3	1	3	3	1
3	3	1	1	1	3	3	3	3	1
1	1	3	2	9	9	1	9	9	9
2	1	3	2	3	2	1	1	9	9
3	3	2	2	3	2	1	1	3	2
2	1	1	1	3	3	1	2	2	3
2	2	9	1	3	2	1	2	3	1
3	3	3	3	2	1	3	2	3	1
1	3	1	1	1	1	1	9	1	1
2	1	1	3	1	2	2	2	1	1
3	3	3	1	1	3	3	3	3	1
3	3	3	1	3	2	2	3	2	2
3	3	3	3	3	1	3	3	3	1
9	9	9	9	9	9	1	9	9	1
9	9	9	9	2	2	2	2	2	3
3	3	3	3	3	3	3	3	3	9
2	2	2	2	3	1	2	2	2	1
3	3	2	1	1	2	1	1	3	1
3	3	3	1	1	3	3	1	3	1
2	2	2	2	1	2	2	2	2	1
9	1	9	9	9	9	1	9	9	9
9	9	9	9	9	9	9	9	9	3
3	3	1	3	1	2	3	3	3	1
3	3	3	3	2	2	1	3	1	2

9	9	9	9	1	2	9	1	3	1
1	1	1	1	3	3	2	3	3	2
3	3	3	3	1	3	3	3	3	1
9	9	9	1	9	9	9	2	9	9
3	3	3	2	3	3	3	1	2	2
9	9	9	9	9	9	9	9	9	9
1	1	2	2	1	3	1	1	1	3
3	3	3	3	2	9	1	1	2	3
3	3	3	3	1	3	3	3	3	1
3	3	3	3	3	1	2	3	3	3
3	3	3	3	3	1	2	3	3	3
3	3	3	3	3	1	2	1	3	3
1	9	9	9	1	9	9	9	9	9
1	9	9	9	1	9	9	9	9	1
9	9	9	9	2	2	1	1	1	3
3	3	2	2	3	2	1	1	1	3
1	3	3	1	3	1	3	3	3	1
3	3	3	3	2	2	3	1	3	2
2	3	2	1	2	2	1	2	3	3
3	3	3	2	2	1	2	2	2	2
2	2	2	2	3	1	1	1	1	1
2	1	2	2	3	1	1	1	1	1
2	2	2	2	2	2	2	2	2	2
2	2	3	3	3	3	2	1	3	3
2	3	2	3	2	1	2	2	2	2
2	3	3	3	9	9	9	9	9	1
1	3	3	1	1	9	9	9	9	9
2	2	2	2	2	2	2	2	2	2
3	2	2	2	9	9	9	9	9	1
3	3	2	2	3	2	1	1	1	3
3	3	3	2	2	1	2	2	2	2
1	9	9	9	1	9	9	9	9	9
3	3	3	3	1	9	9	9	9	1
3	3	3	2	3	2	1	1	2	2
1	1	2	2	2	2	1	1	1	2
9	9	9	1	2	1	2	2	3	9
9	9	9	9	9	9	1	9	9	9
9	9	9	9	9	1	9	9	9	9
3	2	3	3	1	2	1	3	1	1
1	1	3	1	2	2	2	2	2	1
9	9	9	9	9	9	9	1	9	1
1	1	2	1	1	3	3	3	3	1
3	3	3	3	1	3	3	3	3	1
3	3	3	3	3	1	3	3	3	1
1	1	1	1	3	2	1	2	1	1
1	2	2	1	2	1	3	2	3	2
9	9	9	9	9	9	1	9	9	1

1	3	3	1	9	9	9	9	9	9
3	3	3	3	2	2	1	1	1	2
1	2	1	1	2	2	2	1	1	1
9	9	9	9	9	9	1	9	9	9
9	9	9	9	9	1	9	9	9	9
3	3	2	1	3	2	3	3	3	1
1	2	1	3	1	1	1	2	3	1
2	3	2	2	1	2	3	3	3	1
3	1	3	2	1	2	2	3	3	2
1	2	1	3	1	1	2	2	2	1
3	3	3	1	2	1	1	3	3	2
9	9	9	9	2	2	2	2	2	2
3	3	3	3	1	2	3	2	2	2
3	3	3	1	2	2	2	2	2	1
2	2	3	1	3	2	1	2	2	3
3	1	2	1	3	3	3	3	3	3
3	3	3	3	1	3	3	3	3	3
3	3	3	2	1	2	3	3	3	3
3	9	2	3	2	3	3	2	2	1
3	3	3	3	3	1	3	3	3	3
2	2	3	3	3	1	3	3	3	2
9	9	9	9	9	9	9	9	1	9
2	1	2	1	2	2	2	2	2	2
3	2	2	2	3	3	3	3	3	3
1	2	2	1	2	2	2	2	2	2
1	2	2	1	2	2	2	1	2	2
2	2	3	2	3	1	2	2	2	2
3	3	3	3	3	3	1	3	3	1
3	3	2	3	1	1	1	1	1	1
3	3	3	3	3	3	3	1	3	3
9	9	9	9	9	9	1	9	9	1
1	2	9	1	2	1	3	2	2	1
9	9	9	9	9	9	1	9	9	9
3	1	2	1	3	3	2	1	3	2
1	1	2	3	1	2	3	2	2	1
9	1	9	9	1	9	9	9	9	1
9	9	9	9	9	9	9	9	9	9
2	2	2	2	2	2	2	2	2	2
3	3	3	1	2	2	1	2	2	2
1	3	1	2	1	2	1	1	2	1
9	2	1	3	2	3	1	1	3	1
1	1	1	1	1	1	9	9	9	9
2	1	1	1	1	1	1	1	2	1
3	9	1	3	1	2	3	1	3	1
3	1	2	3	3	1	2	2	3	1
9	9	9	9	9	9	9	2	9	1
3	3	3	2	1	1	3	3	3	2

1	1	2	1	2	1	2	3	2	1
3	3	3	2	9	9	1	9	9	9
3	3	3	3	3	3	3	3	3	1
2	3	3	3	3	2	1	1	2	3
3	2	2	2	3	2	2	1	2	2
1	1	2	1	2	1	2	3	2	1
3	3	2	1	1	1	2	3	3	1
2	1	2	1	2	2	2	2	2	2
3	3	3	3	2	2	1	2	2	1
1	1	3	3	3	1	3	3	3	3
2	2	2	2	2	2	2	2	2	2
2	2	2	2	2	2	2	2	2	2
9	9	9	9	9	9	1	9	9	1
2	2	2	2	2	2	2	2	2	1
3	3	3	2	3	2	2	1	2	2
2	2	3	1	3	2	1	2	2	9
3	3	3	3	1	3	3	3	3	3
3	3	3	3	1	3	3	3	3	1
3	3	3	3	2	2	2	1	3	2
2	3	3	1	3	3	1	3	3	1
3	1	2	2	2	2	1	1	1	1
3	3	3	3	3	3	1	3	3	3
1	3	3	2	3	3	3	2	2	1
3	2	2	2	3	1	3	3	3	9
2	2	2	2	2	2	2	2	2	1
2	1	2	2	3	3	1	2	3	3
2	2	2	2	2	2	2	2	2	2
2	2	2	2	1	3	3	3	3	1
1	1	2	1	3	1	1	3	3	2
2	1	1	2	1	1	2	3	3	1
3	3	3	3	1	1	3	3	3	3
3	2	2	2	3	1	3	3	3	1
3	3	2	2	1	3	9	2	1	3
1	2	2	1	3	3	1	1	2	2
2	2	9	3	1	2	1	3	1	1
3	2	3	1	2	2	1	2	2	1
1	1	1	1	3	1	1	1	2	2
3	3	3	2	2	2	2	9	2	2
3	3	3	2	2	3	3	2	3	3
3	2	2	2	3	3	2	1	2	2
1	3	1	3	3	1	3	3	3	3
3	2	2	1	1	3	3	3	3	2
3	3	3	3	9	1	9	9	9	9
3	3	1	1	3	1	1	9	3	3
9	9	9	9	9	1	9	9	9	9
9	9	9	9	9	1	9	9	9	9
9	9	9	9	9	1	9	9	9	9

1	3	3	1	3	1	2	1	2	3
2	2	1	2	2	3	2	2	1	2
3	3	3	3	3	3	1	3	3	3
1	2	3	3	3	3	2	1	2	1
1	3	1	1	1	1	1	3	1	1
9	9	9	9	9	1	9	9	9	9
9	9	9	9	9	1	9	9	9	9
9	9	1	9	9	1	9	9	9	1
9	9	9	9	9	1	9	9	9	1
9	9	9	9	9	9	1	9	9	9
9	9	9	1	9	9	1	9	9	9
1	1	1	1	1	1	1	9	9	9
9	9	9	9	9	1	9	9	9	1
1	3	3	1	3	3	3	1	3	3
3	3	2	3	3	3	2	1	1	3
2	3	3	3	2	1	2	2	2	2
1	1	1	1	2	2	1	2	2	1
1	9	9	9	9	9	9	1	9	9
9	9	9	9	1	9	9	9	9	9
9	9	9	9	9	1	9	9	9	9
3	1	2	2	9	9	9	9	9	9
9	9	9	9	9	1	9	9	9	9
1	1	1	1	2	2	1	1	1	1
9	9	9	9	9	9	1	9	9	1
3	3	3	3	2	2	1	2	2	1
2	2	2	2	2	1	2	2	2	2
3	2	2	1	3	3	1	2	1	3
1	1	1	1	2	1	2	2	2	2
2	2	1	1	1	1	1	2	2	2
3	3	3	3	2	2	1	2	2	1
1	3	3	3	1	1	1	1	1	1
2	2	2	1	3	1	3	3	3	3
1	1	2	2	2	2	2	1	2	1
9	9	9	9	9	9	9	9	9	9
2	2	2	1	1	1	2	1	1	2
2	2	2	1	2	1	2	2	2	2
2	2	2	1	2	1	2	2	2	2
9	9	9	9	9	9	1	9	9	9
3	2	3	1	1	2	2	3	3	1
3	1	3	1	9	9	9	9	9	9
3	9	3	3	2	2	2	2	2	3
1	3	3	1	2	3	3	1	3	2
3	3	3	1	3	3	1	3	3	3
1	1	9	2	2	2	2	2	2	2
2	2	3	3	1	3	3	3	3	9
3	3	3	1	3	2	2	3	1	3
3	3	3	3	3	2	2	1	2	2

2	3	3	3	3	3	3	2	1	1
1	2	3	1	2	3	3	2	2	3
9	9	9	9	3	3	3	3	3	3
3	3	2	1	2	1	2	2	2	2
3	3	3	3	3	3	1	2	3	1
2	9	9	9	9	1	9	9	9	9
3	3	1	1	3	2	1	1	3	1
3	3	3	3	3	1	2	1	3	1
2	9	9	9	9	1	9	9	9	9
2	2	2	3	2	1	2	3	3	2
2	9	9	9	9	1	9	9	9	9
3	3	2	2	3	3	1	2	1	2
1	1	2	1	2	1	2	2	2	2
1	2	9	1	2	1	1	2	3	1
1	9	2	1	3	1	2	2	2	2
2	2	3	3	3	1	3	1	3	1
2	2	2	2	2	2	2	1	1	1
3	3	2	3	2	1	2	3	3	2
3	3	3	3	3	3	3	1	2	2
1	9	9	9	9	9	1	9	9	9
3	3	3	3	3	3	3	1	2	3
3	2	3	1	3	3	3	1	2	2
3	3	3	3	2	2	2	2	2	2
2	3	1	2	1	1	1	1	1	2
3	3	3	3	2	2	1	2	2	2
3	3	3	3	1	1	1	1	1	1
1	3	3	3	3	3	1	3	3	3
3	9	2	1	1	1	3	1	1	1
3	3	3	1	2	1	2	2	2	2
1	1	3	3	3	3	3	1	1	1
1	2	3	1	3	1	2	1	3	3
1	2	2	2	1	2	2	2	2	2
2	1	1	1	2	1	1	1	1	2
1	1	1	1	2	2	2	1	2	1
2	2	2	2	2	2	1	2	2	1
2	9	2	2	2	2	1	2	2	1
2	2	2	1	2	1	2	2	2	1
2	2	1	1	2	2	1	2	2	2
2	2	2	2	2	2	1	2	2	2
9	1	2	2	2	2	2	2	2	2
3	9	3	3	3	3	3	1	3	3
3	3	9	1	2	2	2	2	2	2
3	3	3	3	3	3	2	1	2	3
1	2	2	2	2	1	1	1	2	1
1	3	3	1	3	3	3	3	3	3
3	3	3	3	3	3	3	3	3	3
3	3	3	3	2	2	2	1	3	1

2	2	2	1	1	2	2	3	3	2
3	1	3	1	2	2	1	1	1	2
9	9	1	9	9	9	1	9	9	9
3	3	1	9	9	9	9	3	9	3
9	9	1	9	9	1	9	9	9	9
1	3	3	3	9	9	1	1	9	1
9	9	9	9	9	9	1	9	9	1
3	2	2	3	2	3	2	1	1	1
2	2	2	2	2	2	2	2	2	2
3	3	3	1	3	1	1	3	1	3
1	3	3	1	1	1	1	9	3	9
1	1	1	1	3	3	1	1	1	1
1	1	2	2	2	2	1	1	1	1
1	3	2	2	2	2	2	2	2	2
1	1	1	1	1	1	2	1	1	1
9	9	9	9	9	9	9	9	9	9
3	3	3	3	2	2	2	2	2	2
3	3	3	3	9	9	9	9	3	3
2	3	1	2	9	9	9	9	9	9
1	1	1	1	2	2	2	2	2	2
1	1	1	1	2	1	1	1	1	2
3	2	2	2	3	3	2	1	2	2
2	2	2	2	1	2	2	2	2	1
2	2	3	1	2	2	2	2	2	2
1	2	3	2	2	1	1	1	2	2
3	3	3	3	3	3	1	1	3	2
1	9	9	9	9	9	1	9	9	1
1	2	2	2	2	2	1	2	2	2
3	3	2	3	3	1	2	3	3	2
1	1	3	3	2	2	1	1	1	2
9	9	9	9	9	9	9	9	9	9
2	1	3	2	1	1	2	3	1	2
1	2	1	2	1	1	1	2	2	1
2	2	2	2	2	2	2	2	2	3
2	2	2	2	3	3	3	2	2	2
2	2	2	1	1	2	2	3	3	2
1	1	3	3	3	3	1	3	3	3
3	1	2	3	3	2	1	2	3	1
3	2	2	3	1	1	2	3	3	3
3	3	3	1	1	1	2	3	3	1
1	2	2	2	2	2	1	2	2	2
3	3	3	2	1	1	3	2	3	1
2	3	2	2	3	2	1	1	3	3
9	9	9	9	9	9	9	1	9	9
9	9	9	9	9	9	1	9	9	9
2	3	2	2	3	2	1	1	3	3
3	3	3	3	3	3	1	3	3	1

3	3	3	3	1	3	3	2	3	1
2	2	1	1	2	1	2	2	2	2
9	9	9	9	9	9	1	9	9	9
3	3	3	3	1	9	9	9	9	9
9	9	9	9	9	9	9	9	9	9
2	3	2	1	2	2	2	1	2	1
3	3	3	3	2	2	1	3	3	2
3	3	3	3	3	3	3	1	3	2
2	9	3	2	3	2	1	1	3	3
1	1	2	3	1	3	3	3	3	1
9	9	9	9	9	9	1	9	9	9
9	9	9	9	1	9	9	9	9	1
2	2	2	2	2	2	2	2	2	2
3	2	2	2	1	9	3	9	9	1
9	9	9	9	1	9	9	9	9	9
1	3	3	1	3	3	1	1	1	1
1	1	3	3	3	1	2	3	1	1
3	3	1	2	1	1	1	3	3	1
3	2	3	3	1	3	1	2	3	1
2	2	3	1	3	2	2	1	3	3
2	1	3	3	1	1	2	3	3	1
3	3	1	1	3	3	3	1	3	3
2	1	2	3	1	1	3	3	3	1
9	9	9	9	2	2	2	1	2	3
3	3	3	3	1	2	2	1	1	1
1	3	3	1	3	2	2	2	1	3
9	9	9	9	9	9	9	1	9	9
2	2	3	1	1	3	2	9	1	3
3	3	2	3	2	3	9	9	9	9
2	3	3	2	1	1	1	2	2	1
3	3	3	3	2	2	1	1	3	3
1	1	1	1	2	3	3	3	3	3
1	3	2	1	2	2	1	3	2	2
2	3	3	3	3	1	1	2	2	3
2	3	2	3	3	1	3	2	3	1
1	1	2	1	2	2	2	2	2	2
9	9	9	9	2	2	2	2	2	3
3	3	3	2	2	2	2	2	2	1
9	9	9	1	9	9	1	9	9	1
2	3	2	3	3	1	1	2	3	2
3	3	3	1	2	2	3	2	1	9
1	3	2	1	2	2	2	2	2	2
2	1	1	3	1	2	9	1	3	9
1	3	2	1	1	2	1	2	3	9
3	3	2	3	2	2	2	1	2	2
3	3	3	3	2	2	1	1	3	1
3	3	3	3	2	2	2	1	3	1

3	3	3	3	3	3	3	1	3	3
9	9	9	9	9	9	9	9	9	9
3	3	3	3	9	1	9	9	9	9
3	3	1	1	3	3	1	1	3	3
3	3	1	3	3	1	1	3	3	1
3	3	1	1	3	3	1	3	3	3
3	3	9	9	2	3	9	2	1	2
1	1	1	2	2	2	1	1	1	3
3	3	3	1	3	3	1	3	3	3
3	3	3	3	2	2	1	1	3	1
3	3	3	3	2	2	1	1	3	1
1	2	2	3	1	2	3	2	1	1
3	3	3	2	3	2	3	2	3	3
1	1	1	2	2	2	1	1	1	3
1	2	2	1	2	1	2	1	3	1
2	1	2	3	2	2	2	2	2	2
3	2	2	1	2	2	2	2	2	2
1	1	3	2	3	2	1	1	2	3
1	3	3	3	3	3	1	1	1	3
2	2	2	2	1	2	2	2	2	2
3	3	3	3	1	1	1	3	3	1
1	2	1	1	3	2	1	3	1	2
3	3	3	3	2	2	2	2	2	1
3	3	3	3	2	2	2	2	2	2
2	2	1	3	2	2	1	2	2	2
2	1	2	3	1	2	3	3	2	1
3	3	2	3	3	3	2	1	2	2
3	3	3	3	3	3	3	3	3	2
3	3	3	3	3	3	1	1	3	1
9	9	9	9	9	1	9	9	9	1
2	3	3	3	3	2	1	2	3	2
2	1	3	2	1	1	1	1	1	3
2	2	2	3	2	3	1	2	2	2
3	3	3	1	2	2	2	2	2	1
1	1	1	1	1	1	1	1	1	1
2	2	2	2	2	2	2	2	2	2
3	3	3	1	3	1	3	3	3	3
2	3	2	3	2	1	1	2	2	1
3	3	1	1	3	2	1	2	9	1
1	1	1	2	1	2	2	2	2	2
2	2	2	1	9	9	1	9	9	9
3	3	3	3	3	3	2	3	1	1
3	1	2	3	2	2	1	2	1	1
3	3	3	3	1	3	3	2	3	1
2	2	2	2	2	2	2	2	2	2
3	3	3	3	2	2	2	2	2	3
3	3	3	3	3	3	1	1	2	1

2	3	2	3	2	3	3	1	2	2
3	1	1	3	2	2	1	3	2	2
2	2	2	1	2	2	2	2	2	1
2	2	1	1	1	1	1	1	1	1
2	3	2	2	3	1	1	3	1	3
3	3	3	3	2	2	2	2	2	3
3	3	3	2	3	1	2	2	2	1
3	3	3	2	2	2	2	2	2	2
1	3	3	2	2	2	2	2	2	1
2	2	2	2	2	2	2	2	2	1
9	9	9	9	9	2	9	9	9	2
3	2	3	1	9	1	2	3	3	1
9	9	9	9	9	9	9	9	9	9
2	3	2	2	3	1	1	3	1	3
2	3	3	2	2	2	2	2	2	2
3	3	3	3	9	9	9	9	9	9
2	2	2	3	2	2	1	2	2	2
3	1	1	3	2	1	3	2	3	3
3	3	1	1	3	1	1	3	1	3
3	3	3	3	2	2	2	2	2	1
3	3	9	9	1	1	1	1	2	1
2	3	3	3	3	2	1	2	3	2
9	9	9	9	9	9	1	9	9	1
1	3	3	1	2	2	2	2	2	1
2	3	1	1	3	3	1	1	1	2
3	3	2	1	2	2	2	2	2	2
9	9	1	9	9	1	9	9	9	1
2	2	2	3	2	3	3	3	2	2
3	3	2	1	3	2	2	2	3	3
9	9	9	9	1	9	9	9	9	1
3	3	1	1	2	2	1	1	2	3
2	2	2	2	1	1	1	1	1	2
3	3	1	1	9	9	1	9	9	1
3	2	3	3	1	1	3	3	3	2
3	3	3	3	1	3	3	3	3	1
1	9	9	9	3	3	2	1	2	2
3	3	9	1	3	1	3	3	3	1
3	2	9	3	3	2	1	1	3	1
1	1	1	1	9	9	1	9	9	9
3	3	3	3	3	2	1	2	2	1
3	2	3	1	3	3	2	1	2	3
3	3	2	1	3	2	1	2	3	3
3	3	3	2	2	2	2	2	2	2
2	2	3	1	3	1	2	2	2	3
2	2	2	3	1	3	3	2	3	1
2	3	3	3	2	2	1	2	2	9
2	3	3	3	3	3	1	3	3	1

3	3	3	3	3	3	3	1	2	1
2	2	2	2	2	2	2	2	2	2
9	9	9	9	9	9	1	9	9	2
9	9	1	9	9	9	1	9	9	9
1	2	2	1	1	1	2	2	2	2
3	3	3	3	3	3	3	3	1	1
3	3	3	2	2	2	2	2	2	2
2	3	2	3	3	3	1	2	3	3
3	1	2	1	3	1	2	1	3	1
3	3	3	3	3	1	3	3	3	9
2	2	3	1	3	2	2	1	1	2
1	2	2	1	2	1	3	1	3	2
2	2	2	2	2	2	2	2	2	2
3	3	3	3	3	3	3	3	3	3
2	3	2	3	2	2	1	3	3	2
2	1	2	1	3	3	2	1	1	2
3	9	2	3	3	2	1	3	3	2
3	3	3	1	1	1	3	3	3	3
1	3	3	3	2	2	2	2	2	2
3	1	2	1	2	2	1	1	2	1
2	2	3	2	3	3	1	3	3	3
2	2	2	2	3	1	2	2	2	3
2	2	2	2	3	1	2	2	2	3
1	1	3	1	3	1	3	3	3	1
3	3	3	1	3	3	1	1	3	2
3	3	3	3	3	3	3	1	3	3
3	1	1	3	3	1	2	3	3	3
3	1	1	3	3	1	2	3	3	3
2	2	3	3	2	2	2	2	2	1
2	2	2	2	3	3	3	1	3	2
3	3	2	2	1	2	3	2	2	2
2	2	1	2	3	1	3	2	2	3
3	3	3	3	3	3	3	1	3	3
1	3	3	3	1	2	2	2	2	1
3	3	3	3	2	1	2	2	2	2
3	3	3	3	3	3	3	3	3	3
3	3	3	3	3	3	3	3	3	2
3	1	2	1	3	2	2	1	3	2
2	1	2	2	2	2	2	2	2	2
2	1	2	3	2	2	2	2	2	2
3	1	3	3	3	3	1	2	2	1
3	3	3	1	3	3	3	3	1	1
1	2	1	1	1	1	1	1	1	1
2	2	2	2	1	2	2	2	2	1
2	2	3	2	2	2	2	2	3	2
3	2	2	1	1	3	3	3	3	1
1	3	3	3	2	2	1	2	3	9

1	2	2	2	2	1	2	3	2	2
2	2	2	2	2	9	2	9	2	1
1	1	2	2	2	2	2	2	2	1
2	2	2	2	2	2	2	2	2	2
2	2	2	2	2	2	2	2	2	2
2	2	2	2	1	2	2	2	2	2
1	1	2	1	2	1	3	2	3	1
3	1	3	2	1	2	1	3	2	9
3	2	2	2	2	2	2	2	2	9
3	2	3	3	2	2	2	2	2	2
2	2	2	2	2	2	2	2	2	2
2	2	2	2	2	2	2	2	2	2
2	2	2	2	2	2	2	2	2	2
2	2	2	2	2	2	2	2	2	2
3	3	1	2	2	2	2	2	2	3
2	2	2	2	2	2	1	2	2	9
3	1	2	1	1	2	2	2	2	2
2	2	2	2	2	2	2	2	2	9
1	2	3	1	1	1	1	3	3	9
2	3	2	3	3	1	2	3	2	2
2	2	2	2	2	2	2	2	2	2
2	2	2	2	2	2	2	2	2	1
2	3	3	1	3	3	3	2	3	2
2	2	2	2	2	2	2	2	2	2
2	2	2	2	2	2	2	2	2	9
2	2	2	2	9	9	9	1	9	9
2	2	2	2	2	2	2	2	2	2
2	2	2	2	2	2	2	2	2	2
2	2	2	2	2	2	2	2	2	2
3	3	3	3	3	3	3	1	3	1
3	3	3	3	2	2	2	2	2	2
2	2	2	2	2	2	2	2	2	2
2	2	2	2	2	2	2	2	2	2
3	3	2	2	2	2	2	2	2	2
2	2	2	2	2	2	2	2	2	2
3	2	3	2	3	3	2	1	1	2
2	2	2	2	2	2	2	2	2	2
2	2	2	2	2	2	2	2	2	2
2	2	2	2	3	3	1	2	2	3
2	2	2	2	2	1	2	2	2	9
3	2	1	2	1	1	2	3	3	9
2	2	2	2	2	2	2	2	2	2
2	2	2	2	2	2	2	2	2	9
2	2	2	2	2	2	2	2	2	2
2	2	2	2	2	2	2	2	2	2
2	2	2	2	2	3	2	3	2	1
2	2	2	2	2	2	2	2	2	2

2	2	2	2	2	2	2	2	2	2
2	2	2	2	2	2	2	2	2	9
3	2	2	2	3	2	1	2	3	1
2	2	2	2	2	2	2	2	2	2
2	2	2	2	2	2	2	2	2	2
2	2	2	2	3	3	3	1	2	9
3	3	3	2	2	2	2	2	1	1
9	9	9	9	9	9	1	9	9	9
1	3	3	1	3	1	1	3	3	1
3	3	3	2	2	2	1	3	3	3
2	2	3	1	2	2	1	2	2	1
2	2	2	2	2	2	2	2	2	2
1	2	2	1	2	2	2	2	2	9
1	9	9	9	9	1	9	9	9	9
3	3	3	3	3	3	3	1	3	3
2	2	2	2	2	1	1	2	2	2
2	2	2	2	2	1	2	2	2	2
2	2	2	2	2	2	2	2	2	2
1	3	3	1	1	3	3	3	3	1
2	2	2	2	2	1	2	2	2	1
9	9	9	9	9	1	9	9	9	1
2	2	2	2	2	2	2	2	2	2
2	2	2	2	2	2	2	2	2	9
2	2	2	2	2	2	2	2	2	2
2	2	2	2	2	2	2	2	2	2
2	2	2	2	2	2	2	2	2	2
9	9	9	9	2	9	9	9	9	2
2	2	2	2	2	2	2	2	2	2
2	2	2	2	2	2	2	2	2	2
2	2	2	2	2	2	2	2	2	9
2	2	2	2	2	2	2	2	2	2
3	2	3	2	3	3	2	3	2	1
1	3	3	3	3	3	1	3	3	2
3	3	3	1	3	3	1	3	3	1
2	2	3	1	3	1	1	3	3	1
3	3	3	3	3	2	1	1	3	1
3	3	2	1	3	3	2	1	1	3
3	3	3	3	1	1	1	2	3	1
2	2	3	2	3	1	1	2	3	1
2	3	2	2	3	2	2	2	2	1
3	2	3	2	3	3	1	1	2	1
1	1	2	1	3	3	1	3	3	9
1	1	2	2	2	2	1	1	3	2
2	2	2	2	2	2	2	2	2	2
1	1	1	2	3	3	1	9	9	1
1	2	2	1	2	2	2	1	1	2
3	2	3	3	2	1	2	2	1	1

3	2	2	1	2	1	3	2	1	2
2	9	1	2	1	2	1	3	1	1
1	1	2	2	1	1	1	2	2	1
1	2	1	2	3	2	3	3	3	3
2	3	1	2	2	1	2	3	1	3
1	2	3	1	2	1	3	2	3	2
1	2	3	1	3	1	2	1	2	1
1	9	9	9	9	9	9	2	9	1
2	2	2	2	2	2	2	2	2	2
2	2	2	2	2	2	2	2	2	1
2	2	2	2	2	2	1	2	2	2
2	2	2	2	2	2	1	2	2	2
3	3	3	3	3	3	3	3	3	3
2	2	2	2	3	3	1	2	9	2
3	3	3	3	1	3	3	3	3	2
2	2	2	2	2	1	2	2	2	2
2	2	2	2	1	2	2	2	2	2
3	3	3	3	2	9	1	3	3	1
3	3	3	3	3	2	1	2	3	9
2	3	3	2	3	9	1	1	2	3
3	3	3	3	3	1	2	3	3	9
9	9	9	9	9	9	1	9	9	1
1	3	3	1	3	3	1	1	2	3
9	9	9	9	9	9	9	9	9	9
3	1	2	1	1	2	2	1	3	1
2	3	1	1	1	3	3	1	2	3
3	3	3	3	3	3	1	3	3	2
9	9	9	9	2	9	9	9	9	2
2	2	2	2	1	2	2	2	2	9
1	2	2	1	9	1	9	9	9	9
1	2	2	1	9	1	9	9	9	2
2	2	3	3	3	1	2	3	3	1
2	2	2	1	3	1	1	3	3	3
2	2	2	1	3	1	1	3	3	3
2	2	2	1	3	1	1	3	3	3
2	2	2	2	2	2	2	2	2	2
3	3	3	3	2	1	3	2	3	1
2	2	2	1	3	1	1	3	3	3
2	2	2	1	2	1	1	3	3	2
2	2	2	1	3	1	1	3	3	9
2	1	2	2	2	2	2	2	2	2
3	3	3	2	3	3	3	1	3	2
2	2	2	2	2	2	2	2	2	2
1	2	3	3	2	2	3	1	3	9
1	9	9	9	1	9	9	9	9	9
2	2	2	1	1	1	1	2	2	1
2	2	2	2	2	2	2	1	9	1

2	2	1	2	2	1	2	2	3	1
3	1	2	1	1	2	1	3	2	9
3	3	3	3	3	3	3	1	2	2
3	3	3	3	2	1	2	2	2	9
2	2	2	2	2	2	1	1	1	2
2	2	2	2	2	2	2	2	2	9
2	2	2	2	2	2	2	2	2	2
3	3	3	2	3	3	3	3	3	2
3	3	1	1	3	1	3	3	3	9
2	2	2	2	2	2	2	2	2	2
1	2	2	2	2	2	2	1	2	2
2	1	1	1	1	1	2	1	1	1
3	1	1	3	1	3	3	2	3	1
1	2	3	3	2	2	1	3	2	3
1	3	2	2	3	3	1	3	2	2
1	3	2	3	3	1	2	1	3	3
3	1	1	1	3	1	3	3	3	1
1	3	2	2	1	3	3	2	3	2
2	2	2	2	2	1	2	2	2	2
3	1	1	2	1	3	2	1	3	3
2	3	3	1	2	1	1	2	3	2
1	3	1	3	1	3	3	2	3	3
9	9	9	1	9	1	9	9	9	1
3	3	3	3	3	3	3	1	3	1
2	3	1	1	2	1	1	2	2	1
2	3	3	2	2	2	2	2	2	2
2	2	3	1	3	3	1	3	2	1
2	2	3	3	3	2	1	1	1	3
1	3	2	3	1	2	3	1	2	2
3	3	3	3	1	9	9	9	9	9
2	2	2	2	2	2	2	2	2	2
2	1	2	2	2	1	2	2	2	1
3	1	1	3	1	1	1	1	3	3
3	3	3	3	3	3	3	3	3	3
2	2	2	2	2	2	1	2	2	2
2	2	1	1	2	1	3	3	2	1
3	9	3	1	1	3	3	3	3	3
2	2	2	2	2	2	2	2	2	2
1	2	1	1	2	2	1	1	1	2
9	9	9	1	9	1	9	9	9	9
3	3	3	1	2	2	2	2	2	3
2	3	2	1	1	2	1	3	1	1
3	3	3	3	3	2	1	1	3	2
2	2	3	1	1	2	2	3	3	2
1	1	1	1	3	2	1	1	2	1
3	1	3	1	2	2	2	2	2	3
3	3	3	3	9	9	9	9	9	9

1	2	2	3	2	1	2	2	1	1
3	2	3	1	2	1	1	3	3	9
3	3	3	3	1	3	3	3	3	3
3	3	3	1	3	1	3	3	3	1
3	3	3	3	9	9	9	9	9	1
2	2	2	2	2	3	3	3	1	3
3	2	1	2	1	1	2	2	3	9
2	2	2	1	2	2	9	2	2	2
3	3	3	3	3	2	3	3	3	3
3	9	2	1	2	2	3	2	3	2
3	3	3	1	1	1	2	3	3	1
3	2	2	2	2	2	2	1	2	2
2	2	1	1	3	2	2	1	2	1
1	1	1	1	3	3	1	1	1	3
2	2	2	1	3	3	2	1	3	1
2	2	2	2	2	2	2	2	2	2
1	2	2	2	3	2	2	2	1	1
3	1	2	2	2	2	2	2	2	2
3	3	3	3	3	2	1	1	3	1
2	1	9	1	3	2	1	1	3	1
2	2	2	2	1	2	1	2	2	1
9	1	9	9	9	9	2	9	9	9
2	3	3	3	2	2	2	2	1	1
3	3	3	3	3	3	3	3	3	3
3	2	3	3	3	3	9	9	9	3
2	2	2	2	2	1	2	2	2	1
9	9	9	9	3	1	3	2	2	1
3	3	3	1	2	2	2	2	2	2
3	3	3	3	2	3	3	3	3	3
2	2	3	2	2	1	2	2	3	2
1	1	1	1	3	3	1	1	3	3
9	9	9	9	3	1	3	3	3	2
2	2	3	3	2	3	2	2	3	1
3	2	2	1	3	3	1	1	1	2
1	2	2	1	2	3	2	1	2	1
3	3	2	2	2	2	1	1	1	2
3	1	2	1	3	9	9	1	9	1
1	1	1	1	3	1	2	2	3	3
2	3	3	3	3	2	1	2	3	3
9	9	9	9	9	9	2	9	9	2
2	2	2	1	1	2	2	2	2	2
2	2	1	2	3	3	2	1	1	2
1	1	2	2	3	3	1	2	2	2
1	3	1	1	3	2	1	2	1	2
1	3	3	1	2	1	3	2	3	3
3	3	3	1	2	1	1	3	3	2
2	2	3	1	3	2	1	2	3	3

2	2	3	1	3	2	1	2	3	3
2	2	2	2	2	2	2	2	2	2
2	1	2	3	2	2	3	3	3	1
1	2	9	9	1	2	1	2	1	1
3	3	3	3	2	2	2	1	2	2
1	3	3	3	3	1	1	3	1	1
3	3	3	1	1	9	9	9	9	1
9	9	9	9	9	9	1	9	9	9
1	3	3	1	2	1	2	2	2	2
1	3	1	3	1	9	1	1	1	1
2	2	2	2	2	2	2	2	2	2
3	3	3	2	3	3	1	2	2	3
2	3	3	3	9	1	9	9	9	9
2	2	3	1	1	2	3	3	3	2
2	9	2	9	9	2	2	9	9	9
2	2	1	2	2	2	3	3	3	1
1	1	1	1	1	1	1	1	1	1
3	3	1	2	2	2	1	2	2	2
1	3	3	3	2	3	1	1	9	2
1	3	1	2	3	2	1	2	3	2
1	3	1	1	2	3	1	1	2	1
3	3	3	3	1	3	3	3	3	2
1	1	2	2	2	2	2	1	2	1
1	3	3	3	3	3	2	1	2	3
3	3	3	3	1	3	3	3	3	1
1	1	1	2	2	2	2	1	2	2
3	3	2	3	2	1	2	1	3	2
3	3	3	1	3	3	3	1	3	2
3	3	3	3	3	1	3	3	3	1
3	3	3	1	1	1	3	1	3	1
2	1	2	1	2	1	2	1	1	1
1	1	1	1	1	2	1	1	2	1
1	1	1	2	1	1	1	2	1	1
2	2	2	1	2	2	2	1	2	2
1	1	1	3	3	3	1	2	3	3
3	2	3	1	3	2	1	1	3	1
2	9	1	1	2	2	1	2	2	1
3	3	3	3	3	3	3	1	3	3
1	1	1	2	1	2	2	2	1	1
2	2	1	1	1	2	2	2	1	1
3	3	3	3	3	3	3	3	3	2
1	3	3	3	3	1	3	1	3	3
3	3	3	3	3	3	1	3	3	1
3	3	3	3	3	3	3	3	3	2
3	3	3	2	2	3	1	3	3	1
1	1	1	1	2	2	2	2	2	3
2	2	2	2	2	2	2	2	2	2

2	2	2	2	2	1	3	3	3	1
1	1	3	3	3	3	2	1	3	3
2	2	2	2	2	2	2	2	2	2
1	2	3	2	2	1	1	2	3	2
3	3	3	3	3	3	1	3	3	1
1	1	1	2	2	1	1	3	1	1
3	3	9	2	3	3	1	3	3	1
2	1	2	2	2	2	2	1	2	1

DuringPeri	SeventoTe	MidCycle	SeventoTe	EarlyPerioc	LatePeriod	NoBabies	ManyBabie	HighFat	BirthContr
2	3	9	2	3	2	2	3	2	3
1	3	1	2	2	3	3	1	2	1
2	2	2	2	3	3	3	2	2	2
1	3	1	2	2	3	9	2	3	1
9	9	9	9	9	9	9	9	9	9
1	3	1	2	2	3	3	1	2	1
2	2	2	2	2	2	2	2	2	2
2	1	1	2	1	1	1	1	1	3
9	9	9	9	2	9	9	9	9	9
2	9	9	9	1	9	9	9	9	9
2	2	2	2	9	2	9	9	9	9
1	2	3	3	9	9	9	9	9	9
1	2	2	2	2	2	2	2	2	2
3	1	3	2	2	3	3	3	2	3
2	2	2	2	3	2	3	3	2	2
1	2	2	2	3	3	1	3	2	2
2	9	1	2	2	3	2	9	1	2
2	2	2	2	3	3	1	3	2	2
3	3	2	2	3	3	1	3	2	2
2	2	2	2	3	3	3	3	2	3
2	2	2	2	2	3	3	3	3	3
2	2	2	3	2	3	3	3	1	2
3	3	3	3	9	9	2	9	9	9
2	2	2	3	2	2	2	2	3	3
1	2	2	2	2	2	2	2	2	1
2	2	2	2	2	2	2	2	2	2
2	2	2	2	2	2	2	2	2	2
1	2	2	2	2	2	2	2	2	2
1	2	2	2	1	1	2	2	2	2
2	2	2	2	2	2	2	2	2	2
2	2	2	2	2	2	2	2	2	2
2	2	2	2	3	3	3	3	2	3
2	1	2	2	3	2	2	2	1	2
2	2	2	2	3	3	3	3	2	2
2	2	2	2	3	3	3	3	2	2
2	2	2	1	3	3	3	3	2	2
2	2	2	2	3	3	3	3	2	2
9	1	2	1	1	3	3	1	3	1
1	2	2	2	1	1	9	9	9	9
1	2	2	2	9	9	9	9	9	9
9	1	2	2	2	2	2	2	2	2
9	9	9	9	2	9	9	9	9	9
3	3	3	2	3	3	3	3	3	3
3	3	1	3	3	3	3	3	3	3
2	2	2	2	2	3	3	9	9	9
9	9	9	9	3	1	1	2	3	2

2	1	1	3	3	3	3	3	3	1
9	9	9	9	9	9	9	9	9	1
9	9	9	9	2	9	3	3	9	1
1	2	2	2	2	2	3	3	2	1
9	9	9	9	9	9	9	9	1	9
3	1	3	3	3	3	3	3	1	1
1	1	1	1	3	3	1	3	3	1
1	1	9	9	9	9	9	9	9	1
9	1	9	9	9	9	9	9	9	1
1	2	3	2	1	2	2	3	1	2
9	1	9	9	9	9	9	9	1	9
9	1	9	9	9	9	9	9	9	1
9	9	9	9	2	2	2	3	2	1
2	2	2	2	9	9	9	9	9	1
9	9	9	9	2	2	3	3	2	1
3	1	3	3	3	3	3	3	2	1
2	2	2	2	2	2	2	2	1	2
1	2	1	2	9	9	9	9	1	9
2	3	1	2	3	1	3	3	3	1
2	2	1	2	1	3	3	2	2	2
1	3	1	3	3	3	1	3	3	1
1	3	1	3	3	3	1	3	3	1
1	3	1	3	3	3	1	3	3	1
1	1	1	2	9	9	9	9	3	9
9	3	2	1	3	1	1	2	3	2
9	9	9	9	9	9	9	9	1	9
9	9	9	9	9	9	9	9	9	9
1	2	1	2	3	2	3	2	2	1
3	1	2	1	1	3	3	1	3	1
9	1	9	9	3	3	3	2	3	1
9	9	9	9	2	3	3	3	3	3
1	2	2	2	3	3	3	3	1	2
9	9	9	9	2	2	1	2	2	2
9	1	9	9	3	3	3	9	2	9
1	2	1	1	3	3	3	2	1	2
1	2	1	2	2	2	3	3	3	1
9	9	9	9	2	3	3	3	3	1
2	2	2	2	3	3	3	3	2	2
9	9	9	9	9	9	9	9	9	1
3	3	1	1	3	3	3	3	1	1
9	9	9	9	3	2	1	2	1	1
3	2	3	1	3	3	3	3	1	3
9	9	9	9	3	3	3	3	1	3
1	1	1	1	2	2	3	3	1	1
9	9	9	1	3	9	3	3	2	2
9	9	9	9	9	9	9	9	9	9
1	9	9	9	9	9	9	9	1	9

2	2	2	2	3	3	2	3	2	1
2	2	2	2	3	3	2	2	2	1
2	2	2	2	9	9	9	9	9	9
2	2	1	2	3	3	3	3	2	1
2	2	2	2	9	2	2	2	1	9
1	2	1	2	1	1	2	1	2	2
3	3	3	3	2	2	3	3	2	1
2	1	2	2	3	3	3	1	1	1
2	2	2	2	2	2	3	3	1	2
2	1	1	1	2	1	2	3	1	2
1	2	2	2	3	2	1	2	1	1
2	2	2	2	3	3	3	3	2	2
2	3	3	2	3	3	3	3	1	1
9	1	9	9	9	9	9	9	9	9
2	2	3	1	2	3	3	2	1	2
1	3	2	1	9	9	9	2	9	9
2	1	2	2	2	2	3	3	2	2
2	1	2	2	2	2	2	2	2	2
2	2	2	2	2	2	2	2	2	2
9	9	9	9	2	2	2	2	2	2
1	3	2	2	2	2	2	2	2	2
9	9	9	1	9	9	9	9	9	1
3	1	2	3	2	2	2	2	2	2
3	3	3	1	1	3	3	3	3	3
1	2	2	1	3	2	2	2	1	2
2	2	2	2	2	2	2	2	2	2
9	9	9	1	9	9	9	9	9	1
9	9	9	1	9	9	9	9	9	1
2	2	2	2	2	2	3	3	2	3
9	1	9	9	9	9	9	9	1	9
2	2	2	2	2	2	2	2	2	2
3	2	2	2	2	2	2	2	2	2
1	2	2	2	2	2	2	2	1	2
2	2	2	2	3	3	3	3	3	3
9	1	9	9	9	9	9	9	1	9
1	3	1	3	1	3	3	3	3	3
2	2	9	2	2	2	2	2	2	2
2	1	2	2	2	3	3	1	2	3
9	9	9	9	1	9	9	9	9	9
2	1	2	1	3	3	3	3	3	1
3	1	3	3	2	3	3	3	1	3
3	1	3	3	2	3	3	3	1	3
3	2	2	2	2	2	2	2	2	2
2	2	2	2	2	2	2	2	2	2
2	1	2	1	2	2	2	2	2	2
1	1	2	2	2	2	2	2	1	2
1	3	3	3	2	2	2	2	2	2

1	2	2	2	9	9	9	9	9	9
9	9	9	1	3	3	3	2	2	2
2	1	2	2	3	3	2	2	2	2
3	1	3	3	2	3	3	1	3	3
3	1	3	3	2	3	2	9	3	3
9	9	9	9	3	2	2	3	1	3
1	2	1	2	2	2	1	2	1	3
2	2	2	2	2	2	3	3	2	2
3	3	3	3	2	2	2	2	2	2
2	2	2	2	2	2	2	2	2	2
1	3	2	2	3	3	2	2	3	3
1	3	2	2	3	3	2	2	3	3
9	1	9	9	2	9	9	9	9	9
2	2	2	2	2	2	2	2	2	2
2	1	2	2	3	3	2	2	2	2
2	2	2	2	3	3	2	2	2	2
2	2	2	2	2	2	2	2	2	2
9	1	9	9	3	3	3	2	2	3
9	9	9	1	9	9	9	9	9	9
1	1	2	3	1	2	1	3	2	1
3	1	3	3	9	9	9	9	9	9
3	1	3	1	3	3	3	3	3	2
9	1	2	2	2	1	2	2	2	2
3	3	3	3	3	3	3	3	3	3
3	1	3	3	3	3	3	3	3	3
9	1	9	9	9	9	9	9	9	9
3	1	2	3	3	2	2	3	3	9
9	1	9	9	9	9	9	9	9	9
3	1	3	3	3	3	3	3	3	3
3	1	3	3	3	3	3	3	3	3
1	2	2	2	3	2	2	2	1	1
3	2	2	1	2	2	3	3	1	2
3	1	2	3	9	9	9	9	3	9
3	1	3	3	3	2	3	3	9	1
3	1	3	3	3	2	3	3	2	1
3	1	9	3	3	2	3	3	2	1
3	3	3	3	2	3	2	3	3	1
3	1	3	3	2	3	2	3	3	1
3	1	3	3	3	2	3	3	2	1
3	1	3	3	3	3	3	3	2	1
3	1	3	3	3	3	3	3	3	1
3	3	3	3	3	3	3	3	3	3
9	1	9	9	9	9	9	9	9	9
3	1	3	3	3	3	3	3	3	1
3	1	3	3	1	3	3	3	3	3
3	1	3	3	3	3	3	3	3	1
3	1	3	3	3	3	3	3	3	1

3	1	3	3	3	3	3	3	3	1
3	1	3	3	9	9	9	9	9	1
3	1	3	3	3	9	9	9	9	9
3	3	3	1	9	9	9	9	9	9
3	1	3	3	3	3	3	3	3	3
1	1	1	1	3	1	3	3	3	1
3	1	3	3	3	3	3	3	3	1
2	1	2	2	2	2	3	9	1	2
3	3	1	1	3	3	3	3	1	1
9	9	9	9	9	9	3	9	9	9
9	9	9	9	2	2	3	2	1	2
9	9	9	9	9	9	9	9	9	9
9	9	9	1	3	3	3	3	1	1
3	1	2	3	3	3	3	3	2	1
1	2	1	3	2	2	1	2	2	2
1	1	1	1	2	2	2	2	2	2
3	1	9	2	3	2	2	2	2	2
1	1	1	1	3	3	1	1	1	1
2	2	2	2	2	2	2	2	2	1
1	1	2	1	2	2	2	2	2	2
1	1	2	2	2	1	2	2	1	1
2	2	2	2	2	2	2	2	2	2
3	2	1	2	1	2	2	1	2	2
2	2	2	2	9	9	9	9	9	9
2	2	2	2	2	2	2	2	2	2
9	9	9	9	2	2	2	2	2	2
2	2	2	2	2	2	2	2	2	2
2	2	2	2	2	2	2	2	2	2
2	2	2	2	2	3	3	3	3	3
2	2	2	2	2	2	2	2	2	1
1	2	2	2	2	2	2	2	2	2
1	2	2	2	2	2	2	2	2	2
2	2	2	2	2	2	2	3	2	1
2	2	2	2	2	3	1	1	2	2
2	2	1	2	2	2	2	2	2	2
1	1	1	1	1	2	2	2	1	1
2	1	1	1	2	1	3	3	1	1
3	3	3	1	3	3	2	2	2	1
2	1	2	2	2	2	2	3	2	1
1	2	9	2	2	3	2	3	3	3
2	2	2	2	2	3	2	3	2	2
2	2	2	2	2	2	2	2	2	2
9	9	9	1	1	9	9	9	9	9
9	9	9	1	9	9	2	9	9	9
3	2	2	2	3	3	2	2	3	2
2	1	2	2	2	2	2	3	1	1
2	2	2	2	2	2	2	2	1	1

2	2	2	2	3	3	3	3	2	2
2	2	2	2	3	3	3	3	2	2
2	1	2	2	1	2	2	2	2	2
2	1	1	2	2	2	2	2	1	1
1	2	3	2	3	2	2	3	1	2
3	1	2	2	3	1	2	3	1	2
3	9	9	9	3	3	1	3	3	3
2	2	2	2	2	3	2	3	1	1
2	3	3	2	1	2	3	3	1	2
1	3	3	3	3	3	3	3	3	3
2	2	2	2	3	3	2	9	1	2
3	1	1	2	3	2	3	3	1	1
2	3	3	2	1	2	3	3	1	2
1	2	2	2	1	2	2	2	2	2
2	2	2	2	3	3	2	2	2	2
3	2	2	1	2	3	1	3	3	2
1	2	1	3	3	2	1	2	3	2
2	2	2	2	2	2	3	3	2	3
2	2	2	2	3	3	1	3	3	2
2	1	2	2	3	3	2	1	1	3
2	1	1	2	2	2	1	3	1	1
1	1	2	1	2	2	1	2	1	2
1	2	2	2	3	2	1	2	2	2
2	1	3	2	3	2	2	3	3	2
1	9	9	9	9	9	2	9	9	9
2	2	2	2	2	2	2	2	2	2
2	2	2	2	2	2	2	2	2	2
2	1	3	1	3	2	2	9	1	3
1	2	2	2	2	2	3	3	2	2
1	1	2	2	2	2	2	2	2	2
2	2	2	2	9	1	9	9	9	9
1	2	2	1	2	2	2	2	2	2
1	9	9	9	9	9	9	9	9	9
3	2	2	2	3	3	3	3	2	2
2	2	2	2	1	9	9	9	9	9
3	2	2	2	2	2	2	2	2	2
2	2	1	2	3	3	3	2	2	3
1	2	1	2	2	1	2	2	2	9
1	1	2	1	2	2	2	3	2	2
2	2	1	1	3	3	3	2	3	2
2	3	2	2	2	2	2	2	2	2
1	2	2	2	3	2	2	2	2	1
3	1	2	2	3	3	3	3	2	2
2	2	2	2	2	2	2	2	2	2
9	1	9	9	3	2	2	3	2	1
2	2	2	3	2	3	2	3	2	3
2	1	2	3	3	3	1	3	2	2

1	2	2	1	2	2	3	2	2	2
1	2	2	1	3	3	3	3	2	2
2	1	2	1	2	2	2	2	1	2
2	2	2	1	3	3	2	2	2	1
1	2	1	2	3	3	3	3	2	2
1	2	1	2	3	3	2	2	1	2
2	1	2	2	2	2	3	3	1	1
2	9	9	9	9	9	9	9	1	9
1	2	1	2	3	2	3	3	2	2
2	2	2	2	3	2	2	2	2	2
2	2	2	2	2	2	2	2	2	2
2	2	2	2	2	2	3	2	2	3
1	1	1	1	1	1	1	1	1	1
9	1	9	9	3	3	3	3	2	2
1	1	1	3	3	1	1	3	1	1
2	2	2	2	3	3	3	3	3	3
3	3	3	3	3	3	3	3	2	2
2	3	1	2	1	1	3	2	2	2
1	1	1	2	2	1	3	1	2	1
1	2	1	2	2	1	2	2	1	1
2	2	2	2	2	1	1	2	2	3
1	1	2	3	3	3	3	3	1	1
2	1	2	3	3	3	3	2	1	3
2	2	2	2	2	2	2	2	2	2
2	2	2	2	2	2	2	2	2	2
9	9	9	9	9	9	9	9	9	9
9	9	9	9	1	1	2	3	1	1
2	1	2	3	3	3	3	3	3	3
2	2	2	2	2	2	3	3	1	1
1	1	2	2	2	2	3	3	1	2
2	2	2	2	2	2	2	2	2	2
1	3	2	1	9	9	9	9	9	9
9	9	9	9	9	9	9	9	9	9
2	3	3	3	3	3	3	3	2	3
2	1	2	2	3	3	3	3	1	3
3	3	3	3	3	3	3	2	9	1
1	2	1	2	2	2	3	3	1	3
3	3	3	3	3	3	3	3	3	1
1	1	3	3	3	3	2	2	1	2
2	1	2	2	9	9	9	9	9	9
9	1	9	9	1	2	3	1	2	3
3	3	3	3	1	3	3	9	1	3
2	2	2	2	2	2	3	2	2	2
9	1	3	3	3	3	3	3	2	3
3	3	1	3	2	3	3	3	3	3
1	9	1	3	3	3	1	3	1	3
2	1	2	2	1	2	2	3	2	2

3	2	2	3	2	2	3	2	9	9
3	2	2	3	2	2	3	2	9	9
9	9	9	1	9	9	9	9	1	9
3	1	3	3	1	3	3	3	3	3
9	9	9	9	1	9	9	9	9	9
1	2	1	2	2	3	3	3	1	1
2	2	2	2	2	2	2	2	2	1
9	2	9	9	3	3	3	3	1	3
3	1	3	3	2	2	3	9	2	1
2	1	2	1	3	2	2	2	1	2
2	2	2	2	2	3	3	3	2	3
2	2	2	2	2	2	2	2	2	2
1	2	1	3	2	3	3	3	1	2
2	3	3	3	1	2	3	2	3	2
2	2	2	2	3	2	3	3	2	3
2	3	2	2	2	2	3	3	3	3
3	1	2	2	2	2	2	3	1	3
2	2	2	2	3	2	3	2	2	3
9	9	9	9	3	3	9	3	1	2
3	2	3	2	3	2	3	2	3	3
2	1	2	3	3	2	3	1	3	3
2	3	2	2	1	3	3	3	1	2
2	3	2	2	2	3	3	3	1	2
2	2	1	2	2	1	3	2	2	3
1	2	2	2	2	2	2	2	2	2
2	2	3	3	2	3	9	2	1	2
2	2	2	2	3	2	3	3	2	2
2	1	2	2	1	2	2	2	2	2
1	2	3	1	2	3	3	3	2	2
2	2	1	1	3	3	3	3	1	2
1	3	2	2	3	2	3	3	2	3
1	3	1	2	3	2	3	3	2	1
1	3	2	3	1	3	1	3	3	2
2	2	2	2	2	2	2	2	2	2
2	2	2	2	2	2	2	2	2	2
3	2	1	2	3	3	2	3	2	2
2	3	2	2	2	2	2	3	2	2
2	1	2	2	1	2	2	3	1	2
2	2	2	2	2	2	2	2	2	2
1	1	1	2	3	1	1	2	2	2
1	2	2	3	2	2	2	2	2	2
2	2	2	2	2	2	2	2	2	2
1	1	9	1	2	3	2	1	1	2
3	2	2	2	2	2	2	2	2	2
3	2	3	1	2	2	3	3	3	2
1	3	2	1	3	2	3	3	1	1
2	2	2	2	3	3	2	2	2	2

3	1	2	2	2	2	2	2	2	3
2	2	1	2	3	3	2	2	1	1
2	9	9	9	2	9	9	9	9	9
2	1	2	1	2	2	2	2	2	2
2	2	3	2	3	2	2	3	1	2
2	2	3	1	3	2	3	3	3	2
3	1	2	1	3	3	2	3	2	2
2	2	2	3	2	2	3	3	2	3
2	2	2	3	2	2	3	3	2	3
1	2	1	2	2	2	3	2	1	2
2	1	2	2	2	2	3	3	2	2
2	1	2	2	3	2	3	3	1	2
2	2	2	2	3	3	2	2	2	2
3	2	2	2	3	3	2	2	3	2
3	2	2	2	3	3	2	2	3	2
1	1	2	2	2	2	2	2	2	2
3	1	1	3	3	2	2	3	1	3
2	2	2	2	2	2	1	2	2	2
2	2	2	2	2	2	1	2	3	2
9	9	9	9	9	9	9	9	9	9
3	2	2	2	2	3	1	2	3	1
9	9	9	9	2	2	2	2	2	2
9	9	9	9	3	3	2	2	3	2
3	2	1	2	2	2	3	3	1	1
2	1	2	2	3	3	3	2	2	1
2	2	2	3	2	1	2	2	3	1
9	1	9	9	3	3	3	3	2	2
2	2	2	2	2	2	2	2	2	2
2	2	2	2	2	1	2	1	2	1
9	9	9	9	9	9	1	9	9	9
2	2	2	2	2	2	2	2	2	2
2	2	2	2	2	2	2	1	1	1
2	2	2	2	2	2	2	2	2	2
9	9	9	9	3	3	3	3	1	2
1	1	2	1	3	2	2	3	1	1
3	1	2	2	2	2	2	2	2	2
9	9	9	9	3	2	3	3	2	2
1	1	3	2	3	2	3	2	3	3
2	2	2	1	1	2	3	3	2	2
2	2	1	2	2	1	3	3	1	2
2	2	3	2	2	3	2	3	3	2
1	3	1	2	2	3	3	1	2	1
2	1	2	2	2	2	2	2	3	2
2	2	2	2	3	3	2	3	3	3
3	2	2	2	1	2	3	2	3	3
9	1	9	9	3	3	3	2	3	2
3	3	3	3	3	3	3	2	3	3

9	1	9	9	3	3	3	2	3	2
3	3	3	1	1	3	3	3	3	3
3	1	3	3	3	3	3	3	3	3
1	1	2	2	1	2	1	9	2	9
1	1	3	3	3	3	1	3	9	9
3	1	3	3	3	3	3	3	3	1
2	2	2	2	2	2	2	2	2	2
1	1	3	1	1	3	3	3	3	1
2	1	2	2	2	2	2	2	2	2
3	3	3	1	3	3	3	3	3	3
9	9	9	9	3	2	3	3	2	1
9	1	9	9	3	3	3	2	3	2
3	3	3	1	3	3	3	3	3	3
3	1	3	3	3	2	3	3	3	3
3	1	3	3	3	3	3	3	3	3
1	1	3	3	3	3	3	3	3	3
2	2	2	2	2	2	2	2	2	2
2	2	2	2	2	2	2	2	2	2
1	1	3	3	3	3	3	3	3	3
1	1	3	3	3	3	3	3	9	3
3	1	3	3	3	3	3	3	3	3
3	1	3	3	1	3	3	3	3	3
1	1	3	3	3	3	3	3	3	3
3	1	3	3	3	3	3	3	1	1
3	3	3	1	3	3	3	3	3	1
3	1	3	3	3	3	3	3	9	1
3	1	3	3	3	3	3	3	3	3
3	1	3	3	3	3	3	3	3	3
3	3	3	1	3	3	3	3	3	3
3	1	3	3	3	3	3	3	3	1
9	9	9	9	1	3	3	3	3	3
2	1	3	3	3	3	3	2	3	3
3	1	3	3	3	3	3	3	3	3
3	1	3	3	3	3	3	3	3	9
9	1	9	9	9	9	9	9	9	9
3	1	3	3	3	9	9	9	9	9
2	2	2	2	2	2	1	2	1	2
2	1	1	3	3	1	3	3	1	1
3	1	3	3	3	3	3	3	3	3
3	1	3	3	3	1	3	3	3	1
3	1	3	3	3	9	9	9	9	1
1	1	1	2	1	2	2	2	2	2
9	9	9	9	9	9	9	9	9	1
2	2	2	2	2	2	2	2	2	2
9	9	9	9	2	9	9	9	9	9
2	2	2	1	2	2	2	2	2	2
9	3	2	1	9	2	3	3	1	1

2	2	2	2	2	2	2	2	2	2
1	2	3	2	1	2	2	3	1	2
1	2	3	2	1	2	2	3	1	2
2	2	2	2	3	2	9	2	3	3
2	2	2	2	2	2	1	3	2	2
9	9	1	9	9	9	9	9	9	9
2	2	1	2	1	2	2	2	2	3
1	2	3	1	2	3	2	2	1	2
1	2	2	2	2	2	3	2	2	2
2	2	2	2	2	2	2	2	2	2
9	9	9	9	1	2	1	2	1	1
1	9	9	9	9	9	9	9	9	9
3	1	3	3	9	9	9	9	9	1
1	2	2	2	1	2	2	2	2	2
9	1	9	9	9	1	9	9	9	9
9	1	9	9	9	1	9	9	9	9
1	9	9	9	2	9	9	9	9	9
1	9	9	9	9	9	9	9	1	9
1	9	9	9	9	9	9	9	1	9
9	9	9	9	9	9	9	9	9	9
9	9	9	9	3	1	3	9	1	9
3	3	3	3	3	3	3	3	3	3
1	9	9	9	3	2	3	3	1	3
9	9	9	9	9	9	9	9	9	9
9	9	9	9	2	9	9	9	9	9
9	2	9	9	9	9	9	9	9	9
1	9	9	9	1	9	9	9	9	9
2	2	1	2	1	2	2	2	3	3
9	9	9	9	2	1	3	2	3	1
1	9	9	9	9	9	9	9	9	9
1	9	9	9	1	9	9	9	9	9
2	2	2	2	3	3	2	2	2	2
3	2	2	2	3	3	2	3	2	2
1	1	1	1	3	3	3	3	3	3
2	2	2	2	2	2	2	1	2	2
1	2	2	2	2	2	3	3	2	2
1	2	2	1	3	2	1	3	2	3
3	3	3	1	3	3	3	3	2	3
2	2	2	2	3	3	3	2	2	2
2	3	2	1	2	2	3	1	2	3
1	2	2	3	2	3	2	2	1	1
1	3	2	3	3	3	1	3	2	1
2	3	2	2	3	2	1	2	1	2
2	2	2	1	2	2	3	2	1	1
2	2	2	2	3	2	2	2	2	2
1	2	2	2	3	3	3	3	2	2
1	1	2	1	2	2	2	1	1	1

1	2	2	3	2	2	3	3	2	2
2	2	3	2	9	9	9	9	9	9
2	2	3	3	3	3	3	3	1	2
1	1	3	2	3	1	2	3	1	2
2	3	1	2	3	3	3	3	2	1
2	2	2	2	3	3	3	3	3	2
2	2	2	2	3	3	3	2	3	3
2	3	1	2	3	2	3	3	2	1
2	3	2	2	2	2	3	2	1	2
2	2	2	2	3	3	2	3	3	2
2	2	2	2	3	3	2	3	3	3
1	2	3	2	3	3	3	3	2	2
2	2	2	2	3	2	2	2	2	2
2	2	2	2	3	2	2	2	2	2
1	2	2	2	2	3	3	2	2	3
2	2	2	2	3	3	3	3	2	2
2	2	2	2	3	3	3	2	1	2
1	3	1	3	2	2	2	2	2	2
3	1	3	1	3	3	3	1	3	3
2	2	1	2	3	3	2	3	1	2
3	1	1	3	2	2	2	2	1	1
2	3	2	2	2	3	3	3	1	2
3	2	2	2	2	3	3	3	2	2
3	2	2	2	2	3	3	3	2	2
2	3	3	2	3	2	3	3	2	2
1	2	2	2	2	2	2	2	2	3
1	1	9	3	1	1	3	3	3	9
1	1	1	3	1	1	3	3	1	3
1	3	2	1	2	2	2	2	1	1
1	1	3	2	2	2	3	2	3	3
1	2	3	3	2	3	3	2	2	2
2	2	3	2	2	2	3	3	2	2
2	2	2	2	2	2	2	2	2	2
1	2	2	3	2	3	2	3	2	2
2	1	2	1	2	1	3	1	1	1
9	9	9	2	9	9	9	9	9	9
2	2	2	2	2	2	2	2	2	2
3	1	2	1	3	1	3	2	1	3
3	2	2	2	3	3	3	3	2	2
9	2	2	1	3	9	3	2	1	1
1	9	9	9	2	2	3	3	1	2
3	1	3	3	1	2	3	3	1	1
9	1	9	9	9	9	9	9	1	9
2	2	2	2	3	3	3	3	2	2
3	3	3	3	3	3	3	3	3	2
2	2	2	2	2	2	2	2	3	3
1	9	9	9	9	9	9	9	1	9

9	9	9	9	9	9	9	9	9	9
1	9	9	9	9	9	9	9	9	9
1	9	9	9	2	9	9	9	9	9
1	9	9	9	2	9	9	9	9	9
1	9	9	9	9	9	9	9	9	9
1	9	9	9	9	9	9	9	9	9
1	9	9	9	9	9	9	9	9	9
9	9	9	9	9	9	9	9	9	9
9	9	9	9	9	9	9	9	9	9
9	9	9	9	2	9	9	9	9	9
9	9	9	9	9	9	9	9	9	9
9	9	9	9	2	9	9	9	9	9
9	1	9	9	9	9	9	9	9	9
9	1	9	9	9	1	9	9	9	9
9	9	9	9	9	9	2	9	9	9
9	9	9	9	1	9	9	9	9	9
9	9	9	9	9	9	9	9	9	9
9	9	9	1	9	9	9	9	9	9
2	2	2	2	2	2	2	2	2	2
1	1	1	1	1	1	1	1	2	1
1	1	1	1	9	9	9	9	9	1
1	1	2	3	1	2	3	3	2	2
1	2	1	2	2	2	3	2	2	1
1	1	1	3	1	3	1	3	1	1
1	3	9	2	2	2	2	1	3	3
2	1	3	3	2	3	2	1	2	1
2	2	2	2	2	3	3	3	2	1
1	2	3	2	3	2	3	3	3	2
3	3	1	3	2	2	2	2	1	2
1	2	1	2	2	2	2	2	2	2
1	1	2	1	1	1	2	2	1	1
1	2	2	2	2	2	3	3	3	2
3	2	2	2	2	2	2	2	2	2
3	2	3	3	3	3	1	2	2	3
1	2	2	1	2	2	2	2	1	2
1	2	2	3	1	2	2	2	2	2
3	2	2	2	2	3	2	2	2	1
1	3	2	1	2	2	2	2	1	1
9	9	9	9	1	3	3	1	1	9
1	2	2	2	2	1	2	2	1	2
9	9	9	9	1	1	1	9	9	2
1	3	1	3	3	3	3	1	9	3
9	9	9	9	2	2	1	2	2	2
1	1	1	1	3	1	3	1	1	1
2	3	1	2	3	3	3	2	2	2
9	9	9	9	2	2	1	2	2	2
2	2	2	2	1	2	2	3	1	1

3	2	3	3	1	2	2	2	2	2
1	1	1	3	2	1	3	3	3	1
3	3	1	3	3	1	1	3	1	1
1	1	2	1	1	2	1	2	1	1
2	2	2	2	1	2	2	2	2	2
1	1	2	2	1	2	2	2	2	2
1	2	2	2	2	1	2	2	2	2
3	2	1	1	1	2	2	2	2	2
1	1	1	3	1	1	1	2	1	1
9	9	9	9	2	9	9	9	9	9
9	9	9	9	1	3	2	9	1	1
2	3	9	2	1	1	3	3	3	2
1	1	2	2	1	1	1	2	1	2
3	1	2	2	3	3	2	2	1	2
2	9	3	1	1	1	2	2	2	2
9	9	9	9	3	3	1	1	2	3
9	9	1	9	1	1	1	1	1	1
2	2	2	2	2	2	2	2	2	3
2	2	3	3	2	3	3	1	2	9
3	3	1	3	1	3	3	3	3	3
3	1	2	2	1	3	2	2	3	2
1	1	2	2	1	1	1	2	1	2
3	3	3	3	1	2	2	2	2	2
9	9	9	9	1	3	1	3	2	3
9	9	9	9	1	3	3	2	3	3
3	3	1	3	1	3	1	3	1	3
3	3	2	3	1	3	3	3	3	3
9	9	9	9	3	3	3	3	1	9
2	2	2	2	1	2	2	9	1	1
9	9	9	9	9	9	9	9	9	9
3	1	2	2	2	2	2	2	2	1
1	2	2	2	3	3	1	9	9	9
1	1	1	1	1	2	2	2	3	2
2	1	1	2	2	3	3	3	3	2
9	1	9	9	9	9	9	9	9	9
9	9	9	1	9	9	9	9	9	9
9	9	9	9	9	9	9	9	9	9
2	2	2	2	2	2	2	3	2	2
2	2	2	2	2	1	2	3	2	2
2	2	2	2	2	1	2	3	2	2
2	2	2	2	2	2	2	3	2	2
1	3	2	3	2	2	1	2	2	2
2	3	2	1	1	2	3	1	2	3
2	2	2	2	2	2	2	3	2	2
2	3	2	1	1	2	3	1	2	3
2	2	3	3	1	2	3	2	1	1
3	3	3	3	3	3	3	2	3	3

3	1	3	3	3	3	3	3	3	3
1	1	2	2	2	2	3	3	2	2
3	3	3	3	1	1	1	3	3	2
1	1	2	2	2	2	2	2	2	1
2	2	2	2	2	2	2	2	2	2
1	1	1	1	2	1	2	1	2	3
2	9	1	3	2	2	2	3	2	3
9	9	9	9	9	9	9	9	1	9
9	3	3	3	1	2	3	3	3	3
1	1	2	1	3	3	3	3	2	1
2	2	2	2	3	3	3	3	3	3
9	9	9	9	1	1	1	9	1	9
3	2	2	3	1	2	3	3	2	2
3	2	2	3	1	2	3	2	1	1
2	2	3	3	1	2	3	2	1	1
1	2	3	2	1	2	3	2	1	2
3	2	2	3	1	2	3	2	1	1
3	2	2	3	1	2	3	2	1	1
3	2	2	3	1	2	3	2	1	1
3	1	1	3	1	3	3	3	3	3
2	1	3	2	2	1	3	1	2	1
2	2	3	1	2	2	2	2	1	1
9	9	9	9	3	3	3	1	3	3
3	2	2	1	2	1	2	2	3	3
2	1	3	2	1	3	3	1	1	1
3	1	2	3	2	1	3	3	3	2
3	3	2	3	1	1	1	2	1	1
1	2	2	2	1	2	1	3	1	1
9	9	9	9	9	9	9	9	9	9
2	2	2	2	3	3	3	3	3	3
9	1	3	2	3	3	1	3	2	1
9	9	9	9	2	2	3	2	3	2
2	3	1	2	2	1	2	1	3	2
1	1	2	1	1	1	2	2	1	9
3	3	3	3	3	3	3	3	3	3
3	2	3	3	3	3	3	3	2	3
3	2	2	3	2	2	1	2	2	2
3	2	2	3	2	2	1	2	2	2
3	2	2	3	2	2	1	2	2	2
3	3	2	3	1	3	1	3	3	1
3	1	1	1	2	1	3	2	2	3
1	2	2	9	3	2	1	9	3	3
2	1	3	2	1	2	1	3	2	1
3	1	3	3	3	3	3	3	1	1
9	9	9	9	9	9	1	9	9	9
1	1	2	2	1	1	1	1	1	1
1	2	2	2	1	2	2	2	2	2

2	3	2	3	2	9	9	9	9	9
9	9	9	9	1	9	9	9	9	9
1	9	9	9	1	9	9	9	9	9
3	3	1	3	1	3	1	3	1	3
1	2	2	2	1	2	2	2	2	2
2	2	2	2	1	2	2	2	2	2
3	1	1	2	1	2	1	2	3	1
2	2	2	2	1	2	2	2	2	2
2	2	2	2	1	2	2	2	2	2
9	9	9	9	1	2	2	2	2	2
2	3	3	2	3	3	3	2	2	9
1	2	1	2	3	3	3	3	2	3
2	2	2	2	1	3	2	1	1	2
1	1	1	3	2	1	1	3	3	2
1	2	1	2	2	1	1	3	3	1
3	3	1	2	2	3	3	3	1	2
3	3	3	1	1	2	3	3	3	3
9	9	9	9	3	9	9	9	9	9
9	9	9	9	3	9	9	9	9	9
2	1	3	3	1	2	1	3	1	1
1	1	2	3	1	3	1	2	1	3
2	2	2	2	2	2	3	2	2	3
9	9	9	9	3	3	3	1	3	3
2	2	2	2	2	2	2	2	2	2
3	3	3	3	3	3	3	3	3	3
9	9	9	9	2	9	9	9	9	9
9	9	9	9	2	9	9	9	9	9
1	3	3	3	3	1	3	3	3	1
9	9	9	9	1	3	3	1	3	3
3	3	3	3	2	2	2	2	2	2
9	9	9	9	2	2	2	3	2	1
2	3	2	1	1	1	1	1	9	2
3	3	1	1	1	3	1	1	1	9
9	9	9	9	2	9	9	9	9	9
2	1	3	2	3	2	2	1	1	3
3	3	3	3	3	1	3	3	3	3
9	2	1	1	2	2	2	2	1	2
9	9	9	9	9	9	9	9	9	9
9	9	9	1	9	9	2	9	9	9
2	2	2	2	2	2	2	2	2	2
9	9	9	9	3	9	9	9	9	9
1	2	2	2	2	2	2	2	2	2
2	2	2	2	2	2	2	2	2	2
2	3	2	3	2	3	3	3	2	2
2	2	2	2	2	2	2	2	2	2
2	2	2	2	2	2	2	2	2	2
1	2	2	2	3	3	3	2	2	2

2	2	2	2	2	2	2	2	2	2
2	2	2	2	3	3	3	2	2	2
9	9	9	9	2	2	2	2	1	2
9	9	9	9	3	3	2	2	1	3
9	9	9	9	9	9	9	9	9	9
1	2	3	3	3	1	1	2	3	1
2	2	2	2	2	2	2	2	2	2
1	9	9	9	9	9	9	9	1	9
1	1	1	1	1	2	3	1	2	2
2	9	9	9	2	9	9	9	9	9
2	2	2	2	2	2	2	2	2	2
1	9	9	9	2	2	3	2	2	2
1	9	9	9	9	9	9	9	9	9
2	2	2	2	2	2	2	2	2	2
1	2	1	1	1	3	2	3	1	1
2	2	2	2	2	2	2	2	2	2
2	2	2	2	2	2	2	2	2	2
3	3	3	3	3	9	2	2	1	2
3	3	1	2	9	9	9	9	9	1
9	9	9	9	3	1	9	3	1	1
9	9	9	9	9	9	9	9	2	9
1	3	1	2	3	3	2	2	3	3
2	2	2	2	2	2	2	2	2	2
2	2	2	2	2	2	2	2	2	2
2	2	2	2	2	2	2	2	2	2
3	2	1	1	2	3	2	3	2	3
3	3	1	3	3	3	3	3	3	3
2	2	2	2	2	2	2	2	2	2
2	2	2	2	2	2	2	2	2	2
3	2	2	2	3	3	3	3	3	1
9	9	9	9	9	9	9	9	9	1
2	2	2	2	3	2	3	2	2	2
3	3	3	3	3	3	3	3	3	3
2	2	2	2	2	2	2	2	2	2
2	2	2	3	3	2	3	3	1	2
3	3	1	3	3	2	3	1	1	3
2	2	2	2	2	2	2	2	2	2
2	2	2	3	1	2	2	2	3	1
2	2	1	2	2	2	2	2	2	2
1	2	2	1	1	2	1	3	1	2
1	1	2	1	3	1	2	2	1	2
2	2	2	2	2	2	2	2	2	2
2	3	3	3	3	3	3	3	3	1
1	1	3	2	2	1	2	3	3	3
2	2	2	2	2	2	2	2	2	2
3	3	3	3	3	3	3	3	3	3
2	2	3	2	2	2	2	3	2	3

9	9	9	9	3	3	2	2	3	2
1	1	3	3	3	3	2	2	2	2
3	3	3	3	1	3	3	3	1	3
2	2	2	2	2	2	2	2	2	2
2	2	2	2	1	2	3	3	2	3
3	3	1	3	3	2	1	2	3	2
2	2	2	2	2	2	2	2	2	2
1	1	2	2	2	1	3	1	3	1
1	9	9	9	1	2	3	1	2	3
2	2	2	2	9	9	9	9	9	9
3	1	2	3	2	1	2	3	1	1
2	2	2	2	2	9	9	9	9	9
1	9	9	9	9	9	9	9	9	9
9	1	1	1	2	2	2	3	2	2
1	3	3	3	1	2	2	2	2	2
1	3	3	2	3	3	3	3	3	2
3	3	3	1	2	2	2	2	2	2
1	1	1	1	3	3	1	3	1	2
2	1	3	2	2	1	3	2	2	1
1	2	2	2	2	2	2	3	2	2
3	3	1	3	9	9	9	9	9	9
1	2	2	1	1	1	1	1	9	1
2	2	2	2	2	2	2	2	2	2
1	2	1	1	3	1	2	3	1	1
1	1	3	3	1	3	1	9	3	3
1	2	9	1	9	9	9	9	1	1
2	2	2	2	2	2	2	2	2	2
9	9	9	9	9	9	9	9	1	9
9	9	9	9	2	2	2	2	2	2
2	3	2	2	3	2	2	2	2	3
1	1	1	1	1	1	1	1	1	1
1	1	1	1	1	2	1	3	1	1
1	1	1	1	1	1	1	2	2	3
1	1	1	1	1	1	1	1	1	2
1	2	1	3	9	9	9	9	9	9
9	1	1	1	3	1	3	1	1	1
9	9	1	9	9	9	9	9	9	9
1	1	1	1	1	1	1	1	1	1
2	2	2	2	2	2	2	2	2	2
2	2	2	2	2	2	2	2	2	2
1	2	3	1	1	1	3	3	3	2
1	2	1	2	1	1	1	1	1	1
1	3	1	2	2	1	1	2	3	2
2	2	2	2	3	1	2	2	1	2
2	2	2	2	2	2	2	2	2	2
3	3	1	1	1	2	3	2	2	3
2	2	2	2	2	3	3	2	3	3

1	1	1	2	1	3	3	1	1	3
2	2	3	3	1	3	3	3	3	3
1	2	1	3	2	2	2	2	3	2
2	9	9	9	9	9	9	9	9	9
1	9	9	9	9	9	9	9	9	9
1	2	3	2	1	2	3	1	3	3
9	9	9	9	9	9	9	9	9	9
9	9	9	9	3	3	3	3	3	1
3	3	3	1	1	1	1	3	3	3
2	2	2	2	2	2	2	2	2	2
1	9	9	9	9	9	9	9	9	9
2	2	3	1	3	1	2	9	3	3
2	2	2	2	2	1	1	2	2	2
3	3	2	2	2	2	1	2	2	2
2	2	2	2	1	2	2	2	2	2
3	1	2	3	2	1	2	3	3	9
2	3	2	2	3	1	2	1	2	1
1	9	9	9	9	9	9	9	9	9
1	3	1	2	2	9	9	9	9	9
2	2	1	1	2	2	1	2	1	1
9	9	9	9	2	2	2	2	2	2
1	2	2	2	2	2	2	9	3	3
1	2	9	2	9	9	9	9	9	9
2	2	2	2	3	3	3	1	1	1
3	2	2	3	1	2	3	9	1	2
9	9	9	9	3	3	3	9	3	3
1	2	3	2	2	3	2	3	2	2
3	1	1	3	3	3	3	3	1	1
1	2	2	2	2	1	3	3	3	3
1	3	2	1	3	2	1	2	1	2
2	1	3	2	2	2	3	3	2	3
3	2	2	3	2	3	2	3	2	2
2	2	1	3	3	3	2	9	1	1
3	3	3	3	3	3	3	3	3	3
9	1	9	9	9	1	9	9	9	9
2	3	3	2	1	2	3	3	3	2
1	9	1	1	1	9	1	9	1	1
3	3	3	3	3	3	3	3	3	3
1	3	9	2	2	9	9	1	9	2
3	3	3	3	3	1	3	3	3	3
1	1	3	1	3	1	3	3	3	3
9	9	9	9	9	9	9	9	9	9
1	1	1	1	1	1	1	1	1	1
1	3	9	3	3	3	3	3	1	3
2	2	2	3	9	9	9	9	9	9
9	9	9	9	2	2	2	2	2	2
1	1	2	2	2	1	3	2	1	1

1	9	9	9	3	1	3	3	3	3
3	3	3	3	2	3	3	3	3	3
2	1	2	1	9	9	9	9	9	9
3	3	3	3	1	3	3	3	3	1
2	2	2	2	2	2	2	2	2	2
1	2	3	2	1	1	3	3	3	2
2	2	2	2	3	3	3	3	3	3
2	2	2	2	2	2	2	2	2	2
2	2	2	2	2	2	2	2	2	2
2	2	2	3	2	2	2	2	2	2
1	2	3	2	2	3	2	2	1	1
3	2	2	2	2	2	2	3	3	2
1	3	3	1	3	1	3	1	3	1
1	2	2	3	2	3	2	3	2	3
9	9	9	9	2	2	2	2	2	2
1	2	3	1	3	3	3	3	3	3
2	3	2	3	3	3	3	3	1	3
2	2	2	2	2	2	2	2	2	2
9	9	9	9	3	2	2	2	3	2
9	9	9	9	9	9	9	9	1	9
9	1	9	9	9	9	9	9	9	9
3	3	3	3	3	3	3	3	3	3
3	3	3	3	3	3	3	3	1	3
1	3	1	1	1	2	3	3	1	3
3	3	3	3	1	3	3	9	1	1
1	9	9	9	3	1	3	3	3	3
9	9	9	9	3	3	3	3	2	2
1	3	1	2	1	2	3	2	3	2
2	3	2	1	2	3	2	3	1	1
3	1	1	3	2	2	1	2	2	2
2	2	2	2	3	3	2	3	1	2
1	1	1	1	3	1	3	1	1	1
2	1	2	3	1	2	1	2	1	2
1	2	2	2	3	1	2	2	3	2
9	9	9	9	9	9	9	9	9	9
3	3	3	3	2	2	2	2	2	2
9	9	9	9	2	9	2	2	2	9
2	2	2	2	2	2	2	2	2	2
9	9	9	1	2	3	2	3	1	3
2	2	2	2	3	3	3	3	1	2
2	2	3	1	1	2	3	2	1	2
2	1	2	1	2	2	2	2	2	2
1	1	1	1	1	1	1	1	1	1
9	2	9	9	2	9	9	9	9	9
9	9	9	9	3	9	9	9	9	9
3	2	9	3	3	3	3	3	3	3
1	2	1	2	3	2	2	3	2	2

3	3	3	3	3	3	3	3	1	1
2	2	1	2	3	3	3	3	3	3
3	3	3	3	3	3	3	3	1	3
2	9	9	9	2	2	1	1	1	1
2	2	2	2	3	3	3	3	3	3
9	9	9	9	3	3	3	3	2	3
2	2	2	2	2	2	2	2	3	2
1	2	3	2	3	3	3	3	3	3
1	3	3	3	3	3	3	3	3	3
3	3	3	3	3	2	1	9	9	9
3	1	2	3	9	9	9	9	9	9
3	3	3	3	9	9	9	9	9	9
9	9	9	9	2	9	9	9	9	9
9	9	9	9	3	9	9	9	9	9
1	1	2	1	9	9	9	9	9	9
1	2	2	2	3	3	3	3	2	2
3	3	2	2	1	1	2	1	1	2
2	1	1	2	2	1	2	1	1	2
1	3	3	3	3	3	3	2	2	3
2	2	2	2	3	3	3	3	1	2
1	2	2	2	2	2	2	2	1	9
2	2	2	3	2	2	1	2	2	1
2	2	2	2	2	2	2	2	2	2
2	2	2	1	3	2	2	3	3	1
2	2	2	1	2	2	2	2	1	2
3	2	1	2	1	1	3	3	2	2
9	9	9	9	3	1	9	1	3	3
2	2	2	2	2	2	2	2	2	2
3	2	1	2	1	1	3	3	2	2
1	2	2	2	3	3	3	3	2	2
2	2	2	2	3	3	3	3	1	2
9	9	9	9	2	9	9	9	9	9
1	3	3	2	3	3	3	3	3	3
2	2	2	2	2	2	2	2	2	2
1	1	3	3	1	1	2	1	1	2
9	9	9	9	3	3	3	3	2	2
9	2	9	9	9	9	9	9	9	9
9	2	9	9	9	9	9	9	9	9
2	1	2	2	3	2	2	3	2	2
1	1	1	1	1	2	2	2	2	2
9	9	9	9	9	9	9	9	9	1
3	2	1	1	1	1	1	1	1	1
1	1	1	1	3	3	3	3	1	3
3	3	3	3	3	3	3	3	2	2
2	3	2	3	3	1	1	3	1	3
1	2	2	2	2	2	2	2	2	2
9	9	9	9	9	9	9	9	9	9

9	9	9	9	3	1	1	1	3	1
2	1	1	1	2	2	2	2	2	2
1	1	2	2	1	9	1	3	2	1
1	9	9	9	9	9	9	9	9	9
1	9	9	9	9	9	9	9	9	9
1	2	1	2	1	9	9	9	9	9
1	2	1	2	1	2	1	2	3	1
2	2	2	2	2	2	2	2	2	1
3	2	3	1	1	2	1	3	1	1
3	3	2	2	1	2	1	2	3	1
1	2	1	2	3	2	3	3	1	1
1	1	2	3	1	3	2	2	1	1
3	9	1	1	3	3	1	3	1	1
2	1	2	2	2	2	2	9	2	2
1	2	2	1	3	2	3	2	1	1
3	3	3	3	3	3	3	3	3	3
3	1	3	3	3	3	3	3	1	3
2	1	2	1	2	2	1	2	2	1
1	2	3	1	1	2	3	3	3	1
2	1	1	1	2	3	3	3	3	1
2	3	3	2	2	2	3	3	2	2
9	1	9	9	2	9	9	9	9	9
1	2	1	2	3	3	3	2	2	2
2	3	3	3	2	2	2	3	2	2
1	2	2	2	2	3	2	2	2	2
1	1	2	1	2	2	2	2	2	2
2	2	2	2	3	2	2	2	2	2
2	2	9	1	3	1	3	3	1	1
1	2	2	2	2	2	2	2	1	2
3	1	3	3	3	3	3	3	1	3
9	9	9	9	9	9	9	9	9	1
2	2	2	2	2	2	2	2	1	2
9	1	9	1	2	2	2	2	1	2
1	1	9	3	2	2	1	3	2	2
2	3	2	3	1	2	3	2	1	3
9	9	9	9	9	9	9	9	9	9
9	9	9	9	9	9	9	9	9	9
2	2	2	2	2	2	2	2	2	2
2	2	1	2	3	3	3	3	1	1
1	2	2	1	1	2	2	1	2	2
2	3	1	2	1	1	1	1	1	1
9	1	9	9	1	9	1	9	1	9
1	1	2	1	1	2	1	1	2	1
2	1	2	3	1	2	1	2	1	2
3	2	3	1	2	1	1	2	3	2
9	9	9	9	2	2	3	1	2	3
2	2	2	2	1	2	3	2	1	2

2	2	1	1	1	2	1	2	2	1
9	9	9	9	9	9	9	9	9	9
2	3	3	3	3	3	3	3	3	3
1	2	2	3	2	2	3	3	2	2
1	2	2	1	1	1	3	1	1	3
2	2	1	1	1	2	1	2	2	1
2	3	2	2	3	2	1	1	2	2
2	2	2	2	3	3	3	2	2	2
2	2	2	2	2	2	2	2	2	2
1	1	3	3	3	1	3	1	2	1
1	3	2	2	9	9	9	9	9	9
2	2	2	2	2	2	2	2	2	2
9	9	9	9	9	9	9	9	9	1
1	2	2	2	2	2	2	1	1	2
3	2	2	2	3	3	2	2	3	3
9	9	9	9	9	9	9	9	9	9
2	3	3	3	3	3	2	3	3	3
9	9	9	9	9	9	9	9	9	9
1	1	2	1	2	2	1	1	2	1
2	2	2	2	3	3	3	3	2	2
1	2	2	2	1	1	3	1	2	1
3	1	3	1	2	1	1	3	3	3
3	2	9	3	3	2	1	3	1	2
9	9	9	9	1	2	2	2	2	2
2	2	2	2	2	2	2	2	2	2
2	2	3	2	3	3	2	2	3	2
2	2	2	2	9	9	9	9	9	9
2	2	2	2	2	2	2	2	2	2
3	1	2	1	3	3	3	3	3	3
1	1	1	1	1	2	3	1	1	9
3	3	1	3	3	2	1	2	1	2
3	2	2	1	1	2	2	2	2	2
9	1	2	3	1	3	3	1	2	2
2	2	2	2	1	2	3	2	1	2
1	2	2	2	1	2	2	2	2	2
2	2	2	2	1	2	3	3	1	2
1	1	2	2	2	1	1	2	1	2
3	3	2	2	3	2	2	2	2	1
1	2	3	1	3	2	2	3	1	1
2	1	2	3	2	2	2	2	1	1
2	3	1	3	3	3	2	1	3	2
3	3	3	3	1	3	9	3	3	3
9	9	9	9	3	3	9	3	3	3
1	9	9	9	9	9	9	9	9	9
1	9	9	9	9	9	9	9	9	9
1	9	9	9	9	9	9	9	9	9
1	9	9	9	9	9	9	9	1	9

1	2	2	2	3	3	3	3	2	2
3	2	2	2	2	3	2	3	2	1
9	9	1	9	9	9	9	9	9	9
2	3	3	3	3	2	3	3	3	2
1	1	1	1	3	1	3	1	1	1
1	9	9	9	1	9	9	9	9	9
1	9	9	9	9	9	9	9	9	1
9	9	9	9	9	9	9	9	9	1
9	9	9	9	1	3	3	9	9	9
1	9	9	9	9	9	9	9	9	9
1	9	9	9	9	9	9	9	9	9
9	9	9	9	1	1	2	3	3	9
9	9	9	9	9	9	9	9	9	9
3	3	1	3	3	3	3	3	3	3
1	1	9	3	3	3	1	3	9	1
1	1	1	1	3	2	2	2	1	2
2	2	2	2	2	2	2	2	1	2
9	9	1	9	9	9	9	9	9	9
1	9	9	9	1	9	9	9	9	9
1	9	9	9	1	9	9	9	9	9
9	9	9	9	1	2	3	1	2	3
9	9	9	9	9	9	9	9	9	9
1	1	1	1	1	2	1	1	1	1
9	9	9	9	9	9	9	9	9	9
2	2	1	1	1	1	1	2	2	2
1	2	2	2	1	1	1	2	2	1
1	2	3	1	3	1	2	2	2	2
1	2	2	2	1	1	1	1	1	2
1	2	2	2	1	1	1	2	2	2
2	2	2	2	2	2	1	1	1	1
1	1	2	2	1	1	2	2	1	1
1	3	3	3	2	2	2	2	1	2
1	2	2	2	3	3	3	3	3	3
1	9	9	9	9	9	9	9	9	1
1	2	2	2	3	1	1	1	1	1
1	2	2	2	2	1	1	1	1	1
1	2	2	2	1	1	1	2	1	1
1	9	9	9	9	9	9	9	9	1
2	3	2	3	3	9	3	2	2	1
1	1	3	1	1	3	3	3	1	3
3	3	3	3	3	3	3	3	3	3
1	2	3	1	1	2	3	2	1	1
3	9	1	3	3	3	3	3	3	3
2	2	2	2	2	2	2	2	2	2
1	3	3	3	1	3	1	1	2	2
1	3	2	3	2	2	2	2	2	2
2	2	2	3	3	2	2	2	1	1

3	2	1	3	3	2	3	3	1	3
2	3	1	3	2	1	3	2	2	3
9	9	9	9	3	3	3	3	3	2
1	2	2	3	2	2	3	3	1	2
3	3	3	3	3	3	3	3	1	2
1	9	9	9	9	2	9	9	9	9
3	2	3	2	3	3	3	3	1	2
2	2	2	1	2	1	2	2	1	1
1	9	9	9	9	2	9	9	9	9
1	2	2	3	2	3	2	1	2	3
1	9	9	9	9	2	9	9	9	9
2	1	1	3	3	3	3	3	1	3
2	2	2	2	1	1	1	1	1	1
2	2	2	1	3	3	3	3	3	2
1	3	1	1	3	3	3	3	3	1
1	2	3	3	1	2	1	3	1	2
2	1	2	2	2	2	2	2	1	1
1	2	2	2	2	2	2	2	2	2
1	1	3	1	3	1	1	3	3	3
9	1	9	9	9	9	9	9	9	9
3	3	1	2	3	1	1	3	2	1
2	2	2	2	2	2	1	3	2	2
2	2	2	2	2	2	2	2	2	2
2	2	2	2	3	2	1	1	1	1
2	1	2	2	3	2	2	2	2	2
3	3	3	3	1	1	1	2	2	1
1	3	3	3	1	1	1	1	1	1
1	1	1	1	2	3	3	2	3	2
2	2	2	2	2	2	2	2	2	1
3	1	1	3	3	3	3	3	9	9
1	2	2	1	1	2	2	3	3	1
1	2	2	2	1	2	1	1	1	1
1	1	1	2	2	2	1	1	1	2
1	1	2	1	2	2	2	2	2	2
2	2	2	2	2	2	1	1	2	2
2	2	2	2	3	3	3	3	3	3
2	2	2	2	2	2	2	2	2	2
1	1	1	1	2	2	2	2	1	1
2	2	2	2	2	2	2	2	1	1
2	2	2	2	2	2	2	2	3	3
3	1	3	3	2	3	2	2	2	2
2	2	2	2	3	3	3	3	2	3
2	1	1	2	3	3	1	2	1	1
1	1	1	1	1	2	2	1	2	2
2	3	3	3	9	2	1	2	2	2
3	3	3	3	3	3	3	3	3	3
2	2	2	2	2	2	2	2	2	2

1	2	2	2	3	2	2	2	3	2
1	1	2	1	3	3	3	3	3	9
9	9	1	9	1	9	9	9	9	9
9	9	9	9	9	9	9	9	9	9
9	1	9	9	9	9	9	9	9	9
1	1	9	9	1	1	2	2	2	2
9	9	9	9	9	9	9	9	9	9
2	2	2	2	3	2	2	2	1	2
2	2	2	2	2	2	2	2	2	1
1	1	3	1	2	2	2	2	1	2
9	9	9	9	2	2	2	2	2	2
1	3	1	2	2	2	2	2	1	1
1	1	1	2	1	1	1	1	2	1
2	2	2	2	2	2	2	2	2	2
1	3	1	1	1	1	1	2	2	2
9	9	9	9	2	2	2	2	2	2
2	2	2	2	2	2	2	2	2	2
1	3	9	3	3	3	3	3	3	3
9	9	9	9	9	2	9	2	1	3
2	2	2	2	2	2	2	2	2	2
1	1	1	1	2	2	2	2	2	2
2	1	2	3	2	2	2	2	1	1
2	2	2	2	2	2	2	2	2	2
2	2	2	2	3	3	3	9	2	2
1	2	2	2	2	1	1	1	1	1
2	2	2	2	2	2	2	2	2	2
9	9	9	9	9	9	9	9	9	9
2	1	2	2	3	3	3	3	3	3
1	3	2	2	2	3	2	2	3	3
2	2	2	2	1	1	2	2	2	1
9	9	9	9	9	9	9	9	9	9
1	1	2	3	2	1	2	1	3	1
1	3	1	2	1	3	1	2	1	3
2	2	2	2	3	2	3	3	2	3
2	2	3	3	2	2	2	2	2	3
2	3	1	1	3	2	2	1	1	2
3	3	3	3	1	1	3	3	3	3
1	2	1	3	3	2	3	2	1	1
2	1	2	1	3	2	2	3	2	1
1	1	2	2	1	3	1	3	2	1
2	1	2	2	2	2	2	2	2	2
2	2	9	2	2	2	2	2	3	1
2	2	3	1	2	2	2	2	2	2
9	9	9	1	9	9	9	9	9	9
9	9	9	1	1	9	9	9	9	9
2	2	3	1	2	9	9	9	9	9
2	2	2	2	9	9	9	9	3	3

3	2	3	2	3	2	3	3	1	1
1	2	2	1	2	2	2	2	2	2
1	9	9	9	9	9	9	9	9	9
9	9	9	9	1	3	1	2	3	1
9	9	9	9	3	3	1	2	3	3
2	3	2	1	3	2	2	2	2	2
1	2	2	2	2	2	2	2	2	2
2	2	2	2	2	2	2	2	2	2
3	1	2	1	3	2	3	3	3	2
9	2	3	1	3	3	3	1	3	1
9	1	9	9	9	9	9	9	1	9
9	9	9	9	9	9	9	9	9	9
2	2	2	2	2	2	2	2	2	2
9	9	9	9	9	9	9	9	1	9
1	9	9	9	9	9	9	9	9	9
3	3	1	1	3	2	1	3	3	3
2	1	2	3	2	3	2	1	1	2
2	1	1	3	3	3	1	3	3	3
2	3	1	3	1	1	1	1	1	1
2	1	2	2	2	3	2	2	2	3
2	2	3	1	1	1	3	3	2	2
3	1	1	3	3	3	1	3	1	1
1	2	3	1	1	3	3	1	3	3
2	1	2	2	2	3	3	3	1	2
2	2	2	2	2	2	2	2	2	2
1	2	9	2	1	1	9	9	9	1
9	9	1	9	2	3	3	3	1	2
1	3	1	1	2	1	3	3	2	1
9	9	9	9	1	2	3	3	3	2
1	2	2	3	1	2	2	2	3	2
1	2	3	2	1	3	3	3	1	1
1	1	1	3	2	2	3	2	2	2
1	3	1	1	1	2	2	3	1	1
2	2	1	1	3	2	3	2	3	3
1	2	2	2	2	2	2	2	2	2
1	2	2	2	2	2	2	2	2	2
1	2	1	2	2	1	1	3	3	2
2	2	3	3	2	2	2	2	2	2
9	9	9	9	9	1	9	9	9	9
3	3	2	3	3	2	3	2	3	2
9	2	9	9	9	9	9	1	9	9
2	1	2	2	1	2	2	2	1	2
2	9	9	9	1	9	9	9	9	9
2	9	9	9	1	9	9	9	9	9
2	2	2	2	3	2	3	1	2	3
1	3	1	3	1	3	3	1	1	3
3	3	3	3	3	3	3	3	3	3

3	3	3	3	3	3	3	3	3	3
9	9	9	9	9	9	9	9	9	9
9	9	1	9	3	3	3	3	3	3
3	1	3	3	1	1	1	3	1	1
1	3	1	3	1	3	3	1	3	3
3	1	1	3	1	3	1	3	1	3
3	2	9	2	2	2	2	3	2	3
1	2	1	1	2	1	1	2	2	2
3	1	3	3	1	3	1	3	3	3
1	9	1	3	1	1	3	3	3	3
1	3	1	3	1	3	3	3	1	1
2	3	2	1	1	3	1	2	3	2
2	3	2	2	2	3	2	3	1	2
3	1	3	3	1	3	1	2	3	1
2	1	1	2	1	2	2	2	2	1
2	2	2	2	2	3	1	3	2	3
3	3	2	2	3	2	3	2	3	2
1	2	2	2	2	3	3	2	1	1
1	3	3	3	1	3	3	3	3	3
2	2	2	2	2	2	2	2	2	2
3	3	2	1	2	2	2	2	2	2
1	2	3	2	3	2	2	2	2	1
1	2	2	2	3	3	3	3	3	3
2	2	2	2	3	2	2	2	2	3
2	2	1	2	1	1	1	2	2	1
2	1	2	2	2	1	2	1	2	2
2	2	2	2	3	2	3	9	1	2
2	1	1	3	3	3	2	2	3	2
1	1	1	3	2	3	1	3	1	3
9	9	9	9	1	9	9	9	9	9
3	2	3	3	3	2	2	3	2	1
3	3	3	3	1	3	3	3	2	1
2	3	3	3	1	1	2	3	1	2
3	3	3	3	2	1	2	2	1	2
1	1	1	1	1	1	3	3	1	1
2	2	2	2	2	2	2	2	2	2
3	3	3	3	3	2	2	2	3	3
2	2	3	2	1	3	2	3	2	3
2	2	1	3	2	1	2	3	1	1
2	2	2	2	2	2	2	2	2	2
1	9	9	9	9	9	9	9	9	9
3	3	3	3	9	9	9	9	9	9
3	1	2	3	1	2	3	1	2	1
3	3	3	3	1	3	2	2	1	2
2	2	2	2	1	2	2	2	2	2
3	2	2	2	3	3	2	9	1	2
1	2	2	3	9	9	9	9	9	9

2	3	2	3	1	2	3	3	1	2
1	2	3	2	3	3	3	3	3	3
3	2	2	3	2	2	1	2	3	2
1	2	3	1	2	1	2	1	1	2
2	1	1	2	9	9	9	9	9	9
1	3	3	3	1	1	1	1	1	1
2	2	2	2	2	2	3	2	2	2
2	2	2	2	2	2	2	2	2	2
2	2	2	2	1	2	3	2	2	1
2	1	2	2	2	1	2	2	2	2
9	9	9	9	3	9	9	9	9	9
2	2	3	2	1	2	3	3	3	2
9	9	9	9	1	2	3	3	2	2
9	1	3	1	9	9	9	9	9	9
2	2	2	2	2	2	2	2	3	3
9	9	9	9	9	9	9	9	9	9
2	2	3	3	3	2	2	2	2	3
2	2	1	1	1	1	3	3	3	3
3	1	3	1	3	3	3	2	1	1
3	3	3	3	2	2	2	2	2	2
2	1	1	3	3	3	3	3	1	3
2	2	2	2	2	2	2	2	2	2
9	9	9	9	9	9	9	9	9	9
2	2	2	2	1	3	2	2	2	2
3	2	1	3	2	2	2	2	1	2
2	3	2	2	2	2	2	2	2	2
9	9	9	9	1	9	9	9	9	9
2	3	2	3	2	2	3	2	2	2
2	2	1	2	2	2	3	3	3	3
9	9	9	9	9	9	9	9	1	9
1	1	2	3	2	2	3	3	3	2
2	2	2	1	2	2	2	2	2	3
9	9	9	9	1	3	3	2	3	3
2	2	2	2	2	2	3	3	1	2
3	3	2	2	2	2	3	3	2	2
2	2	2	2	1	2	3	1	1	2
2	2	1	2	2	2	2	2	2	2
3	3	3	2	3	3	3	3	3	3
9	1	9	9	9	9	9	9	9	9
3	2	3	3	3	3	3	3	3	3
1	2	3	2	2	1	2	3	2	3
3	2	1	2	3	2	3	3	2	2
2	2	2	2	2	2	3	3	2	2
3	2	2	1	3	2	3	2	1	1
2	2	2	3	2	2	1	3	1	1
9	9	1	9	2	2	1	1	2	2
2	2	2	2	2	2	3	3	2	2

3	3	2	3	3	3	3	3	3	3
2	2	2	2	2	2	2	2	1	2
9	9	9	9	2	2	2	2	1	1
9	1	9	9	9	9	9	9	9	9
2	2	2	2	2	2	2	2	2	2
3	3	3	3	9	9	9	9	9	9
2	2	2	2	2	2	2	2	2	2
3	1	1	3	3	3	3	3	2	2
3	2	3	2	2	2	3	3	3	2
9	9	9	9	2	2	3	2	3	2
9	1	1	2	3	3	2	2	2	3
1	2	3	1	2	2	1	2	3	2
2	2	2	2	2	2	2	2	2	2
3	3	3	3	3	3	3	3	3	3
1	2	1	3	2	1	2	1	3	2
2	2	1	3	2	2	3	3	2	2
3	2	3	3	3	3	1	3	3	2
3	1	1	3	2	2	2	2	2	2
2	2	2	2	2	2	2	2	2	2
3	2	3	2	2	2	2	2	2	2
2	2	2	2	3	2	2	2	2	2
2	2	1	2	2	2	3	3	2	3
2	2	1	2	2	2	3	3	2	2
3	3	1	3	2	2	2	2	2	2
2	2	2	2	3	3	3	3	1	1
3	3	1	2	1	2	3	2	3	3
1	1	2	1	1	2	1	3	2	3
1	1	2	3	1	2	1	3	2	3
1	2	3	2	3	3	2	2	2	2
2	2	2	2	2	1	2	2	1	2
3	3	3	3	3	2	3	2	2	3
3	3	2	2	2	2	3	3	2	3
3	2	2	2	3	2	3	3	2	3
2	2	2	2	2	2	2	2	2	2
2	2	2	2	3	3	3	3	3	3
3	3	3	3	2	2	2	2	2	2
2	2	2	2	2	2	2	2	2	2
1	3	2	1	1	2	2	3	2	2
3	3	3	3	2	2	3	2	2	1
2	2	3	2	2	2	2	3	2	2
3	3	3	3	2	2	3	3	1	1
3	3	2	3	2	3	3	2	3	3
1	1	2	3	1	1	1	1	1	1
2	2	2	2	1	1	2	2	1	2
2	2	2	3	2	2	3	3	2	3
2	3	3	3	3	3	3	3	9	2
9	9	9	9	9	9	9	9	9	9

1	3	2	3	3	2	1	2	3	3
2	3	2	2	2	2	1	1	2	3
2	2	2	2	9	9	9	9	9	9
2	2	2	2	2	2	1	1	1	1
2	2	2	2	2	2	2	2	2	2
2	2	2	2	2	2	2	2	2	2
1	1	1	2	2	2	3	3	2	1
9	9	9	9	1	2	2	1	1	3
9	9	9	9	2	2	2	2	2	3
2	2	2	2	2	2	2	2	2	2
2	2	2	2	2	2	2	2	2	2
2	2	2	2	2	2	2	2	2	2
2	2	2	2	2	2	2	2	2	2
2	2	2	2	2	2	2	2	2	2
3	3	3	3	2	2	2	2	2	2
9	9	9	9	2	2	2	2	2	2
2	9	9	9	2	2	2	2	2	3
9	9	9	9	2	2	2	2	2	2
9	9	9	9	2	2	3	3	3	3
1	1	2	1	2	2	3	2	3	1
2	2	2	2	2	2	2	2	2	2
2	2	2	2	2	2	2	2	2	2
2	2	2	2	3	3	3	3	2	2
2	2	2	2	2	2	2	2	2	2
9	9	9	9	2	2	2	2	2	2
9	9	9	9	9	9	9	9	9	9
2	2	2	2	2	2	2	2	2	2
2	2	2	2	2	2	2	2	2	2
2	2	2	2	2	2	2	2	2	2
1	1	2	1	3	2	3	3	3	2
2	3	2	2	3	2	2	2	2	2
2	2	2	2	2	2	2	2	2	2
2	2	2	2	2	2	2	2	2	2
2	2	2	2	2	2	2	2	2	2
2	2	2	2	9	9	9	9	9	9
2	2	2	2	1	2	2	2	2	2
2	2	2	2	2	2	2	2	2	2
2	2	2	2	2	2	2	2	2	2
2	2	2	2	2	2	2	2	2	2
9	9	9	9	2	2	2	2	2	2
9	9	9	9	3	3	2	2	2	2
2	2	2	2	2	2	2	2	2	2
9	9	9	9	2	2	2	2	2	2
2	2	2	2	2	2	2	2	2	2
2	2	2	2	2	2	2	2	2	2
2	3	2	2	2	2	2	2	2	3
2	2	2	2	2	2	2	2	2	2

2	2	2	2	2	2	2	2	2	2
9	9	9	9	2	2	2	2	2	2
2	2	2	2	2	3	3	2	1	1
2	2	2	2	2	2	2	2	2	2
2	2	2	2	2	2	2	2	2	2
9	9	9	9	2	2	3	3	3	3
1	2	2	3	9	9	9	9	9	9
2	9	9	9	9	9	9	9	9	9
2	2	2	3	3	1	3	3	1	3
2	2	1	2	3	2	2	2	3	2
2	3	2	3	2	2	2	2	2	2
2	2	2	2	2	2	2	2	2	2
9	9	9	9	2	2	2	2	2	2
1	9	9	9	9	9	1	9	9	9
3	3	3	3	3	3	3	3	3	3
2	2	2	2	2	2	2	2	2	2
2	2	2	2	2	2	2	2	2	2
2	2	2	2	2	2	3	9	9	9
2	2	2	2	9	9	9	9	9	9
2	2	2	2	2	2	2	1	2	2
9	9	9	9	2	9	9	9	9	9
2	2	2	2	2	2	2	2	2	2
9	9	9	9	2	2	2	2	2	2
9	2	2	2	2	2	2	2	2	2
2	2	2	2	2	2	2	2	2	2
2	2	2	2	2	2	2	2	2	2
9	9	9	9	9	9	9	9	9	9
2	2	2	2	2	2	2	2	2	2
2	2	2	2	2	2	2	2	2	2
9	9	9	9	2	1	2	9	9	2
2	2	2	2	2	2	2	2	2	2
2	1	3	3	2	3	1	2	2	3
2	2	2	1	3	3	1	2	3	1
1	1	1	1	2	2	2	2	2	2
2	3	3	2	3	3	3	3	1	1
3	1	3	1	3	3	1	1	2	1
2	2	3	3	3	3	2	3	1	3
2	2	2	2	1	2	1	2	1	2
2	2	1	1	1	1	3	3	1	2
2	2	1	2	2	2	2	2	2	2
2	2	2	2	3	3	3	3	1	3
9	9	1	9	2	2	2	1	2	2
2	1	2	1	1	1	3	3	2	2
2	2	2	2	2	2	2	2	2	2
1	2	1	3	1	1	1	9	1	1
2	2	2	2	2	2	2	2	2	2
2	2	2	2	1	2	2	1	1	2

3	1	2	3	2	3	1	2	1	3
2	1	2	1	1	9	3	1	2	1
1	1	1	1	3	1	2	3	1	2
1	2	1	2	1	3	3	3	2	1
3	2	1	2	9	9	9	9	9	9
1	2	1	3	1	2	3	3	1	2
2	3	1	2	2	1	3	2	3	1
9	9	9	9	2	9	9	9	9	9
2	2	2	2	2	2	2	2	2	2
2	2	2	2	2	2	2	2	2	2
2	2	2	2	3	3	3	3	3	3
2	2	2	2	3	3	3	3	3	3
3	3	3	3	2	2	2	2	2	2
2	2	2	2	2	2	2	2	2	2
1	2	1	1	3	3	3	3	2	2
2	2	2	2	2	2	2	2	2	2
2	2	2	2	2	2	2	2	2	2
3	3	3	3	3	3	3	3	3	3
9	9	9	9	3	3	3	3	2	2
2	1	2	1	1	1	3	2	2	3
9	9	9	9	3	3	2	3	2	1
9	9	9	9	9	9	9	9	9	9
1	1	1	2	2	2	1	3	3	1
9	9	9	9	9	9	9	9	9	9
2	3	1	2	1	2	2	1	3	1
1	2	2	2	2	2	1	1	1	2
2	2	2	2	1	3	3	3	2	2
9	9	9	9	2	9	9	9	9	9
9	9	9	9	2	2	2	2	2	2
9	9	9	9	9	9	9	9	9	9
9	9	9	9	3	3	3	2	3	3
1	2	2	2	9	9	9	9	9	9
1	1	2	1	9	9	9	9	9	9
1	1	2	1	1	2	2	2	1	2
1	1	2	1	1	2	2	2	1	2
2	2	2	2	2	9	9	9	9	9
2	3	1	2	9	9	9	9	9	9
1	1	2	1	1	2	2	2	1	2
1	2	2	1	2	2	2	2	2	2
9	9	9	9	2	2	2	2	2	2
2	2	2	2	2	9	9	9	9	9
2	2	2	2	2	2	2	2	2	2
2	2	2	2	2	2	2	2	2	2
9	9	9	9	9	9	9	9	9	9
2	9	9	9	3	9	9	9	9	9
1	2	2	1	1	1	1	1	1	2
2	2	2	2	2	2	2	2	2	2

2	2	1	1	9	9	9	9	9	9
9	9	9	9	1	2	2	1	1	3
1	3	3	3	3	3	3	3	3	3
2	9	9	9	2	3	1	3	2	1
2	2	2	2	2	2	2	2	2	2
9	9	9	9	3	3	3	3	3	3
2	2	2	2	2	2	2	2	2	2
2	2	2	1	9	9	9	9	9	9
1	9	9	9	9	9	9	9	9	9
2	2	2	2	9	9	9	9	9	9
2	2	1	1	1	1	1	1	1	2
2	2	2	1	2	3	3	2	2	2
2	3	3	3	3	2	2	2	2	2
3	3	3	3	3	3	3	3	3	3
1	3	1	3	3	3	2	2	1	1
2	1	2	2	3	2	3	3	1	2
1	1	2	1	1	2	3	3	3	1
3	1	2	1	3	3	2	3	1	2
2	2	2	1	2	2	2	1	2	2
2	1	3	1	1	1	3	1	3	1
3	1	2	1	1	3	2	1	2	1
1	1	3	3	1	3	3	3	3	3
9	9	9	9	3	2	3	2	1	2
3	3	3	3	3	3	3	3	2	1
1	2	2	1	3	1	2	1	3	3
2	2	2	2	3	2	2	2	2	2
2	2	2	2	2	2	3	3	2	2
9	1	1	2	2	2	3	3	1	2
3	2	3	3	2	1	3	2	3	2
1	3	3	3	3	1	3	3	3	3
1	2	2	2	1	2	3	2	2	3
1	2	1	1	3	3	3	3	3	3
2	2	2	1	1	3	1	3	1	1
3	3	3	3	3	3	3	3	3	3
2	2	2	1	2	3	3	3	3	3
2	3	2	1	2	2	3	1	2	1
3	3	1	3	3	2	1	3	3	3
2	2	2	2	2	2	2	2	2	2
2	1	1	2	1	2	2	3	1	2
9	1	9	9	9	9	9	9	9	9
1	3	3	2	3	3	3	3	3	3
2	3	3	1	2	1	2	3	1	2
3	1	2	2	2	2	3	3	2	3
1	1	2	3	3	3	2	2	1	1
2	1	1	2	2	1	3	3	2	1
3	3	3	3	3	2	3	2	3	2
9	9	9	9	3	2	3	3	3	3

9	9	9	9	1	2	1	2	2	2
9	9	9	9	3	3	2	3	2	1
3	3	3	3	3	3	3	3	3	3
3	3	3	3	3	3	3	3	3	1
1	2	1	2	3	3	3	3	1	1
1	2	2	2	3	3	3	3	1	2
9	9	9	9	1	1	1	1	1	1
2	2	2	2	3	2	2	2	2	2
3	3	1	3	1	3	1	1	3	3
1	2	1	2	2	2	3	3	1	2
3	3	2	3	3	1	3	9	1	1
2	2	2	2	2	2	2	2	2	2
2	2	2	2	2	2	3	2	2	2
1	1	3	1	1	1	1	3	3	3
1	2	2	1	2	2	2	3	2	1
2	2	2	2	2	2	2	2	2	2
2	3	2	2	3	2	2	2	2	1
2	2	2	2	2	2	2	2	1	2
1	2	3	3	3	2	1	3	2	1
2	1	1	2	1	2	3	2	9	9
2	2	3	3	3	3	3	3	2	2
1	9	9	9	9	9	9	9	9	9
3	2	2	2	2	3	2	2	2	3
2	2	2	2	3	3	2	3	3	3
3	3	2	3	3	3	3	3	2	2
2	2	2	1	1	2	2	2	2	2
3	2	2	1	2	2	2	2	2	2
2	2	2	2	2	2	2	2	2	2
3	3	3	3	3	3	3	3	3	3
1	1	1	1	2	2	3	3	1	2
1	1	2	1	1	1	3	3	3	3
1	2	3	1	3	3	2	9	1	3
2	3	2	3	3	2	3	1	3	2
2	3	1	3	2	2	2	2	2	2
1	2	3	1	1	2	3	2	1	1
2	2	2	2	2	2	2	2	2	2
2	2	1	2	3	2	1	3	1	3
1	2	1	1	3	2	3	2	1	2
2	2	1	2	1	2	2	3	9	2
9	9	9	9	2	9	9	9	9	9
2	1	2	2	2	2	2	1	3	3
1	2	2	1	2	3	3	1	1	2
2	2	2	2	2	1	2	2	2	2
1	2	1	2	3	2	2	2	9	1
1	2	3	2	2	3	2	3	3	3
1	3	2	3	9	9	9	1	9	9
1	2	2	1	2	1	3	3	3	3

1	2	2	1	2	1	3	3	2	3
2	2	2	2	2	2	2	2	2	2
1	3	3	3	1	3	3	2	3	3
2	1	3	2	2	1	3	2	1	3
2	2	2	2	3	3	3	3	2	2
3	3	1	1	3	1	3	3	9	1
2	2	1	2	2	2	2	2	1	2
9	1	9	9	1	9	9	9	9	1
2	2	2	2	1	3	2	3	2	2
2	3	1	2	2	1	3	2	3	2
2	2	2	2	2	2	2	2	2	2
3	2	2	1	2	2	2	3	3	2
9	9	9	1	9	9	9	9	9	9
1	1	9	2	2	3	3	2	1	1
9	1	9	9	2	9	2	2	9	9
1	2	2	3	2	1	3	2	2	2
1	1	1	1	9	1	1	1	1	1
2	1	2	2	2	2	1	1	1	2
1	3	2	1	1	3	3	2	1	3
2	3	1	2	2	3	2	3	3	2
1	2	1	1	3	1	3	3	3	1
2	2	2	2	3	2	2	2	2	2
2	2	2	2	2	2	1	2	2	2
2	2	3	2	3	3	3	2	2	2
3	3	1	3	9	9	9	9	1	9
2	1	3	3	1	2	2	2	1	1
9	3	2	1	1	2	2	1	3	3
2	1	2	1	2	2	1	2	1	2
3	3	3	3	2	2	3	2	2	2
2	2	2	2	3	2	2	2	1	1
2	2	1	1	1	2	9	1	2	1
1	1	2	2	2	2	2	2	2	2
1	1	2	1	1	1	1	1	1	2
2	1	2	2	1	1	1	1	1	2
1	3	1	2	1	1	2	3	1	2
1	2	3	2	9	9	9	9	1	9
2	2	2	2	1	1	1	1	2	2
3	3	1	3	3	3	3	3	3	1
1	1	2	2	1	2	1	1	1	3
2	2	1	1	1	1	2	2	2	1
2	3	3	3	3	3	3	3	3	3
3	3	3	3	1	1	2	2	1	1
1	3	2	1	1	3	1	1	1	1
2	2	2	2	2	3	3	3	2	3
2	2	2	3	2	3	2	3	3	3
3	3	3	3	3	3	3	3	3	3
2	2	2	2	2	2	2	2	2	2

2	1	3	3	2	3	1	2	2	1
1	2	3	3	3	1	3	3	3	3
2	2	2	2	2	2	2	2	2	2
2	1	2	2	1	2	3	3	2	1
3	3	3	3	1	3	3	3	3	3
2	3	1	2	1	2	1	2	3	1
3	3	3	3	9	1	9	9	9	9
3	3	2	2	2	2	1	1	1	2

FamilyBrea	EarlyInterc	UTI	BSEnoneec	BSEnecessa	BSEshy	BSEpainful	BSEfearofC	BSEtimewa	BSEdirty
1	1	1	1	1	1	2	3	2	1
3	1	1	1	1	1	1	3	2	1
2	2	2	1	1	3	3	3	3	3
9	1	1	9	1	1	1	3	2	1
9	9	9	1	1	1	9	9	9	9
3	1	1	1	1	1	1	3	2	1
2	1	2	3	1	3	3	3	3	3
1	3	9	1	2	3	1	3	3	3
9	9	9	1	9	3	2	2	3	3
9	9	9	3	9	9	9	9	9	9
9	9	9	2	2	2	2	2	9	9
9	1	9	2	2	1	1	1	1	1
2	2	2	1	1	3	3	3	3	3
3	3	2	2	1	3	2	2	2	3
3	3	2	1	9	3	3	3	3	3
1	3	2	2	1	3	3	3	3	3
2	3	2	3	2	9	1	1	3	2
1	3	2	2	1	3	3	3	3	3
1	3	2	2	1	3	1	3	3	3
3	2	2	3	1	3	3	2	1	1
2	3	3	1	1	3	3	3	3	3
3	3	3	1	3	3	3	3	3	3
3	9	9	2	1	3	3	2	3	3
2	3	3	1	1	3	3	3	3	3
2	2	2	1	1	3	3	2	3	3
2	2	2	1	1	2	1	2	3	2
2	2	2	1	1	3	1	3	3	3
2	1	2	3	1	3	1	3	3	3
3	2	3	3	1	3	3	1	3	3
2	2	2	1	2	1	2	2	2	2
2	2	2	1	2	1	2	2	2	2
3	2	2	3	1	3	3	3	3	3
1	2	1	3	1	3	3	3	3	3
3	2	2	3	1	3	3	3	3	3
3	2	2	3	1	3	3	3	3	3
3	2	2	3	9	3	3	3	3	3
3	2	2	3	1	3	3	3	3	3
1	1	3	3	1	3	3	3	3	3
9	9	9	9	9	9	9	9	9	9
9	9	9	3	1	3	3	1	3	3
1	2	2	2	1	1	3	3	3	3
9	9	9	1	2	3	3	2	3	2
3	3	3	3	1	3	3	9	3	3
3	3	2	9	1	3	9	9	3	3
3	1	3	3	1	3	3	2	3	3
3	1	3	3	1	3	3	3	3	3

3	1	1	3	1	3	2	3	3	3
9	9	9	3	1	1	3	3	3	3
3	3	2	9	1	3	3	3	3	3
3	1	1	2	1	1	2	1	3	3
9	9	9	3	1	3	1	3	3	3
3	3	1	3	1	3	3	3	3	3
3	3	3	3	1	3	3	3	3	3
9	9	9	1	1	3	3	9	3	3
9	9	9	9	1	9	9	9	9	9
3	1	2	2	1	1	2	3	3	2
9	9	9	1	9	9	9	9	9	9
9	9	9	2	9	3	3	3	3	3
3	2	1	2	1	3	3	1	3	3
9	9	9	2	1	3	3	2	3	3
2	1	2	1	1	3	3	3	3	3
3	3	3	2	1	3	3	3	3	3
3	2	2	3	1	3	3	9	3	3
9	9	9	3	1	3	3	3	3	3
3	3	3	3	1	3	3	2	3	3
1	3	3	1	1	1	3	1	1	2
3	3	3	3	3	3	1	3	3	1
3	3	3	3	3	3	1	3	3	1
3	3	3	3	3	3	1	3	3	1
9	9	3	3	1	3	3	3	3	3
3	1	3	9	1	9	1	9	9	9
9	1	2	9	1	1	9	1	9	9
9	9	9	3	1	3	3	3	3	3
3	1	1	1	1	3	1	2	3	3
1	1	3	3	1	3	3	3	3	3
2	1	1	3	1	3	3	1	2	3
1	1	2	9	1	1	1	1	1	9
3	3	1	1	1	3	3	3	3	3
2	1	9	9	1	1	2	2	2	3
1	1	1	3	2	3	3	3	3	3
1	2	1	3	1	1	2	3	3	3
9	9	9	2	1	3	3	3	3	3
1	3	3	3	3	3	1	1	1	1
2	1	2	3	1	3	3	3	3	3
9	9	9	3	1	3	3	3	3	3
3	1	1	3	1	3	3	3	3	3
1	1	2	9	1	3	2	3	3	3
3	2	1	1	1	3	3	3	3	3
3	3	1	1	1	3	3	3	3	3
1	2	1	3	1	3	1	3	3	3
3	2	1	3	1	3	3	2	3	3
9	9	9	1	1	3	3	3	3	3
9	9	9	1	1	3	3	1	1	1

3	3	2	3	1	3	3	3	3	3
2	2	2	3	1	3	1	3	3	3
9	9	9	3	1	3	1	1	3	1
3	2	2	3	1	3	1	3	3	3
2	2	2	3	1	3	3	3	3	3
2	2	2	2	1	9	9	9	9	9
3	2	2	3	1	3	3	3	3	3
3	1	2	3	1	3	3	3	1	3
3	2	2	9	1	9	9	9	9	9
3	1	9	2	1	3	3	3	3	3
2	1	1	1	1	1	1	3	2	3
2	3	2	3	1	3	3	2	3	3
2	3	3	9	9	9	9	9	9	9
9	9	9	9	9	9	9	9	9	9
3	1	2	3	1	3	1	2	3	1
9	9	9	1	1	3	3	2	3	3
3	2	2	3	1	3	2	3	3	3
2	2	2	3	1	3	2	3	3	2
2	2	2	3	3	3	3	3	3	3
2	2	2	3	1	3	2	2	2	1
3	1	2	3	1	3	3	3	3	9
9	9	9	1	1	3	3	3	3	3
2	2	2	3	2	2	2	2	2	2
1	9	1	1	1	3	3	3	3	1
2	2	3	1	3	1	9	9	1	2
2	2	2	3	1	2	2	2	2	2
9	9	9	3	1	3	3	3	3	3
9	9	9	3	9	9	9	9	9	9
2	3	2	3	1	3	3	3	3	3
9	9	9	1	1	3	2	2	2	2
2	2	2	2	1	1	2	2	3	2
2	2	2	1	1	2	1	2	2	1
3	2	2	2	2	3	1	3	1	2
2	3	3	3	1	3	3	1	3	3
9	9	9	1	1	3	2	2	2	2
3	3	3	3	1	1	3	3	1	3
1	2	3	3	1	3	3	1	3	2
2	2	2	3	2	3	2	3	3	1
9	9	9	1	9	9	9	9	9	9
1	1	1	1	1	3	3	3	3	3
2	3	2	2	1	3	3	3	3	3
2	3	2	2	1	3	3	3	3	3
2	2	2	1	1	2	1	2	2	1
2	2	2	3	3	3	2	3	1	1
2	2	2	1	1	3	3	1	3	2
3	2	2	3	1	3	1	1	3	3
1	2	2	1	1	3	3	1	3	1

9	9	1	2	2	1	9	9	9	9
3	2	2	3	1	3	3	3	3	3
3	3	2	3	1	3	1	3	3	3
2	3	2	2	1	3	3	3	3	3
2	3	2	2	2	2	1	3	3	3
2	1	2	2	1	9	1	3	3	3
2	3	1	3	1	2	1	1	3	1
2	2	2	3	1	3	3	3	3	3
2	2	2	1	1	9	2	2	3	2
2	2	2	2	1	2	2	2	2	2
3	2	2	3	1	3	3	2	2	3
3	2	9	3	1	3	3	2	2	3
9	9	9	9	1	9	1	9	9	9
2	2	2	3	1	3	3	3	3	3
3	3	2	3	1	3	1	3	3	3
3	3	2	1	2	3	1	1	1	1
2	2	2	1	2	3	1	3	1	2
2	3	2	1	1	3	3	1	1	3
1	9	9	1	9	9	9	9	9	9
2	1	1	2	1	3	3	1	3	3
9	1	9	3	1	3	3	1	3	3
3	1	1	3	1	3	3	1	3	3
2	1	1	1	1	3	3	3	3	3
3	1	3	2	1	3	3	1	3	3
3	1	3	2	1	3	3	3	3	3
9	1	9	3	1	1	3	1	3	3
1	1	1	2	1	3	3	1	3	3
9	1	9	2	1	3	9	3	3	3
3	1	3	3	1	2	3	1	3	3
3	1	1	3	1	3	3	1	3	3
2	3	1	3	1	3	1	1	3	3
3	1	1	1	2	3	2	2	3	3
1	1	9	2	1	3	3	1	3	3
1	1	9	3	1	9	3	3	1	9
1	1	1	3	1	9	3	3	1	9
1	1	2	3	1	3	3	1	3	3
2	1	2	2	3	3	3	1	3	3
2	1	2	3	1	3	9	1	3	3
1	1	2	3	1	3	3	3	3	3
3	2	3	3	1	3	3	1	3	3
3	1	3	3	1	3	3	1	3	3
9	1	3	2	1	3	3	1	3	3
9	1	9	3	1	1	3	1	3	3
3	1	3	2	1	3	3	1	3	3
3	1	3	3	1	3	3	1	3	3
3	1	3	2	1	3	3	1	3	3
3	1	3	2	1	3	3	1	3	3

3	1	3	9	1	3	3	1	3	3
1	9	9	3	1	3	3	1	3	3
9	1	9	3	1	3	3	1	3	3
9	1	9	3	1	3	3	1	3	3
3	1	3	3	1	3	3	1	3	3
1	1	1	3	1	3	3	1	3	3
3	1	9	3	1	3	3	1	3	3
2	3	3	3	1	3	3	3	3	3
3	1	1	3	1	3	3	3	3	3
9	9	9	3	1	3	3	3	3	3
3	3	3	3	1	1	3	3	3	3
9	9	9	3	1	3	3	2	3	3
3	1	1	3	1	3	3	3	3	3
3	1	1	3	1	1	3	1	3	3
1	1	1	1	1	3	3	3	3	3
1	2	2	1	1	3	3	3	3	3
2	2	9	2	1	1	1	1	2	1
1	1	1	2	2	1	1	1	1	1
3	2	2	2	1	3	3	3	3	3
2	2	2	1	1	3	3	3	3	3
1	2	1	2	1	3	2	2	1	1
3	9	2	2	1	3	3	3	3	3
3	1	2	2	1	1	2	2	2	1
9	1	9	2	1	3	2	2	1	2
2	2	2	2	1	3	3	3	3	3
2	3	9	2	1	2	2	2	2	2
2	2	2	1	1	3	3	3	3	3
2	3	9	2	1	2	2	2	2	2
3	3	3	3	9	1	1	2	2	2
2	3	3	2	1	3	3	3	3	3
2	2	2	3	2	2	2	2	2	2
2	2	2	3	2	2	2	2	2	2
2	3	3	2	1	3	3	3	3	3
1	1	2	1	1	2	3	3	3	1
2	2	2	1	1	2	1	2	2	2
1	2	2	1	1	2	2	2	2	1
2	2	1	2	1	1	1	1	2	1
2	3	2	1	1	2	3	2	3	3
2	3	3	1	1	3	3	3	3	3
3	1	2	2	1	3	3	3	3	2
2	2	2	1	2	2	2	3	2	1
2	2	2	1	1	3	3	3	3	3
9	9	9	3	1	3	3	9	9	9
9	9	9	3	1	3	3	9	9	9
1	1	1	1	3	3	3	1	1	1
2	1	2	1	2	1	1	2	3	1
3	2	2	1	1	1	1	1	2	1

2	3	2	2	1	1	1	2	3	2
3	3	2	2	1	1	3	3	3	2
2	2	2	1	1	3	3	3	3	3
2	2	2	2	2	2	1	2	2	1
2	2	1	1	1	3	3	1	3	3
3	2	2	3	1	3	3	3	3	3
3	3	3	3	1	3	3	3	3	2
3	2	2	3	3	2	1	1	2	2
3	3	1	3	1	3	3	3	3	3
3	3	3	9	1	1	1	3	3	3
2	2	2	1	3	1	2	2	2	2
3	3	2	1	1	3	3	3	3	3
3	3	1	1	1	3	3	3	3	3
2	2	2	2	3	2	2	2	3	2
3	1	2	2	1	3	1	2	3	3
3	3	3	3	1	3	3	3	3	3
3	3	2	9	2	3	3	2	1	2
2	3	2	1	2	2	9	2	2	2
3	2	2	3	3	3	3	3	2	3
3	3	2	1	2	1	1	3	9	1
2	2	1	2	2	1	1	1	1	1
2	2	2	2	1	2	1	2	2	2
3	3	2	9	1	3	3	2	3	2
2	2	2	2	1	3	2	1	3	3
9	9	9	9	1	9	9	9	9	9
2	2	2	2	2	2	2	2	2	2
2	2	2	2	2	2	2	2	2	2
2	3	1	1	3	3	3	3	3	3
2	1	2	1	1	3	2	2	1	2
1	2	2	2	1	3	1	3	2	2
9	9	9	9	1	9	9	9	9	1
2	3	3	3	3	3	3	3	1	3
9	9	2	3	3	3	3	1	1	1
3	2	2	2	1	1	1	9	3	3
9	9	9	1	9	9	9	9	9	1
2	2	2	1	1	2	1	2	2	1
2	3	2	2	1	3	2	3	3	3
1	1	2	1	1	3	3	3	3	2
3	3	1	3	1	1	1	1	1	3
2	2	3	3	2	3	1	3	3	3
2	2	2	2	1	3	3	3	3	3
2	2	2	1	1	3	3	3	2	2
3	3	2	2	2	1	3	3	3	3
2	2	2	1	1	3	2	3	2	2
2	2	1	2	1	2	3	3	2	3
2	3	1	1	3	2	3	3	3	3
3	2	1	1	1	3	2	3	3	2

2	1	1	2	1	1	2	2	2	9
2	1	1	2	1	3	3	3	3	3
2	2	1	3	1	3	3	3	3	3
2	1	2	3	3	3	2	2	3	3
1	2	2	3	2	3	1	3	1	3
2	1	1	1	1	3	2	3	1	3
2	2	1	2	1	3	3	3	3	3
9	9	9	3	1	3	3	3	3	3
2	2	1	3	1	3	1	2	3	3
3	3	2	3	1	3	2	2	2	2
2	2	2	2	2	2	2	2	2	2
3	2	2	3	1	3	3	3	3	3
1	1	1	1	1	1	1	1	1	1
3	3	2	1	1	3	3	3	3	3
1	3	1	1	9	3	1	3	1	1
3	3	2	3	3	3	3	3	3	3
2	3	1	2	1	3	1	3	3	3
3	1	1	2	1	1	1	1	2	1
1	1	1	1	2	1	2	3	1	2
1	1	2	1	1	3	9	9	9	9
3	2	3	3	1	3	3	3	2	3
2	2	2	3	1	3	3	3	3	2
3	1	2	3	1	3	2	3	3	3
2	2	2	1	1	3	1	1	2	3
2	2	2	1	1	3	2	3	2	2
9	9	9	3	1	3	2	3	3	1
2	2	1	1	1	3	3	3	9	2
3	3	1	3	1	3	2	2	3	3
3	2	2	1	1	3	3	3	3	2
3	3	2	9	1	3	3	2	3	2
2	1	2	3	1	3	1	1	3	3
9	9	9	1	3	3	3	3	3	1
9	9	1	3	1	3	3	2	3	3
3	3	2	3	3	3	1	3	3	3
3	3	1	3	1	3	3	3	3	3
3	3	3	3	1	3	3	1	3	1
2	2	2	1	1	1	3	3	3	3
3	3	1	1	1	3	3	3	3	3
3	2	2	3	1	3	3	3	1	3
9	9	9	1	3	9	9	3	3	9
1	2	3	1	1	1	3	1	3	3
2	2	2	3	1	3	3	1	3	1
2	2	1	3	1	3	3	2	2	9
3	3	2	1	3	2	3	3	3	3
3	1	2	1	1	3	3	3	3	3
1	2	2	1	1	3	3	3	3	3
1	2	3	3	1	3	3	3	3	3

9	9	9	3	1	3	3	2	3	1
9	9	9	3	1	3	3	2	3	1
9	9	9	9	1	9	9	9	9	9
3	3	2	3	1	3	3	3	3	3
9	9	9	9	9	9	9	9	9	9
2	2	2	1	1	3	3	3	2	3
1	2	1	3	1	3	2	1	3	3
3	3	3	3	9	3	3	3	3	3
3	1	2	2	1	3	3	1	3	3
1	2	2	1	1	1	2	3	3	3
3	2	2	3	2	2	1	1	3	2
3	1	1	3	1	2	3	3	3	3
3	2	1	3	1	3	2	3	3	3
3	2	2	3	2	2	1	1	3	2
3	2	2	3	2	2	1	1	3	2
3	3	3	2	1	3	1	1	3	1
1	3	2	3	2	2	1	1	3	2
3	1	1	3	1	2	3	3	3	1
3	1	1	3	1	3	3	3	3	1
2	1	1	1	3	3	3	2	3	3
3	2	2	1	3	3	1	3	3	3
2	1	2	3	1	3	3	3	3	3
2	1	2	3	1	3	3	3	3	3
1	1	2	2	1	3	3	3	3	3
1	2	2	1	2	3	3	3	1	2
3	3	2	1	1	2	1	2	2	1
3	3	2	3	1	3	2	2	3	3
3	2	2	1	3	1	2	2	2	2
3	2	2	2	2	3	2	2	3	1
3	3	2	2	1	3	2	1	1	2
3	3	2	3	2	3	1	3	2	2
3	2	2	3	2	3	1	3	2	1
1	2	2	1	1	1	3	3	3	3
2	2	2	2	1	2	2	1	2	1
2	2	2	2	1	2	2	1	2	1
2	3	1	1	3	3	2	3	2	3
3	3	2	3	1	3	3	3	3	3
3	1	1	3	1	3	2	2	3	2
3	1	1	3	1	2	2	2	2	2
3	1	2	3	1	2	2	1	3	3
2	2	2	3	2	2	3	3	3	3
1	1	2	1	2	3	2	2	3	3
2	1	1	3	1	3	3	3	3	3
2	1	2	1	2	3	2	2	3	2
2	1	3	1	3	1	2	2	3	3
2	1	1	2	1	3	3	2	3	3
2	2	1	2	1	3	2	3	2	3

1	2	1	3	3	3	2	2	3	3
2	1	3	1	1	3	1	3	1	3
9	9	9	2	1	1	2	3	3	2
2	2	2	1	1	3	9	1	1	1
1	3	2	3	1	3	3	3	3	3
3	2	2	3	1	3	3	3	3	1
2	2	2	1	1	3	1	2	2	2
3	2	2	3	1	3	3	3	3	3
3	2	2	3	1	3	3	3	3	3
1	2	1	3	1	1	1	1	1	1
1	2	2	3	1	2	2	1	3	1
1	3	1	3	1	1	3	1	3	3
1	2	2	2	1	3	3	3	3	3
3	1	2	1	2	3	3	3	3	2
3	1	2	1	2	3	3	3	3	2
1	1	2	1	1	3	3	3	3	3
1	3	3	1	3	1	1	1	3	1
3	3	2	2	1	1	2	3	3	3
3	3	2	2	1	3	2	3	3	3
1	9	9	2	3	2	2	3	3	2
3	3	3	3	1	3	1	3	3	3
2	2	2	1	1	2	2	1	1	3
2	3	2	2	1	2	3	1	3	3
2	2	1	3	1	3	2	1	2	1
1	1	1	3	1	3	1	2	3	3
2	1	1	2	1	3	2	2	3	2
3	3	2	1	1	3	3	3	3	3
1	2	2	3	1	3	3	3	3	3
3	2	3	1	1	1	2	3	2	1
9	9	9	1	1	3	1	1	3	9
2	2	2	1	2	3	3	3	2	2
3	3	3	1	1	3	3	1	3	3
2	2	2	1	2	3	3	3	2	2
3	3	2	3	1	3	3	3	3	3
3	1	1	3	1	3	3	3	3	3
2	3	1	1	1	1	1	2	1	1
3	3	3	2	2	2	3	3	3	2
1	1	3	1	2	1	3	3	3	3
2	2	2	2	2	2	3	3	3	3
1	1	1	3	1	1	1	1	3	1
2	3	2	1	2	1	1	2	1	2
3	1	1	1	1	1	1	3	2	1
2	3	1	1	1	3	3	2	3	1
2	2	2	3	3	3	1	3	1	1
3	3	3	9	9	9	9	9	9	9
2	1	1	3	1	3	3	1	3	2
3	3	3	3	1	3	3	1	3	3

2	1	1	3	1	3	3	1	3	2
1	1	3	2	1	3	1	1	3	3
3	1	3	3	1	3	3	1	3	3
2	1	1	2	1	3	3	1	3	3
9	9	9	3	1	3	3	1	3	3
3	1	2	1	1	3	3	1	3	3
2	2	2	9	1	3	3	1	3	3
1	1	1	3	1	3	3	3	3	3
2	2	2	3	1	3	3	1	3	3
3	1	3	2	1	3	3	1	3	3
3	1	1	2	1	3	3	3	3	3
2	1	1	3	1	9	9	1	3	3
3	1	3	3	1	3	3	1	3	3
3	1	3	2	1	3	3	1	3	3
3	1	3	3	1	3	3	1	3	3
3	1	3	3	1	1	3	1	3	3
2	2	2	3	2	1	3	1	3	3
2	2	2	3	1	3	3	1	3	3
3	1	3	3	1	3	3	1	3	3
3	1	3	3	1	3	3	1	3	3
3	1	1	2	1	3	1	1	3	1
1	1	1	2	1	1	1	1	3	3
3	1	1	3	1	3	3	1	3	3
3	1	1	2	1	1	1	2	3	3
3	1	3	3	1	3	3	1	3	3
3	1	9	3	1	3	3	1	3	3
3	1	3	2	1	1	3	1	3	3
3	1	1	3	1	3	3	1	3	1
3	3	3	3	1	3	3	9	3	3
3	1	2	2	1	3	3	1	3	3
9	3	3	2	1	3	3	1	3	3
2	1	3	2	1	3	3	1	3	3
1	1	3	2	1	3	3	1	3	3
1	1	3	3	1	3	3	1	3	3
9	1	9	3	1	3	3	1	3	3
9	9	9	3	1	3	3	1	3	3
2	1	2	1	1	3	3	3	3	2
3	1	1	3	1	3	3	1	3	3
3	1	3	3	1	3	3	1	3	3
1	1	1	3	1	3	3	1	3	3
9	1	9	2	1	3	3	1	3	3
2	2	1	3	1	3	3	3	3	3
9	9	9	2	1	3	3	2	2	9
2	2	2	3	1	3	2	2	3	3
9	9	9	3	1	3	3	3	3	3
2	2	2	2	1	3	3	2	3	2
3	9	9	3	1	3	9	2	3	3

2	2	2	3	3	3	2	2	2	2
3	1	1	3	1	3	3	3	2	1
3	1	1	3	1	3	3	3	2	1
9	3	1	3	1	3	3	3	2	3
2	3	1	3	1	3	3	3	3	3
1	9	9	1	2	2	3	3	3	3
3	2	2	1	1	3	1	2	3	3
1	1	2	2	3	2	1	2	2	2
1	1	2	3	1	3	3	3	3	3
2	2	2	2	1	1	1	1	1	1
2	2	1	1	1	1	2	3	2	1
1	9	9	9	9	9	9	9	9	1
9	9	9	9	1	9	9	9	9	9
3	2	2	2	1	3	1	2	3	3
9	9	9	9	9	9	9	9	9	9
9	9	9	9	1	9	9	9	9	9
9	9	9	1	9	9	9	9	9	9
9	9	9	9	9	9	9	9	9	9
9	9	9	9	9	9	9	9	9	9
9	9	9	3	1	3	2	3	3	3
9	9	1	1	9	1	9	1	3	3
3	3	3	1	9	1	3	3	3	3
3	3	2	3	1	2	3	3	3	2
9	1	9	9	1	9	9	9	9	9
9	9	9	9	9	9	9	9	9	9
9	1	1	3	1	3	3	3	3	1
9	9	9	9	9	9	9	9	9	9
2	1	1	3	2	3	1	2	2	3
2	1	1	3	1	3	1	9	9	1
1	9	9	9	9	9	9	9	9	1
9	9	9	9	9	9	9	9	9	9
2	1	2	2	1	3	3	1	3	3
2	2	2	2	1	1	2	2	2	2
1	3	3	1	1	3	3	3	3	3
1	2	2	3	2	3	2	2	1	1
2	3	2	3	1	3	3	3	3	3
3	2	1	3	1	3	2	2	2	2
3	1	3	3	1	3	3	3	3	3
3	3	3	2	2	2	2	2	2	2
3	3	2	2	1	3	2	1	3	3
3	1	2	3	1	3	2	3	3	3
1	1	1	3	1	3	3	3	3	3
1	2	1	3	1	3	3	1	3	2
1	1	1	3	1	2	3	3	3	3
2	1	1	3	1	3	3	2	3	3
3	1	2	2	1	3	3	3	3	3
3	2	2	3	1	3	3	2	3	3

3	1	2	1	1	3	2	2	2	3
9	9	9	3	1	3	3	3	3	3
1	3	2	3	1	3	3	3	3	3
3	2	2	3	1	1	1	2	3	2
3	1	1	3	3	3	3	2	3	2
2	2	2	1	2	3	2	2	2	3
3	3	3	2	1	3	3	1	3	3
2	1	1	2	1	3	3	3	2	1
1	1	1	3	1	3	1	1	2	1
3	2	2	2	1	1	2	2	2	2
3	2	2	2	1	1	2	2	2	2
2	2	2	1	1	3	3	2	3	3
3	3	2	2	2	3	2	1	2	2
3	3	2	2	2	3	2	1	2	2
3	3	2	3	2	1	1	3	3	1
2	2	2	1	1	2	2	2	2	2
2	2	1	1	2	3	2	2	2	3
2	2	2	1	1	3	1	2	1	1
2	2	2	2	1	3	1	1	3	3
3	2	2	3	1	3	2	3	3	2
3	3	2	3	1	3	1	3	3	1
2	1	2	3	1	3	3	3	3	3
3	1	1	3	1	1	3	3	3	3
3	1	2	3	1	1	3	3	3	3
3	2	2	3	2	2	1	1	3	2
3	1	2	2	1	3	2	2	2	3
3	3	3	1	1	3	3	3	3	3
1	3	1	1	1	3	3	3	9	1
1	1	2	3	1	1	2	1	1	1
3	1	3	2	1	2	3	2	3	2
1	3	2	1	1	3	1	1	1	1
2	1	2	9	9	9	9	9	9	9
2	3	2	2	2	1	2	1	2	2
3	2	2	2	3	3	3	3	3	3
3	3	1	3	1	1	1	1	3	2
9	9	2	9	2	9	9	9	9	9
2	2	2	2	2	1	2	1	3	3
3	1	1	3	1	3	1	3	3	3
2	2	2	2	1	2	2	3	3	3
2	1	3	3	1	3	3	3	3	3
3	2	3	3	1	3	3	3	3	2
2	1	2	1	2	3	2	3	1	1
9	9	9	1	1	3	3	3	3	3
2	3	2	3	1	3	3	2	3	3
3	1	2	3	1	3	3	3	3	3
3	2	3	2	2	9	9	9	9	9
9	9	9	9	1	9	9	9	9	9

9	2	9	9	1	9	9	9	9	9
9	1	9	9	9	9	9	9	9	9
9	9	9	9	1	9	9	9	9	9
9	9	9	1	1	3	1	3	3	3
9	9	1	3	1	3	3	3	3	1
1	9	1	1	1	1	9	9	9	9
9	9	1	1	1	1	1	1	9	9
9	1	1	9	1	9	9	9	9	9
9	1	1	9	1	1	9	9	9	9
9	9	9	1	9	9	9	9	9	9
9	9	9	1	9	9	9	3	9	3
9	9	9	1	9	9	9	9	9	9
9	9	1	9	1	9	9	9	9	9
9	9	9	9	1	9	9	9	9	9
9	9	9	1	1	1	1	3	3	3
9	9	9	1	9	9	9	9	9	9
9	1	1	1	9	9	9	9	9	9
9	9	1	1	1	3	1	3	3	3
2	2	2	2	2	2	2	2	2	2
3	1	1	3	2	1	1	2	1	1
9	9	9	1	1	1	1	1	1	1
1	2	3	1	1	3	1	2	3	1
1	1	9	3	1	1	2	2	3	1
3	1	1	1	1	3	3	3	3	3
3	1	2	2	2	3	3	3	3	3
1	3	2	1	3	1	2	2	2	3
1	2	2	1	2	3	2	3	3	2
2	2	3	1	3	3	9	2	3	3
2	2	2	1	3	3	2	2	2	1
2	2	2	1	1	2	2	9	3	3
1	1	1	1	1	3	3	3	3	3
3	2	3	2	1	3	3	3	3	3
2	2	2	1	1	3	1	1	3	3
1	3	3	9	9	9	9	9	9	9
2	1	2	1	1	1	2	1	2	3
2	2	2	1	1	1	1	2	1	2
3	2	1	2	2	2	1	2	3	2
2	3	3	3	3	1	2	1	2	1
9	9	9	1	3	1	1	1	1	1
2	1	2	1	1	3	1	2	1	3
1	1	9	1	3	1	2	2	1	2
1	1	1	9	1	3	3	3	1	3
2	2	2	3	3	3	1	2	1	1
3	1	3	1	3	1	1	1	1	1
1	3	3	3	3	3	3	3	3	3
3	2	2	3	3	3	1	2	1	1
1	1	1	9	2	2	2	3	3	1

3	1	3	1	3	1	3	9	9	1
3	3	3	1	1	2	1	2	2	1
3	3	1	1	9	3	3	1	1	3
1	1	2	2	1	1	2	1	2	1
2	2	2	1	2	3	3	2	1	3
2	2	2	1	2	3	3	2	1	3
1	1	1	1	1	3	2	2	1	2
3	1	3	1	3	1	3	9	9	1
1	1	1	1	1	1	2	1	2	1
9	9	9	1	1	3	3	3	3	3
1	2	3	1	2	3	1	2	1	3
3	1	1	1	1	3	3	3	3	2
2	3	2	2	1	3	2	1	2	2
3	1	2	2	1	1	2	1	2	1
2	2	2	1	2	2	1	3	1	2
1	2	3	3	1	3	2	3	3	2
1	1	1	1	1	1	1	1	1	1
3	3	3	3	3	9	9	9	9	9
1	2	2	1	1	3	3	3	3	3
3	1	3	3	1	3	3	1	3	3
1	1	1	1	3	3	3	3	3	3
2	3	2	2	1	2	3	3	3	3
2	1	3	1	1	1	2	3	2	2
2	3	3	2	1	3	1	3	2	3
3	2	2	3	3	2	2	2	2	2
1	3	3	1	3	1	1	3	1	3
3	1	3	3	1	3	3	1	3	3
3	1	1	3	1	3	3	3	3	3
1	1	1	9	2	2	2	9	3	1
9	1	9	1	3	3	3	3	3	3
2	1	2	3	3	3	3	3	3	3
9	9	9	3	3	3	3	3	9	1
3	1	1	3	1	3	3	3	3	3
3	2	9	3	1	3	3	3	3	3
9	2	9	3	1	3	3	3	3	9
9	1	9	1	3	3	2	3	3	3
9	1	9	1	3	3	3	3	3	3
2	2	2	1	1	3	2	3	3	1
2	2	2	1	1	3	2	2	2	2
2	2	2	1	1	3	2	2	2	2
2	1	2	1	1	3	2	3	2	1
2	2	2	3	3	3	1	2	1	1
2	3	1	3	1	2	1	2	1	2
2	1	2	1	1	3	2	3	2	1
2	3	1	3	1	2	1	2	1	2
1	1	2	1	3	3	1	1	1	2
3	3	3	3	1	1	1	1	3	1

3	3	3	3	1	1	1	3	3	3
3	2	2	3	1	3	3	2	3	2
1	1	1	3	1	3	3	3	3	3
2	1	2	1	2	1	2	2	1	1
2	2	2	3	1	3	3	3	3	3
9	3	3	1	2	1	2	1	3	1
2	3	2	1	3	1	2	2	2	1
1	9	9	1	1	3	3	3	3	3
3	3	3	1	1	3	3	3	3	3
2	2	2	2	1	3	2	3	3	3
3	3	3	3	3	3	3	3	3	3
9	1	9	1	3	1	3	3	3	3
1	2	1	1	3	3	1	1	1	2
1	1	2	1	3	3	1	1	1	2
1	1	2	1	3	3	1	1	1	2
1	1	2	1	3	1	3	2	3	2
1	1	2	1	3	3	1	1	1	2
1	1	2	1	3	3	1	1	1	2
1	1	2	1	3	3	1	1	1	2
3	1	1	1	3	1	1	1	3	1
3	2	1	3	3	1	2	3	1	1
2	3	3	2	2	2	2	2	2	3
1	3	2	1	3	3	9	1	3	1
2	2	3	3	1	3	2	1	2	2
1	1	1	3	9	3	3	1	2	3
3	3	1	1	3	3	3	1	3	3
2	2	2	1	2	1	2	1	2	1
1	3	1	2	1	3	3	1	3	3
9	9	9	1	9	9	9	9	9	9
3	3	3	3	3	3	3	3	3	3
1	1	1	1	1	3	3	3	1	1
3	2	3	2	9	1	3	2	1	3
1	2	3	3	1	2	1	2	1	2
2	2	3	1	2	1	1	1	2	2
2	3	3	1	3	3	2	3	3	3
1	3	2	1	1	1	3	3	3	3
2	9	9	3	2	1	1	1	2	3
2	2	2	1	1	2	1	1	1	2
2	3	2	1	3	2	1	1	1	2
1	1	1	1	1	1	3	3	1	1
1	1	2	1	3	3	1	1	2	1
3	1	1	1	1	1	9	1	3	3
3	2	3	1	2	1	3	2	1	2
3	1	1	3	1	3	3	3	3	3
9	9	9	1	1	3	3	2	3	3
1	1	1	1	9	1	2	1	2	1
2	2	2	1	1	1	1	1	1	2

9	9	9	3	1	3	2	3	3	3
9	9	9	1	9	1	9	9	9	9
9	9	9	1	9	9	9	9	9	9
1	3	3	1	3	1	1	3	1	3
2	2	2	1	1	3	3	2	1	3
2	2	2	1	2	3	3	2	1	3
1	3	3	1	1	3	1	2	1	1
2	2	2	1	2	3	3	2	1	3
2	2	2	1	2	3	3	9	9	3
2	2	2	1	2	3	3	2	1	3
2	3	2	2	1	3	1	1	1	2
2	1	2	3	2	3	1	1	3	3
1	1	2	1	1	1	1	2	1	2
2	1	3	1	1	1	1	3	1	3
2	3	1	3	3	1	1	3	3	1
1	3	1	1	1	1	3	3	2	3
3	3	3	3	1	1	2	3	3	3
9	9	9	3	1	9	9	9	9	9
9	9	9	3	1	9	9	9	9	9
1	1	1	2	1	3	3	1	3	3
2	1	3	1	1	3	1	1	1	1
3	2	2	1	3	3	2	2	2	2
3	3	3	1	1	1	2	1	1	1
2	2	2	2	2	2	2	2	2	2
3	3	3	3	3	3	3	3	3	3
9	9	9	3	9	2	2	2	2	2
9	9	9	3	9	2	2	2	2	2
3	1	2	3	1	2	3	1	3	3
3	3	3	1	1	1	2	1	1	1
2	2	2	2	2	2	2	2	2	2
2	3	3	3	1	9	9	9	9	9
2	1	1	1	3	2	2	1	2	1
1	1	1	1	1	3	9	1	1	2
9	9	9	3	9	2	2	2	2	2
3	2	1	1	3	3	1	1	2	1
3	3	3	1	3	3	9	1	2	2
2	1	1	1	3	1	2	2	2	1
9	9	9	1	3	3	1	1	1	1
1	3	9	1	9	1	9	2	9	9
2	2	2	3	1	1	1	1	1	1
9	9	9	1	3	1	1	1	1	1
2	2	2	3	1	1	2	2	2	2
2	2	2	2	1	3	3	2	2	2
2	2	2	2	2	2	2	2	3	2
2	2	2	2	2	2	1	3	1	1
2	2	2	2	2	2	2	2	2	2
3	2	2	1	3	3	3	3	3	1

2	2	2	1	2	3	3	2	2	2
1	2	2	2	2	2	2	2	2	2
1	2	1	1	3	3	3	3	9	9
1	2	2	9	9	9	9	9	9	9
9	9	9	1	9	9	9	9	9	9
2	1	2	2	1	2	2	1	2	1
2	2	2	1	1	3	2	2	2	2
9	9	9	3	3	3	3	3	3	9
2	1	2	1	1	1	2	1	2	3
9	9	9	9	3	9	9	9	9	9
2	2	2	2	2	2	2	2	2	2
1	1	1	1	1	3	3	1	1	1
9	1	9	1	1	3	3	3	1	1
2	2	2	2	2	2	2	2	2	2
1	1	2	1	2	3	1	1	2	1
2	2	2	3	3	2	2	2	2	2
2	2	2	2	2	2	2	2	2	2
2	1	1	1	1	1	3	1	2	3
9	1	1	1	1	9	9	9	9	9
1	1	2	1	3	3	3	3	3	3
9	9	9	2	9	9	9	9	9	9
1	2	2	1	3	1	2	1	1	1
2	2	2	2	2	2	2	2	2	2
2	2	2	1	2	1	2	2	1	1
2	2	2	1	1	3	3	3	2	2
2	3	3	1	2	1	2	3	1	1
3	3	3	2	9	9	9	9	9	9
2	2	2	9	1	9	9	9	9	9
2	2	2	2	2	2	2	2	2	2
3	3	2	3	1	2	2	3	3	3
9	9	9	9	1	9	9	9	9	9
2	3	2	1	1	1	2	3	3	3
3	1	3	3	3	3	3	3	3	3
2	2	2	1	1	2	2	2	2	3
2	1	2	1	2	1	1	1	1	2
1	1	1	1	1	1	1	1	1	3
2	2	2	2	2	2	2	2	2	2
3	1	2	1	2	2	3	3	1	2
1	1	1	2	2	1	1	2	2	2
1	2	3	1	2	3	3	3	3	3
2	3	2	1	1	3	3	2	3	3
2	2	2	2	2	2	2	2	2	2
2	3	1	3	1	1	3	3	3	3
2	1	1	1	1	3	3	3	3	3
2	2	2	1	2	2	2	2	2	2
3	3	3	3	3	3	3	3	3	3
3	2	2	1	2	3	9	1	2	2

3	1	2	1	1	2	2	2	2	2
2	2	2	2	2	2	2	2	2	2
1	3	3	1	2	2	2	2	2	2
2	1	2	2	2	2	2	2	2	2
2	1	1	1	2	3	3	1	3	3
1	1	2	1	1	3	1	2	1	3
2	2	2	3	2	2	2	2	2	2
1	3	3	2	2	2	2	1	2	3
1	2	3	1	1	3	3	3	3	2
9	9	9	1	1	3	1	2	1	3
3	3	1	1	3	3	1	1	3	2
9	9	9	9	2	9	9	9	9	9
9	1	9	9	1	9	9	9	9	9
2	1	2	1	1	1	2	1	2	2
2	2	2	1	2	1	2	2	3	3
3	1	3	1	3	1	1	2	1	1
2	2	2	9	1	2	2	2	2	2
1	3	1	3	1	3	3	1	1	3
2	1	3	1	2	2	1	1	2	1
2	1	2	1	1	1	2	1	2	2
1	9	9	1	1	1	3	3	3	3
1	1	1	1	2	2	1	2	1	3
2	2	2	1	2	3	3	2	3	2
2	1	2	2	1	1	3	3	3	9
9	3	3	1	3	1	3	3	3	3
2	1	2	2	1	1	3	3	3	9
2	2	2	2	2	2	2	2	2	2
9	9	9	9	1	9	9	9	9	9
2	2	2	1	1	2	2	2	2	2
2	1	2	1	2	2	2	2	3	1
1	1	1	1	1	1	1	1	1	1
1	1	1	1	1	1	1	1	1	1
1	2	1	1	1	1	2	1	1	1
1	2	1	1	2	2	1	1	2	1
9	9	9	1	1	1	3	1	1	2
3	1	1	1	3	1	9	1	1	1
9	1	9	3	1	3	3	3	3	3
1	1	1	1	1	1	1	1	1	1
2	2	2	1	1	2	2	2	2	2
2	2	2	1	1	2	2	2	2	2
2	1	2	3	1	2	3	3	1	2
1	1	1	2	3	3	2	2	1	1
2	1	3	1	9	9	9	9	9	9
2	2	2	1	1	2	1	2	1	1
2	2	2	1	1	2	2	2	2	2
1	1	1	1	1	2	3	1	1	2
3	1	3	2	2	2	2	2	3	2

1	1	1	1	1	3	1	1	1	9
3	3	9	3	1	3	3	3	3	3
2	2	2	3	1	3	3	2	3	3
9	1	9	9	9	1	9	9	9	9
1	9	9	3	9	2	9	9	9	9
2	1	2	1	1	2	3	1	2	3
9	1	9	1	1	2	2	2	2	2
3	1	1	3	1	3	3	3	3	3
3	3	1	1	9	9	9	9	2	2
2	2	2	2	2	2	2	2	2	2
9	1	9	1	9	9	9	9	9	9
2	3	3	1	2	2	2	2	2	3
2	1	2	2	2	2	2	2	2	2
2	1	1	2	1	2	2	2	2	2
2	2	2	1	1	2	2	2	2	2
2	1	3	2	3	2	1	2	3	2
2	3	2	1	2	2	1	3	2	1
9	1	1	1	9	9	9	9	9	9
9	9	9	1	9	9	9	9	9	9
2	1	2	2	2	2	2	2	2	2
2	2	2	1	2	2	2	2	2	2
3	1	2	1	3	9	3	3	3	1
9	9	9	1	3	3	9	9	9	9
1	1	1	1	9	2	2	2	2	2
3	1	2	2	1	2	2	3	1	2
1	3	1	3	3	3	3	3	3	3
2	9	2	1	1	3	3	2	3	3
2	1	3	3	3	3	1	1	3	1
3	3	3	3	1	2	9	2	2	2
3	2	1	1	3	3	2	3	3	3
1	1	1	1	1	3	3	3	2	2
2	1	2	1	1	3	3	2	3	3
3	2	2	3	3	2	3	3	3	3
3	3	3	1	3	3	3	3	3	3
9	9	9	1	1	1	2	2	3	2
2	1	3	1	1	3	1	1	1	2
1	2	1	1	1	1	3	1	1	2
3	3	3	1	3	3	1	1	1	3
1	9	3	2	1	1	9	3	2	9
3	3	3	1	3	3	3	3	3	3
1	1	1	1	3	1	1	3	1	1
9	1	9	1	1	1	2	1	3	1
1	1	1	1	1	1	3	1	1	3
3	1	3	1	1	3	3	3	3	3
9	9	1	3	1	3	1	3	3	1
2	2	2	1	1	2	3	3	2	1
1	1	2	1	1	2	2	1	2	2

1	1	3	1	3	3	3	1	1	1
3	3	3	3	3	3	3	3	3	3
9	1	9	1	1	1	2	1	3	1
1	1	1	2	3	3	1	2	1	1
2	2	2	3	3	2	2	1	1	1
3	1	1	2	2	2	2	2	2	2
3	3	3	2	3	3	1	3	2	1
2	2	2	1	1	1	1	1	1	1
2	2	2	1	1	1	1	1	1	1
1	3	2	2	1	3	1	2	1	1
2	1	2	2	3	1	2	2	3	1
3	2	3	1	1	1	1	2	3	3
3	3	3	3	3	1	1	1	1	1
2	2	2	3	1	3	2	3	2	3
2	2	2	2	1	1	2	2	2	2
3	3	3	2	3	3	1	3	2	1
3	3	1	3	1	1	3	3	3	3
1	1	2	1	3	2	2	2	2	2
1	3	2	2	1	1	2	2	3	2
9	9	9	9	1	9	9	9	9	9
9	1	9	1	9	9	9	9	9	9
3	3	3	1	1	3	1	1	1	1
3	3	3	1	3	3	1	3	3	1
3	3	1	3	3	3	3	3	3	3
3	3	3	1	3	3	3	1	3	3
1	1	3	1	3	3	3	1	1	1
3	1	2	1	1	3	1	1	3	3
1	1	2	3	2	1	2	1	1	2
2	1	1	3	1	1	3	3	1	1
3	1	3	1	3	1	3	3	3	3
3	2	1	3	1	3	2	2	2	2
3	1	3	1	1	1	1	1	1	1
3	1	2	2	1	2	2	1	2	2
1	2	2	1	1	1	1	3	3	3
1	1	9	2	1	3	2	2	3	1
3	2	2	1	2	2	1	2	1	2
1	2	2	1	2	9	1	1	1	2
2	2	2	1	1	3	2	2	2	2
2	2	2	9	1	2	2	1	3	2
2	2	1	1	1	3	3	1	3	3
2	1	3	1	2	1	3	1	2	1
2	2	2	2	2	2	2	2	2	2
1	3	3	1	3	3	3	2	1	1
9	9	9	2	1	9	9	9	9	9
9	9	9	3	9	9	9	9	9	9
3	1	2	3	1	3	3	3	3	3
3	1	1	1	1	3	2	3	3	3

1	1	3	1	9	3	3	3	3	3
3	3	1	3	1	3	2	3	2	3
3	3	2	1	1	3	3	3	3	3
3	2	1	1	2	1	1	2	3	3
3	3	3	2	2	3	3	3	3	3
3	3	3	3	3	3	2	3	3	3
2	2	2	3	9	2	2	2	2	3
3	3	3	2	1	3	3	3	3	3
3	3	2	3	1	3	3	1	3	3
9	9	9	1	3	3	1	3	3	3
9	9	9	1	3	3	2	3	3	3
9	9	9	1	3	3	2	3	3	3
9	9	9	1	3	2	9	9	9	9
9	9	9	1	9	9	9	9	9	9
9	9	9	1	3	3	2	3	3	3
2	3	2	3	1	3	3	3	3	3
9	2	1	1	3	3	3	1	1	2
2	2	2	1	2	2	2	2	2	2
3	1	2	1	1	3	9	1	3	3
1	2	1	1	1	3	3	3	3	3
2	3	1	1	3	3	3	3	3	3
3	2	3	2	1	3	2	9	2	1
2	2	2	1	1	2	2	2	1	2
1	1	2	3	1	3	2	3	3	3
2	2	2	1	1	2	9	1	2	2
2	3	1	1	1	1	3	1	3	3
3	3	2	3	1	3	3	3	3	3
1	1	1	1	3	1	3	3	1	1
2	3	1	1	1	1	3	1	3	3
2	3	2	3	1	3	3	3	3	3
1	2	1	1	1	3	3	3	3	3
9	9	9	1	3	2	9	9	9	9
3	3	1	1	1	1	3	3	3	2
2	2	2	1	1	3	2	3	3	3
1	2	2	1	3	1	1	1	1	1
2	2	2	1	1	3	3	3	3	3
9	9	2	1	2	3	2	3	3	2
9	9	2	1	2	3	3	2	3	3
3	2	2	1	2	3	1	2	2	1
2	2	2	2	2	2	2	2	2	2
9	9	9	3	1	3	3	3	3	1
1	1	2	1	1	3	3	3	3	3
3	3	2	3	1	3	3	3	3	3
2	2	2	2	2	1	2	1	2	2
3	1	3	1	3	1	3	1	3	1
1	1	2	3	2	3	2	2	3	2
9	9	1	9	1	9	9	9	9	9

3	3	1	1	1	2	2	2	2	2
2	2	2	1	1	3	2	2	3	3
9	1	1	1	1	1	1	1	1	2
9	1	9	1	2	1	3	2	3	3
1	9	9	3	1	3	3	1	3	3
9	9	9	9	1	9	9	9	9	9
2	1	3	1	1	1	1	2	1	3
2	3	2	1	1	3	3	2	3	3
2	2	3	1	2	3	2	2	3	2
2	1	3	1	1	1	1	2	3	3
3	2	1	1	1	3	2	3	3	3
3	2	1	1	2	1	2	9	1	2
1	2	2	3	1	3	1	2	2	2
2	2	1	1	1	3	3	3	3	3
2	2	2	2	2	1	3	2	2	3
3	3	3	1	1	3	3	3	3	3
3	3	1	3	1	3	2	3	3	2
3	2	2	1	1	3	3	3	3	3
2	1	2	3	1	3	3	3	3	3
3	1	1	1	2	1	2	1	3	3
2	2	2	1	2	3	3	3	3	3
9	9	9	1	2	2	1	1	2	1
3	1	2	1	2	1	1	1	2	1
3	2	2	2	2	2	2	2	2	2
2	2	2	1	2	3	2	2	3	2
2	2	2	9	1	1	9	9	9	9
2	2	2	1	1	2	3	3	2	2
3	2	1	3	1	1	1	1	2	3
1	2	1	1	3	3	3	3	3	3
3	3	3	3	1	3	3	3	3	1
9	9	9	1	9	9	3	9	9	9
2	2	2	2	2	2	2	2	2	1
2	2	1	1	1	1	9	9	9	9
3	3	3	1	1	1	2	1	2	1
2	1	2	1	3	1	2	3	2	2
9	1	9	1	9	1	9	9	9	9
9	9	9	9	9	9	9	9	9	9
2	2	2	2	2	2	2	2	2	2
3	2	2	2	2	1	2	3	3	1
2	1	3	1	2	1	2	3	2	2
1	1	1	1	2	2	3	1	3	3
1	1	1	1	1	1	1	1	1	1
1	2	1	3	1	2	1	1	1	2
1	2	3	1	2	1	2	3	1	2
1	3	2	1	9	3	2	1	3	2
1	2	3	9	9	9	9	9	9	9
3	2	1	3	3	2	1	3	2	1

1	2	1	2	1	1	1	2	1	2
9	9	9	9	9	9	9	9	9	9
3	3	3	3	3	1	3	3	3	3
3	1	2	1	1	1	1	1	2	1
3	2	3	3	1	3	1	1	3	1
1	2	1	2	1	1	1	2	1	2
1	3	2	1	9	9	9	9	9	9
3	9	2	1	1	3	2	3	3	3
2	2	9	1	1	1	1	1	1	1
3	1	2	1	3	3	1	3	1	2
9	9	9	9	9	9	9	9	9	9
2	2	2	1	9	9	9	9	9	9
9	9	9	9	1	9	9	9	9	9
1	1	1	1	3	3	3	1	1	1
3	2	2	2	1	3	1	2	1	3
9	9	9	1	1	3	2	2	3	3
3	3	3	9	1	3	3	3	3	3
9	9	1	9	1	9	9	9	9	9
3	1	2	2	1	3	3	2	3	3
3	3	3	2	3	2	1	2	3	2
3	2	1	2	1	3	3	1	3	3
3	3	3	1	1	2	2	1	1	1
3	3	1	2	3	1	2	3	1	2
2	2	2	2	1	3	3	2	3	3
2	2	2	3	1	3	2	2	2	2
2	1	1	1	1	2	1	2	2	3
9	9	9	9	9	9	9	9	9	9
2	2	2	1	1	3	3	3	3	3
2	3	1	2	1	3	2	3	2	3
3	3	3	2	2	1	3	2	2	2
3	3	2	3	2	3	3	3	3	3
2	2	2	2	1	1	2	1	3	3
1	3	1	1	3	1	2	9	2	3
3	2	2	1	3	3	3	2	2	2
2	2	2	3	1	2	1	1	1	1
3	1	1	3	1	3	1	2	3	2
1	1	2	1	2	1	2	2	1	1
3	3	3	1	3	1	1	1	1	1
3	1	2	1	3	3	2	2	3	3
2	1	1	1	1	3	1	1	2	1
1	3	3	3	9	2	3	2	3	1
3	3	3	3	3	3	3	3	3	3
3	1	3	1	9	1	9	3	3	3
9	1	9	1	1	1	3	3	3	3
9	9	9	1	1	3	3	3	3	3
9	1	9	1	1	3	3	3	3	3
9	9	9	1	1	1	3	3	3	3

2	1	2	1	1	3	3	2	2	2
3	2	2	2	3	1	2	2	2	3
1	1	9	2	1	3	3	3	3	3
1	2	2	3	1	3	3	3	3	3
3	1	3	1	1	1	1	1	1	1
9	9	9	3	1	3	3	3	3	3
9	9	9	3	3	3	3	3	3	1
9	9	9	1	1	3	3	3	3	3
9	9	1	1	1	1	3	3	3	3
9	1	9	1	1	9	3	3	3	3
9	1	9	9	1	9	3	3	9	9
2	1	2	3	3	3	3	3	3	3
9	9	1	1	1	1	3	3	3	3
1	1	3	1	3	3	1	1	1	1
3	1	3	1	3	3	2	3	3	3
2	1	2	3	1	3	2	3	3	3
2	2	2	3	1	3	9	3	3	3
9	1	9	3	1	3	3	3	9	1
9	9	9	3	1	3	3	3	3	3
9	9	9	1	1	1	1	3	3	3
1	3	2	1	3	2	3	1	1	2
9	9	9	1	1	3	3	3	3	3
1	1	2	1	1	3	3	3	3	3
9	9	1	1	1	3	3	3	3	3
2	2	1	1	1	3	3	3	3	3
1	2	2	1	1	3	3	3	3	3
2	2	2	2	3	1	3	1	3	3
1	1	1	1	1	1	3	3	3	3
1	1	1	1	1	3	3	3	3	3
1	1	1	1	1	3	3	3	3	3
1	1	1	1	1	3	3	3	3	3
2	2	2	3	1	3	3	3	3	3
3	3	3	1	1	9	2	2	2	2
9	9	9	1	1	1	3	3	3	3
1	1	1	1	1	1	3	3	3	3
1	1	1	9	1	3	3	3	3	3
3	3	3	1	1	3	3	3	3	3
9	9	9	1	1	9	3	3	3	3
3	3	3	3	1	3	3	3	3	3
3	3	3	1	1	1	3	9	1	3
3	3	3	2	3	2	3	1	2	2
3	1	3	3	1	3	3	1	3	1
1	3	1	1	9	3	2	3	3	3
2	2	2	1	2	1	1	2	2	2
1	1	1	1	1	1	1	1	3	1
3	2	2	1	1	1	1	1	2	3
3	2	3	1	3	3	2	2	3	3

3	2	3	3	3	3	2	3	3	3
3	3	2	3	3	3	2	3	3	2
2	2	3	1	2	3	1	2	9	2
1	2	2	2	1	3	2	2	3	3
1	3	2	9	1	3	2	3	3	3
9	9	9	2	9	9	9	9	9	9
1	2	3	3	1	3	2	2	3	3
2	2	2	1	3	3	2	2	1	3
9	9	9	1	9	9	9	9	9	9
3	2	2	1	1	9	9	9	9	9
9	9	9	1	9	9	9	9	9	9
2	9	9	9	9	9	9	9	9	9
2	2	2	2	1	9	1	1	2	2
1	2	1	3	2	2	9	1	9	2
1	1	1	1	1	3	3	3	9	3
1	3	3	3	1	3	2	1	1	1
1	2	1	3	2	3	1	2	1	1
2	1	2	3	1	3	3	3	3	3
1	3	1	1	3	1	1	1	1	1
9	1	9	1	9	9	9	9	9	9
2	3	1	1	3	1	1	1	1	1
1	2	3	1	1	3	2	3	3	3
2	2	2	9	1	3	2	2	3	3
1	1	1	1	1	1	1	1	1	1
2	1	2	3	1	3	2	2	3	3
1	1	1	3	1	3	3	3	3	3
2	2	2	1	3	1	1	9	9	3
3	2	3	2	3	2	2	2	2	3
2	2	2	2	1	2	2	1	2	2
1	1	3	2	9	9	9	9	1	9
1	3	1	1	1	1	1	3	1	3
1	1	1	1	1	1	1	1	1	1
2	2	1	1	1	3	1	1	1	1
1	2	2	2	1	1	1	1	1	1
1	1	1	1	1	3	3	3	3	3
3	2	1	3	2	2	2	2	3	3
2	2	2	2	2	1	2	2	2	2
1	1	1	1	2	2	2	1	1	1
1	1	1	1	2	2	2	1	1	1
3	3	3	1	2	2	2	2	2	2
1	2	1	1	3	3	1	3	3	1
2	3	2	1	1	3	2	2	2	2
3	2	2	2	1	3	3	1	3	3
2	2	2	1	1	3	3	2	2	1
3	1	2	3	3	3	3	3	3	2
3	3	3	3	3	3	3	3	3	3
2	2	2	2	1	3	1	2	3	3

2	2	2	9	9	9	9	9	9	9
2	3	1	2	1	3	3	3	3	3
9	9	9	9	9	9	3	9	9	9
9	9	1	1	9	9	9	9	9	9
9	1	9	1	9	9	9	9	9	9
2	2	2	1	1	3	2	9	1	1
9	1	9	9	2	9	9	9	9	9
3	1	2	1	1	3	2	1	3	3
2	2	2	2	2	3	3	2	3	1
2	2	2	1	3	2	2	2	2	2
2	2	2	1	1	3	3	3	3	3
1	3	1	1	3	2	1	1	1	1
1	1	1	1	2	1	1	1	1	1
2	2	2	1	3	1	3	3	3	3
2	1	1	1	3	3	1	1	1	1
2	1	2	1	1	1	2	1	1	2
2	2	2	1	1	1	2	2	2	2
3	3	3	3	3	3	3	3	3	3
2	3	2	3	1	1	3	1	2	2
2	2	2	1	1	1	1	1	1	1
2	2	2	2	3	3	2	3	2	1
2	1	1	1	2	1	1	1	1	2
2	2	2	2	2	1	2	2	2	2
2	2	2	3	3	1	1	1	2	2
1	1	1	2	1	3	3	2	2	2
2	2	2	1	2	2	2	2	2	2
9	9	1	1	2	3	1	1	1	1
3	1	3	1	3	3	3	1	3	3
3	2	3	1	2	3	2	3	2	2
1	1	1	1	2	1	1	2	2	2
9	9	9	2	9	9	9	9	9	9
2	1	3	2	3	2	3	2	3	2
1	2	1	1	1	1	2	1	3	1
3	3	2	2	1	3	3	2	3	2
3	3	3	1	3	3	3	2	2	1
1	3	2	1	2	3	3	3	2	3
3	1	1	1	1	1	1	1	3	1
3	1	1	1	9	1	3	3	1	1
2	3	3	1	2	3	1	2	1	1
2	2	2	2	1	3	1	1	2	3
2	2	2	1	1	3	2	9	2	2
2	2	2	1	1	2	2	2	2	2
2	2	2	3	1	1	3	1	9	9
9	1	9	1	9	9	9	9	9	9
9	9	9	9	1	1	9	9	9	9
9	9	9	9	9	9	9	9	9	9
9	9	3	2	1	3	3	2	3	3

3	2	2	2	1	3	3	2	3	3
2	2	2	2	2	1	1	2	1	1
9	1	9	1	3	1	3	3	1	1
2	3	1	1	3	3	3	3	3	3
1	9	3	1	1	3	9	3	9	9
2	3	2	1	2	2	2	9	3	2
2	2	2	2	1	3	3	3	3	3
2	2	2	2	1	3	2	3	3	3
1	2	2	3	2	2	2	2	2	2
3	1	1	3	3	1	1	3	3	3
9	9	1	9	1	9	9	9	9	9
9	9	1	2	9	1	1	2	3	3
2	2	2	1	1	2	1	2	9	1
9	9	9	2	1	2	1	3	2	3
1	9	9	1	9	1	9	9	9	9
3	1	3	3	1	1	1	3	9	3
2	1	2	2	1	3	1	2	1	3
1	3	2	3	1	3	3	3	3	3
9	1	1	2	9	1	1	1	1	1
3	2	1	1	3	2	1	2	2	2
2	2	1	3	2	2	3	2	1	2
3	1	3	1	3	1	3	1	3	1
3	1	1	1	3	3	1	3	1	1
1	3	3	3	2	3	3	3	2	2
3	1	3	2	2	2	2	2	2	2
9	9	9	3	3	3	3	3	3	3
1	3	3	3	2	3	3	3	2	2
3	1	2	1	1	3	3	1	1	2
1	3	3	3	2	3	2	3	2	3
3	1	2	3	3	2	2	2	2	1
3	3	1	3	1	3	3	3	1	1
3	2	2	3	2	2	2	3	1	1
2	1	3	2	1	1	1	2	1	2
9	2	2	1	1	3	2	2	3	3
3	1	1	2	3	1	3	3	2	1
1	1	9	3	2	2	1	2	2	1
2	2	2	2	2	1	2	2	2	2
2	2	3	1	3	3	1	1	3	3
9	9	9	1	9	9	9	9	9	9
3	3	3	1	1	2	2	3	2	2
9	9	9	3	9	9	9	9	9	9
2	1	2	1	3	9	9	9	9	9
9	9	9	1	3	2	3	2	2	1
9	9	9	1	3	2	3	1	2	2
3	3	3	2	3	1	1	3	3	3
3	1	1	1	3	1	3	1	1	1
3	3	3	2	2	3	3	3	3	3

3	3	3	1	3	3	3	3	3	3
3	9	9	2	1	9	3	9	9	9
3	1	3	3	3	3	3	3	3	3
1	1	1	3	1	3	1	3	3	1
3	1	3	3	1	3	3	3	3	3
3	1	3	1	3	1	1	1	1	1
3	2	1	1	9	2	2	1	1	1
1	1	2	1	1	2	2	2	2	2
1	1	3	1	3	3	3	3	3	3
3	3	1	3	2	3	2	9	1	3
3	3	1	1	1	3	3	1	1	1
3	1	2	3	1	3	3	2	2	1
3	2	3	1	3	1	1	2	1	2
1	2	3	9	1	3	3	3	3	3
1	2	1	1	1	1	1	2	1	2
1	2	2	2	3	3	1	1	2	3
1	1	2	1	3	3	1	2	2	2
3	3	2	1	3	1	3	3	2	3
1	3	1	1	1	3	3	3	3	3
2	2	2	2	2	2	2	2	2	2
2	2	2	3	1	3	1	3	3	3
3	3	1	1	1	2	2	3	1	2
3	3	3	3	3	3	3	3	3	3
1	1	1	1	1	1	3	1	3	3
3	2	2	3	1	3	3	1	2	3
1	2	3	1	1	2	3	3	1	1
3	1	2	1	2	2	3	1	2	3
3	3	3	3	1	2	2	2	3	3
1	1	2	2	1	3	1	2	1	3
9	9	9	9	1	3	9	9	9	9
2	1	3	1	2	3	2	2	3	1
3	1	2	1	1	1	1	1	1	1
1	3	2	3	1	3	3	1	2	3
1	2	2	2	2	3	3	3	1	3
1	1	1	3	3	3	1	2	1	1
2	2	2	2	9	3	3	3	3	3
3	2	2	1	1	3	1	3	3	3
3	1	2	1	3	2	2	2	2	2
1	3	2	1	3	1	2	2	2	1
2	2	1	1	1	3	3	3	3	2
1	9	9	9	9	9	9	9	9	9
9	9	9	3	9	3	2	2	3	9
2	3	2	1	2	1	2	3	1	2
3	1	1	3	1	3	2	1	3	3
3	3	2	1	1	2	2	2	2	2
3	3	1	1	3	1	3	3	3	3
9	9	9	9	9	9	9	9	9	9

1	1	2	1	3	1	1	2	1	1
1	3	3	1	1	1	3	3	3	3
1	2	1	3	3	3	3	3	3	3
3	2	1	1	2	1	2	1	2	3
9	9	9	9	9	9	9	9	9	9
1	1	1	1	9	1	1	1	1	1
2	2	2	1	1	3	2	3	3	3
2	2	2	2	2	2	2	2	2	2
2	1	2	2	3	3	1	1	1	2
1	1	1	1	3	3	2	1	3	2
9	9	9	1	3	3	3	3	3	3
3	2	3	1	2	1	2	2	2	1
3	2	3	1	2	3	2	2	3	1
9	9	9	9	9	9	9	9	9	9
2	3	3	2	1	2	2	2	2	2
9	9	9	9	9	9	9	9	9	9
3	2	3	1	2	2	3	3	2	2
1	1	2	1	3	3	3	3	3	3
2	3	3	1	1	3	2	3	1	1
1	2	2	1	1	2	2	2	2	2
3	3	3	1	1	1	3	1	1	3
2	2	2	3	1	2	2	3	3	3
9	1	9	9	1	9	9	9	9	9
2	2	2	1	3	2	1	3	2	1
2	1	2	1	1	3	2	2	2	2
2	2	2	1	1	2	2	2	2	2
9	9	9	1	9	3	9	3	9	3
2	2	2	2	1	2	2	2	1	2
2	2	2	3	3	1	2	2	2	1
9	9	9	9	1	9	9	9	9	9
1	1	3	3	1	3	2	3	3	1
1	1	1	3	3	2	2	3	1	2
1	3	1	3	3	3	1	1	3	1
1	2	2	1	1	3	2	3	3	2
1	1	1	1	3	3	2	3	3	3
3	2	2	3	3	3	3	3	3	3
3	1	1	1	1	1	2	3	3	1
1	1	3	1	1	3	3	3	3	1
1	9	9	1	2	9	9	9	9	9
3	3	3	2	2	3	3	3	3	3
2	1	2	2	1	3	3	2	1	3
3	1	3	1	1	3	3	3	3	2
3	2	2	1	1	3	3	3	3	3
1	2	1	1	1	3	9	2	2	3
2	1	2	3	3	3	3	3	3	3
3	2	2	2	1	2	1	1	3	2
3	2	2	1	3	3	2	3	2	1

1	2	1	2	2	2	2	2	2	2
2	2	2	3	1	3	2	2	2	3
2	2	1	2	2	2	1	1	1	1
9	1	9	1	1	2	2	2	2	3
2	1	2	2	3	2	1	1	2	1
9	1	9	9	1	9	9	9	9	9
2	2	2	2	2	2	2	2	2	2
1	9	1	3	1	1	3	2	3	3
2	2	3	1	1	2	3	3	3	3
2	1	2	3	1	3	2	3	3	2
3	1	2	9	3	3	2	2	2	1
3	1	1	1	2	1	1	2	1	3
2	2	2	2	2	2	2	2	2	2
3	3	3	3	3	3	3	3	3	3
3	3	3	3	1	3	1	1	2	1
2	1	3	3	3	2	2	3	1	1
3	3	3	2	3	2	1	1	1	1
2	2	2	3	1	1	2	9	3	3
2	3	3	1	1	3	3	3	3	3
2	2	2	1	1	2	2	2	1	2
3	2	2	1	1	1	3	3	1	1
1	3	2	3	1	3	2	3	1	2
3	1	2	3	1	3	2	3	1	2
2	2	2	3	3	3	1	3	1	3
1	1	2	1	1	3	3	3	3	3
1	2	2	2	2	1	1	1	2	3
3	1	2	3	1	3	2	3	3	3
3	1	2	3	1	3	2	3	3	3
2	2	2	2	3	3	2	2	2	2
2	2	2	1	1	3	1	1	3	3
2	3	3	1	3	3	2	2	2	2
2	3	2	3	1	3	2	3	1	2
3	3	1	3	1	3	3	3	3	3
2	2	2	1	1	3	1	3	3	3
3	3	3	1	3	1	1	3	1	1
2	2	2	1	9	3	2	2	1	1
2	2	2	1	3	3	2	2	1	1
2	1	3	1	2	2	1	1	2	3
3	1	2	1	3	1	2	1	1	1
2	1	2	2	2	2	2	2	2	2
2	1	3	1	2	3	2	3	1	3
1	2	2	3	1	3	1	3	3	3
1	1	1	1	1	2	1	2	2	3
1	1	2	1	1	1	2	2	2	1
1	3	3	3	1	3	2	1	3	1
1	3	1	1	1	3	3	3	3	3
9	1	9	3	1	3	3	3	2	2

2	2	1	2	1	3	2	2	1	2
3	3	1	3	2	2	2	2	2	2
9	9	9	1	1	2	2	3	3	3
2	2	2	3	3	2	2	2	2	2
2	2	2	1	1	9	3	3	3	9
2	2	2	2	1	3	2	2	2	2
3	2	2	2	1	3	3	3	3	3
2	1	2	3	1	3	3	3	3	3
2	1	2	1	1	3	2	3	2	1
2	3	2	1	2	3	2	2	3	3
2	2	2	1	2	3	2	2	3	3
2	2	2	1	1	3	1	1	1	2
2	2	2	1	3	3	2	2	2	2
2	2	2	3	1	3	3	1	3	3
2	2	2	1	3	1	1	1	1	1
2	2	2	1	1	2	2	3	3	2
2	1	2	2	2	2	3	3	3	3
2	2	2	1	1	3	1	3	2	2
1	1	2	1	1	1	2	3	3	2
1	2	2	2	1	3	2	1	3	3
2	2	2	1	2	2	2	2	2	2
2	2	2	3	2	2	2	2	2	2
3	3	2	1	2	3	2	2	2	1
2	2	2	2	1	3	2	3	2	2
2	2	2	1	1	2	2	1	3	2
9	1	9	2	1	3	2	1	3	1
2	2	2	1	1	2	2	2	2	2
2	2	2	2	2	2	2	2	2	2
2	2	2	2	1	2	2	2	2	2
3	2	1	3	1	3	3	3	3	3
2	2	2	2	2	2	2	2	2	2
2	2	2	2	2	2	2	2	2	2
2	2	2	2	2	2	2	2	2	2
2	2	2	2	1	2	2	2	2	2
9	9	9	1	9	3	3	9	9	9
2	2	2	1	1	2	2	2	2	2
2	2	2	1	1	2	2	2	2	2
2	2	2	1	2	2	2	2	2	2
2	2	2	2	2	2	2	2	2	2
2	2	2	1	1	3	2	3	3	3
2	2	2	2	2	2	2	2	2	2
2	2	2	1	1	3	3	3	3	1
2	2	2	1	1	3	3	3	3	3
2	2	2	2	2	2	9	2	2	2
2	2	2	1	2	2	2	2	2	2
2	3	1	1	1	3	2	3	2	1
2	2	2	1	1	9	3	3	3	9

2	2	2	2	2	2	2	2	2	2
2	1	2	2	2	2	2	2	2	2
1	1	1	1	1	3	3	3	3	1
2	2	2	2	2	1	2	2	2	2
2	2	2	2	2	2	2	2	2	2
3	2	1	2	3	3	1	3	2	2
9	1	9	1	1	3	3	3	3	3
9	2	9	9	2	3	3	3	3	3
1	1	3	1	1	3	3	1	1	3
2	1	2	2	1	3	3	3	3	3
2	1	1	1	1	3	3	3	1	3
2	1	2	1	3	2	2	2	2	2
2	1	2	1	1	3	3	3	3	2
9	1	1	1	3	1	1	3	9	9
1	1	3	1	1	1	1	1	3	1
2	2	2	2	1	3	2	1	3	3
2	2	2	3	2	2	2	2	2	2
9	9	9	1	1	2	3	2	3	3
9	9	9	1	1	1	1	1	1	1
2	2	2	1	1	3	2	2	3	2
9	9	9	1	1	3	3	3	9	2
2	2	2	2	2	2	2	2	2	2
2	2	2	1	2	3	2	3	3	3
2	2	2	2	2	2	2	2	2	2
2	2	2	1	2	2	2	2	2	2
2	2	2	1	2	2	2	2	2	2
9	1	9	9	1	9	9	9	9	9
2	2	2	1	3	2	2	2	2	2
2	2	2	1	2	2	2	2	2	2
9	1	9	2	1	3	2	1	3	9
2	2	2	2	1	3	2	1	3	3
1	2	2	1	3	1	2	1	3	2
1	3	3	3	3	1	2	3	2	1
2	2	2	1	1	1	2	1	3	2
2	1	1	2	1	3	3	3	3	2
3	2	1	2	2	3	1	1	1	3
3	3	2	3	1	3	2	3	3	3
1	2	2	3	1	3	2	3	3	3
1	1	1	3	1	3	3	3	3	3
3	1	2	1	2	2	2	3	2	1
3	3	3	3	1	3	3	3	3	3
2	2	2	3	2	3	2	2	3	3
3	1	1	1	1	3	2	2	3	3
2	2	2	2	2	2	2	2	2	2
1	1	1	2	3	3	1	2	3	3
1	1	1	1	2	1	1	3	2	1
2	1	1	3	1	3	2	1	1	2

2	1	2	2	1	1	2	9	3	1
1	3	2	1	2	1	2	1	3	3
3	2	1	1	1	3	2	2	3	3
3	2	1	1	1	3	1	3	3	3
9	9	9	3	2	1	2	9	1	3
2	1	1	3	2	1	9	1	2	1
1	1	1	1	1	2	1	1	3	1
9	9	9	3	1	2	9	9	9	9
2	2	2	2	1	2	2	2	2	1
2	2	2	2	2	2	2	2	2	2
3	3	1	1	3	3	1	1	1	1
3	3	1	1	3	3	1	1	1	1
2	2	2	1	2	3	3	3	3	3
1	2	2	3	1	1	2	1	2	2
2	3	3	3	2	3	2	3	2	2
2	2	2	1	2	3	2	2	3	3
2	2	2	2	1	1	2	1	2	1
9	1	9	1	1	3	3	2	2	3
2	1	1	2	2	3	2	2	3	3
1	1	2	1	3	3	3	3	9	3
2	2	2	1	3	3	3	3	3	2
9	9	9	2	2	2	2	2	2	1
1	1	2	1	3	3	2	2	2	3
9	2	9	9	1	9	9	9	9	9
3	2	1	1	3	1	2	3	1	1
1	1	2	1	1	1	2	1	1	3
1	1	1	1	2	2	1	3	2	1
9	9	9	1	3	2	2	2	2	2
2	2	2	2	1	2	2	2	2	2
9	9	9	9	1	9	9	9	9	9
3	1	3	1	3	3	3	3	2	3
9	9	9	9	9	9	9	9	9	9
9	9	9	9	9	9	9	9	9	9
2	1	1	3	1	3	2	2	2	2
2	1	1	3	1	3	2	2	2	2
9	9	9	1	1	3	1	1	1	1
9	9	9	3	3	1	2	3	1	1
2	1	1	3	1	3	2	2	2	2
2	2	2	1	1	1	1	3	1	3
2	2	2	1	1	1	1	3	1	3
9	9	9	9	3	9	9	9	9	9
2	2	2	1	1	3	3	3	3	3
2	2	2	1	2	2	2	2	1	1
9	9	9	9	9	9	9	9	9	9
9	9	9	3	9	9	9	9	9	9
2	2	1	1	2	1	2	2	1	1
2	2	2	2	9	2	2	2	2	2

9	9	9	1	1	3	3	3	1	1
2	1	2	3	1	3	3	3	3	3
3	3	3	2	1	3	3	1	3	3
2	1	1	9	9	9	9	9	9	9
2	2	2	2	2	2	2	2	2	2
3	3	3	1	9	3	3	3	3	3
2	2	2	2	2	2	2	2	2	2
9	9	9	1	1	3	3	3	3	3
9	9	9	1	9	9	9	9	9	9
9	9	9	2	2	2	2	3	2	2
2	2	1	2	2	3	1	3	2	3
2	2	2	3	1	1	3	1	2	2
3	3	1	2	3	3	1	2	1	2
3	3	3	3	2	3	3	3	3	3
3	2	3	1	3	3	2	3	2	2
1	1	2	3	1	3	3	1	3	3
3	1	1	3	3	3	3	3	3	3
1	2	1	2	1	1	1	2	3	2
2	2	2	1	2	2	2	2	2	2
1	3	1	3	1	2	3	1	2	2
3	1	2	3	2	2	3	3	2	2
1	1	1	1	3	1	1	1	1	1
2	1	2	1	1	1	1	1	1	3
2	3	3	1	3	3	2	2	2	2
3	3	3	3	1	1	1	3	3	3
2	2	2	2	2	2	1	2	2	2
2	2	3	1	3	3	2	3	3	3
2	1	2	2	1	3	3	3	3	1
2	1	2	1	3	1	2	2	3	1
3	3	3	2	3	1	2	3	1	1
3	2	2	1	1	1	1	1	1	1
3	3	2	1	2	3	3	3	3	3
3	1	1	2	1	3	3	2	3	3
3	3	3	1	9	3	3	3	3	3
3	1	2	1	2	2	1	2	2	2
2	1	2	3	2	3	1	2	3	2
3	3	3	3	1	1	1	3	3	3
2	2	2	1	1	9	1	1	1	9
1	1	1	1	3	1	1	1	1	1
1	9	9	1	3	3	2	1	1	1
3	3	1	1	1	2	9	9	2	2
3	2	1	2	1	1	2	3	1	2
3	3	2	3	3	3	2	3	2	3
2	3	2	1	1	3	3	1	3	1
1	1	1	1	3	3	2	3	3	1
3	2	2	1	3	2	2	2	2	2
3	3	3	2	2	2	2	2	2	9

1	2	2	1	2	1	3	2	1	2
2	2	3	1	1	1	2	3	3	3
3	3	3	2	1	1	1	1	2	3
3	3	3	1	2	3	3	3	3	2
1	1	1	1	1	1	1	3	3	3
1	3	2	3	3	1	1	2	1	1
1	1	1	9	9	9	9	9	9	9
3	2	2	2	1	3	2	1	2	3
3	3	3	3	1	3	1	3	3	3
3	2	1	1	3	1	1	2	3	1
3	3	3	3	2	3	1	3	3	2
2	2	2	1	3	2	3	2	1	2
2	2	2	2	2	2	2	2	2	2
1	1	1	1	1	3	3	1	3	1
1	1	1	3	1	3	3	3	3	3
2	2	2	2	2	3	2	2	1	2
2	1	2	2	2	2	2	3	2	2
3	2	2	1	1	1	2	1	2	3
1	3	2	3	3	3	1	1	3	1
1	2	2	1	1	3	3	3	2	3
2	1	1	3	1	3	1	1	1	1
9	2	9	3	2	3	2	2	2	3
3	3	3	3	2	1	1	2	3	3
2	2	2	2	3	3	2	2	2	3
2	2	2	3	1	3	2	2	3	2
2	2	2	1	1	3	3	3	3	3
2	2	2	3	9	2	2	2	2	2
1	1	2	1	3	2	1	2	1	2
3	3	3	2	3	3	3	2	1	3
3	2	1	3	1	3	2	2	3	3
3	3	3	1	3	3	3	3	1	1
3	1	1	3	1	3	3	2	3	3
2	2	3	2	1	1	2	3	2	2
2	1	1	1	3	3	3	3	3	3
2	1	3	1	1	1	2	3	1	2
2	2	2	3	1	1	3	1	3	3
1	1	1	3	1	3	1	1	3	3
2	1	1	1	3	3	2	2	3	1
1	3	2	1	3	3	3	2	1	1
9	9	9	3	9	1	9	9	9	9
2	3	2	1	1	3	2	3	2	3
3	2	1	1	1	2	1	3	3	1
2	2	2	1	2	2	2	2	2	3
1	1	1	1	1	3	1	1	3	3
3	3	1	3	1	3	1	3	3	3
9	9	9	3	1	3	2	3	3	3
1	2	2	3	2	2	1	1	2	3

2	2	2	3	2	2	1	2	1	2
2	2	2	2	2	2	2	2	2	2
3	3	1	3	3	3	3	3	3	3
3	2	1	2	1	1	2	1	3	2
2	2	2	9	1	9	9	9	9	9
1	3	3	9	9	9	9	9	9	9
1	3	3	1	9	9	9	9	9	9
1	1	1	1	1	3	2	2	2	2
2	2	2	9	9	9	9	9	9	9
3	1	3	1	3	1	1	1	1	2
1	1	2	1	3	2	1	1	1	2
3	3	2	1	3	3	1	1	3	1
9	1	9	9	1	2	3	1	3	1
2	1	1	9	1	3	3	3	3	3
9	9	9	1	9	9	2	2	2	2
2	2	2	3	1	3	3	2	3	2
1	1	9	9	9	3	2	2	1	1
2	2	1	3	3	1	1	1	1	1
3	1	3	3	3	3	3	3	3	3
2	2	1	3	2	3	1	3	2	3
3	2	1	1	1	1	1	1	1	1
1	2	2	2	1	2	3	3	3	3
2	2	2	1	3	3	1	3	1	2
3	3	2	1	3	3	3	2	3	3
9	9	9	3	1	3	2	3	3	2
1	1	2	2	2	3	3	2	1	1
2	3	2	2	1	2	3	2	3	1
3	2	1	2	3	3	2	2	3	1
3	1	3	3	3	3	3	3	3	3
2	2	2	1	1	9	3	1	2	3
2	1	2	9	2	1	2	2	1	2
2	2	2	1	2	2	2	2	2	2
1	1	1	1	3	1	1	1	1	1
2	2	1	1	1	3	3	3	3	3
3	1	2	3	1	1	2	3	2	1
9	9	9	2	3	1	2	3	3	3
2	2	1	2	1	1	1	1	1	1
1	3	1	1	3	3	3	3	3	3
2	2	1	1	1	3	3	3	3	3
1	1	2	1	1	2	2	2	1	1
3	3	3	2	2	3	3	3	3	3
1	2	2	2	1	1	1	2	2	2
3	3	1	1	3	3	2	1	2	3
3	1	3	1	3	2	1	1	2	2
2	1	1	1	3	1	3	3	3	2
3	3	3	2	3	1	3	3	3	3
2	2	2	1	3	3	2	1	1	2

3	1	1	1	3	3	2	1	1	1
3	1	3	1	2	3	2	2	2	2
2	2	2	2	2	2	2	2	3	1
2	2	1	1	2	2	1	1	2	3
3	3	3	1	1	3	3	1	1	1
3	2	1	1	2	3	1	2	1	2
9	9	9	1	9	2	9	9	9	9
3	2	1	1	3	2	2	2	1	2

BSEdisliket	BSElumpse	CaBreastCo	CaBreastfr	CaBreastTr	BSEdetects	EverDoneB	StandMirro	Liedown	RhandtoLb
1	1	1	1	1	1	1	1	2	3
1	3	1	1	3	1	1	3	3	1
3	1	1	1	2	1	2	3	1	1
1	3	1	1	3	1	1	3	3	1
9	9	9	9	9	1	9	1	9	9
1	3	1	1	3	1	1	1	3	1
3	1	2	1	2	1	1	9	1	1
3	1	1	2	2	1	1	1	2	1
3	2	1	2	3	1	3	1	9	9
9	9	1	1	3	9	9	2	2	9
9	9	2	2	2	2	1	1	9	1
3	3	1	1	2	2	1	2	1	1
2	1	2	2	2	2	1	3	1	1
2	2	1	2	2	1	1	2	1	1
1	1	1	2	2	1	1	1	1	1
3	1	1	1	3	1	2	1	1	2
1	1	1	1	2	2	2	1	2	1
3	1	1	1	3	1	2	1	1	2
3	1	3	1	3	1	2	1	1	2
3	2	1	2	2	2	1	1	2	1
3	1	1	2	3	1	2	1	1	1
3	1	1	2	3	1	2	1	1	1
3	9	2	2	2	1	4	1	1	1
9	1	1	3	3	1	4	9	1	1
3	1	1	1	2	2	3	1	1	2
2	1	2	2	3	1	2	2	1	1
2	1	1	3	3	1	1	3	1	2
3	1	1	1	3	1	2	1	1	1
3	1	1	1	3	1	4	3	1	1
1	1	1	1	3	1	1	3	1	1
1	1	1	1	3	1	1	3	1	1
3	1	1	1	2	1	1	1	2	1
3	1	1	1	1	1	3	2	1	1
3	1	1	1	2	1	3	1	2	1
3	1	1	1	3	1	1	1	3	1
3	1	1	1	2	1	3	1	2	1
3	1	1	1	3	1	1	1	3	1
3	1	1	1	3	1	4	1	1	1
9	9	9	9	9	9	9	3	1	1
3	1	1	1	3	1	1	3	1	1
3	1	1	1	2	1	5	3	1	1
1	1	1	1	2	1	1	1	2	1
3	9	1	1	3	2	2	2	3	2
3	1	1	1	3	1	4	1	3	1
3	1	1	1	3	1	2	3	3	1
3	1	2	1	2	2	2	1	9	9

3	1	1	1	9	1	1	1	1	2
3	1	1	1	3	1	4	3	3	1
3	1	1	1	3	1	4	9	1	1
3	1	1	3	1	1	2	2	1	1
3	1	1	1	3	1	2	1	1	1
3	1	1	1	3	1	5	1	1	1
3	1	1	3	3	1	5	3	1	1
3	1	1	1	3	1	5	1	3	1
9	1	1	1	9	1	4	1	9	1
3	1	2	3	3	1	4	1	3	1
9	9	1	9	9	9	1	9	9	9
3	1	1	1	1	1	3	1	1	1
1	1	2	1	1	1	5	1	3	1
2	1	1	2	1	1	6	1	9	1
1	1	1	1	3	1	5	1	3	2
3	1	1	1	9	1	4	1	2	1
3	1	1	1	3	1	5	1	2	1
3	1	1	1	3	1	4	1	1	1
3	1	1	1	3	2	2	2	3	2
3	3	1	1	3	1	1	1	1	1
3	1	1	1	3	1	5	1	9	9
1	1	1	1	3	1	5	3	1	9
3	1	1	1	3	1	4	9	9	9
3	1	1	1	3	1	2	1	3	1
9	1	9	1	1	1	4	9	1	9
1	1	1	1	9	1	3	1	1	1
3	1	1	1	9	1	6	1	3	1
3	1	1	1	3	1	4	3	3	1
3	1	1	2	3	1	3	1	1	1
3	1	1	1	3	1	3	1	3	1
1	1	3	3	3	3	1	1	1	1
3	1	3	3	3	3	3	1	1	1
1	1	1	2	2	2	9	1	1	1
3	1	1	3	3	1	4	2	3	1
3	1	1	1	1	1	2	1	1	1
3	1	1	1	1	1	3	1	1	1
1	1	1	1	1	1	1	1	1	1
3	1	1	1	3	1	1	2	3	1
3	1	1	1	3	1	3	9	9	1
3	1	1	1	3	1	2	1	3	1
3	1	1	1	2	1	1	1	9	1
3	1	1	1	3	1	5	1	2	1
3	1	1	1	3	1	1	3	3	1
3	1	1	2	3	1	4	1	1	1
3	1	9	9	9	1	4	1	3	1
9	1	9	9	9	9	3	1	1	1
1	1	9	1	9	1	3	1	9	1

3	1	1	2	2	1	1	2	1	1
3	1	1	2	2	1	1	2	1	1
1	1	1	1	3	1	5	1	1	1
3	1	1	2	2	1	1	2	1	1
3	1	1	3	3	1	2	9	9	1
1	1	9	9	9	9	2	1	1	1
3	1	1	1	3	1	3	1	1	1
3	1	1	3	3	1	2	1	3	1
9	1	1	1	3	1	3	1	1	1
3	1	1	1	3	1	4	1	3	1
3	1	1	1	2	1	2	1	2	1
3	1	2	2	2	1	9	2	1	1
9	1	9	9	9	1	3	1	3	1
2	1	1	1	1	1	1	1	3	1
3	1	2	1	1	1	1	1	2	1
1	1	1	2	3	1	4	2	1	1
3	1	1	1	2	2	2	9	1	1
2	1	2	2	3	2	2	1	1	1
3	1	1	1	3	1	2	1	1	1
1	1	1	1	3	1	2	9	9	9
3	1	1	1	3	1	2	3	1	1
3	1	1	1	3	1	5	1	9	1
2	2	2	2	2	2	4	2	2	2
1	1	1	1	3	1	1	3	3	1
1	1	1	2	1	1	9	1	1	1
2	1	1	2	2	1	1	3	1	1
3	1	1	1	3	1	2	1	3	1
9	1	1	9	9	9	2	1	3	1
3	1	2	2	3	2	3	3	2	2
1	1	1	1	1	1	1	2	2	2
2	1	2	2	2	1	1	1	2	2
1	2	2	2	3	2	2	3	2	2
2	1	1	2	9	1	1	2	2	2
2	1	2	3	2	2	3	2	3	2
1	1	1	1	1	1	1	2	2	2
3	1	1	3	3	1	2	3	3	3
1	1	1	1	2	1	1	1	3	2
2	1	2	2	3	1	1	3	1	1
9	1	1	1	9	9	2	9	1	9
3	1	1	1	3	1	6	1	1	1
3	1	1	1	3	1	5	1	1	1
3	1	1	1	3	1	5	1	1	1
1	2	2	2	3	2	2	3	2	2
1	1	1	3	3	1	1	1	2	1
2	2	2	2	2	1	3	2	2	1
3	1	1	1	2	1	4	3	1	1
2	1	1	2	2	1	3	2	2	2

9	1	1	9	9	9	1	2	3	3
3	1	1	1	3	1	1	1	3	1
3	1	1	1	2	1	1	1	2	1
3	9	1	1	3	1	5	1	1	1
3	3	3	2	2	2	5	1	1	1
1	1	1	1	3	1	2	9	1	1
2	3	1	3	2	1	2	3	3	2
3	1	1	2	2	1	4	2	3	1
2	2	2	1	2	2	1	2	2	2
2	1	2	2	2	2	1	2	2	2
2	1	2	1	3	1	1	3	2	2
2	1	2	1	3	1	1	3	2	2
9	9	9	1	3	2	4	1	9	9
3	1	1	1	1	1	2	1	3	1
3	1	1	1	2	1	1	1	2	1
1	1	1	1	2	2	1	1	2	1
3	1	1	1	3	1	1	9	9	1
1	1	1	1	3	1	4	1	3	1
9	9	9	9	1	9	1	9	1	9
3	1	1	1	3	1	1	1	1	1
3	1	1	1	3	1	1	1	9	9
3	1	1	1	3	1	2	1	1	1
3	1	1	1	3	1	1	1	2	1
3	1	1	1	3	1	1	1	1	1
3	1	1	1	3	1	1	1	1	1
3	1	1	1	3	1	1	1	1	1
3	1	1	1	3	1	1	1	1	1
3	1	1	1	3	1	1	1	1	1
3	1	1	1	3	1	1	1	1	1
3	1	1	1	3	1	1	1	1	1
1	1	1	1	3	1	5	1	1	9
1	1	1	1	2	1	1	1	2	3
3	1	1	1	3	1	1	9	9	9
9	1	1	1	3	1	1	1	1	1
9	1	1	1	3	1	1	1	1	1
3	1	1	1	3	1	2	1	1	1
3	3	1	1	3	1	1	1	1	1
3	1	1	1	3	1	1	1	1	1
3	1	1	1	3	1	1	1	9	1
3	1	1	1	3	1	1	1	1	1
3	1	1	1	3	1	1	1	1	1
3	1	1	1	3	1	5	1	1	1
3	1	1	1	3	1	1	9	9	9
3	1	1	1	3	1	1	1	1	1
3	1	1	1	3	1	1	1	1	1
3	1	1	1	3	1	1	1	1	1
3	1	1	1	3	1	1	1	1	1

3	1	1	1	3	1	1	1	1	1
3	1	1	1	3	1	1	1	1	1
3	1	1	1	3	1	1	1	1	1
3	1	1	1	3	1	1	1	1	1
3	1	1	1	3	1	1	1	1	1
3	1	1	1	3	1	2	1	1	1
3	1	1	1	3	1	1	1	1	1
3	1	1	1	9	1	5	1	3	1
3	1	1	1	3	1	2	1	3	1
3	9	1	1	3	1	9	3	1	1
1	1	3	3	3	1	2	1	3	1
3	1	1	1	1	1	3	1	1	1
3	1	1	1	3	1	2	1	3	1
3	1	1	1	3	1	1	9	9	9
2	1	1	1	2	1	5	3	3	1
2	1	1	2	3	1	4	1	2	1
1	1	2	2	2	1	1	1	1	2
1	1	1	1	1	1	1	1	1	1
3	1	1	1	3	1	3	2	1	1
3	1	1	2	2	1	4	2	2	2
1	1	1	1	3	2	1	1	2	1
3	3	1	1	2	1	1	2	2	2
3	1	1	1	2	1	1	1	2	1
2	1	1	1	2	2	3	1	2	1
3	2	1	1	2	1	1	2	2	2
2	2	1	1	2	1	1	2	2	2
3	1	1	2	2	1	4	2	2	2
2	2	1	1	2	1	1	2	2	2
2	2	2	2	2	2	1	2	2	2
3	1	1	1	3	1	2	1	1	1
2	1	1	1	3	2	1	2	2	2
2	1	1	1	3	2	1	2	2	2
3	1	1	1	3	1	2	1	1	1
1	1	1	1	3	1	1	1	3	1
2	1	1	2	2	1	2	1	2	2
1	1	1	1	1	1	1	2	2	2
1	1	2	2	2	1	1	1	1	2
1	1	1	1	2	1	5	1	2	1
3	1	1	1	3	1	2	3	2	2
1	1	3	1	2	1	3	2	2	3
2	3	1	2	2	2	1	1	2	3
3	1	1	2	2	1	1	2	2	2
9	9	1	9	9	9	3	9	9	9
9	9	1	9	9	9	3	9	9	9
1	1	1	1	1	1	1	1	2	2
2	1	1	1	2	1	1	3	2	1
1	1	1	1	3	1	1	2	9	9

3	1	1	1	2	1	1	1	1	1
3	1	1	1	2	1	1	1	1	1
3	1	1	1	2	1	1	2	2	2
1	1	1	3	2	1	1	2	2	2
3	1	3	1	3	1	1	2	1	2
2	1	1	2	3	1	4	1	1	1
2	1	1	1	3	3	3	1	3	1
2	1	1	3	3	2	7	2	3	2
3	1	1	1	3	1	5	1	3	2
3	1	1	3	3	3	3	3	3	3
2	1	1	1	2	2	1	1	2	1
3	1	3	3	2	1	3	3	3	1
3	1	3	3	2	1	3	3	3	1
3	2	2	2	2	2	2	2	3	1
3	1	1	2	2	1	3	1	3	1
3	1	1	1	3	1	2	2	3	1
3	1	1	2	3	2	3	3	3	1
2	2	1	2	3	2	1	2	2	2
2	1	1	1	3	3	1	3	1	1
1	1	1	1	3	2	1	1	2	1
1	1	1	2	1	1	1	1	1	2
2	1	1	1	2	1	2	1	2	1
3	1	1	2	3	1	4	2	3	1
2	1	2	1	3	1	3	1	1	2
9	1	2	1	9	9	1	1	9	1
2	2	2	2	2	2	1	2	2	2
2	2	2	2	2	2	1	2	2	2
3	3	1	9	2	1	1	1	1	1
2	2	2	2	2	2	2	9	9	9
3	1	2	2	2	2	1	9	1	1
9	9	1	9	9	9	2	1	9	9
3	1	2	2	2	1	2	1	1	1
1	1	3	1	1	1	1	2	1	1
3	1	3	2	2	1	2	1	1	1
9	9	9	9	1	9	1	1	9	9
1	2	2	2	3	2	2	3	2	2
3	1	1	1	3	1	1	2	1	1
1	2	1	1	3	1	4	1	3	1
3	1	1	2	2	1	1	2	1	1
3	1	1	3	3	1	2	2	1	1
3	3	1	3	2	1	3	3	2	1
2	1	1	1	2	1	4	1	3	2
3	1	1	2	2	1	5	1	3	1
2	1	1	1	3	1	3	2	2	2
2	1	1	1	2	1	1	1	9	9
9	1	1	1	2	1	3	2	2	2
3	1	2	1	1	2	5	3	2	1

9	9	9	9	9	9	3	9	9	9
3	1	1	1	2	1	2	1	3	2
3	1	1	1	3	1	5	2	2	1
3	1	1	1	2	1	3	1	2	1
3	1	1	3	2	1	1	2	3	2
2	1	1	1	3	1	1	1	2	1
3	1	1	1	1	1	1	1	2	2
3	1	1	1	3	3	3	1	1	1
3	1	1	3	3	1	4	1	9	1
3	1	2	2	2	2	2	2	1	2
2	2	2	2	2	2	1	2	2	2
3	1	1	2	3	1	3	1	2	2
1	1	1	1	1	1	2	9	9	9
1	3	1	1	3	1	2	3	1	1
1	1	3	1	1	1	2	1	1	3
3	1	1	1	3	1	1	1	3	2
3	1	1	1	1	1	1	2	2	1
2	1	1	1	1	2	6	1	1	1
1	2	1	2	1	1	1	1	3	1
9	9	9	9	9	9	9	9	9	9
1	1	2	2	2	3	1	2	3	2
3	1	1	2	2	1	4	1	2	9
3	1	1	1	3	1	4	1	3	1
1	1	2	2	2	2	1	2	2	2
2	1	1	1	3	1	3	2	2	2
2	1	1	3	3	1	4	3	3	1
3	1	1	1	2	1	3	3	2	1
3	1	1	1	3	1	1	2	2	3
3	1	1	1	3	1	3	3	1	1
1	2	1	1	3	1	1	3	2	1
3	1	1	1	3	1	2	1	2	1
3	1	2	3	3	1	1	1	3	1
3	1	1	1	3	1	4	9	9	9
3	1	1	1	3	1	1	3	3	1
3	1	2	1	3	1	1	1	2	1
1	1	1	3	3	1	1	3	1	1
3	1	1	1	3	1	5	3	3	1
3	1	1	1	3	1	4	3	1	1
3	1	1	3	3	1	4	3	1	1
3	1	2	2	2	1	1	2	2	1
1	1	1	3	3	1	2	2	3	2
9	1	1	1	2	1	4	3	1	1
9	9	1	2	2	2	5	9	9	1
3	1	1	2	3	1	1	2	2	2
3	1	3	3	3	1	2	1	3	1
3	1	1	1	2	1	2	2	1	1
3	1	1	2	3	1	5	1	1	1

3	1	1	1	2	1	2	3	2	1
3	1	1	1	2	1	2	3	2	1
9	1	9	9	9	1	2	9	9	9
3	1	1	3	3	1	3	1	1	1
9	1	1	9	9	9	1	9	9	9
3	1	1	2	2	1	4	2	2	1
3	1	1	1	2	1	4	1	2	1
3	9	3	9	3	9	1	9	9	1
3	1	1	1	3	1	1	1	1	1
3	1	1	2	1	1	2	1	2	1
2	1	1	1	3	1	1	1	3	2
1	1	2	2	3	2	1	3	3	2
3	1	2	1	3	1	4	1	3	1
2	1	1	1	3	1	2	1	2	2
2	1	1	1	3	1	1	1	3	1
1	1	1	3	3	1	2	3	2	2
2	1	1	1	3	1	1	1	3	1
2	1	1	2	3	1	2	3	3	2
1	1	1	1	3	1	6	3	3	1
3	1	1	2	2	1	1	2	2	1
1	1	1	3	3	1	1	3	2	1
3	1	1	1	2	1	2	3	3	2
3	1	1	1	3	1	5	3	3	2
3	1	1	2	3	1	4	1	2	3
1	1	1	1	1	2	1	2	2	2
2	1	1	2	3	2	1	2	1	1
3	1	2	1	2	1	1	1	1	1
1	2	2	2	2	2	1	1	1	2
3	1	1	2	2	1	1	2	2	2
2	1	1	3	3	1	2	2	1	1
3	1	1	1	2	1	3	2	3	1
3	1	1	1	2	1	3	2	3	1
3	2	2	3	2	3	1	3	3	2
2	1	2	2	2	2	1	2	2	2
2	1	2	2	2	2	1	2	2	2
3	1	1	1	1	1	1	1	3	2
1	1	2	1	2	1	3	1	3	1
2	1	1	1	1	1	2	9	1	9
2	1	2	2	2	1	1	2	2	2
2	1	1	3	3	3	4	2	3	1
3	1	1	3	1	2	2	2	1	2
3	1	2	2	2	1	2	1	1	2
3	1	1	1	1	1	1	1	2	1
3	1	2	2	2	1	1	1	2	2
3	1	1	1	2	1	1	1	1	2
3	1	1	1	3	1	5	2	1	1
2	1	1	1	3	1	1	2	2	2

3	1	1	1	2	1	1	3	3	2
3	1	1	1	2	1	3	1	2	1
2	1	1	2	3	2	2	1	2	2
1	2	1	1	2	1	1	1	3	1
3	1	1	1	2	1	1	3	1	3
2	1	1	2	2	1	5	3	3	2
2	1	1	2	2	2	1	1	3	2
3	1	1	2	3	1	3	1	2	2
3	1	1	2	3	1	3	3	3	2
1	1	1	1	2	2	1	2	2	1
2	1	1	2	2	1	4	1	1	1
1	1	1	2	2	1	2	1	3	1
3	1	1	1	1	1	2	3	3	1
2	2	2	1	3	2	1	2	2	2
2	2	2	1	3	2	1	2	2	2
1	1	1	1	2	1	1	2	1	1
3	1	3	1	2	1	1	1	2	1
3	1	1	1	2	1	1	1	2	2
3	1	1	1	2	1	1	1	2	2
3	1	1	1	3	1	1	9	1	9
3	1	1	1	1	1	2	1	1	1
3	1	1	1	3	1	1	2	1	2
3	1	1	1	3	1	3	9	9	9
1	1	1	2	1	1	1	2	2	2
3	1	1	1	1	1	2	1	2	1
3	1	2	1	3	1	4	3	3	1
1	2	1	2	3	1	2	3	3	1
1	9	1	3	2	3	9	9	9	9
1	1	2	2	1	1	1	3	3	2
1	1	1	1	2	2	1	2	1	3
2	1	2	2	2	2	1	1	2	1
3	1	2	2	2	1	3	1	2	2
2	1	2	2	2	2	6	2	2	2
3	1	1	1	3	3	9	1	1	1
3	1	1	1	1	1	1	1	1	1
1	1	2	2	1	2	2	1	9	1
3	2	3	3	3	3	1	2	3	3
1	1	1	1	3	1	1	3	2	2
2	2	2	2	2	2	1	3	2	2
1	1	1	1	2	1	4	1	3	1
1	1	2	3	2	3	1	1	2	2
1	3	1	1	3	1	9	1	3	1
1	1	1	1	2	1	3	1	1	2
3	1	1	1	2	3	1	3	1	3
9	9	9	9	9	9	9	9	9	9
2	1	1	1	3	1	5	1	1	1
3	3	1	1	3	1	1	9	9	9

1	9	1	1	3	1	5	1	1	1
3	1	1	1	3	1	2	1	1	1
3	1	1	1	3	1	1	1	1	1
3	1	1	1	3	1	1	1	1	1
3	1	1	1	3	1	1	1	1	1
3	1	1	1	3	1	2	1	1	1
3	1	1	1	3	1	1	1	3	1
3	1	1	1	1	1	1	1	3	1
3	1	1	1	3	1	1	1	3	1
3	1	1	1	3	1	1	1	1	1
9	3	1	3	2	2	1	1	3	1
3	1	9	1	3	1	1	1	1	1
3	1	1	1	3	1	1	1	3	1
3	1	1	1	3	1	1	1	3	1
3	1	1	1	3	1	1	1	1	1
3	1	1	1	3	1	1	1	3	1
3	1	1	1	3	1	1	1	3	1
3	1	1	1	3	1	1	1	3	1
3	1	1	1	3	1	1	1	3	1
3	1	1	1	3	1	1	1	9	1
3	1	1	1	3	1	1	1	3	1
3	1	1	1	3	1	1	1	1	1
3	1	1	1	3	1	1	1	1	1
3	1	1	1	3	1	1	1	1	1
3	1	1	1	3	1	1	1	1	1
3	1	1	1	3	1	1	1	1	1
3	1	1	1	3	1	1	1	1	1
3	1	1	1	3	1	1	1	1	1
3	1	1	1	3	1	1	1	1	1
3	1	1	1	3	1	1	1	1	1
3	1	1	1	3	1	2	1	3	1
3	1	1	1	3	1	1	1	3	1
3	1	1	1	3	1	3	1	1	1
3	1	1	1	3	1	3	1	1	1
3	1	1	1	3	1	1	1	1	1
3	1	1	1	3	1	2	1	1	1
2	1	1	1	3	1	5	1	2	1
3	1	1	1	3	1	1	1	1	1
3	1	1	1	3	1	1	1	1	1
3	1	1	1	3	1	1	1	1	1
3	1	1	1	3	1	2	1	1	1
3	1	2	1	2	1	5	2	2	1
1	1	1	1	2	1	3	1	1	2
3	1	1	1	2	1	1	2	2	1
2	1	1	2	2	2	1	2	9	9
2	1	2	2	2	2	2	1	3	1
3	1	1	1	3	1	4	3	3	1

2	2	1	2	2	2	1	2	2	2
1	1	1	1	2	1	4	1	3	1
1	1	1	1	2	1	4	1	3	1
3	1	1	1	2	1	2	2	1	1
2	1	1	1	2	1	3	1	2	2
1	1	1	2	2	3	1	2	1	2
2	1	1	1	2	3	3	1	3	1
2	2	2	2	1	2	1	2	2	1
3	1	1	3	2	1	3	2	2	1
1	1	1	2	2	2	1	2	2	2
1	1	1	1	2	1	9	1	3	1
9	9	9	9	9	1	1	1	9	9
9	1	1	1	9	9	4	9	9	1
2	1	2	2	2	2	1	2	2	2
1	9	9	9	9	1	1	9	9	9
1	9	9	9	9	1	3	1	9	9
9	9	9	9	9	1	1	1	9	9
9	1	1	9	9	9	1	1	9	9
9	1	1	9	9	9	1	9	9	1
3	1	9	1	3	1	2	1	3	1
3	3	3	3	3	1	3	1	3	3
9	3	9	3	3	1	3	1	3	3
2	1	1	1	3	1	5	1	3	1
9	9	1	9	9	9	3	1	9	9
9	1	9	9	9	1	3	9	9	9
2	1	1	1	3	1	2	1	1	1
9	1	9	9	9	1	3	9	9	1
2	1	1	2	3	1	2	1	3	1
2	1	1	1	3	1	4	1	3	1
9	9	9	9	9	1	1	9	9	1
9	1	1	9	9	1	3	1	9	9
3	2	1	1	3	1	5	1	2	1
2	1	2	3	2	2	1	2	2	2
3	1	1	1	2	1	3	1	3	2
2	1	1	1	1	2	3	1	2	1
2	1	2	1	3	1	2	1	3	1
3	1	1	1	3	1	1	1	2	2
3	1	3	2	3	1	1	3	3	3
2	2	2	2	2	2	1	2	2	2
3	1	1	1	2	1	4	2	3	1
2	3	1	2	3	1	3	9	9	9
3	1	1	1	1	1	3	3	2	1
1	1	1	1	1	1	1	2	1	1
1	1	1	1	3	1	4	2	2	1
2	1	1	1	2	1	3	1	3	1
9	1	1	2	2	2	3	1	3	2
3	1	1	1	3	1	3	1	3	1

1	1	1	2	2	1	2	9	9	9
3	1	1	1	2	1	1	3	3	1
2	1	1	1	1	1	1	3	3	3
3	1	1	1	2	2	1	1	1	2
3	1	1	2	3	2	1	1	3	2
2	1	1	2	2	2	1	2	2	2
3	1	1	3	3	1	1	1	3	2
2	1	1	1	3	1	1	1	1	1
2	1	1	1	1	1	4	1	2	2
2	1	1	2	3	2	1	2	2	2
2	1	1	2	3	2	2	2	3	1
3	1	2	1	2	1	4	1	3	1
2	2	2	3	2	2	1	2	2	2
2	2	2	3	2	2	1	2	2	2
1	2	1	2	3	1	1	1	1	2
2	2	1	2	3	1	1	1	2	2
3	1	2	1	3	1	5	1	2	2
1	1	1	1	1	1	1	2	2	2
3	1	2	1	2	2	3	2	3	1
1	1	1	1	3	1	1	1	3	1
3	1	1	1	3	1	4	3	3	1
3	1	1	1	3	1	2	3	3	2
3	1	1	1	3	1	2	1	3	1
3	1	1	1	3	1	2	1	3	1
2	1	1	1	3	1	2	1	2	1
3	1	1	1	2	1	5	2	2	1
1	1	1	3	3	1	2	1	1	1
2	1	1	3	3	1	2	1	1	1
3	1	1	1	2	1	1	1	2	2
3	1	1	3	3	2	1	2	2	1
1	1	1	1	2	1	9	1	3	1
9	1	1	1	2	1	9	2	9	2
1	2	1	2	3	2	1	3	3	3
1	1	2	3	3	1	4	3	9	1
2	1	1	1	3	3	2	1	2	1
9	9	2	9	9	9	1	2	9	9
2	1	1	3	3	1	1	2	1	1
3	1	1	1	3	1	3	1	1	1
2	2	3	2	2	2	1	2	2	2
3	1	1	1	3	1	3	1	2	1
2	1	1	2	3	1	3	1	2	9
2	1	1	1	2	1	1	1	2	2
3	1	3	1	3	1	4	3	3	3
3	1	2	2	2	1	9	2	1	1
3	1	1	1	1	1	3	1	3	1
9	9	1	2	3	1	3	1	3	3
9	1	1	9	9	9	3	9	9	1

9	1	9	9	9	1	1	1	9	9
9	1	9	9	9	1	1	1	9	9
9	9	1	9	9	9	1	9	9	1
3	1	1	1	3	1	7	1	9	9
3	1	1	1	3	1	3	1	9	9
9	9	9	9	1	9	2	1	9	9
9	9	2	2	2	2	1	1	9	9
9	1	9	9	9	1	1	1	9	9
9	9	9	9	9	1	2	1	9	9
9	9	9	9	9	1	1	1	9	9
9	1	1	3	3	1	1	1	3	3
9	9	9	9	9	1	1	1	9	9
9	9	9	9	9	1	1	1	9	9
9	9	1	9	9	9	3	1	9	9
2	2	1	9	1	1	1	1	9	9
9	9	1	9	9	9	1	1	9	9
9	9	9	9	9	9	1	1	9	9
3	1	1	1	3	1	1	1	9	9
2	2	2	2	2	2	1	2	2	2
2	1	1	1	1	1	1	1	1	1
1	1	1	1	1	1	1	1	1	1
1	1	1	2	2	1	1	3	3	3
1	1	2	1	1	1	1	1	2	2
1	1	1	1	1	1	2	1	3	1
3	1	2	2	3	1	1	2	2	2
2	2	2	1	2	3	5	1	3	2
2	1	1	1	2	1	1	2	2	2
2	2	1	2	2	1	1	1	3	2
9	1	2	2	2	2	1	2	2	2
2	2	3	9	2	2	1	2	2	2
3	3	1	1	1	1	1	3	3	3
3	1	2	2	3	1	4	1	3	2
3	1	1	2	3	1	1	2	2	2
1	9	1	3	3	1	1	1	3	3
3	1	1	1	2	1	1	1	2	1
2	2	1	2	2	2	1	1	2	2
2	2	1	2	3	2	1	2	3	9
1	1	2	3	1	3	2	2	3	1
1	1	1	1	2	1	1	2	1	2
3	1	1	1	2	1	1	1	3	1
1	2	1	2	3	2	9	2	2	9
3	1	1	1	1	1	3	1	3	1
2	2	1	1	3	1	3	3	1	1
3	1	1	1	3	1	2	1	2	1
3	1	1	1	1	1	1	1	3	1
2	2	1	1	3	3	1	9	9	9
2	3	1	2	2	2	1	1	2	2

1	2	1	1	3	2	1	1	3	1
1	1	1	1	2	2	2	1	3	1
1	1	3	1	3	1	1	1	3	1
1	1	1	1	1	1	1	1	2	1
3	1	2	1	3	1	1	3	1	1
3	1	2	1	3	1	1	3	1	1
2	1	1	1	1	2	4	2	1	2
1	2	1	1	3	2	1	1	3	1
1	1	1	2	1	3	1	1	9	1
3	1	1	1	1	1	1	1	3	1
2	1	1	2	1	3	1	2	3	1
2	1	1	1	1	1	3	1	3	1
2	2	2	2	2	2	9	2	2	3
1	1	2	3	1	3	2	1	2	3
2	1	1	3	1	3	1	2	2	2
1	1	1	2	3	1	2	1	2	1
1	1	1	1	1	1	4	1	1	1
9	9	1	9	9	9	1	9	9	9
1	1	1	2	1	1	3	1	3	1
3	1	1	1	3	1	1	3	3	3
3	3	1	2	9	1	1	1	1	3
2	2	2	3	3	3	9	2	2	3
2	3	1	2	3	3	2	1	1	2
3	2	1	3	2	1	2	1	3	1
2	1	1	1	3	1	1	2	2	2
3	1	1	1	3	3	1	1	3	3
3	1	1	1	3	1	1	1	3	3
3	3	1	1	3	1	3	3	1	1
2	3	1	2	2	2	1	1	1	2
1	1	1	1	3	3	3	1	3	1
3	1	1	1	3	1	4	3	1	1
3	1	1	1	3	3	1	3	3	3
2	1	1	1	2	1	1	1	1	2
1	1	1	1	3	3	1	1	3	1
1	1	1	1	3	3	1	1	3	1
1	1	1	1	2	1	1	1	3	1
1	1	1	1	3	2	4	1	3	1
9	9	9	9	9	9	9	9	9	9
1	1	1	1	1	1	1	2	2	2
1	1	1	1	1	1	1	9	9	9
1	1	9	9	9	9	9	9	9	9
2	2	2	9	2	3	9	3	9	3
3	1	1	1	2	3	1	1	3	3
1	1	9	9	9	9	9	9	9	9
3	1	1	1	2	3	1	1	3	3
2	2	1	2	2	2	9	2	3	1
1	1	1	1	1	1	1	1	3	1

3	1	1	1	3	1	1	1	3	3
2	1	1	1	2	1	3	1	1	1
1	1	1	1	1	1	1	1	3	2
1	1	1	1	2	2	1	1	2	2
3	1	3	2	2	1	4	2	2	2
3	2	1	3	2	3	2	3	9	1
2	2	1	2	3	2	1	9	9	9
3	1	1	3	3	1	2	1	3	1
3	3	3	3	2	1	1	3	3	3
3	1	1	1	1	1	5	3	3	1
3	3	3	3	3	3	1	3	3	3
3	1	1	1	3	1	1	1	3	1
2	2	1	2	2	1	1	2	3	1
2	2	1	2	2	2	1	2	3	1
2	2	1	2	2	2	1	2	3	3
3	1	1	2	2	2	1	2	3	2
2	2	1	2	2	2	1	2	3	1
2	2	1	2	2	2	1	2	3	1
2	2	1	2	2	2	1	2	3	1
3	1	1	1	1	1	1	1	3	1
2	3	2	1	3	2	4	1	3	2
3	3	1	2	3	3	9	1	3	2
1	1	1	2	2	2	3	2	2	1
1	1	1	1	2	1	1	1	2	2
2	3	3	3	3	3	1	1	3	3
3	2	1	2	2	2	1	2	1	3
2	1	1	2	2	2	1	1	9	9
3	1	1	1	1	1	1	1	1	1
9	9	9	9	9	1	3	9	9	1
3	3	3	3	3	3	7	1	3	3
1	1	1	2	1	3	1	3	3	1
1	2	2	3	2	3	7	2	9	3
1	3	2	1	3	2	3	2	1	3
3	3	1	2	1	2	1	2	3	3
3	3	3	3	3	3	1	3	3	3
3	1	2	1	1	1	1	3	3	3
1	1	2	1	1	1	1	1	9	9
2	1	1	1	2	1	1	1	3	1
2	1	1	1	2	1	1	2	1	2
1	1	1	1	1	1	1	1	1	1
1	2	1	1	1	2	1	1	2	2
1	1	1	1	2	1	1	1	2	1
1	3	3	1	2	1	1	1	2	3
3	1	1	1	1	1	3	3	1	1
2	1	2	3	3	1	2	1	1	1
2	1	1	2	1	2	1	1	1	1
9	3	1	1	1	1	1	1	1	1

3	1	1	2	3	1	3	1	3	2
9	9	9	9	1	9	1	1	9	9
9	9	1	9	9	9	1	1	9	9
3	1	1	1	3	3	1	3	1	3
3	1	2	1	3	1	1	1	3	2
3	1	2	1	3	1	1	3	1	1
3	1	1	3	1	1	2	2	3	2
3	1	2	1	3	1	1	3	1	1
3	1	2	1	3	1	1	9	1	1
3	1	2	1	3	2	1	1	3	2
2	1	1	1	1	2	1	1	2	3
3	1	2	3	3	1	3	1	1	1
1	1	1	1	2	1	3	1	2	2
3	2	1	1	3	1	2	1	3	1
3	1	1	1	3	1	1	3	3	1
1	1	1	1	3	1	1	2	1	2
3	3	1	2	3	1	1	1	2	3
9	9	9	1	9	9	9	1	9	9
9	9	9	1	9	9	1	1	9	9
3	1	1	1	1	9	1	1	1	2
2	2	3	1	1	1	1	1	1	1
2	2	1	1	1	2	1	2	1	2
1	1	1	1	1	1	9	1	1	1
2	2	2	2	2	2	9	2	2	2
3	3	3	3	3	3	9	3	3	3
2	1	2	1	3	1	1	2	2	3
2	1	2	1	3	1	1	2	2	3
2	1	1	1	3	1	1	3	1	1
1	1	1	1	1	1	1	1	1	1
2	2	2	2	2	2	1	2	2	2
9	9	2	1	2	2	1	2	3	2
1	1	1	1	2	1	1	1	2	1
1	3	1	1	1	1	1	9	1	9
2	1	2	1	3	1	1	2	2	3
1	1	1	1	3	1	4	1	3	1
2	2	2	2	2	2	9	2	2	2
1	2	1	1	2	1	1	1	2	2
3	1	1	1	1	1	1	9	9	9
3	9	9	2	9	1	1	1	9	9
1	1	1	1	3	1	1	1	3	1
1	1	1	1	1	3	1	3	3	3
2	2	2	2	2	2	9	2	2	2
2	2	2	2	2	2	1	1	2	2
2	3	2	3	2	2	1	2	2	2
1	2	1	2	2	2	1	2	2	2
2	2	2	2	2	2	9	2	2	2
2	1	3	1	2	3	9	9	9	9

2	2	2	2	2	2	1	9	9	9
2	2	2	2	3	2	1	2	2	2
9	9	1	1	1	1	9	9	9	9
9	9	1	1	3	1	9	9	9	9
9	9	9	9	9	9	1	1	9	9
2	3	1	2	1	3	1	3	2	1
2	1	1	2	2	2	1	2	2	2
3	1	1	1	3	1	1	3	3	3
1	2	1	2	1	2	1	1	1	1
9	9	9	2	9	9	1	3	9	9
2	2	2	2	2	2	9	2	2	2
2	1	1	1	2	1	4	1	2	1
1	1	1	1	2	1	9	1	2	1
2	2	1	1	2	1	1	2	2	2
1	1	1	1	3	1	1	1	3	3
2	2	2	2	2	2	1	2	2	2
2	2	1	1	2	1	1	2	2	2
2	3	3	3	1	1	1	2	2	2
9	9	1	1	3	1	1	2	2	2
3	2	1	1	1	1	1	3	3	1
9	9	1	9	9	9	1	9	1	9
1	1	1	1	2	3	1	1	3	1
2	2	2	2	2	2	1	2	2	2
1	3	1	2	2	1	1	3	3	1
2	2	1	1	3	1	1	2	2	2
2	2	1	2	3	2	2	2	3	2
9	9	9	9	9	1	1	9	9	9
9	9	9	9	9	9	1	9	1	9
2	2	1	2	2	2	1	3	3	3
3	1	1	2	1	1	1	2	2	2
9	9	1	9	9	1	1	9	1	9
1	1	1	1	2	1	1	1	3	1
3	1	3	3	3	1	2	1	3	1
3	1	2	2	2	2	1	1	2	2
3	1	2	2	2	2	1	1	2	1
1	3	1	1	1	1	2	1	3	1
2	2	2	2	2	2	1	2	2	2
1	1	1	2	2	1	4	1	1	2
2	2	1	1	2	1	1	2	2	2
2	1	1	1	2	1	4	1	2	9
2	1	1	2	2	2	1	2	2	2
2	2	1	2	2	2	1	2	2	2
3	1	1	1	3	2	1	2	3	2
1	1	1	1	3	1	1	1	2	1
2	1	2	2	2	1	1	2	2	2
3	3	3	3	3	3	9	3	3	3
1	2	2	1	2	2	1	2	2	2

2	2	1	2	2	2	1	2	2	2
2	2	2	2	2	2	5	2	2	2
2	2	2	2	1	2	1	2	2	2
2	2	2	2	2	2	1	2	2	2
3	1	1	2	2	2	2	2	2	1
3	1	1	1	1	2	4	3	3	1
2	2	2	2	1	2	1	2	2	2
1	1	1	1	3	1	1	1	2	1
3	1	1	1	1	1	1	2	2	2
3	1	2	2	2	2	1	3	3	1
1	1	1	1	3	1	6	1	3	2
9	9	2	9	9	9	9	9	2	9
9	9	9	1	9	9	1	9	9	9
2	2	2	1	2	1	1	2	2	2
3	3	1	2	2	2	1	1	2	2
1	1	1	3	1	1	1	2	1	3
2	1	2	2	2	1	1	2	1	2
3	1	1	1	1	1	1	1	3	1
2	1	1	2	2	3	1	1	2	2
2	1	2	2	2	3	3	2	3	2
3	2	3	3	1	1	1	3	3	3
3	3	1	1	1	1	1	1	2	2
2	1	1	2	2	1	1	2	2	2
3	1	1	2	2	1	1	1	1	1
3	1	3	3	3	1	1	3	3	3
3	1	1	2	2	1	1	1	9	1
2	2	1	1	2	1	1	2	2	2
9	1	2	1	2	1	1	2	1	1
2	2	1	3	2	1	1	2	2	2
2	1	1	2	3	1	1	2	3	2
1	1	1	1	1	1	2	1	2	1
1	1	1	3	1	1	1	1	1	1
1	1	1	1	1	1	2	3	3	3
1	2	1	1	1	1	1	2	2	1
1	2	1	1	3	2	1	3	3	3
1	1	1	1	3	1	1	3	3	1
3	1	1	1	3	1	2	1	9	9
1	1	1	1	1	1	1	1	1	1
2	2	2	2	2	2	1	2	2	2
2	2	2	2	2	2	1	2	2	2
1	1	2	1	3	1	1	1	3	2
1	1	1	1	1	1	1	1	1	1
9	2	1	1	1	1	1	1	2	3
1	1	1	2	2	1	1	1	2	2
2	2	2	2	2	2	1	2	2	2
3	1	1	2	1	3	1	1	2	3
2	2	2	1	3	2	1	3	3	2

1	9	1	3	1	1	1	3	3	1
3	1	3	3	2	2	1	3	3	3
3	1	1	1	2	1	1	1	3	2
9	9	9	1	9	9	5	9	9	9
9	9	2	9	9	1	2	1	2	9
2	1	3	1	2	3	1	2	2	2
2	1	2	1	2	1	1	2	2	2
3	1	1	1	3	1	1	3	3	3
9	9	2	1	1	1	9	2	3	2
2	2	2	2	2	2	1	2	2	2
9	9	9	9	1	9	1	9	9	1
3	2	1	1	2	3	1	1	2	3
2	2	2	2	2	2	9	2	2	2
2	2	2	2	2	1	1	2	2	2
2	2	2	2	2	2	1	2	2	2
2	3	2	3	1	3	1	2	3	3
2	3	1	2	2	3	4	2	1	2
9	9	9	9	9	1	1	1	9	9
9	9	9	2	9	9	4	9	9	9
2	2	2	2	2	2	9	2	2	2
2	2	2	2	2	2	1	3	2	2
1	2	2	2	2	2	9	2	2	2
9	9	9	9	9	1	1	9	9	9
2	1	1	2	1	1	1	2	9	9
2	3	1	2	3	1	2	1	2	2
3	1	3	1	3	1	1	1	3	1
3	1	3	2	3	1	3	1	3	2
3	1	1	1	3	3	1	1	1	1
2	2	1	3	3	3	1	1	3	1
9	2	1	1	3	2	1	1	2	1
3	1	1	1	3	1	4	1	2	1
3	1	1	1	3	1	3	1	3	1
3	1	1	1	3	1	3	1	3	1
3	3	3	3	9	3	1	3	3	3
3	1	1	1	3	1	1	1	9	1
1	1	1	1	2	1	1	3	2	1
1	1	1	1	1	1	2	1	1	1
3	3	3	3	3	3	1	3	3	3
1	9	2	9	1	9	1	9	1	9
3	3	3	1	3	3	1	3	3	3
3	1	1	3	3	3	6	1	3	3
3	1	1	1	3	1	2	1	9	3
1	1	1	1	1	1	1	1	3	3
1	1	3	1	3	1	1	2	2	2
1	1	1	1	2	1	1	1	2	1
1	1	2	3	2	1	1	3	3	1
2	1	2	1	1	1	1	1	2	1

1	3	2	2	2	2	1	2	2	2
2	2	9	1	3	2	1	1	2	1
3	1	1	1	9	1	2	1	9	9
3	1	1	3	3	1	7	1	1	1
2	1	1	1	2	1	1	3	2	2
2	2	1	3	3	1	1	2	2	2
1	1	2	2	2	2	1	2	2	2
1	1	2	2	2	2	1	9	9	1
1	1	2	2	2	2	1	9	9	1
2	1	1	2	3	2	1	2	2	1
1	1	1	1	2	2	1	1	9	9
1	2	1	1	3	1	1	1	2	1
1	1	1	1	3	3	1	1	3	1
2	3	1	2	3	3	9	2	1	3
2	1	1	2	3	2	2	1	3	1
1	1	2	2	2	2	1	2	2	2
1	1	1	1	3	1	1	1	1	1
2	1	1	2	1	1	1	2	2	2
3	1	1	3	2	1	1	1	2	1
9	1	9	1	9	9	1	9	9	9
9	9	1	9	9	9	1	1	9	9
1	1	1	1	1	1	2	1	3	1
3	1	1	3	3	1	1	3	3	1
3	3	3	3	3	3	1	1	1	3
3	3	3	3	3	3	1	3	3	3
1	3	1	3	3	1	1	2	2	2
1	1	1	1	3	1	1	1	3	1
3	1	1	1	2	1	1	1	2	3
3	1	1	1	1	1	1	1	1	3
2	3	1	1	1	1	1	1	2	2
3	1	1	2	2	2	4	1	2	1
1	1	1	1	3	1	3	3	1	1
1	1	1	1	2	1	3	1	2	2
9	9	1	1	2	2	3	2	1	1
2	1	1	1	2	2	2	1	3	2
1	1	1	1	2	9	2	2	2	2
1	1	1	1	2	1	1	2	2	2
2	2	1	2	3	2	1	2	2	2
2	3	1	3	3	1	1	2	2	3
3	1	1	3	2	1	1	3	3	3
2	1	1	2	1	2	1	1	2	1
2	2	2	2	2	2	1	2	2	2
1	1	1	1	1	1	1	1	1	1
9	9	2	9	9	9	1	2	9	9
9	9	3	9	9	9	1	3	2	2
3	1	3	1	3	1	3	1	3	3
1	1	1	1	2	1	1	1	2	1

3	1	1	1	2	1	4	1	3	2
3	1	2	1	2	2	2	1	3	3
1	1	2	1	3	1	7	1	3	2
3	1	1	1	3	1	5	2	1	1
3	3	1	3	2	2	1	2	2	2
3	2	2	3	2	3	1	3	2	3
2	2	2	9	9	9	1	2	2	2
3	1	1	2	2	1	1	3	3	3
3	3	1	3	3	3	3	1	3	1
3	1	1	1	1	1	1	1	3	2
3	1	1	1	1	1	1	1	3	2
3	1	1	1	1	1	1	1	3	2
9	9	2	9	9	9	1	9	9	1
9	9	1	9	9	9	2	9	9	9
3	1	1	1	1	1	1	1	3	2
1	1	2	3	2	1	1	2	2	2
1	1	1	1	1	1	2	1	1	9
2	2	2	2	2	2	1	2	2	2
3	1	1	1	1	1	5	2	3	1
1	1	1	3	3	1	1	2	2	2
3	9	1	1	2	2	1	2	2	1
2	3	2	2	2	3	2	1	3	2
2	1	9	9	9	9	1	2	2	2
3	1	1	2	3	2	2	2	3	2
2	1	2	1	2	2	1	2	2	2
1	1	2	2	2	1	2	3	3	1
3	1	1	2	3	1	2	1	2	3
1	2	3	3	1	1	4	1	1	1
1	1	2	2	2	1	2	3	3	1
1	1	2	3	2	1	1	2	2	2
1	1	1	3	3	1	1	2	2	2
9	9	2	9	9	9	1	9	9	1
1	1	1	2	3	2	3	1	3	1
3	1	1	1	2	2	1	2	2	2
2	2	1	1	2	1	1	1	3	1
3	1	1	1	2	1	1	1	1	1
2	1	3	2	2	2	2	1	1	1
2	2	1	9	9	9	3	1	9	9
1	1	1	1	3	2	1	1	2	2
2	2	1	2	2	2	2	2	2	2
1	1	1	1	3	1	2	1	3	1
1	1	2	3	1	1	1	1	3	2
3	1	1	1	3	1	3	1	3	2
2	2	1	1	2	2	1	2	2	2
1	1	1	1	1	1	3	1	1	3
2	2	2	2	2	2	1	2	2	2
9	9	9	1	9	9	1	9	9	9

2	2	2	2	2	2	2	3	1	2
1	1	1	1	2	1	1	2	2	2
1	1	1	1	1	1	1	9	9	9
3	1	1	1	2	1	2	1	9	9
3	1	1	1	2	1	2	1	2	1
9	9	1	9	9	9	1	9	9	9
1	2	1	2	1	1	1	1	1	1
3	1	2	1	3	1	3	1	3	1
3	2	1	1	2	3	9	1	2	3
1	2	1	2	3	2	1	1	1	2
2	1	1	1	3	1	4	3	3	2
2	1	1	2	1	2	1	1	3	2
2	1	1	1	3	1	2	2	2	1
3	1	1	1	2	2	1	1	3	1
2	1	1	1	2	1	1	2	2	2
1	9	3	3	3	2	3	2	2	3
3	1	1	1	3	1	1	1	3	1
3	1	1	1	3	1	5	2	2	1
1	1	1	1	2	3	4	1	2	3
3	1	1	1	3	1	2	1	1	1
3	1	1	1	2	1	3	1	3	2
2	1	2	2	2	2	1	2	2	2
1	1	1	1	3	2	3	1	2	3
2	2	3	3	3	3	1	2	2	2
1	1	1	1	1	1	2	2	2	2
9	9	1	1	2	1	1	1	2	2
3	1	1	2	2	1	1	2	2	2
3	1	1	3	1	2	1	2	2	2
3	1	1	2	2	1	3	1	3	2
3	3	1	3	3	3	1	9	9	9
9	9	1	1	1	3	3	9	9	1
3	2	1	1	2	2	1	2	2	2
9	9	1	1	2	1	1	9	1	1
1	1	2	1	2	1	1	1	2	3
3	2	1	2	3	2	1	1	2	3
9	9	9	9	9	9	2	9	9	9
9	9	9	9	9	9	9	9	9	9
2	2	2	2	2	2	1	2	2	2
1	1	1	1	3	1	1	2	2	2
3	1	1	2	1	1	1	1	2	1
3	3	1	1	1	1	1	1	1	1
1	1	2	2	2	2	9	1	1	2
1	1	1	1	1	2	1	3	1	2
1	2	1	2	1	3	2	1	2	1
1	2	1	2	3	1	1	1	3	2
9	9	9	9	9	9	9	9	9	9
1	3	3	3	2	1	1	3	2	2

1	2	1	2	1	2	9	1	2	1
9	9	9	9	9	9	9	9	9	9
3	9	1	1	1	1	2	3	3	9
9	1	2	2	2	2	1	2	2	2
1	1	1	1	3	1	2	2	2	1
1	2	1	2	1	2	9	1	2	1
1	1	1	1	2	2	1	3	1	3
3	1	1	1	2	1	1	1	3	1
1	1	1	2	2	2	2	2	2	2
3	1	1	1	3	1	4	1	3	1
9	9	9	9	9	9	9	9	9	9
9	9	9	9	9	9	9	9	9	9
9	1	9	9	9	1	1	9	9	9
1	1	1	1	2	3	1	1	2	2
1	1	1	1	3	1	2	1	2	2
3	1	1	1	3	2	5	2	3	1
3	3	9	9	9	9	9	9	9	9
9	9	1	1	3	1	2	1	9	9
2	1	1	1	2	1	5	1	3	2
2	1	1	1	3	3	1	1	3	3
1	1	2	2	1	1	4	2	2	1
2	3	1	3	1	1	1	1	1	2
2	2	2	2	2	3	4	3	3	3
1	2	2	2	2	2	1	2	2	2
2	2	1	1	2	1	1	2	2	2
1	1	1	1	3	1	1	3	2	2
9	9	1	2	2	2	1	9	9	9
3	1	2	2	2	1	3	1	3	1
3	1	1	1	2	2	1	1	2	3
2	2	3	3	2	2	2	2	2	2
3	3	3	1	1	1	1	1	3	3
3	1	1	2	3	2	5	1	3	1
9	9	9	9	9	9	9	9	9	9
2	1	3	2	1	3	1	2	2	3
1	1	1	1	2	2	1	1	1	2
3	2	1	1	1	1	1	2	1	2
1	1	1	1	1	1	1	1	1	1
1	1	1	1	3	2	1	1	2	2
3	1	3	1	3	1	1	3	3	2
2	3	1	2	1	3	1	1	2	2
1	3	3	1	2	2	1	1	3	2
3	3	3	3	3	3	9	3	3	3
3	1	1	1	3	1	1	9	9	9
3	1	1	1	3	1	1	9	9	9
3	1	1	1	3	1	2	1	3	2
3	1	1	1	3	1	1	3	3	1
3	1	1	1	3	1	1	9	9	9

2	2	2	2	2	2	1	2	2	2
3	2	2	2	2	2	1	2	2	3
9	1	1	1	1	3	5	1	1	1
3	1	1	1	1	1	1	1	3	1
1	1	1	1	3	1	3	3	1	1
3	1	1	1	3	1	2	1	3	2
3	1	1	1	3	1	2	9	9	1
3	1	1	1	3	1	1	1	9	1
3	1	1	1	3	1	2	9	9	9
3	1	1	1	3	1	3	9	9	9
9	1	1	1	3	1	3	9	9	9
3	1	1	1	3	1	2	9	9	9
3	1	1	1	3	1	1	1	9	9
1	1	1	3	3	1	1	3	3	1
3	1	1	1	3	2	1	1	2	1
3	1	1	1	3	1	7	1	3	3
3	1	1	1	2	1	1	1	1	1
1	1	1	1	1	2	5	9	1	1
3	1	1	1	3	1	5	9	9	9
1	1	1	1	3	1	1	9	9	9
1	2	1	2	1	3	9	1	2	1
3	1	1	1	3	1	3	9	9	9
3	1	1	1	3	1	4	1	1	1
3	1	1	1	3	1	2	9	9	9
3	1	1	1	3	1	2	1	1	1
3	1	1	1	3	1	1	1	1	1
1	1	2	3	2	1	1	3	2	1
3	1	1	1	3	1	4	1	1	1
3	1	1	1	3	1	2	3	2	1
3	1	1	1	3	1	2	1	1	1
3	1	1	1	3	1	1	1	1	1
3	1	1	1	3	1	1	2	1	1
2	1	1	1	3	1	1	3	1	1
3	1	1	1	3	1	1	1	9	9
3	1	1	1	3	1	1	1	1	1
3	1	1	1	3	3	4	1	1	1
3	1	1	1	3	1	4	1	3	1
3	1	1	1	3	1	1	1	9	9
3	1	2	3	3	3	1	1	3	1
1	3	1	3	3	1	2	3	3	1
2	2	2	2	2	2	1	2	3	2
1	1	3	3	2	1	4	1	2	1
2	3	2	1	1	1	1	1	2	3
1	2	2	2	2	2	1	9	9	9
1	1	1	1	3	2	3	1	1	1
1	3	1	3	3	1	1	1	1	3
3	1	1	1	2	1	1	1	3	1

2	1	1	1	3	1	9	9	9	9
3	3	3	1	2	3	2	9	9	9
2	1	1	9	9	9	9	9	9	9
3	1	1	1	2	1	1	9	9	9
3	1	3	1	2	1	1	2	2	1
9	1	1	9	9	9	9	9	9	9
3	1	1	1	2	3	1	2	2	2
1	1	2	2	2	2	2	1	2	1
9	1	1	9	9	9	1	2	9	9
9	9	1	2	9	9	9	9	9	9
9	1	1	9	9	9	1	2	9	9
9	9	9	9	9	9	9	9	9	9
2	1	1	1	1	1	1	2	2	2
9	2	1	2	1	3	4	1	3	2
2	2	1	2	3	1	3	1	3	3
1	2	1	3	3	1	3	1	1	1
1	1	1	1	2	1	3	1	2	2
2	1	1	1	3	1	1	1	2	1
1	1	1	1	1	1	1	1	1	1
9	9	1	9	9	9	1	1	9	9
1	1	1	1	1	1	1	1	1	1
3	1	1	1	2	1	1	2	2	2
3	1	1	1	2	2	1	2	2	1
1	1	1	2	2	1	1	1	1	1
3	1	1	1	3	1	1	1	1	2
3	1	1	1	3	1	3	1	3	1
1	1	1	3	2	2	4	1	9	3
3	3	1	2	1	2	1	2	2	2
2	2	2	2	2	1	1	1	9	9
9	9	2	3	1	9	1	3	9	9
1	1	1	1	1	1	1	1	3	3
1	1	1	1	1	1	9	3	3	3
1	1	1	2	2	2	1	2	3	3
1	1	1	2	2	2	1	2	2	1
3	1	1	1	3	1	1	1	2	2
3	1	1	2	2	1	1	1	3	1
2	1	1	1	2	2	1	1	2	1
1	1	2	2	2	1	1	1	1	1
2	2	2	2	2	1	1	1	1	1
2	2	2	1	1	1	1	1	2	2
3	1	1	2	2	2	1	1	3	1
2	2	1	2	1	2	1	2	2	2
3	1	1	3	3	1	4	9	3	3
2	1	1	1	2	1	1	1	2	2
3	3	1	1	3	3	1	3	3	1
3	3	1	1	1	1	1	1	3	3
3	1	1	1	2	1	1	2	1	2

9	9	9	9	9	9	9	9	9	9
3	1	1	1	2	1	4	1	1	1
9	9	9	2	9	9	1	9	9	9
9	9	9	1	9	9	1	9	9	9
9	9	9	1	9	9	1	9	9	1
1	1	1	1	2	1	1	2	1	1
9	9	9	9	9	1	5	9	9	9
3	1	1	1	2	1	1	1	2	1
2	1	2	2	2	2	1	2	9	9
1	2	2	1	2	2	1	2	3	2
3	3	1	1	2	2	1	9	9	9
3	1	1	2	1	1	1	2	1	2
2	1	1	2	1	1	1	1	1	2
3	3	1	3	1	3	1	2	2	2
1	3	1	1	2	2	1	1	1	1
2	3	2	2	2	1	1	1	9	9
2	2	2	2	2	2	1	2	2	2
3	3	3	3	3	3	2	3	3	3
2	3	2	3	2	1	9	3	1	2
1	1	2	2	2	2	6	3	3	3
2	1	1	1	2	2	1	2	2	2
2	2	1	2	2	1	1	1	1	3
2	2	2	2	2	2	1	2	2	2
2	2	1	2	3	2	1	2	2	2
2	1	1	2	2	2	1	2	2	2
2	2	1	1	1	1	1	2	2	2
1	1	1	1	3	1	1	1	1	3
3	3	1	1	2	1	1	1	3	3
3	2	2	1	2	2	1	1	1	1
2	1	1	1	1	1	1	2	2	2
9	9	9	9	9	9	9	9	9	9
2	1	3	2	3	3	1	2	3	1
2	3	1	2	2	3	9	2	1	3
2	1	1	2	2	1	1	2	2	2
1	1	1	1	1	2	1	2	2	2
3	3	1	1	2	1	1	1	1	1
1	1	1	1	3	1	2	3	1	1
1	1	1	1	3	2	1	1	1	1
1	1	1	1	2	1	1	1	1	1
2	1	1	1	2	1	1	1	3	1
2	2	2	2	2	2	1	2	2	2
2	2	2	2	2	2	1	1	1	1
9	9	9	9	9	9	9	9	9	9
9	9	1	9	9	9	1	9	9	9
9	9	1	9	9	9	1	1	9	9
9	9	9	9	9	9	9	2	2	2
3	1	1	1	3	1	4	2	2	2

3	1	1	3	3	1	3	3	1	1
2	1	1	2	1	1	1	1	2	2
1	1	1	3	2	1	1	1	9	9
3	3	1	2	2	3	3	1	3	3
1	1	1	1	9	1	1	9	9	9
2	1	1	1	3	1	1	2	2	2
3	1	1	2	3	1	5	9	9	9
3	1	1	1	1	1	1	1	9	9
2	2	3	2	3	2	1	2	2	2
1	3	1	1	3	1	1	1	3	3
9	1	9	9	9	1	2	9	9	9
2	2	9	9	9	9	1	2	2	9
2	1	1	2	1	2	1	1	3	2
3	1	1	1	2	1	3	2	2	1
9	9	1	9	9	9	1	1	9	9
1	1	1	1	1	1	5	3	3	1
2	2	2	3	2	2	1	2	3	2
3	1	2	1	2	1	4	2	3	1
1	1	9	1	9	9	2	1	2	2
2	1	1	1	2	1	1	2	2	3
1	2	1	2	2	1	9	1	1	2
1	1	1	1	3	3	1	3	3	1
3	1	2	1	3	2	1	2	2	1
3	1	3	1	3	1	1	1	2	1
2	1	1	1	3	1	1	3	3	2
3	3	1	3	1	1	1	1	2	2
3	1	3	1	3	1	1	1	2	1
1	3	1	2	1	2	1	1	2	1
2	3	9	9	9	9	9	2	3	1
1	2	1	2	3	1	1	1	1	1
1	1	3	2	3	1	3	1	3	1
3	1	1	1	3	1	1	1	3	2
3	1	1	1	1	1	3	2	3	1
2	3	1	1	2	2	4	1	3	1
1	2	1	1	3	1	1	1	3	1
1	1	1	1	2	1	1	2	2	2
2	2	1	1	2	1	1	1	3	1
1	3	1	1	2	1	1	3	3	3
9	9	9	9	1	9	1	9	1	9
3	2	2	2	2	3	1	2	2	2
9	9	9	9	1	9	1	9	9	9
9	9	2	9	9	9	1	9	1	9
3	1	1	2	3	1	1	1	2	1
3	1	1	2	3	1	1	1	2	1
1	1	1	1	3	1	1	1	3	1
1	1	1	3	1	1	9	1	3	1
3	3	1	3	3	3	1	1	1	3

3	2	3	3	3	3	1	3	3	3
9	9	1	9	9	9	1	9	9	9
3	1	1	3	3	1	1	1	3	1
1	3	1	1	3	1	1	1	3	3
3	1	1	1	3	1	1	1	3	3
1	1	1	1	3	1	1	1	3	1
3	1	2	1	2	1	2	2	1	3
2	1	3	2	2	3	1	2	1	2
3	1	1	3	3	1	1	1	3	3
2	1	1	2	1	3	1	3	9	2
3	1	1	3	1	1	1	1	3	1
1	1	1	2	2	3	4	1	2	1
2	1	2	1	1	1	1	2	2	3
3	2	1	3	3	1	2	1	3	3
2	1	2	1	1	1	1	2	1	1
2	1	1	1	2	1	1	1	3	1
3	1	1	1	3	1	1	1	3	1
2	1	1	1	3	2	1	2	3	2
1	1	1	3	3	1	1	1	3	3
2	2	2	2	2	2	1	2	2	2
3	1	3	2	2	2	1	1	2	1
3	2	1	2	1	2	9	2	2	2
3	3	3	3	3	3	1	3	3	3
1	3	1	3	2	1	1	9	9	9
3	1	1	1	1	1	1	2	2	2
2	2	1	2	2	1	9	1	2	1
1	2	1	1	2	1	1	2	1	3
3	1	1	2	1	3	9	2	2	3
3	2	1	1	3	1	1	1	2	1
9	9	9	9	9	1	1	9	1	9
2	1	2	1	9	2	1	2	3	1
1	1	3	1	2	2	2	1	3	2
2	1	2	3	1	2	1	2	2	2
2	1	1	2	1	1	1	2	3	2
3	1	3	1	2	1	1	1	1	1
3	3	3	3	3	3	9	2	2	2
3	1	1	1	2	1	1	2	2	2
2	2	2	3	1	2	1	9	9	9
2	1	1	2	1	3	1	1	2	2
3	3	1	1	3	1	4	1	3	3
9	1	9	9	9	1	1	9	9	9
9	9	9	9	9	9	9	9	9	9
1	3	3	1	3	1	1	2	1	2
3	1	1	1	3	1	1	3	3	3
2	2	1	2	2	2	1	3	3	2
3	1	1	1	3	1	1	1	3	3
9	9	9	9	9	9	9	9	9	9

2	1	1	2	2	2	1	1	3	2
3	1	1	1	1	1	9	9	9	9
1	1	2	1	2	2	1	2	2	2
1	1	1	2	1	9	9	9	9	9
9	9	9	9	9	9	9	9	9	9
1	1	1	1	1	1	1	3	3	3
3	1	2	2	2	2	1	2	2	2
2	2	2	2	2	2	9	2	2	2
1	1	1	2	1	3	1	3	2	3
1	1	2	1	1	2	1	1	2	1
3	1	9	9	9	9	9	9	9	9
9	1	1	2	3	1	1	9	9	9
3	2	2	3	3	3	9	9	9	9
9	9	9	9	9	9	9	1	1	1
3	1	2	2	2	2	1	2	3	2
9	9	9	9	9	9	9	1	1	1
2	3	1	2	2	3	1	1	9	9
3	1	3	3	3	1	2	9	9	9
2	3	3	2	2	1	1	3	3	3
2	2	2	2	2	1	1	2	2	2
1	1	2	3	3	1	2	3	3	3
3	1	3	3	2	1	1	2	2	2
9	1	1	9	9	9	1	1	9	9
3	2	1	3	1	2	1	1	3	2
1	1	1	1	2	1	1	2	3	2
2	2	2	2	2	2	1	2	2	1
9	1	1	9	3	9	1	1	9	9
2	1	1	3	1	3	1	1	2	2
1	2	2	2	2	1	1	2	2	2
9	9	9	9	9	1	3	9	9	9
3	1	1	1	1	1	1	1	2	1
1	1	2	1	2	2	1	2	2	2
1	3	1	1	3	3	1	1	3	1
2	1	1	1	1	1	1	1	3	1
3	1	1	1	1	2	1	1	3	1
3	1	3	1	2	1	2	3	3	2
1	1	1	1	3	1	1	1	3	3
1	1	3	3	3	1	5	3	3	1
9	1	9	9	1	9	1	1	9	9
3	3	3	3	3	3	1	2	2	2
3	1	1	1	3	1	1	2	3	1
3	1	1	1	2	1	1	3	3	2
3	2	2	2	2	2	4	1	3	3
2	1	2	2	2	1	1	3	3	3
3	1	3	1	3	1	3	1	2	2
3	1	1	2	3	2	2	1	3	2
2	1	1	1	3	1	1	9	9	9

2	2	2	2	2	2	1	2	2	2
2	1	2	2	3	2	1	1	3	9
1	1	9	2	2	2	1	2	1	1
2	1	2	1	3	1	1	2	2	1
1	2	2	2	3	2	1	3	3	3
9	9	1	9	9	9	1	9	9	1
2	2	2	2	2	2	2	1	2	2
3	1	9	1	2	1	1	1	3	1
3	1	1	3	3	1	1	1	2	1
2	1	1	2	2	2	1	2	2	2
2	1	1	1	3	1	1	2	2	3
3	1	1	2	2	1	1	1	2	1
2	2	2	2	2	2	1	2	2	2
3	3	3	3	3	3	1	3	3	3
3	2	1	2	1	2	1	2	2	1
2	1	2	2	3	1	1	1	2	2
3	1	1	1	3	2	1	3	3	3
3	1	1	1	2	1	1	2	2	3
3	3	1	1	3	1	1	3	2	2
2	2	1	1	2	2	1	2	3	1
1	1	1	1	3	1	1	1	3	3
2	1	1	1	3	2	1	1	3	1
2	1	1	1	3	2	1	1	3	1
1	1	1	2	2	2	1	1	1	1
3	1	1	1	3	1	2	1	2	2
2	3	2	1	2	1	1	3	3	2
3	1	3	1	3	1	1	1	2	2
3	1	3	1	3	1	1	1	2	2
2	2	1	2	2	2	1	2	2	2
1	1	1	1	2	1	1	1	1	1
2	2	2	2	2	2	1	3	3	3
2	1	1	1	3	2	1	1	3	2
3	1	1	1	2	1	1	1	3	3
3	1	2	2	3	1	4	1	3	1
3	1	1	1	3	1	1	1	1	1
1	1	1	2	2	1	1	2	2	2
1	1	1	2	2	1	1	2	2	2
1	2	1	2	2	2	1	2	2	2
1	1	1	1	3	2	1	2	2	2
2	2	2	2	2	2	1	2	2	2
3	1	2	1	1	1	1	2	3	1
3	1	1	1	2	1	2	2	2	1
2	1	1	1	1	1	1	2	2	2
2	2	1	2	1	1	2	1	2	2
3	2	3	1	1	1	5	1	3	1
3	1	1	3	3	9	1	1	3	1
2	1	1	1	1	1	1	1	3	1

3	1	2	2	2	2	1	2	2	1
2	2	1	1	3	1	1	2	2	2
2	1	1	2	1	2	1	1	3	1
2	2	1	2	1	9	1	2	1	2
1	9	2	2	2	2	9	2	2	2
2	2	2	2	2	2	1	2	2	2
2	1	1	1	2	1	1	1	9	1
1	1	1	1	3	1	1	1	3	1
1	1	2	2	3	1	1	2	2	2
3	1	3	2	3	2	1	2	2	2
3	1	3	2	3	2	1	2	2	2
1	1	2	2	3	2	2	2	2	2
1	2	9	9	9	9	2	2	2	2
3	1	1	1	1	1	1	9	9	9
1	3	1	1	1	2	1	2	2	2
2	1	1	1	1	1	1	1	2	2
1	1	1	1	3	1	1	1	3	1
2	1	1	1	1	1	1	1	2	2
3	1	1	1	1	1	1	1	3	1
2	1	1	1	1	1	1	1	2	2
2	2	2	2	2	2	1	2	2	2
2	2	1	2	2	1	1	2	2	2
2	2	2	3	2	2	1	2	2	2
1	2	2	2	2	2	1	2	2	2
2	1	1	1	1	1	1	1	2	2
1	2	1	1	2	1	1	1	3	1
2	2	1	1	3	1	1	3	3	3
2	2	2	2	2	2	1	2	2	2
2	1	2	2	2	2	1	2	2	2
3	1	1	2	1	1	1	1	2	2
2	2	3	2	2	2	1	3	3	3
2	2	2	2	2	2	1	2	2	2
2	2	2	2	2	2	9	2	2	2
2	2	2	2	2	2	1	2	2	2
9	9	9	9	9	1	1	2	2	2
2	2	2	2	2	2	1	2	2	2
2	2	2	2	2	2	1	2	3	2
2	2	2	2	2	2	1	2	2	2
2	2	2	2	2	2	1	9	9	9
2	1	1	1	2	1	1	1	2	2
2	2	2	2	2	2	9	1	2	1
1	1	2	2	2	2	1	1	2	2
3	1	1	1	2	1	1	2	2	2
2	2	2	2	2	2	1	2	2	2
2	2	2	2	2	2	1	1	2	2
1	1	2	2	3	3	1	2	2	2
1	1	2	2	2	2	1	1	2	2

2	2	2	2	2	2	9	2	2	2
2	2	1	2	2	2	1	1	2	2
2	2	2	3	1	1	5	1	3	3
2	1	1	1	1	2	1	1	2	2
2	2	2	2	2	2	1	2	2	2
2	2	2	2	1	3	1	1	2	3
3	1	3	1	1	1	1	1	9	9
3	1	1	1	2	1	1	1	1	1
1	1	1	1	1	1	2	1	3	1
3	1	1	1	2	1	7	1	3	1
3	1	1	1	2	1	2	1	3	1
2	2	2	1	2	2	1	2	2	2
2	1	2	3	3	1	1	1	3	1
9	9	1	1	3	1	1	2	2	2
1	1	1	1	1	1	1	1	9	1
2	1	3	1	1	1	5	1	2	2
2	1	1	1	1	1	1	1	2	2
2	1	1	1	1	1	1	1	2	1
1	9	1	1	1	1	1	1	1	1
2	1	1	1	2	1	1	1	3	2
2	1	3	1	3	1	1	1	2	1
2	2	2	2	2	2	9	1	2	3
2	1	2	2	2	2	1	1	3	3
2	2	2	2	2	2	1	2	2	2
2	2	2	2	2	2	1	2	2	2
2	2	2	2	2	2	1	2	2	2
9	9	9	9	9	1	2	1	9	9
2	1	1	1	2	2	1	2	2	2
2	2	1	1	1	2	1	1	2	2
9	9	9	9	9	9	9	2	1	1
2	1	2	2	2	1	1	1	2	2
3	2	1	2	3	1	1	1	2	3
2	9	3	2	3	1	1	9	9	9
2	2	1	1	1	1	2	1	2	1
2	1	1	3	1	1	1	9	9	9
3	1	1	3	2	1	1	1	3	1
3	3	1	1	3	1	3	1	3	3
3	1	1	1	3	1	1	2	2	1
3	2	1	2	3	1	1	1	9	3
2	1	3	1	1	2	1	2	2	3
3	1	1	1	3	1	1	1	3	1
1	1	2	1	1	2	1	1	1	1
3	1	1	2	3	1	1	1	3	1
2	2	2	2	2	2	1	2	2	2
3	1	1	2	3	1	4	1	3	1
1	1	1	1	2	1	1	2	2	9
3	1	1	1	1	1	1	1	2	1

1	1	2	1	2	2	1	2	3	3
2	1	1	2	1	3	9	1	2	3
3	1	3	2	3	9	1	1	3	1
3	1	1	1	3	1	1	3	1	1
2	3	3	2	3	1	2	3	2	1
1	1	2	2	3	2	1	1	2	3
1	1	1	3	1	1	1	1	3	3
9	9	2	9	9	9	1	2	9	9
2	1	1	2	2	1	2	2	2	2
2	2	2	2	2	2	2	2	2	2
1	1	1	1	3	2	9	3	2	2
1	1	1	1	3	2	1	3	2	2
3	3	1	1	1	2	1	2	2	2
2	1	2	2	2	2	1	2	2	2
2	2	1	1	3	1	1	2	2	2
3	1	2	1	2	1	1	2	2	2
2	1	2	2	2	2	1	2	2	2
1	1	1	1	2	1	1	3	2	1
3	1	1	1	2	1	1	2	2	2
2	1	1	1	2	1	1	1	3	1
3	3	2	2	1	1	1	9	9	9
2	1	1	1	2	1	1	9	9	9
2	1	1	1	2	2	1	1	3	2
9	1	1	9	9	9	1	1	9	9
2	3	1	2	1	3	3	1	3	1
3	1	1	1	1	1	1	1	2	1
2	1	2	3	1	1	1	1	1	1
2	2	1	1	2	3	1	1	2	2
2	2	1	1	2	1	3	2	2	2
9	9	9	9	9	1	1	1	9	9
2	2	3	3	3	1	1	1	3	3
9	9	9	9	9	9	9	9	9	9
9	9	9	9	9	9	9	9	9	9
2	1	1	1	2	1	1	2	2	2
2	1	1	1	2	1	1	2	2	2
1	1	1	1	2	1	1	1	2	2
1	1	1	3	3	1	1	3	1	2
2	1	1	1	2	1	1	2	2	2
2	1	1	1	1	1	1	1	3	2
2	1	1	1	1	1	1	1	3	2
9	9	9	9	9	9	9	9	9	9
3	3	2	1	3	1	1	2	2	2
2	1	1	2	2	3	2	2	2	2
9	9	9	9	9	9	9	9	9	9
3	9	3	9	9	9	9	3	9	9
1	1	1	1	1	2	1	1	1	2
2	2	1	1	1	1	1	2	1	1

3	1	9	9	9	9	9	9	9	9
1	1	1	1	3	1	1	1	3	1
3	1	1	1	3	1	9	1	3	3
9	9	1	1	2	2	1	1	2	2
2	1	1	1	2	2	1	9	9	9
3	3	3	3	3	3	9	3	3	3
2	2	2	2	2	2	1	2	2	2
3	3	2	1	3	1	1	3	2	3
9	9	9	9	9	1	1	9	9	9
9	1	1	2	2	2	1	2	2	2
3	1	1	2	3	1	3	3	3	1
2	2	2	2	2	2	1	2	2	2
2	1	1	1	3	3	1	2	2	1
3	3	3	3	3	3	1	3	3	3
2	1	2	2	3	2	1	3	3	1
2	1	3	1	1	2	1	3	1	1
1	1	1	1	1	2	1	1	2	2
3	1	3	1	1	2	6	1	1	3
2	2	2	2	2	2	1	9	9	9
1	1	3	1	2	1	1	1	2	1
3	1	1	1	2	1	3	1	3	2
1	1	1	3	1	2	1	2	2	2
2	1	1	1	2	1	1	9	9	9
2	2	1	1	3	3	1	2	2	2
2	3	1	3	1	1	5	1	3	3
2	1	1	2	2	2	1	2	2	2
3	1	1	1	2	2	1	1	2	1
9	3	1	1	2	1	1	1	1	1
3	2	1	3	1	2	2	1	2	3
1	1	1	3	3	3	1	9	9	9
1	1	2	2	2	2	1	2	2	2
3	3	1	2	1	1	1	3	1	3
3	1	1	1	3	1	1	1	3	1
3	1	1	2	2	3	1	3	3	3
3	1	1	1	3	1	1	1	1	1
1	1	1	1	3	2	2	1	2	2
3	1	1	1	1	1	4	1	3	3
3	9	2	3	2	2	1	2	2	2
1	1	1	1	1	1	1	2	2	2
3	1	1	1	1	3	1	9	9	9
2	3	2	3	3	3	1	9	9	9
3	1	1	2	3	1	1	2	1	3
2	1	2	1	3	3	1	1	3	1
1	1	1	3	2	1	1	2	2	1
1	1	1	1	2	3	1	1	3	1
2	2	1	1	2	2	1	2	3	2
9	9	3	3	3	1	1	3	2	2

1	2	1	2	1	2	9	1	3	1
2	1	1	1	2	1	1	1	3	1
3	3	1	1	3	1	1	3	3	1
3	2	1	1	3	3	1	2	3	2
3	1	1	1	3	1	1	1	3	1
2	1	3	1	3	2	1	2	2	2
9	9	9	9	9	9	9	2	2	2
3	1	1	2	3	1	1	2	2	3
3	1	1	1	3	1	1	1	3	1
1	1	1	1	3	1	1	2	1	2
2	1	9	9	9	9	1	1	1	2
2	2	2	2	2	1	1	1	2	2
2	2	2	2	2	2	1	2	2	2
1	1	1	1	1	1	2	1	3	1
3	1	1	2	3	1	1	9	9	9
2	1	1	2	9	1	1	2	2	2
3	1	3	3	3	2	1	2	2	3
3	1	2	1	3	1	1	1	3	1
1	1	1	1	1	1	1	3	1	1
2	1	1	1	2	2	1	1	3	2
3	1	1	1	3	1	2	1	2	1
3	1	1	2	2	1	5	9	9	9
1	1	1	3	3	2	1	2	3	2
1	1	1	1	2	2	1	9	1	9
2	1	3	3	3	3	1	2	2	2
1	1	1	1	2	1	1	2	2	2
2	2	2	2	2	2	1	2	2	3
2	2	1	1	2	2	1	2	2	2
3	3	3	3	3	3	1	3	9	3
3	1	1	1	1	1	1	1	2	1
1	1	3	1	3	1	1	3	3	3
3	1	1	2	3	3	3	3	1	2
2	2	2	1	2	2	1	2	2	2
3	1	1	2	3	1	1	3	3	9
1	1	1	2	2	1	1	1	2	1
3	1	2	1	3	1	1	2	2	2
3	1	1	1	3	1	4	3	1	1
1	1	1	1	3	3	1	2	1	1
1	1	1	1	3	1	1	1	1	2
9	9	9	9	9	1	1	2	9	9
3	1	1	1	3	1	1	2	2	2
2	3	1	1	3	3	1	2	1	1
2	1	2	2	2	2	1	1	3	2
2	1	1	1	3	1	3	1	3	1
3	1	1	3	3	3	5	1	3	1
3	1	1	3	3	1	1	9	9	2
1	1	1	1	2	2	1	2	2	2

2	1	1	2	2	1	1	1	2	1
2	2	2	2	2	2	1	2	3	2
9	9	3	3	3	3	1	3	3	3
3	1	1	1	2	3	1	2	3	2
9	1	1	2	3	1	1	1	3	2
9	9	9	9	9	9	9	2	1	2
9	9	1	1	2	1	1	3	1	1
3	1	1	1	1	1	1	9	9	9
9	9	9	9	9	9	9	1	2	3
1	1	3	1	1	1	2	1	2	1
2	1	1	1	2	2	1	2	3	2
1	1	1	1	3	1	1	1	2	2
1	1	1	1	3	1	4	2	2	3
3	1	1	3	2	1	2	3	1	1
9	9	2	2	9	2	1	9	9	9
2	1	1	2	3	2	1	1	1	1
3	9	9	9	9	9	9	1	1	1
1	1	1	1	2	3	1	3	1	1
3	1	1	1	3	1	1	1	1	3
3	1	1	1	3	1	2	2	3	2
1	1	1	3	3	1	1	1	2	1
3	3	1	1	3	1	2	3	3	1
1	1	1	2	1	3	1	2	2	2
3	1	2	3	2	1	1	1	3	3
2	1	1	1	3	1	3	1	1	1
1	1	1	1	2	1	1	1	2	2
2	3	1	2	3	2	3	3	2	1
1	1	1	3	3	1	1	1	2	2
3	2	2	2	2	2	1	2	2	2
3	1	1	1	2	1	1	1	2	1
1	1	1	2	2	1	1	2	2	1
1	2	2	2	2	2	1	2	2	2
2	1	1	3	3	2	1	2	2	2
3	1	1	1	3	1	1	1	1	1
1	3	1	2	3	2	2	1	2	3
3	1	1	1	3	1	1	1	2	1
1	1	1	1	2	2	4	2	2	2
3	3	1	3	1	3	1	3	3	3
1	3	1	1	2	1	1	1	2	2
1	1	2	2	2	1	1	1	1	2
3	3	2	2	2	2	1	1	1	2
1	1	1	2	2	1	1	1	1	2
3	1	1	1	2	2	1	3	1	2
3	3	1	2	3	3	2	2	2	1
2	2	1	1	2	2	1	2	3	3
3	3	1	1	3	3	1	2	2	2
2	2	1	3	2	2	1	1	1	1

3	1	1	1	3	2	1	1	2	1
3	2	1	1	1	1	1	1	2	2
2	2	1	1	3	2	1	1	1	1
2	1	1	2	3	3	1	1	1	2
1	1	1	1	1	1	4	2	1	2
2	3	1	1	1	2	1	1	2	2
9	9	1	2	1	2	1	1	2	2
3	1	1	2	3	2	2	2	2	2

Fingerpads	Fingernails	Palms	Onefinger	Threemidd	FingersinCi	FingersVer	FingersWe	PressNippl	ExamineAr
2	3	2	2	2	2	3	2	2	2
3	2	1	2	1	1	1	3	2	1
2	1	2	2	2	3	2	2	2	1
3	2	1	2	1	1	1	3	2	9
9	9	9	9	9	9	9	9	9	9
3	2	1	2	1	1	1	3	2	1
2	2	2	2	2	2	2	2	1	2
1	2	3	2	2	1	2	2	1	1
2	9	2	9	9	2	9	9	9	1
1	9	9	9	9	9	9	9	9	9
9	2	9	2	2	1	2	2	2	2
3	3	3	1	2	2	2	2	1	2
1	2	2	1	2	2	2	2	2	2
2	3	2	3	2	1	3	2	1	1
2	2	2	2	2	2	2	2	2	2
2	3	1	3	3	2	2	2	1	1
2	3	2	2	1	1	2	1	1	1
2	3	1	3	3	2	2	2	1	1
2	3	1	3	3	2	2	2	1	2
1	3	2	3	3	2	1	2	3	9
1	2	1	1	3	1	1	1	1	1
1	2	1	1	3	1	1	1	1	1
2	1	9	2	2	9	9	9	1	9
1	3	1	1	2	1	1	2	1	1
2	2	2	2	2	2	2	2	2	2
2	3	2	2	2	2	2	2	2	2
2	2	2	2	2	2	2	2	2	2
2	3	2	3	2	2	1	2	1	1
3	3	1	3	9	1	1	1	1	1
3	3	1	3	2	2	2	2	2	2
3	3	1	3	2	2	2	2	2	2
1	3	1	3	2	9	1	2	1	1
1	3	3	2	1	1	2	2	1	2
1	3	1	3	2	1	2	1	1	1
1	3	3	3	2	9	1	2	1	1
1	3	1	3	2	1	2	1	1	1
1	3	3	3	2	9	1	2	1	1
1	3	3	3	9	1	1	1	1	1
3	3	1	3	1	1	1	1	1	1
3	3	1	3	9	1	1	1	1	1
3	2	1	2	1	1	1	1	1	1
2	2	2	3	9	2	2	2	2	2
1	3	1	3	2	2	1	2	1	3
1	3	1	3	3	1	1	3	1	9
3	3	1	3	1	3	2	1	1	1
9	9	9	9	9	9	9	9	9	9

2	3	3	3	2	3	2	1	1	3
2	1	2	2	1	1	1	1	2	2
9	9	9	9	1	1	1	1	1	1
1	3	2	2	2	1	1	2	1	1
2	3	2	3	2	1	9	1	1	1
1	3	1	3	3	1	1	1	1	1
3	3	3	3	1	1	3	3	1	3
1	3	3	9	1	1	1	1	1	1
9	9	9	9	9	1	9	9	9	1
3	2	3	1	2	1	1	3	2	3
9	9	9	9	9	9	9	9	9	9
3	3	3	1	1	1	1	1	1	1
1	3	2	3	1	1	1	1	2	2
2	9	1	9	3	2	1	9	2	9
3	2	1	2	3	1	1	1	2	1
1	3	3	3	2	1	1	1	3	2
2	2	2	3	1	1	2	2	1	1
2	9	1	1	9	1	1	1	1	1
1	3	1	3	2	2	1	2	1	3
3	2	2	3	1	2	1	2	1	2
9	9	9	9	9	1	9	9	9	9
9	9	9	9	3	1	3	3	1	3
9	9	9	9	9	1	9	9	9	9
2	1	2	3	3	1	2	9	1	1
9	1	9	9	9	9	1	9	9	1
1	3	1	2	3	1	1	1	1	1
1	3	1	3	3	1	1	1	1	1
1	2	1	3	2	1	1	1	2	2
3	3	3	3	1	1	1	1	1	2
1	2	1	2	2	1	1	9	1	1
1	2	1	2	3	1	1	1	1	1
2	1	1	3	3	1	3	1	3	1
1	3	1	2	3	1	1	1	1	1
3	3	1	3	2	1	1	2	3	1
2	2	3	3	2	1	2	2	1	1
3	3	3	2	1	1	1	1	9	1
1	1	1	1	1	1	1	1	1	1
2	2	3	3	1	1	1	1	3	1
1	3	1	3	3	1	1	1	1	1
1	3	1	3	3	1	1	1	1	1
1	2	3	3	1	2	1	9	1	1
3	3	3	3	3	9	9	9	9	1
1	3	1	1	1	1	1	3	3	3
1	1	1	1	2	1	1	1	1	1
3	3	1	3	3	1	1	2	1	3
1	3	3	9	9	1	1	2	2	3
1	9	9	9	9	9	9	9	9	9

2	3	2	2	1	1	1	1	1	1
2	2	2	2	2	1	1	1	2	1
2	3	3	3	1	1	1	1	1	1
2	2	2	2	1	1	1	1	2	1
1	3	3	1	1	1	1	3	2	1
1	2	2	2	2	1	1	2	1	1
1	1	2	3	1	1	1	1	1	1
3	1	3	3	1	1	2	2	1	1
2	3	2	1	1	1	2	2	1	1
1	2	3	3	1	1	1	1	1	1
1	1	1	3	3	1	2	2	2	1
2	3	2	2	2	1	1	1	1	2
1	1	3	3	1	1	1	1	1	2
1	3	3	3	1	1	1	1	1	1
2	3	3	1	2	1	1	2	1	2
2	3	1	2	3	1	2	1	2	1
2	2	1	2	2	2	2	1	2	2
1	3	2	1	1	1	3	3	1	3
2	1	2	1	1	1	1	1	1	1
9	9	9	9	9	9	9	9	9	9
2	2	2	3	2	1	1	1	1	2
1	3	1	3	3	1	1	2	1	3
2	2	3	1	2	2	2	2	2	2
3	3	3	3	1	1	9	9	9	9
1	1	3	3	1	1	1	1	3	3
1	3	2	2	2	1	2	2	2	2
1	9	3	3	2	2	1	2	1	2
3	2	1	3	2	1	2	1	1	2
2	2	2	2	2	2	2	2	2	2
2	2	3	2	2	2	2	2	2	3
2	2	2	2	2	2	2	2	2	2
2	3	2	1	2	2	2	2	2	2
1	3	1	3	2	1	1	3	1	3
2	2	2	2	2	3	2	3	2	2
2	2	2	2	2	2	2	2	2	2
3	2	1	3	9	2	2	1	2	2
1	2	2	2	1	9	1	2	2	2
1	3	1	3	3	3	2	2	2	1
9	1	2	9	9	9	9	9	9	9
3	3	1	3	2	3	2	2	1	2
2	2	3	3	1	1	2	1	3	3
2	2	3	3	1	1	1	2	1	3
2	3	2	2	2	2	2	2	2	2
3	3	2	2	2	1	1	1	1	2
2	2	3	2	2	1	2	2	2	2
1	2	3	2	2	1	1	2	1	1
2	2	1	2	2	1	2	2	1	2

3	3	3	3	3	3	3	3	3	3
1	3	1	2	3	2	1	2	1	2
1	3	2	2	2	2	1	1	1	1
2	2	3	3	1	1	1	2	1	3
1	1	1	1	1	1	1	1	1	1
1	3	3	2	1	1	1	2	1	2
2	3	3	3	3	3	2	2	3	3
2	3	1	3	3	2	2	2	1	2
2	2	2	2	2	2	2	2	2	2
2	2	2	2	2	2	2	2	2	2
3	2	2	9	3	2	2	2	3	2
3	3	2	2	3	2	2	2	3	2
9	9	9	9	9	9	1	9	9	9
1	1	2	2	9	1	1	2	2	2
1	3	2	2	2	2	1	1	1	1
1	3	2	2	2	2	1	1	1	1
9	9	9	9	9	9	9	9	9	9
1	3	3	1	1	1	1	1	3	1
9	9	9	9	9	9	9	9	9	9
1	1	1	3	3	1	1	1	1	1
9	9	9	9	9	9	1	1	1	1
1	1	1	3	3	1	1	1	1	1
1	1	1	3	3	1	1	1	1	1
1	1	1	3	3	1	1	1	1	1
1	1	1	3	3	1	1	1	1	1
1	1	1	3	3	1	1	1	1	1
1	1	1	3	3	1	1	1	1	1
1	1	3	3	9	1	1	1	1	1
1	1	1	3	3	1	1	1	1	1
1	1	1	3	3	1	1	1	1	1
2	3	3	2	2	3	1	3	2	9
2	2	2	2	2	1	1	2	2	3
9	9	9	9	9	9	9	9	9	9
1	1	1	3	3	1	1	1	1	1
1	1	1	3	3	1	1	1	1	1
1	1	1	3	3	1	1	1	1	1
1	1	1	3	3	1	1	1	1	1
1	1	1	3	3	1	1	1	1	1
1	1	3	3	1	1	1	1	1	1
9	1	1	1	3	1	1	1	1	1
1	1	1	3	3	1	1	1	1	1
1	1	1	3	3	1	1	1	1	1
9	9	9	9	9	9	9	9	9	9
1	1	1	1	3	3	1	1	1	1
1	1	1	3	3	1	1	1	1	1
1	1	3	1	1	1	1	1	1	1
1	1	1	3	3	1	1	1	1	1

1	1	1	3	3	1	1	1	1	1
1	1	1	1	3	1	1	1	1	1
1	1	1	1	3	1	1	1	1	1
1	1	1	3	3	1	1	1	1	1
1	1	1	1	1	1	1	1	1	1
1	1	1	2	2	1	1	1	1	1
1	1	1	3	3	1	1	1	1	1
1	3	3	3	1	1	1	1	1	1
1	3	1	3	1	1	1	1	1	1
1	3	2	3	1	1	1	1	1	2
1	3	3	3	1	1	1	1	1	1
1	3	3	3	1	1	3	1	3	1
1	3	1	3	3	1	1	1	1	1
9	9	9	3	3	1	1	1	1	1
2	2	1	1	3	1	2	2	2	2
2	2	1	3	3	2	1	1	1	1
2	2	2	1	1	1	1	1	1	1
1	1	1	1	1	1	1	1	1	1
2	2	1	2	2	1	2	2	1	2
2	2	2	2	2	2	2	2	2	2
1	2	2	3	1	1	1	1	2	1
2	2	2	2	2	2	2	2	2	2
3	2	1	2	2	1	2	1	3	3
1	3	1	2	2	2	1	2	1	2
2	2	2	2	2	2	2	2	2	2
2	2	2	2	2	2	2	2	2	2
2	2	2	2	2	2	2	2	2	2
2	2	2	2	2	2	2	2	2	2
2	2	2	2	2	2	2	2	2	2
2	2	1	2	2	2	2	2	1	2
2	2	2	2	2	2	1	1	2	1
2	2	2	2	2	2	1	1	2	1
2	2	2	2	2	1	2	2	1	2
2	3	3	2	2	2	1	1	3	1
2	2	1	2	2	2	2	1	1	1
2	2	2	2	2	2	2	2	2	2
2	2	2	1	1	1	1	2	2	1
1	2	3	3	3	1	1	1	2	1
2	2	2	2	2	2	1	2	2	2
3	3	1	3	3	2	3	1	1	1
2	1	2	2	2	2	2	2	2	2
2	2	2	2	2	2	2	2	2	2
9	9	9	9	9	9	9	9	9	9
9	9	9	9	9	9	9	9	9	9
3	3	3	2	2	2	3	2	2	1
2	1	3	2	1	3	2	2	1	1
9	9	9	9	9	9	9	9	9	9

2	2	2	2	2	1	2	2	1	2
2	2	2	2	2	1	2	2	1	2
2	2	2	2	2	2	1	2	2	2
2	3	2	2	2	2	2	1	1	2
3	2	1	2	1	2	2	1	2	2
1	2	1	2	3	1	1	2	1	1
1	3	1	3	3	2	1	2	1	1
2	3	2	2	2	2	2	2	1	1
2	2	3	3	3	1	1	2	1	1
3	3	3	3	3	1	1	3	3	3
1	2	2	2	2	2	3	2	2	2
2	3	3	3	1	1	2	1	1	1
3	2	3	3	3	1	1	2	1	1
3	3	3	2	2	2	2	2	1	2
3	3	1	3	2	2	2	2	1	1
3	2	3	3	3	2	3	2	2	2
2	3	3	2	2	2	2	1	2	1
2	2	2	2	2	2	2	2	2	2
2	3	3	3	2	2	3	2	2	2
1	2	2	2	2	2	2	1	1	3
2	2	1	3	3	2	2	2	1	1
2	2	2	2	2	1	1	2	1	1
3	3	3	2	2	2	2	2	1	2
2	2	2	2	2	2	3	3	3	2
9	9	1	9	9	9	9	9	1	9
2	2	2	2	2	2	2	2	2	2
2	2	2	2	2	2	2	2	2	2
2	2	3	9	2	1	9	1	2	9
9	9	9	9	9	9	9	9	9	9
1	2	2	2	2	2	1	2	2	2
9	9	9	9	9	9	1	9	9	9
1	2	2	2	9	9	9	1	1	9
1	3	3	1	3	1	3	2	3	3
2	1	2	2	2	1	2	1	2	1
9	9	9	9	9	9	9	9	9	9
2	3	2	1	2	2	2	2	2	2
2	1	2	2	2	1	2	2	1	1
1	3	2	3	2	3	2	1	2	2
1	2	2	3	1	1	1	1	1	1
1	3	1	3	2	1	1	2	3	1
3	3	1	3	3	2	2	2	1	1
2	3	2	2	2	1	1	1	1	2
2	3	1	2	1	1	1	1	1	1
2	2	2	2	2	2	2	2	1	2
9	9	9	9	9	9	9	9	9	9
2	2	9	1	2	2	2	2	1	1
2	3	2	3	1	1	2	2	1	1

9	9	9	9	9	9	9	9	9	9
1	1	2	3	1	9	9	9	9	9
1	2	1	2	1	1	1	1	1	2
1	2	1	3	9	1	1	1	1	1
2	2	2	2	2	1	3	3	3	2
3	1	2	3	2	1	1	3	1	3
1	1	2	2	2	2	2	1	1	1
1	3	1	2	3	1	1	1	1	1
1	2	2	2	2	2	3	1	1	1
2	1	2	2	2	2	2	2	2	2
2	2	2	2	2	2	2	2	2	2
2	3	1	3	2	1	1	1	2	1
9	9	9	9	9	9	9	9	9	9
1	3	3	1	1	1	1	1	3	3
1	1	3	1	3	3	1	3	3	3
1	2	2	2	9	1	1	2	2	1
1	3	3	1	1	1	1	1	2	2
2	2	1	2	1	1	1	2	1	2
2	2	2	1	1	2	3	1	2	1
9	9	9	9	9	9	9	9	9	9
3	1	2	3	2	1	2	1	3	1
2	3	2	3	1	1	1	1	1	1
3	2	3	3	1	1	2	2	1	1
2	2	2	2	2	2	2	2	2	2
2	2	2	2	2	2	2	2	1	2
9	9	9	9	1	1	1	2	1	1
3	3	3	2	1	2	1	1	1	2
1	3	9	3	9	1	1	2	2	1
3	3	1	3	3	2	1	2	2	1
1	3	3	1	1	1	1	1	2	1
1	1	3	3	1	1	1	1	2	2
2	3	3	3	3	2	3	3	3	2
9	9	9	9	9	1	9	9	9	9
3	2	3	1	1	1	3	3	3	1
3	3	1	2	1	1	1	2	2	2
1	3	3	3	1	3	2	2	1	1
1	3	1	3	3	1	1	1	1	1
1	3	3	3	3	1	1	1	1	1
1	3	3	3	1	1	1	1	1	1
1	3	3	3	1	3	1	2	1	1
3	3	3	2	2	1	3	3	3	3
1	3	3	3	1	1	1	1	1	1
2	2	2	3	2	3	2	2	3	3
2	2	2	2	2	2	2	2	2	2
1	3	3	3	3	3	3	2	1	3
2	2	2	2	2	2	1	2	1	1
3	3	3	3	1	1	1	3	1	1

1	3	3	2	2	1	1	2	2	1
1	3	3	2	2	1	1	2	2	1
9	9	9	9	9	9	1	9	9	9
3	3	3	3	3	1	1	2	1	1
9	9	9	9	9	9	9	9	9	9
2	3	3	3	2	1	2	2	1	1
1	1	2	1	1	1	1	1	1	1
1	3	9	9	9	9	1	9	2	2
9	1	1	3	3	1	1	1	1	1
2	3	1	3	2	3	2	2	1	3
2	2	2	3	3	1	2	2	3	2
3	3	3	2	2	2	2	2	2	2
1	3	2	3	3	2	2	2	1	1
3	2	2	2	2	1	1	2	2	3
3	2	2	3	3	1	2	2	3	2
1	2	2	3	1	2	2	1	1	1
3	2	2	3	3	1	2	2	2	2
1	1	1	1	2	2	2	2	1	3
1	1	1	1	3	1	1	1	1	1
1	2	2	2	2	2	2	2	2	2
2	2	3	9	2	1	1	3	2	1
2	3	2	3	1	1	1	2	1	3
2	3	1	3	3	1	1	2	2	2
3	3	3	3	3	1	1	1	1	2
2	2	2	2	2	2	2	2	2	2
2	2	2	2	2	2	2	2	2	2
2	3	1	2	2	2	2	2	1	1
2	3	2	3	2	2	2	2	2	2
2	3	2	2	3	2	2	1	1	2
1	1	1	1	2	1	1	2	1	1
2	3	2	2	2	2	2	2	2	1
2	3	2	2	2	2	2	2	2	1
3	3	2	2	1	2	3	2	3	1
2	2	2	2	2	2	2	2	2	2
2	2	2	2	2	2	2	2	2	2
3	3	2	2	2	1	1	2	3	2
3	3	2	3	3	2	2	3	1	1
9	9	9	9	9	1	1	9	1	9
2	2	2	2	2	2	2	2	2	2
2	2	3	2	2	2	2	3	2	1
2	2	1	2	2	1	2	2	2	2
2	2	1	2	2	2	1	2	1	2
2	2	2	2	3	1	1	1	1	2
2	2	2	2	2	2	2	2	1	2
2	2	1	2	2	1	2	2	1	2
1	3	1	2	2	1	1	2	1	1
2	2	2	2	2	2	2	2	2	2

1	3	3	3	3	2	2	1	2	3
1	2	3	2	1	1	1	1	2	2
1	2	2	2	2	3	3	2	3	3
2	2	1	2	2	1	2	2	2	2
2	3	2	2	2	1	2	2	1	2
2	3	2	3	3	1	1	2	2	2
2	2	1	2	2	2	1	9	2	9
2	3	1	3	3	1	1	1	1	1
2	3	2	2	1	1	1	1	2	1
2	3	1	2	2	1	1	2	1	3
2	1	2	1	2	1	2	2	2	2
1	1	1	2	2	1	1	1	1	2
3	3	1	3	3	1	1	2	2	1
2	2	2	2	2	2	2	2	2	2
2	2	2	2	2	2	2	2	2	2
1	1	1	1	2	1	1	1	2	1
3	3	1	3	3	1	2	2	1	2
2	2	2	2	2	1	1	1	1	1
2	2	2	2	2	1	1	1	1	1
9	9	9	9	9	9	9	9	9	9
2	2	1	2	2	1	1	2	1	2
2	2	2	2	2	2	2	2	2	2
9	9	9	9	9	9	9	9	9	9
2	2	2	2	2	2	2	2	2	2
1	3	3	2	2	1	1	1	1	1
3	3	9	1	2	9	2	3	1	2
2	2	2	2	2	2	2	2	3	1
9	9	9	9	9	9	9	9	9	9
2	2	2	2	1	2	2	2	2	2
1	2	1	3	2	1	3	1	2	3
2	1	2	2	2	2	2	2	2	2
2	1	3	3	2	3	1	1	2	3
2	1	2	2	2	2	2	2	2	2
1	3	1	1	2	1	1	1	1	1
1	1	2	1	1	1	1	1	1	1
3	2	1	2	1	2	1	1	1	1
3	3	3	3	3	3	3	3	3	2
1	2	1	2	3	2	1	3	2	1
1	2	2	2	2	2	2	1	3	2
1	3	1	2	2	1	1	2	2	1
1	1	2	3	2	2	3	2	1	2
3	2	1	2	1	1	1	3	2	1
1	3	2	3	3	1	1	1	1	1
2	3	3	3	2	3	3	3	3	3
9	9	9	9	9	9	9	9	9	9
1	1	3	3	1	1	1	1	1	1
9	9	9	9	9	1	1	1	9	1

1	1	1	1	1	1	1	1	1	1
1	1	1	3	3	1	1	1	1	1
1	1	1	3	3	1	1	1	1	1
1	1	1	3	3	1	1	1	1	1
1	1	1	1	1	1	1	1	1	1
1	1	1	3	3	1	1	1	1	1
1	1	1	3	3	1	1	1	1	1
1	1	1	3	3	1	1	1	1	1
1	1	1	1	3	3	1	1	1	1
1	1	1	1	3	1	1	1	1	1
2	1	1	2	2	9	1	2	1	1
1	1	1	3	3	1	1	1	1	1
1	1	1	3	3	1	1	1	1	1
1	1	1	3	3	1	1	1	1	1
1	1	1	3	3	1	1	1	1	1
1	1	1	3	3	1	1	1	1	1
1	2	2	2	2	1	2	2	1	2
1	1	3	3	1	1	1	1	1	1
1	1	1	3	3	1	1	1	1	1
1	1	1	3	3	1	1	1	1	1
1	1	1	3	3	1	1	1	1	1
1	1	1	3	3	1	1	1	1	1
1	1	1	3	3	1	1	1	1	1
1	1	1	3	3	1	1	1	1	1
1	1	1	3	3	1	1	1	1	1
1	1	1	3	3	1	1	1	1	1
1	1	1	3	3	1	1	1	1	1
1	1	1	3	3	1	1	1	1	1
1	1	3	1	3	3	1	1	1	1
1	1	1	3	3	1	1	1	1	1
1	1	1	3	3	1	1	1	1	1
1	1	1	3	3	1	1	1	1	1
1	1	1	3	3	1	1	1	1	1
1	1	1	3	3	1	1	1	1	1
1	1	1	3	3	1	1	1	1	1
1	1	1	3	3	1	1	1	1	1
1	3	1	3	2	1	1	1	1	1
1	1	1	1	3	1	1	1	1	1
1	1	1	3	3	1	1	1	1	1
1	1	1	3	3	1	1	1	1	1
9	1	1	3	3	1	1	1	1	1
2	2	1	2	2	2	2	2	1	1
1	2	2	3	2	2	3	1	1	1
2	2	2	2	2	2	2	2	2	2
9	9	9	9	9	9	9	9	9	9
2	2	2	2	2	1	1	2	2	2
1	3	1	3	3	1	1	1	2	1

2	2	2	2	2	2	2	2	2	2
1	2	1	2	2	1	2	1	1	2
1	2	1	2	2	1	2	1	1	2
2	2	3	3	1	1	1	1	1	1
1	1	1	3	1	1	1	1	1	1
2	3	2	2	2	1	2	1	1	1
2	2	2	2	2	2	2	2	2	2
2	2	2	2	2	2	2	2	3	2
2	3	3	2	2	1	1	1	2	1
2	2	2	2	2	1	2	2	2	2
1	2	1	2	2	1	2	1	1	2
9	9	9	9	9	9	9	9	9	9
9	1	2	9	2	1	9	9	9	1
2	2	2	2	2	2	1	2	2	2
9	1	9	9	9	9	9	9	9	9
9	9	9	9	9	9	1	9	9	9
9	9	9	9	9	9	9	9	9	9
9	9	9	9	9	9	9	9	9	9
9	9	9	9	9	9	9	9	9	9
1	3	3	3	2	2	2	1	2	3
3	3	3	3	3	3	3	3	3	9
3	3	3	3	3	3	3	3	3	1
2	3	2	1	3	1	2	1	2	2
9	9	9	9	9	9	9	9	9	9
9	9	9	1	9	9	9	9	9	9
9	9	9	9	1	9	9	9	1	9
9	9	9	9	9	9	9	9	9	9
1	2	3	1	3	3	2	2	1	2
1	3	2	2	1	2	2	2	1	2
9	9	9	9	9	9	9	9	9	9
9	9	9	9	9	9	9	9	9	9
2	2	1	2	2	2	2	2	2	2
2	2	2	2	2	2	2	2	2	2
3	1	1	3	3	1	1	1	1	3
1	2	1	2	2	2	2	1	1	2
2	2	3	3	3	2	2	2	1	1
1	2	1	3	3	2	2	2	9	1
3	3	2	3	3	3	3	2	2	9
2	2	2	2	2	2	2	2	2	2
1	3	1	3	2	1	2	1	1	2
9	9	9	9	9	9	9	9	1	9
1	3	3	3	3	1	1	3	2	3
1	2	1	1	1	1	1	2	2	2
2	1	1	3	2	1	2	1	2	1
3	3	1	3	3	3	2	2	3	3
2	2	2	2	2	1	1	1	2	2
1	3	2	3	3	1	1	1	1	2

9	9	9	9	9	9	9	9	9	9
2	2	2	2	2	2	2	2	2	1
3	3	3	3	3	2	2	2	2	2
3	3	2	2	2	1	1	2	1	1
2	3	1	2	2	1	1	2	1	1
2	2	2	2	2	2	2	2	2	2
2	2	2	2	2	2	2	2	2	2
1	1	1	2	1	1	1	2	1	1
3	3	1	3	3	2	1	1	1	1
2	2	2	2	2	2	2	2	2	2
1	3	2	2	2	2	2	2	2	2
2	3	1	3	2	2	2	1	1	2
2	2	2	2	2	2	2	2	2	2
2	2	2	2	2	2	2	2	2	2
2	2	2	2	2	2	2	2	2	1
2	2	2	2	2	2	2	2	2	2
2	2	2	2	2	1	1	2	2	2
2	2	2	2	2	2	2	2	2	2
2	3	3	3	3	2	2	2	1	1
1	3	2	3	2	1	1	1	1	2
2	3	3	1	1	1	1	1	2	2
2	3	1	3	3	2	2	2	2	2
1	3	1	3	3	1	1	1	1	1
1	3	1	3	3	1	1	1	1	1
2	2	2	2	2	2	1	1	2	2
2	2	2	2	2	1	1	2	2	2
3	3	3	1	1	3	3	1	1	1
3	3	3	2	1	2	3	1	2	1
2	3	1	1	2	1	1	1	1	1
3	2	3	3	3	2	2	2	2	3
1	1	3	1	1	1	2	2	2	2
2	9	9	9	9	9	9	9	9	2
2	3	3	3	2	2	2	2	2	2
3	3	1	3	3	3	2	2	2	2
1	3	1	3	3	2	1	2	1	1
9	9	9	9	9	9	9	9	9	9
2	2	2	2	2	2	2	2	2	2
1	3	3	3	1	3	3	1	1	1
2	2	2	2	2	2	2	2	2	2
2	1	3	3	1	1	1	2	2	1
9	9	9	9	9	1	1	2	9	1
1	2	3	3	2	1	1	1	1	2
1	3	3	3	3	1	1	1	1	1
2	3	2	2	2	1	1	1	1	2
1	3	1	3	3	1	1	1	1	2
2	1	2	1	2	2	2	2	2	2
9	9	9	9	9	9	9	9	9	9

9	9	9	9	9	9	9	9	9	9
9	9	9	9	9	9	9	9	9	9
9	9	9	9	9	9	9	9	9	9
9	9	9	9	9	9	9	9	9	9
9	9	9	9	9	9	9	9	9	9
1	9	9	9	9	9	9	9	9	9
9	9	9	9	9	9	9	9	9	9
9	9	9	9	9	9	9	9	9	9
9	9	9	9	9	9	9	9	9	9
9	9	9	9	9	9	9	9	9	9
1	3	3	1	3	9	9	1	9	9
9	9	9	9	9	9	9	9	9	9
9	9	9	9	9	9	9	9	9	9
9	9	9	9	9	9	9	9	9	9
9	9	9	9	9	9	9	9	9	9
9	9	9	9	9	9	9	9	9	9
9	9	9	9	9	9	9	9	9	9
9	9	9	9	9	9	9	9	9	9
2	2	2	2	2	2	2	2	2	2
1	2	1	1	1	1	2	1	1	1
1	1	1	1	1	1	1	1	1	1
1	2	2	2	2	3	2	1	2	2
9	2	1	1	2	1	2	2	2	3
1	1	1	3	1	3	1	1	1	1
2	2	2	3	3	3	2	3	2	2
2	1	1	2	3	3	2	1	3	1
2	2	2	2	2	2	2	2	2	2
2	2	2	2	3	3	2	2	2	3
2	2	2	2	2	2	2	2	2	2
1	9	2	2	3	2	1	2	2	3
1	2	1	1	1	1	1	1	2	3
2	1	1	3	3	2	2	2	1	3
2	3	3	3	2	2	2	2	2	9
2	3	3	3	3	1	1	3	1	3
2	2	2	2	2	2	2	2	1	1
2	2	1	3	3	3	3	3	2	2
1	2	2	2	2	2	2	2	2	2
2	1	2	2	3	1	3	2	1	2
1	1	1	2	2	2	2	1	1	1
2	1	2	3	3	1	1	1	1	1
2	1	2	2	1	2	1	2	1	2
1	1	3	1	3	1	1	3	1	3
1	1	1	3	1	1	1	1	9	1
1	1	2	3	1	1	1	1	3	1
1	3	2	2	2	2	2	2	2	2
9	9	9	9	9	9	9	9	9	9
2	3	1	1	2	2	3	1	2	3

2	3	3	3	3	1	2	2	1	2
1	1	9	2	9	9	9	9	9	9
3	1	3	1	1	3	1	1	3	1
2	2	9	9	9	9	9	9	9	9
2	1	3	2	3	2	2	2	2	2
2	3	2	2	2	2	2	2	2	2
2	2	9	9	9	9	9	9	9	9
2	3	3	3	1	2	2	1	2	2
3	1	2	1	1	1	3	1	1	1
1	3	3	3	3	1	1	2	1	1
2	9	1	1	2	1	1	1	1	1
1	2	2	1	2	1	1	1	2	2
3	2	2	2	2	2	2	2	2	3
1	2	3	1	2	3	1	2	3	1
2	2	2	2	2	2	2	2	2	2
3	1	1	1	3	2	2	2	1	1
1	1	2	2	1	1	1	1	2	1
9	9	9	9	9	9	9	9	9	9
1	3	9	2	3	1	2	1	2	2
3	3	3	3	3	1	3	3	3	3
2	2	2	3	3	3	3	3	3	3
3	2	2	2	2	2	2	2	2	3
3	3	3	3	3	2	2	2	2	2
3	2	2	9	3	1	3	3	3	3
2	2	2	2	2	2	2	2	2	2
1	1	3	1	3	3	1	1	3	1
3	3	3	3	3	2	3	3	3	3
3	1	3	3	3	3	3	1	1	3
2	1	3	2	1	9	1	1	1	1
2	3	1	3	3	1	1	2	1	1
1	3	1	3	3	1	1	1	1	1
3	3	3	3	3	3	9	9	9	9
1	1	2	2	2	1	3	2	1	1
3	3	2	3	3	1	2	1	2	9
2	3	3	9	3	1	9	9	1	9
2	3	1	3	3	1	2	2	1	1
2	3	1	3	3	1	2	2	1	1
9	9	9	9	9	9	9	9	9	9
2	2	2	2	2	9	9	9	9	9
9	9	9	9	9	9	9	9	9	9
9	9	9	9	9	9	9	9	9	9
2	3	2	1	2	2	3	2	2	3
2	3	1	2	3	1	2	3	1	2
9	9	9	9	9	9	9	9	9	9
2	3	1	2	3	1	2	3	1	2
2	3	1	3	2	1	1	1	2	2
1	3	3	3	1	1	3	3	1	1

3	3	3	3	3	3	3	3	3	3
1	2	1	2	2	1	1	2	1	1
2	2	3	3	3	3	3	2	2	3
2	3	2	2	3	1	2	2	1	1
2	2	2	2	2	2	2	2	2	2
9	1	9	9	1	9	1	9	1	9
9	9	9	9	9	9	9	9	9	9
3	3	1	3	2	2	2	1	1	2
3	3	3	3	3	3	3	3	3	3
1	3	3	3	1	1	1	2	2	2
3	3	3	3	3	3	3	3	3	3
1	3	3	1	3	1	3	1	1	1
2	2	1	2	9	2	2	3	2	2
2	3	1	2	2	1	1	1	1	2
2	3	1	2	2	1	1	1	1	2
2	3	2	2	9	2	2	3	2	2
2	3	1	2	2	1	1	1	1	2
2	3	1	2	2	1	1	1	1	2
2	3	1	2	2	1	1	1	1	2
3	3	3	3	3	3	3	3	1	3
1	1	2	9	1	3	2	1	3	1
3	1	2	3	3	3	3	3	3	3
3	3	1	3	3	1	1	1	1	3
2	2	2	3	2	2	2	3	3	3
3	3	3	3	3	3	3	3	3	3
2	3	1	2	2	1	1	1	1	2
9	9	1	9	9	9	9	9	9	9
2	2	3	3	3	1	1	1	1	3
9	9	9	9	9	9	9	9	9	9
3	3	3	3	3	3	3	3	3	3
2	1	1	2	2	1	1	2	1	1
2	2	3	2	3	2	3	2	2	3
1	2	3	2	1	3	1	3	2	3
3	3	3	3	3	3	3	3	3	3
3	3	3	3	3	3	3	3	3	3
3	3	3	3	9	9	9	9	9	9
9	9	9	9	9	9	9	9	9	9
2	1	1	1	1	1	2	1	1	1
1	2	2	1	1	1	1	2	2	2
1	3	3	3	1	2	2	2	1	2
2	2	3	2	2	9	3	9	1	2
3	3	1	2	2	1	2	1	3	1
9	9	1	9	2	1	1	1	9	2
1	3	1	3	3	3	3	1	1	1
2	1	1	3	3	2	2	1	1	1
1	1	1	1	1	1	1	1	1	1
2	1	2	2	2	2	2	2	2	2

1	3	3	3	3	2	2	1	1	1
9	9	9	9	9	9	9	9	9	9
9	9	9	9	9	9	9	9	9	9
1	3	3	1	3	1	3	1	3	1
2	2	2	2	2	2	2	2	2	2
2	1	3	2	3	2	2	2	2	2
9	3	2	9	2	2	2	2	2	2
2	3	1	3	2	2	2	2	2	2
2	3	1	3	2	1	2	2	2	2
1	3	2	1	2	1	3	2	1	3
2	2	1	3	9	2	3	2	2	2
2	3	3	2	1	1	2	1	1	1
2	3	1	3	2	2	2	1	1	2
1	1	1	3	1	2	3	3	2	1
3	3	3	3	3	1	1	1	1	2
3	2	2	1	2	3	2	1	2	1
1	2	3	1	2	3	1	2	3	1
9	9	9	9	9	9	9	9	9	9
9	9	9	9	9	9	9	9	9	9
2	3	2	3	2	3	1	2	3	1
1	1	1	9	2	2	2	1	1	3
2	2	2	2	2	2	2	2	1	2
1	1	1	1	1	1	1	1	1	1
2	2	2	2	2	2	2	2	2	2
3	3	3	3	3	3	3	3	3	3
2	9	2	2	2	3	2	3	3	9
2	3	2	2	3	2	3	3	9	2
1	3	3	3	1	1	1	1	1	1
1	1	1	1	1	1	1	1	1	1
2	2	2	2	2	2	2	2	2	2
3	1	2	3	2	2	3	3	2	1
1	2	3	3	2	1	2	1	1	1
9	9	9	9	9	9	9	9	9	9
2	9	2	2	3	2	3	3	9	2
2	2	2	2	2	1	2	1	2	1
2	2	2	2	2	2	2	2	2	2
2	2	2	2	2	2	2	1	1	2
9	9	9	9	9	9	9	9	9	9
9	9	9	9	1	3	9	9	2	9
1	1	1	2	2	1	2	2	1	1
3	3	3	3	3	3	3	3	3	3
2	2	2	2	2	2	2	2	2	2
2	2	2	2	2	2	2	2	2	2
2	2	2	2	2	2	2	2	2	2
2	2	2	2	2	2	2	2	2	2
2	2	2	2	2	2	2	2	2	2
9	9	9	9	9	9	9	9	9	9

9	9	9	9	9	9	9	9	9	9
2	2	2	2	2	2	2	2	2	2
9	9	9	9	9	9	9	9	9	9
9	9	9	9	9	9	9	9	9	9
9	9	9	9	9	9	9	9	9	9
1	1	3	3	1	2	1	2	3	1
2	2	2	2	2	2	2	2	2	2
3	3	3	3	3	3	3	3	3	3
1	1	1	1	1	2	3	2	3	2
9	9	9	9	9	9	9	9	9	9
2	2	2	2	2	2	2	2	2	2
2	2	1	2	2	2	2	2	1	2
1	2	2	2	2	2	2	2	1	1
2	2	2	2	2	2	2	2	2	2
1	1	1	3	3	3	1	1	1	2
2	2	2	2	2	2	2	2	2	2
2	2	2	2	2	2	2	2	2	2
2	2	2	2	2	2	2	2	2	2
1	2	1	2	2	1	2	2	1	1
1	1	2	2	3	3	3	3	3	3
9	9	9	9	9	9	9	9	9	9
2	2	2	3	3	3	3	2	2	2
2	2	2	2	2	2	2	2	2	2
1	3	1	2	2	1	1	1	1	2
2	2	2	2	2	2	2	2	2	2
3	3	2	3	3	2	3	2	3	2
9	9	9	9	9	9	9	9	1	9
9	9	9	9	9	9	9	9	9	9
3	3	3	3	3	3	3	3	3	3
2	2	2	2	2	2	2	2	2	2
9	9	9	9	9	9	9	9	9	9
3	3	1	2	2	2	2	2	1	1
1	3	3	3	3	1	1	3	1	3
2	1	2	2	2	2	2	2	2	2
1	1	1	1	1	1	1	1	1	1
1	3	3	3	3	3	3	3	3	3
2	2	2	2	2	2	2	2	2	2
2	1	2	1	2	1	3	2	3	1
2	2	2	2	2	2	2	2	2	2
9	9	1	9	9	9	9	9	9	9
2	2	2	2	2	2	2	2	2	2
2	2	2	2	2	2	2	2	2	2
3	3	3	3	3	3	3	1	1	1
1	3	1	3	2	2	2	2	2	2
2	2	2	2	2	2	2	2	2	2
3	3	3	3	3	3	3	3	3	3
2	2	2	2	2	2	2	2	2	2

2	2	2	2	2	2	2	2	2	2
2	2	2	2	2	2	2	2	2	2
2	2	2	2	2	2	2	2	2	2
2	2	2	2	2	2	2	2	2	2
3	3	3	2	2	2	2	2	2	2
1	3	2	2	2	1	2	3	1	3
2	2	2	2	2	2	2	2	2	2
3	2	2	1	2	1	3	2	1	2
2	2	2	2	2	2	2	2	3	2
1	2	2	2	2	1	2	2	2	2
3	3	3	1	3	3	1	1	1	2
9	9	9	9	9	9	9	9	9	9
9	9	9	9	1	9	9	9	9	9
2	2	2	2	2	2	2	2	2	2
2	2	2	2	2	2	2	2	2	2
2	2	2	3	3	3	3	3	2	2
2	3	3	2	3	2	3	3	2	3
1	1	9	3	3	1	2	2	1	1
1	2	3	2	1	2	3	2	2	1
3	1	2	2	3	2	2	2	3	3
3	3	3	3	3	3	1	1	1	3
2	2	2	2	2	2	2	2	2	2
2	2	2	2	2	2	2	2	2	2
2	2	3	3	3	3	3	3	1	1
3	3	3	1	1	1	1	1	3	3
9	9	9	9	9	9	9	9	1	1
2	2	2	2	2	2	2	2	2	2
1	2	1	2	2	1	1	2	1	1
2	2	2	2	2	2	9	9	9	9
2	2	3	3	3	2	3	3	1	2
9	1	2	2	1	1	1	1	1	1
3	2	1	1	1	2	2	3	3	1
3	3	3	3	3	3	3	1	1	2
1	2	2	2	1	1	1	1	1	1
3	3	3	3	3	3	3	3	3	3
1	1	1	1	1	3	3	1	1	1
9	9	9	9	9	9	9	9	9	9
1	1	1	1	1	1	1	1	1	1
2	2	2	2	2	2	2	2	2	2
2	2	2	2	2	2	2	2	2	2
2	1	3	3	3	1	2	3	1	2
1	2	2	2	2	1	1	2	1	1
2	3	2	2	3	3	2	2	1	2
2	1	2	2	2	2	2	2	2	2
2	2	2	2	2	2	2	2	2	2
2	1	3	2	1	3	2	3	2	3
3	2	2	3	2	3	2	2	2	2

1	2	9	1	3	2	3	2	3	2
3	3	3	3	3	3	3	3	3	3
2	3	1	2	2	3	2	2	3	3
1	9	9	9	9	9	9	9	9	9
2	9	9	9	9	9	9	9	9	9
2	2	3	3	3	2	2	3	2	3
2	2	2	2	2	2	2	2	2	2
3	3	3	3	3	3	3	3	3	3
3	1	2	2	2	2	2	2	2	2
2	2	2	2	2	2	2	2	2	2
9	9	9	9	9	9	9	9	9	9
2	1	2	2	3	1	3	2	3	2
2	2	2	2	2	2	2	2	2	2
2	2	2	2	2	2	1	9	1	2
2	2	2	2	2	2	2	2	2	2
3	3	3	3	9	2	2	2	2	2
3	2	2	1	2	3	2	2	1	2
9	9	9	9	9	9	9	9	9	9
9	9	2	9	9	9	9	9	9	9
2	2	2	2	2	2	1	2	2	1
2	2	2	2	2	2	2	2	2	2
2	2	2	2	2	2	2	2	2	2
9	9	9	9	9	9	9	9	9	9
9	9	9	9	9	9	9	9	9	9
2	3	1	2	3	3	1	2	3	2
3	3	3	1	3	3	3	3	3	3
2	3	3	3	3	2	3	2	3	3
1	3	3	3	2	1	2	1	1	1
3	3	3	3	3	3	3	3	3	3
2	2	3	3	3	1	2	2	1	2
1	1	1	2	3	1	1	1	1	1
2	3	3	3	3	3	3	1	2	2
1	3	2	3	2	1	2	9	2	3
3	3	3	3	3	3	3	3	3	3
1	1	1	1	1	1	1	1	1	1
1	2	3	3	2	1	2	3	1	1
1	1	1	1	1	1	1	1	3	3
3	3	3	3	3	3	3	3	3	3
9	1	9	9	1	9	9	1	9	9
3	3	3	3	3	3	3	3	3	3
3	3	3	3	3	1	1	1	3	1
1	1	3	3	1	1	3	1	1	1
3	3	3	3	3	1	1	1	3	3
2	2	2	2	2	2	2	2	2	2
1	1	2	2	2	1	1	1	1	2
1	1	1	2	1	1	1	1	3	3
1	2	2	2	2	1	1	2	1	2

2	2	2	2	2	2	2	2	2	2
2	1	2	2	2	2	2	2	2	2
1	1	3	1	1	9	9	1	1	1
3	3	1	3	2	1	1	1	2	1
3	3	3	3	3	3	3	3	3	3
2	2	2	2	2	2	2	2	2	2
2	2	2	2	2	2	2	2	2	2
9	9	9	9	9	9	9	9	9	9
9	9	9	9	9	9	9	9	9	9
3	1	2	2	2	2	2	2	2	2
9	9	9	9	9	9	9	9	9	9
1	3	3	3	3	1	2	2	2	3
3	3	1	3	3	9	1	1	1	1
1	3	3	1	3	2	2	3	2	2
2	2	1	2	2	3	2	2	2	1
2	2	2	2	2	2	2	2	2	2
1	3	1	3	3	1	1	1	1	1
2	2	2	2	2	2	2	2	2	2
3	3	2	3	2	2	2	2	3	2
9	1	9	9	9	9	9	9	9	9
9	9	9	9	9	9	9	9	9	9
1	1	1	1	1	3	3	3	3	3
1	1	3	3	3	3	1	1	1	3
2	2	2	2	2	1	1	1	1	1
3	3	3	3	3	3	3	3	3	3
2	2	2	2	2	2	2	2	2	2
1	2	1	3	9	1	2	1	1	1
2	3	3	3	2	2	1	1	1	2
3	2	1	1	1	1	1	3	2	2
1	2	1	3	3	1	2	2	1	3
2	2	2	2	2	2	2	2	2	2
1	1	1	3	1	1	1	1	3	1
2	1	2	2	3	1	2	2	2	1
3	2	1	2	2	2	1	3	2	1
2	1	2	2	2	2	2	1	2	2
2	2	2	2	2	2	2	2	2	2
2	2	2	2	2	2	2	2	2	2
2	2	2	2	2	2	2	2	2	2
2	2	2	3	3	3	9	2	1	1
2	3	1	2	3	1	2	3	1	2
2	3	2	1	3	2	1	2	1	2
2	2	2	2	2	2	2	2	2	2
1	1	1	1	1	1	1	1	1	1
9	9	9	9	9	9	9	9	9	9
2	2	2	2	2	2	2	2	2	2
3	3	1	3	2	2	2	2	2	2
1	3	1	2	3	2	1	1	1	1

3	3	2	2	3	1	1	2	1	2
3	3	3	2	2	2	2	3	1	2
3	3	1	2	3	1	1	3	3	2
2	3	1	3	3	1	1	1	1	2
2	2	2	2	2	2	2	2	2	2
2	2	2	2	2	2	2	3	2	2
3	3	3	2	2	2	2	2	2	2
2	2	2	2	2	2	2	9	1	2
1	1	1	1	1	1	1	1	1	1
2	2	2	3	2	2	2	1	1	2
2	2	2	2	2	3	2	2	2	1
3	3	2	2	2	3	2	2	2	1
9	9	9	9	9	9	9	9	9	9
1	9	9	9	9	9	9	9	9	9
2	3	2	3	3	3	2	2	2	1
3	3	3	3	3	2	2	2	2	3
9	9	9	9	9	9	9	9	9	9
2	2	2	2	2	2	2	1	1	3
2	1	3	1	1	3	2	2	3	1
2	2	2	2	2	2	2	2	2	2
2	1	3	3	3	2	1	1	2	1
1	9	2	1	2	3	2	3	3	2
2	2	2	2	2	2	2	2	2	1
1	3	2	3	1	1	1	1	2	2
2	2	2	2	2	2	2	2	2	2
3	3	2	2	3	2	1	2	2	1
3	3	3	3	3	3	3	3	3	3
1	2	2	2	2	9	2	2	2	9
3	3	2	2	3	2	1	2	2	1
3	3	3	3	2	2	2	2	3	2
2	2	2	2	2	2	2	2	2	2
9	9	9	9	9	9	9	9	9	9
3	1	1	3	3	1	1	3	2	3
2	2	2	2	2	2	2	2	2	2
1	2	3	1	3	3	2	2	2	1
1	2	2	2	2	1	1	1	2	1
1	2	2	1	2	1	1	1	2	1
9	9	9	9	9	9	9	9	9	9
2	2	2	2	2	2	2	2	2	2
2	2	2	2	2	2	2	2	2	2
1	3	3	3	3	1	3	2	1	1
3	2	2	2	3	1	2	3	2	3
2	2	1	2	2	1	1	1	1	1
2	2	2	2	2	2	2	2	2	2
1	1	9	1	1	3	9	2	2	1
2	2	2	2	2	2	2	2	2	2
9	9	9	9	9	9	1	9	9	9

2	2	2	2	2	2	2	2	2	2
2	2	2	2	2	2	1	1	1	2
9	9	9	9	9	1	9	9	9	9
9	9	9	9	9	9	9	9	9	9
2	2	2	2	1	1	1	1	1	1
9	9	9	9	9	9	9	9	9	1
2	2	2	3	3	3	1	1	1	2
1	1	1	2	3	2	1	1	3	2
2	2	2	3	2	2	1	3	1	2
2	3	3	2	2	1	1	2	2	3
2	2	9	1	2	2	1	1	2	2
1	2	1	2	1	2	2	1	2	1
2	2	1	2	2	1	1	1	1	1
1	2	2	2	2	1	1	2	2	2
1	2	1	3	2	1	2	1	1	1
3	3	3	3	3	3	3	3	3	3
1	3	1	3	3	3	3	2	2	2
1	3	2	3	1	1	1	1	1	2
3	3	1	2	2	3	1	1	1	1
1	3	3	3	1	1	1	3	2	3
1	2	2	3	3	2	3	2	2	2
2	2	2	1	2	1	1	1	2	2
3	3	3	3	3	3	2	1	1	1
2	2	2	2	2	2	2	2	2	2
2	2	2	2	2	2	1	2	1	2
1	2	2	2	2	2	2	2	1	1
2	2	2	2	2	2	2	2	2	2
2	2	2	2	2	2	2	2	2	2
1	1	1	3	1	2	3	2	2	2
9	9	9	9	9	9	9	9	9	9
9	9	9	9	9	9	9	9	9	9
1	2	1	2	2	2	2	2	2	2
9	9	9	9	9	9	9	9	9	9
1	2	3	2	1	3	1	3	1	2
2	2	1	2	1	2	3	2	1	2
9	9	9	9	9	9	9	9	9	9
9	9	9	9	9	9	9	9	9	9
2	2	2	2	2	2	2	2	2	2
2	2	2	9	9	9	9	9	9	3
1	2	2	2	1	1	1	3	2	2
1	1	1	1	1	1	1	1	1	1
1	3	1	3	1	2	1	2	1	1
1	1	1	2	1	2	1	1	1	1
2	1	2	2	3	2	1	2	2	3
1	2	3	1	2	2	3	1	2	1
9	9	9	9	9	9	9	9	9	9
1	3	2	1	3	2	2	1	3	2

2	1	2	2	1	2	1	1	2	2
9	9	9	9	9	9	9	9	9	9
9	9	9	9	9	9	9	9	9	9
2	2	2	2	2	2	2	2	2	2
2	3	1	3	2	1	1	1	1	1
2	1	2	2	1	2	1	1	2	2
1	2	2	2	2	1	1	1	1	3
1	2	2	2	2	2	2	2	2	1
2	2	2	2	2	2	2	2	2	2
1	3	9	3	3	1	1	3	1	1
9	9	9	9	9	9	9	9	9	9
9	9	9	9	9	9	9	9	9	9
1	9	9	9	9	9	9	9	9	9
2	2	2	2	2	1	1	3	2	2
1	2	1	2	2	2	1	2	3	3
2	2	2	2	2	9	9	9	9	9
9	9	9	9	9	9	9	9	9	9
9	9	9	9	9	1	9	9	9	9
2	2	9	3	2	1	1	1	1	1
1	1	9	3	3	3	3	2	1	1
1	1	2	1	1	1	1	2	1	2
2	2	3	3	2	2	1	1	1	2
3	3	3	3	3	3	3	3	3	3
2	2	2	2	2	2	2	2	2	2
2	2	2	2	2	2	2	2	2	2
2	2	3	3	2	1	1	2	2	2
9	9	9	9	9	9	9	9	9	9
3	3	2	3	2	2	1	2	1	1
1	2	3	1	2	3	2	1	2	1
2	2	2	2	2	2	2	2	2	2
1	3	3	3	1	1	1	3	1	1
2	2	1	2	2	1	1	1	1	2
9	9	9	9	9	9	9	9	9	9
3	1	1	1	3	2	1	2	1	3
2	2	2	2	2	2	2	2	2	2
2	2	2	2	2	2	2	2	2	2
1	2	2	2	2	1	1	2	2	1
2	2	2	2	2	2	2	2	2	2
2	1	2	1	1	1	1	1	1	2
1	2	3	1	2	1	2	2	1	2
3	2	2	3	1	1	3	2	1	3
3	3	3	3	3	3	3	3	3	3
9	9	9	9	9	9	9	9	9	9
9	9	9	9	9	1	9	9	9	9
2	9	2	2	2	1	2	2	2	2
1	3	1	3	9	9	9	9	9	9
9	9	9	9	9	9	9	9	9	9

2	2	2	2	2	2	2	2	2	2
2	3	3	2	2	2	2	1	2	1
9	3	1	3	3	2	1	2	1	1
1	3	1	2	2	2	2	2	2	1
1	1	1	1	3	1	1	1	3	1
3	3	3	3	3	3	3	9	9	9
9	9	9	9	9	9	9	9	9	9
3	3	3	3	3	1	3	1	1	3
9	9	9	9	9	9	9	9	9	9
9	9	9	9	9	9	9	9	9	9
9	9	9	9	9	9	9	9	9	9
9	9	9	9	9	9	9	9	9	9
9	9	9	9	9	9	9	9	9	9
3	1	3	3	1	3	3	3	3	3
1	2	3	3	1	1	1	1	1	1
2	2	2	2	2	2	1	2	1	1
1	1	1	1	1	1	1	2	2	2
1	3	9	9	9	1	9	9	9	9
9	9	9	9	9	9	9	9	9	9
9	9	9	1	9	9	9	9	9	9
1	1	9	1	9	3	2	9	1	3
9	9	9	9	9	9	9	9	9	9
1	1	1	1	1	1	1	1	1	1
9	9	9	9	9	9	9	9	9	9
1	1	1	1	2	1	1	2	2	2
2	2	1	1	1	1	1	2	2	2
3	2	1	2	2	3	3	3	2	3
1	1	1	1	1	1	2	2	2	2
1	1	1	1	1	1	1	1	1	1
2	2	1	1	1	1	2	2	2	2
1	1	1	1	1	1	1	1	1	2
2	1	1	1	1	2	2	1	1	1
1	1	2	2	2	2	1	1	1	1
9	9	9	9	9	9	9	9	9	9
2	2	2	2	1	1	1	1	1	1
1	1	1	2	2	2	1	1	1	1
3	3	1	2	2	2	3	3	3	3
9	9	9	9	9	9	9	9	9	9
1	1	1	2	2	1	1	2	1	3
3	3	3	3	3	3	1	1	3	3
3	2	2	2	2	2	2	2	2	2
2	3	1	3	2	1	2	1	1	9
1	1	3	9	9	9	9	9	9	9
9	9	9	9	9	9	9	9	9	9
1	1	1	1	3	1	3	3	1	1
1	2	9	1	3	2	9	3	3	3
2	3	2	3	2	3	1	1	1	2

9	9	9	9	9	9	9	9	9	9
9	9	9	9	9	9	9	9	9	9
9	9	9	9	9	9	9	9	9	9
9	9	9	9	9	9	9	9	9	9
2	3	2	3	2	1	1	3	1	2
9	9	9	9	9	9	9	9	9	9
2	2	2	2	2	2	2	2	2	2
1	1	1	2	1	9	9	9	9	9
9	9	9	9	9	9	9	9	9	9
9	9	9	9	9	9	9	9	9	9
9	9	9	9	9	9	9	9	9	9
9	9	9	9	9	9	9	9	9	9
2	2	2	2	2	1	1	1	1	1
2	2	1	3	1	3	2	2	3	1
3	3	3	3	3	1	1	9	2	2
1	1	1	3	2	1	1	2	1	3
1	1	1	1	1	2	2	2	1	1
2	2	1	2	2	1	1	1	1	2
1	1	2	2	2	2	1	1	1	1
9	9	9	9	9	9	9	9	9	9
1	1	1	1	2	2	2	2	2	2
1	2	1	3	3	1	1	3	3	3
2	2	2	2	2	2	2	2	2	2
3	9	1	9	2	1	3	1	1	2
1	2	2	2	1	1	2	1	1	1
1	1	3	3	3	3	3	3	3	3
2	9	9	1	9	9	9	2	9	9
1	1	1	3	3	3	2	2	2	2
9	9	9	9	9	9	9	9	9	9
9	9	9	9	9	9	9	9	9	9
3	3	3	3	1	1	3	1	1	9
3	3	3	3	3	3	3	3	3	3
3	3	3	3	3	3	3	3	3	3
1	1	1	1	1	2	2	1	2	2
2	2	1	1	1	1	2	2	1	1
2	2	2	2	2	1	1	2	2	2
2	2	2	2	2	2	2	2	2	2
1	2	2	2	2	1	1	1	1	1
1	2	2	2	1	1	1	1	1	2
2	2	2	2	2	2	2	2	2	2
1	3	3	3	3	1	1	1	1	1
2	2	2	2	2	2	2	2	2	2
3	3	3	3	3	3	3	1	1	3
2	2	1	2	2	1	2	2	1	1
2	3	1	2	2	3	2	1	1	2
3	3	3	3	3	3	3	3	3	3
2	2	2	2	2	2	2	2	1	2

9	9	9	9	9	9	9	9	9	9
1	1	1	2	2	3	3	2	2	2
9	2	9	9	9	9	9	9	9	9
9	9	9	9	9	9	9	9	9	9
9	9	9	9	9	9	9	9	9	9
1	2	2	1	1	1	1	2	1	1
9	9	9	9	9	9	9	9	9	9
2	3	1	2	3	2	2	2	1	3
9	9	9	9	9	9	9	9	9	9
2	2	3	2	2	2	2	2	2	2
9	9	9	9	9	9	9	9	9	9
2	2	2	2	2	2	2	2	2	2
1	1	1	2	2	2	1	1	1	1
2	2	2	2	2	2	2	2	2	2
1	1	2	2	1	1	2	2	1	1
9	9	9	9	9	9	9	9	9	9
2	2	2	2	2	2	2	2	2	2
3	3	3	3	3	3	3	3	3	3
2	3	1	9	9	9	9	9	9	9
3	3	3	3	3	3	3	3	3	3
2	2	2	2	2	2	2	2	2	2
3	3	1	1	2	2	2	2	2	1
2	2	2	2	2	2	2	2	2	2
2	2	2	2	2	2	2	2	2	2
1	1	1	1	1	1	1	1	1	1
2	2	2	2	2	2	2	2	2	2
1	3	3	1	3	1	3	2	2	3
3	3	1	2	3	1	3	3	3	3
1	2	2	2	2	2	1	1	1	1
2	1	1	1	1	1	2	2	2	2
9	9	9	9	9	9	9	9	9	9
2	3	1	2	2	3	1	2	1	3
1	2	3	2	1	2	2	3	1	2
2	2	2	2	2	2	2	2	2	2
3	3	2	2	2	2	2	2	2	2
2	2	2	1	1	1	2	1	1	1
1	3	3	3	3	3	1	1	1	1
1	2	2	2	2	2	1	1	1	2
2	1	1	1	1	1	1	2	2	2
2	2	2	2	2	1	1	2	2	1
2	2	2	2	2	2	2	2	2	2
1	1	1	1	1	1	1	1	1	1
9	9	9	9	9	9	9	9	9	9
9	9	9	9	9	9	9	9	9	9
9	9	9	9	9	9	9	9	9	9
2	9	1	2	9	2	2	2	1	9
2	2	2	2	2	2	2	2	1	1

1	3	3	3	1	1	1	1	1	1
2	2	2	2	2	2	2	1	2	2
9	9	9	9	9	9	9	9	9	9
2	3	1	2	3	1	2	1	3	2
9	9	9	9	9	9	9	9	9	9
2	2	2	2	2	3	2	3	2	3
1	9	9	9	9	9	9	9	9	9
9	9	9	9	9	9	9	9	9	9
2	2	2	2	2	1	2	2	2	2
3	3	1	1	1	1	1	1	1	2
9	9	9	9	9	9	9	9	9	9
9	9	9	9	9	9	9	9	9	9
1	2	1	1	2	2	2	2	1	2
2	3	1	3	3	2	2	2	1	9
9	9	9	9	9	9	9	9	9	9
1	1	1	1	1	1	3	1	2	9
1	2	3	2	2	1	3	1	2	3
1	3	1	3	2	2	2	3	1	1
2	2	2	2	2	2	2	2	2	3
1	2	3	3	3	2	2	1	1	1
3	3	2	2	1	1	2	2	1	3
1	3	1	3	1	1	1	3	1	1
2	3	3	3	3	1	3	1	1	2
2	2	2	3	3	2	1	1	1	2
2	2	2	2	2	2	2	2	2	2
2	2	9	1	1	1	3	2	2	2
2	2	2	3	3	2	1	1	1	3
1	3	2	2	3	2	1	1	2	2
3	3	9	9	3	2	3	2	2	3
2	2	2	1	2	2	2	1	2	1
3	3	9	1	3	3	1	2	3	1
3	3	3	2	3	2	2	2	3	1
2	9	9	9	9	9	9	9	9	9
3	3	1	3	9	3	3	2	2	3
1	3	2	3	2	3	1	2	1	1
2	2	2	2	2	2	2	2	2	2
3	3	1	3	3	3	2	2	2	2
2	3	3	3	3	1	2	2	1	3
9	9	9	9	9	9	9	9	9	9
2	2	3	2	3	2	3	2	9	2
9	9	9	9	2	9	9	9	9	9
9	9	9	9	9	9	9	9	9	9
2	2	2	2	2	2	3	2	2	2
2	2	1	2	2	1	9	9	9	9
3	3	1	3	3	2	2	3	3	1
1	3	1	1	1	3	1	3	1	1
3	3	3	3	1	1	3	3	9	1

3	3	3	3	3	3	3	3	3	3
9	9	9	9	9	9	9	9	9	9
1	3	3	3	3	1	3	3	3	1
1	3	1	3	3	1	3	1	1	1
3	3	1	3	3	3	3	3	1	3
1	1	3	1	3	3	1	2	2	2
1	3	9	1	2	2	1	3	1	3
2	2	2	2	2	2	2	2	2	2
3	1	1	3	3	3	1	1	3	2
1	3	1	3	2	3	1	2	3	2
3	1	3	1	1	3	1	1	1	2
3	2	3	1	2	3	2	1	3	2
2	2	3	3	3	2	2	3	2	3
3	3	3	2	1	2	1	2	3	1
2	2	2	2	1	1	1	3	1	1
1	3	3	3	3	2	1	1	1	2
1	3	3	2	3	1	1	2	2	3
3	3	2	1	3	2	1	1	1	1
3	3	1	3	3	3	3	1	1	1
2	2	2	2	2	2	2	2	2	2
1	2	1	1	2	2	2	2	2	2
2	2	2	2	2	2	3	3	3	3
3	3	3	3	3	3	3	3	3	3
9	9	9	9	9	9	9	9	9	9
3	3	2	2	2	1	1	1	1	1
2	1	2	2	1	2	1	1	1	2
2	1	3	2	1	3	2	1	3	2
3	3	2	3	3	2	2	3	3	3
1	1	1	3	3	1	1	1	1	1
9	9	9	9	9	9	9	9	9	9
2	3	2	2	2	2	2	2	2	1
1	2	3	1	2	3	2	3	1	2
3	3	3	9	9	1	2	3	1	2
2	3	3	2	3	2	2	2	1	1
3	1	1	1	9	2	9	1	3	3
2	2	2	2	2	2	2	2	2	2
2	2	2	2	9	3	3	3	3	1
9	9	9	9	9	9	9	9	9	9
3	2	2	2	1	2	3	2	2	2
2	3	3	1	2	3	1	2	3	2
9	9	9	9	9	9	9	9	9	9
9	9	9	9	9	9	9	9	9	9
2	3	1	2	1	2	2	3	2	1
3	3	1	3	3	3	1	3	3	3
2	2	2	2	2	2	2	2	2	2
1	9	3	3	3	2	2	2	2	3
9	9	9	9	9	9	9	9	9	9

2	3	2	1	2	2	3	3	3	2
9	9	9	9	9	9	9	9	9	9
3	3	2	3	3	2	2	2	3	1
9	9	9	9	9	9	9	9	9	9
9	9	9	9	9	9	9	9	9	9
3	3	3	3	3	3	3	3	3	3
2	2	2	2	2	2	2	2	2	2
2	2	2	2	2	2	2	2	2	2
3	3	3	3	1	1	1	1	1	1
3	3	3	3	3	2	3	2	3	3
9	9	9	9	9	9	9	9	9	9
9	9	9	9	9	9	9	9	9	9
9	9	9	9	9	9	9	9	9	9
1	2	2	2	2	1	1	2	2	2
2	2	2	2	2	2	2	2	2	2
2	2	2	2	2	2	2	2	2	2
9	9	9	9	9	9	9	9	9	9
9	9	9	9	9	9	9	9	9	9
3	3	3	3	3	3	3	3	3	3
2	2	2	2	2	2	2	2	2	2
3	3	3	3	3	3	3	3	3	3
2	2	2	2	2	2	2	2	2	2
9	9	9	9	9	9	9	9	9	9
1	2	2	1	3	2	1	2	3	1
3	1	2	3	2	2	1	1	2	2
2	1	2	3	3	3	2	2	3	2
9	9	9	9	9	9	9	9	9	9
2	2	2	2	2	2	2	2	2	2
2	2	2	2	2	2	2	2	2	2
9	9	9	9	9	9	9	1	9	9
1	3	3	3	2	2	3	1	1	9
2	2	2	2	2	2	2	2	2	2
3	1	3	1	3	1	3	1	3	1
1	3	2	2	3	1	2	2	2	1
1	3	3	3	3	3	1	1	1	1
3	2	2	1	2	2	2	1	3	2
3	3	3	3	3	3	3	1	1	1
1	3	3	3	3	3	3	3	1	3
9	9	9	9	9	9	9	9	9	9
2	2	2	2	2	2	2	2	2	2
2	3	1	2	2	3	2	3	1	3
2	2	1	2	2	3	3	1	2	2
3	3	3	3	3	3	3	3	3	2
3	3	3	3	3	2	2	2	1	1
2	2	1	2	2	2	2	1	3	1
2	2	2	3	2	3	2	1	2	2
9	9	9	9	9	9	9	9	9	9

2	2	2	2	2	2	2	2	3	3
2	3	2	2	2	2	2	2	2	2
1	2	1	2	2	1	1	1	1	2
3	3	9	2	2	2	2	2	2	2
3	3	3	3	2	3	9	2	1	2
9	9	9	9	9	9	9	9	9	9
2	2	2	2	2	2	2	2	2	2
2	2	1	2	9	2	2	2	2	2
2	2	2	3	1	2	1	2	1	2
2	2	2	2	2	2	2	2	2	2
2	2	3	2	2	2	2	2	2	2
3	1	3	9	3	3	2	2	1	2
2	2	2	2	2	2	2	2	2	2
3	3	3	3	3	3	3	3	3	3
3	3	2	2	3	3	3	3	2	2
3	2	1	2	3	2	2	1	3	2
3	3	2	2	3	3	3	3	3	3
1	2	3	2	3	2	3	3	2	1
2	2	2	2	2	2	2	2	2	2
3	3	3	2	2	2	2	2	2	2
3	3	3	3	3	1	1	2	1	1
2	3	3	3	2	2	3	2	2	1
2	3	3	3	2	2	3	2	2	1
1	2	2	2	1	1	1	1	1	3
1	2	2	2	2	2	2	2	1	1
3	2	3	3	2	2	2	2	2	3
2	2	2	2	1	9	1	2	2	2
2	2	2	2	1	9	1	2	2	2
2	2	2	2	2	2	2	2	2	2
1	2	2	2	2	1	1	1	1	1
2	2	2	2	3	2	3	2	3	2
3	3	2	9	3	2	2	3	2	3
3	3	3	3	3	3	1	1	1	1
2	3	1	2	2	2	2	3	3	3
1	3	3	2	2	2	3	3	2	2
2	2	2	2	2	2	2	2	2	2
2	2	2	2	2	2	2	2	2	2
2	2	2	2	3	1	1	2	3	2
2	2	2	2	2	2	2	2	2	2
2	2	2	2	2	2	2	2	2	2
2	2	3	3	2	1	1	2	1	1
1	3	3	3	3	2	2	3	1	1
2	2	2	2	2	2	2	2	2	2
2	2	2	1	2	2	2	2	2	2
2	3	2	2	2	2	3	2	1	2
1	3	1	1	3	2	1	1	1	1
2	3	1	2	2	2	1	1	1	3

3	2	3	2	2	1	2	3	2	1
2	2	2	2	2	2	2	2	2	2
2	3	2	2	2	2	2	2	1	2
1	2	2	2	2	2	2	2	2	2
2	2	2	2	2	2	2	2	2	2
2	2	2	2	2	2	2	2	2	2
2	3	1	2	2	1	1	1	1	2
1	2	2	3	2	2	1	2	1	1
2	2	2	2	2	2	2	2	2	2
2	3	3	3	2	1	2	2	2	2
2	3	3	3	2	1	2	2	2	2
2	2	2	2	2	2	2	2	2	2
2	2	2	2	2	2	2	2	2	2
9	9	9	9	9	9	9	9	9	9
2	2	2	2	2	2	2	2	2	2
2	1	2	2	2	1	2	2	1	2
1	2	2	3	2	2	1	2	2	2
2	2	2	2	2	1	1	2	2	2
1	1	1	3	3	1	1	1	1	1
1	2	1	3	2	1	2	1	2	1
2	2	2	2	2	2	2	2	2	2
2	2	2	2	2	2	2	2	2	2
2	2	2	3	2	3	2	3	2	2
2	2	2	2	2	2	2	2	2	2
2	2	1	2	2	1	2	2	2	1
1	3	2	3	3	9	2	2	2	2
3	3	3	3	2	2	2	2	2	2
2	2	2	2	2	2	2	2	2	2
2	2	2	2	2	2	2	2	2	2
1	2	2	2	2	2	2	1	1	1
3	3	3	3	3	3	3	3	3	3
2	2	2	2	2	2	2	2	2	2
2	2	2	2	2	2	2	2	2	2
2	2	2	2	2	2	2	2	2	2
2	2	2	2	2	2	2	2	2	2
2	2	2	2	2	2	2	2	2	2
2	2	2	2	2	2	2	2	2	2
2	2	2	2	2	2	2	2	2	2
9	9	9	9	9	9	9	9	9	9
2	1	2	2	2	1	2	2	2	2
2	2	1	2	2	1	1	1	1	2
2	2	1	2	2	2	2	2	2	2
2	2	2	2	2	2	2	2	2	2
2	2	2	2	2	2	2	2	2	2
2	2	2	2	2	2	2	2	2	2
2	2	2	2	2	2	2	2	2	2
2	2	2	2	2	2	2	2	2	2

2	2	2	2	2	2	2	2	2	2
2	2	2	2	2	2	2	2	2	2
1	1	2	3	3	1	1	3	1	1
2	2	2	2	2	2	2	2	2	2
2	2	2	2	2	2	2	2	2	2
2	2	1	2	2	1	2	2	2	2
9	9	9	9	9	9	9	9	9	9
1	3	3	3	3	1	2	1	1	3
1	3	3	3	3	3	1	1	1	1
2	3	3	3	1	2	2	1	1	1
1	2	1	2	2	1	1	1	1	1
3	3	2	3	3	3	2	2	2	2
2	2	2	3	2	1	1	2	1	2
2	2	2	2	2	2	2	2	2	2
1	3	1	3	1	2	2	2	1	1
2	2	2	2	2	2	2	2	2	2
2	2	2	2	2	2	2	2	2	2
1	2	2	2	1	1	1	1	1	2
1	1	1	2	2	1	1	1	1	1
2	2	2	3	1	2	2	2	2	2
1	3	1	3	1	1	1	1	1	1
1	2	2	2	2	3	1	2	2	2
2	2	2	2	2	2	2	2	2	2
2	2	2	2	2	2	2	2	2	2
2	2	2	2	2	2	2	2	2	2
2	2	2	2	2	2	2	2	2	2
9	9	9	9	9	9	9	9	9	9
2	2	2	2	2	2	2	2	2	2
2	2	2	2	2	2	2	2	2	2
2	2	2	2	2	2	2	2	2	2
2	2	2	2	2	2	2	2	2	2
2	2	2	2	2	2	2	2	2	3
9	9	9	9	9	9	9	9	9	9
1	2	1	1	2	3	3	3	3	3
9	9	9	9	9	9	9	9	9	9
1	1	3	3	2	1	1	1	1	1
2	3	3	3	3	3	3	2	2	2
1	2	1	1	2	1	2	1	1	1
2	2	2	2	2	2	2	2	2	2
3	3	3	3	3	3	3	3	3	3
2	2	2	2	1	1	1	1	1	2
2	2	2	1	2	2	2	2	2	2
1	2	1	2	3	1	1	2	1	2
2	2	2	2	2	2	2	2	2	2
2	1	1	1	2	1	2	1	1	1
2	2	2	2	2	2	2	2	2	2
1	1	1	1	2	1	2	1	2	1

2	2	1	2	3	2	2	3	2	1
1	2	1	1	2	3	1	1	2	1
2	2	3	1	9	2	3	3	3	2
3	2	3	3	2	2	1	2	1	2
2	2	3	2	3	2	2	2	2	2
1	2	3	1	2	3	2	2	2	2
2	2	2	1	1	1	1	1	3	2
9	9	9	9	9	9	9	9	9	9
2	2	2	2	2	2	2	2	2	2
2	2	2	2	2	2	2	2	2	2
2	2	2	2	2	2	9	1	2	2
2	2	2	2	2	2	2	1	2	2
2	2	2	9	9	9	9	9	9	9
2	2	2	2	2	2	2	2	2	2
2	2	2	2	2	2	2	2	2	2
2	2	2	2	2	2	2	2	2	2
2	2	1	2	2	1	1	2	1	1
2	3	2	1	2	1	3	3	3	2
2	2	2	2	2	2	2	2	2	2
2	3	1	3	9	2	2	2	2	1
9	9	9	9	9	9	9	9	9	9
9	9	9	9	9	9	9	9	9	9
2	3	3	3	3	3	2	3	1	2
9	9	9	9	9	9	9	9	9	9
2	2	1	3	1	3	1	2	1	2
1	3	1	2	2	1	1	3	1	1
1	1	1	1	1	1	1	1	1	1
2	2	2	2	2	2	2	1	1	1
2	2	2	2	2	2	2	2	2	2
9	9	9	9	9	9	9	9	9	9
2	3	3	3	3	2	2	3	3	3
9	9	9	9	9	9	9	9	9	9
9	9	9	9	9	9	9	9	9	9
2	2	2	2	2	2	2	2	2	2
2	2	2	2	2	2	2	2	2	2
2	2	2	2	2	2	2	2	2	2
2	2	9	1	9	2	3	2	2	3
2	2	2	2	2	2	2	2	2	2
2	2	1	2	2	2	2	2	2	2
2	2	1	2	2	2	2	2	2	2
9	9	9	9	9	9	9	9	9	9
2	2	2	2	2	2	2	2	2	2
2	2	2	2	2	2	2	2	2	2
9	9	9	9	9	9	9	9	9	9
9	9	9	9	9	3	9	3	9	2
2	1	1	1	1	2	1	1	1	2
1	2	2	1	1	1	1	1	2	2

9	9	9	9	9	9	9	9	9	9
1	2	2	3	2	2	1	2	1	1
3	3	3	3	3	3	3	3	1	3
2	2	2	2	2	2	2	2	2	2
9	9	9	9	9	9	9	9	9	9
3	3	3	3	3	3	3	3	3	3
2	2	2	2	2	2	2	2	2	2
3	9	2	3	2	2	2	2	3	3
9	9	9	9	9	9	9	9	9	9
2	2	2	2	2	2	2	2	2	2
3	3	3	3	3	1	1	1	1	1
2	2	2	2	2	2	2	2	2	2
2	2	2	2	2	2	1	2	3	3
3	3	3	3	3	3	3	3	3	3
9	9	9	9	9	9	9	9	9	9
2	3	3	3	3	1	2	1	1	3
2	2	2	2	2	1	1	1	1	1
2	3	2	1	3	1	2	1	3	2
9	1	9	9	9	9	9	9	9	9
3	2	1	3	3	1	1	3	2	2
1	3	1	2	1	3	1	2	1	3
2	2	2	2	2	2	2	2	2	2
9	9	9	9	9	9	9	9	9	9
2	2	2	2	2	2	2	2	2	2
1	1	1	1	1	1	1	3	3	1
2	2	2	2	2	2	2	2	2	2
2	2	2	3	2	2	3	1	1	2
1	3	3	3	3	1	1	1	1	1
2	3	2	1	3	1	2	2	2	3
9	9	9	9	9	9	9	9	9	9
2	2	2	2	2	2	2	2	2	2
1	2	2	1	2	2	2	2	1	2
3	3	3	3	3	3	1	3	1	1
3	3	3	3	3	3	3	3	3	3
1	1	1	1	1	2	1	1	1	1
2	3	2	2	1	1	1	1	2	2
3	3	1	9	3	3	3	3	3	3
2	2	2	2	2	2	2	2	2	9
2	1	2	3	1	1	2	2	1	1
9	9	9	9	9	9	9	9	9	9
9	9	9	9	9	9	9	9	9	9
1	3	1	2	3	1	3	1	2	3
2	3	3	3	2	2	1	1	1	2
2	2	3	2	1	2	1	2	1	2
2	3	2	1	3	1	2	2	2	1
2	2	2	1	1	2	2	2	1	3
2	2	2	3	2	2	3	3	2	2

1	3	3	3	1	1	3	1	1	1
2	1	9	3	2	2	1	3	1	2
2	3	2	2	2	2	2	2	1	1
2	2	2	2	3	3	3	3	3	3
1	3	1	3	3	1	1	3	1	1
2	2	2	2	2	2	2	2	2	2
2	2	2	2	2	2	2	2	2	2
3	2	3	3	3	3	2	2	2	2
1	3	1	3	3	1	1	1	1	1
2	2	2	2	2	2	2	2	2	2
2	1	2	9	1	9	1	2	1	2
2	2	2	1	2	2	1	1	2	1
2	2	2	2	3	2	2	3	2	2
1	3	1	3	3	1	1	1	1	1
9	9	9	9	9	9	9	9	9	9
2	2	2	2	2	2	2	2	2	2
2	3	3	2	3	2	3	3	2	2
3	2	2	2	2	2	2	2	2	2
1	3	3	3	2	2	1	1	3	1
3	2	1	3	1	1	1	2	1	2
2	3	1	3	3	1	2	1	1	2
9	9	9	9	9	9	9	9	9	1
2	2	9	2	2	1	2	2	3	3
9	9	9	9	9	9	9	9	9	9
2	2	2	2	2	2	2	2	2	2
2	2	2	2	9	3	3	3	9	2
2	1	2	3	2	1	2	3	2	2
2	2	2	2	2	2	2	2	2	2
3	3	3	3	3	3	3	3	3	3
2	2	1	2	2	2	2	2	1	1
3	3	3	3	3	3	3	3	1	3
2	3	1	2	3	3	3	2	1	1
3	3	2	3	2	3	3	2	3	2
3	3	3	3	3	2	1	1	2	1
1	2	2	3	3	1	2	1	2	1
2	2	2	2	2	2	2	2	2	2
3	3	3	3	3	1	1	1	1	1
3	3	3	3	3	2	2	2	2	1
2	2	2	2	2	2	2	2	2	2
9	9	9	9	9	9	9	9	9	9
2	2	3	2	2	2	3	3	3	2
1	1	2	3	1	2	1	3	1	2
3	3	3	3	2	3	2	3	2	3
1	3	2	3	2	1	1	1	1	2
3	3	3	3	2	3	3	1	1	3
9	9	9	9	9	9	9	9	9	9
2	2	2	3	3	2	2	3	2	3

2	1	2	1	1	2	1	2	1	2
3	2	2	2	3	2	2	2	2	2
3	3	3	3	3	3	1	1	1	3
1	3	2	2	3	1	2	3	1	2
1	2	2	2	2	2	2	2	1	2
2	1	9	2	1	1	2	2	2	1
2	3	3	3	9	2	2	2	1	3
9	9	9	9	9	9	9	9	9	9
9	9	9	9	9	9	9	9	9	9
3	2	9	1	2	1	2	1	2	1
2	2	2	3	3	2	2	2	2	2
2	3	2	2	2	2	2	2	3	1
2	3	3	3	3	2	1	1	3	2
3	3	1	3	3	3	1	3	1	1
2	9	9	9	9	9	9	9	9	9
3	3	1	2	2	3	3	2	2	1
1	2	1	1	1	2	2	2	2	2
3	2	3	3	3	3	3	2	2	3
3	3	1	3	3	1	1	3	1	3
3	1	3	3	1	2	2	2	3	2
2	3	2	3	2	1	1	1	1	2
1	3	3	3	3	1	1	1	2	1
2	1	2	1	1	1	2	2	1	1
1	3	3	3	3	1	2	3	3	3
2	3	1	2	3	1	1	1	2	1
2	2	2	2	2	1	2	1	1	1
2	3	1	2	3	2	2	3	2	3
1	1	1	2	2	1	1	1	2	1
2	2	2	2	2	2	2	2	2	2
2	2	2	2	2	2	2	2	1	1
2	1	1	1	2	2	2	1	2	2
2	2	2	2	2	2	2	2	2	2
2	2	2	2	2	2	2	2	2	2
1	1	2	2	2	2	1	2	2	2
1	1	1	2	2	2	3	3	3	1
1	3	1	3	2	1	1	2	1	2
2	1	2	1	1	1	2	2	2	1
3	3	3	3	3	3	3	3	3	9
1	1	1	1	2	1	1	2	1	2
2	2	1	1	1	1	1	1	2	2
2	3	3	3	3	2	2	2	2	2
1	1	1	1	2	2	3	1	1	1
1	2	3	3	3	1	3	3	3	2
2	2	3	3	2	2	2	2	2	2
3	3	3	2	2	1	1	2	2	2
2	2	2	2	2	2	2	1	1	2
1	2	2	1	1	1	3	2	2	1

3	3	2	3	3	1	1	2	1	3
2	1	2	2	2	1	1	2	2	2
2	2	2	2	2	3	2	1	2	3
1	1	1	1	2	2	2	1	1	1
3	3	3	3	1	2	2	1	1	3
1	1	2	3	3	2	2	1	1	1
2	3	2	2	1	1	1	1	2	1
1	1	2	2	1	2	2	1	2	2

BreastSize	Nippledevi	Raisopposi	Undressto	HeardCerv	CervicalCA	PapSmear	HPVandVa	Papdetect	PapforMar
2	2	2	3	2	2	2	2	2	2
2	2	1	1	2	2	2	2	2	2
1	2	1	2	2	2	1	2	2	2
1	1	1	1	1	1	2	1	2	2
9	9	9	9	2	2	2	2	2	2
2	2	1	1	1	1	2	2	2	2
2	2	2	2	2	2	2	2	9	9
1	1	1	1	1	2	2	2	1	2
9	9	1	9	2	2	2	2	2	9
9	9	9	9	2	2	2	2	2	9
1	2	2	2	2	2	2	9	2	2
1	2	2	3	2	2	2	3	3	3
2	2	2	2	1	2	2	1	1	2
2	2	2	1	1	3	1	3	1	2
2	2	1	1	2	2	2	2	2	2
1	2	1	2	1	2	2	2	1	3
1	2	1	3	1	1	1	3	2	2
1	2	1	2	1	2	2	2	1	3
1	2	1	2	1	2	2	2	1	3
2	1	1	1	2	2	1	1	2	2
1	1	1	1	1	1	2	2	1	1
1	1	1	1	1	1	2	2	1	1
9	9	1	2	2	2	2	2	3	2
1	2	1	2	1	1	3	3	1	3
2	2	2	2	2	2	2	2	2	2
1	1	1	2	2	2	2	2	2	2
2	2	2	2	3	3	3	3	2	3
2	2	2	3	3	3	1	2	1	1
2	2	1	1	2	2	2	2	2	2
2	2	1	1	2	2	9	2	2	2
2	2	1	1	2	2	2	2	2	2
1	1	1	1	1	1	2	2	1	2
1	1	1	1	2	2	2	2	1	2
1	1	1	1	1	1	1	1	1	2
1	1	1	1	1	1	2	2	1	2
1	1	1	1	1	1	2	1	1	2
1	1	1	1	1	1	2	2	1	2
2	2	1	1	1	1	1	1	1	3
2	2	1	1	9	9	9	9	2	2
1	9	1	1	2	2	2	2	2	2
2	2	1	1	2	2	2	2	2	2
2	1	1	2	1	2	2	3	1	2
1	2	2	3	9	9	9	9	1	1
1	1	1	3	1	1	1	3	1	3
1	1	3	3	2	2	2	2	9	9
9	9	9	9	1	2	2	1	1	1

1	2	1	1	1	1	2	2	1	3
1	2	1	1	1	3	2	1	2	3
9	9	1	9	1	1	1	1	1	2
1	1	1	1	1	1	3	1	1	2
9	1	1	1	1	1	1	1	1	3
1	1	1	3	1	1	1	1	1	3
1	3	1	1	1	1	1	1	1	3
1	1	1	1	1	1	1	2	1	3
1	9	1	9	1	1	2	2	1	9
1	2	1	1	1	1	1	2	1	2
9	9	9	9	2	2	2	2	2	2
1	1	1	1	1	1	2	1	2	2
1	2	1	2	1	1	3	2	1	3
1	9	9	1	2	2	2	2	2	2
2	1	2	2	2	2	2	2	2	2
1	2	1	3	1	1	1	2	1	3
1	2	1	1	1	1	2	1	1	2
1	1	1	1	1	1	1	1	1	2
1	1	2	3	1	2	2	2	1	1
2	2	2	2	1	1	1	2	1	9
9	9	9	9	1	1	2	1	1	3
1	3	3	3	1	1	2	2	1	3
9	9	9	9	1	1	2	1	1	3
1	1	1	1	1	2	2	1	1	3
1	9	1	9	1	1	2	3	9	1
1	1	1	1	1	1	2	1	1	2
2	3	1	1	9	9	9	9	9	9
2	1	2	3	1	2	2	2	1	3
1	2	1	1	2	3	3	3	2	2
1	1	1	3	1	1	1	1	1	3
1	1	1	1	1	1	1	1	1	2
3	1	3	3	1	1	1	2	1	3
1	1	1	1	1	1	9	9	1	2
1	1	1	2	1	1	2	1	9	3
1	2	1	1	1	1	1	1	1	3
2	1	1	1	2	2	2	1	9	9
2	2	2	2	9	9	9	9	9	9
2	2	1	1	9	9	9	9	1	3
1	1	1	3	3	1	1	1	1	3
1	1	1	1	1	1	1	1	1	3
1	1	1	1	1	1	1	1	1	9
1	1	1	3	2	2	3	2	1	3
1	3	1	1	1	1	2	2	1	1
1	1	1	1	1	1	1	1	1	2
2	2	1	1	2	2	2	2	1	2
2	2	3	3	1	1	2	1	9	9
9	9	9	9	2	2	2	9	9	9

2	2	1	1	1	1	2	1	1	2
2	2	1	1	1	1	1	1	1	2
1	1	1	1	1	1	1	1	2	1
2	2	1	1	1	9	1	1	1	3
2	2	1	1	2	2	2	2	9	9
2	2	1	2	2	1	2	2	1	2
1	1	1	1	1	1	1	1	1	3
1	1	1	2	1	1	1	2	1	2
2	2	1	1	1	1	1	1	1	3
1	1	1	1	1	1	1	1	1	3
1	1	1	2	2	2	3	1	1	3
1	2	1	2	1	9	1	3	1	2
2	2	1	1	1	1	3	3	1	2
1	1	1	1	3	3	3	3	1	2
3	2	3	2	2	2	1	3	2	3
2	2	3	3	1	2	2	2	3	2
2	2	1	1	2	2	2	1	2	2
1	2	1	1	2	2	2	2	2	2
2	2	1	2	3	2	2	2	1	2
9	9	9	9	2	2	2	2	2	2
2	1	1	2	2	2	2	2	1	3
1	3	1	3	2	2	2	2	1	3
2	2	2	2	2	2	2	2	2	2
9	9	9	9	2	2	2	2	3	3
2	2	1	1	2	3	3	1	3	1
2	2	1	2	2	2	2	2	2	2
2	1	1	3	1	1	1	2	1	2
2	2	2	2	1	1	1	1	1	2
2	2	2	2	2	2	2	2	2	2
2	2	3	3	2	2	2	2	2	2
2	2	2	2	2	2	2	2	2	2
2	3	2	2	3	3	2	3	2	2
2	2	2	2	2	2	2	2	1	2
2	2	2	2	3	2	2	2	2	2
2	2	2	2	2	2	2	2	2	2
1	1	1	1	2	2	2	2	2	2
1	2	2	2	1	1	2	2	2	2
2	2	1	3	2	2	2	2	2	2
1	9	1	9	1	1	2	2	9	1
1	2	1	2	1	1	3	2	1	3
1	2	1	2	2	2	2	2	2	2
3	2	1	2	3	2	2	2	2	2
2	2	2	2	3	3	2	3	2	2
1	1	2	1	1	1	2	2	1	2
2	2	2	2	3	3	3	3	2	2
1	2	1	1	1	2	2	2	1	2
1	2	3	3	2	2	2	2	2	2

3	3	3	3	2	2	2	2	2	2
2	1	2	1	2	2	2	3	1	2
2	2	1	2	1	1	2	2	9	2
3	2	1	2	3	2	2	2	2	2
1	1	1	1	3	2	2	2	9	9
2	2	1	1	2	2	2	2	2	2
2	2	3	3	2	2	2	2	2	3
1	2	1	1	2	3	2	2	2	2
2	2	2	2	2	2	2	2	2	2
2	2	2	2	2	2	2	2	2	2
3	1	2	3	2	2	2	3	2	1
3	1	2	3	2	2	2	9	2	3
9	9	9	9	2	2	2	2	9	2
2	1	3	2	2	2	2	2	1	2
2	2	1	2	1	1	2	2	2	2
2	2	1	2	1	1	2	2	2	2
9	9	9	9	1	1	3	1	9	9
1	3	1	3	2	2	2	2	2	2
9	9	9	9	1	2	2	2	9	9
1	1	1	1	1	2	2	2	1	3
1	1	1	1	9	1	2	2	1	3
1	1	1	1	2	2	2	2	1	3
1	1	1	1	2	2	2	2	1	3
1	1	1	1	2	2	2	2	1	3
1	1	1	1	2	2	2	2	1	3
1	1	1	1	2	9	2	2	1	3
1	1	1	1	1	2	2	2	1	3
1	1	1	1	1	1	2	2	1	3
1	1	1	1	2	3	2	2	1	3
1	1	1	3	1	2	2	2	1	3
2	3	2	2	1	1	2	2	3	2
1	1	2	2	2	2	2	2	2	2
9	9	9	9	9	9	9	9	1	3
1	1	1	3	2	2	2	2	1	3
1	1	1	3	1	2	2	2	1	3
1	1	1	2	2	2	2	2	1	3
1	1	1	1	1	1	2	2	1	3
1	1	1	1	1	2	2	2	1	3
1	1	1	2	2	2	2	2	1	3
1	1	1	1	2	1	2	2	1	3
1	1	1	1	2	2	2	2	1	3
1	1	1	1	2	2	2	2	1	3
9	9	9	9	9	9	9	9	1	3
1	1	1	3	1	2	2	2	1	3
1	1	1	3	1	2	2	2	1	3
1	1	1	3	1	2	2	2	1	3
1	1	1	3	1	2	2	2	1	3

1	1	1	3	1	2	2	2	1	3
1	1	1	1	2	2	2	2	1	3
1	1	1	1	2	2	2	2	1	3
1	1	1	3	1	1	2	2	1	3
1	1	1	1	2	2	2	2	1	3
1	1	1	1	1	1	1	2	1	3
1	1	1	1	2	1	2	2	1	3
1	1	1	1	1	1	1	1	1	3
1	1	1	1	1	1	1	1	1	3
1	1	1	3	1	1	2	1	1	3
1	1	1	1	9	9	9	9	9	9
1	1	1	1	1	1	1	2	1	3
1	1	1	1	1	1	1	1	1	3
1	1	1	1	1	2	2	2	1	3
1	1	2	2	3	3	3	3	1	2
1	2	1	1	2	2	2	2	1	1
1	1	1	1	1	2	2	2	2	2
1	1	1	1	1	2	9	1	1	1
1	1	1	2	1	2	2	2	2	2
1	2	2	1	1	2	2	2	1	2
1	2	3	3	1	1	3	3	2	2
2	2	2	2	1	2	2	2	2	2
2	1	2	1	2	2	2	2	1	2
1	2	2	2	1	3	3	2	1	2
2	2	2	2	2	2	2	2	2	2
2	2	2	2	2	2	2	2	2	2
2	2	2	1	1	2	2	2	1	2
2	2	2	2	2	2	2	2	2	2
2	2	2	2	2	2	2	2	2	2
2	2	2	2	1	2	2	2	2	2
2	1	2	2	1	1	2	3	1	3
2	1	2	2	1	1	2	3	1	3
1	1	1	2	1	2	2	2	2	2
1	1	1	1	1	1	1	1	1	1
1	2	2	2	1	1	3	1	1	2
2	2	2	2	1	1	1	2	2	2
1	3	2	1	2	2	2	2	2	2
3	1	1	2	1	1	2	2	3	3
2	2	2	2	2	2	2	2	1	2
2	1	2	2	1	2	2	2	1	2
2	2	2	2	1	1	1	1	1	2
2	2	2	1	1	2	2	2	1	2
1	9	9	9	2	2	3	2	1	9
1	9	9	9	2	2	3	2	1	9
1	1	1	1	1	1	2	1	1	3
1	2	3	3	1	1	2	2	1	2
9	9	9	9	3	1	2	2	2	9

1	1	2	3	2	2	2	2	2	2
1	1	2	3	2	2	2	2	2	2
2	2	2	2	2	2	2	2	1	2
2	2	2	2	2	2	1	2	1	2
2	2	2	2	2	2	2	2	2	2
1	1	1	2	1	1	1	1	2	1
1	2	1	1	2	2	2	2	1	2
2	1	2	2	2	2	2	2	2	2
1	1	1	2	1	1	2	2	1	3
3	3	3	3	2	2	2	2	3	3
2	2	2	2	2	2	2	2	2	2
1	1	1	2	1	2	3	1	1	3
1	1	1	2	1	2	3	1	1	3
1	1	1	2	1	3	3	3	2	2
2	2	2	3	2	2	2	2	2	2
1	2	2	3	2	3	3	3	1	2
2	1	3	2	1	3	3	2	2	2
2	2	2	2	2	2	1	1	2	3
3	2	1	3	2	2	2	2	2	9
1	1	3	2	2	1	1	1	1	2
1	1	2	3	2	2	2	1	1	2
1	2	1	2	2	3	2	3	1	2
1	1	1	2	1	3	3	2	2	2
2	2	2	2	2	2	2	2	1	2
1	9	9	9	3	3	3	2	9	2
2	2	2	2	2	2	2	2	2	2
2	2	2	2	2	2	2	9	2	2
1	3	2	3	1	2	3	3	1	9
9	9	9	9	2	2	3	3	2	2
2	2	2	1	2	3	3	3	2	2
9	9	9	9	3	1	1	3	9	9
9	9	1	9	2	2	2	2	2	2
1	1	3	3	2	2	2	2	3	3
1	2	1	2	1	1	2	2	2	2
9	9	9	9	1	3	3	3	1	9
2	3	2	2	3	3	3	3	2	2
1	2	1	2	2	2	2	2	1	2
1	1	3	2	1	2	2	2	1	3
1	1	1	1	1	3	3	3	1	2
1	3	2	3	2	1	2	2	2	2
1	1	3	3	1	1	2	2	1	2
2	1	1	3	1	1	2	2	1	2
1	1	1	1	1	1	1	1	1	1
2	2	2	2	1	2	2	2	2	2
9	9	9	9	1	2	2	1	2	3
2	3	2	3	1	2	2	2	1	2
2	3	1	2	2	2	2	2	2	2

9	9	9	9	9	9	9	9	9	9
9	9	9	9	9	9	9	9	9	9
1	1	1	1	1	1	1	2	1	2
1	1	2	1	1	1	2	1	2	2
1	2	2	2	2	2	2	2	1	3
1	2	1	3	2	2	2	1	3	1
1	1	1	1	1	1	1	1	1	3
3	1	1	3	1	1	1	1	1	1
1	1	1	1	1	2	2	2	2	3
2	2	2	2	1	2	1	2	2	2
2	2	2	2	3	3	3	3	2	2
1	2	1	2	1	1	1	2	1	2
9	9	9	9	9	9	9	9	9	9
1	1	1	3	2	2	1	1	1	1
1	1	1	1	1	1	2	1	1	1
2	2	1	3	9	9	9	9	9	9
2	1	1	1	1	1	1	2	2	1
3	2	2	2	2	2	1	2	1	2
1	1	1	3	1	2	3	1	2	1
9	9	9	9	9	9	9	9	9	9
3	1	2	2	2	2	2	2	9	9
1	2	1	2	1	2	2	2	2	2
2	1	2	2	1	1	2	3	2	2
2	2	2	2	1	2	2	2	2	2
2	2	2	2	1	2	2	2	2	2
1	3	1	1	9	2	3	3	1	2
1	2	1	1	1	1	1	1	1	1
1	1	1	3	3	2	3	2	1	3
1	1	1	1	1	2	1	1	1	3
1	2	1	9	1	2	2	2	1	2
1	2	1	2	1	3	3	1	2	2
1	1	3	3	2	2	2	2	3	3
9	9	9	9	1	1	1	1	9	9
3	3	1	3	2	2	2	2	9	9
1	1	1	1	1	2	2	2	1	2
1	1	1	3	1	1	3	3	2	2
1	2	1	3	1	1	1	2	1	3
1	1	3	3	3	1	1	1	1	2
1	1	1	1	1	1	1	2	1	3
2	2	1	1	1	2	2	2	1	2
3	3	3	3	1	1	1	2	1	2
1	1	1	1	1	1	1	2	1	3
3	3	2	2	1	1	1	1	1	2
2	2	2	2	2	2	2	2	2	2
1	2	2	1	1	1	1	1	2	3
1	1	1	1	1	1	2	2	1	2
1	2	1	3	1	2	2	2	2	2

1	1	1	2	3	2	2	2	2	3
1	1	1	2	3	2	2	2	2	3
9	9	9	9	1	1	1	1	1	9
1	1	1	2	1	2	2	2	2	2
9	9	1	9	1	1	1	3	9	9
1	1	2	2	1	2	2	2	9	9
1	1	1	2	1	1	2	2	2	2
1	9	9	9	1	2	2	1	1	2
1	1	1	1	1	2	2	2	1	3
1	2	1	1	1	2	2	2	1	1
2	2	2	3	2	2	2	2	1	2
2	1	2	2	1	1	1	2	2	2
1	1	1	2	1	1	2	1	1	3
3	2	2	3	2	2	2	2	2	1
2	2	2	1	2	2	2	2	1	2
1	2	1	2	2	9	2	2	2	2
2	2	2	1	2	2	2	2	2	2
2	1	2	3	1	2	2	2	2	2
1	1	1	1	1	1	1	2	2	2
2	1	2	2	2	2	2	2	2	2
1	2	1	2	1	2	1	1	1	1
3	2	1	1	2	2	2	2	1	2
1	1	2	1	1	1	2	2	1	2
1	1	2	2	1	1	2	1	1	3
2	2	2	2	2	2	2	2	2	2
2	2	2	2	2	2	2	2	2	2
1	1	1	2	1	2	2	2	1	2
3	2	2	3	2	3	3	3	2	2
1	1	2	2	1	1	3	3	2	2
1	2	1	2	2	2	2	2	1	2
2	1	2	1	3	3	1	1	1	3
2	1	2	1	2	2	2	2	1	3
1	3	3	3	2	2	2	3	1	1
2	2	2	2	2	2	2	2	2	2
2	2	2	2	2	2	2	2	2	2
1	2	9	1	1	1	2	2	1	3
1	1	1	2	3	2	2	2	2	2
1	9	1	9	3	1	2	3	2	2
2	2	2	2	2	2	2	2	2	2
1	1	3	3	2	2	2	2	2	2
2	2	2	2	3	3	3	2	2	2
1	1	2	1	2	2	2	2	2	2
1	1	1	2	1	2	2	1	1	3
2	2	2	2	2	2	2	2	2	2
1	1	2	1	2	2	2	1	1	2
1	2	1	2	1	1	2	3	1	3
2	2	2	2	1	3	2	2	2	2

1	1	2	3	1	2	2	2	1	3
1	1	2	3	1	1	2	1	1	3
3	3	3	3	1	1	2	1	2	2
1	2	2	2	3	2	3	3	2	2
1	2	2	2	3	2	2	1	2	2
1	2	2	3	1	3	2	1	1	2
2	2	9	9	9	9	9	9	9	9
1	2	1	2	1	1	1	1	1	2
1	2	1	2	1	1	1	3	1	3
1	2	2	1	1	2	2	2	2	3
1	2	2	1	2	2	2	2	3	3
1	1	1	1	2	2	2	2	1	3
1	2	1	2	2	2	2	2	2	2
2	2	2	2	2	2	2	2	1	2
2	2	2	2	2	2	2	2	2	2
1	9	2	2	9	9	9	9	1	2
1	1	2	2	2	2	2	2	1	3
1	2	2	1	3	2	2	2	2	2
1	2	2	1	3	2	2	2	2	2
9	9	9	9	1	2	3	2	2	2
1	1	2	2	1	1	1	2	1	2
2	2	2	2	1	3	2	2	2	2
9	9	9	9	9	9	9	9	9	9
2	2	2	2	2	1	1	1	1	2
1	1	1	1	1	1	2	2	1	3
9	2	9	2	9	9	9	9	9	9
2	1	1	3	3	3	1	1	1	2
9	9	9	9	9	9	9	9	9	9
2	2	2	2	1	1	1	1	2	2
1	1	2	2	1	1	2	2	2	2
2	2	2	2	2	2	2	2	9	9
2	2	3	2	9	9	9	9	9	9
2	2	2	2	2	1	1	2	2	2
1	2	1	3	2	2	2	2	2	1
1	1	1	2	1	2	2	2	2	3
1	1	1	1	1	1	1	1	2	1
3	2	3	2	1	2	3	3	3	3
2	1	2	1	1	2	3	2	2	1
2	2	2	2	9	9	9	9	9	9
1	1	1	2	1	2	2	2	1	3
2	2	1	2	2	3	3	2	2	2
2	2	1	1	2	2	2	2	2	2
1	2	3	1	1	3	3	3	1	2
3	3	3	3	1	2	2	2	3	3
9	9	9	9	9	9	9	9	9	9
1	1	1	9	3	2	2	2	1	3
1	1	1	1	2	2	2	2	1	3

1	1	1	1	3	2	2	2	9	9
1	1	1	1	2	2	2	2	1	3
1	1	1	1	2	2	2	2	1	3
1	1	1	1	1	2	2	2	1	3
1	1	1	1	2	2	2	2	1	3
1	1	1	3	1	1	2	2	1	3
1	1	1	1	2	2	2	2	1	3
1	1	1	1	2	2	2	2	1	3
1	1	1	1	2	2	2	2	1	3
1	1	1	3	1	2	2	2	1	3
1	1	1	1	3	2	2	2	9	1
1	1	1	3	2	2	2	2	1	3
1	1	1	1	1	1	2	2	1	3
1	1	1	1	2	2	2	2	1	3
1	1	1	1	9	2	2	2	1	3
1	1	1	1	1	1	2	2	1	3
1	1	1	2	2	2	2	2	1	3
1	1	1	1	1	2	2	2	1	3
1	1	1	1	1	2	2	2	1	3
1	1	1	1	1	2	2	2	1	3
1	1	1	1	1	2	2	2	1	3
1	1	1	1	1	2	2	2	1	3
1	1	1	1	2	2	2	2	1	3
1	1	1	1	1	1	2	1	1	3
1	1	1	3	2	2	2	2	1	3
1	1	1	3	2	2	2	2	1	3
1	1	1	1	2	2	2	2	1	3
1	1	1	1	9	2	2	2	1	3
1	1	3	1	2	1	2	2	1	3
1	1	1	3	2	2	2	2	1	3
1	1	1	3	2	1	2	2	1	3
1	1	1	1	2	2	2	2	1	3
1	1	1	3	2	1	2	2	1	3
1	1	1	1	2	2	2	2	1	3
1	1	1	3	1	1	2	2	1	3
1	1	1	3	1	1	2	2	1	3
1	1	3	3	1	1	2	2	1	3
1	1	1	1	2	1	2	2	1	3
1	1	1	1	2	2	2	2	1	3
1	1	1	9	1	1	1	2	1	3
1	1	1	1	1	2	2	2	1	3
2	2	2	2	3	3	3	3	2	2
1	2	1	1	1	2	2	1	1	2
2	2	2	2	3	3	3	3	2	2
9	9	9	9	2	2	2	2	2	9
2	2	1	2	2	2	2	2	2	2
1	2	2	2	1	2	1	2	9	2

2	2	2	2	3	2	2	2	2	2
1	1	1	2	1	1	2	1	1	2
1	1	1	2	1	1	2	1	1	2
1	1	1	3	1	1	2	2	1	1
2	2	1	3	3	2	3	3	2	2
1	2	1	2	2	2	2	2	2	2
2	2	2	2	3	1	1	3	2	2
2	2	2	2	3	2	9	3	2	2
2	2	1	1	1	1	2	2	1	2
2	2	2	2	1	2	2	2	2	2
1	1	1	2	1	1	1	1	1	2
9	9	9	9	1	2	1	3	9	9
9	9	1	1	1	1	1	1	1	3
1	2	2	2	2	2	2	2	2	2
9	9	9	9	1	2	2	2	9	9
9	9	9	9	1	1	1	2	9	9
9	9	9	9	2	2	2	2	9	9
9	9	9	9	2	2	2	2	1	9
9	9	9	9	2	2	2	2	9	9
2	2	1	2	1	3	2	2	1	2
3	3	3	3	1	3	1	1	2	9
3	3	1	3	1	2	1	2	9	9
1	2	1	1	1	1	2	2	1	3
9	9	9	9	2	3	2	2	1	9
9	9	9	9	2	2	2	2	9	9
9	9	9	9	9	9	9	9	9	9
9	9	9	9	1	1	1	1	1	9
2	1	1	1	2	2	3	2	9	9
3	1	1	1	2	2	3	1	1	2
9	9	9	9	1	2	1	3	9	9
9	9	9	9	1	1	2	2	1	9
2	2	2	2	2	2	2	2	2	2
2	2	2	2	2	2	2	2	2	2
3	1	1	1	1	1	1	1	1	3
2	1	3	1	3	2	2	2	2	1
1	2	2	3	1	1	2	3	2	2
1	2	2	2	1	1	2	2	1	3
1	1	3	3	1	3	2	2	1	3
2	2	2	2	2	2	3	2	2	2
1	1	3	1	1	3	2	3	1	2
9	9	9	9	3	2	2	2	1	9
1	1	1	1	1	1	1	1	1	3
1	2	1	2	2	2	2	2	2	2
1	1	1	2	3	3	3	3	1	2
1	2	3	3	2	2	2	2	2	2
1	1	3	2	1	1	2	2	2	2
1	1	2	1	2	2	2	2	1	3

1	9	9	9	1	1	1	1	1	3
2	3	3	3	1	1	3	1	1	3
1	1	2	3	2	2	2	2	1	3
1	2	1	1	2	2	2	2	1	2
2	2	3	1	1	3	1	2	2	3
2	2	2	2	3	3	2	2	2	2
2	2	2	2	2	2	2	2	2	2
2	2	2	2	2	1	2	2	2	2
2	1	2	2	2	2	2	2	2	2
2	2	2	2	3	2	2	2	2	2
2	2	2	2	3	3	3	2	2	2
2	2	2	2	1	1	1	2	1	1
2	2	2	2	2	2	2	2	2	2
2	2	2	2	2	2	2	2	2	2
1	2	2	2	1	1	2	2	1	3
2	2	2	2	2	2	2	2	2	2
1	2	1	3	3	3	3	3	2	2
2	2	2	2	2	2	2	2	2	2
2	3	3	1	1	2	2	2	2	2
1	2	2	1	1	1	1	2	1	2
1	2	1	2	2	2	2	1	2	2
1	1	2	1	1	1	2	2	1	2
1	1	1	2	1	1	2	2	1	3
1	1	1	2	2	2	2	2	2	2
2	1	2	3	2	2	2	2	2	2
1	2	2	2	2	2	2	2	2	2
1	1	1	3	1	1	2	1	1	3
1	3	1	3	1	2	1	2	1	3
2	2	2	2	1	1	2	2	1	2
3	3	2	2	1	2	2	2	1	3
1	1	2	2	9	9	9	9	2	2
9	9	2	3	2	2	2	2	2	2
2	2	2	3	2	2	2	2	2	2
2	2	3	2	2	2	2	2	1	2
1	1	1	1	2	2	2	2	2	2
9	9	9	9	3	2	1	2	2	9
2	2	1	2	2	3	2	2	2	2
1	1	1	1	1	1	1	1	1	2
2	2	2	2	2	2	2	2	2	2
1	1	1	2	1	1	2	2	1	3
1	2	1	1	9	9	2	2	1	9
1	1	1	1	2	2	3	1	1	2
3	1	1	1	2	2	2	2	1	2
1	2	1	2	1	9	1	3	1	2
1	1	1	1	1	1	1	2	1	3
2	2	2	2	2	2	2	2	2	2
9	9	9	9	2	2	2	1	1	9

9	9	9	9	1	1	2	2	2	9
9	9	9	9	2	2	2	2	9	9
9	9	9	9	2	2	2	2	1	9
9	9	9	9	9	9	9	9	9	9
9	9	9	9	2	1	2	2	1	9
9	9	9	9	1	2	2	1	1	9
9	9	9	9	2	2	2	2	9	9
9	9	9	9	2	2	2	2	1	9
9	9	9	9	2	2	2	2	1	9
9	9	9	9	1	1	1	1	1	9
9	9	9	9	2	2	2	2	9	9
9	9	9	9	1	1	1	1	1	9
9	9	9	9	2	2	2	2	1	9
9	9	9	9	1	1	1	1	9	9
9	9	9	9	2	1	2	3	9	9
9	9	9	9	2	2	2	2	9	9
9	9	9	9	1	2	2	1	9	2
9	9	9	9	1	2	2	2	1	3
2	2	2	2	3	3	3	3	2	2
1	9	1	2	1	1	1	2	1	9
1	1	1	1	1	1	1	1	1	1
1	3	2	2	2	2	2	2	2	3
3	2	2	2	2	2	9	2	2	2
1	1	1	1	3	1	1	1	1	1
3	2	3	2	2	2	9	2	3	3
1	2	3	3	2	1	2	1	1	3
2	2	2	2	1	1	1	2	2	2
2	2	1	1	3	2	2	2	2	1
2	2	2	2	2	2	2	2	2	2
1	2	1	2	1	3	3	3	2	2
2	2	3	1	9	9	9	9	1	1
1	1	3	1	2	2	2	2	2	2
9	9	9	9	2	2	2	2	1	2
3	1	3	3	1	1	2	2	2	2
1	2	1	1	2	2	2	1	1	1
2	2	2	3	2	2	2	2	1	2
2	2	2	2	2	2	2	2	2	2
3	2	1	2	9	9	9	9	2	3
1	2	1	2	1	1	1	1	1	1
1	1	1	2	1	2	1	9	9	9
2	2	2	2	1	2	1	2	2	2
1	1	3	1	1	1	1	1	1	1
1	1	1	1	9	9	9	9	9	9
2	3	1	1	1	3	1	1	1	1
2	2	2	2	2	2	2	2	2	2
9	9	9	9	1	2	2	2	2	2
1	2	2	2	2	3	3	2	2	2

1	1	1	3	2	2	2	2	2	2
9	9	9	9	9	9	9	9	9	9
3	1	1	1	2	2	2	2	1	1
9	9	9	9	9	9	9	9	9	9
2	2	2	2	1	2	1	1	1	3
2	2	2	2	1	2	1	2	1	3
9	9	9	9	9	9	9	9	9	9
1	1	1	3	2	2	2	2	2	2
1	2	2	3	1	1	1	1	2	1
1	1	1	1	2	2	2	2	1	3
1	1	1	1	1	1	1	1	1	1
1	1	2	1	1	1	2	1	2	3
3	3	2	2	2	3	3	3	3	3
2	3	1	2	9	9	9	9	9	9
2	2	2	2	9	9	9	9	9	9
3	2	3	1	1	3	1	2	9	9
1	1	1	1	1	9	2	2	1	3
9	9	9	9	9	9	9	9	9	9
1	1	2	3	1	2	2	1	1	3
3	3	3	3	1	2	2	2	1	3
3	3	3	3	1	2	2	2	3	3
3	3	2	2	2	3	3	3	3	3
1	2	2	3	1	1	1	1	1	1
1	2	2	3	1	2	2	1	3	1
2	2	2	2	1	2	2	2	2	2
3	1	1	1	2	2	2	2	1	1
3	3	3	3	1	1	1	1	1	2
1	1	1	1	2	2	1	1	3	1
2	2	3	1	2	3	3	3	2	2
1	1	1	1	1	2	2	2	1	2
2	1	3	1	2	2	2	1	9	9
9	9	9	9	1	1	1	2	9	9
2	2	1	1	1	3	3	3	1	2
1	1	2	9	3	2	2	2	2	3
9	1	1	9	3	2	2	9	2	3
1	1	1	1	1	2	2	1	1	2
1	1	1	1	2	2	2	2	2	2
9	9	9	9	9	9	9	9	9	9
9	9	9	9	9	9	9	9	9	9
9	9	9	9	9	9	9	9	9	9
9	9	9	9	9	9	9	9	9	9
2	2	3	2	1	1	1	1	3	2
3	2	1	3	2	2	2	1	1	2
9	9	9	9	9	9	9	9	9	9
1	3	2	3	2	2	2	1	1	2
2	2	2	2	2	3	2	2	2	2
9	1	3	3	2	2	2	2	1	3

3	3	3	3	2	2	2	2	3	3
1	1	1	1	1	3	3	3	2	2
1	3	2	3	1	2	1	1	3	3
1	1	2	2	1	3	3	3	1	2
2	2	2	2	2	2	2	2	2	2
9	1	9	1	1	2	1	1	1	3
9	9	9	9	9	9	9	9	9	9
1	2	1	1	1	1	2	3	1	2
3	3	3	3	2	2	2	2	3	3
1	2	1	2	1	3	2	2	2	2
3	3	3	3	2	2	2	2	3	3
1	1	1	1	1	3	2	2	1	1
2	2	3	3	2	3	2	2	2	2
2	1	2	3	2	3	2	2	2	2
2	2	2	3	2	3	2	2	2	2
2	2	2	3	2	3	2	2	2	2
2	2	2	3	2	3	2	2	2	2
2	1	2	3	2	3	2	2	2	2
2	2	2	3	2	3	2	2	2	2
1	1	3	3	2	2	2	2	1	3
2	3	2	3	2	2	2	2	1	2
3	3	3	3	9	9	9	9	2	3
1	1	1	2	2	2	2	2	2	2
2	2	2	2	2	2	2	2	2	2
3	3	3	3	2	2	2	2	3	3
2	2	2	3	2	3	2	2	2	2
9	9	9	9	2	2	2	2	9	9
3	3	1	1	2	2	2	2	1	3
9	9	9	9	1	1	1	1	1	9
3	3	3	3	2	2	2	2	2	2
2	1	3	1	2	2	2	2	1	3
3	2	3	2	3	3	2	2	3	2
1	2	2	1	1	2	3	2	1	2
3	3	3	3	2	3	2	1	9	9
3	3	3	3	2	2	2	2	3	3
1	1	9	2	2	2	2	2	3	2
9	9	9	9	1	2	2	3	1	9
1	1	2	2	3	3	3	1	2	2
2	2	2	3	2	3	2	2	2	2
1	2	1	2	2	2	2	2	1	1
2	1	2	3	2	2	3	3	1	1
3	1	3	3	2	2	2	2	1	3
1	9	2	2	1	2	1	1	1	1
1	3	1	3	1	1	2	1	3	1
2	3	1	3	2	2	1	2	1	1
1	1	1	1	1	1	1	1	1	1
1	2	1	2	2	2	2	2	2	2

1	1	2	3	2	2	2	2	2	2
9	9	9	9	1	1	1	1	3	9
9	9	9	9	2	2	2	1	1	9
3	1	3	1	2	2	2	2	1	3
2	2	2	2	3	2	2	2	2	2
2	2	2	2	1	2	1	1	1	3
2	2	2	3	3	2	2	2	2	2
2	2	2	2	1	2	1	2	1	3
2	2	2	2	1	2	1	1	1	3
2	3	1	2	1	2	1	2	2	3
3	2	2	3	2	2	2	2	2	2
1	1	2	1	2	2	2	2	1	2
1	2	3	1	2	2	2	2	9	9
1	3	2	3	2	3	3	2	3	2
2	1	2	2	1	2	2	2	1	2
2	2	1	1	1	1	3	1	9	9
2	3	1	2	1	2	3	1	3	3
9	9	9	9	1	1	1	2	9	9
9	9	9	9	1	1	1	2	9	9
3	2	3	2	2	2	2	2	1	2
3	3	1	1	1	2	1	2	1	2
2	2	2	2	1	2	2	2	2	2
1	1	1	1	9	9	9	9	9	9
2	2	2	2	2	2	2	2	2	2
3	3	3	3	9	9	9	9	3	3
2	1	3	2	1	1	1	2	2	2
2	1	3	2	1	1	1	2	2	2
1	1	1	1	1	2	2	2	1	3
1	1	1	1	2	2	9	1	1	1
2	2	2	2	2	2	2	2	2	2
1	1	2	3	1	1	2	1	2	2
1	1	1	1	2	2	2	2	1	2
9	9	9	9	1	2	2	2	1	3
2	1	3	2	1	1	1	2	2	2
2	3	3	2	9	9	9	9	2	1
2	2	2	2	3	3	3	3	2	2
1	2	2	2	3	3	3	1	1	2
9	9	9	9	1	1	1	1	9	9
9	9	9	9	2	2	2	2	9	9
1	1	1	1	1	1	2	2	1	3
3	3	3	3	2	2	2	2	2	2
2	2	2	2	3	3	3	3	2	2
2	2	2	2	2	2	2	2	2	2
2	2	2	2	1	1	1	1	2	2
2	2	2	2	3	3	3	3	2	2
2	2	2	2	2	2	2	2	2	2
9	9	9	9	9	9	9	9	9	9

9	9	9	9	9	9	9	9	9	9
2	2	2	2	3	3	2	3	2	2
9	9	9	9	1	1	2	1	1	3
9	9	9	9	1	1	1	1	1	3
9	9	9	9	1	1	1	1	2	9
2	1	2	2	2	2	2	2	3	3
2	2	2	2	9	9	9	9	9	9
3	3	3	3	2	2	2	2	3	3
3	2	2	3	1	1	1	1	1	2
9	9	9	9	2	2	2	2	2	9
2	2	2	2	2	2	2	2	2	2
2	3	2	1	1	2	2	1	2	2
1	1	1	3	1	2	2	1	2	2
2	2	2	2	2	2	2	2	2	2
1	1	1	2	1	2	2	2	1	1
2	2	2	2	2	2	2	2	2	2
2	2	2	2	2	2	2	2	2	2
2	2	2	2	1	1	1	1	2	2
9	1	2	1	1	2	2	2	1	2
3	3	2	2	2	2	2	2	2	2
9	9	9	9	2	2	2	2	1	9
2	2	3	3	2	1	2	2	3	3
2	2	2	2	3	3	3	3	2	2
2	2	1	3	1	3	2	2	2	2
2	2	2	2	1	1	1	1	2	2
3	2	3	3	2	2	2	2	3	3
9	9	9	9	2	2	2	2	9	9
9	9	9	9	2	2	2	2	2	2
3	3	3	3	2	2	2	2	2	2
2	2	2	2	1	1	1	1	2	3
9	9	9	9	2	9	2	1	2	2
1	2	2	3	2	2	2	2	2	2
1	1	3	1	1	2	1	1	1	3
2	2	2	2	1	1	1	1	2	2
1	1	1	1	1	2	2	2	2	2
2	2	2	2	9	9	9	9	2	2
2	2	2	2	2	2	2	2	2	2
2	3	1	3	1	3	3	2	1	2
2	2	2	2	2	2	2	2	2	2
9	1	9	9	2	2	2	2	2	2
2	2	2	2	3	3	3	3	2	2
2	2	2	2	3	2	2	2	2	2
1	1	3	1	2	2	2	2	1	3
2	2	2	2	9	9	9	9	2	2
2	2	2	2	2	2	2	2	2	2
3	3	3	3	2	2	2	2	3	3
2	2	2	2	2	2	2	2	2	2

2	2	2	2	2	2	2	2	2	2
2	2	2	2	2	2	2	2	2	2
2	2	2	2	2	1	2	2	2	2
2	2	2	2	2	2	2	2	2	2
2	2	2	3	3	3	3	3	3	2
1	1	2	1	2	2	2	2	1	3
2	2	2	2	2	2	2	2	2	2
9	1	3	1	2	2	2	2	2	3
9	1	3	1	2	1	2	2	2	1
2	2	2	2	2	2	2	2	2	2
1	1	2	1	1	1	1	1	1	1
9	9	9	9	1	9	2	2	2	9
9	9	9	9	1	1	3	2	9	9
2	2	2	2	9	9	9	9	2	1
2	2	2	2	1	1	1	1	1	1
2	3	3	3	2	2	2	1	3	2
2	2	2	3	1	2	2	2	2	3
1	1	1	1	2	2	2	2	1	3
2	3	2	1	1	9	9	9	9	9
2	2	2	2	2	2	1	2	2	2
1	1	1	3	1	1	2	2	9	9
2	1	1	1	1	1	1	1	1	3
2	2	2	2	3	1	2	3	2	2
1	3	2	3	1	1	1	1	1	2
3	3	3	3	2	2	2	2	3	3
1	9	9	9	1	1	1	1	1	2
2	2	2	2	2	2	2	2	2	2
1	1	1	1	1	1	1	1	1	3
9	9	9	9	2	2	2	2	2	2
2	1	2	3	2	2	2	2	3	3
1	1	1	1	1	1	1	1	1	1
1	1	1	1	1	3	1	1	1	1
2	1	2	1	9	9	9	9	3	3
1	1	1	1	2	2	1	1	1	1
3	3	3	3	2	2	1	1	3	3
1	3	3	2	2	1	3	1	1	1
9	9	9	9	2	2	2	2	1	9
1	1	1	1	2	2	1	1	1	1
2	2	2	2	3	3	3	3	2	2
2	2	2	2	3	3	3	3	2	2
3	1	3	2	1	1	2	2	1	2
1	1	1	1	2	2	2	2	9	9
3	2	1	3	2	2	2	2	1	2
2	2	2	2	2	2	2	2	2	2
2	2	2	2	3	3	3	3	2	2
1	2	3	1	1	1	1	1	1	2
2	2	2	2	2	2	2	1	2	2

1	9	1	2	1	2	2	2	1	3
3	3	3	3	2	2	2	2	3	3
2	2	3	2	2	1	2	2	1	2
9	9	9	9	3	3	2	3	9	2
9	9	9	9	9	9	9	9	1	9
1	2	2	1	2	2	2	2	1	9
1	2	2	3	3	2	2	2	2	2
3	3	3	3	3	2	3	2	3	3
2	2	2	2	3	1	1	3	3	3
2	2	2	2	2	2	2	2	2	2
9	9	9	9	2	2	2	2	9	9
9	2	9	1	1	1	1	3	1	2
2	2	2	2	9	3	3	3	2	2
2	2	1	2	1	3	3	3	2	2
2	2	2	2	2	3	3	3	2	2
2	1	1	2	3	3	3	3	3	3
3	2	2	3	2	1	3	2	3	2
9	9	9	9	1	1	2	2	9	1
9	9	9	9	1	2	3	2	9	9
2	2	2	2	2	2	2	2	2	2
2	2	2	2	1	1	2	2	2	2
2	2	2	2	2	2	2	2	2	2
9	9	9	9	9	9	9	9	9	9
9	9	9	9	9	9	9	9	9	9
3	2	1	2	2	1	3	1	1	2
1	3	1	3	1	1	2	2	1	1
3	3	3	3	2	1	1	2	1	2
1	1	1	1	1	1	1	2	1	1
3	3	3	3	1	2	2	2	3	1
3	1	2	3	2	2	2	2	2	2
1	1	1	3	1	1	1	2	1	2
2	3	3	2	2	2	2	2	1	2
1	2	2	1	1	1	1	1	1	3
3	3	3	3	2	2	2	2	3	3
1	1	1	9	3	3	3	3	3	3
1	1	1	1	2	1	1	2	2	2
2	2	1	1	2	1	1	1	1	2
3	3	3	3	2	2	2	2	3	3
9	1	2	2	1	1	2	1	1	9
3	3	3	3	2	2	2	2	3	3
3	1	3	1	2	2	2	1	1	3
3	1	2	1	2	1	1	2	1	3
3	3	3	3	2	2	2	2	1	1
2	2	2	2	3	3	3	3	2	2
2	2	1	2	1	2	1	1	1	2
3	3	1	3	9	9	9	9	1	1
1	1	2	3	1	2	2	2	1	2

2	2	2	2	2	2	2	2	2	2
2	2	2	2	2	2	2	2	9	9
3	9	1	1	1	1	2	1	1	2
1	1	1	1	1	1	2	1	1	3
3	3	2	2	2	2	2	2	2	2
2	2	2	2	2	1	2	2	1	2
2	2	3	3	2	3	9	2	3	2
9	9	9	9	2	1	1	2	2	2
9	9	9	9	2	1	1	2	2	2
2	3	2	2	2	2	2	2	3	2
9	9	9	9	3	1	9	2	9	9
1	1	2	1	2	2	2	2	2	2
1	1	3	3	2	2	2	2	1	1
3	1	2	1	1	1	1	2	2	3
1	2	2	2	2	1	1	1	1	1
2	2	3	3	2	3	1	2	3	2
1	1	1	1	2	2	2	2	1	2
1	1	2	2	1	1	2	2	1	2
1	2	2	1	2	3	2	3	2	2
9	9	9	9	1	1	2	2	1	9
9	9	9	9	1	2	1	2	1	3
3	3	3	3	1	1	1	1	1	1
1	1	3	3	1	1	1	1	1	3
1	1	1	1	1	2	1	3	1	3
3	3	3	3	2	2	2	2	3	3
2	2	2	2	3	3	3	3	2	2
1	1	1	1	2	2	2	2	1	2
1	2	1	3	2	2	2	2	2	1
1	1	2	1	2	2	2	1	3	3
1	2	1	1	1	1	1	2	1	1
2	2	2	2	2	2	2	2	2	2
1	1	1	1	1	1	1	1	9	9
2	2	2	1	2	3	1	2	1	1
2	2	2	2	3	3	3	3	9	9
2	1	3	2	1	1	2	2	1	2
2	2	2	2	1	2	2	2	2	2
2	2	2	2	2	2	2	2	2	2
2	2	2	2	3	2	3	2	2	2
1	1	1	1	2	2	2	2	1	2
3	3	3	2	2	2	2	2	2	2
3	2	1	2	1	2	2	2	1	9
2	2	2	2	1	1	1	1	2	2
1	1	1	1	1	2	2	2	2	3
9	9	9	9	2	2	2	2	2	9
2	2	2	2	2	2	2	2	3	3
2	2	2	2	1	1	2	2	2	2
1	2	1	1	2	2	2	2	1	3

1	9	2	2	1	2	2	2	9	9
1	2	3	3	9	9	9	9	2	3
1	1	2	3	2	2	2	2	1	2
1	1	2	2	3	3	3	2	1	3
2	2	2	2	3	1	2	2	3	3
3	3	3	3	1	2	2	2	3	3
2	2	2	2	2	2	2	2	2	2
3	9	1	3	2	1	2	3	2	9
1	1	1	1	1	3	3	2	3	2
2	2	1	1	9	9	9	9	9	9
1	2	2	1	9	9	9	9	9	9
1	2	2	1	9	9	9	9	9	9
9	9	9	9	2	3	2	1	1	9
9	9	9	9	3	2	2	1	3	9
1	2	2	1	9	9	9	9	9	9
2	2	2	3	2	2	2	2	1	2
9	9	9	9	9	9	9	9	1	1
3	1	1	2	1	1	1	1	2	2
1	2	1	3	1	2	2	2	2	1
2	2	2	2	1	1	2	2	1	2
2	1	2	1	2	2	2	2	2	2
1	2	2	2	1	2	2	2	2	2
9	1	1	2	1	2	2	2	2	2
2	3	1	2	2	2	2	2	2	2
2	2	2	2	2	2	2	1	2	2
2	2	1	3	1	2	2	2	2	2
3	2	1	2	1	3	1	3	1	9
2	2	2	2	1	1	1	1	1	1
2	2	1	3	1	2	2	2	2	2
2	2	3	2	2	2	2	2	1	2
2	2	2	2	1	1	2	2	1	2
9	9	9	9	2	3	2	1	1	9
1	1	3	2	1	1	1	1	1	3
2	2	2	2	1	1	2	2	2	2
2	1	1	2	2	2	2	2	2	2
1	1	1	1	2	2	2	2	2	2
1	1	1	1	2	2	2	2	2	2
9	9	9	9	1	3	2	1	1	9
2	2	2	2	2	2	2	2	2	2
2	2	2	2	1	3	3	3	2	2
2	1	1	3	2	2	2	2	2	2
1	2	2	2	1	1	2	1	2	2
1	1	2	1	2	2	2	2	2	2
2	2	2	2	2	2	2	1	2	2
2	1	1	1	1	2	1	1	2	2
2	2	2	2	1	1	3	1	2	2
9	9	9	9	2	2	2	2	1	9

2	2	2	2	9	9	9	9	2	2
1	1	2	2	2	2	2	2	1	3
9	9	9	9	1	2	2	3	9	9
9	9	9	9	2	2	2	2	1	9
1	1	1	3	2	2	2	2	1	2
9	9	9	9	2	2	2	2	1	9
2	2	3	3	2	2	1	2	1	1
1	2	1	3	1	1	1	3	1	1
3	3	2	1	1	2	2	3	1	2
3	2	2	1	2	2	1	2	1	1
1	2	1	3	3	3	2	3	9	9
2	3	3	2	1	1	2	2	1	2
1	1	9	1	2	2	2	2	2	3
2	1	2	2	2	2	2	2	2	2
1	1	3	2	2	2	2	3	1	2
3	3	3	1	2	2	2	2	3	3
2	2	2	2	3	3	3	3	2	2
2	1	2	1	1	1	2	2	1	2
1	1	1	1	2	2	2	2	1	2
1	2	1	1	2	2	2	2	1	3
2	2	3	1	3	2	3	2	2	2
1	1	1	1	3	2	2	2	2	3
1	1	1	1	2	2	2	2	2	2
2	2	2	2	3	3	3	3	2	2
1	2	1	9	3	2	2	2	2	2
1	1	2	1	1	2	2	2	2	2
2	2	2	2	2	2	2	2	2	2
2	2	2	2	2	2	2	2	2	2
9	2	2	2	2	2	2	2	2	2
9	9	9	9	9	9	9	9	9	9
9	9	9	9	1	1	2	2	1	1
2	2	2	2	3	3	3	1	2	2
9	9	9	9	2	2	2	2	1	1
3	2	2	3	9	9	9	9	9	9
3	1	2	1	1	1	1	2	1	2
9	9	9	9	2	2	2	2	9	9
9	9	9	9	9	9	9	9	9	9
2	2	2	2	2	2	2	2	2	2
9	9	9	9	1	1	3	3	1	3
1	2	1	2	2	1	2	1	1	1
1	1	1	1	2	1	1	1	1	1
1	2	3	2	1	2	3	2	2	3
1	1	2	2	2	1	2	3	2	9
1	2	3	2	2	1	2	2	1	2
2	2	2	3	1	1	2	1	3	2
9	9	9	9	9	9	9	9	9	9
2	3	3	3	2	2	2	2	3	2

1	2	2	1	2	2	2	2	1	2
9	9	9	9	9	9	9	9	9	9
9	9	9	9	9	9	9	9	9	9
2	2	2	2	3	3	3	3	2	2
1	1	2	2	1	3	2	2	1	3
1	2	2	1	2	2	2	2	1	2
1	1	1	2	1	1	1	1	2	1
1	2	1	1	2	2	2	2	2	2
2	2	2	2	1	1	1	1	2	2
1	1	3	2	1	1	1	1	1	3
9	9	9	9	9	9	9	9	9	9
9	9	9	9	2	2	2	2	9	9
9	9	9	9	1	2	2	2	1	9
2	2	2	2	1	2	1	1	2	2
1	2	2	2	2	2	2	2	2	2
9	9	9	9	9	9	9	9	9	9
9	9	9	9	9	9	9	9	9	9
9	9	9	9	9	9	9	9	9	9
1	2	2	1	1	2	2	2	1	2
1	1	2	1	1	2	2	2	1	2
3	3	1	2	1	1	9	9	9	9
3	3	2	1	2	1	1	2	1	3
3	3	3	3	2	1	1	3	3	3
2	2	2	2	9	9	9	9	9	9
2	2	2	2	1	1	1	1	2	2
1	2	2	2	2	2	2	2	2	2
9	9	9	9	3	3	3	3	9	9
1	1	2	3	3	2	2	3	9	9
2	1	1	1	2	2	2	2	1	2
2	2	2	2	9	9	9	9	2	2
1	1	1	3	1	2	2	2	2	1
2	2	2	2	3	3	3	3	9	9
9	9	9	9	9	9	9	9	9	9
1	2	3	2	1	2	2	2	1	2
2	2	2	1	2	2	1	1	2	9
2	2	2	2	2	2	2	2	2	2
1	2	1	1	3	3	2	2	1	1
2	2	2	2	2	2	2	2	2	2
1	1	3	2	1	2	2	2	1	9
2	1	2	1	2	2	2	2	1	2
1	1	3	2	2	1	2	1	1	2
3	3	3	3	2	9	2	2	9	9
9	9	9	9	9	9	9	9	9	9
9	9	9	9	2	2	2	2	9	9
2	2	2	2	9	9	9	9	9	9
9	9	9	9	9	9	9	9	9	9
9	9	9	9	9	9	9	9	9	9

2	2	2	2	1	1	2	2	2	2
2	2	2	2	3	2	1	2	2	3
1	1	1	1	1	1	2	2	2	3
1	2	2	2	2	2	2	2	2	2
1	1	1	1	1	1	1	1	9	9
9	9	9	9	9	9	9	9	9	9
9	9	9	9	9	9	9	9	9	9
1	1	3	3	9	9	9	9	9	9
9	9	9	9	9	9	9	9	9	9
9	9	9	9	9	9	9	9	9	9
9	9	9	9	9	9	9	9	9	9
9	9	9	9	9	9	9	9	9	9
9	9	9	9	9	9	9	9	9	9
1	3	3	1	2	2	2	2	1	1
1	1	1	3	2	2	2	2	2	2
1	2	2	1	3	1	2	2	2	2
1	2	2	2	2	2	2	2	2	2
9	9	9	9	9	9	9	9	9	9
9	9	9	9	9	9	9	9	9	9
9	9	9	9	9	9	9	9	9	9
9	2	9	1	1	2	2	2	1	9
9	9	9	9	9	9	9	9	9	9
1	1	1	1	1	1	2	2	1	1
9	9	9	9	9	9	9	9	9	9
2	1	1	1	3	1	1	3	1	1
1	1	1	1	1	1	1	1	1	1
1	2	2	1	1	1	1	1	1	1
1	1	1	1	1	1	1	1	2	2
1	1	1	1	1	1	1	1	1	1
2	1	1	1	1	1	1	1	1	1
2	2	2	2	1	1	1	1	1	1
1	2	2	2	3	3	3	3	1	1
1	1	1	1	1	1	1	1	1	1
9	9	9	9	9	9	9	9	9	9
1	1	1	1	1	1	1	1	1	1
1	1	1	1	1	1	1	1	1	1
3	2	2	2	3	3	3	3	2	2
9	9	9	9	9	9	9	9	9	9
1	2	1	3	2	2	1	1	1	2
1	3	3	3	1	2	2	2	3	3
2	2	2	2	2	1	2	2	2	3
9	9	9	9	9	9	9	9	9	9
9	9	9	9	9	9	9	9	9	9
9	9	9	9	9	9	9	9	9	9
1	1	2	3	3	1	2	1	1	2
2	2	3	2	3	1	1	1	2	2
1	1	2	2	1	2	2	2	9	9

9	9	9	9	9	9	9	9	9	9
9	9	9	9	9	9	9	9	9	9
9	9	9	9	9	9	9	9	9	9
9	9	9	9	9	9	9	9	9	9
1	1	9	1	2	2	2	2	9	9
9	9	9	9	9	9	9	9	9	9
2	2	2	2	9	9	9	9	9	9
9	9	9	9	9	9	9	9	9	9
9	9	9	9	2	2	2	2	9	9
9	9	9	9	9	9	9	9	9	9
9	9	9	9	2	2	2	2	9	9
9	9	9	9	9	9	9	9	9	9
1	3	3	3	1	1	2	1	1	2
2	3	1	2	1	1	1	1	1	3
1	1	1	3	2	2	2	2	2	2
1	1	1	1	1	1	1	2	2	1
1	1	1	2	1	1	3	3	2	2
1	1	1	1	1	1	2	2	1	1
1	1	1	1	1	1	2	2	1	1
9	9	9	9	1	1	1	1	1	9
2	2	2	2	1	1	1	1	1	1
1	2	3	2	1	2	2	2	1	3
2	2	2	2	1	1	3	3	2	2
3	1	1	2	2	2	3	2	2	3
1	1	1	2	1	1	1	1	1	3
3	1	1	3	2	2	2	2	1	3
2	9	9	9	2	2	2	2	1	2
2	2	2	2	2	2	1	1	1	3
9	9	9	9	2	2	2	2	1	9
9	9	9	9	9	2	2	1	9	9
1	1	1	3	9	9	9	9	9	9
3	3	3	3	1	1	9	9	1	1
3	3	3	3	1	2	2	2	2	2
2	2	2	2	2	2	2	2	2	2
1	1	2	2	1	2	2	2	1	2
2	2	2	2	2	3	2	3	2	2
2	2	2	2	1	2	2	2	2	2
1	2	2	2	2	2	2	2	2	2
2	1	1	2	2	2	2	2	2	2
2	2	2	2	2	2	2	2	1	1
1	1	3	1	2	2	2	2	2	2
2	2	2	2	2	2	2	2	1	1
1	1	3	1	1	1	1	2	1	3
2	2	1	1	2	3	2	2	1	1
1	2	2	1	1	1	1	1	1	1
3	3	3	3	1	1	1	1	1	1
1	2	1	2	1	2	2	2	2	2

9	9	9	9	9	9	9	9	9	9
1	1	1	1	1	1	1	1	1	1
9	9	9	9	2	2	2	2	9	9
3	9	9	9	1	2	2	2	9	9
9	9	9	9	1	1	2	3	1	9
1	1	1	2	1	2	2	2	9	9
9	9	9	9	3	3	2	2	9	2
3	3	3	3	2	2	2	2	1	2
9	9	9	9	3	3	3	3	2	9
2	3	1	2	2	2	2	3	2	2
9	9	9	9	9	9	9	9	9	9
1	1	2	2	2	2	2	2	2	2
1	2	2	1	2	2	2	2	1	1
2	2	2	2	1	1	1	1	2	2
1	1	1	1	1	1	1	1	1	1
9	9	9	9	9	9	9	9	9	9
2	2	2	2	2	2	2	2	2	2
3	3	3	3	2	1	2	1	3	3
9	9	9	9	2	2	2	2	2	1
3	3	3	3	1	3	1	2	2	2
2	2	2	2	3	3	3	3	2	2
1	1	1	3	1	1	2	3	3	2
2	2	2	2	9	9	9	9	2	2
2	2	2	2	2	2	2	2	2	2
1	1	1	1	2	2	9	3	2	2
2	2	2	2	2	2	2	2	2	2
1	3	1	2	2	2	2	2	1	2
1	1	1	1	2	2	2	1	1	3
1	1	1	1	1	1	1	1	1	1
2	2	1	1	2	2	2	2	2	2
9	9	9	9	9	9	9	9	9	9
2	3	2	1	3	3	3	3	2	2
3	3	2	3	9	9	9	9	9	9
2	2	2	2	2	2	2	2	2	2
2	2	2	2	2	2	2	2	2	2
1	1	1	1	1	1	1	1	1	1
1	1	2	2	1	2	2	1	3	3
2	2	1	1	1	1	1	2	1	1
2	1	1	1	1	1	1	1	1	1
3	1	3	1	1	1	1	1	1	2
2	2	2	2	1	1	2	2	2	2
1	1	1	1	1	1	1	1	1	2
9	9	9	9	9	9	9	9	9	9
9	1	9	9	2	1	1	3	3	9
9	9	9	9	1	1	1	1	1	1
1	2	2	1	9	9	9	9	9	9
1	1	2	2	1	2	9	2	9	9

1	1	1	1	1	1	1	1	9	9
1	2	2	2	3	1	3	3	2	2
9	9	9	9	2	2	2	2	2	9
1	2	3	2	3	1	2	2	2	3
9	9	9	9	1	1	2	2	9	9
3	2	1	3	2	2	2	2	3	2
9	9	9	9	3	3	3	3	2	3
9	9	9	9	9	9	9	9	9	9
1	2	2	2	9	9	9	9	2	2
2	2	2	1	2	9	9	9	9	9
9	1	9	9	2	2	2	2	1	9
9	9	9	9	3	3	3	3	2	9
1	2	2	2	1	3	1	2	2	2
9	9	9	9	9	9	9	9	9	9
9	9	9	9	2	2	2	2	2	9
2	1	2	3	1	3	3	2	1	2
2	1	2	2	3	2	3	2	2	1
1	2	2	1	2	2	2	2	1	3
3	3	3	3	1	1	2	2	1	1
1	2	2	2	2	1	2	3	1	2
1	2	1	2	2	2	2	2	1	3
3	1	3	1	3	3	3	3	1	3
2	1	3	3	1	2	2	2	2	3
2	2	9	3	2	2	2	2	2	3
2	2	2	2	2	2	2	2	2	2
2	1	1	1	1	1	1	1	1	2
2	2	2	3	2	2	2	2	2	3
1	3	1	1	1	1	1	1	1	2
9	2	2	3	1	9	9	9	2	1
2	1	2	2	1	2	1	3	1	2
3	1	1	3	2	2	1	1	1	1
2	2	2	3	1	2	2	2	2	2
9	9	9	9	9	9	9	9	9	9
3	3	3	2	2	1	1	2	1	2
2	2	2	3	2	2	2	2	1	2
2	2	2	2	2	2	2	2	2	2
2	3	2	3	2	2	2	2	1	1
1	1	2	2	1	2	9	2	2	2
9	9	9	9	2	2	2	2	9	9
2	2	2	2	2	2	2	2	2	2
9	9	9	9	2	2	2	2	9	9
9	9	9	9	2	2	2	2	2	9
2	2	3	2	2	2	2	2	1	2
9	9	9	9	2	2	2	2	1	9
2	2	3	1	1	1	1	1	1	1
3	1	3	1	1	1	2	1	1	1
3	1	1	3	1	1	3	3	1	1

3	3	3	3	2	2	2	2	3	3
3	9	9	9	2	2	2	2	9	9
3	2	2	1	2	2	2	2	3	3
1	1	1	1	1	1	1	2	1	3
1	1	3	1	2	2	2	2	1	3
1	3	1	1	2	2	2	2	1	1
1	2	1	2	1	1	1	1	1	2
2	2	2	2	2	1	1	1	2	2
1	3	3	3	2	2	2	2	2	2
3	1	2	3	1	3	2	3	1	3
2	1	3	3	2	2	2	2	1	3
2	1	2	3	2	2	2	1	1	2
2	3	2	3	3	2	2	1	2	3
1	1	1	2	2	2	2	2	2	2
9	1	2	3	1	1	3	3	1	2
1	1	3	1	2	2	2	3	1	2
2	2	2	1	2	2	2	2	2	2
3	1	2	1	2	2	2	2	1	2
3	3	3	1	2	2	2	2	2	2
2	2	2	2	2	2	2	2	2	2
2	2	2	2	2	2	1	1	2	2
1	2	2	2	2	1	3	1	2	2
3	3	3	3	2	9	2	2	2	2
9	9	9	9	9	9	9	9	9	9
1	1	2	2	3	2	3	2	2	2
3	2	3	1	9	9	9	9	1	2
1	3	2	1	2	2	2	2	1	2
2	3	1	1	9	9	9	9	1	2
1	1	1	2	1	2	2	1	1	2
9	9	9	9	2	2	2	2	1	9
3	2	3	2	1	2	1	2	1	3
1	2	1	3	1	1	2	3	3	1
3	3	2	2	3	2	2	2	2	3
1	1	2	2	3	3	3	3	2	2
9	1	1	1	1	1	1	1	1	1
2	2	2	2	2	2	2	2	2	2
2	2	2	2	2	2	2	2	2	2
9	9	9	9	9	9	9	9	9	9
3	2	1	1	2	2	2	2	2	2
1	3	1	3	1	1	2	2	2	2
9	9	9	9	9	9	9	9	9	9
9	9	9	9	9	9	9	9	9	9
2	3	1	2	3	1	2	3	2	2
3	3	3	1	1	2	2	1	9	9
2	2	2	2	2	2	2	2	2	2
1	1	3	3	2	2	2	2	2	2
9	9	9	9	9	9	9	9	9	9

2	2	2	2	2	2	2	2	2	3
9	9	9	9	9	9	9	9	9	9
1	2	1	1	2	2	2	2	1	2
9	9	9	9	9	9	9	9	9	9
9	9	9	9	9	9	9	9	9	9
3	3	3	3	1	1	1	1	3	3
2	2	2	2	2	2	2	2	2	2
2	2	2	2	3	3	3	3	2	2
2	1	1	2	1	3	1	1	3	3
1	2	2	3	2	2	2	2	2	2
9	9	9	9	9	9	9	9	9	9
9	9	9	9	2	2	2	2	9	9
9	9	9	9	9	9	9	9	9	9
1	1	1	1	1	1	1	1	1	1
2	2	2	2	2	2	2	2	2	2
2	2	2	2	2	2	2	2	1	1
9	9	9	9	2	2	2	2	1	9
9	9	9	9	9	9	9	9	9	9
3	3	3	3	3	3	3	3	3	3
2	2	2	2	2	2	2	2	2	2
3	3	3	3	3	3	3	3	1	1
2	2	2	2	2	2	2	2	2	3
9	9	9	9	2	2	3	2	1	9
2	1	2	3	2	2	3	3	2	3
3	3	3	3	1	2	2	2	2	2
3	2	2	2	3	3	3	3	2	2
9	9	9	9	2	1	2	2	1	3
2	2	2	2	1	1	1	1	1	1
2	2	2	2	2	2	2	2	2	2
9	9	9	9	1	3	3	2	1	9
1	1	2	3	2	2	2	2	2	2
2	2	2	2	3	3	3	2	2	2
3	2	2	2	1	2	2	2	1	3
2	1	2	3	2	2	2	2	2	2
1	1	1	1	2	2	2	2	2	2
2	1	2	3	2	1	1	1	2	1
1	1	1	1	1	1	1	1	1	3
1	1	1	3	2	2	2	2	3	3
9	9	9	9	2	2	2	2	2	2
2	2	2	2	2	2	2	2	3	3
1	1	2	2	2	2	2	2	1	2
3	3	3	3	3	2	2	2	1	3
3	3	3	3	2	1	3	3	2	2
1	2	1	3	2	2	2	2	1	2
3	3	3	1	2	2	2	2	1	2
1	1	2	2	2	2	2	2	2	3
9	9	9	9	9	9	9	9	1	2

1	2	2	2	2	2	2	2	2	2
1	2	2	2	2	2	2	3	1	2
2	2	2	1	1	1	1	1	2	1
2	2	2	2	2	2	2	2	1	3
1	2	3	2	2	2	2	2	2	2
9	9	9	9	2	2	1	1	1	9
2	2	2	2	3	3	3	3	2	2
2	2	2	2	1	1	1	1	1	1
2	2	2	2	2	2	2	2	2	2
2	2	2	2	2	2	2	2	2	2
1	1	2	2	2	2	2	2	2	2
3	2	1	3	2	2	2	2	2	2
2	2	2	2	2	2	2	2	2	2
3	3	3	3	2	2	2	2	3	3
3	2	3	2	1	1	2	2	1	2
1	2	3	1	2	2	2	2	2	1
2	2	2	2	2	2	2	2	2	3
1	1	2	3	2	2	2	2	2	2
2	2	2	2	3	3	3	3	2	2
2	2	2	2	2	1	2	2	1	1
1	1	2	1	1	1	1	2	2	2
1	2	3	1	3	2	3	2	1	1
1	2	3	1	3	2	3	2	1	1
3	3	3	2	9	1	2	2	9	9
1	1	2	2	2	2	2	2	2	2
2	2	2	2	2	2	2	2	2	2
1	2	2	1	2	2	2	2	2	2
2	1	2	1	2	2	2	2	2	2
2	2	2	2	2	2	2	2	2	2
1	1	1	1	1	1	1	1	1	1
2	2	2	2	2	2	2	2	3	3
9	2	2	3	3	2	2	3	1	2
1	1	1	2	2	2	2	2	1	1
3	3	3	3	2	9	2	2	1	2
2	2	2	2	1	2	1	1	1	1
2	2	2	2	2	2	2	2	2	2
2	2	2	2	2	2	2	2	2	2
2	2	2	2	3	3	3	3	2	2
2	2	2	2	2	2	2	2	2	2
2	2	2	2	3	3	3	3	2	2
1	1	2	3	2	2	2	2	1	2
1	3	1	2	1	1	2	2	1	2
1	2	2	2	1	1	1	1	1	2
1	2	2	2	1	2	3	3	2	2
2	3	3	3	2	2	2	2	1	2
1	2	1	1	1	1	3	2	1	3
1	1	3	1	1	2	2	2	1	3

2	2	1	3	2	2	3	3	2	2
2	2	2	2	3	3	1	1	2	2
1	1	1	2	1	2	2	2	1	3
2	2	2	2	2	2	2	2	2	2
2	2	2	2	2	2	2	2	2	2
2	2	2	2	3	2	3	1	2	2
1	1	1	2	2	2	2	2	2	2
1	1	1	2	1	2	2	2	1	2
2	2	2	2	2	3	2	2	2	2
2	2	2	2	2	2	2	2	2	2
2	2	2	2	2	2	2	2	2	2
2	2	2	2	3	2	2	2	2	9
2	2	2	2	9	9	9	9	9	9
1	9	9	9	1	2	2	2	9	9
2	2	2	2	1	2	2	2	2	2
1	1	1	2	1	1	2	2	2	2
2	2	2	2	2	2	2	1	2	3
2	1	1	1	1	1	2	2	2	2
1	1	1	1	1	3	2	2	1	2
1	1	2	2	1	2	1	1	1	2
2	2	2	2	1	1	2	2	2	2
2	2	2	2	3	3	3	3	2	2
1	1	2	1	3	1	2	1	2	2
2	2	2	2	2	2	2	2	2	2
1	1	1	2	1	1	2	2	2	2
2	2	1	1	2	2	2	1	2	2
2	2	2	2	2	2	2	2	2	2
2	2	2	2	2	2	2	2	2	2
2	2	2	2	1	2	2	1	2	2
1	1	1	1	9	9	9	9	9	9
3	3	3	3	3	3	3	3	2	2
2	2	2	2	1	3	3	3	2	2
2	2	2	2	3	1	1	3	2	2
2	2	2	2	3	3	2	2	2	2
2	2	2	2	2	2	2	2	2	2
2	2	2	2	3	3	3	3	2	2
2	2	2	2	2	2	2	2	2	2
2	2	2	2	2	2	2	2	2	2
9	9	9	9	1	2	3	3	2	2
2	2	1	1	1	2	2	1	2	2
2	2	2	2	2	2	2	2	2	2
2	2	2	2	3	3	3	3	2	2
2	2	2	2	1	1	2	2	1	2
2	2	2	2	2	2	2	2	2	2
2	2	2	2	2	2	2	2	2	2
2	2	2	2	3	3	3	2	2	2
2	2	2	2	2	2	2	2	2	2

2	2	2	2	1	1	1	2	2	2
2	2	2	2	1	2	2	2	2	2
1	3	1	1	2	2	2	2	2	2
2	2	2	2	1	2	2	1	2	2
2	2	2	2	2	2	2	2	2	2
2	2	2	2	2	2	1	2	2	2
9	9	9	9	2	2	2	2	9	9
1	2	1	1	1	1	3	2	1	2
1	1	2	3	1	1	1	1	1	3
1	2	2	2	1	2	2	2	2	2
1	1	1	1	1	2	2	2	2	2
2	2	2	3	2	2	2	1	2	2
2	1	3	3	2	2	2	2	2	2
2	2	2	2	1	1	2	2	1	2
1	1	1	1	1	1	2	1	2	2
2	2	2	2	1	2	2	1	1	2
2	2	2	2	1	2	2	1	1	2
2	1	1	1	1	3	2	2	1	2
1	1	1	1	2	2	2	2	1	1
2	1	2	2	1	2	1	1	1	1
1	1	1	1	1	1	1	1	1	1
2	1	3	3	1	1	1	1	1	2
2	2	2	2	3	3	2	2	2	2
2	2	2	2	2	2	2	2	2	2
2	2	2	2	9	9	9	9	2	2
2	2	2	2	2	2	2	2	2	2
9	9	9	9	9	9	9	9	9	9
2	2	2	2	2	2	2	2	2	2
2	2	2	2	3	3	3	2	2	2
2	2	2	2	3	3	3	3	2	2
2	2	2	2	2	2	2	2	2	2
2	1	1	1	1	1	1	1	1	2
9	9	9	9	9	9	9	9	9	9
3	3	3	3	1	1	1	1	9	9
9	9	9	9	9	9	9	9	9	9
1	1	1	1	9	9	9	9	9	9
3	3	2	2	3	1	2	2	2	2
1	1	1	1	1	1	2	1	1	3
1	1	1	9	2	2	2	2	1	2
3	2	2	2	2	2	1	3	2	2
1	1	1	2	1	2	2	2	1	2
2	2	2	2	1	2	2	2	2	2
2	2	2	2	2	2	2	2	1	1
2	2	2	2	3	3	3	3	2	2
1	1	1	2	1	1	2	1	2	2
2	2	2	3	1	1	2	2	2	2
2	1	1	1	2	2	2	2	1	1

2	1	1	2	1	2	2	1	2	1
1	1	2	3	9	9	9	9	3	2
9	1	1	2	3	3	3	2	3	2
1	2	1	2	1	1	2	2	1	2
9	2	1	3	1	1	1	2	1	3
2	2	2	2	2	2	2	3	2	2
9	1	2	3	2	2	2	2	2	1
9	9	9	9	2	2	2	2	2	9
2	2	2	2	2	2	2	2	2	2
2	2	2	2	1	2	2	2	2	2
2	2	2	2	1	2	2	2	2	2
2	2	2	2	1	2	2	2	2	2
9	9	9	9	9	9	9	9	2	2
2	2	2	2	1	2	2	9	1	2
2	2	2	2	1	1	3	1	2	2
2	2	2	2	2	2	2	2	2	2
1	2	2	2	1	1	2	2	2	2
3	2	2	3	2	2	2	1	1	3
2	2	2	2	1	1	1	1	1	2
1	1	3	2	1	1	1	2	1	3
9	9	9	9	1	1	2	2	2	2
9	9	9	9	1	1	2	2	2	2
2	2	2	2	2	2	2	2	2	3
9	9	9	9	2	2	2	2	9	9
1	3	1	3	1	2	1	1	2	1
1	1	1	1	1	1	2	2	1	3
1	1	1	1	2	2	2	2	2	2
1	2	2	1	1	2	2	1	1	2
2	2	2	2	1	1	2	2	2	2
9	9	9	9	1	1	1	1	1	9
3	2	3	3	1	1	1	1	1	2
9	9	9	9	2	2	2	2	9	9
9	9	9	9	9	9	9	9	9	9
2	2	2	2	9	9	9	9	9	9
2	2	2	2	9	9	9	9	9	9
1	2	2	1	2	2	2	2	2	2
2	2	2	2	1	1	1	1	1	3
2	2	2	2	9	9	9	9	9	9
2	2	2	2	2	2	2	2	2	2
2	2	2	2	2	2	2	2	2	2
9	9	9	9	1	1	1	1	1	3
2	2	2	2	2	2	2	2	2	2
2	2	2	2	2	2	2	1	2	2
9	9	9	9	3	3	2	2	3	2
9	9	2	2	9	9	9	9	3	3
1	1	1	1	1	1	1	1	1	1
1	1	1	1	2	2	2	2	3	3

9	9	9	9	2	2	2	2	1	1
1	1	1	2	2	2	2	2	1	2
2	3	3	3	1	2	3	2	1	1
2	2	2	2	2	2	2	1	2	2
9	9	9	9	9	9	9	9	9	9
3	3	3	3	9	9	9	9	9	9
2	2	2	2	2	2	2	2	2	2
3	3	3	3	2	2	2	2	2	2
9	9	9	9	1	2	2	1	1	9
2	2	2	2	2	2	2	2	9	9
1	1	3	3	1	1	1	1	1	1
2	2	2	2	1	2	2	2	2	2
3	2	2	1	3	2	2	2	2	2
3	3	3	3	2	2	2	2	2	2
9	9	9	9	9	9	9	9	9	9
2	3	2	2	2	2	2	2	2	3
3	2	2	2	2	2	2	2	1	3
3	3	1	2	2	3	2	1	1	3
9	9	9	9	3	3	3	2	9	2
1	2	1	3	3	2	1	2	1	2
1	2	1	1	3	1	2	3	1	3
2	2	2	2	1	2	2	1	1	3
9	9	9	9	9	9	9	9	1	3
2	2	2	2	1	2	2	2	2	2
1	3	3	3	2	2	2	2	3	1
2	2	2	2	3	2	2	2	2	2
1	1	2	3	2	2	2	1	2	2
1	1	1	3	2	2	2	2	3	3
2	1	9	3	2	1	3	2	1	2
9	9	9	9	2	2	2	2	1	3
2	2	2	2	2	2	2	2	2	2
2	2	2	2	3	3	2	2	1	1
1	1	3	1	3	3	2	2	1	2
3	3	3	3	1	1	1	1	1	3
1	1	1	1	2	2	2	2	9	9
1	1	1	2	3	2	2	2	1	2
3	3	3	3	2	2	2	2	9	9
1	2	1	1	2	2	2	2	2	2
1	2	1	2	3	2	1	2	2	2
9	9	9	9	9	9	9	9	2	3
9	9	9	9	9	9	9	9	9	9
1	3	1	2	2	1	1	3	2	1
1	1	1	2	2	2	2	2	2	3
1	1	2	1	1	1	2	2	1	3
2	2	2	2	1	1	2	2	1	3
2	3	3	3	9	9	9	9	2	3
3	2	3	2	2	2	3	1	3	2

2	1	2	2	1	2	2	2	1	3
1	3	1	2	2	2	2	2	3	2
1	1	2	1	2	2	2	2	2	2
3	3	3	9	1	2	2	2	3	3
3	1	1	3	2	2	2	2	1	1
2	2	2	2	9	9	9	9	9	9
2	2	2	2	1	9	9	9	9	9
2	2	2	2	2	2	2	2	1	2
1	1	1	1	1	1	1	1	1	3
2	2	2	2	1	2	3	2	1	3
1	2	2	1	1	1	2	3	2	3
2	1	2	1	2	2	2	2	2	2
3	2	3	2	2	2	2	2	2	2
1	1	1	1	3	3	3	3	1	3
9	9	9	9	9	9	9	9	9	9
2	2	2	2	3	1	2	2	2	3
1	2	2	2	2	2	1	1	2	2
2	1	2	2	1	2	2	2	2	2
2	2	3	2	2	2	1	1	1	3
1	1	1	2	1	2	1	2	1	2
9	1	1	2	9	9	9	9	9	9
9	9	9	9	9	9	9	9	9	9
2	2	2	2	2	2	2	2	2	2
9	9	9	9	2	2	2	2	2	9
2	2	2	2	9	9	9	9	9	9
9	1	2	2	2	2	2	2	3	9
1	2	2	2	2	2	2	2	2	2
2	2	2	2	2	2	2	2	1	2
3	3	3	3	2	2	2	2	3	3
1	1	1	2	2	2	2	2	1	2
3	3	3	1	2	2	2	2	3	3
3	3	3	3	2	2	2	2	2	2
2	9	9	9	9	9	9	9	9	9
1	1	1	2	2	2	2	2	9	9
3	1	3	2	9	9	9	9	9	9
2	2	2	2	1	2	2	2	2	2
1	1	1	3	1	1	1	1	1	3
2	3	1	2	2	2	2	2	3	2
1	1	2	2	2	2	2	2	1	2
9	9	9	9	2	2	1	2	2	9
2	2	2	2	3	3	3	3	2	2
2	1	2	3	1	2	2	2	2	2
2	2	2	3	1	2	2	2	3	3
1	1	1	3	2	2	2	2	9	9
3	3	2	2	1	1	1	1	3	1
9	9	9	9	2	2	2	2	9	9
1	2	1	2	2	2	2	2	3	2

3	2	1	2	2	2	1	2	1	2
2	3	2	2	2	2	2	2	2	2
9	3	3	3	2	2	2	2	3	3
3	1	2	3	3	2	1	3	2	3
2	1	2	2	2	2	2	2	2	2
2	2	2	2	1	3	2	1	1	2
1	1	3	1	2	2	2	1	1	3
1	9	9	9	2	2	2	2	1	3
9	9	9	9	9	9	9	9	9	9
2	2	1	2	1	1	1	1	1	2
2	2	3	3	2	3	2	2	2	2
2	2	2	2	2	1	2	2	2	2
1	1	3	3	1	3	2	1	2	2
1	1	1	1	1	1	2	1	1	3
9	9	9	9	2	2	1	2	9	2
1	2	1	2	2	1	2	2	2	2
2	2	2	2	1	1	2	2	2	2
3	3	2	3	2	2	2	2	1	2
3	1	3	3	2	2	2	3	2	3
3	2	2	3	2	2	2	2	2	2
1	1	1	2	2	2	1	2	1	3
3	3	1	3	1	1	2	2	1	2
1	1	2	1	3	3	3	3	2	2
1	3	2	2	2	2	2	2	1	3
1	1	9	9	2	1	2	3	1	3
1	2	2	1	1	1	2	2	2	2
1	2	1	2	1	1	2	2	1	2
2	2	2	2	1	2	2	2	1	2
2	2	2	2	1	2	2	2	2	2
1	1	2	2	3	2	2	2	2	2
2	1	2	2	3	1	3	2	2	2
2	9	1	2	3	1	2	1	2	9
2	2	2	2	2	2	2	1	2	2
1	1	1	1	2	2	2	2	1	1
1	2	2	3	1	2	3	1	1	2
1	1	1	3	2	2	2	2	2	2
1	1	1	2	3	1	2	2	1	2
1	3	2	2	2	9	9	9	1	1
2	2	2	2	1	1	2	2	1	2
1	1	1	1	1	1	1	1	1	1
2	3	3	3	1	2	2	2	2	2
1	1	1	1	1	1	1	2	1	1
2	1	1	1	2	2	2	2	1	1
2	2	2	2	2	2	2	2	1	2
2	1	3	3	2	1	1	2	1	2
2	2	2	2	2	1	1	1	2	2
1	2	1	1	1	1	2	1	1	2

1	2	2	1	1	1	2	2	1	1
1	1	1	2	2	1	2	1	1	2
3	2	1	2	1	2	1	2	1	2
1	2	3	1	2	1	1	2	1	2
2	2	1	1	1	1	1	2	1	1
2	2	2	3	1	1	2	1	3	1
1	1	2	3	2	1	1	2	1	2
1	2	2	3	1	2	1	1	1	2

PapOverEi	PapTwoYe	PapfromM	PapHistory	PapTwoYe	PapOnceo	HPVVaccin	HPVVaccin	EarlyOnset	MultiplePa
2	2	2	2	2	2	2	2	2	2
1	3	3	3	2	1	1	1	2	1
2	2	2	2	2	2	2	2	2	2
1	3	3	3	2	1	1	1	2	1
1	3	3	3	2	1	1	1	2	1
9	3	3	3	2	1	1	1	1	1
2	9	9	9	9	9	9	9	9	9
2	2	2	3	1	2	1	1	1	1
2	9	2	9	2	9	2	2	9	2
9	9	9	9	9	9	9	9	9	2
2	2	3	2	2	2	2	2	3	2
3	3	3	3	3	3	3	2	2	2
2	3	2	1	2	2	1	2	2	2
2	2	2	2	2	2	2	2	2	2
2	2	2	1	2	1	1	1	2	1
2	2	3	3	2	3	1	2	2	2
9	1	2	1	2	2	3	2	1	2
2	2	3	3	2	3	1	2	2	2
2	2	3	3	2	3	1	2	2	2
2	2	2	2	2	2	2	2	3	2
1	1	2	2	1	1	1	1	3	3
1	1	2	2	1	1	1	1	3	3
9	2	2	2	2	2	2	2	2	2
3	3	3	3	1	3	1	1	3	3
2	2	2	2	2	2	2	2	2	2
2	2	2	2	2	2	2	2	2	2
2	1	1	1	1	3	1	1	2	2
1	2	2	2	1	3	1	1	1	1
2	2	2	2	2	2	2	2	2	2
2	2	2	2	2	2	2	2	2	2
2	2	2	2	2	2	2	2	2	2
2	1	2	2	2	2	2	1	2	1
1	1	3	3	2	3	1	1	1	1
2	1	1	2	1	2	1	1	2	1
2	1	2	2	2	2	2	2	2	2
2	1	1	2	1	2	1	1	2	1
2	1	2	2	2	2	2	2	2	2
1	3	3	3	1	3	1	1	1	1
2	2	2	2	2	2	2	2	2	2
2	2	2	2	2	2	2	2	2	2
2	2	2	2	2	2	2	2	2	2
2	1	2	2	2	2	2	2	1	1
2	3	3	9	2	1	1	1	1	1
1	3	3	3	3	3	1	1	3	3
1	2	2	3	3	3	2	3	1	1
2	3	3	9	2	1	1	2	1	1

1	1	2	3	2	3	2	1	1	1
1	2	2	3	1	2	1	1	1	2
2	2	2	2	2	2	1	1	1	1
1	2	2	3	1	2	1	2	1	1
3	2	3	3	2	2	1	1	1	1
3	3	3	3	1	3	1	1	1	1
3	3	3	3	1	3	1	1	1	1
1	9	1	1	9	9	1	1	1	1
1	9	1	1	9	9	1	1	1	1
3	2	1	2	3	1	1	3	1	1
2	2	2	2	2	2	2	2	2	2
1	2	2	2	2	2	1	2	1	1
1	2	3	3	1	3	2	2	1	1
2	2	2	2	2	2	2	2	2	1
2	2	2	2	2	2	2	2	1	1
1	2	3	3	1	3	2	2	1	1
1	2	3	2	1	3	1	1	1	1
1	1	2	1	1	3	1	1	1	1
2	3	3	2	1	1	1	2	1	1
9	9	9	9	9	9	9	9	1	2
3	3	3	3	1	3	1	1	1	1
3	3	3	3	1	3	1	1	1	1
3	3	3	3	1	3	1	1	1	1
1	1	2	2	1	2	1	1	1	1
9	9	1	9	9	1	9	1	1	1
1	1	1	1	1	1	9	1	1	2
9	9	9	9	9	9	9	9	2	2
1	2	2	3	1	2	1	1	1	3
2	2	2	2	2	2	2	2	2	2
2	2	2	1	1	3	1	1	1	1
9	9	9	9	9	9	9	9	1	9
3	3	3	1	3	3	3	3	3	3
9	9	9	9	9	9	9	9	1	9
3	3	3	2	3	3	1	1	1	1
2	2	3	3	1	3	1	1	1	3
9	9	9	9	9	9	9	9	9	9
9	9	9	9	9	9	9	9	9	9
3	2	2	2	2	2	1	1	1	1
1	3	1	3	3	1	1	1	3	3
1	1	3	3	1	3	1	1	1	1
1	1	2	2	1	3	1	1	1	1
9	9	9	9	9	9	9	9	1	9
1	3	3	3	1	3	1	3	1	1
1	2	2	2	1	3	1	1	1	1
1	2	2	2	1	3	1	1	1	1
9	9	9	9	9	9	9	9	9	9
9	9	9	9	9	9	9	9	9	9

2	2	3	1	1	2	1	1	3	2
3	2	3	2	2	3	1	1	1	1
2	2	1	1	1	1	1	1	1	1
2	1	2	2	2	2	2	1	2	2
9	9	9	9	9	9	9	9	9	9
2	2	2	2	2	2	1	1	1	9
3	2	3	1	1	3	1	1	1	1
3	1	2	2	2	3	2	2	1	1
1	1	3	3	1	3	1	1	1	1
1	2	3	3	1	3	1	1	9	9
1	1	1	2	9	2	1	1	1	1
2	1	3	3	3	2	3	1	2	3
1	1	1	1	2	3	9	9	3	1
2	2	2	1	2	9	9	9	1	9
1	2	2	2	1	2	1	2	2	1
2	1	3	2	1	2	2	3	2	2
2	2	2	2	2	2	2	1	2	2
2	2	2	2	2	2	2	1	1	2
1	2	2	1	2	2	9	9	2	2
2	2	2	2	2	2	2	2	2	2
3	3	3	3	1	3	1	1	2	1
1	1	2	2	1	3	2	2	1	1
2	2	2	2	2	2	2	2	1	2
3	3	3	3	3	3	3	3	3	3
2	3	2	2	1	2	2	1	1	1
2	2	2	2	2	2	2	2	2	2
1	2	3	1	2	3	1	2	2	2
1	2	3	2	2	3	1	1	1	2
2	2	2	2	2	2	2	2	2	2
2	2	2	2	2	2	2	2	1	1
2	2	2	2	2	2	2	2	2	2
2	2	2	2	2	2	2	2	2	2
1	2	2	2	2	2	1	1	1	1
3	3	2	2	2	2	2	2	2	2
2	2	2	2	2	2	2	2	1	1
2	2	2	2	2	2	1	2	1	1
2	2	2	2	2	2	2	2	2	2
2	2	2	2	2	2	2	2	2	2
9	9	9	2	9	9	1	9	9	9
1	1	3	3	3	3	1	1	1	1
2	2	2	2	2	2	2	2	2	2
2	2	2	2	2	2	2	2	2	2
2	2	2	2	2	2	2	2	2	2
1	2	1	1	3	2	2	1	2	3
1	2	2	2	2	1	2	2	1	1
2	2	2	2	2	2	1	2	2	2
2	2	2	2	2	2	2	2	2	2

2	2	2	1	1	1	1	2	2	2
1	2	1	2	2	2	2	2	2	2
9	2	1	2	2	2	2	2	2	2
2	2	2	2	2	2	2	2	2	2
9	9	9	9	9	9	9	9	1	1
2	2	2	2	2	2	2	2	1	1
3	3	2	2	3	3	3	2	2	1
2	2	2	2	2	2	2	2	2	2
2	2	2	2	2	2	2	2	2	2
2	2	2	2	2	2	2	2	2	2
2	2	3	2	2	2	2	3	3	2
2	3	2	2	2	2	1	1	2	2
9	9	9	9	9	9	9	9	1	1
2	2	2	2	2	2	2	2	2	1
2	2	2	2	2	2	2	2	1	1
2	2	2	2	2	2	2	2	1	1
9	9	9	9	9	9	9	9	9	9
2	2	2	2	2	2	2	2	2	2
9	9	1	9	9	9	9	9	9	9
3	1	3	3	2	3	1	1	1	1
1	1	3	3	1	3	1	1	1	1
1	1	3	3	2	3	2	1	1	1
1	1	3	3	1	3	1	1	1	1
1	3	3	2	1	3	3	1	1	1
1	1	3	3	2	3	3	1	1	1
1	1	3	9	9	9	9	9	1	1
1	1	3	3	2	3	1	1	1	1
1	1	3	3	2	3	1	1	1	1
1	1	3	3	2	3	1	1	9	1
1	1	3	3	2	3	1	1	1	1
1	2	2	1	2	2	2	2	2	3
2	2	2	2	2	2	2	2	2	2
1	1	3	3	2	3	1	1	1	1
1	1	3	3	2	3	1	1	1	1
1	1	3	2	1	3	1	1	1	1
1	1	3	2	1	3	1	1	1	1
1	1	3	3	2	3	1	1	1	1
1	1	3	3	2	3	1	1	1	1
1	1	3	1	2	3	1	1	1	1
1	1	3	3	2	3	1	1	1	1
1	1	3	3	1	3	1	1	1	1
1	1	3	3	2	3	3	1	1	1
1	1	3	3	2	3	1	1	1	1
1	1	3	3	2	3	1	1	1	1
1	1	3	3	2	3	2	1	1	1
1	1	3	3	2	3	1	1	1	1
1	1	3	3	2	3	1	1	1	1

1	1	3	3	2	3	3	1	1	1
1	1	3	3	2	3	3	1	1	1
1	1	3	3	2	3	3	2	1	1
1	1	3	3	3	3	1	1	1	1
3	1	3	1	2	3	3	1	1	1
1	1	3	3	2	3	1	1	1	1
1	1	3	3	2	3	1	1	1	1
1	3	3	3	1	3	1	1	3	1
1	1	3	3	1	3	1	1	1	1
3	3	3	3	3	3	1	1	1	1
9	9	9	9	9	9	9	9	9	9
1	2	3	3	1	3	1	2	1	1
1	1	3	3	1	3	1	1	1	1
1	1	3	3	2	3	1	1	1	1
2	2	2	1	1	1	1	2	2	1
2	2	2	2	1	2	2	1	1	1
1	2	2	2	2	2	1	1	2	2
1	1	1	1	1	1	1	1	1	1
2	2	2	2	2	2	2	2	1	1
2	2	2	2	2	2	1	1	1	1
2	1	2	1	1	2	1	1	1	1
2	2	2	2	2	2	2	2	2	2
1	2	1	2	3	2	1	2	1	1
1	2	2	2	2	2	1	1	1	1
2	2	2	2	2	2	2	2	2	2
2	2	2	2	2	2	2	2	2	2
2	2	2	2	2	2	1	2	1	1
2	2	2	2	2	2	2	2	2	2
2	2	2	2	2	2	2	2	2	2
2	2	2	2	2	2	2	2	1	1
1	1	2	2	3	3	1	1	1	1
1	1	2	2	3	3	1	1	1	1
2	2	2	2	2	2	2	2	1	1
1	2	2	2	2	2	2	2	1	1
1	2	1	1	1	2	2	2	1	1
2	2	2	2	2	2	2	2	2	2
2	2	2	2	2	2	2	2	2	2
3	3	3	1	1	2	1	1	1	1
1	1	2	2	2	2	2	2	1	1
2	2	2	3	3	2	1	2	1	1
1	2	1	3	3	3	3	3	3	2
2	2	2	2	2	9	1	1	1	1
9	9	9	9	9	9	9	9	2	9
9	9	9	9	9	9	9	9	2	9
3	2	2	3	2	2	1	2	2	1
1	1	3	1	1	2	1	1	1	1
9	9	9	9	9	9	9	9	9	9

2	2	2	2	2	2	2	2	2	2
2	2	2	2	2	2	2	2	2	2
1	2	2	2	1	2	1	2	1	1
2	2	2	2	1	2	1	1	2	2
1	2	1	1	3	3	3	2	1	2
1	2	3	2	3	2	1	2	2	1
2	2	2	2	2	2	1	1	2	2
2	2	2	2	2	2	2	1	2	2
3	3	3	1	1	3	1	1	1	3
3	3	3	3	3	3	3	3	2	2
2	2	2	2	2	2	2	2	2	1
3	3	2	2	2	2	2	2	1	3
3	3	2	2	2	2	2	2	1	3
2	2	2	2	2	3	1	2	2	2
2	2	2	2	2	2	2	2	2	2
2	2	2	1	2	3	1	2	1	2
2	2	2	2	3	3	1	1	1	2
1	3	3	2	1	3	1	2	1	1
9	9	9	9	9	9	9	9	9	9
2	2	2	2	1	3	1	3	9	1
2	2	2	2	1	3	1	1	1	1
2	2	2	2	2	2	2	1	2	1
2	2	2	2	2	3	1	1	2	2
2	2	2	3	1	3	1	2	2	2
9	9	9	9	9	9	9	9	9	9
2	2	2	2	2	2	2	2	2	2
2	2	2	2	2	2	2	2	2	2
1	2	2	2	1	3	2	1	1	9
2	2	2	2	2	2	2	2	2	1
2	2	2	2	2	2	2	2	2	2
1	9	9	9	9	9	9	9	9	1
3	3	3	3	3	3	3	3	3	3
3	3	3	3	3	3	3	3	3	3
2	2	2	2	2	2	2	2	2	2
9	9	9	9	9	9	9	9	1	1
2	2	2	2	2	2	2	2	2	2
2	2	3	3	1	3	1	2	1	2
1	1	3	3	2	2	3	1	1	1
2	2	2	2	2	2	2	2	2	2
2	2	2	3	1	3	2	2	2	2
1	2	2	3	2	2	1	1	1	2
2	2	2	3	1	3	1	1	3	3
1	1	2	2	2	2	2	2	3	3
2	2	2	2	2	2	2	2	2	2
1	2	3	3	2	2	1	2	2	1
2	2	1	1	2	2	2	2	2	2
2	2	2	2	2	2	2	2	2	2

9	9	9	9	9	9	9	9	9	9
9	9	9	9	9	9	9	9	9	9
1	9	9	9	9	9	9	9	9	9
1	1	3	1	1	3	1	1	1	1
3	2	3	2	2	3	2	2	2	2
2	1	2	3	1	2	3	1	2	1
2	1	2	2	1	3	1	1	1	1
1	1	1	1	1	1	1	1	1	1
2	9	3	2	1	1	1	1	1	2
2	2	2	2	2	2	2	2	2	2
2	2	2	2	2	2	2	2	2	2
3	2	2	2	2	3	2	2	9	9
9	9	9	9	9	9	9	9	9	9
1	1	1	1	1	1	1	1	1	3
1	3	3	3	3	1	1	1	3	3
9	9	9	9	9	9	9	9	9	9
1	2	2	2	1	1	2	2	2	2
2	2	1	3	2	1	2	2	2	3
1	1	1	1	1	1	1	3	3	1
9	9	9	9	9	9	9	9	9	9
9	9	9	9	9	9	9	9	9	9
2	2	3	2	2	2	2	2	2	2
3	3	2	2	3	2	1	2	2	1
2	2	2	2	2	2	2	2	1	1
2	2	2	2	2	2	2	2	2	2
2	2	2	3	1	3	2	2	2	1
1	2	2	1	1	2	1	1	2	3
2	2	3	3	2	3	2	2	1	1
2	2	2	3	1	3	1	2	2	3
1	2	2	3	1	3	1	1	2	1
2	2	2	2	2	2	2	2	2	2
2	2	3	3	3	2	3	2	3	1
9	9	9	9	9	9	9	9	9	9
9	9	9	9	9	9	9	9	9	9
2	2	3	3	2	3	1	3	1	1
3	2	3	1	1	3	3	2	1	3
1	1	3	3	1	3	2	2	1	2
3	1	2	1	1	2	1	1	1	3
1	1	2	1	1	3	1	1	1	3
2	2	2	1	2	9	1	2	2	2
3	2	3	2	2	1	1	3	3	2
1	1	3	1	1	3	1	1	3	3
2	2	2	2	2	2	2	2	1	2
2	2	2	2	2	2	2	2	2	2
2	3	3	3	3	3	1	1	3	3
1	9	3	3	2	3	2	2	2	1
2	2	2	2	2	2	2	2	2	2

1	2	3	2	2	2	2	1	9	9
1	2	3	2	2	2	2	1	9	9
9	9	9	9	9	9	9	9	1	9
2	2	2	2	2	2	2	2	2	2
9	9	9	9	9	9	9	1	9	9
9	9	9	9	9	9	9	9	9	9
1	1	2	2	1	3	1	1	1	1
2	2	9	3	9	3	1	9	9	9
1	1	3	3	1	3	1	1	1	1
2	2	3	1	1	2	1	2	1	1
1	2	2	2	3	1	2	1	1	2
2	2	2	2	2	2	2	2	2	2
2	2	3	1	1	3	1	1	1	2
2	2	2	2	3	2	2	2	1	2
1	2	3	3	2	3	1	1	1	1
2	2	2	3	2	3	2	1	2	2
2	2	2	2	2	2	2	2	2	2
2	2	2	2	2	2	2	2	2	1
2	2	2	2	2	2	2	2	2	2
2	2	2	2	2	2	2	2	2	2
2	2	3	1	1	3	1	1	2	3
1	2	3	2	2	3	1	1	1	2
1	2	2	3	2	2	1	2	1	1
2	1	2	3	2	2	1	1	1	1
2	2	2	2	2	2	2	2	2	2
3	2	3	2	3	2	3	2	2	2
2	2	2	2	2	2	2	2	2	2
2	2	2	2	2	2	2	2	2	2
2	2	2	2	2	1	1	1	2	2
2	1	1	2	1	2	1	1	1	1
3	3	2	2	2	3	2	2	2	3
3	3	2	2	2	3	2	2	2	2
2	1	1	3	2	2	1	2	2	2
2	2	2	2	2	2	2	2	2	2
2	2	2	2	2	2	2	2	2	2
1	2	2	1	1	3	1	1	2	1
2	2	2	2	2	2	1	1	1	2
2	2	2	2	2	2	2	2	2	2
2	2	2	2	2	2	2	2	2	2
2	2	2	2	2	2	2	2	2	2
2	2	2	2	2	2	2	2	2	2
2	2	2	2	2	2	2	2	1	1
1	2	1	1	1	2	1	1	1	1
2	2	2	2	2	2	2	2	1	1
1	2	2	2	2	2	1	1	2	2
1	2	2	2	1	3	1	2	1	1
2	2	2	2	2	2	2	2	2	2

3	3	3	2	2	2	1	2	1	1
2	3	3	1	2	2	3	1	1	2
2	2	2	2	2	2	2	2	2	2
2	2	2	2	2	2	2	2	2	2
1	2	2	2	2	2	2	2	2	2
3	3	2	2	1	2	1	2	2	2
9	9	9	9	9	9	9	9	9	9
3	2	2	1	2	2	2	2	2	2
2	2	2	2	2	2	2	2	2	2
1	2	1	2	3	2	2	3	1	2
1	2	1	2	1	2	1	2	1	2
3	3	3	3	1	3	3	1	1	1
2	2	2	2	2	2	2	2	2	1
2	2	2	2	2	2	2	2	2	2
2	2	2	2	2	2	2	2	2	2
1	2	1	1	1	1	1	1	1	1
3	3	3	3	1	3	1	1	3	3
2	2	2	2	2	2	2	2	2	2
2	2	2	2	2	2	2	2	2	2
2	2	2	2	2	2	2	2	2	2
1	2	2	1	1	2	1	2	2	2
2	2	2	2	2	2	2	2	1	1
9	9	9	9	9	9	9	9	9	9
2	2	2	2	2	2	2	2	2	2
1	2	3	3	1	3	1	3	2	1
9	9	9	9	9	9	9	9	9	9
2	2	1	2	1	2	1	1	2	2
9	9	9	9	9	9	9	9	9	9
2	2	2	2	2	2	2	2	1	2
1	1	2	2	1	2	1	1	1	2
9	9	9	9	9	9	9	9	9	9
9	9	9	9	9	9	9	9	9	9
2	2	2	2	2	2	2	2	2	2
2	1	1	1	2	2	1	1	1	1
1	9	1	1	2	9	9	9	9	9
2	1	2	1	1	2	1	2	1	1
3	3	3	3	3	3	3	3	3	3
2	2	2	2	1	2	2	2	2	1
9	9	9	9	9	9	9	9	9	9
1	2	2	3	1	3	1	1	1	1
2	3	2	1	2	2	2	2	2	1
1	3	3	3	2	1	1	1	2	1
2	1	3	2	1	2	3	2	1	2
3	3	3	3	3	3	3	3	2	2
9	9	9	9	9	9	9	9	9	9
1	1	3	3	2	3	1	1	1	1
1	1	3	3	2	3	1	1	1	1

9	9	9	9	9	9	9	9	9	9
1	1	3	3	2	3	1	1	1	1
1	1	3	3	2	3	1	1	1	1
1	1	3	3	2	3	1	1	1	1
3	1	3	3	2	3	1	1	1	1
1	1	3	3	2	3	1	1	1	1
1	1	3	3	2	3	1	1	1	1
1	1	3	3	2	3	1	1	1	1
1	1	3	3	2	3	1	1	1	1
1	1	3	3	2	3	1	1	1	1
3	3	2	2	2	2	2	2	1	1
1	2	3	2	1	1	1	1	1	1
1	1	3	3	1	3	1	1	1	1
1	1	3	3	2	3	3	1	1	1
1	1	3	3	2	3	1	1	1	1
3	1	3	3	2	3	1	1	1	1
1	1	2	3	2	3	2	1	1	1
1	3	3	3	3	3	1	1	1	1
1	1	3	3	2	3	1	1	1	1
1	1	3	3	2	3	1	1	1	1
1	1	3	3	1	3	1	1	1	1
1	1	3	3	2	3	1	1	1	1
1	1	3	3	2	3	1	1	1	1
1	2	3	3	1	3	1	1	1	1
1	1	3	3	2	3	1	1	1	1
1	1	3	3	2	3	1	1	1	1
1	1	3	3	2	3	1	1	1	1
1	1	3	3	2	3	2	1	1	1
1	1	3	3	2	1	1	1	1	1
1	1	3	3	1	3	1	1	1	1
1	1	3	3	2	3	2	1	1	1
1	1	3	3	1	3	1	1	1	1
1	1	3	3	2	3	1	1	1	1
3	1	3	3	2	3	1	1	1	1
1	1	3	3	3	3	1	1	1	1
1	1	3	3	2	3	1	1	1	1
1	1	3	3	2	3	1	1	1	1
1	1	3	3	2	3	1	1	1	1
1	1	3	3	2	3	1	1	1	1
1	1	3	3	2	3	1	1	1	1
1	1	3	3	2	3	1	1	1	1
2	2	2	2	2	2	2	2	2	2
1	2	3	2	1	3	1	1	1	1
2	2	2	2	2	2	2	2	2	2
9	9	9	9	9	9	9	9	2	9
2	2	2	2	2	2	2	2	2	2
9	9	9	9	9	9	9	9	2	9

2	2	2	2	2	2	2	2	2	2
1	2	3	2	3	3	1	1	1	1
1	2	3	2	3	3	1	1	1	1
1	1	3	3	1	2	1	1	1	1
2	2	2	2	2	2	2	2	2	2
1	3	2	1	1	3	2	2	2	2
2	2	2	2	2	2	2	2	2	2
3	2	2	2	2	1	2	2	2	3
2	2	2	3	2	3	1	1	1	1
2	2	2	2	2	2	2	2	2	2
1	2	3	2	3	3	1	1	1	1
9	1	9	9	9	9	9	9	9	9
1	1	3	3	9	9	1	9	1	1
2	2	2	2	2	2	2	2	2	2
9	9	1	9	9	9	9	9	1	9
1	9	9	9	9	9	9	9	1	9
9	9	9	9	9	1	9	9	9	9
9	9	9	9	9	9	9	9	9	9
9	9	9	9	9	9	1	9	9	9
3	2	1	2	1	2	1	2	1	1
2	9	1	1	9	1	9	9	1	1
9	1	9	9	9	9	9	9	1	1
2	3	3	3	1	3	2	2	1	1
9	9	9	9	9	9	9	9	9	9
1	9	9	9	9	9	9	9	1	9
9	9	9	9	9	9	9	9	9	9
9	9	9	9	9	9	9	9	9	9
9	9	9	9	9	9	9	9	9	9
1	2	3	1	2	3	1	1	2	1
9	1	9	9	9	9	9	9	9	9
9	9	9	9	9	9	9	9	1	9
2	2	2	2	2	2	2	1	2	2
2	2	2	2	2	2	2	2	2	2
1	1	3	3	1	3	1	1	1	1
1	1	2	1	1	2	2	1	1	1
1	2	2	2	1	2	2	1	2	2
2	2	2	3	1	3	2	1	1	2
3	2	9	3	3	3	1	1	1	1
2	2	2	2	2	2	2	2	2	2
1	2	2	3	1	3	1	1	1	2
9	9	9	9	9	9	9	9	9	9
2	2	3	2	1	3	1	1	1	1
2	2	2	2	2	2	2	2	2	2
1	1	3	3	1	3	1	1	1	1
2	2	2	2	2	2	2	2	2	2
2	2	2	2	2	2	2	2	2	2
1	2	3	2	2	3	1	1	1	1

2	2	3	2	1	3	1	2	1	3
1	1	3	2	2	3	1	1	1	1
3	3	3	3	1	3	1	2	2	2
2	2	1	1	2	3	1	1	1	1
3	1	3	1	2	2	1	2	1	1
2	2	2	2	2	2	2	2	2	2
3	2	2	3	2	2	1	1	1	1
2	2	2	2	2	2	2	2	2	2
2	2	2	2	2	2	2	2	2	2
2	2	2	2	2	2	2	2	2	2
2	2	2	2	2	2	2	3	2	2
1	2	3	2	2	2	1	2	1	1
2	2	2	2	2	2	2	2	2	2
2	2	2	2	2	2	2	2	2	2
1	2	3	3	1	2	1	2	2	2
2	2	2	2	2	2	2	2	2	2
2	2	2	2	2	2	2	2	2	2
2	2	2	2	2	2	2	2	2	2
2	2	2	2	2	2	2	2	1	1
1	2	3	1	1	3	1	2	1	2
1	2	2	2	2	2	2	2	2	2
1	2	2	3	2	2	2	2	1	1
1	3	2	3	1	3	2	2	1	1
2	2	2	3	1	2	1	2	1	1
2	2	2	2	2	2	2	2	2	2
1	2	2	2	2	2	2	1	2	2
1	1	3	1	1	3	9	1	1	3
1	1	3	1	3	1	3	1	1	3
1	2	3	3	1	3	1	1	1	3
3	3	3	3	3	3	3	3	2	3
2	2	2	2	2	2	2	2	2	2
2	2	2	2	2	2	2	2	9	2
2	2	2	2	2	2	2	2	2	2
2	3	1	3	2	2	3	2	2	2
2	2	2	2	2	2	2	2	2	2
9	9	9	9	9	9	9	9	2	9
2	2	2	2	2	2	2	2	2	2
3	1	2	2	1	3	1	2	1	1
2	2	2	2	2	2	2	2	2	1
1	2	3	3	1	3	2	1	1	1
9	9	9	9	9	9	1	9	2	2
2	2	2	3	1	2	1	1	3	1
2	2	2	3	1	2	1	1	3	1
2	1	3	3	3	2	3	1	1	1
1	2	3	3	1	3	1	1	1	1
2	2	2	2	2	2	2	2	2	2
9	9	9	9	9	9	9	9	2	9

9	9	9	9	9	9	9	9	2	9
9	9	9	1	9	9	9	9	9	9
9	9	9	9	9	9	9	9	9	9
9	9	9	9	9	9	9	9	9	9
9	9	9	9	9	9	9	9	9	9
9	9	9	9	9	9	9	9	1	1
9	2	9	9	9	9	9	9	9	9
9	9	9	9	9	9	9	9	9	1
9	9	9	9	9	9	9	9	9	1
9	9	9	9	9	9	9	9	2	9
9	9	9	9	9	9	9	9	1	9
9	9	9	9	9	9	9	9	2	9
9	9	9	9	9	9	9	9	9	9
1	9	9	9	9	9	9	9	9	1
9	9	9	9	9	9	9	1	9	9
9	9	9	9	9	9	9	1	9	9
9	9	9	9	9	9	9	9	1	9
1	3	3	3	3	3	1	1	1	1
2	2	2	2	2	2	2	2	2	2
9	9	9	9	9	9	9	9	1	9
1	1	1	1	1	1	1	1	1	1
1	2	2	1	3	2	2	3	1	2
1	1	2	2	2	1	1	2	2	3
1	3	2	1	1	1	1	1	1	1
3	2	3	2	3	2	3	2	2	3
2	2	2	1	3	3	3	2	3	1
2	2	2	2	2	2	2	2	2	2
2	2	3	1	2	1	2	3	2	3
2	2	2	2	2	2	2	2	2	2
2	2	2	2	2	2	2	2	2	2
1	1	2	2	2	3	3	3	1	1
2	2	2	2	2	2	2	2	1	1
2	2	2	2	2	2	2	2	2	2
1	3	3	3	2	2	2	2	1	1
2	3	1	2	3	1	2	3	1	2
2	2	3	2	3	2	3	2	1	2
2	2	2	2	2	2	2	2	2	2
2	3	2	3	2	1	1	1	9	9
1	1	1	1	1	1	1	1	1	3
9	9	9	9	9	9	9	9	9	9
2	2	2	2	2	2	2	2	2	2
1	1	1	1	1	1	1	1	3	3
9	9	9	9	9	9	9	9	9	9
1	1	1	3	2	1	1	1	1	1
2	2	2	2	2	2	2	2	2	2
2	2	2	2	2	2	2	2	2	2
2	2	2	3	3	2	2	2	2	2

2	2	2	2	2	2	2	2	1	1
9	9	9	9	9	9	9	9	9	9
3	1	1	3	1	3	1	1	1	3
9	9	9	9	9	9	9	9	9	9
1	2	2	2	2	2	2	2	2	2
1	2	2	2	2	2	2	2	2	2
9	9	9	9	9	9	9	9	9	9
2	2	2	2	2	2	2	2	1	1
3	1	2	1	1	1	1	3	1	1
1	1	2	1	1	3	3	1	2	3
1	1	1	1	1	1	1	1	1	1
1	2	2	2	1	1	1	1	2	2
3	2	2	3	3	2	2	2	2	3
9	9	9	9	9	9	9	9	9	9
9	9	9	9	9	9	9	9	9	9
9	9	9	9	9	9	9	9	9	9
1	1	3	3	2	3	1	1	1	1
9	9	9	9	9	9	9	9	9	9
2	9	3	3	1	3	2	2	2	3
2	1	2	3	1	3	1	2	1	1
3	3	3	2	2	2	1	2	1	1
3	2	2	3	3	3	2	2	2	3
1	1	1	1	1	1	1	1	2	2
3	2	1	3	2	3	1	3	1	2
2	2	2	2	2	2	2	2	2	1
3	1	2	1	2	1	2	1	1	3
3	3	1	2	3	3	3	3	3	9
3	3	1	1	1	3	1	1	1	3
2	2	2	2	2	1	1	2	2	1
2	2	2	3	3	3	2	2	1	1
9	9	9	9	9	9	9	9	9	9
9	1	9	9	9	9	9	9	9	9
1	1	2	2	2	2	2	2	2	1
1	1	1	2	2	9	2	2	1	2
1	1	9	9	2	9	2	9	1	3
2	2	2	1	2	2	1	2	1	1
2	3	2	9	1	3	9	1	1	1
9	9	9	9	9	9	9	9	9	9
9	9	9	9	9	9	9	9	9	9
9	9	9	9	9	9	9	9	9	9
9	9	9	9	9	9	9	9	9	9
3	2	2	1	2	1	2	2	2	1
3	1	2	3	1	2	3	3	2	1
9	9	9	9	9	9	9	9	9	9
3	1	2	1	3	1	2	3	2	2
3	2	2	3	1	2	1	2	1	2
3	3	1	1	3	1	3	3	1	1

3	3	3	3	3	3	3	3	3	3
1	2	2	2	2	2	2	2	2	1
3	3	2	2	3	3	2	2	3	3
2	1	3	2	1	2	2	2	1	2
2	2	2	2	2	2	2	2	2	2
2	9	1	2	3	9	3	2	1	9
9	9	9	9	9	9	9	9	9	9
3	3	9	2	2	3	1	2	3	3
3	3	3	3	3	3	3	3	3	3
1	2	3	3	1	3	1	2	1	1
3	3	3	3	3	3	3	3	3	3
1	1	3	1	1	3	1	1	1	1
3	2	2	3	1	2	1	2	1	2
3	2	2	3	1	2	1	2	1	2
3	2	2	3	1	2	1	2	1	2
3	2	2	3	1	2	1	2	2	1
3	2	2	3	1	2	1	2	1	2
3	2	2	3	1	2	1	2	1	2
3	2	2	3	1	2	1	2	1	2
2	2	1	3	1	2	1	1	1	1
3	2	1	2	1	2	3	3	1	2
2	2	3	2	3	2	9	2	2	3
2	2	2	3	2	3	2	3	2	3
2	2	2	2	2	3	3	3	2	2
3	3	3	3	3	3	3	3	3	3
3	2	2	3	3	2	1	2	1	2
9	9	1	9	9	9	9	9	9	9
1	1	3	3	1	3	3	1	9	9
9	9	9	9	9	9	9	9	9	9
2	2	2	2	2	2	2	2	2	2
2	3	3	3	3	1	1	1	2	1
3	2	3	2	3	2	2	3	2	3
1	3	2	1	3	1	2	1	2	1
9	9	1	9	9	9	9	9	9	9
3	3	3	3	3	3	3	3	3	3
2	2	2	3	3	3	1	2	1	1
9	9	2	9	1	3	9	2	2	9
2	1	2	2	2	1	9	1	2	1
3	2	2	3	1	2	1	2	2	3
2	1	2	3	1	1	1	1	1	1
1	1	2	2	3	1	1	2	2	3
2	1	2	2	9	2	3	2	2	2
2	1	1	3	2	3	1	2	2	1
3	3	1	1	1	3	1	1	1	3
1	2	2	1	1	1	1	2	2	1
1	1	1	1	1	1	1	1	1	1
2	2	2	2	2	2	2	3	2	3

2	2	2	2	2	2	2	2	2	2
9	9	9	9	9	9	9	9	2	9
9	9	9	9	9	9	9	9	9	9
1	1	3	1	1	3	1	3	1	3
9	1	2	1	2	3	1	2	2	2
1	2	2	2	2	2	2	2	2	2
9	1	1	1	1	1	1	1	1	1
1	2	2	2	2	2	2	2	2	2
1	2	2	2	2	2	2	2	2	2
1	2	3	2	1	2	3	3	2	2
3	2	2	3	2	9	9	2	9	9
2	2	1	2	1	3	1	1	1	1
9	9	9	9	9	9	9	9	9	9
3	1	1	3	1	3	1	1	1	1
1	1	1	2	1	3	1	1	1	1
9	9	9	9	9	9	9	9	9	9
3	3	3	3	3	3	3	3	3	3
9	9	9	9	9	9	9	9	9	9
9	9	9	9	9	9	9	9	9	9
3	1	2	3	1	2	3	1	1	2
1	1	1	1	1	1	1	3	1	1
2	2	2	2	2	2	2	2	2	2
9	9	9	9	9	9	9	9	9	9
2	2	2	2	2	2	2	2	2	2
3	3	3	3	3	3	3	3	3	3
3	2	1	2	1	3	1	1	1	1
3	2	1	2	1	3	1	1	1	1
1	2	3	3	1	3	1	3	1	1
1	1	1	1	1	1	1	1	1	1
2	2	2	2	2	2	2	2	2	2
3	2	1	1	1	3	2	2	3	2
2	3	2	2	2	2	3	1	1	1
1	3	2	2	1	2	3	3	1	3
3	2	1	2	1	3	1	1	1	1
1	2	3	2	2	1	1	2	1	1
2	2	2	2	2	2	2	2	2	2
2	2	2	1	1	1	2	2	2	2
9	9	9	9	9	9	9	9	9	9
9	9	2	9	3	9	9	1	9	9
9	1	1	1	2	1	2	1	1	1
2	2	2	2	2	2	2	2	2	2
2	2	2	2	2	2	2	2	2	2
2	2	2	2	2	2	2	2	2	9
2	2	2	2	2	2	2	2	2	2
2	2	2	2	2	2	2	2	2	2
2	2	2	2	2	2	2	2	2	2
9	9	9	9	9	9	9	9	9	9

9	9	9	9	9	9	9	9	9	9
2	2	2	2	2	2	2	2	2	2
1	2	2	3	1	3	1	1	1	1
1	1	2	2	2	3	1	1	1	1
9	9	9	9	9	9	9	9	9	1
2	2	3	2	1	2	3	2	1	2
9	9	9	9	9	9	9	9	9	9
3	3	3	3	3	3	3	3	1	1
1	2	1	1	2	2	1	2	1	2
9	9	9	9	9	9	9	9	9	9
2	2	2	2	2	2	2	2	2	2
2	1	2	2	2	3	2	1	1	1
2	1	2	2	2	3	2	1	1	1
2	2	2	2	2	2	2	2	2	2
3	3	3	2	2	2	1	1	1	1
2	2	2	2	2	2	2	2	2	2
2	2	2	2	2	2	2	2	2	2
2	2	2	2	2	2	2	2	3	3
1	3	2	2	1	3	2	1	1	1
1	1	3	3	1	1	2	2	1	1
9	9	9	9	9	9	9	9	9	9
3	3	3	3	3	3	3	3	2	2
2	2	2	2	2	2	2	9	2	2
2	2	2	2	2	2	2	2	1	1
2	2	2	2	2	2	2	2	2	2
3	3	3	3	3	3	3	3	2	2
9	9	9	9	9	9	2	9	9	1
2	2	2	2	2	2	2	2	2	2
2	2	2	2	2	2	2	2	2	2
2	3	2	1	2	3	2	2	3	2
2	2	2	2	2	2	2	2	2	2
2	2	2	2	2	2	2	2	2	2
1	3	1	1	1	3	3	1	1	1
2	2	2	2	2	2	2	2	2	2
2	2	2	2	2	2	2	2	2	2
2	2	3	3	1	3	1	1	1	1
2	2	2	2	2	2	2	2	2	2
3	1	2	2	2	1	2	1	1	2
2	2	2	2	2	2	2	2	2	2
2	2	2	2	2	2	2	2	2	2
2	2	2	2	2	2	2	2	2	2
2	2	2	2	2	2	2	2	2	2
1	3	3	3	3	3	1	1	1	1
2	2	2	2	2	2	2	2	2	2
2	2	2	2	2	2	2	2	2	2
3	3	3	3	3	3	3	3	3	3
2	2	2	2	2	2	2	2	2	2

2	2	2	2	2	2	2	2	2	2
2	2	2	2	2	2	2	2	2	2
2	2	2	2	2	2	2	2	2	2
2	2	2	2	2	2	2	2	2	2
2	2	2	2	2	2	2	2	2	1
3	1	3	1	2	3	1	1	1	1
2	2	2	2	2	2	2	2	2	2
1	3	2	1	3	2	2	2	2	2
1	2	3	3	1	3	1	1	3	1
2	2	2	2	2	2	2	2	2	2
1	1	3	3	1	3	1	1	1	3
9	9	9	9	9	9	9	9	9	2
9	2	9	9	9	9	9	9	2	9
1	2	2	2	1	3	1	2	2	1
1	1	3	3	3	3	3	3	2	2
2	2	2	2	3	3	3	3	2	2
3	2	2	2	2	3	3	2	2	2
1	9	2	2	2	2	2	2	1	1
9	9	9	9	9	9	9	9	9	9
3	1	2	2	2	3	2	3	3	2
9	9	9	9	9	9	9	9	9	9
2	3	2	1	2	3	2	1	1	2
2	2	2	2	2	2	2	2	1	1
2	2	2	2	2	2	2	2	1	1
3	3	3	3	3	3	3	1	1	1
2	2	3	3	1	3	1	1	1	1
2	2	2	2	2	2	2	2	2	2
1	2	2	3	1	3	1	2	2	1
2	2	2	2	2	2	1	1	2	2
3	2	2	3	2	3	2	3	1	2
1	1	1	1	1	1	1	1	1	1
1	1	1	1	1	1	1	1	3	3
3	3	3	3	3	3	3	3	1	1
1	1	1	1	1	1	1	1	1	1
3	3	3	3	3	3	3	3	3	3
3	3	3	1	1	1	1	3	1	3
9	9	9	9	9	9	9	9	9	9
1	1	1	1	1	1	1	1	1	1
2	2	2	2	2	2	2	2	2	2
2	2	2	2	2	2	2	2	2	2
1	2	2	1	1	3	2	1	2	2
9	9	1	9	9	9	9	9	1	1
3	2	1	3	2	2	2	1	1	2
2	2	2	2	2	2	2	2	2	2
2	2	2	2	2	2	2	2	2	2
2	3	1	2	2	1	3	2	3	3
3	2	2	2	2	1	2	2	1	1

3	1	1	2	1	2	3	1	1	3
3	3	3	3	3	3	3	3	3	3
3	2	1	3	1	2	3	1	3	2
9	9	9	9	9	9	9	9	9	2
9	9	9	9	9	9	9	9	9	9
2	1	2	3	1	2	1	3	3	3
2	2	2	2	2	2	2	2	2	2
3	3	3	3	3	3	3	3	3	1
3	3	3	3	3	3	3	3	2	2
2	2	2	2	2	2	2	2	2	2
9	9	9	9	9	9	9	1	1	9
2	2	3	9	2	3	9	2	2	2
2	2	2	2	2	2	2	2	2	2
2	2	2	2	1	3	3	3	2	2
2	2	2	2	2	2	2	2	2	2
3	3	3	2	2	1	1	1	3	2
1	2	3	2	2	3	3	2	2	3
9	9	9	9	9	9	9	9	1	9
9	9	9	9	9	9	9	2	9	9
2	2	2	2	2	2	2	2	2	2
2	2	2	2	2	2	2	2	2	2
2	2	2	2	2	2	2	2	2	2
9	9	9	9	9	9	9	9	9	9
9	9	9	9	9	9	9	9	9	9
2	3	1	2	3	1	2	3	1	2
1	1	1	1	1	3	1	1	1	1
2	2	2	1	1	1	1	1	2	3
1	1	1	1	1	3	1	1	1	1
3	3	3	1	1	3	3	1	3	1
9	1	2	3	1	2	3	1	2	1
1	2	2	1	1	3	2	1	1	2
3	1	2	2	1	2	3	1	2	3
1	2	2	3	1	3	1	2	1	1
3	3	3	3	3	3	3	3	3	3
3	3	3	3	3	3	3	3	3	3
3	3	3	2	2	3	2	3	1	1
1	3	2	2	2	2	1	2	1	2
3	3	3	3	3	3	3	3	3	3
9	3	1	3	1	9	1	9	1	2
3	3	3	3	3	3	3	3	3	3
3	1	3	1	1	3	1	3	9	1
1	3	1	1	1	2	1	3	1	1
1	1	1	1	1	1	1	1	1	3
2	2	2	2	2	2	2	2	2	2
2	2	1	2	2	2	2	2	2	1
1	1	1	1	1	1	1	1	2	2
1	1	2	2	2	2	1	1	2	1

2	2	2	2	2	2	2	2	2	2
3	2	9	2	9	3	2	3	3	2
1	2	3	1	9	1	9	1	1	2
1	1	3	1	1	1	1	1	3	3
2	2	2	2	2	2	2	2	2	2
1	2	2	2	2	3	2	2	1	3
3	3	2	2	2	2	2	2	2	2
2	2	2	2	2	2	2	2	2	2
2	2	2	2	2	2	2	2	2	2
2	2	2	2	2	2	2	2	2	2
9	9	9	3	9	9	9	9	9	1
2	2	2	2	2	2	2	2	2	2
3	1	1	3	1	3	1	3	1	1
1	2	3	1	2	2	3	3	2	1
1	1	1	1	1	1	1	1	2	2
3	3	2	2	2	2	2	2	1	1
1	2	3	3	1	3	1	1	1	1
2	2	2	2	1	2	1	1	1	1
3	2	2	1	1	3	1	1	1	1
9	9	9	9	9	9	9	9	9	9
1	1	1	3	1	1	1	1	1	1
1	1	1	1	3	2	2	3	1	1
3	3	3	3	3	3	1	1	3	3
3	3	3	3	3	3	3	3	1	1
3	3	3	3	3	3	3	3	3	3
2	2	2	2	2	2	2	2	1	1
1	1	2	1	1	2	1	1	1	1
2	3	1	2	1	2	3	1	1	2
2	1	1	2	3	1	2	2	3	1
2	1	2	1	1	1	1	1	1	1
2	2	2	2	2	2	2	2	2	2
9	9	9	9	9	9	9	9	9	9
2	1	2	2	2	2	1	3	2	3
9	9	9	9	9	9	9	9	9	9
2	2	3	2	1	2	2	2	1	2
2	2	2	2	2	2	2	2	2	2
2	2	2	2	2	2	2	2	2	2
2	2	2	2	2	2	2	2	2	2
2	2	2	2	2	3	3	3	2	2
1	1	2	2	3	1	2	3	1	2
2	2	1	2	1	2	3	1	1	2
2	2	2	2	2	2	2	2	2	2
3	3	2	2	1	2	2	3	1	2
9	9	9	9	9	9	9	9	2	9
3	3	3	3	3	3	3	3	3	3
2	2	2	2	2	2	2	2	2	2
2	2	2	2	1	3	1	1	1	1

9	9	9	9	9	9	9	9	9	9
2	2	1	2	2	9	2	3	2	2
2	3	3	3	3	3	3	3	1	2
1	2	3	2	1	3	1	1	1	1
3	3	3	3	3	3	3	3	2	2
2	2	2	2	9	2	3	2	2	2
2	2	2	9	9	9	9	9	9	9
1	1	3	3	2	3	2	2	2	3
2	2	2	2	2	2	2	2	2	2
9	9	9	9	9	9	9	9	9	9
9	9	9	9	9	9	9	9	9	9
9	9	9	9	9	9	9	9	9	9
9	9	9	9	9	9	9	9	9	2
9	9	9	9	9	9	9	9	3	9
9	9	9	9	9	9	9	9	9	9
2	2	2	2	1	3	1	1	1	2
2	1	1	2	2	1	2	2	1	9
2	2	2	2	2	2	2	2	2	2
3	2	2	2	2	1	3	1	1	2
2	2	2	1	1	2	1	1	1	1
2	2	2	2	2	2	2	2	1	3
1	2	2	2	3	2	2	2	2	3
3	2	1	1	1	3	1	2	1	1
3	3	2	3	2	3	2	1	1	1
2	2	2	2	2	2	2	2	2	2
2	1	2	1	2	3	1	3	2	3
3	9	1	3	9	1	3	3	2	2
1	1	1	1	1	1	1	1	1	1
2	1	2	1	2	3	1	3	2	3
2	2	2	2	1	3	1	1	1	2
2	2	2	1	1	2	1	1	1	1
9	9	9	9	9	9	9	9	9	2
1	1	3	1	2	2	1	3	1	3
2	2	2	2	2	2	2	2	2	2
2	2	2	2	2	2	2	2	2	2
2	2	2	2	2	2	2	2	2	2
2	2	2	2	2	2	2	2	2	2
9	9	9	9	9	9	9	9	2	9
2	2	2	2	2	2	2	2	2	2
2	2	2	2	2	2	2	2	2	2
2	2	2	2	2	2	2	2	2	2
2	2	2	2	2	2	2	2	2	2
2	2	2	2	2	2	2	2	2	2
2	2	2	1	3	3	3	3	1	1
2	2	2	2	2	2	2	2	1	2
2	2	2	2	2	2	2	2	2	2
9	9	9	9	9	9	9	9	9	9

2	2	2	2	2	2	2	2	2	2
2	2	2	2	2	2	2	2	2	2
9	9	1	9	9	9	9	9	9	9
9	9	9	9	9	9	9	9	9	9
1	1	2	1	1	1	2	2	1	2
9	9	9	9	9	9	9	9	1	9
2	2	3	3	1	2	3	1	1	1
2	3	2	3	3	2	1	1	3	2
3	3	2	1	1	2	3	2	9	9
2	2	3	3	2	2	1	1	1	1
9	9	9	9	9	9	9	9	9	9
1	2	1	2	2	1	1	3	2	1
3	3	2	2	2	2	2	2	2	2
2	2	2	2	2	2	2	2	2	2
2	2	2	3	2	2	1	1	2	1
3	3	3	2	3	1	2	3	3	3
2	2	2	2	2	2	2	2	2	2
2	2	2	2	2	2	2	2	2	2
1	2	2	1	3	3	1	2	1	2
1	2	3	3	3	3	3	3	1	1
2	2	2	1	3	3	2	2	3	3
2	2	2	2	2	2	2	2	2	2
1	1	1	2	2	2	1	1	1	3
2	2	2	2	2	2	2	2	3	3
2	2	2	2	2	2	2	2	2	2
2	2	2	2	2	2	2	2	1	1
2	2	2	2	2	2	2	2	2	2
2	2	2	2	2	2	2	2	2	2
2	2	2	2	2	2	2	2	2	2
9	9	9	9	9	9	9	9	9	9
1	1	9	1	9	1	1	1	1	3
2	2	2	2	2	2	2	2	2	2
1	1	1	1	1	1	1	1	9	9
9	9	9	9	9	9	9	9	9	9
3	3	2	1	3	2	1	1	1	2
9	9	9	9	9	9	9	9	9	9
9	9	9	9	9	9	9	9	9	9
2	2	2	2	2	2	2	2	2	2
3	3	3	3	3	3	1	3	9	1
2	1	2	1	1	3	3	2	1	1
1	1	1	1	1	1	1	1	1	1
1	2	3	2	1	1	2	3	1	2
9	9	9	9	9	9	9	9	1	9
2	1	2	2	3	1	2	3	1	2
1	2	2	2	1	3	2	1	2	2
9	9	9	9	9	9	9	9	9	9
2	2	3	1	2	2	3	3	3	3

2	2	1	2	2	2	1	2	1	2
9	9	9	9	9	9	9	9	9	9
9	9	9	9	9	9	9	9	9	9
2	2	2	2	2	2	2	2	2	2
1	2	1	1	1	3	2	1	1	1
2	2	1	2	2	2	1	2	1	2
3	1	2	1	2	3	1	2	1	3
2	2	2	2	2	2	2	2	1	3
2	2	2	2	2	2	2	2	2	2
1	1	3	1	2	1	1	1	1	1
9	9	9	9	9	9	9	9	9	9
9	9	9	9	9	9	9	9	9	9
9	9	9	9	9	9	9	9	1	9
2	2	2	2	2	2	2	2	1	1
1	2	3	2	3	2	3	3	2	1
9	9	9	9	9	9	9	9	9	9
9	9	9	9	9	9	9	9	9	9
9	9	9	9	9	9	9	9	9	9
1	1	2	2	1	2	1	1	1	1
2	2	2	2	2	2	2	2	1	1
9	9	9	9	9	9	9	9	9	9
1	1	1	3	3	2	2	1	1	3
3	3	3	3	3	3	3	3	3	3
9	9	9	9	9	9	9	9	9	9
2	2	2	2	2	2	2	2	2	2
2	2	2	2	2	2	2	2	2	2
9	9	9	9	9	9	9	9	9	9
9	9	9	9	9	9	9	9	9	9
2	2	2	2	2	2	2	2	2	2
2	2	2	2	2	2	2	2	2	2
1	1	2	1	1	2	3	1	1	1
9	9	9	9	9	9	9	9	9	9
9	9	9	9	9	9	9	9	9	9
2	2	2	2	3	3	3	3	1	1
1	2	9	2	1	2	1	2	2	2
2	2	2	2	2	2	2	2	2	2
2	2	2	1	1	1	2	2	2	2
2	2	2	2	2	2	2	2	2	2
2	1	2	1	2	1	2	1	3	2
1	3	1	2	3	1	2	3	1	2
2	3	3	1	2	2	2	3	2	3
9	9	9	9	9	9	9	9	9	9
9	9	9	9	9	9	9	9	9	9
9	9	1	9	9	9	9	9	9	9
9	9	9	9	9	9	9	9	9	9
9	9	9	9	9	9	9	9	9	9
9	9	9	9	9	9	9	9	9	9

2	2	2	2	2	2	2	2	2	2
2	3	2	3	2	3	2	3	2	3
2	2	2	2	2	1	9	1	1	1
2	2	2	2	2	2	2	2	2	2
9	9	9	9	9	9	9	9	9	9
9	9	9	9	9	9	9	9	9	9
9	9	9	9	9	9	9	9	9	9
9	9	9	9	9	9	9	9	9	9
9	9	9	9	9	9	9	9	9	9
9	9	9	9	9	9	9	9	9	9
9	9	9	9	9	9	9	9	9	9
9	9	9	9	9	9	9	9	9	9
9	9	9	9	9	9	9	9	9	9
3	3	1	1	1	1	1	1	1	1
1	1	2	2	2	2	1	1	1	1
2	2	2	2	2	2	2	2	2	2
2	2	2	2	2	2	2	2	2	2
9	9	9	9	9	9	9	9	9	9
9	9	9	9	9	9	9	9	9	9
9	9	9	9	9	9	9	9	9	9
9	9	9	9	2	9	9	9	1	9
9	9	9	9	9	9	9	9	9	9
1	2	2	2	2	2	2	2	2	2
9	9	9	9	9	9	9	9	9	9
1	1	1	1	1	1	1	2	2	2
1	1	1	1	2	2	2	2	1	1
1	1	1	1	1	1	1	1	2	2
2	1	1	1	1	1	1	1	1	1
1	2	2	2	1	1	1	1	2	2
2	2	1	1	1	2	2	1	1	2
1	2	2	2	1	1	1	1	1	1
1	2	2	2	1	1	1	1	2	2
1	1	1	1	1	1	1	1	1	1
9	9	9	9	9	9	9	9	9	9
1	2	1	1	1	1	1	1	2	2
1	1	2	2	2	1	1	1	1	1
2	2	2	2	2	2	2	2	1	1
9	9	9	9	9	9	9	9	9	9
2	2	2	2	2	2	2	2	3	3
1	1	3	1	3	1	1	1	3	3
3	3	3	3	3	3	3	3	3	3
9	9	9	9	9	9	9	9	9	9
9	9	9	9	9	9	9	9	9	9
9	9	9	9	9	9	9	9	9	9
1	9	9	9	9	9	9	9	9	9
2	2	2	2	3	2	1	3	2	3
9	9	9	9	9	9	9	9	9	9

9	9	9	9	9	9	9	9	9	9
9	9	9	9	9	9	9	9	9	9
9	9	9	9	9	9	9	9	9	9
9	9	9	9	9	9	9	9	9	9
9	9	9	9	9	9	9	9	9	9
9	9	9	9	9	9	9	9	9	9
9	9	9	9	9	9	9	9	9	9
9	9	9	9	9	9	9	9	9	9
9	9	9	9	9	9	9	9	9	9
9	9	9	9	9	9	9	9	9	9
2	9	9	9	9	9	9	9	9	9
9	9	9	9	9	9	9	9	9	9
2	2	1	2	2	2	1	1	1	2
1	2	3	2	9	1	1	2	1	1
2	1	1	1	2	2	2	2	1	1
3	2	1	2	3	2	2	1	1	1
2	2	2	2	2	2	2	2	2	2
1	2	2	1	1	1	3	3	3	3
1	2	2	1	1	1	1	2	1	1
9	9	9	9	9	9	9	9	1	9
1	1	1	1	2	2	2	1	1	1
2	1	3	1	1	2	2	1	1	1
2	2	2	2	2	2	2	2	2	2
1	1	1	2	1	1	2	3	1	2
1	2	2	2	1	1	1	1	2	1
3	3	3	3	3	3	1	1	3	3
9	9	2	9	3	2	3	2	1	2
2	2	2	2	2	2	2	2	1	3
9	9	9	9	9	9	9	9	9	1
9	9	9	9	9	9	9	9	3	3
9	9	9	9	9	9	9	9	9	9
1	1	1	1	1	1	1	1	2	2
2	2	2	2	2	2	2	2	2	2
2	1	1	1	1	1	1	2	2	1
1	1	1	1	2	2	1	1	1	2
2	2	2	2	2	2	2	2	2	2
2	2	2	2	2	2	2	2	2	2
2	1	1	1	1	1	1	2	2	1
1	1	2	2	1	2	2	2	2	1
1	2	2	2	1	1	1	2	1	1
2	2	2	2	2	2	2	2	2	2
2	2	1	1	1	2	1	1	1	1
3	3	3	3	1	3	1	1	1	3
1	2	2	2	1	1	1	1	2	1
1	1	2	2	2	1	1	1	1	1
2	2	2	1	1	1	1	1	1	1
2	2	2	2	2	2	2	2	2	2

9	9	9	9	9	9	9	9	9	9
1	3	2	2	2	3	1	1	1	1
9	9	2	9	9	9	9	9	9	9
3	9	9	9	9	9	9	9	9	9
9	9	9	9	9	9	9	9	1	9
9	9	9	9	9	9	9	9	9	9
9	9	9	9	9	9	9	9	9	9
2	2	2	2	2	2	2	2	3	3
9	9	9	9	9	9	9	9	2	9
2	2	2	2	2	2	2	2	2	2
9	9	9	9	9	9	9	9	9	9
2	1	2	1	2	1	2	2	9	9
1	2	2	1	1	1	1	1	1	1
2	2	2	2	2	2	2	2	2	2
1	1	2	2	2	2	2	1	1	1
9	9	9	9	9	9	9	9	9	9
2	2	2	2	2	2	2	2	2	2
3	3	3	3	3	3	3	3	3	3
3	1	2	3	1	2	1	9	9	9
2	2	2	2	2	2	2	2	1	1
2	2	2	2	2	2	2	2	2	2
2	2	3	2	3	2	2	3	3	2
2	2	2	2	2	2	2	2	2	2
2	2	2	2	2	2	2	2	2	2
2	2	2	1	2	2	2	2	2	2
2	2	2	2	2	2	2	2	2	2
3	1	2	2	3	3	1	1	3	3
3	1	1	1	3	3	3	3	1	3
1	1	1	1	1	1	1	1	1	1
2	2	2	1	1	1	1	1	2	2
9	9	9	9	9	9	9	9	9	9
2	2	2	2	2	2	2	2	3	3
9	9	9	9	9	9	9	9	9	9
2	2	2	2	2	2	2	2	2	2
2	2	2	2	2	2	2	2	2	2
2	2	2	1	1	2	1	1	2	2
3	3	3	3	3	3	3	3	3	1
1	2	2	1	1	2	1	1	1	1
1	1	1	1	1	1	1	1	1	1
1	1	1	3	2	2	1	1	2	2
2	2	2	2	2	2	2	2	2	2
2	1	1	2	2	2	2	2	1	1
9	9	9	9	9	9	9	9	9	9
9	9	9	9	9	9	9	9	9	9
9	9	9	9	9	9	9	9	1	9
9	9	9	9	9	9	9	9	9	9
9	9	9	9	9	9	9	9	9	9

9	9	9	9	9	9	9	9	9	9
2	2	2	2	2	2	2	2	2	2
9	9	9	9	9	9	9	9	2	9
1	2	1	1	2	1	2	2	2	1
9	9	9	9	9	9	9	9	9	9
3	3	2	2	1	2	1	3	1	2
3	3	3	3	3	9	2	1	2	2
9	9	9	9	9	9	9	9	9	9
9	9	9	9	9	9	9	9	9	9
9	9	9	9	9	9	9	9	9	9
9	9	9	9	9	9	9	9	9	9
9	9	9	9	9	9	9	9	9	9
1	1	1	2	2	1	2	3	1	2
9	9	9	9	9	9	9	9	9	9
9	9	9	9	9	9	9	9	9	9
1	1	2	3	1	2	3	1	2	1
2	3	2	1	2	3	2	2	1	3
2	2	2	2	1	3	1	2	2	1
1	1	2	2	3	3	3	3	2	3
2	2	1	1	1	2	3	2	1	1
2	1	1	2	2	3	1	2	2	3
1	1	3	3	1	3	1	1	1	3
2	2	1	1	3	1	3	2	1	1
3	1	1	2	1	1	2	2	1	2
2	2	2	2	2	2	2	2	2	2
2	2	2	2	2	2	1	1	1	1
3	3	2	2	2	2	2	2	2	2
1	2	2	2	1	3	1	2	1	2
2	1	1	2	1	2	2	2	2	3
2	1	2	1	2	3	3	3	1	2
3	3	3	1	1	3	1	1	3	3
2	2	2	3	2	2	2	2	1	2
9	9	9	9	9	9	9	9	9	9
3	2	1	3	2	1	1	2	1	2
1	2	2	2	1	3	1	2	1	1
2	2	2	2	2	2	2	2	1	1
1	2	1	2	2	2	2	2	2	2
3	1	2	2	3	1	2	2	1	1
9	9	9	9	9	9	9	1	9	9
2	2	2	2	2	2	2	2	1	2
9	9	9	2	9	9	9	9	9	9
9	9	9	9	9	9	9	9	1	9
2	2	2	3	2	1	2	3	2	3
9	9	9	9	9	9	9	9	1	9
2	1	3	2	1	1	2	1	1	1
1	3	3	1	1	3	3	1	1	1
1	1	1	1	1	1	1	1	2	2

3	3	3	3	3	3	3	3	3	3
9	9	9	9	9	1	9	9	9	9
9	3	3	3	3	3	3	3	2	2
1	3	1	3	1	3	1	1	1	1
1	3	3	3	1	3	1	1	1	1
3	1	3	1	1	3	1	1	1	1
3	1	3	2	3	1	2	1	1	2
2	2	2	2	1	3	1	3	1	2
2	2	2	2	2	2	2	2	2	2
2	3	1	2	3	1	2	3	2	2
1	2	3	1	3	2	1	1	1	2
3	3	2	1	1	3	2	2	2	3
1	2	3	1	3	2	3	1	3	1
2	2	2	2	2	2	2	2	2	2
2	1	3	2	1	2	1	2	1	1
1	2	3	2	3	1	2	1	1	2
2	2	2	2	2	2	2	2	2	2
2	2	2	1	1	3	1	3	1	3
2	2	2	2	2	2	2	2	2	2
2	2	2	2	2	2	2	2	2	2
2	2	2	2	2	2	2	2	2	2
2	1	1	1	1	3	3	3	1	1
2	2	2	2	2	2	2	2	1	2
9	9	9	9	9	9	9	9	9	9
2	2	2	2	2	2	2	2	2	2
2	1	1	1	1	1	2	1	1	2
3	3	2	1	2	3	2	1	2	2
2	1	3	2	2	1	1	2	3	3
1	3	2	1	2	1	1	3	1	1
9	9	9	9	9	9	9	9	9	9
3	2	1	3	2	3	1	2	2	3
1	3	1	2	3	2	1	2	2	3
2	2	3	3	2	1	3	2	3	2
2	2	2	2	2	3	2	2	2	2
2	3	1	2	1	3	1	1	3	3
2	2	2	2	2	2	2	2	2	2
2	2	2	2	2	2	2	2	3	3
9	9	9	9	9	9	9	9	9	9
2	3	3	1	2	2	1	3	1	2
2	2	3	2	2	2	2	2	2	3
9	9	9	9	9	9	9	9	9	9
9	9	9	9	9	9	9	9	9	9
2	2	2	2	2	2	2	2	2	2
9	9	9	9	9	9	9	9	9	9
2	2	2	2	2	2	2	2	2	2
2	2	2	2	2	2	2	2	1	1
9	9	9	9	9	9	9	9	9	9

2	1	2	2	1	2	2	3	2	2
9	9	9	9	9	9	9	9	9	9
2	2	2	2	3	2	1	2	1	3
9	9	9	9	9	9	9	9	9	9
9	9	9	9	9	9	9	9	9	9
3	3	3	3	3	3	3	3	3	3
2	2	2	2	2	2	2	2	2	2
2	2	2	2	2	2	2	2	2	2
3	3	2	2	2	2	2	2	1	1
2	2	2	2	2	2	2	2	2	2
9	9	9	9	9	9	9	9	9	9
9	9	9	9	9	9	9	9	9	9
9	9	9	9	9	9	9	9	9	9
1	1	2	2	2	1	1	1	2	1
2	2	2	2	2	2	2	2	2	2
1	2	2	2	1	1	1	1	1	1
9	9	9	9	9	9	9	9	2	9
9	9	9	9	9	9	9	9	9	9
3	3	3	3	3	3	3	3	1	3
2	2	2	2	2	2	2	2	2	2
1	2	2	1	1	1	1	1	1	1
3	3	3	3	3	3	3	3	3	3
9	9	9	9	9	9	9	9	1	9
2	3	3	2	3	2	2	3	2	3
2	2	3	3	3	3	3	3	1	1
2	2	3	3	2	2	3	3	2	2
9	9	1	9	9	9	1	2	9	9
1	1	1	1	1	1	1	1	1	2
2	2	2	2	2	2	2	2	2	2
9	9	9	9	9	9	9	9	9	9
2	2	1	1	2	3	3	3	3	1
2	2	2	2	2	2	2	2	2	2
1	3	1	2	1	2	2	2	1	1
2	2	2	3	3	2	3	1	2	2
2	2	2	2	2	2	2	2	2	2
3	2	2	2	1	2	9	1	2	1
2	2	2	2	1	3	1	1	1	1
3	3	3	3	3	3	1	3	1	1
2	2	2	2	2	2	2	2	2	2
3	3	3	3	3	3	3	3	3	3
1	1	3	2	1	2	1	2	2	1
1	9	2	2	1	3	1	1	1	1
2	2	2	2	2	2	2	2	2	2
2	2	2	2	2	2	2	3	2	2
1	3	1	3	1	3	1	3	1	3
2	1	1	3	2	1	3	2	1	2
2	2	2	2	2	3	1	1	2	2

2	2	2	2	2	2	2	2	2	3
2	2	2	2	2	2	2	2	2	2
2	1	2	2	1	2	1	1	1	1
2	2	2	1	2	2	1	2	1	2
2	2	2	2	2	2	2	2	1	1
9	9	9	9	9	9	9	9	9	9
2	2	2	2	2	2	2	2	2	2
1	1	2	2	2	2	1	1	1	1
2	2	2	2	2	2	2	2	2	2
2	2	2	2	2	2	2	2	1	1
3	2	2	2	2	2	1	2	1	1
2	2	2	2	2	2	2	2	2	2
2	2	2	2	2	2	2	2	2	2
3	3	3	3	3	3	3	3	3	3
2	3	3	3	2	2	3	2	2	9
2	3	2	1	3	2	1	2	2	1
2	3	2	2	3	2	3	2	3	2
2	1	3	2	3	2	1	3	2	2
2	2	2	2	2	2	2	2	2	2
2	2	1	3	1	2	2	1	2	1
2	2	2	2	2	2	2	2	2	2
2	2	3	2	3	3	1	2	2	2
2	2	3	2	3	3	1	2	2	2
9	9	9	9	9	9	9	9	1	1
2	2	2	2	2	9	1	1	1	1
2	2	2	2	2	2	2	2	2	2
2	2	2	2	2	3	1	1	1	1
2	2	3	3	2	3	2	3	1	1
2	2	2	2	2	2	2	2	2	2
1	1	1	1	1	1	1	1	1	1
3	2	2	2	3	3	3	2	2	2
2	3	3	2	3	2	2	3	2	3
1	1	3	3	3	1	1	1	2	3
2	2	3	2	3	3	3	2	3	3
1	1	3	3	3	1	1	1	2	2
2	2	2	2	2	2	2	2	2	2
2	2	2	2	2	2	2	2	2	2
2	2	2	2	1	2	2	2	2	2
2	2	2	2	2	2	2	2	2	1
2	2	2	2	2	2	2	2	2	9
3	2	3	3	1	3	1	3	1	1
1	2	2	3	1	3	1	2	1	1
2	2	2	2	2	2	2	2	2	1
2	2	2	2	2	2	2	2	2	2
2	3	2	2	2	1	2	3	1	2
1	3	3	3	2	3	1	1	1	1
1	2	2	2	1	3	2	2	1	1

2	2	2	2	2	2	2	2	1	2
2	2	2	2	2	2	2	2	9	2
2	2	2	9	1	3	1	2	1	1
2	2	2	2	2	2	2	2	2	2
2	2	2	2	2	2	2	2	1	3
2	2	2	2	2	2	2	2	2	2
2	2	2	2	2	2	1	1	1	1
1	1	2	2	1	2	1	1	1	1
2	2	2	2	2	2	2	2	2	2
2	2	2	2	2	2	2	2	2	2
2	2	2	2	2	2	2	2	2	2
9	9	9	9	9	9	9	9	9	9
9	9	9	9	9	9	9	9	9	9
2	9	9	9	9	9	9	9	9	1
2	2	2	2	2	2	2	2	1	1
2	2	2	2	2	2	2	2	1	1
1	1	2	2	1	3	1	1	1	1
2	2	2	2	2	2	2	2	1	1
1	1	3	2	1	3	1	1	1	1
1	1	2	1	2	1	1	2	1	1
2	2	2	2	2	2	2	3	1	1
2	2	2	2	2	2	2	2	9	2
2	2	2	2	2	2	2	2	2	2
2	2	2	2	2	2	2	2	2	2
2	2	2	2	2	2	2	2	1	1
2	2	2	1	2	1	1	1	9	9
2	2	2	2	2	2	2	2	1	1
2	2	2	2	2	2	2	2	2	2
2	2	2	2	2	2	2	2	1	1
9	9	9	9	9	9	9	9	9	9
2	2	2	2	2	2	2	2	2	2
2	2	2	2	2	2	2	2	2	2
2	2	2	2	2	2	2	2	1	9
2	2	2	2	2	2	2	2	2	2
2	2	2	2	2	2	2	2	1	1
2	2	2	2	2	2	2	2	2	2
2	2	2	2	2	2	2	2	2	2
2	2	2	2	2	2	2	2	2	2
2	2	2	2	2	2	2	2	2	2
2	2	2	2	2	2	2	2	1	1
2	2	2	2	2	2	2	2	2	2
2	2	2	2	2	2	2	2	2	2
2	2	2	2	2	2	2	2	1	1
2	2	2	2	2	2	2	2	2	1
2	2	2	2	2	2	2	2	2	2
2	2	2	3	2	2	3	2	1	1
2	2	2	2	2	2	2	2	2	2

2	2	2	2	2	2	2	2	1	1
2	2	2	2	2	2	2	2	1	1
2	2	2	2	2	2	2	2	1	1
2	2	2	2	2	2	2	2	2	2
2	2	2	2	2	2	2	2	2	2
1	2	2	2	2	2	2	2	2	2
9	9	9	9	9	9	9	9	9	9
1	2	2	1	1	3	1	1	1	1
1	1	2	1	2	2	2	2	1	2
2	2	2	2	2	2	2	2	1	1
2	2	2	2	2	2	2	2	1	1
2	2	2	2	2	2	2	2	2	2
2	2	2	2	2	2	2	2	1	1
1	9	9	9	9	9	9	1	1	9
2	2	2	2	2	2	1	1	1	1
2	2	2	2	2	2	2	2	1	1
1	1	1	2	2	2	1	1	2	1
1	1	2	2	2	3	2	2	1	1
1	1	1	1	1	1	1	1	1	1
1	2	2	2	2	2	2	2	1	1
1	2	2	3	3	2	2	3	2	1
2	2	9	1	2	2	2	2	1	2
2	2	2	2	2	2	2	2	1	1
2	2	2	2	2	2	2	2	2	2
2	2	2	2	2	2	2	2	2	2
2	2	2	2	2	2	2	2	2	2
9	9	9	9	9	9	9	9	9	9
2	2	2	2	2	2	2	2	2	2
2	2	2	2	2	2	2	2	1	1
2	2	2	2	2	2	2	2	2	2
2	2	2	2	2	2	2	2	2	2
2	2	1	1	2	2	2	2	1	2
9	9	9	9	9	9	9	9	9	9
9	9	9	9	9	9	9	9	9	9
9	9	9	9	9	9	9	9	9	9
9	9	9	9	9	9	9	9	9	9
2	2	2	3	3	3	3	3	1	1
2	3	3	3	3	3	1	1	1	2
1	1	2	2	1	3	3	2	2	1
2	2	2	2	2	2	2	2	2	3
2	3	3	3	2	3	1	2	1	1
2	2	2	2	2	2	2	2	1	1
1	2	2	1	2	1	2	1	1	1
2	2	2	2	2	2	2	2	2	2
2	2	2	2	2	2	2	1	1	1
2	2	2	2	2	2	2	2	1	1
1	1	2	1	2	1	2	1	1	2

3	1	2	3	1	2	9	2	1	1
1	1	2	3	1	9	1	2	1	2
2	9	1	3	1	2	1	1	1	1
1	2	1	2	1	2	1	2	1	2
2	3	2	3	2	1	1	1	2	1
2	2	2	2	3	1	2	2	1	2
1	2	3	2	1	3	1	1	1	1
9	9	9	9	9	9	9	9	2	9
2	2	2	2	2	2	2	2	2	2
2	2	2	2	2	2	2	2	2	2
2	2	2	2	2	2	2	2	2	2
2	2	2	2	2	2	2	2	2	2
2	2	2	2	2	2	2	2	2	2
2	1	2	1	2	3	1	1	1	1
2	2	2	2	2	2	2	2	1	1
2	2	2	2	2	2	2	2	2	2
2	2	2	2	2	2	2	2	2	2
3	2	2	1	1	2	1	1	1	9
1	2	3	3	2	3	1	1	1	1
1	2	3	3	2	3	1	1	1	1
3	3	3	2	3	3	2	2	2	3
2	2	2	2	2	2	2	2	1	2
1	2	3	3	1	3	2	1	1	1
9	9	9	9	9	9	9	9	9	9
3	1	3	1	2	1	3	3	3	1
1	2	2	2	1	3	1	1	1	1
2	2	2	2	2	2	2	2	2	1
1	2	2	2	2	3	2	1	2	2
2	2	2	2	2	2	2	2	2	2
9	9	9	9	9	9	9	9	9	1
2	2	3	3	3	3	3	3	3	1
9	9	9	9	9	9	9	9	9	9
9	9	9	9	9	9	9	9	9	9
9	9	9	9	9	9	9	9	9	9
9	9	9	9	9	9	9	9	9	9
2	2	2	2	2	2	2	2	2	2
2	3	3	3	3	3	3	3	1	3
9	9	9	9	9	9	9	9	9	9
2	2	2	2	2	2	2	2	2	2
2	2	2	2	2	2	2	2	2	2
1	2	3	3	2	3	2	2	3	3
2	2	2	2	2	2	2	2	1	1
2	2	2	2	2	2	1	1	1	1
3	1	2	3	1	2	3	1	3	2
9	9	9	9	9	9	9	9	3	9
2	1	1	1	2	1	1	1	1	2
3	3	3	3	3	3	3	3	3	3

1	1	1	1	1	1	2	1	1	2
1	1	2	2	1	2	1	1	1	1
2	1	1	1	1	1	1	1	1	1
2	2	2	2	2	2	2	2	1	1
9	9	9	9	9	9	9	9	9	9
9	9	9	9	9	9	9	9	9	9
2	2	2	2	2	2	2	2	2	2
2	2	2	2	2	2	2	2	3	3
9	9	9	9	9	9	9	9	9	9
9	9	9	9	9	9	9	9	9	9
2	2	1	2	2	2	1	2	9	1
2	1	1	1	1	1	1	1	2	2
2	2	1	1	3	3	2	2	3	3
2	3	2	2	3	2	3	2	3	3
9	9	2	9	9	9	9	9	3	3
2	3	1	2	1	2	3	1	1	1
1	2	2	1	2	3	1	1	1	1
3	1	1	2	1	2	2	3	2	1
9	9	9	9	9	9	9	9	9	9
3	2	2	1	1	2	3	1	2	9
2	1	2	1	3	1	2	1	1	1
1	1	1	1	2	1	2	1	1	2
1	2	2	1	1	2	1	1	1	2
2	2	2	2	2	2	2	2	2	2
1	1	1	3	3	3	3	3	1	1
2	2	2	2	2	2	2	2	2	2
1	2	3	1	2	3	2	2	2	2
2	2	2	2	2	2	2	2	2	2
3	2	2	2	3	1	2	2	1	2
3	3	1	3	3	9	1	3	3	1
2	2	2	2	2	2	2	2	2	2
2	1	2	2	2	2	1	2	2	2
2	3	3	3	1	3	3	3	3	1
2	2	2	1	1	3	3	2	1	1
1	9	9	9	9	9	9	9	9	9
1	1	1	2	1	2	2	1	2	1
9	9	9	9	9	9	9	9	9	9
2	2	2	2	2	2	2	2	2	2
1	2	2	2	1	2	2	2	9	9
2	1	1	2	1	3	1	1	1	1
9	9	9	9	9	9	9	9	9	9
3	1	3	2	1	3	2	1	1	2
1	2	3	2	3	2	2	2	2	3
1	2	3	2	1	3	1	1	2	2
1	2	3	2	1	3	1	1	2	2
3	2	3	3	3	3	3	3	2	2
2	3	2	3	3	2	2	3	2	3

2	1	2	3	3	2	1	2	2	2
3	3	2	3	3	3	2	3	9	9
2	2	2	2	2	2	2	2	2	2
3	3	3	3	3	3	3	3	3	3
1	1	3	1	1	3	3	1	1	1
9	9	9	9	9	9	9	9	9	9
9	9	9	9	9	9	9	9	9	9
2	2	3	2	2	2	1	2	2	2
1	1	3	3	3	3	1	1	1	1
2	2	2	2	2	2	2	2	3	3
1	2	1	3	2	3	2	2	2	1
3	2	1	2	2	1	2	1	2	1
2	3	2	2	2	2	3	2	2	2
1	1	3	3	1	1	1	1	1	1
9	9	9	9	9	9	9	9	9	9
3	3	3	3	3	3	3	3	3	3
2	2	2	2	2	2	2	2	2	3
2	2	2	2	2	2	2	2	2	2
2	1	2	3	1	1	2	3	1	2
1	2	3	2	1	2	2	2	1	2
9	9	9	9	9	9	9	9	9	9
9	9	9	9	9	2	9	9	9	9
2	2	3	2	2	3	3	2	1	1
9	9	9	9	9	9	9	9	9	9
9	9	9	9	9	9	9	9	9	9
2	2	3	2	3	2	3	2	3	3
3	1	2	3	2	3	3	3	2	3
2	2	2	2	2	2	2	2	2	2
3	3	3	3	3	3	3	3	3	3
1	1	2	2	1	2	1	1	1	1
3	3	3	3	3	3	3	3	3	3
2	2	2	2	2	2	2	2	2	2
9	9	9	9	9	9	9	9	9	9
9	9	9	9	9	9	9	9	9	9
9	9	9	9	9	9	9	9	9	9
2	2	2	2	2	2	2	2	2	2
3	3	3	1	1	3	1	1	1	3
2	2	3	1	1	1	3	1	2	2
1	1	3	3	2	3	1	1	2	1
9	9	9	9	9	9	9	9	9	2
2	2	3	3	2	2	2	3	2	2
2	1	2	1	2	3	3	1	2	1
3	2	2	2	2	3	3	3	2	2
9	9	9	9	9	9	9	9	1	1
2	3	2	3	2	1	2	3	3	2
9	9	9	9	9	9	9	1	1	1
2	2	3	2	3	2	3	2	1	1

1	2	1	2	1	2	1	2	2	1
3	3	3	2	2	2	3	3	2	2
3	3	1	1	3	3	1	1	3	3
1	2	3	1	2	3	1	2	1	2
2	2	2	2	2	2	2	2	3	3
2	2	2	2	2	2	2	2	2	2
3	1	2	2	2	2	1	1	1	2
1	1	3	2	3	3	2	2	1	1
9	9	9	9	9	9	9	9	9	9
1	2	3	2	1	2	2	1	1	3
2	3	3	2	3	2	2	3	1	2
2	2	2	2	2	2	2	2	2	2
2	2	3	3	1	3	1	1	1	1
1	1	3	1	1	3	1	1	1	2
9	9	9	9	9	9	9	9	9	2
3	3	2	2	1	3	2	1	2	2
2	1	1	1	3	2	2	1	1	1
3	1	2	3	1	2	1	2	1	2
1	2	1	3	2	1	3	1	1	3
2	2	2	2	2	2	2	2	2	2
1	1	1	1	1	2	2	1	2	1
2	2	2	2	2	2	2	2	2	2
2	1	2	1	1	1	1	1	1	1
1	2	2	2	2	3	1	1	1	1
1	2	2	2	1	3	2	1	1	1
2	3	2	2	3	3	3	1	1	1
3	2	2	3	2	2	3	2	2	1
2	2	2	2	2	2	2	2	2	1
2	2	2	2	2	2	2	2	2	2
2	2	2	2	2	2	2	2	2	2
2	1	9	1	2	2	1	1	2	2
3	2	9	1	2	3	9	9	2	2
2	2	2	2	2	2	2	2	2	2
1	2	2	1	1	2	2	2	1	2
3	1	3	2	1	2	3	1	1	2
2	2	2	2	2	2	2	2	2	1
1	1	2	2	1	1	2	2	1	1
1	2	2	1	1	1	1	1	1	2
1	2	3	3	2	3	2	1	1	2
2	2	1	1	1	1	1	1	1	1
3	3	3	3	3	3	3	2	3	3
2	1	1	1	1	1	2	2	1	2
2	2	2	1	2	2	2	2	1	1
2	2	1	1	2	1	1	1	2	2
2	2	2	1	1	1	1	2	1	1
2	2	1	1	1	2	2	1	2	2
3	3	2	1	2	1	1	1	1	2

2	2	2	1	1	1	2	2	2	2
2	1	2	2	2	1	2	2	2	3
1	1	2	1	1	2	2	1	1	1
1	3	3	1	1	2	2	1	1	2
1	1	1	2	2	1	2	1	2	1
1	2	3	3	1	1	2	1	1	2
2	3	2	1	2	2	2	1	1	2
2	1	1	2	2	1	1	1	1	1

Smoking	Alcohol	OverFourB	Hightfattyc	BirthPill	Spiritualatt	FamilyHxC	VaginalWa	UrineInfect	STD
2	2	2	2	2	2	2	2	2	2
3	3	3	2	1	2	3	1	1	1
2	2	2	2	2	2	2	2	2	2
3	3	3	2	1	2	3	1	1	1
3	3	3	2	1	2	3	1	1	1
3	3	3	2	1	2	3	1	1	1
2	9	9	9	9	9	9	9	9	9
1	1	2	1	3	2	1	2	1	2
2	9	2	2	9	2	9	2	9	2
9	9	9	9	9	9	9	9	9	9
2	2	2	2	2	2	2	2	3	3
1	1	2	2	2	3	3	3	3	2
2	2	2	2	2	2	2	2	2	2
2	2	2	1	2	2	1	1	2	3
2	1	2	2	2	1	1	1	2	1
2	2	3	2	2	3	1	2	2	2
3	1	2	1	2	3	1	2	1	2
2	2	3	2	2	3	1	2	2	2
2	2	3	2	2	3	1	2	2	2
1	1	1	2	3	3	3	3	3	3
1	1	3	1	2	2	2	2	2	2
1	1	3	2	2	2	2	2	2	2
2	2	2	2	2	2	2	2	2	2
3	3	3	3	3	3	3	3	3	2
2	2	2	2	2	2	2	2	2	2
2	2	2	2	2	2	2	2	2	2
2	2	2	2	2	2	2	2	2	2
1	1	1	1	2	1	2	1	2	2
2	2	2	2	2	2	2	2	2	2
2	2	2	2	2	2	2	2	2	2
2	2	2	2	2	2	2	2	2	2
1	1	2	2	2	1	1	1	1	1
2	2	2	2	2	2	1	1	1	1
1	1	2	2	2	1	1	1	1	1
1	1	2	2	2	3	2	1	1	1
1	1	2	2	2	1	1	1	1	1
1	1	2	2	2	3	2	1	1	1
1	1	1	2	1	2	1	1	2	1
2	2	2	2	2	2	2	2	2	2
2	2	2	2	2	2	2	2	2	2
2	2	2	2	2	2	2	2	2	2
2	2	2	1	2	2	2	2	2	2
3	2	9	2	3	3	3	1	1	1
1	1	3	3	3	3	1	1	3	3
1	1	3	3	2	1	3	1	2	2
3	2	3	3	3	3	3	1	1	2

3	3	3	1	2	3	3	2	1	1
2	2	2	1	2	1	1	9	1	1
2	2	2	3	1	3	3	1	1	1
1	1	3	1	1	3	3	1	1	1
2	1	3	2	2	1	3	1	1	1
3	3	3	3	1	3	3	1	1	1
3	3	3	3	1	3	3	1	1	1
1	1	3	9	1	9	9	1	1	1
9	9	9	9	9	9	9	9	1	1
2	1	3	1	2	3	2	1	1	1
2	2	2	9	9	9	2	2	2	2
2	2	2	2	1	3	2	2	2	2
2	2	3	2	1	2	3	2	1	1
2	1	2	2	1	2	1	2	2	2
1	1	2	2	1	2	2	2	2	1
1	1	3	2	2	3	2	2	2	2
2	2	2	2	1	2	3	1	2	2
2	2	2	2	1	3	2	1	1	1
3	3	2	3	2	3	3	1	1	2
1	1	2	2	3	3	1	2	3	2
3	3	3	3	3	3	3	1	1	1
3	3	3	3	3	3	3	1	1	1
3	3	3	3	3	3	3	1	1	1
3	3	2	2	2	3	3	1	2	2
9	9	1	9	9	9	1	9	9	1
1	1	3	2	2	3	2	1	1	1
1	1	3	1	3	3	1	1	1	1
3	3	2	2	1	3	3	1	1	3
2	2	2	2	2	2	2	2	2	2
1	1	2	2	1	2	2	1	1	1
1	1	3	3	3	3	3	2	9	1
1	1	1	3	3	1	1	1	1	1
1	1	3	2	2	9	2	1	1	1
1	1	3	2	2	3	2	1	1	1
1	1	3	1	2	3	1	1	1	3
9	9	9	9	9	9	9	9	9	9
9	9	9	9	9	9	9	9	9	9
1	1	2	2	2	2	2	2	1	1
3	3	3	3	1	3	3	1	1	1
1	1	3	1	1	1	3	1	1	1
9	1	2	2	1	3	9	1	9	1
1	1	9	1	2	2	3	2	1	1
3	3	3	3	3	3	3	3	1	1
1	1	3	1	1	2	2	1	1	1
1	1	3	3	1	1	2	2	1	1
9	9	9	9	9	9	9	9	9	9
9	9	9	9	9	9	9	9	9	9

2	3	3	2	2	1	2	1	2	2
2	2	2	2	1	2	2	2	1	2
1	2	3	1	2	2	2	1	1	1
2	1	3	3	2	3	2	2	1	2
9	9	9	9	9	9	9	9	9	9
9	1	9	9	9	2	9	9	9	9
1	1	3	2	1	1	3	1	1	1
3	1	3	3	1	3	2	1	1	1
3	3	3	3	3	3	3	1	1	2
1	1	9	1	9	9	9	1	2	9
2	2	3	2	3	3	2	1	1	2
1	3	2	1	2	3	3	2	2	2
3	3	3	3	2	2	2	2	1	1
9	9	9	9	9	9	1	1	9	9
2	3	1	3	2	1	2	2	2	3
1	3	2	3	2	1	2	1	3	2
2	2	2	2	2	2	2	2	2	1
2	2	2	3	2	2	1	2	2	1
2	2	2	2	2	2	2	2	2	2
2	2	3	3	2	1	2	2	1	2
3	1	1	1	2	3	3	2	2	2
2	2	2	3	2	2	2	1	1	1
3	2	2	2	3	3	3	3	3	3
3	3	3	3	3	3	3	3	3	3
3	3	2	1	1	2	2	1	2	1
1	1	2	2	2	2	2	2	2	2
2	2	1	2	1	2	3	1	2	2
2	2	1	2	1	2	2	2	2	2
2	2	2	2	2	2	2	2	2	2
3	2	3	1	2	2	1	1	2	2
2	2	2	2	2	2	2	2	2	2
2	2	2	2	2	2	2	2	2	2
2	2	3	2	2	1	2	2	2	1
2	2	2	2	2	2	2	2	2	2
3	2	3	1	2	2	1	1	2	2
1	1	3	3	2	2	2	2	2	2
2	2	2	2	2	2	2	2	2	2
2	2	2	2	2	2	2	2	2	2
1	9	9	9	9	1	9	9	9	1
3	3	3	3	2	1	3	1	1	3
2	2	2	2	2	2	2	2	2	2
2	2	2	2	2	2	2	2	2	2
2	2	2	2	2	2	2	2	2	2
1	1	2	3	2	3	3	2	2	2
1	1	2	2	2	3	2	2	2	2
1	1	2	2	2	3	3	2	2	2
2	2	2	2	2	2	2	2	2	2

2	2	2	2	2	2	2	2	2	2
2	2	3	1	2	2	2	2	2	2
2	2	3	1	2	2	2	2	2	2
2	2	2	2	2	2	2	2	2	2
1	1	1	1	1	1	1	1	1	1
3	2	3	3	1	2	2	1	1	1
3	9	2	3	3	2	3	2	3	3
2	2	2	2	2	2	2	2	2	2
2	2	2	2	2	2	2	2	2	2
2	2	2	2	2	2	2	2	2	2
2	2	2	9	2	2	3	2	2	2
2	3	2	2	2	2	2	3	3	2
3	3	9	9	9	9	3	3	9	3
2	2	2	2	2	2	2	2	2	2
2	2	2	3	2	1	2	2	1	1
2	2	2	3	2	1	2	2	1	1
9	9	9	9	9	9	9	9	9	9
1	1	3	2	2	3	3	2	2	2
1	9	9	9	9	9	9	9	9	9
1	1	3	2	1	2	1	1	1	1
1	1	3	2	1	2	1	1	1	1
1	1	3	2	1	3	3	1	1	1
1	1	1	3	1	1	1	1	1	1
1	1	3	2	3	2	1	1	1	1
1	1	1	2	1	2	1	1	1	1
1	1	3	2	1	2	1	1	1	1
1	1	3	2	1	2	1	1	1	1
1	1	3	2	1	2	3	1	1	1
1	1	1	2	1	3	1	1	1	1
1	1	1	2	1	2	1	1	1	1
2	2	1	2	2	3	2	2	1	2
2	2	2	2	2	2	2	2	2	2
1	1	3	2	1	2	1	1	1	1
1	1	1	2	1	1	1	1	1	1
1	1	3	2	1	3	1	1	1	1
1	1	3	2	1	2	3	1	1	1
1	1	3	2	1	2	1	1	1	1
1	1	3	2	1	2	1	1	1	1
1	1	3	2	1	2	1	1	1	1
1	1	3	2	1	1	1	1	1	1
1	1	3	2	1	3	1	1	1	1
1	1	1	2	1	2	1	1	1	1
1	1	3	2	1	2	1	1	1	1
1	1	3	2	1	2	1	1	1	1
1	1	3	2	1	1	2	1	1	1
1	1	9	2	1	2	1	1	1	2
1	1	3	2	1	2	1	1	1	1

1	1	3	2	1	2	1	1	1	1
1	1	3	2	1	1	1	1	1	1
1	1	3	2	1	1	1	9	1	1
1	1	3	2	1	2	3	1	1	1
1	1	3	2	1	1	1	1	1	1
1	1	3	1	1	1	1	1	1	1
1	1	3	2	1	3	3	1	1	2
3	3	3	3	2	2	2	2	1	1
1	1	3	1	1	1	3	1	1	1
2	2	2	3	1	3	3	1	3	3
9	9	9	9	9	9	9	9	9	9
1	1	2	2	1	2	2	1	1	1
1	1	3	1	1	1	3	1	1	1
1	1	3	2	1	3	3	1	1	1
2	1	3	2	2	3	2	1	1	1
1	1	3	3	2	1	2	1	1	1
2	2	2	2	2	2	2	2	2	2
1	1	1	1	1	1	1	1	1	1
3	3	2	2	2	1	1	2	1	1
2	2	2	2	2	2	2	2	2	2
2	2	2	1	1	2	1	2	1	1
2	2	2	2	2	2	2	2	2	2
2	2	2	1	2	2	2	2	1	1
2	2	2	2	2	1	2	1	2	2
2	2	2	2	2	2	2	2	2	2
2	2	2	2	2	2	2	2	2	2
2	2	2	2	2	2	2	2	2	2
2	2	2	2	2	2	2	2	2	2
2	2	2	2	2	3	2	1	2	2
3	3	2	2	2	1	1	2	1	1
1	1	2	1	2	2	2	2	2	2
1	1	2	1	2	2	2	2	2	2
3	3	2	2	2	1	1	2	2	1
1	1	2	2	2	1	3	1	2	2
2	2	2	1	1	2	1	2	1	2
2	2	2	2	2	2	2	2	2	2
2	2	2	2	2	2	2	2	2	2
2	2	3	2	1	1	1	1	2	1
1	1	2	2	1	2	2	2	2	2
1	1	2	1	2	3	2	3	2	2
2	2	2	2	2	2	2	2	2	2
2	2	2	2	2	2	2	2	2	2
9	9	9	9	9	9	9	9	9	9
9	9	9	9	9	9	9	9	9	9
2	1	2	3	3	1	1	1	1	1
2	2	2	2	1	2	2	3	1	1
9	9	9	9	1	9	9	9	9	9

2	2	2	2	2	2	2	2	2	2
2	2	2	2	2	2	2	2	2	2
1	1	2	2	1	3	2	1	2	2
2	2	2	2	2	2	3	2	2	2
2	2	2	2	3	3	2	3	3	2
3	3	2	3	2	3	2	2	1	2
1	1	2	2	2	2	2	1	1	2
2	2	2	2	2	2	2	2	2	2
1	1	3	3	2	3	2	2	2	2
2	1	2	2	2	2	2	2	2	2
2	2	2	1	2	2	2	2	2	1
3	3	2	2	2	2	2	2	2	2
3	3	2	2	2	2	2	2	2	2
1	2	3	2	1	2	2	3	2	2
2	2	2	2	2	2	2	2	2	2
1	2	2	2	2	3	3	2	2	2
2	3	1	2	2	2	1	1	3	2
1	1	3	2	2	2	3	2	2	1
9	9	9	9	9	1	9	9	9	1
1	1	1	2	3	3	9	1	2	1
1	1	1	1	3	9	2	1	1	1
1	1	2	2	2	2	2	2	2	2
1	2	3	2	2	2	2	1	1	2
3	2	3	2	3	3	2	3	2	2
2	2	9	9	9	9	2	9	9	9
2	2	2	2	2	2	2	2	2	2
2	2	2	2	2	2	2	2	2	2
2	9	2	9	1	9	2	9	2	9
2	1	3	3	2	2	2	2	2	2
2	2	2	2	2	2	2	2	2	2
9	1	9	9	9	9	9	9	9	9
3	3	3	2	2	2	2	2	2	2
3	3	3	3	3	3	3	3	3	3
1	1	2	2	2	3	3	2	2	2
9	9	9	9	9	9	9	9	9	9
2	2	2	2	2	2	2	2	2	2
2	3	3	3	2	3	3	1	1	2
3	1	2	2	2	1	2	1	1	1
2	2	2	2	2	2	2	2	2	2
2	2	3	3	2	2	3	2	2	2
3	3	3	3	3	2	2	2	2	2
3	2	2	2	1	3	3	1	2	2
1	1	1	1	1	1	1	1	9	9
2	2	2	2	2	2	2	2	2	2
1	2	3	2	2	2	3	1	2	2
2	2	2	2	2	2	3	2	2	2
2	1	2	2	2	2	2	2	2	2

9	9	9	9	9	9	9	9	9	9
9	9	9	9	9	9	9	9	9	9
9	9	9	9	9	9	9	9	9	9
1	1	2	2	2	3	2	1	2	1
1	1	2	2	2	2	9	2	1	2
2	2	2	3	2	2	2	1	2	3
1	1	3	1	1	3	2	2	2	1
1	1	1	1	1	1	1	1	1	1
2	2	3	2	2	2	3	1	1	1
2	2	2	2	2	2	2	2	2	2
2	2	2	2	2	2	2	2	2	2
9	9	9	9	9	9	9	9	9	9
9	9	9	9	9	9	9	9	9	9
1	1	3	3	1	3	3	1	1	1
3	3	9	1	1	1	1	3	3	9
9	9	9	9	9	9	9	9	9	9
3	3	3	3	2	2	2	9	9	9
1	1	2	1	2	1	3	2	3	2
2	3	2	1	3	1	1	1	1	2
9	9	9	9	9	9	9	9	9	9
9	9	9	9	9	9	9	9	9	9
2	2	2	2	2	2	2	2	2	2
1	1	3	2	3	3	3	2	2	2
1	1	3	3	1	2	3	2	2	2
2	2	2	2	2	2	2	2	2	2
1	1	2	1	2	1	1	1	1	3
3	3	1	2	1	1	1	1	2	1
2	3	3	3	3	3	3	1	1	1
3	3	3	1	2	1	3	1	2	1
2	1	3	1	2	3	3	1	2	2
2	2	2	2	2	2	2	2	2	2
1	3	2	3	2	3	2	1	2	2
9	9	9	9	9	9	9	9	9	9
9	9	9	9	9	9	9	9	9	9
2	2	2	2	2	2	2	1	1	1
1	3	3	3	1	3	2	2	3	2
2	1	2	2	2	3	2	2	2	1
1	1	3	3	1	1	3	1	1	1
3	3	3	1	3	3	3	1	1	3
2	2	2	2	2	2	2	2	1	1
1	1	2	1	2	2	2	2	1	1
3	3	9	1	3	3	3	1	1	1
3	3	2	2	2	2	2	2	2	2
2	2	2	2	2	2	2	2	2	2
3	3	3	2	2	3	3	3	2	2
1	2	2	2	2	2	2	2	2	2
2	2	2	2	2	2	1	2	2	2

9	9	9	9	9	9	9	9	9	9
9	9	9	9	9	9	9	9	9	9
9	9	9	9	9	9	9	9	9	9
2	2	2	2	2	2	2	1	1	2
9	9	9	9	1	9	9	9	9	9
9	9	9	9	9	9	9	9	9	9
1	1	2	2	1	2	2	1	1	1
9	9	9	9	9	9	9	9	9	9
1	1	3	2	1	2	3	1	1	1
1	1	2	3	2	1	1	2	2	1
2	2	3	2	2	2	3	2	2	2
2	2	3	2	2	2	2	2	2	2
3	3	3	2	3	2	3	1	1	1
2	2	2	2	2	2	2	3	1	2
2	2	3	2	3	2	2	2	2	1
2	1	2	3	3	3	3	2	2	2
2	2	2	2	2	2	2	2	2	2
1	2	2	2	2	2	2	2	2	2
2	2	2	2	2	2	2	2	2	2
2	2	2	2	2	2	2	2	2	2
1	1	1	2	2	1	1	1	2	2
1	1	3	2	1	2	2	1	2	1
1	1	3	2	2	1	3	2	2	1
2	2	3	2	2	3	1	1	1	1
2	2	2	2	2	2	2	2	2	2
3	2	1	2	3	2	2	2	2	3
2	2	2	2	2	2	2	2	2	2
2	2	2	2	2	2	3	2	2	2
1	2	3	3	2	2	2	1	2	2
1	1	2	2	2	1	2	1	1	1
3	3	2	3	3	1	3	3	3	3
1	2	3	2	1	2	3	2	2	2
1	9	2	3	1	1	1	3	2	2
2	2	2	2	2	2	2	2	2	2
2	2	2	2	2	2	2	2	2	2
3	2	3	2	1	3	2	1	2	1
2	2	2	2	2	2	3	2	2	2
1	1	2	1	2	1	1	2	2	2
2	2	2	2	2	2	2	2	2	2
2	2	2	2	2	2	2	2	2	2
2	1	2	2	2	2	2	2	2	2
1	1	2	2	2	1	2	2	2	2
1	1	1	1	2	2	1	1	1	1
1	1	2	2	2	2	2	2	2	2
3	2	3	3	3	1	2	2	2	1
2	1	2	3	2	3	2	1	1	1
2	2	2	2	2	2	2	2	2	2

3	3	3	3	3	2	1	2	1	3
2	2	1	2	2	1	2	1	2	2
2	2	2	2	2	2	2	2	2	1
2	2	2	2	2	2	2	2	2	2
1	1	2	1	2	2	1	1	2	2
2	2	2	3	2	2	2	2	2	1
9	9	9	9	9	9	9	9	9	9
2	2	2	2	2	2	2	2	2	2
3	3	3	2	2	2	2	2	2	2
3	2	2	2	2	3	3	1	1	1
2	2	1	2	1	2	1	2	1	2
2	2	3	1	1	2	2	1	2	1
1	1	2	2	2	2	2	2	2	2
2	2	2	2	2	2	2	2	2	2
2	2	2	2	2	2	2	2	2	2
1	1	2	1	1	1	1	1	1	1
1	1	3	3	3	1	1	3	3	1
2	2	2	2	2	2	2	2	2	2
2	2	2	2	2	2	2	2	2	2
3	3	3	3	2	2	2	1	2	3
1	1	1	2	2	1	1	1	2	1
3	3	2	2	2	2	2	2	2	2
9	9	9	9	9	9	9	9	9	9
2	2	2	2	2	2	2	2	2	2
1	1	3	2	1	3	1	1	1	1
9	9	9	9	9	9	9	9	9	9
1	3	3	2	2	3	2	3	3	1
9	9	9	9	9	9	9	9	9	9
2	2	2	2	2	2	2	9	2	2
3	2	1	2	3	2	2	1	1	1
9	9	9	9	9	9	9	9	9	9
9	9	9	9	9	9	9	9	9	9
2	2	2	2	2	2	2	2	2	2
2	2	1	1	1	1	2	1	2	1
9	9	9	9	9	9	9	9	9	9
3	2	1	3	1	2	2	1	1	1
3	2	3	3	3	3	3	3	3	3
2	1	2	1	1	2	1	2	2	1
9	9	9	9	9	9	9	9	9	9
1	2	2	1	2	3	2	1	1	2
2	2	2	2	2	2	2	3	2	2
3	3	3	2	1	2	3	1	1	1
2	2	3	3	2	2	3	1	2	3
2	2	2	2	2	2	2	2	2	2
9	9	9	9	9	9	9	9	9	9
1	1	1	2	1	2	2	1	1	1
1	1	1	2	1	1	1	1	1	1

9	9	9	9	9	9	9	9	9	9
1	1	3	1	1	1	1	1	1	1
1	1	1	2	1	9	1	1	1	1
1	1	3	2	1	2	1	1	1	1
1	1	3	1	1	3	1	1	1	1
1	1	3	2	1	2	3	1	1	1
1	1	3	2	2	2	1	1	3	2
1	1	3	1	1	1	3	1	1	1
1	1	2	2	1	2	1	1	1	1
1	1	3	2	1	2	1	1	1	2
1	1	2	2	2	3	2	2	1	2
1	1	1	2	2	2	1	1	1	1
1	1	3	2	1	3	2	1	1	1
1	1	3	2	1	2	1	1	1	1
1	1	1	2	1	2	1	1	1	1
1	1	1	2	1	3	3	1	1	1
1	1	3	2	1	2	1	1	2	1
1	1	3	2	1	2	1	1	1	1
1	1	1	2	1	2	1	1	1	1
1	1	1	2	1	2	1	1	1	1
1	1	3	2	1	1	3	1	1	1
1	1	1	2	1	1	1	1	1	2
1	1	3	2	1	1	3	1	1	1
1	1	3	2	2	2	1	1	1	1
1	1	3	2	1	2	3	1	1	1
1	1	3	2	1	2	3	1	1	1
1	1	3	2	1	1	3	1	1	1
1	1	9	2	1	2	3	1	1	1
1	1	3	2	1	1	2	1	1	1
1	1	3	2	1	2	1	1	1	1
1	1	3	3	1	1	1	1	1	1
1	1	1	2	1	2	2	1	1	1
1	1	3	2	1	1	3	1	1	1
1	1	1	2	1	2	1	1	1	1
1	1	3	2	1	2	1	1	1	1
1	1	3	2	2	2	1	1	1	1
1	1	1	3	9	2	2	1	1	1
1	1	3	2	1	1	1	1	1	1
1	1	1	2	1	1	1	1	1	1
1	1	3	2	1	1	1	1	1	1
1	1	3	2	1	2	3	1	1	1
2	2	2	2	2	2	2	2	2	2
2	2	2	2	1	3	2	1	1	1
2	2	2	2	2	2	2	2	2	2
9	9	9	9	9	9	9	9	9	9
2	2	2	2	2	2	2	2	2	2
9	9	9	9	9	9	9	9	9	9

2	2	2	2	2	2	2	2	2	2
1	1	2	2	1	3	2	1	1	1
1	1	2	2	1	3	2	1	1	1
1	1	3	2	2	2	3	1	1	1
2	2	2	2	2	2	2	2	2	2
1	1	2	3	2	3	1	1	2	1
2	2	2	2	2	2	2	2	2	2
2	1	2	2	2	2	1	2	2	1
2	2	2	2	2	3	1	2	2	1
2	2	2	2	2	2	2	2	2	2
1	1	2	2	1	3	2	1	1	1
9	9	9	9	9	9	9	1	9	9
1	1	3	3	1	2	2	1	1	1
2	2	2	2	2	2	2	2	2	2
9	9	9	9	9	9	9	9	9	9
9	9	9	9	9	9	9	9	9	9
9	9	9	9	9	9	9	1	9	9
9	9	9	9	9	9	9	9	9	9
9	9	9	9	9	9	9	9	9	1
2	1	2	2	1	3	2	1	1	1
3	3	1	1	1	3	1	1	3	3
1	3	9	3	1	1	3	1	9	9
2	2	3	2	2	1	2	2	2	1
9	9	9	9	9	9	9	1	9	9
9	9	9	9	9	9	9	9	9	9
9	9	9	9	9	9	9	9	9	9
9	9	9	9	9	9	9	9	9	9
9	9	9	9	9	9	9	9	9	9
3	3	2	3	1	2	2	1	1	1
9	9	9	9	9	9	9	1	9	9
9	9	9	9	9	9	9	9	9	9
2	2	2	2	2	2	2	2	2	2
2	2	2	2	2	2	2	2	2	2
1	1	3	3	3	3	3	1	3	1
2	2	2	2	2	2	1	1	1	1
2	2	3	1	2	3	2	2	2	1
1	1	3	2	2	2	3	1	2	2
2	2	3	1	1	2	3	1	2	1
2	2	2	2	2	2	2	2	2	2
2	2	3	3	2	1	2	2	2	2
9	9	9	1	9	9	9	9	9	9
2	2	3	2	2	2	1	1	1	1
2	2	2	2	2	2	2	2	2	2
2	2	2	2	1	2	2	2	1	1
1	1	2	2	2	2	2	2	2	1
2	2	2	2	2	2	2	2	2	2
2	1	2	1	1	2	3	2	2	2

2	3	2	2	2	2	9	1	1	1
1	1	2	2	2	2	2	2	2	1
2	2	2	2	2	2	2	2	2	2
1	1	2	2	2	2	2	2	2	1
2	2	2	2	1	3	3	1	1	1
2	2	2	2	2	2	2	2	2	2
2	2	2	2	3	3	3	2	1	1
2	2	2	2	2	2	2	2	2	2
2	2	2	2	2	2	1	2	1	1
2	2	2	2	2	2	2	2	2	2
2	2	2	2	2	2	2	2	2	2
2	1	3	2	2	2	3	2	2	1
2	2	2	2	2	2	2	2	2	2
2	2	2	2	2	2	2	2	2	2
2	2	3	2	2	3	3	1	2	2
2	2	2	2	2	2	2	2	2	2
2	2	2	2	2	2	2	2	2	2
2	2	2	2	2	2	2	2	2	2
2	2	2	2	2	2	2	2	2	1
3	3	3	2	2	3	2	1	2	2
1	1	2	1	1	2	2	2	2	2
1	1	3	2	2	1	2	2	2	1
1	1	3	3	2	3	3	1	1	2
1	1	3	3	2	2	3	1	1	2
3	3	3	3	3	3	2	2	2	2
2	2	2	2	2	2	2	2	2	2
1	1	3	3	1	3	3	9	1	1
1	3	3	1	2	1	1	2	1	3
2	2	3	2	1	1	1	1	1	1
2	2	2	2	3	2	3	2	3	2
2	2	2	2	2	2	2	2	2	2
1	2	2	2	2	2	2	2	2	2
2	2	2	2	2	2	2	2	2	2
2	2	3	2	2	2	3	2	2	2
2	2	2	2	2	2	2	2	2	2
9	9	9	9	9	9	9	9	9	9
2	2	2	2	2	2	2	2	2	2
1	1	2	2	3	2	2	2	1	1
2	1	3	2	2	1	2	2	2	2
3	3	3	1	1	2	2	1	1	2
2	9	3	2	9	2	3	1	1	1
1	1	2	1	2	1	2	3	2	2
1	1	2	1	2	1	2	3	2	2
2	1	2	1	2	3	3	2	2	2
2	1	1	2	1	3	3	1	1	1
2	2	2	2	2	2	2	2	2	2
9	9	9	9	9	9	9	9	9	9

9	9	9	9	9	9	9	9	9	9
9	9	9	9	1	9	9	9	9	9
9	9	9	9	9	9	9	1	9	9
9	9	9	9	9	9	3	3	1	1
9	9	9	9	1	9	9	9	9	9
9	9	9	9	9	9	9	9	9	9
9	9	9	9	2	9	9	9	9	9
9	9	9	9	9	9	9	9	9	9
9	9	9	9	9	9	9	9	9	9
9	9	9	9	9	9	9	9	9	9
9	9	9	9	9	9	9	9	9	9
9	9	9	9	9	9	9	9	9	9
9	9	9	9	9	9	9	2	9	9
9	9	9	9	9	9	9	9	9	9
9	9	9	9	9	9	9	9	9	9
9	9	9	9	9	9	9	9	9	9
9	9	9	9	9	9	9	9	9	9
1	1	3	3	1	1	3	3	1	1
2	2	2	2	2	2	2	2	2	2
9	9	9	9	9	9	9	9	9	9
1	1	1	1	1	1	1	1	1	1
3	1	2	2	1	2	3	3	2	1
3	2	2	2	1	2	1	1	2	2
1	1	1	1	1	1	1	1	1	1
1	2	3	1	2	1	3	2	2	3
2	1	2	3	2	2	2	2	3	2
2	9	1	2	2	2	2	2	2	2
3	2	3	2	1	3	2	3	2	2
2	2	2	2	2	2	2	2	2	2
2	2	2	2	2	2	2	2	2	2
1	1	1	1	1	2	2	2	2	3
1	1	3	3	2	3	3	1	3	1
2	2	2	2	2	2	2	2	2	2
3	3	3	3	3	3	1	1	1	1
3	1	2	3	1	2	3	1	2	3
2	2	2	2	2	2	2	3	3	3
2	2	2	2	2	2	2	2	2	2
9	9	2	3	2	9	9	9	9	9
3	3	1	1	1	3	9	1	9	1
9	9	9	9	9	9	9	9	9	9
2	2	2	2	2	2	2	2	2	2
3	1	2	2	3	3	2	2	2	3
9	9	9	9	9	9	9	9	9	9
1	1	1	1	1	1	3	2	2	1
2	2	2	2	2	2	2	2	2	2
2	2	2	2	2	2	2	2	2	2
1	1	1	2	2	2	2	2	3	3

2	2	3	3	3	3	3	2	2	1
9	9	9	9	9	9	9	9	9	9
1	3	3	1	3	1	3	3	1	3
9	9	9	9	9	9	9	9	9	9
2	2	2	2	2	2	2	2	2	2
2	2	2	2	2	2	2	2	2	2
9	9	9	9	9	9	9	9	9	9
2	2	3	3	3	3	3	2	2	1
1	1	3	1	2	3	1	2	3	2
3	3	3	3	1	3	3	3	3	3
1	1	1	1	1	2	3	1	2	3
1	1	3	2	3	1	1	1	1	1
3	2	2	3	9	2	3	2	3	2
9	9	9	9	9	9	9	9	9	9
9	9	9	9	9	9	9	9	9	9
9	9	9	9	9	9	9	9	9	9
1	3	3	3	1	3	3	1	3	3
9	9	9	9	9	9	9	9	9	9
1	1	3	2	2	3	3	1	1	1
1	1	1	3	3	3	3	1	3	3
2	1	3	2	2	1	1	1	1	1
3	2	2	3	9	2	3	2	3	2
2	2	2	2	2	2	2	2	2	2
3	9	9	9	9	9	9	9	9	9
1	2	2	2	2	2	2	2	2	2
3	1	3	1	3	1	3	1	3	1
1	3	3	3	3	3	3	3	3	3
3	3	3	9	3	3	3	1	1	1
1	1	1	2	1	2	2	2	1	1
2	2	2	2	2	2	2	1	1	1
9	9	9	9	9	9	9	9	9	9
9	9	9	9	1	9	9	9	9	9
1	1	2	2	2	2	2	2	2	1
1	3	3	2	2	3	3	2	1	2
1	9	2	9	9	9	9	9	9	3
2	2	2	2	2	2	2	1	1	1
3	3	3	3	3	3	2	2	2	2
9	9	9	9	9	9	9	9	9	9
9	9	9	9	9	9	9	9	9	9
9	9	9	9	9	9	9	9	9	9
9	9	9	9	9	9	9	9	9	9
2	3	3	1	2	3	2	3	2	9
3	1	2	1	3	2	1	3	1	2
9	9	9	9	9	9	9	9	9	9
3	1	2	1	3	3	2	1	2	3
1	3	3	1	1	1	1	2	2	2
1	1	3	3	3	1	1	3	1	3

3	3	3	3	3	3	3	3	3	3
3	3	3	3	2	1	3	3	3	3
3	3	3	9	3	3	3	3	3	3
1	2	2	2	2	2	2	2	2	2
2	2	2	2	2	2	2	2	2	2
9	9	9	9	1	3	9	2	9	9
9	9	9	9	9	9	9	9	9	9
1	1	3	1	2	3	2	2	1	2
3	3	3	3	3	3	3	3	3	3
1	1	3	3	1	3	3	2	2	2
3	3	3	3	3	3	3	3	3	3
1	1	1	1	9	9	1	1	1	1
1	1	3	1	1	1	1	2	2	2
1	1	3	2	1	1	2	2	2	2
1	1	3	1	1	1	1	2	2	2
1	1	2	2	1	1	1	2	2	2
1	1	3	1	1	1	1	2	2	2
1	1	3	1	1	1	1	2	2	2
1	1	3	1	1	1	1	2	2	2
1	2	3	2	2	3	3	2	2	3
3	2	3	2	3	2	3	1	1	1
3	3	3	9	2	2	2	2	2	9
3	3	3	3	3	2	3	3	3	3
2	3	3	2	2	2	3	3	3	2
3	3	3	3	3	3	3	3	3	3
1	1	3	1	1	1	1	2	2	2
9	2	9	9	9	9	9	9	9	9
9	9	9	9	1	9	9	9	9	9
9	9	9	9	9	9	9	1	9	9
2	2	2	2	2	2	2	2	2	2
3	1	2	1	1	2	1	2	3	2
3	2	3	2	3	2	3	3	2	3
3	2	1	2	1	2	1	3	2	1
9	9	9	9	1	9	9	9	9	9
3	3	3	3	3	3	3	3	3	3
1	2	2	1	2	3	3	2	2	2
9	3	9	9	1	9	9	9	9	2
1	1	2	1	2	1	2	1	2	1
1	1	2	1	1	1	1	2	2	2
1	1	3	2	1	1	1	1	1	1
1	1	2	1	2	3	1	1	1	2
2	2	3	2	2	2	2	2	2	9
1	2	1	2	3	1	9	2	1	2
3	3	3	1	1	3	1	1	1	3
1	1	3	3	1	2	3	2	2	3
1	1	1	1	1	1	1	1	1	1
9	2	3	2	2	9	2	2	2	3

2	2	2	2	2	2	2	2	2	2
9	9	9	9	9	9	9	9	9	9
1	9	9	9	9	9	9	9	9	9
1	1	3	3	1	1	3	1	3	3
2	2	2	2	2	2	2	2	2	2
2	2	2	2	2	2	2	2	2	2
1	1	1	1	1	1	1	1	1	1
2	2	2	2	2	2	2	2	2	2
2	2	2	2	2	2	2	2	2	2
3	1	3	1	1	2	2	2	3	3
9	9	9	9	9	9	9	9	9	9
3	2	2	2	3	3	3	1	2	2
9	9	9	9	9	9	9	9	9	9
3	1	3	3	3	3	1	1	1	1
1	1	3	2	2	2	1	1	1	1
9	9	9	9	9	9	9	9	9	9
3	3	3	3	3	3	2	2	2	2
9	1	9	9	9	9	9	9	9	9
9	1	9	9	9	9	9	9	9	9
3	1	2	3	1	2	3	1	2	3
1	2	1	1	1	1	1	1	1	1
2	2	2	2	2	2	2	2	2	2
9	9	9	9	9	9	9	9	9	9
2	2	2	2	2	2	2	2	2	2
3	3	3	3	3	3	3	3	3	3
1	1	3	3	2	3	1	3	9	1
1	1	3	3	2	3	1	3	9	1
1	1	3	3	1	1	3	2	1	1
1	1	1	1	1	1	1	1	1	1
2	2	2	2	2	2	2	2	2	2
3	3	3	1	3	2	3	3	2	3
1	1	1	1	2	2	1	1	1	1
2	1	1	1	1	2	2	1	1	1
1	1	3	3	2	3	1	3	1	2
2	9	1	1	3	9	2	1	1	1
2	2	2	2	2	2	2	2	2	2
2	2	2	2	2	2	2	2	2	2
9	9	9	9	9	9	9	9	9	9
1	9	2	9	9	3	9	9	9	9
1	1	1	1	3	3	3	1	3	3
2	2	2	2	2	2	2	2	2	2
2	2	2	2	2	2	2	2	2	2
9	9	9	9	9	9	9	9	9	9
2	2	2	2	2	2	2	2	2	2
2	2	2	2	2	2	2	2	2	2
2	2	2	2	2	2	2	2	2	2
9	9	9	9	9	9	9	9	9	9

9	9	9	9	9	9	9	9	9	9
2	2	1	2	2	3	2	2	2	2
2	2	2	2	2	1	2	1	1	1
1	1	2	1	2	2	9	9	1	2
9	9	9	9	9	9	9	9	9	9
3	1	2	3	1	2	3	2	2	2
9	9	9	9	9	9	9	9	9	9
1	1	3	1	1	3	1	1	1	1
2	1	1	2	3	1	2	2	1	2
2	9	9	9	9	9	9	9	9	9
2	2	2	2	2	2	2	2	2	2
1	2	2	2	1	1	2	2	1	1
1	2	2	2	1	1	2	2	1	1
2	2	2	2	2	2	2	2	2	2
1	1	1	2	1	1	1	3	3	3
2	2	2	2	2	2	2	2	2	2
2	2	2	2	2	2	2	2	2	2
3	3	3	3	3	3	3	3	3	3
2	2	2	3	2	2	2	1	1	2
3	3	3	1	2	2	2	2	3	3
1	9	9	9	9	9	9	9	9	9
2	2	2	2	2	2	2	2	3	3
2	2	2	2	2	2	2	2	2	2
2	2	2	2	2	2	2	2	2	2
2	3	3	3	3	3	3	3	3	3
2	2	2	2	2	2	2	2	2	2
9	9	9	9	9	9	9	9	9	9
9	9	9	9	9	9	9	9	9	9
2	2	2	2	2	2	2	2	2	2
3	2	1	3	3	9	2	2	3	3
2	2	2	2	2	2	2	2	2	2
2	2	2	2	2	2	2	2	2	2
3	3	3	3	3	3	3	1	1	1
2	2	2	2	2	2	2	2	2	2
2	2	2	2	2	2	2	2	2	2
2	1	2	2	2	2	2	2	2	2
2	2	2	2	2	2	2	2	2	2
2	2	2	2	3	2	3	2	1	2
2	2	2	2	2	2	2	2	2	2
2	2	2	2	2	2	2	2	2	2
2	2	2	2	2	2	2	2	2	2
2	2	2	2	2	2	2	2	2	2
1	1	3	3	1	3	3	1	1	1
2	2	2	2	2	2	2	2	2	2
2	2	2	2	2	2	2	2	2	2
3	3	3	3	3	3	3	3	3	3
2	2	2	2	2	2	2	2	1	2

2	2	2	2	2	2	2	2	2	2
2	2	2	2	2	2	2	2	2	2
2	2	2	2	2	2	2	2	2	2
2	2	2	2	2	2	2	2	2	2
1	1	2	2	2	1	2	2	2	2
1	1	2	2	1	1	1	9	2	1
2	2	2	2	2	2	2	2	2	2
1	3	1	9	2	1	2	2	1	2
1	1	3	3	1	3	1	1	1	1
2	2	2	2	2	2	2	2	2	2
3	3	3	1	1	3	3	1	1	1
9	9	9	9	9	9	9	9	9	9
9	9	9	9	9	9	9	9	9	9
1	2	2	2	3	9	1	1	2	9
2	2	2	2	2	2	2	2	2	2
2	2	2	2	2	3	3	3	3	3
2	2	2	2	2	3	3	3	3	3
1	1	3	1	3	2	1	1	1	1
9	9	9	9	9	9	9	9	9	9
3	2	2	2	1	2	2	2	2	3
9	9	9	9	9	9	9	9	9	9
2	2	1	1	1	1	1	1	2	9
2	2	2	2	1	1	1	1	1	1
1	1	1	1	1	1	1	1	1	1
1	1	3	3	2	2	2	2	3	3
1	1	1	1	1	1	3	1	1	1
2	2	2	2	2	2	2	2	2	2
2	2	2	2	1	2	3	2	2	1
2	2	2	2	2	2	2	2	2	2
3	1	2	2	3	3	3	3	3	3
1	1	1	1	1	2	1	1	1	1
3	3	3	3	3	3	3	3	3	3
1	1	1	1	1	1	1	1	1	1
1	1	2	2	1	1	2	2	2	1
3	3	3	3	3	3	3	3	3	3
3	3	1	3	1	3	3	3	3	3
9	9	9	9	9	9	9	9	9	1
1	1	1	1	1	1	1	1	1	1
2	2	2	2	2	2	2	2	2	2
2	2	2	2	2	2	2	2	2	2
2	2	3	3	3	3	2	2	2	3
9	9	9	9	9	9	9	9	9	9
3	1	2	1	2	2	1	2	2	3
2	2	2	2	2	2	2	2	2	2
2	2	2	2	2	2	2	2	2	2
3	3	3	3	1	2	2	3	3	2
1	1	2	3	2	2	2	3	2	1

2	1	1	2	3	2	1	1	2	1
3	3	3	3	3	3	3	3	3	3
1	2	2	1	2	3	2	1	2	3
9	9	9	9	9	9	9	9	9	9
9	9	9	9	9	9	9	9	9	9
3	3	3	3	2	3	3	3	2	1
2	2	2	2	2	2	2	2	2	2
1	1	3	1	1	3	1	1	1	1
2	2	2	2	2	2	2	2	2	2
2	2	2	2	2	2	2	2	2	2
9	9	9	9	9	9	9	9	9	9
2	2	2	2	2	2	3	3	3	3
2	2	2	2	2	2	2	2	2	2
9	3	3	1	3	1	2	2	3	2
2	2	2	2	2	2	2	2	2	2
9	1	1	9	1	9	1	2	9	2
2	2	9	3	2	2	2	2	2	2
9	9	9	9	9	9	9	9	9	9
9	9	9	1	9	9	9	9	9	9
2	2	2	2	2	2	2	2	2	2
2	2	2	2	2	2	2	2	2	2
2	2	2	2	2	2	2	2	2	2
9	9	9	9	9	9	9	9	9	9
9	9	9	9	9	9	9	9	9	9
3	1	2	3	1	2	3	1	3	2
1	1	3	3	3	1	3	3	1	1
2	2	2	2	2	2	3	1	1	1
1	1	3	3	3	1	3	1	2	1
1	1	3	3	3	1	3	3	3	1
1	1	2	3	2	3	3	2	2	3
2	2	2	2	2	2	2	1	1	1
1	2	3	3	3	3	2	3	2	1
3	2	1	2	9	3	3	1	2	2
1	1	3	3	3	3	3	3	3	3
3	3	3	3	3	3	3	3	3	3
3	2	2	3	3	2	2	3	3	2
1	1	2	2	2	1	1	1	3	1
1	1	3	3	3	3	3	3	3	3
1	2	3	3	1	3	2	1	9	1
3	3	3	3	3	3	3	3	3	3
1	1	3	9	1	3	1	1	1	1
1	1	3	1	3	3	3	3	1	1
1	1	1	3	1	3	1	3	3	1
2	2	2	2	2	2	2	2	2	2
1	2	2	2	2	2	2	2	2	2
1	1	2	2	2	1	1	2	1	1
1	1	2	2	1	2	1	1	1	1

2	2	2	2	2	2	2	2	2	2
3	3	3	3	3	3	3	3	3	3
1	2	1	2	1	1	2	1	2	9
3	3	3	3	3	3	3	3	3	3
2	2	2	2	2	2	2	2	2	2
2	2	1	2	1	3	2	1	2	2
2	2	2	2	2	2	2	2	2	2
2	2	2	2	2	2	2	2	2	2
2	2	2	2	2	2	2	2	2	2
2	2	2	2	2	2	2	2	2	2
9	9	9	9	9	9	9	9	9	9
2	2	2	2	2	2	2	2	2	2
3	3	3	3	3	3	1	1	1	3
1	1	2	2	2	2	2	1	1	2
1	1	2	2	2	1	2	1	1	2
1	2	1	2	1	2	2	2	2	9
1	1	3	1	3	3	3	3	3	3
2	2	2	2	2	1	1	2	2	2
1	2	3	2	3	2	1	2	2	1
9	9	9	1	9	9	9	9	9	9
1	1	3	1	1	1	1	1	1	1
1	2	3	3	3	2	1	2	1	1
3	3	3	1	1	3	3	3	1	1
1	1	3	3	3	3	3	1	1	1
3	3	3	3	3	3	3	3	3	3
2	2	2	2	2	2	2	2	2	2
1	9	3	2	2	1	2	1	1	1
2	1	2	1	1	2	1	3	1	3
2	2	1	2	1	3	3	2	1	3
2	1	1	1	1	1	1	2	2	1
2	2	2	2	2	2	2	2	2	2
9	9	9	9	9	9	9	9	9	9
1	2	2	2	1	2	1	2	1	2
9	9	9	9	9	9	9	9	9	9
2	2	2	1	2	2	2	2	2	2
2	2	2	2	2	2	2	2	2	2
2	2	2	2	2	2	2	2	2	2
2	2	2	2	2	2	2	2	2	2
2	2	2	2	2	2	2	3	2	2
1	2	3	1	2	3	1	3	1	2
1	3	2	1	2	1	3	1	2	3
2	2	2	2	2	2	2	2	2	2
3	1	2	3	1	2	3	1	2	3
9	9	9	9	9	9	9	9	9	9
3	3	3	3	3	3	3	3	3	3
2	2	2	2	2	2	2	2	2	2
1	1	3	2	2	2	2	3	1	1

9	9	9	9	9	9	9	9	9	9
2	2	2	2	2	2	2	2	2	2
2	3	2	3	3	3	3	3	3	3
2	2	2	2	3	1	3	2	2	1
2	2	2	2	2	3	3	2	2	2
3	3	2	2	2	3	3	3	3	2
9	9	9	9	9	9	9	9	9	9
3	2	9	2	2	2	2	2	2	9
2	2	2	2	2	2	2	2	2	2
9	9	9	9	9	9	9	9	9	9
9	9	9	9	9	9	9	9	9	9
9	9	9	9	9	9	9	9	9	9
9	9	9	9	9	9	9	9	9	9
9	9	9	9	9	9	9	9	9	9
9	9	9	9	9	9	9	9	9	9
2	2	3	2	2	3	2	2	1	1
1	1	2	9	1	9	2	2	9	2
1	1	2	2	2	2	2	2	2	2
1	1	3	2	1	2	3	1	3	2
2	3	2	1	2	2	1	1	1	1
2	3	2	1	2	1	3	1	2	3
2	1	2	2	2	3	2	2	1	2
1	1	3	3	3	1	1	1	1	1
2	2	3	3	1	3	2	1	1	1
2	2	2	2	2	2	2	2	2	2
1	2	2	2	2	2	2	2	1	2
2	2	2	2	2	2	2	2	2	2
1	1	1	1	1	1	1	1	1	1
1	2	2	2	2	2	2	2	1	2
2	2	3	2	2	3	2	2	1	1
2	3	2	1	2	2	1	1	1	1
9	9	9	9	9	9	9	9	9	9
1	1	3	1	3	3	3	3	1	3
2	2	2	2	2	2	2	2	2	2
2	2	2	2	2	2	2	2	2	2
2	2	2	2	2	2	2	2	2	2
2	2	2	2	2	2	2	2	2	2
9	9	9	9	9	9	9	9	9	9
2	2	2	2	2	2	2	2	2	2
2	2	2	2	2	2	2	2	2	2
2	2	2	2	2	2	2	2	2	2
2	2	2	2	2	2	2	2	2	2
2	2	2	2	2	2	2	2	2	2
1	1	1	2	2	2	2	2	3	3
2	2	2	2	2	2	2	2	2	2
2	2	2	2	2	2	2	2	2	2
9	9	9	9	9	9	9	1	9	9

2	2	2	2	2	2	2	2	2	2
2	2	2	2	2	2	2	2	2	2
9	9	9	9	9	9	9	9	9	1
9	9	9	9	9	9	9	9	9	9
2	2	2	2	2	2	2	2	2	2
9	9	9	9	9	9	9	9	9	9
1	1	1	2	3	1	2	3	2	1
1	2	2	2	3	3	2	3	2	2
9	9	9	9	9	9	9	9	9	9
1	1	2	2	2	2	3	3	3	2
9	9	9	9	9	9	9	9	9	9
2	1	1	2	1	2	3	3	2	2
2	2	2	2	2	2	2	2	2	2
2	2	2	2	2	2	2	2	2	2
3	1	2	2	1	2	3	3	1	2
3	3	3	3	3	3	3	3	3	3
2	2	2	2	2	2	2	2	2	2
2	2	2	2	2	2	2	2	2	2
1	1	2	2	1	1	2	3	2	3
1	1	1	2	1	3	3	2	2	1
3	3	3	3	3	3	3	1	1	1
2	2	2	2	2	2	2	2	2	2
3	3	2	2	3	2	1	1	1	1
3	3	3	3	3	3	3	3	3	3
2	2	2	2	2	2	2	2	2	2
1	1	2	2	2	2	3	1	1	1
2	2	2	2	2	2	2	2	2	2
2	2	2	2	2	2	2	2	2	2
2	3	2	2	2	3	2	2	2	2
9	9	9	9	9	9	9	9	9	9
3	3	3	1	1	1	9	1	9	9
2	2	2	2	2	2	2	2	2	2
9	9	9	9	9	9	9	9	9	9
9	9	9	9	9	9	9	9	9	9
3	1	1	3	2	1	2	3	3	2
9	1	9	9	9	9	9	9	9	9
9	9	9	9	9	9	9	9	9	9
2	2	2	2	2	2	2	2	2	2
1	9	9	1	9	9	9	9	9	1
2	1	1	2	2	1	3	3	2	2
1	1	1	1	1	1	1	1	1	1
9	3	2	2	3	1	2	3	1	2
9	9	9	9	9	9	9	9	9	9
2	3	1	2	1	3	2	1	2	3
1	2	2	3	2	2	1	3	2	3
9	9	9	9	9	9	9	9	9	9
2	1	2	3	2	1	1	2	2	3

2	1	1	2	1	2	1	1	1	2
9	9	9	9	9	9	9	9	9	9
9	9	9	9	9	9	9	9	9	9
2	2	2	2	2	2	2	2	2	2
2	2	1	3	1	3	3	3	3	1
2	1	1	2	1	2	1	1	1	2
2	9	1	9	1	9	1	1	1	2
3	3	3	3	3	3	1	1	1	1
2	2	2	2	2	2	2	2	2	2
3	3	1	2	2	2	3	1	1	3
9	9	9	9	9	9	9	9	9	9
9	9	9	9	9	9	9	9	9	9
9	9	9	9	9	9	9	9	9	9
2	1	2	2	2	2	2	2	1	2
2	2	2	2	2	2	2	2	2	2
9	9	9	9	9	9	9	9	9	9
9	9	9	9	9	9	9	9	9	9
9	9	9	9	9	9	9	9	9	9
1	1	1	2	1	2	2	2	2	1
1	1	2	2	2	2	2	2	2	2
9	9	9	9	9	9	9	9	9	9
3	3	3	2	2	1	1	1	3	3
3	3	3	3	3	3	3	3	3	3
9	9	9	9	9	9	9	9	9	9
2	2	2	2	2	2	2	2	2	2
1	1	2	3	2	3	3	2	2	2
9	9	9	9	9	9	9	9	9	9
9	9	9	9	9	9	9	9	9	9
2	2	2	1	2	2	1	2	9	1
2	1	1	2	2	1	1	2	2	2
1	1	1	1	2	3	3	2	2	3
9	9	9	9	9	9	9	9	9	9
9	9	9	9	9	9	9	9	9	9
1	1	1	1	1	1	1	1	1	1
2	2	9	2	9	1	1	1	1	1
2	2	2	2	2	2	2	2	2	2
2	2	2	2	2	1	2	1	1	1
2	2	2	2	2	2	2	2	2	2
9	1	3	3	2	3	1	9	2	1
2	2	1	3	1	2	3	2	3	2
1	1	2	3	3	2	2	2	2	1
9	9	9	9	9	9	9	9	9	9
9	9	9	9	9	9	9	9	9	9
9	9	9	9	9	1	9	9	9	9
9	9	9	9	9	9	9	9	9	9
9	9	9	9	9	9	9	9	9	9
9	9	9	9	9	9	9	9	9	9

2	2	2	2	2	2	2	2	2	2
2	3	2	3	2	3	2	3	2	3
1	1	2	2	2	3	1	1	1	3
2	2	2	2	2	2	2	2	2	2
9	9	9	9	9	9	9	9	9	9
9	9	9	9	9	9	9	9	9	9
9	9	9	9	9	9	9	9	9	9
9	9	9	9	9	9	9	9	9	9
9	9	9	9	9	9	9	9	9	9
9	9	9	9	9	9	9	9	9	9
9	9	9	9	9	9	9	9	9	9
9	9	9	9	9	9	9	9	9	9
9	9	9	9	9	9	9	9	9	9
1	3	3	1	3	3	3	1	3	1
1	1	9	9	9	9	9	9	9	9
1	2	2	1	2	2	2	2	2	2
2	2	2	2	2	2	2	2	2	2
9	9	9	9	9	9	9	9	9	9
9	9	9	9	9	9	9	9	9	9
9	9	9	9	9	9	9	9	9	9
9	9	9	9	2	3	9	9	9	9
9	9	9	9	9	9	9	9	9	9
2	2	1	1	1	1	1	1	1	1
9	9	9	9	9	9	9	9	9	9
2	2	2	1	1	1	1	1	2	1
1	1	2	2	2	2	2	2	2	2
2	2	2	1	1	2	2	2	2	2
1	1	1	1	2	2	2	1	1	1
2	1	1	1	1	2	2	2	1	1
1	1	1	2	2	2	2	1	1	1
1	1	2	2	2	2	2	2	2	2
2	2	1	1	2	2	2	2	2	2
1	1	1	1	1	1	1	1	1	1
9	9	9	9	9	9	9	9	9	9
1	1	1	2	2	2	3	3	2	2
1	1	2	2	2	1	1	1	1	1
1	1	1	1	1	1	1	1	1	1
9	9	9	9	9	9	9	9	9	9
3	3	3	3	1	3	3	3	3	3
3	3	3	3	3	3	3	3	3	3
3	3	3	3	3	3	3	3	3	3
9	9	9	9	9	9	9	9	9	9
9	9	9	9	9	9	9	9	9	9
9	9	9	9	9	9	9	9	9	9
9	9	9	9	9	9	9	9	9	9
9	2	2	2	2	2	2	2	2	2
9	9	9	9	9	9	9	9	9	9

9	9	9	9	9	9	9	9	9	9
9	9	9	9	9	9	9	9	9	9
9	9	9	9	9	9	9	9	9	9
9	9	9	9	9	9	9	9	9	9
9	9	9	9	9	9	9	9	9	9
9	9	9	9	9	9	9	9	9	9
9	9	9	9	9	9	9	9	9	9
9	9	9	9	9	9	9	9	9	9
9	9	9	9	9	9	9	9	9	9
9	9	9	9	9	9	9	9	9	9
3	9	9	9	9	9	9	9	9	9
9	9	9	9	9	9	9	9	9	9
1	1	1	1	1	1	1	1	1	2
1	2	2	1	1	1	1	2	1	1
1	1	2	2	2	2	2	1	1	2
2	2	2	1	2	1	2	1	2	2
2	1	2	1	2	1	1	2	1	1
3	2	1	2	2	2	3	3	3	2
1	1	1	2	2	2	2	2	2	2
9	9	9	9	9	9	9	9	9	9
1	2	2	2	1	1	1	1	1	1
2	2	2	1	2	2	1	2	1	2
2	2	2	2	2	2	2	2	2	2
1	1	1	1	2	1	1	1	1	1
2	2	2	1	1	1	2	1	1	2
1	1	3	3	1	3	3	1	1	1
2	2	1	9	2	2	2	2	2	2
3	3	3	3	3	3	3	3	3	3
9	9	9	9	9	9	9	9	9	9
9	1	3	3	9	9	9	9	9	9
9	9	9	9	9	9	9	9	9	9
1	1	1	2	2	1	1	2	2	1
2	1	1	1	1	2	2	2	2	2
2	2	2	1	1	2	2	1	1	1
2	1	1	1	2	2	2	1	1	2
2	2	2	2	2	2	2	2	2	2
2	2	2	1	1	1	1	1	1	1
1	1	1	1	1	2	2	2	2	2
2	2	1	2	1	1	1	1	2	2
1	1	1	2	2	2	1	1	1	2
2	2	2	2	2	2	2	2	2	2
1	1	2	2	2	1	1	1	1	1
1	1	3	1	1	3	3	1	2	2
1	1	2	1	1	1	2	1	1	1
1	1	1	2	2	2	2	1	1	1
1	1	1	1	1	1	1	1	1	1
2	2	2	2	2	2	2	2	2	2

9	9	9	9	9	9	9	9	9	9
2	2	2	2	2	1	2	2	2	2
9	9	9	2	9	9	9	9	9	9
9	2	9	9	9	9	9	9	9	9
9	9	9	9	9	9	9	9	9	9
9	9	9	9	9	9	9	9	9	9
9	9	9	9	9	9	9	9	1	9
3	3	3	2	2	2	2	9	3	3
9	9	9	9	9	9	9	9	9	9
2	2	2	2	2	2	2	2	2	2
9	9	9	9	9	9	9	9	9	9
9	9	9	9	9	9	9	9	9	9
1	2	2	2	1	1	1	1	2	1
2	2	2	2	2	2	2	2	2	2
1	1	1	1	1	1	1	1	2	2
9	9	9	9	9	9	9	9	9	9
2	2	2	2	2	2	2	2	2	2
3	3	3	3	3	3	3	3	3	3
9	9	9	9	9	9	9	9	9	9
1	1	1	1	1	1	1	1	1	1
2	2	2	2	2	2	2	2	2	2
3	2	3	3	2	2	2	2	3	3
2	2	2	2	2	2	2	2	2	2
2	2	2	2	2	2	2	2	2	2
2	1	1	2	2	2	2	2	2	2
1	1	2	2	2	2	2	2	2	2
3	3	3	3	2	9	2	3	1	2
3	3	3	2	2	2	2	2	1	2
1	1	1	1	1	1	1	1	1	1
2	1	1	1	1	1	1	1	1	1
9	9	9	9	9	9	9	9	9	9
3	3	3	3	2	2	2	2	2	2
9	9	9	9	9	9	9	9	9	9
2	2	2	2	2	2	2	2	2	2
2	2	2	2	2	2	2	2	2	2
2	1	1	1	1	1	2	2	2	2
3	3	3	3	3	3	3	3	3	3
1	1	1	2	2	2	1	1	2	2
1	1	1	1	1	1	1	1	1	1
2	3	3	1	1	1	2	1	2	2
2	2	2	2	2	2	2	2	2	2
1	1	2	2	2	1	1	1	1	1
9	9	9	9	9	9	9	9	9	9
9	9	9	9	9	9	9	9	9	9
9	9	9	9	9	9	9	9	9	9
9	9	9	9	9	9	9	9	9	9
9	9	9	9	9	9	9	9	9	9

9	9	9	9	9	9	9	9	9	9
2	2	2	2	2	2	2	2	2	2
9	9	9	9	9	9	9	9	9	9
2	1	3	1	2	1	2	3	1	2
9	9	9	9	9	9	9	9	9	9
2	1	2	1	1	2	2	3	2	3
2	2	2	2	3	3	2	2	2	3
9	9	9	9	9	9	9	9	9	9
9	9	9	9	9	9	9	9	9	9
9	9	9	9	9	9	9	9	9	9
9	9	9	9	9	9	9	1	9	9
9	9	9	9	9	9	9	9	9	9
2	2	1	2	1	1	1	2	3	2
9	9	9	9	9	9	9	9	9	9
2	9	9	9	9	9	9	9	9	9
3	2	1	2	1	3	2	1	1	3
2	3	3	2	2	3	2	2	1	3
2	2	3	3	2	2	1	2	2	1
3	3	3	2	2	2	2	2	2	2
3	2	2	2	1	1	3	2	2	3
2	1	3	3	2	1	1	2	3	1
3	1	3	3	1	3	3	1	1	1
2	3	1	1	1	2	3	1	1	2
2	3	1	2	1	2	1	2	3	2
2	2	2	2	2	2	2	2	2	2
1	1	1	1	1	2	2	2	1	2
2	2	2	2	2	2	2	2	2	2
2	2	9	2	1	1	1	1	1	1
2	2	2	1	9	1	2	1	2	3
2	2	2	2	1	1	1	2	2	2
3	1	1	2	2	2	2	2	1	9
2	3	2	2	2	2	3	2	2	2
9	9	9	9	9	9	9	9	9	9
3	1	2	9	2	9	1	3	2	1
2	1	2	2	2	2	2	1	1	1
2	2	2	2	2	2	2	1	2	1
2	2	2	2	3	3	3	2	2	2
2	2	2	3	2	3	3	1	2	2
9	9	9	9	9	9	2	9	9	9
3	1	2	3	2	1	2	3	2	1
9	9	9	9	9	9	9	1	9	9
9	9	9	9	9	9	9	9	9	9
2	3	2	1	3	1	2	2	2	2
9	9	9	9	9	9	9	9	9	9
1	1	1	1	1	1	1	1	1	1
3	1	3	1	1	1	3	1	1	3
9	3	3	3	3	3	3	3	3	3

2	2	3	3	3	2	3	3	3	3
1	9	9	9	9	9	9	9	9	9
2	2	2	2	2	2	2	2	2	2
3	1	1	3	1	1	3	3	1	1
3	3	3	3	3	3	3	1	1	1
1	1	3	3	3	1	1	1	3	1
9	1	3	9	1	3	1	2	3	2
9	3	1	2	1	2	3	1	2	1
2	2	2	2	2	2	2	2	2	2
2	2	2	2	2	2	2	2	2	2
1	1	1	1	3	3	2	2	1	1
3	2	1	2	3	2	3	2	2	1
3	2	9	1	2	3	2	1	2	3
2	2	2	2	2	2	2	2	2	2
1	1	3	1	1	2	2	2	2	1
3	1	2	2	1	2	1	2	2	1
2	2	2	2	2	2	2	2	2	2
1	1	3	3	2	2	2	2	1	1
2	2	2	2	2	2	2	2	2	2
2	2	2	2	2	2	2	2	2	2
2	2	2	2	2	2	2	2	2	2
1	1	1	2	3	3	2	3	2	3
9	9	9	9	9	9	9	9	9	9
9	9	9	9	9	9	9	9	9	9
2	2	2	2	2	2	2	2	2	2
3	2	1	2	2	1	2	3	2	1
3	1	3	1	2	2	3	3	2	1
2	2	1	1	1	2	3	3	3	1
1	1	2	1	3	1	1	1	1	1
9	9	9	9	9	1	9	9	9	9
1	2	1	2	3	1	2	2	2	2
2	1	2	2	3	2	3	3	2	1
3	9	2	3	3	2	2	3	2	3
1	1	2	2	2	2	2	3	2	2
3	3	2	1	1	3	3	1	1	3
2	2	2	2	2	2	2	2	2	2
3	3	9	3	3	3	2	2	2	2
9	9	9	9	9	9	9	9	9	9
1	1	1	2	3	3	2	2	1	2
1	1	2	3	1	3	3	3	2	2
9	9	9	9	9	9	9	9	9	9
9	9	9	9	9	9	9	9	9	9
2	9	1	2	2	3	2	1	2	2
9	9	9	9	9	9	9	9	9	9
2	2	2	2	2	2	2	2	2	2
1	1	2	3	2	2	3	1	1	1
9	9	9	9	9	9	9	9	9	9

2	3	3	2	2	2	2	3	2	3
9	9	9	9	9	9	9	9	9	9
1	1	2	3	3	3	2	2	2	1
9	9	9	9	9	9	9	9	9	9
9	9	9	9	9	9	9	9	9	9
3	3	3	3	3	3	3	3	3	3
2	2	2	2	2	2	2	2	2	2
2	2	2	2	2	2	2	2	2	2
1	1	1	1	1	1	1	1	1	1
2	2	2	2	2	2	2	2	2	2
9	9	9	9	9	9	9	9	9	9
9	9	9	9	9	9	9	9	9	9
9	9	9	9	9	9	9	9	9	9
1	1	1	1	1	1	1	1	1	1
2	2	2	2	2	2	2	2	2	2
1	1	1	1	1	1	1	1	1	1
9	9	9	9	9	9	9	9	9	9
9	9	9	9	9	9	9	9	9	9
3	3	3	3	3	3	1	1	1	1
2	2	2	2	2	2	2	2	2	2
1	2	2	2	1	1	1	1	1	1
3	3	3	3	3	3	3	3	2	2
9	9	9	9	9	9	9	9	9	9
2	2	3	3	3	3	2	2	2	2
1	1	1	1	2	2	1	2	2	2
2	2	3	3	3	3	2	2	2	2
9	9	9	1	9	9	1	9	9	9
2	2	1	1	1	1	2	2	2	2
2	2	2	2	2	2	2	2	2	2
1	9	9	9	9	9	9	9	9	9
1	1	3	2	2	3	1	2	2	1
2	2	2	2	2	2	2	2	2	2
3	3	3	3	1	1	1	2	2	2
2	2	2	1	2	3	3	3	3	1
2	2	2	2	2	2	2	2	2	2
2	2	2	1	2	3	3	1	2	2
1	2	3	3	2	2	2	1	1	1
3	3	3	3	3	3	1	1	1	1
2	2	2	2	2	2	2	2	2	2
3	3	3	3	3	3	3	3	3	3
1	1	3	3	2	2	2	1	1	1
2	2	2	2	2	2	2	2	2	3
3	3	3	3	3	3	3	3	3	9
2	2	2	3	3	3	2	2	2	2
2	1	3	1	3	2	3	1	3	1
3	2	1	2	3	2	2	1	1	2
1	1	2	2	2	1	3	2	2	2

9	2	2	3	2	3	2	2	3	3
2	2	2	2	2	2	2	2	2	2
2	1	2	1	1	1	2	2	1	1
3	3	3	3	3	2	2	2	2	2
1	1	2	2	2	1	2	2	2	2
1	9	9	9	9	9	9	9	9	9
2	2	2	2	2	2	2	2	2	2
2	2	1	1	1	1	2	2	2	1
2	2	2	2	2	1	2	2	2	2
2	2	2	3	2	2	2	2	2	2
2	2	2	2	2	2	2	2	2	1
2	2	2	3	2	1	3	1	1	1
2	2	2	2	2	2	2	2	2	2
3	3	3	3	3	3	3	3	3	3
3	2	2	2	3	1	2	2	3	3
3	2	1	3	2	2	1	2	3	1
3	2	3	2	3	2	3	2	3	2
1	1	2	1	3	3	2	2	2	2
2	2	2	2	2	2	2	2	2	2
2	9	2	3	2	2	2	2	1	2
2	2	2	2	2	2	2	2	2	2
3	1	3	2	2	2	2	2	2	2
3	3	3	3	3	3	3	3	3	3
1	1	1	1	9	1	3	3	3	3
1	1	3	3	1	2	1	2	1	1
2	2	2	2	2	2	2	2	2	2
1	2	2	2	2	3	3	2	1	1
1	2	2	2	2	3	3	2	1	1
2	2	2	2	2	2	2	2	2	2
1	1	1	1	1	2	2	1	1	1
2	2	3	3	3	3	2	2	2	2
3	2	2	3	2	2	3	2	3	2
3	3	3	3	2	1	1	1	1	2
3	3	3	3	3	3	3	3	3	2
3	3	1	1	1	2	1	1	1	1
2	2	2	2	2	2	2	2	2	2
2	2	2	2	2	2	2	2	2	2
2	2	2	2	2	3	1	2	1	1
1	1	2	2	2	2	2	2	2	3
1	1	3	2	2	2	2	2	2	2
2	2	2	2	1	3	3	2	2	1
1	1	3	3	3	2	2	1	1	1
2	2	2	2	2	1	1	1	1	1
2	2	2	2	2	2	1	2	2	2
2	2	3	3	3	3	2	2	1	2
1	1	3	2	1	3	1	2	1	1
1	1	2	3	2	3	2	1	2	2

2	2	3	2	2	1	1	2	2	1
2	2	3	3	3	3	3	3	3	3
1	1	2	2	2	3	2	1	2	1
2	2	2	2	2	2	2	2	2	2
3	2	2	2	2	2	2	2	2	2
2	2	2	2	2	2	2	2	2	2
1	1	3	2	1	2	2	2	1	1
2	2	1	1	1	2	2	1	1	1
2	2	2	2	2	2	2	2	2	2
2	2	2	2	2	2	2	2	2	2
2	2	2	2	2	2	2	2	2	2
9	9	9	9	9	9	9	9	9	9
9	9	9	9	9	9	9	9	9	9
9	9	9	9	9	9	9	9	9	9
1	1	2	1	2	2	1	2	1	1
1	2	2	2	2	2	2	2	2	1
2	2	1	1	1	2	2	1	1	1
1	1	2	2	2	2	2	2	2	2
1	1	3	1	1	1	1	1	1	1
1	1	3	2	2	2	1	1	2	1
1	2	3	2	2	1	2	3	2	2
2	2	3	3	3	3	3	3	3	3
2	2	2	2	2	2	2	2	2	2
2	2	2	2	2	2	2	2	2	2
1	1	2	2	2	2	2	2	2	2
1	1	2	3	1	2	1	2	9	2
1	1	2	2	2	2	2	2	1	1
2	2	2	2	2	2	2	2	2	2
2	2	2	2	2	2	2	2	2	2
9	9	9	9	9	9	9	9	9	9
2	2	2	2	2	2	2	2	2	2
2	2	2	2	2	2	2	2	2	2
2	1	1	2	1	3	1	1	2	2
2	2	2	2	2	2	2	2	2	2
1	9	9	9	9	9	9	9	9	9
2	2	2	2	2	2	2	2	2	2
2	2	2	2	2	2	2	2	2	2
2	2	2	2	2	2	2	2	2	2
2	2	2	2	2	2	2	2	2	2
2	2	2	2	2	2	1	1	2	1
2	2	2	2	2	2	2	2	2	2
2	2	2	2	2	2	2	2	2	2
1	1	2	1	1	1	1	1	1	1
1	1	2	2	2	2	1	2	2	1
2	2	2	2	2	2	2	2	2	2
1	1	2	3	2	2	2	2	2	2
2	2	2	2	2	2	2	2	2	2

2	2	2	2	1	1	2	3	3	2
1	1	2	2	2	2	2	2	2	2
1	1	3	3	3	2	1	1	1	1
2	2	2	2	2	2	2	2	2	2
2	2	2	2	2	2	2	2	2	2
2	2	2	3	3	3	2	2	2	2
9	9	9	9	9	9	9	9	9	9
1	1	2	1	2	2	1	1	1	1
3	2	1	1	1	1	1	1	2	2
2	2	2	2	2	3	2	2	1	1
1	1	2	2	2	2	2	1	1	1
2	2	2	2	2	2	2	2	2	2
1	2	2	2	2	2	2	2	1	1
1	1	9	9	9	9	1	9	9	1
1	1	2	2	2	3	1	1	3	1
1	2	2	1	2	2	1	1	2	1
1	2	1	2	2	2	1	1	1	2
1	1	3	2	2	2	2	2	2	2
1	1	1	1	1	1	1	1	1	1
1	2	2	2	2	2	2	3	3	2
1	1	1	1	1	1	2	2	2	2
2	2	1	1	2	2	3	3	2	3
2	2	2	2	2	2	2	2	2	2
2	2	2	2	2	2	2	2	2	2
2	2	2	2	2	2	2	2	2	2
2	2	2	2	2	2	2	2	2	2
9	9	9	9	9	9	9	9	9	9
2	2	2	2	2	2	2	2	2	2
1	1	2	2	2	2	2	2	2	1
2	2	2	2	2	2	2	2	2	2
2	2	2	2	2	2	2	2	2	2
2	1	2	2	2	1	1	2	1	2
9	9	9	9	9	9	9	9	9	9
9	9	9	9	9	9	9	9	9	9
9	9	9	9	9	9	9	9	9	9
9	9	9	9	9	9	9	9	9	9
3	1	2	1	2	3	2	3	2	2
1	1	3	1	2	2	2	1	1	1
1	1	2	1	2	1	1	1	1	1
2	2	2	2	9	2	3	2	2	3
1	3	1	3	1	3	1	2	1	3
1	2	2	2	2	2	2	2	2	2
2	1	2	2	2	1	2	2	2	2
2	2	2	2	2	2	2	2	2	2
1	1	2	2	1	1	3	2	1	1
1	1	2	2	2	2	2	1	2	1
1	1	2	1	2	2	1	1	2	1

2	3	1	2	2	3	1	2	2	1
1	3	2	2	1	1	2	3	2	3
1	2	3	2	1	2	2	2	1	2
1	2	1	1	1	1	1	1	1	1
3	3	2	1	1	2	3	2	3	2
3	1	2	3	3	2	1	2	3	2
1	1	3	3	1	1	1	1	1	1
9	9	9	9	9	9	9	9	9	9
2	2	2	2	2	2	2	2	2	2
2	2	2	2	2	2	2	2	2	2
2	2	2	2	2	2	2	2	2	2
2	2	2	2	2	2	2	2	2	2
2	2	2	2	2	2	2	2	2	2
1	1	2	2	2	1	2	2	2	2
2	2	2	2	1	2	2	1	1	1
2	2	2	2	2	2	2	2	2	2
2	2	2	2	2	2	2	2	2	2
3	2	2	3	3	3	1	1	1	1
2	2	3	2	2	1	2	2	2	1
2	2	9	3	3	3	1	1	2	2
2	2	2	2	3	2	2	2	2	3
2	2	2	2	2	2	2	1	2	1
1	1	3	2	3	2	2	1	1	1
2	2	9	9	9	9	9	9	9	9
2	3	1	1	2	3	1	3	2	9
1	1	2	1	2	2	2	1	1	1
1	1	1	2	2	1	1	2	1	1
1	1	2	2	2	1	2	1	2	2
2	2	2	2	2	2	2	2	2	2
9	9	9	9	9	9	9	9	9	9
3	2	2	3	3	3	3	3	3	3
9	9	9	9	9	9	9	9	9	9
9	9	9	9	9	9	9	9	9	9
9	9	9	9	9	9	9	9	9	9
9	9	9	9	9	9	9	9	9	9
2	2	2	2	2	2	2	2	2	2
3	1	2	2	2	2	2	2	2	2
9	9	9	9	9	9	9	9	9	9
2	2	2	2	2	2	2	2	2	2
2	2	2	2	2	2	2	2	2	2
2	2	2	2	2	3	2	1	2	3
1	2	2	2	2	2	2	2	2	2
2	2	2	2	2	2	2	2	2	2
1	3	2	1	3	2	1	3	2	1
9	9	9	9	9	3	9	3	9	9
1	1	1	1	2	1	1	1	1	1
3	3	3	3	3	3	3	3	3	3

2	1	1	1	2	2	1	1	1	1
2	2	1	1	1	2	2	1	1	1
1	1	2	1	1	1	1	1	1	1
1	2	2	2	2	1	2	2	2	2
9	9	9	9	9	9	9	9	9	9
9	9	9	9	9	9	9	9	9	9
2	2	2	2	2	2	2	2	2	2
3	3	3	3	3	3	3	3	3	3
1	9	9	9	9	9	9	1	9	9
9	9	9	9	9	9	9	9	9	9
1	1	2	2	2	2	3	2	9	1
2	1	1	1	1	2	2	2	1	1
3	2	2	1	1	1	1	1	2	2
3	3	3	3	3	3	3	3	3	3
3	3	3	1	3	3	3	3	3	3
2	2	3	3	3	3	1	2	3	2
1	1	3	2	2	2	3	1	1	2
2	2	1	3	2	3	1	3	2	3
3	9	9	9	9	9	9	9	9	9
1	1	3	2	1	3	3	3	2	1
2	3	1	1	3	1	2	3	1	2
1	2	2	1	2	2	1	2	2	1
3	3	3	2	9	1	1	2	1	9
1	1	2	2	2	2	2	2	2	2
1	1	1	1	3	3	3	3	3	3
3	3	2	2	2	2	2	2	2	2
2	3	3	2	2	2	2	2	1	2
2	2	2	2	2	2	2	2	2	2
9	3	1	9	2	3	1	3	3	1
1	1	3	3	3	3	3	1	3	9
2	2	2	2	2	2	2	2	2	2
2	2	2	2	2	1	1	1	2	1
1	1	3	1	1	1	3	1	1	1
1	1	3	3	1	3	2	3	1	1
1	9	9	9	9	9	9	9	9	9
1	2	2	2	2	2	1	1	1	1
9	9	9	9	9	9	9	9	9	9
2	2	2	2	2	2	2	2	2	2
9	9	9	9	9	9	9	9	9	9
1	1	1	1	1	1	1	1	2	1
9	9	9	9	9	9	9	9	9	9
3	1	2	3	3	3	2	1	2	1
1	3	2	1	2	2	2	3	2	3
1	1	2	1	1	2	1	2	2	2
1	1	2	1	1	2	1	2	2	2
1	3	1	2	3	3	3	2	1	2
2	3	3	2	3	2	3	2	2	2

1	2	2	1	3	1	2	3	1	2
9	9	9	9	9	9	9	9	9	9
1	1	3	2	2	1	2	2	2	2
1	1	3	2	9	3	3	2	3	3
1	1	3	1	1	1	1	1	1	1
9	9	9	9	9	9	9	9	9	9
9	9	9	9	9	9	9	9	9	9
2	2	2	1	2	2	2	2	2	2
3	1	1	1	1	3	1	1	1	3
1	1	3	3	3	3	3	3	9	3
1	1	1	3	1	1	1	2	2	2
3	2	1	2	3	1	2	3	2	1
2	2	2	3	2	3	2	2	3	2
1	1	3	1	1	1	1	1	1	1
9	9	9	9	9	9	9	9	9	9
3	3	3	3	3	3	3	3	3	3
3	3	3	2	2	1	2	3	2	3
2	2	2	2	2	2	2	2	2	2
3	1	2	3	1	1	1	2	3	3
3	2	1	3	2	1	2	3	2	2
9	9	9	9	9	9	9	9	9	9
9	9	9	9	9	9	9	9	9	2
3	3	1	2	3	1	1	2	2	2
9	9	9	9	9	9	2	9	9	9
9	9	9	9	9	9	9	9	9	9
3	3	2	3	9	2	3	3	2	9
3	3	2	2	3	2	3	2	2	2
2	2	2	2	2	2	2	2	2	2
3	3	3	3	3	3	3	3	3	3
1	1	2	1	2	2	1	2	1	1
3	3	3	3	3	3	3	3	3	3
2	2	2	2	2	2	2	1	3	2
9	9	9	9	9	9	9	9	9	9
9	9	9	9	9	9	9	9	9	9
9	9	9	9	9	9	9	9	9	9
1	2	2	2	2	1	2	2	2	2
1	1	3	1	3	3	1	1	1	3
3	1	2	1	3	1	2	1	3	1
2	2	3	2	2	3	2	2	2	2
9	9	9	9	9	9	9	9	9	9
2	2	2	2	2	2	2	2	2	2
2	1	3	1	2	3	1	2	3	1
3	3	2	3	2	3	2	3	2	3
1	1	2	1	1	9	9	9	9	9
1	1	2	2	3	2	1	2	3	2
3	3	1	3	3	3	3	1	3	1
1	1	3	3	2	3	3	3	2	1

3	1	2	3	1	2	3	2	1	2
2	3	3	3	3	2	2	2	2	3
3	3	3	3	9	1	1	1	1	3
3	1	2	3	1	2	3	1	3	1
1	1	3	2	2	3	3	2	2	1
2	2	2	2	2	2	1	1	2	2
2	1	1	9	2	1	2	2	1	2
2	2	2	2	2	2	1	1	1	1
9	9	9	9	9	9	9	9	9	9
2	1	2	1	2	2	1	1	1	1
2	2	2	2	2	2	2	2	2	2
2	2	2	3	3	3	3	3	2	2
1	2	3	2	1	3	3	1	2	1
1	1	3	1	1	1	1	2	3	2
9	9	9	9	9	9	9	9	9	9
2	3	3	3	3	2	2	1	3	2
1	1	2	1	1	1	2	2	3	1
3	1	1	2	2	2	3	3	3	2
2	1	3	2	1	1	3	1	3	2
2	2	2	2	2	2	2	2	2	2
1	1	1	1	1	2	2	1	2	1
2	2	2	2	2	2	1	2	2	2
1	2	1	2	1	1	1	1	2	1
1	1	2	2	2	3	2	2	2	2
3	9	2	1	2	2	2	2	2	2
1	1	2	3	1	3	3	2	2	1
2	2	3	2	2	3	2	1	1	2
1	1	3	3	2	3	2	2	2	1
2	2	2	2	2	2	2	2	2	2
2	2	2	2	2	2	2	2	1	2
2	2	2	2	2	2	9	1	1	2
2	2	2	2	2	2	2	2	2	2
2	2	2	2	2	2	2	2	2	2
1	1	2	3	3	3	3	3	3	3
3	1	3	1	2	3	3	1	1	2
1	1	3	1	1	1	3	2	2	2
2	2	1	1	1	2	2	1	1	1
2	2	2	2	1	1	2	2	1	1
3	3	3	3	1	3	2	1	1	1
1	2	2	2	1	1	1	1	3	3
3	3	3	3	3	9	2	2	2	2
2	2	1	1	2	2	1	1	1	2
1	2	2	2	2	1	2	2	2	2
1	1	1	1	1	1	1	1	1	1
1	2	2	2	2	1	1	1	1	2
1	2	1	1	2	2	2	2	1	1
2	3	3	2	1	1	1	1	1	2

1	1	1	2	2	1	2	2	2	2
3	2	1	1	1	2	2	2	1	1
1	2	2	2	1	1	1	2	1	1
3	3	3	3	2	2	2	3	3	2
1	2	1	2	2	2	1	1	2	1
3	1	2	2	3	3	1	1	2	1
2	1	1	2	2	1	2	3	3	2
1	2	2	2	1	1	1	1	2	2

Poison	InheritedC	OffensiveP	PostCoitalE	Dysmenorr	Menorrhag	Asymptom	VaginalRas	Vaginalswe	WillLikePa
2	2	2	2	2	2	2	2	2	2
2	2	2	1	1	1	2	2	2	2
2	2	1	1	2	1	2	1	1	2
2	2	2	1	1	1	2	1	1	2
2	2	2	1	1	1	2	2	2	2
2	2	2	1	1	1	2	1	1	2
9	9	9	9	9	9	9	9	9	9
3	1	3	3	3	3	1	3	2	2
2	9	2	2	2	2	2	2	2	1
9	9	2	9	9	9	9	9	2	2
3	3	2	9	3	2	2	2	2	2
3	2	2	3	1	1	2	2	3	3
2	1	2	2	2	2	2	2	2	1
2	1	2	3	3	3	2	2	1	2
1	1	2	2	3	2	2	2	2	1
3	1	2	2	3	2	3	2	2	3
1	3	2	3	9	2	1	2	1	1
3	1	2	2	3	2	3	3	1	3
3	1	2	2	3	2	3	2	2	3
3	3	1	2	3	3	3	2	1	1
2	1	2	2	2	2	2	2	2	1
2	1	2	2	2	2	2	2	2	1
2	2	2	2	2	2	2	2	2	2
2	1	2	2	1	2	2	2	2	1
2	2	2	2	2	2	2	2	2	2
3	2	2	2	2	2	2	2	2	1
2	1	2	2	2	2	2	2	2	1
1	1	2	2	2	2	2	1	1	1
2	2	2	2	2	2	2	2	2	2
2	2	2	2	2	2	2	2	2	2
2	2	2	2	2	2	2	2	2	2
1	1	1	2	2	2	3	1	1	1
2	2	1	2	2	2	2	2	1	1
1	1	1	2	2	2	3	1	1	1
3	2	2	2	2	2	2	1	1	2
1	1	1	2	2	2	1	1	1	1
3	2	2	2	2	2	2	1	1	2
3	1	1	1	2	2	2	1	1	1
2	2	2	2	2	2	2	2	2	2
2	2	2	2	2	2	2	2	2	2
2	2	2	2	2	2	2	2	2	2
2	2	2	2	2	2	2	2	2	1
2	1	1	1	2	2	3	2	1	1
1	3	1	3	3	3	3	3	1	1
2	3	1	3	3	3	2	2	2	1
2	1	1	1	2	2	3	2	1	2

3	3	1	1	3	2	3	1	1	1
9	2	1	2	1	2	2	3	1	1
3	2	1	2	3	2	3	1	1	2
2	2	1	2	2	2	2	1	1	1
1	3	1	2	9	3	1	1	1	1
3	3	1	3	3	2	3	1	1	1
3	1	1	1	1	3	3	1	1	1
9	9	1	3	3	3	3	1	1	1
9	9	1	9	9	9	9	1	1	1
3	3	1	1	2	3	2	1	1	1
9	9	2	9	2	9	2	9	2	2
3	2	2	2	2	2	2	2	2	2
3	3	1	1	2	2	3	2	1	1
2	2	1	2	2	2	2	2	1	2
3	3	1	1	1	2	2	1	2	1
2	2	3	3	3	3	3	2	2	3
1	3	1	2	3	3	2	1	1	1
3	2	1	1	2	2	1	1	1	1
2	1	1	1	2	2	3	2	1	1
2	1	9	9	9	9	9	9	1	1
3	3	1	3	3	3	3	1	1	1
3	3	1	1	3	3	3	1	1	1
3	3	1	3	3	3	3	1	1	1
3	3	1	1	1	1	2	2	1	1
9	9	1	9	1	1	9	9	1	1
2	2	1	1	2	2	1	1	1	1
3	2	9	9	9	9	9	9	9	9
1	1	1	1	3	3	3	1	1	1
2	2	9	9	9	9	9	9	9	9
2	3	1	1	2	9	3	1	1	1
1	1	1	1	2	2	2	1	1	2
9	1	3	1	1	1	2	1	1	1
2	2	1	1	2	2	1	1	1	2
3	2	2	1	1	1	2	1	1	1
3	1	2	3	1	1	3	1	1	1
9	9	9	9	9	9	9	9	9	9
9	9	9	9	9	9	9	9	9	9
2	2	2	1	1	1	2	1	1	1
3	3	1	3	3	3	3	3	1	1
1	3	1	1	1	3	3	1	1	1
9	9	1	1	1	9	9	1	1	1
9	9	1	2	2	2	2	2	1	1
3	1	1	1	3	3	1	1	1	1
2	2	1	1	2	2	1	1	1	1
1	2	1	1	2	2	1	1	1	1
9	9	9	9	9	9	9	9	9	9
9	9	9	9	9	9	9	9	9	9

1	3	2	1	3	3	2	1	1	1
2	2	1	2	2	2	2	2	1	1
2	2	1	2	3	2	3	1	1	1
2	1	1	2	3	3	3	2	1	2
9	9	9	9	9	9	9	9	9	9
9	9	1	2	2	1	2	2	1	2
1	1	1	1	2	2	3	2	2	1
3	1	1	2	2	3	2	1	2	1
3	3	1	1	2	2	2	2	2	1
9	9	9	9	9	9	9	9	9	1
2	1	1	3	1	1	3	2	1	1
3	3	1	3	1	1	1	3	3	1
2	3	1	2	3	2	3	1	1	1
9	1	1	2	2	2	2	1	1	1
1	2	2	1	2	3	1	2	2	2
2	3	9	9	9	2	9	9	9	2
2	2	1	2	2	2	2	2	1	1
2	1	1	2	1	1	2	1	1	1
2	1	2	2	2	2	2	2	2	2
1	1	2	1	1	2	2	1	2	1
3	2	1	2	1	2	2	2	2	1
3	2	1	1	1	2	2	1	1	1
3	3	2	2	2	2	2	2	2	2
3	3	9	9	9	9	9	9	9	9
1	1	1	2	1	1	1	2	2	2
2	2	1	2	2	2	2	2	2	1
3	3	2	2	2	2	3	2	1	1
2	2	2	2	2	2	3	2	1	1
2	2	3	2	2	2	2	2	2	2
1	1	1	2	2	3	2	1	1	1
2	2	2	2	2	2	2	2	2	1
2	2	2	2	2	2	2	2	2	2
2	2	1	3	3	2	1	1	1	1
2	2	3	2	2	3	3	2	2	2
1	1	1	2	2	3	2	1	1	1
2	1	2	2	1	2	2	1	1	1
2	2	1	2	1	1	2	1	1	3
2	2	2	2	2	2	2	2	2	2
9	9	1	9	9	9	2	9	1	9
2	1	1	2	2	3	3	1	2	1
2	2	2	2	2	2	2	2	2	2
2	2	2	2	2	2	2	2	2	2
2	2	2	2	2	2	2	2	2	2
3	3	2	2	2	2	3	3	3	1
2	3	2	1	3	3	2	2	2	2
2	2	1	2	2	2	2	2	2	1
3	2	1	1	3	3	2	3	2	2

2	2	9	9	9	9	9	9	9	9
3	2	2	2	2	2	2	2	2	2
3	2	2	2	2	2	2	2	2	2
2	2	2	2	2	2	2	2	2	2
1	1	2	2	2	2	2	2	2	2
2	3	1	1	2	3	2	1	1	1
3	3	2	3	3	2	3	3	3	2
2	2	2	2	2	2	2	2	2	1
2	2	2	2	2	2	2	2	2	2
2	2	2	2	2	2	2	2	2	2
2	2	9	9	9	9	9	9	9	3
2	3	3	2	2	3	3	3	3	3
9	2	9	9	2	9	2	2	2	2
2	2	2	2	3	3	2	2	1	2
2	2	2	1	2	2	2	2	1	3
2	2	2	1	1	2	2	2	1	3
9	9	9	9	9	9	1	9	9	9
2	2	2	1	1	1	1	2	2	1
9	9	1	9	9	9	9	9	9	9
2	2	1	1	1	2	1	1	1	1
1	3	1	1	3	2	1	1	1	1
2	3	1	1	2	1	1	1	1	1
1	2	1	1	1	2	1	1	1	1
2	1	1	1	1	2	1	1	1	1
2	1	1	1	2	2	1	1	1	1
2	2	1	1	1	2	1	1	1	1
2	2	1	1	1	2	1	1	1	1
1	2	1	1	3	2	1	1	1	1
2	3	1	1	2	1	1	1	1	1
2	3	1	1	3	2	1	1	1	1
2	2	2	2	3	2	2	2	1	2
2	2	2	2	2	2	2	2	2	2
2	2	1	1	1	2	1	1	1	1
2	3	1	1	1	2	1	1	1	1
2	3	1	1	2	1	1	1	1	1
2	3	1	1	2	1	1	1	1	1
3	1	1	1	2	2	1	1	1	1
2	1	1	1	2	2	1	1	1	1
2	2	1	1	1	2	1	1	1	1
2	3	1	1	2	3	1	1	1	1
2	3	1	1	1	1	1	1	1	1
2	1	1	1	2	2	1	1	1	1
2	3	1	1	2	2	1	1	1	1
2	3	1	1	2	2	1	1	1	1
2	3	1	1	1	1	1	1	1	1
2	3	1	1	2	2	1	1	1	1
2	3	1	1	2	2	1	1	1	1

2	3	1	1	2	2	1	1	1	1
2	3	1	1	1	1	1	1	1	1
2	3	1	1	1	3	1	1	1	1
3	3	1	1	3	2	1	1	1	1
2	3	1	1	1	1	1	1	1	1
1	3	1	1	1	1	1	1	1	1
3	3	1	1	2	1	1	1	1	1
2	3	1	1	2	2	1	1	1	1
1	3	1	1	1	3	3	1	1	1
2	1	9	9	9	9	9	9	9	1
9	9	9	9	9	9	9	9	9	9
3	2	9	9	9	9	9	9	9	9
1	3	1	1	1	3	1	1	1	1
2	3	1	1	1	1	1	1	1	1
2	2	1	1	2	1	2	1	1	1
2	2	1	1	2	2	1	2	1	1
2	2	2	2	2	2	2	2	2	2
1	1	2	2	2	2	2	2	2	1
2	1	1	1	2	2	2	1	1	1
2	2	1	1	2	2	1	2	1	1
3	2	1	1	2	2	3	1	1	1
2	2	2	2	2	2	2	2	2	2
1	2	1	1	2	2	1	1	2	2
1	2	1	2	3	2	2	2	1	1
2	2	2	2	2	2	2	2	2	2
2	2	2	2	2	2	2	2	2	2
2	1	1	1	2	2	2	2	1	2
2	2	2	2	2	2	2	2	2	2
2	2	2	2	2	2	2	2	2	2
2	2	1	1	2	2	2	1	1	1
3	2	1	1	2	2	2	2	2	1
3	2	1	1	2	2	2	2	2	1
2	1	1	1	2	2	2	1	1	1
1	2	2	1	1	2	2	2	2	1
1	2	1	1	1	1	2	2	1	1
2	2	2	2	2	1	1	1	3	2
2	2	2	2	2	2	2	2	2	1
1	1	2	1	1	1	2	2	3	1
2	2	2	2	2	2	2	2	1	2
2	2	1	1	2	3	3	2	2	2
2	2	1	2	2	2	2	2	2	2
2	1	1	1	2	2	2	2	1	2
9	9	9	2	9	9	9	9	9	9
9	9	9	2	9	9	9	9	9	9
1	1	1	2	2	2	1	2	2	2
1	1	1	1	1	2	1	1	1	1
9	9	9	9	9	9	2	9	9	9

2	2	2	2	2	2	2	2	2	2
2	2	2	2	2	2	2	2	2	2
2	2	2	2	2	2	2	2	1	2
2	1	2	2	2	2	2	2	1	1
2	2	1	2	2	2	2	2	2	2
3	2	2	2	2	2	2	2	2	2
2	2	2	2	2	2	2	1	1	2
2	2	2	2	2	2	2	2	2	2
2	2	1	3	2	2	2	2	2	2
2	2	1	2	2	2	1	2	2	1
2	2	2	2	2	2	2	2	2	2
2	2	2	2	2	2	2	2	2	2
2	2	2	2	2	2	2	2	2	2
2	1	2	2	2	3	3	2	2	2
2	2	2	2	2	2	2	2	2	1
2	3	2	1	2	2	1	3	2	2
2	2	3	3	2	3	3	2	1	3
2	2	1	2	3	3	2	2	1	1
9	9	9	9	9	9	1	9	9	1
3	1	1	2	2	2	1	2	2	1
2	2	1	1	2	2	2	2	2	1
2	1	2	1	2	2	2	2	2	1
2	2	2	2	2	3	3	2	2	3
1	2	2	2	3	3	1	2	2	1
9	9	2	2	9	9	9	2	9	9
2	2	2	2	2	2	2	2	2	2
2	2	2	2	2	2	2	2	2	2
1	2	1	9	2	3	2	2	1	1
2	2	2	2	2	2	2	2	2	2
2	2	2	2	2	2	2	2	2	2
9	9	9	9	1	9	9	9	9	9
2	2	2	2	1	1	2	2	1	1
3	3	3	3	1	1	3	1	3	3
3	2	1	2	2	2	2	2	2	1
9	9	2	9	9	9	9	9	9	2
2	2	2	2	2	2	2	2	2	2
3	3	2	1	3	3	2	2	2	1
2	2	2	1	1	2	3	2	2	2
2	2	2	2	1	1	1	1	9	1
1	3	2	2	2	2	1	1	1	1
2	1	1	2	2	2	3	2	1	1
2	3	1	2	2	2	3	2	2	1
9	9	9	9	9	9	9	9	9	9
2	2	2	2	2	2	2	2	2	2
1	2	2	2	1	2	2	1	1	2
2	2	2	2	2	2	2	2	1	2
2	2	2	2	2	2	2	2	2	2

9	9	9	9	9	9	9	9	9	9
9	9	9	9	9	9	9	9	9	9
9	9	9	9	9	9	9	9	9	9
2	1	1	1	2	2	2	1	1	1
2	3	2	1	3	2	2	1	2	2
2	3	1	1	3	1	2	3	1	1
1	1	1	1	2	2	2	1	1	1
1	3	1	2	2	2	2	1	1	1
3	1	1	9	9	9	9	9	9	9
2	2	2	2	2	2	2	2	2	2
2	2	2	2	2	2	2	2	2	2
9	9	9	9	9	9	9	9	9	1
9	9	9	9	9	9	9	9	9	9
3	1	3	1	1	1	1	1	3	3
1	1	1	1	1	3	3	1	1	1
9	9	9	9	9	9	9	9	9	9
9	9	9	9	9	9	9	9	9	9
2	1	1	1	3	2	2	1	1	1
2	2	1	1	3	2	2	1	1	1
9	9	9	9	9	9	9	9	9	9
9	9	9	9	9	9	9	9	9	9
2	2	1	1	1	2	3	2	2	2
3	2	3	2	2	3	3	1	1	2
2	2	1	1	3	3	2	2	2	2
2	2	2	2	2	2	2	2	2	2
3	1	3	3	3	3	3	1	1	1
2	1	2	1	2	2	3	1	1	1
3	3	1	2	2	3	2	1	1	1
2	1	2	1	1	2	3	1	1	1
2	3	2	2	2	3	3	2	2	1
2	2	2	2	2	2	2	2	2	2
2	2	3	3	2	2	2	2	2	9
9	9	9	9	9	9	9	9	9	9
9	9	9	9	9	9	9	9	9	9
2	2	1	3	3	3	2	1	1	1
1	3	1	3	3	3	1	3	2	3
3	2	2	2	3	2	3	2	2	1
1	3	1	3	3	3	1	3	2	1
3	1	1	3	3	2	3	2	2	1
2	2	1	1	2	1	2	2	2	1
3	2	1	3	2	2	3	2	3	1
3	1	1	3	1	2	3	1	1	1
2	2	9	1	2	2	2	2	2	2
2	2	2	2	2	2	2	2	2	2
3	1	3	3	3	3	3	3	2	2
2	2	2	2	2	2	2	2	2	2
2	2	1	1	2	2	2	2	1	1

9	9	9	9	9	9	9	9	9	9
9	9	9	9	9	9	9	9	9	9
9	9	9	9	9	9	9	9	1	9
2	2	1	2	1	2	2	1	1	1
9	9	9	9	9	9	9	9	9	2
9	9	9	9	9	9	9	9	9	9
2	2	1	1	2	2	2	1	1	1
9	9	3	1	2	3	9	9	2	2
1	3	1	1	2	2	1	1	1	1
1	1	1	2	2	2	3	1	1	3
2	2	2	2	2	2	2	2	2	2
2	2	2	2	2	2	2	2	2	2
2	1	2	2	2	2	2	1	1	1
2	2	2	1	2	2	2	2	2	1
2	2	2	2	2	2	2	2	2	1
3	2	3	2	2	3	2	2	2	2
2	2	2	2	2	2	2	2	2	2
2	2	2	2	2	2	2	2	2	2
2	2	1	2	1	1	2	1	1	2
2	2	2	2	2	2	2	2	2	2
2	2	1	3	2	2	2	2	2	1
1	2	1	1	3	3	2	2	2	1
1	2	1	2	3	3	1	2	2	1
3	3	1	1	1	1	1	1	1	1
2	2	2	2	2	2	2	2	2	2
2	2	2	2	2	3	2	2	2	2
2	2	2	2	2	2	2	2	2	1
2	2	2	2	2	2	2	2	2	2
3	2	2	2	3	2	2	1	1	1
1	2	1	2	2	1	2	1	1	1
3	2	2	2	2	2	1	2	1	2
1	1	2	2	2	2	1	2	1	2
3	2	3	2	2	1	3	2	2	1
2	2	2	2	2	2	2	2	2	2
2	2	2	2	2	2	2	2	2	2
3	2	2	2	2	2	1	1	9	1
2	2	2	2	2	2	2	2	2	2
1	1	2	2	2	2	2	2	2	2
2	2	2	2	2	2	2	2	2	2
2	2	2	2	2	2	2	2	2	2
2	1	2	2	2	2	2	2	2	1
2	1	2	2	2	2	2	2	2	2
3	1	1	2	2	2	1	1	1	1
2	1	2	2	2	2	2	2	2	2
1	2	2	2	2	2	2	2	2	1
3	2	1	2	3	2	1	1	1	1
2	2	2	2	2	2	2	2	2	2

3	2	1	2	1	1	3	1	1	1
2	2	2	2	2	2	2	2	2	1
2	2	2	2	2	2	2	2	2	2
2	2	2	2	2	2	2	2	2	2
1	1	2	2	2	2	2	2	2	2
2	3	1	2	2	2	3	2	1	2
9	9	9	9	9	9	9	9	9	9
2	2	2	2	2	2	2	2	2	2
2	2	2	2	2	2	2	2	2	2
1	1	2	2	2	2	3	1	1	1
2	1	1	2	1	1	2	2	2	1
3	1	1	1	1	2	3	1	1	1
2	2	1	1	1	1	1	1	1	1
2	2	1	1	1	1	2	1	1	1
2	2	2	2	2	2	2	2	2	2
1	1	1	1	1	1	2	1	1	1
1	3	3	3	3	3	3	3	3	1
2	2	2	2	2	2	2	2	2	1
3	2	2	2	2	2	2	2	2	1
2	2	2	2	2	2	2	2	2	3
2	1	1	1	2	2	3	1	1	1
2	2	1	2	2	2	2	1	1	2
9	9	9	9	9	9	9	9	9	9
2	2	2	2	2	2	2	2	2	2
3	1	1	1	2	2	3	1	2	1
9	9	9	9	9	9	9	9	9	9
3	2	2	2	1	2	2	1	1	2
9	9	9	9	9	9	9	9	9	9
2	2	1	2	2	2	2	2	2	1
2	2	1	1	2	2	2	1	1	1
9	9	9	9	9	9	9	9	9	9
9	9	9	9	9	9	9	9	9	9
2	2	2	2	3	2	2	3	2	2
1	1	1	1	1	1	1	1	1	1
9	9	9	9	9	9	9	9	9	9
1	1	1	1	1	2	2	1	2	1
3	2	2	3	3	3	3	3	3	3
1	9	1	2	2	2	2	2	2	9
9	9	9	9	9	9	9	9	9	9
2	1	1	1	2	2	2	1	2	1
2	2	1	2	2	2	1	3	2	9
2	2	2	1	1	1	2	2	2	2
2	1	1	2	2	1	3	2	3	2
2	2	2	2	2	2	2	2	2	1
9	9	3	3	3	3	2	3	3	1
2	2	1	1	2	2	2	1	1	1
3	1	1	1	1	1	3	1	2	1

9	9	9	9	9	9	9	9	9	9
2	3	1	1	2	1	1	1	1	1
3	1	1	1	2	1	1	1	1	1
1	2	1	1	1	2	1	1	1	1
1	3	1	1	2	1	2	1	2	1
2	3	1	1	2	1	1	1	1	1
2	2	1	1	1	2	1	1	1	1
1	3	1	1	2	1	1	1	1	1
2	2	1	1	2	2	1	1	1	1
9	3	1	1	1	2	1	1	1	1
2	2	1	1	2	3	1	1	1	1
2	3	1	1	2	1	1	1	1	1
3	3	1	1	1	2	1	1	1	1
2	3	1	1	1	1	1	1	1	1
3	1	1	1	2	1	1	1	1	1
3	3	1	1	2	1	1	1	1	1
1	2	1	1	1	2	1	1	1	1
2	2	1	1	1	2	1	1	1	1
1	2	1	1	1	1	1	1	1	1
1	2	1	1	1	1	1	1	1	1
1	3	1	1	3	3	3	1	1	1
1	3	1	1	3	3	1	1	1	1
2	3	1	1	3	1	3	2	1	1
1	2	1	1	2	2	1	1	1	1
2	3	1	1	2	1	1	1	1	1
2	3	1	1	2	1	1	1	1	1
1	2	1	1	3	2	1	1	1	1
2	3	1	1	3	3	1	1	1	1
3	1	1	1	2	2	3	1	1	1
2	3	1	1	2	2	1	1	1	1
1	3	1	1	2	2	2	1	1	1
2	3	1	1	2	1	2	1	1	1
1	3	1	1	1	2	2	1	1	1
1	2	1	1	2	2	2	1	1	1
1	3	1	1	1	2	1	1	1	1
2	2	1	1	2	2	1	1	1	1
1	2	1	1	2	1	1	1	1	1
1	3	1	1	2	1	1	1	1	1
1	3	1	1	2	1	1	1	1	1
1	3	1	1	1	1	1	1	1	1
2	3	1	1	2	1	1	1	1	1
2	2	2	2	2	2	2	2	2	2
3	2	1	2	1	2	3	1	1	1
2	2	2	2	2	2	2	2	2	2
9	9	2	9	9	9	9	9	9	2
2	2	2	2	2	2	2	2	2	2
9	9	2	9	9	9	9	9	9	2

2	2	2	2	2	2	2	2	2	2
3	2	1	2	3	2	1	1	1	1
3	2	1	2	3	2	1	1	1	1
2	1	1	1	3	3	1	2	1	1
2	2	2	2	2	2	2	2	2	2
1	1	1	2	2	2	2	1	1	1
2	2	2	2	2	2	2	2	2	2
3	2	2	1	2	2	2	2	3	2
1	1	1	2	2	2	2	1	1	1
2	2	2	2	2	2	2	2	2	2
3	2	1	2	3	2	1	1	1	1
9	9	9	9	3	9	9	9	9	9
1	3	1	3	3	3	3	1	1	1
2	2	2	2	2	2	2	2	2	2
9	9	3	9	9	9	9	9	9	1
9	9	3	9	9	9	9	9	9	1
9	9	3	9	9	9	9	9	9	1
9	1	9	9	3	9	9	9	9	1
9	9	9	9	3	9	9	9	1	9
3	1	1	1	3	1	1	1	1	1
9	9	1	1	9	1	1	3	1	1
9	9	1	1	3	9	1	3	1	9
2	3	3	3	3	2	2	2	3	2
9	9	9	9	9	9	9	1	9	1
9	9	2	9	9	9	9	9	9	9
9	9	9	9	9	9	9	9	9	9
9	2	3	9	9	9	9	9	9	1
9	9	9	9	9	9	9	9	9	9
2	2	1	2	3	2	3	1	1	2
9	9	9	9	3	9	9	9	9	1
9	9	9	9	3	9	9	9	9	9
2	2	2	2	2	2	2	2	2	2
2	2	2	2	2	2	2	2	2	2
3	1	1	1	2	3	2	1	1	1
2	1	1	2	2	2	1	1	1	1
2	1	2	2	2	3	2	2	2	1
2	2	2	2	3	3	2	2	1	9
2	3	2	3	3	2	2	2	1	1
2	2	2	2	2	2	2	2	2	2
3	2	2	2	2	2	3	2	2	1
9	9	9	9	1	9	9	9	9	1
1	2	1	2	1	2	2	1	1	1
2	2	2	2	2	2	2	2	2	2
2	2	1	1	2	1	2	1	1	1
2	2	2	2	2	2	2	2	2	1
2	2	2	2	2	2	2	2	2	2
1	2	2	1	2	2	2	2	1	1

3	1	2	2	2	2	2	2	2	1
3	2	1	1	1	2	3	2	2	1
2	2	2	2	2	2	2	2	2	1
1	2	1	2	2	2	2	1	1	1
2	3	2	2	3	2	3	1	1	1
2	2	2	2	2	2	2	2	2	1
3	3	2	3	2	2	3	2	2	1
2	2	2	2	2	2	2	2	2	2
2	2	2	2	2	2	2	2	2	2
2	2	2	2	2	2	2	2	2	2
2	2	2	2	2	2	2	2	2	2
3	3	2	2	3	2	2	2	1	1
2	2	2	2	2	2	2	2	2	2
2	2	2	2	2	2	2	2	2	2
2	3	2	2	3	2	3	2	2	2
2	2	2	2	2	2	2	2	2	2
2	2	2	2	2	2	9	1	1	2
2	2	2	2	2	2	2	2	2	1
2	2	2	2	2	2	2	2	2	2
3	3	1	2	3	3	3	1	1	1
2	2	2	2	2	2	1	2	2	1
1	2	1	2	3	3	2	2	2	1
3	3	1	3	3	3	2	2	1	1
3	3	1	2	1	1	3	1	1	1
2	3	2	3	2	3	3	2	2	2
2	2	2	2	2	2	2	2	2	2
1	1	1	1	1	1	3	1	3	9
1	2	1	2	1	3	1	2	1	1
2	1	1	2	1	2	2	2	2	1
3	2	2	1	2	2	2	2	3	2
2	2	2	2	2	2	2	2	2	2
2	2	3	2	3	2	2	2	1	2
2	2	2	2	2	2	2	2	2	2
3	2	2	2	3	3	2	2	3	2
2	2	2	2	1	2	2	2	2	2
9	9	2	9	9	9	9	9	9	2
2	2	2	2	2	2	2	2	2	2
2	2	1	1	1	1	2	1	1	1
1	2	2	2	2	2	2	2	2	3
2	2	1	1	2	3	3	1	1	1
2	3	9	9	9	9	9	9	9	1
2	1	1	1	2	2	1	2	2	1
2	1	1	1	3	3	3	1	1	1
3	3	2	1	3	3	2	1	1	1
3	3	1	1	3	1	3	1	1	1
2	2	2	3	3	3	2	2	2	2
9	9	9	9	9	9	9	2	9	1

9	9	9	9	9	9	9	2	9	1
9	9	9	9	3	9	9	9	9	9
9	9	2	9	9	9	9	9	9	1
1	1	9	9	9	9	9	9	9	1
9	9	1	9	9	9	9	9	9	9
9	9	3	1	3	9	9	9	9	9
9	9	9	9	9	9	1	9	9	9
9	9	9	9	3	9	9	9	9	1
9	9	9	9	3	9	9	9	9	9
9	9	3	9	9	9	9	9	9	9
9	9	9	9	3	9	9	9	9	9
9	9	3	9	9	9	9	9	9	9
9	9	9	9	3	9	9	9	9	1
9	9	1	9	3	9	9	9	9	1
9	1	9	9	3	9	9	9	9	9
9	1	9	9	3	9	9	9	9	9
9	9	9	9	9	9	1	9	9	9
1	1	1	1	3	1	1	1	1	1
2	2	2	2	2	2	2	2	2	2
9	9	1	9	9	9	9	9	9	1
1	1	1	1	1	1	1	1	1	1
2	2	2	2	2	2	1	2	3	2
3	2	1	1	2	1	2	1	3	2
1	1	1	1	1	3	1	1	1	1
2	3	1	1	3	2	3	2	3	2
3	2	2	2	2	2	1	2	1	3
2	2	2	2	2	2	2	2	2	2
1	3	2	2	2	3	2	3	1	3
2	2	2	2	2	2	2	2	2	2
2	2	2	2	2	2	2	2	2	2
3	3	3	3	3	3	2	2	2	1
3	2	2	2	2	2	2	2	2	1
2	2	2	2	2	2	2	2	2	2
3	3	1	1	3	3	3	3	1	1
1	2	1	2	3	1	2	3	1	1
3	3	1	2	1	3	3	2	2	1
2	1	3	2	3	2	2	2	2	1
9	9	9	9	9	9	9	9	9	9
1	9	1	1	1	1	1	1	1	1
9	9	9	9	9	9	9	9	9	9
2	2	2	2	2	2	2	2	2	2
2	2	1	2	3	1	1	3	2	2
9	9	9	9	9	9	9	9	9	9
1	1	1	1	1	1	1	1	1	1
2	2	2	2	2	2	2	2	2	2
2	2	2	2	2	2	2	2	2	2
3	3	2	2	2	2	3	2	2	2

3	3	2	2	2	2	2	2	2	2
9	9	9	9	9	9	9	9	9	9
3	1	1	3	1	1	3	3	1	1
9	9	9	9	9	9	9	9	9	9
2	2	1	1	2	2	2	2	2	1
2	2	1	1	2	2	2	2	2	1
9	9	9	9	9	9	9	9	9	9
3	3	2	2	2	2	2	2	2	2
1	2	2	1	2	3	1	1	1	1
3	1	1	1	1	1	1	1	1	2
1	2	1	1	1	1	1	1	1	2
2	1	1	2	1	1	2	1	1	2
2	3	1	1	1	2	2	2	2	2
9	9	9	9	9	9	9	9	9	9
9	9	9	9	9	9	9	9	9	9
9	9	9	9	9	9	9	9	9	9
1	2	2	2	1	3	3	2	1	1
9	9	9	9	9	9	9	9	9	9
2	3	3	3	2	3	9	1	1	1
3	1	1	1	1	1	2	2	2	1
1	1	1	1	1	1	1	1	1	3
2	3	1	1	1	2	2	2	2	2
2	2	3	3	3	3	3	3	3	1
9	9	9	9	9	9	9	9	9	9
2	2	2	2	2	2	2	2	2	2
3	1	1	3	3	1	3	1	3	1
3	3	1	3	3	3	3	3	3	3
3	1	1	3	3	1	1	3	3	1
2	2	1	1	2	2	2	1	1	2
2	2	1	2	1	2	2	1	1	1
9	9	9	9	9	9	9	9	9	9
9	9	9	9	9	2	9	9	9	9
1	1	2	2	1	2	2	2	2	1
3	3	1	3	3	3	2	1	1	3
2	1	1	3	3	3	2	1	9	3
2	2	1	2	1	2	2	1	1	1
3	3	1	2	1	2	2	1	1	1
9	9	9	9	9	9	9	9	9	9
9	9	9	9	9	9	9	9	9	9
9	9	9	9	9	9	9	9	9	9
9	9	9	9	9	9	9	9	9	9
1	1	1	3	2	1	2	1	2	1
3	2	3	3	2	1	1	2	3	1
9	9	9	9	9	9	9	9	9	9
2	3	3	3	2	1	1	2	3	1
2	1	1	2	2	2	1	1	1	2
3	1	1	1	1	3	3	3	3	1

3	3	3	3	3	3	3	3	3	3
3	3	1	1	2	2	3	1	1	2
3	3	1	1	3	3	2	3	3	1
1	2	1	1	1	2	2	2	2	2
2	2	2	2	2	2	2	2	2	2
2	3	2	2	3	9	1	3	1	1
9	9	9	9	9	9	9	9	9	9
3	2	2	2	2	2	2	2	1	2
3	3	3	3	3	3	3	3	3	3
3	2	2	2	2	2	2	2	2	1
3	3	3	3	3	3	3	3	3	3
1	1	3	3	3	3	3	3	3	1
2	1	1	2	2	2	3	1	1	2
1	1	1	2	2	2	3	1	1	2
2	1	1	2	2	2	3	1	1	2
1	1	1	2	2	2	3	1	1	2
2	1	1	2	2	2	3	1	1	2
2	1	1	2	2	2	3	1	1	2
2	1	1	2	2	2	3	1	1	2
2	3	1	2	1	2	3	2	2	1
3	2	2	1	3	2	1	2	3	3
3	3	2	2	2	2	2	2	2	3
2	2	2	3	3	3	2	3	2	2
2	2	2	2	2	2	2	2	2	2
3	3	3	3	3	3	3	3	3	3
2	1	1	2	2	2	3	1	1	2
9	9	9	9	9	2	9	9	9	2
9	9	1	3	1	3	2	1	1	1
9	9	1	9	9	9	9	9	9	1
2	2	3	3	3	3	3	3	3	2
2	1	2	2	1	1	9	2	1	1
2	2	2	2	2	2	2	2	2	3
2	1	1	3	2	2	1	1	1	2
9	9	9	1	9	9	9	9	9	9
3	3	2	3	3	3	3	3	3	2
3	2	1	2	3	3	2	3	3	1
9	9	2	1	3	9	2	9	3	1
1	2	2	2	2	1	1	1	2	1
2	1	2	2	2	1	1	1	2	1
1	1	1	1	1	9	2	1	2	1
3	1	1	1	1	1	2	1	1	1
1	2	2	2	2	2	2	2	2	2
9	2	3	9	1	9	3	9	1	3
3	1	1	3	3	3	3	1	1	1
2	2	2	1	1	3	2	3	2	2
1	1	1	1	1	1	1	1	1	1
2	3	1	2	2	2	2	2	1	2

2	2	1	1	1	1	3	1	1	2
9	9	2	9	9	9	9	9	9	1
9	9	1	9	9	9	9	9	9	1
1	1	1	1	3	1	1	3	1	1
2	2	2	2	2	2	2	2	2	2
2	2	1	1	2	2	2	2	2	1
1	1	1	1	1	1	1	1	1	1
2	2	1	1	2	2	2	2	2	1
2	2	1	1	2	2	2	2	2	1
3	3	1	2	3	1	2	3	1	1
9	9	9	9	9	9	9	9	9	9
3	1	1	1	2	1	3	1	1	1
9	9	9	9	9	9	9	9	9	9
3	1	1	1	1	1	3	1	1	1
2	2	1	1	1	1	3	1	1	1
9	9	9	9	9	9	9	9	9	9
2	2	2	2	2	2	2	2	2	2
9	9	9	9	9	9	9	9	9	9
9	9	1	9	9	9	9	9	9	9
1	2	1	2	3	1	2	3	1	1
1	1	2	1	1	1	1	1	1	1
2	2	2	2	2	2	2	2	2	2
9	9	9	9	9	9	9	9	9	9
2	2	2	2	2	2	2	2	2	2
3	3	3	3	3	3	3	3	3	3
2	1	1	2	1	1	2	3	2	3
2	1	1	2	1	1	2	3	2	3
3	3	1	1	1	1	3	1	1	3
1	1	1	1	1	1	1	1	1	1
2	2	2	2	2	2	2	2	2	2
1	1	1	2	1	1	1	3	2	1
2	2	2	2	1	1	2	2	2	1
2	1	1	9	1	1	1	1	1	2
3	1	1	2	1	1	2	3	2	3
2	3	2	2	2	1	1	1	2	3
2	2	2	2	2	2	2	2	2	2
2	2	2	2	2	2	2	2	2	2
9	9	9	9	9	9	9	9	9	9
9	9	9	1	9	9	9	9	9	1
2	3	1	9	1	1	2	3	1	1
2	2	2	2	2	2	2	2	2	2
2	2	2	2	2	2	2	2	2	2
9	9	2	2	2	2	2	9	2	2
2	2	2	2	2	2	2	2	2	2
2	2	2	2	2	2	2	2	2	2
2	2	2	2	2	2	2	2	2	2
9	9	9	9	9	9	9	9	9	9

9	9	9	9	9	9	9	9	9	9
3	1	2	2	2	2	3	2	2	2
1	2	2	2	2	2	3	1	2	9
3	2	1	2	3	2	3	1	2	9
9	9	9	1	9	9	9	9	9	9
2	2	2	2	2	2	2	2	2	2
9	9	9	9	9	9	9	9	9	9
1	1	1	1	3	1	1	1	1	3
3	1	1	2	2	1	1	2	3	1
9	9	9	2	9	9	9	9	9	9
2	2	2	2	2	2	2	2	2	2
2	2	1	1	2	2	1	2	1	2
2	2	1	1	2	2	1	2	1	2
2	2	2	2	2	2	2	2	2	2
2	2	1	1	1	1	1	1	1	1
2	2	2	2	2	2	2	2	2	2
2	2	2	2	2	2	2	2	2	2
3	3	2	2	2	2	2	2	2	2
1	1	1	2	1	2	3	3	3	1
1	1	1	1	1	1	3	3	1	2
9	9	9	9	1	9	9	9	9	9
3	3	2	2	2	2	2	2	2	2
2	2	2	2	2	2	2	2	2	2
2	2	2	2	2	2	2	2	2	2
3	3	1	1	1	1	1	1	1	3
2	2	2	2	2	3	2	3	2	2
9	9	9	9	9	9	1	9	9	2
9	9	1	9	9	9	9	9	9	9
2	2	2	2	2	2	2	2	2	2
2	2	3	3	3	3	3	3	3	3
2	2	1	9	9	9	9	9	9	2
2	2	2	2	2	2	2	2	2	2
3	1	1	1	1	1	3	1	1	1
2	2	1	2	2	2	2	2	2	2
2	2	2	2	2	2	2	2	2	2
2	1	1	1	1	1	2	1	1	1
2	2	2	2	2	2	2	2	2	2
1	3	1	2	3	1	2	3	2	2
2	2	2	2	2	2	2	2	2	2
2	2	2	2	2	2	2	2	2	2
2	2	2	2	2	2	2	2	2	2
2	2	2	2	2	2	2	2	2	2
3	3	1	1	1	1	2	1	1	3
2	2	2	2	2	2	2	2	2	2
2	2	2	2	2	2	2	2	2	2
3	3	3	3	3	3	3	3	3	3
2	2	2	2	2	2	2	2	2	2

2	2	2	2	2	2	2	2	2	2
2	2	2	2	2	2	2	2	2	2
2	2	2	2	2	2	2	2	2	2
2	2	2	2	2	2	2	2	2	2
3	2	2	3	1	3	2	1	2	1
1	1	1	1	1	1	2	1	1	1
2	2	2	2	2	2	2	2	2	2
3	2	1	1	2	3	2	1	2	2
3	3	1	1	1	1	3	1	1	2
2	2	2	2	2	2	2	2	2	2
3	1	1	1	1	3	1	3	1	1
9	9	2	9	9	9	9	9	9	2
9	9	9	2	9	9	9	9	9	9
9	9	9	9	9	9	9	9	9	9
1	1	1	3	3	3	3	3	3	2
2	2	1	1	2	2	3	3	2	2
2	2	1	1	2	2	3	3	2	2
2	2	1	1	3	2	2	1	1	9
9	9	9	9	9	9	9	9	9	9
2	3	2	1	2	3	2	2	3	2
9	9	9	9	9	9	9	9	9	9
1	2	1	2	2	2	2	2	2	1
2	1	2	2	2	2	2	2	2	2
1	1	1	1	1	1	1	1	1	2
3	3	1	1	1	3	3	3	3	2
3	3	1	2	1	1	3	1	1	1
2	2	2	2	2	2	2	2	2	2
2	3	1	1	1	1	2	1	1	1
3	2	1	2	2	2	2	2	2	2
2	3	3	3	2	2	2	2	2	2
1	1	1	1	1	1	1	1	1	1
3	3	1	1	1	1	1	1	1	3
1	1	1	2	1	2	3	1	2	1
1	1	3	3	3	3	3	3	3	3
3	3	1	1	1	1	1	1	1	1
3	3	1	3	1	1	3	3	3	1
9	9	9	9	9	9	9	9	1	1
1	1	1	1	1	1	1	1	1	1
2	2	2	2	2	2	2	2	2	2
2	2	2	2	2	2	2	2	2	2
2	1	1	1	1	1	1	1	2	1
9	9	1	1	1	2	9	9	1	9
2	3	1	3	2	3	2	2	1	1
2	2	2	2	2	2	2	2	2	2
2	2	2	2	2	2	2	2	2	2
1	1	1	3	2	2	3	1	1	1
2	2	3	2	2	2	2	2	2	2

3	1	1	1	1	1	3	3	1	3
3	3	3	3	3	3	3	3	3	3
1	2	2	1	3	2	1	2	3	1
9	9	9	2	9	9	9	9	9	3
9	1	9	9	9	9	1	9	9	9
1	2	1	1	2	2	2	2	2	2
2	2	2	2	2	2	2	2	2	2
3	3	3	3	3	3	3	3	3	1
2	2	1	2	1	1	2	1	2	2
2	2	2	2	2	2	2	2	2	2
9	9	9	9	9	9	9	9	1	1
3	3	2	2	2	2	2	2	3	2
2	2	2	2	2	2	2	2	2	2
2	2	2	2	2	2	2	2	2	2
2	2	2	2	2	2	2	2	2	2
1	3	2	2	2	2	3	3	2	3
2	2	2	2	2	2	2	2	2	2
9	9	9	9	9	9	9	9	1	1
9	9	9	9	9	1	9	9	9	9
2	2	1	1	1	1	1	1	1	2
2	2	2	2	2	2	2	2	2	2
2	2	2	2	2	2	2	2	2	2
9	9	9	9	9	9	9	9	9	9
9	9	9	9	9	9	9	9	9	9
3	2	1	2	2	3	2	3	3	1
1	2	3	3	1	1	3	1	1	3
2	1	2	3	1	1	1	1	1	1
1	3	1	1	3	3	2	3	1	1
3	3	3	1	3	1	3	3	3	1
3	3	1	2	1	3	3	2	1	1
3	2	1	1	1	2	1	1	1	1
3	3	2	1	2	3	2	1	3	2
3	1	1	2	3	2	1	2	1	1
3	3	3	3	3	3	3	3	3	3
3	3	2	2	2	2	2	2	2	2
3	2	1	2	2	3	3	2	3	2
9	1	1	9	2	9	2	9	9	9
3	3	3	3	3	3	3	3	3	3
9	9	9	2	1	9	2	9	9	1
3	3	3	3	3	3	3	3	3	3
3	1	1	1	1	3	3	1	1	1
1	1	1	1	1	1	2	1	1	1
3	1	3	3	3	3	3	3	3	1
2	2	2	2	2	2	2	2	2	2
2	2	1	1	2	1	2	2	2	2
1	1	2	1	2	2	1	1	2	1
2	1	1	1	1	2	2	1	1	3

2	2	2	2	2	2	2	2	2	2
3	3	3	3	3	3	2	3	3	3
1	3	1	2	1	2	1	2	1	1
3	3	3	1	1	1	1	1	1	1
2	2	2	2	2	2	2	2	2	2
1	2	1	2	2	2	2	2	2	3
2	2	1	2	2	2	2	2	2	2
2	2	2	2	2	2	2	2	2	2
2	2	2	2	2	2	2	2	2	1
2	2	2	2	2	2	2	2	2	2
9	9	1	9	9	9	9	9	9	9
2	2	2	2	2	2	2	2	2	2
3	1	1	1	1	1	3	1	1	3
2	1	1	1	2	1	2	2	1	2
1	1	2	1	2	2	1	1	2	2
2	2	1	2	2	2	2	2	2	1
3	2	1	1	1	1	2	1	1	1
1	2	2	2	2	2	2	1	1	1
2	2	2	2	3	3	3	2	2	1
9	9	9	9	9	9	9	9	1	9
1	1	3	1	1	1	1	1	1	1
3	3	1	1	1	1	1	2	3	1
3	3	3	3	3	3	1	3	3	1
3	1	1	1	1	1	3	1	1	1
3	3	3	3	3	3	3	3	3	3
2	2	2	2	2	2	2	2	2	3
1	1	1	1	1	1	2	2	2	1
1	2	3	1	2	1	2	1	3	1
1	2	1	1	1	2	3	2	1	2
3	3	1	1	1	2	1	3	1	1
2	2	2	2	2	2	2	2	2	2
9	9	9	9	9	9	9	9	9	9
2	2	2	3	2	2	1	2	2	2
9	9	9	9	9	9	9	9	9	9
2	2	1	2	2	2	2	2	3	1
2	2	1	2	1	2	1	2	1	2
2	2	2	2	2	2	2	2	2	2
2	2	2	2	2	2	2	2	2	2
3	2	2	3	2	3	2	3	3	2
3	1	2	1	3	1	2	1	2	1
1	3	1	2	3	2	1	2	3	1
2	2	1	2	2	2	2	2	2	2
1	2	1	2	3	1	2	3	1	1
9	9	9	9	9	9	9	9	9	9
3	3	3	3	3	3	3	3	3	3
2	2	2	2	2	2	2	2	2	2
1	2	1	1	2	2	2	1	1	1

9	9	1	9	9	9	9	9	9	9
2	2	1	2	2	2	2	2	2	2
3	9	1	2	2	2	2	2	2	1
1	1	1	2	3	2	3	3	2	1
2	2	3	3	3	3	3	3	3	2
2	3	2	2	3	3	3	3	3	2
9	9	9	9	9	9	9	9	9	9
2	2	2	2	2	2	2	2	2	2
2	2	2	2	2	2	2	2	2	2
9	9	9	9	9	9	9	9	9	9
9	9	9	9	9	9	9	9	9	9
9	9	9	9	9	9	9	9	9	9
9	9	9	2	9	9	9	9	9	9
9	9	9	9	1	9	9	9	9	3
9	9	9	9	9	9	9	9	9	9
2	2	2	2	3	3	2	2	2	1
9	1	2	2	2	2	2	2	2	2
2	2	1	1	1	1	1	1	1	2
1	2	1	3	1	3	1	2	3	1
2	1	1	2	2	2	2	2	2	1
1	2	1	2	2	2	2	2	2	1
2	1	3	2	2	3	2	2	3	3
1	1	1	1	9	2	9	2	2	2
3	3	1	1	3	3	2	2	2	1
2	2	2	2	2	2	2	2	2	2
2	2	2	2	2	2	1	3	2	2
2	2	2	9	3	2	9	2	9	9
1	1	1	1	1	1	1	1	1	1
2	2	2	2	2	2	1	3	2	2
2	2	2	2	3	3	2	2	2	1
2	1	1	2	2	2	2	2	2	1
9	9	9	2	9	9	9	9	9	9
3	1	1	3	1	1	2	3	1	2
2	2	2	2	2	2	2	2	2	2
2	2	2	2	2	2	2	2	2	2
2	2	2	2	2	2	2	2	2	2
2	2	2	2	2	2	2	2	2	2
9	9	1	9	9	9	9	9	9	1
2	2	2	2	2	2	2	2	2	2
2	2	2	2	2	2	2	2	2	2
2	2	2	2	2	2	2	2	2	2
2	2	2	2	2	2	2	2	2	2
2	2	2	2	2	2	2	2	2	2
3	3	1	2	2	2	2	1	1	2
2	2	1	2	2	2	2	2	2	1
2	2	2	2	2	2	2	2	2	2
9	9	1	9	9	9	9	9	9	9

2	2	2	2	2	2	2	2	2	2
2	2	2	2	2	2	2	2	2	2
9	9	9	1	9	9	9	9	9	9
9	2	9	9	9	9	2	9	9	1
2	2	1	1	2	1	2	2	2	2
9	9	1	9	9	9	9	9	9	1
1	2	1	2	3	2	1	1	2	1
1	2	1	2	3	2	1	2	3	1
9	9	1	2	3	2	3	1	3	1
2	2	1	2	3	2	1	2	3	1
9	9	9	9	9	9	9	9	9	9
3	2	2	1	3	1	1	2	2	1
2	2	2	2	2	2	2	2	2	1
2	2	2	2	2	2	2	2	2	2
1	1	1	2	2	1	2	1	1	1
2	1	3	3	3	3	3	3	3	3
2	2	2	2	2	2	2	2	2	2
2	2	2	2	2	2	2	2	2	2
2	2	1	1	2	1	2	2	2	1
3	3	1	2	3	3	3	1	1	1
3	3	1	1	1	1	3	1	1	1
2	2	2	2	2	2	2	2	2	2
3	3	1	2	1	1	3	1	1	1
3	3	2	2	2	2	2	2	2	2
2	2	2	2	2	2	2	2	2	2
3	2	1	2	2	2	1	1	1	1
2	2	2	2	2	2	2	2	2	2
2	2	2	2	2	2	2	2	2	2
3	2	2	2	2	2	2	2	2	1
9	9	9	9	9	9	9	9	9	9
9	1	9	9	9	9	2	9	9	1
2	2	2	2	2	2	2	2	2	2
9	9	9	9	9	9	9	9	9	9
9	9	9	9	9	9	9	9	9	9
2	3	1	2	3	3	3	3	3	3
9	9	9	9	9	1	9	9	9	9
9	9	9	9	9	9	9	9	9	9
2	2	2	2	2	2	2	2	2	2
1	9	1	2	2	2	2	1	1	1
3	1	1	1	2	1	3	2	2	1
1	1	1	1	1	1	1	1	1	1
2	3	1	3	2	3	1	2	1	1
9	9	2	9	9	9	9	9	9	1
1	2	1	2	1	2	1	2	3	1
1	3	1	2	9	1	3	2	1	2
9	9	9	9	9	9	9	9	9	9
2	1	3	3	2	3	3	1	3	3

1	2	1	2	1	2	1	2	1	1
9	9	9	9	9	9	9	9	9	9
9	9	9	9	9	9	9	9	9	9
2	2	2	2	2	2	2	2	2	2
1	1	1	1	1	1	1	1	1	1
1	2	1	2	1	2	1	2	1	1
1	2	1	1	2	9	1	2	1	1
3	3	1	1	1	1	3	1	1	1
2	2	2	2	2	2	2	2	2	2
3	3	3	1	1	1	2	2	2	1
9	9	9	9	9	9	9	9	9	9
9	9	9	9	9	9	9	9	9	9
9	1	1	9	9	9	9	9	9	9
2	1	1	1	1	2	2	2	2	1
2	2	2	3	1	2	2	2	2	2
9	9	9	9	9	9	9	9	9	9
9	9	9	9	9	9	9	9	9	9
9	9	9	9	9	9	9	9	9	9
2	2	1	2	2	2	2	1	2	2
2	2	1	2	2	2	2	2	2	2
9	9	9	9	9	9	9	9	9	9
2	1	1	3	3	3	3	1	1	1
3	3	3	3	3	3	3	3	3	3
9	9	9	9	9	9	9	9	9	9
2	2	2	2	2	2	2	2	2	2
2	2	2	2	2	2	2	2	2	2
9	9	9	9	9	9	9	9	9	9
9	9	9	9	9	9	9	9	9	9
2	2	2	2	2	2	2	2	2	3
2	2	2	2	9	1	1	1	1	2
2	1	1	1	1	1	1	1	1	1
9	9	9	9	9	9	9	9	9	9
9	9	9	9	9	9	9	9	9	9
1	1	1	3	3	1	3	2	3	1
1	1	3	3	3	3	2	2	2	3
2	2	2	2	2	2	2	2	2	2
2	1	2	2	2	2	2	2	2	2
2	2	2	2	2	2	2	2	2	2
2	1	1	9	1	9	1	3	1	1
3	2	2	9	1	2	3	2	3	1
1	1	1	1	2	2	2	1	3	1
9	9	9	9	9	9	9	9	9	9
9	9	9	9	9	9	9	9	9	9
9	9	9	9	9	9	1	9	9	9
9	9	9	9	9	9	9	9	9	9
9	9	9	9	9	9	9	9	9	9
9	9	9	9	9	9	9	9	9	9

2	2	2	2	2	2	2	2	2	2
2	3	2	2	2	2	2	3	3	3
2	1	1	1	2	2	3	1	1	1
2	2	2	2	2	2	2	2	2	2
9	9	9	9	9	9	9	9	9	9
9	9	9	9	9	9	9	9	9	9
9	9	9	9	9	9	9	9	9	9
9	9	9	9	9	9	9	9	9	9
9	9	9	9	9	9	9	9	9	9
9	9	9	9	9	9	9	9	9	9
9	9	9	9	9	9	9	9	9	9
9	9	9	9	9	9	9	9	9	9
9	9	9	9	9	9	9	9	9	9
1	1	1	9	1	9	1	9	1	1
9	9	9	9	9	9	9	9	9	9
2	2	1	2	2	1	2	2	1	1
2	2	2	2	2	2	2	2	2	2
9	9	9	9	9	9	9	9	9	9
9	9	9	9	9	9	9	9	9	9
9	9	9	9	9	9	9	9	9	9
9	2	2	9	9	9	1	3	9	2
9	9	9	9	9	9	9	9	9	9
1	1	2	2	2	2	1	1	1	2
9	9	9	9	9	9	9	9	9	9
1	1	1	1	1	1	1	1	1	1
2	2	2	2	2	2	2	2	2	1
2	2	3	3	1	1	2	2	2	1
1	1	1	1	1	1	2	2	2	1
1	1	1	1	1	2	1	1	1	2
1	1	1	1	1	1	1	1	1	2
2	2	1	1	1	3	3	2	2	2
2	2	1	1	1	1	1	1	1	1
1	1	1	1	1	1	1	1	1	1
9	9	9	9	9	9	9	9	9	9
2	2	2	1	1	1	2	2	2	1
1	1	1	1	1	1	1	1	1	2
1	1	1	1	1	1	1	1	1	1
9	9	9	9	9	9	9	9	9	9
3	3	2	2	2	2	2	3	3	1
3	3	3	3	1	1	3	1	3	1
3	3	3	3	3	3	3	3	3	3
9	9	9	9	9	9	9	9	9	9
9	9	9	9	9	9	9	9	9	9
9	9	9	9	9	9	9	9	9	9
9	9	9	9	9	9	9	9	9	9
2	2	2	2	2	2	2	2	2	2
9	9	9	9	9	9	9	9	9	9

9	9	9	9	9	9	9	9	9	9
9	9	9	9	9	9	9	9	9	9
9	9	9	9	9	9	9	9	9	9
9	9	9	9	9	9	9	9	9	9
9	9	9	9	9	9	9	9	9	9
9	9	9	9	9	9	9	9	9	9
9	9	9	9	9	9	9	9	9	9
9	9	9	9	9	9	9	9	9	9
9	9	9	9	9	9	9	9	9	9
9	9	9	9	9	9	9	9	9	9
9	9	9	2	9	9	9	9	9	9
9	9	9	9	9	9	9	9	9	9
2	2	2	2	2	2	2	2	2	2
1	1	1	1	1	1	2	2	2	2
2	2	1	1	1	1	2	2	2	2
1	2	1	2	1	1	3	2	1	3
1	1	1	1	2	2	2	2	2	2
2	3	1	1	1	1	2	1	1	1
2	2	2	2	2	2	1	1	1	1
9	9	1	9	9	9	9	9	9	1
2	2	1	1	1	1	1	1	1	1
1	1	1	2	2	2	2	2	2	1
2	2	2	2	2	2	2	2	2	2
1	1	2	2	2	1	2	2	2	1
2	1	1	2	1	1	2	1	1	1
3	3	3	3	3	3	3	1	1	3
2	2	9	9	9	9	9	9	9	1
3	3	2	2	1	1	1	1	1	2
9	9	1	9	9	9	9	9	9	9
9	9	2	9	9	3	1	9	3	9
9	9	9	9	9	9	9	9	9	9
1	1	1	1	1	1	2	2	1	1
2	2	2	2	1	1	1	1	1	2
1	1	1	1	1	1	1	1	1	1
1	1	1	2	2	2	2	2	2	1
2	2	1	2	2	2	2	2	2	2
1	1	1	1	2	2	2	2	2	1
2	2	2	2	2	2	2	2	1	2
2	1	2	2	1	1	1	2	2	2
1	1	1	1	1	1	1	1	1	2
2	2	2	2	2	2	2	2	2	2
1	1	1	1	1	1	1	1	1	1
3	2	1	1	1	3	3	1	1	1
1	1	1	1	1	1	2	2	2	1
1	1	1	1	1	1	1	1	1	2
1	1	1	1	2	2	1	1	1	1
2	2	2	2	2	2	2	2	2	1

9	9	9	9	9	9	9	9	9	9
2	2	1	1	1	1	1	1	1	2
9	9	9	9	9	2	9	9	9	9
9	9	9	9	9	2	9	9	9	9
9	9	9	9	1	9	9	9	9	9
9	9	9	9	9	9	9	9	9	9
9	9	9	9	9	9	9	2	9	9
3	3	1	2	1	1	1	1	1	2
9	9	2	9	9	9	9	9	9	2
2	2	2	2	2	2	2	2	2	2
9	9	9	9	9	9	9	9	9	9
9	9	9	9	9	9	9	9	9	9
1	1	1	1	1	1	2	1	1	1
2	2	2	2	2	2	2	2	2	9
1	1	1	1	1	1	1	1	1	1
9	9	9	9	9	9	9	9	9	9
2	2	2	2	2	2	2	2	2	2
3	3	3	3	3	3	3	3	3	3
9	9	9	9	9	9	9	9	9	9
1	1	2	2	2	2	2	2	2	2
2	2	2	2	2	2	2	2	2	2
3	2	2	2	3	3	3	2	2	3
2	2	2	2	2	2	2	2	2	2
2	2	2	2	2	2	2	2	2	2
2	2	2	1	1	1	1	1	1	2
2	2	2	2	2	2	2	2	2	2
3	1	2	2	2	2	2	2	2	3
2	2	1	1	1	2	2	2	2	1
1	1	1	2	2	2	2	2	2	2
1	2	2	2	2	2	2	1	1	1
9	9	9	9	9	9	9	9	9	9
2	2	3	2	3	2	3	2	3	2
9	9	9	9	9	9	9	9	9	9
2	2	2	2	2	2	2	2	2	2
2	2	2	2	2	2	2	9	2	3
2	2	1	1	1	1	1	1	1	1
3	3	1	1	1	2	2	1	1	1
1	1	1	1	1	1	1	1	1	1
1	1	1	1	1	1	1	1	1	1
2	2	1	1	3	1	3	3	2	1
2	2	2	2	2	2	2	2	2	2
1	1	1	1	1	1	2	2	2	2
9	9	9	9	9	9	9	9	9	9
9	9	9	9	9	9	9	9	9	9
9	9	1	1	9	9	9	9	9	1
9	9	9	9	9	9	9	9	9	9
9	9	9	9	9	9	9	9	9	9

9	9	9	9	9	9	9	9	9	9
2	2	2	2	2	2	2	2	2	2
9	9	9	1	9	9	9	9	9	9
3	1	2	3	1	3	9	1	3	9
9	9	9	9	9	9	9	9	9	9
1	3	2	2	1	2	3	1	2	1
3	3	3	3	3	3	2	2	2	1
9	9	9	9	9	9	9	9	9	9
9	9	9	9	9	9	9	9	9	9
9	9	9	9	9	9	9	9	9	9
9	9	1	9	9	9	9	9	9	9
9	9	9	9	9	9	9	9	9	9
1	2	2	1	2	2	2	2	2	2
9	9	9	9	9	9	9	9	9	9
9	9	9	9	9	2	9	9	9	9
2	1	2	1	2	2	3	2	2	2
1	2	3	2	3	2	1	2	3	3
2	2	2	3	3	2	3	2	2	1
2	2	1	2	3	1	1	2	2	1
2	3	1	2	2	1	3	2	3	2
2	1	1	2	2	1	2	3	1	1
9	3	1	1	3	1	3	3	1	1
1	1	1	2	3	2	1	1	1	1
1	3	1	2	1	3	1	3	1	1
2	2	2	2	2	2	2	2	2	2
1	2	1	1	1	2	2	2	1	2
2	3	3	9	9	9	9	9	9	9
1	9	1	2	9	3	1	2	3	2
2	2	2	3	3	3	9	9	9	9
3	3	1	1	2	2	2	3	3	1
1	2	2	2	2	2	2	2	2	3
2	2	2	2	2	2	2	2	2	2
9	9	9	9	9	9	9	9	9	9
9	1	1	2	2	2	3	3	3	2
2	2	2	2	2	2	3	1	1	1
1	1	2	1	2	2	2	1	1	2
2	2	2	2	2	2	2	2	2	2
2	2	1	1	2	2	3	1	2	2
9	9	9	9	1	9	9	9	9	9
2	3	1	2	2	1	3	1	3	1
9	9	9	9	9	9	9	9	3	9
9	9	1	9	9	9	9	9	9	2
2	2	1	9	9	9	9	9	9	2
9	9	1	9	9	9	9	9	9	2
1	1	3	3	3	3	3	3	3	1
1	3	1	3	1	1	3	1	1	1
3	3	3	3	3	2	2	1	3	3

3	3	3	3	3	3	3	3	3	3
9	9	9	9	9	9	9	9	1	1
2	2	2	2	2	2	2	2	2	2
3	1	1	1	3	1	1	3	1	1
3	3	1	3	1	1	3	1	1	1
1	1	1	1	3	1	1	1	3	1
1	2	2	3	2	3	1	2	3	2
2	3	3	1	1	2	3	1	3	1
2	2	2	2	2	2	2	2	2	2
2	3	2	9	2	3	2	3	2	2
2	3	1	2	2	2	2	2	2	2
2	1	2	1	2	3	3	3	2	2
1	2	1	2	3	1	2	3	2	2
2	2	2	1	3	1	2	1	2	1
2	3	1	2	3	2	2	2	2	1
2	3	2	2	3	2	1	2	3	1
2	2	2	2	2	2	2	2	2	2
2	3	3	3	2	2	1	2	2	2
2	2	2	2	2	2	2	2	2	2
2	2	2	2	2	2	2	2	2	2
2	2	2	2	2	2	2	2	2	3
3	3	1	2	2	2	3	3	3	1
9	9	2	9	9	9	9	9	9	2
9	9	9	9	9	9	9	9	9	9
2	2	2	2	2	2	2	2	2	3
2	3	1	1	1	1	1	1	1	1
2	3	1	2	1	3	2	1	2	1
2	2	1	2	2	3	3	2	1	2
1	1	1	1	1	1	1	1	1	3
9	9	9	9	1	9	9	9	9	9
2	2	2	1	3	1	2	2	2	2
1	1	1	1	1	1	1	1	1	2
9	2	2	3	3	2	3	2	3	2
3	2	2	2	1	2	1	1	1	1
1	1	1	2	9	1	3	1	1	1
2	2	2	2	2	2	2	2	2	2
3	3	3	3	3	3	3	3	2	2
9	9	9	9	9	9	9	9	9	9
1	2	1	2	1	1	3	2	2	2
1	1	2	2	2	2	3	2	2	2
9	9	9	9	9	9	9	9	9	9
9	9	9	9	9	9	9	9	9	9
3	3	2	2	2	2	2	2	2	3
9	9	9	9	9	9	9	9	9	9
2	2	2	2	2	2	2	2	2	2
1	1	2	2	1	2	3	1	1	1
9	9	9	9	9	9	9	9	9	9

2	2	2	2	3	3	2	2	2	2
9	9	9	9	9	9	9	9	9	9
2	2	3	2	2	2	2	2	2	3
9	9	9	9	9	9	9	9	9	9
9	9	9	9	9	9	9	9	9	9
3	3	2	2	2	2	2	2	2	2
2	2	2	2	2	2	2	2	2	1
2	2	2	2	2	2	2	2	2	3
1	1	1	1	1	1	1	1	1	2
2	2	2	2	1	1	2	2	2	2
9	9	9	9	9	9	9	9	9	9
9	9	1	1	1	2	2	3	3	3
9	9	9	9	9	9	9	9	9	9
1	1	1	1	1	1	1	1	1	1
2	2	2	2	2	2	2	2	2	2
1	1	2	2	1	1	2	2	2	3
9	9	2	9	9	9	9	9	9	2
9	9	9	9	9	9	9	9	9	9
1	3	1	1	1	1	1	3	3	1
2	2	2	2	2	2	3	3	2	3
1	1	1	1	1	1	1	1	1	1
3	2	2	2	2	2	2	3	3	2
9	9	1	9	9	9	9	9	9	1
2	3	2	2	2	2	3	3	2	2
2	1	1	1	2	2	2	2	2	3
2	2	2	2	2	2	2	3	3	2
9	9	9	9	9	9	1	9	9	1
2	2	1	1	1	1	1	1	1	1
2	2	2	2	2	2	2	2	2	2
9	9	9	9	1	9	9	9	9	1
3	1	1	2	2	2	3	2	2	1
2	2	2	2	2	2	2	2	2	2
2	2	3	3	3	3	3	3	3	1
2	3	1	1	1	2	3	1	2	2
2	2	2	2	2	2	2	2	2	2
1	3	2	2	2	3	2	1	2	1
1	1	2	2	2	2	2	2	2	2
3	1	9	1	2	2	3	3	3	3
2	2	2	2	2	2	2	2	2	2
3	3	3	3	3	3	3	3	3	3
2	1	1	1	1	2	3	2	1	1
1	1	1	2	2	2	2	1	1	1
3	3	2	2	2	2	2	2	2	2
3	2	2	2	2	2	3	2	2	2
3	1	2	2	2	2	2	2	2	2
3	2	2	3	2	2	2	2	2	2
3	3	2	2	2	2	3	2	2	1

3	2	3	3	2	1	2	2	2	3
2	2	2	2	2	2	2	2	2	1
2	1	1	2	1	1	2	1	1	1
2	3	2	2	2	2	2	2	2	1
2	2	2	1	1	1	2	2	2	2
9	9	9	1	9	9	9	9	9	9
2	2	2	2	2	2	2	2	2	2
1	1	1	1	1	2	2	2	1	1
1	2	2	2	2	2	2	2	2	2
2	2	2	2	2	2	2	2	2	1
1	1	2	2	2	2	1	1	1	2
2	3	1	2	3	1	2	1	2	3
2	2	2	2	2	2	2	2	2	2
3	3	3	3	3	3	3	3	3	3
2	3	2	2	1	3	2	2	1	3
2	3	1	2	3	1	2	3	1	1
3	2	3	2	3	2	3	2	3	2
2	2	2	3	2	3	2	3	2	2
2	2	2	2	2	2	2	2	2	2
1	2	3	2	2	2	2	2	2	2
2	2	2	2	2	2	2	2	2	2
2	2	2	2	2	2	2	2	2	2
3	3	3	2	2	2	2	2	2	2
3	3	3	2	2	2	2	2	2	2
2	2	2	1	1	1	2	1	1	2
2	2	2	2	2	2	2	2	2	2
3	2	2	2	2	2	3	3	3	1
3	2	1	1	1	1	2	3	3	1
2	2	2	2	2	2	2	2	2	2
1	1	1	1	1	1	2	2	2	1
3	3	2	2	2	3	3	3	2	2
3	3	3	2	2	3	3	3	3	3
2	2	1	1	1	3	3	3	3	1
2	2	2	2	2	2	2	2	2	2
1	1	1	1	1	1	2	2	1	1
2	2	2	2	2	2	2	2	2	2
2	2	2	2	2	2	2	2	2	2
1	2	2	1	2	3	2	2	1	2
3	2	2	2	2	2	2	2	2	2
2	2	2	2	2	2	2	2	2	2
2	1	1	1	1	2	2	1	1	1
3	3	1	1	1	1	3	2	2	1
1	1	2	1	2	2	2	2	2	1
2	2	2	2	2	2	1	2	2	2
2	3	2	2	1	2	3	2	3	2
3	1	1	1	2	2	1	3	1	1
2	1	2	2	9	9	9	9	9	9

1	2	2	1	2	2	2	2	1	1
3	3	2	2	2	2	3	9	3	2
3	2	1	2	2	2	2	2	2	1
2	2	2	2	2	2	2	2	2	2
2	2	2	2	2	2	2	2	2	2
2	2	2	2	2	1	2	2	2	2
2	2	1	2	2	2	2	2	2	1
3	2	1	9	1	2	1	1	1	1
2	2	2	2	2	2	2	2	2	2
2	2	2	2	2	2	2	2	2	2
2	2	2	2	2	2	2	2	2	2
9	9	9	9	9	9	9	9	9	9
9	9	9	9	9	9	9	9	9	9
9	9	2	2	2	2	2	2	2	2
1	1	1	1	1	2	2	1	1	2
2	2	2	2	2	2	2	2	2	2
3	2	1	2	1	2	9	1	1	1
2	2	2	2	2	2	2	2	2	1
2	2	1	2	2	2	2	1	1	1
3	2	1	1	2	1	2	2	1	1
2	2	2	2	2	2	3	2	2	2
3	3	1	1	1	1	1	1	1	2
2	2	2	2	2	2	2	2	2	2
2	2	2	2	2	2	2	2	2	2
2	2	2	2	2	2	2	2	2	1
2	2	1	1	1	2	1	2	1	1
1	2	2	2	2	2	2	2	2	2
2	2	2	2	2	2	2	2	2	2
2	2	1	1	1	1	1	1	1	2
9	9	9	9	9	9	9	9	9	9
2	2	3	3	3	3	3	3	3	2
2	2	2	2	2	2	2	2	2	2
2	3	2	2	2	2	2	2	2	2
2	2	2	2	2	2	2	2	2	2
9	9	2	2	2	2	2	2	2	2
2	2	2	2	2	2	2	2	2	2
2	2	2	2	2	2	2	2	2	2
2	2	2	2	2	2	2	2	2	2
2	2	2	2	2	2	2	2	1	2
2	2	2	2	2	2	2	2	2	2
2	2	2	2	2	2	2	2	2	2
2	2	2	2	2	2	2	2	2	2
1	1	1	1	1	1	1	1	1	2
2	2	2	2	2	2	2	2	2	2
2	2	2	2	2	2	2	2	2	2
3	2	2	2	3	2	2	3	3	2
2	2	2	2	2	2	2	2	2	2

1	1	2	2	2	2	2	2	2	1
2	2	1	2	2	2	2	2	2	2
2	3	1	2	1	1	3	1	1	1
2	2	2	2	2	2	2	2	2	2
2	2	2	2	2	2	2	2	2	2
2	2	2	1	1	2	2	2	2	2
9	9	9	9	9	9	9	9	9	9
2	2	1	1	1	1	1	1	1	1
2	2	1	2	1	2	2	2	1	1
3	2	1	1	2	2	3	1	1	2
2	2	1	1	1	2	3	1	1	2
2	2	2	2	2	2	2	2	2	2
2	3	2	2	2	2	2	2	2	2
9	9	1	9	1	9	1	1	1	2
9	3	1	3	3	3	1	1	1	9
2	1	2	2	2	2	2	2	2	1
2	1	1	2	2	2	2	2	2	2
2	2	2	2	2	2	2	2	2	1
1	1	1	1	1	1	1	1	1	1
1	1	1	1	1	1	2	2	3	2
1	1	1	1	1	2	2	1	1	1
3	3	1	1	2	3	3	2	2	1
2	2	2	2	2	2	2	2	2	2
2	2	2	2	2	2	2	2	2	2
2	2	2	2	2	2	2	2	2	2
2	2	2	2	2	2	2	2	2	2
9	9	9	9	9	9	9	9	9	9
2	2	2	2	2	2	2	2	2	2
2	2	2	2	2	2	2	2	2	2
2	2	2	2	2	2	2	2	2	2
2	2	2	2	2	2	2	2	2	2
2	2	1	2	2	2	2	2	2	2
9	9	9	9	9	9	9	9	9	9
9	9	9	9	9	9	9	9	9	9
9	9	9	9	9	9	9	9	9	9
9	9	9	9	9	9	9	9	9	9
3	2	2	3	2	2	3	2	2	1
2	3	1	1	1	1	3	1	1	1
1	1	1	2	1	1	3	1	1	2
2	3	2	2	2	2	2	2	2	2
3	3	1	1	1	2	1	1	1	1
2	2	2	2	2	2	2	2	2	1
1	2	1	1	1	1	2	1	1	2
2	2	2	2	2	2	2	2	2	2
2	3	1	1	2	2	2	2	2	2
2	2	1	1	2	2	2	2	2	2
2	1	1	2	1	3	1	2	2	1

2	2	1	2	3	1	2	3	2	2
1	2	1	2	1	1	2	3	3	3
3	3	1	2	1	3	2	3	2	2
1	1	2	1	1	1	2	1	1	1
2	1	2	1	2	1	3	1	2	3
3	1	2	1	1	1	1	3	2	1
3	1	2	1	1	1	3	1	1	1
9	9	2	9	9	9	9	9	9	2
2	2	2	2	2	2	2	2	2	2
2	2	2	2	2	2	2	2	2	2
2	2	2	2	2	2	2	2	2	2
2	2	2	2	2	2	2	2	2	2
2	2	2	2	2	2	2	2	2	2
1	2	1	1	9	2	2	2	2	2
2	2	1	1	2	1	2	1	1	2
2	2	2	2	2	2	2	2	2	2
2	2	2	2	2	2	2	2	2	2
1	1	1	1	1	1	3	1	1	1
1	2	2	2	2	2	2	2	2	1
2	3	1	1	2	2	1	2	1	1
2	2	2	3	2	2	1	2	2	1
3	2	2	2	2	2	2	2	1	2
1	1	1	1	1	1	2	1	1	1
9	9	9	9	9	9	9	9	9	1
3	1	1	3	2	3	2	3	1	3
1	1	1	1	1	1	2	1	1	1
1	1	2	1	1	1	1	1	1	2
1	3	1	2	1	2	3	2	1	2
2	2	2	2	2	2	2	2	2	2
9	9	9	1	9	9	9	9	9	9
3	3	2	1	3	3	3	3	3	2
9	9	9	9	9	9	9	9	9	9
9	9	9	9	9	9	9	9	9	9
9	9	9	9	9	9	9	9	9	9
9	9	9	9	9	9	9	9	9	9
2	2	2	2	2	2	2	2	2	2
2	2	1	1	1	1	1	1	1	1
9	9	9	9	9	9	9	9	9	9
2	2	2	2	2	2	2	2	2	2
2	2	2	2	2	2	2	2	2	2
2	3	1	1	2	2	2	1	1	9
2	2	2	2	2	2	2	2	2	3
2	2	2	2	2	2	2	2	2	2
3	2	3	3	3	3	3	3	3	9
9	9	9	9	9	9	9	9	9	9
1	1	1	1	2	2	1	1	1	1
3	3	3	3	3	3	3	3	3	3

1	1	1	1	1	1	1	1	1	1
9	2	1	2	1	2	1	1	1	1
2	1	1	1	1	1	1	1	1	1
2	2	2	2	2	2	2	2	2	2
9	9	9	9	9	9	9	9	9	9
9	9	9	9	9	9	9	9	9	9
2	2	2	2	2	2	2	2	2	2
3	3	9	9	9	9	9	9	9	2
9	9	9	9	9	9	9	9	1	1
9	9	9	9	9	9	9	9	9	9
2	3	1	2	2	2	2	2	2	2
1	1	2	1	1	1	2	2	2	2
2	2	3	2	2	2	2	2	2	2
3	3	3	3	3	3	3	3	3	3
3	3	9	9	9	9	9	9	9	9
3	3	2	3	3	2	2	3	2	1
2	2	2	1	1	1	2	1	1	1
2	2	2	2	3	2	1	3	2	2
9	9	1	9	9	9	9	9	9	9
3	2	2	3	1	2	2	1	2	2
1	2	1	2	1	3	1	2	1	1
2	3	1	1	1	2	2	2	1	2
1	1	1	3	2	1	2	2	1	1
2	2	2	2	2	2	2	2	2	1
1	3	9	9	9	9	9	9	9	3
3	3	2	2	2	2	2	2	2	2
2	1	2	1	2	2	2	2	1	2
2	2	1	1	3	3	2	2	2	1
2	3	3	2	3	3	3	3	3	1
3	3	3	2	3	3	3	3	3	3
2	2	2	2	2	2	2	2	2	2
1	1	1	2	2	2	1	1	1	2
1	1	1	1	1	1	1	1	1	1
1	2	3	2	2	2	2	2	2	3
9	9	9	9	9	9	9	9	2	9
2	1	1	1	2	2	1	2	1	1
9	9	9	9	9	9	9	9	9	1
2	2	2	2	2	2	2	2	2	2
9	9	9	9	9	9	9	9	9	9
3	1	1	1	2	2	2	3	1	9
9	9	9	9	9	9	9	9	9	9
3	2	1	2	3	2	2	3	1	1
2	3	2	2	2	2	2	2	2	3
2	1	1	2	2	2	2	1	2	1
2	2	1	2	2	2	2	1	2	1
2	2	2	2	2	2	2	2	2	2
3	3	2	2	2	2	3	3	3	2

2	2	2	2	2	2	2	2	2	2
9	9	9	9	9	9	9	9	9	9
2	2	2	2	2	2	2	2	2	1
3	3	3	3	3	3	3	3	3	2
3	1	1	1	3	3	3	1	1	1
9	9	9	9	9	9	9	9	9	9
9	9	9	9	9	9	9	9	9	9
2	2	2	2	2	2	2	2	2	2
1	1	1	1	1	9	9	1	1	1
1	3	2	2	2	2	2	2	2	2
2	2	1	2	2	2	1	2	2	2
3	1	2	2	2	2	2	2	2	2
2	3	2	2	2	3	9	2	2	2
1	1	1	1	1	1	1	1	1	1
9	9	9	9	9	9	9	9	9	9
3	3	3	3	3	3	3	3	3	3
2	3	2	2	2	2	2	2	2	2
2	2	2	2	2	2	2	2	2	2
3	3	3	1	3	1	2	2	2	1
2	1	1	2	2	1	3	2	1	1
9	9	9	9	9	9	9	9	9	9
9	9	1	9	9	9	9	9	9	9
2	2	2	2	2	1	2	2	2	2
9	9	9	9	9	9	9	9	9	9
9	9	9	9	9	9	9	9	9	9
2	3	2	3	9	2	2	3	3	2
3	3	2	2	2	2	2	2	2	2
2	2	2	2	2	2	2	2	2	2
3	3	3	3	3	3	3	3	3	3
1	1	1	1	1	1	2	1	1	1
3	3	3	3	3	3	3	3	3	3
1	3	1	1	1	1	2	2	2	3
9	9	9	9	9	9	9	9	9	9
9	9	9	9	9	9	9	9	9	9
9	9	9	9	9	9	9	9	9	9
1	1	2	2	2	2	2	2	2	9
1	1	1	1	3	1	3	1	1	1
2	3	2	2	2	3	1	2	3	2
3	1	1	2	1	2	3	1	1	1
9	9	2	9	9	9	9	9	9	2
2	2	2	2	2	2	2	3	3	3
2	2	1	1	2	2	1	2	1	1
2	3	3	3	3	3	3	3	3	3
9	9	9	9	9	9	9	9	9	9
1	2	2	1	3	2	1	2	1	1
3	3	1	1	3	1	3	1	3	9
2	1	1	1	2	2	3	2	2	1

1	2	2	1	3	2	1	2	3	2
3	3	2	2	2	2	2	2	2	3
3	3	1	1	1	1	9	3	3	3
2	3	1	2	3	1	2	3	2	1
3	2	1	2	2	2	2	1	1	1
1	2	1	2	1	1	2	2	1	2
1	2	2	2	2	2	2	2	2	2
2	1	1	1	1	2	2	1	1	1
9	9	9	9	9	9	9	9	9	9
1	1	2	2	2	1	1	1	1	2
1	2	2	2	2	2	2	2	2	2
3	3	2	2	2	2	2	2	2	2
2	2	1	2	1	2	1	1	1	1
2	1	1	2	3	2	3	1	1	1
9	9	9	2	9	9	9	9	9	9
2	2	2	2	1	1	1	9	1	2
1	1	1	1	1	1	2	2	1	1
1	1	1	2	1	2	1	2	3	3
2	2	1	1	3	3	2	2	2	3
2	2	2	2	2	2	2	2	2	2
1	1	2	1	1	2	2	1	1	1
2	2	2	2	2	2	2	2	2	1
1	1	1	3	3	2	1	2	1	1
3	2	2	2	2	2	2	2	2	1
2	1	9	9	9	9	9	9	1	1
3	3	1	1	2	2	2	3	1	1
2	3	2	2	1	1	2	3	3	2
2	2	1	2	2	2	2	2	2	1
2	2	2	2	2	2	2	2	2	2
2	2	2	2	2	2	2	2	1	1
1	1	1	2	1	2	2	2	2	1
2	2	2	2	2	2	2	2	2	2
2	2	2	2	2	2	2	2	2	2
2	2	1	2	1	1	1	2	1	1
2	3	1	2	3	3	3	3	3	1
2	2	1	1	1	2	3	2	2	1
1	1	1	1	2	2	1	1	1	1
1	1	1	1	2	2	2	2	2	1
3	2	1	2	1	1	3	1	1	1
3	3	2	2	3	3	3	3	1	1
2	2	3	3	3	3	2	2	2	2
2	2	1	1	1	1	2	2	2	1
1	1	1	1	2	2	2	1	1	1
1	1	1	1	2	2	2	2	1	1
2	2	2	1	1	1	2	2	2	2
1	1	2	2	2	2	1	1	1	1
1	1	3	3	2	1	1	1	1	3

2	1	2	3	2	1	2	2	1	1
2	2	2	1	2	2	2	1	2	1
1	2	2	1	1	2	2	1	1	1
2	1	2	2	1	2	1	2	1	1
1	1	1	1	2	2	1	1	1	1
1	1	1	2	2	2	1	1	1	2
1	1	1	2	2	1	1	2	2	1
2	2	1	2	2	1	1	1	1	2

AfraidPapS	LiketoKnov	WilldoPapi	WillLikePa	Embarrase	WorriedAb	NoTimefor	PapSmearN	PapAgainst	LikeHPVVa
2	2	2	2	2	2	2	2	2	2
1	3	1	1	1	1	2	2	2	1
1	1	2	1	3	3	3	3	3	9
1	3	1	1	1	1	2	2	2	1
1	1	1	1	1	1	2	2	2	1
1	1	1	1	1	1	2	2	2	1
9	9	9	2	9	9	9	9	9	9
3	1	2	3	3	1	2	2	3	1
9	1	9	1	1	2	2	2	2	1
2	2	2	9	9	9	9	9	9	2
2	3	2	2	2	2	2	2	2	2
3	3	3	3	3	3	3	3	3	2
2	1	1	1	3	3	3	3	3	1
2	1	3	2	2	2	3	2	3	2
3	1	1	1	1	2	3	3	3	1
2	1	3	2	3	2	3	3	3	1
1	1	1	1	1	2	1	1	1	1
2	1	3	2	3	2	3	3	3	1
2	1	3	2	3	2	3	3	3	1
1	1	1	1	1	1	1	1	1	2
1	1	1	3	3	3	3	3	3	1
1	1	1	3	3	3	3	3	3	1
2	1	2	2	2	2	2	2	2	2
1	1	2	1	3	1	3	3	3	1
2	2	2	2	2	2	2	2	2	2
1	1	2	1	3	1	3	1	3	1
1	1	1	1	3	1	3	3	2	1
3	1	3	1	1	3	3	3	3	1
2	2	2	2	2	2	2	2	2	1
2	2	2	2	2	2	2	2	2	1
2	2	2	2	2	2	2	2	9	1
2	1	2	2	1	1	2	1	1	1
3	1	3	1	3	3	3	3	3	1
2	1	2	1	3	1	3	2	3	1
2	2	2	2	2	2	2	2	2	1
2	1	2	1	1	1	2	1	1	1
2	2	2	2	2	2	2	2	2	1
3	1	1	1	3	1	3	1	3	9
2	2	2	2	2	2	2	2	2	2
2	2	2	2	2	2	2	2	2	1
2	2	2	2	2	2	2	2	2	1
2	1	2	2	2	2	3	3	2	2
3	1	3	1	3	1	3	2	3	9
3	1	1	1	3	3	3	2	3	1
3	1	1	1	3	3	3	2	3	1
1	2	3	3	2	2	1	1	2	1

3	1	3	1	3	3	3	3	3	3
1	1	2	1	1	1	1	3	3	1
2	1	3	1	2	2	3	3	3	1
1	1	2	2	1	1	3	2	3	1
1	9	2	1	3	2	3	3	3	1
3	1	3	1	3	3	3	3	3	1
3	1	3	1	3	3	3	3	3	3
3	1	1	1	3	3	3	3	3	1
9	1	9	1	9	9	9	9	9	1
1	1	3	1	1	2	3	3	3	3
9	9	2	9	2	9	2	9	2	2
2	1	3	3	3	2	3	3	3	1
1	1	2	2	3	1	3	3	3	1
2	2	2	2	2	2	2	2	2	9
1	1	1	2	3	2	3	3	3	1
1	1	3	3	3	1	3	3	3	1
3	1	3	1	3	3	3	3	3	1
3	1	3	1	3	3	3	3	3	9
1	1	1	1	2	1	2	3	3	1
9	9	9	9	9	9	9	9	9	1
3	1	3	3	3	3	3	3	3	3
3	1	3	3	3	3	3	3	3	3
3	1	3	3	3	3	3	3	3	3
3	1	3	1	1	1	2	3	3	9
9	1	9	9	9	9	9	9	9	1
1	1	1	1	1	1	2	2	3	9
9	9	9	9	9	9	9	9	9	9
2	1	1	2	3	1	1	3	1	1
9	9	9	9	9	9	9	9	9	1
1	1	2	1	3	3	3	3	3	1
1	1	2	1	1	3	2	1	2	3
3	1	2	1	3	3	1	2	2	1
9	1	9	1	9	1	2	2	3	2
3	1	3	1	3	3	3	3	3	1
1	1	3	1	3	1	3	3	3	1
9	9	9	9	9	9	9	9	9	9
9	9	9	9	9	9	9	9	9	9
3	1	3	1	3	3	3	3	3	1
3	1	3	1	3	3	3	3	3	1
3	1	3	1	3	3	1	3	1	1
9	9	9	9	9	9	9	9	9	1
3	1	9	1	3	3	1	2	3	1
3	1	1	1	3	3	3	3	3	1
2	1	1	1	3	2	3	3	3	1
2	1	1	1	3	2	3	3	3	1
9	9	9	9	9	9	9	9	9	9
9	9	9	9	9	9	9	9	9	9

1	1	1	2	1	3	3	3	3	9
3	1	3	2	3	3	3	3	3	2
3	1	1	2	1	1	1	3	3	1
1	1	2	1	3	3	3	3	3	1
9	9	9	9	9	9	9	9	9	9
1	1	2	2	2	2	2	2	2	2
3	1	3	1	3	3	3	3	3	1
3	1	3	1	3	1	3	3	3	1
3	1	1	2	1	1	2	3	3	1
2	1	2	1	3	3	3	3	3	1
1	1	3	1	2	1	3	2	2	1
1	1	1	2	2	3	3	2	2	1
3	1	3	1	3	3	3	1	3	1
1	1	2	2	2	1	2	2	2	1
1	2	1	2	1	1	2	3	2	2
2	1	1	1	3	2	1	2	2	2
2	1	3	3	3	2	3	3	3	1
2	1	2	1	2	2	2	2	2	1
2	2	2	2	2	2	2	2	2	9
3	1	3	1	3	3	3	3	3	1
3	1	2	1	3	3	3	3	3	1
9	1	3	1	3	3	3	3	3	9
2	2	2	2	2	2	2	2	2	1
9	9	9	9	9	9	9	9	9	1
2	2	1	1	1	1	1	2	2	2
2	1	3	2	1	2	3	3	3	1
3	1	1	2	3	3	3	3	3	1
3	1	1	2	3	3	3	3	3	1
2	2	2	2	2	2	2	2	2	2
2	1	3	1	3	1	2	2	3	1
9	1	2	1	3	2	2	2	3	1
2	2	2	2	2	2	2	2	2	2
2	1	3	1	3	2	3	2	3	1
1	1	2	2	2	2	2	2	2	2
2	1	2	1	3	1	2	3	3	1
2	1	1	2	2	1	2	1	2	1
1	1	2	1	3	3	2	2	2	1
2	2	2	2	2	2	2	2	2	1
2	9	3	9	1	1	9	2	1	9
3	1	3	1	3	3	3	3	3	1
2	2	2	2	2	2	2	2	1	1
2	2	2	2	2	2	2	2	1	1
2	2	2	2	2	2	2	2	2	2
3	1	3	3	3	3	3	1	3	1
1	1	2	2	1	1	2	3	2	2
2	1	1	1	3	3	3	3	3	1
2	2	2	2	2	2	2	2	2	1

9	9	9	9	9	9	9	9	9	3
2	2	1	1	1	2	3	3	2	2
2	2	1	1	1	2	3	3	2	2
2	2	2	2	2	2	2	2	1	1
2	2	2	2	2	2	2	2	1	1
3	1	3	1	3	3	3	3	3	9
1	3	3	3	3	3	3	3	3	3
2	1	3	2	3	3	3	3	3	1
2	2	2	2	2	2	2	2	2	2
2	2	2	2	2	2	2	2	2	2
2	3	2	2	2	3	2	3	2	9
2	2	2	2	2	3	2	3	2	9
2	1	3	2	2	2	2	9	1	2
2	1	2	2	3	3	3	2	3	1
3	3	1	3	1	3	3	1	3	3
3	3	1	3	1	3	3	1	3	3
1	9	9	9	9	9	9	9	9	9
1	1	3	3	3	1	3	3	1	3
9	1	9	9	9	9	9	9	9	9
2	1	3	1	3	1	3	3	3	1
2	1	3	1	3	1	3	3	3	1
2	1	3	1	3	1	3	3	3	1
2	1	3	1	3	3	3	3	3	1
2	1	3	1	3	1	3	3	3	1
2	1	3	1	3	1	3	3	3	1
2	1	3	1	1	1	3	3	3	1
2	1	3	1	1	1	3	3	3	1
3	1	3	1	3	1	3	3	3	1
2	1	3	1	3	1	3	3	3	1
1	1	3	1	3	1	3	3	3	1
2	3	1	3	2	2	2	2	2	1
2	2	2	2	2	2	2	2	2	2
2	1	3	1	1	1	3	3	3	1
9	1	3	1	3	1	3	3	3	1
1	1	3	1	3	1	3	3	3	1
2	1	3	1	3	1	3	3	3	1
2	1	3	1	3	1	3	3	3	1
2	1	3	1	3	1	3	3	3	1
2	1	3	1	3	1	3	3	3	1
3	1	3	1	2	1	3	3	3	1
2	1	1	3	3	1	3	3	3	1
2	1	3	1	3	1	3	3	3	1
2	1	3	1	3	1	3	3	3	1
2	1	3	2	3	1	3	3	3	1
2	1	3	1	1	1	3	3	3	1
2	1	3	2	3	1	3	3	3	1
2	1	3	2	3	1	3	3	3	1

2	1	2	3	1	1	3	3	3	1
9	1	3	1	3	1	3	3	3	1
3	1	1	1	1	1	3	3	3	1
3	1	3	1	1	1	1	3	3	1
2	1	3	1	3	1	3	3	3	1
3	1	3	1	1	1	3	3	9	1
3	3	3	1	1	1	3	3	3	1
3	1	3	1	3	3	3	3	3	1
3	1	3	1	3	3	1	3	1	1
3	1	3	1	3	3	3	3	3	1
9	9	9	9	9	9	9	9	9	9
9	9	9	9	9	9	9	9	9	9
1	1	3	1	3	1	1	1	3	1
1	1	3	1	3	1	3	3	3	1
2	1	1	1	2	1	2	2	2	9
2	1	1	2	2	2	1	3	3	1
2	2	2	2	2	2	2	2	2	2
1	1	1	1	1	1	1	1	1	1
2	1	2	1	3	3	3	2	3	3
3	1	3	1	3	3	3	2	3	1
2	1	1	1	1	2	2	1	1	2
2	2	2	2	2	2	2	2	2	2
1	1	2	2	3	1	3	2	3	1
2	1	1	3	1	1	1	2	3	1
2	2	2	2	2	2	2	2	2	2
2	2	2	2	2	2	2	2	2	2
3	1	3	1	3	3	2	3	3	1
2	2	2	2	2	2	2	2	2	2
2	2	2	2	2	2	2	2	2	2
2	1	2	1	2	3	3	3	3	3
3	1	3	1	3	3	3	3	3	1
3	1	3	1	3	3	3	3	3	1
2	1	1	1	3	3	3	3	3	3
2	2	2	2	1	1	1	2	2	1
2	1	1	1	2	1	2	1	2	1
1	1	1	1	2	2	2	2	1	2
2	2	1	1	2	2	2	1	2	2
1	1	1	1	1	1	1	2	3	1
2	1	2	2	2	2	2	2	2	2
2	3	1	2	3	2	2	2	3	1
2	2	2	2	2	2	2	2	2	1
3	1	3	1	3	3	2	3	3	1
9	9	9	3	9	9	9	9	9	1
9	9	9	3	9	9	9	9	9	1
1	3	1	2	1	2	1	2	1	2
2	1	3	1	2	3	1	1	3	1
1	9	9	9	9	9	9	9	9	1

2	2	2	2	2	2	2	2	2	1
2	2	2	2	2	2	2	2	2	1
2	1	2	2	2	2	2	2	3	2
2	1	1	2	1	2	2	2	2	1
1	1	1	1	1	2	3	1	2	3
2	2	2	2	2	2	2	2	2	2
2	2	2	2	2	2	2	2	2	2
2	1	2	2	1	2	2	2	2	2
2	9	1	2	1	2	2	2	2	1
2	2	1	1	2	2	2	2	2	2
2	1	2	2	2	2	2	2	2	1
2	2	2	2	2	2	2	2	2	2
2	2	2	2	2	2	2	2	2	2
2	3	3	2	2	2	2	2	2	2
2	1	1	1	3	3	3	3	3	1
2	1	3	2	1	2	2	2	2	2
3	3	2	1	2	2	3	2	2	2
2	1	2	2	2	3	3	2	3	1
9	1	9	9	9	9	9	9	9	1
1	1	3	1	3	1	3	3	3	1
1	3	9	1	1	1	1	1	3	1
2	1	1	2	2	2	2	2	2	1
2	3	3	2	2	2	2	2	2	2
9	9	9	9	9	9	9	9	9	1
2	1	9	1	9	1	9	1	9	9
1	1	2	2	2	2	2	2	2	2
1	1	2	2	2	2	2	2	2	2
3	2	2	1	3	2	1	2	2	1
2	2	2	2	2	2	2	2	2	9
2	2	2	2	2	2	2	2	2	1
9	1	9	9	9	9	9	9	2	2
1	1	2	1	3	1	3	3	3	1
3	3	1	3	1	1	1	1	3	1
1	1	3	1	1	1	3	2	3	1
9	9	9	9	9	9	9	9	9	2
2	2	2	2	2	2	2	2	2	2
3	1	3	1	2	1	3	3	3	1
2	1	3	1	3	3	3	3	3	2
1	1	2	1	2	1	2	2	1	1
3	1	2	3	3	2	3	3	3	1
2	1	1	1	1	1	1	1	1	1
3	1	2	1	3	1	3	1	3	1
9	9	9	9	9	9	9	9	9	9
2	2	2	2	2	2	2	2	2	2
3	1	2	2	2	1	1	1	2	2
2	2	2	2	3	3	3	1	1	2
2	2	2	2	2	2	2	2	2	1

9	9	9	9	9	9	9	9	9	9
9	9	9	9	9	9	9	9	9	9
9	9	9	9	9	9	9	9	9	9
1	1	3	1	2	2	3	3	3	1
3	2	2	2	3	2	2	3	3	2
3	1	1	3	3	3	3	2	1	1
3	1	3	1	3	3	2	3	3	1
3	1	1	1	1	1	1	1	3	1
9	9	9	9	9	9	9	9	9	9
2	2	2	2	2	2	2	2	2	2
2	2	2	2	2	2	2	2	2	2
2	1	1	1	2	2	3	2	2	1
9	9	9	9	9	9	9	9	9	9
3	1	3	3	3	3	3	3	3	3
3	3	1	3	1	9	1	9	1	1
9	9	9	9	9	9	9	9	9	9
9	9	9	9	9	9	9	9	9	9
1	1	1	1	3	1	3	2	2	1
1	1	1	1	2	1	1	1	1	1
9	9	9	9	9	9	9	9	9	9
9	9	9	9	9	9	9	9	9	9
3	1	2	1	3	1	3	2	3	1
2	1	2	2	3	2	1	1	2	1
1	1	1	1	1	3	1	1	3	3
2	2	2	2	2	2	2	2	9	2
3	1	3	1	3	3	3	3	3	1
1	3	1	2	1	3	1	2	1	1
3	1	2	1	3	3	3	3	3	1
3	1	3	1	3	1	3	3	3	1
3	1	3	1	3	3	3	3	3	1
2	2	2	2	2	2	2	2	2	2
9	9	9	9	9	9	9	9	9	9
9	9	9	9	9	9	9	9	9	9
9	9	9	9	9	9	9	9	9	2
3	1	3	2	3	3	3	3	3	1
1	1	1	1	1	3	1	2	1	9
1	1	3	1	3	2	3	3	3	1
1	1	1	1	1	1	3	1	3	1
3	1	3	1	3	3	3	3	3	1
2	1	2	1	2	2	3	3	3	2
2	1	1	2	1	2	2	1	2	1
1	1	3	1	1	1	3	3	3	1
2	1	2	2	2	2	2	2	9	1
2	1	2	2	2	2	2	2	2	9
2	1	1	1	3	3	3	3	3	1
2	2	2	2	2	2	2	2	2	2
1	1	3	1	3	1	3	3	3	1

9	9	9	9	9	9	9	9	9	9
9	9	9	9	9	9	9	9	9	1
9	1	9	9	9	9	9	9	9	1
2	1	3	1	3	3	3	3	3	1
9	9	9	9	9	9	9	9	9	1
9	9	9	9	9	9	9	9	9	9
2	1	1	2	2	3	3	3	3	1
9	1	1	2	2	1	9	9	9	2
2	1	3	1	1	1	3	3	3	1
2	1	2	2	3	2	3	3	3	1
2	2	2	2	2	2	2	2	2	2
2	2	2	2	2	2	2	2	2	3
2	1	3	1	3	3	2	2	3	1
1	1	2	3	1	2	3	3	2	3
2	1	3	1	2	2	3	3	2	1
2	1	2	2	2	3	2	3	1	2
2	2	2	2	2	2	9	2	2	2
2	3	3	2	2	2	2	3	2	2
2	2	2	2	2	2	2	2	2	2
2	2	2	2	2	2	2	2	3	2
3	2	2	1	3	3	2	3	3	1
3	1	3	1	3	3	3	3	3	1
2	1	3	1	3	1	3	3	3	1
2	1	2	1	2	2	2	3	3	1
2	2	2	2	2	2	2	2	2	2
2	1	3	2	2	1	1	2	2	2
2	2	2	2	2	2	2	2	3	1
2	2	2	2	2	2	2	2	2	2
1	1	3	2	2	1	2	3	2	1
2	1	1	2	1	1	1	2	2	1
1	1	1	2	3	2	2	3	3	2
1	1	1	2	3	2	2	3	3	2
1	2	2	3	3	3	2	3	1	2
2	2	2	2	2	2	2	2	2	9
2	2	2	2	2	2	2	2	2	9
2	3	2	1	3	3	2	3	3	1
2	1	2	2	2	2	2	2	3	1
2	2	2	2	2	2	2	2	2	9
2	2	2	2	2	2	2	2	2	2
2	2	2	2	2	2	2	2	3	2
2	2	2	1	2	2	3	3	3	2
2	1	2	2	2	2	2	2	2	1
3	1	1	1	3	3	3	3	3	2
2	1	2	2	2	2	2	2	2	1
2	2	1	2	2	1	2	1	2	1
1	1	2	2	1	2	3	3	3	2
2	2	2	2	2	2	2	2	2	1

2	1	3	1	1	3	3	3	2	1
2	9	1	3	3	3	3	3	3	1
2	2	2	2	2	2	2	2	2	2
2	2	2	2	2	2	2	2	2	2
2	2	2	2	2	2	2	2	2	1
2	2	2	2	2	2	2	2	2	2
9	9	9	9	9	9	9	9	9	9
2	2	2	2	2	2	2	2	2	1
2	2	2	2	2	2	2	2	2	1
1	2	2	3	3	1	1	2	2	1
1	1	2	2	1	2	1	2	2	1
1	1	3	1	1	1	3	3	3	1
1	1	1	1	1	1	1	1	1	1
1	1	1	1	3	1	1	2	3	1
2	2	2	2	2	2	2	2	2	2
1	1	1	2	2	1	1	1	1	1
2	1	3	1	3	1	3	3	3	1
2	1	1	1	1	1	2	3	3	1
2	1	1	1	1	1	2	3	3	1
2	9	2	2	2	3	2	3	1	2
3	1	1	1	3	1	2	1	1	2
2	1	2	2	2	2	2	2	2	2
9	9	9	9	9	9	9	9	9	9
2	2	2	2	2	2	2	2	2	2
1	1	2	1	1	1	2	3	3	1
9	9	9	9	9	9	9	9	9	9
1	1	2	3	2	1	2	2	2	1
9	9	9	9	9	9	9	9	9	9
1	2	2	2	2	2	2	2	2	2
2	1	2	1	2	1	2	1	2	1
9	9	9	9	9	9	9	9	9	9
9	9	9	9	9	9	9	9	9	9
2	2	2	2	2	2	2	2	2	2
1	1	1	2	2	1	1	1	1	2
9	9	9	9	9	9	9	9	9	9
1	2	2	1	1	1	1	1	2	1
3	2	3	3	2	3	3	3	3	3
1	1	1	2	1	2	1	9	9	2
9	9	9	9	9	9	9	9	9	9
1	1	3	1	2	1	1	3	3	1
9	9	9	9	9	9	9	9	9	9
1	3	1	1	1	1	1	2	2	1
2	2	2	2	2	2	2	2	2	2
3	1	1	1	1	1	1	1	1	2
1	1	1	9	9	9	9	9	9	1
1	1	3	1	3	1	3	3	3	1
3	1	3	1	3	1	3	3	3	1

9	9	9	9	9	9	9	9	9	9
2	1	3	1	1	1	1	3	3	1
2	1	3	1	3	1	3	3	3	1
1	9	3	1	3	1	3	3	3	1
2	1	3	1	3	1	3	3	3	1
1	1	3	1	1	1	3	3	3	1
2	1	3	1	2	1	3	3	3	1
3	1	3	1	3	1	3	3	3	1
3	1	3	1	3	1	3	3	3	1
2	2	3	1	1	1	3	3	3	1
2	1	2	1	3	1	2	2	3	1
1	1	3	1	1	1	3	3	3	1
3	1	3	1	1	1	3	3	3	1
2	1	3	1	1	1	3	3	3	1
2	1	3	1	3	1	3	3	3	1
2	1	3	1	3	1	3	3	3	1
2	1	3	1	1	1	3	3	3	1
2	1	3	1	1	1	3	3	3	1
2	1	3	1	1	1	3	3	3	1
2	1	3	1	1	1	3	3	3	1
1	1	3	1	3	1	3	3	3	1
2	1	3	1	3	1	3	3	3	1
2	1	3	1	3	1	3	3	3	1
2	1	3	1	1	1	3	3	3	1
2	1	3	1	3	1	3	3	3	1
2	1	3	1	3	1	3	3	3	1
2	1	3	1	3	1	3	3	3	1
1	1	3	1	3	1	3	3	3	1
2	1	3	1	1	1	3	1	3	1
1	1	3	1	2	1	3	3	3	1
2	1	3	1	1	1	3	3	3	1
3	1	3	1	3	1	3	3	3	1
2	1	3	1	3	1	3	3	3	1
2	1	3	1	3	1	3	3	3	1
2	3	1	1	3	1	3	3	3	1
2	1	2	1	3	1	3	3	3	1
3	1	3	1	3	1	3	9	3	1
3	1	3	1	3	1	3	3	3	1
3	1	3	1	1	1	3	3	3	1
3	1	3	1	1	1	3	3	3	1
2	1	3	1	1	1	3	3	3	1
2	2	2	2	2	2	2	2	2	2
1	1	1	1	2	1	3	3	3	1
2	2	2	2	2	2	2	2	2	2
9	9	9	9	9	9	9	9	9	2
2	2	2	2	2	2	2	2	2	2
1	1	2	3	3	1	1	2	3	1

2	2	2	2	2	2	2	2	2	2
2	1	3	1	1	3	2	3	3	1
2	1	3	1	1	3	2	3	3	1
2	1	1	1	2	2	3	2	2	1
2	2	2	2	2	2	2	2	2	1
2	9	9	9	9	9	9	9	9	9
2	2	2	2	2	2	2	2	2	2
1	2	2	2	2	2	2	2	2	1
2	1	1	1	3	1	3	3	3	1
2	1	2	2	2	2	2	2	2	2
2	1	3	1	1	3	2	3	3	1
1	9	9	9	9	9	9	9	9	1
9	9	9	9	9	9	9	9	9	1
2	1	2	1	2	2	2	2	2	2
9	9	9	9	9	9	9	9	9	1
9	9	9	9	9	9	9	9	9	1
9	9	9	9	9	9	9	9	9	1
9	9	9	9	9	9	9	9	9	1
9	9	1	9	9	9	9	9	9	1
3	2	1	1	3	1	3	3	1	1
3	3	9	1	1	9	9	3	3	9
9	9	9	9	1	1	9	9	9	9
2	1	1	1	1	1	3	1	3	1
9	9	9	9	9	9	9	9	9	1
2	9	9	9	9	9	9	9	9	9
9	9	9	9	9	9	9	9	9	9
9	9	9	9	9	9	9	9	9	1
9	9	9	9	9	9	9	9	9	9
3	1	2	1	3	1	3	3	3	1
9	9	9	9	9	9	9	9	9	1
9	9	9	9	9	9	9	3	9	1
2	1	2	2	2	2	2	2	3	1
2	2	2	2	2	2	2	2	2	2
3	1	3	1	3	3	3	3	3	1
2	1	1	2	2	1	1	2	2	1
2	1	2	1	2	2	2	2	3	1
3	1	3	1	3	1	3	2	3	1
3	1	3	1	3	3	3	3	3	1
2	2	2	2	2	2	2	2	2	2
2	1	1	2	3	3	3	2	3	1
2	1	3	1	2	3	2	3	2	1
1	1	3	1	1	1	3	3	3	1
2	2	2	2	2	2	2	2	2	2
3	1	2	1	1	2	2	3	3	9
3	1	1	2	3	1	3	2	3	2
2	2	2	2	2	2	2	2	2	9
1	1	3	1	3	2	3	3	3	1

1	1	1	2	1	2	3	2	2	1
3	1	3	1	3	3	3	3	3	1
2	1	3	1	3	3	3	3	3	1
1	1	1	2	2	1	3	3	3	1
3	1	2	1	3	2	3	1	2	1
2	1	2	2	2	2	2	2	2	1
3	1	2	1	1	1	3	2	3	1
2	2	2	2	2	2	2	2	2	2
2	2	2	2	2	2	2	2	2	2
2	2	2	2	2	2	2	2	2	2
2	2	2	2	2	2	2	2	2	2
2	1	3	2	1	3	3	3	3	2
2	2	2	2	2	2	2	2	2	2
2	2	2	2	2	2	2	2	2	2
2	1	3	2	1	2	3	2	2	2
2	2	2	2	2	2	2	2	2	2
2	2	2	2	2	2	2	2	2	2
2	1	2	2	2	2	2	2	2	2
2	1	1	2	2	2	2	2	3	1
3	1	1	1	3	3	2	3	3	1
2	1	2	2	2	2	3	2	2	2
2	1	3	1	3	1	3	3	3	1
1	1	3	1	3	3	3	3	3	1
2	1	1	1	3	3	2	2	3	1
2	1	2	3	1	1	2	2	3	2
2	1	2	2	2	2	2	2	2	1
1	1	3	1	1	3	1	3	3	9
2	1	2	1	3	1	1	2	1	1
1	1	3	1	3	1	3	3	2	1
2	2	3	3	2	3	2	3	2	2
2	2	2	2	2	2	2	2	2	2
2	1	1	1	1	2	1	2	2	1
1	1	2	2	2	2	2	2	2	2
2	2	2	2	3	3	2	2	2	3
2	2	2	2	2	2	2	2	2	2
9	9	9	9	9	9	9	9	9	2
2	1	3	1	2	1	3	2	3	1
3	1	3	1	3	3	3	3	3	1
2	1	2	2	2	2	2	2	2	3
3	1	2	1	3	3	3	3	3	9
2	1	9	9	9	9	9	9	9	9
1	1	2	1	2	2	2	2	3	1
3	1	2	1	3	3	3	2	3	1
1	1	1	2	2	3	3	2	2	1
1	1	2	1	3	3	3	3	3	1
2	2	2	2	2	2	2	2	2	1
9	9	9	9	9	9	9	9	9	1

9	9	9	9	9	9	9	9	9	1
9	9	9	9	9	9	9	1	9	9
9	9	9	9	9	9	9	9	9	1
1	1	1	1	3	3	3	1	3	1
9	9	1	9	9	9	9	9	9	1
9	9	1	9	9	9	9	9	9	1
9	9	9	9	9	9	9	1	9	1
9	9	9	9	9	9	9	9	9	1
9	9	1	9	9	9	9	9	9	1
9	9	1	9	9	9	9	9	9	1
9	9	9	9	3	9	9	9	9	1
9	1	9	9	9	9	9	9	9	1
9	9	9	9	9	9	9	9	9	1
9	9	9	9	9	9	9	9	9	1
9	9	9	9	9	9	9	9	3	1
9	9	9	9	9	9	9	9	9	1
9	9	9	9	9	9	9	1	9	1
1	1	1	1	3	3	3	1	3	1
2	2	2	2	2	2	2	2	2	2
9	9	9	9	9	9	9	9	9	1
1	1	1	1	1	1	1	1	1	1
1	3	2	2	1	3	1	2	3	2
1	1	2	1	1	2	2	2	2	1
1	1	1	1	3	2	1	1	1	1
2	2	3	3	2	2	3	2	3	1
2	1	2	1	2	1	2	2	2	3
2	2	2	2	2	2	2	2	2	2
3	9	2	1	2	1	1	2	3	3
2	2	2	2	2	2	2	2	2	2
2	2	2	2	2	2	2	2	2	1
1	1	2	2	3	3	3	3	2	1
2	1	2	2	3	2	2	2	3	1
2	2	2	2	2	2	2	2	2	2
2	3	1	3	3	3	3	3	3	1
2	3	1	2	3	1	2	3	1	1
1	2	2	2	2	2	2	2	2	1
2	2	2	2	2	2	2	2	2	2
9	9	9	9	9	9	9	9	9	9
1	1	1	1	1	1	3	1	1	1
9	9	9	9	9	9	9	9	9	9
2	2	3	3	3	3	3	3	3	2
3	2	3	2	2	2	3	2	2	2
9	9	9	9	9	9	9	9	9	9
3	1	1	3	1	1	1	1	1	1
2	2	2	2	2	2	2	2	2	2
2	2	2	2	2	2	2	2	2	9
2	2	2	3	2	3	2	2	2	3

2	2	2	2	2	2	2	2	2	2
9	9	9	9	9	9	9	9	9	9
1	3	1	3	1	3	1	3	1	1
9	9	9	9	9	9	9	9	9	9
1	1	1	1	1	1	1	1	1	1
1	1	1	1	1	1	1	1	1	1
9	9	9	9	9	9	9	9	9	9
2	2	2	2	2	2	2	2	2	2
2	1	1	2	1	2	1	2	3	3
2	2	2	2	2	2	2	2	2	9
2	2	9	3	2	1	1	2	2	9
1	1	2	1	2	1	1	1	3	1
2	3	3	3	3	3	2	2	2	2
9	9	9	9	9	9	9	9	9	9
9	9	9	9	9	9	9	9	9	9
9	9	9	9	9	9	9	9	9	9
2	1	1	2	1	2	3	3	3	1
9	9	9	9	9	9	9	9	9	9
2	1	3	1	3	1	3	2	3	3
3	1	3	2	3	3	3	3	3	1
3	3	3	3	3	3	3	3	3	2
2	3	3	3	3	3	2	2	2	2
1	1	1	1	1	1	1	1	1	3
9	9	9	9	9	9	9	9	9	9
2	2	2	2	2	2	2	2	2	2
3	1	1	3	1	3	1	3	1	1
1	3	3	3	3	3	3	3	3	3
1	1	3	3	1	1	3	1	3	1
2	2	2	1	1	1	1	1	1	2
3	1	3	1	3	3	3	3	3	1
9	9	9	9	9	9	9	9	9	9
9	9	9	1	9	9	9	9	9	9
2	1	2	1	3	3	3	2	3	1
1	1	1	2	1	1	9	3	3	2
9	1	9	2	1	9	1	9	3	2
2	1	3	1	1	3	3	3	3	1
3	1	3	1	3	3	3	3	3	1
9	9	9	9	9	9	9	9	9	9
9	9	9	9	9	9	9	9	9	9
9	9	9	9	9	9	9	9	9	9
9	9	9	9	9	9	9	9	9	9
2	3	2	1	1	1	1	1	1	2
1	2	2	3	3	2	3	1	2	3
9	9	9	9	9	9	9	9	9	9
1	2	2	3	3	2	3	1	2	2
2	1	2	3	1	1	2	1	3	1
1	1	1	1	1	3	3	3	3	1

3	3	3	3	3	3	3	3	3	1
2	1	3	2	2	2	2	2	2	2
3	2	3	3	2	3	3	3	3	3
2	2	2	2	2	2	2	2	2	2
2	2	2	2	2	2	2	2	2	3
1	1	1	1	1	1	9	1	1	1
9	9	9	9	9	9	9	9	9	9
2	1	2	2	2	2	3	3	3	1
3	2	9	2	3	1	1	2	9	2
2	1	3	2	2	2	3	2	3	9
3	3	3	3	3	3	3	3	3	3
3	1	1	1	1	3	3	1	3	1
2	1	3	3	3	1	1	2	3	1
2	1	2	3	1	1	1	2	3	1
2	1	3	2	3	1	1	2	3	1
2	1	2	2	3	2	1	2	1	2
2	1	3	2	3	1	1	2	3	1
2	1	3	2	3	1	1	2	3	1
2	1	3	2	3	1	1	2	3	1
1	3	3	2	1	1	2	1	1	1
1	2	3	1	3	1	3	2	1	3
3	3	3	3	3	3	3	3	3	1
3	2	2	2	2	3	2	2	1	1
2	2	2	2	2	2	2	2	2	2
3	3	3	3	3	3	3	3	3	3
2	1	3	2	3	1	1	2	3	1
1	1	3	9	2	2	9	1	9	9
3	1	3	1	1	1	3	3	3	1
9	9	9	9	9	9	9	9	9	9
2	2	2	2	2	2	2	2	3	2
2	1	2	2	1	3	9	2	1	1
3	2	3	2	3	2	2	3	2	3
9	1	2	3	2	1	2	1	2	1
9	9	9	9	2	9	9	9	9	9
3	3	3	3	3	3	3	3	3	2
3	2	3	3	3	3	3	3	3	1
3	9	2	9	3	1	2	3	3	1
2	1	1	1	2	1	2	2	2	1
2	1	1	1	2	1	2	2	2	1
1	1	2	1	1	1	1	3	1	1
2	1	2	2	1	1	1	1	1	3
2	2	2	2	2	2	2	9	2	2
1	2	2	1	2	1	2	1	2	1
3	1	3	3	1	3	3	3	3	1
3	2	1	2	1	1	1	1	1	9
1	1	1	1	1	1	1	1	9	1
3	3	2	2	3	2	9	2	2	3

2	2	2	2	2	2	2	2	2	1
9	9	9	9	9	9	9	9	9	1
9	9	9	9	9	9	9	9	9	9
3	1	3	1	3	1	3	1	1	1
2	2	2	2	2	2	2	2	2	2
1	1	1	1	1	1	1	1	1	1
1	2	2	2	2	2	2	2	2	1
1	1	1	1	1	1	1	1	1	1
1	1	1	1	1	1	1	1	1	1
2	2	2	2	2	3	3	3	1	9
9	9	9	9	9	9	9	9	9	9
1	1	3	1	2	1	3	3	3	1
9	9	9	9	9	9	9	9	9	9
3	1	3	1	1	1	1	3	2	1
2	1	2	1	3	1	3	3	3	1
9	9	9	9	9	9	9	9	9	9
2	2	2	2	1	1	1	1	2	1
1	9	9	9	9	9	9	9	9	2
1	9	9	9	9	9	9	9	9	2
2	3	1	2	1	2	3	1	2	1
1	1	1	1	1	1	1	3	1	1
2	2	2	2	2	3	3	3	3	2
9	9	9	9	9	9	9	9	9	9
2	2	2	2	2	2	2	2	2	2
3	3	3	3	3	3	3	3	9	3
2	1	3	1	1	2	1	1	3	1
2	1	3	1	1	2	1	1	3	1
2	1	3	3	3	3	3	3	3	1
1	1	1	1	1	1	1	1	1	1
2	2	2	2	2	2	2	2	2	2
2	3	1	3	2	2	1	2	2	2
2	2	1	2	3	3	2	1	2	1
2	1	1	2	2	1	1	2	1	2
2	1	3	1	1	3	1	1	3	1
1	3	2	1	1	3	2	2	1	1
2	2	2	2	2	2	2	2	2	2
2	2	2	2	2	2	2	2	2	1
9	9	9	9	9	9	9	9	9	9
9	9	9	9	9	9	9	9	9	9
2	1	1	1	3	3	1	3	3	1
2	2	2	2	2	2	2	2	2	2
2	2	2	2	2	2	2	2	2	2
2	2	2	2	2	2	2	2	2	2
2	2	2	2	2	2	2	2	2	2
2	2	2	2	2	2	2	2	2	2
2	2	2	2	2	2	2	2	2	2
9	9	9	9	9	9	9	9	9	9

9	9	9	9	9	9	9	9	9	9
2	2	2	2	2	2	2	2	2	2
9	9	9	9	9	9	9	9	9	9
9	9	9	9	9	9	9	9	9	9
1	9	9	9	9	9	9	9	9	9
2	2	2	2	2	2	2	2	2	2
9	9	9	9	9	9	9	9	9	9
3	3	3	3	3	3	3	3	3	3
2	2	3	2	2	1	2	1	1	1
9	2	9	9	9	9	9	9	9	9
2	2	2	2	2	2	2	2	2	2
2	3	2	2	1	2	1	2	1	1
2	3	2	2	1	2	1	2	1	1
2	2	2	2	2	2	2	2	2	2
1	1	1	2	2	2	1	1	1	3
2	2	2	2	2	2	2	2	2	2
2	2	2	2	2	2	2	2	2	2
2	2	2	3	3	3	3	2	1	9
2	2	1	2	2	1	3	3	2	1
3	1	2	3	2	2	2	3	1	3
9	1	9	9	9	9	9	9	9	9
2	2	2	2	2	3	3	3	3	3
2	2	2	2	2	2	2	2	2	2
2	1	3	2	2	2	2	2	2	1
3	3	3	3	3	3	3	3	1	9
3	2	3	2	3	2	3	2	3	2
9	9	9	9	9	9	9	9	9	9
9	1	9	9	9	9	9	9	9	1
2	2	2	2	2	2	2	2	2	2
3	3	3	3	3	3	3	3	2	2
2	2	2	2	2	2	2	2	2	1
2	2	2	2	2	2	2	2	2	1
3	1	1	1	1	3	3	1	3	1
2	2	2	2	2	2	2	2	2	2
2	2	2	2	2	2	2	2	2	2
1	1	1	1	1	1	3	1	1	1
2	2	2	2	2	2	2	2	2	2
2	3	1	3	1	3	2	3	2	1
2	2	2	2	2	2	2	2	2	2
2	2	2	2	2	2	2	2	2	2
2	2	2	2	2	2	2	2	2	9
2	2	2	2	2	2	2	2	2	2
3	3	3	1	2	1	2	3	3	1
2	2	2	2	2	2	2	2	2	2
2	2	2	2	2	2	2	2	2	2
3	3	3	3	3	3	3	3	3	3
2	2	2	2	2	2	2	2	2	2

2	2	2	2	2	2	2	2	2	2
2	2	2	2	2	2	2	2	2	2
2	2	2	2	2	2	2	2	2	2
2	2	2	2	2	2	2	2	2	2
2	2	1	3	2	1	2	1	2	1
1	1	1	2	3	3	2	1	9	2
2	2	2	2	2	2	2	2	2	2
1	2	3	2	1	2	1	1	2	1
2	2	2	2	2	2	2	2	2	2
2	2	2	2	2	2	2	2	2	2
1	1	3	1	3	1	1	1	3	1
9	9	9	9	9	9	9	9	9	2
2	9	9	9	9	9	9	9	9	9
9	9	9	9	9	9	9	9	9	9
2	3	3	3	3	3	3	3	1	1
2	2	3	3	3	1	1	1	2	3
2	2	3	3	3	1	1	1	2	3
1	1	3	1	3	2	3	3	3	1
9	9	9	9	9	9	9	9	9	9
2	1	2	2	3	3	2	3	3	3
9	9	9	9	9	9	9	9	9	9
2	2	2	2	2	2	2	2	2	1
2	2	2	2	2	2	2	2	2	1
2	2	1	1	1	1	1	1	1	9
2	2	2	2	2	2	2	1	1	3
1	9	2	2	2	2	2	2	2	1
2	2	2	2	2	2	2	2	1	2
2	2	2	2	2	2	2	2	3	1
2	2	2	2	2	2	2	2	2	2
3	2	1	2	1	2	2	1	1	1
1	1	1	1	1	1	2	1	9	1
3	3	3	1	1	1	1	1	1	1
2	1	2	1	2	1	1	2	2	9
3	3	3	3	3	3	3	3	3	3
3	1	2	1	1	2	1	3	2	1
3	3	3	1	1	1	1	1	1	1
3	1	3	1	3	3	3	3	3	1
1	1	1	1	1	1	1	1	1	1
2	2	2	2	2	2	2	2	2	2
2	2	2	2	2	2	2	2	2	2
2	3	3	2	1	2	3	1	1	1
1	2	1	9	2	9	2	2	2	1
2	1	2	2	1	2	2	1	1	1
2	2	2	2	2	2	2	2	2	2
2	2	2	2	2	2	2	2	2	2
2	1	2	3	2	3	2	1	2	1
2	2	2	2	2	2	2	2	2	2

1	9	1	1	3	1	2	3	1	1
3	3	3	3	3	3	3	3	3	3
2	3	1	2	3	1	2	3	2	1
9	9	9	9	9	9	9	9	9	9
9	9	9	9	9	1	9	9	9	1
3	1	3	3	2	3	2	1	2	3
2	2	2	2	2	2	2	2	2	2
1	1	1	3	3	3	1	1	2	3
2	2	2	2	2	2	2	9	3	2
2	2	2	2	2	2	2	2	9	2
9	9	9	9	9	9	9	9	9	9
2	2	2	2	3	3	3	3	3	2
2	2	2	2	2	2	2	2	2	2
2	2	2	2	2	2	2	2	2	3
2	2	2	2	2	2	2	2	2	2
2	9	2	2	2	3	3	2	3	2
2	2	2	2	2	2	2	2	2	2
9	9	9	9	9	9	9	9	9	1
9	9	9	2	9	9	9	9	9	9
2	2	2	2	2	2	2	2	2	2
2	2	2	2	2	2	2	2	2	2
2	2	2	2	2	2	2	2	2	2
9	9	9	9	9	9	9	9	9	9
9	9	9	9	9	9	9	9	9	9
2	3	2	2	2	2	2	2	2	1
1	1	1	3	1	3	1	1	3	3
2	1	1	1	1	1	1	1	1	1
3	1	1	1	3	3	2	3	3	1
2	1	2	2	2	2	2	2	2	1
2	9	2	9	1	2	3	1	3	1
2	2	1	1	1	2	1	2	3	1
3	1	2	3	1	2	1	3	1	2
3	3	2	1	3	3	1	3	3	1
3	3	3	3	3	3	3	3	3	3
2	2	2	2	2	2	2	2	9	9
2	2	3	3	3	2	1	3	1	2
1	1	2	1	1	2	1	1	1	1
3	3	3	3	3	3	3	3	3	3
9	3	2	1	9	2	1	9	1	2
3	3	3	3	3	3	3	3	3	3
1	1	1	1	3	1	9	3	3	1
1	2	2	3	3	1	1	3	2	1
1	1	1	1	1	1	1	1	1	3
2	2	2	2	2	2	2	2	2	2
2	1	1	1	2	2	3	3	3	1
3	1	1	2	1	1	3	1	2	1
3	3	2	2	2	2	2	2	2	1

2	2	2	2	2	2	2	2	2	2
3	1	2	1	3	3	3	3	1	2
2	1	3	1	1	1	3	1	1	9
1	1	1	1	1	1	1	1	1	1
2	2	2	2	2	2	2	2	2	2
2	3	2	2	2	2	2	2	2	2
2	2	2	2	2	2	2	2	2	2
2	2	2	2	2	2	2	2	2	2
1	1	1	1	1	1	1	1	1	2
3	2	3	2	2	2	3	2	2	1
1	9	9	9	9	9	9	9	9	1
2	2	2	2	2	2	2	2	2	1
1	1	3	3	3	3	3	3	3	3
1	1	1	2	2	3	2	1	2	1
1	2	1	1	1	3	1	1	1	1
1	1	1	3	2	2	2	2	2	2
3	1	3	1	1	1	3	3	3	1
2	1	2	2	2	2	2	2	2	1
2	1	3	3	3	2	2	3	3	1
9	9	9	1	9	9	9	9	9	1
1	1	3	1	1	1	1	1	1	1
1	3	3	3	3	3	3	3	2	1
3	1	3	1	3	1	1	3	3	1
1	1	1	1	1	1	3	1	1	1
3	3	3	3	3	3	3	3	3	3
3	3	3	3	3	3	3	3	3	3
2	1	2	1	2	1	2	1	1	1
2	3	1	2	1	2	1	2	2	2
1	3	1	2	1	2	3	2	2	1
1	9	1	2	2	1	1	1	1	1
2	2	2	2	2	2	2	2	2	9
9	9	9	9	9	9	9	9	9	9
2	2	1	1	1	2	1	2	2	1
9	9	9	9	9	9	9	9	9	9
2	1	2	2	1	1	2	2	2	2
2	2	2	2	2	2	2	2	2	2
2	2	2	2	2	2	2	2	2	2
2	2	2	2	2	2	2	2	2	1
3	3	2	2	2	3	3	2	3	2
2	2	3	2	1	2	3	2	3	9
2	1	2	2	1	2	3	3	3	1
2	2	2	2	2	2	2	2	2	2
2	3	3	2	1	1	1	2	2	1
9	9	9	9	9	9	9	9	9	9
3	3	3	3	3	3	3	3	3	3
2	2	2	2	2	2	2	2	2	2
2	9	1	1	2	2	2	2	2	9

9	9	9	9	9	9	9	9	9	9
1	1	2	2	2	2	2	2	2	2
1	2	3	2	1	2	2	1	1	1
2	1	1	2	3	3	2	1	3	1
2	2	2	2	2	2	2	2	2	2
2	2	2	2	3	2	2	2	2	9
9	9	9	9	9	9	9	9	9	9
2	2	2	2	3	3	2	3	3	1
2	2	2	2	2	2	2	2	2	2
9	9	9	9	9	9	9	9	9	9
9	9	9	9	9	9	9	9	9	9
9	9	9	9	9	9	9	9	9	9
9	9	1	9	9	9	9	9	9	9
9	9	9	9	9	9	9	9	9	2
9	9	9	9	9	9	9	9	9	9
1	1	2	2	3	1	2	3	3	2
2	2	2	2	2	2	2	2	2	2
2	2	2	2	2	2	2	2	2	2
2	9	1	3	2	1	3	1	2	1
2	1	2	1	1	2	3	1	1	2
2	1	2	3	2	2	2	2	2	1
2	2	1	2	2	2	2	3	2	2
2	2	2	2	1	2	2	2	2	1
3	1	1	3	3	1	3	3	3	1
2	2	2	2	2	2	2	2	2	2
2	2	2	2	2	1	2	3	2	2
9	9	9	9	9	9	9	9	9	9
1	1	1	1	1	1	1	1	1	1
2	2	2	2	2	1	2	3	2	2
1	1	2	2	3	1	2	3	3	2
2	1	2	1	1	2	3	1	1	2
9	9	1	9	9	9	9	9	9	9
1	1	1	1	1	3	3	1	1	1
2	2	2	2	2	2	2	2	2	2
2	2	2	2	2	2	2	2	2	2
2	2	2	2	2	2	2	2	2	2
2	2	2	2	2	2	2	2	2	9
9	9	9	9	9	9	9	9	3	1
2	2	2	2	2	2	2	2	2	2
2	2	2	2	2	2	2	2	2	2
2	2	2	2	2	2	2	2	2	2
2	2	2	2	2	2	2	2	2	9
2	2	2	2	2	2	2	2	2	2
3	3	2	2	2	2	2	2	2	1
1	2	2	2	2	2	2	2	2	9
2	2	2	2	2	2	2	2	2	2
9	9	9	1	9	9	9	9	9	1

2	2	2	2	2	2	2	2	2	3
2	2	2	2	2	2	2	2	2	1
9	9	9	1	9	9	9	9	9	9
9	9	9	9	9	9	9	9	9	1
2	2	2	2	2	2	2	2	2	1
9	9	9	9	9	9	9	9	9	1
1	1	2	2	2	2	2	2	3	1
2	1	2	3	2	3	1	2	1	1
2	3	2	2	1	3	2	1	9	1
2	3	2	1	2	3	2	1	2	1
9	2	2	2	2	2	2	2	2	3
2	1	1	2	2	1	2	1	3	1
1	1	1	2	1	2	2	2	1	2
2	2	2	2	2	2	2	2	2	9
9	9	9	9	9	9	9	9	9	9
3	3	3	3	3	3	3	3	3	3
2	2	2	2	2	2	2	2	2	2
2	2	2	2	2	2	2	2	2	2
2	1	1	2	2	3	1	2	3	1
1	1	3	1	2	2	3	3	3	1
3	1	1	1	3	1	3	1	3	1
2	2	2	2	2	2	2	2	2	2
3	1	1	1	3	1	3	3	3	1
2	2	2	2	2	2	2	2	2	2
2	2	2	2	2	2	2	2	2	2
2	1	2	1	2	1	2	2	3	1
2	2	2	2	2	2	2	2	2	2
2	2	2	2	2	2	2	2	2	2
1	1	1	2	1	1	2	2	2	1
9	9	9	9	9	9	9	9	9	9
9	9	9	9	9	9	9	9	9	1
2	2	2	2	2	2	2	2	2	2
9	9	9	9	9	9	9	9	9	9
9	9	9	9	9	9	9	9	9	9
2	1	2	3	2	2	2	3	3	1
9	9	9	9	9	9	9	9	9	9
9	9	9	9	9	9	9	9	9	9
2	2	2	2	2	2	2	2	2	2
1	1	3	1	2	2	3	3	3	1
1	1	2	2	2	2	2	2	1	1
1	1	1	1	1	1	1	1	1	1
2	3	1	3	1	2	2	1	3	2
9	9	9	9	9	9	9	9	9	2
2	1	2	2	3	2	2	3	1	1
3	1	3	2	1	2	3	1	2	9
9	9	9	9	9	9	9	9	9	9
2	1	2	1	2	1	1	3	3	2

2	1	2	2	1	1	9	1	1	1
9	9	9	9	9	9	9	9	9	9
9	9	9	9	9	9	9	9	9	9
2	2	2	2	2	2	2	2	2	2
1	1	3	3	1	1	1	1	1	1
2	1	2	2	1	1	9	1	1	1
2	9	1	3	9	1	3	1	2	1
3	1	1	1	3	3	3	3	3	1
2	2	2	2	2	2	2	2	2	2
3	1	1	1	3	3	3	3	3	2
9	9	9	9	9	9	9	9	9	9
9	9	9	9	9	9	9	9	9	9
1	9	9	9	9	9	9	9	9	1
9	2	2	2	2	9	1	1	9	9
3	2	2	2	2	2	2	2	2	1
9	9	9	9	9	9	9	9	9	9
9	9	9	9	9	9	9	9	9	9
9	9	9	9	9	9	9	9	9	9
1	2	2	2	2	2	2	2	2	1
2	2	2	2	2	2	2	2	2	1
9	9	9	9	9	9	9	9	9	9
1	1	1	1	3	3	3	2	1	1
3	3	3	3	3	3	3	2	9	2
9	9	9	9	9	9	9	9	9	9
2	2	2	2	2	2	2	2	2	2
2	2	2	2	2	2	2	2	9	2
9	9	9	9	9	9	9	9	9	9
9	9	9	9	9	9	9	9	9	9
2	3	2	1	2	3	3	3	3	3
2	2	2	2	2	2	2	2	2	2
1	1	1	1	1	1	1	1	1	1
9	9	9	9	9	9	9	9	9	9
9	9	9	9	9	9	9	9	9	9
2	1	3	3	1	2	2	2	2	2
2	2	3	3	3	3	2	2	9	2
2	2	2	2	2	2	2	2	2	2
2	3	3	3	3	3	3	2	2	2
2	2	2	2	2	2	2	2	2	2
2	1	9	2	1	2	1	2	2	1
2	1	2	2	1	2	1	2	1	1
2	2	3	3	1	1	1	1	2	2
9	9	9	9	9	9	9	9	9	9
9	9	9	9	9	9	9	9	9	9
9	9	9	9	1	9	9	9	9	9
9	9	9	9	9	9	9	9	9	9
9	9	9	9	9	9	9	9	9	9
9	9	9	9	9	9	9	9	9	9

2	2	2	2	2	2	2	2	2	2
3	3	2	3	2	3	2	3	2	3
1	1	3	1	3	3	3	3	3	1
2	2	2	2	2	2	2	2	2	1
9	9	9	9	9	9	9	9	9	9
9	9	9	9	9	9	9	9	9	9
9	9	9	9	9	9	9	9	9	9
9	9	9	9	9	9	9	9	9	9
9	9	9	9	9	9	9	9	9	9
9	9	9	9	9	9	9	9	9	9
9	9	9	9	9	9	9	9	9	9
9	9	9	9	9	9	9	9	9	9
9	9	9	9	9	9	9	9	9	9
3	3	1	1	1	3	3	1	1	1
9	9	9	9	9	9	9	9	9	9
2	1	1	1	3	1	2	3	2	2
2	2	2	2	2	2	2	2	2	2
9	9	9	9	9	9	9	9	9	9
9	9	9	9	9	9	9	9	9	9
9	9	9	9	9	9	9	9	9	9
9	9	1	9	9	9	9	9	9	9
9	9	9	9	9	9	9	9	9	9
2	2	2	1	1	1	1	1	1	1
9	9	9	9	9	9	9	9	9	9
1	1	1	1	1	1	1	1	1	2
3	3	3	1	1	3	3	3	3	1
1	1	2	2	2	3	3	3	3	2
1	1	1	2	2	2	1	1	1	1
2	1	1	1	1	1	1	1	2	1
1	1	1	1	2	2	1	1	1	1
2	2	2	2	2	2	2	2	2	2
1	1	1	1	1	1	1	1	1	1
1	1	1	1	1	1	1	1	1	1
9	9	9	9	9	9	9	9	9	9
1	1	2	2	2	1	1	1	1	1
1	1	1	2	1	1	2	2	2	2
1	1	1	1	1	1	1	1	1	1
9	9	9	9	9	9	9	9	9	9
2	2	2	2	2	3	3	3	3	1
2	2	2	1	2	3	3	1	1	1
3	3	3	3	3	3	3	3	3	1
9	9	9	9	9	9	9	9	9	9
9	9	9	9	9	9	9	9	9	9
9	9	9	9	9	9	9	9	9	9
9	9	9	9	9	9	9	9	9	9
2	2	2	2	2	2	2	2	2	1
9	9	9	9	9	9	9	9	9	9

9	9	9	9	9	9	9	9	9	9
9	9	9	9	9	9	9	9	9	9
9	9	9	9	9	9	9	9	9	9
9	9	9	9	9	9	9	9	9	9
9	9	9	9	9	9	9	9	9	9
9	9	9	9	9	9	9	9	9	9
9	9	9	9	9	9	9	9	9	9
9	9	9	9	9	9	9	9	9	9
9	9	9	9	9	9	9	9	9	9
9	9	9	9	9	9	9	9	9	9
9	9	1	9	9	9	9	9	9	9
9	9	9	9	9	9	9	9	9	9
2	2	1	2	1	1	1	1	1	1
2	2	2	2	2	2	1	1	1	1
2	2	1	1	1	1	2	2	2	1
3	2	2	1	1	1	2	2	2	1
1	1	2	2	2	2	2	2	2	2
1	1	1	1	1	1	2	1	1	2
2	2	1	1	1	1	1	1	1	1
9	9	9	9	9	9	9	9	9	1
1	1	1	1	1	2	2	2	1	1
3	1	2	2	3	3	3	3	3	1
2	2	2	2	2	2	2	2	2	2
2	2	1	2	2	1	1	1	1	1
2	1	1	2	1	2	1	2	1	1
3	3	3	3	3	3	3	3	3	1
9	1	3	1	2	2	2	9	2	1
2	2	2	2	2	1	1	1	1	2
9	1	9	9	9	9	9	9	9	1
9	9	9	9	9	9	9	9	9	9
9	9	9	9	9	9	9	9	9	9
1	1	1	1	2	2	2	1	1	1
2	1	2	1	1	1	2	2	2	2
1	1	1	1	1	1	1	1	1	2
2	2	1	1	1	1	1	1	1	1
2	2	2	2	2	2	2	2	2	2
2	1	1	2	2	2	2	2	2	1
1	1	1	1	1	1	1	1	1	2
1	2	2	2	1	1	1	1	1	2
1	1	1	1	1	1	1	1	1	1
2	2	2	2	2	2	2	2	2	2
1	2	2	2	1	1	1	1	1	1
1	1	3	1	1	1	3	3	3	1
1	2	2	2	2	1	2	2	3	1
2	2	2	2	1	1	1	1	1	1
2	1	1	1	1	1	1	1	1	1
2	2	2	1	3	2	2	2	3	1

9	9	9	9	9	9	9	9	9	9
2	2	2	2	2	2	2	2	2	1
9	9	9	9	2	9	9	9	9	9
9	9	9	1	9	9	9	9	9	9
1	9	9	9	9	9	9	9	9	9
9	9	9	9	9	9	9	9	9	9
9	9	9	9	9	9	9	9	9	9
2	3	3	3	3	3	3	2	2	1
9	9	9	9	9	9	9	9	2	2
2	2	2	2	2	2	2	2	2	2
9	9	9	9	9	9	9	9	9	9
9	9	9	9	9	9	9	9	9	9
1	1	1	1	1	2	2	2	1	1
9	9	9	9	9	9	9	9	9	9
1	1	1	1	1	1	1	1	1	1
9	9	9	9	9	9	9	9	9	9
2	2	2	2	2	2	2	2	2	2
3	3	3	3	1	3	3	3	3	3
9	9	9	9	9	9	9	9	9	9
2	2	2	2	2	2	2	2	2	2
2	2	2	2	2	2	2	2	2	2
2	2	2	2	2	2	2	3	2	2
2	2	2	2	2	2	2	2	2	2
2	2	2	2	2	2	2	2	2	2
1	1	1	1	1	1	1	1	2	2
2	2	2	2	2	2	2	2	2	2
2	2	9	2	3	1	2	1	2	1
2	1	2	1	2	1	2	2	1	1
2	2	2	2	2	2	2	2	2	2
1	1	1	1	1	1	1	2	2	1
9	9	9	9	9	9	9	9	9	9
3	2	3	2	3	2	2	3	9	2
9	9	9	9	9	9	9	9	9	9
2	2	2	2	2	2	2	2	2	2
3	2	2	2	2	2	2	2	2	2
1	1	1	1	1	1	1	1	1	2
1	2	2	1	1	2	2	2	2	1
1	1	1	1	1	1	1	1	1	1
1	1	1	1	1	1	1	1	1	1
1	1	1	1	1	1	1	1	1	9
2	2	2	2	2	2	2	2	2	1
2	2	1	1	1	2	2	2	2	1
9	9	9	9	9	9	9	9	9	9
9	9	9	9	9	9	9	9	9	9
1	9	9	9	9	9	9	9	9	1
9	9	9	9	9	9	9	9	9	9
9	9	9	9	9	9	9	9	9	9

9	9	9	9	9	9	9	9	9	9
2	2	2	2	2	2	2	2	2	2
9	1	9	9	9	9	9	9	9	1
9	9	9	9	9	9	9	9	9	9
9	9	9	9	9	9	9	9	9	9
2	9	1	2	1	2	3	1	2	2
3	1	2	1	3	3	3	3	3	1
9	9	9	9	9	9	9	9	9	9
9	9	9	9	9	9	9	9	9	9
9	9	9	9	9	9	9	9	9	9
9	1	9	9	9	9	9	9	3	1
9	9	9	9	9	9	9	9	9	9
2	2	2	2	2	2	2	2	2	2
9	9	9	9	9	9	9	9	9	9
9	9	9	9	9	9	9	1	9	9
1	2	2	3	3	3	2	2	3	1
2	1	2	2	3	1	2	2	2	2
3	1	3	1	3	3	3	3	3	1
1	1	1	1	2	2	2	2	2	1
2	1	1	1	3	3	2	2	3	1
2	3	3	2	1	2	3	3	1	1
3	1	1	3	1	1	3	1	1	1
1	9	1	2	1	2	2	1	2	1
2	2	2	3	2	3	3	2	9	9
2	2	2	2	2	2	2	2	2	2
2	2	2	2	1	1	1	1	2	1
2	9	9	9	9	9	9	9	9	9
2	2	2	2	1	1	1	3	3	1
9	9	9	9	9	9	9	9	9	2
1	2	2	2	2	2	2	2	2	1
3	3	3	3	3	3	3	3	1	2
2	2	2	2	2	2	3	2	2	2
9	9	9	9	9	9	9	9	9	9
3	2	3	2	2	3	9	2	3	2
3	1	3	1	3	3	3	1	3	1
2	2	2	2	2	2	2	2	2	1
2	2	2	2	2	2	2	2	2	2
1	3	2	1	1	3	3	2	2	1
9	9	1	9	9	9	9	9	9	9
2	3	2	1	2	3	2	1	2	9
9	9	9	9	9	2	9	9	9	9
9	9	9	9	9	9	9	9	9	1
2	2	2	2	2	2	2	2	3	1
9	9	9	9	9	9	9	9	9	9
1	1	1	1	1	1	1	1	1	1
1	3	3	1	1	3	1	1	3	1
3	3	3	3	3	3	3	3	3	3

3	3	3	3	3	3	3	3	3	3
9	9	9	9	9	9	9	9	9	9
2	2	2	2	2	2	2	1	2	2
1	1	1	1	1	3	3	3	3	1
3	1	3	1	3	3	3	3	3	1
1	3	1	1	3	1	3	1	1	1
3	1	3	1	3	1	2	3	1	1
3	9	2	1	2	1	3	2	3	9
2	2	2	2	2	3	2	3	2	2
3	9	2	3	2	3	2	3	2	2
2	2	2	2	3	3	1	3	1	1
1	2	3	2	1	2	3	2	1	1
1	2	3	1	2	2	3	2	2	2
2	3	1	2	1	2	2	2	2	1
2	2	3	2	1	2	2	2	2	1
2	1	2	3	3	2	1	2	3	1
2	2	2	2	2	2	2	2	2	2
1	2	1	2	1	2	2	1	2	1
2	2	2	2	2	2	2	2	2	2
2	2	2	2	2	2	2	2	2	2
3	9	1	1	3	3	3	3	3	1
2	2	2	1	1	1	3	3	3	1
9	9	9	9	9	9	9	9	9	3
9	9	9	9	9	9	9	9	9	9
3	2	2	2	1	1	1	1	1	2
1	1	1	2	1	2	1	2	2	2
2	3	3	2	1	2	1	2	1	9
3	3	2	2	2	2	1	1	2	1
2	9	1	1	1	1	3	3	1	1
9	1	9	9	9	9	9	9	9	9
3	3	1	2	3	2	1	3	3	1
2	2	2	2	2	2	2	2	2	3
2	3	3	1	2	1	2	3	3	2
2	1	1	1	3	1	3	2	2	1
2	3	1	1	1	1	1	1	3	9
2	2	2	2	2	2	2	2	2	2
2	2	2	2	2	2	2	2	2	2
9	9	9	9	9	9	9	9	9	9
2	2	2	1	1	2	3	3	3	2
2	2	2	2	2	2	2	2	2	1
9	9	9	9	9	9	9	9	9	9
9	9	9	9	9	9	9	9	9	9
3	2	2	2	2	2	1	1	2	1
9	9	9	9	9	9	9	9	9	9
2	2	2	2	2	2	2	2	2	2
3	1	1	1	1	3	1	3	3	2
9	9	9	9	9	9	9	9	9	9

3	2	3	2	2	2	3	2	2	2
9	9	9	9	9	9	9	9	9	9
3	3	3	3	3	3	3	3	3	1
9	9	9	9	9	9	9	9	9	9
9	9	9	9	9	9	9	9	9	9
2	2	2	2	2	2	2	2	2	2
3	1	1	2	3	3	3	3	3	1
2	2	2	2	2	2	2	2	2	2
2	2	2	2	2	2	2	2	2	2
2	2	2	2	2	2	2	2	2	2
9	9	9	9	9	9	9	9	9	9
3	1	3	1	3	3	2	1	1	1
9	9	9	9	9	9	9	9	9	9
1	1	1	2	2	1	1	1	1	1
2	2	2	2	2	2	2	2	2	2
3	3	3	2	2	2	1	1	1	2
9	9	9	9	9	9	9	9	9	2
9	9	9	9	9	9	9	9	9	9
1	1	1	1	1	1	3	3	2	3
3	3	2	2	2	2	3	3	3	3
1	1	1	2	2	1	1	1	1	2
1	1	2	3	3	3	3	3	3	1
9	9	9	9	9	9	9	9	9	9
2	2	2	3	3	3	3	2	2	3
3	2	3	3	3	3	3	3	1	3
2	2	2	2	2	2	3	3	3	2
9	9	9	9	9	9	9	9	9	1
1	1	1	1	1	1	1	1	1	1
2	2	2	2	2	2	2	2	2	2
9	9	9	9	9	9	9	9	9	1
2	3	3	2	1	1	2	3	2	1
2	2	2	2	2	2	2	2	3	1
1	1	1	3	3	3	2	2	1	1
2	2	2	2	2	2	2	2	2	2
2	2	2	2	2	2	2	2	2	2
2	1	2	2	3	1	1	2	3	1
2	1	1	2	2	2	2	3	1	2
1	1	3	2	2	2	2	1	3	1
2	2	2	2	2	2	2	2	2	1
3	3	3	3	3	3	3	3	3	3
1	1	3	1	1	2	3	2	3	1
3	3	1	3	3	3	3	3	3	1
2	1	2	2	3	1	3	2	3	9
2	2	2	2	2	2	2	2	2	2
2	2	2	2	2	2	2	2	2	2
2	2	2	2	2	2	2	2	2	2
2	1	1	3	2	3	1	2	1	1

1	1	2	2	2	2	2	2	2	2
2	2	2	2	2	2	3	2	3	1
1	1	1	2	1	2	1	1	1	1
2	2	2	2	2	2	2	2	2	2
2	2	2	2	3	2	3	2	1	2
1	9	9	9	9	9	9	9	9	1
2	2	2	2	2	2	2	2	2	2
2	2	2	1	1	2	1	1	1	1
2	2	2	2	2	2	2	2	2	2
2	1	2	3	3	2	2	2	2	1
2	2	2	2	2	2	3	2	2	9
1	2	3	1	2	3	1	2	3	1
2	2	2	2	2	2	2	2	2	2
3	3	3	3	3	3	3	3	3	3
3	2	2	2	2	2	2	3	2	2
2	2	1	2	2	1	2	1	2	1
3	2	3	1	2	1	2	3	2	3
1	3	2	3	1	2	3	2	2	1
2	2	2	2	2	2	2	2	2	2
2	1	1	1	1	2	2	2	1	2
2	2	2	2	2	2	2	2	2	1
2	2	2	2	2	2	2	2	2	3
2	2	2	2	1	1	1	3	3	1
2	2	2	2	2	2	2	2	2	1
2	1	2	2	2	2	2	2	2	1
2	1	2	2	2	3	2	2	2	2
2	1	1	1	3	2	3	1	1	2
2	1	1	2	2	3	3	1	1	2
2	2	2	2	2	2	2	2	2	2
1	2	2	2	1	1	1	1	1	1
3	3	3	2	3	2	3	2	2	2
3	3	3	2	2	3	2	3	2	3
1	1	1	1	1	2	3	3	3	1
2	2	2	2	2	2	2	2	2	2
1	1	1	2	2	1	1	1	1	1
2	2	2	2	2	2	2	2	2	2
2	3	3	3	3	3	3	3	1	2
2	2	1	1	1	1	1	2	3	2
2	2	2	2	2	2	2	2	2	2
2	2	2	2	2	2	2	2	2	2
2	3	1	2	2	1	3	2	2	1
2	1	1	2	1	2	1	3	3	2
2	2	2	2	2	2	2	2	9	2
2	2	2	2	2	2	2	2	2	2
2	1	2	2	3	3	1	2	3	2
1	1	1	1	1	1	3	3	3	1
9	9	9	9	9	9	9	9	9	9

1	2	1	2	3	2	2	3	1	1
2	2	2	2	2	3	9	3	1	1
2	1	3	2	3	3	3	3	3	1
2	2	2	2	2	2	2	2	2	2
2	2	2	2	2	2	2	2	2	2
2	2	2	2	2	2	2	2	2	2
3	1	2	2	1	2	2	3	2	1
2	2	2	3	3	1	2	1	2	1
2	2	2	2	2	2	2	2	1	2
2	2	2	2	2	2	2	2	3	2
2	2	2	2	2	2	2	2	2	2
9	9	9	9	9	9	9	9	9	9
9	9	9	9	9	9	9	9	9	9
2	2	2	2	2	2	2	2	2	3
2	1	1	2	2	1	2	1	1	2
2	1	2	2	2	2	2	2	2	1
1	1	1	1	3	3	3	3	3	1
2	1	1	1	1	2	2	2	2	1
1	1	2	2	2	2	3	3	3	1
2	1	3	1	3	3	2	2	3	1
2	2	2	2	2	2	2	2	2	9
2	2	2	2	2	2	2	2	2	2
2	2	2	2	2	2	2	2	3	2
2	2	2	2	2	2	2	2	2	2
2	1	1	1	2	2	2	2	2	1
2	2	2	2	2	2	2	2	2	1
2	2	2	2	2	2	2	2	1	2
2	2	2	2	2	2	2	2	2	2
2	2	2	2	2	2	2	2	2	1
9	9	9	9	9	9	9	9	9	9
2	2	2	2	3	3	3	3	3	3
2	2	2	2	2	2	2	2	2	9
2	2	2	2	2	2	3	2	3	2
2	2	2	2	2	2	2	2	2	3
2	2	1	2	2	2	2	2	2	2
2	2	2	2	2	2	2	2	2	2
2	2	2	2	2	2	2	2	2	9
2	2	2	2	2	2	2	2	2	2
2	2	2	2	2	2	2	2	2	2
2	2	2	2	2	2	2	2	2	1
2	2	2	2	2	2	2	2	2	2
2	2	2	2	2	2	2	2	2	2
2	2	2	2	2	2	2	2	2	2
2	1	2	2	3	1	2	2	3	2
2	2	2	2	2	2	2	2	2	2
2	2	2	2	2	2	2	2	2	2
2	2	2	2	2	2	2	2	2	2

2	3	3	3	1	2	2	1	2	2
2	2	2	2	2	2	2	2	2	1
2	1	2	1	3	1	3	3	3	1
2	2	2	2	2	2	2	2	2	2
2	2	2	2	2	2	2	2	2	2
2	2	2	2	2	2	2	2	2	3
9	9	9	9	9	9	9	9	9	9
3	1	3	1	3	3	3	3	3	1
1	1	1	2	1	1	1	1	2	1
2	1	2	2	3	3	2	3	3	1
2	2	2	2	2	2	2	2	2	1
2	2	2	2	2	2	2	2	2	9
2	2	2	2	2	2	2	2	2	2
2	2	2	2	2	2	2	2	2	1
2	9	9	9	9	9	9	9	9	1
2	2	2	2	2	2	2	2	2	9
2	2	2	2	2	2	2	2	2	9
2	1	1	3	3	1	3	2	3	1
1	1	1	1	1	1	1	1	1	1
2	2	2	2	2	2	2	2	3	9
1	1	1	1	1	2	2	1	1	1
2	2	2	3	3	3	1	1	2	2
2	2	2	2	2	2	2	2	2	2
2	2	2	2	2	2	2	2	2	2
2	2	2	2	2	2	2	2	9	2
2	2	2	2	2	2	2	2	2	2
9	9	9	9	9	9	9	9	9	9
2	2	2	2	2	2	2	2	2	1
2	2	2	2	2	2	2	2	2	9
2	2	2	2	2	2	2	2	2	9
2	2	2	2	2	2	2	2	2	2
2	2	2	2	2	2	2	1	1	1
9	9	9	9	9	9	9	9	9	9
9	9	9	9	9	9	9	9	9	9
9	9	9	9	9	9	9	9	9	9
9	9	9	9	9	9	9	9	9	9
1	1	1	1	2	1	1	1	1	1
2	1	2	2	3	3	3	3	3	1
2	1	2	1	3	1	3	1	3	9
3	3	2	3	2	2	3	2	2	2
1	1	2	3	2	3	3	3	3	2
2	1	2	2	2	2	2	2	2	2
2	2	2	2	2	2	2	2	2	1
2	2	2	2	2	2	2	2	2	2
2	2	2	2	2	2	2	2	2	1
2	2	2	2	2	2	2	2	2	1
1	2	1	2	1	2	1	3	1	1

1	3	2	3	2	1	2	1	1	1
3	2	1	1	2	2	1	2	1	1
3	1	1	2	1	2	2	1	1	2
1	1	1	1	1	1	1	1	1	2
2	1	3	1	2	1	1	2	1	3
2	3	3	2	1	2	3	2	1	1
3	1	1	1	3	1	2	2	3	1
9	9	9	9	9	9	9	9	2	2
2	2	2	2	2	2	2	2	2	2
2	2	2	2	2	2	2	2	2	2
2	2	2	2	2	2	2	2	2	1
2	2	2	2	2	2	2	2	2	1
2	2	2	2	2	2	2	2	2	2
2	2	2	2	2	2	2	2	1	9
2	2	2	2	2	2	2	2	2	2
2	2	2	2	2	2	2	2	2	2
2	2	2	2	2	2	2	2	2	2
3	3	2	1	1	3	2	3	2	1
2	1	1	1	1	1	3	3	2	1
2	1	1	2	3	2	3	3	3	1
3	1	3	2	3	3	3	2	3	1
3	1	3	1	2	2	3	3	2	1
1	1	1	1	3	3	3	3	3	1
9	9	9	9	9	9	9	9	3	9
3	3	3	3	3	3	3	3	3	3
1	1	3	1	1	1	1	1	3	1
2	2	2	2	2	2	2	2	2	1
2	1	1	1	1	1	1	2	2	1
2	2	2	2	2	2	2	2	2	1
9	1	9	9	9	9	9	9	9	9
2	1	3	3	2	2	2	3	3	3
9	9	9	9	9	9	9	9	9	9
9	9	9	9	9	9	9	9	9	9
9	9	9	9	9	9	9	9	9	9
9	9	9	9	9	9	9	9	9	9
2	2	2	2	2	2	2	2	2	2
1	2	2	1	2	9	1	1	1	1
9	9	9	9	9	9	9	9	9	9
2	2	2	2	2	2	2	2	2	1
2	2	2	2	2	2	2	2	2	1
9	9	9	9	9	9	9	9	9	9
3	3	3	3	3	3	3	3	2	3
2	2	2	2	2	2	2	2	2	2
9	9	9	9	9	9	9	9	9	3
9	9	9	9	9	9	9	9	9	9
1	1	1	1	1	1	2	2	2	2
3	3	3	3	3	3	3	3	3	2

1	1	1	1	1	1	1	1	1	1
1	1	1	1	3	3	3	3	3	1
2	2	1	1	1	2	1	1	1	3
2	2	2	2	2	2	2	2	2	2
9	9	9	9	9	9	9	9	9	9
9	9	9	9	9	9	9	9	9	9
2	2	2	2	2	2	2	2	2	2
2	2	2	2	3	3	3	3	2	2
9	9	9	9	9	9	9	9	9	1
9	9	9	9	9	9	9	9	9	9
1	1	3	3	2	1	1	1	1	1
1	1	2	2	1	1	2	1	1	2
3	1	1	1	1	2	2	2	1	9
3	3	3	3	3	3	3	3	3	3
9	9	9	9	9	9	9	9	9	1
2	1	3	3	3	2	1	3	3	2
3	1	1	2	3	3	2	3	3	1
2	2	2	2	2	2	2	2	2	1
9	9	1	9	9	9	9	9	9	9
3	1	1	1	2	1	3	3	2	2
2	3	1	3	1	2	2	1	3	1
3	2	3	2	2	2	2	2	2	1
1	3	1	1	2	1	3	1	2	1
2	1	1	1	2	1	3	2	2	1
3	3	3	3	3	3	3	3	3	1
2	1	2	2	2	2	2	2	2	2
3	2	2	1	2	2	1	3	3	9
3	1	2	1	9	1	3	3	3	1
3	2	1	2	1	3	2	2	2	2
3	1	3	1	3	3	3	3	3	3
2	2	2	2	2	2	2	2	2	2
2	1	1	1	1	1	1	1	3	1
2	3	2	1	2	3	2	1	2	1
3	3	3	1	3	3	1	1	3	3
9	9	9	9	1	9	9	9	9	9
1	1	2	2	1	1	2	2	3	1
3	3	3	3	3	9	9	9	9	1
2	2	2	2	2	2	2	2	2	2
9	9	9	9	9	9	9	9	9	9
9	9	9	9	9	9	9	9	9	9
9	9	9	9	9	9	9	9	9	9
9	1	2	2	3	2	1	2	2	2
1	2	1	2	1	2	2	3	1	3
1	1	2	2	1	1	2	3	3	1
1	9	1	2	2	1	2	3	3	1
2	3	3	2	2	3	2	1	2	3
3	3	3	2	2	3	3	2	3	3

2	2	2	2	2	2	2	2	2	2
9	9	9	9	9	9	9	9	9	9
1	1	3	1	3	1	3	2	3	1
3	3	3	3	3	3	3	3	2	3
3	1	3	1	3	1	1	3	3	9
9	9	9	9	9	9	9	9	9	9
9	9	9	9	9	9	9	9	9	1
2	1	2	2	2	3	3	2	2	9
1	9	9	1	2	9	9	1	2	1
2	2	2	2	2	2	2	2	2	2
2	2	1	1	1	1	1	2	2	2
2	2	2	2	2	3	2	1	9	2
2	2	2	2	2	3	2	3	2	2
1	1	1	1	3	3	3	3	3	1
9	9	9	9	9	9	9	9	9	9
3	1	1	3	3	3	1	3	3	1
2	2	2	2	2	2	2	2	9	2
2	2	2	2	2	2	2	2	2	2
2	3	2	3	3	3	1	2	1	1
2	1	3	2	1	2	1	3	3	1
9	9	9	9	9	9	9	9	9	9
9	9	9	9	2	9	9	9	9	1
2	2	2	1	2	2	2	2	2	2
9	9	9	9	9	9	9	9	9	9
9	9	9	9	9	9	9	9	9	9
2	2	3	3	3	3	2	3	2	3
2	2	2	2	2	2	2	2	2	3
2	1	2	2	3	3	3	3	3	1
3	3	3	3	3	3	3	3	3	3
1	1	1	1	2	1	3	2	3	1
3	3	3	3	3	3	3	3	3	3
1	1	3	3	3	3	3	3	3	1
9	9	9	9	9	9	9	9	9	9
9	9	9	9	9	9	9	9	9	9
9	9	9	9	9	9	9	9	9	9
1	1	1	2	1	2	3	1	3	1
2	1	1	1	2	1	2	1	2	1
2	3	1	2	2	3	1	2	2	1
1	1	3	1	3	1	3	3	3	1
9	9	9	9	9	9	9	9	9	2
3	2	2	2	2	2	2	2	2	2
2	3	3	1	2	3	2	3	2	3
3	3	3	3	3	3	3	3	3	3
9	9	9	9	9	9	9	9	9	9
2	3	2	3	1	2	2	1	1	9
9	9	9	1	9	9	9	9	3	1
2	1	1	2	1	2	3	2	2	2

1	2	1	2	1	2	1	2	1	1
2	2	2	3	3	3	2	2	2	2
1	1	9	2	1	3	1	3	3	3
2	3	1	2	3	1	2	3	1	1
3	1	3	1	3	3	3	3	3	1
2	1	2	1	1	2	2	2	2	1
2	1	2	1	2	1	2	1	1	2
1	1	3	1	3	2	3	3	3	1
9	9	9	9	9	9	9	9	9	9
2	2	2	2	2	2	2	2	2	1
2	2	2	2	2	2	2	2	2	3
2	2	2	2	2	2	2	2	2	2
2	1	3	1	2	3	2	3	3	1
2	1	1	1	3	2	1	2	3	1
2	9	9	9	9	9	9	9	9	2
1	2	2	1	2	1	1	2	1	1
1	1	1	1	2	3	1	2	3	9
2	3	2	2	3	2	3	2	3	2
3	1	2	1	1	3	2	1	3	2
2	1	2	1	3	3	3	3	2	2
2	1	1	1	2	1	2	1	1	1
1	1	1	3	1	2	3	3	3	1
1	1	1	2	1	1	1	1	1	2
3	1	1	1	3	3	3	3	3	1
9	9	9	9	9	9	9	9	9	1
1	1	2	2	1	2	1	2	1	1
1	2	3	1	2	3	1	3	3	2
2	1	2	1	2	2	2	1	3	1
2	2	2	2	2	2	2	2	2	2
1	1	1	2	2	1	3	3	3	1
2	1	2	1	2	2	2	2	2	1
2	2	2	2	2	2	2	2	2	2
2	2	2	2	2	2	2	2	2	1
1	1	1	1	2	2	2	1	1	2
2	3	3	1	1	2	3	3	3	1
2	1	1	2	3	3	3	3	3	1
2	2	1	1	2	1	1	1	2	1
1	2	2	2	3	2	1	1	1	1
1	9	1	2	1	1	1	2	3	1
1	2	2	3	3	2	2	2	2	1
2	3	3	3	3	3	2	2	2	3
1	1	1	1	2	2	2	2	2	1
2	2	2	1	1	2	2	2	2	1
2	2	2	1	1	2	2	1	1	2
2	2	1	1	1	1	1	1	2	2
1	1	1	1	2	2	2	2	1	1
3	3	1	2	2	1	2	2	2	1

1	1	2	2	2	1	1	1	1	1
1	2	2	1	1	1	2	2	1	1
2	2	2	1	1	1	2	1	1	1
2	2	2	1	1	2	2	2	1	2
1	1	2	2	1	1	2	2	1	1
2	1	1	2	1	2	1	2	1	1
3	2	2	1	2	1	2	2	1	1
1	2	2	2	1	2	2	2	1	1

AfraidofHP	KnowMore	HPVVaccin	HPVVaccin	Embarrass	TooBusyfo	HPVVaccin	HPVAgains	HeardCxCA	CxScreen2
2	2	2	2	2	2	2	2	2	2
1	1	1	1	1	2	2	2	2	2
9	9	9	9	9	9	9	9	2	2
1	1	1	1	1	2	2	2	1	1
1	1	1	1	1	2	2	2	2	2
1	1	1	1	1	2	2	2	1	1
9	9	9	2	9	9	9	9	2	2
2	1	1	1	3	2	3	3	1	2
2	2	1	2	2	2	2	2	2	2
9	9	9	9	9	9	9	9	2	2
2	3	2	3	2	2	2	2	2	2
2	2	2	3	1	1	3	1	2	2
3	1	1	1	3	3	3	3	1	2
1	1	3	2	2	3	3	3	1	2
2	1	2	1	3	3	3	3	2	2
2	1	3	2	3	3	3	3	1	2
1	3	2	1	2	2	3	2	1	1
2	1	3	2	3	3	3	3	1	2
2	1	3	2	3	3	3	3	1	2
3	3	1	1	1	1	1	3	2	2
2	1	1	1	1	2	1	3	1	1
2	1	1	1	1	2	1	3	1	1
2	2	2	2	2	2	2	2	2	2
1	1	3	1	1	3	1	3	1	1
2	2	2	2	2	2	2	2	2	2
1	1	2	1	2	3	2	3	2	2
1	1	1	1	3	3	3	2	2	2
1	1	3	1	3	3	3	3	2	2
2	2	2	2	2	2	9	2	2	2
2	2	2	2	2	2	2	2	2	2
2	2	2	2	2	2	2	2	2	2
2	1	2	2	2	2	2	2	1	1
3	1	3	1	3	3	3	3	2	2
2	1	2	1	3	3	3	3	1	1
2	2	2	2	2	2	2	2	1	1
2	1	2	1	3	2	1	1	1	1
3	2	2	2	2	2	2	2	1	1
9	9	9	9	9	9	9	9	1	1
2	2	2	2	2	2	1	2	#NULL!	#NULL!
2	1	1	2	2	2	2	2	2	2
2	2	2	2	2	2	1	2	2	2
2	1	2	2	2	3	2	2	1	2
9	9	9	9	9	9	9	9	#NULL!	#NULL!
3	1	3	1	3	3	1	3	1	1
3	1	1	1	3	3	1	3	2	2
1	2	2	3	1	2	2	1	1	2

3	9	3	3	3	3	3	3	1	1
3	1	1	1	1	1	3	3	1	2
2	2	3	1	2	3	3	3	1	1
1	1	1	2	1	3	2	3	1	1
1	1	3	1	3	3	3	3	1	1
3	1	2	1	3	3	3	3	1	1
3	1	3	1	3	3	3	3	1	1
3	1	1	1	3	3	3	3	1	1
9	1	9	9	9	9	9	9	1	1
2	1	2	1	3	3	2	3	1	1
9	2	2	9	2	2	2	9	2	2
2	1	3	3	3	3	3	3	1	1
2	1	2	2	3	3	3	3	1	1
9	9	9	9	9	9	9	9	2	2
1	1	1	1	3	3	3	3	2	2
1	1	2	2	3	2	3	3	1	1
3	1	3	1	3	3	3	3	1	1
9	9	9	9	9	9	9	9	1	1
3	1	1	2	1	2	3	3	1	2
9	1	2	2	1	1	2	9	1	1
3	1	3	3	1	3	3	3	1	1
3	1	3	3	3	1	1	3	1	1
3	1	3	3	1	3	3	3	1	1
9	9	9	9	9	9	9	9	1	2
9	1	9	9	9	9	9	9	1	1
9	1	9	1	3	1	3	3	1	1
9	9	9	9	9	9	9	9	#NULL!	#NULL!
2	1	1	1	2	3	3	3	1	2
1	1	3	1	3	3	3	3	2	2
2	1	2	1	3	3	3	3	1	1
1	2	1	2	1	2	1	1	1	1
2	1	3	1	3	1	2	3	1	1
9	1	1	1	1	2	2	1	1	1
3	1	3	1	3	3	3	3	1	1
2	1	3	1	3	3	3	3	1	1
9	9	9	9	9	9	9	9	2	2
9	9	9	9	9	9	9	9	#NULL!	#NULL!
3	1	3	1	3	3	3	3	#NULL!	#NULL!
3	1	3	1	3	3	3	3	2	1
1	1	1	1	3	3	1	3	1	1
3	1	2	2	3	3	3	3	1	1
1	1	2	9	3	9	9	3	2	2
3	1	3	1	3	3	1	3	1	1
2	1	1	1	3	2	3	3	1	1
2	1	1	1	3	2	3	3	2	2
9	9	9	9	9	9	9	9	1	1
9	9	9	9	9	9	9	9	2	2

9	9	9	9	9	9	9	9	1	1
3	1	3	2	3	3	9	3	1	1
1	1	1	2	1	3	3	3	1	1
2	1	1	3	3	3	3	1	1	#NULL!
9	9	9	9	9	9	9	9	2	2
1	2	2	2	2	2	2	2	2	1
3	1	3	1	3	3	3	3	1	1
1	1	3	1	3	3	3	3	1	1
1	1	1	2	1	3	3	3	1	1
3	1	3	1	3	3	3	3	1	1
3	1	3	1	2	3	2	2	2	2
3	1	2	1	3	3	3	3	1	#NULL!
1	1	2	2	2	2	3	3	1	1
1	1	2	2	2	2	3	3	2	2
2	1	2	2	3	2	2	1	2	2
2	1	1	2	3	2	2	2	1	2
2	1	3	3	3	3	3	3	2	2
2	1	2	2	2	2	3	3	2	2
9	9	9	9	9	9	9	9	2	2
3	1	3	1	3	3	3	3	2	2
9	1	3	1	3	3	3	3	2	2
9	9	9	9	9	9	9	9	2	2
1	2	2	3	2	2	1	2	2	2
3	1	1	3	1	3	1	1	2	2
1	1	1	2	2	1	2	1	2	2
2	1	3	2	2	3	3	3	2	2
3	1	1	1	3	3	3	3	1	1
3	1	1	2	3	3	3	3	1	1
2	2	2	2	2	2	2	2	2	2
3	1	3	1	3	3	3	3	2	2
2	1	1	3	1	9	3	3	2	2
2	2	2	2	2	2	2	1	2	2
3	1	3	1	3	3	3	3	2	2
2	2	2	2	2	2	2	2	2	2
3	1	3	1	3	2	3	3	2	2
2	1	1	2	2	2	2	2	2	2
1	1	2	1	3	2	2	2	1	1
2	2	3	1	3	2	3	3	2	2
1	1	2	3	2	1	2	9	1	1
3	1	3	3	3	3	3	3	1	1
1	1	2	1	3	3	3	3	2	2
1	1	2	1	3	3	3	3	2	2
2	2	2	2	2	2	2	2	2	2
3	1	3	2	3	3	3	3	1	1
1	1	3	3	2	2	3	2	2	2
1	1	1	1	3	3	3	3	1	2
2	1	2	3	3	1	3	2	2	2

1	2	2	2	3	3	2	3	2	2
2	1	1	2	1	3	3	2	2	2
2	1	1	2	1	3	3	2	1	1
1	1	2	1	3	3	3	3	2	2
1	1	2	1	3	3	3	3	2	2
9	9	9	9	9	9	9	9	2	2
3	2	2	3	3	3	3	2	2	2
3	1	3	1	3	3	3	3	2	2
2	2	2	2	2	2	2	2	2	2
2	2	2	2	2	2	2	2	2	2
9	9	9	9	9	9	9	9	2	2
9	9	9	9	9	9	9	9	2	2
2	2	9	9	2	9	2	9	2	2
1	1	2	2	3	3	3	3	2	2
2	2	2	3	3	3	3	3	1	1
2	2	2	3	3	3	3	3	1	1
9	9	9	9	9	9	9	9	1	1
1	1	3	3	3	3	3	1	2	2
9	9	1	9	9	9	9	9	1	2
2	1	3	1	1	3	3	1	1	2
2	1	3	1	3	3	3	3	#NULL!	1
2	1	3	1	3	3	3	3	2	2
2	1	3	1	3	3	3	3	2	2
2	1	3	1	1	3	3	3	2	2
2	1	3	1	3	3	3	3	2	2
2	1	3	1	3	3	3	3	2	#NULL!
2	1	3	1	3	3	3	3	1	2
2	1	3	1	3	3	3	3	1	1
2	1	3	1	3	3	3	3	2	2
2	1	3	1	3	3	3	3	1	2
2	3	2	2	1	2	9	2	1	1
2	2	2	2	2	2	2	2	2	2
2	1	3	1	3	3	3	3	#NULL!	#NULL!
2	1	3	1	3	3	3	3	2	2
2	1	3	1	3	3	3	3	1	2
2	1	3	1	3	3	3	3	2	2
2	1	3	1	3	3	3	3	1	1
2	1	3	1	3	3	3	3	1	2
2	1	3	1	3	3	3	3	2	2
2	1	3	1	3	3	3	3	2	1
2	1	3	1	3	3	3	3	2	2
2	1	3	1	3	3	3	3	2	2
2	1	3	1	3	3	3	3	#NULL!	#NULL!
2	1	3	1	3	3	3	3	1	2
2	1	3	1	3	3	3	3	1	2
2	1	3	1	3	3	3	3	1	2
2	1	3	1	3	3	3	3	1	2

2	1	3	1	3	3	3	3	1	2
2	1	3	1	1	3	3	3	2	2
2	1	3	1	3	1	3	3	2	2
2	1	3	1	1	3	1	1	1	1
2	1	3	1	1	3	3	3	2	2
2	1	3	1	3	3	3	3	1	1
2	1	3	1	3	3	3	3	2	1
3	1	3	1	3	3	3	3	1	1
1	1	1	1	3	3	1	3	1	1
3	1	3	3	3	3	3	3	1	1
9	9	9	9	9	9	9	9	#NULL!	#NULL!
9	9	9	9	9	9	9	9	1	1
1	9	1	1	3	1	1	3	1	1
2	1	3	1	3	3	3	3	1	2
9	9	9	9	9	9	9	9	2	2
1	1	1	2	1	1	3	3	2	2
2	2	2	2	2	2	2	1	1	2
1	1	1	1	1	1	1	1	1	2
2	1	2	2	1	3	2	3	1	2
2	1	3	1	3	3	3	3	1	2
1	1	1	2	2	2	1	1	1	1
2	2	2	2	2	2	2	2	1	2
2	1	1	1	2	1	2	3	2	2
2	1	1	3	2	1	2	3	1	2
2	2	2	2	2	2	2	2	2	2
2	2	2	2	2	2	2	2	2	2
2	1	2	2	1	3	3	3	1	2
2	2	2	2	2	2	2	2	2	2
2	2	2	2	2	2	2	2	2	2
2	1	2	2	1	3	3	3	1	2
3	1	3	1	3	3	3	3	1	1
3	1	3	1	3	3	3	3	1	1
3	1	2	2	1	3	3	3	1	2
1	1	1	1	2	2	2	3	1	1
1	1	2	1	2	2	2	2	1	1
2	2	2	2	2	2	2	2	1	1
2	2	2	2	2	2	2	1	2	2
1	1	3	2	3	2	2	3	1	1
2	1	2	2	2	2	2	2	2	2
1	1	2	2	1	2	1	1	1	2
1	2	2	2	2	2	2	2	1	1
2	1	2	1	3	3	3	3	1	2
9	9	9	9	9	9	9	9	2	2
9	9	9	9	9	9	9	9	2	2
2	1	1	2	1	2	2	2	1	1
3	1	3	1	3	3	3	3	1	1
9	9	9	9	9	9	9	9	2	1

1	1	1	1	1	1	1	2	2	2
1	1	1	1	1	1	1	2	2	2
2	1	2	2	2	2	2	3	2	2
2	1	1	1	1	2	2	2	2	2
1	3	3	3	1	2	1	1	2	2
2	2	2	2	2	2	2	2	1	1
9	2	9	2	9	9	9	3	2	2
2	1	2	2	1	2	2	2	2	2
2	2	2	2	1	2	3	2	1	1
2	2	2	2	2	2	2	2	2	2
2	1	2	2	2	2	2	2	2	2
2	2	2	2	2	2	2	2	1	2
2	2	2	2	2	2	2	2	1	2
3	1	2	1	1	3	3	2	1	2
3	3	1	1	3	3	3	3	2	2
1	3	2	2	3	1	2	2	2	2
3	1	2	3	2	3	3	2	1	2
3	1	1	2	3	2	1	3	2	2
9	1	9	9	9	9	1	3	2	2
3	1	3	1	3	3	3	3	2	1
1	1	1	1	1	1	1	3	2	2
2	1	1	2	2	2	2	2	2	2
3	1	1	1	3	3	2	3	1	2
9	9	2	2	9	9	9	3	2	2
9	1	9	9	1	9	9	9	2	2
2	2	2	2	2	2	2	2	2	2
2	2	2	2	2	2	2	2	2	2
9	2	9	2	1	3	2	2	1	2
9	9	9	9	9	9	9	9	2	2
2	1	2	1	3	2	3	3	2	2
9	9	9	9	9	9	9	9	2	1
3	3	9	1	3	3	2	3	2	2
1	1	1	1	1	1	2	1	2	2
1	1	3	1	1	3	2	3	1	1
9	9	9	9	9	9	9	9	1	2
2	2	2	2	2	2	2	1	2	2
2	1	3	1	2	3	3	3	2	2
3	1	1	1	2	3	2	3	1	2
1	1	1	1	2	2	2	2	1	2
3	1	1	1	3	3	3	3	2	1
1	1	1	1	1	1	1	1	1	1
2	1	3	1	1	1	3	3	1	1
9	9	9	9	9	9	9	9	1	1
2	2	2	2	2	2	2	2	1	2
2	1	2	1	2	1	2	1	1	2
1	2	3	1	2	2	2	2	1	2
1	1	1	2	3	2	2	3	2	2

9	9	9	9	9	9	9	9	#NULL!	#NULL!
9	9	9	9	9	9	9	9	#NULL!	#NULL!
9	9	9	9	9	9	9	9	1	1
2	1	3	1	3	3	3	3	1	1
1	2	3	2	3	2	3	9	2	2
2	2	3	1	2	3	2	2	2	2
2	1	1	1	2	2	2	3	1	1
3	1	3	1	3	3	1	3	1	1
9	9	9	9	9	9	9	9	1	2
2	2	2	2	2	2	2	2	1	2
2	2	2	2	2	2	2	2	2	2
2	1	3	2	2	2	3	3	1	1
9	9	9	9	9	9	9	9	#NULL!	#NULL!
3	3	3	3	3	3	3	3	2	2
3	9	1	3	1	1	1	3	1	1
9	9	9	9	9	9	9	9	#NULL!	#NULL!
9	9	9	9	9	9	9	9	1	1
1	1	1	1	1	3	2	2	2	2
1	1	1	1	2	2	1	1	1	2
9	9	9	9	9	9	9	9	#NULL!	#NULL!
9	9	9	9	9	9	9	9	2	2
3	1	2	1	2	2	2	2	1	2
1	1	1	1	1	1	3	3	1	1
1	1	2	2	2	2	2	2	1	2
2	2	2	2	2	2	2	2	1	2
3	1	1	1	3	3	3	3	#NULL!	2
2	1	1	1	1	2	1	2	1	1
2	1	2	1	3	3	3	3	2	2
2	1	3	1	1	3	3	1	1	2
3	1	3	1	3	3	3	3	1	2
2	2	2	2	2	2	2	2	1	2
9	9	9	9	9	9	9	9	2	2
9	9	9	9	9	9	9	9	1	1
2	2	2	2	2	2	2	3	2	2
3	1	3	2	3	3	3	3	1	2
1	1	1	2	1	3	1	3	1	1
3	1	3	1	3	3	3	3	1	1
1	1	3	1	3	3	3	3	2	1
3	1	3	1	3	3	1	3	1	1
2	1	3	1	2	3	3	3	1	2
2	1	2	3	2	2	1	9	1	1
1	1	3	1	1	3	3	3	1	1
9	3	2	2	3	9	2	2	1	1
9	9	9	9	9	9	9	9	2	2
3	1	3	1	3	3	3	3	1	1
2	2	2	2	2	2	2	2	1	1
3	1	3	1	1	3	3	3	1	2

9	9	9	9	9	9	9	9	2	2
2	1	3	1	3	2	2	3	2	2
9	9	9	9	9	9	9	9	1	1
3	1	3	1	3	3	3	3	1	2
9	9	9	9	9	9	9	9	1	1
9	9	9	9	9	9	9	9	1	2
3	1	1	2	2	3	3	3	1	1
2	9	3	9	2	1	2	9	1	2
2	1	3	1	3	3	3	3	1	2
2	1	1	1	3	3	3	3	1	2
2	2	2	2	2	2	2	2	2	2
2	1	3	2	3	3	2	2	1	1
2	1	2	1	3	2	3	3	1	1
2	2	1	1	2	3	2	2	2	2
2	1	3	1	3	3	3	2	2	2
3	2	2	2	3	3	2	2	2	#NULL!
2	2	2	2	2	2	2	2	2	2
2	2	2	2	2	2	2	2	1	2
2	2	2	2	2	2	2	2	1	1
2	2	2	2	2	2	2	3	2	2
3	1	3	2	3	3	3	3	1	2
3	1	3	1	3	3	3	3	2	2
3	1	3	1	3	3	3	3	1	1
3	1	2	1	1	3	1	3	1	1
2	2	2	2	2	2	2	2	2	2
1	1	2	2	2	2	2	2	2	2
2	1	2	2	2	3	2	3	1	2
2	2	2	2	2	2	2	2	2	2
2	1	2	3	2	2	2	3	1	1
2	1	1	2	1	1	1	2	2	2
1	1	2	2	3	2	2	2	2	2
1	1	2	2	3	2	3	3	2	2
2	1	1	1	1	1	1	1	2	2
9	9	9	9	9	9	9	9	2	2
9	9	9	9	9	9	9	9	2	2
3	1	2	1	3	2	2	3	1	1
2	1	2	2	2	2	3	9	2	2
9	9	9	9	9	9	9	9	2	1
2	2	2	2	2	2	2	2	2	2
2	2	2	2	2	2	2	2	2	2
2	2	2	2	2	2	2	2	2	2
2	1	2	2	2	2	2	2	2	2
2	1	2	1	3	3	2	2	1	2
2	1	2	2	2	2	2	2	2	2
2	1	2	2	2	1	3	2	2	2
1	1	3	2	1	2	3	3	1	1
2	1	1	1	2	3	2	2	1	2

2	3	3	1	3	3	2	3	1	2
2	1	1	3	3	3	2	3	1	1
2	2	2	2	2	2	2	9	1	1
2	2	2	2	2	2	2	2	2	2
3	1	2	1	3	3	3	3	2	2
2	2	9	2	2	2	2	2	1	2
9	9	9	9	9	9	9	9	#NULL!	#NULL!
3	1	1	1	1	2	3	3	1	1
3	1	1	2	3	3	1	2	1	1
1	1	1	1	3	3	3	3	1	2
2	2	2	2	1	1	2	3	2	2
1	1	1	1	1	3	1	3	2	2
2	2	2	2	2	2	2	2	2	2
1	1	1	1	3	1	2	3	2	2
2	2	2	2	2	2	2	2	2	2
1	2	9	1	1	1	1	1	#NULL!	#NULL!
3	1	1	3	1	1	1	3	2	2
2	1	1	2	1	2	2	3	2	2
2	1	1	1	1	3	2	3	2	2
9	2	2	2	2	2	2	2	1	2
1	1	1	2	1	1	2	2	1	1
2	2	2	2	2	2	2	2	1	2
9	9	9	9	9	9	9	9	#NULL!	#NULL!
2	2	2	2	2	2	2	9	2	1
1	1	1	1	1	1	3	3	1	1
9	9	9	9	9	9	9	9	#NULL!	#NULL!
2	1	1	2	2	3	3	2	2	2
9	9	9	9	9	9	9	9	#NULL!	#NULL!
2	2	2	2	2	2	2	2	1	1
2	1	2	1	2	2	2	2	1	1
9	9	9	9	9	9	9	9	2	2
9	9	9	9	9	9	9	9	#NULL!	#NULL!
2	2	2	2	2	2	2	2	2	1
1	1	1	1	1	2	1	2	2	2
9	9	9	9	9	9	9	9	1	2
1	1	2	2	1	2	2	1	1	1
3	2	2	3	3	3	3	2	1	2
1	2	1	2	1	2	3	1	1	2
9	9	9	9	9	9	9	9	#NULL!	#NULL!
1	1	3	1	1	1	3	3	1	2
9	9	9	9	9	9	9	9	2	2
1	1	1	1	1	2	2	2	2	2
2	2	2	2	2	2	2	3	1	2
2	2	2	2	2	2	2	2	1	2
1	1	1	1	3	3	3	3	#NULL!	#NULL!
2	9	3	1	3	3	3	3	2	2
2	1	3	1	3	9	9	9	2	2

9	9	9	9	9	9	9	9	2	2
2	1	3	1	3	3	1	3	2	2
2	1	3	1	3	3	1	3	2	2
2	1	3	1	3	3	3	1	1	2
2	1	3	1	3	3	3	3	2	2
2	1	3	1	3	3	3	3	1	1
2	1	3	1	3	3	3	3	2	2
2	1	3	1	3	3	1	3	2	2
2	1	3	1	3	3	1	3	2	2
2	1	1	3	3	3	2	3	1	2
2	2	1	1	2	2	1	1	2	2
2	1	3	1	1	3	1	1	2	2
2	1	3	1	3	3	3	3	1	1
2	1	3	1	3	3	1	3	2	2
2	1	3	1	3	3	1	3	#NULL!	2
2	1	3	1	3	3	3	3	1	1
2	1	3	1	3	3	2	2	2	2
2	1	3	1	3	3	1	3	1	2
2	1	3	1	1	3	1	3	1	2
2	1	3	1	1	3	1	3	1	2
1	1	3	1	3	3	3	3	1	2
2	1	3	1	3	3	3	3	1	2
2	1	3	1	1	3	1	3	2	2
2	1	3	1	3	3	1	3	1	1
2	1	3	1	3	3	3	3	2	2
2	1	3	1	3	3	3	3	2	2
2	1	3	1	3	3	3	3	2	2
2	1	3	1	3	3	3	3	#NULL!	2
2	1	3	1	3	3	3	1	2	1
2	1	3	1	2	3	3	3	2	2
2	1	3	1	3	3	3	3	2	1
2	1	3	1	3	3	2	3	2	2
2	1	3	1	3	3	3	3	2	1
2	1	3	1	3	3	3	3	2	2
2	1	3	1	3	3	3	3	1	1
2	1	3	1	3	3	3	3	1	1
3	1	2	1	3	3	2	3	1	1
2	1	3	1	3	3	3	3	2	1
2	1	3	1	3	3	3	3	2	2
2	1	3	1	3	3	3	3	1	1
2	1	3	1	3	3	3	3	1	2
2	2	2	2	2	2	2	2	2	2
2	1	1	1	1	3	3	3	1	2
2	2	2	2	2	2	2	2	2	2
9	9	9	9	9	9	9	9	2	2
2	2	2	2	2	2	2	2	2	2
1	1	1	2	3	3	3	3	1	2

2	2	2	2	2	2	2	2	2	2
3	3	3	1	2	3	3	1	1	1
3	3	3	1	2	3	3	1	1	1
2	2	3	2	2	2	2	2	1	1
2	1	3	1	3	1	2	2	2	2
9	9	9	9	9	9	9	9	2	2
2	2	2	2	2	2	2	2	2	1
2	2	1	2	3	2	2	2	2	2
3	1	2	1	3	3	3	3	1	1
2	1	2	2	2	2	2	2	1	2
3	3	3	1	2	3	3	1	1	1
9	9	9	9	9	9	9	9	1	2
3	1	1	2	1	2	3	3	1	1
1	1	2	2	2	2	2	2	2	2
9	9	9	9	9	9	9	9	1	2
9	9	9	9	9	9	9	9	1	1
9	9	9	9	9	9	9	9	2	2
9	9	9	9	9	9	9	9	2	2
9	9	9	9	9	9	9	9	2	2
3	1	2	1	1	3	1	3	1	2
9	9	9	9	9	9	9	9	1	2
9	9	9	9	9	9	9	9	1	2
3	1	2	1	2	1	1	1	1	1
9	9	9	9	9	9	9	9	2	2
9	1	9	9	9	9	9	9	2	2
9	9	9	9	9	9	9	9	#NULL!	#NULL!
9	9	9	9	9	9	9	9	1	1
9	9	9	9	9	9	9	9	2	2
2	1	2	1	3	3	3	3	2	2
9	9	9	9	9	9	9	9	1	2
3	9	9	9	9	9	9	9	1	1
1	1	2	1	2	2	2	3	2	2
2	2	2	2	2	2	2	2	2	2
3	1	3	1	3	3	3	3	1	1
2	1	1	2	1	2	2	2	2	2
2	1	2	1	2	2	2	2	1	1
3	1	3	1	3	2	2	3	1	1
3	1	3	1	3	3	3	3	1	2
2	2	2	2	2	2	2	2	2	2
2	1	2	1	3	3	3	3	1	2
2	2	2	2	2	3	2	2	2	2
2	1	3	1	3	3	3	3	1	1
2	2	2	2	2	2	2	2	2	2
9	9	9	9	9	9	9	9	2	2
2	1	1	2	2	3	2	3	2	2
9	9	9	9	9	9	9	9	1	1
2	2	3	1	3	3	3	3	2	2

2	1	1	2	2	2	1	2	1	1
3	1	3	1	3	3	3	3	1	1
2	1	3	1	3	3	3	3	2	2
1	1	1	2	2	2	2	3	2	2
3	1	2	1	2	2	1	2	1	2
2	2	2	1	2	2	2	2	2	2
3	1	2	3	2	3	2	3	2	2
2	2	2	2	2	2	2	2	2	1
2	2	2	2	2	2	2	2	2	2
2	2	2	2	2	2	2	2	2	2
2	2	2	2	2	2	2	2	2	2
2	1	3	3	2	3	3	3	1	1
2	2	2	2	2	2	2	2	2	2
2	2	2	2	2	2	2	2	2	2
3	1	3	2	3	3	3	2	1	1
2	2	2	2	2	2	2	2	2	2
2	2	2	2	2	2	2	2	2	2
2	2	2	1	2	2	2	2	2	2
2	1	1	2	2	3	2	3	1	2
3	1	1	1	3	2	3	3	1	1
2	2	2	1	2	2	2	2	2	2
3	1	3	1	3	3	3	3	1	1
2	1	1	1	3	3	3	3	1	1
2	1	1	1	3	3	3	3	2	2
3	2	2	2	1	1	2	3	2	2
2	1	2	1	2	2	2	2	2	2
9	9	9	9	9	9	9	9	1	1
2	1	1	2	1	1	2	1	1	2
1	1	3	1	1	3	1	2	1	1
2	3	3	3	3	3	3	2	1	2
2	2	2	2	2	2	2	9	#NULL!	#NULL!
2	1	1	2	2	1	2	2	2	2
2	1	2	2	2	2	2	2	2	2
2	1	2	2	3	2	2	2	2	2
2	2	2	2	2	2	2	3	2	2
9	9	9	9	9	9	9	9	2	2
2	1	3	1	2	3	3	3	2	2
3	1	3	1	3	3	3	3	1	1
2	1	3	2	2	2	2	2	2	2
9	9	9	9	9	9	9	9	1	1
9	1	9	9	9	9	9	9	#NULL!	#NULL!
1	1	2	1	2	2	2	3	2	2
1	1	2	1	2	2	2	3	2	2
2	1	2	3	2	1	2	3	1	#NULL!
3	1	3	1	3	3	3	3	1	1
2	2	2	2	2	2	2	2	2	2
9	9	9	9	9	9	9	9	2	2

9	9	9	9	9	9	9	9	1	1
9	9	9	1	9	9	9	9	2	2
9	9	9	9	9	9	9	9	2	2
9	9	9	9	9	9	9	9	#NULL!	#NULL!
9	9	9	9	9	9	9	9	2	1
9	9	9	9	9	9	9	9	1	2
9	9	9	9	9	9	9	9	2	2
9	9	9	9	9	9	9	9	2	2
9	9	9	9	9	9	9	9	2	2
9	9	9	9	9	9	9	9	1	1
9	9	9	9	9	9	9	9	2	2
9	9	9	9	9	9	9	9	1	1
9	9	9	9	9	9	9	9	2	2
9	9	9	9	9	9	9	9	1	1
9	9	9	1	9	9	9	9	2	1
9	9	9	9	9	9	9	9	2	2
9	9	9	9	9	9	9	9	1	2
9	9	1	3	3	1	1	3	1	2
2	2	2	2	2	2	2	2	2	2
9	9	9	9	9	9	9	9	1	1
1	1	1	1	1	1	1	1	1	1
2	2	3	3	2	1	2	1	2	2
2	1	1	2	1	2	2	2	2	2
1	1	1	1	1	1	1	1	2	1
2	1	2	2	2	3	2	2	2	2
3	3	3	2	2	2	2	2	2	1
2	2	2	2	2	2	2	2	1	1
2	3	2	1	2	2	1	2	2	2
2	2	2	2	2	2	2	2	2	2
1	1	1	1	1	1	1	1	1	2
1	1	1	2	2	2	3	3	#NULL!	#NULL!
3	1	2	1	3	2	3	3	2	2
2	2	2	2	2	2	2	2	2	2
3	1	1	1	3	3	3	3	1	1
2	1	1	1	2	2	3	3	2	2
1	1	2	2	1	2	1	3	2	2
2	2	2	2	2	2	2	2	2	2
9	9	9	9	9	9	9	9	#NULL!	#NULL!
1	1	1	1	1	1	1	1	1	1
9	9	9	9	9	9	9	9	1	2
3	3	3	2	2	3	2	3	1	2
2	2	3	3	2	9	2	2	1	1
9	9	9	9	9	9	9	9	#NULL!	#NULL!
1	1	1	1	1	1	1	3	1	2
2	2	3	3	2	2	2	2	2	2
9	9	9	9	9	9	9	9	1	2
3	3	3	3	3	3	3	3	2	2

2	2	2	2	2	2	2	2	2	2
9	9	9	9	9	9	9	9	#NULL!	#NULL!
3	1	1	1	3	1	3	1	2	2
9	9	9	9	9	9	9	9	#NULL!	#NULL!
3	1	1	2	3	3	3	3	1	2
3	1	1	2	3	3	3	3	1	2
9	9	9	9	9	9	9	9	#NULL!	#NULL!
2	2	2	2	2	2	2	2	2	2
1	2	1	2	1	2	1	2	1	1
9	9	9	9	9	9	9	9	2	2
9	9	9	9	9	9	9	9	1	1
1	1	1	2	2	1	1	2	1	1
2	3	3	2	9	3	3	2	2	2
9	9	9	9	9	9	9	9	#NULL!	#NULL!
9	9	9	9	9	9	9	9	#NULL!	#NULL!
9	9	9	9	9	9	9	9	1	2
2	1	1	2	3	2	3	3	1	#NULL!
9	9	9	9	9	9	9	9	#NULL!	#NULL!
2	1	3	1	3	3	3	1	1	2
3	1	9	3	3	3	3	3	1	2
3	3	3	3	3	3	3	3	1	2
2	3	3	2	2	3	3	2	2	2
3	3	3	3	3	3	3	2	1	1
9	9	9	9	9	9	9	9	1	2
2	2	2	2	2	2	2	2	1	2
1	3	1	3	1	1	3	1	2	2
1	3	3	3	3	3	3	3	1	1
3	1	1	1	3	3	3	3	2	2
1	2	1	2	1	2	2	1	2	2
3	1	3	1	3	3	3	3	1	2
9	9	9	9	9	9	9	9	2	2
9	9	9	1	9	9	9	9	1	1
2	1	1	1	1	3	3	3	1	2
1	2	1	2	1	2	1	1	2	2
9	1	2	9	1	1	2	2	2	2
3	1	3	1	3	3	3	3	1	2
3	1	3	1	3	3	3	3	2	2
9	9	9	9	9	9	9	9	#NULL!	#NULL!
9	9	9	9	9	9	9	9	#NULL!	#NULL!
9	9	9	9	9	9	9	9	#NULL!	#NULL!
9	9	9	9	9	9	9	9	#NULL!	#NULL!
3	2	2	3	1	1	2	2	1	1
1	1	3	3	1	1	2	1	2	2
9	9	9	9	9	9	9	9	#NULL!	#NULL!
1	1	3	3	2	1	2	3	2	2
2	1	1	1	3	1	1	3	2	2
1	1	3	3	3	3	3	3	2	2

3	1	1	3	3	3	3	3	2	2
2	2	3	2	1	3	1	3	1	2
2	2	3	3	3	3	3	3	1	2
2	2	2	2	2	2	2	2	1	2
3	3	3	2	3	3	3	3	2	2
1	1	1	1	1	1	1	1	1	2
9	9	9	9	9	9	9	9	#NULL!	#NULL!
2	1	1	2	2	3	3	9	1	1
3	3	3	3	2	2	2	3	2	2
9	9	9	9	9	9	9	9	1	2
3	3	3	3	3	3	3	3	2	2
3	3	9	1	2	9	2	9	1	2
2	1	1	1	3	1	1	3	2	2
2	1	1	1	3	1	1	3	2	2
2	1	1	1	3	1	1	3	2	2
2	2	2	3	3	3	1	3	2	2
2	1	1	1	3	1	1	3	2	2
2	1	1	1	3	1	1	3	2	2
2	1	1	1	3	1	1	3	2	2
1	2	3	3	1	1	3	1	2	2
1	2	1	2	1	3	2	3	2	2
1	1	1	1	1	2	2	2	#NULL!	#NULL!
1	1	3	3	3	3	2	1	2	2
2	2	2	2	2	2	2	9	2	2
3	3	3	3	3	3	3	3	2	2
2	2	3	1	1	3	2	1	2	2
9	9	9	9	9	9	9	9	2	2
3	1	3	1	3	3	3	3	2	2
9	9	9	9	9	9	2	9	1	1
2	2	2	2	2	2	2	3	2	2
1	2	1	3	2	9	1	2	2	2
2	3	3	2	3	2	3	2	2	2
2	3	2	1	2	1	2	2	1	2
9	9	9	2	9	9	9	9	2	2
2	2	3	3	3	2	2	2	2	2
3	1	9	2	2	3	2	3	2	2
3	2	9	1	3	9	9	3	1	2
2	1	1	1	2	2	2	3	2	2
2	1	1	1	2	2	2	3	2	2
1	1	1	1	1	3	1	1	2	2
1	1	2	3	1	2	1	1	2	2
2	2	2	2	2	2	2	2	2	2
2	3	1	2	3	1	2	2	1	2
3	1	1	1	3	3	3	3	1	1
9	9	9	9	9	9	9	9	2	2
1	1	1	1	1	1	1	1	1	1
2	3	2	3	2	3	2	2	2	2

2	1	1	3	1	1	3	3	2	2
9	9	9	9	9	9	9	9	1	1
9	9	1	9	9	9	9	9	2	2
3	1	3	1	3	1	3	1	2	2
2	2	2	2	2	2	2	2	2	2
3	1	1	2	3	3	3	3	1	2
1	1	1	2	2	2	2	2	2	2
3	1	1	2	3	3	3	3	1	2
3	1	1	2	3	3	3	3	1	2
9	9	9	9	9	9	9	9	1	2
9	9	9	9	9	9	9	9	2	2
2	1	3	1	3	3	3	3	2	2
9	9	9	9	9	9	9	9	2	2
1	1	1	3	1	3	1	1	2	2
2	1	2	1	2	3	3	3	1	2
9	9	9	9	9	9	9	9	1	1
2	2	2	2	2	2	2	2	1	2
9	9	9	9	9	9	9	9	1	1
9	9	9	9	9	9	9	9	1	1
2	3	1	2	3	1	2	3	2	2
1	1	1	1	1	3	1	1	1	2
2	2	3	3	3	3	2	3	1	2
9	9	9	9	9	9	9	9	#NULL!	#NULL!
2	2	2	2	2	2	2	2	2	2
3	3	3	3	3	3	3	3	#NULL!	#NULL!
2	2	1	1	1	2	3	3	1	1
2	2	3	1	1	1	2	3	1	1
3	1	3	1	3	3	3	3	1	2
1	1	1	1	1	1	1	1	2	2
2	2	2	2	2	2	2	2	2	2
3	3	1	1	1	1	1	2	1	1
2	1	1	2	3	2	1	2	2	2
1	1	1	1	2	2	1	1	1	2
2	2	3	1	1	1	2	2	1	1
1	1	2	2	3	2	1	2	#NULL!	#NULL!
2	2	2	2	2	2	2	2	2	2
2	1	1	1	2	1	3	3	2	2
9	1	9	9	9	9	9	9	1	1
1	9	9	9	9	9	9	9	2	2
1	1	1	3	3	1	2	1	1	1
2	2	2	2	2	2	2	2	2	2
2	2	2	2	2	2	2	2	2	2
2	2	2	2	2	2	2	3	2	2
2	2	2	2	2	2	2	2	1	1
2	2	2	2	2	2	2	2	2	2
2	2	2	2	2	2	2	2	2	2
9	9	9	9	9	9	9	9	#NULL!	#NULL!

9	9	9	9	9	9	9	9	#NULL!	#NULL!
2	2	2	2	2	2	2	2	2	2
9	9	9	9	9	9	9	9	1	1
9	9	9	9	9	9	9	9	1	1
9	9	9	9	9	1	9	9	1	1
2	2	2	2	2	2	2	2	2	2
9	9	9	9	9	9	9	9	#NULL!	#NULL!
3	3	3	3	3	3	3	3	2	2
2	1	2	2	2	2	2	2	1	1
9	2	9	9	9	9	9	9	2	2
2	2	2	2	2	2	2	2	2	2
1	2	2	1	2	1	1	2	1	2
1	2	2	1	2	1	1	2	1	2
2	2	2	2	2	2	2	2	2	2
1	1	1	3	1	1	1	1	1	2
2	2	2	2	2	2	2	2	2	2
2	2	2	2	2	2	2	1	2	2
9	9	9	9	9	9	9	9	1	1
1	1	2	1	2	2	2	3	1	2
3	3	3	3	3	3	3	3	2	2
9	1	9	9	9	9	9	9	2	2
3	3	3	3	3	3	3	3	2	1
2	2	2	2	2	2	2	2	2	2
2	1	3	3	3	3	3	2	1	2
9	9	9	9	9	9	9	9	1	1
3	2	3	2	3	2	3	1	2	2
9	9	1	9	9	9	9	9	2	2
9	9	9	9	9	9	9	9	2	2
2	2	2	2	2	2	2	2	2	2
2	1	1	1	1	3	3	2	1	1
9	9	9	9	9	9	9	9	2	#NULL!
3	1	2	1	3	3	3	3	2	2
3	1	1	1	3	3	1	3	1	2
2	2	2	2	2	2	2	2	1	1
2	2	2	2	2	2	2	2	1	2
1	1	1	1	1	3	3	1	#NULL!	#NULL!
2	2	2	2	2	2	2	2	2	2
2	9	1	3	2	2	2	1	1	2
2	2	2	2	2	2	2	2	2	2
2	2	2	2	2	2	2	2	2	2
9	9	9	9	9	9	9	9	2	2
2	2	2	2	2	2	2	2	2	2
1	3	3	3	1	3	3	3	2	2
2	2	2	2	2	2	2	2	#NULL!	#NULL!
2	2	2	2	2	2	2	2	2	2
3	3	3	3	3	3	3	3	2	2
2	2	2	2	2	2	2	3	2	2

2	2	2	2	2	2	2	2	2	2
2	2	2	2	2	2	2	2	2	2
2	2	2	2	2	2	2	2	2	1
2	2	2	2	2	2	2	2	2	2
2	3	1	2	1	2	3	3	2	2
1	1	1	2	3	1	1	2	2	2
2	2	2	2	2	2	2	2	2	2
2	3	1	3	2	9	2	2	2	2
2	2	2	2	2	2	2	2	2	1
2	2	2	2	2	2	2	2	2	2
1	1	1	1	3	1	1	1	1	1
9	9	9	9	9	9	9	9	1	#NULL!
9	2	9	9	9	9	9	9	1	1
9	9	9	9	9	9	9	9	#NULL!	#NULL!
1	1	1	1	1	1	1	1	1	1
3	3	3	3	3	3	3	3	2	2
3	3	3	3	3	3	3	3	1	2
3	1	3	1	3	3	3	3	2	2
9	9	9	9	9	9	9	9	1	#NULL!
2	2	3	2	2	3	3	1	2	2
9	9	9	9	9	9	9	9	1	1
2	1	2	2	2	2	2	2	1	1
2	1	2	1	2	2	3	2	2	1
9	9	9	9	9	9	9	9	1	1
3	3	2	2	2	2	2	1	2	2
2	1	9	1	2	1	3	3	1	1
2	2	2	2	2	2	2	1	2	2
2	1	2	1	2	3	3	3	1	1
2	2	2	2	2	2	2	2	2	2
1	2	1	2	1	2	2	1	2	2
1	1	2	2	2	1	1	1	1	1
1	1	1	3	3	3	3	3	1	2
9	9	9	9	9	9	9	9	#NULL!	#NULL!
3	3	3	3	3	3	3	3	2	2
1	1	1	1	1	1	3	1	2	2
1	3	3	3	3	3	3	1	2	1
3	1	1	2	3	3	3	3	2	2
1	1	1	1	1	1	1	1	2	2
2	2	2	2	2	2	2	2	2	2
2	2	2	2	2	2	2	2	2	2
3	3	2	1	3	1	2	1	1	1
1	1	1	1	1	1	1	1	2	2
1	1	1	1	3	1	2	2	2	2
2	2	2	2	2	2	2	2	2	2
2	2	2	2	2	2	2	2	2	2
2	1	3	2	1	2	1	1	1	1
2	2	3	2	2	2	2	2	2	2

2	1	2	2	2	1	1	1	1	2
3	3	3	3	3	3	3	3	2	2
2	3	2	1	2	3	1	2	2	1
9	9	9	9	1	9	9	9	2	2
9	9	9	9	9	9	9	9	#NULL!	#NULL!
1	2	1	1	1	2	1	3	2	2
2	2	2	2	2	2	2	2	2	2
9	2	2	9	2	9	1	3	2	2
3	1	2	3	1	3	1	1	2	1
2	2	2	2	2	2	2	2	2	2
9	9	9	9	9	9	1	9	2	2
2	2	2	2	3	3	9	1	1	1
2	2	2	2	2	2	2	2	#NULL!	2
3	2	3	2	3	3	2	2	1	2
2	2	2	2	2	2	2	2	2	2
2	3	9	3	2	3	2	3	2	2
2	2	2	2	2	2	2	2	2	1
9	9	9	9	9	9	9	9	1	1
9	9	9	9	2	9	9	9	1	2
2	2	2	2	2	2	2	2	2	2
2	2	2	2	2	2	2	2	1	1
2	2	2	2	2	2	2	2	2	2
9	9	9	9	9	9	9	9	#NULL!	#NULL!
9	9	9	9	9	9	9	9	#NULL!	#NULL!
1	1	1	1	1	1	1	1	2	1
3	3	3	1	3	3	3	3	1	1
2	2	3	3	3	3	2	2	2	1
3	1	3	1	3	1	3	3	1	1
2	3	1	9	1	2	2	2	1	2
2	3	1	2	3	1	2	3	2	2
2	1	1	2	1	2	9	1	1	1
1	1	3	2	1	2	3	2	2	2
3	3	2	1	3	1	3	3	1	1
3	3	3	3	3	3	3	3	2	2
9	9	9	9	9	9	9	9	2	2
3	2	1	1	2	3	3	2	2	1
3	1	1	1	1	1	1	1	2	1
3	3	3	3	3	3	3	3	2	2
9	1	9	2	1	9	9	3	1	1
3	3	3	3	3	3	3	3	2	2
9	9	9	9	9	9	9	9	2	2
1	1	2	2	3	3	1	1	2	1
3	3	3	3	3	3	3	3	2	2
2	2	2	2	2	2	2	2	2	2
1	1	3	1	3	3	3	3	1	2
2	1	1	2	2	1	1	2	#NULL!	#NULL!
2	1	2	1	1	1	2	3	1	2

2	2	2	2	2	2	2	2	2	2
3	3	3	3	2	2	3	1	2	2
9	9	9	9	9	9	9	9	1	1
1	1	1	1	1	1	1	1	1	1
2	2	2	2	2	2	2	2	2	2
2	2	2	2	2	2	2	2	2	1
2	2	2	2	2	2	2	2	2	2
2	2	2	2	2	2	2	2	2	1
2	2	2	2	2	2	2	2	2	1
2	1	2	1	2	1	3	2	2	2
9	9	9	9	9	9	9	9	2	1
3	1	1	3	1	3	3	2	2	2
1	1	3	3	1	3	3	3	2	2
2	1	1	2	3	1	2	3	1	1
1	9	2	1	2	1	2	1	2	1
2	2	2	2	2	2	2	2	2	2
2	1	3	1	1	3	3	3	2	2
2	1	2	2	2	2	2	2	1	1
2	1	2	1	3	3	3	3	2	2
9	9	9	9	9	9	9	9	1	1
3	3	3	3	3	3	1	3	1	2
1	1	1	1	1	1	1	1	1	1
3	1	3	3	3	1	3	3	1	1
1	1	1	1	1	1	1	1	1	2
3	3	3	3	3	3	3	3	2	2
3	3	3	3	3	3	3	3	2	2
1	1	1	1	1	1	1	3	2	2
2	1	3	1	3	1	2	3	2	2
1	1	2	1	1	1	3	1	2	2
1	2	2	2	1	2	1	1	1	1
9	9	9	9	9	9	9	9	2	2
9	9	9	9	9	9	9	9	1	1
1	3	2	2	1	2	2	1	2	2
9	9	9	9	9	9	9	9	2	2
2	2	2	2	2	2	2	2	1	1
2	2	2	2	2	2	2	2	1	2
2	2	2	2	2	2	2	3	2	2
2	2	2	2	2	2	2	3	2	2
3	2	2	3	3	3	3	3	2	2
9	9	9	9	9	9	9	9	2	2
2	3	2	1	2	2	1	2	1	2
2	2	2	2	2	2	2	2	1	1
2	2	2	2	2	2	1	1	1	2
9	9	9	9	9	9	9	9	2	2
3	3	3	3	3	3	3	3	2	2
2	2	2	2	2	2	2	2	1	1
9	9	9	9	9	9	9	9	2	2

9	9	9	9	9	9	9	9	1	2
1	2	2	2	3	2	2	2	#NULL!	#NULL!
2	2	2	9	1	2	2	2	2	2
2	1	1	2	2	2	1	3	2	2
2	2	2	2	2	2	2	2	2	1
9	9	9	9	9	9	9	9	1	2
9	9	9	9	9	9	9	9	2	2
1	2	2	3	3	3	3	3	2	1
2	2	2	2	2	2	2	2	1	2
9	9	9	9	9	9	9	9	#NULL!	#NULL!
9	9	9	9	9	9	9	9	#NULL!	#NULL!
9	9	9	9	9	9	9	9	#NULL!	#NULL!
1	9	9	9	9	9	9	9	2	2
9	9	9	9	9	9	9	9	2	2
9	9	9	9	9	9	9	9	#NULL!	#NULL!
2	2	9	2	2	2	2	3	2	2
2	2	2	2	2	2	2	2	#NULL!	#NULL!
2	2	2	2	2	2	2	2	1	1
2	3	1	2	1	1	2	2	1	2
2	2	2	2	2	2	2	2	1	1
2	2	2	9	1	2	3	2	2	2
2	1	2	3	3	2	3	1	1	2
1	1	1	1	1	3	2	2	1	2
3	1	1	3	2	3	3	3	2	2
2	2	2	2	2	2	2	2	2	2
2	2	2	2	2	2	2	2	1	2
9	9	9	9	9	9	9	9	1	2
1	1	1	1	1	1	1	1	1	1
2	2	2	2	2	2	2	2	1	2
2	2	9	2	2	2	2	3	2	2
2	2	2	2	2	2	2	2	1	1
1	9	9	9	9	9	9	9	2	2
3	1	1	1	1	3	2	2	1	1
2	2	2	2	2	2	2	2	1	1
2	2	2	2	2	2	2	2	2	2
2	2	2	2	2	2	2	2	2	2
9	9	9	9	9	9	9	9	2	2
9	9	9	9	9	9	9	3	1	2
2	2	2	2	2	2	2	2	2	2
2	2	2	2	2	2	2	2	1	2
2	2	2	2	2	2	2	2	2	2
9	9	9	9	9	9	9	9	1	1
2	2	2	2	2	2	2	2	2	2
1	2	2	2	2	2	2	2	2	2
9	9	9	9	9	9	9	9	1	2
2	2	2	2	2	2	2	2	1	1
9	1	9	9	9	9	9	9	2	2

2	2	2	1	2	2	2	3	#NULL!	#NULL!
2	1	1	1	2	2	1	1	2	2
9	9	9	9	9	9	2	9	1	2
9	9	9	9	9	9	9	9	2	2
2	2	2	2	2	2	2	2	2	2
9	9	9	9	9	9	9	9	2	2
2	3	3	3	2	2	2	2	2	2
1	1	2	2	1	2	2	1	1	1
2	3	2	2	2	3	2	1	1	2
2	3	2	1	2	3	2	1	2	2
2	1	3	2	3	2	3	3	2	2
2	1	2	3	3	3	2	2	1	1
2	2	2	2	2	2	2	2	2	2
9	9	9	9	9	9	9	9	2	2
9	9	9	9	9	9	9	9	2	2
3	3	3	3	3	3	3	3	2	2
2	2	2	2	2	2	2	2	2	2
2	2	2	2	2	2	2	2	1	1
2	1	1	2	1	1	2	3	2	2
1	1	1	1	1	3	3	3	2	2
9	9	9	9	9	9	9	9	2	2
2	2	2	2	2	2	2	2	2	2
3	1	1	1	3	3	3	3	2	2
2	2	2	2	2	2	2	2	2	2
2	2	2	2	2	2	2	2	2	2
3	1	3	1	3	3	1	3	1	2
2	2	2	2	2	2	2	2	2	2
2	2	2	2	2	2	2	2	2	2
1	1	1	2	1	3	3	2	2	2
9	9	9	9	9	9	9	9	#NULL!	#NULL!
9	9	9	9	9	9	9	9	1	1
2	2	2	2	2	2	2	2	2	2
9	9	9	9	9	9	9	9	2	2
9	9	9	9	9	9	9	9	#NULL!	#NULL!
2	3	3	3	2	1	1	2	1	1
9	9	9	9	9	9	9	9	2	2
9	9	9	9	9	9	9	9	#NULL!	#NULL!
2	2	2	2	2	2	2	2	2	2
3	1	3	1	2	3	3	3	1	1
1	1	1	1	2	2	3	9	2	1
1	1	1	1	1	1	1	1	2	1
3	1	2	2	3	1	1	1	1	2
9	9	9	9	9	9	9	9	2	1
2	1	2	1	2	2	2	3	2	1
9	9	9	9	9	9	9	9	1	1
9	9	9	9	9	9	9	9	#NULL!	#NULL!
1	1	2	2	2	2	2	3	2	2

2	2	1	2	2	1	2	1	2	2
9	9	9	9	9	9	9	9	#NULL!	#NULL!
9	9	9	9	9	9	9	9	#NULL!	#NULL!
2	2	2	2	2	2	2	2	2	2
1	1	1	1	1	3	3	3	1	2
2	2	1	2	2	1	2	1	2	2
2	9	2	1	1	2	3	1	1	1
3	1	1	1	3	3	3	3	2	2
2	2	2	2	2	2	2	2	1	1
3	3	3	1	3	3	3	3	1	1
9	9	9	9	9	9	9	9	#NULL!	#NULL!
9	9	9	9	9	9	9	9	2	2
9	9	9	9	9	9	9	9	1	2
9	9	9	9	9	9	9	9	1	2
3	2	2	2	2	3	1	2	2	2
9	9	9	9	9	9	9	9	#NULL!	#NULL!
9	9	9	9	9	9	9	9	#NULL!	#NULL!
9	9	9	9	9	9	9	9	#NULL!	#NULL!
2	1	1	1	2	2	2	1	1	2
1	1	1	1	1	1	2	3	1	2
9	9	9	9	9	9	9	9	1	1
3	3	3	3	3	2	2	1	2	1
2	2	2	2	2	2	2	9	2	1
9	9	9	9	9	9	9	9	#NULL!	#NULL!
2	2	2	2	2	2	2	2	1	1
2	1	2	2	2	2	2	2	2	2
9	9	9	9	9	9	9	9	2	2
9	9	9	9	9	9	9	9	2	2
2	2	2	2	2	2	2	2	2	2
2	2	2	2	2	2	2	2	#NULL!	#NULL!
1	1	1	1	1	1	1	1	1	2
9	9	9	9	9	9	9	9	2	2
9	9	9	9	9	9	9	9	#NULL!	#NULL!
2	3	1	2	3	1	2	2	1	2
1	2	2	2	1	2	1	2	2	2
2	2	1	3	2	1	3	9	2	2
2	2	2	2	2	2	2	2	2	2
2	2	2	2	2	2	2	2	2	2
9	1	2	2	1	2	1	2	1	2
2	3	1	2	3	1	2	2	2	2
1	3	3	3	1	1	1	2	2	1
9	9	9	9	9	9	9	9	2	#NULL!
9	9	9	9	9	9	9	9	#NULL!	#NULL!
9	9	9	1	9	9	9	9	2	2
9	9	9	9	9	9	9	9	#NULL!	#NULL!
9	9	9	9	9	9	9	9	#NULL!	#NULL!
9	9	9	9	9	9	9	9	#NULL!	#NULL!

2	2	2	2	2	2	2	2	1	1
3	2	2	2	2	3	3	3	2	2
3	3	1	3	3	3	3	3	1	1
3	1	3	1	3	3	3	3	2	2
9	9	9	9	9	9	9	9	1	1
9	9	9	9	9	9	9	9	#NULL!	#NULL!
9	9	9	9	9	9	9	9	#NULL!	#NULL!
9	9	9	9	9	9	9	9	#NULL!	#NULL!
9	9	9	9	9	9	9	9	#NULL!	#NULL!
9	9	9	9	9	9	9	9	#NULL!	#NULL!
9	9	9	9	9	9	9	9	#NULL!	#NULL!
9	9	9	9	9	9	9	9	#NULL!	#NULL!
9	9	9	9	9	9	9	9	#NULL!	#NULL!
3	1	3	1	3	1	3	1	2	2
9	9	9	9	9	9	9	9	2	2
2	2	2	1	1	1	1	1	2	1
2	1	2	2	2	2	2	2	2	2
9	9	9	9	9	9	9	9	#NULL!	#NULL!
9	9	9	9	9	9	9	9	#NULL!	#NULL!
9	9	9	9	9	9	9	9	#NULL!	#NULL!
9	9	9	9	9	9	9	9	1	2
9	9	9	9	9	9	9	9	#NULL!	#NULL!
1	1	1	1	1	1	1	1	1	1
9	9	9	9	9	9	9	9	#NULL!	#NULL!
2	2	2	2	1	1	1	1	2	1
1	1	1	1	3	3	1	1	1	1
1	1	1	1	2	2	2	3	1	1
1	1	2	2	1	1	1	1	1	1
1	1	2	2	1	1	1	1	1	1
1	1	1	1	1	2	2	1	1	1
2	2	2	2	2	2	2	2	1	1
1	1	1	1	1	1	1	1	2	2
1	1	1	1	1	1	1	1	1	1
9	9	9	9	9	9	9	9	#NULL!	#NULL!
1	1	2	2	2	1	1	1	1	1
1	1	1	1	1	1	1	1	1	1
2	2	2	2	1	1	1	1	2	2
9	9	9	9	9	9	9	9	#NULL!	#NULL!
3	3	3	3	3	3	3	3	2	2
1	1	1	1	3	3	3	3	1	2
2	3	2	2	2	2	2	2	2	1
9	9	9	9	9	9	9	9	#NULL!	#NULL!
9	9	9	9	9	9	9	9	#NULL!	#NULL!
9	9	9	9	9	9	9	9	#NULL!	#NULL!
9	9	9	9	9	9	9	9	2	1
3	2	2	2	2	2	2	2	2	1
9	9	9	9	9	9	9	9	1	2

9	9	9	9	9	9	9	9	#NULL!	#NULL!
9	9	9	9	9	9	9	9	#NULL!	#NULL!
9	9	9	9	9	9	9	9	#NULL!	#NULL!
9	9	9	9	9	9	9	9	#NULL!	#NULL!
9	9	9	9	9	9	9	9	2	2
9	9	9	9	9	9	9	9	#NULL!	#NULL!
9	9	9	9	9	9	9	9	#NULL!	#NULL!
9	9	9	9	9	9	9	9	#NULL!	#NULL!
9	9	9	9	9	9	9	9	2	2
9	9	9	9	9	9	9	9	#NULL!	#NULL!
2	9	9	9	9	9	9	9	2	2
9	9	9	9	9	9	9	9	#NULL!	#NULL!
2	2	2	2	2	1	1	1	1	1
1	2	2	2	1	1	1	1	1	1
1	1	1	2	2	2	2	1	2	2
3	1	1	1	1	2	3	2	1	1
2	1	1	1	1	1	1	2	1	1
2	1	1	1	2	1	1	1	1	1
1	1	1	1	1	2	2	2	1	1
9	9	9	9	9	9	9	9	1	1
1	1	1	1	1	1	1	1	1	1
3	1	2	2	3	3	1	3	1	2
2	1	1	1	1	1	1	2	1	1
1	1	1	2	1	1	1	1	2	2
1	2	2	1	1	1	1	1	1	1
3	1	1	1	1	1	1	3	2	2
3	1	9	3	2	1	3	9	2	2
1	1	1	2	1	1	1	1	2	2
9	9	9	9	9	9	9	9	2	2
9	9	9	9	1	9	3	3	#NULL!	2
9	9	9	9	9	9	9	9	#NULL!	#NULL!
2	1	1	1	2	2	2	1	1	1
2	1	2	2	2	2	2	2	1	2
2	2	2	1	1	1	1	1	2	2
2	1	1	2	2	1	1	2	1	2
2	2	2	2	2	2	2	2	2	2
1	1	1	2	2	2	2	2	1	2
1	1	1	1	1	1	1	1	2	2
1	1	1	1	1	1	1	1	2	2
1	1	1	1	1	1	1	1	2	2
2	2	2	2	2	2	2	2	2	2
1	1	1	1	1	1	1	9	2	2
1	1	3	1	1	3	3	3	1	1
1	1	1	1	2	2	3	3	2	2
1	1	1	1	2	2	2	1	1	1
1	1	2	2	2	1	1	1	1	1
2	1	2	2	3	3	1	3	1	2

9	9	9	9	9	9	9	9	#NULL!	#NULL!
1	1	1	1	1	1	1	1	1	1
9	9	1	9	9	9	9	9	2	2
9	9	9	2	9	9	9	9	1	2
9	9	9	1	9	9	9	9	1	1
9	9	9	9	9	9	9	9	1	2
9	9	9	9	9	9	9	9	2	2
2	9	3	2	1	2	3	1	2	2
9	9	9	9	9	9	9	9	2	2
2	2	2	2	2	2	2	2	2	2
9	9	9	9	9	9	9	9	#NULL!	#NULL!
9	9	9	9	9	9	9	9	2	2
1	1	1	1	1	1	1	1	2	2
9	9	9	9	9	9	9	9	1	1
1	1	1	1	2	2	1	1	1	1
9	9	9	9	9	9	9	9	#NULL!	#NULL!
2	2	1	1	1	3	3	9	2	2
3	3	3	3	3	3	3	3	2	1
9	9	9	9	9	9	9	9	2	2
2	2	2	2	2	2	2	2	1	2
2	2	2	2	2	2	2	2	2	2
2	2	2	2	2	2	3	2	1	1
2	2	9	2	2	2	2	2	#NULL!	#NULL!
2	2	2	2	2	2	2	2	2	2
1	1	1	1	1	1	1	2	2	2
2	2	2	2	2	2	2	2	2	2
2	3	1	2	3	3	2	2	2	2
2	2	3	1	1	1	2	2	2	2
2	2	2	2	2	2	2	2	1	1
1	1	1	1	2	2	2	2	2	2
9	9	9	9	9	9	9	9	#NULL!	#NULL!
3	3	2	2	3	2	2	2	2	2
9	9	9	9	9	9	9	9	#NULL!	#NULL!
2	2	2	2	2	2	2	2	2	2
2	2	2	2	2	2	2	2	2	2
1	1	1	1	1	1	1	1	1	1
1	1	1	2	2	2	2	2	1	2
1	1	1	1	1	1	1	1	1	1
1	1	1	1	1	1	1	1	1	1
9	9	9	9	9	9	9	9	1	1
9	1	1	1	1	3	3	3	1	1
1	2	2	2	1	1	1	1	1	1
9	9	9	9	9	9	9	9	#NULL!	#NULL!
9	9	9	9	9	9	9	9	2	1
1	9	9	9	9	9	9	9	1	1
9	9	9	9	9	9	9	9	#NULL!	#NULL!
9	9	9	9	9	9	9	9	1	2

9	9	9	9	9	9	9	9	1	1
2	2	2	2	2	2	2	2	2	1
9	9	9	9	9	9	9	9	2	2
9	9	9	9	9	9	9	9	2	1
9	9	9	9	9	9	9	9	1	1
2	1	1	2	3	1	2	2	2	2
3	3	1	1	3	3	3	3	2	2
9	9	9	9	9	9	9	9	#NULL!	#NULL!
9	9	9	9	9	9	9	9	#NULL!	#NULL!
9	9	9	9	9	9	9	9	2	#NULL!
9	9	9	9	9	9	9	3	2	2
9	9	9	9	9	9	9	9	2	2
2	1	2	2	2	2	2	2	1	2
9	9	9	9	9	9	9	9	#NULL!	#NULL!
9	9	9	9	9	9	1	9	2	2
2	1	3	2	1	2	3	2	1	2
1	2	2	3	1	2	3	1	2	2
3	1	3	1	3	3	3	3	2	2
2	2	2	3	3	2	3	3	1	1
2	3	1	1	3	2	3	1	2	1
2	3	3	2	2	1	2	2	2	2
3	1	1	1	1	3	3	1	2	2
1	2	2	3	3	3	2	1	1	2
9	9	9	9	9	9	9	9	2	2
2	2	2	2	2	2	2	2	2	2
1	1	2	1	1	1	3	1	1	1
9	1	9	9	9	9	9	9	2	2
2	2	2	1	1	1	1	2	1	1
1	1	2	9	1	2	2	1	1	#NULL!
1	1	2	2	1	1	2	3	1	2
1	1	1	3	3	1	1	2	2	2
2	3	2	2	3	2	2	2	1	2
9	9	9	9	9	9	9	9	#NULL!	#NULL!
3	1	3	2	1	3	1	2	2	1
3	3	3	1	3	3	3	3	2	2
1	1	1	2	2	2	2	9	2	2
2	3	3	3	2	2	2	2	2	2
1	3	3	3	3	2	2	2	1	2
1	9	9	9	9	9	9	9	2	2
9	9	9	9	9	9	9	9	2	2
9	9	9	9	2	9	9	9	2	2
9	9	9	9	9	9	9	9	2	2
2	2	2	2	2	2	3	3	2	2
9	9	9	2	9	9	9	9	2	2
1	1	1	1	1	1	1	1	1	1
1	1	3	3	1	3	1	3	1	1
1	3	3	3	1	1	1	3	1	1

3	3	3	3	3	3	3	3	2	2
1	9	1	9	9	9	1	9	2	2
2	2	2	2	2	2	1	2	2	2
3	1	3	1	1	1	3	3	1	1
1	3	1	3	1	3	1	3	2	2
1	1	1	3	1	3	1	1	2	2
3	9	2	1	3	2	1	3	1	1
9	9	9	9	9	9	9	9	2	1
3	3	3	3	3	2	2	2	2	2
3	2	3	2	3	2	2	2	1	2
1	2	1	1	1	2	3	3	2	2
2	3	2	1	2	3	2	2	2	2
1	3	2	3	2	1	2	3	2	2
2	3	1	2	3	1	2	3	2	2
2	3	1	2	1	2	3	1	1	1
2	2	2	2	2	2	2	2	2	2
2	2	2	2	2	2	2	2	2	2
2	1	2	1	2	1	3	2	2	2
2	2	2	2	2	2	2	2	2	2
2	2	2	2	2	2	2	2	2	2
3	1	1	3	3	3	2	3	2	2
1	1	2	2	2	2	3	3	2	1
3	3	9	9	9	9	9	9	2	#NULL!
9	9	9	9	9	9	9	9	#NULL!	#NULL!
2	2	2	2	3	3	3	1	2	2
1	2	2	1	2	2	1	1	#NULL!	#NULL!
9	9	9	9	9	9	9	9	2	2
1	1	3	1	2	1	1	3	#NULL!	#NULL!
3	1	2	1	1	3	3	9	1	2
9	1	9	9	9	9	9	9	2	2
2	9	1	3	2	3	2	2	1	2
3	3	3	3	3	3	3	3	1	1
1	3	1	2	2	3	2	2	2	2
2	1	3	2	3	2	2	3	2	2
9	9	9	9	9	9	9	9	1	1
2	2	2	2	2	2	2	2	2	2
2	2	2	2	2	2	2	2	2	2
9	9	9	9	9	9	9	9	#NULL!	#NULL!
2	2	1	1	2	2	2	3	2	2
2	1	2	2	2	2	2	2	1	1
9	9	9	9	9	9	9	9	#NULL!	#NULL!
9	9	9	9	9	9	9	9	#NULL!	#NULL!
2	2	3	1	2	1	2	1	2	1
9	9	9	9	9	9	9	9	1	2
2	2	2	2	2	2	2	2	2	2
3	1	1	1	3	1	3	3	2	2
9	9	9	9	9	9	9	9	#NULL!	#NULL!

2	3	2	2	2	3	2	2	2	2
9	9	9	9	9	9	9	9	#NULL!	#NULL!
1	1	1	1	1	1	1	1	2	2
9	9	9	9	9	9	9	9	#NULL!	#NULL!
9	9	9	9	9	9	9	9	#NULL!	#NULL!
2	2	2	2	2	2	2	2	1	1
3	9	1	2	3	3	3	3	2	2
2	2	2	2	2	2	2	2	2	2
2	2	2	2	2	2	2	1	1	2
2	2	2	2	2	2	2	2	2	2
9	9	9	9	9	9	9	9	#NULL!	#NULL!
2	2	1	2	2	3	3	1	2	2
9	9	9	9	9	9	9	9	#NULL!	#NULL!
1	1	1	1	2	2	2	1	1	1
2	2	2	2	2	2	2	2	2	2
2	2	1	1	1	1	1	1	2	2
9	9	9	9	9	9	9	1	2	2
9	9	9	9	9	9	9	9	#NULL!	#NULL!
3	2	2	2	2	2	3	3	2	2
3	3	2	2	2	3	3	2	2	2
2	2	1	1	1	1	2	2	2	2
1	1	2	2	3	3	3	3	2	2
1	9	9	9	9	9	9	9	2	2
2	3	2	3	2	2	3	3	2	2
3	3	3	3	3	3	3	3	1	2
2	2	2	2	2	2	2	2	2	2
9	9	9	9	9	9	9	9	2	1
1	1	1	1	1	1	1	1	1	1
2	2	2	2	2	2	2	2	2	2
9	9	9	9	9	9	9	9	1	2
3	1	2	3	1	3	1	3	2	2
3	1	1	1	3	2	3	3	2	2
3	1	1	1	2	2	3	3	1	2
2	2	2	2	2	2	2	2	2	2
2	2	2	2	2	2	2	2	2	2
2	3	1	2	3	1	2	3	2	1
1	1	2	2	2	1	2	3	1	1
2	1	2	2	2	2	2	3	2	2
2	2	2	2	2	2	2	3	2	2
3	3	3	3	3	3	3	3	2	2
1	1	3	1	1	3	2	3	2	2
3	1	3	1	3	3	3	3	2	2
9	9	9	9	9	9	9	9	2	1
2	2	2	2	2	2	2	2	2	2
2	2	2	2	2	2	2	3	2	2
2	2	2	2	2	2	3	3	2	2
2	1	1	3	3	1	3	1	#NULL!	#NULL!

3	3	3	3	3	3	2	3	2	2
2	2	2	1	2	2	2	3	2	2
1	1	2	2	1	1	1	1	1	1
2	2	2	2	2	2	2	2	2	2
2	2	1	2	2	2	2	1	2	2
9	9	9	9	9	9	9	9	2	2
2	2	2	2	2	2	2	2	2	2
1	1	1	1	1	1	1	1	1	1
2	2	2	2	2	2	2	3	2	2
2	2	2	2	2	2	2	2	2	2
3	1	2	2	2	2	2	3	2	2
2	3	1	2	3	1	2	3	2	2
2	2	2	2	2	2	2	2	2	2
3	3	3	3	3	3	3	3	2	2
2	3	2	2	2	3	3	1	1	1
2	2	2	3	2	2	3	2	2	2
2	1	2	3	2	2	1	2	2	2
2	1	3	1	1	1	2	2	2	2
2	2	2	2	2	2	2	2	2	2
2	9	1	3	2	1	2	1	2	1
2	2	2	2	2	2	2	2	1	1
3	2	2	2	2	3	2	2	2	2
1	1	3	3	3	2	2	2	2	2
2	2	2	2	2	2	2	2	#NULL!	1
3	1	3	1	3	3	3	3	2	2
2	2	2	2	2	2	2	2	2	2
3	3	3	3	3	1	1	3	2	2
3	3	3	3	3	1	1	3	2	2
2	2	2	2	2	2	2	2	2	2
1	2	2	2	1	1	1	1	1	1
2	2	3	3	3	2	2	2	2	2
3	2	2	3	2	3	2	3	2	2
1	1	1	2	3	3	3	3	2	2
2	2	2	2	2	2	2	2	2	#NULL!
1	1	1	1	1	2	1	1	1	2
2	2	2	2	2	2	2	2	2	2
2	2	2	2	2	2	2	2	2	2
2	2	3	3	3	3	2	3	2	2
2	2	2	2	2	2	2	2	2	2
2	2	2	2	2	2	2	9	2	2
2	2	2	2	2	2	1	3	2	2
3	3	3	3	3	3	2	3	1	1
3	1	2	3	2	3	2	2	1	1
2	2	2	2	2	2	2	2	1	2
1	2	2	3	1	2	3	2	2	2
3	1	1	1	1	2	2	3	1	1
9	9	9	9	9	9	9	9	1	2

2	2	2	3	2	1	2	3	2	2
1	1	2	2	9	3	3	2	2	2
2	1	2	2	3	3	3	3	1	2
2	2	2	2	2	2	2	2	2	2
2	2	2	2	2	2	2	2	2	2
2	2	2	2	2	2	2	2	2	2
2	1	3	9	9	9	9	9	2	2
2	2	2	1	3	3	2	2	1	2
2	2	2	2	2	2	2	1	2	2
2	2	3	1	1	3	2	3	2	2
2	2	2	2	2	2	2	2	2	2
9	9	9	9	9	9	9	9	2	2
9	9	9	9	9	9	9	9	#NULL!	#NULL!
2	2	2	2	2	2	2	2	1	2
2	1	1	1	1	3	3	1	1	2
2	1	1	2	2	2	2	2	1	1
3	1	2	1	3	3	3	3	2	2
2	1	1	2	2	2	2	2	1	1
1	1	2	2	2	3	3	3	1	2
2	1	2	1	3	2	3	2	1	2
9	9	9	9	9	9	9	9	1	1
2	2	2	2	2	3	3	1	2	2
2	2	2	2	2	2	2	3	2	1
2	2	2	2	2	2	2	2	2	2
2	1	1	2	2	2	2	2	1	1
9	9	9	9	9	9	9	9	2	2
2	2	2	2	2	2	2	2	2	2
2	2	2	2	2	2	2	2	2	2
1	2	2	2	2	2	2	2	1	2
9	9	9	9	9	9	9	9	#NULL!	#NULL!
3	3	3	3	3	3	3	3	2	2
9	9	9	9	9	9	9	9	1	2
2	2	3	1	2	2	2	2	2	1
2	2	2	2	2	2	2	2	2	2
2	2	1	2	2	2	2	3	2	2
2	2	2	2	2	2	2	2	2	2
9	9	9	9	9	9	9	9	2	2
2	2	2	2	2	2	2	2	2	2
2	2	2	2	2	2	2	2	1	2
2	2	2	2	2	2	2	2	1	2
2	2	2	2	2	2	2	2	2	2
2	2	2	2	2	2	2	2	2	2
2	2	2	2	2	2	2	2	1	1
2	2	2	2	3	2	2	3	2	2
2	2	2	2	2	2	2	2	2	2
2	3	2	3	3	2	2	2	2	2
2	2	2	2	2	2	2	2	2	2

2	2	2	2	2	2	2	2	1	1
2	1	2	2	2	2	2	2	1	2
3	1	3	1	3	3	3	3	2	2
2	2	2	2	2	2	2	2	1	2
2	2	2	2	2	2	2	2	2	2
2	1	3	2	2	2	2	2	2	2
9	9	9	9	9	9	9	9	2	2
3	1	2	1	3	3	3	3	1	1
1	1	1	2	1	1	2	3	1	1
3	1	3	1	3	3	3	3	1	2
2	1	3	1	3	3	3	3	1	2
9	9	9	9	9	9	9	9	2	2
2	2	2	2	2	2	2	2	2	2
1	1	1	2	2	2	2	9	1	1
9	9	9	9	9	9	9	9	1	1
9	9	9	9	9	9	9	9	1	2
9	9	9	9	9	9	9	9	1	2
1	1	1	3	3	3	2	3	1	2
1	1	1	1	1	1	1	1	2	2
9	9	9	9	9	9	9	9	1	2
1	1	1	1	2	1	1	1	1	1
2	2	1	2	3	3	1	1	1	1
2	2	2	2	2	2	2	2	2	2
2	2	2	2	2	2	2	2	2	2
2	2	2	2	2	2	2	9	#NULL!	#NULL!
2	2	2	2	2	2	2	2	2	2
9	9	9	9	9	9	9	9	#NULL!	#NULL!
3	1	1	2	3	3	2	2	2	2
9	9	9	9	9	9	9	9	2	2
9	9	9	9	9	9	9	9	2	2
2	2	2	2	2	2	2	2	2	2
2	1	1	2	1	1	1	1	1	1
9	9	9	9	9	9	9	9	#NULL!	#NULL!
9	9	9	9	9	9	9	9	1	1
9	9	9	9	9	9	9	9	#NULL!	#NULL!
9	9	9	9	9	9	9	9	#NULL!	#NULL!
2	2	2	2	2	2	3	3	2	1
3	1	2	2	1	3	3	3	1	1
9	9	9	9	9	9	9	9	2	2
2	2	3	3	2	2	2	2	2	2
2	1	2	3	2	2	3	3	1	2
3	1	2	2	2	1	2	3	1	2
2	1	2	1	2	2	1	2	2	2
2	2	2	2	2	2	2	2	2	2
1	2	2	2	2	2	2	1	1	1
2	1	2	1	2	2	2	2	1	1
2	1	2	1	3	1	2	1	2	2

2	1	3	2	1	3	2	2	1	2
2	1	1	2	3	3	3	2	#NULL!	#NULL!
1	9	2	3	1	3	2	1	2	2
2	2	1	2	1	2	1	3	1	1
1	3	2	1	3	2	2	3	1	1
2	2	3	2	3	2	2	2	2	2
3	1	1	1	2	3	3	3	2	2
9	9	9	9	9	9	9	2	2	2
2	2	2	2	2	2	2	2	2	2
2	2	2	2	2	2	2	2	1	2
2	9	1	2	2	2	3	3	1	2
2	1	2	2	2	2	3	3	1	2
2	2	2	1	2	3	2	3	#NULL!	#NULL!
9	9	9	9	9	9	9	9	1	2
2	2	2	2	2	2	2	2	1	1
2	2	2	2	2	2	2	2	2	2
2	2	2	2	2	2	2	2	1	1
3	1	2	1	1	3	3	3	2	2
2	1	1	1	3	3	1	2	1	1
2	1	1	2	2	3	3	3	1	1
2	1	1	1	3	3	2	3	1	1
3	1	3	1	2	3	3	3	1	1
1	1	3	2	3	3	3	3	2	2
9	1	2	3	3	3	3	3	2	2
1	3	3	2	3	9	3	2	1	2
1	1	3	1	1	1	1	3	1	1
1	1	1	1	1	1	1	1	2	2
2	1	1	1	1	1	2	2	1	2
3	1	1	1	3	3	3	2	1	1
9	1	9	9	9	9	9	9	1	1
3	1	3	3	3	3	3	3	1	1
9	9	9	9	9	9	9	9	2	2
9	9	9	9	9	9	9	9	#NULL!	#NULL!
9	9	9	9	9	9	9	9	#NULL!	#NULL!
9	9	9	9	9	9	9	9	#NULL!	#NULL!
2	2	2	2	2	2	2	2	2	2
1	1	1	1	2	2	2	3	1	1
9	9	9	9	9	9	9	9	#NULL!	#NULL!
1	2	2	1	1	2	2	1	2	2
1	2	2	1	1	2	2	1	2	2
9	9	9	9	9	9	9	9	1	1
3	3	3	3	3	3	3	3	2	2
2	2	2	2	2	2	2	2	2	2
3	3	3	2	2	2	3	2	2	2
9	9	9	9	9	9	9	9	#NULL!	#NULL!
1	1	1	1	1	1	1	1	1	1
2	2	2	2	2	2	2	2	2	2

1	1	1	1	1	1	1	1	2	2
3	1	2	1	3	3	3	3	2	2
3	3	2	1	1	1	1	2	1	2
2	2	2	2	2	2	2	2	2	2
9	9	9	9	9	9	9	9	#NULL!	#NULL!
9	9	9	9	9	9	9	9	#NULL!	#NULL!
2	2	2	2	2	2	2	2	2	2
2	2	2	2	2	2	2	2	2	2
9	9	9	9	9	9	9	9	1	2
9	9	9	9	9	9	9	9	2	2
1	1	1	1	2	2	2	1	1	1
1	1	1	1	1	1	1	1	1	2
9	9	9	9	9	9	9	9	2	2
2	3	2	3	2	3	3	2	2	2
1	1	9	9	9	9	9	9	#NULL!	#NULL!
3	1	2	2	3	1	1	2	2	2
3	1	2	2	3	2	3	3	2	2
1	3	2	1	2	2	2	3	2	2
9	9	9	9	9	3	9	9	2	2
3	1	3	3	1	1	2	3	2	2
2	1	3	2	1	1	3	1	2	1
1	1	1	1	1	1	1	1	1	2
2	1	1	1	3	3	3	2	#NULL!	#NULL!
2	1	1	1	2	2	2	2	1	2
1	1	1	3	3	3	3	3	2	2
2	2	9	2	2	2	2	2	2	2
9	9	9	9	9	9	9	9	2	2
3	1	1	1	1	3	3	3	2	2
1	2	2	3	1	1	2	2	2	1
3	3	1	3	1	3	1	3	2	2
2	2	2	2	2	2	2	2	2	2
3	1	3	2	2	2	3	3	2	2
2	3	2	1	2	3	2	1	2	2
3	1	3	2	3	3	3	3	1	1
9	9	9	1	9	9	9	3	2	2
1	2	1	2	1	2	2	3	2	2
3	1	1	3	3	1	3	3	2	2
2	2	2	2	2	2	2	2	2	2
9	9	9	9	9	9	9	9	2	2
9	9	9	9	9	9	9	9	#NULL!	#NULL!
9	9	9	9	9	9	9	9	#NULL!	#NULL!
2	2	3	1	2	3	2	9	2	1
2	2	1	2	2	1	1	2	2	2
1	1	2	1	1	3	3	3	1	1
1	1	2	1	1	3	3	3	1	1
3	3	3	3	2	1	1	2	#NULL!	#NULL!
2	3	2	3	2	3	3	3	2	2

2	2	2	2	2	2	2	2	1	2
9	9	9	9	9	9	9	9	2	2
2	2	2	2	2	2	2	2	2	2
3	3	2	3	2	2	3	3	1	2
9	9	9	9	9	9	9	9	2	2
9	9	9	9	9	9	9	9	#NULL!	#NULL!
1	1	1	1	1	1	3	3	1	#NULL!
9	9	9	9	9	9	9	9	2	2
1	9	3	3	9	1	1	9	1	1
2	2	2	2	2	2	2	2	1	2
2	2	2	1	2	2	2	2	1	1
1	3	1	2	3	1	2	3	2	2
2	2	2	3	2	2	3	2	2	2
3	1	3	3	1	3	3	3	2	2
9	9	9	9	9	9	9	9	#NULL!	#NULL!
3	1	1	3	1	1	3	3	2	1
2	2	2	2	2	2	2	2	2	2
2	2	2	2	2	2	2	2	1	2
3	2	2	2	1	1	1	3	2	2
2	9	1	3	2	2	1	3	1	2
9	9	9	9	9	9	9	9	#NULL!	#NULL!
9	9	9	9	9	9	9	9	#NULL!	#NULL!
3	3	2	3	3	2	2	3	2	2
9	9	9	9	9	9	9	9	2	2
9	9	9	9	9	9	9	9	#NULL!	#NULL!
3	2	3	2	3	2	3	2	2	2
3	2	2	2	3	2	1	2	2	2
2	1	2	1	1	2	2	3	2	2
3	3	3	3	3	3	3	3	2	2
2	1	1	1	2	3	3	3	2	2
3	3	3	3	3	3	3	3	2	2
2	1	1	1	3	3	3	3	2	2
9	9	9	9	9	9	9	9	#NULL!	#NULL!
9	9	9	9	9	9	9	9	2	2
9	9	9	9	9	9	9	9	#NULL!	#NULL!
1	1	1	1	1	3	1	3	1	2
3	1	1	1	1	3	3	1	1	1
2	2	9	3	3	2	2	2	2	2
3	1	3	1	3	3	3	3	2	2
9	9	9	9	9	9	9	9	2	2
2	2	2	3	3	3	2	2	2	2
2	3	2	2	1	2	2	1	1	2
3	3	3	3	3	3	3	9	1	2
9	9	9	9	9	9	9	9	2	2
9	9	9	9	9	9	9	9	1	1
3	1	3	1	3	3	3	3	2	2
1	2	3	2	1	3	2	3	2	2

2	1	3	1	2	1	1	2	2	2
2	3	3	3	3	2	2	2	2	2
3	3	3	3	1	9	3	1	2	2
2	3	1	2	3	1	2	3	2	2
3	1	3	1	3	3	3	3	2	2
1	1	2	2	1	2	2	2	1	2
2	2	2	1	2	2	1	9	2	2
1	1	3	1	3	3	3	3	2	2
9	9	9	9	9	9	9	9	#NULL!	#NULL!
1	2	3	2	2	2	2	9	1	1
2	1	2	2	2	2	2	2	2	2
2	2	2	2	2	2	2	2	2	1
3	1	3	1	3	3	3	3	1	2
2	1	1	1	2	2	3	3	1	1
9	9	9	9	9	9	9	9	2	2
2	3	3	3	3	3	3	3	2	1
1	1	2	1	1	1	1	2	1	1
2	3	3	3	2	2	2	2	2	2
3	1	2	1	3	2	2	2	2	2
2	2	2	2	2	2	2	2	2	2
1	2	1	1	2	1	2	2	2	2
1	1	1	3	1	3	3	3	1	1
3	2	2	1	2	2	1	9	2	2
3	3	3	3	3	3	3	3	2	2
9	9	9	9	9	9	9	9	2	1
2	3	1	1	1	1	1	1	1	1
1	3	2	2	1	3	1	2	1	1
2	1	2	2	2	2	2	2	1	2
2	2	2	2	2	2	2	2	1	2
1	1	3	1	3	3	3	3	2	2
2	1	2	2	2	2	2	2	2	1
2	2	2	2	2	2	2	2	2	1
1	1	3	2	3	3	3	3	2	2
2	1	2	2	2	1	1	1	2	2
2	3	2	3	2	2	3	3	1	2
3	1	1	2	3	2	3	3	2	2
1	1	1	1	2	2	2	1	2	1
1	1	1	1	1	1	2	1	2	#NULL!
3	1	1	9	3	2	1	3	1	1
1	2	2	2	2	3	3	2	1	1
2	3	3	3	3	3	2	2	1	2
1	1	1	2	2	3	3	3	1	1
1	2	2	2	1	1	1	1	2	2
2	2	2	2	1	2	2	2	2	2
2	2	2	1	1	1	1	1	2	1
2	1	1	1	1	2	1	1	2	1
1	1	2	1	1	2	1	1	1	1

1	2	2	1	2	2	2	1	1	1
2	2	1	2	1	1	2	1	2	1
2	1	3	1	1	2	2	1	1	2
1	1	2	1	2	1	3	1	2	1
2	2	2	2	1	1	1	1	1	1
2	1	1	1	2	2	1	1	1	1
2	1	2	1	2	2	3	3	2	1
1	1	1	1	2	2	2	1	1	2

HeardSme	HeardHPV	PadDetCaC	PapMarry2	PapOver18	Pap2yearsS	PapMeno2	PapFromH	Pap2Yearly	PapOnce2
2	2	2	2	2	2	2	2	2	2
2	2	2	2	2	1	2	2	2	1
1	2	2	2	2	2	2	2	2	2
2	1	2	2	2	1	2	2	2	1
2	2	2	2	2	1	2	2	2	1
2	2	2	2	#NULL!	1	2	2	2	1
2	2	#NULL!	#NULL!	2	#NULL!	#NULL!	#NULL!	#NULL!	#NULL!
2	2	2	2	2	2	2	2	2	2
2	2	2	#NULL!	2	#NULL!	2	#NULL!	2	#NULL!
2	2	2	#NULL!	#NULL!	#NULL!	#NULL!	#NULL!	#NULL!	#NULL!
2	#NULL!	2	2	2	2	2	2	2	2
2	2	1	2	1	1	2	2	1	2
2	1	2	2	2	1	2	1	2	2
1	2	2	2	2	2	2	2	2	2
2	2	2	2	2	2	2	1	2	1
2	2	2	2	2	2	2	2	2	2
1	2	2	2	#NULL!	2	2	1	2	2
2	2	2	2	2	2	2	2	2	2
2	2	2	2	2	2	2	2	2	2
1	1	2	2	2	2	2	2	2	2
2	2	2	1	2	2	2	2	2	1
2	2	2	1	2	2	2	2	2	1
2	2	1	2	#NULL!	2	2	2	2	2
2	2	2	2	1	1	2	2	2	2
2	2	2	2	2	2	2	2	2	2
2	2	2	2	2	2	2	2	2	2
2	2	2	2	2	2	1	1	2	2
1	2	2	1	2	2	2	2	2	2
2	2	2	2	2	2	2	2	2	2
#NULL!	2	2	2	2	2	2	2	2	2
2	2	2	2	2	2	2	2	2	2
2	2	2	2	2	2	2	2	2	2
2	2	2	2	2	2	2	2	2	2
1	1	2	2	2	2	1	2	2	2
2	2	2	2	2	2	2	2	2	2
2	1	2	2	2	2	1	2	2	2
2	2	2	2	2	2	2	2	2	2
1	1	2	2	2	1	2	2	2	2
#NULL!	#NULL!	2	2	2	2	2	2	2	2
2	2	2	2	2	2	2	2	2	2
2	2	2	2	2	2	2	2	2	2
2	2	2	2	2	2	2	2	2	2
#NULL!	#NULL!	2	1	2	1	2	#NULL!	2	1
1	2	2	2	2	1	2	2	1	2
2	2	#NULL!	#NULL!	2	2	2	2	1	2
2	1	2	1	2	1	2	#NULL!	2	1

2	2	2	2	2	2	2	2	2	2
2	1	2	2	2	2	2	2	2	2
1	1	2	2	2	2	2	2	2	2
2	1	2	2	2	2	2	2	2	2
1	1	2	2	1	2	2	2	2	2
1	1	2	2	1	1	2	2	2	2
1	1	2	2	1	1	2	2	2	2
1	2	2	2	2	#NULL!	1	1	#NULL!	#NULL!
2	2	2	#NULL!	2	#NULL!	1	1	#NULL!	#NULL!
1	2	2	2	1	2	1	2	1	1
2	2	2	2	2	2	2	2	2	2
2	1	2	2	2	2	2	2	2	2
2	2	2	2	2	2	2	2	2	2
2	2	2	2	2	2	2	2	2	2
2	2	2	2	2	2	2	2	2	2
1	2	2	2	2	2	2	2	2	2
2	1	2	2	2	2	2	2	2	2
1	1	2	2	2	2	2	1	2	2
2	2	2	1	2	1	2	2	2	1
1	2	2	#NULL!	#NULL!	#NULL!	#NULL!	#NULL!	#NULL!	#NULL!
2	1	2	2	1	1	2	2	2	2
2	2	2	2	1	1	2	2	2	2
2	1	2	2	1	1	2	2	2	2
2	1	2	2	2	2	2	2	2	2
2	2	#NULL!	1	#NULL!	#NULL!	1	#NULL!	#NULL!	1
2	1	2	2	2	2	1	1	2	1
#NULL!	#NULL!	#NULL!	#NULL!	#NULL!	#NULL!	#NULL!	#NULL!	#NULL!	#NULL!
2	2	2	2	2	2	2	2	2	2
2	2	2	2	2	2	2	2	2	2
1	1	2	2	2	2	2	1	2	2
1	1	2	2	#NULL!	#NULL!	#NULL!	#NULL!	#NULL!	#NULL!
1	2	2	2	1	1	2	1	1	2
#NULL!	#NULL!	2	2	#NULL!	#NULL!	#NULL!	#NULL!	#NULL!	#NULL!
2	1	#NULL!	2	1	1	2	2	1	2
1	1	2	2	2	2	2	2	2	2
2	1	#NULL!	#NULL!	#NULL!	#NULL!	#NULL!	#NULL!	#NULL!	#NULL!
#NULL!	#NULL!	#NULL!	#NULL!	#NULL!	#NULL!	#NULL!	#NULL!	#NULL!	#NULL!
#NULL!	#NULL!	2	2	1	2	2	2	2	2
1	1	2	2	2	1	1	2	1	1
1	1	2	2	2	2	2	2	2	2
1	1	2	#NULL!	2	2	2	2	2	2
2	2	2	2	#NULL!	#NULL!	#NULL!	#NULL!	#NULL!	#NULL!
2	2	2	1	2	1	2	2	2	2
1	1	2	2	2	2	2	2	2	2
2	2	2	2	2	2	2	2	2	2
2	1	#NULL!	#NULL!	#NULL!	#NULL!	#NULL!	#NULL!	#NULL!	#NULL!
2	#NULL!	#NULL!	#NULL!	#NULL!	#NULL!	#NULL!	#NULL!	#NULL!	#NULL!

2	1	2	2	2	2	2	1	2	2
1	1	2	2	1	2	2	2	2	2
1	1	2	1	2	2	1	1	2	1
1	1	2	2	2	2	2	2	2	2
2	2	#NULL!	#NULL!	#NULL!	#NULL!	#NULL!	#NULL!	#NULL!	#NULL!
2	2	2	2	2	2	2	2	2	2
1	1	2	2	1	2	2	1	2	2
1	2	2	2	1	2	2	2	2	2
1	1	2	2	2	2	2	2	2	2
1	1	2	2	2	2	2	2	2	2
2	1	2	2	2	2	1	2	#NULL!	2
1	2	2	2	2	2	2	2	1	2
2	2	2	2	2	2	1	1	2	2
2	2	2	2	2	2	2	1	2	#NULL!
1	2	2	2	2	2	2	2	2	2
2	2	1	2	2	2	2	2	2	2
2	1	2	2	2	2	2	2	2	2
2	2	2	2	2	2	2	2	2	2
2	2	2	2	2	2	2	1	2	2
2	2	2	2	2	2	2	2	2	2
2	2	2	2	1	1	2	2	2	2
2	2	2	2	2	2	2	2	2	2
2	2	2	2	2	2	2	2	2	2
2	2	1	2	1	1	2	2	1	2
2	1	1	1	2	1	2	2	2	2
2	2	2	2	2	2	2	2	2	2
1	2	2	2	2	2	2	1	2	2
1	1	2	2	2	2	2	2	2	2
2	2	2	2	2	2	2	2	2	2
2	2	2	2	2	2	2	2	2	2
2	2	2	2	2	2	2	2	2	2
2	2	2	2	2	2	2	2	2	2
2	2	2	2	2	2	2	2	2	2
2	2	2	2	1	1	2	2	2	2
2	2	2	2	2	2	2	2	2	2
2	2	2	2	2	2	2	2	2	2
2	2	2	2	2	2	2	2	2	2
2	2	2	2	2	2	2	2	2	2
2	2	#NULL!	1	#NULL!	#NULL!	#NULL!	2	#NULL!	#NULL!
2	2	2	2	2	2	2	2	1	2
2	2	2	2	2	2	2	2	2	2
2	2	2	2	2	2	2	2	2	2
2	2	2	2	2	2	2	2	2	2
2	2	2	2	2	2	1	1	1	2
2	2	2	2	2	2	2	2	2	1
2	2	2	2	2	2	2	2	2	2
2	2	2	2	2	2	2	2	2	2

2	2	2	2	2	2	2	1	2	1
2	2	2	2	2	2	1	2	2	2
2	2	#NULL!	2	#NULL!	2	1	2	2	2
2	2	2	2	2	2	2	2	2	2
2	2	#NULL!	#NULL!	#NULL!	#NULL!	#NULL!	#NULL!	#NULL!	#NULL!
2	2	2	2	2	2	2	2	2	2
2	2	2	2	1	1	2	2	1	2
2	2	2	2	2	2	2	2	2	2
2	2	2	2	2	2	2	2	2	2
2	2	2	2	2	2	2	2	2	2
2	2	2	1	2	2	2	2	2	2
2	#NULL!	2	2	2	1	2	2	2	2
2	2	#NULL!	2	#NULL!	#NULL!	#NULL!	#NULL!	#NULL!	#NULL!
2	2	2	2	2	2	2	2	2	2
2	2	2	2	2	2	2	2	2	2
2	2	2	2	2	2	2	2	2	2
2	1	#NULL!	#NULL!	#NULL!	#NULL!	#NULL!	#NULL!	#NULL!	#NULL!
2	2	2	2	2	2	2	2	2	2
2	2	#NULL!	#NULL!	#NULL!	#NULL!	1	#NULL!	#NULL!	#NULL!
2	2	2	2	1	2	2	2	2	2
2	2	2	2	2	2	2	2	2	2
2	2	2	2	2	2	2	2	2	2
2	2	2	2	2	2	2	2	2	2
2	2	2	2	2	1	2	2	2	2
2	2	2	2	2	2	2	2	2	2
2	2	2	2	2	2	2	#NULL!	#NULL!	#NULL!
2	2	2	2	2	2	2	2	2	2
2	2	2	2	2	2	2	2	2	2
2	2	2	2	2	2	2	2	2	2
2	2	2	2	2	2	2	2	2	2
2	2	1	2	2	2	2	1	2	2
2	2	2	2	2	2	2	2	2	2
#NULL!	#NULL!	2	2	2	2	2	2	2	2
2	2	2	2	2	2	2	2	2	2
2	2	2	2	2	2	2	2	2	2
2	2	2	2	2	2	2	2	2	2
2	2	2	2	2	2	2	2	2	2
2	2	2	2	2	2	2	2	2	2
2	2	2	2	2	2	2	1	2	2
2	2	2	2	2	2	2	2	2	2
2	2	2	2	2	2	2	2	2	2
2	2	2	2	2	2	2	2	2	2
#NULL!	#NULL!	2	2	2	2	2	2	2	2
2	2	2	2	2	2	2	2	2	2
2	2	2	2	2	2	2	2	2	2
2	2	2	2	2	2	2	2	2	2
2	2	2	2	2	2	2	2	2	2

2	2	2	2	2	2	2	2	2	2
2	2	2	2	2	2	2	2	2	2
2	2	2	2	2	2	2	2	2	2
2	2	2	2	2	2	2	2	1	2
2	2	2	2	1	2	2	1	2	2
1	2	2	2	2	2	2	2	2	2
2	2	2	2	2	2	2	2	2	2
1	1	2	2	2	1	2	2	2	2
1	1	2	2	2	2	2	2	2	2
2	1	2	2	1	1	2	2	1	2
#NULL!	#NULL!	#NULL!	#NULL!	#NULL!	#NULL!	#NULL!	#NULL!	#NULL!	#NULL!
1	2	2	2	2	2	2	2	2	2
1	1	2	2	2	2	2	2	2	2
2	2	2	2	2	2	2	2	2	2
2	2	2	2	2	2	2	1	2	1
2	2	2	1	2	2	2	2	2	2
2	2	2	2	2	2	2	2	2	2
#NULL!	1	2	1	2	2	1	1	2	1
2	2	2	2	2	2	2	2	2	2
2	2	2	2	2	2	2	2	2	2
2	2	2	2	2	2	2	1	2	2
2	2	2	2	2	2	2	2	2	2
2	2	2	2	2	2	1	2	1	2
2	2	2	2	2	2	2	2	2	2
2	2	2	2	2	2	2	2	2	2
2	2	2	2	2	2	2	2	2	2
2	2	2	2	2	2	2	2	2	2
2	2	2	2	2	2	2	2	2	2
2	2	2	2	2	2	2	2	2	2
2	2	2	2	2	2	2	2	2	2
2	2	2	2	2	2	2	2	1	2
2	2	2	2	2	2	2	2	1	2
2	2	2	2	2	2	2	2	2	2
1	1	2	1	2	2	2	2	2	2
2	1	2	2	2	2	1	1	2	2
1	2	2	2	2	2	2	2	2	2
2	2	2	2	2	2	2	2	2	2
2	2	1	2	1	1	2	1	2	2
2	2	2	2	2	2	2	2	2	2
2	2	2	2	2	2	2	2	1	2
1	1	2	2	2	2	1	2	1	2
2	2	2	2	2	2	2	2	2	#NULL!
2	2	2	#NULL!	#NULL!	#NULL!	#NULL!	#NULL!	#NULL!	#NULL!
2	2	2	#NULL!	#NULL!	#NULL!	#NULL!	#NULL!	#NULL!	#NULL!
2	1	2	2	1	2	2	2	2	2
2	2	2	2	2	2	2	1	2	2
2	2	2	#NULL!	#NULL!	#NULL!	#NULL!	#NULL!	#NULL!	#NULL!

2	2	2	2	2	2	2	2	2	2
2	2	2	2	2	2	2	2	2	2
2	2	2	2	2	2	2	2	2	2
1	2	2	2	2	2	2	2	2	2
2	2	2	2	2	2	1	1	1	2
1	1	2	1	2	2	2	2	1	2
2	2	2	2	2	2	2	2	2	2
2	2	2	2	2	2	2	2	2	2
2	2	2	2	1	1	2	1	2	2
2	2	1	2	1	1	2	2	1	2
2	2	2	2	2	2	2	2	2	2
2	1	2	2	1	1	2	2	2	2
2	1	2	2	1	1	2	2	2	2
2	2	2	2	2	2	2	2	2	2
2	2	2	2	2	2	2	2	2	2
2	2	2	2	2	2	2	1	2	2
2	2	2	2	2	2	2	2	1	2
1	1	2	2	2	1	2	2	2	2
2	2	2	#NULL!	#NULL!	#NULL!	#NULL!	#NULL!	#NULL!	#NULL!
1	1	2	2	2	2	2	2	2	2
2	1	2	2	2	2	2	2	2	2
2	2	2	2	2	2	2	2	2	2
2	2	2	2	2	2	2	2	2	2
2	2	2	2	2	2	2	2	2	2
2	2	#NULL!	2	#NULL!	#NULL!	#NULL!	#NULL!	#NULL!	#NULL!
2	2	2	2	2	2	2	2	2	2
2	#NULL!	2	2	2	2	2	2	2	2
2	2	2	#NULL!	2	2	2	2	2	2
2	2	2	2	2	2	2	2	2	2
2	2	2	2	2	2	2	2	2	2
1	2	#NULL!	#NULL!	2	#NULL!	#NULL!	#NULL!	#NULL!	#NULL!
2	2	2	2	1	1	2	2	1	2
2	2	1	2	1	1	2	2	1	2
2	2	2	2	2	2	2	2	2	2
2	2	2	#NULL!	#NULL!	#NULL!	#NULL!	#NULL!	#NULL!	#NULL!
2	2	2	2	2	2	2	2	2	2
2	2	2	2	2	2	2	2	2	2
2	2	2	2	2	2	2	2	2	2
2	2	2	2	2	2	2	2	2	2
2	2	2	2	2	2	2	2	2	2
2	2	2	2	2	2	2	2	2	2
2	2	2	2	2	2	2	2	2	2
1	1	2	1	2	2	2	2	2	2
2	2	2	2	2	2	2	2	2	2
2	1	2	2	2	2	2	2	2	2
2	2	2	2	2	2	1	1	2	2
2	2	2	2	2	2	2	2	2	2

#NULL!	#NULL!	#NULL!	#NULL!	#NULL!	#NULL!	#NULL!	#NULL!	#NULL!	#NULL!
#NULL!	#NULL!	#NULL!	#NULL!	#NULL!	#NULL!	#NULL!	#NULL!	#NULL!	#NULL!
1	2	2	2	2	#NULL!	#NULL!	#NULL!	#NULL!	#NULL!
2	1	2	2	2	2	2	1	2	2
2	2	2	2	1	2	2	2	2	2
2	1	1	1	2	2	2	2	2	2
1	1	2	2	2	2	2	2	2	2
1	1	2	1	2	2	1	1	2	1
2	2	2	2	2	#NULL!	2	2	2	1
1	2	2	2	2	2	2	2	2	2
2	2	2	2	2	2	2	2	2	2
1	2	2	2	1	2	2	2	2	2
#NULL!	#NULL!	#NULL!	#NULL!	#NULL!	#NULL!	#NULL!	#NULL!	#NULL!	#NULL!
1	1	2	1	2	2	1	1	2	1
2	1	2	1	2	1	2	2	1	1
#NULL!	#NULL!	#NULL!	#NULL!	#NULL!	#NULL!	#NULL!	#NULL!	#NULL!	#NULL!
1	2	2	1	2	2	2	2	2	1
1	2	2	2	2	2	1	2	2	1
2	1	2	1	2	2	1	1	2	1
#NULL!	#NULL!	#NULL!	#NULL!	#NULL!	#NULL!	#NULL!	#NULL!	#NULL!	#NULL!
2	2	#NULL!	#NULL!	#NULL!	#NULL!	#NULL!	#NULL!	#NULL!	#NULL!
2	2	2	2	2	2	2	2	2	2
2	2	2	2	1	1	2	2	1	2
2	2	2	2	2	2	2	2	2	2
2	2	2	2	2	2	2	2	2	2
2	2	2	2	2	2	2	2	2	2
1	1	2	1	2	2	2	1	2	2
2	2	2	2	2	2	2	2	2	2
1	1	2	2	2	2	2	2	2	2
2	2	2	2	2	2	2	2	2	2
2	1	2	2	2	2	2	2	2	2
2	2	1	2	2	2	2	2	1	2
1	1	#NULL!	#NULL!	#NULL!	#NULL!	#NULL!	#NULL!	#NULL!	#NULL!
2	2	#NULL!	#NULL!	#NULL!	#NULL!	#NULL!	#NULL!	#NULL!	#NULL!
2	2	2	2	2	2	2	2	2	2
2	2	2	2	1	2	2	1	2	2
1	2	2	2	2	2	2	2	2	2
1	1	2	2	1	2	2	1	2	2
1	2	2	2	2	2	2	1	2	2
2	2	2	2	2	2	2	1	2	#NULL!
1	2	2	2	1	2	2	2	2	1
1	2	2	2	2	2	2	1	2	2
1	1	2	2	2	2	2	2	2	2
2	2	2	2	2	2	2	2	2	2
1	1	2	2	2	1	2	2	1	2
2	2	2	2	2	#NULL!	2	2	2	2
2	2	2	2	2	2	2	2	2	2

2	2	2	2	2	2	2	2	2	2
2	2	2	2	2	2	2	2	2	2
1	1	2	#NULL!	#NULL!	#NULL!	#NULL!	#NULL!	#NULL!	#NULL!
2	2	2	2	2	2	2	2	2	2
1	2	#NULL!	#NULL!	#NULL!	#NULL!	#NULL!	#NULL!	#NULL!	#NULL!
2	2	#NULL!	#NULL!	#NULL!	#NULL!	#NULL!	#NULL!	#NULL!	#NULL!
2	2	2	2	2	2	2	2	2	2
2	1	2	2	2	2	#NULL!	2	#NULL!	2
2	2	2	2	2	2	2	2	2	2
2	2	2	1	2	2	2	1	2	2
2	2	2	2	2	2	2	2	1	1
1	2	2	2	2	2	2	2	2	2
2	1	2	2	2	2	2	1	2	2
2	2	2	1	2	2	2	2	1	2
2	2	2	2	2	2	2	2	2	2
2	2	2	2	2	2	2	2	2	2
2	2	2	2	2	2	2	2	2	2
2	2	2	2	2	2	2	2	2	2
1	2	2	2	2	2	2	2	2	2
2	2	2	2	2	2	2	2	2	2
1	1	2	1	2	2	2	1	2	2
2	2	2	2	2	2	2	2	2	2
2	2	2	2	2	2	2	2	2	2
2	1	2	2	2	2	2	2	2	2
2	2	2	2	2	2	2	2	2	2
2	2	2	2	1	2	2	2	1	2
2	2	2	2	2	2	2	2	2	2
2	2	2	2	2	2	2	2	2	2
2	2	2	2	2	2	2	2	2	1
2	2	2	2	2	2	1	2	2	2
1	1	2	2	1	1	2	2	2	2
2	2	2	2	1	1	2	2	2	2
2	2	2	1	2	2	1	2	2	2
2	2	2	2	2	2	2	2	2	2
2	2	2	2	2	2	2	2	2	2
2	2	2	2	2	2	2	1	2	2
2	2	2	2	2	2	2	2	2	2
2	2	2	2	2	2	2	2	2	2
2	2	2	2	2	2	2	2	2	2
2	2	2	2	2	2	2	2	2	2
2	2	2	2	2	2	2	2	2	2
2	2	2	2	2	2	2	2	2	2
2	1	2	2	2	2	1	1	2	2
2	2	2	2	2	2	2	2	2	2
2	1	2	2	2	2	2	2	2	2
2	2	2	2	2	2	2	2	2	2
2	2	2	2	2	2	2	2	2	2

2	2	2	2	1	1	2	2	2	2
2	1	2	2	2	1	2	1	2	2
2	1	2	2	2	2	2	2	2	2
2	2	2	2	2	2	2	2	2	2
2	1	2	2	2	2	2	2	2	2
2	1	2	2	1	1	2	2	2	2
#NULL!	#NULL!	#NULL!	#NULL!	#NULL!	#NULL!	#NULL!	#NULL!	#NULL!	#NULL!
1	1	2	2	1	2	2	1	2	2
1	2	2	2	2	2	2	2	2	2
2	2	2	2	2	2	1	2	1	2
2	2	1	2	2	2	1	2	2	2
2	2	2	2	1	1	2	2	2	2
2	2	2	2	2	2	2	2	2	2
2	2	2	2	2	2	2	2	2	2
2	2	2	2	2	2	2	2	2	2
#NULL!	#NULL!	2	2	2	2	1	1	2	1
2	2	2	2	1	1	2	2	2	2
2	2	2	2	2	2	2	2	2	2
2	2	2	2	2	2	2	2	2	2
2	2	2	2	2	2	2	2	2	2
1	2	2	2	2	2	2	1	2	2
2	2	2	2	2	2	2	2	2	2
#NULL!	#NULL!	#NULL!	#NULL!	#NULL!	#NULL!	#NULL!	#NULL!	#NULL!	#NULL!
1	1	2	2	2	2	2	2	2	2
2	2	2	2	2	2	2	2	2	2
#NULL!	#NULL!	#NULL!	#NULL!	#NULL!	#NULL!	#NULL!	#NULL!	#NULL!	#NULL!
1	1	2	2	2	2	1	2	2	2
#NULL!	#NULL!	#NULL!	#NULL!	#NULL!	#NULL!	#NULL!	#NULL!	#NULL!	#NULL!
1	1	2	2	2	2	2	2	2	2
2	2	2	2	2	2	2	2	2	2
2	2	#NULL!	#NULL!	#NULL!	#NULL!	#NULL!	#NULL!	#NULL!	#NULL!
#NULL!	#NULL!	#NULL!	#NULL!	#NULL!	#NULL!	#NULL!	#NULL!	#NULL!	#NULL!
1	2	2	2	2	2	2	2	2	2
2	2	2	1	2	2	1	1	2	2
2	2	2	2	2	#NULL!	1	1	2	#NULL!
1	1	2	1	2	2	2	1	2	2
2	2	1	2	1	1	2	2	1	2
2	2	2	1	2	2	2	2	2	2
#NULL!	#NULL!	#NULL!	#NULL!	#NULL!	#NULL!	#NULL!	#NULL!	#NULL!	#NULL!
2	2	2	2	2	2	2	2	2	2
2	2	2	2	2	1	2	1	2	2
2	2	2	2	2	1	2	2	2	1
2	2	2	2	2	2	2	2	2	2
2	2	1	2	1	1	2	2	1	2
#NULL!	#NULL!	#NULL!	#NULL!	#NULL!	#NULL!	#NULL!	#NULL!	#NULL!	#NULL!
2	2	2	2	2	2	2	2	2	2
2	2	2	2	2	2	2	2	2	2

2	2	#NULL!	#NULL!	#NULL!	#NULL!	#NULL!	#NULL!	#NULL!	#NULL!
2	2	2	2	2	2	2	2	2	2
2	2	2	2	2	2	2	2	2	2
2	2	2	2	2	2	2	2	2	2
2	2	2	2	1	2	2	2	2	2
2	2	2	2	2	2	2	2	2	2
2	2	2	2	2	2	2	2	2	2
2	2	2	2	2	2	2	2	2	2
2	2	2	2	2	2	2	2	2	2
2	2	2	2	2	2	2	2	2	2
2	2	#NULL!	1	1	1	2	2	2	2
2	2	2	2	2	2	2	2	2	1
2	2	2	2	2	2	2	2	2	2
2	2	2	2	2	2	2	2	2	2
2	2	2	2	2	2	2	2	2	2
2	2	2	2	1	2	2	2	2	2
2	2	2	2	2	2	2	2	2	2
2	2	2	2	2	1	2	2	1	2
2	2	2	2	2	2	2	2	2	2
2	2	2	2	2	2	2	2	2	2
2	2	2	2	2	2	2	2	2	2
2	2	2	2	2	2	2	2	2	2
2	2	2	2	2	2	2	2	2	2
2	1	2	2	2	2	2	2	2	2
2	2	2	2	2	2	2	2	2	2
2	2	2	2	2	2	2	2	2	2
2	2	2	2	2	2	2	2	2	2
2	2	2	2	2	2	2	2	2	2
2	2	2	2	2	2	2	2	2	1
2	2	2	2	2	2	2	2	2	2
2	2	2	2	2	2	2	2	2	2
2	2	2	2	2	2	2	2	2	2
2	2	2	2	2	2	2	2	2	2
2	2	2	2	1	2	2	2	2	2
2	2	2	2	2	2	2	2	1	2
2	2	2	2	2	2	2	2	2	2
2	2	2	2	2	2	2	2	2	2
2	2	2	2	2	2	2	2	2	2
2	2	2	2	2	2	2	2	2	2
1	2	2	2	2	2	2	2	2	2
2	2	2	2	2	2	2	2	2	2
2	2	2	2	2	2	2	2	2	2
2	1	2	2	2	2	2	2	2	2
2	2	2	2	2	2	2	2	2	2
2	2	2	#NULL!	#NULL!	#NULL!	#NULL!	#NULL!	#NULL!	#NULL!
2	2	2	2	2	2	2	2	2	2
1	2	#NULL!	2	#NULL!	#NULL!	#NULL!	#NULL!	#NULL!	#NULL!

2	2	2	2	2	2	2	2	2	2
2	1	2	2	2	2	2	2	1	2
2	1	2	2	2	2	2	2	1	2
2	2	2	1	2	2	2	2	2	2
2	2	2	2	2	2	2	2	2	2
2	2	2	2	2	1	2	1	2	2
1	2	2	2	2	2	2	2	2	2
#NULL!	2	2	2	1	2	2	2	2	1
2	2	2	2	2	2	2	2	2	2
2	2	2	2	2	2	2	2	2	2
1	1	2	2	2	2	2	2	1	2
1	2	#NULL!	#NULL!	#NULL!	2	#NULL!	#NULL!	#NULL!	#NULL!
1	1	2	2	2	2	2	2	#NULL!	#NULL!
2	2	2	2	2	2	2	2	2	2
2	2	#NULL!	#NULL!	#NULL!	#NULL!	1	#NULL!	#NULL!	#NULL!
1	2	#NULL!	#NULL!	2	#NULL!	#NULL!	#NULL!	#NULL!	#NULL!
2	2	#NULL!	#NULL!	#NULL!	#NULL!	#NULL!	#NULL!	#NULL!	1
2	2	2	#NULL!	#NULL!	#NULL!	#NULL!	#NULL!	#NULL!	#NULL!
2	2	#NULL!	#NULL!	#NULL!	#NULL!	#NULL!	#NULL!	#NULL!	#NULL!
2	2	2	2	1	2	1	2	2	2
1	1	2	#NULL!	2	#NULL!	1	1	#NULL!	1
1	2	#NULL!	#NULL!	#NULL!	2	#NULL!	#NULL!	#NULL!	#NULL!
2	2	2	2	2	1	2	2	2	2
2	2	2	#NULL!	#NULL!	#NULL!	#NULL!	#NULL!	#NULL!	#NULL!
2	2	#NULL!	#NULL!	2	#NULL!	#NULL!	#NULL!	#NULL!	#NULL!
#NULL!	#NULL!	#NULL!	#NULL!	#NULL!	#NULL!	#NULL!	#NULL!	#NULL!	#NULL!
1	1	2	#NULL!	#NULL!	#NULL!	#NULL!	#NULL!	#NULL!	#NULL!
2	2	#NULL!	#NULL!	#NULL!	#NULL!	#NULL!	#NULL!	#NULL!	#NULL!
2	1	2	2	2	2	2	1	2	2
1	2	#NULL!	#NULL!	#NULL!	2	#NULL!	#NULL!	#NULL!	#NULL!
2	2	2	#NULL!	#NULL!	#NULL!	#NULL!	#NULL!	#NULL!	#NULL!
2	2	2	2	2	2	2	2	2	2
2	2	2	2	2	2	2	2	2	2
1	1	2	2	2	2	2	2	2	2
2	2	2	1	2	2	2	1	2	2
2	2	2	2	2	2	2	2	2	2
2	2	2	2	2	2	2	2	2	2
2	2	2	2	1	2	#NULL!	2	1	2
2	2	2	2	2	2	2	2	2	2
2	2	2	2	2	2	2	2	2	2
2	2	2	#NULL!	#NULL!	#NULL!	#NULL!	#NULL!	#NULL!	#NULL!
1	1	2	2	2	2	2	2	2	2
2	2	2	2	2	2	2	2	2	2
2	2	2	2	2	2	2	2	2	2
2	2	2	2	2	2	2	2	2	2
2	2	2	2	2	2	2	2	2	2
2	2	2	2	2	2	2	2	2	2

1	1	2	2	2	2	2	2	2	2
2	1	2	2	2	2	2	2	2	2
2	2	2	2	1	1	2	2	2	2
2	2	2	2	2	2	1	1	2	2
1	2	2	2	1	2	2	1	2	2
2	2	2	2	2	2	2	2	2	2
2	2	2	2	1	2	2	2	2	2
2	2	2	2	2	2	2	2	2	2
2	2	2	2	2	2	2	2	2	2
2	2	2	2	2	2	2	2	2	2
2	2	2	2	2	2	2	2	2	2
1	2	2	1	2	2	2	2	2	2
2	2	2	2	2	2	2	2	2	2
2	2	2	2	2	2	2	2	2	2
2	2	2	2	2	2	2	2	2	2
2	2	2	2	2	2	2	2	2	2
2	2	2	2	2	2	2	2	2	2
2	2	2	2	2	2	2	2	2	2
2	2	2	2	2	2	2	2	2	2
1	2	2	2	2	2	2	1	2	2
2	1	2	2	2	2	2	2	2	2
2	2	2	2	2	2	2	2	2	2
2	2	2	2	2	1	2	2	2	2
2	2	2	2	2	2	2	2	2	2
2	2	2	2	2	2	2	2	2	2
2	2	2	2	2	2	2	2	2	2
2	1	2	2	2	2	2	1	2	2
1	2	2	2	2	2	2	1	1	1
2	2	2	2	2	2	2	2	2	2
2	2	2	2	1	1	2	2	1	2
#NULL!	#NULL!	2	2	2	2	2	2	2	2
2	2	2	2	2	2	2	2	2	2
2	2	2	2	2	2	2	2	2	2
2	2	2	2	2	1	1	2	2	2
2	2	2	2	2	2	2	2	2	2
1	2	2	#NULL!	#NULL!	#NULL!	#NULL!	#NULL!	#NULL!	#NULL!
2	2	2	2	2	2	2	2	2	2
1	1	2	2	1	2	2	2	2	2
2	2	2	2	2	2	2	2	2	2
2	2	2	2	2	2	2	2	2	2
2	2	2	#NULL!	#NULL!	#NULL!	#NULL!	#NULL!	#NULL!	#NULL!
2	1	2	2	2	2	2	2	2	2
2	2	2	2	2	2	2	2	2	2
1	2	2	2	2	2	2	2	1	2
1	2	2	2	2	2	2	2	2	2
2	2	2	2	2	2	2	2	2	2
2	1	2	#NULL!	#NULL!	#NULL!	#NULL!	#NULL!	#NULL!	#NULL!

2	2	2	#NULL!	#NULL!	#NULL!	#NULL!	#NULL!	#NULL!	#NULL!
2	2	#NULL!	#NULL!	#NULL!	#NULL!	#NULL!	1	#NULL!	#NULL!
2	2	2	#NULL!	#NULL!	#NULL!	#NULL!	#NULL!	#NULL!	#NULL!
#NULL!	#NULL!	#NULL!	#NULL!	#NULL!	#NULL!	#NULL!	#NULL!	#NULL!	#NULL!
2	2	2	#NULL!	#NULL!	#NULL!	#NULL!	#NULL!	#NULL!	#NULL!
2	1	2	#NULL!	#NULL!	#NULL!	#NULL!	#NULL!	#NULL!	#NULL!
2	2	#NULL!	#NULL!	#NULL!	2	#NULL!	#NULL!	#NULL!	#NULL!
2	2	2	#NULL!	#NULL!	#NULL!	#NULL!	#NULL!	#NULL!	#NULL!
2	2	2	#NULL!	#NULL!	#NULL!	#NULL!	#NULL!	#NULL!	#NULL!
1	1	2	#NULL!	#NULL!	#NULL!	#NULL!	#NULL!	#NULL!	#NULL!
2	2	#NULL!	#NULL!	#NULL!	#NULL!	#NULL!	#NULL!	#NULL!	#NULL!
1	1	2	#NULL!	#NULL!	#NULL!	#NULL!	#NULL!	#NULL!	#NULL!
2	2	2	#NULL!	#NULL!	#NULL!	#NULL!	#NULL!	#NULL!	#NULL!
1	1	#NULL!	#NULL!	2	#NULL!	#NULL!	#NULL!	#NULL!	#NULL!
2	2	#NULL!	#NULL!	#NULL!	#NULL!	#NULL!	#NULL!	#NULL!	#NULL!
2	2	#NULL!	#NULL!	#NULL!	#NULL!	#NULL!	#NULL!	#NULL!	#NULL!
2	1	#NULL!	2	#NULL!	#NULL!	#NULL!	#NULL!	#NULL!	#NULL!
2	2	2	2	2	1	2	2	1	2
2	2	2	2	2	2	2	2	2	2
1	2	2	#NULL!	#NULL!	#NULL!	#NULL!	#NULL!	#NULL!	#NULL!
1	1	2	1	2	2	1	1	2	1
2	2	2	2	2	2	2	1	1	2
#NULL!	2	2	2	2	2	2	2	2	1
1	1	2	1	2	1	2	1	2	1
#NULL!	2	1	2	1	2	2	2	1	2
2	1	2	2	2	2	2	1	1	2
1	2	2	2	2	2	2	2	2	2
2	2	2	1	2	2	2	1	2	1
2	2	2	2	2	2	2	2	2	2
2	2	2	2	2	2	2	2	2	2
#NULL!	#NULL!	2	1	2	2	2	2	2	2
2	2	2	2	2	2	2	2	2	2
2	2	2	2	2	2	2	2	2	2
2	2	2	2	2	1	2	2	2	2
2	1	2	1	2	1	1	2	1	1
2	2	2	2	2	2	2	2	1	2
2	2	2	2	2	2	2	2	2	2
#NULL!	#NULL!	2	2	2	1	2	2	2	1
1	1	2	1	2	2	1	1	2	1
1	#NULL!	#NULL!	#NULL!	#NULL!	#NULL!	#NULL!	#NULL!	#NULL!	#NULL!
1	2	2	2	2	2	2	2	2	2
1	1	2	1	2	2	1	1	2	1
#NULL!	#NULL!	#NULL!	#NULL!	#NULL!	#NULL!	#NULL!	#NULL!	#NULL!	#NULL!
1	1	2	1	2	2	1	2	2	1
2	2	2	2	2	2	2	2	2	2
2	2	2	2	2	2	2	2	2	2
2	2	2	2	2	2	2	2	1	2

2	2	2	2	2	2	2	2	2	2
#NULL!	#NULL!	#NULL!	#NULL!	#NULL!	#NULL!	#NULL!	#NULL!	#NULL!	#NULL!
2	2	2	1	1	2	1	2	2	2
#NULL!	#NULL!	#NULL!	#NULL!	#NULL!	#NULL!	#NULL!	#NULL!	#NULL!	#NULL!
1	1	2	2	2	2	2	2	2	2
1	2	2	2	2	2	2	2	2	2
#NULL!	#NULL!	#NULL!	#NULL!	#NULL!	#NULL!	#NULL!	#NULL!	#NULL!	#NULL!
2	2	2	2	2	2	2	2	2	2
1	1	2	1	1	2	2	1	2	1
2	2	2	2	2	2	2	1	2	2
1	1	2	1	2	2	1	1	2	1
2	1	2	2	2	2	2	2	2	1
2	2	1	2	1	2	2	2	1	2
#NULL!	#NULL!	#NULL!	#NULL!	#NULL!	#NULL!	#NULL!	#NULL!	#NULL!	#NULL!
#NULL!	#NULL!	#NULL!	#NULL!	#NULL!	#NULL!	#NULL!	#NULL!	#NULL!	#NULL!
1	2	#NULL!	#NULL!	#NULL!	#NULL!	#NULL!	#NULL!	#NULL!	#NULL!
2	2	2	2	2	2	2	2	2	2
#NULL!	#NULL!	#NULL!	#NULL!	#NULL!	#NULL!	#NULL!	#NULL!	#NULL!	#NULL!
2	1	2	2	2	#NULL!	2	2	2	2
2	2	2	2	2	2	2	2	2	2
2	2	1	2	1	1	2	2	2	2
2	2	1	2	1	2	2	2	1	2
1	1	2	1	2	2	1	1	2	1
2	1	1	1	1	2	1	2	2	2
2	2	2	2	2	2	2	2	2	2
2	2	2	1	1	2	2	1	2	1
1	1	2	2	1	1	1	2	1	2
1	1	1	1	1	1	1	1	2	2
2	2	2	2	2	2	2	2	2	1
2	2	2	2	2	2	2	2	1	2
2	1	#NULL!	#NULL!	#NULL!	#NULL!	#NULL!	#NULL!	#NULL!	#NULL!
1	2	#NULL!	#NULL!	#NULL!	2	#NULL!	#NULL!	#NULL!	#NULL!
2	2	2	2	2	2	2	2	2	2
2	2	2	2	2	2	1	2	2	#NULL!
2	#NULL!	2	2	2	2	#NULL!	#NULL!	2	#NULL!
2	1	2	2	2	2	2	1	2	2
2	2	2	2	2	1	2	#NULL!	2	2
#NULL!	#NULL!	#NULL!	#NULL!	#NULL!	#NULL!	#NULL!	#NULL!	#NULL!	#NULL!
#NULL!	#NULL!	#NULL!	#NULL!	#NULL!	#NULL!	#NULL!	#NULL!	#NULL!	#NULL!
#NULL!	#NULL!	#NULL!	#NULL!	#NULL!	#NULL!	#NULL!	#NULL!	#NULL!	#NULL!
#NULL!	#NULL!	#NULL!	#NULL!	#NULL!	#NULL!	#NULL!	#NULL!	#NULL!	#NULL!
1	1	1	2	1	2	2	1	2	1
2	1	2	2	1	2	2	2	2	2
#NULL!	#NULL!	#NULL!	#NULL!	#NULL!	#NULL!	#NULL!	#NULL!	#NULL!	#NULL!
2	1	2	2	1	2	2	1	1	1
2	2	2	2	1	2	2	2	2	2
2	2	2	2	1	1	1	1	1	1

2	2	1	2	1	1	2	2	1	2
2	2	2	2	2	2	2	2	2	2
1	1	1	2	1	1	2	2	1	2
2	2	2	2	2	2	2	2	2	2
2	2	2	2	2	2	2	2	2	2
1	1	2	2	2	#NULL!	1	2	1	#NULL!
#NULL!	#NULL!	#NULL!	#NULL!	#NULL!	#NULL!	#NULL!	#NULL!	#NULL!	#NULL!
2	2	2	2	1	1	#NULL!	2	2	2
2	2	1	2	1	1	2	2	1	2
2	2	2	2	2	2	2	2	2	2
2	2	1	2	1	1	2	2	1	2
2	2	2	1	2	2	2	1	2	2
2	2	2	2	1	2	2	2	2	2
2	2	2	2	1	2	2	2	2	2
2	2	2	2	1	2	2	2	2	2
2	2	2	2	1	2	2	2	2	2
2	2	2	2	1	2	2	2	2	2
2	2	2	2	1	2	2	2	2	2
2	2	2	2	1	2	2	2	2	2
2	2	2	2	2	2	1	2	2	2
2	2	2	2	1	2	1	2	2	2
#NULL!	#NULL!	2	2	2	2	2	2	1	2
2	2	2	2	2	2	2	2	2	2
2	2	2	2	2	2	2	2	2	2
2	2	1	2	1	1	2	2	1	2
2	2	2	2	1	2	2	2	1	2
2	2	#NULL!	#NULL!	#NULL!	#NULL!	1	#NULL!	#NULL!	#NULL!
2	2	2	2	2	2	2	2	2	2
1	1	2	#NULL!	#NULL!	#NULL!	#NULL!	#NULL!	#NULL!	#NULL!
2	2	2	2	2	2	2	2	2	2
2	2	2	2	2	1	2	2	1	1
2	2	1	2	1	2	2	2	1	2
2	2	2	2	2	1	2	1	1	1
2	1	#NULL!	#NULL!	#NULL!	#NULL!	1	#NULL!	#NULL!	#NULL!
2	2	1	2	1	1	2	2	1	2
2	2	1	2	2	2	2	2	1	2
2	2	2	#NULL!	#NULL!	#NULL!	2	#NULL!	2	2
2	1	2	2	2	2	2	2	2	1
2	2	2	2	1	2	2	2	2	2
2	2	2	1	2	2	2	2	2	1
2	2	2	1	2	2	2	2	1	1
2	2	2	2	2	2	2	2	#NULL!	2
1	1	2	1	2	2	1	2	2	2
2	1	1	1	1	1	1	1	2	2
1	2	2	1	2	2	2	1	2	1
1	1	2	1	2	2	1	1	2	1
2	2	2	2	2	2	2	2	2	2

2	2	2	2	2	2	2	2	2	2
1	1	1	#NULL!	#NULL!	#NULL!	#NULL!	#NULL!	#NULL!	#NULL!
2	1	2	#NULL!	#NULL!	#NULL!	#NULL!	#NULL!	#NULL!	#NULL!
2	2	2	2	2	2	2	1	2	2
2	2	2	2	#NULL!	2	2	1	2	2
1	1	2	2	2	2	2	2	2	2
2	2	2	2	#NULL!	2	1	1	2	1
1	2	2	2	2	2	2	2	2	2
1	1	2	2	2	2	2	2	2	2
1	2	2	2	2	2	2	2	2	2
2	2	2	2	1	2	2	2	2	#NULL!
2	2	2	2	2	2	1	2	2	2
2	2	#NULL!	#NULL!	#NULL!	#NULL!	#NULL!	#NULL!	#NULL!	#NULL!
2	2	1	2	1	2	1	2	2	2
2	2	2	2	2	2	1	2	2	2
2	1	#NULL!	#NULL!	#NULL!	#NULL!	#NULL!	#NULL!	#NULL!	#NULL!
2	1	1	2	1	1	2	2	1	2
1	2	#NULL!	#NULL!	#NULL!	#NULL!	#NULL!	#NULL!	#NULL!	#NULL!
1	2	#NULL!	#NULL!	#NULL!	#NULL!	#NULL!	#NULL!	#NULL!	#NULL!
2	2	2	2	1	2	2	2	2	2
1	2	2	2	2	2	1	1	2	1
2	2	2	2	2	2	2	2	2	2
#NULL!	#NULL!	#NULL!	#NULL!	#NULL!	#NULL!	#NULL!	#NULL!	#NULL!	#NULL!
2	2	2	2	2	2	2	2	2	2
#NULL!	#NULL!	1	2	1	1	2	2	1	2
1	2	2	2	1	2	1	2	2	2
1	2	2	2	1	2	1	2	2	2
2	2	2	2	2	2	2	2	2	2
#NULL!	1	2	1	2	2	1	1	2	1
2	2	2	2	2	2	2	2	2	2
2	1	2	2	1	2	1	1	2	2
2	2	2	2	2	1	2	2	2	2
2	2	2	2	2	1	2	2	2	2
1	2	2	2	1	2	1	2	2	2
#NULL!	#NULL!	2	1	2	2	2	2	2	1
2	2	2	2	2	2	2	2	2	2
2	1	2	2	2	2	2	1	2	1
1	1	#NULL!	#NULL!	#NULL!	#NULL!	#NULL!	#NULL!	#NULL!	#NULL!
2	2	#NULL!	#NULL!	#NULL!	#NULL!	2	#NULL!	1	#NULL!
2	2	2	2	#NULL!	2	1	1	2	1
2	2	2	2	2	2	2	2	2	2
2	2	2	2	2	2	2	2	2	2
2	2	2	2	2	2	2	2	2	2
1	1	2	2	2	2	2	2	2	2
2	2	2	2	2	2	2	2	2	2
2	2	2	2	2	2	2	2	2	2
#NULL!	#NULL!	#NULL!	#NULL!	#NULL!	#NULL!	#NULL!	#NULL!	#NULL!	#NULL!

#NULL!	#NULL!	#NULL!	#NULL!	#NULL!	#NULL!	#NULL!	#NULL!	#NULL!	#NULL!
2	2	2	2	2	2	2	2	2	2
2	1	2	2	2	2	2	2	2	2
1	1	2	2	2	2	2	2	2	2
1	1	2	#NULL!	#NULL!	#NULL!	#NULL!	#NULL!	#NULL!	#NULL!
2	2	1	2	2	2	2	2	2	2
#NULL!	#NULL!	#NULL!	#NULL!	#NULL!	#NULL!	#NULL!	#NULL!	#NULL!	#NULL!
2	2	1	2	1	1	2	2	1	2
1	1	2	2	2	2	1	1	2	2
2	2	2	#NULL!	#NULL!	#NULL!	#NULL!	#NULL!	#NULL!	#NULL!
2	2	2	2	2	2	2	2	2	2
2	1	2	2	2	2	2	2	2	2
2	1	2	2	2	2	2	2	2	2
2	2	2	2	2	2	2	2	2	2
2	2	2	1	1	1	2	2	2	2
2	2	2	2	2	2	2	2	2	2
2	2	2	2	2	2	2	2	2	2
1	1	2	2	2	2	2	2	2	2
2	2	2	2	2	1	2	2	2	2
2	2	2	2	2	2	2	2	2	1
2	2	2	#NULL!	#NULL!	#NULL!	#NULL!	#NULL!	#NULL!	#NULL!
2	2	1	2	1	1	2	2	1	2
2	2	2	2	2	2	2	2	2	2
2	2	2	2	2	2	2	2	2	2
1	1	2	2	2	2	2	2	2	2
2	2	1	2	1	1	2	2	1	2
2	2	#NULL!	#NULL!	#NULL!	#NULL!	#NULL!	#NULL!	#NULL!	#NULL!
2	2	2	2	2	2	2	2	2	2
2	2	2	2	2	2	2	2	2	2
1	1	2	2	2	1	2	1	2	2
2	1	2	2	2	2	2	2	2	2
2	2	2	2	2	2	2	2	2	2
1	1	2	2	2	1	1	1	2	2
1	1	2	2	2	2	2	2	2	2
2	2	2	2	2	2	2	2	2	2
#NULL!	#NULL!	2	2	2	2	2	2	2	2
2	2	2	2	2	2	2	2	2	2
2	2	2	2	1	2	2	2	2	1
2	2	2	2	2	2	2	2	2	2
2	2	2	2	2	2	2	2	2	2
2	2	2	2	2	2	2	2	2	2
2	2	2	2	2	2	2	2	2	2
2	2	2	2	2	1	2	2	1	2
#NULL!	#NULL!	2	2	2	2	2	2	2	2
2	2	2	2	2	2	2	2	2	2
2	2	1	2	1	1	2	2	1	2
2	2	2	2	2	2	2	2	2	2

2	2	2	2	2	2	2	2	2	2
2	2	2	2	2	2	2	2	2	2
2	2	2	2	2	2	2	2	2	2
2	2	2	2	2	2	2	2	2	2
2	2	1	2	2	2	2	2	2	2
2	2	2	2	1	2	2	1	2	2
2	2	2	2	2	2	2	2	2	2
2	2	2	2	2	1	2	1	1	2
2	2	2	1	2	2	2	2	2	2
2	2	2	2	2	2	2	2	2	2
1	1	2	1	2	2	2	2	2	2
2	2	2	#NULL!	#NULL!	#NULL!	#NULL!	#NULL!	#NULL!	#NULL!
2	2	#NULL!	#NULL!	#NULL!	2	#NULL!	#NULL!	#NULL!	#NULL!
#NULL!	#NULL!	2	1	2	2	2	2	2	2
1	1	2	1	2	2	2	2	1	2
2	1	1	2	2	2	2	2	1	2
2	2	2	2	1	2	2	2	2	2
2	2	2	2	2	#NULL!	2	2	2	2
#NULL!	#NULL!	#NULL!	#NULL!	#NULL!	#NULL!	#NULL!	#NULL!	#NULL!	#NULL!
1	2	2	2	1	2	2	2	2	2
2	2	#NULL!	#NULL!	#NULL!	#NULL!	#NULL!	#NULL!	#NULL!	#NULL!
1	1	2	2	2	1	2	1	2	2
2	2	2	2	2	2	2	2	2	2
1	1	2	2	2	2	2	2	2	2
2	2	1	2	1	1	2	2	1	2
1	1	2	2	2	2	2	2	2	2
2	2	2	2	2	2	2	2	2	2
1	1	2	2	2	2	2	2	2	2
2	2	2	2	2	2	2	2	2	2
2	2	1	2	1	2	2	2	2	2
1	1	2	1	2	2	1	1	2	1
1	1	2	1	2	2	1	1	2	1
#NULL!	#NULL!	1	2	1	1	2	2	1	2
1	1	2	1	2	2	1	1	2	1
1	1	1	2	1	1	2	2	1	2
2	1	2	1	1	1	2	1	2	1
2	2	2	#NULL!	#NULL!	#NULL!	#NULL!	#NULL!	#NULL!	#NULL!
1	1	2	1	2	2	1	1	2	1
2	2	2	2	2	2	2	2	2	2
2	2	2	2	2	2	2	2	2	2
2	2	2	2	2	2	2	1	2	2
2	2	#NULL!	#NULL!	#NULL!	#NULL!	1	#NULL!	#NULL!	#NULL!
2	2	2	2	1	2	1	2	2	2
2	2	2	2	2	2	2	2	2	2
2	2	2	2	2	2	2	2	2	2
1	1	2	2	2	1	1	2	2	1
2	1	2	2	1	2	2	2	2	1

2	2	2	2	1	2	1	2	2	2
2	2	1	2	1	1	2	2	1	2
2	2	2	2	1	2	1	2	2	2
2	2	#NULL!	2	#NULL!	#NULL!	#NULL!	#NULL!	#NULL!	#NULL!
#NULL!	#NULL!	2	#NULL!	#NULL!	#NULL!	#NULL!	#NULL!	#NULL!	#NULL!
2	2	2	#NULL!	2	2	2	2	2	2
2	2	2	2	2	2	2	2	2	2
2	2	1	2	1	1	2	2	1	2
1	2	1	2	1	1	2	2	1	2
2	2	2	2	2	2	2	2	2	2
2	2	#NULL!	#NULL!	#NULL!	#NULL!	#NULL!	#NULL!	#NULL!	#NULL!
1	2	2	2	2	2	2	#NULL!	2	2
2	2	2	2	2	2	2	2	2	2
2	2	2	2	2	2	2	2	2	2
2	2	2	2	2	2	2	2	2	2
2	2	1	2	1	1	2	2	2	1
2	2	1	2	2	2	2	2	2	2
2	2	#NULL!	1	#NULL!	#NULL!	#NULL!	#NULL!	#NULL!	#NULL!
2	2	#NULL!	#NULL!	#NULL!	#NULL!	#NULL!	#NULL!	#NULL!	#NULL!
2	2	2	2	2	2	2	2	2	2
2	2	2	2	2	2	2	2	2	2
2	2	2	2	2	2	2	2	2	2
#NULL!	#NULL!	#NULL!	#NULL!	#NULL!	#NULL!	#NULL!	#NULL!	#NULL!	#NULL!
#NULL!	#NULL!	#NULL!	#NULL!	#NULL!	#NULL!	#NULL!	#NULL!	#NULL!	#NULL!
2	1	2	2	2	1	1	2	1	1
2	2	2	1	2	2	1	1	2	2
1	2	2	2	2	2	2	1	2	1
1	2	2	1	2	2	1	1	2	2
2	2	1	1	1	1	2	1	2	2
2	2	2	2	#NULL!	2	2	2	2	2
1	2	2	2	2	2	2	1	2	2
2	2	2	2	1	2	2	2	2	2
1	1	2	2	2	2	2	2	2	2
2	2	1	2	1	1	2	2	1	2
2	2	1	2	1	1	2	2	1	2
1	2	2	2	1	1	2	2	2	2
1	1	2	2	2	1	2	2	2	2
2	2	1	2	1	1	2	2	1	2
2	1	2	#NULL!	#NULL!	1	1	2	2	#NULL!
2	2	1	2	1	1	2	2	1	2
2	1	2	2	1	2	2	1	2	2
1	2	2	2	2	1	1	1	2	2
2	2	2	1	2	2	1	1	2	1
2	2	2	2	2	2	2	2	2	2
1	1	2	2	2	2	1	2	2	2
#NULL!	#NULL!	2	1	2	2	1	1	2	1
2	2	2	2	2	2	2	2	2	2

2	2	2	2	2	2	2	2	2	2
2	2	#NULL!	#NULL!	1	2	#NULL!	2	#NULL!	2
2	1	2	2	2	2	2	1	#NULL!	1
2	1	2	2	2	2	2	1	2	1
2	2	2	2	2	2	2	2	2	2
2	2	2	2	2	2	2	2	2	2
#NULL!	2	1	2	1	1	2	2	2	2
1	2	2	2	2	2	2	2	2	2
1	2	2	2	2	2	2	2	2	2
2	2	1	2	2	2	2	2	2	2
#NULL!	2	#NULL!	#NULL!	#NULL!	#NULL!	#NULL!	2	#NULL!	#NULL!
2	2	2	2	2	2	2	2	2	2
2	2	2	1	1	2	1	2	2	2
1	2	2	2	2	2	2	1	2	2
1	1	2	1	2	2	1	1	2	1
1	2	1	2	1	1	2	2	2	2
2	2	2	2	2	2	2	2	2	2
2	2	2	2	2	2	2	2	2	2
2	2	2	2	1	2	2	1	2	2
2	2	2	#NULL!	#NULL!	#NULL!	#NULL!	#NULL!	#NULL!	#NULL!
1	2	2	2	2	2	1	2	2	1
1	1	2	1	2	2	1	1	1	2
1	1	2	2	1	1	2	2	1	2
1	2	2	2	1	1	2	2	1	2
2	2	1	2	1	1	2	2	1	2
2	2	2	2	2	2	2	2	2	2
2	2	2	2	2	2	2	1	2	2
2	2	2	1	2	1	1	2	2	2
2	1	1	2	2	2	1	2	1	1
1	2	2	1	2	2	2	1	2	1
2	2	2	2	2	2	2	2	2	2
1	1	#NULL!	#NULL!	#NULL!	#NULL!	#NULL!	#NULL!	#NULL!	#NULL!
1	2	2	1	2	2	2	2	2	2
2	2	#NULL!	#NULL!	#NULL!	#NULL!	#NULL!	#NULL!	#NULL!	#NULL!
2	2	2	2	2	2	2	2	2	2
2	2	2	2	2	2	2	2	2	2
2	2	2	2	2	2	2	2	2	2
2	2	2	2	2	2	2	2	2	2
2	2	2	2	2	2	2	2	2	2
2	2	2	2	2	2	2	2	1	1
2	2	2	#NULL!	2	2	1	2	2	2
1	1	2	2	2	2	2	2	2	2
2	2	2	2	1	1	2	2	2	2
2	2	2	#NULL!	#NULL!	#NULL!	#NULL!	#NULL!	#NULL!	#NULL!
2	2	1	2	1	1	2	2	1	2
2	2	2	2	2	2	2	2	2	2
2	2	2	2	2	2	2	2	2	2

2	2	#NULL!	#NULL!	#NULL!	#NULL!	#NULL!	#NULL!	#NULL!	#NULL!
#NULL!	#NULL!	2	2	2	2	1	2	2	#NULL!
2	2	2	2	2	1	2	2	1	2
2	2	2	2	2	2	2	2	2	2
2	2	1	2	1	1	2	2	1	2
2	2	1	2	2	2	2	2	#NULL!	2
2	2	2	2	2	2	2	#NULL!	#NULL!	#NULL!
2	2	2	#NULL!	2	2	2	2	2	2
2	2	1	2	2	2	2	2	2	2
#NULL!	#NULL!	#NULL!	#NULL!	#NULL!	#NULL!	#NULL!	#NULL!	#NULL!	#NULL!
#NULL!	#NULL!	#NULL!	#NULL!	#NULL!	#NULL!	#NULL!	#NULL!	#NULL!	#NULL!
#NULL!	#NULL!	#NULL!	#NULL!	#NULL!	#NULL!	#NULL!	#NULL!	#NULL!	#NULL!
2	1	2	#NULL!	#NULL!	#NULL!	#NULL!	#NULL!	#NULL!	#NULL!
2	1	1	#NULL!	#NULL!	#NULL!	#NULL!	#NULL!	#NULL!	#NULL!
#NULL!	#NULL!	#NULL!	#NULL!	#NULL!	#NULL!	#NULL!	#NULL!	#NULL!	#NULL!
2	2	2	2	2	2	2	2	2	2
#NULL!	#NULL!	2	1	2	2	1	2	2	1
1	1	2	2	2	2	2	2	2	2
2	2	2	1	1	2	2	2	2	1
2	2	2	2	2	2	2	1	2	2
2	2	2	2	2	2	2	2	2	2
2	2	2	2	2	2	2	2	1	2
2	2	2	2	1	2	1	1	2	2
2	2	2	2	1	1	2	2	2	2
2	1	2	2	2	2	2	2	2	2
2	2	2	2	2	2	2	1	2	2
1	2	2	#NULL!	1	#NULL!	1	2	#NULL!	1
1	1	2	1	2	2	1	1	2	1
2	2	2	2	2	2	2	1	2	2
2	2	2	2	2	2	2	2	2	2
2	2	2	2	2	2	2	1	2	2
2	1	2	#NULL!	#NULL!	#NULL!	#NULL!	#NULL!	#NULL!	#NULL!
1	1	2	2	2	2	2	1	2	2
2	2	2	2	2	2	2	2	2	2
2	2	2	2	2	2	2	2	2	2
2	2	2	2	2	2	2	2	2	2
2	2	2	2	2	2	2	2	2	2
2	1	2	#NULL!	#NULL!	#NULL!	#NULL!	#NULL!	#NULL!	#NULL!
2	2	2	2	2	2	2	2	2	2
2	2	2	2	2	2	2	2	2	2
2	2	2	2	2	2	2	2	2	2
2	1	2	2	2	2	2	2	2	2
2	2	2	2	2	2	2	2	2	2
2	1	2	2	2	2	2	1	1	2
1	1	2	2	2	2	2	2	2	2
2	1	2	2	2	2	2	2	2	2
2	2	2	#NULL!	#NULL!	#NULL!	#NULL!	#NULL!	#NULL!	#NULL!

#NULL!	#NULL!	2	2	2	2	2	2	2	2
2	2	2	2	2	2	2	2	2	2
2	2	#NULL!	#NULL!	#NULL!	#NULL!	1	#NULL!	#NULL!	#NULL!
2	2	2	#NULL!	#NULL!	#NULL!	#NULL!	#NULL!	#NULL!	#NULL!
2	2	2	2	2	2	2	1	2	1
2	2	2	#NULL!	#NULL!	#NULL!	#NULL!	#NULL!	#NULL!	#NULL!
1	2	2	1	2	2	2	2	2	2
1	2	2	1	2	1	2	2	1	2
2	2	2	2	1	1	2	1	2	2
1	2	2	1	2	2	2	2	2	2
2	2	#NULL!	#NULL!	#NULL!	#NULL!	#NULL!	#NULL!	#NULL!	#NULL!
2	2	2	2	2	2	1	2	2	1
2	2	2	2	1	1	2	2	2	2
2	2	2	2	2	2	2	2	2	2
2	2	2	2	2	2	2	2	2	2
2	2	1	2	1	1	2	2	1	1
2	2	2	2	2	2	2	2	2	2
2	2	2	2	2	2	2	2	2	2
2	2	2	2	2	2	2	1	1	2
2	2	2	2	2	2	2	2	1	2
2	2	2	2	2	2	2	1	1	2
2	2	2	2	2	2	2	2	2	2
2	2	2	2	2	2	1	2	2	2
2	2	2	2	2	2	2	2	2	2
2	2	2	2	2	2	2	2	2	2
2	2	2	2	2	2	2	2	2	2
2	2	2	2	2	2	2	2	2	2
2	2	2	2	2	2	2	2	2	2
2	2	2	2	2	2	2	2	2	2
#NULL!	#NULL!	#NULL!	#NULL!	#NULL!	#NULL!	#NULL!	#NULL!	#NULL!	#NULL!
2	2	2	1	2	2	#NULL!	1	#NULL!	1
2	1	2	2	2	2	2	2	2	2
2	2	2	1	2	2	1	1	2	1
#NULL!	#NULL!	#NULL!	#NULL!	#NULL!	#NULL!	#NULL!	#NULL!	#NULL!	#NULL!
1	2	2	2	1	1	2	1	1	2
2	2	#NULL!	#NULL!	#NULL!	#NULL!	#NULL!	#NULL!	#NULL!	#NULL!
#NULL!	#NULL!	#NULL!	#NULL!	#NULL!	#NULL!	#NULL!	#NULL!	#NULL!	#NULL!
2	2	2	2	2	2	2	2	2	2
2	2	2	2	1	1	2	2	1	2
2	1	2	1	2	2	2	1	2	2
1	1	2	1	2	2	1	1	2	1
2	2	2	2	2	2	2	2	2	1
2	2	2	#NULL!	#NULL!	#NULL!	#NULL!	#NULL!	#NULL!	#NULL!
2	2	2	2	2	2	2	2	1	1
2	1	1	2	2	2	2	2	2	2
#NULL!	#NULL!	#NULL!	#NULL!	#NULL!	#NULL!	#NULL!	#NULL!	#NULL!	#NULL!
2	2	1	2	2	2	2	1	2	2

2	2	2	2	2	2	1	2	2	2
#NULL!	#NULL!	#NULL!	#NULL!	#NULL!	#NULL!	#NULL!	#NULL!	#NULL!	#NULL!
#NULL!	#NULL!	#NULL!	#NULL!	#NULL!	#NULL!	#NULL!	#NULL!	#NULL!	#NULL!
2	2	2	2	2	2	2	2	2	2
2	2	2	2	2	2	1	1	2	2
2	2	2	2	2	2	1	2	2	2
1	1	2	1	1	2	2	1	2	2
2	2	2	2	2	2	2	2	2	2
1	1	2	2	2	2	2	2	2	2
1	1	2	2	2	2	2	1	2	1
#NULL!	#NULL!	#NULL!	#NULL!	#NULL!	#NULL!	#NULL!	#NULL!	#NULL!	#NULL!
2	2	#NULL!	#NULL!	#NULL!	#NULL!	#NULL!	#NULL!	#NULL!	#NULL!
2	2	2	#NULL!	#NULL!	#NULL!	#NULL!	#NULL!	#NULL!	#NULL!
1	1	2	2	2	2	2	2	2	2
2	2	2	2	2	2	2	2	1	2
#NULL!	#NULL!	#NULL!	#NULL!	#NULL!	#NULL!	#NULL!	#NULL!	#NULL!	#NULL!
#NULL!	#NULL!	#NULL!	#NULL!	#NULL!	#NULL!	#NULL!	#NULL!	#NULL!	#NULL!
#NULL!	#NULL!	#NULL!	#NULL!	#NULL!	#NULL!	#NULL!	#NULL!	#NULL!	#NULL!
2	2	2	2	2	2	2	2	2	2
2	2	2	2	2	2	2	2	2	2
#NULL!	#NULL!	#NULL!	#NULL!	#NULL!	#NULL!	#NULL!	#NULL!	#NULL!	#NULL!
1	2	2	2	2	2	1	2	1	2
1	2	1	2	1	1	2	2	1	2
#NULL!	#NULL!	#NULL!	#NULL!	#NULL!	#NULL!	#NULL!	#NULL!	#NULL!	#NULL!
1	1	2	2	2	2	2	2	2	2
2	2	2	2	2	2	2	2	2	2
2	2	#NULL!	#NULL!	#NULL!	#NULL!	#NULL!	#NULL!	#NULL!	#NULL!
2	2	#NULL!	#NULL!	#NULL!	#NULL!	#NULL!	#NULL!	#NULL!	#NULL!
2	2	2	2	2	2	2	2	2	2
#NULL!	#NULL!	2	2	2	2	2	2	2	2
2	2	2	1	2	2	2	1	2	2
2	2	#NULL!	#NULL!	#NULL!	#NULL!	#NULL!	#NULL!	#NULL!	#NULL!
#NULL!	#NULL!	#NULL!	#NULL!	#NULL!	#NULL!	#NULL!	#NULL!	#NULL!	#NULL!
2	2	2	2	2	2	2	2	1	2
1	1	2	#NULL!	2	2	#NULL!	2	2	2
2	2	2	2	2	2	2	2	2	2
2	2	2	1	2	2	2	1	2	1
2	2	2	2	2	2	2	2	2	2
2	2	2	#NULL!	2	2	2	1	2	1
2	2	2	2	2	1	1	2	1	1
2	1	2	2	2	1	2	1	2	2
2	2	#NULL!	#NULL!	#NULL!	#NULL!	#NULL!	#NULL!	#NULL!	#NULL!
#NULL!	#NULL!	#NULL!	#NULL!	#NULL!	#NULL!	#NULL!	#NULL!	#NULL!	#NULL!
2	2	#NULL!	#NULL!	#NULL!	#NULL!	1	#NULL!	#NULL!	#NULL!
#NULL!	#NULL!	#NULL!	#NULL!	#NULL!	#NULL!	#NULL!	#NULL!	#NULL!	#NULL!
#NULL!	#NULL!	#NULL!	#NULL!	#NULL!	#NULL!	#NULL!	#NULL!	#NULL!	#NULL!
#NULL!	#NULL!	#NULL!	#NULL!	#NULL!	#NULL!	#NULL!	#NULL!	#NULL!	#NULL!

2	2	2	2	2	2	2	2	2	2
1	2	2	2	2	1	2	2	2	2
2	2	2	2	2	2	2	2	2	1
2	2	2	2	2	2	2	2	2	2
1	1	#NULL!	#NULL!	#NULL!	#NULL!	#NULL!	#NULL!	#NULL!	#NULL!
#NULL!	#NULL!	#NULL!	#NULL!	#NULL!	#NULL!	#NULL!	#NULL!	#NULL!	#NULL!
#NULL!	#NULL!	#NULL!	#NULL!	#NULL!	#NULL!	#NULL!	#NULL!	#NULL!	#NULL!
#NULL!	#NULL!	#NULL!	#NULL!	#NULL!	#NULL!	#NULL!	#NULL!	#NULL!	#NULL!
#NULL!	#NULL!	#NULL!	#NULL!	#NULL!	#NULL!	#NULL!	#NULL!	#NULL!	#NULL!
#NULL!	#NULL!	#NULL!	#NULL!	#NULL!	#NULL!	#NULL!	#NULL!	#NULL!	#NULL!
#NULL!	#NULL!	#NULL!	#NULL!	#NULL!	#NULL!	#NULL!	#NULL!	#NULL!	#NULL!
#NULL!	#NULL!	#NULL!	#NULL!	#NULL!	#NULL!	#NULL!	#NULL!	#NULL!	#NULL!
#NULL!	#NULL!	#NULL!	#NULL!	#NULL!	#NULL!	#NULL!	#NULL!	#NULL!	#NULL!
2	2	2	1	1	1	1	1	2	1
2	2	2	2	2	2	2	2	2	2
2	2	2	2	2	2	2	2	2	2
2	2	2	2	2	2	2	2	2	2
#NULL!	#NULL!	#NULL!	#NULL!	#NULL!	#NULL!	#NULL!	#NULL!	#NULL!	#NULL!
#NULL!	#NULL!	#NULL!	#NULL!	#NULL!	#NULL!	#NULL!	#NULL!	#NULL!	#NULL!
#NULL!	#NULL!	#NULL!	#NULL!	#NULL!	#NULL!	#NULL!	#NULL!	#NULL!	#NULL!
2	2	2	#NULL!	#NULL!	#NULL!	#NULL!	#NULL!	2	#NULL!
#NULL!	#NULL!	#NULL!	#NULL!	#NULL!	#NULL!	#NULL!	#NULL!	#NULL!	#NULL!
2	2	2	1	2	2	2	2	2	2
#NULL!	#NULL!	#NULL!	#NULL!	#NULL!	#NULL!	#NULL!	#NULL!	#NULL!	#NULL!
1	2	2	1	2	2	1	1	2	1
1	1	2	1	2	2	1	1	2	2
1	1	2	1	2	2	1	1	2	1
1	1	2	2	2	2	1	1	2	1
1	1	2	1	2	2	2	2	2	1
1	1	2	1	2	2	1	1	2	2
1	1	2	1	2	2	2	2	2	1
2	2	2	1	2	2	2	2	2	1
1	1	2	1	2	2	1	1	2	1
#NULL!	#NULL!	#NULL!	#NULL!	#NULL!	#NULL!	#NULL!	#NULL!	#NULL!	#NULL!
1	1	2	1	2	2	1	1	2	1
1	1	2	1	2	2	2	2	2	1
2	2	2	2	2	2	2	2	2	2
#NULL!	#NULL!	#NULL!	#NULL!	#NULL!	#NULL!	#NULL!	#NULL!	#NULL!	#NULL!
1	1	2	2	2	2	2	2	2	2
2	2	1	2	2	2	2	1	1	1
2	2	2	2	1	1	2	2	1	2
#NULL!	#NULL!	#NULL!	#NULL!	#NULL!	#NULL!	#NULL!	#NULL!	#NULL!	#NULL!
#NULL!	#NULL!	#NULL!	#NULL!	#NULL!	#NULL!	#NULL!	#NULL!	#NULL!	#NULL!
#NULL!	#NULL!	#NULL!	#NULL!	#NULL!	#NULL!	#NULL!	#NULL!	#NULL!	#NULL!
2	1	2	2	2	#NULL!	#NULL!	#NULL!	#NULL!	#NULL!
1	1	2	2	2	2	2	2	1	2
2	2	#NULL!	#NULL!	#NULL!	#NULL!	#NULL!	#NULL!	#NULL!	#NULL!

#NULL!	#NULL!	#NULL!	#NULL!	#NULL!	#NULL!	#NULL!	#NULL!	#NULL!	#NULL!
#NULL!	#NULL!	#NULL!	#NULL!	#NULL!	#NULL!	#NULL!	#NULL!	#NULL!	#NULL!
#NULL!	#NULL!	#NULL!	#NULL!	#NULL!	#NULL!	#NULL!	#NULL!	#NULL!	#NULL!
#NULL!	#NULL!	#NULL!	#NULL!	#NULL!	#NULL!	#NULL!	#NULL!	#NULL!	#NULL!
2	2	#NULL!	#NULL!	#NULL!	#NULL!	#NULL!	#NULL!	#NULL!	#NULL!
#NULL!	#NULL!	#NULL!	#NULL!	#NULL!	#NULL!	#NULL!	#NULL!	#NULL!	#NULL!
#NULL!	#NULL!	#NULL!	#NULL!	#NULL!	#NULL!	#NULL!	#NULL!	#NULL!	#NULL!
#NULL!	#NULL!	#NULL!	#NULL!	#NULL!	#NULL!	#NULL!	#NULL!	#NULL!	#NULL!
2	2	#NULL!	#NULL!	#NULL!	#NULL!	#NULL!	#NULL!	#NULL!	#NULL!
#NULL!	#NULL!	#NULL!	#NULL!	#NULL!	#NULL!	#NULL!	#NULL!	#NULL!	#NULL!
2	2	#NULL!	#NULL!	2	#NULL!	#NULL!	#NULL!	#NULL!	#NULL!
#NULL!	#NULL!	#NULL!	#NULL!	#NULL!	#NULL!	#NULL!	#NULL!	#NULL!	#NULL!
2	1	2	2	2	2	1	2	2	2
1	1	2	2	2	2	2	2	#NULL!	1
2	2	2	2	2	2	1	1	2	2
1	2	2	1	1	2	1	2	1	2
2	2	2	2	2	2	2	2	2	2
2	2	2	1	2	2	2	1	2	1
2	2	2	1	2	2	2	1	2	1
1	1	2	#NULL!	#NULL!	#NULL!	#NULL!	#NULL!	#NULL!	#NULL!
1	1	2	1	2	2	1	1	2	2
2	2	2	2	2	2	2	1	2	2
2	2	2	2	2	2	2	2	2	2
2	2	2	2	2	2	1	2	2	1
1	1	2	2	2	2	2	2	2	1
2	2	2	2	1	1	2	2	1	2
2	2	2	2	#NULL!	#NULL!	2	#NULL!	1	2
1	1	2	2	2	2	2	2	2	2
2	2	2	#NULL!	#NULL!	#NULL!	#NULL!	#NULL!	#NULL!	#NULL!
2	1	#NULL!	#NULL!	#NULL!	#NULL!	#NULL!	#NULL!	#NULL!	#NULL!
#NULL!	#NULL!	#NULL!	#NULL!	#NULL!	#NULL!	#NULL!	#NULL!	#NULL!	#NULL!
#NULL!	#NULL!	2	1	2	2	1	1	2	1
2	2	2	2	2	2	2	2	2	2
2	2	2	2	2	2	1	1	2	1
2	2	2	2	2	2	1	1	2	2
2	2	2	2	2	2	2	2	2	2
2	2	2	2	2	2	2	2	2	2
2	2	2	2	2	2	1	1	2	1
2	2	2	2	2	2	2	2	2	2
2	2	2	1	2	2	2	2	2	1
2	2	2	2	2	2	2	2	2	2
2	2	2	1	2	2	1	1	2	2
1	2	2	2	1	1	2	2	2	2
2	2	2	1	2	2	2	2	2	1
1	1	2	1	2	2	2	2	2	1
1	1	2	1	2	2	2	1	2	1
2	2	2	2	2	2	2	2	2	2

#NULL!	#NULL!	#NULL!	#NULL!	#NULL!	#NULL!	#NULL!	#NULL!	#NULL!	#NULL!
1	1	2	1	2	1	2	2	2	2
2	2	#NULL!	#NULL!	#NULL!	#NULL!	2	#NULL!	#NULL!	#NULL!
2	2	#NULL!	#NULL!	1	#NULL!	#NULL!	#NULL!	#NULL!	#NULL!
2	2	2	#NULL!	#NULL!	#NULL!	#NULL!	#NULL!	#NULL!	#NULL!
2	2	#NULL!	#NULL!	#NULL!	#NULL!	#NULL!	#NULL!	#NULL!	#NULL!
2	2	#NULL!	2	#NULL!	#NULL!	#NULL!	#NULL!	#NULL!	#NULL!
2	2	2	2	2	2	2	2	2	2
2	2	2	#NULL!	#NULL!	#NULL!	#NULL!	#NULL!	#NULL!	#NULL!
2	2	2	2	2	2	2	2	2	2
#NULL!	#NULL!	#NULL!	#NULL!	#NULL!	#NULL!	#NULL!	#NULL!	#NULL!	#NULL!
2	2	2	2	2	2	2	1	2	1
2	2	2	1	2	2	2	1	2	1
1	1	2	2	2	2	2	2	2	2
1	1	2	1	2	2	2	2	2	2
#NULL!	#NULL!	#NULL!	#NULL!	#NULL!	#NULL!	#NULL!	#NULL!	#NULL!	#NULL!
2	2	2	2	2	2	2	2	2	2
2	1	1	2	1	1	2	2	1	2
2	2	2	1	1	2	2	2	2	2
1	2	2	2	2	2	2	2	2	2
2	2	2	2	2	2	2	2	2	2
2	2	1	2	2	2	2	2	1	2
#NULL!	#NULL!	2	2	2	2	2	2	2	2
2	2	2	2	2	2	2	2	2	2
#NULL!	2	2	2	2	2	2	1	2	2
2	2	2	2	2	2	2	2	2	2
2	2	2	2	1	2	2	2	1	2
2	1	2	2	1	2	1	1	1	2
1	1	2	1	2	2	1	1	2	1
2	2	2	2	2	2	2	1	2	1
#NULL!	#NULL!	#NULL!	#NULL!	#NULL!	#NULL!	#NULL!	#NULL!	#NULL!	#NULL!
2	2	2	2	2	2	2	2	2	2
#NULL!	#NULL!	#NULL!	#NULL!	#NULL!	#NULL!	#NULL!	#NULL!	#NULL!	#NULL!
2	2	2	2	2	2	2	2	2	2
2	2	2	2	2	2	2	2	2	2
1	1	2	1	2	2	2	1	2	2
2	1	1	2	1	1	2	2	1	2
1	2	2	1	2	2	2	1	2	2
1	1	2	1	2	2	1	1	2	1
1	1	2	2	2	2	1	2	2	2
2	2	2	2	2	2	2	2	2	2
1	1	2	2	2	2	1	2	2	2
#NULL!	#NULL!	#NULL!	#NULL!	#NULL!	#NULL!	#NULL!	#NULL!	#NULL!	#NULL!
1	2	1	#NULL!	#NULL!	#NULL!	#NULL!	#NULL!	#NULL!	#NULL!
1	1	2	1	#NULL!	#NULL!	#NULL!	#NULL!	#NULL!	#NULL!
#NULL!	#NULL!	#NULL!	#NULL!	#NULL!	#NULL!	#NULL!	#NULL!	#NULL!	#NULL!
#NULL!	2	#NULL!	#NULL!	#NULL!	#NULL!	#NULL!	#NULL!	#NULL!	#NULL!

1	1	#NULL!	#NULL!	#NULL!	#NULL!	#NULL!	#NULL!	#NULL!	#NULL!
2	2	2	2	2	2	2	2	2	2
2	2	2	#NULL!	#NULL!	#NULL!	#NULL!	#NULL!	#NULL!	#NULL!
2	2	2	2	2	2	1	1	2	1
2	2	#NULL!	#NULL!	#NULL!	#NULL!	#NULL!	#NULL!	#NULL!	#NULL!
2	2	1	2	1	1	2	2	2	2
2	2	2	2	1	1	2	2	1	#NULL!
#NULL!	#NULL!	#NULL!	#NULL!	#NULL!	#NULL!	#NULL!	#NULL!	#NULL!	#NULL!
#NULL!	#NULL!	2	2	#NULL!	#NULL!	#NULL!	#NULL!	#NULL!	#NULL!
#NULL!	#NULL!	#NULL!	#NULL!	#NULL!	#NULL!	#NULL!	#NULL!	#NULL!	#NULL!
2	2	2	#NULL!	#NULL!	#NULL!	#NULL!	#NULL!	#NULL!	#NULL!
2	2	2	#NULL!	#NULL!	#NULL!	#NULL!	#NULL!	#NULL!	#NULL!
1	2	2	2	2	2	1	2	2	1
#NULL!	#NULL!	#NULL!	#NULL!	#NULL!	#NULL!	#NULL!	#NULL!	#NULL!	#NULL!
2	2	2	#NULL!	#NULL!	#NULL!	#NULL!	#NULL!	#NULL!	#NULL!
2	2	2	2	2	2	2	2	2	2
2	2	2	1	2	1	2	1	2	2
2	2	2	2	2	2	2	2	2	2
2	2	2	1	2	2	2	2	1	2
2	2	2	2	2	2	1	1	2	2
2	2	2	2	2	2	1	2	2	2
2	2	2	2	2	2	2	2	2	2
2	2	2	2	2	2	1	1	1	1
2	2	2	2	1	2	1	2	2	1
2	2	2	2	2	2	2	2	2	2
1	1	2	2	2	2	2	2	2	2
2	2	2	2	1	1	2	2	2	2
1	1	2	2	2	2	2	2	2	2
#NULL!	#NULL!	2	1	2	2	1	2	2	2
1	2	2	2	2	2	2	1	2	2
1	1	2	1	1	1	2	1	2	2
2	2	2	2	2	2	2	2	2	2
#NULL!	#NULL!	#NULL!	#NULL!	#NULL!	#NULL!	#NULL!	#NULL!	#NULL!	#NULL!
1	2	2	2	1	2	1	2	2	1
2	2	2	2	2	2	2	2	2	2
2	2	2	2	2	2	2	2	2	2
2	2	2	1	2	2	1	2	2	2
#NULL!	2	2	2	1	2	2	2	1	1
2	2	#NULL!	#NULL!	#NULL!	#NULL!	#NULL!	#NULL!	#NULL!	#NULL!
2	2	2	2	2	2	2	2	2	2
2	2	#NULL!	#NULL!	#NULL!	#NULL!	#NULL!	2	#NULL!	#NULL!
2	2	2	#NULL!	#NULL!	#NULL!	#NULL!	#NULL!	#NULL!	#NULL!
2	2	2	2	2	2	2	2	2	1
2	2	2	#NULL!	#NULL!	#NULL!	#NULL!	#NULL!	#NULL!	#NULL!
1	1	2	1	2	2	2	2	2	1
2	1	2	1	2	1	2	1	2	2
2	2	2	1	2	2	1	1	2	1

2	2	1	2	1	1	2	2	1	2
2	2	#NULL!	#NULL!	#NULL!	#NULL!	#NULL!	#NULL!	#NULL!	1
2	2	1	2	#NULL!	1	2	2	1	2
1	2	2	2	2	1	1	2	2	2
2	2	2	2	2	1	2	2	2	2
2	2	2	1	1	2	2	1	2	2
1	1	2	2	1	2	2	2	1	1
1	1	2	2	2	2	2	2	2	2
2	2	2	2	2	2	2	2	2	2
2	2	2	2	2	1	1	2	1	1
2	2	2	2	2	2	2	1	1	2
2	1	2	2	1	1	2	1	2	2
2	1	2	2	2	2	2	1	1	2
2	2	2	2	2	2	2	2	2	2
2	2	2	2	2	2	2	2	2	2
2	2	2	2	2	2	2	2	1	1
2	2	2	2	2	2	2	2	2	2
2	2	2	2	2	2	2	1	2	2
2	2	2	2	2	2	2	2	2	2
2	2	2	2	2	2	2	2	2	2
1	1	2	2	2	2	2	2	2	2
2	1	2	2	2	2	1	1	2	2
2	2	2	2	2	2	2	2	2	2
#NULL!	#NULL!	#NULL!	#NULL!	#NULL!	#NULL!	#NULL!	#NULL!	#NULL!	#NULL!
2	2	2	2	2	2	2	2	2	2
#NULL!	#NULL!	2	2	2	2	1	1	2	1
2	2	2	2	1	1	2	1	2	2
#NULL!	#NULL!	2	2	2	2	2	2	2	1
2	1	2	2	2	1	2	1	2	1
2	2	2	#NULL!	#NULL!	#NULL!	#NULL!	#NULL!	#NULL!	#NULL!
1	2	2	2	1	2	1	2	2	2
2	2	1	1	2	1	1	2	1	2
2	2	2	2	2	2	2	2	2	1
2	2	2	2	2	2	2	2	2	2
1	1	2	1	2	1	1	2	2	2
2	2	2	2	2	2	2	2	2	2
2	2	2	2	2	2	2	2	2	2
#NULL!	#NULL!	#NULL!	#NULL!	#NULL!	#NULL!	#NULL!	#NULL!	#NULL!	#NULL!
2	2	2	2	2	1	2	1	2	2
2	2	2	2	2	2	2	2	2	2
#NULL!	#NULL!	#NULL!	#NULL!	#NULL!	#NULL!	#NULL!	#NULL!	#NULL!	#NULL!
#NULL!	#NULL!	#NULL!	#NULL!	#NULL!	#NULL!	#NULL!	#NULL!	#NULL!	#NULL!
2	2	2	2	2	2	2	2	2	2
2	1	#NULL!	#NULL!	#NULL!	#NULL!	#NULL!	#NULL!	#NULL!	#NULL!
2	2	2	2	2	2	2	2	2	2
2	2	2	2	2	2	2	2	2	2
#NULL!	#NULL!	#NULL!	#NULL!	#NULL!	#NULL!	#NULL!	#NULL!	#NULL!	#NULL!

2	2	2	2	2	2	2	2	2	2
#NULL!	#NULL!	#NULL!	#NULL!	#NULL!	#NULL!	#NULL!	#NULL!	#NULL!	#NULL!
2	2	2	2	2	2	2	2	1	2
#NULL!	#NULL!	#NULL!	#NULL!	#NULL!	#NULL!	#NULL!	#NULL!	#NULL!	#NULL!
#NULL!	#NULL!	#NULL!	#NULL!	#NULL!	#NULL!	#NULL!	#NULL!	#NULL!	#NULL!
1	1	1	2	1	1	2	2	1	2
2	2	2	2	2	2	2	2	2	2
2	2	2	2	2	2	2	2	2	2
1	1	1	2	1	1	2	2	2	2
2	2	2	2	2	2	2	2	2	2
#NULL!	#NULL!	#NULL!	#NULL!	#NULL!	#NULL!	#NULL!	#NULL!	#NULL!	#NULL!
2	2	#NULL!	#NULL!	#NULL!	#NULL!	#NULL!	#NULL!	#NULL!	#NULL!
#NULL!	#NULL!	#NULL!	#NULL!	#NULL!	#NULL!	#NULL!	#NULL!	#NULL!	#NULL!
1	1	2	1	2	2	2	2	2	1
2	2	2	2	2	2	2	2	2	2
2	2	2	1	2	2	2	2	2	1
2	2	2	#NULL!	#NULL!	#NULL!	#NULL!	#NULL!	#NULL!	#NULL!
#NULL!	#NULL!	#NULL!	#NULL!	#NULL!	#NULL!	#NULL!	#NULL!	#NULL!	#NULL!
2	2	1	2	1	1	2	2	1	2
2	2	2	2	2	2	2	2	2	2
2	2	2	1	2	2	2	1	2	1
2	2	2	2	1	1	2	2	1	2
2	2	2	#NULL!	#NULL!	#NULL!	#NULL!	#NULL!	#NULL!	#NULL!
2	2	2	2	2	1	2	2	1	2
2	2	2	2	2	2	2	2	1	2
2	2	2	2	2	2	2	2	2	2
2	2	2	2	#NULL!	#NULL!	1	#NULL!	#NULL!	#NULL!
1	1	2	1	2	2	1	1	2	1
2	2	2	2	2	2	2	2	2	2
2	2	2	#NULL!	#NULL!	#NULL!	#NULL!	#NULL!	#NULL!	#NULL!
2	2	2	2	2	2	1	1	2	2
2	2	2	2	2	2	2	2	2	2
2	2	2	2	2	1	1	2	2	2
2	2	2	2	2	2	2	2	1	2
2	2	2	2	2	2	2	2	2	2
1	1	2	1	1	2	2	2	2	2
1	1	2	2	2	2	2	2	2	2
2	2	1	2	1	1	2	2	1	2
2	2	2	2	2	2	2	2	2	2
2	2	1	2	1	1	2	2	1	2
2	2	2	2	2	2	2	2	2	2
2	2	2	2	2	#NULL!	2	2	2	2
2	2	2	2	2	2	2	2	2	2
2	2	2	2	2	2	2	2	2	2
2	2	2	2	2	1	1	2	2	2
2	2	2	2	2	2	1	2	2	1
#NULL!	#NULL!	2	2	2	2	2	2	2	2

2	2	2	2	2	2	2	2	2	2
2	2	2	2	2	2	2	2	2	2
1	1	2	1	2	2	2	2	2	2
2	2	2	2	2	2	2	1	2	2
2	2	2	2	2	2	2	2	2	2
1	1	2	#NULL!	#NULL!	#NULL!	#NULL!	#NULL!	#NULL!	#NULL!
2	2	2	2	2	2	2	2	2	2
1	1	2	1	2	2	2	2	2	2
2	2	2	2	2	2	2	2	2	2
2	2	2	2	2	2	2	2	2	2
2	2	2	2	1	2	2	2	2	2
2	2	2	2	2	2	2	2	2	2
2	2	2	2	2	2	2	2	2	2
2	2	1	2	1	1	2	2	1	2
2	2	2	2	2	1	2	2	2	2
2	2	2	1	2	1	2	1	1	2
2	2	2	2	2	1	2	2	1	2
2	2	2	2	2	2	2	2	1	2
2	2	2	2	2	2	2	2	2	2
2	2	2	1	2	2	1	2	2	2
1	2	2	2	2	2	2	2	2	2
2	2	2	1	2	2	2	2	1	2
2	2	2	1	2	2	2	2	1	2
2	2	#NULL!	#NULL!	#NULL!	#NULL!	#NULL!	#NULL!	#NULL!	#NULL!
2	2	2	2	2	2	2	2	2	#NULL!
2	2	2	2	2	2	2	2	2	2
2	2	2	2	2	2	2	2	2	2
2	2	2	2	2	2	2	2	2	2
2	2	2	2	2	2	2	2	2	2
1	1	2	1	2	2	1	1	2	1
2	2	1	2	1	2	2	2	1	2
2	2	2	2	2	1	2	2	1	2
2	2	2	1	2	2	2	2	1	1
2	2	2	2	2	2	2	2	1	2
1	1	2	1	2	2	2	2	1	1
2	2	2	2	2	2	2	2	2	2
2	2	2	2	2	2	2	2	2	2
2	2	2	2	2	2	2	2	2	2
2	2	2	2	2	2	2	2	2	2
2	2	2	2	2	2	2	2	2	2
2	2	2	2	1	2	2	2	2	2
2	2	2	2	2	2	2	2	2	2
1	1	2	2	2	2	2	2	2	2
2	2	2	2	2	2	2	2	2	2
2	2	2	2	2	1	2	2	2	1
2	2	2	2	2	1	2	2	2	2
2	2	2	2	2	2	2	2	2	2

2	2	2	2	2	2	2	2	2	2
1	1	2	2	2	2	2	2	2	2
2	2	2	2	2	2	2	#NULL!	2	2
2	2	2	2	2	2	2	2	2	2
2	2	2	2	2	2	2	2	2	2
2	1	2	2	2	2	2	2	2	2
2	2	2	2	2	2	2	2	2	2
2	2	2	2	2	2	2	2	2	2
2	2	2	2	2	2	2	2	2	2
2	2	2	2	2	2	2	2	2	2
2	2	2	2	2	2	2	2	2	2
2	2	2	#NULL!	#NULL!	#NULL!	#NULL!	#NULL!	#NULL!	#NULL!
#NULL!	#NULL!	#NULL!	#NULL!	#NULL!	#NULL!	#NULL!	#NULL!	#NULL!	#NULL!
2	2	#NULL!	#NULL!	2	#NULL!	#NULL!	#NULL!	#NULL!	#NULL!
2	2	2	2	2	2	2	2	2	2
2	2	2	2	2	2	2	2	2	2
2	1	2	2	2	2	2	2	2	2
2	2	2	2	2	2	2	2	2	2
2	2	2	2	2	2	2	2	2	2
1	1	2	2	2	2	2	1	2	1
2	2	2	2	2	2	2	2	2	2
2	2	2	2	2	2	2	2	2	2
2	1	2	2	2	2	2	2	2	2
2	2	2	2	2	2	2	2	2	2
2	2	2	2	2	2	2	2	2	2
2	1	2	2	2	2	2	1	2	1
2	2	2	2	2	2	2	2	2	2
2	2	2	2	2	2	2	2	2	2
2	1	2	2	2	2	2	2	2	2
#NULL!	#NULL!	#NULL!	#NULL!	#NULL!	#NULL!	#NULL!	#NULL!	#NULL!	#NULL!
2	2	2	2	2	2	2	2	2	2
2	2	2	2	2	2	2	2	2	2
1	2	2	2	2	2	2	2	2	2
2	2	2	2	2	2	2	2	2	2
2	2	2	2	2	2	2	2	2	2
2	2	2	2	2	2	2	2	2	2
2	2	2	2	2	2	2	2	2	2
2	2	2	2	2	2	2	2	2	2
2	2	2	2	2	2	2	2	2	2
2	1	2	2	2	2	2	2	2	2
2	2	2	2	2	2	2	2	2	2
2	2	2	2	2	2	2	2	2	2
2	2	2	2	2	2	2	2	2	2
2	2	2	2	2	2	2	2	2	2
2	2	2	2	2	2	2	2	2	2
2	2	2	2	2	2	2	2	2	2
2	2	2	2	2	2	2	2	2	2

1	2	2	2	2	2	2	2	2	2
2	2	2	2	2	2	2	2	2	2
2	2	2	2	2	2	2	2	2	2
2	1	2	2	2	2	2	2	2	2
2	2	2	2	2	2	2	2	2	2
1	2	2	2	2	2	2	2	2	2
2	2	#NULL!	#NULL!	#NULL!	#NULL!	#NULL!	#NULL!	#NULL!	#NULL!
2	2	2	2	2	2	2	1	2	2
1	1	2	2	2	2	2	1	2	2
2	2	2	2	2	2	2	2	2	2
2	2	2	2	2	2	2	2	2	2
2	1	2	2	2	2	2	2	2	2
2	2	2	2	2	2	2	2	2	2
2	2	2	2	2	#NULL!	#NULL!	#NULL!	#NULL!	#NULL!
2	1	2	2	2	2	2	2	2	2
2	1	2	2	2	2	2	2	2	2
2	1	2	2	2	2	1	2	2	2
2	2	2	2	2	2	2	2	2	2
2	2	2	1	2	2	1	1	2	1
1	1	2	1	2	2	2	2	2	2
1	1	2	1	2	2	2	2	1	2
1	1	2	2	2	2	#NULL!	1	2	2
2	2	2	2	2	2	2	2	2	2
2	2	2	2	2	2	2	2	2	2
#NULL!	#NULL!	2	2	2	2	2	2	2	2
2	2	2	2	2	2	2	2	2	2
#NULL!	#NULL!	#NULL!	#NULL!	#NULL!	#NULL!	#NULL!	#NULL!	#NULL!	#NULL!
2	2	2	2	2	2	2	2	2	2
2	2	2	2	2	2	2	2	2	2
2	2	2	2	2	2	2	2	2	2
2	2	2	2	2	2	2	2	2	2
1	1	2	2	2	2	1	1	2	2
#NULL!	#NULL!	#NULL!	#NULL!	#NULL!	#NULL!	#NULL!	#NULL!	#NULL!	#NULL!
1	1	#NULL!	#NULL!	#NULL!	#NULL!	#NULL!	#NULL!	#NULL!	#NULL!
#NULL!	#NULL!	#NULL!	#NULL!	#NULL!	#NULL!	#NULL!	#NULL!	#NULL!	#NULL!
#NULL!	#NULL!	#NULL!	#NULL!	#NULL!	#NULL!	#NULL!	#NULL!	#NULL!	#NULL!
2	2	2	2	2	2	2	2	1	2
2	1	2	2	2	1	2	2	1	2
2	2	2	2	2	2	2	2	2	2
1	2	2	2	2	2	2	2	2	2
2	2	2	2	2	1	2	2	2	2
2	2	2	2	2	2	2	2	2	2
2	2	2	1	2	2	2	1	2	1
2	2	2	2	2	2	2	2	2	2
2	1	2	2	2	2	2	2	2	2
2	2	2	2	2	2	2	2	2	2
2	2	2	1	2	2	2	1	2	1

2	1	2	1	1	2	2	2	2	2
#NULL!	#NULL!	1	2	2	2	2	2	2	#NULL!
2	2	1	2	2	#NULL!	1	2	2	2
2	2	2	2	2	2	1	2	2	2
1	2	2	2	2	1	2	2	2	1
2	2	2	2	2	2	2	2	1	1
2	2	2	1	2	2	2	2	2	2
2	2	2	#NULL!	#NULL!	#NULL!	#NULL!	#NULL!	#NULL!	#NULL!
2	2	2	2	2	2	2	2	2	2
2	2	2	2	2	2	2	2	2	2
2	2	2	2	2	2	2	2	2	2
2	2	2	2	2	2	2	2	2	2
#NULL!	#NULL!	2	2	2	2	2	2	2	2
2	#NULL!	2	2	2	2	2	1	2	2
2	1	2	2	2	2	2	2	2	2
2	2	2	2	2	2	2	2	2	2
2	2	2	2	2	2	2	2	2	2
2	1	2	2	1	2	2	1	2	2
1	1	2	2	2	2	2	2	2	2
1	2	2	2	2	2	2	2	2	2
2	2	2	2	1	1	2	2	1	2
2	2	2	2	2	2	2	2	2	2
2	2	2	2	2	2	2	2	2	2
2	2	#NULL!	#NULL!	#NULL!	#NULL!	#NULL!	#NULL!	#NULL!	#NULL!
1	1	2	1	1	2	2	1	2	1
2	2	2	2	2	2	2	2	2	2
2	2	2	2	2	2	2	2	2	2
2	1	2	2	2	2	2	2	2	2
2	2	2	2	2	2	2	2	2	2
1	1	2	#NULL!	#NULL!	#NULL!	#NULL!	#NULL!	#NULL!	#NULL!
1	1	2	2	2	2	2	2	1	2
2	2	#NULL!	#NULL!	#NULL!	#NULL!	#NULL!	#NULL!	#NULL!	#NULL!
#NULL!	#NULL!	#NULL!	#NULL!	#NULL!	#NULL!	#NULL!	#NULL!	#NULL!	#NULL!
#NULL!	#NULL!	#NULL!	#NULL!	#NULL!	#NULL!	#NULL!	#NULL!	#NULL!	#NULL!
#NULL!	#NULL!	#NULL!	#NULL!	#NULL!	#NULL!	#NULL!	#NULL!	#NULL!	#NULL!
2	2	2	2	2	2	2	2	2	2
1	1	2	2	2	1	2	2	1	2
#NULL!	#NULL!	#NULL!	#NULL!	#NULL!	#NULL!	#NULL!	#NULL!	#NULL!	#NULL!
2	2	2	2	2	2	2	2	2	2
2	2	2	2	2	2	2	2	2	2
1	1	2	2	2	2	2	2	2	2
2	2	2	2	2	2	2	2	2	2
2	1	2	2	2	2	2	2	2	2
2	2	1	2	1	2	2	2	2	2
#NULL!	#NULL!	1	2	#NULL!	#NULL!	#NULL!	#NULL!	#NULL!	#NULL!
1	1	2	1	2	2	1	1	2	1
2	2	1	2	1	1	2	2	1	2

2	2	2	1	2	2	1	1	2	1
2	2	2	2	2	2	2	2	2	2
2	2	2	1	2	2	1	1	2	1
2	1	2	2	2	2	2	2	2	2
#NULL!	#NULL!	#NULL!	#NULL!	#NULL!	#NULL!	#NULL!	#NULL!	#NULL!	#NULL!
#NULL!	#NULL!	#NULL!	#NULL!	#NULL!	#NULL!	#NULL!	#NULL!	#NULL!	#NULL!
2	2	2	2	2	2	2	2	2	2
2	2	2	2	2	2	2	2	2	2
2	1	2	#NULL!	#NULL!	#NULL!	#NULL!	#NULL!	#NULL!	#NULL!
2	2	#NULL!	#NULL!	#NULL!	#NULL!	#NULL!	#NULL!	#NULL!	#NULL!
1	1	2	1	2	2	1	2	2	2
2	2	2	2	2	2	1	1	2	1
2	2	2	2	2	2	1	1	1	2
2	2	2	2	2	1	2	2	1	2
#NULL!	#NULL!	#NULL!	#NULL!	#NULL!	#NULL!	2	#NULL!	#NULL!	#NULL!
2	2	2	2	2	1	1	2	2	2
2	2	2	2	2	2	2	1	2	2
2	1	2	2	1	2	1	2	2	2
2	2	#NULL!	2	#NULL!	#NULL!	#NULL!	#NULL!	#NULL!	#NULL!
1	2	2	2	1	2	2	1	2	2
2	2	2	2	2	2	2	1	1	1
2	1	2	2	2	2	1	1	2	1
#NULL!	#NULL!	2	2	2	2	2	1	2	2
2	2	2	2	2	2	2	2	2	2
2	2	1	1	2	2	1	2	1	2
2	2	2	2	2	2	2	2	2	2
2	1	2	2	2	2	2	1	2	2
2	2	1	2	2	2	2	2	2	2
2	2	2	2	1	2	2	2	1	1
2	2	2	2	1	1	1	2	1	#NULL!
2	2	2	2	2	2	2	2	2	2
2	2	2	1	2	2	2	2	2	2
2	2	2	2	2	1	2	2	2	2
1	1	2	2	2	2	2	1	2	2
2	2	#NULL!	#NULL!	2	#NULL!	#NULL!	#NULL!	#NULL!	#NULL!
2	2	2	2	2	2	1	2	2	2
2	2	#NULL!	#NULL!	#NULL!	#NULL!	#NULL!	#NULL!	#NULL!	#NULL!
2	2	2	2	2	2	2	2	2	2
1	2	2	2	2	2	2	2	2	2
#NULL!	#NULL!	2	2	2	2	1	2	2	2
#NULL!	#NULL!	#NULL!	#NULL!	#NULL!	#NULL!	#NULL!	#NULL!	#NULL!	#NULL!
1	2	2	1	1	2	2	2	2	2
2	2	2	2	2	2	2	2	1	2
2	2	2	2	2	2	2	2	2	2
2	2	2	2	2	2	2	2	2	2
#NULL!	#NULL!	2	2	1	2	2	2	1	2
2	1	1	2	2	1	2	2	1	2

2	2	2	2	2	2	2	2	1	2
2	2	1	2	1	1	2	2	1	2
2	2	2	2	2	2	2	2	2	2
2	2	1	2	1	1	2	2	1	2
2	2	2	1	2	2	2	1	2	2
#NULL!	#NULL!	#NULL!	#NULL!	#NULL!	#NULL!	#NULL!	#NULL!	#NULL!	#NULL!
#NULL!	#NULL!	#NULL!	#NULL!	#NULL!	#NULL!	#NULL!	#NULL!	#NULL!	#NULL!
2	2	2	2	2	2	2	2	2	2
1	1	2	2	2	2	2	2	1	2
2	2	2	2	2	2	2	2	2	2
2	2	2	2	2	2	1	2	2	2
2	2	2	2	1	2	1	2	2	1
2	2	2	2	2	1	2	2	2	2
2	2	2	2	2	2	2	2	2	1
#NULL!	#NULL!	#NULL!	#NULL!	#NULL!	#NULL!	#NULL!	#NULL!	#NULL!	#NULL!
2	2	2	2	1	1	2	2	1	2
1	1	2	2	2	2	2	2	2	2
2	2	2	2	2	2	2	2	2	2
1	1	2	2	2	2	2	2	2	1
1	2	2	2	2	2	2	2	2	2
#NULL!	#NULL!	#NULL!	#NULL!	#NULL!	#NULL!	#NULL!	#NULL!	#NULL!	#NULL!
#NULL!	#NULL!	#NULL!	#NULL!	#NULL!	#NULL!	#NULL!	#NULL!	#NULL!	2
2	2	2	2	2	2	2	2	2	2
2	2	2	#NULL!	#NULL!	#NULL!	#NULL!	#NULL!	#NULL!	#NULL!
#NULL!	#NULL!	#NULL!	#NULL!	#NULL!	#NULL!	#NULL!	#NULL!	#NULL!	#NULL!
2	2	1	#NULL!	2	2	2	2	1	2
2	2	2	2	1	2	2	2	2	2
2	2	2	2	2	2	2	2	2	2
2	2	1	2	1	1	2	2	1	2
2	2	2	2	2	2	2	2	2	2
2	2	1	2	1	1	2	2	1	2
2	2	2	2	2	2	2	2	2	2
#NULL!	#NULL!	#NULL!	#NULL!	#NULL!	#NULL!	#NULL!	#NULL!	#NULL!	#NULL!
2	2	#NULL!	#NULL!	#NULL!	#NULL!	#NULL!	#NULL!	#NULL!	#NULL!
#NULL!	#NULL!	#NULL!	#NULL!	#NULL!	#NULL!	#NULL!	#NULL!	#NULL!	#NULL!
2	2	2	2	2	2	2	2	2	2
1	1	2	2	1	1	2	1	2	2
2	2	1	2	2	2	2	1	2	1
2	2	2	2	2	2	2	2	2	2
1	2	2	#NULL!	#NULL!	#NULL!	#NULL!	#NULL!	#NULL!	#NULL!
2	2	2	2	2	2	2	2	2	2
2	2	2	2	2	2	2	1	2	2
2	2	1	2	1	2	2	2	2	2
2	2	#NULL!	#NULL!	#NULL!	#NULL!	#NULL!	#NULL!	#NULL!	#NULL!
1	1	1	1	2	1	2	2	2	1
2	2	#NULL!	#NULL!	#NULL!	#NULL!	#NULL!	#NULL!	#NULL!	#NULL!
2	2	1	2	2	2	2	2	1	2

1	2	2	2	2	2	1	2	2	2
2	2	2	2	1	1	2	2	2	2
2	2	1	2	1	1	1	1	1	2
1	2	2	2	2	2	2	1	2	2
2	2	2	2	2	2	2	2	2	2
2	1	2	2	2	2	2	2	2	2
2	1	2	2	1	2	2	2	2	2
2	2	2	2	2	2	2	2	1	2
#NULL!	#NULL!	#NULL!	#NULL!	#NULL!	#NULL!	#NULL!	#NULL!	#NULL!	#NULL!
1	1	2	2	2	2	2	2	2	2
2	2	2	2	2	1	2	2	1	2
2	2	2	2	2	2	2	2	2	2
2	1	2	2	2	2	2	2	2	2
2	1	2	2	2	2	2	1	2	2
1	2	#NULL!	2	#NULL!	#NULL!	#NULL!	#NULL!	#NULL!	#NULL!
2	2	2	2	1	1	2	2	2	2
2	2	2	2	2	2	1	1	1	2
2	2	2	2	1	2	2	2	2	2
2	2	2	2	2	2	1	2	2	1
2	2	2	2	2	2	2	2	2	2
1	2	2	2	2	2	1	1	2	2
2	2	2	2	2	2	2	2	2	2
2	2	2	2	2	2	2	1	2	1
2	2	2	2	2	2	2	2	2	2
2	2	2	2	2	2	2	2	2	2
2	2	2	2	2	1	2	2	1	2
2	2	2	2	1	2	2	2	2	2
2	2	2	2	2	2	2	2	2	2
2	2	2	2	2	2	2	2	2	2
2	2	2	2	2	2	2	2	2	2
2	2	2	2	2	2	#NULL!	1	2	2
2	1	2	#NULL!	1	2	#NULL!	1	2	2
2	1	2	2	2	2	2	2	2	2
2	2	2	1	2	2	2	1	2	2
2	1	2	2	1	2	2	2	2	2
2	2	2	2	2	2	2	2	2	2
2	2	2	2	2	2	2	2	2	1
#NULL!	#NULL!	2	1	2	2	2	1	2	1
2	2	2	2	2	2	2	2	2	2
1	1	2	1	2	2	1	1	2	1
2	2	2	2	1	1	2	2	1	2
1	2	2	1	2	2	1	1	2	1
2	2	2	1	2	2	2	1	2	2
2	2	2	2	2	2	1	1	2	1
1	2	2	2	2	2	2	1	2	1
1	1	2	2	2	2	1	1	2	2
2	1	2	2	1	1	2	1	2	1

2	2	2	1	2	2	2	1	2	1
2	1	2	2	2	2	2	2	2	1
1	2	2	2	2	2	2	1	2	2
1	2	2	2	2	1	2	1	2	2
1	2	2	1	2	2	1	2	2	1
2	1	1	1	2	2	2	2	2	1
1	2	2	2	2	1	2	1	2	2
1	1	2	2	2	2	1	2	2	1

HPVPrever	HPVb4Sex2	EarlySex2	Multipartn	Smoke2	Alcohol2	Over4Baby	HighFat2	BirthPill2	Spirit2
2	2	2	2	2	2	2	2	2	2
2	2	2	2	1	2	1	2	2	2
2	2	2	2	2	2	2	2	2	2
2	2	2	2	1	2	1	2	2	2
2	2	2	2	1	2	1	2	2	2
2	2	2	2	1	2	1	2	2	2
#NULL!	#NULL!	#NULL!	#NULL!	2	#NULL!	#NULL!	#NULL!	#NULL!	#NULL!
2	2	2	2	2	1	2	1	1	2
2	2	#NULL!	2	2	#NULL!	2	2	#NULL!	2
#NULL!	#NULL!	#NULL!	2	#NULL!	#NULL!	#NULL!	#NULL!	#NULL!	#NULL!
2	2	1	2	2	2	2	2	2	2
1	2	2	2	2	1	2	2	2	2
2	2	2	2	2	2	2	2	2	2
2	2	2	2	2	2	2	1	2	2
2	2	2	2	2	1	2	2	2	1
2	2	2	2	2	2	1	2	2	2
1	2	2	2	1	1	2	1	2	2
2	2	2	2	2	2	1	2	2	2
2	2	2	2	2	2	1	2	2	2
2	2	1	2	2	1	2	2	1	2
2	2	1	1	2	1	1	1	2	2
2	2	1	1	2	1	1	2	2	2
2	2	2	2	2	2	2	2	2	2
2	2	1	1	1	2	1	2	1	2
2	2	2	2	2	2	2	2	2	2
2	2	2	2	2	2	2	2	2	2
2	2	2	2	2	2	2	2	2	2
2	2	2	2	2	1	2	1	2	1
2	2	2	2	2	2	2	2	2	2
2	2	2	2	2	2	2	2	2	2
2	2	2	2	2	2	2	2	2	2
2	2	2	2	2	1	2	2	2	1
2	2	2	2	2	2	2	2	2	2
2	2	2	2	2	1	2	2	2	1
2	2	2	2	2	1	2	2	2	2
2	2	2	2	2	1	2	2	2	1
2	2	2	2	2	1	2	2	2	2
2	2	2	2	2	1	2	2	2	2
2	2	2	2	2	2	2	2	2	2
2	2	2	2	2	2	2	2	2	2
2	2	2	2	2	2	2	2	2	2
2	2	2	2	2	2	2	1	2	2
2	2	2	2	1	2	#NULL!	2	1	2
2	2	1	1	2	1	1	2	1	2
2	1	2	2	2	1	1	2	2	1
2	2	2	2	1	2	1	2	1	2

2	2	2	2	1	2	1	1	2	2
2	2	2	2	2	2	2	1	2	1
2	2	2	2	2	2	2	2	2	2
2	2	2	2	2	1	1	1	2	2
2	2	2	2	2	1	1	2	2	1
2	2	2	2	1	2	1	2	2	2
2	2	2	2	1	2	1	2	2	2
2	2	2	2	2	1	1	#NULL!	2	#NULL!
2	2	2	2	#NULL!	#NULL!	#NULL!	#NULL!	#NULL!	#NULL!
2	1	2	2	2	1	1	1	2	2
2	2	2	2	2	2	2	#NULL!	#NULL!	#NULL!
2	2	2	2	2	2	2	2	2	2
2	2	2	2	2	2	1	2	2	2
2	2	2	2	2	1	2	2	2	2
2	2	2	2	2	1	2	2	2	2
2	2	2	2	2	1	1	2	2	2
2	2	2	2	2	2	2	2	2	2
2	2	2	2	2	2	2	2	2	2
2	2	2	2	1	2	2	2	2	2
#NULL!	#NULL!	2	2	2	1	2	2	1	2
2	2	2	2	1	2	1	2	1	2
2	2	2	2	1	2	1	2	1	2
2	2	2	2	1	2	1	2	1	2
2	2	2	2	1	2	2	2	2	2
#NULL!	2	2	2	#NULL!	#NULL!	2	#NULL!	#NULL!	#NULL!
#NULL!	2	2	2	2	1	1	2	2	2
#NULL!	#NULL!	2	2	2	1	1	1	1	2
2	2	2	1	1	2	2	2	2	2
2	2	2	2	2	2	2	2	2	2
2	2	2	2	2	1	2	2	2	2
#NULL!	#NULL!	2	#NULL!	2	1	1	2	1	2
1	1	1	1	2	1	2	2	1	1
#NULL!	#NULL!	2	#NULL!	2	1	1	2	2	#NULL!
2	2	2	2	2	1	1	2	2	2
2	2	2	1	2	1	1	1	2	2
#NULL!	#NULL!	#NULL!	#NULL!	#NULL!	#NULL!	#NULL!	#NULL!	#NULL!	#NULL!
#NULL!	#NULL!	#NULL!	#NULL!	#NULL!	#NULL!	#NULL!	#NULL!	#NULL!	#NULL!
2	2	2	2	2	1	2	2	2	2
2	2	1	1	1	2	1	2	2	2
2	2	2	2	2	1	1	1	2	1
2	2	2	2	#NULL!	1	2	2	2	2
#NULL!	#NULL!	2	#NULL!	2	1	#NULL!	1	2	2
2	1	2	2	1	2	1	2	1	2
2	2	2	2	2	1	1	1	2	2
2	2	2	2	2	1	1	2	2	1
#NULL!	#NULL!	#NULL!	#NULL!	#NULL!	#NULL!	#NULL!	#NULL!	#NULL!	#NULL!
#NULL!	#NULL!	#NULL!	#NULL!	#NULL!	#NULL!	#NULL!	#NULL!	#NULL!	#NULL!

2	2	1	2	2	2	1	2	2	1
2	2	2	2	2	2	2	2	2	2
2	2	2	2	2	2	1	1	2	2
2	2	2	2	2	1	1	2	2	2
#NULL!	#NULL!	#NULL!	#NULL!	#NULL!	#NULL!	#NULL!	#NULL!	#NULL!	#NULL!
2	2	2	#NULL!	#NULL!	1	#NULL!	#NULL!	#NULL!	2
2	2	2	2	2	1	1	2	2	1
2	2	2	2	1	1	1	2	2	2
2	2	2	2	1	2	1	2	1	2
2	2	#NULL!	#NULL!	2	1	#NULL!	1	#NULL!	#NULL!
2	2	2	2	2	2	1	2	1	2
1	2	2	1	2	2	2	1	2	2
#NULL!	#NULL!	1	2	1	2	1	2	2	2
#NULL!	#NULL!	2	#NULL!	#NULL!	#NULL!	#NULL!	#NULL!	#NULL!	#NULL!
2	2	2	2	2	2	2	2	2	1
2	1	2	2	2	2	2	2	2	1
2	2	2	2	2	2	2	2	2	2
2	2	2	2	2	2	2	2	2	2
#NULL!	#NULL!	2	2	2	2	2	2	2	2
2	2	2	2	2	2	1	2	2	1
2	2	2	2	1	1	2	1	2	2
2	2	2	2	2	2	2	2	2	2
2	2	2	2	1	2	2	2	1	2
1	1	1	1	1	2	1	2	1	2
2	2	2	2	1	2	2	1	2	2
2	2	2	2	2	1	2	2	2	2
2	2	2	2	2	2	2	2	2	2
2	2	2	2	2	2	2	2	2	2
2	2	2	2	2	2	2	2	2	2
2	2	2	2	1	2	1	1	2	2
2	2	2	2	2	2	2	2	2	2
2	2	2	2	2	2	2	2	2	2
2	2	2	2	2	2	1	2	2	1
2	2	2	2	2	2	2	2	2	2
2	2	2	2	1	2	1	1	2	2
2	2	2	2	2	1	1	2	2	2
2	2	2	2	2	2	2	2	2	2
2	2	2	2	2	2	2	2	2	2
2	#NULL!	#NULL!	#NULL!	2	#NULL!	#NULL!	#NULL!	#NULL!	1
2	2	2	2	1	2	1	2	2	1
2	2	2	2	2	2	2	2	2	2
2	2	2	2	2	2	2	2	2	2
2	2	2	2	2	2	2	2	2	2
2	2	2	1	2	1	2	2	2	2
2	2	2	2	2	1	2	2	2	2
2	2	2	2	2	1	2	2	2	2
2	2	2	2	2	2	2	2	2	2

2	2	2	2	2	2	2	2	2	2
2	2	2	2	2	2	1	1	2	2
2	2	2	2	2	2	1	1	2	2
2	2	2	2	2	2	2	2	2	2
#NULL!	#NULL!	2	2	2	1	2	1	2	1
2	2	2	2	1	2	1	2	2	2
1	2	2	2	1	#NULL!	2	2	1	2
2	2	2	2	2	2	2	2	2	2
2	2	2	2	2	2	2	2	2	2
2	2	2	2	2	2	2	2	2	2
2	1	1	2	2	2	2	#NULL!	2	2
2	2	2	2	2	2	2	2	2	2
#NULL!	#NULL!	2	2	1	2	#NULL!	#NULL!	#NULL!	#NULL!
2	2	2	2	2	2	2	2	2	2
2	2	2	2	2	2	2	2	2	1
2	2	2	2	2	2	2	2	2	1
#NULL!	#NULL!	#NULL!	#NULL!	#NULL!	#NULL!	#NULL!	#NULL!	#NULL!	#NULL!
2	2	2	2	2	1	1	2	2	2
#NULL!	#NULL!	#NULL!	#NULL!	2	#NULL!	#NULL!	#NULL!	#NULL!	#NULL!
2	2	2	2	2	1	1	2	2	2
2	2	2	2	2	1	1	2	2	2
2	2	2	2	2	1	1	2	2	2
2	2	2	2	2	1	2	2	2	1
1	2	2	2	2	1	1	2	1	2
1	2	2	2	2	1	2	2	2	2
#NULL!	#NULL!	2	2	2	1	1	2	2	2
2	2	2	2	2	1	1	2	2	2
2	2	2	2	2	1	1	2	2	2
2	2	#NULL!	2	2	1	2	2	2	2
2	2	2	2	2	1	2	2	2	2
2	2	2	1	2	2	2	2	2	2
2	2	2	2	2	2	2	2	2	2
2	2	2	2	2	1	1	2	2	2
2	2	2	2	2	1	2	2	2	1
2	2	2	2	2	1	1	2	2	2
2	2	2	2	2	1	1	2	2	2
2	2	2	2	2	1	1	2	2	2
2	2	2	2	2	1	1	2	2	2
2	2	2	2	2	1	1	2	2	2
2	2	2	2	2	1	1	2	2	1
2	2	2	2	2	1	1	2	2	2
1	2	2	2	2	1	2	2	2	2
2	2	2	2	2	1	1	2	2	2
2	2	2	2	2	1	1	2	2	2
2	2	2	2	2	1	1	2	2	1
2	2	2	2	2	1	#NULL!	2	2	2
2	2	2	2	2	1	1	2	2	2

1	2	2	2	2	1	1	2	2	2
1	2	2	2	2	1	1	2	2	1
1	2	2	2	2	1	1	2	2	1
2	2	2	2	2	1	1	2	2	2
1	2	2	2	2	1	1	2	2	1
2	2	2	2	2	1	1	1	2	1
2	2	2	2	2	1	1	2	2	2
2	2	1	2	1	2	1	2	2	2
2	2	2	2	2	1	1	1	2	1
2	2	2	2	2	2	2	2	2	2
#NULL!	#NULL!	#NULL!	#NULL!	#NULL!	#NULL!	#NULL!	#NULL!	#NULL!	#NULL!
2	2	2	2	2	1	2	2	2	2
2	2	2	2	2	1	1	1	2	1
2	2	2	2	2	1	1	2	2	2
2	2	2	2	2	1	1	2	2	2
2	2	2	2	2	1	1	2	2	1
2	2	2	2	2	2	2	2	2	2
2	2	2	2	2	1	2	1	2	1
2	2	2	2	1	2	2	2	2	1
2	2	2	2	2	2	2	2	2	2
2	2	2	2	2	2	2	1	2	2
2	2	2	2	2	2	2	2	2	2
2	2	2	2	2	2	2	1	2	2
2	2	2	2	2	2	2	2	2	1
2	2	2	2	2	2	2	2	2	2
2	2	2	2	2	2	2	2	2	2
2	2	2	2	2	2	2	2	2	2
2	2	2	2	2	2	2	2	2	2
2	2	2	2	2	2	2	2	2	2
2	2	2	2	1	2	2	2	2	1
2	2	2	2	2	1	2	1	2	2
2	2	2	2	2	1	2	1	2	2
2	2	2	2	1	2	2	2	2	1
2	2	2	2	2	1	2	2	2	1
2	2	2	2	2	2	2	1	2	2
2	2	2	2	2	2	2	2	2	2
2	2	2	2	2	2	2	2	2	2
2	2	2	2	2	2	1	2	2	1
2	2	2	2	2	1	2	2	2	2
2	2	2	2	2	1	2	1	2	2
1	1	1	2	2	2	2	2	2	2
2	2	2	2	2	2	2	2	2	2
#NULL!	#NULL!	2	#NULL!	#NULL!	#NULL!	#NULL!	#NULL!	#NULL!	#NULL!
#NULL!	#NULL!	2	#NULL!	#NULL!	#NULL!	#NULL!	#NULL!	#NULL!	#NULL!
2	2	2	2	2	1	2	2	1	1
2	2	2	2	2	2	2	2	2	2
#NULL!	#NULL!	#NULL!	#NULL!	#NULL!	#NULL!	#NULL!	#NULL!	2	#NULL!

2	2	2	2	2	2	2	2	2	2
2	2	2	2	2	2	2	2	2	2
2	2	2	2	2	1	2	2	2	2
2	2	2	2	2	2	2	2	2	2
1	2	2	2	2	2	2	2	1	2
2	2	2	2	1	2	2	2	2	2
2	2	2	2	2	1	2	2	2	2
2	2	2	2	2	2	2	2	2	2
2	2	2	1	2	1	1	2	2	2
1	1	2	2	2	1	2	2	2	2
2	2	2	2	2	2	2	1	2	2
2	2	2	1	1	2	2	2	2	2
2	2	2	1	1	2	2	2	2	2
2	2	2	2	2	2	1	2	2	2
2	2	2	2	2	2	2	2	2	2
2	2	2	2	2	2	2	2	2	2
2	2	2	2	2	2	2	2	2	2
2	2	2	2	2	1	1	2	2	2
#NULL!	#NULL!	#NULL!	#NULL!	#NULL!	#NULL!	#NULL!	#NULL!	#NULL!	1
2	1	#NULL!	2	2	1	2	2	1	2
2	2	2	2	2	1	2	1	1	#NULL!
2	2	2	2	2	1	2	2	2	2
2	2	2	2	2	2	1	2	2	2
2	2	2	2	1	2	1	2	1	2
#NULL!	#NULL!	#NULL!	#NULL!	2	2	#NULL!	#NULL!	#NULL!	#NULL!
2	2	2	2	2	2	2	2	2	2
2	2	2	2	2	2	2	2	2	2
2	2	2	#NULL!	2	#NULL!	2	#NULL!	2	#NULL!
2	2	2	2	2	1	1	2	2	2
2	2	2	2	2	2	2	2	2	2
#NULL!	#NULL!	#NULL!	2	#NULL!	1	#NULL!	#NULL!	#NULL!	#NULL!
1	1	1	1	1	2	1	2	2	2
1	1	1	1	1	2	1	2	1	2
2	2	2	2	2	1	2	2	2	2
#NULL!	#NULL!	2	2	#NULL!	#NULL!	#NULL!	#NULL!	#NULL!	#NULL!
2	2	2	2	2	2	2	2	2	2
2	2	2	2	2	2	1	2	2	2
1	2	2	2	1	1	2	2	2	1
2	2	2	2	2	2	2	2	2	2
2	2	2	2	2	2	1	2	2	2
2	2	2	2	1	2	1	2	1	2
2	2	1	1	1	2	2	2	2	2
2	2	1	1	2	1	2	1	2	1
2	2	2	2	2	2	2	2	2	2
2	2	2	2	2	2	1	2	2	2
2	2	2	2	2	2	2	2	2	2
2	2	2	2	2	1	2	2	2	2

#NULL!	#NULL!	#NULL!	#NULL!	#NULL!	#NULL!	#NULL!	#NULL!	#NULL!	#NULL!
#NULL!	#NULL!	#NULL!	#NULL!	#NULL!	#NULL!	#NULL!	#NULL!	#NULL!	#NULL!
#NULL!	#NULL!	#NULL!	#NULL!	#NULL!	#NULL!	#NULL!	#NULL!	#NULL!	#NULL!
2	2	2	2	2	1	2	2	2	2
2	2	2	2	2	1	2	2	2	2
1	2	2	2	2	2	2	2	2	2
2	2	2	2	2	1	1	1	2	2
2	2	2	2	2	1	2	1	2	1
2	2	2	2	2	2	1	2	2	2
2	2	2	2	2	2	2	2	2	2
2	2	2	2	2	2	2	2	2	2
2	2	#NULL!	#NULL!	#NULL!	#NULL!	#NULL!	#NULL!	#NULL!	#NULL!
#NULL!	#NULL!	#NULL!	#NULL!	#NULL!	#NULL!	#NULL!	#NULL!	#NULL!	#NULL!
2	2	2	1	2	1	1	2	2	2
2	2	1	1	1	2	#NULL!	1	2	1
#NULL!	#NULL!	#NULL!	#NULL!	#NULL!	#NULL!	#NULL!	#NULL!	#NULL!	#NULL!
2	2	2	2	1	2	1	2	2	2
2	2	2	1	2	1	2	1	2	1
2	1	1	2	2	2	2	1	1	1
#NULL!	#NULL!	#NULL!	#NULL!	#NULL!	#NULL!	#NULL!	#NULL!	#NULL!	#NULL!
#NULL!	#NULL!	#NULL!	#NULL!	#NULL!	#NULL!	#NULL!	#NULL!	#NULL!	#NULL!
2	2	2	2	2	2	2	2	2	2
2	2	2	2	2	1	1	2	1	2
2	2	2	2	2	1	1	2	2	2
2	2	2	2	2	2	2	2	2	2
2	2	2	2	2	1	2	1	2	1
2	2	2	1	1	2	2	2	2	1
2	2	2	2	2	2	1	2	1	2
2	2	2	1	1	2	1	1	2	1
2	2	2	2	2	1	1	1	2	2
2	2	2	2	2	2	2	2	2	2
1	2	1	2	2	2	2	2	2	2
#NULL!	#NULL!	#NULL!	#NULL!	#NULL!	#NULL!	#NULL!	#NULL!	#NULL!	#NULL!
#NULL!	#NULL!	#NULL!	#NULL!	#NULL!	#NULL!	#NULL!	#NULL!	#NULL!	#NULL!
2	1	2	2	2	2	2	2	2	2
1	2	2	1	2	2	1	2	2	2
2	2	2	2	2	1	2	2	2	2
2	2	2	1	2	1	1	2	2	1
2	2	2	1	1	2	1	1	1	2
2	2	2	2	2	2	2	2	2	2
2	1	1	2	2	1	2	1	2	2
2	2	1	1	1	2	#NULL!	1	1	2
2	2	2	2	1	2	2	2	2	2
2	2	2	2	2	2	2	2	2	2
2	2	1	1	1	2	1	2	2	2
2	2	2	2	2	2	2	2	2	2
2	2	2	2	2	2	2	2	2	2

2	2	#NULL!	#NULL!	#NULL!	#NULL!	#NULL!	#NULL!	#NULL!	#NULL!
2	2	#NULL!	#NULL!	#NULL!	#NULL!	#NULL!	#NULL!	#NULL!	#NULL!
#NULL!	#NULL!	2	#NULL!	#NULL!	#NULL!	#NULL!	#NULL!	#NULL!	#NULL!
2	2	2	2	2	2	2	2	2	2
#NULL!	2	#NULL!	#NULL!	#NULL!	#NULL!	#NULL!	#NULL!	2	#NULL!
#NULL!	#NULL!	#NULL!	#NULL!	#NULL!	#NULL!	#NULL!	#NULL!	#NULL!	#NULL!
2	2	2	2	2	1	2	2	2	2
2	#NULL!	#NULL!	#NULL!	#NULL!	#NULL!	#NULL!	#NULL!	#NULL!	#NULL!
2	2	2	2	2	1	1	2	2	2
2	2	2	2	2	1	2	2	2	1
2	2	2	2	2	2	1	2	2	2
2	2	2	2	2	2	1	2	2	2
2	2	2	2	1	2	1	2	1	2
2	2	2	2	2	2	2	2	2	2
2	2	2	2	2	2	1	2	1	2
2	2	2	2	2	1	2	2	1	2
2	2	2	2	2	2	2	2	2	2
2	2	2	2	2	2	2	2	2	2
2	2	2	2	2	2	2	2	2	2
2	2	2	2	2	2	2	2	2	2
2	2	2	1	2	1	2	2	2	1
2	2	2	2	2	1	1	2	2	2
2	2	2	2	2	1	1	2	2	1
2	2	2	2	2	2	1	2	2	2
2	2	2	2	2	2	2	2	2	2
1	2	2	2	1	2	2	2	1	2
2	2	2	2	2	2	2	2	2	2
2	2	2	2	2	2	2	2	2	2
2	2	2	2	2	2	1	2	2	2
2	2	2	2	2	1	2	2	2	1
2	2	2	1	1	2	2	2	1	1
2	2	2	2	2	2	1	2	2	2
2	2	2	2	2	#NULL!	2	2	2	1
2	2	2	2	2	2	2	2	2	2
2	2	2	2	2	2	2	2	2	2
2	2	2	2	1	2	1	2	2	2
2	2	2	2	2	2	2	2	2	2
2	2	2	2	2	1	2	1	2	1
2	2	2	2	2	2	2	2	2	2
2	2	2	2	2	2	2	2	2	2
2	2	2	2	2	1	2	2	2	2
2	2	2	2	2	1	2	2	2	1
2	2	2	2	2	1	2	1	2	2
2	2	2	2	2	1	2	2	2	2
2	2	2	2	1	2	1	2	1	1
2	2	2	2	2	1	2	2	2	2
2	2	2	2	2	2	2	2	2	2

2	2	2	2	1	2	1	2	1	2
1	2	2	2	2	2	2	2	2	1
2	2	2	2	2	2	2	2	2	2
2	2	2	2	2	2	2	2	2	2
2	2	2	2	2	1	2	1	2	2
2	2	2	2	2	2	2	2	2	2
#NULL!	#NULL!	#NULL!	#NULL!	#NULL!	#NULL!	#NULL!	#NULL!	#NULL!	#NULL!
2	2	2	2	2	2	2	2	2	2
2	2	2	2	1	2	1	2	2	2
2	1	2	2	1	2	2	2	2	2
2	2	2	2	2	2	2	2	2	2
1	2	2	2	2	2	1	1	2	2
2	2	2	2	2	1	2	2	2	2
2	2	2	2	2	2	2	2	2	2
2	2	2	2	2	2	2	2	2	2
2	2	2	2	2	1	2	1	2	1
2	2	1	1	2	1	1	2	1	1
2	2	2	2	2	2	2	2	2	2
2	2	2	2	2	2	2	2	2	2
2	2	2	2	1	2	1	2	2	2
2	2	2	2	2	1	2	2	2	1
2	2	2	2	1	2	2	2	2	2
#NULL!	#NULL!	#NULL!	#NULL!	#NULL!	#NULL!	#NULL!	#NULL!	#NULL!	#NULL!
2	2	2	2	2	2	2	2	2	2
2	1	2	2	2	1	1	2	2	2
#NULL!	#NULL!	#NULL!	#NULL!	#NULL!	#NULL!	#NULL!	#NULL!	#NULL!	#NULL!
2	2	2	2	2	2	1	2	2	2
#NULL!	#NULL!	#NULL!	#NULL!	#NULL!	#NULL!	#NULL!	#NULL!	#NULL!	#NULL!
2	2	2	2	2	2	2	2	2	2
2	2	2	2	1	2	2	2	1	2
#NULL!	#NULL!	#NULL!	#NULL!	#NULL!	#NULL!	#NULL!	#NULL!	#NULL!	#NULL!
#NULL!	#NULL!	#NULL!	#NULL!	#NULL!	#NULL!	#NULL!	#NULL!	#NULL!	#NULL!
2	2	2	2	2	2	2	2	2	2
2	2	2	2	2	2	2	1	2	1
#NULL!	#NULL!	#NULL!	#NULL!	#NULL!	#NULL!	#NULL!	#NULL!	#NULL!	#NULL!
2	2	2	2	1	2	2	2	2	2
1	1	1	1	1	2	1	2	1	2
2	2	2	2	2	1	2	1	2	2
#NULL!	#NULL!	#NULL!	#NULL!	#NULL!	#NULL!	#NULL!	#NULL!	#NULL!	#NULL!
2	2	2	2	2	2	2	1	2	2
2	2	2	2	2	2	2	2	2	2
2	2	2	2	1	2	1	2	2	2
1	2	2	2	2	2	1	2	2	2
1	1	2	2	2	2	2	2	2	2
#NULL!	#NULL!	#NULL!	#NULL!	#NULL!	#NULL!	#NULL!	#NULL!	#NULL!	#NULL!
2	2	2	2	2	1	2	2	2	2
2	2	2	2	2	1	2	2	2	1

#NULL!	#NULL!	#NULL!	#NULL!	#NULL!	#NULL!	#NULL!	#NULL!	#NULL!	#NULL!
2	2	2	2	2	1	1	1	2	1
2	2	2	2	2	1	2	2	2	#NULL!
2	2	2	2	2	1	1	2	2	2
2	2	2	2	2	1	1	1	2	2
2	2	2	2	2	1	1	2	2	2
2	2	2	2	2	1	1	2	2	2
2	2	2	2	2	1	1	1	2	1
2	2	2	2	2	1	2	2	2	2
2	2	2	2	2	1	1	2	2	2
2	2	2	2	2	1	2	2	2	2
2	2	2	2	2	1	2	2	2	2
2	2	2	2	2	1	1	2	2	2
1	2	2	2	2	1	1	2	2	2
2	2	2	2	2	1	2	2	2	2
2	2	2	2	2	1	2	2	2	2
2	2	2	2	2	1	1	2	2	2
2	2	2	2	2	1	1	2	2	2
2	2	2	2	2	1	2	2	2	2
2	2	2	2	2	1	2	2	2	2
2	2	2	2	2	1	1	2	2	1
2	2	2	2	2	1	2	2	2	1
2	2	2	2	2	1	1	2	2	1
2	2	2	2	2	1	1	2	2	2
2	2	2	2	2	1	1	2	2	2
2	2	2	2	2	1	1	2	2	2
2	2	2	2	2	1	1	2	2	1
2	2	2	2	2	1	#NULL!	2	2	2
2	2	2	2	2	1	1	2	2	1
2	2	2	2	2	1	1	2	2	2
2	2	2	2	2	1	1	2	2	1
2	2	2	2	2	1	2	2	2	2
2	2	2	2	2	1	1	2	2	1
2	2	2	2	2	1	2	2	2	2
2	2	2	2	2	1	1	2	2	2
2	2	2	2	2	1	1	2	2	2
2	2	2	2	2	1	2	2	#NULL!	2
2	2	2	2	2	1	1	2	2	1
2	2	2	2	2	1	2	2	2	1
2	2	2	2	2	1	1	2	2	1
2	2	2	2	2	1	1	2	2	2
2	2	2	2	2	2	2	2	2	2
2	2	2	2	2	2	2	2	2	2
2	2	2	2	2	2	2	2	2	2
#NULL!	#NULL!	2	#NULL!	#NULL!	#NULL!	#NULL!	#NULL!	#NULL!	#NULL!
2	2	2	2	2	2	2	2	2	2
#NULL!	#NULL!	2	#NULL!	#NULL!	#NULL!	#NULL!	#NULL!	#NULL!	#NULL!

2	2	2	2	2	2	2	2	2	2
2	2	2	2	2	1	2	2	2	2
2	2	2	2	2	1	2	2	2	2
2	2	2	2	2	1	1	2	2	2
2	2	2	2	2	2	2	2	2	2
2	2	2	2	2	1	2	2	2	2
2	2	2	2	2	2	2	2	2	2
2	2	2	1	2	1	2	2	2	2
2	2	2	2	2	2	2	2	2	2
2	2	2	2	2	2	2	2	2	2
2	2	2	2	2	1	2	2	2	2
#NULL!	#NULL!	#NULL!	#NULL!	#NULL!	#NULL!	#NULL!	#NULL!	#NULL!	#NULL!
2	#NULL!	2	2	2	1	1	2	2	2
2	2	2	2	2	2	2	2	2	2
#NULL!	#NULL!	2	#NULL!	#NULL!	#NULL!	#NULL!	#NULL!	#NULL!	#NULL!
#NULL!	#NULL!	2	#NULL!	#NULL!	#NULL!	#NULL!	#NULL!	#NULL!	#NULL!
#NULL!	#NULL!	#NULL!	#NULL!	#NULL!	#NULL!	#NULL!	#NULL!	#NULL!	#NULL!
#NULL!	#NULL!	#NULL!	#NULL!	#NULL!	#NULL!	#NULL!	#NULL!	#NULL!	#NULL!
2	#NULL!	#NULL!	#NULL!	#NULL!	#NULL!	#NULL!	#NULL!	#NULL!	#NULL!
2	2	2	2	2	1	2	2	2	2
#NULL!	#NULL!	2	2	1	2	2	1	2	2
#NULL!	#NULL!	2	2	2	2	#NULL!	2	2	1
2	2	2	2	2	2	1	2	2	1
#NULL!	#NULL!	#NULL!	#NULL!	#NULL!	#NULL!	#NULL!	#NULL!	#NULL!	#NULL!
#NULL!	#NULL!	2	#NULL!	#NULL!	#NULL!	#NULL!	#NULL!	#NULL!	#NULL!
#NULL!	#NULL!	#NULL!	#NULL!	#NULL!	#NULL!	#NULL!	#NULL!	#NULL!	#NULL!
#NULL!	#NULL!	#NULL!	#NULL!	#NULL!	#NULL!	#NULL!	#NULL!	#NULL!	#NULL!
#NULL!	#NULL!	#NULL!	#NULL!	#NULL!	#NULL!	#NULL!	#NULL!	#NULL!	#NULL!
2	2	2	2	1	2	2	2	2	2
#NULL!	#NULL!	#NULL!	#NULL!	#NULL!	#NULL!	#NULL!	#NULL!	#NULL!	#NULL!
#NULL!	#NULL!	2	#NULL!	#NULL!	#NULL!	#NULL!	#NULL!	#NULL!	#NULL!
2	2	2	2	2	2	2	2	2	2
2	2	2	2	2	2	2	2	2	2
2	2	2	2	2	1	1	2	1	2
2	2	2	2	2	2	2	2	2	2
2	2	2	2	2	2	1	1	2	2
2	2	2	2	2	1	1	2	2	2
2	2	2	2	2	2	1	1	2	2
2	2	2	2	2	2	2	2	2	2
2	2	2	2	2	2	1	2	2	1
#NULL!	#NULL!	#NULL!	#NULL!	#NULL!	#NULL!	#NULL!	1	#NULL!	#NULL!
2	2	2	2	2	2	1	2	2	2
2	2	2	2	2	2	2	2	2	2
2	2	2	2	2	2	2	2	2	2
2	2	2	2	2	1	2	2	2	2
2	2	2	2	2	2	2	2	2	2
2	2	2	2	2	1	2	1	2	2

2	2	2	1	2	2	2	2	2	2
2	2	2	2	2	1	2	2	2	2
2	2	2	2	2	2	2	2	2	2
2	2	2	2	2	1	2	2	2	2
2	2	2	2	2	2	2	2	2	2
2	2	2	2	2	2	2	2	2	2
2	2	2	2	2	2	2	2	1	2
2	2	2	2	2	2	2	2	2	2
2	2	2	2	2	2	2	2	2	2
2	2	2	2	2	2	2	2	2	2
2	1	2	2	2	2	2	2	2	2
2	2	2	2	2	1	1	2	2	2
2	2	2	2	2	2	2	2	2	2
2	2	2	2	2	2	2	2	2	2
2	2	2	2	2	2	1	2	2	2
2	2	2	2	2	2	2	2	2	2
2	2	2	2	2	2	2	2	2	2
2	2	2	2	2	2	2	2	2	2
2	2	2	2	2	2	2	2	2	2
2	2	2	2	1	2	1	2	2	2
2	2	2	2	2	1	2	1	2	2
2	2	2	2	2	1	1	2	2	1
2	2	2	2	2	1	1	2	2	2
2	2	2	2	2	1	1	2	2	2
2	2	2	2	1	2	1	2	1	2
2	2	2	2	2	2	2	2	2	2
#NULL!	2	2	1	2	1	1	2	2	2
1	2	2	1	2	2	1	1	2	1
2	2	2	1	2	2	1	2	2	1
1	1	2	1	2	2	2	2	1	2
2	2	2	2	2	2	2	2	2	2
2	2	#NULL!	2	2	2	2	2	2	2
2	2	2	2	2	2	2	2	2	2
1	2	2	2	2	2	1	2	2	2
2	2	2	2	2	2	2	2	2	2
#NULL!	#NULL!	2	#NULL!	#NULL!	#NULL!	#NULL!	#NULL!	#NULL!	#NULL!
2	2	2	2	2	2	2	2	2	2
2	2	2	2	2	1	2	2	1	2
2	2	2	2	2	1	1	2	2	1
2	2	2	2	1	2	1	1	2	2
2	#NULL!	2	2	2	#NULL!	1	2	#NULL!	2
2	2	1	2	2	1	2	1	2	1
2	2	1	2	2	1	2	1	2	1
1	2	2	2	2	1	2	1	2	2
2	2	2	2	2	1	2	2	2	2
2	2	2	2	2	2	2	2	2	2
#NULL!	#NULL!	2	#NULL!	#NULL!	#NULL!	#NULL!	#NULL!	#NULL!	#NULL!

#NULL!	#NULL!	2	#NULL!	#NULL!	#NULL!	#NULL!	#NULL!	#NULL!	#NULL!
#NULL!	#NULL!	#NULL!	#NULL!	#NULL!	#NULL!	#NULL!	#NULL!	2	#NULL!
#NULL!	#NULL!	#NULL!	#NULL!	#NULL!	#NULL!	#NULL!	#NULL!	#NULL!	#NULL!
#NULL!	#NULL!	#NULL!	#NULL!	#NULL!	#NULL!	#NULL!	#NULL!	#NULL!	#NULL!
#NULL!	#NULL!	#NULL!	#NULL!	#NULL!	#NULL!	#NULL!	#NULL!	2	#NULL!
#NULL!	#NULL!	2	2	#NULL!	#NULL!	#NULL!	#NULL!	#NULL!	#NULL!
#NULL!	#NULL!	#NULL!	#NULL!	#NULL!	#NULL!	#NULL!	#NULL!	2	#NULL!
#NULL!	#NULL!	#NULL!	2	#NULL!	#NULL!	#NULL!	#NULL!	#NULL!	#NULL!
#NULL!	#NULL!	#NULL!	2	#NULL!	#NULL!	#NULL!	#NULL!	#NULL!	#NULL!
#NULL!	#NULL!	2	#NULL!	#NULL!	#NULL!	#NULL!	#NULL!	#NULL!	#NULL!
#NULL!	#NULL!	2	#NULL!	#NULL!	#NULL!	#NULL!	#NULL!	#NULL!	#NULL!
#NULL!	#NULL!	2	#NULL!	#NULL!	#NULL!	#NULL!	#NULL!	#NULL!	#NULL!
#NULL!	#NULL!	#NULL!	#NULL!	#NULL!	#NULL!	#NULL!	#NULL!	#NULL!	#NULL!
#NULL!	#NULL!	#NULL!	2	#NULL!	#NULL!	#NULL!	#NULL!	#NULL!	#NULL!
#NULL!	2	#NULL!	#NULL!	#NULL!	#NULL!	#NULL!	#NULL!	#NULL!	#NULL!
#NULL!	2	#NULL!	#NULL!	#NULL!	#NULL!	#NULL!	#NULL!	#NULL!	#NULL!
#NULL!	#NULL!	2	#NULL!	#NULL!	#NULL!	#NULL!	#NULL!	#NULL!	#NULL!
2	2	2	2	2	1	1	2	2	1
2	2	2	2	2	2	2	2	2	2
#NULL!	#NULL!	2	#NULL!	#NULL!	#NULL!	#NULL!	#NULL!	#NULL!	#NULL!
2	2	2	2	2	1	2	1	2	1
2	1	2	2	1	1	2	2	2	2
2	2	2	1	1	2	2	2	2	2
2	2	2	2	2	1	2	1	2	1
1	2	2	1	2	2	1	1	2	1
1	2	1	2	2	1	2	2	2	2
2	2	2	2	2	#NULL!	2	2	2	2
2	1	2	1	1	2	1	2	2	2
2	2	2	2	2	2	2	2	2	2
2	2	2	2	2	2	2	2	2	2
1	1	2	2	2	1	2	1	2	2
2	2	2	2	2	1	1	2	2	2
2	2	2	2	2	2	2	2	2	2
2	2	2	2	1	2	1	2	1	2
2	1	2	2	1	1	2	2	2	2
1	2	2	2	2	2	2	2	2	2
2	2	2	2	2	2	2	2	2	2
2	2	#NULL!	#NULL!	#NULL!	#NULL!	2	2	2	#NULL!
2	2	2	1	1	2	2	1	2	2
#NULL!	#NULL!	#NULL!	#NULL!	#NULL!	#NULL!	#NULL!	#NULL!	#NULL!	#NULL!
2	2	2	2	2	2	2	2	2	2
2	2	1	1	1	1	2	2	1	2
#NULL!	#NULL!	#NULL!	#NULL!	#NULL!	#NULL!	#NULL!	#NULL!	#NULL!	#NULL!
2	2	2	2	2	1	2	1	2	1
2	2	2	2	2	2	2	2	2	2
2	2	2	2	2	2	2	2	2	2
2	2	2	2	2	1	2	2	2	2

2	2	2	2	2	2	1	2	1	2
#NULL!	#NULL!	#NULL!	#NULL!	#NULL!	#NULL!	#NULL!	#NULL!	#NULL!	#NULL!
2	2	2	1	2	2	1	1	1	1
#NULL!	#NULL!	#NULL!	#NULL!	#NULL!	#NULL!	#NULL!	#NULL!	#NULL!	#NULL!
2	2	2	2	2	2	2	2	2	2
2	2	2	2	2	2	2	2	2	2
#NULL!	#NULL!	#NULL!	#NULL!	#NULL!	#NULL!	#NULL!	#NULL!	#NULL!	#NULL!
2	2	2	2	2	2	1	2	1	2
2	1	2	2	2	1	1	1	2	2
1	2	2	1	1	2	1	2	2	2
2	2	2	2	2	1	2	1	2	2
2	2	2	2	2	1	1	2	1	1
2	2	2	1	1	2	2	2	#NULL!	2
#NULL!	#NULL!	#NULL!	#NULL!	#NULL!	#NULL!	#NULL!	#NULL!	#NULL!	#NULL!
#NULL!	#NULL!	#NULL!	#NULL!	#NULL!	#NULL!	#NULL!	#NULL!	#NULL!	#NULL!
#NULL!	#NULL!	#NULL!	#NULL!	#NULL!	#NULL!	#NULL!	#NULL!	#NULL!	#NULL!
2	2	2	2	2	2	1	2	2	2
#NULL!	#NULL!	#NULL!	#NULL!	#NULL!	#NULL!	#NULL!	#NULL!	#NULL!	#NULL!
2	2	2	1	2	1	1	2	2	2
2	2	2	2	2	1	2	2	1	2
2	2	2	2	2	1	1	2	2	1
2	2	2	1	1	2	2	2	#NULL!	2
2	2	2	2	2	2	2	2	2	2
2	1	2	2	1	#NULL!	#NULL!	#NULL!	#NULL!	#NULL!
2	2	2	2	2	2	2	2	2	2
2	2	2	1	1	1	1	1	1	1
1	1	1	#NULL!	2	2	1	2	1	2
2	2	2	1	1	2	1	#NULL!	1	2
2	2	2	2	2	1	2	2	2	2
2	2	2	2	2	2	2	2	2	2
#NULL!	#NULL!	#NULL!	#NULL!	#NULL!	#NULL!	#NULL!	#NULL!	#NULL!	#NULL!
#NULL!	#NULL!	#NULL!	#NULL!	#NULL!	#NULL!	#NULL!	#NULL!	2	#NULL!
2	2	2	2	2	1	2	2	2	2
2	2	2	2	2	2	1	2	2	2
2	#NULL!	2	1	2	#NULL!	2	#NULL!	#NULL!	#NULL!
2	2	2	2	2	2	2	2	2	2
#NULL!	2	2	2	1	2	1	2	1	2
#NULL!	#NULL!	#NULL!	#NULL!	#NULL!	#NULL!	#NULL!	#NULL!	#NULL!	#NULL!
#NULL!	#NULL!	#NULL!	#NULL!	#NULL!	#NULL!	#NULL!	#NULL!	#NULL!	#NULL!
#NULL!	#NULL!	#NULL!	#NULL!	#NULL!	#NULL!	#NULL!	#NULL!	#NULL!	#NULL!
#NULL!	#NULL!	#NULL!	#NULL!	#NULL!	#NULL!	#NULL!	#NULL!	#NULL!	#NULL!
2	2	2	2	2	2	1	1	2	2
1	1	2	2	1	1	2	1	1	2
#NULL!	#NULL!	#NULL!	#NULL!	#NULL!	#NULL!	#NULL!	#NULL!	#NULL!	#NULL!
2	1	2	2	1	1	2	1	1	2
2	2	2	2	2	2	1	1	2	1
1	1	2	2	2	1	1	2	1	1

1	1	1	1	1	2	1	2	1	2
2	2	2	2	1	2	1	2	2	1
2	2	1	1	1	2	1	#NULL!	1	2
2	2	2	2	2	2	2	2	2	2
2	2	2	2	2	2	2	2	2	2
1	2	2	#NULL!	#NULL!	#NULL!	#NULL!	#NULL!	2	2
#NULL!	#NULL!	#NULL!	#NULL!	#NULL!	#NULL!	#NULL!	#NULL!	#NULL!	#NULL!
2	2	1	1	2	1	1	1	2	2
1	1	1	1	1	2	1	2	1	2
2	2	2	2	2	1	1	2	2	2
1	1	1	1	1	2	1	2	1	2
2	2	2	2	2	1	2	1	#NULL!	#NULL!
2	2	2	2	2	1	1	1	2	1
2	2	2	2	2	1	1	2	2	1
2	2	2	2	2	1	1	1	2	1
2	2	2	2	2	1	2	2	2	1
2	2	2	2	2	1	1	1	2	1
2	2	2	2	2	1	1	1	2	1
2	2	2	2	2	1	1	1	2	1
2	2	2	2	2	2	1	2	2	2
1	1	2	2	1	2	1	2	1	2
#NULL!	2	2	1	1	2	1	#NULL!	2	2
2	1	2	1	1	2	1	2	1	2
1	1	2	2	2	2	1	2	2	2
1	1	1	1	1	2	1	2	1	2
2	2	2	2	2	1	1	1	2	1
#NULL!	#NULL!	#NULL!	#NULL!	#NULL!	2	#NULL!	#NULL!	#NULL!	#NULL!
1	2	#NULL!	#NULL!	#NULL!	#NULL!	#NULL!	#NULL!	2	#NULL!
#NULL!	#NULL!	#NULL!	#NULL!	#NULL!	#NULL!	#NULL!	#NULL!	#NULL!	#NULL!
2	2	2	2	2	2	2	2	2	2
2	2	2	2	1	1	2	1	2	2
2	1	2	1	1	2	1	2	1	2
2	2	2	2	1	2	2	2	2	2
#NULL!	#NULL!	#NULL!	#NULL!	#NULL!	#NULL!	#NULL!	#NULL!	2	#NULL!
1	1	1	1	1	2	1	2	1	2
2	2	2	2	2	2	2	1	2	2
#NULL!	2	2	#NULL!	#NULL!	2	#NULL!	#NULL!	2	#NULL!
#NULL!	2	2	2	2	1	2	1	2	1
2	2	2	1	2	1	2	1	2	1
2	2	2	2	2	1	1	2	2	1
2	2	2	1	2	1	2	1	2	2
1	2	2	2	2	2	1	2	2	2
2	2	2	2	2	2	2	2	1	1
2	2	2	1	1	2	1	1	2	2
2	2	2	2	2	1	1	2	2	2
2	2	2	2	2	1	2	1	2	1
2	1	2	1	#NULL!	2	1	2	2	#NULL!

2	2	2	2	2	2	2	2	2	2
#NULL!	#NULL!	2	#NULL!	#NULL!	#NULL!	#NULL!	#NULL!	#NULL!	#NULL!
#NULL!	#NULL!	#NULL!	#NULL!	2	#NULL!	#NULL!	#NULL!	#NULL!	#NULL!
2	1	2	1	2	1	1	2	2	1
2	2	2	2	2	2	2	2	2	2
2	2	2	2	2	2	2	2	2	2
2	2	2	2	2	1	2	1	2	1
2	2	2	2	2	2	2	2	2	2
2	2	2	2	2	2	2	2	2	2
1	1	2	2	1	1	1	1	2	2
#NULL!	2	#NULL!	#NULL!	#NULL!	#NULL!	#NULL!	#NULL!	#NULL!	#NULL!
2	2	2	2	1	2	2	2	1	2
#NULL!	#NULL!	#NULL!	#NULL!	#NULL!	#NULL!	#NULL!	#NULL!	#NULL!	#NULL!
2	2	2	2	1	1	1	2	1	2
2	2	2	2	2	1	1	2	2	2
#NULL!	#NULL!	#NULL!	#NULL!	#NULL!	#NULL!	#NULL!	#NULL!	#NULL!	#NULL!
1	1	1	1	1	2	1	2	1	2
#NULL!	#NULL!	#NULL!	#NULL!	#NULL!	1	#NULL!	#NULL!	#NULL!	#NULL!
#NULL!	#NULL!	#NULL!	#NULL!	#NULL!	1	#NULL!	#NULL!	#NULL!	#NULL!
1	2	2	2	1	1	2	2	2	2
2	1	2	2	2	2	2	1	2	1
2	2	2	2	2	2	2	2	2	2
#NULL!	#NULL!	#NULL!	#NULL!	#NULL!	#NULL!	#NULL!	#NULL!	#NULL!	#NULL!
2	2	2	2	2	2	2	2	2	2
1	1	1	1	1	2	1	2	1	2
2	2	2	2	2	1	1	2	2	2
2	2	2	2	2	1	1	2	2	2
2	1	2	2	2	1	1	2	2	1
2	2	2	2	2	1	2	1	2	1
2	2	2	2	2	2	2	2	2	2
2	2	1	2	1	2	1	1	1	2
1	2	2	2	2	1	2	1	2	2
1	1	2	1	2	1	2	1	2	2
2	2	2	2	2	1	1	2	2	2
2	2	2	2	2	#NULL!	2	1	1	#NULL!
2	2	2	2	2	2	2	2	2	2
2	2	2	2	2	2	2	2	2	2
#NULL!	#NULL!	#NULL!	#NULL!	#NULL!	#NULL!	#NULL!	#NULL!	#NULL!	#NULL!
#NULL!	2	#NULL!	#NULL!	2	#NULL!	2	#NULL!	#NULL!	2
2	2	2	2	2	1	2	1	1	2
2	2	2	2	2	2	2	2	2	2
2	2	2	2	2	2	2	2	2	2
2	2	2	#NULL!	#NULL!	#NULL!	#NULL!	#NULL!	#NULL!	#NULL!
2	2	2	2	2	2	2	2	2	2
2	2	2	2	2	2	2	2	2	2
2	2	2	2	2	2	2	2	2	2
#NULL!	#NULL!	#NULL!	#NULL!	#NULL!	#NULL!	#NULL!	#NULL!	#NULL!	#NULL!

#NULL!	#NULL!	#NULL!	#NULL!	#NULL!	#NULL!	#NULL!	#NULL!	#NULL!	#NULL!
2	2	2	2	2	2	2	2	2	2
2	2	2	2	2	2	2	2	2	1
2	2	2	2	2	1	2	1	2	2
#NULL!	#NULL!	#NULL!	2	#NULL!	#NULL!	#NULL!	#NULL!	#NULL!	#NULL!
1	2	2	2	1	1	2	2	2	2
#NULL!	#NULL!	#NULL!	#NULL!	#NULL!	#NULL!	#NULL!	#NULL!	#NULL!	#NULL!
1	1	2	2	2	1	1	1	2	2
2	2	2	2	2	1	2	2	1	1
#NULL!	#NULL!	#NULL!	#NULL!	2	#NULL!	#NULL!	#NULL!	#NULL!	#NULL!
2	2	2	2	2	2	2	2	2	2
2	2	2	2	2	2	2	2	2	1
2	2	2	2	2	2	2	2	2	1
2	2	2	2	2	2	2	2	2	2
2	2	2	2	2	1	2	2	2	1
2	2	2	2	2	2	2	2	2	2
2	2	2	2	2	2	2	2	2	2
2	2	1	1	1	2	1	2	1	2
2	2	2	2	2	2	2	2	2	2
2	2	2	2	1	2	1	1	2	2
#NULL!	#NULL!	#NULL!	#NULL!	2	#NULL!	#NULL!	#NULL!	#NULL!	#NULL!
1	1	2	2	2	2	2	2	2	2
2	#NULL!	2	2	2	2	2	2	2	2
2	2	2	2	2	2	2	2	2	2
2	2	2	2	2	2	1	2	1	2
1	1	2	2	2	2	2	2	2	2
2	#NULL!	#NULL!	2	#NULL!	#NULL!	#NULL!	#NULL!	#NULL!	#NULL!
2	2	2	2	#NULL!	#NULL!	#NULL!	#NULL!	#NULL!	#NULL!
2	2	2	2	2	2	2	2	2	2
2	2	1	2	1	2	2	2	1	#NULL!
2	2	2	2	2	2	2	2	2	2
2	2	2	2	2	2	2	2	2	2
1	2	2	2	1	2	1	2	1	2
2	2	2	2	2	2	2	2	2	2
2	2	2	2	2	2	2	2	2	2
2	2	2	2	2	1	2	2	2	2
2	2	2	2	2	2	2	2	2	2
2	2	2	2	2	2	2	2	1	2
2	2	2	2	2	2	2	2	2	2
2	2	2	2	2	2	2	2	2	2
2	2	2	2	2	2	2	2	2	2
2	2	2	2	2	2	2	2	2	2
2	2	2	2	2	1	1	2	2	2
2	2	2	2	2	2	2	2	2	2
2	2	2	2	2	2	2	2	2	2
1	1	1	1	1	2	1	2	1	2
2	2	2	2	2	2	2	2	2	2

2	2	2	2	2	2	2	2	2	2
2	2	2	2	2	2	2	2	2	2
2	2	2	2	2	2	2	2	2	2
2	2	2	2	2	2	2	2	2	2
2	2	2	2	2	1	2	2	2	1
2	2	2	2	2	1	2	2	2	1
2	2	2	2	2	2	2	2	2	2
2	2	2	2	2	2	2	#NULL!	2	1
2	2	1	2	2	1	1	2	2	2
2	2	2	2	2	2	2	2	2	2
2	2	2	1	1	2	1	1	2	2
#NULL!	#NULL!	#NULL!	2	#NULL!	#NULL!	#NULL!	#NULL!	#NULL!	#NULL!
#NULL!	#NULL!	2	#NULL!	#NULL!	#NULL!	#NULL!	#NULL!	#NULL!	#NULL!
2	2	2	2	2	2	2	2	1	#NULL!
1	1	2	2	2	2	2	2	2	2
1	1	2	2	2	2	2	2	2	2
1	2	2	2	2	2	2	2	2	2
2	2	2	2	2	1	1	1	1	2
#NULL!	#NULL!	#NULL!	#NULL!	#NULL!	#NULL!	#NULL!	#NULL!	#NULL!	#NULL!
2	1	1	2	1	2	2	2	2	2
#NULL!	#NULL!	#NULL!	#NULL!	#NULL!	#NULL!	#NULL!	#NULL!	#NULL!	#NULL!
2	2	2	2	2	2	2	1	2	1
2	2	2	2	2	2	2	2	2	1
2	2	2	2	2	1	2	1	2	1
1	2	2	2	2	1	1	2	2	2
2	2	2	2	2	1	2	1	2	1
2	2	2	2	2	2	2	2	2	2
2	2	2	2	2	2	2	2	2	2
2	2	2	2	2	2	2	2	2	2
2	1	2	2	1	1	2	2	1	2
2	2	2	2	2	1	2	1	2	2
2	2	1	1	1	2	1	2	1	2
1	1	2	2	2	1	2	1	2	1
2	2	2	2	2	1	2	2	2	1
1	1	1	1	1	2	1	2	1	2
2	1	2	1	1	2	2	2	2	2
#NULL!	#NULL!	#NULL!	#NULL!	#NULL!	#NULL!	#NULL!	#NULL!	#NULL!	#NULL!
2	2	2	2	2	1	2	1	2	1
2	2	2	2	2	2	2	2	2	2
2	2	2	2	2	2	2	2	2	2
2	2	2	2	2	2	1	2	1	2
#NULL!	#NULL!	2	2	#NULL!	#NULL!	#NULL!	#NULL!	#NULL!	#NULL!
2	2	2	2	1	1	2	1	2	2
2	2	2	2	2	2	2	2	2	2
2	2	2	2	2	2	2	2	2	2
1	2	1	1	1	2	1	2	2	2
2	2	2	2	2	1	2	2	2	2

1	2	2	1	2	1	2	2	1	2
1	1	1	1	1	2	1	2	1	2
1	2	1	2	2	2	2	1	2	2
#NULL!	#NULL!	#NULL!	2	#NULL!	#NULL!	#NULL!	#NULL!	#NULL!	#NULL!
#NULL!	#NULL!	#NULL!	#NULL!	#NULL!	#NULL!	#NULL!	#NULL!	#NULL!	#NULL!
2	1	1	1	1	2	1	2	2	2
2	2	2	2	2	2	2	2	2	2
1	1	1	2	2	1	1	1	2	2
1	1	2	2	2	2	2	2	2	2
2	2	2	2	2	2	2	2	2	2
#NULL!	2	2	#NULL!	#NULL!	#NULL!	#NULL!	#NULL!	#NULL!	#NULL!
#NULL!	2	2	2	2	2	2	2	2	2
2	2	2	2	2	2	2	2	2	2
1	1	2	2	#NULL!	2	1	1	1	1
2	2	2	2	2	2	2	2	2	2
2	2	1	2	#NULL!	1	2	#NULL!	2	#NULL!
1	2	2	1	2	2	#NULL!	2	2	2
#NULL!	#NULL!	2	#NULL!	#NULL!	#NULL!	#NULL!	#NULL!	#NULL!	#NULL!
#NULL!	2	#NULL!	#NULL!	#NULL!	#NULL!	#NULL!	1	#NULL!	#NULL!
2	2	2	2	2	2	2	2	2	2
2	2	2	2	2	2	2	2	2	2
2	2	2	2	2	2	2	2	2	2
#NULL!	#NULL!	#NULL!	#NULL!	#NULL!	#NULL!	#NULL!	#NULL!	#NULL!	#NULL!
#NULL!	#NULL!	#NULL!	#NULL!	#NULL!	#NULL!	#NULL!	#NULL!	#NULL!	#NULL!
2	1	2	2	1	1	2	2	2	2
2	2	2	2	2	1	1	2	1	1
2	2	2	1	2	2	2	2	2	2
2	2	2	2	2	1	1	2	1	1
1	2	1	2	2	1	1	2	1	1
1	2	2	2	2	1	2	2	2	2
2	2	2	2	2	2	2	2	2	2
1	2	2	1	2	2	1	2	1	2
2	2	2	2	1	2	2	2	#NULL!	2
1	1	1	1	2	1	1	2	1	2
1	1	1	1	1	2	1	2	1	2
2	1	2	2	1	2	2	2	1	2
2	2	2	2	2	1	2	2	2	1
1	1	1	1	2	1	1	2	1	2
2	#NULL!	2	2	2	2	1	2	2	2
1	1	1	1	1	2	1	2	1	2
2	1	#NULL!	2	2	1	1	#NULL!	2	2
2	1	2	2	2	1	1	1	1	2
2	2	2	1	2	1	2	2	2	2
2	2	2	2	2	2	2	2	2	2
2	2	2	2	2	2	2	2	2	2
2	2	2	2	2	1	2	2	2	1
2	2	2	2	2	1	2	2	2	2

2	2	2	2	2	2	2	2	2	2
2	1	1	2	1	2	1	2	1	2
#NULL!	2	2	2	2	2	2	2	2	1
2	2	1	1	1	2	1	2	1	2
2	2	2	2	2	2	2	2	2	2
2	2	2	1	2	2	2	2	2	2
2	2	2	2	2	2	2	2	2	2
2	2	2	2	2	2	2	2	2	2
2	2	2	2	2	2	2	2	2	2
2	2	2	2	2	2	2	2	2	2
#NULL!	#NULL!	#NULL!	2	#NULL!	#NULL!	#NULL!	#NULL!	#NULL!	#NULL!
2	2	2	2	2	2	2	2	2	2
2	1	2	2	1	2	1	2	1	2
1	1	2	2	2	1	2	2	2	2
2	2	2	2	2	1	2	2	2	1
2	2	2	2	2	2	2	2	2	2
2	2	2	2	2	1	1	1	1	2
2	2	2	2	2	2	2	2	2	1
2	2	2	2	2	2	1	2	1	2
#NULL!	#NULL!	#NULL!	#NULL!	#NULL!	#NULL!	#NULL!	1	#NULL!	#NULL!
2	2	2	2	2	1	1	1	2	1
2	1	2	2	2	2	1	2	1	2
2	2	1	1	1	2	1	1	2	2
1	1	2	2	2	1	1	2	1	2
1	1	1	1	1	2	1	2	1	2
2	2	2	2	2	2	2	2	2	2
2	2	2	2	2	#NULL!	1	2	2	1
1	2	2	2	2	1	2	1	2	2
2	2	1	2	2	2	2	2	2	2
2	2	2	2	2	1	2	1	2	1
2	2	2	2	2	2	2	2	2	2
#NULL!	#NULL!	#NULL!	#NULL!	#NULL!	#NULL!	#NULL!	#NULL!	#NULL!	#NULL!
2	1	2	1	2	2	2	2	2	2
#NULL!	#NULL!	#NULL!	#NULL!	#NULL!	#NULL!	#NULL!	#NULL!	#NULL!	#NULL!
2	2	2	2	2	2	2	1	2	2
2	2	2	2	2	2	2	2	2	2
2	2	2	2	2	2	2	2	2	2
2	2	2	2	2	2	2	2	2	2
1	1	2	2	2	2	2	2	2	2
2	1	2	2	2	2	1	1	2	2
1	2	2	2	2	2	2	1	2	1
2	2	2	2	2	2	2	2	2	2
2	1	2	2	1	1	2	2	2	2
#NULL!	#NULL!	2	#NULL!	#NULL!	#NULL!	#NULL!	#NULL!	#NULL!	#NULL!
1	1	1	1	1	2	1	2	1	2
2	2	2	2	2	2	2	2	2	2
2	2	2	2	2	1	1	2	2	2

#NULL!	#NULL!	#NULL!	#NULL!	#NULL!	#NULL!	#NULL!	#NULL!	#NULL!	#NULL!
2	1	2	2	2	2	2	2	2	2
1	1	2	2	2	2	2	2	1	2
2	2	2	2	2	2	2	2	1	1
1	1	2	2	2	2	2	2	2	2
1	2	2	2	1	2	2	2	2	2
#NULL!	#NULL!	#NULL!	#NULL!	#NULL!	#NULL!	#NULL!	#NULL!	#NULL!	#NULL!
2	2	2	1	1	2	#NULL!	2	2	2
2	2	2	2	2	2	2	2	2	2
#NULL!	#NULL!	#NULL!	#NULL!	#NULL!	#NULL!	#NULL!	#NULL!	#NULL!	#NULL!
#NULL!	#NULL!	#NULL!	#NULL!	#NULL!	#NULL!	#NULL!	#NULL!	#NULL!	#NULL!
#NULL!	#NULL!	#NULL!	#NULL!	#NULL!	#NULL!	#NULL!	#NULL!	#NULL!	#NULL!
#NULL!	#NULL!	#NULL!	2	#NULL!	#NULL!	#NULL!	#NULL!	#NULL!	#NULL!
#NULL!	#NULL!	1	#NULL!	#NULL!	#NULL!	#NULL!	#NULL!	#NULL!	#NULL!
#NULL!	#NULL!	#NULL!	#NULL!	#NULL!	#NULL!	#NULL!	#NULL!	#NULL!	#NULL!
2	2	2	2	2	2	1	2	2	2
2	2	2	#NULL!	2	1	2	#NULL!	2	#NULL!
2	2	2	2	2	1	2	2	2	2
1	2	2	2	2	1	1	2	2	2
2	2	2	2	2	2	2	1	2	2
2	2	2	1	2	2	2	1	2	1
2	2	2	1	2	1	2	2	2	2
2	2	2	2	2	1	1	2	1	1
2	2	2	2	2	2	1	2	2	2
2	2	2	2	2	2	2	2	2	2
2	1	2	1	2	2	2	2	2	2
1	1	2	2	2	2	2	2	2	2
2	2	2	2	2	1	2	1	2	1
2	1	2	1	2	2	2	2	2	2
2	2	2	2	2	2	1	2	2	2
2	2	2	2	2	2	2	1	2	2
#NULL!	#NULL!	#NULL!	2	#NULL!	#NULL!	#NULL!	#NULL!	#NULL!	#NULL!
2	1	2	1	2	1	1	1	1	2
2	2	2	2	2	2	2	2	2	2
2	2	2	2	2	2	2	2	2	2
2	2	2	2	2	2	2	2	2	2
2	2	2	2	2	2	2	2	2	2
#NULL!	#NULL!	2	#NULL!	#NULL!	#NULL!	#NULL!	#NULL!	#NULL!	#NULL!
2	2	2	2	2	2	2	2	2	2
2	2	2	2	2	2	2	2	2	2
2	2	2	2	2	2	2	2	2	2
2	2	2	2	2	2	2	2	2	2
2	2	2	2	2	2	2	2	2	2
1	1	2	2	2	1	2	2	2	2
2	2	2	2	2	2	2	2	2	2
2	2	2	2	2	2	2	2	2	2
#NULL!	#NULL!	#NULL!	#NULL!	#NULL!	#NULL!	#NULL!	#NULL!	#NULL!	#NULL!

2	2	2	2	2	2	2	2	2	2
2	2	2	2	2	2	2	2	2	2
#NULL!	#NULL!	#NULL!	#NULL!	#NULL!	#NULL!	#NULL!	#NULL!	#NULL!	#NULL!
#NULL!	#NULL!	#NULL!	#NULL!	#NULL!	#NULL!	#NULL!	#NULL!	#NULL!	#NULL!
2	2	2	2	2	2	2	2	2	2
#NULL!	#NULL!	2	#NULL!	#NULL!	#NULL!	#NULL!	#NULL!	#NULL!	#NULL!
1	2	2	2	2	1	2	2	1	1
2	2	1	2	2	2	2	2	1	2
1	2	#NULL!	#NULL!	#NULL!	#NULL!	#NULL!	#NULL!	#NULL!	#NULL!
2	2	2	2	2	1	2	2	2	2
#NULL!	#NULL!	#NULL!	#NULL!	#NULL!	#NULL!	#NULL!	#NULL!	#NULL!	#NULL!
2	1	2	2	2	1	2	2	2	2
2	2	2	2	2	2	2	2	2	2
2	2	2	2	2	2	2	2	2	2
2	2	2	2	1	1	2	2	2	2
2	1	1	1	1	2	1	2	1	2
2	2	2	2	2	2	2	2	2	2
2	2	2	2	2	2	2	2	2	2
2	2	2	2	2	1	2	2	2	1
1	1	2	2	2	1	2	2	2	2
2	2	1	1	1	2	1	2	1	2
2	2	2	2	2	2	2	2	2	2
2	2	2	1	1	2	2	2	1	2
2	2	1	1	1	2	1	2	1	2
2	2	2	2	2	2	2	2	2	2
2	2	2	2	2	1	2	2	2	2
2	2	2	2	2	2	2	2	2	2
2	2	2	2	2	2	2	2	2	2
2	2	2	2	2	2	2	2	2	2
#NULL!	#NULL!	#NULL!	#NULL!	#NULL!	#NULL!	#NULL!	#NULL!	#NULL!	#NULL!
2	2	2	1	1	2	1	1	2	1
2	2	2	2	2	2	2	2	2	2
2	2	#NULL!	#NULL!	#NULL!	#NULL!	#NULL!	#NULL!	#NULL!	#NULL!
#NULL!	#NULL!	#NULL!	#NULL!	#NULL!	#NULL!	#NULL!	#NULL!	#NULL!	#NULL!
2	2	2	2	1	1	2	2	2	1
#NULL!	#NULL!	#NULL!	#NULL!	#NULL!	1	#NULL!	#NULL!	#NULL!	#NULL!
#NULL!	#NULL!	#NULL!	#NULL!	#NULL!	#NULL!	#NULL!	#NULL!	#NULL!	#NULL!
2	2	2	2	2	2	2	2	2	2
2	1	#NULL!	2	2	#NULL!	#NULL!	1	#NULL!	#NULL!
1	2	2	2	2	1	2	2	2	1
2	2	2	2	2	1	2	1	2	1
2	1	2	2	#NULL!	2	2	2	1	1
#NULL!	#NULL!	2	#NULL!	#NULL!	#NULL!	#NULL!	#NULL!	#NULL!	#NULL!
2	1	2	2	2	2	2	2	2	2
2	2	2	2	2	2	2	2	2	2
#NULL!	#NULL!	#NULL!	#NULL!	#NULL!	#NULL!	#NULL!	#NULL!	#NULL!	#NULL!
1	1	1	1	2	1	2	2	2	1

2	2	2	2	2	1	2	2	2	2
#NULL!	#NULL!	#NULL!	#NULL!	#NULL!	#NULL!	#NULL!	#NULL!	#NULL!	#NULL!
#NULL!	#NULL!	#NULL!	#NULL!	#NULL!	#NULL!	#NULL!	#NULL!	#NULL!	#NULL!
2	2	2	2	2	2	2	2	2	2
2	2	2	2	2	2	2	2	2	2
2	2	2	2	2	1	2	2	2	2
2	2	2	1	2	#NULL!	2	#NULL!	2	#NULL!
2	2	2	1	1	2	1	2	1	2
2	2	2	2	2	2	2	2	2	2
2	2	2	2	1	2	2	2	2	2
#NULL!	#NULL!	#NULL!	#NULL!	#NULL!	#NULL!	#NULL!	#NULL!	#NULL!	#NULL!
#NULL!	#NULL!	#NULL!	#NULL!	#NULL!	#NULL!	#NULL!	#NULL!	#NULL!	#NULL!
#NULL!	#NULL!	2	#NULL!	#NULL!	#NULL!	#NULL!	#NULL!	#NULL!	#NULL!
2	2	2	2	2	1	2	2	2	2
1	1	2	2	2	2	2	2	2	2
#NULL!	#NULL!	#NULL!	#NULL!	#NULL!	#NULL!	#NULL!	#NULL!	#NULL!	#NULL!
#NULL!	#NULL!	#NULL!	#NULL!	#NULL!	#NULL!	#NULL!	#NULL!	#NULL!	#NULL!
#NULL!	#NULL!	#NULL!	#NULL!	#NULL!	#NULL!	#NULL!	#NULL!	#NULL!	#NULL!
2	2	2	2	2	1	2	2	2	2
2	2	2	2	2	1	2	2	2	2
#NULL!	#NULL!	#NULL!	#NULL!	#NULL!	#NULL!	#NULL!	#NULL!	#NULL!	#NULL!
2	2	2	1	1	2	1	2	2	1
1	1	1	1	1	2	1	2	1	2
#NULL!	#NULL!	#NULL!	#NULL!	#NULL!	#NULL!	#NULL!	#NULL!	#NULL!	#NULL!
2	2	2	2	2	2	2	2	2	2
2	2	2	2	2	1	2	2	2	2
#NULL!	#NULL!	#NULL!	#NULL!	#NULL!	#NULL!	#NULL!	#NULL!	#NULL!	#NULL!
#NULL!	#NULL!	#NULL!	#NULL!	#NULL!	#NULL!	#NULL!	#NULL!	#NULL!	#NULL!
2	2	2	2	2	2	2	1	2	2
2	2	2	2	2	1	2	2	2	1
1	2	2	2	2	1	2	1	2	2
#NULL!	#NULL!	#NULL!	#NULL!	#NULL!	#NULL!	#NULL!	#NULL!	#NULL!	#NULL!
#NULL!	#NULL!	#NULL!	#NULL!	#NULL!	#NULL!	#NULL!	#NULL!	#NULL!	#NULL!
1	1	2	2	2	1	2	1	2	1
2	2	2	2	2	2	#NULL!	2	#NULL!	1
2	2	2	2	2	2	2	2	2	2
2	2	2	2	2	2	2	2	2	1
2	2	2	2	2	2	2	2	2	2
2	2	1	2	#NULL!	1	1	2	2	2
2	1	2	2	2	2	2	2	2	2
2	1	2	1	2	1	2	2	1	2
#NULL!	#NULL!	#NULL!	#NULL!	#NULL!	#NULL!	#NULL!	#NULL!	#NULL!	#NULL!
#NULL!	#NULL!	#NULL!	#NULL!	#NULL!	#NULL!	#NULL!	#NULL!	#NULL!	#NULL!
#NULL!	#NULL!	#NULL!	#NULL!	#NULL!	#NULL!	#NULL!	#NULL!	#NULL!	1
#NULL!	#NULL!	#NULL!	#NULL!	#NULL!	#NULL!	#NULL!	#NULL!	#NULL!	#NULL!
#NULL!	#NULL!	#NULL!	#NULL!	#NULL!	#NULL!	#NULL!	#NULL!	#NULL!	#NULL!
#NULL!	#NULL!	#NULL!	#NULL!	#NULL!	#NULL!	#NULL!	#NULL!	#NULL!	#NULL!

2	2	2	2	2	2	2	2	2	2
2	1	2	1	2	2	2	2	2	2
#NULL!	2	2	2	2	1	2	2	2	2
2	2	2	2	2	2	2	2	2	2
#NULL!	#NULL!	#NULL!	#NULL!	#NULL!	#NULL!	#NULL!	#NULL!	#NULL!	#NULL!
#NULL!	#NULL!	#NULL!	#NULL!	#NULL!	#NULL!	#NULL!	#NULL!	#NULL!	#NULL!
#NULL!	#NULL!	#NULL!	#NULL!	#NULL!	#NULL!	#NULL!	#NULL!	#NULL!	#NULL!
#NULL!	#NULL!	#NULL!	#NULL!	#NULL!	#NULL!	#NULL!	#NULL!	#NULL!	#NULL!
#NULL!	#NULL!	#NULL!	#NULL!	#NULL!	#NULL!	#NULL!	#NULL!	#NULL!	#NULL!
#NULL!	#NULL!	#NULL!	#NULL!	#NULL!	#NULL!	#NULL!	#NULL!	#NULL!	#NULL!
#NULL!	#NULL!	#NULL!	#NULL!	#NULL!	#NULL!	#NULL!	#NULL!	#NULL!	#NULL!
#NULL!	#NULL!	#NULL!	#NULL!	#NULL!	#NULL!	#NULL!	#NULL!	#NULL!	#NULL!
#NULL!	#NULL!	#NULL!	#NULL!	#NULL!	#NULL!	#NULL!	#NULL!	#NULL!	#NULL!
2	2	2	2	2	2	1	1	1	2
2	2	2	2	2	1	#NULL!	#NULL!	#NULL!	#NULL!
2	2	2	2	2	2	2	1	2	2
2	2	2	2	2	2	2	2	2	2
#NULL!	#NULL!	#NULL!	#NULL!	#NULL!	#NULL!	#NULL!	#NULL!	#NULL!	#NULL!
#NULL!	#NULL!	#NULL!	#NULL!	#NULL!	#NULL!	#NULL!	#NULL!	#NULL!	#NULL!
#NULL!	#NULL!	#NULL!	#NULL!	#NULL!	#NULL!	#NULL!	#NULL!	#NULL!	#NULL!
#NULL!	#NULL!	2	#NULL!	#NULL!	#NULL!	#NULL!	#NULL!	2	2
#NULL!	#NULL!	#NULL!	#NULL!	#NULL!	#NULL!	#NULL!	#NULL!	#NULL!	#NULL!
2	2	2	2	2	2	2	1	2	1
#NULL!	#NULL!	#NULL!	#NULL!	#NULL!	#NULL!	#NULL!	#NULL!	#NULL!	#NULL!
2	2	2	2	2	2	2	1	2	1
2	2	2	2	2	1	2	2	2	2
2	2	2	2	2	2	2	1	2	2
2	2	2	2	2	1	2	1	2	2
2	2	2	2	2	1	2	1	2	2
2	2	2	2	2	1	2	2	2	2
2	2	2	2	2	1	2	2	2	2
2	2	2	2	2	2	2	1	2	2
2	2	2	2	2	1	2	1	2	1
#NULL!	#NULL!	#NULL!	#NULL!	#NULL!	#NULL!	#NULL!	#NULL!	#NULL!	#NULL!
2	2	2	2	2	1	2	2	2	2
2	2	2	2	2	1	2	2	2	1
2	2	2	2	2	1	2	1	2	1
#NULL!	#NULL!	#NULL!	#NULL!	#NULL!	#NULL!	#NULL!	#NULL!	#NULL!	#NULL!
2	2	1	1	1	2	1	2	2	2
2	2	1	1	1	2	1	2	1	2
1	1	1	1	1	2	1	2	1	2
#NULL!	#NULL!	#NULL!	#NULL!	#NULL!	#NULL!	#NULL!	#NULL!	#NULL!	#NULL!
#NULL!	#NULL!	#NULL!	#NULL!	#NULL!	#NULL!	#NULL!	#NULL!	#NULL!	#NULL!
#NULL!	#NULL!	#NULL!	#NULL!	#NULL!	#NULL!	#NULL!	#NULL!	#NULL!	#NULL!
#NULL!	#NULL!	#NULL!	#NULL!	#NULL!	#NULL!	#NULL!	#NULL!	#NULL!	#NULL!
2	1	2	1	#NULL!	2	2	2	2	2
#NULL!	#NULL!	#NULL!	#NULL!	#NULL!	#NULL!	#NULL!	#NULL!	#NULL!	#NULL!

#NULL!	#NULL!	#NULL!	#NULL!	#NULL!	#NULL!	#NULL!	#NULL!	#NULL!	#NULL!
#NULL!	#NULL!	#NULL!	#NULL!	#NULL!	#NULL!	#NULL!	#NULL!	#NULL!	#NULL!
#NULL!	#NULL!	#NULL!	#NULL!	#NULL!	#NULL!	#NULL!	#NULL!	#NULL!	#NULL!
#NULL!	#NULL!	#NULL!	#NULL!	#NULL!	#NULL!	#NULL!	#NULL!	#NULL!	#NULL!
#NULL!	#NULL!	#NULL!	#NULL!	#NULL!	#NULL!	#NULL!	#NULL!	#NULL!	#NULL!
#NULL!	#NULL!	#NULL!	#NULL!	#NULL!	#NULL!	#NULL!	#NULL!	#NULL!	#NULL!
#NULL!	#NULL!	#NULL!	#NULL!	#NULL!	#NULL!	#NULL!	#NULL!	#NULL!	#NULL!
#NULL!	#NULL!	#NULL!	#NULL!	#NULL!	#NULL!	#NULL!	#NULL!	#NULL!	#NULL!
#NULL!	#NULL!	#NULL!	#NULL!	#NULL!	#NULL!	#NULL!	#NULL!	#NULL!	#NULL!
#NULL!	#NULL!	#NULL!	#NULL!	#NULL!	#NULL!	#NULL!	#NULL!	#NULL!	#NULL!
#NULL!	#NULL!	#NULL!	#NULL!	1	#NULL!	#NULL!	#NULL!	#NULL!	#NULL!
#NULL!	#NULL!	#NULL!	#NULL!	#NULL!	#NULL!	#NULL!	#NULL!	#NULL!	#NULL!
2	2	2	2	2	1	2	1	2	1
2	2	2	2	2	2	2	1	2	1
2	2	2	2	2	1	2	2	2	2
2	2	2	2	2	2	2	1	2	1
2	2	2	2	2	1	2	1	2	1
1	1	1	1	1	2	2	2	2	2
2	2	2	2	2	1	2	2	2	2
#NULL!	#NULL!	2	#NULL!	#NULL!	#NULL!	#NULL!	#NULL!	#NULL!	#NULL!
2	2	2	2	2	2	2	2	2	1
2	2	2	2	2	2	2	1	2	2
2	2	2	2	2	2	2	2	2	2
2	1	2	2	2	1	2	1	2	1
2	2	2	2	2	2	2	1	2	1
2	2	1	1	2	1	1	2	2	2
1	2	2	2	2	2	2	#NULL!	2	2
2	2	2	1	1	2	1	2	1	2
#NULL!	#NULL!	#NULL!	2	#NULL!	#NULL!	#NULL!	#NULL!	#NULL!	#NULL!
#NULL!	#NULL!	1	1	#NULL!	1	1	2	#NULL!	#NULL!
#NULL!	#NULL!	#NULL!	#NULL!	#NULL!	#NULL!	#NULL!	#NULL!	#NULL!	#NULL!
2	2	2	2	2	1	2	2	2	1
2	2	2	2	2	1	2	1	2	2
2	2	2	2	2	2	2	1	2	2
2	2	2	2	2	1	2	1	2	2
2	2	2	2	2	2	2	2	2	2
2	2	2	2	2	2	2	1	2	1
2	2	2	2	2	1	2	1	2	2
2	2	2	2	2	2	2	2	2	1
2	2	2	2	2	1	2	2	2	2
2	2	2	2	2	2	2	2	2	2
2	2	2	2	2	1	2	2	2	1
2	2	2	1	2	1	1	1	2	2
2	2	2	2	2	1	2	1	2	1
2	2	2	2	2	1	2	2	2	2
2	2	2	2	2	1	2	1	2	1
2	2	2	2	2	2	2	2	2	2

#NULL!	#NULL!	#NULL!	#NULL!	#NULL!	#NULL!	#NULL!	#NULL!	#NULL!	#NULL!
2	2	2	2	2	2	2	2	2	1
#NULL!	#NULL!	#NULL!	#NULL!	#NULL!	#NULL!	#NULL!	2	#NULL!	#NULL!
#NULL!	#NULL!	#NULL!	#NULL!	#NULL!	2	#NULL!	#NULL!	#NULL!	#NULL!
#NULL!	#NULL!	2	#NULL!	#NULL!	#NULL!	#NULL!	#NULL!	#NULL!	#NULL!
#NULL!	#NULL!	#NULL!	#NULL!	#NULL!	#NULL!	#NULL!	#NULL!	#NULL!	#NULL!
#NULL!	#NULL!	#NULL!	#NULL!	#NULL!	#NULL!	#NULL!	#NULL!	#NULL!	#NULL!
2	2	1	1	1	2	1	2	2	2
#NULL!	#NULL!	2	#NULL!	#NULL!	#NULL!	#NULL!	#NULL!	#NULL!	#NULL!
2	2	2	2	2	2	2	2	2	2
#NULL!	#NULL!	#NULL!	#NULL!	#NULL!	#NULL!	#NULL!	#NULL!	#NULL!	#NULL!
2	2	#NULL!	#NULL!	#NULL!	#NULL!	#NULL!	#NULL!	#NULL!	#NULL!
2	2	2	2	2	2	2	2	2	1
2	2	2	2	2	2	2	2	2	2
2	2	2	2	2	1	2	1	2	1
#NULL!	#NULL!	#NULL!	#NULL!	#NULL!	#NULL!	#NULL!	#NULL!	#NULL!	#NULL!
2	2	2	2	2	2	2	2	2	2
1	1	1	1	1	2	1	2	1	2
2	#NULL!	#NULL!	#NULL!	#NULL!	#NULL!	#NULL!	#NULL!	#NULL!	#NULL!
2	2	2	2	2	1	2	1	2	1
2	2	2	2	2	2	2	2	2	2
2	1	1	2	1	2	1	2	2	2
2	2	2	2	2	2	2	2	2	2
2	2	2	2	2	2	2	2	2	2
2	2	2	2	2	1	2	2	2	2
2	2	2	2	2	1	2	2	2	2
2	2	1	1	1	2	1	2	2	#NULL!
1	1	2	1	1	2	1	2	2	2
2	2	2	2	2	1	2	1	2	1
2	2	2	2	2	1	2	1	2	1
#NULL!	#NULL!	#NULL!	#NULL!	#NULL!	#NULL!	#NULL!	#NULL!	#NULL!	#NULL!
2	2	1	1	1	2	1	2	2	2
#NULL!	#NULL!	#NULL!	#NULL!	#NULL!	#NULL!	#NULL!	#NULL!	#NULL!	#NULL!
2	2	2	2	2	2	2	2	2	2
2	2	2	2	2	2	2	2	2	2
2	2	2	2	2	1	2	1	2	1
1	1	1	2	1	2	1	2	1	2
2	2	2	2	2	1	2	2	2	2
2	2	2	2	2	1	2	1	2	1
2	2	2	2	2	2	1	1	2	1
2	2	2	2	2	2	2	2	2	2
2	2	2	2	2	1	2	2	2	1
#NULL!	#NULL!	#NULL!	#NULL!	#NULL!	#NULL!	#NULL!	#NULL!	#NULL!	#NULL!
#NULL!	#NULL!	#NULL!	#NULL!	#NULL!	#NULL!	#NULL!	#NULL!	#NULL!	#NULL!
#NULL!	#NULL!	2	#NULL!	#NULL!	#NULL!	#NULL!	#NULL!	#NULL!	#NULL!
#NULL!	#NULL!	#NULL!	#NULL!	#NULL!	#NULL!	#NULL!	#NULL!	#NULL!	#NULL!
#NULL!	#NULL!	#NULL!	#NULL!	#NULL!	#NULL!	#NULL!	#NULL!	#NULL!	#NULL!

#NULL!	#NULL!	#NULL!	#NULL!	#NULL!	#NULL!	#NULL!	#NULL!	#NULL!	#NULL!
2	2	2	2	2	2	2	2	2	2
#NULL!	#NULL!	2	#NULL!	#NULL!	#NULL!	#NULL!	#NULL!	#NULL!	#NULL!
2	2	2	2	2	1	1	1	2	1
#NULL!	#NULL!	#NULL!	#NULL!	#NULL!	#NULL!	#NULL!	#NULL!	#NULL!	#NULL!
2	1	2	2	2	1	2	1	2	2
2	2	2	2	2	2	2	2	1	2
#NULL!	#NULL!	#NULL!	#NULL!	#NULL!	#NULL!	#NULL!	#NULL!	#NULL!	#NULL!
#NULL!	#NULL!	#NULL!	#NULL!	#NULL!	#NULL!	#NULL!	#NULL!	#NULL!	#NULL!
#NULL!	#NULL!	#NULL!	#NULL!	#NULL!	#NULL!	#NULL!	#NULL!	#NULL!	#NULL!
#NULL!	#NULL!	#NULL!	#NULL!	#NULL!	#NULL!	#NULL!	#NULL!	#NULL!	#NULL!
#NULL!	#NULL!	#NULL!	#NULL!	#NULL!	#NULL!	#NULL!	#NULL!	#NULL!	#NULL!
2	1	2	2	2	2	2	2	2	1
#NULL!	#NULL!	#NULL!	#NULL!	#NULL!	#NULL!	#NULL!	#NULL!	#NULL!	#NULL!
#NULL!	#NULL!	#NULL!	#NULL!	2	#NULL!	#NULL!	#NULL!	#NULL!	#NULL!
1	2	2	2	1	2	2	2	2	2
2	2	2	1	2	2	1	2	2	2
2	2	2	2	2	2	1	2	2	2
1	1	2	1	1	2	1	2	2	2
1	2	2	2	1	2	2	2	2	1
2	2	2	1	2	1	1	2	2	1
2	2	2	1	1	1	1	2	2	2
1	2	2	2	2	2	2	1	2	2
2	2	2	2	2	2	2	2	2	2
2	2	2	2	2	2	2	2	2	2
2	2	2	2	2	1	2	1	2	2
2	2	2	2	2	2	2	2	2	2
2	2	2	2	2	2	#NULL!	2	2	1
2	2	2	1	2	2	2	1	#NULL!	1
1	1	2	2	2	2	2	2	2	1
2	2	1	1	1	1	2	2	2	2
2	2	2	2	2	2	2	2	2	2
#NULL!	#NULL!	#NULL!	#NULL!	#NULL!	#NULL!	#NULL!	#NULL!	#NULL!	#NULL!
2	2	2	2	1	1	2	#NULL!	2	#NULL!
2	2	2	2	2	1	2	2	2	2
2	2	2	2	2	2	2	2	2	2
2	2	2	2	2	2	2	2	1	2
2	2	2	2	2	2	2	2	2	2
#NULL!	2	#NULL!	#NULL!	#NULL!	#NULL!	#NULL!	#NULL!	#NULL!	#NULL!
2	2	2	2	1	1	2	2	2	1
#NULL!	#NULL!	#NULL!	#NULL!	#NULL!	#NULL!	#NULL!	#NULL!	#NULL!	#NULL!
#NULL!	#NULL!	2	#NULL!	#NULL!	#NULL!	#NULL!	#NULL!	#NULL!	#NULL!
2	1	2	1	2	2	2	1	1	1
#NULL!	#NULL!	2	#NULL!	#NULL!	#NULL!	#NULL!	#NULL!	#NULL!	#NULL!
2	2	2	2	2	1	2	1	2	1
1	2	2	2	1	1	1	1	2	1
2	2	2	2	#NULL!	2	1	2	1	2

1	1	1	1	2	2	1	2	1	2
#NULL!	#NULL!	#NULL!	#NULL!	2	#NULL!	#NULL!	#NULL!	#NULL!	#NULL!
1	1	2	2	2	2	2	2	2	2
2	2	2	2	1	1	2	2	2	1
2	2	2	2	1	2	1	2	1	2
2	2	2	2	2	1	1	2	1	1
2	2	2	2	#NULL!	1	1	#NULL!	2	2
2	1	2	2	#NULL!	2	2	2	2	2
2	2	2	2	2	2	2	2	2	2
2	1	2	2	2	2	2	2	2	2
2	2	2	2	2	1	2	1	1	2
2	2	2	1	1	2	2	2	1	2
1	2	1	2	1	2	#NULL!	1	2	2
2	2	2	2	2	2	2	2	2	2
2	2	2	2	2	1	1	1	2	2
2	2	2	2	1	1	2	2	2	2
2	2	2	2	2	2	2	2	2	2
2	1	2	1	2	1	1	2	2	2
2	2	2	2	2	2	2	2	2	2
2	2	2	2	2	2	2	2	2	2
2	2	2	2	2	2	2	2	2	2
1	1	2	2	2	1	2	2	1	2
2	2	2	2	#NULL!	#NULL!	#NULL!	#NULL!	#NULL!	#NULL!
#NULL!	#NULL!	#NULL!	#NULL!	#NULL!	#NULL!	#NULL!	#NULL!	#NULL!	#NULL!
2	2	2	2	2	2	2	2	2	2
2	2	2	2	1	2	2	2	2	1
2	2	2	2	1	1	1	1	2	2
2	2	1	1	2	2	2	1	2	2
2	1	2	2	2	1	2	1	1	1
#NULL!	#NULL!	#NULL!	#NULL!	#NULL!	#NULL!	#NULL!	#NULL!	#NULL!	1
2	2	2	1	2	2	2	2	1	1
2	2	2	1	2	1	2	2	1	2
1	2	1	2	1	#NULL!	2	2	1	2
2	2	2	2	2	1	2	2	2	2
2	2	1	1	1	2	2	1	2	2
2	2	2	2	2	2	2	2	2	2
2	2	1	1	1	2	#NULL!	2	1	2
#NULL!	#NULL!	#NULL!	#NULL!	#NULL!	#NULL!	#NULL!	#NULL!	#NULL!	#NULL!
2	1	2	2	2	1	2	2	1	2
2	2	2	1	2	1	2	2	2	2
#NULL!	#NULL!	#NULL!	#NULL!	#NULL!	#NULL!	#NULL!	#NULL!	#NULL!	#NULL!
#NULL!	#NULL!	#NULL!	#NULL!	#NULL!	#NULL!	#NULL!	#NULL!	#NULL!	#NULL!
2	2	2	2	2	#NULL!	2	2	2	2
#NULL!	#NULL!	#NULL!	#NULL!	#NULL!	#NULL!	#NULL!	#NULL!	#NULL!	#NULL!
2	2	2	2	2	2	2	2	2	2
2	2	2	2	2	1	2	2	2	2
#NULL!	#NULL!	#NULL!	#NULL!	#NULL!	#NULL!	#NULL!	#NULL!	#NULL!	#NULL!

2	1	2	2	2	2	1	2	2	2
#NULL!	#NULL!	#NULL!	#NULL!	#NULL!	#NULL!	#NULL!	#NULL!	#NULL!	#NULL!
2	2	2	1	2	1	2	2	1	2
#NULL!	#NULL!	#NULL!	#NULL!	#NULL!	#NULL!	#NULL!	#NULL!	#NULL!	#NULL!
#NULL!	#NULL!	#NULL!	#NULL!	#NULL!	#NULL!	#NULL!	#NULL!	#NULL!	#NULL!
1	1	1	1	1	2	1	2	1	2
2	2	2	2	2	2	2	2	2	2
2	2	2	2	2	2	2	2	2	2
2	2	2	2	2	1	2	1	2	1
2	2	2	2	2	2	2	2	2	2
#NULL!	#NULL!	#NULL!	#NULL!	#NULL!	#NULL!	#NULL!	#NULL!	#NULL!	#NULL!
#NULL!	#NULL!	#NULL!	#NULL!	#NULL!	#NULL!	#NULL!	#NULL!	#NULL!	#NULL!
#NULL!	#NULL!	#NULL!	#NULL!	#NULL!	#NULL!	#NULL!	#NULL!	#NULL!	#NULL!
2	2	2	2	2	1	2	1	2	1
2	2	2	2	2	2	2	2	2	2
2	2	2	2	2	1	2	1	2	1
#NULL!	#NULL!	2	#NULL!	#NULL!	#NULL!	#NULL!	#NULL!	#NULL!	#NULL!
#NULL!	#NULL!	#NULL!	#NULL!	#NULL!	#NULL!	#NULL!	#NULL!	#NULL!	#NULL!
1	1	2	1	1	2	1	2	1	2
2	2	2	2	2	2	2	2	2	2
2	2	2	2	2	2	2	2	2	1
1	1	1	1	1	2	1	2	1	2
#NULL!	#NULL!	2	#NULL!	#NULL!	#NULL!	#NULL!	#NULL!	#NULL!	#NULL!
2	1	2	1	2	2	1	2	1	2
1	1	2	2	2	1	2	1	2	2
1	1	2	2	2	2	1	2	1	2
2	2	#NULL!	#NULL!	#NULL!	#NULL!	#NULL!	1	#NULL!	#NULL!
2	2	2	2	2	2	2	1	2	1
2	2	2	2	2	2	2	2	2	2
#NULL!	#NULL!	#NULL!	#NULL!	2	#NULL!	#NULL!	#NULL!	#NULL!	#NULL!
1	1	1	2	2	1	1	2	2	2
2	2	2	2	2	2	2	2	2	2
2	2	2	2	1	2	1	2	2	1
1	2	2	2	2	2	2	1	2	2
2	2	2	2	2	2	2	2	2	2
#NULL!	2	2	2	2	2	2	1	2	2
2	2	2	2	2	2	1	2	2	2
2	1	2	2	1	2	1	2	1	2
2	2	2	2	2	2	2	2	2	2
1	1	1	1	1	2	1	2	1	2
2	2	2	2	2	1	1	2	2	2
2	2	2	2	2	2	2	2	2	2
2	2	2	2	1	2	1	2	1	2
2	1	2	2	2	2	2	2	1	2
2	1	2	1	2	1	1	1	1	2
1	2	2	2	1	2	2	2	1	2
2	2	2	2	2	1	2	2	2	1

2	2	2	1	#NULL!	2	2	2	2	2
2	2	2	2	2	2	2	2	2	2
2	2	2	2	2	1	2	1	2	1
2	2	2	2	1	2	1	2	1	2
2	2	2	2	2	1	2	2	2	1
#NULL!	#NULL!	#NULL!	#NULL!	2	#NULL!	#NULL!	#NULL!	#NULL!	#NULL!
2	2	2	2	2	2	2	2	2	2
2	2	2	2	2	2	2	1	2	1
2	2	2	2	2	2	2	2	2	1
2	2	2	2	2	2	2	2	2	2
2	2	2	2	2	2	2	2	2	2
2	2	2	2	2	2	2	2	2	1
2	2	2	2	2	2	2	2	2	2
1	1	1	1	1	2	1	2	1	2
1	2	2	#NULL!	1	2	2	2	1	1
2	2	2	2	1	2	2	2	2	2
1	2	1	2	1	2	1	2	1	2
2	1	2	2	2	1	2	1	1	2
2	2	2	2	2	2	2	2	2	2
2	2	2	2	2	#NULL!	2	2	2	2
2	2	2	2	2	2	2	2	2	2
2	2	2	2	1	1	1	2	2	2
2	2	2	2	1	2	1	2	1	2
#NULL!	#NULL!	2	2	2	1	2	1	#NULL!	1
2	2	2	2	2	1	1	2	2	2
2	2	2	2	2	2	2	2	2	2
2	2	2	2	2	2	2	2	2	2
2	1	2	2	2	2	2	2	2	2
2	2	2	2	2	2	2	2	2	2
2	2	2	2	2	1	2	1	2	2
1	2	2	2	2	2	1	2	1	2
2	1	2	1	1	2	2	2	2	2
2	2	2	1	1	2	1	2	2	1
1	2	1	1	1	2	1	2	1	2
2	2	2	2	1	2	2	1	2	2
2	2	2	2	2	2	2	2	2	2
2	2	2	2	2	2	2	2	2	2
2	2	2	2	2	2	2	2	2	2
2	2	2	2	2	1	2	2	2	2
2	2	2	#NULL!	2	1	1	2	2	2
2	1	2	2	2	2	2	2	2	2
2	2	2	2	2	1	1	2	1	2
2	2	2	2	2	2	2	2	2	1
2	2	2	2	2	2	2	2	2	2
2	1	2	2	2	2	1	2	1	2
2	2	2	2	2	1	1	2	2	2
2	2	2	2	2	1	2	2	2	2

2	2	2	2	2	2	1	2	2	1
2	2	#NULL!	2	2	2	1	2	1	2
2	2	2	2	2	1	2	2	2	2
2	2	2	2	2	2	2	2	2	2
2	2	2	1	1	2	2	2	2	2
2	2	2	2	2	2	2	2	2	2
2	2	2	2	2	1	1	2	2	2
2	2	2	2	2	2	2	1	2	2
2	2	2	2	2	2	2	2	2	2
2	2	2	2	2	2	2	2	2	2
2	2	2	2	2	2	2	2	2	2
#NULL!	#NULL!	#NULL!	#NULL!	#NULL!	#NULL!	#NULL!	#NULL!	#NULL!	#NULL!
#NULL!	#NULL!	#NULL!	#NULL!	#NULL!	#NULL!	#NULL!	#NULL!	#NULL!	#NULL!
#NULL!	#NULL!	#NULL!	2	#NULL!	#NULL!	#NULL!	#NULL!	#NULL!	#NULL!
2	2	2	2	2	1	2	1	2	2
2	2	2	2	2	2	2	2	2	2
2	2	2	2	2	2	2	1	2	2
2	2	2	2	2	1	2	2	2	2
2	2	2	2	2	1	1	1	2	1
2	2	2	2	2	1	1	2	2	2
2	1	2	2	2	2	1	2	2	1
2	2	#NULL!	2	2	2	1	2	1	2
2	2	2	2	2	2	2	2	2	2
2	2	2	2	2	2	2	2	2	2
2	2	2	2	2	1	2	2	2	2
2	2	#NULL!	#NULL!	2	1	2	2	2	2
2	2	2	2	2	1	2	2	2	2
2	2	2	2	2	2	2	2	2	2
2	2	2	2	2	2	2	2	2	2
#NULL!	#NULL!	#NULL!	#NULL!	#NULL!	#NULL!	#NULL!	#NULL!	#NULL!	#NULL!
2	2	2	2	2	2	2	2	2	2
2	2	2	2	2	2	2	2	2	2
2	2	2	#NULL!	2	1	2	2	2	2
2	2	2	2	2	2	2	2	2	2
2	2	2	2	2	#NULL!	#NULL!	#NULL!	#NULL!	#NULL!
2	2	2	2	2	2	2	2	2	2
2	2	2	2	2	2	2	2	2	2
2	2	2	2	2	2	2	2	2	2
2	2	2	2	2	2	2	2	2	2
2	2	2	2	2	2	2	2	2	2
2	2	2	2	2	2	2	2	2	2
2	2	2	2	2	2	2	2	2	2
2	2	2	2	2	1	2	1	2	1
2	2	2	2	2	1	2	2	2	2
2	2	2	2	2	2	2	2	2	2
1	2	2	2	2	1	2	2	2	2
2	2	2	2	2	2	2	2	2	2

2	2	2	2	2	2	2	2	2	1
2	2	2	2	2	1	2	2	2	2
2	2	2	2	2	1	1	2	1	2
2	2	2	2	2	2	2	2	2	2
2	2	2	2	2	2	2	2	2	2
2	2	2	2	2	2	2	2	1	2
#NULL!	#NULL!	#NULL!	#NULL!	#NULL!	#NULL!	#NULL!	#NULL!	#NULL!	#NULL!
2	2	2	2	2	1	2	1	2	2
2	2	2	2	1	2	2	1	2	1
2	2	2	2	2	2	2	2	2	2
2	2	2	2	2	1	2	2	2	2
2	2	2	2	2	2	2	2	2	2
2	2	2	2	2	2	2	2	2	2
#NULL!	2	2	#NULL!	2	1	#NULL!	#NULL!	#NULL!	#NULL!
2	2	2	2	2	1	2	2	2	2
2	2	2	2	2	2	2	1	2	2
2	2	2	2	2	2	2	2	2	2
2	2	2	2	2	1	1	2	2	2
2	2	2	2	2	1	2	1	2	1
2	2	2	2	2	2	2	2	2	2
2	1	2	2	2	1	2	1	2	1
2	2	2	2	2	2	2	1	2	2
2	2	2	2	2	2	2	2	2	2
2	2	2	2	2	2	2	2	2	2
2	2	2	2	2	2	2	2	2	2
2	2	2	2	2	2	2	2	2	2
#NULL!	#NULL!	#NULL!	#NULL!	#NULL!	#NULL!	#NULL!	#NULL!	#NULL!	#NULL!
2	2	2	2	2	2	2	2	2	2
2	2	2	2	2	1	2	2	2	2
2	2	2	2	2	2	2	2	2	2
2	2	2	2	2	2	2	2	2	2
2	2	2	2	2	1	2	2	2	1
#NULL!	#NULL!	#NULL!	#NULL!	#NULL!	#NULL!	#NULL!	#NULL!	#NULL!	#NULL!
#NULL!	#NULL!	#NULL!	#NULL!	#NULL!	#NULL!	#NULL!	#NULL!	#NULL!	#NULL!
#NULL!	#NULL!	#NULL!	#NULL!	#NULL!	#NULL!	#NULL!	#NULL!	#NULL!	#NULL!
#NULL!	#NULL!	#NULL!	#NULL!	#NULL!	#NULL!	#NULL!	#NULL!	#NULL!	#NULL!
1	1	2	2	1	1	2	1	2	2
2	2	2	2	2	1	1	1	2	2
1	2	2	2	2	1	2	1	2	1
2	2	2	1	2	2	2	2	#NULL!	2
2	2	2	2	2	2	2	2	2	2
2	2	2	2	2	2	2	2	2	2
2	2	2	2	2	1	2	2	2	1
2	2	2	2	2	2	2	2	2	2
2	2	2	2	2	1	2	2	2	1
2	2	2	2	2	1	2	2	2	2
2	2	2	2	2	1	2	1	2	2

#NULL!	2	2	2	2	2	2	2	2	2
2	2	2	2	2	2	2	2	2	1
2	2	2	2	2	2	1	2	2	2
2	2	2	2	2	2	2	1	2	1
2	2	2	2	1	2	2	1	2	2
2	2	2	2	1	1	2	2	1	2
2	2	2	2	2	1	1	2	2	1
#NULL!	#NULL!	2	#NULL!	#NULL!	#NULL!	#NULL!	#NULL!	#NULL!	#NULL!
2	2	2	2	2	2	2	2	2	2
2	2	2	2	2	2	2	2	2	2
2	2	2	2	2	2	2	2	2	2
2	2	2	2	2	2	2	2	2	2
2	2	2	2	2	2	2	2	2	2
2	2	2	2	2	1	2	2	2	1
2	2	2	2	2	2	2	2	2	2
2	2	2	2	2	2	2	2	2	2
2	2	2	2	2	2	2	2	2	2
2	2	2	#NULL!	1	2	2	2	1	2
2	2	2	2	2	2	1	2	2	1
2	2	2	2	2	2	#NULL!	2	1	2
2	2	2	1	2	2	2	2	1	2
2	2	2	2	2	2	2	2	2	2
2	2	2	2	2	1	1	2	1	2
#NULL!	#NULL!	#NULL!	#NULL!	2	2	#NULL!	#NULL!	#NULL!	#NULL!
1	1	1	2	2	2	2	1	2	2
2	2	2	2	2	1	2	1	2	2
2	2	2	2	2	1	2	2	2	1
2	2	2	2	2	1	2	2	2	1
2	2	2	2	2	2	2	2	2	2
#NULL!	#NULL!	#NULL!	2	#NULL!	#NULL!	#NULL!	#NULL!	#NULL!	#NULL!
1	1	1	2	1	2	2	2	1	2
#NULL!	#NULL!	#NULL!	#NULL!	#NULL!	#NULL!	#NULL!	#NULL!	#NULL!	#NULL!
#NULL!	#NULL!	#NULL!	#NULL!	#NULL!	#NULL!	#NULL!	#NULL!	#NULL!	#NULL!
#NULL!	#NULL!	#NULL!	#NULL!	#NULL!	#NULL!	#NULL!	#NULL!	#NULL!	#NULL!
#NULL!	#NULL!	#NULL!	#NULL!	#NULL!	#NULL!	#NULL!	#NULL!	#NULL!	#NULL!
2	2	2	2	2	2	2	2	2	2
1	1	2	1	1	1	2	2	2	2
#NULL!	#NULL!	#NULL!	#NULL!	#NULL!	#NULL!	#NULL!	#NULL!	#NULL!	#NULL!
2	2	2	2	2	2	2	2	2	2
2	2	2	2	2	2	2	2	2	2
2	2	1	1	2	2	2	2	2	2
2	2	2	2	2	2	2	2	2	2
2	2	2	2	2	2	2	2	2	2
1	2	1	2	2	2	2	1	1	2
#NULL!	#NULL!	1	#NULL!	#NULL!	#NULL!	#NULL!	#NULL!	#NULL!	2
2	2	2	2	2	1	2	1	2	1
1	1	1	1	1	2	1	2	1	2

2	2	2	2	2	1	2	1	2	2
2	2	2	2	2	2	2	1	2	2
2	2	2	2	2	1	2	1	2	1
2	2	2	2	2	2	2	2	2	1
#NULL!	#NULL!	#NULL!	#NULL!	#NULL!	#NULL!	#NULL!	#NULL!	#NULL!	#NULL!
#NULL!	#NULL!	#NULL!	#NULL!	#NULL!	#NULL!	#NULL!	#NULL!	#NULL!	#NULL!
2	2	2	2	2	2	2	2	2	2
2	2	1	1	1	2	1	2	1	2
#NULL!	#NULL!	#NULL!	#NULL!	2	#NULL!	#NULL!	#NULL!	#NULL!	#NULL!
#NULL!	#NULL!	#NULL!	#NULL!	#NULL!	#NULL!	#NULL!	#NULL!	#NULL!	#NULL!
2	2	#NULL!	2	2	1	2	2	2	2
2	2	2	2	2	1	2	1	2	2
2	2	1	1	1	2	2	1	2	1
1	2	1	1	1	2	1	2	1	2
#NULL!	#NULL!	1	1	1	2	1	1	1	2
1	2	2	2	2	2	1	2	1	2
2	2	2	2	2	1	1	2	2	2
2	1	2	2	2	2	2	2	2	2
#NULL!	#NULL!	#NULL!	#NULL!	1	#NULL!	#NULL!	#NULL!	#NULL!	#NULL!
1	2	2	#NULL!	2	1	1	2	2	2
2	2	2	2	2	2	2	1	1	1
2	2	2	2	2	2	2	1	2	2
2	2	2	2	1	2	1	2	#NULL!	1
2	2	2	2	2	1	2	2	2	2
1	1	2	2	2	1	2	1	1	2
2	2	2	2	1	2	2	2	2	2
2	2	2	2	2	2	1	2	2	2
2	2	2	2	2	2	2	2	2	2
2	2	2	2	#NULL!	2	2	#NULL!	2	2
2	1	1	2	2	1	1	2	1	2
2	2	2	2	2	2	2	2	2	2
2	2	2	2	2	2	2	2	2	1
1	1	1	2	2	1	1	1	2	1
1	2	2	2	2	1	1	2	2	2
#NULL!	#NULL!	#NULL!	#NULL!	2	#NULL!	#NULL!	#NULL!	#NULL!	#NULL!
2	2	2	2	2	2	2	2	2	2
#NULL!	#NULL!	#NULL!	#NULL!	#NULL!	#NULL!	#NULL!	#NULL!	#NULL!	#NULL!
2	2	2	2	2	2	2	2	2	2
2	2	#NULL!	#NULL!	#NULL!	#NULL!	#NULL!	#NULL!	#NULL!	#NULL!
2	2	2	2	2	1	2	1	2	1
#NULL!	#NULL!	#NULL!	#NULL!	#NULL!	#NULL!	#NULL!	#NULL!	#NULL!	#NULL!
2	2	2	2	1	1	2	2	1	2
2	2	2	1	2	2	2	1	2	2
2	2	2	2	2	1	2	1	2	2
2	2	2	2	2	1	2	1	2	2
1	1	2	2	2	2	2	2	1	2
2	1	2	1	2	2	1	2	1	2

2	2	2	2	2	2	2	1	1	1
2	1	#NULL!	#NULL!	#NULL!	#NULL!	#NULL!	#NULL!	#NULL!	#NULL!
2	2	2	2	2	1	1	2	2	1
1	1	1	1	2	1	1	2	#NULL!	2
1	2	2	2	2	1	1	1	2	1
#NULL!	#NULL!	#NULL!	#NULL!	#NULL!	#NULL!	#NULL!	#NULL!	#NULL!	#NULL!
#NULL!	#NULL!	#NULL!	#NULL!	#NULL!	#NULL!	#NULL!	#NULL!	#NULL!	#NULL!
2	2	2	2	2	2	2	1	2	2
2	2	2	2	1	1	2	1	2	2
2	2	1	1	2	1	1	2	1	2
2	2	2	2	2	1	2	2	2	1
2	2	2	2	1	2	2	2	1	1
1	2	2	2	2	2	2	2	2	2
2	2	2	2	2	1	1	1	2	1
#NULL!	#NULL!	#NULL!	#NULL!	#NULL!	#NULL!	#NULL!	#NULL!	#NULL!	#NULL!
1	1	1	1	1	2	1	2	1	2
2	2	2	1	1	2	1	2	2	1
2	2	2	2	2	2	2	2	2	2
2	1	2	2	1	1	2	2	2	1
2	2	2	2	1	2	2	2	2	1
#NULL!	#NULL!	#NULL!	#NULL!	#NULL!	#NULL!	#NULL!	#NULL!	#NULL!	#NULL!
#NULL!	#NULL!	#NULL!	#NULL!	#NULL!	#NULL!	#NULL!	#NULL!	#NULL!	#NULL!
1	2	2	2	1	2	2	2	1	1
#NULL!	#NULL!	#NULL!	#NULL!	#NULL!	#NULL!	#NULL!	#NULL!	#NULL!	#NULL!
#NULL!	#NULL!	#NULL!	#NULL!	#NULL!	#NULL!	#NULL!	#NULL!	#NULL!	#NULL!
1	2	1	1	1	2	2	2	#NULL!	2
1	1	2	1	1	2	2	2	1	2
2	2	2	2	2	2	2	2	2	2
1	1	1	1	1	2	1	2	1	2
2	2	2	2	2	1	2	1	2	2
1	1	1	1	1	2	1	2	1	2
2	2	2	2	2	2	2	2	2	2
#NULL!	#NULL!	#NULL!	#NULL!	#NULL!	#NULL!	#NULL!	#NULL!	#NULL!	#NULL!
#NULL!	#NULL!	#NULL!	#NULL!	#NULL!	#NULL!	#NULL!	#NULL!	#NULL!	#NULL!
#NULL!	#NULL!	#NULL!	#NULL!	#NULL!	#NULL!	#NULL!	#NULL!	#NULL!	#NULL!
2	2	2	2	2	2	2	2	2	1
2	2	2	1	2	1	1	1	1	2
1	2	2	2	1	1	2	1	1	1
2	2	2	2	2	2	1	2	2	2
#NULL!	#NULL!	#NULL!	2	#NULL!	#NULL!	#NULL!	#NULL!	#NULL!	#NULL!
2	1	2	2	2	2	2	2	2	2
1	2	2	2	2	1	1	1	2	2
1	1	2	2	1	2	2	2	2	2
#NULL!	#NULL!	2	2	2	1	2	1	2	#NULL!
2	1	1	2	2	1	2	2	1	2
#NULL!	2	2	2	1	2	2	2	1	2
1	2	2	2	2	1	1	2	2	2

2	2	2	2	1	1	2	2	2	2
1	1	2	2	2	2	1	2	1	2
2	2	1	1	1	2	1	2	#NULL!	1
2	2	2	2	1	1	2	2	2	2
2	2	1	1	2	1	1	2	2	2
2	2	2	2	2	2	2	2	2	2
2	2	2	2	2	1	2	#NULL!	2	1
2	2	2	2	2	2	2	2	2	2
#NULL!	#NULL!	#NULL!	#NULL!	#NULL!	#NULL!	#NULL!	#NULL!	#NULL!	#NULL!
2	2	2	1	2	1	2	1	2	2
2	1	2	2	2	2	2	2	2	2
2	2	2	2	2	2	2	2	1	2
2	2	2	2	2	2	1	2	2	2
2	2	2	2	2	1	1	1	2	1
#NULL!	#NULL!	#NULL!	2	#NULL!	#NULL!	#NULL!	#NULL!	#NULL!	#NULL!
2	2	2	2	2	2	1	2	1	2
2	2	2	2	2	1	2	1	2	1
2	2	2	2	1	1	2	2	2	2
1	2	2	1	2	1	1	2	2	1
2	2	2	2	2	2	2	2	2	2
2	2	2	2	2	1	2	1	2	2
2	2	2	2	2	2	2	2	2	2
2	2	2	2	2	2	2	2	2	1
2	2	2	2	2	1	2	2	2	2
2	2	2	2	1	#NULL!	2	1	2	2
1	2	2	2	2	1	2	2	2	2
1	2	2	2	2	2	1	2	2	2
2	2	2	2	2	1	1	2	2	2
2	2	2	2	2	2	2	2	2	2
2	2	2	2	2	2	2	2	2	2
2	2	2	2	2	2	2	2	2	2
#NULL!	#NULL!	2	2	2	2	2	2	2	2
2	2	2	2	2	2	2	2	2	2
2	2	2	2	2	1	2	2	1	2
1	2	2	2	1	1	1	1	2	2
2	2	2	2	2	1	1	1	2	1
2	2	2	2	2	2	2	1	2	2
2	2	2	2	2	2	2	2	2	1
2	2	2	2	1	2	1	2	2	2
2	2	2	2	2	2	2	2	2	1
1	2	1	1	1	2	1	2	1	#NULL!
2	2	2	2	2	2	2	1	2	2
2	2	2	2	2	2	2	2	2	1
2	2	2	2	2	1	2	1	2	1
2	2	2	2	2	2	2	2	2	1
2	2	2	2	2	2	2	1	2	2
2	2	2	2	2	2	1	2	2	1

2	2	2	2	2	1	2	2	2	1
2	2	2	1	1	2	2	1	2	2
2	2	2	2	2	2	2	2	2	1
2	2	2	2	1	2	1	2	2	2
2	2	2	2	2	2	2	2	2	2
2	2	2	2	1	1	2	2	1	2
2	2	2	2	2	1	2	2	2	1
2	2	2	2	2	2	2	2	2	1

FamHx2	Warts2	UTI2	STD2	Poison2	Inherit2	Offensive2	SexBleed2	DysMeno2	Menorrhag
2	2	2	2	2	2	2	2	2	2
2	1	1	1	2	2	2	2	1	1
2	2	2	2	2	2	1	2	2	1
2	1	1	1	2	2	2	2	1	1
2	1	1	1	2	2	2	2	1	1
2	1	1	1	2	2	2	2	1	1
#NULL!	#NULL!	#NULL!	#NULL!	#NULL!	#NULL!	#NULL!	#NULL!	#NULL!	#NULL!
1	2	1	2	2	1	2	1	2	2
#NULL!	2	#NULL!	2	2	#NULL!	2	2	2	2
#NULL!	#NULL!	#NULL!	#NULL!	#NULL!	#NULL!	2	#NULL!	#NULL!	#NULL!
2	2	2	2	2	2	2	#NULL!	2	2
2	2	2	2	2	2	2	1	1	1
2	2	2	2	2	1	2	2	2	2
1	1	2	2	2	1	2	1	2	2
1	1	2	1	1	1	2	2	2	2
1	2	2	2	2	1	2	2	2	2
1	2	1	2	1	2	2	1	#NULL!	2
1	2	2	2	2	1	2	2	2	2
1	2	2	2	2	1	2	2	2	2
2	2	2	2	2	2	1	2	2	2
2	2	2	2	2	1	2	2	2	2
2	2	2	2	2	1	2	2	2	2
2	2	2	2	2	2	2	2	2	2
2	2	2	2	2	1	2	2	1	2
2	2	2	2	2	2	2	2	2	2
2	2	2	2	2	2	2	2	2	2
2	2	2	2	2	1	2	2	2	2
2	1	2	2	1	1	2	2	2	2
2	2	2	2	2	2	2	2	2	2
2	2	2	2	2	2	2	2	2	2
2	2	2	2	2	2	2	2	2	2
1	1	1	1	1	1	1	2	2	2
1	1	1	1	2	2	1	2	2	2
1	1	1	1	1	1	1	2	2	2
2	1	1	1	2	2	2	2	2	2
1	1	1	1	1	1	1	2	2	2
2	1	1	1	2	2	2	2	2	2
1	1	2	1	2	1	1	2	2	2
2	2	2	2	2	2	2	2	2	2
2	2	2	2	2	2	2	2	2	2
2	2	2	2	2	2	2	2	2	2
2	2	2	2	2	2	2	2	2	2
2	1	1	1	2	1	1	2	2	2
1	1	2	2	1	2	1	1	2	2
2	1	2	2	2	2	1	1	2	2
2	1	1	2	2	1	1	2	2	2

2	2	1	1	2	2	1	2	2	2
1	#NULL!	1	1	#NULL!	2	1	2	1	2
2	1	1	1	2	2	1	2	2	2
2	1	1	1	2	2	1	2	2	2
2	1	1	1	1	2	1	2	#NULL!	2
2	1	1	1	2	2	1	1	2	2
2	1	1	1	2	1	1	2	1	2
#NULL!	1	1	1	#NULL!	#NULL!	1	1	2	2
#NULL!	#NULL!	1	1	#NULL!	#NULL!	1	#NULL!	#NULL!	#NULL!
2	1	1	1	2	2	1	2	2	2
2	2	2	2	#NULL!	#NULL!	2	#NULL!	2	#NULL!
2	2	2	2	2	2	2	2	2	2
2	2	1	1	2	2	1	2	2	2
1	2	2	2	2	2	1	2	2	2
2	2	2	1	2	2	1	2	1	2
2	2	2	2	2	2	2	1	2	2
2	1	2	2	1	2	1	2	2	2
2	1	1	1	2	2	1	2	2	2
2	1	1	2	2	1	1	2	2	2
1	2	2	2	2	1	#NULL!	#NULL!	#NULL!	#NULL!
2	1	1	1	2	2	1	1	2	2
2	1	1	1	2	2	1	2	2	2
2	1	1	1	2	2	1	1	2	2
2	1	2	2	2	2	1	2	1	1
1	#NULL!	#NULL!	1	#NULL!	#NULL!	1	#NULL!	1	1
2	1	1	1	2	2	1	2	2	2
1	1	1	1	2	2	#NULL!	#NULL!	#NULL!	#NULL!
2	1	1	2	1	1	1	2	2	2
2	2	2	2	2	2	#NULL!	#NULL!	#NULL!	#NULL!
2	1	1	1	2	2	1	2	2	#NULL!
2	2	#NULL!	1	1	1	1	2	2	2
1	1	1	1	#NULL!	1	2	2	1	1
2	1	1	1	2	2	1	2	2	2
2	1	1	1	2	2	2	2	1	1
1	1	1	2	2	1	2	1	1	1
#NULL!	#NULL!	#NULL!	#NULL!	#NULL!	#NULL!	#NULL!	#NULL!	#NULL!	#NULL!
#NULL!	#NULL!	#NULL!	#NULL!	#NULL!	#NULL!	#NULL!	#NULL!	#NULL!	#NULL!
2	2	1	1	2	2	2	2	1	1
2	1	1	1	2	2	1	1	2	2
2	1	1	1	1	2	1	2	1	2
#NULL!	1	#NULL!	1	#NULL!	#NULL!	1	2	1	#NULL!
2	2	1	1	#NULL!	#NULL!	1	2	2	2
2	2	1	1	2	1	1	2	2	2
2	1	1	1	2	2	1	2	2	2
2	2	1	1	1	2	1	2	2	2
#NULL!	#NULL!	#NULL!	#NULL!	#NULL!	#NULL!	#NULL!	#NULL!	#NULL!	#NULL!
#NULL!	#NULL!	#NULL!	#NULL!	#NULL!	#NULL!	#NULL!	#NULL!	#NULL!	#NULL!

2	1	2	2	1	2	2	2	2	2
2	2	1	2	2	2	1	2	2	2
2	1	1	1	2	2	1	2	2	2
2	2	1	2	2	1	1	2	2	2
#NULL!	#NULL!	#NULL!	#NULL!	#NULL!	#NULL!	#NULL!	#NULL!	#NULL!	#NULL!
#NULL!	#NULL!	#NULL!	#NULL!	#NULL!	#NULL!	1	2	2	1
2	1	1	1	1	1	1	2	2	2
2	1	1	1	2	1	1	2	2	2
2	1	1	2	2	2	1	2	2	2
#NULL!	1	2	#NULL!	#NULL!	#NULL!	#NULL!	#NULL!	#NULL!	#NULL!
2	1	1	2	2	1	1	1	1	1
2	2	2	2	2	2	1	1	1	1
2	2	1	1	2	2	1	2	2	2
1	1	#NULL!	#NULL!	#NULL!	1	1	2	2	2
2	2	2	2	1	2	2	2	2	2
2	1	2	2	2	2	#NULL!	#NULL!	#NULL!	2
2	2	2	1	2	2	1	2	2	2
1	2	2	1	2	1	1	2	1	1
2	2	2	2	2	1	2	2	2	2
2	2	1	2	1	1	2	2	1	2
2	2	2	2	2	2	1	2	1	2
2	1	1	1	2	2	1	2	1	2
2	2	2	2	2	2	2	2	2	2
2	2	2	2	2	2	#NULL!	#NULL!	#NULL!	#NULL!
2	1	2	1	1	1	1	2	1	1
2	2	2	2	2	2	1	2	2	2
2	1	2	2	2	2	2	2	2	2
2	2	2	2	2	2	2	2	2	2
2	2	2	2	2	2	2	2	2	2
1	1	2	2	1	1	1	2	2	2
2	2	2	2	2	2	2	2	2	2
2	2	2	2	2	2	2	2	2	2
2	2	2	1	2	2	1	1	2	2
2	2	2	2	2	2	2	2	2	2
1	1	2	2	1	1	1	2	2	2
2	2	2	2	2	1	2	2	1	2
2	2	2	2	2	2	1	2	1	1
2	2	2	2	2	2	2	2	2	2
#NULL!	#NULL!	#NULL!	1	#NULL!	#NULL!	1	#NULL!	#NULL!	#NULL!
2	1	1	2	2	1	1	2	2	2
2	2	2	2	2	2	2	2	2	2
2	2	2	2	2	2	2	2	2	2
2	2	2	2	2	2	2	2	2	2
2	2	2	2	2	2	2	2	2	2
2	2	2	2	2	2	2	2	2	2
2	2	2	2	2	2	1	2	2	2
2	2	2	2	2	2	1	2	2	2

2	2	2	2	2	2	#NULL!	#NULL!	#NULL!	#NULL!
2	2	2	2	2	2	2	2	2	2
2	2	2	2	2	2	2	2	2	2
2	2	2	2	2	2	2	2	2	2
1	1	1	1	1	1	2	2	2	2
2	1	1	1	2	2	1	2	2	2
2	2	2	2	2	2	2	1	2	2
2	2	2	2	2	2	2	2	2	2
2	2	2	2	2	2	2	2	2	2
2	2	2	2	2	2	2	2	2	2
2	2	2	2	2	2	#NULL!	#NULL!	#NULL!	#NULL!
2	2	2	2	2	2	2	2	2	2
2	2	#NULL!	2	#NULL!	2	#NULL!	#NULL!	2	#NULL!
2	2	2	2	2	2	2	2	2	2
2	2	1	1	2	2	2	2	2	2
2	2	1	1	2	2	2	2	1	2
#NULL!	#NULL!	#NULL!	#NULL!	#NULL!	#NULL!	#NULL!	#NULL!	#NULL!	#NULL!
2	2	2	2	2	2	2	2	1	1
#NULL!	#NULL!	#NULL!	#NULL!	#NULL!	#NULL!	1	#NULL!	#NULL!	#NULL!
1	1	1	1	2	2	1	2	1	2
1	1	1	1	1	2	1	2	2	2
2	1	1	1	2	2	1	2	2	1
1	1	1	1	1	2	1	2	1	2
1	1	1	1	2	1	1	2	1	2
1	1	1	1	2	1	1	2	2	2
1	1	1	1	2	2	1	2	1	2
1	1	1	1	2	2	1	2	1	2
2	1	1	1	1	2	1	2	2	2
1	1	1	1	2	2	1	2	2	1
1	1	1	1	2	2	1	2	2	2
2	2	1	2	2	2	2	2	2	2
2	2	2	2	2	2	2	2	2	2
1	1	1	1	2	2	1	2	1	2
1	1	1	1	2	2	1	2	1	2
1	1	1	1	2	2	1	2	2	1
2	1	1	1	2	2	1	2	2	1
1	1	1	1	2	1	1	2	2	2
1	1	1	1	2	1	1	2	2	2
1	1	1	1	2	2	1	2	1	2
1	1	1	1	2	2	1	2	2	2
1	1	1	1	2	2	1	2	1	1
1	1	1	1	2	1	1	2	2	2
1	1	1	1	2	2	1	2	2	2
1	1	1	1	2	2	1	2	2	2
2	1	1	1	2	2	1	2	1	1
1	1	1	2	2	2	1	2	2	2
1	1	1	1	2	2	1	2	2	2

1	1	1	1	2	2	1	2	2	2
1	1	1	1	2	2	1	2	1	1
1	#NULL!	1	1	2	2	1	2	1	2
2	1	1	1	2	2	1	2	2	2
1	1	1	1	2	2	1	2	1	1
1	1	1	1	1	2	1	2	1	1
2	1	1	2	2	2	1	2	2	1
2	2	1	1	2	2	1	2	2	2
2	1	1	1	1	2	1	2	1	2
2	1	2	2	2	1	#NULL!	#NULL!	#NULL!	#NULL!
#NULL!	#NULL!	#NULL!	#NULL!	#NULL!	#NULL!	#NULL!	#NULL!	#NULL!	#NULL!
2	1	1	1	2	2	#NULL!	#NULL!	#NULL!	#NULL!
2	1	1	1	1	2	1	2	1	2
2	1	1	1	2	2	1	2	1	1
2	1	1	1	2	2	1	2	2	1
2	1	1	1	2	2	1	2	2	2
2	2	2	2	2	2	2	2	2	2
1	1	1	1	1	1	2	2	2	2
1	2	1	1	2	1	1	2	2	2
2	2	2	2	2	2	1	2	2	2
1	2	1	1	2	2	1	2	2	2
2	2	2	2	2	2	2	2	2	2
2	2	1	1	1	2	1	2	2	2
2	1	2	2	1	2	1	2	2	2
2	2	2	2	2	2	2	2	2	2
2	2	2	2	2	2	2	2	2	2
2	2	2	2	2	1	1	2	2	2
2	2	2	2	2	2	2	2	2	2
2	1	2	2	2	2	2	2	2	2
1	2	1	1	2	2	1	2	2	2
2	2	2	2	2	2	1	2	2	2
2	2	2	2	2	2	1	2	2	2
1	2	2	1	2	1	1	2	2	2
2	1	2	2	1	2	2	2	1	2
1	2	1	2	1	2	1	2	1	1
2	2	2	2	2	2	2	2	2	1
2	2	2	2	2	2	2	2	2	2
1	1	2	1	1	1	2	2	1	1
2	2	2	2	2	2	2	2	2	2
2	2	2	2	2	2	1	2	2	2
2	2	2	2	2	2	1	2	2	2
2	2	2	2	2	1	1	2	2	2
#NULL!	#NULL!	#NULL!	#NULL!	#NULL!	#NULL!	#NULL!	2	#NULL!	#NULL!
#NULL!	#NULL!	#NULL!	#NULL!	#NULL!	#NULL!	#NULL!	2	#NULL!	#NULL!
1	1	1	1	1	1	1	2	2	2
2	2	1	1	1	1	1	2	1	2
#NULL!	#NULL!	#NULL!	#NULL!	#NULL!	#NULL!	#NULL!	#NULL!	#NULL!	#NULL!

2	2	2	2	2	2	2	2	2	2
2	2	2	2	2	2	2	2	2	2
2	1	2	2	2	2	2	2	2	2
2	2	2	2	2	1	2	2	2	2
2	2	2	2	2	2	1	2	2	2
2	2	1	2	2	2	2	2	2	2
2	1	1	2	2	2	2	2	2	2
2	2	2	2	2	2	2	2	2	2
2	2	2	2	2	2	1	1	2	2
2	2	2	2	2	2	1	2	2	2
2	2	2	1	2	2	2	2	2	2
2	2	2	2	2	2	2	2	2	2
2	2	2	2	2	2	2	2	2	2
2	2	2	2	2	1	2	2	2	2
2	2	2	2	2	2	2	2	2	2
2	2	2	2	2	2	2	2	2	2
1	1	2	2	2	2	2	1	2	2
2	2	2	1	2	2	1	2	2	2
#NULL!	#NULL!	#NULL!	1	#NULL!	#NULL!	#NULL!	#NULL!	#NULL!	#NULL!
#NULL!	1	2	1	2	1	1	2	2	2
2	1	1	1	2	2	1	2	2	2
2	2	2	2	2	1	2	2	2	2
2	1	1	2	2	2	2	2	2	2
2	2	2	2	1	2	2	2	2	2
2	#NULL!	#NULL!	#NULL!	#NULL!	#NULL!	2	2	#NULL!	#NULL!
2	2	2	2	2	2	2	2	2	2
2	2	2	2	2	2	2	2	2	2
2	#NULL!	2	#NULL!	1	2	1	#NULL!	2	2
2	2	2	2	2	2	2	2	2	2
2	2	2	2	2	2	2	2	2	2
#NULL!	#NULL!	#NULL!	#NULL!	#NULL!	#NULL!	#NULL!	#NULL!	1	#NULL!
2	2	2	2	2	2	2	2	1	1
2	2	2	2	2	2	2	1	1	1
2	2	2	2	2	2	1	2	2	2
#NULL!	#NULL!	#NULL!	#NULL!	#NULL!	#NULL!	2	#NULL!	#NULL!	#NULL!
2	2	2	2	2	2	2	2	2	2
2	1	1	2	2	2	2	2	2	2
2	1	1	1	2	2	2	2	1	2
2	2	2	2	2	2	2	2	1	1
2	2	2	2	1	2	2	2	2	2
2	2	2	2	2	1	1	2	2	2
2	1	2	2	2	2	1	2	2	2
1	1	#NULL!	#NULL!	#NULL!	#NULL!	#NULL!	#NULL!	#NULL!	#NULL!
2	2	2	2	2	2	2	2	2	2
2	1	2	2	1	2	2	2	1	2
2	2	2	2	2	2	2	2	2	2
2	2	2	2	2	2	2	2	2	2

#NULL!	#NULL!	#NULL!	#NULL!	#NULL!	#NULL!	#NULL!	#NULL!	#NULL!	#NULL!
#NULL!	#NULL!	#NULL!	#NULL!	#NULL!	#NULL!	#NULL!	#NULL!	#NULL!	#NULL!
#NULL!	#NULL!	#NULL!	#NULL!	#NULL!	#NULL!	#NULL!	#NULL!	#NULL!	#NULL!
2	1	2	1	2	1	1	2	2	2
#NULL!	2	1	2	2	2	2	2	2	2
2	1	2	2	2	2	1	2	2	1
2	2	2	1	1	1	1	2	2	2
1	1	1	1	1	2	1	2	2	2
2	1	1	1	2	1	1	#NULL!	#NULL!	#NULL!
2	2	2	2	2	2	2	2	2	2
2	2	2	2	2	2	2	2	2	2
#NULL!	#NULL!	#NULL!	#NULL!	#NULL!	#NULL!	#NULL!	#NULL!	#NULL!	#NULL!
#NULL!	#NULL!	#NULL!	#NULL!	#NULL!	#NULL!	#NULL!	#NULL!	#NULL!	#NULL!
2	1	1	1	2	1	2	2	1	1
1	2	2	#NULL!	1	1	1	2	1	2
#NULL!	#NULL!	#NULL!	#NULL!	#NULL!	#NULL!	#NULL!	#NULL!	#NULL!	#NULL!
2	#NULL!	#NULL!	#NULL!	#NULL!	#NULL!	#NULL!	#NULL!	#NULL!	#NULL!
2	2	2	2	2	1	1	2	2	2
1	1	1	2	2	2	1	2	2	2
#NULL!	#NULL!	#NULL!	#NULL!	#NULL!	#NULL!	#NULL!	#NULL!	#NULL!	#NULL!
#NULL!	#NULL!	#NULL!	#NULL!	#NULL!	#NULL!	#NULL!	#NULL!	#NULL!	#NULL!
2	2	2	2	2	2	1	2	1	2
2	2	2	2	2	2	2	2	2	2
2	2	2	2	2	2	1	2	2	2
2	2	2	2	2	2	2	2	2	2
1	1	1	2	2	1	2	1	2	2
1	1	2	1	2	1	2	2	2	2
2	1	1	1	2	2	1	2	2	2
2	1	2	1	2	1	2	2	1	2
2	1	2	2	2	2	2	2	2	2
2	2	2	2	2	2	2	2	2	2
2	1	2	2	2	2	2	1	2	2
#NULL!	#NULL!	#NULL!	#NULL!	#NULL!	#NULL!	#NULL!	#NULL!	#NULL!	#NULL!
#NULL!	#NULL!	#NULL!	#NULL!	#NULL!	#NULL!	#NULL!	#NULL!	#NULL!	#NULL!
2	1	1	1	2	2	1	1	2	2
2	2	2	2	1	2	1	1	2	2
2	2	2	1	2	2	2	2	2	2
2	1	1	1	1	2	1	1	2	2
2	1	1	2	2	1	1	1	2	2
2	2	1	1	2	2	1	2	2	1
2	2	1	1	2	2	1	1	2	2
2	1	1	1	2	1	1	1	1	2
2	2	2	2	2	2	#NULL!	2	2	2
2	2	2	2	2	2	2	2	2	2
2	2	2	2	2	1	2	1	2	2
2	2	2	2	2	2	2	2	2	2
1	2	2	2	2	2	1	2	2	2

#NULL!	#NULL!	#NULL!	#NULL!	#NULL!	#NULL!	#NULL!	#NULL!	#NULL!	#NULL!
#NULL!	#NULL!	#NULL!	#NULL!	#NULL!	#NULL!	#NULL!	#NULL!	#NULL!	#NULL!
#NULL!	#NULL!	#NULL!	#NULL!	#NULL!	#NULL!	#NULL!	#NULL!	#NULL!	#NULL!
2	1	1	2	2	2	1	2	1	2
#NULL!	#NULL!	#NULL!	#NULL!	#NULL!	#NULL!	#NULL!	#NULL!	#NULL!	#NULL!
#NULL!	#NULL!	#NULL!	#NULL!	#NULL!	#NULL!	#NULL!	#NULL!	#NULL!	#NULL!
2	1	1	1	2	2	1	2	2	2
#NULL!	#NULL!	#NULL!	#NULL!	#NULL!	#NULL!	2	2	2	2
2	1	1	1	1	2	1	2	2	2
1	2	2	1	1	1	1	2	2	2
2	2	2	2	2	2	2	2	2	2
2	2	2	2	2	2	2	2	2	2
2	1	1	1	2	1	2	2	2	2
2	2	1	2	2	2	2	2	2	2
2	2	2	1	2	2	2	2	2	2
2	2	2	2	2	2	2	2	2	2
2	2	2	2	2	2	2	2	2	2
2	2	2	2	2	2	2	2	2	2
2	2	2	2	2	2	1	2	1	1
2	2	2	2	2	2	2	2	2	2
1	1	2	2	2	2	1	1	2	2
2	1	2	1	1	2	1	2	2	2
2	2	2	1	1	2	1	2	2	2
1	1	1	1	2	2	1	2	1	1
2	2	2	2	2	2	2	2	2	2
2	2	2	2	2	2	2	2	2	2
2	2	2	2	2	2	2	2	2	2
2	2	2	2	2	2	2	2	2	2
2	1	2	2	2	2	2	2	2	2
2	1	1	1	1	2	1	2	2	1
2	2	2	2	2	2	2	2	2	2
2	2	2	2	1	1	2	2	2	2
1	2	2	2	2	2	2	2	2	1
2	2	2	2	2	2	2	2	2	2
2	2	2	2	2	2	2	2	2	2
2	1	2	1	2	2	2	2	2	2
2	2	2	2	2	2	2	2	2	2
1	2	2	2	1	1	2	2	2	2
2	2	2	2	2	2	2	2	2	2
2	2	2	2	2	2	2	2	2	2
2	2	2	2	2	1	2	2	2	2
2	2	2	2	2	1	2	2	2	2
1	1	1	1	2	1	1	2	2	2
2	2	2	2	2	1	2	2	2	2
2	2	2	1	1	2	2	2	2	2
2	1	1	1	2	2	1	2	2	2
2	2	2	2	2	2	2	2	2	2

1	2	1	2	2	2	1	2	1	1
2	1	2	2	2	2	2	2	2	2
2	2	2	1	2	2	2	2	2	2
2	2	2	2	2	2	2	2	2	2
1	1	2	2	1	1	2	2	2	2
2	2	2	1	2	2	1	2	2	2
#NULL!	#NULL!	#NULL!	#NULL!	#NULL!	#NULL!	#NULL!	#NULL!	#NULL!	#NULL!
2	2	2	2	2	2	2	2	2	2
2	2	2	2	2	2	2	2	2	2
2	1	1	1	1	1	2	2	2	2
1	2	1	2	2	1	1	2	1	1
2	1	2	1	2	1	1	2	1	2
2	2	2	2	2	2	1	2	1	1
2	2	2	2	2	2	1	2	1	1
2	2	2	2	2	2	2	2	2	2
1	1	1	1	1	1	1	2	1	1
1	2	2	1	1	2	2	1	2	2
2	2	2	2	2	2	2	2	2	2
2	2	2	2	2	2	2	2	2	2
2	1	2	2	2	2	2	2	2	2
1	1	2	1	2	1	1	2	2	2
2	2	2	2	2	2	1	2	2	2
#NULL!	#NULL!	#NULL!	#NULL!	#NULL!	#NULL!	#NULL!	#NULL!	#NULL!	#NULL!
2	2	2	2	2	2	2	2	2	2
1	1	1	1	2	1	1	2	2	2
#NULL!	#NULL!	#NULL!	#NULL!	#NULL!	#NULL!	#NULL!	#NULL!	#NULL!	#NULL!
2	2	2	1	2	2	2	2	1	2
#NULL!	#NULL!	#NULL!	#NULL!	#NULL!	#NULL!	#NULL!	#NULL!	#NULL!	#NULL!
2	#NULL!	2	2	2	2	1	2	2	2
2	1	1	1	2	2	1	2	2	2
#NULL!	#NULL!	#NULL!	#NULL!	#NULL!	#NULL!	#NULL!	#NULL!	#NULL!	#NULL!
#NULL!	#NULL!	#NULL!	#NULL!	#NULL!	#NULL!	#NULL!	#NULL!	#NULL!	#NULL!
2	2	2	2	2	2	2	2	2	2
2	1	2	1	1	1	1	2	1	1
#NULL!	#NULL!	#NULL!	#NULL!	#NULL!	#NULL!	#NULL!	#NULL!	#NULL!	#NULL!
2	1	1	1	1	1	1	2	1	2
2	2	2	2	2	2	2	1	2	2
1	2	2	1	1	#NULL!	1	2	2	2
#NULL!	#NULL!	#NULL!	#NULL!	#NULL!	#NULL!	#NULL!	#NULL!	#NULL!	#NULL!
2	1	1	2	2	1	1	2	2	2
2	2	2	2	2	2	1	2	2	2
2	1	1	1	2	2	2	2	1	1
2	1	2	2	2	1	1	2	2	1
2	2	2	2	2	2	2	2	2	2
#NULL!	#NULL!	#NULL!	#NULL!	#NULL!	#NULL!	2	1	2	2
2	1	1	1	2	2	1	2	2	2
1	1	1	1	2	1	1	2	1	1

#NULL!	#NULL!	#NULL!	#NULL!	#NULL!	#NULL!	#NULL!	#NULL!	#NULL!	#NULL!
1	1	1	1	2	2	1	2	2	1
1	1	1	1	2	1	1	2	2	1
1	1	1	1	1	2	1	2	1	2
1	1	1	1	1	2	1	2	2	1
2	1	1	1	2	2	1	2	2	1
1	1	2	2	2	2	1	2	1	2
2	1	1	1	1	2	1	2	2	1
1	1	1	1	2	2	1	2	2	2
1	1	1	2	#NULL!	2	1	2	1	2
2	2	1	2	2	2	1	2	2	2
1	1	1	1	2	2	1	2	2	1
2	1	1	1	2	2	1	2	1	2
1	1	1	1	2	2	1	2	1	1
1	1	1	1	2	1	1	2	2	1
2	1	1	1	2	2	1	2	2	1
1	1	2	1	1	2	1	2	1	2
1	1	1	1	2	2	1	2	1	2
1	1	1	1	1	2	1	2	1	1
1	1	1	1	1	2	1	2	1	1
2	1	1	1	1	2	1	2	2	2
1	1	1	2	1	2	1	2	2	2
2	1	1	1	2	2	1	2	2	1
1	1	1	1	1	2	1	2	2	2
2	1	1	1	2	2	1	2	2	1
2	1	1	1	2	2	1	2	2	1
2	1	1	1	1	2	1	2	2	2
2	1	1	1	2	2	1	2	2	2
2	1	1	1	2	1	1	2	2	2
1	1	1	1	2	2	1	2	2	2
1	1	1	1	1	2	1	2	2	2
2	1	1	1	2	2	1	2	2	1
2	1	1	1	1	2	1	2	1	2
1	1	1	1	1	2	1	2	2	2
1	1	1	1	1	2	1	2	1	2
1	1	1	1	2	2	1	2	2	2
2	1	1	1	1	2	1	2	2	1
1	1	1	1	1	2	1	2	2	1
1	1	1	1	1	2	1	2	2	1
1	1	1	1	1	2	1	2	1	1
2	1	1	1	2	2	1	2	2	1
2	2	2	2	2	2	2	2	2	2
2	1	1	1	2	2	1	2	1	2
2	2	2	2	2	2	2	2	2	2
#NULL!	#NULL!	#NULL!	#NULL!	#NULL!	#NULL!	2	#NULL!	#NULL!	#NULL!
2	2	2	2	2	2	2	2	2	2
#NULL!	#NULL!	#NULL!	#NULL!	#NULL!	#NULL!	2	#NULL!	#NULL!	#NULL!

2	2	2	2	2	2	2	2	2	2
2	1	1	1	2	2	1	2	2	2
2	1	1	1	2	2	1	2	2	2
2	1	1	1	2	1	1	2	2	2
2	2	2	2	2	2	2	2	2	2
1	1	2	1	1	1	1	2	2	2
2	2	2	2	2	2	2	2	2	2
1	2	2	1	2	2	2	2	2	2
1	2	2	1	1	1	1	2	2	2
2	2	2	2	2	2	2	2	2	2
2	1	1	1	2	2	1	2	2	2
#NULL!	1	#NULL!	#NULL!	#NULL!	#NULL!	#NULL!	#NULL!	2	#NULL!
2	1	1	1	1	2	1	1	2	2
2	2	2	2	2	2	2	2	2	2
#NULL!	#NULL!	#NULL!	#NULL!	#NULL!	#NULL!	2	#NULL!	#NULL!	#NULL!
#NULL!	#NULL!	#NULL!	#NULL!	#NULL!	#NULL!	2	#NULL!	#NULL!	#NULL!
#NULL!	1	#NULL!	#NULL!	#NULL!	#NULL!	2	#NULL!	#NULL!	#NULL!
#NULL!	#NULL!	#NULL!	#NULL!	#NULL!	1	#NULL!	#NULL!	2	#NULL!
#NULL!	#NULL!	#NULL!	1	#NULL!	#NULL!	#NULL!	#NULL!	2	#NULL!
2	1	1	1	2	1	1	2	2	1
1	1	2	2	#NULL!	#NULL!	1	2	#NULL!	1
2	1	#NULL!	#NULL!	#NULL!	#NULL!	1	2	2	#NULL!
2	2	2	1	2	2	2	1	2	2
#NULL!	1	#NULL!	#NULL!	#NULL!	#NULL!	#NULL!	#NULL!	#NULL!	#NULL!
#NULL!	#NULL!	#NULL!	#NULL!	#NULL!	#NULL!	2	#NULL!	#NULL!	#NULL!
#NULL!	#NULL!	#NULL!	#NULL!	#NULL!	#NULL!	#NULL!	#NULL!	#NULL!	#NULL!
#NULL!	#NULL!	#NULL!	#NULL!	#NULL!	2	2	#NULL!	#NULL!	#NULL!
#NULL!	#NULL!	#NULL!	#NULL!	#NULL!	#NULL!	#NULL!	#NULL!	#NULL!	#NULL!
2	1	1	1	2	2	1	2	2	2
#NULL!	1	#NULL!	#NULL!	#NULL!	#NULL!	#NULL!	#NULL!	2	#NULL!
#NULL!	#NULL!	#NULL!	#NULL!	#NULL!	#NULL!	#NULL!	#NULL!	2	#NULL!
2	2	2	2	2	2	2	2	2	2
2	2	2	2	2	2	2	2	2	2
2	1	2	1	2	1	1	2	2	2
1	1	1	1	2	1	1	2	2	2
2	2	2	1	2	1	2	2	2	2
2	1	2	2	2	2	2	2	2	2
2	1	2	1	2	2	2	1	2	2
2	2	2	2	2	2	2	2	2	2
2	2	2	2	2	2	2	2	2	2
#NULL!	#NULL!	#NULL!	#NULL!	#NULL!	#NULL!	#NULL!	#NULL!	1	#NULL!
1	1	1	1	1	2	1	2	1	2
2	2	2	2	2	2	2	2	2	2
2	2	1	1	2	2	1	2	2	1
2	2	2	1	2	2	2	2	2	2
2	2	2	2	2	2	2	2	2	2
2	2	2	2	1	2	2	2	2	2

#NULL!	1	1	1	2	1	2	2	2	2
2	2	2	1	2	2	1	2	1	2
2	2	2	2	2	2	2	2	2	2
2	2	2	1	1	2	1	2	2	2
2	1	1	1	2	2	2	2	2	2
2	2	2	2	2	2	2	2	2	2
2	2	1	1	2	2	2	1	2	2
2	2	2	2	2	2	2	2	2	2
1	2	1	1	2	2	2	2	2	2
2	2	2	2	2	2	2	2	2	2
2	2	2	2	2	2	2	2	2	2
2	2	2	1	2	2	2	2	2	2
2	2	2	2	2	2	2	2	2	2
2	2	2	2	2	2	2	2	2	2
2	1	2	2	2	2	2	2	2	2
2	2	2	2	2	2	2	2	2	2
2	2	2	2	2	2	2	2	2	2
2	2	2	2	2	2	2	2	2	2
2	2	2	1	2	2	2	2	2	2
2	1	2	2	2	2	1	2	2	2
2	2	2	2	2	2	2	2	2	2
2	2	2	1	1	2	1	2	2	2
2	1	1	2	2	2	1	1	2	2
2	1	1	2	2	2	1	2	1	1
2	2	2	2	2	2	2	1	2	2
2	2	2	2	2	2	2	2	2	2
2	#NULL!	1	1	1	1	1	2	1	1
1	2	1	2	1	2	1	2	1	2
1	1	1	1	2	1	1	2	1	2
2	2	2	2	2	2	2	2	2	2
2	2	2	2	2	2	2	2	2	2
2	2	2	2	2	2	2	2	2	2
2	2	2	2	2	2	2	2	2	2
2	2	2	2	2	2	2	2	2	2
2	2	2	2	2	2	2	2	1	2
#NULL!	#NULL!	#NULL!	#NULL!	#NULL!	#NULL!	2	#NULL!	#NULL!	#NULL!
2	2	2	2	2	2	2	2	2	2
2	2	1	1	2	2	1	2	1	1
2	2	2	2	1	2	2	2	2	2
2	1	1	2	2	2	1	2	2	2
2	1	1	1	2	2	#NULL!	#NULL!	#NULL!	#NULL!
2	2	2	2	2	1	1	2	2	2
2	2	2	2	2	1	1	2	2	2
2	2	2	2	2	2	2	2	2	2
2	1	1	1	2	2	1	2	2	1
2	2	2	2	2	2	2	1	2	2
#NULL!	#NULL!	#NULL!	#NULL!	#NULL!	#NULL!	#NULL!	#NULL!	#NULL!	#NULL!

#NULL!	#NULL!	#NULL!	#NULL!	#NULL!	#NULL!	#NULL!	#NULL!	#NULL!	#NULL!
#NULL!	#NULL!	#NULL!	#NULL!	#NULL!	#NULL!	#NULL!	#NULL!	2	#NULL!
#NULL!	1	#NULL!	#NULL!	#NULL!	#NULL!	2	#NULL!	#NULL!	#NULL!
2	2	1	1	1	1	#NULL!	#NULL!	#NULL!	#NULL!
#NULL!	#NULL!	#NULL!	#NULL!	#NULL!	#NULL!	1	#NULL!	#NULL!	#NULL!
#NULL!	#NULL!	#NULL!	#NULL!	#NULL!	#NULL!	2	2	2	#NULL!
#NULL!	#NULL!	#NULL!	#NULL!	#NULL!	#NULL!	#NULL!	#NULL!	#NULL!	#NULL!
#NULL!	#NULL!	#NULL!	#NULL!	#NULL!	#NULL!	#NULL!	#NULL!	2	#NULL!
#NULL!	#NULL!	#NULL!	#NULL!	#NULL!	#NULL!	#NULL!	#NULL!	2	#NULL!
#NULL!	#NULL!	#NULL!	#NULL!	#NULL!	#NULL!	2	#NULL!	#NULL!	#NULL!
#NULL!	#NULL!	#NULL!	#NULL!	#NULL!	#NULL!	#NULL!	#NULL!	2	#NULL!
#NULL!	#NULL!	#NULL!	#NULL!	#NULL!	#NULL!	2	#NULL!	#NULL!	#NULL!
#NULL!	2	#NULL!	#NULL!	#NULL!	#NULL!	#NULL!	#NULL!	2	#NULL!
#NULL!	#NULL!	#NULL!	#NULL!	#NULL!	#NULL!	1	#NULL!	2	#NULL!
#NULL!	#NULL!	#NULL!	#NULL!	#NULL!	1	#NULL!	#NULL!	2	#NULL!
#NULL!	#NULL!	#NULL!	#NULL!	#NULL!	1	#NULL!	#NULL!	2	#NULL!
#NULL!	#NULL!	#NULL!	#NULL!	#NULL!	#NULL!	#NULL!	#NULL!	#NULL!	#NULL!
2	2	1	1	1	1	1	2	2	1
2	2	2	2	2	2	2	2	2	2
#NULL!	#NULL!	#NULL!	#NULL!	#NULL!	#NULL!	1	#NULL!	#NULL!	#NULL!
1	1	1	1	1	1	1	2	1	1
2	2	2	1	2	2	2	2	2	2
1	1	2	2	2	2	1	2	2	1
1	1	1	1	1	1	1	2	1	2
2	2	2	2	2	2	1	2	2	2
2	2	2	2	2	2	2	2	2	2
2	2	2	2	2	2	2	2	2	2
2	2	2	2	1	2	2	2	2	2
2	2	2	2	2	2	2	2	2	2
2	2	2	2	2	2	2	2	2	2
2	2	2	2	2	2	2	1	2	2
2	1	2	1	2	2	2	2	2	2
2	2	2	2	2	2	2	2	2	2
1	1	1	1	2	2	1	2	2	2
2	1	2	2	1	2	1	2	2	1
2	2	2	2	2	2	1	2	1	2
2	2	2	2	2	1	2	2	2	2
#NULL!	#NULL!	#NULL!	#NULL!	#NULL!	#NULL!	#NULL!	#NULL!	#NULL!	#NULL!
#NULL!	1	#NULL!	1	1	#NULL!	1	2	1	1
#NULL!	#NULL!	#NULL!	#NULL!	#NULL!	#NULL!	#NULL!	#NULL!	#NULL!	#NULL!
2	2	2	2	2	2	2	2	2	2
2	2	2	2	2	2	1	2	2	1
#NULL!	#NULL!	#NULL!	#NULL!	#NULL!	#NULL!	#NULL!	#NULL!	#NULL!	#NULL!
2	2	2	1	1	1	1	2	1	1
2	2	2	2	2	2	2	2	2	2
2	2	2	2	2	2	2	2	2	2
2	2	2	2	2	2	2	2	2	2

2	2	2	1	2	2	2	2	2	2
#NULL!	#NULL!	#NULL!	#NULL!	#NULL!	#NULL!	#NULL!	#NULL!	#NULL!	#NULL!
2	2	1	2	2	1	1	1	1	1
#NULL!	#NULL!	#NULL!	#NULL!	#NULL!	#NULL!	#NULL!	#NULL!	#NULL!	#NULL!
2	2	2	2	2	2	1	2	2	2
2	2	2	2	2	2	1	2	2	2
#NULL!	#NULL!	#NULL!	#NULL!	#NULL!	#NULL!	#NULL!	#NULL!	#NULL!	#NULL!
2	2	2	1	2	2	2	2	2	2
1	2	2	2	1	2	2	2	2	2
2	2	2	2	2	1	1	2	1	1
2	1	2	2	1	2	1	2	1	1
1	1	1	1	2	1	1	2	1	1
2	2	2	2	2	2	1	2	1	2
#NULL!	#NULL!	#NULL!	#NULL!	#NULL!	#NULL!	#NULL!	#NULL!	#NULL!	#NULL!
#NULL!	#NULL!	#NULL!	#NULL!	#NULL!	#NULL!	#NULL!	#NULL!	#NULL!	#NULL!
#NULL!	#NULL!	#NULL!	#NULL!	#NULL!	#NULL!	#NULL!	#NULL!	#NULL!	#NULL!
2	1	2	2	1	2	2	2	1	2
#NULL!	#NULL!	#NULL!	#NULL!	#NULL!	#NULL!	#NULL!	#NULL!	#NULL!	#NULL!
2	1	1	1	2	2	2	1	2	2
2	1	2	2	2	1	1	2	1	1
1	1	1	1	1	1	1	2	1	1
2	2	2	2	2	2	1	2	1	2
2	2	2	2	2	2	2	1	2	2
#NULL!	#NULL!	#NULL!	#NULL!	#NULL!	#NULL!	#NULL!	#NULL!	#NULL!	#NULL!
2	2	2	2	2	2	2	2	2	2
2	1	2	1	2	1	1	1	2	1
2	2	2	2	2	2	1	1	2	2
2	1	1	1	2	1	1	1	2	1
2	2	1	1	2	2	1	2	2	2
2	1	1	1	2	2	1	2	1	2
#NULL!	#NULL!	#NULL!	#NULL!	#NULL!	#NULL!	#NULL!	#NULL!	#NULL!	#NULL!
#NULL!	#NULL!	#NULL!	#NULL!	#NULL!	#NULL!	#NULL!	#NULL!	#NULL!	2
2	2	2	1	1	1	2	2	1	2
2	2	1	2	2	2	1	1	2	2
#NULL!	#NULL!	#NULL!	2	2	1	1	1	2	2
2	1	1	1	2	2	1	2	1	2
2	2	2	2	2	2	1	2	1	2
#NULL!	#NULL!	#NULL!	#NULL!	#NULL!	#NULL!	#NULL!	#NULL!	#NULL!	#NULL!
#NULL!	#NULL!	#NULL!	#NULL!	#NULL!	#NULL!	#NULL!	#NULL!	#NULL!	#NULL!
#NULL!	#NULL!	#NULL!	#NULL!	#NULL!	#NULL!	#NULL!	#NULL!	#NULL!	#NULL!
#NULL!	#NULL!	#NULL!	#NULL!	#NULL!	#NULL!	#NULL!	#NULL!	#NULL!	#NULL!
2	2	2	#NULL!	1	1	1	1	2	1
1	2	1	2	2	2	2	1	2	1
#NULL!	#NULL!	#NULL!	#NULL!	#NULL!	#NULL!	#NULL!	#NULL!	#NULL!	#NULL!
2	1	2	2	2	2	2	1	2	1
1	2	2	2	2	1	1	2	2	2
1	2	1	2	2	1	1	2	1	2

2	2	2	2	2	2	2	1	2	2
2	2	2	2	2	2	1	2	2	2
2	2	2	2	2	2	1	2	2	2
2	2	2	2	1	2	1	2	1	2
2	2	2	2	2	2	2	2	2	2
#NULL!	2	#NULL!	#NULL!	2	2	2	2	2	#NULL!
#NULL!	#NULL!	#NULL!	#NULL!	#NULL!	#NULL!	#NULL!	#NULL!	#NULL!	#NULL!
2	2	1	2	2	2	2	2	2	2
2	2	2	2	2	2	2	1	2	2
2	2	2	2	2	2	2	2	2	2
2	2	2	2	2	2	2	1	2	2
1	1	1	1	1	1	2	1	2	2
1	2	2	2	2	1	1	2	2	2
2	2	2	2	1	1	1	2	2	2
1	2	2	2	2	1	1	2	2	2
1	2	2	2	1	1	1	2	2	2
1	2	2	2	2	1	1	2	2	2
1	2	2	2	2	1	1	2	2	2
1	2	2	2	2	1	1	2	2	2
2	2	2	2	2	2	1	2	1	2
2	1	1	1	2	2	2	2	2	2
2	2	2	#NULL!	2	2	2	2	2	2
2	2	2	2	2	2	2	1	2	2
2	2	2	2	2	2	2	2	2	2
2	2	2	2	2	2	2	1	2	2
1	2	2	2	2	1	1	2	2	2
#NULL!	#NULL!	#NULL!	#NULL!	#NULL!	#NULL!	#NULL!	#NULL!	#NULL!	2
#NULL!	#NULL!	#NULL!	#NULL!	#NULL!	#NULL!	1	1	1	2
#NULL!	1	#NULL!	#NULL!	#NULL!	#NULL!	1	#NULL!	#NULL!	#NULL!
2	2	2	2	2	2	2	1	2	2
1	2	2	2	2	1	2	2	1	1
2	2	2	2	2	2	2	2	2	2
1	2	2	1	2	1	1	1	2	2
#NULL!	#NULL!	#NULL!	#NULL!	#NULL!	#NULL!	#NULL!	2	#NULL!	#NULL!
2	2	2	2	2	2	2	1	2	2
2	2	2	2	2	2	1	2	2	2
#NULL!	#NULL!	#NULL!	2	#NULL!	#NULL!	2	2	2	#NULL!
2	1	2	1	1	2	2	2	2	1
1	2	2	2	2	1	2	2	2	1
1	1	1	1	1	1	1	2	1	#NULL!
1	1	1	2	2	1	1	2	1	1
2	2	2	#NULL!	1	2	2	2	2	2
#NULL!	2	1	2	#NULL!	2	2	#NULL!	1	#NULL!
1	1	1	2	2	1	1	1	2	2
2	2	2	2	2	2	2	2	1	2
1	1	1	1	1	1	1	2	1	1
2	2	2	2	2	2	1	2	2	2

2	2	2	2	2	2	1	2	1	1
#NULL!	#NULL!	#NULL!	#NULL!	#NULL!	#NULL!	2	#NULL!	#NULL!	#NULL!
#NULL!	#NULL!	#NULL!	#NULL!	#NULL!	#NULL!	1	#NULL!	#NULL!	#NULL!
2	1	2	2	1	1	1	2	2	1
2	2	2	2	2	2	2	2	2	2
2	2	2	2	2	2	1	2	2	2
1	1	1	1	1	1	1	2	1	1
2	2	2	2	2	2	1	2	2	2
2	2	2	2	2	2	1	2	2	2
2	2	2	2	2	2	1	2	2	1
#NULL!	#NULL!	#NULL!	#NULL!	#NULL!	#NULL!	#NULL!	#NULL!	#NULL!	#NULL!
2	1	2	2	2	1	1	2	2	1
#NULL!	#NULL!	#NULL!	#NULL!	#NULL!	#NULL!	#NULL!	#NULL!	#NULL!	#NULL!
1	1	1	1	2	1	1	2	1	1
1	1	1	1	2	2	1	2	1	1
#NULL!	#NULL!	#NULL!	#NULL!	#NULL!	#NULL!	#NULL!	#NULL!	#NULL!	#NULL!
2	2	2	2	2	2	2	2	2	2
#NULL!	#NULL!	#NULL!	#NULL!	#NULL!	#NULL!	#NULL!	#NULL!	#NULL!	#NULL!
#NULL!	#NULL!	#NULL!	#NULL!	#NULL!	#NULL!	1	#NULL!	#NULL!	#NULL!
2	1	2	2	1	2	1	2	2	1
1	1	1	1	1	1	2	2	1	1
2	2	2	2	2	2	2	2	2	2
#NULL!	#NULL!	#NULL!	#NULL!	#NULL!	#NULL!	#NULL!	#NULL!	#NULL!	#NULL!
2	2	2	2	2	2	2	2	2	2
2	2	2	2	2	2	2	1	2	2
1	2	#NULL!	1	2	1	1	2	1	1
1	2	#NULL!	1	2	1	1	2	1	1
2	2	1	1	2	2	1	2	1	1
1	1	1	1	1	1	1	2	1	1
2	2	2	2	2	2	2	2	2	2
2	2	2	2	1	1	1	2	1	1
1	1	1	1	2	2	2	2	1	1
2	1	1	1	2	1	1	#NULL!	1	1
1	2	1	2	2	1	1	2	1	1
2	1	1	1	2	2	2	2	2	1
2	2	2	2	2	2	2	2	2	2
2	2	2	2	2	2	2	2	2	2
#NULL!	#NULL!	#NULL!	#NULL!	#NULL!	#NULL!	#NULL!	#NULL!	#NULL!	#NULL!
#NULL!	#NULL!	#NULL!	#NULL!	#NULL!	#NULL!	#NULL!	2	#NULL!	#NULL!
2	1	2	2	2	2	1	#NULL!	1	1
2	2	2	2	2	2	2	2	2	2
2	2	2	2	2	2	2	2	2	2
#NULL!	#NULL!	#NULL!	#NULL!	#NULL!	#NULL!	2	2	2	2
2	2	2	2	2	2	2	2	2	2
2	2	2	2	2	2	2	2	2	2
2	2	2	2	2	2	2	2	2	2
#NULL!	#NULL!	#NULL!	#NULL!	#NULL!	#NULL!	#NULL!	#NULL!	#NULL!	#NULL!

#NULL!	#NULL!	#NULL!	#NULL!	#NULL!	#NULL!	#NULL!	#NULL!	#NULL!	#NULL!
2	2	2	2	2	1	2	2	2	2
2	1	1	1	1	2	2	2	2	2
#NULL!	#NULL!	1	2	2	2	1	2	2	2
#NULL!	#NULL!	#NULL!	#NULL!	#NULL!	#NULL!	#NULL!	2	#NULL!	#NULL!
2	2	2	2	2	2	2	2	2	2
#NULL!	#NULL!	#NULL!	#NULL!	#NULL!	#NULL!	#NULL!	#NULL!	#NULL!	#NULL!
1	1	1	1	1	1	1	2	2	1
2	2	1	2	2	1	1	2	2	1
#NULL!	#NULL!	#NULL!	#NULL!	#NULL!	#NULL!	#NULL!	2	#NULL!	#NULL!
2	2	2	2	2	2	2	2	2	2
2	2	1	1	2	2	1	2	2	2
2	2	1	1	2	2	1	2	2	2
2	2	2	2	2	2	2	2	2	2
1	2	2	2	2	2	1	2	1	1
2	2	2	2	2	2	2	2	2	2
2	2	2	2	2	2	2	2	2	2
2	2	2	2	2	2	2	2	2	2
2	1	1	2	1	1	1	2	1	2
2	2	2	2	1	1	1	2	1	1
#NULL!	#NULL!	#NULL!	#NULL!	#NULL!	#NULL!	#NULL!	#NULL!	1	#NULL!
2	2	2	2	2	2	2	2	2	2
2	2	2	2	2	2	2	2	2	2
2	2	2	2	2	2	2	2	2	2
2	2	2	2	2	2	1	2	1	1
2	2	2	2	2	2	2	2	2	2
#NULL!	#NULL!	#NULL!	#NULL!	#NULL!	#NULL!	#NULL!	#NULL!	#NULL!	#NULL!
#NULL!	#NULL!	#NULL!	#NULL!	#NULL!	#NULL!	1	#NULL!	#NULL!	#NULL!
2	2	2	2	2	2	2	2	2	2
2	2	2	2	2	2	2	1	2	2
2	2	2	2	2	2	1	#NULL!	#NULL!	#NULL!
2	2	2	2	2	2	2	2	2	2
2	1	1	1	2	1	1	2	1	1
2	2	2	2	2	2	1	2	2	2
2	2	2	2	2	2	2	2	2	2
2	2	2	2	2	1	1	2	1	1
2	2	2	2	2	2	2	2	2	2
2	2	1	2	1	2	1	2	2	1
2	2	2	2	2	2	2	2	2	2
2	2	2	2	2	2	2	2	2	2
2	2	2	2	2	2	2	2	2	2
2	2	2	2	2	2	2	2	2	2
2	1	1	1	2	2	1	2	1	1
2	2	2	2	2	2	2	2	2	2
2	2	2	2	2	2	2	2	2	2
2	2	2	2	2	2	2	1	2	2
2	2	1	2	2	2	2	2	2	2

2	2	2	2	2	2	2	2	2	2
2	2	2	2	2	2	2	2	2	2
2	2	2	2	2	2	2	2	2	2
2	2	2	2	2	2	2	2	2	2
2	2	2	2	2	2	2	1	1	2
1	#NULL!	2	1	1	1	1	2	1	1
2	2	2	2	2	2	2	2	2	2
2	2	1	2	2	2	1	2	2	2
1	1	1	1	2	2	1	2	1	1
2	2	2	2	2	2	2	2	2	2
2	1	1	1	2	1	1	2	1	2
#NULL!	#NULL!	#NULL!	#NULL!	#NULL!	#NULL!	2	#NULL!	#NULL!	#NULL!
#NULL!	#NULL!	#NULL!	#NULL!	#NULL!	#NULL!	#NULL!	2	#NULL!	#NULL!
1	1	2	#NULL!	#NULL!	#NULL!	#NULL!	#NULL!	#NULL!	#NULL!
2	2	2	2	1	1	1	1	2	2
2	2	2	2	2	2	1	2	2	2
2	2	2	2	2	2	1	2	2	2
1	1	1	1	2	2	1	2	2	2
#NULL!	#NULL!	#NULL!	#NULL!	#NULL!	#NULL!	#NULL!	#NULL!	#NULL!	#NULL!
2	2	2	2	2	2	2	2	2	2
#NULL!	#NULL!	#NULL!	#NULL!	#NULL!	#NULL!	#NULL!	#NULL!	#NULL!	#NULL!
1	1	2	#NULL!	1	2	1	2	2	2
1	1	1	1	2	1	2	2	2	2
1	1	1	1	1	1	1	2	1	1
2	2	2	2	2	2	1	2	1	2
2	1	1	1	2	2	1	2	1	1
2	2	2	2	2	2	2	2	2	2
2	2	2	1	2	2	1	2	1	1
2	2	2	2	2	2	1	2	2	2
2	2	2	2	2	2	2	1	2	2
1	1	1	1	1	1	1	2	1	1
2	2	2	2	2	2	1	2	1	1
1	1	1	1	1	1	1	2	1	2
2	2	2	1	1	1	2	1	2	2
2	2	2	2	2	2	1	2	1	1
2	2	2	2	2	2	1	1	1	1
#NULL!	#NULL!	#NULL!	1	#NULL!	#NULL!	#NULL!	#NULL!	#NULL!	#NULL!
1	1	1	1	1	1	1	2	1	1
2	2	2	2	2	2	2	2	2	2
2	2	2	2	2	2	2	2	2	2
2	2	2	2	2	1	1	2	1	1
#NULL!	#NULL!	#NULL!	#NULL!	#NULL!	#NULL!	1	2	1	2
1	2	2	2	2	2	1	1	2	2
2	2	2	2	2	2	2	2	2	2
2	2	2	2	2	2	2	2	2	2
2	2	2	2	1	1	1	1	2	2
2	2	2	1	2	2	2	2	2	2

1	1	2	1	2	1	1	2	1	1
2	2	2	2	2	2	2	1	2	2
2	1	2	2	1	2	2	2	2	2
#NULL!	#NULL!	#NULL!	#NULL!	#NULL!	#NULL!	#NULL!	2	#NULL!	#NULL!
#NULL!	#NULL!	#NULL!	#NULL!	#NULL!	1	#NULL!	#NULL!	#NULL!	#NULL!
2	2	2	1	1	2	1	2	2	2
2	2	2	2	2	2	2	2	2	2
1	1	1	1	2	2	2	1	2	2
2	2	2	2	2	2	1	2	1	1
2	2	2	2	2	2	2	2	2	2
#NULL!	#NULL!	#NULL!	#NULL!	#NULL!	#NULL!	#NULL!	#NULL!	#NULL!	#NULL!
2	2	2	2	2	2	2	2	2	2
2	2	2	2	2	2	2	2	2	2
2	2	2	2	2	2	2	2	2	2
2	2	2	2	2	2	2	2	2	2
1	2	#NULL!	2	1	2	2	2	2	2
2	2	2	2	2	2	2	2	2	2
#NULL!	#NULL!	#NULL!	#NULL!	#NULL!	#NULL!	#NULL!	#NULL!	#NULL!	#NULL!
#NULL!	#NULL!	#NULL!	#NULL!	#NULL!	#NULL!	#NULL!	#NULL!	#NULL!	1
2	2	2	2	2	2	1	2	1	1
2	2	2	2	2	2	2	2	2	2
2	2	2	2	2	2	2	2	2	2
#NULL!	#NULL!	#NULL!	#NULL!	#NULL!	#NULL!	#NULL!	#NULL!	#NULL!	#NULL!
#NULL!	#NULL!	#NULL!	#NULL!	#NULL!	#NULL!	#NULL!	#NULL!	#NULL!	#NULL!
2	1	2	2	2	2	1	2	2	2
2	2	1	1	1	2	2	1	1	1
2	1	1	1	2	1	2	1	1	1
2	1	2	1	1	2	1	2	2	2
2	2	2	1	2	2	2	2	2	1
2	2	2	2	2	2	1	2	1	2
2	1	1	1	2	2	1	2	1	2
2	2	2	1	2	2	2	2	2	2
2	1	2	2	2	1	1	2	2	2
2	2	2	2	2	2	2	1	2	2
2	2	2	2	2	2	2	2	2	2
2	2	2	2	2	2	1	2	2	2
1	1	2	1	#NULL!	1	1	#NULL!	2	#NULL!
2	2	2	2	2	2	2	1	2	2
2	1	#NULL!	1	#NULL!	#NULL!	#NULL!	2	1	#NULL!
2	2	2	2	2	2	2	1	2	2
1	1	1	1	2	1	1	2	1	2
2	2	1	1	1	1	1	2	1	1
1	2	2	1	2	1	2	1	2	2
2	2	2	2	2	2	2	2	2	2
2	2	2	2	2	2	1	2	2	1
1	2	1	1	1	1	2	2	2	2
1	1	1	1	2	1	1	2	1	2

2	2	2	2	2	2	2	2	2	2
2	2	2	2	2	2	2	1	2	2
2	1	2	#NULL!	1	2	1	2	1	2
2	2	2	2	2	2	2	2	1	1
2	2	2	2	2	2	2	2	2	2
2	1	2	2	1	2	1	2	2	2
2	2	2	2	2	2	1	2	2	2
2	2	2	2	2	2	2	2	2	2
2	2	2	2	2	2	2	2	2	2
2	2	2	2	2	2	2	2	2	2
#NULL!	#NULL!	#NULL!	#NULL!	#NULL!	#NULL!	1	#NULL!	#NULL!	#NULL!
2	2	2	2	2	2	2	2	2	2
1	1	1	2	2	1	1	2	1	1
2	1	1	2	2	1	1	2	2	1
2	1	1	2	1	1	2	2	2	2
2	2	2	#NULL!	2	2	1	2	2	2
2	2	2	2	2	2	1	2	1	1
1	2	2	2	1	2	2	2	2	2
1	2	2	1	2	2	2	2	2	2
#NULL!	#NULL!	#NULL!	#NULL!	#NULL!	#NULL!	#NULL!	#NULL!	#NULL!	#NULL!
1	1	1	1	1	1	2	2	1	1
1	2	1	1	2	2	1	2	1	1
2	2	1	1	2	2	2	1	2	2
2	1	1	1	2	1	1	2	1	1
2	2	2	2	2	2	2	1	2	2
2	2	2	2	2	2	2	2	2	2
2	1	1	1	1	1	1	2	1	1
1	2	1	2	1	2	2	2	2	1
2	2	1	2	1	2	1	2	1	2
1	2	2	1	2	2	1	2	1	2
2	2	2	2	2	2	2	2	2	2
#NULL!	#NULL!	#NULL!	#NULL!	#NULL!	#NULL!	#NULL!	#NULL!	#NULL!	#NULL!
1	2	1	2	2	2	2	1	2	2
#NULL!	#NULL!	#NULL!	#NULL!	#NULL!	#NULL!	#NULL!	#NULL!	#NULL!	#NULL!
2	2	2	2	2	2	1	2	2	2
2	2	2	2	2	2	1	2	1	2
2	2	2	2	2	2	2	2	2	2
2	2	2	2	2	2	2	2	2	2
2	2	2	2	2	2	2	1	2	2
1	2	1	2	2	1	2	2	2	1
2	1	2	2	1	2	1	2	2	2
2	2	2	2	2	2	1	2	2	2
2	1	2	2	1	2	1	2	2	1
#NULL!	#NULL!	#NULL!	#NULL!	#NULL!	#NULL!	#NULL!	#NULL!	#NULL!	#NULL!
2	2	2	2	2	2	2	1	2	2
2	2	2	2	2	2	2	2	2	2
2	2	1	1	1	2	1	2	2	2

#NULL!	#NULL!	#NULL!	#NULL!	#NULL!	#NULL!	1	#NULL!	#NULL!	#NULL!
2	2	2	2	2	2	1	2	2	2
2	2	2	2	2	#NULL!	1	2	2	2
2	2	2	1	1	1	1	2	2	2
2	2	2	2	2	2	2	1	2	2
2	2	2	2	2	2	2	2	2	2
#NULL!	#NULL!	#NULL!	#NULL!	#NULL!	#NULL!	#NULL!	#NULL!	#NULL!	#NULL!
2	2	2	#NULL!	2	2	2	2	2	2
2	2	2	2	2	2	2	2	2	2
#NULL!	#NULL!	#NULL!	#NULL!	#NULL!	#NULL!	#NULL!	#NULL!	#NULL!	#NULL!
#NULL!	#NULL!	#NULL!	#NULL!	#NULL!	#NULL!	#NULL!	#NULL!	#NULL!	#NULL!
#NULL!	#NULL!	#NULL!	#NULL!	#NULL!	#NULL!	#NULL!	#NULL!	#NULL!	#NULL!
#NULL!	#NULL!	#NULL!	#NULL!	#NULL!	#NULL!	#NULL!	2	#NULL!	#NULL!
#NULL!	#NULL!	#NULL!	#NULL!	#NULL!	#NULL!	#NULL!	#NULL!	1	#NULL!
#NULL!	#NULL!	#NULL!	#NULL!	#NULL!	#NULL!	#NULL!	#NULL!	#NULL!	#NULL!
2	2	1	1	2	2	2	2	2	2
2	2	#NULL!	2	#NULL!	1	2	2	2	2
2	2	2	2	2	2	1	2	1	1
2	1	2	2	1	2	1	1	1	2
1	1	1	1	2	1	1	2	2	2
2	1	2	2	1	2	1	2	2	2
2	2	1	2	2	1	2	2	2	2
1	1	1	1	1	1	1	2	#NULL!	2
2	1	1	1	2	2	1	2	2	2
2	2	2	2	2	2	2	2	2	2
2	2	1	2	2	2	2	2	2	2
2	2	2	2	2	2	2	#NULL!	2	2
1	1	1	1	1	1	1	2	1	1
2	2	1	2	2	2	2	2	2	2
2	2	1	1	2	2	2	2	2	2
1	1	1	1	2	1	1	2	2	2
#NULL!	#NULL!	#NULL!	#NULL!	#NULL!	#NULL!	#NULL!	2	#NULL!	#NULL!
2	2	1	2	2	1	1	1	1	1
2	2	2	2	2	2	2	2	2	2
2	2	2	2	2	2	2	2	2	2
2	2	2	2	2	2	2	2	2	2
2	2	2	2	2	2	2	2	2	2
#NULL!	#NULL!	#NULL!	#NULL!	#NULL!	#NULL!	1	#NULL!	#NULL!	#NULL!
2	2	2	2	2	2	2	2	2	2
2	2	2	2	2	2	2	2	2	2
2	2	2	2	2	2	2	2	2	2
2	2	2	2	2	2	2	2	2	2
2	2	2	2	2	2	2	2	2	2
2	2	2	2	2	2	1	2	2	2
2	2	2	2	2	2	1	2	2	2
2	2	2	2	2	2	2	2	2	2
#NULL!	1	#NULL!	#NULL!	#NULL!	#NULL!	1	#NULL!	#NULL!	#NULL!

2	2	2	2	2	2	2	2	2	2
2	2	2	2	2	2	2	2	2	2
#NULL!	#NULL!	#NULL!	1	#NULL!	#NULL!	#NULL!	2	#NULL!	#NULL!
#NULL!	#NULL!	#NULL!	#NULL!	#NULL!	2	#NULL!	#NULL!	#NULL!	#NULL!
2	2	2	2	2	2	1	2	2	1
#NULL!	#NULL!	#NULL!	#NULL!	#NULL!	#NULL!	1	#NULL!	#NULL!	#NULL!
2	2	2	1	1	2	1	2	2	2
2	2	2	2	1	2	1	2	2	2
#NULL!	#NULL!	#NULL!	#NULL!	#NULL!	#NULL!	1	2	2	2
2	2	2	2	2	2	1	2	2	2
#NULL!	#NULL!	#NULL!	#NULL!	#NULL!	#NULL!	#NULL!	#NULL!	#NULL!	#NULL!
2	2	2	2	2	2	2	2	2	1
2	2	2	2	2	2	2	2	2	2
2	2	2	2	2	2	2	2	2	2
2	2	1	2	1	1	1	2	2	1
2	2	2	2	2	1	2	1	2	2
2	2	2	2	2	2	2	2	2	2
2	2	2	2	2	2	2	2	2	2
2	2	2	2	2	2	1	2	2	1
2	2	2	1	2	2	1	2	2	2
2	1	1	1	2	2	1	2	1	1
2	2	2	2	2	2	2	2	2	2
1	1	1	1	2	2	1	2	1	1
2	2	2	2	2	2	2	2	2	2
2	2	2	2	2	2	2	2	2	2
2	1	1	1	2	2	1	2	2	2
2	2	2	2	2	2	2	2	2	2
2	2	2	2	2	2	2	2	2	2
2	2	2	2	2	2	2	2	2	2
#NULL!	#NULL!	#NULL!	#NULL!	#NULL!	#NULL!	#NULL!	#NULL!	#NULL!	#NULL!
#NULL!	1	#NULL!	#NULL!	#NULL!	1	#NULL!	#NULL!	#NULL!	#NULL!
2	2	2	2	2	2	2	2	2	2
#NULL!	#NULL!	#NULL!	#NULL!	#NULL!	#NULL!	#NULL!	#NULL!	#NULL!	#NULL!
#NULL!	#NULL!	#NULL!	#NULL!	#NULL!	#NULL!	#NULL!	#NULL!	#NULL!	#NULL!
2	2	2	2	2	2	1	2	2	2
#NULL!	#NULL!	#NULL!	#NULL!	#NULL!	#NULL!	#NULL!	#NULL!	#NULL!	1
#NULL!	#NULL!	#NULL!	#NULL!	#NULL!	#NULL!	#NULL!	#NULL!	#NULL!	#NULL!
2	2	2	2	2	2	2	2	2	2
#NULL!	#NULL!	#NULL!	1	1	#NULL!	1	2	2	2
2	2	2	2	2	1	1	2	2	1
1	1	1	1	1	1	1	2	1	1
2	2	1	2	2	2	1	1	2	2
#NULL!	#NULL!	#NULL!	#NULL!	#NULL!	#NULL!	2	#NULL!	#NULL!	#NULL!
2	1	2	2	1	2	1	2	1	2
1	2	2	2	1	2	1	2	#NULL!	1
#NULL!	#NULL!	#NULL!	#NULL!	#NULL!	#NULL!	#NULL!	#NULL!	#NULL!	#NULL!
1	2	2	2	2	1	2	1	2	2

1	1	1	2	1	2	1	2	1	2
#NULL!	#NULL!	#NULL!	#NULL!	#NULL!	#NULL!	#NULL!	#NULL!	#NULL!	#NULL!
#NULL!	#NULL!	#NULL!	#NULL!	#NULL!	#NULL!	#NULL!	#NULL!	#NULL!	#NULL!
2	2	2	2	2	2	2	2	2	2
2	2	2	1	1	1	1	2	1	1
1	1	1	2	1	2	1	2	1	2
1	1	1	2	1	2	1	2	2	#NULL!
1	1	1	1	2	2	1	2	1	1
2	2	2	2	2	2	2	2	2	2
2	1	1	2	2	2	2	2	1	1
#NULL!	#NULL!	#NULL!	#NULL!	#NULL!	#NULL!	#NULL!	#NULL!	#NULL!	#NULL!
#NULL!	#NULL!	#NULL!	#NULL!	#NULL!	#NULL!	#NULL!	#NULL!	#NULL!	#NULL!
#NULL!	#NULL!	#NULL!	#NULL!	#NULL!	1	1	#NULL!	#NULL!	#NULL!
2	2	1	2	2	1	1	2	1	2
2	2	2	2	2	2	2	1	1	2
#NULL!	#NULL!	#NULL!	#NULL!	#NULL!	#NULL!	#NULL!	#NULL!	#NULL!	#NULL!
#NULL!	#NULL!	#NULL!	#NULL!	#NULL!	#NULL!	#NULL!	#NULL!	#NULL!	#NULL!
#NULL!	#NULL!	#NULL!	#NULL!	#NULL!	#NULL!	#NULL!	#NULL!	#NULL!	#NULL!
2	2	2	1	2	2	1	2	2	2
2	2	2	2	2	2	1	2	2	2
#NULL!	#NULL!	#NULL!	#NULL!	#NULL!	#NULL!	#NULL!	#NULL!	#NULL!	#NULL!
1	1	2	2	2	1	1	1	2	2
2	2	2	2	2	2	2	1	2	2
#NULL!	#NULL!	#NULL!	#NULL!	#NULL!	#NULL!	#NULL!	#NULL!	#NULL!	#NULL!
2	2	2	2	2	2	2	2	2	2
2	2	2	2	2	2	2	2	2	2
#NULL!	#NULL!	#NULL!	#NULL!	#NULL!	#NULL!	#NULL!	#NULL!	#NULL!	#NULL!
#NULL!	#NULL!	#NULL!	#NULL!	#NULL!	#NULL!	#NULL!	#NULL!	#NULL!	#NULL!
1	2	#NULL!	1	2	2	2	2	2	2
1	2	2	2	2	2	2	2	#NULL!	1
2	2	2	2	2	1	1	2	1	1
#NULL!	#NULL!	#NULL!	#NULL!	#NULL!	#NULL!	#NULL!	#NULL!	#NULL!	#NULL!
#NULL!	#NULL!	#NULL!	#NULL!	#NULL!	#NULL!	#NULL!	#NULL!	#NULL!	#NULL!
1	1	1	1	1	1	1	1	2	1
1	1	1	1	1	1	2	1	2	2
2	2	2	2	2	2	2	2	2	2
2	1	1	1	2	1	2	2	2	2
2	2	2	2	2	2	2	2	2	2
1	#NULL!	2	1	2	1	1	#NULL!	1	#NULL!
2	2	2	2	2	2	2	#NULL!	1	2
2	2	2	1	1	1	1	2	2	2
#NULL!	#NULL!	#NULL!	#NULL!	#NULL!	#NULL!	#NULL!	#NULL!	#NULL!	#NULL!
#NULL!	#NULL!	#NULL!	#NULL!	#NULL!	#NULL!	#NULL!	#NULL!	#NULL!	#NULL!
#NULL!	#NULL!	#NULL!	#NULL!	#NULL!	#NULL!	#NULL!	#NULL!	#NULL!	#NULL!
#NULL!	#NULL!	#NULL!	#NULL!	#NULL!	#NULL!	#NULL!	#NULL!	#NULL!	#NULL!
#NULL!	#NULL!	#NULL!	#NULL!	#NULL!	#NULL!	#NULL!	#NULL!	#NULL!	#NULL!
#NULL!	#NULL!	#NULL!	#NULL!	#NULL!	#NULL!	#NULL!	#NULL!	#NULL!	#NULL!

2	2	2	2	2	2	2	2	2	2
2	2	2	2	2	2	2	2	2	2
1	1	1	2	2	1	1	2	2	2
2	2	2	2	2	2	2	2	2	2
#NULL!	#NULL!	#NULL!	#NULL!	#NULL!	#NULL!	#NULL!	#NULL!	#NULL!	#NULL!
#NULL!	#NULL!	#NULL!	#NULL!	#NULL!	#NULL!	#NULL!	#NULL!	#NULL!	#NULL!
#NULL!	#NULL!	#NULL!	#NULL!	#NULL!	#NULL!	#NULL!	#NULL!	#NULL!	#NULL!
#NULL!	#NULL!	#NULL!	#NULL!	#NULL!	#NULL!	#NULL!	#NULL!	#NULL!	#NULL!
#NULL!	#NULL!	#NULL!	#NULL!	#NULL!	#NULL!	#NULL!	#NULL!	#NULL!	#NULL!
#NULL!	#NULL!	#NULL!	#NULL!	#NULL!	#NULL!	#NULL!	#NULL!	#NULL!	#NULL!
#NULL!	#NULL!	#NULL!	#NULL!	#NULL!	#NULL!	#NULL!	#NULL!	#NULL!	#NULL!
#NULL!	#NULL!	#NULL!	#NULL!	#NULL!	#NULL!	#NULL!	#NULL!	#NULL!	#NULL!
#NULL!	#NULL!	#NULL!	#NULL!	#NULL!	#NULL!	#NULL!	#NULL!	#NULL!	#NULL!
2	1	2	1	1	1	1	#NULL!	1	#NULL!
#NULL!	#NULL!	#NULL!	#NULL!	#NULL!	#NULL!	#NULL!	#NULL!	#NULL!	#NULL!
2	2	2	2	2	2	1	2	2	1
2	2	2	2	2	2	2	2	2	2
#NULL!	#NULL!	#NULL!	#NULL!	#NULL!	#NULL!	#NULL!	#NULL!	#NULL!	#NULL!
#NULL!	#NULL!	#NULL!	#NULL!	#NULL!	#NULL!	#NULL!	#NULL!	#NULL!	#NULL!
#NULL!	#NULL!	#NULL!	#NULL!	#NULL!	#NULL!	#NULL!	#NULL!	#NULL!	#NULL!
#NULL!	#NULL!	#NULL!	#NULL!	#NULL!	2	2	#NULL!	#NULL!	#NULL!
#NULL!	#NULL!	#NULL!	#NULL!	#NULL!	#NULL!	#NULL!	#NULL!	#NULL!	#NULL!
1	1	1	1	1	1	2	2	2	2
#NULL!	#NULL!	#NULL!	#NULL!	#NULL!	#NULL!	#NULL!	#NULL!	#NULL!	#NULL!
1	1	2	1	1	1	1	2	1	1
2	2	2	2	2	2	2	2	2	2
2	2	2	2	2	2	2	1	1	1
2	1	1	1	1	1	1	2	1	1
2	2	1	1	1	1	1	2	1	2
2	1	1	1	1	1	1	2	1	1
2	2	2	2	2	2	1	2	1	2
2	2	2	2	2	2	1	2	1	1
1	1	1	1	1	1	1	2	1	1
#NULL!	#NULL!	#NULL!	#NULL!	#NULL!	#NULL!	#NULL!	#NULL!	#NULL!	#NULL!
2	2	2	2	2	2	2	2	1	1
1	1	1	1	1	1	1	2	1	1
1	1	1	1	1	1	1	2	1	1
#NULL!	#NULL!	#NULL!	#NULL!	#NULL!	#NULL!	#NULL!	#NULL!	#NULL!	#NULL!
2	2	2	2	2	2	2	2	2	2
2	2	2	2	2	2	2	1	1	1
2	2	2	2	2	2	2	1	2	2
#NULL!	#NULL!	#NULL!	#NULL!	#NULL!	#NULL!	#NULL!	#NULL!	#NULL!	#NULL!
#NULL!	#NULL!	#NULL!	#NULL!	#NULL!	#NULL!	#NULL!	#NULL!	#NULL!	#NULL!
#NULL!	#NULL!	#NULL!	#NULL!	#NULL!	#NULL!	#NULL!	#NULL!	#NULL!	#NULL!
#NULL!	#NULL!	#NULL!	#NULL!	#NULL!	#NULL!	#NULL!	#NULL!	#NULL!	#NULL!
2	2	2	2	2	2	2	2	2	2
#NULL!	#NULL!	#NULL!	#NULL!	#NULL!	#NULL!	#NULL!	#NULL!	#NULL!	#NULL!

#NULL!	#NULL!	#NULL!	#NULL!	#NULL!	#NULL!	#NULL!	#NULL!	#NULL!	#NULL!
#NULL!	#NULL!	#NULL!	#NULL!	#NULL!	#NULL!	#NULL!	#NULL!	#NULL!	#NULL!
#NULL!	#NULL!	#NULL!	#NULL!	#NULL!	#NULL!	#NULL!	#NULL!	#NULL!	#NULL!
#NULL!	#NULL!	#NULL!	#NULL!	#NULL!	#NULL!	#NULL!	#NULL!	#NULL!	#NULL!
#NULL!	#NULL!	#NULL!	#NULL!	#NULL!	#NULL!	#NULL!	#NULL!	#NULL!	#NULL!
#NULL!	#NULL!	#NULL!	#NULL!	#NULL!	#NULL!	#NULL!	#NULL!	#NULL!	#NULL!
#NULL!	#NULL!	#NULL!	#NULL!	#NULL!	#NULL!	#NULL!	#NULL!	#NULL!	#NULL!
#NULL!	#NULL!	#NULL!	#NULL!	#NULL!	#NULL!	#NULL!	#NULL!	#NULL!	#NULL!
#NULL!	#NULL!	#NULL!	#NULL!	#NULL!	#NULL!	#NULL!	#NULL!	#NULL!	#NULL!
#NULL!	#NULL!	#NULL!	#NULL!	#NULL!	#NULL!	#NULL!	#NULL!	#NULL!	#NULL!
#NULL!	#NULL!	#NULL!	#NULL!	#NULL!	#NULL!	#NULL!	2	#NULL!	#NULL!
#NULL!	#NULL!	#NULL!	#NULL!	#NULL!	#NULL!	#NULL!	#NULL!	#NULL!	#NULL!
1	1	1	2	2	2	2	2	2	2
1	2	1	1	1	1	1	2	1	1
2	1	1	2	2	2	1	2	1	1
2	1	2	2	1	2	1	2	1	1
1	2	1	1	1	1	1	2	2	2
2	2	2	2	2	2	1	2	1	1
2	2	2	2	2	2	2	2	2	2
#NULL!	#NULL!	#NULL!	#NULL!	#NULL!	#NULL!	1	#NULL!	#NULL!	#NULL!
1	1	1	1	2	2	1	2	1	1
1	2	1	2	1	1	1	2	2	2
2	2	2	2	2	2	2	2	2	2
1	1	1	1	1	1	2	2	2	1
2	1	1	2	2	1	1	2	1	1
2	1	1	1	2	2	2	1	2	2
2	2	2	2	2	2	#NULL!	#NULL!	#NULL!	#NULL!
2	2	2	2	2	2	2	2	1	1
#NULL!	#NULL!	#NULL!	#NULL!	#NULL!	#NULL!	1	#NULL!	#NULL!	#NULL!
#NULL!	#NULL!	#NULL!	#NULL!	#NULL!	#NULL!	2	#NULL!	#NULL!	2
#NULL!	#NULL!	#NULL!	#NULL!	#NULL!	#NULL!	#NULL!	#NULL!	#NULL!	#NULL!
1	2	2	1	1	1	1	2	1	1
2	2	2	2	2	2	2	2	1	1
2	1	1	1	1	1	1	2	1	1
2	1	1	2	1	1	1	2	2	2
2	2	2	2	2	2	1	2	2	2
1	1	1	1	1	1	1	2	2	2
2	2	2	2	2	2	2	2	2	2
1	1	2	2	2	1	2	2	1	1
1	1	1	2	1	1	1	2	1	1
2	2	2	2	2	2	2	2	2	2
1	1	1	1	1	1	1	2	1	1
2	1	2	2	2	2	1	2	1	2
2	1	1	1	1	1	1	2	1	1
2	1	1	1	1	1	1	2	1	1
1	1	1	1	1	1	1	2	2	2
2	2	2	2	2	2	2	2	2	2

#NULL!	#NULL!	#NULL!	#NULL!	#NULL!	#NULL!	#NULL!	#NULL!	#NULL!	#NULL!
2	2	2	2	2	2	1	2	1	1
#NULL!	#NULL!	#NULL!	#NULL!	#NULL!	#NULL!	#NULL!	#NULL!	#NULL!	2
#NULL!	#NULL!	#NULL!	#NULL!	#NULL!	#NULL!	#NULL!	#NULL!	#NULL!	2
#NULL!	#NULL!	#NULL!	#NULL!	#NULL!	#NULL!	#NULL!	#NULL!	1	#NULL!
#NULL!	#NULL!	#NULL!	#NULL!	#NULL!	#NULL!	#NULL!	#NULL!	#NULL!	#NULL!
#NULL!	#NULL!	1	#NULL!	#NULL!	#NULL!	#NULL!	#NULL!	#NULL!	#NULL!
2	#NULL!	2	2	2	2	1	2	1	1
#NULL!	#NULL!	#NULL!	#NULL!	#NULL!	#NULL!	2	#NULL!	#NULL!	#NULL!
2	2	2	2	2	2	2	2	2	2
#NULL!	#NULL!	#NULL!	#NULL!	#NULL!	#NULL!	#NULL!	#NULL!	#NULL!	#NULL!
#NULL!	#NULL!	#NULL!	#NULL!	#NULL!	#NULL!	#NULL!	#NULL!	#NULL!	#NULL!
1	1	2	1	1	1	1	2	1	1
2	2	2	2	2	2	2	2	2	2
1	1	2	2	1	1	1	2	1	1
#NULL!	#NULL!	#NULL!	#NULL!	#NULL!	#NULL!	#NULL!	#NULL!	#NULL!	#NULL!
2	2	2	2	2	2	2	2	2	2
2	2	2	2	2	2	2	1	2	2
#NULL!	#NULL!	#NULL!	#NULL!	#NULL!	#NULL!	#NULL!	#NULL!	#NULL!	#NULL!
1	1	1	1	1	1	2	2	2	2
2	2	2	2	2	2	2	2	2	2
2	2	2	2	2	2	2	2	2	2
2	2	2	2	2	2	2	2	2	2
2	2	2	2	2	2	2	2	2	2
2	2	2	2	2	2	2	2	1	1
2	2	2	2	2	2	2	2	2	2
2	2	1	2	2	1	2	2	2	2
2	2	1	2	2	2	1	2	1	2
1	1	1	1	1	1	1	2	2	2
1	1	1	1	1	2	2	2	2	2
#NULL!	#NULL!	#NULL!	#NULL!	#NULL!	#NULL!	#NULL!	#NULL!	#NULL!	#NULL!
2	2	2	2	2	2	2	2	2	2
#NULL!	#NULL!	#NULL!	#NULL!	#NULL!	#NULL!	#NULL!	#NULL!	#NULL!	#NULL!
2	2	2	2	2	2	2	2	2	2
2	2	2	2	2	2	2	2	2	2
2	2	2	2	2	2	1	2	1	1
2	2	2	2	2	2	1	2	1	2
1	1	2	2	1	1	1	2	1	1
1	1	1	1	1	1	1	2	1	1
2	1	2	2	2	2	1	2	2	1
2	2	2	2	2	2	2	2	2	2
1	1	1	1	1	1	1	2	1	1
#NULL!	#NULL!	#NULL!	#NULL!	#NULL!	#NULL!	#NULL!	#NULL!	#NULL!	#NULL!
#NULL!	#NULL!	#NULL!	#NULL!	#NULL!	#NULL!	#NULL!	#NULL!	#NULL!	#NULL!
#NULL!	#NULL!	#NULL!	#NULL!	#NULL!	#NULL!	1	2	#NULL!	#NULL!
#NULL!	#NULL!	#NULL!	#NULL!	#NULL!	#NULL!	#NULL!	#NULL!	#NULL!	#NULL!
#NULL!	#NULL!	#NULL!	#NULL!	#NULL!	#NULL!	#NULL!	#NULL!	#NULL!	#NULL!

#NULL!	#NULL!	#NULL!	#NULL!	#NULL!	#NULL!	#NULL!	#NULL!	#NULL!	#NULL!
2	2	2	2	2	2	2	2	2	2
#NULL!	#NULL!	#NULL!	#NULL!	#NULL!	#NULL!	#NULL!	2	#NULL!	#NULL!
2	2	1	2	2	1	2	1	1	2
#NULL!	#NULL!	#NULL!	#NULL!	#NULL!	#NULL!	#NULL!	#NULL!	#NULL!	#NULL!
2	2	2	2	1	2	2	2	1	2
2	2	2	2	2	2	2	1	2	2
#NULL!	#NULL!	#NULL!	#NULL!	#NULL!	#NULL!	#NULL!	#NULL!	#NULL!	#NULL!
#NULL!	#NULL!	#NULL!	#NULL!	#NULL!	#NULL!	#NULL!	#NULL!	#NULL!	#NULL!
#NULL!	#NULL!	#NULL!	#NULL!	#NULL!	#NULL!	#NULL!	#NULL!	#NULL!	#NULL!
#NULL!	1	#NULL!	#NULL!	#NULL!	#NULL!	1	#NULL!	#NULL!	#NULL!
#NULL!	#NULL!	#NULL!	#NULL!	#NULL!	#NULL!	#NULL!	#NULL!	#NULL!	#NULL!
1	2	2	2	1	2	2	2	2	2
#NULL!	#NULL!	#NULL!	#NULL!	#NULL!	#NULL!	#NULL!	#NULL!	#NULL!	#NULL!
#NULL!	#NULL!	#NULL!	#NULL!	#NULL!	#NULL!	#NULL!	#NULL!	#NULL!	2
2	1	1	2	2	1	2	2	2	2
2	2	1	2	1	2	2	2	2	2
1	2	2	1	2	2	2	1	2	2
2	2	2	2	2	2	1	2	2	1
2	2	2	2	2	2	1	2	2	1
1	2	2	1	2	1	1	2	2	1
2	1	1	1	#NULL!	2	1	2	2	1
2	1	1	2	1	1	1	2	2	2
1	2	2	2	1	2	1	2	1	2
2	2	2	2	2	2	2	2	2	2
2	2	1	2	1	2	1	2	1	2
2	2	2	2	2	2	2	#NULL!	#NULL!	#NULL!
1	1	1	1	1	#NULL!	1	2	#NULL!	2
2	1	2	2	2	2	2	1	2	2
1	2	2	2	2	2	1	2	2	2
2	2	1	#NULL!	1	2	2	2	2	2
2	2	2	2	2	2	2	2	2	2
#NULL!	#NULL!	#NULL!	#NULL!	#NULL!	#NULL!	#NULL!	#NULL!	#NULL!	#NULL!
1	2	2	1	#NULL!	1	1	2	2	2
2	1	1	1	2	2	2	2	2	2
2	1	2	1	1	1	2	2	2	2
2	2	2	2	2	2	2	2	2	2
2	1	2	2	2	2	1	2	2	2
2	#NULL!	#NULL!	#NULL!	#NULL!	#NULL!	#NULL!	#NULL!	1	#NULL!
2	2	2	1	2	2	1	2	2	1
#NULL!	1	#NULL!	#NULL!	#NULL!	#NULL!	#NULL!	#NULL!	#NULL!	#NULL!
#NULL!	#NULL!	#NULL!	#NULL!	#NULL!	#NULL!	1	#NULL!	#NULL!	#NULL!
2	2	2	2	2	2	1	#NULL!	#NULL!	#NULL!
#NULL!	#NULL!	#NULL!	#NULL!	#NULL!	#NULL!	1	#NULL!	#NULL!	#NULL!
1	1	1	1	1	1	2	1	2	2
2	1	1	2	1	2	1	1	1	1
2	2	2	2	2	2	2	1	2	2

2	2	2	2	2	2	2	1	2	2
#NULL!	#NULL!	#NULL!	#NULL!	#NULL!	#NULL!	#NULL!	#NULL!	#NULL!	#NULL!
2	2	2	2	2	2	2	2	2	2
2	2	1	1	2	1	1	2	2	1
2	1	1	1	2	2	1	1	1	1
1	1	2	1	1	1	1	2	2	1
1	2	2	2	1	2	2	1	2	2
2	1	2	1	2	2	2	2	1	2
2	2	2	2	2	2	2	2	2	2
2	2	2	2	2	2	2	#NULL!	2	2
2	2	1	1	2	2	1	2	2	2
2	2	2	1	2	1	2	2	2	2
2	1	2	2	1	2	1	2	2	1
2	2	2	2	2	2	2	2	2	1
2	2	2	1	2	2	1	2	2	2
1	2	2	1	2	2	2	2	2	2
2	2	2	2	2	2	2	2	2	2
2	2	1	1	2	2	2	1	2	2
2	2	2	2	2	2	2	2	2	2
2	2	2	2	2	2	2	2	2	2
2	2	2	2	2	2	2	2	2	2
2	2	2	2	2	2	1	2	2	2
#NULL!	#NULL!	#NULL!	#NULL!	#NULL!	#NULL!	2	#NULL!	#NULL!	#NULL!
#NULL!	#NULL!	#NULL!	#NULL!	#NULL!	#NULL!	#NULL!	#NULL!	#NULL!	#NULL!
2	2	2	2	2	2	2	2	2	2
2	2	2	1	2	2	1	2	1	1
2	2	2	1	2	2	1	2	1	2
2	2	2	1	2	2	1	2	2	2
1	1	1	1	1	1	1	2	1	1
#NULL!	#NULL!	#NULL!	#NULL!	#NULL!	#NULL!	#NULL!	#NULL!	1	#NULL!
2	2	2	2	2	2	2	2	2	1
2	2	2	1	1	1	1	2	1	1
2	2	2	2	#NULL!	2	2	1	2	2
2	2	2	2	2	2	2	2	1	2
2	1	1	2	1	1	1	2	#NULL!	1
2	2	2	2	2	2	2	2	2	2
2	2	2	2	2	2	2	1	2	2
#NULL!	#NULL!	#NULL!	#NULL!	#NULL!	#NULL!	#NULL!	#NULL!	#NULL!	#NULL!
2	2	1	2	1	2	1	2	1	1
2	2	2	2	1	1	2	2	2	2
#NULL!	#NULL!	#NULL!	#NULL!	#NULL!	#NULL!	#NULL!	#NULL!	#NULL!	#NULL!
#NULL!	#NULL!	#NULL!	#NULL!	#NULL!	#NULL!	#NULL!	#NULL!	#NULL!	#NULL!
2	1	2	2	2	2	2	2	2	2
#NULL!	#NULL!	#NULL!	#NULL!	#NULL!	#NULL!	#NULL!	#NULL!	#NULL!	#NULL!
2	2	2	2	2	2	2	2	2	2
2	1	1	1	1	1	2	2	1	2
#NULL!	#NULL!	#NULL!	#NULL!	#NULL!	#NULL!	#NULL!	#NULL!	#NULL!	#NULL!

2	2	2	2	2	2	2	2	2	2
#NULL!	#NULL!	#NULL!	#NULL!	#NULL!	#NULL!	#NULL!	#NULL!	#NULL!	#NULL!
2	2	2	1	2	2	2	2	2	2
#NULL!	#NULL!	#NULL!	#NULL!	#NULL!	#NULL!	#NULL!	#NULL!	#NULL!	#NULL!
#NULL!	#NULL!	#NULL!	#NULL!	#NULL!	#NULL!	#NULL!	#NULL!	#NULL!	#NULL!
2	2	2	2	2	2	2	2	2	2
2	2	2	2	2	2	2	2	2	2
2	2	2	2	2	2	2	2	2	2
1	1	1	1	1	1	1	2	1	1
2	2	2	2	2	2	2	2	1	1
#NULL!	#NULL!	#NULL!	#NULL!	#NULL!	#NULL!	#NULL!	#NULL!	#NULL!	#NULL!
#NULL!	#NULL!	#NULL!	#NULL!	#NULL!	#NULL!	1	2	1	2
#NULL!	#NULL!	#NULL!	#NULL!	#NULL!	#NULL!	#NULL!	#NULL!	#NULL!	#NULL!
1	1	1	1	1	1	1	2	1	1
2	2	2	2	2	2	2	2	2	2
1	1	1	1	1	1	2	2	1	1
#NULL!	#NULL!	#NULL!	#NULL!	#NULL!	#NULL!	2	#NULL!	#NULL!	#NULL!
#NULL!	#NULL!	#NULL!	#NULL!	#NULL!	#NULL!	#NULL!	#NULL!	#NULL!	#NULL!
1	1	1	1	1	2	1	2	1	1
2	2	2	2	2	2	2	2	2	2
1	1	1	1	1	1	1	2	1	1
2	2	2	2	2	2	2	2	2	2
#NULL!	#NULL!	#NULL!	#NULL!	#NULL!	#NULL!	1	#NULL!	#NULL!	#NULL!
2	2	2	2	2	2	2	2	2	2
1	2	2	2	2	1	1	2	2	2
2	2	2	2	2	2	2	2	2	2
1	#NULL!	#NULL!	#NULL!	#NULL!	#NULL!	#NULL!	#NULL!	#NULL!	#NULL!
2	2	2	2	2	2	1	2	1	1
2	2	2	2	2	2	2	2	2	2
#NULL!	#NULL!	#NULL!	#NULL!	#NULL!	#NULL!	#NULL!	#NULL!	1	#NULL!
1	2	2	1	2	1	1	2	2	2
2	2	2	2	2	2	2	2	2	2
1	2	2	2	2	2	2	1	2	2
2	2	2	1	2	2	1	2	1	2
2	2	2	2	2	2	2	2	2	2
2	1	2	2	1	2	2	2	2	2
2	1	1	1	1	1	2	2	2	2
1	1	1	1	2	1	#NULL!	2	2	2
2	2	2	2	2	2	2	2	2	2
2	2	2	2	2	2	2	1	2	2
2	1	1	1	2	1	1	2	1	2
2	2	2	2	1	1	1	2	2	2
2	2	2	#NULL!	2	2	2	2	2	2
2	2	2	2	2	2	2	2	2	2
2	1	2	1	2	1	2	2	2	2
2	1	1	2	2	2	2	1	2	2
2	2	2	2	2	2	2	2	2	2

2	2	2	2	2	2	2	1	2	1
2	2	2	2	2	2	2	2	2	2
2	2	1	1	2	1	1	2	1	1
2	2	2	2	2	2	2	2	2	2
2	2	2	2	2	2	2	2	1	1
#NULL!	#NULL!	#NULL!	#NULL!	#NULL!	#NULL!	#NULL!	2	#NULL!	#NULL!
2	2	2	2	2	2	2	2	2	2
2	2	2	1	1	1	1	2	1	2
2	2	2	2	1	2	2	2	2	2
2	2	2	2	2	2	2	2	2	2
2	2	2	1	1	1	2	2	2	2
2	1	1	1	2	2	1	2	2	1
2	2	2	2	2	2	2	2	2	2
2	2	2	2	2	2	2	1	2	2
2	2	2	2	2	2	2	2	1	2
1	2	2	1	2	2	1	2	2	1
2	2	2	2	2	2	2	2	2	2
2	2	2	2	2	2	2	1	2	2
2	2	2	2	2	2	2	2	2	2
2	2	1	2	1	2	2	2	2	2
2	2	2	2	2	2	2	2	2	2
2	2	2	2	2	2	2	2	2	2
2	2	2	2	2	2	2	2	2	2
2	2	2	2	2	2	2	2	2	2
1	2	1	1	2	2	2	2	1	1
2	2	2	2	2	2	2	2	2	2
2	2	1	1	2	2	2	2	2	2
2	2	1	1	2	2	1	2	1	1
2	2	2	2	2	2	2	2	2	2
2	1	1	1	1	1	1	2	1	1
2	2	2	2	2	2	2	2	2	2
2	2	2	2	2	2	2	2	2	2
1	1	1	2	2	2	1	2	1	2
2	2	2	2	2	2	2	2	2	2
1	1	1	1	1	1	1	2	1	1
2	2	2	2	2	2	2	2	2	2
2	2	2	2	2	2	2	2	2	2
1	2	1	1	1	2	2	2	2	2
2	2	2	2	2	2	2	2	2	2
2	2	2	2	2	2	2	2	2	2
2	2	2	1	2	1	1	2	1	2
2	1	1	1	2	2	1	2	1	1
1	1	1	1	1	1	2	2	2	2
1	2	2	2	2	2	2	2	2	2
2	2	1	2	2	2	2	2	1	2
1	2	1	1	2	1	1	2	2	2
2	1	2	2	2	1	2	2	#NULL!	#NULL!

1	2	2	1	1	2	2	2	2	2
2	2	2	2	2	2	2	2	2	2
2	1	2	1	2	2	1	2	2	2
2	2	2	2	2	2	2	2	2	2
2	2	2	2	2	2	2	2	2	2
2	2	2	2	2	2	2	2	2	1
2	2	1	1	2	2	1	2	2	2
2	1	1	1	2	2	1	#NULL!	1	2
2	2	2	2	2	2	2	2	2	2
2	2	2	2	2	2	2	2	2	2
2	2	2	2	2	2	2	2	2	2
#NULL!	#NULL!	#NULL!	#NULL!	#NULL!	#NULL!	#NULL!	#NULL!	#NULL!	#NULL!
#NULL!	#NULL!	#NULL!	#NULL!	#NULL!	#NULL!	#NULL!	#NULL!	#NULL!	#NULL!
#NULL!	#NULL!	#NULL!	#NULL!	#NULL!	#NULL!	2	2	2	2
1	2	1	1	1	1	1	2	1	2
2	2	2	1	2	2	2	2	2	2
2	1	1	1	2	2	1	2	1	2
2	2	2	2	2	2	2	2	2	2
1	1	1	1	2	2	1	2	2	2
1	1	2	1	2	2	1	2	2	1
2	2	2	2	2	2	2	2	2	2
2	2	2	2	2	2	1	2	1	1
2	2	2	2	2	2	2	2	2	2
2	2	2	2	2	2	2	2	2	2
2	2	2	2	2	2	2	2	2	2
1	2	#NULL!	2	2	2	1	2	1	2
2	2	1	1	1	2	2	2	2	2
2	2	2	2	2	2	2	2	2	2
2	2	2	2	2	2	1	2	1	1
#NULL!	#NULL!	#NULL!	#NULL!	#NULL!	#NULL!	#NULL!	#NULL!	#NULL!	#NULL!
2	2	2	2	2	2	2	1	2	2
2	2	2	2	2	2	2	2	2	2
1	1	2	2	2	2	2	2	2	2
2	2	2	2	2	2	2	2	2	2
#NULL!	#NULL!	#NULL!	#NULL!	#NULL!	#NULL!	2	2	2	2
2	2	2	2	2	2	2	2	2	2
2	2	2	2	2	2	2	2	2	2
2	2	2	2	2	2	2	2	2	2
2	2	2	2	2	2	2	2	2	2
1	1	2	1	2	2	2	2	2	2
2	2	2	2	2	2	2	2	2	2
2	2	2	2	2	2	2	2	2	2
1	1	1	1	1	1	1	2	1	1
1	2	2	1	2	2	2	2	2	2
2	2	2	2	2	2	2	2	2	2
2	2	2	2	2	2	2	2	2	2
2	2	2	2	2	2	2	2	2	2

2	2	2	2	1	1	2	2	2	2
2	2	2	2	2	2	1	2	2	2
1	1	1	1	2	2	1	2	1	1
2	2	2	2	2	2	2	2	2	2
2	2	2	2	2	2	2	2	2	2
2	2	2	2	2	2	2	2	1	2
#NULL!	#NULL!	#NULL!	#NULL!	#NULL!	#NULL!	#NULL!	#NULL!	#NULL!	#NULL!
1	1	1	1	2	2	1	2	1	1
1	1	2	2	2	2	1	2	1	2
2	2	1	1	2	2	1	2	2	2
2	1	1	1	2	2	1	2	1	2
2	2	2	2	2	2	2	2	2	2
2	2	1	1	2	2	2	2	2	2
1	#NULL!	#NULL!	1	#NULL!	#NULL!	1	#NULL!	1	#NULL!
1	1	2	1	#NULL!	2	1	1	2	2
1	1	2	1	2	1	2	2	2	2
1	1	1	2	2	1	1	2	2	2
2	2	2	2	2	2	2	2	2	2
1	1	1	1	1	1	1	2	1	1
2	2	2	2	1	1	1	2	1	1
2	2	2	2	1	1	1	2	1	2
2	2	2	2	2	2	1	2	2	2
2	2	2	2	2	2	2	2	2	2
2	2	2	2	2	2	2	2	2	2
2	2	2	2	2	2	2	2	2	2
2	2	2	2	2	2	2	2	2	2
#NULL!	#NULL!	#NULL!	#NULL!	#NULL!	#NULL!	#NULL!	#NULL!	#NULL!	#NULL!
2	2	2	2	2	2	2	2	2	2
2	2	2	1	2	2	2	2	2	2
2	2	2	2	2	2	2	2	2	2
2	2	2	2	2	2	2	2	2	2
1	2	1	2	2	2	1	2	2	2
#NULL!	#NULL!	#NULL!	#NULL!	#NULL!	#NULL!	#NULL!	#NULL!	#NULL!	#NULL!
#NULL!	#NULL!	#NULL!	#NULL!	#NULL!	#NULL!	#NULL!	#NULL!	#NULL!	#NULL!
#NULL!	#NULL!	#NULL!	#NULL!	#NULL!	#NULL!	#NULL!	#NULL!	#NULL!	#NULL!
#NULL!	#NULL!	#NULL!	#NULL!	#NULL!	#NULL!	#NULL!	#NULL!	#NULL!	#NULL!
2	2	2	2	2	2	2	1	2	2
2	1	1	1	2	2	1	2	1	1
1	1	1	1	1	1	1	2	1	1
2	2	2	2	2	2	2	2	2	2
1	2	1	2	2	2	1	2	1	2
2	2	2	2	2	2	2	2	2	2
2	2	2	2	1	2	1	2	1	1
2	2	2	2	2	2	2	2	2	2
2	2	1	1	2	2	1	2	2	2
2	1	2	1	2	2	1	2	2	2
1	1	2	1	2	1	1	2	1	2

1	2	2	1	2	2	1	2	2	1
2	2	2	2	1	2	1	2	1	1
2	2	1	2	2	2	1	2	1	2
1	1	1	1	1	1	2	2	1	1
2	2	2	2	2	1	2	2	2	1
1	2	2	2	2	1	2	2	1	1
1	1	1	1	2	1	2	2	1	1
#NULL!	#NULL!	#NULL!	#NULL!	#NULL!	#NULL!	2	#NULL!	#NULL!	#NULL!
2	2	2	2	2	2	2	2	2	2
2	2	2	2	2	2	2	2	2	2
2	2	2	2	2	2	2	2	2	2
2	2	2	2	2	2	2	2	2	2
2	2	2	2	2	2	2	2	2	2
2	2	2	2	1	2	1	2	#NULL!	2
2	1	1	1	2	2	1	2	2	1
2	2	2	2	2	2	2	2	2	2
2	2	2	2	2	2	2	2	2	2
1	1	1	1	1	1	1	2	1	1
2	2	2	1	1	2	2	2	2	2
1	1	2	2	2	2	1	2	2	2
2	2	2	2	2	2	2	1	2	2
2	1	2	1	2	2	2	2	2	2
2	1	1	1	1	1	1	2	1	1
#NULL!	#NULL!	#NULL!	#NULL!	#NULL!	#NULL!	#NULL!	#NULL!	#NULL!	#NULL!
1	2	2	#NULL!	2	1	1	1	2	2
2	1	1	1	1	1	1	2	1	1
1	2	1	1	1	1	2	2	1	1
2	1	2	2	1	2	1	2	1	2
2	2	2	2	2	2	2	2	2	2
#NULL!	#NULL!	#NULL!	#NULL!	#NULL!	#NULL!	#NULL!	2	#NULL!	#NULL!
2	2	2	2	2	2	2	2	2	2
#NULL!	#NULL!	#NULL!	#NULL!	#NULL!	#NULL!	#NULL!	#NULL!	#NULL!	#NULL!
#NULL!	#NULL!	#NULL!	#NULL!	#NULL!	#NULL!	#NULL!	#NULL!	#NULL!	#NULL!
#NULL!	#NULL!	#NULL!	#NULL!	#NULL!	#NULL!	#NULL!	#NULL!	#NULL!	#NULL!
#NULL!	#NULL!	#NULL!	#NULL!	#NULL!	#NULL!	#NULL!	#NULL!	#NULL!	#NULL!
2	2	2	2	2	2	2	2	2	2
2	2	2	2	2	2	1	2	1	1
#NULL!	#NULL!	#NULL!	#NULL!	#NULL!	#NULL!	#NULL!	#NULL!	#NULL!	#NULL!
2	2	2	2	2	2	2	2	2	2
2	2	2	2	2	2	2	2	2	2
2	1	2	2	2	2	1	2	2	2
2	2	2	2	2	2	2	2	2	2
2	2	2	2	2	2	2	2	2	2
1	2	2	1	2	2	2	1	2	2
#NULL!	2	#NULL!	#NULL!	#NULL!	#NULL!	#NULL!	#NULL!	#NULL!	#NULL!
1	1	1	1	1	1	1	2	2	2
2	2	2	2	2	2	2	1	2	2

1	1	1	1	1	1	1	2	1	1
2	1	1	1	#NULL!	2	1	2	1	2
1	1	1	1	2	1	1	2	1	1
2	2	2	2	2	2	2	2	2	2
#NULL!	#NULL!	#NULL!	#NULL!	#NULL!	#NULL!	#NULL!	#NULL!	#NULL!	#NULL!
#NULL!	#NULL!	#NULL!	#NULL!	#NULL!	#NULL!	#NULL!	#NULL!	#NULL!	#NULL!
2	2	2	2	2	2	2	2	2	2
2	2	2	2	2	2	#NULL!	#NULL!	#NULL!	#NULL!
#NULL!	1	#NULL!	#NULL!	#NULL!	#NULL!	#NULL!	#NULL!	#NULL!	#NULL!
#NULL!	#NULL!	#NULL!	#NULL!	#NULL!	#NULL!	#NULL!	#NULL!	#NULL!	#NULL!
2	2	#NULL!	1	2	2	1	2	2	2
2	2	1	1	1	1	2	2	1	1
1	1	2	2	2	2	2	2	2	2
2	2	2	2	2	2	2	1	2	2
2	2	2	2	2	2	#NULL!	#NULL!	#NULL!	#NULL!
1	2	2	2	2	2	2	1	2	2
2	1	1	2	2	2	2	2	1	1
1	2	2	2	2	2	2	2	2	2
#NULL!	#NULL!	#NULL!	#NULL!	#NULL!	#NULL!	1	#NULL!	#NULL!	#NULL!
2	2	2	1	2	2	2	1	1	2
2	2	1	2	1	2	1	2	1	2
1	2	2	1	2	2	1	2	1	2
1	2	1	#NULL!	1	1	1	1	2	1
2	2	2	2	2	2	2	2	2	2
2	2	2	2	1	2	#NULL!	#NULL!	#NULL!	#NULL!
2	2	2	2	2	2	2	2	2	2
2	2	1	2	2	1	2	2	2	2
2	2	2	2	2	2	1	2	2	2
1	2	2	1	2	2	2	2	2	2
2	1	2	#NULL!	2	2	2	2	2	2
2	2	2	2	2	2	2	2	2	2
1	1	2	1	1	1	1	2	2	2
2	1	1	1	1	1	1	2	1	1
2	2	1	1	1	2	2	2	2	2
#NULL!	#NULL!	#NULL!	#NULL!	#NULL!	#NULL!	#NULL!	#NULL!	#NULL!	#NULL!
1	1	1	1	2	1	1	2	2	2
#NULL!	#NULL!	#NULL!	#NULL!	#NULL!	#NULL!	#NULL!	#NULL!	#NULL!	#NULL!
2	2	2	2	2	2	2	2	2	2
#NULL!	#NULL!	#NULL!	#NULL!	#NULL!	#NULL!	#NULL!	#NULL!	#NULL!	#NULL!
1	1	2	1	2	1	1	2	2	2
#NULL!	#NULL!	#NULL!	#NULL!	#NULL!	#NULL!	#NULL!	#NULL!	#NULL!	#NULL!
2	1	2	1	2	2	1	2	2	2
2	2	2	2	2	2	2	2	2	2
1	2	2	2	2	1	1	2	2	2
1	2	2	2	2	2	1	2	2	2
2	2	1	2	2	2	2	2	2	2
2	2	2	2	2	2	2	2	2	2

2	2	1	2	2	2	2	2	2	2
#NULL!	#NULL!	#NULL!	#NULL!	#NULL!	#NULL!	#NULL!	#NULL!	#NULL!	#NULL!
2	2	2	2	2	2	2	2	2	2
2	2	2	2	2	2	2	1	2	2
1	1	1	1	2	1	1	2	2	2
#NULL!	#NULL!	#NULL!	#NULL!	#NULL!	#NULL!	#NULL!	#NULL!	#NULL!	#NULL!
#NULL!	#NULL!	#NULL!	#NULL!	#NULL!	#NULL!	#NULL!	#NULL!	#NULL!	#NULL!
2	2	2	2	2	2	2	2	2	2
1	1	1	2	1	1	1	2	1	#NULL!
2	2	#NULL!	2	1	2	2	2	2	2
1	2	2	2	2	2	1	2	2	2
2	2	2	1	2	1	2	2	2	2
2	2	2	2	2	2	2	2	2	2
1	1	1	1	1	1	1	2	1	1
#NULL!	#NULL!	#NULL!	#NULL!	#NULL!	#NULL!	#NULL!	#NULL!	#NULL!	#NULL!
2	2	2	2	2	2	2	1	2	2
2	2	2	2	2	2	2	2	2	2
2	2	2	2	2	2	2	2	2	2
1	2	2	2	2	2	2	2	2	1
2	2	2	2	2	1	1	2	2	1
#NULL!	#NULL!	#NULL!	#NULL!	#NULL!	#NULL!	#NULL!	#NULL!	#NULL!	#NULL!
#NULL!	#NULL!	#NULL!	2	#NULL!	#NULL!	1	#NULL!	#NULL!	#NULL!
1	2	2	2	2	2	2	2	2	1
2	#NULL!	#NULL!	#NULL!	#NULL!	#NULL!	#NULL!	#NULL!	#NULL!	#NULL!
#NULL!	#NULL!	#NULL!	#NULL!	#NULL!	#NULL!	#NULL!	#NULL!	#NULL!	#NULL!
2	2	2	#NULL!	2	2	2	1	#NULL!	2
2	2	2	2	2	2	2	2	2	2
2	2	2	2	2	2	2	2	2	2
2	2	2	2	2	2	2	1	2	2
1	2	1	1	1	1	1	2	1	1
2	2	2	2	2	2	2	1	2	2
2	1	2	2	1	2	1	2	1	1
#NULL!	#NULL!	#NULL!	#NULL!	#NULL!	#NULL!	#NULL!	#NULL!	#NULL!	#NULL!
#NULL!	#NULL!	#NULL!	#NULL!	#NULL!	#NULL!	#NULL!	#NULL!	#NULL!	#NULL!
#NULL!	#NULL!	#NULL!	#NULL!	#NULL!	#NULL!	#NULL!	#NULL!	#NULL!	#NULL!
2	2	2	2	1	1	2	2	2	2
1	1	1	2	1	1	1	2	2	1
2	1	2	1	2	2	2	2	2	2
2	2	2	2	2	1	1	2	1	2
#NULL!	#NULL!	#NULL!	#NULL!	#NULL!	#NULL!	2	#NULL!	#NULL!	#NULL!
2	2	2	2	2	2	2	2	2	2
1	2	2	1	2	2	1	2	2	2
2	2	2	2	2	2	2	1	2	2
#NULL!	#NULL!	#NULL!	#NULL!	#NULL!	#NULL!	#NULL!	#NULL!	#NULL!	#NULL!
1	2	2	2	1	2	2	2	2	2
2	1	2	1	2	2	1	2	2	1
2	2	2	1	2	1	1	2	2	2

2	2	1	2	1	2	2	2	2	2
2	2	2	2	2	2	2	2	2	2
1	1	1	2	2	2	1	2	1	1
2	1	2	1	2	2	1	2	2	1
2	2	2	1	2	2	1	2	2	2
1	1	2	2	1	2	1	2	1	1
2	2	1	2	1	2	2	2	2	2
1	1	1	1	2	1	1	2	1	2
#NULL!	#NULL!	#NULL!	#NULL!	#NULL!	#NULL!	#NULL!	#NULL!	#NULL!	#NULL!
1	1	1	1	1	1	2	2	2	1
2	2	2	2	1	2	2	2	2	2
2	2	2	2	2	2	2	2	2	2
2	1	2	1	2	2	1	2	1	2
1	2	2	2	2	1	1	2	2	2
#NULL!	#NULL!	#NULL!	#NULL!	#NULL!	#NULL!	#NULL!	2	#NULL!	#NULL!
2	1	2	2	2	2	2	2	1	1
2	2	2	1	1	1	1	2	1	1
2	2	2	2	1	1	1	2	1	2
2	1	2	2	2	2	1	2	2	2
2	2	2	2	2	2	2	2	2	2
2	1	2	1	1	1	2	2	1	2
1	2	2	2	2	2	2	2	2	2
1	1	2	1	1	1	1	1	2	2
2	2	2	2	2	2	2	2	2	2
2	2	2	2	2	1	#NULL!	#NULL!	#NULL!	#NULL!
2	2	2	1	2	2	1	2	2	2
2	1	1	2	2	2	2	2	1	1
2	2	2	1	2	2	1	2	2	2
2	2	2	2	2	2	2	2	2	2
2	2	1	2	2	2	2	2	2	2
#NULL!	1	1	2	1	1	1	2	1	2
2	2	2	2	2	2	2	2	2	2
2	2	2	2	2	2	2	2	2	2
2	2	2	2	2	2	1	2	1	1
2	1	1	2	2	2	1	2	2	2
2	2	2	2	2	2	1	2	1	2
2	1	1	1	1	1	1	2	2	2
2	2	1	1	1	1	1	2	2	2
2	1	1	1	2	2	1	2	1	1
1	1	2	2	2	2	2	2	2	2
2	2	2	2	2	2	2	1	2	2
1	1	1	2	2	2	1	2	1	1
2	2	2	2	1	1	1	2	2	2
1	1	1	1	1	1	1	2	2	2
1	1	1	2	2	2	2	2	1	1
2	2	1	1	1	1	2	2	2	2
1	1	1	2	1	1	2	1	2	1

2	2	2	2	2	1	2	1	2	1
2	2	1	1	2	2	2	2	2	2
1	2	1	1	1	2	2	2	1	2
2	2	2	2	2	1	2	2	1	2
1	1	2	1	1	1	1	2	2	2
1	1	2	1	1	1	1	2	2	2
2	2	2	2	1	1	1	2	2	1
1	1	2	2	2	2	1	2	2	1

Asymptom	VagRash2	VagSwell2
2	2	2
2	2	2
2	1	1
2	1	1
2	2	2
2	1	1
#NULL!	#NULL!	#NULL!
2	2	2
2	2	2
#NULL!	#NULL!	2
2	2	2
2	2	2
2	2	2
2	2	1
2	2	2
1	2	2
2	2	1
1	2	1
1	2	2
1	2	1
2	2	2
2	2	2
2	2	2
2	2	2
2	2	2
2	2	2
2	2	2
2	1	1
2	2	2
2	2	2
2	2	2
1	1	1
2	2	1
1	1	1
2	1	1
2	1	1
2	1	1
2	1	1
2	2	2
2	2	2
2	2	2
2	2	2
1	2	1
1	2	1
2	2	2
1	2	1

1	1	1
2	2	1
1	1	1
2	1	1
2	1	1
1	1	1
1	1	1
1	1	1
#NULL!	1	1
2	1	1
2	#NULL!	2
2	2	2
1	2	1
2	2	1
2	1	2
1	2	2
2	1	1
2	1	1
1	2	1
#NULL!	#NULL!	1
1	1	1
1	1	1
1	1	1
2	2	1
#NULL!	#NULL!	1
2	1	1
#NULL!	#NULL!	#NULL!
1	1	1
#NULL!	#NULL!	#NULL!
1	1	1
2	1	1
2	1	1
2	1	1
2	1	1
1	1	1
#NULL!	#NULL!	#NULL!
#NULL!	#NULL!	#NULL!
2	1	1
1	2	1
1	1	1
#NULL!	1	1
2	2	1
2	1	1
2	1	1
2	1	1
#NULL!	#NULL!	#NULL!
#NULL!	#NULL!	#NULL!

2	1	1
2	2	1
1	1	1
1	2	1
#NULL!	#NULL!	#NULL!
2	2	1
1	2	2
2	1	2
2	2	2
#NULL!	#NULL!	#NULL!
1	2	1
2	2	2
1	1	1
2	1	1
2	2	2
#NULL!	#NULL!	#NULL!
2	2	1
2	1	1
2	2	2
2	1	2
2	2	2
2	1	1
2	2	2
#NULL!	#NULL!	#NULL!
2	2	2
2	2	2
1	2	1
1	2	1
2	2	2
2	1	1
2	2	2
2	2	2
2	1	1
1	2	2
2	1	1
2	1	1
2	1	1
2	2	2
2	#NULL!	1
1	1	2
2	2	2
2	2	2
2	2	2
1	2	2
2	2	2
2	2	2
2	2	2

#NULL!	#NULL!	#NULL!
2	2	2
2	2	2
2	2	2
2	2	2
2	1	1
1	2	2
2	2	2
2	2	2
2	2	2
#NULL!	#NULL!	#NULL!
1	2	2
2	2	2
2	2	1
2	2	1
2	2	1
2	#NULL!	#NULL!
2	2	2
#NULL!	#NULL!	#NULL!
2	1	1
2	1	1
2	1	1
2	1	1
2	1	1
2	1	1
2	1	1
2	1	1
2	1	1
2	1	1
2	1	1
2	2	1
2	2	2
2	1	1
2	1	1
2	1	1
2	1	1
2	1	1
2	1	1
2	1	1
2	1	1
2	1	1
2	1	1
2	1	1
2	1	1
2	1	1
2	1	1
2	1	1

2	1	1
2	1	1
2	1	1
2	1	1
2	1	1
2	1	1
2	1	1
2	1	1
1	1	1
#NULL!	#NULL!	#NULL!
#NULL!	#NULL!	#NULL!
#NULL!	#NULL!	#NULL!
2	1	1
2	1	1
2	1	1
2	2	1
2	2	2
2	2	2
2	1	1
2	2	1
1	1	1
2	2	2
2	1	2
2	2	1
2	2	2
2	2	2
2	2	1
2	2	2
2	2	2
2	1	1
2	2	2
2	2	2
2	1	1
2	2	2
2	2	1
2	1	2
2	2	2
2	2	2
2	2	1
1	2	2
2	2	2
2	2	1
#NULL!	#NULL!	#NULL!
#NULL!	#NULL!	#NULL!
2	2	2
2	1	1
2	#NULL!	#NULL!

2	2	2
2	2	2
2	2	1
2	2	1
2	2	2
2	2	2
2	1	1
2	2	2
2	2	2
2	2	2
2	2	2
2	2	2
2	2	2
1	2	2
2	2	2
2	2	2
1	2	1
2	2	1
2	#NULL!	#NULL!
2	2	2
2	2	2
2	2	2
1	2	2
2	2	2
#NULL!	2	#NULL!
2	2	2
2	2	2
2	2	1
2	2	2
2	2	2
#NULL!	#NULL!	#NULL!
2	2	1
1	1	2
2	2	2
#NULL!	#NULL!	#NULL!
2	2	2
2	2	2
1	2	2
2	1	#NULL!
2	1	1
1	2	1
1	2	2
#NULL!	#NULL!	#NULL!
2	2	2
2	1	1
2	2	1
2	2	2

#NULL!	#NULL!	#NULL!
#NULL!	#NULL!	#NULL!
#NULL!	#NULL!	#NULL!
2	1	1
2	1	2
2	2	1
2	1	1
2	1	1
#NULL!	#NULL!	#NULL!
2	2	2
2	2	2
#NULL!	#NULL!	#NULL!
#NULL!	#NULL!	#NULL!
2	1	2
1	1	1
#NULL!	#NULL!	#NULL!
#NULL!	#NULL!	#NULL!
2	1	1
2	1	1
#NULL!	#NULL!	#NULL!
#NULL!	#NULL!	#NULL!
1	2	2
1	1	1
2	2	2
2	2	2
1	1	1
1	1	1
2	1	1
1	1	1
1	2	2
2	2	2
2	2	2
#NULL!	#NULL!	#NULL!
#NULL!	#NULL!	#NULL!
2	1	1
2	2	2
1	2	2
2	2	2
1	2	2
2	2	2
1	2	2
1	1	1
2	2	2
2	2	2
1	2	2
2	2	2
2	2	1

#NULL!	#NULL!	#NULL!
#NULL!	#NULL!	#NULL!
#NULL!	#NULL!	1
2	1	1
#NULL!	#NULL!	#NULL!
#NULL!	#NULL!	#NULL!
2	1	1
#NULL!	#NULL!	2
2	1	1
1	1	1
2	2	2
2	2	2
2	1	1
2	2	2
2	2	2
2	2	2
2	2	2
2	2	2
2	1	1
2	2	2
2	2	2
2	2	2
2	2	2
2	1	1
2	2	2
2	2	2
2	2	2
2	2	2
2	1	1
2	1	1
2	2	1
2	2	1
1	2	2
2	2	2
2	2	2
2	1	#NULL!
2	2	2
2	2	2
2	2	2
2	2	2
2	2	2
2	2	2
2	1	1
2	2	2
2	2	2
2	1	1
2	2	2

1	1	1
2	2	2
2	2	2
2	2	2
2	2	2
1	2	1
#NULL!	#NULL!	#NULL!
2	2	2
2	2	2
1	1	1
2	2	2
1	1	1
2	1	1
2	1	1
2	2	2
2	1	1
1	2	2
2	2	2
2	2	2
2	2	2
1	1	1
2	1	1
#NULL!	#NULL!	#NULL!
2	2	2
1	1	2
#NULL!	#NULL!	#NULL!
2	1	1
#NULL!	#NULL!	#NULL!
2	2	2
2	1	1
#NULL!	#NULL!	#NULL!
#NULL!	#NULL!	#NULL!
2	2	2
2	1	1
#NULL!	#NULL!	#NULL!
2	1	2
1	2	2
2	2	2
#NULL!	#NULL!	#NULL!
2	1	2
2	2	2
2	2	2
1	2	2
2	2	2
2	2	2
2	1	1
1	1	2

#NULL!	#NULL!	#NULL!
2	1	1
2	1	1
2	1	1
2	1	2
2	1	1
2	1	1
2	1	1
2	1	1
2	1	1
2	1	1
2	1	1
2	1	1
2	1	1
2	1	1
2	1	1
2	1	1
2	1	1
2	1	1
2	1	1
1	1	1
2	1	1
1	2	1
2	1	1
2	1	1
2	1	1
2	1	1
2	1	1
1	1	1
2	1	1
2	1	1
2	1	1
2	1	1
2	1	1
2	1	1
2	1	1
2	1	1
2	1	1
2	1	1
2	1	1
2	1	1
2	2	2
1	1	1
2	2	2
#NULL!	#NULL!	#NULL!
2	2	2
#NULL!	#NULL!	#NULL!

2	2	2
2	1	1
2	1	1
2	2	1
2	2	2
2	1	1
2	2	2
2	2	2
2	1	1
2	2	2
2	1	1
#NULL!	#NULL!	#NULL!
1	1	1
2	2	2
#NULL!	#NULL!	#NULL!
#NULL!	#NULL!	#NULL!
#NULL!	#NULL!	#NULL!
#NULL!	#NULL!	#NULL!
#NULL!	#NULL!	1
2	1	1
2	2	1
2	2	1
2	2	2
#NULL!	1	#NULL!
#NULL!	#NULL!	#NULL!
#NULL!	#NULL!	#NULL!
#NULL!	#NULL!	#NULL!
#NULL!	#NULL!	#NULL!
1	1	1
#NULL!	#NULL!	#NULL!
#NULL!	#NULL!	#NULL!
2	2	2
2	2	2
2	1	1
2	1	1
2	2	2
2	2	1
2	2	1
2	2	2
1	2	2
#NULL!	#NULL!	#NULL!
2	1	1
2	2	2
2	1	1
2	2	2
2	2	2
2	2	1

2	2	2
1	2	2
2	2	2
2	1	1
1	1	1
2	2	2
1	2	2
2	2	2
2	2	2
2	2	2
2	2	2
2	2	1
2	2	2
2	2	2
1	2	2
2	2	2
#NULL!	1	1
2	2	2
2	2	2
1	1	1
2	2	2
2	2	2
2	2	1
1	1	1
1	2	2
2	2	2
1	1	2
2	2	1
2	2	2
2	2	2
2	2	2
2	2	1
2	2	2
2	2	2
2	2	2
#NULL!	#NULL!	#NULL!
2	2	2
2	1	1
2	2	2
1	1	1
#NULL!	#NULL!	#NULL!
2	2	2
1	1	1
2	1	1
1	1	1
2	2	2
#NULL!	2	#NULL!

#NULL!	2	#NULL!
#NULL!	#NULL!	#NULL!
#NULL!	#NULL!	#NULL!
#NULL!	#NULL!	#NULL!
#NULL!	#NULL!	#NULL!
#NULL!	#NULL!	#NULL!
2	#NULL!	#NULL!
#NULL!	#NULL!	#NULL!
#NULL!	#NULL!	#NULL!
#NULL!	#NULL!	#NULL!
#NULL!	#NULL!	#NULL!
#NULL!	#NULL!	#NULL!
#NULL!	#NULL!	#NULL!
#NULL!	#NULL!	#NULL!
#NULL!	#NULL!	#NULL!
#NULL!	#NULL!	#NULL!
2	#NULL!	#NULL!
2	1	1
2	2	2
#NULL!	#NULL!	#NULL!
2	1	1
2	2	2
2	1	2
2	1	1
1	2	2
2	2	1
2	2	2
2	2	1
2	2	2
2	2	2
2	2	2
2	2	2
2	2	2
1	2	1
2	2	1
1	2	2
2	2	2
#NULL!	#NULL!	#NULL!
2	1	1
#NULL!	#NULL!	#NULL!
2	2	2
2	2	2
#NULL!	#NULL!	#NULL!
2	1	1
2	2	2
2	2	2
1	2	2

2	2	2
#NULL!	#NULL!	#NULL!
1	2	1
#NULL!	#NULL!	#NULL!
2	2	2
2	2	2
#NULL!	#NULL!	#NULL!
2	2	2
2	1	1
2	1	1
2	1	1
2	1	1
2	2	2
#NULL!	#NULL!	#NULL!
#NULL!	#NULL!	#NULL!
#NULL!	#NULL!	#NULL!
1	2	1
#NULL!	#NULL!	#NULL!
#NULL!	1	1
2	2	2
2	1	1
2	2	2
1	2	2
#NULL!	#NULL!	#NULL!
2	2	2
1	1	2
1	2	2
2	2	2
2	1	1
2	1	1
#NULL!	#NULL!	#NULL!
#NULL!	#NULL!	#NULL!
2	2	2
2	1	1
2	1	#NULL!
2	1	1
2	1	1
#NULL!	#NULL!	#NULL!
#NULL!	#NULL!	#NULL!
#NULL!	#NULL!	#NULL!
#NULL!	#NULL!	#NULL!
2	1	2
2	2	2
#NULL!	#NULL!	#NULL!
2	2	2
2	1	1
1	2	2

1	2	2
1	1	1
2	2	2
2	2	2
2	2	2
2	2	1
#NULL!	#NULL!	#NULL!
2	2	1
1	2	2
2	2	2
1	2	2
1	2	2
1	1	1
1	1	1
1	1	1
1	1	1
1	1	1
1	1	1
1	1	1
1	2	2
2	2	2
2	2	2
2	2	2
2	2	2
1	2	2
1	1	1
#NULL!	#NULL!	#NULL!
2	1	1
#NULL!	#NULL!	#NULL!
1	2	2
#NULL!	2	1
2	2	2
2	1	1
#NULL!	#NULL!	#NULL!
1	2	2
2	2	2
2	#NULL!	2
2	1	2
2	1	2
2	1	2
2	1	1
2	2	2
1	#NULL!	1
1	1	1
2	2	2
2	1	1
2	2	1

1	1	1
#NULL!	#NULL!	#NULL!
#NULL!	#NULL!	#NULL!
2	2	1
2	2	2
2	2	2
2	1	1
2	2	2
2	2	2
2	2	1
#NULL!	#NULL!	#NULL!
1	1	1
#NULL!	#NULL!	#NULL!
1	1	1
1	1	1
#NULL!	#NULL!	#NULL!
2	2	2
#NULL!	#NULL!	#NULL!
#NULL!	#NULL!	#NULL!
2	2	1
2	1	1
2	2	2
#NULL!	#NULL!	#NULL!
2	2	2
1	2	2
2	2	2
2	2	2
1	1	1
2	1	1
2	2	2
2	2	2
2	2	2
2	1	1
2	2	2
2	1	2
2	2	2
2	2	2
#NULL!	#NULL!	#NULL!
#NULL!	#NULL!	#NULL!
2	2	1
2	2	2
2	2	2
2	#NULL!	2
2	2	2
2	2	2
2	2	2
#NULL!	#NULL!	#NULL!

#NULL!	#NULL!	#NULL!
1	2	2
1	1	2
1	1	2
#NULL!	#NULL!	#NULL!
2	2	2
#NULL!	#NULL!	#NULL!
2	1	1
2	2	2
#NULL!	#NULL!	#NULL!
2	2	2
2	2	1
2	2	1
2	2	2
2	1	1
2	2	2
2	2	2
2	2	2
1	2	2
1	2	1
#NULL!	#NULL!	#NULL!
2	2	2
2	2	2
2	2	2
2	1	1
2	2	2
2	#NULL!	#NULL!
#NULL!	#NULL!	#NULL!
2	2	2
1	2	2
#NULL!	#NULL!	#NULL!
2	2	2
1	1	1
2	2	2
2	2	2
2	1	1
2	2	2
2	2	2
2	2	2
2	2	2
2	2	2
2	2	2
2	1	1
2	2	2
2	2	2
1	2	2
2	2	2

2	2	2
2	2	2
2	2	2
2	2	2
2	1	2
2	1	1
2	2	2
2	1	2
1	1	1
2	2	2
2	2	1
#NULL!	#NULL!	#NULL!
#NULL!	#NULL!	#NULL!
#NULL!	#NULL!	#NULL!
1	2	2
1	2	2
1	2	2
2	1	1
#NULL!	#NULL!	#NULL!
2	2	2
#NULL!	#NULL!	#NULL!
2	2	2
2	2	2
2	1	1
1	2	2
1	1	1
2	2	2
2	1	1
2	2	2
2	2	2
2	1	1
2	1	1
1	1	2
1	2	2
2	1	1
1	2	2
#NULL!	#NULL!	1
2	1	1
2	2	2
2	2	2
2	1	2
#NULL!	#NULL!	1
2	2	1
2	2	2
2	2	2
1	1	1
2	2	2

1	2	1
1	2	2
2	2	2
#NULL!	#NULL!	#NULL!
2	#NULL!	#NULL!
2	2	2
2	2	2
1	2	2
2	1	2
2	2	2
#NULL!	#NULL!	1
2	2	2
2	2	2
2	2	2
2	2	2
1	2	2
2	2	2
#NULL!	#NULL!	1
#NULL!	#NULL!	#NULL!
2	1	1
2	2	2
2	2	2
#NULL!	#NULL!	#NULL!
#NULL!	#NULL!	#NULL!
2	2	2
1	1	1
2	1	1
2	2	1
1	2	2
1	2	1
2	1	1
2	1	2
2	2	1
1	2	2
2	2	2
1	2	2
2	#NULL!	#NULL!
1	2	2
2	#NULL!	#NULL!
1	2	2
1	1	1
2	1	1
1	2	2
2	2	2
2	2	2
2	1	2
2	1	1

2	2	2
2	2	2
2	2	1
2	1	1
2	2	2
2	2	2
2	2	2
2	2	2
2	2	2
2	2	2
#NULL!	#NULL!	#NULL!
2	2	2
1	1	1
2	2	1
2	1	2
2	2	2
2	1	1
2	1	1
1	2	2
#NULL!	#NULL!	1
2	1	1
2	2	2
2	2	2
1	1	1
1	2	2
2	2	2
2	2	2
2	1	2
1	2	1
2	2	1
2	2	2
#NULL!	#NULL!	#NULL!
2	2	2
#NULL!	#NULL!	#NULL!
2	2	2
2	2	1
2	2	2
2	2	2
2	2	2
2	1	2
2	2	2
2	2	2
2	2	1
#NULL!	#NULL!	#NULL!
1	2	2
2	2	2
2	1	1

#NULL!	#NULL!	#NULL!
2	2	2
2	2	2
1	2	2
1	2	2
1	2	2
#NULL!	#NULL!	#NULL!
2	2	2
2	2	2
#NULL!	#NULL!	#NULL!
#NULL!	#NULL!	#NULL!
#NULL!	#NULL!	#NULL!
#NULL!	#NULL!	#NULL!
#NULL!	#NULL!	#NULL!
#NULL!	#NULL!	#NULL!
2	2	2
2	2	2
2	1	1
2	2	2
2	2	2
2	2	2
2	2	2
#NULL!	2	2
2	2	2
2	2	2
2	2	2
#NULL!	2	#NULL!
2	1	1
2	2	2
2	2	2
2	2	2
#NULL!	#NULL!	#NULL!
2	2	1
2	2	2
2	2	2
2	2	2
2	2	2
#NULL!	#NULL!	#NULL!
2	2	2
2	2	2
2	2	2
2	2	2
2	2	2
2	1	1
2	2	2
2	2	2
#NULL!	#NULL!	#NULL!

2	2	2
2	2	2
#NULL!	#NULL!	#NULL!
2	#NULL!	#NULL!
2	2	2
#NULL!	#NULL!	#NULL!
2	1	2
2	2	2
1	1	2
2	2	2
#NULL!	#NULL!	#NULL!
2	2	2
2	2	2
2	2	2
2	1	1
1	2	2
2	2	2
2	2	2
2	2	2
1	1	1
1	1	1
2	2	2
1	1	1
2	2	2
2	2	2
2	1	1
2	2	2
2	2	2
2	2	2
#NULL!	#NULL!	#NULL!
2	#NULL!	#NULL!
2	2	2
#NULL!	#NULL!	#NULL!
#NULL!	#NULL!	#NULL!
1	2	2
#NULL!	#NULL!	#NULL!
#NULL!	#NULL!	#NULL!
2	2	2
2	1	1
1	2	2
2	1	1
2	2	1
#NULL!	#NULL!	#NULL!
2	2	2
1	2	1
#NULL!	#NULL!	#NULL!
1	1	2

2	2	1
#NULL!	#NULL!	#NULL!
#NULL!	#NULL!	#NULL!
2	2	2
2	1	1
2	2	1
2	2	1
1	1	1
2	2	2
2	2	2
#NULL!	#NULL!	#NULL!
#NULL!	#NULL!	#NULL!
#NULL!	#NULL!	#NULL!
2	2	2
2	2	2
#NULL!	#NULL!	#NULL!
#NULL!	#NULL!	#NULL!
#NULL!	#NULL!	#NULL!
2	1	2
2	2	2
#NULL!	#NULL!	#NULL!
1	1	1
1	2	2
#NULL!	#NULL!	#NULL!
2	2	2
2	2	2
#NULL!	#NULL!	#NULL!
#NULL!	#NULL!	#NULL!
2	2	2
2	1	1
2	1	1
#NULL!	#NULL!	#NULL!
#NULL!	#NULL!	#NULL!
1	2	2
2	2	2
2	2	2
2	2	2
2	2	2
2	2	1
1	2	2
2	1	2
#NULL!	#NULL!	#NULL!
#NULL!	#NULL!	#NULL!
2	#NULL!	#NULL!
#NULL!	#NULL!	#NULL!
#NULL!	#NULL!	#NULL!
#NULL!	#NULL!	#NULL!

2	2	2
2	2	2
1	1	1
2	2	2
#NULL!	#NULL!	#NULL!
#NULL!	#NULL!	#NULL!
#NULL!	#NULL!	#NULL!
#NULL!	#NULL!	#NULL!
#NULL!	#NULL!	#NULL!
#NULL!	#NULL!	#NULL!
#NULL!	#NULL!	#NULL!
#NULL!	#NULL!	#NULL!
#NULL!	#NULL!	#NULL!
2	#NULL!	1
#NULL!	#NULL!	#NULL!
2	2	1
2	2	2
#NULL!	#NULL!	#NULL!
#NULL!	#NULL!	#NULL!
#NULL!	#NULL!	#NULL!
2	2	#NULL!
#NULL!	#NULL!	#NULL!
2	1	1
#NULL!	#NULL!	#NULL!
2	1	1
2	2	2
2	2	2
2	2	2
2	1	1
2	1	1
1	2	2
2	1	1
2	1	1
#NULL!	#NULL!	#NULL!
2	2	2
2	1	1
2	1	1
#NULL!	#NULL!	#NULL!
2	2	2
1	1	2
1	2	2
#NULL!	#NULL!	#NULL!
#NULL!	#NULL!	#NULL!
#NULL!	#NULL!	#NULL!
#NULL!	#NULL!	#NULL!
2	2	2
#NULL!	#NULL!	#NULL!

#NULL!	#NULL!	#NULL!
#NULL!	#NULL!	#NULL!
#NULL!	#NULL!	#NULL!
#NULL!	#NULL!	#NULL!
#NULL!	#NULL!	#NULL!
#NULL!	#NULL!	#NULL!
#NULL!	#NULL!	#NULL!
#NULL!	#NULL!	#NULL!
#NULL!	#NULL!	#NULL!
#NULL!	#NULL!	#NULL!
#NULL!	#NULL!	#NULL!
#NULL!	#NULL!	#NULL!
2	2	2
2	2	2
2	2	2
1	2	1
2	2	2
2	1	1
2	1	1
#NULL!	#NULL!	#NULL!
2	1	1
2	2	2
2	2	2
2	2	2
2	1	1
1	1	1
#NULL!	#NULL!	#NULL!
2	1	1
#NULL!	#NULL!	#NULL!
2	#NULL!	2
#NULL!	#NULL!	#NULL!
2	2	1
2	1	1
2	1	1
2	2	2
2	2	2
2	2	2
2	2	1
2	2	2
2	1	1
2	2	2
2	1	1
1	1	1
2	2	2
2	1	1
2	1	1
2	2	2

#NULL!	#NULL!	#NULL!
2	1	1
#NULL!	#NULL!	#NULL!
#NULL!	#NULL!	#NULL!
#NULL!	#NULL!	#NULL!
#NULL!	#NULL!	#NULL!
#NULL!	2	#NULL!
2	1	1
#NULL!	#NULL!	#NULL!
2	2	2
#NULL!	#NULL!	#NULL!
#NULL!	#NULL!	#NULL!
2	1	1
2	2	2
2	1	1
#NULL!	#NULL!	#NULL!
2	2	2
1	2	2
#NULL!	#NULL!	#NULL!
2	2	2
2	2	2
1	2	2
2	2	2
2	2	2
2	1	1
2	2	2
2	2	2
2	2	2
2	2	2
2	1	1
#NULL!	#NULL!	#NULL!
1	2	2
#NULL!	#NULL!	#NULL!
2	2	2
2	#NULL!	2
2	1	1
2	1	1
2	1	1
2	1	1
1	2	2
2	2	2
2	2	2
#NULL!	#NULL!	#NULL!
#NULL!	#NULL!	#NULL!
#NULL!	#NULL!	#NULL!
#NULL!	#NULL!	#NULL!
#NULL!	#NULL!	#NULL!

#NULL!	#NULL!	#NULL!
2	2	2
#NULL!	#NULL!	#NULL!
#NULL!	1	2
#NULL!	#NULL!	#NULL!
1	1	2
2	2	2
#NULL!	#NULL!	#NULL!
#NULL!	#NULL!	#NULL!
#NULL!	#NULL!	#NULL!
#NULL!	#NULL!	#NULL!
#NULL!	#NULL!	#NULL!
2	2	2
#NULL!	#NULL!	#NULL!
#NULL!	#NULL!	#NULL!
1	2	2
2	2	2
1	2	2
2	2	2
1	2	2
2	2	1
1	2	1
2	1	1
2	2	1
2	2	2
2	2	1
#NULL!	#NULL!	#NULL!
2	2	2
#NULL!	#NULL!	#NULL!
2	2	2
2	2	2
2	2	2
#NULL!	#NULL!	#NULL!
1	2	2
1	1	1
2	1	1
2	2	2
1	1	2
#NULL!	#NULL!	#NULL!
1	1	2
#NULL!	#NULL!	2
#NULL!	#NULL!	#NULL!
#NULL!	#NULL!	#NULL!
#NULL!	#NULL!	#NULL!
1	2	2
1	1	1
2	1	2

1	2	2
#NULL!	#NULL!	1
2	2	2
2	2	1
1	1	1
2	1	2
2	2	2
1	1	2
2	2	2
2	2	2
2	2	2
1	2	2
2	2	2
2	1	2
2	2	2
2	2	2
2	2	2
2	2	2
2	2	2
2	2	2
2	2	2
1	2	2
#NULL!	#NULL!	#NULL!
#NULL!	#NULL!	#NULL!
2	2	2
2	1	1
2	1	2
1	2	1
2	1	1
#NULL!	#NULL!	#NULL!
2	2	2
2	1	1
1	2	2
2	1	1
1	1	1
2	2	2
1	2	2
#NULL!	#NULL!	#NULL!
1	2	2
1	2	2
#NULL!	#NULL!	#NULL!
#NULL!	#NULL!	#NULL!
2	2	2
#NULL!	#NULL!	#NULL!
2	2	2
1	1	1
#NULL!	#NULL!	#NULL!

2	2	2
#NULL!	#NULL!	#NULL!
2	2	2
#NULL!	#NULL!	#NULL!
#NULL!	#NULL!	#NULL!
2	2	2
2	2	2
2	2	2
2	1	1
2	2	2
#NULL!	#NULL!	#NULL!
2	2	2
#NULL!	#NULL!	#NULL!
2	1	1
2	2	2
2	2	2
#NULL!	#NULL!	#NULL!
#NULL!	#NULL!	#NULL!
2	2	2
1	2	2
2	1	1
2	2	2
#NULL!	#NULL!	#NULL!
1	2	2
2	2	2
2	2	2
2	#NULL!	#NULL!
2	1	1
2	2	2
#NULL!	#NULL!	#NULL!
1	2	2
2	2	2
1	2	2
1	1	2
2	2	2
2	1	2
2	2	2
1	2	2
2	2	2
1	2	2
1	2	1
2	1	1
2	2	2
1	2	2
2	2	2
2	2	2
1	2	2

2	2	2
2	2	2
2	1	1
2	2	2
2	2	2
#NULL!	#NULL!	#NULL!
2	2	2
2	2	1
2	2	2
2	2	2
2	1	1
2	1	2
2	2	2
1	2	2
2	2	1
2	2	1
1	2	2
2	2	2
2	2	2
2	2	2
2	2	2
2	2	2
2	2	2
2	2	2
2	1	1
2	2	2
1	2	2
2	2	2
2	2	2
2	2	2
1	2	2
1	2	2
1	2	2
2	2	2
2	2	1
2	2	2
2	2	2
2	2	1
2	2	2
2	2	2
2	1	1
1	2	2
2	2	2
2	2	2
1	2	2
2	2	1
#NULL!	#NULL!	#NULL!

2	2	1
1	#NULL!	2
2	2	2
2	2	2
2	2	2
2	2	2
2	2	2
2	1	1
2	2	2
2	2	2
2	2	2
#NULL!	#NULL!	#NULL!
#NULL!	#NULL!	#NULL!
2	2	2
2	1	1
2	2	2
#NULL!	1	1
2	2	2
2	1	1
2	2	1
1	2	2
2	1	1
2	2	2
2	2	2
2	2	2
2	2	1
2	2	2
2	2	2
2	1	1
#NULL!	#NULL!	#NULL!
1	2	2
2	2	2
2	2	2
2	2	2
2	2	2
2	2	2
2	2	2
2	2	2
2	2	1
2	2	2
2	2	2
2	2	2
2	1	1
2	2	2
2	2	2
2	2	2
2	2	2

2	2	2
2	2	2
1	1	1
2	2	2
2	2	2
2	2	2
#NULL!	#NULL!	#NULL!
2	1	1
2	2	1
1	1	1
1	1	1
2	2	2
2	2	2
2	1	1
2	1	1
2	2	2
2	2	2
2	2	2
2	1	1
2	2	2
2	1	1
1	2	2
2	2	2
2	2	2
2	2	2
2	2	2
#NULL!	#NULL!	#NULL!
2	2	2
2	2	2
2	2	2
2	2	2
2	2	2
#NULL!	#NULL!	#NULL!
#NULL!	#NULL!	#NULL!
#NULL!	#NULL!	#NULL!
#NULL!	#NULL!	#NULL!
1	2	2
1	1	1
1	1	1
2	2	2
2	1	1
2	2	2
2	1	1
2	2	2
2	2	2
2	2	2
2	2	2

2	2	2
2	2	2
2	2	2
2	1	1
1	1	2
2	2	2
1	1	1
#NULL!	#NULL!	#NULL!
2	2	2
2	2	2
2	2	2
2	2	2
2	2	2
2	2	2
2	1	1
2	2	2
2	2	2
1	1	1
2	2	2
2	2	1
2	2	2
2	2	1
2	1	1
#NULL!	#NULL!	#NULL!
2	2	1
2	1	1
2	1	1
1	2	1
2	2	2
#NULL!	#NULL!	#NULL!
1	2	2
#NULL!	#NULL!	#NULL!
#NULL!	#NULL!	#NULL!
#NULL!	#NULL!	#NULL!
#NULL!	#NULL!	#NULL!
2	2	2
2	1	1
#NULL!	#NULL!	#NULL!
2	2	2
2	2	2
2	1	1
2	2	2
2	2	2
1	2	2
#NULL!	#NULL!	#NULL!
2	1	1
1	2	2

2	1	1
2	1	1
2	1	1
2	2	2
#NULL!	#NULL!	#NULL!
#NULL!	#NULL!	#NULL!
2	2	2
#NULL!	#NULL!	#NULL!
#NULL!	#NULL!	1
#NULL!	#NULL!	#NULL!
2	2	2
2	2	2
2	2	2
1	2	2
#NULL!	#NULL!	#NULL!
2	2	2
2	1	1
2	2	2
#NULL!	#NULL!	#NULL!
2	1	2
2	2	1
2	2	1
2	2	1
2	2	2
#NULL!	#NULL!	#NULL!
2	2	2
2	2	1
2	2	2
1	2	2
1	2	2
2	2	2
2	1	1
2	1	1
2	2	2
#NULL!	#NULL!	2
2	2	1
#NULL!	#NULL!	#NULL!
2	2	2
#NULL!	#NULL!	#NULL!
2	2	1
#NULL!	#NULL!	#NULL!
2	2	1
2	2	2
2	1	2
2	1	2
2	2	2
1	2	2

2	2	2
#NULL!	#NULL!	#NULL!
2	2	2
1	2	2
1	1	1
#NULL!	#NULL!	#NULL!
#NULL!	#NULL!	#NULL!
2	2	2
#NULL!	1	1
2	2	2
2	2	2
2	2	2
#NULL!	2	2
2	1	1
#NULL!	#NULL!	#NULL!
1	2	2
2	2	2
2	2	2
2	2	2
1	2	1
#NULL!	#NULL!	#NULL!
#NULL!	#NULL!	#NULL!
2	2	2
#NULL!	#NULL!	#NULL!
#NULL!	#NULL!	#NULL!
2	2	2
2	2	2
2	2	2
1	2	2
2	1	1
1	2	2
2	2	2
#NULL!	#NULL!	#NULL!
#NULL!	#NULL!	#NULL!
#NULL!	#NULL!	#NULL!
2	2	2
1	1	1
2	2	2
1	1	1
#NULL!	#NULL!	#NULL!
2	2	2
2	2	1
1	2	2
#NULL!	#NULL!	#NULL!
2	2	1
1	1	2
1	2	2

2	2	2
2	2	2
#NULL!	2	2
2	2	2
2	1	1
2	2	1
2	2	2
2	1	1
#NULL!	#NULL!	#NULL!
2	1	1
2	2	2
2	2	2
2	1	1
1	1	1
#NULL!	#NULL!	#NULL!
2	#NULL!	1
2	2	1
2	2	2
2	2	2
2	2	2
2	1	1
2	2	2
2	2	1
2	2	2
#NULL!	#NULL!	1
2	2	1
2	2	2
2	2	2
2	2	2
2	2	1
2	2	2
2	2	2
2	2	2
2	2	1
1	2	2
1	2	2
2	1	1
2	2	2
1	1	1
1	2	1
2	2	2
2	2	2
2	1	1
2	2	1
2	2	2
2	1	1
2	1	1

2	2	1
2	1	2
2	1	1
2	2	1
2	1	1
2	1	1
2	2	2
2	1	1
