# Supplementary material for: Outcomes from integrating anti-cervical cancer teachings into the curriculum of high schools in a South-Eastern Nigerian State
Source: BMC Public Health. 2022 Oct 14;22:1914. doi: 10.1186/s12889-022-14231-4 (PMC9562070; doi:10.1186/s12889-022-14231-4)
Supplement: Supplementary file 6 — Additional file 6. [file 12889_2022_14231_MOESM6_ESM.pdf]

Table: Phase 2 Data (1 month Post Intervention for anti-cervical cancer teachings for high schools in SE Nigeria

ID	Gender	Age	Age2	StateofSch	ClassofStu	SchoolGen	HeardofBr	HeardofBS	PainBreast
1	1	16	2	9	2	3	2	2	2
2	1	16	2	1	2	3	2	2	2
3	1	15	2	9	1	3	2	2	2
4	2	15	2	9	2	3	2	2	3
5	2	18	2	9	3	3	9	1	1
6	2	16	2	9	2	3	1	1	2
7	2	16	2	9	2	1	2	2	1
8	2	15	2	9	2	1	1	1	2
9	2	15	2	9	2	1	1	1	2
10	2	15	2	9	2	1	1	1	1
11	2	14	1	9	2	1	1	1	2
12	2	15	2	9	2	1	2	1	1
13	2	14	1	9	2	1	1	2	1
14	2	17	2	9	2	1	1	2	3
15	2	15	2	9	2	1	1	2	2
16	2	16	2	9	2	1	2	1	2
17	2	0	#NULL!	9	2	1	1	1	1
18	2	16	2	9	2	1	1	2	1
19	2	17	2	9	2	1	1	2	1
20	2	15	2	9	2	1	1	1	1
21	2	17	2	9	3	1	1	1	1
22	2	17	2	9	3	1	1	1	1
23	2	17	2	9	3	1	1	1	1
24	2	17	2	9	3	1	2	1	1
25	2	14	1	9	1	1	3	3	2
26	2	14	1	9	1	1	2	2	1
27	2	13	1	9	1	1	1	1	1
28	2	15	2	9	1	1	1	1	1
29	2	14	1	9	1	1	1	1	1
30	2	15	2	9	1	1	1	2	1
31	2	13	1	9	1	1	1	3	3
32	2	15	2	9	1	1	1	1	1
33	2	14	1	9	1	3	1	2	1
34	2	16	2	9	1	1	1	2	1
35	2	13	1	9	1	1	1	2	1
36	2	15	2	9	1	1	1	2	3
37	2	15	2	9	1	1	1	1	1
38	2	15	2	9	1	1	1	2	2
39	2	15	2	9	1	1	1	1	1
40	2	15	2	9	2	1	1	2	9
41	2	15	2	9	3	3	1	1	1
42	2	16	2	9	3	3	1	2	1
43	2	15	2	9	3	3	1	1	9
44	2	17	2	9	3	3	1	1	1
45	2	13	1	9	1	3	1	1	1
46	9	14	1	9	1	3	1	1	3

47	2	14	1	9	1	3	1	1	1
48	2	10	1	9	1	9	1	1	1
49	2	14	1	9	1	1	1	1	1
50	2	13	1	9	1	3	1	1	2
51	2	16	2	9	1	3	1	1	1
52	2	14	1	9	1	3	1	2	1
53	1	16	2	9	1	3	1	2	2
54	2	18	2	9	1	3	2	2	1
55	2	29	3	9	1	3	1	1	1
56	1	15	2	9	1	3	1	1	3
57	2	13	1	9	1	3	1	2	9
58	2	14	1	9	1	3	2	1	1
59	1	16	2	9	2	2	2	2	2
60	1	15	2	9	1	3	1	2	1
61	2	15	2	9	2	3	1	1	1
62	2	14	1	9	2	3	2	1	1
63	1	15	2	9	1	3	1	2	2
64	2	16	2	9	1	3	1	2	3
65	2	14	1	9	1	3	1	2	1
66	1	0	#NULL!	9	1	3	3	3	2
67	2	16	2	9	1	3	3	3	3
68	2	16	2	9	1	3	1	1	1
69	2	16	2	9	2	3	1	1	1
70	1	16	2	9	2	3	1	2	1
71	1	15	2	9	2	3	2	2	1
72	9	0	#NULL!	9	1	3	2	2	2
73	2	15	2	9	1	3	1	2	1
74	1	15	2	9	1	3	1	2	1
75	2	15	2	9	1	3	1	2	1
76	2	15	2	9	1	3	1	1	1
77	2	13	1	9	1	3	1	1	2
78	2	14	1	9	1	3	1	2	3
79	2	15	2	9	1	3	1	2	1
80	1	13	1	9	1	3	1	1	1
81	2	0	#NULL!	9	1	3	1	2	1
82	1	16	2	9	1	3	1	2	1
83	2	14	1	9	1	3	1	1	2
84	2	14	1	9	1	3	1	2	1
85	2	15	2	9	1	3	1	2	1
86	1	15	2	9	1	3	1	1	2
87	2	16	2	9	1	3	1	1	2
88	9	15	2	9	1	3	1	1	1
89	1	16	2	9	1	3	1	1	9
90	2	13	1	9	1	3	1	2	2
91	1	16	2	9	1	3	1	2	1
92	1	18	2	9	1	3	1	1	1
93	2	16	2	9	1	3	1	1	1

94	2	19	2	9	1	3	1	2	2
95	2	15	2	9	1	3	1	3	3
96	1	16	2	9	1	3	1	3	1
97	2	11	1	9	1	3	1	2	2
98	1	19	2	9	1	3	1	1	2
99	2	16	2	9	1	3	1	1	1
100	2	15	2	9	1	3	1	2	2
101	1	19	2	9	1	3	1	1	3
102	2	15	2	9	1	3	1	1	1
103	2	16	2	9	1	3	1	3	1
104	2	14	1	9	1	3	1	2	1
105	2	15	2	9	2	3	1	1	1
106	2	15	2	9	2	3	2	1	1
107	2	16	2	9	1	3	1	1	1
108	2	16	2	9	1	3	2	2	1
109	2	15	2	9	1	3	2	9	1
110	1	17	2	9	2	3	1	3	3
111	2	16	2	9	1	3	1	2	2
112	2	17	2	9	1	3	1	1	1
113	1	14	1	9	1	3	1	1	2
114	2	14	1	9	1	3	1	2	1
115	2	15	2	9	1	3	1	1	1
116	1	15	2	9	2	3	1	1	1
117	9	17	2	9	1	3	3	3	1
118	2	15	2	9	1	3	2	2	3
119	2	16	2	9	1	3	1	2	2
120	1	15	2	9	2	3	1	2	3
121	2	16	2	9	1	3	1	2	1
122	2	15	2	9	1	3	1	1	1
123	1	14	1	9	1	3	1	1	1
124	2	15	2	9	2	3	2	1	1
125	2	16	2	9	2	3	2	2	3
126	2	16	2	9	2	3	2	1	2
127	2	17	2	9	2	1	1	1	1
128	2	16	2	9	2	3	1	1	1
129	2	15	2	9	2	3	1	1	2
130	1	16	2	9	2	3	1	1	1
131	2	15	2	9	1	3	1	2	1
132	2	14	1	9	1	3	1	2	1
133	2	13	1	9	1	3	3	3	3
134	2	16	2	9	2	3	2	1	1
135	9	0	#NULL!	9	9	9	9	9	9
136	1	15	2	9	2	3	2	1	1
137	1	15	2	9	2	3	2	1	2
138	9	14	1	9	1	3	1	1	1
139	2	14	1	9	1	3	1	1	1
140	2	16	2	9	1	3	1	2	1

141	2	19	2	9	1	3	1	2	1
142	1	13	1	9	1	3	1	2	3
143	1	15	2	9	2	3	2	1	3
144	1	15	2	9	1	3	1	1	1
145	1	17	2	9	1	3	1	2	1
146	1	17	2	9	1	3	1	2	3
147	2	16	2	9	1	9	1	2	1
148	2	19	2	9	1	3	2	2	1
149	1	15	2	9	1	3	1	2	9
150	1	16	2	9	1	3	1	2	1
151	1	16	2	9	1	3	1	9	1
152	2	14	1	9	1	3	1	1	1
153	2	0	#NULL!	9	1	3	1	2	2
154	1	20	3	9	1	3	2	2	9
155	1	16	2	9	2	3	2	1	1
156	1	16	2	9	2	3	2	1	2
157	1	16	2	9	2	3	2	1	1
158	1	16	2	9	2	3	2	1	1
159	1	17	2	9	1	3	1	2	1
160	2	15	2	9	1	3	1	2	1
161	2	16	2	9	1	3	2	2	1
162	1	17	2	9	3	2	1	1	1
163	1	19	2	9	3	2	2	2	3
164	1	19	2	9	3	2	3	3	1
165	1	18	2	9	3	2	1	3	1
166	1	15	2	9	1	3	1	2	3
167	1	19	2	9	3	2	1	2	1
168	1	15	2	9	1	3	1	2	1
169	1	16	2	9	1	3	1	2	1
170	1	16	2	9	2	3	1	2	1
171	9	0	#NULL!	9	9	9	9	9	9
172	1	14	1	9	1	3	2	2	2
173	1	18	2	9	1	3	1	3	1
174	1	19	2	9	2	9	9	9	1
175	1	13	1	9	1	3	1	1	1
176	2	18	2	9	1	3	1	2	1
177	1	40	3	9	1	2	1	1	1
178	2	14	1	9	1	3	1	1	1
179	2	15	2	9	2	3	1	1	1
180	2	17	2	1	3	1	2	1	1
181	1	17	2	1	1	1	1	1	2
182	2	14	1	9	1	3	1	1	2
183	1	19	2	9	1	3	1	2	1
184	9	0	#NULL!	9	9	9	9	9	9
185	9	0	#NULL!	9	9	9	9	9	9
186	2	15	2	9	2	3	2	1	1
187	1	17	2	9	1	2	1	3	2

188	1	17	2	9	1	2	3	2	9
189	1	0	#NULL!	9	3	2	1	1	9
190	1	18	2	9	3	2	1	1	1
191	1	17	2	9	3	2	1	3	9
192	1	17	2	9	3	2	1	2	2
193	1	18	2	9	3	2	1	3	1
194	1	17	2	9	3	2	1	1	2
195	1	18	2	9	3	2	2	2	1
196	9	0	#NULL!	9	3	2	1	3	1
197	1	18	2	9	1	2	2	2	9
198	1	14	1	9	1	2	1	2	1
199	9	0	#NULL!	9	1	1	1	2	1
200	1	17	2	9	1	2	1	2	1
201	1	18	2	9	9	9	9	9	2
202	1	20	3	9	2	2	3	1	9
203	1	16	2	9	1	2	1	1	1
204	9	0	#NULL!	9	2	9	1	9	3
205	1	18	2	9	1	2	1	1	2
206	1	18	2	9	3	2	3	2	1
207	1	18	2	9	3	3	1	2	2
208	1	17	2	9	3	2	1	2	2
209	1	17	2	9	3	2	1	2	1
210	1	17	2	9	3	2	1	2	3
211	9	19	2	9	3	2	1	2	1
212	1	19	2	9	3	2	1	2	2
213	1	17	2	9	1	2	1	2	2
214	9	9	1	9	1	2	1	1	1
215	1	17	2	9	1	2	1	1	1
216	9	0	#NULL!	9	9	9	9	9	9
217	1	16	2	9	1	2	1	1	1
218	1	18	2	9	1	2	1	2	1
219	1	18	2	9	3	2	1	1	1
220	1	18	2	9	3	2	1	2	3
221	1	19	2	9	3	2	2	2	1
222	1	18	2	9	3	3	2	2	1
223	1	14	1	9	1	2	2	1	1
224	1	14	1	9	1	2	1	2	1
225	1	15	2	9	1	2	1	3	1
226	1	14	1	9	1	2	1	2	1
227	1	19	2	9	1	1	1	2	1
228	1	17	2	9	1	2	1	1	1
229	1	13	1	9	1	2	1	2	1
230	1	15	2	9	1	2	1	2	3
231	1	15	2	9	1	2	2	2	1
232	1	17	2	9	1	2	1	3	2
233	9	0	#NULL!	9	1	2	2	1	1
234	1	0	#NULL!	9	1	2	2	2	3

235	9	0	#NULL!	9	3	2	1	1	1
236	1	19	2	9	3	2	1	1	1
237	1	22	3	9	3	2	1	1	1
238	1	17	2	9	3	2	1	1	1
239	1	14	1	9	3	2	1	3	1
240	1	17	2	9	3	2	1	2	1
241	1	17	2	9	3	2	9	1	1
242	1	40	3	9	1	2	2	1	1
243	1	16	2	9	1	2	1	2	1
244	1	14	1	9	1	2	1	2	1
245	1	17	2	9	1	2	1	3	1
246	1	15	2	9	1	2	1	3	1
247	1	14	1	9	1	2	1	2	2
248	1	18	2	9	3	2	1	2	2
249	1	16	2	9	2	3	2	2	9
250	9	0	#NULL!	9	9	9	9	9	9
251	1	14	1	9	1	2	2	2	9
252	1	14	1	9	1	2	2	2	9
253	1	16	2	9	2	2	2	2	2
254	9	0	#NULL!	9	9	9	9	9	9
255	9	0	#NULL!	9	9	9	9	9	9
256	1	14	1	9	2	2	2	2	2
257	1	16	2	9	2	2	2	2	2
258	1	17	2	9	3	2	3	3	3
259	1	16	2	9	2	2	2	2	2
260	1	16	2	9	2	2	2	2	9
261	1	16	2	9	2	2	2	2	2
262	9	0	#NULL!	9	9	9	9	9	9
263	9	0	#NULL!	9	9	9	9	9	9
264	1	16	2	9	2	2	1	2	1
265	9	0	#NULL!	9	9	9	9	9	9
266	1	18	2	9	3	2	2	2	9
267	1	16	2	9	2	3	2	2	9
268	1	19	2	9	3	2	2	2	9
269	1	15	2	9	1	2	2	2	9
270	1	17	2	9	2	2	2	2	9
271	1	18	2	9	3	2	2	2	9
272	1	19	2	9	3	2	2	2	9
273	1	17	2	9	3	2	2	2	9
274	1	0	#NULL!	9	2	9	1	1	3
275	9	0	#NULL!	9	9	9	9	9	9
276	9	0	#NULL!	9	2	2	1	2	1
277	1	10	1	1	1	1	1	1	3
278	1	17	2	9	2	2	1	2	3
279	1	17	2	1	2	2	1	1	1
280	1	17	2	9	2	2	1	2	1
281	9	0	#NULL!	9	2	2	1	2	9

282	1	18	2	9	2	2	1	2	9
283	1	18	2	9	2	2	1	2	1
284	1	17	2	9	2	2	1	3	1
285	1	19	2	9	2	2	1	1	1
286	1	0	#NULL!	1	3	1	1	1	2
287	1	13	1	9	1	2	1	1	1
288	1	15	2	9	1	2	1	2	1
289	1	15	2	9	1	2	1	2	1
290	9	0	#NULL!	9	9	9	9	9	1
291	1	20	3	1	1	2	2	2	1
292	9	0	#NULL!	9	9	9	9	9	9
293	9	0	#NULL!	9	9	9	9	9	9
294	2	16	2	9	3	1	1	1	1
295	1	19	2	9	2	2	1	1	1
296	2	16	2	9	2	1	1	1	1
297	1	18	2	9	2	2	2	2	1
298	2	15	2	9	3	1	1	1	9
299	1	18	2	9	3	2	1	2	1
300	1	17	2	9	3	2	1	3	1
301	1	19	2	9	3	2	2	2	1
302	1	27	3	9	3	2	1	1	1
303	1	18	2	9	3	2	1	2	2
304	1	20	3	9	3	2	1	1	3
305	1	18	2	9	3	2	1	1	1
306	1	15	2	9	3	2	1	1	2
307	1	20	3	9	3	2	1	1	2
308	1	12	1	9	3	2	1	2	2
309	1	18	2	9	3	2	1	1	1
310	1	19	2	9	3	2	3	2	2
311	1	18	2	9	3	2	1	2	1
312	1	17	2	9	3	2	1	2	2
313	1	18	2	9	3	3	1	1	2
314	1	19	2	9	3	2	3	2	2
315	1	20	3	9	3	2	3	3	2
316	1	20	3	9	3	2	2	2	3
317	1	20	3	9	3	2	1	1	1
318	1	16	2	9	3	2	3	2	3
319	1	18	2	9	2	2	1	2	2
320	9	19	2	9	2	2	2	2	3
321	9	18	2	9	2	2	2	2	3
322	1	18	2	9	2	2	2	1	2
323	9	0	#NULL!	9	2	2	1	2	2
324	1	19	2	9	2	2	2	2	1
325	1	18	2	9	2	2	1	2	2
326	1	17	2	9	2	2	1	2	2
327	2	13	1	9	2	2	2	2	2
328	1	15	2	9	2	2	1	1	2

329	1	13	1	9	2	2	1	2	1
330	1	18	2	9	1	2	1	2	1
331	9	16	2	9	2	2	1	2	9
332	1	18	2	9	2	2	1	2	1
333	1	21	3	9	2	2	1	1	1
334	1	18	2	9	2	2	2	9	2
335	1	18	2	9	1	2	1	2	1
336	1	17	2	9	1	2	1	2	2
337	1	15	2	9	1	2	2	1	2
338	9	28	3	9	1	2	1	1	2
339	1	16	2	9	2	2	1	2	1
340	1	17	2	9	2	2	1	2	1
341	1	16	2	9	2	2	2	2	2
342	1	17	2	9	2	2	2	2	9
343	1	19	2	9	2	2	1	1	1
344	1	17	2	9	2	2	1	2	9
345	1	16	2	9	2	2	2	2	2
346	1	18	2	9	1	3	2	2	1
347	1	16	2	9	1	3	1	1	1
348	1	15	2	9	1	3	1	2	1
349	2	14	1	9	1	3	1	1	1
350	2	14	1	9	1	3	1	1	1
351	1	14	1	9	1	3	2	2	1
352	2	14	1	9	1	3	2	2	2
353	2	15	2	9	1	3	1	1	2
354	2	16	2	9	1	1	1	2	3
355	2	17	2	9	1	3	9	2	2
356	1	16	2	9	1	3	1	2	1
357	1	14	1	9	1	3	1	3	1
358	1	17	2	9	1	3	1	1	1
359	1	14	1	9	1	3	1	3	1
360	1	15	2	9	1	3	1	2	2
361	9	17	2	9	1	3	1	1	1
362	2	16	2	9	3	3	2	2	1
363	1	19	2	9	3	3	2	2	9
364	2	16	2	9	3	3	1	3	2
365	1	0	#NULL!	9	3	3	1	3	3
366	2	18	2	9	3	3	3	2	1
367	2	16	2	9	3	3	1	1	1
368	1	12	1	9	3	3	1	1	2
369	2	22	3	9	3	3	1	2	1
370	2	16	2	9	2	3	9	1	3
371	2	16	2	9	2	3	1	1	9
372	2	17	2	9	2	3	1	3	1
373	1	16	2	9	2	3	1	1	1
374	2	17	2	9	2	3	1	1	1
375	1	16	2	9	2	3	1	1	1

376	1	16	2	9	2	3	1	1	1
377	1	15	2	9	2	3	1	1	2
378	2	14	1	1	1	3	1	1	1
379	2	14	1	9	2	3	2	2	1
380	1	20	3	1	3	2	2	2	2
381	2	18	2	9	3	3	1	2	1
382	1	0	#NULL!	9	2	3	1	3	1
383	2	0	#NULL!	9	2	3	1	1	1
384	1	18	2	9	2	3	2	1	9
385	2	15	2	9	2	3	1	1	1
386	2	16	2	9	2	3	1	1	1
387	2	16	2	9	2	3	1	1	1
388	2	16	2	9	2	3	1	1	1
389	2	15	2	9	2	3	1	1	1
390	2	15	2	9	2	3	1	1	1
391	2	16	2	9	2	3	3	2	1
392	9	0	#NULL!	9	9	9	9	9	9
393	2	15	2	9	2	1	2	2	2
394	2	15	2	9	1	1	1	2	9
395	2	14	1	9	2	3	1	1	1
396	2	16	2	9	2	3	1	1	2
397	2	14	1	9	2	3	2	2	1
398	2	15	2	9	1	1	1	2	1
399	2	15	2	9	1	1	1	2	9
400	2	14	1	9	1	1	1	1	1
401	2	15	2	9	1	1	1	2	2
402	2	15	2	9	1	1	1	1	2
403	2	19	2	9	1	1	1	2	1
404	2	15	2	9	1	1	2	2	2
405	2	15	2	9	1	1	1	2	1
406	9	0	#NULL!	9	9	9	9	9	9
407	2	14	1	9	1	1	1	1	1
408	2	15	2	9	1	1	1	2	1
409	2	17	2	9	2	1	1	2	1
410	2	16	2	9	2	1	1	2	1
411	2	16	2	9	2	1	2	1	1
412	2	17	2	9	2	1	1	2	1
413	2	16	2	9	2	1	1	3	1
414	2	17	2	9	2	1	1	1	3
415	2	16	2	9	1	1	1	2	2
416	2	15	2	9	1	1	1	1	2
417	2	15	2	9	1	1	1	1	1
418	2	15	2	9	1	1	1	1	3
419	2	15	2	9	1	1	1	2	2
420	9	0	#NULL!	9	9	9	9	9	1
421	2	15	2	9	1	1	1	1	1
422	2	15	2	9	1	1	1	2	3

423	2	14	1	9	1	1	1	1	1
424	2	14	1	9	1	1	1	1	2
425	2	15	2	9	1	1	1	1	3
426	2	15	2	9	1	1	1	1	9
427	2	18	2	9	3	1	2	1	1
428	2	18	2	9	3	1	1	1	1
429	1	16	2	9	2	3	2	2	2
430	1	16	2	9	2	3	2	2	2
431	1	17	2	9	2	3	3	3	9
432	1	14	1	9	1	3	9	9	2
433	1	14	1	9	1	3	1	2	1
434	1	14	1	9	1	3	2	2	2
435	1	15	2	9	1	3	1	2	1
436	1	14	1	9	1	3	2	2	2
437	1	15	2	9	1	3	1	2	1
438	1	20	3	9	3	3	2	2	2
439	1	19	2	9	3	3	3	3	3
440	1	19	2	9	3	3	1	1	1
441	2	18	2	9	3	3	1	1	2
442	1	18	2	9	3	3	1	1	2
443	1	18	2	9	3	3	9	9	9
444	1	18	2	9	3	3	2	2	2
445	2	19	2	9	3	3	2	2	2
446	1	19	2	9	3	3	2	2	2
447	9	0	#NULL!	9	9	9	9	9	9
448	2	18	2	9	2	3	1	1	1
449	2	17	2	9	2	3	1	1	2
450	2	18	2	9	2	3	1	1	1
451	2	18	2	9	2	3	1	1	1
452	2	17	2	9	2	3	2	2	2
453	2	17	2	9	2	3	3	3	2
454	2	17	2	9	2	3	1	1	1
455	2	16	2	9	2	3	2	2	1
456	2	17	2	9	2	3	3	3	2
457	2	18	2	9	2	3	2	2	2
458	9	0	#NULL!	9	9	9	9	9	3
459	2	17	2	9	2	3	3	3	2
460	1	14	1	9	1	3	2	2	2
461	2	15	2	9	1	1	1	1	1
462	2	15	2	9	1	1	1	2	2
463	2	13	1	9	1	1	1	3	9
464	2	17	2	9	1	1	1	1	1
465	2	15	2	9	1	1	1	1	1
466	2	15	2	9	1	1	1	2	1
467	9	0	#NULL!	9	1	1	1	2	3
468	2	15	2	9	1	1	1	1	1
469	2	15	2	9	1	1	1	1	1

470	2	16	2	9	1	1	1	1	1
471	2	14	1	9	1	1	1	2	1
472	2	14	1	9	1	1	1	1	1
473	2	13	1	9	1	1	1	1	1
474	2	14	1	9	1	1	1	2	1
475	2	15	2	9	1	1	1	3	1
476	9	0	#NULL!	9	9	9	9	9	1
477	2	0	#NULL!	9	1	1	1	3	1
478	2	15	2	9	1	1	1	1	1
479	2	16	2	9	1	1	1	2	1
480	2	14	1	9	1	1	1	2	1
481	2	14	1	9	1	1	1	2	9
482	2	13	1	9	1	1	1	2	2
483	9	14	1	9	1	1	2	2	1
484	9	18	2	9	1	1	1	2	9
485	2	17	2	9	3	1	1	1	1
486	2	15	2	9	1	1	1	2	2
487	2	16	2	9	1	1	1	2	2
488	2	16	2	9	1	1	1	2	1
489	2	14	1	9	1	1	1	3	1
490	2	15	2	9	1	1	2	2	2
491	2	14	1	9	1	1	1	3	2
492	2	15	2	9	1	1	1	2	3
493	2	12	1	9	1	1	1	1	1
494	2	16	2	9	1	1	1	3	1
495	2	16	2	9	1	1	1	1	1
496	2	14	1	9	1	1	1	1	9
497	2	14	1	9	1	1	1	2	1
498	2	15	2	1	1	1	1	1	9
499	2	17	2	9	1	1	1	1	1
500	2	18	2	9	1	1	1	2	1
501	9	0	#NULL!	9	1	1	2	2	3
502	2	17	2	9	1	1	1	1	1
503	2	16	2	9	1	1	1	1	1
504	2	16	2	9	1	1	1	1	1
505	2	16	2	9	1	1	1	1	2
506	2	15	2	9	1	1	9	1	2
507	2	15	2	9	1	1	1	2	1
508	2	15	2	9	1	1	1	3	1
509	2	13	1	9	1	1	1	1	2
510	2	15	2	9	1	1	1	3	1
511	2	15	2	9	1	3	1	3	1
512	2	14	1	9	1	1	1	2	1
513	2	15	2	9	1	1	1	2	1
514	2	16	2	9	1	1	1	1	1
515	2	16	2	9	1	1	2	2	1
516	2	16	2	9	1	1	1	1	1

517	2	14	1	9	1	1	1	3	1
518	2	14	1	9	1	1	1	1	1
519	2	15	2	9	1	1	1	2	1
520	2	16	2	9	1	1	1	1	1
521	2	14	1	9	1	1	1	2	1
522	2	0	#NULL!	9	2	9	1	9	1
523	9	0	#NULL!	9	2	9	1	9	1
524	2	16	2	9	3	1	1	1	1
525	2	18	2	9	3	1	1	1	1
526	2	0	#NULL!	9	3	1	1	1	1
527	2	15	2	9	3	1	1	3	2
528	2	12	1	9	3	1	1	1	1
529	2	17	2	9	3	1	1	1	1
530	2	17	2	9	3	1	2	2	2
531	2	17	2	9	3	1	2	1	1
532	1	16	2	9	2	3	2	2	2
533	1	17	2	9	2	3	3	3	9
534	1	0	#NULL!	9	3	3	9	9	9
535	1	18	2	9	3	3	2	2	1
536	1	18	2	9	3	3	3	3	2
537	1	19	2	9	3	3	2	2	2
538	1	18	2	9	3	3	9	9	9
539	1	19	2	9	3	3	2	2	2
540	1	0	#NULL!	9	2	3	2	2	2
541	1	16	2	9	2	3	2	2	9
542	1	16	2	9	2	3	2	2	3
543	1	17	2	9	2	3	9	9	9
544	2	16	2	9	2	3	1	1	1
545	1	17	2	9	2	3	2	2	3
546	1	16	2	9	2	3	2	2	3
547	1	17	2	9	2	3	2	2	3
548	1	17	2	9	2	3	2	2	2
549	1	16	2	9	2	3	2	2	1
550	1	17	2	9	2	3	2	2	3
551	1	19	2	9	3	3	3	3	3
552	1	20	3	9	3	3	2	2	9
553	1	19	2	9	3	3	2	2	2
554	2	18	2	9	3	3	9	9	9
555	2	17	2	9	3	3	2	2	2
556	2	18	2	9	3	3	3	3	1
557	2	18	2	9	3	3	1	1	2
558	2	20	3	9	3	3	1	1	2
559	2	19	2	9	3	3	2	2	2
560	2	18	2	9	3	3	3	3	2
561	1	14	1	9	1	3	2	2	2
562	1	15	2	9	1	3	2	2	2
563	9	0	#NULL!	9	9	9	9	9	9

564	2	18	2	9	2	3	2	2	1
565	2	17	2	9	2	3	3	3	2
566	2	17	2	9	2	3	2	2	2
567	2	16	2	9	2	3	1	1	1
568	2	13	1	9	1	3	2	2	2
569	2	14	1	9	1	3	2	2	2
570	2	15	2	9	1	3	2	2	2
571	2	15	2	9	1	3	1	2	3
572	9	0	#NULL!	9	9	9	9	9	9
573	9	0	#NULL!	9	9	9	9	9	9
574	9	0	#NULL!	9	9	9	9	9	9
575	9	0	#NULL!	9	9	9	9	9	9
576	9	0	#NULL!	9	9	9	9	9	9
577	9	0	#NULL!	9	9	9	9	9	9
578	1	14	1	9	1	3	2	2	2
579	1	15	2	9	1	3	2	2	2
580	1	15	2	9	1	3	1	2	1
581	2	16	2	9	2	3	2	2	2
582	1	14	1	9	1	3	2	2	2
583	1	15	2	9	1	3	2	2	2
584	1	15	2	9	1	3	2	2	2
585	2	14	1	9	1	3	1	2	2
586	2	15	2	9	1	3	1	2	1
587	1	15	2	9	1	3	2	2	2
588	1	14	1	9	1	3	2	2	2
589	1	15	2	9	1	3	1	2	1
590	2	18	2	9	3	3	3	3	9
591	2	19	2	9	2	3	2	2	2
592	2	0	#NULL!	9	2	3	2	2	9
593	2	18	2	9	2	3	1	1	1
594	2	16	2	9	2	3	1	1	1
595	2	17	2	9	2	3	2	2	2
596	2	17	2	9	2	3	3	3	2
597	2	17	2	9	2	3	1	1	2
598	2	18	2	9	2	3	1	1	2
599	1	14	1	9	1	3	1	2	1
600	9	18	2	9	3	3	2	2	3
601	2	0	#NULL!	9	3	3	2	2	2
602	2	19	2	9	3	3	3	3	1
603	2	19	2	9	3	3	2	2	3
604	2	18	2	9	3	3	2	2	2
605	2	18	2	9	3	3	2	2	9
606	2	18	2	9	3	3	2	2	2
607	2	18	2	9	3	3	1	1	1
608	1	19	2	9	3	3	2	2	1
609	2	17	2	9	3	3	2	2	2
610	1	18	2	9	3	3	2	2	2

611	2	18	2	9	3	3	3	3	3
612	1	19	2	9	3	3	9	9	9
613	1	17	2	9	3	3	2	2	3
614	1	20	3	9	3	3	2	2	3
615	1	18	2	9	3	3	9	9	9
616	1	19	2	9	3	3	2	2	2
617	2	18	2	9	3	3	1	1	1
618	2	18	2	9	3	3	2	2	2
619	2	18	2	9	3	3	1	1	2
620	1	19	2	9	3	3	1	1	1
621	1	18	2	9	3	3	2	2	2
622	1	19	2	9	3	3	2	2	9
623	2	17	2	9	3	1	1	2	1
624	2	16	2	9	3	1	2	2	9
625	2	15	2	9	1	1	1	2	1
626	2	15	2	9	1	1	1	1	3
627	2	16	2	9	1	1	2	2	9
628	2	15	2	9	1	1	1	2	9
629	2	14	1	9	1	1	1	2	1
630	2	16	2	9	3	1	2	2	2
631	2	17	2	9	2	1	1	1	1
632	2	15	2	9	2	1	1	2	9
633	2	14	1	9	2	1	1	3	1
634	2	16	2	9	2	1	1	3	1
635	2	15	2	9	3	1	1	1	1
636	2	17	2	9	3	1	1	1	1
637	2	17	2	9	3	1	1	1	1
638	2	16	2	9	3	1	1	1	2
639	2	16	2	9	3	1	2	1	2
640	2	16	2	9	2	1	1	2	1
641	2	16	2	9	2	1	1	3	2
642	2	16	2	9	2	1	2	2	2
643	2	15	2	9	2	1	1	1	1
644	2	14	1	9	1	1	1	2	9
645	2	14	1	9	2	1	1	9	1
646	9	0	#NULL!	9	9	9	9	9	9
647	9	0	#NULL!	9	9	9	9	9	9
648	1	19	2	9	3	3	1	1	9
649	1	15	2	9	1	3	2	2	9
650	1	18	2	9	3	3	2	2	2
651	2	16	2	9	2	3	1	1	9
652	2	18	2	9	3	3	1	1	2
653	1	18	2	9	3	3	1	1	2
654	1	16	2	9	2	3	2	2	2
655	2	14	1	9	1	3	2	2	2
656	1	16	2	9	2	3	2	2	2
657	2	16	2	9	2	3	1	3	3

658	2	15	2	9	2	3	1	1	1
659	2	16	2	9	2	3	1	2	2
660	2	15	2	9	2	3	1	1	1
661	2	16	2	9	2	3	1	2	1
662	2	16	2	9	9	9	9	9	3
663	2	15	2	9	2	3	1	1	1
664	2	15	2	9	2	3	1	2	1
665	1	18	2	9	3	3	2	2	3
666	1	17	2	9	2	3	2	2	2
667	1	18	2	9	3	3	1	1	2
668	2	18	2	9	3	3	1	1	2
669	2	16	2	9	2	3	1	1	2
670	1	14	1	9	2	3	1	1	1
671	1	17	2	9	2	3	1	2	2
672	9	0	#NULL!	9	9	9	9	9	1
673	1	16	2	9	2	3	1	3	2
674	1	16	2	9	2	3	1	2	3
675	2	17	2	9	2	3	1	3	1
676	2	14	1	9	2	3	1	1	1
677	2	16	2	9	2	3	1	1	1
678	2	16	2	9	2	3	1	3	2
679	2	15	2	9	9	9	9	9	1
680	9	16	2	9	2	3	1	1	2
681	2	16	2	9	2	3	1	2	1
682	1	15	2	9	2	3	1	3	2
683	2	16	2	9	2	3	1	2	1
684	2	16	2	9	2	3	1	1	2
685	2	15	2	9	2	3	1	3	3
686	2	15	2	9	2	3	1	3	1
687	2	17	2	9	2	3	1	1	1
688	2	15	2	9	2	3	1	2	1
689	2	16	2	9	9	9	9	9	2
690	1	16	2	9	2	3	1	2	3
691	9	0	#NULL!	9	9	9	9	9	1
692	1	16	2	9	2	3	1	3	9
693	2	15	2	9	2	3	1	2	2
694	1	14	1	9	2	3	1	1	1
695	1	18	2	9	3	3	1	1	1
696	1	16	2	9	2	3	1	2	1
697	1	17	2	9	2	3	2	2	3
698	2	16	2	9	1	3	2	2	3
699	2	14	1	9	1	3	1	3	3
700	1	16	2	9	2	3	1	1	1
701	2	16	2	9	2	3	1	2	1
702	1	15	2	9	2	3	1	3	3
703	1	16	2	9	2	3	1	3	3
704	1	18	2	9	2	3	1	3	3

705	1	15	2	9	2	3	2	2	3
706	1	20	3	9	2	2	1	1	3
707	9	0	#NULL!	9	2	3	2	2	1
708	2	16	2	9	9	9	9	9	1
709	9	16	2	9	2	3	1	1	1
710	1	17	2	9	2	3	2	1	1
711	1	17	2	9	2	2	1	1	2
712	1	19	2	9	1	3	2	2	2
713	2	16	2	9	9	9	9	9	1
714	2	14	1	9	1	3	1	1	1
715	2	17	2	9	1	3	1	2	2
716	2	15	2	9	1	3	1	9	3
717	1	17	2	9	1	3	1	3	1
718	2	15	2	9	2	3	1	1	2
719	2	16	2	9	2	3	1	9	1
720	2	16	2	9	1	3	2	2	2
721	2	16	2	9	1	3	2	2	2
722	1	16	2	9	1	2	1	2	2
723	1	15	2	9	1	1	2	2	3
724	2	15	2	9	1	3	1	2	3
725	2	16	2	9	9	9	9	9	1
726	1	17	2	9	2	3	1	1	1
727	1	17	2	9	2	3	1	1	3
728	1	15	2	9	9	9	9	9	1
729	1	17	2	9	2	3	1	1	3
730	1	15	2	9	2	3	1	1	3
731	2	15	2	9	1	3	1	1	2
732	1	15	2	9	1	3	1	2	3
733	1	15	2	9	1	3	1	2	2
734	1	15	2	9	1	3	3	3	2
735	1	12	1	9	1	3	1	1	3
736	1	16	2	9	1	3	2	2	3
737	1	0	#NULL!	9	9	9	9	9	1
738	1	15	2	9	1	3	1	3	1
739	2	15	2	9	1	3	1	2	1
740	9	16	2	9	9	9	9	9	3
741	1	14	1	9	1	3	1	1	1
742	1	10	1	9	9	9	9	9	3
743	2	0	#NULL!	9	9	9	9	9	1
744	2	0	#NULL!	9	1	3	1	3	1
745	2	14	1	9	1	3	1	2	1
746	2	10	1	9	1	3	1	2	2
747	2	14	1	9	1	3	1	2	2
748	1	15	2	9	1	3	1	3	2
749	2	15	2	9	9	9	9	9	1
750	2	15	2	9	1	3	1	2	1
751	2	15	2	9	1	3	1	3	2

752	2	16	2	9	1	3	1	1	2
753	2	15	2	9	1	3	1	2	1
754	2	15	2	9	1	3	1	3	2
755	1	16	2	9	1	3	1	2	1
756	2	14	1	9	1	3	1	2	1
757	2	15	2	9	1	3	1	2	1
758	2	15	2	9	1	3	1	2	1
759	1	15	2	9	1	3	1	3	1
760	1	15	2	9	1	3	1	3	3
761	9	0	#NULL!	9	9	9	9	9	2
762	1	16	2	9	1	2	1	2	1
763	1	17	2	9	1	2	1	2	1
764	1	15	2	9	1	2	3	2	9
765	1	16	2	9	1	2	3	1	9
766	1	10	1	9	1	2	1	2	1
767	1	12	1	9	1	2	2	1	9
768	1	0	#NULL!	9	1	2	1	1	9
769	1	17	2	9	1	2	1	1	1
770	9	0	#NULL!	9	9	9	9	9	1
771	1	16	2	9	1	2	1	2	3
772	1	17	2	9	1	2	1	3	2
773	1	17	2	9	1	2	1	2	2
774	1	16	2	9	1	2	2	2	1
775	1	17	2	9	9	9	9	9	1
776	1	18	2	9	1	2	1	1	2
777	1	17	2	9	1	2	1	1	1
778	9	0	#NULL!	9	1	2	1	2	1
779	1	16	2	9	1	2	1	1	3
780	1	18	2	9	1	2	1	9	2
781	1	18	2	9	1	2	1	2	2
782	1	18	2	9	1	2	1	3	3
783	1	17	2	9	1	2	1	1	1
784	1	18	2	9	1	2	1	2	1
785	1	18	2	9	1	2	1	3	2
786	1	16	2	9	1	2	1	2	2
787	1	15	2	9	1	2	1	1	1
788	1	17	2	9	2	2	2	2	9
789	1	17	2	9	2	2	1	2	3
790	1	0	#NULL!	9	2	2	1	1	1
791	1	14	1	9	1	2	2	3	3
792	1	0	#NULL!	9	1	1	1	1	1
793	1	15	2	9	1	2	2	2	1
794	1	15	2	9	1	2	3	2	2
795	1	16	2	9	1	2	1	2	2
796	1	15	2	9	1	2	3	3	3
797	1	16	2	9	1	2	1	2	9
798	1	17	2	9	3	2	1	2	2

799	1	15	2	9	3	2	2	2	2
800	1	18	2	9	3	2	1	2	1
801	1	19	2	9	3	2	3	3	2
802	1	16	2	9	3	2	1	9	2
803	1	18	2	9	3	2	2	2	2
804	1	18	2	9	3	2	1	3	9
805	1	18	2	9	3	2	1	2	9
806	1	20	3	9	3	2	3	3	3
807	1	15	2	9	2	2	1	2	2
808	1	16	2	9	2	2	2	2	1
809	2	18	2	9	1	3	1	1	9
810	2	15	2	9	1	3	1	2	1
811	2	14	1	9	3	3	1	1	2
812	2	17	2	9	3	3	1	1	9
813	2	17	2	9	3	3	1	2	9
814	2	16	2	9	3	3	1	2	9
815	2	16	2	9	3	3	1	2	2
816	2	17	2	9	3	3	1	1	1
817	2	17	2	9	3	3	1	3	1
818	2	16	2	9	3	3	1	1	2
819	2	17	2	9	3	3	3	2	1
820	2	17	2	9	3	3	1	1	1
821	2	0	#NULL!	9	3	3	1	2	1
822	2	17	2	9	3	3	1	2	1
823	2	16	2	9	3	3	1	1	1
824	2	19	2	9	3	3	1	1	3
825	2	16	2	9	3	3	1	2	2
826	2	17	2	9	3	3	1	2	2
827	2	15	2	9	1	3	1	2	2
828	9	0	#NULL!	9	1	3	1	2	3
829	9	0	#NULL!	9	9	9	9	9	1
830	2	15	2	9	1	3	9	9	1
831	2	11	1	9	1	3	1	1	1
832	2	15	2	9	1	3	1	1	1
833	2	15	2	9	1	3	1	1	1
834	1	14	1	9	1	3	1	2	2
835	1	14	1	9	1	3	1	1	2
836	1	14	1	9	1	3	1	1	1
837	1	0	#NULL!	9	1	3	1	1	1
838	2	15	2	9	1	3	1	3	1
839	1	14	1	9	1	3	1	1	1
840	2	16	2	9	1	3	1	1	2
841	1	16	2	9	1	3	2	2	1
842	2	15	2	9	1	3	1	1	1
843	2	14	1	9	1	3	1	1	2
844	2	14	1	9	1	3	1	1	1
845	2	13	1	9	1	3	1	2	3

846	2	14	1	9	1	3	1	2	1
847	2	15	2	9	1	3	1	1	1
848	2	14	1	9	1	3	1	2	3
849	1	0	#NULL!	9	1	2	1	2	1
850	2	16	2	9	1	1	1	1	2
851	2	15	2	9	2	1	1	2	1
852	2	14	1	9	2	1	1	1	1
853	2	15	2	9	2	1	1	2	1
854	2	16	2	9	2	1	1	2	1
855	2	14	1	9	2	1	1	1	9
856	2	15	2	9	2	1	1	2	1
857	2	16	2	9	2	1	1	1	1
858	2	15	2	9	2	1	1	1	1
859	2	15	2	9	2	1	2	2	1
860	2	16	2	9	2	1	1	2	1
861	2	16	2	9	2	1	1	1	2
862	2	16	2	9	9	9	9	9	1
863	2	16	2	9	2	1	2	2	9
864	2	15	2	9	2	1	2	2	9
865	2	15	2	9	2	1	2	2	1
866	2	0	#NULL!	9	3	1	1	1	1
867	2	15	2	9	2	3	2	2	1
868	1	19	2	9	3	2	1	9	3
869	1	23	3	9	3	9	9	9	3
870	1	15	2	9	2	2	1	2	1
871	1	15	2	9	1	2	1	3	1
872	1	15	2	9	1	2	2	2	1
873	1	16	2	9	1	2	1	2	2
874	1	16	2	9	1	2	1	3	2
875	1	16	2	9	1	2	2	2	1
876	1	15	2	9	1	2	2	2	3
877	1	0	#NULL!	9	9	9	9	9	2
878	1	19	2	9	2	2	1	3	1
879	1	17	2	9	2	2	1	2	1
880	1	16	2	1	1	3	1	1	1
881	1	18	2	9	2	2	1	1	1
882	1	17	2	9	2	2	1	1	1
883	1	19	2	9	2	9	9	9	1
884	1	16	2	9	2	2	1	2	1
885	1	16	2	9	1	3	1	3	2
886	1	15	2	9	1	3	1	1	2
887	1	17	2	9	2	3	3	3	2
888	1	16	2	9	2	3	2	2	3
889	2	15	2	9	1	3	1	1	2
890	2	16	2	9	2	3	1	1	1
891	1	15	2	9	1	3	1	1	1
892	1	16	2	9	2	3	2	2	2

893	1	15	2	9	1	3	2	2	9
894	2	18	2	9	3	3	1	1	2
895	2	17	2	9	3	3	1	1	2
896	1	15	2	9	9	3	9	9	9
897	2	14	1	9	1	3	2	2	9
898	1	16	2	9	2	3	2	2	1
899	2	14	1	9	1	3	2	2	9
900	2	15	2	9	1	3	2	2	9
901	1	18	2	9	3	3	2	2	9
902	2	15	2	9	2	3	1	1	1
903	2	16	2	9	2	3	2	2	2
904	2	18	2	9	3	3	1	1	2
905	2	18	2	9	3	3	1	1	2
906	1	17	2	9	3	3	2	2	3
907	2	14	1	9	1	3	1	1	2
908	1	17	2	9	3	3	2	2	9
909	2	15	2	9	2	3	1	1	2
910	1	16	2	9	2	3	2	2	2
911	2	13	1	9	1	3	2	2	9
912	2	16	2	9	2	3	1	1	2
913	2	16	2	9	1	3	1	2	2
914	2	15	2	9	1	3	1	1	2
915	2	15	2	9	1	3	1	1	1
916	1	16	2	9	2	3	1	2	1
917	9	0	#NULL!	9	1	3	1	2	3
918	9	0	#NULL!	9	9	9	9	9	3
919	1	15	2	9	1	3	2	2	2
920	2	14	1	9	1	3	1	2	1
921	2	15	2	9	1	2	1	1	1
922	2	15	2	9	1	3	1	2	1
923	1	16	2	9	1	3	1	2	3
924	1	15	2	9	1	3	3	3	1
925	1	25	3	9	3	2	1	2	2
926	9	17	2	9	3	2	1	1	1
927	1	15	2	9	1	2	1	2	1
928	9	0	#NULL!	9	1	2	1	1	9
929	1	15	2	9	1	2	3	3	3
930	1	16	2	9	1	2	1	2	1
931	1	14	1	9	1	2	1	2	2
932	1	15	2	9	1	2	1	2	2
933	1	14	1	9	1	2	1	1	1
934	1	0	#NULL!	9	1	2	1	2	1
935	1	18	2	9	1	2	1	2	1
936	1	16	2	9	1	2	2	1	9
937	1	18	2	9	1	2	1	2	1
938	1	19	2	9	1	1	9	9	1
939	1	16	2	9	1	2	1	1	1

940	1	17	2	9	1	2	3	2	3
941	1	17	2	9	1	2	1	3	3
942	2	15	2	9	1	3	1	1	1
943	1	16	2	9	1	3	1	1	2
944	1	17	2	9	3	3	1	1	1
945	1	15	2	9	1	3	1	1	2
946	2	16	2	9	1	3	1	1	1
947	1	16	2	9	2	3	1	1	1
948	1	14	1	9	1	3	2	2	3
949	2	15	2	9	1	3	1	1	1
950	1	15	2	9	1	3	1	1	1
951	2	16	2	9	2	3	1	1	1
952	2	16	2	9	2	3	1	1	1
953	2	18	2	9	3	3	1	1	1
954	1	17	2	9	3	3	1	1	1
955	2	16	2	9	2	3	1	1	1
956	2	14	1	9	1	3	1	1	1
957	2	16	2	9	1	3	1	1	1
958	2	16	2	9	2	3	1	1	1
959	1	16	2	9	1	3	2	2	2
960	2	15	2	9	1	3	1	1	1
961	1	17	2	9	2	3	2	2	1
962	9	0	#NULL!	9	9	9	9	9	9
963	1	18	2	9	3	3	1	1	1
964	2	16	2	9	2	3	1	1	1
965	2	14	1	9	1	3	1	1	1
966	2	15	2	9	1	3	1	1	1
967	2	15	2	9	1	3	1	1	1
968	2	15	2	9	1	3	3	3	2
969	2	16	2	9	3	3	1	1	1
970	1	16	2	9	3	3	3	3	2
971	1	18	2	9	3	3	3	3	2
972	2	17	2	9	3	3	1	1	1
973	2	16	2	9	2	3	1	1	1
974	1	15	2	9	1	3	1	1	1
975	1	16	2	9	2	3	3	3	2
976	2	15	2	9	1	3	1	1	1
977	2	14	1	9	1	3	1	1	1
978	1	16	2	9	9	3	9	9	9
979	1	16	2	9	3	3	2	2	2
980	1	16	2	9	2	3	1	1	2
981	1	17	2	9	2	3	2	2	2
982	2	17	2	9	3	3	1	1	1
983	1	17	2	9	2	3	2	2	2
984	2	15	2	9	1	3	1	1	1
985	1	16	2	9	2	3	2	2	2
986	2	18	2	9	3	3	1	1	1

987	2	15	2	1	1	3	1	1	1
988	1	15	2	9	1	3	2	2	2
989	2	16	2	9	2	3	1	1	1
990	2	15	2	9	2	1	1	1	1
991	2	19	2	9	3	3	1	1	1
992	1	17	2	9	2	3	2	2	2
993	2	17	2	9	3	3	1	1	1
994	2	18	2	9	3	3	1	1	1
995	1	16	2	9	3	3	1	1	2
996	2	16	2	9	2	3	1	1	1
997	1	17	2	9	3	3	3	3	2
998	1	16	2	9	2	3	1	1	1
999	1	15	2	9	1	3	3	3	2
1000	2	15	2	9	1	1	3	1	1
1001	2	16	2	9	2	3	1	1	1
1002	1	16	2	9	2	3	3	3	2
1003	2	15	2	9	1	3	1	1	1
1004	9	0	#NULL!	9	9	9	9	9	9
1005	1	16	2	9	2	2	1	2	2
1006	2	15	2	1	1	1	1	1	1
1007	2	16	2	1	1	1	1	1	1
1008	2	16	2	1	1	1	1	1	1
1009	2	15	2	1	1	1	1	1	1
1010	2	15	2	1	1	1	1	1	1
1011	2	15	2	1	1	1	1	1	1
1012	2	19	2	1	1	1	1	1	1
1013	2	14	1	1	1	1	1	1	1
1014	2	15	2	1	1	1	1	1	1
1015	2	15	2	1	1	1	1	1	1
1016	2	14	1	1	1	1	1	1	1
1017	2	17	2	1	1	1	1	1	1
1018	2	15	2	1	1	1	1	1	1
1019	2	13	1	1	1	1	1	1	1
1020	2	15	2	1	1	1	1	1	1
1021	2	0	#NULL!	1	1	1	1	1	1
1022	2	17	2	1	1	1	1	1	3
1023	2	14	1	1	1	1	1	1	1
1024	2	14	1	1	1	1	1	1	1
1025	2	14	1	1	1	1	1	1	1
1026	2	13	1	1	1	1	1	1	1
1027	2	13	1	1	1	1	1	1	3
1028	2	15	2	1	1	1	1	1	1
1029	2	15	2	1	1	1	1	1	1
1030	2	15	2	1	1	1	1	1	9
1031	2	0	#NULL!	9	9	9	9	9	9
1032	2	17	2	1	1	1	1	3	9
1033	2	15	2	9	1	1	1	1	9

1034	2	15	2	1	1	1	3	3	9
1035	2	14	1	1	1	1	1	1	9
1036	9	0	#NULL!	9	9	9	9	9	9
1037	2	15	2	1	1	1	1	2	9
1038	2	16	2	1	1	1	2	2	9
1039	2	13	1	1	1	1	1	1	9
1040	2	14	1	1	1	1	1	2	9
1041	2	14	1	1	1	1	1	1	9
1042	9	0	#NULL!	9	9	9	9	9	9
1043	2	15	2	9	1	1	1	1	9
1044	2	15	2	1	1	1	1	2	9
1045	2	16	2	1	1	1	1	1	9
1046	2	14	1	1	1	1	1	1	9
1047	2	14	1	1	1	1	1	1	9
1048	2	12	1	1	1	1	1	1	9
1049	2	16	2	1	1	1	1	9	9
1050	2	15	2	1	1	1	2	2	9
1051	2	13	1	9	1	1	1	9	9
1052	2	16	2	9	1	1	9	9	9
1053	2	16	2	1	1	1	1	1	1
1054	2	17	2	1	1	1	1	1	1
1055	2	15	2	1	1	1	1	1	1
1056	2	14	1	1	1	1	1	1	1
1057	2	16	2	1	1	1	1	1	1
1058	2	15	2	1	1	1	1	1	1
1059	2	13	1	1	1	1	1	1	1
1060	2	14	1	1	1	1	1	1	1
1061	2	13	1	1	1	1	1	1	1
1062	2	14	1	1	1	1	1	1	3
1063	2	15	2	1	1	1	1	1	3
1064	2	16	2	1	1	1	1	1	1
1065	2	13	1	1	1	1	1	1	1
1066	2	15	2	1	1	1	1	1	1
1067	2	15	2	1	1	1	1	1	1
1068	2	15	2	1	1	1	1	1	1
1069	2	16	2	1	1	1	1	2	2
1070	2	15	2	1	1	1	1	1	3
1071	2	13	1	9	1	1	1	1	1
1072	2	13	1	1	1	1	1	1	1
1073	2	15	2	1	1	1	1	1	9
1074	2	15	2	1	1	1	1	1	3
1075	2	14	1	1	1	1	1	1	1
1076	2	15	2	1	1	1	1	1	3
1077	2	14	1	1	1	1	1	1	1
1078	2	15	2	9	1	1	1	2	3
1079	2	15	2	1	1	1	1	1	1
1080	2	15	2	1	1	1	1	1	1

1081	2	15	2	1	1	1	1	1	1
1082	2	15	2	1	1	1	1	1	1
1083	2	16	2	1	1	1	1	3	1
1084	2	14	1	1	1	1	1	1	1
1085	2	15	2	1	1	1	1	1	1
1086	2	14	1	1	1	1	1	1	1
1087	2	15	2	1	1	1	1	3	1
1088	9	0	#NULL!	9	9	9	9	9	1
1089	2	14	1	1	1	1	1	1	1
1090	2	15	2	1	1	1	1	1	3
1091	2	14	1	1	1	1	1	1	1
1092	2	14	1	1	1	1	1	1	3
1093	2	15	2	1	1	1	1	1	1
1094	2	15	2	1	1	1	1	1	9
1095	2	15	2	1	1	1	1	1	3
1096	2	13	1	1	1	1	1	1	1
1097	2	16	2	1	1	1	1	1	1
1098	2	15	2	1	1	1	1	1	1
1099	2	15	2	1	1	1	1	1	1
1100	2	14	1	1	1	1	1	1	1
1101	2	14	1	1	1	1	1	1	1
1102	9	0	#NULL!	9	9	9	9	9	1
1103	2	15	2	1	1	1	1	1	1
1104	2	14	1	1	1	1	1	1	3
1105	2	14	1	1	1	1	1	1	1
1106	2	15	2	1	1	1	1	1	1
1107	2	16	2	1	1	1	1	1	3
1108	2	15	2	1	1	1	1	1	1
1109	2	16	2	1	1	1	1	1	1
1110	2	15	2	1	1	1	1	1	1
1111	2	14	1	1	1	1	1	1	3
1112	2	15	2	1	1	1	1	1	3
1113	2	16	2	1	1	1	1	2	3
1114	2	16	2	1	1	1	1	1	1
1115	2	15	2	1	1	1	1	1	9
1116	2	0	#NULL!	1	1	1	1	1	3
1117	2	15	2	1	1	1	1	1	1
1118	2	15	2	1	1	1	1	1	3
1119	2	16	2	1	1	1	1	1	9
1120	2	15	2	1	1	1	1	1	9
1121	2	16	2	1	1	1	1	1	3
1122	2	16	2	1	1	1	1	1	1
1123	2	14	1	1	1	1	1	1	3
1124	2	14	1	1	1	1	1	1	1
1125	2	15	2	1	1	1	1	1	1
1126	2	0	#NULL!	1	1	1	1	1	1
1127	2	14	1	1	1	1	1	1	1

1128	2	15	2	1	1	1	1	1	1
1129	2	13	1	1	1	1	1	1	1
1130	2	14	1	1	1	1	1	1	1
1131	2	15	2	1	1	1	1	1	1
1132	2	15	2	1	1	1	1	1	1
1133	2	15	2	1	1	1	1	1	1
1134	2	13	1	1	1	1	1	1	1
1135	2	18	2	1	1	1	1	1	1
1136	2	14	1	1	1	1	1	1	1
1137	2	14	1	1	1	1	1	1	1
1138	2	13	1	1	1	1	1	1	1
1139	2	17	2	1	1	1	1	1	1
1140	2	16	2	1	1	1	1	1	1
1141	2	17	2	1	1	1	1	1	1
1142	2	15	2	1	1	1	1	1	1
1143	2	0	#NULL!	1	1	1	1	1	1
1144	2	14	1	1	1	1	1	1	1
1145	2	17	2	1	1	1	1	1	1
1146	2	17	2	1	1	1	1	1	1
1147	9	0	#NULL!	9	9	9	9	9	9
1148	2	14	1	1	1	1	1	1	1
1149	2	15	2	1	1	1	1	1	1
1150	2	15	2	1	1	1	1	1	1
1151	2	13	1	1	1	1	1	1	1
1152	2	14	1	1	1	1	1	2	9
1153	2	16	2	1	1	1	1	1	1
1154	2	14	1	1	1	1	1	1	1
1155	2	15	2	1	1	1	1	1	1
1156	2	13	1	1	1	1	1	1	1
1157	2	16	2	1	1	1	1	1	1
1158	2	15	2	1	1	1	1	1	1
1159	2	14	1	1	1	1	1	1	1
1160	2	15	2	1	1	1	1	1	1
1161	2	14	1	1	1	1	1	1	1
1162	2	14	1	1	1	1	1	1	1
1163	2	14	1	1	1	1	1	1	1
1164	2	13	1	1	1	1	1	1	1
1165	2	13	1	1	1	1	1	1	1
1166	2	14	1	1	1	1	1	1	1
1167	2	16	2	1	1	1	1	1	1
1168	2	16	2	1	1	1	1	1	1
1169	9	0	#NULL!	9	9	9	9	9	9
1170	2	15	2	1	1	1	1	1	1
1171	2	16	2	1	1	1	1	1	1
1172	2	15	2	1	1	1	1	1	1
1173	2	17	2	1	1	1	1	1	1
1174	2	17	2	1	1	1	1	1	1

1175	2	18	2	1	1	1	1	1	1
1176	2	15	2	1	1	1	1	1	1
1177	2	14	1	1	1	1	1	1	1
1178	2	15	2	1	1	1	1	1	1
1179	2	15	2	1	1	1	1	1	1
1180	2	15	2	1	1	1	1	1	1
1181	2	14	1	1	1	1	1	1	1
1182	2	19	2	1	1	1	1	1	1
1183	2	15	2	1	1	1	1	1	1
1184	2	13	1	1	1	1	1	1	1
1185	2	15	2	1	1	1	1	1	1
1186	2	13	1	1	1	1	1	1	1
1187	2	15	2	1	1	1	1	1	1
1188	2	17	2	1	1	1	1	1	1
1189	2	16	2	1	1	1	1	1	3
1190	2	14	1	1	1	1	1	1	1
1191	2	14	1	1	1	1	1	1	1
1192	2	16	2	1	1	1	1	1	1
1193	2	14	1	1	1	1	1	1	1
1194	2	14	1	1	1	1	1	1	1
1195	2	15	2	1	1	1	1	1	1
1196	2	14	1	1	1	1	1	1	1
1197	2	16	2	9	1	1	1	1	3
1198	2	13	1	1	1	1	1	1	3
1199	2	14	1	1	1	1	1	1	1
1200	2	14	1	1	1	1	1	1	1
1201	2	14	1	1	1	1	1	1	1
1202	2	15	2	1	1	1	1	2	9
1203	9	0	#NULL!	1	1	1	1	9	9
1204	2	14	1	1	1	1	1	3	9
1205	2	14	1	1	1	1	1	1	9
1206	2	15	2	9	1	1	1	1	9
1207	2	16	2	1	1	1	1	2	9
1208	2	17	2	9	1	1	1	1	9
1209	2	15	2	1	1	1	1	2	9
1210	2	16	2	1	1	1	1	1	9
1211	2	14	1	1	1	1	1	3	9
1212	2	15	2	1	1	1	1	3	9
1213	2	14	1	9	1	1	9	9	9
1214	2	16	2	1	1	1	1	2	9
1215	2	15	2	9	1	1	1	2	9
1216	2	14	1	1	1	1	1	2	9
1217	2	14	1	1	1	1	1	1	1
1218	2	17	2	1	1	1	1	1	9
1219	2	14	1	1	1	1	1	1	9
1220	2	15	2	1	1	1	1	1	9
1221	9	16	2	1	1	1	2	2	9

1222	2	15	2	1	1	1	1	1	9
1223	2	14	1	1	1	1	1	1	9
1224	2	15	2	1	1	1	1	2	9
1225	9	14	1	9	1	1	1	1	9
1226	2	15	2	1	1	1	1	1	9
1227	2	15	2	1	1	1	1	2	9
1228	2	13	1	1	1	1	1	1	9
1229	2	16	2	1	1	1	1	1	3
1230	2	14	1	1	1	1	1	1	9
1231	2	0	#NULL!	1	1	1	1	1	9
1232	9	9	1	9	9	9	9	9	9
1233	2	16	2	1	1	3	9	9	9
1234	2	15	2	1	1	1	1	1	9
1235	2	17	2	1	1	3	1	1	1
1236	1	15	2	1	1	3	1	2	1
1237	2	16	2	1	1	3	1	2	1
1238	2	22	3	1	1	3	1	2	1
1239	2	16	2	1	1	3	1	1	2
1240	2	15	2	1	1	3	2	3	1
1241	1	15	2	1	1	3	1	2	2
1242	2	15	2	1	1	3	1	1	1
1243	2	15	2	1	1	3	2	2	9
1244	2	14	1	1	1	3	1	2	1
1245	2	14	1	1	1	3	1	3	1
1246	2	15	2	1	1	3	1	2	9
1247	2	15	2	1	1	3	1	1	1
1248	2	17	2	1	1	3	1	2	9
1249	2	13	1	1	1	3	1	2	1
1250	2	14	1	9	1	3	1	1	1
1251	2	16	2	1	1	3	1	1	3
1252	1	16	2	1	1	3	2	2	2
1253	2	15	2	1	1	3	1	2	1
1254	2	15	2	1	1	3	1	2	9
1255	2	15	2	1	1	3	1	2	1
1256	1	14	1	1	1	3	1	1	1
1257	2	15	2	9	1	3	1	2	1
1258	2	14	1	9	1	3	1	3	1
1259	2	15	2	1	1	3	1	1	1
1260	2	14	1	1	1	3	1	1	1
1261	2	16	2	1	1	3	1	2	3
1262	9	0	#NULL!	1	1	3	2	3	9
1263	1	17	2	1	1	3	2	2	1
1264	2	15	2	9	1	3	2	1	1
1265	9	16	2	9	1	3	1	1	9
1266	2	15	2	1	1	1	1	2	9
1267	2	15	2	1	1	1	1	2	9
1268	2	14	1	1	1	3	2	3	1

1269	2	17	2	9	1	3	1	2	9
1270	1	15	2	1	1	3	2	2	9
1271	2	20	3	1	1	3	1	2	2
1272	2	15	2	1	1	3	1	1	1
1273	1	15	2	1	1	3	1	2	1
1274	2	13	1	1	1	3	2	2	9
1275	2	14	1	1	1	3	1	1	1
1276	2	14	1	1	1	3	2	2	2
1277	2	14	1	1	1	3	2	2	2
1278	2	15	2	1	1	3	1	2	2
1279	2	14	1	1	1	3	1	1	1
1280	1	15	2	1	1	3	2	2	9
1281	1	14	1	1	1	3	1	1	9
1282	2	14	1	1	1	3	2	2	2
1283	2	19	2	1	1	3	1	2	1
1284	2	16	2	1	1	3	1	1	1
1285	2	14	1	1	1	3	2	2	9
1286	1	15	2	9	1	3	2	2	2
1287	1	18	2	1	1	3	3	2	1
1288	1	14	1	1	1	3	1	3	1
1289	1	17	2	1	1	3	1	2	1
1290	2	14	1	1	1	3	2	2	2
1291	1	13	1	1	1	3	1	1	2
1292	2	14	1	1	1	3	1	2	2
1293	1	14	1	1	1	3	1	3	2
1294	2	14	1	1	1	3	1	2	1
1295	2	17	2	1	1	3	2	2	2
1296	2	15	2	9	1	3	2	1	1
1297	2	19	2	1	1	3	1	3	1
1298	2	15	2	1	1	3	1	1	1
1299	2	14	1	1	1	3	1	1	3
1300	9	0	#NULL!	9	9	9	9	9	1
1301	2	15	2	1	1	3	2	2	1
1302	2	14	1	1	1	3	1	1	1
1303	2	14	1	1	1	3	1	2	1
1304	9	0	#NULL!	9	9	9	9	9	1
1305	2	16	2	1	1	3	1	1	1
1306	2	15	2	1	1	1	1	1	9
1307	9	0	#NULL!	9	9	9	9	9	2
1308	2	15	2	9	1	3	1	1	1
1309	1	15	2	1	1	3	2	2	1
1310	2	16	2	1	1	3	1	1	1
1311	1	17	2	1	1	3	1	1	1
1312	2	15	2	1	1	3	1	1	1
1313	1	15	2	1	1	3	1	1	1
1314	2	14	1	1	1	1	1	1	1
1315	2	12	1	1	1	1	1	3	1

1316	2	13	1	1	1	3	1	1	1
1317	1	14	1	1	1	3	1	1	1
1318	2	16	2	1	1	3	1	3	2
1319	9	0	#NULL!	9	9	9	9	9	1
1320	2	15	2	1	1	3	1	1	1
1321	2	16	2	1	1	3	1	1	1
1322	2	14	1	1	1	3	1	1	1
1323	1	15	2	1	1	3	3	3	2
1324	1	14	1	1	1	3	1	3	2
1325	2	15	2	1	1	3	3	2	1
1326	9	0	#NULL!	9	9	9	9	9	2
1327	1	19	2	1	1	3	3	3	2
1328	1	14	1	1	1	3	2	2	2
1329	1	17	2	1	1	3	1	2	2
1330	9	0	#NULL!	9	9	9	9	9	2
1331	2	15	2	1	1	3	3	1	2
1332	1	17	2	1	1	3	1	1	3
1333	2	14	1	1	1	3	1	1	2
1334	2	14	1	1	1	3	2	2	2
1335	1	18	2	1	1	3	1	2	1
1336	2	14	1	1	1	3	1	1	1
1337	2	16	2	1	1	3	1	2	1
1338	9	0	#NULL!	9	9	9	9	9	2
1339	9	0	#NULL!	9	9	9	9	9	1
1340	2	16	2	1	1	3	1	1	1
1341	2	15	2	1	1	3	1	3	9
1342	1	14	1	1	1	3	1	2	1
1343	2	13	1	1	1	3	1	3	1
1344	2	15	2	1	1	3	1	1	3
1345	2	14	1	1	1	3	1	1	1
1346	2	12	1	1	1	1	1	1	1
1347	1	15	2	1	1	3	2	2	1
1348	1	14	1	1	1	3	2	2	2
1349	1	19	2	1	1	9	2	1	3
1350	1	15	2	1	1	3	2	1	1
1351	2	15	2	1	1	3	9	9	9
1352	1	15	2	1	1	3	1	1	1
1353	1	14	1	1	1	3	1	1	2
1354	2	15	2	1	1	3	1	1	1
1355	2	15	2	1	1	3	1	2	1
1356	2	17	2	1	1	3	1	1	1
1357	2	14	1	1	1	3	1	1	1
1358	2	16	2	1	1	3	1	3	1
1359	1	14	1	1	1	3	1	2	9
1360	1	15	2	1	1	3	2	2	9
1361	1	14	1	1	1	2	2	2	9
1362	1	14	1	1	1	3	2	1	1

1363	2	15	2	1	1	3	3	2	1
1364	2	16	2	1	1	3	1	2	1
1365	2	15	2	1	1	3	1	2	1
1366	1	17	2	1	1	3	1	2	1
1367	2	18	2	1	1	3	2	2	9
1368	2	14	1	1	1	3	1	1	1
1369	2	19	2	1	1	3	1	3	3
1370	1	16	2	1	1	3	1	1	1
1371	2	15	2	1	1	3	1	1	1
1372	2	16	2	1	1	3	2	3	1
1373	1	18	2	1	1	3	1	2	1
1374	1	17	2	1	1	3	1	1	2
1375	1	19	2	9	1	3	2	2	2
1376	1	16	2	1	1	3	3	3	1
1377	1	18	2	1	1	3	1	2	2
1378	2	14	1	1	1	3	1	1	1
1379	2	15	2	1	1	3	2	2	1
1380	1	15	2	1	1	3	1	2	1
1381	2	16	2	1	1	3	2	2	2
1382	2	15	2	1	1	3	1	2	1
1383	1	12	1	1	1	3	1	2	1
1384	2	14	1	1	1	3	2	3	1
1385	2	15	2	1	1	3	2	9	1
1386	1	15	2	1	1	3	1	1	1
1387	2	17	2	1	1	3	1	3	3
1388	9	0	#NULL!	9	9	9	9	9	9
1389	2	14	1	1	1	3	1	2	1
1390	1	16	2	1	1	3	2	2	1
1391	1	19	2	1	1	3	1	1	1
1392	2	16	2	1	1	3	1	3	1
1393	2	14	1	1	1	3	1	2	2
1394	2	14	1	1	1	3	1	2	1
1395	2	15	2	1	1	3	1	2	1
1396	1	16	2	1	1	3	1	1	1
1397	1	17	2	1	1	3	1	1	1
1398	2	50	3	9	9	9	9	9	2
1399	1	15	2	1	1	3	2	2	3
1400	2	14	1	1	1	3	1	9	1
1401	2	0	#NULL!	1	1	3	1	1	1
1402	2	14	1	1	1	3	1	2	1
1403	1	16	2	1	1	3	1	1	2
1404	1	15	2	1	1	3	3	3	2
1405	2	14	1	1	1	3	1	1	3
1406	1	12	1	1	1	3	1	3	2
1407	1	14	1	1	1	3	1	3	2
1408	2	14	1	1	1	1	1	1	1
1409	1	16	2	9	1	3	2	3	2

1410	2	14	1	1	1	3	1	1	1
1411	1	15	2	1	1	3	1	2	2
1412	2	14	1	1	1	3	1	1	1
1413	2	14	1	1	1	3	1	2	1
1414	1	17	2	1	1	3	1	3	2
1415	1	2	1	1	1	3	1	3	3
1416	1	14	1	1	1	3	1	1	1
1417	1	14	1	1	1	3	1	1	1
1418	2	13	1	1	1	3	1	1	1
1419	2	14	1	1	1	3	1	1	1
1420	2	15	2	1	1	3	1	2	2
1421	1	14	1	1	1	3	3	3	2
1422	2	15	2	1	1	3	1	2	2
1423	2	16	2	1	1	3	1	1	1
1424	1	17	2	1	1	3	1	2	2
1425	2	14	1	1	1	3	1	1	1
1426	2	15	2	1	1	3	3	2	2
1427	2	14	1	1	1	3	1	1	1
1428	2	15	2	1	1	3	1	3	3
1429	2	16	2	1	1	3	1	2	1
1430	2	15	2	1	1	3	1	1	1
1431	1	17	2	1	3	3	1	1	1
1432	2	17	2	1	3	3	1	1	9
1433	1	16	2	1	3	3	1	1	2
1434	1	18	2	1	3	3	3	2	2
1435	2	17	2	1	3	3	1	1	1
1436	2	16	2	1	3	3	1	1	2
1437	2	16	2	1	3	3	1	1	2
1438	2	16	2	1	3	3	1	1	3
1439	2	16	2	1	3	3	1	1	1
1440	2	15	2	1	3	3	1	1	1
1441	2	16	2	1	3	3	1	2	1
1442	2	17	2	1	3	3	1	2	1
1443	2	16	2	1	3	3	1	1	9
1444	1	14	1	9	3	3	1	1	2
1445	2	17	2	1	3	3	1	1	1
1446	2	17	2	1	3	3	1	3	2
1447	2	16	2	1	3	3	1	1	1
1448	1	35	3	1	1	3	1	1	2
1449	1	10	1	1	2	3	3	1	2
1450	1	20	3	1	1	3	1	1	1
1451	9	0	#NULL!	9	9	9	9	9	9
1452	9	0	#NULL!	9	9	9	9	9	9
1453	2	25	3	9	1	1	1	1	1
1454	1	30	3	9	1	2	1	1	1
1455	9	0	#NULL!	9	9	9	9	9	9
1456	1	28	3	1	1	3	1	1	2

1457	9	0	#NULL!	9	9	9	9	9	2
1458	1	0	#NULL!	1	3	3	1	1	1
1459	1	25	3	1	3	3	2	2	1
1460	1	30	3	1	3	3	2	2	1
1461	2	17	2	1	3	3	1	1	1
1462	2	17	2	1	3	3	1	1	1
1463	1	16	2	9	3	3	1	3	2
1464	1	29	3	1	3	3	1	1	2
1465	1	13	1	1	3	3	1	1	1
1466	9	0	#NULL!	9	9	9	9	9	9
1467	2	20	3	1	1	1	1	1	1
1468	2	15	2	1	3	3	2	1	1
1469	2	14	1	1	3	1	9	9	1
1470	1	20	3	1	3	3	1	1	1
1471	1	18	2	1	3	3	1	1	1
1472	9	0	#NULL!	9	9	9	9	9	9
1473	9	0	#NULL!	9	9	9	9	9	9
1474	2	13	1	1	3	3	1	1	1
1475	2	13	1	9	3	3	1	2	2
1476	2	17	2	1	3	3	1	1	2
1477	1	8	1	9	1	1	2	3	2
1478	2	19	2	1	3	3	1	1	1
1479	2	16	2	1	3	3	1	3	3
1480	2	18	2	1	3	3	3	1	2
1481	1	17	2	1	3	3	2	2	2
1482	2	18	2	1	3	1	1	1	9
1483	2	16	2	1	3	3	1	1	1
1484	2	17	2	1	3	3	9	1	2
1485	9	0	#NULL!	9	9	9	9	9	2
1486	2	17	2	1	3	3	2	2	2
1487	1	19	2	1	3	3	1	1	2
1488	2	16	2	1	3	3	1	2	2
1489	2	18	2	1	3	3	3	3	2
1490	1	16	2	9	3	3	1	1	2
1491	2	14	1	1	3	3	1	1	1
1492	2	13	1	1	3	3	1	2	2
1493	2	13	1	1	3	1	2	2	3
1494	2	17	2	9	3	3	1	2	9
1495	1	40	3	1	3	3	2	2	9
1496	2	15	2	1	3	3	1	1	2
1497	1	20	3	1	3	1	3	3	2
1498	2	14	1	1	3	3	1	1	9
1499	1	12	1	1	3	3	2	3	1
1500	2	14	1	9	3	3	1	1	2
1501	2	12	1	1	3	3	3	3	2
1502	9	0	#NULL!	1	2	2	1	1	2
1503	2	15	2	1	3	3	1	1	2

1504	2	16	2	1	3	3	2	3	2
1505	2	16	2	1	3	3	1	2	1
1506	1	17	2	1	3	3	3	3	2
1507	2	17	2	1	3	3	1	2	1
1508	9	0	#NULL!	9	1	3	1	2	2
1509	2	18	2	1	3	3	2	2	2
1510	2	17	2	1	3	3	2	1	1
1511	2	17	2	1	3	3	1	2	1
1512	2	19	2	9	3	3	1	2	2
1513	2	16	2	1	3	3	1	2	9
1514	2	16	2	1	3	3	1	2	9
1515	2	14	1	1	3	3	1	1	2
1516	9	0	#NULL!	1	3	3	1	1	1
1517	2	15	2	1	3	3	2	2	1
1518	2	18	2	1	3	3	1	2	1
1519	2	17	2	1	3	3	1	2	1
1520	2	16	2	1	3	3	1	2	2
1521	2	17	2	1	3	3	1	2	2
1522	2	16	2	9	1	3	1	1	3
1523	2	17	2	1	3	3	1	2	2
1524	2	19	2	1	3	3	3	1	1
1525	2	16	2	1	3	3	1	1	1
1526	1	15	2	1	3	3	1	2	1
1527	9	0	#NULL!	9	9	9	9	9	9
1528	1	17	2	1	3	3	9	9	3
1529	1	18	2	1	3	3	1	2	9
1530	1	18	2	1	3	3	1	2	9
1531	1	19	2	1	3	3	1	2	9
1532	1	20	3	1	3	3	1	1	1
1533	2	19	2	1	3	3	1	1	1
1534	2	17	2	1	3	3	2	2	2
1535	2	16	2	1	3	3	1	1	1
1536	1	20	3	1	3	3	2	2	1
1537	2	18	2	1	3	3	3	3	2
1538	2	17	2	1	3	3	1	1	1
1539	2	16	2	1	3	3	1	1	1
1540	1	15	2	1	3	3	1	2	1
1541	9	18	2	1	3	3	1	2	2
1542	1	13	1	1	3	3	1	1	1
1543	1	18	2	1	3	3	1	1	1
1544	1	16	2	9	3	3	1	3	3
1545	1	16	2	1	3	3	2	2	2
1546	2	19	2	1	3	3	1	1	9
1547	2	16	2	1	3	3	1	1	9
1548	2	17	2	1	3	3	2	2	1
1549	2	18	2	1	3	3	1	2	2
1550	1	0	#NULL!	1	3	3	1	2	2

1551	1	19	2	1	3	3	1	1	3
1552	1	17	2	1	3	3	3	9	9
1553	1	18	2	1	3	3	1	1	9
1554	9	0	#NULL!	9	9	9	9	9	9
1555	1	17	2	1	3	3	3	2	9
1556	2	0	#NULL!	1	3	3	1	2	9
1557	9	0	#NULL!	9	9	9	9	9	9
1558	9	0	#NULL!	9	9	9	9	9	9
1559	9	0	#NULL!	9	9	9	9	9	9
1560	2	14	1	1	3	3	1	1	9
1561	2	18	2	1	3	3	1	1	2
1562	2	17	2	1	3	3	1	1	1
1563	2	17	2	1	3	3	1	1	1
1564	1	17	2	1	3	3	3	3	3
1565	2	13	1	1	3	3	1	1	2
1566	1	17	2	1	3	3	1	1	1
1567	2	17	2	1	3	3	1	1	2
1568	2	16	2	9	3	3	1	2	1
1569	2	16	2	1	3	3	2	1	2
1570	2	16	2	1	3	3	1	2	2
1571	2	15	2	1	3	3	1	2	1
1572	2	17	2	1	3	3	1	2	3
1573	2	15	2	9	3	3	1	2	1
1574	2	16	2	1	3	3	1	1	2
1575	1	17	2	1	3	3	1	1	2
1576	2	18	2	9	3	3	2	2	3
1577	2	16	2	9	3	3	2	1	3
1578	2	15	2	1	3	3	1	1	1
1579	9	0	#NULL!	9	9	9	9	9	2
1580	1	17	2	1	3	3	2	1	2
1581	9	0	#NULL!	9	9	9	9	9	9
1582	1	17	2	1	3	3	1	1	2
1583	1	17	2	1	3	3	3	3	1
1584	1	19	2	1	2	3	1	1	9
1585	2	17	2	1	2	1	2	2	3
1586	1	16	2	1	2	3	2	2	1
1587	2	16	2	1	2	3	1	2	2
1588	2	16	2	9	2	3	1	1	2
1589	9	16	2	1	2	3	1	1	1
1590	9	0	#NULL!	9	9	9	9	9	9
1591	2	16	2	1	2	3	1	1	1
1592	1	18	2	1	2	3	1	2	2
1593	2	0	#NULL!	1	2	3	2	2	2
1594	2	17	2	1	2	3	1	1	1
1595	2	16	2	1	2	3	1	1	1
1596	2	16	2	1	2	3	2	2	9
1597	2	14	1	1	2	3	1	2	2

1598	9	0	#NULL!	9	9	9	9	9	9
1599	2	0	#NULL!	1	2	3	1	2	3
1600	2	17	2	1	2	3	1	1	2
1601	9	0	#NULL!	9	9	9	9	9	9
1602	9	0	#NULL!	9	9	9	9	9	9
1603	9	0	#NULL!	9	9	9	9	9	9
1604	9	0	#NULL!	9	9	9	9	9	9
1605	9	0	#NULL!	9	9	9	9	9	9
1606	9	0	#NULL!	9	9	9	9	9	9
1607	9	0	#NULL!	9	9	9	9	9	9
1608	9	0	#NULL!	9	9	9	9	9	9
1609	9	0	#NULL!	9	9	9	9	9	9
1610	9	0	#NULL!	9	9	9	9	9	9
1611	9	0	#NULL!	9	9	9	9	9	9
1612	9	0	#NULL!	9	9	9	9	9	9
1613	9	0	#NULL!	9	9	9	9	9	9
1614	9	0	#NULL!	9	9	9	9	9	9
1615	9	0	#NULL!	9	9	9	9	9	9
1616	9	0	#NULL!	9	9	9	9	9	9
1617	9	0	#NULL!	9	9	9	9	9	9
1618	9	0	#NULL!	9	9	9	9	9	9
1619	9	0	#NULL!	9	9	9	9	9	9
1620	9	0	#NULL!	9	9	9	9	9	9
1621	1	16	2	1	2	3	1	1	2
1622	2	15	2	1	2	3	1	2	2
1623	2	15	2	1	2	3	1	1	1
1624	2	15	2	1	2	3	1	1	1
1625	2	14	1	1	2	3	1	2	1
1626	2	15	2	1	2	3	1	1	1
1627	1	15	2	1	2	3	1	1	2
1628	2	17	2	1	2	3	1	1	1
1629	2	18	2	1	2	3	1	3	1
1630	1	16	2	1	2	3	2	2	2
1631	2	15	2	1	2	3	1	1	1
1632	2	16	2	1	2	3	1	1	1
1633	2	14	1	1	2	3	1	1	1
1634	2	15	2	1	2	3	1	1	1
1635	2	16	2	1	2	3	1	1	1
1636	2	16	2	1	2	3	1	1	1
1637	1	16	2	1	2	3	1	1	1
1638	2	19	2	1	2	3	1	1	1
1639	1	18	2	1	2	3	1	1	1
1640	2	14	1	1	2	3	1	1	1
1641	2	17	2	1	2	3	1	1	1
1642	2	15	2	1	2	3	1	2	1
1643	1	17	2	1	2	2	1	2	1
1644	1	16	2	1	2	3	1	1	1

1645	2	14	1	1	2	3	1	1	1
1646	2	17	2	1	2	3	1	1	1
1647	2	16	2	1	2	3	1	1	3
1648	2	14	1	1	2	3	1	1	1
1649	2	15	2	9	2	2	1	1	1
1650	2	15	2	1	2	3	1	2	1
1651	2	15	2	1	2	3	1	1	1
1652	2	17	2	1	2	3	1	1	9
1653	2	16	2	1	2	3	1	2	1
1654	2	18	2	1	2	3	1	2	1
1655	2	16	2	1	2	3	1	1	9
1656	2	16	2	1	2	3	1	1	9
1657	2	16	2	1	2	3	1	1	9
1658	1	16	2	1	2	3	1	1	9
1659	2	14	1	1	2	3	1	1	1
1660	2	15	2	1	2	3	1	2	1
1661	2	16	2	1	2	3	1	1	1
1662	1	16	2	1	2	3	1	2	1
1663	2	16	2	1	2	3	1	1	1
1664	1	14	1	1	2	3	1	1	1
1665	2	17	2	1	2	3	1	1	9
1666	1	17	2	1	2	3	1	3	1
1667	2	15	2	1	2	3	1	1	9
1668	2	17	2	1	2	3	1	1	9
1669	1	17	2	1	2	3	1	1	9
1670	2	19	2	9	2	1	1	1	1
1671	1	0	#NULL!	1	2	3	1	1	1
1672	2	15	2	1	2	3	1	2	1
1673	2	16	2	1	2	3	1	1	1
1674	1	16	2	1	2	3	1	1	2
1675	2	16	2	1	2	1	1	2	1
1676	2	16	2	9	2	3	1	1	1
1677	2	15	2	1	2	3	1	2	3
1678	2	17	2	1	2	3	1	2	1
1679	2	16	2	1	2	3	1	1	1
1680	1	17	2	1	2	3	1	2	1
1681	2	18	2	1	2	3	1	1	2
1682	2	17	2	1	2	3	3	2	2
1683	9	16	2	1	2	2	2	1	2
1684	1	16	2	1	2	3	1	1	2
1685	2	14	1	1	2	3	1	1	1
1686	2	15	2	1	2	3	1	1	1
1687	2	16	2	1	2	3	1	2	1
1688	2	17	2	1	2	3	1	2	3
1689	2	17	2	1	2	3	1	1	1
1690	2	15	2	1	2	3	1	1	9
1691	2	16	2	1	2	3	1	1	9

1692	2	15	2	1	2	3	1	1	2
1693	2	17	2	1	3	3	1	1	2
1694	2	15	2	1	2	3	1	2	1
1695	2	16	2	1	2	3	1	1	9
1696	1	15	2	1	2	3	1	1	2
1697	2	9	1	1	2	3	1	2	1
1698	2	18	2	1	2	3	1	1	1
1699	2	16	2	1	2	3	1	1	9
1700	2	15	2	1	2	3	3	3	2
1701	1	18	2	1	2	3	1	1	2
1702	1	16	2	1	2	3	1	1	2
1703	2	16	2	1	2	3	1	1	9
1704	9	15	2	1	2	3	1	1	1
1705	2	16	2	1	2	3	1	1	2
1706	2	19	2	1	2	3	1	1	9
1707	1	14	1	1	2	3	1	1	3
1708	2	17	2	1	2	3	1	1	9
1709	2	17	2	1	2	3	1	1	9
1710	1	18	2	1	2	3	1	1	2
1711	2	15	2	1	2	3	1	1	1
1712	2	14	1	1	2	3	1	1	1
1713	2	14	1	1	2	3	1	1	1
1714	1	16	2	1	2	3	2	3	3
1715	2	17	2	1	2	3	1	1	1
1716	2	15	2	1	2	3	1	1	1
1717	2	16	2	1	2	3	3	9	9
1718	2	0	#NULL!	9	2	3	1	2	9
1719	2	16	2	1	2	3	1	1	1
1720	2	17	2	1	2	3	1	2	3
1721	2	14	1	1	2	3	1	1	1
1722	9	0	#NULL!	9	9	9	9	9	9
1723	9	0	#NULL!	9	9	9	9	9	9
1724	9	0	#NULL!	9	9	9	9	9	9
1725	9	0	#NULL!	9	9	9	9	9	9
1726	9	0	#NULL!	9	9	9	9	9	9
1727	9	0	#NULL!	9	9	9	9	9	9
1728	9	0	#NULL!	9	9	9	9	9	9
1729	9	0	#NULL!	9	9	9	9	9	9
1730	9	0	#NULL!	9	9	9	9	9	9
1731	9	0	#NULL!	9	9	9	9	9	9
1732	9	0	#NULL!	9	9	9	9	9	9
1733	9	0	#NULL!	9	9	9	9	9	9
1734	9	0	#NULL!	9	9	9	9	9	9
1735	9	0	#NULL!	9	9	9	9	9	9
1736	9	0	#NULL!	9	9	9	9	9	9
1737	9	0	#NULL!	9	9	9	9	9	9
1738	9	0	#NULL!	9	9	9	9	9	9

1739	9	0	#NULL!	9	9	9	9	9	9
1740	9	0	#NULL!	9	9	9	9	9	9
1741	9	0	#NULL!	9	9	9	9	9	9
1742	9	0	#NULL!	9	9	9	9	9	9
1743	9	0	#NULL!	9	9	9	9	9	9
1744	9	0	#NULL!	9	9	9	9	9	9
1745	9	0	#NULL!	9	9	9	9	9	9
1746	9	0	#NULL!	9	9	9	9	9	9
1747	9	0	#NULL!	1	2	3	1	1	9
1748	9	0	#NULL!	9	9	9	9	9	9
1749	9	0	#NULL!	9	9	9	9	9	9
1750	9	0	#NULL!	9	9	9	9	9	9
1751	9	0	#NULL!	9	9	9	9	9	9
1752	9	0	#NULL!	9	9	9	9	9	9
1753	9	0	#NULL!	9	9	9	9	9	9
1754	9	0	#NULL!	9	9	9	9	9	9
1755	9	0	#NULL!	9	9	9	9	9	9
1756	9	0	#NULL!	9	9	9	9	9	9
1757	9	0	#NULL!	9	9	9	9	9	9
1758	2	0	#NULL!	1	2	3	1	1	1
1759	9	0	#NULL!	9	9	9	9	9	9
1760	9	0	#NULL!	9	9	9	9	9	9
1761	9	0	#NULL!	9	9	9	9	9	9
1762	9	0	#NULL!	9	9	9	9	9	9
1763	9	0	#NULL!	9	9	9	9	9	9
1764	9	0	#NULL!	9	9	9	9	9	9
1765	9	0	#NULL!	9	9	9	9	9	9
1766	9	0	#NULL!	9	9	9	9	9	9
1767	9	0	#NULL!	9	9	9	9	9	9
1768	9	0	#NULL!	9	9	9	9	9	9
1769	9	0	#NULL!	9	9	9	9	9	9
1770	9	0	#NULL!	9	9	9	9	9	9
1771	9	0	#NULL!	9	9	9	9	9	9
1772	9	0	#NULL!	9	9	9	9	9	9
1773	9	0	#NULL!	9	9	9	9	9	9
1774	1	16	2	1	2	3	1	1	2
1775	2	17	2	1	2	3	1	1	1
1776	2	16	2	1	2	3	1	1	1
1777	2	17	2	1	2	3	1	1	1
1778	1	17	2	1	2	3	3	3	3
1779	2	17	2	1	2	3	1	1	1
1780	1	15	2	1	2	3	1	1	9
1781	2	17	2	1	2	3	1	1	1
1782	1	15	2	1	2	3	2	2	9
1783	1	15	2	1	2	3	1	3	2
1784	1	16	2	1	2	2	1	2	1
1785	2	16	2	1	2	3	1	1	1

1786	2	15	2	1	2	3	1	1	1
1787	2	0	#NULL!	1	2	3	1	1	1
1788	2	16	2	1	2	3	1	1	1
1789	2	14	1	1	2	3	1	1	1
1790	2	15	2	1	2	3	1	1	2
1791	2	14	1	1	2	3	1	1	1
1792	1	14	1	1	2	3	1	1	1
1793	2	16	2	1	2	3	1	1	1
1794	1	17	2	1	2	3	2	2	2
1795	1	16	2	1	2	3	1	2	1
1796	2	14	1	1	2	3	1	1	9
1797	2	17	2	1	2	3	1	1	1
1798	1	19	2	1	2	3	1	1	1
1799	2	16	2	1	2	3	1	1	1
1800	2	16	2	1	2	3	1	1	2
1801	1	18	2	1	2	3	1	1	1
1802	1	0	#NULL!	1	2	3	1	1	1
1803	1	16	2	9	2	3	1	3	1
1804	2	14	1	1	2	3	1	3	1
1805	2	15	2	1	2	3	1	1	1
1806	2	17	2	1	2	3	1	2	1
1807	2	15	2	9	2	3	1	1	1
1808	2	16	2	1	2	3	1	1	1
1809	2	15	2	1	2	3	1	1	9
1810	1	17	2	1	2	3	2	2	3
1811	1	16	2	1	3	2	1	2	1
1812	2	16	2	1	3	2	2	2	1
1813	1	15	2	1	3	2	2	2	3
1814	1	15	2	1	3	2	1	1	2
1815	1	0	#NULL!	1	3	2	1	1	1
1816	1	17	2	1	3	2	1	1	2
1817	1	0	#NULL!	1	3	2	1	1	1
1818	1	0	#NULL!	1	3	2	1	1	1
1819	1	18	2	1	3	2	1	2	9
1820	1	18	2	1	3	2	1	1	9
1821	1	18	2	1	3	2	1	2	9
1822	1	18	2	1	3	2	1	1	9
1823	1	18	2	1	3	2	1	1	9
1824	1	18	2	1	3	2	1	1	9
1825	1	18	2	1	3	2	1	2	9
1826	1	18	2	1	3	2	1	1	1
1827	2	18	2	9	3	1	1	1	1
1828	1	16	2	1	3	2	1	1	1
1829	1	17	2	1	2	2	1	2	1
1830	1	14	1	9	3	2	2	2	1
1831	1	14	1	1	3	2	2	1	3
1832	1	12	1	1	3	2	2	2	1

1833	1	16	2	1	3	2	2	2	2
1834	1	16	2	1	3	2	2	2	2
1835	1	30	3	1	3	2	2	2	2
1836	1	15	2	1	3	2	2	2	1
1837	1	17	2	1	3	2	1	1	2
1838	1	16	2	1	3	2	2	2	1
1839	1	14	1	1	3	2	1	2	1
1840	1	18	2	1	3	2	1	3	1
1841	1	14	1	1	3	2	2	2	1
1842	1	16	2	1	3	2	1	2	9
1843	1	18	2	1	3	2	1	1	2
1844	1	17	2	1	3	2	2	2	2
1845	1	14	1	1	3	2	2	2	1
1846	1	14	1	1	3	2	1	2	1
1847	1	18	2	1	3	2	2	2	1
1848	1	18	2	1	3	2	2	2	1
1849	1	18	2	1	3	2	1	1	3
1850	1	19	2	1	3	2	1	2	1
1851	1	18	2	1	3	2	1	2	1
1852	1	18	2	1	3	2	3	2	9
1853	1	18	2	1	3	2	2	1	3
1854	1	18	2	1	3	2	2	2	2
1855	1	16	2	1	3	2	1	2	1
1856	1	19	2	1	3	2	1	2	1
1857	1	14	1	1	3	2	2	2	1
1858	1	18	2	1	3	2	2	2	1
1859	1	18	2	1	3	2	1	3	1
1860	1	19	2	1	3	2	1	2	3
1861	1	17	2	1	3	2	2	2	1
1862	1	18	2	1	3	2	1	2	1
1863	1	18	2	1	3	2	1	2	1
1864	1	16	2	1	3	2	2	2	1
1865	1	16	2	1	3	2	2	2	1
1866	1	18	2	1	3	2	2	2	1
1867	1	16	2	1	3	2	1	2	1
1868	1	13	1	1	3	2	2	2	1
1869	1	20	3	1	3	2	1	9	1
1870	1	14	1	1	3	2	1	2	9
1871	1	14	1	1	3	2	1	2	1
1872	1	14	1	1	3	2	1	2	1
1873	1	15	2	1	3	2	2	2	1
1874	1	14	1	1	3	2	2	2	1
1875	1	12	1	1	3	2	2	2	1
1876	1	14	1	1	3	2	2	2	2
1877	1	13	1	1	3	2	3	3	2
1878	1	18	2	1	3	2	2	2	1
1879	2	18	2	1	3	1	2	2	3

1880	2	18	2	1	3	1	1	1	1
1881	1	15	2	1	3	2	2	2	1
1882	1	17	2	1	3	2	1	1	1
1883	1	23	3	1	3	2	1	2	2
1884	1	20	3	1	3	2	1	2	1
1885	1	29	3	9	3	2	2	2	2
1886	1	19	2	1	3	2	1	2	2
1887	1	12	1	1	3	1	3	3	9
1888	9	0	#NULL!	1	3	2	9	9	9
1889	1	19	2	1	3	2	2	3	1
1890	1	17	2	1	3	2	1	1	2
1891	1	18	2	1	3	2	1	2	2
1892	1	15	2	1	3	2	2	2	2
1893	1	20	3	1	3	2	1	1	2
1894	2	19	2	1	3	1	2	2	2
1895	2	19	2	1	3	1	2	2	2
1896	1	19	2	1	3	9	9	9	2
1897	1	20	3	1	3	2	1	1	1
1898	1	19	2	1	3	2	1	1	2
1899	1	18	2	1	3	2	1	1	3
1900	1	14	1	1	3	2	2	2	2
1901	1	17	2	1	3	2	1	2	2
1902	1	19	2	1	3	2	1	2	2
1903	1	17	2	1	3	2	2	2	2
1904	1	18	2	1	3	2	1	2	2
1905	1	18	2	1	3	2	1	2	2
1906	1	17	2	1	3	2	1	1	2
1907	1	19	2	9	3	2	1	3	1
1908	2	23	3	9	3	2	2	2	1
1909	1	18	2	1	3	2	2	1	2
1910	1	18	2	9	9	9	9	9	2
1911	1	17	2	1	3	2	1	1	2
1912	1	19	2	9	9	9	9	9	2
1913	1	17	2	1	3	2	2	2	2
1914	1	17	2	1	3	2	1	1	2
1915	1	17	2	1	3	2	1	1	2
1916	1	19	2	1	3	2	1	1	2
1917	1	16	2	1	3	2	1	2	2
1918	1	17	2	9	9	9	9	9	2
1919	1	16	2	1	3	2	1	2	3
1920	1	17	2	1	3	2	1	2	2
1921	1	17	2	1	3	2	1	2	2
1922	1	20	3	9	2	2	1	1	1
1923	1	14	1	1	2	2	1	1	2
1924	1	20	3	1	2	2	1	1	2
1925	1	15	2	1	2	9	9	9	2
1926	1	18	2	1	3	2	1	2	2

1927	1	14	1	1	2	2	1	1	2
1928	1	19	2	1	2	2	1	1	1
1929	1	16	2	1	3	2	2	2	1
1930	1	18	2	1	2	2	1	2	2
1931	1	21	3	1	2	2	1	1	1
1932	1	16	2	9	9	9	9	9	2
1933	1	15	2	1	2	2	2	1	9
1934	1	18	2	1	2	2	1	1	1
1935	1	18	2	1	2	2	1	1	1
1936	9	0	#NULL!	1	2	2	2	2	2
1937	1	19	2	1	2	2	2	2	3
1938	1	19	2	9	2	1	2	1	2
1939	1	17	2	1	2	2	2	2	1
1940	1	18	2	1	2	2	1	1	3
1941	1	17	2	9	2	2	1	1	2
1942	1	17	2	1	2	2	1	2	1
1943	1	17	2	1	2	2	1	1	1
1944	1	18	2	1	2	2	1	1	1
1945	1	16	2	1	2	2	1	1	1
1946	1	15	2	1	2	2	1	1	2
1947	1	16	2	1	2	2	1	1	2
1948	1	16	2	1	2	3	1	1	9
1949	1	16	2	1	2	2	1	1	1
1950	1	17	2	1	2	2	1	2	2
1951	1	30	3	1	2	3	1	1	1
1952	1	17	2	1	2	2	2	2	2
1953	1	14	1	1	2	2	2	2	1
1954	1	15	2	1	2	2	1	1	1
1955	1	18	2	1	2	2	2	1	1
1956	1	18	2	1	3	3	1	1	1
1957	9	0	#NULL!	9	9	9	9	9	1
1958	1	25	3	1	3	3	2	2	2
1959	1	18	2	1	2	2	2	2	2
1960	1	16	2	1	2	2	1	2	1
1961	1	17	2	1	2	2	1	2	1
1962	1	16	2	1	2	3	1	1	1
1963	1	16	2	1	2	2	2	1	1
1964	1	19	2	1	2	2	1	1	2
1965	1	18	2	1	2	2	3	3	2
1966	1	29	3	1	2	2	3	3	2
1967	1	18	2	1	3	2	1	1	2
1968	1	22	3	1	2	2	1	1	1
1969	1	18	2	1	2	2	1	2	9
1970	1	16	2	1	2	2	2	2	2
1971	1	17	2	1	2	2	1	1	1
1972	1	16	2	1	2	2	1	1	1
1973	1	18	2	1	2	2	1	1	1

1974	1	15	2	1	2	3	1	1	1
1975	1	16	2	1	2	2	1	1	2
1976	1	15	2	1	2	2	1	1	1
1977	1	27	3	1	3	2	1	1	2
1978	1	16	2	1	2	2	1	1	2
1979	1	16	2	1	2	2	1	1	1
1980	1	18	2	1	2	2	1	1	3
1981	1	16	2	1	1	1	1	1	1
1982	1	16	2	1	2	2	1	2	1
1983	1	17	2	1	2	2	1	1	1
1984	1	12	1	1	2	2	1	2	3
1985	1	25	3	9	3	3	3	1	2
1986	1	15	2	1	2	2	1	2	2
1987	1	15	2	1	2	2	1	2	2
1988	1	50	3	9	3	1	2	2	2
1989	1	20	3	9	3	1	2	2	1
1990	1	18	2	1	2	2	2	2	1
1991	1	16	2	1	2	2	1	1	2
1992	1	5	1	9	3	1	9	9	9
1993	9	17	2	1	2	2	1	2	1
1994	1	15	2	1	2	2	1	1	1
1995	1	17	2	1	2	2	1	1	1
1996	1	13	1	1	2	2	2	1	1
1997	1	16	2	1	2	2	1	2	1
1998	1	13	1	1	2	2	2	2	9
1999	1	17	2	1	2	2	1	1	2
2000	1	16	2	1	2	2	1	3	2
2001	1	16	2	1	2	2	1	2	3
2002	1	18	2	1	2	2	1	1	2
2003	1	15	2	1	2	2	2	2	2
2004	9	0	#NULL!	9	9	9	9	9	3
2005	1	16	2	1	2	2	1	2	2
2006	1	39	3	1	2	2	3	3	3
2007	1	25	3	1	2	2	1	1	1
2008	1	15	2	1	2	2	1	1	2
2009	9	0	#NULL!	9	9	9	9	9	3
2010	1	16	2	1	2	2	3	2	2
2011	1	18	2	1	2	3	3	3	3
2012	1	16	2	1	2	2	1	3	1
2013	1	19	2	1	2	2	1	1	3
2014	1	16	2	1	2	2	1	1	1
2015	1	12	1	1	2	2	2	2	2
2016	9	0	#NULL!	9	9	9	9	9	9
2017	1	13	1	1	2	2	2	2	3
2018	1	14	1	1	2	2	1	3	2
2019	1	15	2	1	2	2	1	1	2
2020	1	15	2	1	2	2	2	1	1

2021	1	17	2	1	2	2	1	1	2
2022	1	16	2	1	2	2	1	2	1
2023	1	12	1	1	2	2	2	3	3
2024	1	16	2	1	2	2	1	1	1
2025	1	14	1	9	1	2	1	2	1
2026	1	13	1	9	1	2	2	2	2
2027	1	14	1	1	1	2	1	1	2
2028	1	13	1	1	1	2	1	2	2
2029	9	0	#NULL!	9	9	9	9	9	2
2030	1	13	1	1	1	2	1	2	3
2031	1	15	2	1	1	2	1	2	2
2032	1	14	1	1	1	2	2	2	1
2033	1	14	1	1	1	2	1	2	2
2034	1	13	1	1	1	2	1	2	1
2035	1	16	2	1	1	2	2	2	2
2036	1	12	1	1	1	2	1	2	1
2037	1	14	1	1	1	2	2	3	2
2038	1	13	1	1	1	2	1	2	1
2039	1	14	1	1	1	2	1	2	1
2040	1	14	1	1	1	2	1	1	1
2041	1	14	1	1	1	2	1	1	2
2042	1	15	2	1	1	2	2	2	2
2043	1	12	1	1	1	2	2	2	1
2044	1	15	2	9	1	2	1	1	2
2045	1	14	1	1	1	1	1	1	1
2046	9	0	#NULL!	9	9	9	9	9	1
2047	1	13	1	1	1	2	1	2	1
2048	1	14	1	1	1	2	1	1	3
2049	1	14	1	1	1	2	1	2	1
2050	1	14	1	1	1	2	1	2	1
2051	1	13	1	1	1	2	1	2	1
2052	9	0	#NULL!	9	9	9	9	9	2
2053	1	14	1	9	1	2	1	2	2
2054	1	14	1	1	1	2	1	2	1
2055	1	11	1	1	1	2	1	2	1
2056	1	13	1	1	1	2	1	2	1
2057	9	0	#NULL!	1	1	2	2	2	2
2058	1	15	2	1	1	2	2	2	9
2059	1	13	1	1	3	2	3	3	2
2060	1	15	2	1	1	2	1	2	1
2061	1	14	1	1	1	2	1	2	2
2062	1	15	2	1	1	2	2	2	2
2063	1	15	2	1	1	2	1	2	2
2064	1	14	1	1	9	2	2	2	2
2065	1	14	1	1	9	2	2	2	2
2066	1	14	1	1	1	3	1	2	1
2067	1	15	2	9	3	3	2	2	2

2068	1	14	1	9	9	9	9	9	9
2069	1	15	2	1	1	2	1	1	1
2070	1	14	1	1	1	2	1	2	1
2071	1	15	2	1	1	2	1	2	1
2072	1	16	2	1	1	2	1	2	1
2073	1	16	2	1	1	2	1	2	1
2074	1	16	2	1	1	2	1	2	1
2075	1	0	#NULL!	9	1	2	1	2	9
2076	1	16	2	1	1	2	1	2	1
2077	1	14	1	1	1	2	1	1	1
2078	1	16	2	1	1	2	1	1	1
2079	1	14	1	9	1	2	2	2	1
2080	1	14	1	1	1	2	1	2	1
2081	1	15	2	1	1	2	1	1	1
2082	9	0	#NULL!	1	1	1	1	1	2
2083	1	15	2	1	1	2	3	3	2
2084	1	15	2	9	1	2	1	1	2
2085	1	14	1	1	1	2	1	2	1
2086	1	16	2	1	1	2	1	2	9
2087	1	15	2	1	1	2	1	1	2
2088	9	0	#NULL!	9	9	9	9	9	9
2089	9	0	#NULL!	1	1	2	1	1	1
2090	1	14	1	1	1	2	1	3	2
2091	9	0	#NULL!	9	9	9	9	9	3
2092	1	16	2	1	1	2	2	2	1
2093	1	14	1	1	1	2	2	2	2
2094	1	15	2	1	1	2	2	1	1
2095	1	16	2	1	1	2	1	2	1
2096	1	15	2	1	1	2	1	1	1
2097	1	15	2	1	1	2	1	1	1
2098	1	15	2	1	1	2	1	2	3
2099	1	15	2	1	1	2	1	2	2
2100	9	18	2	1	1	2	1	1	1
2101	1	15	2	1	1	2	2	2	3
2102	1	13	1	1	1	2	3	3	2
2103	9	0	#NULL!	1	1	2	2	2	2
2104	9	0	#NULL!	1	1	2	1	2	9
2105	1	15	2	1	1	2	1	2	2
2106	1	16	2	1	1	2	1	1	2
2107	1	16	2	1	1	2	1	1	2
2108	1	16	2	1	1	2	1	1	2
2109	1	15	2	1	1	2	1	2	1
2110	1	15	2	1	1	2	1	1	1
2111	1	15	2	1	1	2	1	2	2
2112	1	16	2	1	1	2	1	2	9
2113	9	17	2	1	2	2	1	1	3
2114	1	18	2	1	1	2	1	2	1

2115	9	13	1	1	1	2	1	2	3
2116	1	14	1	1	1	2	1	1	1
2117	1	15	2	1	1	2	1	1	1
2118	9	0	#NULL!	1	1	2	2	2	9
2119	1	15	2	1	1	2	2	2	1
2120	1	15	2	1	1	2	2	2	3
2121	1	15	2	1	1	1	1	1	1
2122	1	15	2	1	1	2	2	2	3
2123	1	13	1	1	1	2	1	3	2
2124	1	13	1	1	1	2	1	1	1
2125	1	13	1	1	1	2	2	2	2
2126	1	13	1	1	1	2	1	2	1
2127	1	18	2	1	1	2	2	2	1
2128	1	18	2	9	1	2	1	2	9
2129	1	19	2	9	1	1	2	2	2
2130	1	15	2	1	1	2	2	3	3
2131	1	15	2	1	1	2	1	2	1
2132	1	13	1	1	1	2	1	2	2
2133	1	14	1	1	1	2	3	3	2
2134	1	15	2	1	1	2	1	2	1
2135	1	14	1	1	1	2	1	2	1
2136	1	12	1	1	1	2	2	2	3
2137	1	17	2	1	1	2	2	2	1
2138	1	14	1	1	1	2	1	3	1
2139	1	14	1	1	1	2	1	2	1
2140	1	14	1	1	1	2	2	2	1
2141	1	14	1	9	1	2	1	2	1
2142	1	15	2	1	1	2	1	2	1
2143	1	14	1	1	1	2	1	2	2
2144	1	29	3	9	1	2	1	2	1
2145	1	15	2	1	1	2	1	1	3
2146	1	0	#NULL!	1	1	2	3	3	1
2147	1	14	1	1	1	1	1	1	1
2148	1	19	2	9	1	2	1	1	1
2149	1	13	1	1	1	2	1	2	1
2150	1	15	2	1	1	2	1	2	1
2151	1	14	1	1	1	2	1	1	1
2152	1	14	1	1	1	2	1	2	1
2153	1	15	2	1	1	2	1	2	1
2154	1	15	2	1	1	2	1	2	1
2155	1	15	2	1	1	2	2	2	3
2156	1	14	1	1	1	2	1	2	1
2157	1	14	1	1	1	2	1	1	3
2158	1	15	2	1	1	2	1	2	1
2159	9	0	#NULL!	9	9	9	9	9	2
2160	1	16	2	1	9	9	1	1	2
2161	1	19	2	9	1	2	1	1	1

2162	1	15	2	1	1	2	1	2	1
2163	1	16	2	1	1	2	1	3	1
2164	1	15	2	1	1	2	1	3	2
2165	1	14	1	1	1	2	1	2	1
2166	1	14	1	1	1	2	1	2	1
2167	1	13	1	1	1	2	1	2	3
2168	1	13	1	1	1	2	2	3	1
2169	1	14	1	1	1	2	1	1	1
2170	1	13	1	1	1	2	1	2	1
2171	1	14	1	1	1	2	1	2	1
2172	1	14	1	1	1	2	1	3	1
2173	1	17	2	1	1	2	1	2	1
2174	1	14	1	1	1	2	1	1	1
2175	1	14	1	1	1	2	1	2	1
2176	1	14	1	9	1	2	1	2	1
2177	1	13	1	1	1	2	1	2	1
2178	1	12	1	1	1	2	3	3	1
2179	1	14	1	1	1	2	1	1	1
2180	1	14	1	1	1	2	1	2	3
2181	1	13	1	1	1	2	1	2	1
2182	1	14	1	1	1	2	1	2	1
2183	1	14	1	1	1	2	1	1	1
2184	1	0	#NULL!	1	1	2	1	2	1
2185	1	15	2	1	1	2	1	2	1
2186	1	15	2	1	1	2	2	2	1
2187	1	13	1	1	1	2	2	2	2
2188	1	15	2	1	1	2	2	1	1
2189	1	14	1	1	1	2	1	1	1
2190	1	14	1	1	1	2	1	1	2
2191	1	14	1	1	1	2	1	2	9
2192	1	15	2	1	1	2	1	2	9
2193	1	15	2	1	1	2	1	2	9
2194	1	30	3	1	3	2	1	1	2
2195	1	14	1	1	1	2	2	2	9
2196	9	0	#NULL!	1	1	2	1	2	2
2197	9	0	#NULL!	1	1	2	1	1	2
2198	1	14	1	1	1	2	2	3	2
2199	1	15	2	1	1	2	2	2	1
2200	1	14	1	1	1	2	1	2	1
2201	1	14	1	1	1	2	1	1	2
2202	1	17	2	1	1	2	1	1	2
2203	1	15	2	1	1	2	1	2	1
2204	1	14	1	1	1	2	1	2	3
2205	1	15	2	1	1	2	1	2	3
2206	1	13	1	1	1	2	1	1	1
2207	1	16	2	1	1	2	2	1	2
2208	1	17	2	1	1	2	2	1	1

2209	1	2	1	1	1	2	2	2	1
2210	1	13	1	1	1	2	1	2	1
2211	1	16	2	1	1	2	1	1	2
2212	1	16	2	1	1	2	1	2	1
2213	1	15	2	1	1	2	1	1	2
2214	1	17	2	1	1	2	1	2	2
2215	1	14	1	1	1	2	1	2	9
2216	1	14	1	1	1	2	1	2	1
2217	1	13	1	1	1	2	1	2	1
2218	1	14	1	1	1	2	1	1	3
2219	1	12	1	1	1	2	2	2	2
2220	1	13	1	9	1	2	1	1	1
2221	1	13	1	1	1	2	1	1	1
2222	1	15	2	1	1	2	2	2	2
2223	1	15	2	1	1	2	2	2	2
2224	1	0	#NULL!	1	1	2	1	2	1
2225	1	14	1	1	1	2	2	2	2
2226	1	13	1	1	1	2	2	2	2
2227	1	15	2	1	1	2	2	2	2
2228	1	14	1	1	1	2	1	2	9
2229	2	0	#NULL!	1	3	1	1	1	1
2230	2	19	2	1	3	1	1	1	1
2231	2	18	2	1	3	1	1	1	1
2232	2	16	2	1	3	1	1	1	1
2233	2	16	2	1	3	1	1	1	1
2234	2	0	#NULL!	1	3	1	1	1	1
2235	2	16	2	1	3	1	1	1	1
2236	2	16	2	1	3	1	1	1	1
2237	2	16	2	1	3	1	1	1	1
2238	9	0	#NULL!	9	9	9	9	9	9
2239	2	17	2	1	3	1	1	1	1
2240	9	0	#NULL!	1	3	1	1	1	1
2241	2	15	2	1	3	1	1	1	1
2242	2	16	2	1	3	1	1	1	1
2243	2	16	2	1	3	1	1	1	1
2244	2	16	2	1	3	1	1	1	1
2245	2	0	#NULL!	1	3	1	1	1	1
2246	2	16	2	1	3	1	1	1	1
2247	2	18	2	1	3	1	1	1	1
2248	2	18	2	1	3	1	1	1	1
2249	2	17	2	1	3	1	1	1	1
2250	2	16	2	1	3	1	1	1	1
2251	2	17	2	1	3	1	1	1	1
2252	2	20	3	1	3	1	1	1	9
2253	2	17	2	1	3	1	1	1	1
2254	2	17	2	1	3	1	1	1	1
2255	2	17	2	1	3	1	1	1	1

2256	2	17	2	9	3	1	1	1	1
2257	2	17	2	1	3	1	1	1	1
2258	2	19	2	1	3	1	1	1	1
2259	2	16	2	1	3	1	1	1	1
2260	2	17	2	1	3	1	1	1	1
2261	2	16	2	1	3	1	1	1	1
2262	2	18	2	1	3	1	1	1	1
2263	2	16	2	1	3	1	1	1	1
2264	2	16	2	1	3	1	1	1	1
2265	2	16	2	1	3	1	1	1	1
2266	2	17	2	1	3	9	1	1	1
2267	2	18	2	1	3	1	1	1	1
2268	2	17	2	1	3	1	1	1	1
2269	9	0	#NULL!	9	9	9	1	1	1
2270	9	0	#NULL!	1	9	9	1	1	1
2271	2	16	2	1	3	1	1	1	1
2272	2	18	2	1	3	1	1	1	1
2273	2	18	2	1	3	1	1	1	1
2274	2	15	2	1	9	9	1	1	1
2275	2	15	2	1	3	1	1	1	1
2276	2	16	2	1	3	1	1	1	9
2277	2	17	2	1	3	1	1	1	9
2278	2	17	2	1	3	1	1	1	1
2279	2	18	2	1	3	1	1	1	1
2280	9	0	#NULL!	1	3	1	1	1	1
2281	2	16	2	1	3	1	1	1	1
2282	2	16	2	1	3	1	1	1	1
2283	2	17	2	1	3	1	1	1	1
2284	2	17	2	1	3	1	1	1	1
2285	2	17	2	1	3	1	1	1	1
2286	9	0	#NULL!	1	3	9	1	2	1
2287	2	17	2	1	3	1	1	1	1
2288	2	17	2	1	3	1	1	1	1
2289	2	16	2	1	3	1	1	1	1
2290	2	17	2	1	3	1	1	1	1
2291	2	16	2	1	3	1	1	1	1
2292	2	18	2	1	3	1	1	1	9
2293	2	18	2	1	3	1	1	1	1
2294	2	17	2	1	3	1	1	1	1
2295	2	17	2	1	3	1	1	1	1
2296	2	16	2	1	3	1	1	1	1
2297	2	17	2	1	3	1	1	1	1
2298	2	16	2	1	3	1	1	1	1
2299	2	15	2	1	3	1	1	1	1
2300	9	0	#NULL!	9	9	9	9	9	1
2301	2	15	2	1	3	1	1	1	1
2302	2	16	2	1	3	1	1	1	1

2303	2	17	2	1	3	1	1	1	1
2304	2	16	2	1	3	1	1	1	1
2305	2	16	2	1	3	1	1	1	1
2306	2	17	2	1	3	1	1	1	1
2307	2	16	2	1	3	1	1	1	1
2308	2	17	2	1	3	1	1	2	9
2309	2	17	2	1	3	1	1	1	1
2310	2	19	2	1	3	1	1	1	1
2311	2	18	2	1	3	1	1	1	1
2312	2	19	2	1	3	1	1	1	1
2313	2	17	2	1	3	1	1	2	2
2314	2	20	3	1	3	1	1	1	1
2315	2	17	2	1	3	1	2	1	3
2316	2	17	2	1	3	1	1	2	1
2317	2	18	2	1	3	1	1	1	2
2318	2	17	2	1	3	1	1	1	1
2319	2	16	2	1	3	1	1	1	1
2320	2	16	2	1	3	1	1	1	9
2321	2	17	2	1	3	1	1	1	1
2322	2	16	2	1	3	1	1	1	1
2323	2	17	2	1	3	1	1	1	1
2324	2	17	2	1	3	1	1	1	2
2325	2	15	2	9	3	1	1	1	1
2326	2	18	2	1	3	1	1	1	1
2327	2	16	2	1	3	1	1	1	1
2328	2	16	2	1	3	1	1	1	1
2329	2	17	2	1	3	1	1	2	9
2330	2	17	2	1	3	1	9	1	1
2331	9	18	2	1	3	1	1	1	1
2332	2	0	#NULL!	1	3	1	1	1	2
2333	2	17	2	1	3	1	1	1	2
2334	2	18	2	1	3	1	1	2	1
2335	2	17	2	1	3	1	2	1	2
2336	2	17	2	1	3	1	1	1	9
2337	2	17	2	1	3	1	1	1	1
2338	2	17	2	1	3	1	1	1	1
2339	9	16	2	9	3	1	1	1	1
2340	9	17	2	1	3	1	1	1	1
2341	9	17	2	1	3	1	1	1	1
2342	2	17	2	1	3	1	2	1	1
2343	2	16	2	1	3	1	1	1	1
2344	2	16	2	1	3	1	1	1	1
2345	2	17	2	1	3	1	1	1	1
2346	2	16	2	9	3	1	1	1	3
2347	2	15	2	1	3	1	2	2	1
2348	2	16	2	1	3	1	1	1	1
2349	2	18	2	1	3	1	1	1	9

2350	2	16	2	1	3	1	1	1	1
2351	2	18	2	1	3	1	1	1	2
2352	2	17	2	1	3	1	1	1	1
2353	2	16	2	1	3	1	1	1	2
2354	2	15	2	1	3	1	1	1	1
2355	2	0	#NULL!	9	3	1	1	1	2
2356	2	16	2	1	3	1	9	1	2
2357	2	16	2	1	3	1	1	1	1
2358	2	17	2	1	3	1	1	1	1
2359	2	16	2	1	3	1	1	1	1
2360	2	15	2	1	3	1	1	1	2
2361	2	15	2	1	3	1	1	1	3
2362	2	17	2	1	3	1	1	1	1
2363	2	15	2	1	3	1	1	1	1
2364	2	16	2	1	3	1	1	1	1
2365	2	16	2	1	3	1	1	1	9
2366	2	16	2	1	3	1	1	1	1
2367	2	16	2	1	3	1	1	1	1
2368	2	17	2	1	3	1	1	1	1
2369	2	16	2	1	3	1	1	1	1
2370	2	16	2	1	3	1	1	1	3
2371	2	17	2	1	3	1	1	1	1
2372	9	0	#NULL!	1	3	1	1	1	1
2373	9	19	2	1	3	1	1	1	1
2374	2	16	2	1	3	1	1	1	1
2375	2	16	2	1	3	1	1	1	1
2376	2	15	2	1	3	1	1	1	9
2377	2	16	2	1	3	1	1	1	9
2378	2	15	2	1	3	1	1	1	9
2379	2	17	2	1	3	1	1	1	1
2380	2	15	2	9	3	1	1	1	1
2381	2	18	2	1	3	1	1	1	1
2382	2	15	2	1	3	1	1	1	1
2383	2	16	2	1	3	1	1	1	1
2384	2	16	2	1	3	1	1	1	9
2385	2	0	#NULL!	1	3	1	1	1	1
2386	2	17	2	1	3	1	1	1	1
2387	9	17	2	1	3	1	1	1	3
2388	2	17	2	1	3	1	1	1	1
2389	2	15	2	1	3	1	1	1	1
2390	2	17	2	9	3	1	1	1	1
2391	2	16	2	1	3	1	1	1	1
2392	2	16	2	1	3	1	1	1	1
2393	2	16	2	1	3	1	1	1	1
2394	2	16	2	1	3	1	1	1	1
2395	2	18	2	9	3	1	1	1	1
2396	2	16	2	1	3	1	1	1	1

2397	2	0	#NULL!	1	3	1	1	1	1
2398	2	17	2	1	3	1	1	1	1
2399	9	0	#NULL!	9	9	9	9	9	9
2400	2	18	2	1	3	1	1	1	9
2401	2	18	2	1	3	1	1	1	1
2402	2	16	2	1	3	1	1	1	1
2403	2	18	2	1	3	1	1	1	9
2404	2	16	2	1	3	1	1	1	1
2405	2	18	2	1	3	1	1	1	1
2406	2	17	2	1	3	1	1	1	9
2407	2	17	2	9	3	1	1	1	1
2408	2	17	2	1	3	1	1	1	1
2409	2	19	2	1	3	1	1	1	1
2410	2	17	2	1	3	1	1	1	9
2411	2	18	2	9	3	1	1	1	1
2412	2	17	2	1	3	1	1	1	1
2413	2	18	2	1	3	1	1	1	9
2414	2	16	2	1	3	1	1	1	9
2415	2	17	2	1	3	1	1	1	9
2416	2	17	2	1	3	1	1	1	3
2417	2	17	2	9	3	1	1	1	1
2418	2	15	2	1	3	1	1	1	9
2419	2	16	2	1	3	1	1	1	9
2420	2	19	2	1	3	1	1	1	1
2421	2	17	2	1	3	1	1	1	1
2422	2	17	2	1	3	1	1	1	1
2423	2	16	2	1	3	1	1	1	3
2424	2	15	2	1	3	1	1	1	1
2425	2	18	2	1	3	1	1	1	1
2426	2	16	2	1	3	1	1	1	1
2427	2	15	2	9	3	1	1	1	1
2428	2	17	2	1	3	1	1	1	1
2429	2	0	#NULL!	9	3	1	1	1	1
2430	2	17	2	1	3	1	1	1	1
2431	2	17	2	1	3	1	2	1	1
2432	2	16	2	1	3	1	1	1	9
2433	2	17	2	1	3	1	2	1	9
2434	2	17	2	1	3	1	1	1	1
2435	2	17	2	1	3	1	1	1	1
2436	2	17	2	1	3	1	1	1	1
2437	2	17	2	1	3	9	1	1	2
2438	2	16	2	1	3	1	1	1	1
2439	2	17	2	1	3	1	1	1	9
2440	2	17	2	1	3	1	1	1	9
2441	2	16	2	1	3	1	1	1	9
2442	2	18	2	1	3	1	1	1	9
2443	2	15	2	1	3	1	1	1	2

2444	2	17	2	1	3	1	1	1	1
2445	2	16	2	1	3	1	1	1	1
2446	2	16	2	1	3	1	1	1	9
2447	2	18	2	1	3	1	1	1	2
2448	2	17	2	1	3	1	1	1	1
2449	2	16	2	1	3	1	1	1	1
2450	2	15	2	1	3	1	1	1	2
2451	2	17	2	9	3	1	1	1	1
2452	2	16	2	1	3	1	2	1	1
2453	2	18	2	1	3	1	1	1	1
2454	2	15	2	1	3	1	1	1	2
2455	2	17	2	1	3	1	1	1	1
2456	2	17	2	1	3	1	1	1	1
2457	2	16	2	1	3	1	1	1	9
2458	2	17	2	1	3	1	1	1	9
2459	2	18	2	1	3	1	1	1	9
2460	2	16	2	1	3	1	1	1	3
2461	2	18	2	1	3	1	1	1	1
2462	2	18	2	1	3	1	1	1	1
2463	2	18	2	1	3	1	1	1	1
2464	2	15	2	1	3	1	1	1	1
2465	2	15	2	1	3	1	1	1	1
2466	2	16	2	1	3	1	1	1	2
2467	2	19	2	1	3	1	1	1	2
2468	2	17	2	1	3	1	1	1	2
2469	2	17	2	1	3	1	1	1	9
2470	2	15	2	1	3	1	1	1	1
2471	2	17	2	1	3	1	1	1	1
2472	2	18	2	1	3	1	1	1	1
2473	2	15	2	1	3	1	1	1	9
2474	2	17	2	1	3	1	1	1	1
2475	2	16	2	1	3	1	1	1	1
2476	2	15	2	1	3	1	1	1	9
2477	2	14	1	1	3	1	1	2	1
2478	2	17	2	1	3	1	1	1	1
2479	2	17	2	1	3	1	1	1	3
2480	2	15	2	1	3	1	1	1	1
2481	2	15	2	1	3	1	1	1	1
2482	9	0	#NULL!	1	3	1	1	1	9
2483	2	18	2	1	3	1	1	2	9
2484	2	17	2	1	3	1	1	1	1
2485	9	18	2	1	3	1	1	1	1
2486	2	16	2	1	3	1	2	1	1
2487	2	16	2	1	3	1	1	1	1
2488	2	19	2	1	3	1	1	1	1
2489	2	17	2	1	3	1	1	1	9
2490	2	14	1	1	2	1	1	1	1

2491	2	16	2	1	2	1	1	1	1
2492	2	15	2	1	2	1	1	1	2
2493	2	17	2	1	2	1	1	1	1
2494	2	14	1	1	2	1	1	3	1
2495	2	15	2	1	2	1	1	3	1
2496	2	12	1	1	2	1	1	1	1
2497	2	15	2	1	2	1	1	1	1
2498	2	14	1	1	2	1	1	1	1
2499	2	16	2	1	2	1	1	1	2
2500	2	15	2	1	2	1	1	1	1
2501	2	15	2	1	2	1	1	1	1
2502	2	17	2	1	2	1	1	1	1
2503	2	17	2	1	2	1	1	1	1
2504	2	15	2	1	2	1	2	1	1
2505	2	14	1	1	2	1	1	2	1
2506	2	17	2	1	2	1	1	1	1
2507	2	16	2	1	2	1	1	1	1
2508	2	15	2	1	2	1	1	1	2
2509	2	16	2	1	2	1	1	1	2
2510	2	15	2	1	2	1	1	1	1
2511	2	15	2	1	2	1	1	1	1
2512	2	17	2	1	2	1	1	1	2
2513	2	16	2	1	2	1	1	1	2
2514	2	16	2	1	2	1	1	1	1
2515	2	15	2	1	2	1	1	1	1
2516	2	16	2	1	2	1	1	1	2
2517	2	15	2	1	2	1	2	2	1
2518	2	15	2	1	2	1	1	1	1
2519	2	16	2	1	2	1	1	1	1
2520	2	18	2	9	2	1	2	9	1
2521	2	15	2	1	2	1	1	1	1
2522	2	14	1	1	2	1	1	1	1
2523	2	16	2	1	2	1	1	2	1
2524	2	16	2	1	2	1	1	1	1
2525	2	16	2	1	2	1	2	1	9
2526	2	17	2	1	2	1	1	1	9
2527	2	16	2	1	2	1	2	1	1
2528	2	17	2	1	2	1	2	1	1
2529	2	17	2	1	2	1	1	1	9
2530	2	16	2	1	2	1	1	1	9
2531	2	16	2	1	2	1	1	1	1
2532	2	15	2	1	2	1	1	1	1
2533	2	15	2	1	2	1	1	1	1
2534	2	16	2	1	2	1	1	1	2
2535	2	17	2	9	2	1	1	1	9
2536	2	17	2	1	2	1	2	1	1
2537	2	18	2	1	2	1	1	1	1

2538	2	16	2	1	2	1	1	1	1
2539	2	15	2	1	2	1	1	9	2
2540	2	19	2	1	2	1	1	1	1
2541	2	16	2	1	2	1	1	1	9
2542	2	16	2	1	2	1	1	1	9
2543	2	18	2	1	2	1	1	1	1
2544	2	18	2	9	9	9	9	9	9
2545	2	17	2	1	2	1	1	1	9
2546	2	15	2	1	2	1	1	1	9
2547	2	16	2	1	2	1	1	1	1
2548	2	17	2	1	2	1	1	1	1
2549	2	15	2	1	2	1	1	1	1
2550	2	16	2	1	2	1	1	1	1
2551	2	15	2	1	2	1	1	1	1
2552	2	16	2	1	2	1	1	1	1
2553	2	15	2	1	2	1	1	1	1
2554	2	15	2	1	2	1	1	1	1
2555	2	16	2	9	2	1	1	3	1
2556	2	15	2	1	2	1	1	1	1
2557	2	17	2	1	2	1	2	2	1
2558	2	15	2	1	2	1	1	1	1
2559	2	17	2	1	2	1	1	2	1
2560	2	16	2	1	2	1	1	1	9
2561	2	14	1	1	2	1	1	1	1
2562	2	15	2	1	2	1	1	1	1
2563	2	15	2	1	2	1	1	1	1
2564	2	14	1	9	2	3	2	1	3
2565	2	15	2	1	2	1	1	1	1
2566	2	15	2	1	2	1	1	1	1
2567	2	16	2	1	2	1	1	1	1
2568	2	15	2	1	2	1	1	1	1
2569	2	15	2	1	2	1	1	1	1
2570	2	17	2	1	2	1	1	3	1
2571	2	16	2	1	2	1	1	1	1
2572	2	16	2	1	2	1	1	1	1
2573	2	16	2	1	2	1	1	1	1
2574	2	14	1	1	2	1	1	1	1
2575	2	15	2	1	2	1	1	1	1
2576	2	15	2	1	2	1	1	1	1
2577	2	15	2	1	2	1	1	1	1
2578	2	16	2	1	2	1	1	1	1
2579	2	17	2	1	2	1	1	2	1
2580	2	20	3	1	2	1	1	2	1
2581	2	17	2	1	2	1	3	3	1
2582	2	15	2	1	2	1	3	1	1
2583	2	17	2	1	2	1	1	1	1
2584	2	16	2	1	2	1	1	1	1

2585	2	16	2	9	2	1	1	1	1
2586	2	15	2	1	2	1	1	1	1
2587	2	18	2	1	2	1	1	1	1
2588	2	14	1	1	2	1	3	1	1
2589	2	15	2	1	2	1	1	1	1
2590	2	17	2	1	2	1	3	3	1
2591	2	16	2	1	2	1	1	1	1
2592	2	15	2	1	2	1	1	1	1
2593	2	16	2	1	2	1	1	1	1
2594	2	16	2	1	2	1	1	1	1
2595	2	16	2	1	2	1	1	1	1
2596	2	21	3	1	2	1	1	1	1
2597	2	15	2	1	2	1	1	1	1
2598	2	16	2	1	2	1	1	1	1
2599	2	16	2	1	2	1	1	1	1
2600	2	16	2	1	2	1	1	1	2
2601	2	15	2	1	2	1	1	1	1
2602	2	13	1	1	2	1	1	1	2
2603	2	15	2	1	2	1	1	2	1
2604	2	15	2	1	2	1	1	1	1
2605	2	15	2	1	2	1	1	1	1
2606	2	16	2	1	2	1	1	1	1
2607	2	16	2	1	2	1	1	2	1
2608	2	15	2	1	2	1	1	2	1
2609	2	15	2	1	2	9	1	2	1
2610	2	14	1	1	2	1	1	1	1
2611	2	14	1	1	2	1	1	2	1
2612	2	15	2	1	2	1	1	2	1
2613	2	15	2	1	2	1	1	1	1
2614	2	16	2	1	2	1	1	1	1
2615	2	14	1	9	2	1	1	2	3
2616	2	15	2	1	2	1	1	1	2
2617	2	0	#NULL!	1	2	1	1	1	1
2618	2	16	2	1	2	1	1	1	1
2619	2	16	2	1	2	1	1	2	1
2620	2	17	2	1	2	1	1	1	1
2621	2	15	2	1	2	1	1	3	2
2622	2	14	1	9	2	1	1	1	2
2623	2	15	2	1	2	1	1	1	1
2624	2	17	2	1	2	1	1	1	1
2625	2	15	2	1	2	1	1	1	1
2626	2	14	1	9	2	1	1	1	1
2627	2	17	2	1	2	1	1	1	1
2628	2	15	2	9	2	1	1	1	1
2629	2	16	2	1	2	1	1	1	1
2630	2	15	2	1	2	1	1	1	1
2631	2	0	#NULL!	9	2	1	1	1	1

2632	2	15	2	1	2	1	1	1	1
2633	2	14	1	1	2	1	1	2	2
2634	2	15	2	1	2	1	1	1	1
2635	2	15	2	1	2	1	1	1	1
2636	2	15	2	1	2	1	1	1	1
2637	2	16	2	1	2	1	1	1	1
2638	2	14	1	1	2	1	1	1	1
2639	2	13	1	1	2	1	1	1	1
2640	2	16	2	1	2	1	1	1	1
2641	2	15	2	1	2	1	1	1	1
2642	2	14	1	1	2	1	1	1	1
2643	2	14	1	1	2	1	1	1	2
2644	2	15	2	1	2	1	1	1	9
2645	2	16	2	1	2	1	1	1	1
2646	2	14	1	1	2	1	1	1	1
2647	2	17	2	1	2	1	1	2	1
2648	2	15	2	1	2	1	1	1	1
2649	2	14	1	1	2	1	1	1	1
2650	2	16	2	1	2	1	1	1	1
2651	2	15	2	1	2	1	1	1	1
2652	2	16	2	9	2	1	1	1	1
2653	2	15	2	1	2	1	1	1	1
2654	2	16	2	1	2	1	1	1	1
2655	2	16	2	9	2	1	1	1	1
2656	2	14	1	1	2	1	1	1	1
2657	2	16	2	1	2	1	1	1	1
2658	2	15	2	1	2	1	1	1	1
2659	2	17	2	1	2	1	1	1	2
2660	2	16	2	9	2	1	1	1	1
2661	2	16	2	1	2	1	1	1	9
2662	2	15	2	1	2	1	1	1	2
2663	2	17	2	1	2	1	1	1	1
2664	2	15	2	1	2	1	1	2	2
2665	2	15	2	1	2	1	1	2	1
2666	2	16	2	1	2	1	1	1	9
2667	2	15	2	1	2	1	1	2	1
2668	2	16	2	1	2	1	1	2	2
2669	2	20	3	1	2	1	1	2	1
2670	2	12	1	1	2	1	1	1	1
2671	2	18	2	1	2	1	1	1	1
2672	2	16	2	1	2	1	1	1	9
2673	2	16	2	1	2	1	1	1	1
2674	2	15	2	1	2	1	1	1	1
2675	2	18	2	9	2	1	1	1	9
2676	2	17	2	1	1	1	2	2	1
2677	2	15	2	1	2	1	1	3	1
2678	2	16	2	1	2	1	1	1	1

2679	2	14	1	1	2	1	1	1	1
2680	2	14	1	1	2	1	1	1	1
2681	2	16	2	1	2	1	1	1	1
2682	2	14	1	1	2	1	1	1	2
2683	2	15	2	1	2	1	1	2	1
2684	2	15	2	1	2	1	1	1	1
2685	2	16	2	1	2	1	1	1	1
2686	2	15	2	1	2	1	1	1	1
2687	2	15	2	1	2	1	1	2	1
2688	2	16	2	1	2	1	1	2	1
2689	2	0	#NULL!	1	2	1	1	1	1
2690	2	17	2	1	2	1	1	1	1
2691	2	18	2	1	2	1	1	1	1
2692	9	15	2	1	2	1	1	1	1
2693	2	16	2	1	2	1	1	1	2
2694	2	15	2	1	2	1	1	1	2
2695	2	17	2	1	2	1	1	1	1
2696	2	19	2	1	2	1	1	1	1
2697	2	16	2	1	2	1	1	1	1
2698	2	16	2	1	2	1	1	1	1
2699	2	16	2	1	2	1	1	1	1
2700	2	15	2	1	2	1	1	1	1
2701	2	16	2	1	2	1	1	1	1
2702	2	16	2	1	2	1	1	1	1
2703	2	15	2	1	2	1	1	1	1
2704	9	18	2	1	2	1	1	1	1
2705	2	16	2	1	2	1	1	1	1
2706	2	19	2	1	2	1	1	1	1
2707	2	16	2	1	2	1	1	1	1
2708	2	15	2	1	2	1	1	1	1
2709	2	15	2	1	2	1	1	1	1
2710	2	17	2	1	2	1	1	1	1
2711	2	15	2	1	2	1	1	1	1
2712	2	15	2	1	2	1	1	1	1
2713	9	16	2	1	2	1	1	1	1
2714	2	17	2	1	2	1	1	1	1
2715	2	16	2	9	2	1	1	1	1
2716	2	17	2	1	2	1	1	1	9
2717	2	15	2	1	2	1	1	1	1
2718	2	15	2	1	2	1	1	1	1
2719	2	15	2	1	2	1	1	1	1
2720	2	15	2	1	2	1	1	1	1
2721	2	15	2	1	2	1	1	2	1
2722	2	17	2	1	2	1	2	2	1
2723	2	16	2	1	2	1	1	1	1
2724	2	15	2	1	2	1	1	1	1
2725	2	15	2	1	2	1	1	1	1

2726	2	16	2	1	2	1	1	1	1
2727	2	15	2	1	2	1	1	1	1
2728	2	16	2	1	2	1	1	1	1
2729	2	16	2	1	2	1	1	1	1
2730	2	16	2	1	2	1	1	1	1
2731	2	17	2	1	2	1	1	1	1
2732	2	16	2	1	2	1	1	1	1
2733	2	16	2	1	2	1	1	1	1
2734	2	16	2	1	2	1	1	1	1
2735	2	15	2	1	2	1	9	9	1
2736	2	15	2	1	2	1	1	1	1
2737	2	16	2	1	2	1	1	1	1
2738	2	15	2	1	2	1	1	1	1
2739	2	16	2	1	2	1	1	1	1
2740	2	16	2	1	2	1	1	1	1
2741	2	16	2	1	2	1	1	1	1
2742	2	14	1	1	2	1	1	2	1
2743	2	15	2	1	1	1	1	1	2
2744	2	16	2	1	2	1	1	1	1
2745	2	14	1	1	2	1	1	9	1
2746	2	18	2	1	2	3	3	1	1
2747	2	15	2	9	2	1	1	1	3
2748	2	15	2	1	2	1	1	1	9
2749	2	15	2	1	2	1	1	1	9
2750	2	15	2	9	2	1	1	1	1
2751	2	17	2	1	2	1	1	1	9
2752	2	16	2	1	2	1	1	2	3
2753	2	18	2	1	2	1	2	2	9
2754	2	17	2	1	2	1	1	1	2
2755	1	18	2	9	2	1	1	2	9
2756	2	16	2	1	2	1	1	2	9
2757	2	16	2	9	2	1	1	1	9
2758	2	16	2	1	2	1	1	3	1
2759	2	15	2	1	2	1	1	1	9
2760	2	16	2	1	2	1	1	1	2
2761	2	15	2	1	2	1	1	1	1
2762	2	15	2	1	2	1	1	1	1
2763	2	18	2	1	2	1	1	1	1
2764	2	16	2	9	2	1	1	1	1
2765	2	15	2	1	2	1	1	1	1
2766	2	17	2	1	2	1	1	1	1
2767	2	20	3	1	2	1	1	1	1
2768	2	16	2	1	2	1	1	2	1
2769	2	16	2	1	2	1	1	1	1
2770	2	18	2	1	2	1	1	2	2
2771	2	18	2	1	2	1	1	1	1
2772	2	16	2	1	2	1	1	1	1

2773	2	15	2	1	2	1	1	1	1
2774	2	16	2	1	2	1	1	1	1
2775	2	15	2	1	2	1	1	1	2
2776	2	16	2	1	2	1	1	2	1
2777	2	16	2	9	2	1	1	2	1
2778	2	16	2	1	2	1	1	1	1
2779	2	16	2	1	2	1	1	2	2
2780	2	15	2	1	2	1	1	1	1
2781	2	15	2	1	2	1	1	1	1
2782	2	19	2	1	2	1	2	2	2
2783	2	17	2	1	2	1	1	1	1
2784	2	13	1	1	2	1	1	2	1
2785	2	17	2	1	2	1	1	2	1
2786	2	14	1	1	2	1	1	2	3
2787	2	17	2	1	2	1	1	3	1
2788	2	15	2	1	2	1	1	2	1
2789	2	16	2	1	2	1	1	1	2
2790	2	15	2	1	2	1	1	1	1
2791	2	15	2	1	2	1	1	1	1
2792	2	15	2	1	2	1	1	1	1
2793	2	17	2	9	2	1	1	1	1
2794	2	16	2	1	2	1	1	1	1
2795	2	18	2	1	2	1	1	1	1
2796	2	16	2	1	2	1	1	2	1
2797	2	14	1	1	2	1	1	1	1
2798	2	15	2	1	2	1	1	1	2
2799	2	15	2	1	2	1	1	1	1
2800	2	16	2	1	2	1	1	1	1
2801	2	16	2	1	2	1	1	2	2
2802	2	16	2	1	2	1	1	1	2
2803	2	16	2	9	2	1	1	1	9
2804	2	17	2	1	2	1	1	2	2
2805	2	17	2	1	2	1	1	1	2
2806	2	15	2	1	2	1	1	2	2
2807	2	14	1	1	2	1	1	1	9
2808	2	18	2	1	2	1	1	2	9
2809	2	16	2	1	2	1	1	2	2
2810	2	16	2	1	2	1	1	1	2
2811	2	15	2	1	2	1	1	3	1
2812	9	0	#NULL!	9	9	9	9	9	1
2813	2	14	1	1	2	1	1	1	1
2814	2	15	2	1	2	1	1	1	1

ChestPain	Headache	BreastLum	ArmpitLum	DischargeC	DischargeP	DischargeS	Women20	WomenMa	WomenInt
2	2	2	2	2	2	2	2	2	2
2	2	2	2	2	2	2	2	2	2
2	2	2	2	2	2	2	2	2	2
3	3	3	3	3	3	3	2	2	2
1	2	2	2	3	3	3	1	2	2
2	2	2	2	2	2	2	2	2	2
2	2	3	2	3	1	2	3	2	2
1	1	1	2	2	2	2	1	3	3
1	1	2	9	1	2	2	1	2	1
1	1	1	2	1	1	2	2	1	3
2	2	2	2	2	2	2	3	3	3
1	2	2	2	3	2	1	1	1	2
2	1	1	2	1	2	2	2	2	2
2	1	1	2	2	1	1	3	3	2
2	2	2	2	2	2	2	2	2	2
2	1	2	2	2	2	2	2	2	2
2	1	3	2	2	2	2	2	2	2
3	3	3	3	3	3	3	3	3	3
2	2	1	3	2	2	2	2	2	2
2	2	1	2	3	2	1	2	3	3
2	2	1	1	1	3	1	1	3	1
1	1	1	1	1	2	2	1	3	2
1	2	1	1	1	1	1	1	3	3
1	1	1	2	2	2	1	1	2	2
2	2	2	2	2	2	2	2	2	1
3	3	1	3	1	3	3	3	3	3
9	9	9	9	9	9	9	9	9	9
1	2	1	1	1	1	3	1	3	3
2	3	1	2	2	2	2	1	3	3
1	3	3	3	1	1	1	1	2	3
3	3	1	2	2	2	3	3	3	3
3	3	1	3	2	2	2	3	3	3
1	2	1	2	1	9	2	3	3	3
1	1	1	2	1	3	3	1	2	9
2	1	1	1	9	9	9	1	2	1
1	1	2	2	9	9	2	3	3	2
1	3	2	3	2	1	2	1	1	2
3	3	1	3	2	2	1	2	3	1
2	2	1	2	2	2	2	1	3	3
1	3	2	2	1	3	2	2	2	2
2	3	1	2	2	2	2	3	3	2
9	9	9	9	9	9	9	3	9	9
9	9	9	9	9	9	9	1	9	9
2	2	1	1	2	2	2	1	3	3
2	2	1	2	2	3	2	3	3	3
2	1	3	3	3	3	3	9	9	9

1	2	2	2	1	1	2	1	1	2
2	2	2	2	1	1	2	1	2	2
2	2	2	2	2	2	2	1	2	2
2	2	1	2	2	1	2	2	1	1
3	3	1	3	1	1	9	3	3	3
3	3	1	3	1	1	3	1	3	3
2	2	3	2	2	2	3	2	2	2
2	3	1	2	3	3	2	2	1	3
1	1	1	3	3	3	1	1	3	3
2	1	3	3	3	2	1	3	1	2
9	9	1	9	9	9	9	9	2	9
1	2	2	2	2	3	2	1	1	2
2	2	1	1	1	1	1	1	1	2
1	1	1	1	1	1	1	1	1	1
1	1	2	2	1	1	2	1	1	2
1	2	2	1	1	1	2	1	1	2
2	2	2	9	1	2	2	3	2	3
1	3	1	9	3	3	1	1	2	3
3	2	1	3	2	2	2	2	2	2
3	3	3	3	3	3	3	2	2	2
1	3	3	3	3	3	9	3	3	3
2	1	1	2	2	1	2	2	3	1
1	2	2	2	2	1	1	1	2	2
1	2	2	3	3	3	3	2	1	1
2	2	3	1	2	2	2	1	1	2
2	2	1	1	1	2	1	2	2	2
1	2	9	2	2	1	1	3	1	3
1	1	1	2	2	1	2	2	3	3
1	2	1	2	2	1	1	1	1	3
3	3	1	2	9	1	9	1	3	3
2	2	2	2	2	2	2	9	9	9
3	3	2	1	3	3	3	1	3	3
3	3	1	3	1	1	1	3	3	9
2	2	2	1	1	1	1	1	2	2
2	2	1	1	9	9	9	2	3	3
1	3	1	2	1	1	1	1	3	3
3	3	1	3	3	2	1	3	2	3
2	2	1	1	2	2	2	1	2	2
3	3	1	3	1	3	3	1	3	3
2	2	1	1	2	2	2	1	2	2
3	3	1	3	2	2	3	1	2	3
9	1	1	1	1	1	1	3	3	3
9	9	9	9	9	9	9	1	3	1
2	2	1	2	2	2	2	1	2	2
1	2	1	2	2	3	3	1	2	2
1	1	1	1	1	1	1	1	3	1
2	2	1	2	2	2	2	1	3	9

1	3	2	1	2	1	3	1	2	1
1	1	3	3	3	1	3	3	3	3
1	2	1	1	2	2	2	1	2	2
1	1	1	2	3	3	2	1	2	3
2	2	3	2	1	1	1	1	2	2
2	2	1	2	2	3	2	1	2	2
2	1	2	2	2	2	2	1	2	3
3	3	3	3	3	3	3	1	3	3
2	3	2	2	2	2	2	2	3	1
2	2	1	9	2	3	1	1	2	3
2	2	1	3	1	2	3	3	1	2
3	3	1	2	2	1	1	3	3	3
2	2	1	2	2	2	2	1	1	2
2	2	1	2	2	2	2	2	2	2
2	2	1	2	1	1	1	2	1	1
2	2	1	2	1	1	1	2	1	1
3	3	1	3	2	2	1	1	3	3
3	3	3	3	2	2	2	2	2	2
1	3	1	3	1	9	2	2	2	2
2	2	1	2	2	1	1	1	2	2
1	3	3	3	3	3	3	3	1	1
1	2	1	1	1	2	2	1	3	3
1	1	1	1	2	1	1	1	1	2
2	1	2	1	2	9	2	1	2	2
3	1	3	3	3	3	3	3	3	3
1	3	2	2	1	3	2	3	3	3
2	1	1	2	2	1	1	1	1	1
2	1	2	1	2	2	2	1	1	2
2	1	2	2	2	2	2	1	1	1
2	3	1	2	2	2	3	1	3	3
1	1	2	2	1	1	1	1	1	1
3	3	3	3	3	3	3	1	2	1
1	2	3	1	2	2	9	3	3	3
2	1	1	2	2	2	2	1	2	3
2	1	3	3	3	3	1	1	3	2
2	2	2	2	2	2	2	1	1	1
1	2	2	1	2	9	2	1	9	9
1	9	1	9	1	9	1	1	3	3
1	1	1	3	2	1	3	1	3	3
3	3	3	3	3	3	3	1	3	3
1	2	2	2	2	1	2	1	1	2
9	9	9	9	9	9	9	9	9	9
2	2	1	1	2	2	2	1	1	2
2	2	1	1	2	1	1	2	1	1
3	3	1	3	2	3	1	1	3	3
2	2	1	2	2	2	1	2	3	3
1	9	1	2	2	2	1	1	3	9

3	3	1	1	3	1	3	1	1	1
3	3	3	3	3	3	3	3	3	3
3	3	3	3	3	3	3	3	3	3
2	2	2	2	2	2	2	1	3	3
1	2	1	2	1	2	2	1	2	1
3	3	3	3	3	3	3	3	3	1
3	3	1	3	1	9	2	1	9	9
1	3	2	2	3	2	1	3	3	3
9	9	9	9	9	9	9	2	2	2
2	3	2	2	2	2	2	2	2	2
1	1	1	1	1	1	1	2	3	1
3	3	1	3	1	3	2	2	2	2
2	2	9	2	2	2	2	2	2	2
1	3	2	2	2	2	2	3	3	3
1	2	3	2	2	2	2	1	1	2
1	1	1	1	1	1	1	1	1	2
1	1	1	2	2	1	1	1	1	2
1	2	2	2	2	1	1	1	1	2
1	2	1	2	2	2	2	2	2	2
1	3	2	2	1	2	2	3	3	3
9	9	9	9	9	9	9	2	9	9
2	3	9	1	2	2	2	2	3	3
3	2	2	2	2	3	2	2	3	3
1	2	1	1	2	9	2	2	1	1
2	2	1	2	3	3	3	1	2	3
3	1	3	3	3	3	3	3	3	1
2	3	1	2	1	2	2	1	3	3
1	3	1	1	2	2	2	3	3	1
1	2	1	1	2	2	2	2	3	3
2	2	2	1	1	1	1	1	1	1
9	9	9	9	9	9	9	9	9	9
2	2	2	2	2	2	2	2	2	2
2	2	2	1	1	1	2	1	2	2
1	1	1	1	1	1	1	1	1	1
1	2	1	2	2	2	2	1	2	2
1	1	1	1	1	1	1	1	1	1
1	1	1	1	1	1	1	1	1	1
2	3	1	1	2	2	1	2	3	2
3	3	3	3	1	3	1	1	3	3
1	1	1	2	2	2	2	2	3	3
1	2	1	2	1	2	1	3	9	3
2	2	2	2	2	2	2	2	2	2
2	2	3	1	1	1	1	1	1	2
9	9	9	9	9	9	9	9	9	9
9	9	9	9	9	9	9	9	9	9
1	2	2	1	1	2	2	1	1	2
2	1	1	1	2	2	2	1	2	2

9	1	9	9	9	9	9	9	9	9
9	9	1	9	9	9	9	1	9	9
1	2	1	2	1	1	1	1	3	3
9	9	1	9	9	9	9	1	9	9
3	3	1	1	2	3	2	3	3	3
1	1	2	2	2	2	2	1	3	3
2	9	1	2	2	2	2	1	3	3
2	1	3	3	1	3	1	3	1	2
1	3	1	2	1	1	1	2	1	2
9	9	9	9	1	9	9	9	9	1
9	9	9	9	9	9	9	9	9	9
9	9	9	9	9	9	9	9	2	9
1	1	2	2	1	1	1	1	1	1
3	1	1	2	2	3	3	2	2	3
2	9	9	9	9	9	9	9	9	2
1	2	1	1	2	2	2	1	3	3
3	3	3	3	3	3	3	3	3	3
2	2	1	1	1	1	1	2	1	1
1	2	1	2	1	1	1	1	2	2
3	2	1	2	2	2	3	1	2	2
2	2	1	2	2	2	1	1	3	1
2	3	1	3	1	2	2	3	2	2
1	1	3	9	3	3	3	2	2	2
1	3	1	2	2	1	1	1	3	3
3	3	2	1	2	2	2	2	3	2
3	1	2	2	2	2	2	2	1	2
1	1	2	2	2	2	2	2	2	1
2	2	2	2	3	3	3	2	2	1
9	9	9	9	9	9	9	9	9	9
1	2	1	1	1	2	2	1	2	1
2	2	2	2	2	2	2	2	2	2
2	1	2	1	1	2	1	1	1	1
3	3	2	3	3	9	2	1	2	3
1	1	2	2	2	2	1	1	2	3
2	9	1	3	1	3	1	1	3	3
1	1	2	1	1	1	1	2	1	2
3	1	2	3	2	1	1	3	1	3
2	2	1	1	1	1	1	2	1	1
3	1	2	3	2	1	1	3	1	1
1	1	1	1	1	2	2	2	2	1
1	1	2	2	1	1	2	2	1	2
2	2	2	1	1	3	3	1	3	2
3	3	3	3	3	3	3	1	2	1
1	1	2	2	1	1	1	1	1	2
2	2	2	2	2	2	2	1	2	1
1	3	1	3	2	1	2	1	1	1
3	3	3	3	3	3	3	9	9	9

1	1	1	2	1	3	1	1	3	3
1	1	1	2	1	1	2	3	3	3
1	1	1	2	2	3	3	1	1	1
3	2	1	3	1	2	1	1	3	3
1	2	2	2	2	2	2	3	3	3
2	3	2	2	2	2	2	2	3	3
1	3	2	2	1	1	1	1	2	3
1	2	2	2	2	2	2	3	2	1
2	2	1	1	1	1	1	2	2	2
2	1	1	1	1	1	1	1	1	1
1	1	1	1	1	1	1	1	1	1
1	1	1	1	1	1	1	2	3	2
9	9	9	9	9	9	9	2	9	9
2	2	9	2	2	2	2	2	2	2
9	3	1	9	9	2	1	2	9	2
9	9	9	9	9	9	9	9	9	9
1	9	9	1	9	9	9	2	9	9
2	1	9	9	9	9	9	9	2	9
9	2	9	9	2	9	2	9	1	9
9	9	9	9	9	9	9	9	9	9
9	9	9	9	9	9	9	9	9	9
2	2	2	2	2	2	2	2	2	2
2	2	2	2	2	2	2	2	2	2
3	3	3	3	3	3	3	2	3	3
2	1	1	2	2	2	2	2	2	2
2	9	9	2	9	2	2	9	9	2
2	2	2	2	2	2	2	2	2	2
9	9	9	9	9	9	9	9	9	9
9	9	9	9	9	9	9	9	9	9
1	2	2	2	2	2	2	2	2	2
9	2	9	9	9	9	9	9	9	9
9	9	9	9	9	9	9	9	9	9
9	9	9	9	9	9	9	9	9	9
2	9	9	2	9	9	2	2	9	2
9	2	1	9	3	1	9	9	2	9
9	1	9	9	3	1	9	9	3	1
9	9	9	9	9	9	9	9	9	9
2	9	9	9	2	9	9	9	2	9
9	3	1	3	9	2	1	9	2	9
9	9	9	9	9	9	9	3	9	9
9	9	9	2	9	9	9	9	9	1
9	9	9	9	9	9	9	1	9	9
3	3	3	3	3	3	3	3	3	3
1	3	1	1	1	1	1	2	2	2
2	3	1	2	3	1	1	2	1	2
1	1	1	1	1	1	1	1	1	9
1	9	9	9	9	9	9	9	9	1

9	9	1	9	9	9	9	1	9	9
9	9	9	9	9	9	9	1	9	9
1	2	1	3	2	3	2	3	2	1
3	9	3	2	9	2	3	3	2	9
2	2	2	2	2	2	2	1	1	1
2	2	1	2	2	2	2	2	2	1
1	2	1	2	1	3	2	1	3	3
3	1	1	1	1	1	1	3	3	1
2	1	1	1	1	1	1	9	9	9
1	2	1	2	2	2	2	1	2	3
9	9	9	9	9	9	9	9	9	9
9	9	9	9	9	9	9	9	9	9
9	9	9	9	9	9	9	9	1	9
1	2	2	1	2	2	1	1	1	1
1	1	1	1	1	1	1	1	1	1
1	1	2	2	1	2	1	2	1	2
9	9	9	9	9	9	9	9	9	9
1	2	1	2	2	2	2	2	2	1
1	2	3	1	1	1	1	1	1	1
1	1	1	1	1	1	1	1	3	1
3	3	1	3	1	1	3	3	3	1
2	3	2	2	2	2	2	1	3	3
3	3	2	2	1	1	2	1	3	3
1	1	1	1	1	1	1	2	2	2
3	1	1	1	1	1	1	2	2	2
2	2	2	2	2	2	2	2	2	2
2	2	2	2	2	2	2	2	2	2
2	3	1	2	3	1	2	1	2	3
2	2	2	2	2	2	2	3	3	3
1	1	1	1	1	1	1	2	2	2
2	2	2	2	2	2	2	3	2	2
2	2	2	2	2	2	2	2	2	2
3	2	1	2	3	2	3	2	2	2
2	2	2	2	2	2	2	2	2	2
3	9	3	3	3	3	3	3	3	3
1	2	2	2	2	2	2	1	3	3
2	2	2	2	1	1	1	9	9	9
1	2	3	3	2	3	2	3	1	2
3	3	3	3	3	3	3	3	3	3
3	3	3	3	3	3	3	3	3	3
2	2	2	2	2	2	9	2	2	2
1	2	1	3	1	2	3	1	1	2
1	1	1	1	1	1	2	1	3	2
1	1	3	1	2	3	3	3	1	1
2	2	2	2	2	2	2	1	1	1
2	2	2	2	2	2	2	2	2	2
2	2	2	2	2	2	2	1	1	1

1	2	1	3	2	1	2	2	2	1
1	3	1	1	2	2	2	9	9	1
9	9	1	9	9	9	9	9	1	9
3	2	1	2	3	2	3	1	2	3
9	9	9	9	9	9	9	9	9	2
2	1	2	3	2	1	2	2	2	1
9	9	9	9	9	9	9	9	9	1
9	9	9	9	9	9	9	3	9	9
2	2	2	2	2	2	2	2	2	2
9	9	9	9	9	9	9	1	9	9
9	9	9	9	9	9	9	9	1	9
9	9	9	9	9	9	9	9	2	9
9	9	9	9	9	9	9	9	9	2
9	2	9	9	9	9	9	9	1	9
9	9	9	9	9	9	9	1	9	9
9	2	9	9	9	9	9	9	9	2
2	1	2	1	3	2	1	2	3	2
9	2	1	3	2	1	3	2	1	3
2	1	1	2	3	1	2	1	3	2
2	2	2	1	1	2	2	2	2	2
2	3	1	2	1	2	3	3	3	3
2	2	1	2	1	2	1	1	3	2
1	3	1	3	1	1	2	1	2	3
1	2	1	2	3	2	2	2	3	1
9	2	1	2	3	3	3	1	3	3
3	3	3	3	3	3	3	3	3	3
2	2	2	2	2	2	2	9	3	1
2	2	2	3	2	3	2	1	3	3
1	2	1	2	2	2	2	2	2	1
2	2	1	1	1	1	1	1	2	1
1	2	1	1	2	1	1	1	3	2
2	2	2	2	2	2	2	2	2	2
2	2	1	1	1	1	2	1	1	1
1	1	1	1	1	1	1	1	9	9
9	9	9	9	9	9	9	2	3	1
2	3	1	2	1	2	2	1	3	3
3	3	3	3	3	3	3	9	9	9
1	1	2	2	2	2	2	1	2	1
1	9	2	2	2	2	2	2	9	1
2	2	2	2	2	2	2	2	2	2
9	9	9	9	9	9	9	1	9	9
3	3	2	3	2	3	3	3	3	3
9	9	1	9	9	9	9	1	9	9
3	2	1	2	1	3	1	2	3	2
1	2	2	2	2	2	2	2	2	2
1	2	9	2	2	1	3	1	2	3
1	1	1	1	1	1	1	2	2	2

1	1	2	2	3	2	1	3	2	1
9	9	1	9	9	9	9	9	9	9
9	9	2	2	9	9	9	2	9	9
2	9	3	2	3	2	3	1	2	3
2	2	2	2	2	2	2	3	3	3
9	2	2	2	2	2	2	2	2	2
3	3	1	3	2	2	2	1	2	1
1	2	1	2	2	2	1	2	2	1
9	9	9	9	9	9	9	9	9	9
1	3	1	2	2	3	2	2	3	3
1	3	1	2	1	2	1	1	3	3
9	9	9	9	9	9	9	1	9	9
1	1	1	1	2	2	1	3	2	2
9	9	9	9	9	9	9	1	9	9
2	2	1	2	1	2	2	1	3	2
1	2	1	2	1	2	2	2	3	3
9	9	9	9	9	9	9	9	9	9
9	9	9	9	9	9	9	2	9	9
9	9	1	9	9	9	9	1	9	9
3	3	1	3	2	3	3	3	3	2
2	2	2	2	2	2	2	2	2	2
3	1	3	3	3	2	2	2	2	3
2	1	2	2	2	2	2	2	1	2
9	9	9	1	9	9	9	1	9	9
2	1	2	2	1	2	2	1	3	9
2	2	2	1	2	2	2	1	1	1
2	2	1	2	2	1	1	1	1	1
1	1	2	1	2	1	2	2	1	2
2	1	2	3	3	3	3	3	2	2
9	9	9	9	9	9	9	1	9	9
9	9	9	9	9	9	9	9	9	9
2	1	1	1	1	1	1	1	1	2
1	1	1	1	1	2	2	1	3	2
1	1	1	1	1	9	9	9	3	3
2	1	1	1	1	2	2	1	3	3
2	2	1	1	1	1	1	2	2	1
1	2	1	1	1	9	9	1	3	2
2	2	1	1	1	1	1	2	1	2
2	9	3	1	2	2	9	2	3	2
2	9	1	1	2	2	1	2	1	2
2	2	1	2	2	2	2	1	3	3
3	3	1	1	2	3	2	2	2	2
3	3	3	3	3	2	2	2	2	3
2	1	1	2	1	2	1	2	3	3
1	1	1	1	1	1	1	2	2	2
1	1	1	1	1	1	1	2	2	2
3	3	3	3	3	3	3	2	2	2

2	2	2	2	2	2	2	2	2	2
9	9	1	9	9	9	9	1	9	9
1	9	9	9	9	9	9	9	9	9
9	9	1	9	9	9	9	1	9	9
1	2	2	3	1	2	9	1	2	2
1	1	2	1	1	1	1	3	3	3
2	2	9	9	2	9	2	2	2	2
9	2	2	9	2	9	2	2	9	2
9	9	9	9	9	9	9	9	9	9
9	9	9	2	9	9	9	9	9	2
2	2	1	2	2	2	2	1	2	2
2	2	2	2	2	2	2	2	2	2
2	2	2	2	2	2	2	2	2	2
2	2	2	2	2	2	2	2	2	2
9	9	1	9	9	9	9	2	2	2
9	9	9	9	9	9	9	2	9	9
3	3	3	3	3	3	3	3	3	3
9	9	9	9	9	9	9	1	9	9
9	9	9	9	9	9	9	2	9	9
9	9	9	9	9	9	9	2	9	9
9	9	9	9	9	9	9	9	9	9
9	9	9	9	9	9	9	2	9	9
2	2	2	2	2	2	2	2	2	2
2	2	2	2	2	2	2	2	2	2
9	9	9	9	9	9	9	9	9	9
9	9	9	9	9	9	9	1	9	9
2	1	1	1	1	1	1	1	9	1
1	9	1	1	9	9	9	1	9	9
3	3	1	1	3	3	3	1	3	3
2	2	2	2	2	2	2	2	9	2
2	2	2	9	9	9	9	1	9	2
2	3	1	1	3	3	3	1	3	3
9	1	9	1	9	1	9	1	9	1
9	2	9	9	9	1	9	1	9	1
2	2	2	2	2	2	2	2	2	2
3	3	3	2	2	2	2	1	9	9
2	2	2	2	2	2	2	2	2	9
2	2	2	2	2	2	2	2	2	2
1	1	1	1	2	2	2	1	2	3
1	1	2	2	2	9	2	2	2	2
9	9	9	9	1	9	9	1	9	9
3	3	1	1	2	2	1	2	3	3
3	3	1	2	1	2	3	2	1	1
1	1	2	2	3	9	3	3	3	3
3	3	1	1	1	1	1	3	3	3
2	3	1	2	2	1	2	2	1	2
2	3	1	2	2	3	2	1	3	3

9	9	9	9	9	9	9	1	9	9
3	3	9	1	1	2	1	3	1	3
2	2	2	2	2	2	2	2	2	2
9	9	1	9	1	9	1	1	9	9
2	2	2	2	2	2	2	2	2	2
1	3	3	2	1	3	2	1	3	2
2	1	2	2	1	1	1	3	3	3
1	3	3	2	1	3	2	1	3	2
3	3	1	3	1	1	1	3	3	2
2	2	1	1	1	9	1	2	2	2
2	2	2	2	2	2	2	2	2	3
9	9	9	9	9	9	9	1	2	3
9	9	1	3	9	9	9	9	9	9
2	3	1	2	2	1	1	1	2	3
9	9	2	9	2	9	9	9	2	9
1	2	2	2	2	1	1	2	2	2
3	3	1	1	2	2	3	1	2	3
2	2	1	2	2	2	2	2	3	3
1	3	1	1	3	9	2	2	3	3
1	2	2	2	1	1	2	1	3	3
9	9	9	9	9	9	9	2	9	9
3	1	3	3	2	3	1	1	3	3
2	3	1	3	3	3	3	3	3	9
9	9	9	9	9	9	9	1	9	9
9	9	1	2	1	9	9	9	9	9
1	1	1	1	1	1	1	1	1	1
9	9	9	1	9	9	9	1	9	9
2	2	1	1	1	1	1	2	2	2
9	9	9	9	9	9	9	9	9	9
9	9	9	9	9	9	9	9	1	9
1	2	2	2	2	2	2	2	2	3
2	1	2	1	3	2	3	2	3	3
2	1	1	1	1	2	3	2	2	2
1	2	1	2	1	1	1	1	1	1
1	2	2	2	1	3	1	1	3	2
2	3	2	3	2	9	9	2	3	2
3	3	2	2	2	2	3	3	1	1
1	1	1	1	1	1	1	1	1	1
2	2	1	2	2	2	2	3	3	3
2	2	2	2	2	2	2	2	2	2
9	3	1	2	2	2	2	1	9	9
9	9	1	2	2	2	2	1	3	3
2	1	3	1	1	2	3	3	3	2
2	3	2	1	1	2	2	3	3	3
9	1	3	3	3	3	3	3	2	2
1	1	3	2	1	2	2	2	1	3
1	3	3	3	3	1	1	3	3	1

9	9	9	9	9	9	9	9	9	9
2	3	1	2	2	2	2	2	2	2
3	3	1	2	2	3	2	1	2	2
2	3	1	1	1	2	1	3	3	3
2	2	2	2	2	2	2	1	2	1
1	3	9	9	9	9	9	9	9	9
9	1	9	9	9	9	9	9	9	9
2	2	2	1	2	2	2	2	2	2
1	2	1	1	2	2	2	2	2	2
2	2	1	2	2	2	2	2	2	2
2	3	2	2	2	2	2	2	2	2
2	3	2	2	2	2	2	3	2	2
2	2	1	2	1	2	2	2	3	3
2	2	2	2	2	2	2	2	2	2
3	3	3	3	2	3	2	3	2	2
9	9	2	9	2	9	2	2	9	2
9	9	9	9	2	9	9	2	9	9
9	9	9	9	9	9	9	9	9	9
9	9	9	9	9	9	9	1	9	9
2	2	2	2	2	2	2	2	9	2
9	9	9	9	9	9	9	2	9	9
9	9	9	9	9	9	9	9	9	9
2	2	2	2	2	2	2	2	2	2
9	9	9	9	9	9	9	2	9	9
9	9	9	9	9	9	9	9	9	9
9	9	9	9	9	9	9	3	9	9
9	9	9	9	9	9	9	9	9	9
1	1	1	1	1	1	1	1	1	1
9	9	9	9	9	9	2	2	9	9
9	9	9	9	9	9	3	3	9	9
9	9	9	9	9	9	9	3	9	9
2	2	2	2	2	2	2	2	2	2
1	1	1	1	1	1	1	1	1	1
9	9	9	9	9	9	9	3	9	9
3	3	3	3	3	3	3	2	2	2
9	9	9	9	9	9	9	9	9	9
9	9	9	9	9	9	9	2	9	9
9	9	9	9	9	9	9	9	9	9
9	1	9	9	2	9	9	1	9	9
9	9	9	9	9	9	9	1	9	9
2	2	2	2	2	2	2	2	2	2
9	9	9	9	9	9	9	2	9	9
9	9	9	9	9	9	9	2	9	9
9	9	9	9	9	9	9	9	9	9
2	2	2	2	2	2	2	2	2	2
2	2	2	2	2	2	2	2	2	2
9	9	9	9	9	9	9	9	9	9

2	1	1	9	1	1	1	1	1	1
1	9	1	2	1	2	2	2	2	2
9	9	9	2	9	9	9	1	9	9
3	3	1	2	2	2	2	1	2	3
9	9	9	9	9	9	9	2	9	9
9	9	9	9	9	9	9	2	9	9
9	9	9	9	9	9	9	2	9	9
9	9	9	9	9	9	9	2	9	9
9	9	9	9	9	9	9	9	9	9
9	9	9	9	9	9	9	9	9	9
9	9	9	9	9	9	9	9	9	9
9	9	9	9	9	9	9	9	9	9
9	9	9	9	9	9	9	9	9	9
9	9	9	9	9	9	9	9	9	9
2	2	2	2	2	2	2	2	2	2
2	2	2	2	2	2	2	2	2	2
2	2	1	2	2	2	2	1	2	2
2	2	2	2	2	2	2	2	2	2
2	2	2	2	2	2	2	2	2	2
2	2	2	2	2	2	2	2	2	2
2	2	2	2	2	2	2	2	2	2
9	9	9	9	9	9	9	2	9	9
9	9	9	9	9	9	9	1	9	9
2	2	2	2	2	2	2	2	2	2
2	2	2	2	2	2	2	2	2	2
9	9	1	2	2	2	2	1	2	2
9	9	9	9	9	9	9	9	9	9
2	2	2	2	2	2	2	3	3	3
9	9	9	9	9	9	9	9	9	9
1	1	1	1	1	1	1	2	2	2
1	1	1	1	1	1	1	1	1	1
2	3	2	1	2	3	2	1	2	1
1	1	2	2	2	2	2	1	1	1
3	3	1	9	2	2	2	1	1	1
2	2	2	2	2	2	2	2	1	1
9	9	1	9	9	9	9	1	9	9
9	9	9	9	9	9	3	3	9	9
9	9	9	9	9	9	9	2	9	2
1	1	1	1	1	1	1	1	1	1
9	9	9	9	9	9	3	3	9	9
9	9	9	9	9	9	9	2	9	9
9	9	9	9	9	9	9	9	9	9
2	2	2	2	2	2	2	2	2	2
9	9	1	1	9	9	9	1	9	9
9	1	9	1	9	1	2	9	1	9
9	9	9	9	9	9	2	2	9	9
9	9	9	2	9	9	2	2	9	9

9	9	9	9	9	9	9	2	9	9
9	9	9	9	9	9	9	9	9	9
9	9	9	9	9	9	3	3	9	9
9	9	9	9	9	9	9	3	9	9
9	9	9	9	9	9	9	9	9	9
9	9	9	9	9	9	9	2	9	9
9	9	1	1	9	9	9	1	9	9
9	9	9	9	9	9	9	2	9	9
2	2	9	2	2	2	2	2	9	9
9	9	9	9	9	9	9	1	1	1
9	9	9	9	9	9	9	2	9	9
9	9	9	9	9	9	9	9	9	9
3	3	1	1	1	3	3	3	3	1
9	9	9	9	9	9	9	9	9	9
1	2	1	2	3	1	1	3	3	3
2	3	1	2	1	2	2	2	3	1
9	9	1	9	9	9	9	9	9	9
9	9	1	9	9	9	9	9	9	9
3	2	2	2	2	9	1	1	3	2
2	2	1	2	2	9	9	2	2	2
1	3	2	2	2	3	2	1	3	3
9	1	9	9	9	9	9	2	9	9
2	2	3	2	3	1	1	3	3	3
2	2	3	2	3	1	2	2	2	2
2	9	1	2	2	2	2	2	3	3
2	2	1	2	2	2	2	2	3	3
2	2	1	1	2	2	2	3	3	3
2	1	2	2	1	2	2	3	3	3
3	3	9	9	9	9	9	1	1	2
2	1	1	1	2	9	1	2	2	2
9	9	9	9	9	9	9	2	9	9
9	9	9	9	9	9	9	1	9	9
2	1	1	3	1	1	1	1	3	3
9	9	9	9	9	2	9	9	9	9
3	3	1	2	9	9	9	1	9	9
9	9	9	9	9	9	9	9	9	9
9	9	9	9	9	9	9	9	9	9
2	9	2	9	9	2	9	9	2	9
9	9	9	9	9	9	9	9	9	9
2	9	2	9	2	9	2	2	9	2
2	9	9	2	9	9	2	2	9	9
2	2	2	2	2	2	2	2	2	2
2	2	2	2	2	2	2	2	2	2
2	2	2	2	2	2	2	2	2	2
2	2	2	2	2	2	2	2	2	2
2	2	2	2	2	2	2	2	2	2
1	1	2	3	2	1	2	2	2	1

2	1	1	3	1	2	3	2	2	2
3	2	1	2	2	2	2	2	2	2
2	1	1	1	1	2	2	2	3	2
2	1	3	2	1	2	1	3	3	3
3	1	3	3	3	3	3	3	3	3
1	2	3	2	2	2	1	1	3	3
2	2	1	2	2	2	3	1	2	2
1	1	3	3	3	3	3	3	3	3
2	2	2	2	2	2	2	2	2	2
2	2	2	2	2	2	2	2	2	2
2	2	2	2	2	2	2	2	2	2
2	2	2	2	2	2	2	2	2	2
2	1	2	3	3	2	1	1	3	3
1	3	1	3	2	1	2	1	3	2
2	2	2	2	2	3	3	3	2	1
2	2	2	2	2	2	2	2	2	2
1	3	3	3	3	3	3	2	3	2
2	3	1	3	2	3	2	1	2	2
2	2	9	1	2	2	3	1	3	3
3	2	1	2	1	3	2	3	2	2
1	1	2	1	2	1	2	2	1	3
3	2	1	1	1	2	2	2	3	1
1	1	2	1	3	2	1	2	3	1
2	2	1	2	1	2	2	3	1	2
2	1	1	2	2	1	1	1	3	3
2	3	2	2	2	3	3	2	3	2
2	3	2	3	3	3	3	2	3	3
2	3	2	3	2	2	3	2	3	3
3	3	1	3	2	2	3	1	1	3
1	2	1	3	1	3	3	1	3	3
1	2	2	2	1	3	3	3	3	3
1	1	2	2	3	2	2	1	2	2
2	3	3	3	3	3	3	3	3	3
1	3	1	1	1	2	3	3	3	3
9	9	1	9	9	9	9	9	9	1
1	3	3	3	2	3	3	2	3	3
2	2	1	3	3	2	2	1	3	3
1	1	1	1	1	1	1	1	1	1
2	2	1	3	3	2	2	1	3	3
2	1	3	2	9	9	9	9	9	9
2	9	2	2	2	2	2	2	2	2
3	1	3	3	3	3	3	1	9	9
1	2	2	3	2	1	3	1	3	2
2	2	2	3	2	1	3	1	3	2
3	3	2	2	3	2	3	2	3	2
3	1	3	3	3	3	3	2	3	2
2	3	2	2	3	2	3	2	1	2

3	3	3	3	3	3	3	2	1	1
3	3	3	3	3	3	3	3	3	3
1	2	2	2	2	3	3	1	1	3
2	1	3	3	2	3	2	3	3	3
9	2	9	1	2	2	3	2	1	3
1	3	1	2	1	1	2	1	3	3
3	2	1	1	1	2	2	1	2	2
3	2	3	9	2	3	1	3	1	2
3	3	1	3	1	1	1	1	3	3
2	2	2	2	1	3	3	1	3	3
2	2	1	2	1	2	2	2	3	3
2	3	2	2	2	2	3	3	2	3
2	9	2	1	9	1	2	2	2	2
2	1	1	3	2	2	2	1	1	2
3	2	2	1	1	2	3	2	1	3
2	2	2	2	2	2	2	2	3	2
2	2	2	9	1	2	2	2	3	3
2	2	2	2	2	2	2	3	3	3
2	2	3	3	3	3	3	2	9	2
3	1	3	3	3	3	3	2	3	2
2	1	1	2	1	2	2	1	1	2
1	2	2	1	1	2	2	2	1	1
3	2	3	3	3	3	2	2	3	3
2	1	3	2	3	2	1	1	1	2
2	3	3	3	3	3	3	3	3	3
3	1	2	1	2	3	2	1	2	3
3	1	2	3	2	2	1	2	2	2
2	2	2	1	2	2	2	2	2	2
3	9	2	3	2	2	2	3	3	2
2	2	2	2	2	2	2	2	2	2
3	3	3	3	3	3	3	2	3	1
9	9	9	9	9	9	9	3	9	9
2	2	2	1	2	1	2	2	3	3
2	1	1	2	2	2	1	3	3	3
3	2	1	3	1	2	3	1	3	3
1	3	3	2	3	2	2	3	1	3
2	1	1	1	1	2	1	3	3	3
1	2	3	1	2	2	1	2	3	2
3	9	1	1	1	3	3	3	3	3
2	2	1	2	2	1	2	2	3	3
2	1	1	1	2	2	2	1	3	1
2	2	2	2	2	2	2	2	2	2
2	2	2	2	2	2	2	2	2	2
2	2	2	2	2	2	2	2	2	2
2	2	2	1	2	1	2	2	3	3
1	1	2	2	2	2	1	1	2	3
2	2	2	3	2	2	2	3	3	3

3	2	2	2	2	3	2	2	2	3
1	2	2	2	1	2	2	1	2	3
2	2	2	2	2	2	2	3	3	3
1	1	2	1	1	3	1	3	3	3
1	1	9	2	1	2	2	9	9	9
1	2	1	2	1	2	2	1	2	3
1	2	2	2	1	2	2	1	2	3
2	1	1	2	1	2	9	1	3	2
3	2	1	2	2	3	2	3	3	3
9	9	9	9	9	9	9	9	9	9
1	3	1	2	2	1	2	3	2	3
1	3	1	2	2	1	2	3	2	3
9	9	1	9	9	9	9	9	9	1
9	9	1	9	9	9	9	2	9	1
1	2	2	2	3	1	2	3	2	1
9	9	9	9	9	9	9	9	9	9
3	1	3	3	3	3	3	1	9	1
9	9	9	9	9	9	9	1	9	9
9	9	9	9	9	9	9	9	9	9
3	3	1	3	2	3	2	3	3	2
9	3	2	2	2	2	2	1	3	3
2	9	9	9	1	9	9	1	9	9
2	1	1	2	3	1	2	1	1	2
1	1	2	2	2	2	3	2	2	2
2	2	2	2	2	2	2	1	2	2
1	1	2	2	2	2	3	3	2	2
1	2	1	1	2	2	3	2	2	2
9	9	9	9	9	9	9	1	3	2
2	2	2	2	2	2	2	2	2	2
2	3	1	2	1	2	2	2	3	2
3	3	1	1	9	9	9	9	9	9
1	2	3	3	2	2	2	1	1	3
2	3	1	2	1	3	2	1	3	3
2	2	2	2	2	2	2	3	2	2
2	2	2	2	2	2	2	2	2	1
1	1	2	9	2	9	3	3	2	2
1	9	9	9	9	9	9	1	9	9
3	1	2	2	2	2	2	1	3	2
1	2	2	1	2	2	2	2	3	3
3	3	3	3	3	3	3	1	1	1
2	2	1	1	1	2	2	2	2	1
2	9	1	2	1	2	3	2	3	2
1	1	2	2	2	2	2	2	2	1
1	1	1	2	2	2	2	2	2	9
2	3	1	3	3	3	2	1	3	3
9	9	9	9	9	9	9	9	9	9
2	1	2	2	2	3	3	3	3	3

3	1	2	2	1	1	2	3	1	2
1	1	3	3	3	3	3	2	2	2
3	3	1	1	2	2	3	3	9	3
2	3	1	2	1	2	1	1	2	1
3	9	3	3	3	3	3	3	2	3
9	9	9	1	1	1	1	1	1	1
9	9	1	9	9	9	9	1	9	9
3	3	3	3	3	3	3	3	3	3
3	3	3	3	3	3	3	3	3	3
2	2	2	2	2	2	2	3	3	2
1	1	2	2	2	2	2	2	2	2
3	3	1	1	3	3	3	1	2	3
3	3	2	2	2	1	1	1	2	2
9	9	9	9	1	9	9	3	3	3
9	9	2	9	9	9	9	1	9	9
9	9	1	9	9	9	9	9	9	9
2	2	1	1	1	1	1	3	3	2
1	1	1	3	9	1	1	9	3	3
2	3	1	2	2	2	2	2	3	3
2	2	2	2	2	2	2	2	3	3
1	1	2	2	3	3	2	2	2	2
1	1	1	1	1	1	9	1	3	3
2	2	1	1	1	2	1	2	2	2
2	2	1	2	2	2	2	2	2	2
1	3	1	1	1	3	1	1	2	2
2	2	2	2	2	2	2	2	2	2
2	2	2	2	2	2	2	2	3	3
9	9	9	9	9	9	9	2	9	9
9	2	3	2	3	2	1	1	3	3
3	3	3	3	3	3	3	2	2	2
1	1	1	1	1	1	1	1	1	1
1	2	1	2	2	1	2	3	3	9
1	2	1	2	1	1	9	1	2	2
2	2	1	1	1	2	2	2	2	2
1	2	2	1	1	1	1	2	2	3
2	2	2	2	2	2	2	1	2	3
2	2	2	2	2	2	2	1	1	1
3	2	1	3	3	3	1	1	3	3
2	3	1	3	2	3	2	3	3	3
1	1	1	2	2	3	3	3	3	3
9	9	9	9	9	9	9	9	9	9
1	2	1	2	1	2	1	2	2	2
2	2	1	2	2	2	2	1	3	2
2	2	1	2	2	2	2	3	3	3
1	1	2	2	2	2	2	2	2	3
2	1	1	2	2	2	2	1	1	1
9	9	9	9	9	9	9	3	9	9

2	1	2	3	2	1	2	3	3	3
2	1	1	2	9	1	2	3	9	3
3	1	3	3	3	3	3	1	2	3
2	2	1	2	2	2	2	1	3	3
2	9	1	3	1	2	1	3	2	3
3	3	1	2	1	3	2	1	3	2
2	3	1	1	1	2	3	1	3	2
9	9	9	9	9	9	9	1	9	9
9	9	9	9	9	9	9	1	9	9
9	9	1	9	9	9	9	9	1	9
2	9	1	2	2	2	1	2	2	2
1	1	1	1	1	1	1	3	3	3
1	1	1	1	1	2	2	1	2	3
9	9	9	9	9	9	9	2	9	9
1	1	3	2	2	2	2	2	2	2
2	2	1	1	1	9	9	1	9	9
2	9	2	2	2	9	1	3	2	1
9	9	1	9	9	9	9	9	9	1
9	9	1	9	9	9	9	9	9	1
9	9	9	9	9	9	9	9	9	2
1	1	1	1	1	1	1	1	1	1
1	2	2	2	1	1	1	3	3	3
2	2	3	2	3	2	2	1	2	2
3	3	3	3	3	3	3	2	2	3
1	9	9	9	9	1	1	2	1	2
1	3	3	3	1	1	2	1	3	3
1	1	3	3	3	3	3	1	3	3
9	9	9	9	9	9	9	2	9	9
2	2	2	2	2	2	3	1	2	2
3	9	1	3	1	3	1	1	3	3
2	1	3	3	3	3	3	3	1	1
9	9	9	9	9	9	9	1	9	9
1	1	1	1	2	2	3	3	3	3
9	9	9	9	9	9	9	3	9	9
9	9	9	9	9	9	9	1	9	9
1	2	2	1	2	2	2	2	2	2
9	9	9	9	9	9	9	1	9	9
1	1	1	1	1	1	9	1	2	3
2	2	1	2	3	2	2	3	3	3
2	2	2	2	2	2	2	2	9	2
2	2	2	2	2	2	2	2	2	2
2	2	2	2	2	2	2	2	2	2
3	3	3	3	3	3	3	3	3	3
2	2	2	2	2	2	2	2	2	2
1	1	1	1	1	1	1	2	2	1
1	1	1	1	1	1	9	1	1	1
2	2	2	2	2	2	2	1	3	1

3	1	3	9	1	3	1	9	1	3
9	2	2	9	2	2	2	9	2	9
9	2	8	2	2	2	9	9	2	9
9	9	9	9	9	9	9	9	9	9
1	3	1	9	1	9	1	1	9	3
1	3	2	1	1	9	1	1	2	1
3	1	9	9	3	1	9	9	2	9
2	9	2	9	9	2	9	2	3	1
2	9	9	9	2	9	2	2	9	2
1	1	1	1	1	1	1	1	1	1
2	2	2	2	2	2	9	2	2	2
9	9	2	9	9	2	9	2	9	9
2	2	2	2	2	2	2	2	2	2
3	3	3	3	3	3	3	3	3	3
2	2	2	2	2	2	2	2	2	2
9	9	9	9	9	9	9	9	9	9
9	2	9	9	2	9	2	2	9	9
2	2	2	2	2	2	2	2	2	2
2	1	9	2	1	2	9	9	1	2
2	2	2	2	2	2	2	2	2	2
2	2	2	2	2	2	2	2	2	2
2	2	2	2	2	2	2	2	2	2
2	1	3	1	1	2	3	1	2	9
1	9	2	1	1	1	1	1	2	9
3	3	3	3	3	3	3	2	2	2
3	3	3	3	3	3	3	3	3	3
3	1	3	1	2	1	2	3	3	3
2	2	1	2	2	3	2	2	3	3
2	1	3	2	1	2	3	3	3	3
2	2	1	2	2	2	2	2	3	2
3	3	3	3	3	3	3	2	2	3
2	2	2	2	2	2	2	3	2	2
3	3	2	2	2	2	1	2	2	1
1	1	2	2	1	2	2	3	3	9
2	2	1	2	1	1	1	1	3	3
1	9	9	9	9	9	9	1	1	1
2	3	1	3	3	3	2	2	3	3
3	3	1	3	1	2	2	1	3	2
2	2	2	2	2	2	3	2	1	2
2	3	2	2	2	2	2	2	3	3
1	1	3	3	3	3	3	1	3	1
1	3	2	2	3	2	3	3	3	2
1	3	1	2	3	1	2	3	2	3
9	9	9	9	9	9	9	9	9	9
3	3	2	1	2	1	1	1	9	9
1	1	2	2	9	9	3	3	2	2
3	1	3	3	3	3	3	1	3	1

1	2	1	2	2	2	2	2	1	1
9	3	3	2	2	2	2	2	2	2
1	1	1	1	1	1	1	1	1	1
2	2	2	2	2	2	2	2	2	2
1	1	1	2	2	2	2	1	1	1
2	2	2	2	2	2	2	2	2	2
1	1	1	1	1	2	1	1	1	1
1	9	1	1	3	3	1	1	3	3
3	3	3	3	3	3	3	3	3	3
2	1	1	1	1	1	2	1	2	1
1	1	1	1	1	9	9	1	1	1
1	1	1	1	1	1	1	1	1	1
1	1	1	1	1	1	1	1	1	1
1	1	1	1	1	1	1	1	1	2
1	1	1	2	2	2	2	1	1	1
1	1	1	1	1	1	1	2	2	2
1	1	2	2	2	1	1	1	2	1
1	2	2	1	2	2	2	1	1	2
1	1	1	1	1	1	1	1	1	1
2	2	2	2	2	2	2	2	2	2
9	9	9	9	9	9	9	1	2	1
1	1	2	2	2	2	2	2	2	2
9	9	9	9	9	9	9	9	9	9
1	1	1	1	1	1	1	1	1	1
1	9	1	1	1	1	1	1	9	1
2	1	1	1	1	1	1	2	1	2
2	1	1	1	1	2	1	1	1	1
1	1	2	1	1	1	1	1	1	1
2	9	2	2	2	2	2	2	9	2
1	1	1	9	1	1	1	1	9	1
2	2	2	2	2	2	2	2	2	2
2	2	2	2	2	2	2	2	2	2
1	1	1	1	1	9	1	1	1	1
1	1	1	1	1	1	1	1	1	1
1	1	1	1	2	2	2	1	1	1
2	2	2	2	2	2	2	2	2	2
2	1	2	1	2	1	2	1	2	2
1	1	1	2	2	1	1	1	1	1
9	9	9	9	9	9	9	9	9	9
2	2	2	2	2	2	2	2	2	2
2	2	2	2	2	2	2	2	2	2
2	2	2	2	2	2	2	2	2	2
1	1	1	1	1	1	1	1	1	1
2	2	2	2	2	2	2	2	2	2
1	1	1	2	1	2	1	1	1	1
2	2	2	2	2	2	2	2	2	2
1	1	1	1	1	1	1	1	1	1

2	1	1	2	1	1	1	1	2	1
2	2	2	2	2	2	2	2	9	2
1	1	1	1	1	1	1	1	1	1
1	1	9	1	1	1	9	1	9	1
1	1	1	1	1	1	1	1	1	1
2	2	2	2	2	2	2	2	2	2
1	1	1	1	1	1	1	1	1	1
1	1	1	1	1	1	1	1	1	1
9	2	2	9	2	2	2	2	9	2
1	1	1	1	1	1	1	1	1	1
2	9	2	2	2	2	2	9	2	2
9	1	1	1	1	9	1	1	9	1
2	2	2	2	2	2	2	2	9	2
1	1	1	1	1	1	1	1	1	1
1	1	1	1	1	1	1	1	1	1
2	2	2	2	2	2	2	2	2	2
1	1	1	1	1	9	9	1	1	1
9	9	9	9	9	9	9	9	9	9
2	2	2	2	2	2	2	2	2	2
3	1	1	1	1	3	3	1	3	3
3	2	1	1	1	1	3	1	3	3
3	2	1	1	1	1	3	1	3	3
3	2	1	1	1	3	1	1	3	3
3	2	1	1	1	3	3	1	3	3
3	2	1	1	1	3	3	1	3	3
3	2	1	1	1	3	3	1	9	3
3	2	1	1	1	9	3	1	3	3
3	1	1	1	1	3	3	1	9	9
3	2	1	1	1	3	3	1	3	3
3	1	1	1	1	3	3	1	3	3
3	2	1	1	1	3	1	1	3	3
3	2	1	1	1	3	3	1	3	3
3	2	1	1	1	3	3	1	3	3
3	2	1	1	1	3	3	1	3	3
3	1	9	9	1	9	3	1	3	3
3	1	2	1	1	2	3	1	3	3
3	2	1	1	1	3	3	1	3	3
1	2	1	1	1	3	3	1	3	3
3	2	1	1	1	3	3	1	3	3
3	2	1	1	1	3	3	1	3	3
3	2	1	1	1	3	3	1	3	3
3	2	1	1	1	3	3	1	3	3
9	1	1	1	3	3	3	1	3	3
9	9	9	9	9	9	9	9	9	9
9	9	9	9	9	9	9	9	9	9
9	9	9	9	9	9	9	9	9	9

9	9	9	9	9	9	9	9	9	9
9	9	9	9	9	9	9	3	3	2
9	9	9	9	9	9	9	9	9	9
9	9	9	9	9	9	9	9	9	9
9	9	9	9	9	9	9	9	9	9
9	9	9	9	9	9	9	9	9	9
9	9	9	9	9	9	9	9	9	9
9	9	9	9	9	9	9	9	9	9
9	9	9	9	9	9	9	9	9	9
9	9	9	9	9	9	9	1	9	9
9	9	9	9	9	9	9	1	9	9
9	9	9	9	9	9	9	9	9	9
9	9	9	9	9	9	9	1	1	2
9	9	9	9	9	9	9	1	9	9
9	9	9	9	9	9	9	9	9	9
9	9	9	9	9	9	9	9	9	9
9	9	9	9	9	9	9	1	1	1
9	9	9	9	9	9	9	1	9	9
9	9	9	9	9	9	9	9	9	9
3	2	1	1	1	3	3	1	3	3
3	2	1	1	1	3	3	1	3	3
3	2	1	1	1	3	1	1	3	1
3	2	1	9	1	3	3	1	3	3
3	1	1	1	1	3	3	1	3	3
3	2	1	1	1	3	3	1	3	3
3	2	1	1	1	3	3	1	3	3
3	1	1	1	1	3	3	1	3	3
3	2	1	1	1	3	3	1	3	3
2	1	1	9	9	3	3	1	3	3
3	9	9	1	1	1	1	1	3	3
3	1	1	1	1	3	3	1	3	3
3	3	9	9	1	3	3	1	3	3
3	2	1	1	1	9	3	1	3	3
3	3	1	1	1	3	3	1	3	3
3	1	1	1	1	3	3	1	3	3
9	1	1	9	2	9	3	3	9	9
2	2	1	1	1	3	9	1	3	3
3	3	3	9	1	3	3	1	3	3
3	3	9	9	1	3	3	1	3	3
9	9	9	9	1	9	9	1	3	3
3	1	1	1	1	3	3	1	3	3
3	2	1	1	1	3	3	1	3	3
3	2	1	9	1	3	1	1	3	3
3	2	1	1	1	3	3	1	3	3
3	2	1	1	1	3	3	1	3	3
3	1	1	1	1	3	3	1	3	3
3	1	1	1	1	3	3	1	3	3

3	2	1	1	1	1	1	1	3	3
3	1	1	1	1	3	3	1	3	3
3	1	1	1	1	3	3	1	3	3
3	2	1	1	1	3	3	1	3	3
3	1	1	1	1	3	3	1	3	3
3	2	1	1	1	3	3	1	3	3
3	1	1	1	1	3	3	1	3	3
3	3	1	1	1	1	3	1	3	3
3	3	1	1	1	1	3	1	3	3
2	1	9	9	1	3	9	1	3	3
3	2	1	1	1	3	1	1	3	3
2	1	3	9	9	9	9	1	3	3
3	2	1	1	1	3	3	1	3	3
3	2	1	1	3	3	3	1	3	3
3	2	1	9	9	9	9	1	3	3
3	2	3	2	3	3	3	1	3	3
3	2	1	1	1	3	3	1	3	3
3	2	1	1	1	3	3	1	3	3
2	1	1	1	2	3	3	1	3	3
3	2	1	1	1	3	3	1	3	3
3	2	1	1	1	3	3	3	3	3
3	2	1	1	1	9	3	1	3	3
2	1	1	1	1	3	3	1	3	3
3	2	1	1	1	3	3	1	3	3
3	2	1	1	1	3	3	1	3	3
3	2	1	1	1	3	3	1	3	3
1	1	1	3	3	3	1	1	3	3
3	2	1	1	1	3	3	3	3	3
3	3	1	1	1	1	3	1	3	3
3	3	9	1	1	1	3	1	3	3
2	1	1	9	9	3	3	1	3	3
3	2	1	1	1	9	3	1	3	3
3	2	1	1	1	9	3	1	3	3
3	1	1	1	1	3	3	1	3	3
9	9	9	9	9	9	9	1	3	3
2	3	1	1	1	3	3	1	3	3
3	2	1	1	1	3	3	1	3	3
3	1	1	1	3	3	3	1	3	3
9	1	9	9	9	9	9	1	3	3
9	9	9	9	9	9	9	1	3	3
2	1	1	1	3	3	3	1	3	3
3	2	1	1	1	1	1	1	3	3
3	2	1	1	1	1	1	1	3	3
3	1	1	1	1	3	3	1	3	3
3	1	1	1	1	3	3	1	3	3
3	2	1	1	1	3	3	1	3	3
3	1	1	1	1	3	3	1	3	3

3	1	1	2	3	9	3	1	3	3
3	2	1	1	1	3	3	1	3	3
3	1	1	1	1	3	3	1	3	3
3	2	1	1	1	3	3	1	3	3
3	2	1	1	1	3	3	1	3	3
3	2	1	1	1	3	3	1	3	3
3	2	1	1	1	3	3	1	3	3
3	2	1	1	1	3	3	1	3	3
2	1	1	1	1	3	3	1	3	3
3	2	1	1	1	3	3	1	3	3
3	1	1	1	1	3	3	1	3	3
3	1	1	1	1	3	3	1	3	3
3	2	1	1	1	3	3	1	3	3
3	2	1	1	1	3	3	1	3	1
9	3	1	1	1	1	1	3	3	3
3	2	1	1	1	1	1	3	3	3
3	2	1	1	1	3	3	1	3	3
3	1	1	1	1	3	3	3	3	3
3	2	1	1	1	3	3	1	3	3
9	9	9	9	9	9	9	9	9	9
3	2	1	1	3	3	3	1	3	3
3	2	9	1	1	1	1	1	3	3
3	2	1	1	1	3	1	1	3	3
3	1	1	1	1	3	3	1	3	3
9	9	9	9	9	9	9	3	3	3
3	2	1	1	1	3	3	3	3	3
3	2	1	1	1	3	3	1	3	3
3	3	1	1	1	3	3	1	3	3
3	2	1	1	1	3	3	1	3	3
3	2	1	1	1	3	3	1	3	3
3	2	1	1	1	3	3	1	3	3
3	1	1	1	1	3	1	1	3	3
3	2	1	1	3	3	3	1	3	3
3	2	1	1	3	3	3	1	3	3
3	1	1	1	1	3	3	1	3	3
3	1	1	1	3	3	3	1	3	3
3	1	1	1	3	3	3	1	3	3
3	2	1	1	1	3	3	1	3	3
3	1	1	1	1	3	3	1	3	3
3	1	1	1	1	3	3	1	3	3
3	2	1	1	1	3	3	1	3	3
9	9	9	9	9	9	9	9	9	9
3	1	1	1	1	3	3	1	3	3
3	3	1	1	1	3	3	1	3	3
3	2	1	1	1	3	3	1	3	3
3	1	1	1	1	3	3	1	3	3
3	1	1	1	1	3	3	1	3	3

3	2	1	1	1	3	3	1	3	3
3	2	1	1	1	3	3	1	3	3
3	1	1	1	1	3	3	1	3	3
3	2	1	1	1	3	3	1	3	3
3	1	1	1	1	3	3	1	3	3
3	3	1	1	1	3	3	1	3	3
3	2	1	1	1	3	3	1	3	3
3	2	1	9	1	9	3	1	3	3
3	2	1	1	1	3	3	1	3	3
3	1	1	1	3	3	3	1	3	3
9	9	9	9	1	3	3	1	3	3
3	2	1	1	1	3	3	1	3	3
3	1	1	1	1	3	3	1	3	3
3	2	1	1	2	3	3	1	3	3
2	1	1	1	1	3	3	1	3	3
3	1	1	1	1	3	3	1	3	3
3	2	1	1	1	3	3	1	3	3
3	2	1	1	1	3	3	1	3	3
3	2	1	1	1	3	3	1	3	3
3	2	1	1	1	3	3	1	3	3
3	2	1	1	1	3	3	1	3	3
3	2	1	1	1	1	3	1	3	3
1	9	1	1	1	3	3	1	3	3
1	2	1	1	1	3	3	1	3	3
3	2	1	1	1	1	3	1	3	3
3	2	1	1	1	1	1	1	3	3
2	3	1	1	1	1	1	1	3	3
9	9	9	9	9	9	9	9	9	2
9	9	9	9	9	9	9	9	9	2
9	9	9	9	9	9	9	9	9	2
9	9	9	9	9	9	9	9	9	2
9	9	9	9	9	9	9	9	9	9
9	9	9	9	9	9	9	2	2	2
9	9	9	9	9	9	9	9	9	9
9	9	9	9	9	9	9	2	9	9
9	9	9	9	9	9	9	9	9	9
9	9	9	9	9	9	9	9	9	9
9	9	9	9	9	9	9	9	9	9
9	9	9	9	9	9	9	9	9	9
9	9	9	9	9	9	9	9	9	9
9	9	9	9	9	9	9	9	9	9
9	9	9	9	9	9	9	1	3	3
2	2	1	1	2	2	2	2	2	1
9	9	9	9	9	9	9	1	1	1
9	9	9	9	9	9	9	9	9	9
9	9	9	9	9	9	9	9	9	9
9	9	9	9	9	9	9	9	9	9

9	9	9	9	9	9	9	9	9	9
9	9	9	9	9	9	9	1	3	3
9	9	9	9	9	9	9	9	9	9
9	9	9	9	9	9	9	9	9	9
9	9	9	9	9	9	9	9	9	9
9	9	9	9	9	9	9	9	9	1
9	9	9	9	9	9	9	1	3	3
2	2	2	2	2	2	2	2	2	2
9	9	9	9	9	9	9	9	9	9
9	9	9	9	9	9	9	9	9	9
9	9	9	9	9	9	9	9	9	9
9	9	9	9	9	9	9	9	9	9
9	9	9	9	9	9	9	9	9	9
1	1	3	2	1	2	2	1	3	3
2	9	1	2	2	2	2	2	2	2
3	3	1	3	2	3	3	2	3	3
1	1	1	3	3	2	3	3	3	3
2	2	1	1	1	2	2	1	2	3
1	1	1	3	1	3	3	2	3	3
2	1	2	1	3	2	1	2	1	3
2	2	1	1	1	2	2	1	3	1
9	9	9	9	9	9	9	3	3	3
2	2	1	1	1	3	3	3	3	2
2	2	9	2	2	2	2	2	3	3
9	9	1	9	9	9	9	1	9	9
2	2	2	2	2	2	2	2	2	2
9	9	9	9	9	9	9	2	3	2
1	2	1	1	1	2	3	2	1	1
3	1	1	1	3	9	2	1	3	3
1	3	1	1	1	2	2	2	3	2
3	2	3	2	3	2	2	3	3	3
2	1	2	1	3	2	1	1	2	3
9	9	1	9	9	9	9	9	1	9
2	1	3	2	2	3	1	1	2	2
9	9	9	9	9	9	9	1	9	9
9	9	9	9	9	9	9	2	9	9
2	2	1	2	1	1	1	1	2	1
2	1	1	1	3	2	2	1	2	9
9	9	1	1	1	9	9	1	9	9
1	1	3	3	3	3	3	9	9	9
9	9	9	9	9	9	9	9	9	9
1	3	1	1	2	1	1	1	3	1
2	1	1	1	1	1	1	1	2	1
9	9	9	9	9	9	9	3	3	3
9	9	9	9	9	9	9	9	9	9
9	9	9	9	9	9	9	2	2	2
2	2	2	2	2	2	2	2	2	2

9	9	1	9	9	9	9	1	9	9
9	9	9	9	9	9	9	9	9	9
3	2	3	2	1	3	3	3	3	3
1	1	1	1	1	1	1	2	1	2
9	9	9	9	9	9	9	1	9	9
2	9	9	9	9	9	9	2	9	9
1	1	1	2	2	2	2	1	2	2
2	2	2	2	2	2	2	2	2	2
2	2	2	2	2	2	2	2	2	2
2	2	2	1	1	1	1	1	1	1
2	2	1	1	1	1	1	2	2	1
9	9	9	9	9	9	9	9	9	9
9	9	9	1	9	9	1	1	9	9
2	2	2	2	2	2	2	2	2	2
2	2	1	2	2	2	2	3	2	3
2	3	1	2	3	2	3	2	3	3
9	9	9	9	9	9	9	3	3	3
1	2	3	3	1	2	1	1	2	2
9	9	1	3	1	2	3	1	1	9
2	2	3	2	1	2	3	9	9	9
1	1	1	1	1	1	1	2	3	3
2	2	2	2	2	2	2	1	2	2
2	2	2	2	2	2	2	2	2	2
2	2	2	2	2	2	2	2	3	2
2	2	2	2	2	2	2	2	2	2
3	3	1	1	2	2	1	2	2	1
2	2	2	2	2	2	2	3	2	2
1	1	1	1	1	1	1	3	3	3
2	2	1	2	1	2	2	2	2	3
1	2	1	1	1	1	1	1	2	1
3	2	2	2	3	3	3	3	3	3
1	2	1	1	1	1	2	2	3	3
1	1	1	1	1	1	1	2	2	2
2	2	1	2	1	1	1	1	2	2
2	9	1	1	2	3	1	2	1	3
3	2	3	1	1	2	3	9	9	9
1	9	1	3	3	2	2	2	2	2
9	1	1	1	2	9	9	9	2	2
9	9	9	9	9	9	9	9	9	9
1	2	1	1	2	2	2	1	3	3
2	1	2	2	2	2	2	2	3	3
1	2	2	2	2	2	2	2	2	2
2	3	1	2	3	1	2	1	2	3
2	1	2	2	1	2	2	1	3	3
1	1	1	1	1	1	1	1	3	1
1	2	2	2	3	2	2	1	2	2
3	3	1	1	2	2	2	1	2	2

1	2	1	1	2	1	1	3	3	2
1	1	1	2	2	3	1	2	1	2
3	2	1	2	3	2	3	2	2	2
2	2	1	2	2	2	2	9	9	9
2	1	2	2	1	2	2	1	3	3
2	2	1	2	1	2	2	1	2	2
2	3	1	3	1	3	3	1	2	2
3	2	9	1	2	2	2	1	2	2
2	2	2	2	2	2	2	2	2	2
1	2	3	3	2	1	2	2	2	1
2	2	2	2	2	2	2	2	2	2
2	2	2	2	2	2	2	2	2	2
2	2	2	2	2	2	2	1	1	1
2	2	2	2	2	2	2	2	2	2
2	2	2	2	2	2	2	9	9	9
3	2	2	2	2	2	2	2	1	2
3	3	3	3	3	3	3	3	3	3
2	2	2	2	2	2	2	2	2	2
2	2	2	2	2	2	2	1	2	2
2	3	1	2	3	1	2	1	2	3
2	1	2	2	2	2	2	1	1	1
1	1	1	1	1	1	1	1	1	1
2	2	2	2	2	2	2	9	9	9
1	1	1	1	1	1	1	9	9	9
1	3	1	3	1	3	1	1	3	3
9	9	9	9	9	9	9	1	3	1
1	1	1	9	3	3	1	2	1	2
2	2	1	1	2	2	2	3	3	3
2	3	1	3	1	3	2	1	3	3
2	1	1	1	2	3	2	3	3	3
2	3	1	1	1	2	3	1	3	3
2	9	1	2	3	1	2	9	9	9
2	2	1	1	1	1	2	1	1	2
2	2	3	3	3	3	3	2	1	3
9	2	3	9	2	1	2	1	1	1
9	9	9	9	9	9	9	9	9	9
1	2	1	1	1	1	1	1	2	1
2	1	1	1	2	1	1	2	2	2
3	3	1	3	2	2	9	2	3	2
2	2	2	2	2	2	2	2	2	2
2	3	1	2	3	2	3	2	3	2
1	2	1	1	2	1	2	1	3	1
2	3	1	1	1	2	3	1	3	3
9	9	9	9	9	9	9	1	3	2
9	9	9	9	9	9	9	9	9	9
9	9	9	9	9	9	9	9	2	9
1	1	1	1	1	1	1	1	1	1

1	2	1	2	3	2	2	3	3	2
2	2	1	2	1	3	1	1	3	2
2	2	1	2	1	2	3	1	3	3
3	1	3	3	1	3	1	1	3	3
9	9	9	9	9	9	9	1	2	2
2	2	1	1	1	3	2	2	3	3
1	3	3	2	3	3	3	3	3	3
2	2	1	2	2	2	3	1	3	3
1	2	1	1	1	2	1	1	3	1
2	3	1	2	3	1	2	3	2	2
2	3	2	1	2	3	1	1	2	1
2	2	2	2	2	2	2	2	2	2
2	2	2	2	2	2	2	2	2	2
1	3	1	1	3	3	3	3	3	3
2	2	2	2	2	2	2	2	2	2
2	9	1	1	2	2	2	3	3	3
3	3	1	1	1	1	1	2	3	1
3	3	2	3	2	2	3	3	3	1
1	3	2	1	1	1	1	1	3	3
2	3	1	1	2	3	1	1	3	3
3	3	2	3	1	2	3	2	1	3
3	3	3	2	1	1	1	1	3	3
3	3	2	2	2	2	2	1	3	3
3	3	2	3	2	2	3	2	3	3
3	3	3	3	3	3	3	1	3	2
9	9	9	9	9	9	9	9	9	9
2	3	1	2	3	1	2	2	1	2
1	2	2	2	2	2	2	3	3	3
2	3	1	2	1	1	1	2	2	2
2	1	1	3	1	1	2	1	2	1
2	2	2	2	2	2	2	2	2	2
9	9	9	9	9	9	9	9	9	9
2	1	1	2	2	2	2	1	3	3
2	3	2	3	1	3	2	3	1	2
1	1	1	2	1	3	2	3	2	3
1	2	1	3	2	1	3	1	9	1
3	3	3	3	3	3	3	1	3	9
3	3	1	3	1	1	2	1	2	2
1	2	1	2	2	2	2	3	3	2
9	9	9	9	9	9	9	2	9	9
2	1	1	2	1	3	2	1	2	3
2	2	2	2	2	2	2	2	2	2
3	3	1	1	1	3	3	3	3	3
2	2	2	2	2	2	2	2	2	2
2	2	2	2	2	2	2	2	2	2
3	2	2	2	2	2	2	1	2	2
2	2	3	3	3	2	2	2	2	2

1	1	1	1	1	1	1	1	3	3
2	2	2	2	2	2	2	2	2	2
3	3	1	3	2	3	2	2	3	3
2	2	1	2	1	2	2	2	2	3
2	2	2	2	2	2	2	1	3	3
3	2	2	2	2	2	2	2	2	2
1	3	1	1	1	1	3	1	3	3
1	3	1	2	1	1	1	1	3	3
1	3	1	1	1	9	1	1	3	3
3	3	1	1	1	3	3	3	3	3
1	3	1	3	1	3	2	3	3	3
2	2	2	2	2	2	2	2	2	2
1	3	1	3	1	3	2	3	3	3
2	3	1	3	2	1	1	1	3	3
2	2	2	2	2	2	2	2	2	2
3	3	1	1	1	2	2	1	3	2
1	3	2	3	2	2	2	9	9	9
2	2	2	2	1	2	2	2	3	2
3	2	1	2	2	2	2	1	2	3
2	3	2	1	1	1	1	2	3	3
3	3	1	1	1	1	1	1	2	2
2	3	1	3	3	3	1	2	3	1
9	9	9	9	9	9	1	9	9	9
2	2	2	2	2	2	2	2	2	2
2	2	2	2	2	2	2	2	2	2
1	2	1	1	1	1	1	3	1	2
2	3	2	2	2	2	2	3	3	2
2	2	2	2	2	2	2	2	3	2
3	3	1	1	1	3	1	1	3	3
1	2	1	1	1	2	2	3	3	1
2	2	2	3	1	3	1	1	3	3
1	2	1	2	2	2	1	1	2	2
2	1	2	1	1	1	1	3	9	2
9	9	9	9	9	9	9	1	2	2
9	9	9	9	9	9	9	2	9	9
1	2	1	1	1	3	1	3	3	3
2	2	2	2	2	2	2	2	2	1
1	1	3	3	3	2	2	1	2	1
2	2	1	1	1	1	1	1	1	1
2	2	2	2	2	2	2	1	1	1
2	3	2	1	3	1	3	1	2	2
9	9	9	9	9	9	9	9	9	9
9	9	9	9	9	9	9	9	9	9
1	1	1	1	1	1	1	1	1	1
2	2	3	2	3	2	3	1	2	3
9	9	9	9	9	9	9	9	9	9
2	2	2	2	2	9	2	1	2	2

2	2	2	2	2	2	2	2	2	2
3	3	3	3	2	2	2	1	2	1
2	3	1	2	3	1	2	1	3	2
9	1	3	1	9	1	3	1	2	3
2	3	3	3	2	2	1	1	3	3
2	3	1	2	3	1	2	1	2	3
2	2	1	2	2	1	2	2	2	1
9	9	9	9	9	9	9	2	9	9
1	1	1	1	2	3	3	2	1	3
9	9	9	9	9	9	9	9	9	9
1	1	1	1	1	1	1	2	2	2
3	3	2	1	1	3	1	3	3	3
2	1	3	1	1	2	2	1	2	2
2	2	3	2	3	2	2	1	2	2
1	1	1	1	1	1	1	1	1	1
9	9	9	9	9	9	9	9	9	9
9	9	9	9	9	9	9	9	9	9
2	2	1	2	1	2	2	2	2	1
2	2	2	2	2	2	2	2	2	2
2	2	1	2	3	3	3	1	1	1
2	2	2	2	2	2	2	2	2	2
9	1	1	1	1	1	1	2	2	2
3	3	2	2	2	2	2	1	3	3
3	2	1	2	3	1	3	2	1	3
2	2	2	9	2	2	2	1	2	9
9	9	1	9	9	9	9	9	1	9
1	1	1	1	1	2	2	1	9	1
1	2	1	1	1	1	1	2	2	2
2	2	2	2	2	2	2	9	9	9
2	3	2	2	2	2	2	3	3	3
1	2	3	1	1	2	2	1	1	2
2	2	2	2	2	2	2	2	2	2
2	2	2	2	2	2	2	2	2	2
2	2	2	2	2	2	2	2	2	2
2	2	1	2	2	2	2	1	1	2
2	2	2	2	2	2	2	2	2	2
3	3	3	3	3	3	3	2	2	2
9	9	1	9	9	9	9	1	9	9
9	9	9	9	9	2	9	9	9	9
2	2	2	2	2	2	2	2	2	2
2	2	2	2	2	2	2	2	2	2
9	9	1	9	9	9	9	9	9	1
1	1	1	1	2	2	2	1	2	1
9	9	9	9	9	9	9	2	9	9
2	2	2	2	2	2	2	2	2	2
1	2	2	1	2	2	1	1	2	1
2	2	2	2	2	2	2	2	2	2

3	1	2	3	1	3	2	2	1	2
9	9	9	9	9	9	9	1	9	9
2	2	2	2	2	2	2	2	2	2
1	2	1	2	3	2	2	2	3	3
2	2	2	2	2	2	3	2	2	2
2	1	2	1	1	2	2	1	3	2
1	2	1	2	1	1	1	2	1	1
2	2	3	1	2	2	2	1	2	3
2	2	2	2	2	2	2	2	2	2
9	9	9	9	9	9	9	1	1	1
9	9	1	9	9	9	9	3	3	3
2	2	2	2	2	2	2	2	2	2
1	9	1	9	1	2	3	2	2	3
2	2	1	3	2	3	1	2	3	2
2	1	1	2	1	1	1	1	3	1
1	1	1	1	1	1	1	2	1	2
2	2	2	2	2	2	2	2	2	2
2	2	2	2	2	2	2	2	2	2
3	1	1	1	1	2	3	3	3	3
2	2	1	2	2	2	1	1	3	3
1	2	1	1	1	2	1	2	2	2
2	2	1	2	2	2	2	2	3	3
1	3	3	3	2	1	2	1	2	3
9	9	9	9	9	9	9	9	9	9
3	3	3	3	3	3	3	3	2	3
9	9	1	9	9	9	9	1	9	9
9	9	9	9	9	9	9	9	9	9
9	9	9	9	9	9	9	1	9	9
1	1	2	2	2	2	3	3	1	2
1	2	1	1	1	1	1	1	3	3
2	2	2	2	2	2	2	3	3	3
2	9	9	1	9	2	1	1	3	1
2	1	1	2	2	2	2	1	2	1
2	2	2	2	2	2	2	2	2	2
1	2	1	1	1	1	1	2	2	2
1	1	1	1	1	2	2	1	3	1
3	1	2	1	2	1	3	2	3	3
3	2	1	2	9	3	2	2	9	1
2	2	1	1	1	2	1	2	3	1
1	2	1	2	2	2	3	3	3	3
3	2	2	3	3	2	2	1	2	3
1	9	2	3	9	2	1	3	2	3
9	9	9	9	9	9	1	1	9	9
9	2	9	9	9	9	9	3	9	9
2	1	3	2	3	1	2	2	3	1
2	2	2	2	2	2	2	2	2	2
2	2	2	2	2	2	2	2	2	2

3	3	1	1	1	2	2	1	3	2
9	9	9	9	9	9	9	9	9	9
9	9	9	9	9	9	9	9	9	9
9	9	9	9	9	9	9	9	9	9
9	9	9	9	9	9	9	2	2	2
9	1	9	9	9	9	9	1	9	9
9	9	9	9	9	9	9	9	9	9
9	9	9	9	9	9	9	9	9	9
9	9	9	9	9	9	9	9	9	9
9	9	1	9	9	9	9	9	9	1
2	2	2	2	2	2	2	2	2	2
2	2	2	2	3	3	3	1	1	2
2	2	1	1	1	1	2	1	2	2
2	2	3	3	2	3	2	2	2	2
2	2	2	2	2	2	2	1	1	2
2	3	2	3	2	3	2	1	2	2
3	2	3	2	2	2	2	1	1	1
2	2	2	2	2	2	2	2	9	2
1	1	2	1	1	1	1	2	3	2
3	3	2	2	1	2	2	3	2	1
2	2	3	2	1	3	2	3	3	2
3	2	1	2	9	1	1	3	3	2
1	1	1	1	1	1	1	1	1	1
2	2	2	2	2	2	2	2	2	2
2	2	2	2	2	2	2	2	2	2
3	3	3	3	3	3	3	2	2	2
3	3	3	3	3	3	3	2	2	2
1	1	1	1	1	2	2	1	1	1
2	2	2	2	2	2	2	9	9	9
2	2	2	2	2	2	2	3	2	2
9	9	9	9	9	9	9	9	9	9
2	2	2	2	2	2	2	2	2	2
2	1	1	1	1	1	1	1	1	1
9	9	1	9	9	9	9	1	9	9
3	3	3	3	3	3	3	3	3	3
1	1	1	1	1	1	1	1	3	1
2	2	2	2	2	2	2	2	2	2
2	2	2	2	2	2	2	2	2	2
1	3	1	2	3	1	3	1	3	2
9	9	9	9	9	9	9	9	9	9
2	3	1	2	2	3	2	1	3	2
2	2	2	2	2	2	2	2	2	2
2	2	2	2	2	2	2	2	2	2
2	2	1	1	1	2	1	3	3	3
2	2	1	1	1	1	2	1	3	3
9	9	9	9	9	9	9	9	9	9
2	2	1	2	2	2	2	2	2	2

9	9	9	9	9	9	9	9	9	9
3	3	1	1	2	2	2	3	3	1
3	3	1	1	1	1	1	3	3	3
9	9	9	9	9	9	9	9	9	9
9	9	9	9	9	9	9	9	9	9
9	9	9	9	9	9	9	9	9	9
9	9	9	9	9	9	9	9	9	9
9	9	9	9	9	9	9	9	9	9
9	9	9	9	9	9	9	9	9	9
9	9	9	9	9	9	9	9	9	9
9	9	9	9	9	9	9	9	9	9
9	9	9	9	9	9	9	9	9	9
9	9	9	9	9	9	9	9	9	9
9	9	9	9	9	9	9	9	9	9
9	9	9	9	9	9	9	9	9	9
9	9	9	9	9	9	9	9	9	9
9	9	9	9	9	9	9	9	9	9
9	9	9	9	9	9	9	9	9	9
9	9	9	9	9	9	9	9	9	9
9	9	9	9	9	9	9	9	9	9
9	9	9	9	9	9	9	9	9	9
9	9	9	9	9	9	9	9	9	9
9	9	9	9	9	9	9	9	9	9
1	1	2	1	3	2	1	1	2	3
2	1	2	2	2	2	2	2	2	2
1	2	1	1	2	2	2	2	2	2
2	2	1	2	2	2	2	1	2	3
3	3	2	2	2	3	2	1	3	3
9	9	9	9	9	9	9	1	9	9
2	2	2	2	2	2	2	2	2	2
2	2	1	1	1	9	2	1	3	9
2	2	2	2	2	2	2	1	1	1
2	1	3	1	2	1	3	1	2	2
1	1	1	1	1	1	1	2	3	3
2	2	1	2	2	2	2	1	9	9
2	1	1	1	1	2	1	1	3	2
2	2	1	1	1	3	1	1	3	1
1	1	1	1	2	2	2	1	3	3
9	2	1	1	1	2	1	1	3	2
9	9	9	9	9	9	9	1	9	9
2	2	1	2	1	2	2	1	3	3
1	3	1	1	1	3	1	1	2	3
3	9	9	3	9	3	9	1	9	9
2	2	1	1	2	2	2	1	3	1
2	2	1	1	3	3	1	1	3	3
2	2	2	2	2	2	2	2	2	2
3	3	2	3	2	3	2	2	2	2

1	2	1	1	1	2	1	1	2	1
1	1	1	1	1	2	1	1	3	3
3	3	1	1	1	3	1	1	3	3
2	2	1	1	2	2	2	3	3	3
2	1	2	2	1	2	2	2	1	2
9	9	9	9	9	9	9	1	9	9
2	1	1	1	1	2	1	1	3	2
9	9	1	9	9	9	9	1	9	9
9	9	9	9	9	9	9	1	9	9
9	9	9	9	9	9	9	1	9	9
9	9	1	9	9	9	9	1	9	9
9	9	1	9	9	9	9	1	9	9
9	9	1	9	9	9	9	9	1	9
9	9	1	9	9	9	9	1	9	9
9	9	9	9	9	9	9	9	1	9
1	3	1	3	1	3	3	3	3	3
3	3	3	3	1	1	3	3	3	3
3	1	2	2	2	2	1	1	3	2
9	9	9	9	9	9	9	9	9	1
3	3	1	3	1	3	1	3	3	1
9	9	1	9	9	9	9	1	9	9
9	9	9	9	9	9	9	3	3	3
9	9	1	9	9	9	9	1	9	9
9	9	1	9	9	9	9	3	9	9
9	9	1	9	9	9	9	1	9	9
2	3	2	1	2	3	2	1	2	1
1	3	3	1	1	3	3	1	9	1
2	3	1	2	1	2	3	2	3	3
1	2	1	1	1	2	1	1	3	3
2	2	2	2	2	2	2	2	2	2
2	3	1	1	1	1	1	1	3	3
1	3	1	9	1	3	3	1	3	3
3	3	3	3	3	3	3	1	3	3
3	1	1	3	1	3	1	9	9	9
2	3	1	2	1	2	1	2	3	2
9	9	9	9	9	9	9	1	9	9
2	2	2	2	2	2	2	2	2	2
2	2	2	2	2	2	2	3	3	3
3	3	2	1	2	3	2	3	2	2
1	2	1	1	1	2	3	2	3	2
2	2	1	1	1	2	2	1	2	2
1	2	9	1	2	2	2	9	3	2
1	1	1	1	3	3	1	1	9	9
3	3	2	2	2	1	1	2	2	2
1	2	1	1	2	2	2	2	1	3
9	9	1	9	9	9	9	2	9	9
9	9	9	1	9	9	9	1	9	9

1	1	1	1	1	2	2	3	3	1
2	2	1	9	3	2	3	1	3	3
1	2	1	1	1	1	1	1	3	3
9	9	1	9	9	9	1	1	9	9
9	9	1	2	9	9	3	2	9	1
1	3	1	1	1	2	1	1	3	1
1	2	1	1	1	3	2	1	3	3
9	9	9	9	9	9	9	1	3	3
9	9	9	9	9	9	9	2	9	9
2	2	2	2	3	3	3	1	3	3
2	2	2	2	2	2	2	2	2	2
9	9	9	9	9	9	9	1	1	3
1	2	1	1	1	2	2	1	3	1
3	9	1	9	9	1	3	3	9	1
9	9	9	9	1	9	9	9	9	9
3	3	3	3	3	3	3	1	3	2
9	9	1	9	9	9	9	9	9	9
9	9	9	9	9	9	9	1	3	3
2	2	2	2	2	2	2	3	3	3
2	1	1	1	1	2	2	1	2	1
3	3	1	1	1	3	2	3	3	3
1	1	1	1	1	1	1	1	3	3
1	3	3	3	3	3	3	3	3	1
1	2	1	1	1	2	1	1	3	3
2	2	1	2	1	2	2	2	2	2
9	9	9	9	9	9	9	9	9	9
9	9	1	9	9	9	9	1	3	1
3	3	1	1	1	3	1	1	3	1
3	3	3	3	3	3	3	1	3	3
3	3	1	1	1	1	1	3	1	2
9	9	9	9	9	9	9	9	9	9
9	9	9	9	9	9	9	9	9	9
9	9	9	9	9	9	9	9	9	9
9	9	9	9	9	9	9	9	9	9
9	9	9	9	9	9	9	9	9	9
9	9	9	9	9	9	9	9	9	9
9	9	9	9	9	9	9	9	9	9
9	9	9	9	9	9	9	9	9	9
9	9	9	9	9	9	9	9	9	9
9	9	9	9	9	9	9	9	9	9
9	9	9	9	9	9	9	9	9	9
9	9	9	9	9	9	9	9	9	9
9	9	9	9	9	9	9	9	9	9
9	9	9	9	9	9	9	9	9	9
9	9	9	9	9	9	9	9	9	9
9	9	9	9	9	9	9	9	9	9
9	9	9	9	9	9	9	9	9	9

9	9	9	9	9	9	9	9	9	9
9	9	9	9	9	9	9	9	9	9
9	9	9	9	9	9	9	9	9	9
9	9	9	9	9	9	9	9	9	9
9	9	9	9	9	9	9	9	9	9
9	9	9	9	9	9	9	9	9	9
9	9	9	9	9	9	9	9	9	9
9	9	9	9	9	9	9	9	9	9
9	9	9	9	9	9	9	9	9	9
9	9	9	9	9	9	9	9	9	9
9	9	9	9	9	9	9	9	9	9
9	9	9	9	9	9	9	9	9	9
9	9	9	9	9	9	9	9	9	9
9	9	9	9	9	9	9	9	9	9
9	9	9	9	9	9	9	9	9	9
9	9	9	9	9	9	9	9	9	9
9	9	9	9	9	9	9	9	9	9
9	9	9	9	9	9	9	9	9	9
9	9	9	9	9	9	9	9	9	9
2	1	1	2	1	2	2	1	2	2
9	9	9	9	9	9	9	9	9	9
9	9	9	9	9	9	9	9	9	9
9	9	9	9	9	9	9	9	9	9
9	9	9	9	9	9	9	9	9	9
9	9	9	9	9	9	9	9	9	9
9	9	9	9	9	9	9	9	9	9
9	9	9	9	9	9	9	9	9	9
9	9	9	9	9	9	9	9	9	9
9	9	9	9	9	9	9	9	9	9
9	9	9	9	9	9	9	9	9	9
9	9	9	9	9	9	9	9	9	9
9	9	9	9	9	9	9	9	9	9
9	9	9	9	9	9	9	9	9	9
9	9	9	9	9	9	9	9	9	9
9	9	9	9	9	9	9	9	9	9
2	2	2	2	2	2	2	2	9	9
3	3	3	3	3	3	3	1	1	1
3	3	1	1	2	3	3	2	3	3
1	1	1	1	3	3	1	3	3	1
3	3	3	3	3	3	3	3	3	3
1	1	1	1	1	1	1	3	3	3
9	9	9	9	9	9	9	1	3	3
9	9	9	9	9	9	9	3	9	9
9	9	9	9	9	9	9	1	3	3
2	2	2	1	3	1	2	2	2	2
1	2	1	1	1	1	1	1	2	1
2	3	1	2	2	2	2	1	3	3

9	2	1	1	1	2	1	1	3	2
1	2	1	1	1	2	2	1	1	1
1	2	1	1	9	2	2	9	9	9
3	3	1	2	1	2	2	1	3	3
2	2	1	1	1	2	1	2	3	3
2	1	1	1	1	1	1	1	2	9
1	2	1	1	1	2	1	1	2	2
1	1	1	1	2	2	2	1	3	2
1	2	9	3	2	2	1	2	3	3
3	3	1	1	2	1	1	3	2	1
9	9	1	1	9	9	9	1	9	9
3	3	1	3	1	1	1	2	3	3
1	2	1	1	1	2	2	3	3	3
1	2	1	2	1	2	2	2	2	3
1	3	1	1	1	2	1	2	2	3
3	1	1	1	1	2	1	3	3	3
1	2	1	1	2	2	1	2	2	2
3	3	1	3	3	3	3	2	2	2
1	1	1	1	1	2	1	2	1	1
1	1	1	1	1	1	1	1	2	3
2	1	1	9	2	3	2	2	9	9
2	2	1	1	1	2	2	1	3	2
1	2	1	1	1	2	2	1	3	3
9	9	9	9	1	9	9	1	9	9
3	3	3	3	3	3	3	2	2	2
1	1	2	1	1	2	1	2	1	2
2	1	1	9	1	9	1	2	2	2
3	1	1	1	2	2	2	3	3	1
2	2	2	2	2	2	2	2	2	2
2	1	1	1	1	2	1	2	2	2
2	3	2	3	3	3	3	1	2	1
2	1	1	1	1	2	2	2	2	2
2	1	1	1	1	2	1	2	2	2
9	9	9	9	9	9	9	9	9	9
9	9	9	9	9	9	9	9	9	9
9	9	9	9	9	9	9	2	2	2
9	9	9	9	9	9	9	2	1	2
9	9	9	9	9	9	9	3	1	2
9	9	9	9	9	9	9	2	1	1
9	9	9	9	9	9	9	9	9	9
1	2	3	1	2	3	2	1	1	1
2	2	1	2	2	2	2	1	3	1
1	1	1	2	9	1	1	1	3	3
3	3	1	3	1	3	1	3	3	3
1	1	1	1	1	1	1	1	3	1
1	3	3	3	3	3	3	1	1	1
3	3	1	3	1	3	1	1	3	3

2	2	2	2	2	2	2	1	2	2
3	3	1	2	3	2	1	2	3	2
2	2	2	2	2	2	2	1	2	2
1	3	1	3	3	9	1	1	3	1
2	2	2	3	1	1	1	1	2	1
2	2	1	2	3	9	2	3	3	2
9	3	1	9	1	9	1	1	1	3
1	2	3	2	1	2	2	3	2	1
1	3	1	2	1	1	1	2	3	3
9	9	1	9	9	9	9	1	1	1
2	2	1	2	2	2	2	1	3	3
1	1	3	2	1	1	3	1	3	1
1	3	1	2	3	2	2	1	1	1
3	3	1	3	1	1	1	1	3	1
1	1	3	1	2	2	2	3	1	1
1	1	1	2	1	1	2	1	3	3
2	2	2	2	2	3	2	1	2	2
9	9	9	9	9	9	9	9	9	9
2	3	1	1	2	3	1	1	3	3
9	9	1	9	9	9	9	9	1	9
2	1	1	1	2	3	2	3	3	2
2	2	1	2	1	2	1	2	2	3
3	3	1	9	1	1	1	1	1	1
3	1	1	3	1	2	2	1	3	1
2	1	1	2	2	1	1	1	2	2
3	3	1	3	1	3	3	1	3	3
2	1	1	3	1	2	3	1	2	3
3	3	3	3	3	3	3	1	3	1
1	2	2	2	2	2	2	1	1	1
1	3	2	3	1	2	3	3	3	2
1	3	1	3	2	2	2	1	3	3
2	3	2	1	2	3	1	3	2	1
1	1	1	2	3	1	1	1	1	1
3	3	1	3	1	3	3	1	3	3
1	1	1	1	1	1	1	1	1	3
1	1	1	3	3	1	3	3	3	3
2	3	1	2	3	2	1	1	2	1
9	9	9	9	9	9	9	9	9	9
9	1	1	1	3	1	1	1	3	3
3	3	1	1	3	1	2	1	3	1
1	1	3	3	1	1	1	1	3	1
2	3	1	2	3	1	2	2	2	2
3	1	1	3	1	1	3	1	3	3
2	2	1	2	3	2	2	3	1	3
2	2	2	2	2	2	2	2	2	2
3	9	2	2	1	1	2	2	3	2
3	3	3	3	3	3	3	2	2	3

3	3	3	3	3	2	2	1	2	2
1	1	2	9	1	1	2	9	9	9
2	2	2	2	2	2	2	1	3	3
1	3	3	2	2	2	1	2	2	2
1	2	2	2	2	2	1	2	2	2
2	2	3	3	3	2	2	1	1	1
2	2	2	2	2	2	2	2	2	2
9	9	9	9	9	9	9	3	3	2
9	9	9	9	9	9	9	9	9	9
2	3	2	1	2	1	3	1	9	2
2	2	2	2	2	2	2	2	2	2
2	2	2	2	2	2	2	2	2	2
2	2	2	2	2	2	2	2	2	2
2	1	1	1	1	1	1	2	2	2
2	2	2	2	2	2	2	2	2	2
2	2	2	2	2	2	2	2	2	2
2	2	2	2	2	2	2	2	2	2
2	2	2	2	2	2	2	2	2	2
2	3	2	2	2	2	3	2	2	2
2	2	2	2	2	2	2	2	2	2
2	2	1	1	2	2	2	2	3	2
2	2	2	2	2	2	2	2	2	2
2	2	2	2	2	2	2	2	2	2
2	1	2	3	3	2	2	2	2	2
2	2	2	2	2	2	2	2	2	2
2	2	2	2	2	2	2	2	2	2
2	2	2	2	2	2	2	2	2	2
2	3	1	1	2	2	3	1	1	1
2	3	2	2	3	1	1	2	2	2
2	2	2	2	2	2	2	2	2	2
2	2	2	2	2	2	2	2	2	2
2	2	2	2	2	2	2	2	2	2
2	2	2	2	2	2	2	2	2	2
2	2	2	2	2	2	2	2	2	2
2	2	2	2	2	2	2	2	2	2
2	2	2	2	2	2	2	2	2	2
2	2	2	2	2	9	2	1	9	2
2	2	2	2	2	2	2	2	2	2
2	2	2	2	2	2	2	2	2	2
3	1	1	1	1	3	3	1	1	1
2	2	2	2	2	2	2	2	2	2
2	2	2	2	2	2	2	2	2	2
2	2	1	3	3	3	3	2	1	1
2	2	2	2	2	2	2	2	2	2
1	1	2	2	3	2	2	1	1	2
2	2	2	2	2	2	2	2	2	2
2	2	2	2	2	2	2	2	2	2

2	2	2	2	2	2	2	1	2	2
2	2	1	1	2	2	2	1	2	1
1	2	1	1	2	2	2	2	2	2
2	2	2	2	2	2	2	2	2	2
2	3	2	1	2	2	3	1	2	3
2	2	2	2	2	2	2	2	2	2
9	9	9	9	9	9	9	1	9	9
1	1	3	1	3	1	1	1	1	1
9	9	9	9	9	9	9	1	9	9
2	2	2	2	2	2	2	2	2	2
3	3	3	3	3	3	3	2	2	2
2	2	2	2	2	1	1	1	2	2
1	1	1	1	1	1	1	3	2	3
9	9	9	9	9	9	9	3	9	9
2	2	2	2	2	2	2	2	2	9
2	2	1	2	1	2	1	1	2	1
1	2	2	2	2	2	2	2	2	2
1	1	1	1	1	1	1	1	9	1
1	9	9	9	9	9	9	1	2	1
2	2	2	2	2	2	2	2	2	2
3	1	1	1	1	1	2	1	9	2
9	9	9	9	9	9	1	1	9	9
9	9	9	9	9	9	9	1	1	9
2	9	3	1	2	3	2	2	9	9
2	3	2	3	2	3	1	1	2	3
1	1	1	1	2	2	2	2	1	2
2	3	1	1	2	2	1	2	3	3
1	3	3	9	1	1	1	3	2	2
2	1	2	1	9	1	3	2	2	2
1	1	1	1	1	1	1	1	9	9
1	1	1	1	1	1	1	9	9	9
2	3	3	3	3	3	3	2	2	2
2	2	2	2	2	2	2	2	2	2
9	9	9	9	9	2	9	2	9	2
2	2	2	2	2	2	2	1	1	1
9	9	9	9	9	9	9	9	9	1
1	1	1	3	1	1	3	1	3	1
2	2	2	2	2	2	2	2	2	2
2	2	2	2	2	2	2	2	2	2
2	2	2	2	2	2	2	2	2	2
3	9	1	1	1	2	9	1	1	9
1	2	1	2	1	2	1	2	2	1
2	9	9	9	9	9	9	9	9	9
2	1	9	2	1	1	1	1	1	1
2	2	2	2	2	2	2	1	1	1
1	1	1	1	1	1	1	2	2	3
9	9	9	9	9	9	9	2	9	9

1	1	1	1	1	1	1	1	1	1
2	2	2	2	2	2	2	1	2	2
1	1	1	1	1	1	1	1	1	1
2	2	2	2	2	2	2	2	2	2
9	9	9	9	9	9	9	2	9	9
2	1	3	2	2	3	1	2	3	2
3	3	3	3	3	3	3	1	3	3
1	1	1	1	1	1	1	1	1	1
1	1	1	1	1	1	1	2	2	2
1	1	3	3	3	3	3	1	1	1
3	3	3	3	3	3	3	2	2	2
2	2	2	2	2	2	2	1	1	1
2	2	2	2	2	2	2	2	2	2
2	2	2	2	2	2	2	2	2	2
2	2	2	2	2	2	2	2	2	2
1	2	1	2	1	2	1	2	2	2
1	1	1	1	1	1	1	1	1	1
1	1	1	1	1	1	1	3	1	3
9	9	9	9	9	9	9	9	9	9
2	1	2	1	2	1	2	1	2	9
1	1	1	1	1	2	2	1	1	2
1	3	1	1	2	2	2	1	3	3
1	1	1	1	1	1	1	1	3	3
2	9	1	2	1	2	2	3	3	3
9	9	9	9	9	9	9	3	3	3
1	1	1	1	1	1	1	2	2	1
1	1	2	3	2	1	2	1	2	1
2	2	2	1	2	1	2	2	2	2
1	1	1	1	2	9	3	1	9	9
2	2	2	2	2	2	2	2	2	3
3	3	3	3	3	3	3	3	3	3
2	2	2	2	2	2	2	1	3	3
3	3	3	3	3	3	3	3	3	3
2	1	3	2	1	2	3	1	2	9
2	2	2	2	2	2	2	2	2	2
3	3	3	3	3	3	3	2	2	2
3	2	3	2	1	2	1	2	3	9
3	3	3	3	3	2	2	2	2	2
3	2	1	2	3	1	2	3	2	2
3	3	3	3	3	3	3	3	3	3
1	1	1	1	1	1	1	1	2	1
1	2	1	3	2	1	2	2	1	3
9	9	9	9	9	9	9	9	9	9
3	3	3	3	3	3	3	3	3	3
2	1	1	1	1	1	1	1	1	1
2	2	2	2	2	2	9	2	2	3
2	2	1	1	1	2	3	1	3	3

2	2	2	2	2	2	2	1	1	1
1	3	3	3	1	3	3	3	3	3
3	3	3	3	3	3	3	3	3	3
1	3	3	3	1	3	3	3	3	3
1	2	1	3	1	1	9	2	1	3
2	2	2	9	9	2	2	2	2	2
2	2	2	2	2	2	2	1	2	1
2	2	2	2	2	2	2	2	2	2
2	2	2	2	2	2	2	9	9	9
3	2	1	3	2	1	2	3	3	9
2	2	2	2	2	2	2	2	2	2
3	2	3	2	3	2	3	3	3	1
2	2	2	2	2	2	2	2	2	2
2	2	1	3	1	1	1	3	3	3
2	2	2	2	2	2	2	2	2	2
2	2	1	1	2	2	2	2	2	2
2	2	2	2	2	2	2	2	2	2
1	3	1	3	2	3	2	2	1	3
1	2	9	2	2	2	2	2	1	2
2	2	3	2	2	2	2	1	9	9
1	2	1	2	1	2	2	2	2	2
2	2	2	2	2	2	2	3	3	1
1	2	1	1	2	2	2	3	3	1
2	2	2	2	2	2	2	1	2	3
1	1	1	1	1	1	1	1	1	1
3	3	1	1	3	2	1	9	9	9
1	3	3	2	1	1	1	1	2	3
3	3	1	3	1	3	1	1	2	3
3	2	2	3	1	2	3	1	1	3
2	2	1	2	1	2	3	2	3	3
3	3	1	1	2	2	3	2	3	2
2	2	2	2	2	2	2	9	9	9
2	2	2	2	2	2	2	1	3	1
9	2	3	1	3	2	3	3	3	2
3	3	1	3	1	3	3	1	1	1
2	2	1	2	1	2	1	1	2	1
2	2	2	2	2	2	2	1	1	1
9	9	9	9	9	9	9	2	2	2
2	2	2	2	2	2	1	2	2	2
3	2	2	1	2	3	2	2	3	3
2	1	2	1	2	3	2	2	3	3
2	2	2	2	2	2	2	2	2	2
2	2	2	2	2	2	2	2	2	2
2	2	2	2	2	2	2	2	2	2
1	1	1	1	2	3	1	3	1	9
1	2	1	3	1	1	9	1	3	1
2	2	2	2	2	2	2	1	3	1

9	9	9	9	9	9	9	9	9	9
2	1	2	1	2	3	1	1	2	2
1	2	9	3	1	1	2	2	1	3
1	1	1	3	1	3	3	2	1	3
2	2	1	1	2	3	1	2	1	3
1	1	1	2	2	2	2	2	2	3
3	2	2	1	2	1	3	2	3	2
9	9	9	9	9	9	9	1	1	1
1	2	2	2	2	2	2	1	3	2
1	2	1	3	3	3	1	1	3	2
1	2	1	1	1	2	2	2	3	3
1	2	2	2	3	2	3	3	2	3
2	2	2	2	2	2	2	2	2	2
1	1	1	1	1	1	1	1	1	1
2	2	3	1	2	2	1	1	2	3
2	2	2	2	2	2	2	2	2	2
1	2	3	1	2	1	2	3	1	3
1	9	1	2	3	3	3	3	1	1
9	9	9	9	9	9	1	1	3	9
2	2	2	2	2	2	2	3	3	3
9	9	9	9	9	9	9	9	9	9
2	1	3	1	2	3	2	1	2	9
2	2	1	1	3	3	3	2	3	1
3	2	2	1	1	2	3	1	2	1
1	2	2	2	2	3	3	1	1	2
2	2	2	3	3	2	3	2	2	3
2	2	2	3	2	3	2	3	2	1
2	1	3	2	1	1	2	2	2	1
3	3	1	3	1	3	3	2	2	2
2	3	1	1	1	2	2	1	1	1
3	2	2	2	1	1	1	1	2	3
3	1	2	3	2	1	3	1	2	1
1	3	3	3	1	3	3	1	3	3
3	3	3	3	3	3	3	2	2	2
2	2	2	2	2	2	2	2	2	2
2	2	2	2	2	2	2	2	2	2
9	9	9	9	9	9	9	3	3	3
2	2	2	2	2	2	2	2	2	2
2	2	1	1	1	1	1	2	2	1
2	2	1	1	1	1	1	2	3	1
2	2	2	2	2	2	2	1	1	1
2	3	2	1	2	3	2	2	3	1
1	1	1	1	1	2	3	2	3	2
3	3	2	3	2	2	2	2	2	2
9	9	9	9	9	9	9	2	9	9
3	3	3	3	3	3	3	1	3	3
1	3	1	2	1	2	2	2	3	2

3	3	3	3	3	3	3	3	3	1
1	1	1	3	3	9	2	3	3	1
2	3	2	3	2	1	1	2	3	2
9	9	9	9	9	9	9	9	9	9
1	1	1	2	2	3	2	2	3	2
3	3	3	3	3	3	3	3	3	3
1	1	1	1	1	1	1	1	1	1
3	3	3	3	3	3	3	2	2	2
2	3	2	1	2	2	3	3	2	2
9	1	1	2	2	1	2	1	3	2
2	2	2	2	2	2	2	2	2	2
1	2	1	2	2	2	2	1	3	3
1	1	2	1	2	1	2	1	1	1
9	9	9	1	9	9	9	1	9	9
2	2	2	2	2	2	2	2	2	2
3	3	2	2	3	2	3	2	2	2
1	2	1	1	1	1	2	3	3	3
2	2	2	2	2	2	2	2	2	2
2	2	2	2	2	2	2	2	2	2
1	1	1	1	1	2	2	2	2	1
2	2	1	2	1	3	2	3	3	1
3	3	3	3	2	3	2	2	2	1
2	2	1	2	2	2	1	2	2	3
1	2	1	3	2	2	2	1	2	3
2	3	1	2	3	1	2	1	2	3
2	3	3	3	3	3	3	1	2	1
2	3	2	2	1	2	1	3	2	3
1	2	3	2	1	2	3	2	3	3
3	2	1	3	2	1	2	3	1	2
1	1	1	1	1	1	1	2	2	2
3	3	3	3	3	3	3	3	1	2
1	2	2	2	2	2	2	3	2	2
1	1	1	1	1	1	1	1	2	3
1	1	1	1	2	2	2	1	1	1
3	3	3	1	1	2	2	3	3	1
3	1	3	1	2	9	2	1	3	3
3	1	3	2	3	2	3	1	3	3
2	3	1	3	1	1	1	3	1	1
1	3	2	2	2	1	2	3	3	1
1	1	3	1	1	3	2	1	3	3
3	3	3	3	3	3	3	2	2	2
3	3	1	1	1	1	1	1	1	2
2	3	1	2	1	2	3	2	1	2
1	2	1	9	1	9	3	3	2	3
3	9	2	3	2	9	2	9	9	9
1	3	2	3	2	1	2	2	1	1
3	3	1	3	1	3	1	1	1	3

2	2	1	2	2	2	2	1	2	2
3	2	1	2	1	2	3	2	3	1
1	1	2	1	2	2	2	1	2	2
1	1	1	1	1	1	1	1	1	1
2	3	1	2	3	1	3	1	3	1
2	2	2	2	2	2	2	1	1	1
2	3	1	2	2	1	3	1	3	3
2	3	1	2	3	3	2	1	3	3
3	3	1	3	1	3	1	1	2	3
3	3	1	3	1	3	2	2	3	3
2	3	2	3	2	3	1	1	2	3
2	3	2	1	2	2	1	3	1	1
1	1	1	1	1	1	1	1	1	1
3	9	3	3	1	1	1	3	1	1
1	3	1	1	3	3	2	3	1	2
1	3	3	1	1	3	3	1	1	3
1	2	2	3	3	2	2	1	3	1
1	1	1	1	1	1	1	3	3	3
2	1	3	2	1	1	1	3	2	1
1	2	1	1	1	1	1	1	3	2
2	1	2	2	1	2	2	2	2	3
1	3	3	3	3	1	3	3	3	3
1	1	1	1	1	3	1	3	3	3
2	2	1	2	1	1	2	1	2	1
1	1	2	2	2	2	2	1	2	2
2	2	2	2	2	2	2	2	2	2
1	3	2	1	2	2	1	1	1	3
2	3	1	2	1	2	3	1	2	3
2	2	2	2	2	2	2	3	2	2
9	9	9	9	9	9	9	9	9	9
9	9	9	9	9	9	9	1	2	1
9	9	9	9	9	9	9	2	1	9
2	2	2	2	1	1	1	2	2	2
9	9	1	9	9	9	9	1	9	9
2	2	2	2	2	2	2	2	2	2
2	2	2	2	2	2	2	1	1	1
2	3	2	2	2	3	2	3	2	2
2	2	1	2	2	2	2	9	1	9
9	9	9	9	9	9	9	9	9	9
1	2	2	2	2	3	2	3	1	3
3	3	1	2	2	2	3	2	3	2
1	3	1	1	2	1	2	2	2	3
3	3	3	3	3	3	3	1	3	3
3	3	3	3	3	3	3	1	3	3
1	3	2	3	2	1	1	1	2	3
2	2	2	2	2	2	2	2	2	2
1	3	2	1	2	2	1	1	1	3

2	2	2	1	1	3	3	1	9	9
2	2	2	2	2	1	1	2	2	1
2	2	2	2	2	2	2	3	2	2
2	3	1	3	2	2	1	1	2	3
2	3	2	2	2	3	2	3	2	2
2	3	2	2	2	3	2	3	2	2
9	9	1	9	2	9	2	3	1	3
1	2	1	3	2	2	2	3	2	3
3	3	1	2	1	2	3	1	3	3
3	3	3	3	3	3	3	2	3	1
3	2	1	3	2	2	1	2	3	2
3	2	1	3	2	3	2	3	2	9
3	1	2	9	1	3	2	1	1	1
2	2	2	2	2	2	2	2	2	2
2	2	2	2	2	2	2	2	2	2
1	2	2	2	2	2	2	1	3	3
2	2	2	2	2	2	2	2	2	2
2	2	2	2	2	2	2	2	2	2
2	2	2	2	2	2	2	2	2	2
2	9	9	9	9	9	9	1	3	2
3	1	1	1	1	1	1	1	9	9
3	1	1	1	1	1	1	1	9	9
9	9	1	1	1	1	1	1	9	9
2	2	1	1	1	2	2	1	2	2
3	3	1	1	1	1	1	1	3	3
9	9	1	9	1	1	1	1	9	9
3	3	1	1	1	1	1	1	2	1
3	3	1	1	1	1	1	1	2	1
2	2	1	1	1	1	1	1	2	1
9	9	1	9	1	1	9	9	9	9
9	9	9	1	1	1	1	1	9	9
9	9	9	9	9	9	9	1	9	9
3	3	1	1	1	1	1	1	2	2
3	3	1	1	1	1	1	1	3	3
9	9	1	1	1	1	1	1	9	9
3	3	1	1	1	1	1	1	3	2
9	9	1	1	1	1	1	1	9	9
3	9	1	1	1	1	1	1	9	9
3	3	1	1	1	1	1	1	9	9
3	3	1	1	1	1	1	1	3	1
3	2	1	1	1	1	1	1	3	3
9	9	1	9	9	9	9	9	9	9
3	3	1	1	1	1	1	1	9	9
9	9	1	9	9	9	9	3	1	2
9	9	1	9	1	1	1	1	9	9
3	3	1	1	1	1	1	1	3	1
3	9	1	1	1	1	1	1	2	2

3	1	1	9	1	1	1	1	9	9
3	1	1	1	1	1	1	1	2	2
3	3	1	1	1	1	1	1	3	3
1	1	1	1	1	1	1	1	1	1
3	3	1	1	1	1	1	1	9	9
3	3	1	1	1	1	1	1	9	9
1	1	1	1	1	1	1	1	1	1
1	2	1	2	1	2	2	1	1	2
3	3	9	1	1	1	1	1	9	9
9	9	1	1	1	1	1	1	9	9
3	2	3	1	1	1	1	1	2	2
3	1	1	1	1	2	2	1	2	2
3	3	1	1	1	1	1	1	9	9
3	1	1	1	1	1	1	1	9	9
9	1	1	1	1	1	1	1	9	9
3	3	1	1	1	1	1	1	9	9
3	1	1	1	1	9	9	1	9	9
3	1	1	1	1	9	9	1	9	9
3	3	1	1	1	1	1	1	9	9
1	1	1	1	9	9	9	1	2	2
9	9	9	9	9	2	9	1	9	9
9	9	9	9	9	9	9	1	9	9
2	3	1	1	1	1	1	1	3	3
3	3	1	1	1	1	1	1	9	9
3	3	1	1	1	1	1	1	2	1
3	3	1	3	1	1	1	1	3	3
3	1	1	1	1	3	3	1	3	3
3	1	3	3	3	3	3	1	9	9
3	3	1	1	1	1	1	1	3	3
9	9	9	1	1	1	1	1	3	3
1	3	1	1	1	1	1	1	2	2
3	3	1	3	1	1	3	1	1	3
9	9	1	1	1	1	1	1	9	9
9	9	1	1	1	1	1	1	9	9
3	3	1	1	1	1	1	1	9	9
3	3	1	3	1	1	1	1	3	3
9	9	1	9	1	1	9	1	9	9
3	3	1	1	1	1	1	1	9	9
3	3	1	3	1	3	1	1	3	3
3	3	1	3	1	1	1	1	3	3
3	3	1	1	1	1	1	1	3	3
3	1	1	1	1	1	1	1	2	3
9	9	1	9	1	1	1	1	9	9
9	1	1	1	1	1	1	1	9	9
3	3	1	1	1	1	1	9	9	9
3	3	1	1	1	1	1	3	3	3
9	9	1	9	1	1	9	1	9	9

3	3	1	1	1	1	1	1	3	1
3	3	1	1	1	1	1	1	3	3
3	3	1	2	1	1	1	1	3	3
3	3	1	1	1	1	1	1	3	1
9	9	1	1	1	9	9	1	9	9
9	9	1	9	1	1	9	1	9	9
3	3	1	2	3	3	3	1	3	3
3	1	1	1	1	9	9	1	3	3
3	1	1	1	1	9	9	1	3	3
3	3	1	1	1	3	2	1	3	3
2	3	1	2	1	2	2	2	3	3
9	9	1	9	9	9	9	1	3	3
3	3	2	1	1	3	9	1	9	9
2	2	1	2	2	2	2	3	3	2
2	2	2	2	2	2	2	2	2	2
1	9	1	1	1	2	2	1	2	1
9	9	9	9	9	9	9	1	3	3
9	9	1	9	9	9	9	1	9	9
1	1	1	1	1	1	1	1	3	3
2	1	1	1	1	1	3	1	3	3
1	3	1	1	1	2	2	1	3	1
2	3	1	1	2	3	2	1	3	2
2	3	1	2	2	3	2	1	3	3
1	2	1	1	2	2	2	1	3	3
9	9	9	9	9	9	9	1	3	3
2	3	1	2	1	3	2	1	3	3
9	9	9	9	9	9	9	1	3	2
1	3	2	1	2	1	3	3	3	2
2	2	1	1	2	2	2	1	3	3
2	3	1	1	1	2	2	1	2	2
2	2	1	2	2	2	2	1	2	2
2	2	1	1	2	3	2	1	3	1
2	3	1	2	2	2	2	2	1	3
9	9	1	9	9	9	9	1	9	9
1	3	1	2	2	3	2	1	3	3
1	1	1	2	2	2	2	1	3	3
1	1	1	9	1	2	2	1	9	9
1	1	1	2	9	1	2	1	9	9
1	1	1	2	1	1	2	1	9	9
3	3	1	3	1	3	2	3	3	2
1	3	1	1	1	3	1	1	3	3
2	3	1	1	1	2	1	1	3	3
1	2	1	2	2	2	2	1	3	3
3	3	3	3	3	3	3	2	2	2
1	1	2	9	2	2	2	1	3	3
2	3	1	1	1	2	1	1	9	9
9	9	1	9	9	9	9	2	3	3

3	3	1	1	1	1	1	1	9	9
2	2	1	1	2	2	2	1	1	2
2	2	1	2	1	2	1	1	3	3
2	2	1	2	2	2	2	1	2	2
9	1	1	1	1	9	1	1	2	3
1	1	1	2	2	2	2	1	2	2
2	2	2	2	2	2	2	9	9	9
2	2	1	2	2	2	2	2	3	3
2	3	1	1	1	3	2	1	9	9
2	3	1	1	2	2	2	3	3	3
2	3	1	3	1	1	1	2	3	3
3	3	1	3	3	3	3	1	3	3
3	2	1	1	2	2	2	3	3	3
1	2	1	1	1	2	2	1	3	3
9	1	2	9	1	2	2	1	9	9
9	9	9	1	9	9	9	1	9	9
1	2	1	1	1	3	1	1	2	1
2	2	1	1	2	2	1	1	2	2
1	1	1	1	1	3	3	1	3	3
1	2	1	1	1	2	1	1	3	2
9	9	9	9	9	9	9	1	9	9
1	2	9	1	1	2	1	1	9	9
2	2	2	2	2	2	2	1	2	2
3	3	3	3	3	3	3	1	3	3
2	1	1	3	2	1	3	1	3	3
2	1	1	3	2	1	3	1	3	3
9	9	1	1	9	9	1	1	9	9
9	9	9	9	9	9	9	9	9	9
9	9	9	9	9	9	9	1	9	9
2	3	1	2	3	1	2	1	3	2
9	9	9	1	1	9	9	1	9	9
1	2	1	1	2	2	1	1	3	3
9	3	1	1	1	1	1	1	9	9
9	9	9	1	1	1	1	1	3	3
3	3	1	1	1	1	1	1	3	3
9	9	1	1	1	1	1	1	9	9
1	3	1	9	1	1	1	1	2	3
3	3	1	1	1	3	1	1	3	3
3	3	1	1	1	1	1	1	3	3
2	3	1	1	1	1	1	1	2	3
3	1	1	1	1	1	2	1	1	1
9	9	1	9	1	1	1	1	9	9
3	3	1	2	1	1	1	1	3	3
9	9	1	9	1	1	9	1	9	9
3	3	1	1	1	1	1	1	3	2
3	3	1	1	1	1	1	3	9	9
9	9	1	9	9	9	9	1	9	9

3	3	1	1	1	3	1	1	3	3
9	9	1	9	1	1	9	1	9	9
9	9	9	9	9	9	9	9	9	9
9	9	9	9	9	9	9	1	9	9
1	1	9	2	9	2	9	1	3	9
3	3	1	1	1	3	3	1	3	3
9	9	1	9	9	9	9	1	2	1
9	9	9	9	9	9	9	1	9	9
9	9	9	9	9	9	9	9	9	9
9	9	1	9	9	9	9	1	9	9
3	1	1	3	3	3	3	1	3	3
2	2	2	2	2	2	2	1	1	9
2	2	1	1	2	2	1	1	3	3
9	9	1	9	9	9	9	1	9	9
1	1	1	2	1	2	2	1	1	3
1	2	1	1	2	2	2	1	3	3
9	9	1	9	9	9	1	1	9	9
9	9	1	9	9	9	9	1	9	9
9	9	1	9	9	9	9	1	9	9
3	3	3	1	3	3	3	1	3	3
1	3	1	2	1	2	1	1	3	3
9	9	1	9	9	9	9	1	9	9
9	9	1	9	9	9	9	1	9	9
1	2	1	1	1	1	1	1	3	3
9	9	1	9	1	9	1	1	9	9
1	2	1	1	1	2	1	1	2	2
2	9	1	9	9	9	1	1	3	3
2	1	1	2	3	3	3	2	3	2
1	2	2	3	1	2	2	1	3	3
1	2	2	3	1	2	2	1	3	3
1	2	1	1	2	3	2	1	3	3
2	1	1	1	1	2	3	1	2	2
1	9	1	1	1	2	2	1	3	3
3	3	1	1	3	3	3	1	3	3
1	2	1	1	2	2	2	2	3	2
9	9	1	9	9	9	9	1	9	9
9	9	1	9	9	9	9	1	9	9
9	9	1	1	9	9	9	1	9	1
1	1	1	1	1	2	3	1	3	3
2	2	1	1	1	1	1	1	3	3
3	2	1	2	3	2	3	2	2	2
2	3	1	2	3	2	2	1	3	3
9	9	1	9	9	9	9	1	9	9
9	9	1	9	9	9	9	1	9	9
9	9	1	1	9	9	9	1	9	9
9	9	1	9	9	9	9	1	3	3
9	9	1	1	1	9	1	1	3	2

3	1	1	1	1	3	1	1	3	3
1	2	1	1	3	3	2	1	3	1
9	9	1	9	9	9	9	1	9	9
2	1	1	2	2	2	2	1	3	3
9	1	1	3	1	3	3	1	3	3
9	9	9	9	9	9	9	1	9	9
3	3	2	2	2	2	2	1	2	2
2	2	1	2	1	2	2	1	3	2
2	2	1	1	1	2	2	1	3	3
1	1	1	2	2	1	2	1	3	3
1	2	1	2	1	2	1	1	3	2
9	9	9	9	9	9	9	1	3	3
9	9	9	9	9	9	9	1	9	9
9	9	1	9	9	9	9	1	9	9
9	9	1	9	9	9	9	1	9	9
9	9	1	9	9	9	9	1	9	9
9	3	1	3	3	3	3	1	3	3
2	2	1	2	2	2	2	1	3	2
9	9	9	9	9	9	9	1	3	3
1	2	1	1	1	2	2	1	9	9
1	1	1	1	2	2	1	1	3	3
2	2	1	1	1	2	2	1	3	3
2	2	1	1	1	2	2	1	3	3
3	3	1	2	1	2	3	1	2	3
3	3	2	2	1	3	3	1	2	2
9	9	1	9	9	9	9	1	3	3
2	2	1	1	1	9	3	1	3	2
2	9	1	2	2	2	2	1	3	3
9	9	1	1	1	9	9	1	3	3
9	9	1	9	9	9	9	1	9	9
2	2	1	1	2	3	2	1	3	3
9	9	9	9	9	9	9	1	9	9
9	9	9	9	1	9	9	1	9	9
2	3	1	1	2	2	2	1	2	3
1	2	1	2	1	2	1	1	2	3
3	3	1	3	3	3	3	1	3	3
2	2	1	2	1	2	2	2	3	3
3	3	1	1	1	1	1	1	3	3
9	9	1	1	1	1	1	9	9	9
3	3	1	9	1	1	9	1	9	9
3	3	3	2	2	3	3	1	3	2
2	2	2	2	9	1	2	1	3	3
2	2	1	1	2	2	2	1	9	9
1	2	1	1	1	2	2	1	2	1
1	2	2	2	2	1	2	2	2	3
9	9	1	9	9	9	9	1	9	9
1	3	1	1	1	9	1	2	3	3

1	3	1	1	1	3	1	1	3	1
2	3	1	1	1	2	2	2	1	1
1	2	1	2	1	2	2	1	3	2
2	2	1	1	1	2	2	1	2	2
1	2	1	2	1	3	2	1	2	2
2	3	1	1	3	3	3	1	2	2
3	2	1	3	1	3	1	1	2	1
2	3	1	1	1	1	2	1	2	2
3	3	1	3	1	2	1	1	2	3
2	2	1	1	1	3	3	3	3	3
2	2	1	1	2	2	2	1	2	2
2	2	1	2	2	2	2	1	3	2
2	2	1	1	1	1	1	1	3	3
1	3	1	1	1	2	2	2	2	2
1	1	1	1	2	2	2	2	2	2
2	3	1	1	1	1	1	1	3	1
2	1	2	2	2	2	2	1	2	2
2	2	2	2	2	2	2	2	2	2
2	2	2	2	2	2	2	1	3	3
1	1	1	1	1	2	2	1	3	2
2	3	1	2	1	2	3	1	2	3
2	3	2	2	3	2	2	2	2	3
2	2	1	1	1	2	2	1	9	9
2	2	1	2	1	2	2	3	3	3
2	9	1	2	1	2	2	2	2	1
2	3	1	1	1	2	2	9	9	9
1	2	1	1	1	1	1	1	3	3
2	9	1	2	1	2	2	2	2	1
2	2	1	2	1	2	2	2	2	1
1	1	1	2	3	3	1	1	2	2
2	2	1	2	1	2	2	1	2	3
1	2	1	2	1	2	1	1	3	2
9	9	9	9	9	9	9	1	2	2
9	9	9	9	9	9	9	1	2	2
9	9	1	9	9	9	9	1	9	9
9	9	1	9	9	9	9	9	9	9
9	9	9	9	9	9	9	2	2	1
9	9	9	9	9	9	9	9	9	1
9	9	1	9	9	9	9	9	9	9
9	9	1	9	3	9	9	2	2	1
3	1	1	2	1	2	2	2	3	2
9	9	9	9	9	9	9	1	9	9
9	9	9	9	9	9	9	1	9	9
2	3	1	2	2	2	2	2	2	3
9	9	9	9	1	9	9	9	9	1
9	9	9	9	9	9	9	1	9	9
9	9	9	9	9	9	9	1	9	9

1	1	1	1	1	2	1	1	3	1
1	1	3	2	3	2	3	3	1	2
9	9	9	9	9	9	9	1	9	9
9	9	1	9	9	9	9	3	2	1
9	9	1	9	9	9	9	1	3	2
9	9	9	9	9	9	9	1	9	9
9	9	1	9	9	9	9	1	9	9
9	9	1	9	9	9	9	1	9	9
9	9	9	9	1	9	9	9	9	9
9	9	9	9	9	9	9	1	9	9
1	1	1	1	3	9	1	1	3	3
3	3	1	1	1	3	2	1	2	1
3	3	1	1	1	3	2	1	3	3
1	1	1	1	1	3	3	1	2	3
1	1	1	1	1	1	1	1	3	3
9	9	1	1	1	1	3	1	3	3
9	9	1	1	1	1	3	1	3	3
1	2	1	1	1	9	3	1	3	2
3	3	1	1	1	3	2	1	2	1
2	2	1	2	2	2	2	2	2	1
2	2	1	1	2	2	2	1	2	1
3	3	1	1	1	1	1	2	2	2
9	9	1	9	9	9	9	1	9	9
1	3	2	3	1	2	9	2	2	1
1	3	9	1	2	1	1	1	3	1
1	2	1	2	2	1	1	1	3	1
3	3	3	3	3	3	3	3	3	3
1	1	1	1	1	1	2	3	3	3
1	3	1	1	1	1	1	1	3	3
1	1	2	2	2	3	2	2	2	1
1	1	1	1	1	1	2	1	3	1
1	3	1	1	2	2	2	1	3	2
2	2	1	1	1	3	3	1	3	1
2	2	1	2	1	2	2	1	2	1
1	1	1	1	1	1	1	3	3	3
1	1	1	1	1	1	1	2	1	3
1	3	1	1	1	3	1	3	3	1
1	3	1	1	1	3	1	3	3	1
1	3	1	3	1	3	3	3	3	1
3	3	1	2	1	2	2	3	3	3
1	1	1	2	1	1	2	1	3	1
1	3	1	1	1	1	1	1	3	1
1	1	1	1	2	2	1	3	3	1
3	3	1	1	1	1	1	3	3	1
3	3	1	1	1	3	1	3	3	1
3	3	1	1	1	3	1	3	3	1
2	2	1	1	1	2	1	3	3	3

1	1	1	1	2	2	2	1	2	3
1	1	1	1	1	1	1	1	2	3
1	1	1	1	1	2	2	3	3	1
3	3	1	1	1	3	1	3	3	1
3	3	1	1	1	3	1	3	3	1
3	3	1	1	1	3	1	3	3	1
2	2	1	1	1	2	1	3	3	3
2	2	1	1	1	2	1	1	3	3
2	2	1	1	1	3	1	2	3	3
1	9	1	1	1	9	1	3	9	9
1	3	1	1	1	2	2	1	3	2
9	9	1	1	1	1	1	1	9	9
2	2	1	1	2	2	1	1	1	2
2	3	1	1	1	2	1	1	2	3
3	3	1	3	1	3	1	1	3	3
2	2	2	2	2	2	2	2	2	2
1	9	1	2	1	9	1	1	1	9
2	2	1	2	2	2	2	1	1	2
3	1	1	3	3	1	3	1	3	3
1	2	1	1	1	2	1	1	2	1
3	3	1	3	2	3	3	1	3	3
1	3	1	3	1	1	1	1	3	3
1	2	1	1	1	1	1	2	9	1
1	2	1	1	1	1	1	2	2	1
1	2	1	1	1	1	1	2	2	1
1	2	1	2	1	2	2	3	3	3
2	2	1	2	1	2	3	1	3	3
1	2	1	2	1	1	2	2	2	2
2	2	1	1	1	2	2	2	2	2
2	3	1	2	2	3	2	1	3	2
3	2	2	2	3	3	3	2	3	3
3	3	2	2	3	2	3	2	3	3
2	1	1	1	1	1	1	1	3	3
1	3	1	1	1	1	1	1	1	1
1	2	1	1	1	1	1	9	9	1
3	3	1	1	1	3	1	3	3	1
3	3	1	3	3	2	2	1	3	3
2	2	2	2	2	2	2	1	9	3
3	3	1	1	1	1	1	1	9	9
1	3	1	1	1	2	2	1	3	3
2	1	2	3	2	2	2	1	3	3
1	1	1	3	1	1	1	9	9	9
1	1	1	3	1	1	1	1	9	3
1	1	1	1	1	3	3	3	3	3
1	1	1	1	3	9	2	1	3	3
1	2	1	2	1	2	2	1	3	2
1	2	1	1	2	2	2	3	3	1

1	2	2	3	1	2	1	1	3	2
2	2	2	2	2	2	2	2	2	2
1	1	1	1	1	3	3	3	3	3
2	2	1	2	1	2	1	1	3	2
2	1	1	1	1	2	2	1	3	3
2	2	1	2	1	2	2	3	3	2
2	3	1	2	3	3	3	1	3	3
2	2	1	2	2	2	2	2	3	3
1	2	1	3	3	2	2	2	1	3
1	1	1	1	1	3	3	1	3	3
1	2	1	2	2	2	2	3	3	1
2	2	2	2	3	2	3	1	3	2
9	9	1	9	9	9	9	1	3	2
2	1	2	1	2	2	2	1	3	3
1	3	1	3	3	3	3	1	3	3
2	2	1	2	1	1	1	2	2	2
2	1	1	3	1	2	3	1	2	1
2	2	1	1	1	2	2	2	3	3
2	9	1	2	1	3	2	2	3	3
1	1	1	1	2	2	2	1	3	1
1	1	1	1	2	2	1	1	2	3
1	2	1	1	2	3	2	1	2	2
3	3	1	1	1	1	1	1	3	3
1	1	1	2	2	2	1	1	9	9
3	3	1	1	1	1	1	2	3	3
1	3	1	1	1	3	1	3	3	2
3	3	1	1	1	1	3	1	3	1
1	2	2	1	3	2	3	2	2	1
9	9	9	9	9	9	9	2	9	9
2	9	1	9	9	9	9	9	2	9
2	3	2	2	3	2	2	2	2	3
1	2	1	1	1	2	2	1	1	2
2	2	1	2	2	3	1	1	3	2
1	2	1	1	1	2	1	3	3	3
9	9	1	9	9	9	1	2	3	2
1	1	1	1	1	2	2	1	2	2
2	2	1	1	2	2	2	2	2	2
9	9	9	9	9	9	9	9	9	1
3	1	1	2	1	3	2	1	2	3
2	1	1	1	3	3	3	2	3	3
9	9	1	9	9	9	9	2	3	3
1	2	1	1	1	1	2	2	3	2
2	1	1	1	1	2	1	1	1	2
9	9	1	9	9	9	9	2	3	2
2	2	1	1	1	2	2	2	3	2
3	3	1	2	3	2	2	2	2	3
2	2	1	1	1	2	1	2	3	3

2	2	1	2	1	2	2	2	3	2
1	1	1	1	2	2	2	1	3	3
1	2	1	1	2	2	3	1	3	3
3	3	1	1	2	2	2	2	2	1
2	1	2	2	3	2	3	1	2	3
1	2	1	2	1	2	2	1	3	3
2	1	1	2	1	2	1	1	3	3
9	9	1	9	9	9	9	9	9	9
2	2	2	2	2	2	2	1	3	3
2	2	1	2	1	1	2	3	3	2
2	2	1	1	2	2	2	1	2	2
9	9	9	9	9	9	9	2	3	2
1	1	2	1	2	2	2	2	2	2
2	2	2	2	2	2	2	1	2	1
3	3	1	3	1	1	1	1	3	3
3	3	1	3	3	2	2	1	3	3
1	1	1	1	1	1	1	1	2	2
1	3	1	1	1	3	1	1	1	2
2	2	1	2	2	1	2	1	9	9
9	9	1	1	2	3	1	3	3	3
3	3	1	1	2	3	1	3	3	3
1	2	1	1	1	1	1	1	3	1
2	2	1	2	2	2	2	1	2	2
2	3	2	1	1	3	2	1	3	3
3	2	1	2	1	3	3	3	3	3
1	1	1	9	1	1	1	1	9	1
2	2	1	1	2	3	2	2	1	2
2	3	1	1	1	1	1	2	1	2
1	1	1	1	1	1	1	2	1	2
9	9	9	9	9	9	9	1	9	9
2	3	1	1	1	1	1	2	1	2
2	1	3	2	2	2	1	1	2	3
2	2	1	1	1	1	1	1	9	9
2	2	1	2	1	2	2	1	3	3
1	2	1	2	1	3	3	1	3	3
2	1	1	1	2	1	2	1	2	2
1	2	1	1	1	2	2	1	2	2
9	9	9	9	9	9	9	1	9	9
2	3	1	1	1	3	1	3	3	3
3	3	1	1	1	3	2	1	1	2
2	2	1	1	1	2	2	1	3	2
1	3	1	1	1	3	1	1	3	1
2	2	1	2	2	2	2	2	2	2
3	3	1	3	1	2	3	1	3	3
3	3	1	3	1	3	1	1	1	3
3	3	1	3	1	3	1	1	1	2
2	3	1	1	1	1	1	1	3	3

3	3	1	3	1	3	1	1	1	3
3	3	1	3	1	3	1	1	1	3
3	3	1	1	1	3	2	1	3	2
2	2	1	1	1	1	1	1	2	3
3	3	1	3	1	3	1	1	3	3
3	3	1	3	1	3	1	1	2	1
1	3	1	1	1	3	1	1	1	1
1	3	1	2	1	2	3	1	3	3
3	3	1	1	1	1	2	1	3	1
3	3	1	1	1	3	2	1	2	1
3	3	1	9	1	3	9	1	3	1
1	3	1	1	1	3	2	1	3	1
1	1	1	1	1	2	1	1	3	1
1	1	1	2	3	2	1	3	3	3
1	1	1	1	2	2	1	3	1	3
1	1	1	1	1	2	3	2	3	3
1	3	1	2	1	1	2	1	1	2
2	3	1	1	1	2	2	1	3	2
1	2	1	2	1	1	1	1	9	9
1	1	9	3	9	9	9	1	3	1
1	1	2	2	1	1	2	2	1	2
3	3	1	1	1	3	3	1	2	2
9	9	9	1	9	9	9	1	3	2
9	9	1	9	9	9	9	3	2	1
9	9	9	9	9	9	9	2	9	9
9	9	9	9	1	9	9	9	9	9
3	3	1	1	2	3	3	1	9	9
9	9	1	9	9	1	9	9	9	9
9	9	9	9	9	9	9	3	9	9
9	9	9	9	1	9	9	9	9	9
9	9	1	9	9	9	9	9	9	9
9	9	1	9	9	9	9	9	9	9
9	9	9	9	9	9	9	2	9	9
9	9	1	9	9	9	9	1	9	9
2	3	1	1	9	9	9	9	9	9
1	3	1	1	1	2	2	1	3	1
1	2	1	1	1	1	1	2	2	2
1	3	1	1	1	3	1	3	1	2
2	2	1	1	2	9	2	3	3	3
2	2	1	1	2	3	2	2	1	2
9	1	1	1	1	1	1	1	2	2
3	3	1	1	1	3	2	2	3	3
2	1	1	2	3	3	2	1	2	1
2	2	1	1	1	1	2	2	3	3
2	3	2	3	2	2	2	1	2	2
3	1	3	1	1	1	3	1	3	1
1	2	1	1	2	2	1	1	2	3

1	1	2	2	1	1	2	3	2	1
1	2	1	2	1	2	2	2	3	2
2	3	1	2	2	2	2	1	2	2
1	3	1	1	1	1	1	2	2	3
1	2	2	2	3	2	2	1	2	2
3	1	1	1	1	1	1	1	9	9
2	2	2	2	2	2	9	9	9	9
3	1	1	3	2	2	2	2	2	1
2	1	1	2	2	2	2	1	2	3
2	2	2	2	2	2	2	1	2	2
2	1	1	2	2	2	2	3	3	3
2	1	1	1	1	2	2	2	3	3
1	1	1	2	3	3	3	2	1	2
3	3	3	3	3	3	3	9	9	9
2	2	2	2	2	2	2	2	2	2
2	2	1	2	1	1	2	3	1	1
1	9	2	1	2	2	2	2	1	2
2	1	2	9	1	2	1	3	3	1
1	1	1	1	1	1	1	2	1	1
1	1	1	1	1	1	1	2	2	2
1	1	1	1	1	1	1	3	3	3
1	1	3	3	2	2	2	1	2	2
2	1	1	2	2	2	2	1	2	3
1	2	1	1	1	1	1	2	2	2
1	2	1	1	1	2	2	3	3	3
2	1	3	1	2	3	1	1	2	3
1	2	1	2	2	2	1	1	3	2
1	1	1	1	1	1	1	1	1	1
2	2	2	2	2	2	2	1	1	1
2	2	2	2	2	2	2	2	2	2
9	9	1	9	9	9	9	9	1	9
9	1	3	2	9	1	3	1	2	2
2	2	2	2	2	2	2	1	9	9
2	3	1	9	2	2	2	1	3	3
9	9	1	9	9	9	9	9	9	1
2	2	2	2	2	2	2	1	3	2
2	2	2	2	2	2	2	2	2	2
2	3	2	2	1	2	2	1	3	3
2	3	1	2	2	2	2	1	1	2
2	2	1	1	1	2	1	1	3	3
2	2	1	2	1	2	2	1	3	3
2	2	1	2	1	2	2	1	3	3

WomenPre	WomenMe	CABreastH	Daily	Weekly	Monthly	SixMonthly	Yearly	Anytime	DuringPeri
2	2	2	3	3	3	3	3	3	3
2	2	2	2	2	2	2	2	2	2
2	2	2	2	2	2	2	2	2	2
2	2	2	2	2	2	2	2	2	2
2	1	2	1	2	2	2	3	1	2
2	2	2	2	2	2	2	2	2	2
2	2	3	3	3	3	1	2	2	2
2	3	2	3	2	1	1	3	2	2
2	2	2	1	1	2	2	2	1	1
2	1	2	2	2	2	2	2	3	2
3	3	1	3	3	1	3	3	3	2
3	2	1	1	1	2	2	3	1	2
2	2	2	1	1	1	1	1	2	2
2	2	1	2	1	2	2	2	3	1
2	2	2	2	2	2	2	2	2	2
2	2	2	3	3	3	2	2	3	2
2	2	2	2	2	2	2	2	1	2
3	3	3	1	3	3	1	3	3	3
2	2	2	2	2	2	2	2	1	2
3	3	2	2	1	1	2	1	1	2
1	3	3	3	3	1	3	3	3	2
3	2	3	2	3	1	3	3	3	2
3	3	3	2	3	1	3	3	3	3
2	2	2	2	2	1	2	3	2	2
2	2	2	2	2	2	2	2	2	2
3	3	1	3	3	3	1	3	3	3
9	9	1	9	9	1	9	9	1	9
3	3	3	3	3	1	1	1	1	3
3	3	3	3	2	1	1	2	2	2
2	1	3	3	3	1	2	1	3	2
3	3	1	3	3	1	2	2	1	2
3	3	1	2	1	2	2	2	2	2
3	3	3	1	3	3	3	3	1	3
1	3	1	3	1	1	1	2	3	1
9	1	9	1	2	2	1	1	2	1
2	3	2	3	3	2	3	3	3	3
3	3	2	1	1	1	2	3	1	1
2	3	3	2	1	2	1	1	1	2
3	3	3	1	1	1	2	3	3	2
2	3	2	1	1	1	2	2	1	2
2	1	3	2	1	1	2	3	2	1
9	9	9	9	1	9	9	9	9	2
9	9	9	9	1	9	9	9	1	9
3	2	1	3	1	9	2	3	1	2
3	3	3	3	3	3	1	3	2	2
9	9	9	9	9	9	9	9	9	9

2	2	2	1	1	2	3	3	1	2
2	3	2	1	1	2	2	3	1	2
2	1	2	2	2	1	2	2	2	1
2	2	2	2	2	1	2	2	2	1
3	3	3	3	3	3	3	1	1	1
1	3	1	3	1	1	3	3	3	1
2	2	1	2	2	2	2	2	3	2
3	3	1	3	3	2	1	1	3	2
3	9	9	3	3	3	1	2	9	1
3	3	1	3	3	3	3	1	2	2
9	9	9	9	9	9	1	9	9	9
2	2	3	1	2	2	2	3	1	1
2	2	2	1	1	2	2	2	2	2
1	2	2	1	1	2	2	2	2	1
2	2	1	1	1	2	2	2	1	2
2	2	2	1	1	1	1	2	1	1
2	2	3	3	1	2	2	2	3	2
3	2	3	3	3	1	1	1	2	3
2	2	1	2	2	2	2	2	2	2
2	2	2	2	2	2	2	2	3	3
3	3	3	3	3	3	3	3	3	3
2	3	2	2	2	1	2	3	2	1
2	2	2	1	2	2	2	2	2	2
1	2	3	1	2	2	3	3	1	2
2	2	1	1	2	2	3	3	1	2
2	2	2	1	2	2	2	2	2	1
3	3	1	2	1	1	2	3	1	1
2	2	1	1	2	2	3	2	2	1
3	3	3	2	1	1	3	3	3	1
2	1	1	3	3	3	3	1	1	1
9	9	9	9	9	9	9	9	9	9
3	3	3	3	3	3	1	3	2	2
3	2	2	3	1	1	2	3	1	1
2	2	2	2	2	2	2	2	2	1
9	1	2	3	1	3	3	3	3	3
2	1	1	3	3	3	2	1	2	2
3	3	2	2	1	2	1	2	2	3
2	2	3	2	2	2	1	3	1	2
3	3	3	3	3	1	1	3	3	1
2	1	1	2	2	2	1	2	1	2
3	3	2	3	2	1	1	2	2	3
3	3	3	2	2	2	2	2	2	2
1	3	1	1	2	1	1	1	1	1
3	2	2	3	2	2	1	1	1	2
3	3	3	3	3	3	2	1	1	2
2	3	2	1	2	2	2	2	3	2
9	9	9	2	2	1	2	2	2	2

3	3	1	2	2	3	1	1	2	3
3	3	1	3	3	1	3	3	3	2
3	3	2	3	3	3	2	1	2	2
1	2	3	1	2	2	1	3	1	2
1	2	2	1	1	2	2	2	2	2
3	2	1	2	2	1	3	2	3	2
3	2	3	1	3	3	2	2	1	2
2	3	3	1	3	9	2	2	3	2
2	3	1	1	3	3	3	3	1	2
3	1	2	2	9	1	9	9	3	1
1	1	1	9	9	9	9	9	9	9
3	3	1	3	3	1	1	2	1	1
2	3	2	1	2	2	3	3	1	2
2	2	2	2	2	2	2	2	1	2
1	3	3	3	3	1	3	3	2	2
1	3	3	3	3	1	3	3	2	2
3	2	1	3	1	2	1	3	1	1
2	2	2	3	2	1	9	1	1	3
2	9	1	9	1	9	9	9	9	9
3	2	2	1	2	2	2	3	2	1
3	3	3	3	3	3	3	3	3	3
3	3	1	3	3	3	1	2	3	3
2	3	2	1	1	2	2	3	2	2
2	2	2	1	1	1	1	1	2	2
3	3	3	2	2	2	2	2	2	2
3	3	3	1	2	2	3	3	1	2
2	2	1	1	1	2	2	2	1	2
1	2	1	1	2	1	1	1	2	1
1	2	1	2	2	2	1	3	2	2
2	3	3	1	1	2	2	3	2	1
2	1	1	1	1	2	2	2	1	1
2	2	1	2	1	3	3	3	1	2
3	3	3	3	2	1	2	1	2	2
1	2	1	1	1	1	1	2	1	2
3	3	3	2	1	3	1	1	1	2
1	1	1	2	2	1	2	2	2	9
9	9	9	9	9	1	9	9	2	9
2	2	1	3	2	2	1	2	3	2
3	3	3	3	3	1	3	3	1	3
3	3	1	3	2	3	9	2	3	2
2	2	2	1	2	1	1	1	1	2
9	9	9	9	9	9	9	9	9	9
2	2	2	1	2	2	1	1	1	2
9	2	2	2	2	2	2	3	1	2
3	3	3	1	2	3	3	3	3	3
3	3	2	2	3	2	1	3	1	3
3	2	1	1	1	2	2	3	1	2

2	2	3	1	9	3	2	3	3	3
3	3	3	3	3	3	3	3	3	3
3	3	3	3	3	3	3	3	3	3
3	3	1	1	3	3	1	3	1	1
2	3	1	3	3	1	2	2	1	2
2	2	1	3	1	2	3	2	3	3
9	9	1	9	2	9	1	2	2	9
2	1	2	2	1	2	1	3	1	2
2	2	2	2	2	2	2	2	2	2
2	2	1	2	2	2	2	2	2	2
1	2	1	2	3	1	3	1	1	1
2	3	1	1	9	9	3	3	9	9
9	2	1	2	2	2	2	2	2	2
3	3	3	3	3	3	3	3	2	2
1	1	2	2	2	1	1	1	1	2
1	1	1	2	2	1	1	1	1	1
1	1	1	1	1	2	2	2	1	2
2	2	1	2	2	1	1	1	1	1
2	2	1	1	2	2	3	3	1	2
3	2	1	2	2	3	2	3	1	2
9	9	9	9	9	2	9	9	9	2
3	2	2	3	1	2	2	2	3	3
3	3	3	2	1	2	2	2	2	2
1	1	1	3	3	2	1	1	3	2
3	3	3	2	2	1	2	2	3	1
1	2	3	3	3	2	3	3	1	3
2	3	3	3	1	1	3	3	1	3
2	2	3	2	1	1	1	2	1	2
3	3	1	2	2	2	1	1	1	2
2	2	1	2	1	1	1	2	1	2
9	9	9	9	9	9	9	9	9	9
2	2	2	2	2	2	2	2	2	2
1	1	2	2	2	1	2	2	1	1
1	1	1	1	1	1	1	1	1	1
2	2	2	3	3	3	3	3	2	3
1	1	1	2	1	1	1	1	2	2
1	1	1	2	2	2	2	2	3	3
3	3	2	3	1	1	2	3	3	1
3	3	1	9	1	1	9	9	1	9
3	9	3	3	3	3	3	3	3	3
9	3	9	1	3	1	1	1	2	1
2	2	2	2	2	2	2	2	2	2
2	3	2	3	1	1	2	3	2	1
9	9	9	9	9	9	9	9	9	9
9	9	9	9	9	9	9	9	9	9
2	1	1	1	2	2	1	1	2	1
2	3	2	2	1	2	2	2	1	2

2	9	9	9	9	9	1	9	9	9
9	9	9	2	9	9	9	9	1	9
3	3	3	9	9	1	9	9	1	3
9	9	9	1	9	9	9	9	1	9
3	3	3	3	3	2	1	3	2	2
3	3	3	2	2	1	2	3	2	1
3	3	3	3	2	2	1	3	2	2
1	3	2	9	9	9	9	9	9	9
1	1	1	3	3	1	3	3	2	1
9	9	9	9	9	2	9	9	9	9
9	2	9	9	9	1	9	9	9	2
9	9	9	9	9	9	9	1	3	9
1	1	2	2	2	2	2	2	2	2
3	1	1	2	2	3	3	1	3	2
9	9	9	9	1	9	9	9	9	9
1	2	2	3	3	3	2	3	2	2
3	3	3	3	3	3	3	3	3	3
1	1	2	2	2	1	1	2	2	1
2	2	3	2	2	1	1	1	1	2
2	2	2	9	9	2	9	9	2	2
2	3	2	1	2	3	3	3	1	2
2	3	2	3	2	2	2	2	1	2
2	2	2	2	2	1	1	2	2	2
3	3	3	2	3	3	3	3	1	2
1	2	2	2	2	2	2	2	2	2
2	2	2	3	2	2	1	2	2	1
1	1	3	1	1	2	2	3	2	1
2	1	1	2	2	2	1	1	2	2
9	9	9	9	9	9	9	9	9	9
2	2	2	1	2	1	2	2	1	1
2	1	2	2	2	2	1	2	2	2
1	1	1	1	1	1	1	1	2	2
2	3	2	3	2	1	3	3	2	3
2	2	3	1	3	3	3	3	3	1
3	3	1	3	3	3	3	1	3	1
1	1	2	2	2	2	2	2	1	1
3	2	2	1	1	2	2	2	1	2
2	2	2	2	2	2	1	2	9	2
2	3	2	1	2	3	2	2	1	2
2	2	2	2	1	2	2	2	2	2
2	2	1	1	2	2	2	2	1	1
1	3	2	3	1	1	2	1	1	1
3	3	3	3	2	1	1	1	1	2
2	2	2	2	2	2	2	2	2	2
3	1	3	2	2	1	2	2	2	2
2	3	3	3	3	2	1	2	3	1
9	9	9	9	9	9	9	9	9	9

3	3	3	1	2	3	9	3	1	1
3	2	1	1	3	2	2	3	1	1
1	2	1	2	2	2	2	2	1	1
3	3	3	3	3	1	3	3	1	2
3	3	3	2	2	2	2	2	2	2
2	2	3	3	9	2	1	3	3	3
1	1	3	1	1	1	3	1	1	2
1	1	1	1	2	2	2	2	2	3
2	2	2	2	2	2	9	9	1	2
1	2	2	1	1	1	1	1	3	2
2	2	1	1	1	1	2	2	2	1
2	1	1	1	1	1	1	1	1	2
9	9	9	2	9	9	9	9	2	9
2	2	2	2	2	2	2	2	2	2
2	2	9	9	2	9	2	2	9	1
9	9	9	9	9	9	9	9	2	9
2	9	2	2	9	9	2	9	2	9
9	2	9	9	2	1	2	9	9	3
2	9	2	2	2	9	2	2	2	9
9	9	9	9	9	9	9	9	9	9
9	9	9	9	9	9	9	9	9	9
2	2	2	2	2	2	2	2	2	2
2	2	2	2	2	2	2	2	2	2
3	3	3	2	2	2	2	3	2	2
2	2	2	2	2	2	2	2	2	2
9	9	2	9	2	9	2	2	2	9
2	2	2	2	2	2	2	2	2	2
9	9	9	9	9	9	9	9	9	9
9	9	9	9	9	9	9	9	9	9
2	2	2	2	2	2	2	2	1	2
9	9	9	9	9	9	9	9	2	9
9	9	9	9	9	9	9	9	9	9
9	9	9	9	9	9	9	9	9	9
9	2	9	2	9	2	9	2	9	2
9	3	9	9	9	2	1	9	9	9
9	3	1	3	1	9	1	2	9	1
9	9	9	9	9	9	9	9	9	9
9	9	9	9	9	2	9	9	9	2
9	2	9	9	2	9	9	2	9	9
9	9	9	3	9	9	9	9	3	9
9	9	9	1	9	9	9	9	9	9
9	9	9	9	9	1	9	9	1	9
3	3	3	3	3	3	3	3	3	3
1	1	2	2	1	1	2	1	1	1
1	2	1	1	2	1	3	2	1	2
2	1	9	9	1	2	1	1	9	1
9	9	9	1	9	9	9	9	9	9

9	9	9	9	9	1	9	9	9	9
9	9	9	9	9	1	9	9	2	9
1	1	2	2	9	9	9	9	3	9
1	2	3	3	2	1	2	3	3	1
1	1	1	1	2	1	2	1	2	1
2	1	1	2	2	2	2	2	2	2
3	3	3	2	1	1	3	3	2	3
1	1	1	3	1	2	2	3	1	2
9	9	9	9	9	9	9	9	9	9
3	3	3	3	3	3	3	3	3	3
9	9	9	9	9	9	9	9	9	9
9	9	9	9	9	9	9	9	9	9
9	9	9	9	9	9	1	9	9	1
1	1	9	1	1	1	1	2	1	2
1	1	1	1	1	1	1	1	1	1
1	1	1	1	2	2	2	2	1	1
9	9	9	9	9	9	9	9	9	9
2	2	1	1	2	2	2	2	1	2
1	1	1	1	1	1	1	1	1	1
3	3	1	3	3	1	2	3	1	2
3	2	2	9	9	1	9	9	3	1
3	3	1	2	3	1	2	3	1	1
3	2	3	3	2	3	1	1	1	2
2	2	2	2	2	2	2	2	2	2
2	2	2	2	2	2	1	2	2	2
2	2	2	2	2	2	2	2	2	2
2	2	2	2	2	2	2	2	2	2
1	2	3	1	1	1	1	1	1	2
2	2	2	2	2	3	3	3	2	2
2	2	2	1	2	2	1	2	1	2
2	2	2	2	2	2	2	2	2	2
2	2	2	2	2	2	2	2	2	2
2	2	3	3	3	2	2	3	2	2
9	3	3	2	2	2	2	2	2	3
3	3	3	2	2	2	2	3	2	3
3	3	2	2	2	3	1	2	1	2
9	9	9	9	9	9	9	9	9	9
1	1	2	3	2	1	2	2	2	2
3	3	3	3	3	3	3	3	3	3
3	3	3	3	3	3	3	3	3	3
2	2	2	2	2	1	1	1	2	1
3	1	2	3	2	1	3	1	2	3
3	3	3	1	3	3	3	1	2	2
2	2	2	1	2	2	2	3	2	2
1	1	1	3	3	3	2	3	2	3
2	2	2	2	2	2	2	2	2	2
1	9	9	2	2	2	9	9	2	2

2	2	2	2	3	1	2	2	2	3
9	9	9	1	9	9	9	9	1	9
9	9	9	9	1	9	9	9	9	1
1	2	3	1	2	3	1	2	1	9
9	9	9	2	9	9	9	9	9	9
1	2	3	3	3	2	9	1	2	2
9	9	9	9	2	9	9	9	3	9
9	9	9	9	9	9	1	9	9	9
2	2	9	2	2	2	2	2	2	2
9	9	9	2	9	9	9	9	9	2
9	9	9	9	2	9	9	9	9	9
9	9	9	9	9	2	9	9	1	9
9	9	9	9	2	9	9	9	9	2
9	9	9	9	9	3	9	9	1	9
9	9	9	9	1	9	9	9	1	9
9	9	9	2	9	9	9	9	9	9
1	2	3	2	3	2	1	3	3	2
2	1	1	3	2	1	3	2	1	2
1	1	2	1	2	1	1	2	1	2
2	3	2	2	2	2	2	2	2	2
3	2	3	3	2	1	2	3	1	1
2	3	3	1	1	1	2	3	1	2
2	1	1	1	1	1	1	1	1	1
2	2	2	3	1	2	2	1	1	2
3	9	3	2	2	1	3	3	2	1
3	3	3	1	3	3	3	3	3	3
3	3	2	2	2	2	2	2	2	2
1	3	2	1	3	3	3	3	1	2
2	3	2	9	2	1	1	3	9	1
3	3	3	3	1	2	2	2	2	2
1	2	1	1	1	3	1	1	1	3
2	2	2	2	2	2	2	2	2	2
2	2	3	1	1	1	1	1	2	2
9	9	9	2	9	9	9	9	1	1
3	3	3	9	9	9	9	9	9	9
3	3	3	2	2	2	2	2	1	2
9	9	9	9	9	9	9	9	9	9
2	2	1	2	1	1	1	1	1	1
1	2	2	2	2	2	2	2	2	2
2	2	2	2	2	2	2	2	2	2
9	9	9	9	2	9	9	9	1	9
3	3	3	1	2	3	3	3	3	3
9	9	9	9	9	9	1	9	1	9
3	2	1	3	3	1	1	2	3	2
2	2	2	2	2	2	2	2	2	2
1	2	3	3	3	3	1	2	1	2
2	2	2	2	2	2	9	2	2	2

2	3	3	3	2	2	1	2	3	1
9	9	1	9	9	1	9	9	9	9
9	9	9	9	9	1	9	9	9	9
1	2	3	1	2	1	3	2	1	2
3	3	3	3	3	3	3	3	1	1
2	2	2	9	9	1	9	9	2	9
2	2	2	3	2	2	1	2	2	1
2	2	2	2	2	2	1	2	1	1
9	9	9	9	9	9	9	9	9	9
3	3	2	3	3	2	1	2	1	2
3	3	1	3	3	3	1	3	3	3
9	9	9	9	9	2	9	9	1	9
2	2	1	2	1	1	2	3	9	9
9	9	9	1	9	9	9	9	2	9
3	3	2	9	9	9	1	9	9	9
3	2	2	3	3	3	1	3	3	2
9	9	9	9	9	9	9	9	9	9
9	9	9	3	9	9	9	9	1	9
9	9	9	9	9	1	9	9	9	9
9	2	3	2	2	2	3	2	3	2
2	2	2	2	2	2	2	2	2	2
3	2	1	3	2	2	2	1	2	2
3	2	1	2	2	2	2	2	2	1
9	9	9	9	9	9	1	9	9	9
9	9	9	9	9	9	1	9	9	9
2	2	1	1	1	1	1	1	2	1
1	1	1	1	1	1	1	1	1	1
3	1	2	1	2	3	1	1	2	1
3	3	3	3	2	2	2	2	2	2
9	9	9	3	9	9	9	9	2	9
9	9	9	9	9	9	9	9	9	9
2	1	1	1	2	1	1	1	1	2
2	2	2	2	2	1	2	2	3	1
3	3	3	1	1	2	2	2	1	1
3	3	3	3	1	3	1	1	1	3
2	1	2	1	2	2	1	2	1	2
3	3	3	1	2	2	1	3	9	9
1	1	2	1	1	2	2	2	3	3
1	2	1	2	3	2	3	1	3	1
2	1	2	1	1	1	1	1	2	2
2	2	3	2	2	2	2	2	1	2
2	3	3	2	2	2	2	2	2	2
3	2	2	3	3	3	2	2	2	2
2	3	1	3	2	1	1	1	1	2
2	2	2	9	3	1	9	9	3	2
2	2	2	2	2	2	2	2	2	2
2	2	2	2	2	1	2	2	3	3

2	2	2	1	2	2	2	2	2	2
9	9	9	1	9	9	9	9	9	1
9	1	9	9	9	9	9	1	9	9
9	9	9	9	1	9	9	9	9	1
2	2	2	3	2	1	1	1	2	2
3	2	2	2	3	3	3	2	3	2
2	2	2	2	2	2	2	2	2	2
2	2	2	2	9	2	2	2	2	9
9	9	9	9	9	9	9	9	9	9
9	9	9	9	9	2	9	9	9	9
2	2	2	2	2	2	2	2	2	2
2	2	2	2	2	2	2	2	2	2
9	9	9	2	2	2	9	9	2	2
2	2	2	2	2	2	2	2	2	2
2	2	2	2	2	2	2	2	2	2
9	9	9	2	9	9	9	9	2	9
3	3	3	3	3	3	3	3	3	3
9	9	9	1	9	9	9	9	1	9
9	9	9	2	9	9	9	9	2	9
9	9	9	2	9	9	9	9	2	9
9	9	9	9	9	9	9	9	9	9
9	9	9	2	9	9	9	9	2	9
2	2	2	3	3	3	3	3	2	2
2	2	2	2	2	2	2	2	2	2
9	9	9	9	9	9	9	9	9	9
9	9	9	9	9	1	9	9	9	9
9	9	9	1	9	1	9	9	1	9
9	9	9	9	1	1	9	9	9	9
3	3	3	3	3	2	3	3	3	3
9	2	2	2	9	2	2	2	2	9
1	2	2	2	9	2	9	2	2	9
3	3	3	3	3	1	3	3	3	3
9	1	9	3	1	3	2	1	1	9
9	9	9	9	9	9	9	1	1	9
2	2	2	2	2	2	2	2	3	3
9	1	9	9	1	9	2	1	2	9
2	2	2	2	9	9	2	2	2	2
2	2	2	2	2	2	2	2	1	2
2	3	3	2	1	3	1	3	1	1
2	2	2	2	2	2	2	2	2	2
9	9	9	9	1	9	9	9	1	9
2	3	3	1	2	3	3	3	1	2
2	3	2	1	1	2	9	3	2	3
2	2	2	2	3	2	3	1	1	2
3	3	1	3	3	1	3	3	3	1
3	2	1	1	2	1	2	1	1	2
3	2	2	1	2	3	3	3	2	2

9	9	9	9	9	1	9	9	9	9
2	1	3	2	1	3	2	1	1	3
2	2	2	2	2	2	2	2	9	9
9	9	9	1	9	9	9	9	1	9
2	1	2	2	2	2	2	2	2	2
3	3	2	3	2	2	1	1	2	3
3	3	3	3	3	3	3	3	3	3
3	3	2	3	2	2	1	1	2	3
3	3	1	3	2	2	3	3	1	3
2	2	1	1	9	9	1	9	2	2
2	3	3	2	2	9	2	2	1	2
3	2	2	2	2	2	2	3	1	2
9	9	9	9	1	9	9	9	9	9
2	2	2	1	3	2	1	3	9	9
9	9	9	2	9	9	9	9	9	9
2	2	2	2	1	1	1	1	1	1
2	3	2	2	2	1	3	3	2	2
3	3	3	2	3	3	3	3	1	2
3	3	3	2	1	2	3	2	3	1
3	3	2	2	2	1	2	3	1	2
9	9	9	9	9	1	9	9	1	9
3	3	3	3	3	9	1	3	3	3
3	2	2	3	2	2	3	2	1	3
9	9	9	9	9	9	1	9	1	9
9	9	9	9	9	1	9	9	1	9
1	1	1	1	2	2	2	2	2	2
9	9	9	9	9	2	9	9	9	2
1	2	9	1	1	1	2	1	2	2
9	9	9	9	9	9	9	9	9	9
9	9	9	9	9	1	9	9	9	9
3	3	3	3	3	3	1	3	3	2
2	3	2	2	3	2	1	3	2	3
3	1	2	9	9	9	9	9	9	9
2	9	9	9	9	1	9	9	9	1
1	1	1	2	1	1	2	2	2	2
3	2	3	9	9	1	9	9	9	9
3	1	2	2	1	9	9	9	1	1
1	1	1	1	1	1	1	1	1	1
3	3	3	3	3	3	1	3	2	1
2	2	2	2	2	2	2	2	2	2
9	9	9	9	9	1	9	9	9	9
3	3	3	9	9	9	9	3	1	3
3	1	3	9	9	9	1	9	1	9
3	3	3	3	3	2	2	2	1	3
3	3	1	2	3	1	2	2	2	1
1	2	3	2	2	3	2	2	2	2
1	1	3	3	3	3	3	3	1	3

9	1	9	9	9	9	2	9	9	9
2	2	2	2	2	2	2	2	1	2
1	1	2	3	1	1	1	1	2	1
3	3	3	2	2	2	2	2	1	2
2	1	1	2	2	2	2	2	2	2
9	9	9	9	9	9	9	9	9	9
9	9	9	9	9	9	9	9	9	9
2	2	2	9	9	1	9	9	1	9
2	2	2	1	2	1	2	2	2	1
2	2	2	9	9	2	9	9	9	9
2	2	2	2	2	2	2	2	3	3
2	2	2	2	2	2	2	2	9	2
3	2	3	2	1	1	3	3	2	2
2	2	2	2	2	2	2	2	2	2
2	2	2	3	3	1	2	3	3	1
9	2	2	2	9	2	9	2	2	9
9	2	9	2	9	9	2	9	2	9
9	9	9	9	9	9	9	9	9	9
9	9	9	1	9	9	9	9	1	9
9	2	2	2	9	2	9	9	2	9
9	9	9	2	9	9	9	9	2	9
9	9	9	9	9	9	9	9	9	9
2	2	2	2	2	2	2	2	2	2
9	9	9	2	9	9	9	9	2	9
9	9	9	9	9	9	9	9	9	9
9	9	9	3	9	9	9	9	3	9
9	9	9	9	9	9	9	9	9	9
1	1	1	1	1	1	1	1	1	1
9	3	3	2	9	9	2	3	2	9
9	9	3	3	9	9	9	3	3	9
9	9	9	3	9	9	9	9	3	9
2	2	2	2	2	2	2	2	2	2
1	1	1	1	1	1	1	1	1	1
9	9	9	3	9	9	9	9	3	9
2	2	2	2	2	2	2	2	2	2
9	9	9	9	9	9	9	9	9	9
9	9	9	2	9	9	9	9	2	9
9	9	9	9	9	9	9	9	9	9
2	9	3	1	9	2	9	3	3	9
9	9	9	1	9	9	9	9	1	9
2	2	2	2	2	2	2	2	2	2
9	9	9	2	9	9	9	9	2	9
9	9	9	2	9	9	9	9	2	9
2	9	9	9	1	9	9	2	2	9
2	2	2	2	2	2	2	2	2	2
2	2	2	2	2	2	2	2	2	2
9	9	9	9	9	9	9	9	9	9

1	1	1	1	1	1	1	1	1	1
2	2	2	2	2	2	2	2	2	2
9	9	1	2	9	9	1	2	1	9
1	2	2	2	2	1	3	3	2	2
9	9	9	2	9	9	9	9	2	9
9	9	9	2	9	9	9	9	2	9
9	9	9	2	9	9	9	9	2	9
9	9	9	1	9	9	9	9	2	9
9	9	9	9	9	9	9	9	9	9
9	9	9	9	9	9	9	9	9	9
9	9	9	9	9	9	9	9	9	9
9	9	9	9	9	9	9	9	9	9
9	9	9	9	9	9	9	9	9	9
9	9	9	9	9	9	9	9	9	9
2	2	2	2	2	2	2	2	2	2
2	2	2	2	2	2	2	2	2	2
2	2	2	2	2	2	2	2	2	2
2	2	2	2	2	2	2	2	2	2
2	2	2	2	2	2	2	2	2	2
2	2	2	2	2	2	2	2	2	2
2	2	2	2	2	2	2	2	2	2
9	9	9	2	9	9	9	9	2	9
9	9	9	1	9	9	9	9	9	9
2	2	2	2	2	2	2	2	2	2
2	2	2	2	2	2	2	2	2	2
2	2	2	2	2	1	2	2	1	2
9	9	9	9	9	9	9	9	9	9
3	3	3	2	1	9	2	2	1	2
9	9	9	9	9	9	9	9	9	9
2	2	2	3	3	3	3	3	2	2
1	1	1	1	1	1	1	1	1	1
1	2	1	2	1	2	3	2	2	1
1	1	1	1	1	1	1	1	1	2
1	1	1	1	1	3	3	3	1	2
1	1	1	1	1	1	1	1	1	1
9	9	9	9	9	1	9	9	1	9
9	9	3	3	9	9	9	3	3	9
2	2	2	2	2	2	2	2	2	2
1	1	1	1	1	1	1	1	1	1
9	9	3	3	9	9	9	3	3	9
9	9	9	2	9	9	9	9	2	9
9	9	9	9	9	9	9	9	9	9
2	2	2	2	2	2	2	2	2	2
9	9	9	9	9	1	9	9	9	9
1	9	1	1	9	1	9	1	1	9
9	9	2	2	9	9	9	2	2	9
2	9	2	2	9	2	9	2	2	9

9	9	9	2	9	1	9	9	2	9
9	9	9	9	9	9	9	9	9	9
3	9	1	2	9	9	1	3	2	9
9	9	9	3	9	9	9	9	3	9
9	9	9	9	9	9	9	9	9	9
9	9	9	2	9	9	9	9	2	9
9	9	9	9	1	1	9	9	9	1
9	9	9	2	9	9	9	9	2	9
2	9	2	1	1	9	9	1	9	3
9	9	9	2	1	1	1	2	1	1
9	9	9	2	9	9	9	9	9	9
9	9	9	9	9	9	9	9	9	9
3	3	3	3	3	3	1	3	3	3
9	9	9	9	9	9	9	9	9	9
3	3	3	2	1	1	9	3	2	3
9	2	2	2	2	3	1	1	3	2
1	9	9	9	9	9	9	1	2	9
9	9	1	1	2	2	2	2	2	2
3	3	2	2	2	2	2	2	2	2
2	2	2	2	2	9	2	9	9	1
3	3	2	1	2	2	2	2	1	9
9	9	9	9	1	9	9	9	9	9
3	3	3	3	3	3	1	2	1	2
3	2	1	2	1	2	1	2	1	2
3	3	3	2	2	1	2	2	3	3
3	2	3	2	2	1	2	2	2	2
3	3	3	3	1	3	3	3	1	1
3	3	3	2	1	3	3	3	1	3
3	2	2	2	1	1	2	3	3	1
2	2	2	2	2	2	2	2	1	2
9	9	9	2	9	9	9	9	1	2
9	9	9	1	9	9	9	9	1	9
3	3	3	2	3	2	2	3	1	2
9	2	9	9	9	3	9	9	9	9
9	9	9	2	9	9	9	9	1	9
9	9	9	9	9	9	9	9	9	9
9	9	9	9	9	9	9	9	9	9
9	2	2	9	2	9	9	2	9	9
9	9	9	9	9	9	9	9	9	9
9	2	2	2	9	2	9	2	2	9
2	9	2	2	9	9	2	2	2	9
2	2	2	2	2	2	2	2	2	2
2	2	2	2	2	2	2	2	2	2
2	2	2	2	2	2	2	2	2	2
2	2	2	2	2	2	2	2	2	2
2	2	2	2	2	2	2	2	2	2
2	1	2	2	2	2	2	1	2	1

3	2	2	3	2	1	1	3	2	2
2	2	1	2	2	2	1	3	2	2
2	2	1	9	9	9	1	9	9	9
3	3	3	3	1	3	1	2	1	2
3	3	3	3	3	3	3	3	3	3
3	9	1	2	2	2	1	1	1	2
2	2	1	2	2	1	1	2	2	2
3	3	3	3	3	3	3	3	3	3
2	2	2	2	2	2	2	2	2	2
2	2	2	2	2	2	2	2	2	2
2	2	2	2	2	2	2	2	2	2
2	2	2	2	2	2	2	2	2	2
3	3	3	3	1	3	3	3	1	1
2	3	3	1	1	1	2	3	2	1
1	2	3	2	2	1	2	2	1	2
2	2	2	2	2	2	2	2	2	2
3	2	2	2	3	2	3	2	2	1
2	3	3	2	2	1	2	3	2	2
3	3	2	1	2	2	3	3	2	2
3	2	1	2	2	1	3	3	1	1
3	2	2	2	1	1	1	2	1	1
2	3	2	3	2	2	1	3	3	2
2	2	2	2	1	3	1	2	1	2
1	3	1	3	3	2	1	2	3	2
3	3	3	3	3	1	3	3	1	3
2	2	2	2	3	2	2	2	9	1
3	3	2	1	2	2	2	3	1	2
3	2	2	1	2	3	3	2	2	9
3	2	1	3	1	1	2	3	3	2
3	3	3	1	1	1	1	1	1	2
3	3	1	3	3	3	1	2	1	3
1	1	2	2	1	2	2	2	2	2
3	3	3	3	3	3	3	3	3	3
3	3	2	2	2	2	2	2	2	2
9	9	9	9	1	9	9	9	9	1
2	3	3	3	2	1	3	2	2	3
2	2	3	1	1	1	1	1	2	2
1	1	1	1	1	1	1	1	1	1
2	2	3	1	1	1	1	1	2	2
9	9	9	9	9	9	9	9	9	9
2	3	1	3	2	2	2	3	1	3
9	9	9	9	1	9	9	9	9	9
2	3	3	3	3	1	2	3	3	2
2	3	3	3	3	1	2	3	3	2
3	2	2	2	3	2	3	2	2	3
3	2	3	3	3	2	2	2	2	2
2	3	2	2	3	2	3	3	2	3

1	2	2	1	2	2	2	2	2	1
3	3	3	3	3	3	3	3	3	3
1	3	1	2	3	9	1	1	1	1
3	3	3	3	2	2	2	3	1	3
1	2	2	1	2	1	2	3	1	1
3	3	3	3	3	3	3	3	1	2
2	1	9	1	2	2	2	2	3	3
3	1	2	1	2	3	2	1	1	2
3	3	1	3	2	1	1	2	3	3
3	3	3	3	3	3	1	3	2	2
2	3	2	3	2	2	2	2	2	1
3	3	1	3	3	2	2	3	1	3
9	2	2	1	9	1	2	3	1	9
1	2	3	2	2	1	1	1	3	9
2	2	1	2	1	1	1	3	1	2
3	2	2	2	2	2	2	2	1	2
3	2	3	3	3	3	3	3	3	2
3	3	3	2	2	2	2	2	2	3
2	2	2	3	3	3	3	3	2	2
2	2	3	2	9	9	9	9	2	9
1	1	2	2	2	2	1	2	2	1
1	2	1	2	1	2	2	9	1	2
2	3	3	3	2	3	2	3	3	3
2	2	1	1	1	1	2	3	3	2
3	3	3	3	3	3	3	3	3	3
2	1	2	2	1	3	2	1	1	2
2	2	2	9	9	9	9	1	1	9
2	2	2	3	3	3	3	3	2	2
2	2	2	2	3	2	2	2	2	2
2	2	2	2	2	2	2	2	2	2
3	2	3	2	1	2	2	1	1	3
9	9	9	3	9	9	9	9	2	9
2	3	2	3	1	2	2	2	1	2
3	3	3	2	2	2	2	2	1	3
3	2	1	1	1	2	2	3	1	3
3	2	1	3	1	2	3	2	1	2
3	3	3	1	1	3	3	3	3	1
3	1	1	2	3	2	2	3	2	3
3	3	3	3	3	1	2	2	3	3
2	3	2	2	1	2	2	2	1	1
3	3	1	3	1	3	3	3	1	2
2	2	2	2	2	2	2	2	2	2
2	2	2	2	2	2	2	2	2	2
2	2	2	2	2	2	2	2	2	2
2	3	2	3	1	2	2	2	1	2
3	3	2	2	1	1	1	2	2	2
3	3	2	2	2	2	2	2	1	2

2	2	2	2	2	2	2	2	3	1
3	2	3	9	1	2	2	2	1	2
3	3	2	2	2	2	2	2	1	3
3	3	3	2	2	1	3	3	2	2
9	9	9	9	9	9	9	9	9	9
3	2	3	3	1	2	2	2	1	2
3	2	3	3	1	2	2	2	1	2
3	2	1	2	2	2	2	2	2	2
2	2	1	2	2	2	2	2	2	3
9	9	9	9	9	9	9	9	9	9
3	2	2	3	2	1	3	2	1	1
3	2	2	3	2	1	3	2	1	1
9	9	9	9	9	9	1	9	9	9
9	9	9	9	9	9	9	9	9	9
2	3	2	3	3	3	2	2	2	3
9	9	1	9	9	1	9	9	9	9
3	1	1	9	9	1	9	9	9	1
9	9	9	9	1	9	9	9	1	9
9	9	9	9	9	9	9	9	9	9
3	2	1	2	2	2	2	1	2	2
2	3	1	1	3	1	3	3	1	2
9	9	9	9	9	9	9	1	9	1
1	1	1	2	1	2	3	1	1	2
2	2	1	1	2	2	2	2	1	2
2	2	2	3	3	1	3	3	3	3
2	2	2	2	2	2	2	2	1	2
2	9	1	3	2	3	1	1	3	1
3	2	3	9	1	9	9	9	9	9
2	2	2	2	2	2	2	2	2	2
9	1	2	2	1	3	2	1	2	2
9	9	9	9	9	9	9	9	9	9
9	2	1	1	2	1	2	2	1	2
3	3	3	3	3	2	2	2	1	2
2	2	9	3	3	1	3	3	2	2
2	2	1	2	2	2	2	2	2	2
2	2	2	2	2	2	2	2	1	2
9	9	9	9	9	9	1	9	9	1
3	2	2	2	2	2	2	2	2	2
3	2	2	2	2	2	2	2	2	2
1	1	1	1	1	1	1	1	2	2
2	2	2	3	3	2	1	1	1	1
3	3	3	2	2	2	2	2	2	1
1	1	1	1	1	1	2	3	1	2
9	9	9	1	9	9	9	9	9	9
3	3	3	3	2	3	3	2	2	3
9	9	9	9	9	9	9	9	9	9
2	3	3	3	2	2	2	3	3	3

2	1	3	2	2	1	2	3	1	1
2	2	2	2	2	2	2	2	2	2
2	1	2	3	3	3	3	3	2	3
2	3	2	2	2	3	2	3	1	2
2	3	2	2	3	1	3	2	1	3
1	9	1	3	3	3	3	3	2	2
9	9	9	9	9	1	9	9	9	9
3	3	3	3	3	3	3	3	3	3
3	3	9	2	3	2	3	9	9	3
2	1	3	2	2	3	1	2	2	1
2	2	2	2	2	2	2	2	2	2
3	3	3	3	3	3	1	3	9	9
1	1	2	2	2	1	1	3	2	3
3	3	3	1	9	9	9	9	1	9
9	9	9	9	9	1	9	9	1	9
9	1	9	1	9	9	9	9	9	9
2	3	3	2	1	1	3	3	1	2
1	1	1	3	3	1	3	3	1	3
3	3	2	3	2	1	2	3	1	3
3	3	3	1	2	3	3	3	1	2
2	2	2	2	2	2	2	2	2	9
3	3	1	1	2	3	2	3	1	1
2	2	2	2	2	2	2	2	2	2
2	2	2	2	2	2	2	2	2	2
2	2	2	1	3	3	3	3	1	1
2	2	2	2	2	2	2	2	2	2
3	3	3	2	2	2	2	2	2	2
9	9	9	2	9	9	9	9	2	9
3	3	3	2	2	2	2	2	2	2
2	2	2	2	2	2	2	2	2	2
1	1	1	1	1	1	1	1	1	1
9	9	9	1	2	1	2	3	3	2
1	2	1	2	2	2	9	1	1	2
2	2	2	2	2	2	2	2	1	2
2	2	2	3	3	2	3	3	2	1
2	2	2	2	3	1	2	2	2	1
1	1	1	2	2	2	2	2	1	1
3	9	3	1	3	3	3	3	1	3
2	2	3	1	2	3	2	3	3	9
3	3	2	1	2	3	3	3	1	2
9	9	1	9	9	1	9	9	1	9
2	2	2	1	2	1	2	2	1	2
2	2	2	3	2	1	2	2	1	2
3	3	3	2	2	3	3	3	1	1
3	3	3	3	3	3	3	1	3	3
2	3	3	1	3	2	2	3	1	1
9	9	9	3	9	9	9	9	3	9

3	9	2	3	3	1	3	2	2	3
2	2	1	2	1	3	2	3	3	2
2	3	2	2	3	2	3	2	3	2
3	3	2	1	2	2	2	3	1	2
2	1	2	1	2	2	2	1	1	2
1	2	3	3	2	1	1	1	1	9
3	3	1	3	2	1	3	2	3	2
9	9	9	1	9	9	9	9	1	9
9	9	9	1	9	9	9	9	1	9
9	9	9	1	9	9	9	9	9	9
2	3	1	2	2	2	1	2	2	2
3	9	9	3	9	9	9	9	3	9
3	3	1	3	2	2	1	2	1	2
9	9	9	1	9	9	9	9	2	9
2	2	2	1	2	2	2	2	2	2
9	9	9	9	9	9	1	9	9	9
2	1	2	3	1	1	3	3	3	1
9	9	9	9	9	1	9	9	9	9
9	9	9	9	1	9	9	9	9	9
9	9	9	9	9	2	9	9	9	9
1	1	1	1	1	1	1	1	1	1
3	3	3	2	2	1	1	3	2	2
2	2	2	2	3	2	2	2	2	2
2	3	3	3	2	2	3	2	1	2
3	3	3	1	3	3	3	3	2	3
3	3	2	2	1	3	3	3	1	3
1	3	3	3	3	3	3	1	3	3
9	9	9	9	9	9	1	9	9	9
1	2	2	2	2	2	2	2	2	2
9	3	3	9	9	9	9	9	1	1
1	3	1	3	3	3	3	1	3	1
9	9	9	1	9	9	9	9	1	9
3	3	3	3	3	3	3	3	3	3
9	9	1	2	9	9	9	9	2	9
9	9	9	1	9	9	9	9	1	9
2	2	2	1	1	2	1	2	1	2
9	9	9	9	9	9	9	9	1	9
1	1	9	1	1	1	1	1	2	3
3	3	3	3	2	2	3	3	1	3
2	2	2	2	2	2	2	2	2	2
2	2	2	2	2	2	2	2	2	2
2	2	2	2	2	2	2	2	2	2
3	3	3	3	3	3	3	3	3	3
2	2	2	9	2	9	9	2	9	2
1	1	1	2	2	2	2	2	1	2
1	1	1	2	2	2	2	2	2	2
3	1	1	1	3	1	3	1	1	9

2	1	1	1	9	3	1	9	3	1
2	2	2	2	2	2	2	2	1	1
2	9	2	9	2	2	2	9	9	2
9	9	9	9	9	9	9	9	9	9
2	1	1	1	9	2	1	9	1	9
2	1	1	1	3	1	3	1	1	9
2	2	9	9	2	9	2	2	3	1
9	2	9	9	1	3	2	1	3	1
9	2	2	9	2	2	2	2	1	9
1	1	1	2	2	2	2	2	1	1
2	2	2	2	2	2	2	2	2	2
9	2	2	2	9	9	2	9	2	9
2	2	2	2	2	2	2	2	2	2
3	3	3	3	3	3	3	3	3	3
2	2	2	2	2	2	2	2	2	2
9	9	9	9	9	9	9	9	9	9
2	9	2	2	9	2	9	2	9	2
2	2	2	2	2	2	2	2	2	2
9	2	1	3	1	9	3	1	9	1
2	2	2	2	2	2	2	2	2	2
2	2	2	2	2	2	2	2	2	2
2	2	2	2	2	2	2	2	2	2
2	1	3	2	2	1	9	1	2	1
1	2	9	2	1	1	3	1	1	1
2	3	2	3	2	2	2	3	3	2
3	3	3	3	3	3	3	3	3	3
2	1	1	1	2	3	2	1	2	9
2	2	1	2	2	1	2	2	1	2
3	1	2	1	3	1	2	1	1	1
3	2	2	2	2	1	2	3	1	2
2	2	2	2	2	3	2	3	2	9
2	3	2	9	9	9	9	9	2	2
2	2	2	3	3	1	1	1	1	1
3	3	3	3	1	9	9	9	2	9
3	3	3	1	1	1	3	3	2	1
1	1	2	1	9	2	1	2	1	1
3	3	3	3	1	3	3	3	1	3
2	2	1	2	2	1	2	2	2	1
2	3	2	1	2	2	2	3	2	2
2	3	3	3	3	2	2	3	3	2
3	1	1	3	3	3	3	1	1	2
3	2	3	3	9	1	2	3	1	3
3	2	2	3	2	2	1	2	3	2
9	9	9	9	9	9	9	9	9	9
9	9	9	1	2	2	1	2	1	2
2	9	2	2	2	2	2	2	1	2
3	1	1	9	9	1	9	9	9	1

1	2	2	3	2	3	1	1	3	9
2	2	2	9	9	9	9	9	9	9
1	1	1	2	2	2	2	2	1	2
2	2	2	2	2	2	2	2	2	2
1	2	2	1	1	1	1	1	1	1
2	2	2	2	2	2	2	2	2	2
2	2	2	1	1	1	9	9	2	2
3	3	3	3	1	3	3	3	1	1
3	3	3	3	3	3	3	3	3	3
1	2	2	1	1	1	1	1	1	2
1	1	2	1	1	1	1	2	1	1
1	1	1	9	1	9	2	2	1	9
1	1	1	9	1	2	2	2	2	2
2	2	1	9	1	2	2	2	1	2
1	2	2	2	2	1	1	9	2	2
1	1	1	9	2	2	2	2	1	9
1	1	1	1	2	1	1	2	1	2
2	1	1	1	1	1	2	2	1	1
1	1	1	9	1	2	2	2	1	1
2	2	2	2	2	2	2	2	2	2
2	1	2	1	1	1	1	1	2	1
2	2	2	2	2	2	2	2	2	2
9	9	9	9	9	9	9	9	9	9
1	1	1	9	1	2	2	2	1	9
1	1	1	1	1	1	1	1	1	1
1	1	1	1	2	1	1	2	2	1
1	1	1	1	2	1	2	1	1	1
1	1	2	1	1	1	2	2	1	2
9	2	2	2	9	2	2	2	1	2
1	1	1	1	1	1	1	1	1	1
2	2	2	2	2	2	2	2	2	2
2	2	2	2	2	2	2	2	2	2
1	1	1	1	1	1	1	1	1	1
1	1	1	1	1	1	1	1	2	2
1	1	1	2	2	2	2	2	2	2
2	2	2	2	2	2	2	2	2	2
2	1	1	1	1	1	1	1	1	1
1	1	1	1	1	2	2	1	1	2
9	9	9	9	9	9	9	9	9	9
2	2	2	2	2	9	2	2	2	2
2	2	2	2	2	2	2	2	2	2
2	2	2	2	2	2	2	2	2	2
1	1	1	9	1	2	2	2	2	2
2	2	2	2	2	2	2	2	2	2
1	2	1	1	1	1	2	1	1	1
2	2	2	2	2	2	2	2	2	2
1	1	1	9	1	9	9	9	2	1

1	1	2	2	2	2	2	2	2	2
2	2	2	2	2	2	2	2	2	2
1	1	1	9	1	2	2	2	1	1
1	1	9	1	1	2	2	1	1	1
1	1	1	1	1	1	1	1	1	1
2	2	2	2	2	2	2	2	2	2
1	1	1	1	1	1	1	1	1	2
2	2	1	9	1	2	2	2	1	2
9	2	2	2	9	2	2	2	9	2
1	1	1	1	1	1	1	1	1	1
9	2	2	2	2	2	9	2	9	9
9	1	1	9	1	1	1	1	1	9
2	2	2	2	9	2	2	2	9	9
1	1	1	1	1	1	1	1	1	1
1	1	1	9	1	9	9	9	1	9
2	2	2	2	2	2	2	2	2	2
1	1	1	2	1	2	2	2	1	1
9	9	9	9	9	9	9	9	9	9
2	2	2	2	2	2	2	2	2	2
3	3	3	3	2	1	3	3	1	2
3	3	3	3	2	1	3	3	1	2
3	3	3	3	2	1	3	3	1	2
3	3	3	3	2	1	3	3	1	2
3	3	3	3	2	1	3	3	1	2
3	3	3	3	2	1	3	3	1	2
3	3	3	2	2	1	3	3	1	2
3	3	3	3	2	1	3	3	1	2
9	9	9	3	2	1	9	3	1	2
3	3	3	3	2	1	3	3	1	2
3	3	3	3	2	1	3	3	1	3
9	3	3	3	2	1	3	3	1	2
9	3	3	3	2	1	3	3	1	2
3	3	3	3	3	2	1	3	1	2
9	3	3	3	2	1	3	3	1	2
3	3	3	3	2	1	3	3	1	2
3	3	3	3	2	1	3	3	1	2
3	3	3	3	2	1	3	3	1	2
3	3	3	3	2	1	3	3	1	2
3	3	3	3	2	1	3	3	1	2
3	3	3	3	2	1	3	3	1	2
3	3	3	3	2	1	3	3	1	2
3	3	3	3	2	1	3	3	1	2
3	3	3	3	2	1	3	3	1	2
3	3	3	3	2	1	3	3	1	2
9	9	9	9	9	9	9	9	9	9
9	9	9	9	9	9	9	9	9	9
9	9	9	9	9	9	9	9	9	9

9	9	9	9	9	9	9	9	9	9
2	3	3	3	2	1	2	2	9	9
9	9	9	9	9	9	9	9	9	9
9	9	9	9	9	9	9	9	9	9
9	9	9	9	9	9	9	9	9	9
9	9	9	9	9	9	9	9	9	9
9	9	9	9	9	9	9	9	9	9
9	9	9	9	9	9	9	9	9	9
9	9	9	9	9	9	9	9	9	9
9	9	9	9	9	9	9	9	9	9
9	9	9	9	9	9	9	9	9	9
9	9	9	9	9	9	9	9	9	9
2	2	1	1	2	2	2	3	1	2
9	9	9	9	9	9	9	9	9	9
9	9	9	9	9	9	9	9	9	9
9	9	9	9	9	9	9	9	9	9
1	1	1	1	2	1	1	3	9	9
9	9	9	9	9	9	9	9	9	9
9	9	9	9	9	9	9	9	9	9
3	3	3	3	2	1	3	3	1	2
3	3	3	3	2	1	3	3	1	2
3	3	3	3	2	1	3	3	1	2
3	3	3	3	2	1	3	3	1	2
3	3	3	3	2	1	3	3	1	2
3	3	3	3	2	1	3	3	1	2
3	3	3	3	2	1	3	3	1	2
3	3	3	3	2	1	3	3	1	2
3	3	3	3	2	1	3	3	1	2
3	3	3	3	2	1	3	3	1	3
3	3	3	3	1	3	3	3	1	3
3	3	3	3	2	1	3	3	1	2
3	3	3	3	3	1	3	3	1	2
3	3	3	3	2	1	3	3	1	2
3	3	9	3	2	1	3	3	1	2
3	3	3	3	3	1	3	3	1	2
9	9	9	3	9	9	9	9	1	2
3	3	3	3	3	1	3	3	1	2
3	3	3	3	3	1	3	3	1	2
3	3	3	3	3	1	3	3	1	2
3	3	3	3	2	1	3	3	1	2
3	3	9	3	9	1	3	3	1	2
3	3	3	3	2	1	3	3	1	2
3	3	3	3	2	1	3	3	1	2
3	3	3	3	2	1	3	3	1	2
3	3	3	3	3	1	3	3	1	3
3	3	3	3	2	1	3	3	1	2
3	3	3	3	2	1	3	3	1	2

3	3	3	3	2	1	3	3	1	2
3	3	3	3	2	1	3	3	1	2
3	3	3	3	2	1	3	3	1	2
3	3	3	3	2	1	3	3	1	2
3	3	3	3	2	1	3	3	3	3
3	3	3	3	2	1	3	3	1	2
3	3	3	3	2	1	3	3	1	2
3	3	3	3	3	2	3	3	1	9
3	3	3	3	3	2	3	3	1	2
3	3	3	3	2	1	3	3	1	2
3	3	3	3	2	1	3	3	1	2
3	3	3	3	2	1	3	3	1	3
3	3	3	3	2	1	3	3	1	2
3	3	3	3	3	1	3	3	1	2
3	3	3	3	3	1	3	3	1	2
3	3	3	3	2	1	3	3	1	2
3	3	3	3	3	1	3	3	1	2
3	3	3	3	2	1	3	3	1	2
3	3	3	9	2	1	3	3	1	2
3	3	3	3	2	1	3	3	1	2
3	3	3	3	2	1	3	3	1	2
3	3	3	3	2	1	3	3	1	2
3	3	3	3	3	1	3	3	1	2
3	3	3	3	3	1	3	3	1	2
3	3	3	3	2	1	3	3	1	2
3	3	3	3	3	1	3	3	1	2
3	3	3	3	2	1	3	3	1	2
3	3	3	1	3	1	3	3	1	2
3	3	3	3	3	1	3	3	1	2
3	3	3	3	3	1	3	3	1	9
3	3	3	3	2	1	3	3	1	2
3	3	3	3	2	1	3	3	1	2
3	3	3	9	9	9	9	9	1	2
3	3	3	3	2	1	3	3	1	2
3	3	3	2	3	1	3	3	9	9
3	3	3	3	2	1	3	3	1	2
3	3	3	3	3	1	3	3	1	3
3	3	3	3	3	1	3	3	1	3
3	3	3	3	3	3	3	3	1	3
3	3	3	3	2	1	3	3	1	2
3	3	3	1	2	1	3	3	1	3
3	3	3	3	2	1	3	3	1	2
3	3	3	3	3	1	3	3	1	3
3	3	3	3	3	1	3	3	1	2
3	3	3	3	2	1	3	3	1	2
3	3	3	3	2	1	3	3	1	2
3	3	3	3	2	1	3	3	1	2

3	3	3	3	2	1	3	3	1	2
3	3	3	3	2	1	3	3	1	2
3	3	3	3	2	1	3	3	1	2
3	3	3	3	2	1	3	3	1	2
3	3	3	3	2	1	3	3	1	2
3	3	3	3	2	1	3	3	1	2
3	3	3	3	2	1	3	3	1	2
3	3	3	3	2	1	3	3	1	2
3	3	3	3	2	1	3	3	1	2
3	3	3	3	2	1	3	3	1	2
3	3	3	3	2	1	3	3	1	2
3	3	3	3	2	1	3	3	1	2
3	3	3	3	2	1	3	3	1	2
3	3	3	3	2	3	3	3	1	2
3	3	3	3	3	1	3	3	1	2
3	3	3	3	2	1	3	3	1	2
3	3	3	3	2	1	3	3	1	2
3	3	3	3	2	1	3	3	1	2
3	3	3	3	2	1	3	3	1	2
9	9	9	9	9	9	9	9	9	9
3	3	3	3	3	1	3	3	1	2
3	3	3	3	2	1	3	3	1	2
3	3	3	3	2	1	3	3	1	2
3	3	3	3	2	1	3	3	1	2
3	1	9	9	9	9	9	9	1	2
3	3	3	3	2	1	3	3	1	2
3	3	3	3	2	1	3	3	1	2
9	3	3	3	2	1	3	3	1	2
3	3	3	3	2	1	3	3	1	2
3	3	3	3	2	1	3	3	1	2
3	3	3	3	2	1	3	3	1	2
3	3	3	3	2	1	3	3	1	2
3	3	3	3	3	1	3	3	1	2
3	3	3	3	3	1	3	3	1	2
3	3	3	3	2	1	3	3	1	2
3	3	3	3	2	1	3	3	1	2
3	3	3	1	2	1	3	3	1	2
3	3	3	3	2	1	3	3	1	2
3	3	3	3	2	1	3	3	1	2
3	3	3	3	2	1	3	3	1	2
3	3	3	3	2	1	3	3	1	2
9	9	9	9	9	9	9	9	9	9
3	3	3	3	2	1	3	3	1	2
3	3	3	3	2	1	3	3	1	2
3	3	3	3	2	1	3	3	1	2
3	3	3	3	2	1	3	3	1	2
3	3	3	3	2	1	3	3	1	2

3	3	3	3	2	1	3	3	1	2
3	3	3	3	2	1	3	3	1	2
3	3	3	3	2	1	3	3	1	2
3	3	3	3	2	1	3	3	1	2
3	3	3	3	2	1	3	3	1	2
3	3	3	3	2	1	3	3	1	2
3	3	3	3	2	1	3	3	1	2
3	3	3	3	9	1	3	3	1	3
3	3	3	3	3	1	3	3	1	2
3	3	3	3	2	1	3	3	1	2
3	3	3	3	2	1	3	3	1	2
3	3	3	3	2	1	3	3	1	2
3	3	3	3	2	1	3	3	1	2
3	3	3	3	2	1	3	3	1	2
3	3	3	3	2	1	3	3	1	2
3	3	3	3	2	1	3	3	1	2
3	3	3	3	2	1	3	3	1	2
3	3	3	3	2	1	3	3	1	2
3	3	3	3	2	1	3	3	1	3
3	3	3	3	2	1	3	3	1	2
3	3	3	3	2	1	3	3	1	2
3	3	3	3	2	1	3	3	1	2
3	3	3	3	2	1	3	3	1	2
3	3	3	3	2	1	3	3	1	2
3	3	3	3	2	1	3	3	1	2
3	3	3	3	2	1	3	3	1	2
3	3	3	3	2	1	3	3	3	2
9	9	9	9	9	9	9	9	9	9
9	9	9	9	9	9	9	9	9	9
9	9	9	9	9	9	9	9	9	9
9	9	9	9	9	9	9	9	9	9
9	9	9	9	9	9	9	9	9	9
2	2	2	2	2	9	9	9	9	9
9	9	9	9	9	9	9	9	9	9
9	9	9	9	9	9	9	9	9	9
9	9	9	9	9	9	9	9	9	9
9	9	9	9	9	9	9	9	9	9
9	9	9	9	9	9	9	9	9	9
9	9	9	9	9	9	9	9	9	9
9	9	9	9	9	9	9	9	9	9
9	9	9	9	9	9	9	9	9	9
3	9	9	9	9	9	9	9	9	9
3	3	1	3	2	1	2	3	1	2
1	1	1	1	1	1	1	1	1	1
9	9	9	9	9	9	9	9	9	9
9	9	9	9	9	9	9	9	9	9
9	9	9	9	9	9	9	9	9	9

9	9	9	9	9	9	9	9	9	9
3	3	9	9	9	9	9	9	9	9
9	9	9	9	9	9	9	9	9	9
1	9	9	9	9	9	1	9	1	9
9	9	9	9	9	9	9	9	9	9
9	9	9	9	2	9	9	9	9	9
3	1	2	1	1	1	1	3	1	9
2	2	1	3	3	1	3	3	2	2
9	9	9	9	9	9	9	9	9	9
9	9	9	9	9	9	9	9	9	9
9	9	9	9	9	9	9	9	9	9
9	9	9	9	9	9	9	9	9	9
9	9	9	9	9	9	9	9	9	9
1	1	3	3	1	1	2	1	9	9
2	2	2	2	2	2	2	2	2	2
3	3	3	2	2	1	2	2	2	1
1	3	1	3	3	2	1	1	3	3
3	3	3	1	3	2	2	3	1	2
3	9	1	2	3	2	3	3	1	2
1	3	1	1	3	1	1	3	1	2
3	3	1	2	1	2	1	3	2	2
3	3	3	1	1	1	1	1	1	3
3	2	2	3	2	3	2	3	1	3
3	3	3	3	3	3	3	3	3	2
9	9	9	9	9	1	9	9	2	9
2	2	2	2	2	2	2	2	2	2
2	2	2	2	2	2	2	2	3	2
1	1	3	3	1	3	3	3	1	1
3	9	1	2	3	1	3	2	1	3
2	2	2	2	2	2	3	3	1	3
3	3	3	3	3	3	3	3	2	2
1	2	1	1	2	3	1	2	1	2
9	9	9	9	9	1	9	9	9	9
3	2	2	3	2	2	2	2	3	2
9	9	9	1	9	9	9	9	9	1
9	9	9	9	9	9	1	9	2	9
2	2	1	2	2	2	1	2	1	2
3	3	1	3	3	2	1	2	3	3
9	9	1	9	1	9	9	9	1	9
9	9	9	1	2	2	2	2	1	1
9	9	9	9	9	9	9	9	9	9
3	3	1	1	9	1	2	3	1	9
2	2	1	3	3	1	3	3	2	1
3	3	3	9	9	9	9	9	1	1
9	9	1	1	1	1	1	9	9	9
2	2	1	9	9	9	1	9	2	1
2	2	2	2	2	2	2	2	2	2

9	9	9	2	9	9	9	9	9	9
9	9	9	9	9	9	9	9	9	9
3	3	3	2	3	1	2	2	1	2
2	2	1	1	1	1	3	3	1	3
9	9	9	1	9	9	9	9	2	9
9	9	9	9	9	2	9	9	9	2
2	2	2	2	2	1	2	2	2	2
2	2	2	2	2	2	2	2	2	2
2	2	2	2	2	2	2	2	2	2
1	1	1	1	2	2	2	2	2	2
1	2	2	2	2	2	2	2	2	3
9	9	9	9	9	9	9	9	9	9
9	9	9	9	9	1	9	9	1	9
2	2	2	2	2	2	2	2	2	2
2	2	2	2	2	2	3	3	1	2
3	3	3	2	2	2	3	2	3	1
3	3	3	1	1	1	1	1	1	3
3	2	1	3	9	1	2	1	1	1
1	2	1	1	2	1	1	1	1	2
9	9	9	2	1	3	2	3	1	2
3	2	2	2	2	2	2	2	2	2
2	2	2	2	2	2	2	2	2	2
2	2	2	2	2	2	2	2	2	2
2	2	2	3	2	1	2	2	2	2
2	2	2	2	2	2	2	2	3	3
1	2	3	3	2	2	2	9	1	1
3	2	2	3	3	2	2	2	3	3
3	2	2	2	2	2	2	2	3	2
2	2	2	2	2	2	2	2	1	2
1	1	2	1	1	1	2	2	2	2
3	3	3	1	3	3	3	3	3	3
3	3	3	3	1	2	3	3	2	3
2	2	1	2	1	1	1	1	1	2
2	2	2	3	2	2	1	1	1	2
2	1	1	2	2	2	2	2	2	1
9	9	9	9	9	9	9	9	3	3
2	2	2	2	3	2	2	2	3	2
2	9	9	3	3	3	3	3	3	1
9	9	9	9	9	9	9	9	2	9
3	3	3	1	3	3	3	3	1	3
2	2	3	3	2	2	2	3	3	3
3	3	2	2	2	2	2	2	1	2
1	2	3	1	2	3	1	2	1	2
3	9	3	2	3	3	3	3	3	2
3	3	1	1	1	3	3	3	1	1
2	2	2	1	1	1	1	1	1	3
2	2	2	1	2	2	2	2	1	2

2	2	2	2	2	2	2	3	2	2
3	2	3	3	2	3	2	3	1	2
2	2	2	1	1	1	1	1	2	3
9	9	9	9	9	9	9	9	2	2
3	3	3	2	3	3	3	3	2	1
2	2	2	1	2	2	1	2	1	2
2	3	2	1	2	2	3	2	2	2
2	2	2	2	1	2	2	2	2	1
2	2	2	2	2	2	2	2	2	2
3	2	3	1	3	2	3	2	1	2
2	2	2	2	2	2	2	2	2	2
2	2	2	2	2	2	2	2	2	2
1	1	1	2	2	2	2	2	1	1
2	2	2	2	2	2	2	2	2	2
9	9	9	9	9	9	9	9	2	2
2	2	2	1	2	2	2	2	2	1
3	3	1	3	3	3	3	3	3	3
2	2	2	2	2	2	2	2	2	2
2	2	2	2	2	2	2	2	2	2
1	2	1	1	2	3	1	2	1	2
3	3	3	2	2	2	2	2	2	2
1	2	1	2	2	2	1	1	1	1
9	9	9	9	9	9	9	9	2	2
9	9	9	9	9	9	9	9	1	3
3	3	3	3	3	3	3	3	3	3
1	1	1	2	1	1	2	1	9	9
3	1	1	1	1	2	3	3	2	1
3	3	3	1	3	3	3	3	3	1
1	1	1	1	3	1	3	2	1	2
3	2	2	1	2	2	2	2	1	1
2	2	2	1	2	2	2	3	1	2
9	1	9	9	9	9	9	2	2	9
3	3	3	3	2	2	2	3	2	2
2	1	2	2	2	2	2	2	1	1
1	1	1	9	9	9	1	9	3	2
9	9	9	9	9	9	9	9	9	9
2	3	1	1	1	3	3	3	1	1
1	2	3	2	2	2	2	2	1	2
3	2	3	3	1	2	1	1	2	3
2	2	2	2	2	2	2	2	2	1
3	3	2	2	1	3	3	2	1	2
3	1	1	3	2	1	2	3	1	3
2	2	2	1	2	2	2	3	1	2
3	9	9	9	9	9	9	9	9	9
9	9	9	9	9	9	9	9	9	9
9	9	9	9	9	9	9	9	9	9
1	1	1	9	9	9	1	9	3	2

3	2	2	1	2	2	3	3	1	2
3	3	2	2	1	1	3	3	3	3
3	1	2	3	2	2	1	2	3	2
3	3	1	1	3	3	3	3	1	3
2	2	2	2	2	2	1	2	2	2
2	3	2	2	1	3	3	3	1	3
3	3	1	3	3	3	3	3	3	3
2	2	3	3	3	3	3	3	1	1
1	1	1	1	2	1	1	2	1	1
2	2	2	2	3	1	2	1	1	2
3	1	2	1	2	3	1	2	1	3
2	2	2	1	1	3	3	3	2	2
2	2	2	2	2	2	2	2	2	2
3	3	1	1	1	2	3	3	1	3
2	2	2	2	2	2	2	2	2	2
3	3	3	1	2	2	2	2	1	2
1	3	1	3	3	3	1	1	1	2
3	3	1	2	1	3	3	3	3	2
3	3	3	1	1	1	3	3	3	3
2	3	1	9	9	9	9	9	1	3
1	2	3	3	3	2	1	1	3	2
1	2	1	3	1	2	3	3	1	3
3	3	3	1	3	3	3	3	2	2
3	3	1	1	3	3	3	3	3	2
2	3	3	1	3	2	1	3	1	3
9	9	9	9	9	9	9	9	9	9
1	2	1	1	2	2	1	2	2	2
3	3	1	2	2	1	3	3	2	3
2	2	2	2	2	3	3	2	3	3
2	2	3	3	3	1	2	2	2	3
2	2	2	3	2	2	2	2	2	2
9	9	1	9	9	1	9	9	1	9
3	1	2	3	2	1	3	3	3	2
2	3	2	3	3	3	1	1	1	1
1	2	2	1	2	3	3	3	3	2
2	3	2	2	1	2	3	2	2	3
3	9	9	1	2	2	2	2	2	2
2	2	2	3	2	1	1	2	2	2
3	3	3	1	3	3	3	3	1	2
9	9	9	2	9	9	9	9	9	9
1	2	1	1	2	3	2	1	3	2
2	2	2	2	2	2	2	2	2	2
3	3	3	2	2	2	2	2	2	2
2	2	2	2	2	2	2	2	2	2
2	2	2	2	2	2	2	2	2	2
3	9	1	2	2	1	1	1	2	3
3	2	3	2	2	2	1	1	2	2

3	2	3	1	1	2	2	3	1	3
2	2	2	2	2	2	2	2	2	2
3	2	3	3	1	2	3	3	1	3
3	3	2	2	2	1	2	3	2	2
1	3	3	2	2	2	2	2	2	2
3	3	3	2	2	2	2	2	3	3
3	2	3	1	1	3	3	3	1	3
3	2	3	2	1	2	2	1	1	2
3	3	1	1	1	3	3	3	1	1
3	3	3	3	3	3	3	3	1	3
3	3	3	1	3	3	3	2	2	3
2	2	2	3	3	3	3	3	1	2
3	3	3	3	3	3	3	3	2	3
3	3	1	3	2	1	1	3	1	1
2	2	2	1	1	1	1	1	2	2
3	2	2	2	2	2	2	2	2	2
9	9	3	9	9	9	1	9	1	2
3	2	2	2	2	2	2	2	1	1
2	2	1	2	2	2	2	2	2	2
2	1	1	2	1	1	1	3	2	2
2	1	1	2	2	1	1	1	1	1
3	2	2	3	3	1	3	3	3	3
9	9	1	9	2	9	9	9	2	9
2	2	2	3	3	1	3	3	3	3
2	2	2	9	9	9	1	9	1	2
2	2	1	2	2	2	2	2	1	2
2	2	3	1	1	3	3	3	1	2
2	2	2	2	2	2	2	2	1	2
3	3	3	3	3	1	3	3	3	3
2	2	1	3	2	1	2	2	2	2
3	3	3	3	3	1	3	3	3	3
2	2	2	1	2	2	2	2	1	1
1	1	1	3	2	2	2	2	1	2
2	2	2	1	2	2	2	2	2	2
9	9	9	2	9	9	9	9	2	9
9	1	3	3	3	1	3	3	3	3
2	2	2	2	2	2	2	2	2	2
1	2	1	1	1	1	1	1	2	3
1	1	1	1	1	1	1	1	1	1
2	2	2	2	2	2	2	2	2	2
3	2	1	1	2	3	2	1	1	2
9	9	9	9	9	9	9	9	9	9
9	9	9	9	9	9	9	9	9	9
1	1	1	1	1	1	1	1	1	1
2	2	3	1	2	3	2	3	1	2
9	9	9	9	9	9	9	9	9	9
2	2	2	1	1	1	1	1	1	2

2	2	2	2	2	2	2	2	2	2
2	2	1	1	1	1	1	1	1	3
3	2	2	1	2	3	1	2	1	2
2	1	2	1	2	3	2	1	1	2
3	3	3	3	3	1	3	3	2	2
1	2	3	1	2	3	1	2	1	2
2	2	2	1	2	2	2	2	1	2
9	9	9	1	9	9	9	9	1	9
3	2	1	1	1	1	2	3	1	1
9	9	9	9	9	9	9	9	9	9
2	2	2	1	1	1	1	1	1	1
2	2	3	2	2	1	3	3	3	3
2	2	2	2	2	1	3	1	1	1
2	2	2	2	2	2	2	2	1	2
1	1	1	1	1	1	1	1	1	1
9	9	9	9	9	9	9	9	9	9
9	9	9	9	9	9	9	9	9	9
1	1	2	2	3	3	3	3	3	3
2	2	2	2	2	2	2	2	2	2
2	2	2	2	9	9	9	9	2	2
2	2	2	2	2	2	2	2	2	2
2	2	2	9	9	9	9	9	2	2
3	3	2	3	3	3	3	1	2	2
2	1	2	2	1	2	3	2	2	1
2	3	2	2	9	1	3	3	1	9
9	9	9	9	9	1	9	9	1	9
9	2	2	2	2	1	2	2	2	1
2	2	2	9	1	9	9	9	2	2
9	9	9	9	9	9	9	9	2	2
3	3	2	3	2	1	2	2	3	2
1	2	1	1	2	1	2	1	1	2
2	2	2	2	2	2	2	2	2	2
2	2	2	2	2	2	2	2	2	2
2	2	2	2	2	2	2	2	2	2
1	2	1	1	2	2	2	2	1	2
2	2	2	2	2	2	2	2	2	2
2	2	2	3	3	3	3	3	3	3
9	9	9	9	9	1	9	9	1	9
9	9	2	9	9	9	9	2	9	9
2	2	2	2	2	2	2	2	2	2
2	2	2	2	2	2	2	2	2	2
9	9	9	9	1	9	9	9	1	9
2	1	2	2	2	3	1	2	1	2
9	9	9	2	9	9	9	9	2	9
2	2	2	2	2	2	2	2	2	2
2	2	1	2	1	2	1	2	1	2
2	2	2	2	2	2	2	2	2	2

1	2	3	3	9	9	9	9	3	2
9	9	9	9	9	1	9	9	1	9
2	2	2	2	2	2	2	2	2	2
2	2	1	3	3	1	2	3	2	1
2	2	2	3	3	3	3	3	2	3
3	3	3	3	3	2	2	1	2	2
2	3	1	2	2	1	1	2	2	1
1	2	3	2	1	2	3	1	1	2
2	2	2	2	2	2	2	2	2	2
1	1	1	9	9	9	9	1	3	3
3	3	1	3	3	3	3	1	9	9
2	2	2	2	2	2	2	2	2	2
2	3	2	3	2	2	2	2	1	2
3	3	3	3	2	2	3	3	2	1
2	2	2	1	2	3	2	2	2	2
3	2	2	1	2	3	2	2	2	2
2	2	2	2	1	2	2	2	2	2
2	2	2	2	2	2	2	2	2	2
3	2	1	3	2	3	1	1	3	2
2	2	2	1	2	3	1	3	1	2
2	2	2	2	1	2	3	3	2	1
3	2	3	2	2	1	2	2	1	2
1	3	2	1	2	1	3	2	2	1
9	9	9	9	9	9	9	9	9	9
3	3	1	2	2	2	2	2	2	2
9	9	9	9	9	9	1	9	1	9
9	9	9	9	9	9	9	1	9	9
9	9	9	9	9	9	1	9	9	9
3	3	3	2	2	2	3	3	1	2
3	3	3	2	2	2	2	2	2	2
3	3	3	2	2	2	2	2	2	2
1	3	9	1	9	9	9	9	1	9
1	3	1	2	2	2	2	2	1	2
2	2	2	2	2	2	2	2	2	2
9	9	9	9	9	1	9	9	9	9
3	2	3	2	2	1	1	2	1	1
3	2	1	1	2	9	3	1	1	2
2	3	3	1	2	1	2	1	1	2
2	2	1	2	1	3	3	3	2	3
3	3	3	3	3	1	3	3	3	3
2	2	2	1	2	3	2	1	1	2
1	2	1	9	9	9	9	1	1	2
9	9	9	9	9	1	9	9	9	9
9	9	9	9	9	1	9	9	9	9
2	1	3	1	3	2	3	1	2	3
2	2	2	2	2	2	2	2	2	2
2	2	2	2	2	2	2	2	2	2

1	3	2	1	2	3	1	2	2	1
9	9	9	9	9	9	9	9	9	9
9	9	9	9	9	9	9	9	9	9
9	9	9	9	9	9	9	9	9	9
2	2	2	9	9	1	9	9	1	9
9	9	1	9	9	1	9	9	9	9
9	9	9	9	9	9	9	9	9	9
9	9	9	9	9	9	9	9	9	9
9	9	9	9	9	9	9	9	9	9
9	9	9	1	9	9	9	9	9	9
1	2	1	1	2	1	2	1	2	2
1	1	2	1	1	1	1	1	1	2
1	2	1	1	1	2	3	3	1	3
3	2	2	2	2	2	2	2	1	2
1	2	1	2	2	2	2	2	1	2
2	2	2	1	2	2	2	2	1	2
1	1	2	1	2	1	9	9	1	3
2	2	2	1	2	2	2	2	1	2
2	3	2	1	1	2	3	3	1	9
2	2	1	3	3	2	9	1	2	2
2	2	3	3	2	1	3	3	2	1
2	2	3	3	2	3	1	3	2	1
1	1	1	3	9	3	3	3	2	2
2	2	2	3	3	3	3	3	2	2
2	2	2	2	2	2	2	2	2	2
2	2	2	2	2	2	2	2	3	3
2	2	2	2	2	2	2	2	2	2
1	2	2	2	1	1	2	2	2	3
9	9	9	9	9	9	9	9	2	2
2	2	2	2	2	2	2	2	2	2
9	9	9	9	9	9	9	9	9	9
2	2	2	2	2	2	2	2	2	1
1	1	1	1	1	1	1	1	1	1
9	9	9	1	9	9	9	9	9	9
3	3	3	3	3	3	3	3	3	3
2	1	1	1	1	1	3	3	1	1
2	2	2	2	2	2	2	2	2	2
2	2	2	2	2	2	2	2	2	2
1	1	3	3	1	3	3	3	1	2
9	9	9	9	9	9	9	9	9	9
3	1	1	2	1	1	2	1	2	2
2	2	2	3	1	3	3	3	1	1
2	2	2	2	2	2	2	2	2	2
3	3	3	2	1	3	3	3	2	2
3	2	2	2	2	1	2	2	2	2
9	9	9	9	9	9	9	9	9	9
2	2	2	2	2	2	2	2	2	2

9	9	9	9	9	9	9	9	9	9
2	1	3	2	2	2	2	2	2	2
3	3	3	1	1	1	9	1	9	1
9	9	9	9	9	9	9	9	9	9
9	9	9	9	9	9	9	9	9	9
9	9	9	9	9	9	9	9	9	9
9	9	9	9	9	9	9	9	9	9
9	9	9	9	9	9	9	9	9	9
9	9	9	9	9	9	9	9	9	9
9	9	9	9	9	9	9	9	9	9
9	9	9	9	9	9	9	9	9	9
9	9	9	9	9	9	9	9	9	9
9	9	9	9	9	9	9	9	9	9
9	9	9	9	9	9	9	9	9	9
9	9	9	9	9	9	9	9	9	9
9	9	9	9	9	9	9	9	9	9
9	9	9	9	9	9	9	9	9	9
9	9	9	9	9	9	9	9	9	9
9	9	9	9	9	9	9	9	9	9
9	9	9	9	9	9	9	9	9	9
9	9	9	9	9	9	9	9	9	9
9	9	9	9	9	9	9	9	9	9
9	9	9	9	9	9	9	9	9	9
9	9	9	9	9	9	9	9	9	9
2	1	3	1	2	3	1	2	1	2
2	2	2	2	2	2	2	2	2	2
2	2	2	1	2	2	2	2	1	2
2	3	3	2	1	1	2	2	2	2
3	3	3	3	3	1	3	3	3	3
9	9	9	9	9	1	9	9	2	3
2	2	2	2	2	2	2	2	2	2
3	3	3	3	3	1	3	3	3	3
1	1	3	9	9	1	9	9	1	3
2	2	2	3	3	1	2	2	1	2
3	3	3	1	3	3	3	3	1	3
9	9	9	9	9	1	9	9	9	9
3	2	3	1	1	3	3	3	3	3
1	1	1	3	3	1	3	3	3	3
2	2	2	1	1	2	2	2	2	3
3	2	3	1	1	3	3	3	3	3
9	9	9	9	9	1	9	9	9	1
3	3	3	1	2	3	2	3	1	2
3	1	3	3	3	1	3	3	1	3
2	9	9	3	9	1	9	9	1	9
3	3	3	3	3	1	3	1	3	3
3	3	3	3	3	1	3	3	3	3
2	2	2	1	2	3	2	3	2	2
2	2	2	2	2	1	2	2	3	3

1	1	1	1	2	2	1	3	1	1
3	3	1	3	1	1	1	1	3	3
3	3	3	9	9	1	9	9	3	1
3	3	1	3	2	2	3	1	3	3
1	2	3	2	1	1	9	2	3	2
9	9	9	1	9	9	9	9	1	9
9	2	3	1	1	2	3	3	3	1
9	9	9	9	9	9	9	1	1	9
9	9	9	2	9	9	9	9	1	9
9	9	9	2	9	9	9	9	1	9
9	9	9	9	9	1	9	9	1	9
9	9	9	9	9	1	9	9	9	9
9	9	9	9	9	9	1	9	9	9
9	9	9	9	9	9	1	9	9	9
9	9	9	9	1	9	9	9	9	9
3	1	3	3	3	3	1	3	3	3
3	3	3	3	3	3	3	3	3	3
2	3	3	2	2	2	2	2	2	2
9	9	9	9	9	9	1	9	9	9
3	3	3	1	3	3	3	3	3	3
9	9	9	9	9	1	9	9	9	9
3	1	2	3	3	1	3	3	1	9
9	9	9	9	9	1	9	9	9	9
9	9	9	9	1	9	9	9	1	9
9	9	9	9	9	1	9	9	9	9
2	3	3	1	2	3	2	1	1	2
1	3	3	3	3	1	1	3	1	3
3	3	2	1	2	3	2	3	1	2
3	3	2	3	1	2	2	3	1	3
2	2	2	2	2	2	2	2	2	2
3	1	1	2	2	9	2	1	3	3
3	3	1	1	3	1	9	3	3	3
3	3	3	3	3	1	3	3	1	3
9	9	9	1	3	1	1	3	1	3
3	3	2	2	3	1	1	3	2	2
9	9	9	9	1	9	9	9	9	9
2	2	2	2	2	2	2	2	2	2
3	3	2	2	2	2	2	2	2	2
2	2	1	2	2	2	2	2	2	2
2	2	1	2	1	1	2	3	2	2
2	2	3	2	2	1	2	3	2	2
2	2	1	2	1	2	2	2	2	2
9	9	9	3	1	3	3	3	3	3
2	2	2	3	3	3	3	1	2	2
2	2	3	3	1	2	1	2	2	3
9	9	9	9	1	9	9	9	1	9
9	9	9	9	9	1	9	9	9	9

1	1	2	2	2	2	1	3	2	1
3	3	3	3	3	1	3	3	3	2
3	3	3	3	3	1	3	3	1	3
9	9	9	1	9	9	9	9	1	9
9	1	3	1	9	9	9	2	2	9
3	2	3	3	3	1	1	1	2	1
3	3	2	3	2	1	1	3	3	1
2	2	3	2	9	9	9	9	9	9
9	9	9	2	9	9	9	9	2	9
2	2	2	1	9	9	9	9	1	2
2	2	2	2	2	2	2	2	2	2
1	1	1	1	9	9	9	9	3	3
3	3	2	3	1	2	3	3	9	1
3	1	9	3	9	1	9	2	3	9
9	9	1	9	9	9	1	9	1	9
2	2	1	3	2	1	3	3	1	1
9	1	9	9	1	9	9	9	9	9
9	1	9	3	1	2	9	3	3	1
3	3	1	3	3	3	3	3	2	3
2	1	2	2	2	2	2	2	2	1
3	3	1	3	3	2	2	1	3	3
3	3	3	1	3	3	2	3	3	2
3	3	2	3	3	3	3	3	3	3
3	3	3	2	2	2	2	2	3	3
2	2	2	1	2	2	2	2	2	2
9	9	9	9	9	9	9	9	9	9
3	1	1	1	9	9	9	9	1	3
2	1	1	3	3	1	3	3	1	3
3	3	3	3	2	2	2	2	3	3
2	2	1	9	9	1	9	9	2	3
9	9	9	9	9	9	9	9	9	9
9	9	9	9	9	9	9	9	9	9
9	9	9	9	9	9	9	9	9	9
9	9	9	9	9	9	9	9	9	9
9	9	9	9	9	9	9	9	9	9
9	9	9	9	9	9	9	9	9	9
9	9	9	9	9	9	9	9	9	9
9	9	9	9	9	9	9	9	9	9
9	9	9	9	9	9	9	9	9	9
9	9	9	9	9	9	9	9	9	9
9	9	9	9	9	9	9	9	9	9
9	9	9	9	9	9	9	9	9	9
9	9	9	9	9	9	9	9	9	9
9	9	9	9	9	9	9	9	9	9
9	9	9	9	9	9	9	9	9	9
9	9	9	9	9	9	9	9	9	9
9	9	9	9	9	9	9	9	9	9

9	9	9	9	9	9	9	9	9	9
9	9	9	9	9	9	9	9	9	9
9	9	9	9	9	9	9	9	9	9
9	9	9	9	9	9	9	9	9	9
9	9	9	9	9	9	9	9	9	9
9	9	9	9	9	9	9	9	9	9
9	9	9	9	9	9	9	9	9	9
9	9	9	9	9	9	9	9	9	9
9	9	9	9	9	9	9	9	9	9
9	9	9	9	9	9	9	9	9	9
9	9	9	9	9	9	9	9	9	9
9	9	9	9	9	9	9	9	9	9
9	9	9	9	9	9	9	9	9	9
9	9	9	9	9	9	9	9	9	9
9	9	9	9	9	9	9	9	9	9
9	9	9	9	9	9	9	9	9	9
9	9	9	9	9	9	9	9	9	9
9	9	9	9	9	9	9	9	9	9
9	9	9	9	9	9	9	9	9	9
2	2	2	2	2	1	2	2	2	2
9	9	9	9	9	9	9	9	9	9
9	9	9	9	9	9	9	9	9	9
9	9	9	9	9	9	9	9	9	9
9	9	9	9	9	9	9	9	9	9
9	9	9	9	9	9	9	9	9	9
9	9	9	9	9	9	9	9	9	9
9	9	9	9	9	9	9	9	9	9
9	9	9	9	9	9	9	9	9	9
9	9	9	9	9	9	9	9	9	9
9	9	9	9	9	9	9	9	9	9
9	9	9	9	9	9	9	9	9	9
9	9	9	9	9	9	9	9	9	9
9	9	9	9	9	9	9	9	9	9
9	9	9	9	9	9	9	9	9	9
9	9	9	9	9	9	9	9	9	9
9	9	9	2	2	9	9	9	2	2
1	1	1	1	3	3	3	3	1	3
3	3	3	1	1	2	1	3	1	2
3	3	2	3	1	2	3	3	1	3
3	3	3	3	3	3	3	3	3	3
3	3	3	3	3	3	3	3	1	3
3	1	1	2	1	3	3	3	1	2
9	9	9	2	9	9	9	9	2	9
3	1	1	2	1	3	3	3	1	2
2	1	3	2	2	1	2	3	2	2
1	3	1	1	2	3	1	2	1	2
3	3	3	3	2	2	2	2	2	2

3	2	3	1	1	3	3	3	3	3
1	1	1	3	3	1	3	3	1	2
9	9	9	1	1	2	3	3	1	2
3	3	3	3	1	3	3	3	2	2
3	3	3	2	2	2	1	2	2	2
1	1	3	2	1	1	3	3	3	3
2	1	3	2	2	1	2	2	3	2
2	2	2	3	2	1	3	3	3	3
2	2	2	3	2	1	2	2	2	3
1	2	3	3	2	1	2	1	2	1
9	9	9	9	9	1	9	9	9	9
3	2	2	2	2	2	2	3	1	2
3	3	3	1	2	3	2	3	1	2
2	2	2	2	2	2	2	1	2	1
2	2	2	2	2	2	1	3	2	1
3	3	3	3	3	1	3	3	2	2
2	2	3	3	3	2	1	2	2	2
2	2	2	1	2	3	1	2	1	2
1	3	1	1	1	1	3	3	9	1
1	1	1	1	1	1	1	1	1	2
9	9	9	9	2	9	9	9	1	2
2	2	1	3	2	1	2	3	2	2
3	2	3	3	3	2	1	3	3	1
9	9	9	9	9	1	9	9	9	9
2	2	2	3	1	3	3	3	3	3
1	2	1	3	3	3	1	2	3	3
2	1	1	2	2	2	2	2	1	1
3	2	1	1	3	1	3	1	3	2
2	2	2	2	2	2	2	2	1	2
2	2	2	2	2	2	2	2	2	2
2	3	1	2	9	9	9	9	9	9
2	2	2	1	2	2	2	2	2	2
2	2	2	2	2	2	2	2	2	2
9	9	9	9	9	1	9	9	9	9
9	9	9	9	9	1	9	9	9	9
2	2	2	9	9	1	9	9	9	9
1	2	2	9	9	9	9	9	9	9
2	2	2	2	2	2	2	2	2	1
2	2	1	2	2	2	2	2	9	9
9	9	9	9	9	9	9	9	9	9
1	3	2	3	3	1	1	2	3	2
2	3	2	2	2	1	1	3	2	2
3	3	3	1	1	1	1	3	1	1
3	3	1	3	3	1	1	3	1	3
1	1	1	1	1	1	1	1	1	1
3	3	1	3	1	3	3	3	1	3
1	1	1	3	1	1	1	3	3	1

2	2	2	3	2	1	1	1	1	1
2	3	2	2	3	1	3	2	1	2
2	2	1	1	2	3	1	2	1	2
3	1	1	1	1	9	9	9	1	1
2	2	2	1	2	3	2	1	1	1
3	3	2	3	2	3	3	3	1	2
3	1	1	1	1	1	3	3	1	1
3	2	1	1	2	3	2	3	3	1
3	3	3	2	1	2	2	3	1	1
1	1	1	1	9	9	9	9	1	9
3	3	3	2	3	1	3	3	1	3
1	3	1	1	1	3	3	2	1	3
1	1	1	2	2	2	2	2	1	1
1	3	1	1	1	3	3	2	1	3
1	1	1	1	1	1	1	1	3	1
3	3	3	3	1	1	1	1	1	3
3	2	3	2	2	2	3	3	1	2
1	9	9	9	1	9	9	9	9	9
2	2	1	1	2	2	3	1	1	2
9	9	9	9	1	9	9	9	9	9
1	3	2	2	3	3	2	2	1	3
2	3	1	9	9	1	9	9	2	2
1	3	1	1	3	2	1	1	9	3
2	3	1	3	3	1	2	2	1	3
3	3	1	1	3	1	1	2	1	2
3	3	3	1	3	3	3	3	3	3
1	1	1	1	1	1	1	3	1	1
3	1	3	1	3	3	1	3	1	1
3	1	1	1	1	1	3	3	3	1
3	2	1	3	2	2	1	2	1	2
3	3	3	2	3	3	1	3	1	1
2	3	1	1	2	3	3	2	1	3
3	3	1	1	3	3	3	3	3	3
3	3	1	1	3	3	3	3	3	3
3	1	1	1	1	1	9	3	1	1
3	3	1	3	3	1	3	3	1	3
3	2	1	1	2	3	3	2	1	2
9	9	9	9	9	9	9	9	9	9
3	3	9	3	3	3	1	3	1	3
2	3	1	3	1	1	2	3	1	2
3	3	1	1	1	1	1	3	1	3
2	2	2	1	1	1	1	1	1	1
3	3	1	3	1	1	1	3	1	3
3	1	1	2	3	3	3	3	3	2
2	2	2	2	2	2	2	2	2	2
3	2	3	1	3	3	2	2	1	1
3	9	9	3	3	2	9	3	3	3

1	2	3	3	2	1	1	2	1	1
9	9	9	9	9	9	9	9	1	3
3	3	3	2	2	1	3	3	2	3
2	2	2	2	2	2	2	2	1	1
2	2	2	1	1	2	2	2	2	2
2	2	3	2	2	2	1	1	2	2
2	2	2	2	2	2	2	2	2	2
2	2	2	3	1	2	2	2	9	9
9	9	9	9	9	9	9	9	9	9
2	3	1	1	2	2	2	3	1	1
2	2	2	2	2	2	2	2	2	2
2	2	2	2	2	2	2	2	2	2
2	2	2	2	2	2	2	2	2	2
2	2	2	1	1	2	2	2	1	1
2	2	2	2	2	2	2	2	2	2
2	2	2	2	2	2	2	2	2	2
2	2	2	2	2	2	2	2	2	2
2	2	2	2	2	2	2	2	2	2
2	2	2	1	2	2	2	2	2	2
2	2	2	2	1	2	2	2	1	2
2	2	3	2	2	2	1	1	2	2
2	2	2	2	2	2	2	2	2	2
2	1	2	2	2	2	2	1	2	2
2	2	2	2	2	2	2	2	2	2
2	2	2	2	2	2	2	2	2	2
2	2	2	2	2	2	2	2	2	2
2	2	2	2	2	2	2	2	2	2
1	1	1	2	2	1	2	2	1	2
2	9	1	1	2	2	3	1	1	1
2	2	2	2	2	2	2	2	2	2
2	2	2	2	2	2	2	2	2	2
2	2	2	2	2	2	2	2	2	2
2	2	2	2	2	2	2	2	2	2
2	2	2	2	2	2	2	2	2	2
2	2	2	2	2	2	2	2	1	2
2	2	2	2	2	2	2	2	2	2
2	2	2	2	2	2	2	2	2	2
2	2	2	2	2	2	2	2	2	2
2	2	2	2	2	2	2	2	2	2
1	1	1	3	3	1	3	1	1	3
2	2	2	2	2	2	2	2	2	2
2	2	2	9	9	9	9	9	2	2
3	2	2	1	2	3	3	3	2	1
2	2	2	2	2	2	2	2	2	2
2	3	2	1	2	9	9	9	1	9
2	2	2	2	2	2	2	2	3	3
2	2	2	2	2	2	2	2	2	2

3	2	2	3	3	2	1	2	2	2
2	2	3	3	3	1	2	1	2	2
2	2	2	2	2	2	2	2	2	2
2	2	2	2	2	2	2	2	2	2
2	1	2	1	2	3	2	1	1	2
2	2	2	2	2	2	2	2	2	2
9	9	9	9	9	9	9	9	3	3
2	3	2	1	2	3	1	2	1	1
9	9	9	9	9	1	9	9	1	9
2	2	2	2	2	2	2	2	2	2
2	2	2	2	2	2	2	2	3	3
2	2	2	2	2	1	2	2	1	1
3	2	3	3	3	1	2	3	3	3
9	9	9	3	9	9	9	9	3	9
9	9	1	2	2	2	2	1	2	2
1	2	2	2	1	2	2	2	2	1
2	2	2	2	2	1	2	2	1	2
1	1	1	1	1	9	2	1	1	1
9	9	9	9	9	1	9	9	1	9
2	2	2	2	2	1	2	2	1	2
3	1	1	2	1	9	2	1	1	2
9	9	9	9	9	1	9	9	9	1
9	9	9	9	9	1	9	9	1	9
9	9	9	3	3	9	2	3	2	2
1	2	1	3	1	3	1	2	1	2
2	1	2	3	2	2	2	3	2	1
1	1	3	3	3	2	1	2	2	1
2	1	1	3	1	3	9	1	1	1
3	2	3	3	3	3	1	1	1	2
9	9	9	1	9	9	1	9	1	1
9	9	9	9	9	9	9	9	3	3
2	2	2	2	2	2	2	2	2	2
2	2	2	2	2	2	2	2	2	2
9	9	9	2	9	2	9	9	1	9
1	1	1	9	9	9	9	9	1	3
9	9	9	9	9	1	9	9	9	9
1	1	1	3	1	3	1	3	1	3
2	2	2	2	2	2	2	2	3	3
2	2	2	2	2	2	2	2	2	2
2	2	2	2	2	2	2	2	2	2
2	3	1	2	3	9	1	2	2	9
2	2	2	2	2	2	2	2	1	2
9	9	2	9	1	9	9	9	9	9
1	1	1	1	1	1	1	1	3	3
1	1	1	3	3	1	3	3	1	3
2	3	2	2	2	2	2	2	3	2
9	9	9	9	9	9	9	1	2	2

1	1	1	1	1	1	1	1	1	1
2	2	2	2	2	1	2	2	2	2
1	1	1	1	1	1	1	1	1	1
2	2	2	2	2	2	2	2	2	2
9	9	9	9	9	2	9	9	2	9
1	2	3	2	1	2	1	3	1	2
3	3	3	3	3	3	3	3	3	9
1	1	1	1	1	1	1	1	1	1
2	2	2	2	2	1	1	1	1	2
1	1	1	3	1	1	1	1	1	1
2	2	2	2	2	2	2	2	2	2
1	1	1	1	1	1	1	1	2	2
2	2	2	2	2	2	2	2	2	2
2	2	2	2	2	2	2	2	2	2
2	2	2	2	2	2	2	2	2	2
2	2	2	2	2	2	2	2	2	2
1	1	1	1	1	1	1	1	1	1
3	2	3	3	1	2	2	2	2	3
9	9	9	9	9	9	9	9	9	9
1	2	1	1	2	1	2	1	1	2
2	1	1	1	1	2	2	3	1	1
3	3	3	3	3	1	3	3	1	3
3	3	3	3	3	3	3	3	3	3
3	3	1	2	2	1	1	3	2	2
3	3	3	3	3	3	3	3	3	3
2	1	2	1	2	2	1	2	2	2
1	1	1	2	1	1	1	1	1	2
2	2	2	2	2	2	2	2	2	2
9	9	9	2	9	9	9	9	3	2
2	1	1	2	2	2	2	2	2	2
3	3	3	2	2	2	2	2	1	1
3	3	3	1	2	3	2	2	2	2
3	3	3	3	3	3	3	3	3	3
1	2	3	2	1	3	2	1	1	1
2	2	2	2	2	2	2	2	2	2
3	3	2	2	2	2	2	2	2	2
1	2	3	1	2	3	2	1	2	3
2	2	2	2	2	2	2	2	3	3
3	3	2	1	2	3	1	2	1	2
3	3	3	3	3	3	3	3	3	3
3	3	1	1	2	3	1	2	1	1
2	1	2	1	2	2	3	1	1	3
9	9	9	9	9	9	9	9	9	9
3	3	3	3	3	3	3	3	3	3
1	1	2	2	2	3	1	1	1	1
2	2	2	2	2	2	2	3	2	2
3	2	2	1	2	3	3	3	1	2

1	1	1	2	2	2	2	2	1	1
3	3	3	3	3	1	3	3	1	3
3	3	3	2	2	2	2	2	3	3
3	3	3	3	3	1	3	3	1	3
2	1	2	3	1	2	2	3	3	1
2	2	2	2	2	2	2	2	2	2
2	3	2	2	2	2	2	2	2	2
2	2	2	2	2	2	2	2	2	2
9	9	9	9	9	9	9	9	2	2
1	3	3	1	3	2	1	3	1	1
2	2	2	2	2	2	2	2	2	2
3	2	1	1	3	1	2	3	3	2
2	2	2	2	2	2	2	2	2	2
1	3	3	3	3	1	1	3	9	9
2	2	2	2	2	2	2	2	2	2
2	2	2	2	2	2	2	2	2	2
2	2	2	2	2	2	2	2	2	2
3	3	3	2	1	1	2	3	2	2
3	1	2	2	2	2	2	2	2	2
9	9	9	3	2	2	2	1	2	1
2	2	1	3	3	1	2	3	1	2
2	1	2	2	1	3	2	1	2	1
2	1	2	2	1	3	2	1	2	1
2	2	1	2	2	2	2	2	2	2
1	1	1	1	1	1	1	1	1	1
9	9	9	9	9	9	9	9	1	1
1	3	3	3	2	1	1	3	1	1
3	3	3	3	2	3	1	1	3	3
9	2	3	9	9	1	9	9	1	3
2	3	3	2	1	2	3	3	1	2
1	3	1	3	1	1	1	1	9	1
9	9	9	9	9	9	9	9	2	2
2	2	2	2	2	2	2	2	1	2
2	3	3	2	3	2	3	2	3	3
2	1	3	2	2	1	3	1	1	1
1	2	9	2	2	2	1	2	2	2
1	1	1	2	2	2	2	2	2	2
2	2	2	9	9	9	9	9	9	9
2	2	1	1	2	2	2	2	2	2
3	3	2	3	2	2	3	3	3	2
3	3	2	3	2	9	3	3	3	2
2	2	2	2	2	2	2	2	2	2
2	2	2	2	2	2	2	2	2	2
2	2	2	2	2	2	2	2	2	2
9	1	3	3	2	9	3	2	2	9
2	1	2	9	9	9	9	9	3	1
2	3	3	2	2	2	2	2	2	1

9	9	9	9	9	9	9	9	9	9
3	1	3	1	2	2	9	2	1	2
2	1	2	3	1	2	2	3	1	1
3	3	3	3	3	1	3	3	1	3
2	2	3	2	3	9	1	3	1	3
3	3	2	2	3	2	2	2	3	2
3	1	2	3	3	3	1	1	1	2
1	1	2	2	2	2	2	2	2	1
2	2	1	2	1	2	2	2	3	3
3	3	3	3	2	1	3	3	3	3
3	3	2	2	2	3	3	3	1	2
3	1	2	3	3	3	1	3	1	3
2	2	3	2	2	2	2	2	2	2
1	1	1	9	9	1	9	9	1	2
9	1	2	3	2	2	1	3	1	2
2	2	2	2	2	2	2	2	2	2
1	2	1	1	2	3	2	3	1	2
3	3	1	3	3	3	3	3	1	1
3	3	3	3	2	9	1	9	9	9
3	3	3	2	2	2	2	2	2	2
9	9	9	9	9	9	9	9	9	9
2	1	3	3	2	1	3	1	1	2
1	1	2	1	2	2	2	2	1	3
2	2	2	1	1	2	2	3	1	1
2	3	3	3	3	1	2	1	2	2
3	3	3	3	2	2	2	2	3	3
3	2	3	9	9	9	9	1	3	1
1	3	2	1	1	2	1	1	1	1
1	3	2	2	2	1	3	3	3	1
1	2	1	1	1	2	2	2	1	2
2	1	2	1	2	3	2	2	2	2
3	2	2	3	2	2	1	1	2	2
3	3	3	3	2	3	3	3	1	3
2	2	2	2	2	2	2	2	2	2
2	2	2	2	2	2	2	2	2	2
2	2	2	2	2	2	2	2	2	2
3	3	3	3	3	3	3	3	3	3
2	2	2	3	3	1	2	2	1	2
2	1	1	2	3	2	1	2	3	2
2	1	1	2	3	2	1	2	3	2
1	1	1	2	2	2	2	2	3	3
3	2	1	2	2	2	2	2	2	2
2	1	3	2	3	1	1	2	1	2
2	2	2	2	2	2	2	2	2	2
9	9	9	3	3	3	1	2	1	9
3	3	3	3	3	3	3	3	3	3
3	3	3	2	2	2	3	1	2	2

2	3	2	2	1	1	3	2	2	3
3	3	3	3	3	3	1	3	1	1
1	2	2	2	2	3	1	2	1	2
9	9	9	9	9	9	9	9	9	9
3	2	3	2	3	2	3	2	2	3
3	3	3	3	3	3	3	3	3	3
3	1	1	1	1	1	1	1	1	1
2	2	2	3	3	3	3	3	3	3
3	1	2	2	3	3	2	1	1	2
3	1	2	2	3	1	9	3	1	2
2	2	2	2	2	2	2	2	2	2
3	3	3	3	3	3	1	2	1	3
1	1	1	1	1	1	1	1	1	1
9	9	9	9	9	1	9	9	1	9
9	1	1	1	1	1	1	1	2	1
2	2	2	3	3	3	3	3	2	3
3	1	1	1	1	1	1	1	3	3
2	2	2	1	9	1	9	1	2	2
9	2	2	1	2	3	2	3	1	1
1	3	1	2	2	3	2	3	1	1
2	3	9	3	1	2	3	2	1	3
1	1	2	2	2	2	3	3	3	3
3	3	3	2	2	2	2	2	2	2
1	2	1	2	2	1	3	3	2	2
1	2	3	1	2	3	1	2	1	2
3	3	3	2	2	1	2	1	3	2
1	2	1	3	3	3	1	3	2	1
3	3	3	3	3	2	1	2	1	3
3	2	1	1	2	3	1	2	1	3
2	2	2	2	2	2	2	2	2	2
1	1	2	3	3	2	2	3	1	1
2	2	2	1	1	1	1	1	2	2
2	3	2	1	3	3	3	3	1	2
3	1	2	1	3	3	1	1	1	2
1	3	2	3	1	1	3	3	2	3
3	3	1	1	3	3	3	3	3	3
3	2	3	1	1	1	3	3	1	3
3	3	1	3	3	3	3	3	3	3
3	1	1	1	3	3	3	3	3	1
3	2	3	1	1	2	2	2	1	3
2	2	2	2	2	2	2	2	2	1
3	1	3	3	1	3	3	3	3	1
2	3	2	3	2	1	3	2	1	2
2	2	1	1	1	2	3	2	3	2
9	9	9	9	9	9	9	9	1	2
2	3	2	3	3	3	3	3	2	1
1	9	3	1	3	1	3	1	1	3

2	3	2	2	1	2	2	2	1	1
2	3	1	1	2	3	2	3	1	2
2	2	2	2	2	2	2	2	2	2
3	1	1	1	1	1	1	1	1	1
2	1	3	1	2	3	1	2	1	2
3	2	2	3	1	2	2	2	1	1
2	3	1	1	1	2	3	3	1	3
3	3	2	3	2	1	2	1	2	3
1	2	1	1	2	1	2	1	1	2
1	3	1	3	2	1	3	3	3	1
1	2	3	1	2	3	2	3	1	2
3	3	1	3	3	9	1	2	1	3
1	1	1	3	1	1	1	1	1	1
3	1	1	3	1	3	1	3	3	3
2	3	2	3	2	2	2	3	1	2
1	1	1	3	3	1	1	1	9	1
2	3	1	2	2	2	1	3	1	1
3	3	3	3	3	3	3	3	1	3
3	3	3	3	1	2	3	3	1	1
9	1	2	3	3	3	1	2	1	3
2	3	2	2	2	2	2	2	1	2
2	3	1	3	1	1	1	3	3	1
9	3	3	3	1	3	1	3	3	1
2	2	1	2	2	2	2	2	1	2
1	1	2	2	1	1	3	3	2	2
2	2	2	2	2	2	2	2	2	2
3	3	3	1	3	1	3	2	1	1
1	9	9	1	3	1	2	2	2	1
2	2	2	3	2	2	2	3	2	2
9	9	9	9	3	9	9	9	9	9
3	3	1	9	9	9	9	9	9	9
9	9	9	9	9	9	9	9	9	9
2	2	2	1	1	2	2	2	1	2
9	9	9	9	9	1	9	9	9	1
2	2	2	3	3	3	3	3	3	3
1	1	1	2	2	2	2	2	2	2
3	3	2	3	3	2	2	2	3	3
9	9	9	1	9	9	9	9	2	2
9	9	9	3	3	3	2	2	3	2
2	3	3	2	2	2	2	2	3	2
3	2	1	2	1	3	2	3	1	3
1	2	1	1	2	1	1	3	2	1
3	3	1	3	2	2	1	1	1	3
3	2	2	1	1	2	1	1	1	3
1	2	2	1	2	1	2	3	1	2
2	2	2	1	2	1	2	1	1	1
3	3	3	1	3	3	3	2	1	1

9	9	9	9	9	1	9	9	2	2
2	1	1	1	1	1	1	1	1	2
3	2	3	3	2	2	2	3	2	2
1	2	1	2	1	1	3	3	2	1
2	2	2	3	3	2	2	3	2	2
3	3	2	3	2	3	3	3	3	3
3	3	3	9	9	9	1	9	1	2
2	3	3	3	2	3	1	1	1	3
3	2	1	3	2	2	1	2	1	3
1	3	3	3	2	3	1	3	1	3
1	2	3	2	3	2	1	3	3	1
2	1	1	3	2	1	1	3	3	1
1	3	2	1	1	1	1	1	1	1
2	2	2	2	2	2	2	2	2	2
2	2	2	2	2	2	2	2	2	2
3	2	2	9	9	9	9	9	2	3
1	2	1	3	2	2	2	2	2	2
2	2	2	2	2	2	2	2	2	2
2	2	2	2	2	2	2	2	2	2
3	3	1	3	2	3	1	2	1	2
9	9	9	1	2	3	9	9	9	9
9	9	9	1	9	9	9	9	9	9
9	9	9	1	9	9	9	9	9	9
2	2	1	1	1	2	2	2	1	1
3	3	3	1	3	3	3	3	3	3
9	9	9	1	9	9	9	9	9	9
2	2	1	1	9	9	9	9	9	9
2	2	2	1	2	2	2	2	1	2
2	2	2	2	2	2	2	2	1	2
9	9	9	9	9	9	9	9	9	9
9	9	9	1	9	9	9	9	9	9
9	9	9	9	1	9	9	9	1	9
2	1	1	1	3	3	3	3	2	2
3	3	3	1	3	3	3	3	1	3
9	9	9	1	9	9	9	9	9	9
2	3	3	1	9	9	9	9	1	3
9	9	9	1	1	9	9	9	9	9
9	9	9	1	9	9	9	9	9	9
9	9	9	1	9	9	9	9	9	9
3	3	9	1	3	3	3	3	9	9
3	3	1	1	3	3	3	3	1	3
9	1	9	1	9	9	9	9	1	9
9	9	9	1	9	9	9	9	9	9
3	9	9	1	9	9	1	9	9	9
9	9	9	1	9	9	9	9	9	9
1	2	1	1	1	1	3	3	1	3
3	2	2	1	2	2	2	2	3	2

9	9	1	1	9	9	9	9	9	9
3	3	2	1	2	2	2	2	2	2
3	3	3	1	2	2	2	2	1	2
1	1	1	1	1	1	1	1	1	1
9	9	9	1	9	9	9	9	9	9
9	9	9	1	9	9	9	9	9	9
1	1	1	1	9	9	9	9	1	1
2	2	2	2	2	1	2	2	2	2
9	9	9	1	9	9	9	9	9	9
9	9	9	1	9	9	9	9	9	9
2	2	2	1	9	9	9	9	2	2
2	2	2	1	9	9	9	9	2	2
9	9	9	1	9	9	9	9	9	9
9	9	9	1	9	9	9	9	9	9
9	9	9	1	9	9	9	9	1	9
9	9	9	1	9	9	9	9	9	9
9	9	9	1	9	9	9	9	9	9
9	9	9	1	9	9	9	9	9	9
9	9	9	1	9	9	9	9	9	9
3	3	3	1	9	9	9	9	1	9
9	9	9	1	9	9	9	9	1	9
9	9	9	1	9	9	9	9	9	9
3	3	3	3	3	3	3	3	1	1
9	9	9	1	9	9	9	9	9	9
2	2	1	1	9	9	9	9	1	1
3	3	9	3	3	9	9	1	3	3
3	3	3	1	3	3	3	3	3	3
9	9	9	1	9	9	9	9	9	9
3	3	3	1	3	3	3	3	9	9
3	3	1	1	9	9	9	9	9	9
2	1	1	1	2	2	2	2	2	2
3	3	1	1	3	3	3	3	3	3
9	9	9	1	9	9	9	9	9	9
9	9	9	9	9	9	9	9	9	9
9	9	9	1	9	9	9	9	9	9
3	3	3	1	3	3	3	3	3	3
9	9	9	1	9	9	9	9	9	9
9	9	9	1	9	9	9	9	9	9
3	3	3	1	3	3	3	3	9	9
3	3	3	1	2	2	1	2	1	3
3	3	3	3	1	1	1	3	1	1
3	3	3	1	1	3	3	3	1	2
9	9	9	1	9	9	9	9	9	9
9	9	9	1	9	9	9	9	9	9
9	9	9	9	9	9	9	9	9	9
3	2	3	1	9	9	9	9	9	9
3	9	9	1	9	9	9	9	9	9

3	1	3	1	9	9	9	9	9	9
3	3	1	3	3	3	3	1	3	3
3	2	3	1	9	9	9	9	9	9
3	1	1	1	9	9	9	9	1	3
9	9	9	1	9	9	9	9	9	9
9	9	9	1	9	9	9	9	9	1
3	1	1	3	1	1	1	3	3	3
3	3	3	3	3	1	3	3	9	9
3	3	3	3	3	1	3	3	9	9
3	3	3	3	3	1	3	3	3	3
3	3	3	2	2	1	1	1	1	1
3	3	3	9	9	9	1	9	9	1
9	9	9	9	9	9	9	1	9	9
2	3	3	3	2	2	1	2	2	1
2	2	2	2	2	2	2	2	1	1
2	2	2	3	3	1	1	2	2	1
9	9	9	9	9	9	1	9	9	9
9	9	9	9	9	9	1	9	9	9
2	9	3	1	1	1	1	2	1	2
2	3	2	1	1	1	1	2	1	1
3	3	2	3	1	1	3	3	1	3
2	2	2	2	1	3	2	3	2	2
3	3	3	3	3	3	2	1	3	2
3	3	3	3	3	3	1	3	1	3
3	3	3	3	3	3	2	1	9	9
3	3	3	9	9	1	9	9	2	2
3	2	3	2	3	1	1	2	2	3
9	1	2	3	2	2	3	3	3	3
3	3	3	3	2	3	3	3	2	1
2	1	1	2	2	2	1	1	1	2
2	2	2	2	2	1	2	2	1	2
2	2	3	2	1	1	1	3	1	2
2	1	3	2	2	1	2	1	3	2
9	9	9	9	9	9	1	9	9	9
3	3	3	3	3	3	2	1	3	2
3	2	1	3	3	2	2	1	1	2
9	9	9	9	1	9	9	9	2	1
9	9	9	9	1	9	9	9	2	1
9	9	9	9	1	9	9	9	2	1
9	1	3	1	1	3	2	3	1	1
3	3	3	3	3	3	3	1	3	3
3	3	3	1	1	3	3	3	3	3
3	3	2	2	2	2	3	3	2	1
2	2	2	2	2	2	2	2	1	1
3	3	3	2	2	2	3	3	2	9
9	9	9	9	9	3	9	9	9	9
3	3	3	9	9	9	1	9	2	9

9	9	9	1	9	9	9	9	9	9
2	3	2	3	2	2	1	2	1	2
3	3	1	2	2	2	2	1	2	2
2	2	2	2	2	2	2	1	1	2
2	9	3	3	3	2	1	1	1	1
3	1	9	3	3	3	3	3	1	2
9	9	9	2	1	2	2	2	1	2
3	3	1	3	3	1	3	3	1	2
9	9	9	9	9	9	9	1	9	2
3	3	3	9	9	1	9	9	1	9
3	3	3	2	2	2	2	3	3	2
3	3	3	3	1	3	3	3	3	3
3	3	3	3	1	2	3	3	3	9
2	3	3	9	1	9	9	9	3	3
9	9	9	9	1	9	9	9	3	2
9	9	9	9	1	9	9	9	9	9
2	3	3	1	1	3	3	3	1	2
2	2	2	2	1	2	2	2	2	9
3	3	3	3	1	3	3	3	3	3
3	1	3	9	1	9	9	9	9	9
9	9	9	9	1	9	9	9	3	9
9	9	9	1	9	9	9	9	1	9
2	2	3	9	1	9	9	9	2	2
3	3	1	1	3	3	3	3	3	3
2	9	3	2	1	3	3	3	9	3
2	9	3	2	1	3	3	3	9	3
9	9	9	9	1	9	9	9	9	9
9	9	9	9	9	9	9	9	9	9
9	9	9	1	9	9	9	9	9	9
2	2	9	1	1	1	3	2	2	3
9	9	9	3	1	3	3	3	9	9
9	2	3	2	1	3	3	3	3	2
9	9	9	1	9	9	9	9	9	9
3	3	3	1	9	3	9	9	3	9
3	3	3	1	2	2	2	2	3	2
9	9	9	1	9	9	9	9	1	9
3	3	3	1	1	1	1	3	2	2
3	3	3	1	3	3	3	3	1	3
3	3	3	1	1	3	3	3	3	3
3	3	3	1	1	1	1	3	2	2
1	3	1	1	9	9	9	9	1	2
9	9	9	1	9	9	1	9	9	9
3	3	3	1	3	3	3	3	3	1
9	9	9	1	9	9	1	9	9	9
3	3	3	1	3	3	3	3	3	3
9	9	9	9	1	9	9	9	9	9
9	9	9	1	9	9	9	9	9	9

3	3	3	1	3	3	3	3	3	3
9	9	9	1	9	9	9	9	9	9
9	9	9	9	9	9	9	9	9	9
9	9	9	9	9	9	9	1	1	9
9	9	9	9	1	9	9	9	1	2
3	3	1	3	3	1	1	3	2	2
2	1	2	2	1	2	9	2	9	9
9	9	9	9	1	9	9	9	9	9
9	9	9	9	9	9	9	9	9	9
9	9	9	9	1	9	9	9	9	9
3	3	3	1	3	3	3	3	1	3
9	9	9	1	9	9	9	9	2	2
3	3	3	1	2	2	2	3	3	9
9	9	9	9	1	9	9	9	9	9
1	1	1	9	1	2	1	1	1	3
3	3	3	3	1	2	3	3	3	2
9	9	9	9	1	9	9	9	9	9
9	9	9	9	1	9	9	9	9	1
9	9	9	9	1	9	9	9	9	9
3	3	3	3	1	3	3	3	3	3
3	3	1	1	3	3	9	3	3	3
9	9	9	9	1	9	9	9	9	9
9	9	9	9	1	9	9	9	9	9
3	3	3	1	9	9	9	9	1	3
9	9	9	9	1	9	9	9	9	1
3	1	1	2	1	1	3	1	2	9
3	3	3	9	1	9	9	9	9	9
3	1	3	2	1	2	3	3	1	2
1	1	2	9	1	9	9	9	1	1
3	3	3	1	1	3	3	3	1	2
3	3	3	2	3	3	3	3	9	1
1	1	2	3	1	3	3	3	2	9
3	2	2	1	1	1	1	1	1	1
3	3	3	3	1	3	3	3	3	3
3	3	3	1	2	1	1	3	2	2
9	9	9	9	1	9	9	9	9	9
9	9	9	9	1	9	9	9	9	9
9	9	9	9	1	9	9	9	9	9
3	3	1	3	1	2	3	3	3	3
3	1	1	3	1	3	3	3	1	3
3	2	2	2	1	2	3	3	2	3
2	3	3	2	1	3	3	3	1	3
9	9	9	9	9	1	9	9	9	9
9	9	9	9	1	1	9	9	9	9
9	9	9	1	9	9	9	9	1	9
3	3	3	1	9	9	9	9	1	9
3	2	1	9	1	9	9	9	9	9

3	3	1	3	1	3	3	3	3	3
1	2	1	9	1	9	9	9	1	9
9	9	9	9	1	9	9	9	9	9
3	3	2	2	1	1	2	2	2	1
3	3	3	9	1	9	9	9	3	3
9	9	9	9	1	9	9	9	1	9
2	2	2	2	1	2	2	3	3	2
2	2	1	2	1	2	2	2	1	2
3	3	3	3	1	3	2	3	2	2
2	3	2	3	1	2	2	3	2	1
2	2	3	3	1	2	2	3	3	2
3	3	3	9	1	9	9	9	9	1
9	9	9	9	1	9	9	9	9	9
9	9	9	9	1	9	9	9	9	9
9	9	9	9	1	9	9	9	9	9
9	9	9	9	1	9	9	9	9	9
3	3	3	3	1	3	3	3	3	3
2	1	3	2	1	2	2	2	3	3
3	3	3	9	9	9	9	9	9	1
9	9	9	9	1	9	9	9	3	3
3	3	3	2	1	3	3	3	2	1
2	2	2	3	1	2	9	9	3	2
3	3	2	2	1	1	2	2	2	2
3	3	3	1	1	2	2	3	2	3
2	2	2	3	1	3	3	3	3	3
9	9	9	9	1	9	9	9	9	9
3	3	3	3	1	1	1	3	3	3
3	3	3	2	1	2	2	2	3	3
3	3	3	1	1	9	9	9	9	9
9	9	9	9	1	9	9	9	9	9
3	3	3	3	1	3	3	3	2	2
9	9	9	9	1	9	9	9	9	9
9	9	9	9	1	9	9	9	9	9
3	2	1	2	1	2	2	3	1	2
3	3	3	2	1	2	2	1	9	9
3	1	1	2	1	2	2	2	3	3
3	3	2	2	1	3	3	3	2	2
3	3	3	3	3	1	2	2	2	2
9	9	3	9	9	9	1	9	3	9
9	9	9	1	9	9	9	9	9	9
3	3	3	3	2	2	3	2	3	2
3	2	3	2	2	2	2	2	2	3
9	9	9	9	9	9	1	9	9	9
1	2	1	2	2	2	1	1	1	1
3	2	2	1	1	2	3	2	2	1
9	9	9	9	1	9	9	9	9	9
3	3	1	3	3	1	1	3	3	2

3	3	3	3	3	1	3	3	3	3
2	2	2	3	2	1	2	2	3	3
2	2	2	3	3	2	1	2	1	2
2	2	2	2	2	1	3	3	2	2
2	2	1	3	2	1	2	3	2	2
1	2	2	3	3	1	3	3	3	1
3	2	1	1	1	3	1	2	1	3
2	2	2	2	2	1	2	3	2	2
2	3	3	2	1	1	2	2	2	3
3	3	3	3	3	2	1	2	1	2
2	2	3	2	2	1	2	2	1	1
2	2	2	9	2	9	9	9	2	2
3	3	3	3	2	1	2	2	3	1
2	2	2	2	2	2	2	2	1	1
2	2	2	2	2	2	2	2	1	2
2	2	1	3	2	2	1	1	2	2
2	2	2	2	2	2	2	2	1	2
2	2	2	2	2	2	2	2	2	2
3	3	3	2	2	2	2	2	2	2
2	3	2	3	1	2	2	3	1	1
2	3	3	2	3	1	2	1	2	3
3	3	2	2	1	2	2	1	1	2
9	9	9	9	9	1	9	9	1	2
2	2	3	1	1	2	3	3	1	3
1	1	2	2	2	1	1	1	2	2
9	9	9	9	9	9	9	9	1	2
1	1	1	2	3	3	3	3	1	2
1	1	2	9	1	9	9	9	2	2
1	1	2	9	9	1	9	9	2	2
2	9	3	1	9	2	9	3	1	1
2	2	3	2	3	1	1	2	2	1
2	2	1	2	1	2	2	2	2	2
2	3	1	2	2	1	2	1	2	1
2	3	1	2	2	1	2	1	2	1
9	9	9	9	9	9	1	9	9	9
9	1	9	9	9	9	9	1	9	1
1	9	2	1	9	9	9	9	1	9
9	9	9	1	9	9	9	9	1	9
9	9	9	9	1	9	9	9	9	1
1	1	9	9	9	1	9	9	1	2
2	3	2	1	2	2	3	3	1	2
9	9	9	9	9	9	9	1	1	9
9	9	9	9	9	9	9	1	1	9
3	3	2	2	1	2	2	2	1	2
9	9	9	1	9	9	9	9	1	9
9	9	9	9	1	9	9	9	1	9
9	9	9	9	1	9	9	9	1	9

3	3	3	1	1	1	3	3	1	2
3	1	2	1	3	2	1	2	1	3
9	9	9	9	1	9	9	9	1	9
3	2	3	3	2	1	2	3	1	9
2	2	1	2	2	1	2	3	1	9
9	9	9	9	9	1	9	9	2	9
9	9	9	9	9	1	9	9	9	9
9	9	9	9	9	1	9	9	9	9
9	9	9	9	9	1	9	9	9	9
9	9	9	1	9	9	9	9	1	9
3	3	3	2	1	2	3	3	3	3
2	3	3	3	1	3	3	3	1	3
3	3	3	2	1	3	3	3	1	3
3	3	3	1	2	2	2	2	3	3
3	3	3	3	3	1	3	3	1	1
3	3	3	3	3	3	1	3	3	3
3	3	3	3	3	3	1	3	3	3
3	3	3	3	3	3	1	3	3	3
2	3	3	3	1	3	3	3	1	3
2	3	1	2	1	1	2	1	2	2
2	2	2	1	1	2	2	2	1	1
2	2	1	2	2	2	2	2	1	3
9	9	9	9	9	1	9	9	9	9
1	2	1	1	2	3	2	1	1	1
3	3	3	3	2	3	3	3	3	3
3	2	3	3	1	1	1	2	3	3
3	3	3	9	9	9	9	9	9	1
3	3	3	2	2	2	2	2	2	1
3	3	3	3	3	1	3	3	2	3
2	2	2	9	9	9	9	9	9	1
3	3	3	2	2	1	1	3	3	2
2	2	2	3	3	1	2	2	9	9
3	3	3	3	1	1	3	3	1	3
2	3	3	2	2	1	2	2	3	3
3	3	3	2	3	1	3	3	1	1
3	2	3	3	1	1	1	2	1	3
3	3	3	1	3	3	3	3	3	3
3	3	3	3	1	3	3	3	3	3
3	3	3	3	3	1	3	3	3	3
3	2	3	2	1	1	2	3	3	1
3	3	3	1	9	9	9	9	3	2
3	3	3	3	1	1	1	3	1	1
2	2	3	3	2	1	2	3	3	3
3	3	3	3	9	1	3	3	1	3
3	3	3	3	9	1	3	3	1	3
3	3	3	3	3	1	3	3	1	3
2	3	3	1	2	1	1	2	1	1

2	3	2	1	2	2	3	2	2	3
1	2	1	1	1	1	2	3	1	1
2	2	1	2	2	2	2	2	1	1
3	3	3	3	3	1	3	3	1	3
3	3	3	3	3	1	3	3	1	3
3	3	3	3	3	1	3	3	1	3
2	3	3	1	3	1	3	3	1	1
3	3	3	3	2	1	3	3	1	1
3	2	2	2	1	1	3	3	3	2
9	9	9	9	9	9	1	9	1	1
2	1	9	1	1	3	3	2	3	1
9	9	9	9	9	9	1	9	1	9
2	1	1	2	2	1	2	2	1	2
2	2	2	3	3	1	2	2	1	2
3	1	1	3	2	1	1	3	3	1
2	2	2	2	2	2	2	2	2	2
1	2	2	2	1	9	1	9	1	1
2	2	2	3	3	1	2	2	1	2
1	1	3	1	3	2	1	3	1	3
1	3	1	1	1	3	2	2	1	1
3	3	1	1	3	3	3	3	1	2
3	3	3	1	3	1	1	3	1	1
2	2	9	2	1	2	1	3	2	3
2	3	1	2	1	2	2	3	2	3
2	3	1	3	1	2	2	3	3	2
2	2	3	2	1	1	3	2	2	2
3	2	1	3	2	3	3	3	1	2
2	2	2	1	2	2	2	2	2	2
3	2	2	1	2	2	3	3	1	2
2	2	2	2	2	1	2	2	2	2
2	2	3	2	2	2	2	2	2	3
2	2	3	3	3	1	1	2	2	2
3	3	3	2	2	9	1	2	9	9
9	3	2	1	3	1	1	3	3	1
2	2	9	2	1	2	1	3	2	3
3	3	3	3	3	1	3	3	1	3
3	3	3	3	3	1	2	3	3	2
3	3	3	2	1	1	2	3	3	2
9	9	9	9	9	9	1	9	1	9
3	2	2	3	3	1	3	2	2	2
3	3	3	1	2	2	2	2	1	2
9	9	9	9	9	9	9	9	1	1
3	1	1	1	3	2	1	3	1	1
3	3	2	3	3	1	3	3	3	1
3	3	3	3	3	2	2	2	2	2
3	3	3	3	3	3	3	2	2	2
2	1	3	3	3	1	3	3	2	1

2	1	3	3	1	1	1	2	2	1
2	2	2	2	2	2	2	2	2	2
3	3	2	3	3	1	3	3	3	1
2	3	3	2	2	2	2	1	2	2
3	2	3	3	3	1	1	2	1	2
3	3	2	2	3	3	3	3	1	1
3	2	9	9	9	9	1	9	1	9
3	3	3	3	3	2	2	2	2	2
2	2	2	1	1	2	2	2	1	2
3	3	2	3	3	1	2	2	2	2
3	3	3	2	3	3	2	1	1	1
2	3	3	3	1	3	3	3	2	1
2	3	3	9	9	1	9	9	2	9
2	2	2	1	2	3	3	3	1	2
3	3	3	3	1	2	3	3	1	3
2	2	2	2	2	2	2	2	2	2
1	3	1	3	1	2	1	2	2	2
3	3	3	1	2	2	3	3	1	2
3	3	3	1	2	1	2	2	1	1
3	3	3	3	3	1	3	3	1	2
2	3	1	2	2	1	1	3	2	1
2	2	3	9	1	9	9	9	1	1
3	3	3	3	3	1	3	3	3	3
9	9	9	3	3	1	1	3	3	3
3	3	3	3	3	2	3	3	3	3
1	2	3	3	1	1	3	3	3	3
3	3	3	3	3	3	1	3	3	3
1	1	2	2	9	1	2	2	1	2
9	9	9	1	9	9	9	9	2	2
2	9	9	3	1	9	9	9	9	2
3	3	2	2	1	2	2	1	1	2
2	3	2	1	2	3	3	3	1	1
2	3	2	3	2	1	1	3	2	1
3	3	3	9	9	1	9	9	2	2
3	3	2	9	9	9	9	1	9	9
2	3	2	2	2	1	2	2	1	2
2	2	2	2	2	2	2	2	2	2
9	9	9	9	3	9	9	9	9	1
2	3	2	2	1	1	1	1	2	3
2	3	2	2	1	2	3	3	2	1
3	3	3	9	9	1	9	9	3	9
3	2	3	3	2	1	1	3	1	2
1	2	1	2	1	1	2	2	2	1
3	2	2	2	1	2	2	3	2	1
2	2	1	3	2	3	3	3	3	2
3	3	2	2	2	2	2	2	2	2
3	3	3	1	1	2	2	3	1	3

2	2	2	1	3	3	3	3	1	2
3	3	2	1	1	1	1	1	1	2
3	3	3	2	1	1	2	3	2	2
2	2	1	3	2	1	2	3	1	2
2	2	3	1	2	3	1	3	1	2
3	2	3	1	3	3	2	3	2	2
3	3	1	2	2	1	3	3	2	2
9	9	1	9	9	9	1	9	3	9
3	3	1	2	2	2	2	2	2	2
2	2	2	3	2	2	2	2	2	2
2	2	2	2	2	1	3	3	2	1
2	3	2	3	2	9	9	9	9	9
2	2	2	2	2	2	2	2	2	1
2	2	2	1	2	2	2	2	1	2
3	3	3	3	3	1	2	3	3	9
3	3	3	3	3	1	2	3	3	2
2	3	1	9	9	9	1	9	1	2
1	2	1	3	1	1	1	1	3	3
9	9	9	9	9	1	9	9	9	1
3	3	3	3	3	1	3	3	1	1
3	3	3	3	3	1	3	3	3	1
2	1	1	3	2	1	1	2	2	1
2	2	2	2	2	1	2	1	2	1
3	3	2	3	3	1	3	3	2	3
3	3	3	3	2	1	3	3	1	2
2	2	1	1	1	1	1	2	2	1
3	3	1	3	1	1	1	2	1	1
3	3	1	2	2	2	2	2	1	1
3	3	1	3	3	1	1	1	1	1
9	9	9	9	9	1	9	9	9	9
1	1	1	3	3	1	1	2	1	1
1	1	2	1	1	2	1	2	2	3
9	9	1	1	1	1	2	2	1	1
3	2	1	2	1	1	2	2	1	2
3	2	1	3	3	3	1	2	3	1
2	2	2	1	1	1	2	2	1	1
2	2	2	1	1	1	2	2	1	1
9	9	9	9	9	9	1	9	1	2
3	3	3	3	3	1	3	3	3	3
2	1	1	2	1	1	2	3	1	1
3	3	3	1	1	1	1	1	1	2
1	1	1	3	1	1	1	1	3	3
2	2	2	2	2	2	2	2	1	2
3	2	3	3	3	1	2	3	1	1
1	1	1	3	1	1	1	3	1	1
3	1	1	2	2	1	1	1	1	1
2	1	1	3	1	3	3	3	1	3

2	1	1	2	1	1	2	2	1	1
2	1	1	2	1	1	2	2	1	1
2	3	3	3	3	1	3	1	3	1
2	2	1	2	2	1	1	1	3	1
3	1	1	2	1	1	2	2	1	1
2	1	3	2	1	1	1	2	1	2
3	3	1	3	2	1	3	1	1	1
2	3	3	1	1	3	2	3	1	1
3	3	3	3	3	1	3	3	1	3
2	3	3	3	1	3	3	3	1	3
3	3	3	3	3	1	3	3	1	1
3	3	3	3	3	1	3	3	1	1
2	3	3	3	3	1	2	2	1	1
3	2	3	3	2	3	2	3	2	3
3	3	3	3	3	3	1	3	3	2
3	9	2	1	2	3	3	3	1	1
2	2	1	1	2	2	2	2	1	2
2	3	2	3	1	1	2	1	1	1
9	9	9	9	9	1	9	9	9	9
3	3	3	9	9	9	9	9	3	3
1	1	3	2	1	3	2	1	2	1
2	3	2	1	1	2	3	3	1	2
2	2	1	2	1	2	2	2	1	9
3	2	3	3	2	1	2	3	1	9
9	9	9	9	1	9	9	9	1	9
9	9	9	9	9	9	1	9	9	9
9	9	9	9	9	1	9	9	1	2
9	9	1	9	9	9	9	1	9	9
9	9	9	3	9	9	9	9	2	9
9	9	1	9	9	1	9	9	9	9
9	9	1	9	9	9	1	9	1	9
9	9	1	9	9	9	9	1	1	9
9	9	9	1	9	9	9	9	2	9
9	9	9	9	1	9	9	9	1	9
9	9	9	2	1	1	2	2	1	2
3	3	3	3	3	1	3	3	3	3
2	1	1	2	1	1	2	2	1	2
1	2	1	3	1	1	1	1	3	3
3	3	2	1	1	1	2	2	1	1
3	3	1	3	1	1	1	2	1	1
1	1	1	9	9	9	1	9	1	2
3	3	3	1	9	9	9	9	1	3
2	2	3	2	2	9	1	1	2	2
3	3	3	2	2	1	2	2	1	2
1	3	2	2	2	2	2	2	3	3
3	3	3	3	3	3	1	3	1	3
2	1	1	3	3	1	3	3	2	1

2	2	3	2	2	1	2	3	1	2
2	3	2	2	3	3	2	2	2	2
2	2	2	1	2	2	2	2	1	2
3	3	2	1	1	2	1	2	3	3
3	2	2	2	2	1	1	1	1	1
9	9	9	9	9	1	9	9	2	1
9	9	9	9	9	9	9	9	2	2
3	3	2	2	2	2	2	2	2	2
3	3	2	3	3	2	2	2	2	2
2	2	2	2	2	3	2	2	2	3
3	3	3	2	2	2	2	2	1	2
3	3	3	3	2	1	1	2	2	2
2	2	2	2	2	2	2	1	1	2
1	9	9	9	9	1	9	9	3	3
2	2	2	2	2	2	2	2	2	2
2	3	1	2	2	2	2	2	2	2
1	9	1	2	2	2	2	2	1	2
3	3	2	3	3	1	1	3	1	2
1	2	3	3	3	1	3	3	2	2
2	2	2	2	2	2	2	2	1	1
3	3	1	3	3	3	3	3	1	1
2	2	2	2	2	2	2	2	2	2
3	3	2	3	3	2	2	2	2	2
2	2	2	3	3	1	3	3	1	3
3	3	1	3	3	1	3	3	1	1
3	3	3	1	9	9	9	9	2	2
3	2	3	2	1	1	3	3	1	2
1	1	1	2	1	2	3	3	1	2
1	1	1	2	2	2	2	2	2	2
2	2	2	2	2	2	2	2	2	2
9	9	9	9	9	9	1	9	9	9
1	1	1	1	1	1	1	1	1	2
9	9	9	3	3	1	3	3	3	3
3	2	2	1	3	3	3	3	1	1
9	9	9	1	9	9	9	9	1	3
2	2	2	2	1	1	2	2	9	1
2	2	2	2	2	2	2	2	1	1
2	2	3	3	2	1	2	1	3	2
3	3	3	2	9	1	2	3	1	2
3	3	3	3	3	3	2	3	1	2
2	2	3	2	1	1	2	2	1	2
2	2	3	2	1	1	2	3	1	2

SeventoTe	MidCycle	SeventoTe	EarlyPerio	LatePeriod	NoBabies	ManyBabie	HighFat	BirthContr	FamilyBrea
3	3	3	2	2	2	2	2	2	2
2	2	2	2	2	2	2	2	2	2
2	2	2	2	2	2	2	2	2	2
2	2	2	2	2	2	2	2	2	2
2	3	3	1	2	3	2	2	2	1
2	2	2	2	2	2	2	2	2	2
2	2	2	2	2	2	2	2	2	2
2	2	2	2	2	2	2	2	2	2
1	1	2	1	2	2	2	2	2	2
2	3	2	2	1	2	3	1	2	3
1	3	1	3	3	3	3	1	1	1
2	3	3	1	1	2	2	3	3	2
2	2	2	3	2	1	2	2	2	2
1	2	2	1	2	3	2	2	1	2
2	2	2	2	2	2	2	2	2	2
3	3	3	2	2	2	2	2	2	2
2	2	2	2	3	3	3	2	9	3
1	3	3	2	2	2	2	2	2	2
2	2	3	2	3	3	3	1	2	2
2	1	1	2	2	2	2	2	2	2
1	2	2	2	2	2	3	2	3	3
1	3	3	2	2	2	2	2	2	2
1	2	3	2	2	3	3	2	2	3
1	3	1	1	2	2	2	2	2	2
2	2	2	2	2	2	2	3	2	2
1	3	3	3	1	3	1	3	3	3
9	9	9	9	9	9	9	9	9	1
3	3	3	3	3	3	1	1	2	3
2	2	2	2	2	3	2	2	2	3
1	1	3	2	1	2	3	2	1	2
2	2	2	3	3	3	3	3	3	1
2	2	2	2	2	2	2	2	1	1
2	3	3	9	9	9	9	9	9	9
1	2	1	9	1	3	1	2	1	3
2	1	1	2	9	1	2	9	3	9
3	2	3	1	1	1	2	3	3	3
2	3	2	2	2	2	2	1	3	2
3	3	2	3	3	3	3	2	1	2
2	2	2	3	3	2	3	2	2	3
2	3	3	1	2	1	2	2	3	1
2	2	2	2	2	2	3	2	2	2
9	9	9	3	9	2	9	9	9	2
9	9	9	9	9	9	9	9	9	9
3	2	2	3	3	3	3	2	1	2
2	1	3	2	2	1	1	1	2	2
9	9	9	9	9	9	9	9	9	9

2	3	3	1	2	2	3	2	2	3
2	3	3	1	2	2	1	2	2	3
2	2	2	2	1	2	2	2	2	2
1	2	2	2	2	1	2	2	2	2
3	9	3	3	3	3	3	3	1	3
3	2	3	3	3	3	3	1	3	1
3	2	3	3	2	2	2	2	2	2
9	9	3	1	3	3	3	3	3	1
1	1	1	9	9	1	1	1	9	9
1	2	1	2	2	1	2	3	9	1
9	2	9	2	9	2	9	9	9	9
2	2	2	1	1	2	2	2	2	2
1	1	1	1	1	1	1	2	2	2
1	1	1	1	1	3	3	2	2	2
2	2	2	1	1	2	2	2	1	1
1	2	2	1	1	1	2	2	2	1
2	2	1	2	3	3	1	1	2	2
2	1	1	9	9	2	9	1	3	1
2	2	2	2	2	2	2	2	2	2
3	3	3	2	3	3	2	9	2	3
3	3	3	3	3	3	3	3	3	3
1	2	2	1	2	3	3	2	2	2
2	1	1	1	1	1	1	2	2	1
2	3	2	1	2	2	3	2	1	2
2	3	2	1	2	2	1	1	2	2
3	2	2	2	2	3	2	1	2	1
3	2	1	3	1	3	3	1	2	1
1	2	2	1	3	2	3	1	2	1
2	1	2	3	3	3	3	1	2	1
3	3	3	3	3	3	3	9	1	3
9	9	9	9	9	9	9	9	9	9
2	2	2	2	2	2	2	2	2	2
2	3	3	3	1	2	9	9	9	9
2	2	2	2	1	2	2	2	2	2
1	3	2	3	3	1	9	1	2	9
2	2	1	2	2	2	2	2	2	1
1	1	1	1	3	3	3	2	1	2
2	3	2	2	1	1	2	2	1	2
1	1	3	1	3	3	2	2	2	2
2	2	2	1	2	2	1	1	2	2
1	3	2	2	1	3	2	1	2	3
2	2	2	9	9	9	9	9	9	9
3	2	3	1	2	3	3	1	1	1
2	2	2	1	2	2	3	3	2	2
2	3	2	2	2	3	3	2	2	1
3	1	2	2	2	2	2	2	2	2
2	2	2	2	2	3	3	2	2	2

1	1	2	1	2	1	2	3	1	1
2	3	2	1	3	9	1	9	3	3
2	2	3	2	2	2	2	2	2	2
2	3	2	1	1	1	1	2	2	2
1	1	3	1	1	2	3	2	1	1
2	2	1	2	1	1	1	1	1	1
2	2	2	2	2	3	2	2	2	2
3	2	1	3	2	3	1	2	3	3
2	2	2	2	2	2	2	2	1	2
3	2	3	3	3	3	3	2	2	3
9	9	9	9	9	9	9	9	9	9
2	2	1	1	2	2	1	1	2	2
1	1	1	1	1	1	2	3	2	2
2	2	2	2	2	2	2	2	2	2
2	2	2	2	2	3	1	2	1	1
2	2	2	2	2	3	1	9	1	1
2	3	2	1	2	1	3	1	1	3
3	3	3	3	3	2	2	2	2	2
1	2	1	3	3	3	3	3	3	3
2	2	1	1	1	1	2	2	1	1
3	3	3	1	3	3	3	2	3	1
2	1	2	2	2	3	3	3	2	1
2	1	1	1	1	1	1	1	2	2
2	2	2	2	2	2	2	2	2	2
2	2	2	2	2	2	2	2	2	2
3	2	2	3	3	3	3	3	2	3
2	2	2	1	1	2	2	3	1	1
2	1	2	2	1	2	1	2	1	2
1	2	1	1	2	1	3	3	2	2
1	1	2	3	2	3	3	3	2	3
2	3	2	1	2	1	2	2	2	2
2	1	2	2	2	2	2	2	1	2
3	2	1	1	2	3	2	2	1	3
9	1	2	1	2	1	2	1	2	1
3	3	1	3	2	2	1	2	1	3
1	3	1	1	2	3	2	9	2	2
9	9	9	2	9	9	9	9	9	9
1	2	3	2	3	2	2	1	1	1
3	2	2	3	1	9	9	9	9	9
9	2	2	1	3	9	1	3	3	3
2	3	2	1	1	2	1	1	1	1
9	9	9	9	9	9	9	9	9	9
1	1	1	1	2	2	1	2	2	2
2	3	3	1	2	2	3	2	2	2
2	2	1	2	2	2	2	1	2	2
2	2	3	2	2	2	2	2	2	2
2	1	1	2	1	2	3	1	2	1

9	1	2	3	2	3	9	2	1	1
3	3	3	3	3	3	3	3	3	3
3	3	3	3	3	3	2	9	3	3
2	2	3	2	2	2	2	2	2	2
2	3	1	2	1	1	3	3	1	1
3	3	3	3	3	1	2	3	3	9
1	9	9	2	3	1	2	1	9	1
3	1	2	1	2	3	1	2	3	1
2	2	2	1	9	9	9	9	9	9
2	2	2	3	1	2	2	2	2	2
1	1	1	2	1	2	3	2	1	2
9	9	9	2	2	2	2	2	3	1
2	2	2	2	2	2	2	2	2	2
2	2	2	1	1	2	2	2	3	3
2	1	1	1	1	1	1	1	1	2
2	2	2	1	2	1	2	2	2	2
2	1	1	2	2	2	2	2	2	2
2	2	2	1	2	2	3	2	2	2
2	3	2	2	2	3	3	2	2	1
3	1	3	2	1	1	3	1	2	2
9	9	9	9	9	9	9	9	9	9
3	3	3	3	3	2	3	2	2	2
2	2	2	3	3	3	3	3	3	2
1	1	1	2	3	2	2	2	1	1
2	2	2	3	3	2	3	1	2	3
9	2	3	3	3	1	2	3	3	2
3	2	2	2	3	3	3	2	2	2
2	2	3	9	9	9	9	9	9	9
3	2	2	3	3	2	3	2	2	2
2	1	1	1	1	2	2	1	1	1
9	9	9	2	2	2	3	1	2	2
2	2	2	2	2	2	2	2	2	2
2	2	2	1	2	2	1	1	2	2
1	1	1	1	3	3	3	3	3	3
2	2	2	2	2	2	2	2	3	2
2	1	1	2	1	1	1	1	1	1
3	3	3	1	2	2	2	2	2	2
2	3	3	2	2	3	3	3	1	3
9	9	9	9	9	9	9	9	9	1
3	3	3	3	3	3	3	3	3	3
1	2	1	3	2	2	1	2	1	2
2	2	2	2	2	2	2	2	2	2
1	1	3	1	3	2	3	1	2	2
9	9	9	9	9	9	9	9	9	9
9	9	9	9	9	9	9	9	9	9
1	1	1	1	2	2	1	1	2	2
2	3	3	1	1	1	1	1	2	2

9	1	9	9	9	9	9	9	2	9
9	9	9	9	9	9	9	9	2	9
2	2	2	1	1	3	2	1	1	1
9	9	9	1	9	9	9	9	9	9
2	2	2	3	2	2	3	3	2	3
2	2	2	9	9	9	9	9	9	9
2	2	2	3	3	3	2	2	2	1
9	9	9	3	1	2	3	1	2	3
3	2	1	1	2	1	3	2	2	2
9	1	9	9	2	9	9	9	9	9
9	9	9	9	9	9	9	1	9	9
9	9	9	9	2	9	9	9	9	9
2	2	2	2	2	1	2	2	1	1
3	1	2	2	2	3	2	1	3	1
3	9	9	2	9	9	9	9	9	9
3	3	3	3	3	3	3	3	3	3
3	3	3	3	3	3	3	3	3	3
1	1	1	2	1	1	1	2	2	2
1	2	2	1	2	2	2	1	2	2
2	2	3	3	3	3	3	3	2	2
2	3	2	2	2	2	2	1	9	2
3	1	2	3	2	2	2	3	2	2
2	2	2	3	3	2	2	2	2	3
1	9	2	1	3	3	3	2	1	2
2	2	2	2	2	2	2	2	2	2
1	1	1	2	1	2	2	3	3	2
1	1	3	2	2	1	2	2	2	2
1	3	2	2	2	2	1	2	2	2
9	9	9	3	3	3	3	3	3	3
2	2	1	1	1	2	2	2	2	1
2	2	1	2	2	2	2	2	2	2
2	2	2	1	1	2	2	2	3	3
3	2	1	3	9	9	9	9	9	2
2	2	2	2	2	2	3	3	2	1
2	9	1	3	1	3	3	3	2	1
2	2	2	2	2	2	2	2	1	2
2	2	3	2	2	3	1	1	1	1
9	9	9	2	1	1	1	1	2	2
2	3	3	2	2	3	2	2	1	1
1	2	3	1	1	1	1	2	2	2
2	2	1	2	1	2	2	1	1	1
1	1	1	1	1	3	9	2	1	1
3	1	2	1	1	3	3	3	2	2
2	2	2	9	9	9	9	9	9	9
2	2	2	2	2	2	2	1	3	1
2	9	3	1	1	3	3	1	1	2
9	9	9	9	9	9	9	9	9	9

3	3	3	3	3	2	3	1	1	3
3	3	2	1	3	3	3	1	3	1
1	1	2	1	1	1	1	1	2	2
2	1	3	1	3	3	3	1	1	3
2	2	2	3	3	3	3	2	2	2
2	3	2	3	2	3	3	3	3	2
1	2	1	3	3	3	3	3	3	3
2	2	2	1	2	2	2	1	1	1
2	1	2	1	1	1	2	1	2	2
2	1	1	2	2	1	1	1	1	1
9	2	2	2	2	2	2	3	2	2
2	2	2	2	9	9	9	9	9	9
9	9	9	2	9	9	9	9	9	9
2	2	2	2	2	2	2	2	2	2
9	9	2	9	3	1	9	9	3	1
9	9	2	2	2	9	2	9	2	9
9	9	1	9	1	1	9	2	3	1
1	9	9	9	1	9	9	9	1	9
2	9	2	2	9	2	9	2	9	2
9	9	9	9	9	9	9	9	9	9
9	9	9	9	9	9	9	9	9	9
2	2	2	2	2	2	2	2	2	2
2	2	2	2	2	2	2	2	2	2
3	3	3	3	3	3	3	3	3	3
2	2	2	1	1	2	2	2	2	1
2	9	2	2	9	2	9	9	2	9
2	2	2	2	2	2	2	2	2	2
9	9	9	9	9	9	9	9	9	9
9	9	9	9	9	9	9	9	9	9
1	1	1	2	2	2	2	2	2	2
9	9	9	9	2	9	9	9	9	9
9	9	9	9	9	9	9	9	9	9
9	9	9	9	9	9	9	9	9	9
2	9	2	2	9	2	9	9	2	9
3	1	9	9	3	1	9	9	3	1
9	3	1	9	9	1	9	9	1	9
9	9	9	9	9	9	9	9	9	9
9	9	9	9	2	9	9	9	9	9
2	1	9	9	9	1	9	3	2	9
9	9	9	3	9	9	9	9	9	9
1	9	9	2	9	9	9	9	9	9
9	9	9	9	9	9	9	9	1	9
3	3	3	3	3	3	3	3	3	3
2	1	1	2	1	1	1	1	9	1
3	1	2	1	2	1	3	1	2	1
2	9	9	1	1	1	9	1	1	1
1	9	9	3	9	9	9	9	9	9

2	9	9	2	9	9	9	9	9	9
9	9	9	9	9	9	9	2	9	9
9	9	9	9	2	9	3	3	3	9
2	1	3	3	2	1	3	2	2	1
3	3	2	2	3	3	2	2	2	2
1	1	2	2	2	2	2	2	2	1
3	3	2	2	2	3	3	3	2	2
3	1	1	2	2	1	1	2	1	2
9	9	9	9	9	9	9	9	9	9
3	3	3	3	3	3	3	3	3	3
9	9	9	9	9	9	9	9	9	9
9	9	9	9	9	9	9	9	9	9
9	9	9	1	9	9	9	9	9	9
1	3	1	1	1	1	1	2	2	3
1	1	1	1	1	1	1	1	1	1
2	1	1	1	2	1	1	1	2	2
9	9	9	9	9	9	9	9	9	9
2	2	2	1	2	2	2	1	1	2
1	1	1	1	1	1	1	1	1	1
2	2	2	3	3	3	9	2	3	3
2	1	3	3	1	2	1	2	3	1
2	2	3	2	2	3	3	3	2	1
1	3	2	2	3	3	2	3	2	2
2	2	2	2	2	2	2	2	2	2
2	2	2	2	2	2	2	2	2	2
2	2	2	2	2	2	2	2	2	2
2	2	2	2	2	2	2	2	2	2
3	1	2	1	2	3	1	2	3	1
2	2	2	2	2	2	2	2	2	2
3	2	3	1	1	1	2	2	2	3
2	2	2	2	2	2	2	2	2	2
2	2	2	2	2	2	2	2	2	2
1	2	1	2	2	3	2	3	2	3
3	3	2	2	2	2	2	2	2	2
2	3	1	3	2	1	3	3	3	3
9	3	3	1	2	3	2	3	1	2
9	9	9	9	9	9	9	9	9	9
2	2	2	2	2	2	2	2	2	2
3	3	3	3	3	3	3	3	3	3
3	3	3	3	3	3	3	3	3	3
1	2	2	2	9	9	9	9	9	9
1	2	3	2	3	1	3	2	1	2
1	1	1	3	3	1	1	1	2	2
1	1	1	1	1	1	1	1	1	1
3	3	3	1	1	1	2	2	2	2
2	2	2	2	2	2	2	2	2	2
2	2	2	2	2	2	2	2	2	2

1	2	3	2	3	1	2	3	1	2
9	9	9	1	9	9	9	9	9	9
9	9	9	9	9	9	9	9	1	9
9	9	9	1	9	9	9	9	9	9
9	9	1	2	9	9	9	9	9	9
2	1	1	1	1	1	1	1	1	1
9	9	9	2	9	9	9	9	9	9
2	9	9	9	9	9	9	2	9	9
2	2	2	2	2	2	2	2	1	2
9	9	9	9	9	9	9	9	9	9
2	9	9	9	9	9	9	2	9	9
9	9	9	9	9	9	9	9	2	9
9	9	9	2	9	9	9	9	9	9
9	9	9	9	9	2	9	9	9	9
9	9	9	1	9	9	9	9	9	9
9	9	2	9	9	2	9	9	9	9
9	9	9	9	2	9	9	9	9	9
3	1	3	2	1	3	1	2	3	1
2	1	2	1	2	1	3	1	2	2
2	2	2	2	2	2	2	2	3	3
2	2	3	3	2	2	2	1	1	3
1	1	2	3	2	3	3	1	2	3
1	1	1	1	2	1	3	1	2	1
3	2	1	2	3	2	9	2	2	3
3	2	2	3	3	3	2	3	2	2
3	3	3	3	3	3	3	3	3	3
2	2	2	2	2	2	1	2	1	3
2	3	2	3	2	3	3	3	3	1
2	1	2	1	1	2	2	1	2	2
2	1	1	1	1	1	1	2	9	1
2	1	3	1	2	2	2	2	2	1
2	2	2	3	3	1	1	2	1	3
3	3	2	2	2	1	1	2	1	1
1	1	1	1	1	2	2	1	1	1
9	9	9	9	9	9	9	9	9	9
2	2	2	3	3	3	3	2	2	3
9	9	9	9	9	9	9	9	9	9
1	1	2	2	2	1	1	2	1	1
1	2	2	1	1	2	2	2	1	2
2	2	2	2	2	2	2	2	2	2
9	1	9	9	9	9	9	9	9	9
3	3	3	3	3	3	3	3	3	3
9	9	9	3	3	3	3	1	3	3
2	1	1	3	3	2	2	3	2	3
2	2	2	2	2	2	2	2	2	2
3	1	2	1	2	2	3	2	2	3
2	2	2	2	2	2	2	2	2	2

2	2	3	1	2	2	2	2	2	3
2	9	9	9	9	9	9	9	9	9
2	9	9	2	2	2	2	2	2	9
3	1	2	1	2	2	9	1	2	1
1	1	2	2	2	2	2	2	2	2
9	9	9	2	9	9	9	9	9	9
1	2	2	1	1	2	2	2	2	1
2	2	2	1	2	2	9	3	2	2
9	9	9	9	9	9	9	9	9	9
2	2	2	3	3	2	3	2	3	1
1	3	3	3	3	3	3	3	3	3
9	9	9	9	9	9	9	9	9	9
9	9	9	2	9	9	9	9	9	9
9	9	9	2	1	2	2	3	2	2
2	9	9	2	9	9	9	9	9	9
2	1	2	2	3	1	9	3	2	2
9	9	9	9	9	9	9	9	9	9
9	9	9	2	9	9	9	9	9	9
1	9	9	9	9	9	2	9	9	9
2	2	3	3	3	3	3	3	3	3
2	2	2	2	2	2	2	2	2	2
2	2	2	3	3	3	3	2	2	1
2	2	2	2	2	2	1	2	2	2
9	1	9	9	9	9	9	1	9	9
1	9	9	9	9	9	9	9	9	1
1	1	1	1	2	2	2	2	1	1
1	1	1	1	1	1	1	1	1	1
1	2	1	2	3	1	2	3	2	1
2	2	2	2	2	2	3	3	3	3
9	9	9	9	9	9	9	9	9	1
9	9	9	2	1	1	2	2	2	2
1	2	1	2	1	2	3	2	1	2
1	2	2	2	2	3	2	2	2	2
2	3	3	2	2	2	2	2	2	1
3	2	2	3	3	3	1	1	1	1
1	2	3	1	2	1	2	1	1	2
9	9	9	9	9	9	9	9	9	9
2	2	1	1	1	2	2	2	2	2
2	2	2	2	3	3	2	3	9	2
2	2	2	1	1	1	1	1	1	1
2	1	3	2	2	2	2	2	2	2
2	2	2	2	2	2	2	2	2	2
2	2	2	2	2	2	2	2	2	2
3	2	1	2	1	2	1	2	2	2
3	2	3	3	3	3	3	3	3	3
2	2	2	2	2	2	2	2	2	2
3	3	3	3	3	3	3	3	3	3

2	2	2	2	9	9	9	9	9	9
9	9	9	9	9	9	9	9	9	1
1	9	9	9	9	9	1	9	9	9
9	9	9	1	9	9	9	9	9	9
2	1	2	9	9	9	9	9	9	9
2	3	2	3	2	2	9	1	3	3
2	2	2	2	2	2	2	2	2	2
2	2	2	2	2	9	2	2	2	2
9	9	9	9	9	9	9	9	9	9
1	9	9	9	9	9	9	9	9	9
2	2	2	2	2	2	2	2	2	2
2	2	2	2	2	2	2	2	2	2
2	2	9	2	2	2	2	2	2	2
2	2	2	2	2	2	2	2	2	2
2	2	2	2	2	2	2	2	2	2
9	9	9	2	9	9	9	9	9	9
3	3	3	3	3	3	3	3	3	3
9	9	9	1	9	9	9	9	9	9
9	9	9	2	9	9	9	9	9	9
9	9	9	2	9	9	9	9	9	9
9	9	9	9	9	9	9	9	9	9
9	9	9	2	9	9	9	9	9	9
2	2	2	2	2	2	2	2	2	2
2	2	2	2	2	2	2	2	9	2
9	9	9	9	9	9	9	9	9	9
1	9	9	2	9	9	9	9	9	9
2	9	9	9	9	9	9	9	9	9
1	9	9	2	2	2	9	2	9	9
1	3	3	2	2	2	2	2	2	2
2	9	2	9	9	9	9	9	9	9
2	9	2	2	9	2	9	2	9	2
1	9	3	2	2	2	2	2	2	2
3	1	9	1	9	1	9	1	9	1
9	9	2	9	9	9	9	9	9	9
3	3	3	3	3	3	3	3	3	3
1	9	1	9	9	9	9	9	9	9
2	2	2	1	1	9	2	9	2	9
2	2	2	9	9	9	9	9	9	9
3	2	1	2	1	3	3	1	1	3
2	2	2	2	2	2	2	2	2	2
9	9	9	9	9	9	9	2	9	9
2	2	2	2	1	3	3	1	2	3
2	3	2	2	3	2	2	1	3	1
3	2	3	2	3	2	3	2	3	1
3	3	3	3	3	3	3	3	3	3
2	9	1	1	1	1	2	2	2	1
2	2	2	9	9	9	9	9	1	9

9	1	9	9	9	9	9	1	9	9
3	2	1	1	2	2	3	3	2	3
9	9	9	2	2	2	2	2	2	2
9	9	9	1	1	1	1	1	1	1
2	2	2	2	2	2	2	2	2	2
2	1	3	2	3	2	2	3	1	2
3	3	3	3	3	3	3	3	3	3
2	1	3	2	3	2	2	3	1	3
2	3	3	2	3	3	3	1	3	1
2	2	2	2	2	2	2	2	2	2
2	2	2	2	3	3	3	3	3	3
9	2	3	2	1	3	2	2	2	2
1	9	9	3	3	3	3	3	3	3
1	2	2	2	2	2	2	2	2	2
9	9	2	9	9	9	2	9	9	9
1	2	2	2	2	1	1	1	2	1
2	2	2	2	2	3	3	2	3	2
2	2	2	2	2	2	2	1	2	2
2	3	1	3	3	3	3	3	2	3
3	2	3	2	2	2	2	2	2	2
9	9	9	2	2	2	2	2	2	3
1	3	3	1	1	1	1	2	2	1
2	9	1	1	2	1	9	3	2	1
9	9	9	9	9	1	9	9	9	9
9	9	9	9	9	9	9	9	9	1
2	2	2	1	1	1	1	1	1	1
9	9	9	9	2	2	2	2	2	2
1	1	1	3	3	3	3	3	3	3
9	9	9	9	9	9	9	9	9	9
9	1	9	9	9	9	9	1	9	9
3	2	1	2	2	2	2	1	2	2
1	3	2	3	2	3	2	3	1	2
9	9	9	9	9	9	9	9	9	9
9	9	9	9	9	9	9	9	1	9
1	1	2	1	2	2	2	1	1	1
9	9	9	9	9	9	9	9	9	9
2	2	1	1	3	3	2	2	1	3
1	1	1	3	3	3	3	3	3	3
2	2	2	3	1	2	2	3	2	3
2	2	2	2	2	2	2	2	2	2
9	1	9	2	1	2	3	2	1	1
3	3	3	3	3	3	3	3	3	3
9	9	9	2	9	9	9	9	9	9
3	2	3	2	2	2	3	3	3	3
2	9	1	2	1	1	2	3	3	3
2	2	2	2	2	2	2	2	2	2
3	3	2	2	3	3	3	2	2	2

9	9	2	9	9	9	9	9	9	9
2	2	2	2	2	2	2	2	2	2
1	2	2	3	3	2	3	1	2	2
2	2	2	2	2	2	2	2	2	2
2	2	2	2	3	3	3	2	3	2
9	9	9	9	9	9	9	9	9	9
9	9	9	1	1	2	1	1	2	2
9	9	9	2	2	2	2	2	2	2
2	1	1	2	2	2	1	1	2	2
9	9	1	2	2	2	2	2	2	2
3	3	3	2	2	2	2	2	2	2
9	9	9	3	1	3	2	2	3	2
1	1	2	3	3	3	3	2	2	3
2	2	2	2	2	2	2	2	2	2
3	1	1	3	1	3	3	3	3	3
2	9	2	3	3	3	3	3	3	3
2	2	2	9	9	9	9	9	9	9
9	9	9	9	9	9	9	9	9	9
9	9	9	1	9	9	9	9	9	9
2	9	2	9	9	9	2	9	9	9
9	9	9	2	9	9	9	9	9	9
9	9	9	9	9	9	9	9	9	9
2	2	2	2	2	2	2	2	2	2
9	9	9	2	9	9	9	9	9	9
9	9	9	9	9	9	9	9	9	9
9	9	9	3	9	9	9	9	9	9
9	9	9	9	9	9	9	9	9	9
1	1	1	1	9	1	1	1	9	2
3	9	3	3	3	3	9	9	9	2
9	9	3	9	9	9	9	9	9	9
9	9	9	3	9	9	3	9	9	9
2	2	2	2	2	2	2	2	2	2
1	1	1	1	1	1	1	1	1	1
9	9	9	3	9	9	9	9	9	9
9	2	2	2	1	9	2	1	9	1
9	9	9	9	9	9	9	9	9	9
9	9	9	2	9	9	9	9	9	9
9	9	9	9	9	9	9	9	9	9
2	9	1	1	9	9	1	9	2	9
9	9	9	1	9	9	9	9	9	9
2	2	2	2	2	2	2	2	2	2
9	9	9	2	9	9	9	9	9	9
9	9	9	2	9	9	9	9	9	9
9	9	9	2	9	9	9	9	9	9
2	2	2	2	2	2	2	2	2	2
2	2	2	9	9	9	9	9	9	9
9	9	9	9	9	9	9	9	9	9

1	3	2	9	9	9	9	9	9	9
2	2	2	1	2	2	2	2	2	2
2	9	1	9	2	9	9	9	2	9
2	2	2	2	9	2	9	9	2	9
9	9	9	9	9	9	9	9	9	9
9	9	9	9	9	9	9	9	9	9
9	9	9	9	9	9	9	9	9	9
9	9	9	2	9	9	9	9	9	9
9	9	9	9	9	9	9	9	9	9
9	9	9	9	9	9	9	9	9	9
9	9	9	9	9	9	9	9	9	9
9	9	9	9	9	9	9	9	9	9
9	9	9	9	9	9	9	9	9	9
9	9	9	9	9	9	9	9	9	9
2	2	2	2	2	2	2	2	2	2
2	2	2	2	2	2	2	2	2	2
2	2	2	2	2	2	2	2	2	2
2	2	2	2	2	2	2	2	2	2
2	2	2	9	9	9	9	9	9	9
2	2	2	2	2	2	2	2	2	2
2	2	2	9	9	9	9	9	9	9
9	9	9	2	2	9	9	9	9	9
9	9	9	9	9	9	9	9	9	9
2	2	2	9	9	9	9	9	9	9
2	2	2	2	2	2	2	2	2	2
2	2	2	2	2	2	2	2	2	2
9	9	9	9	9	9	9	9	9	9
2	2	2	1	2	1	2	1	2	1
9	9	9	9	9	9	9	9	9	9
2	2	2	2	2	2	2	2	2	2
1	1	1	1	1	1	1	1	1	1
3	1	2	2	2	2	2	2	2	2
2	2	2	9	9	9	9	9	9	9
1	2	1	2	1	2	3	2	2	1
1	1	1	9	9	9	9	9	9	9
9	9	9	2	2	2	2	2	2	2
9	9	3	2	9	9	9	9	9	9
2	2	2	2	2	9	2	9	2	2
1	1	1	1	1	1	1	1	1	1
9	9	3	3	9	9	9	9	9	9
9	9	9	9	9	9	9	9	9	9
9	9	9	9	9	9	9	9	9	9
2	2	2	2	2	2	2	2	2	2
1	9	9	9	2	9	2	9	9	9
1	9	1	1	9	3	1	9	1	9
9	9	2	2	9	9	9	9	9	9
2	9	2	2	9	9	2	9	9	2

9	1	9	2	9	9	9	9	9	9
9	9	9	9	9	9	9	9	9	9
1	9	3	2	9	9	9	1	9	9
9	9	9	3	9	9	9	9	9	9
9	9	9	9	9	9	9	9	9	9
9	9	9	9	9	9	9	9	9	9
1	1	9	2	9	9	9	9	9	9
9	9	9	2	9	9	9	9	9	9
1	9	9	1	2	9	2	9	2	9
2	2	2	1	1	1	9	1	9	9
9	9	9	9	9	9	9	9	9	9
9	9	9	9	9	9	9	9	9	9
3	1	3	9	9	9	9	9	9	9
9	9	9	9	9	9	9	9	9	9
2	9	2	3	3	3	2	2	9	1
3	1	2	3	3	2	1	3	1	2
9	9	9	9	9	9	9	9	9	9
1	9	9	2	2	2	2	2	2	2
2	2	2	2	2	2	2	2	2	2
2	9	9	2	2	9	2	9	9	9
9	9	9	2	9	9	9	9	9	9
2	9	9	1	9	9	9	9	9	9
2	2	2	3	3	3	3	1	2	3
2	3	3	1	2	3	1	2	3	1
1	2	2	2	2	3	2	2	2	3
1	2	2	2	3	3	3	2	2	2
3	3	2	3	3	3	3	2	2	2
2	2	2	2	2	2	2	2	2	2
1	3	2	3	2	3	3	2	2	3
2	2	2	2	2	2	2	2	2	2
2	2	2	2	9	9	9	9	9	9
9	9	9	2	9	9	9	9	9	9
2	9	3	3	2	3	3	1	3	3
9	2	9	9	9	9	9	9	9	2
9	9	9	1	9	9	9	9	9	9
9	9	9	9	9	9	9	9	9	9
9	9	9	9	9	9	9	9	9	9
1	9	1	9	2	9	9	2	9	9
9	9	9	9	9	9	9	9	9	9
2	9	2	2	9	2	9	9	2	9
9	2	9	2	9	9	2	9	9	2
2	2	2	2	2	2	2	2	2	2
2	2	2	2	2	2	2	2	2	2
2	2	2	2	2	2	2	2	2	2
2	2	2	9	9	9	9	9	9	9
2	2	2	2	2	2	2	2	2	2
3	2	2	2	3	2	2	3	2	2

1	2	2	2	1	3	3	2	2	2
2	3	2	2	2	3	3	3	2	2
9	9	1	9	9	9	9	1	9	9
1	2	2	2	2	3	2	3	2	2
3	3	3	3	3	3	3	3	3	3
2	2	2	2	3	3	2	2	2	1
1	2	2	3	3	2	2	2	1	1
3	3	3	3	3	3	3	3	3	3
2	2	2	2	2	2	2	2	2	2
2	2	2	2	2	2	2	2	2	2
2	2	2	2	9	9	2	9	9	9
2	2	2	2	2	2	2	2	2	2
2	2	1	2	2	2	2	2	2	2
2	3	2	3	2	1	2	3	1	2
2	3	3	2	1	2	3	3	2	3
2	2	2	2	2	2	2	2	2	2
2	3	1	2	2	2	1	2	1	2
2	3	2	2	2	3	2	2	2	3
2	2	2	2	2	3	3	3	1	3
1	1	3	3	3	1	2	3	3	2
2	2	2	2	1	2	2	3	3	3
1	3	2	2	2	1	3	1	2	1
3	1	2	2	2	3	2	2	3	1
2	2	1	2	3	3	3	2	2	3
3	3	3	2	2	2	2	1	2	1
2	2	2	3	3	3	3	2	2	2
2	2	2	2	2	2	2	2	2	1
2	2	3	2	3	2	2	2	3	3
2	2	1	3	3	9	2	9	1	2
1	1	1	1	1	2	3	1	2	3
3	3	3	3	3	3	3	3	3	3
1	2	2	3	3	3	3	3	3	3
3	3	3	3	3	3	3	3	3	3
2	2	2	2	2	2	2	2	2	2
9	9	9	9	1	9	9	9	9	9
2	1	2	3	2	3	2	1	3	3
2	2	2	3	3	2	2	2	2	2
1	1	1	2	2	2	2	2	2	2
2	2	2	3	3	2	2	2	2	2
9	9	9	9	9	9	9	9	9	9
2	2	2	2	2	2	3	2	2	2
9	1	9	9	9	9	9	9	1	9
2	2	3	2	1	2	1	2	1	2
1	2	3	2	9	9	1	9	9	9
3	2	2	2	1	2	1	2	3	2
2	3	2	2	2	3	2	3	3	3
2	3	3	2	3	1	2	1	2	3

1	1	2	2	1	2	2	1	2	1
3	3	3	3	3	3	3	3	3	3
1	3	1	3	1	3	1	1	3	1
3	2	3	2	2	1	3	3	3	3
2	1	3	1	1	2	3	3	1	3
1	1	3	2	3	3	3	3	3	2
3	3	3	2	2	2	2	2	2	2
1	3	3	2	1	2	2	1	2	9
2	1	2	3	3	3	3	1	2	1
2	2	2	2	2	2	2	2	2	2
2	1	2	3	2	2	3	3	3	2
2	2	2	2	2	2	3	2	2	2
2	1	2	2	1	2	1	2	2	2
3	2	2	1	1	3	2	1	3	3
2	2	2	3	2	3	3	2	2	2
2	2	2	3	2	2	3	2	2	2
2	3	2	1	2	3	3	3	3	2
2	3	3	2	3	2	3	2	3	3
2	2	2	2	2	2	2	2	2	2
9	9	9	9	2	9	9	9	9	9
2	2	2	2	1	2	1	1	2	2
1	1	2	2	1	2	2	1	2	2
2	2	3	2	3	3	2	3	2	3
1	2	1	2	2	1	3	2	2	3
3	3	3	3	3	3	3	3	3	3
1	3	2	1	1	2	3	1	2	2
9	9	9	9	9	9	9	9	9	1
2	2	2	2	2	2	2	2	2	2
2	2	2	2	2	2	2	2	2	2
2	2	2	2	2	2	2	2	2	2
2	2	1	2	1	3	9	2	3	2
9	9	9	2	3	2	9	1	2	9
2	2	2	3	3	3	3	2	2	2
2	2	2	3	3	3	3	3	2	1
2	3	2	3	2	3	3	2	3	1
2	3	2	2	2	3	2	9	1	2
1	1	1	3	2	2	2	2	2	1
2	3	2	2	3	2	3	2	3	2
3	3	1	3	3	3	3	2	1	3
2	2	2	3	3	3	2	1	2	2
2	2	2	2	2	2	2	2	2	2
2	2	2	2	2	2	2	2	2	2
2	2	2	2	2	2	2	2	2	2
2	2	2	2	2	2	2	2	2	2
2	2	2	3	3	3	3	2	2	2
1	1	2	3	2	3	3	2	3	3
2	2	2	2	2	2	2	2	2	2

2	2	2	3	2	2	1	2	3	2
2	2	2	3	3	3	3	2	2	2
3	3	3	2	2	2	2	2	2	2
2	2	2	2	2	3	3	1	2	2
9	9	9	2	2	2	2	2	2	1
2	2	2	3	3	3	3	2	2	2
2	2	2	3	3	3	3	2	2	2
2	2	2	2	2	2	2	2	2	2
2	2	2	2	2	3	2	2	2	2
9	9	9	9	9	9	9	9	9	9
3	2	2	1	2	3	2	2	1	3
3	2	2	1	2	3	2	1	2	1
9	2	9	9	9	9	9	9	1	9
9	9	9	9	9	9	9	9	2	9
2	2	2	3	1	3	2	2	2	2
9	9	1	9	9	9	9	9	9	9
9	9	9	9	9	9	9	9	9	9
9	9	9	9	9	9	1	9	9	9
9	9	9	9	9	9	9	9	9	9
2	2	2	2	2	2	2	2	2	2
3	2	3	3	3	3	9	1	1	1
9	9	9	9	9	9	9	9	9	9
1	2	1	9	9	9	9	9	9	9
2	2	2	2	2	2	2	2	2	2
3	1	3	2	2	2	1	3	2	2
2	2	2	2	2	2	2	2	2	2
2	9	3	2	3	3	1	9	2	2
9	9	9	9	9	9	9	9	9	9
2	2	2	2	2	2	2	2	2	2
2	1	2	2	2	2	2	3	2	1
9	9	9	9	9	9	9	9	9	9
2	1	3	1	2	3	1	2	1	2
2	2	2	1	1	2	3	2	3	3
2	2	2	2	1	2	2	2	2	2
2	2	1	2	1	2	2	2	1	2
2	2	2	2	2	2	2	2	2	2
9	9	9	9	1	9	9	9	9	9
2	2	2	1	3	3	2	1	1	2
2	2	2	2	2	2	2	2	2	2
2	2	2	3	2	1	1	2	1	2
1	1	1	2	2	1	1	1	2	3
3	2	3	2	3	3	3	1	1	1
2	3	2	2	2	2	3	1	1	2
9	9	9	3	3	3	2	2	1	1
1	2	3	3	2	3	2	3	1	3
9	9	9	2	1	9	3	1	3	1
3	3	3	3	3	3	3	3	3	3

3	3	2	3	3	1	2	2	3	2
2	2	2	2	2	2	2	2	2	2
2	3	1	2	2	9	3	3	3	3
2	2	2	2	1	2	2	2	2	2
2	2	3	3	2	3	2	3	2	3
2	2	2	2	1	2	2	2	2	2
1	9	9	9	9	9	9	9	9	9
3	3	3	3	3	3	3	3	3	3
2	3	9	3	3	3	3	3	3	3
2	2	2	2	1	2	2	3	2	2
2	2	2	2	1	3	9	3	2	2
9	9	9	9	9	9	9	9	9	9
1	1	1	1	1	2	2	2	1	1
9	9	9	9	9	9	9	1	9	9
9	9	9	9	9	9	9	9	9	9
9	9	9	9	9	9	9	9	9	9
1	2	1	2	3	1	1	2	1	2
9	2	3	1	3	3	3	1	1	3
3	2	3	3	3	3	3	3	2	2
2	2	2	2	2	3	3	2	2	2
3	2	1	2	3	9	2	2	2	2
1	1	1	1	1	1	3	1	3	3
2	2	2	2	2	2	2	2	2	3
2	2	2	2	2	2	2	2	2	2
1	1	1	3	3	3	3	1	3	3
2	2	2	2	2	2	2	2	2	2
2	2	2	2	2	2	2	2	3	2
9	9	9	2	9	9	9	9	9	9
2	2	2	2	2	2	2	2	2	2
2	2	2	2	2	2	2	9	2	3
1	1	1	1	1	1	1	1	1	1
1	3	2	2	3	2	1	3	1	2
2	2	1	2	2	1	1	1	1	2
2	2	2	2	2	2	2	2	2	2
2	2	2	2	1	3	9	2	2	2
2	3	1	2	9	1	3	2	1	2
1	1	1	1	1	1	1	1	1	1
3	3	3	2	2	3	3	2	2	2
3	2	1	1	3	2	2	2	1	3
2	3	2	3	2	3	2	2	3	2
9	9	9	9	9	9	9	9	9	9
2	1	1	2	2	2	9	1	1	2
2	2	2	2	2	2	2	2	2	2
2	2	2	2	2	2	2	2	2	2
3	3	3	3	3	3	3	3	3	3
2	1	2	3	3	1	3	3	2	3
9	9	9	3	3	3	3	3	3	3

9	1	2	3	3	3	3	3	3	3
1	2	2	3	2	3	2	3	2	1
3	2	3	3	3	2	2	3	2	3
2	2	2	2	2	2	2	1	2	2
3	1	2	1	2	9	9	1	3	2
9	9	9	9	9	1	9	9	9	9
1	2	3	3	3	3	3	2	2	3
9	9	9	9	9	9	9	9	9	9
9	9	9	9	9	9	9	9	9	9
9	9	9	9	9	9	9	9	9	9
1	9	2	2	1	2	2	1	2	1
9	9	9	9	9	9	2	9	9	9
3	2	2	3	3	3	3	2	2	3
9	9	9	2	9	9	9	9	9	9
2	2	2	2	2	2	2	1	2	1
9	9	9	9	9	2	9	9	9	9
3	2	1	2	3	3	2	2	2	3
1	9	9	9	9	1	9	9	9	9
1	9	9	9	9	1	9	9	9	9
2	9	9	9	9	2	9	9	9	9
1	1	1	1	1	1	1	1	1	1
1	2	3	2	2	1	1	2	2	2
2	2	2	2	2	2	2	2	2	2
1	1	2	3	3	3	2	3	2	2
3	3	3	1	1	1	1	2	9	2
2	2	2	3	3	3	2	3	3	3
3	3	1	3	1	1	1	3	3	1
1	9	9	9	9	9	9	1	9	9
2	2	2	2	2	2	2	2	2	2
3	3	3	9	1	3	3	3	3	1
3	1	3	1	1	3	1	1	3	1
9	9	9	3	9	9	9	9	9	9
3	3	3	3	3	3	3	3	3	3
9	9	9	3	9	9	9	9	9	9
9	9	9	1	9	9	9	9	9	9
9	9	9	1	1	2	1	1	2	2
9	9	9	1	1	1	1	1	1	1
1	1	1	9	9	9	9	9	9	9
2	2	2	9	9	9	9	9	9	9
2	2	2	2	2	2	2	2	2	2
2	2	2	2	2	2	2	2	2	2
2	2	2	2	2	2	2	2	2	2
3	3	3	3	3	3	3	3	3	3
9	2	9	2	9	9	9	2	9	9
2	2	2	2	2	2	2	2	2	2
2	2	2	2	2	2	2	2	2	2
1	9	1	1	9	1	3	1	3	1

3	1	9	9	1	9	9	9	1	3
1	1	1	2	2	9	9	2	9	2
2	2	2	2	2	2	9	2	9	9
9	9	9	1	1	1	1	1	1	1
9	1	9	1	2	9	3	1	9	3
1	3	1	1	9	1	9	3	1	9
9	2	9	9	1	9	9	9	2	1
3	2	1	2	1	3	9	1	3	9
1	1	1	1	9	2	9	2	9	2
1	1	1	2	2	2	2	2	2	2
2	2	2	2	2	2	2	2	2	2
9	2	9	2	9	2	9	9	2	9
2	2	2	2	2	2	2	2	2	2
3	3	3	9	9	9	9	9	9	9
2	2	2	2	2	2	2	2	2	2
9	9	9	9	9	9	9	9	9	9
9	2	2	2	9	9	9	2	9	9
2	2	2	9	9	9	9	9	9	9
9	3	1	3	1	3	2	1	9	3
2	2	2	2	2	2	2	2	2	2
2	2	2	2	2	2	2	2	2	2
2	2	2	2	2	2	2	2	2	2
1	3	2	1	2	2	1	3	2	1
2	9	1	1	2	1	1	1	9	2
3	2	3	2	3	2	3	2	2	2
3	3	3	3	3	3	3	3	3	3
1	1	2	3	1	2	1	2	3	1
2	2	2	2	2	2	2	1	2	2
3	1	3	1	3	2	1	1	1	3
2	2	2	2	2	3	3	1	1	2
2	3	2	2	2	2	2	2	2	2
3	3	3	3	2	1	2	3	3	2
2	2	2	2	2	2	2	2	2	2
1	1	1	1	2	1	2	1	1	1
1	3	3	1	2	1	2	2	2	2
3	2	3	1	1	1	1	1	1	1
3	3	3	3	1	2	3	1	1	3
2	2	2	3	3	9	1	1	1	1
2	1	3	1	2	3	3	2	2	2
2	9	2	2	2	3	3	9	2	3
3	1	1	3	1	1	1	3	3	1
2	1	2	3	2	2	3	2	3	3
2	2	2	2	3	2	2	2	3	3
9	9	9	9	9	9	9	9	9	9
1	1	2	9	9	9	9	9	9	9
2	2	2	2	2	2	2	2	2	2
9	9	9	3	1	1	3	2	9	1

9	9	9	1	3	3	1	2	2	2
9	9	9	1	3	3	2	2	2	3
2	2	2	2	2	2	2	2	2	2
2	2	2	2	2	2	2	2	2	2
1	2	2	2	2	2	2	2	2	2
2	2	2	2	2	2	2	2	2	2
2	2	2	1	1	1	1	1	1	1
1	1	1	1	2	1	1	1	1	1
3	3	3	3	3	3	3	3	3	3
1	2	1	1	1	2	1	2	1	2
1	1	1	1	1	1	1	1	1	1
9	9	9	2	2	2	2	2	2	2
2	2	2	2	2	2	2	2	2	2
2	2	2	2	2	2	2	2	2	2
2	2	2	2	2	2	2	2	2	2
9	9	9	2	2	2	2	2	2	2
1	2	1	1	1	2	1	1	1	1
2	2	2	1	1	1	2	1	1	1
1	1	1	2	2	2	2	2	2	2
2	2	2	2	2	2	2	2	2	2
2	1	2	2	3	2	1	9	1	1
2	2	2	2	2	2	2	2	2	2
9	9	9	9	9	9	9	9	9	9
9	9	9	2	2	2	2	2	2	2
1	1	1	1	1	1	9	1	9	1
1	1	1	1	2	1	2	1	1	1
1	2	1	1	1	2	2	1	2	1
1	1	2	1	2	1	1	2	9	1
2	9	2	2	2	2	9	2	2	2
1	1	1	1	1	1	1	1	1	1
2	2	2	2	2	2	2	2	2	2
2	2	2	2	2	2	2	2	2	2
1	1	1	2	2	2	2	2	2	2
2	2	2	2	2	2	2	2	2	2
2	2	2	2	2	2	2	2	2	2
2	9	2	9	9	9	9	9	9	9
1	1	2	1	1	1	1	1	1	1
2	9	1	1	2	1	1	1	1	1
9	9	9	2	2	2	2	9	2	2
2	9	2	2	2	2	2	2	2	2
2	2	2	2	2	2	2	2	2	2
2	2	2	2	2	2	2	2	2	2
2	9	2	2	2	2	2	2	2	2
2	2	2	2	2	2	2	2	2	2
1	2	1	1	1	2	1	1	2	1
2	2	2	2	2	2	2	2	2	2
1	9	2	9	1	2	2	2	2	2

2	2	9	1	2	1	1	1	1	1
2	2	2	2	2	2	2	9	2	9
2	2	2	2	2	2	2	2	2	2
1	2	9	1	1	1	2	1	1	1
1	1	1	1	1	1	1	1	1	1
2	2	2	2	2	2	2	2	2	2
2	2	2	2	2	2	2	2	2	2
2	2	2	1	1	1	1	2	2	1
2	9	2	9	9	9	9	9	9	9
9	1	1	2	2	2	9	2	9	2
9	9	9	2	2	9	2	2	2	9
1	9	1	1	1	9	1	1	9	1
9	9	9	9	9	9	9	9	9	9
1	1	1	1	1	1	1	1	2	2
9	9	9	1	1	1	1	9	1	9
2	2	2	2	2	2	2	2	2	2
1	1	1	9	9	9	9	9	9	9
9	9	9	9	9	9	9	9	9	9
2	2	2	2	2	2	2	2	2	2
3	2	3	1	3	1	3	1	1	1
3	2	3	1	3	1	3	1	1	1
3	2	3	1	3	1	3	1	1	1
3	2	3	1	3	1	3	1	1	1
2	2	3	1	3	1	9	1	1	1
3	2	3	1	3	1	9	1	1	1
3	2	3	2	3	1	3	1	1	1
3	2	3	1	3	1	3	1	1	1
3	2	3	1	3	1	1	1	1	1
3	2	3	1	3	1	3	1	1	1
3	2	3	1	3	2	3	1	1	1
3	2	3	1	3	1	3	1	1	1
2	2	3	1	3	1	3	1	1	1
3	2	3	1	3	1	3	1	1	1
3	2	3	1	3	1	3	1	1	1
3	2	3	1	3	1	3	1	1	1
3	2	3	1	3	1	3	1	1	1
3	2	3	1	3	1	3	1	1	1
3	2	3	1	3	1	1	1	1	1
3	2	3	1	3	1	1	1	1	1
3	2	3	1	3	1	1	1	1	1
3	2	3	1	3	1	3	1	1	1
3	2	3	1	3	1	3	1	1	1
3	2	3	1	3	1	3	1	1	1
3	2	3	1	3	3	3	1	1	1
9	9	9	9	9	9	9	9	9	9
9	9	9	9	9	9	9	9	9	9
9	9	9	9	9	9	9	9	9	9

9	9	9	9	9	9	9	9	9	9
9	9	9	9	9	9	9	9	9	9
9	9	9	9	9	9	9	9	9	9
9	9	9	9	9	9	9	9	9	9
9	9	9	9	9	9	9	9	9	9
9	9	9	9	9	9	9	9	9	9
9	9	9	9	9	9	9	9	9	9
9	9	9	9	9	9	9	9	9	9
9	9	9	9	9	9	9	9	9	9
9	9	9	9	9	9	9	9	9	9
9	9	9	9	9	9	9	9	9	9
9	9	9	9	9	9	9	9	9	9
9	2	2	9	9	9	9	9	9	9
9	9	9	9	9	9	9	9	9	9
9	9	9	9	9	9	9	9	9	9
9	9	9	9	9	9	9	9	9	9
9	9	9	9	9	9	9	9	9	9
9	9	9	9	9	9	9	9	9	9
9	9	9	9	9	9	9	9	9	9
3	2	3	1	3	1	3	1	1	1
3	2	3	1	3	1	3	1	1	1
3	2	3	1	3	1	3	1	1	1
3	2	3	1	3	1	3	1	1	1
2	2	2	1	3	1	3	1	1	1
3	2	3	1	3	1	3	1	1	1
3	2	3	1	3	1	3	1	1	1
3	2	3	1	3	1	3	1	1	1
3	2	3	1	3	1	3	1	1	1
2	2	3	1	3	1	3	1	1	1
2	2	3	1	3	1	3	1	1	1
3	2	3	1	3	1	3	1	1	1
3	2	3	1	3	1	3	1	1	1
3	2	3	1	3	1	3	1	1	3
2	2	3	1	3	1	3	1	1	1
2	2	2	1	3	1	3	1	1	1
9	2	3	2	9	9	3	3	9	9
3	2	3	1	3	1	3	1	1	1
3	2	3	1	3	1	3	1	9	1
3	2	3	1	3	1	3	1	1	1
3	2	3	3	9	1	3	1	1	1
3	2	3	1	3	1	3	1	9	9
3	2	3	1	3	1	3	1	1	1
3	2	3	1	3	3	9	1	1	3
3	2	3	1	1	3	3	1	1	1
3	2	3	1	3	1	9	3	1	1
3	2	3	1	3	1	3	1	1	1
3	2	3	1	3	1	3	1	1	1

3	2	3	1	3	1	3	1	1	1
3	2	3	1	1	1	9	9	9	9
3	2	3	1	3	1	3	1	1	1
3	2	3	1	3	1	3	1	1	1
2	9	3	1	3	1	3	1	1	1
9	2	3	1	1	3	3	1	1	1
3	2	3	1	3	1	1	1	1	1
3	2	9	1	3	1	3	1	1	1
3	2	3	1	3	1	3	1	1	1
3	2	3	1	3	1	3	1	1	1
3	2	3	1	3	1	3	1	1	1
2	3	2	1	3	1	3	1	1	1
3	2	3	1	3	1	3	1	1	1
3	2	3	1	3	1	3	1	1	1
3	2	3	1	3	1	3	1	1	3
3	2	3	1	3	1	3	1	1	1
3	2	3	1	3	1	1	1	1	1
3	2	3	1	3	1	3	1	1	1
3	2	3	3	1	3	1	1	1	1
3	2	3	1	3	1	3	1	1	1
3	2	3	1	3	1	3	1	1	1
3	2	3	1	1	3	1	1	1	1
3	2	3	1	3	1	3	1	1	1
3	2	3	1	3	1	3	1	1	1
2	2	3	1	3	2	3	1	1	1
3	2	3	1	3	1	3	1	1	9
3	9	2	1	1	3	1	1	1	1
3	2	3	1	3	1	3	1	1	1
3	3	3	1	3	1	3	1	1	1
3	9	3	9	3	1	3	1	1	1
3	2	3	1	3	1	3	1	1	1
9	2	3	1	3	1	3	1	1	1
9	2	3	1	3	1	3	1	1	1
3	2	3	1	3	1	3	1	1	1
9	9	9	9	9	9	9	9	9	9
2	2	3	1	9	1	3	1	1	1
3	2	3	1	3	3	3	1	1	1
3	3	3	1	3	1	3	1	1	1
3	9	3	9	3	1	1	1	1	1
9	2	3	1	9	9	9	9	9	9
2	3	2	1	3	1	3	1	1	1
3	9	3	1	3	1	3	1	1	1
3	3	3	3	3	1	3	1	1	1
3	2	3	1	3	1	3	1	9	1
3	2	3	1	3	1	3	1	1	1
3	2	3	1	3	1	3	1	1	1
3	2	3	1	3	1	3	1	1	1

3	2	3	1	3	1	3	1	1	1
3	2	3	1	3	1	3	1	1	1
3	2	3	1	3	1	3	1	1	1
3	2	3	1	3	1	3	1	1	1
3	2	3	1	3	1	3	1	1	1
3	2	3	1	3	1	3	1	1	1
3	2	3	1	3	1	3	1	1	1
3	2	3	1	3	1	9	1	1	1
3	2	3	1	3	1	3	1	1	1
3	2	3	1	3	1	3	1	1	1
3	2	3	1	3	1	3	1	1	1
3	2	3	1	3	1	3	1	1	1
3	2	3	1	3	1	3	1	1	1
3	2	3	1	3	1	3	1	1	1
3	2	3	1	3	1	9	1	1	1
3	2	3	1	3	1	3	1	1	1
3	2	3	1	3	1	3	1	1	1
3	2	3	1	3	1	3	1	1	1
3	2	3	1	3	1	3	1	1	1
9	9	9	9	9	9	9	9	9	9
3	2	3	1	3	1	3	1	1	1
3	2	3	1	3	1	3	1	1	1
3	2	3	1	3	1	3	1	1	1
3	2	3	1	3	1	3	1	1	1
3	1	3	1	3	2	9	9	9	9
3	2	3	1	3	1	3	1	1	1
3	2	3	1	3	1	1	1	1	1
3	2	3	1	3	1	3	1	1	1
3	3	3	1	3	1	3	1	1	1
3	2	3	1	3	1	3	1	1	1
3	2	3	1	3	1	3	1	1	1
3	2	3	1	3	1	3	1	1	1
3	2	3	1	1	3	3	1	1	1
3	2	3	1	1	3	3	1	1	1
2	2	3	1	3	1	3	1	1	1
2	2	3	1	3	1	3	1	1	1
3	2	3	1	3	3	1	3	1	1
3	2	3	1	3	1	1	1	1	1
3	2	3	1	3	1	3	1	1	1
3	2	3	1	3	1	3	1	1	1
3	2	3	1	3	1	3	1	1	1
9	9	9	9	9	9	9	9	9	9
3	2	3	1	3	1	3	1	9	1
3	2	3	1	3	1	3	1	1	1
3	2	3	1	3	9	3	1	1	1
3	2	3	1	3	1	3	1	1	1
3	2	3	1	3	1	3	1	1	1

3	2	3	1	3	1	3	1	1	1
3	2	3	1	3	1	3	1	1	1
3	2	3	1	3	1	3	1	1	1
3	2	3	1	3	1	3	1	1	1
3	2	3	1	3	1	3	1	1	1
3	2	3	1	3	1	3	1	1	1
3	2	3	1	3	1	3	1	1	1
2	2	3	1	3	9	3	3	9	2
3	2	3	1	3	1	3	1	1	1
3	2	3	1	3	1	3	1	1	1
3	2	3	1	3	1	3	1	1	1
3	2	3	1	3	1	3	1	1	1
3	2	3	1	3	1	3	1	1	1
2	3	3	1	3	1	3	1	1	1
3	2	3	1	3	1	3	3	1	1
3	2	3	1	3	1	3	1	1	1
3	2	3	1	3	1	3	1	1	1
3	2	3	1	3	1	3	1	1	1
2	2	3	1	3	1	3	1	1	1
3	2	3	1	3	1	3	1	1	1
3	2	3	1	3	1	3	1	1	1
3	2	3	1	3	1	3	1	1	1
3	2	3	1	3	1	3	1	1	1
3	2	3	1	3	1	3	1	3	1
3	2	3	1	3	1	3	1	1	1
3	2	3	1	3	1	3	1	1	1
3	2	3	1	3	1	3	1	1	1
9	9	9	9	9	9	9	9	9	9
9	9	9	9	9	9	9	9	9	9
9	9	9	9	9	9	9	9	9	9
9	9	9	9	9	9	9	9	9	9
9	9	9	9	9	9	9	9	9	9
9	9	9	9	9	9	9	9	9	9
9	9	9	9	9	9	9	9	9	9
9	9	9	9	9	9	9	9	9	9
9	9	9	9	9	9	9	9	9	9
9	9	9	9	9	9	9	9	9	9
9	9	9	9	9	9	9	9	9	9
9	9	9	9	9	9	9	9	9	9
9	9	9	9	9	9	9	9	9	9
9	9	9	9	9	9	9	9	9	9
9	9	9	9	9	9	9	9	9	9
2	3	1	1	2	1	2	1	2	1
1	1	1	1	9	1	1	1	9	9
9	9	9	9	9	9	9	9	9	9
9	9	9	9	9	9	9	9	9	9
9	9	9	9	9	9	9	9	9	9

9	9	9	9	9	9	9	9	9	9
9	9	9	9	9	9	9	9	9	9
9	9	9	9	9	9	9	9	9	9
9	9	9	9	9	9	9	9	9	9
9	9	9	9	9	9	9	9	9	9
9	9	9	9	9	9	9	9	9	9
9	9	9	9	9	9	9	9	9	9
2	2	2	2	3	3	3	1	2	3
9	9	9	9	9	9	9	9	9	9
9	9	9	9	9	9	9	9	9	9
9	9	9	9	9	9	9	9	9	9
9	9	9	9	9	9	9	9	9	9
9	9	9	9	9	9	9	9	9	9
9	9	9	1	2	3	1	1	1	1
2	2	2	2	2	2	2	2	2	2
2	2	2	2	2	2	2	2	2	1
9	2	1	3	1	1	3	3	1	3
2	3	2	2	2	2	2	2	2	3
2	2	2	3	1	1	3	3	3	3
1	2	3	2	1	1	2	3	3	3
1	2	1	1	1	2	1	1	2	2
1	3	1	1	3	1	3	1	3	1
3	2	3	1	3	2	2	3	3	3
3	3	3	2	2	3	3	9	3	3
9	9	9	9	9	9	1	9	9	9
2	2	2	2	2	2	2	2	2	2
2	2	2	2	9	9	9	9	9	9
1	2	2	3	2	3	3	1	3	2
2	3	1	3	3	3	3	2	1	1
3	3	3	3	3	3	3	2	2	2
3	3	2	2	2	3	2	2	3	1
1	3	2	1	2	1	2	1	2	1
1	9	9	9	9	9	9	9	9	9
2	2	2	3	3	2	3	1	1	2
9	9	9	9	9	1	9	9	9	9
9	9	9	2	9	9	9	9	9	9
2	1	1	1	2	2	2	1	2	1
2	1	1	3	1	2	3	1	2	1
1	9	9	9	9	9	9	1	9	9
1	9	3	1	1	2	2	3	2	3
9	9	9	9	9	9	9	9	9	9
2	1	3	1	3	3	1	1	2	3
2	2	2	2	2	2	3	9	2	2
1	1	1	1	1	1	1	1	1	1
9	9	9	9	9	9	9	9	9	9
2	2	2	9	9	9	9	9	9	9
2	2	2	2	2	2	3	2	2	2

9	2	9	9	9	9	9	9	9	9
9	9	9	9	9	9	9	9	9	9
2	1	2	2	2	1	2	2	2	3
3	2	2	2	2	2	2	2	1	3
9	9	9	9	9	9	9	9	9	1
9	9	9	9	9	9	9	2	9	9
2	2	2	2	2	2	2	2	2	2
2	2	2	2	2	2	2	2	2	2
2	2	2	2	2	2	2	2	2	2
3	1	1	2	2	2	1	1	1	3
1	2	3	1	2	3	2	3	2	3
9	9	9	9	9	9	9	9	9	9
9	9	9	9	9	9	9	9	9	9
2	2	2	2	2	2	2	2	2	2
2	2	2	2	2	2	2	2	2	2
2	2	2	2	1	3	2	3	3	1
1	3	1	1	3	1	3	1	3	1
1	1	1	2	2	2	2	3	2	3
2	1	1	2	1	1	1	2	1	1
1	2	3	9	9	2	2	9	2	2
2	2	2	2	2	2	2	2	2	2
1	2	2	2	2	2	2	2	2	2
2	2	2	2	2	2	2	2	2	2
2	2	2	2	2	2	2	2	2	2
3	2	2	3	2	2	2	2	2	3
1	2	2	2	2	2	1	3	3	2
2	2	2	2	2	2	2	2	2	2
2	2	2	2	2	2	3	3	3	3
2	2	2	2	2	3	2	2	2	9
1	1	1	2	1	3	2	1	1	2
2	1	2	3	3	3	3	3	3	3
2	2	2	1	1	1	3	2	1	1
1	2	1	1	1	1	1	1	1	1
1	2	1	2	2	1	1	1	1	3
2	1	2	2	2	1	2	1	2	1
3	3	3	2	2	2	3	2	3	1
2	2	2	3	2	2	2	2	2	2
3	3	3	2	2	2	9	1	2	9
9	9	9	2	9	9	9	9	9	9
3	3	3	3	3	3	3	3	3	3
3	2	2	2	2	2	2	2	3	3
2	3	2	2	2	2	2	2	2	2
3	1	2	1	2	3	1	2	3	1
2	2	3	2	1	1	3	3	2	3
1	1	1	1	1	1	1	1	1	1
2	2	2	1	1	3	9	2	2	2
2	2	2	2	2	2	2	2	2	2

2	2	2	2	2	2	2	2	2	3
3	1	3	2	2	2	3	1	2	3
2	2	2	2	2	2	2	9	1	1
3	1	2	2	3	3	3	2	3	2
2	2	2	3	3	2	3	3	2	2
2	2	2	2	2	2	2	2	1	2
3	2	3	2	2	1	3	3	3	3
2	3	1	3	2	1	3	2	1	1
2	2	2	2	2	2	2	2	2	2
3	2	2	3	2	1	3	2	3	2
2	2	2	2	2	2	2	2	2	2
2	2	2	2	2	2	2	2	2	2
1	1	1	2	2	2	2	2	2	2
2	2	2	2	2	2	2	2	2	2
2	2	2	2	2	2	2	2	2	2
1	2	1	2	1	2	1	2	2	2
3	3	3	3	3	3	3	3	3	3
2	2	2	2	2	2	2	2	2	2
2	2	2	2	2	2	2	2	2	2
3	1	2	1	2	3	1	2	3	1
2	1	2	1	2	3	1	1	2	3
1	3	3	1	1	1	1	1	1	1
2	2	2	2	2	2	2	2	2	2
1	1	1	1	1	1	1	1	1	1
3	3	3	3	3	3	3	1	1	3
9	9	9	9	9	9	9	9	9	9
3	2	1	1	2	3	3	2	1	1
3	3	2	2	2	2	2	3	2	2
1	2	1	1	1	1	2	2	1	2
2	2	3	3	2	1	3	2	1	3
2	2	2	2	2	3	3	2	1	3
2	9	1	1	3	2	9	1	1	2
2	3	3	2	2	2	3	3	3	3
1	1	1	1	2	2	1	2	2	2
1	2	2	1	1	2	3	1	2	1
9	9	9	9	9	9	9	9	9	9
3	2	3	1	3	3	1	1	1	1
1	2	3	2	2	2	2	2	2	1
3	3	2	2	2	3	3	3	2	3
1	2	1	2	2	1	2	1	2	1
3	3	2	2	2	3	2	3	3	2
2	1	2	2	1	2	1	2	1	1
2	2	2	2	2	3	3	2	1	3
9	9	9	9	9	9	9	9	9	9
9	9	9	9	9	9	9	9	9	9
9	9	9	9	9	9	9	9	9	9
1	2	2	1	2	3	2	2	3	2

3	2	2	3	2	3	2	2	3	3
2	9	2	3	9	3	9	2	1	2
1	2	2	3	2	3	3	2	3	2
3	2	3	3	3	3	3	1	1	3
2	1	2	9	9	9	9	9	9	9
3	3	2	2	2	1	1	3	3	3
3	3	3	2	3	3	2	1	3	2
2	2	2	2	2	1	2	1	1	2
1	1	1	2	1	2	3	1	1	1
3	1	2	1	2	3	1	2	3	1
1	2	1	2	2	2	1	1	3	2
2	2	2	2	2	2	2	2	2	2
2	2	2	2	2	2	2	2	2	2
1	3	1	1	1	3	3	1	1	1
2	2	2	2	2	2	2	2	2	2
2	2	2	2	2	2	2	1	2	2
1	1	3	3	3	1	1	3	3	1
3	2	2	3	2	3	2	1	2	1
1	2	2	3	1	1	2	2	1	2
3	2	3	3	1	1	2	3	1	3
3	1	2	3	2	3	2	1	2	1
3	2	3	3	1	1	2	3	1	2
2	2	2	2	2	2	2	2	2	2
3	2	2	1	3	3	3	2	3	2
2	1	2	3	3	3	3	3	3	3
9	9	9	9	9	9	9	9	9	9
3	1	2	2	2	1	3	2	2	1
3	2	1	3	3	1	3	3	3	3
3	3	3	2	2	2	2	2	2	2
1	2	3	1	2	1	2	1	2	3
2	2	2	2	2	2	2	2	2	2
9	9	9	9	2	9	9	9	9	1
1	2	2	3	2	2	1	2	2	2
3	2	2	2	2	2	2	1	3	3
3	3	2	2	1	3	3	2	1	1
1	2	1	2	1	2	1	3	2	1
2	2	2	2	2	2	2	2	2	2
1	3	1	2	2	1	1	1	1	3
2	2	2	3	2	1	2	3	2	3
2	9	9	2	9	9	9	9	9	9
1	2	3	1	2	2	1	2	2	3
2	2	2	2	2	2	2	2	2	2
2	2	2	2	2	2	2	2	2	2
2	2	2	2	2	2	2	2	2	2
2	2	2	2	2	2	2	2	2	2
2	2	2	1	2	3	1	1	1	2
2	2	2	2	2	2	2	2	2	2

2	1	2	1	3	1	2	2	1	1
2	2	2	2	2	2	2	2	2	2
2	2	3	3	3	3	3	3	3	3
2	2	2	2	2	1	3	2	2	2
2	2	2	2	2	2	2	2	2	2
3	3	3	2	2	2	2	2	2	2
2	2	3	1	1	3	3	3	3	3
1	1	2	1	1	3	3	3	2	1
1	1	1	3	3	3	3	1	1	1
3	3	3	3	3	3	3	3	2	3
3	2	1	3	3	2	3	1	1	2
2	2	2	2	2	2	2	2	2	2
3	2	1	3	3	2	3	1	1	2
2	2	2	3	3	3	2	1	1	3
2	2	2	2	2	2	2	2	2	2
2	2	2	2	2	2	2	2	2	3
2	2	2	2	2	2	9	2	3	2
2	2	2	2	2	2	2	2	2	2
2	2	2	2	2	2	2	2	2	2
2	1	2	2	2	2	2	2	2	2
1	2	1	1	1	1	3	1	1	1
1	3	3	1	3	1	1	3	3	3
9	9	9	9	9	9	9	2	9	9
1	3	3	1	2	2	2	2	2	2
2	2	2	2	2	2	2	2	2	2
2	2	2	2	2	3	2	2	2	2
2	2	2	3	3	3	2	2	2	3
2	2	2	3	3	3	2	2	2	2
1	3	3	1	3	1	1	1	3	1
1	2	1	1	1	1	1	1	1	1
1	3	3	1	3	1	1	3	3	3
1	2	2	3	3	3	3	1	1	1
2	3	2	1	2	2	2	3	2	2
1	2	2	1	2	2	2	2	2	2
9	9	9	2	9	9	9	9	9	9
1	3	3	1	3	1	1	3	1	1
2	2	2	2	2	2	2	2	2	2
1	1	1	1	1	1	1	3	3	3
2	2	2	1	1	2	2	2	2	2
2	2	2	2	2	2	2	2	2	2
3	2	2	1	2	2	3	2	2	1
9	9	9	9	9	9	9	9	9	9
9	9	9	9	9	9	9	9	9	9
1	1	1	1	1	1	1	1	1	1
2	3	3	1	2	2	3	3	2	2
9	9	9	9	9	9	9	9	9	9
2	2	2	1	1	1	1	1	2	2

2	2	2	2	2	2	2	2	2	2
3	3	2	1	1	1	1	1	1	1
3	1	2	1	2	3	1	2	3	1
3	2	1	1	2	3	2	1	2	3
1	3	3	1	2	3	1	2	3	2
3	1	2	1	2	3	1	2	3	1
3	2	2	2	2	3	2	2	2	2
9	9	9	1	9	9	9	9	9	9
1	1	1	2	1	3	1	1	1	2
9	9	9	9	9	9	9	9	9	9
1	1	1	2	2	2	2	2	2	2
1	2	2	2	3	3	3	2	2	2
1	1	1	2	1	1	2	3	1	3
2	3	2	2	2	2	2	2	2	2
1	1	1	1	1	1	1	1	1	1
9	9	9	9	9	9	9	9	9	9
9	9	9	9	9	9	9	9	9	9
3	3	3	2	2	2	2	2	2	2
2	2	2	2	2	2	2	2	2	2
2	1	3	1	2	2	1	2	1	2
2	2	2	2	2	2	2	2	2	2
2	2	2	2	2	2	9	9	2	2
9	1	2	2	2	1	2	3	2	1
2	3	2	1	2	3	2	1	2	3
2	9	2	2	2	2	2	9	2	9
9	9	9	9	9	9	9	9	1	9
2	2	2	2	2	2	2	1	1	1
2	2	2	2	2	2	9	1	2	2
2	2	2	1	1	2	2	1	1	1
1	2	2	3	2	3	3	2	2	2
1	2	3	1	1	2	2	3	3	2
2	2	2	2	2	2	2	2	2	2
2	2	2	2	2	2	2	2	2	2
2	2	2	2	2	2	2	2	2	2
2	2	2	2	2	2	2	2	2	2
2	2	2	2	2	2	2	2	2	2
3	3	3	3	3	3	3	3	3	3
9	9	9	9	9	9	9	9	1	9
9	9	2	9	9	9	9	9	9	9
2	2	2	2	2	2	2	2	2	2
2	2	2	2	2	2	2	2	2	2
9	9	9	9	9	9	9	1	9	9
2	2	1	1	2	1	2	1	2	2
9	9	9	2	9	9	9	9	9	9
2	2	2	2	2	2	2	2	2	2
1	2	2	2	1	2	1	1	9	1
2	2	2	2	2	2	2	2	2	2

9	2	3	1	2	1	2	1	2	1
9	9	9	9	9	9	9	1	9	9
2	2	2	1	1	1	1	1	1	1
2	1	2	2	1	3	2	2	2	2
2	3	2	2	2	3	3	3	3	3
2	1	2	2	1	1	2	2	2	1
1	2	2	2	2	1	1	1	1	2
3	2	2	2	2	1	1	3	3	2
2	2	2	2	2	2	2	2	2	2
3	3	1	9	9	9	1	9	9	9
9	9	1	9	9	9	9	9	9	1
2	2	2	2	2	2	2	2	2	2
2	3	3	9	3	2	2	2	2	2
2	3	3	2	2	2	2	2	3	3
2	1	2	2	2	1	2	2	1	2
9	1	2	2	2	1	2	2	9	1
2	2	2	2	2	2	2	2	2	2
2	2	2	2	2	2	2	2	2	2
2	2	3	2	3	2	3	2	3	2
1	3	1	1	3	1	2	2	1	2
2	1	2	2	1	1	2	1	2	1
2	2	2	2	2	1	3	3	3	2
3	2	2	1	2	1	3	3	2	3
9	9	9	9	9	9	9	9	9	9
1	2	2	3	3	3	3	3	3	3
9	9	9	9	9	9	9	1	9	9
9	9	9	9	9	9	9	9	9	9
1	9	9	9	9	9	9	9	9	9
2	2	1	1	2	2	2	3	3	3
2	2	2	3	3	3	3	3	3	2
2	2	2	2	2	2	2	2	2	2
9	9	9	1	9	9	9	9	9	9
3	3	2	2	2	2	3	1	1	3
2	2	2	2	2	2	2	2	2	2
1	9	9	9	9	9	9	1	9	9
1	2	2	2	2	2	2	1	1	1
2	3	1	1	3	1	2	3	1	1
9	2	1	1	2	9	9	3	1	2
1	2	3	1	2	3	1	2	3	2
1	3	3	1	3	1	1	1	2	1
3	2	2	2	2	2	2	2	2	2
3	9	1	1	2	2	2	2	3	1
1	9	9	1	9	9	9	9	9	9
9	9	1	9	9	9	1	9	9	9
1	2	3	1	2	1	3	1	2	3
2	2	2	2	2	2	2	2	2	2
2	2	2	2	2	2	2	2	2	2

3	1	2	2	2	9	1	1	1	3
9	9	9	9	9	9	9	9	9	9
9	9	9	9	9	9	9	9	9	9
9	9	9	9	9	9	9	9	9	9
9	9	9	9	9	9	9	9	9	9
1	9	9	9	9	9	9	9	1	9
9	9	9	9	9	9	9	9	9	9
9	9	9	9	9	9	9	9	9	9
9	9	9	9	9	9	9	9	9	9
1	9	9	9	9	9	9	1	9	9
2	1	2	2	2	1	2	2	2	2
1	1	1	1	1	1	2	9	1	2
1	9	3	2	1	2	2	1	1	1
2	2	2	2	2	2	2	2	2	2
2	2	2	2	2	2	2	2	2	2
2	2	2	1	2	2	2	2	2	1
3	3	3	1	1	1	1	2	3	1
2	2	2	2	2	2	2	1	2	2
1	2	2	1	1	2	9	1	3	2
2	3	3	2	3	2	3	2	1	2
2	1	3	1	3	2	3	2	9	1
2	1	3	1	3	2	3	1	2	1
2	2	2	3	3	3	3	3	3	3
2	2	2	2	2	2	2	2	2	2
2	2	2	3	3	3	3	3	3	3
3	3	3	3	3	3	3	3	3	3
2	2	2	2	2	2	2	2	2	2
1	2	3	1	2	3	1	2	1	3
2	2	2	2	2	2	2	2	2	2
2	2	2	2	2	2	2	2	2	2
9	9	9	9	9	9	9	9	9	9
2	2	2	2	2	2	1	2	2	2
1	1	1	2	1	1	1	1	1	1
1	9	9	1	9	9	9	9	9	9
3	3	3	3	3	3	3	3	3	3
1	1	1	2	2	2	2	2	2	2
2	2	2	2	2	2	2	2	2	2
2	2	2	2	2	2	2	2	2	2
1	2	3	1	1	3	1	3	3	2
9	9	9	9	9	9	9	9	9	9
1	1	1	1	1	2	3	2	3	3
2	2	2	2	2	2	2	2	2	2
2	2	2	2	2	2	2	2	2	2
1	2	1	2	2	2	2	2	2	2
1	2	2	3	3	2	1	2	2	3
9	9	9	9	9	9	9	9	9	9
2	2	2	2	2	2	1	2	2	2

9	9	9	9	9	9	9	9	9	9
2	2	2	3	2	2	2	2	2	2
9	9	9	1	9	9	9	9	9	9
9	9	9	9	9	9	9	9	9	9
9	9	9	9	9	9	9	9	9	9
9	9	9	9	9	9	9	9	9	9
9	9	9	9	9	9	9	9	9	9
9	9	9	9	9	9	9	9	9	9
9	9	9	9	9	9	9	9	9	9
9	9	9	9	9	9	9	9	9	9
9	9	9	9	9	9	9	9	9	9
9	9	9	9	9	9	9	9	9	9
9	9	9	9	9	9	9	9	9	9
9	9	9	9	9	9	9	9	9	9
9	9	9	9	9	9	9	9	9	9
9	9	9	9	9	9	9	9	9	9
9	9	9	9	9	9	9	9	9	9
9	9	9	9	9	9	9	9	9	9
9	9	9	9	9	9	9	9	9	9
9	9	9	9	9	9	9	9	9	9
9	9	9	9	9	9	9	9	9	9
9	9	9	9	9	9	9	9	9	9
9	9	9	9	9	9	9	9	9	9
9	9	9	9	9	9	9	9	9	9
9	9	9	9	9	9	9	9	9	9
3	2	2	1	2	1	3	1	2	1
2	2	2	2	2	2	2	2	2	2
3	3	3	2	1	1	2	1	3	3
1	1	2	1	1	1	2	1	2	2
3	1	3	1	1	3	2	2	2	3
1	2	2	1	2	2	1	2	2	2
2	2	2	2	2	2	2	2	2	2
1	9	3	1	3	3	2	3	2	2
2	2	2	1	2	2	2	2	2	2
2	2	2	2	2	2	2	2	2	2
2	2	2	3	3	1	2	1	1	3
1	9	9	1	3	1	3	2	2	1
1	3	2	1	2	1	1	2	3	1
1	3	3	1	3	1	1	1	3	2
2	2	1	2	2	2	1	1	2	2
1	3	2	1	2	1	1	1	1	2
9	9	9	1	9	9	9	9	9	9
1	2	2	1	2	2	1	3	2	2
1	3	3	1	3	1	3	1	3	3
9	9	9	9	9	9	9	1	9	9
1	3	3	1	1	1	1	3	1	1
1	3	1	1	3	1	3	2	1	2
2	2	2	1	2	2	2	2	2	2
2	2	1	2	2	2	2	2	2	2

1	2	2	1	1	1	2	1	2	2
1	2	2	1	2	1	2	2	2	1
1	3	1	1	1	1	1	1	1	1
1	2	2	1	1	1	2	1	2	2
3	2	2	2	1	2	1	2	3	2
9	9	9	1	9	9	9	9	9	9
2	2	3	1	2	1	1	1	2	2
9	9	9	1	9	9	9	9	9	9
9	9	9	2	9	9	9	9	9	9
9	9	9	2	9	9	9	9	9	9
9	9	9	9	9	1	9	9	9	9
1	9	9	1	9	9	9	9	9	9
1	9	9	9	9	1	9	9	9	9
1	9	9	9	9	9	1	9	9	9
1	9	9	9	9	9	9	9	9	9
3	3	1	1	1	3	3	3	3	3
3	3	3	3	3	3	3	3	1	9
2	2	2	2	2	2	2	2	2	2
1	9	9	9	1	9	9	9	9	9
3	3	3	1	3	3	1	1	3	1
1	9	9	1	9	9	9	9	9	9
9	9	9	9	9	9	1	9	9	9
1	9	9	1	9	9	9	9	9	9
9	9	9	9	9	9	9	9	9	9
1	9	9	1	9	9	9	9	9	9
3	2	1	2	2	3	2	1	1	2
3	3	1	1	1	3	1	1	3	3
3	2	2	2	2	3	2	2	2	3
1	3	3	2	2	2	2	3	1	1
2	2	2	2	2	2	2	2	2	2
2	9	9	3	3	2	2	3	1	1
1	3	1	1	1	9	9	9	9	9
3	3	3	1	3	3	3	1	3	3
1	2	1	3	1	2	2	1	1	1
2	2	3	3	3	2	3	3	3	2
1	9	9	9	1	9	9	9	9	9
2	2	2	2	2	2	2	2	2	2
2	2	2	2	2	2	2	2	2	2
2	2	2	3	3	1	2	3	1	2
2	2	2	2	1	3	2	2	2	1
1	2	1	1	2	1	1	1	2	2
1	2	1	1	2	1	1	1	2	2
1	1	3	1	1	3	3	3	1	3
3	2	1	2	2	2	2	2	2	2
1	1	1	1	3	3	1	3	3	3
9	9	9	9	9	9	9	1	9	9
1	9	9	9	9	1	9	9	9	9

2	9	3	1	1	1	1	1	1	1
1	3	3	2	3	1	3	3	3	3
1	3	3	1	3	3	3	1	3	3
9	9	9	9	9	9	1	9	9	9
1	9	1	3	2	1	9	9	2	9
1	2	1	3	1	2	3	1	2	3
1	3	2	1	2	1	1	1	1	1
9	9	9	9	9	9	9	9	9	9
9	9	9	2	9	9	9	9	9	9
1	1	2	3	3	3	3	3	3	3
2	2	2	2	2	2	2	2	2	2
1	3	2	3	3	3	1	1	3	1
1	1	2	1	1	2	1	1	2	2
1	9	2	1	3	1	2	9	1	9
9	9	9	1	9	9	9	9	9	9
2	2	2	2	1	2	2	2	2	2
1	9	9	9	9	9	9	9	9	1
1	9	1	9	9	9	9	9	9	9
2	3	2	3	3	3	3	3	3	3
1	2	2	1	2	1	1	1	2	1
1	3	2	3	3	3	3	2	2	1
3	3	2	3	1	3	2	3	1	1
2	3	3	3	2	3	3	3	3	1
1	2	2	1	1	1	1	1	2	1
1	2	2	2	2	2	2	2	2	2
9	9	9	9	9	9	9	9	9	9
1	2	2	1	2	3	1	3	1	9
3	1	3	3	3	3	3	1	3	1
3	3	3	3	3	3	3	3	3	3
1	2	3	1	2	3	2	2	3	2
9	9	9	9	9	9	9	9	9	9
9	9	9	9	9	9	9	9	9	9
9	9	9	9	9	9	9	9	9	9
9	9	9	9	9	9	9	9	9	9
9	9	9	9	9	9	9	9	9	9
9	9	9	9	9	9	9	9	9	9
9	9	9	9	9	9	9	9	9	9
9	9	9	9	9	9	9	9	9	9
9	9	9	9	9	9	9	9	9	9
9	9	9	9	9	9	9	9	9	9
9	9	9	9	9	9	9	9	9	9
9	9	9	9	9	9	9	9	9	9
9	9	9	9	9	9	9	9	9	9
9	9	9	9	9	9	9	9	9	9
9	9	9	9	9	9	9	9	9	9
9	9	9	9	9	9	9	9	9	9
9	9	9	9	9	9	9	9	9	9

9	9	9	9	9	9	9	9	9	9
9	9	9	9	9	9	9	9	9	9
9	9	9	9	9	9	9	9	9	9
9	9	9	9	9	9	9	9	9	9
9	9	9	9	9	9	9	9	9	9
9	9	9	9	9	9	9	9	9	9
9	9	9	9	9	9	9	9	9	9
9	9	9	9	9	9	9	9	9	9
9	9	9	9	9	9	9	9	9	9
9	9	9	9	9	9	9	9	9	9
9	9	9	9	9	9	9	9	9	9
9	9	9	9	9	9	9	9	9	9
9	9	9	9	9	9	9	9	9	9
9	9	9	9	9	9	9	9	9	9
9	9	9	9	9	9	9	9	9	9
9	9	9	9	9	9	9	9	9	9
9	9	9	9	9	9	9	9	9	9
9	9	9	9	9	9	9	9	9	9
9	9	9	9	9	9	9	9	9	9
1	2	2	1	2	2	2	2	2	2
9	9	9	9	9	9	9	9	9	9
9	9	9	9	9	9	9	9	9	9
9	9	9	9	9	9	9	9	9	9
9	9	9	9	9	9	9	9	9	9
9	9	9	9	9	9	9	9	9	9
9	9	9	9	9	9	9	9	9	9
9	9	9	9	9	9	9	9	9	9
9	9	9	9	9	9	9	9	9	9
9	9	9	9	9	9	9	9	9	9
9	9	9	9	9	9	9	9	9	9
9	9	9	9	9	9	9	9	9	9
9	9	9	9	9	9	9	9	9	9
9	9	9	9	9	9	9	9	9	9
9	9	9	9	9	9	9	9	9	9
9	9	9	9	9	9	9	9	9	9
2	2	2	2	2	2	2	2	2	2
3	3	3	3	3	3	3	3	3	3
9	9	3	3	2	3	3	2	3	3
1	3	1	1	1	1	1	1	1	3
3	3	3	3	3	3	3	3	3	3
3	3	3	1	1	1	1	1	1	1
2	3	2	9	9	1	9	9	9	9
9	9	9	1	9	9	9	9	9	9
2	3	2	9	9	1	9	9	9	9
2	2	3	2	2	2	3	1	2	3
1	1	1	1	2	1	1	1	2	3
2	2	2	3	2	3	2	2	2	2

1	3	2	1	2	1	1	1	1	2
2	2	1	1	2	3	2	1	2	3
1	1	3	1	2	1	1	1	2	1
1	2	2	1	3	1	3	2	2	1
2	2	2	1	2	2	1	2	2	1
1	2	1	1	2	1	9	3	9	1
1	2	2	1	1	1	1	1	2	3
1	3	3	1	2	2	2	2	2	2
2	1	2	2	1	3	2	2	2	1
1	3	2	1	2	1	3	2	1	1
1	9	9	1	9	9	9	9	9	9
3	3	3	2	2	3	2	2	9	2
1	2	2	1	2	1	1	3	2	2
2	2	2	1	3	3	2	2	2	1
2	1	2	2	2	3	3	2	2	2
2	9	1	3	3	2	2	2	2	2
1	2	2	1	2	1	2	1	2	1
3	2	1	2	1	1	2	3	2	1
1	1	1	1	1	3	3	1	2	1
1	2	1	2	3	3	3	2	1	1
3	9	1	2	1	2	2	2	2	3
1	2	1	1	2	1	1	1	2	2
1	9	3	1	1	2	2	2	1	1
1	9	9	9	9	9	9	9	9	9
3	3	3	3	3	3	3	3	3	3
3	1	1	1	2	3	1	2	2	1
1	1	1	1	1	1	2	9	1	1
1	3	1	3	2	1	2	2	1	3
2	2	2	2	2	2	2	2	2	2
2	2	2	2	2	2	2	2	2	2
9	9	9	2	2	3	2	2	2	3
2	2	2	2	2	2	2	2	2	2
2	2	2	2	2	2	2	2	2	2
9	9	9	9	9	9	9	9	9	9
9	9	9	9	9	9	9	9	9	9
9	9	9	9	9	9	9	9	9	9
9	9	9	9	9	9	9	9	9	9
2	2	2	9	9	9	9	9	9	9
9	9	9	9	9	9	9	9	9	9
9	9	9	9	9	9	9	9	9	9
2	2	2	2	2	1	3	2	1	2
2	1	1	2	2	2	2	2	2	2
1	1	1	1	2	2	2	1	1	2
3	1	3	3	3	3	3	1	1	1
1	2	3	1	3	1	3	1	3	1
3	3	3	3	1	3	3	3	3	3
3	1	3	1	3	3	1	3	1	3

2	1	2	2	2	2	2	2	2	2
3	2	1	1	2	3	1	2	1	2
2	2	2	2	2	2	2	2	2	2
1	1	3	1	3	1	9	1	1	1
1	2	2	1	3	3	1	2	2	2
3	1	2	3	2	1	3	2	1	2
1	1	1	1	1	9	1	1	1	3
2	3	1	2	1	3	2	1	9	2
1	3	2	3	1	2	2	2	2	1
9	9	9	1	9	9	9	9	9	9
3	3	3	2	2	2	3	3	1	3
1	1	2	1	2	3	1	1	3	3
1	1	1	3	1	3	1	1	1	1
1	1	2	1	3	3	3	2	3	3
3	2	1	1	2	1	3	1	1	2
1	1	1	3	1	2	3	2	3	2
2	1	1	2	2	2	2	2	1	2
9	9	1	9	9	1	9	9	9	9
3	2	1	1	2	1	2	1	3	3
1	9	9	9	9	9	9	1	9	9
2	3	2	1	1	2	3	2	1	2
1	2	2	2	2	1	2	2	3	1
1	1	1	1	9	9	3	2	3	3
1	2	2	1	1	1	2	2	1	1
3	1	2	1	3	2	3	3	1	2
3	3	1	3	1	3	3	3	3	3
2	3	1	1	2	1	2	2	2	1
3	1	1	1	3	3	1	1	1	3
2	2	1	1	1	2	2	1	2	2
2	3	2	1	2	3	3	2	3	1
3	3	3	2	1	3	1	3	3	3
2	3	1	2	1	2	3	1	2	1
1	1	1	1	3	2	2	2	2	1
3	3	1	3	3	1	3	3	3	3
1	1	3	1	1	3	1	1	1	3
9	1	1	1	3	1	3	3	3	3
3	1	3	1	2	1	3	2	3	1
9	9	9	9	9	9	9	9	9	9
3	3	3	1	3	3	3	1	3	3
1	2	3	3	1	3	1	3	1	1
1	3	1	1	3	3	3	1	3	1
2	2	3	1	2	2	1	3	3	2
1	3	1	3	1	3	3	1	3	3
2	1	1	1	2	2	3	1	2	2
2	2	2	2	2	2	2	2	2	2
2	2	3	9	9	2	9	9	9	9
2	2	2	3	3	3	3	3	3	3

2	3	1	2	1	2	2	2	1	2
1	1	1	3	1	2	3	2	3	2
3	1	1	2	2	9	2	9	1	2
1	2	3	2	2	2	2	1	1	3
2	2	2	1	1	1	1	1	2	2
2	3	3	2	2	3	3	2	1	1
2	2	2	2	2	2	2	2	2	2
9	9	9	9	9	9	9	9	9	9
9	9	9	9	9	9	9	9	9	9
2	1	3	1	2	3	2	1	2	3
2	2	2	2	2	2	2	2	2	2
2	2	2	2	2	2	2	2	2	2
2	2	2	2	2	2	2	2	2	2
1	1	2	2	2	2	2	2	2	2
2	2	2	2	2	2	2	2	2	2
2	2	2	2	2	2	2	2	2	2
2	2	2	2	2	2	2	2	2	2
2	2	2	2	3	2	3	2	1	2
2	2	2	2	2	2	2	2	2	2
2	2	2	2	2	2	2	2	2	2
2	3	1	2	2	2	2	2	2	2
2	2	2	2	2	2	2	2	2	2
2	2	2	2	2	2	2	2	2	2
2	2	2	2	2	2	2	2	2	2
2	2	2	2	2	2	2	2	2	2
2	2	2	2	2	2	2	2	2	2
2	2	2	2	2	2	2	2	2	2
3	1	2	1	2	1	2	9	1	2
2	2	2	2	2	1	1	3	3	3
2	2	9	2	2	2	2	2	2	2
2	2	2	2	2	2	2	2	2	2
2	2	2	2	2	2	2	2	2	2
2	2	2	2	2	2	2	2	2	2
2	2	2	2	2	2	2	2	2	2
2	2	2	2	2	2	2	2	2	2
2	2	2	2	2	2	2	2	2	2
2	2	2	2	2	2	2	2	2	2
2	2	2	2	2	2	2	2	2	2
2	2	2	2	2	2	2	2	2	2
3	1	1	3	3	3	3	1	1	3
2	2	2	2	2	2	2	2	2	2
2	2	2	2	2	2	2	2	2	2
1	2	3	2	2	1	1	3	3	3
2	2	2	2	2	2	2	2	2	2
9	9	9	2	1	2	1	2	1	1
3	3	3	3	3	3	3	3	3	3
2	2	2	2	9	2	2	2	2	2

2	2	2	2	2	2	2	2	2	2
2	2	2	2	2	2	2	2	2	2
2	2	2	2	2	2	2	2	2	2
2	2	2	2	2	2	2	2	2	2
3	2	1	2	3	2	1	2	3	2
2	2	2	2	2	2	2	2	2	2
1	1	3	1	9	9	9	9	9	9
1	1	1	1	2	3	1	3	3	3
9	9	9	9	9	9	9	9	9	9
2	2	2	2	2	2	2	2	2	2
3	3	3	3	3	3	3	3	3	2
1	1	1	1	1	1	1	1	1	1
2	1	2	2	2	3	3	3	3	3
9	9	9	3	9	9	9	9	9	9
2	2	2	2	2	2	2	2	2	2
1	2	2	2	1	2	2	2	1	2
2	2	2	2	2	2	2	2	2	2
1	2	1	1	1	1	1	1	9	3
9	9	9	9	9	9	9	9	9	9
3	3	3	2	2	2	2	2	2	1
1	1	2	1	2	1	3	2	2	1
9	9	9	9	9	9	9	9	9	9
9	9	9	1	9	9	9	9	9	9
9	2	2	2	2	2	2	2	2	2
3	1	2	1	3	2	3	1	3	2
3	2	2	2	2	3	3	3	3	3
2	2	2	1	1	3	1	2	9	2
3	9	1	1	1	1	3	9	1	9
9	1	3	3	1	2	9	1	2	1
1	1	1	1	1	1	1	1	1	1
3	3	3	3	3	3	3	3	3	3
2	2	2	2	2	2	2	2	2	2
2	2	2	2	2	2	2	2	2	2
1	9	9	9	3	2	9	9	9	9
3	2	2	2	2	2	2	2	2	2
1	9	9	9	9	9	9	9	9	9
1	1	3	1	3	1	1	1	1	3
3	3	3	2	2	2	2	2	2	2
2	2	2	2	2	2	2	2	2	2
2	2	2	2	2	2	2	2	2	2
3	1	2	2	2	2	2	2	3	3
2	2	2	2	2	2	2	2	2	2
9	9	2	9	9	3	9	9	9	9
3	1	1	1	1	1	1	1	1	1
3	2	2	2	2	2	2	2	2	2
2	3	3	2	2	2	2	2	2	2
2	2	2	2	2	2	2	2	2	2

1	1	1	1	1	1	1	1	1	1
2	2	2	2	2	2	2	2	2	2
1	1	1	1	1	1	1	1	1	1
2	2	2	2	2	2	2	2	2	2
9	9	9	9	9	9	9	9	9	9
2	1	3	2	3	2	1	2	3	1
9	9	9	3	3	3	3	3	3	3
1	1	1	1	1	1	1	1	1	1
3	9	1	1	1	2	2	1	3	1
1	1	1	3	3	3	3	3	3	3
2	2	2	1	1	1	1	1	1	1
2	2	2	2	2	2	2	2	2	2
2	2	2	2	2	2	2	2	2	2
2	2	2	2	2	2	2	2	2	2
2	2	2	2	2	2	2	2	2	2
2	2	2	2	2	2	2	2	3	3
1	1	1	1	1	1	1	1	1	1
3	3	3	1	1	3	3	2	3	3
9	9	9	9	9	9	9	9	9	9
1	2	1	1	2	1	2	3	1	2
1	2	2	1	1	1	1	1	2	2
3	3	3	3	3	3	2	2	2	3
3	3	3	2	2	3	2	9	2	3
1	2	2	1	1	1	3	3	3	3
3	3	3	3	3	3	3	3	3	3
2	1	2	2	2	1	1	1	1	2
3	2	1	1	1	1	1	2	2	2
2	2	2	2	1	2	2	2	1	2
2	2	3	1	2	1	2	1	2	2
2	2	2	2	2	2	2	2	2	2
1	1	1	2	2	2	2	2	2	2
2	2	2	2	2	2	2	2	2	2
3	3	3	3	3	3	3	3	3	3
1	3	3	2	1	3	1	2	1	3
2	2	2	2	2	2	2	2	2	2
2	2	2	2	2	2	2	2	2	2
1	2	3	2	1	3	2	2	2	3
3	3	3	2	2	2	2	2	2	2
3	1	2	1	2	3	1	2	3	1
3	3	3	3	3	3	3	3	3	3
1	1	1	1	2	1	2	1	2	3
2	3	2	1	1	1	1	1	1	1
9	9	9	9	9	9	9	9	9	9
3	3	3	3	3	9	9	9	3	3
1	1	1	1	1	1	1	1	1	1
2	2	2	2	2	2	2	2	2	2
1	3	3	3	3	3	2	1	2	2

1	1	9	1	1	1	1	1	1	1
3	3	3	3	3	3	3	1	3	1
3	3	3	2	2	2	2	9	1	1
3	3	3	3	3	3	3	1	3	1
2	2	2	1	1	2	1	1	1	1
2	2	2	2	2	2	2	2	2	2
2	2	2	2	2	2	2	2	2	2
2	2	2	2	2	2	2	2	2	2
2	2	2	1	1	1	1	1	1	1
1	1	1	2	3	1	1	2	9	1
2	2	2	1	1	1	1	1	1	1
1	2	1	1	2	3	3	2	1	2
2	2	2	2	2	2	2	2	2	2
9	9	9	1	3	2	1	3	2	1
2	2	2	2	2	2	2	2	2	2
2	2	2	2	2	2	2	2	2	2
2	2	2	2	2	2	2	2	2	2
2	2	2	3	2	3	2	2	1	3
2	2	2	2	2	2	2	2	2	2
3	3	2	3	3	1	2	1	2	2
2	2	1	2	2	2	2	2	2	2
1	2	3	2	2	1	2	1	1	2
1	2	3	1	3	3	2	2	1	1
2	2	2	2	2	2	2	2	2	2
1	1	1	1	1	1	1	1	1	1
1	3	1	3	1	3	1	3	3	3
3	1	1	1	1	2	1	1	2	3
3	3	2	1	2	3	2	1	3	2
3	1	2	1	3	2	1	2	2	1
3	2	3	3	3	3	2	2	1	2
9	2	2	2	2	2	2	2	2	2
2	2	2	2	2	2	2	2	2	1
2	2	1	2	2	2	2	2	1	2
3	3	2	1	2	3	2	1	3	2
2	1	3	1	2	3	1	3	1	3
2	2	2	2	2	2	2	2	2	1
2	2	2	2	2	2	2	2	2	2
9	9	9	9	9	9	9	9	9	9
2	2	1	1	2	2	1	2	1	2
3	1	2	2	3	2	2	2	9	1
3	2	1	2	2	1	2	3	2	3
2	2	2	2	2	2	2	2	2	2
2	2	2	2	2	2	2	2	2	2
2	2	2	2	2	2	2	2	2	2
1	2	3	2	3	1	2	3	3	9
2	2	2	1	1	2	1	1	1	1
2	2	2	2	2	2	2	2	2	2

9	9	9	9	9	9	9	9	9	9
9	3	9	1	2	3	1	2	3	1
2	2	2	1	1	2	1	1	1	1
1	9	1	1	3	3	3	3	3	3
1	1	3	3	1	2	2	3	1	1
1	2	2	3	1	3	2	2	2	3
3	2	1	1	2	2	1	1	3	2
2	2	2	9	9	9	9	9	9	9
3	3	3	3	3	3	3	1	3	2
3	3	3	3	3	3	3	1	1	3
2	2	2	2	2	2	2	1	2	2
2	2	3	1	2	2	2	2	2	2
2	2	2	2	2	2	2	2	2	2
2	1	1	1	1	1	1	1	1	1
3	2	3	1	2	3	2	1	3	1
2	2	2	2	2	2	2	2	2	2
1	1	2	1	2	2	1	1	2	3
3	1	9	3	1	2	2	1	2	2
9	9	1	9	9	9	9	9	9	9
2	2	2	2	2	2	2	2	2	2
9	9	9	9	9	9	9	9	9	9
3	1	2	1	2	3	3	2	1	2
3	2	2	3	2	1	2	1	3	3
3	3	3	1	1	3	3	3	2	2
1	1	3	3	3	2	2	2	1	1
3	2	2	2	2	3	3	3	3	3
2	2	2	1	2	2	3	2	1	2
2	3	2	1	1	2	2	2	1	2
2	2	1	2	2	3	1	3	2	2
2	2	3	2	2	3	3	1	2	1
2	2	2	1	1	1	2	2	2	3
1	2	2	2	1	3	1	3	2	9
3	3	3	1	3	3	3	1	3	3
2	2	2	2	2	2	2	2	2	2
2	2	2	2	2	2	2	2	2	2
2	2	2	2	2	2	2	3	3	3
3	3	3	3	3	3	3	3	3	3
1	3	2	2	2	2	3	1	2	3
1	2	2	2	1	1	1	1	1	1
1	1	2	2	1	1	1	1	1	1
3	3	3	1	1	1	1	1	1	1
3	3	3	1	1	1	2	3	3	3
1	3	2	2	3	2	1	1	2	3
2	3	3	2	2	2	2	2	2	3
9	9	9	9	9	9	9	9	9	9
3	3	3	3	3	3	3	3	3	3
2	3	3	2	2	2	2	2	2	3

3	3	3	1	1	2	3	3	3	3
3	2	3	3	3	3	3	3	3	1
3	1	2	2	3	2	2	2	3	2
9	9	9	9	9	9	9	9	9	9
2	3	2	2	2	3	2	2	2	3
3	3	3	3	3	3	3	3	3	3
9	2	2	2	2	2	2	2	2	2
3	3	3	3	3	3	3	3	3	3
2	2	3	3	3	3	3	2	2	2
3	2	1	1	2	3	3	3	1	2
2	2	2	2	2	2	2	2	2	2
2	3	2	2	2	3	1	3	2	3
1	1	1	1	2	1	2	1	2	1
9	9	9	1	9	9	9	9	9	9
1	1	2	2	2	1	1	1	1	1
3	3	3	2	3	3	2	2	3	3
3	1	3	1	1	1	1	1	2	1
2	2	2	2	2	2	2	2	2	2
2	2	2	3	3	2	2	3	2	2
2	9	1	3	2	3	2	1	3	1
2	3	2	3	1	2	3	2	3	1
9	3	3	3	3	3	3	3	3	3
2	2	2	2	2	2	2	2	2	2
2	2	2	2	1	3	2	2	2	3
3	1	2	1	2	3	1	2	3	1
2	3	1	1	3	3	3	3	3	3
2	2	3	3	1	2	3	3	3	1
3	2	2	2	2	3	9	1	2	3
1	2	3	2	3	9	3	1	2	1
2	2	2	2	2	2	2	2	2	2
1	2	3	1	2	1	2	1	2	3
2	2	2	2	2	2	2	2	2	2
3	2	1	1	2	3	1	2	3	1
3	3	3	1	1	2	3	3	3	3
1	2	3	1	2	3	1	2	3	1
3	1	1	1	3	2	3	2	3	1
3	9	1	1	2	2	2	3	2	3
3	3	3	2	2	2	1	2	1	1
3	1	1	1	1	1	2	2	2	2
3	1	2	1	1	2	2	3	9	1
2	2	2	2	1	2	2	1	2	2
2	3	2	1	3	1	3	1	3	1
2	2	1	1	1	2	3	2	3	2
3	1	3	3	2	1	3	9	2	2
3	2	2	2	2	3	2	2	3	2
2	1	2	1	3	2	2	1	2	1
3	3	3	1	3	9	1	3	1	2

2	2	2	3	3	3	3	2	2	3
3	1	3	1	3	2	1	3	1	2
2	2	2	2	2	2	2	2	2	2
3	1	3	1	1	3	1	1	1	3
3	1	2	1	2	3	1	2	3	1
3	1	2	2	2	2	2	2	2	2
2	3	1	2	1	2	3	2	1	2
2	1	3	2	1	2	3	1	2	3
2	1	3	1	1	2	1	2	1	2
3	3	3	3	3	3	3	1	1	2
3	2	3	1	2	3	2	3	2	3
2	1	3	1	2	3	1	2	3	2
1	1	1	1	1	1	1	1	1	1
1	1	3	1	3	1	1	1	3	3
2	3	2	1	2	3	2	2	1	1
3	1	3	3	1	1	1	3	3	3
2	2	3	1	1	3	3	2	1	1
2	9	9	3	3	3	3	3	3	3
3	2	3	1	2	3	1	2	1	1
1	2	1	3	1	1	1	2	1	1
2	2	2	2	2	2	2	2	2	1
3	3	3	2	1	3	3	3	1	3
3	2	1	1	3	2	1	1	3	1
2	2	3	2	2	3	3	1	2	1
2	2	2	2	2	3	1	3	2	3
2	2	2	2	2	2	2	2	2	2
1	3	3	1	1	3	2	1	1	3
3	2	3	2	1	2	1	2	1	3
2	2	2	2	2	2	2	2	2	2
9	9	9	9	9	9	9	9	9	9
9	9	9	9	9	9	9	9	9	9
9	9	9	9	9	9	9	9	9	9
2	2	2	2	2	2	2	2	2	2
9	9	9	1	9	9	9	9	9	9
3	3	3	2	2	2	2	2	2	2
2	2	2	2	2	2	2	2	2	2
3	1	2	2	2	3	2	2	2	3
2	2	2	2	2	2	2	2	2	2
9	2	3	9	2	9	2	9	9	2
3	2	2	2	2	3	2	3	2	3
2	1	2	3	2	3	2	3	2	3
1	2	2	3	1	1	2	2	2	3
3	2	3	3	3	2	2	2	1	3
3	2	3	3	3	2	2	2	1	3
3	2	1	1	2	3	3	3	2	1
1	1	1	2	2	2	2	2	2	2
1	3	3	1	1	3	2	1	1	3

2	2	2	9	9	9	9	9	9	9
1	2	2	1	2	3	2	2	2	1
2	2	2	2	2	2	2	2	2	2
2	1	1	1	3	3	2	1	1	1
2	2	2	3	2	2	2	2	2	2
3	1	2	2	2	3	2	2	2	3
1	3	2	1	2	1	1	2	3	9
2	2	3	2	3	3	1	2	3	1
1	2	1	1	2	1	3	3	3	3
3	3	2	3	3	3	3	2	2	3
2	3	2	2	3	2	1	2	3	2
2	3	2	3	2	1	2	3	2	3
2	1	1	1	1	3	1	1	9	3
2	2	2	2	2	2	2	2	2	2
2	2	2	2	2	2	2	2	2	2
3	3	3	3	3	2	2	2	3	9
2	2	2	3	1	2	2	2	2	2
2	2	2	2	2	2	2	2	2	2
2	2	2	2	2	2	2	2	2	2
3	1	2	3	2	3	1	1	9	9
2	9	9	9	1	1	9	9	9	1
1	9	9	9	1	1	9	9	9	1
1	9	9	9	1	1	9	9	9	1
2	2	2	1	1	1	1	1	1	1
1	3	3	3	1	1	3	3	3	1
1	9	9	9	1	1	9	9	9	1
1	9	9	2	1	1	2	2	2	2
1	2	1	1	1	1	9	9	9	1
1	9	2	1	2	1	2	1	2	1
9	9	9	9	9	1	9	9	9	1
1	9	9	1	9	1	9	9	9	1
9	9	9	9	9	1	9	9	9	9
1	3	3	3	3	1	3	3	3	1
3	3	3	3	3	1	3	3	3	1
1	9	9	9	1	1	9	9	9	1
1	2	2	2	1	1	3	3	3	1
1	9	9	9	1	1	9	9	9	1
1	9	9	9	1	1	9	9	9	1
1	9	9	9	1	1	9	9	9	1
1	9	9	9	1	1	9	9	9	1
1	3	3	1	3	1	3	3	3	1
9	9	9	1	9	1	1	9	9	2
1	9	9	9	1	1	9	9	9	1
9	9	9	1	9	9	9	9	9	9
1	9	9	9	1	1	9	9	9	1
1	3	2	3	1	1	3	3	2	1
1	2	2	9	1	1	9	9	9	1

1	9	9	9	1	1	9	9	9	1
1	2	9	3	1	1	1	3	2	1
2	2	1	1	1	1	1	1	2	1
1	1	1	1	1	1	1	1	1	1
1	9	9	9	9	1	9	9	9	1
1	9	9	9	9	1	9	9	9	1
1	1	1	1	1	1	1	1	1	1
2	2	2	1	3	1	1	1	1	1
1	9	9	9	1	1	9	9	9	1
1	9	9	3	1	1	3	9	9	1
1	2	2	2	1	1	2	2	2	1
1	2	2	2	1	1	2	2	1	2
1	9	9	1	9	1	9	9	9	1
1	9	9	9	1	1	9	9	9	1
1	9	9	9	1	1	9	9	9	1
1	9	9	9	1	1	9	9	9	1
1	9	9	9	9	1	9	9	9	1
1	9	9	9	9	1	9	9	9	1
1	9	9	9	1	1	9	9	9	1
9	9	9	1	1	1	1	9	9	9
9	9	9	9	1	9	9	9	9	9
1	9	9	9	9	1	9	9	9	1
1	1	1	3	3	1	1	1	1	1
9	9	1	9	9	1	9	9	9	1
1	1	1	3	3	1	3	3	1	1
1	3	3	3	3	1	3	3	3	3
1	3	3	3	3	1	3	3	3	3
1	9	9	3	3	1	9	3	3	1
1	9	9	3	1	1	3	3	3	1
1	9	9	9	9	1	9	9	9	9
1	2	2	9	1	1	1	1	1	1
1	3	3	3	1	1	3	3	3	1
1	9	9	9	1	1	9	9	9	1
1	9	9	9	1	1	9	9	9	1
1	9	9	9	1	1	9	9	9	1
1	3	3	3	3	1	1	1	2	1
1	9	9	9	9	1	9	9	9	1
1	9	9	9	9	1	9	9	9	1
1	9	9	3	1	1	3	3	3	1
2	3	2	3	1	1	1	1	1	1
1	3	2	3	1	1	3	3	1	1
2	2	2	1	3	1	3	1	1	1
1	9	9	9	1	1	9	9	9	1
1	9	9	9	9	1	3	1	1	1
1	9	9	3	1	1	3	3	3	1
1	9	9	1	3	1	3	3	3	1
1	9	9	9	1	1	9	9	9	1

1	9	9	9	1	1	9	9	9	1
1	3	3	1	1	1	9	9	9	1
1	9	9	9	1	1	9	9	9	1
1	3	1	1	1	1	1	3	1	1
1	9	9	9	1	1	1	9	9	1
9	9	9	9	1	9	9	9	9	1
1	3	1	1	3	9	9	9	9	9
1	9	9	9	9	1	9	9	9	1
1	9	9	9	9	1	9	9	9	1
1	3	3	1	3	1	1	1	3	3
2	2	2	1	3	1	1	1	2	2
9	9	9	9	9	9	9	1	9	9
9	1	9	9	9	1	9	1	9	2
2	2	2	3	2	1	2	2	2	2
2	2	2	1	3	1	2	2	2	2
2	1	2	1	2	1	1	1	2	2
9	1	9	9	9	9	9	1	9	9
9	1	9	1	9	9	9	9	9	9
2	1	2	2	2	2	1	1	2	1
2	1	1	1	1	1	1	1	1	1
3	3	3	3	3	3	3	2	2	3
2	2	1	3	3	2	9	1	3	2
2	9	2	1	3	1	1	1	2	1
3	3	3	1	2	1	1	1	1	1
9	9	9	9	3	9	9	9	9	9
1	2	2	1	1	1	1	1	2	2
2	1	3	1	2	1	3	2	1	2
2	2	2	2	3	1	1	1	2	3
1	2	1	2	2	2	1	1	2	1
2	2	2	1	3	1	1	1	3	1
2	2	2	2	2	1	2	2	2	2
2	1	3	1	2	2	1	2	1	1
2	9	2	1	1	1	1	1	1	1
1	9	9	9	9	9	9	9	9	9
2	2	2	1	3	1	1	1	2	1
2	2	1	1	1	1	2	1	1	1
1	2	2	1	9	1	1	2	2	1
1	2	2	1	2	1	1	2	2	2
1	2	2	1	9	1	1	2	2	1
2	3	2	1	3	1	1	1	3	3
1	2	3	1	3	1	1	1	2	1
1	3	1	1	3	1	1	1	2	1
2	2	2	1	2	3	3	2	2	2
2	2	2	1	1	2	9	2	2	2
2	2	2	1	2	3	3	9	2	2
9	1	9	1	1	1	1	1	1	1
9	9	9	9	9	9	9	1	9	9

1	9	9	3	1	1	3	1	2	1
1	2	1	1	2	1	1	1	1	1
2	2	2	1	1	1	9	1	2	1
2	2	2	2	2	2	2	2	2	2
2	3	2	9	9	9	1	9	9	9
2	2	2	1	2	3	1	2	2	3
2	2	2	2	2	2	2	2	2	2
3	3	3	1	2	1	1	2	2	1
9	9	9	3	1	1	3	1	3	1
9	9	9	1	3	3	1	1	2	1
3	9	3	1	3	1	1	1	2	1
3	3	1	3	3	3	3	3	3	1
1	2	2	2	2	3	2	2	9	1
1	3	3	9	9	9	9	9	9	9
1	2	2	2	9	9	1	2	2	2
1	9	9	9	9	9	9	9	9	9
1	2	2	2	2	3	1	1	2	2
1	2	1	9	9	9	9	9	9	9
1	3	3	3	2	3	1	1	1	1
1	9	9	1	2	3	3	2	2	1
9	9	9	2	9	9	9	9	9	9
9	9	9	9	9	9	9	9	9	9
2	2	1	2	2	2	1	2	2	2
3	3	3	1	3	3	3	3	3	1
1	2	3	2	2	3	1	2	1	3
1	2	2	1	2	3	1	2	9	3
1	9	9	9	9	9	1	1	9	9
1	9	9	9	9	9	9	9	9	9
1	9	9	9	9	9	9	9	9	9
1	2	2	3	2	3	2	1	2	2
1	9	9	3	3	3	1	9	9	9
1	3	1	2	3	3	1	1	1	3
1	9	9	9	1	1	9	9	9	1
1	3	3	1	9	1	3	9	3	1
2	2	1	1	3	1	3	3	3	1
9	9	9	9	9	9	9	9	9	1
1	2	2	1	1	1	3	1	3	1
1	3	2	2	2	3	3	3	3	2
1	3	3	3	1	1	3	3	3	1
1	2	2	1	1	1	3	1	3	1
1	2	1	2	1	1	3	2	2	1
1	9	9	9	9	1	9	9	9	1
1	3	1	1	1	1	3	3	3	1
1	9	9	9	9	1	9	9	9	9
1	3	3	3	1	1	3	3	3	1
1	9	9	9	1	1	9	9	9	1
1	9	9	1	9	1	9	9	9	1

1	3	3	3	3	1	3	3	3	1
1	9	1	9	9	1	9	9	9	1
9	9	9	9	9	9	9	9	9	9
9	9	9	9	9	9	9	9	9	9
9	9	9	9	1	9	3	3	3	3
1	2	2	2	2	3	1	1	1	3
1	9	9	9	9	9	1	9	9	9
1	9	9	9	1	9	9	9	9	9
1	9	9	9	9	9	9	1	9	9
1	9	9	9	9	9	9	9	9	9
3	3	1	3	3	3	3	3	3	1
1	2	2	2	2	2	1	2	2	1
1	3	1	3	3	3	1	1	2	3
1	9	9	9	9	9	9	9	9	9
1	1	1	1	1	3	1	1	3	1
1	2	1	3	2	2	1	1	2	3
1	9	9	9	9	9	9	9	9	9
9	9	9	2	2	3	1	3	9	3
1	9	9	9	9	9	1	9	9	9
1	3	3	3	3	3	1	2	3	3
1	3	3	1	2	1	3	3	1	1
1	9	9	1	9	9	9	9	9	9
1	9	9	1	9	9	9	9	9	9
1	1	3	2	2	2	2	2	1	1
9	9	9	9	9	1	1	1	9	1
1	1	2	2	1	2	1	1	2	2
1	3	9	9	9	9	1	9	9	9
1	3	2	3	2	3	1	2	3	3
2	2	1	1	3	3	2	2	2	9
2	2	1	1	2	3	2	2	2	3
2	1	1	2	3	3	3	1	2	3
1	1	2	2	2	2	1	2	1	2
1	9	9	3	3	3	2	1	3	2
1	3	3	3	3	3	1	1	3	3
1	3	1	3	3	3	2	2	3	3
1	9	9	9	1	9	9	9	9	9
1	9	9	3	3	3	3	9	9	9
1	9	9	9	9	9	9	9	1	9
3	2	1	2	2	2	9	1	1	1
1	3	2	3	3	3	3	9	2	3
1	3	2	2	3	3	1	2	3	3
1	3	3	3	3	3	3	3	2	2
1	9	9	9	1	9	9	9	9	9
1	9	9	9	1	9	9	9	9	9
9	9	9	9	9	9	1	9	1	9
9	9	9	9	9	9	9	9	9	9
1	9	9	1	9	9	1	9	9	9

3	3	1	3	3	1	1	3	3	3
1	9	9	2	2	3	1	1	1	3
1	9	9	9	9	9	9	9	9	9
1	2	2	3	2	1	1	3	3	3
1	3	3	3	3	1	1	1	1	1
9	9	9	2	9	9	9	9	9	9
1	2	2	2	2	2	1	3	3	2
2	2	2	2	2	2	2	2	2	2
1	2	2	3	3	3	3	3	3	3
1	2	1	2	2	2	1	1	2	3
1	3	2	3	3	3	1	1	2	3
9	9	9	9	9	9	9	9	1	9
1	9	9	9	9	9	9	9	1	9
1	9	1	9	9	9	1	9	9	9
1	9	9	9	9	9	9	9	9	9
1	9	9	9	9	9	1	9	9	9
1	3	3	9	9	9	9	9	9	9
1	3	2	2	2	2	2	2	2	2
9	9	9	9	9	9	9	9	1	9
1	2	2	2	1	2	2	2	1	1
2	3	1	3	3	3	1	1	1	3
1	2	2	2	2	3	2	3	2	3
1	2	2	2	2	3	2	2	3	3
1	2	3	3	2	3	1	2	2	2
1	3	3	3	3	3	2	3	3	3
1	9	9	9	9	9	1	9	9	9
1	3	3	2	2	3	3	3	3	3
1	3	3	2	2	2	1	2	2	2
1	9	9	9	9	9	1	9	9	9
1	9	9	9	9	9	9	1	9	9
1	2	1	3	2	3	1	2	2	3
1	1	9	9	9	9	9	9	9	9
1	9	9	9	9	9	9	1	9	9
9	2	1	3	1	1	3	1	1	2
1	9	9	3	9	1	9	9	9	9
1	3	3	2	3	3	2	2	2	1
2	2	2	1	3	1	2	1	2	1
1	2	2	3	1	1	1	1	9	1
9	9	9	9	9	3	1	9	9	1
1	9	9	9	1	1	9	9	9	1
1	2	3	3	3	1	2	2	2	2
3	2	2	1	2	3	3	3	3	3
9	1	9	1	2	1	1	1	2	1
1	1	1	1	2	1	2	2	2	1
2	2	1	2	1	2	2	1	2	1
1	9	9	9	9	9	9	9	1	9
1	1	1	3	3	3	2	3	9	1

1	3	3	3	3	3	1	2	3	3
1	3	3	2	3	3	3	2	1	2
2	1	2	3	2	1	2	2	1	1
2	2	1	3	3	3	1	1	1	3
2	1	2	3	3	2	1	2	2	2
2	1	2	2	2	2	1	1	3	3
2	2	3	3	3	3	1	3	2	3
2	2	2	9	2	2	1	2	2	2
2	1	3	3	3	3	1	1	1	3
2	2	2	3	2	1	1	2	1	2
2	2	2	2	2	3	3	1	2	2
2	2	2	2	2	2	2	2	2	2
1	2	2	2	3	3	1	3	3	3
2	2	2	3	1	1	2	2	1	3
2	2	2	2	2	2	1	2	1	2
2	2	2	3	3	2	2	2	3	3
2	2	2	2	2	2	2	2	2	2
2	2	2	2	2	2	2	2	2	2
2	2	2	2	2	2	2	2	2	2
1	1	1	2	2	3	1	1	2	2
1	2	3	3	2	3	1	2	1	2
2	3	2	3	3	3	1	2	3	2
2	2	2	9	1	3	2	2	3	3
2	2	2	2	2	2	1	2	1	1
1	2	1	2	2	2	1	2	2	2
2	3	2	3	3	3	1	2	3	1
2	3	2	2	2	1	1	2	2	1
1	9	1	2	2	2	1	2	2	2
1	2	1	2	2	2	1	2	2	2
3	9	3	3	2	9	1	2	9	1
2	2	2	3	3	3	1	1	2	2
1	2	1	3	3	2	2	3	3	1
2	2	1	9	9	9	9	9	9	9
2	2	1	1	9	9	9	9	9	9
1	9	9	9	9	9	9	9	9	9
9	9	9	1	9	9	9	9	9	9
9	9	9	9	9	9	9	9	9	9
9	9	9	9	9	9	9	9	9	9
9	9	9	9	9	9	1	9	9	9
2	3	1	3	3	3	1	2	2	3
3	2	1	3	2	3	1	2	1	2
9	9	9	9	9	9	9	1	9	9
9	9	9	9	9	9	9	1	9	9
2	3	2	3	3	3	1	2	3	3
9	9	9	9	9	9	9	9	9	9
9	9	9	1	9	9	9	9	9	9
9	9	9	1	9	9	9	9	9	9

2	2	2	3	3	3	2	2	2	3
2	2	3	3	1	3	2	3	3	1
9	9	9	1	9	9	9	9	9	9
9	9	9	9	9	9	9	9	9	9
9	9	9	9	9	9	9	9	9	9
9	9	9	9	9	9	9	9	9	9
1	9	9	9	9	9	1	9	9	9
1	9	9	9	9	9	1	9	9	9
9	9	1	9	9	9	1	9	9	9
9	9	9	9	9	9	9	9	1	9
1	3	1	3	3	3	3	1	3	3
3	3	3	3	3	3	3	3	3	3
1	3	3	3	3	3	3	1	1	2
1	3	3	3	3	3	2	3	1	3
1	2	1	3	3	3	3	3	1	3
1	3	3	3	3	3	1	3	1	3
1	3	3	3	3	3	1	3	1	3
1	3	3	3	3	3	1	3	1	1
3	3	3	3	3	3	3	3	1	1
2	2	1	2	2	2	2	2	1	1
1	2	2	1	2	3	1	2	2	1
1	2	3	1	1	3	3	1	1	1
9	9	1	9	9	9	9	1	9	9
1	2	1	2	3	1	2	2	1	3
3	3	1	3	3	3	3	3	3	3
1	1	3	3	3	2	1	1	1	2
9	9	9	9	1	9	1	3	3	3
1	2	3	3	3	3	1	3	3	3
1	1	3	3	3	1	3	3	3	3
2	2	3	2	1	2	1	3	2	3
1	3	1	3	2	2	3	2	3	3
9	9	9	2	2	1	9	1	1	3
1	1	3	2	2	3	3	3	2	3
1	3	3	2	2	3	3	3	1	3
2	2	3	2	2	2	3	1	1	2
1	1	1	1	3	3	3	3	3	3
1	3	3	3	3	3	1	3	1	3
1	3	1	3	3	3	3	3	1	3
3	3	1	3	3	3	3	9	3	3
1	1	1	3	3	3	1	2	1	3
1	3	3	1	1	2	1	3	3	2
1	9	1	3	3	1	3	3	1	1
1	2	3	2	3	2	3	3	2	2
3	3	3	3	3	3	1	1	3	3
3	3	3	3	3	3	1	1	3	3
3	3	3	3	3	1	3	3	3	3
1	2	2	2	3	3	2	2	1	2

1	2	3	3	2	1	1	9	9	9
1	1	1	3	3	1	1	1	1	2
1	2	1	1	2	3	2	9	1	3
3	3	3	3	3	3	1	3	3	3
3	3	3	3	3	3	1	1	3	3
3	3	3	3	3	3	1	3	3	3
1	2	2	2	3	3	2	2	1	2
1	9	1	3	2	2	2	2	9	9
1	2	2	2	2	2	2	2	2	3
1	1	1	1	1	9	9	9	1	9
2	2	2	1	2	2	2	2	2	1
2	2	2	1	1	1	1	9	1	1
9	9	9	9	1	1	2	2	2	1
2	3	2	1	3	1	3	2	2	1
1	1	1	1	1	1	3	1	3	1
2	2	2	2	2	2	2	2	2	2
1	9	1	2	2	9	1	9	9	1
2	2	2	2	2	3	3	2	2	2
1	3	1	1	3	2	1	1	2	3
1	2	2	2	3	3	2	2	2	2
2	2	2	3	3	1	1	2	3	1
1	1	1	3	3	3	3	1	3	3
1	2	3	2	2	2	2	1	2	1
1	2	3	2	2	2	2	1	2	1
1	2	3	2	2	2	2	1	3	1
1	1	2	1	2	2	1	2	3	3
2	2	2	3	2	3	2	2	2	3
2	2	2	3	3	1	1	2	2	3
2	2	2	2	2	2	1	2	2	2
2	1	2	2	2	2	2	1	2	2
2	2	2	2	3	2	2	3	2	3
1	2	2	2	3	3	3	2	2	2
9	9	9	2	2	1	1	1	3	3
2	3	3	3	3	3	1	1	3	3
1	2	3	2	2	2	2	1	2	1
3	3	3	3	3	1	3	3	3	3
1	2	9	3	3	2	2	3	3	3
2	2	2	3	3	2	2	3	1	3
9	9	9	3	3	3	1	3	3	3
1	1	2	1	3	3	1	3	1	3
2	2	2	1	2	3	1	2	2	3
3	3	3	3	9	1	1	2	3	3
3	3	3	3	1	1	1	2	3	3
2	2	1	1	1	2	1	1	2	3
2	2	2	3	3	3	2	1	3	3
2	2	2	3	3	3	3	2	2	3
2	2	2	3	1	3	1	3	1	3

2	9	1	1	1	2	2	2	3	3
2	2	2	2	2	2	2	2	2	1
2	2	1	1	1	2	1	1	2	3
2	2	1	2	2	2	1	3	1	3
2	1	3	1	2	1	1	1	1	3
2	2	3	2	2	1	1	2	2	2
9	9	9	3	3	3	9	2	2	2
2	2	2	3	3	3	3	2	2	3
2	2	2	1	2	2	2	2	2	2
1	1	1	3	3	3	1	3	3	3
2	3	3	1	3	3	3	1	1	3
2	1	2	3	1	1	1	3	1	2
9	9	9	9	9	9	9	9	9	9
2	2	2	2	2	3	2	2	1	1
3	3	3	1	3	3	1	3	3	3
2	2	2	2	2	2	2	2	2	2
1	2	1	3	3	2	1	3	1	2
2	2	2	2	2	1	1	2	1	1
2	2	2	2	2	2	1	2	1	1
9	1	3	2	2	2	1	1	1	2
1	3	1	3	3	3	1	1	1	3
1	2	2	1	2	3	2	1	2	3
1	3	3	3	3	3	1	2	3	1
1	3	3	3	2	3	2	3	1	2
1	3	3	3	3	3	3	3	3	3
1	3	3	3	3	3	3	3	1	3
1	3	3	3	3	3	1	3	1	3
1	3	2	3	3	3	1	2	3	1
1	1	1	1	1	1	1	1	2	9
1	9	9	9	9	2	9	9	9	9
2	3	2	3	3	3	1	2	3	2
3	2	3	3	3	3	1	2	1	1
2	2	1	2	2	3	1	1	2	3
2	2	2	2	2	3	1	1	3	2
1	9	9	9	9	9	1	9	9	9
2	2	2	3	3	3	1	3	1	3
2	2	2	2	2	2	2	2	2	2
9	9	9	9	9	2	9	9	9	9
1	1	1	2	1	1	2	3	2	1
2	2	1	3	3	3	3	1	2	1
9	9	9	9	9	9	9	9	9	9
2	2	2	1	3	3	1	2	3	2
1	2	1	1	2	2	1	1	2	2
2	3	3	2	3	2	9	2	1	2
3	2	2	3	3	3	3	1	1	3
2	1	3	3	3	3	2	2	3	3
2	2	2	3	2	3	2	2	3	3

3	3	3	1	2	3	1	2	2	3
2	2	2	2	2	2	1	2	2	2
2	2	2	2	2	2	2	2	2	2
1	2	2	2	2	2	2	2	2	1
2	1	2	1	2	2	1	2	2	3
3	2	2	2	3	3	3	2	3	9
2	2	2	3	3	3	1	1	1	1
9	9	9	9	9	9	9	9	9	1
2	2	2	2	2	2	1	2	2	3
3	9	2	3	3	3	2	2	2	2
1	2	1	2	2	2	1	2	2	2
1	9	9	9	9	9	9	9	9	9
1	2	1	1	1	3	3	3	9	1
2	2	2	2	2	2	2	2	2	2
9	3	9	3	3	2	2	3	3	3
1	2	1	3	3	2	2	3	3	3
1	1	1	1	2	2	2	2	2	2
1	1	1	2	1	1	9	1	9	9
9	9	9	9	9	9	9	9	9	9
1	3	1	1	1	1	1	1	1	1
1	3	1	1	1	1	1	1	2	1
2	2	2	1	1	2	2	1	1	1
2	2	2	1	2	2	2	2	2	2
2	2	2	1	2	2	2	2	2	1
3	3	3	1	1	2	3	1	2	2
2	2	2	1	1	1	9	1	9	1
2	2	2	1	2	1	1	1	2	1
1	2	2	1	2	1	1	1	2	1
1	1	2	1	2	2	1	1	2	1
9	9	1	3	9	9	9	9	9	9
1	2	2	1	2	1	1	1	2	1
3	2	1	2	2	1	1	1	2	3
1	1	1	1	2	1	2	1	2	1
2	2	2	3	3	1	1	2	2	1
1	2	1	1	2	3	1	2	2	1
1	2	1	1	2	1	2	2	2	1
1	2	2	1	1	1	2	2	1	1
2	2	2	1	1	1	1	2	2	1
1	3	3	1	1	1	3	1	3	1
2	2	2	1	3	1	2	2	2	1
2	2	2	2	2	1	1	2	2	1
1	1	1	1	1	1	1	1	3	1
2	2	2	2	2	1	2	1	2	2
3	2	3	3	3	3	1	1	3	1
2	1	1	1	1	1	3	3	3	1
1	2	2	1	2	1	1	3	3	1
1	2	1	1	1	1	1	1	2	1

2	3	3	1	3	1	3	3	3	1
2	3	3	1	3	1	3	3	3	1
1	2	2	1	3	1	3	1	3	3
1	2	1	1	3	1	1	1	2	1
2	3	3	1	3	1	3	3	3	1
2	2	2	1	1	1	1	2	2	1
3	1	3	3	1	3	1	3	3	1
1	2	2	3	2	1	1	2	2	3
3	3	3	3	3	3	3	3	3	3
1	3	3	3	3	3	3	1	1	2
3	3	3	3	3	3	3	3	3	3
3	3	3	3	3	3	3	3	3	9
1	1	2	2	2	2	1	1	2	1
1	2	3	3	2	3	2	2	3	2
1	3	3	1	2	3	1	1	2	3
2	2	2	2	2	2	1	1	2	3
2	2	2	2	2	3	1	2	3	2
2	2	2	2	1	1	2	1	1	2
1	9	9	3	3	3	3	3	1	3
3	3	1	3	3	3	3	3	3	3
2	2	1	3	2	1	3	1	2	1
2	2	2	9	1	9	9	9	9	9
9	9	9	9	9	9	9	9	9	9
9	9	9	9	9	9	9	9	9	9
9	9	9	9	9	9	9	1	9	9
9	9	1	9	9	9	1	9	9	9
2	2	2	3	3	3	1	2	2	3
1	9	9	9	9	9	9	9	9	9
9	9	9	2	9	9	9	9	9	9
1	9	9	9	9	9	9	9	9	9
9	9	9	9	9	9	9	9	9	9
9	9	9	9	9	9	9	9	9	9
9	9	9	3	9	9	9	9	9	9
9	9	9	3	2	2	3	2	1	1
2	3	2	3	3	3	1	2	3	1
1	3	3	2	3	3	1	2	2	2
2	2	2	1	1	1	1	2	2	1
1	1	1	1	1	1	1	1	3	1
1	2	2	1	2	1	1	1	2	1
1	2	2	1	2	1	1	1	2	1
2	2	2	1	1	1	1	2	2	1
3	3	3	2	2	3	1	3	3	3
1	1	2	1	2	2	1	2	2	2
2	2	2	2	2	3	1	2	2	2
3	2	3	3	3	2	1	3	2	2
3	3	1	1	3	3	1	3	3	1
2	1	1	3	2	2	1	1	2	2

2	1	3	2	2	2	1	2	2	1
2	2	2	2	2	3	2	2	2	2
2	3	2	3	3	3	3	3	3	3
3	3	1	3	1	2	2	1	2	1
1	1	2	1	2	2	2	3	2	1
1	1	1	2	2	2	1	1	1	2
2	2	2	2	2	3	2	2	2	2
2	2	2	2	1	2	2	2	2	2
2	3	2	2	2	3	1	1	2	2
1	2	3	2	2	2	2	2	2	2
2	2	2	3	3	2	2	1	2	2
2	1	2	3	2	3	2	2	1	2
1	2	2	2	1	2	1	1	1	3
3	1	3	3	3	3	3	3	3	3
2	2	2	2	2	2	2	2	2	2
2	2	2	2	2	2	2	2	2	2
2	2	2	3	3	2	1	2	1	1
1	2	2	3	1	3	9	2	3	1
1	3	1	1	1	1	1	1	1	1
1	1	1	1	1	1	1	1	1	1
2	2	2	1	1	1	1	1	1	3
2	2	2	1	1	1	1	2	3	3
2	3	2	2	2	3	1	1	2	2
3	3	3	2	1	2	1	3	1	2
2	2	2	1	1	2	1	2	2	1
1	2	1	2	3	2	1	2	3	1
2	2	2	2	1	2	1	1	1	2
1	1	1	2	2	3	1	1	1	1
2	2	2	2	2	2	1	2	2	1
2	2	2	9	9	9	1	9	9	9
9	9	1	9	9	9	9	9	9	9
1	2	1	1	2	2	1	1	1	1
1	3	3	1	1	1	1	1	1	2
2	2	3	3	3	3	1	3	2	2
3	3	3	3	3	3	3	3	3	3
2	2	2	9	2	2	2	2	2	2
2	3	2	2	2	2	2	2	2	2
1	2	2	3	3	2	2	2	2	3
3	2	3	3	3	3	2	1	1	3
1	2	1	2	2	2	2	2	1	2
2	2	2	2	2	2	1	2	2	2
2	2	2	2	2	2	1	2	2	2

EarlyInterc	UTI	BSEnoneec	BSEnecessa	BSEshy	BSEpainful	BSEfearofC	BSEtimewa	BSEdirty	BSEdisliket
2	2	2	2	2	2	2	9	3	3
2	2	2	2	2	2	2	2	2	2
2	2	2	2	2	2	2	2	2	2
2	2	2	2	2	3	3	3	3	3
2	2	1	3	2	2	1	1	2	2
2	2	2	2	2	2	2	2	2	2
2	2	2	2	2	2	2	2	2	2
2	2	1	1	3	3	3	3	3	1
2	2	1	2	3	3	3	2	2	1
2	2	3	2	1	2	2	3	1	2
1	2	3	1	3	3	3	3	1	2
2	2	1	3	2	2	2	2	1	2
2	2	1	1	1	2	2	2	1	2
2	2	3	3	3	3	3	2	2	1
2	2	3	2	1	2	2	2	2	2
2	2	3	2	3	3	3	3	3	3
2	2	1	2	1	2	2	2	1	1
1	2	2	1	3	3	1	2	1	1
2	3	1	3	3	2	2	1	3	1
2	2	2	1	1	2	3	2	1	3
2	2	3	1	3	1	3	3	3	3
2	2	3	1	3	2	1	3	3	3
1	1	3	1	3	3	3	3	3	3
2	2	2	1	2	2	3	3	3	1
2	2	2	2	2	2	2	2	2	2
3	3	3	3	3	3	3	9	1	9
9	9	3	1	3	3	3	3	3	3
2	2	1	1	3	3	9	3	3	3
3	2	2	2	1	2	3	3	2	1
3	1	1	9	1	1	1	1	1	1
2	2	2	2	1	1	1	1	2	2
2	2	2	1	3	3	3	3	3	3
1	9	3	3	3	1	3	3	3	3
1	1	1	2	3	1	1	3	3	1
1	1	3	1	3	1	1	1	9	1
3	3	3	3	3	2	3	2	3	3
2	2	3	1	3	3	3	3	2	1
3	1	1	2	3	2	1	2	3	2
2	3	3	1	3	3	3	3	3	3
2	3	2	3	3	2	1	2	3	1
2	2	2	2	1	2	3	3	2	2
1	9	3	3	2	9	9	9	9	9
9	9	9	9	9	9	9	9	9	9
2	2	1	1	2	2	3	2	2	2
2	2	1	1	3	3	3	1	2	2
9	9	9	9	9	9	9	9	9	9

3	2	1	2	1	2	2	2	2	1
2	2	1	3	2	2	1	1	1	1
2	2	2	1	1	1	1	1	1	1
2	1	3	3	3	2	3	1	3	2
3	3	3	1	3	3	3	3	3	3
3	3	3	1	3	1	3	3	3	3
3	2	3	2	1	3	2	9	1	2
1	1	2	1	3	1	1	3	3	3
9	1	1	3	1	1	1	1	1	1
3	2	3	1	2	2	2	2	3	1
9	9	1	9	9	2	9	9	9	9
1	1	1	3	1	2	2	3	2	2
1	1	2	2	2	2	1	1	1	2
1	1	1	2	1	1	2	2	1	1
1	1	1	1	1	1	1	2	2	1
1	1	1	3	1	2	2	1	2	2
2	2	3	2	2	2	2	3	3	3
1	3	3	1	3	3	1	3	2	3
2	2	2	2	2	2	9	2	2	2
3	3	3	3	3	3	3	3	3	3
3	2	1	3	2	3	2	2	3	2
3	2	2	3	3	1	2	2	2	1
1	1	1	2	1	1	1	2	2	1
2	2	1	3	1	2	2	3	2	2
2	2	1	2	1	2	2	3	2	1
1	1	3	9	9	2	2	3	3	3
1	1	3	3	1	3	2	3	1	3
1	1	3	3	3	1	1	1	1	2
1	1	2	1	1	1	1	3	1	1
3	3	3	1	3	3	3	3	3	3
9	9	9	9	9	9	9	9	9	9
2	2	2	2	3	2	3	2	2	3
9	9	9	9	9	9	1	9	9	9
1	1	2	3	1	3	3	3	2	3
1	1	3	3	1	9	1	1	1	1
1	2	1	2	1	2	2	2	2	2
1	1	1	3	3	1	2	1	1	1
1	3	3	1	3	3	3	2	2	2
3	2	3	1	2	3	1	3	2	2
1	2	3	1	2	1	1	1	2	2
2	1	3	3	1	1	1	1	1	1
9	9	9	9	9	9	9	9	9	9
1	1	1	1	2	1	1	3	3	3
1	2	3	1	3	3	2	1	1	2
2	1	1	2	3	9	2	1	3	1
2	2	1	1	1	1	1	1	1	2
2	2	1	1	1	2	3	3	3	3

2	3	1	2	1	2	3	2	1	1
3	3	1	1	3	1	1	1	1	1
2	2	1	3	3	3	2	3	2	2
1	3	3	1	1	1	2	2	1	1
1	1	1	1	1	2	2	1	1	2
2	1	1	2	2	2	1	1	1	1
2	2	3	1	3	2	3	2	2	2
1	1	1	3	1	1	1	3	3	3
1	2	2	1	1	3	3	3	3	2
1	1	1	1	3	2	2	3	3	2
9	9	1	3	3	1	2	1	1	1
2	2	2	1	1	1	2	2	2	1
1	1	1	2	1	1	1	2	2	1
2	1	2	1	2	3	1	3	3	3
1	3	1	1	3	2	3	3	3	3
1	3	1	1	3	2	3	3	3	3
1	1	1	2	1	1	1	3	2	1
2	2	2	9	2	3	3	3	3	3
3	1	3	1	3	3	3	1	3	3
1	2	1	3	2	2	2	1	1	1
1	1	1	1	3	3	3	3	3	1
2	3	1	1	1	3	1	3	3	3
1	1	2	1	1	1	1	2	2	1
2	2	1	1	2	2	2	2	2	2
2	2	2	2	2	2	2	2	2	2
3	3	1	1	3	3	3	3	3	3
1	1	3	2	2	2	2	2	2	1
2	1	2	1	2	9	1	2	2	1
2	2	1	2	2	1	2	2	2	1
1	2	3	3	1	2	3	1	1	3
1	1	1	2	1	1	1	1	2	1
1	1	3	1	3	2	2	2	1	2
2	2	3	1	2	2	1	1	2	2
2	1	1	9	2	1	2	1	1	1
3	2	3	1	3	2	3	9	3	2
2	3	1	3	1	2	2	2	2	3
9	9	2	2	2	2	2	2	2	2
1	3	1	2	1	1	1	1	2	1
9	9	9	9	9	9	9	9	9	9
3	3	1	1	3	1	1	1	1	1
1	1	1	2	1	1	1	2	1	1
9	9	9	9	9	9	9	9	9	9
1	1	1	2	2	2	2	2	2	1
2	2	1	2	1	2	1	1	2	1
2	2	3	1	3	1	3	3	3	3
2	2	3	1	3	1	3	3	3	1
1	2	1	3	3	3	1	2	1	1

3	2	1	1	2	2	2	9	1	3
3	3	9	3	3	3	3	3	3	3
9	3	1	1	1	1	1	1	1	1
2	2	3	2	3	2	2	2	2	2
1	2	1	1	3	3	1	1	1	3
1	3	1	2	2	2	3	2	1	2
9	9	1	9	9	9	9	9	9	9
2	3	1	2	2	2	3	3	1	3
9	9	1	3	1	1	1	2	1	1
2	2	9	9	9	9	9	9	9	9
3	1	1	2	2	1	3	3	2	1
1	1	1	2	3	3	3	3	3	3
2	2	2	2	2	2	2	2	2	2
3	3	1	1	2	2	2	2	2	2
2	1	1	2	1	2	2	1	2	1
1	2	2	1	1	1	1	1	2	2
1	1	1	2	1	2	2	1	1	2
2	2	1	2	2	2	2	2	2	1
2	1	1	2	3	9	2	1	3	1
3	1	1	3	1	9	1	1	1	1
2	9	9	9	9	9	1	9	9	9
3	1	1	1	3	3	3	3	3	3
3	3	1	3	3	3	3	1	1	1
2	1	3	3	1	2	1	2	2	2
3	2	1	1	3	3	3	3	3	3
1	3	1	2	2	9	1	3	3	1
3	2	3	1	3	3	3	3	3	3
1	9	1	3	3	2	3	3	3	2
1	1	9	9	9	9	9	9	9	9
1	1	2	1	1	1	1	1	1	2
2	2	2	2	2	2	2	2	2	2
2	2	1	1	2	2	2	2	2	2
2	2	1	2	3	3	2	2	2	2
1	3	1	1	1	1	1	1	1	1
3	2	3	1	1	1	2	2	2	2
1	1	1	1	1	1	3	1	1	1
2	2	1	1	1	1	1	1	1	1
1	2	3	1	1	1	1	2	2	3
1	9	3	1	3	3	3	3	3	3
3	3	1	2	2	2	2	2	2	2
1	1	2	1	2	9	9	2	2	1
2	2	2	2	2	2	2	2	2	2
2	2	1	3	3	1	3	2	2	2
9	9	9	9	9	9	9	9	9	9
9	9	9	9	9	9	9	9	9	9
1	1	1	2	1	1	1	1	2	1
2	2	2	2	1	1	1	1	2	1

9	9	9	9	9	9	9	9	1	9
9	9	2	1	3	3	3	3	3	3
1	3	3	1	3	3	3	3	3	3
9	9	2	1	3	3	2	3	3	3
1	3	1	1	3	3	9	9	9	9
9	9	9	9	9	9	9	9	9	9
1	1	3	3	3	2	1	2	1	1
2	1	3	3	3	1	1	2	3	1
2	3	1	2	3	2	2	3	2	3
9	9	9	9	9	9	1	9	9	9
9	9	9	1	9	9	9	9	9	9
9	9	9	9	2	9	9	9	9	9
2	3	1	3	1	2	3	2	1	2
3	2	2	3	3	3	1	1	2	2
9	9	9	1	9	9	9	9	9	9
3	3	2	2	2	2	2	2	2	2
3	3	3	3	3	3	3	3	3	3
2	2	2	1	1	1	2	2	1	2
2	2	1	1	3	2	2	3	3	3
3	1	3	3	2	2	2	2	2	2
1	2	1	2	3	3	3	3	3	1
2	1	2	2	9	2	3	1	2	2
2	3	1	1	2	1	1	1	3	1
3	2	1	1	3	2	3	3	1	3
2	2	1	2	2	2	2	2	2	2
2	2	3	1	3	3	3	2	3	3
2	2	1	2	2	2	1	2	2	2
2	2	2	3	1	2	2	2	2	2
3	3	3	1	1	3	3	2	3	3
1	1	3	1	2	3	1	3	1	3
1	2	3	1	1	1	1	2	3	2
1	3	3	1	3	3	3	3	3	3
9	9	1	2	1	1	2	9	9	9
2	1	1	1	1	3	1	2	1	1
3	1	3	1	2	1	9	1	1	1
2	2	3	3	3	3	2	3	2	2
1	9	1	2	2	1	2	3	2	1
2	2	3	1	3	2	1	1	1	1
1	2	1	2	2	1	3	2	1	2
1	1	1	3	1	2	2	1	1	2
1	1	1	2	9	1	1	1	1	2
1	2	1	1	3	1	2	1	1	3
2	1	2	1	3	2	1	2	1	1
9	9	2	1	1	1	1	3	1	1
2	3	9	2	2	2	2	2	2	2
3	3	3	1	3	1	3	2	1	1
9	9	9	9	9	9	9	9	9	9

3	1	3	1	3	3	3	3	3	3
3	1	1	1	3	1	3	3	3	1
1	2	3	3	3	3	1	3	3	3
2	3	1	1	3	2	3	3	3	3
3	3	2	1	3	2	3	3	3	3
2	2	2	3	1	2	2	9	1	2
3	3	1	1	3	3	3	3	2	3
1	1	1	2	2	1	1	2	1	2
2	2	2	1	2	1	2	2	1	2
2	2	1	1	1	1	1	2	3	2
2	2	2	2	2	2	2	2	2	1
9	9	1	9	9	9	9	9	9	9
9	9	2	9	9	9	9	9	9	9
2	2	2	2	2	2	2	2	2	2
9	9	3	1	9	9	3	1	9	3
2	9	2	2	2	9	2	9	9	2
9	9	9	1	1	9	9	1	9	1
9	9	9	1	9	9	1	2	9	1
9	2	9	2	2	9	9	2	9	2
9	9	9	9	9	9	9	9	9	9
9	9	9	9	9	9	9	9	9	9
2	2	2	2	2	2	2	2	2	2
2	2	2	2	2	2	2	2	2	2
3	3	1	2	1	1	1	1	2	1
2	2	2	2	2	2	2	2	2	2
2	9	9	2	9	9	2	9	2	2
2	2	2	2	2	2	2	2	2	2
9	9	9	9	9	9	9	9	9	9
9	9	9	9	9	9	9	9	9	9
2	2	1	1	2	2	1	2	2	2
9	9	9	2	9	9	9	9	9	9
9	9	9	9	9	9	9	9	9	9
9	9	9	9	9	9	9	9	9	9
9	2	9	9	9	9	9	9	9	9
9	9	3	1	9	9	1	2	9	1
9	9	1	2	9	1	2	9	9	1
9	9	9	9	9	9	9	9	9	9
2	9	9	2	9	9	9	2	9	9
1	2	1	2	9	9	9	1	2	9
9	9	3	9	9	9	9	9	9	9
9	9	9	3	9	9	9	9	9	9
9	9	1	9	9	9	9	9	9	9
3	3	3	3	3	3	3	3	3	3
3	3	3	3	3	3	3	3	1	2
2	1	1	2	1	3	1	2	1	2
1	1	1	9	9	9	1	9	3	9
9	9	1	9	9	9	9	9	9	9

9	9	9	1	9	9	9	9	9	9
9	9	2	9	9	9	9	9	9	9
2	2	1	3	2	2	2	1	1	3
3	2	2	1	2	3	1	2	3	1
3	3	1	2	2	1	1	2	1	1
2	2	1	2	2	2	2	2	2	2
3	2	1	1	3	1	3	3	2	3
1	1	2	1	1	2	2	3	1	1
9	9	9	9	9	9	9	9	9	9
3	3	3	3	3	3	3	3	3	3
9	9	9	9	9	9	9	9	9	9
9	9	9	9	9	9	9	9	9	9
9	9	9	1	9	9	9	9	9	9
1	2	1	9	9	9	1	9	9	9
1	1	1	1	1	1	1	1	1	1
2	2	3	3	1	1	1	2	2	3
9	9	9	9	9	9	9	9	9	9
1	1	1	2	2	2	3	3	2	3
1	1	1	1	1	1	1	1	1	1
1	2	3	3	1	1	1	1	1	3
2	1	3	1	1	3	1	3	1	1
2	1	1	1	3	1	3	3	3	2
2	3	3	1	2	1	2	3	2	2
2	2	2	2	2	2	2	2	2	2
2	2	1	2	2	2	2	2	2	2
2	2	2	2	2	2	2	2	2	2
2	2	2	2	2	2	2	2	2	2
2	3	1	2	1	2	3	1	2	3
2	2	2	3	2	3	9	2	3	2
3	3	1	1	2	2	2	1	1	2
2	2	1	2	2	2	2	2	2	2
2	2	2	2	2	2	2	2	2	2
1	2	2	1	2	3	2	3	3	3
2	2	3	2	2	2	2	2	2	2
3	3	1	3	1	1	1	1	1	3
3	2	2	2	2	2	2	2	2	2
9	9	9	9	9	9	9	9	9	9
1	2	3	3	3	2	2	2	2	2
3	3	3	3	3	3	3	3	3	3
3	3	3	3	3	3	3	3	3	3
9	9	2	9	2	9	9	9	9	9
1	3	1	1	2	1	2	1	2	2
1	1	9	1	9	9	9	9	2	9
1	1	2	2	2	2	2	2	2	2
1	1	3	3	3	2	2	1	1	2
2	2	2	2	2	2	2	2	2	2
2	2	2	3	2	2	2	1	1	2

3	1	2	1	2	3	1	2	3	1
9	9	1	9	1	9	9	9	9	9
9	9	9	9	9	9	9	1	9	9
9	9	9	2	9	9	9	1	9	9
9	9	2	9	9	9	1	9	9	9
1	1	1	2	1	1	1	2	1	1
9	9	1	9	9	9	9	9	9	9
9	9	9	1	9	9	9	9	9	9
2	2	2	1	1	2	2	1	1	2
1	9	1	9	9	9	9	9	9	9
9	9	3	9	9	9	9	3	9	9
9	9	3	9	9	3	9	9	9	9
9	9	2	9	9	9	9	9	9	9
9	9	9	3	9	9	1	9	9	9
9	9	2	9	9	9	9	9	9	9
9	9	9	9	9	9	9	9	9	9
9	9	3	9	9	9	2	9	9	9
2	3	2	3	2	3	1	2	3	1
1	2	1	2	1	1	3	1	2	1
3	3	1	2	2	2	2	2	2	2
1	2	3	1	1	2	1	2	3	3
1	1	2	1	3	1	3	3	3	2
3	2	1	2	1	2	3	1	2	3
2	2	2	1	3	1	2	2	2	1
1	2	1	1	3	2	2	3	3	3
3	3	2	3	2	9	2	3	3	3
1	2	2	3	2	1	1	2	1	1
1	1	1	1	3	1	2	3	3	3
2	2	3	2	1	2	2	2	1	3
1	1	2	1	1	1	1	1	1	2
2	1	2	3	1	2	1	3	1	2
2	2	1	2	2	2	2	2	2	2
1	1	2	2	1	1	2	2	2	2
1	1	2	2	1	2	2	2	2	1
9	9	9	9	9	9	9	9	9	9
3	2	3	9	3	3	2	3	3	3
9	9	9	9	9	9	9	9	9	9
2	1	1	1	1	1	2	2	2	2
2	2	3	2	2	1	2	2	2	1
2	2	2	2	2	2	2	2	2	2
9	9	1	1	3	3	2	3	3	3
3	3	1	3	3	2	3	3	3	3
3	3	1	3	3	3	1	3	3	3
2	1	3	1	3	3	2	3	3	3
2	2	2	2	2	2	2	2	2	2
3	1	3	1	1	1	2	2	2	2
2	2	2	2	2	2	2	2	2	2

3	2	3	1	2	1	2	3	2	2
1	9	1	1	1	9	9	9	9	9
3	3	9	2	2	2	2	2	2	2
3	2	2	3	1	2	3	1	2	3
2	2	3	3	3	3	3	3	3	3
9	9	2	2	9	9	9	9	9	9
1	1	3	1	3	1	1	3	3	3
1	1	3	2	3	1	1	3	3	3
9	9	9	9	9	9	9	9	9	9
3	2	3	1	3	3	1	3	3	3
1	3	3	1	3	3	1	3	2	3
9	2	9	1	9	9	9	9	9	9
9	9	2	9	9	9	9	9	9	9
2	2	1	2	1	2	2	3	3	3
9	9	9	1	9	9	9	9	9	9
3	1	2	2	3	2	2	1	2	2
9	9	9	9	9	9	9	9	9	9
9	9	9	9	9	9	9	9	9	9
9	9	9	1	9	9	2	9	9	9
3	3	1	3	3	2	1	1	1	2
2	2	2	2	2	2	2	2	2	2
1	1	1	3	1	1	1	3	1	1
3	2	3	2	2	2	9	2	2	2
9	9	9	9	9	9	9	9	9	9
9	9	2	1	3	3	3	3	3	3
1	2	3	1	1	2	2	1	1	1
1	1	2	1	1	1	1	1	1	1
1	1	1	2	1	2	3	1	2	3
3	3	2	2	2	2	2	2	2	2
9	9	9	2	9	9	9	9	1	9
1	2	3	2	2	2	2	2	2	2
2	2	1	1	1	2	3	1	2	3
2	2	1	3	1	2	2	2	2	1
2	2	1	1	2	2	2	2	2	1
1	1	1	1	3	3	1	1	1	1
2	2	1	2	2	2	1	2	1	2
9	9	9	9	9	9	9	9	9	9
2	2	9	9	9	9	9	9	9	9
3	2	2	9	2	3	2	3	1	2
1	1	2	1	1	9	2	1	2	1
2	2	2	1	1	9	1	9	1	9
2	2	3	1	3	3	3	3	3	3
2	1	2	2	2	2	2	3	3	3
1	1	1	2	1	1	2	3	2	1
3	3	2	2	2	2	2	2	2	2
2	2	1	1	3	3	2	3	2	1
3	3	1	1	2	2	2	2	2	2

9	9	3	3	3	3	3	3	3	3
9	9	2	1	3	3	3	2	2	2
9	9	1	9	9	9	1	9	9	9
9	9	9	1	9	9	9	9	9	9
9	1	2	3	1	1	2	2	1	1
3	3	1	1	3	2	2	3	3	3
2	2	2	2	2	2	2	2	2	2
9	2	2	2	2	9	2	9	2	2
9	9	9	9	9	9	9	9	9	9
9	9	9	9	9	9	9	9	9	9
2	2	9	3	2	2	2	2	2	2
2	2	9	3	2	2	2	2	2	2
2	9	9	3	2	2	2	2	2	2
2	2	9	9	9	9	9	9	9	9
2	2	9	3	2	2	2	2	2	2
9	9	2	9	9	9	9	9	9	9
3	3	3	3	3	3	3	3	3	3
9	9	1	9	9	9	9	9	9	9
9	9	2	9	9	9	9	9	9	9
9	2	2	9	9	9	9	9	9	9
9	9	9	9	9	9	9	9	9	9
9	9	2	9	9	9	9	9	9	9
2	2	2	2	2	2	2	2	2	2
2	2	9	9	9	9	9	9	9	9
9	9	9	9	9	9	9	9	9	9
9	9	9	9	9	9	9	9	9	9
9	9	9	9	9	9	9	9	9	9
2	9	3	3	3	3	3	3	3	2
2	2	1	1	3	3	3	3	3	3
9	9	9	9	9	9	9	9	9	9
9	2	2	9	2	9	2	9	2	9
2	2	2	2	2	2	2	2	2	2
9	1	1	9	1	9	1	9	9	1
9	9	9	9	9	9	9	9	9	9
3	3	3	3	3	3	3	3	3	3
9	9	9	9	9	9	9	9	9	9
2	2	1	9	9	9	9	9	9	9
9	9	2	2	2	2	2	2	2	2
2	1	2	1	1	1	3	2	3	3
2	2	2	1	1	2	3	2	2	3
9	9	9	1	1	9	9	9	9	9
2	2	3	1	1	3	2	9	1	2
2	1	1	1	3	2	3	1	2	3
2	3	1	2	1	2	3	1	2	3
3	1	1	1	1	3	1	3	2	3
2	1	1	2	1	2	1	2	1	3
9	9	2	1	1	3	2	3	3	1

9	9	9	1	9	9	9	9	9	9
3	2	1	1	2	3	2	1	2	1
2	2	3	3	9	1	1	1	1	1
1	1	1	1	1	1	1	1	1	1
2	2	2	2	2	2	2	2	2	2
3	2	1	2	2	1	3	3	2	1
3	3	3	3	3	3	3	3	3	3
2	2	3	2	2	2	2	2	1	1
1	2	3	1	3	3	3	3	3	3
1	1	3	3	3	3	9	1	1	1
2	2	2	2	2	2	2	2	2	2
1	2	2	2	2	2	2	2	2	2
3	3	3	3	3	3	1	2	3	3
2	2	2	2	2	2	2	2	2	2
9	9	9	9	2	9	9	9	9	9
1	2	2	3	3	2	2	3	2	2
2	2	2	1	1	2	2	2	3	2
2	2	3	1	3	1	3	1	1	3
3	2	2	3	1	3	3	3	1	9
2	2	2	2	2	2	2	2	2	2
3	2	2	1	3	2	2	9	3	1
3	1	1	1	3	3	3	1	1	1
2	1	2	3	3	1	2	9	3	3
9	9	1	9	9	9	9	9	9	9
9	9	9	1	9	9	9	9	9	9
1	1	1	1	1	1	1	1	1	1
2	2	9	9	1	9	9	9	9	1
3	3	2	1	3	3	3	3	3	1
9	9	9	9	9	9	9	9	9	9
9	9	9	1	9	9	9	9	9	9
2	2	3	1	3	1	1	1	3	1
3	2	2	3	3	2	3	2	3	2
9	9	9	9	9	9	9	9	9	9
9	9	1	9	9	9	9	9	9	9
9	1	1	1	3	2	1	3	2	2
9	9	9	9	9	9	9	9	9	9
3	2	2	2	2	3	2	3	2	3
3	3	1	1	1	1	1	1	2	1
2	2	1	1	1	1	2	2	2	2
2	2	2	1	3	2	2	2	2	2
2	1	1	1	3	3	2	3	3	3
3	3	2	2	2	2	2	2	2	2
9	9	2	9	3	2	1	2	3	2
3	3	3	2	2	2	3	3	3	3
3	3	1	1	2	2	3	3	2	2
3	3	3	2	2	3	2	2	2	2
3	1	1	1	1	3	2	2	3	2

2	9	9	1	2	9	9	9	9	9
2	2	3	1	3	1	3	1	1	1
2	2	1	3	3	2	3	3	3	1
2	2	9	9	9	9	9	9	9	9
1	1	2	1	3	2	1	3	3	3
9	9	9	9	9	9	9	9	9	9
2	2	9	9	9	9	9	9	9	9
2	2	2	2	2	2	2	2	2	2
2	1	1	2	3	3	3	3	3	3
2	2	2	1	3	2	2	3	2	2
2	2	1	1	1	1	1	1	1	1
3	2	2	2	3	3	3	3	2	2
2	3	2	1	3	2	2	3	3	3
2	2	2	2	2	2	2	2	2	2
3	2	1	1	3	1	2	3	2	1
3	3	3	3	3	3	3	3	3	3
9	9	9	9	9	9	9	9	9	9
9	9	9	9	9	9	9	9	9	9
9	9	1	9	9	9	9	9	9	9
9	9	2	9	9	9	9	9	9	9
9	9	9	9	9	9	9	9	9	9
9	9	9	9	9	9	9	9	9	9
2	2	2	2	2	9	2	2	2	2
9	9	2	9	9	9	9	9	9	9
9	9	9	9	9	9	9	9	9	9
9	9	3	9	9	9	9	9	9	9
9	9	9	9	9	9	9	9	9	9
2	2	1	1	1	1	1	1	1	1
2	2	2	9	2	2	3	9	9	2
9	9	3	9	9	9	3	9	9	9
9	3	9	9	9	9	9	9	9	9
2	2	2	2	2	2	2	2	2	2
1	1	9	9	9	9	9	9	9	9
9	9	3	9	9	9	3	9	9	9
9	2	1	9	9	1	2	2	1	1
9	9	9	9	9	9	9	9	9	9
9	9	2	9	9	9	9	9	9	9
9	9	9	9	9	9	9	9	9	9
9	3	1	9	2	9	9	1	9	2
9	9	1	9	9	9	9	9	9	9
2	2	2	2	2	2	2	2	2	2
9	9	2	9	9	9	9	9	9	9
9	9	2	9	9	9	9	9	9	9
9	9	2	9	9	9	9	9	9	9
2	2	9	9	9	9	9	9	9	9
9	9	9	3	2	2	2	2	2	2
9	9	9	9	9	9	9	9	9	9

9	9	9	9	9	9	9	9	9	9
2	2	2	2	2	2	2	2	2	2
9	9	2	9	9	2	9	9	9	2
9	9	2	9	9	2	9	9	2	9
9	9	9	9	9	9	9	9	9	9
9	9	9	9	9	9	9	9	9	9
9	9	9	9	9	9	9	9	9	9
9	9	2	9	9	9	9	9	9	9
9	9	9	9	9	9	9	9	9	9
9	9	9	9	9	9	9	9	9	9
9	9	9	9	9	9	9	9	9	9
9	9	9	9	9	9	9	9	9	9
9	9	9	9	9	9	9	9	9	9
9	9	9	9	9	9	9	9	9	9
2	2	9	3	2	2	2	2	2	2
2	2	2	2	2	2	2	2	2	2
2	2	9	3	2	2	2	2	2	2
2	2	2	2	2	2	2	2	2	2
9	9	2	2	2	2	2	2	2	2
2	2	2	2	2	2	2	2	2	2
9	9	9	9	9	9	9	9	9	9
9	9	1	9	9	9	9	9	9	9
9	9	9	1	2	2	2	9	9	9
9	9	9	2	2	2	9	2	2	2
2	2	2	2	2	2	2	2	2	2
2	2	9	9	9	9	9	9	9	9
9	9	9	9	9	9	9	9	9	9
1	1	1	2	3	1	2	2	9	3
9	9	9	9	9	9	9	9	9	9
2	2	9	9	9	9	9	9	9	9
1	1	1	1	1	1	1	1	1	1
2	2	3	2	1	3	2	9	1	2
9	9	9	9	9	9	9	9	9	9
1	1	1	2	2	2	2	2	2	2
9	9	9	9	9	9	9	9	9	9
2	2	1	9	1	9	9	9	1	9
2	2	2	9	9	9	2	9	9	9
9	2	2	2	2	2	2	2	2	2
1	1	1	1	1	1	1	1	1	1
9	3	3	9	9	9	9	9	9	9
9	9	9	9	9	9	9	9	9	9
9	9	9	9	9	9	9	9	9	9
2	2	2	2	2	2	2	2	2	2
9	9	3	1	3	9	9	9	9	9
3	1	1	9	9	1	9	9	1	9
9	2	2	9	9	9	9	9	9	9
9	2	2	9	9	9	2	9	9	9

9	9	2	9	9	9	9	9	9	9
9	9	9	9	9	9	9	9	9	9
9	3	2	9	9	1	9	3	2	1
9	9	3	9	9	9	9	9	9	9
9	9	9	9	9	9	9	9	9	9
9	9	9	9	9	9	9	9	9	9
9	9	2	9	9	9	9	9	9	9
9	9	2	9	9	9	9	9	9	9
2	9	2	9	9	2	9	9	2	9
9	9	2	2	2	2	2	9	9	9
9	9	9	9	9	9	9	9	9	9
9	9	9	9	9	9	9	9	9	9
9	9	9	9	9	9	9	9	9	9
9	9	9	9	9	9	9	9	9	9
2	1	1	1	3	3	3	2	3	3
1	1	1	1	3	1	1	3	2	1
2	9	1	9	9	9	9	9	9	9
2	2	3	1	1	3	1	3	2	2
2	2	1	1	3	3	3	3	2	2
9	9	2	2	2	2	2	2	2	2
9	9	9	1	9	9	9	9	9	9
9	9	3	9	9	9	9	9	3	9
3	3	2	3	3	2	2	2	3	2
2	3	1	2	3	1	2	3	1	2
2	2	2	1	3	3	3	3	3	1
2	2	2	1	3	3	1	3	3	3
3	3	1	9	3	3	3	3	3	3
2	2	3	1	3	3	3	3	3	2
1	1	2	1	3	3	2	3	3	2
2	2	1	1	3	3	3	3	3	1
9	9	1	9	9	9	9	9	9	9
9	9	3	9	9	9	9	9	9	9
1	1	1	9	9	9	9	9	9	9
9	9	9	9	9	9	9	9	9	1
9	9	2	9	9	9	9	9	9	9
9	9	9	9	9	9	9	9	9	9
9	9	9	9	9	9	9	9	9	9
9	2	9	2	9	2	9	9	2	9
9	9	9	9	9	9	9	9	9	9
9	2	9	2	9	9	2	9	2	9
9	2	2	9	2	9	2	9	2	9
2	2	2	2	2	2	2	2	2	2
2	2	2	2	2	2	2	2	2	2
2	2	2	2	2	2	2	2	2	2
9	9	9	9	9	9	9	9	9	9
2	2	1	3	2	2	2	2	2	2
3	2	1	1	2	3	2	2	1	2

1	1	2	2	3	2	3	3	2	2
1	2	2	1	3	3	2	3	2	2
9	9	3	3	3	3	3	1	3	3
3	2	3	2	3	3	1	2	1	2
3	3	3	1	3	3	3	3	3	3
2	2	3	1	3	1	2	3	3	3
2	1	2	1	3	1	2	1	1	2
3	3	3	3	3	3	3	3	3	3
2	2	2	2	2	2	2	2	2	2
2	2	2	2	2	2	2	2	2	2
2	9	9	2	9	2	9	9	2	9
2	2	2	2	2	2	2	2	2	2
1	2	1	3	3	1	1	2	2	2
1	3	3	1	2	3	1	2	2	1
2	2	2	2	2	3	3	3	3	3
2	2	2	2	2	2	2	2	2	2
1	2	2	2	1	2	1	2	3	1
3	3	1	2	3	3	3	2	3	3
2	2	9	1	3	3	3	3	3	3
2	3	2	1	1	3	1	3	2	2
2	2	1	3	3	2	3	2	1	2
3	1	1	2	3	1	2	3	1	2
1	2	1	3	2	1	1	2	2	1
1	2	3	1	1	2	3	3	3	2
2	1	1	2	3	3	3	2	3	3
2	2	3	1	2	2	2	3	3	3
1	1	3	1	2	1	2	3	3	3
2	2	3	1	3	2	9	2	2	3
3	2	1	1	3	2	2	3	2	2
3	3	2	1	3	3	3	3	3	3
3	2	2	3	3	1	3	3	3	2
1	2	2	2	3	1	3	3	3	2
3	3	3	3	3	3	3	3	3	3
2	2	1	1	3	3	3	3	3	2
9	9	1	9	9	9	9	9	9	9
3	3	3	1	2	3	1	2	3	3
2	2	3	3	1	1	1	2	1	1
2	2	2	2	2	2	2	2	2	2
2	2	3	3	1	1	1	1	1	1
9	9	9	9	9	9	9	9	9	9
2	2	1	1	3	3	3	3	3	3
9	9	1	1	3	3	3	3	3	3
1	2	1	1	2	1	3	2	2	1
1	2	1	1	1	2	3	2	1	1
3	1	2	2	2	3	2	3	2	2
3	3	3	3	3	3	3	3	3	3
2	3	2	2	3	3	2	3	2	2

1	2	1	2	1	3	2	2	2	2
3	3	3	3	3	3	3	3	3	3
3	3	3	1	3	3	9	3	1	1
3	2	1	1	2	3	3	3	3	3
2	2	1	3	3	3	2	2	3	1
3	2	1	1	3	1	3	3	3	3
3	3	3	2	3	2	2	3	3	3
1	3	2	2	2	3	1	3	1	3
1	1	1	3	1	1	2	1	1	2
2	2	2	2	1	2	2	2	2	2
2	3	9	2	2	2	2	3	3	2
2	2	1	1	3	3	3	3	3	3
2	1	1	3	2	1	2	2	2	2
2	3	1	1	1	2	3	2	2	2
1	2	3	1	3	2	3	3	3	2
2	2	1	9	3	3	3	9	1	3
2	2	3	3	1	1	1	2	2	2
2	2	1	2	3	2	1	2	1	2
2	2	1	2	2	2	2	2	2	2
9	9	1	9	9	9	9	9	9	9
2	1	1	1	1	2	2	2	2	2
2	2	1	1	1	1	2	1	2	1
2	2	2	2	2	3	2	3	2	2
1	3	2	3	2	3	1	3	2	3
3	3	3	3	3	3	3	3	3	3
3	3	1	1	2	3	2	1	3	1
9	9	2	2	1	2	2	2	1	2
2	2	2	2	2	2	2	2	2	2
2	2	3	3	2	2	2	2	2	2
2	2	2	2	2	2	2	2	2	2
3	1	3	1	1	3	1	3	2	2
9	1	1	3	2	1	2	2	2	2
1	1	1	1	3	2	2	1	1	1
3	2	1	1	2	3	3	1	3	3
2	2	1	1	3	2	3	3	3	3
3	3	2	3	2	3	9	2	9	1
2	2	1	1	3	3	3	3	3	3
1	2	1	1	2	2	3	1	2	2
3	2	2	1	3	3	3	3	3	3
1	1	2	3	2	3	2	2	1	1
1	2	1	1	3	2	2	3	3	3
2	2	2	2	2	2	2	2	2	2
2	2	1	2	2	2	2	2	2	2
2	2	2	2	2	2	2	2	2	2
1	1	1	1	3	2	2	1	1	1
3	3	3	1	1	1	1	3	3	3
2	2	2	1	2	2	1	3	3	3

1	1	1	2	1	3	1	1	2	2
1	1	1	1	3	2	2	1	1	1
2	2	2	1	2	2	1	3	3	3
3	2	1	3	2	2	1	3	1	1
2	1	2	1	1	2	2	9	2	2
1	1	1	1	3	2	2	1	1	1
1	1	1	1	3	2	2	1	1	1
2	2	2	2	2	2	2	2	2	2
2	2	1	1	2	2	2	2	2	2
9	9	9	9	9	9	9	9	9	9
1	2	1	3	3	3	3	3	3	3
3	1	1	3	3	3	3	3	3	3
9	9	2	1	3	3	1	2	3	3
1	9	1	1	1	2	2	3	2	2
2	2	2	3	3	2	2	2	1	1
9	1	9	1	9	9	9	9	9	9
9	9	9	9	9	9	9	9	9	9
9	9	1	9	9	9	2	9	9	9
9	9	9	9	9	9	9	9	9	9
2	2	3	3	3	3	3	3	3	3
3	3	1	1	1	1	3	3	2	1
1	9	9	9	9	9	9	9	9	9
9	9	9	9	9	9	9	9	9	9
2	2	3	3	2	2	2	2	2	2
3	3	1	1	2	1	2	2	2	1
2	2	3	3	2	2	2	2	2	2
3	3	3	3	2	1	2	1	3	1
9	9	1	3	2	1	9	3	1	1
2	2	1	2	3	2	1	1	3	1
3	1	1	3	2	2	3	1	2	2
9	9	9	9	9	9	9	9	9	9
1	3	1	1	2	1	2	3	1	1
3	3	3	1	3	2	2	1	2	3
1	1	2	2	2	2	2	2	2	2
3	3	2	2	2	2	9	2	9	2
2	2	3	3	2	2	2	2	2	2
9	9	1	1	9	9	9	9	9	9
2	2	1	1	2	2	2	2	2	2
2	2	1	1	3	3	3	2	3	2
3	3	2	2	1	2	1	1	1	1
1	1	2	1	1	1	1	1	3	2
1	1	1	1	3	3	1	3	1	3
3	2	9	1	3	1	2	1	1	1
2	2	1	2	2	1	9	1	1	1
3	3	3	1	2	3	1	2	3	2
1	3	1	1	2	1	1	1	9	2
3	3	3	2	3	3	2	3	2	3

1	9	2	2	1	2	3	2	3	2
2	2	2	2	2	2	2	2	2	2
1	3	3	3	3	3	2	2	2	2
2	2	2	2	1	2	2	2	2	2
1	2	2	3	2	2	1	2	3	1
2	2	2	2	2	2	2	2	2	2
9	1	9	1	3	9	9	9	9	9
3	2	1	1	3	3	1	2	3	2
3	3	3	9	9	9	9	9	9	9
2	2	3	1	2	2	2	1	3	2
2	3	1	1	2	2	2	2	2	2
9	9	1	2	1	1	1	1	1	1
1	2	2	3	1	1	1	2	2	2
9	9	1	9	9	1	9	1	9	9
9	9	2	9	9	9	9	9	9	9
9	9	1	1	1	1	1	9	3	1
2	2	1	1	3	3	3	3	3	3
3	2	1	1	1	3	3	3	3	2
2	2	3	3	3	1	3	2	1	1
3	2	3	1	3	3	3	3	3	3
2	2	1	2	2	2	3	2	2	2
3	9	3	3	1	1	3	3	3	3
1	2	2	1	3	3	1	3	3	3
2	2	2	2	2	2	2	2	2	2
9	1	3	1	3	3	3	3	3	3
2	2	2	2	2	2	2	2	2	2
2	2	1	1	2	2	2	2	2	2
9	9	9	1	9	9	9	9	1	9
3	2	2	2	2	3	2	3	2	1
2	2	2	1	3	3	3	3	3	3
1	1	1	1	1	1	1	1	1	1
1	3	2	1	1	2	3	1	2	1
1	3	1	2	2	2	1	2	1	1
2	2	3	1	3	1	2	2	2	2
2	2	1	3	1	3	9	1	1	1
2	1	1	2	2	3	1	3	1	2
1	1	1	1	1	1	1	1	1	1
3	2	3	1	3	3	3	3	3	3
3	2	1	1	1	2	1	3	2	3
3	2	2	2	3	3	1	1	2	1
1	9	1	3	3	1	1	1	1	1
1	2	1	2	3	3	9	3	3	3
2	2	1	1	3	3	2	9	3	2
2	2	1	1	3	3	2	3	3	3
3	3	9	9	9	9	9	9	9	9
3	2	2	1	3	9	3	3	3	1
3	3	3	3	3	3	3	3	3	3

3	1	3	1	3	1	2	3	2	2
2	1	3	1	3	3	2	3	1	2
2	3	2	2	2	2	2	2	2	2
2	2	2	2	2	2	2	2	2	2
1	9	3	9	1	2	9	3	2	9
9	9	9	1	9	9	9	9	9	9
2	2	1	1	3	3	3	3	2	3
1	9	1	9	9	9	9	9	9	9
1	9	1	9	9	9	9	9	9	9
9	1	1	1	3	3	3	3	3	3
9	9	1	9	3	9	9	9	9	9
9	9	9	1	9	9	9	9	9	9
3	2	1	1	3	3	1	9	9	9
9	9	9	3	9	9	9	9	9	9
1	2	1	1	2	2	2	3	3	2
9	9	9	9	9	9	9	9	9	9
2	3	1	2	9	9	9	9	9	9
9	9	1	9	9	9	9	9	9	9
9	9	1	9	9	9	9	9	9	9
9	9	9	2	9	2	9	9	9	9
1	1	3	1	1	1	1	1	1	1
1	2	2	3	1	1	2	2	2	1
2	2	1	1	2	2	2	3	3	3
3	1	1	2	3	3	3	2	3	3
1	1	3	3	3	9	3	1	2	3
2	1	3	1	3	3	3	3	3	3
1	3	1	3	1	3	1	1	1	1
9	9	2	9	9	9	9	9	9	9
2	2	1	2	3	1	2	1	2	2
1	1	3	1	3	3	3	1	1	1
1	3	3	1	3	3	2	3	3	1
9	9	1	9	9	9	9	9	9	9
3	3	1	1	1	1	1	1	1	1
9	9	9	1	9	9	9	9	9	9
9	9	1	9	9	9	9	9	9	9
2	1	1	2	1	1	1	1	2	2
1	1	1	1	1	1	1	1	1	1
9	9	9	9	9	9	9	9	9	9
9	9	9	9	9	9	9	9	9	9
2	2	2	2	2	2	2	2	2	2
2	2	2	2	2	2	2	2	2	2
2	2	2	2	2	2	2	2	2	2
3	3	3	3	3	3	3	3	3	3
2	9	9	2	9	9	2	9	9	2
2	2	2	2	2	2	2	2	2	2
2	2	2	2	2	2	2	2	2	2
9	1	9	1	3	1	3	2	1	1

1	9	9	1	9	3	1	9	9	1
2	9	1	2	9	9	1	2	9	1
2	9	2	9	9	1	9	9	1	2
1	1	2	2	2	2	2	2	2	2
9	1	1	9	1	3	3	1	9	1
1	9	9	1	9	3	1	9	9	1
3	1	1	2	9	3	1	3	9	1
1	2	9	1	9	9	9	2	1	2
9	2	2	2	9	2	9	2	9	2
2	9	2	2	2	2	2	2	2	2
2	2	2	2	2	2	2	2	2	2
9	2	2	9	9	2	9	9	2	9
2	2	2	2	2	2	2	2	2	2
9	9	9	9	9	9	9	9	9	9
2	2	2	2	2	2	2	2	2	2
9	9	9	9	9	9	9	9	9	9
2	9	9	9	9	9	9	9	9	9
9	9	9	9	9	9	9	9	9	9
1	9	9	1	9	3	1	9	9	3
2	2	2	2	2	2	2	2	2	2
2	2	2	1	3	2	2	2	2	2
2	2	2	2	2	2	2	2	2	2
3	2	1	2	2	9	1	3	2	2
1	2	1	2	1	3	1	2	9	2
3	3	3	3	2	2	2	2	3	3
3	3	2	2	1	1	2	3	3	2
2	3	2	3	3	2	1	3	1	3
2	2	1	2	3	3	3	3	3	3
3	1	1	1	3	3	1	2	1	1
2	2	1	1	3	3	2	3	3	2
2	2	2	2	3	3	9	2	2	3
1	2	3	2	2	3	1	2	3	2
2	2	1	2	2	2	2	2	2	2
1	1	2	1	2	2	2	2	2	2
1	3	1	2	1	1	2	3	1	1
1	1	2	2	2	2	2	2	2	2
3	3	3	3	3	3	3	3	3	2
1	3	1	1	1	1	1	1	1	1
2	3	1	3	3	3	3	3	3	1
2	2	3	3	1	2	2	3	2	3
1	3	1	3	1	3	1	1	1	1
2	3	3	3	9	9	9	9	9	9
2	2	1	3	2	3	3	2	3	3
9	9	2	2	2	1	1	3	3	1
9	9	9	9	9	9	9	9	9	9
2	2	3	3	2	2	2	2	2	2
2	3	3	9	2	3	1	1	3	1

3	3	3	2	2	2	1	2	2	2
2	2	1	1	2	1	3	9	1	1
2	2	2	2	2	2	2	2	2	2
2	2	2	2	2	2	2	2	2	2
2	2	2	2	2	2	2	2	2	2
2	2	2	2	2	2	2	2	2	2
1	1	1	1	1	1	1	1	1	1
2	3	1	1	1	2	3	3	3	1
3	3	3	3	3	3	3	3	3	3
1	2	1	2	1	1	1	1	1	1
9	9	1	1	1	1	1	1	9	9
2	2	2	2	2	2	2	2	2	2
2	2	2	2	2	2	2	2	2	2
2	2	2	2	2	2	2	2	2	2
2	2	2	2	2	2	2	2	2	2
2	2	1	1	1	1	9	1	1	1
9	1	1	1	1	1	1	1	1	1
2	2	1	1	1	2	2	1	2	1
2	2	2	2	2	2	2	2	2	2
2	2	2	2	2	2	2	2	2	2
1	1	1	2	1	2	3	2	1	2
2	2	2	2	2	2	2	2	2	2
9	9	9	9	9	9	9	9	9	9
2	2	2	2	2	2	2	2	2	2
1	1	9	9	9	9	9	9	9	9
1	2	1	2	1	2	1	2	1	1
1	1	1	1	1	1	1	1	1	2
1	1	9	1	1	1	1	1	1	1
2	2	2	2	2	9	2	2	2	2
9	1	1	1	1	9	1	1	1	1
2	2	2	2	2	2	2	2	2	2
2	2	2	2	2	2	2	2	2	2
2	9	2	2	2	2	2	2	2	2
2	2	3	1	2	3	3	2	3	2
2	2	2	2	2	2	2	2	2	2
9	9	9	9	9	9	9	9	9	9
1	2	1	1	1	1	1	2	2	2
1	1	1	1	2	1	1	1	1	1
9	2	9	1	1	1	1	1	1	1
2	2	2	2	2	2	2	2	2	2
2	2	2	2	2	2	2	2	2	2
2	2	1	1	1	1	1	1	2	2
2	2	2	2	2	2	2	2	2	2
2	2	2	2	2	2	2	2	2	2
1	2	1	2	1	1	1	1	1	1
2	2	2	2	2	2	2	2	2	2
2	2	3	1	2	2	2	9	9	1

1	1	1	1	2	1	1	2	1	1
2	2	9	9	1	1	9	1	1	1
2	2	3	1	1	1	1	1	1	1
1	1	9	1	1	2	1	1	1	2
1	1	2	1	1	1	1	2	2	1
2	2	2	2	2	2	2	2	2	2
2	2	3	1	2	2	2	2	2	2
2	2	3	1	2	2	2	2	2	2
9	9	9	9	9	9	9	9	9	9
2	2	1	1	1	9	1	9	1	1
2	2	9	9	2	2	9	2	2	2
1	1	1	1	1	1	1	9	1	1
9	9	9	9	9	9	9	9	9	9
2	2	1	2	1	1	1	1	1	1
9	9	1	1	1	1	1	1	1	1
2	2	2	2	2	2	9	9	2	2
9	9	9	9	9	9	9	9	9	9
9	9	9	9	9	9	9	9	9	9
2	2	2	2	2	2	2	2	2	2
1	1	3	1	3	3	3	3	3	1
1	1	3	1	3	3	3	3	3	1
1	1	3	1	3	3	3	3	3	1
1	1	3	1	3	3	3	3	3	1
1	1	3	1	3	3	3	3	3	1
1	1	3	1	3	3	3	3	3	1
1	1	3	1	3	3	3	3	3	1
1	1	9	1	3	3	3	3	3	1
1	1	3	1	3	3	3	3	3	3
1	1	3	1	3	3	3	3	3	1
1	1	3	1	3	3	3	3	3	1
1	1	3	1	3	3	3	3	3	1
1	1	3	1	3	3	3	3	3	1
1	1	3	1	3	3	3	3	3	1
1	1	3	1	3	3	3	3	3	1
1	1	3	1	3	3	3	3	3	1
1	1	1	3	1	3	3	3	3	1
1	1	3	1	3	3	3	3	3	1
1	1	3	1	3	3	3	3	3	1
1	1	9	1	3	3	3	3	3	1
1	1	3	1	3	3	3	3	3	1
1	1	3	1	3	3	3	3	3	1
1	1	3	1	3	3	3	3	3	1
1	1	3	1	3	3	3	3	3	1
1	1	3	9	1	3	3	3	3	1
9	9	9	9	9	9	9	9	9	9
9	9	9	9	9	9	9	9	9	9
9	9	9	9	9	9	9	9	9	9

9	9	9	9	9	9	9	9	9	9
9	9	9	9	9	9	9	9	9	9
9	9	9	9	9	9	9	9	9	9
9	9	9	9	9	9	9	9	9	9
9	9	9	9	9	9	9	9	9	9
9	9	9	9	9	9	9	9	9	9
9	9	9	9	9	9	9	9	9	9
9	9	9	9	9	9	9	9	9	9
9	9	9	9	9	9	9	9	9	9
9	9	9	9	9	9	9	9	9	9
9	9	9	9	9	9	9	9	9	9
9	9	9	9	9	9	9	9	9	9
9	9	9	9	9	9	9	9	9	9
9	9	9	9	9	9	9	9	9	9
9	9	9	9	9	9	9	9	9	9
9	9	9	9	9	9	9	9	9	9
9	9	9	9	9	9	9	9	9	9
9	9	9	9	9	9	9	9	9	9
9	9	9	9	9	9	9	9	9	9
1	1	3	1	3	3	3	3	3	1
1	1	3	1	3	3	3	3	3	1
1	1	3	1	3	3	3	3	3	1
1	1	3	1	3	3	3	3	3	1
1	1	3	1	3	3	3	3	3	1
1	1	3	1	3	3	3	3	3	1
1	1	3	1	3	3	3	3	3	1
1	1	3	1	3	3	3	3	3	1
1	1	3	1	3	3	3	3	3	1
1	1	3	1	3	3	3	3	3	1
1	1	3	1	3	3	3	3	3	1
1	1	3	1	3	3	3	3	3	1
1	1	3	1	3	3	3	3	3	1
1	1	3	1	3	3	3	3	3	1
1	1	3	1	3	3	3	3	3	1
1	1	3	1	3	3	3	3	3	1
9	9	9	1	9	9	9	9	9	9
1	1	3	1	3	3	3	3	3	1
1	1	3	1	3	3	3	3	3	1
1	1	3	1	3	3	3	3	3	1
9	9	3	1	3	3	3	3	3	1
9	1	3	1	3	3	3	3	3	3
1	1	3	1	3	3	3	3	3	1
1	1	3	3	3	3	3	3	3	1
1	1	3	1	3	3	3	3	3	1
1	1	3	1	3	3	3	3	3	3
1	1	3	1	3	3	3	3	3	1
1	1	3	1	3	3	3	3	3	1

1	1	3	1	3	3	3	3	3	1
9	9	3	1	3	3	3	3	3	1
1	1	3	1	3	3	3	3	1	1
1	1	3	1	3	3	3	3	3	1
1	1	3	1	3	3	3	3	3	1
1	1	3	1	3	3	3	3	3	1
1	1	3	1	3	3	3	3	1	1
1	1	3	1	3	3	3	3	3	1
1	1	3	1	3	3	3	3	3	1
1	1	3	1	3	3	3	3	3	1
1	1	3	1	3	3	3	3	3	1
1	1	3	1	3	3	3	3	3	1
1	1	3	1	3	3	3	3	3	1
1	1	3	1	3	3	3	3	3	1
1	1	3	1	3	3	3	3	3	1
1	1	3	1	3	3	3	3	3	1
1	1	3	1	3	3	3	3	3	1
1	1	3	1	3	3	3	3	3	1
1	1	3	1	3	3	3	3	3	1
1	1	3	1	3	3	3	3	3	1
1	1	3	1	3	3	3	3	3	1
1	1	3	1	3	3	3	3	3	1
1	1	1	3	1	3	3	3	3	1
1	1	3	1	3	3	3	3	3	1
1	1	3	1	3	3	3	3	3	1
1	1	3	1	3	3	3	3	3	1
1	1	3	1	3	3	3	3	3	1
1	1	3	1	3	3	3	3	3	1
1	1	3	1	3	3	3	3	3	1
1	1	3	1	3	3	3	3	3	1
1	1	3	1	3	3	3	3	3	1
1	1	3	1	3	3	3	3	3	1
1	1	3	1	3	3	3	3	3	1
1	1	3	1	3	3	3	3	3	1
9	9	9	9	9	9	9	9	9	9
1	1	3	1	3	3	3	3	3	1
3	1	3	1	3	3	3	3	3	1
1	1	3	1	3	3	3	3	3	1
1	1	3	1	3	3	3	3	3	1
9	9	3	1	3	3	3	3	3	1
1	1	3	1	3	3	3	3	3	1
1	1	3	1	3	3	3	3	3	1
1	1	1	1	3	3	3	3	3	3
1	9	3	1	3	3	3	3	3	1
1	1	3	1	3	3	3	1	3	1
1	1	3	1	3	3	3	3	3	1
1	1	3	1	3	3	3	3	3	1

1	1	1	1	3	3	3	3	3	1
1	1	3	1	3	3	3	3	3	1
1	1	3	1	3	3	3	3	3	1
1	1	3	1	3	3	3	3	3	1
1	1	3	1	3	3	3	3	3	1
1	1	3	1	3	3	3	3	3	1
1	1	3	1	3	3	3	3	3	1
1	1	3	1	3	3	3	3	3	1
1	1	3	1	3	3	3	3	3	1
1	1	3	1	3	3	3	3	3	1
1	1	9	1	3	3	3	3	3	1
1	1	3	1	3	3	3	3	3	1
1	1	3	1	3	3	3	3	3	1
1	1	3	1	3	3	3	3	3	1
1	1	9	9	9	9	9	9	9	9
1	1	3	1	3	3	3	3	3	1
1	1	3	1	3	3	3	3	3	1
1	1	3	1	3	3	3	3	3	1
1	1	3	1	3	3	3	3	3	1
9	9	9	9	9	9	9	9	9	9
1	1	3	1	3	3	3	3	3	1
1	1	3	1	3	3	3	3	3	1
1	1	3	1	3	3	3	3	3	1
1	1	3	1	3	3	3	3	3	1
9	9	9	9	9	9	9	9	9	9
1	1	3	1	3	3	3	3	3	1
1	1	3	1	3	3	3	3	3	1
1	1	3	1	3	3	3	3	3	1
1	1	3	1	3	3	3	3	3	1
1	1	3	1	3	3	3	3	3	1
1	1	3	1	3	3	3	3	3	1
1	1	3	1	3	3	3	3	3	1
1	1	3	9	1	3	3	3	3	1
1	1	3	9	1	3	3	3	3	1
1	1	3	1	3	3	3	3	3	1
1	1	3	1	3	3	3	3	3	1
1	1	3	1	3	3	3	3	3	1
1	1	3	1	3	3	3	3	3	1
1	1	3	1	3	3	3	3	3	1
1	1	3	1	3	3	3	3	3	1
1	1	3	1	3	3	3	3	3	1
9	9	9	9	9	9	9	9	9	9
1	1	3	1	3	3	3	3	3	1
1	1	9	1	3	3	3	3	3	1
1	1	3	1	3	3	3	3	3	1
1	1	3	1	3	3	3	3	3	1
1	1	3	1	3	3	3	3	3	1

1	1	3	1	3	3	3	3	3	1
1	1	3	1	3	3	3	3	3	1
1	1	3	1	3	3	3	3	3	1
1	1	3	1	3	3	3	3	3	1
1	1	3	1	3	3	3	3	3	1
1	1	3	1	3	3	3	3	3	1
1	1	3	1	3	3	3	3	3	1
3	3	3	1	3	3	3	3	3	1
1	1	3	1	3	3	3	3	3	1
1	1	3	1	3	3	3	3	3	1
1	1	3	1	3	3	3	3	3	1
1	1	3	1	3	3	3	3	3	1
1	1	3	1	3	3	3	3	3	1
1	1	3	1	3	3	3	3	3	1
1	1	3	1	3	3	3	3	3	1
1	1	3	1	3	3	3	3	3	1
1	1	3	1	3	3	3	3	3	1
1	1	3	1	3	3	3	3	3	1
1	1	3	1	3	3	3	3	3	1
1	1	3	1	3	3	3	3	3	1
1	1	3	1	3	3	3	3	3	1
1	1	3	1	3	3	3	3	3	1
1	1	3	1	3	3	3	3	3	1
1	1	3	1	3	3	3	3	3	1
1	1	3	1	3	3	3	3	3	1
1	1	3	1	3	3	3	3	3	1
1	1	3	1	3	3	1	3	3	1
9	9	9	9	9	9	9	9	9	9
9	9	9	9	9	9	9	9	9	9
9	9	9	9	9	9	9	9	9	9
9	9	9	9	9	9	9	9	9	9
9	9	9	9	9	9	9	9	9	9
9	9	9	9	9	9	9	9	9	9
9	9	9	9	9	9	9	9	9	9
9	9	9	9	9	9	9	9	9	9
9	9	9	9	9	9	9	9	9	9
9	9	9	9	9	9	9	9	9	9
9	9	9	9	9	9	9	9	9	9
9	9	9	9	9	9	9	9	9	9
9	9	9	9	9	9	9	9	9	9
9	9	9	9	9	9	9	9	9	9
9	9	9	9	9	9	9	9	9	9
1	1	1	1	1	3	3	3	2	2
9	9	1	1	1	1	1	1	1	1
9	9	9	9	9	9	9	9	9	9
9	9	9	9	9	9	9	9	9	9
9	9	9	9	9	9	9	9	9	9

9	9	9	9	9	9	9	9	9	9
9	9	9	9	9	9	9	9	9	9
9	9	9	9	9	9	9	9	9	9
9	9	9	9	9	9	9	9	9	9
9	9	9	9	9	9	9	9	9	9
9	9	9	9	9	9	9	9	9	9
9	9	9	9	9	9	9	9	9	9
3	2	1	1	3	9	9	9	9	9
9	9	9	9	9	9	9	9	9	9
9	9	9	9	9	9	9	9	9	9
9	9	9	9	9	9	9	9	9	9
9	9	9	9	9	9	9	9	9	9
9	9	9	9	9	9	9	9	9	9
1	2	3	3	3	3	1	3	3	1
2	2	1	2	2	2	2	3	2	2
2	1	1	3	3	3	3	1	3	3
3	3	1	2	2	3	3	2	1	1
2	2	1	1	1	2	3	3	3	3
3	3	3	3	1	1	1	3	3	1
3	3	1	3	1	2	1	2	3	3
1	1	2	1	3	3	3	3	3	2
3	1	1	3	1	3	1	3	1	3
2	2	2	1	3	1	9	2	2	3
3	3	2	3	2	2	3	2	3	3
9	9	2	1	3	3	2	3	3	3
2	2	2	2	2	2	2	2	2	2
9	9	9	9	9	9	9	9	9	9
1	1	3	1	3	3	3	3	3	3
1	1	1	1	3	3	1	3	1	1
2	2	3	1	3	3	3	3	3	3
3	1	2	3	2	3	9	2	2	3
2	1	1	2	1	2	1	3	1	2
1	9	9	1	9	9	9	9	9	9
2	2	1	1	3	2	3	3	3	2
9	9	3	1	3	1	3	3	3	3
9	9	2	9	2	9	9	9	9	9
2	1	1	2	2	2	2	2	2	2
1	3	1	1	2	1	1	2	2	3
1	1	9	1	3	3	1	2	3	3
2	1	1	1	3	9	1	9	1	1
9	9	9	9	9	9	9	9	9	9
1	1	1	2	2	1	1	1	1	1
2	2	2	1	2	2	2	2	2	2
1	1	1	1	1	1	1	1	1	1
9	9	9	9	9	9	9	9	9	9
9	9	9	9	9	9	9	9	9	9
2	2	1	3	2	2	1	2	2	2

1	9	1	9	9	1	9	9	9	9
9	9	9	9	9	9	9	9	9	9
3	2	1	3	2	2	3	2	1	2
2	1	1	1	3	2	3	3	3	3
9	9	1	1	3	3	1	1	3	3
9	9	1	9	9	9	9	9	9	9
2	2	1	1	9	9	9	9	9	9
2	2	2	2	2	2	2	2	2	2
2	2	2	2	2	2	2	2	2	2
3	2	3	3	1	1	1	1	1	2
2	3	2	3	3	3	1	2	1	2
9	9	9	9	9	9	9	9	9	9
1	9	1	1	9	9	9	9	9	9
2	2	3	2	2	2	1	2	2	2
1	2	2	2	1	2	2	3	1	1
1	2	1	1	2	3	3	3	1	3
3	1	1	3	1	3	1	3	1	3
1	2	1	1	3	1	1	3	3	3
1	1	1	9	1	1	2	1	3	1
1	1	3	1	1	2	3	9	2	2
2	2	2	2	2	2	2	2	2	2
2	2	2	2	2	2	2	2	2	2
2	2	2	2	2	2	2	2	2	2
2	2	2	2	2	2	2	2	2	2
2	2	2	2	2	2	2	2	2	2
2	1	2	1	3	3	3	3	3	1
2	2	2	2	2	2	2	2	2	2
3	3	3	1	1	2	3	3	2	3
3	2	2	3	1	2	3	3	1	1
1	1	3	1	2	3	3	1	3	3
2	2	3	1	3	3	3	3	3	3
2	2	2	1	3	2	3	3	1	2
1	1	2	1	3	2	3	3	3	3
3	3	3	1	2	1	1	2	3	2
2	2	3	1	3	2	2	2	2	2
2	1	3	2	2	3	3	3	3	2
2	2	1	1	2	3	3	3	3	2
9	9	1	1	1	1	1	1	2	9
9	9	2	9	9	9	9	9	9	9
3	3	3	1	3	3	3	3	3	3
3	3	2	2	2	3	3	3	3	3
2	2	2	1	2	1	2	1	2	2
2	3	1	2	1	2	3	1	2	3
3	3	2	1	3	2	2	3	2	3
1	1	1	1	1	1	1	1	1	1
3	3	1	1	1	3	3	3	3	2
2	2	2	1	2	2	2	2	2	2

2	2	2	2	2	2	2	2	2	2
2	3	2	1	2	2	2	3	3	1
1	1	2	2	1	1	1	1	1	2
3	3	1	2	1	2	3	2	2	3
2	1	3	1	3	3	3	3	3	3
2	2	1	1	3	3	3	3	2	3
2	3	1	1	3	3	3	3	3	3
2	3	1	3	2	3	3	2	1	3
2	2	2	2	2	2	2	2	2	2
3	2	1	1	1	2	2	1	2	2
2	2	2	2	2	2	2	2	2	2
2	2	1	2	2	2	2	2	2	2
2	2	2	3	3	3	3	3	3	3
2	2	2	2	2	2	2	2	2	2
2	2	2	2	2	2	2	2	2	2
2	2	2	2	2	2	2	2	2	2
3	3	3	3	3	3	3	3	3	3
2	2	2	2	2	2	2	2	2	2
2	2	2	2	2	2	2	2	2	2
2	3	1	2	1	2	3	1	2	3
2	3	2	2	2	2	2	2	2	2
1	3	1	1	2	2	2	2	1	1
2	2	9	2	2	2	2	2	2	2
1	1	3	3	1	1	1	1	1	1
3	3	9	1	3	3	3	3	3	3
9	9	9	9	9	9	9	9	9	9
2	3	1	1	3	1	1	2	3	1
1	2	2	2	3	2	2	2	2	2
3	2	2	1	1	2	1	2	1	1
3	2	3	1	2	3	3	3	3	3
2	2	2	1	2	2	2	3	3	2
3	2	1	1	1	2	9	2	1	3
2	2	3	3	3	3	2	2	3	3
2	2	1	1	1	1	1	1	1	1
2	1	1	9	1	2	9	1	9	9
9	9	9	9	9	9	9	9	9	9
1	1	1	1	1	3	2	3	3	3
1	1	3	1	3	2	2	1	2	2
3	9	9	9	9	9	9	9	9	9
2	1	1	1	1	1	1	1	1	1
3	2	1	1	3	3	3	3	3	2
1	2	3	1	3	1	1	3	1	1
2	2	2	1	2	2	2	3	3	2
9	9	9	9	9	9	9	9	9	9
9	9	9	9	9	9	9	9	9	9
9	9	9	9	9	9	9	9	9	9
1	1	9	9	9	9	9	9	9	9

2	1	3	1	1	3	1	3	1	2
3	2	9	9	9	9	9	9	9	9
2	2	2	1	3	3	2	3	3	3
1	3	1	3	1	1	3	9	3	1
9	9	9	9	9	9	9	9	9	9
2	2	3	1	3	3	3	3	3	3
2	3	3	1	3	2	3	3	3	3
2	2	3	1	3	3	3	2	2	3
1	1	3	1	3	3	2	1	3	3
2	3	2	2	2	2	2	2	2	2
1	2	9	2	2	2	2	1	3	1
2	2	2	2	2	2	2	2	2	2
2	2	1	2	2	2	2	2	2	2
3	1	1	1	1	1	3	1	1	1
2	2	2	2	2	2	2	2	2	2
1	1	3	1	3	3	3	3	3	3
1	3	1	3	2	3	1	1	3	3
1	2	3	1	3	3	1	3	3	2
3	9	9	1	3	9	1	9	1	1
2	1	1	2	1	3	1	3	1	1
1	2	3	1	3	3	1	3	3	2
3	1	1	1	2	1	3	3	3	2
2	2	3	1	3	3	3	3	3	3
2	3	3	1	9	9	9	9	9	9
3	3	1	1	3	2	3	3	3	3
9	9	9	9	9	9	9	9	9	9
1	1	1	2	1	2	1	3	1	3
3	3	1	1	1	1	1	1	1	1
2	2	1	3	3	3	3	3	3	3
1	1	1	3	2	1	3	2	2	1
2	2	3	2	3	2	3	1	2	2
9	9	9	1	9	9	9	9	9	9
2	2	2	1	3	3	2	3	3	3
3	1	2	1	1	3	2	2	2	3
3	1	3	1	2	9	1	1	1	2
2	3	1	2	3	1	2	1	3	2
2	2	2	2	2	2	2	2	2	2
3	2	3	1	3	1	2	1	3	3
2	2	3	1	3	3	3	3	1	3
9	9	3	9	1	9	9	9	9	9
3	2	9	9	9	9	9	9	9	9
2	2	1	2	1	2	2	2	2	2
2	2	3	2	3	3	3	3	3	1
2	2	1	2	2	2	2	2	2	2
2	2	1	2	1	2	2	2	2	2
2	9	1	1	3	1	1	3	3	9
2	2	2	2	2	2	2	2	2	2

3	1	3	2	1	1	1	2	3	3
2	2	1	2	2	2	2	2	2	2
1	3	1	1	3	2	2	1	3	1
1	1	3	1	3	3	3	3	3	3
2	2	2	2	2	2	2	2	2	2
2	2	2	2	2	2	2	3	3	2
3	1	1	3	3	2	2	2	2	2
1	3	1	1	3	3	3	3	3	2
1	1	1	1	1	1	3	1	3	1
3	3	3	1	3	3	3	3	3	3
1	2	2	2	3	3	3	3	3	3
2	2	3	3	3	3	3	3	3	2
1	2	2	2	3	3	3	3	3	3
3	2	1	2	3	3	3	3	3	9
2	2	1	1	3	3	3	3	3	3
2	2	2	1	2	2	2	2	2	2
3	1	2	1	9	2	3	2	3	2
2	2	2	2	2	3	2	2	2	1
2	2	1	2	2	2	2	3	2	2
2	1	1	1	3	1	3	3	3	2
1	1	3	1	3	3	1	3	3	3
1	3	3	1	1	2	2	3	3	2
9	9	3	1	3	3	2	3	3	2
2	2	2	2	2	2	2	2	2	2
2	2	2	2	2	2	2	2	2	2
1	1	3	1	3	3	3	3	3	1
2	2	1	1	3	3	3	3	2	2
2	2	1	1	3	3	3	3	3	3
1	3	3	1	3	3	3	3	3	1
1	1	2	1	3	2	2	3	3	2
1	2	3	1	3	1	3	3	3	3
1	1	2	2	2	2	2	2	2	1
2	1	1	3	1	2	1	2	1	2
2	2	1	2	2	2	2	2	2	2
9	9	2	2	2	2	2	2	2	2
1	1	2	1	3	3	9	3	3	3
2	2	2	2	2	2	2	2	2	2
3	3	1	2	1	2	2	2	1	2
3	2	1	2	2	2	2	2	2	1
2	2	2	2	2	2	2	2	2	2
2	3	1	3	2	2	1	3	2	1
9	9	9	9	9	9	9	9	9	9
9	9	9	9	9	9	9	9	9	9
1	1	1	1	1	1	1	1	1	1
3	2	1	2	3	2	3	1	2	3
9	9	9	9	9	9	9	9	9	9
2	2	9	9	9	9	9	9	9	9

2	2	2	2	2	2	2	2	2	2
1	1	3	1	1	3	2	3	1	1
2	3	1	2	1	2	3	1	2	3
2	1	1	2	2	3	1	2	2	2
3	3	1	2	2	2	1	1	1	3
2	3	1	2	1	2	3	1	2	3
2	2	1	2	1	2	3	2	2	2
9	9	1	9	1	9	9	9	9	9
2	2	1	1	1	2	2	1	2	1
9	9	9	9	9	9	9	9	9	9
2	2	1	1	1	1	1	1	1	1
3	3	3	3	3	3	3	3	3	3
2	1	1	1	1	2	3	2	3	3
3	2	1	2	2	2	2	2	3	2
1	1	1	1	1	1	1	1	1	1
9	9	9	9	9	9	9	9	9	9
9	9	9	9	9	9	9	9	9	9
2	2	1	1	2	3	3	3	3	3
2	2	2	2	2	2	2	2	2	2
1	3	2	2	1	1	1	2	2	1
2	2	2	2	2	2	2	2	2	2
2	2	2	2	2	2	2	2	2	2
2	2	3	1	1	2	2	1	3	1
1	2	1	2	2	1	3	2	3	1
2	9	1	2	2	2	9	9	1	9
9	9	9	1	9	9	9	9	9	9
1	1	3	1	3	3	3	3	3	3
2	1	1	1	3	3	9	2	9	3
1	1	1	1	1	1	1	1	1	1
2	2	3	1	9	9	9	9	9	9
1	1	1	1	1	2	1	2	2	3
2	2	2	2	2	2	2	2	2	2
2	2	2	2	2	2	2	2	2	2
2	2	2	2	2	2	2	2	2	2
2	2	3	1	1	1	1	1	1	1
2	2	2	2	2	2	2	2	2	2
3	3	3	3	3	3	3	2	2	2
9	9	1	9	9	9	9	9	9	9
2	9	9	9	9	9	9	9	9	9
2	2	2	2	2	2	2	2	2	2
2	2	2	2	2	2	2	2	2	2
9	9	9	1	9	9	9	9	9	9
2	1	1	2	1	2	1	2	1	2
9	9	1	1	2	2	9	9	1	1
2	2	2	2	2	2	2	2	2	2
2	2	2	2	1	2	2	1	2	2
2	2	2	2	2	2	2	2	2	2

3	2	2	1	2	3	1	3	1	2
9	9	1	9	9	9	9	9	9	9
1	1	2	2	2	2	2	2	2	2
2	2	3	3	3	3	3	3	3	3
3	3	2	2	2	2	2	2	2	2
2	9	3	3	2	2	2	1	1	1
1	1	2	1	3	2	2	2	3	2
1	2	2	1	1	2	2	2	2	3
2	2	2	2	2	2	2	2	2	2
9	9	1	1	3	3	3	3	3	3
9	9	1	1	3	3	3	3	3	1
2	2	2	2	2	2	2	2	2	2
3	1	2	2	9	3	1	1	1	1
2	3	3	1	2	2	1	3	3	2
1	2	1	2	1	1	1	2	1	1
1	2	1	2	1	1	1	2	1	1
2	2	1	1	2	2	2	2	2	2
2	2	2	2	2	2	2	2	2	2
3	2	1	1	1	2	1	1	1	2
1	2	2	1	2	2	1	2	1	3
1	1	1	1	1	2	2	2	2	2
2	2	1	1	2	2	2	2	3	3
1	2	1	2	2	1	1	2	1	3
9	9	9	9	9	9	9	9	9	9
3	3	2	9	2	2	2	2	2	2
9	9	9	1	9	9	9	9	9	9
9	9	9	9	9	9	9	9	9	9
9	9	9	9	9	9	9	9	9	9
3	3	1	2	2	2	2	2	1	1
3	3	3	1	1	3	1	3	3	3
2	2	2	2	2	2	2	2	2	2
9	9	1	9	9	9	1	3	1	1
2	1	1	1	2	2	2	2	2	2
2	2	2	2	2	2	2	2	2	2
9	9	1	1	2	3	3	3	3	2
1	1	2	1	3	3	3	3	3	3
2	1	1	2	1	2	1	3	3	2
3	1	1	2	2	1	2	1	2	2
1	1	1	1	2	2	3	2	1	3
1	1	3	1	3	3	3	3	3	3
2	2	1	2	2	2	3	3	2	2
2	1	2	2	2	2	2	2	2	2
9	9	1	9	9	9	9	9	9	1
9	9	2	9	9	9	9	9	1	9
1	2	1	2	1	2	1	3	1	2
2	2	2	2	2	2	2	2	2	2
2	2	2	2	2	2	2	2	2	2

3	3	1	1	1	1	1	3	3	2
9	9	9	9	9	9	9	9	9	9
9	9	9	9	9	9	9	9	9	9
9	9	9	9	9	9	9	9	9	9
9	9	9	9	9	9	9	9	9	9
9	9	1	9	9	9	9	9	9	9
9	9	9	9	9	9	9	9	9	9
9	9	9	9	9	9	9	9	9	9
9	9	9	9	9	9	9	9	9	9
9	9	3	1	3	3	3	3	2	2
2	2	2	2	2	2	2	2	2	2
1	2	1	2	1	2	1	3	1	2
1	1	3	1	3	3	3	3	3	3
2	2	1	2	2	2	2	2	2	2
2	2	2	1	3	3	3	2	2	2
2	1	1	2	2	3	1	3	1	2
3	2	2	2	1	1	2	2	1	1
2	2	3	1	3	3	2	3	3	3
2	3	2	1	1	1	1	1	2	1
1	2	3	2	1	2	1	1	2	1
2	1	1	2	1	2	9	2	3	2
2	3	1	2	1	2	3	2	1	2
3	3	1	1	1	1	1	1	1	1
2	2	2	2	2	2	2	2	2	2
3	3	2	2	2	2	2	2	2	2
3	3	2	2	2	2	2	2	2	2
2	2	3	3	3	3	3	3	3	3
2	1	2	1	1	2	1	2	1	2
2	2	2	2	2	2	2	2	2	2
2	2	2	2	2	2	2	2	2	2
9	9	9	9	9	9	9	9	9	9
2	2	2	2	2	2	2	2	2	2
1	1	1	1	1	1	1	1	1	1
9	9	9	1	1	9	9	9	9	9
3	3	3	3	2	2	2	2	2	2
2	2	3	2	2	2	2	2	2	2
2	2	2	2	2	2	2	2	2	2
2	2	2	2	2	2	2	2	2	2
2	3	3	1	1	1	3	1	2	2
9	9	9	9	9	9	9	9	9	9
1	2	2	1	3	1	1	3	3	2
2	2	2	2	2	2	2	2	2	2
2	2	2	2	2	2	2	2	2	2
2	2	2	1	3	3	3	3	3	3
1	1	1	1	3	3	3	3	3	3
9	9	9	9	9	9	9	9	9	9
2	2	2	2	2	2	2	2	2	2

9	9	9	9	9	9	9	9	9	9
2	2	2	2	3	3	3	3	2	2
9	9	9	1	9	9	9	9	9	9
9	9	9	9	9	9	9	9	9	9
9	9	9	9	9	9	9	9	9	9
9	9	9	9	9	9	9	9	9	9
9	9	9	9	9	9	9	9	9	9
9	9	9	9	9	9	9	9	9	9
9	9	9	9	9	9	9	9	9	9
9	9	9	9	9	9	9	9	9	9
9	9	9	9	9	9	9	9	9	9
9	9	9	9	9	9	9	9	9	9
9	9	9	9	9	9	9	9	9	9
9	9	9	9	9	9	9	9	9	9
9	9	9	9	9	9	9	9	9	9
9	9	9	9	9	9	9	9	9	9
9	9	9	9	9	9	9	9	9	9
9	9	9	9	9	9	9	9	9	9
9	9	9	9	9	9	9	9	9	9
9	9	9	9	9	9	9	9	9	9
9	9	9	9	9	9	9	9	9	9
9	9	9	9	9	9	9	9	9	9
9	9	9	9	9	9	9	9	9	9
3	2	1	2	1	2	3	2	1	2
2	2	1	2	2	2	2	2	2	2
3	3	3	1	3	3	3	3	3	3
2	2	3	1	3	3	3	3	3	3
2	1	3	1	3	3	3	3	3	3
1	1	9	1	3	3	3	3	3	3
2	2	1	1	3	2	2	2	2	2
2	2	3	1	3	3	3	3	3	2
2	2	2	1	1	1	1	1	1	2
2	2	1	2	2	3	2	3	1	1
3	3	3	1	3	3	3	3	3	3
1	2	3	1	3	3	3	3	3	3
1	1	3	1	3	2	3	3	3	3
1	3	3	1	3	2	2	3	3	3
2	2	3	1	3	2	2	3	3	3
9	1	1	1	1	3	3	3	1	3
9	9	1	9	9	9	9	9	9	9
1	2	1	1	3	1	3	3	3	3
1	3	1	1	3	3	3	3	3	3
9	9	1	9	1	9	9	2	9	9
1	3	3	1	3	3	3	1	3	3
1	3	3	1	3	3	1	3	3	3
2	2	3	3	3	2	2	2	2	2
2	2	3	1	3	3	3	3	9	2

1	2	2	1	3	3	3	3	3	3
2	1	2	1	3	3	3	3	3	3
1	1	1	1	3	3	3	3	3	3
2	2	3	1	3	3	2	3	3	3
1	3	2	2	3	2	2	2	3	2
9	3	1	9	9	9	9	9	9	9
1	2	3	1	3	3	3	3	3	3
9	9	9	1	9	9	9	9	9	9
9	9	3	1	3	3	2	3	3	3
9	9	3	1	3	3	2	3	3	3
9	9	2	1	9	9	9	9	9	9
9	9	2	1	9	9	9	9	9	9
9	9	1	9	9	1	9	9	9	9
9	9	1	9	9	9	9	9	9	9
1	9	1	1	3	3	3	3	3	3
3	3	1	1	3	3	3	3	3	3
1	1	1	1	3	1	3	3	3	1
2	2	1	2	2	2	2	2	2	2
9	9	9	1	9	9	9	9	9	9
1	3	3	1	3	3	3	3	3	3
9	9	2	1	1	2	3	3	3	1
9	9	1	9	9	9	9	9	9	9
9	9	1	1	1	3	1	3	3	3
1	9	9	1	9	9	9	9	9	9
9	9	9	1	9	9	9	9	9	9
3	3	1	2	2	2	1	2	3	2
1	3	3	1	1	3	3	1	3	3
3	1	3	1	3	3	3	3	3	3
3	3	2	1	3	3	2	3	3	3
2	2	2	2	2	2	2	2	2	2
3	1	3	1	1	3	3	3	3	3
9	9	3	1	3	3	3	3	3	3
3	3	2	1	1	2	3	1	2	1
1	1	3	1	1	1	1	3	3	1
3	1	3	1	3	1	1	3	3	1
9	9	1	9	9	9	9	9	9	9
2	2	2	2	2	2	2	2	2	2
2	2	3	9	3	3	3	3	3	3
2	2	2	3	2	2	3	2	3	2
3	2	2	2	2	2	2	2	2	2
1	2	2	1	2	3	2	2	3	3
1	2	2	1	3	1	2	2	2	3
1	1	3	3	3	3	3	3	3	9
2	3	2	2	2	1	1	3	3	3
1	1	3	1	3	3	3	3	3	3
9	9	3	1	3	3	2	3	3	3
9	9	3	1	3	3	3	3	3	3

1	1	1	1	1	1	3	3	3	3
3	3	2	1	2	2	3	2	2	2
1	3	1	1	3	3	3	3	3	3
9	9	3	1	3	1	1	1	1	1
1	9	2	9	9	2	9	9	9	9
3	2	2	3	1	1	2	3	1	3
1	1	3	1	3	3	3	3	3	3
9	9	9	9	9	9	9	9	9	9
9	9	2	9	2	9	9	9	9	9
3	3	1	1	1	1	1	1	1	1
2	2	2	2	2	2	2	2	2	2
3	3	3	1	3	3	3	3	3	3
1	1	2	1	2	3	2	3	2	1
9	9	9	9	9	9	9	9	9	9
9	9	9	9	9	9	9	9	9	9
1	3	3	3	3	3	3	3	3	3
9	9	9	1	9	9	9	1	9	9
9	9	9	9	9	9	9	9	9	9
3	3	2	3	3	3	3	2	2	2
2	2	2	1	3	3	1	2	3	3
3	1	1	1	3	3	3	3	3	3
1	1	1	1	1	1	1	1	1	1
3	1	3	2	3	3	3	1	1	1
1	1	3	1	3	3	3	3	3	3
2	2	3	1	1	1	2	2	1	1
9	9	9	9	9	9	9	9	9	9
9	9	2	9	9	9	9	9	9	9
1	1	3	1	3	3	3	3	3	1
3	3	3	3	3	3	3	3	3	3
3	2	2	3	3	3	3	1	3	3
9	9	9	9	9	9	9	9	9	9
9	9	9	9	9	9	9	9	9	9
9	9	9	9	9	9	9	9	9	9
9	9	9	9	9	9	9	9	9	9
9	9	9	9	9	9	9	9	9	9
9	9	9	9	9	9	9	9	9	9
9	9	9	9	9	9	9	9	9	9
9	9	9	9	9	9	9	9	9	9
9	9	9	9	9	9	9	9	9	9
9	9	9	9	9	9	9	9	9	9
9	9	9	9	9	9	9	9	9	9
9	9	9	9	9	9	9	9	9	9
9	9	9	9	9	9	9	9	9	9
9	9	9	9	9	9	9	9	9	9
9	9	9	9	9	9	9	9	9	9
9	9	9	9	9	9	9	9	9	9
9	9	9	9	9	9	9	9	9	9

9	9	9	9	9	9	9	9	9	9
9	9	9	9	9	9	9	9	9	9
9	9	9	9	9	9	9	9	9	9
9	9	9	9	9	9	9	9	9	9
9	9	9	9	9	9	9	9	9	9
9	9	9	9	9	9	9	9	9	9
9	9	9	9	9	9	9	9	9	9
9	9	9	9	9	9	9	9	9	9
9	9	9	9	9	9	9	9	9	9
9	9	9	9	9	9	9	9	9	9
9	9	9	9	9	9	9	9	9	9
9	9	9	9	9	9	9	9	9	9
9	9	9	9	9	9	9	9	9	9
9	9	9	9	9	9	9	9	9	9
9	9	9	9	9	9	9	9	9	9
9	9	9	9	9	9	9	9	9	9
9	9	9	9	9	9	9	9	9	9
9	9	9	9	9	9	9	9	9	9
9	9	9	9	9	9	9	9	9	9
1	2	2	1	2	2	2	2	2	2
9	9	9	9	9	9	9	9	9	9
9	9	9	9	9	9	9	9	9	9
9	9	9	9	9	9	9	9	9	9
9	9	9	9	9	9	9	9	9	9
9	9	9	9	9	9	9	9	9	9
9	9	9	9	9	9	9	9	9	9
9	9	9	9	9	9	9	9	9	9
9	9	9	9	9	9	9	9	9	9
9	9	9	9	9	9	9	9	9	9
9	9	9	9	9	9	9	9	9	9
9	9	9	9	9	9	9	9	9	9
9	9	9	9	9	9	9	9	9	9
9	9	9	9	9	9	9	9	9	9
9	9	9	9	9	9	9	9	9	9
9	9	9	9	9	9	9	9	9	9
2	2	2	9	2	9	9	9	9	9
3	3	1	1	3	1	3	3	1	3
2	3	3	1	3	9	1	3	3	3
1	1	3	1	3	3	3	3	3	3
3	3	2	2	2	2	2	2	2	2
1	1	1	1	1	1	1	1	1	1
9	9	1	1	3	2	3	3	2	3
9	9	9	1	9	9	9	9	9	9
9	9	1	1	3	2	3	3	2	3
2	1	2	2	2	2	2	2	2	1
1	1	1	1	1	3	3	3	3	1
2	2	3	1	3	3	3	3	3	3

9	1	1	1	1	3	3	3	1	3
1	1	3	1	3	3	3	3	3	3
1	2	1	1	3	1	2	3	3	2
1	3	2	1	3	2	2	3	3	3
1	2	3	1	3	1	3	3	3	3
1	1	2	2	1	2	3	3	3	3
2	2	3	1	3	3	3	3	3	3
2	2	2	1	3	3	3	3	3	2
3	2	2	2	1	2	3	2	1	3
2	1	1	3	3	3	3	1	3	3
9	9	9	1	9	9	1	9	9	9
3	3	3	1	3	2	1	3	2	2
1	1	1	1	3	3	3	3	3	3
1	2	2	1	3	1	2	3	3	3
2	1	2	1	3	1	2	3	3	2
2	1	3	1	3	3	3	3	3	1
2	2	9	9	9	9	9	9	9	9
3	3	3	1	3	1	1	3	3	9
1	2	1	1	1	2	1	2	2	1
1	1	2	9	1	1	2	1	3	1
1	9	3	1	3	3	1	3	3	3
1	2	2	1	3	3	3	2	3	3
1	1	3	1	1	3	3	3	3	3
1	9	3	1	3	3	3	3	3	3
3	3	3	3	3	3	3	3	3	3
2	1	1	3	1	1	1	1	2	1
1	2	1	1	1	1	1	1	1	1
1	2	1	2	3	1	3	2	1	2
2	2	2	2	2	2	2	2	2	2
2	2	3	1	2	2	2	2	2	2
3	2	2	2	2	3	2	3	2	2
2	2	2	1	3	2	2	2	2	2
2	2	3	1	2	2	2	2	2	2
9	9	9	9	9	9	9	9	9	9
9	1	9	9	9	9	9	9	9	9
9	9	9	9	9	9	9	9	9	9
9	9	1	1	2	3	1	2	1	2
9	9	1	1	2	2	1	2	1	1
9	9	1	1	9	9	9	9	9	9
9	9	9	9	9	9	9	9	9	9
2	2	1	2	3	1	2	2	2	3
2	2	3	2	1	2	1	3	1	2
1	2	3	1	3	3	3	3	3	3
1	3	1	3	3	1	3	3	3	3
1	1	3	1	1	1	3	3	3	3
3	3	3	1	3	1	3	3	3	3
1	3	3	1	3	3	3	9	3	1

2	2	2	2	2	2	2	2	2	2
3	1	1	3	2	1	1	3	2	2
2	2	1	2	2	1	2	2	1	2
1	3	1	3	3	1	1	1	1	1
3	2	1	1	2	3	3	3	2	2
1	2	1	3	1	2	3	1	2	1
3	3	1	1	3	1	9	3	9	1
3	1	3	2	2	3	1	2	1	2
1	1	3	1	3	3	3	3	3	3
9	9	1	9	9	9	9	9	9	9
3	3	3	1	3	1	3	3	3	3
1	1	1	3	1	3	1	2	1	3
3	3	3	1	1	3	1	3	2	2
1	1	1	1	3	3	3	3	3	3
1	3	3	1	1	1	1	1	1	1
3	2	1	9	3	1	1	1	3	1
1	2	2	2	2	1	3	2	2	2
9	9	9	1	9	9	9	9	9	9
2	1	1	1	3	3	1	1	1	2
9	9	1	1	3	3	3	3	3	3
3	1	1	2	1	1	2	2	3	3
1	2	1	3	2	1	1	1	1	2
1	1	1	1	3	3	3	3	3	3
1	3	1	3	3	1	1	3	1	3
1	2	1	2	2	3	1	1	2	2
1	3	1	1	3	3	3	3	3	1
1	1	1	1	3	3	3	3	3	3
3	3	1	1	1	2	1	3	2	3
2	2	1	1	1	3	3	3	1	3
2	1	1	3	1	2	3	1	3	2
3	3	2	3	1	2	1	1	1	1
2	3	1	1	1	2	3	1	2	2
1	1	1	1	2	1	3	3	3	9
1	3	1	1	3	3	3	3	3	1
3	3	1	1	1	1	1	1	1	1
1	1	3	1	3	3	3	3	1	1
2	1	1	2	1	2	3	1	2	1
9	9	9	9	9	9	9	9	9	9
3	3	3	1	3	3	1	3	3	1
3	2	3	1	1	9	3	3	3	1
1	1	1	1	1	1	3	3	3	3
3	2	1	2	1	2	3	1	2	3
1	1	1	1	1	1	1	3	1	1
2	2	2	2	2	1	2	3	2	2
2	2	2	2	2	2	2	2	2	2
9	9	3	3	2	1	2	3	3	2
3	3	3	3	3	3	3	3	3	3

3	1	1	1	2	3	1	2	2	1
3	2	1	3	1	1	1	1	1	1
2	2	3	1	2	2	2	2	2	3
2	3	1	2	1	2	2	3	2	2
2	2	1	1	2	2	2	2	2	2
1	2	1	1	2	2	2	1	1	1
2	2	2	2	2	2	2	2	2	2
9	9	9	9	9	9	9	9	9	9
9	9	9	9	9	9	9	9	9	9
2	1	1	2	1	2	1	2	1	2
2	2	1	1	2	2	2	2	2	2
2	2	2	2	2	2	2	2	2	2
2	2	1	3	2	2	2	2	2	2
2	2	2	2	1	1	1	1	1	1
2	2	2	2	3	2	2	2	2	2
2	2	2	2	2	2	2	2	2	2
2	2	2	2	2	2	2	3	3	2
2	2	2	2	2	2	2	2	2	2
2	2	1	2	1	3	2	2	2	2
2	2	3	2	2	2	2	2	2	2
3	2	1	1	2	2	2	2	2	2
2	2	2	2	2	2	2	2	2	2
2	2	2	2	2	2	2	2	2	2
2	2	1	3	2	2	1	2	2	2
2	2	2	2	2	2	2	2	2	2
2	2	1	2	2	1	2	2	2	2
2	2	1	1	2	2	2	2	2	2
3	2	1	2	1	2	3	1	2	1
3	3	2	2	2	2	2	2	2	2
2	2	2	2	2	2	2	2	2	2
2	2	2	2	2	2	2	2	2	2
2	2	1	1	2	2	2	2	2	2
2	2	2	2	2	2	2	2	2	2
2	2	2	2	2	2	2	2	2	2
2	2	1	2	2	2	2	2	2	2
2	2	1	1	2	2	2	2	2	2
2	2	2	2	2	2	2	2	2	2
2	2	2	2	2	2	2	2	2	2
2	2	2	2	2	2	2	2	2	2
1	1	1	1	3	3	3	1	3	2
2	2	1	1	2	2	2	2	3	3
2	2	2	2	2	2	2	2	2	2
1	2	2	2	1	1	3	1	3	2
2	2	2	2	2	2	2	2	2	2
2	2	1	2	2	2	1	1	1	1
3	3	2	2	2	2	2	2	2	2
2	2	3	3	3	3	3	3	3	3

2	2	2	2	2	2	2	2	2	2
2	2	1	3	2	2	2	2	2	2
2	2	1	2	2	2	2	2	2	2
2	2	2	2	2	2	2	2	2	2
1	2	1	2	3	2	1	2	3	2
2	2	2	2	2	2	2	2	2	2
9	9	3	1	1	1	1	1	1	1
9	1	1	1	1	1	1	1	1	1
1	9	1	9	9	9	9	9	9	9
2	2	2	2	2	2	2	2	2	2
2	2	2	2	2	2	3	3	3	3
1	1	1	1	1	1	1	1	1	1
3	3	2	2	2	3	3	2	3	3
9	9	1	1	1	3	9	9	9	9
2	2	1	3	2	2	2	2	2	2
1	2	1	9	2	2	2	1	2	2
2	2	1	1	3	2	2	2	2	2
2	1	1	1	1	1	1	1	1	1
1	9	1	9	9	9	9	9	9	9
3	3	1	1	2	2	1	1	3	3
1	2	1	1	1	2	1	2	2	2
9	1	1	9	9	9	9	9	9	9
9	9	9	1	9	9	9	9	9	9
2	2	1	3	2	3	3	1	1	2
2	3	1	2	1	3	2	9	1	1
3	3	2	2	1	1	1	1	2	3
2	1	3	1	2	2	1	2	3	1
2	1	1	1	1	3	3	3	1	1
2	3	1	2	1	2	9	1	3	1
1	1	1	1	1	1	1	1	1	1
3	3	3	3	1	2	1	1	2	2
2	2	2	3	3	2	1	3	2	2
2	2	2	2	2	2	2	2	2	2
9	9	1	2	9	2	9	9	9	2
2	1	1	1	3	3	3	3	3	3
9	1	1	9	9	9	9	9	9	9
3	3	1	1	1	1	3	3	3	3
2	2	1	1	1	1	1	1	1	1
2	2	2	2	2	2	2	2	2	2
2	2	2	2	2	2	2	2	2	2
1	1	9	9	9	9	9	9	9	9
2	2	1	1	2	2	2	2	2	2
9	9	9	1	9	9	9	9	9	9
1	1	1	1	1	1	1	1	1	1
2	1	1	1	2	3	2	2	2	2
2	2	2	1	2	2	2	2	2	2
2	2	2	1	2	1	2	2	2	2

1	1	1	1	1	1	1	1	1	1
2	2	1	1	2	2	2	2	2	2
1	1	1	1	1	1	1	1	1	1
2	2	2	2	2	2	2	2	2	2
2	9	9	1	9	9	1	9	9	9
2	3	3	3	1	2	2	3	2	1
3	3	3	3	3	3	3	3	3	3
1	1	1	2	2	2	2	2	2	2
2	3	1	1	2	2	2	2	2	2
3	3	1	1	1	1	1	1	1	1
1	1	1	2	1	2	3	2	1	2
2	2	3	3	3	3	3	3	3	3
2	2	1	3	1	1	1	1	1	1
2	2	1	3	1	1	1	1	1	1
2	2	2	2	2	2	2	2	2	2
2	2	3	2	3	2	2	2	2	2
1	1	1	1	1	1	1	1	1	1
3	3	1	1	3	3	3	3	3	3
9	9	9	9	9	9	9	9	9	9
1	2	1	2	2	1	2	1	3	1
2	1	3	3	1	1	1	1	1	2
1	2	1	3	2	2	2	2	2	2
3	3	1	1	1	1	1	1	1	1
2	2	2	1	1	2	9	1	1	1
3	3	9	3	9	3	9	3	9	9
2	2	1	2	2	1	1	1	2	2
3	3	1	1	1	2	9	1	2	1
1	2	2	2	2	2	1	2	1	2
2	3	1	9	2	9	9	9	9	9
2	2	2	3	1	2	1	2	2	1
2	2	1	2	1	1	9	2	2	2
2	2	2	2	2	2	2	2	2	2
3	3	3	3	3	3	3	3	3	3
1	2	1	1	2	2	3	1	2	3
2	2	2	2	2	2	2	2	2	2
2	2	3	2	3	2	3	2	3	2
1	3	1	3	2	3	9	2	3	3
2	2	2	2	3	3	9	2	2	2
2	3	1	2	3	1	2	1	2	1
3	3	3	3	3	3	3	3	3	3
1	1	1	1	1	2	1	2	3	1
1	1	2	2	3	2	3	2	3	2
9	9	9	9	9	9	9	9	9	9
3	3	3	3	3	3	3	3	3	3
1	1	1	2	1	1	2	2	1	1
2	2	2	2	2	2	2	2	2	2
2	1	2	1	3	3	3	3	3	3

1	1	1	1	1	1	1	1	1	1
3	3	1	1	3	3	3	3	1	1
1	1	1	1	1	1	1	1	1	1
3	3	1	1	3	3	3	3	1	1
1	1	1	3	1	1	2	3	1	1
2	2	2	2	2	2	2	2	2	2
2	2	2	2	2	2	2	2	2	2
2	2	2	2	2	2	2	2	2	2
1	1	1	1	2	2	2	2	1	1
3	2	1	3	1	2	1	1	2	1
1	1	2	2	2	2	2	2	2	1
3	1	1	3	2	3	2	1	2	3
2	2	2	3	2	3	2	2	2	2
3	1	2	1	2	3	2	2	3	2
2	2	2	2	2	2	2	2	2	2
2	2	1	1	3	3	3	3	2	2
2	2	2	2	2	2	2	2	2	2
2	2	2	3	2	1	3	1	1	2
1	2	1	2	2	1	2	2	2	2
3	3	3	1	3	3	2	3	1	1
2	2	1	1	1	1	1	1	1	2
3	2	1	2	3	1	1	1	1	1
1	1	1	2	3	1	1	1	3	1
2	2	2	2	2	2	2	2	2	2
1	1	1	1	1	1	1	1	1	1
3	3	2	3	2	2	2	2	2	2
3	3	1	2	1	1	1	1	1	1
3	1	3	1	1	3	3	3	3	1
2	2	1	9	2	3	2	2	2	3
2	3	2	1	2	3	2	3	3	3
2	2	1	1	3	1	2	3	1	1
2	2	1	3	3	2	1	2	3	2
2	2	1	1	1	1	1	1	1	2
2	3	2	2	2	2	2	2	2	2
3	3	1	2	1	1	1	3	1	1
2	2	1	2	1	2	3	2	1	2
2	2	1	2	2	2	2	2	2	2
9	9	9	9	9	9	9	9	9	9
2	2	1	2	2	2	2	2	1	1
3	3	1	3	2	3	3	1	1	2
3	2	1	3	2	3	3	1	1	2
2	2	2	2	2	2	2	2	2	2
2	2	2	2	2	2	2	2	2	2
2	2	2	2	2	2	2	2	2	2
3	2	9	9	9	9	9	9	9	9
1	1	1	3	1	1	2	3	1	1
2	2	2	2	2	2	2	2	2	2

9	9	9	9	9	9	9	9	9	9
3	1	1	2	2	3	1	3	1	3
1	1	1	1	2	3	1	2	3	1
1	3	2	1	3	3	1	1	1	1
9	1	3	3	3	3	3	3	3	3
3	3	1	1	3	2	1	3	3	3
3	1	1	1	3	3	3	3	3	3
9	9	9	9	9	9	9	9	9	9
1	2	1	2	2	1	1	2	1	1
3	1	1	1	3	1	3	3	3	3
3	2	2	2	2	2	2	1	2	2
2	3	1	3	1	2	3	2	3	3
2	2	2	1	9	9	9	9	9	9
1	1	1	1	3	1	2	3	2	3
2	2	1	2	3	1	3	2	2	2
2	2	2	2	2	2	2	2	2	2
2	3	1	2	2	1	2	3	2	2
3	2	1	9	3	3	3	2	2	1
1	9	1	9	9	9	9	9	9	9
2	2	2	2	2	2	2	2	2	2
9	9	9	9	9	9	9	9	9	9
3	2	1	2	1	2	2	1	2	3
3	2	1	2	2	2	1	1	2	3
2	1	1	2	2	3	3	2	1	1
3	3	1	1	2	2	2	1	1	2
3	3	3	3	3	3	3	2	3	2
2	3	1	2	2	3	2	1	3	1
1	2	1	1	2	2	2	2	2	2
1	2	1	1	3	2	2	3	1	3
1	2	1	1	3	3	3	3	3	3
3	3	1	1	2	2	3	3	3	2
2	1	1	2	2	1	2	3	2	1
3	3	3	3	3	1	3	3	3	3
2	2	2	2	2	2	2	2	2	2
2	2	2	2	2	2	2	2	2	2
3	3	2	2	2	9	1	2	2	2
3	3	1	3	3	3	3	3	3	3
2	1	2	2	2	3	3	3	1	2
1	1	1	2	1	2	3	1	2	3
1	1	1	2	2	1	3	2	2	3
1	1	1	3	1	1	2	1	1	1
2	1	2	2	2	2	2	2	2	2
2	1	3	1	3	3	2	2	1	2
3	3	1	2	3	3	2	2	2	3
9	9	9	9	9	9	9	9	9	9
3	3	3	3	3	3	3	3	3	3
3	2	1	1	3	3	3	3	3	2

3	3	3	3	3	3	3	2	2	2
9	1	1	1	1	1	3	3	1	2
1	2	3	1	2	1	2	3	1	2
9	9	1	2	2	2	2	2	2	2
3	3	1	3	2	2	3	2	3	2
3	3	3	3	3	3	3	3	3	3
2	2	1	1	1	1	1	1	1	1
3	3	3	3	3	3	3	3	3	3
2	1	2	3	1	2	1	2	1	1
3	1	1	1	3	2	3	1	3	3
2	2	2	2	2	2	2	2	2	2
2	3	2	2	2	2	2	2	2	2
2	1	1	2	2	2	2	1	9	1
9	9	9	1	9	9	9	9	9	9
1	1	1	1	9	1	9	2	2	2
3	3	3	3	3	3	3	3	3	3
1	1	1	1	1	1	1	1	1	1
2	2	2	2	2	2	2	2	2	2
2	2	2	2	2	2	2	2	2	2
1	1	1	1	1	2	2	2	3	1
2	1	1	3	1	3	1	1	1	3
3	3	9	9	9	9	9	9	9	9
2	2	3	3	3	1	3	3	1	2
3	1	2	1	2	3	1	3	2	2
2	3	1	2	3	1	2	3	1	2
1	1	1	3	3	1	1	3	1	1
2	2	1	1	1	1	1	1	1	1
2	1	1	3	1	2	3	1	2	1
3	2	2	1	2	1	3	1	2	1
2	2	1	1	3	3	3	3	3	3
3	2	1	1	2	3	2	3	2	1
2	2	3	3	3	3	3	3	3	3
2	3	1	2	2	2	1	2	2	2
3	3	1	3	2	2	3	3	3	1
2	3	1	1	1	3	1	1	3	1
1	2	1	1	1	2	1	3	2	3
2	3	1	1	1	2	2	1	2	2
1	1	1	1	3	3	9	2	3	1
1	1	1	3	1	1	1	1	1	1
1	2	3	1	1	2	1	2	1	2
2	2	2	2	2	2	2	2	2	3
1	3	1	1	1	1	1	1	1	1
1	2	1	2	1	2	1	2	1	2
2	1	1	1	3	9	2	9	3	1
2	3	1	2	2	1	2	1	2	2
3	2	2	2	1	2	1	3	2	1
3	3	3	1	1	3	3	3	1	1

1	3	2	3	3	3	1	3	1	1
1	2	1	3	1	2	3	2	1	2
2	9	1	2	2	2	2	2	2	2
3	1	3	1	1	1	3	1	3	3
2	3	1	2	1	2	3	1	2	3
2	2	1	1	1	2	2	3	3	2
2	1	1	3	2	1	2	3	1	2
1	2	1	2	1	3	9	1	2	2
1	3	3	3	1	1	1	3	1	1
2	2	2	3	1	2	2	2	1	1
2	2	1	2	1	2	1	2	3	1
1	2	1	1	2	3	1	1	2	1
1	1	3	1	1	1	1	3	3	3
1	3	3	1	3	1	1	3	1	3
2	2	3	3	1	1	9	9	2	1
3	3	3	3	3	3	3	1	1	1
1	1	1	3	1	3	1	1	1	1
3	3	1	1	1	3	9	2	3	1
1	1	1	1	3	2	2	2	2	1
1	1	1	1	1	1	1	2	1	1
2	2	1	9	1	1	1	2	1	1
1	1	3	3	3	3	3	3	3	3
1	2	1	1	3	3	3	3	3	1
1	1	1	1	1	1	1	1	3	1
2	2	1	3	2	2	2	2	2	2
2	2	2	2	2	2	2	2	2	2
3	3	1	3	3	1	1	2	1	1
2	1	1	9	2	1	3	2	1	2
2	2	2	2	2	2	2	2	2	2
9	9	9	9	9	9	9	9	9	9
9	9	9	9	9	9	9	9	9	9
9	9	9	9	9	9	9	9	9	9
2	2	1	1	2	2	2	2	2	2
9	9	1	1	9	9	9	9	9	9
2	2	2	2	2	2	2	2	2	2
2	2	1	1	1	3	3	3	3	3
2	3	3	2	2	1	1	2	1	1
2	2	1	3	3	3	3	3	9	9
2	2	3	1	2	9	9	9	9	1
3	2	1	1	2	2	1	1	1	2
2	3	3	1	3	3	3	3	3	2
3	1	1	1	1	1	3	1	1	1
3	3	3	1	3	3	2	3	2	2
3	3	3	1	3	3	2	3	2	2
3	2	1	1	3	1	2	1	3	1
2	2	1	3	1	2	1	2	1	3
3	3	1	3	3	1	1	2	1	1

2	9	3	3	3	2	1	1	1	9
1	2	1	2	1	2	2	2	2	2
2	2	3	3	2	2	3	2	2	2
2	1	3	3	3	1	1	1	3	3
2	2	3	1	3	3	9	3	3	1
2	3	3	2	2	1	2	1	2	1
9	9	1	9	1	9	2	9	9	9
2	1	1	3	2	3	1	1	1	1
1	1	3	1	3	3	2	3	3	3
2	1	3	3	3	3	2	3	3	3
2	1	1	2	3	2	1	2	3	3
2	3	1	3	3	3	3	3	1	3
1	2	1	1	1	2	9	2	3	1
2	2	2	2	2	2	2	2	2	2
2	2	2	2	2	2	2	2	2	2
9	9	1	9	9	2	2	2	2	9
2	2	1	1	3	3	3	3	3	3
2	2	2	2	2	2	2	2	2	2
2	2	2	2	2	2	2	2	2	2
9	9	2	2	2	2	2	2	2	2
3	9	1	1	1	2	1	3	1	1
3	9	1	1	3	9	9	9	9	9
3	9	9	1	9	9	9	9	9	9
1	2	3	1	3	3	3	3	3	3
3	3	3	1	3	3	3	3	3	3
9	9	9	1	9	9	9	9	9	9
2	2	3	1	3	3	3	3	3	3
3	3	2	1	3	9	9	9	9	9
1	2	2	1	3	2	2	2	2	1
3	3	3	1	3	3	3	3	3	3
3	3	3	1	3	9	9	9	9	9
9	9	9	1	3	9	9	9	9	9
3	3	3	1	3	3	3	3	3	3
3	3	1	1	3	3	3	3	3	3
9	9	3	1	3	9	9	9	9	9
3	3	3	1	3	3	3	3	3	3
9	9	3	1	3	9	9	9	9	9
9	9	9	1	3	9	9	9	9	9
3	3	3	1	3	3	3	9	9	9
3	9	1	1	3	9	9	9	9	9
3	3	3	1	3	3	3	3	3	3
1	9	9	1	9	9	9	9	9	9
3	3	2	1	3	3	3	3	3	3
9	9	1	1	3	9	3	3	3	9
3	3	3	1	3	9	9	9	9	9
3	3	3	1	3	3	3	3	3	3
3	3	3	1	3	9	9	9	9	9

9	9	9	1	9	9	9	9	9	9
9	2	3	1	3	2	2	2	2	2
3	3	2	1	3	2	3	2	3	3
1	1	1	1	1	1	1	1	1	1
3	3	3	1	3	9	9	9	9	9
3	3	3	1	3	9	9	9	9	9
1	1	2	1	3	1	1	1	1	1
1	2	2	1	2	2	2	2	2	2
3	3	2	1	3	9	9	9	9	9
9	9	3	1	3	3	3	3	3	3
3	3	3	1	3	3	3	3	3	3
1	2	3	1	3	3	2	3	3	3
3	3	3	1	3	9	9	9	9	9
3	3	3	1	3	9	9	9	9	9
3	3	3	1	3	9	9	9	9	9
3	9	3	1	3	9	9	9	9	9
9	9	3	1	3	3	2	9	9	9
9	9	9	1	3	9	9	9	9	9
3	3	3	1	3	9	9	9	9	9
9	9	3	1	9	9	9	9	9	9
9	9	9	1	3	1	3	2	1	1
1	9	2	1	3	9	9	9	9	9
1	2	3	1	3	2	2	3	3	3
3	3	2	1	3	9	9	9	9	9
2	1	1	1	3	3	3	3	3	3
3	3	1	1	1	1	1	1	1	1
3	3	1	1	3	3	1	3	3	3
3	3	1	1	3	3	2	3	3	3
3	3	2	1	3	2	3	3	3	3
9	9	3	1	3	9	9	9	9	9
3	3	3	1	3	1	1	1	2	1
3	3	3	1	3	2	3	3	3	3
3	3	3	1	3	9	9	9	9	9
9	9	2	1	3	9	9	9	9	9
9	9	3	1	3	9	9	3	9	9
1	1	3	1	3	3	2	3	3	3
9	9	2	1	3	2	2	2	2	2
3	1	2	1	3	3	3	3	3	3
9	9	3	1	3	3	3	3	3	3
1	3	3	1	3	3	3	3	3	3
1	3	3	1	3	3	3	3	3	3
3	3	2	1	3	3	3	3	3	3
1	9	2	1	3	9	9	9	9	9
1	1	3	1	3	3	3	9	9	9
3	3	3	1	3	2	3	3	3	3
3	3	3	1	1	3	3	3	3	3
9	9	3	1	3	3	3	3	3	3

3	3	9	1	3	3	1	3	3	3
1	3	3	1	3	3	3	3	3	3
3	3	2	1	3	9	3	3	3	3
1	1	9	1	3	3	3	3	3	3
1	9	3	1	3	3	3	3	3	3
9	9	3	1	3	3	3	3	3	9
9	9	3	1	1	3	3	3	3	3
9	9	1	9	3	9	9	9	9	9
9	9	1	9	3	9	9	9	9	9
3	3	2	1	3	3	3	3	3	1
1	2	3	1	3	1	2	3	3	3
9	9	9	1	9	9	9	9	9	9
9	9	3	1	3	3	3	3	3	3
2	2	2	1	1	2	3	3	3	2
2	2	3	3	3	3	3	2	3	2
1	1	3	1	3	3	3	3	3	3
9	9	9	9	9	9	9	9	9	9
9	9	1	1	3	9	3	3	3	3
2	3	3	1	3	3	3	3	2	1
1	1	1	1	3	3	3	1	1	3
1	3	2	1	3	3	3	3	3	3
2	3	3	1	3	3	2	1	2	2
2	2	2	1	3	2	2	3	3	3
1	1	3	1	3	3	3	3	3	3
9	9	1	1	3	3	3	3	3	1
3	1	1	1	3	3	3	3	3	1
2	1	3	2	2	3	3	2	2	2
1	2	3	1	1	1	1	3	3	2
2	2	3	1	3	3	3	3	3	3
1	2	2	1	3	3	2	9	3	2
2	2	3	1	3	3	1	2	3	3
3	2	1	1	9	9	9	9	9	9
1	2	3	1	2	3	3	2	2	3
1	9	1	1	3	3	3	3	3	1
2	2	2	1	3	2	2	3	3	3
2	2	1	1	3	3	1	3	3	3
9	9	1	1	3	1	3	1	3	1
1	2	1	1	3	1	3	3	3	1
9	9	1	1	3	1	3	1	3	1
1	2	1	1	3	3	3	3	3	3
1	1	3	1	3	3	3	3	3	3
1	2	3	1	3	3	3	3	3	3
2	2	3	1	3	3	2	2	3	1
2	2	1	1	2	2	2	2	2	2
2	2	3	1	3	3	2	2	1	1
1	2	3	1	3	3	3	3	3	3
9	9	1	1	9	9	1	3	1	2

3	3	3	1	3	3	9	3	3	3
1	2	1	1	3	2	2	2	3	3
1	2	3	1	3	3	3	3	3	3
2	2	3	1	3	2	2	3	3	3
1	1	3	1	3	1	3	3	3	1
3	3	1	3	1	1	1	1	2	1
2	2	2	1	3	3	2	3	3	3
1	2	3	2	2	3	3	2	2	2
1	2	1	1	3	3	3	3	3	3
1	3	3	1	3	3	3	3	3	3
1	2	3	1	2	3	3	3	3	3
3	3	3	1	3	3	3	3	3	3
1	2	3	1	3	3	3	3	3	3
9	9	2	1	3	3	2	3	3	3
1	2	2	1	3	3	3	3	3	2
9	9	9	1	9	9	9	9	9	9
1	2	3	1	3	3	3	3	3	3
9	9	2	1	1	2	2	2	2	1
3	3	3	1	3	3	3	3	3	3
1	2	3	1	3	3	2	3	3	3
9	9	1	9	9	9	9	9	9	9
9	9	9	1	3	3	3	3	3	1
2	2	3	1	3	3	9	9	9	9
1	3	1	1	1	1	3	3	3	3
2	3	3	1	3	3	2	3	3	3
2	3	9	1	3	3	9	3	3	3
1	9	3	1	3	1	1	3	3	1
9	9	9	9	9	9	9	9	9	9
9	9	9	9	9	9	9	9	9	9
3	1	3	1	3	2	2	3	2	2
9	1	3	1	3	3	3	3	3	3
1	3	3	1	3	3	3	3	3	3
9	9	3	1	3	3	3	3	3	3
1	9	9	1	3	3	3	3	3	3
3	3	2	1	3	9	9	9	9	9
9	1	2	1	3	3	3	3	2	3
1	1	3	1	3	3	3	3	3	3
3	3	2	1	3	2	3	3	2	3
3	3	3	1	3	3	3	3	3	3
1	1	3	1	3	3	3	3	3	3
3	2	3	1	3	1	3	3	3	1
9	9	3	1	3	2	3	3	2	3
1	3	3	1	3	3	3	3	3	3
3	9	2	9	3	9	9	9	9	9
3	3	3	1	3	3	3	3	3	3
3	3	3	1	3	3	3	3	9	9
9	9	3	1	3	3	3	3	3	3

3	3	3	1	3	3	3	3	3	3
1	3	2	1	3	9	3	9	3	3
9	9	9	9	9	9	9	9	9	9
1	9	3	1	3	2	3	3	1	3
2	9	1	9	1	1	3	3	3	3
1	1	3	1	3	3	3	3	1	3
9	9	9	1	1	1	2	2	2	1
9	9	3	1	3	3	2	3	3	2
9	9	1	9	9	9	9	9	9	9
9	9	9	1	9	9	9	9	9	9
1	1	1	1	3	3	3	3	3	1
1	1	3	1	3	2	3	2	3	9
1	1	3	1	3	2	3	3	3	3
9	9	9	1	9	9	9	9	9	9
1	1	3	1	1	1	3	1	1	3
1	1	3	1	3	3	3	3	3	3
9	9	9	1	9	9	9	9	9	9
2	9	3	1	3	3	3	3	3	3
9	9	3	1	3	3	3	3	3	3
3	2	3	1	3	3	1	3	3	3
1	1	3	1	3	3	3	3	3	3
9	9	3	1	3	3	3	3	3	3
9	9	3	1	3	3	3	3	3	3
1	1	3	1	3	3	3	1	3	3
9	9	1	1	9	9	3	9	9	9
1	2	1	1	1	1	1	1	1	1
9	9	9	1	3	2	3	3	3	3
1	1	2	1	1	1	2	3	2	3
1	1	1	1	1	1	1	1	1	1
1	1	1	1	1	1	1	1	1	1
1	2	2	1	3	3	2	3	2	3
1	1	3	1	3	3	2	1	2	3
2	3	3	1	3	2	3	3	2	2
1	3	3	1	3	3	3	3	3	3
1	1	3	1	3	3	3	3	3	3
9	9	9	1	9	9	9	9	9	9
1	9	9	1	3	3	3	3	3	3
1	9	9	1	9	9	9	9	9	9
2	2	2	1	3	9	1	3	3	3
3	2	1	1	3	3	3	3	3	3
1	2	3	1	3	3	3	2	3	3
2	2	9	9	3	3	3	3	3	3
9	9	9	1	9	9	9	9	9	9
9	9	9	1	9	9	9	9	9	9
1	9	3	1	3	2	3	3	3	3
1	9	9	1	3	9	1	9	9	1
1	9	3	1	3	9	9	9	9	9

3	3	3	1	3	3	3	3	3	3
1	1	9	1	9	9	9	9	9	9
1	9	1	1	1	1	3	3	3	9
1	1	2	1	3	2	1	3	3	2
1	3	3	1	3	3	3	3	3	3
9	9	2	1	3	2	3	3	3	3
1	2	3	1	3	3	3	3	3	2
2	2	1	1	3	3	1	3	3	2
3	3	3	1	3	9	3	3	3	3
1	1	2	1	3	2	2	2	3	3
3	3	3	1	3	1	3	3	3	3
9	9	3	1	9	9	9	9	9	9
9	9	3	1	3	3	3	3	9	9
9	9	9	1	9	9	9	9	9	9
1	9	9	1	9	9	9	9	9	9
9	9	9	1	9	9	9	9	9	9
3	3	3	1	3	3	3	3	3	3
1	2	1	1	3	2	2	2	3	2
9	9	3	1	3	3	3	3	3	3
2	2	3	1	3	3	3	3	3	1
1	1	3	1	1	2	1	3	3	3
2	2	3	1	3	3	2	3	2	2
2	2	3	1	3	3	2	3	1	2
3	3	2	1	3	2	2	3	3	3
1	2	1	1	3	2	3	3	3	3
1	9	9	1	9	9	9	9	9	9
9	3	3	1	3	3	3	3	3	1
2	2	1	1	3	3	3	3	3	1
1	1	3	1	3	3	3	3	3	3
9	9	3	1	3	3	3	3	3	3
2	3	3	1	3	2	3	3	3	3
1	9	9	1	9	9	9	9	9	9
9	9	3	1	3	3	3	3	3	3
2	3	1	1	1	3	3	2	1	3
9	9	2	1	3	3	3	3	3	3
3	3	2	1	3	3	3	3	3	3
2	2	3	1	3	3	3	3	3	3
3	3	2	1	3	2	2	2	3	2
9	9	3	1	3	3	9	9	9	9
3	3	3	1	3	3	3	3	3	9
3	2	2	1	2	3	2	3	2	3
1	2	2	1	2	3	2	9	2	2
1	1	3	1	3	3	2	3	3	2
1	2	3	1	3	3	3	3	3	1
1	1	3	1	3	3	2	3	3	1
9	9	9	1	9	9	9	9	9	9
1	1	3	1	3	3	1	3	3	3

1	3	3	1	3	3	3	3	3	3
1	2	3	1	2	3	3	3	3	3
1	1	3	1	3	3	3	3	3	3
1	1	3	1	3	3	3	3	3	3
2	2	1	1	1	2	3	3	2	2
1	2	2	2	1	2	2	3	3	3
1	1	1	1	3	3	3	2	3	2
1	2	3	1	3	1	2	3	3	2
1	2	3	1	3	2	3	3	3	2
1	2	1	1	1	1	1	2	2	3
1	2	3	1	3	3	3	3	3	2
1	2	1	2	2	2	2	2	2	2
1	1	3	1	3	1	1	3	3	1
1	2	3	1	3	3	3	3	3	3
1	2	1	1	2	2	2	2	2	2
1	1	1	1	3	3	3	3	3	3
2	2	1	2	2	2	3	3	3	3
2	2	2	2	2	2	2	2	2	2
2	2	2	2	2	2	2	2	2	2
1	1	3	1	3	3	3	1	3	3
1	2	2	1	1	3	3	1	3	3
3	2	3	1	3	3	3	1	2	3
2	1	3	1	3	3	2	3	3	3
1	1	3	1	3	3	3	3	3	3
1	1	2	1	3	2	3	3	3	3
3	2	3	1	1	1	2	2	2	3
2	1	2	2	2	2	2	2	2	2
1	1	9	1	3	2	3	3	1	9
1	1	3	1	3	2	3	3	1	9
1	1	3	1	1	3	3	3	3	3
1	3	2	1	2	2	3	3	2	2
1	1	2	1	3	3	3	3	3	3
1	9	9	1	9	9	9	9	9	9
9	9	9	1	9	9	9	9	9	9
1	9	9	9	9	9	9	9	9	9
9	9	9	1	9	9	9	9	9	9
1	9	9	1	9	9	9	9	9	9
1	9	9	1	9	9	9	9	9	9
9	9	9	1	9	9	9	9	9	9
1	3	3	9	9	9	9	9	9	9
1	1	2	1	3	3	2	3	3	3
9	9	9	1	9	9	9	9	9	9
9	9	9	1	9	9	9	9	9	9
1	2	3	1	1	3	3	3	2	3
1	9	9	1	9	9	9	9	9	9
9	9	9	1	9	9	9	9	9	9
9	9	9	1	9	9	9	9	9	9

1	1	3	1	3	3	3	3	3	3
2	3	1	3	2	1	1	3	2	3
9	9	9	1	9	9	9	9	9	9
1	9	9	1	9	9	9	9	9	9
1	9	9	1	9	9	9	9	9	9
1	9	1	9	9	9	9	9	9	9
9	9	9	1	9	9	9	9	9	9
9	9	9	1	9	9	9	9	9	9
9	9	3	1	3	3	3	3	3	3
9	9	9	1	9	9	9	9	9	9
1	1	3	1	3	3	1	3	3	3
1	1	3	1	3	3	3	3	3	3
1	1	2	1	3	3	3	3	3	3
1	2	3	1	3	3	3	3	3	3
1	1	3	1	3	3	1	3	3	3
1	1	3	1	3	3	3	3	3	3
1	1	3	1	3	3	3	3	1	3
1	1	3	3	1	1	1	2	1	1
1	1	3	1	3	3	3	3	3	3
1	1	3	1	3	3	9	3	3	3
1	1	1	1	3	1	3	3	3	3
1	1	1	1	3	1	3	3	3	3
9	9	9	1	9	9	9	9	9	9
1	2	3	2	2	9	1	2	2	3
1	1	1	2	3	1	2	9	1	3
1	1	3	1	3	3	3	3	3	1
3	3	9	9	9	9	9	9	9	9
1	2	3	1	1	3	3	3	3	3
1	1	2	1	3	3	2	3	3	3
1	2	3	1	3	2	3	3	2	2
1	1	3	1	3	2	3	3	3	3
1	3	1	1	3	3	2	3	2	3
1	1	3	1	3	1	3	3	3	3
1	2	1	1	3	3	2	3	3	3
1	1	1	1	1	3	3	3	3	1
1	1	3	1	3	3	3	3	3	3
1	1	3	1	3	3	3	3	3	1
1	3	3	1	3	3	3	3	3	3
1	1	3	1	3	3	1	3	3	3
1	2	3	1	1	1	2	3	3	3
1	3	1	1	3	3	3	3	3	3
1	1	2	1	3	1	3	3	3	3
1	1	3	1	2	3	1	3	3	1
3	3	3	1	3	3	3	3	3	3
3	3	3	1	3	3	3	3	3	3
1	3	3	1	3	3	3	3	3	3
1	2	2	1	3	3	3	3	3	3

9	9	3	1	3	3	3	3	3	3
1	1	3	1	3	1	1	3	3	3
1	1	3	1	1	1	1	3	3	1
1	3	3	1	3	3	3	3	3	3
3	3	3	1	3	3	3	3	3	3
1	3	3	1	3	3	3	3	3	3
1	2	2	1	3	3	3	3	3	3
1	1	3	1	3	3	3	3	3	3
1	2	3	1	3	1	1	3	3	3
1	9	3	1	3	3	1	3	3	3
1	2	3	1	3	3	3	3	3	3
9	9	3	1	3	9	3	3	3	1
1	2	3	1	1	1	3	3	1	1
1	2	3	1	1	1	3	3	1	1
1	3	3	1	3	3	3	3	3	3
2	2	2	2	3	3	3	3	3	3
1	1	1	2	2	3	3	3	3	2
1	2	1	2	1	1	2	2	2	2
2	1	1	3	1	1	3	2	3	1
1	2	3	1	3	3	3	3	3	3
1	1	3	1	3	1	3	3	3	3
1	1	3	1	3	3	3	3	3	3
1	1	3	1	3	1	3	3	3	3
1	1	3	1	3	1	3	3	3	3
1	1	3	1	3	1	3	3	3	3
1	1	3	1	3	2	3	3	3	3
3	2	3	1	3	3	3	3	3	1
9	2	3	1	2	1	3	2	2	2
2	1	3	1	3	3	3	3	3	3
1	2	2	1	3	2	3	3	3	2
2	2	2	2	3	3	2	3	3	3
2	2	3	1	3	1	3	3	3	3
1	1	3	1	1	3	1	2	9	1
1	2	3	1	1	1	3	3	3	3
1	1	3	1	3	1	3	3	3	3
1	3	3	1	3	3	3	3	3	3
1	2	3	1	3	3	3	3	1	2
1	2	2	2	1	3	1	3	3	1
1	1	1	1	3	1	3	3	1	3
1	1	1	1	3	3	3	3	3	1
1	1	3	2	3	2	2	2	2	2
1	3	3	1	3	3	3	3	3	3
1	3	3	1	3	3	3	3	3	3
1	1	3	2	1	2	2	3	2	2
1	1	3	1	3	3	2	3	3	3
3	3	2	1	3	2	2	3	3	3
1	3	1	1	1	3	3	3	3	1

2	2	1	1	3	3	3	3	3	1
2	2	2	2	2	2	2	2	2	1
1	1	3	2	1	2	3	2	1	1
1	1	1	1	3	3	3	3	3	1
1	1	1	1	3	3	2	3	3	3
2	2	3	1	3	3	3	3	3	3
1	2	3	3	3	3	3	9	2	3
1	2	2	1	3	2	3	2	3	3
2	2	2	1	3	3	3	3	2	3
1	1	2	1	3	3	2	3	3	3
1	2	3	1	3	3	3	3	3	3
1	1	3	1	3	3	3	3	3	3
1	9	1	9	9	9	9	9	9	9
2	2	1	1	3	3	1	3	3	1
1	1	1	1	1	3	3	3	3	3
2	2	3	1	3	1	2	2	2	2
1	2	3	1	3	2	1	3	3	3
1	1	3	1	3	3	3	3	3	3
1	2	3	1	3	3	3	3	3	3
1	1	1	3	3	3	3	3	3	3
1	1	2	1	3	3	3	3	3	1
1	1	3	1	1	1	1	3	3	3
1	1	3	1	3	3	3	3	3	3
1	1	3	1	3	3	2	3	3	3
1	3	2	1	1	9	3	9	3	2
1	1	3	1	3	3	3	3	3	3
1	1	3	1	3	3	3	3	3	3
2	1	9	9	9	9	9	9	9	9
2	9	1	9	2	9	9	9	9	9
2	1	2	1	2	9	2	9	2	9
3	2	3	1	3	3	3	1	2	3
1	1	3	1	3	3	3	3	3	3
1	1	3	1	3	3	3	3	3	1
1	1	1	1	3	2	3	3	3	3
1	9	9	1	9	1	9	9	9	9
1	1	2	1	1	1	1	3	1	1
2	2	2	1	1	2	2	2	2	2
9	9	9	2	9	1	9	9	9	9
2	1	1	1	1	2	2	1	1	1
2	2	3	1	3	3	3	3	3	3
1	9	3	1	3	3	3	3	3	2
1	1	3	1	3	3	3	3	3	3
1	1	3	1	1	3	3	3	2	2
9	9	9	1	9	9	9	9	9	9
1	1	1	2	1	2	1	3	3	3
1	2	3	1	3	2	2	2	2	2
1	1	3	1	1	1	1	3	3	1

1	1	3	1	3	3	3	3	3	3
1	1	2	1	3	3	2	2	2	2
2	2	1	1	3	2	3	3	3	3
1	2	3	1	3	2	1	2	2	1
2	2	1	2	2	2	1	2	3	2
1	2	3	1	3	3	3	3	3	3
1	2	1	1	3	1	1	3	3	3
9	9	9	9	9	9	9	9	9	9
1	1	1	2	1	2	2	2	2	2
1	1	3	1	3	3	2	3	3	2
2	3	2	1	1	2	2	2	3	3
1	1	3	1	3	2	2	3	3	2
1	1	3	3	3	3	3	3	3	3
2	2	2	1	2	2	2	2	2	2
2	3	3	1	3	1	3	2	2	1
1	3	3	1	3	3	3	3	1	1
1	1	2	1	3	3	3	3	3	3
9	9	1	1	1	9	1	1	2	1
1	9	3	1	3	3	3	3	3	3
1	3	2	1	3	1	2	9	1	1
1	3	2	1	3	1	2	3	1	1
1	1	1	1	3	1	1	3	3	1
2	2	2	1	3	3	3	3	3	3
1	2	3	1	3	3	3	3	3	2
1	3	3	1	3	3	3	3	3	2
9	2	1	1	3	1	1	1	1	1
2	2	1	1	3	3	3	3	3	3
2	2	1	1	3	2	3	3	3	2
2	2	1	1	3	2	3	3	3	2
9	9	1	9	2	9	9	1	9	9
2	2	1	1	3	2	3	3	3	2
2	1	1	1	2	1	2	3	3	3
1	1	3	1	3	3	2	3	3	2
2	3	3	1	3	3	3	3	3	3
1	2	1	1	9	3	3	3	1	2
1	1	2	1	2	2	2	2	2	1
1	2	2	1	3	3	3	3	3	1
1	2	9	9	9	9	9	9	9	9
1	1	3	1	3	3	3	3	3	3
1	3	2	1	3	3	1	1	3	3
2	2	3	1	3	3	3	3	3	3
1	1	3	1	1	3	3	3	3	3
2	2	3	1	1	2	2	2	2	1
1	2	3	1	3	3	3	3	3	3
2	3	3	1	3	3	1	3	3	1
1	3	3	1	1	3	3	3	3	3
1	1	3	3	1	1	1	1	1	1

1	3	3	1	1	1	1	3	1	1
1	3	3	1	1	1	1	3	1	1
3	3	1	1	3	3	1	3	3	3
1	1	3	1	3	3	2	3	3	2
1	3	3	1	1	1	1	3	1	1
1	2	3	1	1	1	1	3	1	1
1	1	9	1	3	3	3	3	3	9
2	2	2	1	3	3	3	3	3	1
1	1	3	1	3	3	3	3	3	3
1	1	2	1	3	3	3	3	3	3
1	1	3	1	3	3	3	3	3	3
1	1	3	1	3	3	3	3	3	3
1	1	3	1	3	3	2	3	3	3
1	2	3	1	3	3	2	3	3	3
1	1	3	1	3	3	3	3	3	3
1	1	3	1	3	3	3	3	3	3
1	1	3	1	2	3	2	1	1	2
1	2	2	1	1	1	2	3	1	1
1	3	3	1	3	3	3	3	3	3
3	1	3	1	1	3	3	3	3	3
1	1	2	1	3	1	2	1	2	1
1	9	3	1	3	3	2	3	3	3
1	9	9	1	9	9	9	9	9	9
1	9	9	1	9	9	9	9	9	9
9	9	9	1	9	9	9	9	9	9
9	9	3	1	3	3	3	3	3	3
1	1	9	1	3	3	3	3	3	1
1	9	9	9	9	9	9	9	9	9
9	9	1	9	9	9	9	9	9	9
1	9	9	9	9	9	1	9	9	9
1	9	9	9	9	9	9	9	9	9
1	9	9	9	9	9	9	9	9	9
9	9	9	9	9	9	9	9	9	9
1	1	2	3	3	3	3	3	2	1
3	2	3	1	1	1	2	2	2	3
1	1	2	1	1	3	1	3	3	3
1	2	3	1	3	3	3	3	3	3
1	1	3	1	1	3	3	3	3	3
2	2	1	1	3	3	3	9	3	3
2	2	1	1	3	3	3	3	3	3
1	2	3	1	3	9	3	3	3	1
1	1	3	1	3	3	3	3	3	3
1	1	2	1	3	1	9	3	2	3
1	1	1	1	3	2	3	3	3	3
1	1	1	2	3	2	2	2	3	3
1	1	3	3	1	1	1	1	1	1
1	1	3	1	3	3	3	3	3	3

1	2	1	1	1	2	3	3	1	1
2	3	3	3	1	1	1	3	3	3
3	3	3	1	3	3	3	3	3	9
3	2	2	3	3	3	3	3	3	3
1	1	1	1	3	1	1	3	1	1
2	1	2	1	3	1	2	3	3	3
2	2	9	9	9	9	9	9	9	9
2	2	2	1	1	2	2	3	3	2
1	1	2	1	3	3	1	2	2	3
2	2	3	3	1	2	3	1	2	3
2	2	1	2	1	3	3	3	3	3
1	1	3	1	3	3	3	3	3	3
3	3	1	1	1	1	2	1	1	1
3	2	2	2	3	3	1	2	3	2
2	2	3	1	2	1	3	3	3	3
1	1	2	3	3	3	3	3	1	1
1	1	3	2	2	3	3	3	3	3
2	1	1	1	1	2	1	2	1	2
1	1	2	2	3	2	2	1	2	1
1	1	1	1	1	1	1	1	1	1
3	1	1	1	1	1	1	1	2	1
2	2	2	2	2	2	2	2	2	2
1	1	9	9	9	9	9	9	9	9
1	1	3	1	2	3	9	1	2	2
1	1	3	1	1	1	1	3	1	1
3	2	2	2	3	3	2	2	1	1
1	1	3	1	3	2	3	2	3	3
1	1	1	1	3	1	3	3	3	3
2	2	1	2	2	2	2	2	2	2
1	9	1	1	3	3	1	3	3	3
1	9	3	1	3	9	3	3	3	3
3	2	1	1	1	1	1	1	1	1
3	2	3	2	2	9	1	2	3	1
1	1	3	1	3	3	3	3	3	3
3	3	9	1	1	1	3	2	3	2
2	2	9	9	9	9	9	9	9	9
2	2	2	2	2	2	2	2	2	2
1	2	1	1	3	1	2	1	3	1
3	1	1	2	2	3	2	2	3	2
1	1	3	1	3	3	3	3	3	3
2	2	1	1	3	2	2	3	3	2
2	2	1	1	3	2	2	3	3	2

BSElumpse	CaBreastC	CaBreastfr	CaBreastTr	BSEdetects	StandMirr	Liedown	RhandtoLb	Fingerpads	Fingernails
3	1	1	2	2	2	2	2	3	3
2	2	2	2	2	3	2	3	2	2
2	3	3	3	3	2	2	2	2	2
3	2	2	2	2	2	2	2	2	2
3	3	2	2	1	1	2	2	2	1
2	2	2	2	2	2	2	2	2	2
2	1	2	2	2	2	2	2	2	2
3	3	3	3	3	1	2	2	3	2
1	3	2	1	3	1	2	3	2	2
3	1	2	2	3	2	1	2	1	2
1	3	3	1	3	2	2	2	2	2
1	3	2	2	1	1	1	2	2	1
1	1	2	1	3	1	1	1	1	1
1	1	2	2	2	2	2	1	2	2
1	2	2	2	2	2	2	2	2	2
3	2	2	2	2	2	2	2	2	2
1	1	1	1	1	9	9	9	9	9
3	1	3	2	1	2	1	1	1	3
1	1	1	1	1	2	3	2	2	2
1	2	1	3	1	1	2	1	2	2
1	1	1	3	1	1	1	1	1	2
1	1	1	2	1	1	1	1	1	3
1	1	1	3	1	1	1	1	1	2
1	2	1	2	1	1	1	1	1	2
2	3	3	2	2	2	2	2	2	2
1	1	2	3	2	2	1	3	9	3
1	1	1	1	1	9	9	9	9	9
1	1	1	3	1	3	1	1	2	3
2	1	2	3	2	1	2	3	2	3
1	2	1	3	1	1	2	1	1	3
2	1	2	2	1	2	3	2	2	2
1	1	2	2	1	3	1	1	1	1
1	1	2	2	1	9	9	9	9	9
1	1	1	2	1	1	9	1	3	3
2	1	2	1	1	1	2	1	2	3
2	3	3	3	3	3	2	3	1	3
1	1	1	3	1	1	3	1	2	2
1	1	1	3	1	1	2	1	2	1
1	1	1	2	1	3	1	1	1	3
3	1	2	2	3	1	2	2	2	3
1	2	2	2	2	9	9	9	9	9
9	9	9	9	2	9	9	9	9	9
9	9	9	9	9	9	9	9	9	9
2	1	2	2	1	2	2	2	2	2
1	1	1	2	1	2	1	2	2	2
9	9	9	9	9	9	9	9	9	9

1	1	2	2	1	1	1	2	2	3
1	1	1	2	1	1	1	2	2	1
1	2	2	1	1	1	1	2	2	2
1	1	1	2	1	1	1	2	1	3
1	3	1	9	1	3	1	1	3	3
1	3	1	9	1	3	1	1	3	3
1	3	2	1	2	3	2	3	3	2
1	1	1	1	1	2	2	2	2	2
3	1	1	1	1	1	9	1	9	1
2	1	3	1	1	2	1	3	3	1
9	2	9	9	9	9	1	9	9	9
1	1	2	2	2	1	2	2	2	1
2	2	2	1	1	3	3	3	3	3
2	1	1	2	2	1	1	2	2	1
1	1	2	2	2	1	1	2	2	2
2	1	2	2	2	1	2	2	1	2
2	2	2	3	1	1	2	2	3	3
1	3	1	2	1	2	3	2	3	2
1	1	2	2	1	2	2	2	2	2
1	3	3	3	3	9	9	9	9	9
2	2	2	2	2	2	2	2	2	2
1	2	2	2	3	2	2	1	2	3
1	2	2	1	1	1	1	2	2	1
2	1	2	2	2	1	2	2	3	2
2	1	2	3	2	1	2	3	1	2
1	2	2	2	2	1	3	2	2	1
1	1	1	1	3	3	1	1	1	3
1	1	2	3	2	1	2	2	1	2
1	1	1	1	1	2	2	1	1	2
1	3	1	1	1	3	3	1	3	3
9	9	9	9	9	9	9	9	9	9
1	1	3	3	1	3	1	1	1	3
1	9	9	9	1	1	2	1	9	9
1	2	1	3	3	2	2	2	2	2
9	1	3	3	3	3	3	1	3	3
1	1	1	1	2	2	2	2	2	2
1	1	2	2	3	1	3	2	1	3
2	2	2	1	2	1	1	2	2	1
1	1	1	2	1	3	3	3	3	3
1	2	2	2	1	1	1	2	2	1
1	1	1	3	3	2	3	1	2	3
9	9	9	9	9	9	9	9	9	9
1	1	1	1	1	1	2	1	3	1
2	2	2	1	2	2	2	2	2	2
1	2	1	3	1	1	3	1	2	3
3	3	3	2	2	3	3	2	2	2
2	1	1	2	1	2	2	2	2	2

3	1	2	3	1	2	1	3	1	2
1	1	1	1	1	3	1	1	1	1
1	1	1	1	2	1	2	2	2	2
2	2	2	2	1	2	1	1	1	1
3	1	2	2	2	1	2	2	1	2
1	1	1	2	1	1	2	1	2	2
1	2	2	2	1	1	2	2	2	2
3	1	3	3	1	1	3	3	1	3
1	1	1	2	1	2	3	1	2	2
1	1	1	2	1	3	3	1	2	9
1	1	2	2	3	1	3	2	1	3
1	1	2	2	1	1	1	2	2	3
1	1	2	1	1	1	1	1	1	2
1	1	1	2	1	1	1	1	2	2
1	1	2	1	1	1	3	2	1	1
1	1	1	1	1	1	3	2	1	1
1	1	2	1	1	1	2	2	1	2
3	1	1	3	1	1	1	2	2	3
1	1	9	9	9	1	3	1	1	1
1	1	2	2	1	3	1	2	2	2
1	1	1	1	1	3	3	1	1	1
1	2	1	2	1	1	2	1	2	2
1	2	2	1	1	1	1	1	1	1
2	2	2	2	2	1	2	2	1	3
2	2	2	2	2	2	2	2	2	2
1	1	1	3	1	1	3	1	2	2
1	1	1	3	1	1	3	1	1	3
1	1	2	2	1	1	1	1	2	1
2	1	1	2	2	1	2	1	1	1
1	1	1	3	2	1	3	1	2	3
1	1	2	1	1	2	2	1	2	1
1	1	1	2	1	3	3	2	1	1
3	1	2	1	3	3	3	2	3	1
1	2	2	1	2	1	9	1	2	2
1	1	1	3	1	1	2	1	1	1
3	1	2	3	2	1	2	2	2	1
2	3	3	3	3	2	2	2	2	2
1	1	1	2	1	1	1	2	1	1
9	9	9	9	9	3	3	9	9	9
1	1	1	1	1	3	1	1	1	1
1	1	1	2	1	2	2	1	2	2
9	9	9	9	9	9	9	9	9	9
1	1	2	2	3	1	2	3	2	2
1	2	2	1	1	1	2	2	1	2
1	1	1	3	1	2	1	1	2	3
1	1	1	2	2	1	3	3	3	2
1	1	1	2	1	1	3	1	1	2

1	1	1	1	2	1	3	1	2	9
3	3	3	3	3	3	3	3	3	3
1	3	3	3	3	3	3	3	3	3
2	1	2	1	1	3	2	2	1	1
3	1	2	1	2	2	3	1	2	2
3	1	3	1	2	2	3	2	3	2
9	1	2	3	1	2	9	2	1	2
2	1	2	3	1	1	9	2	1	2
1	1	1	3	1	1	9	9	9	9
9	1	3	3	2	2	2	2	2	2
1	2	1	1	3	1	2	1	3	1
1	1	1	2	1	1	3	1	2	2
1	1	2	2	1	2	2	2	2	2
2	3	3	3	3	1	1	1	2	2
1	2	2	1	2	1	1	2	2	3
1	1	2	1	1	1	1	2	2	1
1	1	2	2	1	1	1	2	2	2
1	1	2	1	1	1	2	2	1	1
1	2	1	3	1	1	3	9	9	9
1	1	3	1	1	2	3	3	3	3
9	9	1	9	9	2	2	2	2	3
2	1	2	2	1	1	3	1	1	2
1	2	1	3	3	3	3	3	3	3
1	2	2	1	2	1	3	2	2	2
3	1	1	1	1	3	3	1	2	3
1	2	1	3	2	1	3	1	3	2
1	2	1	3	1	1	3	1	2	2
1	2	1	1	1	3	3	3	3	3
9	9	9	9	9	1	2	1	1	1
1	1	1	2	1	1	1	2	2	2
2	2	2	2	2	1	2	1	2	1
2	2	1	1	1	1	1	1	1	1
2	2	2	2	2	1	1	2	2	2
1	1	1	1	1	1	1	1	1	1
2	1	2	2	2	1	2	2	2	3
1	3	3	3	1	3	3	3	1	1
1	3	3	3	3	2	2	2	2	2
1	1	1	3	1	1	1	1	2	3
3	1	1	1	1	1	3	1	3	1
2	3	3	3	3	2	2	2	2	2
1	2	2	2	2	2	2	1	1	2
2	2	2	2	2	1	3	2	2	2
2	3	1	1	2	1	2	2	2	2
9	9	9	9	9	9	9	9	9	9
9	9	9	9	9	9	9	9	9	9
1	1	1	2	1	1	2	1	1	1
1	2	2	1	1	1	1	2	2	1

9	9	3	9	9	9	9	9	9	9
1	1	1	3	1	3	1	2	2	1
1	1	1	1	1	1	1	1	1	1
1	1	1	2	1	1	9	9	9	9
9	9	9	9	1	9	9	9	9	9
9	9	9	9	9	9	9	9	9	9
1	1	1	1	2	2	2	2	3	3
1	9	9	9	9	9	9	9	9	9
1	2	1	2	2	1	3	2	1	2
9	9	1	9	9	9	9	9	1	9
9	9	2	9	9	1	9	9	9	9
9	9	2	9	9	1	9	9	9	9
2	1	2	2	2	2	2	2	1	2
1	2	2	1	1	2	1	2	3	2
9	9	3	9	9	1	9	9	9	9
2	3	3	3	3	2	2	2	2	3
3	3	3	3	3	3	3	3	3	3
2	1	2	2	2	2	1	1	2	2
1	2	1	2	2	1	2	1	1	1
2	3	3	2	2	2	2	2	2	2
1	2	1	2	3	1	3	3	1	3
9	1	2	2	2	9	9	9	9	9
1	2	1	2	2	1	2	1	1	1
2	1	1	2	1	3	1	1	1	1
2	2	2	2	2	2	2	2	2	2
3	2	3	2	2	2	1	1	2	2
2	2	2	2	1	3	2	1	1	2
2	1	2	2	3	1	1	1	2	2
3	3	3	3	3	3	3	3	3	3
1	1	1	1	1	1	3	1	1	1
3	1	2	2	2	1	2	2	2	2
1	1	1	3	1	2	2	2	2	1
9	1	9	9	9	9	9	9	9	9
1	1	1	1	3	2	2	1	3	3
3	3	1	3	3	3	1	3	3	3
2	1	1	2	3	3	1	1	1	1
3	2	1	2	2	2	3	1	2	3
1	1	1	2	1	1	2	2	1	1
3	2	1	3	2	2	1	2	2	1
2	2	1	2	2	1	1	1	1	1
2	2	3	1	1	1	2	2	1	1
1	2	3	2	1	1	3	2	2	1
1	1	2	3	1	1	2	1	1	2
3	9	9	2	9	2	2	2	2	2
2	3	2	2	2	1	2	3	2	1
2	1	1	3	1	1	3	1	1	1
9	9	9	9	9	9	9	9	9	9

1	1	1	3	1	1	2	1	2	2
1	1	1	3	1	3	3	3	3	3
3	1	1	1	1	1	3	1	3	2
1	1	1	3	1	1	1	2	3	2
1	3	1	2	1	1	1	2	3	3
3	2	3	2	1	2	2	2	1	2
3	1	1	3	1	1	3	3	1	1
2	2	3	2	2	2	2	2	2	2
2	1	2	3	2	2	2	2	2	1
2	2	2	2	2	2	1	2	1	1
2	1	1	1	1	2	2	2	2	2
9	1	9	9	9	2	9	9	9	9
9	2	9	9	9	2	9	9	9	9
2	2	2	2	2	2	2	2	2	2
1	9	1	3	1	2	9	2	9	9
2	2	2	2	2	2	9	9	2	9
1	1	9	1	1	9	9	9	9	9
9	9	2	1	9	9	9	9	9	9
2	2	2	2	2	9	2	9	2	9
9	9	9	9	9	9	9	9	9	9
9	9	9	9	9	9	9	9	9	9
2	2	2	2	2	2	2	2	2	2
2	2	2	2	2	2	2	2	2	2
1	1	1	1	2	1	1	1	2	2
2	2	2	2	2	1	1	2	2	1
2	2	9	2	2	9	9	9	9	9
2	2	2	2	2	2	2	2	2	2
9	9	9	9	9	9	9	9	9	9
9	9	9	9	9	9	9	9	9	9
1	1	1	2	2	1	2	2	1	2
9	9	2	9	9	9	2	9	9	9
9	9	9	9	9	9	9	9	9	9
9	9	9	9	9	9	9	9	9	9
9	9	9	9	9	9	9	9	9	9
2	1	3	2	1	9	9	1	9	9
9	9	9	1	9	9	1	9	9	2
9	9	9	9	9	9	9	9	9	9
2	2	9	9	2	9	2	9	9	9
9	9	1	9	9	9	9	9	9	9
9	3	9	9	9	3	9	9	9	9
1	2	9	9	1	1	9	9	9	9
9	1	2	2	2	9	9	9	9	9
3	3	3	3	3	3	3	3	3	3
3	1	2	2	1	1	2	3	1	2
3	2	1	2	1	2	1	2	3	1
3	1	1	1	2	1	1	2	1	1
9	1	9	9	9	1	9	9	9	9

9	1	9	9	9	2	9	9	9	9
9	9	1	9	9	2	9	9	9	9
3	2	2	3	1	3	2	1	3	3
2	3	1	2	3	2	3	1	2	3
2	2	3	3	2	2	2	2	2	2
2	1	1	1	2	2	2	2	2	2
2	2	1	1	1	2	3	1	2	3
2	1	2	3	1	1	3	1	1	1
9	9	9	9	9	2	3	2	3	2
3	3	3	3	3	3	3	3	3	3
9	9	9	9	9	9	9	9	9	9
9	9	9	9	9	9	9	9	9	9
9	1	9	9	9	9	1	9	9	9
9	9	2	9	9	9	2	9	9	9
1	1	1	1	1	1	1	1	1	1
3	1	1	1	1	1	2	1	1	3
9	9	9	9	9	9	9	9	9	9
2	1	1	1	1	1	2	2	2	2
1	1	1	1	1	1	1	1	1	1
3	1	1	1	2	1	2	2	2	2
3	1	3	1	2	3	3	3	3	3
1	1	1	2	1	2	2	2	2	2
3	1	1	2	3	3	3	2	1	2
2	2	2	2	2	2	2	2	2	2
2	1	2	1	2	2	2	2	2	2
2	1	1	1	1	2	2	2	2	2
2	2	2	2	2	2	2	2	2	2
1	1	2	3	1	1	2	1	3	3
3	3	3	3	3	2	2	2	3	2
1	2	1	2	2	2	3	2	1	2
2	2	2	2	2	2	2	2	2	2
2	2	2	2	2	2	2	2	2	2
2	2	2	3	1	2	3	2	2	2
2	2	3	3	3	2	2	3	3	9
2	2	3	1	3	1	1	1	1	3
2	1	2	3	9	1	2	3	1	2
9	9	9	9	9	9	9	9	9	9
2	9	1	3	2	2	2	2	2	2
3	3	3	3	3	3	3	3	3	3
3	3	3	3	3	3	3	3	3	3
9	2	9	9	9	2	9	9	9	9
1	2	2	1	1	2	1	2	1	2
9	9	2	9	9	9	9	9	9	9
2	1	1	1	1	1	1	1	1	1
2	1	2	2	2	1	2	2	1	1
2	2	2	2	2	2	2	2	2	2
1	2	1	2	2	2	2	2	2	2

2	2	3	1	3	9	9	9	9	9
9	2	9	9	9	1	9	9	9	9
9	9	1	9	9	9	9	1	9	9
9	9	2	9	9	3	9	9	9	9
9	9	9	2	9	9	9	1	9	9
1	1	1	1	1	1	1	1	1	1
9	3	9	9	9	2	9	9	9	9
9	1	9	9	9	9	9	9	9	1
2	1	1	2	2	2	2	2	2	1
9	1	9	9	9	2	9	9	9	9
9	9	2	9	9	9	2	9	9	9
9	9	9	2	9	9	9	9	9	2
9	2	9	9	9	2	9	9	9	9
9	9	2	9	9	9	9	9	2	9
9	1	9	9	9	1	9	9	9	9
9	9	9	9	9	9	9	9	9	9
9	9	2	9	9	9	9	9	9	9
2	3	1	2	1	2	1	2	3	2
1	1	1	2	2	1	1	2	2	2
2	2	2	2	2	2	2	2	2	2
1	1	2	3	1	2	1	1	2	3
1	1	1	3	1	1	2	3	2	3
1	1	1	1	1	1	2	1	2	3
2	2	2	3	1	2	3	1	2	3
1	2	2	3	3	2	2	2	2	2
3	3	3	3	3	2	2	2	9	2
1	1	3	3	2	2	2	2	2	2
1	1	1	1	1	1	3	1	3	3
2	1	1	2	3	1	2	2	2	2
1	1	1	2	1	1	1	1	1	1
2	1	2	3	1	1	2	2	2	3
2	1	1	2	1	1	2	1	1	1
3	2	2	2	2	1	1	1	1	1
1	1	1	1	1	1	1	1	2	2
9	9	9	9	9	9	9	9	9	9
2	1	2	3	2	2	2	2	2	2
9	9	9	9	9	9	9	9	9	9
2	2	2	2	1	1	1	1	1	2
2	3	3	3	3	3	3	3	2	3
2	2	2	2	2	2	2	2	2	2
1	1	1	2	1	2	2	1	2	2
1	1	3	3	1	1	1	1	3	1
1	1	3	3	1	1	1	2	1	3
1	2	1	3	1	1	2	1	1	2
2	2	2	2	2	2	2	2	2	2
1	1	1	2	1	1	2	3	2	2
2	2	2	2	2	2	2	2	2	2

3	2	3	2	3	2	1	3	2	9
9	9	1	9	9	2	2	2	2	2
2	1	9	9	9	3	3	2	2	2
2	1	2	9	3	2	2	1	2	3
3	3	3	3	3	3	3	2	2	2
9	2	9	9	9	3	3	3	2	2
1	1	1	1	1	1	1	1	2	2
1	1	1	2	1	1	1	1	1	1
9	9	9	9	9	9	9	9	9	9
1	1	1	2	2	2	2	1	2	2
1	1	1	3	1	1	1	1	1	2
9	1	9	9	9	9	9	9	9	9
9	1	9	9	9	9	9	9	9	9
3	3	3	3	3	1	9	1	9	1
1	1	1	2	1	9	9	9	9	9
2	2	1	2	2	2	3	2	1	1
9	9	9	9	9	9	9	9	9	9
9	9	9	2	9	9	9	2	9	9
9	9	1	9	9	9	9	9	9	9
2	3	2	3	2	2	2	3	3	2
2	2	2	2	2	2	2	2	2	2
1	1	3	1	2	2	2	2	2	2
1	2	1	3	2	2	1	2	2	2
1	1	9	9	9	9	9	9	9	9
1	1	1	2	2	1	9	9	9	9
1	1	2	2	1	2	2	1	1	2
1	1	1	1	1	1	1	1	1	2
1	1	2	2	3	1	2	3	1	1
2	3	3	3	3	1	2	2	2	2
9	9	2	9	9	2	9	9	9	9
2	2	2	2	2	2	2	2	2	2
1	2	3	1	2	1	2	3	1	2
1	2	3	1	1	3	1	2	2	1
1	1	2	2	2	1	1	1	1	3
1	1	1	3	1	1	3	1	1	3
3	2	1	3	2	9	9	9	9	9
9	9	9	9	9	9	9	9	9	9
9	9	9	9	9	9	9	9	9	9
2	2	3	1	3	3	2	1	2	3
2	1	2	1	2	2	1	2	1	2
1	1	2	1	2	9	1	1	2	3
3	1	2	3	2	1	1	1	1	3
1	1	3	2	2	3	3	3	3	2
2	1	2	3	1	1	3	2	1	2
2	3	3	3	3	2	2	2	2	2
2	1	1	1	2	1	2	2	2	2
2	2	2	2	2	2	2	2	3	3

3	1	2	2	2	1	1	1	1	1
1	2	1	2	1	1	2	1	2	3
9	9	2	9	9	9	2	9	9	9
9	9	9	9	1	9	9	9	9	9
9	1	2	2	1	9	9	9	9	9
3	1	3	2	2	3	2	2	2	3
2	2	2	2	2	2	9	2	2	9
2	2	2	2	2	2	2	9	2	2
9	9	9	9	9	9	9	9	9	9
9	9	9	9	9	9	9	9	9	9
2	2	2	2	2	2	2	2	2	2
2	2	2	2	2	9	9	9	9	9
2	2	2	2	2	2	2	2	2	2
9	9	9	9	9	9	9	9	9	9
2	2	9	9	9	2	2	2	2	2
9	2	9	9	9	2	9	9	9	9
3	3	3	3	3	3	3	3	3	3
9	1	9	9	9	1	9	9	9	9
2	2	9	9	2	2	9	9	9	9
2	2	2	2	2	2	9	9	2	9
9	9	9	9	9	9	9	9	9	9
9	2	9	9	9	2	9	9	9	9
2	2	2	2	2	2	2	2	2	2
9	9	9	9	9	9	9	9	9	9
9	9	9	9	9	9	9	9	9	9
9	9	9	9	9	9	9	9	9	9
9	9	9	9	9	9	9	9	9	9
2	2	2	2	2	3	3	2	9	2
3	1	1	3	1	1	3	1	1	3
9	9	9	9	9	9	9	9	9	9
2	2	2	2	2	2	9	9	9	9
2	2	2	2	2	2	9	2	9	2
9	1	9	1	1	1	9	9	2	1
9	9	9	9	9	9	9	9	9	9
3	3	3	3	3	2	2	2	9	2
9	9	9	9	9	9	9	9	9	9
9	1	9	9	9	1	9	9	9	9
2	2	2	2	2	9	9	9	9	9
1	1	2	2	3	1	1	1	1	1
1	1	3	2	3	2	1	2	3	1
9	1	9	9	9	2	9	9	9	9
9	9	9	9	9	9	9	9	9	9
1	1	3	3	2	2	1	3	2	2
1	1	2	3	2	1	2	1	2	2
1	3	3	3	3	3	3	3	3	3
3	1	2	1	2	1	2	2	1	2
1	1	1	2	2	2	1	1	9	9

9	9	9	9	1	9	1	9	9	9
1	1	2	2	3	2	1	2	3	3
1	1	1	2	2	2	2	2	2	2
1	1	1	3	1	1	1	1	1	1
2	2	1	2	2	2	2	3	2	2
2	2	2	1	3	2	3	1	2	2
3	1	1	1	1	3	3	3	3	3
1	2	2	1	2	2	3	3	2	2
1	3	2	2	1	2	2	2	2	2
1	1	1	2	1	2	2	1	2	2
2	2	2	2	2	2	2	2	3	3
2	1	2	3	9	3	2	9	9	9
1	1	2	3	1	1	3	2	2	2
2	2	2	2	2	2	2	2	2	2
9	9	2	9	9	1	9	9	9	9
1	1	2	1	1	1	2	1	1	3
1	1	1	2	1	3	1	1	2	3
1	1	1	2	3	1	2	2	1	2
9	1	1	3	1	3	1	1	3	2
2	2	2	2	2	2	2	2	2	2
1	1	2	3	3	1	1	2	2	2
1	1	1	1	1	1	9	1	3	3
3	1	1	3	3	1	3	3	2	3
9	1	9	9	9	9	1	9	9	9
1	9	9	9	1	9	9	1	9	9
1	1	1	1	1	1	1	1	1	1
9	9	1	9	9	9	9	2	1	9
1	1	1	3	2	1	1	1	1	2
9	9	9	9	9	9	9	9	9	9
9	9	9	9	1	9	9	9	9	9
2	1	3	2	2	2	3	2	3	2
3	2	3	2	1	3	2	3	2	3
9	9	9	9	9	9	9	9	9	9
9	1	9	9	9	1	9	9	9	9
1	1	2	1	1	1	1	2	2	3
9	9	9	9	9	9	9	9	9	9
2	3	3	2	2	2	3	2	3	2
1	1	1	1	1	1	1	1	1	1
2	2	2	2	2	2	2	2	2	2
1	2	2	2	2	1	1	1	2	2
1	2	2	2	1	2	1	2	2	2
2	2	2	2	2	2	2	2	2	2
3	2	3	2	2	2	2	2	2	2
3	3	3	3	3	2	2	2	2	2
2	1	2	3	3	2	2	2	2	2
2	1	2	2	2	2	2	9	2	9
2	1	9	2	2	2	3	1	2	3

9	9	9	9	1	9	9	9	9	9
1	1	2	2	1	2	2	2	2	2
1	1	1	2	1	1	3	2	2	3
1	9	9	9	1	9	1	9	9	9
1	1	3	2	2	1	9	9	9	9
9	9	9	9	9	9	9	9	9	9
9	9	9	9	9	9	9	9	9	9
2	2	2	2	2	1	9	1	1	2
3	2	2	2	1	2	2	2	2	2
1	1	1	2	1	3	1	1	9	9
1	1	2	2	2	1	2	1	3	3
1	1	1	2	1	9	1	1	2	2
1	1	2	2	1	1	3	1	2	2
2	2	2	2	2	2	2	2	2	2
1	1	3	3	3	2	2	1	3	2
3	3	3	3	3	9	9	9	9	9
9	9	9	9	9	9	9	9	9	9
9	9	9	9	9	9	9	9	9	9
9	1	9	9	9	1	9	9	9	9
9	2	9	9	9	2	9	9	9	9
9	9	9	9	9	9	9	9	9	9
9	9	9	9	9	9	9	9	9	9
2	2	2	2	2	2	2	2	2	2
9	2	9	9	9	2	9	9	9	9
9	9	9	9	9	9	9	9	9	9
9	3	9	9	9	3	9	9	9	9
9	9	9	9	9	9	9	9	9	9
1	1	1	1	1	1	9	1	9	2
2	2	9	2	9	2	9	9	9	9
3	3	9	9	3	3	9	9	9	9
9	9	9	9	9	9	9	9	9	9
2	2	2	2	2	2	2	2	2	2
9	9	9	9	9	9	9	9	9	9
3	3	3	3	3	9	9	9	9	9
1	1	1	9	1	9	9	9	9	9
9	9	9	9	9	9	9	9	9	9
9	2	9	9	9	2	9	9	9	9
9	9	9	9	9	9	9	9	9	9
3	1	2	3	2	9	9	9	9	9
9	1	9	9	9	1	9	9	9	9
2	2	2	2	2	2	2	2	2	2
9	2	9	9	9	2	9	9	9	9
9	2	9	9	9	9	9	9	9	9
9	2	9	9	9	2	9	9	9	9
9	9	9	9	9	9	9	9	9	9
2	2	2	2	2	9	9	9	9	9
9	9	9	9	9	9	9	9	9	9

9	9	9	9	9	9	9	9	9	9
2	9	9	9	9	9	9	9	9	9
9	2	9	9	9	3	9	9	9	9
9	2	9	9	9	9	9	9	9	9
9	9	9	9	9	9	9	9	9	9
9	9	9	9	9	9	9	9	9	9
9	9	9	9	9	9	9	9	9	9
9	1	9	9	9	9	9	9	9	9
9	9	9	9	9	9	9	9	9	9
9	9	9	9	9	9	9	9	9	9
9	9	9	9	9	9	9	9	9	9
9	9	9	9	9	9	9	9	9	9
9	9	9	9	9	9	9	9	9	9
9	9	9	9	9	9	9	9	9	9
2	2	2	2	2	9	9	9	9	9
2	2	2	2	2	9	9	9	9	9
9	2	2	2	2	9	9	9	9	9
2	2	2	2	2	2	2	2	2	2
9	2	9	9	9	9	9	9	9	9
2	2	2	2	2	2	2	2	2	2
9	9	9	9	9	9	9	9	9	9
9	1	9	9	9	2	9	9	9	9
9	2	9	9	9	9	9	9	9	9
9	2	2	2	2	9	9	9	9	9
2	2	2	2	2	9	9	9	9	9
1	9	9	9	1	9	9	9	9	9
9	9	9	9	9	9	9	9	9	9
2	1	2	2	2	2	2	9	2	9
9	9	9	9	9	9	9	9	9	9
9	9	9	9	9	9	9	9	9	9
1	1	1	1	1	1	1	1	1	1
2	1	2	3	2	1	3	3	2	2
9	9	9	9	9	9	9	9	9	9
2	2	1	1	1	2	2	2	2	2
9	9	9	9	9	9	9	9	9	9
9	1	9	9	9	9	9	1	9	9
2	2	9	2	2	9	9	9	9	9
2	2	2	2	2	2	9	9	9	9
1	1	1	1	1	1	1	1	1	1
3	3	9	9	3	3	9	9	9	9
9	9	9	9	9	9	9	9	9	9
9	9	9	9	9	9	9	9	9	9
2	2	2	2	2	2	2	2	2	2
9	1	1	9	9	2	9	9	2	9
1	1	9	1	9	1	9	1	9	9
2	2	9	9	2	2	9	9	9	9
2	2	9	2	2	9	9	9	9	9

9	2	9	9	9	2	9	9	9	9
9	9	9	9	9	9	9	9	9	9
3	1	3	9	2	2	9	9	1	9
9	3	9	9	9	9	9	9	9	9
9	9	9	9	9	9	9	9	9	9
9	9	9	9	9	9	9	9	9	9
9	2	9	9	9	2	9	9	9	9
9	2	9	9	9	2	9	9	9	9
9	2	9	2	9	2	9	9	9	9
9	2	2	2	2	2	2	2	2	2
9	9	9	9	9	9	9	9	9	9
9	9	9	9	9	9	9	9	9	9
9	9	9	9	9	9	9	9	9	9
9	9	9	9	9	9	9	9	9	9
1	3	2	1	1	1	2	2	2	2
1	1	2	2	1	1	1	1	2	1
9	2	9	9	9	2	9	9	9	9
1	2	2	2	2	2	2	2	2	2
2	2	2	2	2	2	2	2	2	2
2	2	2	2	2	2	9	9	3	9
9	1	9	9	9	9	1	9	9	9
9	9	9	1	9	9	9	9	9	9
3	1	1	3	1	1	2	1	3	3
3	1	2	3	1	1	2	3	1	2
1	1	2	3	2	1	1	1	1	1
1	2	2	3	1	1	3	1	2	3
1	1	1	3	1	3	3	3	1	1
3	1	1	3	9	1	2	2	1	2
1	1	1	2	2	9	1	2	3	2
9	1	1	2	1	1	1	1	1	1
9	9	9	9	9	1	1	1	1	1
9	1	9	9	9	1	9	9	9	9
9	1	9	9	9	9	1	9	9	9
9	9	1	9	9	3	9	9	9	9
9	1	9	9	9	2	9	9	9	9
9	9	9	9	9	9	9	9	9	9
9	9	9	9	9	9	9	9	9	9
2	2	9	2	9	9	2	9	9	2
9	9	9	9	9	9	9	9	9	9
2	2	9	2	2	2	9	9	2	9
2	2	9	2	2	9	9	9	9	9
2	2	2	2	2	2	2	2	2	2
2	2	2	2	2	9	9	9	9	9
2	2	2	2	2	9	9	9	9	9
9	9	9	9	9	9	9	9	9	9
2	2	2	2	2	2	2	2	2	2
1	2	2	2	2	3	3	2	2	3

1	1	2	2	1	1	3	1	1	2
1	1	1	3	1	1	2	2	2	2
1	1	2	2	1	1	1	1	1	1
3	2	2	2	2	3	1	1	1	2
3	3	3	3	3	1	1	2	2	3
1	1	1	2	1	1	2	1	2	2
1	1	1	2	1	1	2	2	2	2
3	3	3	3	3	3	3	3	3	3
2	2	2	2	2	2	2	2	2	2
2	2	2	2	2	2	2	2	2	2
2	2	9	9	2	2	9	9	9	2
2	2	2	2	2	2	9	2	9	9
2	2	2	2	2	2	2	2	2	2
1	1	1	1	2	1	3	2	2	1
3	3	3	1	2	1	1	1	1	1
2	2	2	2	2	2	2	2	2	2
2	2	2	1	1	1	1	2	2	1
1	2	1	2	1	2	2	2	2	2
1	1	1	3	1	9	9	9	9	9
1	1	2	2	1	1	3	3	2	2
3	3	2	2	3	2	1	2	3	2
1	1	1	1	1	3	3	1	1	2
1	3	2	2	1	3	1	1	2	3
1	1	1	3	1	1	2	1	2	3
1	1	1	2	2	1	2	2	2	2
1	1	2	3	1	2	2	2	2	2
2	1	1	2	1	1	2	1	1	2
1	2	1	3	1	2	3	1	2	2
1	1	2	2	1	1	3	2	2	2
1	3	1	3	1	3	1	1	1	3
1	1	3	3	1	2	2	3	3	3
3	2	1	3	1	3	3	1	2	2
1	3	3	3	3	3	3	3	3	3
1	2	2	3	3	1	3	2	2	2
9	1	9	9	9	9	9	9	9	1
3	3	3	3	3	3	3	2	2	3
1	9	9	9	9	1	2	1	2	3
2	2	2	2	2	2	2	2	2	2
2	1	1	1	1	3	1	2	2	2
9	9	9	9	9	9	9	9	9	9
1	2	2	2	2	2	2	2	2	2
1	1	1	9	1	1	3	9	9	9
2	1	3	1	2	2	2	1	3	3
2	2	2	3	2	2	2	2	2	2
3	2	3	2	2	2	3	2	3	2
3	2	2	3	2	3	2	3	2	3
3	1	2	3	2	2	3	2	3	3

2	1	1	2	1	9	9	9	9	9
2	3	3	3	3	3	3	3	3	3
1	1	1	1	3	1	1	1	3	3
1	1	1	3	3	3	1	2	2	2
1	2	3	2	1	1	2	1	2	1
1	9	9	9	9	1	3	1	1	3
2	3	3	3	3	2	2	3	2	2
1	2	2	3	3	2	2	2	2	2
1	1	1	1	1	3	1	3	3	3
2	1	1	2	1	2	2	2	2	2
2	3	3	3	3	3	2	2	2	2
1	2	2	2	2	2	2	2	2	2
2	2	3	2	2	9	9	9	9	9
2	2	3	2	2	2	3	2	2	2
1	1	2	2	1	2	1	2	1	2
3	1	1	3	1	2	2	2	2	2
2	2	3	3	3	3	3	2	2	2
2	1	2	2	2	2	2	2	2	2
2	2	2	2	2	2	2	2	2	2
9	1	9	9	9	9	1	9	9	9
1	1	1	1	1	1	9	1	2	9
2	2	1	2	1	1	3	1	2	1
3	2	3	2	3	2	3	3	3	3
2	2	2	2	1	2	2	3	2	3
3	1	1	1	1	3	3	3	3	3
3	1	2	3	2	1	2	3	2	1
2	1	3	2	1	1	9	9	9	9
2	2	2	2	2	2	2	2	2	2
2	2	2	2	2	2	2	2	2	2
2	2	2	2	2	2	2	2	2	2
3	1	3	1	2	2	3	2	2	1
2	2	9	2	2	9	9	9	9	9
2	1	1	2	2	9	9	9	9	9
1	1	2	1	1	1	3	2	2	2
1	1	1	2	1	1	3	3	2	3
2	2	3	2	1	1	9	9	9	9
1	1	1	3	1	1	1	1	2	2
1	1	2	1	2	2	2	2	2	2
1	1	1	3	3	3	2	1	3	3
1	1	1	3	1	1	2	1	2	1
1	1	1	2	1	1	9	9	9	9
2	2	2	2	2	2	2	2	2	2
2	2	2	2	2	2	2	2	2	2
2	2	2	2	2	2	2	2	2	2
2	1	1	2	2	2	2	2	2	2
1	1	1	3	1	1	3	2	2	2
1	1	1	2	1	2	2	2	2	2

2	2	2	3	3	3	2	3	3	3
2	1	1	2	1	2	2	2	2	2
1	1	1	2	1	2	2	2	2	2
3	2	1	3	1	1	2	2	2	2
2	2	1	2	2	2	2	2	2	2
2	1	1	2	1	2	2	2	2	2
2	1	1	2	1	2	2	2	2	2
2	2	2	2	2	2	2	2	2	2
2	2	2	2	2	2	2	2	2	2
9	9	9	9	9	9	9	9	9	9
3	2	1	1	1	2	2	3	3	3
3	1	2	2	1	2	2	1	3	2
1	1	1	3	1	1	2	1	2	2
1	1	3	3	1	1	9	2	1	2
1	3	2	1	3	3	3	3	3	3
9	9	9	9	1	1	9	1	1	1
9	9	9	9	9	9	9	9	9	9
9	9	1	9	9	1	9	9	9	9
9	9	9	9	9	9	9	9	9	9
1	1	2	2	2	2	2	2	2	2
1	1	1	1	1	1	3	1	3	2
9	1	1	3	9	9	9	9	9	9
9	9	9	9	9	9	9	9	9	9
2	1	2	2	1	2	2	2	2	2
1	3	2	1	2	1	1	1	1	1
2	1	2	2	1	2	2	2	2	2
2	1	3	2	1	2	1	3	2	1
1	1	1	1	1	3	1	3	2	3
1	1	3	3	1	1	2	1	2	1
2	2	2	2	2	2	1	3	2	2
9	9	9	9	9	9	9	9	9	9
1	1	2	1	3	1	2	1	3	1
2	1	2	2	3	1	2	3	1	2
3	3	3	1	1	2	2	2	1	2
2	1	2	2	2	2	2	2	2	2
2	1	2	2	1	2	2	2	2	2
9	1	1	2	1	2	2	1	1	1
2	2	2	2	2	2	2	2	2	2
2	2	2	2	2	1	1	2	2	2
1	3	3	3	3	3	3	3	2	2
2	1	2	2	2	1	1	1	1	1
1	1	1	1	1	1	3	1	2	3
1	1	1	2	1	2	2	1	2	2
9	1	1	2	1	2	2	1	2	2
2	3	3	3	3	3	3	2	3	2
1	1	1	1	3	2	1	1	2	3
3	3	2	2	3	3	3	3	2	3

2	2	1	1	3	2	2	1	3	3
2	2	2	2	2	2	2	2	2	2
1	2	2	2	3	2	2	2	2	3
2	2	2	2	2	2	2	2	2	2
3	2	2	1	3	3	3	3	3	2
2	2	2	2	2	2	2	2	2	2
9	9	9	9	1	9	9	9	9	9
1	3	3	3	3	3	3	3	3	3
1	1	2	2	1	9	9	9	9	9
2	1	2	2	2	2	2	1	2	2
2	2	2	2	2	2	2	2	2	2
1	1	1	1	1	1	2	1	1	3
1	2	1	1	2	1	2	2	1	1
9	1	9	9	9	9	9	9	9	9
1	9	9	9	1	9	9	9	9	9
1	1	1	1	1	9	9	1	9	9
1	1	1	3	1	1	2	1	2	3
1	2	3	1	1	3	3	1	1	1
1	1	1	2	1	3	3	3	3	3
1	2	1	2	1	1	2	1	1	2
2	2	1	2	2	1	1	9	2	9
1	1	1	3	1	3	3	9	1	3
1	1	1	2	1	1	3	1	1	3
2	1	2	2	2	2	2	2	2	2
1	1	1	3	1	1	1	1	1	1
2	2	2	2	2	2	2	2	2	2
2	2	2	2	2	2	2	2	2	2
9	2	9	9	9	2	2	2	2	2
2	2	3	2	2	2	2	3	2	3
3	2	2	2	2	3	3	3	3	3
1	1	1	1	1	2	2	2	2	2
3	1	2	2	3	1	3	2	1	3
1	1	1	2	2	2	2	2	2	2
2	2	2	2	2	2	2	2	2	2
1	1	1	2	1	2	2	2	2	1
1	2	3	1	2	2	1	3	2	3
1	1	1	1	1	1	1	1	1	1
1	1	1	2	1	1	3	1	2	2
2	2	3	2	3	1	2	3	2	3
2	1	1	2	1	1	3	2	2	2
1	1	1	1	1	9	1	9	9	9
3	3	3	3	1	1	2	2	2	1
2	2	2	2	2	2	2	2	2	2
1	1	2	2	2	1	2	1	2	3
9	9	9	9	9	2	2	2	2	2
9	2	2	2	1	1	3	2	2	3
3	2	2	2	2	2	2	2	2	2

1	1	2	2	3	1	1	1	1	1
1	1	1	3	1	1	3	1	1	3
2	2	2	3	2	2	3	2	3	2
2	2	2	2	2	2	2	2	2	2
9	2	3	2	3	2	1	2	1	2
9	9	1	9	9	9	1	9	9	9
3	3	2	3	2	2	2	3	2	2
9	9	9	9	1	9	1	9	9	9
9	9	9	9	1	9	1	9	9	9
1	3	3	3	3	9	1	1	1	1
1	9	9	9	2	9	1	9	9	9
9	9	9	9	2	9	9	9	9	9
9	9	9	9	9	9	9	2	9	9
9	9	1	9	9	3	9	9	9	9
1	1	1	2	1	1	3	2	2	2
2	9	9	9	2	2	9	9	9	9
9	9	9	9	9	9	9	9	9	9
9	9	1	9	9	1	9	9	9	9
9	9	1	9	9	1	9	9	9	9
9	9	2	9	9	9	9	9	9	2
1	1	1	1	1	9	9	9	9	9
1	1	1	2	2	2	2	2	2	2
1	1	1	3	1	1	2	2	2	2
2	3	2	2	2	3	3	3	3	3
9	1	1	3	2	1	1	1	3	2
2	1	1	3	1	1	2	1	2	2
1	3	1	1	1	3	1	3	1	1
9	9	9	9	1	2	9	9	9	9
2	2	1	2	2	2	3	2	2	2
1	1	1	1	1	1	3	1	1	1
3	3	1	1	3	3	3	3	3	3
9	1	9	9	9	2	9	9	9	9
1	2	3	2	1	3	3	2	1	2
9	1	9	9	9	9	9	2	9	9
9	1	9	9	9	1	9	9	9	9
2	2	3	2	3	1	1	2	1	2
1	1	1	1	1	1	1	1	1	1
9	9	9	9	9	9	9	9	9	9
9	9	9	9	9	9	9	9	9	9
2	2	2	2	2	2	2	2	2	2
2	2	2	2	2	2	9	2	9	2
2	2	2	2	2	2	2	2	2	2
3	3	3	3	3	3	3	3	3	3
9	2	9	9	2	9	2	9	9	9
2	2	2	2	2	1	1	1	1	1
2	2	2	2	2	2	2	2	2	2
1	1	3	1	1	9	1	3	1	9

9	1	9	1	9	9	9	9	9	9
9	1	2	2	9	9	1	9	9	3
9	9	1	9	1	9	1	9	9	9
2	2	2	2	2	2	2	2	2	2
9	1	9	3	1	9	3	1	3	9
1	2	9	2	2	9	1	9	9	3
2	2	1	3	1	9	9	9	9	9
9	1	9	3	1	9	9	9	9	9
2	2	9	2	2	2	9	9	2	9
2	2	2	2	2	2	2	2	2	2
2	2	2	2	2	9	9	9	9	9
2	9	2	9	2	9	2	9	9	2
2	2	2	2	2	2	2	2	2	2
9	9	9	9	9	9	9	9	9	9
2	2	2	2	2	2	2	2	2	2
9	9	9	9	9	9	9	9	9	9
9	9	9	9	9	9	9	9	9	9
9	9	9	9	9	9	9	9	9	9
1	3	1	3	1	9	9	9	9	9
2	2	2	2	2	9	9	9	9	9
2	2	2	2	2	2	2	2	2	2
2	2	2	2	2	2	2	2	2	2
1	2	2	3	1	1	3	2	1	3
3	1	1	2	3	1	2	2	1	3
3	2	2	2	3	2	2	2	2	2
1	2	2	2	2	1	3	2	2	2
1	2	3	2	1	1	1	1	1	1
1	2	1	2	1	3	3	3	9	2
3	1	1	3	2	1	3	1	1	3
1	2	1	2	1	3	3	2	2	2
3	3	2	2	3	2	2	3	2	3
2	2	1	3	2	2	3	1	2	1
2	1	1	2	2	2	2	2	2	2
2	2	2	2	2	9	9	9	9	9
3	1	1	3	2	1	3	1	2	2
2	3	3	3	3	3	3	3	3	3
3	2	1	2	1	1	3	2	2	2
1	3	3	3	3	1	1	3	1	3
1	1	2	2	2	2	2	2	2	2
2	3	2	3	2	2	2	3	2	2
1	3	1	1	1	3	1	3	1	1
9	9	9	9	9	9	9	9	9	9
2	2	1	3	2	2	2	2	2	2
1	9	9	9	9	9	9	9	9	9
9	9	9	9	9	1	2	2	1	2
2	1	2	2	1	2	2	2	2	2
2	2	3	1	3	3	3	2	3	3

1	1	1	2	2	2	2	1	2	3
2	1	3	2	1	1	1	3	3	3
9	1	1	1	1	2	2	2	2	2
2	2	2	2	2	2	2	2	9	9
2	2	2	2	2	1	1	1	1	1
2	2	2	2	2	1	1	1	1	1
1	1	1	1	1	1	1	1	1	1
2	2	1	1	1	1	2	1	3	3
3	3	3	3	3	3	3	3	3	3
2	2	3	2	1	1	3	1	1	3
9	1	1	1	1	2	2	2	2	2
2	1	1	1	1	2	2	2	2	2
2	2	2	2	2	2	2	2	2	2
2	2	2	2	2	2	2	2	2	2
2	2	2	2	2	9	9	9	9	9
1	1	1	1	1	1	1	1	1	9
2	2	1	1	1	1	1	1	1	1
2	2	1	1	1	1	1	1	2	2
2	2	2	2	2	9	9	9	9	9
2	2	2	2	2	2	2	2	2	2
1	1	2	3	2	1	2	3	1	1
2	2	2	2	2	1	1	1	1	1
9	9	9	9	9	9	9	9	9	9
2	2	2	2	2	2	2	2	2	2
9	9	9	9	9	9	9	9	9	9
1	1	1	2	1	1	1	1	1	1
1	2	1	1	1	1	2	1	1	1
1	1	9	1	1	1	1	1	1	1
2	2	2	2	2	2	2	9	2	9
1	1	1	1	1	2	2	2	2	2
2	2	2	2	2	2	2	2	2	2
2	2	2	2	2	2	2	2	2	2
9	2	2	2	2	2	2	2	2	2
1	1	1	1	1	1	1	1	1	1
2	2	2	2	2	2	2	2	2	2
9	9	9	9	9	9	9	9	9	9
1	1	2	2	2	1	1	1	2	2
2	1	1	1	1	1	2	1	1	1
1	1	1	1	1	9	9	9	9	9
2	2	2	2	2	2	2	2	2	2
2	2	2	2	2	2	2	2	2	2
2	1	1	1	1	9	9	9	9	9
2	2	2	2	2	2	2	2	2	2
2	2	2	2	2	2	2	2	2	2
1	1	2	1	1	1	1	1	1	1
2	2	2	2	2	2	2	2	2	2
1	1	1	3	1	1	1	9	9	9

2	1	2	1	1	1	2	1	1	2
9	1	1	1	9	2	2	9	2	9
1	1	1	1	1	1	1	1	1	1
1	1	2	1	2	1	1	1	1	1
1	1	1	1	1	2	2	2	2	2
2	2	2	2	2	2	2	2	2	2
2	1	1	1	1	1	1	1	1	1
1	2	2	2	2	2	2	2	2	2
9	9	9	9	9	9	9	9	9	9
1	1	1	1	1	9	1	1	1	1
2	2	2	2	2	2	2	9	9	2
1	1	1	1	1	2	2	9	2	2
9	9	9	9	9	9	9	9	9	9
1	1	2	2	2	9	9	9	9	9
9	1	1	1	1	9	9	9	9	9
2	3	3	3	9	9	9	9	9	9
9	9	9	9	9	2	2	2	2	2
9	9	9	9	9	9	9	9	9	9
2	2	2	2	2	2	2	2	2	2
1	1	1	3	1	1	1	1	1	3
1	1	1	3	1	1	1	1	1	3
1	1	1	3	1	1	1	1	1	3
1	1	1	3	1	1	1	1	1	9
1	1	1	3	1	1	1	1	1	3
1	1	1	3	1	1	1	1	1	3
1	1	1	3	1	1	1	1	1	3
1	1	1	3	1	1	1	1	9	3
1	1	1	3	1	1	1	9	9	3
1	1	1	3	1	1	1	1	1	3
1	1	1	3	1	1	1	1	1	3
1	1	1	3	1	1	1	1	1	3
1	1	1	3	1	1	1	1	1	3
1	1	1	3	1	9	1	1	1	3
1	1	1	3	1	1	1	1	1	3
1	1	1	3	1	1	1	1	1	3
1	1	1	3	1	1	1	1	1	3
1	1	1	3	1	1	1	1	1	3
1	1	1	3	1	1	1	1	1	3
1	1	1	3	1	1	1	1	1	9
1	1	1	3	1	1	1	1	9	3
1	1	1	3	1	1	1	1	1	3
1	1	1	3	1	1	1	1	1	3
1	1	1	3	1	1	1	1	1	3
1	1	1	3	1	1	1	1	1	3
9	9	9	9	9	9	9	9	9	9
9	9	9	9	9	9	9	9	9	9
9	9	9	9	9	9	9	9	9	9

9	9	9	9	9	9	9	9	9	9
9	9	9	9	9	9	9	9	9	9
9	9	9	9	9	9	9	9	9	9
9	9	9	9	9	9	9	9	9	9
9	9	9	9	9	9	9	9	9	9
9	9	9	9	9	9	9	9	9	9
9	9	9	9	9	9	9	9	9	9
9	9	9	9	9	9	9	9	9	9
9	9	9	9	9	9	9	9	9	9
9	9	9	9	9	9	9	9	9	9
9	9	9	9	9	9	9	9	9	9
9	9	9	9	9	9	9	9	9	9
9	9	9	9	9	9	9	9	9	9
9	9	9	9	9	9	9	9	9	9
9	9	9	9	9	9	9	9	9	9
9	9	9	9	9	9	9	9	9	9
9	9	9	9	9	9	9	9	9	9
9	9	9	9	9	9	9	9	9	9
9	9	9	9	9	9	9	9	9	9
1	1	1	3	1	1	1	1	3	3
1	1	1	3	1	1	1	1	1	3
1	1	1	3	1	1	1	1	1	3
1	1	1	3	1	1	1	1	1	3
1	1	1	3	1	1	1	1	1	3
1	1	1	3	1	1	1	1	9	3
1	1	1	3	1	1	1	1	1	2
1	1	1	3	1	1	1	1	1	3
1	1	1	3	1	1	1	1	1	3
1	1	1	3	1	1	1	1	1	3
1	1	1	3	1	1	1	1	1	3
1	1	1	3	9	1	1	1	1	3
1	1	1	3	1	1	1	1	1	3
1	1	1	3	1	1	1	1	1	3
1	1	1	3	1	1	1	1	1	3
1	1	1	3	1	1	1	1	1	3
1	9	1	9	1	1	9	1	9	9
1	1	1	3	1	1	1	1	1	3
1	1	1	3	1	1	1	1	1	3
1	1	1	3	1	1	1	1	1	3
1	1	1	3	1	1	1	1	1	3
1	1	1	3	1	1	1	1	1	3
1	1	1	3	1	1	1	1	1	3
1	1	1	3	1	1	1	1	1	3
1	1	1	3	1	1	1	1	9	3
1	1	1	3	1	1	1	1	1	1
1	1	1	3	1	1	1	1	3	3
1	1	1	3	1	1	1	1	1	3

1	1	1	3	1	1	1	1	1	3
1	1	1	3	1	1	1	1	3	3
1	1	3	3	1	1	1	1	1	3
1	1	1	3	1	1	1	1	1	3
1	1	1	3	3	1	1	1	1	3
1	1	1	3	1	1	1	1	1	3
1	1	1	3	1	1	1	1	1	1
1	1	1	3	1	1	1	1	1	3
1	1	1	3	1	1	1	1	1	3
1	1	1	3	1	1	1	1	1	3
1	1	1	3	1	1	1	1	1	3
1	1	1	3	1	1	1	1	1	3
1	1	1	3	1	1	1	1	3	3
1	1	1	3	1	1	1	1	1	3
1	1	1	3	1	1	1	1	1	3
1	1	1	3	1	1	1	1	1	3
1	1	1	3	1	1	1	1	1	3
1	1	1	3	1	1	1	1	1	3
1	1	1	3	1	1	1	1	1	3
1	1	1	3	1	1	1	1	1	3
1	1	1	3	1	1	1	1	1	3
1	1	3	3	1	1	1	1	3	3
1	1	1	3	1	1	1	1	3	3
1	1	1	3	1	1	1	1	1	1
1	1	1	3	1	1	1	1	1	3
1	1	1	3	1	1	1	1	3	3
1	1	1	3	1	1	1	1	1	3
1	1	1	3	1	1	1	1	1	1
1	1	1	3	1	1	1	1	3	3
1	1	1	3	1	1	1	1	1	3
1	1	1	3	1	1	1	1	1	3
1	1	1	3	1	1	1	1	1	3
1	1	1	3	1	1	9	9	9	9
1	1	1	3	1	1	1	1	1	3
9	9	9	9	9	9	9	9	9	9
1	1	1	3	1	1	1	1	1	3
1	1	1	3	1	1	1	1	1	1
1	9	1	3	1	1	1	1	1	3
1	1	1	3	1	1	1	1	1	1
1	1	1	3	1	1	1	1	1	3
1	1	1	3	1	1	1	1	3	3
1	1	1	3	1	1	1	1	3	3
1	1	1	3	1	1	1	1	1	3
1	1	1	3	1	1	1	1	1	3
1	1	1	3	1	1	1	1	1	3
1	1	1	3	1	1	1	1	1	3
1	1	1	3	1	1	1	1	1	3

1	1	1	3	1	1	1	1	1	3
1	1	1	3	1	1	1	1	1	3
1	1	1	3	1	1	1	1	1	3
1	1	1	3	1	1	1	1	1	3
1	1	1	3	1	1	1	1	1	3
1	1	1	3	1	1	1	1	3	1
1	1	1	3	1	1	1	1	1	3
1	1	1	3	1	1	1	1	1	3
1	1	1	3	1	1	1	1	1	1
1	1	1	3	1	1	1	1	1	3
1	1	1	3	1	1	1	1	1	3
1	1	1	3	1	1	1	1	1	3
1	1	1	3	1	1	1	1	3	3
1	1	1	3	1	1	1	1	1	3
9	9	9	9	9	1	1	1	3	3
1	1	1	3	1	1	1	1	1	3
1	1	1	3	1	1	1	1	1	3
1	1	1	3	1	1	1	1	3	3
1	1	1	3	1	1	1	1	3	3
9	9	9	9	9	9	9	9	9	9
1	1	1	3	1	1	1	1	3	3
1	1	1	3	1	1	1	1	1	3
1	1	1	3	1	1	1	1	1	3
1	1	1	3	1	1	1	1	3	3
9	9	9	9	9	1	1	1	2	1
1	1	1	3	1	1	1	1	1	3
1	1	1	3	1	1	1	1	1	3
1	1	1	3	1	1	1	1	1	1
1	1	1	3	1	1	1	1	1	3
1	1	1	3	1	1	1	1	1	3
1	1	1	3	1	1	1	1	1	3
1	1	1	3	1	1	1	1	1	3
1	1	1	3	1	1	1	1	3	3
1	1	1	3	1	1	1	1	1	3
1	1	1	3	1	1	1	1	1	3
1	1	1	3	1	1	1	1	1	3
1	1	1	3	1	1	1	1	1	3
1	1	1	3	1	1	1	1	1	3
1	1	1	3	1	1	1	1	1	3
1	1	1	1	3	1	1	1	1	3
1	1	1	3	1	1	1	1	1	3
9	9	9	9	9	9	9	9	9	9
1	1	1	3	1	1	1	1	1	3
1	1	1	3	1	1	1	1	1	3
1	1	1	3	1	1	1	1	1	3
1	1	1	3	1	1	1	1	1	3
1	1	1	3	1	1	1	1	1	3

1	1	1	3	1	1	1	1	1	3
1	1	1	3	1	1	1	1	1	3
1	1	1	3	1	1	1	1	9	3
1	1	1	3	1	1	1	1	1	3
1	1	1	3	1	1	1	1	1	3
1	1	1	3	1	1	1	1	1	3
1	1	1	3	1	1	1	1	1	3
1	1	1	3	1	1	1	1	1	3
1	1	1	3	1	1	1	1	1	3
1	1	1	9	1	1	1	1	3	3
1	1	1	3	1	1	1	1	1	3
1	1	1	3	1	1	1	1	1	3
1	1	1	3	1	1	1	1	1	3
1	1	1	3	1	1	1	1	1	3
1	1	1	3	1	1	1	1	1	3
1	1	1	3	1	1	1	1	1	3
1	1	1	3	1	1	1	1	1	3
1	1	1	3	1	1	1	1	1	3
1	1	1	3	1	1	1	1	1	3
1	1	1	3	1	1	1	1	1	3
1	1	1	3	1	1	1	1	1	3
1	1	1	3	1	1	1	1	1	1
1	1	1	3	1	1	1	1	1	3
1	1	1	3	1	1	1	1	1	3
1	1	1	3	1	1	1	1	1	3
1	1	1	3	1	1	1	1	1	1
1	1	1	3	1	1	1	1	1	1
9	9	9	9	9	9	9	9	9	9
9	9	9	9	9	9	9	9	9	9
9	9	9	9	9	9	9	9	9	9
9	9	9	9	9	9	9	9	9	9
9	9	9	9	9	9	9	9	9	9
9	9	9	9	9	9	9	9	9	9
9	9	9	9	9	9	9	9	9	9
9	9	9	9	9	9	9	9	9	9
9	9	9	9	9	9	9	9	9	9
9	9	9	9	9	9	9	9	9	9
9	9	9	9	9	9	9	9	9	9
9	9	9	9	9	9	9	9	9	9
9	9	9	9	9	9	9	9	9	9
9	9	9	9	9	9	9	9	9	9
9	9	9	9	9	9	9	9	9	9
1	9	9	9	9	9	9	9	9	9
1	1	1	1	1	9	9	9	9	9
9	9	9	9	9	9	9	9	9	9
9	9	9	9	9	9	9	9	9	9
9	9	9	9	9	9	9	9	9	9

9	9	9	9	9	9	9	9	9	9
9	9	9	9	9	9	9	9	9	9
9	9	9	9	9	9	9	9	9	9
9	9	9	9	9	9	9	9	9	9
9	9	9	9	9	9	9	9	9	9
9	9	9	9	9	9	9	9	9	9
9	9	9	9	9	9	9	9	9	9
9	9	9	9	9	9	9	9	9	9
9	9	9	9	9	9	9	9	9	9
9	9	9	9	9	9	9	9	9	9
9	9	9	9	9	9	9	9	9	9
9	9	9	9	9	9	9	9	9	9
9	9	9	9	9	9	9	9	9	9
1	1	1	3	2	1	2	1	2	2
2	2	2	2	2	2	2	2	2	2
1	1	1	2	1	1	3	1	1	2
3	2	3	1	1	1	1	1	2	3
1	1	1	3	1	1	3	1	2	3
1	1	1	3	1	1	1	3	3	3
2	1	2	3	2	1	2	1	2	3
1	2	1	2	1	1	1	1	1	1
1	1	3	1	3	1	3	1	1	1
1	1	1	3	1	3	3	1	2	3
2	2	3	2	3	2	3	2	2	3
1	1	1	2	1	9	9	9	9	9
2	2	2	2	2	2	2	2	2	3
9	9	9	3	3	9	9	9	9	9
1	1	1	3	3	3	1	3	1	1
1	2	1	3	2	2	2	2	1	3
1	3	2	2	1	2	2	1	1	3
2	2	2	2	3	1	3	3	3	3
1	3	2	1	2	2	1	3	1	2
9	1	9	9	9	1	9	9	9	9
1	1	2	3	3	2	1	2	1	2
1	1	1	3	3	1	3	1	1	3
9	2	9	9	9	1	9	9	9	9
1	1	1	2	1	2	2	1	1	2
3	1	2	2	1	2	1	1	2	3
1	1	3	3	1	3	1	1	3	3
1	1	1	1	1	3	1	1	3	1
9	9	9	9	9	9	9	9	9	9
1	1	1	1	1	9	9	9	9	9
2	1	1	1	1	2	1	1	2	2
1	9	9	9	9	1	1	1	1	1
9	9	9	9	9	9	9	9	9	9
9	9	9	9	9	9	9	9	9	9
1	1	2	2	2	3	3	2	2	2

9	9	9	9	1	9	9	9	9	9
9	9	9	9	9	9	9	9	9	9
3	3	2	2	2	2	1	2	2	3
1	1	1	3	1	3	2	2	1	2
1	1	1	1	1	1	2	2	2	1
9	9	9	9	2	2	9	9	9	9
9	2	2	2	2	2	2	2	2	2
2	2	2	2	2	2	2	2	2	2
2	2	2	2	2	2	2	2	2	2
2	2	2	1	1	1	2	2	2	3
3	1	1	3	3	3	2	1	2	9
9	9	9	9	9	9	9	9	9	9
9	9	9	1	9	1	9	2	9	9
2	2	2	2	2	2	2	2	2	2
1	2	3	2	2	3	1	2	2	2
1	1	2	1	1	2	1	1	3	2
1	1	3	1	3	1	3	1	1	1
1	2	2	2	2	2	2	2	2	2
1	1	2	1	1	2	9	1	1	1
1	1	2	1	3	2	1	9	3	9
2	2	2	2	2	2	2	2	2	2
2	2	2	2	2	2	2	2	2	2
2	2	2	2	2	2	2	2	2	2
2	2	2	2	2	2	2	2	2	2
2	2	2	2	2	2	2	2	2	2
3	2	2	3	1	3	3	1	1	2
2	2	2	2	2	2	2	2	2	2
1	2	2	2	2	2	2	2	2	2
1	2	2	2	1	3	1	2	2	2
1	1	1	2	1	1	1	1	1	2
1	1	1	1	1	3	1	2	3	3
1	1	1	2	1	3	1	1	2	3
1	1	1	1	1	2	1	1	1	1
1	1	1	3	1	1	1	1	1	2
1	1	2	2	1	1	1	2	2	2
2	3	3	3	2	3	2	3	2	3
1	1	2	2	2	2	1	1	1	1
9	2	2	2	2	2	2	2	2	2
9	2	9	9	9	2	9	9	9	9
3	1	1	2	1	3	1	1	2	2
3	2	2	2	2	1	1	1	1	1
2	2	2	2	2	1	1	1	1	2
1	1	2	3	1	1	2	3	1	2
1	3	2	3	1	3	1	2	1	3
1	1	1	1	1	1	1	1	1	3
1	3	1	3	2	1	1	1	1	1
2	1	1	3	1	3	1	1	2	3

2	2	2	2	2	2	1	1	2	2
1	2	3	2	2	2	2	2	3	3
2	2	2	2	1	2	9	1	1	2
2	2	3	2	3	2	3	3	2	3
1	1	1	3	1	2	1	1	3	1
1	1	1	2	1	3	1	1	1	1
1	1	1	2	1	2	1	1	3	3
3	1	1	2	3	2	3	1	2	2
2	2	2	2	2	2	2	2	2	2
1	2	1	2	3	1	2	1	3	1
2	2	2	2	2	2	2	2	2	2
2	2	2	2	2	2	2	2	2	2
3	2	2	2	2	1	1	1	1	1
2	2	2	2	2	2	2	2	2	2
2	2	2	2	2	2	2	2	2	2
2	2	3	2	3	2	3	2	3	2
3	3	3	3	3	3	3	3	3	3
2	2	2	2	2	2	2	2	2	2
2	2	2	2	2	2	2	2	2	2
1	1	2	3	1	1	2	3	1	2
2	1	2	1	2	1	2	1	2	1
1	1	1	1	2	1	1	1	1	1
2	2	2	2	2	2	2	2	2	2
1	1	1	1	1	1	1	1	1	3
1	1	1	3	1	1	1	1	3	1
9	9	9	9	9	9	9	9	9	9
2	2	3	1	3	3	1	2	2	3
1	1	2	2	2	2	2	1	2	2
3	1	2	1	1	1	2	2	1	3
1	1	2	9	1	2	1	1	2	2
1	1	1	3	1	2	1	1	2	3
2	2	2	1	2	1	2	2	3	1
3	2	2	2	2	2	2	2	2	2
1	2	1	2	1	3	1	1	1	1
2	1	2	1	1	1	1	1	1	1
9	9	9	9	9	2	2	2	2	2
1	9	9	9	9	9	9	9	9	9
1	1	2	2	2	1	2	1	2	2
9	9	9	9	9	9	9	9	9	9
1	1	1	2	1	1	1	1	1	2
1	2	1	2	1	1	1	1	2	2
1	3	1	2	1	1	1	1	2	2
1	1	1	3	1	2	1	1	2	3
9	9	9	9	9	9	9	9	9	9
9	9	9	9	9	9	9	9	9	9
9	9	9	9	9	9	9	9	9	9
9	9	2	9	9	1	2	3	2	2

2	1	2	2	3	1	1	1	2	1
9	9	9	9	9	9	9	9	9	9
1	1	2	3	1	2	2	1	3	2
3	1	1	3	3	1	1	1	1	1
9	9	9	9	9	9	9	9	9	9
1	1	1	3	1	3	1	1	2	3
2	2	3	3	3	3	3	2	3	3
1	2	2	2	1	1	2	2	3	2
1	1	1	2	1	1	1	1	1	2
2	1	2	3	2	1	2	3	1	2
2	1	2	1	3	1	2	1	2	1
2	2	2	2	2	2	2	2	2	2
2	2	2	2	2	2	2	2	2	2
3	1	1	3	1	9	9	9	9	9
2	2	2	2	2	2	2	2	2	2
1	1	1	2	1	2	1	1	1	2
3	3	3	3	3	3	3	1	1	1
3	1	3	2	3	3	1	1	2	3
1	1	2	3	2	1	2	1	3	1
2	1	2	1	3	9	9	9	9	9
3	3	1	2	3	3	1	1	2	3
2	2	1	3	2	1	1	1	2	2
1	1	1	3	1	1	1	1	1	3
9	9	9	9	9	3	1	2	3	3
2	2	2	3	2	2	3	3	3	2
9	9	9	9	9	9	9	9	9	9
1	1	1	3	1	1	1	1	1	1
1	2	1	1	1	2	2	2	2	2
1	2	2	3	1	2	2	2	2	2
1	1	2	2	3	1	3	1	2	2
1	2	1	2	2	1	1	2	2	2
9	1	9	9	9	1	9	9	9	9
1	1	2	2	1	3	1	1	2	3
1	1	1	3	2	3	1	1	2	2
1	3	1	2	1	1	3	1	2	3
1	2	1	3	1	1	2	1	2	3
2	2	2	2	2	9	9	9	9	9
1	1	1	3	1	1	2	1	1	3
1	1	1	2	1	3	1	1	2	3
9	9	9	1	9	2	9	9	9	9
9	9	9	9	9	1	3	2	2	2
2	2	2	2	2	2	2	2	2	2
1	1	1	3	1	3	1	2	2	2
2	2	2	2	2	2	2	2	2	2
2	2	2	2	2	2	2	2	2	2
9	1	9	9	9	1	9	2	9	9
2	2	2	2	2	2	2	2	2	2

1	1	1	3	1	1	1	1	3	3
2	2	2	2	2	2	2	2	2	2
1	1	2	3	1	1	3	2	2	2
1	1	1	2	1	3	3	1	2	2
2	2	2	2	2	1	3	1	1	3
2	2	2	3	3	2	2	2	2	2
2	1	1	3	1	1	1	1	1	1
1	1	1	3	1	1	2	1	2	3
1	1	1	3	1	1	1	1	1	1
1	1	1	3	1	2	3	1	3	3
1	3	1	3	3	2	3	1	2	2
2	2	3	2	3	3	3	3	3	3
1	1	1	3	1	2	2	2	2	2
1	1	1	1	1	1	2	1	1	2
1	9	9	9	9	9	9	9	9	9
1	2	2	2	1	1	1	1	1	2
3	2	2	2	3	9	2	2	2	2
1	1	2	2	1	1	2	2	2	2
2	2	2	2	2	2	2	3	3	2
1	1	1	3	1	1	2	2	2	2
1	1	1	2	1	1	3	1	1	3
1	1	1	3	1	1	1	3	1	3
1	1	2	3	2	3	3	3	3	3
2	3	2	3	2	1	1	2	2	2
2	2	2	2	2	2	2	2	2	2
1	1	1	3	1	1	3	2	1	1
1	1	1	3	1	1	3	2	2	2
1	1	1	2	1	1	2	2	2	2
1	1	1	3	1	1	1	1	1	3
1	1	1	3	1	1	1	1	1	3
1	1	1	3	1	1	1	1	1	2
1	1	1	1	1	3	1	1	1	1
1	2	1	1	2	1	2	2	1	1
2	1	2	2	2	9	9	9	9	9
2	2	2	2	2	2	9	9	9	9
1	1	1	3	1	1	1	1	1	3
2	2	2	2	2	2	2	2	2	2
3	1	2	1	2	1	2	1	1	1
1	1	1	1	1	1	1	1	1	1
2	2	2	2	2	2	2	2	2	2
2	1	2	3	2	1	2	3	2	3
9	9	9	9	9	9	9	9	9	9
9	9	9	9	9	9	9	9	9	9
1	1	1	1	1	1	1	1	1	1
2	1	2	3	2	1	1	3	2	3
9	9	9	9	9	9	9	9	9	9
9	9	9	9	9	9	9	9	9	9

2	2	2	2	2	2	2	2	2	2
1	2	3	2	1	2	3	2	3	1
1	1	2	3	1	1	2	3	1	2
3	1	2	3	2	1	2	3	2	1
3	1	2	3	3	1	2	2	2	3
1	1	2	3	1	1	2	3	1	2
2	1	2	3	2	1	2	2	2	2
9	2	9	9	9	1	9	9	9	9
2	2	2	2	2	1	1	2	1	1
9	9	9	9	9	9	9	9	9	9
1	1	1	1	1	1	1	1	1	1
3	1	1	2	3	1	3	2	1	1
1	1	2	2	2	9	9	9	9	9
2	2	3	2	2	9	9	9	9	9
1	1	1	1	1	2	2	2	2	2
9	9	9	9	9	9	9	9	9	9
9	9	9	9	9	9	9	9	9	9
3	1	1	2	1	2	2	2	2	2
2	2	2	2	2	2	2	2	2	2
2	2	2	2	2	3	3	1	2	2
2	2	2	2	2	2	2	2	2	2
2	2	2	2	2	2	9	2	2	2
3	1	1	2	2	1	1	3	3	3
2	2	3	1	2	2	3	1	2	3
2	1	2	2	2	1	1	9	2	9
1	9	9	9	1	9	9	9	9	1
1	1	1	1	1	1	1	1	1	2
3	1	2	2	1	1	1	1	1	1
1	3	3	3	3	1	2	1	2	1
9	1	1	2	2	1	2	1	1	1
1	1	1	1	1	1	1	2	1	2
2	9	9	9	9	2	2	2	2	2
2	2	2	2	2	2	2	2	2	2
2	2	2	2	2	2	2	2	2	2
1	1	1	3	1	1	1	1	1	1
2	2	2	2	2	2	2	2	2	2
2	2	2	2	2	2	2	2	2	2
9	1	9	9	9	1	9	9	9	9
2	9	9	9	2	9	9	9	9	9
2	2	2	2	2	2	2	2	2	2
2	2	2	2	2	2	2	2	2	2
9	1	2	3	1	9	9	9	9	9
2	1	2	3	2	1	2	2	2	2
1	2	9	9	9	1	9	9	9	9
2	2	2	2	2	3	3	3	3	3
9	1	2	2	1	9	9	9	9	9
2	2	2	2	2	2	2	2	2	2

3	3	2	3	1	1	3	2	1	3
9	9	1	9	9	1	9	9	9	9
2	2	2	2	2	2	2	2	2	2
1	1	1	2	2	2	3	3	3	3
2	3	3	3	3	1	1	1	2	2
2	3	3	2	1	2	2	2	2	2
1	1	1	3	1	1	1	1	1	1
2	2	2	1	2	1	2	3	1	2
2	2	2	2	2	2	2	2	2	2
3	9	3	3	3	1	1	1	3	3
1	1	1	2	1	1	3	1	1	3
2	2	2	2	2	2	2	2	2	2
2	2	2	2	2	2	2	2	2	2
3	1	2	3	1	3	3	1	1	2
1	1	1	2	1	1	1	1	2	2
1	1	1	2	1	1	1	1	2	2
2	1	2	2	2	2	2	2	2	2
2	2	2	2	2	2	2	2	2	2
3	2	3	2	2	1	2	2	3	2
2	2	2	1	1	2	2	9	1	9
2	1	2	3	1	1	2	1	1	1
1	1	2	2	1	1	2	1	2	2
3	2	1	2	2	1	1	2	3	3
9	9	9	9	9	9	9	9	9	9
2	2	2	2	2	3	3	3	3	3
9	9	1	9	9	9	1	9	9	9
9	9	9	9	9	9	9	9	9	9
9	9	9	9	9	9	9	9	9	9
1	3	3	3	1	1	2	2	2	2
3	1	1	1	1	1	1	1	1	1
2	2	2	2	2	2	2	2	2	2
3	1	2	9	1	1	3	2	1	1
2	2	2	2	2	2	2	2	2	2
2	2	2	2	2	2	2	2	2	2
1	1	1	1	1	1	9	1	1	1
1	1	1	1	1	1	1	1	1	3
1	1	2	2	1	1	2	1	1	3
3	3	1	2	1	1	9	3	2	9
3	2	3	1	2	1	2	3	2	3
1	1	1	2	1	1	1	1	1	3
1	1	1	1	1	1	1	1	1	1
2	2	2	2	3	2	2	2	2	2
9	9	9	9	1	1	9	9	9	9
9	9	1	9	9	9	9	9	2	9
3	1	3	2	1	1	2	2	3	1
2	2	2	2	2	2	2	2	2	2
2	2	2	2	2	2	2	2	2	2

2	1	1	2	3	1	2	3	2	1
9	9	9	9	9	9	9	9	9	9
9	9	9	9	9	9	9	9	9	9
9	9	9	9	9	9	9	9	9	9
9	9	9	9	9	9	9	9	9	9
1	1	9	9	9	1	9	9	9	9
9	9	9	9	9	9	9	9	9	9
9	9	9	9	9	9	9	9	9	9
9	9	9	9	9	9	9	9	9	9
1	1	2	3	1	9	9	9	9	9
9	2	2	2	2	1	2	2	2	2
1	2	2	2	2	1	2	2	2	2
1	1	1	1	1	1	1	1	1	1
2	2	2	2	2	2	1	2	2	2
2	1	1	2	1	2	2	2	2	2
2	2	1	3	2	1	3	2	3	2
2	1	1	2	1	2	1	1	1	1
1	2	1	3	1	2	2	2	2	2
2	1	1	3	1	1	3	1	1	1
1	1	1	2	1	1	1	1	2	3
1	1	2	1	3	1	2	1	3	2
1	1	2	1	3	1	2	1	3	2
1	2	2	2	2	3	3	3	3	9
2	2	2	2	2	2	2	2	2	2
2	2	2	2	2	2	2	2	2	2
2	2	2	2	2	2	2	2	2	2
3	3	3	3	3	2	2	2	2	2
1	1	2	1	2	1	1	1	2	2
2	2	2	2	2	2	2	2	2	2
2	2	2	2	2	2	2	2	2	2
9	9	9	9	9	9	9	9	9	9
2	2	2	2	2	2	2	2	2	2
1	2	2	2	2	1	1	1	1	1
9	1	9	9	9	1	9	9	9	9
2	3	3	3	3	9	9	9	9	9
2	2	2	2	2	2	2	2	2	2
2	2	2	2	2	2	2	2	2	2
2	2	2	2	2	2	2	2	2	2
1	1	1	1	3	3	3	1	1	1
9	9	9	9	9	9	9	9	9	9
2	1	1	1	1	2	2	1	2	2
2	2	2	2	2	2	2	2	2	2
2	2	2	2	2	2	2	2	2	2
1	1	1	3	1	1	1	1	1	3
1	1	1	3	1	1	1	1	1	3
9	9	9	9	9	9	9	9	9	9
2	1	2	2	1	2	1	2	2	2

9	9	9	9	9	9	9	9	9	9
2	1	2	3	2	3	1	1	1	1
9	9	9	1	9	1	9	9	9	9
9	9	9	9	9	9	9	9	9	9
9	9	9	9	9	9	9	9	9	9
9	9	9	9	9	9	9	9	9	9
9	9	9	9	9	9	9	9	9	9
9	9	9	9	9	9	9	9	9	9
9	9	9	9	9	9	9	9	9	9
9	9	9	9	9	9	9	9	9	9
9	9	9	9	9	9	9	9	9	9
9	9	9	9	9	9	9	9	9	9
9	9	9	9	9	9	9	9	9	9
9	9	9	9	9	9	9	9	9	9
9	9	9	9	9	9	9	9	9	9
9	9	9	9	9	9	9	9	9	9
9	9	9	9	9	9	9	9	9	9
9	9	9	9	9	9	9	9	9	9
9	9	9	9	9	9	9	9	9	9
9	9	9	9	9	9	9	9	9	9
9	9	9	9	9	9	9	9	9	9
9	9	9	9	9	9	9	9	9	9
9	9	9	9	9	9	9	9	9	9
9	9	9	9	9	9	9	9	9	9
3	1	2	3	2	1	2	3	2	1
2	1	1	2	1	2	2	2	2	2
1	3	1	1	1	1	1	1	1	3
1	1	2	2	1	1	1	1	1	3
1	1	1	3	1	3	1	1	1	3
1	1	9	9	1	1	1	1	1	3
2	1	2	2	1	1	1	1	1	1
1	1	1	2	1	1	1	1	1	3
1	1	2	2	1	1	1	2	2	1
1	2	1	2	1	1	1	2	2	2
1	1	9	1	1	2	1	1	1	3
1	1	2	2	1	1	1	1	1	2
1	1	1	2	1	1	3	1	1	3
1	1	1	3	1	1	1	1	1	3
1	1	1	2	1	1	1	1	1	3
1	1	1	2	1	1	1	1	1	3
9	1	9	9	9	9	9	9	9	9
1	1	1	2	1	1	1	1	1	2
3	1	1	3	1	1	1	1	2	2
9	1	2	9	9	1	9	9	9	9
1	1	1	2	1	1	1	1	1	3
1	1	1	1	1	1	1	1	1	3
2	1	2	2	2	1	2	2	2	2
1	3	3	2	1	1	1	1	1	2

1	1	1	2	1	1	1	1	1	2
1	1	1	1	1	3	1	1	1	1
1	1	1	3	1	1	1	1	2	1
1	2	1	2	1	1	1	1	1	3
3	2	1	2	3	2	1	2	3	2
9	1	9	9	9	1	9	9	9	9
1	1	1	2	1	1	1	1	1	1
9	1	9	9	9	1	9	9	9	9
1	1	1	2	1	1	2	1	1	1
1	1	1	2	1	1	2	1	1	1
1	1	9	9	9	1	9	9	9	9
1	9	9	9	1	9	9	9	9	1
9	1	9	9	9	1	9	9	9	9
9	9	9	9	1	1	9	9	9	9
1	1	3	1	1	1	9	9	9	9
1	1	3	1	3	9	9	9	9	9
3	1	1	1	1	1	1	1	1	3
2	1	1	2	1	1	2	1	2	2
9	9	9	9	1	1	9	9	9	9
1	1	1	3	1	3	1	3	3	3
1	1	1	3	1	1	1	9	9	9
1	1	9	9	1	3	1	9	9	9
1	1	1	3	1	1	9	9	9	9
9	1	9	9	9	1	9	9	9	9
9	1	9	9	9	1	9	9	9	9
1	1	2	3	2	1	2	2	1	2
1	3	1	3	1	1	1	1	1	3
1	2	2	2	1	1	1	1	2	3
1	1	2	1	1	1	1	1	1	2
2	2	2	2	2	2	2	2	2	2
1	1	1	1	1	1	1	1	1	1
1	3	9	9	1	1	1	1	1	3
3	1	3	3	3	1	2	1	1	3
1	1	3	3	1	1	3	1	3	1
1	1	1	3	1	1	2	1	2	2
9	1	9	9	9	1	9	9	9	9
2	1	2	2	2	1	2	9	1	1
3	2	1	3	2	2	2	3	2	2
2	2	3	2	3	2	3	2	1	2
2	2	2	2	2	2	2	2	2	2
3	1	1	3	3	1	1	1	1	3
1	1	1	3	1	1	1	1	1	1
1	1	1	3	1	1	1	1	1	1
2	2	2	1	3	2	2	2	1	1
1	1	2	3	1	1	1	1	1	3
1	1	1	2	1	9	1	1	9	9
1	1	1	3	1	1	1	1	1	3

1	1	1	2	1	1	1	1	1	3
3	2	1	3	3	1	2	3	2	3
1	1	3	2	1	1	1	1	1	3
3	1	1	3	1	1	9	3	3	3
9	2	3	1	2	1	9	9	9	9
1	1	1	2	1	1	3	2	2	3
1	1	1	3	1	1	1	1	1	3
9	9	9	9	9	9	9	9	9	9
9	2	9	9	9	2	9	9	9	9
1	2	2	2	2	2	2	3	3	2
2	2	2	2	2	2	1	2	2	2
3	1	1	3	1	1	3	3	3	3
1	1	1	1	1	1	1	1	1	2
9	9	9	9	9	9	9	9	9	9
9	9	9	9	9	9	9	9	9	9
3	3	3	3	3	2	2	3	3	3
9	1	9	9	9	1	9	9	9	9
9	9	9	9	9	9	9	9	9	9
1	3	3	3	3	1	1	1	1	1
1	1	1	2	1	1	1	1	1	3
1	2	3	3	2	1	1	1	1	3
1	1	1	1	1	1	1	1	1	1
3	3	3	3	3	3	1	2	2	2
1	1	1	2	1	1	1	1	1	3
1	1	1	1	1	1	1	1	1	1
9	9	9	9	9	9	9	9	9	9
9	9	9	9	9	1	9	9	9	9
1	1	3	3	1	1	1	3	1	3
3	3	3	3	3	3	3	2	2	2
1	1	2	3	2	1	1	1	1	3
9	9	9	9	9	9	9	9	9	9
9	9	9	9	9	9	9	9	9	9
9	9	9	9	9	9	9	9	9	9
9	9	9	9	9	9	9	9	9	9
9	9	9	9	9	9	9	9	9	9
9	9	9	9	9	9	9	9	9	9
9	9	9	9	9	9	9	9	9	9
9	9	9	9	9	9	9	9	9	9
9	9	9	9	9	9	9	9	9	9
9	9	9	9	9	9	9	9	9	9
9	9	9	9	9	9	9	9	9	9
9	9	9	9	9	9	9	9	9	9
9	9	9	9	9	9	9	9	9	9
9	9	9	9	9	9	9	9	9	9
9	9	9	9	9	9	9	9	9	9
9	9	9	9	9	9	9	9	9	9
9	9	9	9	9	9	9	9	9	9

9	9	9	9	9	9	9	9	9	9
9	9	9	9	9	9	9	9	9	9
9	9	9	9	9	9	9	9	9	9
9	9	9	9	9	9	9	9	9	9
9	9	9	9	9	9	9	9	9	9
9	9	9	9	9	9	9	9	9	9
9	9	9	9	9	9	9	9	9	9
9	9	9	9	9	9	9	9	9	9
9	9	9	9	9	9	9	9	9	9
9	9	9	9	9	9	9	9	9	9
9	9	9	9	9	9	9	9	9	9
9	9	9	9	9	9	9	9	9	9
9	9	9	9	9	9	9	9	9	9
9	9	9	9	9	9	9	9	9	9
9	9	9	9	9	9	9	9	9	9
9	9	9	9	9	9	9	9	9	9
9	9	9	9	9	9	9	9	9	9
9	9	9	9	9	9	9	9	9	9
9	9	9	9	9	9	9	9	9	9
2	2	2	2	2	1	1	1	2	2
9	9	9	9	9	9	9	9	9	9
9	9	9	9	9	9	9	9	9	9
9	9	9	9	9	9	9	9	9	9
9	9	9	9	9	9	9	9	9	9
9	9	9	9	9	9	9	9	9	9
9	9	9	9	9	9	9	9	9	9
9	9	9	9	9	9	9	9	9	9
9	9	9	9	9	9	9	9	9	9
9	9	9	9	9	9	9	9	9	9
9	9	9	9	9	9	9	9	9	9
9	9	9	9	9	9	9	9	9	9
9	9	9	9	9	9	9	9	9	9
9	9	9	9	9	9	9	9	9	9
9	9	9	9	9	9	9	9	9	9
9	9	9	9	9	9	9	9	9	9
9	2	9	9	9	2	2	2	2	2
1	1	1	1	1	3	1	1	3	3
1	1	1	3	1	1	1	1	2	2
1	1	1	3	1	1	3	1	1	3
2	3	3	3	3	2	3	3	3	3
1	1	1	1	1	3	1	1	1	1
1	1	2	3	1	1	3	1	1	3
1	1	9	9	9	1	9	9	9	9
1	1	2	3	1	1	3	1	1	3
2	2	2	1	2	2	2	2	1	3
1	1	1	2	2	1	1	1	1	2
1	1	1	2	1	3	2	3	2	2

1	1	1	2	1	1	1	1	1	3
1	1	1	3	1	1	1	1	1	2
1	1	3	3	1	1	1	1	1	1
1	1	1	1	1	1	1	1	1	3
1	1	1	3	1	1	1	1	2	2
1	1	1	2	1	1	1	1	1	2
1	1	1	3	1	1	9	1	1	3
1	1	1	3	1	1	1	1	1	3
2	3	2	1	2	3	2	2	2	1
1	1	1	1	1	1	1	1	1	1
9	9	9	9	9	1	1	1	1	9
1	2	2	3	1	1	2	2	2	3
3	1	1	2	1	1	1	1	1	3
1	2	2	2	1	1	1	1	1	2
1	2	2	2	1	1	1	1	2	2
1	1	1	2	1	1	1	1	1	2
9	1	1	2	1	1	1	1	1	2
1	1	1	3	1	1	1	1	2	2
1	1	1	3	1	1	1	1	1	2
3	1	2	1	1	3	1	2	1	2
1	1	1	3	1	1	1	1	9	2
1	1	1	3	1	1	1	1	1	3
1	1	1	3	1	1	1	1	1	1
1	3	1	1	1	1	1	3	3	3
3	3	3	3	3	3	3	3	3	3
2	1	2	1	2	1	2	1	2	1
1	2	2	2	2	1	1	1	1	1
1	3	2	1	1	1	2	3	1	3
2	2	2	2	2	2	2	2	2	2
2	2	2	2	2	2	2	2	2	2
1	2	2	3	2	2	2	2	1	1
2	1	1	2	1	2	2	2	2	2
2	1	2	2	1	2	2	2	2	2
9	9	9	9	9	9	9	9	9	9
9	9	9	9	9	9	9	9	9	9
9	9	9	9	9	9	9	9	9	9
3	2	1	2	1	9	9	9	9	9
3	9	9	9	9	9	9	9	9	9
9	2	1	3	1	9	9	9	9	9
9	9	9	9	9	9	9	9	9	9
1	1	1	2	1	1	2	3	2	1
1	1	1	2	1	9	9	9	9	9
1	1	1	3	1	1	2	1	1	1
1	1	1	3	1	3	3	3	3	3
3	3	1	1	1	1	1	1	1	3
9	3	3	3	1	3	3	3	3	3
1	1	1	1	3	1	3	1	1	3

2	3	2	3	2	2	3	2	3	2
3	2	3	2	2	1	1	1	1	1
1	1	2	2	2	2	1	3	1	2
3	1	1	1	1	1	3	1	1	1
2	2	1	2	1	2	2	1	2	1
2	1	2	1	3	1	2	3	1	2
1	1	1	3	1	3	3	1	1	1
1	2	3	2	1	3	2	3	1	2
1	1	1	1	1	1	1	1	1	1
9	1	9	9	9	1	9	9	2	9
1	1	1	3	1	3	3	1	3	3
1	1	2	1	2	1	2	1	2	1
1	1	1	2	1	1	2	1	2	1
1	1	1	3	1	3	1	1	1	3
1	1	1	1	1	3	1	1	1	1
9	2	2	2	3	1	1	2	3	1
2	2	2	3	2	2	2	2	3	2
1	9	9	9	1	9	9	9	9	9
2	1	1	1	2	2	3	1	2	2
1	3	1	3	1	3	3	2	2	1
1	3	2	3	2	3	2	1	1	1
1	3	1	1	2	3	3	3	3	3
1	1	1	3	9	3	1	1	1	3
1	1	1	2	1	3	3	1	2	3
1	1	2	3	1	1	2	1	1	1
3	1	1	1	1	1	3	1	1	1
1	1	1	3	1	1	2	1	1	2
3	1	2	3	1	1	1	2	3	1
1	1	3	1	1	1	1	3	3	3
1	1	1	2	1	1	3	2	3	3
1	1	1	2	1	1	2	1	1	2
1	1	2	3	1	1	2	3	1	2
1	1	1	1	3	2	3	2	2	2
3	1	1	1	1	1	3	1	1	3
1	1	3	1	1	1	1	1	1	1
1	1	3	3	1	1	1	1	3	3
3	1	2	1	3	1	2	3	1	2
9	9	9	9	9	9	9	9	9	9
1	1	3	3	1	1	3	3	3	9
1	1	3	3	1	1	3	1	1	3
1	1	1	1	1	1	3	1	1	1
1	1	3	2	3	1	2	3	1	2
1	9	1	1	1	1	3	1	1	1
2	3	2	1	1	2	3	3	2	1
2	2	2	2	2	2	2	2	2	2
1	2	3	2	1	1	1	1	3	3
3	3	3	3	9	3	3	3	3	3

2	1	1	2	1	1	2	1	2	2
1	1	1	1	1	9	9	9	9	9
1	2	2	2	2	2	2	3	1	3
1	2	2	3	3	1	1	1	2	2
2	1	1	1	2	3	3	3	3	3
3	1	2	3	1	1	1	2	2	3
2	2	2	2	2	2	2	2	2	2
9	9	9	9	9	9	9	9	9	9
9	9	9	9	9	9	9	9	9	9
2	1	2	2	1	1	2	2	1	2
2	2	2	2	2	1	2	2	2	2
2	2	2	2	2	2	2	2	2	2
3	2	3	3	3	2	2	2	2	2
2	2	2	2	2	2	1	1	1	1
1	2	2	2	2	1	1	1	1	1
2	2	2	2	2	2	2	1	1	1
3	2	2	2	2	2	2	2	2	2
2	2	2	2	2	1	2	2	2	2
2	2	2	2	2	1	2	1	2	2
2	2	2	2	2	2	2	2	2	2
2	1	1	2	2	2	2	2	2	2
2	2	2	2	2	2	2	2	2	2
2	2	2	2	2	2	2	2	2	2
2	2	2	2	2	1	1	2	2	2
2	2	2	2	2	2	2	2	2	2
2	2	2	2	2	2	2	2	2	2
2	2	2	2	2	1	2	2	2	2
2	1	2	3	2	1	2	2	1	2
2	2	2	2	2	1	1	1	2	2
2	2	2	2	2	2	2	2	2	2
2	2	2	2	2	2	2	2	2	2
2	2	2	2	2	1	2	2	2	2
2	2	2	2	2	2	2	2	2	2
2	2	2	2	2	2	2	2	2	2
1	2	2	2	2	1	2	2	2	2
1	2	2	2	2	1	2	2	2	2
2	2	2	2	2	2	2	2	2	2
2	2	2	2	2	2	2	2	2	2
2	2	2	2	2	2	2	2	2	2
1	3	3	3	1	1	3	1	1	1
1	2	2	2	2	1	3	2	2	2
2	2	2	2	2	2	2	2	2	2
2	2	1	2	2	2	2	1	1	1
2	2	2	2	2	2	2	2	2	2
3	1	2	3	3	2	1	1	2	2
2	2	2	2	2	3	3	3	3	3
3	9	3	3	3	3	3	3	3	3

2	2	2	2	2	2	2	2	2	2
2	1	1	2	2	2	2	2	2	2
2	2	2	2	2	1	2	2	2	2
2	2	2	2	2	2	2	2	2	2
1	2	3	3	2	1	2	3	3	3
2	2	2	2	2	2	2	2	2	2
3	1	1	3	3	9	9	9	9	9
1	1	2	3	1	1	1	1	1	1
9	1	1	3	1	1	9	9	9	9
2	2	2	2	2	2	2	2	2	2
2	2	2	2	2	2	3	2	3	2
1	2	2	1	1	3	3	3	2	2
2	2	2	2	2	2	2	3	2	2
9	1	9	9	9	3	9	9	9	9
2	1	1	1	1	2	2	2	2	2
1	2	1	2	2	1	1	1	1	2
2	1	1	1	1	1	1	2	2	2
1	1	2	1	1	1	1	1	1	1
9	9	9	9	1	9	1	9	9	9
2	3	1	1	1	1	1	1	2	2
2	2	2	2	2	1	2	2	2	1
9	1	9	9	9	1	9	9	9	9
9	1	9	9	9	1	9	9	9	9
2	1	1	2	2	1	3	1	3	3
2	1	2	3	1	2	3	9	1	2
3	1	2	1	1	1	9	3	3	3
1	1	3	1	1	1	2	3	3	3
1	1	1	3	1	1	9	3	9	1
3	1	2	3	1	1	2	3	1	2
1	1	1	1	1	1	1	1	1	1
2	2	2	2	2	3	3	3	3	2
3	2	2	2	2	2	2	2	2	2
2	2	2	2	2	2	2	2	2	2
9	1	2	2	9	1	9	9	9	1
1	1	1	1	1	1	2	2	2	2
9	1	9	9	9	9	9	9	9	9
1	1	1	3	3	1	3	1	1	3
1	3	3	3	3	2	2	2	2	2
2	2	2	2	2	2	2	2	2	2
2	2	2	2	2	2	2	2	2	2
9	9	9	9	9	1	3	3	2	2
2	2	2	2	2	2	2	2	2	2
9	9	1	9	9	9	9	9	9	9
1	2	2	3	1	2	2	2	2	3
2	1	2	2	2	1	1	1	1	1
2	1	1	3	1	3	9	2	3	3
2	1	1	1	1	2	2	2	2	2

1	1	1	1	2	1	1	1	1	1
2	2	2	2	2	2	2	2	2	2
1	1	1	1	1	1	1	1	1	1
2	2	2	2	2	2	2	2	2	2
9	1	9	9	9	9	9	1	9	9
1	3	2	2	1	1	1	2	3	2
3	3	9	9	9	3	3	3	3	3
2	1	1	1	1	1	1	1	1	1
2	2	2	2	2	2	2	2	2	2
1	1	1	3	3	1	1	1	1	1
3	2	2	2	2	3	3	3	3	3
3	3	3	3	3	1	1	1	1	1
2	2	2	2	2	2	2	2	2	2
2	2	2	2	2	2	2	2	2	2
2	2	2	2	2	2	2	2	2	2
2	2	2	2	2	1	1	1	1	1
1	1	1	1	1	1	1	1	1	1
3	1	2	2	3	1	3	3	3	3
9	9	9	9	9	9	9	9	9	9
1	1	2	1	3	9	9	9	9	9
2	1	1	2	2	1	1	2	2	1
2	2	2	2	2	2	2	2	2	2
1	1	1	1	1	1	1	1	1	1
1	1	2	2	2	1	2	2	2	2
3	9	3	9	3	3	3	3	3	3
1	1	2	2	2	1	1	1	1	1
2	1	1	2	3	1	2	2	1	1
1	2	2	2	2	2	1	2	1	2
9	2	9	2	9	1	2	1	9	9
1	2	2	2	2	2	2	3	3	3
1	1	2	1	2	2	9	1	9	2
2	2	2	2	2	2	2	2	2	2
3	3	3	3	3	1	9	1	1	1
1	2	3	1	2	1	2	3	1	3
2	2	2	2	2	2	2	2	2	2
3	2	2	3	2	2	2	2	2	3
3	2	2	2	2	1	2	3	3	3
2	3	2	2	2	2	2	2	2	2
2	1	2	3	1	1	2	3	1	2
3	3	3	3	3	3	3	3	3	3
2	1	2	3	1	1	2	1	2	9
1	2	1	3	1	2	2	2	2	2
9	9	9	9	9	9	9	9	9	9
3	3	3	3	3	3	9	9	9	9
2	1	1	2	2	1	1	1	1	2
2	2	2	2	2	2	2	2	2	2
1	1	1	2	1	1	1	2	2	2

1	2	2	2	9	1	1	1	1	1
1	3	3	3	1	1	3	3	1	1
1	2	1	1	1	2	2	2	2	2
1	3	3	3	1	1	3	3	1	1
1	1	1	1	3	1	1	2	1	2
2	2	2	2	2	2	2	2	2	2
2	2	2	2	2	2	2	2	2	2
2	2	2	2	2	2	2	2	2	2
1	1	1	1	1	2	2	2	2	2
2	2	1	2	2	2	1	2	2	1
2	2	2	2	2	2	2	2	2	2
1	1	2	2	3	1	2	3	1	3
2	1	2	3	1	1	2	2	1	2
1	3	3	3	3	1	1	1	1	1
2	2	2	2	2	2	2	2	2	2
1	1	2	3	2	3	2	1	2	2
2	1	2	2	2	1	2	2	2	2
1	1	1	1	2	2	2	2	2	2
1	1	2	2	2	2	2	2	2	2
1	1	1	3	1	2	2	1	1	2
1	2	2	2	1	1	2	2	2	2
1	2	2	2	2	2	2	2	2	2
1	1	2	1	1	3	1	1	1	9
2	2	2	2	2	2	2	2	2	2
1	1	1	1	1	1	1	1	1	1
2	2	2	2	2	2	2	2	2	2
3	1	1	9	3	1	1	3	3	2
3	1	1	1	1	2	3	3	3	3
2	2	2	3	1	1	2	2	2	3
1	1	2	3	1	1	3	1	2	2
1	1	3	2	2	1	3	3	1	2
2	2	1	3	2	1	2	2	3	1
1	2	2	2	2	2	2	2	2	2
2	2	2	2	2	2	2	2	2	2
1	1	1	3	3	1	2	2	1	2
3	1	2	2	1	3	3	3	3	3
2	2	2	2	2	1	9	2	2	2
9	9	9	9	9	9	9	9	9	9
9	2	2	1	2	2	2	2	2	2
1	1	1	2	1	2	2	1	2	3
1	1	1	2	1	2	2	1	2	3
2	2	2	2	2	2	2	2	2	2
2	2	2	2	2	2	2	2	2	2
2	2	2	2	2	2	2	2	2	2
9	1	1	1	3	9	9	9	9	9
1	1	1	1	3	1	1	2	1	2
2	9	1	3	2	2	2	2	2	2

9	9	9	9	9	9	9	9	9	9
1	1	1	2	3	1	2	2	3	1
2	1	1	1	2	3	3	1	2	3
1	1	1	3	2	1	3	1	3	3
1	1	1	3	1	1	3	3	1	3
1	1	1	3	1	1	3	9	3	3
1	1	1	3	1	1	3	1	3	3
9	9	9	9	9	9	9	9	9	9
2	2	2	3	2	3	3	3	3	2
3	3	2	3	2	3	3	3	2	1
1	1	2	2	2	2	2	2	2	2
2	1	2	2	3	1	3	2	2	3
9	9	9	9	9	1	2	2	2	2
1	1	1	3	1	1	1	1	1	1
3	1	2	3	2	1	2	3	2	2
2	2	2	2	2	2	2	2	2	2
1	2	3	2	2	1	2	2	1	3
2	2	2	2	2	2	2	1	3	2
9	9	1	9	9	1	9	9	9	9
2	2	2	2	2	9	9	9	9	9
9	9	9	9	9	9	9	9	9	9
3	1	2	2	3	3	2	3	1	3
2	1	2	3	2	2	1	1	1	2
2	3	2	1	2	1	1	2	2	1
3	1	2	3	1	1	1	2	2	2
2	3	3	3	3	2	3	3	3	3
2	2	1	2	2	2	2	2	3	1
2	1	1	1	2	2	2	2	2	1
2	1	1	2	3	2	3	2	1	2
1	2	1	1	1	1	3	1	2	1
2	2	2	3	1	3	3	3	2	2
2	2	3	2	1	1	3	2	2	2
1	1	1	3	3	3	3	2	2	3
2	3	3	3	3	2	2	2	2	2
2	2	2	2	2	2	2	2	2	2
2	2	2	2	2	2	2	2	2	2
3	3	3	3	3	3	3	3	3	3
3	9	9	9	9	2	2	2	2	2
1	1	2	3	3	9	9	9	9	9
2	1	1	2	3	9	9	9	9	9
1	3	3	3	3	1	2	3	1	2
2	2	2	2	2	2	2	2	2	2
3	1	2	3	1	3	2	1	2	3
2	1	3	3	2	2	1	2	3	2
9	9	9	9	9	9	9	9	9	9
3	3	3	3	3	3	3	3	3	3
3	1	2	3	2	1	3	1	1	3

2	1	1	1	1	1	1	1	1	1
1	1	1	3	1	2	2	1	1	3
3	2	1	2	1	1	2	3	1	2
2	1	1	2	1	2	2	2	2	2
3	2	3	2	3	1	2	1	3	2
3	3	3	3	3	1	3	3	3	3
1	1	1	1	1	1	1	1	1	1
3	3	3	3	3	3	3	3	3	3
1	2	2	1	2	2	2	3	3	3
1	2	1	3	1	1	1	1	2	3
2	2	2	2	2	2	2	2	2	2
2	2	2	2	2	1	1	1	1	1
2	1	2	1	2	9	9	2	3	2
9	9	1	9	9	1	2	3	3	3
1	2	2	3	3	2	2	2	2	2
3	2	2	2	2	3	3	3	3	3
1	1	1	1	1	3	1	1	1	1
2	1	2	3	1	1	2	3	1	2
2	1	2	2	2	2	2	2	2	2
2	1	1	2	2	2	3	2	1	2
1	1	1	3	3	1	2	3	2	1
9	9	9	9	9	3	3	3	3	3
1	1	3	3	2	3	3	3	3	3
1	1	1	2	2	2	2	3	3	2
3	1	2	3	2	1	2	3	1	2
1	1	1	1	3	2	2	2	3	3
1	1	1	2	3	2	2	3	3	3
2	1	2	3	2	1	2	3	1	2
1	2	1	3	1	2	3	1	2	9
3	1	1	2	2	1	1	1	1	1
3	1	2	3	1	1	2	3	1	2
3	2	2	2	2	1	1	1	1	1
2	2	2	2	2	2	2	2	2	2
1	1	1	3	3	1	1	1	1	3
2	1	2	3	1	1	2	3	1	2
2	1	1	3	1	3	2	3	2	1
1	1	3	2	3	2	1	3	2	2
1	1	3	3	3	3	2	1	2	3
1	1	1	3	1	1	1	1	1	2
3	1	1	1	3	1	2	1	2	1
3	3	3	2	2	1	2	2	2	2
1	1	1	3	1	3	3	1	1	1
3	1	2	3	2	1	2	1	2	3
1	2	1	2	2	1	2	1	1	2
1	1	2	3	1	1	2	3	2	2
2	3	2	1	2	1	2	3	1	2
3	3	3	1	1	1	1	3	3	3

2	1	1	1	1	2	3	3	2	2
3	1	2	3	1	1	3	1	2	3
2	3	3	1	2	2	2	2	2	2
3	1	1	1	1	1	1	1	1	1
1	1	2	3	1	1	2	3	1	2
2	1	1	2	2	1	3	2	1	3
1	1	2	1	3	1	1	2	3	2
1	1	3	2	1	1	2	1	2	1
3	3	1	1	1	1	3	1	3	1
1	3	1	1	1	3	3	1	1	3
3	1	2	3	1	1	2	3	1	3
3	2	2	3	1	1	2	3	3	1
3	1	1	1	1	1	1	1	1	1
1	1	1	3	3	1	1	3	3	1
2	3	3	2	2	1	1	1	2	2
1	1	1	1	1	1	3	1	1	1
1	1	1	1	1	1	3	1	2	3
1	1	1	1	3	3	3	3	2	2
1	1	1	2	3	1	2	1	1	1
1	1	2	2	1	2	1	1	1	1
1	1	2	2	1	1	1	2	2	3
1	1	1	3	3	1	1	1	1	2
1	1	1	1	1	1	3	1	1	3
1	1	1	1	1	1	9	1	1	3
2	1	1	2	2	2	2	2	2	2
2	2	2	2	2	2	2	2	2	2
1	1	3	3	1	3	3	2	3	3
1	3	2	1	1	1	2	3	1	2
2	2	2	2	2	2	2	2	2	2
9	9	9	9	9	9	9	9	9	9
9	9	9	9	9	9	9	9	9	9
9	9	9	9	9	9	9	9	9	9
2	1	1	2	2	2	2	2	1	1
9	9	9	9	1	9	1	9	9	9
2	3	3	3	3	2	2	2	2	2
3	2	2	2	2	9	9	9	9	9
2	1	2	2	1	1	2	1	2	2
9	9	9	9	2	1	9	9	9	9
1	9	1	1	1	1	2	1	1	1
2	1	3	3	2	2	2	3	2	3
1	1	2	3	1	9	9	9	9	9
1	3	1	3	1	1	3	3	1	3
2	3	1	2	1	3	1	2	2	2
2	3	3	2	1	3	1	2	2	2
3	1	2	3	1	1	3	1	2	3
2	1	2	3	2	1	2	3	2	1
1	1	3	3	1	3	3	2	3	3

9	1	1	2	2	1	2	2	2	1
2	1	1	2	2	2	2	2	1	2
3	3	2	3	2	2	2	2	2	2
1	1	3	1	1	1	2	1	1	2
1	1	1	9	1	2	2	2	2	2
2	2	1	3	1	1	2	1	2	3
1	9	9	9	1	9	9	9	9	9
2	2	2	1	3	2	2	1	1	3
1	1	1	3	1	3	1	2	2	2
3	3	3	3	3	3	3	3	3	3
1	1	1	2	3	2	3	1	2	3
1	1	3	2	3	3	2	2	1	2
2	1	2	2	2	1	3	2	1	1
2	2	2	2	2	2	2	2	2	2
2	2	2	2	2	2	2	2	2	2
9	1	2	9	2	9	1	2	2	9
3	2	2	2	2	2	2	2	2	2
2	2	2	2	2	2	2	2	2	2
2	2	2	2	2	2	2	2	2	2
2	2	2	2	2	9	2	2	2	2
1	1	1	1	1	1	1	1	1	1
1	1	1	1	1	1	1	1	1	1
1	1	1	1	1	1	1	1	1	1
1	1	1	1	1	1	1	1	1	2
1	1	1	1	1	1	1	1	1	3
9	1	9	9	9	1	1	1	1	3
9	1	1	2	2	3	3	1	3	3
1	1	1	1	1	1	1	1	1	2
1	1	1	1	1	1	2	1	2	2
1	1	9	1	1	1	1	1	1	3
9	1	1	1	1	1	1	1	1	3
9	9	1	9	2	1	9	9	9	9
1	1	1	1	1	1	1	1	1	3
1	1	1	1	1	1	1	1	1	3
9	1	1	1	1	1	1	1	1	3
1	1	1	1	1	1	1	1	1	3
1	1	1	1	1	1	1	1	1	3
9	1	1	1	1	9	1	1	1	3
9	1	9	1	1	1	1	1	1	3
9	1	1	1	1	1	1	1	1	3
1	1	1	1	1	1	1	1	1	1
1	1	1	1	1	1	9	1	9	9
3	1	1	1	1	1	1	1	1	1
9	9	9	9	9	1	1	1	1	3
9	1	1	1	1	9	1	1	1	3
1	1	3	1	1	1	1	1	1	3
9	1	9	1	1	3	1	1	3	1

1	1	1	1	1	1	1	1	1	3
1	1	1	1	1	3	3	2	2	2
1	1	1	1	1	1	1	1	1	3
1	1	1	1	1	1	1	1	1	1
9	1	1	1	1	1	1	1	1	3
9	1	1	1	1	1	1	1	1	3
1	1	1	1	1	1	1	1	1	1
1	1	1	1	1	1	3	1	1	1
9	1	1	1	1	1	1	1	1	3
1	1	1	1	1	1	1	1	1	3
1	1	1	1	1	1	1	1	1	3
1	1	1	1	1	1	1	1	3	1
9	1	1	1	1	1	1	1	1	3
9	1	1	1	1	1	1	1	1	3
9	1	1	1	1	1	1	1	3	1
9	1	1	1	1	9	1	1	1	3
9	1	1	1	1	1	1	1	1	1
9	1	1	1	1	1	1	1	1	1
9	1	1	1	1	1	1	1	1	3
9	1	9	9	1	1	3	1	1	1
1	1	9	9	9	1	1	9	1	9
9	1	9	9	1	1	1	1	3	9
1	1	1	1	1	1	1	1	1	3
1	1	1	1	1	1	1	1	1	3
1	1	1	1	1	3	1	1	1	3
1	1	1	1	1	1	3	1	1	3
3	1	3	1	1	1	1	1	1	1
2	1	1	1	1	1	3	1	1	3
1	1	1	1	1	1	1	1	1	3
9	1	1	1	1	9	1	9	9	9
1	1	1	1	1	1	1	1	3	3
1	1	1	1	1	1	1	1	1	3
9	1	1	1	1	9	1	1	1	3
9	1	9	1	1	1	1	1	1	3
1	1	1	1	1	1	1	1	1	3
1	1	1	1	1	1	1	1	1	3
1	1	9	1	9	1	1	1	9	9
1	1	1	1	1	1	1	1	1	3
1	1	1	1	1	1	1	1	1	3
1	1	1	1	1	1	1	1	1	1
1	1	1	1	1	1	1	1	1	3
1	1	1	2	1	1	1	1	1	1
9	1	9	9	1	1	1	1	3	9
1	1	1	1	1	1	1	1	1	3
1	1	1	1	1	1	1	1	1	3
1	1	1	1	1	1	1	1	1	3
1	1	1	1	1	1	1	1	1	2

1	1	1	1	1	1	1	1	1	3
1	1	1	1	1	1	1	1	1	3
1	1	1	1	1	1	1	1	1	3
1	1	1	1	1	1	1	1	1	3
1	1	1	1	1	1	1	1	1	3
1	1	1	1	1	1	1	1	1	1
3	1	1	1	2	9	9	1	3	3
9	1	1	1	1	1	1	1	1	1
9	1	1	1	1	1	1	1	1	1
1	1	1	1	1	1	1	1	1	3
1	3	1	2	1	1	3	1	2	3
9	9	1	9	9	1	9	9	9	9
1	1	1	3	1	1	1	1	1	1
1	2	2	3	1	1	2	2	1	1
2	1	1	1	2	2	2	2	2	9
1	1	1	2	1	1	2	1	1	3
1	9	9	9	1	9	9	9	9	9
1	1	1	1	1	9	9	9	9	9
1	1	1	3	1	1	1	1	1	1
1	1	1	1	1	1	1	1	1	1
1	1	1	3	1	1	3	1	1	3
1	1	1	3	2	2	2	1	1	2
2	1	2	3	2	3	3	3	3	3
1	1	1	1	1	1	3	1	3	1
1	1	1	3	1	1	1	1	1	3
1	1	1	2	1	1	3	9	1	2
1	2	1	1	1	1	3	1	2	1
2	1	1	3	2	3	1	1	2	1
1	1	1	2	1	1	1	1	1	2
1	1	1	1	1	1	1	1	1	2
1	1	1	2	1	2	1	1	1	2
1	9	1	9	9	1	9	9	9	9
1	1	1	3	1	1	2	1	1	3
1	1	1	2	1	1	3	1	1	3
2	1	2	3	2	3	3	3	3	3
1	1	1	2	1	1	2	1	1	2
1	1	1	3	1	2	1	9	3	3
1	1	1	3	1	2	1	1	3	3
1	1	1	3	1	2	1	1	3	3
1	1	1	3	1	1	1	1	1	1
1	1	1	1	1	1	1	1	1	3
1	1	1	3	1	1	1	1	1	3
1	1	1	2	1	1	2	1	2	1
2	3	3	3	3	2	2	2	2	1
1	1	9	1	1	1	2	1	2	1
3	1	1	1	1	1	3	1	1	3
1	1	1	3	9	1	9	9	9	9

1	1	1	1	1	1	1	1	1	3
1	1	1	3	1	1	2	1	1	1
1	1	1	2	1	1	3	1	1	2
1	1	2	2	1	9	1	2	2	2
1	1	1	3	1	9	9	9	9	9
3	3	1	1	1	1	1	2	2	2
3	1	2	2	2	1	1	1	1	1
1	1	1	1	1	1	2	1	2	2
1	1	1	3	1	1	3	1	3	3
1	1	1	3	1	1	1	1	1	3
1	1	1	1	1	1	1	1	1	2
3	1	1	3	1	1	3	1	1	3
1	1	2	2	1	1	1	1	2	1
3	1	1	3	2	1	3	1	1	3
1	1	1	2	2	2	1	1	1	2
9	1	9	9	9	9	9	9	9	9
1	1	1	3	1	1	1	2	2	2
1	1	1	2	1	1	2	1	2	1
1	1	1	1	1	1	1	1	1	3
1	1	1	3	1	1	1	1	2	2
9	1	9	9	9	9	9	9	1	9
1	1	1	3	1	1	1	1	1	1
9	9	9	9	9	9	1	2	2	2
3	3	3	3	3	3	3	1	3	3
1	1	1	3	1	1	3	1	1	2
1	1	1	3	1	1	3	1	1	2
1	1	1	9	1	9	9	1	1	2
9	9	9	9	9	9	9	9	9	9
9	9	9	9	9	9	9	9	9	9
1	1	1	2	1	1	3	1	2	3
1	1	1	3	1	1	3	1	3	3
1	1	1	3	1	1	2	1	1	2
1	1	1	1	1	1	1	1	1	1
3	1	9	1	1	1	1	1	9	9
9	1	1	1	1	1	1	1	1	3
1	1	1	1	1	1	1	1	1	1
1	1	1	1	1	1	1	1	1	3
1	1	1	1	2	1	1	1	1	1
1	1	3	1	1	1	1	1	3	3
1	1	1	1	1	1	1	1	1	1
1	1	1	1	1	1	1	1	2	1
1	1	1	1	1	1	1	1	1	3
1	1	1	1	1	1	1	1	1	3
9	1	1	1	1	1	1	1	1	3
1	1	1	1	1	1	1	1	1	3
1	1	1	1	1	1	1	1	1	1
1	1	9	9	9	1	1	1	1	3

1	1	1	1	1	1	1	1	1	1
3	1	1	1	1	1	1	1	3	1
9	9	9	9	9	9	9	9	9	9
1	1	1	3	1	3	3	1	1	3
1	1	1	3	1	1	1	1	1	2
1	1	1	3	1	1	1	1	1	3
1	1	1	2	1	9	9	1	1	1
1	9	9	9	1	1	3	1	1	3
9	1	9	9	9	1	9	9	9	9
1	1	1	3	1	9	9	9	9	9
3	1	1	3	1	1	1	1	1	1
9	1	1	2	1	1	1	1	1	3
1	1	1	2	1	2	3	2	2	3
1	9	9	9	1	9	9	9	9	9
1	1	1	3	1	1	2	1	1	2
1	1	1	3	1	2	1	1	1	3
9	1	9	9	1	1	1	1	1	9
1	1	1	3	1	3	1	1	1	2
1	1	1	2	1	1	1	1	1	1
1	1	1	3	1	2	2	1	1	2
1	1	1	3	1	1	3	1	3	3
1	1	1	3	1	1	3	2	1	1
1	1	1	3	1	1	3	2	1	1
1	1	1	1	1	1	1	1	1	3
1	1	1	2	1	1	9	1	1	9
1	1	2	2	1	1	1	1	1	1
1	1	1	3	1	9	9	1	1	9
1	1	1	3	1	1	2	1	2	1
1	1	2	2	1	1	1	1	1	1
1	1	2	3	1	1	1	1	1	1
1	2	3	3	1	1	1	1	3	3
1	1	1	2	1	1	2	1	1	2
1	1	1	2	1	1	9	1	2	2
1	1	1	3	1	1	3	1	1	1
1	1	1	3	1	1	1	1	1	3
9	1	9	9	1	1	3	1	2	3
1	1	3	3	1	3	1	3	1	3
1	1	9	9	1	1	9	9	1	9
1	1	1	2	1	1	2	1	1	1
1	1	1	3	1	1	1	1	1	1
1	1	2	2	2	1	3	1	2	1
3	1	1	2	1	2	1	1	2	3
9	1	9	9	9	9	9	9	9	9
9	1	9	9	9	9	9	9	9	9
1	1	1	3	1	1	3	1	1	3
1	1	1	3	1	1	1	1	1	3
1	1	1	3	1	9	9	9	9	9

1	1	1	3	1	1	3	1	3	3
1	1	1	3	1	2	1	1	1	1
1	1	1	1	1	2	2	1	2	2
1	1	1	3	1	3	1	1	1	2
1	1	1	3	1	3	3	1	1	1
1	1	1	2	1	1	1	2	2	1
2	1	2	2	1	2	1	2	2	2
1	1	1	3	1	1	1	1	1	1
1	1	1	3	1	1	1	1	1	2
1	1	1	2	1	1	2	2	3	2
1	1	1	2	1	1	1	1	3	1
9	1	9	9	9	1	9	9	9	9
9	1	9	9	9	1	9	9	9	9
9	1	9	9	9	9	9	9	9	9
9	1	9	9	9	1	9	9	9	9
9	1	9	9	9	9	9	9	9	9
3	3	1	3	1	3	1	1	9	1
1	1	1	3	1	3	9	1	3	3
1	1	1	3	1	9	9	9	9	9
1	1	1	3	1	3	1	1	1	2
1	1	1	1	1	1	2	9	1	3
1	1	1	2	1	3	3	1	1	3
1	1	1	2	1	3	2	1	1	3
1	1	2	3	2	1	2	3	1	2
1	1	1	2	1	3	3	1	3	2
1	1	1	9	1	2	9	1	1	3
1	1	1	3	1	1	2	1	1	3
1	1	1	3	1	1	3	1	2	3
1	1	1	2	1	9	9	1	9	9
1	1	1	3	1	3	3	1	1	3
1	1	1	2	1	1	2	1	1	2
1	9	1	9	9	1	1	1	1	1
1	1	9	9	9	1	1	1	1	3
1	1	1	2	1	1	2	1	1	2
1	1	1	3	1	1	1	1	2	2
3	2	2	2	1	1	2	1	1	3
1	1	2	1	1	1	3	1	2	3
1	1	1	1	1	1	1	1	1	3
9	1	1	1	1	1	1	3	1	3
1	1	1	1	1	1	1	1	1	3
2	1	2	1	2	1	3	1	3	3
2	1	2	1	2	1	3	1	2	2
1	1	1	2	1	1	3	1	2	3
1	1	1	1	1	1	2	2	1	2
1	1	1	1	1	1	1	1	1	1
1	1	1	2	1	1	1	1	1	9
3	1	1	3	1	1	1	1	1	1

1	1	1	3	1	1	1	1	1	3
1	1	1	2	1	1	9	1	1	1
1	1	1	2	1	1	2	1	1	1
1	1	1	2	1	1	2	2	2	2
1	2	1	1	1	1	3	1	3	3
1	1	2	3	1	2	2	2	2	2
1	1	1	2	2	1	3	1	1	2
1	1	1	3	1	1	2	1	2	2
1	1	1	2	2	1	2	1	1	2
1	1	1	3	1	1	1	1	2	2
1	1	1	2	1	1	3	1	2	3
2	2	2	2	2	2	2	2	2	2
2	1	1	3	1	2	3	2	2	2
1	1	1	3	1	1	3	1	1	3
2	1	1	2	2	2	2	2	2	2
1	1	1	2	1	1	3	1	1	3
2	1	1	2	1	2	2	2	2	3
2	2	2	2	2	2	2	2	2	2
2	2	2	2	2	1	2	1	1	2
1	1	1	3	1	1	2	1	1	2
1	1	1	3	1	1	2	1	2	2
1	1	1	3	2	1	9	9	9	9
1	1	2	2	1	1	3	1	1	3
1	1	2	1	1	1	3	1	3	3
1	1	2	2	1	1	1	1	1	1
1	1	1	2	1	1	1	1	3	3
2	1	1	1	1	1	1	2	2	1
9	1	2	9	1	1	1	1	2	1
9	1	1	3	1	1	3	3	3	3
1	3	1	3	1	3	3	1	1	2
1	1	1	2	1	1	2	1	1	2
1	2	1	2	1	1	2	1	1	2
9	9	9	9	1	1	9	9	9	9
1	9	9	9	1	9	9	9	9	9
1	9	9	9	1	9	9	9	9	9
1	9	9	9	1	9	9	9	9	9
9	9	9	9	1	1	9	9	9	9
9	9	9	9	1	1	9	9	9	9
9	1	9	9	9	1	9	9	9	9
9	1	1	1	1	9	9	9	9	9
1	1	1	2	1	1	3	1	3	3
9	9	9	9	1	1	9	9	9	9
9	9	9	9	1	1	9	9	9	9
1	1	2	3	1	1	1	1	1	2
9	1	9	9	9	1	9	9	9	9
9	1	9	9	9	1	9	9	9	9
9	1	9	9	9	1	9	9	9	9

1	1	1	3	1	1	2	1	1	1
2	2	1	2	3	2	3	1	3	2
9	1	9	9	9	1	9	9	9	9
9	9	9	9	1	1	9	9	9	9
9	9	9	9	1	1	9	9	9	9
1	9	9	9	1	1	9	9	9	9
9	1	9	9	9	9	9	1	9	9
9	1	9	9	9	1	9	1	9	9
1	1	1	3	1	1	9	9	9	9
9	1	9	9	9	1	9	9	9	9
1	1	1	3	1	1	3	1	1	1
1	1	1	1	1	1	3	1	1	3
1	1	1	1	1	1	3	1	1	3
1	1	1	3	1	1	1	1	1	2
1	1	1	3	1	1	1	1	1	1
1	1	1	3	1	1	1	1	1	1
1	1	1	3	1	1	1	1	1	1
1	1	1	3	2	1	1	1	1	1
1	1	1	1	1	1	3	1	1	3
1	1	1	1	1	1	1	1	1	1
1	1	1	3	1	1	1	1	1	1
2	1	3	1	1	1	3	1	3	3
9	9	9	9	1	9	9	1	9	9
1	1	2	3	1	2	1	2	2	2
1	3	1	2	2	3	2	1	1	2
1	1	1	2	2	1	3	1	1	3
9	9	1	1	9	3	3	3	3	3
1	1	1	3	1	1	1	1	1	3
1	1	1	3	1	1	3	1	1	1
2	1	1	3	1	1	3	1	1	2
3	1	1	3	1	1	3	1	1	1
1	1	2	3	1	1	3	2	2	3
1	1	1	2	1	1	3	1	1	3
1	1	2	3	1	1	3	1	1	1
2	1	1	1	1	1	3	1	3	3
1	1	1	3	1	1	1	1	1	1
1	1	1	3	1	1	3	1	1	1
1	1	1	3	1	1	3	1	1	1
1	1	1	3	1	1	3	1	1	1
1	1	3	3	1	1	2	1	1	3
1	1	1	3	1	1	3	1	1	3
1	3	1	3	1	1	1	1	1	3
1	1	1	2	3	1	2	2	2	1
1	1	3	3	1	1	3	1	1	1
1	1	3	3	1	1	3	1	1	1
1	9	3	3	1	1	3	1	1	1
1	1	2	3	1	1	2	1	1	1

1	2	2	3	1	1	1	1	1	1
1	1	1	3	1	1	1	1	1	1
1	1	1	2	1	1	2	1	1	3
1	1	3	3	1	1	3	1	1	1
1	1	3	3	1	1	3	1	1	1
1	1	3	3	1	1	3	1	1	1
1	1	2	3	1	1	2	1	1	1
1	1	2	3	1	1	1	1	3	1
1	1	1	3	1	1	2	1	1	2
3	1	1	3	1	1	1	1	1	1
1	1	1	2	1	1	1	1	1	1
1	1	1	2	1	1	1	1	3	3
1	1	1	3	1	1	1	1	1	2
1	1	1	3	1	1	1	1	1	1
1	3	1	3	1	1	1	1	1	3
1	1	1	1	1	2	1	1	1	1
1	1	1	3	2	1	1	1	3	3
2	1	2	2	2	1	2	1	2	2
1	3	3	3	1	3	1	3	1	2
1	1	1	3	1	1	2	1	1	1
1	1	1	2	1	1	3	1	1	3
1	1	1	1	1	1	1	1	1	1
1	1	1	1	1	1	3	1	1	1
1	1	1	1	1	2	3	1	1	1
1	1	1	1	1	9	1	1	1	1
1	1	1	2	1	2	2	1	2	3
1	1	2	3	1	2	2	2	2	2
1	2	1	2	2	2	2	2	2	2
1	1	2	2	1	1	1	1	3	2
1	1	1	2	1	2	2	2	2	1
2	1	2	2	2	2	2	2	3	2
1	1	2	3	1	1	2	1	3	3
9	1	1	3	1	1	3	1	2	2
1	1	1	3	1	1	2	1	1	9
1	1	1	1	1	1	3	1	1	1
1	1	3	3	1	1	3	1	1	1
1	1	1	3	1	1	3	3	3	3
1	3	3	3	2	1	3	2	2	2
1	1	1	3	1	3	3	1	1	1
1	1	1	3	1	1	3	1	3	3
1	1	1	3	1	2	2	2	2	2
1	1	1	3	1	1	2	1	1	1
1	1	1	3	1	1	2	1	1	1
1	1	1	2	2	1	3	1	2	2
1	1	1	1	1	1	2	1	1	3
1	1	2	2	1	1	2	1	2	2
2	1	1	3	1	3	3	2	2	2

1	1	1	3	1	2	3	1	3	3
2	1	1	1	1	2	2	2	2	2
1	1	1	3	1	1	2	1	1	2
1	1	1	3	1	1	3	1	3	3
1	1	1	2	1	1	1	1	2	2
1	1	1	1	2	2	1	1	2	2
1	1	1	2	1	3	3	3	3	3
1	1	1	3	2	2	2	2	2	2
3	3	3	3	1	1	2	2	2	2
1	1	1	3	1	2	1	1	1	3
1	1	2	2	1	2	1	1	2	1
1	1	1	3	1	1	2	1	1	1
9	1	9	9	9	9	9	9	9	9
1	1	1	2	1	1	3	2	2	3
1	1	1	3	3	1	1	3	1	3
2	1	1	1	1	3	3	1	2	2
1	1	1	2	1	1	3	1	2	3
1	1	1	1	1	1	1	1	3	2
1	1	1	1	1	1	1	1	1	2
1	1	1	2	2	1	2	1	2	3
1	1	3	3	1	1	3	1	2	1
1	1	2	2	1	2	1	1	1	1
3	1	3	3	1	1	1	1	1	3
1	1	1	3	1	1	3	1	1	1
2	1	1	3	3	1	1	1	2	2
1	1	1	3	1	1	1	1	3	3
1	1	1	3	1	1	1	1	1	1
9	9	9	9	9	1	1	1	3	3
9	1	9	9	9	1	1	1	1	2
2	1	2	2	1	1	9	9	9	2
1	1	1	3	2	2	3	2	2	2
1	1	1	3	1	1	2	1	3	3
1	1	1	2	1	1	2	2	2	3
1	1	1	2	1	1	2	1	1	2
1	1	3	3	1	1	9	9	9	9
1	1	1	3	1	1	2	1	1	2
2	1	2	2	2	2	2	2	2	2
9	3	9	9	9	9	9	9	2	9
1	1	1	2	1	1	1	1	2	3
1	1	1	2	1	1	2	1	1	3
1	1	1	1	1	9	9	9	9	9
1	3	1	3	1	1	2	1	2	2
1	1	1	2	1	1	2	1	1	2
1	9	9	9	1	9	9	9	9	9
2	1	1	1	1	2	2	2	2	2
2	2	2	2	2	2	3	1	2	2
1	1	2	3	1	1	1	1	1	1

1	1	1	3	1	3	2	2	2	3
1	1	1	2	1	1	1	1	2	2
1	1	2	2	1	1	2	1	2	2
1	1	2	2	1	1	2	2	2	2
2	1	2	2	2	1	2	1	1	2
1	1	1	3	1	3	2	2	2	3
1	2	1	3	3	1	1	1	1	2
1	1	9	9	9	9	1	9	9	9
2	2	1	2	2	2	2	2	2	2
1	1	3	1	1	1	2	3	2	3
1	1	1	2	1	1	1	1	2	2
1	2	1	2	2	2	2	1	1	2
1	1	1	1	1	1	3	1	2	3
2	2	2	2	2	2	2	2	2	2
1	1	1	3	1	1	3	3	2	3
1	1	1	3	1	1	3	3	3	3
1	1	1	3	1	1	2	1	1	1
1	1	1	1	1	1	1	1	1	3
1	1	1	2	1	1	1	2	2	2
1	1	1	1	1	1	1	1	9	3
1	1	1	1	1	1	1	1	9	3
1	1	1	2	1	1	1	1	1	2
1	2	1	2	2	1	1	2	1	2
1	3	1	3	1	3	1	1	3	3
1	1	1	2	1	2	1	2	1	3
2	1	1	1	2	1	1	1	1	2
1	1	1	3	1	1	1	1	2	2
1	1	1	2	1	1	1	1	1	3
1	1	1	2	1	1	1	1	1	3
9	1	9	2	9	9	9	9	9	9
1	1	1	2	1	1	1	1	1	3
1	1	1	1	1	1	1	1	1	2
1	1	1	1	1	1	1	1	1	2
1	1	1	1	1	1	1	2	1	3
1	1	2	3	1	1	2	1	3	2
1	1	1	2	1	1	1	1	1	2
1	1	1	2	1	1	1	1	1	2
9	9	9	9	9	1	1	1	3	3
1	1	1	3	1	1	1	1	1	3
1	1	1	3	1	1	1	1	2	3
1	1	1	1	1	2	1	1	1	2
1	1	1	3	1	1	1	1	1	3
1	1	1	3	1	1	1	1	2	2
1	1	1	1	1	1	1	1	1	3
1	1	1	1	1	1	1	1	3	3
1	1	1	3	1	3	1	1	3	3
1	1	1	1	1	2	1	1	1	3

1	1	1	1	1	1	1	1	3	3
1	1	1	1	1	1	1	1	3	3
1	1	1	3	1	1	1	1	1	3
1	1	1	2	1	1	1	1	1	3
1	1	1	1	1	1	1	1	1	3
1	1	1	1	1	1	1	1	1	3
1	1	1	3	1	3	1	1	3	9
1	3	1	3	1	1	1	1	1	2
1	1	1	1	1	1	3	1	1	3
1	1	1	1	1	1	3	1	1	3
1	1	1	3	1	1	3	1	1	1
1	1	1	3	1	1	3	1	1	1
1	1	1	2	1	3	3	1	1	3
1	3	1	1	3	1	3	1	3	3
1	1	1	3	1	1	3	1	1	3
1	1	1	3	1	1	3	1	1	3
1	1	1	2	2	1	2	2	1	2
1	1	1	3	1	1	3	1	1	3
1	1	1	3	1	1	3	1	1	1
1	1	1	1	1	1	3	3	1	1
1	1	3	1	1	1	1	1	2	2
1	1	2	2	1	1	3	3	3	3
9	9	9	9	1	1	9	9	9	9
9	9	9	9	1	1	9	9	9	9
9	9	9	1	9	9	9	1	9	9
1	1	1	3	1	1	9	9	9	9
1	1	1	3	1	1	3	3	3	3
1	9	9	9	1	9	9	9	9	9
9	2	9	9	9	2	9	9	9	9
9	1	9	9	9	9	9	9	9	9
1	9	1	9	9	1	9	9	9	9
1	9	1	9	9	1	9	9	9	9
1	9	9	2	9	1	9	9	9	9
2	1	2	1	1	1	9	9	9	9
1	1	1	3	1	1	1	1	3	3
1	1	1	3	1	1	1	1	1	1
1	1	1	1	1	2	1	1	1	1
1	1	1	3	1	1	3	1	1	1
1	1	1	3	1	1	1	1	2	2
1	1	1	3	1	1	1	1	2	2
1	1	1	2	1	1	1	1	3	3
1	1	1	3	1	1	3	1	1	1
1	1	1	2	2	2	3	1	1	2
1	2	1	2	1	2	3	1	1	3
1	1	1	2	2	1	2	3	3	3
1	1	1	1	3	1	1	3	3	3
1	1	1	1	1	1	3	2	1	3

1	2	2	1	2	1	2	2	2	2
1	1	1	2	2	2	1	1	2	2
1	1	3	2	1	2	3	1	1	3
1	1	1	3	1	3	3	1	3	1
1	1	1	1	1	1	1	1	1	1
1	1	1	3	1	1	1	1	1	1
9	9	9	9	9	9	9	9	9	9
3	1	1	3	2	2	2	1	2	2
1	1	1	1	1	1	3	2	2	2
1	2	2	2	2	3	3	3	3	3
3	1	1	1	1	1	1	2	2	2
1	1	1	2	1	1	1	1	1	2
1	2	2	2	1	1	3	1	2	2
2	3	3	3	3	2	2	3	3	3
1	1	1	3	1	1	3	1	1	1
1	1	9	3	1	2	2	2	2	2
1	1	1	1	1	1	1	1	1	1
1	9	9	9	9	1	2	3	1	2
2	1	2	3	1	1	2	3	2	2
1	1	1	1	1	1	1	1	1	1
2	1	1	1	1	1	2	1	1	1
2	3	3	2	2	2	2	2	2	2
9	9	9	9	9	1	3	2	2	2
1	1	1	1	1	2	9	1	1	1
1	1	1	1	1	1	1	1	1	1
3	2	3	2	1	1	2	3	2	2
1	2	1	2	3	1	2	1	2	3
1	1	1	1	1	1	1	1	1	1
2	2	2	2	2	2	2	2	2	2
1	1	1	3	2	2	2	2	2	2
1	1	1	3	1	1	1	1	1	1
1	1	1	1	1	1	1	1	1	1
2	2	2	1	3	2	2	2	2	2
1	1	2	3	1	2	1	1	2	1
1	1	1	3	2	2	2	1	2	2
9	9	9	9	9	1	1	2	2	3
2	2	2	2	2	2	2	2	2	2
1	1	1	3	1	1	3	1	2	3
1	1	1	3	1	3	3	2	3	2
1	1	1	1	1	2	1	1	1	1
1	1	1	2	1	1	2	1	2	1
1	1	1	2	1	1	2	1	2	1

Palms	Onefinger	Threemidd	FingersinCi	FingersVer	FingersWe	PressNippl	ExamineAr	BreastSizea	Nippledevi
3	1	1	1	2	2	2	2	2	2
3	9	3	9	2	3	2	2	3	2
2	2	2	2	2	2	2	2	2	2
2	2	2	2	2	2	2	2	2	2
1	2	2	2	2	2	2	2	2	2
3	3	3	9	3	3	3	3	3	3
2	2	2	2	2	2	2	2	2	2
2	1	2	2	2	2	2	1	1	2
2	9	1	1	3	3	3	3	3	3
3	2	2	1	3	2	1	3	2	1
2	2	2	2	2	2	2	2	2	2
2	2	2	2	2	2	1	2	2	2
1	1	1	2	2	2	2	2	2	2
2	1	2	2	2	1	2	2	1	2
2	2	2	2	2	2	2	2	2	2
2	2	2	2	2	2	2	2	2	2
9	9	9	9	9	9	9	9	9	9
2	2	2	1	1	1	1	1	1	1
2	2	2	2	2	2	2	3	9	9
1	2	3	3	2	2	2	3	9	2
2	2	1	1	1	1	1	1	1	9
3	2	2	1	2	2	9	1	1	1
3	1	2	1	1	3	1	1	1	1
1	2	2	2	2	1	1	1	1	1
2	2	2	2	2	2	2	2	2	2
2	2	2	2	3	2	2	2	2	2
9	9	9	9	9	1	9	9	9	9
2	2	9	1	1	1	1	1	3	2
2	9	1	2	1	1	1	2	3	2
9	2	3	1	2	1	3	1	2	1
2	2	2	2	2	2	2	2	2	2
2	3	3	2	2	2	1	3	3	2
9	9	9	9	9	1	9	9	9	9
2	3	3	1	1	9	1	1	2	1
1	2	9	1	2	3	1	9	1	2
2	2	3	2	3	2	3	1	3	2
2	2	2	2	2	2	2	2	2	2
2	2	1	1	1	1	1	2	2	1
2	2	1	1	3	1	2	1	3	2
3	2	3	2	9	1	3	1	1	2
9	9	9	9	9	9	9	9	9	9
9	9	9	9	9	9	9	9	9	2
9	9	9	9	9	9	9	9	9	9
1	2	2	2	1	2	2	2	2	2
2	2	1	2	1	2	2	2	1	2
9	9	9	9	9	9	9	9	9	9

2	2	2	2	3	2	2	3	2	2
1	2	2	3	2	2	2	2	1	2
1	1	2	2	2	1	2	1	2	2
2	2	1	1	2	2	1	1	2	2
1	1	3	1	1	1	1	1	1	3
1	1	3	1	1	1	1	1	9	3
3	3	9	9	3	3	3	2	3	2
2	2	2	2	2	2	2	2	2	2
9	9	1	1	1	1	1	1	2	1
3	2	1	2	2	3	1	3	1	2
9	9	9	9	1	9	9	9	9	9
1	2	2	2	1	1	1	2	2	2
3	3	3	3	2	2	1	1	2	2
1	2	2	2	1	1	2	2	1	1
2	3	2	1	2	2	2	1	2	2
2	2	3	2	2	2	3	2	2	2
2	3	3	2	3	3	3	2	2	2
1	3	2	1	1	1	1	2	3	1
2	2	2	2	2	2	2	2	2	2
9	9	9	9	9	9	9	9	9	9
2	2	2	2	2	2	2	2	2	2
2	3	9	2	3	1	2	9	2	2
1	2	2	2	2	2	2	2	2	2
2	2	3	2	1	2	2	3	1	2
2	1	2	1	2	1	2	2	1	2
3	3	9	2	2	3	2	3	2	2
3	2	1	1	2	9	3	1	1	3
3	2	1	2	3	3	3	2	1	1
2	3	2	3	2	1	1	1	1	1
1	3	3	3	1	1	1	3	1	1
9	9	9	9	9	9	9	9	9	9
3	3	1	1	2	2	1	1	1	3
1	3	3	9	2	2	1	1	1	1
2	2	2	2	2	2	2	2	2	2
3	3	3	3	3	3	3	1	1	3
2	2	2	2	2	2	2	2	2	2
2	2	2	3	3	1	1	3	1	1
1	1	1	1	2	3	2	1	1	2
1	2	3	2	3	2	1	1	2	2
2	1	2	2	2	1	1	2	2	2
2	2	3	1	2	1	1	1	1	1
9	9	9	9	9	9	9	9	9	9
1	9	9	9	9	9	9	9	9	9
2	1	2	2	1	1	2	2	3	2
2	1	1	1	1	2	2	2	1	1
2	3	3	3	3	3	2	2	2	3
2	2	2	2	2	1	2	2	2	1

3	1	2	3	1	2	3	2	1	2
1	3	9	2	1	2	3	3	3	3
2	2	2	2	2	2	2	1	2	2
2	1	1	1	2	1	1	2	3	2
2	1	2	2	3	2	1	2	2	2
1	1	2	2	2	1	1	2	2	3
2	2	2	2	2	2	2	2	2	2
1	3	3	3	1	9	1	2	9	1
3	2	2	1	1	2	2	1	2	2
2	9	3	1	2	2	1	2	1	1
1	2	1	3	3	9	9	9	9	9
2	1	2	2	3	2	1	2	2	2
1	1	1	2	1	1	1	1	1	2
2	2	2	2	2	2	1	1	1	1
2	3	3	1	2	1	1	1	1	1
2	3	3	1	2	3	1	2	2	1
2	1	2	2	1	2	2	3	2	2
3	3	3	3	3	3	1	1	3	1
3	9	9	1	1	1	1	1	1	1
2	2	3	2	2	2	2	3	2	2
1	1	1	3	1	1	1	3	3	1
1	3	3	2	2	2	1	2	1	2
1	2	1	1	1	2	1	1	1	2
2	2	2	3	2	1	2	2	2	2
2	2	2	2	2	2	2	2	2	2
3	3	3	9	2	1	1	2	2	2
3	1	9	1	1	1	1	1	1	1
2	1	2	1	2	1	2	1	1	2
2	2	2	2	2	1	2	2	1	2
1	2	3	2	3	2	1	1	1	2
1	2	2	1	1	2	1	1	3	2
1	1	2	3	2	1	2	1	1	1
2	9	1	1	3	1	1	2	1	2
1	2	2	1	1	2	1	2	1	2
1	2	3	2	1	2	1	2	1	1
3	2	1	1	2	2	3	1	2	2
2	2	2	2	2	2	2	2	2	3
1	2	2	1	1	2	2	2	3	2
9	9	9	9	9	9	9	9	9	9
2	3	9	2	1	2	3	3	3	3
2	2	1	1	3	2	2	2	3	2
9	9	9	9	9	9	9	9	9	9
2	1	2	2	1	2	2	1	2	2
3	2	1	2	2	2	2	2	2	2
3	3	9	1	1	1	1	1	1	3
1	2	1	3	2	1	1	1	1	2
1	3	2	1	1	1	1	2	1	1

1	2	9	3	1	2	2	3	1	2
3	3	3	3	3	3	3	3	3	3
3	3	3	3	3	3	3	3	3	3
1	1	2	2	2	2	2	2	2	2
1	1	2	3	3	2	1	3	1	3
2	3	2	2	2	2	1	2	3	2
1	3	1	9	3	1	9	1	9	9
9	1	1	2	2	1	1	9	1	2
9	9	9	9	9	9	9	9	9	9
2	2	2	2	2	2	2	2	2	2
2	2	2	2	2	2	1	1	1	1
2	2	2	2	2	1	1	3	3	3
2	2	9	2	2	2	2	2	2	2
2	3	3	3	3	3	3	2	2	2
3	2	2	2	2	2	2	1	1	2
1	2	2	2	2	1	2	2	3	3
2	1	2	2	3	2	2	1	1	1
1	2	2	1	1	2	2	1	1	1
9	9	9	9	9	9	9	9	9	9
3	3	2	3	3	3	1	1	1	1
2	3	2	1	2	1	2	3	1	2
2	2	2	2	1	2	2	1	1	1
3	3	3	3	3	3	3	3	2	3
2	2	2	1	2	2	1	2	1	2
1	1	1	1	1	2	3	3	3	3
3	3	1	2	1	1	1	3	3	1
3	2	2	1	2	2	1	2	1	1
3	3	3	3	2	2	2	2	1	2
2	3	3	2	2	2	2	2	2	9
2	2	2	2	1	2	3	2	1	2
2	1	1	1	2	2	1	2	1	2
1	2	2	2	2	1	1	1	2	2
2	2	2	2	2	2	2	2	1	2
1	3	3	3	1	1	3	3	3	3
2	2	2	2	1	2	2	1	2	3
2	2	1	1	1	1	2	1	1	1
2	2	2	2	2	2	2	2	2	2
1	3	3	1	2	3	1	1	2	1
1	3	3	3	1	1	1	1	1	1
2	2	2	2	2	2	2	2	2	2
2	1	1	2	2	2	2	2	1	1
2	2	2	2	2	2	2	2	2	2
2	2	2	2	2	2	2	3	3	1
9	9	9	9	9	9	9	9	9	9
9	9	9	9	9	9	9	9	9	9
2	2	1	1	1	2	1	1	1	1
1	1	2	2	2	2	2	2	2	2

9	9	9	9	1	9	9	9	9	9
2	3	3	2	2	2	3	3	3	3
1	2	1	1	1	1	1	1	1	1
9	9	9	9	9	9	9	9	9	9
9	9	9	9	9	9	1	9	9	9
9	9	9	9	9	9	9	9	9	9
3	3	2	2	2	3	1	1	1	1
9	9	9	9	9	9	9	9	9	9
2	3	2	2	3	2	1	2	3	3
9	9	9	9	9	9	9	9	9	9
9	9	9	9	9	9	9	9	9	9
9	9	9	9	9	9	9	9	9	9
2	1	1	2	2	1	2	2	2	2
1	2	3	1	2	2	2	2	1	1
9	9	9	9	9	9	9	9	9	9
3	3	3	3	2	3	3	3	3	2
3	3	3	3	3	3	3	3	3	3
1	1	2	2	2	1	2	2	2	2
2	1	2	1	1	2	1	2	1	1
2	3	2	2	3	2	2	2	2	2
1	3	3	3	2	2	2	2	9	9
9	9	9	9	9	9	9	9	9	9
1	1	1	1	1	1	1	1	1	1
1	2	2	1	1	1	1	1	1	1
2	2	2	2	2	2	2	2	2	2
1	1	1	1	1	1	1	1	1	1
1	1	1	1	1	1	1	1	2	2
2	1	1	1	1	1	1	1	1	1
3	2	2	2	2	2	2	3	3	3
3	3	3	1	3	3	3	3	3	3
2	2	2	2	2	2	2	2	2	2
2	2	2	2	1	2	2	2	1	1
9	9	9	9	9	9	9	9	9	9
2	2	2	3	3	2	3	2	2	2
1	1	1	1	1	2	1	1	1	1
1	1	1	1	1	1	1	2	2	2
9	2	1	2	2	1	2	1	2	1
2	1	1	1	2	1	1	2	2	2
1	1	3	3	3	1	2	3	2	3
1	2	3	2	1	1	2	2	2	1
1	2	2	2	2	2	2	2	2	2
3	2	1	3	1	3	2	1	3	2
3	3	2	2	1	2	1	2	3	3
2	2	2	2	2	2	2	2	2	2
2	2	1	3	3	3	3	3	3	2
2	9	1	3	2	1	2	3	2	1
9	9	9	9	9	9	9	9	9	9

1	3	1	1	1	2	3	3	2	2
3	3	3	3	3	1	2	1	1	1
3	3	3	1	1	1	1	3	1	1
2	2	1	2	3	3	2	2	9	2
2	2	2	2	2	2	2	2	2	2
2	2	2	2	3	2	3	2	3	2
3	1	3	3	3	3	3	1	2	3
2	2	1	1	2	2	2	2	2	2
2	1	1	1	1	1	1	1	1	1
2	1	1	1	1	2	2	2	1	1
2	2	2	2	2	1	1	1	1	1
9	9	9	9	9	9	9	9	9	9
9	9	9	9	9	9	9	9	9	9
2	2	2	2	2	2	2	2	2	2
2	9	2	9	9	2	9	9	2	9
9	9	9	9	2	9	9	9	9	2
9	9	9	9	9	9	9	9	9	9
9	9	9	9	9	9	9	9	9	9
9	9	9	9	2	9	9	9	2	9
9	9	9	9	9	9	9	9	9	9
9	9	9	9	9	9	9	9	9	9
2	2	2	2	2	2	2	2	2	2
2	2	2	2	2	2	2	2	2	2
2	2	2	2	2	2	1	1	1	2
1	1	1	1	2	2	2	2	1	1
9	9	9	9	9	9	9	9	9	9
2	2	2	2	2	2	2	2	2	2
9	9	9	9	9	9	9	9	9	9
9	9	9	9	9	9	9	9	9	9
2	1	2	2	1	2	2	1	1	2
2	9	9	9	9	2	9	9	9	9
9	9	9	9	9	9	9	9	9	9
9	9	9	9	9	9	9	9	9	9
9	9	9	9	9	9	9	9	9	9
3	1	9	9	3	1	9	3	1	9
1	9	2	1	9	2	1	9	1	9
9	9	9	9	9	9	9	9	9	9
9	9	2	9	9	9	9	9	2	9
9	9	9	9	9	9	9	9	9	9
9	9	9	9	9	9	9	9	9	9
9	9	9	2	9	9	9	9	9	9
9	9	9	9	9	9	9	9	1	9
3	3	3	3	3	3	3	3	3	3
1	2	1	1	3	1	2	1	2	3
2	1	3	1	2	2	1	3	2	1
1	1	9	1	9	9	2	9	9	1
9	9	9	9	9	9	9	9	9	9

9	9	9	9	9	9	9	9	9	9
9	9	9	9	9	9	9	9	9	9
1	2	1	1	1	1	1	2	2	2
1	2	3	1	2	3	1	2	3	1
3	3	3	3	3	3	3	3	3	3
2	3	3	3	3	3	3	2	2	2
2	2	3	2	2	2	1	2	1	2
3	2	1	3	3	2	1	1	1	1
3	2	3	2	3	2	3	2	2	2
3	3	3	3	3	3	3	3	3	3
9	9	9	9	9	9	9	9	9	9
9	9	9	9	9	9	9	9	9	9
9	9	9	9	9	9	9	9	9	9
9	9	9	9	9	2	9	9	9	9
1	1	1	1	1	1	1	1	1	1
3	3	3	3	2	2	1	1	2	3
9	9	9	9	9	9	9	9	9	9
2	2	2	2	1	2	1	2	2	2
1	1	1	1	1	1	1	1	1	1
2	2	2	2	2	2	2	2	2	2
3	3	3	3	1	2	2	3	1	2
2	2	2	2	2	2	2	2	2	2
3	1	2	3	2	1	3	1	2	3
2	2	2	2	2	2	2	2	2	2
2	2	2	2	2	2	2	2	2	2
2	2	2	2	2	2	2	2	2	2
2	2	2	2	2	2	2	2	2	2
3	1	2	1	2	3	1	2	3	2
2	2	3	3	3	9	2	2	2	2
1	2	1	1	1	2	1	1	2	1
2	2	2	2	2	2	2	2	2	2
2	2	2	2	2	2	2	2	2	2
2	2	3	2	1	2	2	3	2	1
2	2	2	2	2	3	3	3	3	9
3	9	3	1	3	1	2	9	1	3
3	1	2	3	2	2	2	2	2	2
9	9	9	9	9	9	9	9	9	9
2	2	2	2	2	2	2	2	2	2
3	3	3	3	3	3	3	3	3	3
3	3	3	3	3	3	3	3	3	3
9	9	9	9	2	9	9	9	9	9
3	1	2	3	2	3	9	9	9	9
9	1	9	9	9	9	9	9	9	9
1	1	1	1	1	1	1	1	1	1
2	2	2	2	2	2	2	2	2	3
2	2	2	2	2	2	2	2	2	2
3	3	3	3	3	2	3	3	3	3

9	9	2	9	9	9	9	9	9	9
9	9	9	9	9	9	9	9	9	9
9	9	9	9	9	9	9	9	9	9
9	9	9	1	9	9	9	9	9	9
9	9	9	9	9	9	9	9	9	9
1	1	1	1	1	1	1	1	1	1
9	9	9	9	9	9	9	9	9	9
9	9	9	2	9	9	9	9	9	9
1	2	2	1	2	2	1	1	1	1
9	9	9	9	9	9	9	9	9	9
9	9	9	9	9	9	9	9	9	9
9	9	9	9	9	9	9	9	2	9
9	9	9	9	9	9	9	9	9	9
9	9	9	9	9	9	9	9	9	9
9	9	9	9	9	9	9	9	9	9
9	9	9	9	9	9	9	9	9	9
2	9	9	9	9	9	2	9	3	9
1	2	3	1	2	3	1	2	3	2
3	2	2	2	1	1	1	2	3	1
2	2	2	2	2	2	2	2	2	2
2	3	2	1	2	2	1	1	2	1
3	3	2	1	1	2	1	2	1	2
2	1	2	2	2	2	2	2	2	2
2	2	2	3	2	2	2	1	2	2
2	2	2	2	2	2	2	3	9	1
2	2	9	2	2	2	2	2	2	2
2	2	2	2	2	2	2	2	2	2
3	3	3	2	2	2	2	2	2	2
3	1	2	1	2	3	1	2	1	3
1	1	1	1	1	1	1	1	1	1
2	1	2	1	1	1	1	2	3	2
3	3	2	1	1	3	3	3	3	3
2	2	2	2	2	2	3	3	3	3
2	2	2	2	2	1	1	1	2	2
9	9	9	9	9	9	9	9	9	9
2	2	2	2	2	2	2	2	2	2
9	9	9	9	9	9	9	9	9	9
2	2	1	2	2	2	2	2	2	2
3	2	3	2	2	3	2	2	3	3
2	2	2	2	2	2	2	2	2	2
2	2	9	1	2	2	2	1	1	2
2	2	1	1	3	3	2	3	1	1
3	3	2	2	3	1	1	3	1	1
2	3	2	1	1	2	1	1	1	1
2	2	2	2	2	2	2	2	2	2
1	2	2	3	1	2	2	2	2	2
2	2	2	2	2	2	2	2	2	2

1	2	2	1	2	3	1	2	2	1
2	2	2	2	2	2	2	2	2	2
9	1	1	1	1	2	2	1	1	1
1	2	3	1	2	3	1	2	3	1
2	2	2	2	2	2	2	2	2	2
2	2	2	2	2	2	3	3	2	2
2	1	1	1	2	2	3	3	9	2
2	3	1	1	1	1	1	1	1	2
9	9	9	9	9	9	9	9	9	9
2	2	2	2	2	1	3	3	3	1
1	3	1	2	1	1	1	1	1	1
9	9	9	9	9	9	9	9	9	9
9	9	9	9	9	9	9	9	9	9
9	1	1	1	9	1	9	1	1	9
9	9	2	9	9	9	9	9	9	9
2	2	2	3	3	1	1	1	2	2
9	9	9	9	9	9	9	9	9	9
9	9	9	9	9	9	9	9	9	9
9	9	9	9	1	9	9	9	9	9
3	2	2	3	2	3	2	3	2	3
2	2	2	2	2	2	2	2	2	2
2	2	2	2	2	2	2	2	3	3
2	1	2	2	2	2	2	2	2	2
9	9	9	9	9	9	9	9	9	9
9	9	9	9	9	9	9	9	9	9
2	1	1	1	1	1	1	2	2	2
3	1	1	2	1	1	1	1	1	1
1	2	2	2	3	3	3	2	1	3
3	3	2	3	2	3	3	2	2	2
9	9	9	9	9	9	9	9	9	9
2	2	2	2	2	2	2	2	2	2
3	1	2	3	1	2	3	1	2	3
1	1	2	1	1	1	1	1	1	1
3	3	3	3	3	3	3	3	3	3
1	1	3	1	1	1	1	1	1	1
9	9	9	9	9	9	9	9	9	9
9	9	9	9	9	9	9	9	9	9
9	9	9	9	9	9	9	9	9	9
1	9	3	2	1	2	3	2	2	1
1	2	1	2	1	2	1	2	1	2
2	3	2	1	1	1	1	3	1	1
3	3	3	1	2	3	2	1	3	3
2	2	2	2	2	2	2	2	2	2
1	1	2	3	2	3	1	2	2	1
2	2	2	2	3	3	3	3	3	3
2	2	2	2	2	2	2	2	2	2
2	2	2	2	2	2	3	3	3	3

1	1	2	2	2	2	2	2	2	2
9	2	3	2	1	2	3	1	2	3
9	9	9	9	9	9	9	9	9	9
9	9	9	9	9	1	9	9	9	9
9	9	9	1	9	9	9	9	9	9
2	3	2	2	3	1	2	2	1	2
2	9	9	2	9	2	9	2	9	9
9	9	2	2	9	2	9	2	9	2
9	9	9	9	9	9	9	9	9	9
9	9	9	9	9	9	9	9	9	9
2	2	2	2	2	2	2	2	2	2
9	9	9	9	9	9	9	9	9	9
2	2	9	9	9	9	9	9	9	9
9	9	9	9	9	9	9	9	9	9
2	9	9	9	9	9	9	9	9	9
9	9	9	9	9	9	9	9	9	9
3	3	3	3	3	3	3	3	3	3
9	9	9	9	9	9	9	9	9	9
9	9	9	9	9	9	9	9	9	9
9	2	9	2	9	2	9	2	9	2
9	9	9	9	9	9	9	9	9	9
9	9	9	9	9	9	9	9	9	9
2	2	2	2	2	2	2	2	2	2
9	9	9	9	9	9	9	9	9	9
9	9	9	9	9	9	9	9	9	9
9	9	9	9	9	9	9	9	9	9
9	9	9	9	9	9	9	9	9	9
9	9	9	2	9	9	9	9	9	9
3	3	1	1	9	9	9	9	9	9
9	9	9	9	9	9	9	9	9	9
9	2	9	9	9	9	9	9	9	9
9	2	2	2	9	2	9	2	9	2
9	1	9	9	1	9	1	9	1	9
9	9	9	9	9	9	9	9	9	9
9	2	2	2	2	2	2	2	2	2
9	9	9	9	9	9	9	9	9	9
2	9	9	9	9	9	9	9	9	2
9	9	9	9	9	9	9	9	9	9
2	3	1	1	1	3	1	1	1	1
3	2	2	2	1	2	1	3	2	1
9	9	9	9	9	9	9	9	9	9
9	9	9	9	9	9	9	9	9	9
1	3	2	1	2	3	2	1	3	2
3	2	3	2	1	2	3	1	2	3
3	3	3	3	3	3	3	3	3	3
2	1	2	2	1	2	1	2	1	3
9	9	9	9	9	9	9	9	9	9

9	9	9	9	9	9	9	9	9	9
2	1	1	3	2	1	1	2	2	3
2	2	2	2	2	2	2	2	2	2
1	1	1	1	1	1	1	1	1	1
2	2	2	2	2	2	2	2	2	2
2	3	2	1	2	1	1	3	3	2
3	3	3	3	3	3	3	3	3	3
1	1	2	3	2	3	2	2	2	2
2	2	2	3	3	3	2	2	2	2
2	2	2	2	2	2	1	2	1	1
3	3	3	3	3	2	2	2	2	3
9	9	9	9	9	9	1	9	9	9
1	3	3	9	9	9	9	9	9	9
2	2	2	2	2	2	2	2	2	2
9	9	9	9	9	9	9	9	9	9
2	3	3	1	1	2	2	2	2	2
2	2	2	2	1	2	1	1	1	2
2	3	3	1	1	1	1	1	1	1
1	3	2	1	2	3	1	1	1	1
2	2	2	2	2	2	2	2	2	2
2	2	2	2	2	2	2	2	2	2
3	3	3	3	2	2	1	3	2	3
3	3	3	2	3	2	1	1	1	1
9	9	9	9	9	9	9	9	9	9
9	9	9	9	9	9	9	9	9	9
2	2	2	2	2	2	2	2	2	2
9	3	9	9	9	9	3	9	9	9
2	2	2	2	1	2	2	2	2	2
9	9	9	9	9	9	9	9	9	9
9	9	9	9	9	9	2	9	9	9
9	2	9	2	2	3	9	2	2	2
1	3	1	3	1	2	3	2	3	2
9	9	9	9	9	9	9	9	9	9
9	9	9	9	9	9	9	9	9	9
3	3	2	1	2	2	3	2	1	2
9	9	9	9	9	9	9	9	9	9
1	1	2	3	2	1	2	3	2	3
1	1	1	1	1	1	1	1	1	1
2	2	2	2	2	2	2	2	2	2
2	2	2	2	2	2	2	2	2	2
2	2	2	2	2	2	2	2	2	2
2	2	2	2	2	2	2	2	2	2
2	2	2	2	2	2	2	2	2	2
2	2	2	2	2	2	2	2	2	2
2	2	2	2	2	2	2	2	2	2
2	9	9	9	2	9	2	9	2	2
2	2	2	2	2	2	3	3	2	3

2	9	9	9	9	9	9	9	9	9
2	2	2	2	2	2	2	2	2	2
1	3	3	3	2	1	1	3	1	1
9	9	9	9	9	9	9	9	9	9
9	9	9	9	9	9	9	9	9	9
9	9	9	9	9	9	9	9	9	9
9	9	9	9	9	9	9	9	9	9
2	2	2	1	1	2	2	2	2	2
2	2	2	2	2	2	2	2	2	2
9	9	9	9	9	9	9	9	9	9
3	3	3	2	2	2	2	2	2	2
1	2	1	2	1	2	2	2	3	2
2	2	3	1	2	2	1	1	2	1
2	2	2	2	2	2	2	2	2	2
1	2	3	1	3	3	2	3	1	2
9	9	9	9	9	9	9	9	9	9
9	9	9	9	9	9	9	9	9	9
9	9	9	9	9	9	9	9	9	9
9	9	9	9	9	9	9	9	9	9
9	9	9	9	9	9	9	9	9	9
9	9	9	9	9	9	9	9	9	9
9	9	9	9	9	9	9	9	9	9
2	2	2	2	2	2	2	2	2	2
9	9	9	9	9	9	9	9	9	9
9	9	9	9	9	9	9	9	9	9
9	9	9	9	9	9	9	9	9	9
9	9	9	9	9	9	9	9	9	9
9	9	1	9	9	1	9	9	9	9
2	9	9	9	9	2	9	9	9	2
9	9	9	3	9	9	9	9	9	9
9	9	9	9	9	9	9	9	9	9
2	9	2	9	9	9	9	2	9	2
9	9	9	9	9	9	9	9	9	9
9	9	9	9	9	9	9	9	9	9
9	9	9	9	9	9	9	9	9	9
9	9	9	9	9	9	9	9	9	9
9	9	9	9	9	9	9	9	9	9
9	9	9	9	9	9	9	9	9	9
9	9	9	9	9	9	9	9	9	9
9	9	9	9	9	9	9	9	9	9
2	2	2	2	2	2	2	2	2	2
9	9	9	9	9	9	9	9	9	9
9	9	9	9	9	9	9	9	9	9
9	9	9	9	9	9	9	9	9	9
9	9	9	9	9	9	9	9	9	9
9	9	9	9	9	9	9	9	9	9
9	9	9	9	9	9	9	9	9	9

9	9	9	9	9	9	9	9	9	9
9	9	9	9	9	9	9	9	9	9
9	9	9	9	9	9	9	9	9	9
9	9	9	9	9	9	9	9	9	9
9	9	9	9	9	9	9	9	9	9
9	9	9	9	9	9	9	9	9	9
9	9	9	9	9	9	9	9	9	9
9	9	9	9	9	9	9	9	9	9
9	9	9	9	9	9	9	9	9	9
9	9	9	9	9	9	9	9	9	9
9	9	9	9	9	9	9	9	9	9
9	9	9	9	9	9	9	9	9	9
9	9	9	9	9	9	9	9	9	9
9	9	9	9	9	9	9	9	9	9
9	9	9	9	9	9	9	9	9	9
9	9	9	9	9	9	9	9	9	9
9	9	9	9	9	9	9	9	9	9
2	2	2	2	2	2	2	2	2	2
9	9	9	9	9	9	9	9	9	9
2	2	2	2	2	2	2	2	2	2
9	9	9	9	9	9	9	9	9	9
9	9	9	9	9	9	9	9	9	9
9	9	9	9	9	9	9	9	9	9
9	9	9	9	9	9	9	9	9	9
9	9	9	9	9	9	9	9	9	9
9	9	9	9	9	9	9	9	9	9
9	9	9	9	9	9	9	9	9	9
3	2	9	9	2	9	3	9	9	2
9	9	9	9	9	9	9	9	9	9
9	9	9	9	9	9	9	9	9	9
1	1	1	1	1	1	1	1	1	1
1	1	2	2	3	2	9	1	1	3
9	9	9	9	9	9	9	9	9	9
2	2	2	2	2	1	1	1	1	2
9	9	9	9	9	9	9	9	9	9
9	9	9	9	9	9	9	9	1	9
9	9	9	9	9	9	9	9	9	9
9	9	9	9	9	9	9	9	9	9
1	1	1	1	1	1	1	1	1	1
9	9	9	9	9	9	9	9	9	9
9	9	9	9	9	9	9	9	9	9
9	9	9	9	9	9	9	9	9	9
2	2	2	2	2	2	3	3	3	3
9	9	9	9	9	9	9	9	9	9
9	1	9	1	2	9	1	9	9	9
9	9	9	9	9	9	9	9	9	9
9	9	9	9	9	9	9	9	9	9

9	9	9	2	9	9	9	9	9	9
9	9	9	9	9	9	9	9	9	9
3	9	9	1	9	2	3	9	1	9
9	9	9	9	9	9	9	9	9	9
9	9	9	9	9	9	9	9	9	9
9	9	9	9	9	9	9	9	9	9
9	9	9	9	9	9	9	9	9	9
9	9	9	9	9	9	9	9	9	9
2	9	9	9	2	9	9	9	9	9
2	2	2	9	9	9	9	9	9	9
9	9	9	9	9	9	9	9	9	9
9	9	9	9	9	9	9	9	9	9
9	9	9	9	9	9	9	9	9	9
9	9	9	9	9	9	9	9	9	9
2	2	2	2	2	2	2	2	2	2
3	1	1	1	1	1	2	1	1	2
9	9	9	9	9	9	9	9	9	9
2	2	2	2	2	2	2	2	2	2
2	2	2	2	2	2	2	2	2	2
9	9	9	2	9	9	3	9	9	9
9	9	9	9	9	9	9	9	9	9
1	9	9	9	9	9	9	9	9	9
2	1	2	3	1	2	1	3	3	2
3	1	2	3	1	2	3	1	2	3
2	3	2	1	1	1	1	2	1	2
2	2	2	2	2	2	1	2	2	2
1	1	1	1	1	3	3	1	1	3
1	3	2	2	2	2	2	2	2	2
2	2	2	2	2	2	2	2	2	2
1	1	1	1	1	1	1	1	1	1
1	1	1	9	9	9	9	9	9	9
9	9	9	9	9	9	9	9	9	9
9	9	9	9	9	9	9	9	9	9
9	9	9	9	9	9	9	9	9	9
9	9	9	9	9	9	9	9	9	9
9	9	9	9	9	9	9	9	9	9
9	9	9	9	9	9	9	9	9	9
9	9	9	2	9	9	2	9	9	9
9	9	9	9	9	9	9	9	9	9
9	9	2	9	2	9	2	9	9	2
9	9	9	9	9	9	9	9	9	9
2	2	2	2	2	2	2	2	2	2
9	9	9	9	9	9	9	9	9	9
9	9	9	9	9	9	9	9	9	9
9	9	9	9	9	9	9	9	9	9
2	2	2	2	2	2	2	2	2	2
2	2	2	3	2	3	2	3	3	2

1	9	2	1	1	1	1	2	1	1
2	2	2	2	2	2	2	1	1	1
1	2	2	1	1	1	1	1	1	1
1	3	2	2	3	2	3	2	1	3
3	3	3	3	3	3	3	3	3	3
1	2	1	2	1	1	1	2	2	2
1	3	3	2	1	1	1	1	1	1
3	3	3	3	3	3	3	3	3	3
2	2	2	2	2	2	2	2	2	2
2	2	2	2	2	2	2	2	2	2
9	9	9	2	9	9	2	9	9	9
9	9	1	9	2	9	1	9	1	9
2	2	2	2	2	2	2	2	2	2
3	2	2	2	2	2	3	1	3	9
2	2	1	3	2	3	3	2	2	1
2	2	2	2	2	2	2	2	2	2
3	2	3	2	3	1	2	1	3	2
2	2	2	2	2	2	2	2	2	2
9	9	9	9	9	9	9	9	9	9
2	2	2	2	2	2	1	3	2	2
2	2	3	3	2	3	3	3	3	2
3	2	1	1	2	3	1	1	2	3
1	2	3	2	2	3	1	2	1	2
2	1	2	2	2	2	1	2	1	1
1	2	2	1	1	1	1	1	1	2
2	2	2	2	2	2	2	1	1	1
2	3	1	1	1	2	1	1	1	2
3	2	2	2	2	2	2	2	2	3
2	2	2	1	1	2	1	3	1	2
2	3	1	1	3	2	1	3	1	2
3	3	3	2	2	3	3	2	2	3
3	2	2	2	2	2	2	2	2	2
3	3	3	3	3	3	3	3	3	3
2	2	2	2	2	2	2	2	2	3
9	9	9	9	9	9	9	9	9	9
2	2	3	3	3	3	2	2	2	3
2	1	2	1	1	2	2	2	1	1
2	2	2	2	2	2	2	2	2	2
2	2	1	1	2	1	1	2	3	3
9	9	9	9	9	9	9	9	9	9
2	2	2	2	2	2	2	2	2	2
9	9	9	9	9	9	9	9	1	9
3	3	2	1	3	3	2	2	1	2
2	2	2	2	2	2	2	2	2	2
3	1	2	1	1	1	2	3	1	3
2	3	2	3	2	3	2	3	3	2
1	2	3	2	3	2	2	2	2	3

9	9	9	9	9	9	9	9	9	9
3	3	3	3	3	3	3	3	3	3
3	1	1	1	1	1	3	3	1	3
2	3	2	1	1	9	1	2	2	3
2	3	3	3	2	1	1	2	1	1
3	3	2	1	1	1	1	1	1	9
2	2	2	2	2	2	2	2	2	2
2	2	2	2	2	3	3	9	1	3
3	3	2	2	2	1	1	1	3	1
2	2	2	2	2	2	2	1	1	2
2	2	2	3	3	3	2	2	3	3
9	2	9	2	2	2	2	2	2	2
9	9	9	9	9	9	9	9	9	9
2	2	2	2	3	3	2	3	2	2
2	2	2	1	1	1	2	2	1	2
2	2	2	2	2	2	2	2	2	2
2	2	2	2	2	3	3	3	1	1
2	2	2	2	2	2	2	2	2	2
2	2	2	2	2	2	2	2	2	2
9	9	9	9	9	9	9	9	9	9
1	2	2	2	2	2	1	2	1	2
2	1	2	2	1	1	1	1	2	2
3	3	2	3	2	3	2	2	2	2
1	2	3	2	3	2	2	2	9	1
3	3	3	3	3	3	3	3	3	3
2	3	3	2	2	1	1	2	3	2
9	9	9	9	9	9	9	9	9	9
2	2	2	2	2	2	2	2	2	2
2	2	2	2	2	2	2	2	2	2
2	2	2	2	2	2	2	2	2	2
3	2	3	2	3	1	1	2	2	1
9	9	9	9	9	9	9	9	9	9
9	9	9	9	9	9	9	9	9	9
2	2	2	2	1	1	1	1	1	1
1	3	3	1	2	2	1	2	3	3
9	9	9	9	9	9	9	9	9	9
2	2	2	3	3	3	3	3	3	3
2	2	2	2	2	1	1	1	1	2
3	3	3	1	2	1	2	1	3	2
1	2	2	2	2	1	1	2	2	1
9	9	9	9	9	9	9	9	9	9
2	2	2	2	2	2	2	2	2	2
2	2	2	2	2	2	2	2	2	2
2	2	2	2	2	2	2	2	2	2
2	2	2	2	2	2	2	2	2	2
2	2	2	2	2	1	1	2	3	2
2	2	2	2	2	2	2	2	2	2

3	2	1	3	2	2	1	1	3	3
2	2	2	2	2	2	2	2	2	2
2	2	2	2	2	2	2	2	2	2
2	2	2	2	2	2	2	2	2	2
2	2	2	2	2	1	2	2	2	2
2	2	2	2	2	2	2	2	2	2
2	2	2	2	2	2	2	2	2	2
2	2	2	2	2	2	2	2	2	2
2	2	2	2	2	2	2	2	2	2
9	9	9	9	9	9	9	9	9	9
3	3	3	3	3	2	1	1	1	1
3	1	2	2	1	3	1	1	2	3
2	2	2	1	2	2	2	2	1	1
2	1	2	1	2	2	2	1	2	1
3	3	3	3	3	3	2	2	2	2
2	1	2	1	9	1	2	1	1	2
9	9	9	9	9	9	9	9	9	9
9	2	9	9	9	9	2	9	9	9
9	9	9	9	9	9	9	9	9	9
2	2	2	2	2	2	2	2	2	2
2	2	2	2	2	2	3	9	2	2
9	9	9	9	9	9	9	9	9	9
9	9	9	9	9	9	9	9	9	9
2	2	2	2	2	2	2	2	2	2
1	2	2	1	1	2	2	2	1	1
2	2	2	2	2	2	2	2	2	2
2	2	1	3	3	1	1	2	3	1
3	3	2	3	3	3	3	2	2	2
1	2	1	1	2	1	3	1	2	1
2	2	3	2	1	2	3	1	2	3
9	9	9	9	9	9	9	9	9	9
1	1	3	1	2	1	3	1	2	1
3	2	1	3	2	1	3	1	3	2
2	2	2	2	2	3	3	3	2	2
2	2	2	2	2	2	2	2	2	2
2	2	2	2	2	2	2	2	2	2
2	9	9	9	9	9	9	9	9	9
2	2	2	2	2	2	2	2	2	2
2	2	2	2	2	2	2	2	2	2
2	2	3	3	3	3	3	3	3	3
3	1	1	3	1	1	1	1	1	1
2	2	2	3	1	1	1	1	1	1
2	2	2	2	2	2	2	2	2	2
2	2	2	2	2	2	2	2	2	2
3	1	2	2	2	3	2	3	1	2
1	3	1	3	1	1	2	1	2	2
3	2	3	2	2	2	3	3	3	3

2	3	1	1	2	2	2	1	2	1
2	2	2	2	2	2	2	2	2	2
9	1	9	1	3	2	9	2	2	9
2	2	2	2	2	2	2	2	2	2
3	2	3	1	2	3	3	3	3	2
2	2	2	2	2	2	2	2	2	2
9	9	9	9	1	9	9	9	9	9
3	3	3	3	3	3	3	3	3	3
9	9	9	9	9	9	9	9	9	9
2	3	3	3	3	2	2	2	2	2
2	2	2	2	2	2	2	2	2	2
3	2	9	1	1	2	1	1	1	1
2	2	1	1	2	3	3	2	2	1
9	9	9	9	9	9	9	9	1	9
9	9	1	9	1	9	9	9	9	9
9	9	9	9	9	9	9	9	9	9
1	2	2	1	2	2	1	1	1	1
1	3	1	1	1	1	1	1	1	1
1	3	9	2	2	2	1	2	1	1
2	3	2	1	1	2	1	1	1	1
2	2	2	2	2	2	2	2	2	9
1	3	1	3	1	3	1	3	1	1
2	2	1	9	2	2	1	2	1	1
2	2	2	2	2	2	2	2	2	2
1	3	1	1	1	1	1	1	1	1
2	2	2	2	2	2	2	2	2	2
2	2	2	2	2	2	2	2	2	2
2	2	2	2	2	2	2	2	2	2
2	3	2	2	1	2	3	2	3	9
3	3	3	3	3	3	3	3	3	3
2	1	2	1	2	2	1	2	3	1
2	1	2	3	2	2	3	1	2	1
2	2	2	2	2	2	2	3	3	3
2	2	2	2	2	2	2	2	2	2
1	1	2	2	2	2	2	1	2	2
2	2	2	3	2	2	2	3	2	1
1	1	1	1	1	1	1	1	1	1
2	2	2	2	2	2	2	2	2	2
2	3	1	3	1	3	2	3	2	3
2	2	2	2	2	2	2	2	2	2
9	9	9	1	3	1	3	1	1	1
1	2	1	2	2	2	2	2	2	2
2	2	2	2	2	2	2	2	2	2
1	3	2	1	1	1	1	2	1	2
2	2	2	2	2	2	2	2	2	2
2	3	1	2	2	2	3	2	2	2
2	2	2	2	2	2	2	2	2	2

1	2	3	2	1	3	1	2	2	2
3	3	1	1	1	2	1	2	1	2
3	2	3	2	3	2	3	2	3	2
2	2	2	2	2	2	2	2	2	2
3	1	3	3	1	9	2	1	2	1
9	9	9	9	9	9	9	9	9	9
2	2	3	2	2	2	3	2	2	2
9	9	9	9	9	9	9	9	9	9
9	9	9	9	9	9	9	9	9	9
1	3	3	3	3	3	3	3	3	3
9	9	9	9	9	9	9	9	9	9
2	9	9	9	9	9	9	9	9	9
9	9	9	9	9	9	9	9	9	9
9	9	9	9	9	9	9	9	9	9
1	2	3	2	2	2	2	1	1	1
9	9	9	9	9	9	9	9	9	9
9	9	9	9	9	9	9	9	9	9
9	9	9	9	9	9	9	9	9	9
9	9	9	9	9	9	9	9	9	9
9	9	9	9	9	9	9	9	9	9
9	9	9	9	9	9	9	9	9	9
2	2	2	2	2	2	2	2	2	2
2	2	2	2	2	2	2	2	2	2
3	3	2	2	2	3	1	3	2	1
2	2	1	2	1	1	3	3	3	2
3	2	2	2	3	2	2	1	1	1
3	1	1	1	3	3	3	1	2	2
9	9	9	9	9	9	9	9	9	9
2	2	2	2	2	2	2	3	2	2
3	9	1	1	1	1	3	3	1	1
1	3	1	3	3	2	1	3	3	3
9	9	9	9	9	9	9	9	9	9
2	2	2	2	2	2	2	2	2	2
9	9	9	9	9	9	9	9	9	9
9	9	9	9	9	9	9	9	9	9
1	2	2	1	1	2	3	2	1	1
1	1	1	1	1	1	1	1	1	1
9	9	9	9	9	9	9	9	9	9
9	9	9	9	9	9	9	9	9	9
2	2	2	2	2	2	2	2	2	2
9	9	2	9	2	9	2	2	2	2
2	2	2	2	2	2	2	2	2	2
3	3	3	3	3	3	3	3	3	3
9	2	9	9	9	9	9	2	9	9
1	1	1	1	1	1	1	2	2	2
2	2	2	2	2	2	2	2	2	2
3	1	9	3	1	9	3	1	3	1

9	9	9	9	9	9	9	9	9	9
1	9	9	9	3	1	9	3	1	9
3	1	9	9	9	1	9	9	9	1
2	2	2	2	2	2	2	2	2	2
2	1	2	1	9	3	2	1	9	3
1	3	1	3	2	1	9	9	2	1
9	9	9	9	9	9	9	9	9	9
9	9	9	9	9	9	9	9	9	9
2	9	9	2	9	2	9	2	9	2
2	2	2	2	2	2	2	2	2	2
9	9	9	9	9	9	9	9	9	9
9	9	2	9	9	2	9	9	2	9
2	2	2	2	2	2	2	2	2	2
9	9	9	9	9	9	9	9	9	9
2	2	2	2	2	2	2	2	2	2
9	9	9	9	9	9	9	9	9	9
9	9	9	9	9	9	9	9	9	9
9	9	9	9	9	9	9	9	9	9
9	9	9	9	9	9	9	9	9	9
9	9	9	9	9	9	9	9	9	9
2	2	2	2	2	2	2	2	2	2
2	2	2	2	2	2	2	2	2	2
2	2	3	2	1	2	1	3	9	2
1	1	1	2	3	1	1	2	1	2
2	2	2	2	3	3	3	3	3	3
2	2	2	2	2	2	2	2	2	2
1	1	1	1	1	1	1	1	1	1
3	2	2	3	3	1	1	1	2	2
3	1	1	1	1	2	3	1	2	3
2	2	2	2	2	2	2	2	2	2
2	2	2	2	2	3	3	3	2	2
2	3	3	2	2	3	3	2	2	2
2	2	2	2	2	2	2	2	2	1
9	9	9	9	9	9	9	9	9	9
1	2	1	1	2	1	1	2	1	1
3	3	3	3	3	3	3	3	3	3
2	2	2	2	2	2	2	2	2	2
1	2	1	2	1	2	2	2	1	1
2	2	2	2	2	2	2	2	2	2
3	3	2	3	3	2	2	2	2	2
9	3	2	1	1	1	2	2	1	1
9	9	9	9	9	9	9	9	9	9
2	2	2	2	2	2	2	2	2	2
9	9	9	9	9	9	9	9	9	9
1	2	1	2	2	1	2	1	2	1
2	2	2	2	2	2	2	2	2	2
2	3	2	2	2	2	2	2	2	2

2	2	2	1	3	2	1	1	1	1
3	2	9	1	1	2	1	1	3	1
2	2	2	2	2	2	2	2	2	2
2	2	2	2	2	2	2	2	2	2
1	1	2	2	2	2	2	2	2	2
1	1	9	2	2	2	2	2	2	2
1	1	1	1	1	1	1	1	1	1
3	1	1	2	1	2	1	2	1	2
3	3	3	3	3	3	3	3	3	3
3	3	1	1	1	2	1	1	1	1
2	2	2	2	2	2	2	2	2	2
2	2	2	2	2	2	2	1	1	1
2	2	2	2	2	2	1	1	1	1
2	1	1	1	1	1	1	1	1	1
9	9	9	9	9	9	9	9	9	9
1	9	9	1	1	1	2	2	2	2
1	1	1	1	2	2	2	2	9	1
2	2	1	1	1	1	2	1	1	1
9	9	9	9	9	9	9	9	9	9
2	2	2	2	2	2	2	2	2	2
2	2	1	1	2	1	2	1	1	1
2	2	2	2	2	2	2	2	2	2
9	9	9	9	9	9	9	9	9	9
2	2	2	2	2	2	2	2	2	2
9	9	9	9	9	9	9	9	9	9
1	1	1	1	2	2	1	1	1	1
1	1	2	2	1	2	1	1	1	1
1	9	1	1	1	2	2	2	2	1
2	2	9	2	2	2	9	2	9	2
2	2	2	2	2	9	2	2	9	2
2	2	2	2	2	9	2	2	2	2
2	2	2	2	2	2	2	2	2	2
2	2	2	2	2	2	2	2	2	2
1	1	1	1	1	1	1	1	1	1
2	2	2	2	2	2	2	2	2	2
9	9	9	9	9	9	9	9	9	9
1	1	1	1	2	2	2	2	1	1
1	1	1	2	2	1	1	1	1	1
9	9	9	9	9	9	9	9	9	9
2	2	2	2	2	2	2	2	2	2
2	2	2	2	9	2	9	9	9	9
9	9	9	9	9	9	9	9	9	9
2	2	2	2	2	2	2	2	2	2
2	2	2	2	2	2	2	2	2	2
1	1	1	1	2	1	1	2	3	2
2	2	2	2	9	2	2	9	2	9
1	9	9	9	9	9	9	9	9	9

1	2	1	2	2	1	1	1	1	1
2	9	9	2	2	9	9	2	9	2
1	1	1	1	1	1	1	1	1	1
1	1	1	2	1	2	1	1	1	1
2	2	2	2	2	2	2	2	2	2
2	2	2	2	2	2	2	2	2	2
1	1	1	1	1	1	1	1	1	1
2	2	2	2	2	2	2	2	2	2
9	9	9	9	9	9	9	9	9	9
9	1	9	1	9	9	2	2	9	2
9	2	9	2	9	2	2	9	2	2
9	2	9	2	2	2	2	9	2	9
9	9	9	9	9	9	9	9	9	9
9	9	9	9	9	9	9	9	9	9
9	9	9	9	9	9	9	9	9	9
9	9	9	9	9	9	9	9	9	9
9	2	2	9	9	9	9	9	9	9
9	9	9	9	9	9	9	9	9	9
2	2	2	2	2	2	2	2	2	2
3	3	1	1	1	1	3	1	1	2
3	3	1	1	1	1	3	1	1	2
3	3	1	1	1	1	3	1	1	2
3	3	1	1	1	1	1	1	1	2
3	1	3	1	1	1	3	1	1	2
3	3	1	1	1	1	3	1	1	2
3	3	1	1	1	1	1	1	1	2
3	3	1	1	1	1	3	1	1	2
3	1	3	1	1	1	3	1	1	2
9	3	1	1	1	1	3	1	1	2
3	3	1	1	1	1	3	1	1	2
3	3	1	1	1	1	3	1	1	1
3	3	1	1	1	3	1	1	1	2
3	3	9	1	1	1	3	1	1	2
3	3	1	1	1	1	3	1	1	2
3	3	1	1	1	1	3	1	1	2
3	3	3	1	1	3	3	1	1	2
3	3	3	1	1	1	3	1	1	2
3	3	1	1	1	1	3	1	1	2
3	3	1	1	9	1	3	1	1	2
3	3	1	1	1	1	3	1	1	2
3	3	1	1	1	1	3	1	1	2
3	3	1	1	1	1	3	1	1	2
3	3	1	1	1	1	3	1	1	2
3	3	1	1	1	3	1	1	1	2
9	9	9	9	9	9	9	9	9	9
9	9	9	9	9	9	9	9	9	9
9	9	9	9	9	9	9	9	9	9

9	9	9	9	9	9	9	9	9	9
9	9	9	9	9	9	9	9	9	9
9	9	9	9	9	9	9	9	9	9
9	9	9	9	9	9	9	9	9	9
9	9	9	9	9	9	9	9	9	9
9	9	9	9	9	9	9	9	9	9
9	9	9	9	9	9	9	9	9	9
9	9	9	9	9	9	9	9	9	9
9	9	9	9	9	9	9	9	9	9
9	9	9	9	9	9	9	9	9	9
9	9	9	9	9	9	9	9	9	9
9	9	9	9	9	9	9	9	9	9
9	9	9	9	9	9	9	9	9	9
9	9	9	9	9	9	9	9	9	9
9	9	9	9	9	9	9	9	9	9
9	9	9	9	9	9	9	9	9	9
9	9	9	9	9	9	9	9	9	9
9	9	9	9	9	9	9	9	9	9
9	9	9	9	9	9	9	9	9	9
3	3	1	1	1	1	3	1	1	2
3	3	1	1	1	1	3	1	1	2
3	3	1	1	1	1	3	1	1	2
3	3	1	1	1	1	3	1	1	2
3	3	1	1	3	1	3	1	1	2
3	3	3	1	3	1	3	1	1	2
2	3	1	1	1	1	3	1	1	2
3	3	1	1	1	1	3	1	1	2
3	3	1	1	1	1	1	3	1	2
3	3	1	1	1	1	3	1	1	2
3	1	1	1	1	1	3	1	1	2
3	3	1	1	1	3	1	1	2	1
3	3	1	1	1	1	1	1	1	2
3	3	1	1	1	1	3	1	1	2
3	3	1	1	1	3	3	1	1	2
3	9	1	1	1	1	3	1	1	2
9	9	9	9	9	9	9	9	9	9
3	3	1	1	1	1	3	1	1	2
3	3	1	1	1	1	1	1	1	2
3	3	1	1	1	1	1	1	1	2
3	3	1	1	1	9	3	1	1	2
3	1	3	1	1	1	3	1	1	2
3	3	1	1	1	1	3	1	1	2
3	9	1	1	1	1	3	1	1	2
3	3	1	1	1	1	3	1	1	2
9	3	1	1	1	3	1	1	1	2
9	9	1	1	1	1	1	1	1	2
3	3	1	1	1	1	1	1	1	2

3	1	3	1	1	3	1	1	1	2
3	3	1	1	1	1	1	1	2	2
3	3	1	1	1	1	3	1	1	2
3	3	1	1	1	1	3	1	1	2
3	3	1	1	1	1	3	1	1	2
3	3	1	1	1	1	3	1	1	2
3	3	3	1	1	1	1	1	1	2
3	3	1	1	1	3	3	1	1	2
3	3	1	1	1	1	3	1	1	2
3	3	1	1	1	1	3	1	1	2
3	3	1	1	1	3	3	1	1	2
9	3	1	1	1	1	3	1	1	2
3	3	1	1	1	1	3	1	1	2
3	3	1	1	1	1	3	1	1	2
3	9	1	1	1	1	3	1	1	2
3	3	1	1	1	1	3	1	1	2
3	3	1	1	1	1	3	1	1	1
3	3	1	1	1	1	3	1	1	2
3	3	1	1	1	1	3	2	1	2
3	3	1	1	1	1	3	1	1	2
3	3	1	1	1	1	3	1	1	2
3	1	1	1	1	1	3	1	1	2
3	3	3	1	1	3	1	1	1	2
3	3	1	1	1	1	3	1	1	2
3	3	1	1	1	1	3	1	1	2
3	9	1	1	1	1	3	1	1	2
3	3	1	1	1	1	3	1	1	2
3	3	3	1	1	1	9	1	1	2
3	3	1	1	3	1	3	1	1	2
3	3	1	1	1	1	3	1	1	9
3	3	1	1	1	1	3	1	1	2
3	3	3	1	1	1	1	3	1	2
9	9	9	9	9	9	9	9	9	9
3	9	1	1	1	1	3	1	1	2
9	9	9	9	9	9	9	9	9	9
3	3	1	1	3	1	3	1	1	2
3	3	1	1	1	1	3	9	3	1
3	3	1	1	1	1	3	1	1	2
3	3	1	1	1	1	3	1	1	2
3	3	1	1	1	3	1	3	1	2
3	1	1	1	3	3	1	1	1	2
3	3	1	1	1	1	1	1	1	2
3	3	1	1	1	1	3	1	1	2
3	3	1	1	3	1	3	1	1	2
3	3	1	1	1	1	3	1	1	2
3	3	1	1	1	1	3	1	1	2
3	3	1	1	1	1	3	1	1	2

3	3	1	1	1	1	3	1	1	2
3	3	1	1	1	1	3	1	1	2
3	3	1	1	1	1	3	1	1	2
3	3	1	1	1	1	3	1	1	2
3	3	1	1	1	3	1	1	2	1
3	3	1	1	1	1	3	1	1	2
3	3	1	1	1	1	3	1	1	2
3	3	1	1	3	3	3	1	2	2
3	3	1	1	1	1	3	1	1	2
3	3	1	1	1	1	3	1	1	2
3	3	1	1	1	1	3	1	1	2
3	3	1	1	1	1	3	1	1	2
3	3	1	1	1	1	3	1	1	2
3	3	1	1	1	1	3	1	1	2
3	3	9	9	9	9	9	9	9	9
3	3	1	1	1	1	3	1	1	2
3	3	1	1	1	1	1	1	1	2
3	3	1	1	1	1	1	1	1	2
3	3	1	1	1	1	1	1	1	2
9	9	9	9	9	9	9	9	9	9
3	3	1	1	1	1	3	1	1	2
3	3	1	1	1	3	1	1	1	2
3	3	1	1	1	1	3	1	1	2
9	3	1	1	1	1	3	1	1	2
2	2	1	9	9	9	9	1	1	1
3	9	3	1	1	1	3	1	9	1
3	3	1	1	1	1	3	1	1	2
3	3	1	1	1	1	3	1	1	2
3	3	1	1	1	1	3	1	2	1
1	3	1	1	1	1	1	1	1	3
3	3	1	1	1	1	3	1	1	2
3	3	1	1	1	1	3	1	1	2
3	1	1	1	1	3	1	1	2	1
3	3	1	1	1	1	3	1	1	2
3	3	1	1	1	1	3	1	1	2
3	3	1	1	1	9	3	1	1	2
3	3	1	1	1	1	3	1	1	2
3	3	1	1	1	1	3	1	1	2
3	3	1	1	1	1	3	1	1	2
3	3	1	1	1	1	3	1	1	2
3	3	1	1	1	1	3	1	1	2
9	9	9	9	9	9	9	9	9	9
3	3	1	1	1	1	3	1	1	2
3	3	1	1	1	1	3	1	1	2
3	1	1	1	1	1	3	1	1	2
3	3	1	1	1	1	3	1	1	2
3	3	1	1	1	1	3	1	1	2

3	3	1	1	1	1	3	1	1	2
3	3	1	1	1	1	3	1	1	2
3	3	1	1	1	1	3	1	1	2
3	1	1	1	3	1	3	1	1	2
3	3	1	1	1	1	3	1	1	2
3	3	1	1	3	1	3	1	1	2
3	3	1	1	3	3	3	1	1	2
3	3	1	1	1	1	3	1	1	2
3	3	1	1	3	3	3	1	1	2
1	1	1	1	1	3	1	1	1	1
3	3	1	1	1	1	3	1	1	2
3	3	1	1	1	1	3	1	1	2
3	3	1	1	1	1	3	1	1	2
3	3	1	1	1	1	3	1	1	2
1	3	9	1	1	1	3	1	1	2
3	3	1	1	1	1	3	1	1	2
3	3	1	1	1	1	3	1	1	2
3	3	1	1	1	1	3	1	1	2
3	3	1	1	1	1	3	1	1	2
3	3	1	1	1	1	1	1	1	2
3	3	1	1	1	1	3	1	1	2
3	3	3	1	1	1	3	1	1	2
3	3	1	1	1	1	3	1	1	2
3	3	1	1	1	1	3	1	1	2
3	3	1	1	1	1	1	1	1	2
3	3	1	1	1	1	3	1	1	2
3	3	1	1	1	1	1	1	1	2
9	9	9	9	9	9	9	9	9	9
9	9	9	9	9	9	9	9	9	9
9	9	9	9	9	9	9	9	9	9
9	9	9	9	9	9	9	9	9	9
9	9	9	9	9	9	9	9	9	9
9	9	9	9	9	9	9	9	9	9
9	9	9	9	9	9	9	9	9	9
9	9	9	9	9	9	9	9	9	9
9	9	9	9	9	9	9	9	9	9
9	9	9	9	9	9	9	9	9	9
9	9	9	9	9	9	9	9	9	9
9	9	9	9	9	9	9	9	9	9
9	9	9	9	9	9	9	9	9	9
9	9	9	9	9	9	9	9	9	9
9	9	9	9	9	9	9	9	9	9
9	9	9	9	9	9	9	9	9	9
9	9	9	9	9	9	9	9	9	9
9	9	9	9	9	9	9	9	9	9
9	9	9	9	9	9	9	9	9	9
9	9	9	9	9	9	9	9	9	9

9	9	9	9	9	9	9	9	9	9
9	9	9	9	9	9	9	9	9	9
9	9	9	9	9	9	9	9	9	9
9	9	9	9	9	9	9	9	9	9
9	9	9	9	9	9	9	9	9	9
9	9	9	9	9	9	9	9	9	9
9	9	9	9	9	9	9	9	9	9
9	9	9	9	9	9	9	9	9	9
9	9	9	9	9	9	9	9	9	9
9	9	9	9	9	9	9	9	9	9
9	9	9	9	9	9	9	9	9	9
9	9	9	9	9	9	9	9	9	9
9	9	9	9	9	9	9	9	9	9
1	1	2	1	1	2	3	1	2	1
2	2	2	2	2	2	2	2	2	2
2	2	2	2	2	2	3	2	2	2
1	2	3	2	1	3	1	1	3	3
1	2	1	3	2	1	1	1	1	1
3	3	3	1	1	1	1	1	1	3
1	2	2	1	3	2	1	1	1	3
3	3	1	1	1	1	1	1	1	2
1	1	3	2	1	2	3	1	2	3
9	9	9	9	9	9	9	9	9	9
3	2	2	2	3	3	3	2	2	2
1	9	9	9	9	9	9	9	9	9
3	3	3	3	3	2	2	2	3	3
9	9	9	9	9	9	9	9	9	9
1	3	3	3	3	3	3	1	1	1
2	3	1	2	1	1	1	1	2	3
3	3	1	1	1	2	2	1	2	2
3	3	3	3	3	3	3	3	3	3
3	1	2	3	1	2	3	2	3	1
9	9	9	9	9	9	9	9	9	9
3	1	2	1	2	3	2	3	1	3
1	3	1	1	1	1	1	1	1	1
9	9	9	9	9	9	9	9	9	9
2	2	2	2	2	2	2	2	2	2
2	1	1	1	3	3	2	2	2	1
1	3	3	3	2	1	1	1	1	1
1	1	1	1	1	3	2	1	1	1
9	9	9	9	9	9	9	9	9	9
9	9	9	9	9	9	9	9	9	9
2	1	2	2	2	2	2	2	2	2
9	1	9	1	1	1	9	1	1	1
9	9	9	9	9	9	9	9	9	9
9	9	9	9	9	9	9	9	9	9
2	3	3	2	2	2	2	2	2	2

1	9	9	9	9	9	9	9	9	9
9	9	9	9	9	9	9	9	9	9
2	2	3	2	3	2	3	2	1	2
2	2	2	2	2	1	2	1	2	1
1	2	2	1	1	1	1	1	1	1
9	9	9	9	9	9	9	9	9	9
2	2	2	2	2	2	2	2	2	2
2	2	2	2	2	2	2	2	2	2
2	2	2	2	2	2	2	2	2	2
3	3	1	1	1	1	2	2	3	3
2	9	2	1	2	2	1	3	3	3
9	9	9	9	9	9	9	9	9	9
9	9	9	9	9	9	9	9	9	9
2	2	2	2	2	2	2	2	2	2
2	2	2	2	2	2	2	2	2	2
1	2	3	1	1	2	1	3	1	2
1	1	3	2	1	2	3	1	2	3
2	2	2	2	2	2	2	2	2	2
1	3	2	2	1	1	1	3	3	2
3	9	9	9	9	9	9	9	9	9
3	2	2	2	2	2	3	2	2	2
2	2	2	2	2	2	2	2	2	2
2	2	2	2	2	2	2	2	2	2
2	2	2	2	2	2	2	2	2	2
2	2	2	2	2	2	2	2	2	2
2	1	1	3	3	2	2	1	1	3
2	2	2	2	2	2	2	2	2	2
2	2	2	2	2	2	2	2	2	2
1	2	2	2	2	2	2	2	2	2
1	2	2	1	1	1	1	1	1	2
3	3	3	1	1	2	2	2	1	1
2	3	2	1	2	3	1	1	1	1
1	2	1	1	1	2	1	1	1	1
1	3	3	1	1	1	1	2	1	1
2	2	1	1	2	1	2	1	2	1
2	3	1	3	2	3	2	3	2	2
1	2	2	2	2	2	2	2	2	2
2	2	2	2	2	2	2	2	2	2
9	9	9	9	9	9	9	9	9	9
1	3	3	1	1	1	2	1	3	2
1	2	2	2	2	2	2	2	2	1
1	2	2	2	2	2	2	1	2	2
3	1	2	3	1	2	3	1	2	3
2	3	2	2	2	2	1	2	3	2
1	2	1	1	1	1	1	1	1	1
1	2	2	2	3	3	3	2	9	3
3	3	2	1	2	2	1	1	1	2

2	2	2	2	2	2	2	1	1	1
2	2	2	3	2	2	3	2	3	9
2	2	2	2	2	2	2	2	2	2
2	3	2	3	2	2	3	2	9	3
1	1	1	2	3	2	2	2	2	2
1	1	1	1	1	2	2	2	2	2
1	3	3	1	3	3	1	3	2	2
3	2	2	1	2	2	2	1	3	3
2	2	2	2	2	2	2	2	2	2
2	1	2	3	1	2	3	2	2	3
2	2	2	2	2	2	2	2	2	2
2	2	2	2	2	2	2	2	2	2
1	1	1	1	1	1	1	1	1	1
3	2	2	2	2	2	2	2	3	2
2	2	2	2	2	2	2	2	2	2
3	2	2	3	2	2	3	2	3	2
3	3	3	3	3	3	3	3	3	3
2	2	2	2	2	2	2	2	2	2
2	2	2	2	2	2	2	2	2	2
3	1	2	3	1	2	3	1	2	1
2	1	2	1	1	2	1	1	2	1
2	2	2	2	2	2	1	1	1	1
2	2	2	2	2	2	2	2	2	2
3	3	3	3	3	1	1	1	1	1
3	3	1	1	1	1	1	1	1	1
9	9	9	9	9	9	9	9	9	9
2	3	1	2	1	2	1	2	3	2
2	2	2	2	2	2	2	2	2	2
1	2	3	2	1	1	1	1	1	3
1	2	1	1	2	1	2	1	1	2
2	3	2	1	2	2	1	1	1	2
2	1	2	2	1	2	2	3	2	2
2	3	3	3	3	3	3	3	3	3
1	1	1	2	2	1	2	1	3	2
2	2	9	9	1	1	1	3	3	9
2	2	2	2	9	9	9	9	9	9
9	9	9	9	9	9	9	9	9	9
2	2	2	2	1	1	2	2	1	2
9	9	9	9	9	9	9	9	9	9
1	2	2	2	2	2	1	2	2	2
2	3	3	9	2	2	2	2	2	3
1	1	2	1	2	1	1	2	1	1
2	1	2	2	1	1	1	2	1	2
9	9	9	9	9	9	9	9	9	9
9	9	9	9	9	9	9	9	9	9
9	9	9	9	9	9	9	9	9	9
2	1	1	1	1	1	3	3	3	3

3	3	9	9	9	9	9	9	9	9
9	9	9	9	9	9	9	9	9	9
2	3	3	2	3	2	1	2	2	2
1	1	1	3	3	3	3	3	3	3
9	9	9	9	9	9	9	9	9	9
1	3	3	3	2	2	3	3	3	3
2	2	2	3	2	3	3	3	2	3
2	3	3	3	2	2	2	2	2	2
1	3	2	1	1	1	1	1	1	2
3	1	2	3	1	2	3	1	2	3
2	3	1	2	1	3	2	1	2	3
2	2	2	2	2	2	2	2	2	2
2	2	2	2	2	2	2	2	2	2
9	9	9	9	9	9	9	9	9	9
2	2	2	2	2	2	2	2	2	2
2	2	2	1	9	1	1	1	1	1
1	1	1	1	3	1	1	3	1	1
3	2	3	3	1	1	2	2	2	1
3	1	2	2	2	1	2	3	1	2
9	9	9	9	9	9	9	9	9	9
3	3	3	1	1	2	2	1	3	1
3	3	1	3	2	1	2	3	1	2
1	2	3	3	3	3	3	2	2	2
2	1	3	1	2	3	2	2	3	1
3	2	9	3	2	3	3	2	2	3
9	9	9	9	9	9	9	9	9	9
1	1	2	1	3	2	3	2	2	2
2	2	2	2	2	2	2	2	2	2
2	2	3	3	3	3	3	3	3	2
1	2	2	3	1	2	1	2	3	1
2	2	2	2	2	2	2	2	2	2
9	9	9	9	9	9	9	9	9	9
3	1	3	1	1	3	3	2	2	2
3	3	2	2	2	2	3	2	2	2
1	9	3	1	2	2	2	3	1	2
1	2	1	2	1	3	1	2	1	3
9	9	9	9	9	9	9	9	9	9
1	3	2	1	1	1	1	2	1	1
1	3	3	1	1	1	2	1	1	2
9	9	9	9	9	9	9	9	9	9
1	1	2	3	2	1	2	3	1	3
2	2	2	2	2	2	2	2	2	2
2	2	2	2	2	2	2	2	2	2
2	2	2	2	2	2	2	2	2	2
2	2	2	2	2	2	2	2	2	2
9	9	9	9	9	9	9	9	9	9
2	2	2	2	2	2	2	2	9	3

2	3	3	1	1	1	1	1	1	1
2	2	2	2	2	2	2	2	2	2
2	2	2	2	2	2	2	2	2	3
1	2	3	3	3	2	2	2	2	2
3	3	3	3	3	1	1	3	1	1
2	2	3	3	2	3	3	3	2	2
1	3	1	1	1	1	1	1	1	1
1	3	3	1	2	1	2	2	2	2
1	3	1	1	1	1	1	1	1	1
1	3	3	1	1	2	3	3	2	1
3	3	2	2	2	2	2	2	2	2
3	3	3	3	3	3	2	2	2	2
2	2	2	2	2	2	2	2	2	2
1	3	2	2	1	1	1	2	1	1
9	9	9	9	9	9	9	9	9	9
2	2	2	1	2	1	1	1	1	1
3	1	3	3	2	2	1	2	3	1
2	2	2	2	2	1	2	2	1	1
2	2	2	2	3	2	2	3	2	3
2	2	2	2	1	1	1	1	1	1
1	3	3	1	1	1	1	1	1	1
3	3	1	9	9	9	1	3	1	1
3	3	3	1	2	2	3	3	2	2
2	2	2	2	2	2	2	2	2	2
2	2	2	2	9	3	2	9	2	2
2	2	3	2	2	1	1	1	1	1
1	3	2	2	3	1	1	2	1	1
2	3	3	2	2	2	2	2	2	1
3	3	1	1	3	3	1	1	3	1
3	3	1	1	2	2	1	1	1	1
3	3	1	1	1	1	1	1	1	2
2	2	2	2	2	1	1	1	2	1
2	2	2	2	2	1	1	1	1	2
9	9	9	9	9	9	9	9	9	9
9	9	9	9	9	9	9	9	9	9
3	3	1	1	3	1	1	1	1	3
2	2	2	2	2	2	2	2	2	2
1	1	1	2	2	3	3	3	3	3
1	1	1	2	2	2	2	2	1	1
2	2	2	2	2	2	2	2	2	2
1	2	3	1	1	2	2	3	2	3
9	9	9	9	9	9	9	9	9	9
9	9	9	9	9	9	9	9	9	9
1	1	1	1	1	1	1	1	1	1
1	2	2	3	2	3	2	2	2	2
9	9	9	9	9	9	9	9	9	9
9	9	9	9	9	9	9	9	9	9

2	2	2	2	2	2	2	2	2	2
3	1	1	1	1	1	1	1	1	1
3	1	2	3	1	2	3	1	2	3
2	3	1	2	3	1	3	2	1	2
3	3	1	1	2	2	2	2	2	2
3	1	2	3	1	2	3	1	2	3
3	2	1	2	3	2	1	2	2	1
9	9	9	9	9	9	9	9	9	9
2	3	2	2	1	2	2	2	2	1
9	9	9	9	9	9	9	9	9	9
1	1	1	1	1	1	1	1	1	1
1	3	1	1	1	1	1	1	1	1
9	9	9	9	9	9	9	9	9	9
9	9	9	9	9	9	9	9	9	9
1	2	2	2	2	2	2	2	2	2
9	9	9	9	9	9	9	9	9	9
9	9	9	9	9	9	9	9	9	9
2	2	2	2	2	2	2	2	2	2
2	2	2	2	2	2	2	2	2	2
2	2	2	2	9	1	1	1	2	3
2	2	2	2	2	2	2	2	2	2
2	2	2	2	2	2	2	2	2	2
3	2	3	3	2	2	3	3	2	1
2	1	2	1	2	3	2	1	2	3
2	2	2	9	9	3	3	9	9	9
9	9	9	9	9	9	9	9	9	9
2	2	1	1	1	1	1	1	1	1
1	1	1	1	1	1	1	1	1	1
1	1	1	1	1	1	1	1	1	1
1	3	1	1	1	1	1	1	1	1
2	2	1	1	2	1	2	1	1	1
2	2	2	2	2	2	2	2	2	2
2	2	2	2	2	2	2	2	2	2
2	2	2	2	2	2	2	2	2	2
1	1	1	1	1	1	1	1	1	1
2	2	2	2	2	2	2	2	2	2
2	2	2	2	2	2	2	2	2	2
9	9	9	9	9	9	9	9	9	9
9	9	9	9	9	9	9	9	9	2
2	2	2	2	2	2	2	2	2	2
2	2	2	2	2	2	2	2	2	2
9	9	9	9	9	9	9	9	9	9
2	2	2	2	2	2	2	2	2	2
9	9	9	9	9	9	9	9	9	9
3	3	3	3	3	3	3	3	3	3
9	9	9	9	9	9	9	9	9	9
2	2	2	2	2	2	2	2	2	2

1	2	1	2	1	3	1	2	3	1
9	9	9	9	9	9	9	9	9	9
2	2	2	2	2	2	2	2	2	2
3	3	2	2	2	3	2	2	1	2
2	2	2	2	2	2	2	3	3	3
2	1	1	3	1	2	2	2	2	2
1	2	2	2	2	2	1	1	2	1
2	2	3	2	2	2	2	3	1	1
2	2	2	2	2	2	2	2	2	2
3	3	3	3	2	1	1	1	1	1
3	3	3	1	1	3	1	1	1	1
2	2	2	2	2	2	2	2	2	2
3	3	2	2	2	2	2	2	2	2
3	2	2	3	2	3	2	2	2	3
1	1	1	2	1	1	1	1	2	1
1	1	1	2	1	1	1	1	2	1
2	2	2	2	1	2	2	2	2	2
2	2	2	2	2	2	2	2	2	2
3	2	3	2	2	2	3	3	3	2
2	9	2	3	2	1	2	2	2	9
2	3	2	1	2	1	1	1	2	1
1	2	2	2	2	2	1	1	1	2
2	1	2	3	2	2	1	1	2	2
9	9	9	9	9	9	9	9	9	9
3	3	3	3	3	3	3	3	3	3
9	9	9	9	9	9	9	9	9	9
9	9	9	9	9	9	9	9	9	9
9	9	9	9	9	9	9	9	9	9
2	2	2	2	3	3	3	3	3	1
1	1	1	1	1	1	1	1	1	1
2	2	2	2	2	2	2	2	2	2
1	9	1	3	1	2	1	1	1	1
2	2	2	2	2	2	2	2	2	2
2	2	2	2	2	2	2	2	2	2
1	1	1	1	1	1	1	1	1	1
3	3	1	1	1	1	1	1	1	1
1	1	3	2	2	1	1	2	1	9
2	1	2	1	3	2	1	2	3	2
1	2	1	1	1	3	2	2	3	1
3	3	1	1	2	1	1	1	1	1
1	1	1	1	1	1	1	1	1	1
2	2	2	2	2	2	2	2	2	2
9	9	9	9	9	9	9	9	9	9
9	9	9	9	9	9	9	9	9	9
2	3	2	3	1	2	1	2	3	2
2	2	2	2	2	2	2	2	2	2
2	2	2	2	2	2	2	2	2	2

2	2	2	3	3	1	1	1	2	3
9	9	9	9	9	9	9	9	9	9
9	9	9	9	9	9	9	9	9	9
9	9	9	9	9	9	9	9	9	9
9	9	9	9	9	9	9	9	9	9
9	9	9	9	9	9	9	9	9	9
9	9	9	9	9	9	9	9	9	9
9	9	9	9	9	9	9	9	9	9
9	9	9	9	9	9	9	9	9	9
9	9	9	9	9	9	9	9	9	9
2	2	2	2	2	2	2	2	2	2
2	3	2	2	3	2	2	2	1	2
3	3	3	1	1	2	1	1	1	1
2	2	2	2	3	2	3	2	2	3
2	2	2	2	2	2	2	2	2	2
2	2	3	2	3	2	3	2	2	1
1	2	2	1	9	2	1	2	3	3
2	2	2	2	2	2	2	2	2	2
1	3	2	1	1	2	2	2	1	1
3	3	3	3	2	2	1	1	1	2
2	2	3	2	1	2	3	1	3	2
2	3	2	1	2	3	1	2	3	1
3	3	3	3	3	3	3	3	3	3
2	2	2	2	2	2	2	2	2	2
2	2	2	2	2	2	2	2	2	2
2	2	2	2	2	2	2	2	2	2
2	2	2	2	9	3	3	3	3	3
3	2	1	1	2	1	1	2	2	1
2	2	2	2	2	2	2	2	2	2
2	2	2	2	2	2	2	2	2	2
9	9	9	9	9	9	9	9	9	9
2	2	2	2	2	2	2	2	2	2
1	1	1	1	1	1	1	1	1	1
9	9	9	9	9	9	9	9	9	9
9	9	9	9	9	9	9	9	9	9
2	2	2	2	2	2	2	2	2	2
2	2	2	2	2	2	2	2	2	2
2	2	2	2	2	2	2	2	2	2
1	3	2	1	3	2	1	1	3	2
9	9	9	9	9	9	9	9	9	9
1	2	1	1	1	2	1	2	1	2
2	2	2	2	2	2	2	2	2	2
2	2	3	3	3	3	2	2	2	2
3	3	1	1	1	1	1	1	1	1
3	9	2	2	2	1	1	1	1	1
9	9	9	9	9	9	9	9	9	9
2	1	2	1	2	2	2	1	2	2

9	9	9	9	9	9	9	9	9	9
2	1	1	1	1	1	2	2	2	2
9	9	9	9	9	9	9	9	9	9
9	9	9	9	9	9	9	9	9	9
9	9	9	9	9	9	9	9	9	9
9	9	9	9	9	9	9	9	9	9
9	9	9	9	9	9	9	9	9	9
9	9	9	9	9	9	9	9	9	9
9	9	9	9	9	9	9	9	9	9
9	9	9	9	9	9	9	9	9	9
9	9	9	9	9	9	9	9	9	9
9	9	9	9	9	9	9	9	9	9
9	9	9	9	9	9	9	9	9	9
9	9	9	9	9	9	9	9	9	9
9	9	9	9	9	9	9	9	9	9
9	9	9	9	9	9	9	9	9	9
9	9	9	9	9	9	9	9	9	9
9	9	9	9	9	9	9	9	9	9
9	9	9	9	9	9	9	9	9	9
9	9	9	9	9	9	9	9	9	9
9	9	9	9	9	9	9	9	9	9
9	9	9	9	9	9	9	9	9	9
9	9	9	9	9	9	9	9	9	9
2	3	2	1	2	3	2	1	2	3
2	2	2	2	2	2	2	2	2	2
3	3	3	1	1	1	1	2	2	2
3	3	2	2	1	1	1	1	1	2
9	3	3	2	1	2	1	1	9	2
2	2	1	1	2	3	2	2	1	1
1	2	1	1	1	2	1	2	1	2
1	3	3	3	1	1	2	2	3	1
2	2	2	1	2	2	1	1	1	1
2	2	9	2	1	1	1	1	1	1
3	1	1	1	1	1	1	1	1	1
1	2	3	1	2	3	1	2	1	2
2	3	3	1	1	1	1	1	2	2
3	3	2	1	2	2	1	1	1	2
3	3	1	1	1	1	1	1	2	2
1	2	1	1	1	2	1	1	1	1
9	9	9	9	9	9	9	9	9	9
2	3	3	1	1	1	1	2	1	2
3	3	1	1	1	2	1	1	1	1
9	9	9	9	1	9	9	9	9	9
1	3	3	1	1	2	2	1	1	2
1	3	1	1	3	3	1	1	1	1
2	2	2	2	3	2	3	3	3	3
3	1	9	1	2	2	3	2	3	2

2	2	2	1	1	1	1	1	1	2
1	1	1	1	1	1	1	1	1	1
3	3	2	1	1	2	1	1	2	2
3	2	2	1	2	2	1	1	1	2
1	2	3	2	3	2	1	2	1	2
9	9	9	9	9	9	9	9	9	9
1	1	2	1	1	1	1	1	1	1
9	9	9	9	9	9	9	9	9	9
1	2	3	1	1	1	1	1	1	2
1	2	9	1	1	1	1	1	1	2
9	9	9	9	9	9	9	9	9	9
9	9	9	9	9	9	9	9	9	9
9	9	9	9	9	9	9	9	9	9
9	9	9	9	9	9	9	9	9	9
9	9	9	9	9	9	9	9	9	9
9	9	9	9	9	9	9	9	9	9
3	3	3	3	3	1	1	1	1	1
2	2	2	2	2	2	2	2	2	2
9	9	9	9	9	9	9	9	9	9
1	3	1	1	1	3	3	3	3	3
9	9	9	9	9	9	9	9	9	9
9	9	9	9	9	9	9	9	9	9
9	9	9	9	9	9	9	9	9	9
9	9	9	9	9	9	9	9	9	9
9	9	9	9	9	9	9	9	9	9
3	3	2	1	2	2	3	3	2	3
3	3	1	3	1	3	1	1	1	3
2	2	2	1	9	2	2	3	2	2
1	2	1	1	1	2	1	1	1	1
2	2	2	2	2	2	2	2	2	2
1	1	2	1	2	1	1	1	1	2
3	3	1	1	1	3	1	1	1	2
3	3	2	1	3	3	3	3	1	3
1	3	1	2	3	1	1	1	1	1
2	3	2	2	2	3	2	9	2	3
9	9	9	9	9	9	9	9	9	9
2	1	2	2	2	2	2	1	2	2
2	2	2	2	2	2	2	2	2	2
1	2	3	1	2	3	3	1	2	2
2	2	2	2	2	2	2	2	2	2
3	3	3	3	3	1	1	1	1	1
3	1	2	3	3	1	1	1	1	1
1	3	3	1	1	1	1	1	1	1
1	3	3	3	2	2	1	1	2	3
3	3	1	1	1	1	1	1	2	2
9	9	9	9	1	9	1	1	1	9
2	3	1	1	1	1	3	1	1	1

3	3	1	1	1	2	3	1	1	2
2	3	9	3	3	3	3	3	3	3
3	3	1	1	1	2	2	1	1	2
3	3	1	9	3	3	3	1	1	1
9	9	1	9	9	2	9	9	9	9
1	3	2	2	2	2	1	1	1	1
1	3	1	1	1	1	1	1	1	1
9	9	9	9	9	9	9	9	9	9
9	9	9	9	9	9	9	9	9	9
2	2	2	2	2	2	2	2	2	2
2	2	2	2	2	2	2	2	2	2
3	3	3	1	2	3	3	3	3	3
2	2	1	1	1	1	1	1	1	2
9	9	9	9	9	9	9	9	9	9
9	9	9	9	9	9	9	9	9	9
3	9	9	1	1	1	1	1	1	2
9	9	9	9	9	9	9	9	9	9
9	9	9	9	9	9	9	9	9	9
1	1	1	1	1	1	1	1	1	1
3	3	3	3	3	1	1	1	1	1
1	2	1	1	1	2	1	1	1	1
1	1	1	1	1	1	1	1	1	1
2	3	2	2	2	2	1	3	1	2
2	3	1	2	1	2	3	1	3	1
1	1	1	1	1	1	1	1	1	1
9	9	9	9	9	9	9	9	9	9
9	9	9	9	9	9	9	9	9	9
3	1	1	1	1	1	1	1	1	1
2	2	2	2	1	1	1	1	2	2
2	3	1	1	3	3	1	1	1	1
9	9	9	9	9	9	9	9	9	9
9	9	9	9	9	9	9	9	9	9
9	9	9	9	9	9	9	9	9	9
9	9	9	9	9	9	9	9	9	9
9	9	9	9	9	9	9	9	9	9
9	9	9	9	9	9	9	9	9	9
9	9	9	9	9	9	9	9	9	9
9	9	9	9	9	9	9	9	9	9
9	9	9	9	9	9	9	9	9	9
9	9	9	9	9	9	9	9	9	9
9	9	9	9	9	9	9	9	9	9
9	9	9	9	9	9	9	9	9	9
9	9	9	9	9	9	9	9	9	9
9	9	9	9	9	9	9	9	9	9
9	9	9	9	9	9	9	9	9	9
9	9	9	9	9	9	9	9	9	9
9	9	9	9	9	9	9	9	9	9

9	9	9	9	9	9	9	9	9	9
9	9	9	9	9	9	9	9	9	9
9	9	9	9	9	9	9	9	9	9
9	9	9	9	9	9	9	9	9	9
9	9	9	9	9	9	9	9	9	9
9	9	9	9	9	9	9	9	9	9
9	9	9	9	9	9	9	9	9	9
9	9	9	9	9	9	9	9	9	9
9	9	9	9	9	9	9	9	9	9
9	9	9	9	9	9	9	9	9	9
9	9	9	9	9	9	9	9	9	9
9	9	9	9	9	9	9	9	9	9
9	9	9	9	9	9	9	9	9	9
9	9	9	9	9	9	9	9	9	9
9	9	9	9	9	9	9	9	9	9
9	9	9	9	9	9	9	9	9	9
9	9	9	9	9	9	9	9	9	9
9	9	9	9	9	9	9	9	9	9
9	9	9	9	9	9	9	9	9	9
2	2	2	2	2	2	2	2	2	2
9	9	9	9	9	9	9	9	9	9
9	9	9	9	9	9	9	9	9	9
9	9	9	9	9	9	9	9	9	9
9	9	9	9	9	9	9	9	9	9
9	9	9	9	9	9	9	9	9	9
9	9	9	9	9	9	9	9	9	9
9	9	9	9	9	9	9	9	9	9
9	9	9	9	9	9	9	9	9	9
9	9	9	9	9	9	9	9	9	9
9	9	9	9	9	9	9	9	9	9
9	9	9	9	9	9	9	9	9	9
9	9	9	9	9	9	9	9	9	9
9	9	9	9	9	9	9	9	9	9
9	9	9	9	9	9	9	9	9	9
9	9	9	9	9	9	9	9	9	9
2	2	2	2	2	2	2	2	2	2
3	3	3	3	3	3	3	3	3	3
3	2	2	1	3	2	1	1	1	2
1	3	1	1	1	3	1	1	1	1
3	3	3	3	3	3	3	3	3	2
1	1	1	1	1	1	1	1	1	1
3	2	1	3	1	3	1	1	1	1
9	9	9	9	9	9	9	9	9	9
3	2	1	3	1	3	1	1	1	1
1	2	2	2	2	1	3	2	2	2
2	2	2	2	2	1	1	1	2	3
2	2	2	2	2	2	2	2	2	2

1	2	1	1	1	2	1	1	1	1
3	3	1	1	1	3	1	1	1	1
3	3	1	1	1	2	2	1	1	2
2	3	9	1	2	2	1	1	1	1
2	2	2	1	2	2	1	1	1	2
2	2	1	1	1	1	1	1	1	1
3	3	3	1	1	1	1	1	2	2
1	3	3	2	1	1	1	1	2	2
9	2	1	2	3	1	1	3	2	1
2	3	1	1	2	2	1	2	2	2
9	9	1	1	9	9	9	1	9	9
2	2	2	3	1	2	2	3	3	2
3	3	1	2	1	2	2	1	1	1
1	2	2	1	2	2	1	1	2	2
1	2	3	2	2	2	1	1	1	1
2	2	2	1	1	1	1	1	1	1
2	2	2	2	2	2	2	9	9	9
2	2	2	1	1	3	9	1	1	2
1	2	2	1	1	1	1	1	1	1
1	1	1	2	2	1	1	3	3	2
2	2	2	2	9	2	1	1	1	2
3	3	3	3	3	1	1	1	1	1
1	3	3	1	1	1	2	2	1	1
9	9	1	1	1	1	1	1	1	1
3	3	3	3	3	3	3	3	3	3
2	1	2	1	2	1	2	1	2	1
1	1	1	1	2	2	2	2	2	2
1	2	3	1	1	2	3	3	2	2
2	2	2	2	2	2	2	2	2	2
2	2	2	2	9	9	9	9	9	9
2	2	2	2	1	2	3	2	3	3
2	2	2	2	2	2	2	2	2	2
2	2	2	2	2	2	2	2	2	2
9	9	9	9	9	9	9	9	9	9
9	9	9	9	9	9	9	9	9	9
9	9	9	9	9	9	9	9	9	9
9	9	9	9	9	9	9	9	9	9
9	9	9	9	9	9	9	9	9	9
9	9	9	9	9	9	9	9	9	9
9	9	9	9	9	9	9	9	9	9
2	2	2	2	1	1	1	2	3	2
9	9	9	9	9	9	9	9	9	9
2	1	2	1	1	1	1	1	1	1
3	3	3	1	1	1	3	3	3	3
1	1	1	1	1	1	1	1	1	1
3	3	3	1	3	3	3	3	3	3
1	3	1	1	1	3	1	1	1	3

3	2	3	2	3	2	3	2	3	2
1	1	1	2	1	2	2	3	3	2
3	2	2	2	2	2	3	2	2	3
1	3	3	1	3	1	3	9	1	1
9	3	3	2	2	2	1	2	2	1
3	2	1	2	3	2	1	2	3	1
3	3	1	3	1	3	3	1	1	2
1	2	1	2	3	3	2	1	2	3
3	3	3	1	1	2	2	1	1	1
9	9	9	2	9	1	9	2	9	3
3	3	3	3	3	3	3	3	3	3
1	3	9	1	3	1	1	2	1	1
1	1	2	2	2	1	2	2	2	2
3	3	3	1	1	1	1	3	2	1
1	1	1	1	1	1	1	1	1	1
9	1	9	1	3	2	3	2	3	3
2	2	2	2	9	2	3	2	2	2
9	9	1	9	9	9	9	9	9	9
1	2	3	2	1	2	1	2	3	2
1	2	1	1	1	3	1	2	1	1
2	3	2	2	1	2	3	2	3	2
3	3	3	3	3	3	3	3	3	3
3	3	1	1	1	1	9	9	1	2
3	1	2	1	1	1	1	3	1	1
2	3	1	1	2	2	1	1	2	3
3	1	3	3	3	1	3	1	1	1
3	1	9	1	3	1	1	2	1	1
1	3	1	1	1	1	2	2	1	3
2	2	2	2	2	2	2	1	1	2
3	2	2	1	2	2	1	3	1	1
2	2	2	2	2	2	2	1	1	2
3	1	2	3	1	2	3	1	2	3
2	2	2	2	2	2	2	2	2	2
1	3	3	3	3	3	1	3	1	1
3	1	1	1	1	1	1	1	9	1
1	3	3	3	3	3	3	1	1	3
3	1	1	2	2	1	2	3	3	2
9	9	9	9	9	9	9	9	9	9
1	3	3	3	3	1	1	1	1	1
9	2	1	1	3	1	1	1	1	3
3	1	3	9	1	1	1	3	1	1
3	9	1	2	3	1	2	3	1	2
3	1	1	1	9	1	1	3	1	1
2	3	3	1	1	1	1	1	1	3
2	2	2	2	2	2	2	2	2	2
3	3	3	3	3	3	3	3	3	3
3	3	3	3	3	3	3	3	3	3

3	3	3	2	1	2	3	2	3	3
9	9	9	9	9	9	9	9	9	9
2	2	2	2	2	2	2	2	3	2
2	2	3	3	3	3	2	2	1	1
2	2	2	2	2	1	1	3	2	2
3	2	2	1	2	3	2	1	2	3
2	2	2	2	2	2	2	2	2	2
9	9	9	9	9	9	9	9	9	9
9	9	9	9	9	9	9	9	9	9
3	1	2	3	2	1	2	3	2	1
2	2	2	2	2	2	2	2	2	2
2	2	2	2	2	2	2	2	2	2
2	1	1	1	2	2	1	1	2	2
2	2	2	2	2	2	2	2	2	2
1	1	1	1	1	1	1	1	1	1
1	1	1	1	1	1	1	1	1	1
2	2	2	3	3	3	2	2	2	2
2	2	2	2	2	2	2	2	2	2
2	2	2	2	1	2	3	3	3	3
2	2	2	2	3	2	2	2	3	3
2	1	1	1	3	2	2	2	3	3
2	2	2	2	2	2	2	2	2	2
2	2	2	2	2	2	2	2	2	2
2	2	2	2	2	2	2	1	2	2
2	2	2	2	2	2	2	2	2	2
2	2	2	2	2	2	2	2	2	2
2	2	2	2	2	2	2	2	2	2
2	1	2	1	3	1	2	3	1	2
2	1	2	1	1	1	1	1	2	2
2	2	2	2	2	2	2	2	2	2
2	2	2	2	2	2	2	2	2	2
2	2	2	2	2	2	2	2	2	2
2	2	2	2	2	2	2	2	2	2
2	2	2	2	2	2	2	2	2	2
2	2	2	2	2	2	2	2	2	2
2	2	2	2	2	2	2	2	2	2
2	2	2	2	2	2	2	2	2	2
2	2	2	2	2	2	2	2	2	2
2	2	2	2	2	2	2	2	2	2
1	3	3	1	1	1	1	1	1	1
2	2	2	2	2	2	2	2	2	2
2	2	2	2	2	2	2	2	2	2
2	3	2	2	1	1	3	2	2	3
2	2	2	2	2	2	2	2	2	2
1	1	2	3	3	3	2	2	3	3
3	3	3	3	3	3	3	3	3	3
3	3	3	3	3	3	3	3	3	3

2	2	2	2	2	2	2	2	2	2
2	2	2	2	2	2	2	2	2	2
2	2	2	2	2	2	2	2	2	2
2	2	2	2	2	2	2	2	2	2
3	2	2	2	1	1	1	1	2	2
2	2	2	2	2	2	2	2	2	2
9	9	9	9	9	9	9	9	9	9
1	1	1	1	1	1	3	1	3	1
9	9	9	9	9	1	9	9	9	9
2	2	2	2	2	2	2	2	2	2
3	2	3	2	2	1	2	2	2	2
2	1	1	1	3	3	3	2	2	2
2	3	2	3	3	3	3	2	3	2
9	9	9	9	9	9	9	9	9	9
2	2	2	2	2	2	2	2	2	2
2	9	9	9	9	9	9	9	9	9
2	2	2	2	2	2	2	2	2	2
1	1	1	1	1	1	1	1	1	1
9	9	9	9	9	9	9	9	9	9
2	2	2	2	2	2	2	2	2	2
1	2	2	2	2	2	1	9	2	2
9	9	9	9	9	9	9	9	9	9
9	9	9	9	9	9	9	9	9	9
3	3	3	3	3	2	3	3	2	3
3	1	2	3	1	2	1	2	3	1
3	3	3	2	3	3	3	3	3	3
2	3	3	2	3	3	3	3	3	3
3	1	1	1	1	1	1	1	1	9
3	9	1	3	1	2	9	1	2	9
1	1	1	1	1	1	1	1	1	1
2	2	2	2	2	2	2	2	2	2
3	3	3	3	2	2	2	3	2	2
2	2	2	2	2	2	2	2	2	2
9	2	9	2	9	9	9	9	2	9
2	2	2	2	2	2	2	2	2	2
9	9	9	9	9	9	9	9	9	1
3	3	1	1	1	1	1	1	3	3
2	2	2	2	2	2	2	2	2	2
2	2	2	2	2	2	2	2	2	2
2	2	2	2	2	2	2	2	2	2
1	2	3	1	3	2	1	2	3	1
2	2	2	2	2	2	2	2	2	2
9	9	2	9	9	9	9	9	9	9
3	2	1	1	1	3	2	2	1	1
3	2	2	1	1	2	2	2	2	2
3	3	9	2	3	3	3	2	3	3
2	2	2	1	2	1	2	1	2	2

1	1	1	1	1	1	1	1	1	1
2	2	2	2	2	2	2	2	2	2
1	1	1	1	1	1	1	1	1	1
2	2	2	2	2	2	2	2	2	2
9	9	9	9	9	9	9	9	9	9
1	2	2	1	1	2	3	2	3	2
3	3	3	3	3	3	3	3	3	3
1	1	1	1	1	2	9	2	2	3
2	2	2	2	2	2	2	2	2	2
1	1	1	1	1	1	1	1	3	3
3	3	3	3	3	3	3	3	3	3
1	1	1	1	1	1	1	1	1	1
2	2	2	2	2	2	2	2	2	2
2	2	2	2	2	2	2	2	2	2
2	2	2	2	2	2	2	2	2	2
1	1	1	1	1	1	1	1	1	1
1	1	1	1	1	1	1	1	1	1
3	1	1	1	1	1	3	3	3	3
9	9	9	9	9	9	9	9	9	9
9	9	9	9	9	9	9	9	9	9
1	1	2	1	1	1	9	2	2	2
2	2	2	2	2	2	2	2	2	2
1	1	1	1	1	1	1	1	1	1
2	2	2	2	2	2	2	2	2	2
3	3	3	3	3	3	3	3	9	3
2	1	1	1	1	1	1	1	1	1
3	1	2	2	1	1	1	2	3	2
2	2	2	2	2	1	2	1	2	1
9	9	2	9	9	2	9	9	9	2
3	3	3	3	3	3	3	3	3	3
9	1	2	1	2	9	2	1	2	9
2	2	2	2	3	3	2	2	2	2
1	1	1	1	1	1	1	1	1	1
2	1	2	2	2	2	2	2	2	2
2	2	2	2	2	2	2	2	2	2
9	2	3	3	2	2	2	2	3	3
3	3	3	2	2	2	2	2	2	2
2	2	2	2	2	2	2	2	2	2
3	1	2	3	1	2	3	1	2	3
3	3	3	3	3	3	3	3	3	3
1	2	9	1	1	2	1	1	3	1
2	2	2	2	3	3	3	3	1	1
9	9	9	9	9	9	9	9	9	9
9	9	9	9	9	9	9	9	3	3
2	2	2	2	1	1	1	1	1	1
2	2	2	2	2	2	2	2	2	2
2	2	1	2	2	2	1	1	9	2

1	1	1	1	1	1	1	1	1	1
3	3	3	1	3	1	1	1	3	3
2	1	1	1	1	1	3	3	3	3
3	3	3	1	3	1	1	1	3	3
3	9	9	1	2	1	2	1	1	1
2	2	2	2	2	2	2	2	2	2
2	2	2	2	2	2	2	2	2	2
2	2	2	2	2	2	2	2	2	2
2	2	2	2	1	1	1	1	1	1
9	2	2	1	1	1	2	3	1	2
2	2	2	2	1	2	3	2	2	1
3	2	3	1	3	1	3	1	2	2
2	2	2	2	2	2	2	2	2	2
3	3	2	2	2	3	2	1	1	2
2	2	2	2	2	2	2	2	2	2
2	2	2	2	2	2	2	2	2	2
2	2	2	2	2	2	2	2	2	2
2	2	2	2	2	2	2	2	2	2
2	9	2	2	2	2	2	2	2	2
2	2	2	2	2	2	3	2	2	2
2	2	2	2	2	2	2	2	2	2
2	2	2	2	2	2	2	2	2	2
2	3	3	1	2	1	3	1	1	3
2	2	2	2	2	2	2	2	2	2
1	1	1	1	1	1	1	1	1	1
2	2	2	2	2	2	2	2	2	2
2	2	3	1	1	1	2	3	1	1
3	3	3	3	2	2	2	2	2	2
2	3	2	2	3	1	3	2	1	2
9	3	3	2	1	2	1	2	1	2
2	2	2	2	1	1	2	2	2	3
1	2	3	3	2	2	2	3	3	2
2	2	2	2	2	2	2	2	2	2
2	2	2	2	2	2	2	2	2	2
2	1	2	2	2	1	1	1	1	1
3	3	3	3	3	3	3	3	3	3
2	2	2	2	2	2	2	2	2	2
9	9	9	9	9	9	9	9	9	9
2	2	2	2	2	2	2	2	2	2
3	2	3	3	2	1	1	2	1	2
3	2	3	2	1	1	2	1	2	2
2	2	2	2	2	2	2	2	2	2
2	2	2	2	2	2	2	2	2	2
2	2	2	2	2	2	2	2	2	2
9	9	9	9	9	9	9	9	9	9
3	9	9	1	2	1	2	1	1	1
2	2	2	2	2	2	2	2	2	2

9	9	9	9	9	9	9	9	9	9
2	1	1	2	1	3	1	3	1	2
2	1	2	3	1	1	3	2	1	2
2	2	3	1	1	1	1	3	2	1
1	3	3	1	1	3	1	1	1	1
2	2	2	1	2	2	1	2	1	1
3	3	3	3	3	3	3	3	3	3
9	9	9	9	9	9	9	9	9	9
2	2	2	2	3	3	3	3	3	3
2	3	2	3	2	3	2	3	2	2
2	1	2	2	2	2	1	1	1	1
2	3	2	3	1	1	2	2	1	3
2	2	2	2	2	2	1	2	1	1
1	1	1	1	1	1	1	1	1	1
1	2	3	2	1	2	2	1	2	3
2	2	2	2	2	2	2	2	2	2
2	1	1	2	1	2	3	3	3	2
3	2	2	2	2	2	2	2	2	2
9	9	9	9	9	9	9	9	9	9
9	9	9	9	9	9	9	9	9	9
9	9	9	9	9	9	9	9	9	9
2	3	1	3	2	2	2	1	2	3
3	2	1	2	2	2	1	2	3	2
1	3	2	2	2	1	1	1	1	2
1	1	1	2	3	2	1	1	2	2
3	3	3	3	3	3	3	3	3	3
3	2	2	1	2	3	1	2	2	1
2	2	2	1	2	1	2	3	3	3
3	1	3	2	2	2	2	3	3	1
3	2	2	1	2	1	1	1	1	1
2	2	1	1	1	1	3	3	3	2
2	2	2	1	1	1	1	3	3	3
3	3	3	2	2	2	2	3	3	3
2	2	2	2	2	2	2	2	2	2
2	2	2	2	2	2	2	2	2	2
2	2	2	2	2	2	2	2	2	2
3	3	3	3	3	3	3	3	3	3
2	2	2	2	2	2	3	3	1	2
9	9	9	9	9	9	9	9	9	9
9	9	9	9	9	9	9	9	9	9
3	1	2	3	1	2	3	1	2	3
2	2	2	2	2	2	2	2	2	2
2	1	2	3	2	1	2	1	3	1
2	2	3	3	3	2	1	1	2	3
9	9	9	9	9	9	9	9	9	9
3	3	3	3	3	3	3	3	3	3
3	3	3	3	3	3	3	3	2	1	2

1	1	1	1	1	1	1	1	1	1
1	9	2	1	2	3	1	1	1	1
3	1	2	3	1	2	3	3	2	1
1	2	2	2	2	2	2	2	2	2
2	2	2	3	2	3	2	3	2	2
3	3	3	3	3	3	3	3	3	3
1	1	1	1	1	1	1	1	1	1
3	3	3	3	3	3	3	3	3	3
2	2	3	2	2	2	2	2	2	3
2	3	2	3	2	1	1	1	2	1
2	2	2	2	2	2	2	2	2	2
1	3	3	3	2	1	2	3	1	2
2	2	2	2	2	2	2	3	2	2
9	1	2	1	1	1	2	1	9	1
2	2	2	2	2	2	2	2	2	2
3	3	3	3	3	3	3	2	2	2
2	3	1	1	1	3	3	1	1	1
1	2	3	1	2	3	1	2	3	1
2	2	2	2	2	2	2	2	2	2
1	1	2	2	3	1	2	2	2	3
3	2	2	1	3	2	1	3	3	2
3	3	3	3	3	3	3	3	3	3
3	3	3	3	3	3	3	3	3	3
1	1	1	1	1	2	1	3	3	3
3	1	2	3	1	2	3	1	2	3
3	3	3	2	3	3	2	3	3	3
3	3	3	3	3	2	3	2	3	3
3	2	1	2	3	2	1	2	2	1
3	1	2	1	2	1	3	1	2	3
1	2	2	2	2	2	2	2	2	2
3	1	2	3	2	2	1	2	3	2
1	1	1	1	2	2	2	2	2	2
2	2	2	2	2	2	2	2	2	2
3	3	2	2	1	1	1	1	1	1
3	1	2	1	2	3	1	2	3	1
1	1	1	2	3	2	2	1	1	2
1	1	2	3	3	3	1	2	1	3
2	3	3	3	3	3	3	3	3	3
2	1	1	2	2	1	1	2	2	1
2	3	3	2	1	2	2	2	3	2
2	2	2	2	2	2	2	2	2	2
1	1	1	1	1	1	1	1	1	1
2	1	2	1	2	1	2	1	2	1
2	2	2	1	2	1	2	2	1	2
2	2	2	1	2	2	2	2	2	2
3	1	2	2	3	2	1	2	2	1
3	1	1	1	2	1	2	1	3	3

2	1	2	1	2	2	1	1	1	1
1	2	3	2	3	2	2	1	2	3
1	1	1	2	2	2	2	2	2	2
3	3	3	1	1	1	1	1	1	1
3	1	2	3	1	2	3	1	2	3
1	3	2	1	1	1	2	3	3	3
1	2	3	2	2	1	3	2	1	2
3	1	2	3	2	3	2	3	3	2
3	3	2	1	1	1	1	1	1	1
3	3	3	3	3	2	1	2	1	1
2	3	2	2	1	2	3	2	3	1
2	2	3	2	1	2	3	1	2	3
1	1	1	1	1	1	1	1	1	1
3	1	1	1	3	3	1	1	1	1
3	2	3	2	3	1	2	3	2	3
1	3	3	1	1	1	1	3	1	1
1	2	2	1	2	1	1	1	1	3
2	3	2	2	2	1	2	9	1	1
1	3	2	2	2	2	1	1	1	2
2	2	9	1	1	1	1	1	1	3
3	3	3	2	2	3	3	9	2	2
1	2	2	3	3	3	1	3	3	3
1	3	3	3	1	1	1	1	3	9
3	1	2	1	1	2	1	2	1	2
2	2	2	2	2	2	2	2	2	2
2	2	2	2	2	2	2	2	2	2
2	2	2	1	2	1	1	3	2	1
1	2	1	3	2	1	3	1	2	1
2	2	2	2	2	2	2	2	2	2
9	9	9	9	9	9	9	9	9	9
9	9	9	9	9	9	9	9	9	9
9	9	9	9	9	9	9	9	9	9
1	1	1	2	2	2	2	1	1	2
9	9	9	9	9	9	9	9	9	9
2	2	2	2	2	2	2	2	2	2
9	9	9	9	9	9	9	9	9	9
2	2	2	1	2	3	2	2	1	3
9	9	9	9	9	9	9	9	9	9
9	9	9	1	2	2	1	2	9	1
2	1	2	3	2	3	3	3	2	1
9	9	9	9	9	9	9	9	9	9
1	2	2	1	1	1	2	3	1	2
2	2	3	2	3	2	1	3	3	2
2	2	2	2	3	2	3	1	3	2
3	2	2	1	2	3	2	3	1	2
2	3	2	2	2	1	2	3	2	3
2	2	2	1	2	1	1	3	2	1

2	2	3	2	1	2	1	9	2	1
2	2	1	2	1	2	2	2	2	2
2	2	2	2	2	2	2	2	2	2
2	1	2	1	1	2	1	2	1	1
2	2	2	2	2	2	2	2	2	2
1	3	1	2	2	1	2	3	1	2
9	9	9	9	9	9	1	9	9	9
1	2	3	2	1	2	3	2	1	1
2	3	2	2	1	1	1	2	3	1
3	3	3	3	3	3	2	2	2	3
1	2	3	1	2	3	1	2	3	1
3	1	2	3	1	2	3	2	3	2
3	9	2	1	2	1	3	1	3	2
2	2	2	2	2	2	2	2	2	2
2	2	2	2	2	2	2	2	2	2
9	9	9	9	3	9	2	9	9	1
2	2	2	2	2	2	2	2	2	2
2	2	2	2	2	2	2	2	2	2
2	2	2	2	2	2	2	2	2	2
2	2	2	2	2	2	2	2	2	2
1	1	9	1	1	1	1	1	1	1
1	9	9	1	1	1	1	1	1	1
1	9	9	1	1	1	1	1	1	1
2	2	2	1	1	1	1	1	1	1
1	3	3	1	1	1	1	1	1	1
1	3	3	1	1	1	1	1	1	1
3	3	1	1	1	1	1	1	1	1
2	2	2	1	1	2	1	1	1	2
2	2	1	1	1	2	1	1	1	2
1	3	3	1	1	1	1	1	1	1
1	3	3	1	1	1	1	1	1	1
9	9	9	9	9	9	9	9	9	9
1	3	3	1	1	1	1	1	1	1
1	3	3	1	1	1	1	1	1	1
1	3	3	1	1	1	1	1	1	1
1	3	3	1	1	1	1	1	1	1
1	3	3	1	1	1	1	1	1	1
1	3	3	1	1	1	1	1	1	1
1	3	3	1	1	1	1	1	1	1
1	3	3	1	1	1	1	1	1	1
3	3	9	1	1	1	1	1	1	1
9	9	9	9	9	9	9	9	1	9
1	3	3	1	1	1	1	1	1	1
3	9	9	9	1	1	1	1	1	1
1	3	3	1	1	1	1	1	1	1
1	3	3	1	1	1	1	1	1	1
1	3	1	1	9	9	1	1	1	1

1	3	3	1	1	1	1	1	1	1
2	9	3	1	1	1	1	1	1	1
1	3	3	1	1	1	1	1	1	1
1	1	1	1	1	1	1	1	1	1
1	3	3	1	1	1	1	1	1	1
1	3	3	1	1	1	1	1	1	1
1	2	2	1	1	1	1	1	1	1
2	2	2	1	1	1	1	1	1	2
1	3	3	1	1	1	1	1	1	1
1	3	3	1	1	1	1	1	1	1
1	3	3	1	1	1	1	1	1	1
3	3	2	1	1	1	1	1	1	1
3	3	3	1	1	1	1	1	1	1
1	3	3	1	1	1	1	1	1	1
3	3	1	1	1	1	1	1	1	1
1	3	3	1	1	1	1	1	1	1
1	1	3	1	1	1	1	1	1	1
3	1	1	1	1	1	1	1	1	1
1	3	3	1	1	1	1	1	1	1
1	1	1	1	1	1	1	1	1	1
3	2	2	2	2	3	3	1	9	2
1	1	2	1	1	3	3	3	3	3
1	1	3	1	1	1	1	1	1	1
1	3	3	1	1	1	1	1	1	1
3	3	1	1	1	1	1	1	1	1
1	3	3	1	1	1	1	1	1	1
1	3	3	1	1	1	1	1	1	1
9	1	3	2	2	1	3	3	3	3
1	3	3	1	1	1	1	1	1	1
9	1	3	1	9	9	1	1	1	1
1	3	3	1	1	1	1	1	1	1
1	3	3	1	1	1	1	1	1	1
1	3	3	1	1	1	1	1	1	1
1	3	3	1	1	1	1	1	1	1
1	3	3	1	1	1	1	1	1	1
1	3	3	1	1	1	1	1	1	1
9	9	9	9	9	9	9	1	9	1
1	3	3	1	1	1	1	1	1	1
1	3	3	1	1	1	1	1	1	1
1	1	1	1	1	1	1	2	1	1
1	3	3	1	1	1	1	1	1	1
1	3	3	1	1	1	1	1	1	1
1	3	3	1	1	1	1	1	1	1
1	3	3	1	1	1	1	1	1	1
1	3	3	1	1	1	1	1	1	1
1	3	3	1	1	1	1	1	1	1
1	2	2	1	1	1	1	1	1	1

1	3	3	1	1	1	1	1	1	1
1	3	3	1	1	1	1	1	1	1
1	1	3	1	1	1	1	1	1	1
1	3	3	1	1	1	1	1	1	1
3	3	1	1	1	1	1	1	1	1
1	1	1	1	1	1	1	1	1	3
1	3	3	2	1	1	1	1	1	1
1	1	1	1	1	1	1	1	1	1
3	1	1	1	1	1	1	1	1	1
3	3	1	1	1	3	1	1	1	1
1	2	2	1	1	3	2	2	1	1
9	9	9	9	9	9	9	9	9	9
1	3	3	1	1	1	1	1	1	1
3	2	2	2	2	2	2	2	2	2
3	1	3	1	2	2	2	2	2	2
2	1	1	1	1	1	1	1	1	1
9	9	9	9	9	9	9	9	9	9
9	9	9	9	9	9	9	9	9	9
1	1	1	1	1	1	1	1	1	1
1	1	1	1	1	1	1	1	1	1
1	3	3	1	1	1	1	1	1	1
2	3	9	2	1	2	2	1	1	2
3	3	3	2	3	2	2	2	1	2
3	1	3	2	1	2	1	3	2	2
1	1	3	3	3	3	3	1	1	3
2	2	2	1	1	2	1	1	2	1
2	3	2	1	3	2	1	2	1	2
1	1	1	2	1	1	1	1	1	1
2	2	1	1	1	2	2	1	1	1
1	3	1	1	1	2	2	1	2	1
2	1	1	2	2	2	2	2	2	1
1	9	9	9	1	9	9	1	9	9
2	1	2	2	1	2	2	2	2	2
1	3	3	2	1	2	1	2	1	2
3	3	3	2	3	1	1	1	2	2
2	3	1	1	1	1	1	2	1	1
3	2	1	1	1	1	2	2	2	2
3	2	2	2	2	2	2	2	2	2
3	2	2	2	1	2	3	1	1	1
2	2	1	1	2	1	2	3	2	2
9	3	1	1	1	1	1	1	1	1
3	3	1	1	1	1	1	1	1	2
1	2	2	2	1	2	1	1	2	2
2	2	2	1	1	1	1	2	2	2
1	2	2	2	1	2	1	1	2	9
3	3	3	3	1	1	1	1	1	1
9	9	9	9	2	1	1	1	1	1

1	3	3	1	1	1	1	1	1	1
1	1	1	1	2	1	1	1	3	2
1	2	2	1	1	1	1	2	2	2
2	2	2	2	2	2	2	2	2	2
9	9	9	9	9	9	9	9	9	9
3	3	3	2	1	1	2	2	2	2
1	1	1	1	1	1	9	1	1	1
2	2	2	2	9	9	9	1	1	2
1	3	3	2	2	1	2	2	2	2
2	3	2	1	2	1	1	1	2	2
1	2	3	1	3	1	1	3	3	3
3	3	1	1	1	1	1	1	1	1
2	2	2	2	1	1	1	1	1	2
3	3	1	1	3	2	1	1	1	2
2	2	1	2	1	1	2	2	1	2
9	9	9	9	1	9	9	9	9	9
2	3	1	1	1	1	1	1	1	1
2	2	2	1	2	2	1	2	1	2
1	1	1	1	1	1	1	1	1	1
2	3	1	1	1	1	1	1	1	1
9	9	9	9	9	9	9	9	9	9
2	3	9	1	1	2	1	1	1	2
2	2	2	2	2	2	2	2	2	2
3	3	3	3	1	1	1	1	1	1
3	3	2	1	1	1	1	2	1	1
3	3	2	2	1	1	1	1	1	1
2	2	2	2	1	1	1	1	1	9
9	9	9	9	9	9	9	9	9	9
9	9	9	9	9	9	9	9	9	9
1	3	2	1	1	3	2	3	1	3
3	3	1	1	3	3	1	1	1	1
1	2	1	1	1	1	2	1	1	2
1	1	9	1	1	1	1	1	1	1
9	9	9	1	1	1	1	1	1	1
1	3	3	1	1	1	1	1	1	1
1	1	3	2	1	1	1	1	1	1
1	3	3	1	1	1	1	1	1	1
1	3	1	1	1	1	1	1	1	1
1	3	3	1	1	1	1	1	1	1
1	3	1	1	1	1	1	1	1	1
3	2	3	1	1	1	1	1	1	1
1	3	3	1	1	1	1	1	1	1
1	3	3	1	1	1	1	1	1	1
9	3	3	1	1	1	1	1	1	1
1	3	3	1	1	1	1	1	1	1
1	1	3	3	1	1	1	1	1	1
3	9	9	1	1	1	1	1	1	1

1	3	9	1	1	1	1	1	1	1
3	3	3	3	1	1	1	1	1	1
9	9	9	9	9	9	9	9	9	9
3	9	1	1	1	1	1	9	1	1
9	9	1	9	1	9	9	9	1	9
3	3	3	1	1	1	1	1	1	1
2	1	2	1	2	3	1	2	1	3
3	3	1	1	1	2	1	1	1	2
9	9	9	9	9	9	9	9	9	9
9	9	9	9	9	9	9	9	9	9
3	1	3	1	3	3	3	3	3	3
1	9	2	1	1	1	1	2	2	1
3	1	1	2	2	2	1	1	1	1
9	9	9	9	9	9	9	9	9	9
1	2	2	2	1	2	1	1	1	1
1	3	3	2	1	2	1	1	1	2
9	9	1	1	9	1	1	1	1	9
2	9	1	2	1	1	1	1	1	2
3	3	1	1	1	1	1	1	1	1
3	2	2	1	1	2	3	1	2	2
1	3	3	1	1	3	3	3	1	1
1	3	1	1	1	1	1	1	1	1
1	1	3	1	1	1	1	1	1	1
3	1	1	1	1	1	1	1	1	1
9	9	9	1	9	1	1	9	1	1
3	1	2	1	1	1	1	1	1	1
2	2	1	1	9	9	1	1	1	2
1	3	3	1	1	1	3	2	2	3
1	3	1	1	1	1	1	1	1	1
3	1	1	1	1	1	1	1	1	1
3	2	2	1	1	1	2	3	1	2
1	2	3	1	1	1	1	1	1	1
2	2	2	1	1	1	3	1	1	2
1	3	3	1	1	1	3	1	1	1
3	3	2	1	3	2	3	1	1	2
1	3	2	1	1	1	1	3	1	2
3	3	1	1	1	3	1	1	1	1
9	9	9	9	9	9	9	1	9	1
2	2	2	2	2	2	1	1	2	2
1	3	2	1	1	1	1	1	1	1
3	3	2	2	1	2	2	1	3	2
1	3	3	1	1	3	2	3	1	3
9	9	9	9	9	9	9	9	9	9
9	9	9	9	9	9	9	9	9	9
2	3	2	1	1	1	1	1	1	1
1	3	9	3	3	1	1	1	1	1
9	9	9	9	9	9	9	9	9	9

1	3	1	1	1	1	1	1	1	3
3	3	1	1	1	1	1	2	1	2
2	2	2	1	2	1	1	1	2	2
2	2	2	9	1	1	2	1	1	2
1	3	1	1	1	3	1	1	1	3
2	2	2	2	2	1	1	1	1	2
1	2	2	1	2	2	3	2	2	3
2	1	1	1	1	2	1	2	1	2
2	2	1	1	1	1	1	1	1	1
2	2	2	1	2	2	2	3	1	1
1	2	2	1	1	2	1	1	1	3
9	9	9	9	9	9	9	9	9	9
9	9	9	9	9	9	9	9	9	9
9	9	9	9	9	9	9	1	9	9
9	9	9	9	9	9	9	9	9	9
9	9	9	9	9	9	9	1	9	9
3	3	1	1	3	3	3	1	3	3
3	3	3	1	1	3	1	1	1	1
9	9	9	1	9	9	9	9	9	9
3	3	1	1	1	2	2	2	1	2
2	3	3	1	1	2	1	1	1	1
3	1	2	3	2	2	1	1	1	2
3	1	1	1	1	1	1	1	1	2
1	3	1	1	2	2	1	2	3	2
3	3	1	2	1	2	1	1	2	2
3	3	2	1	1	3	1	1	1	1
2	2	2	1	3	1	1	1	1	1
2	3	3	1	1	2	3	3	1	1
9	9	1	1	9	9	9	1	1	9
9	9	9	1	1	1	2	1	1	2
2	2	2	1	1	2	1	1	1	1
9	9	9	1	9	9	9	1	9	1
3	3	3	1	1	2	1	3	1	2
3	3	2	1	1	1	1	1	2	1
2	2	2	1	1	2	1	2	1	2
3	3	1	1	2	2	1	3	3	2
1	3	3	1	2	2	1	2	1	2
1	3	3	1	1	1	1	1	1	1
3	9	3	9	9	1	1	1	1	1
1	3	3	1	1	1	1	1	1	1
2	3	2	3	2	3	2	3	2	3
2	2	2	2	1	2	2	2	2	2
3	2	1	1	2	1	1	1	1	2
2	2	2	2	2	2	1	1	1	1
1	2	2	1	1	1	1	1	2	2
9	9	1	1	9	1	1	1	1	9
1	1	1	1	1	1	1	1	1	1

1	3	3	1	1	1	1	1	1	1
1	2	1	1	1	1	1	1	1	1
2	2	2	2	2	2	2	1	2	1
1	2	3	1	2	3	1	1	1	1
1	2	3	2	2	2	2	2	1	2
2	2	3	2	2	1	2	2	2	2
2	2	2	2	2	9	1	2	1	1
2	2	2	1	2	2	1	1	1	1
1	1	1	2	2	2	2	2	2	2
1	2	2	1	1	1	1	1	1	1
1	2	2	2	3	2	2	1	1	2
2	2	2	2	2	2	2	2	2	2
2	2	2	1	2	2	1	1	1	1
1	2	2	1	1	2	1	1	1	1
2	2	2	2	2	2	2	2	2	2
1	2	2	1	1	1	1	1	1	1
2	2	2	2	2	2	2	2	2	2
2	2	2	2	2	2	2	2	2	2
1	2	2	2	2	2	2	2	2	2
2	2	2	2	2	2	2	2	2	2
2	2	2	2	2	2	2	2	2	2
9	9	9	9	9	9	9	9	9	9
3	1	3	1	2	1	1	1	1	1
3	1	3	2	2	2	3	3	1	2
2	1	1	2	1	2	1	1	1	2
3	3	1	2	2	1	1	2	1	2
1	1	2	2	1	1	1	1	2	1
2	1	1	1	9	1	9	9	9	9
3	3	9	9	9	2	2	1	1	2
3	2	3	1	2	2	2	3	3	3
2	2	2	1	1	1	1	1	1	1
2	2	2	1	1	1	1	1	1	1
9	9	9	9	9	9	9	9	9	9
9	9	9	9	9	9	9	9	9	9
9	9	9	9	9	9	9	9	9	9
9	9	9	9	9	9	9	9	9	9
9	9	9	9	9	9	9	9	9	9
9	9	9	9	9	9	9	9	9	9
9	9	9	9	9	9	9	9	9	9
9	9	9	9	9	9	9	9	9	9
3	3	2	2	3	2	1	2	1	1
9	9	9	9	9	9	9	9	9	9
9	9	9	9	9	9	9	9	9	9
2	3	3	2	2	2	2	2	1	1
9	9	9	9	9	9	9	9	9	9
9	9	9	9	9	9	9	9	9	9
9	9	9	9	9	9	9	9	9	9

1	1	1	1	1	1	2	1	1	1
1	3	2	3	3	3	3	3	2	1
9	9	9	9	9	9	9	9	9	9
9	9	9	9	9	9	9	9	9	9
9	9	9	9	9	9	9	9	9	9
9	9	9	9	9	9	9	9	9	9
9	9	9	9	9	9	9	9	9	9
9	9	9	9	9	9	9	9	9	9
9	9	9	9	9	9	9	9	9	9
9	9	9	9	9	9	9	9	9	9
1	3	3	1	1	3	1	1	1	1
2	3	3	1	1	1	1	1	1	1
2	3	3	1	1	1	1	1	1	1
1	2	2	1	1	1	1	1	1	1
1	1	1	1	1	1	1	1	1	1
1	3	1	1	1	1	1	1	1	1
1	3	1	1	1	1	1	1	1	1
1	1	1	1	1	1	1	1	1	1
2	3	3	1	1	1	1	1	1	1
1	2	1	1	1	2	1	1	1	1
1	2	1	1	1	2	1	1	1	1
3	2	2	2	3	2	1	1	2	2
9	9	9	9	9	9	9	9	9	9
1	1	3	1	1	1	1	2	1	1
3	9	1	3	1	2	2	9	9	9
3	3	1	1	1	1	1	2	1	1
3	3	3	3	1	2	1	2	1	2
1	3	3	1	1	2	1	1	1	1
1	1	1	1	1	1	1	1	1	1
1	2	2	1	1	2	2	2	1	2
1	2	2	1	1	9	1	1	1	1
3	2	2	1	2	2	1	1	1	2
3	1	3	1	1	1	1	2	2	2
1	3	2	1	1	1	1	1	1	1
1	2	2	2	2	2	1	1	2	1
1	3	1	1	1	1	1	1	1	1
1	3	9	1	1	1	1	1	1	1
1	3	3	1	1	1	1	1	1	1
1	3	3	1	1	1	1	1	1	1
1	2	2	1	1	1	1	2	1	1
3	9	1	1	1	2	1	1	1	1
1	3	1	1	1	1	1	1	1	1
3	1	1	1	2	3	3	3	2	2
3	2	9	2	3	1	1	2	1	1
1	2	3	1	1	2	1	1	1	1
3	1	3	1	3	1	1	1	1	1
1	1	1	1	1	1	1	1	1	1

1	1	1	1	1	1	1	1	1	1
1	1	1	1	1	1	1	1	1	1
1	1	1	1	1	1	1	1	1	1
9	3	2	1	3	2	1	2	1	1
1	2	3	1	1	2	1	1	1	1
1	3	9	2	1	9	2	1	2	1
1	1	1	1	1	1	1	1	1	1
9	2	3	1	1	2	1	1	9	1
1	3	2	1	1	2	1	1	1	1
1	1	1	1	1	1	1	1	1	1
2	2	2	1	1	1	1	1	1	1
3	1	1	1	1	1	1	1	1	1
2	2	2	1	1	1	1	1	1	1
2	2	2	1	1	1	1	1	1	1
3	3	1	1	1	1	1	1	3	1
1	2	1	1	1	1	1	1	1	1
1	2	2	2	2	2	1	1	1	1
2	2	2	2	2	2	2	2	1	2
1	3	9	1	3	1	1	2	1	3
1	3	3	3	3	1	1	1	1	1
3	3	3	1	1	2	3	3	2	3
1	1	3	1	1	1	1	1	1	1
1	1	2	1	2	2	1	2	1	1
1	2	2	1	1	2	1	1	1	2
1	1	1	1	1	1	1	1	1	2
3	2	2	2	2	2	1	1	1	1
1	1	2	1	1	1	1	1	1	1
2	2	2	2	2	2	2	2	2	2
2	2	2	2	1	1	1	1	1	1
1	2	2	1	1	1	2	2	1	2
2	2	3	3	3	3	2	2	2	3
2	2	1	2	2	2	1	1	3	2
2	2	2	2	2	2	1	1	1	1
3	2	1	1	1	2	1	1	1	1
1	9	9	9	9	9	9	9	9	9
1	1	2	1	2	2	1	2	1	1
1	3	3	2	2	2	1	3	1	1
2	2	2	2	2	2	2	2	2	2
3	3	3	1	1	1	1	1	1	1
1	3	3	2	1	1	1	1	1	1
2	2	2	2	2	2	2	2	2	2
3	3	1	1	1	1	1	3	1	1
3	3	1	1	1	1	1	3	1	1
2	2	2	1	2	2	1	2	1	2
3	1	2	1	1	2	1	1	1	1
2	1	2	2	2	1	1	2	1	1
2	2	2	2	2	2	2	2	2	2

3	3	2	3	2	2	3	3	2	3
2	2	2	2	2	2	2	2	2	2
1	3	3	1	2	2	3	1	1	2
1	3	3	3	3	1	1	3	1	1
1	3	2	2	2	2	1	1	2	1
1	3	3	3	2	2	2	2	2	2
3	3	3	1	2	2	3	3	3	2
2	2	2	2	2	2	2	2	2	2
1	2	2	2	2	3	2	3	1	1
2	2	1	1	1	1	1	1	1	2
1	2	2	2	2	1	2	1	1	2
2	3	2	1	1	1	1	3	1	1
9	9	9	9	9	9	9	9	1	9
3	3	3	2	2	2	1	2	1	1
3	3	3	1	3	3	3	3	3	3
2	2	2	2	2	2	2	2	2	2
3	1	3	2	2	1	1	1	1	2
2	2	2	1	1	1	1	1	1	1
2	3	1	1	1	1	1	2	3	2
1	1	2	1	2	1	1	1	1	1
1	3	3	1	3	1	1	1	1	1
1	3	2	2	1	1	1	1	1	1
1	3	1	1	1	1	1	1	1	3
3	3	1	1	1	2	2	1	3	2
1	2	2	3	2	2	1	1	1	1
1	2	3	1	1	2	1	1	1	1
1	3	1	1	1	1	1	1	1	1
3	3	1	2	2	1	1	2	1	2
2	3	3	2	2	2	2	2	1	1
9	1	9	9	9	9	9	9	9	9
2	2	1	3	1	1	1	2	3	2
2	2	3	3	3	3	3	1	1	2
2	2	2	2	3	3	2	1	1	2
1	2	2	2	2	2	1	1	1	1
9	9	9	1	9	1	1	1	9	9
1	1	2	1	1	2	2	1	1	2
2	2	9	1	3	2	2	2	1	1
9	9	9	9	9	9	9	9	9	9
1	3	3	3	2	1	1	1	3	2
3	3	1	1	2	2	1	1	2	2
1	9	9	9	9	1	9	9	9	9
1	3	1	1	2	1	1	1	1	1
1	3	3	1	1	1	1	1	1	1
9	9	9	1	9	9	9	9	9	1
3	2	2	2	2	1	1	3	2	1
2	3	3	2	2	2	2	2	2	2
1	2	2	1	1	1	1	1	1	1

1	3	2	1	3	1	1	1	2	2
2	2	2	1	1	1	1	1	1	2
2	2	1	1	1	1	1	1	1	1
1	2	2	1	1	2	1	1	2	2
2	2	3	3	3	2	2	2	2	3
1	3	2	2	1	2	1	1	1	2
2	2	1	1	1	1	1	1	2	1
9	9	9	9	9	9	9	9	9	9
2	2	2	2	2	2	2	2	2	2
2	2	2	2	2	2	2	2	2	2
1	2	3	2	2	2	2	1	1	1
2	2	2	2	1	2	1	2	2	3
3	1	1	2	2	1	1	1	2	1
2	2	2	2	2	2	2	2	2	2
1	3	2	2	2	2	2	2	2	2
1	3	3	2	2	2	1	3	1	1
2	2	1	1	1	1	1	1	1	1
3	1	9	1	1	1	1	1	1	1
1	3	3	2	1	1	1	1	1	1
2	2	2	2	9	1	1	1	1	1
2	2	2	1	2	1	1	1	1	1
1	1	1	1	1	1	1	1	1	1
2	2	2	1	1	1	1	2	2	2
3	3	3	1	1	1	1	2	2	2
3	3	3	2	1	1	1	1	2	3
1	1	1	1	1	1	1	1	1	1
2	3	2	1	2	2	1	1	1	1
1	1	1	1	1	1	1	1	1	1
1	1	1	1	1	1	1	1	1	1
9	9	9	9	9	9	9	9	9	9
1	3	1	1	1	1	1	1	1	1
1	2	2	1	1	1	1	1	1	1
1	2	2	1	1	1	1	1	1	1
2	3	3	1	1	2	1	2	2	2
1	2	1	9	9	9	9	9	9	9
1	2	1	1	1	1	1	1	1	1
1	2	1	1	1	1	1	1	1	1
3	1	1	1	1	1	1	1	1	1
3	3	1	1	1	1	1	1	1	1
3	3	2	1	1	1	1	1	2	2
1	2	2	1	2	2	1	1	1	1
3	1	9	1	1	1	1	1	1	1
2	2	2	3	3	3	2	2	2	2
3	3	3	1	3	3	1	3	1	1
1	3	3	1	1	1	1	3	1	1
3	3	3	1	1	1	3	3	3	1
1	3	1	1	1	1	1	1	1	1

1	3	3	1	1	1	1	3	1	1
1	3	3	1	1	1	1	3	1	1
3	3	3	1	1	1	1	1	1	9
1	3	2	1	1	1	1	1	1	1
3	3	1	1	1	1	1	1	1	1
3	3	1	1	1	1	1	1	1	1
3	3	3	1	1	1	1	1	1	1
1	1	1	1	1	2	1	2	1	3
2	3	3	1	1	1	1	1	1	1
2	3	3	1	1	1	1	1	1	1
1	3	3	1	1	1	1	1	1	1
1	3	3	1	1	1	1	1	1	1
1	3	3	1	1	1	1	1	1	1
1	3	1	3	2	1	1	3	1	1
1	3	3	1	1	2	1	9	1	1
1	3	3	1	1	1	1	1	1	1
1	1	2	2	3	2	1	2	1	1
2	2	2	1	1	1	1	1	2	1
1	1	1	1	1	1	1	1	1	1
1	1	1	1	1	1	1	1	1	1
2	2	1	1	1	1	1	2	2	1
3	3	3	1	1	3	3	1	3	3
9	9	9	9	9	9	9	9	9	9
9	9	9	9	9	9	9	9	9	9
9	9	9	9	9	9	9	9	9	9
9	9	9	9	9	9	9	9	9	9
3	3	3	1	1	1	3	1	9	9
9	9	9	9	9	9	9	9	9	9
9	9	9	9	9	9	9	9	9	9
9	9	9	9	9	9	1	9	9	9
9	9	9	9	9	9	9	9	9	9
9	9	9	9	9	9	9	9	9	9
9	9	9	9	9	9	9	9	9	9
9	9	9	9	9	9	9	9	9	9
3	3	1	2	2	1	1	2	1	2
1	3	3	1	1	1	1	1	1	1
1	2	2	1	1	1	1	1	1	1
1	1	3	1	1	1	1	1	1	1
3	2	1	2	2	2	1	1	1	1
2	3	2	1	2	2	1	1	1	1
3	1	1	1	1	1	1	1	1	1
3	2	2	1	2	1	2	2	1	1
1	3	1	1	1	2	2	1	1	2
3	3	1	1	3	2	2	2	2	2
1	3	2	1	2	2	3	3	2	3
3	3	1	1	1	1	1	3	1	1
2	1	2	1	1	1	2	1	1	1

1	1	1	2	2	2	2	1	1	2
2	2	2	2	2	2	2	2	2	1
1	3	3	1	1	1	1	1	1	2
1	1	1	3	2	2	3	2	3	1
1	2	2	1	1	1	1	1	2	1
2	2	2	1	1	1	2	2	2	2
9	9	9	9	9	9	9	9	9	9
2	2	2	2	2	2	2	2	2	2
1	2	3	3	2	1	1	1	2	2
1	2	3	1	1	1	1	1	2	2
2	2	2	2	2	2	2	2	2	2
2	2	2	1	1	1	1	1	1	1
2	3	1	2	2	2	2	1	1	2
3	3	3	3	2	1	2	3	3	2
2	2	2	2	2	2	2	2	2	2
2	2	2	2	2	2	2	2	2	2
1	1	2	2	2	2	2	2	2	2
1	2	2	1	2	1	2	1	3	1
1	2	2	1	1	1	1	1	1	1
1	1	1	1	1	1	1	1	1	1
1	2	2	3	3	1	2	3	1	1
2	2	2	3	3	2	2	2	2	2
1	2	3	3	2	1	1	1	2	2
1	1	1	1	1	2	1	1	1	1
1	1	1	1	1	1	1	1	1	1
3	2	3	1	2	3	2	1	2	2
1	2	1	2	1	2	1	2	2	2
1	2	2	1	1	1	1	1	1	1
2	2	2	2	2	2	2	2	2	2
2	2	2	2	2	2	2	2	2	2
1	1	1	1	1	1	1	1	1	1
1	1	1	1	1	1	1	1	1	1
2	2	2	2	2	2	2	2	2	3
2	3	1	1	1	1	1	1	2	2
2	2	2	1	1	3	3	2	2	2
3	3	3	3	3	2	2	2	2	2
2	2	2	2	2	2	2	2	2	2
1	3	2	2	2	2	2	1	2	2
2	2	2	2	2	2	2	2	2	2
1	3	1	1	1	1	1	1	1	1
1	2	2	1	1	2	1	1	1	1
1	2	2	1	1	2	1	1	1	1

Raisopposi	Undressto	EverDoneB	HeardCerv	CervicalCA	PapSmear	HPVandVa	Papdetect	PapforMar	PapOverEi
2	3	1	2	2	2	2	2	2	2
3	3	1	2	2	2	2	2	3	2
2	2	1	2	2	2	2	2	2	2
2	2	1	2	2	2	2	3	2	2
2	1	2	2	1	1	2	1	2	2
3	2	1	2	2	2	2	2	2	2
2	2	1	2	2	2	2	2	2	2
2	2	1	1	2	2	1	1	1	2
3	3	1	2	2	1	1	1	1	2
3	2	2	3	2	1	3	2	3	1
2	2	1	2	2	2	1	2	2	2
2	3	2	2	1	2	1	1	2	2
2	2	1	2	1	1	1	1	1	2
2	2	2	2	2	2	2	1	2	2
2	2	1	3	3	2	2	2	2	2
2	2	1	3	1	3	3	2	2	2
9	9	1	9	9	9	9	9	9	9
1	1	2	3	1	3	2	2	2	2
9	9	1	9	9	9	9	9	9	9
3	3	2	1	2	2	3	9	9	9
1	1	4	1	1	1	2	1	3	1
1	1	1	1	1	1	1	1	3	2
1	1	5	1	1	1	1	1	3	1
1	1	5	1	1	1	1	1	2	1
2	2	2	3	2	2	2	2	9	9
2	2	1	9	9	9	9	2	2	1
9	9	1	1	1	1	1	1	3	3
1	1	7	1	1	1	1	2	2	2
2	1	1	2	2	2	3	1	2	3
1	3	4	1	2	2	2	9	9	9
2	2	1	3	1	1	1	1	2	3
1	1	2	1	1	2	2	1	3	2
9	9	3	2	2	2	1	1	2	2
1	2	2	1	1	3	2	1	2	9
3	1	9	2	2	1	1	1	2	1
2	2	1	1	1	2	1	3	2	3
2	2	5	2	1	1	2	2	1	2
2	1	1	2	2	2	2	3	2	1
1	1	4	1	1	1	3	1	3	2
9	2	9	9	9	9	9	1	2	1
9	9	9	9	9	9	9	1	2	2
9	9	2	1	2	2	1	9	9	9
9	9	9	2	2	2	2	9	9	9
2	2	1	1	2	2	2	2	2	2
2	2	2	2	2	3	2	2	2	2
9	9	9	9	9	9	9	9	9	9

2	2	2	1	1	1	1	1	1	2
2	2	2	2	1	1	1	1	1	2
2	2	1	1	2	1	2	2	2	2
1	2	3	1	1	2	2	2	2	2
3	3	5	3	2	1	1	2	3	3
3	3	5	3	2	1	1	2	3	3
3	2	1	2	2	2	3	2	3	9
2	2	1	2	2	2	2	2	2	2
1	1	2	2	1	1	1	1	1	1
1	3	1	2	2	1	2	3	2	3
1	9	1	9	9	9	9	1	9	9
1	1	1	1	2	1	1	1	1	2
1	1	1	2	2	2	2	1	2	2
2	2	1	1	2	1	1	1	1	2
2	2	1	2	1	1	2	1	2	2
2	2	1	2	1	1	1	1	1	2
3	3	9	2	2	2	2	2	2	2
3	2	1	2	2	2	2	1	3	1
2	2	1	2	2	2	2	2	2	2
9	9	9	9	9	9	9	2	2	2
2	2	1	1	2	2	2	3	2	2
2	2	1	3	2	2	1	2	2	2
2	2	9	2	1	1	1	1	1	2
2	2	1	1	1	2	1	1	1	2
2	2	9	1	2	2	1	1	1	2
3	2	1	2	2	2	2	1	2	1
1	3	1	3	3	1	1	1	3	3
2	3	9	2	2	1	2	1	2	3
1	2	1	2	2	2	2	1	3	1
1	3	5	3	1	3	3	3	3	3
9	9	9	9	9	9	9	9	9	9
1	3	1	2	2	2	2	2	2	3
1	9	2	2	2	2	2	1	3	3
2	2	1	2	2	2	2	2	2	2
3	3	1	3	2	2	2	2	2	2
2	2	1	2	2	2	2	2	2	2
3	3	1	1	1	3	1	1	2	2
2	1	1	1	2	2	2	1	2	2
3	3	7	2	2	2	2	3	3	2
2	1	1	1	2	2	2	1	2	2
1	2	1	2	3	1	2	1	2	2
9	9	9	9	9	9	9	9	9	9
9	9	1	2	2	3	2	1	2	2
2	1	1	1	2	2	2	1	1	1
2	2	1	3	2	3	3	2	2	1
2	3	9	1	1	1	1	3	2	2
3	3	1	3	2	2	2	1	2	2

3	1	3	1	2	3	1	1	2	1
3	3	1	3	3	3	3	3	3	3
2	2	1	2	3	2	2	2	2	2
1	1	1	1	2	2	2	1	2	2
2	2	1	1	2	1	1	1	1	2
3	2	1	2	2	2	2	3	2	2
2	2	2	1	2	1	2	1	2	2
3	3	9	9	9	9	9	9	9	9
2	2	1	2	2	2	2	2	2	2
1	2	4	1	3	1	2	1	2	1
9	2	9	1	1	9	2	1	2	2
3	3	1	1	2	1	1	1	2	2
1	1	1	1	1	2	1	1	1	2
2	2	2	3	3	2	2	2	2	2
2	2	1	1	2	2	1	2	2	2
2	2	1	1	9	2	2	2	2	2
2	1	1	2	1	1	1	1	1	1
1	3	1	2	2	2	2	1	2	2
3	3	3	2	9	2	2	2	9	2
1	1	1	2	2	2	2	3	1	2
1	3	9	1	2	2	1	1	3	1
1	3	1	2	2	1	3	1	2	1
1	1	1	2	1	1	2	2	2	2
2	2	1	1	1	1	1	2	2	2
2	2	2	2	3	3	2	1	2	3
2	2	1	2	2	2	2	1	2	3
1	3	2	1	1	2	2	1	3	1
1	2	2	1	1	1	2	1	2	1
2	1	1	2	2	2	2	1	2	2
1	3	1	1	1	3	2	2	2	2
1	1	1	1	2	1	2	1	1	2
1	2	1	2	2	2	2	1	2	2
2	1	1	3	2	2	3	3	9	3
1	1	7	1	1	2	2	1	2	2
1	2	1	2	2	2	2	1	2	2
1	3	9	1	1	2	2	1	1	1
3	3	1	2	2	2	9	2	3	2
2	2	1	2	1	1	1	1	2	2
9	9	9	9	9	9	9	9	9	9
3	3	1	2	2	2	2	3	3	3
1	1	1	2	1	1	1	1	2	3
9	9	9	9	9	9	9	9	9	9
2	2	1	2	1	1	1	1	2	2
1	2	2	2	1	1	1	1	2	2
3	3	3	2	2	2	2	9	9	9
1	1	3	1	1	1	3	1	3	2
1	1	1	2	2	2	2	1	2	2

3	1	1	2	2	2	2	2	2	9
3	3	9	2	2	2	2	1	1	1
3	3	1	1	1	1	1	3	3	3
2	2	1	3	2	2	2	1	2	2
2	1	1	2	2	1	2	1	2	2
3	2	1	2	3	2	2	2	3	2
9	2	9	2	1	2	1	2	9	1
9	3	9	1	1	2	2	3	2	9
9	9	1	3	2	2	2	2	9	9
2	2	9	2	2	2	2	2	2	2
3	3	2	1	1	2	3	1	2	3
3	3	1	2	2	2	1	1	2	2
2	2	9	1	2	2	2	2	2	2
2	2	1	2	2	2	1	2	2	2
2	3	1	2	1	1	1	1	2	2
3	3	1	1	1	1	1	1	2	3
1	1	1	2	1	1	1	1	2	3
2	2	1	2	1	1	1	1	2	2
9	9	1	3	2	2	2	2	9	9
3	2	1	2	2	2	2	3	9	2
1	2	1	1	2	3	3	2	3	1
2	2	1	3	2	2	3	2	2	2
3	2	1	3	3	2	2	2	2	2
2	2	1	3	2	2	2	1	2	1
2	2	5	1	1	2	2	2	2	2
3	1	1	2	2	2	2	3	3	3
2	2	1	2	2	2	2	2	3	1
2	3	1	3	1	1	1	2	2	2
2	2	9	2	2	2	2	2	2	2
1	1	1	1	1	2	2	1	2	2
1	2	7	1	1	1	2	1	2	2
3	3	7	2	2	2	2	1	1	2
1	1	2	1	1	1	1	1	1	1
2	3	2	1	3	1	2	3	3	3
3	3	1	1	1	1	1	2	2	2
1	1	7	1	1	1	1	3	3	3
2	2	1	1	1	1	1	1	1	1
1	1	2	1	1	1	1	1	3	1
1	3	2	2	2	2	2	1	3	1
2	2	9	1	2	1	1	1	3	3
1	1	9	9	2	2	1	9	9	9
2	2	4	1	1	1	2	2	2	2
2	2	1	1	2	2	2	2	2	2
9	9	9	9	9	9	9	9	9	9
9	9	9	9	9	9	9	9	9	9
1	2	1	2	1	1	2	1	2	2
1	1	2	1	2	1	1	1	2	2

9	9	2	1	1	3	2	1	9	9
3	3	1	2	2	2	2	2	2	2
1	1	5	1	2	2	2	2	9	9
9	9	3	1	2	2	2	1	9	9
9	9	1	2	2	2	2	9	9	2
9	9	9	9	9	9	9	9	9	9
2	2	1	1	1	2	2	2	2	2
9	9	9	9	9	9	9	9	9	9
2	3	1	1	3	2	2	1	2	1
9	9	4	2	3	1	1	9	9	9
9	9	1	2	2	2	2	9	9	9
9	9	4	2	2	3	3	9	9	9
2	1	2	1	2	3	3	1	1	2
2	3	9	9	9	9	9	1	2	2
9	9	9	2	1	1	1	3	9	9
2	3	1	2	1	2	1	3	2	3
3	3	9	1	1	1	1	3	3	3
2	2	2	1	2	1	1	3	3	2
1	1	1	3	3	3	3	2	2	2
3	2	1	2	2	2	2	2	2	2
9	9	1	2	1	2	2	1	1	1
9	9	9	9	9	9	9	9	9	9
1	1	1	3	2	2	2	3	3	3
1	2	1	2	2	2	2	1	3	1
2	2	1	3	2	2	2	2	2	2
1	1	1	2	2	2	9	1	1	2
1	1	5	1	2	2	2	2	2	2
1	1	1	1	2	2	3	3	3	2
3	3	1	1	1	1	1	1	3	3
3	3	2	1	1	1	1	1	3	3
2	2	1	3	2	2	2	2	2	2
2	2	1	2	2	1	2	1	1	2
9	9	9	9	9	9	9	9	9	9
2	2	1	2	2	2	2	3	2	1
1	3	2	2	2	2	1	2	3	1
2	2	2	1	2	2	1	1	1	2
3	3	9	2	1	2	1	2	9	1
2	2	1	1	2	2	3	2	2	1
1	9	1	3	2	1	2	2	9	9
1	3	1	2	2	2	2	1	1	1
2	2	1	1	2	1	3	1	2	2
1	3	1	1	1	1	1	3	1	3
2	2	3	1	2	1	3	1	1	1
2	2	1	1	1	2	2	2	2	2
1	1	1	3	3	3	3	2	2	2
2	3	2	1	1	1	2	3	2	2
9	9	9	9	9	9	9	9	9	9

2	2	3	2	2	2	1	1	1	1
1	1	1	1	2	2	2	1	3	2
1	1	1	2	2	2	2	2	1	1
3	2	3	2	9	9	9	9	9	9
2	2	2	1	2	2	2	2	2	2
2	2	1	2	2	3	3	2	3	2
3	3	5	1	1	1	2	1	3	1
2	2	1	3	2	1	3	3	2	2
1	1	1	2	1	2	2	1	1	1
1	1	1	1	2	2	2	3	3	1
1	1	9	2	2	2	3	2	2	1
9	9	9	1	1	2	3	3	9	9
9	9	1	2	2	2	2	2	9	9
2	2	1	1	1	1	1	1	9	9
2	2	9	2	2	2	2	2	9	9
9	2	9	2	2	2	2	9	9	9
9	9	9	9	9	9	9	9	9	9
9	9	9	9	9	9	9	9	9	9
2	2	1	2	2	2	2	2	9	2
9	9	9	9	9	9	9	9	9	9
9	9	9	9	9	9	9	9	9	9
2	2	1	1	2	1	1	1	1	1
2	2	1	2	1	2	1	1	2	2
1	1	1	2	1	1	1	1	1	2
2	2	1	2	2	1	1	1	1	2
9	9	9	9	9	9	9	9	9	9
2	2	1	1	2	1	2	1	2	2
9	9	9	9	9	9	9	9	9	9
9	9	9	9	9	9	9	9	9	9
2	2	1	1	1	2	1	1	2	2
9	9	1	2	2	2	2	9	9	9
9	9	9	9	9	9	9	9	9	9
9	9	9	9	9	9	9	9	9	9
9	9	9	9	9	9	9	9	9	9
3	1	1	2	2	2	2	9	3	2
3	1	1	2	2	2	2	9	1	9
9	9	9	9	9	9	9	9	9	9
9	9	1	9	9	9	9	9	9	9
9	9	9	9	9	9	9	9	9	9
9	9	1	9	9	9	9	3	9	9
9	2	9	9	9	9	9	3	9	9
9	9	1	2	2	2	2	9	9	9
3	3	1	1	1	1	1	3	3	3
2	2	9	2	2	2	1	1	3	1
3	1	1	1	1	1	1	3	2	1
2	1	2	1	1	9	9	1	9	9
9	9	1	1	2	2	1	1	9	9

9	9	1	3	2	2	2	1	9	9
9	9	1	1	2	2	2	9	9	9
3	3	4	1	1	2	2	2	2	2
3	2	1	1	2	1	1	3	1	2
3	2	9	9	9	9	9	2	2	1
2	2	1	1	2	3	9	2	2	2
1	2	1	1	1	2	1	2	1	3
1	1	1	2	2	2	2	2	1	3
2	2	9	3	3	3	3	2	2	2
3	3	1	1	1	1	1	3	3	3
9	9	9	9	9	9	9	9	9	9
9	9	9	9	9	9	9	9	9	9
9	9	2	2	2	2	2	9	9	9
9	9	9	1	1	1	1	9	1	9
1	1	1	1	2	1	1	2	9	2
2	2	9	1	1	1	2	1	2	2
9	9	9	9	9	9	9	9	9	9
1	2	1	1	2	2	2	2	2	1
1	1	9	1	1	1	1	1	1	1
2	2	1	2	2	2	2	1	3	3
3	3	4	1	2	2	2	3	1	3
2	2	1	1	2	2	2	2	2	2
3	3	1	2	2	2	1	2	3	2
2	2	1	1	2	1	1	2	2	2
2	2	1	2	1	2	2	2	2	2
2	2	1	1	1	1	1	2	2	2
2	2	1	2	2	2	2	2	2	2
3	1	2	1	1	1	1	1	1	1
2	2	9	2	3	3	3	2	2	2
2	1	1	1	2	2	1	1	2	1
2	2	1	3	3	3	3	2	2	2
2	2	1	3	3	3	3	2	2	2
2	3	4	2	2	2	2	2	1	3
2	2	9	2	2	2	3	3	3	3
3	3	9	1	1	1	2	1	3	2
2	2	9	2	1	3	3	1	2	3
9	9	9	9	9	9	9	9	9	9
2	2	1	2	2	2	2	2	2	2
3	3	1	2	2	2	2	3	3	3
3	3	9	2	2	2	2	3	3	3
9	9	9	3	3	3	3	2	9	9
9	9	9	9	9	9	9	9	9	9
9	9	9	2	2	2	9	9	9	9
1	1	7	1	1	2	3	2	2	2
2	2	2	2	2	1	3	2	2	2
2	2	9	2	2	2	2	2	2	2
3	3	4	1	1	1	1	2	2	1

9	9	9	1	2	3	2	9	9	9
9	9	1	2	2	1	1	2	9	9
9	9	1	2	2	9	2	9	9	1
9	9	3	2	3	1	1	9	9	2
9	9	1	1	3	3	3	9	9	9
1	1	1	1	1	1	1	3	3	9
9	9	1	3	2	3	3	9	9	9
9	9	1	2	2	2	1	2	9	9
1	2	1	2	2	2	2	2	9	9
9	9	1	2	2	2	2	2	9	9
9	9	9	2	3	2	2	9	9	9
9	9	9	1	9	2	2	9	9	9
9	9	9	2	2	2	2	2	9	9
9	9	9	3	2	3	1	9	2	9
9	9	2	2	2	2	3	2	9	9
9	9	9	9	9	9	9	9	9	9
9	2	3	2	2	2	2	9	9	2
1	2	5	2	2	1	1	2	9	2
3	3	1	1	1	1	1	1	2	2
2	2	1	2	2	2	2	2	2	2
1	1	2	1	1	1	2	1	3	1
2	3	5	9	9	9	9	1	3	2
2	2	1	2	2	2	2	2	2	2
3	2	7	3	3	1	3	2	3	2
2	3	7	2	3	3	3	2	3	9
2	2	1	9	9	9	9	9	9	9
2	2	1	3	2	2	2	2	2	2
2	2	1	2	2	2	2	2	2	2
2	2	1	1	3	1	3	1	2	2
1	1	1	1	1	1	1	1	1	1
2	1	1	1	1	1	1	1	2	2
3	3	1	2	2	2	2	2	2	2
3	3	2	2	1	1	2	2	3	3
1	1	1	2	2	2	2	1	2	2
9	9	9	9	9	9	9	9	9	9
2	2	1	1	2	2	2	2	2	2
9	9	9	9	9	9	9	9	9	9
2	2	3	2	2	1	2	1	2	2
2	3	2	2	2	2	2	9	9	9
2	2	9	1	1	1	1	2	2	2
1	2	1	2	2	2	2	1	2	2
2	3	2	2	2	2	2	3	3	3
1	3	1	2	2	2	1	1	9	2
1	2	1	2	3	2	2	2	2	2
2	2	1	2	2	2	2	3	3	3
2	2	1	1	1	2	2	1	1	1
2	2	1	1	1	1	1	2	2	2

1	3	1	1	1	1	1	1	3	1
2	2	1	2	9	2	2	2	9	9
2	2	1	1	1	1	1	1	1	2
2	3	1	2	2	2	3	2	1	2
2	2	1	2	2	2	2	2	2	2
3	2	1	2	2	2	2	2	2	2
2	2	9	1	1	2	2	1	2	1
2	2	1	1	1	3	2	1	3	2
9	9	9	9	9	9	9	9	9	9
2	2	1	2	2	2	2	2	2	2
1	2	2	1	2	2	2	2	2	2
9	2	5	2	2	2	2	1	9	9
9	9	1	9	9	9	9	1	9	9
1	9	9	2	2	2	1	1	1	1
9	2	1	2	1	2	2	1	9	9
2	2	1	2	2	2	2	2	2	3
9	9	9	9	9	9	9	9	9	9
9	9	1	9	3	2	1	2	9	9
9	9	1	2	3	3	3	9	9	2
2	3	1	3	2	2	2	2	3	2
2	2	9	3	3	3	3	2	2	2
2	3	1	2	2	2	2	2	2	2
2	2	1	2	2	2	2	2	2	2
9	1	1	2	2	2	2	9	9	9
9	9	1	2	2	2	2	2	9	9
2	2	3	2	2	2	2	1	1	1
1	1	3	1	1	1	1	1	2	2
1	1	2	1	1	1	1	2	3	1
2	2	1	9	9	9	9	9	9	9
9	9	4	2	3	1	1	3	9	9
2	2	9	2	2	2	2	2	2	2
1	2	1	2	2	2	2	1	2	3
1	1	1	2	2	2	2	2	2	2
3	3	2	1	2	2	2	3	2	1
1	3	1	1	2	2	2	1	1	1
9	9	9	9	9	9	9	1	1	1
9	9	9	9	9	9	9	9	9	9
9	9	9	9	9	9	9	9	9	9
1	3	2	1	1	1	3	3	1	3
1	2	1	1	1	1	2	1	2	1
1	1	1	1	2	1	2	2	2	2
3	3	6	2	2	2	2	2	2	2
2	2	1	2	2	2	2	2	2	2
1	2	1	2	2	2	2	1	2	1
3	3	9	9	9	9	9	3	3	3
2	2	2	1	2	3	3	2	2	2
3	3	6	1	2	3	3	3	3	2

2	2	2	2	2	2	3	3	3	2
1	2	4	1	1	1	1	1	3	2
9	9	2	3	1	2	1	9	9	1
9	9	2	2	2	2	2	1	9	9
9	9	1	2	2	3	3	2	2	3
3	2	1	1	1	1	1	3	3	2
2	9	1	2	2	2	2	2	9	9
2	2	1	2	2	2	2	3	9	9
9	9	9	9	9	9	9	9	9	9
9	9	9	9	9	9	9	9	9	9
2	2	1	2	2	2	2	9	9	9
9	9	1	2	2	2	2	9	9	9
9	9	1	2	2	2	2	9	9	9
9	9	9	9	9	9	9	9	9	9
9	9	1	2	2	2	2	9	9	9
9	9	1	2	2	2	2	2	9	9
3	3	1	3	3	3	3	3	3	3
9	9	1	1	1	1	1	1	9	9
9	9	1	1	1	1	1	2	9	9
9	2	1	1	1	1	1	2	9	9
9	9	9	9	9	9	9	9	9	9
9	9	1	2	2	2	2	2	9	9
2	2	9	2	2	2	2	2	2	2
9	9	9	9	9	9	9	9	9	9
9	9	9	9	9	9	9	9	9	9
9	9	9	1	1	1	1	2	9	2
9	9	9	9	9	9	9	9	9	9
9	9	1	1	1	1	1	1	2	2
9	9	5	1	1	1	1	1	3	1
9	9	9	9	9	9	9	9	9	9
9	2	1	9	9	9	9	9	9	9
2	2	1	1	1	1	1	2	2	2
1	1	1	9	9	9	9	9	9	9
9	9	9	9	9	9	9	9	9	9
2	1	9	9	9	9	9	9	9	9
9	9	9	9	9	9	9	9	9	9
1	9	1	2	2	2	2	9	9	9
9	9	1	9	9	9	9	9	9	9
2	2	1	2	2	2	2	2	3	2
2	1	4	1	2	3	2	3	1	2
9	9	1	2	2	2	2	9	9	9
9	9	9	9	9	9	9	9	9	9
1	2	1	2	2	2	2	2	3	1
1	2	1	2	2	2	2	1	2	3
3	3	1	1	2	2	2	3	3	3
2	1	9	1	2	1	1	1	2	2
9	9	2	9	9	9	9	2	3	1

9	9	9	1	1	2	2	1	9	9
3	1	2	1	1	3	2	2	3	3
2	2	1	2	2	2	2	2	2	2
1	1	4	1	1	1	1	1	1	1
2	2	1	2	2	2	2	2	2	2
2	2	5	3	3	3	1	2	2	2
3	3	2	2	1	1	1	9	9	9
2	2	1	3	3	2	2	3	3	3
2	2	1	3	3	3	3	2	2	2
1	2	3	1	2	2	2	1	2	1
2	2	4	2	2	9	2	2	2	9
9	9	1	3	3	2	2	9	9	9
9	9	1	2	2	2	2	9	9	9
2	2	1	1	2	1	1	9	9	9
9	9	2	1	2	2	2	2	9	9
2	2	5	3	3	1	2	2	2	2
1	3	1	2	2	2	2	3	3	2
1	2	3	2	2	2	2	1	3	3
1	1	1	2	2	2	2	1	1	2
2	2	1	1	1	1	3	2	2	2
2	2	1	2	2	2	2	2	2	2
2	3	1	9	9	9	9	9	9	9
1	1	1	2	2	2	1	3	3	3
9	9	2	1	2	2	2	2	9	9
9	9	2	2	3	2	2	9	9	9
2	2	1	1	1	1	1	1	1	1
9	9	9	1	9	2	2	9	9	9
2	1	3	2	2	2	2	2	3	3
9	9	9	9	9	9	9	9	9	9
9	9	1	1	1	1	1	9	9	9
2	2	1	1	1	1	2	2	3	2
1	3	1	1	2	2	2	2	3	2
9	9	9	9	9	9	9	9	9	9
9	9	4	1	1	1	1	1	9	9
2	1	1	1	2	2	1	1	2	1
9	9	9	9	9	9	9	9	9	9
2	1	1	3	3	2	9	2	3	1
1	1	3	1	1	1	1	1	1	1
2	2	1	3	3	3	3	2	2	2
2	2	2	1	1	2	2	9	9	9
2	2	1	2	2	2	2	2	2	2
2	2	9	2	2	2	2	1	2	1
2	2	1	2	2	2	2	2	2	2
2	2	1	2	2	2	2	2	2	2
2	2	1	2	2	2	2	2	3	3
2	2	1	2	2	2	2	3	3	2
2	2	2	3	3	3	3	2	2	2

9	9	1	2	3	3	2	2	9	9
2	2	5	3	1	3	3	2	2	2
3	3	1	2	2	2	2	2	2	2
9	9	5	1	1	2	1	9	9	9
9	9	1	2	2	2	2	3	9	9
9	9	9	9	9	9	9	9	9	9
9	9	9	9	9	9	9	9	9	9
2	2	9	2	2	2	2	2	2	2
2	2	1	1	1	1	1	2	2	2
9	9	1	2	2	2	2	9	9	9
2	2	1	1	1	1	1	2	2	2
3	3	1	1	1	1	2	2	3	3
2	2	4	1	1	1	1	1	2	2
2	2	1	2	2	2	2	2	2	2
2	3	1	2	2	3	1	9	9	9
9	9	1	9	9	9	9	9	9	9
9	9	9	9	9	9	9	9	9	9
9	9	9	9	9	9	9	9	9	9
9	9	1	2	2	2	2	1	9	9
9	9	1	2	2	2	2	2	9	9
9	9	9	9	9	9	9	9	9	9
9	9	9	9	9	9	9	9	9	9
2	2	1	2	2	2	2	2	2	2
9	9	1	2	2	2	2	2	9	9
9	9	9	9	9	9	9	9	9	9
9	9	9	2	2	2	2	3	9	9
9	9	9	9	9	9	9	9	9	9
9	1	1	2	2	2	2	9	9	9
9	2	1	2	2	2	2	2	9	9
9	3	1	2	9	2	2	3	9	9
9	9	9	9	9	9	9	9	9	9
2	2	1	2	2	2	2	2	2	2
9	9	9	9	9	9	9	9	9	9
9	9	1	9	9	9	9	9	9	9
9	9	1	2	2	2	2	9	9	9
9	9	9	9	9	9	9	9	9	9
9	9	1	2	2	2	2	9	9	9
9	9	9	9	9	9	9	9	9	9
9	9	1	9	9	9	9	9	9	9
9	9	1	9	9	9	9	9	9	9
2	2	1	1	1	1	1	2	2	2
9	9	1	1	1	1	1	2	9	9
9	9	1	9	9	9	9	9	9	9
9	9	1	2	2	2	2	9	9	9
9	9	9	9	9	9	9	9	9	9
9	9	1	2	2	2	2	9	9	9
9	9	9	9	9	9	9	9	9	9

9	9	9	9	9	9	9	9	9	9
9	9	9	9	9	9	9	3	3	3
9	9	1	2	2	2	2	2	9	9
9	9	1	9	9	9	9	9	9	9
9	9	9	9	9	9	9	9	9	9
9	9	9	9	9	9	9	9	9	9
9	9	9	9	9	9	9	9	9	9
9	9	1	9	9	9	9	9	9	9
9	9	9	9	9	9	9	9	9	9
9	9	9	9	9	9	9	9	9	9
9	9	9	9	9	9	9	9	9	9
9	9	9	9	9	9	9	9	9	9
9	9	9	9	9	9	9	9	9	9
9	9	9	9	9	9	9	9	9	9
9	9	1	2	2	2	2	9	9	9
9	9	1	2	2	2	2	9	9	9
9	9	1	9	9	9	9	9	9	9
2	2	1	2	2	2	2	2	2	2
9	9	1	9	9	9	9	9	9	9
2	2	1	2	2	2	2	9	9	9
9	9	9	9	9	9	9	9	9	9
9	9	1	2	2	9	2	9	9	9
9	9	1	9	9	9	9	9	9	9
9	9	1	2	2	2	2	9	9	9
9	9	1	2	2	2	2	2	2	2
9	9	1	2	2	2	2	9	9	9
9	9	9	9	9	9	9	9	9	9
2	1	1	2	2	3	2	1	9	2
9	9	9	9	9	9	9	9	9	9
9	9	9	9	9	9	9	9	9	9
1	1	1	1	1	1	1	1	1	1
2	1	1	2	2	2	2	2	2	2
9	9	9	9	9	9	9	9	9	9
1	2	1	2	2	2	2	3	3	3
9	9	9	9	9	9	9	9	9	9
1	9	1	2	2	2	2	2	2	2
9	9	1	1	1	1	1	2	9	9
9	9	1	9	9	9	9	9	9	9
1	1	1	2	2	2	2	1	1	1
9	3	1	2	2	2	2	3	9	9
9	9	9	9	9	9	9	9	9	9
9	9	9	9	9	9	9	9	9	9
3	3	2	2	2	2	2	2	2	2
9	9	1	1	1	1	1	1	2	2
9	1	1	2	2	2	2	9	1	9
9	2	1	9	9	9	9	9	9	9
9	9	1	9	9	9	9	9	9	9

9	2	1	3	3	3	3	2	9	9
9	9	9	9	9	9	9	9	9	9
2	3	1	2	2	2	2	2	9	9
9	9	1	9	9	9	9	9	9	9
9	9	9	9	9	9	9	9	9	9
9	9	9	9	9	9	9	9	9	9
9	9	1	1	1	1	1	2	9	9
9	9	9	2	2	2	2	2	9	9
9	2	1	1	1	1	1	2	9	9
9	9	1	2	2	2	2	9	9	9
9	9	9	2	2	2	2	9	9	9
9	9	9	9	9	9	9	9	9	9
9	9	9	9	9	9	9	9	9	9
9	9	9	9	9	9	9	9	9	9
2	2	1	2	2	2	2	1	2	2
1	1	1	2	2	2	2	3	2	1
9	9	1	2	2	2	2	9	9	9
2	2	1	2	2	2	2	2	2	2
2	2	1	2	2	2	2	2	2	2
9	9	3	1	1	1	1	1	9	9
9	9	3	1	1	2	2	1	9	9
9	9	2	1	1	9	1	1	9	9
2	1	1	2	2	2	2	3	2	2
1	2	1	2	2	2	2	1	2	3
1	2	2	2	2	2	2	2	2	2
1	2	1	1	1	2	2	2	2	2
1	1	2	2	2	2	2	1	3	3
2	2	2	3	3	3	1	2	2	3
2	2	3	2	2	3	1	2	1	1
1	1	1	2	2	2	2	1	2	2
9	9	1	2	2	2	2	1	2	2
9	9	1	1	1	9	3	2	9	9
9	9	4	1	2	1	1	9	9	9
9	9	5	2	2	3	2	2	9	9
9	9	1	1	9	2	1	2	9	9
9	9	9	9	9	9	9	9	9	9
9	9	9	9	9	9	9	9	9	9
2	9	1	1	1	1	1	9	2	9
9	9	9	9	9	9	9	9	9	9
9	2	9	2	2	2	2	2	9	2
9	9	1	9	9	9	9	9	9	9
2	2	1	1	1	1	1	2	2	2
9	9	1	9	9	9	9	9	9	9
9	9	1	9	9	9	9	9	9	9
9	9	9	9	9	9	9	9	9	9
2	2	1	2	2	2	2	2	2	2
3	3	1	3	3	2	2	1	2	3

2	2	2	1	3	3	3	1	1	1
2	2	1	1	1	2	2	2	2	2
1	1	2	1	1	3	3	2	1	2
2	1	1	9	9	9	9	3	3	3
3	3	4	3	2	2	2	3	2	3
2	1	1	3	1	2	2	1	2	1
9	1	1	2	2	2	2	2	2	2
3	3	1	2	2	2	2	3	3	3
2	2	1	2	2	2	2	9	9	9
2	2	1	1	1	1	1	2	2	2
2	9	1	2	2	2	2	2	9	2
9	1	1	2	2	2	2	9	9	1
2	2	9	1	2	2	1	2	2	2
1	2	1	1	2	2	1	1	2	3
2	3	1	2	2	3	3	3	2	3
2	2	1	2	2	2	2	2	2	2
1	2	9	2	2	2	2	2	1	3
2	2	9	3	3	3	3	2	3	3
9	9	4	9	9	9	9	9	9	9
3	2	2	1	1	2	2	2	2	2
2	2	1	1	1	2	2	3	1	2
2	3	2	2	1	2	2	1	1	2
3	3	5	1	3	2	2	2	1	1
1	2	1	1	3	1	2	1	2	2
2	2	1	2	2	2	2	1	3	1
1	1	2	2	2	2	2	2	2	2
1	1	2	1	3	2	2	2	3	1
2	2	1	2	2	2	2	2	2	2
2	3	1	2	1	2	2	2	2	2
1	1	2	2	2	2	2	1	1	1
3	3	1	3	2	2	2	3	3	3
2	2	3	2	3	3	3	9	9	9
3	3	1	2	2	2	2	3	3	3
3	2	1	3	3	2	2	2	2	2
9	9	1	3	3	3	3	9	3	9
3	2	1	3	3	3	3	3	3	3
1	2	1	2	2	2	2	2	2	2
2	2	3	1	1	1	1	1	1	1
3	3	1	2	2	1	2	2	2	3
9	9	9	9	9	9	9	9	9	9
2	2	3	1	2	2	2	2	2	2
9	9	5	3	1	2	1	9	9	9
3	1	1	2	1	2	3	1	2	1
2	2	1	9	2	2	2	2	2	2
2	3	1	2	3	3	3	2	3	2
3	2	1	3	2	2	1	9	9	9
3	1	1	2	3	3	2	3	3	3

9	9	2	9	9	9	9	9	9	9
3	3	3	2	2	2	2	3	3	3
1	3	1	1	2	2	1	1	3	1
3	3	3	2	2	2	2	2	2	3
2	2	2	3	1	3	1	1	2	1
9	9	1	9	9	9	9	9	9	9
2	2	4	1	2	2	2	2	2	2
1	2	3	3	3	3	3	1	2	3
2	1	1	2	2	2	1	1	3	3
2	2	1	2	3	3	3	1	2	2
2	3	9	2	3	3	3	1	2	1
2	2	3	1	2	2	2	2	2	2
9	9	1	9	9	9	9	9	9	9
2	3	1	2	2	2	2	3	2	2
3	3	2	1	1	3	1	1	2	2
2	2	1	2	2	2	2	2	2	2
1	1	1	2	2	2	2	2	2	2
2	2	1	1	3	2	2	2	2	2
2	2	1	9	9	9	9	2	2	2
9	9	3	1	3	3	1	2	9	9
1	1	2	9	9	9	9	9	9	9
1	2	9	9	9	9	9	1	2	1
3	3	1	2	2	2	2	2	2	2
3	2	1	2	2	2	3	2	3	1
3	3	9	9	9	9	9	9	9	9
1	2	1	2	3	3	2	1	2	3
9	9	1	2	2	2	2	2	9	9
2	2	9	2	2	3	2	2	2	2
2	2	1	3	3	3	3	2	2	2
2	2	7	3	3	3	3	2	2	2
3	1	2	2	3	1	2	3	2	1
9	9	1	9	9	9	9	9	9	9
9	9	9	9	9	9	9	9	9	9
1	2	1	2	1	1	2	1	3	2
3	2	3	1	1	2	2	1	3	2
9	9	9	9	9	9	9	9	9	9
3	3	1	9	9	9	9	9	9	9
2	2	1	2	2	2	2	2	3	3
1	1	5	1	1	1	2	1	3	2
1	2	3	2	2	2	2	1	2	1
9	9	2	2	2	2	2	2	2	2
2	2	1	2	2	2	2	2	2	2
2	2	1	2	2	2	2	2	2	2
2	2	1	3	3	3	3	2	2	2
2	2	1	2	2	2	2	2	2	2
3	3	1	3	2	2	2	2	2	2
2	2	1	2	2	1	2	2	2	2

3	3	5	1	2	1	2	3	3	3
2	2	1	2	2	2	2	2	2	2
2	2	1	2	2	1	2	2	2	2
2	2	1	1	1	1	1	1	1	1
2	2	4	3	2	2	2	2	2	2
2	2	1	2	2	2	2	2	2	2
2	2	1	2	2	2	2	2	2	2
2	2	9	2	2	2	2	2	2	2
2	2	1	2	2	2	2	2	2	2
9	9	9	9	9	9	9	9	9	9
2	2	1	1	1	2	1	1	1	3
3	3	1	2	2	3	2	2	2	2
2	2	1	2	2	2	2	1	2	1
1	2	1	1	3	1	2	3	2	1
2	2	1	2	2	2	2	3	3	3
1	2	1	1	1	2	1	9	9	9
9	9	9	9	9	9	9	9	9	9
9	2	3	2	3	3	3	9	2	9
9	9	9	9	9	9	9	9	9	9
2	2	9	9	9	9	9	2	2	2
1	1	1	2	2	3	2	1	3	2
9	9	9	9	9	9	9	9	9	9
9	9	9	9	9	9	9	9	9	9
2	2	9	3	3	3	3	2	2	2
1	1	2	1	2	2	1	2	1	2
2	2	1	3	3	3	3	2	2	2
2	3	9	1	2	1	2	1	1	2
3	2	1	1	3	3	2	3	2	9
2	3	1	2	2	2	2	2	1	2
2	3	2	2	1	3	1	2	2	1
9	9	9	9	9	9	9	9	9	9
3	1	3	9	9	9	9	1	2	1
1	2	2	1	2	2	1	2	3	3
2	1	2	1	9	1	1	2	2	2
2	2	1	1	2	2	2	2	2	1
2	2	1	3	3	3	3	2	2	2
9	9	1	9	9	9	9	9	9	9
2	2	9	2	2	2	2	2	2	2
2	2	1	3	3	3	3	2	2	2
3	3	1	1	2	3	2	3	1	2
1	1	1	1	2	2	1	1	2	2
1	1	1	1	2	2	1	2	1	2
2	2	1	2	2	2	2	1	2	2
2	2	1	1	3	3	3	1	2	2
2	3	1	2	2	2	2	2	3	2
2	1	1	1	2	2	2	1	2	1
2	3	1	1	2	2	2	3	3	2

3	3	9	9	9	9	9	2	9	1
2	2	1	2	3	3	3	2	2	2
3	3	1	2	2	1	2	2	2	2
2	2	1	2	3	3	3	2	2	2
3	2	1	1	3	3	3	3	2	3
2	2	1	9	9	9	9	9	9	9
9	9	1	9	9	9	2	9	9	9
3	3	2	2	2	2	2	2	2	3
9	9	9	9	9	9	9	9	9	9
3	1	9	9	9	9	9	9	9	9
2	2	1	2	2	2	2	2	2	2
1	2	9	3	1	2	2	1	2	2
1	2	2	1	1	1	1	2	1	1
9	9	1	3	3	1	1	9	9	1
9	9	1	2	2	2	2	9	9	2
9	9	1	2	2	2	2	1	3	2
2	1	3	1	1	2	3	1	2	1
1	1	4	9	9	9	9	9	9	9
2	2	1	1	2	3	3	1	3	2
1	1	3	1	1	1	3	2	2	2
1	2	3	1	2	3	9	2	1	2
3	1	1	2	2	2	2	9	9	9
2	2	1	2	2	2	2	2	2	2
2	2	1	3	2	2	2	2	2	2
1	1	3	2	2	2	2	2	2	2
2	2	1	3	3	3	3	2	2	2
2	2	1	3	2	2	2	2	2	2
2	2	1	2	2	2	2	2	2	2
1	2	1	3	3	3	3	2	3	2
3	3	1	2	2	2	2	3	3	2
2	1	2	2	2	1	3	2	1	2
2	2	3	1	1	1	1	1	2	3
3	3	1	2	2	2	2	2	2	1
2	2	1	2	2	3	3	2	2	2
2	2	1	1	2	9	2	2	2	2
2	1	1	2	3	1	2	2	2	1
1	1	3	1	1	2	1	1	1	1
2	2	1	2	2	2	2	2	2	2
1	1	1	1	1	2	1	2	9	2
2	2	3	2	2	2	2	2	3	2
1	9	1	1	1	1	1	1	1	1
2	2	2	1	2	2	2	2	2	2
2	2	9	2	2	2	2	2	2	2
1	1	3	1	2	2	2	2	2	2
2	2	9	1	2	2	2	2	2	2
9	2	4	2	2	2	1	1	2	2
2	2	9	2	2	2	2	2	2	2

3	2	1	3	1	2	3	2	3	2
1	3	5	1	2	2	1	1	2	1
3	2	1	1	1	2	2	2	3	2
2	2	1	1	1	1	1	2	2	2
2	2	4	2	3	3	1	2	9	1
9	9	2	2	1	2	2	9	9	9
2	2	1	3	3	3	3	3	2	3
9	9	1	1	1	1	1	1	9	9
9	9	1	1	1	1	1	1	9	9
3	3	4	9	9	9	9	9	9	9
9	9	2	2	2	2	2	9	9	9
9	9	1	2	1	9	3	9	9	2
9	9	9	2	1	2	1	2	9	9
9	9	1	2	3	2	1	9	9	9
2	2	1	2	2	2	2	2	2	2
9	9	1	2	2	9	9	2	9	9
9	9	9	9	9	9	9	9	9	9
9	9	1	2	2	2	2	9	9	1
9	9	1	2	2	2	2	9	9	1
9	9	3	9	9	9	9	9	9	9
9	9	1	9	9	9	9	9	9	9
2	2	1	2	3	3	3	2	2	2
3	3	1	2	2	2	2	2	2	2
2	3	1	2	2	2	2	2	2	2
2	1	2	2	2	2	1	1	2	9
2	2	1	1	2	2	9	9	9	9
2	2	1	2	2	2	2	1	3	2
9	9	1	2	2	2	2	1	9	9
2	2	1	2	2	2	2	3	3	3
3	3	2	1	2	1	2	1	1	1
3	3	2	1	2	2	2	3	3	3
9	9	1	2	2	2	2	3	9	9
2	2	7	2	2	1	1	2	2	2
9	9	1	2	2	2	2	2	9	9
9	9	9	1	1	1	1	1	9	9
3	2	9	9	9	9	9	3	2	1
1	1	9	1	1	1	1	1	1	1
9	9	9	9	9	9	9	9	9	9
9	9	9	2	2	2	2	9	9	9
2	2	9	3	3	3	3	2	2	2
2	2	9	3	3	3	3	9	9	9
2	2	9	1	1	1	3	2	2	2
3	3	1	2	2	2	2	3	3	3
2	9	1	2	2	2	2	9	9	2
9	1	3	1	1	1	1	2	2	2
2	2	3	1	1	1	1	2	2	2
9	1	9	2	2	2	2	9	1	9

9	9	1	9	9	9	9	9	9	9
3	1	1	2	2	2	2	2	2	9
9	9	1	9	9	9	9	9	9	9
2	2	3	1	1	1	1	2	2	2
1	9	1	2	2	2	2	9	3	1
9	2	1	2	2	2	2	9	1	9
9	9	1	2	2	2	2	9	9	9
9	9	1	9	9	9	9	9	9	9
9	2	1	2	2	2	2	2	9	9
2	2	3	1	1	1	1	2	2	2
9	9	1	9	9	9	9	9	9	9
2	9	1	2	2	2	2	2	9	9
2	2	1	2	2	2	2	2	2	2
9	9	9	9	9	9	9	9	9	9
2	2	1	1	1	1	1	2	2	2
9	9	9	9	9	9	9	9	9	9
9	9	9	9	9	9	9	9	9	9
9	9	9	9	9	9	9	9	9	9
9	9	1	9	9	9	9	9	9	9
9	9	1	9	9	9	9	9	9	9
2	2	1	2	2	2	1	2	2	2
2	2	1	2	2	2	2	2	2	2
1	2	2	2	2	2	2	1	2	3
1	1	1	1	1	2	1	1	1	1
3	3	1	2	1	2	2	2	3	3
2	2	1	3	2	3	3	2	2	2
1	1	1	1	1	1	1	1	1	1
2	3	1	2	2	2	2	2	2	2
1	3	2	2	3	3	1	1	3	1
2	2	9	2	2	2	2	2	2	2
3	2	9	1	2	2	2	2	2	2
2	3	1	3	3	2	2	2	3	2
2	2	1	3	3	3	3	2	2	2
9	9	2	9	9	9	9	9	9	9
1	1	1	2	2	2	2	3	3	3
3	3	2	1	1	1	1	3	1	1
2	2	1	2	2	2	2	2	2	2
1	3	1	1	2	1	1	2	2	2
2	2	1	2	2	2	2	2	2	2
2	2	1	2	2	2	2	2	2	2
1	1	1	1	1	2	2	2	2	2
9	9	9	9	9	9	9	9	9	9
2	2	1	1	2	2	2	2	2	2
9	9	9	9	9	9	9	9	9	9
2	1	9	2	2	1	1	9	9	9
2	2	1	3	3	3	3	2	2	2
3	3	5	1	3	2	2	9	9	9

2	1	2	2	3	3	3	2	2	2
2	2	9	9	9	9	9	1	1	1
2	2	5	1	1	1	1	1	1	1
2	2	3	1	1	1	1	1	1	1
2	2	3	1	1	1	1	2	2	2
2	2	9	1	1	1	1	1	1	1
1	1	3	1	1	1	1	1	1	1
1	2	3	1	1	1	1	1	2	1
3	3	1	2	2	2	2	3	3	3
1	1	5	1	1	1	1	1	1	1
2	2	4	3	3	3	3	9	9	9
1	1	4	1	1	1	1	2	2	2
9	1	5	1	1	1	1	2	2	2
1	1	4	1	1	1	1	2	2	2
9	9	9	1	1	1	1	2	2	2
2	1	3	1	1	1	1	1	1	1
1	1	4	1	1	1	1	1	2	1
2	2	4	1	1	1	1	1	2	1
9	9	9	1	1	1	1	1	1	1
2	2	9	3	3	3	3	9	9	9
2	1	9	1	1	1	1	1	2	1
2	2	1	2	2	2	3	2	2	2
9	9	9	9	9	9	9	9	9	9
2	2	1	1	1	1	1	1	1	1
9	9	9	9	9	9	9	9	9	9
1	2	9	1	1	1	1	1	1	2
1	2	1	1	1	1	1	1	2	1
1	1	1	1	1	3	1	1	1	2
2	2	9	1	1	1	1	9	9	9
9	2	4	1	1	1	1	2	2	2
2	2	2	3	3	3	3	9	9	9
2	2	1	1	1	2	2	9	9	9
2	2	2	1	1	1	1	2	2	2
1	1	4	1	1	1	1	1	1	1
2	2	1	1	1	2	3	2	2	2
9	9	9	9	9	9	9	9	9	9
1	1	9	1	1	1	1	1	1	1
1	1	9	1	1	1	1	1	1	1
9	9	2	9	9	9	9	9	9	9
2	2	9	3	3	3	3	9	9	9
9	9	9	1	1	1	1	2	2	2
9	9	9	9	9	9	9	9	9	9
2	2	9	1	1	1	1	2	2	2
2	2	9	1	1	1	1	2	2	2
1	1	1	1	1	1	1	1	1	2
2	2	1	2	2	2	2	2	2	2
9	9	2	1	1	1	1	1	2	1

1	1	1	1	1	1	1	1	2	1
2	2	1	2	2	2	2	2	9	2
1	1	2	1	1	1	1	3	3	3
1	2	9	1	1	1	1	1	1	1
2	2	3	1	1	1	1	2	2	2
2	2	1	3	9	9	9	9	9	9
1	1	3	1	1	1	1	2	2	2
2	2	1	1	1	1	1	2	2	2
9	9	9	3	3	3	3	2	2	9
2	9	9	1	1	1	1	1	1	9
2	9	4	3	3	3	3	9	9	9
2	2	2	1	1	1	1	9	9	9
9	9	9	9	9	9	9	9	9	9
9	9	9	1	1	1	1	9	9	9
9	9	9	3	3	3	3	9	9	9
9	9	9	9	9	9	9	9	9	9
9	9	9	1	1	1	1	1	1	1
9	9	9	9	9	9	9	9	9	9
2	2	9	3	3	3	3	2	2	2
1	1	4	1	1	1	1	1	3	3
1	1	2	1	1	1	1	1	3	3
1	1	4	1	1	1	1	1	3	3
1	1	4	1	1	1	1	1	3	3
1	1	4	1	1	1	1	1	3	3
1	1	4	1	1	1	1	1	3	3
1	1	2	1	1	1	1	1	3	3
1	1	4	2	1	1	1	1	3	3
1	1	1	1	1	1	1	1	3	3
1	1	4	1	1	1	1	1	3	3
1	1	1	2	1	1	1	1	3	3
9	1	9	2	1	1	1	1	3	3
1	1	9	2	1	1	1	1	3	3
1	1	4	1	1	1	1	1	3	3
1	1	4	2	1	1	1	1	3	3
1	1	4	1	1	1	1	1	3	3
1	1	4	2	1	1	1	1	3	3
1	1	4	1	1	1	1	1	3	3
1	1	4	9	9	9	9	1	3	3
1	1	4	1	1	1	1	1	3	3
1	1	4	1	1	1	1	1	3	3
1	1	1	1	1	1	1	1	3	3
1	1	4	1	1	1	1	1	3	3
1	1	4	1	1	1	1	1	3	3
1	1	4	1	1	1	1	3	1	3
9	9	9	9	9	9	9	9	9	9
9	9	9	9	9	9	9	9	9	9
9	9	9	9	9	9	9	9	9	9

9	9	9	9	9	9	9	9	9	9
9	9	9	9	9	9	9	9	9	9
9	9	9	9	9	9	9	9	9	9
9	9	9	9	9	9	9	9	9	9
9	9	9	9	9	9	9	9	9	9
9	9	9	9	9	9	9	9	9	9
9	9	9	9	9	9	9	9	9	9
9	9	9	9	9	9	9	9	9	9
9	9	9	9	9	9	9	9	9	9
9	9	9	9	9	9	9	9	9	9
9	9	9	9	9	9	9	9	9	9
9	9	9	9	9	9	9	9	9	9
9	9	9	9	9	9	9	9	9	9
9	9	9	9	9	9	9	9	9	9
9	9	9	9	9	9	9	9	9	9
9	9	9	9	9	9	9	9	9	9
9	9	9	9	9	9	9	9	9	9
9	9	9	9	9	9	9	9	9	9
9	9	9	9	9	9	9	9	9	9
1	1	4	1	9	1	1	1	3	3
1	1	4	1	1	1	1	1	3	3
1	1	1	1	1	1	1	1	3	3
1	1	1	1	1	1	1	1	3	3
1	1	1	1	1	1	1	1	3	3
1	1	1	1	1	2	1	1	3	3
1	1	1	1	2	1	1	1	3	3
1	1	1	1	1	1	1	1	3	3
1	1	4	1	2	1	1	1	3	3
1	1	2	1	1	1	1	1	1	3
1	1	1	1	1	1	1	1	1	3
1	1	1	1	1	1	1	1	3	3
1	1	4	1	1	1	1	1	3	3
1	1	4	1	1	1	1	1	3	3
1	1	5	1	1	1	1	1	3	3
1	1	1	1	1	1	1	1	3	3
1	9	1	2	2	2	2	1	9	9
1	1	1	1	1	1	1	1	3	3
1	1	4	1	1	1	1	1	3	3
1	1	4	1	1	1	1	1	3	3
1	1	1	2	1	1	1	1	3	3
1	1	4	1	1	1	1	1	3	3
1	1	4	1	2	1	1	1	3	3
1	1	4	1	1	1	1	1	3	3
1	1	9	1	1	1	1	1	1	3
1	1	1	1	1	1	3	1	3	3
1	1	1	1	1	1	1	1	3	3
1	1	1	9	1	1	1	1	3	3

1	1	1	1	1	1	1	1	3	3
1	1	1	9	1	1	1	1	3	3
1	1	4	3	1	1	1	1	3	3
1	1	1	1	1	1	1	1	3	3
1	1	1	1	1	1	1	1	3	3
1	1	4	1	1	1	1	1	3	3
1	1	1	1	1	1	1	1	3	3
1	1	1	1	1	1	1	1	3	1
1	1	1	1	1	1	1	1	3	3
1	1	4	1	1	1	1	1	3	3
1	1	1	1	1	1	1	1	3	3
1	1	1	1	1	1	1	1	3	3
1	1	4	1	1	1	1	1	3	3
1	1	3	1	1	1	1	1	3	3
1	1	9	2	1	1	1	1	3	3
1	1	4	1	1	1	1	1	3	3
1	1	9	2	1	1	1	1	3	3
1	1	4	1	1	1	1	1	3	3
1	1	4	1	1	1	1	1	3	3
1	1	4	1	1	1	1	1	3	3
1	1	4	1	1	1	1	1	3	3
1	3	4	2	1	1	1	1	3	3
1	1	1	1	1	1	1	1	3	3
1	1	1	1	1	1	1	1	3	3
1	1	1	1	1	1	1	1	3	3
1	1	4	1	1	1	1	1	3	3
1	1	1	1	1	1	1	1	3	3
1	1	4	1	1	1	1	1	3	3
1	1	1	1	1	1	1	1	3	3
1	1	1	1	1	1	1	1	3	3
1	1	2	1	1	1	1	1	3	3
1	1	1	1	9	9	9	1	1	3
9	9	9	9	9	9	9	1	1	1
1	1	4	1	1	1	1	1	3	3
9	9	9	9	9	9	9	9	9	9
1	1	1	2	1	1	1	1	3	3
2	1	1	1	9	1	1	1	3	3
1	1	4	9	9	1	1	1	3	3
1	1	4	1	9	1	1	1	3	3
1	1	9	2	1	1	1	1	3	3
1	1	4	1	1	1	1	1	3	3
1	1	1	1	1	1	1	1	3	3
1	1	1	2	2	1	1	1	3	3
1	1	4	1	1	1	1	1	3	3
1	1	4	1	1	1	1	1	3	3
1	1	4	1	1	1	1	1	3	3
1	1	4	1	1	1	1	1	3	3

1	1	4	1	1	1	1	1	3	3
1	1	4	1	1	1	1	1	3	3
1	1	4	1	1	1	1	1	3	3
1	1	4	1	1	1	1	1	3	3
1	1	4	1	1	1	1	1	3	3
1	1	4	1	1	1	9	1	3	3
1	1	4	1	1	1	1	1	3	3
1	1	9	1	1	1	1	1	3	3
1	1	4	1	1	1	1	1	3	3
1	1	4	1	1	1	1	1	3	3
1	1	4	1	1	1	1	1	3	3
1	1	4	1	1	1	1	1	3	3
1	1	4	1	1	1	1	1	3	3
1	1	4	1	1	1	1	1	3	3
9	9	4	1	1	1	1	1	3	3
1	1	4	1	1	1	1	1	3	3
1	1	4	1	1	1	1	1	3	3
1	1	4	1	1	1	1	1	3	3
1	1	4	1	1	1	1	1	3	3
9	9	9	9	9	9	9	1	3	3
1	1	4	2	1	1	1	1	3	3
1	1	4	1	1	1	1	1	3	3
1	1	4	1	1	1	1	1	3	3
1	1	4	1	1	1	1	1	3	3
1	1	4	1	1	1	1	1	3	9
2	1	1	1	1	1	1	1	3	3
1	1	9	1	1	1	1	1	3	3
1	1	4	1	1	1	1	1	3	3
1	1	1	1	1	1	1	1	3	3
1	1	1	1	1	1	1	1	3	3
1	1	1	1	1	1	1	1	3	3
1	1	1	1	1	1	1	1	3	3
1	2	1	1	1	1	1	3	1	3
1	1	1	1	1	1	1	3	1	3
1	1	1	1	1	1	1	1	3	3
1	1	2	1	1	1	1	1	3	3
1	1	1	1	1	1	1	1	3	3
1	1	4	1	1	1	1	1	3	3
1	1	4	1	1	1	1	1	3	3
1	1	4	1	1	1	1	1	3	3
1	1	3	1	1	1	1	1	3	3
9	9	9	9	9	9	9	9	9	9
1	1	4	1	1	1	1	1	3	3
1	1	3	1	1	1	1	1	3	3
1	1	4	1	1	1	1	1	3	3
1	1	4	1	1	1	1	1	3	3
1	1	4	1	1	1	1	1	3	3

1	1	4	1	1	1	1	1	3	3
1	1	4	1	1	1	1	1	3	3
1	1	1	1	1	1	1	1	3	3
1	1	4	1	1	1	1	1	3	3
1	1	4	1	1	1	1	1	3	3
1	1	4	1	1	1	1	1	3	3
1	1	1	1	1	1	1	1	3	3
1	1	1	9	1	2	1	1	3	3
1	1	1	1	1	1	1	1	3	3
1	1	9	9	9	9	9	9	9	9
1	1	4	1	1	1	1	1	3	3
1	1	4	1	1	1	1	1	3	3
1	1	4	1	1	1	1	1	3	3
1	1	4	1	1	1	1	1	3	3
1	1	4	1	1	1	1	1	3	3
1	1	4	1	1	1	1	1	3	3
1	1	4	1	1	1	1	1	3	3
1	1	4	1	1	1	1	1	3	3
1	1	4	1	1	1	1	1	3	3
1	1	4	1	1	1	1	1	3	3
1	1	4	1	1	1	1	1	3	3
1	1	4	1	1	1	1	1	3	3
1	1	4	1	1	1	1	1	3	3
1	1	1	1	1	1	1	1	3	3
1	1	4	1	1	1	1	1	3	3
1	1	4	1	1	1	1	1	3	3
1	1	4	9	9	9	9	1	3	3
9	9	9	9	9	9	9	9	9	9
9	9	9	9	9	9	9	9	9	9
9	9	9	9	9	9	9	9	9	9
9	9	9	9	9	9	9	9	9	9
9	9	9	9	9	9	9	9	9	9
9	9	9	9	9	9	9	9	9	9
9	9	9	9	9	9	9	9	9	9
9	9	9	9	9	9	9	9	9	9
9	9	9	9	9	9	9	9	9	9
9	9	9	9	9	9	9	9	9	9
9	9	9	9	9	9	9	9	9	9
9	9	9	9	9	9	9	9	9	9
9	9	9	9	9	9	9	9	9	9
9	9	9	9	9	9	9	9	9	9
9	9	9	9	9	9	9	9	9	9
9	9	9	9	9	9	9	9	9	9
9	9	9	9	9	9	9	9	9	9
9	9	9	9	9	9	9	9	9	9
9	9	9	9	9	9	9	9	9	9
9	9	9	9	9	9	9	9	9	9

9	9	9	9	9	9	9	9	9	9
9	9	9	9	9	9	9	9	9	9
9	9	9	9	9	9	9	9	9	9
9	9	9	9	9	9	9	9	9	9
9	9	9	9	9	9	9	9	9	9
9	9	9	9	9	9	9	9	9	9
9	9	9	9	9	9	9	9	9	9
9	9	9	9	9	9	9	9	9	9
9	9	9	9	9	9	9	9	9	9
9	9	9	9	9	9	9	9	9	9
9	9	9	9	9	9	9	9	9	9
9	9	9	9	9	9	9	9	9	9
9	9	9	9	9	9	9	9	9	9
3	3	1	1	1	1	1	3	3	1
2	2	9	3	3	3	3	2	2	2
2	2	2	2	2	2	2	2	2	2
2	2	9	9	9	9	9	1	1	3
1	1	5	1	1	2	1	1	2	1
3	3	9	9	9	9	9	1	1	1
2	2	9	1	1	1	1	1	2	3
1	1	4	1	1	2	1	1	2	1
1	3	5	2	2	3	3	2	2	2
9	9	9	9	9	9	9	9	9	9
2	3	1	3	3	3	3	2	3	2
9	9	1	2	2	2	2	9	9	9
3	3	9	1	1	1	1	2	2	3
9	9	9	9	9	9	9	9	9	9
3	3	2	2	2	2	2	1	1	1
3	2	9	2	3	1	2	3	2	1
2	3	2	1	1	2	2	2	2	2
3	3	1	2	2	2	2	3	3	3
2	3	1	2	2	2	3	2	1	2
9	9	3	1	1	2	2	1	9	9
3	3	9	1	1	2	2	1	3	2
1	1	2	1	1	1	1	1	1	1
9	9	1	1	1	2	1	2	9	9
2	2	1	1	3	1	2	2	2	2
1	1	2	3	2	1	1	1	2	1
1	1	3	1	1	2	1	2	2	1
1	1	9	1	1	1	1	1	1	1
9	9	9	9	9	9	9	9	9	9
9	9	9	9	9	9	9	9	9	9
1	1	2	1	1	1	1	1	1	2
1	1	9	1	1	3	2	1	1	1
9	9	9	9	9	9	9	9	9	9
9	9	9	9	9	9	9	9	9	9
2	2	1	3	3	9	9	9	9	9

9	9	5	1	2	1	2	9	9	9
9	9	9	9	9	9	9	9	9	9
3	2	1	3	3	2	1	2	2	1
2	2	1	1	1	2	2	2	2	2
2	3	2	1	1	2	2	1	2	2
9	9	3	1	1	2	1	9	2	9
2	2	9	2	2	1	1	2	2	2
2	2	9	2	1	1	1	2	2	2
2	2	9	2	2	2	2	2	2	2
3	2	9	1	2	2	2	2	2	2
3	3	1	1	2	3	3	3	3	2
9	9	9	9	9	9	9	9	9	9
9	9	4	2	2	2	2	1	9	9
2	2	1	3	3	3	3	2	2	2
2	2	1	3	3	2	2	2	2	2
3	3	1	2	2	2	2	2	2	2
1	3	1	2	2	3	3	2	2	2
2	2	9	1	1	1	1	2	2	2
1	9	9	1	1	1	1	1	1	1
9	2	9	9	9	9	9	9	9	9
2	2	3	1	2	2	2	2	2	2
2	2	9	9	9	9	9	2	2	2
2	2	9	2	3	3	3	2	2	2
2	2	9	2	2	1	3	2	2	2
2	2	9	9	9	9	9	2	2	2
1	1	1	2	1	1	2	2	2	1
2	2	9	2	2	2	2	2	2	2
2	2	5	2	2	2	3	2	2	2
2	2	1	2	2	2	2	2	2	2
1	2	1	1	1	1	1	1	3	1
2	3	2	1	1	3	1	1	3	3
2	3	1	1	2	2	2	1	2	2
1	1	1	1	2	3	3	1	2	1
1	1	4	1	1	1	1	1	2	2
2	2	9	1	1	2	2	2	1	2
3	1	1	2	2	1	2	1	2	2
2	2	1	2	2	2	2	2	2	2
2	2	9	1	3	3	1	1	1	1
9	9	1	3	9	3	2	2	9	9
1	1	2	2	2	2	2	2	2	2
1	1	2	1	2	2	2	3	3	3
2	2	1	1	2	2	2	2	2	2
1	2	1	1	1	2	2	1	2	3
1	1	1	1	1	2	2	1	2	2
1	1	1	1	1	1	1	1	1	1
2	2	2	1	1	1	1	1	1	1
1	2	2	2	2	2	9	2	2	2

2	1	1	2	2	2	2	2	2	2
1	1	2	2	2	2	2	1	2	3
2	2	9	1	2	2	2	2	2	2
3	2	9	3	1	2	2	2	2	3
2	2	2	1	1	1	1	2	2	2
2	2	2	1	1	1	1	2	2	2
1	3	1	2	2	2	2	1	2	2
1	3	9	3	3	3	3	2	3	2
2	2	1	3	3	3	3	2	2	2
1	3	1	1	2	2	3	1	2	3
2	2	2	2	2	2	2	2	2	2
2	2	1	3	3	3	3	2	2	2
1	1	9	2	2	2	1	2	2	2
2	2	9	9	9	9	9	2	2	2
2	2	9	1	1	1	1	2	2	2
2	2	1	2	2	2	3	2	2	1
3	3	4	1	1	1	1	2	2	2
2	2	1	1	1	2	2	2	2	2
2	2	9	9	9	9	9	2	2	2
2	1	2	2	1	1	3	1	2	3
2	2	2	2	2	2	2	2	2	2
1	2	1	2	2	2	2	1	1	1
2	2	3	2	2	2	2	2	2	2
1	1	9	1	1	1	1	1	1	1
1	1	2	1	1	1	2	1	3	3
9	9	9	9	9	9	9	9	9	9
1	2	2	2	2	2	9	9	9	9
2	1	1	2	2	2	2	2	2	2
1	3	9	1	1	1	1	9	9	9
1	1	2	3	2	2	1	2	1	1
1	1	2	2	2	2	2	1	2	1
1	2	9	1	2	1	2	1	3	2
3	3	9	2	2	2	1	2	2	2
2	2	9	1	1	1	1	2	2	2
2	1	9	1	1	1	1	9	9	1
9	9	9	9	9	9	9	2	2	2
9	9	9	9	9	9	9	9	9	9
2	2	9	9	9	9	9	1	2	1
9	9	9	9	9	9	9	9	9	9
2	2	1	1	1	2	2	2	2	2
3	2	1	2	2	2	2	2	3	3
1	3	3	1	1	1	2	1	1	1
1	2	2	2	2	2	2	1	2	1
9	9	9	9	9	9	9	9	9	9
9	9	9	9	9	9	9	9	9	9
9	9	9	9	9	9	9	9	9	9
3	3	3	1	1	1	1	1	1	1

9	9	9	9	9	9	9	9	9	9
9	9	9	9	9	9	9	9	9	9
2	2	3	1	2	2	1	1	2	2
3	3	9	9	9	9	9	9	9	9
9	9	9	9	9	9	9	9	9	9
3	1	1	1	1	3	3	2	2	2
2	3	1	2	1	3	2	3	1	2
2	2	9	9	9	9	9	9	9	9
1	2	1	1	1	1	1	1	2	1
1	2	2	3	3	3	3	1	2	3
1	2	2	1	2	3	2	2	3	1
2	2	9	3	3	3	3	2	2	2
2	2	1	3	3	3	3	2	2	2
9	9	9	9	9	9	9	9	9	9
2	2	9	3	3	3	3	2	2	2
9	3	1	2	2	2	3	2	2	2
1	3	5	1	3	1	2	3	3	2
2	3	1	2	2	3	2	2	3	1
3	3	9	1	1	1	1	1	2	3
9	9	9	9	9	9	9	9	9	9
2	3	1	2	2	3	2	2	3	1
3	1	2	1	3	1	1	2	1	1
2	2	1	2	2	2	2	2	2	2
2	3	1	2	2	3	2	9	2	1
2	3	9	9	9	9	9	3	3	3
9	9	9	9	9	9	9	9	9	9
2	2	1	2	2	2	2	3	3	2
2	2	9	2	2	2	2	9	9	9
2	2	4	2	2	2	1	2	2	2
1	2	4	2	2	2	3	1	2	1
2	2	1	2	2	2	2	2	2	2
9	9	2	2	2	2	2	1	9	9
2	2	1	2	2	2	2	1	9	9
2	2	5	9	9	9	9	2	2	2
1	2	2	2	1	2	2	2	1	2
1	2	9	9	9	9	9	2	1	3
9	9	9	9	9	9	9	2	2	2
1	1	3	1	1	2	3	1	2	2
1	1	2	2	2	2	2	2	2	2
9	9	1	3	2	2	2	2	9	9
2	2	9	9	9	9	9	1	2	2
2	2	1	1	3	3	3	2	2	2
2	2	1	2	2	2	2	3	3	1
2	2	1	2	2	3	2	2	2	2
2	2	1	1	3	3	3	2	2	2
9	9	9	2	2	1	2	1	9	9
3	3	1	3	3	3	3	2	2	3

1	3	2	1	1	1	1	1	3	1
2	2	9	3	3	3	3	2	2	2
1	1	1	1	2	2	2	2	3	1
1	2	1	2	2	2	2	1	2	2
1	2	1	2	2	2	2	2	2	2
2	2	9	2	2	3	3	2	2	2
1	1	1	2	2	2	2	1	1	1
1	2	1	1	1	2	2	2	1	3
1	3	1	1	1	2	2	1	3	1
3	3	2	2	2	2	3	3	3	3
2	2	1	1	1	2	2	2	2	2
2	2	2	3	2	2	2	2	2	2
2	2	1	1	1	2	2	2	2	2
1	3	1	1	1	2	2	1	3	1
9	9	9	2	2	2	2	2	1	9
1	2	3	2	2	2	2	2	2	2
2	3	1	3	2	2	2	2	2	3
2	2	9	9	9	9	9	1	2	2
2	2	1	2	2	2	3	3	2	2
1	2	1	2	2	2	2	1	1	1
1	1	1	2	2	2	2	1	3	1
3	3	9	1	1	1	1	9	9	9
3	3	1	2	2	2	2	2	2	2
2	2	9	1	1	1	1	2	2	2
9	2	1	1	1	2	2	2	2	2
1	1	2	2	2	2	2	1	3	1
2	1	2	3	3	3	1	2	2	3
2	1	2	1	3	3	1	2	2	2
1	1	4	1	1	1	1	1	3	1
1	2	1	1	1	1	1	1	3	1
3	1	1	1	1	1	1	1	3	3
1	1	3	2	2	2	1	2	3	2
1	1	2	1	1	1	2	1	2	1
9	9	9	9	9	9	9	9	9	9
9	9	2	3	3	3	3	3	9	9
1	3	1	1	1	1	1	1	3	1
2	2	9	9	9	9	9	2	2	2
1	1	2	1	1	1	1	1	1	1
1	1	9	1	1	1	1	1	1	1
2	2	4	1	1	1	3	2	2	2
2	2	2	1	1	1	1	1	2	3
9	9	9	9	9	9	9	9	9	9
9	9	9	9	9	9	9	9	9	9
1	1	9	1	1	1	1	1	1	1
2	2	2	1	1	1	1	1	2	3
9	9	9	9	9	9	9	9	9	9
9	9	9	9	9	9	9	9	9	9

2	2	9	9	9	9	9	2	2	2
1	1	1	1	1	1	1	1	1	1
1	2	3	1	1	1	1	1	2	3
1	2	2	2	2	2	2	2	3	2
1	1	1	2	2	2	2	1	2	2
1	2	1	1	2	3	1	1	2	3
2	2	9	2	2	1	1	1	2	2
9	9	9	2	9	1	3	1	9	9
2	1	1	1	1	1	1	2	3	1
9	9	9	9	9	9	9	9	9	9
1	1	9	1	1	1	1	2	2	2
1	1	4	3	3	3	3	2	2	3
9	9	9	9	9	9	9	1	1	1
9	9	9	9	9	9	9	9	9	9
2	2	9	1	1	1	1	2	2	2
9	9	9	9	9	9	9	9	9	9
9	9	9	9	9	9	9	9	9	9
2	2	2	2	1	1	1	2	2	2
2	2	9	2	3	3	3	2	2	2
3	2	3	1	1	2	2	2	2	1
2	2	1	2	2	2	2	2	2	2
2	2	9	1	1	1	1	2	2	2
2	3	5	1	3	3	3	2	3	3
2	3	4	1	3	2	1	2	3	1
9	9	9	3	2	2	2	1	1	1
9	9	3	1	2	2	2	1	9	9
1	1	4	2	9	2	2	2	2	2
1	1	2	9	9	9	9	2	3	2
1	1	1	1	1	1	2	1	1	1
1	1	5	3	3	3	3	1	2	3
1	9	3	2	1	1	1	1	1	1
2	2	1	2	3	3	3	2	2	2
2	2	9	9	9	9	9	2	2	2
2	2	9	3	3	3	3	2	2	2
1	1	2	1	1	1	1	2	2	2
2	2	9	3	3	3	3	2	2	2
2	2	9	2	2	2	2	3	3	3
9	9	2	1	2	2	2	9	9	9
9	9	9	2	2	2	2	9	9	9
2	2	2	9	9	9	9	2	2	2
2	3	9	3	2	2	2	3	3	3
1	9	3	1	3	3	3	2	2	2
2	2	9	9	9	9	9	1	2	2
9	9	2	2	2	1	3	2	9	9
3	3	1	1	1	2	2	2	2	2
9	9	9	9	9	9	9	9	9	9
2	2	2	2	2	2	2	2	2	2

2	1	1	3	3	3	3	1	2	1
9	9	1	2	2	2	2	9	9	9
2	2	9	9	9	9	9	2	2	2
2	2	1	2	2	2	2	2	2	2
3	3	9	1	2	1	1	3	3	3
2	2	9	1	2	3	3	2	2	2
2	1	4	2	2	2	2	1	2	1
3	2	1	9	9	9	9	9	9	9
2	2	9	1	2	2	2	2	2	2
1	1	9	2	2	2	2	1	1	1
3	3	2	2	2	2	2	2	2	2
2	2	9	3	3	3	3	2	2	2
2	2	2	1	2	1	2	2	3	9
2	3	1	2	2	2	2	2	3	3
1	1	1	1	2	1	2	2	1	2
1	1	1	1	2	1	2	2	1	2
2	2	9	2	2	2	2	2	2	2
2	2	9	1	2	2	2	2	2	2
2	3	5	9	9	9	9	9	9	9
3	1	1	9	9	9	9	9	9	9
2	2	1	2	2	2	2	3	2	2
2	2	1	1	1	2	2	1	2	2
1	1	1	1	2	1	2	9	9	9
9	9	9	9	9	9	9	9	9	9
3	3	9	3	3	3	3	3	3	3
9	9	1	3	3	3	3	9	9	9
9	9	9	9	9	9	9	9	9	9
9	9	9	9	9	9	9	9	9	9
1	1	1	9	9	9	9	1	2	3
1	1	4	2	2	2	2	1	1	1
2	2	4	2	2	2	2	2	2	2
1	1	4	2	2	2	1	1	2	1
2	2	1	2	2	2	2	1	2	2
2	2	9	3	3	3	3	2	2	2
1	1	3	2	2	2	2	1	2	2
1	1	4	1	2	2	2	2	2	2
3	3	9	1	2	1	3	1	2	2
1	2	2	2	2	1	2	1	2	3
1	1	1	3	3	1	3	2	1	3
1	1	4	1	1	1	1	1	3	1
1	1	9	1	1	2	2	1	1	2
2	2	2	2	2	2	1	2	2	2
9	9	1	1	1	2	1	1	9	9
9	9	2	1	1	1	3	9	9	9
2	1	3	1	2	1	3	1	3	2
2	2	9	3	3	3	3	2	2	2
2	2	1	3	3	2	2	2	2	2

1	1	2	1	2	3	1	1	1	1
9	9	9	9	9	9	9	9	9	9
9	9	9	9	9	9	9	9	9	9
9	9	9	9	9	9	9	9	9	9
9	9	9	9	9	9	9	9	9	9
9	9	3	1	1	2	1	9	9	9
9	9	9	9	9	9	9	9	9	9
9	9	9	9	9	9	9	9	9	9
9	9	9	9	9	9	9	9	9	9
9	1	3	3	3	3	3	9	9	9
2	2	4	1	2	3	3	1	2	2
1	2	4	1	2	1	2	1	2	2
1	1	1	9	9	9	9	9	9	9
2	9	4	1	2	2	2	1	2	2
2	2	1	1	1	2	2	2	2	2
1	3	2	9	9	9	9	9	9	9
2	3	4	1	1	1	1	1	1	1
2	2	3	2	2	2	2	2	2	2
1	2	1	2	1	1	2	2	2	1
2	2	1	3	2	2	2	2	2	2
1	3	3	1	2	3	2	1	3	2
2	3	1	1	2	2	2	3	3	2
3	3	9	1	2	2	1	3	3	3
2	2	9	1	1	1	2	2	2	2
2	2	1	2	2	2	2	2	2	2
2	2	9	1	2	2	1	2	2	2
3	3	1	1	1	2	2	2	2	2
1	1	3	1	1	1	1	1	2	2
2	2	1	2	2	2	2	2	2	2
2	2	9	3	3	3	3	2	2	2
9	9	9	9	9	9	9	9	9	9
2	2	9	3	3	3	3	2	2	2
1	1	9	1	1	1	1	3	2	2
9	9	1	1	1	1	1	1	9	9
9	9	9	9	9	9	9	3	3	3
2	2	9	3	3	3	3	2	2	2
2	2	9	3	9	3	3	2	2	2
2	2	1	1	2	1	2	2	2	2
1	3	4	1	1	1	2	1	3	3
9	9	9	9	9	9	9	9	9	9
1	2	3	3	2	2	1	1	2	1
2	2	3	3	3	3	3	2	2	2
2	2	1	2	2	3	2	2	2	2
1	3	2	1	1	1	1	1	3	2
2	2	4	1	1	1	1	1	2	1
9	9	9	9	9	9	9	9	9	9
2	2	9	1	2	2	9	9	9	9

9	9	9	9	9	9	9	9	9	9
2	2	1	1	2	2	3	2	2	2
9	9	5	1	1	1	2	1	9	9
9	9	9	9	9	9	9	9	9	9
9	9	9	9	9	9	9	9	9	9
9	9	9	9	9	9	9	9	9	9
9	9	9	9	9	9	9	9	9	9
9	9	9	9	9	9	9	9	9	9
9	9	9	9	9	9	9	9	9	9
9	9	9	9	9	9	9	9	9	9
9	9	9	9	9	9	9	9	9	9
9	9	9	9	9	9	9	9	9	9
9	9	9	9	9	9	9	9	9	9
9	9	9	9	9	9	9	9	9	9
9	9	9	9	9	9	9	9	9	9
9	9	9	9	9	9	9	9	9	9
9	9	9	9	9	9	9	9	9	9
9	9	9	9	9	9	9	9	9	9
9	9	9	9	9	9	9	9	9	9
9	9	9	9	9	9	9	9	9	9
9	9	9	9	9	9	9	9	9	9
9	9	9	9	9	9	9	9	9	9
9	9	9	9	9	9	9	9	9	9
2	1	3	3	2	1	2	1	2	3
2	2	1	1	2	2	2	2	2	2
2	2	2	1	1	1	1	1	2	1
1	1	3	1	1	2	1	1	2	2
3	2	4	1	3	2	1	2	2	1
1	1	3	1	1	3	3	1	9	9
1	2	3	1	1	2	2	2	2	2
1	1	4	1	1	1	1	1	1	1
2	3	1	3	2	3	3	2	2	2
2	1	1	1	3	2	2	2	2	1
1	1	4	1	1	2	2	9	9	9
2	1	4	1	1	3	3	1	3	3
1	2	1	1	1	1	1	1	3	1
2	1	4	1	1	2	2	9	9	9
1	2	1	1	1	1	1	2	2	2
1	1	1	1	1	1	1	1	3	1
1	9	1	1	9	2	1	1	9	9
1	2	3	1	1	1	1	1	2	1
1	1	1	1	1	2	1	1	1	2
9	9	1	1	2	1	1	1	9	9
1	1	1	1	1	2	1	2	2	1
1	1	1	1	1	1	1	1	3	3
3	3	1	2	2	2	2	2	2	2
2	1	3	1	1	1	2	2	2	2

1	1	3	1	1	1	1	1	2	1
1	1	3	1	1	1	1	1	2	2
1	1	4	1	1	2	2	1	3	2
1	1	5	1	1	1	1	1	2	2
3	1	2	2	1	2	1	2	1	2
9	9	1	2	1	2	1	1	9	9
1	2	3	1	1	2	1	2	2	3
9	9	2	1	1	1	1	1	9	9
2	2	1	2	2	2	2	2	2	2
2	2	9	2	2	2	2	2	2	2
9	9	1	1	1	2	1	9	9	9
9	9	1	1	1	1	1	1	9	9
9	9	5	1	1	1	2	9	9	9
9	9	1	9	9	9	9	9	9	9
9	9	4	1	1	1	1	9	9	9
9	9	9	9	9	9	9	9	9	9
3	3	2	9	9	9	9	9	9	9
2	2	9	2	1	2	2	2	2	2
9	9	9	1	1	2	1	9	9	9
3	3	3	1	1	1	1	1	1	1
9	9	4	1	1	1	1	1	2	1
9	9	4	1	3	2	1	9	9	9
9	9	3	1	1	1	1	1	9	9
9	9	3	1	2	2	1	9	9	9
9	9	4	1	1	1	1	1	9	9
1	2	9	1	1	1	1	2	3	1
3	1	4	1	1	1	1	1	3	1
1	2	2	2	2	1	1	2	2	3
1	1	1	2	1	2	2	1	2	2
2	2	1	9	9	9	9	9	9	9
1	2	1	1	1	2	2	2	2	3
1	3	3	1	1	1	1	3	3	1
2	1	4	3	2	2	2	3	3	3
1	3	1	2	2	2	1	1	1	3
1	1	3	1	1	2	2	1	3	1
9	9	3	1	2	2	2	9	1	9
1	2	1	1	1	2	2	2	2	2
2	2	1	1	2	1	2	1	3	1
3	2	1	2	3	2	3	2	3	2
2	2	1	2	2	2	2	2	1	2
1	2	1	1	1	1	1	1	3	1
1	1	1	1	1	1	1	1	3	1
1	3	1	1	1	2	1	1	3	1
2	1	1	3	3	3	3	2	3	3
1	1	2	9	1	1	1	1	3	1
1	1	3	1	1	2	2	1	2	9
1	1	4	1	1	1	1	1	3	1

1	1	5	1	1	1	1	1	2	1
3	3	1	1	1	1	1	1	2	3
1	1	3	1	1	2	2	9	9	9
1	1	2	1	1	2	1	1	3	3
9	2	1	9	9	9	9	9	9	9
1	1	4	1	1	1	1	1	3	1
1	1	1	1	1	1	1	2	2	1
9	9	9	9	9	9	9	9	9	9
9	9	2	1	1	2	2	2	9	9
2	2	2	3	3	3	3	2	2	2
2	2	1	1	1	2	2	2	2	1
3	3	3	1	1	2	2	1	3	3
1	3	3	1	1	1	1	1	3	1
9	9	1	9	9	9	9	9	9	9
9	9	9	9	9	9	9	9	9	9
2	2	1	1	2	1	1	3	9	2
9	9	1	1	1	1	1	1	9	9
9	9	9	9	9	9	9	9	9	9
1	1	1	3	3	3	3	3	2	9
1	3	1	1	1	1	1	1	3	1
1	1	1	1	1	1	1	2	2	2
1	1	2	1	1	1	1	1	1	1
2	3	3	3	3	1	3	2	1	1
1	1	1	1	1	1	1	1	3	1
1	1	1	1	3	3	3	2	2	2
9	9	9	9	9	9	9	9	9	9
9	9	9	1	1	1	1	1	9	9
1	1	1	1	1	1	1	1	3	1
2	2	1	2	2	1	1	3	3	3
1	1	1	1	1	2	1	2	1	2
9	9	9	9	9	9	9	9	9	9
9	9	9	9	9	9	9	9	9	9
9	9	9	9	9	9	9	9	9	9
9	9	9	9	9	9	9	9	9	9
9	9	9	9	9	9	9	9	9	9
9	9	9	9	9	9	9	9	9	9
9	9	9	9	9	9	9	9	9	9
9	9	9	9	9	9	9	9	9	9
9	9	9	9	9	9	9	9	9	9
9	9	9	9	9	9	9	9	9	9
9	9	9	9	9	9	9	9	9	9
9	9	9	9	9	9	9	9	9	9
9	9	9	9	9	9	9	9	9	9
9	9	9	9	9	9	9	9	9	9
9	9	9	9	9	9	9	9	9	9
9	9	9	9	9	9	9	9	9	9
9	9	9	9	9	9	9	9	9	9

9	9	9	9	9	9	9	9	9	9
9	9	9	9	9	9	9	9	9	9
9	9	9	9	9	9	9	9	9	9
9	9	9	9	9	9	9	9	9	9
9	9	9	9	9	9	9	9	9	9
9	9	9	9	9	9	9	9	9	9
9	9	9	9	9	9	9	9	9	9
9	9	9	9	9	9	9	9	9	9
9	9	9	9	9	9	9	9	9	9
9	9	9	9	9	9	9	9	9	9
9	9	9	9	9	9	9	9	9	9
9	9	9	9	9	9	9	9	9	9
9	9	9	9	9	9	9	9	9	9
9	9	9	9	9	9	9	9	9	9
9	9	9	9	9	9	9	9	9	9
9	9	9	9	9	9	9	9	9	9
9	9	9	9	9	9	9	9	9	9
9	9	9	9	9	9	9	9	9	9
9	9	9	9	9	9	9	9	9	9
2	2	5	1	1	1	1	2	2	2
9	9	9	9	9	9	9	9	9	9
9	9	9	9	9	9	9	9	9	9
9	9	9	9	9	9	9	9	9	9
9	9	9	9	9	9	9	9	9	9
9	9	9	9	9	9	9	9	9	9
9	9	9	9	9	9	9	9	9	9
9	9	9	9	9	9	9	9	9	9
9	9	9	9	9	9	9	9	9	9
9	9	9	9	9	9	9	9	9	9
9	9	9	9	9	9	9	9	9	9
9	9	9	9	9	9	9	9	9	9
9	9	9	9	9	9	9	9	9	9
9	9	9	9	9	9	9	9	9	9
9	9	9	9	9	9	9	9	9	9
9	9	9	9	9	9	9	9	9	9
2	2	2	3	3	3	3	2	2	2
3	3	2	9	9	9	9	9	9	9
2	3	1	1	1	3	1	1	1	1
1	1	3	1	1	1	1	1	3	1
2	3	9	3	3	3	3	3	3	3
1	1	2	9	9	9	9	9	9	9
1	3	9	1	2	3	1	9	9	9
9	9	3	1	1	1	1	1	9	9
1	3	3	1	2	3	1	9	9	9
3	1	1	2	2	3	3	2	2	2
2	2	1	2	2	2	2	9	9	9
2	2	3	1	1	2	1	2	2	2

1	1	2	1	1	2	1	1	3	1
1	1	9	1	1	2	2	2	2	2
1	1	3	1	1	1	1	1	2	2
1	1	5	1	1	2	2	1	2	2
1	2	1	1	1	1	1	1	2	2
1	1	3	1	1	1	2	1	2	1
1	1	1	1	1	1	2	1	2	1
1	3	4	1	1	2	2	1	2	2
2	1	2	2	2	2	1	2	2	1
1	2	1	1	1	3	3	2	1	2
1	9	4	1	1	2	2	1	9	9
3	2	1	1	2	3	2	2	2	3
1	1	1	9	9	9	9	9	9	9
2	2	3	1	3	2	2	2	2	3
2	2	3	1	3	2	2	2	2	3
1	1	4	1	1	2	2	1	3	2
9	9	9	9	9	9	9	9	9	9
1	2	1	1	1	2	2	1	2	9
2	1	3	3	1	1	2	1	3	1
2	3	9	1	2	3	1	1	1	1
1	2	2	1	1	1	2	1	2	1
1	3	1	1	1	1	1	1	3	1
1	3	1	1	1	1	1	1	3	1
1	1	4	1	1	1	1	1	3	1
3	3	9	1	2	1	1	3	3	3
2	1	2	2	2	2	2	1	2	1
2	2	9	2	2	2	2	1	1	1
3	2	9	9	9	9	9	1	3	2
2	2	9	2	1	1	2	2	2	2
9	9	9	9	9	9	9	9	9	9
2	3	1	1	9	9	9	9	9	9
2	2	2	1	1	3	1	2	2	2
2	2	1	1	1	1	1	2	2	2
9	9	9	2	2	2	2	9	9	9
9	9	9	9	9	9	9	9	9	9
9	9	9	2	2	2	2	9	9	9
9	9	9	1	1	1	1	9	9	9
9	9	9	1	1	1	1	2	1	3
9	9	9	1	1	1	1	9	9	9
9	9	9	9	9	9	9	9	9	9
1	3	1	1	1	1	1	1	2	2
9	9	4	1	1	2	2	9	9	9
1	1	1	1	1	1	1	1	1	1
3	1	1	1	1	2	1	3	3	3
1	3	1	1	2	3	2	1	1	1
3	3	3	1	2	1	2	3	3	3
1	3	1	2	2	2	2	1	3	1

3	2	9	1	1	2	2	2	3	3
2	2	9	9	9	9	9	1	2	1
2	2	2	2	2	2	1	1	2	1
1	1	2	1	3	1	1	1	1	1
2	3	5	1	3	3	3	2	2	2
2	2	1	1	2	2	1	2	3	2
2	3	1	1	9	1	1	1	1	1
1	2	3	2	1	1	3	2	3	1
1	1	4	1	2	2	1	1	1	1
2	9	9	9	9	9	9	1	9	2
3	3	2	1	1	1	1	1	3	3
3	1	9	1	2	2	2	1	1	2
1	2	1	1	2	1	1	2	1	1
3	2	3	2	2	2	2	1	2	3
1	1	9	2	2	2	2	1	1	1
3	3	1	1	1	3	1	1	3	2
3	2	1	1	3	1	1	2	2	2
9	9	2	1	2	2	2	1	9	9
1	2	9	1	3	2	3	1	2	1
1	1	2	3	3	3	2	1	2	2
2	1	9	1	3	3	3	1	1	2
3	3	4	1	1	2	2	1	1	3
1	2	9	1	1	1	1	1	3	2
3	1	1	2	2	2	2	2	2	2
1	2	1	2	1	3	3	1	2	2
3	3	1	2	1	1	2	3	2	1
1	1	1	2	2	2	2	1	2	1
1	1	2	1	1	1	1	1	1	2
2	1	2	2	1	2	2	2	1	1
2	3	2	2	2	2	2	2	3	2
1	2	4	3	2	2	2	1	3	2
1	2	2	1	1	1	1	1	2	3
2	2	1	1	2	2	2	2	2	2
3	3	1	2	2	2	2	2	1	1
3	1	5	1	1	1	1	1	1	3
1	3	4	2	2	2	2	1	3	3
1	2	1	2	3	1	2	1	1	2
9	9	9	9	9	9	9	9	9	9
3	3	5	1	1	2	1	1	3	1
3	3	1	1	2	1	2	1	3	9
1	3	1	1	2	2	2	1	3	3
3	1	1	1	1	1	1	1	2	3
1	3	1	1	2	2	2	1	3	1
1	2	2	1	1	1	1	1	3	2
2	2	9	1	1	1	1	2	2	2
3	3	1	2	2	2	2	2	3	3
3	3	1	2	2	2	2	3	3	3

2	2	2	1	1	1	1	1	1	2
9	9	1	9	9	9	9	1	1	1
3	3	1	1	1	2	1	1	2	2
1	1	2	1	2	1	3	1	2	2
2	2	3	1	1	1	1	1	1	1
2	1	2	2	2	1	1	1	1	1
2	2	9	2	2	2	2	2	2	2
9	9	9	9	9	9	9	9	9	9
9	9	9	9	9	9	9	9	9	9
2	3	1	2	2	1	1	1	3	2
2	1	1	1	1	1	1	2	2	2
2	2	9	2	2	2	2	2	2	2
2	2	1	2	2	2	2	2	2	2
2	2	3	1	1	1	1	1	1	2
1	1	1	2	2	2	2	2	2	2
1	1	1	2	2	2	2	2	2	2
2	2	1	2	2	2	2	2	2	2
2	2	1	2	3	3	3	2	2	2
2	2	2	1	1	1	1	2	2	2
3	3	1	2	2	2	2	2	2	2
2	2	9	2	2	2	2	2	3	3
2	2	1	1	1	2	2	2	2	2
2	2	1	1	1	2	2	2	2	2
2	2	9	9	9	9	9	2	2	2
2	2	9	3	3	3	3	2	2	2
2	2	1	2	2	3	1	2	2	2
2	1	1	1	1	1	1	1	2	2
1	2	2	1	1	1	2	1	2	9
2	2	3	1	1	1	1	1	1	1
2	2	9	2	9	2	2	2	2	2
2	2	9	2	2	2	2	2	2	2
2	1	1	1	1	1	1	1	2	2
2	2	9	2	2	2	2	2	2	2
2	2	1	2	2	2	2	2	2	2
2	1	1	1	1	1	1	2	2	2
2	1	1	1	1	1	1	2	2	2
2	2	9	2	1	2	1	2	2	2
2	2	1	2	2	1	1	2	2	2
2	2	9	2	2	2	2	2	2	2
3	1	2	9	9	9	9	1	3	3
2	1	1	1	2	1	1	2	2	2
2	2	1	1	1	2	2	2	2	2
2	1	1	1	1	1	1	2	3	2
2	2	9	9	9	9	9	2	2	2
3	3	1	1	1	1	2	1	2	3
3	3	9	2	2	1	1	2	2	2
3	3	1	3	3	2	2	2	2	2

2	2	9	1	2	2	2	2	2	2
2	2	1	2	1	2	2	2	2	2
2	2	1	1	2	2	2	1	2	2
2	2	1	2	1	1	1	2	2	2
3	3	1	1	1	2	1	2	2	2
2	2	9	2	2	2	2	2	2	2
9	9	1	1	2	2	2	9	9	9
3	1	2	1	1	1	1	1	1	1
9	9	3	1	1	2	2	9	9	9
2	2	3	2	2	2	2	2	2	2
1	2	9	1	1	1	1	1	2	1
1	1	9	1	1	1	1	1	1	1
1	2	1	3	3	3	3	2	3	2
9	9	1	1	2	2	2	9	9	9
2	2	1	2	2	3	2	2	2	2
9	9	1	2	2	2	2	2	3	1
2	2	1	1	1	1	1	1	2	2
1	1	9	1	1	1	1	1	1	1
9	9	1	1	1	1	1	2	9	9
2	2	9	2	2	1	3	1	1	9
2	1	1	1	1	2	2	1	2	2
9	9	1	1	1	1	1	9	9	9
9	9	4	3	3	1	9	9	9	1
3	3	1	1	2	2	2	3	3	3
2	3	9	1	1	1	1	1	2	3
2	2	9	1	2	2	2	3	3	3
2	2	1	1	2	9	9	2	2	2
3	3	1	1	1	1	2	1	1	1
1	3	2	1	1	2	2	1	1	1
1	1	1	1	1	1	1	1	1	1
2	2	9	2	3	3	3	2	2	2
2	2	1	2	2	2	2	2	2	2
2	2	1	3	3	3	3	2	2	2
9	9	1	1	3	2	2	1	9	9
2	2	9	1	3	2	2	2	2	2
9	9	1	1	1	1	1	1	9	9
3	3	9	1	2	2	2	3	1	1
2	2	9	1	1	2	3	1	1	1
2	2	9	3	3	3	3	2	2	2
2	2	9	3	3	3	3	2	2	2
2	3	1	9	3	2	3	2	3	9
2	2	9	1	2	2	2	2	2	2
9	9	3	3	9	2	3	9	9	9
1	1	1	1	1	1	1	1	1	1
2	2	1	1	1	2	2	1	1	1
3	3	9	2	3	3	3	3	3	3
1	2	1	2	3	3	1	1	1	1

1	1	1	2	1	1	2	9	9	9
2	2	1	3	3	3	3	2	2	2
1	1	9	1	1	1	1	1	1	1
2	2	1	3	3	3	3	2	2	2
9	9	4	3	3	3	3	9	1	9
3	2	4	1	1	1	1	1	2	2
3	3	1	2	2	2	2	3	3	3
9	2	9	1	2	1	1	2	2	2
2	2	9	1	2	2	1	2	2	2
3	3	1	1	1	1	1	1	2	2
3	3	3	2	1	3	3	9	9	9
1	1	3	1	1	1	1	1	1	1
2	2	1	1	2	2	1	2	2	2
2	2	9	1	2	2	2	2	2	2
2	2	9	2	2	2	2	2	2	2
1	1	1	2	2	2	2	3	3	3
1	1	9	1	2	2	2	1	1	1
1	3	3	1	1	1	1	3	3	3
9	9	9	9	9	9	9	9	9	9
9	9	9	9	9	9	9	1	2	1
2	2	3	1	1	3	3	2	2	2
2	2	1	1	2	2	1	2	2	2
1	1	1	1	1	1	1	1	1	1
2	2	9	2	2	2	1	2	2	2
9	3	9	2	2	2	2	3	3	9
1	1	3	1	2	2	1	1	2	2
2	1	9	1	1	1	1	1	9	1
2	3	1	3	1	1	1	2	2	2
9	1	2	1	1	2	2	1	2	2
3	3	1	2	2	2	2	2	2	2
2	1	9	1	2	2	1	1	2	9
2	3	2	2	2	2	2	2	2	2
1	1	9	1	3	3	3	1	1	1
2	2	1	2	2	1	1	1	1	1
2	2	3	3	3	3	3	2	2	2
3	3	9	3	3	3	3	2	2	2
2	2	1	1	1	2	1	1	1	1
2	2	2	2	1	1	1	3	3	3
1	2	9	1	2	3	1	1	2	3
3	3	9	2	2	2	2	3	3	3
1	1	3	1	1	1	1	1	1	1
1	1	9	9	9	9	9	2	2	9
9	9	9	9	9	9	9	9	9	9
3	3	9	2	2	2	9	3	3	3
2	2	1	1	2	1	1	9	9	9
2	2	9	3	3	3	3	2	2	2
2	1	5	1	1	1	1	1	2	2

1	1	1	1	1	1	1	1	1	1
3	3	4	1	1	1	1	1	3	3
3	3	3	9	9	9	9	9	9	9
3	3	4	1	1	1	1	1	3	3
1	1	9	1	2	1	1	1	1	9
2	2	5	3	3	3	3	2	2	2
2	2	1	3	3	3	3	2	2	2
2	2	5	9	9	9	9	2	2	2
1	1	5	2	1	1	3	3	2	1
2	3	1	1	2	1	1	1	3	2
2	2	5	2	2	3	2	2	2	2
3	2	1	1	2	2	2	2	1	2
2	2	3	1	1	1	1	2	2	2
2	2	5	1	1	1	1	1	3	2
2	2	5	3	3	3	3	2	2	2
2	2	1	1	2	2	2	2	2	2
2	2	4	2	3	2	2	2	2	2
2	2	1	3	3	3	3	2	2	2
2	2	1	3	3	3	3	2	2	2
2	1	4	1	1	3	3	2	2	1
2	2	1	1	1	3	3	2	2	2
2	2	1	2	2	2	2	3	3	3
2	3	1	1	2	2	1	2	2	2
2	2	1	2	2	2	2	2	2	2
1	1	1	1	1	1	1	1	1	1
2	2	5	1	3	3	3	2	2	2
2	2	2	1	2	1	2	1	1	3
2	2	1	1	2	3	1	2	2	2
2	2	3	2	1	3	3	2	2	3
3	2	1	2	2	2	2	2	2	2
3	1	3	3	3	2	2	2	2	2
1	1	5	1	1	1	2	1	2	3
2	2	1	2	2	2	3	2	2	2
2	2	5	9	9	9	9	2	2	2
1	2	5	1	1	1	2	1	2	2
3	3	5	2	1	2	1	1	2	3
2	2	1	2	2	2	2	9	9	9
9	9	9	9	9	9	9	9	9	9
2	2	1	1	3	1	2	2	2	2
3	1	2	2	2	2	2	2	2	2
3	1	1	9	9	9	9	2	2	3
2	2	1	2	1	2	2	2	2	2
2	2	1	2	2	2	2	2	2	2
2	2	5	9	9	9	9	2	2	2
9	9	9	9	9	9	9	9	9	9
1	1	1	1	2	1	1	1	1	9
2	2	1	3	3	3	3	2	2	2

9	9	9	9	9	9	9	9	9	9
2	2	1	1	1	2	1	1	2	2
3	2	1	3	2	1	2	2	1	3
1	3	1	9	9	9	9	1	3	2
3	3	1	2	2	2	2	1	3	1
2	1	1	1	1	2	2	1	1	3
3	3	9	9	9	9	9	2	2	3
9	9	9	9	9	9	9	9	9	9
3	3	3	2	2	2	2	2	2	2
2	3	1	2	2	3	2	3	2	2
1	1	1	1	2	3	3	2	2	2
2	1	9	1	1	1	1	1	1	1
1	1	1	1	1	2	2	2	2	2
1	1	4	2	2	2	2	1	1	1
2	1	9	1	3	3	1	1	2	3
2	2	9	3	3	3	3	2	2	2
2	1	9	3	3	2	2	1	2	2
2	2	1	2	2	2	2	2	2	2
9	9	1	9	9	9	9	9	9	9
9	9	9	9	9	9	9	9	9	9
9	9	9	9	9	9	9	9	9	9
2	1	1	1	2	3	3	1	2	3
1	2	1	3	2	2	2	2	2	2
2	1	9	9	9	9	9	1	1	2
3	1	9	9	9	9	9	1	1	2
3	2	9	2	2	2	2	3	3	3
3	2	4	3	3	1	2	1	3	2
2	2	1	1	2	2	3	2	1	2
2	1	4	2	1	3	2	1	2	3
2	3	1	3	2	2	2	1	2	2
2	2	9	2	2	2	3	9	9	9
2	1	1	2	2	2	3	2	2	2
3	3	1	1	1	2	1	1	3	3
2	2	9	2	2	2	3	2	2	2
2	2	9	1	1	1	1	3	3	3
2	2	2	2	2	2	2	2	2	2
3	3	9	2	2	1	2	3	3	3
2	3	9	1	1	1	2	2	2	2
9	9	9	9	9	9	9	9	9	9
9	9	9	9	9	9	9	9	9	9
1	2	9	1	2	1	3	1	2	3
2	2	9	2	2	2	2	2	2	2
2	2	2	1	2	1	1	2	3	1
2	1	1	2	2	2	2	1	2	3
9	9	9	2	2	2	2	9	9	9
3	3	9	1	2	2	1	3	3	3
3	1	9	2	2	2	2	2	2	2

1	1	9	9	9	9	9	1	1	1
2	2	1	2	2	2	2	2	2	2
2	3	1	2	1	3	1	2	1	3
2	2	9	2	2	2	2	1	9	9
2	3	1	2	2	2	2	2	3	2
3	3	9	1	2	1	2	3	3	3
1	1	9	1	3	3	1	1	1	1
3	3	9	1	1	1	1	3	3	3
2	2	1	2	2	2	2	2	3	3
1	1	9	9	9	9	9	2	1	2
2	2	1	3	3	3	3	2	2	2
2	2	5	3	3	2	2	1	2	1
2	2	9	9	9	9	9	1	1	1
2	1	3	1	1	2	2	1	2	1
2	2	9	1	1	2	2	1	2	2
2	2	9	2	3	2	2	3	3	3
1	3	1	1	1	2	2	3	3	1
2	1	1	3	3	3	3	1	2	3
2	2	1	3	3	3	3	9	9	9
2	1	9	1	2	2	2	2	3	2
2	1	9	9	9	9	9	1	2	3
3	3	3	2	2	1	2	3	3	3
3	3	1	2	2	2	1	2	2	2
3	3	1	2	1	2	3	1	2	1
1	2	9	2	2	2	2	1	2	3
3	2	1	2	2	2	2	1	2	2
3	2	1	3	2	2	2	2	2	2
2	3	1	2	2	2	2	1	2	3
1	2	5	1	1	1	3	1	2	1
2	2	1	2	2	2	2	2	2	2
2	2	5	2	3	1	2	1	1	3
2	2	9	2	2	2	2	2	2	2
2	2	2	3	3	3	3	2	2	2
1	1	9	1	1	2	1	1	2	3
2	3	9	1	2	2	2	1	2	3
3	1	1	1	1	1	1	1	2	2
1	2	4	1	1	1	2	1	2	3
3	3	9	2	2	2	2	3	3	3
1	2	1	1	2	2	2	1	1	1
1	2	1	1	2	1	2	2	1	1
2	2	9	2	2	2	2	2	2	2
1	1	1	1	2	1	1	1	2	3
2	1	3	1	2	2	3	1	2	2
1	2	2	1	1	1	1	1	1	1
3	3	9	1	2	3	3	1	2	2
2	2	1	3	3	2	2	2	1	2
1	3	9	1	1	2	2	3	1	3

1	3	1	1	2	2	2	2	2	1
1	2	3	1	1	3	3	1	3	3
2	2	9	1	2	2	2	2	2	2
1	1	2	1	1	1	1	1	1	1
1	2	3	2	2	2	2	1	2	3
3	2	1	1	1	1	1	1	2	3
3	2	4	1	1	2	2	1	2	3
2	1	4	1	1	1	2	2	1	2
3	2	3	1	1	1	2	1	2	1
2	3	1	2	2	2	2	3	3	3
2	3	9	1	3	2	1	1	2	3
2	1	1	2	2	2	2	2	1	3
1	1	5	1	1	1	1	1	1	1
3	3	1	1	2	1	2	1	3	1
2	3	1	2	2	2	2	2	2	3
1	3	1	1	2	2	1	1	3	1
1	3	1	2	2	2	2	1	1	1
3	3	1	1	2	2	2	1	1	3
1	1	4	1	1	2	3	1	2	3
3	2	5	2	1	2	1	2	3	2
3	3	1	1	2	3	3	3	3	3
1	3	1	2	2	2	2	3	3	3
3	1	3	1	2	2	2	3	2	3
1	1	4	1	1	2	1	1	3	1
2	2	2	2	2	2	2	2	2	2
2	2	9	9	9	9	9	2	2	2
3	1	4	1	1	1	1	1	3	3
3	2	1	1	2	3	1	2	1	3
2	2	9	9	9	9	9	2	2	2
9	9	9	9	9	9	9	9	9	9
9	9	9	9	9	9	9	9	9	9
9	9	9	9	9	9	9	9	9	9
2	2	3	1	1	1	1	2	1	1
9	9	1	9	9	9	9	9	9	9
2	2	9	9	9	9	9	3	3	3
9	9	9	9	9	9	9	9	9	9
2	3	1	1	2	2	2	2	2	2
9	9	1	2	2	2	2	9	9	9
9	1	9	9	9	9	9	1	1	1
3	2	3	1	1	1	1	2	3	2
9	9	9	9	9	9	9	9	9	9
2	2	2	1	2	2	2	2	2	2
3	1	1	1	2	1	1	1	9	1
3	1	9	9	9	9	9	1	3	3
1	1	9	1	3	2	1	1	3	2
1	2	1	1	2	1	1	1	1	2
3	1	4	1	1	1	1	1	3	3

2	1	9	1	2	2	1	1	2	1
2	1	3	2	2	2	9	1	2	2
2	2	9	9	9	9	9	9	9	9
1	1	3	2	2	2	1	2	1	3
2	2	1	2	9	2	1	2	1	3
2	1	1	1	2	1	1	1	2	2
9	9	1	2	1	3	1	9	9	2
2	3	1	1	2	3	3	1	2	3
2	3	4	1	2	1	2	1	1	1
2	3	1	1	1	1	1	3	3	3
2	3	1	2	2	1	2	2	3	1
2	2	9	3	2	2	1	2	1	3
3	1	9	1	1	1	3	1	3	1
2	2	1	2	2	2	2	2	2	2
2	2	1	2	2	2	2	2	2	2
1	1	9	9	9	9	9	9	9	9
2	2	3	2	3	3	3	2	2	2
2	2	1	3	3	3	3	2	2	2
2	2	1	2	2	2	2	2	2	2
2	2	9	2	2	2	2	2	2	2
1	9	1	1	1	1	1	1	3	1
1	9	1	1	1	1	1	1	3	1
1	9	1	1	1	1	1	1	3	1
1	1	1	1	1	1	1	1	2	1
1	1	3	1	1	1	1	1	3	1
1	3	4	1	1	1	1	1	3	1
1	3	2	1	1	1	1	1	3	1
1	2	1	1	1	1	1	1	3	3
1	2	1	1	1	1	1	1	2	1
1	9	1	1	1	2	1	1	3	1
1	9	2	1	1	1	1	1	3	1
9	9	3	3	1	2	3	9	2	9
1	1	3	1	1	1	1	1	2	1
1	3	1	1	1	1	1	1	3	1
1	9	1	1	2	2	1	2	2	2
1	9	2	1	1	1	1	1	2	2
1	1	9	1	1	3	1	1	2	2
1	9	1	1	1	1	1	1	3	2
1	1	1	1	1	1	1	9	9	1
1	3	1	1	1	2	1	1	3	1
1	3	3	1	1	1	1	1	1	1
1	1	1	1	1	1	1	1	9	9
1	3	1	1	1	1	1	1	1	1
1	1	1	1	1	1	1	1	3	1
1	1	2	1	1	1	1	1	1	1
1	3	4	1	1	1	1	1	3	1
1	9	9	1	1	1	1	1	2	2

1	9	2	1	1	1	1	1	2	2
1	3	1	1	1	1	1	1	2	2
1	1	1	1	1	1	1	1	3	1
1	1	1	1	1	1	1	1	1	1
1	9	1	1	1	1	1	1	3	2
1	9	1	1	1	1	1	1	3	2
1	1	1	1	1	2	2	2	2	2
1	1	1	1	1	1	1	1	2	2
1	3	1	1	1	1	1	9	9	9
1	1	1	1	1	1	1	1	3	1
1	2	1	1	1	2	2	9	3	1
1	2	2	1	3	3	1	2	2	2
1	9	1	1	1	1	1	1	3	2
1	3	1	1	1	1	1	1	3	2
1	3	1	1	1	1	1	1	3	2
1	9	1	1	1	1	1	1	3	2
1	9	1	1	1	3	1	1	3	1
1	1	1	1	1	2	2	1	9	1
1	9	1	1	1	1	1	1	3	2
1	1	1	1	1	3	1	2	9	2
1	2	2	1	2	2	2	3	1	3
3	3	9	1	2	2	2	1	3	3
1	3	1	1	1	1	1	1	1	1
1	9	1	1	1	1	1	1	3	1
1	1	1	1	2	2	2	3	2	1
1	1	1	1	1	1	1	1	3	3
1	9	1	1	3	2	1	1	1	1
3	2	1	1	1	1	1	1	3	1
1	3	1	1	1	1	2	1	3	1
1	9	3	1	1	2	1	1	3	1
1	2	1	1	1	2	2	1	2	2
1	3	1	1	1	1	1	1	3	1
1	9	2	1	1	1	1	9	9	9
1	9	4	1	1	1	1	1	3	1
1	1	1	1	1	1	1	1	3	1
1	1	1	1	1	1	1	1	3	1
9	9	2	1	1	1	1	1	3	1
1	9	1	1	1	1	1	1	3	1
1	3	1	1	1	1	1	1	3	1
1	1	1	1	1	1	1	1	3	1
1	3	4	1	1	1	1	1	3	1
1	1	1	1	1	1	1	1	3	1
1	9	1	1	1	1	1	1	3	1
1	1	1	1	1	1	1	1	3	1
1	2	1	1	1	1	1	1	3	2
1	1	1	1	1	1	1	1	2	1
1	9	1	1	1	1	1	1	3	9

1	9	1	1	2	2	1	1	3	1
1	9	1	1	1	1	1	1	3	1
1	2	2	1	1	1	1	1	3	1
1	9	1	1	1	1	1	1	1	1
1	1	1	1	1	1	1	1	3	1
1	1	1	1	2	2	1	1	1	1
1	1	5	1	1	2	1	3	1	1
1	1	9	9	9	9	9	1	9	1
1	1	9	1	1	9	9	1	9	1
1	1	4	1	1	1	1	1	3	1
2	1	4	2	2	1	1	1	3	2
9	9	1	1	1	1	1	9	9	9
3	1	5	1	1	2	1	1	3	1
2	1	1	1	1	2	1	1	3	3
3	1	1	2	2	3	3	2	9	1
1	3	4	1	1	2	2	1	2	2
9	9	1	1	1	1	1	9	9	9
9	9	1	1	1	1	1	9	9	9
1	1	4	1	1	1	1	1	2	2
1	1	3	1	1	2	1	2	2	2
1	1	3	1	1	1	1	1	3	1
2	3	3	1	1	1	1	1	3	2
3	3	9	1	1	1	1	1	3	2
3	3	3	1	1	1	1	1	3	2
3	1	4	1	1	2	2	1	3	3
1	1	1	1	1	2	2	1	3	3
2	3	1	1	1	2	3	2	3	1
1	2	1	1	1	1	1	2	2	2
1	1	1	1	1	1	1	1	2	2
1	1	1	1	1	1	1	1	2	1
1	1	1	1	1	1	1	1	2	2
9	9	2	1	1	1	2	2	3	1
1	1	1	1	1	2	1	2	2	2
1	1	5	1	1	1	1	1	3	1
1	1	5	1	1	1	1	1	1	1
2	1	3	1	1	1	1	2	2	2
1	1	9	1	1	3	9	9	9	9
2	2	9	1	1	3	1	1	1	1
1	2	9	1	1	3	1	1	1	1
1	1	2	1	1	1	1	1	3	2
1	1	4	1	1	1	1	1	3	1
1	1	3	1	1	1	1	1	3	1
1	1	3	1	1	2	2	1	2	1
2	2	9	9	9	9	9	2	2	2
1	1	3	1	1	2	2	1	2	1
1	1	1	1	2	2	1	1	3	1
1	2	5	1	1	1	1	1	3	1

1	1	1	1	1	1	1	1	3	1
1	1	1	1	1	2	1	1	2	1
1	1	1	1	1	3	1	1	3	1
2	2	1	1	1	2	1	1	2	2
9	9	4	1	1	1	1	1	1	1
3	3	2	2	1	2	2	2	3	2
1	1	2	1	1	1	1	1	2	2
1	1	4	1	1	2	1	9	9	9
1	1	5	1	1	1	1	9	9	9
1	1	1	1	1	1	1	1	2	2
1	1	1	1	1	1	1	1	3	2
1	1	4	1	1	1	1	1	3	1
1	1	1	1	1	2	2	2	2	2
2	1	3	1	1	1	1	1	2	1
1	2	3	3	1	2	1	2	2	2
9	9	3	1	1	1	1	9	9	9
1	2	3	1	1	1	1	1	2	1
1	1	1	1	1	1	1	1	2	1
1	1	2	1	1	1	1	1	3	1
1	1	3	1	1	1	1	1	3	2
9	9	3	1	1	2	1	9	9	9
1	2	3	1	1	1	1	1	1	1
3	2	3	1	1	1	1	9	9	9
1	1	2	1	1	1	1	1	2	1
1	1	2	1	1	1	1	1	3	1
1	1	9	1	1	1	1	1	3	1
1	9	9	1	1	1	1	2	2	1
9	9	9	9	9	9	9	9	9	9
9	9	9	9	9	9	9	9	9	9
1	1	2	1	1	2	1	1	3	3
1	1	3	1	1	1	1	1	2	2
1	1	4	1	1	1	1	1	2	1
1	9	1	1	1	1	1	9	9	1
1	9	9	1	1	1	1	9	9	9
1	3	1	1	1	1	1	1	3	1
1	9	1	1	1	1	1	1	2	1
1	1	1	1	1	1	1	1	3	1
1	1	2	1	1	1	1	1	3	1
1	1	1	1	1	1	1	1	3	1
1	2	4	1	1	1	1	1	3	1
1	9	1	1	1	2	1	1	1	2
1	9	1	1	1	2	1	1	3	1
1	3	1	1	1	1	1	1	3	1
1	1	1	1	1	3	1	3	1	1
1	1	1	1	1	1	1	1	2	2
1	1	9	1	1	2	2	3	3	3
1	9	1	1	1	1	3	1	3	1

1	1	2	1	1	1	1	1	3	1
1	9	1	1	1	2	2	1	3	1
9	9	9	9	9	9	9	9	9	9
1	9	3	1	9	1	1	1	3	1
1	9	1	1	1	1	1	1	3	1
3	3	3	1	1	1	1	1	2	1
1	1	1	1	1	3	1	1	1	1
1	1	5	1	1	1	1	1	3	2
9	9	3	1	1	1	1	2	9	9
9	9	9	1	1	1	1	1	3	1
3	3	9	1	1	9	9	1	3	3
1	1	2	1	1	3	1	1	1	1
1	2	1	1	1	1	1	1	2	1
1	9	4	1	1	1	1	9	9	1
2	1	3	1	1	1	1	1	2	1
1	1	3	1	1	1	1	1	3	1
1	1	1	1	1	9	1	9	9	9
1	3	3	1	2	1	1	9	9	9
1	2	3	1	1	1	1	1	2	1
1	2	1	1	1	1	1	1	3	3
1	3	1	1	1	1	1	1	3	1
1	3	3	1	1	1	1	1	3	3
1	3	3	1	1	1	1	1	3	3
1	1	2	1	1	1	1	1	3	1
1	1	9	1	1	9	1	1	9	1
1	3	3	1	1	1	1	1	2	2
1	1	1	1	1	1	1	1	3	1
1	1	3	1	2	1	1	1	3	1
1	3	3	1	1	1	1	1	2	2
1	3	3	1	1	1	1	1	2	2
1	1	3	1	2	2	1	1	2	3
1	2	2	1	1	1	1	1	2	1
1	1	3	1	1	1	1	1	3	1
1	1	3	1	1	1	1	1	2	1
1	2	4	1	1	1	1	1	3	1
1	1	3	1	1	1	1	9	9	9
1	1	4	1	1	1	1	1	3	1
1	9	3	1	1	1	1	1	9	1
2	2	3	1	1	2	1	1	1	2
1	1	2	3	1	3	1	1	3	1
2	3	2	1	1	3	3	2	3	2
3	1	3	1	1	1	1	1	3	1
1	9	1	1	1	1	1	1	9	9
1	9	1	1	1	1	1	9	9	1
1	1	3	1	1	1	1	1	2	1
1	1	2	1	1	1	1	1	3	1
9	9	9	1	1	1	1	1	9	9

1	3	3	1	1	1	1	1	3	1
1	1	3	1	1	1	1	1	2	1
1	2	1	1	1	1	1	2	2	2
1	1	1	1	1	1	1	1	3	1
1	1	1	1	1	1	1	1	2	2
1	3	1	1	1	1	1	1	2	1
2	3	3	1	1	9	1	2	2	2
1	1	1	1	1	1	1	1	2	1
2	2	3	1	1	1	3	1	2	1
2	2	1	1	1	1	1	1	2	1
2	2	3	1	1	1	1	1	2	1
9	9	4	1	1	1	1	1	9	9
9	9	3	1	1	1	1	1	9	9
9	9	1	1	1	1	1	1	9	9
9	9	1	1	1	1	1	1	9	9
9	9	1	1	1	1	1	1	9	9
3	3	3	1	1	9	1	1	3	1
1	1	4	2	2	2	2	1	2	2
9	9	3	1	1	1	1	9	9	1
1	1	3	1	1	1	1	1	3	1
1	1	3	1	1	1	1	1	3	1
1	3	3	1	1	2	1	9	9	9
1	2	4	1	1	2	1	1	3	1
2	3	3	1	1	2	1	2	3	2
1	1	3	1	1	1	1	1	2	2
1	1	3	1	1	1	1	1	2	1
1	1	3	1	1	3	1	1	2	1
1	1	2	1	1	1	1	1	3	3
1	1	4	1	1	1	1	1	2	2
1	1	3	1	1	1	1	1	3	9
1	1	3	1	1	1	1	1	2	1
1	9	9	1	1	1	1	1	9	1
2	3	3	1	1	1	1	1	3	1
1	2	3	1	1	1	1	1	3	1
1	1	3	1	1	1	1	1	2	1
2	1	3	1	1	1	1	1	2	2
1	1	3	1	1	2	1	2	2	2
1	1	1	1	1	1	1	1	1	3
1	1	1	1	2	1	1	9	9	9
1	1	1	1	1	1	1	1	3	1
2	3	3	1	1	3	3	1	2	3
2	2	9	1	1	3	3	1	2	3
1	1	5	1	1	1	1	1	3	1
1	1	5	1	1	1	1	1	2	1
2	2	2	1	1	1	1	1	3	1
1	1	2	1	1	1	1	1	9	1
1	1	1	1	1	1	1	1	3	1

1	1	3	1	1	1	1	1	3	1
1	1	4	1	1	1	1	1	3	1
1	1	5	1	1	1	1	1	2	1
2	2	4	1	1	1	1	1	2	1
3	3	4	1	1	1	1	1	1	1
2	2	3	1	1	1	1	1	3	1
2	2	2	1	1	1	1	1	3	2
2	1	3	1	1	1	1	1	2	1
2	2	4	1	1	1	1	2	1	2
1	2	4	1	1	1	1	1	2	2
1	3	3	1	1	1	1	1	3	1
2	2	9	1	1	1	1	2	2	2
2	2	1	1	1	1	1	1	3	1
2	1	1	1	1	1	1	1	3	1
2	2	1	1	1	1	1	1	1	1
1	1	3	1	1	1	1	1	1	1
2	2	2	1	1	2	1	2	2	2
2	2	1	1	1	2	1	2	2	2
2	2	4	3	3	3	3	2	3	2
3	3	3	1	1	1	1	1	3	1
3	3	4	1	1	1	1	1	3	1
9	9	9	9	9	9	9	1	3	3
2	1	4	1	1	1	1	1	3	2
1	3	3	1	1	1	1	1	3	3
1	2	2	1	1	1	1	1	2	1
1	1	1	1	9	9	9	9	9	9
1	1	1	9	9	9	9	9	9	9
9	9	3	1	1	1	1	1	9	9
3	9	1	9	9	9	9	1	3	2
3	3	2	1	1	1	1	3	9	1
1	1	4	1	1	1	1	1	2	1
1	1	4	1	1	1	1	1	2	1
9	9	9	1	1	1	1	9	9	9
1	9	1	9	9	9	9	9	9	9
1	9	1	1	2	1	1	9	9	9
1	9	1	1	1	1	1	9	9	9
9	9	1	1	1	1	1	9	9	9
9	9	1	1	1	1	1	9	9	9
9	9	3	1	1	1	1	1	9	9
9	9	9	1	1	1	3	3	2	2
1	1	3	1	1	1	1	1	3	2
9	9	3	1	1	1	1	1	9	9
9	9	5	1	1	1	1	1	9	9
2	2	2	1	1	1	1	2	2	2
9	9	1	1	1	1	1	9	9	9
9	9	3	1	1	1	1	1	9	9
9	9	3	1	1	1	1	1	9	9

1	1	2	1	1	1	1	1	3	1
2	1	9	1	1	2	1	9	9	9
9	9	3	1	1	1	1	9	1	9
9	9	1	1	1	1	1	1	9	2
9	9	1	1	1	1	1	1	9	2
9	9	1	1	1	1	1	1	9	9
9	9	9	1	1	1	1	1	9	9
9	9	3	1	1	1	1	1	9	9
9	9	4	1	1	1	1	9	9	1
9	9	1	1	1	1	1	1	9	9
3	3	3	1	1	1	1	1	3	1
1	1	3	1	1	1	1	1	3	3
1	1	3	1	1	1	1	1	3	1
1	1	4	1	1	2	1	1	2	2
1	1	4	1	1	1	1	1	1	9
3	1	1	1	1	2	1	9	9	9
3	1	1	1	1	2	1	1	1	1
1	1	1	1	2	1	1	1	1	1
1	1	3	1	1	1	1	1	3	3
1	1	3	1	9	1	1	1	3	2
1	1	3	1	1	1	1	1	3	2
9	2	1	1	1	2	1	1	1	1
9	9	1	1	1	1	1	1	9	9
1	1	9	1	1	1	1	1	9	1
9	9	9	1	1	1	1	3	1	2
1	1	3	1	1	1	1	1	3	1
1	2	1	2	1	2	1	2	3	3
1	1	2	1	1	1	1	1	3	2
1	2	1	1	1	1	1	1	3	1
1	2	1	1	1	2	2	2	2	1
1	2	3	1	1	1	1	1	2	1
1	1	3	1	1	3	1	1	2	1
2	1	1	1	1	1	1	1	1	1
1	1	4	1	1	1	1	1	2	1
2	2	3	1	1	1	1	1	2	2
1	1	2	1	1	1	1	1	3	2
1	3	2	1	1	1	1	1	3	1
1	3	3	1	1	1	1	1	3	1
1	1	1	1	1	1	1	1	3	1
1	3	1	1	1	1	1	2	3	3
1	2	4	1	1	3	1	1	3	1
1	1	3	1	2	2	1	1	2	1
1	3	1	1	3	2	1	1	2	1
1	1	3	1	1	1	1	1	3	2
1	1	9	1	1	1	1	1	9	2
3	3	3	1	1	1	1	1	3	1
1	1	2	1	1	1	1	1	3	1

1	1	2	1	1	1	1	1	3	2
1	1	2	1	1	1	1	1	3	1
1	1	3	1	1	1	1	1	2	2
1	1	4	1	3	3	1	1	3	2
1	1	3	1	1	1	1	1	3	2
1	1	4	1	3	1	1	1	3	2
1	1	2	1	1	1	1	1	3	1
1	1	4	1	1	1	1	1	3	2
1	3	2	1	1	1	1	1	2	1
1	1	9	1	1	1	1	1	3	1
1	1	2	1	1	1	1	1	2	2
1	1	4	1	1	1	1	1	3	3
1	3	3	1	1	1	1	1	3	1
1	3	4	1	1	1	1	1	3	1
1	1	1	1	1	1	1	1	3	1
1	1	2	1	1	1	1	1	3	3
1	3	3	1	2	2	2	1	2	1
2	2	2	1	1	1	1	1	2	2
1	3	1	2	1	1	2	3	1	3
1	2	3	1	1	1	1	1	1	1
1	1	2	1	1	1	1	1	3	1
1	1	2	1	1	1	1	1	3	1
1	1	4	1	3	3	1	1	3	2
1	1	1	1	1	2	1	1	2	1
1	1	1	1	1	2	1	1	2	1
3	2	3	1	1	1	1	1	3	1
2	2	1	1	1	2	2	2	2	1
2	2	1	1	2	2	2	2	2	2
1	1	3	1	1	1	1	1	2	1
2	3	3	1	1	1	1	1	2	1
3	2	1	1	1	2	2	2	2	3
2	2	1	1	1	1	1	1	2	2
1	2	1	1	1	1	1	1	3	1
1	2	1	1	1	1	1	1	1	1
9	9	9	1	3	3	1	1	3	2
1	1	4	1	3	3	1	1	3	2
2	3	5	1	1	1	1	3	3	3
2	2	1	1	1	1	1	3	3	3
1	3	4	1	1	1	1	1	1	3
2	2	2	1	1	1	1	1	3	1
2	2	1	1	1	1	1	1	3	1
1	3	5	1	1	1	1	1	1	1
1	3	9	1	1	1	1	1	1	1
2	2	3	1	1	1	1	1	2	2
1	1	1	1	1	1	1	1	3	3
2	1	2	1	1	1	1	1	2	2
2	2	1	1	1	1	1	1	3	1

3	3	5	1	1	1	1	2	2	2
2	2	1	1	1	1	1	1	3	2
1	2	1	1	1	1	1	1	3	2
1	1	1	1	1	1	1	3	3	1
1	1	4	1	1	1	1	1	3	1
2	2	1	1	1	1	1	1	2	2
3	3	4	1	1	1	1	1	3	1
2	1	1	1	1	1	1	2	2	2
2	2	3	1	1	1	1	3	1	3
1	1	4	1	1	1	1	1	3	1
2	1	5	1	1	2	1	2	2	2
3	2	3	1	1	1	1	1	3	3
9	9	9	1	1	1	9	1	9	9
2	3	1	1	1	1	1	1	3	3
3	1	1	1	1	1	1	1	3	1
2	2	1	2	2	2	2	1	2	2
2	1	4	1	1	1	1	1	2	1
1	1	3	1	1	1	1	1	3	1
1	1	2	1	1	1	1	1	3	1
2	2	1	1	1	2	1	1	2	2
1	1	4	1	1	1	1	1	3	1
1	1	3	1	1	1	1	1	3	2
1	1	4	1	1	1	1	1	1	1
1	1	4	1	1	1	1	1	3	1
1	2	4	1	1	1	1	1	2	1
1	1	4	1	1	1	1	1	3	1
3	1	1	1	1	2	1	1	3	1
1	1	9	1	1	1	3	3	2	2
2	2	9	1	1	1	1	2	2	2
9	9	9	1	1	1	1	2	9	9
3	2	1	1	1	1	1	1	2	2
3	3	2	1	1	1	1	9	9	9
2	2	1	1	9	2	1	1	2	2
1	1	1	1	1	2	1	1	2	1
9	9	1	1	1	1	1	1	3	3
1	2	1	1	3	3	1	1	2	1
1	3	9	3	2	2	3	2	2	2
9	9	3	1	1	3	1	9	9	9
1	2	5	1	1	1	1	1	2	3
1	2	3	1	1	2	2	2	2	2
1	9	4	1	2	2	1	2	2	2
2	3	3	1	1	1	1	3	3	1
1	1	3	1	1	2	1	3	3	1
9	9	4	1	1	2	1	1	9	2
2	3	1	1	1	2	2	2	2	2
2	2	4	1	1	1	1	2	2	2
1	1	5	1	1	2	1	1	3	2

2	2	2	1	1	2	1	1	3	2
2	3	3	1	1	1	1	1	2	2
2	1	1	1	1	2	1	2	2	2
2	2	2	1	1	2	1	2	2	2
1	1	5	1	1	2	2	1	2	2
2	2	2	1	1	2	1	1	3	2
1	1	5	1	1	2	1	1	3	1
9	9	1	1	1	1	1	1	9	9
2	2	1	1	1	2	1	1	3	1
2	2	1	1	1	2	1	1	2	2
1	2	4	1	1	1	1	1	3	1
2	2	1	1	1	1	1	2	2	2
2	2	2	1	1	2	1	1	2	2
2	2	2	1	1	1	1	2	2	2
2	2	1	1	1	1	1	1	3	3
3	3	4	1	1	1	1	1	3	3
1	1	1	1	1	1	1	2	2	1
1	3	1	1	1	1	1	1	3	1
1	2	4	1	1	1	1	1	3	3
1	2	9	1	1	1	1	1	2	2
1	2	4	1	1	1	1	1	2	1
1	2	3	1	1	1	1	1	2	2
2	2	4	3	1	3	1	1	2	2
2	2	4	1	1	1	1	1	3	1
2	3	4	1	1	1	1	1	2	2
1	2	1	1	1	1	1	1	2	2
1	2	4	1	1	1	1	1	2	1
1	3	4	1	1	1	1	1	2	1
1	3	4	1	1	1	1	1	2	1
9	9	9	1	2	1	1	9	9	9
1	3	4	1	1	1	1	1	2	1
2	1	2	1	1	1	1	1	3	3
2	1	2	1	1	1	1	1	1	1
2	9	3	1	1	1	1	1	1	1
9	9	4	1	9	1	1	9	9	9
1	2	4	1	1	1	1	1	2	1
1	1	4	1	1	1	1	1	2	1
1	1	4	9	9	9	9	1	9	9
1	1	4	1	1	1	1	1	3	3
2	3	1	1	1	1	1	1	2	2
2	1	9	1	1	1	1	1	3	2
1	3	1	1	1	1	1	1	3	1
2	3	3	1	1	2	2	2	2	2
3	3	1	1	1	1	1	1	3	1
1	1	4	1	1	1	1	1	3	1
3	1	4	1	1	1	1	1	3	1
1	1	1	1	1	1	1	1	3	3

1	1	3	1	1	1	1	1	3	1
1	1	3	1	1	1	1	1	3	1
2	1	1	1	1	1	1	1	3	1
1	1	3	1	1	1	1	1	3	1
1	1	9	1	1	1	1	1	3	3
1	1	4	1	1	1	1	1	3	3
3	1	4	1	1	1	1	1	3	1
2	1	3	1	1	1	1	2	1	1
1	1	3	1	1	1	1	1	3	3
1	1	3	1	1	1	1	1	3	1
1	3	1	1	1	1	1	1	3	1
1	3	4	1	1	3	1	1	3	1
1	3	4	1	1	1	1	1	3	1
1	1	4	1	1	1	1	1	2	3
1	1	3	1	1	1	1	1	3	1
1	1	2	1	1	1	1	1	3	1
2	3	1	1	1	1	3	1	1	1
1	2	3	1	1	1	1	1	2	1
1	1	4	1	1	1	1	1	3	1
1	1	2	1	1	3	1	1	3	1
1	2	9	1	1	3	1	1	2	2
3	3	1	1	1	1	1	1	3	3
9	9	1	1	1	1	1	1	9	9
9	9	9	1	1	1	1	9	2	9
9	9	2	1	1	1	1	1	9	9
9	9	4	1	1	1	1	9	9	1
9	9	4	1	1	1	1	1	3	3
1	9	1	1	2	1	1	9	9	9
9	9	1	1	1	1	1	1	9	9
9	9	1	2	2	2	2	9	9	1
9	9	1	1	1	1	1	9	9	1
9	9	1	1	1	1	1	9	9	1
9	9	1	1	1	2	1	2	9	9
9	9	3	1	1	1	1	1	9	9
1	1	3	1	1	1	1	2	2	1
1	2	4	1	1	1	1	1	3	1
1	2	2	1	1	1	1	1	2	1
1	3	1	1	1	1	1	1	3	1
1	3	4	1	1	1	1	1	2	1
1	3	4	1	1	1	1	1	2	1
1	1	9	1	1	1	1	1	3	3
1	2	5	1	1	1	1	2	2	2
1	2	1	1	1	2	1	1	2	1
2	2	1	1	3	1	1	1	3	2
2	2	9	1	1	2	1	2	2	1
1	3	5	1	1	1	1	1	3	1
1	3	4	1	1	1	1	1	2	1

2	2	3	1	1	2	1	2	2	1
2	1	1	1	1	1	1	2	2	2
1	3	1	1	1	1	1	1	2	3
1	3	9	1	1	2	1	3	2	1
2	2	3	2	2	2	2	1	1	2
2	2	3	1	1	1	1	1	3	1
9	9	9	9	9	9	9	9	9	9
2	2	1	1	1	2	1	2	2	2
3	2	4	1	1	2	1	1	2	1
2	2	4	1	1	2	1	2	2	2
1	1	9	1	2	2	1	2	2	2
1	1	3	1	1	1	1	1	2	1
1	2	1	1	2	1	1	1	2	2
2	3	1	1	1	2	2	3	3	3
2	2	5	1	1	1	1	1	2	1
2	2	5	1	1	1	1	1	1	1
2	2	9	9	9	9	9	9	9	9
2	2	1	1	2	1	1	1	2	1
1	1	9	1	1	2	1	2	2	1
1	1	1	1	1	1	1	1	1	1
1	2	9	1	1	1	1	1	1	1
2	2	1	1	1	1	1	2	2	2
2	2	9	1	1	2	1	1	2	1
1	1	1	2	1	2	2	2	2	2
1	1	1	1	1	1	1	1	1	1
1	2	2	1	2	2	2	2	3	1
3	1	3	1	1	1	1	1	2	1
1	1	3	1	1	1	1	1	2	1
2	2	1	2	2	2	2	2	2	2
2	2	1	1	1	1	1	2	2	2
1	1	2	1	1	1	1	1	1	1
1	1	1	1	1	2	2	2	2	2
2	1	9	1	1	2	1	1	3	1
2	2	4	1	1	1	1	1	2	2
2	2	1	1	2	2	1	9	9	9
2	2	2	1	1	3	9	2	2	1
2	2	1	1	1	1	1	2	2	2
2	2	3	1	1	2	1	2	2	2
2	2	1	1	1	2	1	2	2	2
9	2	1	1	1	2	1	1	3	2
2	1	1	1	1	2	1	2	2	2
2	1	1	1	1	2	1	2	2	2

PapTwoYe	PapfromM	PapHistory	PapTwoYe	PapOnceor	HPVVaccin	HPVVaccin	EarlyOnset	MultiplePa	Smoking
2	2	2	2	2	2	2	1	2	2
2	2	3	2	3	2	3	2	3	2
2	2	2	2	2	2	2	2	2	2
2	2	2	2	2	2	3	2	2	2
1	2	2	1	2	2	2	1	2	2
2	2	2	2	2	2	2	2	2	2
2	2	2	2	2	2	2	1	2	3
2	3	3	2	2	2	2	1	2	2
3	3	3	3	3	3	3	2	1	1
2	3	1	3	2	1	2	3	2	1
2	2	2	2	2	2	2	2	2	2
2	3	3	2	2	2	2	1	1	2
2	2	1	2	1	2	2	2	2	2
2	1	2	2	2	1	1	3	2	1
2	2	2	2	2	2	2	2	2	2
2	2	2	2	2	2	2	2	2	2
9	9	9	9	9	9	9	9	9	9
2	2	2	2	2	2	2	1	1	1
9	9	9	9	9	9	9	9	9	9
9	9	9	9	9	9	9	9	9	9
1	2	3	2	3	1	1	1	2	1
3	3	3	3	3	2	2	2	1	2
2	3	2	2	3	1	1	1	2	2
2	1	1	1	2	1	2	1	1	1
9	9	9	9	9	9	9	2	9	9
2	2	2	2	1	1	1	1	1	1
3	3	1	9	3	3	1	1	3	3
2	2	2	2	2	2	2	2	2	2
2	1	3	2	1	2	3	1	2	1
9	9	9	9	9	9	9	2	1	3
3	3	3	3	3	1	3	3	2	2
2	2	2	2	2	2	2	1	1	1
3	3	3	3	3	3	3	3	3	3
2	9	1	1	1	2	1	1	9	1
2	3	1	2	3	2	1	1	1	1
3	2	2	3	2	2	2	3	3	2
2	2	2	2	2	2	2	1	2	2
2	3	2	2	2	2	2	2	9	1
2	2	2	1	3	1	1	2	2	1
2	3	2	3	3	2	2	3	1	2
2	2	3	1	3	1	2	2	2	2
9	2	9	9	9	3	9	2	9	9
9	9	9	9	9	9	9	3	9	9
2	2	2	2	2	2	2	2	2	1
2	2	2	2	2	2	2	2	2	2
9	9	9	9	9	9	9	9	9	9

2	1	2	1	2	2	2	2	2	2
2	1	2	1	2	2	2	1	2	2
2	1	1	2	2	2	2	2	2	2
1	1	2	1	1	2	2	2	1	2
3	3	3	1	3	3	3	1	9	1
3	3	3	1	1	3	3	1	9	9
2	9	2	2	2	3	2	2	3	3
2	2	2	2	2	2	2	2	2	2
1	2	2	2	9	3	3	1	1	1
2	1	3	3	3	3	1	1	3	1
9	9	9	9	9	9	9	9	2	9
2	1	1	2	2	2	2	1	1	2
1	2	2	2	3	3	3	2	2	2
2	1	1	1	2	2	2	1	1	2
1	1	2	2	1	1	1	1	1	2
2	3	2	1	2	2	3	1	2	2
2	3	3	3	3	3	3	2	2	1
1	3	2	3	1	1	2	1	1	3
2	2	2	2	2	2	2	1	2	2
2	2	2	2	2	2	2	2	2	2
2	2	2	2	2	2	2	2	2	2
2	2	2	2	2	2	2	1	2	2
2	2	1	1	1	1	1	1	1	1
2	1	2	1	2	2	2	1	1	2
2	3	2	1	2	2	1	1	2	2
1	2	2	1	2	2	2	2	1	1
3	3	3	3	3	1	3	3	3	3
3	3	2	2	1	1	2	1	2	2
2	2	3	1	3	1	1	1	1	2
3	3	3	3	3	3	3	3	3	1
9	9	9	9	9	9	9	9	9	9
3	3	2	2	2	1	2	2	1	1
2	2	9	9	9	9	1	1	3	9
2	2	2	2	2	2	2	2	2	2
2	3	2	1	3	3	1	1	1	2
2	2	2	2	2	2	2	1	2	1
9	9	9	9	9	9	9	1	1	1
2	1	2	2	3	2	2	2	1	2
2	2	2	2	2	2	2	1	3	3
1	2	2	1	2	3	3	2	1	3
2	3	2	1	2	1	2	1	1	1
9	9	9	9	9	9	9	9	9	9
3	2	3	1	2	3	1	1	1	1
1	1	1	2	2	1	1	3	1	2
2	3	2	2	3	3	2	1	1	1
3	1	3	2	3	2	3	3	2	1
2	2	2	2	2	1	2	2	3	2

3	2	1	3	2	1	2	1	2	3
3	3	3	3	3	3	3	3	3	3
2	2	2	2	2	2	2	1	1	1
1	1	2	2	2	2	3	2	1	2
3	2	1	1	1	1	1	1	1	2
2	2	2	1	2	2	3	2	2	2
2	2	2	2	2	2	2	2	2	1
9	9	9	9	9	9	9	9	9	9
2	2	2	2	2	2	2	9	1	1
3	3	2	2	3	1	2	1	1	1
1	1	3	2	1	3	1	1	1	1
1	2	2	1	2	2	2	1	1	2
2	3	3	3	3	2	2	1	2	3
2	2	2	2	2	2	2	1	2	1
2	2	2	2	2	2	2	3	1	2
2	2	2	2	2	2	2	3	9	2
1	2	2	1	2	2	1	1	1	2
2	3	3	3	2	2	2	2	1	1
2	2	1	9	2	9	1	1	1	2
2	1	2	2	1	1	1	1	1	2
3	1	3	1	3	1	3	1	3	1
1	2	1	1	3	1	1	1	2	1
2	1	1	2	2	1	1	1	2	3
2	2	2	2	2	2	2	2	2	2
2	2	1	1	2	2	3	2	2	2
3	2	3	9	3	1	1	1	2	1
1	3	3	3	3	1	1	1	1	1
2	1	2	2	1	1	2	2	1	1
2	2	3	3	3	3	3	1	2	1
2	3	2	3	3	2	1	2	3	2
1	1	2	3	3	3	3	2	2	1
1	2	3	2	1	2	2	1	2	3
2	2	3	2	2	2	2	3	2	2
1	1	1	1	1	1	2	2	1	1
1	3	2	1	3	1	3	1	3	2
1	1	1	1	1	1	1	1	1	1
3	2	3	2	3	2	3	2	2	1
1	2	2	3	2	2	2	1	2	2
9	9	9	9	9	9	9	9	9	9
3	3	3	3	3	3	3	3	3	3
2	2	1	2	2	2	2	1	2	2
9	9	9	9	9	9	9	9	9	9
1	2	2	2	2	1	1	1	1	2
1	2	3	2	2	1	2	2	2	1
9	9	9	9	9	9	9	9	9	9
2	2	3	1	3	1	2	3	2	1
1	2	2	1	1	1	1	1	1	2

1	3	2	9	1	3	1	3	3	3
1	3	2	1	3	1	1	1	1	1
3	3	3	3	3	3	3	3	3	3
2	2	2	2	2	2	2	2	2	2
2	3	2	2	2	2	3	2	2	2
2	1	2	3	2	2	3	2	2	9
2	3	2	9	1	3	3	1	9	3
1	3	2	2	2	1	3	1	2	3
9	9	9	9	9	9	9	2	9	9
2	2	2	2	2	2	2	2	2	2
1	2	3	1	1	2	3	1	3	1
1	2	2	1	3	1	1	1	1	2
2	2	2	2	2	2	2	2	2	2
2	2	2	1	1	1	1	1	1	1
3	2	1	2	2	2	2	1	2	2
2	2	1	2	1	1	1	1	1	1
2	2	1	1	1	1	1	1	2	2
2	1	1	1	2	1	1	1	2	3
9	9	9	9	9	9	9	2	9	9
3	2	1	1	2	2	1	1	1	1
2	2	3	1	2	2	1	1	1	1
3	2	2	2	2	3	2	1	1	1
2	2	3	2	3	2	9	3	3	3
2	1	2	1	1	2	1	1	1	1
2	2	9	9	9	9	9	3	2	1
3	3	3	3	3	3	3	3	9	1
3	2	2	3	2	1	3	1	2	2
2	2	2	2	2	2	2	1	2	2
2	2	2	2	2	2	2	1	2	2
1	2	2	2	2	2	1	1	1	2
2	1	1	2	2	1	2	1	2	2
2	2	1	1	1	3	3	2	2	2
2	2	2	2	1	2	2	1	2	2
1	1	1	3	3	3	3	3	3	3
2	3	2	2	2	2	2	2	2	1
3	1	1	1	1	1	1	3	3	3
1	1	1	1	1	1	1	3	3	3
3	3	3	2	3	1	1	1	1	3
1	3	3	1	1	1	1	1	1	1
3	3	3	3	3	3	3	2	2	2
9	9	9	9	9	9	9	9	9	9
2	2	2	2	2	2	2	2	2	2
2	2	2	2	2	2	2	2	2	2
9	9	9	9	9	9	9	9	9	9
9	9	9	9	9	9	9	9	9	9
2	1	1	2	2	1	2	1	2	2
1	2	2	3	3	3	3	1	2	2

9	9	9	9	9	9	9	9	9	9
2	9	9	9	9	9	9	9	9	9
9	9	9	9	9	9	9	9	1	9
9	9	9	9	9	9	9	9	9	9
9	9	9	9	9	9	9	1	9	9
9	9	9	9	9	9	9	9	9	9
2	2	2	2	2	2	2	1	1	1
9	9	9	9	9	9	9	9	9	9
1	2	2	1	2	1	2	1	1	1
2	9	9	9	9	9	9	9	9	9
9	1	9	9	9	9	9	9	9	9
2	9	9	9	9	9	9	9	9	9
2	1	1	1	1	2	1	1	1	1
2	2	1	1	2	2	1	1	2	3
9	9	9	9	9	9	9	9	1	9
2	3	2	3	3	3	2	2	2	2
3	3	3	3	3	3	3	3	3	3
1	1	1	1	1	1	1	3	1	1
2	2	2	2	2	2	2	1	1	1
2	2	2	2	2	2	2	2	2	2
2	2	2	2	2	2	2	1	2	2
9	9	9	9	9	9	9	9	9	9
3	3	3	3	3	2	2	2	2	2
2	3	1	2	2	1	3	1	1	1
2	2	2	2	2	2	2	2	2	2
2	1	1	1	1	1	1	1	2	1
2	2	2	3	2	1	1	1	1	2
2	3	1	1	1	1	1	1	1	1
3	3	3	3	3	3	3	3	3	3
1	1	1	1	3	1	1	1	1	1
2	2	2	2	2	2	2	2	2	2
2	1	1	2	2	2	2	1	1	2
9	9	9	9	9	9	9	9	9	9
1	1	2	3	3	3	3	2	2	2
3	1	3	1	3	3	1	1	1	1
2	1	1	1	1	1	1	1	1	1
2	3	2	2	3	1	2	2	3	1
1	1	2	1	1	1	1	1	2	2
9	9	9	9	9	9	9	9	1	9
1	1	2	1	1	2	1	2	2	1
1	1	2	1	1	1	1	1	1	1
1	2	1	3	9	1	3	1	2	1
2	2	2	3	3	3	2	2	2	1
2	2	2	2	2	2	2	2	2	2
2	2	2	2	2	2	2	1	1	1
2	2	2	2	2	2	2	1	1	2
9	9	9	9	9	9	9	9	9	9

1	1	1	1	1	1	1	1	1	1
2	1	1	2	1	1	2	1	1	1
1	1	1	1	3	1	2	1	1	1
9	9	9	9	9	9	9	9	9	9
2	2	2	2	2	2	2	2	2	2
9	1	2	2	3	2	1	2	2	1
1	2	2	1	3	1	1	2	3	3
1	1	1	1	1	1	1	2	1	2
1	2	3	2	3	2	2	2	2	1
2	1	1	1	1	1	1	1	2	2
2	2	2	2	2	2	2	1	1	2
9	9	9	9	9	9	9	3	9	9
9	9	9	9	9	9	9	2	9	9
9	9	9	9	9	9	9	1	9	9
9	2	9	9	2	9	2	2	9	9
9	9	9	9	9	9	9	9	9	9
9	9	9	9	9	9	9	9	9	9
9	9	9	9	9	9	9	9	9	9
9	2	9	2	9	2	2	9	2	9
9	9	9	9	9	9	9	9	9	9
9	9	9	9	9	9	9	9	9	9
2	2	2	2	2	2	2	2	2	2
1	1	2	2	2	2	2	2	2	2
2	1	1	1	1	1	1	1	1	1
2	1	1	1	1	1	1	1	1	2
9	9	9	9	9	9	9	9	9	9
1	2	2	2	1	2	2	2	2	2
9	9	9	9	9	9	9	9	9	9
9	9	9	9	9	9	9	9	9	9
3	2	1	2	2	2	1	1	2	2
9	9	9	9	9	9	9	9	9	9
9	9	9	9	9	9	9	9	9	9
9	9	9	9	9	9	9	9	9	9
9	9	9	9	9	9	9	9	9	9
1	3	1	9	1	3	1	9	3	1
3	1	9	1	9	9	1	9	1	9
9	9	9	9	9	9	9	9	9	9
9	9	9	9	9	9	9	9	9	9
9	9	9	9	9	9	9	9	9	9
9	9	9	9	9	9	9	3	9	9
9	9	9	9	9	9	9	1	9	9
9	9	9	9	9	9	9	9	9	9
3	3	3	3	3	3	3	3	3	3
3	2	2	1	2	2	1	1	2	3
2	1	2	1	2	3	1	1	2	3
9	9	9	9	2	9	9	2	9	9
9	9	9	9	9	9	9	1	9	9

9	9	9	9	9	9	9	9	2	9
2	9	9	9	9	9	9	3	9	9
2	3	3	3	3	3	3	3	3	3
3	1	2	3	1	2	3	3	1	2
1	1	1	1	1	1	1	3	2	2
2	3	3	3	3	2	2	2	2	2
2	1	1	2	3	2	2	2	1	3
1	1	2	2	2	3	1	1	3	1
2	2	2	2	2	2	2	2	2	2
3	3	3	3	3	3	3	3	3	3
9	9	9	9	9	9	9	9	9	9
9	9	9	9	9	9	9	9	9	9
9	9	9	9	9	9	9	9	9	9
9	9	9	9	9	9	9	9	1	9
2	1	1	1	3	3	3	1	1	1
3	3	3	2	1	2	2	1	1	1
9	9	9	9	9	9	9	9	9	9
2	1	1	1	2	2	2	1	1	1
1	1	1	1	1	1	1	1	1	1
2	3	2	2	2	2	2	1	1	1
2	1	2	3	1	2	1	1	2	1
2	2	2	2	2	2	2	2	2	2
3	3	2	1	2	2	3	3	9	9
2	2	2	2	2	2	2	2	2	2
2	2	2	2	2	2	2	2	2	2
2	2	2	2	2	2	2	2	2	2
2	2	2	2	2	2	2	2	2	2
2	3	1	1	1	1	3	3	2	2
2	2	3	3	3	3	3	2	2	2
2	1	2	1	1	1	1	1	2	2
2	2	2	2	2	2	2	2	2	2
2	2	2	2	2	2	2	2	2	2
2	2	1	2	2	2	3	3	3	3
3	3	3	3	3	3	3	3	2	9
1	3	1	1	3	3	2	3	3	2
1	2	3	1	2	3	2	2	2	2
9	9	9	9	9	9	9	9	9	9
2	2	2	2	2	2	2	2	2	2
3	3	3	3	3	3	3	3	3	3
3	3	3	3	3	3	3	3	3	3
9	9	2	9	9	9	9	2	9	9
9	9	9	9	9	9	9	9	9	9
3	9	9	9	9	9	9	9	9	9
2	2	2	2	2	2	2	1	1	1
1	2	2	1	2	2	2	1	2	2
2	2	2	2	2	2	2	2	2	2
1	1	1	2	3	3	3	1	1	1

9	2	9	9	9	9	9	9	9	9
9	9	9	9	9	9	9	1	1	9
9	9	9	9	9	9	9	9	9	9
9	9	9	9	9	9	9	9	9	1
9	3	9	9	9	9	9	9	9	9
2	2	2	2	2	1	1	3	3	2
9	9	9	2	9	9	9	9	9	9
9	9	9	9	9	9	9	2	9	9
9	9	9	9	9	9	9	2	9	9
9	9	9	9	9	9	9	2	9	9
2	9	9	9	9	9	9	9	9	9
9	2	9	9	9	9	9	9	9	9
9	9	9	9	9	9	9	2	9	9
9	9	9	9	9	9	9	9	9	9
9	9	9	9	9	9	9	9	9	1
9	9	9	9	9	9	9	9	9	9
9	2	9	9	3	9	9	9	9	9
1	2	3	1	2	2	9	2	1	3
1	2	2	1	2	1	2	1	2	2
2	2	2	2	2	2	2	2	2	2
2	3	3	3	3	1	1	1	1	2
2	2	1	3	2	3	2	2	2	1
2	2	2	2	2	2	2	2	2	2
1	3	2	2	3	2	3	2	3	2
2	3	3	2	3	2	3	2	3	1
9	9	9	9	9	9	9	9	9	9
2	2	2	2	2	2	2	1	1	1
2	2	2	2	2	2	2	3	3	3
2	1	2	3	1	2	2	2	1	2
1	1	1	1	1	1	2	2	1	1
2	1	3	2	1	2	1	1	1	1
2	2	2	2	2	2	2	2	2	2
2	2	2	2	1	1	1	1	1	2
2	1	1	1	1	1	1	2	2	2
9	9	9	9	9	9	9	9	9	9
2	2	2	2	2	2	2	2	2	2
9	9	9	9	9	9	9	9	9	9
2	1	1	1	2	2	2	2	2	2
9	9	9	9	9	9	9	9	9	9
2	2	2	2	2	2	2	2	2	2
2	2	1	2	3	1	2	1	2	2
3	3	3	3	3	3	3	3	3	3
9	2	3	1	3	3	3	1	1	1
2	2	2	2	2	2	2	2	2	1
3	3	2	2	2	2	2	3	3	3
2	2	3	2	2	2	2	1	3	2
2	2	2	2	2	2	2	2	2	2

2	2	2	3	2	3	2	1	1	1
9	9	9	9	9	9	9	9	9	9
2	9	1	1	3	3	3	9	9	9
3	2	1	2	1	3	2	2	3	2
2	2	2	2	2	2	2	3	3	2
2	2	2	2	3	3	3	3	3	2
1	3	1	3	1	1	1	1	1	1
1	2	1	1	3	1	1	1	1	1
9	9	9	9	9	9	9	9	9	9
2	2	2	2	2	2	2	2	2	2
2	2	2	2	2	2	2	1	1	2
9	9	9	9	9	9	9	1	9	9
9	9	9	9	9	9	9	1	9	9
1	9	1	1	1	9	1	2	2	2
9	9	9	9	9	9	9	9	9	9
3	3	2	2	2	2	2	1	1	2
9	9	9	9	9	9	9	9	9	9
9	9	9	9	9	9	9	2	9	9
9	9	9	9	9	9	9	9	9	9
3	2	3	2	3	2	3	3	2	3
2	2	2	2	2	2	2	2	2	2
2	2	2	2	2	2	2	1	1	2
2	2	2	2	2	2	2	2	2	1
9	9	9	9	9	1	9	9	9	9
9	9	9	9	9	9	9	2	9	9
1	1	1	2	2	2	1	2	2	2
1	2	2	2	2	2	2	1	2	3
2	3	2	1	2	2	1	1	2	3
9	9	9	9	9	9	9	9	9	9
9	9	9	9	9	9	9	1	2	1
2	2	2	2	2	2	2	2	2	2
1	2	3	1	2	3	1	2	3	1
3	3	2	1	1	1	1	1	1	2
1	2	2	1	1	1	1	1	2	2
1	1	1	1	1	1	1	1	1	1
2	1	2	2	2	1	2	2	1	2
9	9	9	9	9	9	9	9	9	9
9	9	9	9	9	9	9	9	9	9
2	3	1	3	2	1	3	2	2	3
2	1	2	1	2	2	1	1	2	1
2	2	2	2	2	2	2	2	2	2
2	2	2	2	2	2	2	2	2	2
2	2	2	2	2	2	2	2	2	2
3	1	2	1	3	1	2	1	2	1
3	3	3	3	3	3	3	2	2	2
2	2	2	2	2	2	2	2	2	2
2	2	2	2	2	3	3	3	3	3

3	3	3	3	3	3	3	1	1	2
1	2	3	1	2	3	1	1	2	3
9	9	9	9	9	9	9	9	9	9
9	9	9	9	9	9	9	9	9	9
2	3	2	2	3	1	2	1	9	9
3	2	1	2	3	2	3	2	2	9
9	2	9	9	9	2	9	2	9	9
3	9	3	9	3	3	3	3	9	3
9	9	9	9	9	9	9	9	9	9
9	9	9	9	9	9	9	9	9	9
9	9	9	9	9	9	9	9	9	9
9	9	9	9	9	9	9	9	9	9
9	9	9	9	9	9	9	9	9	9
9	9	9	9	9	9	9	9	9	9
9	9	9	9	9	9	9	9	9	9
9	9	9	9	9	9	9	2	9	9
3	3	3	3	3	3	3	3	3	3
9	9	9	9	9	9	9	1	9	9
9	9	9	9	9	9	9	2	9	9
9	2	9	9	2	9	2	2	9	9
9	9	9	9	9	9	9	9	9	9
9	9	9	9	9	9	9	2	9	9
2	2	2	2	2	2	2	2	2	2
9	9	9	9	9	9	9	9	9	9
9	9	9	9	9	9	9	9	9	9
9	9	9	9	9	9	9	2	9	9
9	9	9	9	9	9	9	9	9	9
2	9	2	9	2	9	2	2	9	2
2	2	2	2	2	2	2	2	2	2
9	9	9	9	9	9	9	9	9	9
9	9	9	9	9	9	9	9	9	9
2	2	2	2	2	2	2	2	2	2
9	9	9	9	9	9	9	9	9	9
9	9	9	9	9	9	9	9	9	9
9	9	9	9	9	9	9	9	9	9
9	9	9	9	9	9	9	9	9	9
9	9	9	9	9	9	9	9	9	9
9	9	9	9	9	9	9	9	9	9
3	1	2	1	3	1	2	2	1	3
3	1	2	2	1	2	1	2	1	3
9	9	9	9	9	2	9	1	3	3
9	9	9	9	9	9	9	9	9	9
2	3	1	2	3	3	2	2	1	3
1	2	3	1	2	3	2	1	2	3
3	3	3	3	1	1	1	3	3	3
1	2	3	2	3	2	2	2	1	2
2	3	2	9	9	9	9	1	1	1

9	9	9	9	9	9	9	9	9	9
2	1	2	1	1	3	2	2	3	2
2	2	2	2	2	2	2	2	2	2
1	1	1	1	1	1	1	1	1	1
2	2	2	2	2	2	2	2	2	2
2	2	3	3	3	3	3	2	2	3
9	9	9	9	9	9	9	9	9	9
3	2	2	2	2	2	2	1	3	2
2	2	2	2	2	2	2	2	2	2
2	2	2	2	2	2	2	1	1	1
3	2	2	2	2	2	2	2	2	9
9	9	2	9	9	9	9	9	9	9
9	9	9	1	9	9	9	9	9	9
9	9	9	9	9	1	9	9	9	1
9	9	9	9	9	9	9	9	9	1
2	2	2	2	2	2	2	1	1	1
2	2	2	2	2	2	2	2	2	2
3	3	3	2	3	1	2	2	2	2
3	3	3	3	3	3	2	3	3	1
2	2	2	2	2	2	2	2	2	2
2	2	2	2	2	2	2	2	2	2
9	9	9	9	9	9	9	9	9	9
3	1	1	1	1	3	1	2	3	1
9	9	9	9	9	9	9	9	9	9
9	9	9	9	9	1	9	9	9	9
1	1	1	1	1	1	1	2	2	3
1	9	9	2	9	9	9	9	9	9
2	2	1	1	1	2	2	2	2	1
9	9	9	9	9	9	9	9	9	9
9	9	9	9	9	1	9	9	9	9
3	3	3	2	2	1	3	1	1	2
3	1	3	1	3	2	3	2	1	3
9	9	9	9	9	9	9	9	9	9
9	9	9	9	9	9	9	1	9	9
2	2	3	3	1	2	2	1	2	1
9	9	9	9	9	9	9	9	9	9
2	3	2	3	2	1	2	1	2	3
1	1	1	1	1	1	1	1	1	2
2	2	2	2	2	2	2	2	2	2
9	9	9	9	9	9	9	9	9	9
2	2	2	2	2	2	2	2	2	2
2	2	2	2	2	2	2	1	1	1
2	2	2	2	2	2	2	2	2	2
2	2	2	2	2	2	2	2	2	2
2	3	2	3	2	2	3	2	3	3
2	2	2	2	2	2	2	2	2	2
2	3	3	3	3	3	2	2	2	2

9	9	9	9	9	9	9	9	1	9
2	2	2	2	2	2	2	2	2	2
2	2	2	2	2	2	2	2	2	2
9	2	9	9	9	9	9	1	9	9
9	9	9	9	9	9	9	2	9	9
9	9	9	9	9	9	9	9	9	9
9	9	9	9	9	9	9	9	9	9
2	2	2	2	2	2	2	1	1	2
2	2	2	2	2	2	2	2	2	1
9	9	9	9	9	9	9	9	9	1
2	2	2	2	2	2	2	2	2	1
3	2	2	2	3	2	3	2	2	2
2	2	2	2	2	2	2	1	1	2
2	2	2	2	2	2	2	2	2	2
9	9	9	9	9	9	9	9	9	9
9	9	9	9	9	9	9	9	9	9
9	9	9	9	9	9	9	9	9	9
9	9	9	9	9	9	9	9	9	9
9	9	9	9	9	9	9	1	9	9
9	9	9	9	9	9	9	2	9	9
9	9	9	9	9	9	9	9	9	9
9	9	9	9	9	9	9	9	9	9
2	2	2	2	2	2	2	2	9	2
9	9	9	9	9	9	9	2	9	9
9	9	9	9	9	9	9	9	9	9
9	9	9	9	9	9	9	3	9	9
9	9	9	9	9	9	9	9	9	9
9	9	9	9	9	9	9	9	9	9
9	9	2	9	9	9	2	2	9	9
9	9	9	9	9	9	9	3	9	9
9	9	9	9	9	9	9	9	9	9
2	2	2	2	2	2	2	2	2	2
9	9	9	9	9	9	9	9	9	9
9	9	9	9	9	9	9	9	9	9
9	9	9	9	9	9	9	9	9	9
9	9	9	9	9	9	9	9	9	9
9	9	9	9	9	9	9	9	9	9
9	9	9	9	9	9	9	9	9	9
9	9	9	9	9	9	9	9	9	9
9	9	9	9	9	9	9	9	9	9
2	2	2	2	2	2	2	2	9	2
9	9	9	9	9	9	9	2	9	9
9	9	9	9	9	9	9	9	9	9
9	9	9	9	9	9	9	9	9	9
9	9	9	9	9	9	9	9	9	9
9	9	9	9	9	9	9	9	9	9
9	9	9	9	9	9	9	9	9	9

9	9	9	9	9	9	9	9	9	9
3	3	3	3	3	3	3	3	3	3
9	9	9	9	9	9	9	9	9	9
9	9	9	9	9	9	9	9	9	9
9	9	9	9	9	9	9	9	9	9
9	9	9	9	9	9	9	9	9	9
9	9	9	9	9	9	9	9	9	9
9	9	9	9	9	9	9	9	9	9
9	9	9	9	9	9	9	9	9	9
9	9	9	9	9	9	9	9	9	9
9	9	9	9	9	9	9	9	9	9
9	9	9	9	9	9	9	9	9	9
9	9	9	9	9	9	9	9	9	9
9	9	9	9	9	9	9	9	9	9
9	9	9	9	9	9	9	9	9	9
9	9	9	9	9	9	9	9	9	9
9	9	9	9	9	9	9	9	9	9
2	2	2	2	2	2	2	2	2	2
9	9	9	9	9	9	9	9	9	9
9	9	9	9	9	9	9	9	9	9
9	9	9	9	9	9	9	9	9	9
9	9	9	9	9	9	9	9	9	9
9	9	9	9	9	9	9	9	9	9
9	9	9	9	9	9	9	9	9	9
2	2	2	2	2	2	2	2	2	2
9	9	9	9	9	9	9	9	9	9
9	9	9	9	9	9	9	9	9	9
2	1	1	2	2	1	2	1	9	9
9	9	9	9	9	9	9	9	9	9
9	9	9	9	9	9	9	9	9	9
1	1	1	1	1	1	1	1	1	1
2	1	1	3	3	1	1	2	2	1
9	9	9	9	9	9	9	9	9	9
3	3	3	3	3	3	3	3	3	2
9	9	9	9	9	9	9	9	9	9
2	2	2	2	2	2	2	2	2	2
9	9	9	9	9	9	9	2	9	9
9	9	9	9	9	9	9	9	9	9
1	1	1	1	1	1	1	1	1	1
9	9	9	9	9	9	3	3	9	9
9	9	9	9	9	9	9	9	9	9
9	9	9	9	9	9	9	9	9	9
2	2	2	2	2	2	2	2	2	2
2	2	2	2	2	2	2	2	2	2
1	9	1	9	1	9	1	9	1	9
9	9	9	9	9	9	9	9	9	9
9	9	9	9	9	9	9	9	9	9

9	9	2	9	9	9	2	2	9	9
9	9	9	9	9	9	9	9	9	9
9	1	3	9	1	2	3	2	9	9
9	9	9	9	9	9	9	9	9	9
9	9	9	9	9	9	9	9	9	9
9	9	9	9	9	9	9	9	9	9
9	9	9	9	9	9	9	2	9	9
9	9	9	9	9	9	9	2	9	9
2	9	9	2	9	9	2	2	9	9
9	9	9	9	9	9	9	9	9	9
9	9	9	9	9	9	9	9	9	9
9	9	9	9	9	9	9	9	9	9
9	9	9	9	9	9	9	9	9	9
9	9	9	9	9	9	9	9	9	9
1	3	3	3	3	2	2	1	1	1
2	3	1	2	3	1	2	1	2	3
9	9	9	2	9	9	9	9	9	2
2	2	2	2	2	2	2	2	2	2
3	2	2	3	2	2	2	2	2	2
9	9	9	9	9	9	9	1	9	9
9	9	9	9	9	9	9	9	9	9
9	9	9	9	9	9	9	1	9	9
2	2	2	2	2	2	2	3	3	3
1	2	3	1	2	3	1	2	3	1
2	2	2	1	3	2	2	2	2	3
2	2	2	2	2	2	2	2	2	2
3	2	9	3	3	3	3	2	2	2
3	3	2	2	2	2	2	1	1	1
3	3	3	2	1	1	3	2	2	1
2	2	2	2	2	2	2	2	2	2
2	2	2	2	2	2	2	2	2	2
9	9	9	9	9	9	9	2	9	9
9	9	9	9	9	9	9	1	9	9
9	9	9	9	9	9	9	2	9	9
9	9	9	9	9	9	9	1	9	9
9	9	9	9	9	9	9	9	9	9
9	9	9	9	9	9	9	9	9	9
9	9	2	9	9	9	2	9	9	2
9	9	9	9	9	9	9	9	9	9
9	2	9	2	9	2	2	2	9	9
9	9	9	9	9	9	9	9	9	9
2	2	2	2	2	2	2	2	2	2
9	9	9	9	9	9	9	9	9	9
9	9	9	9	9	9	9	9	9	9
9	9	9	9	9	9	9	9	9	9
2	2	2	2	2	2	2	2	2	2
2	1	3	2	3	2	2	2	3	2

2	2	2	2	2	2	2	2	2	2
2	2	2	2	2	2	2	2	1	2
2	2	2	2	2	2	2	2	2	2
3	3	1	3	1	2	3	1	1	3
3	3	3	3	3	3	3	3	3	3
2	2	2	1	3	1	1	1	2	1
2	2	2	2	2	2	2	2	2	2
3	3	3	3	3	3	3	3	3	3
9	9	9	9	9	9	9	9	9	9
2	2	2	2	2	2	2	2	2	2
9	2	9	2	9	9	2	9	2	9
9	9	1	9	1	9	1	9	2	1
2	2	2	2	2	2	2	2	2	2
2	2	1	1	1	3	3	2	1	2
3	3	3	3	3	3	3	3	2	2
2	2	2	2	2	2	2	2	2	2
2	3	2	1	2	9	1	2	3	1
3	3	3	2	3	3	3	2	3	1
9	9	9	9	9	9	9	9	9	9
1	3	2	1	2	2	1	1	2	2
1	3	2	3	1	2	1	2	3	3
3	1	3	1	3	1	1	1	1	1
2	2	2	2	1	2	2	2	1	2
1	2	1	2	3	1	1	1	1	2
2	2	3	2	3	1	1	1	1	1
2	2	2	2	2	2	2	2	2	1
2	3	2	1	3	2	1	1	1	1
2	2	2	3	3	3	2	2	3	2
2	2	2	2	2	2	2	2	2	2
2	3	2	2	3	1	1	1	1	2
3	3	3	3	3	3	3	3	3	3
9	9	9	9	9	9	9	9	9	9
3	3	3	3	3	3	3	3	3	3
2	2	2	2	2	2	2	2	2	2
9	9	9	9	9	9	9	9	1	9
3	3	3	2	2	2	2	3	2	2
2	2	2	2	2	2	2	2	2	2
1	1	1	1	1	1	1	1	1	1
2	2	2	2	2	3	2	1	2	2
9	9	9	9	9	9	9	9	9	9
2	2	2	2	2	2	2	2	2	2
9	9	9	9	9	1	9	9	9	9
3	1	2	1	2	1	1	2	2	3
2	2	2	2	2	2	2	2	2	2
3	2	3	2	3	2	3	2	3	2
9	9	9	9	9	9	9	9	9	9
2	2	1	2	2	2	2	3	2	2

9	9	9	9	9	9	9	9	9	9
3	3	3	3	3	3	3	3	3	3
1	1	3	3	1	1	3	1	1	1
2	2	3	2	2	2	3	2	3	3
1	3	2	1	3	1	3	1	1	1
9	9	9	9	9	9	9	9	9	9
2	2	2	2	2	2	2	2	2	2
3	1	2	3	2	1	3	3	3	3
3	1	3	2	3	1	1	1	1	1
2	2	2	2	2	1	1	2	2	2
2	3	2	2	2	1	2	2	1	1
2	2	2	2	2	2	2	2	2	2
9	9	9	9	9	9	9	9	9	9
2	3	3	3	3	3	2	2	2	3
2	2	2	1	3	1	1	1	1	1
2	2	2	2	2	2	2	2	2	2
2	2	2	2	2	2	2	2	2	2
2	2	2	2	3	2	2	3	2	2
2	2	2	2	2	2	2	2	2	2
9	9	9	9	9	9	9	9	2	9
9	9	9	9	9	9	9	9	9	9
3	2	2	1	2	1	2	2	1	1
3	3	3	2	3	2	3	2	3	2
2	2	3	2	3	2	3	2	3	2
9	9	9	9	9	9	9	9	9	9
3	3	2	3	2	1	1	1	2	2
9	9	9	9	9	9	9	9	9	1
2	2	2	2	2	2	2	2	2	2
2	2	2	2	2	2	2	2	2	2
2	2	2	2	2	2	2	2	2	2
3	2	1	2	2	1	1	3	3	3
9	9	9	9	9	9	9	9	9	9
9	9	9	9	9	9	9	9	9	9
2	2	2	2	2	1	2	2	1	1
2	3	1	3	3	1	2	1	1	2
9	9	9	9	9	9	9	9	9	9
9	9	9	9	9	9	9	9	9	9
3	3	2	2	2	2	2	2	3	2
2	3	3	9	2	1	2	2	2	2
2	2	2	1	2	2	2	1	1	1
2	2	2	2	2	2	2	2	2	2
2	2	2	2	2	2	2	2	2	2
2	2	2	2	2	2	2	2	2	2
2	2	2	2	2	2	2	2	2	2
2	2	2	2	2	2	2	2	2	1
2	3	2	1	2	3	2	2	2	2
2	2	2	2	2	2	2	2	2	2

2	1	2	3	3	3	3	3	3	2
2	2	2	2	2	2	2	2	2	1
2	2	2	2	2	2	2	2	2	2
1	1	1	1	2	3	2	2	1	2
2	2	2	2	2	2	2	2	2	2
2	2	2	2	2	2	2	2	2	1
2	2	2	2	2	2	2	2	2	1
2	2	2	2	2	2	2	2	2	2
2	2	2	2	2	2	2	2	2	2
9	9	9	9	9	9	9	9	9	9
1	2	2	2	2	2	2	3	3	3
1	3	2	3	3	1	2	1	3	1
1	2	1	1	3	1	1	1	1	1
2	2	1	1	2	2	1	1	1	2
3	3	3	3	3	3	3	3	3	3
9	9	9	1	9	9	9	9	9	9
9	9	9	9	9	9	9	9	9	9
9	9	9	1	9	9	9	9	9	9
9	9	9	9	9	9	9	9	9	9
2	2	2	2	2	2	2	2	2	2
2	1	2	9	1	3	2	1	2	3
9	9	9	9	9	9	9	9	9	9
9	9	9	9	9	9	9	9	9	9
2	2	2	2	2	2	2	2	2	2
1	3	1	1	1	1	1	1	1	1
2	2	2	2	2	2	2	2	2	2
2	3	2	2	1	1	2	2	2	3
3	3	2	2	2	2	2	2	1	1
1	3	1	2	1	2	2	2	1	1
2	1	2	2	1	3	2	2	1	2
9	9	9	9	9	9	9	9	9	9
2	1	3	1	3	1	2	1	2	1
3	3	1	1	1	2	2	2	1	2
2	1	2	2	2	2	2	3	3	3
1	2	2	2	2	2	2	1	2	2
2	2	2	2	2	2	2	2	2	2
9	9	9	9	9	9	9	9	9	9
2	2	2	2	2	2	2	2	2	2
2	2	2	2	2	2	2	2	2	2
1	2	3	2	2	3	2	1	3	2
3	3	3	3	3	3	3	3	3	3
3	1	2	1	2	2	2	1	1	2
2	2	2	2	1	1	2	2	2	2
2	2	2	2	2	2	2	1	1	1
2	1	2	2	2	3	3	2	1	9
1	1	1	2	2	1	3	1	1	1
2	2	2	3	3	2	2	3	1	1

3	2	2	1	1	3	3	2	2	3
2	2	2	2	2	2	2	2	2	2
2	2	3	3	3	3	3	2	2	2
2	2	2	2	2	2	2	2	2	2
3	3	3	3	2	3	2	3	3	3
9	9	9	9	9	9	9	9	9	9
9	1	9	9	9	9	9	1	9	9
3	3	3	3	3	3	3	3	3	3
9	9	9	9	9	9	9	9	9	9
9	9	9	9	9	9	9	9	9	9
2	2	2	2	2	2	2	2	2	2
2	2	1	2	2	1	2	1	1	1
2	2	1	1	2	1	2	2	1	2
9	9	9	9	9	9	1	9	9	9
9	9	9	9	9	9	1	1	2	9
1	2	3	3	1	9	1	2	2	3
2	2	2	1	3	1	1	1	1	1
9	9	9	9	9	9	9	9	9	9
2	2	2	1	3	2	2	2	1	1
2	2	2	2	2	2	2	2	2	2
2	2	2	2	2	2	2	2	3	2
9	9	9	9	9	9	9	9	9	9
2	2	2	2	2	2	2	2	2	2
2	2	2	2	2	2	2	2	2	2
2	2	2	2	2	2	2	2	2	2
2	2	2	2	2	2	2	2	2	2
2	2	2	2	2	2	2	2	2	2
2	2	2	2	2	2	2	2	2	2
2	3	2	3	2	3	1	2	2	9
3	2	3	2	3	3	2	2	2	2
2	3	1	2	1	2	2	2	3	2
2	2	3	3	2	3	2	1	2	2
1	2	2	2	2	1	1	1	2	2
2	2	2	2	2	2	2	2	2	2
2	1	2	3	2	2	2	2	1	1
3	2	1	2	2	3	1	2	2	1
1	1	1	1	1	1	1	2	2	2
2	3	3	3	3	3	3	2	2	2
1	2	2	3	2	3	2	1	1	1
3	3	2	1	3	1	1	3	3	9
1	1	1	3	3	1	1	1	1	3
2	2	2	3	3	3	3	1	1	1
2	2	2	2	2	2	2	2	2	2
2	2	2	2	2	2	2	1	2	2
2	2	2	2	2	2	2	2	2	2
2	3	2	3	2	3	1	1	2	2
2	2	2	2	2	2	2	2	2	2

2	3	2	1	3	1	1	2	3	1
1	2	2	1	2	1	1	1	1	2
3	2	3	2	3	2	3	2	3	2
2	2	2	2	2	2	2	2	2	2
9	9	9	9	9	9	9	3	9	9
9	1	9	9	9	9	9	9	9	9
2	3	2	3	2	3	2	3	2	3
9	9	9	9	9	9	9	1	9	9
9	9	9	9	9	9	9	1	9	9
9	9	9	9	9	9	9	9	9	9
9	9	3	9	9	9	9	9	9	9
9	9	9	9	9	9	9	9	9	2
9	9	9	9	9	9	9	1	9	9
9	1	9	9	9	9	9	9	9	9
2	2	2	2	2	2	2	1	1	1
9	9	9	9	9	9	9	1	9	9
9	9	9	9	9	9	9	9	9	9
9	9	9	9	9	9	9	9	9	9
9	9	9	9	9	9	9	9	9	9
3	9	9	9	9	9	9	9	9	9
9	9	9	9	9	9	9	9	9	9
2	2	2	2	2	2	2	2	2	2
2	2	2	2	2	2	2	2	2	2
3	1	1	1	3	9	2	2	2	3
1	3	3	3	3	3	3	2	2	2
9	9	9	9	9	9	9	9	9	9
1	2	1	1	1	3	2	2	1	1
9	9	9	9	9	9	9	9	9	9
2	2	2	3	3	3	2	1	1	1
1	1	1	1	3	1	1	1	1	1
3	3	3	3	3	3	3	3	1	9
9	9	9	9	9	9	9	1	9	9
2	2	2	2	2	3	2	2	2	2
9	9	9	9	9	9	9	9	9	2
9	9	9	9	9	9	9	1	9	9
1	2	1	1	1	2	1	1	2	3
1	9	1	2	1	2	1	1	3	3
9	9	9	9	9	9	9	9	9	9
9	9	9	9	9	9	9	9	9	9
2	2	2	2	2	2	2	2	2	2
9	9	9	9	9	9	9	9	9	9
2	2	2	2	2	2	2	2	2	2
3	3	3	3	3	3	3	3	3	3
9	9	9	2	9	9	9	9	9	9
2	2	2	2	2	2	2	2	2	2
2	2	2	2	2	2	2	2	2	2
3	1	9	9	1	9	9	9	9	9

9	9	9	9	9	9	9	9	9	9
9	2	9	2	9	2	2	9	2	9
9	9	9	9	9	9	9	9	9	9
2	2	2	2	2	2	2	2	2	2
9	3	1	3	1	3	1	9	1	9
3	1	3	1	9	1	2	3	1	3
9	9	9	9	9	9	9	9	9	9
9	9	9	9	9	9	9	9	9	9
2	9	2	9	9	2	2	9	2	9
2	2	2	2	2	2	2	2	2	2
9	9	9	9	9	9	9	9	9	9
2	9	2	9	2	9	2	2	9	9
2	2	2	2	2	2	2	2	2	2
9	9	9	9	9	9	9	9	9	9
2	2	2	2	2	2	9	2	2	2
9	9	9	9	9	9	9	9	9	9
9	9	9	9	9	9	9	9	9	9
9	9	9	9	9	9	9	9	9	9
9	9	9	9	9	9	9	9	9	9
9	9	9	9	9	9	9	9	9	9
2	2	2	2	2	2	2	2	2	2
2	2	2	2	2	2	2	2	2	2
1	3	2	1	2	1	3	1	3	2
1	1	1	1	1	1	1	2	1	2
3	3	3	3	3	3	3	2	2	2
2	2	2	2	2	2	2	2	2	2
1	1	1	1	1	1	1	1	1	1
2	2	2	2	2	2	2	2	2	2
3	1	1	1	1	2	3	1	2	3
2	2	2	2	2	2	2	2	2	2
3	3	3	2	2	3	2	2	2	2
3	2	3	2	3	3	2	2	2	2
2	2	2	2	2	2	2	1	1	1
9	9	9	9	9	9	9	9	9	9
2	1	3	1	2	3	2	1	2	3
1	1	1	1	1	1	1	2	2	2
2	2	2	2	2	2	2	2	2	2
2	2	2	2	2	2	2	1	1	1
2	2	3	2	3	2	3	1	2	3
2	2	2	2	2	2	2	1	2	1
3	3	3	3	2	2	2	1	1	1
9	9	9	9	9	9	9	9	9	9
9	9	9	9	9	9	9	2	2	2
9	9	9	9	9	9	9	9	9	9
9	9	9	9	9	9	9	1	1	1
2	2	2	2	2	2	2	2	2	2
9	9	9	9	9	9	9	9	9	9

2	2	1	1	3	2	3	2	2	2
2	3	3	3	3	3	9	2	2	2
1	1	2	2	2	2	2	2	2	2
1	9	9	2	2	2	2	2	2	2
2	2	2	2	2	9	9	1	1	1
1	1	1	2	2	2	2	1	1	1
1	9	1	1	1	1	1	1	1	1
2	1	3	3	3	2	1	1	9	1
3	3	3	3	3	3	3	3	3	3
1	1	1	1	2	1	3	1	1	1
9	9	9	9	9	9	9	9	9	9
2	2	2	2	2	2	2	2	2	2
2	2	2	2	2	2	2	2	2	2
2	2	2	2	2	2	2	2	2	2
2	2	2	2	2	2	2	2	2	2
1	1	1	1	1	2	2	1	1	1
1	2	1	1	2	1	2	2	1	1
1	1	1	2	2	1	2	1	1	1
1	1	1	1	1	1	1	1	1	1
9	9	9	9	9	9	9	9	9	9
2	1	1	1	1	2	1	1	2	1
2	2	2	2	2	2	2	2	2	2
9	9	9	9	9	9	9	9	9	9
1	1	2	2	2	2	2	1	1	1
9	9	9	9	9	9	9	9	9	9
1	2	1	1	1	1	1	1	1	2
1	1	1	2	2	2	1	1	1	1
2	2	2	2	2	2	2	1	1	1
9	9	9	9	9	9	9	9	9	9
2	2	2	9	2	2	2	2	9	2
9	9	9	9	9	9	9	9	9	9
9	9	9	9	9	9	9	9	9	9
2	2	2	2	2	2	2	2	2	2
1	1	1	1	1	1	1	1	1	2
2	2	2	2	2	2	2	2	2	2
9	9	9	9	9	9	9	9	9	9
2	1	1	2	1	2	1	1	1	1
1	1	1	2	1	1	1	1	2	1
9	9	9	9	9	9	9	9	9	9
9	9	9	9	9	9	9	9	9	9
2	2	2	2	2	2	2	2	2	9
9	9	9	9	9	9	9	9	9	9
2	2	2	2	2	2	9	2	2	2
2	2	2	2	2	2	2	9	9	9
1	1	1	2	2	2	1	1	2	1
2	2	2	2	2	2	2	2	2	2
1	1	1	1	1	1	1	1	1	9

1	2	1	1	1	1	2	1	1	1
2	2	9	2	9	9	9	2	9	2
9	3	3	3	3	3	3	2	2	2
1	2	2	2	2	1	1	1	1	1
2	2	2	2	2	2	2	2	2	2
9	9	9	9	9	9	9	9	9	9
2	2	2	2	2	2	2	1	1	1
2	2	2	2	2	2	2	2	2	2
2	9	2	2	9	2	2	9	9	2
1	1	1	1	1	2	2	9	1	1
9	9	9	9	9	9	9	9	9	9
9	9	9	9	9	9	9	9	9	9
9	9	9	9	9	9	9	9	9	9
9	9	9	9	9	9	9	9	9	9
9	9	9	9	9	9	9	9	9	9
9	9	9	9	9	9	9	9	9	9
1	1	1	1	1	1	1	2	2	2
9	9	9	9	9	9	9	9	9	9
2	2	2	2	2	2	2	2	2	2
3	3	3	1	3	3	1	1	1	1
3	3	3	1	2	1	1	1	1	1
3	3	3	1	2	1	1	1	1	1
3	3	3	1	2	1	1	1	1	1
9	3	3	1	2	1	1	1	1	1
1	3	3	1	2	1	1	1	1	1
3	3	3	1	2	1	1	1	1	1
3	3	3	1	2	1	1	1	1	1
3	3	3	1	2	1	1	1	1	1
1	3	3	1	9	1	1	1	1	1
3	3	3	1	2	1	1	1	1	1
3	3	3	1	2	1	1	1	1	1
3	3	3	1	2	1	1	1	1	1
3	3	3	1	2	1	1	1	1	1
3	3	3	1	2	1	1	1	1	1
9	3	3	1	2	1	1	1	1	1
3	3	3	1	2	3	1	1	1	1
3	3	3	1	2	1	1	1	1	1
1	3	3	1	2	1	1	1	1	1
1	3	3	1	2	1	1	1	1	1
3	3	3	1	2	1	1	1	1	1
3	3	3	1	2	1	1	1	1	1
3	3	3	1	2	1	1	1	1	1
3	3	3	1	2	1	1	1	1	1
3	3	3	1	2	1	1	1	1	1
9	9	9	9	9	9	9	9	9	9
9	9	9	9	9	9	9	9	9	9
9	9	9	9	9	9	9	9	9	9

9	9	9	9	9	9	9	9	9	9
9	9	9	9	9	9	9	9	9	9
9	9	9	9	9	9	9	9	9	9
9	9	9	9	9	9	9	9	9	9
9	9	9	9	9	9	9	9	9	9
9	9	9	9	9	9	9	9	9	9
9	9	9	9	9	9	9	9	9	9
9	9	9	9	9	9	9	9	9	9
9	9	9	9	9	9	9	9	9	9
9	9	9	9	9	9	9	9	9	9
9	9	9	9	9	9	9	9	9	9
9	9	9	9	9	9	9	9	9	9
9	9	9	9	9	9	9	9	9	9
9	9	9	9	9	9	9	9	9	9
9	9	9	9	9	9	9	9	9	9
9	9	9	9	9	9	9	9	9	9
9	9	9	9	9	9	9	9	9	9
9	9	9	9	9	9	9	9	9	9
9	9	9	9	9	9	9	9	9	9
3	3	3	1	2	1	1	1	1	1
3	3	3	1	2	1	1	1	1	1
3	3	3	1	2	1	1	1	1	1
3	3	3	1	2	1	1	1	1	1
3	3	3	1	2	1	1	1	1	1
3	3	3	1	2	1	1	1	1	1
1	3	3	1	2	1	1	1	1	1
1	3	3	1	2	1	1	1	1	1
3	3	3	1	2	1	1	1	1	1
3	3	3	1	2	1	1	1	1	1
3	3	3	1	2	1	1	1	1	1
1	3	3	3	2	1	1	1	1	1
3	3	3	1	2	1	1	1	1	1
1	3	3	1	2	1	1	1	1	1
3	3	9	9	2	1	1	1	1	1
3	3	3	1	3	1	1	1	1	1
9	9	9	9	9	1	9	9	1	9
3	3	3	1	2	1	1	1	1	1
3	3	3	1	2	1	1	1	1	1
3	3	3	1	2	1	1	1	1	1
3	3	3	1	2	1	1	1	1	1
3	3	3	1	2	1	1	1	1	1
3	3	3	1	2	1	1	1	1	1
1	3	3	1	2	1	1	1	1	1
3	3	3	9	2	1	1	1	1	1
1	3	3	1	2	1	1	1	1	1
3	3	3	1	2	1	1	1	1	1
3	3	3	1	2	1	1	1	1	1

9	3	3	1	2	1	1	1	1	1
3	3	1	1	2	1	1	1	1	1
3	3	3	1	2	1	1	1	1	1
9	3	1	1	2	1	1	1	1	1
3	3	3	1	2	1	1	1	1	1
3	3	3	1	2	1	1	1	1	1
3	3	1	2	2	1	1	1	1	1
3	3	3	3	2	1	1	1	1	1
3	3	3	1	2	1	1	1	1	1
3	3	3	1	2	1	1	1	1	1
3	3	1	1	2	1	1	1	1	1
3	3	3	1	2	1	1	1	1	1
1	3	3	1	2	1	1	1	1	1
3	3	1	2	2	1	1	1	1	1
3	3	3	1	2	1	1	1	1	1
1	3	3	1	2	1	1	1	1	1
3	3	3	1	2	1	1	1	1	1
3	3	3	1	2	1	1	1	1	1
3	3	3	1	2	1	1	1	1	1
9	3	3	1	2	1	1	1	1	1
3	3	3	1	2	1	1	1	1	1
3	3	3	3	2	3	1	1	1	1
3	3	3	1	2	1	1	1	1	1
3	3	3	1	2	1	1	1	1	1
9	3	3	1	2	1	1	1	1	1
3	3	3	1	2	1	1	1	1	1
3	1	3	1	2	1	1	1	1	1
3	3	3	1	2	1	1	1	1	1
3	3	3	1	2	1	1	1	1	1
3	3	3	1	9	1	1	1	1	1
9	3	3	1	2	1	1	1	1	1
3	3	3	9	9	1	1	1	1	1
1	9	9	9	9	9	9	9	9	9
3	3	3	1	2	1	1	1	1	1
9	9	9	9	9	9	9	9	9	9
3	3	3	1	2	1	1	1	1	1
9	3	3	1	2	1	1	1	1	1
9	3	1	9	2	1	1	1	1	1
3	3	3	1	2	1	1	1	1	1
3	1	3	1	2	1	1	1	1	1
3	3	3	1	2	1	1	1	1	1
1	3	3	1	2	1	1	1	1	1
3	3	3	1	2	1	1	1	1	1
3	3	3	1	2	1	1	1	1	1
3	3	3	1	2	1	1	1	1	1
1	3	3	1	2	1	1	1	1	1
3	3	3	1	2	1	1	1	1	1

3	3	3	1	2	1	1	1	1	1
3	3	3	1	2	1	1	1	1	1
1	3	3	1	2	1	1	1	1	1
3	3	3	1	2	1	1	1	1	1
1	3	3	1	2	1	1	1	1	1
1	3	3	1	2	1	1	1	1	1
1	3	3	1	2	1	1	1	1	1
3	3	3	1	2	1	1	1	1	1
3	3	3	1	2	1	1	1	1	1
1	3	3	1	2	1	1	1	1	1
9	3	3	1	2	1	1	1	1	1
3	3	3	1	2	1	1	1	1	1
3	3	3	1	2	1	1	1	1	1
3	3	3	1	2	1	1	1	1	1
9	3	9	9	9	1	1	1	1	1
3	3	3	1	2	1	1	1	1	1
3	3	3	1	2	1	1	1	1	1
3	3	3	1	2	1	1	1	1	1
3	3	3	1	2	1	1	1	1	1
9	3	3	1	2	1	1	1	1	1
9	3	3	1	2	1	1	1	1	1
3	3	3	1	2	1	1	1	1	1
3	3	3	1	2	1	1	1	1	1
3	3	3	1	2	1	1	1	1	1
3	1	9	9	9	9	1	1	1	1
1	3	3	1	3	1	1	1	1	1
1	3	3	1	2	1	1	1	1	1
3	1	3	1	2	1	1	1	1	1
3	3	3	1	3	1	1	1	1	1
3	3	3	1	2	1	1	1	1	1
3	3	3	1	3	1	1	1	1	1
3	3	3	1	2	1	1	1	1	1
3	3	3	1	2	1	1	1	1	1
3	3	3	1	2	1	1	1	1	1
3	3	3	1	2	1	1	1	1	1
3	3	3	1	2	1	1	1	1	1
3	3	3	1	2	1	1	1	1	1
9	9	3	1	2	9	9	1	1	1
3	3	3	1	2	1	1	1	1	1
3	3	3	1	2	1	1	1	1	1
1	3	3	1	2	1	1	1	1	1
9	9	9	9	9	9	9	9	9	9
1	3	3	1	2	1	1	1	1	1
1	3	3	1	3	1	1	1	1	1
1	3	3	1	2	1	1	1	1	1
1	3	3	3	2	1	1	1	1	1
3	3	3	3	2	1	1	1	1	1

1	1	3	1	3	1	1	1	1	1
1	3	3	1	2	1	1	1	1	1
3	3	3	1	2	1	1	1	1	1
3	3	3	1	2	1	1	1	1	1
3	3	3	1	2	1	1	1	1	1
3	3	3	1	2	1	1	1	1	1
3	3	3	1	2	1	1	1	1	1
1	3	3	1	2	1	1	1	1	1
3	3	3	1	2	1	1	1	1	1
9	9	9	9	9	9	9	9	9	9
3	3	3	1	2	1	1	1	1	1
1	3	3	1	2	1	1	1	1	1
1	3	3	1	2	1	1	1	1	1
3	3	3	1	2	1	1	1	1	1
3	3	3	1	2	1	1	1	1	1
3	3	3	1	2	1	1	1	1	1
3	3	3	1	2	1	1	1	1	1
3	3	3	3	2	2	1	1	1	1
3	3	3	1	2	1	1	1	1	1
1	3	3	1	2	1	1	1	1	1
3	3	3	1	2	1	1	1	1	1
3	3	3	1	2	1	1	1	1	1
3	3	3	1	2	1	1	1	1	1
3	3	3	1	2	1	1	1	1	1
1	3	3	1	2	1	1	1	1	1
1	3	3	1	2	1	1	1	1	1
1	3	3	1	2	1	1	1	1	1
9	9	9	9	9	9	9	9	9	9
9	9	9	9	9	9	9	9	9	9
9	9	9	9	9	9	9	9	9	9
9	9	9	9	9	9	9	9	9	9
9	9	9	9	9	9	9	9	9	9
9	9	9	9	9	9	9	9	9	9
9	9	9	9	9	9	9	9	9	9
9	9	9	9	9	9	9	9	9	9
9	9	9	9	9	9	9	9	9	9
9	9	9	9	9	9	9	9	9	9
9	9	9	9	9	9	9	9	9	9
9	9	9	9	9	9	9	9	9	9
9	9	9	9	9	9	9	9	9	9
9	9	9	9	9	9	9	9	9	9
9	9	9	9	9	9	9	9	9	9
9	9	9	9	9	9	9	9	9	9
9	9	9	9	9	9	9	9	9	9
9	9	9	9	9	9	9	9	9	9
9	9	9	9	9	9	9	9	9	9
9	9	9	9	9	9	9	9	9	9

9	9	9	9	9	9	9	9	9	9
9	9	9	9	9	9	9	9	9	9
9	9	9	9	9	9	9	9	9	9
9	9	9	9	9	9	9	9	9	9
9	9	9	9	9	9	9	9	9	9
9	9	9	9	9	9	9	9	9	9
9	9	9	9	9	9	9	9	9	9
9	9	9	9	9	9	9	9	9	9
9	9	9	9	9	9	9	9	9	9
9	9	9	9	9	9	9	9	9	9
9	9	9	9	9	9	9	9	9	9
9	9	9	9	9	9	9	9	9	9
9	9	9	9	9	9	9	9	9	9
2	1	2	2	3	2	2	1	1	1
2	2	2	2	2	2	2	2	2	2
2	2	2	2	2	2	2	2	2	2
1	3	2	1	2	1	1	1	3	1
3	2	1	2	3	2	1	1	2	2
1	1	1	2	2	2	2	2	2	2
1	1	1	1	2	3	3	1	2	1
1	2	1	2	2	1	1	1	1	2
2	2	2	2	2	2	1	3	2	3
9	9	9	9	9	9	9	9	9	9
2	2	2	3	2	2	3	2	3	1
9	9	9	9	9	9	1	9	9	9
3	3	3	3	2	2	2	2	2	2
9	9	9	9	9	9	9	9	9	9
1	1	1	1	1	1	1	1	1	1
3	2	1	3	1	1	1	1	1	1
2	2	2	2	2	2	2	2	2	2
3	3	3	3	3	3	1	1	1	1
3	2	1	3	2	3	2	2	3	1
9	9	9	9	9	9	9	9	1	9
2	2	1	2	1	2	2	2	2	1
1	1	1	1	1	1	1	1	1	3
9	9	9	9	9	9	9	1	9	9
2	2	2	2	2	2	2	2	1	2
3	2	1	2	3	1	1	2	1	1
2	2	3	1	1	1	2	3	1	1
1	1	1	1	1	1	1	1	3	2
9	9	9	9	9	9	9	9	9	9
9	9	9	9	9	9	9	9	9	9
2	2	2	2	2	2	2	1	2	1
1	1	1	1	1	1	1	1	1	1
9	9	9	9	9	9	9	9	9	9
9	9	9	9	9	9	9	9	9	9
9	9	9	9	9	9	9	9	9	9

9	9	9	9	9	9	1	9	9	9
9	9	9	9	9	9	9	9	9	9
3	2	2	1	2	2	3	2	2	3
2	2	2	2	2	2	2	1	2	2
3	3	3	1	3	1	1	1	2	3
9	9	9	9	9	9	9	9	9	9
2	2	2	2	2	2	2	2	2	2
2	2	2	2	2	2	2	2	2	2
2	2	2	2	2	2	2	2	2	2
2	2	1	1	1	2	2	2	2	3
2	3	3	3	3	3	3	3	9	2
9	9	9	9	9	9	9	9	9	9
9	9	9	9	9	9	9	9	3	9
2	2	2	2	2	2	2	2	2	2
2	2	2	2	2	2	2	1	1	2
2	2	2	2	2	2	2	2	2	2
2	2	2	2	2	2	1	1	2	3
2	2	2	2	2	2	2	2	2	2
1	3	1	3	1	1	1	1	2	9
9	9	9	9	9	9	9	9	9	9
2	2	2	2	2	2	2	2	2	2
2	2	2	2	2	2	2	2	2	2
2	2	2	2	2	2	2	2	2	2
2	2	2	2	2	2	2	2	2	2
2	2	2	2	2	2	2	2	2	2
1	2	2	1	1	2	2	1	1	2
2	2	2	2	2	2	2	2	2	2
2	2	2	2	2	2	2	2	2	2
2	2	2	2	2	2	2	1	9	9
1	2	2	1	2	1	1	1	1	1
3	3	3	2	3	1	1	3	3	1
2	2	2	2	3	1	2	1	1	1
1	2	1	1	1	1	1	1	1	2
2	2	2	1	3	3	1	1	1	2
1	3	2	1	3	2	3	2	2	2
2	1	1	1	2	2	2	1	2	2
2	2	1	2	2	2	2	3	3	3
9	9	9	9	9	9	9	2	2	2
9	9	9	9	9	9	9	2	9	9
2	2	2	2	2	2	2	2	2	2
3	3	1	1	1	1	1	1	2	2
2	2	2	1	2	2	2	2	2	2
1	2	3	1	2	3	1	1	2	3
3	3	3	2	2	2	9	9	9	9
1	1	1	1	1	1	1	1	1	1
1	2	2	2	1	1	1	1	3	3
2	2	2	2	2	2	2	1	1	3

2	2	2	2	2	2	2	2	2	2
1	2	3	1	2	3	1	1	1	1
2	2	2	2	2	2	2	3	3	3
3	2	3	2	2	3	2	2	3	2
2	2	2	2	2	2	2	2	2	2
2	2	2	2	2	2	2	2	2	2
2	2	2	2	2	1	2	2	2	2
2	3	1	2	2	3	3	2	2	3
2	2	2	2	2	2	2	2	2	2
1	2	1	3	1	2	3	3	1	2
2	2	2	2	2	2	2	2	2	2
2	2	2	2	2	2	2	2	2	2
2	2	2	2	2	2	2	2	2	2
3	3	3	3	3	3	3	3	3	3
2	2	2	2	2	2	2	2	2	2
2	1	2	2	1	2	2	2	2	2
2	2	2	2	2	2	2	2	2	2
2	2	2	2	2	2	2	2	2	2
2	2	2	2	2	2	2	2	2	2
1	2	3	1	2	3	1	1	2	3
2	1	2	1	2	1	2	1	2	1
1	1	1	1	3	3	3	1	1	1
2	2	2	2	2	2	2	2	2	2
3	3	3	3	3	3	3	1	1	1
1	3	3	1	3	1	1	1	1	1
9	9	9	9	9	9	9	9	9	9
9	9	9	9	9	9	9	9	9	9
2	2	2	2	2	2	2	2	2	2
9	9	9	9	9	9	9	9	9	9
1	3	3	2	1	1	2	3	3	2
1	2	2	2	2	1	2	2	1	2
1	2	2	2	1	2	2	1	3	2
2	3	3	3	3	3	3	9	9	9
2	2	2	2	2	2	2	1	1	1
9	9	9	9	9	9	9	9	2	1
2	2	2	2	2	2	2	2	2	2
9	9	9	9	9	9	9	9	9	9
2	2	1	2	2	1	1	3	3	2
9	9	9	9	9	9	9	9	9	9
2	2	2	2	2	2	2	2	2	2
2	2	2	3	2	3	2	2	3	2
1	2	1	1	2	2	2	2	2	1
1	2	1	2	2	1	2	2	3	2
9	9	9	9	9	9	9	9	9	9
9	9	9	9	9	9	9	9	9	9
9	9	9	9	9	9	9	9	9	9
1	1	1	1	2	2	2	1	1	1

9	9	9	9	9	9	9	9	9	9
9	9	9	9	9	9	9	9	9	9
2	2	3	2	3	1	1	3	1	2
9	9	9	9	9	9	9	9	9	9
9	9	9	9	9	9	9	9	9	9
2	3	3	2	2	2	2	3	3	3
2	2	3	3	2	2	3	2	2	2
9	9	9	9	9	9	9	9	9	9
1	2	2	1	3	1	1	2	1	1
1	2	3	1	2	3	1	1	2	3
1	2	3	1	2	3	1	1	2	3
2	2	2	2	2	2	2	2	2	2
2	2	2	2	2	2	2	2	2	2
9	9	9	9	9	9	9	9	9	9
2	2	2	2	2	2	2	2	2	2
2	2	2	2	2	2	2	2	2	2
3	3	3	1	3	1	1	1	1	1
3	3	3	2	1	2	2	2	3	2
3	2	1	3	2	1	2	1	2	2
9	9	9	9	9	9	9	9	9	9
3	3	3	2	1	2	2	2	3	2
3	2	1	1	1	1	1	3	3	3
2	2	2	2	2	2	2	2	2	2
3	3	3	2	2	1	2	2	3	2
3	3	3	3	3	3	3	2	3	3
9	9	9	9	9	9	9	9	9	9
3	3	2	2	2	2	1	1	2	3
9	9	9	9	9	9	9	9	9	9
2	2	2	2	2	2	2	3	3	3
2	3	2	1	2	3	2	1	1	2
2	2	2	2	2	2	2	2	2	2
9	9	9	9	9	9	9	9	9	9
9	9	9	9	9	9	9	9	9	9
2	2	2	2	2	2	2	2	2	2
1	2	2	2	1	2	2	1	2	2
1	3	1	2	1	2	3	2	1	3
2	2	2	2	2	2	9	2	2	2
2	2	2	1	3	2	2	1	1	2
2	2	2	2	2	2	2	2	2	2
9	9	9	9	9	9	9	2	9	9
2	2	1	1	2	2	2	1	1	1
2	2	2	2	2	2	2	2	2	2
2	2	2	2	2	2	2	2	2	2
2	2	2	2	2	2	2	2	2	2
2	2	2	2	2	2	2	2	2	2
9	9	9	9	9	9	9	1	9	9
3	2	2	3	2	2	3	2	3	3

2	1	1	1	1	1	1	1	1	1
2	2	2	2	2	2	2	2	2	2
1	3	3	1	3	2	2	1	1	3
2	2	2	2	2	1	2	2	2	2
2	2	2	2	2	2	2	2	2	2
2	2	3	3	3	2	2	2	2	2
3	2	2	1	2	3	1	1	2	3
2	1	2	2	1	2	2	1	2	2
1	3	3	1	3	1	1	1	1	1
2	3	3	3	3	1	1	1	1	2
2	2	2	2	2	2	2	2	2	2
2	2	2	2	2	2	2	2	2	2
2	2	2	2	2	2	2	2	2	2
2	3	3	1	3	1	1	1	1	1
2	1	3	1	9	3	1	3	2	1
2	2	2	2	2	2	2	2	2	2
2	3	2	3	2	3	2	2	9	2
2	1	2	1	2	1	2	2	2	2
2	2	3	2	2	3	2	2	2	2
1	2	2	1	1	1	1	1	1	1
1	2	1	1	3	1	1	1	1	1
9	9	9	9	9	9	9	9	9	9
2	2	2	2	2	2	2	2	2	2
2	2	2	2	2	2	2	2	2	2
2	2	2	2	2	2	2	2	2	2
1	2	2	2	2	2	2	2	1	2
2	2	2	2	2	2	1	1	2	1
2	2	2	2	2	1	1	1	2	1
3	3	3	3	3	1	1	1	1	1
1	2	2	2	2	1	1	2	1	1
3	3	3	3	3	1	1	1	1	1
1	2	2	1	1	1	1	2	1	1
2	1	2	1	2	2	2	3	2	2
9	9	9	9	9	9	9	9	9	9
9	9	9	9	9	9	9	9	9	9
3	3	3	1	3	1	1	1	1	1
2	2	2	2	2	2	2	2	2	2
2	1	2	1	2	1	1	1	1	1
1	1	1	1	1	1	1	1	1	1
2	2	2	2	2	2	2	2	2	2
1	2	1	2	2	2	2	1	2	2
9	9	9	9	9	9	9	9	9	9
9	9	9	9	9	9	9	9	9	9
1	1	1	1	1	1	1	1	1	1
1	2	2	1	3	2	3	1	2	2
9	9	9	9	9	9	9	9	9	9
9	9	9	9	9	9	9	9	9	9

2	2	2	2	2	2	2	2	2	2
1	1	2	2	2	3	3	3	3	3
1	2	3	1	2	3	1	1	2	3
2	3	2	2	3	2	2	2	2	2
2	3	3	3	3	1	1	1	2	2
1	2	3	1	2	3	1	1	2	3
3	2	2	2	2	2	2	2	2	1
9	9	9	9	9	9	9	9	9	9
1	1	2	2	1	2	2	1	2	2
9	9	9	9	9	9	9	9	9	9
2	2	2	2	2	2	2	1	1	1
3	3	3	3	2	2	2	2	1	1
1	2	2	3	3	2	1	2	2	1
9	9	9	9	9	9	9	9	9	9
2	2	2	2	2	2	2	1	1	1
9	9	9	9	9	9	9	9	9	9
9	9	9	9	9	9	9	9	9	9
2	2	2	2	2	2	2	2	2	2
2	2	2	2	2	2	2	2	2	2
2	1	2	2	2	1	2	2	2	2
2	2	2	2	2	2	2	2	2	2
2	2	2	2	2	2	2	2	2	2
3	3	3	3	3	3	3	2	2	2
2	3	2	3	2	1	2	1	2	1
1	9	2	9	9	3	9	3	3	3
9	9	9	9	9	9	9	2	9	9
2	2	2	2	2	2	2	2	2	2
2	2	2	2	2	2	2	1	1	1
1	1	1	1	1	1	1	2	2	1
3	3	3	3	3	1	1	3	1	1
1	2	1	1	2	1	2	1	1	1
2	2	2	2	2	2	2	2	2	2
2	2	2	2	2	2	2	2	2	2
2	2	2	2	2	2	2	2	2	2
2	2	2	2	2	2	2	2	2	2
2	2	2	2	2	2	2	2	2	2
3	3	3	3	3	3	3	2	3	2
9	9	1	9	9	9	9	9	9	9
9	9	9	9	9	9	2	9	9	9
2	2	2	2	2	2	2	2	2	2
3	3	3	3	3	3	3	3	3	3
2	2	2	2	2	2	2	9	1	9
2	2	2	2	2	2	2	1	2	2
9	9	9	9	9	9	9	2	9	9
2	2	2	2	2	2	2	3	3	3
9	9	9	9	9	9	9	9	9	9
2	2	2	2	2	2	2	2	2	2

3	1	3	1	2	9	3	3	1	2
9	9	9	9	9	1	9	9	9	9
2	2	2	2	2	2	2	2	2	2
2	2	2	2	2	2	2	1	2	1
2	2	2	2	2	2	2	2	2	2
2	2	2	2	2	2	2	2	2	2
2	2	2	1	3	1	2	1	2	2
9	9	9	9	9	9	9	9	9	9
2	2	2	2	2	2	2	2	2	2
1	2	2	2	2	2	2	2	2	2
2	2	2	2	2	2	2	2	2	2
2	2	2	2	2	2	2	2	2	2
2	3	9	2	3	3	2	2	2	2
2	2	3	3	3	2	2	1	2	3
1	1	1	2	2	1	3	2	3	1
1	1	2	1	1	2	1	2	3	1
2	2	2	2	2	2	2	2	2	2
2	2	2	2	2	2	2	2	2	2
9	9	9	9	9	9	9	9	9	9
9	9	9	9	9	9	9	9	9	9
3	2	3	1	2	2	3	1	1	1
2	2	2	2	2	1	1	1	1	2
9	9	9	9	9	9	9	9	9	9
9	9	9	9	9	9	9	9	9	9
3	3	3	3	3	3	3	3	3	3
9	9	9	9	9	9	9	9	9	9
9	9	9	9	9	9	9	9	9	9
9	9	9	9	9	9	9	9	9	9
1	2	2	2	3	1	1	1	2	1
1	1	1	1	2	1	1	2	2	2
2	2	2	2	2	2	2	2	2	2
9	1	2	1	9	1	1	1	1	1
1	2	3	1	2	2	3	3	2	1
2	2	2	2	2	2	2	2	2	2
2	2	2	2	2	2	2	2	2	2
2	2	2	2	2	2	2	1	1	1
1	2	2	1	3	3	2	1	2	1
2	3	2	3	1	2	3	1	2	3
2	1	3	1	1	2	2	2	2	1
2	2	2	2	2	2	2	1	1	1
2	3	3	2	2	1	1	2	2	1
2	2	2	2	2	2	1	2	2	2
9	9	9	9	9	9	9	1	9	9
1	9	9	9	9	9	9	9	9	9
1	2	1	3	2	2	3	1	2	1
2	2	2	2	2	2	2	2	2	2
2	2	2	2	2	2	2	2	2	2

2	2	2	2	3	3	3	2	2	2
9	9	9	9	9	9	9	9	9	9
9	9	9	9	9	9	9	9	9	9
9	9	9	9	9	9	9	9	9	9
9	9	9	9	9	9	9	9	9	9
9	9	9	9	9	9	1	9	9	9
9	9	9	9	9	9	9	9	9	9
9	9	9	9	9	9	9	9	9	9
9	9	9	9	9	9	9	9	9	9
9	9	9	9	1	9	9	1	9	9
2	2	2	2	2	2	2	2	2	2
2	2	2	3	2	3	2	2	1	3
9	9	9	9	1	1	9	1	2	1
2	2	2	2	2	2	2	2	3	2
2	2	2	2	2	2	2	2	2	2
9	9	9	9	9	9	9	9	9	9
2	2	2	2	3	3	3	2	2	2
2	2	2	2	2	2	2	2	2	2
2	2	2	2	2	2	9	2	2	2
2	2	2	2	2	2	2	1	1	2
1	2	3	1	2	3	1	1	2	3
3	2	1	2	3	2	3	1	3	2
3	3	3	3	3	3	3	2	2	2
2	2	2	2	2	2	2	2	2	2
2	2	2	2	2	2	2	2	2	2
2	2	2	2	2	2	2	2	2	2
2	2	2	2	2	2	2	3	3	3
1	2	1	2	1	2	1	1	1	1
2	2	2	2	2	2	2	2	2	2
2	2	2	2	2	2	2	2	2	2
9	9	9	9	9	9	9	9	9	9
2	2	2	2	2	2	2	2	2	2
2	1	1	1	1	2	2	2	2	2
9	9	9	9	9	9	9	1	9	9
3	3	3	3	3	3	3	9	9	9
2	2	2	2	2	2	2	2	2	2
2	2	2	2	2	2	2	2	2	2
2	2	2	2	2	2	2	2	2	2
2	3	2	3	1	3	1	1	3	3
9	9	9	9	9	9	9	9	9	9
2	3	2	3	1	3	3	1	1	2
2	2	2	2	2	2	2	2	2	2
2	2	2	2	2	2	2	2	2	2
2	2	2	1	3	1	1	1	1	1
2	2	2	1	1	1	1	2	2	1
9	9	9	9	9	9	9	9	9	9
9	9	9	9	9	9	9	9	9	9

9	9	9	9	9	9	9	9	9	9
2	2	2	2	2	2	2	1	1	1
9	9	9	9	9	9	9	2	9	9
9	9	9	9	9	9	9	9	9	9
9	9	9	9	9	9	9	9	9	9
9	9	9	9	9	9	9	9	9	9
9	9	9	9	9	9	9	9	9	9
9	9	9	9	9	9	9	9	9	9
9	9	9	9	9	9	9	9	9	9
9	9	9	9	9	9	9	9	9	9
9	9	9	9	9	9	9	9	9	9
9	9	9	9	9	9	9	9	9	9
9	9	9	9	9	9	9	9	9	9
9	9	9	9	9	9	9	9	9	9
9	9	9	9	9	9	9	9	9	9
9	9	9	9	9	9	9	9	9	9
9	9	9	9	9	9	9	9	9	9
9	9	9	9	9	9	9	9	9	9
9	9	9	9	9	9	9	9	9	9
9	9	9	9	9	9	9	9	9	9
9	9	9	9	9	9	9	9	9	9
9	9	9	9	9	9	9	9	9	9
9	9	9	9	9	9	9	9	9	9
9	9	9	9	9	9	9	9	9	9
9	9	9	9	9	9	9	9	9	9
1	2	3	2	1	3	2	1	1	1
2	2	2	2	2	2	2	2	2	2
1	2	2	2	2	2	2	1	1	1
2	2	2	2	2	2	2	1	1	1
2	1	3	2	2	1	2	1	2	3
9	9	9	9	9	9	9	1	2	1
2	2	2	2	2	2	2	2	2	2
1	1	1	2	2	3	3	1	1	1
2	2	2	2	2	2	2	3	2	2
1	2	2	3	3	3	3	1	1	1
9	9	9	9	9	9	9	1	1	1
3	2	2	2	2	2	2	2	1	2
2	3	3	1	3	1	1	1	1	2
9	9	9	9	9	9	9	1	1	1
2	2	2	2	2	2	2	1	1	1
1	2	1	1	3	1	2	1	1	2
9	9	9	9	9	9	9	1	9	9
2	2	2	1	2	2	1	1	1	1
1	2	2	2	3	1	1	2	1	1
9	9	9	9	9	9	9	1	9	9
2	2	3	1	2	1	2	1	1	1
2	3	3	1	3	1	1	1	1	1
2	2	2	2	2	2	2	1	2	2
3	2	3	2	3	2	3	3	1	2

2	2	2	2	3	1	1	1	1	1
2	2	2	2	2	1	2	1	1	1
2	3	3	1	3	1	1	1	1	1
2	2	1	1	2	1	2	1	1	1
3	2	3	1	3	2	1	3	2	1
9	9	9	9	9	9	9	3	9	9
2	1	1	2	1	1	2	1	1	2
9	9	9	9	9	9	9	1	9	9
2	2	2	2	2	2	2	2	2	2
2	2	2	2	2	2	2	2	2	2
1	9	9	9	9	9	9	9	9	9
9	9	9	9	9	9	9	1	9	9
9	1	9	9	9	9	9	1	9	9
1	9	9	9	9	9	9	1	9	9
9	9	9	9	9	9	9	9	1	9
9	9	9	9	9	9	9	9	9	9
9	9	9	9	9	9	9	9	9	9
2	2	2	2	2	2	2	9	9	9
9	9	9	9	9	1	9	9	9	1
3	1	3	1	3	3	1	1	1	1
1	2	2	1	2	2	2	9	1	1
9	1	9	9	1	9	9	9	1	9
9	9	9	9	9	9	9	9	1	9
9	9	2	9	9	9	9	9	1	9
9	9	9	9	9	9	9	1	9	9
2	3	1	3	1	2	3	1	2	3
1	3	1	1	1	3	1	3	1	3
2	1	2	2	3	3	2	2	3	1
2	2	2	2	2	1	2	2	2	2
9	9	9	9	9	9	9	9	9	9
3	2	1	2	1	2	1	2	1	1
1	3	1	3	1	1	1	1	1	3
3	3	3	3	3	3	3	3	3	3
1	3	1	1	3	1	1	1	3	1
1	3	3	1	3	1	1	1	2	3
9	9	9	9	9	9	9	3	9	9
2	3	2	2	2	2	2	2	2	2
1	3	3	1	1	1	1	1	1	1
3	2	3	1	2	3	2	2	2	2
1	1	3	1	2	1	1	2	1	1
2	3	3	2	3	1	2	1	1	1
2	2	3	2	3	1	1	1	1	1
1	3	3	1	3	1	1	1	3	1
3	3	2	2	1	1	2	1	1	2
9	2	1	1	3	2	2	2	1	1
9	9	9	9	9	9	9	2	2	1
1	1	2	1	2	1	1	1	1	1

2	1	2	2	1	1	1	1	1	1
2	3	2	3	3	3	3	2	1	2
9	9	9	9	9	9	9	9	9	9
3	3	3	3	3	1	1	9	1	1
9	9	9	9	9	9	9	9	9	9
1	2	1	1	3	1	1	1	1	1
1	2	3	1	3	1	1	1	1	1
9	9	9	9	9	9	9	9	9	9
9	9	9	9	9	9	9	2	9	9
2	2	2	2	2	2	2	2	2	2
3	2	3	2	1	1	2	2	3	1
3	3	1	3	3	3	1	9	1	1
2	3	3	1	1	1	1	1	2	2
9	9	9	9	9	9	9	9	9	9
9	9	9	9	9	9	9	9	9	9
2	9	3	3	3	2	2	2	2	1
9	9	9	9	9	9	9	1	9	9
9	9	9	9	9	9	9	9	9	9
2	3	2	2	2	3	3	3	3	3
2	3	3	3	2	3	2	1	1	1
2	2	2	2	2	2	2	2	2	2
1	1	1	1	1	1	1	1	1	1
3	3	2	3	1	3	3	2	1	3
3	3	3	2	3	2	1	1	1	1
2	2	2	2	2	2	2	2	2	2
9	9	9	9	9	9	9	9	9	9
9	9	9	9	9	9	9	2	9	9
1	3	1	1	3	3	1	1	1	1
3	3	2	1	1	2	2	3	3	2
3	2	1	3	2	1	3	2	3	2
9	9	9	9	9	9	9	9	9	9
9	9	9	9	9	9	9	9	9	9
9	9	9	9	9	9	9	9	9	9
9	9	9	9	9	9	9	9	9	9
9	9	9	9	9	9	9	9	9	9
9	9	9	9	9	9	9	9	9	9
9	9	9	9	9	9	9	9	9	9
9	9	9	9	9	9	9	9	9	9
9	9	9	9	9	9	9	9	9	9
9	9	9	9	9	9	9	9	9	9
9	9	9	9	9	9	9	9	9	9
9	9	9	9	9	9	9	9	9	9
9	9	9	9	9	9	9	9	9	9
9	9	9	9	9	9	9	9	9	9
9	9	9	9	9	9	9	9	9	9
9	9	9	9	9	9	9	9	9	9
9	9	9	9	9	9	9	9	9	9

9	9	9	9	9	9	9	9	9	9
9	9	9	9	9	9	9	9	9	9
9	9	9	9	9	9	9	9	9	9
9	9	9	9	9	9	9	9	9	9
9	9	9	9	9	9	9	9	9	9
9	9	9	9	9	9	9	9	9	9
9	9	9	9	9	9	9	9	9	9
9	9	9	9	9	9	9	9	9	9
9	9	9	9	9	9	9	9	9	9
9	9	9	9	9	9	9	9	9	9
9	9	9	9	9	9	9	9	9	9
9	9	9	9	9	9	9	9	9	9
9	9	9	9	9	9	9	9	9	9
9	9	9	9	9	9	9	9	9	9
9	9	9	9	9	9	9	9	9	9
9	9	9	9	9	9	9	9	9	9
9	9	9	9	9	9	9	9	9	9
9	9	9	9	9	9	9	9	9	9
9	9	9	9	9	9	9	9	9	9
2	2	2	2	2	2	2	9	9	9
9	9	9	9	9	9	9	9	9	9
9	9	9	9	9	9	9	9	9	9
9	9	9	9	9	9	9	9	9	9
9	9	9	9	9	9	9	9	9	9
9	9	9	9	9	9	9	9	9	9
9	9	9	9	9	9	9	9	9	9
9	9	9	9	9	9	9	9	9	9
9	9	9	9	9	9	9	9	9	9
9	9	9	9	9	9	9	9	9	9
9	9	9	9	9	9	9	9	9	9
9	9	9	9	9	9	9	9	9	9
9	9	9	9	9	9	9	9	9	9
9	9	9	9	9	9	9	9	9	9
9	9	9	9	9	9	9	9	9	9
9	9	9	9	9	9	9	9	9	9
2	2	2	2	2	2	2	2	2	2
9	9	9	9	9	9	9	9	9	9
1	2	3	1	3	1	1	3	3	1
3	1	1	1	1	1	1	1	1	1
3	3	3	3	3	3	3	3	3	3
9	9	9	9	9	9	9	9	9	9
9	9	9	9	9	9	9	2	2	1
9	9	9	9	9	9	9	9	1	9
9	9	9	9	9	9	9	2	2	1
2	2	1	3	2	2	2	2	2	2
1	9	9	9	9	9	9	9	1	9
2	2	2	2	2	2	2	2	2	2

1	2	1	1	3	1	2	1	1	2
1	2	2	2	2	2	2	1	1	1
2	2	2	2	2	1	2	1	1	1
2	2	2	2	3	1	1	1	1	3
1	3	3	1	3	1	1	1	1	1
2	2	3	1	3	3	1	1	1	1
1	2	2	2	2	2	2	1	1	1
2	2	2	2	2	2	2	1	2	1
3	1	2	2	2	3	2	2	3	2
1	3	3	1	2	2	1	1	1	1
9	9	9	9	9	9	9	9	1	9
2	2	3	1	2	3	3	2	9	2
9	9	9	9	9	9	9	9	9	9
2	2	2	2	2	2	2	1	1	1
2	2	2	2	2	2	2	2	1	1
2	2	3	2	2	1	2	2	2	2
9	9	9	9	9	9	9	9	9	9
9	9	9	9	9	9	9	1	1	9
1	2	1	1	2	1	1	1	1	1
1	1	1	1	3	1	1	3	1	2
3	3	1	9	3	1	1	1	1	1
2	3	3	2	3	1	2	1	1	1
2	2	2	1	2	2	1	1	1	1
3	3	3	1	3	1	1	1	1	1
3	3	3	3	3	3	3	3	3	3
2	1	2	1	2	1	2	1	3	2
1	1	1	1	1	1	1	1	1	1
3	1	2	3	2	3	1	1	2	1
2	2	2	2	2	2	2	2	2	2
9	9	9	9	9	9	9	9	9	9
9	9	9	9	9	9	9	9	9	9
2	2	2	2	2	2	2	1	1	2
2	2	2	2	2	2	2	2	2	2
9	9	9	9	9	9	9	9	9	9
9	9	9	9	9	9	9	9	9	9
9	9	9	9	9	9	9	9	9	9
9	9	9	9	9	9	9	1	1	1
2	1	2	3	2	1	2	1	1	1
9	9	9	9	9	9	9	9	9	9
9	9	9	9	9	9	9	9	9	9
2	1	1	2	1	2	1	1	1	1
9	9	9	9	9	9	9	9	9	9
3	3	3	2	3	1	1	1	1	1
3	3	3	1	3	3	1	3	3	1
1	2	1	1	3	2	3	1	1	1
3	3	3	3	3	1	3	3	3	3
1	3	1	1	1	3	1	1	1	3

2	3	2	3	2	3	2	2	3	2
2	1	2	1	2	3	2	1	1	2
2	2	1	2	2	1	3	1	2	3
2	2	2	3	3	1	1	1	2	1
3	3	2	2	3	2	1	1	2	3
2	3	2	1	2	3	2	1	2	3
1	1	2	2	3	3	3	1	2	3
2	3	1	2	3	1	2	1	3	1
1	1	1	2	1	1	2	1	1	1
9	1	9	2	3	9	2	1	2	9
3	3	3	3	3	3	3	3	3	3
1	1	1	2	3	1	1	1	1	2
2	2	1	2	1	2	2	3	2	3
1	2	3	1	2	3	1	1	2	3
1	1	1	1	1	1	1	1	1	3
2	3	3	3	3	1	2	1	1	1
3	2	3	2	2	3	2	2	2	2
9	9	9	9	9	9	9	9	1	9
1	2	2	2	1	3	2	1	2	3
3	3	3	3	3	3	1	1	2	2
2	3	3	2	2	2	2	1	2	3
2	1	1	2	2	2	2	1	1	1
1	1	3	1	2	2	1	1	1	1
2	2	2	2	2	2	2	2	2	2
1	1	2	2	2	1	1	2	3	3
1	1	3	1	1	1	1	1	1	3
2	3	2	1	3	1	1	1	1	1
2	2	1	3	1	2	1	1	3	1
2	2	2	2	1	3	2	1	1	1
2	3	1	2	2	1	1	1	1	2
2	2	2	2	2	2	3	3	3	1
1	2	3	1	2	3	1	1	2	1
2	2	2	2	2	2	2	1	1	2
3	3	1	3	3	1	3	1	1	1
1	2	3	1	2	3	1	1	2	3
3	3	3	3	3	1	1	1	1	1
2	1	1	3	2	1	2	1	2	3
9	9	9	9	9	9	9	9	9	9
1	3	1	3	3	1	9	1	1	1
1	2	1	3	1	1	3	1	2	1
3	1	3	1	3	1	1	1	3	1
1	2	3	1	2	3	1	1	2	3
1	1	3	1	1	1	1	1	1	3
2	3	2	1	3	1	2	2	2	3
2	2	2	2	2	2	2	2	2	2
3	3	3	3	3	3	3	3	1	3
3	3	3	3	3	9	9	9	9	9

3	1	2	3	2	1	2	1	2	9
1	1	1	1	1	1	1	1	1	1
2	2	2	3	3	3	3	1	1	2
3	2	2	1	2	3	2	1	2	2
2	2	2	2	2	1	1	1	1	1
2	2	3	3	3	2	2	1	1	2
2	2	2	2	2	2	2	2	2	2
9	9	9	9	9	9	9	9	9	9
9	9	9	9	9	9	9	9	9	9
3	2	1	2	3	2	1	1	2	3
2	2	2	2	2	2	2	1	1	1
2	2	2	2	2	2	2	2	2	2
2	2	2	1	1	1	1	1	1	2
2	2	1	2	1	2	2	2	1	1
2	2	2	2	2	2	2	2	2	2
2	2	2	2	2	2	2	2	2	2
2	2	2	2	2	2	2	2	2	2
2	2	2	2	2	2	2	3	2	2
2	3	1	1	1	2	3	2	2	2
2	2	2	2	2	2	2	2	2	2
2	2	2	2	2	2	2	2	2	1
2	2	2	2	2	2	2	2	2	2
2	2	2	2	2	2	2	2	2	2
1	2	2	2	3	2	2	3	2	2
2	2	2	2	2	2	2	2	2	2
2	2	2	2	2	2	2	2	2	2
2	2	2	2	2	2	2	1	1	1
1	2	1	2	3	1	2	1	2	2
1	1	2	2	2	2	2	2	3	3
2	2	2	2	2	2	2	2	2	2
2	2	2	2	2	2	2	2	2	2
2	2	2	2	2	2	2	1	1	1
2	2	2	2	2	2	2	2	2	2
2	2	2	2	2	2	2	2	2	2
2	2	2	2	2	2	2	2	2	2
2	2	2	2	2	2	2	2	1	1
2	2	2	2	2	2	2	2	2	2
2	2	2	2	2	2	2	2	2	2
2	2	2	2	2	2	2	2	2	2
1	1	1	3	3	1	1	1	1	1
2	2	2	2	2	2	2	2	2	2
2	2	2	2	2	2	2	2	2	2
2	2	1	1	3	3	9	1	1	1
2	2	2	2	2	2	2	2	2	2
3	3	2	2	3	3	2	2	1	1
2	2	2	2	2	2	2	1	1	1
2	2	2	2	2	2	2	2	2	2

2	2	2	2	2	2	2	2	2	2
2	2	2	2	2	2	2	2	2	2
2	2	2	2	2	2	2	1	1	1
2	2	2	2	2	2	2	2	2	2
2	2	2	2	2	2	2	2	2	2
2	2	2	2	2	2	2	2	2	2
9	9	9	9	9	9	9	9	9	9
1	1	1	1	1	1	1	1	3	1
9	9	9	9	9	9	1	9	1	9
2	2	2	2	2	2	2	3	3	3
1	3	2	2	3	2	1	1	2	2
1	1	1	1	1	1	1	2	2	2
2	3	2	3	3	2	2	3	2	2
9	9	9	9	9	9	9	3	3	3
2	2	2	2	2	2	2	9	9	9
2	2	1	2	2	1	2	1	1	2
2	2	2	2	2	2	2	1	2	2
1	1	1	1	1	1	1	1	1	1
9	9	9	9	9	9	9	2	9	9
2	2	2	1	1	2	2	3	3	3
2	2	2	2	2	2	2	2	3	2
9	9	9	9	9	9	1	9	9	9
9	9	9	9	9	9	9	9	1	9
3	3	3	3	3	3	3	9	1	1
1	2	3	1	2	3	2	1	2	3
3	3	2	1	1	3	1	1	1	2
2	2	2	2	2	2	3	2	3	3
3	3	3	2	9	2	1	1	1	2
1	1	1	1	1	9	2	1	1	1
2	2	2	2	2	2	2	1	9	1
2	2	2	2	2	2	2	2	2	2
2	3	3	3	3	3	3	3	2	2
2	2	2	2	2	2	2	2	2	2
9	1	9	9	9	9	9	1	9	9
2	2	2	2	2	2	2	2	2	2
9	9	9	9	9	9	9	1	9	9
3	3	1	1	3	3	3	1	3	2
1	1	1	1	1	1	1	3	3	3
2	2	2	2	2	2	2	2	2	2
2	2	2	2	2	2	2	2	2	2
3	9	2	9	2	1	9	2	9	2
2	2	2	2	2	2	2	2	2	2
9	2	9	9	9	9	9	9	9	9
1	1	1	2	3	3	3	3	3	3
1	2	2	2	2	2	2	1	2	1
3	3	3	3	3	3	3	2	2	2
2	2	1	1	2	2	2	1	1	1

9	9	9	9	1	9	9	9	9	1
2	2	2	2	2	2	2	2	2	2
1	1	1	1	1	1	1	1	1	1
2	2	2	2	2	2	2	2	2	2
9	9	9	9	9	9	9	1	9	9
2	3	3	2	1	1	1	1	1	1
3	3	3	3	3	3	3	3	3	3
2	2	2	2	2	2	2	2	2	2
2	2	2	2	2	2	2	2	2	2
2	2	2	3	3	3	3	1	1	1
9	9	9	9	9	9	9	9	9	9
1	1	1	1	1	1	1	1	1	1
2	2	2	2	2	2	2	2	2	2
2	2	2	2	2	2	2	2	2	2
2	2	2	2	2	2	2	2	2	2
3	3	3	3	3	3	3	2	2	2
1	1	1	1	1	1	1	1	1	1
3	1	1	1	1	2	2	1	1	1
9	9	9	9	9	9	9	9	9	9
3	1	3	1	2	1	2	1	2	1
2	2	2	2	1	1	1	1	1	1
2	2	2	2	2	2	2	1	1	3
1	1	1	1	1	1	1	1	1	1
2	2	2	2	2	2	2	2	2	2
3	3	3	3	3	3	3	3	3	9
2	1	2	1	2	2	1	1	1	1
2	3	2	1	2	3	1	1	1	2
2	2	2	2	2	2	2	2	2	2
9	2	2	1	9	2	2	1	2	1
2	2	2	2	2	2	2	2	2	2
3	1	2	3	1	3	2	1	3	2
2	3	3	3	2	2	2	2	2	2
1	1	1	1	1	1	1	1	1	1
2	2	2	2	2	2	3	2	9	2
2	2	2	2	2	2	2	2	2	2
2	2	2	2	2	2	2	2	2	2
1	1	1	1	1	1	1	1	1	1
3	2	2	2	2	1	1	1	1	2
1	2	3	1	2	3	1	1	2	3
3	3	3	3	3	3	3	3	3	3
1	2	1	2	3	1	2	1	2	3
3	3	3	3	1	1	1	2	2	2
9	9	9	9	9	9	9	9	9	9
3	3	3	3	3	3	3	3	3	3
9	9	9	9	9	9	9	9	9	9
2	2	2	2	2	2	2	2	2	2
2	2	1	2	3	2	1	1	1	1

1	1	1	1	1	1	9	2	2	2
3	3	3	3	3	1	1	3	3	3
9	9	9	9	9	9	9	9	9	9
3	3	3	3	3	1	1	3	3	3
1	2	9	1	1	1	9	1	2	3
2	2	2	2	2	2	2	2	2	2
2	2	2	2	2	2	2	2	2	2
2	2	2	2	2	2	2	2	2	2
2	2	2	2	3	3	3	1	1	1
2	1	2	1	1	2	2	2	1	2
2	2	2	2	2	2	2	1	2	2
3	1	3	2	1	3	2	1	2	3
2	2	2	2	2	2	2	2	2	2
3	2	3	2	3	1	2	1	3	2
2	2	2	2	2	2	2	2	2	2
2	2	2	2	2	2	2	2	2	1
2	2	2	2	2	2	2	2	2	2
2	2	2	2	2	2	2	2	2	2
2	2	2	2	2	2	2	2	2	2
3	3	2	1	9	2	3	3	2	1
2	2	2	2	2	2	2	2	2	2
3	3	3	3	3	3	3	2	2	2
2	2	2	2	2	2	2	2	2	2
2	2	2	2	2	2	2	2	2	2
1	1	1	1	1	1	1	1	1	1
2	2	2	2	2	2	2	2	2	2
1	1	2	2	1	2	2	1	3	3
2	2	2	2	2	2	2	2	2	2
2	1	1	1	2	3	2	1	1	1
2	2	2	2	2	2	2	1	1	2
3	2	1	2	2	2	2	1	1	1
2	2	3	3	2	3	2	1	1	2
2	2	2	2	2	2	2	2	2	2
2	2	2	2	2	2	2	3	2	3
2	2	1	1	3	3	3	3	1	3
3	2	2	3	2	3	2	1	2	2
9	9	9	9	9	9	9	9	9	9
9	9	9	9	9	9	9	9	9	9
2	2	2	2	2	2	2	3	3	3
2	2	2	2	2	2	2	2	2	2
2	2	2	2	2	2	3	9	2	1
2	2	2	2	2	2	2	2	2	2
2	2	2	2	2	2	2	2	2	2
2	2	2	2	2	2	2	2	2	2
9	9	9	9	9	9	9	9	9	9
1	2	1	1	1	1	3	1	2	3
2	2	2	2	2	2	2	2	2	2

9	9	9	9	9	9	9	9	9	9
3	1	2	2	3	2	1	1	2	2
1	3	2	1	3	2	3	3	1	2
1	3	1	1	3	2	2	1	1	3
1	3	3	1	9	1	1	1	1	3
2	1	2	1	1	2	3	1	2	2
1	2	2	1	3	2	3	1	1	1
9	9	9	9	9	9	9	9	9	9
2	2	2	2	2	2	2	2	2	2
2	3	3	3	2	2	3	3	2	2
2	2	2	2	2	2	2	2	3	1
2	2	3	9	3	3	3	3	1	1
1	2	2	3	3	1	1	1	1	2
2	1	1	1	1	1	2	1	1	1
2	1	3	2	2	2	3	2	1	3
2	2	2	2	2	2	2	2	2	2
1	1	1	2	2	1	2	3	2	1
2	2	2	2	2	2	2	2	2	2
9	9	9	9	9	9	9	9	9	9
9	9	9	9	9	9	9	9	9	9
9	9	9	9	9	9	9	9	9	9
2	1	3	2	1	1	2	1	2	3
3	3	1	2	2	3	2	2	2	2
2	2	3	3	3	2	1	3	3	3
2	3	3	3	2	1	1	1	1	1
2	2	2	2	2	2	3	3	3	3
1	1	2	2	3	2	1	2	3	2
3	3	2	2	2	2	1	1	2	1
3	2	1	2	2	1	3	1	2	3
2	3	1	2	3	1	2	1	2	1
9	9	9	9	9	9	9	1	1	1
2	2	2	2	2	2	2	1	1	1
3	3	3	3	3	3	2	3	3	2
2	2	2	2	2	2	2	3	3	3
3	3	3	3	3	3	3	3	3	3
2	2	2	2	2	2	2	2	2	2
3	3	3	3	3	3	3	3	3	3
2	2	2	2	2	2	2	2	2	2
9	9	9	9	9	9	9	9	9	9
9	9	9	9	9	9	9	9	9	9
1	1	1	2	2	3	3	1	1	1
2	2	2	2	2	2	2	2	2	2
2	1	1	2	3	1	2	2	1	1
2	3	2	2	3	2	1	3	1	3
9	9	9	9	9	9	9	9	9	1
3	3	3	3	3	3	3	3	3	3
2	3	2	1	3	2	2	2	2	2

1	1	1	1	1	1	1	1	1	1
2	2	2	2	2	2	2	2	1	2
2	1	2	3	1	2	3	2	2	3
3	9	9	1	9	3	2	1	1	1
3	2	3	2	9	3	1	1	1	2
3	3	3	3	3	3	3	2	3	9
1	1	1	1	1	1	1	2	2	2
3	3	3	3	3	3	3	3	3	3
3	2	2	2	2	3	2	1	2	2
3	1	2	3	2	1	2	1	3	2
2	2	2	2	2	2	2	2	2	2
2	1	3	1	2	3	2	1	2	3
2	2	3	2	3	2	3	1	9	9
3	1	2	3	1	1	3	1	1	1
2	2	2	2	2	2	2	2	2	2
3	2	2	2	3	3	3	3	3	3
1	1	1	1	1	1	1	1	1	1
1	2	3	1	2	3	1	2	2	2
9	9	9	9	9	9	9	9	9	9
1	2	1	2	3	1	2	3	1	2
2	3	2	1	2	1	2	1	3	2
3	3	3	2	3	2	3	2	3	2
2	2	2	2	2	2	2	1	1	1
3	3	1	2	2	2	3	1	1	1
1	2	3	1	2	3	1	1	2	3
2	2	2	3	2	3	3	2	1	1
2	2	2	2	2	2	2	1	1	2
2	1	3	1	2	3	2	1	2	3
3	2	1	2	1	3	1	1	2	3
2	2	2	2	2	2	2	2	2	2
3	1	2	3	1	1	1	3	3	3
2	2	2	2	2	2	2	2	2	2
2	2	2	2	2	2	2	2	2	2
3	3	1	1	2	3	3	1	1	1
1	2	3	1	2	3	1	1	2	3
1	2	2	1	2	2	1	1	2	3
1	3	2	1	2	3	1	1	1	2
3	3	3	3	3	3	3	3	3	3
2	2	1	1	1	1	9	1	2	2
2	3	2	3	1	2	1	2	1	3
2	2	2	2	2	2	2	1	1	1
1	2	3	1	2	3	1	1	2	3
1	1	2	2	3	2	3	1	2	3
1	1	1	1	1	1	1	1	1	1
3	3	2	3	3	3	2	2	3	3
2	1	2	3	1	2	3	1	2	2
3	1	3	1	1	3	1	1	2	1

1	1	2	2	2	2	2	2	2	2
3	1	2	3	2	1	2	1	2	3
2	1	2	1	2	1	2	2	2	3
1	1	1	1	1	1	1	1	1	1
1	2	3	1	2	3	1	1	2	1
2	1	3	2	1	3	2	1	1	1
2	1	2	2	1	2	1	1	2	1
1	2	1	2	1	2	2	1	2	3
2	1	1	1	2	3	3	1	1	2
3	3	3	3	3	3	3	3	3	2
2	3	2	3	1	2	3	1	3	2
2	1	2	1	1	3	1	1	2	2
1	1	1	1	1	1	1	1	1	1
1	1	3	1	1	1	3	1	3	1
2	3	1	1	2	2	3	1	2	3
1	1	1	1	3	1	1	1	1	3
1	2	1	2	3	2	3	1	1	1
3	3	3	3	3	3	3	1	1	1
2	1	3	2	1	3	2	1	2	3
2	1	2	3	1	2	3	2	3	1
3	3	3	3	3	3	3	2	2	2
3	3	3	3	3	3	3	3	3	3
2	1	3	1	2	1	3	3	3	2
1	1	2	1	1	1	1	1	1	2
2	2	2	2	2	2	2	2	2	2
2	2	2	2	2	2	2	2	2	2
3	1	1	2	2	1	3	1	1	1
2	1	3	2	1	3	2	2	1	1
2	2	2	2	2	2	2	2	2	2
9	9	9	9	9	9	9	9	9	9
9	9	9	9	9	9	9	9	9	9
9	9	9	9	9	9	9	9	9	9
1	3	3	3	3	3	3	2	2	3
9	9	9	9	9	9	9	9	9	9
3	3	2	2	2	2	2	2	2	2
9	9	9	9	9	9	9	9	9	9
2	2	2	2	2	2	2	1	1	1
9	9	9	9	9	9	1	9	1	9
9	9	9	1	1	2	1	9	9	1
3	2	3	2	3	1	2	2	2	2
9	9	9	9	9	9	9	9	9	9
2	2	2	1	2	2	1	1	1	1
3	2	3	2	3	2	2	3	3	3
2	3	2	1	2	2	1	3	3	3
1	2	2	1	2	3	1	1	1	1
1	3	1	2	1	2	1	3	1	2
3	1	1	2	2	1	3	1	1	1

1	2	2	1	2	3	2	1	1	1
2	2	2	2	1	1	2	1	2	2
9	9	9	9	9	9	9	9	9	9
2	1	2	3	3	2	1	2	1	2
9	2	1	3	2	1	9	2	1	1
1	3	2	1	2	1	1	1	2	1
9	9	9	9	2	9	9	9	9	1
3	2	1	2	3	2	1	1	2	3
2	3	3	1	1	1	1	1	2	1
3	3	3	3	3	3	3	3	3	3
2	3	1	2	3	1	2	2	3	1
1	2	1	2	1	3	2	3	2	1
2	1	3	1	2	1	3	1	3	2
2	2	2	2	2	2	2	2	2	2
2	2	2	2	2	2	2	2	2	2
9	9	9	9	9	9	1	3	9	9
2	2	2	2	2	2	2	2	2	2
2	2	2	2	2	2	2	2	2	2
2	2	2	2	2	2	2	2	2	2
2	2	2	2	2	2	2	2	2	2
1	1	1	1	9	1	1	1	1	1
1	1	1	1	9	1	1	1	1	1
1	1	1	1	9	1	1	1	1	1
2	2	3	1	3	1	1	1	1	1
1	3	3	1	3	1	1	1	1	1
1	3	3	1	3	1	1	1	1	1
2	2	2	2	1	2	1	2	2	2
2	3	2	2	1	3	1	1	1	3
2	2	1	2	2	1	1	1	1	1
2	2	1	1	3	1	1	1	1	1
1	9	2	1	9	1	1	1	1	1
9	9	9	9	9	9	9	9	9	1
2	2	1	1	2	1	1	1	1	1
3	3	3	1	3	1	1	1	1	1
2	2	2	2	2	2	2	2	2	2
3	3	2	1	1	2	2	3	3	2
3	3	2	1	1	2	2	3	3	2
1	1	3	9	2	1	9	9	1	1
3	1	9	1	9	1	1	1	1	1
2	2	1	1	1	1	3	1	1	1
1	3	3	1	3	1	1	1	1	1
9	1	1	1	9	1	9	1	1	1
2	2	2	2	2	9	1	1	1	1
1	3	3	1	1	1	1	1	1	1
2	2	2	2	1	2	2	2	1	1
1	2	1	1	1	9	1	1	1	1
1	2	1	2	3	1	2	2	1	1

3	3	2	1	1	2	2	3	3	2
1	2	1	2	2	1	2	1	9	3
1	3	3	1	3	1	1	1	1	1
1	1	1	1	1	1	1	1	1	1
2	3	2	3	9	1	1	1	1	1
2	3	2	3	3	1	1	1	1	1
2	1	2	2	2	2	2	2	2	2
2	1	1	1	3	1	1	1	1	1
9	9	9	9	9	9	9	9	9	9
2	2	2	1	2	1	1	2	2	1
1	3	1	1	2	2	2	1	1	1
2	2	2	2	2	2	2	1	1	2
2	3	2	3	3	1	1	1	1	1
2	3	3	2	3	1	1	1	1	1
2	3	3	2	3	1	1	1	1	1
2	3	3	2	3	1	1	1	1	1
2	3	3	1	3	2	1	1	1	2
3	3	1	1	1	9	3	1	3	2
2	3	3	2	3	1	1	1	1	1
2	2	2	3	2	1	1	1	2	1
1	1	1	3	2	1	3	1	2	3
3	3	3	3	2	2	2	1	2	9
1	1	1	1	3	1	1	1	1	1
3	3	1	1	3	1	1	3	3	1
2	1	1	1	1	1	1	3	1	1
3	3	2	1	3	1	1	1	1	1
3	3	3	3	3	3	3	3	3	3
3	3	3	3	3	1	1	1	1	1
2	2	3	1	3	1	2	1	1	2
2	1	3	2	2	1	1	1	1	1
2	1	2	2	1	1	1	1	1	1
3	3	3	3	3	1	3	1	1	3
9	9	9	9	9	9	9	1	1	9
2	2	9	9	3	1	1	1	1	1
3	3	3	2	3	1	1	1	1	1
3	3	3	2	3	1	1	1	1	1
2	2	9	1	3	1	9	9	1	1
3	9	3	1	3	1	1	1	1	1
3	3	3	1	3	9	1	1	1	1
1	3	3	1	3	1	1	1	1	1
2	3	3	1	3	1	1	1	1	1
3	3	3	3	1	1	1	1	3	1
9	1	1	9	9	9	9	3	3	1
3	3	3	1	3	1	1	1	1	1
3	2	2	2	1	1	1	1	1	1
2	2	3	1	3	1	1	1	1	1
9	9	1	1	3	1	1	1	1	1

1	2	3	1	3	1	1	1	1	3
3	3	3	1	9	1	1	1	1	1
1	2	3	1	3	1	1	1	1	1
1	1	1	1	1	3	3	1	1	1
1	3	3	1	3	1	1	1	1	1
1	1	1	1	1	2	1	1	1	1
3	1	1	1	1	1	1	9	9	9
3	3	9	1	1	1	9	1	3	9
9	3	3	1	1	1	3	1	3	2
1	3	3	1	3	1	1	1	1	1
3	3	3	1	3	2	2	1	1	1
9	9	9	9	9	1	9	1	1	1
1	1	3	1	3	1	1	1	1	1
3	2	2	2	3	2	1	1	1	1
3	2	1	2	2	2	2	2	2	2
2	2	3	2	2	1	1	1	1	1
9	9	9	9	9	1	9	9	1	1
9	9	9	9	9	1	9	9	1	1
3	2	2	2	1	1	2	1	1	1
2	2	2	2	2	2	2	1	1	1
2	3	3	1	3	1	1	1	1	1
1	2	2	3	3	1	2	1	1	1
3	3	3	2	3	1	3	3	1	1
9	1	2	2	9	1	1	1	1	1
3	1	3	1	1	1	1	3	1	1
3	1	3	1	1	1	1	3	1	1
3	2	2	1	3	2	1	1	2	3
2	2	2	2	2	2	2	2	2	1
1	2	2	2	2	2	2	1	2	1
2	1	2	1	3	1	1	1	1	1
2	2	2	2	2	1	2	2	1	2
2	3	1	3	1	1	1	1	1	9
2	1	2	1	3	1	2	1	1	1
1	3	3	1	3	1	1	1	1	2
1	1	3	3	1	1	1	3	3	3
1	2	1	2	2	3	2	1	1	1
9	9	9	9	9	9	9	1	1	1
1	2	1	1	1	1	1	1	1	2
1	1	2	1	1	1	1	1	1	2
1	1	3	2	2	1	1	1	1	1
1	3	3	1	3	1	1	1	1	1
1	3	1	3	3	1	1	1	1	1
2	2	2	2	3	2	2	1	2	2
2	2	2	2	2	2	2	9	9	9
2	2	2	2	3	2	2	9	2	2
1	1	3	3	3	1	1	1	1	1
1	2	1	1	2	1	1	1	1	1

1	2	1	1	3	1	1	1	1	1
2	3	2	1	1	1	1	1	1	1
2	2	2	2	2	1	1	1	1	1
2	2	2	1	2	1	1	1	1	1
1	1	1	1	1	1	9	1	1	1
3	2	3	3	2	1	1	2	2	3
2	2	2	2	2	2	2	9	1	1
9	9	9	9	9	9	9	1	1	1
9	9	9	9	9	9	9	9	9	9
2	2	3	2	3	1	1	1	1	1
3	3	1	1	3	1	1	1	1	1
3	3	3	3	3	1	1	1	1	1
2	2	2	2	2	2	2	2	2	2
3	3	1	2	1	2	1	1	1	1
2	2	2	2	2	2	2	9	1	1
9	9	9	9	9	1	9	1	9	9
1	3	2	1	3	1	1	1	1	2
2	2	2	2	2	1	1	1	1	1
2	3	3	1	3	1	1	1	1	1
2	1	2	2	3	1	1	1	1	1
9	9	9	9	9	2	9	1	9	9
1	3	3	1	3	1	1	1	1	3
9	9	9	9	9	9	9	1	2	2
1	2	3	1	2	1	1	1	1	1
2	2	3	2	3	1	1	1	1	1
2	3	2	2	2	1	1	1	1	1
1	2	2	1	9	1	1	1	1	1
9	9	9	9	9	9	9	9	9	9
9	9	9	9	9	9	9	9	9	9
1	2	3	1	3	1	2	1	1	2
1	1	3	1	3	1	1	1	1	1
1	3	3	1	3	1	3	1	1	1
3	1	9	9	9	9	9	1	1	1
9	9	9	9	9	9	9	1	1	1
1	3	3	1	3	1	1	1	1	1
3	3	2	1	3	1	2	2	1	1
1	2	3	1	3	1	1	1	1	1
1	1	1	1	1	1	1	1	1	1
3	3	3	1	3	1	1	1	1	1
1	2	3	1	3	1	1	1	1	1
1	2	3	1	3	2	2	2	2	2
1	2	3	1	3	1	2	2	1	1
1	3	1	1	3	1	1	1	1	1
3	9	9	9	9	9	9	9	9	9
3	3	2	2	2	1	1	1	1	2
3	3	3	3	3	3	3	2	2	1
3	3	3	2	2	2	1	9	3	1

1	1	1	1	1	1	1	1	1	1
3	9	9	9	9	9	9	1	1	1
9	9	9	9	9	9	9	9	9	9
3	3	3	2	3	1	1	1	1	1
1	3	9	1	1	1	2	9	9	1
2	2	2	1	2	1	1	1	1	2
1	2	2	2	3	1	1	9	1	9
2	3	2	1	3	1	1	1	1	3
9	2	9	9	9	9	9	9	2	9
3	3	3	2	2	3	2	1	1	2
3	1	1	1	3	1	1	1	1	3
2	2	2	2	2	2	2	2	2	2
1	2	3	2	2	1	1	1	1	1
9	1	9	9	9	9	1	9	1	1
1	2	1	2	2	1	1	1	1	2
2	3	3	2	3	1	2	1	1	2
9	9	9	9	9	9	9	1	1	9
9	9	9	9	9	1	9	1	1	1
1	3	3	1	3	1	1	1	1	1
2	2	2	2	3	1	2	2	1	1
1	3	3	1	3	1	1	1	1	1
3	3	3	3	3	1	3	1	1	1
3	3	3	3	3	1	3	1	1	1
1	3	3	1	2	1	1	1	1	1
2	2	2	1	3	1	1	1	1	9
3	1	1	2	2	1	1	1	1	3
2	2	3	1	2	1	1	1	1	1
1	1	3	2	1	1	1	2	1	3
3	1	1	2	2	1	1	1	1	3
2	1	1	1	1	1	1	1	1	3
1	2	2	3	3	1	1	2	1	1
1	2	2	1	1	3	1	1	1	1
1	3	3	1	9	1	1	1	1	3
1	2	2	1	1	1	1	1	1	2
1	1	3	2	3	1	1	1	1	1
9	9	9	9	9	1	9	9	1	9
3	3	3	2	9	1	1	1	1	3
9	1	9	9	9	1	9	1	1	9
2	2	2	2	2	2	2	2	1	1
2	2	2	2	2	1	2	2	2	1
2	3	2	2	3	3	2	1	3	2
3	3	3	1	1	3	1	1	1	3
9	9	9	9	9	9	9	9	1	9
9	9	9	9	9	9	9	9	1	9
1	3	3	1	3	1	1	1	1	1
1	3	3	3	3	1	1	1	1	1
9	9	9	9	9	3	1	9	1	1

1	3	3	3	3	1	1	1	1	1
2	1	1	1	3	1	1	1	1	1
2	2	2	1	9	1	1	1	1	9
1	2	3	1	2	1	1	1	1	1
2	2	3	3	3	2	2	1	1	1
2	2	3	1	3	1	1	1	1	1
2	2	2	3	2	2	2	1	1	2
2	2	2	2	2	2	2	2	1	2
2	2	2	2	2	2	2	2	1	1
2	2	2	2	2	1	1	1	1	2
2	2	2	1	3	1	1	1	1	1
9	9	9	9	9	9	9	9	1	9
9	9	9	9	9	9	9	1	9	9
9	9	9	9	9	9	9	9	9	9
9	9	9	9	9	9	9	9	9	9
9	9	9	9	9	9	9	9	9	9
3	2	3	2	3	1	3	9	3	1
3	3	3	2	2	3	2	1	3	2
9	9	9	9	9	9	9	9	9	1
2	2	3	2	3	1	1	1	1	1
2	3	3	1	2	1	1	1	1	1
9	9	9	9	9	9	9	1	1	2
3	2	2	1	1	1	1	1	1	2
3	3	2	2	3	2	3	2	1	1
3	2	2	1	3	1	1	1	1	2
1	3	3	1	3	1	1	1	1	1
1	2	9	3	3	1	1	1	1	1
3	3	3	2	3	2	2	3	1	2
2	2	2	1	3	1	1	1	1	1
1	3	3	1	3	1	1	1	1	1
2	2	2	2	2	1	1	1	1	1
9	1	9	1	9	1	1	9	1	1
3	3	3	1	3	1	2	1	1	1
2	3	1	2	2	1	1	1	1	1
2	2	3	1	3	1	1	1	1	3
1	1	1	3	3	2	1	2	1	1
2	2	2	2	2	1	2	2	1	1
1	3	3	1	3	1	2	1	1	1
9	9	9	9	9	9	9	9	9	9
1	3	3	1	3	1	1	9	1	1
2	3	2	3	2	2	3	2	3	2
2	3	2	2	3	1	1	2	3	2
2	2	3	1	3	1	2	1	1	1
1	2	2	1	2	1	1	1	1	1
2	3	3	2	3	1	2	1	1	1
1	3	3	1	2	1	1	1	1	1
3	3	3	1	1	1	1	1	1	1

3	3	3	3	3	1	1	1	1	1
2	2	1	1	1	1	1	1	1	1
2	2	3	3	3	1	1	1	1	2
1	3	3	1	3	1	1	1	1	1
2	2	3	3	3	1	1	9	9	9
2	2	2	1	3	1	1	1	1	1
3	3	3	2	2	1	1	1	1	3
2	3	3	1	3	1	1	1	1	1
2	2	2	2	2	3	3	2	2	2
2	3	1	2	1	1	1	1	1	1
2	2	3	2	2	1	1	1	1	1
2	2	2	2	2	2	2	2	2	2
1	3	3	1	2	1	1	1	1	2
2	2	2	2	2	1	2	1	1	1
2	1	2	2	2	1	1	1	1	2
1	2	2	1	2	1	1	1	1	2
2	2	2	2	2	2	2	1	1	1
2	2	2	2	2	2	2	2	2	9
2	3	3	2	2	2	2	1	2	2
3	3	3	3	3	1	1	1	1	1
3	3	3	3	3	1	1	1	1	1
1	3	3	1	3	1	1	1	1	2
1	2	3	2	3	1	1	1	1	1
2	2	2	2	1	2	2	3	3	1
2	2	2	1	2	1	1	1	1	1
9	9	9	9	9	9	9	9	9	9
9	9	9	9	9	9	9	9	9	9
9	9	9	9	9	9	9	1	9	9
2	3	3	1	3	1	1	1	1	2
9	2	3	9	2	3	3	1	1	1
1	2	3	1	3	1	1	1	1	1
1	2	3	1	3	1	1	1	1	1
9	9	9	9	9	1	9	9	1	9
9	9	9	9	9	9	1	9	1	9
9	9	9	9	9	9	1	9	1	9
9	9	9	9	9	9	1	9	1	9
9	9	9	9	9	1	9	9	1	9
9	9	9	9	9	1	9	9	1	9
9	9	9	9	9	9	9	1	9	9
3	2	3	1	3	1	1	1	1	2
2	3	3	1	3	1	1	1	1	2
9	9	9	9	9	9	9	9	1	9
9	9	9	9	9	9	9	9	1	9
2	3	2	2	2	1	2	1	1	1
9	9	9	9	9	9	9	9	9	9
9	9	9	9	9	9	9	9	9	9
9	9	9	9	9	9	9	9	9	9

1	2	3	1	3	1	1	1	1	1
9	9	9	9	9	9	9	9	9	9
9	9	9	9	9	9	9	9	9	9
3	3	3	2	2	1	2	9	1	3
3	3	3	2	2	1	2	9	1	3
9	9	9	9	9	9	9	9	9	9
9	9	9	9	9	9	9	9	1	9
9	9	9	9	9	9	9	9	1	9
9	9	9	9	9	9	9	9	1	9
9	9	9	9	9	9	9	9	1	9
1	3	3	3	3	1	1	1	1	1
3	3	1	1	1	1	3	1	1	1
1	3	1	1	3	1	1	1	1	1
2	2	2	2	2	1	2	1	2	1
3	3	3	3	3	1	1	1	1	1
9	9	9	9	9	9	9	1	1	3
2	2	2	3	3	1	1	1	1	3
2	2	2	3	3	1	1	1	1	3
3	3	1	1	1	1	3	1	1	1
3	3	3	2	2	1	1	1	2	1
3	3	3	2	2	1	1	1	2	1
1	1	1	1	1	1	1	2	1	2
9	9	9	9	9	9	9	1	9	9
1	2	2	2	1	1	1	1	1	2
3	2	2	3	3	3	3	3	2	9
1	2	3	1	1	1	1	1	1	1
3	2	3	3	3	3	9	2	2	2
2	3	3	2	3	1	1	1	1	1
2	2	2	3	3	1	1	1	1	1
2	2	1	1	3	2	1	1	1	3
2	2	3	2	3	1	1	1	1	1
1	3	2	1	3	2	2	1	1	3
2	2	2	3	3	1	1	1	1	3
1	2	3	2	3	1	1	1	2	2
2	2	2	1	1	1	1	1	1	1
2	3	3	3	2	1	1	1	1	1
3	3	3	3	3	1	1	1	1	3
1	3	3	1	3	1	1	1	1	3
3	3	3	1	3	1	1	1	1	3
3	3	3	2	3	1	1	1	1	1
1	2	9	9	9	9	9	9	9	9
1	1	1	1	3	1	1	1	1	1
3	3	3	2	2	1	2	1	2	2
2	2	2	1	3	2	2	1	1	1
2	2	2	1	3	2	2	1	1	1
1	1	3	1	1	1	1	1	1	1
2	2	2	2	3	1	1	1	1	2

2	3	3	2	3	1	2	2	1	1
1	3	1	1	3	1	1	1	1	1
2	2	3	3	3	1	1	1	1	1
2	2	2	1	3	2	2	1	1	1
2	2	2	1	3	2	2	1	1	1
2	2	2	1	3	2	2	1	1	1
2	2	2	2	3	1	1	1	1	2
2	3	3	1	3	1	1	1	1	1
1	2	3	1	3	1	1	1	1	2
1	3	3	1	1	1	1	1	1	3
2	2	1	2	2	1	1	1	1	1
2	9	3	3	1	1	1	1	1	1
2	2	3	2	3	1	1	9	1	1
2	2	3	2	3	1	1	1	1	1
1	3	3	3	3	1	3	1	1	3
2	3	3	3	1	1	1	1	1	2
2	2	2	2	2	2	2	2	2	2
2	2	2	2	2	2	2	2	1	2
1	3	3	2	3	1	3	1	3	3
1	2	1	2	3	1	1	1	1	1
2	3	2	1	3	1	1	1	1	1
1	3	3	1	3	1	1	1	1	1
2	2	1	3	3	2	2	1	1	1
1	2	2	2	2	1	1	1	1	1
1	2	2	2	2	1	1	1	1	1
2	3	3	2	3	2	1	1	1	1
2	3	3	3	3	1	1	2	3	3
2	2	2	2	2	2	2	3	1	3
2	2	2	1	3	1	1	1	1	1
1	2	2	1	2	1	2	1	1	2
3	2	3	2	2	3	2	2	2	3
2	3	2	3	3	1	1	1	1	1
1	3	3	1	2	1	1	1	1	1
2	2	2	1	1	1	1	1	1	3
2	2	1	3	2	2	2	1	1	1
2	2	1	3	3	2	2	1	1	1
3	2	2	2	2	2	2	2	2	1
3	2	2	2	2	2	2	2	2	1
1	3	1	1	1	1	1	1	1	1
2	3	1	3	3	1	1	1	1	1
2	3	2	1	1	1	1	1	1	1
1	3	3	1	3	1	1	1	1	3
1	3	3	1	3	1	1	1	1	3
2	2	2	2	2	1	1	1	1	1
3	3	1	1	3	1	1	1	1	1
2	2	2	1	3	2	1	1	1	1
3	3	3	3	3	9	1	1	1	1

2	2	2	2	2	2	2	3	2	2
2	2	1	1	2	2	2	1	1	9
3	3	3	3	3	1	1	1	1	1
3	3	2	1	2	1	2	1	1	2
2	3	2	1	3	1	1	1	1	1
2	3	2	2	3	2	2	1	1	2
3	3	2	1	3	1	2	1	1	3
2	2	2	2	2	2	2	1	1	1
3	2	1	2	1	1	1	2	1	2
2	3	3	1	3	1	1	1	1	3
2	2	2	2	2	2	2	1	1	2
3	3	3	1	3	1	2	1	1	3
9	9	9	9	9	9	9	1	9	9
3	2	2	1	2	1	2	1	1	3
3	3	3	3	3	1	1	1	1	1
2	2	2	2	2	2	2	2	2	2
2	3	3	1	3	1	1	1	1	3
2	3	3	1	3	1	1	1	1	1
2	3	1	1	3	1	1	1	1	1
2	2	2	2	3	1	1	1	1	2
3	1	1	3	3	1	1	1	1	1
3	3	3	2	2	1	1	1	2	1
1	3	1	1	3	1	1	1	1	1
2	3	3	1	3	1	2	1	1	2
2	2	2	2	2	1	1	2	1	1
2	3	3	2	3	1	1	1	1	1
1	3	3	1	3	1	1	1	1	3
3	2	3	1	3	1	1	1	1	2
2	3	2	2	2	1	2	1	1	1
9	9	9	9	9	9	9	9	1	9
1	3	1	2	2	2	1	1	1	2
9	9	9	9	9	9	9	1	1	1
3	1	2	2	2	2	2	1	1	1
1	2	1	2	3	2	1	2	1	1
2	3	3	2	1	1	1	1	1	1
1	2	1	1	1	1	1	1	1	1
2	2	2	2	1	2	1	9	9	9
9	9	9	2	9	9	9	9	9	9
1	2	1	3	2	3	1	2	3	1
2	2	2	2	2	2	1	2	2	2
2	2	2	2	2	2	2	1	1	1
3	3	3	1	3	3	1	1	1	1
1	3	3	1	2	1	1	1	1	1
2	9	3	9	9	1	2	2	1	1
2	2	2	1	2	2	3	2	2	2
2	2	2	2	2	1	1	2	1	1
2	1	3	3	1	1	1	1	1	2

3	3	2	1	2	1	1	1	1	1
2	2	2	2	1	1	1	1	1	1
2	2	2	2	2	2	2	1	1	1
2	2	2	1	2	1	2	2	1	1
1	2	2	2	1	2	2	1	2	2
2	3	3	1	3	2	2	1	1	1
2	3	1	1	2	1	1	1	1	1
9	9	9	9	9	9	9	9	9	9
3	3	2	2	2	1	1	1	1	1
2	2	2	2	2	1	1	1	1	1
1	2	1	1	2	3	2	1	1	1
1	2	3	2	3	2	3	2	3	2
1	2	2	2	2	2	2	1	1	1
2	2	2	2	2	2	2	2	2	2
3	3	3	1	3	3	1	1	1	2
3	3	3	1	3	3	1	1	1	2
1	2	2	2	2	1	1	1	1	1
1	1	1	3	1	1	2	1	1	1
2	3	2	1	3	1	3	9	9	9
2	2	1	1	9	1	1	1	1	1
2	2	3	1	3	3	1	1	1	2
1	1	1	1	1	1	1	1	1	1
2	2	2	2	2	2	2	2	2	2
1	1	1	1	3	1	1	1	1	1
2	3	3	1	3	1	2	1	1	2
1	1	1	1	1	1	1	1	1	1
2	1	1	2	1	1	1	1	1	1
2	1	1	1	2	1	1	1	2	3
2	1	1	1	2	1	1	1	2	3
9	1	9	9	9	9	9	9	9	1
2	1	1	1	2	1	1	2	1	3
1	1	1	1	2	1	1	1	1	1
1	1	1	1	2	1	1	1	1	1
2	2	2	2	3	1	2	1	1	1
9	9	9	9	9	9	9	9	9	9
1	2	1	1	1	1	1	1	1	1
1	2	1	1	1	1	1	1	1	1
2	9	9	9	1	1	1	1	1	1
3	2	3	3	1	1	1	1	1	1
1	2	1	1	3	1	1	1	1	1
2	2	2	1	3	1	2	1	1	1
1	1	1	1	3	1	2	1	1	1
2	2	2	2	2	2	2	2	1	2
1	3	3	3	1	3	1	1	1	1
3	3	3	1	3	1	1	1	1	3
1	1	1	1	1	1	1	1	1	1
2	3	3	1	3	2	2	1	1	1

3	3	1	1	3	1	1	1	1	1
3	3	1	1	3	1	1	1	1	1
2	3	2	1	3	1	1	1	1	3
1	1	1	1	2	1	1	1	1	1
2	3	3	1	1	1	1	1	1	1
2	3	3	3	1	1	1	1	1	1
1	1	1	3	3	1	1	9	1	1
2	2	2	2	2	1	1	1	1	1
3	3	1	9	1	1	9	1	1	1
1	1	1	3	1	1	1	1	1	1
1	3	3	3	3	1	1	1	1	1
1	1	1	1	3	3	1	1	1	1
1	1	1	1	3	3	1	1	1	1
3	3	1	1	1	1	3	1	1	1
1	2	3	1	3	1	2	1	1	2
1	3	3	1	3	1	1	1	1	1
1	2	1	1	3	2	1	2	2	1
1	2	3	1	2	1	1	1	1	1
1	3	3	1	3	1	1	1	1	1
1	3	3	3	3	1	3	9	9	9
1	1	1	2	2	1	1	1	1	1
1	3	3	1	3	1	1	1	1	2
3	3	3	2	2	1	2	9	1	3
3	3	3	2	2	1	2	9	1	3
9	9	9	9	9	9	9	9	9	9
9	9	9	9	9	9	9	9	1	9
1	3	3	1	3	1	1	1	1	1
9	9	9	9	9	9	1	9	1	9
9	9	9	9	9	9	9	1	9	9
9	9	9	9	9	9	9	9	9	1
9	9	9	9	9	9	9	1	9	9
9	9	9	9	9	9	9	1	9	9
9	9	9	9	9	9	9	3	9	9
9	9	9	9	9	9	9	1	9	9
2	1	2	1	3	3	1	1	1	1
1	3	3	1	3	1	2	1	1	1
1	2	2	1	2	1	1	1	1	1
1	1	1	3	1	1	2	1	1	1
2	1	1	2	1	1	1	1	1	1
2	1	1	2	1	1	1	1	1	1
2	9	3	3	1	1	1	9	9	9
3	1	1	1	1	1	1	9	9	2
2	3	2	2	2	1	1	1	1	1
2	2	2	1	2	2	2	1	1	1
1	1	3	2	2	2	2	1	1	2
1	3	1	1	3	1	1	1	1	1
1	9	9	2	2	9	1	1	1	1

2	2	1	1	2	1	1	1	1	1
2	2	2	2	2	2	2	1	1	1
3	3	2	2	2	1	1	1	1	3
2	2	2	3	1	2	1	2	2	3
1	1	2	2	2	1	1	1	1	2
2	3	3	1	3	1	1	1	1	1
9	9	9	9	9	9	9	9	9	9
2	2	2	2	2	2	2	1	1	2
1	2	2	2	2	1	3	1	1	1
2	2	2	2	3	3	1	2	2	2
2	2	2	2	2	2	2	1	1	1
1	3	3	1	3	1	1	1	1	1
2	3	2	3	2	2	2	2	3	2
3	3	3	3	3	3	3	3	2	2
1	2	2	2	2	2	2	1	2	1
1	3	1	1	1	1	1	1	1	1
9	9	9	9	9	9	9	9	9	9
3	2	1	2	3	2	2	1	2	1
2	1	1	2	1	2	1	1	2	1
1	1	1	1	1	1	1	1	1	1
1	1	1	1	1	1	1	1	1	1
2	2	2	2	2	2	2	2	2	2
1	2	2	2	2	1	3	2	1	1
2	2	2	2	2	2	2	2	2	2
1	1	1	1	1	1	1	1	1	1
2	3	2	1	3	2	1	1	2	3
2	3	1	2	3	2	1	1	1	1
1	2	1	2	2	1	1	1	1	1
2	2	2	2	2	2	2	1	1	1
2	2	2	2	2	2	2	1	1	1
3	1	1	1	1	1	1	1	1	1
2	2	2	2	2	2	2	1	1	1
1	2	2	2	2	2	2	1	2	1
1	2	2	1	2	1	2	1	1	1
9	9	9	9	9	1	1	9	9	9
1	1	1	1	1	1	1	1	1	1
2	2	2	2	2	2	2	2	2	2
2	2	2	2	2	1	2	1	1	1
1	3	3	1	2	1	2	2	1	1
2	2	3	2	3	9	1	1	1	1
2	2	2	2	2	1	2	1	1	1
2	2	2	2	2	1	2	1	1	1

Alcohol	OverFourB	Hightfatty	BirthPill	Spiritualat	FamilyHxC	VaginalWa	UrineInfect	STD	Poison
2	2	2	2	2	2	2	2	2	2
3	2	3	2	3	2	3	2	3	2
2	2	2	2	2	2	2	2	2	2
2	2	2	2	2	2	2	2	2	2
2	1	1	2	2	2	3	2	2	3
2	2	2	2	2	2	2	2	2	2
2	1	2	3	2	1	3	2	1	2
3	3	2	2	2	2	3	2	2	2
9	1	1	2	9	1	2	1	2	3
9	1	3	1	2	3	2	3	2	1
2	2	2	2	2	1	2	2	2	2
2	2	2	3	2	2	2	2	2	2
3	3	3	1	1	1	1	2	1	2
2	3	3	3	3	2	2	2	2	2
2	2	2	2	2	2	2	2	2	2
2	2	2	2	2	2	2	2	2	2
9	9	9	9	9	9	9	9	9	9
1	2	3	2	2	2	2	2	2	2
9	9	9	9	9	9	9	9	9	9
9	9	9	9	9	9	9	9	9	9
1	1	1	1	1	1	1	1	1	1
2	2	2	2	2	2	1	2	1	1
2	1	2	2	2	3	1	1	1	2
1	1	1	1	1	1	1	1	1	1
9	9	9	9	9	9	9	9	9	9
1	2	2	1	1	1	1	2	2	3
1	3	3	1	3	1	3	3	1	3
2	2	2	2	2	2	2	2	2	2
2	1	2	3	1	2	3	1	2	3
2	1	1	2	2	3	1	2	3	1
2	2	3	3	3	1	2	1	3	3
1	3	2	2	2	2	2	2	1	2
3	3	3	3	3	3	3	3	3	1
2	1	1	1	1	1	1	2	1	1
1	2	2	2	1	1	1	2	2	2
2	2	3	3	3	3	2	2	2	2
2	2	2	2	2	2	2	2	2	2
1	2	1	3	3	3	1	1	2	3
1	2	2	1	3	3	1	1	1	3
2	2	2	2	2	3	3	9	1	3
2	2	2	2	2	2	2	2	2	3
9	9	1	9	2	9	9	9	9	9
9	9	9	9	9	9	9	9	9	9
2	2	2	2	2	2	2	1	2	2
1	2	2	2	2	3	2	2	2	2
9	9	9	9	9	9	9	9	9	9

2	2	2	2	2	3	3	2	2	3
2	2	2	2	1	2	3	2	3	1
2	1	1	2	2	2	2	2	2	1
2	2	1	2	2	2	1	1	2	2
3	3	3	3	3	3	1	1	1	3
3	3	3	3	3	3	1	1	1	3
2	2	2	3	2	2	3	2	2	3
2	2	2	2	2	2	2	2	2	2
1	1	1	1	1	1	1	1	1	1
2	3	1	2	3	1	2	3	1	2
9	9	9	9	9	9	1	9	9	9
2	2	2	1	1	2	3	2	2	2
2	2	1	1	1	1	1	2	2	2
2	2	3	3	2	2	2	1	1	1
2	2	1	1	1	2	3	2	3	2
3	2	2	1	1	2	2	3	2	2
1	1	2	3	3	3	3	2	2	2
3	2	3	2	9	1	1	1	3	1
2	2	2	2	2	2	2	2	2	2
2	2	3	9	2	2	3	3	9	2
2	2	2	2	2	2	2	2	2	2
1	2	3	3	3	2	3	2	1	3
1	1	2	2	3	3	2	2	2	2
2	1	1	2	2	1	2	2	1	1
1	2	2	1	2	2	3	3	2	2
1	2	2	2	1	2	1	1	1	2
3	3	3	1	3	3	1	1	1	3
3	3	1	1	1	1	2	2	1	1
2	3	1	2	2	1	1	1	1	2
3	3	3	1	3	3	3	1	1	3
9	9	9	9	9	9	9	9	9	9
2	3	3	2	1	3	2	2	2	3
9	9	9	9	9	2	1	9	1	9
2	2	2	2	2	2	2	2	2	2
3	3	3	9	9	3	1	2	2	9
1	2	2	2	2	2	2	2	2	2
1	2	2	2	9	2	1	1	1	1
2	3	2	2	1	2	2	3	2	2
2	2	1	2	3	2	2	1	3	3
2	2	2	1	1	2	2	2	2	2
1	2	2	2	1	2	1	2	1	1
9	9	9	9	9	9	9	9	9	9
3	9	9	9	1	1	1	1	1	1
2	2	2	3	2	2	1	1	2	2
1	2	2	2	1	1	2	2	1	1
1	2	3	2	3	2	3	2	1	2
3	3	2	2	2	2	2	2	3	3

1	2	3	1	1	2	2	1	3	2
3	3	3	3	3	3	3	3	3	3
1	2	1	2	1	1	2	2	1	1
2	1	1	2	1	2	3	2	1	2
1	1	1	1	1	1	1	1	1	1
2	1	1	2	1	3	2	1	1	2
1	2	2	2	2	2	2	2	2	3
9	9	9	9	9	9	9	9	9	9
1	1	2	2	2	2	1	1	2	2
1	3	2	2	3	2	1	1	1	3
1	2	3	2	2	3	1	1	1	1
2	1	1	2	3	2	2	1	2	2
2	2	2	1	1	2	2	1	2	2
2	2	2	2	1	2	2	2	2	2
2	2	3	3	1	1	2	2	1	1
2	2	1	1	3	2	2	1	9	2
2	1	1	1	1	2	1	1	1	1
1	3	3	2	3	2	2	2	2	3
2	2	9	2	2	9	1	2	1	2
2	1	1	2	2	1	1	2	2	2
3	1	3	1	3	1	3	1	3	1
1	2	2	2	2	1	1	2	2	2
1	1	1	2	1	1	1	2	1	1
2	2	2	2	2	2	2	2	2	2
2	2	2	2	2	2	2	2	2	2
2	2	1	3	3	3	1	2	2	3
1	3	1	3	1	3	1	1	1	1
2	1	2	1	2	1	2	1	2	1
2	2	1	2	2	1	1	1	1	2
1	3	2	2	1	3	2	2	1	3
1	2	3	2	1	2	2	3	3	3
1	2	2	2	2	2	3	3	3	3
2	3	1	1	2	1	9	1	2	2
2	1	2	1	2	1	2	1	1	2
2	2	1	9	3	2	1	9	3	2
1	1	1	3	1	2	1	3	2	3
1	2	1	2	9	1	2	2	9	3
2	1	1	2	3	3	2	2	2	2
9	9	9	9	9	9	9	9	9	9
3	3	3	3	3	3	3	3	3	3
2	3	2	1	2	1	2	2	1	1
9	9	9	9	9	9	9	9	9	9
2	1	1	2	2	2	1	1	2	2
2	2	3	2	2	2	1	2	2	2
9	9	9	9	9	9	9	9	9	9
1	2	3	3	3	3	2	2	2	2
9	1	1	2	1	1	1	2	1	1

3	3	9	1	1	1	2	3	2	3
1	1	1	1	1	3	1	1	1	1
3	3	3	3	3	3	3	3	3	3
2	2	2	2	2	2	2	2	2	2
2	2	2	2	3	3	2	2	2	3
2	3	2	3	2	3	2	3	2	2
1	3	1	3	1	3	3	1	9	1
1	3	2	1	3	1	3	1	9	1
9	9	9	9	9	9	9	9	9	9
2	2	2	2	2	2	2	2	2	2
2	2	2	2	3	3	3	3	2	2
2	2	2	2	2	1	1	1	1	2
2	2	2	2	2	2	2	2	2	2
1	2	1	2	3	2	2	2	2	2
3	2	2	2	2	1	2	2	2	2
2	3	2	1	2	3	2	2	2	2
1	1	1	2	2	1	1	2	1	1
2	1	2	2	1	2	1	2	2	2
9	9	9	9	9	9	9	9	9	9
1	3	1	2	2	1	2	3	2	1
1	2	2	2	1	2	2	1	1	1
1	2	2	2	3	2	2	1	1	2
3	3	3	3	3	3	3	3	3	3
1	9	2	1	1	1	1	1	1	2
1	1	2	3	3	3	2	2	2	1
2	1	3	2	3	3	3	3	1	1
2	3	3	2	9	9	9	9	9	9
2	3	3	3	3	3	3	3	3	3
1	2	2	3	3	2	2	1	2	2
2	3	3	3	3	3	3	3	3	2
1	1	1	2	2	2	1	1	2	2
2	2	1	1	2	2	1	1	2	2
2	1	1	2	2	2	1	1	2	1
3	3	1	1	1	1	3	3	3	3
1	1	1	1	1	3	3	2	2	2
3	3	1	1	1	1	1	1	1	1
3	3	3	3	3	3	3	3	3	3
3	3	3	1	3	3	1	1	1	3
1	3	3	3	1	1	1	3	2	1
2	2	2	2	2	2	2	2	2	2
9	9	9	9	9	9	9	9	9	9
2	2	2	2	2	2	2	2	2	2
2	2	2	2	2	2	2	2	2	2
9	9	9	9	9	9	9	9	9	9
9	9	9	9	9	9	9	9	9	9
3	3	3	3	2	2	1	2	1	2
3	3	3	3	3	2	1	2	2	3

1	9	9	9	9	9	9	9	9	9
9	9	9	2	9	9	9	9	9	9
9	9	9	9	9	9	9	9	9	9
9	9	9	1	9	9	9	9	9	9
9	9	9	9	9	9	9	9	9	9
9	9	9	9	9	9	9	9	9	9
1	2	2	2	2	1	1	2	1	2
9	9	9	9	9	9	9	9	9	9
2	3	3	3	2	2	2	3	1	2
9	1	9	9	9	9	9	9	9	9
9	9	2	9	9	9	9	9	9	9
9	1	9	9	9	9	9	9	9	9
1	1	1	1	1	1	1	1	2	1
1	2	3	1	2	3	1	2	1	3
9	9	9	9	9	9	9	9	9	9
2	2	2	3	3	3	3	3	3	3
3	3	3	3	3	3	3	3	3	3
2	2	2	2	2	2	2	2	2	2
2	1	2	2	9	2	2	2	2	2
2	2	2	2	2	2	2	2	2	2
2	3	1	2	2	2	1	2	2	2
9	9	9	9	9	9	9	9	9	9
2	3	1	1	2	2	1	1	1	1
2	3	2	1	2	2	2	1	1	2
2	2	2	2	2	2	2	2	2	2
1	1	2	1	1	2	2	2	2	2
2	3	2	2	2	2	2	1	1	1
2	2	1	1	1	1	2	1	1	1
3	3	3	3	3	3	3	3	3	3
1	3	3	3	1	3	3	1	1	1
2	2	2	2	2	2	2	2	2	2
2	2	2	2	1	1	1	2	1	1
9	9	9	9	9	9	9	9	9	9
2	2	2	2	1	1	1	1	2	2
1	1	3	3	1	1	1	1	1	1
2	2	3	2	1	2	3	2	1	2
2	3	1	2	3	2	1	2	1	2
1	1	1	1	1	1	1	1	1	1
9	9	9	9	9	9	9	9	9	9
1	2	2	2	1	2	2	2	2	2
2	2	3	2	2	2	2	2	1	1
3	1	3	2	2	1	1	1	1	3
1	2	3	3	3	2	1	1	1	1
2	2	2	2	2	2	2	2	2	2
1	1	1	1	1	1	1	1	1	1
2	3	3	2	2	1	1	3	2	2
9	9	9	9	9	9	9	9	9	9

9	1	1	1	1	1	1	2	9	2
1	3	3	3	2	1	1	1	9	2
1	1	1	1	1	1	1	1	1	1
9	9	9	9	9	9	9	9	9	9
2	2	2	2	2	2	2	2	2	2
1	3	2	2	2	2	3	2	3	2
3	3	3	3	2	1	1	1	2	2
2	3	2	2	2	2	1	1	2	2
2	2	3	2	2	2	2	2	2	2
1	1	1	1	1	2	1	2	3	2
2	2	2	2	2	1	2	2	1	1
9	9	9	9	9	9	9	9	9	9
9	9	9	9	9	9	9	9	9	9
9	9	9	9	9	9	9	9	9	9
9	9	2	9	9	2	9	9	2	9
9	9	9	9	9	9	9	9	9	9
9	9	9	9	9	9	9	9	9	9
9	9	9	9	9	9	9	9	9	9
2	9	9	2	9	2	9	9	2	9
9	9	9	9	9	9	9	9	9	9
9	9	9	9	9	9	9	9	9	9
1	1	1	1	1	1	1	1	1	1
2	1	1	2	2	1	1	2	2	2
2	2	1	1	1	1	1	1	1	1
1	1	1	1	1	1	2	2	1	1
9	9	9	9	9	9	9	9	9	9
1	1	2	3	2	1	2	1	2	2
9	9	9	9	9	9	9	9	9	9
9	9	9	9	9	9	9	9	9	9
1	2	1	2	2	1	2	2	2	2
9	9	9	9	9	9	9	9	9	9
9	9	9	9	9	9	9	9	9	9
9	9	9	9	9	9	9	9	9	9
9	9	9	9	9	9	9	9	9	9
9	1	9	2	9	3	1	2	9	9
9	9	1	9	9	9	9	1	9	9
9	9	9	9	9	9	9	9	9	9
9	9	9	9	9	9	9	9	9	9
9	9	9	9	9	9	9	9	9	9
9	9	9	9	9	9	9	9	9	9
9	9	9	9	9	9	9	9	9	9
9	9	9	9	9	9	9	9	9	9
3	3	3	3	3	3	3	3	3	3
1	2	3	1	2	1	2	3	2	1
1	2	3	1	1	2	1	3	1	3
9	9	9	1	9	9	9	9	2	9
9	9	9	9	9	9	9	9	9	9

9	9	9	9	9	9	9	9	9	9
9	9	9	9	9	9	9	9	9	9
3	3	3	2	2	2	2	2	1	1
3	1	2	3	1	2	3	1	2	3
2	3	2	3	2	3	2	2	3	2
2	2	2	2	3	3	3	3	3	2
3	2	1	2	1	3	2	1	2	3
2	2	2	3	1	2	1	2	2	2
2	2	2	3	3	3	3	2	2	2
3	3	3	3	3	3	3	3	3	3
9	9	9	9	9	9	9	9	9	9
9	9	9	9	9	9	9	9	9	9
9	9	9	9	9	9	9	9	9	9
9	9	9	9	9	9	9	9	9	9
1	1	1	1	1	1	1	1	1	1
2	3	3	3	3	2	3	1	1	1
9	9	9	9	9	9	9	9	9	9
1	2	1	1	1	2	1	1	1	1
1	1	1	1	1	1	1	1	1	1
1	1	1	1	1	1	1	1	1	1
3	1	2	3	1	2	1	2	3	1
2	2	2	2	2	1	2	1	1	2
9	9	9	9	9	9	9	9	9	9
2	2	2	2	2	2	2	2	2	2
2	2	2	2	3	3	3	2	2	3
2	2	2	2	2	2	2	2	2	2
2	2	2	2	2	2	2	2	2	2
2	2	1	3	3	3	2	2	2	2
2	2	2	2	2	2	9	3	3	3
1	1	1	1	2	1	2	1	1	2
2	2	2	2	2	2	2	2	2	2
2	2	2	2	2	2	2	2	2	2
3	3	3	3	3	3	2	3	2	3
2	2	2	2	2	2	2	2	2	2
2	1	1	3	3	3	1	1	3	1
3	9	1	2	3	2	3	2	2	2
9	9	9	9	9	9	9	9	9	9
9	2	1	2	2	2	2	2	2	2
3	3	3	3	3	3	3	3	3	3
3	3	3	3	3	3	3	3	3	3
9	9	9	9	9	9	9	9	9	9
9	9	9	9	9	9	9	9	9	9
3	9	9	9	9	9	9	9	9	9
1	1	2	1	1	1	2	1	1	1
2	2	1	1	2	2	1	1	1	2
2	2	2	2	2	2	2	2	2	2
2	2	2	2	2	3	3	3	3	3

9	9	9	2	9	9	9	9	9	9
9	9	9	9	9	9	9	9	9	9
9	9	1	9	9	9	9	9	9	9
9	9	9	9	9	9	9	9	9	9
9	9	2	9	9	9	9	1	9	9
2	2	2	1	1	2	2	1	2	1
9	9	9	2	9	9	9	9	9	9
9	9	9	9	9	9	9	9	9	9
9	9	9	9	9	9	9	9	9	9
9	9	9	9	9	9	9	9	9	9
9	9	9	9	2	9	9	9	9	9
9	9	9	9	9	3	9	9	9	9
9	9	9	9	9	9	9	9	9	9
9	9	9	3	9	9	9	9	9	9
9	9	9	9	9	9	9	9	9	9
9	9	9	9	9	9	9	9	9	9
2	9	9	9	9	9	1	9	9	9
2	3	2	1	2	1	2	9	2	1
1	2	3	2	2	1	2	1	2	2
2	2	2	2	2	2	2	2	2	2
3	3	2	1	3	3	1	2	2	3
2	2	2	1	2	2	1	3	2	3
2	2	2	2	2	2	2	2	2	2
2	2	3	2	3	2	3	2	1	2
2	1	2	2	3	2	1	2	1	2
9	9	9	9	9	9	9	9	9	9
2	2	3	2	1	3	2	2	2	2
3	3	3	3	3	2	2	2	2	2
2	2	2	2	1	3	2	1	2	3
1	1	1	1	1	2	2	2	1	1
1	1	1	2	9	3	2	2	1	2
2	2	2	2	2	2	2	2	2	2
2	2	2	2	3	3	3	3	3	3
2	2	2	2	1	1	1	1	1	1
9	9	9	9	9	9	9	9	9	9
2	2	2	2	2	2	2	2	2	2
9	9	9	9	9	9	9	9	9	9
2	1	1	1	2	2	1	1	2	2
9	9	9	9	9	9	9	9	9	9
2	2	2	2	2	2	2	2	2	2
2	2	3	3	1	1	2	2	3	2
3	3	3	3	3	3	3	3	3	3
1	9	2	1	3	2	1	1	2	3
1	2	2	2	3	3	2	1	2	2
9	2	2	2	2	2	3	9	2	2
2	2	3	3	1	2	2	3	1	3
2	2	2	2	2	2	2	2	2	2

2	2	3	3	3	2	2	2	2	2
9	2	9	9	9	9	9	9	9	9
1	2	2	1	1	2	2	2	3	3
1	3	2	3	1	3	2	3	2	3
9	1	1	1	1	1	1	1	1	1
2	2	2	2	2	3	3	3	3	3
1	2	2	1	2	1	2	2	2	2
1	2	2	1	2	2	2	2	2	2
9	9	9	9	9	9	9	9	9	9
2	2	2	2	2	2	2	2	2	2
2	3	3	2	3	3	2	2	3	2
9	9	9	9	9	9	9	9	9	9
9	9	9	9	9	9	9	9	9	9
2	2	1	1	1	1	1	3	3	3
9	9	9	9	9	9	9	9	9	9
2	3	3	3	3	3	2	2	2	2
9	9	9	9	9	9	9	9	9	9
9	9	9	9	9	9	9	9	9	9
9	9	1	9	9	9	9	9	9	9
2	3	2	3	2	3	2	3	2	3
2	2	2	2	2	2	2	2	2	2
2	2	2	2	2	1	2	2	2	1
2	2	2	2	2	2	2	2	2	2
9	9	9	9	9	9	1	9	9	9
9	9	9	9	9	9	9	9	9	9
1	1	1	2	1	1	2	2	1	2
2	1	2	3	3	3	3	3	3	3
1	2	3	2	1	1	1	2	3	3
9	9	9	9	9	9	9	9	9	9
2	3	9	9	9	9	9	9	9	9
2	2	2	2	2	2	2	2	2	2
2	3	1	2	3	1	2	3	1	2
3	1	1	1	1	2	2	2	2	2
3	2	2	1	1	2	2	2	2	2
1	3	9	1	3	3	1	1	1	3
1	2	2	2	3	3	3	3	3	2
9	9	9	9	9	9	9	9	9	9
9	9	9	9	9	9	9	9	9	9
1	3	9	1	2	2	2	3	2	1
2	1	2	1	2	1	2	1	2	1
2	2	2	2	2	2	2	2	2	2
2	2	2	2	2	2	2	2	2	2
2	2	2	2	2	2	2	2	2	2
3	1	2	1	2	2	1	2	3	2
2	2	2	2	2	2	2	2	2	2
2	2	2	2	2	2	2	2	2	2
2	2	2	2	2	3	3	3	3	3

2	2	1	1	1	1	1	1	1	1
1	2	1	2	3	1	2	3	1	3
9	9	9	9	9	9	9	1	9	9
9	9	9	9	9	9	9	1	9	9
9	9	9	9	9	9	9	9	9	9
1	3	2	2	3	2	2	3	2	2
9	2	9	9	2	9	9	2	9	9
9	9	3	9	9	3	9	3	9	3
9	9	9	9	9	9	9	9	9	9
9	9	9	9	9	9	9	9	9	9
9	9	9	9	9	9	9	9	9	9
9	9	9	9	9	9	9	9	9	9
9	9	9	9	9	9	9	9	9	9
9	9	9	9	9	9	9	9	9	9
9	9	9	9	9	9	9	9	9	9
9	9	9	9	9	9	9	9	9	9
3	3	3	3	3	3	3	3	3	3
9	9	9	9	9	9	9	9	9	9
9	9	9	9	9	9	9	9	9	9
2	9	9	2	9	2	9	2	9	9
9	9	9	9	9	9	9	9	9	9
9	9	9	9	9	9	9	9	9	9
2	2	2	2	2	2	2	2	2	2
9	9	9	9	9	9	9	9	9	9
9	9	9	9	9	9	9	9	9	9
9	9	9	9	9	9	9	9	9	9
9	9	9	9	9	9	9	9	9	9
9	9	2	9	9	2	9	2	9	9
2	2	9	2	9	2	9	2	9	2
9	9	9	9	9	9	9	9	9	9
9	9	9	9	9	9	9	9	9	9
9	2	9	9	2	9	2	2	2	9
9	9	9	9	9	9	9	9	9	9
9	9	9	9	9	9	9	9	9	9
9	9	9	9	9	9	9	9	9	9
9	9	9	9	9	9	9	9	9	9
9	9	9	9	9	9	9	9	9	9
9	9	9	9	9	9	9	9	9	9
1	2	1	3	2	1	2	1	3	2
2	2	3	2	1	3	2	3	1	2
2	2	9	9	2	9	9	2	9	2
9	9	9	9	9	9	9	9	9	9
2	1	3	2	2	1	2	1	3	2
2	3	1	2	1	2	3	1	2	3
3	3	3	3	1	1	1	1	1	1
2	3	1	2	2	1	2	1	2	3
2	2	2	1	2	3	2	2	3	1

9	9	9	9	9	9	1	9	9	9
1	1	2	1	1	2	3	3	3	1
2	2	2	2	2	2	2	2	2	2
1	1	1	1	1	1	1	1	1	1
2	2	2	3	3	3	3	3	3	3
2	1	2	3	1	2	2	2	2	2
9	9	9	9	9	9	9	9	9	9
2	2	2	3	2	1	1	2	1	2
2	2	2	2	2	2	2	2	2	2
1	2	2	1	2	2	1	1	1	2
2	9	2	2	2	2	2	2	2	2
9	9	9	9	9	9	9	9	2	9
9	9	9	9	9	9	1	9	9	9
9	9	9	9	9	9	9	9	9	9
9	9	9	9	9	9	9	9	9	9
1	1	1	1	1	1	2	1	1	1
2	2	2	2	2	9	9	9	9	9
3	3	2	2	3	3	2	2	2	3
3	3	3	3	3	3	3	3	2	3
2	2	2	2	2	2	2	2	2	2
2	2	2	2	2	2	2	2	2	2
9	9	9	9	9	9	9	9	9	9
3	2	1	3	1	1	1	1	1	1
2	9	9	9	9	9	9	9	9	9
9	9	9	9	9	9	1	9	9	9
3	3	2	1	2	2	2	2	3	3
2	9	9	9	9	9	9	9	9	9
1	3	3	3	2	2	2	1	1	1
9	9	9	9	9	9	9	9	9	9
9	9	9	9	9	9	1	9	9	9
3	2	3	2	3	1	3	2	1	2
2	3	2	3	2	3	2	3	2	3
9	9	9	9	9	9	9	9	9	9
9	9	9	9	9	9	9	9	9	9
3	2	3	1	2	2	3	2	2	3
9	9	9	9	9	9	9	9	9	9
2	2	3	2	3	2	1	3	1	2
9	9	9	9	9	9	9	9	9	9
2	2	2	2	2	2	2	2	2	2
9	9	9	9	9	9	9	9	9	9
2	2	2	2	2	2	1	2	2	2
1	2	2	1	2	2	2	2	2	2
2	2	2	2	2	2	2	2	2	2
2	2	2	2	2	2	2	2	2	2
3	2	3	2	3	3	3	2	3	3
2	2	2	2	2	2	2	2	2	2
2	3	3	2	3	2	3	2	2	2

9	9	9	9	9	9	9	9	9	9
2	2	2	2	2	2	2	2	2	2
2	2	2	2	2	2	2	2	2	2
9	9	9	9	9	9	9	9	9	9
9	9	9	9	9	9	9	9	9	9
9	9	9	9	9	9	9	9	9	9
9	9	9	9	9	9	9	9	9	9
2	2	2	2	2	2	2	2	2	2
1	2	1	2	1	2	2	1	1	1
1	9	9	9	9	9	9	9	9	9
1	1	1	2	2	2	2	2	2	2
2	2	2	2	3	3	1	1	1	1
1	2	2	2	2	2	2	2	2	2
2	2	2	2	2	2	2	2	2	2
9	9	9	9	9	9	9	9	9	9
9	9	9	9	9	9	9	9	9	9
9	9	9	9	9	9	9	9	9	9
9	9	9	9	9	9	9	9	9	9
9	9	9	9	9	9	9	9	9	9
9	9	9	9	9	9	9	9	9	9
9	9	9	9	9	9	9	9	9	9
9	9	9	9	9	9	9	9	9	9
2	2	2	9	2	2	2	2	2	2
9	9	9	9	9	9	9	9	9	9
9	9	9	9	9	9	9	9	9	9
9	9	9	9	9	9	9	9	9	9
9	9	9	9	9	9	9	9	9	9
9	9	9	9	9	9	9	9	9	9
9	9	9	9	9	2	9	9	9	9
9	9	9	9	9	9	9	9	9	9
9	9	9	9	9	9	9	9	9	9
2	2	2	2	2	2	2	2	2	2
9	9	9	9	9	9	9	9	9	9
9	9	9	9	9	9	9	9	9	9
9	9	9	9	9	9	9	9	9	9
9	9	9	9	9	9	9	9	9	9
9	9	9	9	9	9	9	9	9	9
9	9	9	9	9	9	9	9	9	9
9	9	9	9	9	9	9	9	9	9
9	9	9	9	9	9	9	9	9	9
9	2	2	2	2	2	2	2	2	2
9	9	9	9	9	9	9	9	9	9
9	9	9	9	9	9	9	9	9	9
9	9	9	9	9	9	9	9	9	9
9	9	9	9	9	9	9	9	9	9
9	9	9	9	9	9	9	9	9	9
9	9	9	9	9	9	9	9	9	9

9	9	9	9	9	9	9	9	9	9
3	3	3	3	3	3	3	3	3	3
9	9	9	9	9	9	9	9	9	9
9	9	9	9	9	9	9	9	9	9
9	9	9	9	9	9	9	9	9	9
9	9	9	9	9	9	9	9	9	9
9	9	9	9	9	9	9	9	9	9
9	9	9	9	9	9	9	9	9	9
9	9	9	9	9	9	9	9	9	9
9	9	9	9	9	9	9	9	9	9
9	9	9	9	9	9	9	9	9	9
9	9	9	9	9	9	9	9	9	9
9	9	9	9	9	9	9	9	9	9
9	9	9	9	9	9	9	9	9	9
9	9	9	9	9	9	9	9	9	9
9	9	9	9	9	9	9	9	9	9
9	9	9	9	9	9	9	9	9	9
2	2	2	2	2	2	2	2	2	2
9	9	9	9	9	9	9	9	9	9
9	9	9	9	9	9	9	9	9	9
9	9	9	9	9	9	9	9	9	9
9	9	9	9	9	9	9	9	9	9
9	9	9	9	9	9	9	9	9	9
9	9	9	9	9	9	9	9	9	9
2	2	2	2	2	2	2	2	2	2
9	9	9	9	9	9	9	9	9	9
9	9	9	9	9	9	9	9	9	9
9	1	9	9	9	1	9	2	2	2
9	9	9	9	9	9	9	9	9	9
9	9	9	9	9	9	9	9	9	9
1	1	1	1	1	1	1	1	1	1
2	1	1	1	1	1	2	2	2	2
9	9	9	9	9	9	9	9	9	9
2	2	2	2	2	2	2	2	2	2
9	9	9	9	9	9	9	9	9	9
2	2	2	2	2	2	2	2	2	2
9	9	9	9	9	9	9	9	9	9
9	9	9	9	9	9	9	9	9	9
1	1	1	1	1	1	1	1	1	1
9	9	9	9	9	9	9	9	9	9
9	9	9	9	9	9	9	9	9	9
9	9	9	9	9	9	9	9	9	9
2	2	2	2	2	2	2	2	2	2
2	2	2	2	2	2	2	2	2	2
3	1	9	3	1	9	1	9	1	9
9	9	9	9	9	9	9	9	9	9
9	9	9	9	9	9	9	9	9	9

9	9	9	2	9	9	9	9	9	9
9	9	9	9	9	9	9	9	9	9
1	9	2	3	1	9	2	3	1	2
9	9	9	9	9	9	9	9	9	9
9	9	9	9	9	9	9	9	9	9
9	9	9	9	9	9	9	9	9	9
9	9	9	9	9	9	9	9	9	9
9	9	9	9	9	9	9	9	9	9
9	9	2	9	9	9	2	9	9	2
9	9	9	9	9	9	9	9	9	9
9	9	9	9	9	9	9	9	9	9
9	9	9	9	9	9	9	9	9	9
9	9	9	9	9	9	9	9	9	9
9	9	9	9	9	9	9	9	9	9
1	3	2	2	1	2	2	1	3	2
2	2	3	1	3	2	3	1	1	3
9	9	9	9	9	9	9	9	9	9
2	2	2	2	2	2	2	2	2	2
3	2	2	2	2	3	3	3	3	2
9	9	9	9	9	9	9	9	9	9
2	9	9	9	9	9	9	9	9	9
9	9	9	9	9	9	9	9	9	9
3	3	3	3	3	3	3	3	3	3
2	3	1	2	3	1	2	3	1	2
3	3	3	2	3	2	2	2	2	3
2	2	2	2	2	2	2	2	2	2
2	2	2	2	3	3	3	3	3	3
1	2	2	2	3	9	2	2	1	2
3	3	1	2	2	3	3	2	2	9
2	2	2	2	2	2	2	2	2	2
2	2	2	2	2	2	2	2	2	2
9	9	9	9	9	9	9	9	9	9
9	9	9	9	9	9	9	9	9	9
9	9	9	9	9	9	9	9	9	9
9	9	9	9	9	9	9	9	9	9
9	9	9	9	9	9	9	9	9	9
9	9	9	9	9	9	9	9	9	9
9	9	9	9	2	9	9	9	9	2
9	9	9	9	9	9	9	9	9	9
2	9	2	9	9	2	2	9	2	9
9	9	9	9	9	9	9	9	9	9
2	2	2	2	2	2	2	2	2	2
9	9	9	9	9	9	9	9	9	9
9	9	9	9	9	9	9	9	9	9
9	9	9	9	9	9	9	9	9	9
2	2	2	2	2	2	2	2	2	2
3	2	3	2	3	3	2	2	9	1

2	2	2	2	2	2	2	2	2	2
2	2	2	2	2	2	2	2	1	2
2	2	2	2	2	2	2	2	2	2
1	3	2	1	2	1	1	1	1	3
3	3	3	3	3	3	3	3	3	3
1	2	2	2	1	1	1	2	2	1
2	2	2	2	2	2	2	2	2	2
3	3	3	3	3	3	3	3	3	3
9	9	9	9	9	9	9	9	9	9
2	2	2	2	2	2	2	2	2	2
9	9	2	9	9	9	9	2	9	9
9	1	9	3	1	9	3	1	9	3
2	2	2	2	2	2	2	2	2	2
1	2	1	2	3	1	2	3	2	1
2	2	2	2	2	2	2	3	3	3
2	2	2	2	2	2	2	2	2	2
2	2	1	2	3	3	3	2	2	2
1	3	2	2	3	3	3	2	3	3
9	9	9	9	9	9	9	9	9	9
3	1	1	2	1	1	2	3	2	2
3	3	2	2	1	2	2	2	2	2
1	2	2	3	2	1	1	2	2	1
1	2	2	1	1	3	2	3	2	1
2	3	2	2	3	1	1	2	3	2
1	3	1	1	2	2	2	2	1	2
1	2	2	2	2	2	2	2	2	2
1	2	2	2	2	2	1	2	1	1
3	2	2	2	3	2	2	3	2	2
2	2	2	2	2	2	2	2	2	2
2	2	2	2	2	3	2	1	2	3
3	3	3	3	3	3	3	3	3	3
9	9	9	9	9	9	9	9	9	9
3	3	3	3	3	3	3	3	3	3
2	2	2	2	2	2	2	2	2	2
9	9	9	9	9	9	9	9	9	9
3	2	3	2	2	2	2	2	3	3
2	2	1	1	1	1	2	2	2	2
1	1	1	1	1	1	1	1	1	1
2	1	1	1	1	1	1	2	2	2
9	9	9	9	9	9	9	9	9	9
2	2	2	2	2	2	2	2	2	2
9	9	9	9	9	9	9	9	9	9
1	2	1	3	3	2	2	3	2	1
2	2	2	2	2	2	2	2	2	2
3	2	3	2	3	2	3	2	3	2
9	9	9	9	9	9	9	9	9	9
3	3	3	2	3	2	3	3	2	2

9	9	9	9	9	9	9	9	9	9
3	3	3	3	3	3	3	3	3	3
1	1	3	3	1	3	1	1	1	2
3	3	3	2	3	2	3	2	2	3
1	2	2	2	2	3	3	3	1	2
9	9	9	9	9	9	9	9	9	9
2	2	2	2	2	2	2	2	2	2
3	3	3	3	3	2	1	1	2	3
1	3	1	1	2	2	1	1	2	3
2	2	2	2	2	1	2	2	2	2
3	3	2	2	2	2	2	2	2	2
2	2	2	2	2	2	2	2	2	2
9	9	9	9	9	9	9	9	9	9
3	2	2	2	2	3	2	2	3	3
2	3	2	2	2	3	2	1	1	3
2	2	2	2	2	2	2	2	2	2
3	3	3	1	3	3	3	3	3	3
3	9	2	3	2	3	2	3	2	3
2	2	2	2	2	2	2	2	2	2
9	9	9	9	9	9	9	9	9	9
9	9	9	9	9	9	9	9	9	9
1	2	1	2	1	2	1	2	1	2
2	2	2	2	2	3	2	3	3	3
3	2	3	2	3	2	3	2	3	2
9	9	9	9	9	9	9	9	9	9
2	3	2	1	2	2	3	1	3	2
9	9	9	9	9	9	9	9	9	9
2	2	2	2	2	2	2	2	2	2
2	2	2	2	2	2	2	2	2	2
2	2	2	2	2	2	2	2	2	2
2	1	2	3	2	1	2	1	3	1
9	9	9	9	9	9	9	9	9	9
9	9	9	9	9	9	9	9	9	9
2	2	3	2	2	2	1	2	1	2
3	3	2	2	3	1	1	1	2	3
9	9	9	9	9	9	9	9	9	9
9	9	9	9	9	9	9	9	9	9
2	2	2	3	2	3	2	3	2	3
1	2	1	1	3	3	2	1	3	3
1	2	2	2	1	2	1	1	2	2
2	2	2	2	2	2	2	2	2	2
2	2	2	2	2	2	2	2	2	2
2	2	2	2	2	2	2	2	2	2
2	2	2	2	2	2	2	2	2	2
1	2	3	2	3	3	2	1	1	3
2	3	3	2	3	2	2	3	2	3
2	2	2	2	2	2	2	2	2	2

3	2	1	1	1	3	3	3	3	3
1	3	2	2	2	2	2	1	1	3
2	2	2	2	2	2	2	2	2	2
2	1	2	3	2	1	3	1	1	1
2	2	2	2	2	2	2	2	2	2
1	3	3	2	3	3	2	1	1	3
1	3	3	2	3	2	2	1	1	3
2	2	2	2	2	2	2	2	2	2
2	2	2	2	2	2	2	2	2	2
9	9	9	9	9	9	9	9	9	9
3	3	3	3	3	3	3	3	3	3
2	1	1	1	2	3	1	2	1	1
2	2	3	2	1	2	1	1	1	1
1	1	2	2	1	1	2	2	2	1
3	1	3	3	3	1	2	3	9	2
9	9	9	9	9	9	9	9	9	9
9	9	9	9	9	9	9	9	9	9
9	9	9	1	9	9	9	3	9	9
9	9	9	9	9	9	9	9	9	9
9	9	9	9	9	9	9	9	9	9
1	2	9	3	2	2	1	1	1	1
9	9	9	9	9	9	9	9	9	9
9	9	9	9	9	9	9	9	9	9
2	2	2	2	2	2	2	2	2	2
9	1	1	1	1	1	1	1	1	1
2	2	2	2	2	2	2	2	2	2
3	1	1	2	3	3	3	2	2	2
1	3	2	3	2	3	2	9	9	9
2	3	1	1	2	2	1	1	2	1
3	1	2	3	1	2	2	2	3	1
9	9	9	9	9	9	9	9	9	9
3	1	2	1	2	3	2	1	2	3
1	1	2	3	2	3	1	2	3	2
1	1	1	1	1	1	2	2	2	9
2	2	2	2	2	1	1	3	3	2
2	2	2	2	2	2	2	2	2	2
9	9	9	9	9	9	9	9	9	9
2	2	2	2	2	2	2	2	2	2
2	2	2	2	2	2	2	2	2	2
3	3	3	2	3	2	1	1	1	1
3	3	3	3	3	3	3	3	9	2
3	2	1	2	2	3	1	2	1	3
1	1	1	1	2	2	2	2	1	1
1	1	1	2	2	2	2	3	3	3
3	2	3	1	3	2	1	2	3	2
2	3	2	1	1	1	1	1	1	1
1	2	2	2	3	3	3	3	3	3

1	2	2	1	1	1	2	2	2	2
2	2	2	2	2	2	2	2	2	2
2	2	2	2	2	3	3	3	3	3
2	2	2	2	2	2	2	2	2	2
3	2	3	3	2	3	3	2	3	2
9	9	9	9	9	9	9	9	9	9
9	9	9	9	9	9	9	9	9	9
3	3	3	3	3	3	3	3	3	3
9	9	9	9	9	9	9	9	9	9
9	9	9	9	9	9	9	9	9	9
2	2	2	2	2	2	2	2	2	2
1	2	2	2	2	1	2	1	1	1
2	1	1	1	2	2	2	2	2	2
1	9	9	9	9	9	9	9	9	9
9	9	9	9	9	9	9	9	9	9
9	3	3	1	3	3	3	1	1	3
1	1	2	2	2	2	2	2	1	1
9	9	9	9	9	9	9	9	9	9
1	2	2	2	2	2	2	2	1	1
2	2	2	2	2	2	2	2	2	2
2	3	2	2	3	2	3	2	2	2
9	9	9	9	9	9	9	9	9	9
2	2	2	2	2	2	2	2	2	2
2	2	2	2	2	2	2	2	2	2
2	2	2	2	2	2	2	2	2	2
2	2	2	2	2	2	2	2	2	2
2	2	2	2	2	2	2	2	2	2
2	2	2	2	2	2	2	2	2	2
2	9	1	2	3	2	3	2	3	2
2	2	2	2	2	2	2	2	2	2
1	2	2	3	1	2	2	1	2	2
3	2	3	2	2	3	3	2	2	1
2	1	2	1	1	2	2	3	3	1
2	2	2	2	2	2	2	2	2	2
1	2	2	1	1	1	1	1	1	1
2	2	2	3	3	1	1	2	2	2
2	2	2	2	2	2	2	2	2	2
2	2	2	2	2	2	2	2	2	2
1	9	1	9	2	3	2	3	3	2
2	2	2	2	3	2	2	2	2	3
3	3	1	1	3	3	1	1	1	3
1	2	1	1	1	2	3	1	1	2
2	2	2	2	2	2	2	2	2	2
2	2	2	2	2	2	2	2	2	2
2	2	2	2	2	2	2	2	2	2
2	2	3	2	2	2	2	2	1	2
2	2	2	2	2	2	2	2	2	2

1	2	1	3	2	1	3	1	3	2
2	3	2	3	2	2	3	1	1	3
3	2	3	2	3	2	3	2	3	2
2	2	2	2	2	2	2	2	2	2
9	9	9	9	9	9	9	9	9	9
9	1	9	9	9	9	9	9	9	9
2	3	2	3	2	3	2	2	3	2
9	9	9	9	9	9	9	9	9	9
9	9	9	9	9	9	9	9	9	9
9	9	9	9	9	9	9	9	9	9
9	9	9	9	3	9	9	9	9	9
9	9	9	9	9	9	9	9	9	9
9	9	9	9	9	9	9	9	9	9
9	9	9	9	1	9	9	9	9	9
1	3	2	2	2	1	1	2	1	2
9	9	9	9	9	9	9	9	9	9
9	9	9	9	9	9	9	9	9	9
9	9	1	9	9	9	9	9	9	9
9	9	1	9	9	9	9	9	9	9
2	9	9	9	9	9	9	9	9	9
9	9	9	9	9	9	9	9	9	9
2	2	2	2	2	2	2	2	2	2
2	2	2	2	2	2	2	2	2	2
3	3	3	2	2	1	1	1	2	2
2	2	2	3	1	3	1	2	1	3
9	9	9	9	9	9	9	9	9	9
2	2	2	1	3	2	2	2	2	2
9	9	2	9	9	9	9	9	9	9
1	1	1	2	2	2	3	3	3	2
1	3	1	1	1	3	1	1	1	1
1	9	2	3	2	1	3	1	1	3
9	9	9	9	9	9	9	9	9	9
2	2	2	2	2	2	2	2	2	2
9	9	9	9	9	9	9	9	9	9
9	9	9	9	9	9	9	9	9	9
9	1	1	1	1	2	1	1	1	1
2	1	1	1	1	1	1	1	1	1
9	9	9	9	9	9	9	9	9	9
9	9	9	9	9	9	9	9	9	9
2	2	2	2	2	2	2	2	2	2
9	9	9	9	9	9	9	9	9	9
2	2	2	2	2	2	2	2	2	2
3	3	3	3	3	3	3	3	3	3
2	9	9	9	9	9	9	2	9	9
2	2	2	2	2	2	2	2	2	2
2	2	2	2	2	2	2	2	2	2
9	9	9	9	9	9	9	9	9	9

9	9	9	9	9	9	9	9	9	9
9	2	9	9	2	9	2	9	9	2
9	9	9	9	9	9	9	9	9	9
2	2	2	2	2	2	2	2	2	2
9	9	2	1	9	9	9	9	9	9
2	1	3	1	3	1	9	3	1	3
9	9	9	9	9	9	9	9	9	9
9	9	9	9	9	9	9	9	9	9
9	2	9	9	9	2	9	9	2	9
2	2	2	2	2	2	2	2	2	2
9	9	9	9	9	9	9	9	9	9
2	9	9	2	9	9	2	9	9	2
2	2	2	2	2	2	2	2	2	2
9	9	9	9	9	9	9	9	9	9
2	2	2	2	2	2	2	2	2	2
9	9	9	9	9	9	9	9	9	9
9	9	9	9	9	9	9	9	9	9
9	9	9	9	9	9	9	9	9	9
9	9	9	9	9	9	9	9	9	9
9	9	9	9	9	9	9	9	9	9
2	2	2	2	2	2	2	2	2	2
2	2	2	2	2	2	2	2	2	2
3	2	1	1	2	3	1	2	2	3
2	3	3	2	1	2	3	1	3	2
2	2	2	3	3	2	2	3	3	3
2	2	2	2	2	2	2	2	2	2
1	1	1	1	1	1	1	1	1	1
2	2	2	2	2	2	2	2	2	2
1	1	1	1	2	3	2	1	2	1
2	2	2	2	2	2	2	2	2	2
2	2	3	3	3	3	3	2	2	2
3	3	2	1	2	2	1	3	2	1
1	2	2	2	2	2	2	2	2	2
9	9	9	9	9	9	9	9	9	9
2	1	1	2	2	3	2	1	2	3
2	2	2	2	2	2	2	2	2	2
2	2	2	2	2	2	2	2	2	2
1	1	1	1	1	1	1	1	1	1
1	2	3	2	3	2	3	2	3	2
3	1	3	2	1	2	3	1	2	3
2	2	2	2	1	1	1	1	1	1
9	9	9	9	9	9	9	9	9	9
2	3	9	9	9	9	9	9	9	9
9	9	9	9	9	9	9	9	9	9
2	1	2	1	2	1	2	1	2	1
2	2	2	2	2	2	2	2	2	2
9	9	9	9	9	9	9	9	9	9

2	2	2	2	2	2	1	2	1	3
2	2	1	1	1	2	2	2	1	1
2	2	2	9	3	9	1	1	1	1
2	2	2	2	2	2	2	2	2	2
9	1	9	1	9	9	9	9	1	1
1	1	1	1	1	1	2	2	2	2
1	1	1	1	1	2	2	3	3	3
1	1	1	1	3	3	3	1	1	1
3	3	3	3	3	3	3	3	3	3
1	1	2	1	1	3	1	2	1	3
9	9	9	9	9	9	9	9	9	9
2	2	2	2	2	2	2	2	2	2
2	2	2	2	2	2	2	2	2	2
2	2	2	2	2	2	2	2	2	2
2	2	2	2	2	2	2	2	2	2
1	1	1	1	1	1	1	1	1	1
1	1	2	1	2	1	1	1	1	1
1	2	2	2	2	2	1	1	1	1
1	1	1	1	1	1	1	1	1	1
9	9	9	9	9	9	9	9	9	9
1	1	2	2	1	1	2	2	1	1
2	2	9	9	2	2	2	9	9	2
9	9	9	9	9	9	9	9	9	9
1	1	1	1	1	1	1	1	1	1
9	9	9	9	9	9	9	9	9	9
2	2	1	1	1	1	1	2	1	2
2	1	1	1	1	1	1	2	9	1
1	1	1	1	1	1	1	1	1	1
9	9	9	9	9	9	9	9	9	9
2	9	2	9	2	2	2	2	2	9
9	9	9	9	9	9	9	9	9	9
9	9	9	9	9	9	9	9	9	9
2	2	2	2	2	2	2	2	2	2
2	9	2	2	2	2	2	2	2	2
2	2	2	2	2	2	2	2	2	2
9	9	9	9	9	9	9	9	9	9
1	1	1	1	1	1	1	1	1	1
1	2	1	1	1	1	1	1	1	1
9	9	9	9	9	9	9	9	9	9
9	9	9	9	9	9	9	9	9	9
2	9	2	2	9	2	2	2	9	9
9	9	9	9	9	9	9	9	9	9
9	2	2	2	2	2	9	2	2	2
9	9	9	9	9	9	9	9	9	9
1	1	1	1	1	1	2	1	2	1
2	9	9	2	9	2	9	2	9	2
1	1	9	9	1	9	1	1	1	1

1	1	2	1	1	1	1	2	2	2
2	9	2	2	2	9	2	9	2	2
2	2	2	2	2	2	2	2	2	2
1	2	2	2	1	1	1	1	1	1
2	2	2	2	2	2	2	2	2	2
9	9	9	9	9	9	9	9	9	9
1	1	1	1	1	1	1	1	1	3
2	2	2	2	2	2	2	2	2	2
9	2	9	2	9	9	2	9	9	2
1	9	1	1	9	1	1	2	2	2
9	9	9	9	9	9	9	9	9	9
9	9	9	9	9	9	9	9	9	9
9	9	9	9	9	9	9	9	9	9
9	9	9	9	9	9	9	9	9	9
9	9	9	9	9	9	9	9	9	9
9	9	9	9	9	9	9	9	9	9
2	2	2	2	2	2	2	2	2	2
9	9	9	9	9	9	9	9	9	9
2	2	2	2	2	2	2	2	2	2
1	2	2	1	3	3	1	1	1	3
1	2	2	1	3	3	1	1	1	3
1	2	2	1	3	3	1	1	1	3
1	2	2	1	3	3	1	1	1	3
1	2	2	1	3	3	1	1	1	3
1	2	2	1	3	3	1	1	1	3
1	2	2	1	3	3	1	1	1	3
1	2	2	1	3	3	1	1	1	3
1	2	2	3	3	3	1	1	1	3
1	2	2	1	3	3	1	1	1	3
1	2	1	1	3	3	1	1	3	3
1	2	2	1	3	3	1	1	1	3
1	2	2	1	3	3	1	1	1	3
1	2	2	1	3	3	1	1	1	3
1	2	2	1	3	3	1	1	1	3
1	2	2	1	3	3	1	1	1	3
1	2	2	1	3	3	1	1	1	3
1	2	2	1	3	3	1	1	1	3
1	2	2	1	3	3	1	1	1	3
1	2	2	1	3	3	1	1	1	3
1	2	2	1	3	3	1	1	1	3
1	2	2	1	3	3	1	1	1	3
1	2	2	1	3	3	1	1	1	3
1	2	2	1	3	3	1	1	1	3
1	2	2	1	3	3	1	1	1	3
9	9	9	9	9	9	9	9	9	9
9	9	9	9	9	9	9	9	9	9
9	9	9	9	9	9	9	9	9	9

9	9	9	9	9	9	9	9	9	9
9	9	9	9	9	9	9	9	9	9
9	9	9	9	9	9	9	9	9	9
9	9	9	9	9	9	9	9	9	9
9	9	9	9	9	9	9	9	9	9
9	9	9	9	9	9	9	9	9	9
9	9	9	9	9	9	9	9	9	9
9	9	9	9	9	9	9	9	9	9
9	9	9	9	9	9	9	9	9	9
9	9	9	9	9	9	9	9	9	9
9	9	9	9	9	9	9	9	9	9
9	9	9	9	9	9	9	9	9	9
9	9	9	9	9	9	9	9	9	9
9	9	9	9	9	9	9	9	9	9
9	9	9	9	9	9	9	9	9	9
9	9	9	9	9	9	9	9	9	9
9	9	9	9	9	9	9	9	9	9
9	9	9	9	9	9	9	9	9	9
9	9	9	9	9	9	9	9	9	9
1	2	2	1	3	3	1	1	1	3
1	2	2	1	3	3	1	1	1	3
1	2	2	1	3	3	1	1	1	3
1	2	2	1	3	3	1	1	1	3
1	2	2	1	3	3	1	1	1	3
1	2	2	1	3	3	1	1	1	3
1	2	2	1	3	3	1	1	1	3
1	2	2	1	3	3	1	1	1	3
1	2	2	1	3	3	1	1	1	3
1	2	1	1	3	3	1	1	1	3
2	2	3	3	9	1	1	1	1	3
1	2	2	1	3	3	1	1	1	3
2	2	1	1	3	3	1	1	1	3
1	2	2	1	3	3	1	1	1	3
2	2	2	1	3	3	1	1	1	3
1	2	2	1	3	3	1	1	1	3
9	3	9	9	3	1	9	1	1	9
1	2	2	1	3	3	1	1	1	3
2	2	1	1	3	3	1	1	1	3
2	2	1	1	3	3	1	1	1	3
1	2	2	1	3	3	1	1	3	3
1	2	2	1	3	3	1	1	1	3
1	2	2	1	3	3	1	1	1	3
1	2	2	1	3	3	1	1	1	3
2	9	2	1	3	3	1	1	1	3
1	2	2	1	3	3	1	1	1	3
1	2	2	1	3	3	1	1	1	3
1	2	2	1	3	3	1	1	1	3

1	2	2	1	3	3	1	1	1	3
1	2	2	1	3	1	9	9	9	9
1	2	2	1	3	3	1	1	1	3
1	2	2	9	3	3	1	1	1	3
1	2	2	1	3	3	1	1	1	3
1	2	2	1	3	3	1	1	1	3
1	2	2	1	3	3	1	1	1	3
1	2	2	1	3	3	1	1	1	3
1	2	2	1	3	3	1	1	1	3
1	2	2	1	3	3	1	1	1	3
1	2	2	1	3	3	1	1	1	3
1	2	2	1	3	3	1	1	1	3
1	2	2	1	3	3	1	1	1	3
2	2	1	3	3	1	1	1	1	3
1	2	2	1	3	3	1	2	1	3
1	2	2	1	3	3	1	1	1	3
2	2	1	9	1	3	1	3	1	3
1	2	2	1	3	3	1	1	1	3
1	2	2	1	3	3	1	1	1	3
1	2	2	1	3	3	1	1	1	3
1	2	2	1	3	3	1	1	1	3
1	2	2	3	3	1	1	3	1	3
1	2	2	3	3	1	1	1	1	3
1	2	2	1	3	3	1	1	1	3
1	2	2	1	3	3	1	1	1	3
1	2	2	1	3	3	1	1	1	3
1	2	2	1	1	3	1	1	1	3
1	2	2	1	3	3	1	1	1	3
1	2	2	1	3	3	1	1	1	3
1	9	1	1	3	3	1	1	1	3
1	2	2	2	3	3	1	1	1	3
1	2	2	1	3	3	1	1	1	3
9	9	9	9	3	3	1	9	1	9
1	2	2	1	3	3	1	1	1	3
9	9	9	9	9	9	9	9	9	9
1	2	2	1	3	3	1	1	1	3
1	2	2	1	3	3	1	1	1	3
1	2	2	1	3	3	1	1	1	3
1	2	2	1	3	3	1	1	1	3
1	9	9	9	9	9	9	9	9	9
1	2	2	1	3	3	1	1	1	3
1	2	2	1	3	1	1	1	1	1
1	2	2	1	3	3	2	1	1	3
1	2	2	1	3	3	1	1	1	3
1	2	2	1	3	3	1	1	1	3
1	2	2	1	3	3	1	1	1	3
1	2	2	1	3	3	1	1	1	3

1	2	2	1	3	3	1	1	1	3
1	2	2	1	3	3	1	1	1	3
1	2	2	1	3	3	1	1	1	3
1	2	2	1	3	3	1	1	1	3
1	2	2	1	3	3	1	1	1	3
1	2	2	1	3	3	1	1	1	3
1	2	2	1	3	3	1	1	1	3
1	2	2	1	3	3	1	1	1	3
1	2	2	1	3	3	1	1	1	3
1	2	2	1	3	3	1	1	1	3
1	2	2	1	3	3	1	1	1	3
1	2	2	1	3	1	1	1	1	3
1	2	2	1	3	3	1	1	1	3
1	2	2	1	3	3	1	1	1	3
2	1	2	1	3	3	1	1	1	3
1	2	2	1	3	3	1	1	1	3
1	2	2	1	3	3	1	1	1	3
1	2	2	1	3	3	1	1	1	3
1	2	2	1	3	3	1	1	1	3
1	1	2	3	3	9	1	1	1	3
1	2	2	1	3	3	1	1	1	3
1	2	2	1	3	3	1	1	1	3
1	2	2	1	3	3	1	1	1	3
1	2	3	1	3	3	1	1	1	3
1	2	2	1	3	3	1	1	1	3
9	1	2	2	2	9	1	1	1	3
1	2	2	1	3	3	1	1	1	3
1	2	2	1	3	3	1	1	1	3
1	2	2	1	3	3	1	1	1	3
1	2	2	1	3	3	1	1	1	3
1	2	2	2	3	3	1	1	1	3
1	2	2	1	3	3	1	1	1	3
1	1	2	1	3	3	1	1	1	3
1	2	2	1	3	3	1	1	1	3
1	2	2	1	3	3	1	1	1	3
1	2	1	3	3	9	1	1	1	3
1	2	2	1	3	3	1	1	1	3
1	2	2	1	3	3	1	1	1	3
1	2	2	1	3	3	1	1	1	3
1	2	2	1	3	3	1	1	1	3
1	2	2	1	3	3	1	1	1	3
9	9	9	9	9	9	9	9	9	9
1	2	2	1	1	3	3	2	9	1
1	2	2	1	3	3	1	1	1	3
1	2	2	1	3	3	1	1	1	3
2	2	1	3	3	1	1	1	1	3
2	2	1	3	3	1	1	3	3	3

1	2	3	1	3	3	1	1	1	3
1	2	2	1	3	3	1	1	1	3
1	2	2	1	3	3	1	1	1	3
1	2	2	1	3	3	1	1	1	3
1	2	2	1	3	3	1	1	1	3
1	2	2	1	3	3	1	1	1	3
1	2	2	3	3	3	1	1	1	3
1	2	2	1	3	3	1	1	1	3
1	2	1	1	3	3	1	1	1	3
9	9	9	9	9	9	9	9	9	9
1	2	2	1	3	3	1	1	1	3
1	2	1	1	3	3	1	1	1	3
1	2	2	1	3	3	1	1	1	3
1	2	2	1	3	3	1	1	1	3
1	2	2	1	3	3	1	1	1	3
1	2	2	1	3	3	1	1	1	3
1	2	2	1	3	3	1	1	1	3
1	2	2	1	3	3	1	1	1	3
1	2	2	1	3	3	1	1	1	3
1	2	2	1	3	3	1	1	1	3
1	2	2	9	3	1	1	1	1	3
2	1	1	2	3	3	1	1	1	3
1	2	2	1	3	3	1	1	1	3
1	2	2	1	3	3	1	1	1	3
1	2	2	1	3	3	1	1	1	3
1	2	2	1	3	3	1	1	1	3
1	2	2	1	3	3	1	1	1	3
9	9	9	9	9	9	9	9	9	9
9	9	9	9	9	9	9	9	9	9
9	9	9	9	9	9	9	9	9	9
9	9	9	9	9	9	9	9	9	9
9	9	9	9	9	9	9	9	9	9
9	9	9	9	9	9	9	9	9	9
9	9	9	9	9	9	9	9	9	9
9	9	9	9	9	9	9	9	9	9
9	9	9	9	9	9	9	9	9	9
9	9	9	9	9	9	9	9	9	9
9	9	9	9	9	9	9	9	9	9
9	9	9	9	9	9	9	9	9	9
9	9	9	9	9	9	9	9	9	9
9	9	9	9	9	9	9	9	9	9
9	9	9	9	9	9	9	9	9	9
9	9	9	9	9	9	9	9	9	9
9	9	9	9	9	9	9	9	9	9
9	9	9	9	9	9	9	9	9	9
9	9	9	9	9	9	9	9	9	9
9	9	9	9	9	9	9	9	9	9

9	9	9	9	9	9	9	9	9	9
9	9	9	9	9	9	9	9	9	9
9	9	9	9	9	9	9	9	9	9
9	9	9	9	9	9	9	9	9	9
9	9	9	9	9	9	9	9	9	9
9	9	9	9	9	9	9	9	9	9
9	9	9	9	9	9	9	9	9	9
9	9	9	9	9	9	9	9	9	9
9	9	9	9	9	9	9	9	9	9
9	9	9	9	9	9	9	9	9	9
9	9	9	9	9	9	9	9	9	9
9	9	9	9	9	9	9	9	9	9
9	9	9	9	9	9	9	9	9	9
2	2	1	1	1	1	1	2	9	1
2	2	2	2	2	2	2	2	2	2
2	2	2	2	2	2	2	2	2	2
9	1	2	1	1	1	1	3	3	2
1	2	2	2	1	2	2	2	1	1
2	2	2	2	2	2	2	2	2	2
3	2	1	1	1	2	2	2	2	2
2	1	1	2	2	2	2	1	1	2
2	3	3	3	3	3	3	3	3	3
9	9	9	9	9	9	9	9	9	9
2	3	2	3	3	3	3	3	3	3
9	9	1	9	9	9	9	9	9	9
2	2	2	3	3	3	3	3	2	2
9	9	9	9	9	9	9	9	9	9
1	1	1	1	1	1	1	1	1	1
1	2	3	2	2	2	1	3	2	1
2	2	2	2	2	2	2	2	2	2
2	2	1	3	3	3	1	1	1	2
2	3	2	1	2	1	2	3	1	2
9	9	9	9	9	9	9	9	9	9
1	2	1	1	2	1	2	2	2	2
9	1	1	1	1	9	9	1	1	1
9	9	9	9	9	9	9	9	9	9
2	1	1	1	1	1	3	3	3	3
3	3	3	1	1	1	2	2	3	2
1	2	1	2	1	2	2	1	2	1
1	3	1	2	2	2	1	2	3	3
9	9	9	9	9	9	9	9	9	9
9	9	9	9	9	9	9	9	9	9
1	2	2	2	2	2	2	2	2	2
1	1	1	1	1	1	1	1	1	1
9	9	9	9	9	9	9	9	9	9
9	9	9	9	9	9	9	9	9	9
9	9	9	9	9	9	9	9	9	9

9	9	1	9	9	9	9	9	9	9
9	9	9	9	9	9	9	9	9	9
2	2	3	2	3	2	1	2	3	2
2	2	2	2	2	1	2	1	1	1
3	3	3	2	2	1	1	1	1	2
9	9	9	1	9	9	9	9	9	9
2	2	2	2	2	2	2	2	2	2
2	2	2	2	2	2	2	2	2	2
2	2	2	2	2	2	2	2	2	2
3	3	3	1	1	1	1	2	2	1
3	2	3	3	3	3	3	3	2	2
9	9	9	9	9	9	9	9	9	9
9	9	9	9	9	9	9	9	9	9
2	2	2	2	2	2	2	2	2	2
2	2	2	2	2	2	2	2	2	3
2	2	2	2	2	2	2	2	2	2
1	2	3	1	2	3	1	2	3	1
2	2	2	2	2	2	2	2	2	2
1	9	1	1	1	1	1	9	1	1
9	9	9	9	9	9	9	9	9	9
2	2	2	2	2	2	2	2	2	2
2	2	2	2	2	2	2	2	2	2
2	2	2	2	2	2	2	2	2	2
2	2	2	2	2	2	2	2	2	2
2	2	2	2	2	2	2	2	2	2
2	2	1	1	3	3	1	1	3	3
2	2	2	2	2	2	2	2	2	2
2	2	2	2	2	2	2	2	2	2
9	9	9	9	9	9	9	9	9	9
1	2	1	2	1	3	1	1	1	1
1	3	3	2	3	3	3	2	2	3
1	2	2	2	2	2	2	2	2	2
2	2	1	1	2	1	1	1	1	2
2	1	1	1	2	2	2	2	2	2
2	2	2	2	2	2	2	2	2	2
2	2	1	1	3	3	3	3	2	2
2	2	2	2	2	2	2	2	2	2
2	1	1	1	9	9	9	9	9	9
9	9	9	9	9	9	9	9	9	9
2	2	2	2	2	2	2	2	2	2
2	2	2	2	3	3	3	3	3	3
2	2	2	3	3	2	2	3	2	2
1	2	3	1	2	3	1	2	3	1
9	9	9	9	9	9	9	9	9	9
1	1	1	1	1	1	1	1	1	1
3	3	3	3	3	9	2	2	2	3
3	3	2	2	3	3	2	2	3	3

2	2	2	2	2	2	2	2	2	2
1	2	3	2	3	2	2	2	3	2
3	3	3	3	3	3	3	3	3	3
3	2	3	3	3	2	1	2	3	1
2	2	2	2	2	2	2	2	2	2
2	2	2	2	2	2	2	2	2	2
2	2	2	2	2	2	2	2	2	2
3	3	3	2	2	1	1	1	2	1
2	2	2	2	2	2	2	2	2	2
3	2	3	2	1	2	3	1	1	2
2	2	2	2	2	2	2	2	2	2
2	2	2	2	2	2	2	2	2	2
2	2	2	2	2	2	2	2	2	2
3	3	3	3	2	3	3	3	3	3
2	2	2	2	2	2	2	2	2	2
2	3	2	3	2	3	2	3	2	3
2	2	2	2	2	2	2	2	2	2
2	2	2	2	2	2	2	2	2	2
2	2	2	2	2	2	2	2	2	2
1	2	3	1	2	3	1	2	3	1
2	1	2	1	2	1	2	1	2	1
1	1	1	1	1	2	2	2	2	2
2	2	2	2	2	2	2	2	2	2
1	1	3	3	3	3	3	1	1	1
1	1	1	3	3	9	1	1	1	3
9	9	9	9	9	9	9	9	9	9
9	9	9	9	9	9	9	9	9	9
2	2	2	2	2	2	2	2	2	2
9	9	9	9	9	9	9	9	9	9
3	1	1	1	3	3	2	1	2	2
2	2	2	2	3	3	2	2	2	3
1	2	3	1	2	3	2	2	2	2
9	9	9	9	9	9	9	9	9	9
1	1	1	1	1	1	1	1	1	1
1	2	9	1	1	2	2	2	3	1
2	2	2	2	2	2	2	2	2	2
9	9	9	9	9	9	9	9	9	9
2	1	2	1	1	1	2	2	2	3
9	9	9	9	9	9	9	9	9	9
2	2	2	2	2	2	2	2	2	2
3	2	3	2	3	2	2	3	2	3
1	2	1	1	1	2	2	2	1	2
2	3	1	2	2	1	2	2	3	2
9	9	9	9	9	9	9	9	9	9
9	9	9	9	9	9	9	9	9	9
9	9	9	9	9	9	9	9	9	9
1	1	1	1	2	2	2	2	2	2

9	9	9	9	9	9	9	9	9	9
9	9	9	9	9	9	9	9	9	9
3	2	1	2	2	2	2	2	2	3
9	9	9	9	9	9	9	9	9	9
9	9	9	9	9	9	9	9	9	9
3	3	3	3	3	3	2	2	2	2
3	3	2	2	1	1	2	3	2	2
9	9	9	9	9	9	9	9	9	9
1	2	1	1	1	2	1	1	1	1
1	2	3	1	2	3	1	2	3	1
1	2	3	1	2	3	1	2	3	1
2	2	2	2	2	2	2	2	2	2
2	2	2	2	2	2	2	2	2	2
9	9	9	9	9	9	9	9	9	9
2	2	2	2	2	2	2	2	2	2
2	2	2	2	2	2	2	2	2	2
1	1	1	1	1	1	1	1	1	1
2	1	2	3	3	2	2	1	2	3
3	3	2	1	1	1	2	2	2	2
9	9	9	9	9	9	9	9	9	9
2	1	2	3	3	2	2	3	2	3
3	2	9	1	9	1	3	2	1	3
2	2	2	2	2	2	2	2	2	2
2	2	1	2	3	3	2	2	3	2
3	2	3	3	3	2	2	3	3	2
9	9	9	9	9	9	9	9	9	9
3	3	1	3	3	1	2	1	2	1
9	9	9	9	9	9	9	9	9	9
3	3	3	3	3	3	3	3	3	3
1	2	2	2	1	1	2	3	2	1
2	2	2	2	2	2	2	2	2	2
9	9	9	9	9	1	9	9	9	9
9	9	9	9	9	1	9	9	9	9
2	2	2	2	2	2	2	2	2	2
2	2	1	1	3	3	3	9	2	2
2	1	2	3	2	1	3	1	2	2
2	2	9	9	9	9	9	9	9	9
2	3	3	3	2	2	1	1	1	2
2	2	2	2	2	2	2	2	2	2
9	9	9	9	9	9	9	9	9	9
1	2	2	2	1	2	2	1	2	1
2	2	2	2	2	2	2	2	2	2
2	2	1	1	2	2	2	2	2	2
2	2	2	2	2	2	2	2	2	2
2	2	2	2	2	2	2	2	2	2
9	9	9	9	9	9	9	9	9	9
2	2	2	2	2	2	2	2	2	2

1	3	1	1	1	1	1	1	1	1
2	2	2	2	2	2	2	2	2	2
3	3	3	3	3	3	2	2	2	2
2	2	2	2	2	2	2	2	2	2
2	2	2	2	2	2	2	2	2	2
2	2	2	2	2	3	3	3	3	3
3	3	1	1	2	2	2	1	1	3
3	2	1	2	3	2	1	2	1	2
1	2	1	1	2	1	1	1	1	2
2	3	3	3	3	2	2	1	1	3
2	3	2	3	2	2	2	2	2	2
2	2	2	2	2	1	1	1	1	1
2	2	2	2	2	2	2	2	2	2
1	2	1	1	2	3	1	2	3	2
9	9	2	9	1	2	1	9	1	3
2	2	2	2	2	2	2	2	2	2
9	2	9	2	2	2	2	3	2	3
2	2	1	1	9	2	9	1	2	9
3	2	2	2	3	2	2	2	2	3
1	2	2	2	2	1	1	1	1	1
1	2	1	1	1	1	1	1	1	2
9	9	9	9	9	9	9	9	9	9
2	2	2	2	2	2	2	2	2	2
2	2	2	2	2	2	2	2	2	2
2	9	1	2	9	2	2	2	2	2
2	2	2	2	2	2	1	1	1	3
1	2	2	2	1	2	2	2	2	2
1	2	2	2	2	2	2	2	2	2
1	1	9	3	3	3	1	3	1	3
1	1	1	1	3	1	1	1	2	3
1	1	3	1	3	3	1	1	1	3
2	1	2	1	2	1	2	1	2	1
2	2	2	2	1	1	1	3	2	1
9	9	9	9	9	9	9	9	9	9
9	9	2	9	9	9	9	9	9	9
1	1	3	1	3	3	1	1	1	3
2	2	2	2	2	2	2	2	2	2
1	1	2	3	2	2	1	1	1	1
1	1	1	1	9	2	2	2	2	2
2	2	2	2	2	2	2	2	2	2
2	3	2	3	2	3	2	2	3	3
9	9	9	9	9	9	9	9	9	9
9	9	9	9	9	9	9	9	9	9
1	1	1	1	1	1	1	1	1	1
3	3	3	2	2	2	2	2	2	2
9	9	9	9	9	9	9	9	9	9
9	9	9	9	9	9	9	9	9	9

2	2	2	2	2	2	2	2	2	2
3	3	2	2	2	1	1	1	1	1
1	2	3	1	2	3	1	2	3	1
2	2	2	2	2	2	2	2	2	2
2	2	1	1	1	1	1	1	3	3
1	2	3	1	2	3	1	2	3	1
2	2	2	1	2	1	2	2	1	2
9	9	9	1	9	9	9	9	9	9
2	3	3	3	1	1	2	1	3	1
9	9	9	9	9	9	9	9	9	9
1	1	1	1	1	1	1	1	1	1
1	2	2	2	1	2	1	1	1	2
1	1	1	1	2	2	2	2	1	3
9	9	9	9	9	9	9	9	9	9
1	1	1	1	1	1	1	1	1	1
9	9	9	9	9	9	9	9	9	9
9	9	9	9	9	9	9	9	9	9
2	2	2	2	2	2	2	2	2	2
2	2	2	2	2	2	2	2	2	2
1	2	2	1	2	1	2	2	2	2
2	2	2	2	2	2	2	2	2	2
2	2	2	2	2	2	2	2	2	2
2	2	2	2	2	2	2	2	2	2
3	2	1	2	3	2	1	2	3	2
9	3	3	3	3	9	3	3	3	3
9	9	9	9	9	9	9	9	9	9
2	2	2	2	2	2	2	2	2	2
1	1	9	2	2	2	2	2	2	2
1	1	1	1	1	1	1	1	1	1
1	3	2	2	1	1	1	1	1	1
1	1	2	2	2	2	2	2	2	2
2	2	2	2	2	2	2	2	2	2
2	2	2	2	2	2	2	2	2	2
2	2	2	2	2	2	2	2	2	2
2	2	2	2	2	2	2	2	2	2
2	2	2	2	2	2	2	2	2	2
1	3	1	3	1	2	3	2	1	3
9	9	9	1	9	9	9	9	9	9
9	9	9	2	9	9	9	9	9	9
2	2	2	2	2	2	2	2	2	2
3	3	3	3	3	3	3	3	2	3
9	9	9	9	9	9	9	9	9	1
2	2	2	2	2	2	2	2	2	2
9	9	9	9	9	9	9	9	9	9
3	3	3	3	3	3	3	3	3	3
9	9	9	9	9	9	9	9	9	9
2	2	2	2	2	2	2	2	2	2

1	2	1	3	1	3	2	3	2	3
9	9	9	9	9	9	9	9	9	9
2	2	2	2	2	2	2	2	2	2
1	2	1	2	2	2	2	2	2	2
2	2	2	2	2	2	2	2	2	2
3	2	2	2	2	2	2	2	2	2
2	1	1	1	1	1	1	1	1	1
9	9	9	9	9	9	9	9	9	9
2	2	2	2	2	2	2	2	2	2
2	2	2	2	2	2	2	2	2	2
2	2	2	2	2	2	2	2	2	2
2	2	2	2	2	2	9	2	2	2
2	2	2	2	2	2	2	3	3	2
2	2	2	3	3	3	2	2	1	2
3	2	2	1	3	2	2	3	1	3
9	3	1	2	1	1	1	1	1	1
2	2	2	2	2	2	2	2	2	2
2	2	2	2	2	2	2	2	2	2
9	9	9	9	9	9	9	9	9	9
9	9	9	9	9	9	9	9	9	9
1	2	1	2	1	2	1	1	1	1
2	2	2	2	3	2	1	1	1	3
9	9	9	9	9	9	9	9	9	9
9	9	9	9	9	9	9	9	9	9
3	3	3	3	3	3	3	3	3	3
9	9	9	9	9	9	9	9	9	9
9	9	9	9	9	9	9	9	9	9
9	9	9	9	9	9	9	9	9	9
1	1	3	3	1	2	2	2	3	1
2	2	2	2	2	2	2	2	2	2
2	2	2	2	2	2	2	2	2	2
9	2	2	1	1	1	1	1	1	1
1	2	3	1	2	3	3	2	1	1
2	2	2	2	2	2	2	2	2	2
2	2	2	2	2	2	2	2	2	2
2	1	1	1	2	1	1	1	1	2
1	2	2	1	2	3	3	3	1	2
9	2	3	9	2	3	1	2	1	3
1	2	3	1	1	2	1	1	3	2
1	1	1	2	3	3	1	1	2	3
1	2	2	3	3	2	2	1	1	2
2	2	2	2	2	2	2	2	2	2
9	9	9	9	9	9	9	9	9	9
9	1	9	9	9	9	9	9	9	9
2	3	2	1	2	2	3	1	2	3
2	2	2	2	2	2	2	2	2	2
2	2	2	2	2	2	2	2	2	2

2	9	1	1	1	1	3	3	3	3
9	9	9	9	9	9	9	9	9	9
9	9	9	9	9	9	9	9	9	9
9	9	9	9	9	9	9	9	9	9
9	9	9	9	9	9	9	9	9	9
9	9	1	9	9	9	9	9	9	9
9	9	9	9	9	9	9	9	9	9
9	9	9	9	9	9	9	9	9	9
9	9	9	9	9	9	9	9	9	9
9	9	9	9	9	9	9	9	9	9
2	2	2	2	2	2	2	2	2	2
2	1	2	3	3	2	3	2	2	2
1	2	1	1	2	1	1	1	1	2
3	2	2	2	2	2	2	2	2	2
2	2	2	2	2	2	2	2	2	2
9	9	9	9	9	9	9	9	9	9
2	2	2	2	2	2	2	3	3	3
2	2	2	2	2	2	2	2	2	2
2	2	2	2	2	2	2	2	2	2
2	2	2	3	2	3	3	2	2	2
2	2	3	1	3	1	2	2	1	3
3	1	2	3	1	1	3	2	1	3
2	2	2	2	2	2	2	2	2	2
2	2	2	2	2	2	2	2	2	2
2	2	2	2	2	2	2	2	2	2
2	2	2	2	2	2	2	2	2	2
3	3	3	3	3	3	3	3	3	3
2	2	2	2	2	2	2	1	1	2
2	2	2	2	2	2	2	2	2	2
2	2	2	2	2	2	2	2	2	2
9	9	9	9	9	9	9	9	9	9
2	2	2	2	2	2	2	2	2	2
1	1	1	1	2	2	2	3	3	3
9	9	9	9	9	9	9	9	9	9
9	9	9	9	9	9	9	9	9	9
2	2	2	2	2	2	2	2	2	2
2	2	2	2	2	2	2	2	2	2
2	2	2	2	2	2	2	2	2	2
3	3	2	3	3	3	3	2	1	1
9	9	9	9	9	9	9	9	9	9
1	1	1	1	2	2	3	3	1	2
2	2	2	2	2	2	2	2	2	2
2	2	2	2	2	2	2	2	2	2
1	1	2	1	1	2	1	2	1	2
1	1	1	2	2	2	1	1	2	2
9	9	9	9	9	9	9	9	9	9
9	9	9	9	9	9	9	9	9	9

9	9	9	9	9	9	9	9	9	9
1	1	2	2	3	3	2	2	1	3
9	9	9	9	9	9	9	9	9	9
9	9	9	9	9	9	9	9	9	9
9	9	9	9	9	9	9	9	9	9
9	9	9	9	9	9	9	9	9	9
9	9	9	9	9	9	9	9	9	9
9	9	9	9	9	9	9	9	9	9
9	9	9	9	9	9	9	9	9	9
9	9	9	9	9	9	9	9	9	9
9	9	9	9	9	9	9	9	9	9
9	9	9	9	9	9	9	9	9	9
9	9	9	9	9	9	9	9	9	9
9	9	9	9	9	9	9	9	9	9
9	9	9	9	9	9	9	9	9	9
9	9	9	9	9	9	9	9	9	9
9	9	9	9	9	9	9	9	9	9
9	9	9	9	9	9	9	9	9	9
9	9	9	9	9	9	9	9	9	9
9	9	9	9	9	9	9	9	9	9
9	9	9	9	9	9	9	9	9	9
9	9	9	9	9	9	9	9	9	9
9	9	9	9	9	9	9	9	9	9
1	1	1	1	2	2	2	1	2	3
2	2	2	2	2	2	2	2	2	2
1	2	1	2	2	1	2	2	2	2
1	1	1	2	2	2	2	2	2	2
3	2	2	3	1	2	1	1	2	3
2	2	2	2	3	3	1	2	2	3
2	2	2	2	2	2	2	2	2	2
1	2	2	3	9	1	9	2	2	2
2	2	2	2	2	2	2	2	2	2
2	2	2	3	3	3	2	2	1	3
1	1	1	1	3	2	1	1	1	3
2	2	2	2	3	2	1	1	1	3
1	1	2	2	2	1	1	1	1	3
1	1	1	2	2	2	2	1	1	2
1	1	1	1	2	1	1	1	1	2
1	1	2	2	3	2	1	1	1	3
9	9	9	9	9	9	9	9	9	9
2	1	3	2	2	2	2	1	1	2
1	2	2	2	1	3	3	2	1	2
9	9	1	9	9	9	9	9	9	9
1	1	1	2	2	2	2	2	1	2
1	1	1	1	3	1	1	1	1	3
2	2	2	2	2	2	2	2	2	2
1	2	1	1	9	1	2	3	9	2

1	1	1	1	2	2	1	1	1	2
1	2	1	1	3	1	1	2	1	3
1	1	1	1	2	2	1	1	1	3
1	1	1	2	2	2	2	2	2	2
3	2	1	1	1	1	2	2	3	2
9	9	9	9	9	9	9	9	9	9
1	1	1	3	3	2	2	2	1	3
9	9	9	9	9	9	9	9	9	9
2	2	2	2	2	2	2	2	2	2
2	2	2	2	2	2	2	2	2	2
1	9	9	9	9	9	9	9	9	9
9	9	9	9	9	9	9	9	9	9
9	9	9	9	9	9	9	9	9	9
9	9	9	9	9	9	9	9	9	9
9	9	9	9	9	9	9	9	9	9
9	9	9	9	9	9	9	9	9	9
9	9	9	9	9	9	9	9	9	9
9	9	9	9	9	9	9	9	9	9
9	9	9	9	9	9	9	9	9	9
3	1	1	3	3	3	1	3	1	3
1	1	1	1	2	2	2	2	1	2
9	9	9	9	9	9	9	9	9	9
9	9	9	9	9	9	9	9	9	9
9	9	9	9	9	9	9	9	9	9
9	9	9	9	9	9	9	9	9	9
2	1	2	3	1	2	3	1	3	2
1	1	1	3	3	3	3	1	3	3
1	2	2	2	3	3	2	1	2	2
2	2	2	2	2	2	2	2	2	2
9	9	9	9	9	9	9	9	9	9
1	2	3	2	2	1	1	2	2	2
3	1	3	3	3	3	1	3	3	9
3	3	3	3	3	3	3	3	3	3
3	1	1	2	3	2	3	1	1	3
3	3	3	3	3	1	1	1	1	3
9	9	9	9	9	9	9	9	9	9
2	2	2	2	2	2	2	2	2	2
1	1	2	2	3	2	2	2	2	3
2	2	2	3	3	3	3	3	3	1
1	2	3	1	3	3	3	1	1	3
1	1	1	2	2	2	2	2	1	2
1	1	1	2	2	2	2	2	1	3
1	1	1	1	3	3	1	1	1	3
2	3	3	3	3	2	2	1	2	2
1	1	1	3	3	2	2	2	3	3
1	3	1	1	3	2	1	2	2	3
1	1	2	1	3	2	1	1	1	3

1	1	1	1	3	1	1	1	1	3
9	2	2	3	9	2	3	2	3	3
9	9	9	9	9	9	9	9	9	9
1	1	1	1	3	9	9	1	1	3
9	9	9	9	9	9	9	9	9	9
1	1	1	2	3	2	1	2	2	3
2	1	1	1	2	3	1	1	1	3
9	9	9	9	9	9	9	9	9	9
9	9	9	9	9	9	9	9	9	9
2	2	2	2	2	2	2	2	2	2
1	2	1	2	3	2	2	2	3	2
1	1	1	3	3	1	1	3	3	3
1	1	2	9	1	2	1	3	1	2
9	9	9	9	9	9	9	9	9	9
9	9	9	9	9	9	9	9	9	9
2	1	1	2	1	2	2	2	2	3
9	9	9	9	9	9	9	9	9	9
9	9	9	9	9	9	9	9	9	9
3	3	3	3	3	3	3	3	3	3
1	1	1	1	1	1	1	1	1	1
2	2	2	2	2	2	2	2	2	2
1	1	1	1	1	1	1	1	1	1
3	3	2	1	1	1	3	3	2	1
1	1	1	2	3	1	2	1	1	2
2	2	2	2	2	2	2	2	2	2
9	9	9	9	9	9	9	9	9	9
9	9	9	9	9	9	9	9	9	9
1	3	1	3	3	1	1	1	3	3
2	2	2	2	2	3	3	3	3	3
3	2	3	3	9	1	2	3	3	2
9	9	9	9	9	9	9	9	9	9
9	9	9	9	9	9	9	9	9	9
9	9	9	9	9	9	9	9	9	9
9	9	9	9	9	9	9	9	9	9
9	9	9	9	9	9	9	9	9	9
9	9	9	9	9	9	9	9	9	9
9	9	9	9	9	9	9	9	9	9
9	9	9	9	9	9	9	9	9	9
9	9	9	9	9	9	9	9	9	9
9	9	9	9	9	9	9	9	9	9
9	9	9	9	9	9	9	9	9	9
9	9	9	9	9	9	9	9	9	9
9	9	9	9	9	9	9	9	9	9
9	9	9	9	9	9	9	9	9	9
9	9	9	9	9	9	9	9	9	9
9	9	9	9	9	9	9	9	9	9
9	9	9	9	9	9	9	9	9	9

9	9	9	9	9	9	9	9	9	9
9	9	9	9	9	9	9	9	9	9
9	9	9	9	9	9	9	9	9	9
9	9	9	9	9	9	9	9	9	9
9	9	9	9	9	9	9	9	9	9
9	9	9	9	9	9	9	9	9	9
9	9	9	9	9	9	9	9	9	9
9	9	9	9	9	9	9	9	9	9
9	9	9	9	9	9	9	9	9	9
9	9	9	9	9	9	9	9	9	9
9	9	9	9	9	9	9	9	9	9
9	9	9	9	9	9	9	9	9	9
9	9	9	9	9	9	9	9	9	9
9	9	9	9	9	9	9	9	9	9
9	9	9	9	9	9	9	9	9	9
9	9	9	9	9	9	9	9	9	9
9	9	9	9	9	9	9	9	9	9
9	9	9	9	9	9	9	9	9	9
9	9	9	9	9	9	9	9	9	9
9	9	9	9	9	9	9	9	9	9
9	9	9	9	9	9	9	9	9	9
9	9	9	9	9	9	9	9	9	9
9	9	9	9	9	9	9	9	9	9
9	9	9	9	9	9	9	9	9	9
9	9	9	9	9	9	9	9	9	9
9	9	9	9	9	9	9	9	9	9
9	9	9	9	9	9	9	9	9	9
9	9	9	9	9	9	9	9	9	9
9	9	9	9	9	9	9	9	9	9
9	9	9	9	9	9	9	9	9	9
9	9	9	9	9	9	9	9	9	9
9	9	9	9	9	9	9	9	9	9
9	9	9	9	9	9	9	9	9	9
9	9	9	9	9	9	9	9	9	9
9	9	9	9	9	9	9	9	9	9
2	2	2	2	2	2	2	2	2	2
9	9	9	9	9	9	9	9	9	9
1	3	2	3	3	3	2	2	3	9
1	1	1	1	1	3	1	1	1	3
3	3	3	3	3	3	3	3	3	3
9	9	9	9	9	9	9	9	9	9
1	2	3	2	3	3	1	1	1	2
9	9	9	9	9	9	9	9	9	9
1	2	3	2	3	3	1	1	1	2
2	1	3	2	2	2	2	2	1	2
9	1	1	9	9	9	9	9	9	1
2	2	2	2	2	2	2	2	2	2

1	1	2	2	3	2	1	1	1	3
2	2	1	2	1	3	2	2	2	2
1	2	1	2	1	1	1	2	2	2
3	1	9	2	2	1	1	1	1	2
1	1	2	2	3	2	2	1	1	3
1	1	2	3	3	2	1	1	1	3
1	1	1	2	2	3	2	2	2	3
2	2	2	2	2	3	1	2	1	3
1	3	9	2	3	1	2	3	2	2
2	3	2	3	9	2	1	2	3	1
9	9	9	9	9	9	9	9	9	9
3	2	2	9	2	2	1	1	2	3
9	9	9	9	9	9	9	9	9	9
2	9	9	2	2	2	1	1	1	2
2	3	3	2	2	2	1	1	1	2
2	2	2	2	2	2	2	2	2	2
9	9	9	9	9	9	9	9	9	9
9	9	9	9	9	9	9	9	9	9
1	1	1	1	2	1	1	1	1	1
1	1	1	2	2	2	1	9	3	3
1	1	2	2	3	2	2	1	1	3
1	1	1	2	2	2	2	2	1	2
1	1	3	1	1	1	1	2	9	2
1	1	3	3	3	1	1	1	1	1
3	3	2	3	2	3	1	2	2	3
2	1	3	1	2	1	2	1	2	1
1	1	1	1	1	1	1	1	1	1
3	3	3	1	1	3	2	2	1	2
2	2	2	2	2	2	2	2	2	2
9	9	9	9	9	9	9	9	9	9
9	9	9	9	9	9	9	9	9	9
2	2	2	2	2	2	1	1	1	2
2	2	2	2	2	2	2	2	2	2
9	9	9	9	9	9	9	9	9	9
9	9	9	9	9	9	9	9	9	9
9	9	9	9	9	9	9	9	9	9
1	3	1	1	3	2	3	2	1	2
1	2	2	2	2	2	2	2	9	2
9	9	9	9	9	9	9	9	9	9
9	9	9	9	9	9	9	9	9	9
2	2	2	3	3	3	3	3	2	2
9	9	9	9	9	9	9	9	9	9
1	2	1	2	2	3	1	2	2	2
1	3	1	3	1	1	1	3	3	1
3	3	1	2	1	1	1	1	1	1
3	1	3	3	3	3	3	3	3	3
1	3	3	1	1	3	1	3	1	1

3	2	3	2	3	2	3	2	3	2
2	2	2	3	3	2	2	1	1	2
1	3	2	1	2	1	2	2	2	2
1	1	1	2	1	1	1	1	1	1
2	1	2	2	1	2	2	3	2	1
2	1	3	1	2	3	2	1	2	2
1	1	1	1	1	1	1	1	2	3
2	3	1	2	2	3	2	1	2	1
1	1	1	3	1	1	1	1	1	1
9	1	9	2	9	9	9	2	1	9
3	3	3	1	1	3	1	3	3	1
3	1	1	1	2	1	1	3	3	1
9	1	1	1	3	1	2	2	2	3
3	2	1	2	3	3	2	1	1	2
3	1	3	1	3	1	3	1	1	1
2	3	2	2	2	2	2	2	2	2
2	2	3	2	2	1	2	2	2	2
9	9	9	9	9	9	9	9	9	9
2	1	2	3	2	1	2	2	9	2
2	3	3	1	2	2	2	2	1	2
1	2	3	1	1	1	1	2	3	3
1	1	2	2	2	1	1	1	1	1
1	2	3	3	3	2	2	1	1	1
2	2	2	2	2	2	2	2	2	2
2	3	1	2	3	1	2	3	1	2
3	3	1	3	3	1	1	3	1	1
1	2	2	2	1	2	1	1	1	2
1	1	2	3	3	3	3	2	2	2
1	2	2	2	2	2	2	2	1	2
1	3	2	2	3	1	2	1	2	3
1	1	1	3	1	2	2	2	2	1
3	2	1	2	3	2	1	2	3	2
2	3	3	1	1	1	2	2	1	1
3	9	9	9	1	1	3	1	1	1
1	2	3	1	2	3	1	2	3	1
1	3	3	1	1	3	1	1	1	1
3	2	1	2	3	2	2	3	1	1
9	9	9	9	9	9	9	9	9	9
1	3	1	1	3	3	1	1	1	3
1	3	1	2	2	1	1	1	3	1
1	3	3	3	3	3	3	2	1	3
1	2	3	1	2	3	1	2	3	1
3	3	3	1	1	1	1	3	3	1
2	3	2	3	2	2	2	3	1	1
2	2	2	2	2	2	2	2	2	2
3	3	3	3	3	3	3	2	3	3
9	9	9	9	9	9	9	9	9	9

2	1	1	2	1	1	2	3	2	2
1	1	1	1	1	1	1	1	1	1
2	2	2	1	1	3	3	3	2	2
2	1	1	3	3	2	2	1	2	2
1	2	2	2	2	2	1	2	2	2
2	3	2	1	2	3	2	1	1	2
2	2	2	2	2	2	2	2	2	2
9	9	9	9	9	9	9	9	9	9
9	9	9	9	9	9	9	9	9	9
2	3	1	2	2	1	1	2	3	2
1	2	2	2	3	2	2	2	2	2
2	2	2	2	2	2	2	2	2	2
2	2	2	2	2	1	1	1	1	1
1	1	1	1	1	1	2	2	2	2
2	2	2	2	2	2	2	2	2	2
2	2	2	2	2	2	2	2	2	2
2	2	2	2	2	2	3	2	2	2
3	2	3	2	2	2	2	2	2	2
2	2	2	2	2	2	2	2	2	2
2	2	2	2	2	2	3	3	2	2
2	2	2	1	2	2	1	1	1	2
2	2	2	2	2	2	2	2	2	2
2	2	2	2	2	2	2	2	2	2
1	2	3	2	2	9	2	1	2	1
2	2	2	2	2	2	2	2	2	2
2	2	2	2	2	2	2	2	2	2
1	2	2	2	3	2	2	2	2	2
9	1	9	2	9	2	3	1	2	3
3	3	1	1	1	2	2	2	2	2
2	2	2	2	2	2	2	2	2	2
2	2	2	2	2	2	2	2	2	2
1	2	2	2	3	2	2	2	2	3
2	2	2	2	2	2	2	2	2	2
2	2	2	2	2	2	2	2	2	2
2	2	2	2	2	2	2	2	1	3
1	2	2	2	2	2	2	2	2	2
2	2	2	2	2	2	2	2	2	2
2	2	2	2	2	2	2	2	2	2
2	2	2	2	2	2	2	2	2	2
1	3	1	1	2	3	3	1	1	1
2	2	2	2	2	2	2	2	2	2
2	2	2	2	2	2	2	2	2	2
3	3	3	2	2	2	1	1	3	2
2	2	2	2	2	2	2	2	2	2
1	1	1	2	3	2	3	1	1	1
1	1	1	1	1	1	1	1	1	1
2	2	2	2	2	2	2	2	2	2

2	2	2	2	2	2	2	2	2	2
2	2	2	2	2	2	2	2	2	2
1	3	2	2	3	2	2	2	2	2
2	2	2	2	2	2	2	2	2	2
2	2	2	2	2	2	2	2	2	2
2	2	2	2	2	2	2	2	2	2
9	9	9	9	9	9	9	9	9	9
3	1	1	1	1	1	1	1	1	1
9	9	9	9	9	9	9	9	9	9
3	3	3	3	3	3	3	3	3	3
2	2	1	3	3	3	1	2	1	3
2	2	2	2	2	2	2	2	2	2
2	3	3	2	2	2	2	2	3	2
3	3	3	3	3	3	3	3	3	3
9	9	9	9	9	9	9	9	9	9
2	2	2	1	2	1	1	1	1	2
2	2	2	2	2	2	2	2	2	2
1	1	1	1	1	1	1	1	1	1
9	9	9	9	9	9	9	9	9	9
2	2	2	2	2	2	2	2	2	2
2	1	1	2	2	2	2	1	3	3
9	9	1	9	9	9	9	9	9	9
9	9	9	9	9	9	9	9	9	9
3	3	3	3	3	9	3	3	3	3
3	2	1	2	1	2	1	2	3	2
9	1	1	1	1	1	1	1	1	1
3	3	3	3	3	3	3	3	3	3
9	1	3	9	1	2	1	1	1	1
1	3	2	3	9	1	2	1	2	1
1	1	1	3	3	3	3	3	3	3
2	2	2	2	2	2	2	2	2	2
2	2	2	2	2	2	2	2	2	2
2	2	2	2	2	2	2	2	2	2
9	1	9	9	9	1	9	1	9	9
2	2	2	2	2	2	2	2	2	1
9	9	9	9	9	9	9	9	9	9
2	2	1	2	9	1	1	1	1	1
3	3	3	3	3	3	3	3	3	3
2	2	2	2	2	2	2	2	2	2
9	2	2	2	2	2	2	2	2	2
2	2	2	2	2	2	9	1	1	1
2	2	2	2	2	2	2	2	2	2
1	9	9	9	9	9	9	9	9	9
3	3	2	1	1	1	1	1	2	2
1	2	2	2	2	2	2	2	2	2
2	2	2	2	2	2	2	2	2	2
1	2	2	2	2	2	2	2	2	2

9	9	9	9	9	9	9	9	9	9
2	2	2	2	2	2	2	2	2	2
1	1	1	1	1	1	1	1	1	1
2	2	2	2	2	2	2	2	2	2
9	9	9	9	9	9	9	9	9	9
2	2	3	3	3	2	2	1	1	2
3	3	3	3	3	3	3	3	3	3
2	2	2	2	2	2	2	2	2	2
2	2	2	2	2	2	2	2	2	2
1	1	3	3	3	3	3	3	3	3
9	9	9	9	9	9	9	9	9	9
1	1	1	1	1	1	1	1	1	1
2	2	2	2	2	2	2	2	2	2
2	2	2	2	2	2	2	2	2	2
2	2	2	2	2	2	2	2	2	2
2	2	2	2	2	2	2	2	2	2
1	1	1	1	1	1	1	1	1	1
1	1	1	1	1	1	1	3	3	3
9	9	9	9	9	9	9	9	9	9
2	3	1	2	1	3	1	2	1	2
1	1	1	1	1	1	1	1	1	1
3	3	3	3	1	1	1	1	1	1
1	1	1	1	1	1	1	1	1	1
1	2	2	2	1	2	2	2	2	2
3	9	3	9	3	3	3	3	3	3
1	1	1	2	1	1	2	1	1	1
2	3	1	1	1	2	3	1	2	1
2	2	2	2	2	2	2	2	2	2
2	1	9	1	3	1	1	2	1	2
2	2	2	2	2	2	2	2	2	2
9	1	2	3	1	2	1	2	9	1
2	2	2	3	3	3	3	2	2	2
3	3	3	3	3	3	3	3	3	3
3	2	2	2	9	3	3	9	2	3
2	2	2	2	2	2	2	2	2	2
2	2	2	2	2	2	2	2	2	2
1	1	1	1	1	1	1	1	1	1
2	2	2	2	2	2	2	2	2	2
1	2	3	1	2	1	2	1	2	1
3	3	3	3	3	3	3	3	3	3
1	2	1	3	1	3	1	2	1	2
2	2	2	2	2	1	1	3	3	3
9	9	9	9	9	9	9	9	9	9
3	3	3	3	3	9	9	9	9	9
9	9	9	9	9	9	9	9	9	9
2	2	2	2	2	2	2	2	2	2
2	1	1	2	3	3	3	2	2	2

2	2	2	2	2	2	2	2	2	2
3	3	1	3	1	3	1	3	3	1
9	9	9	9	9	9	9	9	9	9
3	3	1	3	1	3	1	3	3	1
1	2	1	1	3	2	1	3	3	1
2	2	2	2	2	2	2	2	2	2
2	2	2	2	2	2	2	2	2	2
2	2	2	2	2	2	2	2	2	2
1	1	1	1	1	1	1	1	1	1
3	2	3	2	1	2	2	3	1	2
1	2	3	2	1	2	2	2	3	2
1	3	9	1	3	2	2	3	1	2
2	2	2	2	2	2	2	2	2	2
3	2	1	2	3	2	3	3	3	3
2	2	2	2	2	2	2	2	2	2
1	2	2	2	2	2	2	2	2	2
2	2	2	2	2	2	2	2	2	2
2	2	2	2	2	2	2	2	2	2
2	2	2	2	2	2	2	2	2	2
2	1	2	2	2	2	2	2	2	2
2	2	2	2	2	2	2	2	2	2
2	2	2	2	2	2	2	2	2	2
2	2	2	2	2	2	2	1	2	1
2	2	2	2	2	2	2	2	2	2
1	1	1	1	1	1	1	1	1	1
2	2	2	2	2	2	2	2	2	2
1	1	1	2	2	2	2	1	1	1
2	2	2	2	2	2	2	2	2	2
1	3	3	2	3	2	3	1	1	3
3	3	2	2	1	1	2	3	2	2
1	3	9	2	2	1	1	1	1	1
3	3	3	3	3	2	2	1	1	1
2	2	2	2	2	2	2	2	2	2
2	3	3	2	3	2	3	2	3	2
3	3	3	2	1	2	2	2	3	1
1	2	3	3	2	3	2	1	3	2
9	9	9	9	9	9	9	9	9	9
9	9	9	9	9	9	9	9	9	9
3	3	3	3	3	3	3	3	3	3
2	2	2	2	2	2	2	2	2	2
2	2	2	2	2	2	2	3	1	2
2	2	2	2	2	2	2	2	2	2
2	2	2	2	2	2	2	2	2	2
2	2	2	2	2	2	2	2	2	2
9	9	9	9	9	9	9	9	9	9
1	2	1	1	1	2	1	2	2	1
2	2	2	2	2	2	2	2	2	2

9	9	9	9	9	9	9	9	9	9
2	2	1	2	3	1	2	2	9	1
3	2	1	1	3	2	1	3	3	1
3	2	2	2	3	1	9	2	2	3
3	3	3	3	3	3	3	3	1	3
3	1	1	1	2	3	1	1	2	2
2	2	3	3	3	3	2	2	1	1
9	9	9	9	9	9	9	9	9	9
2	2	2	2	2	2	2	2	2	2
3	2	2	3	3	2	2	1	1	2
1	2	1	2	2	2	2	2	1	1
2	3	1	2	2	3	2	1	1	1
2	3	1	2	1	2	1	2	1	2
1	1	1	1	1	1	1	1	1	1
2	2	3	1	2	3	1	2	3	1
2	2	2	2	2	2	2	2	2	2
2	2	1	2	3	2	1	2	2	3
2	2	2	2	2	2	2	2	2	2
9	9	9	9	9	9	9	9	9	9
9	9	9	9	9	9	9	9	9	9
9	9	9	9	9	9	9	9	9	9
1	2	3	2	1	2	3	2	1	3
3	3	3	1	1	2	1	2	3	2
3	2	2	1	1	1	1	1	2	2
2	2	2	2	3	3	3	2	2	1
3	3	3	3	3	3	3	3	3	3
2	1	2	1	3	2	1	2	3	2
2	9	2	3	3	3	3	3	3	2
2	3	2	3	1	2	2	1	2	3
1	2	2	2	2	1	1	2	1	2
3	2	2	2	2	2	3	3	3	3
1	2	2	2	2	3	3	3	3	2
2	3	3	9	2	3	3	9	1	3
3	3	3	3	3	3	3	3	3	3
3	3	3	3	3	3	3	3	3	3
2	2	2	2	2	2	2	2	2	2
3	3	3	3	3	3	3	3	3	3
2	2	2	2	2	2	2	2	2	2
9	9	9	9	9	9	9	9	9	9
9	9	9	9	9	9	9	9	9	9
1	1	1	1	1	1	1	1	1	1
2	2	2	2	2	2	2	2	2	2
2	1	3	3	2	1	2	3	2	1
2	2	3	2	3	2	2	3	3	2
1	9	9	9	9	9	9	9	9	9
3	3	3	3	3	3	3	3	3	3
2	2	1	1	2	2	2	2	3	3

1	1	1	1	1	1	1	1	1	1
3	2	2	3	1	2	1	1	3	2
1	2	1	3	2	1	2	3	1	2
3	9	2	2	2	2	2	1	2	3
3	2	3	2	1	2	2	1	3	2
2	2	2	3	3	3	3	3	3	3
2	2	2	2	2	2	2	2	2	2
3	3	3	3	3	3	3	3	3	3
3	2	2	3	2	2	3	3	2	2
1	2	2	1	3	1	1	3	2	3
2	2	2	2	2	2	2	2	2	2
3	2	3	3	3	3	3	1	3	3
2	2	2	3	2	3	2	2	2	3
1	1	2	3	1	1	1	1	2	2
2	1	1	1	1	1	1	1	1	1
3	3	3	3	3	2	2	3	3	3
1	1	1	1	1	3	3	3	3	3
2	2	2	2	2	2	2	2	2	2
9	9	9	9	9	9	9	9	9	9
3	1	2	2	1	2	1	2	1	2
3	1	2	3	1	2	1	2	1	3
2	3	3	2	3	2	3	2	3	2
1	2	2	2	2	3	2	2	2	2
2	2	3	2	2	1	2	2	1	3
1	2	3	1	2	3	1	2	3	1
2	3	2	2	3	2	3	3	3	1
2	2	2	2	2	2	2	2	2	2
1	2	2	1	2	3	2	2	1	1
1	2	1	3	1	2	3	1	2	1
2	2	2	2	2	2	2	2	2	2
1	2	1	1	1	2	2	3	2	1
2	2	2	2	2	2	2	2	2	2
2	2	2	2	2	2	2	2	2	2
3	3	2	2	3	3	2	2	3	2
1	2	3	1	2	3	1	2	3	1
2	2	1	1	1	1	2	3	1	2
2	3	3	1	2	3	1	2	1	1
3	3	3	3	3	3	3	3	3	3
2	2	3	3	3	3	3	3	3	1
1	3	1	2	1	3	2	3	1	1
1	1	1	1	2	2	2	2	2	2
1	2	3	1	2	1	2	3	1	2
2	3	2	3	2	9	2	2	2	3
1	1	1	1	1	1	1	1	1	1
3	3	3	3	3	2	2	2	2	2
3	2	2	3	2	3	2	2	1	2
1	2	1	1	2	1	2	1	1	2

2	3	3	3	3	2	2	2	3	3
1	2	1	2	3	2	3	1	2	1
2	3	2	2	2	2	2	2	2	2
1	1	1	1	1	1	1	1	1	1
2	3	1	2	3	1	2	3	1	2
2	2	2	2	3	3	3	3	2	2
2	2	1	2	3	2	1	2	2	1
1	2	1	2	3	1	2	3	1	2
1	2	1	3	1	1	3	1	3	1
3	3	2	2	3	2	3	2	3	3
3	2	3	2	3	2	3	2	3	1
2	1	2	1	3	2	1	2	1	2
1	1	1	1	1	1	1	1	1	1
3	3	3	1	1	1	3	1	1	1
1	2	3	1	2	3	2	1	3	2
3	3	3	3	3	1	1	1	1	3
1	1	2	1	1	1	2	2	3	3
1	3	3	3	1	1	1	1	1	2
2	2	3	1	2	1	3	3	2	2
2	1	3	2	2	2	2	2	2	2
3	2	3	2	3	2	3	2	3	2
3	3	3	3	3	3	3	3	3	3
1	3	3	1	2	3	1	3	1	3
2	2	2	2	2	1	1	1	1	1
2	2	2	2	2	2	2	2	2	2
2	2	2	2	2	2	2	2	2	2
1	3	3	3	2	3	2	2	1	2
1	2	3	2	3	1	2	2	3	1
2	2	2	2	2	2	2	2	2	2
9	9	9	9	9	9	9	9	9	9
9	9	9	9	9	9	9	9	9	9
9	9	9	9	9	9	9	9	9	9
3	3	3	3	3	3	3	3	3	2
9	9	9	9	9	9	9	9	9	9
2	2	2	2	2	2	2	2	2	2
9	9	9	9	9	9	9	9	9	9
2	1	1	2	1	2	2	2	2	1
9	9	9	9	9	9	9	9	9	9
2	2	2	1	1	1	9	1	9	1
2	2	3	2	3	2	2	2	2	2
9	9	9	9	9	9	9	9	9	9
1	1	1	1	1	3	1	1	3	1
3	3	3	3	3	3	3	3	3	3
3	3	3	3	3	3	3	3	3	3
3	2	2	2	1	2	1	9	1	2
1	2	3	2	3	1	2	3	1	2
1	3	3	3	2	3	2	2	1	2

1	3	3	3	1	3	2	2	3	1
2	2	2	2	2	2	2	2	2	2
9	9	9	9	9	9	9	9	9	9
2	1	1	2	3	2	1	2	1	1
2	2	1	3	9	9	2	9	9	9
2	1	3	1	2	1	2	1	3	1
9	9	9	9	9	9	9	9	9	9
2	1	2	1	3	2	3	1	2	1
1	2	3	2	2	3	3	3	2	2
3	3	3	3	3	3	3	3	3	3
2	3	1	2	3	1	2	3	1	2
2	3	3	2	1	2	3	2	1	2
1	1	2	9	1	2	2	1	2	9
2	2	2	2	2	2	2	2	2	2
2	2	2	2	2	2	2	2	2	2
9	2	9	9	9	9	9	9	9	9
2	2	2	2	2	2	2	2	2	2
2	2	2	2	2	2	2	2	2	2
2	2	2	2	2	2	2	2	2	2
2	2	2	2	2	2	2	2	2	2
1	9	9	9	1	1	1	1	1	9
1	9	9	9	1	1	1	1	1	9
1	1	9	9	9	1	1	1	1	9
1	1	1	1	2	1	1	2	2	2
1	1	1	2	2	1	1	2	1	3
9	9	9	9	9	9	9	9	9	9
1	2	3	3	2	3	3	2	1	2
1	1	1	3	3	1	2	3	1	3
1	2	1	3	2	1	1	2	2	2
1	1	1	1	3	1	1	1	1	3
1	1	1	3	3	3	1	1	9	9
1	9	9	9	9	1	9	9	9	9
1	1	1	2	2	2	1	1	1	3
1	1	2	3	2	1	1	1	1	3
2	3	2	1	2	1	1	2	2	2
2	3	2	2	3	1	2	3	3	3
2	3	2	2	3	1	2	3	3	3
1	1	9	9	9	1	9	9	1	9
1	1	3	3	3	1	1	1	1	3
1	1	1	1	1	1	1	1	1	1
1	1	3	3	3	1	1	1	1	3
1	1	9	9	9	9	9	9	1	9
2	2	2	2	9	1	1	1	1	1
1	1	1	1	1	1	1	1	1	1
1	1	3	3	3	1	1	1	1	3
2	1	3	2	3	1	1	3	3	2
1	2	3	3	3	2	1	1	2	3

2	3	2	2	3	1	2	3	3	3
3	9	9	9	9	9	1	9	3	9
1	1	2	2	2	2	1	2	1	2
1	1	1	1	1	1	1	1	1	1
9	1	2	2	3	9	1	1	9	3
1	1	2	2	3	9	1	1	9	3
2	2	2	2	2	2	2	2	2	2
1	1	1	1	1	1	1	1	1	3
9	9	9	9	9	9	9	9	9	9
1	1	1	2	3	1	2	2	2	3
1	1	3	2	2	1	2	2	2	2
2	1	2	2	2	2	2	2	2	2
9	1	2	2	3	9	1	1	9	3
9	1	2	2	3	9	1	1	3	3
9	1	2	2	9	3	1	1	3	3
1	1	2	2	9	9	1	1	3	3
2	1	2	2	3	3	1	1	1	3
1	1	1	2	3	3	1	1	3	1
1	1	2	2	3	9	1	1	3	3
2	3	2	3	1	2	1	3	1	2
1	2	3	1	2	1	3	2	1	2
2	9	2	2	1	1	3	3	3	3
1	1	1	1	3	1	1	1	1	1
1	1	1	3	3	1	2	1	1	3
1	1	1	2	2	1	1	1	1	2
1	3	1	1	1	1	1	1	1	1
3	1	3	3	3	3	3	3	3	3
1	1	1	1	3	1	1	1	1	3
2	1	3	2	3	3	2	2	1	2
1	1	1	1	1	1	1	1	1	1
1	3	2	2	2	2	2	2	2	2
3	1	3	3	3	1	1	3	3	3
9	1	1	9	9	1	9	9	9	9
9	1	1	9	9	9	9	9	1	9
1	1	1	9	3	1	1	9	1	3
1	1	1	2	3	2	1	2	1	3
1	1	1	2	9	1	1	1	1	2
1	1	1	3	3	1	9	1	1	3
1	1	1	3	9	1	9	1	1	9
1	1	1	3	3	1	1	1	1	3
1	1	1	1	3	1	1	1	1	3
1	3	1	1	3	3	3	1	1	3
1	9	1	2	3	1	1	2	1	3
1	1	1	1	3	1	1	3	1	3
1	1	1	2	3	1	1	1	1	3
1	1	2	3	3	1	1	2	1	3
9	1	3	9	9	9	9	9	9	9

3	1	3	1	3	2	1	1	1	2
1	1	1	3	3	1	1	1	1	3
2	1	3	2	3	2	1	2	1	3
3	1	3	3	3	1	1	1	1	1
1	1	1	1	3	1	1	1	1	3
1	1	1	3	3	1	1	1	1	1
1	9	9	9	9	9	9	9	9	9
1	1	1	9	1	1	1	1	1	1
1	1	1	3	1	1	1	1	1	1
1	1	1	1	3	1	1	1	1	3
1	1	1	1	2	2	2	2	1	2
1	1	1	9	9	9	9	9	9	9
1	1	1	1	3	3	1	1	1	3
1	1	3	2	3	3	1	1	3	3
2	2	2	1	1	1	1	3	3	2
1	1	1	1	2	2	1	1	1	2
1	1	1	9	9	9	9	9	9	9
1	1	1	9	9	9	9	9	9	9
1	1	1	1	1	3	1	1	1	3
1	1	1	1	2	1	2	2	1	2
1	3	3	3	1	2	1	2	1	2
2	1	2	2	3	3	2	1	2	2
1	1	1	1	3	1	2	2	1	3
1	1	1	1	2	2	1	1	1	3
1	3	1	2	2	2	1	1	1	2
1	3	1	3	3	3	1	1	3	1
3	3	3	2	2	2	2	3	2	3
1	2	2	2	2	2	2	2	2	2
1	1	1	1	1	1	1	1	1	1
1	1	1	3	3	1	1	2	3	3
2	1	2	2	2	2	2	2	2	2
1	2	2	3	1	1	2	2	2	2
1	1	1	2	3	1	2	1	1	3
3	1	2	2	1	3	1	1	1	2
3	1	1	1	1	1	1	1	1	1
1	1	2	1	2	2	1	1	1	2
1	1	1	2	2	3	3	3	3	3
1	2	1	2	3	3	3	3	3	3
1	1	9	2	3	3	3	3	3	3
1	1	1	3	3	3	2	2	2	2
1	1	1	3	3	1	1	1	1	3
1	1	1	3	3	3	1	1	1	3
2	3	2	2	3	2	2	2	1	2
9	9	9	9	9	9	9	9	9	9
2	2	3	2	2	2	2	1	9	2
1	1	1	3	3	3	1	1	1	3
1	1	2	1	2	2	1	2	1	3

1	1	1	2	3	1	1	1	1	2
1	1	1	1	3	2	1	2	1	3
1	1	1	2	2	2	2	2	1	2
1	1	1	2	2	1	1	2	1	2
1	2	2	2	1	2	1	1	1	3
3	2	3	2	2	3	1	2	1	1
1	9	9	1	9	9	9	9	9	9
1	1	1	2	2	2	2	2	2	2
9	9	9	9	9	9	9	9	9	9
1	1	1	2	2	1	2	2	9	3
1	1	1	2	2	1	1	2	2	2
1	1	2	2	3	2	1	1	1	3
2	2	2	2	2	2	2	2	2	2
1	1	2	2	3	3	3	1	1	3
2	2	2	9	1	2	2	2	1	2
9	9	9	9	9	9	9	9	9	9
2	1	2	2	2	2	1	2	2	2
1	1	2	1	2	2	1	2	1	2
1	1	1	1	1	1	1	1	1	1
2	1	2	2	2	2	1	1	1	3
9	9	9	9	9	9	9	9	9	9
3	1	3	1	3	3	1	3	1	3
2	2	2	2	3	3	2	2	2	2
1	1	1	1	3	1	1	1	1	3
1	1	1	1	2	2	1	1	1	1
1	3	2	1	1	2	2	1	1	1
1	1	2	2	2	2	1	1	1	9
9	9	9	9	9	9	9	9	9	9
9	9	9	9	9	9	9	9	9	9
3	2	2	1	3	2	1	1	2	3
1	1	1	1	3	3	1	1	1	3
1	1	1	1	2	2	2	1	1	2
1	1	1	3	1	3	1	1	1	3
1	1	1	3	3	1	9	9	9	9
1	1	3	3	3	1	1	1	1	3
2	1	1	2	3	1	2	2	1	2
1	1	1	3	2	1	1	1	1	2
1	1	3	3	1	1	1	1	1	1
1	1	1	3	3	1	3	1	3	3
1	1	1	3	2	1	1	1	1	2
2	1	3	2	1	1	1	1	2	1
1	1	2	2	3	1	1	2	3	3
1	1	1	3	3	1	1	2	1	3
9	9	9	9	9	9	9	9	9	9
2	1	2	2	3	1	1	1	1	1
2	2	2	2	2	2	1	1	2	2
9	2	1	2	2	2	2	1	1	1

1	1	3	3	3	1	1	1	1	3
2	1	2	2	2	1	1	2	2	3
9	9	9	9	9	9	9	9	9	9
1	1	1	2	3	3	1	1	1	3
1	1	9	9	9	3	9	9	9	9
2	1	1	1	2	2	1	1	1	2
9	1	1	2	3	2	1	2	1	2
3	1	3	1	3	1	1	2	1	3
9	9	9	2	9	9	9	9	9	9
2	1	2	2	2	2	1	2	1	2
1	1	3	3	2	3	3	3	1	3
2	1	2	2	2	2	2	2	2	3
1	1	2	1	3	3	1	2	1	2
1	1	9	9	9	1	9	9	1	9
1	2	1	2	1	1	1	1	1	2
2	1	2	3	3	3	1	1	1	2
9	1	9	9	9	9	1	9	1	9
9	1	9	9	9	9	9	9	1	9
1	1	2	1	2	2	1	2	1	2
1	1	3	3	2	3	3	2	2	2
3	3	3	2	9	1	1	3	1	3
1	1	1	1	3	3	1	1	1	3
1	1	1	1	3	3	1	1	1	3
1	1	1	1	3	1	1	1	1	3
1	1	1	1	2	2	1	1	1	2
3	1	1	2	3	3	1	2	1	3
2	1	2	1	9	3	1	1	1	2
3	1	2	2	3	3	1	1	1	3
3	1	1	2	1	3	2	1	1	2
3	1	1	2	1	3	1	1	1	2
1	1	1	3	2	3	1	2	1	3
1	1	2	1	2	2	1	1	1	2
3	1	3	3	2	2	1	2	1	2
2	2	1	2	2	2	2	3	1	3
1	1	2	2	3	3	1	1	2	3
9	1	9	9	9	1	1	9	9	9
3	1	3	1	3	3	1	3	3	3
9	1	9	9	9	9	9	9	9	9
1	2	2	2	1	9	1	1	1	1
1	2	2	2	2	2	2	2	1	2
3	2	3	2	2	3	2	3	2	3
2	3	3	2	2	3	3	2	2	2
9	9	9	9	9	9	9	9	9	9
9	9	9	9	9	9	9	9	9	9
1	1	1	1	2	3	1	1	1	2
1	1	1	1	3	3	1	1	1	3
9	1	9	9	9	9	1	9	1	9

3	1	3	3	3	3	1	1	1	3
1	1	1	1	2	2	1	1	1	2
1	1	1	1	9	9	1	1	1	9
1	1	1	3	3	3	2	1	1	2
1	1	1	1	2	2	1	1	1	2
2	1	2	2	2	3	2	2	1	3
2	2	2	2	2	2	2	2	2	2
2	2	2	2	2	2	1	1	1	2
2	2	2	2	2	2	2	2	2	2
2	1	1	2	2	3	1	2	1	3
1	2	2	2	2	2	3	2	1	3
9	9	9	9	9	9	9	9	9	9
9	9	9	9	9	9	9	9	9	9
1	9	9	9	9	9	9	9	9	9
9	9	9	9	9	9	9	1	9	9
9	1	9	9	9	9	9	9	9	9
1	3	3	2	2	3	1	3	3	3
3	2	1	2	1	2	3	2	2	1
9	9	9	9	9	9	9	9	9	9
1	1	1	2	2	2	1	1	2	2
1	1	1	1	3	3	1	1	1	2
2	9	2	2	3	2	1	2	1	2
2	1	2	2	3	2	1	2	1	2
1	1	2	2	3	3	2	1	1	3
2	1	2	2	2	3	2	1	1	3
1	1	1	1	3	2	1	1	1	3
9	1	3	3	3	3	1	1	1	3
2	1	2	3	3	3	1	2	2	3
1	1	9	9	9	9	1	1	1	9
1	1	9	9	9	1	1	1	1	3
1	1	2	1	1	2	1	1	1	2
1	9	1	9	9	9	1	1	1	9
1	1	2	2	2	3	1	1	1	2
1	1	3	3	3	1	1	1	1	3
3	1	2	2	3	3	1	1	1	3
1	1	2	1	2	1	1	1	1	2
1	1	1	2	2	2	1	2	1	2
3	1	3	3	3	2	2	2	1	3
9	9	9	9	9	9	9	9	9	9
1	1	1	3	3	1	1	1	2	3
2	2	2	3	3	3	1	2	1	1
2	2	2	2	9	1	3	1	1	2
1	1	1	1	3	1	1	1	1	3
2	1	2	2	2	1	1	1	1	2
1	1	1	2	2	1	1	2	1	2
1	1	2	1	2	2	1	1	1	2
1	1	3	1	1	3	1	1	1	1

3	1	3	3	3	3	1	3	1	3
1	1	2	1	2	1	1	1	1	3
1	3	2	3	1	2	1	1	1	3
1	1	1	2	2	3	3	1	1	2
9	9	9	9	9	9	9	9	9	9
1	1	3	3	3	3	1	1	1	3
3	1	3	3	3	3	1	1	1	3
1	1	1	2	2	3	1	1	1	3
1	1	2	2	2	9	1	1	3	3
1	1	2	1	1	2	2	1	1	3
1	1	1	2	2	3	1	1	1	3
2	2	2	1	2	1	1	2	2	1
1	1	2	3	2	3	1	1	1	2
1	1	3	3	3	3	1	1	1	3
3	1	2	1	3	3	1	3	2	2
1	1	2	2	2	2	1	1	1	2
1	1	2	2	2	2	1	1	2	2
2	1	2	1	2	2	1	1	1	2
1	1	3	3	3	3	1	2	2	3
1	1	1	2	2	2	2	1	1	2
1	1	1	2	2	2	2	1	1	2
2	1	2	1	3	9	9	9	9	9
1	2	2	2	3	2	1	1	2	3
1	1	2	2	3	1	1	1	1	2
1	1	2	2	2	2	1	1	2	2
9	9	9	9	9	9	9	9	9	9
9	9	9	9	9	9	9	9	9	9
9	9	9	9	9	9	9	9	9	9
2	1	2	1	3	2	1	1	1	3
2	1	1	2	3	3	2	9	2	9
1	1	2	2	1	2	2	1	1	2
1	1	2	2	1	9	2	1	1	2
9	9	9	9	9	9	9	9	9	9
9	9	9	9	9	9	9	9	9	9
9	9	9	9	9	9	9	9	9	9
9	9	9	9	9	9	9	9	9	9
9	9	9	9	9	9	9	9	9	9
9	9	9	9	9	9	9	9	9	9
9	9	9	9	9	9	9	9	9	9
2	1	1	3	2	1	1	1	3	3
2	1	2	1	3	2	1	1	1	3
9	9	9	9	9	9	9	9	9	9
9	9	9	9	9	9	9	9	9	9
1	1	1	2	3	3	1	1	1	2
9	9	9	9	9	9	9	9	9	9
9	1	9	9	9	9	9	9	9	9
9	1	9	9	9	9	9	9	9	9

1	1	2	3	3	3	1	1	1	3
9	9	9	9	9	9	9	9	9	9
1	9	9	9	9	9	9	9	9	9
9	1	3	2	2	3	1	1	1	1
9	1	3	2	2	3	1	1	1	1
1	9	9	9	9	9	9	9	9	9
9	9	9	9	9	9	9	9	9	9
9	9	9	9	9	9	9	9	9	9
9	9	9	9	9	9	9	9	9	9
9	9	9	9	9	9	9	9	9	9
1	1	1	2	3	3	3	1	3	3
1	1	1	1	3	1	1	3	1	3
3	1	1	1	3	1	1	1	1	3
2	2	2	2	2	2	1	1	1	2
1	1	3	1	3	3	1	3	3	3
3	1	3	1	1	3	1	1	1	1
3	3	3	3	1	1	1	1	1	3
3	1	2	2	2	1	1	1	1	3
1	1	1	1	3	1	1	3	1	3
1	2	1	2	1	2	1	1	1	1
1	2	1	2	1	2	1	1	1	1
2	2	2	2	2	2	1	1	1	1
9	9	9	9	9	9	9	9	9	9
1	1	1	1	1	1	1	2	3	2
1	1	2	3	3	3	3	3	2	2
1	1	1	2	3	3	2	1	1	3
2	2	3	3	3	3	3	3	9	3
1	1	3	2	2	3	1	1	1	1
1	3	2	2	2	3	1	1	1	2
2	2	3	2	3	2	1	2	3	2
1	2	2	2	3	3	1	1	1	3
3	2	2	1	3	3	2	2	1	3
3	1	2	2	2	3	1	1	1	3
2	2	2	2	2	3	2	1	1	2
2	2	2	2	2	3	1	1	1	2
1	1	2	1	1	3	1	1	1	2
3	1	9	9	9	9	1	9	1	9
3	3	3	1	3	3	3	1	1	3
3	3	3	3	3	3	1	1	1	3
1	3	2	1	1	3	1	2	1	2
9	9	9	9	9	9	9	9	9	9
1	3	3	1	1	1	1	1	1	1
3	3	3	3	2	3	2	2	2	2
1	1	2	2	2	2	1	2	2	2
1	1	2	2	2	2	1	1	2	2
1	1	1	1	1	1	1	1	1	1
2	2	2	1	3	3	1	1	2	3

1	2	3	1	3	3	1	1	1	1
1	1	1	1	1	1	1	1	1	1
1	1	1	1	1	1	1	1	1	1
1	1	2	2	2	2	1	1	1	2
1	1	2	2	2	2	1	1	2	2
1	1	2	2	2	2	1	1	9	2
2	2	2	1	3	3	1	1	2	3
1	2	2	1	1	3	2	1	1	1
2	2	2	1	2	2	1	1	1	2
3	3	3	1	3	3	1	9	1	3
1	1	2	2	2	1	1	1	1	3
1	3	3	3	9	1	9	1	1	3
1	1	2	3	2	1	2	3	3	2
1	2	3	2	2	1	2	3	3	2
3	3	1	1	1	1	1	1	1	3
2	3	3	3	1	2	2	9	1	2
2	2	2	2	2	2	2	2	2	2
2	2	2	2	2	2	2	2	2	2
1	1	1	3	1	3	3	1	3	3
1	2	2	2	3	2	2	2	1	3
1	1	2	3	3	2	1	1	1	3
1	1	3	3	3	1	1	1	1	3
1	2	2	2	2	1	1	1	1	2
1	2	1	1	2	1	1	1	1	2
1	2	1	2	1	1	1	1	1	2
1	1	1	2	3	3	2	1	1	3
2	2	3	3	1	1	2	2	2	1
1	1	1	1	2	3	1	2	1	3
1	1	2	2	2	2	1	1	1	2
2	2	1	3	3	2	1	1	1	3
3	3	3	3	2	2	3	3	2	2
1	1	3	3	3	3	3	2	2	2
1	1	1	3	3	3	1	1	1	3
3	1	2	3	3	3	1	2	2	3
1	2	2	2	2	2	1	1	1	2
1	2	2	2	2	1	1	1	1	2
3	2	3	3	3	3	3	3	2	2
3	1	3	3	3	3	3	3	3	3
1	1	2	3	3	3	1	2	2	3
2	1	3	1	3	3	1	1	2	3
1	1	3	2	3	3	1	1	1	3
3	1	2	3	1	3	1	1	1	3
3	1	2	3	9	3	1	1	1	3
1	2	2	1	2	1	2	2	2	2
1	1	3	3	3	1	1	1	1	3
1	2	2	2	2	2	1	2	2	2
1	1	1	1	3	1	3	1	1	1

2	3	2	2	2	3	2	2	2	2
9	1	9	9	9	9	9	9	1	9
1	1	1	2	2	3	3	1	1	2
2	1	1	2	3	2	2	2	2	3
1	1	1	1	2	2	2	1	1	2
1	1	2	2	2	1	2	2	1	9
3	1	2	1	3	3	2	2	1	3
1	2	2	1	2	2	1	2	2	2
3	3	3	3	3	2	2	1	1	2
1	1	3	2	3	2	1	1	1	3
2	1	2	2	2	2	1	2	1	2
3	1	3	3	3	3	1	3	1	3
9	9	9	9	9	9	9	9	9	9
3	3	3	1	3	2	2	1	1	3
1	1	1	3	3	3	3	1	1	3
2	2	2	2	2	2	2	2	2	2
3	1	3	1	2	1	1	3	2	3
1	1	2	2	2	1	1	1	1	3
1	1	2	1	2	1	1	1	1	3
1	1	1	1	2	1	1	1	1	9
1	1	2	1	1	2	1	2	1	2
1	2	1	2	1	2	1	1	1	1
1	1	1	3	3	3	1	1	1	3
2	2	1	1	2	3	2	2	2	2
9	1	1	1	1	2	1	2	1	2
3	1	3	1	3	3	1	1	1	1
3	1	3	1	1	3	1	1	1	1
2	1	1	3	2	1	1	1	3	3
1	1	2	3	3	9	1	1	1	2
9	9	9	9	9	9	9	9	9	9
2	1	3	1	2	3	2	1	2	3
1	1	3	2	1	2	1	1	1	1
1	1	2	2	2	2	2	2	1	2
1	1	1	2	1	2	1	1	1	2
1	1	2	2	2	2	1	1	1	2
1	1	2	2	1	2	1	1	2	1
9	9	9	9	9	9	9	9	9	9
1	9	9	9	9	9	9	9	9	9
1	2	2	2	2	2	3	2	1	1
2	2	2	1	2	2	1	1	1	2
1	2	3	2	2	1	1	2	1	2
1	1	2	2	3	2	1	1	1	2
1	1	1	2	2	2	1	1	1	2
9	1	2	3	9	2	9	9	1	9
2	2	2	2	2	2	3	2	9	2
2	1	2	2	2	2	1	2	2	1
2	1	1	3	3	2	1	1	1	3

2	1	2	1	3	2	1	1	1	3
1	1	2	1	2	2	1	1	1	2
1	1	2	2	2	2	2	2	2	2
2	2	1	2	2	1	1	2	1	2
2	1	2	2	2	1	1	1	1	1
1	1	2	3	9	3	1	1	1	3
1	1	1	1	3	1	1	1	1	3
9	9	1	9	9	9	9	9	9	9
1	1	2	2	3	1	1	1	1	3
1	2	2	2	2	2	1	1	1	2
1	1	2	2	3	9	1	2	1	3
3	2	2	3	2	2	3	2	3	2
1	2	1	3	2	1	1	1	2	9
2	2	2	2	2	2	2	2	2	2
3	1	3	1	3	3	1	1	1	3
3	1	3	1	3	3	1	1	1	3
2	1	1	1	1	2	1	1	1	1
1	1	1	2	1	1	1	1	1	3
1	9	9	9	9	9	9	9	9	9
1	3	3	3	2	1	1	1	1	9
2	1	2	2	3	1	1	1	1	3
1	2	1	1	2	1	1	1	1	2
1	2	2	2	1	2	2	2	2	2
1	1	1	2	3	1	1	1	1	3
1	1	1	2	3	3	1	3	1	3
1	9	1	1	9	1	1	1	1	1
1	1	2	1	3	1	1	1	1	3
1	1	1	1	3	1	1	1	2	2
1	1	1	1	3	1	1	1	2	2
9	9	9	9	9	9	9	9	9	9
1	1	1	1	3	1	1	1	2	2
1	2	9	1	1	2	1	1	1	2
1	2	1	1	3	1	1	1	1	2
2	1	2	2	2	1	2	1	1	2
9	9	9	9	9	9	9	9	9	9
1	2	2	2	2	1	1	1	1	2
1	2	2	1	2	1	1	1	1	2
1	3	3	3	9	1	9	1	1	1
1	3	3	3	3	1	1	1	1	3
1	2	2	2	3	3	1	2	1	3
1	1	2	2	3	1	1	2	2	2
1	1	1	2	3	1	1	1	1	3
1	2	2	2	2	2	2	2	2	2
1	1	1	3	3	1	1	1	1	3
3	3	3	3	3	3	1	3	3	3
1	1	1	3	3	1	3	3	1	3
1	1	1	1	3	1	1	1	1	2

3	3	3	3	3	1	1	1	1	3
3	3	3	3	3	1	1	1	1	3
3	3	3	3	3	3	1	2	2	3
1	2	1	2	3	1	1	1	1	1
1	3	3	3	3	1	1	1	1	3
1	3	3	3	3	2	1	1	1	1
1	1	3	1	3	1	1	1	1	3
1	1	1	1	1	1	1	2	1	2
1	1	1	9	2	3	1	3	1	3
3	1	1	1	3	1	1	1	1	3
1	1	1	1	3	3	1	1	1	3
1	1	1	2	2	1	1	1	1	1
1	1	1	1	1	1	1	1	1	1
3	3	3	1	1	1	1	3	3	3
1	1	1	1	3	3	1	1	1	1
1	1	1	3	3	3	1	1	1	3
2	1	2	1	2	2	1	2	2	1
2	1	1	2	2	2	1	1	1	2
1	1	3	1	3	3	1	1	1	3
9	9	9	9	9	9	9	9	9	9
1	3	1	1	1	1	2	1	1	1
2	1	2	1	3	2	1	1	1	3
9	1	3	9	9	3	9	1	1	1
9	1	3	2	2	3	9	1	1	1
9	9	1	9	9	9	9	9	9	9
9	9	9	9	9	9	9	9	9	9
1	1	2	2	3	3	1	1	1	3
9	9	9	9	9	9	9	9	9	9
9	9	9	9	9	9	9	9	9	9
9	9	9	9	9	9	9	9	9	9
9	9	9	9	9	9	9	9	9	9
9	9	9	9	9	9	9	9	9	9
9	9	9	9	9	9	9	9	9	9
9	9	9	9	9	9	9	9	9	9
1	1	1	1	3	2	2	2	1	2
2	1	2	1	2	2	2	3	1	3
1	1	1	2	2	1	2	1	1	2
1	1	1	2	1	1	1	1	1	3
1	1	2	1	3	1	1	1	1	3
1	1	2	1	3	1	1	1	1	3
9	9	9	9	9	9	9	9	9	9
2	3	2	2	2	2	2	1	1	9
1	1	2	1	2	2	1	1	1	2
1	1	2	2	3	2	2	2	2	2
2	1	2	2	3	3	2	2	1	2
1	1	3	1	3	1	1	1	1	3
1	1	1	2	2	1	1	1	1	2

1	2	2	2	2	2	2	2	2	2
2	1	2	2	2	2	1	2	1	2
1	2	2	9	3	2	1	1	1	1
2	3	2	9	1	2	1	2	3	2
1	2	2	1	1	1	2	1	2	1
1	1	3	3	2	3	1	1	1	2
9	9	9	9	9	9	9	9	9	9
2	2	2	2	2	3	2	2	2	2
1	1	1	2	2	2	2	1	1	2
2	2	2	2	2	2	2	2	2	2
1	1	2	2	2	2	1	1	1	2
1	1	1	1	1	2	1	1	1	1
3	2	2	3	1	2	3	2	1	2
3	3	3	2	3	3	3	2	3	2
9	1	3	1	3	3	1	2	1	3
1	1	1	3	3	3	1	1	1	3
9	9	9	9	9	9	9	9	9	9
2	1	3	2	1	2	2	3	2	2
2	1	1	1	2	2	1	1	1	3
1	1	1	1	1	1	1	1	1	1
1	1	1	1	1	1	1	1	1	1
2	3	3	3	2	2	2	2	2	2
3	2	2	1	3	2	2	3	3	1
2	2	2	2	2	2	2	2	2	2
1	1	1	1	1	1	1	1	1	1
2	1	2	2	2	1	2	1	3	3
2	2	1	2	3	2	2	1	1	2
1	2	1	1	2	1	1	1	1	2
1	1	2	2	2	2	2	2	2	2
1	1	2	2	2	2	2	2	2	2
1	1	2	2	3	1	1	1	1	2
1	1	1	1	1	1	1	1	1	1
2	1	3	2	3	1	1	2	2	2
1	1	3	1	2	2	1	1	1	2
9	1	9	9	9	9	9	9	9	9
2	2	2	2	2	1	1	1	1	1
2	2	2	2	2	2	2	2	2	2
1	1	2	1	2	2	2	2	2	2
1	1	1	1	1	3	1	3	2	2
1	1	2	2	2	1	1	1	1	2
1	1	1	2	2	2	1	2	1	2
1	1	1	2	2	2	1	2	1	3

InheritedC	OffensiveP	PostCoitalB	Dysmenorr	Menorrhag	Asymptom	VaginalRas	Vaginalswe	WillLikePa	AfraidPapS
2	2	2	2	2	2	2	2	2	2
3	2	2	2	2	2	2	2	3	3
2	2	2	2	2	2	2	2	2	2
2	2	1	1	9	3	3	3	3	3
3	1	2	2	2	3	2	2	1	2
3	3	3	3	3	3	3	3	2	2
2	1	2	3	1	3	2	1	2	1
2	1	2	2	2	3	2	2	1	2
1	2	2	2	2	2	2	2	3	3
2	1	1	2	2	3	1	2	2	3
2	2	2	2	2	2	2	2	2	2
2	1	1	2	2	2	2	3	1	2
1	1	1	2	2	3	3	1	1	2
2	2	2	2	2	2	2	1	1	1
2	2	2	2	2	2	2	2	2	2
2	2	2	2	2	2	2	2	2	2
9	9	9	9	9	9	9	9	9	9
2	2	2	2	2	2	2	2	1	1
9	9	9	9	9	9	9	9	9	9
9	9	9	9	9	9	9	9	9	9
1	1	1	1	1	1	1	1	1	1
2	1	2	1	1	2	2	1	2	2
2	1	1	1	1	2	1	1	1	2
1	1	1	1	1	1	1	1	1	1
9	2	9	9	9	9	9	9	9	9
3	1	2	2	2	2	2	2	1	1
3	1	3	1	1	1	1	1	1	1
2	2	2	2	2	2	2	2	1	1
1	1	2	3	3	2	3	1	1	3
3	1	3	3	2	1	1	1	2	1
2	1	2	3	3	1	1	1	1	1
2	2	2	2	2	2	2	2	1	2
3	3	3	3	1	3	3	3	3	1
1	1	1	2	1	1	1	1	3	1
9	2	1	1	1	1	2	3	1	2
3	3	3	2	3	2	3	1	2	2
2	2	2	2	2	2	2	2	2	1
2	2	1	2	2	2	1	1	2	1
3	2	2	2	2	2	1	1	1	3
1	1	3	3	1	1	2	3	3	3
2	2	2	3	2	2	2	2	1	2
3	2	2	1	3	2	1	2	9	2
9	9	9	9	9	9	9	9	9	9
2	2	2	2	2	2	2	2	2	2
2	2	2	2	1	2	2	2	2	2
9	9	9	9	9	9	9	9	9	9

3	1	1	2	2	3	2	2	2	2
2	1	2	2	1	1	2	2	2	2
1	2	2	2	1	3	2	2	1	2
2	1	1	1	2	2	1	1	2	2
1	3	3	3	3	3	3	3	3	3
1	3	3	3	3	3	3	3	3	3
3	2	2	3	2	3	3	2	2	2
2	2	1	3	1	2	1	1	1	3
1	1	2	1	2	3	1	2	3	2
1	1	9	9	9	9	9	9	9	9
9	1	9	9	9	9	9	9	1	9
2	2	2	2	2	1	1	1	1	1
2	1	1	1	1	1	1	1	1	1
1	1	1	2	2	2	3	3	1	1
2	3	2	1	2	2	1	1	1	2
2	1	1	2	2	3	3	3	1	1
3	9	9	9	9	9	9	9	3	1
1	1	3	3	3	2	3	3	3	2
2	2	1	2	2	2	2	2	1	1
3	9	9	9	9	9	9	9	2	2
2	2	3	3	3	3	3	3	3	2
3	1	2	2	2	2	1	2	2	3
2	1	1	2	2	3	3	3	1	2
1	1	1	2	2	1	1	1	1	1
2	2	2	2	2	1	1	1	1	2
2	2	2	2	1	2	1	1	1	2
3	3	3	3	3	3	1	1	3	1
2	1	2	3	3	2	2	1	1	1
1	1	1	2	2	3	1	1	1	2
3	3	3	3	3	3	3	3	3	1
9	9	9	9	9	9	9	9	9	9
1	1	2	1	1	2	2	1	3	2
1	3	1	1	9	9	1	1	1	9
2	2	2	2	2	2	2	2	2	2
2	2	2	1	9	1	3	1	1	2
2	2	2	2	2	2	2	2	2	2
1	3	9	9	9	9	9	9	9	9
2	1	1	1	2	2	1	1	2	2
2	1	2	2	2	2	2	2	2	2
2	2	2	2	2	2	1	2	1	2
2	2	2	1	2	3	2	1	1	2
9	9	9	9	9	9	9	9	9	9
3	1	1	2	3	1	1	1	2	1
2	1	1	1	1	1	1	1	1	1
1	2	1	2	2	2	2	2	3	2
2	1	2	1	2	3	3	3	3	2
2	2	2	3	2	2	2	2	1	2

1	1	2	1	2	1	2	3	1	2
3	3	3	3	3	3	3	3	3	3
1	2	2	2	2	2	2	2	2	2
2	2	2	2	2	2	1	2	2	1
1	1	2	1	1	2	3	2	1	1
1	2	2	2	1	1	1	1	1	1
3	2	2	2	2	2	2	2	2	2
9	9	9	9	9	9	9	9	9	9
2	2	1	1	1	2	1	2	2	2
2	1	2	3	3	2	1	1	1	1
1	1	2	1	3	2	2	3	1	1
3	1	1	2	2	2	1	1	2	2
2	1	2	2	3	3	3	3	1	2
2	1	2	2	2	2	2	2	2	2
2	2	3	3	1	2	1	1	2	3
1	3	1	2	1	2	1	1	2	3
1	1	1	2	3	2	1	1	1	1
3	2	2	2	2	2	2	2	3	2
9	1	2	2	2	2	1	1	1	3
2	1	1	1	2	2	2	1	2	2
3	1	1	1	1	1	1	1	1	3
1	2	3	3	2	2	2	2	1	1
1	1	2	3	2	1	1	2	1	2
2	2	2	2	2	2	2	2	1	2
2	2	2	2	2	2	2	2	2	2
3	2	2	2	2	2	2	2	1	2
3	1	1	3	3	3	1	1	1	3
2	1	2	1	2	1	2	1	2	1
2	1	2	2	2	1	1	2	1	2
1	1	3	2	2	1	2	3	1	1
3	2	2	2	3	2	1	1	1	1
1	1	2	3	3	2	1	2	3	2
1	2	2	2	1	9	1	3	3	2
2	2	1	1	2	1	2	1	2	1
3	1	2	3	2	2	1	2	1	2
1	1	1	1	3	1	1	1	1	1
3	2	2	2	2	2	2	2	3	3
2	1	1	1	2	2	1	1	1	1
9	9	9	9	9	9	9	9	9	9
3	3	3	3	3	3	3	3	3	3
1	1	2	1	1	2	1	1	1	1
9	9	9	9	9	9	9	9	9	9
2	1	1	2	2	2	1	1	1	1
2	1	2	2	3	2	2	2	1	2
9	9	9	9	9	9	9	9	9	9
3	1	3	2	2	3	2	1	2	1
1	1	1	1	1	2	1	1	1	1

1	1	3	3	1	2	2	1	2	3
2	1	1	1	1	2	1	1	3	1
3	3	3	3	3	3	3	3	3	3
2	1	1	1	1	1	1	1	2	2
3	1	2	2	2	2	3	3	1	2
2	2	3	2	3	2	3	2	2	3
2	1	2	9	1	9	3	1	2	9
2	1	2	9	1	2	3	1	2	9
9	1	9	9	9	9	9	9	1	9
2	2	2	2	2	2	2	2	2	2
1	1	1	2	3	2	1	3	2	2
1	2	1	2	2	2	2	1	2	2
2	2	2	2	2	2	2	2	2	2
1	2	2	2	2	3	2	2	1	2
2	1	2	2	2	2	1	1	1	2
2	1	2	2	1	1	1	1	1	2
1	1	1	2	2	1	1	2	1	1
2	2	2	2	2	1	1	1	1	1
9	1	9	9	9	9	9	9	1	9
1	2	1	2	3	2	2	1	2	3
2	2	1	2	2	3	3	2	1	1
2	2	2	2	2	2	2	2	2	2
3	3	3	3	3	3	3	3	3	3
2	2	2	2	2	2	2	2	2	2
3	1	2	1	1	3	2	1	1	3
3	1	3	3	1	1	3	3	2	2
9	9	9	9	9	9	9	9	9	9
3	2	2	2	2	2	2	2	2	2
2	2	3	1	1	3	2	2	3	1
2	1	2	2	2	1	1	2	1	2
2	2	1	1	1	2	2	2	2	2
1	1	1	1	2	2	2	2	2	2
1	1	1	1	2	3	3	3	1	1
3	3	3	3	3	3	2	3	3	3
2	2	2	1	2	2	2	2	2	1
1	3	3	3	1	1	1	1	1	1
3	2	2	2	2	2	2	2	3	3
3	1	1	2	2	3	1	1	1	1
1	1	1	1	1	2	2	2	1	1
2	2	2	2	2	2	2	2	2	2
9	9	9	9	9	9	9	9	9	9
2	2	2	2	2	2	2	2	2	2
2	2	2	2	2	2	2	2	2	2
9	9	9	9	9	9	9	9	9	9
9	9	9	9	9	9	9	9	9	9
2	1	2	2	2	2	2	2	1	2
3	1	1	2	2	2	2	2	1	2

9	2	9	9	9	9	9	9	9	9
1	9	9	2	9	9	9	9	9	9
1	2	1	1	3	1	1	1	2	1
9	2	9	9	9	9	9	9	1	9
9	3	3	3	3	3	3	3	9	9
9	9	9	9	9	9	9	9	9	9
1	1	1	1	1	1	2	2	9	9
9	9	9	9	9	9	9	9	9	9
2	3	2	1	2	2	1	1	2	3
9	9	9	9	9	1	9	9	9	9
9	1	9	9	9	9	9	9	9	9
9	9	9	9	9	9	2	9	9	9
1	2	2	1	1	2	2	3	2	1
2	2	2	2	3	3	3	3	3	3
9	9	9	2	9	9	9	9	9	9
3	2	3	2	2	3	3	3	2	2
3	1	1	1	1	1	1	1	1	1
2	1	2	2	2	2	2	2	1	2
2	2	1	1	1	2	2	2	2	2
2	2	2	2	2	2	2	3	2	2
2	1	1	2	3	1	1	1	1	1
9	9	9	9	9	9	9	9	9	9
1	2	2	1	1	1	1	1	2	1
2	1	2	1	1	1	1	1	1	3
2	2	2	2	2	2	2	2	2	2
2	1	1	1	2	2	3	3	2	1
1	1	1	1	1	2	2	1	1	1
1	1	1	2	3	2	2	2	2	1
3	3	3	3	3	3	3	3	3	3
1	1	1	3	1	3	1	1	1	3
2	2	2	2	2	2	2	2	2	2
1	1	2	1	1	2	2	1	1	1
9	9	9	9	9	9	9	9	9	9
1	1	2	2	2	1	1	1	1	1
1	1	3	1	1	1	3	3	1	1
2	1	2	3	2	2	1	1	2	1
3	1	2	3	9	9	9	9	9	2
1	2	1	1	2	2	1	1	2	1
9	2	9	9	9	9	9	9	2	9
2	2	1	1	1	2	2	3	2	2
1	1	1	2	2	1	1	1	2	2
2	1	3	2	2	1	1	1	1	1
1	2	2	2	2	2	2	2	1	1
2	2	2	2	2	2	2	2	2	2
1	3	3	3	3	3	3	3	2	2
1	2	2	2	2	2	2	2	2	1
9	9	9	9	9	9	9	9	9	9

2	1	1	1	1	1	1	1	1	1
2	1	2	1	1	1	1	1	1	3
1	1	1	1	1	1	1	1	1	1
9	9	9	9	9	9	9	9	9	9
2	2	2	2	2	2	2	2	2	2
3	1	1	1	1	1	2	2	2	3
3	1	1	3	2	2	1	1	3	3
2	1	3	2	2	1	1	1	3	2
2	1	2	1	1	1	1	1	2	1
1	2	1	1	1	2	2	2	1	1
2	2	2	2	3	2	2	1	2	2
9	2	9	9	9	9	9	9	3	9
9	2	9	9	9	9	9	9	2	9
9	1	9	9	9	9	9	9	1	9
2	2	9	9	9	2	9	9	9	2
9	9	9	9	9	9	9	9	9	9
9	9	9	9	9	9	9	9	9	9
9	9	9	9	9	9	9	9	9	9
2	9	2	2	9	9	2	2	9	2
9	9	9	9	9	9	9	9	9	9
9	9	9	9	9	9	9	9	9	9
2	1	2	2	1	1	1	1	1	2
2	2	2	2	2	2	2	2	1	1
1	2	1	1	1	1	1	1	1	1
1	2	2	2	1	1	1	1	1	2
9	9	9	9	9	9	9	9	9	9
2	1	1	2	2	2	2	2	1	2
9	9	9	9	9	9	9	9	9	9
9	9	9	9	9	9	9	9	9	9
2	1	1	2	2	2	2	2	1	2
9	9	9	9	9	9	9	9	9	9
9	9	9	9	9	9	9	9	9	9
9	9	9	9	9	9	9	9	9	9
9	9	9	9	9	9	9	9	9	9
9	9	9	9	9	9	9	9	9	9
9	9	2	1	9	3	1	9	9	9
9	9	9	9	9	9	9	9	9	9
9	9	9	9	9	9	9	9	9	9
9	9	9	9	9	9	9	9	9	9
9	3	9	9	9	9	9	9	3	9
9	2	9	9	9	9	9	9	1	9
9	9	9	9	9	9	9	9	9	9
3	3	3	3	3	3	3	3	3	3
9	1	1	1	1	1	1	1	3	3
1	2	1	2	1	2	1	3	1	2
1	1	1	1	1	9	2	3	1	1
9	2	9	9	9	9	9	9	1	9

9	9	2	9	9	9	9	9	1	9
9	9	9	3	9	9	9	9	3	9
1	3	3	1	2	2	2	3	2	2
1	1	2	3	1	2	3	1	2	3
3	2	1	2	2	2	2	2	2	2
2	2	2	2	3	3	3	3	2	2
2	1	1	1	1	1	1	1	1	1
2	1	1	2	2	3	2	1	1	2
2	2	2	2	2	2	2	2	2	2
3	3	3	3	3	3	3	3	3	3
9	9	9	9	9	9	9	9	9	9
9	9	9	9	9	9	9	9	9	9
9	9	9	9	9	9	9	9	9	9
9	1	9	9	9	9	9	9	9	1
1	2	2	2	2	2	2	2	3	3
1	1	1	1	2	3	3	3	1	1
9	9	9	9	9	9	9	9	9	9
1	1	2	1	1	2	1	1	2	2
1	1	1	1	1	1	1	1	1	1
1	1	1	1	1	1	1	1	2	2
2	1	2	2	2	2	2	2	2	2
2	2	2	2	2	2	2	2	1	3
9	1	9	9	9	9	9	9	1	9
2	2	2	2	2	2	2	2	2	2
3	2	2	2	2	2	2	2	2	2
2	1	2	1	2	1	2	1	2	2
2	2	2	2	2	2	2	2	2	2
1	1	1	1	1	1	1	1	3	2
3	2	2	2	2	2	3	3	3	2
1	2	2	2	2	1	1	1	1	2
2	2	2	2	2	2	2	2	2	2
2	2	2	2	2	2	2	2	2	2
1	1	2	3	1	2	3	2	2	2
2	2	2	2	2	3	3	3	2	2
2	1	1	1	1	1	1	1	3	3
3	3	3	2	2	2	2	2	1	2
9	9	9	9	9	9	9	9	9	9
2	2	2	2	2	2	2	2	2	2
3	3	3	3	3	3	3	3	3	3
3	3	3	3	3	3	3	3	3	3
9	2	9	9	9	9	9	9	2	9
9	9	9	9	9	9	9	9	9	9
9	9	9	9	1	9	9	9	9	9
1	1	1	1	1	1	1	1	2	2
1	1	2	2	2	2	2	2	2	2
2	2	2	2	2	2	2	2	2	2
3	3	3	2	1	1	1	1	2	2

9	2	9	9	9	9	9	9	9	9
9	1	9	9	9	9	9	9	2	9
9	9	9	1	9	9	9	9	1	9
9	9	9	9	1	9	9	9	9	9
3	9	1	9	9	9	9	9	9	1
2	3	3	2	1	1	1	2	3	2
9	2	9	9	9	9	9	9	2	9
9	1	9	9	9	9	9	9	3	9
9	2	9	9	9	9	9	9	2	9
9	2	9	9	9	9	9	9	2	9
9	2	9	9	9	9	9	9	9	9
9	9	9	2	9	9	9	9	9	9
9	2	9	9	9	9	9	9	2	9
9	9	9	2	9	9	9	9	9	9
9	1	9	9	9	9	9	9	1	9
9	9	9	9	9	9	9	9	9	9
9	2	9	9	2	9	9	2	9	2
3	3	2	1	3	9	2	1	1	2
2	1	2	3	2	2	2	2	1	1
2	2	2	2	2	2	2	2	2	2
3	1	2	2	2	3	1	2	2	1
2	1	1	2	2	2	2	2	9	1
2	2	2	2	2	2	2	2	2	2
3	2	3	1	9	2	3	2	2	3
2	2	3	1	3	2	2	1	3	1
9	9	9	9	9	9	9	9	9	9
3	2	1	1	2	2	2	2	2	1
2	2	2	2	2	2	2	2	9	9
2	1	2	3	1	2	2	3	1	2
2	1	1	1	1	1	2	2	1	1
3	1	2	1	3	2	2	1	1	2
2	2	2	2	2	2	2	2	2	2
3	1	1	1	1	1	1	1	2	2
1	1	1	1	1	1	1	1	1	1
9	9	9	9	9	9	9	9	9	9
2	2	2	2	2	2	2	2	2	2
9	9	9	9	9	9	9	9	9	9
2	1	2	2	1	1	1	2	1	2
9	9	9	9	9	9	9	9	9	9
2	2	2	2	2	2	2	2	2	2
2	1	2	2	2	1	2	3	1	2
3	3	3	3	3	3	3	3	3	1
3	2	3	9	3	2	2	2	1	1
2	2	2	3	2	3	2	2	2	2
2	2	2	2	2	2	2	2	2	2
2	1	1	2	2	2	2	3	1	2
2	2	2	2	2	2	2	2	2	3

1	1	1	1	2	2	2	3	1	2
9	3	9	9	9	9	9	9	9	9
2	2	2	2	2	2	2	2	2	2
1	2	9	1	3	1	2	3	2	1
1	1	1	3	2	2	2	2	3	2
2	2	2	2	3	3	3	3	2	2
2	1	2	2	1	2	2	2	1	1
2	1	1	2	2	2	2	2	1	2
9	9	9	9	9	9	9	9	9	9
2	2	2	2	2	2	2	2	2	3
3	2	2	2	2	2	2	2	2	2
9	1	9	9	9	9	9	9	1	9
9	1	9	9	9	9	9	9	1	9
3	3	3	3	1	1	1	1	2	2
2	9	9	9	9	9	9	2	1	9
2	1	1	1	1	2	2	2	2	2
9	9	9	9	9	9	9	9	9	9
9	1	9	9	9	9	9	9	1	9
9	9	9	9	1	9	9	9	9	9
2	3	2	3	2	3	2	3	2	3
2	2	2	2	2	2	2	2	2	2
2	2	2	3	3	1	2	2	3	1
2	2	2	2	2	2	2	2	2	2
9	9	9	9	9	9	1	9	9	9
9	2	9	9	9	9	9	9	2	9
2	1	2	2	2	2	2	2	1	2
3	1	1	1	1	1	1	1	1	1
1	2	2	1	1	3	3	1	2	1
9	9	9	9	9	9	9	9	9	9
9	1	9	9	9	9	9	9	3	9
2	2	2	2	2	2	2	2	2	2
3	1	2	3	1	2	3	1	2	3
2	1	2	2	2	2	2	3	2	2
2	2	2	1	2	2	2	2	1	1
1	1	1	3	3	3	1	1	1	1
1	1	2	3	1	2	1	3	1	2
9	9	9	9	9	9	9	9	9	9
9	9	9	9	9	9	9	9	9	9
2	3	2	1	3	1	2	3	3	2
2	1	2	1	2	1	2	1	1	2
2	2	2	2	2	2	2	2	2	2
2	2	2	2	2	2	2	2	2	2
2	2	2	2	2	2	2	2	1	2
1	1	2	3	1	2	3	1	1	2
2	1	1	1	1	1	1	1	2	2
2	2	2	2	2	2	2	2	2	2
3	3	3	3	3	2	2	2	2	2

1	2	2	2	2	2	2	2	1	2
1	1	2	3	9	2	2	2	1	2
9	9	9	9	1	9	9	9	9	9
9	9	9	9	9	9	9	1	9	9
9	2	9	9	9	9	9	9	9	9
3	3	2	9	2	3	1	2	2	9
2	2	9	2	9	9	2	2	2	9
9	2	9	9	2	9	2	2	2	9
9	9	9	9	9	9	9	9	9	9
9	9	9	9	9	9	9	9	9	9
9	9	9	9	9	9	9	9	9	9
9	9	9	9	9	9	9	9	9	9
9	9	9	9	9	9	9	9	9	9
9	9	9	9	9	9	9	9	9	9
9	9	9	9	9	9	9	9	9	9
9	2	9	9	9	9	9	9	2	9
3	3	3	3	3	3	3	3	3	3
9	1	9	9	9	9	9	9	1	9
9	2	9	9	9	9	9	9	2	9
2	2	9	9	2	9	9	2	2	9
9	9	9	9	9	9	9	9	9	9
9	2	9	9	9	9	9	9	2	9
2	2	2	2	2	2	2	2	2	2
9	9	9	9	9	9	9	9	9	9
9	9	9	9	9	9	9	9	9	9
9	2	9	9	9	9	9	9	2	9
9	9	9	9	9	9	9	9	9	9
2	2	9	2	9	2	9	2	2	9
9	2	1	3	2	3	3	2	9	9
9	9	9	9	9	9	9	9	9	9
9	9	9	9	9	9	9	9	9	9
2	2	9	2	9	2	9	2	2	9
9	9	9	9	9	9	9	9	9	9
9	9	9	9	9	9	9	9	9	9
9	9	9	9	9	9	9	9	9	9
9	9	9	9	9	9	9	9	9	9
9	9	9	9	9	9	9	9	9	9
9	9	9	9	9	9	9	9	9	9
2	1	2	3	1	2	3	2	1	2
3	2	3	1	2	3	1	2	2	2
1	2	9	9	9	9	9	9	9	9
9	9	9	9	9	9	9	9	9	9
1	2	1	3	2	1	3	2	2	1
2	1	2	2	2	2	2	2	3	3
1	1	1	1	3	3	3	3	1	1
2	1	2	1	1	3	2	1	2	2
1	2	3	3	3	3	2	1	1	2

9	9	9	9	9	9	9	1	9	9
3	1	2	2	1	3	3	2	1	2
2	2	2	2	2	2	2	2	2	2
1	1	1	1	1	1	1	1	1	1
3	2	3	2	3	2	3	2	2	2
2	2	2	2	1	2	1	2	2	1
9	9	9	9	9	9	9	9	9	9
1	1	2	3	1	2	2	1	3	3
2	2	2	2	2	2	2	2	2	2
2	1	1	1	1	2	1	1	1	1
2	2	3	3	2	3	2	3	2	2
9	9	9	1	9	9	9	9	9	9
9	9	9	9	9	9	1	9	9	9
9	2	9	9	9	9	9	9	1	9
9	9	9	9	9	9	9	2	9	9
1	1	1	1	1	2	1	1	9	9
9	3	2	2	2	2	2	2	2	2
1	2	2	2	2	2	2	1	1	1
3	1	3	3	3	3	2	3	1	1
2	2	2	2	2	2	2	2	2	2
2	2	2	2	2	2	2	2	2	2
9	9	9	9	9	9	9	9	9	9
1	3	1	1	3	3	1	1	3	1
9	9	2	9	9	9	9	9	9	2
9	9	9	9	9	9	1	1	9	9
3	1	1	1	1	1	1	1	2	2
9	9	9	2	9	9	9	9	9	9
1	1	1	2	2	2	2	2	3	3
9	9	9	9	9	9	9	9	9	9
9	9	9	9	9	9	9	1	1	9
3	3	2	3	1	2	9	9	1	3
2	3	2	3	2	3	2	1	3	3
9	9	9	9	9	9	9	9	9	9
9	1	9	9	9	9	9	9	1	9
2	1	2	3	2	2	2	2	1	2
9	9	9	9	9	9	9	9	9	9
3	9	9	9	9	9	9	9	9	9
9	9	1	9	9	9	9	9	1	9
2	2	2	2	2	2	2	2	2	2
9	9	9	9	9	9	9	9	9	9
2	2	2	2	2	2	2	2	2	2
2	1	1	1	1	2	1	1	1	1
2	2	2	3	3	3	3	3	2	2
2	2	2	2	2	2	2	2	2	2
2	3	2	3	9	2	3	3	2	2
2	2	2	2	2	2	2	2	2	2
2	2	2	2	2	2	2	2	3	3

9	2	9	9	9	9	9	9	9	9
2	2	2	2	2	2	2	2	2	2
2	2	2	2	2	2	2	2	2	2
9	9	9	9	9	9	9	9	9	9
9	2	9	9	9	9	9	9	9	9
9	9	9	9	9	9	9	9	9	9
9	9	9	9	9	9	9	9	9	9
2	2	2	2	2	2	2	2	2	2
2	1	1	2	2	2	1	1	1	2
9	9	9	9	9	9	9	9	9	9
2	2	2	2	2	2	2	2	1	1
1	2	2	2	2	2	2	3	1	1
2	1	1	1	2	2	2	2	2	2
2	2	2	2	2	2	2	2	2	2
9	9	9	9	9	9	9	9	9	9
9	9	9	9	9	9	9	9	9	9
9	9	9	9	9	9	9	9	9	9
9	9	9	9	9	9	9	9	9	9
9	1	9	9	9	9	9	9	1	9
9	2	9	9	9	9	9	9	2	9
9	9	9	9	9	9	9	9	9	9
9	9	9	9	9	9	9	9	9	9
2	2	2	2	2	2	2	2	2	2
9	2	9	9	9	9	9	9	2	9
9	9	9	9	9	9	9	9	9	9
9	9	9	9	9	9	9	9	9	9
9	9	9	9	9	9	9	9	9	9
9	9	9	9	9	9	9	9	9	9
2	9	9	9	9	9	9	9	9	9
9	3	9	9	9	9	9	9	3	9
9	9	9	9	9	9	9	9	9	9
2	9	9	9	9	9	9	9	9	9
9	9	9	9	9	9	9	9	9	9
9	9	9	9	9	9	9	9	9	9
9	9	9	9	9	9	9	9	9	9
9	9	9	9	9	9	9	9	9	9
9	9	9	9	9	9	9	9	9	9
9	9	9	9	9	9	9	9	9	9
9	9	9	9	9	9	9	9	9	9
9	9	9	9	9	9	9	9	9	9
2	2	2	2	2	2	2	2	2	2
9	2	9	9	9	9	9	9	2	9
9	9	9	9	9	9	9	9	9	9
9	9	9	9	9	9	9	9	9	9
9	9	9	9	9	9	9	9	9	9
9	9	9	9	9	9	9	9	9	9
9	9	9	9	9	9	9	9	9	9

9	9	9	9	9	9	9	9	9	9
3	2	2	2	2	2	2	2	2	2
9	9	9	9	9	9	9	9	9	9
9	9	9	9	9	9	9	9	9	9
9	9	9	9	9	9	9	9	9	9
9	9	9	9	9	9	9	9	9	9
9	9	9	9	9	9	9	9	9	9
9	9	9	9	9	9	9	9	9	9
9	9	9	9	9	9	9	9	9	9
9	9	9	9	9	9	9	9	9	9
9	9	9	9	9	9	9	9	9	9
9	9	9	9	9	9	9	9	9	9
9	9	9	9	9	9	9	9	9	9
9	9	9	9	9	9	9	9	9	9
9	9	9	9	9	9	9	9	9	9
9	9	9	9	9	9	9	9	9	9
9	9	9	9	9	9	9	9	9	9
2	2	2	2	2	2	2	2	2	2
9	9	9	9	9	9	9	9	9	9
9	9	9	9	9	9	9	9	9	9
9	9	9	9	9	9	9	9	9	9
9	9	9	9	9	9	9	9	9	9
9	9	9	9	9	9	9	9	9	9
9	9	9	9	9	9	9	9	9	9
2	2	2	2	2	2	2	2	2	2
9	9	9	9	9	9	9	9	9	9
9	9	9	9	9	9	9	9	9	9
2	1	1	1	1	1	1	1	1	1
9	9	9	9	9	9	9	9	9	9
9	9	9	9	9	9	9	9	9	9
1	1	1	1	1	1	1	1	1	1
2	1	2	3	2	1	2	2	1	1
9	9	9	9	9	9	9	9	9	9
2	2	2	2	2	2	2	2	2	2
9	9	9	9	9	9	9	9	9	9
2	2	2	2	2	2	2	2	2	2
9	9	9	9	9	9	9	9	9	9
9	9	9	9	9	9	9	9	9	9
1	1	1	1	1	1	1	1	1	1
3	3	9	9	9	9	9	9	3	9
9	9	9	9	9	9	9	9	9	9
9	9	9	9	9	9	9	9	9	9
2	2	2	2	2	2	2	2	2	2
2	2	2	2	2	2	2	2	2	2
1	1	9	1	9	3	1	1	1	9
9	9	9	9	9	9	9	9	9	9
9	9	9	9	9	9	9	9	9	9

2	2	9	9	2	9	9	2	2	9
9	9	9	9	9	9	9	9	9	9
3	9	9	9	9	9	9	9	9	9
9	9	9	9	9	9	9	9	9	9
9	9	9	9	9	9	9	9	9	9
9	9	9	9	9	9	9	9	9	9
9	2	9	9	9	9	9	9	2	9
9	2	9	9	9	9	9	9	2	9
9	2	9	2	9	2	9	2	2	9
9	9	9	9	9	9	9	9	9	9
9	9	9	9	9	9	9	9	9	9
9	9	9	9	9	9	9	9	9	9
9	9	9	9	9	9	9	9	9	9
9	9	9	9	9	9	9	9	9	9
3	2	3	3	2	2	2	2	1	1
2	1	2	3	1	2	1	3	1	3
9	9	9	9	9	2	9	9	9	9
2	2	2	2	2	2	2	2	2	2
2	2	2	2	3	3	2	2	2	2
9	1	9	9	9	9	9	9	2	9
9	2	9	9	9	9	9	9	1	9
9	1	9	9	9	9	9	9	1	9
3	1	1	1	1	1	1	1	2	2
3	1	2	3	1	2	3	1	2	3
3	2	2	2	2	2	2	2	2	2
2	2	2	2	2	2	2	2	2	2
3	9	9	9	9	9	9	9	9	9
3	2	2	2	2	2	2	2	1	3
2	2	2	2	2	2	2	2	1	2
2	2	2	2	2	2	2	9	2	2
2	2	2	2	2	2	2	2	2	2
9	9	9	9	9	9	9	9	9	9
9	1	9	9	9	9	9	9	1	9
9	9	9	9	9	9	9	1	9	9
9	1	9	9	9	9	9	9	9	2
9	9	9	9	9	9	9	9	9	9
9	9	9	9	9	9	9	9	9	9
9	9	2	9	9	2	9	2	9	2
9	9	9	9	9	9	9	9	9	9
2	2	9	9	2	9	9	2	2	9
9	9	9	9	9	9	9	9	9	9
2	2	2	2	2	2	2	2	2	2
9	9	9	9	9	9	9	9	9	9
9	9	9	9	9	9	9	9	9	9
9	9	9	9	9	9	9	9	9	9
2	1	1	2	2	2	2	2	2	2
3	2	1	2	1	2	2	1	2	2

2	2	9	1	1	1	1	1	2	1
2	1	2	2	2	3	2	2	1	2
2	1	2	2	2	2	2	2	1	2
3	1	1	2	1	2	2	3	1	1
3	3	3	3	3	3	3	3	3	3
1	2	2	2	2	2	2	2	1	1
2	2	2	2	2	2	1	1	1	2
3	3	3	3	3	3	3	3	3	3
9	9	9	9	9	9	9	9	9	9
2	2	2	2	2	2	2	2	2	2
2	2	9	9	2	9	9	2	1	9
1	3	1	1	2	2	1	1	3	1
2	2	2	2	2	2	2	2	2	2
2	1	1	2	2	3	3	1	1	1
3	3	3	3	3	3	3	3	3	3
2	2	2	2	2	2	2	2	2	2
1	2	1	2	3	1	2	3	1	2
3	2	3	3	3	3	2	2	2	2
9	9	9	9	9	9	9	9	9	9
2	1	2	2	1	1	2	2	2	2
2	2	2	3	1	2	2	2	2	2
3	1	1	2	2	3	1	1	1	1
2	2	2	2	1	3	1	1	2	2
3	2	1	1	2	2	3	2	2	1
2	2	2	2	2	1	2	2	1	3
2	2	2	2	2	2	2	2	1	2
2	2	2	2	2	2	2	2	2	2
2	2	2	2	2	2	3	2	3	2
2	2	2	2	2	2	2	1	2	2
3	1	2	1	1	3	3	3	3	3
3	3	3	3	3	3	3	3	3	3
9	9	9	1	1	9	9	9	9	9
3	3	3	3	3	3	3	3	3	3
2	2	2	2	2	2	2	2	2	2
9	9	2	9	9	9	9	9	9	1
3	3	2	2	3	3	2	3	3	2
2	3	3	3	3	3	3	3	3	3
1	1	1	1	1	1	1	1	1	1
2	2	2	1	2	1	2	2	2	2
9	9	9	9	9	9	9	9	9	9
2	2	2	2	2	2	2	2	2	2
9	9	9	9	9	9	9	9	9	9
1	2	3	2	2	3	2	3	2	1
2	2	2	2	2	2	2	2	2	2
3	1	2	3	1	2	2	2	2	2
9	9	9	2	9	9	9	9	2	1
2	2	2	3	2	3	3	3	1	2

9	9	9	9	9	9	9	9	9	9
3	3	3	3	3	3	3	3	3	3
1	9	3	3	1	3	3	3	1	3
3	3	3	2	2	2	2	2	2	2
1	1	1	2	2	1	2	3	1	2
9	9	9	9	9	9	9	9	9	9
2	3	3	3	3	3	3	3	2	2
3	2	3	2	3	1	2	3	1	2
1	2	1	2	2	2	1	1	1	2
2	2	2	2	2	2	2	2	2	2
2	2	2	2	2	2	2	2	1	1
2	2	2	2	2	2	2	2	2	2
9	9	9	9	9	9	9	9	9	9
3	3	3	2	3	2	3	2	2	3
2	2	2	2	2	2	2	2	1	3
2	2	2	2	2	2	2	2	2	2
3	2	2	2	2	2	2	2	2	2
2	2	2	2	2	2	2	2	3	3
2	2	2	2	2	2	2	2	2	2
9	9	9	9	9	2	9	9	1	9
9	9	9	9	9	9	9	9	9	9
2	2	2	2	2	2	2	2	1	1
2	2	2	2	2	2	2	2	2	3
3	2	3	2	2	3	2	2	1	2
9	9	9	9	9	9	9	9	9	9
1	1	3	2	3	1	2	1	1	2
9	1	9	9	9	9	9	9	1	9
2	2	2	2	2	2	2	2	2	2
2	2	2	2	2	2	2	2	2	2
2	2	2	2	2	2	2	2	2	2
2	1	2	3	2	3	2	3	1	2
9	9	9	9	9	9	9	9	9	9
9	9	9	9	9	9	9	9	9	9
2	1	2	2	2	2	2	1	1	1
2	2	2	2	3	3	2	1	3	9
9	9	9	9	9	9	9	9	9	9
9	9	9	9	9	9	9	9	9	9
2	2	2	2	2	1	1	1	2	2
3	2	2	2	2	3	2	2	1	3
1	1	1	2	2	1	1	2	2	1
2	2	2	2	2	2	2	2	2	2
2	2	2	2	2	2	2	2	2	2
2	2	2	2	2	2	2	2	2	2
2	2	2	2	2	2	2	2	2	2
2	2	2	2	3	2	2	2	2	1
2	2	1	1	1	2	1	1	2	3
2	2	2	2	2	2	2	2	2	2

3	3	3	2	1	2	3	3	3	2
2	2	2	2	3	2	2	2	2	1
2	2	2	2	2	2	2	2	2	2
1	1	1	1	1	1	1	1	2	1
2	2	2	2	2	2	2	2	2	2
2	2	2	2	3	2	2	2	2	1
2	2	2	2	2	2	2	2	2	1
2	2	2	2	2	2	2	2	2	2
2	2	2	2	2	3	2	2	1	2
9	9	9	9	9	9	9	9	9	9
3	2	2	2	2	2	2	2	2	2
3	1	2	1	1	2	3	2	1	2
2	1	1	1	1	1	2	1	1	3
1	1	1	1	1	1	1	1	1	2
3	3	3	3	3	3	3	3	2	2
1	9	9	1	9	2	9	2	1	3
9	9	9	9	9	9	9	9	9	9
9	9	9	9	9	1	9	9	9	9
9	9	9	9	9	9	9	9	9	9
9	9	9	9	9	9	9	9	9	9
1	2	1	3	3	1	1	2	3	3
9	9	9	9	9	9	9	9	9	9
9	9	9	9	9	9	9	9	9	9
2	2	2	2	2	2	2	2	2	1
1	2	2	2	2	2	2	2	2	2
2	2	2	2	2	2	2	2	2	1
3	2	2	3	3	1	1	2	1	1
9	9	9	9	9	9	9	9	9	9
2	2	1	2	2	2	2	1	2	3
3	2	3	2	2	2	2	1	1	2
9	9	9	9	9	9	9	9	9	9
1	1	2	1	2	1	2	1	1	1
1	1	2	3	2	1	2	2	1	2
1	2	2	2	3	3	1	1	3	3
2	1	1	2	2	2	1	1	2	1
2	2	2	2	2	2	2	2	2	1
9	9	9	9	9	9	9	9	9	9
2	2	2	2	2	2	2	2	2	2
2	2	2	2	2	2	2	2	2	2
1	1	2	3	2	1	1	1	2	2
1	2	2	2	2	2	2	2	2	2
2	2	2	1	1	1	1	1	2	2
2	1	1	1	2	2	2	3	1	1
3	1	1	1	1	2	2	2	1	1
3	1	2	3	1	3	2	2	2	3
2	1	1	1	1	1	1	1	2	1
3	3	3	3	2	2	2	2	3	3

2	2	2	3	3	3	2	2	1	1
2	2	2	2	2	2	2	2	2	2
3	2	2	3	1	9	1	2	3	3
2	2	2	2	2	2	2	2	2	2
3	3	3	3	2	3	2	3	3	3
9	9	9	9	9	9	9	9	9	9
9	1	9	9	9	9	9	9	9	9
3	2	2	2	2	2	2	2	2	3
9	9	9	9	9	9	9	9	9	9
9	9	9	9	9	9	9	9	9	9
2	2	3	1	1	2	2	2	2	2
1	2	2	2	2	2	2	2	1	1
1	1	2	2	2	3	3	3	2	3
9	2	9	1	9	9	9	9	1	1
9	2	2	2	2	2	2	2	9	9
1	3	3	3	3	1	3	3	1	3
2	1	2	2	2	1	2	1	1	1
9	9	9	9	9	9	9	9	9	9
2	2	2	2	2	2	2	2	1	2
2	1	1	3	3	2	2	2	2	2
3	2	2	2	2	2	2	2	3	3
9	9	9	9	9	9	9	9	9	9
2	2	2	2	2	2	2	2	2	2
2	2	2	2	2	2	2	2	2	1
2	2	2	2	2	2	2	2	2	2
2	2	2	2	2	2	2	2	2	2
2	2	2	2	2	2	2	2	2	1
2	2	2	2	2	2	2	2	2	2
1	2	1	2	2	2	3	2	2	2
2	2	2	2	2	2	2	2	2	2
3	1	1	1	1	1	2	2	1	3
2	1	2	2	3	3	2	3	1	2
2	2	3	3	9	1	1	1	1	2
2	2	2	2	2	2	2	2	2	2
1	2	1	1	1	2	1	1	1	1
2	1	2	3	2	1	2	2	1	2
2	2	2	2	2	2	2	2	1	1
2	2	2	2	2	2	2	2	2	2
2	2	2	2	2	2	2	2	1	2
2	2	2	2	2	3	2	2	1	1
1	1	1	1	1	1	1	1	1	1
2	2	3	2	2	1	3	3	2	2
2	2	2	2	2	2	2	2	2	2
2	2	2	2	2	2	2	2	2	2
2	2	2	2	2	2	2	2	2	2
2	1	1	2	2	2	2	2	1	2
2	2	2	2	2	2	2	2	2	2

3	2	1	3	2	3	1	2	3	1
2	2	1	2	2	3	2	2	1	1
3	2	3	2	3	2	3	2	2	3
2	2	2	2	2	2	2	2	2	2
9	2	9	9	9	9	9	9	1	9
9	9	9	1	9	9	9	9	9	9
3	2	3	2	3	2	3	2	3	2
9	1	9	9	9	9	9	9	1	9
9	1	9	9	9	9	9	9	1	9
9	9	9	9	9	9	9	9	9	9
9	9	9	9	9	3	9	9	9	9
9	9	9	9	9	2	9	9	9	9
9	9	9	9	9	9	9	2	1	9
9	9	1	9	9	9	9	9	9	3
3	3	3	3	3	1	2	1	2	2
9	1	9	9	9	9	9	9	9	9
9	9	9	9	9	9	9	9	9	9
9	9	9	9	1	9	9	9	9	9
9	9	9	9	1	9	9	9	9	9
9	9	9	9	9	2	9	9	9	9
9	9	9	9	9	9	9	9	9	9
2	2	2	2	2	2	2	2	2	2
2	2	3	2	2	2	2	2	1	2
2	2	1	1	1	1	1	1	1	1
2	1	1	2	2	2	1	1	1	2
9	9	9	9	9	9	9	9	9	9
2	1	2	2	2	3	3	3	3	2
9	9	9	9	9	9	9	2	2	9
3	2	2	2	2	2	2	2	1	2
1	1	1	1	1	1	1	1	1	1
2	1	3	3	3	3	3	3	3	3
9	2	9	9	9	9	9	9	1	9
2	2	2	2	2	2	2	2	2	2
9	2	9	9	9	9	9	9	2	9
9	3	9	9	9	9	9	9	3	9
1	1	1	2	2	1	1	1	1	1
9	1	1	1	1	1	1	1	1	1
9	9	9	9	9	9	9	9	9	9
9	9	9	9	9	9	9	9	9	9
2	2	2	2	2	2	2	2	2	2
9	9	9	9	9	9	9	9	9	9
2	2	2	2	2	2	2	2	2	2
3	3	3	3	3	3	3	3	3	3
9	9	9	2	9	9	9	9	9	2
2	2	2	2	2	2	2	2	2	2
2	2	2	2	2	2	2	2	2	2
9	9	9	9	9	9	9	9	9	9

9	9	9	9	9	9	9	9	9	9
9	2	9	2	9	9	2	2	9	2
9	9	9	9	9	9	9	9	9	9
2	2	2	2	2	2	2	2	2	2
9	9	9	9	9	9	9	9	9	9
1	3	1	3	2	1	3	1	9	1
9	9	9	9	9	9	9	9	9	9
9	9	9	9	9	9	9	9	9	9
2	9	2	9	9	2	9	2	9	2
2	9	9	9	9	9	9	9	9	9
9	9	9	9	9	9	9	9	9	9
9	9	2	9	9	2	9	2	2	9
2	2	2	2	2	2	2	2	2	2
9	9	9	9	9	9	9	9	9	9
2	2	2	2	2	2	2	2	2	2
9	9	9	9	9	9	9	9	9	9
9	9	9	9	9	9	9	9	9	9
9	9	9	9	9	9	9	9	9	9
9	9	9	9	9	9	9	9	9	9
9	9	9	9	9	9	9	9	9	9
2	2	2	2	2	2	2	2	2	2
2	2	2	2	2	2	2	2	2	2
2	3	3	3	3	3	3	3	3	3
2	2	1	1	1	1	2	3	1	3
3	2	2	2	2	2	2	2	2	2
2	2	2	2	2	2	2	2	2	2
1	1	1	1	1	1	1	1	1	1
2	1	2	2	2	2	2	2	2	2
1	1	1	1	2	3	3	1	1	2
2	1	2	2	2	2	2	2	2	2
3	2	2	2	1	1	1	1	2	2
3	2	2	2	1	3	2	3	2	3
2	2	2	2	2	2	2	2	2	2
9	9	9	9	9	9	9	9	9	9
2	1	1	1	2	2	2	2	1	2
2	2	2	2	2	2	2	2	2	2
2	2	2	2	2	2	2	2	2	2
1	1	1	1	1	1	1	1	3	3
3	1	2	3	1	2	3	1	1	2
1	2	2	2	9	2	2	2	2	2
1	9	9	9	9	9	9	9	9	9
9	9	9	9	9	9	9	9	9	9
9	3	2	2	2	2	2	2	2	2
9	9	9	9	9	9	9	9	9	9
2	9	9	1	9	2	1	9	1	2
2	2	2	2	2	2	2	2	2	1
9	9	9	9	9	9	9	9	9	9

3	2	2	3	3	3	3	3	3	2
3	1	1	1	2	2	3	3	1	1
1	9	9	9	9	9	9	9	9	9
2	2	2	2	2	2	2	2	2	2
1	9	9	9	9	9	9	9	9	9
2	1	1	1	1	1	1	1	2	2
3	1	1	1	1	2	2	2	1	1
1	1	2	9	3	2	2	2	1	2
3	3	3	3	3	3	3	3	3	3
2	1	1	1	1	1	1	2	1	1
9	9	9	9	9	9	9	9	9	9
2	2	2	2	2	2	2	2	2	2
2	2	2	2	2	2	2	2	2	2
2	2	2	2	2	2	2	2	2	2
2	9	9	9	9	9	9	9	2	2
1	2	2	2	9	1	1	1	1	1
1	2	1	2	1	2	1	1	1	1
2	1	1	2	1	2	1	2	1	1
1	1	1	1	1	1	1	1	1	1
9	9	9	9	9	9	9	9	9	9
2	1	1	2	2	1	2	1	1	1
2	2	2	2	2	2	2	2	2	2
9	9	9	9	9	9	9	9	9	9
1	2	2	2	2	2	2	2	2	2
9	9	9	9	9	9	9	9	9	9
1	1	1	1	1	1	1	1	1	2
1	1	1	1	1	1	9	2	1	1
9	1	1	9	1	1	1	1	1	1
9	9	9	9	9	9	9	9	9	9
2	9	9	9	9	9	9	9	9	9
9	9	9	9	9	9	9	9	9	9
9	9	9	9	9	9	9	9	9	9
2	1	9	1	1	1	1	1	1	1
2	1	1	1	1	1	1	1	1	2
2	2	2	2	2	2	2	2	2	2
9	9	9	9	9	9	9	9	9	9
1	1	2	2	2	2	3	1	1	1
1	1	1	1	1	1	1	1	1	1
9	9	9	9	9	9	9	9	9	9
9	9	9	9	9	9	9	9	9	9
9	9	9	9	9	9	9	9	9	9
9	9	9	9	9	9	9	9	9	9
9	1	1	1	1	1	1	1	2	2
9	9	9	9	9	9	9	9	9	9
2	1	1	2	1	2	1	2	1	2
2	2	2	2	2	2	2	2	2	2
1	1	1	1	1	1	1	1	1	9

2	1	1	1	1	1	2	2	1	1
9	2	2	9	2	2	2	2	9	2
2	2	2	2	2	2	2	2	2	2
1	1	1	1	1	2	1	1	1	1
2	2	2	2	2	2	2	2	2	2
9	9	9	9	9	9	9	9	9	9
1	3	2	2	2	2	9	1	2	2
2	2	2	2	2	2	2	2	2	2
9	9	9	9	9	9	9	9	9	9
2	9	9	9	9	9	9	9	9	9
9	9	9	9	9	9	9	9	9	9
9	9	9	9	9	9	9	9	9	9
9	9	9	9	9	9	9	9	9	9
9	9	9	9	9	9	9	9	9	9
9	9	9	9	9	9	9	9	9	9
9	9	9	9	9	9	9	9	9	9
2	9	9	9	9	9	9	9	9	9
9	9	9	9	9	9	9	9	9	9
2	2	2	2	2	2	2	2	2	2
3	1	1	3	3	2	2	1	1	3
3	1	1	3	3	2	1	2	1	3
3	1	1	3	3	2	1	2	1	3
3	1	1	3	3	2	1	2	1	3
3	1	1	3	3	2	2	1	1	3
3	1	1	3	3	2	2	1	1	3
3	1	1	1	3	2	2	1	1	3
3	1	1	3	3	2	1	1	1	3
3	1	1	3	3	2	1	1	1	3
3	1	1	3	3	2	2	1	1	3
3	1	1	3	3	2	1	1	1	3
3	1	1	3	3	2	1	1	1	3
3	1	1	3	3	2	1	1	1	3
3	1	1	3	3	2	2	1	1	3
3	1	1	3	9	2	1	1	1	3
3	1	1	3	3	2	2	9	1	3
3	1	1	3	3	2	1	1	1	3
3	1	1	3	3	2	2	2	1	3
3	1	1	3	3	2	2	1	1	3
3	1	1	3	3	2	1	1	1	3
3	1	1	3	3	2	1	1	1	3
3	1	1	3	3	2	1	1	1	3
3	1	1	3	3	2	2	3	1	3
3	1	1	3	3	2	2	2	1	3
3	1	1	1	3	3	2	1	1	3
9	9	9	9	9	9	9	9	9	9
9	9	9	9	9	9	9	9	9	9
9	9	9	9	9	9	9	9	9	9

9	9	9	9	9	9	9	9	9	9
9	9	9	9	9	9	9	9	9	9
9	9	9	9	9	9	9	9	9	9
9	9	9	9	9	9	9	9	9	9
9	9	9	9	9	9	9	9	9	9
9	9	9	9	9	9	9	9	9	9
9	9	9	9	9	9	9	9	9	9
9	9	9	9	9	9	9	9	9	9
9	9	9	9	9	9	9	9	9	9
9	9	9	9	9	9	9	9	9	9
9	9	9	9	9	9	9	9	9	9
9	9	9	9	9	9	9	9	9	9
9	9	9	9	9	9	9	9	9	9
9	9	9	9	9	9	9	9	9	9
9	9	9	9	9	9	9	9	9	9
9	9	9	9	9	9	9	9	9	9
9	9	9	9	9	9	9	9	9	9
9	9	9	9	9	9	9	9	9	9
9	9	9	9	9	9	9	9	9	9
3	1	1	3	3	2	2	1	1	3
3	1	1	3	3	2	1	1	1	3
3	1	1	3	3	2	2	1	1	3
3	1	1	3	3	2	2	1	1	3
3	1	1	3	3	2	2	1	1	3
3	1	1	3	3	2	2	9	1	3
3	1	1	3	3	2	1	2	2	3
3	1	1	3	3	2	2	2	1	3
3	1	1	3	3	2	2	2	1	3
3	1	1	3	3	2	1	2	1	3
3	1	1	3	3	3	1	2	1	3
3	1	1	3	3	2	2	1	3	1
3	1	1	3	3	2	1	1	1	3
3	1	1	3	3	2	2	1	1	3
3	1	1	3	3	1	2	1	1	3
3	1	1	3	3	2	1	1	1	3
1	1	9	3	3	9	1	1	1	9
3	1	1	3	3	2	1	2	1	3
3	1	1	3	3	2	1	1	1	3
3	1	1	3	3	2	1	1	1	3
3	1	1	3	3	2	1	1	1	3
3	1	1	3	2	9	1	1	1	3
3	1	1	3	3	2	2	1	1	3
3	1	1	3	3	2	1	1	1	3
3	1	1	3	3	2	2	9	1	3
3	1	1	3	3	2	2	9	1	3
3	1	1	3	3	2	1	2	1	3
3	1	1	3	3	2	1	1	1	3

3	1	1	3	3	2	1	1	1	2
9	1	1	3	3	2	1	1	1	3
3	1	1	3	3	3	2	2	1	3
3	1	1	3	3	2	1	3	1	3
3	1	1	3	3	2	2	1	1	3
3	1	1	3	3	2	1	1	1	3
3	1	1	3	3	2	1	2	1	3
3	1	1	3	3	2	1	2	1	3
3	1	1	3	3	9	1	2	1	3
3	1	1	3	3	2	3	1	1	3
3	1	1	3	3	2	1	1	1	3
3	1	1	3	3	2	1	3	1	1
3	1	1	3	3	2	2	1	1	3
3	1	1	1	3	1	1	2	1	3
3	1	1	3	3	2	1	2	1	3
3	1	1	3	3	2	1	2	1	3
3	1	9	3	3	2	1	2	1	3
3	1	1	3	3	2	1	2	1	3
3	1	1	3	3	2	2	1	1	3
3	1	1	3	3	2	2	1	1	3
3	1	1	3	3	2	2	1	1	3
3	1	1	3	3	2	1	1	1	3
3	1	1	1	2	2	1	1	1	3
3	1	1	3	3	2	2	1	1	3
3	1	1	3	3	2	2	1	3	1
3	1	1	3	3	2	1	2	1	3
3	1	1	3	3	1	1	2	1	3
3	1	1	3	3	2	2	1	1	1
3	1	1	1	3	2	1	9	1	3
3	1	1	1	3	9	3	1	1	3
3	1	1	3	3	2	2	2	1	3
3	1	1	3	3	2	1	1	1	3
9	1	3	3	3	2	1	1	1	3
3	1	1	3	3	2	2	1	1	3
9	9	9	9	9	9	9	9	9	9
3	1	1	3	3	2	1	1	1	3
3	1	1	3	3	2	2	1	1	3
3	1	1	3	3	2	1	1	1	3
3	1	1	3	3	2	1	2	1	3
9	1	1	3	3	2	1	2	1	3
3	1	1	3	3	2	1	2	1	3
3	1	1	3	3	2	1	2	1	3
3	1	1	3	3	2	2	1	1	3
3	1	1	3	3	2	1	1	1	3
3	1	1	3	3	2	2	1	1	3
3	1	1	3	3	2	1	2	1	3
3	1	1	3	3	2	2	9	1	3

3	1	1	1	3	3	2	1	1	3
3	1	1	3	3	2	2	1	1	3
3	1	1	3	3	2	2	1	1	3
3	1	1	3	3	2	2	1	3	3
3	1	1	3	3	2	2	1	1	1
3	1	1	3	3	2	1	2	1	3
3	1	1	3	3	2	2	1	1	3
3	1	1	3	3	2	1	1	1	3
3	1	1	3	3	2	2	1	1	3
3	1	1	3	3	2	2	1	1	3
3	1	1	3	3	2	2	1	1	3
1	1	1	3	3	2	2	1	1	3
3	1	1	3	3	2	1	1	1	3
3	1	1	3	3	2	2	1	1	3
3	1	1	3	3	9	2	1	1	3
3	1	1	3	3	2	2	1	1	3
3	1	1	3	3	2	1	2	1	3
3	1	1	3	3	2	1	2	1	1
3	1	1	3	3	2	1	2	1	3
3	1	3	3	9	3	1	1	1	3
3	1	1	3	3	2	1	1	1	3
3	1	1	3	3	2	2	1	1	3
3	1	1	3	3	2	2	1	1	3
3	1	1	3	3	2	2	1	3	3
3	1	1	3	3	2	2	1	3	2
3	1	1	3	3	2	1	1	1	3
3	1	1	3	3	2	1	2	1	3
3	1	1	3	3	2	2	9	1	3
3	1	1	3	3	2	1	2	1	3
3	1	1	3	3	2	2	1	1	3
3	1	1	3	3	2	1	2	1	3
3	1	1	3	3	2	1	1	1	2
3	1	1	1	3	3	2	1	3	1
3	1	1	1	3	3	2	1	3	1
3	1	1	3	3	2	2	3	1	3
3	1	1	3	3	2	2	3	1	3
3	1	1	3	3	2	2	3	1	3
3	1	1	3	3	2	2	1	1	3
3	1	1	3	3	2	2	1	1	3
3	1	1	3	3	2	2	1	1	3
3	1	1	3	3	2	2	1	1	3
9	9	9	9	9	9	9	9	9	9
3	1	1	3	3	2	1	1	1	3
3	1	1	3	3	2	2	1	1	3
3	1	1	3	3	2	2	1	1	3
3	1	1	3	3	2	2	1	3	1
3	1	1	3	3	2	2	1	3	1

3	1	1	3	3	2	2	1	1	3
3	1	1	3	3	2	2	1	1	3
3	1	1	3	1	2	2	1	1	3
3	1	1	3	3	2	2	1	1	3
3	1	1	3	3	2	2	1	1	3
3	1	1	3	3	2	2	1	1	3
3	1	1	3	3	2	1	1	1	3
3	1	1	3	3	2	2	3	1	3
3	1	1	3	3	2	1	1	1	3
9	9	9	9	9	9	9	9	9	9
3	1	1	3	3	2	2	1	1	3
3	1	1	3	3	2	2	1	1	3
3	1	1	3	3	2	1	1	1	3
3	1	1	3	3	2	2	9	1	3
3	1	1	3	3	2	2	9	1	3
3	1	1	3	3	2	2	1	1	3
3	1	1	3	3	2	2	3	1	3
3	1	1	3	3	2	1	3	1	3
3	1	1	3	3	2	2	1	1	3
3	1	1	3	3	2	2	1	1	3
3	1	1	3	3	2	2	1	1	3
3	1	1	3	3	2	1	2	1	3
3	1	1	3	3	2	1	2	1	3
3	1	1	3	3	2	1	1	1	3
3	1	1	3	3	2	2	1	1	3
3	1	1	3	3	2	2	1	1	3
3	1	1	3	3	2	2	1	1	3
9	9	9	9	9	9	9	9	9	9
9	9	9	9	9	9	9	9	9	9
9	9	9	9	9	9	9	9	9	9
9	9	9	9	9	9	9	9	9	9
9	9	9	9	9	9	9	9	9	9
9	9	9	9	9	9	9	9	9	9
9	9	9	9	9	9	9	9	9	9
9	9	9	9	9	9	9	9	9	9
9	9	9	9	9	9	9	9	9	9
9	9	9	9	9	9	9	9	9	9
9	9	9	9	9	9	9	9	9	9
9	9	9	9	9	9	9	9	9	9
9	9	9	9	9	9	9	9	9	9
9	9	9	9	9	9	9	9	9	9
9	9	9	9	9	9	9	9	9	9
9	9	9	9	9	9	9	9	9	9
9	9	9	9	9	9	9	9	9	9
9	9	9	9	9	9	9	9	9	9
9	9	9	9	9	9	9	9	9	9
9	9	9	9	9	9	9	9	9	9

9	9	9	9	9	9	9	9	9	9
9	9	9	9	9	9	9	9	9	9
9	9	9	9	9	9	9	9	9	9
9	9	9	9	9	9	9	9	9	9
9	9	9	9	9	9	9	9	9	9
9	9	9	9	9	9	9	9	9	9
9	9	9	9	9	9	9	9	9	9
9	9	9	9	9	9	9	9	9	9
9	9	9	9	9	9	9	9	9	9
9	9	9	9	9	9	9	9	9	9
9	9	9	9	9	9	9	9	9	9
9	9	9	9	9	9	9	9	9	9
9	9	9	9	9	9	9	9	9	9
1	1	3	3	2	2	1	1	1	1
2	2	2	2	2	2	2	2	2	2
2	2	2	2	2	2	2	2	2	2
2	1	1	1	1	2	2	2	1	2
1	1	2	2	2	2	1	1	2	2
1	2	3	3	3	3	3	3	2	2
2	9	9	9	9	9	9	9	2	1
2	2	2	2	2	3	1	1	1	2
3	2	3	3	3	2	1	1	2	3
9	9	9	9	9	9	9	9	9	9
3	2	2	2	3	2	2	3	2	3
9	9	9	9	1	9	9	9	9	9
2	2	2	2	3	3	3	3	2	2
9	9	9	9	9	9	9	9	9	9
1	1	1	1	1	1	1	1	1	1
2	1	2	1	3	3	3	2	2	2
2	2	2	2	2	2	2	2	1	1
2	1	1	2	2	2	3	3	3	3
2	3	2	2	3	1	2	3	1	2
9	1	9	9	9	9	9	9	1	9
2	2	2	3	2	3	3	2	1	1
1	1	9	3	1	1	1	1	1	1
9	2	9	9	9	9	9	9	2	9
3	1	2	1	2	2	2	2	1	1
1	2	1	1	3	2	1	2	1	2
2	1	2	2	2	2	2	1	1	2
3	1	1	1	1	1	1	1	1	1
9	9	9	9	9	9	9	9	9	9
9	9	9	9	9	9	9	9	9	9
2	1	2	3	3	2	2	2	2	2
1	9	9	9	9	9	9	9	9	9
9	9	9	9	9	9	9	9	9	9
9	9	9	9	9	9	9	9	9	9
9	9	9	9	9	9	9	9	9	9

9	9	9	1	9	9	9	9	9	9
9	9	9	9	9	9	9	9	9	9
1	2	2	2	2	3	3	3	3	1
1	1	1	3	3	2	2	2	1	2
1	1	1	1	1	3	1	1	3	2
9	1	9	9	9	9	9	9	2	9
2	2	2	2	2	2	2	2	2	2
2	2	2	2	2	2	2	2	2	2
2	2	2	2	2	2	2	2	2	2
1	1	1	1	2	2	3	3	1	1
2	3	3	3	3	3	3	3	2	2
9	9	9	9	9	9	9	9	9	9
9	1	9	9	9	9	9	9	9	2
2	2	2	2	2	2	2	2	2	2
2	2	2	2	2	2	2	2	1	2
2	1	1	1	2	2	1	2	2	2
3	1	1	1	1	1	1	1	1	1
2	9	9	9	9	9	9	9	1	1
1	2	1	1	1	1	2	1	1	1
9	9	9	9	9	9	9	9	9	9
2	2	2	2	2	2	2	2	2	2
2	2	2	2	2	2	2	2	2	2
2	2	2	2	2	2	2	2	2	2
2	2	2	2	2	2	2	2	2	2
2	2	2	2	2	2	2	2	2	2
1	1	2	1	2	3	2	1	1	1
2	2	2	2	2	2	2	2	2	2
2	2	3	1	2	3	1	2	1	2
9	9	9	9	9	9	9	9	9	9
1	1	1	1	1	3	1	1	1	3
3	2	3	3	3	2	3	1	1	3
2	1	3	2	2	2	1	1	3	3
1	1	1	1	1	1	1	1	1	2
2	2	2	2	2	2	2	2	1	1
2	2	1	3	2	3	2	1	2	1
2	1	1	1	2	9	2	2	2	2
2	2	2	2	2	2	2	2	1	1
9	1	1	1	9	9	9	9	2	2
9	2	9	9	9	9	9	9	2	9
2	2	2	2	2	2	2	2	2	2
3	3	3	3	3	3	3	3	3	3
2	2	2	2	2	2	1	2	2	2
2	1	2	3	1	2	3	1	1	2
9	9	9	9	9	9	9	9	9	9
1	1	1	1	1	1	1	1	1	1
3	1	1	2	2	2	2	2	1	1
3	2	3	2	2	3	2	2	2	2

2	2	2	2	2	2	2	2	2	2
3	2	2	2	3	3	3	3	2	2
3	1	1	1	1	1	1	1	2	2
9	2	3	3	3	2	2	3	2	2
2	2	2	2	2	2	2	2	2	2
2	2	2	2	2	2	2	2	2	2
2	2	2	2	2	2	1	3	2	3
3	2	2	3	3	3	2	2	3	2
2	2	2	2	2	2	2	2	2	2
3	2	1	3	2	2	3	1	2	1
2	2	2	2	2	2	2	2	2	2
2	2	2	2	2	2	2	2	2	2
2	1	1	1	1	1	1	1	1	1
3	2	2	2	2	2	2	2	2	2
2	2	2	2	2	2	2	2	2	2
2	2	2	2	2	2	2	2	3	2
2	3	3	3	3	3	3	3	3	3
2	2	2	2	2	2	2	2	2	2
2	2	2	2	2	2	2	2	2	2
2	1	2	3	1	2	3	1	1	2
2	1	2	1	2	1	2	1	1	2
2	1	1	1	1	1	1	2	1	1
2	2	9	1	3	1	1	3	1	2
1	1	1	1	1	3	3	3	1	1
1	1	1	3	3	3	1	1	1	1
9	9	9	9	9	9	9	9	9	9
9	9	9	9	9	9	9	9	9	9
2	2	2	2	2	2	2	2	2	2
9	1	1	2	3	2	2	1	1	2
2	1	3	2	1	1	2	1	1	1
3	2	2	2	2	2	2	2	2	2
2	2	9	1	2	1	2	2	1	1
9	2	2	2	2	2	2	2	1	1
1	1	1	1	1	1	1	1	3	3
9	1	1	1	3	2	1	1	9	9
2	9	2	2	2	2	2	9	2	2
9	9	9	9	9	9	9	9	9	9
1	9	9	9	9	9	9	9	9	9
9	9	9	9	9	9	9	9	9	9
2	2	2	2	2	2	2	2	1	1
2	2	3	3	2	3	2	2	3	2
1	3	1	1	3	3	1	1	1	2
1	2	1	1	1	2	2	2	2	1
9	9	9	9	9	9	9	9	9	9
9	9	9	9	9	9	9	9	9	9
9	9	9	9	9	9	9	9	9	9
3	1	1	1	2	2	2	3	1	1

9	9	9	9	9	9	9	9	9	9
9	9	9	9	9	9	9	9	9	9
2	2	2	1	2	2	1	1	3	2
9	9	9	9	9	9	9	9	9	9
9	9	9	9	9	9	9	9	9	9
2	3	3	3	3	3	3	3	2	3
2	1	2	3	2	2	2	2	1	1
9	9	9	9	9	9	9	9	9	9
1	1	1	2	2	3	1	1	1	1
2	1	1	3	1	2	3	1	2	1
2	1	2	3	1	2	3	2	2	3
2	2	2	2	2	2	2	2	2	2
2	2	2	2	2	2	2	2	2	2
9	9	9	9	9	9	9	9	9	9
2	2	2	2	2	2	2	2	2	2
2	2	2	2	2	2	2	2	2	2
1	1	1	1	2	3	3	3	1	3
3	3	3	3	1	2	3	2	2	3
2	2	2	2	9	1	1	3	1	3
9	9	9	9	9	9	9	9	9	9
3	3	3	3	1	2	3	2	2	3
1	1	3	1	3	2	3	2	1	3
2	2	2	2	2	2	2	2	2	2
3	3	3	3	2	1	3	2	2	3
3	2	3	2	3	3	2	2	3	3
9	9	9	9	9	9	9	9	9	9
1	1	1	1	1	1	1	1	1	2
9	9	9	9	9	9	9	9	9	9
3	3	3	3	3	3	3	3	3	3
2	2	1	3	1	2	3	1	1	2
2	2	2	2	2	2	2	2	2	2
9	9	9	9	9	9	9	1	9	9
9	9	9	9	9	9	9	1	9	9
2	2	2	2	2	2	2	2	2	2
2	1	1	9	2	2	2	2	2	2
3	2	2	1	2	3	2	2	2	3
9	9	9	9	9	9	9	9	9	9
1	1	2	2	2	2	2	2	1	2
2	2	2	2	2	2	2	2	2	2
9	2	9	9	9	9	9	9	2	9
2	9	9	9	9	9	9	9	9	9
2	2	2	2	2	2	2	2	2	2
2	2	3	3	3	1	3	3	2	2
2	2	2	2	2	2	2	2	2	2
2	2	2	2	2	2	2	2	2	2
9	1	9	9	9	9	9	9	3	1
2	2	2	2	2	2	2	2	2	2

1	2	2	2	2	2	2	2	2	2
2	2	2	2	2	2	2	2	2	2
3	3	2	3	2	3	2	2	1	3
2	2	2	2	2	2	2	2	1	3
2	2	2	2	2	2	2	2	2	2
3	2	2	2	2	2	2	3	2	2
3	3	2	2	2	3	2	3	1	1
3	1	1	1	2	2	1	2	1	2
1	1	1	2	3	3	1	1	1	2
3	2	3	3	3	3	2	2	1	3
2	3	2	2	3	1	2	3	2	2
1	3	3	3	3	3	3	3	3	3
2	2	2	2	2	2	2	2	1	1
2	1	1	1	2	3	1	2	1	2
1	1	9	1	1	9	1	1	2	9
2	2	2	2	2	2	2	2	2	2
2	2	3	1	2	2	1	2	2	2
2	2	1	2	3	3	2	1	2	1
2	2	2	2	2	3	2	2	2	2
2	1	1	1	1	2	2	2	2	1
1	1	1	1	1	1	1	1	1	3
9	9	9	9	9	9	9	9	9	9
2	2	2	2	2	2	2	2	2	2
2	2	2	2	2	2	2	2	2	2
9	1	9	2	9	2	9	2	2	2
3	1	1	2	1	2	1	1	1	2
2	2	3	3	3	2	2	2	2	1
2	2	3	2	2	2	2	2	2	1
3	1	3	3	3	1	3	1	1	3
2	2	2	2	2	2	2	2	1	3
3	1	1	3	3	3	1	3	1	1
3	1	1	1	2	1	2	1	2	2
2	3	2	1	1	1	1	2	2	1
9	9	9	9	9	9	9	9	9	9
9	2	9	9	9	9	9	9	2	9
3	1	2	3	3	3	1	2	1	3
2	2	2	2	2	2	2	2	2	2
2	1	1	1	2	1	2	1	1	2
2	1	1	1	1	1	1	1	2	2
2	2	2	2	2	2	2	2	2	2
2	1	2	3	2	3	2	3	1	2
9	9	9	9	9	9	9	9	9	9
9	9	9	9	9	9	9	9	9	9
1	1	1	1	1	1	1	1	1	1
2	1	2	2	2	2	2	2	2	2
9	9	9	9	9	9	9	9	9	9
9	9	9	9	9	9	9	9	9	9

2	2	2	2	2	2	2	2	2	2
1	1	1	1	1	1	1	1	1	1
2	9	9	9	9	9	9	9	9	9
2	1	1	1	1	1	1	1	3	3
3	1	2	3	3	3	3	3	2	2
2	1	2	3	1	2	3	1	1	2
3	1	2	3	2	2	1	2	1	2
9	1	9	9	9	9	9	9	9	9
2	1	2	2	2	2	2	3	1	1
9	9	9	9	9	9	9	9	9	9
1	1	1	1	1	1	1	1	1	1
1	1	3	3	3	2	1	1	1	1
3	1	1	1	1	1	1	1	2	2
9	9	9	9	9	9	9	9	9	9
1	1	1	1	1	1	1	1	1	1
9	9	9	9	9	9	9	9	9	9
9	9	9	9	9	9	9	9	9	9
2	2	2	2	2	2	2	2	2	2
2	2	2	2	2	2	2	2	2	2
2	2	2	1	3	2	2	2	2	2
2	2	2	2	2	2	2	2	2	2
2	2	2	2	2	2	2	2	2	2
2	2	2	2	3	3	3	3	3	2
1	2	3	1	2	3	1	2	1	2
9	1	9	2	2	3	9	2	2	9
9	1	9	9	9	9	9	9	2	9
2	1	1	1	2	2	2	2	1	3
2	1	2	2	2	2	2	2	3	3
1	1	1	1	1	1	1	1	1	3
2	1	3	3	3	2	1	1	3	1
2	1	1	2	1	2	2	1	1	1
2	2	2	2	2	2	2	2	2	2
2	2	2	2	2	2	2	2	2	2
2	2	2	2	2	2	2	2	2	2
2	2	2	2	2	2	2	2	2	2
2	2	2	2	2	2	2	2	2	2
2	2	3	1	2	3	2	1	1	2
9	9	9	9	9	1	9	9	1	9
9	9	9	9	9	9	1	9	1	9
2	2	2	2	2	2	2	2	2	2
2	2	2	2	2	2	2	2	3	3
9	2	9	9	9	9	9	9	2	1
2	1	2	1	2	2	2	3	1	2
9	2	9	9	9	9	9	9	2	9
3	2	2	2	2	2	2	2	2	2
9	9	9	9	9	9	9	9	9	9
2	2	2	2	2	2	2	2	2	2

2	3	2	1	2	3	2	1	9	9
9	1	9	9	9	9	9	9	1	9
2	1	1	1	1	1	1	1	2	2
2	2	2	2	2	2	2	2	2	2
2	2	2	2	2	2	2	2	2	2
2	2	2	2	2	2	2	2	2	2
2	2	1	1	2	3	1	1	1	2
9	9	9	9	9	9	9	9	9	9
2	2	2	2	2	2	2	2	2	2
2	2	2	2	2	2	2	2	2	2
2	2	2	2	2	2	2	2	2	2
2	2	2	2	2	2	2	2	2	2
2	2	2	2	2	2	2	2	3	3
2	1	2	3	3	3	2	2	1	2
1	1	2	1	1	2	1	3	2	1
1	1	2	1	1	2	1	1	2	1
2	2	2	2	2	2	2	2	2	2
2	2	2	2	2	2	2	2	2	2
9	9	9	9	9	9	9	9	9	9
9	9	9	9	9	9	9	9	9	9
2	3	2	3	2	3	2	3	3	2
3	1	2	2	2	2	2	2	1	2
9	9	9	9	9	9	9	9	9	9
9	9	9	9	9	9	9	9	9	9
3	3	3	3	3	3	3	3	3	3
9	9	9	9	9	9	9	9	9	9
9	9	9	9	9	9	9	9	9	9
9	9	9	9	9	9	9	9	9	9
1	1	1	1	1	3	1	1	1	2
2	1	1	1	1	1	1	1	1	1
2	2	2	2	2	2	2	2	2	2
1	1	2	2	9	2	1	9	1	1
2	2	2	2	2	2	2	2	2	2
2	2	2	2	2	2	2	2	2	2
2	2	2	2	2	2	2	2	2	2
1	1	2	2	2	2	2	2	1	3
3	1	2	2	1	3	3	2	1	3
2	1	3	2	1	2	2	1	3	2
3	2	1	2	3	2	1	2	1	1
3	1	1	1	1	1	1	1	1	3
2	1	2	3	9	3	9	3	1	2
2	2	2	2	2	2	2	2	2	2
9	1	9	9	9	9	9	9	9	9
9	9	2	9	9	9	9	9	9	2
2	1	3	2	3	2	3	2	1	2
2	2	2	2	2	2	2	2	2	2
2	2	2	2	2	2	2	2	2	2

3	1	1	1	1	2	9	3	2	2
9	9	9	9	9	9	9	9	9	9
9	9	9	9	9	9	9	9	9	9
9	9	9	9	9	9	9	9	9	9
9	9	9	9	9	9	9	9	9	9
9	9	9	9	9	1	9	9	9	1
9	9	9	9	9	9	9	9	9	9
9	9	9	9	9	9	9	9	9	9
9	9	9	9	9	9	9	9	9	9
9	9	1	9	9	9	9	9	3	9
2	2	2	2	2	2	2	2	2	2
2	1	2	2	3	3	2	2	2	2
1	1	1	2	2	2	1	1	1	3
2	2	2	2	2	2	2	2	2	2
2	2	2	2	2	2	2	2	2	2
9	9	9	9	9	9	9	9	9	9
3	2	2	2	2	2	3	2	2	2
2	2	2	2	2	2	2	2	2	2
2	2	2	2	2	2	2	2	2	2
2	2	3	2	3	1	2	2	2	2
2	1	3	1	2	1	3	2	1	3
2	3	2	1	3	3	2	1	2	3
2	2	2	2	2	2	2	2	2	2
2	2	2	2	2	2	2	2	2	2
2	2	2	2	2	2	2	2	2	2
2	1	1	1	1	1	1	1	2	2
3	3	3	3	3	3	3	3	3	3
2	1	2	2	2	1	2	1	1	2
2	2	2	2	2	2	2	2	2	2
2	2	2	2	2	2	2	2	2	2
9	9	9	9	9	9	9	9	9	9
2	2	2	2	2	2	2	2	2	2
3	1	1	1	1	1	1	1	2	2
9	9	9	9	9	1	9	9	9	9
9	3	3	3	3	3	3	3	3	3
2	2	2	2	2	2	2	2	2	2
2	2	2	2	2	2	2	2	2	2
2	2	2	2	2	2	2	2	2	2
1	1	2	1	2	3	1	2	1	3
9	9	9	9	9	9	9	9	9	9
2	1	1	1	1	2	2	2	1	1
2	2	2	2	2	2	2	2	2	2
2	2	2	2	2	2	2	2	2	2
2	1	2	1	2	2	2	1	1	3
2	1	2	1	2	2	1	1	1	2
9	9	9	9	9	9	9	9	9	9
9	9	9	9	9	9	9	9	9	9

9	9	9	9	9	9	9	9	9	9
2	2	2	2	2	2	2	2	2	2
9	2	9	9	9	9	9	9	1	9
9	9	9	9	9	9	9	9	9	9
9	9	9	9	9	9	9	9	9	9
9	9	9	9	9	9	9	9	9	9
9	9	9	9	9	9	9	9	9	9
9	9	9	9	9	9	9	9	9	9
9	9	9	9	9	9	9	9	9	9
9	9	9	9	9	9	9	9	9	9
9	9	9	9	9	9	9	9	9	9
9	9	9	9	9	9	9	9	9	9
9	9	9	9	9	9	9	9	9	9
9	9	9	9	9	9	9	9	9	9
9	9	9	9	9	9	9	9	9	9
9	9	9	9	9	9	9	9	9	9
9	9	9	9	9	9	9	9	9	9
9	9	9	9	9	9	9	9	9	9
9	9	9	9	9	9	9	9	9	9
9	9	9	9	9	9	9	9	9	9
9	9	9	9	9	9	9	9	9	9
9	9	9	9	9	9	9	9	9	9
9	9	9	9	9	9	9	9	9	9
9	9	9	9	9	9	9	9	9	9
9	9	9	9	9	9	9	9	9	9
2	1	1	1	1	2	1	2	1	2
2	2	1	2	2	2	2	2	2	2
2	1	1	1	2	2	1	2	2	2
1	1	1	1	1	2	2	2	1	3
2	1	1	1	1	2	2	1	1	2
2	1	2	1	2	2	1	1	1	2
2	2	2	2	2	2	2	2	2	2
2	9	3	1	2	3	1	1	1	1
2	1	9	1	2	9	1	2	2	2
2	1	2	1	2	2	3	3	2	2
2	1	1	1	3	3	1	1	1	1
3	1	1	3	3	3	1	1	1	2
1	1	1	1	1	2	1	1	1	3
2	1	1	2	2	1	2	2	1	2
2	2	2	2	2	2	2	2	2	2
1	1	1	2	2	3	1	1	1	2
9	3	1	2	1	3	1	2	1	9
2	1	1	1	1	2	1	1	1	2
3	1	1	1	1	1	1	1	2	1
9	1	9	9	9	9	9	9	1	9
2	2	1	1	2	2	2	1	1	2
1	1	1	1	3	3	1	1	1	1
2	2	2	2	2	2	2	2	2	2
2	1	1	1	1	1	1	1	2	1

2	1	1	2	1	2	1	1	1	2
1	1	2	3	2	3	3	3	1	2
1	1	1	3	3	3	1	1	1	3
2	9	9	9	9	9	9	9	2	2
1	2	3	3	2	2	1	2	3	2
9	1	9	9	9	9	9	9	1	9
2	1	2	2	2	1	1	1	2	2
9	1	9	9	9	9	1	3	1	9
2	2	2	2	2	2	2	2	2	2
2	2	2	2	2	2	2	2	2	2
9	9	9	9	9	1	9	9	9	9
9	9	9	9	9	1	9	9	9	9
9	1	9	9	9	9	9	9	1	9
9	9	9	9	9	1	9	9	9	9
9	1	9	9	9	9	9	9	1	1
9	9	9	9	9	9	9	9	9	9
9	9	9	9	9	9	9	9	9	9
9	9	9	9	9	9	9	9	9	9
9	9	1	9	9	9	9	9	9	1
3	3	1	3	3	3	3	3	1	3
2	1	9	9	9	9	9	9	1	9
9	1	9	9	9	9	9	9	9	9
9	1	9	9	9	9	9	9	1	9
9	9	9	9	9	9	9	1	9	9
9	1	9	9	9	9	9	9	1	9
1	1	2	3	2	1	3	2	1	2
3	3	9	9	9	9	9	9	9	9
3	2	3	2	2	2	1	1	1	3
2	2	2	2	2	2	2	2	1	2
9	9	9	9	9	9	9	9	9	9
1	1	2	2	2	2	2	2	2	2
3	3	3	3	3	3	3	3	1	3
3	2	9	9	9	9	9	9	3	2
1	9	9	9	9	9	9	9	1	1
3	3	3	3	3	3	3	3	1	1
9	2	9	9	9	9	9	9	3	2
2	2	2	2	2	2	2	2	2	2
2	2	2	2	2	2	2	1	1	2
2	2	3	1	1	2	3	2	1	2
3	3	1	1	1	2	1	1	1	1
3	1	1	1	2	3	2	1	1	2
3	1	1	1	1	3	2	1	1	1
3	3	3	1	9	3	3	1	1	3
2	2	2	1	3	3	2	1	3	1
3	2	2	2	2	2	1	9	2	3
2	2	2	3	2	3	2	1	1	2
2	1	2	3	3	2	1	1	1	2

2	9	9	9	9	9	9	9	9	9
3	1	9	9	9	9	9	9	2	9
9	9	9	9	9	9	9	9	9	9
3	1	1	3	3	1	1	1	1	9
9	9	9	9	9	9	9	9	9	9
3	1	1	2	2	2	1	1	1	2
3	1	1	2	2	1	1	1	1	3
9	9	9	9	9	9	9	9	9	9
9	2	9	9	9	9	9	9	2	9
2	2	2	2	2	2	2	2	2	2
2	2	2	1	2	3	1	1	2	1
1	1	1	3	3	1	1	1	1	3
2	1	2	2	2	3	1	2	1	2
9	9	9	9	9	9	9	9	9	9
9	9	9	9	9	9	9	9	9	9
3	2	3	3	2	3	2	1	1	1
9	9	9	9	9	1	9	9	9	9
9	9	9	9	9	9	9	9	9	9
3	3	3	3	3	3	3	3	3	3
1	1	1	1	1	2	2	1	1	1
2	2	2	2	2	2	2	2	2	2
1	1	1	1	1	1	1	1	2	1
3	2	1	3	3	3	3	2	1	2
1	1	1	3	2	1	1	2	1	2
2	2	2	2	2	2	2	2	2	2
9	9	9	9	9	9	9	9	9	9
9	1	9	9	9	9	9	9	1	9
1	1	1	1	1	1	3	1	3	3
3	2	2	3	1	1	1	1	3	3
1	1	2	1	1	3	1	1	2	3
9	9	9	9	9	9	9	9	9	9
9	9	9	9	9	9	9	9	9	9
9	9	9	9	9	9	9	9	9	9
9	9	9	9	9	9	9	9	9	9
9	9	9	9	9	9	9	9	9	9
9	9	9	9	9	9	9	9	9	9
9	9	9	9	9	9	9	9	9	9
9	9	9	9	9	9	9	9	9	9
9	9	9	9	9	9	9	9	9	9
9	9	9	9	9	9	9	9	9	9
9	9	9	9	9	9	9	9	9	9
9	9	9	9	9	9	9	9	9	9
9	9	9	9	9	9	9	9	9	9
9	9	9	9	9	9	9	9	9	9
9	9	9	9	9	9	9	9	9	9
9	9	9	9	9	9	9	9	9	9
9	9	9	9	9	9	9	9	9	9
9	9	9	9	9	9	9	9	9	9

9	9	9	9	9	9	9	9	9	9
9	9	9	9	9	9	9	9	9	9
9	9	9	9	9	9	9	9	9	9
9	9	9	9	9	9	9	9	9	9
9	9	9	9	9	9	9	9	9	9
9	9	9	9	9	9	9	9	9	9
9	9	9	9	9	9	9	9	9	9
9	9	9	9	9	9	9	9	9	9
9	9	9	9	9	9	9	9	9	9
9	9	9	9	9	9	9	9	9	9
9	9	9	9	9	9	9	9	9	9
9	9	9	9	9	9	9	9	9	9
9	9	9	9	9	9	9	9	9	9
9	9	9	9	9	9	9	9	9	9
9	9	9	9	9	9	9	9	9	9
9	9	9	9	9	9	9	9	9	9
9	9	9	9	9	9	9	9	9	9
9	9	9	9	9	9	9	9	9	9
9	9	9	9	9	9	9	9	9	9
9	9	9	9	9	9	9	9	9	9
9	9	9	9	9	9	9	9	9	9
9	9	9	9	9	9	9	9	9	9
9	9	9	9	9	9	9	9	9	9
9	9	9	9	9	9	9	9	9	9
9	9	9	9	9	9	9	9	9	9
9	9	9	9	9	9	9	9	9	9
9	9	9	9	9	9	9	9	9	9
9	9	9	9	9	9	9	9	9	9
9	9	9	9	9	9	9	9	9	9
9	9	9	9	9	9	9	9	9	9
9	9	9	9	9	9	9	9	9	9
9	9	9	9	9	9	9	9	9	9
9	9	9	9	9	9	9	9	9	9
9	9	9	9	9	9	9	9	9	9
9	9	9	9	9	9	9	9	9	9
2	2	2	2	2	2	2	2	2	2
9	9	9	9	9	9	9	9	9	9
3	2	3	3	3	3	1	1	1	2
3	1	1	3	3	3	1	1	1	3
3	3	3	3	3	3	3	3	3	3
9	9	9	9	9	9	9	9	9	9
3	9	9	9	9	9	9	9	9	9
9	1	9	9	9	9	9	9	2	9
3	9	9	9	9	9	9	9	9	9
3	2	2	2	1	3	1	3	9	9
1	9	9	9	9	9	9	9	9	9
2	2	2	2	2	2	2	2	2	2

1	1	1	2	2	3	1	1	1	2
2	2	2	2	2	2	2	2	2	2
1	2	1	1	2	3	2	2	1	2
1	1	2	3	2	2	2	1	1	2
2	1	1	2	2	2	1	1	1	2
2	1	1	3	3	3	1	1	1	1
2	1	1	1	1	2	1	1	1	2
2	1	2	1	2	3	1	1	2	2
1	2	1	2	2	1	3	2	2	2
1	2	3	1	2	3	2	1	2	2
9	9	9	9	9	9	1	9	1	9
3	2	3	2	3	3	2	3	1	2
9	9	9	9	9	9	9	9	9	9
2	2	1	2	2	2	1	1	2	1
2	2	1	2	2	2	1	1	2	1
2	1	1	1	2	2	1	1	1	3
9	9	9	9	9	9	9	9	9	9
9	1	1	2	2	2	1	1	1	2
1	1	1	1	1	1	1	1	1	1
3	1	2	1	2	1	2	3	1	3
2	1	1	2	2	2	1	1	1	9
3	1	1	1	1	3	2	1	1	2
2	1	1	1	1	2	1	1	1	1
1	1	3	3	3	3	1	1	1	3
3	3	3	3	3	3	3	3	3	3
2	3	2	3	2	3	2	3	2	2
1	2	2	2	2	2	2	2	2	2
1	3	2	1	3	1	1	3	1	2
2	2	2	2	2	2	2	2	2	2
9	9	9	9	9	9	9	9	9	9
9	9	9	9	9	9	9	9	9	9
1	2	2	2	2	2	2	2	2	2
2	2	2	2	2	2	2	2	2	2
9	9	9	9	9	9	9	9	9	9
9	9	9	9	9	9	9	9	9	9
9	9	9	9	9	9	9	9	9	9
3	2	2	3	2	3	1	3	1	3
3	1	1	2	1	9	9	9	1	2
9	1	1	1	9	1	2	2	1	2
9	9	9	9	9	9	9	9	9	9
2	2	2	2	2	2	1	1	1	1
9	9	9	9	9	9	9	9	9	9
3	1	1	1	1	3	1	1	1	3
1	1	1	3	3	3	1	1	1	1
1	1	1	1	3	3	3	3	1	3
3	3	3	1	3	3	3	3	3	1
3	1	1	1	1	3	1	1	1	3

3	2	3	2	3	3	2	2	3	2
2	1	2	3	1	2	3	2	1	2
2	2	2	1	2	3	1	2	2	2
1	1	2	1	1	1	1	1	1	1
2	2	2	3	3	3	3	2	1	2
1	1	2	3	1	2	3	1	1	2
1	1	2	3	1	2	3	1	1	2
3	2	1	2	1	2	3	2	2	3
1	1	1	1	1	3	1	1	1	3
2	1	2	9	1	2	2	2	1	2
1	1	3	1	3	3	3	3	1	1
2	1	3	2	1	1	2	1	1	2
2	2	1	1	2	2	2	2	2	2
3	1	2	2	3	2	1	1	1	1
1	1	3	9	2	1	1	3	1	1
2	2	3	1	1	1	1	1	1	1
2	2	2	2	3	2	3	2	2	2
9	9	1	9	9	9	9	9	1	9
1	1	2	2	1	2	2	3	1	2
2	3	2	3	3	1	1	1	3	3
3	1	1	2	2	2	2	2	3	3
1	1	1	2	2	2	1	2	1	1
2	1	1	1	2	3	1	2	1	2
2	2	2	2	2	2	2	2	1	1
2	1	2	2	2	3	3	3	3	2
3	1	3	3	3	1	1	1	1	9
2	1	1	1	2	3	2	2	1	3
2	1	1	1	1	2	3	3	3	1
3	1	1	1	1	1	1	1	2	2
2	1	2	1	2	3	1	1	1	2
1	3	2	2	2	2	2	2	1	1
1	1	2	1	2	3	2	1	1	2
1	2	3	3	3	2	1	1	1	1
3	2	3	3	3	1	2	2	1	1
2	1	2	3	1	2	3	1	1	2
1	1	1	1	1	3	1	1	1	1
2	1	2	3	2	1	2	3	1	3
9	9	9	9	9	9	9	9	9	9
1	1	1	1	1	3	1	1	1	1
3	1	3	3	1	1	3	1	1	1
1	1	3	1	1	3	1	1	1	3
2	1	2	3	1	2	3	1	1	2
3	1	1	1	1	3	1	1	1	1
3	2	1	2	3	1	2	2	1	1
2	2	2	2	2	2	2	2	2	2
3	2	3	3	2	3	3	1	3	3
9	9	9	9	9	9	9	9	9	9

2	2	1	2	3	1	2	2	1	2
9	1	1	1	1	1	1	1	1	1
1	2	2	2	2	2	2	1	1	3
3	1	2	2	2	1	2	3	1	1
2	2	2	2	2	2	2	2	1	1
3	1	1	1	1	2	2	3	2	2
2	2	2	2	2	2	2	2	2	2
9	9	9	9	9	9	9	9	9	9
9	9	9	9	9	9	9	9	9	9
1	1	2	3	2	1	2	3	1	2
2	1	2	2	2	2	1	1	1	2
2	2	2	2	2	2	2	2	2	2
1	1	1	2	2	2	2	2	1	1
2	2	2	2	2	2	2	2	2	2
2	2	2	2	2	2	2	2	2	2
2	2	2	2	2	2	2	2	2	2
2	2	2	2	2	2	2	2	2	2
2	2	2	2	2	2	2	2	2	2
2	2	3	3	3	3	2	2	2	2
2	2	2	2	2	2	2	2	3	2
2	2	2	2	2	2	3	1	2	2
2	2	2	2	2	2	2	2	2	2
2	2	2	2	2	2	2	2	2	2
2	1	2	2	3	2	2	2	2	1
2	2	2	2	2	2	2	2	2	2
2	2	2	2	2	2	2	2	2	2
3	1	2	2	2	2	1	1	1	2
2	1	2	1	2	1	2	3	1	2
2	1	2	2	2	2	2	1	1	1
2	2	2	2	2	2	2	2	2	2
2	2	2	2	2	2	2	2	2	2
2	1	2	2	2	2	1	1	1	2
2	2	2	2	2	2	2	2	2	2
2	2	2	2	2	2	2	2	2	2
3	2	2	2	2	2	2	2	1	2
2	1	2	2	2	2	1	1	1	2
2	2	2	2	2	2	2	2	2	2
2	2	2	2	2	2	2	2	2	2
2	2	2	2	2	2	2	2	2	2
2	1	1	2	2	3	1	1	1	3
2	2	2	2	2	2	2	2	1	2
2	2	2	2	2	2	2	2	2	2
3	3	2	1	2	3	3	2	1	2
2	2	2	2	2	2	2	2	2	2
1	2	1	1	2	3	3	2	2	1
1	2	2	2	2	2	2	2	2	2
2	3	3	3	3	3	3	3	2	2

2	2	2	2	2	2	2	2	2	2
3	2	2	2	2	2	2	2	3	2
2	2	2	2	2	2	2	2	2	2
2	2	2	2	2	2	2	2	2	2
2	2	2	2	2	2	2	2	2	2
2	2	2	2	2	2	2	2	2	2
9	1	3	1	3	3	1	1	1	3
1	1	1	1	1	1	1	1	1	1
9	1	9	9	9	9	9	9	9	1
3	2	2	2	2	2	2	2	2	2
3	1	1	1	1	1	1	1	2	3
2	1	1	1	1	1	1	1	2	2
3	2	2	2	2	2	2	2	2	3
3	3	3	3	3	3	3	3	9	9
9	2	2	2	2	2	2	2	2	2
1	1	2	2	1	2	2	2	2	2
2	1	1	1	1	1	1	1	2	2
1	1	1	1	1	1	1	1	1	1
9	2	9	9	9	9	9	9	2	9
2	1	3	3	1	2	2	2	2	2
2	1	9	2	2	2	2	2	3	1
9	9	9	9	9	1	9	9	9	9
9	1	9	9	9	9	9	9	1	9
3	2	2	2	2	2	2	2	2	2
1	1	2	3	1	2	3	2	1	2
1	3	1	1	2	2	1	1	1	2
3	1	1	1	1	2	2	2	1	2
1	1	1	1	2	2	9	1	1	1
2	1	2	9	2	9	2	3	9	9
3	1	1	1	1	1	1	1	1	1
2	3	3	3	3	3	3	3	2	2
2	2	2	2	2	2	2	2	2	2
2	2	2	2	2	2	2	2	2	2
9	1	9	9	9	2	9	9	9	1
2	2	2	2	2	2	2	2	2	2
9	9	9	1	9	9	9	9	9	9
1	1	1	1	3	9	3	3	1	3
3	2	2	2	2	2	2	2	3	3
2	2	2	2	2	2	2	2	2	2
2	2	2	2	2	2	2	2	2	2
1	2	9	9	2	9	2	3	1	2
2	2	2	2	2	2	2	2	2	2
9	9	9	9	2	9	9	9	9	9
2	1	1	1	2	2	2	3	2	2
2	2	2	2	2	2	2	2	2	2
2	3	3	3	3	3	3	3	2	2
2	1	1	2	2	2	2	2	1	1

9	9	9	1	9	9	9	9	9	9
2	2	2	2	2	2	2	2	2	2
1	1	1	1	1	1	1	1	1	1
2	2	2	2	2	2	2	2	2	2
9	9	9	9	9	9	9	1	9	9
2	1	1	2	2	3	3	2	1	1
3	3	3	3	3	3	3	3	3	3
2	1	1	1	1	1	1	1	1	1
2	2	2	2	2	2	2	2	2	2
3	1	1	1	3	3	3	3	1	1
9	9	9	9	9	9	9	9	9	9
1	1	1	1	1	1	1	1	1	1
2	2	2	2	2	2	2	2	2	2
2	2	2	2	2	2	2	2	2	2
2	2	2	2	2	2	2	2	2	2
2	2	2	2	2	2	2	2	2	2
1	1	1	1	1	1	1	1	1	1
3	1	1	1	1	3	3	3	1	1
9	9	9	9	9	9	9	9	9	9
3	1	2	3	1	2	1	3	2	1
1	2	2	2	2	2	2	2	1	1
2	1	1	1	1	2	1	1	2	2
1	1	1	1	1	1	1	1	1	1
2	1	1	1	1	1	1	1	2	2
3	3	3	3	3	3	3	3	3	3
1	1	1	1	2	2	2	2	1	1
2	1	1	2	9	1	2	1	1	1
2	2	2	2	2	2	2	2	2	2
2	1	2	2	2	2	3	3	2	3
2	2	2	2	2	2	2	2	2	2
3	1	2	9	9	2	1	3	1	1
2	2	2	2	2	2	2	2	2	2
3	3	3	3	3	3	3	3	3	3
1	1	2	9	3	2	2	1	2	2
2	2	2	2	2	2	2	2	2	2
2	3	3	3	3	3	3	3	3	3
1	1	1	1	1	1	1	1	2	2
2	2	2	2	2	2	2	2	2	2
2	1	2	3	1	3	1	2	1	2
3	3	3	3	3	3	3	3	3	3
1	1	1	1	1	1	1	1	1	1
2	2	2	2	2	2	2	2	2	2
9	9	9	9	9	9	9	9	9	9
9	9	3	3	3	3	3	3	3	3
9	9	9	9	9	9	9	9	9	9
2	2	2	2	2	2	2	2	2	2
2	1	1	1	1	1	2	2	1	3

2	2	2	2	2	2	2	2	3	3
1	1	1	1	3	3	1	1	1	1
9	9	9	9	9	9	9	9	9	9
1	1	1	1	3	3	1	1	1	1
2	1	2	2	3	2	3	2	1	2
2	2	2	2	2	2	2	2	2	2
2	2	2	2	2	2	2	2	2	2
2	2	2	2	2	2	2	2	2	2
1	2	2	2	2	2	2	2	1	1
1	9	9	9	9	9	9	9	2	2
3	2	2	2	2	2	2	2	2	3
1	3	2	1	3	1	1	3	1	2
2	2	2	2	2	2	2	2	2	2
2	1	2	3	3	3	2	1	1	2
2	2	2	2	2	2	2	2	2	2
2	2	2	2	2	2	2	2	2	2
2	2	2	2	2	2	2	2	2	2
2	2	2	2	2	2	2	2	2	2
2	2	2	2	2	2	2	2	2	2
1	1	2	2	2	2	2	2	2	2
1	2	2	2	2	2	2	2	2	2
2	2	2	2	2	2	2	2	2	2
2	2	2	2	2	2	2	2	2	2
2	2	2	2	2	2	2	2	2	2
1	1	1	1	1	1	1	1	1	1
2	2	2	2	2	2	2	2	2	2
2	1	1	1	1	3	3	3	1	3
2	2	2	2	2	2	2	2	2	2
1	3	1	2	2	1	1	1	1	2
3	1	1	2	2	2	1	3	1	2
1	1	1	1	1	1	1	1	1	1
1	1	1	1	2	2	3	3	3	3
2	2	2	2	2	2	2	2	2	2
3	2	3	1	2	3	1	3	2	2
3	1	2	1	2	2	2	3	1	2
1	1	2	3	2	3	3	2	2	3
9	9	9	9	9	9	9	9	9	9
9	9	9	9	9	9	9	9	9	9
3	3	3	3	1	3	2	1	2	1
2	2	2	2	2	2	2	2	2	2
1	2	1	3	2	2	3	2	2	9
2	2	2	2	2	2	2	2	2	2
2	2	2	2	2	2	2	2	2	2
2	2	2	2	2	2	2	2	2	2
9	9	9	9	9	9	9	9	9	9
2	1	1	2	1	2	2	1	1	2
2	2	2	2	2	2	2	2	2	2

9	9	9	9	9	9	9	9	9	9
3	1	2	2	1	2	3	3	9	9
2	1	2	2	3	2	3	2	1	2
3	9	9	9	9	9	9	9	9	9
3	3	9	3	3	3	3	3	1	3
2	1	1	1	3	1	2	2	1	1
2	2	2	1	1	3	3	2	1	2
9	9	9	9	9	9	9	9	9	9
2	2	1	2	2	2	1	1	2	2
3	1	1	1	1	2	1	1	2	2
1	2	2	2	2	2	2	2	2	2
3	1	1	1	2	2	3	3	1	1
2	1	1	2	2	2	2	1	1	2
3	1	1	1	1	3	1	1	1	2
1	1	1	2	3	1	3	2	1	2
2	2	2	2	2	2	2	2	2	2
2	2	2	1	1	2	2	1	1	2
2	3	3	3	2	2	2	2	2	2
9	9	9	9	9	9	9	9	9	9
9	9	9	9	9	9	9	9	9	9
9	9	9	9	9	9	9	9	9	9
2	1	2	3	2	3	1	2	1	2
1	2	3	3	3	3	3	2	2	1
3	1	2	2	2	1	1	3	1	2
3	1	2	2	3	3	3	2	3	3
2	3	2	2	2	3	2	3	3	3
1	1	2	3	1	2	2	3	3	1
2	1	1	1	1	2	9	3	1	2
2	3	3	2	2	1	3	2	1	2
1	1	2	1	2	3	2	1	1	3
3	3	9	2	2	2	2	2	2	2
1	1	1	2	2	3	3	2	1	1
3	1	3	9	2	2	3	3	2	1
3	2	2	2	2	2	2	2	3	3
3	2	2	2	2	2	2	2	2	2
2	2	2	2	2	2	2	2	2	2
3	3	3	3	3	3	3	3	3	3
2	2	2	2	2	2	2	2	2	2
9	9	9	9	9	9	9	9	9	9
9	9	9	9	9	9	9	9	9	9
1	2	2	2	2	2	2	2	3	3
2	2	2	3	3	3	3	3	3	3
2	2	2	2	3	2	2	2	2	3
3	1	1	1	2	3	2	2	3	3
9	2	2	2	2	9	9	9	9	9
3	1	3	3	2	3	9	2	2	9
2	1	1	1	2	2	2	2	2	2

1	1	1	1	1	1	9	1	1	1
2	1	1	3	2	3	2	1	1	2
3	2	3	1	3	2	1	2	9	9
1	1	3	2	2	2	3	1	1	9
3	1	2	2	2	3	1	3	2	3
3	3	3	3	3	3	3	3	3	3
2	9	1	1	1	1	1	1	1	1
3	3	3	3	3	3	3	3	3	3
2	1	2	2	2	3	2	2	2	2
1	1	2	2	1	3	2	1	1	2
2	2	2	2	2	2	2	2	2	2
3	1	2	1	2	3	1	2	1	2
2	2	2	3	2	2	2	2	2	2
2	1	1	1	1	1	1	1	1	1
1	1	1	1	1	1	2	2	2	2
3	3	3	3	3	3	2	2	2	2
1	1	3	1	1	1	3	3	1	1
2	1	1	1	1	1	1	1	3	3
9	9	9	9	9	9	9	9	9	9
3	1	1	2	1	3	3	2	1	2
2	1	1	1	1	1	1	1	1	1
2	2	1	2	3	3	2	3	2	3
2	1	1	1	1	1	1	1	1	1
2	2	2	2	2	2	2	1	1	2
2	1	2	3	1	2	3	1	1	2
3	3	2	2	3	3	2	2	1	2
2	1	1	1	1	2	3	2	3	3
2	1	2	3	2	2	2	3	1	2
3	2	1	3	1	3	2	1	1	2
2	2	2	2	2	2	2	2	1	3
2	1	2	1	2	1	2	1	1	2
2	2	2	2	2	2	2	2	2	2
2	2	2	2	2	2	2	2	2	2
2	1	2	3	3	3	3	3	1	1
2	1	2	3	1	2	3	1	1	2
3	1	1	2	2	3	2	1	1	2
1	1	2	3	1	3	1	2	2	2
3	3	3	3	3	3	3	3	3	3
1	1	1	2	2	3	3	1	1	1
3	2	1	2	3	3	2	1	1	2
2	3	3	2	2	2	2	2	2	2
3	1	2	3	1	2	3	1	1	2
2	1	2	3	3	2	2	1	1	2
1	1	1	1	1	1	1	1	1	1
2	1	2	2	2	3	3	2	1	2
2	1	2	3	1	2	1	2	1	2
2	1	1	2	3	1	2	1	1	3

2	2	1	1	1	2	1	2	1	1
2	1	2	3	1	2	2	1	1	3
2	1	2	2	2	2	2	2	2	2
1	1	1	1	1	1	1	1	1	1
3	1	2	3	1	2	3	1	1	2
2	1	2	3	2	3	1	2	1	2
3	1	2	3	2	3	1	2	1	2
3	1	2	3	1	2	3	2	1	2
3	1	1	2	1	2	1	2	1	2
3	2	2	2	2	2	3	3	3	3
2	1	2	3	1	2	3	1	1	2
1	2	1	2	1	3	2	1	1	2
1	1	1	1	1	1	1	1	3	3
3	1	3	3	3	1	3	1	1	3
3	2	3	2	3	1	2	3	2	3
1	1	1	1	3	3	3	3	3	1
1	1	1	1	1	2	2	3	1	1
1	1	1	1	1	1	1	1	1	3
2	1	1	3	2	3	2	2	1	1
2	2	2	2	2	2	2	2	3	2
3	3	3	3	3	3	3	3	2	2
3	3	3	3	3	3	3	3	3	3
2	3	3	2	3	1	3	2	2	1
1	1	2	1	1	2	2	2	1	1
2	2	2	2	2	2	2	2	2	2
2	2	2	2	2	2	2	2	2	2
2	3	3	2	2	1	1	1	1	1
2	1	3	1	2	1	3	2	3	2
2	2	2	2	2	2	2	2	2	2
9	9	9	9	9	9	9	9	9	9
9	9	9	9	9	9	9	9	9	9
9	9	9	9	9	9	9	9	9	9
2	1	1	1	1	2	2	2	2	2
9	9	9	9	9	9	9	9	9	9
2	1	1	1	1	1	1	1	2	2
9	9	9	9	9	9	9	9	9	9
1	1	2	2	3	2	3	3	2	2
9	9	9	9	9	9	9	9	9	9
2	1	9	9	1	9	1	1	9	1
2	1	2	1	2	1	2	1	1	2
9	9	9	9	9	9	9	9	9	9
1	1	1	1	1	3	1	1	1	1
3	3	3	3	3	3	3	3	1	1
3	3	3	3	3	3	3	3	3	2
1	1	3	2	2	2	1	2	1	3
3	1	2	3	2	1	3	2	1	2
2	3	3	2	2	1	1	1	1	1

2	2	2	2	2	1	2	2	1	3
2	1	1	1	2	2	2	1	1	1
9	9	9	9	9	9	9	9	9	9
1	1	1	1	2	9	1	1	1	2
1	2	3	1	9	2	3	2	1	9
2	1	1	2	1	2	2	1	2	1
9	9	9	9	1	9	9	9	9	9
2	1	2	3	2	1	2	2	2	2
1	1	2	3	3	1	1	2	1	1
3	3	3	3	3	3	3	3	3	3
3	1	2	3	1	2	3	1	1	2
3	3	2	1	2	3	2	1	1	3
1	1	2	3	2	1	2	1	1	2
2	2	2	2	2	2	2	2	2	2
2	2	2	2	2	2	2	2	2	2
9	1	9	9	2	9	9	2	3	9
2	2	2	2	2	2	2	2	2	2
2	2	2	2	2	2	3	2	2	3
2	2	2	2	2	2	2	2	2	2
2	2	2	2	2	2	2	2	2	2
1	1	1	1	3	3	1	1	1	3
1	1	1	1	3	3	1	1	1	3
1	1	1	1	1	3	1	1	1	9
1	1	1	2	2	2	1	1	1	3
3	1	1	3	3	3	1	1	1	3
9	1	9	3	3	3	1	1	1	9
2	2	1	2	2	2	1	1	2	2
1	3	1	3	3	2	1	1	1	2
1	1	1	2	1	3	1	1	1	2
1	1	1	1	1	1	1	1	1	1
3	9	1	9	9	9	9	9	1	9
9	9	9	9	9	2	9	9	9	9
2	1	3	3	1	3	1	1	1	3
1	2	1	1	2	3	1	1	1	3
1	9	2	3	3	1	9	9	9	9
2	2	2	1	1	1	1	2	2	2
2	2	1	1	1	1	2	2	2	2
1	1	9	9	9	9	1	1	1	3
1	1	1	1	1	1	3	1	1	3
1	1	1	1	1	1	1	1	1	1
1	1	1	1	1	3	3	1	1	3
9	9	1	9	9	9	9	9	1	9
1	1	1	1	1	1	1	1	1	1
1	1	1	3	3	1	1	1	1	3
1	1	1	1	1	3	1	1	1	2
1	2	1	2	2	1	2	2	1	3
3	2	2	2	2	2	2	2	2	3

2	2	2	1	1	1	1	2	2	2
1	9	2	9	3	2	3	1	2	2
1	1	1	2	1	2	1	1	1	3
1	1	1	1	1	1	1	1	1	1
1	1	1	9	2	1	2	2	1	3
1	1	1	9	2	1	2	2	1	3
2	1	1	1	1	1	1	1	2	2
1	1	1	2	2	2	1	1	1	2
9	9	9	9	9	9	9	9	9	9
1	3	3	3	3	3	1	1	1	3
1	2	2	2	2	2	2	2	2	2
2	2	2	2	2	2	2	2	2	2
1	1	1	9	2	1	2	2	1	3
1	1	1	9	2	1	2	2	1	3
1	1	1	9	2	1	2	2	1	3
1	1	1	9	2	1	2	2	1	3
2	9	9	9	9	9	1	1	1	2
1	1	2	1	1	1	1	1	1	3
1	1	1	9	2	1	2	2	1	3
1	2	1	3	2	2	1	2	1	3
3	9	9	1	9	9	9	9	1	9
3	1	3	2	2	1	2	1	1	9
1	1	1	2	2	3	2	1	1	2
1	1	1	3	3	3	2	1	1	3
1	1	1	2	2	1	1	1	1	3
1	1	1	1	1	1	1	1	1	1
3	1	1	1	1	1	1	1	3	3
1	1	1	1	1	3	1	1	2	2
2	2	1	2	2	2	2	2	1	3
1	1	1	1	3	3	1	1	1	3
2	1	1	2	1	1	1	2	1	1
1	1	1	3	3	3	3	1	1	3
1	1	1	9	9	9	1	9	1	9
9	1	1	9	1	9	9	9	1	3
1	1	1	2	2	2	1	1	1	1
1	1	1	3	3	3	1	1	1	3
9	9	1	9	1	9	1	1	2	9
1	1	1	1	1	3	3	1	1	3
3	1	1	1	1	3	3	1	1	3
1	1	1	3	3	3	1	1	1	3
1	1	1	3	3	3	1	1	1	3
1	1	1	1	3	3	1	1	1	3
1	1	1	2	2	3	2	1	1	2
1	3	1	1	1	3	1	1	1	3
2	1	1	3	3	3	1	1	1	3
2	1	1	3	3	3	1	1	1	3
9	2	3	1	3	3	2	2	1	3

1	1	1	1	1	3	1	1	1	1
1	1	1	3	1	3	1	1	1	3
2	2	1	2	2	3	2	2	1	3
1	1	1	1	3	3	3	3	1	3
1	1	1	3	3	3	1	1	1	3
1	1	1	3	2	2	1	1	1	1
9	9	9	9	9	9	9	1	1	9
1	1	1	1	1	1	1	1	9	1
1	1	1	1	1	9	1	1	1	3
1	1	1	3	1	3	1	1	1	3
1	2	2	2	2	2	2	2	1	2
1	9	1	9	9	9	9	9	1	9
3	1	1	3	3	1	1	1	3	1
3	1	1	1	2	3	1	1	1	2
2	2	2	2	2	2	2	2	3	3
1	1	1	1	1	2	1	1	1	3
1	9	1	9	9	9	9	9	1	9
1	9	1	9	9	9	9	9	1	9
3	1	1	1	1	1	1	1	1	1
1	1	1	1	1	1	1	1	2	2
2	1	2	3	3	3	2	1	1	3
3	9	9	9	9	9	9	9	9	9
1	3	1	1	2	3	2	1	1	1
1	1	1	2	1	3	1	1	1	3
2	3	1	1	1	3	3	3	3	3
3	3	1	1	1	3	3	3	3	3
1	1	2	3	2	1	3	1	1	3
2	2	2	2	2	2	2	2	2	2
1	1	1	2	2	2	2	2	1	3
1	1	1	2	2	2	2	2	1	2
2	1	1	2	2	2	2	2	1	2
1	1	1	2	2	1	1	2	1	1
1	1	1	3	1	2	1	1	2	3
2	1	2	3	2	3	1	1	1	3
1	3	3	1	1	3	1	1	1	1
1	1	1	1	2	2	2	1	2	1
3	9	9	9	9	9	9	9	1	2
3	2	2	3	3	3	3	3	1	1
3	1	2	1	2	2	1	2	1	2
3	2	3	3	3	2	2	1	1	3
1	1	1	3	1	3	1	1	1	3
1	1	1	3	3	3	1	1	1	3
3	1	2	2	2	3	1	1	1	1
9	2	2	2	2	1	1	1	9	1
3	1	2	2	9	3	1	1	1	1
1	1	1	3	3	3	1	1	1	3
3	9	9	9	9	1	9	9	1	1

1	1	1	3	1	3	2	2	1	3
2	2	1	3	2	2	2	2	2	2
1	2	2	2	2	2	2	2	1	2
1	2	1	2	2	3	2	2	1	2
1	1	1	3	3	1	2	1	9	1
1	1	2	2	2	2	3	3	1	2
9	9	9	9	9	9	9	1	9	9
2	2	1	2	2	2	2	2	9	9
9	9	9	9	9	9	9	9	9	9
1	1	2	2	2	3	1	1	1	2
1	1	1	2	2	3	2	1	1	3
2	1	1	2	9	2	1	1	1	3
2	2	2	3	3	3	2	2	2	2
2	1	1	2	2	2	1	2	1	2
2	2	2	1	2	2	2	2	2	9
9	1	9	9	9	9	9	9	9	9
2	1	1	2	1	1	1	1	1	2
2	1	1	2	2	2	1	1	1	1
1	1	1	2	1	1	1	1	1	3
3	1	1	2	2	2	1	2	1	3
9	1	9	9	1	9	9	9	9	9
3	1	1	1	1	3	1	1	1	3
2	1	2	2	2	2	2	2	1	2
1	1	1	1	1	1	1	1	1	2
3	1	1	1	1	2	1	1	1	3
3	1	1	1	1	1	1	1	1	2
9	1	1	2	2	2	1	1	1	1
9	9	9	9	9	9	9	9	9	9
9	9	9	9	9	9	9	9	9	9
1	1	2	2	2	3	1	1	1	3
3	1	1	1	2	3	1	1	1	3
3	1	1	1	2	3	2	1	1	3
1	1	1	1	9	9	9	9	9	9
9	1	1	9	3	3	3	1	9	9
1	1	3	1	2	3	1	1	1	3
1	1	1	3	3	3	1	1	1	1
1	1	1	3	2	3	1	1	1	3
1	1	1	1	1	3	1	1	1	9
1	9	1	3	3	3	3	1	1	3
1	1	1	3	2	3	1	1	1	3
2	1	2	2	2	2	2	9	2	1
1	2	1	1	2	3	2	2	1	3
1	1	1	3	1	3	1	1	1	3
9	9	9	9	9	9	9	9	9	9
3	1	2	1	1	1	1	1	1	3
1	2	2	2	2	2	2	2	1	2
1	1	2	9	1	2	1	1	1	3

1	1	1	1	1	3	1	1	1	9
2	2	1	2	2	1	2	2	9	2
9	9	9	9	9	9	9	9	9	9
1	1	1	2	2	1	1	1	1	1
9	9	1	1	9	9	9	9	1	3
2	1	2	2	2	1	1	1	1	1
1	2	3	3	2	1	1	2	1	1
2	1	1	2	2	3	2	2	9	1
9	2	9	9	9	9	2	9	9	9
2	1	1	2	2	1	2	2	1	2
3	1	1	1	1	3	1	3	1	1
2	2	2	2	1	2	1	2	2	2
3	1	1	1	2	2	1	1	1	2
9	1	9	9	9	9	1	1	1	9
1	1	1	1	2	2	1	2	1	1
2	1	2	2	2	3	2	1	1	3
9	1	1	9	1	9	1	9	9	9
9	1	1	9	9	9	1	1	1	9
2	1	1	1	2	2	1	1	1	1
2	1	3	2	2	3	1	1	2	2
1	2	1	3	2	1	2	2	3	1
1	1	1	3	1	1	1	1	1	3
1	1	1	3	1	1	1	1	1	3
2	1	1	1	1	2	1	1	1	1
2	1	1	1	1	2	1	1	2	3
2	1	1	1	1	2	1	3	1	1
3	1	2	2	2	1	1	1	1	3
3	2	1	1	2	2	1	1	1	2
3	1	1	1	1	2	1	3	1	1
3	1	1	1	1	2	3	3	1	1
2	1	1	1	2	1	1	1	2	3
3	1	1	2	1	1	1	1	1	2
3	1	1	1	1	2	1	1	1	2
3	1	3	3	3	2	1	1	1	3
1	1	2	3	2	3	1	1	1	3
9	1	1	2	2	9	1	1	1	3
3	1	2	2	2	3	3	1	1	3
9	1	1	1	9	9	9	9	1	9
2	1	1	1	1	2	2	1	1	1
2	2	2	1	1	1	1	1	1	2
2	2	2	3	2	1	2	3	2	2
2	1	1	3	3	2	3	2	2	2
9	1	9	9	9	9	9	9	1	9
9	1	9	9	9	9	9	9	1	9
2	1	1	1	1	2	1	1	2	2
1	1	1	1	1	3	1	1	1	1
9	1	1	9	9	9	1	1	1	9

3	1	1	3	3	3	1	1	1	1
2	1	1	2	1	1	1	1	1	3
9	1	1	2	2	2	9	1	1	9
3	1	1	2	2	2	2	1	1	2
2	1	1	1	1	2	1	1	1	3
3	1	2	2	1	2	1	1	1	2
2	2	2	2	2	2	2	2	2	2
3	1	2	1	2	1	1	1	2	2
2	1	2	3	3	2	3	3	9	9
2	1	2	1	2	2	1	1	1	2
3	1	1	1	1	3	1	1	1	3
9	1	9	9	9	9	9	9	1	9
9	9	9	9	9	9	1	9	1	9
9	9	9	9	9	9	9	1	1	9
9	9	9	9	9	9	9	1	1	9
9	9	9	9	9	9	9	1	1	9
3	3	3	9	2	2	3	1	2	3
2	2	3	3	2	1	3	1	1	2
9	1	9	9	9	9	9	9	1	3
1	1	2	1	1	1	1	1	1	2
2	1	1	1	2	1	1	1	1	2
2	1	2	2	2	3	1	1	1	2
2	1	2	2	2	3	1	1	1	2
3	1	1	2	2	2	2	1	1	2
2	1	1	1	2	2	1	2	1	2
2	1	1	1	1	3	1	1	1	3
3	1	3	3	2	1	1	2	1	3
3	1	1	1	3	3	1	1	1	3
2	1	1	2	2	1	1	1	1	2
3	1	1	1	2	3	1	1	1	3
3	1	1	2	2	2	1	1	1	2
9	1	1	9	9	9	1	1	1	9
3	1	2	3	2	3	1	1	1	3
2	1	1	1	1	3	2	1	1	1
3	1	3	2	2	1	1	1	1	1
2	1	1	2	2	3	1	1	1	3
2	1	2	2	1	3	2	2	1	3
2	1	1	9	1	3	2	2	2	2
9	9	9	9	9	9	9	9	9	9
1	2	2	2	2	1	1	2	1	2
2	1	2	2	1	1	2	3	1	2
1	1	2	2	1	1	2	3	1	2
1	2	1	3	3	3	1	1	1	3
1	1	1	1	1	2	1	1	1	2
2	1	1	2	2	2	2	2	1	2
2	1	1	2	2	3	1	2	1	3
1	1	1	3	1	3	1	1	1	1

3	1	1	1	1	1	1	1	1	3
1	1	1	3	1	3	1	1	1	2
2	1	1	3	1	2	2	1	1	1
3	1	1	3	2	3	1	1	1	3
9	1	2	3	1	3	1	1	3	2
3	1	1	3	1	3	2	1	1	2
2	1	1	2	1	2	1	1	1	2
2	1	1	3	1	3	1	1	1	2
3	1	1	3	1	3	1	1	1	3
3	1	1	2	1	3	1	1	1	2
2	1	1	1	1	2	1	1	1	2
2	2	1	2	2	2	2	2	2	2
3	1	1	1	1	3	2	1	2	1
3	1	1	3	1	3	1	1	1	1
2	2	1	1	1	2	2	1	1	1
2	1	1	2	2	2	1	1	1	1
2	2	2	2	2	2	2	2	2	2
2	2	2	2	2	2	2	2	2	2
3	1	3	2	2	2	2	1	2	2
2	1	1	1	2	2	2	2	1	2
2	1	1	1	2	2	2	2	1	2
9	1	1	3	3	3	1	1	1	2
2	1	2	2	2	2	1	1	1	2
2	1	2	2	2	3	1	1	1	3
2	1	1	1	1	2	1	1	1	3
9	9	9	9	9	9	9	9	9	9
9	9	9	9	9	9	9	9	9	9
9	1	9	9	9	9	9	9	9	9
2	1	1	3	3	3	1	1	1	9
2	3	3	9	2	9	1	1	3	9
2	1	1	1	1	2	1	1	1	3
2	1	1	1	1	2	1	1	1	3
9	1	9	9	9	9	9	9	1	9
9	9	1	9	9	9	9	9	1	9
9	9	1	9	9	9	9	9	1	9
9	9	1	9	9	9	9	9	1	9
9	1	9	9	9	9	9	9	1	9
9	1	9	9	9	9	9	9	1	9
9	1	9	9	9	9	9	9	1	9
2	1	2	3	3	1	1	1	1	2
2	1	1	3	3	3	1	1	1	2
9	1	9	9	9	9	9	9	9	1
9	1	9	9	9	9	9	9	1	9
3	1	1	3	2	3	2	2	1	2
9	1	9	9	9	9	9	9	2	9
9	1	9	9	9	9	9	9	9	9
9	1	9	9	9	9	9	9	9	9

3	1	1	3	1	3	1	1	1	2
9	9	9	9	9	9	9	9	9	9
9	1	9	9	9	9	9	9	9	9
3	1	2	2	2	3	1	1	1	3
3	1	2	2	2	3	1	1	1	3
9	1	9	9	9	9	9	9	9	9
9	9	9	9	9	9	1	9	1	9
9	9	9	9	9	9	1	9	1	9
9	1	9	9	9	9	9	9	9	9
9	1	9	9	9	9	9	9	1	9
3	1	1	2	2	1	1	1	1	1
3	1	1	9	1	3	1	1	1	3
2	1	1	1	3	1	1	1	1	3
2	1	2	2	1	2	1	1	1	3
3	1	1	1	1	1	1	1	1	3
3	1	1	3	3	1	1	1	1	1
1	1	1	3	3	3	3	3	1	9
1	1	1	3	1	1	1	1	1	9
3	1	1	9	1	3	1	1	1	3
3	1	1	2	1	1	1	1	1	1
3	1	1	2	1	1	1	1	1	1
1	1	1	1	1	1	1	1	2	1
9	1	9	9	9	9	9	9	9	9
3	1	1	1	1	3	1	1	1	1
1	1	2	3	3	3	3	3	2	1
2	1	1	3	3	1	1	1	1	1
9	3	3	3	3	3	3	3	3	3
3	1	1	3	1	1	1	1	1	3
2	1	1	3	2	3	1	1	1	1
2	2	1	1	1	2	1	1	2	1
2	1	1	2	1	2	1	1	1	1
2	1	1	2	2	3	1	1	1	1
1	1	1	3	1	1	1	1	1	3
2	1	2	2	2	3	2	1	1	2
3	1	1	1	1	1	1	1	1	3
2	1	1	1	3	2	1	1	1	3
3	1	1	3	1	3	1	1	1	3
3	1	1	3	1	3	1	1	1	3
3	1	1	3	1	3	1	1	1	3
3	1	1	2	3	1	1	1	1	3
9	3	3	3	1	1	1	1	1	3
1	1	1	3	1	2	1	1	1	2
3	3	2	3	1	1	2	2	1	1
2	1	1	1	1	3	1	1	1	3
2	1	1	1	1	3	1	1	1	3
1	3	3	3	1	1	1	1	1	3
3	1	1	1	1	2	1	1	1	3

1	1	1	3	3	2	1	1	1	3
1	1	1	3	1	1	1	1	1	1
1	1	1	1	1	1	1	1	1	1
2	1	1	1	1	2	1	1	1	3
2	1	1	1	1	3	1	1	1	3
2	1	1	1	1	3	1	1	1	3
3	1	1	1	1	2	1	1	1	2
3	1	1	2	1	1	1	1	1	3
2	1	1	2	1	2	1	1	1	3
2	1	1	3	1	1	1	1	1	3
1	1	2	3	2	2	2	2	1	2
1	1	1	3	1	3	1	1	1	3
1	1	1	2	9	2	1	2	1	2
1	2	1	2	2	2	2	2	1	1
1	1	1	3	3	3	1	1	1	3
1	1	1	3	1	3	1	1	1	2
2	2	2	2	2	2	2	2	2	2
2	2	2	2	1	2	2	2	1	2
3	1	3	3	1	1	3	1	3	1
3	1	1	3	1	3	2	1	1	2
2	1	1	2	1	2	1	1	1	3
3	1	1	1	1	1	1	1	1	3
2	1	1	1	1	1	2	1	1	3
1	1	1	1	1	2	1	1	1	2
1	1	1	1	1	2	1	1	1	2
3	1	1	3	1	2	1	1	1	3
2	2	3	2	2	3	3	3	1	1
1	1	1	1	1	2	3	2	2	2
1	1	1	3	1	3	2	2	1	3
2	1	1	2	1	2	1	1	1	3
3	2	2	2	3	3	3	3	2	2
3	1	2	2	2	3	3	2	1	2
3	1	1	1	1	3	1	1	1	2
2	1	1	3	1	2	1	1	3	2
2	1	1	1	1	2	1	1	1	3
2	1	1	1	1	2	1	1	1	3
2	2	1	1	2	2	2	2	2	2
3	2	1	1	2	2	2	2	2	2
3	1	1	1	1	3	1	1	3	3
3	1	1	3	1	2	2	1	1	3
2	2	2	2	1	3	1	1	1	2
3	1	1	1	1	3	3	1	1	3
3	1	1	1	1	3	3	1	1	3
1	2	2	2	2	2	2	2	1	2
3	2	2	3	2	2	2	2	1	3
1	1	2	2	1	3	2	1	9	3
1	1	1	2	1	1	1	1	1	3

2	2	2	2	2	2	2	2	2	2
9	1	2	3	2	2	1	1	1	1
3	1	1	1	1	2	1	1	1	1
2	1	1	1	1	2	2	2	2	2
1	2	2	2	2	3	1	2	1	1
1	2	1	2	1	2	1	1	2	2
3	2	1	3	2	3	1	1	1	3
2	2	2	2	1	3	2	2	1	2
2	2	1	1	2	2	2	1	2	1
3	1	1	3	1	3	1	1	1	3
2	1	1	1	1	2	1	1	1	2
3	1	1	3	1	3	1	1	1	3
9	9	9	9	1	9	9	9	1	9
2	2	1	1	3	2	2	2	1	3
3	1	1	1	1	3	1	1	1	1
2	2	2	2	2	2	2	2	2	2
2	1	1	1	1	3	2	2	1	3
1	1	1	3	3	1	2	2	1	3
1	1	1	3	1	2	2	2	1	3
1	1	2	1	2	2	1	1	1	2
1	1	1	1	1	1	1	1	1	3
3	1	1	2	1	1	1	1	1	1
1	1	1	3	1	3	1	1	1	3
3	1	1	2	1	3	1	1	1	2
2	1	1	1	1	2	1	1	2	1
1	1	1	1	1	3	1	1	1	3
3	1	1	3	3	3	1	1	1	1
2	1	2	3	3	1	1	1	1	2
3	1	1	3	2	3	2	2	1	2
9	1	9	9	9	9	9	9	1	9
2	2	2	3	9	2	2	3	1	2
1	1	2	3	3	2	1	1	9	9
2	1	2	2	2	2	2	2	1	2
3	1	1	2	1	2	1	1	1	3
2	1	2	2	2	2	1	1	1	1
2	1	2	2	2	3	1	1	1	1
9	2	2	2	2	2	2	2	2	2
9	9	9	9	1	9	9	9	9	9
1	1	1	2	3	1	3	3	1	1
1	1	2	2	2	2	1	1	1	3
2	1	2	1	2	2	1	1	1	1
2	1	1	2	2	2	1	1	1	2
2	1	2	2	2	2	1	1	1	2
9	1	9	9	9	1	1	9	9	9
3	2	2	2	2	2	2	2	2	2
2	1	2	2	2	2	2	2	2	2
2	1	1	2	2	3	1	1	1	1

3	1	1	1	2	3	1	1	1	2
2	1	1	1	2	2	1	1	1	2
3	1	2	2	2	2	2	1	2	2
2	1	2	2	2	2	1	1	1	2
2	1	2	2	1	2	2	2	1	1
2	1	2	1	2	9	2	2	1	3
1	1	3	3	1	2	1	1	1	1
9	9	1	9	9	9	9	9	9	9
1	1	3	3	1	2	1	1	2	1
2	1	2	2	2	2	1	1	1	1
2	1	2	3	3	3	1	1	1	2
3	2	3	2	3	2	3	2	2	3
1	1	1	2	2	2	2	2	1	2
2	2	2	2	2	2	2	1	2	2
2	3	1	3	1	3	1	1	1	3
2	3	1	3	1	3	1	1	1	3
2	1	1	1	1	1	1	1	1	2
1	1	1	3	3	2	2	1	1	3
9	9	9	9	9	9	1	9	1	9
1	1	1	3	1	3	1	1	1	3
1	1	3	3	2	3	1	1	1	2
1	1	1	2	2	2	1	1	1	1
2	2	2	2	2	2	2	2	3	2
2	1	2	2	2	2	1	1	2	3
3	1	1	1	2	3	1	1	1	3
1	1	1	1	1	1	1	1	1	2
1	1	1	3	2	2	1	1	1	3
1	1	1	3	3	2	1	1	2	3
1	1	1	3	3	2	1	1	2	3
9	9	9	1	9	9	9	9	9	9
1	1	1	3	3	2	1	1	2	3
1	1	1	3	3	3	1	1	1	1
1	1	1	3	3	3	1	1	1	1
1	1	1	3	2	3	1	1	1	2
9	9	9	9	9	9	9	9	9	9
1	1	1	1	1	1	1	1	1	2
1	1	1	1	1	1	1	1	1	2
1	3	1	3	9	3	3	1	9	9
1	1	1	3	1	3	1	1	1	3
1	1	1	2	2	3	1	1	1	3
2	1	1	2	2	2	3	2	1	3
1	1	1	3	3	2	2	1	1	3
2	2	1	2	2	2	2	2	2	2
3	1	3	3	3	1	1	1	1	3
1	1	3	3	3	3	3	3	1	1
1	1	1	3	3	3	1	1	1	3
1	1	1	3	2	2	3	3	1	1

1	3	1	3	3	3	3	1	1	1
1	3	1	3	3	3	3	1	1	1
3	1	2	3	2	2	1	1	1	3
1	1	2	1	1	3	1	1	1	1
1	1	1	3	1	3	1	1	1	1
1	1	1	1	1	3	1	1	1	3
1	1	1	3	1	3	1	1	3	1
1	1	1	1	1	2	1	2	9	9
3	1	2	3	1	1	1	1	1	3
2	1	1	1	1	1	1	1	1	3
3	1	1	1	1	3	1	1	1	3
1	1	2	3	1	1	1	1	1	3
1	1	2	3	1	1	1	1	1	3
3	1	1	1	3	3	1	3	9	9
2	1	1	2	1	1	1	1	1	2
3	1	1	1	1	2	1	1	1	2
2	1	1	1	2	3	1	1	2	2
2	1	1	2	1	3	2	1	1	1
3	1	1	3	3	1	1	1	1	3
9	9	9	9	9	9	9	9	9	9
1	1	1	1	2	2	1	1	1	1
2	1	2	2	2	3	1	1	1	3
1	1	2	2	2	3	1	1	1	3
3	1	2	2	2	3	1	1	1	3
9	9	1	9	9	9	9	9	9	9
9	1	9	9	9	9	9	9	9	9
3	1	1	3	3	3	1	1	1	3
9	9	1	9	9	9	9	9	1	9
9	1	9	9	9	9	9	9	9	9
9	1	9	9	9	9	9	9	9	9
9	9	9	9	1	9	9	9	9	9
9	9	9	9	1	9	9	9	9	9
9	1	9	9	9	9	9	9	1	9
9	1	9	9	9	9	9	9	9	9
3	9	9	9	9	9	9	9	9	9
2	1	1	2	1	1	1	1	1	1
2	1	1	1	1	1	1	1	2	2
1	1	1	3	3	2	2	1	1	3
1	1	1	3	3	2	1	1	1	3
1	1	1	3	3	2	1	1	1	3
9	1	1	3	1	3	1	1	1	3
3	1	1	3	3	3	1	1	1	1
1	1	2	2	1	3	1	1	1	1
2	1	1	2	2	2	2	2	2	2
3	1	1	2	2	2	2	2	2	2
3	1	1	3	1	3	1	1	1	3
1	1	1	1	1	2	1	1	1	3

2	2	1	2	2	1	2	1	2	2
2	2	2	9	1	2	2	2	1	1
3	2	2	2	2	2	2	2	1	2
1	3	2	2	2	2	1	1	2	2
1	1	1	2	1	1	2	1	1	1
2	1	2	2	1	2	1	1	1	3
9	9	9	9	9	9	9	9	9	9
2	2	2	2	2	2	2	2	2	2
2	1	2	2	2	2	1	1	1	2
2	2	2	2	2	1	3	1	1	2
3	1	2	1	2	2	1	1	1	2
2	1	1	1	1	2	1	1	1	3
3	2	2	2	3	2	2	1	1	1
1	3	3	3	3	3	3	3	3	3
1	2	2	2	2	2	2	2	2	2
3	2	1	1	1	2	1	1	1	2
9	9	9	9	9	9	9	9	9	9
3	1	2	1	2	1	2	1	2	1
3	1	2	2	2	3	1	1	1	1
1	1	1	1	1	1	1	1	1	1
1	3	1	1	1	1	1	1	1	2
2	2	2	2	2	2	2	2	3	3
1	2	2	1	1	3	3	2	1	2
2	2	2	2	2	2	2	2	1	2
1	1	1	1	1	1	1	1	1	1
2	1	2	2	2	2	2	2	2	3
2	3	3	2	2	1	1	1	2	2
1	1	1	1	1	1	1	1	1	3
2	2	2	2	2	2	2	2	2	2
2	2	2	2	2	2	2	2	2	2
3	1	1	3	3	3	1	1	1	3
1	3	3	3	3	3	3	3	1	1
2	1	2	3	2	1	2	2	1	2
2	1	1	2	1	2	1	1	1	2
9	1	9	9	9	9	9	1	9	9
2	1	1	1	1	1	1	1	1	1
2	2	2	2	2	2	2	2	2	2
2	2	2	2	2	2	2	2	9	9
1	2	2	2	2	3	1	1	2	2
1	1	1	1	2	2	1	1	1	1
2	1	1	2	2	2	2	1	1	2
3	1	1	2	2	2	2	1	1	2

LiketoKnov	WilldoPap	WillLikePa	Embarrase	WorriedAb	NoTimefor	PapSmearN	PapAgainst	LikeHPVVa	AfraidofHP
2	2	2	2	2	2	2	2	2	2
3	3	3	3	3	3	3	3	2	2
2	2	2	2	2	2	2	2	2	2
3	3	2	2	2	2	2	2	2	2
2	3	2	2	2	2	3	3	1	2
2	2	2	3	3	3	3	3	3	3
3	1	2	3	1	3	2	2	2	1
2	3	3	2	2	1	2	2	1	2
9	3	3	3	3	3	3	3	3	3
2	3	2	3	3	2	2	2	3	2
1	2	3	2	3	1	2	2	2	2
2	2	2	3	1	2	2	2	1	2
3	1	2	3	1	2	3	2	1	2
2	2	2	1	2	2	2	2	1	2
2	2	2	2	2	2	2	2	9	9
2	2	2	2	2	2	2	2	2	2
9	9	9	9	9	9	9	9	9	9
1	1	3	1	1	1	1	1	1	1
9	9	9	9	9	9	9	9	9	9
9	9	9	9	9	9	9	9	9	9
1	1	1	1	1	1	3	3	1	1
2	2	2	2	2	2	2	2	2	2
1	1	1	3	3	3	1	3	1	2
1	1	1	2	1	1	1	1	1	2
9	2	9	9	9	9	9	9	9	9
1	2	2	2	1	1	3	1	1	1
1	3	3	3	1	1	1	3	1	3
1	1	2	1	1	2	3	3	1	2
2	1	2	3	3	2	1	1	1	2
2	9	1	1	3	1	3	2	2	3
1	1	3	1	1	2	2	3	1	1
1	2	1	2	3	1	2	3	1	2
1	1	1	1	1	1	1	3	1	1
1	1	1	2	1	1	1	1	1	3
2	1	1	2	1	1	1	1	1	1
1	3	1	2	1	3	1	2	3	2
1	3	2	2	2	2	3	3	2	2
1	1	2	1	1	3	2	3	1	1
1	2	1	3	3	3	3	3	1	3
9	2	3	2	2	3	3	3	3	2
1	2	1	2	2	3	3	3	1	2
9	9	9	1	3	9	9	1	1	2
9	9	9	9	9	9	9	9	9	9
2	2	2	2	2	2	2	2	2	2
1	2	2	2	2	1	2	2	1	2
9	9	9	9	9	9	9	9	3	2

2	3	3	2	2	2	1	2	1	1
2	1	1	2	2	2	3	3	1	2
2	2	2	1	1	2	2	2	1	2
2	2	1	1	2	1	2	2	2	2
1	3	3	3	1	3	3	3	1	1
1	3	3	1	3	3	3	3	1	1
2	2	3	3	3	3	2	2	2	3
1	1	1	3	3	3	3	3	1	3
1	3	2	1	3	2	1	3	1	9
9	9	9	3	9	9	9	1	3	9
9	9	9	9	9	9	9	1	2	9
2	2	3	3	3	2	2	2	2	2
2	2	1	2	1	1	2	1	1	1
1	2	2	3	3	3	3	3	1	1
2	3	2	1	2	2	1	2	1	1
2	2	1	2	2	2	2	3	1	1
2	3	2	1	2	3	2	2	1	2
1	2	1	3	3	1	2	1	1	2
1	2	2	3	2	2	3	3	1	2
2	2	3	3	3	3	3	2	3	3
1	2	2	1	2	3	3	3	1	2
1	2	1	1	2	2	2	2	2	2
2	3	3	2	2	2	2	2	1	1
1	2	2	1	2	2	2	2	1	2
2	3	2	2	2	2	1	1	1	2
2	2	1	1	3	1	2	1	2	2
1	3	3	1	1	3	1	3	1	1
2	2	3	3	1	2	3	2	1	2
9	1	2	3	2	3	3	3	1	2
9	3	3	3	3	3	3	3	3	1
9	9	9	9	9	9	9	9	9	9
1	3	3	3	3	3	3	3	3	3
1	2	2	3	3	3	2	3	1	2
2	2	2	2	2	2	2	2	1	3
1	1	3	2	3	2	3	2	1	1
2	2	2	2	2	2	2	2	2	2
9	9	9	9	9	9	9	9	1	3
1	2	2	1	2	2	3	2	1	2
2	2	2	2	2	2	2	2	2	2
2	1	1	2	1	2	2	1	2	1
1	1	3	2	3	2	9	2	1	2
9	9	9	9	9	9	9	9	9	9
1	3	2	3	2	1	1	1	2	2
1	1	2	3	2	2	2	2	1	1
2	2	3	2	1	2	3	2	2	1
2	3	2	2	3	1	2	2	2	2
1	3	2	2	1	2	3	3	1	3

3	1	1	2	3	1	2	1	1	2
3	3	3	3	3	3	3	3	1	3
2	2	2	2	2	2	2	2	1	1
1	2	2	2	1	2	2	2	2	2
1	1	1	2	2	1	1	2	1	2
1	1	1	2	1	1	1	1	2	2
1	1	2	2	2	2	2	3	1	2
9	9	9	9	9	9	9	9	9	9
2	2	2	2	2	2	2	2	2	2
1	2	1	3	3	2	2	3	1	2
1	9	2	2	3	1	2	1	9	9
2	2	3	2	2	2	1	1	2	2
2	3	3	3	3	3	2	2	1	2
2	2	2	2	2	2	2	2	1	1
1	3	2	3	3	3	2	3	2	3
1	3	2	2	3	2	3	3	2	3
2	2	1	1	2	2	2	1	1	1
1	1	2	1	2	1	1	1	1	2
3	3	1	3	1	3	1	3	1	3
1	2	1	2	2	2	2	1	2	2
1	3	1	3	1	3	1	3	1	3
1	2	3	2	1	2	2	3	1	1
2	2	1	1	2	2	2	1	1	1
3	1	2	2	2	2	2	2	2	1
2	2	2	2	2	2	2	2	2	2
1	1	2	2	1	2	1	3	1	2
1	3	1	3	3	3	3	3	1	3
1	1	2	2	2	2	2	2	1	1
2	2	2	1	1	2	1	1	2	2
3	1	2	1	3	3	1	2	1	2
2	2	3	2	1	2	2	2	2	3
2	1	3	2	2	1	1	1	1	2
9	2	2	1	1	2	3	2	3	2
2	2	1	1	2	1	2	2	1	2
3	3	3	2	2	1	2	3	1	2
1	1	1	9	2	1	1	1	1	1
3	3	3	3	3	3	2	2	2	2
2	2	1	2	1	1	1	1	1	2
9	9	9	9	9	9	9	9	9	9
3	3	3	3	3	3	3	3	1	3
2	2	3	2	1	2	1	1	1	2
9	9	9	9	9	9	9	9	9	9
2	1	1	1	1	2	1	1	1	2
2	3	2	1	1	1	1	1	1	1
9	9	9	9	9	9	9	9	1	3
1	3	1	3	2	3	3	3	1	3
1	3	2	1	3	3	1	2	1	2

2	3	2	3	1	2	3	1	2	3
1	1	1	3	3	3	3	3	3	3
3	3	3	3	3	3	3	3	3	3
2	2	2	2	2	2	2	2	2	2
2	2	3	3	2	2	2	2	2	2
1	2	3	2	3	2	3	2	2	3
1	9	1	2	3	9	2	1	9	9
1	9	1	2	1	9	2	1	9	9
9	9	9	9	9	9	9	9	2	9
2	2	2	2	2	2	2	2	2	2
2	1	1	1	3	3	2	3	1	2
1	2	2	2	3	3	3	3	1	9
2	2	2	2	2	2	2	9	2	2
1	2	1	1	2	2	3	3	1	2
3	2	1	2	2	2	2	2	1	2
2	1	1	1	1	2	2	3	1	2
2	2	1	2	2	1	1	2	1	2
2	2	3	2	1	2	2	2	1	2
9	9	9	9	9	9	9	9	2	9
1	2	2	1	2	3	1	3	2	3
1	1	1	2	1	3	2	2	2	1
2	2	2	1	1	2	2	2	1	1
3	3	3	3	3	3	3	3	9	9
2	2	2	2	2	2	2	1	2	2
1	1	2	3	3	3	3	3	1	3
2	1	3	3	3	1	3	2	2	1
9	9	9	9	9	9	9	9	9	9
2	2	2	2	2	2	2	2	3	3
1	2	2	2	2	1	2	3	3	3
2	1	2	2	2	2	2	2	1	2
2	1	1	1	2	1	2	1	9	9
1	1	1	1	2	2	2	2	2	1
1	1	2	2	2	1	1	1	1	1
3	1	3	3	1	3	2	3	3	3
1	1	1	3	3	3	3	2	1	1
1	1	1	1	1	1	1	1	1	1
3	3	3	3	3	3	3	3	3	3
1	2	2	1	1	2	3	3	1	3
1	3	1	3	3	3	1	2	1	2
2	2	2	2	2	2	2	2	2	2
9	9	9	9	9	9	9	9	9	9
2	2	2	2	2	2	2	2	2	2
2	2	2	2	2	2	2	3	2	2
9	9	9	9	9	9	9	9	9	9
9	9	9	9	9	9	9	9	9	9
2	3	2	1	2	2	2	2	1	2
2	3	2	2	2	1	1	1	1	2

9	3	9	9	9	9	9	9	9	9
9	9	9	9	9	9	9	9	9	9
2	3	3	3	3	3	3	3	1	3
9	9	9	9	9	9	9	9	1	9
9	9	9	9	9	9	9	9	1	9
9	9	9	9	9	9	9	9	9	9
9	9	9	9	9	9	9	9	9	9
9	9	9	9	9	9	9	9	9	9
2	2	2	1	2	1	3	2	2	2
9	9	9	9	1	9	9	9	9	9
2	9	9	9	9	9	9	9	9	9
9	9	9	9	9	9	2	9	9	9
1	2	2	1	1	2	1	2	1	1
9	3	2	2	1	3	1	3	1	2
9	9	9	1	9	9	9	9	9	1
2	2	2	3	3	3	3	3	3	3
1	1	1	1	1	1	1	1	1	1
2	2	2	2	2	2	2	2	2	1
2	2	2	2	2	2	2	2	2	2
2	2	2	2	2	2	2	2	2	2
2	1	1	1	1	1	1	1	1	1
9	9	9	9	9	9	9	9	9	9
1	1	1	2	1	1	1	2	2	2
1	2	1	2	1	3	3	3	1	3
2	2	2	2	2	2	2	2	2	2
1	2	2	2	2	2	2	2	1	1
1	1	1	1	2	3	3	2	2	2
2	2	1	1	2	2	2	2	1	1
3	3	3	3	3	3	3	3	9	9
1	1	1	3	3	3	1	1	1	3
2	2	2	2	2	2	2	2	2	2
2	2	1	1	1	1	1	1	1	1
9	9	9	9	9	9	9	9	9	9
2	2	2	1	1	1	1	1	2	2
1	3	1	1	1	3	1	1	3	3
1	2	2	1	2	2	1	2	1	2
9	9	9	9	9	9	9	9	2	9
1	1	1	1	2	2	1	1	3	2
9	9	9	9	9	9	9	9	3	9
1	1	1	1	2	2	2	2	2	1
1	1	3	2	2	2	2	2	1	1
1	1	2	2	3	3	3	1	1	1
1	1	2	2	2	2	1	1	1	1
2	2	2	2	2	2	2	2	2	2
2	2	2	2	2	2	2	2	1	2
2	3	3	2	1	2	3	1	1	2
9	9	9	9	9	9	9	9	9	9

9	2	1	1	1	1	3	2	1	1
2	2	1	1	2	1	1	1	1	2
1	1	1	1	1	1	1	1	1	1
9	9	9	9	9	9	9	9	9	9
2	2	2	2	2	2	2	2	2	2
3	2	2	2	2	2	1	1	2	3
1	1	3	1	3	2	1	3	3	3
2	1	2	2	2	2	2	2	2	1
1	1	2	1	1	1	1	1	1	1
1	3	1	1	1	1	1	3	1	3
2	2	1	2	2	1	1	2	1	2
9	9	9	9	9	9	9	9	3	9
9	9	9	9	9	9	9	9	1	9
9	9	9	9	9	9	9	9	1	9
9	2	9	9	2	9	9	2	2	9
9	9	9	9	9	9	9	9	9	9
9	9	9	9	9	9	9	9	9	9
9	9	9	9	9	9	9	9	9	9
9	2	9	2	2	9	2	2	9	9
9	9	9	9	9	9	9	9	9	9
9	9	9	9	9	9	9	9	9	9
2	2	2	2	2	2	2	2	1	1
2	2	1	2	2	2	1	2	2	2
2	2	1	1	1	1	1	1	2	2
1	1	2	2	1	1	1	1	2	1
9	9	9	9	9	9	9	9	9	9
2	1	1	2	2	2	2	2	2	2
9	9	9	9	9	9	9	9	9	9
9	9	9	9	9	9	9	9	9	9
2	3	2	2	2	2	2	2	2	2
9	9	9	9	9	9	9	9	9	9
9	9	9	9	9	9	9	9	9	9
9	9	9	9	9	9	9	9	9	9
9	9	9	9	9	9	9	9	9	9
9	9	9	9	9	9	9	9	9	9
1	9	9	3	1	9	9	1	9	1
9	9	9	9	9	9	9	9	9	9
9	9	9	9	9	9	9	9	9	9
9	9	9	9	9	9	9	9	9	9
9	9	9	9	9	9	9	9	3	9
9	9	9	9	9	9	9	2	3	9
9	9	9	9	9	9	9	9	9	9
3	3	3	3	3	3	3	3	3	3
3	3	3	3	3	3	3	3	3	3
1	2	1	3	1	3	1	2	1	2
1	1	1	9	9	9	3	2	2	2
9	9	9	9	9	9	9	9	1	9

9	9	9	9	9	9	9	9	1	9
9	9	9	9	9	9	9	9	3	9
2	2	1	1	3	3	3	3	3	2
1	2	3	1	3	1	2	3	3	2
2	2	2	2	2	2	2	2	9	9
2	2	2	3	3	3	3	3	2	2
1	1	1	1	1	1	1	1	9	9
2	2	1	2	3	1	3	2	2	2
3	2	3	1	2	2	2	2	2	2
3	3	3	3	3	3	3	3	3	3
9	9	9	9	9	9	9	9	9	9
9	9	9	9	9	9	9	9	9	9
9	9	9	9	9	9	9	9	9	9
9	9	9	9	9	9	9	9	9	9
3	3	3	3	3	3	3	3	2	2
1	1	1	1	2	2	2	3	1	2
9	9	9	9	9	9	9	9	9	9
1	1	2	2	2	2	3	3	1	1
1	1	1	1	1	1	1	1	1	1
2	2	2	2	2	2	2	2	2	2
2	2	2	2	2	2	2	3	2	2
9	1	2	1	3	3	3	1	1	3
9	9	9	9	9	9	9	9	1	9
2	2	2	2	2	2	2	2	2	2
2	2	2	3	3	3	3	3	2	2
2	2	2	2	2	2	2	2	1	2
2	2	2	2	2	2	2	2	2	2
2	2	2	1	1	1	1	1	1	2
2	2	2	2	2	2	2	2	3	2
1	1	1	1	2	1	1	2	1	1
2	2	2	2	2	2	2	2	2	2
2	2	2	2	2	2	2	2	2	2
2	2	3	3	3	3	3	3	3	2
2	2	2	2	2	2	2	2	3	3
1	1	1	3	3	3	3	3	1	1
3	1	2	3	1	2	3	2	1	2
9	9	9	9	9	9	9	9	9	9
2	1	2	2	2	2	2	2	3	2
3	3	3	3	3	3	3	3	3	3
3	3	3	3	3	3	3	3	3	3
9	9	9	9	9	9	9	9	2	9
9	9	9	9	9	9	9	9	9	9
9	9	9	9	1	9	9	9	2	1
2	2	2	2	2	2	3	3	1	1
2	2	2	2	1	1	1	1	1	2
2	2	2	2	2	2	2	2	2	2
3	2	2	1	1	2	2	2	1	2

9	9	1	9	9	9	9	9	9	9
9	9	9	9	9	9	9	9	1	9
9	9	9	9	9	9	9	9	9	9
2	9	9	9	9	9	9	1	9	9
9	9	9	9	9	9	9	1	9	2
2	2	2	1	1	1	2	2	1	1
9	9	9	9	9	9	9	9	2	9
9	9	9	9	9	9	9	9	2	9
9	9	9	9	9	9	9	9	2	9
9	9	9	9	9	9	9	9	2	9
1	9	9	9	9	9	9	9	9	9
9	9	2	9	9	9	9	2	9	9
9	9	9	9	9	9	9	9	2	9
9	9	3	9	9	9	9	9	9	9
9	9	9	9	9	9	9	9	1	9
9	9	9	9	9	9	9	9	9	9
9	9	9	9	9	9	9	2	9	9
3	2	1	2	3	9	2	1	3	2
2	2	1	1	2	2	1	2	1	1
2	2	3	3	3	2	3	3	3	3
1	2	2	1	1	2	1	3	1	2
2	1	2	1	2	9	2	2	1	2
2	1	2	2	2	2	3	2	2	2
2	3	2	1	2	3	3	3	9	9
2	2	3	2	2	9	1	2	2	2
9	9	9	9	9	9	9	9	9	9
1	2	2	2	2	2	2	2	1	1
9	9	9	9	9	9	9	1	9	9
2	2	3	2	2	3	1	1	1	3
2	2	1	1	1	1	1	2	9	9
2	1	2	2	1	2	2	1	1	1
2	2	2	2	2	2	2	2	1	1
2	2	3	3	3	3	3	3	1	2
1	1	1	1	1	1	1	1	1	1
9	9	9	9	9	9	9	9	9	9
2	2	2	2	2	2	2	2	2	2
9	9	9	9	9	9	9	9	9	9
2	1	1	3	2	2	2	2	1	1
9	9	9	9	9	9	9	9	9	9
2	2	2	2	2	2	2	2	2	2
1	1	2	1	2	2	1	1	1	2
3	3	3	3	3	3	3	3	3	3
1	1	1	3	3	3	3	3	1	1
2	2	2	3	3	3	3	3	2	2
2	2	2	2	2	2	2	2	3	3
2	1	2	2	2	2	2	2	2	2
3	3	3	9	2	2	2	3	2	2

2	1	1	1	2	2	3	3	1	2
3	9	9	9	9	9	9	9	9	9
2	2	2	2	2	2	2	2	1	2
2	1	2	1	2	1	2	1	1	3
2	2	2	2	1	1	1	1	1	1
2	2	2	2	2	3	3	3	2	2
1	1	2	2	1	3	3	3	1	1
1	2	1	2	1	3	3	3	9	9
9	9	9	9	9	9	9	9	9	9
1	1	1	2	2	3	3	3	2	2
2	2	2	2	2	2	2	2	2	2
9	9	9	9	9	9	9	9	1	9
9	9	9	9	9	9	9	9	1	9
1	1	9	1	1	9	9	9	1	1
9	9	9	9	9	9	9	9	1	9
2	2	2	2	2	2	2	2	2	2
9	9	9	9	9	9	9	9	9	9
9	9	9	9	9	9	9	9	2	9
9	9	9	9	9	2	9	9	9	9
2	3	2	3	2	3	2	3	2	3
2	2	2	2	2	2	2	2	2	2
3	3	3	2	2	2	1	1	2	1
2	3	3	2	2	2	2	2	2	2
9	1	9	9	9	9	9	9	9	9
9	9	9	9	9	9	9	9	9	3
2	1	1	2	2	2	2	2	1	2
1	2	2	2	1	1	1	1	1	1
2	1	2	1	1	2	3	2	2	2
9	9	9	9	9	9	9	9	9	9
9	9	9	9	9	9	9	9	1	9
2	2	2	2	2	2	2	2	9	9
1	2	3	1	2	3	1	2	3	1
2	2	1	2	2	3	3	3	2	2
2	2	1	1	2	2	2	2	1	2
1	1	2	2	2	2	2	2	1	1
2	1	2	2	2	1	2	2	1	1
9	9	9	9	9	9	9	9	9	9
9	9	9	9	9	9	9	9	9	9
3	9	1	3	2	3	1	2	3	2
1	2	1	2	1	2	1	2	1	2
2	2	2	2	2	2	2	2	2	2
2	2	2	2	2	2	2	3	1	2
1	1	3	2	3	3	3	3	1	2
3	2	1	3	2	3	2	2	1	2
9	3	3	3	2	2	3	2	2	2
2	2	2	2	2	2	2	2	2	2
2	2	2	3	3	3	3	3	2	2

1	1	1	1	1	1	1	2	1	2
9	2	2	3	2	3	2	2	2	2
1	9	9	9	9	9	9	9	1	9
9	9	1	9	9	9	9	9	1	9
1	9	9	9	9	9	9	9	9	9
2	9	1	2	1	2	1	2	1	1
2	9	9	2	9	9	9	2	2	9
2	9	2	2	2	2	2	2	2	2
9	9	9	9	9	9	9	9	9	9
9	9	9	9	9	9	9	9	9	9
9	9	9	9	9	9	9	9	9	9
9	9	9	9	9	9	9	9	9	9
9	9	9	9	9	9	9	9	9	9
9	9	9	9	9	9	9	9	9	9
9	9	9	9	9	9	9	9	9	9
9	9	9	9	9	9	9	9	2	9
3	3	3	3	3	3	3	3	3	3
9	9	9	9	9	9	9	9	1	9
9	9	9	9	9	9	9	9	2	9
9	2	9	2	9	9	2	2	9	9
9	9	9	9	9	9	9	9	9	9
9	9	9	9	9	9	9	9	2	9
2	2	2	2	2	2	2	2	2	2
9	9	9	9	9	9	9	9	9	9
9	9	9	9	9	9	9	9	9	9
9	9	9	9	9	9	9	9	2	9
9	9	9	9	9	9	9	9	9	9
9	2	9	9	2	9	2	9	2	9
9	9	9	9	9	9	9	9	9	9
9	9	9	9	9	9	9	9	9	9
9	9	9	9	9	9	9	9	9	9
2	9	2	9	2	9	2	9	9	9
9	9	9	9	9	9	9	9	9	9
9	9	9	9	9	9	9	9	9	9
9	9	9	9	9	9	9	9	9	9
9	9	9	9	9	9	9	9	9	9
9	9	9	9	9	9	9	9	9	9
9	9	9	9	9	9	9	9	9	9
3	2	1	2	3	2	2	2	2	3
2	3	2	3	1	2	3	2	2	2
1	9	9	9	9	9	9	9	9	9
9	9	9	9	9	9	9	9	9	9
3	1	2	1	3	1	3	2	1	3
3	3	3	2	2	2	2	2	1	2
1	1	3	3	3	3	3	3	1	3
1	1	1	3	2	1	2	2	2	1
9	2	3	3	2	1	3	9	9	9

1	9	9	9	9	9	9	9	1	9
2	1	3	3	3	2	3	3	1	3
2	2	2	2	2	2	2	2	9	9
1	1	1	1	1	1	1	1	1	1
2	2	2	2	2	2	2	2	2	2
2	1	2	2	2	2	2	2	2	1
9	9	9	9	9	9	9	9	9	9
2	2	3	2	1	2	3	2	1	2
2	2	2	2	2	2	2	2	2	2
1	1	1	3	1	3	3	3	1	1
1	2	1	1	2	2	3	3	1	3
2	9	9	9	9	9	9	9	9	9
1	9	9	9	9	9	9	9	9	9
9	9	9	9	9	9	9	9	1	9
9	2	9	9	9	9	9	9	2	9
1	9	9	9	9	9	9	9	9	1
2	2	2	2	2	2	2	2	2	2
1	2	9	3	1	3	3	3	1	1
1	2	3	3	9	2	3	3	3	3
2	2	2	2	2	2	2	2	2	2
2	2	2	2	2	2	2	2	2	2
9	9	9	9	9	9	9	9	9	9
2	1	2	1	2	2	1	1	3	1
9	9	9	9	9	9	9	9	9	9
1	9	9	9	9	9	9	9	1	9
3	3	2	2	3	2	3	2	1	1
9	1	9	9	9	9	9	9	9	9
2	2	2	2	2	2	2	2	2	2
9	9	9	9	9	9	9	9	9	9
9	9	9	9	9	9	9	9	1	9
2	3	3	3	2	3	2	9	3	2
2	3	2	3	2	1	3	3	3	2
9	9	9	9	9	9	9	9	9	9
9	9	9	9	9	9	9	9	9	9
3	2	2	1	2	2	3	1	1	2
9	9	9	9	9	9	9	9	9	9
9	9	9	9	9	9	9	9	9	9
1	9	1	1	1	1	1	1	1	1
2	2	2	2	2	2	2	2	2	2
9	9	9	9	9	9	9	9	9	9
2	2	2	2	2	2	2	2	9	9
1	1	1	2	1	2	2	2	1	1
2	2	2	2	2	2	2	2	3	3
2	2	2	2	2	2	2	2	2	2
1	3	2	3	2	3	2	3	1	2
2	2	2	2	2	2	2	9	2	2
3	3	3	2	2	1	2	2	2	3

1	9	9	9	9	9	9	9	1	9
2	2	2	2	2	2	2	2	2	2
2	2	2	2	2	2	2	2	1	2
1	9	9	9	9	9	9	9	9	9
1	9	9	9	9	9	9	9	9	9
9	9	9	9	9	9	9	9	9	9
9	9	9	9	9	9	9	9	9	9
2	2	2	2	2	2	2	2	2	2
1	2	1	2	2	2	2	3	1	2
9	9	9	9	9	9	9	9	9	9
1	1	2	1	1	2	1	2	1	1
1	1	2	2	2	3	3	3	2	3
2	2	2	2	2	2	2	3	2	2
2	2	2	2	2	2	2	2	2	2
9	9	9	9	9	9	9	9	9	9
9	9	9	9	9	9	9	9	9	9
9	9	9	9	9	9	9	9	9	9
9	9	9	9	9	9	9	9	9	9
9	9	9	9	9	9	9	9	1	9
9	9	9	9	9	9	9	9	2	9
9	9	9	9	9	9	9	9	9	9
9	9	9	9	9	9	9	9	9	9
9	2	2	2	9	2	2	2	2	2
9	9	9	9	9	9	9	2	2	9
9	9	9	9	9	9	9	9	9	9
9	9	9	9	9	9	9	9	9	9
9	9	9	9	9	9	9	9	9	9
9	9	9	9	9	9	9	9	9	9
9	9	9	9	9	9	9	9	9	9
9	9	9	9	9	9	9	9	3	9
9	9	9	9	9	9	9	9	9	9
9	9	9	9	9	9	9	9	9	9
9	9	9	9	9	9	9	9	9	9
9	9	9	9	9	9	9	9	9	9
9	9	9	9	9	9	9	9	9	9
9	9	9	9	9	9	9	9	9	9
9	9	9	9	9	9	9	9	9	9
9	9	9	9	9	9	9	9	9	9
9	9	9	9	9	9	9	9	9	9
9	9	9	9	9	9	9	9	9	9
2	2	2	2	2	2	2	2	2	2
9	9	9	9	9	9	9	9	2	9
9	9	9	9	9	9	9	9	9	9
9	9	9	9	9	9	9	9	9	9
9	9	9	9	9	9	9	9	9	9
9	9	9	9	9	9	9	9	9	9
9	9	9	9	9	9	9	9	9	9

9	9	9	9	9	9	9	9	9	9
2	2	2	2	2	2	2	2	2	2
9	9	9	9	9	9	9	9	9	9
9	9	9	9	9	9	9	9	9	9
9	9	9	9	9	9	9	9	9	9
9	9	9	9	9	9	9	9	9	9
9	9	9	9	9	9	9	9	9	9
9	9	9	9	9	9	9	9	9	9
9	9	9	9	9	9	9	9	9	9
9	9	9	9	9	9	9	9	9	9
9	9	9	9	9	9	9	9	9	9
9	9	9	9	9	9	9	9	9	9
9	9	9	9	9	9	9	9	9	9
9	9	9	9	9	9	9	9	9	9
9	9	9	9	9	9	9	9	9	9
9	9	9	9	9	9	9	9	9	9
9	9	9	9	9	9	9	9	9	9
2	2	2	2	2	2	2	2	9	9
9	9	9	9	9	9	9	9	9	9
9	9	9	9	9	9	9	9	9	9
9	9	9	9	9	9	9	9	9	9
9	9	9	9	9	9	9	9	9	9
9	9	9	9	9	9	9	9	9	9
9	9	9	9	9	9	9	9	9	9
2	2	2	2	2	2	2	2	2	2
9	9	9	9	9	9	9	9	9	9
9	9	9	9	9	9	9	9	9	9
1	1	1	1	1	1	1	1	1	1
9	9	9	9	9	9	9	9	9	9
9	9	9	9	9	9	9	9	9	9
1	1	1	1	1	1	1	1	1	1
9	1	1	1	1	1	1	1	3	2
9	9	9	9	9	9	9	9	9	9
2	2	2	2	2	2	2	2	9	9
9	9	9	9	9	9	9	9	9	9
2	2	2	2	2	2	2	2	2	9
9	9	9	9	9	9	9	9	9	9
9	9	9	9	9	9	9	9	9	9
1	1	1	1	1	1	1	1	1	1
9	9	9	9	9	9	9	3	3	9
9	9	9	9	9	9	9	9	9	9
9	9	9	9	9	9	9	9	9	9
2	2	2	2	2	2	2	2	2	2
2	2	2	2	2	2	2	2	2	2
9	1	9	1	9	1	9	1	1	9
9	9	9	9	9	9	9	9	9	9
9	9	9	9	9	9	9	9	9	9

9	9	2	9	9	9	2	2	9	9
9	9	9	9	9	9	9	9	9	9
9	9	9	9	9	9	9	9	9	9
9	9	9	9	9	9	9	9	9	9
9	9	9	9	9	9	9	9	9	9
9	9	9	9	9	9	9	9	9	9
9	9	9	9	9	9	9	9	9	9
9	9	9	9	9	9	9	9	2	9
2	9	2	9	2	9	2	2	2	9
9	9	9	9	9	9	9	9	9	9
9	9	9	9	9	9	9	9	9	9
9	9	9	9	9	9	9	9	9	9
9	9	9	9	9	9	9	9	9	9
9	9	9	9	9	9	9	9	9	9
2	2	2	1	1	2	2	1	2	2
2	1	2	1	1	2	2	3	2	1
1	9	9	9	9	9	9	9	9	9
2	2	2	2	2	2	2	2	2	2
2	2	2	3	3	3	2	2	2	3
9	9	9	9	9	9	9	9	1	9
9	9	9	9	9	9	9	9	1	9
9	9	9	9	9	9	9	9	1	9
2	2	2	2	2	2	2	2	3	3
1	2	3	1	2	3	1	2	1	2
2	2	2	2	2	2	2	2	1	2
2	2	2	2	2	2	2	2	1	3
1	3	2	3	3	3	3	3	1	3
1	3	1	1	1	3	3	3	1	1
1	2	1	2	3	3	3	3	1	1
2	2	2	2	2	2	2	2	2	2
2	2	2	2	2	2	2	9	2	9
9	9	9	9	9	9	9	2	9	9
9	9	9	9	9	9	9	9	1	9
9	9	9	9	9	9	9	1	9	9
9	9	9	9	9	9	9	9	9	2
9	9	9	9	9	9	9	9	9	9
9	9	9	9	9	9	9	9	9	9
9	9	9	2	9	9	2	9	9	2
9	9	9	9	9	9	9	9	9	9
9	9	2	9	2	9	2	2	2	9
9	9	9	9	9	9	9	9	9	9
2	2	2	2	2	2	2	2	2	2
9	9	9	9	9	9	9	9	9	9
9	9	9	9	9	9	9	9	9	9
9	9	9	9	9	9	9	9	9	9
2	2	2	2	2	2	2	2	2	2
3	9	1	1	3	2	2	2	1	2

1	1	1	1	1	1	1	2	1	2
1	2	2	1	2	3	2	2	2	2
1	3	2	1	2	2	2	2	2	2
1	3	3	3	2	2	1	3	1	2
3	3	3	3	3	3	3	3	3	3
1	2	2	2	2	3	3	3	1	1
2	2	2	2	2	2	2	2	1	2
3	3	3	3	3	3	3	3	3	3
9	9	9	9	9	9	9	9	9	9
2	2	2	2	2	2	2	2	2	2
2	9	9	2	9	2	9	2	2	9
9	1	2	3	1	3	1	1	1	9
2	2	2	2	2	2	2	2	1	2
2	2	1	2	3	3	2	2	1	2
3	2	2	3	3	3	3	3	2	3
2	2	2	2	2	2	2	2	2	2
2	3	1	2	3	1	3	2	2	1
2	2	2	2	2	2	2	2	9	9
9	9	9	9	9	9	9	9	9	9
1	2	3	1	2	2	1	2	1	1
2	2	2	2	2	2	2	2	2	1
1	2	2	3	1	2	3	2	1	1
2	2	2	2	2	2	2	2	1	2
2	3	2	2	2	2	2	1	2	3
9	2	2	3	2	2	3	3	2	2
1	2	2	2	2	2	2	2	1	2
2	2	2	2	2	2	2	2	2	2
1	3	2	3	3	2	2	2	2	2
2	2	2	2	2	2	2	2	1	2
9	2	2	9	2	9	2	2	3	9
2	3	3	3	3	3	3	3	1	3
9	9	9	9	9	9	9	9	9	9
3	3	3	3	3	3	3	3	3	3
2	2	2	2	2	2	2	2	2	2
9	9	9	9	9	9	9	9	9	2
2	3	2	2	2	3	3	2	2	2
3	3	2	1	1	2	2	3	2	1
1	1	1	1	1	1	1	1	1	1
2	2	2	2	2	2	2	2	1	2
9	9	9	9	9	9	9	9	9	9
2	2	2	2	2	2	2	2	2	2
9	9	9	9	9	9	9	9	9	9
2	2	1	3	2	1	3	2	2	2
2	2	2	2	2	2	2	2	2	2
2	2	3	1	2	3	2	3	2	3
2	2	2	2	2	3	2	3	2	3
3	2	2	3	2	3	3	2	2	3

9	9	9	9	9	9	9	9	9	9
3	3	3	3	3	3	3	3	3	3
1	1	3	3	1	3	3	1	1	3
1	2	2	3	3	3	3	3	2	3
2	2	1	1	3	3	3	3	1	9
9	9	9	9	9	9	9	9	9	9
2	2	2	2	2	2	2	2	2	3
2	1	2	3	2	3	1	2	2	2
1	1	1	1	1	2	2	1	1	3
2	2	2	2	2	2	2	2	1	1
1	1	1	1	1	1	2	3	1	2
2	2	2	2	2	2	2	2	2	2
9	9	9	9	9	9	9	9	9	9
3	9	2	3	2	3	2	3	3	3
1	1	2	1	2	3	2	3	1	3
2	2	2	2	2	2	2	2	2	2
2	2	2	2	2	2	2	2	2	2
3	3	3	3	2	2	9	2	2	2
2	2	2	2	2	2	2	2	2	2
9	9	9	9	9	9	9	9	1	9
9	9	9	9	9	9	9	9	9	9
1	2	1	2	1	2	1	2	2	1
3	3	3	3	2	3	2	2	2	2
3	1	2	2	3	3	2	2	2	3
9	9	9	9	9	9	9	9	9	9
1	3	2	2	1	3	2	1	1	2
9	9	9	9	9	9	9	9	9	9
2	2	2	2	2	2	2	2	2	2
2	2	2	2	2	2	2	2	2	2
2	2	2	2	2	2	2	2	2	2
2	3	1	2	3	2	3	2	9	9
9	9	9	9	9	9	9	9	9	9
9	9	9	9	9	9	9	9	9	9
1	1	1	2	1	3	3	3	2	3
9	9	9	9	9	9	9	9	9	9
9	9	9	9	9	9	9	9	9	9
9	9	9	9	9	9	9	9	9	9
1	2	2	2	1	2	2	2	2	2
1	3	1	3	3	2	2	3	9	9
1	1	2	2	1	1	2	2	1	2
2	2	2	2	2	2	2	2	1	1
2	2	2	2	2	2	2	2	2	2
2	2	2	2	2	2	2	2	2	2
2	2	2	2	2	2	2	2	2	2
2	2	2	2	2	2	2	2	2	1
2	1	2	3	2	3	1	2	2	2
2	2	2	2	2	2	2	2	2	2

3	3	3	3	3	2	1	2	2	3
2	2	2	2	2	2	2	9	2	1
2	2	2	2	2	2	2	2	2	2
1	1	2	1	1	1	2	1	2	2
2	2	2	2	2	2	2	2	2	2
2	2	2	2	2	2	2	2	2	1
2	2	2	2	2	2	2	2	2	1
2	2	2	2	2	2	2	2	2	2
2	2	2	2	2	2	2	2	2	2
9	9	9	9	9	9	9	9	9	9
2	2	2	2	2	2	2	2	2	2
9	2	1	2	3	1	1	3	1	2
1	3	1	3	3	3	3	3	9	9
9	1	2	1	2	1	2	1	1	3
2	2	2	2	2	2	2	2	2	2
2	9	1	1	2	1	3	1	9	9
9	9	9	9	9	9	9	9	9	9
9	9	2	9	9	9	1	9	9	1
9	9	9	9	9	9	9	9	9	9
9	9	9	9	9	9	9	9	9	9
2	2	2	2	2	1	1	9	1	1
9	9	9	9	9	9	9	9	9	9
9	9	9	9	9	9	9	9	9	9
1	1	2	1	1	2	2	2	1	2
2	2	2	2	2	2	2	2	3	3
1	1	2	2	1	1	2	2	1	2
2	3	9	2	1	1	2	1	3	3
9	9	9	9	9	9	9	9	9	9
2	3	1	2	1	3	1	2	2	2
1	1	2	2	1	2	2	2	2	1
9	9	9	9	9	9	9	9	9	9
2	3	2	1	2	3	1	2	1	2
3	2	1	3	2	3	1	9	2	2
3	3	2	2	2	2	2	2	1	1
2	2	1	2	2	1	2	2	1	1
1	1	2	2	1	1	2	2	1	2
9	9	9	9	9	9	9	9	9	9
2	2	2	2	2	2	2	2	1	1
2	2	2	2	2	2	2	2	2	2
2	2	2	2	2	2	2	2	1	1
2	2	2	2	2	2	2	2	2	2
2	2	3	3	3	1	1	1	1	3
1	1	2	2	2	3	3	3	1	1
1	1	2	2	2	3	3	2	2	2
3	3	3	2	2	3	3	2	3	3
1	1	1	1	2	2	2	3	1	1
2	2	2	3	2	3	2	3	3	2

2	2	1	1	2	3	2	9	2	2
2	2	2	2	2	2	2	9	2	2
2	3	3	9	2	2	3	2	3	3
2	2	2	2	2	2	2	2	2	2
3	3	2	3	2	3	3	2	2	3
9	9	9	9	9	9	9	9	9	9
9	9	9	1	9	9	9	9	1	9
3	2	3	3	3	3	3	2	3	3
9	9	9	9	9	9	9	9	9	9
9	9	9	9	9	9	9	9	9	9
2	2	2	2	2	2	2	2	2	2
2	1	1	2	2	1	2	2	9	9
2	2	2	2	1	1	1	2	2	1
1	9	9	9	9	9	9	9	2	1
1	9	9	9	9	9	9	9	1	9
1	1	1	1	1	3	1	1	1	1
1	3	3	3	1	3	3	3	1	2
9	9	9	9	9	9	9	9	9	9
1	3	1	1	2	3	3	3	1	2
2	2	2	2	2	2	2	2	1	1
3	3	3	3	3	3	3	2	2	2
9	9	9	9	9	9	9	9	9	9
2	2	2	2	2	2	2	2	2	2
1	2	2	2	2	2	2	2	2	1
2	2	2	2	2	2	2	2	2	2
2	2	2	2	2	2	2	2	2	2
1	1	2	2	2	2	2	2	2	2
2	2	2	2	2	2	2	2	2	2
2	9	3	3	2	3	2	2	2	3
2	2	2	2	2	2	2	2	2	2
2	1	2	3	2	1	2	2	1	1
3	2	1	2	2	2	3	2	1	2
1	2	2	1	1	2	1	3	1	1
2	2	2	2	2	2	2	2	1	1
2	1	1	1	1	2	1	1	1	2
3	1	2	3	1	2	3	2	3	2
1	1	1	1	1	1	1	1	3	1
2	2	2	2	2	2	2	2	2	2
3	3	2	3	2	3	2	3	1	1
1	1	3	1	1	1	1	1	1	1
1	1	1	3	3	1	3	3	3	3
2	3	3	1	1	3	3	2	2	1
2	2	2	2	2	2	2	2	2	2
2	2	2	2	2	2	2	2	2	2
2	2	2	2	2	2	2	2	2	2
1	2	2	3	3	3	3	3	1	3
2	2	2	2	2	2	2	2	2	2

2	1	3	2	1	3	2	1	2	1
1	1	1	3	1	3	2	3	1	1
2	3	2	3	2	3	2	3	2	2
2	2	2	2	2	2	2	2	2	2
9	9	9	9	9	9	9	2	2	9
9	9	2	9	9	9	9	9	9	9
3	2	3	2	3	2	3	2	1	2
9	9	9	9	9	9	9	9	1	9
9	9	9	9	9	9	9	9	1	9
9	9	9	9	9	9	9	9	9	9
9	9	9	3	9	9	9	9	9	9
9	9	2	9	9	9	9	9	9	9
9	9	9	9	9	9	9	9	9	9
9	9	9	9	9	9	9	9	3	9
2	2	2	2	2	2	2	2	1	2
9	9	9	9	9	9	9	1	1	9
9	9	9	9	9	9	9	9	9	9
9	9	9	1	9	9	9	9	9	9
9	9	9	1	9	9	9	9	9	9
9	9	9	2	9	9	9	9	9	9
9	9	9	9	9	9	9	9	9	9
2	2	2	2	2	2	2	2	2	2
1	1	1	1	1	1	2	3	1	2
2	2	3	2	1	2	3	2	3	3
3	1	2	3	2	1	2	1	3	3
9	9	9	9	9	9	9	9	9	9
2	2	2	2	2	2	2	2	2	2
9	9	9	9	9	9	9	9	1	9
1	2	3	2	2	2	3	3	2	2
1	1	1	1	1	1	1	1	1	1
3	3	3	3	3	3	3	9	3	3
9	9	9	9	9	9	9	9	1	9
2	3	3	2	3	1	3	3	1	1
9	9	9	9	9	9	9	9	9	1
9	9	9	9	9	9	9	9	1	9
2	2	2	2	2	2	2	1	1	2
1	1	1	1	1	1	1	1	1	1
9	9	9	9	9	9	9	9	9	9
9	9	9	9	9	9	9	9	9	9
2	2	2	2	2	2	2	2	9	9
9	9	9	9	9	9	9	9	9	9
2	2	2	2	2	2	2	2	2	2
3	3	3	3	3	3	3	3	3	3
9	9	9	9	2	9	9	9	9	9
2	2	2	2	2	2	2	2	9	9
2	2	2	2	2	2	2	2	9	9
9	9	9	9	9	9	9	9	9	9

9	9	9	9	9	9	9	9	9	9
9	2	9	2	2	9	9	2	9	2
9	9	9	9	9	9	9	9	9	9
2	2	2	2	2	2	2	2	9	9
9	9	9	9	9	9	9	9	9	9
3	9	1	9	3	1	1	1	9	9
9	9	9	9	9	9	9	9	9	9
9	9	9	9	9	9	9	9	9	9
9	2	9	2	9	2	2	2	2	9
9	9	9	9	9	9	9	9	9	9
9	9	9	9	9	9	9	9	9	9
9	2	9	2	9	9	2	2	9	2
2	2	2	2	2	2	2	2	2	2
9	9	9	9	9	9	9	9	9	9
2	2	2	2	2	2	2	2	2	2
9	9	9	9	9	9	9	9	9	9
9	9	9	9	9	9	9	9	9	9
9	9	9	9	9	9	9	9	9	9
9	9	9	9	9	9	9	9	9	9
9	9	9	9	9	9	9	9	9	9
2	2	2	2	2	2	2	2	2	2
2	2	2	2	2	2	2	2	2	2
9	2	3	3	3	1	3	3	3	3
2	9	1	2	3	1	3	1	1	2
2	2	2	2	2	2	2	2	2	2
2	2	2	2	2	2	2	2	1	1
1	1	1	1	1	1	1	1	1	1
2	2	2	2	2	2	2	2	2	2
3	1	1	2	3	1	1	1	1	3
2	2	2	2	2	2	2	2	2	2
2	9	1	1	1	3	3	2	3	9
1	3	2	3	2	2	3	2	2	1
2	2	2	2	2	2	2	2	2	1
9	9	9	9	9	9	9	9	9	9
3	9	1	3	1	3	1	2	1	2
2	2	2	2	2	2	2	2	3	3
2	2	2	2	2	2	2	2	2	2
3	3	3	1	1	1	1	1	2	2
3	1	2	3	1	3	2	2	1	2
2	2	2	2	1	1	1	1	2	2
9	9	9	9	9	9	9	9	9	9
9	9	9	9	9	9	9	9	9	9
2	2	2	2	2	2	2	2	2	2
9	9	9	9	9	9	9	9	9	9
1	2	1	2	1	2	1	1	9	9
1	1	2	2	1	1	2	2	1	2
9	9	9	9	9	9	9	9	9	9

3	2	1	1	1	2	2	2	1	2
1	1	1	3	3	3	3	2	1	1
9	9	9	9	9	9	9	9	9	9
2	2	2	2	2	2	2	9	9	9
9	9	9	9	9	9	9	9	9	9
2	2	2	2	2	2	2	2	1	1
1	1	1	2	2	2	2	2	2	2
1	1	1	1	1	1	2	1	1	2
3	3	3	3	3	3	3	3	3	3
1	2	1	2	3	2	1	2	1	1
9	9	9	9	9	9	9	9	9	9
2	2	2	2	2	2	2	2	2	2
2	2	2	2	2	2	2	2	9	9
2	2	2	2	2	2	2	2	2	2
2	2	2	2	2	2	2	2	9	9
1	1	1	1	1	9	9	9	1	1
9	2	1	1	2	9	1	1	1	1
1	2	1	2	1	1	2	1	1	1
1	1	1	1	1	1	1	1	2	2
9	9	9	9	9	9	9	9	9	9
1	1	2	1	1	2	1	1	1	2
2	2	2	2	2	2	2	9	2	2
9	9	9	9	9	9	9	9	9	9
2	2	2	2	2	2	2	2	2	2
9	9	9	9	9	9	9	9	9	9
1	2	2	1	1	2	2	1	1	1
1	1	1	1	2	1	2	1	1	1
1	1	1	1	1	1	1	9	1	1
9	9	9	9	9	9	9	9	9	9
9	9	9	9	9	9	9	9	9	9
9	9	9	9	9	9	9	9	9	9
9	9	9	9	9	9	9	9	9	9
1	1	1	1	1	1	1	9	9	9
2	2	2	2	2	2	2	2	1	1
2	2	2	2	2	2	2	2	2	2
9	9	9	9	9	9	9	9	9	9
1	1	1	1	1	1	1	2	1	1
1	2	1	1	1	1	1	1	1	1
9	9	9	9	9	9	9	9	9	9
9	9	9	9	9	9	9	9	9	9
9	9	9	9	9	9	9	9	9	9
9	9	9	9	9	9	9	9	9	9
2	9	1	1	1	1	1	9	1	1
9	9	9	9	9	9	9	9	9	9
1	9	1	2	1	1	2	2	1	1
2	2	2	2	2	2	2	2	2	2
9	9	9	9	9	9	9	9	1	9

1	1	1	2	2	2	2	2	1	1
9	2	9	2	2	9	2	2	9	9
2	2	2	2	2	2	2	2	3	3
1	2	2	1	2	1	2	1	1	1
2	2	2	2	2	2	2	2	9	9
9	9	9	9	9	9	9	9	9	9
2	2	2	2	2	2	2	2	9	9
2	2	2	2	2	2	2	2	2	2
9	9	9	9	9	9	9	9	9	9
9	9	9	9	9	9	9	9	9	9
9	9	9	9	9	9	9	9	9	9
9	9	9	9	9	9	9	9	9	9
9	9	9	9	9	9	9	9	9	9
9	9	9	9	9	9	9	9	9	9
9	9	9	9	9	9	9	9	9	9
9	9	9	9	9	9	9	9	9	9
9	9	9	9	9	9	9	9	9	9
9	9	9	9	9	9	9	9	9	9
2	2	2	2	2	2	2	2	2	2
1	1	2	2	2	3	3	3	1	3
1	2	2	2	2	3	3	3	1	3
1	1	2	2	2	3	3	3	1	3
1	2	2	2	2	3	3	3	1	3
1	2	2	2	2	3	3	3	1	3
1	1	2	2	2	3	3	3	1	3
1	2	2	2	2	3	3	3	1	3
1	1	2	2	2	3	3	3	1	3
1	1	1	2	2	3	3	3	1	3
1	1	2	2	2	3	3	3	1	3
1	3	2	2	2	3	3	3	1	3
1	2	2	2	2	3	3	3	2	3
1	1	2	2	2	3	3	3	1	3
1	1	2	2	2	3	3	3	1	3
1	3	1	2	2	3	3	3	1	3
9	1	2	3	2	3	3	3	1	3
1	2	2	2	3	3	3	3	1	3
1	1	2	2	2	3	3	3	1	3
1	2	2	2	2	3	3	3	1	3
1	1	2	2	2	3	3	3	3	3
1	2	2	2	2	3	3	3	1	3
1	1	2	2	2	3	3	3	3	1
1	1	2	2	2	3	3	3	1	3
1	2	2	2	2	3	3	3	1	3
1	1	2	2	2	3	3	3	1	3
9	9	9	9	9	9	9	9	9	9
9	9	9	9	9	9	9	9	9	9
9	9	9	9	9	9	9	9	9	9

9	9	9	9	9	9	9	9	9	9
9	9	9	9	9	9	9	9	9	9
9	9	9	9	9	9	9	9	9	9
9	9	9	9	9	9	9	9	9	9
9	9	9	9	9	9	9	9	9	9
9	9	9	9	9	9	9	9	9	9
9	9	9	9	9	9	9	9	9	9
9	9	9	9	9	9	9	9	9	9
9	9	9	9	9	9	9	9	9	9
9	9	9	9	9	9	9	9	9	9
9	9	9	9	9	9	9	9	9	9
9	9	9	9	9	9	9	9	9	9
9	9	9	9	9	9	9	9	9	9
9	9	9	9	9	9	9	9	9	9
9	9	9	9	9	9	9	9	9	9
9	9	9	9	9	9	9	9	9	9
9	9	9	9	9	9	9	9	9	9
9	9	9	9	9	9	9	9	9	9
9	9	9	9	9	9	9	9	9	9
1	1	2	2	2	3	3	3	1	3
1	2	2	2	2	3	3	3	1	3
1	1	2	2	2	3	3	3	1	3
1	1	2	2	2	3	3	3	1	3
1	1	2	2	2	3	3	3	1	3
1	1	2	2	2	3	3	3	1	3
1	2	2	2	2	3	3	3	1	3
1	2	2	2	2	3	3	3	1	3
1	1	2	2	2	1	3	3	1	3
1	1	2	2	2	3	3	3	1	3
1	1	2	2	2	3	3	3	1	3
1	2	2	2	2	3	3	3	1	3
1	1	2	2	2	1	3	3	1	3
1	1	2	2	2	3	3	3	1	3
1	1	2	2	2	9	3	3	1	3
1	1	2	2	2	3	3	3	1	3
9	9	9	9	9	9	9	9	1	3
1	1	2	2	2	3	3	3	1	3
1	1	2	2	2	1	1	3	1	3
1	1	2	2	2	1	1	3	1	3
1	1	2	2	3	3	3	3	1	3
1	1	2	2	2	3	3	3	1	3
1	1	2	2	2	3	3	3	1	3
1	1	2	2	2	3	3	3	1	3
1	1	3	2	2	3	3	3	1	3
1	1	2	2	2	3	3	3	1	3
1	1	2	2	2	3	3	3	1	3
1	3	1	2	2	2	3	3	1	1

1	1	2	2	2	3	3	3	1	3
1	3	1	2	2	2	3	3	1	2
1	3	1	1	2	3	3	3	1	3
1	1	2	2	3	3	3	3	1	3
1	1	2	2	2	3	3	3	1	3
1	2	2	2	2	3	3	3	1	3
1	3	1	2	2	2	3	3	1	3
1	1	2	2	2	3	3	3	1	3
1	1	2	2	2	3	3	3	1	3
1	3	2	2	2	3	3	3	1	3
1	1	2	2	2	3	3	3	1	3
2	2	2	3	3	3	3	3	1	3
1	2	2	2	2	3	3	3	1	3
1	1	2	2	2	3	3	3	1	3
1	1	2	2	2	3	3	3	1	3
1	2	2	2	2	3	3	3	1	3
1	1	2	2	2	3	3	3	1	3
1	1	2	2	2	3	3	3	1	3
1	1	2	2	2	3	3	3	1	3
1	2	2	2	2	3	3	3	1	3
1	2	2	2	2	3	3	3	1	3
1	1	2	2	2	3	3	3	1	3
1	2	2	2	3	3	3	3	1	3
1	1	2	2	2	3	3	3	1	3
9	1	2	2	2	3	3	3	1	3
1	2	2	2	2	2	3	3	1	3
1	1	2	2	2	3	3	3	1	3
1	2	2	2	3	9	3	3	1	3
1	1	2	2	2	2	3	3	1	3
1	1	9	9	9	3	3	3	1	3
1	1	2	2	2	3	3	3	3	1
1	1	2	2	2	3	3	3	1	3
1	1	2	2	2	3	3	3	1	3
1	1	2	2	2	3	3	3	1	3
9	9	9	9	9	9	9	9	9	9
1	1	2	2	2	3	3	3	1	3
1	2	2	3	3	3	3	3	3	3
1	1	2	2	2	3	3	3	9	3
1	2	2	3	3	3	3	3	1	3
1	1	2	2	2	3	3	3	1	3
1	1	2	2	3	3	3	3	1	3
1	1	2	2	2	3	3	3	1	3
1	1	2	2	2	3	3	9	1	3
1	1	2	2	2	9	9	3	1	3
1	2	2	2	2	3	3	3	1	3
1	2	2	2	2	3	3	3	1	3
1	1	2	2	2	3	3	3	1	3

1	1	2	2	2	3	3	3	1	3
1	1	2	2	2	3	3	3	1	3
1	1	2	2	2	3	3	3	1	3
1	2	2	2	2	3	3	3	1	3
1	2	2	2	2	2	3	3	1	3
1	2	2	2	2	3	3	3	1	3
1	1	2	2	2	3	3	3	1	2
1	2	2	2	2	3	3	3	1	3
1	1	2	2	2	3	3	3	1	3
1	1	2	2	2	3	3	3	1	3
1	1	1	2	2	3	3	3	1	3
1	1	2	2	2	3	3	3	1	3
1	1	1	2	2	3	3	3	1	3
1	1	2	2	2	2	3	3	1	3
1	1	2	2	9	9	9	3	1	3
9	2	2	2	2	3	3	3	1	3
1	1	2	2	2	3	3	3	1	3
2	2	2	2	3	3	2	3	1	3
1	1	1	2	2	3	3	3	1	3
1	1	2	2	2	3	3	3	1	3
1	1	2	2	2	3	3	3	1	3
1	1	2	2	2	3	3	3	1	3
1	2	2	2	2	3	3	3	1	3
1	1	1	2	2	3	3	3	1	3
1	2	1	1	1	1	1	9	1	9
1	2	2	2	2	3	3	3	3	3
1	1	2	2	2	3	3	3	3	3
1	2	2	2	2	3	3	3	1	3
1	1	2	2	2	3	3	3	2	3
1	1	2	2	2	3	3	3	3	1
1	1	2	2	2	3	3	3	2	3
1	1	2	2	2	3	3	3	1	3
3	1	2	2	2	3	3	3	1	3
3	1	2	2	2	3	3	3	1	3
1	1	2	2	2	3	3	3	1	3
1	1	2	2	9	3	3	3	1	3
1	1	2	2	2	3	3	3	1	3
1	1	2	2	2	3	3	3	1	3
1	1	2	2	2	3	3	3	1	3
1	1	2	2	2	3	3	3	1	1
1	2	2	2	2	3	3	3	1	1
9	9	9	9	9	9	9	9	9	9
1	1	2	2	2	3	3	3	1	3
1	1	2	2	2	3	3	3	1	3
1	1	2	2	2	3	3	3	1	3
2	2	2	2	3	3	3	3	1	3
2	2	2	2	3	3	3	3	1	3

1	1	2	2	2	3	3	3	1	3
1	1	2	2	2	3	3	3	1	3
1	1	2	2	2	3	3	3	1	3
1	1	2	2	2	3	3	3	1	3
1	1	2	2	2	3	3	3	1	3
1	1	2	2	2	3	3	3	1	3
1	1	2	2	2	3	3	3	1	3
1	1	2	2	2	3	3	3	1	3
1	1	2	2	2	3	3	3	1	3
9	9	9	9	9	9	9	9	9	9
1	2	2	2	2	3	3	3	1	3
1	1	2	2	2	3	3	3	1	3
1	2	2	2	2	3	3	3	1	3
1	2	2	2	2	3	3	3	1	3
1	2	2	2	2	3	3	3	1	3
1	2	2	2	2	3	3	3	1	3
1	2	2	2	2	3	3	3	1	3
1	1	2	2	2	3	3	3	1	3
1	2	2	2	2	3	3	3	1	3
1	1	2	2	2	3	3	3	1	3
1	1	2	2	2	3	3	3	1	3
1	1	2	2	2	3	3	3	1	3
1	2	2	2	2	3	3	3	1	3
1	2	2	2	2	3	3	3	1	3
1	2	2	2	2	3	3	3	1	3
1	1	2	2	2	3	3	3	1	3
1	1	2	2	2	3	3	3	3	3
9	9	9	9	9	9	9	9	9	9
9	9	9	9	9	9	9	9	9	9
9	9	9	9	9	9	9	9	9	9
9	9	9	9	9	9	9	9	9	9
9	9	9	9	9	9	9	9	9	9
9	9	9	9	9	9	9	9	9	9
9	9	9	9	9	9	9	9	9	9
9	9	9	9	9	9	9	9	9	9
9	9	9	9	9	9	9	9	9	9
9	9	9	9	9	9	9	9	9	9
9	9	9	9	9	9	9	9	9	9
9	9	9	9	9	9	9	9	9	9
9	9	9	9	9	9	9	9	9	9
9	9	9	9	9	9	9	9	9	9
9	9	9	9	9	9	9	9	9	9
9	9	9	9	9	9	9	9	9	9
9	9	9	9	9	9	9	9	9	9
9	9	9	9	9	9	9	9	9	9
9	9	9	9	9	9	9	9	9	9
9	9	9	9	9	9	9	9	9	9

9	9	9	9	9	9	9	9	9	9
9	9	9	9	9	9	9	9	9	9
9	9	9	9	9	9	9	9	9	9
9	9	9	9	9	9	9	9	9	9
9	9	9	9	9	9	9	9	9	9
9	9	9	9	9	9	9	9	9	9
9	9	9	9	9	9	9	9	9	9
9	9	9	9	9	9	9	9	9	9
9	9	9	9	9	9	9	9	9	9
9	9	9	9	9	9	9	9	9	9
9	9	9	9	9	9	9	9	9	9
9	9	9	9	9	9	9	9	9	9
9	9	9	9	9	9	9	9	9	9
9	1	2	1	2	3	2	3	3	2
2	2	2	2	2	2	2	2	2	2
2	2	2	2	2	2	2	2	2	2
9	2	1	1	1	3	3	1	3	1
2	2	2	2	1	1	1	1	1	1
2	2	2	2	2	2	2	2	1	1
2	1	1	2	2	1	2	1	3	3
1	3	1	2	2	2	2	3	9	9
2	3	2	1	2	2	2	3	2	2
9	9	9	9	9	9	9	9	9	9
2	3	2	2	2	2	2	2	3	2
1	9	9	9	9	9	9	9	9	9
2	2	3	3	3	3	3	2	2	2
9	9	9	9	9	9	9	9	9	9
1	1	1	1	1	1	1	1	1	1
2	1	1	1	3	3	3	1	2	3
1	1	3	1	1	3	3	3	1	1
2	9	1	2	2	3	2	1	3	3
1	2	3	2	3	2	3	1	1	2
9	9	9	9	9	9	9	9	1	9
1	2	1	2	1	3	3	3	1	1
1	1	9	1	1	9	1	1	9	9
9	9	9	9	9	9	9	9	2	9
1	2	2	2	2	2	2	3	1	1
2	1	1	3	3	1	1	2	1	2
1	1	2	2	1	2	2	2	1	2
1	1	1	1	1	3	1	2	1	3
9	9	9	9	9	9	9	9	9	9
9	9	9	9	9	9	9	9	9	9
2	2	2	2	2	2	2	2	9	9
9	9	9	9	9	9	9	9	1	1
9	9	9	9	9	9	9	9	9	9
9	9	9	9	9	9	9	9	9	9
9	9	9	9	9	9	9	9	9	9

9	9	9	9	9	9	1	9	1	1
9	9	9	9	9	9	9	9	9	9
2	3	2	3	2	1	3	9	2	2
2	2	2	1	1	2	2	2	1	2
1	1	2	1	1	1	1	2	1	3
9	9	9	9	9	9	9	9	3	9
2	2	2	2	2	2	2	2	2	2
2	2	2	2	2	2	2	2	2	2
2	2	2	2	2	2	2	2	2	2
1	1	2	2	3	3	3	1	1	1
2	2	2	2	2	2	2	2	2	2
9	9	9	9	9	9	9	9	9	9
9	9	9	9	9	9	9	9	1	9
2	2	2	2	2	2	2	2	2	2
1	1	2	1	1	3	3	3	1	2
1	1	1	2	3	1	2	3	1	1
1	1	1	1	1	1	1	1	1	1
1	3	1	3	3	2	2	2	2	2
1	1	2	1	1	1	1	2	1	2
9	9	9	9	9	9	9	9	9	9
2	2	2	2	2	2	2	2	2	2
2	2	2	2	2	2	2	2	2	2
2	2	2	2	2	2	2	2	2	2
2	2	2	2	2	2	2	2	2	2
2	2	2	2	2	2	2	2	2	2
1	2	1	1	2	2	3	1	1	1
2	2	2	2	2	2	2	2	2	2
3	9	2	1	2	3	2	1	1	2
9	9	9	9	9	9	9	9	9	9
1	1	3	3	1	3	3	3	1	3
1	3	1	3	3	3	3	3	1	3
9	2	3	1	1	1	2	2	1	1
1	2	1	2	2	2	2	2	1	2
1	1	1	1	1	2	3	3	1	1
3	3	2	9	1	2	2	2	1	2
2	2	2	2	1	1	1	2	9	9
1	1	2	3	3	3	3	3	1	3
2	2	9	9	9	9	9	9	1	1
9	9	9	9	9	9	9	3	9	9
2	2	2	2	2	2	2	2	2	2
3	3	2	2	2	2	2	2	3	3
2	2	2	2	1	2	3	3	1	2
3	1	2	3	1	2	3	1	1	2
9	9	9	9	9	9	9	9	9	9
1	1	1	1	1	1	1	1	1	1
1	1	2	2	2	2	2	1	1	1
1	2	3	2	2	2	3	3	1	2

2	2	2	2	2	2	2	2	2	2
2	2	3	2	1	2	1	2	1	2
2	2	2	2	2	2	2	2	2	2
2	2	2	2	2	2	2	2	9	9
2	2	2	2	2	2	2	2	9	9
2	2	2	2	2	2	2	2	2	2
1	2	2	3	3	3	3	3	1	3
1	2	3	2	1	3	2	2	2	2
2	2	2	2	2	2	2	2	9	9
3	1	2	3	2	3	1	2	1	2
2	2	2	2	2	2	2	2	2	2
2	2	2	2	2	2	2	2	2	2
1	1	1	1	1	1	1	1	1	1
2	3	3	3	3	3	3	3	2	2
2	2	2	2	2	2	2	2	9	9
2	2	2	2	2	2	2	2	2	2
3	3	3	3	3	3	3	3	3	3
2	2	2	2	2	2	2	2	2	2
2	2	2	2	2	2	2	2	2	2
3	1	2	3	1	2	3	1	1	2
1	2	1	2	1	2	1	2	1	2
1	2	2	2	2	1	1	3	1	1
1	3	1	1	2	1	3	1	9	9
1	1	1	1	1	1	1	3	9	9
1	3	3	3	3	3	3	3	1	3
9	9	9	9	9	9	9	9	9	9
9	9	9	9	9	9	9	9	9	9
1	1	1	1	1	2	2	2	2	2
3	1	1	2	2	2	2	1	1	9
1	2	1	3	1	1	1	3	1	1
2	2	2	2	2	2	2	3	1	2
2	1	2	2	2	2	2	1	1	1
1	1	1	1	1	1	1	1	2	2
3	3	3	3	3	3	3	3	1	9
9	9	9	9	9	9	9	9	9	9
2	2	2	2	2	2	2	2	2	2
9	9	9	9	9	9	9	9	9	9
9	9	9	9	9	9	9	9	9	9
9	9	9	9	9	9	9	9	9	9
1	1	1	1	1	1	1	1	1	1
3	3	9	2	3	2	2	2	2	3
2	9	1	1	3	1	3	3	3	3
2	1	2	2	2	2	1	2	2	2
9	9	9	9	9	9	9	9	9	9
9	9	9	9	9	9	9	9	9	9
9	9	9	9	9	9	9	9	9	9
1	1	1	2	2	2	2	3	1	1

9	9	9	9	9	9	9	9	9	9
9	9	9	9	9	9	9	9	9	9
9	2	2	2	2	2	2	2	9	9
9	9	9	9	9	9	9	9	9	9
9	9	9	9	9	9	9	9	9	9
3	3	2	2	2	3	3	3	9	9
1	2	3	2	2	2	2	3	1	3
9	9	9	9	9	9	9	9	9	9
1	1	2	2	1	3	3	3	1	1
3	2	3	1	2	3	1	2	1	2
1	2	1	3	2	1	2	1	1	2
2	2	2	2	2	2	2	2	2	2
2	2	2	2	2	2	2	2	2	2
9	9	9	9	9	9	9	9	9	9
2	2	2	2	2	2	2	9	2	2
2	2	2	2	2	2	2	2	2	2
3	3	3	1	1	2	2	1	1	1
1	3	3	2	1	2	3	2	2	3
2	1	3	2	2	3	2	2	1	2
9	9	9	9	9	9	9	9	9	9
1	3	3	2	1	2	3	2	2	3
3	2	2	1	2	1	2	3	1	3
2	2	2	2	2	2	2	2	2	2
1	3	3	1	2	3	2	2	2	2
3	2	2	3	2	3	2	3	3	2
9	9	9	9	9	9	9	9	9	9
3	2	1	1	2	1	2	1	1	1
9	9	9	9	9	9	9	9	9	9
3	3	3	3	3	3	3	3	3	3
3	2	2	2	2	2	2	2	1	2
2	2	2	2	2	2	2	2	2	2
1	9	9	9	9	9	9	9	9	9
1	9	9	9	9	9	9	9	9	9
2	2	2	2	2	2	2	2	2	2
2	3	3	3	3	3	3	2	2	2
1	2	2	1	2	1	2	1	2	1
9	9	9	9	9	9	9	9	2	2
1	1	1	3	2	2	3	3	1	2
2	2	2	2	2	2	2	2	2	2
9	9	9	9	9	9	9	1	2	9
9	9	9	9	9	9	9	9	1	2
2	2	2	2	2	2	2	2	2	2
2	2	2	2	2	2	2	2	2	2
2	2	2	2	2	2	2	2	2	2
2	2	2	2	2	2	2	2	2	2
9	9	9	1	9	9	9	9	1	9
2	2	2	2	2	2	2	2	2	2

2	2	2	2	2	2	2	2	2	2
2	2	2	2	2	2	2	2	2	2
1	3	1	3	3	3	3	3	1	3
1	2	2	3	3	3	3	3	1	1
2	2	2	2	2	2	2	2	2	2
2	2	3	3	3	3	3	2	2	2
2	3	1	1	1	3	2	2	1	2
3	2	1	2	1	2	1	3	1	2
1	2	2	3	3	3	2	3	1	3
1	3	1	1	3	3	3	3	1	3
2	2	2	2	2	2	2	2	3	2
3	3	2	2	2	2	2	2	2	2
1	1	2	2	2	2	2	2	1	1
1	1	3	3	3	3	3	3	1	2
1	2	3	1	2	3	1	3	3	1
2	2	2	2	2	2	2	2	1	2
2	2	2	3	3	3	3	3	2	2
2	2	2	2	1	3	2	1	2	1
2	2	2	3	2	2	2	3	2	1
1	1	3	3	3	3	3	3	1	3
1	2	1	1	2	3	2	2	1	3
9	9	9	9	9	9	9	9	9	9
2	2	2	2	2	2	2	3	2	2
2	2	2	2	2	2	2	2	2	2
2	2	2	2	2	2	2	2	2	2
1	1	1	3	1	3	2	3	1	3
1	1	2	1	1	2	3	3	2	1
1	1	2	1	1	2	1	3	2	1
1	3	1	3	3	3	3	3	1	3
1	3	2	3	3	3	3	3	1	2
1	3	3	3	3	3	3	3	1	1
2	2	2	2	2	2	2	1	1	1
2	2	2	2	2	2	2	2	2	1
9	9	9	9	9	9	9	9	9	9
9	9	9	9	9	9	9	9	1	9
9	3	1	3	3	3	1	3	1	3
2	2	2	2	2	2	2	2	2	2
2	1	2	1	2	1	1	1	1	2
1	2	2	1	2	2	9	2	1	1
2	2	2	2	2	2	2	2	2	2
3	2	1	2	3	1	3	2	2	1
9	9	9	9	9	9	9	9	9	9
9	9	9	9	9	9	9	9	9	9
1	1	1	1	1	1	1	1	1	1
2	9	9	9	9	9	9	9	9	9
9	9	9	9	9	9	9	9	9	9
9	9	9	9	9	9	9	9	9	9

2	2	2	2	2	2	2	2	2	2
3	1	3	3	3	2	1	2	1	1
9	9	9	9	9	9	9	9	9	9
3	3	3	3	3	3	3	2	1	1
1	1	1	2	1	2	3	1	2	2
3	1	2	3	1	2	3	1	1	2
2	3	2	1	2	2	2	1	1	1
9	9	1	9	9	9	9	9	1	9
2	1	3	3	2	1	2	2	1	1
9	9	9	9	9	9	9	9	9	9
1	1	1	1	1	1	1	1	1	1
1	1	3	1	1	3	3	3	1	1
1	1	2	9	1	9	9	9	9	9
9	9	9	9	9	9	9	9	9	9
1	1	1	1	1	1	1	1	1	1
9	9	9	9	9	9	9	9	9	9
9	9	9	9	9	9	9	9	9	9
2	2	2	2	2	2	2	2	2	2
2	2	2	2	2	2	2	2	2	2
3	2	3	2	2	2	2	2	1	2
2	2	2	2	2	2	2	2	2	2
2	2	2	2	2	2	2	2	2	2
2	2	2	2	2	2	2	2	1	2
3	2	1	3	2	1	3	2	1	3
2	2	9	2	2	2	2	1	1	1
9	9	9	9	9	9	9	9	1	9
1	3	1	1	3	3	3	3	1	3
3	9	2	2	2	2	2	2	3	1
3	3	3	2	1	1	1	1	9	9
1	1	1	1	1	3	2	3	2	2
1	1	2	1	2	1	1	2	1	1
2	2	2	2	2	2	2	2	2	2
2	2	2	2	2	2	2	9	2	2
2	2	2	2	2	2	2	2	2	2
2	2	2	2	2	2	2	2	2	2
2	2	2	2	2	2	2	2	2	2
1	3	2	1	3	2	3	2	2	3
9	9	9	9	9	9	9	9	1	9
9	9	9	9	9	9	9	9	9	9
2	2	2	2	2	2	2	2	1	3
3	3	3	3	3	3	3	2	2	3
9	9	9	9	9	3	9	9	9	9
1	2	2	2	2	2	2	2	1	2
9	9	9	9	9	9	9	9	1	9
2	2	2	3	3	3	3	3	3	3
9	9	9	9	9	9	9	9	9	9
2	2	2	2	2	2	2	2	2	2

9	9	9	9	9	9	9	9	2	1
9	9	9	9	9	9	9	9	1	9
2	2	2	2	2	2	2	2	1	1
2	2	2	3	3	2	1	3	3	3
2	2	2	2	2	2	2	2	1	2
2	2	2	2	2	2	2	2	2	2
2	2	1	1	2	2	3	3	1	1
9	9	9	9	9	9	9	9	9	9
2	2	2	2	2	2	2	2	2	2
2	2	2	2	2	2	2	2	3	3
2	2	2	2	2	2	2	2	2	2
2	2	2	2	2	2	2	2	2	2
3	3	3	3	3	3	3	3	1	2
2	1	3	3	3	2	2	3	2	2
1	2	1	2	2	3	1	3	3	3
1	2	1	2	2	3	1	3	3	3
2	2	2	2	2	2	2	2	2	2
2	2	2	2	2	2	2	2	2	2
9	9	9	9	9	9	9	9	9	9
9	9	9	9	9	9	9	9	9	9
3	3	3	3	3	3	3	3	2	2
1	2	1	2	2	3	3	3	1	2
9	9	9	9	9	9	9	9	9	9
9	9	9	9	9	9	9	9	9	9
3	3	3	3	3	3	3	3	3	3
9	9	9	9	9	9	9	9	9	9
9	9	9	9	9	9	9	9	9	9
9	9	9	9	9	9	9	9	9	9
3	1	2	3	1	2	3	1	3	2
1	1	1	1	1	1	1	1	1	1
2	2	2	2	2	2	2	2	2	2
2	9	1	2	1	1	1	9	1	1
2	2	2	2	2	2	2	2	2	2
2	2	2	2	2	2	2	2	2	2
2	2	2	2	2	2	2	2	2	9
1	3	1	3	3	3	3	3	1	3
1	2	2	1	2	3	3	1	3	3
1	3	2	1	3	1	2	2	2	1
2	3	3	1	2	2	2	2	1	2
1	2	2	3	1	2	3	2	1	3
9	2	2	3	3	2	2	2	1	2
2	2	2	2	2	2	2	2	3	3
1	9	9	9	9	9	9	9	1	9
9	9	9	9	9	9	9	9	9	9
1	3	2	2	3	2	1	2	2	3
2	2	2	2	2	2	2	2	2	2
2	2	2	2	2	2	2	2	2	2

2	3	3	3	1	1	1	2	1	2
9	9	9	9	9	9	9	9	9	9
9	9	9	9	9	9	9	9	9	9
9	9	9	9	9	9	9	9	9	9
9	9	9	9	9	9	9	9	9	9
9	9	9	9	9	9	9	9	1	9
9	9	9	9	9	9	9	9	9	9
9	9	9	9	9	9	9	9	9	9
9	9	9	9	9	9	9	9	9	9
9	9	9	9	3	9	9	9	3	9
2	2	2	2	2	2	2	2	2	2
2	3	2	3	2	3	2	2	1	2
1	3	1	3	3	3	3	3	1	3
2	2	2	2	2	2	2	2	2	2
2	2	2	2	2	2	2	2	2	2
9	9	9	9	9	9	9	9	9	9
2	2	2	2	2	2	2	2	1	1
2	2	2	2	2	2	2	2	2	2
2	2	2	2	2	2	2	2	2	2
1	1	2	2	1	1	2	2	3	3
1	2	1	2	3	1	3	2	1	2
1	2	3	2	1	3	2	1	3	1
2	2	2	2	2	2	2	2	1	1
2	2	2	2	2	2	2	2	2	2
2	2	2	2	2	2	2	2	2	2
2	2	2	2	2	2	2	2	2	2
3	3	3	3	3	3	3	3	2	2
2	2	1	1	2	2	1	1	1	2
2	2	2	2	2	2	2	2	9	9
2	2	2	2	2	2	2	2	2	2
9	9	9	9	9	9	9	9	9	9
2	2	2	2	2	2	2	2	2	2
9	1	1	1	1	1	1	1	1	2
9	9	9	1	9	9	9	9	9	9
3	3	3	3	3	3	9	3	3	3
2	2	2	2	2	2	2	2	2	2
2	2	2	2	2	2	2	2	2	2
2	2	2	2	2	2	2	2	2	2
1	1	1	1	2	1	1	2	3	2
9	9	9	9	9	9	9	9	9	9
1	1	2	1	1	1	1	2	2	1
2	2	2	2	2	2	2	2	2	2
2	2	2	2	2	2	2	2	2	2
1	2	1	2	2	2	2	3	1	2
1	1	2	3	2	2	2	3	1	2
9	9	9	9	9	9	9	9	9	9
9	9	9	9	9	9	9	9	9	9

9	9	9	9	9	9	9	9	9	9
2	2	2	2	2	9	2	3	2	2
9	9	9	9	9	9	9	9	1	9
9	9	9	9	9	9	9	9	9	9
9	9	9	9	9	9	9	9	9	9
9	9	9	9	9	9	9	9	9	9
9	9	9	9	9	9	9	9	9	9
9	9	9	9	9	9	9	9	9	9
9	9	9	9	9	9	9	9	9	9
9	9	9	9	9	9	9	9	9	9
9	9	9	9	9	9	9	9	9	9
9	9	9	9	9	9	9	9	9	9
9	9	9	9	9	9	9	9	9	9
9	9	9	9	9	9	9	9	9	9
9	9	9	9	9	9	9	9	9	9
9	9	9	9	9	9	9	9	9	9
9	9	9	9	9	9	9	9	9	9
9	9	9	9	9	9	9	9	9	9
9	9	9	9	9	9	9	9	9	9
9	9	9	9	9	9	9	9	9	9
9	9	9	9	9	9	9	9	9	9
9	9	9	9	9	9	9	9	9	9
9	9	9	9	9	9	9	9	9	9
9	9	9	9	9	9	9	9	9	9
9	9	9	9	9	9	9	9	9	9
3	2	1	2	3	2	1	2	1	2
2	2	2	2	2	2	2	2	2	2
2	2	2	2	2	2	2	2	2	2
1	2	1	3	2	1	3	3	1	2
3	2	2	1	2	2	1	1	9	9
1	1	1	2	2	3	2	3	1	2
2	2	2	2	2	2	2	2	2	2
1	1	2	1	1	1	2	2	1	3
2	2	2	2	2	2	2	3	1	1
1	2	1	3	2	3	1	1	1	2
1	3	1	3	1	3	3	3	1	1
1	3	1	3	3	3	3	3	1	3
1	1	2	2	1	1	3	3	1	2
1	2	2	2	2	2	2	3	1	2
2	2	2	2	2	2	2	2	1	2
1	1	2	3	3	3	3	3	2	2
9	9	9	9	9	9	9	1	1	9
1	3	1	3	3	2	3	3	1	1
2	1	3	2	2	2	2	2	1	2
9	9	9	9	9	9	9	9	1	9
1	3	1	1	3	3	3	3	1	2
1	3	1	1	3	3	3	3	1	1
2	2	2	2	2	2	2	2	2	2
2	1	3	2	3	2	1	3	1	2

1	3	1	3	3	3	3	3	1	2
1	1	3	2	1	2	3	3	1	3
1	1	2	1	3	3	3	3	1	3
1	1	2	3	1	3	3	3	1	2
1	2	3	2	1	2	1	2	2	2
9	9	9	9	9	9	9	9	1	9
1	3	2	3	3	3	3	3	1	3
9	9	9	9	9	9	9	9	1	9
2	2	2	2	2	2	2	2	2	2
2	2	2	2	2	2	2	2	2	2
1	9	9	9	9	9	9	9	9	9
1	9	9	9	9	9	9	9	9	9
9	9	9	9	9	9	9	9	1	2
9	9	9	1	9	9	9	9	1	3
1	1	3	1	1	1	1	3	9	9
9	9	9	9	9	9	9	9	9	9
9	9	9	9	9	9	9	9	9	9
9	9	9	9	9	9	9	9	9	9
9	9	9	9	9	9	9	9	9	1
3	3	1	3	3	3	9	3	3	3
9	9	9	9	9	9	9	9	1	9
1	9	9	9	9	9	9	9	9	2
9	9	9	9	9	9	9	9	1	9
9	9	1	9	9	9	9	9	1	9
9	9	9	9	9	9	9	9	1	9
3	1	2	3	1	3	2	1	2	1
9	9	9	9	9	9	9	9	9	9
1	2	1	3	3	3	3	3	1	3
1	3	2	2	3	3	2	3	1	3
9	9	9	9	9	9	9	9	9	9
2	1	2	2	2	2	2	3	1	2
3	1	3	3	3	3	1	3	1	3
1	3	3	3	3	3	3	3	3	3
1	3	1	3	1	3	3	3	1	9
1	1	1	1	1	1	1	3	1	1
1	2	3	1	2	3	2	3	3	9
2	2	2	3	2	2	2	3	2	2
1	1	1	2	1	2	2	3	1	1
3	3	3	3	2	1	2	2	2	3
1	1	1	3	3	3	3	1	1	1
1	2	1	2	2	2	3	3	1	2
1	1	1	2	1	2	3	3	1	1
1	3	1	3	3	3	3	3	9	9
2	1	1	3	3	2	2	2	2	3
1	3	1	3	1	3	3	3	2	1
1	3	3	3	3	3	3	3	3	2
1	3	1	3	3	3	3	3	1	3

9	9	9	9	9	9	9	9	9	9
1	9	2	2	1	1	2	9	1	2
9	9	9	9	9	9	9	9	9	9
1	3	1	2	3	3	1	3	1	3
9	9	9	9	9	9	9	9	9	9
1	3	1	3	3	3	3	3	1	1
1	3	3	1	1	1	3	3	1	3
9	9	9	9	9	9	9	9	9	9
9	9	9	9	9	9	9	2	2	9
2	2	2	2	2	2	2	2	2	2
1	1	1	3	1	1	1	2	2	1
1	3	1	3	3	3	3	3	9	9
1	2	2	1	2	1	1	1	1	3
9	9	9	9	9	9	9	9	3	2
9	9	9	9	9	9	9	9	9	9
2	3	1	2	1	2	3	3	3	3
1	9	9	9	9	9	9	9	1	9
9	9	9	9	9	9	9	9	9	9
2	2	2	2	2	2	2	3	2	2
1	1	1	1	2	1	1	2	1	2
2	2	2	2	2	2	2	2	9	9
3	1	1	1	1	1	1	2	1	1
3	2	2	3	2	1	3	3	2	2
1	1	9	9	9	9	9	9	9	9
2	2	2	2	2	2	2	2	1	1
9	9	9	9	9	9	9	9	9	9
9	9	9	9	9	9	9	9	1	9
9	3	1	1	1	1	1	1	1	3
3	3	3	2	1	1	1	2	1	2
9	1	3	3	1	3	3	3	1	3
9	9	9	9	9	9	9	9	9	9
9	9	9	9	9	9	9	9	9	9
9	9	9	9	9	9	9	9	9	9
9	9	9	9	9	9	9	9	9	9
9	9	9	9	9	9	9	9	9	9
9	9	9	9	9	9	9	9	9	9
9	9	9	9	9	9	9	9	9	9
9	9	9	9	9	9	9	9	9	9
9	9	9	9	9	9	9	9	9	9
9	9	9	9	9	9	9	9	9	9
9	9	9	9	9	9	9	9	9	9
9	9	9	9	9	9	9	9	9	9
9	9	9	9	9	9	9	9	9	9
9	9	9	9	9	9	9	9	9	9
9	9	9	9	9	9	9	9	9	9
9	9	9	9	9	9	9	9	9	9
9	9	9	9	9	9	9	9	9	9

9	9	9	9	9	9	9	9	9	9
9	9	9	9	9	9	9	9	9	9
9	9	9	9	9	9	9	9	9	9
9	9	9	9	9	9	9	9	9	9
9	9	9	9	9	9	9	9	9	9
9	9	9	9	9	9	9	9	9	9
9	9	9	9	9	9	9	9	9	9
9	9	9	9	9	9	9	9	9	9
9	9	9	9	9	9	9	9	9	9
9	9	9	9	9	9	9	9	9	9
9	9	9	9	9	9	9	9	9	9
9	9	9	9	9	9	9	9	9	9
9	9	9	9	9	9	9	9	9	9
9	9	9	9	9	9	9	9	9	9
9	9	9	9	9	9	9	9	9	9
9	9	9	9	9	9	9	9	9	9
9	9	9	9	9	9	9	9	9	9
9	9	9	9	9	9	9	9	9	9
9	9	9	9	9	9	9	9	9	9
9	9	9	9	9	9	9	9	9	9
9	9	9	9	9	9	9	9	9	9
9	9	9	9	9	9	9	9	9	9
9	9	9	9	9	9	9	9	9	9
9	9	9	9	9	9	9	9	9	9
9	9	9	9	9	9	9	9	9	9
9	9	9	9	9	9	9	9	9	9
9	9	9	9	9	9	9	9	9	9
9	9	9	9	9	9	9	9	9	9
9	9	9	9	9	9	9	9	9	9
9	9	9	9	9	9	9	9	9	9
9	9	9	9	9	9	9	9	9	9
9	9	9	9	9	9	9	9	9	9
9	9	9	9	9	9	9	9	9	9
9	9	9	9	9	9	9	9	9	9
9	9	9	9	9	9	9	9	9	9
2	2	2	2	2	2	2	2	2	2
9	9	9	9	9	9	9	9	9	9
9	2	2	3	3	3	3	3	9	9
1	3	1	3	3	3	1	3	1	3
3	3	3	3	3	3	3	3	3	3
9	9	9	9	9	9	9	9	9	9
9	9	9	9	9	9	9	9	9	9
9	9	9	9	9	9	9	9	1	9
9	9	9	9	9	9	9	9	9	9
9	9	9	9	9	9	9	9	2	2
9	9	9	9	9	9	9	9	1	2
2	2	2	2	2	2	2	2	1	3

1	1	2	3	3	3	3	3	2	2
2	2	2	2	2	2	2	2	1	1
1	1	3	1	1	3	3	3	1	3
1	2	2	3	2	3	3	3	1	2
1	2	3	3	2	3	3	3	1	2
1	1	1	1	1	3	2	3	1	1
1	2	1	3	3	3	3	3	2	3
1	2	1	3	2	3	2	3	1	2
9	3	2	2	1	2	3	2	2	2
2	1	1	3	1	2	3	3	1	2
9	1	9	9	9	9	9	9	1	9
2	2	1	2	3	3	1	2	2	2
9	9	9	9	9	9	9	9	9	9
1	1	1	3	1	3	3	3	1	2
1	1	1	3	1	3	3	3	1	2
1	3	1	3	3	3	3	3	1	3
9	9	9	9	9	9	9	9	9	9
1	2	3	3	2	3	3	3	1	2
1	2	1	2	1	2	3	3	1	2
2	2	2	3	3	1	2	2	2	2
1	9	2	3	3	3	3	3	1	2
1	3	1	2	2	2	3	3	1	2
1	1	2	1	1	2	2	1	1	3
1	3	1	3	3	3	3	3	1	3
3	3	3	3	3	3	3	3	3	3
2	1	2	1	2	1	2	1	1	2
1	1	1	1	1	1	1	2	1	2
1	3	1	3	2	3	1	3	1	2
2	2	2	2	2	2	2	2	2	2
9	9	9	9	9	9	9	9	9	9
9	9	9	9	9	9	9	9	9	9
2	2	2	2	2	2	2	2	9	9
2	2	2	2	2	2	2	2	9	9
9	9	9	9	9	9	9	9	9	9
9	9	9	9	9	9	9	9	9	9
9	9	9	9	9	9	9	9	9	9
1	1	1	2	3	2	1	2	1	3
3	1	2	3	2	3	2	1	9	9
1	2	1	2	1	2	1	2	9	9
9	9	9	9	9	9	9	9	9	9
1	1	1	1	1	3	2	3	1	2
9	9	9	9	9	9	9	9	9	9
1	3	1	3	3	3	3	3	1	3
1	3	1	3	1	3	3	3	3	3
3	3	1	1	1	1	2	1	1	2
3	3	3	3	3	3	3	3	3	3
1	1	3	1	1	3	1	1	1	3

3	2	3	3	3	2	1	1	2	3
1	9	2	2	2	1	2	2	1	2
1	2	1	2	1	2	2	1	2	2
1	2	3	3	3	3	3	3	1	1
2	9	1	2	2	1	2	2	1	2
3	1	2	3	1	2	3	2	1	2
1	1	1	2	1	2	2	1	3	3
2	1	2	1	3	2	1	2	2	1
1	1	1	3	1	1	2	3	1	3
9	1	2	3	3	3	2	1	1	2
1	1	1	1	1	1	2	3	1	1
3	1	2	1	2	3	1	2	1	2
2	2	1	1	2	1	2	2	2	2
1	2	3	3	2	1	2	2	1	1
1	1	1	1	1	1	1	2	3	3
1	1	1	1	1	1	1	3	1	3
3	2	2	2	2	2	2	2	2	3
9	9	9	9	9	9	9	9	9	9
3	1	2	2	1	2	2	3	1	1
1	3	3	3	3	3	1	3	2	1
3	3	2	2	1	2	2	2	1	2
2	2	3	2	2	2	1	2	3	1
3	1	2	3	1	2	3	1	1	2
1	3	1	1	1	1	2	3	1	1
2	2	3	3	3	3	2	2	2	2
3	3	3	3	2	2	2	1	1	3
1	3	1	3	3	3	1	3	1	3
3	3	1	1	2	1	1	1	1	1
2	2	2	2	2	2	2	2	2	2
2	3	1	1	2	2	3	3	1	3
1	1	2	2	2	2	2	3	1	1
3	1	2	2	2	3	2	1	1	2
2	2	3	3	3	2	1	2	2	2
3	3	3	3	2	2	2	1	2	2
3	9	1	2	3	1	2	3	1	2
1	1	1	3	3	3	1	3	3	3
2	1	3	2	1	2	3	2	2	3
9	9	9	9	9	9	9	9	9	9
1	1	1	3	1	1	1	3	1	1
3	1	1	3	1	3	3	1	1	3
1	3	1	1	1	1	1	1	1	1
3	9	1	2	3	1	2	3	1	2
1	1	1	3	3	3	2	2	1	3
1	3	1	3	1	1	1	2	2	1
2	2	2	2	2	2	2	2	2	2
3	3	3	3	3	3	3	1	3	3
9	9	9	9	9	9	9	9	9	9

3	3	2	2	2	1	2	2	1	2
9	3	3	3	3	3	3	3	9	9
3	3	3	3	3	3	3	2	3	3
1	1	3	2	2	2	3	3	2	2
2	2	2	2	2	1	1	2	1	1
2	1	2	2	1	1	1	2	1	1
2	2	2	2	2	2	2	2	2	2
9	9	9	9	9	9	9	9	9	9
9	9	9	9	9	9	9	9	9	9
2	2	3	3	2	1	1	1	1	2
2	2	2	2	2	2	2	3	1	2
2	2	2	2	2	2	2	2	2	2
2	2	2	2	2	1	2	2	2	2
2	2	2	2	2	2	2	2	1	1
2	2	2	2	2	2	2	2	2	2
2	2	2	2	2	2	2	2	2	2
2	2	2	2	2	2	2	2	2	2
2	1	2	2	2	2	3	2	3	1
1	1	2	2	2	2	2	1	2	2
2	2	2	2	2	2	2	2	1	2
2	2	2	2	2	2	2	2	2	2
2	2	2	2	2	2	2	2	2	2
2	2	2	2	2	2	2	2	2	2
1	2	2	1	2	2	2	2	1	1
2	2	2	2	2	2	2	2	2	2
2	2	2	2	2	2	2	2	2	2
2	2	2	2	2	2	2	3	1	2
1	2	3	2	1	2	1	2	9	9
1	1	1	1	1	1	1	1	2	2
2	2	2	2	2	2	2	2	2	2
2	2	2	2	2	2	2	2	2	2
2	2	2	2	2	2	2	3	1	2
2	2	2	2	2	2	2	2	9	9
2	2	2	2	2	2	2	2	2	2
2	2	2	2	2	2	2	3	2	2
2	2	2	2	2	2	2	3	2	2
2	2	2	2	2	2	2	2	2	2
2	2	2	2	2	2	2	2	2	2
2	2	2	2	2	2	2	2	2	2
1	3	1	3	1	1	3	3	9	9
2	2	2	2	2	2	2	3	2	2
2	2	2	2	2	2	2	2	2	2
3	2	1	2	3	2	2	2	1	1
2	2	2	2	2	2	2	2	2	2
1	2	1	1	3	2	2	3	1	2
2	2	2	2	2	2	2	2	9	9
2	2	2	2	2	2	2	2	3	3

2	2	2	2	2	2	2	2	2	2
2	3	2	3	2	2	3	2	3	2
2	2	2	2	2	2	2	2	2	2
2	2	2	2	2	2	2	2	2	2
2	2	2	2	2	2	2	2	2	2
2	2	2	2	2	2	2	2	2	2
1	1	3	3	3	3	3	3	1	3
1	1	1	1	1	1	1	1	1	1
9	9	9	9	9	9	9	9	1	9
2	2	2	2	2	2	2	2	2	2
2	3	2	1	1	1	1	1	1	2
2	2	2	9	1	1	1	1	1	1
2	3	2	3	2	2	2	3	1	1
9	9	9	9	9	9	9	9	9	9
2	2	2	2	2	2	2	2	2	2
1	2	2	3	2	2	1	2	1	2
2	2	2	2	2	2	2	3	1	1
1	2	2	2	3	2	1	1	1	1
9	9	9	9	9	9	9	9	1	9
2	2	2	2	2	2	2	1	1	1
2	2	1	3	2	2	2	2	1	2
9	9	9	9	1	9	9	9	9	9
9	9	9	9	9	9	9	9	1	9
2	2	2	2	2	2	2	2	2	1
1	2	3	2	2	1	3	2	1	3
2	1	1	1	2	2	1	1	1	1
2	2	1	2	2	2	2	1	1	1
9	1	1	1	2	2	2	1	1	1
9	9	9	9	9	9	9	9	9	2
1	1	1	1	1	2	2	2	9	9
2	2	2	2	2	2	2	2	9	9
2	2	2	2	2	2	2	2	3	3
2	2	2	2	2	2	2	2	2	2
9	9	9	2	9	9	9	9	1	9
9	2	2	3	3	3	2	3	1	3
9	9	9	1	9	9	9	9	1	9
3	1	2	2	1	1	3	3	1	1
3	3	3	3	3	3	3	9	1	1
2	2	2	2	2	2	2	2	2	2
2	2	2	2	2	2	2	2	2	2
9	2	1	3	1	2	1	3	2	2
2	2	2	2	2	2	2	2	2	2
9	9	9	9	3	9	9	9	9	9
2	2	2	2	2	2	2	2	2	2
2	2	2	2	2	2	2	2	1	1
2	2	1	3	3	3	2	3	3	3
1	2	1	2	1	2	1	2	3	2

9	1	9	9	9	9	9	9	9	1
2	2	2	2	2	2	2	2	2	2
1	1	1	1	1	1	1	1	1	1
2	2	2	2	2	2	2	2	2	2
9	9	9	9	9	1	9	9	1	9
1	1	2	2	2	2	2	3	1	1
3	3	3	3	3	3	3	3	3	3
1	1	1	1	1	1	1	1	1	1
2	2	2	2	2	2	2	2	1	1
1	1	1	3	3	3	3	3	1	1
9	9	9	9	9	9	9	9	2	2
1	1	1	1	1	1	1	1	1	1
2	2	2	2	2	2	2	2	2	2
2	2	2	2	2	2	2	2	2	2
2	2	2	2	2	2	2	2	2	2
2	2	2	2	2	2	2	2	2	2
1	1	1	1	1	1	1	1	1	1
1	3	3	3	3	3	3	3	1	1
9	9	9	9	9	9	9	9	9	9
2	3	1	2	3	2	1	2	1	3
1	1	1	1	1	1	1	1	2	2
2	2	2	2	2	2	2	2	1	9
1	1	1	1	1	1	1	9	9	9
2	2	2	2	2	2	2	2	1	1
3	3	3	3	3	3	3	3	3	3
1	1	1	1	1	1	1	1	1	2
1	1	1	2	3	2	1	2	1	1
2	2	2	2	2	2	2	2	2	2
2	2	2	1	1	1	1	2	1	2
2	2	2	2	2	2	2	2	2	2
1	2	2	3	1	1	2	2	1	2
2	3	3	3	3	2	2	2	2	2
3	3	3	3	3	3	3	3	3	3
3	3	3	1	1	1	9	1	1	2
2	2	2	2	2	2	2	2	2	2
3	3	3	3	3	3	2	2	2	3
2	2	2	3	3	3	3	3	1	9
2	2	2	2	2	2	2	2	2	2
3	1	2	3	1	2	3	1	1	2
3	3	3	3	3	3	3	3	3	3
1	1	1	1	3	1	2	1	1	2
2	2	2	2	2	2	2	2	2	2
9	9	9	9	9	9	9	9	9	9
3	3	3	3	3	3	3	3	9	9
9	9	9	9	9	9	9	9	9	9
2	2	2	2	2	2	2	2	2	2
9	3	3	2	1	1	2	3	1	2

3	3	3	3	3	3	3	3	1	1
1	1	1	3	3	3	3	3	1	1
9	9	9	9	9	9	9	9	9	9
1	1	3	3	3	3	3	3	1	1
3	1	2	3	1	2	3	2	1	2
2	2	2	2	2	2	2	2	2	2
2	2	2	2	2	2	2	2	2	2
2	2	2	2	2	2	2	2	2	2
1	1	1	1	1	1	1	1	9	9
1	1	2	1	2	1	2	1	1	2
3	2	3	2	3	2	3	2	2	2
1	3	1	3	2	3	1	3	1	2
2	2	2	2	2	2	2	2	2	2
3	3	2	2	3	2	3	2	1	3
2	2	2	2	2	2	2	2	2	2
2	2	2	2	2	2	2	2	2	2
2	2	2	2	2	2	2	9	2	2
2	2	2	2	2	2	2	2	2	2
2	2	2	2	2	2	2	2	2	2
2	3	1	2	2	1	3	2	2	1
2	2	2	2	2	2	2	1	1	1
2	2	2	2	2	2	2	2	2	2
2	2	2	2	2	2	2	2	9	9
2	2	2	2	2	2	2	2	2	2
1	1	1	1	1	1	1	1	1	1
2	2	2	2	2	2	2	2	9	9
2	3	1	1	1	1	1	1	1	1
2	2	2	2	2	2	2	2	2	2
2	1	2	2	3	2	1	1	2	2
2	2	2	2	1	2	2	2	1	2
1	9	2	1	1	2	2	2	2	2
2	2	3	3	2	2	1	2	9	9
2	2	2	2	2	2	2	2	2	2
2	2	2	2	2	2	2	2	3	2
1	3	2	1	1	2	3	3	1	3
2	2	3	2	2	1	2	1	1	2
9	9	9	9	9	9	9	9	9	9
9	9	9	9	9	9	9	9	9	9
9	2	3	2	2	1	3	1	2	1
2	2	2	2	2	2	2	2	2	2
2	3	2	1	2	1	2	2	2	3
2	2	2	2	2	2	2	2	9	9
2	2	2	2	2	2	2	2	2	2
2	2	2	2	2	2	2	2	2	2
9	9	9	9	9	9	9	9	9	9
1	1	1	1	2	2	1	2	9	9
2	2	2	2	2	2	2	3	2	2

9	9	9	9	9	9	9	9	9	9
9	9	9	9	9	9	9	9	1	2
3	1	2	3	1	2	3	2	1	2
9	9	9	9	9	9	9	9	9	9
1	1	1	1	3	1	3	1	1	3
2	2	3	1	2	1	2	3	1	2
3	2	1	2	3	2	1	3	1	2
9	9	9	9	9	9	9	9	9	9
2	2	2	2	2	2	2	2	2	2
2	2	3	3	2	2	2	3	2	2
2	2	2	2	2	2	2	2	1	2
1	2	2	1	2	3	3	1	1	2
2	2	2	2	2	2	2	2	2	1
2	1	1	1	3	3	3	3	1	1
3	1	2	3	1	2	3	1	1	2
2	2	2	2	2	2	2	2	2	2
1	2	2	3	2	2	3	2	1	2
2	2	2	2	2	2	3	2	2	2
9	9	9	9	9	9	9	9	9	9
9	9	9	9	9	9	9	9	9	9
9	9	9	9	9	9	9	9	9	9
3	2	3	1	2	1	2	1	1	2
2	3	2	1	3	1	3	1	1	1
3	3	2	2	3	1	2	3	1	2
3	1	1	2	3	3	3	1	3	2
3	3	2	2	2	3	3	3	3	3
2	2	1	2	2	2	2	2	1	3
3	3	3	3	3	9	2	2	2	2
3	3	2	2	1	2	1	1	1	1
1	1	1	3	2	3	2	3	1	2
2	1	1	1	1	1	1	1	1	1
1	1	2	2	2	2	3	1	2	1
2	3	3	3	2	3	2	2	2	2
3	3	3	3	3	3	3	3	2	3
2	2	2	2	2	2	2	2	2	2
2	2	2	2	2	2	2	2	2	2
3	3	3	3	3	3	3	3	3	3
2	2	2	2	2	2	2	2	3	3
9	9	9	9	9	9	9	9	9	9
9	9	9	9	9	9	9	9	9	9
3	3	3	3	3	3	3	3	1	2
3	3	2	2	2	2	1	1	2	1
2	3	1	2	3	1	2	3	1	2
2	3	3	2	3	2	2	1	3	1
9	9	9	9	9	9	9	9	9	9
3	9	3	3	3	3	3	3	3	3
2	2	2	1	3	3	2	2	1	2

1	1	1	1	1	1	1	1	1	1
3	2	2	3	3	1	1	2	1	1
9	9	9	9	9	9	9	9	2	1
9	2	9	9	2	9	1	3	2	3
2	3	2	3	1	2	3	2	3	2
3	3	3	3	3	3	3	3	3	3
1	1	1	1	1	1	1	1	1	1
3	3	3	3	3	3	3	3	3	3
2	3	3	3	2	2	2	2	2	2
2	2	1	1	2	2	3	3	1	3
2	2	2	2	2	2	2	2	2	2
2	1	2	3	1	2	3	2	3	1
3	3	3	3	2	2	3	2	2	2
1	1	1	1	1	1	1	1	1	1
1	1	1	1	1	2	2	2	1	1
2	2	3	3	3	3	3	3	2	2
1	1	3	3	3	3	3	1	1	1
3	3	3	3	3	3	3	3	2	3
9	9	9	9	9	9	9	9	9	9
3	1	2	3	1	2	2	1	2	1
1	1	1	1	1	1	1	1	1	2
2	3	2	3	2	3	2	3	2	3
1	1	1	1	1	1	1	1	1	1
2	2	1	3	3	2	1	9	1	2
3	1	2	3	1	2	3	1	1	2
2	3	3	3	3	2	2	3	2	1
2	3	2	2	3	3	3	1	1	1
3	2	1	2	2	3	2	2	1	2
1	2	3	1	1	2	1	2	1	2
1	1	1	3	3	3	3	3	1	3
3	2	3	2	2	3	3	2	1	3
2	2	2	2	2	2	2	2	2	2
2	2	2	2	2	2	2	9	2	2
1	1	1	2	1	2	2	3	1	3
3	1	2	3	1	2	3	1	1	2
3	1	2	1	2	1	2	3	3	3
2	2	1	1	3	3	9	1	1	3
3	3	3	3	3	3	3	3	3	3
1	2	2	3	3	1	9	1	1	1
1	3	2	1	2	3	1	2	2	1
2	2	2	2	2	2	2	2	2	2
3	1	2	1	2	1	2	1	1	2
1	2	1	2	1	3	2	2	1	2
1	1	1	1	1	1	1	1	1	1
2	2	2	9	3	2	2	2	9	9
3	1	2	2	2	1	2	2	1	2
1	1	2	1	1	3	2	1	3	1

2	2	1	2	2	3	3	2	1	1
2	3	2	3	2	1	2	1	1	2
2	2	2	1	1	1	1	2	2	2
1	1	1	1	1	1	1	1	1	1
3	1	2	3	1	2	3	1	1	2
3	1	2	3	2	1	3	2	1	2
3	1	3	2	1	2	3	3	1	2
2	2	1	2	1	1	2	2	1	2
1	2	1	2	1	2	2	1	1	2
3	3	3	3	3	3	3	1	3	2
2	3	2	3	2	2	3	1	1	2
3	2	2	1	3	2	3	1	1	2
2	1	1	1	2	1	3	3	1	1
3	3	1	1	3	3	1	1	1	1
2	1	2	3	2	1	2	2	2	2
3	3	3	3	3	3	1	3	1	1
1	1	3	3	3	2	3	2	1	1
2	1	1	2	1	1	1	3	1	1
1	3	2	2	2	1	1	2	2	1
1	2	2	2	3	3	2	1	1	1
1	1	3	2	2	3	1	1	1	2
3	3	3	1	3	3	1	3	1	1
3	1	3	2	3	1	9	1	1	2
1	1	2	1	2	1	1	1	1	2
2	2	2	2	2	2	2	2	2	2
2	2	2	2	2	2	2	2	2	2
2	2	1	1	3	3	2	3	1	2
1	2	3	2	1	3	2	1	2	1
2	2	2	2	2	2	2	2	2	2
9	9	9	9	9	9	9	9	9	9
9	9	9	9	9	9	9	9	9	9
9	9	9	9	9	9	9	9	9	9
2	2	3	3	3	3	3	2	1	2
9	9	9	9	9	9	9	9	9	9
2	2	2	2	2	2	2	2	9	9
9	9	9	9	9	9	9	9	9	9
2	2	2	1	2	2	2	3	2	2
1	9	9	9	9	9	9	9	3	9
2	3	3	3	1	1	2	1	2	2
2	2	3	3	3	3	3	2	2	2
9	9	9	9	9	9	9	9	9	9
1	1	1	1	1	1	1	3	2	1
2	3	2	1	2	3	2	1	3	3
1	3	2	1	1	2	3	3	1	2
2	1	3	2	2	3	1	1	1	3
3	2	1	2	3	1	3	2	1	2
2	2	1	1	3	3	2	3	1	2

1	2	2	2	2	2	2	1	2	2
1	2	2	2	2	2	2	1	1	2
9	9	9	9	9	9	9	9	9	9
1	1	1	2	1	1	1	3	1	2
9	9	9	3	9	2	2	2	1	9
2	1	2	1	2	3	1	1	1	1
9	9	1	9	9	9	9	9	9	9
1	2	3	1	2	3	2	2	2	2
2	1	2	3	3	3	2	3	1	2
3	3	3	3	3	3	3	9	3	3
3	1	2	3	1	2	3	2	2	3
2	3	1	2	1	2	1	3	1	3
1	1	3	3	1	1	3	1	1	3
2	2	2	2	2	2	2	2	2	2
2	2	2	2	2	2	2	2	2	2
9	9	1	9	9	9	9	1	9	9
2	2	2	2	2	2	2	2	2	2
9	2	1	2	1	3	2	2	2	2
2	2	2	2	2	2	2	2	2	2
2	2	2	2	2	2	2	2	2	2
1	9	1	9	9	9	9	9	1	9
1	9	1	9	9	9	9	9	1	9
1	9	1	9	9	9	9	9	1	9
1	3	1	3	3	3	3	3	1	3
1	3	1	3	3	3	3	3	1	3
9	9	1	3	3	3	3	3	1	9
1	2	1	3	3	3	2	3	1	2
1	2	1	3	2	3	2	3	1	2
1	2	1	3	2	3	2	3	1	2
1	1	1	1	1	1	1	9	9	9
9	9	9	9	9	9	9	9	9	9
1	9	9	9	9	9	9	3	9	3
1	3	1	3	3	3	3	3	1	3
1	3	1	3	3	2	3	3	1	3
1	1	3	9	9	9	9	9	1	2
1	1	3	2	2	2	2	3	1	3
1	1	3	3	2	2	2	3	1	3
1	9	1	9	9	9	9	9	9	9
1	1	1	1	1	3	3	9	1	3
1	1	3	3	3	3	3	3	1	1
1	3	1	3	3	3	3	3	1	3
9	9	1	9	9	9	9	9	1	9
1	1	1	1	1	1	1	1	1	3
1	3	1	3	3	3	3	3	1	3
2	2	2	3	3	3	2	2	1	1
1	3	1	3	3	3	3	3	1	3
1	3	2	3	3	3	3	2	3	2

1	1	3	2	2	2	2	3	1	3
1	1	1	3	1	2	2	2	1	2
1	3	1	3	3	3	3	3	1	3
1	1	1	1	1	1	1	1	1	1
1	3	1	3	1	3	3	3	1	1
1	3	1	3	1	3	3	3	1	1
1	2	2	1	2	2	2	2	1	1
1	1	1	1	1	2	2	2	1	2
9	9	9	9	9	9	9	9	1	1
1	3	1	3	3	3	3	3	1	3
1	1	2	2	1	2	3	2	1	1
1	2	2	2	2	2	2	2	2	2
1	3	1	3	1	3	3	3	1	1
1	3	1	3	1	3	3	3	1	1
1	3	1	3	1	3	3	3	1	1
1	3	1	3	1	3	3	1	1	1
1	3	1	3	1	3	3	3	1	1
9	1	2	1	1	1	3	1	1	1
1	3	1	3	1	3	3	3	1	1
2	3	2	3	1	3	2	2	3	2
2	9	3	3	3	3	3	3	1	9
2	3	2	2	3	3	3	2	3	3
1	2	1	3	2	3	3	3	1	1
1	3	1	3	3	3	3	3	2	3
1	1	1	1	1	2	1	3	1	3
1	1	1	1	1	1	1	3	1	1
3	3	3	3	3	3	3	3	1	1
1	2	2	2	2	2	2	3	1	2
1	3	1	3	1	3	2	3	1	2
1	3	1	3	1	3	3	3	1	1
1	1	2	2	3	2	2	1	2	2
1	1	1	3	3	3	3	3	1	3
9	9	9	9	9	9	9	9	9	9
1	3	1	3	3	3	3	3	1	3
1	2	1	3	1	3	3	3	1	1
1	3	1	3	3	3	3	3	1	3
1	1	1	1	1	3	2	9	1	1
1	3	1	3	1	3	3	3	1	3
1	3	1	3	3	3	3	3	1	3
1	1	1	3	3	3	3	3	1	3
1	3	1	3	3	3	3	3	1	3
1	3	2	3	3	3	3	3	1	3
1	1	1	1	1	1	1	3	1	3
1	3	1	1	3	3	1	3	1	3
1	2	1	3	3	3	3	3	9	9
3	1	1	3	3	3	3	3	1	3
3	1	3	3	3	3	3	3	1	3

1	3	1	3	1	3	3	3	1	1
1	1	1	3	3	3	3	3	1	3
1	3	1	1	2	3	3	3	1	1
1	1	1	1	3	1	2	2	3	1
1	3	1	3	3	3	3	3	1	3
1	1	1	1	1	1	1	1	3	1
9	9	9	9	9	9	9	9	1	9
1	1	9	3	3	3	3	3	1	3
1	1	1	3	3	3	3	3	1	3
1	1	3	1	3	3	3	3	1	3
1	3	1	3	3	3	3	3	1	3
9	9	9	9	9	9	9	9	1	9
1	1	1	3	3	3	3	3	1	3
1	3	1	3	3	2	3	3	1	2
3	3	3	3	3	3	3	3	2	2
1	3	1	3	3	3	3	3	1	3
9	9	9	9	9	9	9	9	1	9
9	9	9	9	9	9	9	9	1	9
1	1	1	2	2	2	2	3	2	2
2	2	2	2	2	2	2	9	3	3
1	2	1	3	1	3	1	3	1	3
9	9	9	9	9	9	9	9	9	9
1	2	1	3	2	3	2	1	1	1
1	2	1	3	3	3	2	3	1	3
3	3	1	1	1	1	1	1	1	2
3	3	1	1	1	1	1	1	3	3
1	1	1	3	1	2	1	2	2	1
2	2	2	2	2	2	2	2	2	2
1	1	1	3	3	3	3	3	1	3
1	2	1	2	2	2	2	3	1	3
1	2	1	2	2	2	2	3	1	3
1	9	3	1	2	1	2	2	2	1
9	1	9	3	3	2	2	3	1	3
1	1	2	3	3	3	3	3	1	2
3	3	3	2	2	1	1	1	1	1
1	1	1	3	3	2	2	3	1	1
1	3	3	3	3	3	3	3	2	2
1	1	1	1	3	3	3	3	1	1
1	2	3	3	3	3	3	3	1	1
2	3	1	1	1	3	3	3	1	3
1	1	1	3	3	3	3	3	1	3
1	1	3	3	3	3	3	3	1	3
1	3	1	3	1	3	3	3	1	1
1	1	1	1	1	1	1	9	1	2
1	3	9	1	9	3	3	3	2	1
1	1	3	3	3	3	3	3	1	3
9	3	1	3	1	3	3	3	1	3

1	3	1	3	3	3	3	3	1	3
1	1	1	2	2	3	2	3	1	2
1	1	2	3	1	2	3	3	1	3
2	2	2	2	2	2	2	3	1	2
1	3	3	1	1	2	3	1	1	2
1	3	3	3	2	2	2	2	1	2
9	1	9	9	9	9	9	9	1	9
9	9	9	9	9	9	9	9	2	2
9	9	9	9	9	9	9	9	9	9
1	1	3	3	3	3	3	3	1	1
1	2	1	2	2	2	3	3	1	2
1	3	1	3	3	3	3	3	1	3
2	2	2	2	2	2	2	2	2	2
1	2	3	2	2	3	1	2	1	2
9	1	2	1	2	2	2	2	2	1
9	9	1	9	9	9	9	9	1	9
1	3	1	3	3	3	3	3	1	3
1	1	1	1	1	2	2	2	1	1
1	3	1	3	3	3	3	3	1	3
1	3	1	3	3	3	3	3	1	3
9	1	9	9	9	9	9	9	1	9
1	2	1	2	2	2	1	2	1	3
2	2	2	2	3	2	2	9	9	9
9	3	1	3	3	3	3	3	1	3
1	1	2	3	1	1	3	3	1	2
1	1	2	3	1	1	3	3	1	2
1	3	1	3	1	3	2	3	1	1
9	9	9	9	9	9	9	9	9	9
9	9	9	9	9	9	9	9	9	9
1	3	1	2	3	3	1	3	1	2
1	3	1	3	3	3	3	3	1	3
1	3	1	3	3	3	3	3	1	3
9	9	9	9	9	9	9	9	9	9
9	9	9	9	9	9	9	9	1	3
1	3	1	3	3	3	3	3	1	3
1	1	1	2	1	3	3	3	2	2
1	3	1	3	3	3	3	3	1	3
1	9	1	2	2	1	1	2	1	2
3	1	1	3	3	3	3	3	1	3
1	3	1	3	3	3	3	3	1	3
1	2	1	3	3	3	2	3	1	2
1	2	1	3	3	3	3	3	1	3
1	3	1	3	3	3	3	3	1	3
9	9	9	9	9	9	9	9	9	9
1	1	1	3	1	3	3	3	3	3
1	3	1	3	3	3	3	3	1	3
1	1	3	3	3	3	3	3	1	3

1	9	1	9	9	9	9	9	1	9
1	2	9	1	1	1	3	2	3	1
9	9	9	9	9	9	9	9	9	9
1	1	1	1	1	2	1	1	1	3
1	9	1	1	1	9	9	9	9	9
1	2	2	1	2	3	3	3	1	2
1	1	1	2	1	3	2	3	1	2
1	2	2	3	2	3	3	3	1	1
2	9	9	9	9	9	9	9	9	9
1	2	1	2	3	3	3	3	1	2
1	1	1	1	1	1	1	3	1	1
2	2	2	2	2	2	2	3	1	2
1	3	2	3	3	3	3	3	3	1
1	1	1	9	1	9	9	9	1	3
2	2	3	1	1	2	1	2	1	1
1	3	1	3	1	3	3	3	1	3
9	9	9	9	9	9	9	9	9	9
1	9	1	9	3	9	9	9	1	9
1	1	1	1	3	3	3	3	1	1
2	2	2	2	2	2	2	2	2	2
2	3	1	2	3	1	2	3	1	3
1	3	1	1	3	3	1	3	1	3
1	3	1	1	3	3	1	3	1	3
1	3	1	3	3	3	3	3	1	3
1	2	3	3	3	3	3	3	1	1
1	1	1	1	1	1	1	2	1	1
1	1	2	3	3	3	3	3	1	3
1	1	1	2	2	3	2	3	1	2
1	1	1	1	1	1	1	3	1	1
1	1	1	1	1	1	1	1	1	1
1	3	1	3	3	3	3	3	3	3
1	2	1	2	3	2	3	3	3	3
1	1	2	2	2	3	3	2	1	2
1	1	1	3	3	3	3	3	1	3
1	2	1	3	3	3	3	3	1	3
1	1	2	2	1	3	3	3	1	2
1	3	1	3	3	3	3	3	1	3
1	1	1	9	9	9	9	9	9	9
1	1	2	1	2	1	1	2	1	3
1	1	1	1	1	1	1	1	1	1
3	2	1	2	3	2	2	2	1	2
3	1	1	1	1	3	2	3	9	9
9	9	9	9	9	9	9	9	1	9
9	9	9	9	9	9	9	9	1	9
1	1	2	2	3	2	3	3	1	3
1	1	9	2	1	1	2	3	1	3
1	9	9	9	9	9	9	9	1	9

1	3	3	3	3	3	3	3	1	3
1	1	1	3	3	3	3	3	1	3
1	9	1	9	1	9	9	9	1	2
1	3	1	3	1	3	3	3	1	3
1	1	1	3	3	3	3	3	1	3
1	2	1	3	3	3	3	3	1	9
2	2	2	2	2	2	2	2	2	2
1	2	2	2	2	2	2	2	1	1
9	9	9	9	9	9	9	9	9	9
1	1	1	2	1	3	3	3	1	2
1	3	1	3	3	3	3	3	1	3
9	9	9	9	9	9	9	9	9	1
9	9	9	9	9	9	9	9	9	9
9	9	9	9	9	9	9	9	1	9
9	9	9	9	9	9	9	9	1	9
9	9	9	9	9	9	9	9	1	9
1	2	2	2	3	3	2	3	3	3
3	3	2	1	2	3	2	1	1	2
1	3	1	3	3	3	3	3	1	2
1	2	2	1	2	1	2	2	1	2
1	9	1	3	1	2	3	3	1	3
1	1	1	3	3	3	1	3	1	3
1	2	2	1	1	3	1	3	1	2
1	1	2	9	2	2	3	2	1	2
1	2	1	3	2	3	3	3	1	3
1	1	1	3	1	3	3	3	1	3
1	3	1	3	3	3	2	3	1	3
1	3	1	3	3	3	3	3	1	3
1	2	3	3	3	3	9	3	1	3
1	3	1	3	3	3	3	3	1	3
1	2	1	2	3	3	2	2	1	2
1	9	1	9	1	9	9	9	1	9
1	3	1	3	1	3	3	3	1	3
1	3	1	1	1	3	3	3	1	1
1	1	2	3	1	3	2	3	1	2
1	1	1	3	1	3	3	3	1	3
1	3	2	3	2	3	3	3	1	3
1	3	2	2	2	3	3	3	2	2
9	9	9	9	9	9	9	9	9	9
1	1	1	3	3	3	3	3	9	9
3	2	2	2	1	2	1	2	2	1
3	2	2	2	1	2	1	2	2	1
1	2	2	3	3	3	3	3	1	2
1	2	1	2	1	2	3	3	1	2
2	2	1	1	2	3	3	3	1	2
1	1	2	3	3	3	3	3	1	3
1	3	1	1	1	3	3	3	1	1

1	3	1	3	3	3	3	3	1	3
1	2	1	3	2	3	3	3	1	3
1	3	1	1	1	3	3	3	1	1
1	3	1	3	3	3	3	3	1	3
2	3	3	2	2	3	2	3	1	3
1	1	2	2	2	2	3	3	1	2
1	2	1	2	2	1	2	2	1	2
1	2	3	2	1	3	3	3	1	2
2	2	2	2	2	3	2	9	9	9
1	1	1	1	1	3	3	2	1	3
1	2	2	2	2	2	2	2	1	2
2	2	2	2	2	2	2	2	2	2
1	3	1	1	1	3	3	3	1	3
1	1	3	1	1	3	2	3	1	1
1	2	2	2	2	2	2	2	1	1
1	1	1	3	1	3	2	3	1	1
1	1	2	2	2	2	2	2	2	2
2	2	2	2	2	2	2	2	1	2
2	2	2	2	2	2	2	2	2	2
1	3	1	3	3	3	3	3	1	3
1	3	1	3	3	3	3	3	1	3
1	2	1	3	2	3	3	3	1	9
1	3	1	3	1	2	3	3	1	2
1	3	1	3	3	3	1	3	2	3
1	1	2	3	3	3	3	3	1	3
9	9	9	9	9	9	9	9	9	9
9	9	9	9	9	9	9	9	9	9
1	9	9	9	9	9	9	9	9	9
1	9	9	9	9	9	9	9	1	3
2	9	2	3	3	3	9	3	3	9
1	3	1	3	3	3	3	3	1	3
1	3	3	3	3	3	3	3	1	3
9	9	9	9	9	9	9	9	1	9
9	9	9	9	9	9	9	9	1	9
9	9	9	9	9	9	9	9	1	9
9	9	9	9	9	9	9	9	1	9
9	9	9	9	9	9	9	9	1	9
9	9	9	9	9	9	9	9	1	9
9	9	9	9	9	9	9	9	9	9
1	2	3	3	2	3	3	3	1	2
1	2	1	3	2	3	3	3	1	3
9	9	9	9	9	9	9	9	9	9
9	9	9	9	9	9	9	9	9	9
1	1	1	3	3	3	2	3	1	3
9	9	9	9	9	9	9	9	1	9
9	9	9	9	9	9	9	1	1	9
9	9	9	9	9	9	9	1	1	9

1	1	1	2	3	3	3	3	1	3
9	9	9	9	9	9	9	9	1	3
1	9	9	9	9	9	9	9	9	9
3	1	1	3	1	3	3	3	1	3
3	1	1	3	1	3	3	3	1	3
9	9	9	9	9	9	9	1	1	9
9	9	9	9	9	9	9	9	1	9
9	9	9	9	9	9	9	9	1	9
9	9	1	9	9	9	9	9	1	9
9	9	9	9	9	9	9	9	1	9
1	3	1	3	1	3	3	3	1	1
1	3	1	3	1	3	3	3	1	3
1	3	1	1	3	3	3	3	1	3
1	9	1	3	3	3	9	3	1	3
1	3	1	3	1	3	3	3	1	3
1	1	3	1	3	3	1	3	1	3
1	1	1	3	1	3	1	3	1	3
1	1	1	3	1	3	1	3	1	3
1	3	1	3	1	3	3	3	1	3
1	1	1	1	1	2	3	3	1	1
1	1	1	1	1	2	3	3	1	1
2	2	2	2	2	2	2	1	1	1
9	1	9	9	9	9	9	9	9	9
1	1	1	3	1	1	3	3	1	1
3	3	3	2	2	2	2	1	2	2
1	1	2	1	1	1	1	3	1	1
3	9	2	3	3	3	2	3	2	3
1	3	1	1	3	3	3	3	1	1
1	1	1	1	1	3	3	3	1	1
1	1	1	3	3	3	3	2	2	2
1	3	3	3	3	3	3	3	2	3
1	3	1	1	3	3	3	3	3	3
1	1	1	3	1	3	1	3	1	3
1	2	1	1	1	3	3	3	1	2
1	1	1	3	3	3	3	3	1	3
1	3	1	3	1	3	3	3	1	3
1	3	1	3	3	3	3	3	1	3
1	3	1	3	3	3	3	3	1	3
1	3	1	3	1	3	1	3	1	3
2	3	1	3	1	3	1	3	1	2
1	3	1	3	1	3	3	3	3	9
1	3	1	3	1	3	3	3	1	2
1	3	3	3	2	2	2	2	1	1
1	3	1	3	1	3	3	3	1	3
1	3	1	9	1	3	3	3	1	3
1	3	1	1	1	3	1	3	1	1
1	3	1	3	3	3	3	3	1	3

1	3	3	1	1	3	3	3	1	3
1	3	1	1	1	1	3	3	1	1
2	3	3	3	2	2	2	2	9	9
1	3	1	3	3	3	3	3	1	2
1	3	1	3	1	3	3	3	1	2
1	3	1	3	3	3	3	3	1	2
1	3	1	3	3	3	2	3	1	3
1	3	1	3	2	3	3	3	1	3
1	3	1	3	1	3	3	3	1	3
1	3	3	1	1	1	1	1	1	2
1	2	1	3	1	3	3	3	1	3
1	3	1	3	3	3	3	3	9	9
1	1	1	3	3	3	3	3	1	1
1	1	1	3	3	3	3	3	1	1
3	3	1	3	3	3	3	3	1	3
2	2	2	2	2	2	2	2	2	2
2	2	2	2	2	2	2	2	2	2
2	2	2	2	2	2	2	9	1	9
2	1	1	1	3	1	3	1	3	2
1	3	1	3	1	3	3	3	1	3
1	3	1	3	3	3	3	3	1	3
1	1	1	3	3	3	3	3	1	3
3	3	1	3	3	3	3	3	1	2
2	2	1	2	2	2	3	2	1	2
2	2	1	2	2	2	3	2	1	2
3	3	2	3	3	2	1	3	1	3
1	3	1	2	1	3	3	3	1	2
2	2	2	2	2	2	2	3	2	2
1	3	1	1	3	3	3	3	1	3
1	3	1	2	2	2	3	3	1	2
1	2	2	2	2	2	2	2	2	3
1	3	1	1	3	2	2	3	1	2
1	2	1	1	1	3	3	3	1	2
1	2	9	9	9	9	9	9	9	9
1	3	1	3	3	3	3	3	1	2
1	3	1	3	3	3	3	3	1	2
2	2	2	2	2	2	2	2	2	2
2	2	2	2	2	2	2	2	2	2
1	3	1	3	3	3	1	3	1	1
9	2	2	1	3	3	2	3	1	3
1	2	1	3	3	3	3	3	1	3
3	1	1	3	3	3	3	3	1	3
3	1	1	3	3	3	3	3	1	3
2	2	2	2	2	2	2	2	1	3
1	3	1	3	3	3	3	3	1	3
1	3	1	2	2	3	3	3	1	2
1	3	1	3	3	3	1	3	1	3

2	2	2	2	2	2	2	2	1	2
1	3	1	1	2	3	1	2	1	2
1	1	3	2	1	3	3	3	1	1
1	1	1	2	1	2	2	3	1	2
1	2	1	3	3	3	2	3	1	2
1	2	1	3	3	3	3	3	3	3
1	3	1	3	3	3	1	3	1	3
1	3	1	2	2	3	3	3	1	2
2	1	2	2	2	2	2	2	1	2
1	2	2	1	2	3	3	3	1	3
1	2	1	2	1	2	2	2	1	2
2	3	1	3	3	3	3	3	1	3
9	9	9	9	9	9	9	9	1	9
1	1	1	3	3	3	3	3	1	3
1	3	1	1	1	3	3	3	1	3
2	2	2	2	2	2	2	2	2	2
1	2	1	3	2	3	2	3	1	3
1	3	1	3	3	3	3	3	1	3
1	3	1	3	3	3	3	3	1	3
1	2	1	3	3	3	3	3	1	3
1	9	1	3	1	3	3	3	1	3
1	1	1	1	1	2	3	3	1	1
1	3	1	3	3	3	3	3	1	3
1	3	1	3	2	3	3	3	9	9
1	2	2	1	2	2	3	3	1	2
1	2	1	3	3	3	3	3	1	3
1	3	1	3	3	3	3	3	1	3
1	2	3	3	2	3	3	3	1	2
1	1	1	3	3	3	2	3	1	3
9	9	9	9	9	9	9	9	1	9
2	2	3	1	2	2	3	3	9	9
9	9	9	9	9	9	9	9	9	9
1	1	2	2	2	2	2	3	1	2
1	2	1	3	3	3	3	3	1	3
1	2	1	1	1	2	3	3	1	1
1	1	1	2	2	2	3	3	1	3
2	2	2	2	1	1	2	2	2	2
1	9	9	9	9	9	9	2	9	9
2	2	1	3	2	3	1	2	2	3
1	2	2	3	3	3	3	3	1	3
1	1	1	3	3	3	1	3	1	9
1	3	1	1	3	3	3	3	1	3
1	2	1	2	2	3	3	3	1	2
9	1	9	9	1	9	2	9	1	3
2	2	2	2	2	2	2	2	2	2
2	2	2	2	2	2	2	2	9	9
1	3	9	1	1	3	3	3	1	1

1	1	2	2	2	3	3	3	1	2
1	1	2	2	1	2	2	2	1	2
2	2	2	2	2	2	2	2	1	2
1	2	2	2	2	2	3	2	1	2
2	2	3	1	3	2	3	2	2	2
1	1	1	3	3	3	3	3	1	3
1	1	2	1	1	3	1	3	1	1
9	9	1	9	9	9	9	9	9	9
1	2	2	1	1	3	1	3	1	1
1	1	2	2	2	3	3	3	1	1
1	1	1	1	1	1	1	3	1	2
2	3	2	2	1	3	2	2	3	3
2	1	2	1	2	3	1	2	1	3
2	2	2	2	2	2	2	2	2	2
1	1	2	1	3	3	3	3	1	3
1	1	2	1	3	3	3	3	1	3
1	3	1	3	3	3	1	3	1	3
1	3	1	3	3	3	3	3	1	3
9	9	9	9	9	9	9	9	9	9
1	9	1	2	2	3	3	3	1	3
1	1	2	2	2	3	3	3	1	3
1	1	1	2	1	1	1	1	1	1
3	2	2	2	3	3	3	3	3	3
1	1	1	3	3	3	3	3	1	3
1	2	1	3	3	3	3	3	1	3
1	1	1	1	1	1	1	2	1	2
1	1	1	3	3	3	3	3	1	3
1	3	1	3	1	3	3	3	1	3
1	3	1	3	1	3	3	3	1	3
2	9	9	9	9	9	9	1	1	9
1	3	1	3	1	3	3	3	1	3
1	1	2	2	2	1	3	3	1	1
1	1	2	2	2	1	3	3	1	1
1	3	1	2	3	3	3	3	1	2
9	9	9	9	9	9	9	9	2	1
1	2	1	1	1	2	1	9	1	2
1	2	1	2	1	2	2	2	1	2
9	9	9	9	9	9	9	9	9	9
1	3	1	3	3	3	3	3	1	1
1	1	1	1	1	3	3	3	1	3
1	3	1	3	3	3	3	3	1	3
1	3	1	3	3	3	3	3	1	3
2	2	2	2	2	2	2	2	2	2
3	3	1	3	3	3	3	3	1	3
1	3	3	3	1	3	1	3	1	1
3	1	1	3	1	3	3	3	1	3
1	1	1	3	1	3	3	3	1	1

1	1	1	1	1	3	3	3	1	1
1	1	1	1	1	3	3	3	1	1
1	3	1	3	3	3	3	3	1	3
1	2	1	2	2	2	2	2	1	1
1	1	1	1	1	3	3	3	1	1
1	9	1	1	1	3	3	3	1	1
1	3	1	3	1	3	3	3	1	1
9	9	9	9	9	9	9	9	9	9
1	1	1	3	3	3	3	3	1	1
1	9	9	9	9	9	9	9	1	3
1	3	1	3	3	3	3	3	1	3
1	3	1	3	3	3	3	3	1	3
1	3	1	3	3	3	3	3	1	3
9	9	9	9	9	9	9	9	9	9
3	3	1	3	1	3	3	3	1	3
2	3	1	3	3	3	3	3	1	3
1	2	2	1	1	2	1	1	1	2
1	2	1	1	1	1	2	3	1	2
1	3	1	3	1	3	3	3	1	3
9	9	9	9	9	9	9	9	9	9
2	1	2	2	1	1	1	1	1	2
1	3	1	3	3	3	3	3	1	3
3	1	1	1	1	3	3	3	1	3
3	1	1	1	1	3	3	3	1	3
1	9	9	9	9	9	9	9	1	9
9	9	1	9	9	9	9	9	1	9
1	1	1	3	2	3	3	3	1	3
9	9	9	9	9	9	9	9	1	9
1	9	9	9	9	9	9	9	9	9
1	9	9	9	9	9	9	9	9	9
1	9	9	9	9	9	9	9	1	9
1	9	9	9	9	9	9	9	1	9
9	9	9	9	9	9	9	9	1	9
1	9	9	9	9	9	9	9	9	9
9	9	9	9	9	9	9	9	9	9
1	1	2	1	1	3	3	3	1	1
1	2	2	2	2	2	2	2	1	3
1	3	1	3	3	3	3	3	1	3
1	1	1	3	3	3	3	3	1	3
1	1	1	3	3	3	3	3	1	3
1	3	1	3	3	3	3	3	9	9
1	1	1	2	2	2	3	3	1	3
1	1	2	1	1	2	2	3	1	2
2	2	2	2	2	2	2	2	1	3
1	2	2	3	2	2	3	1	2	2
1	3	1	3	3	3	3	3	1	3
1	2	1	2	2	3	3	3	1	3

2	2	2	2	2	2	2	2	1	1
2	2	2	2	2	2	2	2	2	2
1	2	1	2	2	1	1	1	1	3
1	2	3	1	3	2	3	2	1	3
1	1	1	2	1	2	1	1	1	1
1	1	1	1	1	1	1	3	1	3
9	9	9	9	9	9	9	9	9	9
2	2	2	2	2	2	2	2	2	2
3	2	2	2	1	3	3	3	1	1
2	3	2	3	2	3	3	2	2	2
1	1	1	1	1	2	3	1	1	2
1	3	1	2	3	3	3	3	1	3
1	2	3	2	2	2	3	2	1	1
3	3	3	3	3	2	2	1	3	3
2	2	2	2	2	2	2	2	2	2
1	1	2	3	1	3	1	3	1	1
9	9	9	9	9	9	9	9	9	9
2	1	2	1	2	1	2	3	1	1
1	2	2	2	2	2	2	3	1	3
1	1	1	1	1	1	1	1	1	1
1	2	1	2	1	2	1	2	1	1
9	1	2	2	2	1	1	1	2	2
2	3	3	3	2	2	1	1	2	1
1	1	1	1	2	2	3	3	1	2
1	1	1	1	1	1	1	1	1	1
2	2	2	2	2	2	3	3	1	2
1	2	3	1	2	2	2	1	3	2
9	1	2	1	1	2	1	2	1	1
2	2	2	2	2	2	2	2	2	2
2	2	2	2	2	2	2	2	1	2
9	3	1	3	2	3	1	3	1	3
1	1	1	1	1	1	1	3	1	2
2	2	1	2	2	2	1	1	2	2
1	1	1	2	2	2	2	3	1	2
1	9	9	9	9	9	9	9	1	9
1	1	1	1	1	1	1	1	1	1
2	2	2	2	2	2	2	2	2	2
9	9	9	9	9	9	9	9	9	9
3	9	2	1	9	2	3	1	1	1
1	1	1	1	1	1	1	1	1	1
1	2	1	2	2	3	3	3	1	2
1	2	1	2	2	3	3	3	1	2

KnowMore	HPVVaccin	HPVVaccin	Embarrass	TooBusyfo	HPVVaccin	HPVAgains	CervCA2	CervScreer	PapSmear2
2	2	2	2	2	2	2	2	2	2
2	2	2	2	2	2	2	2	2	2
2	2	2	2	2	3	2	2	2	2
2	2	2	2	2	2	2	2	2	2
2	2	3	2	2	2	3	2	1	1
3	3	3	3	3	3	2	2	2	2
3	2	3	2	1	3	2	2	2	2
2	1	1	2	2	2	2	1	2	2
3	3	3	3	1	1	1	2	2	1
1	2	3	2	1	2	2	2	2	1
2	2	2	2	2	2	2	2	2	2
2	1	2	2	3	2	3	2	1	2
9	2	2	2	3	3	3	2	1	1
2	2	2	3	2	2	2	2	2	2
9	9	9	9	9	9	9	2	2	2
2	2	2	2	2	2	2	2	1	2
9	9	9	9	9	9	9	#NULL!	#NULL!	#NULL!
2	2	2	1	1	1	1	2	1	2
9	9	9	9	9	9	9	#NULL!	#NULL!	#NULL!
9	9	9	9	9	9	9	1	2	2
1	3	1	3	3	3	3	1	1	1
2	2	2	2	2	2	3	1	1	1
1	1	1	2	1	2	2	1	1	1
9	1	2	1	2	1	1	1	1	1
9	9	2	9	9	9	9	2	2	2
1	3	3	2	2	1	1	#NULL!	#NULL!	#NULL!
9	1	1	1	3	3	3	1	1	1
1	2	2	1	3	3	3	1	1	1
3	3	2	1	1	2	3	2	2	2
1	2	3	1	1	2	1	1	2	2
1	1	2	1	2	3	3	2	1	1
1	2	1	2	1	3	3	1	1	2
1	1	1	1	1	1	1	2	2	2
2	1	1	1	2	1	9	1	1	2
2	1	2	1	2	2	1	2	2	1
3	9	2	2	3	3	3	1	1	2
2	2	2	2	9	2	2	2	1	1
1	1	1	1	1	2	3	2	2	2
1	1	2	3	3	3	3	1	1	1
3	2	3	2	1	1	2	#NULL!	#NULL!	#NULL!
1	2	1	2	3	3	3	#NULL!	#NULL!	#NULL!
3	9	2	1	9	1	2	1	2	2
9	9	9	9	9	9	9	2	2	2
2	2	2	2	2	2	2	1	2	2
2	3	2	2	2	2	2	2	2	2
1	2	3	2	1	3	1	#NULL!	#NULL!	#NULL!

2	2	3	2	2	1	1	1	1	1
2	1	2	2	2	3	3	2	1	1
2	2	1	2	2	2	2	1	2	1
1	2	2	2	1	1	2	1	1	2
1	3	3	1	3	3	3	2	2	1
1	3	3	1	3	3	3	2	2	1
2	3	2	3	2	3	2	2	2	2
1	1	1	3	3	3	3	2	2	2
1	9	1	1	9	1	2	2	1	1
9	9	1	3	9	9	1	2	2	1
9	9	9	9	9	9	3	#NULL!	#NULL!	#NULL!
2	2	3	3	2	2	1	1	2	1
1	1	1	1	1	1	1	2	2	2
2	2	2	3	3	2	2	1	2	1
2	2	2	2	2	3	3	2	1	1
2	2	2	3	3	3	2	2	1	1
2	3	2	3	3	2	2	2	2	2
1	1	2	1	1	3	3	2	2	2
1	2	1	2	3	3	3	2	2	2
3	3	3	3	3	3	3	#NULL!	#NULL!	#NULL!
1	1	2	2	2	2	2	1	2	2
1	3	2	2	2	2	3	2	2	2
2	2	1	1	2	2	1	2	1	1
2	2	3	1	2	2	2	1	1	2
2	1	2	2	2	2	2	1	2	2
3	2	1	2	2	3	1	2	2	2
1	9	3	3	1	9	3	2	2	1
3	1	2	3	3	2	1	2	2	1
9	1	2	1	3	3	2	2	2	2
1	3	3	3	3	3	3	2	1	2
9	9	9	9	9	9	9	#NULL!	#NULL!	#NULL!
1	3	3	2	3	3	3	2	2	2
1	3	2	2	3	9	3	2	2	2
1	1	2	2	2	2	2	2	2	2
1	2	3	2	9	2	3	2	2	2
2	2	2	2	2	2	2	2	2	2
1	3	1	2	3	2	3	1	1	2
2	1	2	2	3	2	2	1	2	2
2	2	2	2	2	2	9	2	2	2
2	2	3	2	1	1	2	1	2	2
1	2	1	3	1	2	3	2	2	1
9	9	9	9	9	9	9	#NULL!	#NULL!	#NULL!
1	3	2	1	3	2	1	2	2	2
1	2	2	1	1	1	2	1	2	2
1	1	1	1	2	2	3	2	2	2
1	2	3	1	2	1	2	1	1	1
1	1	1	2	2	3	3	2	2	2

3	1	2	3	1	2	1	1	2	2
2	3	3	2	3	3	1	2	2	2
1	2	3	1	1	2	3	2	2	2
1	2	2	1	1	1	1	1	2	2
1	2	1	2	2	2	2	1	2	1
2	1	2	2	2	2	2	2	2	2
1	1	2	2	2	2	3	1	2	1
9	9	9	9	9	9	9	#NULL!	#NULL!	#NULL!
1	2	1	3	3	2	2	2	2	2
1	2	2	1	2	2	3	1	2	1
9	9	9	9	9	9	9	1	1	#NULL!
1	1	2	2	2	2	1	1	2	1
2	3	2	2	2	2	2	1	1	2
1	2	1	3	3	3	3	2	2	2
3	2	2	3	3	2	3	1	2	2
3	2	2	3	3	2	3	1	#NULL!	2
1	2	2	1	2	2	1	2	1	1
1	1	3	1	2	1	3	2	2	2
1	9	1	3	3	1	3	2	#NULL!	2
1	2	2	1	1	1	1	2	2	2
1	3	1	3	1	3	1	1	2	2
1	1	2	3	2	3	3	2	2	1
2	2	2	1	2	1	1	2	1	1
2	1	2	2	2	2	2	1	1	1
2	2	2	2	3	2	2	2	2	2
1	1	1	2	2	2	3	2	2	2
1	3	1	3	3	3	3	1	1	2
2	1	2	1	2	2	9	1	1	1
2	1	1	2	1	2	1	2	2	2
1	1	3	3	1	3	3	1	1	2
3	2	2	2	1	2	2	1	2	1
3	1	2	3	1	2	2	2	2	2
2	1	2	2	3	2	2	2	2	2
1	2	1	2	1	1	2	1	1	2
1	2	2	1	2	3	9	2	2	2
1	1	1	9	1	1	1	1	1	2
2	2	2	3	3	3	3	2	2	2
2	1	2	2	1	2	2	2	1	1
9	9	9	9	9	9	9	#NULL!	#NULL!	#NULL!
2	3	3	2	3	3	1	2	2	2
2	1	2	2	2	1	2	2	1	1
9	9	9	9	9	9	9	#NULL!	#NULL!	#NULL!
1	1	2	3	2	2	1	2	1	1
2	3	2	1	2	2	1	2	1	1
1	9	3	3	3	3	3	2	2	2
1	3	1	1	3	3	3	1	1	1
1	1	2	2	3	3	3	2	2	2

2	3	2	3	1	2	2	2	2	2
1	3	3	3	3	3	3	2	2	2
3	3	3	3	3	3	3	1	1	1
2	2	2	2	2	2	2	2	2	2
1	2	2	2	2	2	3	2	2	1
2	3	2	3	2	3	2	2	2	2
9	9	9	9	9	9	9	2	1	2
9	9	9	9	9	9	9	1	1	2
9	9	9	9	9	9	9	2	2	2
2	2	2	2	2	2	2	2	2	2
1	2	3	2	1	1	2	1	1	2
1	3	3	3	3	3	3	2	2	2
2	2	2	2	2	2	9	1	2	2
3	2	2	1	2	1	3	2	2	2
2	1	2	1	1	1	1	2	1	1
3	2	1	2	2	2	2	1	1	1
3	2	2	1	1	1	1	2	1	1
3	2	2	1	1	1	1	2	1	1
9	9	9	9	9	9	9	2	2	2
2	2	3	2	3	1	3	2	2	2
1	1	2	2	3	1	2	1	2	2
1	1	1	2	2	2	2	2	2	2
9	9	9	9	9	9	9	2	2	2
2	2	2	2	2	2	2	2	2	2
1	1	2	3	3	3	3	1	1	2
2	1	2	3	2	1	3	2	2	2
9	9	9	9	9	9	9	2	2	2
3	3	3	3	3	3	3	2	1	1
1	1	3	3	1	3	3	2	2	2
3	2	2	2	2	2	2	1	1	2
9	9	9	9	9	9	9	1	1	1
1	1	3	3	3	3	2	2	2	2
1	2	2	1	2	1	1	1	1	1
1	3	3	1	3	2	2	1	2	1
2	1	2	2	1	3	2	1	1	1
1	1	1	1	1	1	1	1	1	1
3	3	3	3	3	3	3	1	1	1
1	3	2	1	1	3	1	1	1	1
1	1	1	3	3	3	3	2	2	2
2	2	2	2	2	2	2	1	2	1
9	9	9	9	9	9	9	#NULL!	2	2
2	2	2	2	2	2	2	1	1	1
2	2	2	2	2	2	9	1	2	2
9	9	9	9	9	9	9	#NULL!	#NULL!	#NULL!
9	9	9	9	9	9	9	#NULL!	#NULL!	#NULL!
2	3	2	1	2	2	2	2	1	1
2	3	3	2	2	1	2	1	2	1

9	1	9	9	9	9	9	1	1	2
9	9	9	9	9	9	9	2	2	2
1	3	1	3	3	3	3	1	2	2
9	9	9	9	9	9	9	1	2	2
9	9	9	9	9	9	9	2	2	2
9	9	9	9	9	9	9	#NULL!	#NULL!	#NULL!
9	9	9	9	9	9	9	1	1	2
9	9	9	9	9	9	9	#NULL!	#NULL!	#NULL!
1	3	1	2	2	1	2	1	2	2
9	9	9	9	9	9	9	2	2	1
9	9	2	9	9	9	9	2	2	2
9	9	9	9	9	1	9	2	2	2
2	3	2	1	1	2	1	1	2	2
3	3	2	1	3	2	1	#NULL!	#NULL!	#NULL!
9	9	9	9	9	9	2	2	1	1
3	2	3	3	2	2	3	2	1	2
2	1	1	1	1	1	1	1	1	1
1	2	2	1	1	1	1	1	2	1
2	2	2	2	2	2	2	2	2	2
1	2	2	2	2	2	2	2	2	2
2	1	1	1	1	1	1	2	1	2
9	9	9	9	9	9	9	#NULL!	#NULL!	#NULL!
3	1	1	1	1	1	2	2	2	2
1	3	1	3	3	3	3	2	2	2
2	2	2	2	2	2	2	2	2	2
1	1	2	1	1	1	2	2	2	2
2	1	1	2	2	2	2	1	2	2
2	2	3	2	2	2	2	1	2	2
9	9	9	9	9	9	9	1	1	1
1	1	1	3	3	3	3	1	1	1
2	2	2	2	2	2	2	2	2	2
2	2	1	1	1	1	1	2	2	1
9	9	9	9	9	9	9	#NULL!	#NULL!	#NULL!
2	2	1	1	1	2	2	2	2	2
1	3	3	3	3	2	3	2	2	2
9	3	2	1	2	2	2	1	2	2
9	9	9	9	9	9	9	2	1	2
1	1	1	2	2	2	2	1	2	2
9	9	9	9	9	9	9	2	2	1
2	1	2	1	2	2	3	2	2	2
1	1	1	2	3	3	2	1	2	1
1	3	2	2	1	3	1	1	1	1
1	2	2	1	1	2	2	1	2	1
2	2	2	2	2	2	2	1	1	2
3	1	2	2	2	2	2	2	2	2
3	2	3	2	1	2	9	1	1	1
9	9	9	9	9	9	9	#NULL!	#NULL!	#NULL!

1	3	2	2	2	2	2	2	2	2
1	1	1	2	3	1	3	1	2	2
1	1	1	1	1	1	1	2	2	2
9	9	9	9	9	9	9	2	#NULL!	#NULL!
2	2	2	2	2	2	2	1	2	2
2	2	3	2	2	2	3	2	2	2
3	1	2	3	1	2	3	1	1	1
2	1	1	2	1	1	2	2	2	1
1	1	1	1	2	1	1	2	1	2
3	2	1	1	2	1	2	1	2	2
2	2	2	2	2	2	2	2	2	2
9	9	9	9	9	9	9	1	1	2
9	9	9	9	9	9	9	2	2	2
9	9	9	9	9	9	9	1	1	1
9	2	9	9	2	2	2	2	2	2
9	9	9	9	9	9	9	2	2	2
9	9	9	9	9	9	9	#NULL!	#NULL!	#NULL!
9	9	9	9	9	9	9	#NULL!	#NULL!	#NULL!
9	9	9	9	9	9	9	2	2	2
9	9	9	9	9	9	9	#NULL!	#NULL!	#NULL!
9	9	9	9	9	9	9	#NULL!	#NULL!	#NULL!
2	2	1	1	1	1	1	1	2	1
2	2	2	2	2	2	2	2	1	2
2	2	2	1	1	2	1	2	1	1
2	1	1	2	1	1	1	2	2	1
9	9	9	9	9	9	9	#NULL!	#NULL!	#NULL!
2	2	2	1	2	2	2	1	2	1
9	9	9	9	9	9	9	#NULL!	#NULL!	#NULL!
9	9	9	9	9	9	9	#NULL!	#NULL!	#NULL!
1	2	2	2	2	2	2	1	1	2
9	9	9	9	9	9	9	2	2	2
9	9	9	9	9	9	9	#NULL!	#NULL!	#NULL!
9	9	9	9	9	9	9	#NULL!	#NULL!	#NULL!
9	9	9	9	9	9	9	#NULL!	#NULL!	#NULL!
9	9	9	9	9	9	9	2	2	2
9	9	1	9	9	1	1	2	2	2
9	9	9	9	9	9	9	#NULL!	#NULL!	#NULL!
9	9	9	9	9	9	9	#NULL!	#NULL!	#NULL!
9	9	9	9	9	9	9	#NULL!	#NULL!	#NULL!
9	9	9	9	9	9	9	#NULL!	#NULL!	#NULL!
9	9	9	9	9	9	2	#NULL!	#NULL!	#NULL!
9	9	9	9	9	9	9	2	2	2
3	3	3	3	3	3	3	1	1	1
3	3	3	3	3	3	3	2	2	2
1	3	2	1	2	1	2	1	1	1
2	1	1	1	1	1	1	1	1	#NULL!
9	9	9	9	9	9	9	1	2	2

9	9	9	9	9	9	9	2	2	2
9	9	9	9	9	9	9	1	2	2
2	1	2	3	2	2	3	1	1	2
3	2	3	1	3	2	3	1	2	1
9	9	9	9	9	9	9	#NULL!	#NULL!	#NULL!
3	3	2	2	3	2	3	1	2	2
9	9	9	9	9	9	9	1	1	2
1	3	2	1	1	2	1	2	2	2
2	2	2	1	3	2	2	2	2	2
3	3	3	3	3	3	3	1	1	1
9	9	9	9	9	9	9	#NULL!	#NULL!	#NULL!
9	9	9	9	9	9	9	#NULL!	#NULL!	#NULL!
9	9	9	9	9	9	9	2	2	2
2	9	9	9	9	9	2	1	1	1
2	2	2	2	2	2	2	1	2	1
2	2	2	2	1	1	2	1	1	1
9	9	9	9	9	9	9	#NULL!	#NULL!	#NULL!
1	2	3	2	2	2	3	1	2	2
1	1	1	1	1	1	1	1	1	1
2	2	2	2	2	2	2	2	2	2
2	2	2	2	2	2	2	1	2	2
1	1	3	3	2	2	3	1	2	2
9	9	9	9	9	9	9	2	2	2
2	2	2	2	2	2	2	1	2	1
2	2	2	2	2	2	2	2	1	2
2	2	2	2	2	2	2	1	1	1
2	2	2	2	2	2	2	2	2	2
1	1	1	1	1	1	1	1	1	1
3	9	2	3	9	2	2	2	2	2
2	1	1	2	1	2	2	1	2	2
2	2	2	2	2	2	2	2	2	2
2	2	2	2	2	2	2	2	2	2
2	3	2	3	2	3	2	2	2	2
3	3	9	9	2	2	2	2	2	2
1	1	3	3	3	3	3	1	1	1
3	1	2	3	1	2	2	2	1	2
9	9	9	9	9	9	9	#NULL!	#NULL!	#NULL!
1	3	2	3	3	3	2	2	2	2
3	3	3	3	3	3	3	2	2	2
3	3	3	3	3	3	3	2	2	2
9	9	9	9	9	9	9	2	2	2
9	9	9	9	9	9	9	#NULL!	#NULL!	#NULL!
1	2	1	1	1	1	3	2	2	2
2	2	3	2	2	2	2	1	1	2
1	1	1	1	1	1	1	2	2	1
2	2	2	2	2	2	2	2	2	2
2	2	2	3	2	2	2	1	1	1

9	9	2	9	9	9	2	1	2	2
9	9	9	9	9	9	9	2	2	1
9	1	9	9	9	9	9	2	2	#NULL!
9	9	1	9	9	9	3	2	2	1
9	9	9	9	9	1	9	1	2	2
1	1	2	2	2	1	9	1	1	1
9	9	9	9	9	9	9	2	2	2
9	9	9	9	9	9	9	2	2	2
9	9	9	9	9	9	9	2	2	2
9	9	9	9	9	9	9	2	2	2
9	1	9	9	9	9	2	2	2	2
9	2	9	9	9	3	2	1	#NULL!	2
9	9	9	9	9	9	2	2	2	2
2	9	9	9	9	9	9	2	2	2
9	9	9	9	9	9	9	2	2	2
9	9	9	9	9	9	9	#NULL!	#NULL!	#NULL!
9	9	2	9	9	9	2	2	2	2
3	2	1	2	9	2	1	2	2	1
2	1	1	2	3	1	1	1	1	1
2	2	2	2	2	2	2	2	2	2
1	3	2	1	2	1	3	1	1	1
1	3	2	3	2	2	3	#NULL!	#NULL!	#NULL!
2	2	2	2	2	2	2	2	2	2
9	9	9	9	9	9	9	2	2	1
3	1	2	3	2	2	2	2	2	2
9	9	9	9	9	9	9	#NULL!	#NULL!	#NULL!
1	1	3	2	3	1	2	2	2	2
9	9	9	9	9	9	1	2	2	2
2	1	2	1	3	1	2	1	2	1
9	9	9	9	9	9	9	1	1	1
2	2	3	2	2	1	2	1	1	1
1	1	3	1	1	3	2	2	2	2
2	2	1	1	1	1	2	2	1	1
1	1	1	1	1	1	1	2	2	2
9	9	9	9	9	9	9	#NULL!	#NULL!	#NULL!
2	2	2	2	2	2	2	1	2	2
9	9	9	9	9	9	9	#NULL!	#NULL!	#NULL!
2	2	1	2	2	2	2	2	2	1
9	9	9	9	9	9	9	2	2	2
2	2	2	2	2	2	2	1	1	1
2	1	2	2	1	2	9	2	2	2
3	3	3	3	3	3	3	2	2	2
1	1	3	3	3	3	3	2	2	2
2	2	2	2	2	3	3	2	2	2
3	3	3	3	3	3	2	2	2	2
2	2	2	2	2	2	2	1	1	2
2	2	2	2	2	2	2	1	1	1

2	1	2	2	1	3	3	1	1	1
9	9	9	9	9	9	3	2	#NULL!	2
9	2	2	2	3	2	2	1	1	1
2	9	1	2	3	1	3	2	2	2
1	1	1	1	2	2	2	2	2	2
2	2	2	2	2	2	2	2	2	2
1	2	2	2	3	3	3	1	1	2
9	9	9	9	9	9	9	1	1	2
9	9	9	9	9	9	9	#NULL!	#NULL!	#NULL!
1	1	1	2	3	3	3	2	2	2
2	2	2	2	2	2	1	1	2	2
9	9	9	9	9	9	9	2	2	2
9	9	9	9	9	9	9	#NULL!	#NULL!	#NULL!
1	1	1	9	1	1	1	2	2	2
9	9	9	9	9	9	9	2	1	2
3	3	2	1	3	2	2	2	2	2
9	9	9	9	9	9	9	#NULL!	#NULL!	#NULL!
9	9	9	9	9	9	9	#NULL!	2	2
9	9	9	1	9	9	9	2	2	2
2	3	2	3	2	3	2	2	2	2
2	2	2	2	2	2	2	2	2	2
3	3	3	2	2	1	1	2	2	2
2	2	2	1	2	2	2	2	2	2
1	9	9	9	9	9	9	2	2	2
9	9	9	9	9	9	9	2	2	2
2	2	2	2	2	2	2	2	2	2
1	1	1	1	1	2	3	1	1	1
1	2	3	2	3	1	2	1	1	1
9	9	9	9	9	9	9	#NULL!	#NULL!	#NULL!
9	9	9	9	9	9	9	2	2	1
9	9	9	9	9	9	9	2	2	2
2	3	1	2	3	1	2	2	2	2
2	2	1	3	3	3	3	2	2	2
2	1	2	2	2	2	2	1	2	2
1	1	2	2	1	1	1	1	2	2
2	1	2	2	3	2	2	#NULL!	#NULL!	#NULL!
9	9	9	9	9	9	9	#NULL!	#NULL!	#NULL!
9	9	9	9	9	9	9	#NULL!	#NULL!	#NULL!
3	1	3	1	1	2	2	1	1	1
1	2	1	2	1	2	2	1	1	1
2	2	2	2	2	2	2	1	2	1
1	1	2	2	2	3	3	2	2	2
1	1	3	2	3	3	3	2	2	2
2	3	2	1	2	3	3	2	2	2
3	2	2	1	3	1	3	#NULL!	#NULL!	#NULL!
2	2	2	2	2	2	2	1	2	2
2	2	2	3	3	3	3	1	2	2

2	2	2	1	1	1	1	2	2	2
1	2	2	3	3	2	2	1	1	1
9	9	9	9	9	9	9	2	1	2
9	9	9	9	9	9	9	2	2	2
1	9	9	9	9	9	9	2	2	2
3	3	1	3	9	1	3	1	1	1
2	2	9	2	9	2	2	2	2	2
2	2	2	2	2	2	2	2	2	2
9	9	9	9	9	9	9	#NULL!	#NULL!	#NULL!
9	9	9	9	9	9	9	#NULL!	#NULL!	#NULL!
9	9	9	9	9	9	9	2	2	2
9	9	9	9	9	9	9	2	2	2
9	9	9	9	9	9	9	2	2	2
9	9	9	9	9	9	9	#NULL!	#NULL!	#NULL!
9	9	9	9	9	9	9	2	2	2
9	9	9	9	9	9	9	2	2	2
3	3	3	3	3	3	3	2	2	2
9	9	9	9	9	9	9	1	1	1
9	9	9	9	9	9	9	1	1	1
9	9	9	9	9	9	9	1	1	1
9	9	9	9	9	9	9	#NULL!	#NULL!	#NULL!
9	9	9	9	9	9	9	2	2	2
2	2	2	2	2	2	2	2	2	2
9	9	9	9	9	9	9	#NULL!	#NULL!	#NULL!
9	9	9	9	9	9	9	#NULL!	#NULL!	#NULL!
9	9	9	9	9	9	9	1	1	1
9	9	9	9	9	9	9	#NULL!	#NULL!	#NULL!
9	2	9	2	9	9	9	1	1	1
9	9	9	9	9	9	9	1	1	1
9	9	9	9	9	9	9	#NULL!	#NULL!	#NULL!
9	9	9	9	9	9	9	#NULL!	#NULL!	#NULL!
9	9	9	9	9	9	9	1	1	1
9	9	9	9	9	9	9	#NULL!	#NULL!	#NULL!
9	9	9	9	9	9	9	#NULL!	#NULL!	#NULL!
9	9	9	9	9	9	9	#NULL!	#NULL!	#NULL!
9	9	9	9	9	9	9	#NULL!	#NULL!	#NULL!
9	9	9	9	9	9	9	2	2	2
9	9	9	9	9	9	9	#NULL!	#NULL!	#NULL!
1	2	2	2	2	2	2	2	2	2
1	2	3	2	1	2	2	1	2	2
2	9	9	9	9	9	9	2	2	2
9	9	9	9	9	9	9	#NULL!	#NULL!	#NULL!
1	3	1	3	3	2	2	2	2	2
3	2	3	2	2	2	2	2	2	2
1	1	1	1	1	1	9	1	2	2
2	2	1	2	1	2	2	1	2	1
1	9	9	9	9	9	9	#NULL!	#NULL!	#NULL!

9	9	9	9	9	9	9	1	1	2
2	3	3	1	2	2	1	1	1	2
9	9	9	9	9	9	9	2	2	2
1	1	1	1	1	1	9	1	1	1
2	2	2	2	2	2	2	2	2	2
2	2	1	2	2	2	2	2	2	2
9	9	9	9	9	9	9	2	1	1
2	3	2	1	1	1	2	2	2	2
2	2	2	2	2	2	2	2	2	2
1	1	1	1	3	9	3	1	2	2
2	1	1	3	2	2	1	2	2	#NULL!
9	1	9	9	9	9	9	2	2	2
9	9	9	9	9	9	9	2	2	2
9	9	9	9	9	9	9	1	2	1
9	9	9	9	9	9	9	1	2	2
9	9	9	9	9	9	9	2	2	1
2	2	2	2	2	2	2	2	2	2
1	1	1	3	2	3	3	2	2	2
2	9	3	1	3	3	9	2	2	2
2	2	2	2	2	2	2	1	1	1
2	2	2	2	2	2	2	2	2	2
9	9	9	9	9	9	9	#NULL!	#NULL!	#NULL!
1	1	3	3	1	1	1	2	2	2
9	9	2	9	9	9	9	1	2	2
9	9	9	9	9	9	9	2	2	2
1	1	1	1	1	1	1	1	1	1
2	9	9	9	9	2	9	1	#NULL!	2
3	3	3	1	1	1	1	2	2	2
9	9	9	9	9	9	9	#NULL!	#NULL!	#NULL!
9	9	9	9	9	9	9	1	1	1
1	1	3	1	2	1	2	1	1	1
3	2	2	3	2	3	2	1	2	2
9	9	9	9	9	9	9	#NULL!	#NULL!	#NULL!
9	9	9	9	9	9	1	1	1	1
3	2	2	3	3	2	2	1	2	2
9	9	9	9	9	9	9	#NULL!	#NULL!	#NULL!
9	9	9	9	9	9	9	2	2	2
1	1	1	1	1	1	1	1	1	1
2	2	2	2	2	2	2	2	2	2
9	9	9	9	9	9	9	1	1	2
9	9	9	9	9	9	9	2	2	2
1	1	1	1	9	3	3	2	2	2
2	2	2	2	2	2	2	2	2	2
2	2	2	2	2	2	2	2	2	2
9	3	2	3	3	2	2	2	2	2
2	2	2	2	2	2	2	2	2	2
2	3	2	3	3	2	2	2	2	2

9	9	9	9	9	9	9	2	2	2
2	2	2	2	2	2	2	2	1	2
2	1	2	2	3	2	3	2	2	2
9	9	1	9	9	9	9	1	1	2
9	1	9	9	9	9	9	2	2	2
9	9	9	9	9	9	9	#NULL!	#NULL!	#NULL!
9	9	9	9	9	9	9	#NULL!	#NULL!	#NULL!
1	1	2	3	3	3	3	2	2	2
1	2	1	2	2	2	3	1	1	1
9	9	9	9	9	9	9	2	2	2
1	1	2	1	2	1	2	1	1	1
2	3	2	2	1	1	1	1	1	1
2	2	2	2	2	2	2	1	1	1
2	9	9	9	9	9	9	2	2	2
9	9	9	9	9	9	9	2	2	2
9	9	9	9	9	9	9	#NULL!	#NULL!	#NULL!
9	9	9	9	9	9	9	#NULL!	#NULL!	#NULL!
9	9	9	9	9	9	9	#NULL!	#NULL!	#NULL!
9	9	9	9	9	9	9	2	2	2
9	9	9	9	9	9	9	2	2	2
9	9	9	9	9	9	9	#NULL!	#NULL!	#NULL!
9	9	9	9	9	9	9	#NULL!	#NULL!	#NULL!
2	2	2	2	2	2	2	2	2	2
9	9	9	9	9	9	2	2	2	2
9	9	9	9	9	9	9	#NULL!	#NULL!	#NULL!
9	9	9	9	9	9	9	2	2	2
9	9	9	9	9	9	9	#NULL!	#NULL!	#NULL!
9	9	9	9	9	9	9	2	2	2
9	9	9	9	9	9	9	2	2	2
9	9	9	9	3	9	9	2	#NULL!	2
9	9	9	9	9	9	9	#NULL!	#NULL!	#NULL!
9	9	9	9	9	9	9	2	2	2
9	9	9	9	9	9	9	#NULL!	#NULL!	#NULL!
9	9	9	9	9	9	9	#NULL!	#NULL!	#NULL!
9	9	9	9	9	9	9	2	2	2
9	9	9	9	9	9	9	#NULL!	#NULL!	#NULL!
9	9	9	9	9	9	9	2	2	2
9	9	9	9	9	9	9	#NULL!	#NULL!	#NULL!
9	9	9	9	9	9	9	#NULL!	#NULL!	#NULL!
9	9	9	9	9	9	9	#NULL!	#NULL!	#NULL!
2	2	2	2	2	2	2	1	1	1
9	9	9	9	9	9	2	1	1	1
9	9	9	9	9	9	9	#NULL!	#NULL!	#NULL!
9	9	9	9	9	9	9	2	2	2
9	9	9	9	9	9	9	#NULL!	#NULL!	#NULL!
9	9	9	9	9	9	9	2	2	2
9	9	9	9	9	9	9	#NULL!	#NULL!	#NULL!

9	9	9	9	9	9	9	#NULL!	#NULL!	#NULL!
2	2	2	2	2	2	2	#NULL!	#NULL!	#NULL!
9	9	9	9	9	9	9	2	2	2
9	9	9	9	9	9	9	#NULL!	#NULL!	#NULL!
9	9	9	9	9	9	9	#NULL!	#NULL!	#NULL!
9	9	9	9	9	9	9	#NULL!	#NULL!	#NULL!
9	9	9	9	9	9	9	#NULL!	#NULL!	#NULL!
9	9	9	9	9	9	9	#NULL!	#NULL!	#NULL!
9	9	9	9	9	9	9	#NULL!	#NULL!	#NULL!
9	9	9	9	9	9	9	#NULL!	#NULL!	#NULL!
9	9	9	9	9	9	9	#NULL!	#NULL!	#NULL!
9	9	9	9	9	9	9	#NULL!	#NULL!	#NULL!
9	9	9	9	9	9	9	#NULL!	#NULL!	#NULL!
9	9	9	9	9	9	9	#NULL!	#NULL!	#NULL!
9	9	9	9	9	9	9	2	2	2
9	9	9	9	9	9	9	2	2	2
9	9	9	9	9	9	9	#NULL!	#NULL!	#NULL!
9	9	9	9	9	9	9	2	2	2
9	9	9	9	9	9	9	#NULL!	#NULL!	#NULL!
9	9	9	9	9	9	9	2	2	2
9	9	9	9	9	9	9	#NULL!	#NULL!	#NULL!
9	9	9	9	9	9	9	2	2	#NULL!
9	9	9	9	9	9	9	#NULL!	#NULL!	#NULL!
9	9	9	9	9	9	9	2	2	2
2	2	2	2	2	2	2	2	2	2
9	9	9	9	9	9	9	2	2	2
9	9	9	9	9	9	9	#NULL!	#NULL!	#NULL!
1	1	1	1	1	1	1	2	2	2
9	9	9	9	9	9	9	#NULL!	#NULL!	#NULL!
9	9	9	9	9	9	9	#NULL!	#NULL!	#NULL!
1	1	1	1	1	1	1	1	1	1
3	1	1	3	2	1	1	2	2	2
9	9	9	9	9	9	9	#NULL!	#NULL!	#NULL!
9	9	9	9	9	9	9	2	2	2
9	9	9	9	9	9	9	#NULL!	#NULL!	#NULL!
9	2	9	2	9	9	2	2	2	2
9	9	9	9	9	9	9	1	1	1
9	9	9	9	9	9	9	#NULL!	#NULL!	#NULL!
1	1	1	1	1	1	1	2	2	2
9	9	9	9	9	9	2	2	2	2
9	9	9	9	9	9	9	#NULL!	#NULL!	#NULL!
9	9	9	9	9	9	9	#NULL!	#NULL!	#NULL!
2	2	2	2	2	2	2	2	2	2
2	2	2	2	2	2	2	1	1	1
9	1	9	1	9	1	1	2	2	2
9	9	9	9	9	9	9	#NULL!	#NULL!	#NULL!
9	9	9	9	9	9	9	#NULL!	#NULL!	#NULL!

9	9	9	9	9	9	9	2	2	2
9	9	9	9	9	9	9	#NULL!	#NULL!	#NULL!
9	9	9	9	9	9	9	2	2	2
9	9	9	9	9	9	9	#NULL!	#NULL!	#NULL!
9	9	9	9	9	9	9	#NULL!	#NULL!	#NULL!
9	9	9	9	9	9	9	#NULL!	#NULL!	#NULL!
9	9	9	9	9	9	9	1	1	1
9	9	9	9	9	9	9	2	2	2
9	2	9	2	9	2	2	1	1	1
9	9	9	9	9	9	9	2	2	2
9	9	9	9	9	9	9	2	2	2
9	9	9	9	9	9	9	#NULL!	#NULL!	#NULL!
9	9	9	9	9	9	9	#NULL!	#NULL!	#NULL!
9	9	9	9	9	9	9	#NULL!	#NULL!	#NULL!
2	2	2	2	1	9	2	2	2	2
2	1	3	3	1	2	3	2	2	2
1	9	9	9	9	9	9	2	2	2
2	1	1	1	2	2	2	2	2	2
3	3	2	2	2	2	3	2	2	2
9	9	9	9	9	9	1	1	1	1
9	9	9	9	9	9	9	1	1	2
9	9	9	9	9	9	9	1	1	#NULL!
2	3	1	2	3	1	2	2	2	2
3	1	2	3	1	2	3	2	2	2
2	2	2	2	2	2	2	2	2	2
1	1	3	3	2	2	3	1	1	2
1	9	1	3	3	3	3	2	2	2
1	1	1	2	3	3	3	2	2	2
1	2	1	1	2	2	3	2	2	2
2	2	2	2	2	2	2	2	2	2
9	9	9	9	9	9	9	2	2	2
9	9	2	9	9	9	9	1	1	#NULL!
9	9	9	9	9	9	9	1	2	1
9	9	9	9	2	9	9	2	2	2
9	9	9	9	9	9	9	1	#NULL!	2
9	9	9	9	9	9	9	#NULL!	#NULL!	#NULL!
9	9	9	9	9	9	9	#NULL!	#NULL!	#NULL!
9	9	9	2	9	2	2	1	1	1
9	9	9	9	9	9	9	#NULL!	#NULL!	#NULL!
2	9	2	9	9	2	2	2	2	2
9	9	9	9	9	9	9	#NULL!	#NULL!	#NULL!
2	2	2	2	2	2	2	1	1	1
9	9	9	9	9	9	9	#NULL!	#NULL!	#NULL!
9	9	9	9	9	9	9	#NULL!	#NULL!	#NULL!
9	9	9	9	9	9	9	#NULL!	#NULL!	#NULL!
2	2	2	2	2	2	2	2	2	2
1	2	2	3	2	2	2	2	2	2

1	1	2	2	2	1	3	1	2	2
1	1	1	2	2	2	2	1	1	2
2	2	2	2	2	2	2	1	1	2
1	2	2	3	1	2	3	#NULL!	#NULL!	#NULL!
3	3	3	3	3	3	3	2	2	2
1	2	2	3	3	3	3	2	1	2
1	1	1	1	2	1	1	2	2	2
3	3	3	3	3	3	3	2	2	2
9	9	9	9	9	9	9	2	2	2
2	2	2	2	2	2	2	1	1	1
9	2	9	2	9	9	2	2	2	2
3	1	9	1	9	1	1	2	2	2
1	1	2	3	2	3	3	1	2	2
1	2	3	3	2	3	3	1	2	2
3	2	3	2	3	3	3	2	2	2
2	2	2	2	2	2	2	2	2	2
2	1	2	1	2	1	2	2	2	2
9	9	9	9	9	9	9	2	2	2
9	9	9	9	9	9	9	#NULL!	#NULL!	#NULL!
2	2	2	3	1	3	2	1	1	2
1	3	3	3	3	2	3	1	1	2
1	1	1	1	2	2	2	2	1	2
2	3	3	3	2	2	2	1	2	2
2	2	2	1	2	3	2	1	2	1
1	2	2	3	3	3	3	2	2	2
2	2	3	2	2	2	2	2	2	2
2	2	2	2	2	2	3	1	2	2
2	2	2	3	2	2	3	2	2	2
1	2	1	2	1	2	3	2	1	2
3	3	2	9	9	1	1	2	2	2
1	1	1	2	3	2	3	2	2	2
9	9	9	9	9	9	9	2	2	2
3	3	3	3	3	3	3	2	2	2
2	2	2	2	2	2	2	2	2	2
9	9	9	9	9	9	9	2	2	2
3	2	3	2	2	2	2	2	2	2
2	2	3	3	3	3	3	2	2	2
1	1	1	1	1	1	1	1	1	1
1	2	2	1	1	1	1	2	2	1
9	9	9	9	9	9	9	#NULL!	#NULL!	#NULL!
2	2	2	2	2	2	2	1	2	2
9	9	9	9	9	9	9	2	1	2
3	2	1	2	2	2	3	2	1	2
2	2	2	2	2	2	2	#NULL!	2	2
2	3	2	3	2	3	2	2	2	2
1	1	1	2	3	3	3	2	2	2
3	3	2	2	1	3	2	2	2	2

9	9	9	9	9	9	9	#NULL!	#NULL!	#NULL!
3	3	3	3	3	3	3	2	2	2
1	1	3	3	3	3	1	1	2	2
2	3	2	3	2	3	2	2	2	2
1	2	1	1	1	2	2	2	1	2
9	9	9	9	9	9	9	#NULL!	#NULL!	#NULL!
3	2	3	2	2	2	2	1	2	2
3	1	3	2	2	3	2	2	2	2
1	3	1	3	3	3	3	2	2	2
1	1	3	2	1	3	3	2	2	2
1	1	1	1	1	1	3	2	2	2
2	2	2	2	2	2	2	1	2	2
9	9	9	9	9	9	9	#NULL!	#NULL!	#NULL!
2	2	2	3	2	3	3	2	2	2
1	2	1	3	3	2	3	1	1	2
2	2	2	2	2	2	2	2	2	2
3	3	3	2	1	1	2	2	2	2
2	2	2	2	2	2	2	1	2	2
2	2	2	2	2	2	2	#NULL!	#NULL!	#NULL!
9	9	9	9	9	9	9	1	2	2
9	9	9	9	9	9	9	#NULL!	#NULL!	#NULL!
2	1	1	2	1	2	1	#NULL!	#NULL!	#NULL!
3	3	3	2	3	2	2	2	2	2
2	3	2	3	2	3	2	2	2	2
9	9	9	9	9	9	9	#NULL!	#NULL!	#NULL!
3	2	1	2	3	2	2	2	2	2
9	9	9	9	9	1	9	2	2	2
2	2	2	2	2	2	2	2	2	2
2	2	2	2	2	2	2	2	2	2
2	2	2	2	2	2	2	2	2	2
9	9	9	9	9	9	9	2	2	1
9	9	9	9	9	9	9	#NULL!	#NULL!	#NULL!
9	9	9	9	9	9	9	#NULL!	#NULL!	#NULL!
2	2	2	3	3	3	3	2	1	1
9	9	9	9	9	9	9	1	1	2
9	9	9	9	9	9	9	#NULL!	#NULL!	#NULL!
9	9	9	9	9	9	9	#NULL!	#NULL!	#NULL!
3	2	2	2	2	2	2	2	2	2
9	9	9	9	9	9	9	1	1	1
1	1	2	2	2	2	2	2	2	2
1	1	3	3	1	2	3	2	2	2
2	2	2	2	2	2	2	2	2	2
2	2	2	2	2	2	2	2	2	2
2	2	2	2	2	2	2	2	2	2
2	2	2	2	2	2	2	2	2	2
3	9	2	1	2	3	3	2	2	2
2	2	2	2	2	2	2	2	2	1

3	3	2	2	1	1	3	1	2	1
2	1	1	2	2	2	2	2	2	2
2	2	2	2	2	2	2	2	2	1
2	9	1	1	1	1	1	1	1	1
2	2	2	2	2	2	2	2	2	2
2	2	2	2	2	2	2	2	2	2
2	2	2	2	2	2	2	2	2	2
2	2	2	2	2	2	2	2	2	2
2	2	2	2	1	1	3	2	2	2
9	9	9	9	9	9	9	#NULL!	#NULL!	#NULL!
2	2	2	2	2	2	3	1	1	2
2	1	3	1	1	2	3	2	2	2
9	9	9	9	9	9	9	2	2	2
2	1	2	1	2	1	1	1	2	1
2	2	2	2	2	3	3	2	2	2
9	9	9	9	9	9	9	1	1	2
9	9	9	9	9	9	9	#NULL!	#NULL!	#NULL!
9	9	9	9	9	2	2	2	2	2
9	9	9	9	9	9	9	#NULL!	#NULL!	#NULL!
9	9	9	9	9	9	9	#NULL!	#NULL!	#NULL!
2	2	2	2	2	2	1	2	2	2
9	9	9	9	9	9	9	#NULL!	#NULL!	#NULL!
9	9	9	9	9	9	9	#NULL!	#NULL!	#NULL!
1	2	2	2	1	2	2	2	2	2
3	3	3	3	3	3	3	1	2	2
1	2	2	2	1	2	2	2	2	2
2	2	1	1	2	1	2	1	2	1
9	9	9	9	9	9	9	1	2	2
2	2	2	2	2	2	2	2	2	2
3	1	2	2	3	1	2	2	1	2
9	9	9	9	9	9	9	#NULL!	#NULL!	#NULL!
1	3	1	2	1	2	2	#NULL!	#NULL!	#NULL!
3	2	3	2	2	3	2	1	2	2
1	1	1	1	2	2	2	1	#NULL!	1
1	1	2	2	2	3	3	1	2	2
1	2	2	2	1	2	2	2	2	2
9	9	9	9	9	9	9	#NULL!	#NULL!	#NULL!
2	2	2	1	2	1	2	2	2	2
2	2	2	2	2	2	2	2	2	2
1	1	2	2	2	3	3	1	2	2
2	2	2	2	2	2	2	1	2	2
1	2	1	3	1	1	3	1	2	2
1	1	2	2	2	2	3	2	2	2
2	1	1	1	1	2	2	1	2	2
2	3	2	3	3	2	2	2	2	2
1	1	2	2	2	2	2	1	2	2
3	3	3	2	2	3	3	1	2	2

2	2	2	2	2	2	2	#NULL!	#NULL!	#NULL!
2	2	2	2	2	2	2	2	2	2
2	2	2	2	2	2	2	2	2	1
2	2	2	2	2	2	2	2	2	2
2	2	3	3	3	2	2	1	2	2
9	9	9	9	9	9	9	#NULL!	#NULL!	#NULL!
9	9	9	9	9	9	9	#NULL!	#NULL!	#NULL!
3	3	3	3	3	3	3	2	2	2
9	9	9	9	9	9	9	#NULL!	#NULL!	#NULL!
9	9	9	9	9	9	9	#NULL!	#NULL!	#NULL!
2	2	2	2	2	2	2	2	2	2
9	9	9	9	9	9	9	2	1	2
2	2	1	1	2	2	2	1	1	1
9	9	9	9	9	9	3	2	2	1
1	9	9	9	9	9	9	2	2	2
1	1	1	1	3	3	3	2	2	2
1	1	2	3	1	3	3	1	1	2
9	9	9	9	9	9	9	#NULL!	#NULL!	#NULL!
1	3	1	1	3	3	3	1	2	2
1	1	2	2	3	3	3	1	1	1
2	2	2	2	2	2	2	1	2	2
9	9	9	9	9	9	9	2	2	2
2	2	2	2	2	2	2	2	2	2
1	2	2	2	2	2	2	2	2	2
2	2	2	2	2	2	9	2	2	2
2	2	2	2	2	2	2	2	2	2
1	1	2	2	2	9	3	2	2	2
2	2	2	2	2	2	2	2	2	2
2	2	3	2	2	2	2	2	2	2
2	2	2	2	2	2	2	2	2	2
2	1	1	2	1	1	1	2	2	1
3	1	2	2	3	1	2	1	1	1
1	1	2	3	2	2	3	2	2	2
1	1	2	2	2	3	3	2	2	2
1	1	3	1	1	1	2	1	2	#NULL!
1	2	2	3	2	1	2	2	2	1
1	1	1	1	1	1	3	1	1	2
2	2	2	2	2	2	2	2	2	2
1	2	2	2	9	2	3	1	1	2
1	1	3	1	1	2	2	2	2	2
1	3	1	3	3	3	3	1	1	1
1	3	1	1	3	3	3	1	2	2
2	2	2	2	2	2	2	2	2	2
2	2	2	2	2	2	2	1	2	2
2	2	2	2	2	2	2	1	2	2
1	3	3	3	3	3	3	2	2	2
2	2	2	2	2	2	2	2	2	2

3	2	1	3	2	1	2	2	1	2
1	1	1	3	3	2	3	1	2	2
9	3	3	2	3	2	2	1	1	2
2	2	2	2	2	2	9	1	1	1
9	9	9	9	9	9	1	2	2	2
9	9	9	9	9	2	9	2	1	2
3	2	3	1	2	2	3	2	2	2
9	9	9	9	9	9	9	1	1	1
9	9	9	9	9	9	9	1	1	1
9	9	9	9	9	9	9	#NULL!	#NULL!	#NULL!
9	9	9	9	2	9	9	2	2	2
9	9	9	9	9	2	9	2	1	#NULL!
9	9	9	2	9	9	9	2	1	2
9	9	9	9	9	9	9	2	2	2
1	1	1	3	3	3	3	2	2	2
9	9	9	9	9	9	9	2	2	#NULL!
9	9	9	9	9	9	9	#NULL!	#NULL!	#NULL!
9	9	9	1	9	9	9	2	2	2
9	9	9	1	9	9	9	2	2	2
9	9	9	1	9	9	9	#NULL!	#NULL!	#NULL!
9	9	9	9	9	9	9	#NULL!	#NULL!	#NULL!
2	2	2	2	2	2	2	2	2	2
2	2	2	2	3	3	3	2	2	2
2	2	2	2	3	1	1	2	2	2
1	3	3	3	2	3	3	2	2	2
9	9	9	9	9	9	9	1	2	2
2	2	2	2	2	2	2	2	2	2
9	9	9	9	9	9	9	2	2	2
3	9	2	2	2	1	1	2	2	2
1	1	1	1	1	1	1	1	2	1
3	3	3	3	2	2	9	1	2	2
9	9	9	9	9	9	9	2	2	2
1	1	1	1	1	1	1	2	2	1
9	9	9	9	9	9	9	2	2	2
9	9	9	9	9	9	9	1	1	1
1	2	1	2	1	1	2	#NULL!	#NULL!	#NULL!
1	1	1	1	1	1	1	1	1	1
9	9	9	9	9	9	9	#NULL!	#NULL!	#NULL!
9	9	9	9	9	9	9	2	2	2
9	9	9	9	9	9	9	2	2	2
9	9	9	9	9	9	9	2	2	2
2	2	2	2	2	2	2	1	1	1
3	3	3	3	3	3	3	2	2	2
9	9	9	9	9	9	9	2	2	2
9	9	9	9	9	9	9	1	1	1
9	9	9	9	9	9	9	1	1	1
9	9	9	9	9	9	9	2	2	2

9	9	9	9	9	9	9	#NULL!	#NULL!	#NULL!
9	2	9	2	2	2	2	2	2	2
9	9	9	9	9	9	9	#NULL!	#NULL!	#NULL!
9	9	9	9	9	9	9	1	1	1
9	9	9	9	9	9	9	2	2	2
1	2	9	9	1	9	1	2	2	2
9	9	9	9	9	9	9	2	2	2
9	9	9	9	9	9	9	#NULL!	#NULL!	#NULL!
9	2	9	2	9	2	2	2	2	2
9	9	9	9	9	9	9	1	1	1
9	9	9	9	9	9	9	#NULL!	#NULL!	#NULL!
9	2	9	9	2	9	2	2	2	2
2	2	2	2	2	2	2	2	2	2
9	9	9	9	9	9	9	#NULL!	#NULL!	#NULL!
2	2	2	2	2	2	2	1	1	1
9	9	9	9	9	9	9	#NULL!	#NULL!	#NULL!
9	9	9	9	9	9	9	#NULL!	#NULL!	#NULL!
9	9	9	9	9	9	9	#NULL!	#NULL!	#NULL!
9	9	9	9	9	9	9	#NULL!	#NULL!	#NULL!
9	9	9	9	9	9	9	#NULL!	#NULL!	#NULL!
2	2	2	2	2	2	2	2	2	2
2	2	2	2	2	2	2	2	2	2
3	3	3	3	3	3	3	2	2	2
1	2	1	2	1	2	1	1	1	2
3	3	3	3	2	2	2	2	1	2
1	1	3	3	3	3	3	2	2	2
1	1	1	1	1	1	1	1	1	1
2	2	2	2	2	2	2	2	2	2
1	1	1	2	3	1	1	2	2	2
2	2	2	2	2	2	2	2	2	2
2	2	3	3	9	2	2	1	2	2
2	3	1	3	2	2	3	2	2	2
2	2	2	2	2	2	2	2	2	2
9	9	9	9	9	9	9	#NULL!	#NULL!	#NULL!
3	3	3	2	1	2	1	2	2	2
3	3	3	3	3	3	3	1	1	1
2	2	2	2	2	2	2	2	2	2
2	2	2	2	2	2	2	1	2	1
9	2	2	3	2	2	2	2	2	2
2	2	2	2	2	2	2	2	2	2
9	9	9	9	9	9	9	1	1	2
9	9	9	9	9	9	9	#NULL!	#NULL!	#NULL!
2	2	2	2	2	2	2	1	2	2
9	9	9	9	9	9	9	#NULL!	#NULL!	#NULL!
9	9	9	9	9	9	9	2	2	1
1	2	2	2	1	2	2	2	2	2
9	9	9	9	9	9	9	1	2	2

2	2	1	1	2	2	2	2	2	2
2	2	2	3	3	3	3	#NULL!	#NULL!	#NULL!
9	9	9	9	9	9	9	1	1	1
9	9	9	9	9	9	9	1	1	1
9	9	9	9	9	9	9	1	1	1
1	1	9	9	9	9	9	1	1	1
2	2	2	2	2	2	2	1	1	1
2	2	2	2	2	2	2	1	1	1
3	3	3	3	3	3	3	2	2	2
1	1	1	1	1	1	2	1	1	1
9	9	9	9	9	9	9	2	2	2
2	2	2	2	2	2	2	1	1	1
9	9	9	9	9	9	9	1	1	1
2	2	2	2	2	2	2	1	1	1
9	9	9	9	9	9	9	1	1	1
1	1	1	1	1	1	1	1	1	1
1	2	1	2	1	2	1	1	1	1
2	2	1	1	1	1	2	1	1	1
2	2	2	2	2	2	9	1	1	1
9	9	9	9	9	9	9	2	2	2
2	2	1	1	1	2	1	1	1	1
2	9	2	2	2	2	9	2	2	2
9	9	9	9	9	9	9	#NULL!	#NULL!	#NULL!
2	2	2	2	2	2	9	1	1	1
9	9	9	9	9	9	9	#NULL!	#NULL!	#NULL!
1	1	2	2	2	1	1	1	1	1
2	2	2	1	1	1	2	1	1	1
1	1	1	1	1	1	9	1	1	2
9	9	9	9	9	9	9	1	1	1
9	9	9	9	9	9	9	1	1	1
9	9	9	9	9	9	9	2	2	2
9	9	9	9	9	9	9	1	1	2
9	9	9	9	9	9	9	1	1	1
1	1	1	3	3	1	3	1	1	1
2	2	2	2	2	2	2	1	1	2
9	9	9	9	9	9	9	#NULL!	#NULL!	#NULL!
2	1	1	2	1	2	1	1	1	1
2	1	1	1	1	1	2	1	1	1
9	9	9	9	9	9	9	#NULL!	#NULL!	#NULL!
9	9	9	9	9	9	9	2	2	2
9	9	9	9	9	9	9	1	1	1
9	9	9	9	9	9	9	#NULL!	#NULL!	#NULL!
1	1	2	2	2	2	9	1	1	1
9	9	9	9	9	9	9	1	1	1
1	1	1	1	2	2	1	1	1	1
2	2	2	2	2	2	2	2	2	2
9	9	9	9	9	9	9	1	1	1

1	1	1	1	1	2	1	1	1	1
9	9	9	9	9	9	9	2	2	2
3	3	3	2	2	2	2	1	1	1
1	1	1	1	1	1	1	1	1	1
9	9	9	9	9	9	9	1	1	1
9	9	9	9	9	9	9	2	#NULL!	#NULL!
9	9	9	9	9	9	9	1	1	1
2	2	2	2	2	2	2	1	1	1
9	9	9	9	9	9	9	2	2	2
9	9	9	9	9	9	9	1	1	1
9	9	9	9	9	9	9	2	2	2
9	9	9	9	9	9	9	1	1	1
9	9	9	9	9	9	9	#NULL!	#NULL!	#NULL!
9	9	9	9	9	9	9	1	1	1
9	9	9	9	9	9	9	2	2	2
9	9	9	9	9	9	9	#NULL!	#NULL!	#NULL!
9	9	9	9	9	9	9	1	1	1
9	9	9	9	9	9	9	#NULL!	#NULL!	#NULL!
2	2	2	2	2	2	2	2	2	2
1	1	2	2	3	3	3	1	1	1
1	2	2	2	3	3	3	1	1	1
1	1	2	2	3	3	3	1	1	1
1	1	2	2	2	9	3	1	1	1
1	2	2	2	2	3	3	1	1	1
1	2	2	2	3	3	3	1	1	1
1	1	2	2	2	3	3	1	1	1
1	1	2	2	3	3	3	2	1	1
1	3	2	3	1	2	3	1	1	1
1	1	2	2	3	3	3	1	1	1
1	1	2	2	3	3	3	2	1	1
1	1	2	3	3	3	3	2	1	1
1	1	2	2	3	3	3	2	1	1
1	1	2	2	3	3	3	1	1	1
1	3	2	2	3	3	3	2	1	1
1	1	2	2	3	3	3	1	1	1
1	2	2	2	3	3	3	2	1	1
1	2	2	3	3	3	3	1	1	1
1	2	2	2	3	3	3	#NULL!	#NULL!	#NULL!
1	1	2	2	3	3	3	1	1	1
1	2	2	9	3	3	3	1	1	1
1	2	2	2	3	3	3	1	1	1
1	2	2	2	3	3	3	1	1	1
1	2	2	2	3	3	3	1	1	1
1	1	2	2	3	3	3	1	1	1
9	9	9	9	9	9	9	#NULL!	#NULL!	#NULL!
9	9	9	9	9	9	9	#NULL!	#NULL!	#NULL!
9	9	9	9	9	9	9	#NULL!	#NULL!	#NULL!

9	9	9	9	9	9	9	#NULL!	#NULL!	#NULL!
9	9	9	9	9	9	9	#NULL!	#NULL!	#NULL!
9	9	9	9	9	9	9	#NULL!	#NULL!	#NULL!
9	9	9	9	9	9	9	#NULL!	#NULL!	#NULL!
9	9	9	9	9	9	9	#NULL!	#NULL!	#NULL!
9	9	9	9	9	9	9	#NULL!	#NULL!	#NULL!
9	9	9	9	9	9	9	#NULL!	#NULL!	#NULL!
9	9	9	9	9	9	9	#NULL!	#NULL!	#NULL!
9	9	9	9	9	9	9	#NULL!	#NULL!	#NULL!
9	9	9	9	9	9	9	#NULL!	#NULL!	#NULL!
9	9	9	9	9	9	9	#NULL!	#NULL!	#NULL!
9	9	9	9	9	9	9	#NULL!	#NULL!	#NULL!
9	9	9	9	9	9	9	#NULL!	#NULL!	#NULL!
9	9	9	9	9	9	9	#NULL!	#NULL!	#NULL!
9	9	9	9	9	9	9	#NULL!	#NULL!	#NULL!
9	9	9	9	9	9	9	#NULL!	#NULL!	#NULL!
9	9	9	9	9	9	9	#NULL!	#NULL!	#NULL!
9	9	9	9	9	9	9	#NULL!	#NULL!	#NULL!
9	9	9	9	9	9	9	#NULL!	#NULL!	#NULL!
1	1	2	2	3	2	2	1	#NULL!	1
1	1	2	2	3	3	3	1	1	1
1	1	2	2	3	3	3	1	1	1
1	1	2	2	3	3	3	1	1	1
1	1	2	2	3	3	3	1	1	1
1	1	2	2	1	2	3	1	1	2
1	2	2	2	3	3	3	1	2	1
1	2	2	2	3	3	3	1	1	1
1	2	2	2	3	3	3	1	2	1
1	2	2	3	3	3	3	1	1	1
1	2	2	3	3	3	3	1	1	1
1	1	2	2	3	3	3	1	1	1
1	1	2	2	3	3	3	1	1	1
1	1	2	2	3	3	3	1	1	1
1	1	2	2	3	3	3	1	1	1
1	1	2	2	3	3	3	1	1	1
1	1	2	9	3	3	3	2	2	2
1	1	2	2	3	3	3	1	1	1
1	1	2	2	3	3	3	1	1	1
1	1	2	2	3	3	3	1	1	1
1	1	9	2	2	3	3	2	1	1
1	1	2	2	3	3	3	1	1	1
1	1	2	2	3	3	3	1	2	1
1	1	2	2	3	3	3	1	1	1
1	1	2	2	3	3	3	1	1	1
1	1	2	2	3	3	3	1	1	1
1	1	2	2	3	3	3	1	1	1
1	2	2	3	3	3	3	#NULL!	1	1

1	1	2	2	3	3	3	1	1	1
3	3	1	2	1	2	3	#NULL!	1	1
1	1	1	3	3	3	3	2	1	1
1	2	2	3	3	3	3	1	1	1
1	1	2	3	3	3	3	1	1	1
1	1	2	2	3	3	3	1	1	1
1	1	2	2	3	3	3	1	1	1
1	1	2	2	3	3	3	1	1	1
1	1	2	2	3	3	3	1	1	1
1	1	2	2	3	3	3	1	1	1
1	1	2	2	3	3	3	1	1	1
1	3	2	2	3	3	3	1	1	1
1	2	2	2	3	3	3	1	1	1
1	2	2	1	3	3	3	1	1	1
1	1	2	2	3	3	3	2	1	1
1	1	2	2	3	3	3	1	1	1
1	1	2	2	3	3	3	2	1	1
1	1	2	2	3	3	3	1	1	1
1	2	2	2	3	3	3	1	1	1
1	1	2	2	3	3	3	1	1	1
1	1	2	2	2	3	3	1	1	1
1	1	9	2	2	3	3	2	1	1
1	1	2	2	3	3	3	1	1	1
1	1	2	2	3	3	3	1	1	1
1	1	2	2	3	3	3	1	1	1
1	1	1	2	3	3	3	1	1	1
1	1	2	2	3	3	3	1	1	1
1	1	2	2	3	3	9	1	1	1
1	1	2	2	3	3	3	1	1	1
1	1	9	9	3	3	3	1	1	1
9	2	2	3	3	3	3	1	1	1
1	1	2	2	3	3	3	1	#NULL!	#NULL!
1	1	2	2	3	3	3	#NULL!	#NULL!	#NULL!
1	1	2	2	3	3	3	1	1	1
9	9	9	9	9	9	9	#NULL!	#NULL!	#NULL!
1	1	2	2	3	3	3	2	1	1
1	1	2	2	3	3	3	1	#NULL!	1
1	1	2	2	3	3	3	#NULL!	#NULL!	1
1	1	2	2	3	3	3	1	#NULL!	1
1	1	2	2	3	3	3	2	1	1
1	1	2	2	3	3	3	1	1	1
1	1	2	2	3	3	3	1	1	1
1	1	2	2	3	3	3	2	2	1
1	1	2	2	3	3	3	1	1	1
1	1	2	2	3	3	3	1	1	1
1	2	2	2	3	3	3	1	1	1
1	2	2	2	3	3	3	1	1	1

1	1	2	2	3	3	3	1	1	1
1	2	2	2	3	3	3	1	1	1
1	1	2	2	3	3	3	1	1	1
1	1	2	2	3	3	3	1	1	1
1	2	2	2	3	3	3	1	1	1
1	2	2	2	3	3	3	1	1	1
1	2	2	2	2	3	3	1	1	1
1	2	2	2	3	3	9	1	1	1
1	9	2	2	3	3	3	1	1	1
1	1	2	2	3	3	3	1	1	1
1	1	2	2	3	3	3	1	1	1
1	1	2	2	3	3	3	1	1	1
1	1	2	2	3	3	3	1	1	1
1	2	2	2	3	3	3	1	1	1
1	1	2	2	3	3	3	1	1	1
1	1	2	2	3	3	3	1	1	1
1	1	2	2	3	3	3	1	1	1
1	1	2	2	3	3	3	1	1	1
1	1	2	2	3	3	3	1	1	1
1	2	2	2	3	3	3	#NULL!	#NULL!	#NULL!
1	2	2	2	3	3	3	2	1	1
1	1	2	2	3	3	3	1	1	1
1	1	2	2	3	3	3	1	1	1
1	2	1	3	3	3	3	1	1	1
1	1	2	2	3	3	3	1	1	1
1	1	2	2	3	3	3	1	1	1
1	1	2	2	3	3	3	1	1	1
1	3	2	2	3	3	3	1	1	1
1	2	2	2	3	3	3	1	1	1
1	2	2	3	3	3	3	1	1	1
1	2	2	2	3	3	3	1	1	1
1	1	2	2	3	3	3	1	1	1
1	1	2	2	3	3	3	1	1	1
1	1	2	2	3	3	3	1	1	1
1	1	2	2	3	3	3	1	1	1
1	1	2	3	3	3	3	1	1	1
1	1	2	3	3	3	3	1	1	1
1	1	2	2	3	3	3	1	1	1
1	1	2	2	3	3	3	1	1	1
1	9	2	2	3	3	3	1	1	1
1	2	2	2	3	3	3	1	1	1
9	9	9	9	9	9	9	#NULL!	#NULL!	#NULL!
1	2	2	2	3	3	3	1	1	1
1	1	2	2	3	3	3	1	1	1
1	1	2	2	3	3	3	1	1	1
1	2	2	2	3	3	3	1	1	1
1	1	2	2	3	3	3	1	1	1

1	1	2	2	3	3	3	1	1	1
1	1	2	2	3	3	3	1	1	1
1	1	2	2	3	3	3	1	1	1
1	1	3	2	3	3	3	1	1	1
1	1	2	2	3	3	3	1	1	1
1	1	2	2	3	3	3	1	1	1
1	2	2	2	3	3	3	1	1	1
1	1	2	2	3	3	3	#NULL!	1	2
1	2	2	2	3	3	3	1	1	1
9	9	9	9	9	9	9	#NULL!	#NULL!	#NULL!
1	1	2	2	3	3	3	1	1	1
1	1	2	2	3	3	3	1	1	1
1	1	2	2	3	3	3	1	1	1
1	2	2	3	3	3	3	1	1	1
1	2	2	2	3	3	3	1	1	1
1	2	2	2	3	3	3	1	1	1
1	2	2	2	3	3	3	1	1	1
1	2	2	2	3	3	3	1	1	1
1	2	1	2	2	3	3	1	1	1
1	1	2	2	3	3	3	1	1	1
1	1	2	2	3	3	3	1	1	1
1	1	2	2	3	3	3	1	1	1
1	1	2	2	3	3	3	1	1	1
1	1	2	2	3	3	3	1	1	1
1	2	2	2	3	3	3	1	1	1
1	1	2	2	3	3	3	1	1	1
1	2	2	2	3	3	3	#NULL!	#NULL!	#NULL!
9	9	9	9	9	9	9	#NULL!	#NULL!	#NULL!
9	9	9	9	9	9	9	#NULL!	#NULL!	#NULL!
9	9	9	9	9	9	9	#NULL!	#NULL!	#NULL!
9	9	9	9	9	9	9	#NULL!	#NULL!	#NULL!
9	9	9	9	9	9	9	#NULL!	#NULL!	#NULL!
9	9	9	9	9	9	9	#NULL!	#NULL!	#NULL!
9	9	9	9	9	9	9	#NULL!	#NULL!	#NULL!
9	9	9	9	9	9	9	#NULL!	#NULL!	#NULL!
9	9	9	9	9	9	9	#NULL!	#NULL!	#NULL!
9	9	9	9	9	9	9	#NULL!	#NULL!	#NULL!
9	9	9	9	9	9	9	#NULL!	#NULL!	#NULL!
9	9	9	9	9	9	9	#NULL!	#NULL!	#NULL!
9	9	9	9	9	9	9	#NULL!	#NULL!	#NULL!
9	9	9	9	9	9	9	#NULL!	#NULL!	#NULL!
9	9	9	9	9	9	9	#NULL!	#NULL!	#NULL!
9	9	9	9	9	9	9	#NULL!	#NULL!	#NULL!
9	9	9	9	9	9	9	#NULL!	#NULL!	#NULL!
9	9	9	9	9	9	9	#NULL!	#NULL!	#NULL!
9	9	9	9	9	9	9	#NULL!	#NULL!	#NULL!
9	9	9	9	9	9	9	#NULL!	#NULL!	#NULL!

9	9	9	9	9	9	9	#NULL!	#NULL!	#NULL!
9	9	9	9	9	9	9	#NULL!	#NULL!	#NULL!
9	9	9	9	9	9	9	#NULL!	#NULL!	#NULL!
9	9	9	9	9	9	9	#NULL!	#NULL!	#NULL!
9	9	9	9	9	9	9	#NULL!	#NULL!	#NULL!
9	9	9	9	9	9	9	#NULL!	#NULL!	#NULL!
9	9	9	9	9	9	9	#NULL!	#NULL!	#NULL!
9	9	9	9	9	9	9	#NULL!	#NULL!	#NULL!
9	9	9	9	9	9	9	#NULL!	#NULL!	#NULL!
9	9	9	9	9	9	9	#NULL!	#NULL!	#NULL!
9	9	9	9	9	9	9	#NULL!	#NULL!	#NULL!
9	9	9	9	9	9	9	#NULL!	#NULL!	#NULL!
9	9	9	9	9	9	9	#NULL!	#NULL!	#NULL!
3	2	2	1	2	2	3	1	1	1
2	2	2	2	2	2	2	2	2	2
2	2	2	2	2	2	2	2	2	2
2	1	1	1	3	3	2	#NULL!	#NULL!	#NULL!
2	2	2	2	1	2	1	1	1	2
1	1	1	1	1	1	1	#NULL!	#NULL!	#NULL!
2	1	1	2	1	1	1	1	1	1
9	9	9	9	9	9	9	1	1	2
3	2	2	2	2	2	2	2	2	2
9	9	9	9	9	9	9	#NULL!	#NULL!	#NULL!
2	2	2	2	2	3	2	2	2	2
1	9	9	9	9	9	9	2	2	2
2	3	3	3	2	2	2	1	1	1
9	9	9	9	9	9	9	#NULL!	#NULL!	#NULL!
1	1	1	1	1	1	1	2	2	2
2	3	2	3	2	2	2	2	2	1
1	1	3	1	2	1	3	1	1	2
3	3	3	3	3	3	3	2	2	2
3	2	2	2	3	2	2	2	2	2
9	9	9	9	9	9	9	1	1	2
1	1	1	1	3	1	3	1	1	2
9	9	9	9	9	9	9	1	1	1
9	9	9	9	9	9	9	1	1	2
2	2	2	1	3	3	1	1	2	1
1	2	1	2	1	2	1	2	2	1
1	2	1	2	1	1	2	1	1	2
1	2	2	2	2	1	1	1	1	1
9	9	9	9	9	9	9	#NULL!	#NULL!	#NULL!
9	9	9	9	9	9	9	#NULL!	#NULL!	#NULL!
9	9	9	9	9	9	9	1	1	1
1	1	1	1	1	1	9	1	1	2
9	9	9	9	9	9	9	#NULL!	#NULL!	#NULL!
9	9	9	9	9	9	9	#NULL!	#NULL!	#NULL!
9	9	9	9	9	9	9	2	2	#NULL!

1	1	1	1	1	1	1	1	2	1
9	9	9	9	9	9	9	#NULL!	#NULL!	#NULL!
2	2	3	3	1	3	2	2	2	2
1	1	2	3	2	2	2	1	1	2
1	1	1	3	1	2	2	1	1	2
9	9	9	9	9	9	9	1	1	2
2	2	2	2	2	2	2	2	2	1
2	2	2	2	2	2	2	2	1	1
2	2	2	2	2	2	2	2	2	2
1	1	1	1	2	2	2	1	2	2
2	2	2	2	2	2	2	1	2	2
9	9	9	9	9	9	9	#NULL!	#NULL!	#NULL!
9	9	9	9	9	9	9	2	2	2
2	2	2	2	2	2	2	2	2	2
1	1	2	3	3	1	3	2	2	2
1	2	1	3	2	3	3	2	2	2
1	1	1	1	1	1	3	2	2	2
2	2	2	2	2	2	2	1	1	1
1	9	1	1	2	1	1	1	1	1
9	9	9	9	9	9	9	#NULL!	#NULL!	#NULL!
2	2	2	2	2	2	2	1	2	2
2	2	2	2	2	2	2	#NULL!	#NULL!	#NULL!
2	2	2	2	2	2	2	2	2	2
2	2	2	2	2	2	2	2	2	1
2	2	2	2	2	2	2	#NULL!	#NULL!	#NULL!
1	2	2	2	2	1	1	2	1	1
2	2	2	2	2	2	2	2	2	2
3	2	1	2	3	2	1	2	2	2
9	9	9	9	9	9	9	2	2	2
1	1	2	2	3	3	3	1	1	1
1	1	1	3	3	3	3	1	1	2
1	1	1	2	2	3	3	1	2	2
1	3	2	3	3	2	2	1	2	2
1	1	1	3	3	3	3	1	1	1
9	3	2	3	2	1	3	1	1	2
9	9	9	9	9	9	9	2	2	1
1	1	2	3	2	2	3	2	2	2
1	1	9	9	9	9	9	1	2	2
9	9	9	9	9	9	9	2	#NULL!	2
2	2	2	2	2	2	2	2	2	2
3	3	3	3	3	3	3	1	2	2
2	2	2	2	2	2	2	1	2	2
3	1	2	3	1	2	3	1	1	2
9	9	9	9	9	9	9	1	1	2
1	1	1	1	1	1	1	1	1	1
2	2	2	2	2	2	1	1	1	1
1	3	3	2	3	3	3	2	2	2

2	2	2	2	2	2	2	2	2	2
3	2	2	2	3	2	9	2	2	2
2	2	2	2	2	2	2	1	2	2
9	9	9	9	9	9	9	2	1	2
9	9	9	9	9	9	9	1	1	1
2	2	2	2	2	2	2	1	1	1
1	3	2	3	3	3	3	2	2	2
2	3	3	1	1	1	1	2	2	2
9	9	9	9	9	9	9	2	2	2
2	3	1	2	2	3	2	1	2	2
2	2	2	2	2	2	2	2	2	2
2	2	2	2	2	2	2	2	2	2
1	1	1	1	1	1	1	2	2	2
2	2	2	2	2	2	2	#NULL!	#NULL!	#NULL!
9	9	9	9	9	9	9	1	1	1
2	2	2	2	3	2	2	2	2	2
3	3	3	3	3	3	3	1	1	1
2	2	2	2	2	2	2	1	1	2
2	2	2	2	2	2	2	#NULL!	#NULL!	#NULL!
3	1	2	3	1	2	3	2	1	1
1	2	1	2	1	1	1	2	2	2
1	1	1	1	1	2	2	2	2	2
9	9	9	9	9	9	9	2	2	2
9	9	9	9	9	9	9	1	1	1
1	3	3	3	3	3	3	1	1	1
9	9	9	9	9	9	9	#NULL!	#NULL!	#NULL!
9	9	9	9	9	9	9	2	2	2
2	2	2	2	2	2	2	2	2	2
2	1	2	1	1	1	1	1	1	1
3	2	2	3	1	2	3	2	2	2
1	2	3	2	2	3	3	2	2	2
1	1	1	1	1	1	1	1	2	1
2	2	2	2	2	2	3	2	2	2
1	2	2	1	9	2	1	1	1	1
9	9	9	9	9	9	9	1	1	1
2	2	2	2	2	2	9	#NULL!	#NULL!	#NULL!
9	9	9	9	9	9	9	#NULL!	#NULL!	#NULL!
9	9	9	9	9	9	9	#NULL!	#NULL!	#NULL!
9	9	9	9	9	9	9	#NULL!	#NULL!	#NULL!
1	1	1	1	1	1	1	1	1	2
3	3	2	3	2	2	2	2	2	2
1	3	3	3	3	2	3	1	1	1
2	2	1	2	3	3	2	2	2	2
9	9	9	9	9	9	9	#NULL!	#NULL!	#NULL!
9	9	9	9	9	9	9	#NULL!	#NULL!	#NULL!
9	9	9	9	9	9	9	#NULL!	#NULL!	#NULL!
1	1	1	1	1	1	1	1	1	1

9	9	9	9	9	9	9	#NULL!	#NULL!	#NULL!
9	9	9	9	9	9	9	#NULL!	#NULL!	#NULL!
9	9	9	9	9	9	9	1	2	2
9	9	9	9	9	9	9	#NULL!	#NULL!	#NULL!
9	9	9	9	9	9	9	#NULL!	#NULL!	#NULL!
9	9	9	9	9	9	9	1	1	2
2	2	3	1	2	1	3	2	1	2
9	9	9	9	9	9	9	#NULL!	#NULL!	#NULL!
1	1	3	3	3	3	3	1	1	1
3	1	2	3	1	2	3	2	2	2
3	2	1	2	3	1	2	1	2	2
2	2	2	2	2	2	2	2	2	2
2	2	2	2	2	2	2	2	2	2
9	9	9	9	9	9	9	#NULL!	#NULL!	#NULL!
2	2	2	2	2	2	2	2	2	2
2	2	2	2	2	2	9	2	2	2
1	1	1	1	1	1	1	1	2	1
2	1	3	3	2	1	3	2	2	2
3	2	1	2	2	2	2	1	1	1
9	9	9	9	9	9	9	#NULL!	#NULL!	#NULL!
1	2	3	2	1	2	3	2	2	2
1	2	1	3	1	3	3	1	2	1
2	2	2	2	2	2	2	2	2	2
2	2	3	3	3	2	2	2	2	2
3	3	2	3	3	3	2	#NULL!	#NULL!	#NULL!
9	9	9	9	9	9	9	#NULL!	#NULL!	#NULL!
1	1	1	1	1	1	1	2	2	2
9	9	9	9	9	9	9	2	2	2
3	3	3	3	3	3	3	2	2	2
2	9	2	3	2	2	3	2	2	2
2	2	2	2	2	2	2	2	2	2
1	9	9	9	9	9	9	2	2	2
1	9	9	9	9	9	3	2	2	2
2	2	2	2	2	2	2	#NULL!	#NULL!	#NULL!
2	2	2	2	3	3	3	2	1	2
2	1	3	2	1	2	1	#NULL!	#NULL!	#NULL!
2	2	2	2	2	2	2	#NULL!	#NULL!	#NULL!
1	1	1	2	2	2	3	1	1	2
2	2	2	2	2	2	2	2	2	2
9	9	9	9	9	9	9	2	2	2
2	2	1	2	2	1	2	#NULL!	#NULL!	#NULL!
2	2	2	2	2	2	2	1	2	2
2	2	2	2	2	2	3	2	2	2
2	2	2	2	2	2	2	2	2	2
2	2	2	2	2	2	2	1	2	2
1	9	1	3	9	9	9	2	2	1
2	2	2	2	2	2	2	2	2	2

2	2	2	2	2	2	2	1	1	1
2	2	2	2	2	2	2	2	2	2
1	3	1	3	3	3	3	1	2	2
1	2	2	3	3	3	3	2	2	2
2	2	2	2	2	2	2	2	2	2
2	2	3	3	3	2	2	2	2	2
3	1	2	2	1	1	2	2	2	2
2	1	2	2	2	2	9	1	1	2
1	3	3	3	3	3	3	1	1	2
3	3	3	3	3	3	3	2	2	2
2	2	2	2	2	2	2	1	1	2
2	2	2	2	3	3	3	2	2	2
1	1	3	3	3	3	3	1	1	2
1	1	3	3	3	3	3	1	1	2
1	2	1	2	1	2	3	2	2	2
1	2	1	2	2	2	2	2	2	2
2	2	2	3	3	3	3	2	2	2
3	2	1	2	1	3	2	#NULL!	#NULL!	#NULL!
2	1	2	1	2	1	2	2	2	2
1	3	1	3	3	3	3	2	2	2
1	3	1	3	3	3	3	2	2	2
9	9	9	9	9	9	9	1	1	1
2	2	2	2	2	2	2	2	2	2
2	2	2	2	2	2	2	1	1	1
2	2	2	2	2	2	2	1	1	2
1	1	1	3	3	3	3	2	2	2
1	1	2	1	2	2	3	2	2	2
1	1	2	1	2	2	3	1	2	2
1	3	1	3	3	3	1	1	1	1
1	3	1	3	3	3	3	1	1	1
2	9	3	3	3	3	3	1	1	1
1	2	2	3	1	2	2	2	2	2
1	1	1	2	1	2	1	1	1	1
9	9	9	9	9	9	9	#NULL!	#NULL!	#NULL!
9	9	9	9	9	9	9	2	2	2
3	9	1	3	9	1	3	1	1	1
2	2	1	2	2	2	2	#NULL!	#NULL!	#NULL!
1	2	1	2	1	2	1	1	1	1
2	2	2	2	2	2	2	1	1	1
2	2	3	3	2	3	2	1	1	1
2	3	2	1	2	3	2	1	1	1
9	9	9	9	9	9	9	#NULL!	#NULL!	#NULL!
9	9	9	9	9	9	9	#NULL!	#NULL!	#NULL!
1	1	1	1	1	1	1	1	1	1
9	9	9	9	9	9	9	1	1	1
9	9	9	9	9	9	9	#NULL!	#NULL!	#NULL!
9	9	9	9	9	9	9	#NULL!	#NULL!	#NULL!

9	9	9	9	9	9	9	#NULL!	#NULL!	#NULL!
2	3	1	1	2	1	1	1	1	1
9	9	9	9	9	9	9	1	1	1
2	2	3	3	2	2	2	2	2	2
3	3	3	3	2	2	2	2	2	2
3	1	2	3	1	2	3	1	2	2
1	2	2	2	2	2	2	2	2	1
9	9	9	9	9	9	9	2	#NULL!	1
2	2	1	2	2	2	2	1	1	1
9	9	9	9	9	9	9	#NULL!	#NULL!	#NULL!
1	1	1	1	1	1	1	1	1	1
1	1	3	1	3	3	3	2	2	2
9	9	9	9	9	9	9	#NULL!	#NULL!	#NULL!
9	9	9	9	9	9	9	#NULL!	#NULL!	#NULL!
1	1	1	1	1	1	1	1	1	1
9	9	9	9	9	9	9	#NULL!	#NULL!	#NULL!
9	9	9	9	9	9	9	#NULL!	#NULL!	#NULL!
2	2	2	2	2	2	2	2	1	1
2	2	2	2	2	2	2	2	2	2
1	2	2	1	2	1	1	1	1	2
2	2	2	2	2	2	2	2	2	2
2	2	2	2	2	2	2	1	1	1
1	1	2	2	2	3	1	1	2	2
2	1	2	1	2	3	2	1	2	2
1	1	1	1	1	2	2	2	2	2
9	9	9	9	9	9	9	1	2	2
1	3	3	3	3	3	3	2	#NULL!	2
2	3	1	1	2	2	3	#NULL!	#NULL!	#NULL!
9	9	9	9	9	9	9	1	1	1
2	2	3	2	2	2	3	2	2	2
1	2	1	2	1	2	2	2	1	1
2	2	2	2	2	2	9	2	2	2
2	2	2	2	2	2	2	#NULL!	#NULL!	#NULL!
2	2	2	2	2	2	2	2	2	2
2	2	2	2	2	2	2	1	1	1
2	2	2	2	2	2	2	2	2	2
1	3	2	3	2	1	2	2	2	2
9	9	9	9	9	9	9	1	2	2
9	9	9	9	2	9	9	2	2	2
1	1	3	3	3	3	3	#NULL!	#NULL!	#NULL!
2	3	2	3	2	3	2	2	2	2
1	9	1	9	9	9	3	1	2	2
2	2	1	2	2	2	1	#NULL!	#NULL!	#NULL!
9	9	9	9	9	9	9	2	2	1
3	3	2	2	2	2	2	1	1	2
9	9	9	9	9	9	9	#NULL!	#NULL!	#NULL!
2	2	2	2	2	2	2	2	2	2

3	1	3	1	2	2	1	2	2	2
9	9	9	9	9	9	9	2	2	2
1	1	1	1	1	1	1	#NULL!	#NULL!	#NULL!
9	2	2	3	3	3	3	2	2	2
3	9	1	3	2	1	9	1	2	1
1	2	2	2	2	2	2	1	2	2
1	1	3	1	2	3	3	2	2	2
9	9	9	9	9	9	9	#NULL!	#NULL!	#NULL!
2	2	2	2	2	2	2	1	2	2
3	3	3	3	3	3	3	2	2	2
2	2	2	2	2	2	2	2	2	2
2	2	2	2	2	2	2	2	2	2
9	3	2	3	1	2	3	1	2	1
3	3	3	2	2	3	9	2	2	2
2	2	2	1	2	1	1	1	2	1
2	2	2	1	2	1	1	1	2	1
2	2	2	2	2	2	2	2	2	2
2	2	2	2	2	2	2	1	2	2
9	9	9	9	9	9	9	#NULL!	#NULL!	#NULL!
9	9	9	9	9	9	9	#NULL!	#NULL!	#NULL!
2	3	3	3	3	3	3	2	2	2
1	2	3	3	3	1	3	1	1	2
9	9	9	9	9	9	9	1	2	1
9	9	9	9	9	9	9	#NULL!	#NULL!	#NULL!
3	3	3	3	3	3	3	2	2	2
9	9	9	9	9	9	9	2	2	2
9	9	9	9	9	9	9	#NULL!	#NULL!	#NULL!
9	9	9	9	9	9	9	#NULL!	#NULL!	#NULL!
1	3	2	1	3	1	2	#NULL!	#NULL!	#NULL!
1	1	1	1	1	1	3	2	2	2
2	2	2	2	2	2	2	2	2	2
1	1	2	3	1	2	2	2	2	2
2	2	2	2	2	2	2	2	2	2
2	2	2	2	2	2	2	2	2	2
9	3	3	3	3	3	3	2	2	2
1	3	1	3	3	3	3	1	2	2
1	2	1	3	3	1	2	1	2	1
2	9	1	2	2	2	2	2	2	1
3	1	2	1	1	1	2	2	2	1
1	2	2	3	3	3	2	1	1	1
3	3	3	2	2	2	2	1	1	2
3	3	3	3	9	3	3	2	2	2
9	9	9	9	9	9	9	1	1	2
1	9	9	9	9	9	9	1	1	1
1	2	3	2	1	3	2	1	2	1
2	2	2	2	2	2	2	2	2	2
2	2	2	2	2	2	2	2	2	2

3	1	1	2	9	2	2	1	2	2
9	9	9	9	9	9	9	#NULL!	#NULL!	#NULL!
9	9	9	9	9	9	9	#NULL!	#NULL!	#NULL!
9	9	9	9	9	9	9	#NULL!	#NULL!	#NULL!
9	9	9	9	9	9	9	#NULL!	#NULL!	#NULL!
9	9	9	9	9	9	9	1	1	2
9	9	9	9	9	9	9	#NULL!	#NULL!	#NULL!
9	9	9	9	9	9	9	#NULL!	#NULL!	#NULL!
9	9	9	9	9	9	9	#NULL!	#NULL!	#NULL!
9	9	3	9	9	3	3	2	2	2
2	2	2	2	2	2	2	1	2	2
2	1	1	2	1	1	2	1	2	1
1	3	1	3	3	3	3	#NULL!	#NULL!	#NULL!
2	2	2	2	2	2	2	1	2	2
2	2	2	2	2	2	2	1	1	2
9	9	9	9	9	9	9	#NULL!	#NULL!	#NULL!
1	1	1	1	1	2	2	1	1	1
2	2	2	2	2	2	2	2	2	2
2	2	2	2	2	2	2	2	1	1
3	1	3	1	3	1	2	2	2	2
1	3	2	1	3	1	2	1	2	2
3	3	3	3	2	3	3	1	2	2
1	1	1	1	1	1	1	1	2	2
2	2	2	2	2	2	9	1	1	1
2	2	2	2	2	2	9	2	2	2
2	2	2	2	2	2	2	1	2	2
2	2	2	2	2	2	2	1	1	2
1	2	1	2	1	2	1	1	1	1
9	9	9	9	9	9	9	2	2	2
2	2	2	2	2	2	2	2	2	2
9	9	9	9	9	9	9	#NULL!	#NULL!	#NULL!
2	2	2	2	2	2	2	2	2	2
2	3	3	2	1	1	1	1	1	1
1	9	9	9	9	9	9	1	1	1
3	3	3	3	3	3	3	#NULL!	#NULL!	#NULL!
2	2	2	2	2	2	2	2	2	2
2	2	2	2	2	2	2	2	#NULL!	2
2	2	2	2	2	2	2	1	2	1
1	3	3	3	3	1	1	1	1	1
9	9	9	9	9	9	9	#NULL!	#NULL!	#NULL!
1	9	1	1	2	2	2	2	2	2
2	2	2	2	2	2	2	2	2	2
2	2	2	2	2	2	2	2	2	2
1	2	1	2	2	1	3	1	1	1
1	1	2	2	2	2	3	1	1	1
9	9	9	9	9	9	9	#NULL!	#NULL!	#NULL!
9	9	9	9	9	9	9	1	2	2

9	9	9	9	9	9	9	#NULL!	#NULL!	#NULL!
2	2	2	2	2	2	2	1	2	2
9	9	9	9	9	9	9	1	1	1
9	9	9	9	9	9	9	#NULL!	#NULL!	#NULL!
9	9	9	9	9	9	9	#NULL!	#NULL!	#NULL!
9	9	9	9	9	9	9	#NULL!	#NULL!	#NULL!
9	9	9	9	9	9	9	#NULL!	#NULL!	#NULL!
9	9	9	9	9	9	9	#NULL!	#NULL!	#NULL!
9	9	9	9	9	9	9	#NULL!	#NULL!	#NULL!
9	9	9	9	9	9	9	#NULL!	#NULL!	#NULL!
9	9	9	9	9	9	9	#NULL!	#NULL!	#NULL!
9	9	9	9	9	9	9	#NULL!	#NULL!	#NULL!
9	9	9	9	9	9	9	#NULL!	#NULL!	#NULL!
9	9	9	9	9	9	9	#NULL!	#NULL!	#NULL!
9	9	9	9	9	9	9	#NULL!	#NULL!	#NULL!
9	9	9	9	9	9	9	#NULL!	#NULL!	#NULL!
9	9	9	9	9	9	9	#NULL!	#NULL!	#NULL!
9	9	9	9	9	9	9	#NULL!	#NULL!	#NULL!
9	9	9	9	9	9	9	#NULL!	#NULL!	#NULL!
9	9	9	9	9	9	9	#NULL!	#NULL!	#NULL!
9	9	9	9	9	9	9	#NULL!	#NULL!	#NULL!
9	9	9	9	9	9	9	#NULL!	#NULL!	#NULL!
9	9	9	9	9	9	9	#NULL!	#NULL!	#NULL!
3	2	1	2	3	2	1	2	2	1
1	2	2	2	2	2	2	1	2	2
2	2	2	2	2	2	9	1	1	1
1	2	1	2	1	2	3	1	1	2
9	9	9	9	9	9	9	1	2	2
9	2	1	2	3	3	3	1	1	2
2	2	2	2	2	2	2	1	1	2
1	2	1	1	2	1	3	1	1	1
1	2	1	1	1	2	3	2	2	2
1	1	3	3	2	1	3	1	2	2
1	3	1	1	3	3	3	1	1	2
1	3	1	3	3	3	3	1	1	2
1	1	2	2	1	3	3	1	1	1
1	2	2	3	3	3	3	1	1	2
1	1	2	2	2	2	2	1	1	1
1	2	2	3	3	2	3	1	1	1
9	9	9	9	9	9	2	1	#NULL!	2
1	3	1	3	2	3	3	1	1	1
2	2	2	2	2	2	2	1	1	2
9	2	9	9	9	9	9	1	2	1
1	3	1	1	3	3	3	1	1	2
1	3	1	3	3	3	3	1	1	1
2	2	2	2	2	2	2	2	2	2
3	9	1	3	1	3	1	1	1	1

1	3	1	3	3	3	3	1	1	1
1	1	3	3	3	3	3	1	1	1
1	1	2	1	3	3	3	1	1	2
1	3	1	2	2	3	3	1	1	1
3	1	2	3	1	2	3	2	1	2
9	9	9	9	9	9	9	2	1	2
1	3	1	3	3	2	3	1	1	2
9	9	9	9	9	9	9	1	1	1
2	2	2	2	2	2	2	2	2	2
2	2	2	2	2	2	2	2	2	2
9	1	9	9	9	9	9	1	1	2
9	1	9	9	9	9	9	1	1	1
3	1	1	2	3	1	1	1	1	1
1	3	9	2	9	9	9	#NULL!	#NULL!	#NULL!
9	9	9	9	9	9	9	1	1	1
9	9	9	9	9	9	9	#NULL!	#NULL!	#NULL!
9	9	9	9	9	9	9	#NULL!	#NULL!	#NULL!
9	9	9	9	9	9	9	2	1	2
9	9	9	9	9	9	9	1	1	2
9	1	1	3	3	3	3	1	1	1
9	9	9	9	9	9	9	1	1	1
1	9	9	3	9	9	2	1	2	2
9	9	9	9	9	9	9	1	1	1
9	9	9	9	9	9	9	1	2	2
9	9	9	9	9	9	9	1	1	1
2	3	9	1	2	3	2	1	1	1
9	9	9	9	9	9	9	1	1	1
1	9	1	3	3	3	3	2	2	1
1	2	2	3	3	3	3	2	1	2
9	9	9	9	9	9	9	#NULL!	#NULL!	#NULL!
1	1	2	3	2	2	3	1	1	2
1	1	3	3	3	3	3	1	1	1
3	3	3	3	3	3	3	2	2	2
1	3	1	3	3	3	1	2	2	2
1	1	1	3	3	1	3	1	1	2
9	9	9	9	9	9	3	1	2	2
2	1	1	2	2	2	3	1	1	2
1	1	1	2	2	2	3	1	2	1
3	2	3	2	3	2	3	2	2	2
1	1	1	3	3	3	1	2	2	2
1	2	2	2	2	3	3	1	1	1
1	1	1	1	1	1	1	1	1	1
9	9	9	9	9	9	9	1	1	2
2	1	1	2	2	3	3	2	2	2
1	2	1	3	3	3	3	#NULL!	1	1
1	3	2	3	3	3	3	1	1	2
1	3	1	3	3	3	3	1	1	1

9	9	9	9	9	9	9	1	1	1
3	2	2	2	2	2	2	1	1	1
9	9	9	9	9	9	9	1	1	2
1	1	3	2	3	3	3	1	1	2
9	9	9	9	9	9	9	#NULL!	#NULL!	#NULL!
1	3	1	3	3	3	3	1	1	1
1	1	1	3	3	3	3	1	1	1
9	9	9	9	9	9	9	#NULL!	#NULL!	#NULL!
9	9	9	9	9	9	2	1	1	2
2	2	2	2	2	2	2	2	2	2
1	1	1	3	1	2	1	1	1	2
9	9	9	9	9	9	9	1	1	2
2	3	1	2	2	2	1	1	1	1
9	1	9	1	9	9	2	#NULL!	#NULL!	#NULL!
9	9	9	9	9	9	9	#NULL!	#NULL!	#NULL!
3	9	2	2	2	1	2	1	2	1
9	9	9	9	9	9	9	1	1	1
9	9	9	9	9	9	9	#NULL!	#NULL!	#NULL!
3	3	3	3	3	3	9	2	2	2
1	2	2	2	2	3	3	1	1	1
9	9	9	9	9	9	9	1	1	1
1	1	1	2	1	1	3	1	1	1
2	1	2	3	1	3	2	2	2	1
9	9	9	9	9	9	9	1	1	1
1	2	2	2	2	2	2	1	2	2
9	9	9	9	9	9	9	#NULL!	#NULL!	#NULL!
9	9	9	9	9	9	9	1	1	1
1	3	1	1	1	1	1	1	1	1
1	1	2	2	2	3	3	2	2	1
1	1	2	3	3	3	3	1	1	2
9	9	9	9	9	9	9	#NULL!	#NULL!	#NULL!
9	9	9	9	9	9	9	#NULL!	#NULL!	#NULL!
9	9	9	9	9	9	9	#NULL!	#NULL!	#NULL!
9	9	9	9	9	9	9	#NULL!	#NULL!	#NULL!
9	9	9	9	9	9	9	#NULL!	#NULL!	#NULL!
9	9	9	9	9	9	9	#NULL!	#NULL!	#NULL!
9	9	9	9	9	9	9	#NULL!	#NULL!	#NULL!
9	9	9	9	9	9	9	#NULL!	#NULL!	#NULL!
9	9	9	9	9	9	9	#NULL!	#NULL!	#NULL!
9	9	9	9	9	9	9	#NULL!	#NULL!	#NULL!
9	9	9	9	9	9	9	#NULL!	#NULL!	#NULL!
9	9	9	9	9	9	9	#NULL!	#NULL!	#NULL!
9	9	9	9	9	9	9	#NULL!	#NULL!	#NULL!
9	9	9	9	9	9	9	#NULL!	#NULL!	#NULL!
9	9	9	9	9	9	9	#NULL!	#NULL!	#NULL!
9	9	9	9	9	9	9	#NULL!	#NULL!	#NULL!
9	9	9	9	9	9	9	#NULL!	#NULL!	#NULL!

9	9	9	9	9	9	9	#NULL!	#NULL!	#NULL!
9	9	9	9	9	9	9	#NULL!	#NULL!	#NULL!
9	9	9	9	9	9	9	#NULL!	#NULL!	#NULL!
9	9	9	9	9	9	9	#NULL!	#NULL!	#NULL!
9	9	9	9	9	9	9	#NULL!	#NULL!	#NULL!
9	9	9	9	9	9	9	#NULL!	#NULL!	#NULL!
9	9	9	9	9	9	9	#NULL!	#NULL!	#NULL!
9	9	9	9	9	9	9	#NULL!	#NULL!	#NULL!
9	9	9	9	9	9	9	#NULL!	#NULL!	#NULL!
9	9	9	9	9	9	9	#NULL!	#NULL!	#NULL!
9	9	9	9	9	9	9	#NULL!	#NULL!	#NULL!
9	9	9	9	9	9	9	#NULL!	#NULL!	#NULL!
9	9	9	9	9	9	9	#NULL!	#NULL!	#NULL!
9	9	9	9	9	9	9	#NULL!	#NULL!	#NULL!
9	9	9	9	9	9	9	#NULL!	#NULL!	#NULL!
9	9	9	9	9	9	9	#NULL!	#NULL!	#NULL!
9	9	9	9	9	9	9	#NULL!	#NULL!	#NULL!
9	9	9	9	9	9	9	#NULL!	#NULL!	#NULL!
9	9	9	9	9	9	9	#NULL!	#NULL!	#NULL!
9	9	9	9	9	9	9	1	1	1
9	9	9	9	9	9	9	#NULL!	#NULL!	#NULL!
9	9	9	9	9	9	9	#NULL!	#NULL!	#NULL!
9	9	9	9	9	9	9	#NULL!	#NULL!	#NULL!
9	9	9	9	9	9	9	#NULL!	#NULL!	#NULL!
9	9	9	9	9	9	9	#NULL!	#NULL!	#NULL!
9	9	9	9	9	9	9	#NULL!	#NULL!	#NULL!
9	9	9	9	9	9	9	#NULL!	#NULL!	#NULL!
9	9	9	9	9	9	9	#NULL!	#NULL!	#NULL!
9	9	9	9	9	9	9	#NULL!	#NULL!	#NULL!
9	9	9	9	9	9	9	#NULL!	#NULL!	#NULL!
9	9	9	9	9	9	9	#NULL!	#NULL!	#NULL!
9	9	9	9	9	9	9	#NULL!	#NULL!	#NULL!
9	9	9	9	9	9	9	#NULL!	#NULL!	#NULL!
9	9	9	9	9	9	9	#NULL!	#NULL!	#NULL!
9	9	9	9	9	9	9	#NULL!	#NULL!	#NULL!
2	2	2	2	2	2	2	2	2	2
9	9	9	9	9	9	9	#NULL!	#NULL!	#NULL!
9	9	9	9	9	9	9	1	1	2
3	3	3	3	3	3	3	1	1	1
3	3	3	3	3	3	3	2	2	2
9	9	9	9	9	9	9	#NULL!	#NULL!	#NULL!
9	9	9	9	9	9	9	1	2	2
9	9	9	9	9	9	9	1	1	1
9	9	9	9	9	9	9	1	2	2
2	2	2	2	2	2	2	2	2	2
9	1	1	1	1	1	2	2	2	2
9	2	2	3	2	2	3	1	1	2

1	2	2	3	3	2	3	1	1	2
1	2	2	2	2	2	1	1	1	2
1	1	3	1	3	3	3	1	1	1
1	1	2	3	3	3	3	1	1	2
1	3	3	3	3	3	3	1	1	1
1	1	3	1	3	2	3	1	1	1
1	2	2	3	3	3	3	1	1	1
1	2	1	3	3	3	3	1	1	2
2	3	2	1	2	3	2	2	2	2
1	3	1	3	2	3	3	1	1	2
9	9	9	9	9	9	9	1	1	2
2	2	2	2	2	2	2	1	2	2
9	9	9	9	9	9	9	#NULL!	#NULL!	#NULL!
1	1	3	3	3	2	3	1	2	2
1	1	3	3	3	2	3	1	2	2
1	3	1	3	3	3	3	1	1	2
9	9	9	9	9	9	9	#NULL!	#NULL!	#NULL!
1	3	3	3	3	3	3	1	1	2
1	2	1	1	1	1	3	2	1	1
3	3	3	1	1	1	1	1	2	2
9	3	3	3	3	3	3	1	1	1
1	2	2	2	2	3	3	1	1	1
1	1	1	3	3	3	1	1	1	1
1	3	1	3	3	3	3	1	1	1
3	3	3	3	3	3	3	1	2	1
1	2	1	2	1	2	1	2	2	2
9	1	1	1	1	1	1	2	2	2
1	1	3	3	1	2	1	#NULL!	#NULL!	#NULL!
2	2	2	2	2	2	2	2	1	1
9	9	9	9	9	9	9	#NULL!	#NULL!	#NULL!
9	9	9	9	9	9	9	1	#NULL!	#NULL!
9	9	9	9	9	9	9	1	1	2
9	9	9	9	9	9	9	1	1	1
9	9	9	9	9	9	9	2	2	2
9	9	9	9	9	9	9	#NULL!	#NULL!	#NULL!
9	9	9	9	9	9	9	2	2	2
1	1	1	1	2	3	2	1	1	1
9	9	9	9	9	9	9	1	1	1
9	9	9	9	9	9	9	1	1	1
9	9	9	9	9	9	9	#NULL!	#NULL!	#NULL!
3	2	3	2	1	1	1	1	1	1
9	9	9	9	9	9	9	1	1	2
1	3	1	3	3	3	3	1	1	1
1	3	1	3	3	3	1	1	1	2
3	3	3	3	3	3	1	1	2	2
3	1	3	3	3	3	3	1	2	1
1	1	1	3	3	3	1	2	2	2

2	2	3	3	2	3	9	1	1	2
2	1	3	2	3	1	2	#NULL!	#NULL!	#NULL!
2	2	2	2	2	2	2	2	2	2
1	1	1	1	1	1	3	1	2	1
2	3	2	2	2	2	2	1	2	2
3	1	2	3	1	2	3	1	2	2
3	3	1	1	2	2	1	1	#NULL!	1
2	3	2	3	2	1	2	2	1	1
1	3	1	3	3	3	3	1	2	2
9	1	9	2	3	2	2	#NULL!	#NULL!	#NULL!
1	1	1	3	3	2	3	1	1	1
3	1	2	3	1	2	3	1	2	2
2	1	2	1	2	1	2	1	2	1
2	3	2	2	3	2	9	2	2	2
3	2	1	2	2	1	1	2	2	2
2	2	2	2	2	3	1	1	1	2
2	3	2	2	2	2	2	1	2	1
1	9	9	9	9	9	9	1	2	2
2	3	2	2	1	2	2	1	2	2
2	2	2	1	9	1	2	2	2	2
3	3	2	3	2	1	2	1	2	2
1	1	1	3	3	3	3	1	1	2
3	1	2	3	1	2	3	1	1	1
1	1	1	3	3	3	3	2	2	2
1	1	2	1	2	2	1	2	1	2
3	1	1	3	2	2	3	2	1	1
1	3	1	3	3	1	3	2	2	2
2	3	3	3	3	3	2	1	1	1
2	2	2	2	2	2	2	2	1	2
1	2	3	3	2	3	2	2	2	2
3	2	3	3	2	3	3	2	2	2
3	2	1	2	3	1	2	1	1	1
2	2	2	2	3	3	1	1	2	2
2	3	3	3	2	2	1	2	2	2
3	1	2	3	1	2	3	1	1	1
1	1	3	3	3	3	3	2	2	2
2	1	1	2	3	2	2	2	2	1
9	9	9	9	9	9	9	#NULL!	#NULL!	#NULL!
1	1	1	1	1	1	1	1	1	2
1	1	3	1	3	1	3	1	2	1
1	1	1	3	1	1	1	1	2	2
3	1	2	3	1	2	3	1	1	1
1	1	1	3	1	2	3	1	2	2
1	3	1	3	2	2	2	1	1	1
2	2	2	2	2	2	2	1	1	1
2	2	1	1	3	3	1	2	2	2
9	9	9	9	9	9	9	2	2	2

1	3	2	3	2	2	2	1	1	1
9	9	9	9	9	9	9	#NULL!	#NULL!	#NULL!
3	3	2	2	2	3	3	1	1	2
2	3	3	3	3	2	2	1	2	1
1	1	1	1	1	1	2	1	1	1
2	2	2	3	3	2	2	2	2	1
2	2	2	2	2	2	2	2	2	2
9	9	9	9	9	9	9	#NULL!	#NULL!	#NULL!
9	9	9	9	9	9	9	#NULL!	#NULL!	#NULL!
2	2	2	1	1	1	3	2	2	1
2	2	2	2	2	2	3	1	1	1
2	2	2	2	2	2	2	2	2	2
2	2	2	2	2	2	1	2	2	2
2	2	2	2	2	2	2	1	1	1
2	2	2	2	2	2	2	2	2	2
2	2	2	2	2	2	2	2	2	2
2	2	2	2	2	2	9	2	2	2
2	2	1	2	2	1	3	2	2	2
2	2	2	2	1	1	3	1	1	1
2	2	2	2	2	2	2	2	2	2
2	1	1	2	2	2	2	2	2	2
2	2	2	2	2	2	2	1	1	2
2	2	2	2	2	2	2	1	1	2
2	2	1	1	2	2	2	#NULL!	#NULL!	#NULL!
2	2	2	2	2	2	9	2	2	2
2	2	2	2	2	2	2	2	2	2
2	2	2	2	2	2	3	1	1	1
9	9	9	9	9	9	9	1	1	1
2	2	2	2	2	2	2	1	1	1
2	2	2	2	2	2	2	2	#NULL!	2
2	2	2	2	2	2	9	2	2	2
2	2	2	2	2	2	3	1	1	1
9	9	9	9	9	9	9	2	2	2
2	2	2	2	2	2	2	2	2	2
2	2	2	2	2	2	3	1	1	1
2	2	2	2	2	2	3	1	1	1
2	2	2	2	2	2	2	2	1	2
2	2	2	2	2	2	2	2	2	1
2	2	2	2	2	2	2	2	2	2
9	9	9	9	9	9	9	#NULL!	#NULL!	#NULL!
2	2	2	2	2	2	2	1	2	1
2	2	2	2	2	2	2	1	1	2
2	3	2	1	2	3	2	1	1	1
2	2	2	2	2	2	2	#NULL!	#NULL!	#NULL!
3	9	2	2	3	2	1	1	1	1
9	9	9	9	9	9	9	2	2	1
3	3	3	3	3	3	3	2	2	2

2	2	2	2	2	2	2	1	2	2
3	2	3	3	2	2	3	2	1	2
2	2	2	2	2	2	2	1	2	2
2	2	2	2	2	2	2	2	1	1
2	2	2	2	2	2	2	1	1	2
2	2	2	2	2	2	2	2	2	2
1	1	3	3	3	3	3	1	2	2
3	1	3	1	3	1	3	1	1	1
9	9	9	9	9	9	9	1	1	2
2	2	2	2	2	2	2	2	2	2
3	3	2	2	2	2	1	1	1	1
1	1	2	2	2	2	2	1	1	1
2	2	3	2	1	1	2	2	2	2
9	9	9	9	9	9	9	1	2	2
2	2	2	2	2	2	2	2	2	2
9	1	1	2	2	2	2	2	2	2
1	1	3	2	2	2	3	1	1	1
1	1	1	1	1	1	1	1	1	1
9	9	9	9	9	9	9	1	1	1
2	2	2	2	2	2	2	2	2	1
2	1	2	2	2	2	2	1	1	2
9	9	9	1	9	9	9	1	1	1
9	9	9	9	9	9	9	2	2	1
1	1	2	1	3	3	9	1	2	2
2	1	2	1	2	3	2	1	1	1
1	1	2	3	2	1	3	1	2	2
1	2	3	2	3	1	9	1	2	#NULL!
1	9	2	2	3	3	1	1	1	1
2	1	1	1	1	1	1	1	1	2
1	9	9	9	9	9	2	1	1	1
9	9	9	9	9	9	9	2	2	2
3	3	3	3	3	3	3	2	2	2
2	2	2	2	2	2	2	2	2	2
2	9	2	9	9	2	9	1	2	2
1	2	3	3	3	3	3	1	2	2
9	9	9	9	9	9	9	1	1	1
3	2	2	2	2	2	2	1	2	2
1	1	1	1	1	1	1	1	1	2
2	2	2	2	2	2	2	2	2	2
2	2	2	2	2	2	2	2	2	2
3	9	3	3	9	2	3	#NULL!	2	2
2	2	2	2	2	2	2	1	2	2
9	9	9	9	9	1	9	2	#NULL!	2
2	2	3	3	3	2	2	1	1	1
1	1	1	1	1	1	3	1	1	2
3	3	3	2	3	2	2	2	2	2
3	2	3	2	3	2	2	2	2	2

9	9	9	9	9	9	1	2	1	1
2	2	2	2	2	2	2	2	2	2
1	1	1	1	1	1	1	1	1	1
2	2	2	2	2	2	2	2	2	2
9	9	9	9	9	9	9	2	2	2
1	2	2	2	3	3	3	1	1	1
3	3	3	3	3	3	3	2	2	2
1	1	1	1	1	1	1	1	2	1
1	1	1	1	1	1	1	1	2	2
1	1	1	1	1	1	1	1	1	1
2	2	2	2	2	2	9	2	1	2
1	1	1	1	1	1	1	1	1	1
2	2	2	2	2	2	2	1	2	2
2	2	2	2	2	2	2	1	2	2
2	2	2	2	2	2	2	2	2	2
2	2	2	2	2	2	2	2	2	2
1	1	1	1	1	1	1	1	2	2
1	1	1	1	1	1	3	1	1	1
9	9	9	9	9	9	9	#NULL!	#NULL!	#NULL!
1	3	1	3	1	3	1	#NULL!	#NULL!	#NULL!
1	1	1	1	1	1	3	1	1	2
2	1	2	2	2	2	2	1	2	2
9	9	9	9	9	9	9	1	1	1
1	1	1	1	1	1	9	2	2	2
3	3	3	3	3	3	3	2	2	2
2	1	1	1	1	2	2	1	2	2
1	1	1	2	2	9	2	1	1	1
2	1	2	1	2	2	1	2	1	1
3	3	2	2	2	2	2	1	1	2
2	2	2	2	2	2	2	2	2	2
9	1	2	9	1	2	2	1	2	2
2	2	2	2	2	2	3	2	2	2
3	3	3	3	3	3	9	1	2	2
1	2	3	2	3	1	2	2	2	1
2	2	2	2	2	2	2	2	2	2
3	3	3	3	3	3	2	2	2	2
3	9	2	2	1	2	2	1	1	2
2	2	2	2	2	2	2	2	1	1
3	1	2	3	1	2	3	1	2	2
3	3	3	3	3	3	3	2	2	2
9	1	2	3	1	2	3	1	1	1
2	2	2	2	2	2	2	#NULL!	#NULL!	#NULL!
9	9	9	9	9	9	9	#NULL!	#NULL!	#NULL!
9	9	9	9	9	9	9	2	2	2
9	9	9	9	9	9	9	1	2	1
2	2	2	2	2	2	2	2	2	2
2	2	1	3	3	3	3	1	1	1

1	1	1	1	1	1	9	1	1	1
1	3	1	3	3	1	3	1	1	1
9	9	9	9	9	9	9	#NULL!	#NULL!	#NULL!
1	3	1	3	3	1	3	1	1	1
3	2	1	2	1	3	9	1	2	1
2	2	2	2	2	2	2	2	2	2
2	2	2	2	2	2	2	2	2	2
2	2	2	2	2	2	9	#NULL!	#NULL!	#NULL!
9	9	9	9	9	9	9	2	1	1
2	2	1	2	2	3	9	1	2	1
1	2	2	2	2	2	2	2	2	2
1	1	3	3	1	2	1	1	2	2
2	2	2	2	2	2	2	1	1	1
2	1	2	3	3	2	1	1	1	1
2	2	2	2	2	2	9	2	2	2
2	2	2	2	2	2	2	1	2	2
2	2	2	2	2	2	9	2	2	2
2	2	2	2	2	2	2	2	2	2
2	2	2	2	2	2	2	2	2	2
3	1	3	2	2	2	1	1	1	2
1	1	2	2	1	2	1	1	1	2
2	2	2	2	2	2	2	2	2	2
9	9	9	9	9	9	9	1	2	2
2	2	2	2	2	2	2	2	2	2
1	1	1	1	1	1	1	1	1	1
9	9	9	9	9	9	9	1	2	2
3	2	2	1	1	2	3	1	2	1
2	3	3	3	3	3	3	1	2	2
2	1	1	2	3	1	2	2	1	2
1	1	3	2	2	2	2	2	2	2
1	1	2	3	2	2	2	2	2	2
9	9	9	9	9	9	9	1	1	1
2	2	2	2	2	2	2	2	2	2
3	2	2	2	2	2	2	#NULL!	#NULL!	#NULL!
2	1	2	1	2	2	1	1	1	1
2	2	3	2	2	2	2	2	1	2
9	9	9	9	9	9	9	2	2	2
9	9	9	9	9	9	9	#NULL!	#NULL!	#NULL!
3	1	3	3	2	2	1	1	2	1
2	2	2	2	2	2	2	2	2	2
2	1	2	3	1	2	2	#NULL!	#NULL!	#NULL!
9	9	9	9	9	9	9	2	1	2
2	2	2	2	2	2	2	2	2	2
2	2	2	2	2	2	2	#NULL!	#NULL!	#NULL!
9	9	9	9	9	9	9	#NULL!	#NULL!	#NULL!
9	9	9	9	9	9	9	1	2	1
2	2	2	2	2	2	3	2	2	2

9	9	9	9	9	9	9	#NULL!	#NULL!	#NULL!
3	1	2	2	1	2	2	1	1	2
3	2	1	2	1	3	9	2	2	1
9	9	9	9	9	9	9	#NULL!	#NULL!	#NULL!
1	1	1	1	1	3	3	2	2	2
2	3	1	1	2	2	1	1	1	2
3	2	1	2	3	2	1	#NULL!	#NULL!	#NULL!
9	9	9	9	9	9	9	#NULL!	#NULL!	#NULL!
1	2	2	2	2	2	2	2	2	2
3	2	3	2	3	2	1	2	2	2
1	1	2	2	2	2	2	1	2	2
3	2	2	2	3	3	1	1	1	1
2	2	2	1	1	2	9	1	1	2
1	1	1	2	2	3	3	2	2	2
3	1	2	3	1	2	1	1	2	2
2	2	2	2	2	2	2	2	2	2
2	3	2	2	1	2	3	2	2	2
2	2	2	2	2	2	2	2	2	2
9	9	9	9	9	9	9	#NULL!	#NULL!	#NULL!
9	9	9	9	9	9	9	#NULL!	#NULL!	#NULL!
9	9	9	9	9	9	9	#NULL!	#NULL!	#NULL!
3	2	2	3	2	1	2	1	2	2
2	2	3	3	3	3	1	2	2	2
2	2	1	1	1	2	3	#NULL!	#NULL!	#NULL!
2	2	1	1	3	3	1	#NULL!	#NULL!	#NULL!
3	3	2	2	3	3	3	2	2	2
2	2	2	3	2	2	2	2	2	1
2	2	3	2	1	1	3	1	2	2
1	1	1	1	1	3	3	2	1	2
1	1	1	3	3	3	3	2	2	2
1	1	2	2	2	2	2	2	2	2
2	3	2	1	2	3	2	2	2	2
2	9	1	3	3	3	1	1	1	2
2	2	3	3	3	3	2	2	2	2
2	2	2	2	2	2	2	1	1	1
2	2	2	2	2	2	2	2	2	2
3	3	3	3	3	3	3	2	2	1
3	3	3	3	2	2	2	1	1	1
9	9	9	9	9	9	9	#NULL!	#NULL!	#NULL!
9	9	9	9	9	9	9	#NULL!	#NULL!	#NULL!
3	1	2	3	1	2	3	1	2	1
2	3	3	3	2	1	1	2	2	2
3	2	1	3	2	2	3	1	2	1
1	3	2	3	2	1	1	2	2	2
9	9	9	9	9	9	9	2	2	2
3	3	3	3	3	3	3	1	2	2
3	2	1	2	1	2	3	2	2	2

1	1	1	1	1	1	1	#NULL!	#NULL!	#NULL!
1	2	3	2	2	2	3	2	2	2
3	2	2	1	3	2	2	2	1	2
1	3	1	3	2	2	3	2	2	2
3	2	3	2	3	2	1	2	2	2
3	3	3	3	3	3	3	1	2	1
1	1	1	1	1	1	1	1	2	2
3	3	3	3	3	3	3	1	1	1
3	3	2	2	3	2	2	2	2	2
1	3	1	3	2	3	2	#NULL!	#NULL!	#NULL!
2	2	2	2	2	2	2	2	2	2
3	2	1	3	2	3	1	2	2	2
2	2	1	3	2	1	1	#NULL!	#NULL!	#NULL!
2	2	2	2	1	1	1	1	1	2
1	1	2	2	2	2	2	1	1	2
2	2	3	3	3	3	3	2	2	2
1	1	1	1	1	1	3	1	1	2
9	2	1	1	1	2	3	2	2	2
9	9	9	9	9	9	9	2	2	2
2	3	1	2	2	3	2	1	2	2
3	1	3	1	2	3	2	#NULL!	#NULL!	#NULL!
2	3	3	2	3	2	3	2	2	1
1	1	1	1	1	1	1	2	2	2
3	2	2	2	2	2	9	2	1	2
3	1	2	3	1	2	3	2	2	2
2	3	2	2	3	3	3	2	2	2
1	1	1	1	1	1	1	2	2	2
2	2	3	2	1	2	2	2	2	2
2	1	3	2	2	1	3	1	1	1
1	1	1	3	3	3	3	2	2	2
1	2	2	2	2	2	1	2	2	1
2	2	2	2	2	2	2	2	2	2
2	2	2	2	2	2	2	2	2	2
2	1	2	2	1	2	1	1	1	2
3	1	2	3	1	2	1	1	2	2
2	3	3	3	2	3	3	1	1	1
9	1	2	2	1	2	2	1	1	1
3	3	3	3	3	3	3	2	2	2
2	2	2	2	1	1	2	1	2	2
1	1	2	3	2	3	2	1	2	1
2	2	2	2	2	2	9	2	2	2
3	1	2	3	1	2	3	1	2	1
3	2	1	2	3	2	2	1	2	2
1	1	1	1	1	1	1	1	1	1
9	9	9	9	9	9	9	1	2	2
3	2	2	3	2	2	2	2	2	2
1	1	3	2	1	1	1	1	1	2

2	2	2	1	3	3	3	1	2	2
3	1	2	3	1	2	3	1	1	2
1	2	2	2	2	2	2	1	2	2
1	1	1	1	1	1	1	1	1	1
3	1	2	3	1	2	3	2	2	2
1	2	2	2	1	3	3	1	1	1
3	1	2	1	2	3	3	1	1	2
3	2	1	2	1	2	2	1	1	1
1	2	2	1	1	1	1	1	1	1
1	1	3	2	1	3	3	2	2	2
3	2	1	2	3	1	1	1	2	2
2	1	1	2	3	2	2	2	2	2
1	1	1	1	1	1	1	1	1	1
1	3	3	1	3	3	1	1	2	1
3	3	2	2	3	1	9	2	2	2
1	3	1	1	3	1	1	1	2	2
1	1	3	3	2	3	2	2	2	2
1	1	3	3	2	3	2	1	2	2
1	2	2	2	2	2	3	1	1	2
1	1	2	2	1	3	1	2	1	2
1	1	2	3	3	3	3	1	2	2
3	3	3	1	3	3	3	2	2	2
2	1	1	2	1	2	1	1	2	2
3	1	1	2	2	1	2	1	1	2
2	2	2	2	2	2	2	2	2	2
2	2	2	2	2	2	2	#NULL!	#NULL!	#NULL!
3	1	3	1	3	2	3	1	1	1
3	1	2	3	3	1	2	1	2	2
2	2	2	2	2	2	2	#NULL!	#NULL!	#NULL!
9	9	9	9	9	9	9	#NULL!	#NULL!	#NULL!
9	9	9	9	9	9	9	#NULL!	#NULL!	#NULL!
9	9	9	9	9	9	9	#NULL!	#NULL!	#NULL!
3	3	2	1	2	3	2	1	1	1
9	9	9	9	9	9	9	#NULL!	#NULL!	#NULL!
9	9	9	9	9	9	9	#NULL!	#NULL!	#NULL!
9	9	9	9	9	9	9	#NULL!	#NULL!	#NULL!
2	2	2	2	2	2	2	1	2	2
9	9	9	9	9	9	9	2	2	2
9	2	9	9	2	2	1	#NULL!	#NULL!	#NULL!
3	3	3	3	3	3	3	1	1	1
9	9	9	9	9	9	9	#NULL!	#NULL!	#NULL!
1	1	1	1	1	1	3	1	2	2
3	3	3	3	3	3	3	1	2	1
3	3	2	1	1	3	1	#NULL!	#NULL!	#NULL!
2	1	3	2	1	3	1	1	2	2
1	3	2	1	2	1	2	1	2	1
3	1	3	1	3	2	3	1	1	1

2	2	2	1	2	3	2	1	2	2
2	2	2	1	2	2	1	2	2	2
9	9	9	9	9	9	9	#NULL!	#NULL!	#NULL!
1	2	1	1	1	2	1	2	2	2
3	2	9	2	2	3	1	2	#NULL!	2
1	2	1	2	3	9	2	1	2	1
9	9	1	9	9	9	9	2	1	2
3	2	3	2	2	2	9	1	2	2
3	3	3	1	2	2	2	1	2	1
3	3	3	3	3	3	3	1	1	1
1	2	3	1	2	3	2	2	2	1
2	3	2	3	2	3	3	2	2	2
1	2	2	1	2	1	3	1	1	1
2	2	2	2	2	2	2	2	2	2
2	2	2	2	2	2	2	2	2	2
2	9	9	9	9	2	2	#NULL!	#NULL!	#NULL!
2	2	2	2	2	2	2	2	2	2
2	2	2	2	2	2	2	2	2	2
2	2	2	2	2	2	2	2	2	2
2	2	2	2	2	2	2	2	2	2
1	9	1	9	9	9	9	1	1	1
1	9	1	9	9	9	9	1	1	1
1	9	1	9	9	9	9	1	1	1
1	3	1	3	3	3	3	1	1	1
1	3	1	3	3	3	3	1	1	1
1	9	1	3	3	3	3	1	1	1
1	2	1	3	2	1	1	1	1	1
1	2	1	3	3	3	3	1	1	1
1	2	1	3	3	3	3	1	1	1
9	9	9	9	9	9	9	1	1	2
9	9	9	9	9	9	9	1	1	1
9	9	9	9	9	9	9	2	1	2
1	3	1	3	3	3	3	1	1	1
1	3	1	3	2	3	3	1	1	1
1	1	3	3	3	3	1	1	2	2
1	1	1	3	3	3	3	1	1	1
1	1	1	3	3	3	3	1	1	2
9	9	9	9	9	9	9	1	1	1
3	3	1	3	3	3	3	1	1	1
1	1	1	3	3	3	3	1	1	2
1	3	3	3	3	3	3	1	1	1
1	9	1	9	9	9	9	1	1	1
1	1	1	3	3	3	3	1	1	1
1	1	1	3	3	3	3	1	1	1
2	2	2	2	2	2	2	1	1	1
1	3	1	3	3	3	3	1	1	1
1	3	3	3	3	2	2	1	1	1

1	1	1	3	3	3	3	1	1	1
1	1	2	9	9	2	2	1	1	1
1	3	1	3	3	3	3	1	1	1
1	1	1	1	1	1	1	1	1	1
1	3	1	3	3	3	3	1	1	1
1	3	1	3	3	3	3	1	1	1
1	1	1	1	1	1	1	1	1	2
1	1	1	2	3	3	3	1	1	1
1	1	1	3	3	3	3	1	1	1
1	3	1	3	3	3	3	1	1	1
1	2	1	1	3	2	2	1	1	2
1	2	2	2	2	2	2	1	2	2
1	3	1	3	3	3	3	1	1	1
1	3	1	3	3	3	3	1	1	1
1	3	1	3	3	3	3	1	1	1
1	3	1	3	3	3	3	1	1	1
1	3	1	2	3	3	3	1	1	2
1	1	2	1	9	1	3	1	1	2
1	3	1	3	3	3	3	1	1	1
3	2	3	1	2	3	2	1	1	2
2	9	9	9	9	9	9	1	2	2
3	3	3	3	3	3	3	1	2	2
1	1	1	1	1	1	1	1	1	1
1	3	1	9	3	1	3	1	1	1
1	1	2	1	2	1	3	1	2	2
1	1	1	1	1	3	3	1	1	1
3	3	3	3	3	3	3	1	2	2
1	2	1	2	3	3	3	1	1	1
1	2	1	3	3	3	3	1	1	1
1	3	1	3	3	3	3	1	1	2
2	2	2	2	2	2	2	1	1	2
1	3	1	3	3	3	3	1	1	1
1	9	9	9	9	9	9	1	1	1
1	3	1	3	3	3	3	1	1	1
1	2	1	2	3	3	3	1	1	1
1	3	1	3	3	3	3	1	1	1
1	1	1	1	2	2	2	1	1	1
1	3	1	3	3	3	3	1	1	1
1	3	1	3	3	3	3	1	1	1
1	1	1	3	3	3	3	1	1	1
1	3	1	3	3	3	3	1	1	1
1	1	1	1	1	1	3	1	1	1
1	9	2	3	3	1	3	1	1	1
1	3	1	3	3	3	3	1	1	1
9	9	9	9	9	9	9	1	1	1
3	1	1	3	3	3	3	1	1	1
3	1	3	3	3	3	3	1	1	1

1	3	1	3	3	3	3	1	2	2
1	1	1	3	3	3	3	1	1	1
1	3	1	3	3	3	3	1	1	1
1	1	1	1	1	1	1	1	1	1
1	1	1	3	3	3	3	1	1	1
1	3	3	1	1	1	1	1	2	2
9	9	9	9	9	9	9	1	1	2
1	1	1	3	3	3	3	#NULL!	#NULL!	#NULL!
1	1	1	3	3	3	3	1	1	#NULL!
1	1	3	1	3	1	3	1	1	1
1	2	1	3	3	3	3	2	2	1
9	9	9	9	9	9	9	1	1	1
1	1	1	3	3	3	3	1	1	2
2	3	1	2	1	3	3	1	1	2
2	1	1	1	3	3	3	2	2	2
1	3	1	3	3	3	3	1	1	2
9	9	9	9	9	9	9	1	1	1
9	9	9	9	9	9	9	1	1	1
2	2	2	2	2	2	2	1	1	1
3	3	3	3	3	3	3	1	1	2
1	2	1	3	3	1	3	1	1	1
9	9	9	9	9	9	9	1	1	1
1	1	1	1	1	1	3	1	1	1
1	3	1	3	3	3	3	1	1	1
1	1	1	2	3	1	3	1	1	2
3	1	3	1	3	1	3	1	1	2
1	1	2	1	1	1	2	1	1	2
2	2	2	2	2	2	2	1	1	1
1	1	1	3	3	3	3	1	1	1
1	3	1	3	2	3	3	1	1	1
1	3	1	3	2	3	3	1	1	1
3	2	1	2	3	1	2	1	1	1
1	3	1	3	2	2	3	1	1	2
1	1	2	3	3	1	3	1	1	1
1	1	1	1	1	1	1	1	1	1
1	1	2	3	2	2	3	1	1	1
9	3	3	3	3	3	3	1	1	2
1	1	1	3	3	3	3	1	1	2
3	3	3	3	3	3	3	1	1	2
1	1	1	3	3	1	3	1	1	1
1	1	1	3	3	3	3	1	1	1
1	3	1	3	3	3	3	1	1	1
1	3	1	3	3	3	3	1	1	2
9	2	2	9	2	2	2	#NULL!	#NULL!	#NULL!
9	2	3	3	3	3	3	1	1	2
1	3	1	3	3	3	3	1	2	2
1	3	1	3	3	3	3	1	1	1

1	3	1	3	3	3	3	1	1	1
1	2	1	3	2	3	3	1	1	2
1	1	2	3	2	9	3	1	1	2
2	2	2	2	2	2	3	1	1	2
1	2	3	2	3	2	3	1	1	1
1	2	3	3	2	3	2	2	1	2
9	9	9	9	9	9	9	1	1	1
1	2	2	2	2	2	2	1	1	2
9	9	9	9	9	9	9	1	1	1
1	1	3	3	3	3	3	1	1	1
1	2	1	2	2	2	3	1	1	1
1	3	1	3	3	3	3	1	1	1
2	2	2	2	2	2	2	1	1	2
1	1	1	1	3	2	1	1	1	1
1	2	2	2	2	2	2	2	1	2
9	9	9	9	9	9	9	1	1	1
1	3	1	3	3	3	3	1	1	1
1	1	1	1	2	2	2	1	1	1
1	3	1	3	3	3	3	1	1	1
1	3	1	3	3	3	3	1	1	1
9	9	9	9	9	9	9	1	1	2
1	3	1	3	3	3	3	1	1	1
9	9	9	9	9	9	9	1	1	1
3	3	3	3	3	3	3	1	1	1
1	1	2	2	3	3	3	1	1	1
1	1	2	2	3	3	3	1	1	1
1	2	1	3	3	3	3	1	1	1
9	9	9	9	9	9	9	#NULL!	#NULL!	#NULL!
9	9	9	9	9	9	9	#NULL!	#NULL!	#NULL!
1	3	1	3	3	2	3	1	1	2
1	3	1	3	3	3	3	1	1	1
1	3	1	3	3	3	3	1	1	1
9	9	9	9	9	9	9	1	1	1
1	1	3	3	9	9	9	1	1	1
1	3	2	3	3	3	3	1	1	1
1	2	1	3	2	1	3	1	1	1
1	3	1	3	3	3	3	1	1	1
1	1	1	1	1	1	1	1	1	1
1	1	3	3	3	3	3	1	1	1
1	3	1	3	3	3	3	1	1	1
1	2	1	3	2	2	3	1	1	2
1	3	1	3	3	3	3	1	1	2
1	3	1	3	3	3	3	1	1	1
9	9	9	9	9	9	9	1	1	2
1	1	1	3	1	2	2	1	1	1
1	3	1	3	3	3	1	1	1	2
1	2	2	3	3	3	3	1	1	1

1	1	9	9	9	9	9	1	1	1
1	2	3	1	1	9	9	1	1	2
9	9	9	9	9	9	9	#NULL!	#NULL!	#NULL!
1	1	1	1	2	1	1	1	#NULL!	1
9	9	9	9	9	9	9	1	1	1
1	1	3	3	3	3	3	1	1	1
1	1	1	1	3	1	3	1	1	2
1	2	2	2	3	3	3	1	1	1
9	9	9	9	9	9	9	1	1	1
1	2	1	2	3	3	3	1	1	1
1	1	1	1	1	1	1	1	1	#NULL!
1	1	2	2	2	2	3	1	1	2
1	1	2	2	2	2	3	1	1	1
1	1	1	3	1	3	9	1	1	1
2	1	1	2	2	1	1	1	1	1
1	3	1	3	3	3	3	1	1	1
9	9	9	9	9	9	9	1	1	#NULL!
9	9	9	9	9	9	9	1	2	1
1	1	1	1	3	3	3	1	1	1
2	2	2	2	2	2	2	1	1	1
1	1	2	2	3	2	1	1	1	1
1	3	3	3	3	3	3	1	1	1
1	3	3	3	3	3	3	1	1	1
1	3	1	3	3	3	3	1	1	1
1	1	2	3	3	3	3	1	1	#NULL!
1	1	1	1	1	1	2	1	1	1
1	1	2	3	3	3	3	1	1	1
1	1	1	1	3	1	2	1	2	1
1	1	1	1	1	1	3	1	1	1
1	1	1	1	1	1	9	1	1	1
1	3	3	3	3	2	3	1	2	2
1	1	1	3	1	3	3	1	1	1
1	1	2	2	3	3	2	1	1	1
1	1	1	3	3	3	3	1	1	1
1	2	1	3	3	3	3	1	1	1
1	3	1	3	3	3	3	1	1	1
1	3	1	3	3	3	3	1	1	1
1	1	9	9	9	9	9	1	1	1
1	1	2	1	1	2	1	1	1	2
1	1	1	1	1	1	1	2	1	2
1	2	2	2	3	2	3	1	1	2
9	9	9	9	9	9	9	1	1	1
9	9	9	9	9	9	9	1	1	1
9	9	9	9	9	9	9	1	1	1
1	2	2	3	2	3	3	1	1	1
1	1	2	1	2	1	3	1	1	1
1	9	9	9	9	9	9	1	1	1

1	3	1	3	3	3	3	1	1	1
1	1	1	3	3	3	3	1	1	1
1	9	1	9	2	9	3	1	1	1
1	3	1	3	3	3	3	1	1	1
1	3	1	3	3	3	3	1	1	1
9	9	9	9	9	9	9	1	1	1
2	2	2	2	2	2	9	1	1	#NULL!
1	2	2	2	2	2	2	1	1	1
9	9	9	9	9	9	9	1	1	1
1	2	1	3	3	2	3	1	1	1
1	3	1	3	3	3	3	1	1	1
9	9	9	9	9	9	9	1	1	1
9	9	9	9	9	9	9	1	1	1
9	9	9	9	9	9	9	1	1	1
9	9	9	9	9	9	9	1	1	1
9	9	9	9	9	9	9	1	1	1
1	1	2	3	1	1	3	1	1	#NULL!
2	3	3	1	2	3	1	2	2	2
1	3	1	3	3	3	3	1	1	1
1	2	2	2	1	3	3	1	1	1
1	1	1	3	3	3	3	1	1	1
1	1	1	3	3	3	3	1	1	2
1	2	1	3	2	3	3	1	1	2
1	1	2	2	1	2	3	1	1	2
1	2	1	3	3	3	3	1	1	1
1	1	3	3	3	3	3	1	1	1
1	1	1	3	1	3	3	1	1	2
1	3	1	3	3	3	3	1	1	1
1	2	3	3	3	3	3	1	1	1
1	3	1	3	3	3	3	1	1	1
1	2	1	2	2	1	3	1	1	1
1	9	1	9	9	9	9	1	1	1
1	3	1	3	3	3	3	1	1	1
1	3	1	1	3	3	3	1	1	1
1	1	2	3	3	1	3	1	1	1
1	1	1	3	3	1	3	1	1	1
1	3	2	3	3	3	3	1	1	2
2	3	2	2	3	3	3	1	1	1
9	9	9	9	9	9	9	1	2	1
9	9	9	9	9	9	9	1	1	1
2	1	2	2	1	3	3	1	1	2
2	1	2	2	1	3	3	1	1	2
1	2	2	3	3	3	3	1	1	1
1	2	1	3	3	3	3	1	1	1
1	1	1	1	3	2	3	1	1	1
1	1	2	3	3	3	3	1	1	1
1	3	1	1	3	3	3	1	1	1

1	3	1	3	3	3	3	1	1	1
1	2	1	3	3	3	3	1	1	1
1	9	1	3	3	3	3	1	1	1
1	3	1	3	3	3	3	1	1	1
1	2	1	3	2	1	3	1	1	1
1	1	2	2	2	3	3	1	1	1
1	2	1	2	2	2	2	1	1	1
1	2	1	2	3	3	3	1	1	1
9	9	9	9	9	9	9	1	1	1
1	2	1	3	3	3	3	1	1	1
2	1	1	2	2	2	2	1	1	1
2	2	2	2	2	2	2	1	1	1
1	3	1	1	3	3	3	1	1	1
1	1	3	1	3	3	3	1	1	1
1	1	2	2	2	2	2	1	1	1
1	1	1	3	3	3	3	1	1	1
2	2	2	2	2	2	3	1	1	2
1	1	1	2	2	2	3	1	1	2
2	2	2	2	2	2	2	2	2	2
1	3	1	3	3	3	3	1	1	1
1	3	1	3	3	3	3	1	1	1
9	9	9	9	9	9	9	#NULL!	#NULL!	#NULL!
1	3	1	3	2	3	3	1	1	1
1	3	1	3	3	1	3	1	1	1
1	1	3	3	3	3	3	1	1	1
9	9	9	9	9	9	9	1	#NULL!	#NULL!
9	9	9	9	9	9	9	#NULL!	#NULL!	#NULL!
1	9	9	9	9	9	9	1	1	1
1	2	1	3	3	3	3	#NULL!	#NULL!	#NULL!
3	1	1	1	3	3	9	1	1	1
1	3	1	3	3	3	3	1	1	1
1	3	1	3	3	3	3	1	1	1
9	9	9	9	9	9	9	1	1	1
9	9	9	9	9	9	9	#NULL!	#NULL!	#NULL!
9	9	9	9	9	9	9	1	2	1
9	9	9	9	9	9	9	1	1	1
9	9	9	9	9	9	9	1	1	1
9	9	9	9	9	9	9	1	1	1
9	9	1	9	9	9	9	1	1	1
2	2	3	1	2	2	1	1	1	1
1	2	1	3	3	3	3	1	1	1
1	9	9	9	9	9	9	1	1	1
1	9	9	9	9	9	9	1	1	1
1	1	1	3	3	1	3	1	1	1
9	9	9	9	9	9	9	1	1	1
9	9	9	9	9	9	9	1	1	1
9	9	9	9	9	9	9	1	1	1

1	3	1	3	3	3	3	1	1	1
1	3	1	1	3	3	1	1	1	2
9	1	9	9	9	9	9	1	1	1
1	3	1	3	3	3	3	1	1	1
1	3	1	3	3	3	3	1	1	1
9	9	9	9	9	9	9	1	1	1
9	9	9	9	9	9	9	1	1	1
9	9	9	9	9	9	9	1	1	1
9	9	9	9	9	9	9	1	1	1
9	9	9	9	9	9	9	1	1	1
1	3	1	1	3	3	3	1	1	1
1	3	1	3	3	3	3	1	1	1
9	3	1	1	3	3	3	1	1	1
9	9	9	9	9	9	9	1	1	2
1	3	1	3	3	3	3	1	1	1
1	3	1	3	3	3	3	1	1	2
1	1	1	9	1	1	1	1	1	2
1	1	1	9	3	1	1	1	2	1
1	3	1	3	3	3	3	1	1	1
2	1	1	1	3	3	3	1	#NULL!	1
2	1	1	1	3	3	3	1	1	1
1	1	1	1	1	1	3	1	1	2
9	1	9	9	9	9	9	1	1	1
1	1	1	3	1	3	1	1	1	1
3	2	3	3	2	1	2	1	1	1
1	1	1	1	1	1	3	1	1	1
2	3	3	3	3	3	3	2	1	2
1	3	1	1	3	3	3	1	1	1
1	1	1	2	3	3	3	1	1	1
2	3	3	3	3	3	3	1	1	2
1	3	3	3	3	3	3	1	1	1
1	1	2	2	3	1	3	1	1	2
1	1	1	3	3	1	1	1	1	1
1	3	1	3	3	3	3	1	1	1
1	1	2	3	3	3	3	1	1	1
1	3	1	3	3	3	3	1	1	1
1	3	1	3	3	3	3	1	1	1
1	3	1	3	3	3	3	1	1	1
1	3	1	3	3	1	3	1	1	1
1	1	1	2	3	1	1	1	1	1
9	9	9	9	9	9	9	1	1	2
3	3	1	3	3	3	3	1	2	2
2	2	2	2	3	1	1	1	2	2
1	3	1	3	3	3	3	1	1	1
1	3	1	3	3	1	3	1	1	1
1	3	1	3	3	1	3	1	1	1
1	3	1	3	3	3	3	1	1	1

1	3	1	1	3	3	1	1	1	1
1	3	1	1	1	3	3	1	1	1
9	9	9	9	9	9	9	1	1	1
1	3	2	3	3	1	3	1	2	2
1	3	1	3	3	1	3	1	1	1
1	3	2	3	3	1	3	1	2	1
1	3	1	3	3	3	3	1	1	1
3	3	1	3	3	3	3	1	1	1
1	3	1	3	3	3	3	1	1	1
1	3	1	1	1	1	1	1	1	1
1	2	1	3	3	2	3	1	1	1
9	9	9	9	9	9	9	1	1	1
1	1	1	1	3	3	3	1	1	1
1	1	1	3	3	3	3	1	1	1
1	1	1	3	3	3	3	1	1	1
2	2	2	2	2	2	2	1	1	1
2	2	2	2	2	2	2	1	2	2
2	9	2	2	2	2	2	1	1	1
3	1	1	2	3	1	2	2	1	1
1	3	1	3	3	3	3	1	1	1
1	3	1	3	3	3	3	1	1	1
1	1	1	3	3	3	3	1	1	1
1	3	2	3	3	1	3	1	2	2
1	1	2	2	2	3	3	1	1	2
1	1	2	2	2	3	3	1	1	2
2	1	3	3	2	3	2	1	1	1
1	2	1	3	3	3	3	1	1	2
2	2	2	2	2	2	3	1	2	2
1	3	1	3	3	3	3	1	1	1
1	2	1	2	2	3	3	1	1	1
1	2	2	2	2	2	2	1	1	2
1	3	1	1	3	3	3	1	1	1
1	2	1	1	2	3	3	1	1	1
9	9	9	9	9	9	9	1	1	1
1	3	2	3	3	1	3	1	2	2
1	3	2	3	3	1	3	1	2	2
2	2	2	2	2	2	2	1	1	1
2	2	2	2	2	2	2	1	1	1
1	3	1	3	3	3	3	1	1	1
1	3	1	3	2	1	2	1	1	1
1	3	1	3	3	3	3	1	1	1
1	1	3	3	3	3	3	1	1	1
1	1	3	3	3	3	3	1	1	1
1	1	3	3	2	2	2	1	1	1
1	3	1	3	3	3	3	1	1	1
1	2	1	3	3	3	3	1	1	1
1	3	1	1	3	3	3	1	1	1

2	2	2	3	2	3	3	1	1	1
1	2	1	1	3	3	2	1	1	1
1	1	2	1	2	3	3	1	1	1
1	1	2	2	1	1	1	1	1	1
1	3	1	3	3	2	3	1	1	1
1	2	3	3	3	3	3	1	1	1
1	2	3	3	2	3	3	1	1	1
1	2	1	2	3	3	3	1	1	1
1	1	1	1	3	1	2	1	1	1
1	1	2	3	3	3	3	1	1	1
1	2	2	2	2	2	2	1	1	2
1	3	1	3	3	3	3	1	1	1
9	9	9	9	9	9	9	1	1	1
1	1	3	3	3	1	3	1	1	1
1	1	1	3	3	1	3	1	1	1
2	2	2	2	2	2	2	2	2	2
1	2	1	3	3	3	3	1	1	1
1	3	1	3	3	3	3	1	1	1
1	3	1	3	3	3	3	1	1	1
1	2	1	3	3	3	3	1	1	2
1	3	3	3	3	1	3	1	1	1
2	1	1	1	3	3	3	1	1	1
1	3	1	3	3	3	3	1	1	1
9	9	9	9	9	9	9	1	1	1
1	3	1	1	2	1	3	1	1	1
1	3	1	3	3	3	3	1	1	1
1	3	1	3	3	3	3	1	1	2
2	2	3	1	2	2	1	1	1	1
1	1	1	3	3	1	3	1	1	1
9	9	9	9	9	9	9	1	1	1
9	9	9	9	9	9	9	1	1	1
9	9	9	9	9	9	9	1	1	1
1	3	1	3	3	3	3	1	#NULL!	2
1	2	1	3	3	3	3	1	1	2
1	2	1	1	2	1	3	1	1	1
1	1	1	3	3	3	3	1	2	2
2	2	2	3	3	2	2	2	2	2
9	1	9	9	9	9	2	1	1	2
2	1	3	2	1	2	1	1	1	1
1	1	2	3	3	3	3	1	1	2
1	9	2	9	2	9	2	1	2	2
1	3	1	1	3	3	3	1	1	1
1	3	1	3	3	2	2	1	1	2
9	9	2	9	2	1	9	1	1	2
2	2	2	2	2	2	2	1	1	2
9	9	9	9	9	9	9	1	1	1
1	3	1	1	3	9	3	1	1	2

1	1	1	2	3	3	3	1	1	2
1	1	1	1	2	2	2	1	1	1
1	1	1	3	3	3	3	1	1	2
1	1	2	2	2	3	2	1	1	2
3	3	3	2	2	3	2	1	1	2
9	3	1	3	3	3	3	1	1	2
1	1	1	1	3	3	3	1	1	2
9	9	1	9	9	9	9	1	1	1
1	2	2	1	3	2	3	1	1	2
1	2	1	1	3	3	3	1	1	2
1	1	1	2	1	3	1	1	1	1
2	3	2	3	2	1	3	1	1	1
9	3	3	3	3	1	3	1	1	2
2	2	2	2	2	2	2	1	1	1
1	1	3	1	3	3	3	1	1	1
1	1	3	1	3	3	3	1	1	1
1	3	1	3	3	3	3	1	1	1
1	9	1	3	3	3	3	1	1	1
1	9	9	9	9	9	9	1	1	1
1	1	3	9	9	3	3	1	1	1
1	1	2	3	3	3	3	1	1	1
1	2	1	1	1	1	1	1	1	1
2	3	3	2	3	2	3	2	1	2
1	2	2	3	3	3	3	1	1	1
1	3	3	3	3	3	3	1	1	1
1	1	2	1	1	1	1	1	1	1
1	1	1	1	3	3	3	1	1	1
1	1	1	1	3	3	3	1	1	1
1	1	1	1	3	3	3	1	1	1
9	9	2	9	9	9	2	1	2	1
1	3	3	3	3	3	3	1	1	1
1	1	2	2	1	3	3	1	1	1
1	1	2	2	1	3	3	1	1	1
1	3	1	3	3	3	3	1	1	1
3	1	2	2	1	2	1	1	#NULL!	1
1	1	2	1	1	1	1	1	1	1
1	2	1	2	2	2	2	1	1	1
9	9	9	9	9	9	9	#NULL!	#NULL!	#NULL!
1	3	1	3	3	3	3	1	1	1
1	1	1	3	3	3	3	1	1	1
1	3	1	3	3	3	3	1	1	1
1	3	1	3	3	3	3	1	1	1
2	2	2	2	2	2	9	1	1	2
1	3	1	3	3	3	3	1	1	1
1	3	3	3	3	3	3	1	1	1
1	1	1	3	3	3	3	1	1	1
1	1	1	3	3	2	3	1	1	1

1	3	3	1	3	3	3	1	1	1
1	3	3	1	3	3	3	1	1	1
1	3	1	3	3	3	3	1	1	1
1	2	1	2	2	2	2	1	1	1
1	3	3	1	3	3	3	1	1	1
1	3	3	1	3	3	3	1	1	1
1	3	1	3	3	3	3	1	1	1
9	9	9	9	9	9	9	1	1	1
3	3	1	3	3	3	1	1	1	1
3	1	1	3	3	9	9	1	1	1
1	3	1	3	3	3	3	1	1	1
1	3	1	3	3	3	1	1	1	2
1	3	1	3	3	3	1	1	1	1
9	9	9	9	9	9	9	1	1	1
3	3	1	3	3	2	2	1	1	1
3	3	1	3	3	3	3	1	1	1
2	1	2	1	1	2	3	1	1	1
1	2	1	3	3	3	3	1	1	1
1	3	1	1	3	3	3	1	1	1
9	9	9	9	9	9	9	1	1	2
3	1	1	1	1	1	1	1	1	2
1	2	1	3	3	3	3	1	1	1
1	3	1	3	3	3	3	1	1	1
1	3	1	3	3	3	3	1	1	1
9	9	9	9	9	9	9	1	1	1
9	9	9	9	9	9	9	1	1	1
1	1	1	3	3	3	3	1	1	1
9	9	9	9	9	9	9	1	2	1
1	9	9	9	9	9	9	1	1	1
1	9	9	9	9	9	9	2	2	2
9	9	9	9	9	9	9	1	1	1
9	9	9	9	9	9	9	1	1	1
9	9	9	9	9	9	9	1	1	2
1	9	9	9	9	9	9	1	1	1
9	9	9	9	9	9	9	1	1	1
1	1	2	1	3	3	3	1	1	1
1	1	1	3	2	2	2	1	1	1
1	3	1	3	3	3	3	1	1	1
1	1	2	1	3	1	2	1	1	1
1	1	1	1	3	3	3	1	1	1
9	9	9	9	9	9	9	1	1	1
1	3	1	3	3	3	3	1	1	1
1	1	1	3	3	3	3	1	1	2
1	3	2	2	2	2	2	1	2	1
1	2	2	3	2	3	2	1	1	2
1	3	1	3	3	3	3	1	1	1
1	1	2	3	3	3	3	1	1	1

2	2	2	2	2	2	2	1	1	2
2	2	2	2	2	2	2	1	1	1
3	1	2	2	2	2	3	1	1	1
1	1	3	3	2	2	2	1	1	2
1	2	1	2	1	2	1	2	2	2
1	1	1	2	3	3	3	1	1	1
9	9	9	9	9	9	9	#NULL!	#NULL!	#NULL!
2	2	2	2	2	2	2	1	1	2
2	2	2	1	2	2	3	1	1	2
3	2	1	2	3	1	2	1	1	2
1	1	1	2	3	2	1	1	2	2
1	3	1	2	3	3	3	1	1	1
2	3	2	3	2	2	2	1	2	1
3	3	3	3	3	3	3	1	1	2
2	2	2	2	2	2	2	1	1	1
1	1	1	3	3	3	3	1	1	1
9	9	9	9	9	9	9	#NULL!	#NULL!	#NULL!
2	1	2	2	3	3	3	1	2	1
1	1	3	1	3	1	3	1	1	2
1	1	1	1	1	1	1	1	1	1
1	1	1	1	1	1	1	1	1	1
2	2	2	2	2	2	2	1	1	1
3	2	2	1	2	2	3	1	1	2
2	2	2	2	2	2	2	2	1	2
1	1	1	1	1	1	1	1	1	1
3	2	1	2	2	2	2	1	2	2
1	3	2	2	1	2	2	1	1	1
2	2	2	1	2	2	1	1	1	1
2	2	2	2	2	2	2	2	2	2
2	2	2	2	2	2	2	1	1	1
1	3	1	3	3	3	3	1	1	1
9	1	2	1	2	3	2	1	1	2
2	1	2	2	2	2	3	1	1	2
1	1	1	2	1	2	2	1	1	1
1	1	1	9	9	9	3	1	2	2
1	1	1	1	1	1	9	1	1	2
2	2	2	2	2	2	2	1	1	1
9	9	9	9	9	9	9	1	1	2
1	2	2	1	2	3	2	1	1	2
1	1	1	1	1	1	1	1	1	2
1	2	1	2	3	3	3	1	1	2
1	2	1	2	3	3	3	1	1	2

HPVVacc2	Pap2Detec	Married2	PapOver18	pap2years	menopaus	historyCAC	pap2yearly	onceonly2	hpvpreven
2	2	2	2	2	2	2	2	2	2
2	2	1	2	2	2	1	2	1	2
2	2	2	2	2	2	2	2	2	2
2	1	2	2	2	2	2	2	2	2
2	2	2	2	2	2	2	2	2	2
2	2	2	2	2	2	2	2	2	2
2	2	2	2	2	2	2	2	2	2
1	2	2	2	2	1	1	2	2	2
1	2	2	2	1	1	1	1	1	1
2	2	1	2	2	1	2	1	2	2
1	2	2	2	2	2	2	2	2	2
1	2	2	2	2	1	1	2	2	2
1	2	2	2	2	2	2	2	2	2
2	2	2	2	2	2	2	2	2	2
2	2	2	2	2	2	2	2	2	2
2	2	2	2	2	2	2	2	2	2
#NULL!	#NULL!	#NULL!	#NULL!	#NULL!	#NULL!	#NULL!	#NULL!	#NULL!	#NULL!
2	2	2	2	2	2	2	2	2	2
#NULL!	#NULL!	#NULL!	#NULL!	#NULL!	#NULL!	#NULL!	#NULL!	#NULL!	#NULL!
2	#NULL!	#NULL!	#NULL!	#NULL!	#NULL!	#NULL!	#NULL!	#NULL!	#NULL!
2	2	1	2	2	2	1	2	1	2
1	2	1	2	1	1	1	1	1	2
1	2	1	2	2	1	2	2	1	2
1	2	2	2	2	2	2	2	2	2
2	2	#NULL!	#NULL!	#NULL!	#NULL!	#NULL!	#NULL!	#NULL!	#NULL!
#NULL!	2	2	2	2	2	2	2	2	2
1	2	1	1	1	1	2	#NULL!	1	1
1	2	2	2	2	2	2	2	2	2
2	2	2	1	2	2	1	2	2	2
2	#NULL!	#NULL!	#NULL!	#NULL!	#NULL!	#NULL!	#NULL!	#NULL!	#NULL!
1	2	2	1	1	1	1	1	1	2
2	2	1	2	2	2	2	2	2	2
1	2	2	2	1	1	1	1	1	1
2	2	2	#NULL!	2	#NULL!	2	2	2	2
1	2	2	2	2	1	2	2	1	2
1	1	2	1	1	2	2	1	2	2
2	2	2	2	2	2	2	2	2	2
2	1	2	2	2	1	2	2	2	2
2	2	1	2	2	2	2	2	1	2
#NULL!	2	2	2	2	1	2	1	1	2
#NULL!	2	2	2	2	2	1	2	1	2
1	#NULL!	#NULL!	#NULL!	#NULL!	2	#NULL!	#NULL!	#NULL!	1
2	#NULL!	#NULL!	#NULL!	#NULL!	#NULL!	#NULL!	#NULL!	#NULL!	#NULL!
2	2	2	2	2	2	2	2	2	2
2	2	2	2	2	2	2	2	2	2
#NULL!	#NULL!	#NULL!	#NULL!	#NULL!	#NULL!	#NULL!	#NULL!	#NULL!	#NULL!

1	2	2	2	2	2	2	2	2	2
1	2	2	2	2	2	2	2	2	2
2	2	2	2	2	2	2	2	2	2
2	2	2	2	2	2	2	2	2	2
1	2	1	1	1	1	1	2	1	1
1	2	1	1	1	1	1	2	2	1
2	2	1	#NULL!	2	#NULL!	2	2	2	1
2	2	2	2	2	2	2	2	2	2
1	2	2	2	2	2	2	2	#NULL!	1
2	1	2	1	2	2	1	1	1	1
#NULL!	2	#NULL!	#NULL!	#NULL!	#NULL!	#NULL!	#NULL!	#NULL!	#NULL!
1	2	2	2	2	2	2	2	2	2
2	2	2	2	2	2	2	2	1	1
1	2	2	2	2	2	2	2	2	2
2	2	2	2	2	2	2	2	2	2
1	2	2	2	2	1	2	2	2	2
2	2	2	2	2	1	1	1	1	1
2	2	1	2	2	1	2	1	2	2
2	2	2	2	2	2	2	2	2	2
#NULL!	2	2	2	2	2	2	2	2	2
2	1	2	2	2	2	2	2	2	2
1	2	2	2	2	2	2	2	2	2
1	2	2	2	2	2	2	2	2	2
1	2	2	2	2	2	2	2	2	2
1	2	2	2	2	1	2	2	2	2
2	2	2	2	2	2	2	2	2	2
1	2	1	1	1	1	1	1	1	2
2	2	2	1	1	1	2	2	2	2
2	2	1	2	2	2	1	2	1	2
2	1	1	1	1	1	1	1	1	1
#NULL!	#NULL!	#NULL!	#NULL!	#NULL!	#NULL!	#NULL!	#NULL!	#NULL!	#NULL!
2	2	2	1	1	1	2	2	2	2
2	2	1	1	2	2	#NULL!	#NULL!	#NULL!	#NULL!
2	2	2	2	2	2	2	2	2	2
2	2	2	2	2	1	2	2	1	1
2	2	2	2	2	2	2	2	2	2
1	2	2	2	#NULL!	#NULL!	#NULL!	#NULL!	#NULL!	#NULL!
2	2	2	2	2	2	2	2	1	2
2	1	1	2	2	2	2	2	2	2
2	2	2	2	2	2	2	2	2	1
2	2	2	2	2	1	2	2	2	2
#NULL!	#NULL!	#NULL!	#NULL!	#NULL!	#NULL!	#NULL!	#NULL!	#NULL!	#NULL!
2	2	2	2	1	2	1	2	2	1
2	2	2	2	2	2	2	2	2	2
2	2	2	2	2	1	2	2	1	1
1	1	2	2	1	2	1	2	1	2
2	2	2	2	2	2	2	2	2	2

1	2	2	2	1	2	2	1	2	2
2	1	1	1	1	1	1	1	1	1
2	2	2	2	2	2	2	2	2	2
2	2	2	2	2	2	2	2	2	2
1	2	2	2	1	2	2	2	2	2
2	1	2	2	2	2	2	2	2	2
2	2	2	2	2	2	2	2	2	2
#NULL!	#NULL!	#NULL!	#NULL!	#NULL!	#NULL!	#NULL!	#NULL!	#NULL!	#NULL!
2	2	2	2	2	2	2	2	2	2
2	2	2	2	1	1	2	2	1	2
2	2	2	2	2	2	1	2	2	1
1	2	2	2	2	2	2	2	2	2
1	2	2	2	2	1	1	1	1	2
2	2	2	2	2	2	2	2	2	2
1	2	2	2	2	2	2	2	2	2
2	2	2	2	2	2	2	2	2	2
1	2	2	2	2	2	2	2	2	2
2	2	2	2	2	1	1	1	2	2
2	2	#NULL!	2	2	2	2	#NULL!	2	#NULL!
2	1	2	2	2	2	2	2	2	2
1	2	1	2	1	2	1	2	1	2
2	2	2	2	2	2	2	2	1	2
2	2	2	2	2	2	2	2	2	2
1	2	2	2	2	2	2	2	2	2
2	2	2	1	2	2	2	2	2	2
2	2	2	1	1	2	1	#NULL!	1	2
2	2	1	2	2	1	1	1	1	2
2	2	2	2	2	2	2	2	2	2
2	2	2	2	2	2	1	1	1	1
2	2	2	2	2	1	2	1	1	2
2	2	2	2	2	2	2	1	1	1
2	2	2	2	2	2	1	2	2	2
2	1	#NULL!	1	2	2	1	2	2	2
2	2	2	2	2	2	2	2	2	2
2	2	2	2	2	1	2	2	1	2
2	2	2	2	2	2	2	2	2	2
#NULL!	2	1	2	1	2	1	2	1	2
1	2	2	2	2	2	2	1	2	2
#NULL!	#NULL!	#NULL!	#NULL!	#NULL!	#NULL!	#NULL!	#NULL!	#NULL!	#NULL!
2	1	1	1	1	1	1	1	1	1
1	2	2	1	2	2	2	2	2	2
#NULL!	#NULL!	#NULL!	#NULL!	#NULL!	#NULL!	#NULL!	#NULL!	#NULL!	#NULL!
1	2	2	2	2	2	2	2	2	2
1	2	2	2	2	2	1	2	2	2
2	#NULL!	#NULL!	#NULL!	#NULL!	#NULL!	#NULL!	#NULL!	#NULL!	#NULL!
2	2	1	2	2	2	1	2	1	2
2	2	2	2	2	2	2	2	2	2

2	2	2	#NULL!	2	1	2	#NULL!	2	1
2	2	2	2	2	1	2	2	1	2
1	1	1	1	1	1	1	1	1	1
2	2	2	2	2	2	2	2	2	2
2	2	2	2	2	1	2	2	2	2
2	2	1	2	2	2	2	1	2	2
1	2	#NULL!	2	2	1	2	#NULL!	2	1
2	1	2	#NULL!	2	1	2	2	2	2
2	2	#NULL!	#NULL!	#NULL!	#NULL!	#NULL!	#NULL!	#NULL!	#NULL!
2	2	2	2	2	2	2	2	2	2
2	2	2	1	2	2	1	2	2	2
1	2	2	2	2	2	2	2	1	2
2	2	2	2	2	2	2	2	2	2
1	2	2	2	2	2	2	2	2	2
1	2	2	2	1	2	2	2	2	2
1	2	2	1	2	2	2	2	2	2
1	2	2	1	2	2	2	2	2	2
1	2	2	2	2	2	2	2	2	2
2	2	#NULL!	#NULL!	#NULL!	#NULL!	#NULL!	#NULL!	#NULL!	#NULL!
2	1	#NULL!	2	1	2	2	2	2	2
2	2	1	2	2	2	1	2	2	2
2	2	2	2	1	2	2	2	2	1
2	2	2	2	2	2	1	2	1	2
2	2	2	2	2	2	2	2	2	2
2	2	2	2	2	2	#NULL!	#NULL!	#NULL!	#NULL!
2	1	1	1	1	1	1	1	1	1
2	2	1	2	1	2	2	1	2	2
1	2	2	2	2	2	2	2	2	2
2	2	2	2	2	2	2	2	2	2
2	2	2	2	2	2	2	2	2	2
2	2	2	2	2	2	2	2	2	2
2	2	2	2	2	2	2	2	2	1
1	2	2	2	2	2	2	2	2	2
2	1	1	1	2	2	2	1	1	1
1	2	2	2	2	1	2	2	2	2
1	1	1	1	1	2	2	2	2	2
1	2	2	2	2	2	2	2	2	2
1	2	1	2	1	1	1	2	1	2
2	2	1	2	2	1	1	2	2	2
1	2	1	1	1	1	1	1	1	1
1	#NULL!	#NULL!	#NULL!	#NULL!	#NULL!	#NULL!	#NULL!	#NULL!	#NULL!
2	2	2	2	2	2	2	2	2	2
2	2	2	2	2	2	2	2	2	2
#NULL!	#NULL!	#NULL!	#NULL!	#NULL!	#NULL!	#NULL!	#NULL!	#NULL!	#NULL!
#NULL!	#NULL!	#NULL!	#NULL!	#NULL!	#NULL!	#NULL!	#NULL!	#NULL!	#NULL!
2	2	2	2	2	2	2	2	2	2
1	2	2	2	2	2	2	1	1	1

2	2	#NULL!	#NULL!	#NULL!	#NULL!	#NULL!	#NULL!	#NULL!	#NULL!
2	2	2	2	2	#NULL!	#NULL!	#NULL!	#NULL!	#NULL!
2	2	#NULL!	#NULL!	#NULL!	#NULL!	#NULL!	#NULL!	#NULL!	#NULL!
2	2	#NULL!	#NULL!	#NULL!	#NULL!	#NULL!	#NULL!	#NULL!	#NULL!
2	#NULL!	#NULL!	2	#NULL!	#NULL!	#NULL!	#NULL!	#NULL!	#NULL!
#NULL!	#NULL!	#NULL!	#NULL!	#NULL!	#NULL!	#NULL!	#NULL!	#NULL!	#NULL!
2	2	2	2	2	2	2	2	2	2
#NULL!	#NULL!	#NULL!	#NULL!	#NULL!	#NULL!	#NULL!	#NULL!	#NULL!	#NULL!
2	2	2	2	2	2	2	2	2	2
1	#NULL!	#NULL!	#NULL!	2	#NULL!	#NULL!	#NULL!	#NULL!	#NULL!
2	#NULL!	#NULL!	#NULL!	#NULL!	2	#NULL!	#NULL!	#NULL!	#NULL!
2	#NULL!	#NULL!	#NULL!	2	#NULL!	#NULL!	#NULL!	#NULL!	#NULL!
2	2	2	2	2	2	2	2	2	2
#NULL!	2	2	2	2	2	2	2	2	2
1	1	#NULL!	#NULL!	#NULL!	#NULL!	#NULL!	#NULL!	#NULL!	#NULL!
1	1	2	1	2	1	2	1	1	1
1	1	1	1	1	1	1	1	1	1
1	1	1	2	2	2	2	2	2	2
2	2	2	2	2	2	2	2	2	2
2	2	2	2	2	2	2	2	2	2
2	2	2	2	2	2	2	2	2	2
#NULL!	#NULL!	#NULL!	#NULL!	#NULL!	#NULL!	#NULL!	#NULL!	#NULL!	#NULL!
2	1	1	1	1	1	1	1	1	2
2	2	1	2	2	1	2	2	2	2
2	2	2	2	2	2	2	2	2	2
#NULL!	2	2	2	2	2	2	2	2	2
2	2	2	2	2	2	2	1	2	2
2	1	1	2	2	1	2	2	2	2
1	2	1	1	1	1	1	1	1	1
1	2	1	1	2	2	2	2	1	2
2	2	2	2	2	2	2	2	2	2
2	2	2	2	2	2	2	2	2	2
#NULL!	#NULL!	#NULL!	#NULL!	#NULL!	#NULL!	#NULL!	#NULL!	#NULL!	#NULL!
2	1	2	2	2	2	2	1	1	1
1	2	1	2	1	2	1	2	1	1
1	2	2	2	2	2	2	2	2	2
1	2	#NULL!	2	2	1	2	2	1	2
2	2	2	2	2	2	2	2	2	2
2	2	#NULL!	#NULL!	#NULL!	#NULL!	#NULL!	#NULL!	#NULL!	#NULL!
2	2	2	2	2	2	2	2	2	2
2	2	2	2	2	2	2	2	2	2
1	1	2	1	2	2	2	1	#NULL!	2
2	2	2	2	2	2	2	1	1	1
2	2	2	2	2	2	2	2	2	2
2	2	2	2	2	2	2	2	2	2
2	1	2	2	2	2	2	2	2	2
#NULL!	#NULL!	#NULL!	#NULL!	#NULL!	#NULL!	#NULL!	#NULL!	#NULL!	#NULL!

1	2	2	2	2	2	2	2	2	2
2	2	1	2	2	2	2	2	2	2
2	2	2	2	2	2	2	2	1	2
#NULL!	#NULL!	#NULL!	#NULL!	#NULL!	#NULL!	#NULL!	#NULL!	#NULL!	#NULL!
2	2	2	2	2	2	2	2	2	2
2	2	1	2	#NULL!	2	2	2	1	2
2	2	1	2	2	2	2	2	1	2
2	1	2	2	2	2	2	2	2	2
2	2	2	2	2	2	1	2	1	2
2	1	1	2	2	2	2	2	2	2
2	2	2	2	2	2	2	2	2	2
2	1	#NULL!	#NULL!	#NULL!	#NULL!	#NULL!	#NULL!	#NULL!	#NULL!
2	2	#NULL!	#NULL!	#NULL!	#NULL!	#NULL!	#NULL!	#NULL!	#NULL!
1	2	#NULL!	#NULL!	#NULL!	#NULL!	#NULL!	#NULL!	#NULL!	#NULL!
2	2	#NULL!	#NULL!	#NULL!	2	#NULL!	#NULL!	2	#NULL!
2	#NULL!	#NULL!	#NULL!	#NULL!	#NULL!	#NULL!	#NULL!	#NULL!	#NULL!
#NULL!	#NULL!	#NULL!	#NULL!	#NULL!	#NULL!	#NULL!	#NULL!	#NULL!	#NULL!
#NULL!	#NULL!	#NULL!	#NULL!	#NULL!	#NULL!	#NULL!	#NULL!	#NULL!	#NULL!
2	2	#NULL!	2	#NULL!	2	#NULL!	2	#NULL!	2
#NULL!	#NULL!	#NULL!	#NULL!	#NULL!	#NULL!	#NULL!	#NULL!	#NULL!	#NULL!
#NULL!	#NULL!	#NULL!	#NULL!	#NULL!	#NULL!	#NULL!	#NULL!	#NULL!	#NULL!
1	2	2	2	2	2	2	2	2	2
1	2	2	2	2	2	2	2	2	2
1	2	2	2	2	2	2	2	2	2
1	2	2	2	2	2	2	2	2	2
#NULL!	#NULL!	#NULL!	#NULL!	#NULL!	#NULL!	#NULL!	#NULL!	#NULL!	#NULL!
2	2	2	2	2	2	2	2	2	2
#NULL!	#NULL!	#NULL!	#NULL!	#NULL!	#NULL!	#NULL!	#NULL!	#NULL!	#NULL!
#NULL!	#NULL!	#NULL!	#NULL!	#NULL!	#NULL!	#NULL!	#NULL!	#NULL!	#NULL!
1	2	2	2	1	2	2	2	2	2
2	#NULL!	#NULL!	#NULL!	#NULL!	#NULL!	#NULL!	#NULL!	#NULL!	#NULL!
#NULL!	#NULL!	#NULL!	#NULL!	#NULL!	#NULL!	#NULL!	#NULL!	#NULL!	#NULL!
#NULL!	#NULL!	#NULL!	#NULL!	#NULL!	#NULL!	#NULL!	#NULL!	#NULL!	#NULL!
#NULL!	#NULL!	#NULL!	#NULL!	#NULL!	#NULL!	#NULL!	#NULL!	#NULL!	#NULL!
2	#NULL!	1	2	2	1	2	#NULL!	2	1
2	#NULL!	2	#NULL!	1	2	#NULL!	2	#NULL!	#NULL!
#NULL!	#NULL!	#NULL!	#NULL!	#NULL!	#NULL!	#NULL!	#NULL!	#NULL!	#NULL!
#NULL!	#NULL!	#NULL!	#NULL!	#NULL!	#NULL!	#NULL!	#NULL!	#NULL!	#NULL!
#NULL!	#NULL!	#NULL!	#NULL!	#NULL!	#NULL!	#NULL!	#NULL!	#NULL!	#NULL!
#NULL!	1	#NULL!	#NULL!	#NULL!	#NULL!	#NULL!	#NULL!	#NULL!	#NULL!
#NULL!	1	#NULL!	#NULL!	#NULL!	#NULL!	#NULL!	#NULL!	#NULL!	#NULL!
2	#NULL!	#NULL!	#NULL!	#NULL!	#NULL!	#NULL!	#NULL!	#NULL!	#NULL!
1	1	1	1	1	1	1	1	1	1
1	2	1	2	1	2	2	2	2	2
1	1	2	2	2	2	2	2	2	1
#NULL!	2	#NULL!	#NULL!	#NULL!	#NULL!	#NULL!	#NULL!	2	#NULL!
1	2	#NULL!	#NULL!	#NULL!	#NULL!	#NULL!	#NULL!	#NULL!	#NULL!

2	2	#NULL!	#NULL!	#NULL!	#NULL!	#NULL!	#NULL!	#NULL!	#NULL!
2	#NULL!	#NULL!	#NULL!	2	#NULL!	#NULL!	#NULL!	#NULL!	#NULL!
2	2	2	2	2	1	1	1	1	1
1	1	2	2	1	2	2	1	2	2
#NULL!	2	2	2	2	2	2	2	2	2
#NULL!	2	2	2	2	1	1	1	1	2
1	2	2	1	2	2	2	2	1	2
2	2	2	1	2	2	2	2	2	1
2	2	2	2	2	2	2	2	2	2
1	1	1	1	1	1	1	1	1	1
#NULL!	#NULL!	#NULL!	#NULL!	#NULL!	#NULL!	#NULL!	#NULL!	#NULL!	#NULL!
#NULL!	#NULL!	#NULL!	#NULL!	#NULL!	#NULL!	#NULL!	#NULL!	#NULL!	#NULL!
2	#NULL!	#NULL!	#NULL!	#NULL!	#NULL!	#NULL!	#NULL!	#NULL!	#NULL!
1	#NULL!	2	#NULL!	#NULL!	#NULL!	#NULL!	#NULL!	#NULL!	#NULL!
1	2	#NULL!	2	2	2	2	2	1	1
2	2	2	2	1	1	1	2	2	2
#NULL!	#NULL!	#NULL!	#NULL!	#NULL!	#NULL!	#NULL!	#NULL!	#NULL!	#NULL!
2	2	2	2	2	2	2	2	2	2
1	2	2	2	2	2	2	2	2	2
2	2	1	1	2	1	2	2	2	2
2	1	2	1	2	2	2	1	2	2
2	2	2	2	2	2	2	2	2	2
1	2	1	2	1	1	2	2	2	2
1	2	2	2	2	2	2	2	2	2
2	2	2	2	2	2	2	2	2	2
1	2	2	2	2	2	2	2	2	2
2	2	2	2	2	2	2	2	2	2
1	2	2	2	2	1	2	2	2	2
2	2	2	2	2	2	1	1	1	1
1	2	2	2	2	2	2	2	2	2
2	2	2	2	2	2	2	2	2	2
2	2	2	2	2	2	2	2	2	2
2	2	2	1	2	2	2	2	2	2
2	1	1	1	1	1	1	1	1	1
2	2	1	2	2	1	2	2	1	1
2	2	2	1	2	2	1	2	2	1
#NULL!	#NULL!	#NULL!	#NULL!	#NULL!	#NULL!	#NULL!	#NULL!	#NULL!	#NULL!
2	2	2	2	2	2	2	2	2	2
2	1	1	1	1	1	1	1	1	1
2	1	1	1	1	1	1	1	1	1
2	2	#NULL!	#NULL!	#NULL!	#NULL!	2	#NULL!	#NULL!	#NULL!
#NULL!	#NULL!	#NULL!	#NULL!	#NULL!	#NULL!	#NULL!	#NULL!	#NULL!	#NULL!
#NULL!	#NULL!	#NULL!	#NULL!	1	#NULL!	#NULL!	#NULL!	#NULL!	#NULL!
2	2	2	2	2	2	2	2	2	2
2	2	2	2	2	2	2	2	2	2
2	2	2	2	2	2	2	2	2	2
1	2	2	2	2	2	2	2	1	1

2	#NULL!	#NULL!	#NULL!	#NULL!	2	#NULL!	#NULL!	#NULL!	#NULL!
1	2	#NULL!	#NULL!	#NULL!	#NULL!	#NULL!	#NULL!	#NULL!	#NULL!
2	#NULL!	#NULL!	2	#NULL!	#NULL!	#NULL!	#NULL!	#NULL!	#NULL!
1	#NULL!	#NULL!	2	#NULL!	#NULL!	#NULL!	#NULL!	#NULL!	#NULL!
2	#NULL!	#NULL!	#NULL!	#NULL!	1	#NULL!	#NULL!	#NULL!	#NULL!
1	1	1	#NULL!	2	2	2	2	2	2
2	#NULL!	#NULL!	#NULL!	#NULL!	#NULL!	#NULL!	2	#NULL!	#NULL!
1	2	#NULL!	#NULL!	#NULL!	#NULL!	#NULL!	#NULL!	#NULL!	#NULL!
2	2	#NULL!	#NULL!	#NULL!	#NULL!	#NULL!	#NULL!	#NULL!	#NULL!
2	2	#NULL!	#NULL!	#NULL!	#NULL!	#NULL!	#NULL!	#NULL!	#NULL!
2	#NULL!	#NULL!	#NULL!	2	#NULL!	#NULL!	#NULL!	#NULL!	#NULL!
2	#NULL!	#NULL!	#NULL!	#NULL!	2	#NULL!	#NULL!	#NULL!	#NULL!
2	2	#NULL!	#NULL!	#NULL!	#NULL!	#NULL!	#NULL!	#NULL!	#NULL!
1	#NULL!	2	#NULL!	#NULL!	#NULL!	#NULL!	#NULL!	#NULL!	#NULL!
2	2	#NULL!	#NULL!	#NULL!	#NULL!	#NULL!	#NULL!	#NULL!	#NULL!
#NULL!	#NULL!	#NULL!	#NULL!	#NULL!	#NULL!	#NULL!	#NULL!	#NULL!	#NULL!
2	#NULL!	#NULL!	2	#NULL!	2	#NULL!	#NULL!	1	#NULL!
1	2	#NULL!	2	2	2	1	2	2	2
1	2	2	2	2	2	2	2	2	2
2	2	2	2	2	2	2	2	2	2
2	2	1	2	2	1	1	1	1	2
#NULL!	2	1	2	2	2	2	1	2	1
2	2	2	2	2	2	2	2	2	2
2	2	1	2	2	1	2	2	1	2
2	2	1	#NULL!	2	1	1	2	1	2
#NULL!	#NULL!	#NULL!	#NULL!	#NULL!	#NULL!	#NULL!	#NULL!	#NULL!	#NULL!
2	2	2	2	2	2	2	2	2	2
2	2	2	2	2	2	2	2	2	2
2	2	2	2	2	2	2	1	2	2
1	2	2	2	2	2	2	2	2	2
1	2	2	2	2	2	1	2	2	2
2	2	2	2	2	2	2	2	2	2
2	2	1	1	2	2	2	2	2	2
2	2	2	2	2	2	2	2	2	2
#NULL!	#NULL!	#NULL!	#NULL!	#NULL!	#NULL!	#NULL!	#NULL!	#NULL!	#NULL!
2	2	2	2	2	2	2	2	2	2
#NULL!	#NULL!	#NULL!	#NULL!	#NULL!	#NULL!	#NULL!	#NULL!	#NULL!	#NULL!
2	2	2	2	2	2	2	2	2	2
2	#NULL!	#NULL!	#NULL!	#NULL!	#NULL!	#NULL!	#NULL!	#NULL!	#NULL!
1	2	2	2	2	2	2	2	2	2
2	2	2	2	2	2	2	2	1	2
2	1	1	1	1	1	1	1	1	1
1	2	#NULL!	2	#NULL!	2	1	2	1	1
2	2	2	2	2	2	2	2	2	2
2	1	1	1	1	1	2	2	2	2
2	2	2	2	2	2	1	2	2	2
1	2	2	2	2	2	2	2	2	2

1	2	1	2	2	2	2	1	2	1
2	2	#NULL!	#NULL!	#NULL!	#NULL!	#NULL!	#NULL!	#NULL!	#NULL!
1	2	2	2	2	#NULL!	2	2	1	1
2	2	2	2	1	2	2	2	2	1
2	2	2	2	2	2	2	2	2	2
2	2	2	2	2	2	2	2	1	1
2	2	2	2	2	1	2	1	2	2
2	2	1	2	2	2	2	2	1	2
#NULL!	#NULL!	#NULL!	#NULL!	#NULL!	#NULL!	#NULL!	#NULL!	#NULL!	#NULL!
2	2	2	2	2	2	2	2	2	2
2	2	2	2	2	2	2	2	2	2
2	2	#NULL!	#NULL!	#NULL!	#NULL!	#NULL!	#NULL!	#NULL!	#NULL!
#NULL!	2	#NULL!	#NULL!	#NULL!	#NULL!	#NULL!	#NULL!	#NULL!	#NULL!
1	2	2	2	2	#NULL!	2	2	2	#NULL!
2	2	#NULL!	#NULL!	#NULL!	#NULL!	#NULL!	#NULL!	#NULL!	#NULL!
2	2	2	1	1	1	2	2	2	2
#NULL!	#NULL!	#NULL!	#NULL!	#NULL!	#NULL!	#NULL!	#NULL!	#NULL!	#NULL!
1	2	#NULL!	#NULL!	#NULL!	#NULL!	#NULL!	#NULL!	#NULL!	#NULL!
2	#NULL!	#NULL!	2	#NULL!	#NULL!	#NULL!	#NULL!	#NULL!	#NULL!
2	2	1	2	1	2	1	2	1	2
2	2	2	2	2	2	2	2	2	2
2	2	2	2	2	2	2	2	2	2
2	2	2	2	2	2	2	2	2	2
2	#NULL!	#NULL!	#NULL!	#NULL!	#NULL!	#NULL!	#NULL!	#NULL!	2
2	2	#NULL!	#NULL!	#NULL!	#NULL!	#NULL!	#NULL!	#NULL!	#NULL!
2	2	2	2	2	2	2	2	2	2
1	2	2	2	2	2	2	2	2	2
1	2	1	2	2	1	2	2	2	2
#NULL!	#NULL!	#NULL!	#NULL!	#NULL!	#NULL!	#NULL!	#NULL!	#NULL!	#NULL!
1	1	#NULL!	#NULL!	#NULL!	#NULL!	#NULL!	#NULL!	#NULL!	#NULL!
2	2	2	2	2	2	2	2	2	2
2	2	2	1	2	2	1	2	2	1
2	2	2	2	1	1	2	2	2	2
2	1	2	2	2	2	2	2	2	2
2	2	2	2	2	2	2	2	2	2
#NULL!	2	2	2	2	2	2	2	2	2
#NULL!	#NULL!	#NULL!	#NULL!	#NULL!	#NULL!	#NULL!	#NULL!	#NULL!	#NULL!
#NULL!	#NULL!	#NULL!	#NULL!	#NULL!	#NULL!	#NULL!	#NULL!	#NULL!	#NULL!
2	1	2	1	2	1	2	1	2	2
2	2	2	2	2	2	2	2	2	2
2	2	2	2	2	2	2	2	2	2
2	2	2	2	2	2	2	2	2	2
2	2	2	2	2	2	2	2	2	2
2	2	2	2	1	2	2	2	1	2
#NULL!	1	1	1	1	1	1	1	1	1
2	2	2	2	2	2	2	2	2	2
2	1	1	2	2	2	2	2	2	1

2	1	1	2	1	1	1	1	1	1
1	2	1	2	2	2	1	2	2	1
1	#NULL!	#NULL!	2	#NULL!	#NULL!	#NULL!	#NULL!	#NULL!	#NULL!
2	2	#NULL!	#NULL!	#NULL!	#NULL!	#NULL!	#NULL!	#NULL!	#NULL!
2	2	2	1	2	1	2	2	1	2
1	1	1	2	1	2	2	2	1	2
2	2	#NULL!	#NULL!	#NULL!	2	#NULL!	#NULL!	#NULL!	2
2	1	#NULL!	#NULL!	1	#NULL!	1	#NULL!	1	1
#NULL!	#NULL!	#NULL!	#NULL!	#NULL!	#NULL!	#NULL!	#NULL!	#NULL!	#NULL!
#NULL!	#NULL!	#NULL!	#NULL!	#NULL!	#NULL!	#NULL!	#NULL!	#NULL!	#NULL!
2	#NULL!	#NULL!	#NULL!	#NULL!	#NULL!	#NULL!	#NULL!	#NULL!	#NULL!
2	#NULL!	#NULL!	#NULL!	#NULL!	#NULL!	#NULL!	#NULL!	#NULL!	#NULL!
2	#NULL!	#NULL!	#NULL!	#NULL!	#NULL!	#NULL!	#NULL!	#NULL!	#NULL!
#NULL!	#NULL!	#NULL!	#NULL!	#NULL!	#NULL!	#NULL!	#NULL!	#NULL!	#NULL!
2	#NULL!	#NULL!	#NULL!	#NULL!	#NULL!	#NULL!	#NULL!	#NULL!	#NULL!
2	2	#NULL!	#NULL!	#NULL!	#NULL!	#NULL!	#NULL!	#NULL!	#NULL!
2	1	1	1	1	1	1	1	1	1
1	2	#NULL!	#NULL!	#NULL!	#NULL!	#NULL!	#NULL!	#NULL!	#NULL!
1	2	#NULL!	#NULL!	#NULL!	#NULL!	#NULL!	#NULL!	#NULL!	#NULL!
1	2	#NULL!	#NULL!	#NULL!	2	#NULL!	#NULL!	2	#NULL!
#NULL!	#NULL!	#NULL!	#NULL!	#NULL!	#NULL!	#NULL!	#NULL!	#NULL!	#NULL!
2	2	#NULL!	#NULL!	#NULL!	#NULL!	#NULL!	#NULL!	#NULL!	#NULL!
2	2	2	2	2	2	2	2	2	2
#NULL!	#NULL!	#NULL!	#NULL!	#NULL!	#NULL!	#NULL!	#NULL!	#NULL!	#NULL!
#NULL!	#NULL!	#NULL!	#NULL!	#NULL!	#NULL!	#NULL!	#NULL!	#NULL!	#NULL!
1	2	#NULL!	2	#NULL!	#NULL!	#NULL!	#NULL!	#NULL!	#NULL!
#NULL!	#NULL!	#NULL!	#NULL!	#NULL!	#NULL!	#NULL!	#NULL!	#NULL!	#NULL!
1	2	2	2	2	#NULL!	2	#NULL!	2	#NULL!
1	2	1	2	2	2	2	2	2	2
#NULL!	#NULL!	#NULL!	#NULL!	#NULL!	#NULL!	#NULL!	#NULL!	#NULL!	#NULL!
#NULL!	#NULL!	#NULL!	#NULL!	#NULL!	#NULL!	#NULL!	#NULL!	#NULL!	#NULL!
1	2	2	2	2	2	2	2	2	2
#NULL!	#NULL!	#NULL!	#NULL!	#NULL!	#NULL!	#NULL!	#NULL!	#NULL!	#NULL!
#NULL!	#NULL!	#NULL!	#NULL!	#NULL!	#NULL!	#NULL!	#NULL!	#NULL!	#NULL!
#NULL!	#NULL!	#NULL!	#NULL!	#NULL!	#NULL!	#NULL!	#NULL!	#NULL!	#NULL!
#NULL!	#NULL!	#NULL!	#NULL!	#NULL!	#NULL!	#NULL!	#NULL!	#NULL!	#NULL!
2	#NULL!	#NULL!	#NULL!	#NULL!	#NULL!	#NULL!	#NULL!	#NULL!	#NULL!
#NULL!	#NULL!	#NULL!	#NULL!	#NULL!	#NULL!	#NULL!	#NULL!	#NULL!	#NULL!
2	2	1	2	1	2	2	2	1	2
2	1	2	2	1	2	2	2	2	2
2	#NULL!	#NULL!	#NULL!	#NULL!	#NULL!	#NULL!	#NULL!	#NULL!	2
#NULL!	#NULL!	#NULL!	#NULL!	#NULL!	#NULL!	#NULL!	#NULL!	#NULL!	#NULL!
2	2	1	2	2	1	2	2	1	1
2	2	2	1	2	2	1	2	2	1
2	1	1	1	1	1	1	1	2	2
1	2	2	2	2	2	1	2	1	2
#NULL!	2	1	2	2	1	2	#NULL!	#NULL!	#NULL!

2	2	#NULL!	#NULL!	#NULL!	#NULL!	#NULL!	#NULL!	#NULL!	#NULL!
2	2	1	1	2	2	2	2	2	1
2	2	2	2	2	2	2	2	2	2
1	2	2	2	2	2	2	2	2	2
2	2	2	2	2	2	2	2	2	2
1	2	2	2	2	2	1	1	1	1
1	#NULL!	#NULL!	#NULL!	#NULL!	#NULL!	#NULL!	#NULL!	#NULL!	#NULL!
2	1	1	1	1	2	2	2	2	2
2	2	2	2	2	2	2	2	2	2
2	2	2	2	2	2	2	2	2	2
2	2	2	#NULL!	1	2	2	2	2	2
2	#NULL!	#NULL!	#NULL!	#NULL!	#NULL!	2	#NULL!	#NULL!	#NULL!
2	#NULL!	#NULL!	#NULL!	#NULL!	#NULL!	#NULL!	2	#NULL!	#NULL!
1	#NULL!	#NULL!	#NULL!	#NULL!	#NULL!	#NULL!	#NULL!	#NULL!	2
2	2	#NULL!	#NULL!	#NULL!	#NULL!	#NULL!	#NULL!	#NULL!	#NULL!
2	2	2	2	2	2	2	2	2	2
2	1	1	2	2	2	2	2	2	2
2	2	1	1	1	1	1	2	1	2
2	2	2	2	1	1	1	1	1	1
2	2	2	2	2	2	2	2	2	2
2	2	2	2	2	2	2	2	2	2
#NULL!	#NULL!	#NULL!	#NULL!	#NULL!	#NULL!	#NULL!	#NULL!	#NULL!	#NULL!
1	1	1	1	1	2	2	2	2	1
2	2	#NULL!	#NULL!	#NULL!	#NULL!	#NULL!	#NULL!	#NULL!	#NULL!
2	#NULL!	#NULL!	#NULL!	#NULL!	#NULL!	#NULL!	#NULL!	#NULL!	2
1	2	2	2	2	2	2	2	2	2
2	#NULL!	#NULL!	#NULL!	2	#NULL!	#NULL!	2	#NULL!	#NULL!
2	2	1	1	2	2	2	2	2	2
#NULL!	#NULL!	#NULL!	#NULL!	#NULL!	#NULL!	#NULL!	#NULL!	#NULL!	#NULL!
1	#NULL!	#NULL!	#NULL!	#NULL!	#NULL!	#NULL!	#NULL!	#NULL!	2
2	2	1	2	1	1	1	2	2	2
2	2	1	2	1	2	1	2	1	2
#NULL!	#NULL!	#NULL!	#NULL!	#NULL!	#NULL!	#NULL!	#NULL!	#NULL!	#NULL!
1	2	#NULL!	#NULL!	#NULL!	#NULL!	#NULL!	#NULL!	#NULL!	#NULL!
1	2	2	2	2	2	1	1	2	2
#NULL!	#NULL!	#NULL!	#NULL!	#NULL!	#NULL!	#NULL!	#NULL!	#NULL!	#NULL!
#NULL!	2	1	2	2	1	2	1	2	2
1	2	2	2	2	2	2	2	2	2
2	2	2	2	2	2	2	2	2	2
2	#NULL!	#NULL!	#NULL!	#NULL!	#NULL!	#NULL!	#NULL!	#NULL!	#NULL!
2	2	2	2	2	2	2	2	2	2
2	2	2	2	2	2	2	2	2	2
2	2	2	2	2	2	2	2	2	2
2	2	2	2	2	2	2	2	2	2
2	2	1	1	2	1	2	1	2	2
2	1	1	2	2	2	2	2	2	2
2	2	2	2	2	1	1	1	1	1

2	2	#NULL!	#NULL!	#NULL!	#NULL!	#NULL!	#NULL!	#NULL!	#NULL!
2	2	2	2	2	2	2	2	2	2
2	2	2	2	2	2	2	2	2	2
1	#NULL!	#NULL!	#NULL!	#NULL!	2	#NULL!	#NULL!	#NULL!	#NULL!
2	1	#NULL!	#NULL!	#NULL!	#NULL!	#NULL!	#NULL!	#NULL!	#NULL!
#NULL!	#NULL!	#NULL!	#NULL!	#NULL!	#NULL!	#NULL!	#NULL!	#NULL!	#NULL!
#NULL!	#NULL!	#NULL!	#NULL!	#NULL!	#NULL!	#NULL!	#NULL!	#NULL!	#NULL!
2	2	2	2	2	2	2	2	2	2
1	2	2	2	2	2	2	2	2	2
2	#NULL!	#NULL!	#NULL!	#NULL!	#NULL!	#NULL!	#NULL!	#NULL!	#NULL!
1	2	2	2	2	2	2	2	2	2
2	2	1	1	1	2	2	2	1	2
1	2	2	2	2	2	2	2	2	2
2	2	2	2	2	2	2	2	2	2
1	#NULL!	#NULL!	#NULL!	#NULL!	#NULL!	#NULL!	#NULL!	#NULL!	#NULL!
#NULL!	#NULL!	#NULL!	#NULL!	#NULL!	#NULL!	#NULL!	#NULL!	#NULL!	#NULL!
#NULL!	#NULL!	#NULL!	#NULL!	#NULL!	#NULL!	#NULL!	#NULL!	#NULL!	#NULL!
#NULL!	#NULL!	#NULL!	#NULL!	#NULL!	#NULL!	#NULL!	#NULL!	#NULL!	#NULL!
2	2	#NULL!	#NULL!	#NULL!	#NULL!	#NULL!	#NULL!	#NULL!	#NULL!
2	2	#NULL!	#NULL!	#NULL!	#NULL!	#NULL!	#NULL!	#NULL!	#NULL!
#NULL!	#NULL!	#NULL!	#NULL!	#NULL!	#NULL!	#NULL!	#NULL!	#NULL!	#NULL!
#NULL!	#NULL!	#NULL!	#NULL!	#NULL!	#NULL!	#NULL!	#NULL!	#NULL!	#NULL!
2	2	2	2	2	2	2	2	2	2
2	2	#NULL!	#NULL!	#NULL!	#NULL!	#NULL!	#NULL!	#NULL!	#NULL!
#NULL!	#NULL!	#NULL!	#NULL!	#NULL!	#NULL!	#NULL!	#NULL!	#NULL!	#NULL!
2	1	#NULL!	#NULL!	#NULL!	#NULL!	#NULL!	#NULL!	#NULL!	#NULL!
#NULL!	#NULL!	#NULL!	#NULL!	#NULL!	#NULL!	#NULL!	#NULL!	#NULL!	#NULL!
2	#NULL!	#NULL!	#NULL!	#NULL!	#NULL!	#NULL!	#NULL!	#NULL!	#NULL!
2	2	#NULL!	#NULL!	#NULL!	#NULL!	2	#NULL!	#NULL!	#NULL!
2	1	#NULL!	#NULL!	#NULL!	#NULL!	#NULL!	#NULL!	#NULL!	#NULL!
#NULL!	#NULL!	#NULL!	#NULL!	#NULL!	#NULL!	#NULL!	#NULL!	#NULL!	#NULL!
2	2	2	2	2	2	2	2	2	2
#NULL!	#NULL!	#NULL!	#NULL!	#NULL!	#NULL!	#NULL!	#NULL!	#NULL!	#NULL!
#NULL!	#NULL!	#NULL!	#NULL!	#NULL!	#NULL!	#NULL!	#NULL!	#NULL!	#NULL!
2	#NULL!	#NULL!	#NULL!	#NULL!	#NULL!	#NULL!	#NULL!	#NULL!	#NULL!
#NULL!	#NULL!	#NULL!	#NULL!	#NULL!	#NULL!	#NULL!	#NULL!	#NULL!	#NULL!
2	#NULL!	#NULL!	#NULL!	#NULL!	#NULL!	#NULL!	#NULL!	#NULL!	#NULL!
#NULL!	#NULL!	#NULL!	#NULL!	#NULL!	#NULL!	#NULL!	#NULL!	#NULL!	#NULL!
#NULL!	#NULL!	#NULL!	#NULL!	#NULL!	#NULL!	#NULL!	#NULL!	#NULL!	#NULL!
#NULL!	#NULL!	#NULL!	#NULL!	#NULL!	#NULL!	#NULL!	#NULL!	#NULL!	#NULL!
1	2	2	2	2	2	2	2	2	2
1	2	#NULL!	#NULL!	#NULL!	#NULL!	#NULL!	#NULL!	#NULL!	#NULL!
#NULL!	#NULL!	#NULL!	#NULL!	#NULL!	#NULL!	#NULL!	#NULL!	#NULL!	#NULL!
2	#NULL!	#NULL!	#NULL!	#NULL!	#NULL!	#NULL!	#NULL!	#NULL!	#NULL!
#NULL!	#NULL!	#NULL!	#NULL!	#NULL!	#NULL!	#NULL!	#NULL!	#NULL!	#NULL!
2	#NULL!	#NULL!	#NULL!	#NULL!	#NULL!	#NULL!	#NULL!	#NULL!	#NULL!
#NULL!	#NULL!	#NULL!	#NULL!	#NULL!	#NULL!	#NULL!	#NULL!	#NULL!	#NULL!

#NULL!	#NULL!	#NULL!	#NULL!	#NULL!	#NULL!	#NULL!	#NULL!	#NULL!	#NULL!
#NULL!	1	1	1	1	1	1	1	1	1
2	2	#NULL!	#NULL!	#NULL!	#NULL!	#NULL!	#NULL!	#NULL!	#NULL!
#NULL!	#NULL!	#NULL!	#NULL!	#NULL!	#NULL!	#NULL!	#NULL!	#NULL!	#NULL!
#NULL!	#NULL!	#NULL!	#NULL!	#NULL!	#NULL!	#NULL!	#NULL!	#NULL!	#NULL!
#NULL!	#NULL!	#NULL!	#NULL!	#NULL!	#NULL!	#NULL!	#NULL!	#NULL!	#NULL!
#NULL!	#NULL!	#NULL!	#NULL!	#NULL!	#NULL!	#NULL!	#NULL!	#NULL!	#NULL!
#NULL!	#NULL!	#NULL!	#NULL!	#NULL!	#NULL!	#NULL!	#NULL!	#NULL!	#NULL!
#NULL!	#NULL!	#NULL!	#NULL!	#NULL!	#NULL!	#NULL!	#NULL!	#NULL!	#NULL!
#NULL!	#NULL!	#NULL!	#NULL!	#NULL!	#NULL!	#NULL!	#NULL!	#NULL!	#NULL!
#NULL!	#NULL!	#NULL!	#NULL!	#NULL!	#NULL!	#NULL!	#NULL!	#NULL!	#NULL!
#NULL!	#NULL!	#NULL!	#NULL!	#NULL!	#NULL!	#NULL!	#NULL!	#NULL!	#NULL!
#NULL!	#NULL!	#NULL!	#NULL!	#NULL!	#NULL!	#NULL!	#NULL!	#NULL!	#NULL!
#NULL!	#NULL!	#NULL!	#NULL!	#NULL!	#NULL!	#NULL!	#NULL!	#NULL!	#NULL!
2	#NULL!	#NULL!	#NULL!	#NULL!	#NULL!	#NULL!	#NULL!	#NULL!	#NULL!
2	#NULL!	#NULL!	#NULL!	#NULL!	#NULL!	#NULL!	#NULL!	#NULL!	#NULL!
#NULL!	#NULL!	#NULL!	#NULL!	#NULL!	#NULL!	#NULL!	#NULL!	#NULL!	#NULL!
2	2	2	2	2	2	2	2	2	2
#NULL!	#NULL!	#NULL!	#NULL!	#NULL!	#NULL!	#NULL!	#NULL!	#NULL!	#NULL!
2	#NULL!	#NULL!	#NULL!	#NULL!	#NULL!	#NULL!	#NULL!	#NULL!	#NULL!
#NULL!	#NULL!	#NULL!	#NULL!	#NULL!	#NULL!	#NULL!	#NULL!	#NULL!	#NULL!
2	#NULL!	#NULL!	#NULL!	#NULL!	#NULL!	#NULL!	#NULL!	#NULL!	#NULL!
#NULL!	#NULL!	#NULL!	#NULL!	#NULL!	#NULL!	#NULL!	#NULL!	#NULL!	#NULL!
2	#NULL!	#NULL!	#NULL!	#NULL!	#NULL!	#NULL!	#NULL!	#NULL!	#NULL!
2	2	2	2	2	2	2	2	2	2
2	#NULL!	#NULL!	#NULL!	#NULL!	#NULL!	#NULL!	#NULL!	#NULL!	#NULL!
#NULL!	#NULL!	#NULL!	#NULL!	#NULL!	#NULL!	#NULL!	#NULL!	#NULL!	#NULL!
2	2	#NULL!	2	2	2	2	2	2	2
#NULL!	#NULL!	#NULL!	#NULL!	#NULL!	#NULL!	#NULL!	#NULL!	#NULL!	#NULL!
#NULL!	#NULL!	#NULL!	#NULL!	#NULL!	#NULL!	#NULL!	#NULL!	#NULL!	#NULL!
1	2	2	2	2	2	2	2	2	2
2	2	2	2	2	2	2	1	1	2
#NULL!	#NULL!	#NULL!	#NULL!	#NULL!	#NULL!	#NULL!	#NULL!	#NULL!	#NULL!
2	1	1	1	1	1	1	1	1	1
#NULL!	#NULL!	#NULL!	#NULL!	#NULL!	#NULL!	#NULL!	#NULL!	#NULL!	#NULL!
2	2	2	2	2	2	2	2	2	2
1	2	#NULL!	#NULL!	#NULL!	#NULL!	#NULL!	#NULL!	#NULL!	#NULL!
#NULL!	#NULL!	#NULL!	#NULL!	#NULL!	#NULL!	#NULL!	#NULL!	#NULL!	#NULL!
2	2	2	2	2	2	2	2	2	2
2	1	#NULL!	#NULL!	#NULL!	#NULL!	#NULL!	#NULL!	#NULL!	#NULL!
#NULL!	#NULL!	#NULL!	#NULL!	#NULL!	#NULL!	#NULL!	#NULL!	#NULL!	#NULL!
#NULL!	#NULL!	#NULL!	#NULL!	#NULL!	#NULL!	#NULL!	#NULL!	#NULL!	#NULL!
2	2	2	2	2	2	2	2	2	2
1	2	2	2	2	2	2	2	2	2
2	#NULL!	2	#NULL!	2	#NULL!	2	#NULL!	2	#NULL!
#NULL!	#NULL!	#NULL!	#NULL!	#NULL!	#NULL!	#NULL!	#NULL!	#NULL!	#NULL!
#NULL!	#NULL!	#NULL!	#NULL!	#NULL!	#NULL!	#NULL!	#NULL!	#NULL!	#NULL!

2	2	#NULL!	#NULL!	#NULL!	#NULL!	2	#NULL!	#NULL!	#NULL!
#NULL!	#NULL!	#NULL!	#NULL!	#NULL!	#NULL!	#NULL!	#NULL!	#NULL!	#NULL!
2	2	#NULL!	#NULL!	#NULL!	2	1	#NULL!	2	2
#NULL!	#NULL!	#NULL!	#NULL!	#NULL!	#NULL!	#NULL!	#NULL!	#NULL!	#NULL!
#NULL!	#NULL!	#NULL!	#NULL!	#NULL!	#NULL!	#NULL!	#NULL!	#NULL!	#NULL!
#NULL!	#NULL!	#NULL!	#NULL!	#NULL!	#NULL!	#NULL!	#NULL!	#NULL!	#NULL!
1	2	#NULL!	#NULL!	#NULL!	#NULL!	#NULL!	#NULL!	#NULL!	#NULL!
2	2	#NULL!	#NULL!	#NULL!	#NULL!	#NULL!	#NULL!	#NULL!	#NULL!
1	2	#NULL!	#NULL!	2	#NULL!	#NULL!	2	#NULL!	#NULL!
2	#NULL!	#NULL!	#NULL!	#NULL!	#NULL!	#NULL!	#NULL!	#NULL!	#NULL!
2	#NULL!	#NULL!	#NULL!	#NULL!	#NULL!	#NULL!	#NULL!	#NULL!	#NULL!
#NULL!	#NULL!	#NULL!	#NULL!	#NULL!	#NULL!	#NULL!	#NULL!	#NULL!	#NULL!
#NULL!	#NULL!	#NULL!	#NULL!	#NULL!	#NULL!	#NULL!	#NULL!	#NULL!	#NULL!
#NULL!	#NULL!	#NULL!	#NULL!	#NULL!	#NULL!	#NULL!	#NULL!	#NULL!	#NULL!
2	2	2	2	2	1	1	1	1	2
2	1	2	2	2	1	2	2	1	2
2	#NULL!	#NULL!	#NULL!	#NULL!	#NULL!	#NULL!	2	#NULL!	#NULL!
2	2	2	2	2	2	2	2	2	2
2	2	2	2	1	2	2	1	2	2
1	2	#NULL!	#NULL!	#NULL!	#NULL!	#NULL!	#NULL!	#NULL!	#NULL!
2	2	#NULL!	#NULL!	#NULL!	#NULL!	#NULL!	#NULL!	#NULL!	#NULL!
1	2	#NULL!	#NULL!	#NULL!	#NULL!	#NULL!	#NULL!	#NULL!	#NULL!
2	1	2	2	2	2	2	2	2	2
2	2	2	1	2	2	1	2	2	1
2	2	2	2	2	2	2	2	1	2
2	2	2	2	2	2	2	2	2	2
2	2	1	1	1	2	#NULL!	1	1	1
1	2	2	1	1	1	2	2	2	2
1	2	2	2	1	1	1	2	2	2
2	2	2	2	2	2	2	2	2	2
2	2	2	2	2	2	2	2	2	2
2	2	#NULL!	#NULL!	#NULL!	#NULL!	#NULL!	#NULL!	#NULL!	#NULL!
1	#NULL!	#NULL!	#NULL!	#NULL!	#NULL!	#NULL!	#NULL!	#NULL!	#NULL!
2	2	#NULL!	#NULL!	#NULL!	#NULL!	#NULL!	#NULL!	#NULL!	#NULL!
1	2	#NULL!	#NULL!	#NULL!	#NULL!	#NULL!	#NULL!	#NULL!	#NULL!
#NULL!	#NULL!	#NULL!	#NULL!	#NULL!	#NULL!	#NULL!	#NULL!	#NULL!	#NULL!
#NULL!	#NULL!	#NULL!	#NULL!	#NULL!	#NULL!	#NULL!	#NULL!	#NULL!	#NULL!
1	#NULL!	2	#NULL!	#NULL!	#NULL!	2	#NULL!	#NULL!	#NULL!
#NULL!	#NULL!	#NULL!	#NULL!	#NULL!	#NULL!	#NULL!	#NULL!	#NULL!	#NULL!
2	2	#NULL!	2	#NULL!	2	#NULL!	2	#NULL!	2
#NULL!	#NULL!	#NULL!	#NULL!	#NULL!	#NULL!	#NULL!	#NULL!	#NULL!	#NULL!
1	2	2	2	2	2	2	2	2	2
#NULL!	#NULL!	#NULL!	#NULL!	#NULL!	#NULL!	#NULL!	#NULL!	#NULL!	#NULL!
#NULL!	#NULL!	#NULL!	#NULL!	#NULL!	#NULL!	#NULL!	#NULL!	#NULL!	#NULL!
#NULL!	#NULL!	#NULL!	#NULL!	#NULL!	#NULL!	#NULL!	#NULL!	#NULL!	#NULL!
2	2	2	2	2	2	2	2	2	2
2	2	2	1	2	2	1	2	1	2

2	2	2	2	2	2	2	2	2	2
2	2	2	2	2	2	2	2	2	2
2	2	2	2	2	2	2	2	2	2
#NULL!	1	1	1	1	1	2	1	2	2
2	1	2	1	1	1	1	1	1	1
2	2	2	2	2	2	2	2	1	2
2	2	2	2	2	2	2	2	2	2
2	1	1	1	1	1	1	1	1	1
2	#NULL!	#NULL!	#NULL!	#NULL!	#NULL!	#NULL!	#NULL!	#NULL!	#NULL!
1	2	2	2	2	2	2	2	2	2
2	2	#NULL!	2	#NULL!	2	#NULL!	2	#NULL!	#NULL!
2	#NULL!	#NULL!	2	#NULL!	#NULL!	2	#NULL!	2	#NULL!
1	2	2	2	2	2	2	2	2	2
1	2	2	1	2	2	2	2	2	1
2	1	2	1	1	1	1	1	1	1
2	2	2	2	2	2	2	2	2	2
2	2	2	1	2	1	2	2	2	#NULL!
2	2	1	1	1	1	1	2	1	1
#NULL!	#NULL!	#NULL!	#NULL!	#NULL!	#NULL!	#NULL!	#NULL!	#NULL!	#NULL!
2	2	2	2	2	1	2	2	2	2
2	1	2	2	2	1	2	1	2	2
2	2	2	2	1	2	1	2	1	2
2	2	2	2	2	2	2	2	2	2
2	2	2	2	2	2	2	2	1	2
2	2	1	2	2	2	1	2	1	2
2	2	2	2	2	2	2	2	2	2
2	2	1	2	2	1	2	2	1	2
2	2	2	2	2	2	2	1	1	1
2	2	2	2	2	2	2	2	2	2
2	2	2	2	2	1	2	2	1	2
2	1	1	1	1	1	1	1	1	1
2	#NULL!	#NULL!	#NULL!	#NULL!	#NULL!	#NULL!	#NULL!	#NULL!	#NULL!
2	1	1	1	1	1	1	1	1	1
2	2	2	2	2	2	2	2	2	2
2	#NULL!	1	#NULL!	#NULL!	#NULL!	#NULL!	#NULL!	#NULL!	#NULL!
2	1	1	1	1	1	1	2	2	2
2	2	2	2	2	2	2	2	2	2
1	2	2	2	2	2	2	2	2	2
2	2	2	1	2	2	2	2	2	1
#NULL!	#NULL!	#NULL!	#NULL!	#NULL!	#NULL!	#NULL!	#NULL!	#NULL!	#NULL!
2	2	2	2	2	2	2	2	2	2
1	#NULL!	#NULL!	#NULL!	#NULL!	#NULL!	#NULL!	#NULL!	#NULL!	2
2	2	2	2	1	2	2	2	2	2
2	2	2	2	2	2	2	2	2	2
2	2	1	2	1	2	1	2	1	2
1	#NULL!	#NULL!	#NULL!	#NULL!	#NULL!	#NULL!	#NULL!	#NULL!	#NULL!
2	1	1	1	2	2	2	2	2	2

#NULL!	#NULL!	#NULL!	#NULL!	#NULL!	#NULL!	#NULL!	#NULL!	#NULL!	#NULL!
2	1	1	1	1	1	1	1	1	1
1	2	1	2	2	2	1	1	2	2
2	2	2	1	2	2	1	2	2	2
1	2	2	2	2	1	2	2	1	2
#NULL!	#NULL!	#NULL!	#NULL!	#NULL!	#NULL!	#NULL!	#NULL!	#NULL!	#NULL!
2	2	2	2	2	2	2	2	2	2
2	2	2	1	1	2	2	1	2	2
1	2	1	1	1	2	1	2	1	2
2	2	2	2	2	2	2	2	2	2
2	2	2	2	2	1	2	2	2	2
2	2	2	2	2	2	2	2	2	2
#NULL!	#NULL!	#NULL!	#NULL!	#NULL!	#NULL!	#NULL!	#NULL!	#NULL!	#NULL!
2	1	2	2	2	1	1	1	1	1
1	2	2	2	2	2	2	2	1	2
2	2	2	2	2	2	2	2	2	2
2	2	2	2	2	2	2	2	2	2
2	2	2	2	2	2	2	2	1	2
#NULL!	2	2	2	2	2	2	2	2	2
1	2	#NULL!	#NULL!	#NULL!	#NULL!	#NULL!	#NULL!	#NULL!	#NULL!
#NULL!	#NULL!	#NULL!	#NULL!	#NULL!	#NULL!	#NULL!	#NULL!	#NULL!	#NULL!
#NULL!	2	2	2	1	2	2	2	2	2
2	2	2	2	1	1	1	2	1	2
2	2	1	2	2	2	1	2	1	2
#NULL!	#NULL!	#NULL!	#NULL!	#NULL!	#NULL!	#NULL!	#NULL!	#NULL!	#NULL!
2	2	2	1	1	1	2	1	2	2
2	2	#NULL!	#NULL!	#NULL!	#NULL!	#NULL!	#NULL!	#NULL!	#NULL!
2	2	2	2	2	2	2	2	2	2
2	2	2	2	2	2	2	2	2	2
2	2	2	2	2	2	2	2	2	2
2	1	2	2	1	2	2	2	2	2
#NULL!	#NULL!	#NULL!	#NULL!	#NULL!	#NULL!	#NULL!	#NULL!	#NULL!	#NULL!
#NULL!	#NULL!	#NULL!	#NULL!	#NULL!	#NULL!	#NULL!	#NULL!	#NULL!	#NULL!
2	2	1	2	2	2	2	2	2	2
2	2	1	2	2	1	2	1	1	2
#NULL!	#NULL!	#NULL!	#NULL!	#NULL!	#NULL!	#NULL!	#NULL!	#NULL!	#NULL!
#NULL!	#NULL!	#NULL!	#NULL!	#NULL!	#NULL!	#NULL!	#NULL!	#NULL!	#NULL!
2	2	1	1	1	1	2	2	2	2
2	2	1	2	2	1	1	#NULL!	2	2
2	2	2	2	2	2	2	2	2	2
2	2	2	2	2	2	2	2	2	2
2	2	2	2	2	2	2	2	2	2
2	2	2	2	2	2	2	2	2	2
2	2	2	2	2	2	2	2	2	2
2	2	2	2	2	2	2	2	2	2
2	2	2	2	2	1	2	2	2	1
2	2	2	2	2	2	2	2	2	2

2	1	1	1	2	2	2	1	1	1
2	2	2	2	2	2	2	2	2	2
2	2	2	2	2	2	2	2	2	2
1	2	2	2	2	2	2	2	2	1
2	2	2	2	2	2	2	2	2	2
2	2	2	2	2	2	2	2	2	2
2	2	2	2	2	2	2	2	2	2
2	2	2	2	2	2	2	2	2	2
2	2	2	2	2	2	2	2	2	2
#NULL!	#NULL!	#NULL!	#NULL!	#NULL!	#NULL!	#NULL!	#NULL!	#NULL!	#NULL!
1	2	2	1	2	2	2	2	2	2
2	2	2	2	2	1	2	1	1	2
2	2	2	2	2	2	2	2	1	2
2	1	2	2	2	2	2	2	2	2
2	1	1	1	1	1	1	1	1	1
1	#NULL!	#NULL!	#NULL!	#NULL!	#NULL!	#NULL!	2	#NULL!	#NULL!
#NULL!	#NULL!	#NULL!	#NULL!	#NULL!	#NULL!	#NULL!	#NULL!	#NULL!	#NULL!
2	#NULL!	2	#NULL!	#NULL!	#NULL!	#NULL!	2	#NULL!	#NULL!
#NULL!	#NULL!	#NULL!	#NULL!	#NULL!	#NULL!	#NULL!	#NULL!	#NULL!	#NULL!
#NULL!	2	2	2	2	2	2	2	2	2
2	2	1	2	2	2	2	#NULL!	2	1
#NULL!	#NULL!	#NULL!	#NULL!	#NULL!	#NULL!	#NULL!	#NULL!	#NULL!	#NULL!
#NULL!	#NULL!	#NULL!	#NULL!	#NULL!	#NULL!	#NULL!	#NULL!	#NULL!	#NULL!
2	2	2	2	2	2	2	2	2	2
1	2	2	2	2	1	2	2	2	2
2	2	2	2	2	2	2	2	2	2
2	2	2	2	2	1	2	2	2	2
2	1	2	#NULL!	1	1	2	2	2	2
2	2	2	2	2	1	2	2	2	2
1	2	2	2	2	2	2	2	2	1
#NULL!	#NULL!	#NULL!	#NULL!	#NULL!	#NULL!	#NULL!	#NULL!	#NULL!	#NULL!
#NULL!	2	2	2	2	2	1	2	1	2
1	2	1	1	1	1	2	2	2	2
1	2	2	2	2	2	2	2	2	2
2	2	2	2	2	2	2	2	2	2
2	2	2	2	2	2	2	2	2	2
#NULL!	#NULL!	#NULL!	#NULL!	#NULL!	#NULL!	#NULL!	#NULL!	#NULL!	#NULL!
2	2	2	2	2	2	2	2	2	2
2	2	2	2	2	2	2	2	2	2
2	1	2	2	2	2	1	2	2	1
1	2	2	2	1	1	1	1	1	1
1	2	2	2	1	2	2	2	2	2
2	2	2	2	2	2	2	2	2	2
2	2	2	2	2	2	2	2	2	2
2	2	1	2	2	2	2	2	2	1
2	2	2	2	2	2	2	2	2	2
2	1	1	2	2	2	2	1	1	2

#NULL!	2	#NULL!	2	1	2	2	2	2	1
2	2	2	2	2	2	2	2	2	2
2	2	2	2	2	2	1	1	1	1
2	2	2	2	2	2	2	2	2	2
2	1	2	1	1	1	1	1	2	1
#NULL!	#NULL!	#NULL!	#NULL!	#NULL!	#NULL!	#NULL!	#NULL!	#NULL!	#NULL!
2	#NULL!	#NULL!	#NULL!	#NULL!	2	#NULL!	#NULL!	#NULL!	#NULL!
2	2	2	1	1	1	1	1	1	1
#NULL!	#NULL!	#NULL!	#NULL!	#NULL!	#NULL!	#NULL!	#NULL!	#NULL!	#NULL!
#NULL!	#NULL!	#NULL!	#NULL!	#NULL!	#NULL!	#NULL!	#NULL!	#NULL!	#NULL!
2	2	2	2	2	2	2	2	2	2
2	2	2	2	2	2	2	2	2	2
1	2	2	2	2	2	2	2	2	2
1	#NULL!	#NULL!	2	#NULL!	#NULL!	#NULL!	#NULL!	#NULL!	#NULL!
2	#NULL!	#NULL!	2	#NULL!	#NULL!	#NULL!	#NULL!	#NULL!	#NULL!
2	2	1	2	2	2	1	1	2	#NULL!
2	2	2	2	2	2	2	2	1	2
#NULL!	#NULL!	#NULL!	#NULL!	#NULL!	#NULL!	#NULL!	#NULL!	#NULL!	#NULL!
2	2	1	2	2	2	2	2	1	2
2	2	2	2	2	2	2	2	2	2
#NULL!	2	2	2	2	2	2	2	2	2
2	#NULL!	#NULL!	#NULL!	#NULL!	#NULL!	#NULL!	#NULL!	#NULL!	#NULL!
2	2	2	2	2	2	2	2	2	2
2	2	2	2	2	2	2	2	2	2
2	2	2	2	2	2	2	2	2	2
2	2	2	2	2	2	2	2	2	2
2	2	2	2	2	2	2	2	2	2
2	2	2	2	2	2	2	2	2	2
2	2	1	2	2	1	2	1	2	1
2	1	1	2	1	2	1	2	1	1
2	2	2	2	2	1	2	2	2	2
1	2	2	1	2	2	1	1	2	1
2	2	2	2	2	2	2	2	2	2
2	2	2	2	2	2	2	2	2	2
2	2	2	2	2	2	2	1	2	2
2	2	2	2	1	2	2	2	2	1
1	2	2	2	2	2	2	2	2	2
2	2	2	2	2	1	1	1	1	1
1	2	#NULL!	2	2	2	2	1	2	1
2	2	1	2	1	1	2	2	1	2
1	2	2	2	2	2	2	1	1	2
2	2	2	2	2	2	2	1	1	1
2	2	2	2	2	2	2	2	2	2
2	2	2	2	2	2	2	2	2	2
2	2	2	2	2	2	2	2	2	2
1	2	2	2	2	1	2	1	2	1
2	2	2	2	2	2	2	2	2	2

2	2	1	2	2	1	2	2	1	2
1	2	2	2	2	2	2	2	2	2
2	2	1	2	1	2	1	2	1	2
1	2	2	2	2	2	2	2	2	2
1	2	#NULL!	2	#NULL!	#NULL!	#NULL!	#NULL!	#NULL!	#NULL!
2	#NULL!	#NULL!	#NULL!	#NULL!	2	#NULL!	#NULL!	#NULL!	#NULL!
2	1	2	1	2	1	2	1	2	1
1	2	#NULL!	#NULL!	#NULL!	#NULL!	#NULL!	#NULL!	#NULL!	#NULL!
1	2	#NULL!	#NULL!	#NULL!	#NULL!	#NULL!	#NULL!	#NULL!	#NULL!
#NULL!	#NULL!	#NULL!	#NULL!	#NULL!	#NULL!	#NULL!	#NULL!	#NULL!	#NULL!
2	#NULL!	#NULL!	#NULL!	#NULL!	#NULL!	1	#NULL!	#NULL!	#NULL!
2	#NULL!	#NULL!	2	#NULL!	#NULL!	#NULL!	#NULL!	#NULL!	#NULL!
1	2	#NULL!	#NULL!	#NULL!	#NULL!	#NULL!	#NULL!	#NULL!	#NULL!
1	#NULL!	#NULL!	#NULL!	#NULL!	2	#NULL!	#NULL!	#NULL!	#NULL!
2	2	2	2	2	2	2	2	2	2
#NULL!	2	#NULL!	#NULL!	#NULL!	#NULL!	#NULL!	#NULL!	#NULL!	#NULL!
#NULL!	#NULL!	#NULL!	#NULL!	#NULL!	#NULL!	#NULL!	#NULL!	#NULL!	#NULL!
2	#NULL!	#NULL!	2	#NULL!	#NULL!	#NULL!	#NULL!	#NULL!	#NULL!
2	#NULL!	#NULL!	2	#NULL!	#NULL!	#NULL!	#NULL!	#NULL!	#NULL!
#NULL!	#NULL!	#NULL!	#NULL!	1	#NULL!	#NULL!	#NULL!	#NULL!	#NULL!
#NULL!	#NULL!	#NULL!	#NULL!	#NULL!	#NULL!	#NULL!	#NULL!	#NULL!	#NULL!
2	2	2	2	2	2	2	2	2	2
2	2	2	2	2	2	2	2	2	2
2	2	2	2	1	2	2	2	1	#NULL!
1	2	2	#NULL!	2	1	1	1	1	1
#NULL!	#NULL!	#NULL!	#NULL!	#NULL!	#NULL!	#NULL!	#NULL!	#NULL!	#NULL!
2	2	1	2	2	2	2	2	2	1
2	2	#NULL!	#NULL!	#NULL!	#NULL!	#NULL!	#NULL!	#NULL!	#NULL!
2	1	1	1	2	2	2	1	1	1
2	2	2	2	2	2	2	2	1	2
2	1	1	1	1	1	1	1	1	1
2	1	#NULL!	#NULL!	#NULL!	#NULL!	#NULL!	#NULL!	#NULL!	#NULL!
1	2	2	2	2	2	2	2	2	1
2	2	#NULL!	#NULL!	#NULL!	#NULL!	#NULL!	#NULL!	#NULL!	#NULL!
1	2	#NULL!	#NULL!	#NULL!	#NULL!	#NULL!	#NULL!	#NULL!	#NULL!
#NULL!	1	2	2	2	2	2	2	2	2
1	2	2	2	2	#NULL!	2	2	2	2
#NULL!	#NULL!	#NULL!	#NULL!	#NULL!	#NULL!	#NULL!	#NULL!	#NULL!	#NULL!
2	#NULL!	#NULL!	#NULL!	#NULL!	#NULL!	#NULL!	#NULL!	#NULL!	#NULL!
2	2	2	2	2	2	2	2	2	2
2	#NULL!	#NULL!	#NULL!	#NULL!	#NULL!	#NULL!	#NULL!	#NULL!	#NULL!
2	2	2	2	2	2	2	2	2	2
2	1	1	1	1	1	1	1	1	1
2	#NULL!	#NULL!	2	#NULL!	#NULL!	#NULL!	2	#NULL!	#NULL!
1	2	2	2	2	2	2	2	2	2
1	2	2	2	2	2	2	2	2	2
2	#NULL!	2	#NULL!	1	2	#NULL!	#NULL!	2	#NULL!

#NULL!	#NULL!	#NULL!	#NULL!	#NULL!	#NULL!	#NULL!	#NULL!	#NULL!	#NULL!
2	2	2	#NULL!	#NULL!	2	#NULL!	2	#NULL!	2
#NULL!	#NULL!	#NULL!	#NULL!	#NULL!	#NULL!	#NULL!	#NULL!	#NULL!	#NULL!
1	2	2	2	2	2	2	2	2	2
2	#NULL!	1	2	#NULL!	1	2	1	2	1
2	#NULL!	2	#NULL!	1	2	1	2	#NULL!	2
2	#NULL!	#NULL!	#NULL!	#NULL!	#NULL!	#NULL!	#NULL!	#NULL!	#NULL!
#NULL!	#NULL!	#NULL!	#NULL!	#NULL!	#NULL!	#NULL!	#NULL!	#NULL!	#NULL!
2	2	#NULL!	#NULL!	2	#NULL!	2	#NULL!	#NULL!	2
1	2	2	2	2	2	2	2	2	2
#NULL!	#NULL!	#NULL!	#NULL!	#NULL!	#NULL!	#NULL!	#NULL!	#NULL!	#NULL!
2	2	#NULL!	#NULL!	2	#NULL!	2	#NULL!	2	#NULL!
2	2	2	2	2	2	2	2	2	2
#NULL!	#NULL!	#NULL!	#NULL!	#NULL!	#NULL!	#NULL!	#NULL!	#NULL!	#NULL!
1	2	2	2	2	2	2	2	2	2
#NULL!	#NULL!	#NULL!	#NULL!	#NULL!	#NULL!	#NULL!	#NULL!	#NULL!	#NULL!
#NULL!	#NULL!	#NULL!	#NULL!	#NULL!	#NULL!	#NULL!	#NULL!	#NULL!	#NULL!
#NULL!	#NULL!	#NULL!	#NULL!	#NULL!	#NULL!	#NULL!	#NULL!	#NULL!	#NULL!
#NULL!	#NULL!	#NULL!	#NULL!	#NULL!	#NULL!	#NULL!	#NULL!	#NULL!	#NULL!
#NULL!	#NULL!	#NULL!	#NULL!	#NULL!	#NULL!	#NULL!	#NULL!	#NULL!	#NULL!
1	2	2	2	2	2	2	2	2	2
2	2	2	2	2	2	2	2	2	2
2	2	2	1	2	1	2	2	2	2
1	2	2	2	2	2	2	2	2	2
2	2	1	1	1	1	1	1	1	1
2	2	2	2	2	2	2	2	2	2
1	2	2	2	2	2	2	2	2	2
2	2	2	2	2	2	2	2	2	2
1	2	1	2	1	2	2	2	2	2
2	2	2	2	2	2	2	2	2	2
2	2	2	2	1	1	1	2	2	1
2	2	1	2	1	2	1	2	1	1
2	2	2	2	2	2	2	2	2	2
#NULL!	#NULL!	#NULL!	#NULL!	#NULL!	#NULL!	#NULL!	#NULL!	#NULL!	#NULL!
2	1	1	1	2	2	1	2	2	1
1	1	2	2	2	2	2	2	2	2
2	2	2	2	2	2	2	2	2	2
1	2	2	2	2	2	2	2	2	2
2	2	2	2	2	2	1	2	1	2
2	2	2	2	2	2	2	2	2	2
2	2	2	2	1	1	1	1	2	2
#NULL!	#NULL!	#NULL!	#NULL!	#NULL!	#NULL!	#NULL!	#NULL!	#NULL!	#NULL!
2	2	2	2	#NULL!	#NULL!	#NULL!	#NULL!	#NULL!	#NULL!
#NULL!	#NULL!	#NULL!	#NULL!	#NULL!	#NULL!	#NULL!	#NULL!	#NULL!	#NULL!
1	#NULL!	#NULL!	#NULL!	#NULL!	#NULL!	#NULL!	#NULL!	#NULL!	#NULL!
2	2	2	2	2	2	2	2	2	2
2	#NULL!	#NULL!	#NULL!	#NULL!	#NULL!	#NULL!	#NULL!	#NULL!	#NULL!

2	2	2	2	2	2	2	2	1	2
#NULL!	2	2	2	2	1	1	1	1	1
1	2	2	2	2	2	2	2	2	2
1	2	2	2	2	#NULL!	#NULL!	2	2	2
1	2	2	2	2	2	2	2	2	#NULL!
1	2	2	2	2	2	2	2	2	2
1	2	2	2	2	#NULL!	2	2	2	2
1	2	2	2	2	2	1	1	1	2
2	1	1	1	1	1	1	1	1	1
1	2	2	2	2	2	2	2	2	2
2	#NULL!	#NULL!	#NULL!	#NULL!	#NULL!	#NULL!	#NULL!	#NULL!	#NULL!
1	2	2	2	2	2	2	2	2	2
1	2	2	2	2	2	2	2	2	2
1	2	2	2	2	2	2	2	2	2
1	2	2	2	2	2	2	2	2	2
1	2	2	2	2	2	2	2	2	2
1	2	2	2	2	2	2	2	2	2
1	2	2	2	2	2	2	2	2	2
1	2	2	2	2	2	2	2	2	2
2	#NULL!	#NULL!	#NULL!	#NULL!	#NULL!	#NULL!	#NULL!	#NULL!	#NULL!
1	2	2	2	2	2	2	2	2	2
2	2	2	2	2	2	2	2	2	2
#NULL!	#NULL!	#NULL!	#NULL!	#NULL!	#NULL!	#NULL!	#NULL!	#NULL!	#NULL!
1	2	2	2	2	2	2	2	2	2
#NULL!	#NULL!	#NULL!	#NULL!	#NULL!	#NULL!	#NULL!	#NULL!	#NULL!	#NULL!
1	2	2	2	2	2	2	2	2	2
1	2	2	2	2	2	2	2	2	2
1	2	2	2	2	2	2	2	2	2
1	#NULL!	#NULL!	#NULL!	#NULL!	#NULL!	#NULL!	#NULL!	#NULL!	#NULL!
1	2	2	2	2	2	2	#NULL!	2	2
2	#NULL!	#NULL!	#NULL!	#NULL!	#NULL!	#NULL!	#NULL!	#NULL!	#NULL!
2	#NULL!	#NULL!	#NULL!	#NULL!	#NULL!	#NULL!	#NULL!	#NULL!	#NULL!
1	2	2	2	2	2	2	2	2	2
1	2	2	2	2	2	2	2	2	2
2	2	2	2	2	2	2	2	2	2
#NULL!	#NULL!	#NULL!	#NULL!	#NULL!	#NULL!	#NULL!	#NULL!	#NULL!	#NULL!
1	2	2	2	2	2	2	2	2	2
1	2	2	2	2	2	2	2	2	2
#NULL!	#NULL!	#NULL!	#NULL!	#NULL!	#NULL!	#NULL!	#NULL!	#NULL!	#NULL!
2	#NULL!	#NULL!	#NULL!	#NULL!	#NULL!	#NULL!	#NULL!	#NULL!	#NULL!
1	2	2	2	2	2	2	2	2	2
#NULL!	#NULL!	#NULL!	#NULL!	#NULL!	#NULL!	#NULL!	#NULL!	#NULL!	#NULL!
1	2	2	2	2	2	2	2	2	2
1	2	2	2	2	2	2	2	2	2
1	2	2	2	2	2	2	2	2	2
2	2	2	2	2	2	2	2	2	2
1	2	2	2	2	2	2	2	2	2

1	2	2	2	2	2	2	2	2	2
2	2	#NULL!	2	2	2	#NULL!	2	#NULL!	#NULL!
1	1	1	1	#NULL!	1	1	1	1	1
1	2	2	2	2	2	2	2	2	2
1	2	2	2	2	2	2	2	2	2
#NULL!	#NULL!	#NULL!	#NULL!	#NULL!	#NULL!	#NULL!	#NULL!	#NULL!	#NULL!
1	2	2	2	2	2	2	2	2	2
1	2	2	2	2	2	2	2	2	2
2	2	2	#NULL!	2	#NULL!	2	2	#NULL!	2
1	2	2	#NULL!	2	2	2	2	2	2
2	#NULL!	#NULL!	#NULL!	#NULL!	#NULL!	#NULL!	#NULL!	#NULL!	#NULL!
1	#NULL!	#NULL!	#NULL!	#NULL!	#NULL!	#NULL!	#NULL!	#NULL!	#NULL!
#NULL!	#NULL!	#NULL!	#NULL!	#NULL!	#NULL!	#NULL!	#NULL!	#NULL!	#NULL!
1	#NULL!	#NULL!	#NULL!	#NULL!	#NULL!	#NULL!	#NULL!	#NULL!	#NULL!
2	#NULL!	#NULL!	#NULL!	#NULL!	#NULL!	#NULL!	#NULL!	#NULL!	#NULL!
#NULL!	#NULL!	#NULL!	#NULL!	#NULL!	#NULL!	#NULL!	#NULL!	#NULL!	#NULL!
1	2	2	2	2	2	2	2	2	2
#NULL!	#NULL!	#NULL!	#NULL!	#NULL!	#NULL!	#NULL!	#NULL!	#NULL!	#NULL!
2	2	2	2	2	2	2	2	2	2
1	2	1	1	1	1	1	2	1	1
1	2	1	1	1	1	1	2	2	2
1	2	1	1	1	1	1	2	2	2
1	2	1	1	1	1	1	2	2	2
1	2	1	1	#NULL!	1	1	2	2	2
1	2	1	1	2	1	1	2	2	2
1	2	1	1	1	1	1	2	2	2
1	2	1	1	1	1	1	2	2	2
1	2	1	1	1	1	1	2	2	2
1	2	1	1	2	1	1	2	#NULL!	2
1	2	1	1	1	1	1	2	2	2
1	2	1	1	1	1	1	2	2	2
1	2	1	1	1	1	1	2	2	2
1	2	1	1	1	1	1	2	2	2
1	2	1	1	1	1	1	2	2	2
1	2	1	1	#NULL!	1	1	2	2	2
1	2	1	1	1	1	1	2	2	1
1	2	1	1	1	1	1	2	2	2
#NULL!	2	1	1	2	1	1	2	2	2
1	2	1	1	2	1	1	2	2	2
1	2	1	1	1	1	1	2	2	2
1	2	1	1	1	1	1	2	2	2
1	2	1	1	1	1	1	2	2	2
1	2	1	1	1	1	1	2	2	2
1	1	2	1	1	1	1	2	2	2
#NULL!	#NULL!	#NULL!	#NULL!	#NULL!	#NULL!	#NULL!	#NULL!	#NULL!	#NULL!
#NULL!	#NULL!	#NULL!	#NULL!	#NULL!	#NULL!	#NULL!	#NULL!	#NULL!	#NULL!
#NULL!	#NULL!	#NULL!	#NULL!	#NULL!	#NULL!	#NULL!	#NULL!	#NULL!	#NULL!

#NULL!	#NULL!	#NULL!	#NULL!	#NULL!	#NULL!	#NULL!	#NULL!	#NULL!	#NULL!
#NULL!	#NULL!	#NULL!	#NULL!	#NULL!	#NULL!	#NULL!	#NULL!	#NULL!	#NULL!
#NULL!	#NULL!	#NULL!	#NULL!	#NULL!	#NULL!	#NULL!	#NULL!	#NULL!	#NULL!
#NULL!	#NULL!	#NULL!	#NULL!	#NULL!	#NULL!	#NULL!	#NULL!	#NULL!	#NULL!
#NULL!	#NULL!	#NULL!	#NULL!	#NULL!	#NULL!	#NULL!	#NULL!	#NULL!	#NULL!
#NULL!	#NULL!	#NULL!	#NULL!	#NULL!	#NULL!	#NULL!	#NULL!	#NULL!	#NULL!
#NULL!	#NULL!	#NULL!	#NULL!	#NULL!	#NULL!	#NULL!	#NULL!	#NULL!	#NULL!
#NULL!	#NULL!	#NULL!	#NULL!	#NULL!	#NULL!	#NULL!	#NULL!	#NULL!	#NULL!
#NULL!	#NULL!	#NULL!	#NULL!	#NULL!	#NULL!	#NULL!	#NULL!	#NULL!	#NULL!
#NULL!	#NULL!	#NULL!	#NULL!	#NULL!	#NULL!	#NULL!	#NULL!	#NULL!	#NULL!
#NULL!	#NULL!	#NULL!	#NULL!	#NULL!	#NULL!	#NULL!	#NULL!	#NULL!	#NULL!
#NULL!	#NULL!	#NULL!	#NULL!	#NULL!	#NULL!	#NULL!	#NULL!	#NULL!	#NULL!
#NULL!	#NULL!	#NULL!	#NULL!	#NULL!	#NULL!	#NULL!	#NULL!	#NULL!	#NULL!
#NULL!	#NULL!	#NULL!	#NULL!	#NULL!	#NULL!	#NULL!	#NULL!	#NULL!	#NULL!
#NULL!	#NULL!	#NULL!	#NULL!	#NULL!	#NULL!	#NULL!	#NULL!	#NULL!	#NULL!
#NULL!	#NULL!	#NULL!	#NULL!	#NULL!	#NULL!	#NULL!	#NULL!	#NULL!	#NULL!
#NULL!	#NULL!	#NULL!	#NULL!	#NULL!	#NULL!	#NULL!	#NULL!	#NULL!	#NULL!
#NULL!	#NULL!	#NULL!	#NULL!	#NULL!	#NULL!	#NULL!	#NULL!	#NULL!	#NULL!
#NULL!	#NULL!	#NULL!	#NULL!	#NULL!	#NULL!	#NULL!	#NULL!	#NULL!	#NULL!
1	2	1	1	1	1	1	2	2	2
1	2	1	1	1	1	1	2	2	2
1	2	1	1	1	1	1	2	2	2
1	2	1	1	1	1	1	2	2	2
1	2	1	1	1	1	1	2	2	2
1	2	1	1	1	1	1	2	2	2
1	2	1	1	2	1	1	2	2	2
1	2	1	1	2	1	1	2	2	2
1	2	1	1	1	1	1	2	2	2
1	2	2	1	1	1	1	2	2	2
1	2	2	1	1	1	1	2	2	2
1	2	1	1	2	1	1	1	2	2
1	2	1	1	1	1	1	2	2	2
1	2	1	1	2	1	1	2	2	2
1	2	1	1	1	1	#NULL!	#NULL!	2	2
1	2	1	1	1	1	1	2	1	2
2	2	#NULL!	#NULL!	#NULL!	#NULL!	#NULL!	#NULL!	#NULL!	2
1	2	1	1	1	1	1	2	2	2
1	2	1	1	1	1	1	2	2	2
1	2	1	1	1	1	1	2	2	2
1	2	1	1	1	1	1	2	2	2
1	2	1	1	1	1	1	2	2	2
1	2	1	1	1	1	1	2	2	2
1	2	1	1	2	1	1	2	2	2
1	2	2	1	1	1	1	#NULL!	2	2
2	2	1	1	2	1	1	2	2	2
1	2	1	1	1	1	1	2	2	2
1	2	1	1	1	1	1	2	2	2

1	2	1	1	#NULL!	1	1	2	2	2
1	2	1	1	1	1	2	2	2	2
1	2	1	1	1	1	1	2	2	2
1	2	1	1	#NULL!	1	2	2	2	2
1	2	1	1	1	1	1	2	2	2
1	2	1	1	1	1	1	2	2	2
1	2	1	1	1	1	2	2	2	2
1	2	1	2	1	1	1	1	2	2
1	2	1	1	1	1	1	2	2	2
1	2	1	1	1	1	1	2	2	2
1	2	1	1	1	1	2	2	2	2
1	2	1	1	1	1	1	2	2	2
1	2	1	1	2	1	1	2	2	2
1	2	1	1	1	1	2	2	2	2
1	2	1	1	1	1	1	2	2	2
1	2	1	1	2	1	1	2	2	2
1	2	1	1	1	1	1	2	2	2
1	2	1	1	1	1	1	2	2	2
1	2	1	1	1	1	1	2	2	2
1	2	1	1	#NULL!	1	1	2	2	2
1	2	1	1	1	1	1	2	2	2
1	2	1	1	1	1	1	1	2	1
1	2	1	1	1	1	1	2	2	2
1	2	1	1	1	1	1	2	2	2
1	2	1	1	#NULL!	1	1	2	2	2
1	2	1	1	1	1	1	2	2	2
1	2	1	1	1	2	1	2	2	2
1	2	1	1	1	1	1	2	2	2
1	2	1	1	1	1	1	2	2	2
1	2	1	1	1	1	1	2	#NULL!	2
1	2	1	1	#NULL!	1	1	2	2	2
#NULL!	2	2	1	1	1	1	#NULL!	#NULL!	2
#NULL!	2	2	2	2	#NULL!	#NULL!	#NULL!	#NULL!	#NULL!
1	2	1	1	1	1	1	2	2	2
#NULL!	#NULL!	#NULL!	#NULL!	#NULL!	#NULL!	#NULL!	#NULL!	#NULL!	#NULL!
1	2	1	1	1	1	1	2	2	2
1	2	1	1	#NULL!	1	1	2	2	2
1	2	1	1	#NULL!	1	2	#NULL!	2	2
1	2	1	1	1	1	1	2	2	2
1	2	1	1	1	2	1	2	2	2
1	2	1	1	1	1	1	2	2	2
1	2	1	1	2	1	1	2	2	2
1	2	1	1	1	1	1	2	2	2
1	2	1	1	1	1	1	2	2	2
1	2	1	1	1	1	1	2	2	2
1	2	1	1	2	1	1	2	2	2
1	2	1	1	1	1	1	2	2	2

1	2	1	1	1	1	1	2	2	2
1	2	1	1	1	1	1	2	2	2
1	2	1	1	2	1	1	2	2	2
1	2	1	1	1	1	1	2	2	2
1	2	1	1	2	1	1	2	2	2
#NULL!	2	1	1	2	1	1	2	2	2
1	2	1	1	2	1	1	2	2	2
1	2	1	1	1	1	1	2	2	2
1	2	1	1	1	1	1	2	2	2
1	2	1	1	2	1	1	2	2	2
1	2	1	1	#NULL!	1	1	2	2	2
1	2	1	1	1	1	1	2	2	2
1	2	1	1	1	1	1	2	2	2
1	2	1	1	1	1	1	2	2	2
1	2	1	1	#NULL!	1	#NULL!	#NULL!	#NULL!	2
1	2	1	1	1	1	1	2	2	2
1	2	1	1	1	1	1	2	2	2
1	2	1	1	1	1	1	2	2	2
1	2	1	1	1	1	1	2	2	2
#NULL!	2	1	1	#NULL!	1	1	2	2	2
1	2	1	1	#NULL!	1	1	2	2	2
1	2	1	1	1	1	1	2	2	2
1	2	1	1	1	1	1	2	2	2
1	2	1	1	1	1	1	2	2	2
1	2	1	#NULL!	1	2	#NULL!	#NULL!	#NULL!	#NULL!
1	2	1	1	2	1	1	2	1	2
1	2	1	1	2	1	1	2	2	2
1	2	1	1	1	2	1	2	2	2
1	2	1	1	1	1	1	2	1	2
1	2	1	1	1	1	1	2	2	2
1	2	1	1	1	1	1	2	1	2
1	2	1	1	1	1	1	2	2	2
1	1	2	1	1	1	1	2	2	2
1	1	2	1	1	1	1	2	2	2
1	2	1	1	1	1	1	2	2	2
1	2	1	1	1	1	1	2	2	2
1	2	1	1	1	1	1	2	2	2
1	2	1	1	#NULL!	#NULL!	1	2	2	#NULL!
1	2	1	1	1	1	1	2	2	2
1	2	1	1	1	1	1	2	2	2
1	2	1	1	2	1	1	2	2	2
#NULL!	#NULL!	#NULL!	#NULL!	#NULL!	#NULL!	#NULL!	#NULL!	#NULL!	#NULL!
1	2	1	1	2	1	1	2	2	2
1	2	1	1	2	1	1	2	1	2
1	2	1	1	2	1	1	2	2	2
1	2	1	1	2	1	1	1	2	2
1	2	1	1	1	1	1	1	2	2

1	2	1	1	2	2	1	2	1	2
1	2	1	1	2	1	1	2	2	2
1	2	1	1	1	1	1	2	2	2
1	2	1	1	1	1	1	2	2	2
1	2	1	1	1	1	1	2	2	2
1	2	1	1	1	1	1	2	2	2
1	2	1	1	1	1	1	2	2	2
1	2	1	1	2	1	1	2	2	2
1	2	1	1	1	1	1	2	2	2
#NULL!	#NULL!	#NULL!	#NULL!	#NULL!	#NULL!	#NULL!	#NULL!	#NULL!	#NULL!
1	2	1	1	1	1	1	2	2	2
1	2	1	1	2	1	1	2	2	2
1	2	1	1	2	1	1	2	2	2
1	2	1	1	1	1	1	2	2	2
1	2	1	1	1	1	1	2	2	2
1	2	1	1	1	1	1	2	2	2
1	2	1	1	1	1	1	2	2	2
1	2	1	1	1	1	1	1	2	2
1	2	1	1	1	1	1	2	2	2
1	2	1	1	2	1	1	2	2	2
1	2	1	1	1	1	1	2	2	2
1	2	1	1	1	1	1	2	2	2
1	2	1	1	1	1	1	2	2	2
1	2	1	1	1	1	1	2	2	2
1	2	1	1	2	1	1	2	2	2
1	2	1	1	2	1	1	2	2	2
#NULL!	2	1	1	2	1	1	2	2	2
#NULL!	#NULL!	#NULL!	#NULL!	#NULL!	#NULL!	#NULL!	#NULL!	#NULL!	#NULL!
#NULL!	#NULL!	#NULL!	#NULL!	#NULL!	#NULL!	#NULL!	#NULL!	#NULL!	#NULL!
#NULL!	#NULL!	#NULL!	#NULL!	#NULL!	#NULL!	#NULL!	#NULL!	#NULL!	#NULL!
#NULL!	#NULL!	#NULL!	#NULL!	#NULL!	#NULL!	#NULL!	#NULL!	#NULL!	#NULL!
#NULL!	#NULL!	#NULL!	#NULL!	#NULL!	#NULL!	#NULL!	#NULL!	#NULL!	#NULL!
#NULL!	#NULL!	#NULL!	#NULL!	#NULL!	#NULL!	#NULL!	#NULL!	#NULL!	#NULL!
#NULL!	#NULL!	#NULL!	#NULL!	#NULL!	#NULL!	#NULL!	#NULL!	#NULL!	#NULL!
#NULL!	#NULL!	#NULL!	#NULL!	#NULL!	#NULL!	#NULL!	#NULL!	#NULL!	#NULL!
#NULL!	#NULL!	#NULL!	#NULL!	#NULL!	#NULL!	#NULL!	#NULL!	#NULL!	#NULL!
#NULL!	#NULL!	#NULL!	#NULL!	#NULL!	#NULL!	#NULL!	#NULL!	#NULL!	#NULL!
#NULL!	#NULL!	#NULL!	#NULL!	#NULL!	#NULL!	#NULL!	#NULL!	#NULL!	#NULL!
#NULL!	#NULL!	#NULL!	#NULL!	#NULL!	#NULL!	#NULL!	#NULL!	#NULL!	#NULL!
#NULL!	#NULL!	#NULL!	#NULL!	#NULL!	#NULL!	#NULL!	#NULL!	#NULL!	#NULL!
#NULL!	#NULL!	#NULL!	#NULL!	#NULL!	#NULL!	#NULL!	#NULL!	#NULL!	#NULL!
#NULL!	#NULL!	#NULL!	#NULL!	#NULL!	#NULL!	#NULL!	#NULL!	#NULL!	#NULL!
#NULL!	#NULL!	#NULL!	#NULL!	#NULL!	#NULL!	#NULL!	#NULL!	#NULL!	#NULL!
#NULL!	#NULL!	#NULL!	#NULL!	#NULL!	#NULL!	#NULL!	#NULL!	#NULL!	#NULL!
#NULL!	#NULL!	#NULL!	#NULL!	#NULL!	#NULL!	#NULL!	#NULL!	#NULL!	#NULL!
#NULL!	#NULL!	#NULL!	#NULL!	#NULL!	#NULL!	#NULL!	#NULL!	#NULL!	#NULL!
#NULL!	#NULL!	#NULL!	#NULL!	#NULL!	#NULL!	#NULL!	#NULL!	#NULL!	#NULL!

#NULL!	#NULL!	#NULL!	#NULL!	#NULL!	#NULL!	#NULL!	#NULL!	#NULL!	#NULL!
#NULL!	#NULL!	#NULL!	#NULL!	#NULL!	#NULL!	#NULL!	#NULL!	#NULL!	#NULL!
#NULL!	#NULL!	#NULL!	#NULL!	#NULL!	#NULL!	#NULL!	#NULL!	#NULL!	#NULL!
#NULL!	#NULL!	#NULL!	#NULL!	#NULL!	#NULL!	#NULL!	#NULL!	#NULL!	#NULL!
#NULL!	#NULL!	#NULL!	#NULL!	#NULL!	#NULL!	#NULL!	#NULL!	#NULL!	#NULL!
#NULL!	#NULL!	#NULL!	#NULL!	#NULL!	#NULL!	#NULL!	#NULL!	#NULL!	#NULL!
#NULL!	#NULL!	#NULL!	#NULL!	#NULL!	#NULL!	#NULL!	#NULL!	#NULL!	#NULL!
#NULL!	#NULL!	#NULL!	#NULL!	#NULL!	#NULL!	#NULL!	#NULL!	#NULL!	#NULL!
#NULL!	#NULL!	#NULL!	#NULL!	#NULL!	#NULL!	#NULL!	#NULL!	#NULL!	#NULL!
#NULL!	#NULL!	#NULL!	#NULL!	#NULL!	#NULL!	#NULL!	#NULL!	#NULL!	#NULL!
#NULL!	#NULL!	#NULL!	#NULL!	#NULL!	#NULL!	#NULL!	#NULL!	#NULL!	#NULL!
#NULL!	#NULL!	#NULL!	#NULL!	#NULL!	#NULL!	#NULL!	#NULL!	#NULL!	#NULL!
#NULL!	#NULL!	#NULL!	#NULL!	#NULL!	#NULL!	#NULL!	#NULL!	#NULL!	#NULL!
1	1	1	2	2	2	2	2	1	2
2	2	2	2	2	2	2	2	2	2
2	2	2	2	2	2	2	2	2	2
#NULL!	2	2	1	2	1	2	2	2	2
1	2	2	2	1	2	2	2	1	2
#NULL!	2	2	2	2	2	2	2	2	2
1	2	2	1	2	2	2	2	2	1
1	2	2	2	2	2	2	2	2	2
2	2	2	2	2	2	2	2	2	2
#NULL!	#NULL!	#NULL!	#NULL!	#NULL!	#NULL!	#NULL!	#NULL!	#NULL!	#NULL!
2	2	1	2	2	2	2	1	2	2
2	#NULL!	#NULL!	#NULL!	#NULL!	#NULL!	#NULL!	#NULL!	#NULL!	#NULL!
1	2	2	1	1	1	1	1	2	2
#NULL!	#NULL!	#NULL!	#NULL!	#NULL!	#NULL!	#NULL!	#NULL!	#NULL!	#NULL!
2	2	2	2	2	2	2	2	2	2
2	1	2	2	1	2	2	1	2	2
2	2	2	2	2	2	2	2	2	2
2	1	1	1	1	1	1	1	1	1
2	2	2	2	1	2	2	1	2	1
2	2	#NULL!	#NULL!	#NULL!	#NULL!	#NULL!	#NULL!	#NULL!	#NULL!
2	2	1	2	2	2	2	2	2	2
1	2	2	2	2	2	2	2	2	2
1	2	#NULL!	#NULL!	#NULL!	#NULL!	#NULL!	#NULL!	#NULL!	#NULL!
2	2	2	2	2	2	2	2	2	2
1	2	2	2	1	2	2	2	1	2
1	2	2	2	2	2	1	2	2	2
1	2	2	2	2	2	2	2	2	2
#NULL!	#NULL!	#NULL!	#NULL!	#NULL!	#NULL!	#NULL!	#NULL!	#NULL!	#NULL!
#NULL!	#NULL!	#NULL!	#NULL!	#NULL!	#NULL!	#NULL!	#NULL!	#NULL!	#NULL!
1	2	2	2	2	2	2	2	2	2
2	2	2	2	2	2	2	2	2	2
#NULL!	#NULL!	#NULL!	#NULL!	#NULL!	#NULL!	#NULL!	#NULL!	#NULL!	#NULL!
#NULL!	#NULL!	#NULL!	#NULL!	#NULL!	#NULL!	#NULL!	#NULL!	#NULL!	#NULL!
#NULL!	#NULL!	#NULL!	#NULL!	#NULL!	#NULL!	#NULL!	#NULL!	#NULL!	#NULL!

2	#NULL!	#NULL!	#NULL!	#NULL!	#NULL!	#NULL!	#NULL!	#NULL!	#NULL!
#NULL!	#NULL!	#NULL!	#NULL!	#NULL!	#NULL!	#NULL!	#NULL!	#NULL!	#NULL!
1	2	2	2	1	2	2	2	2	2
2	2	2	2	2	2	2	2	2	2
2	2	2	2	1	1	1	2	1	2
1	#NULL!	2	#NULL!	#NULL!	#NULL!	#NULL!	#NULL!	#NULL!	#NULL!
1	2	2	2	2	2	2	2	2	2
1	2	2	2	2	2	2	2	2	2
2	2	2	2	2	2	2	2	2	2
2	2	2	2	2	2	2	2	2	2
2	1	1	2	2	1	1	1	1	1
#NULL!	#NULL!	#NULL!	#NULL!	#NULL!	#NULL!	#NULL!	#NULL!	#NULL!	#NULL!
2	2	#NULL!	#NULL!	#NULL!	#NULL!	#NULL!	#NULL!	#NULL!	#NULL!
2	2	2	2	2	2	2	2	2	2
2	2	2	2	2	2	2	2	2	2
2	2	2	2	2	2	2	2	2	2
2	2	2	2	2	2	2	2	2	2
1	2	2	2	2	2	2	2	2	2
1	2	2	2	2	1	2	1	2	2
#NULL!	#NULL!	#NULL!	#NULL!	#NULL!	#NULL!	#NULL!	#NULL!	#NULL!	#NULL!
2	2	2	2	2	2	2	2	2	2
#NULL!	2	2	2	2	2	2	2	2	2
2	2	2	2	2	2	2	2	2	2
2	2	2	2	2	2	2	2	2	2
#NULL!	2	2	2	2	2	2	2	2	2
2	2	2	2	2	2	2	2	2	2
2	2	2	2	2	2	2	2	2	2
2	2	2	2	2	2	2	2	2	2
2	2	2	2	2	2	2	2	2	2
1	2	1	2	2	2	2	2	2	2
1	2	1	1	1	1	1	2	1	2
2	2	2	2	2	2	2	2	1	2
2	2	2	2	2	2	2	2	2	2
1	2	2	2	2	2	2	2	1	1
2	2	2	2	2	1	2	2	1	2
2	2	2	2	2	2	2	2	2	2
2	2	2	2	2	2	2	2	2	2
1	2	2	2	#NULL!	#NULL!	#NULL!	#NULL!	#NULL!	#NULL!
2	2	#NULL!	#NULL!	#NULL!	#NULL!	#NULL!	#NULL!	#NULL!	#NULL!
2	2	2	2	2	2	2	2	2	2
2	1	1	1	1	1	2	2	2	2
2	2	2	2	2	2	2	2	2	2
2	2	2	1	2	2	1	2	2	1
2	2	2	2	1	1	1	2	2	2
1	2	2	2	2	2	2	2	2	2
1	2	2	2	2	2	2	2	2	2
#NULL!	2	2	2	2	2	2	2	2	2

2	2	2	2	2	2	2	2	2	2
2	2	2	1	2	2	1	2	2	1
2	2	2	2	2	2	2	2	2	2
2	2	2	1	1	2	1	2	2	1
1	2	2	2	2	2	2	2	2	2
1	2	2	2	2	2	2	2	2	2
2	2	2	2	2	2	2	2	2	2
2	2	1	2	2	1	2	2	2	1
2	2	2	2	2	2	2	2	2	2
2	2	2	1	2	2	2	1	2	2
2	2	2	2	2	2	2	2	2	2
2	2	2	2	2	2	2	2	2	2
1	2	2	2	2	2	2	2	2	2
#NULL!	2	2	2	1	1	1	1	1	1
1	2	2	2	2	2	2	2	2	2
2	2	2	2	2	2	2	2	2	2
1	2	2	2	2	2	2	2	2	2
2	2	2	2	2	2	2	2	2	2
#NULL!	2	2	2	2	2	2	2	2	2
2	2	2	1	2	2	1	2	2	1
2	2	2	2	2	2	2	2	2	2
2	2	2	2	2	2	2	2	1	1
2	2	2	2	2	2	2	2	2	2
1	2	2	2	1	1	1	1	1	1
2	2	1	1	2	1	1	2	1	2
#NULL!	#NULL!	#NULL!	#NULL!	#NULL!	#NULL!	#NULL!	#NULL!	#NULL!	#NULL!
#NULL!	#NULL!	#NULL!	#NULL!	#NULL!	#NULL!	#NULL!	#NULL!	#NULL!	#NULL!
2	2	2	2	2	2	2	2	2	2
1	#NULL!	#NULL!	#NULL!	#NULL!	#NULL!	#NULL!	#NULL!	#NULL!	#NULL!
1	2	2	2	2	1	1	2	2	2
2	2	2	2	2	2	2	2	2	2
2	2	1	2	2	2	2	2	2	2
1	2	2	2	2	1	1	1	1	1
1	2	2	2	2	2	2	2	2	2
1	#NULL!	#NULL!	2	#NULL!	#NULL!	#NULL!	#NULL!	#NULL!	#NULL!
#NULL!	2	2	2	2	2	2	2	2	2
#NULL!	#NULL!	#NULL!	#NULL!	#NULL!	#NULL!	#NULL!	#NULL!	#NULL!	#NULL!
#NULL!	2	2	2	2	2	2	2	2	2
#NULL!	#NULL!	#NULL!	#NULL!	#NULL!	#NULL!	#NULL!	#NULL!	#NULL!	#NULL!
2	2	2	2	2	2	2	2	2	2
2	2	1	1	2	2	2	1	2	1
2	2	2	2	2	2	2	2	2	2
2	2	2	2	2	2	2	2	2	2
#NULL!	#NULL!	#NULL!	#NULL!	#NULL!	#NULL!	#NULL!	#NULL!	#NULL!	#NULL!
#NULL!	#NULL!	#NULL!	#NULL!	#NULL!	#NULL!	#NULL!	#NULL!	#NULL!	#NULL!
#NULL!	#NULL!	#NULL!	#NULL!	#NULL!	#NULL!	#NULL!	#NULL!	#NULL!	#NULL!
1	2	2	2	2	2	2	2	2	2

#NULL!	#NULL!	#NULL!	#NULL!	#NULL!	#NULL!	#NULL!	#NULL!	#NULL!	#NULL!
#NULL!	#NULL!	#NULL!	#NULL!	#NULL!	#NULL!	#NULL!	#NULL!	#NULL!	#NULL!
1	2	2	2	2	2	1	2	1	2
#NULL!	#NULL!	#NULL!	#NULL!	#NULL!	#NULL!	#NULL!	#NULL!	#NULL!	#NULL!
#NULL!	#NULL!	#NULL!	#NULL!	#NULL!	#NULL!	#NULL!	#NULL!	#NULL!	#NULL!
2	2	2	2	2	1	1	2	2	2
2	1	2	2	2	2	1	1	2	2
#NULL!	#NULL!	#NULL!	#NULL!	#NULL!	#NULL!	#NULL!	#NULL!	#NULL!	#NULL!
1	2	2	2	2	2	2	2	1	2
2	2	2	1	2	2	1	2	2	1
2	2	1	2	2	2	1	2	2	1
2	2	2	2	2	2	2	2	2	2
2	2	2	2	2	2	2	2	2	2
#NULL!	#NULL!	#NULL!	#NULL!	#NULL!	#NULL!	#NULL!	#NULL!	#NULL!	#NULL!
2	2	2	2	2	2	2	2	2	2
2	2	2	2	2	2	2	2	2	2
2	1	1	2	1	1	1	2	1	2
2	2	1	2	1	1	1	2	2	2
1	2	2	1	1	2	2	1	2	2
#NULL!	#NULL!	#NULL!	#NULL!	#NULL!	#NULL!	#NULL!	#NULL!	#NULL!	#NULL!
2	2	1	2	1	1	1	2	2	2
1	2	2	2	1	2	2	2	2	2
2	2	2	2	2	2	2	2	2	2
2	#NULL!	2	2	1	1	1	2	2	2
#NULL!	1	1	1	1	1	1	1	1	1
#NULL!	#NULL!	#NULL!	#NULL!	#NULL!	#NULL!	#NULL!	#NULL!	#NULL!	#NULL!
2	1	1	2	1	1	2	2	2	2
2	#NULL!	#NULL!	#NULL!	#NULL!	#NULL!	#NULL!	#NULL!	#NULL!	#NULL!
1	2	2	2	2	2	2	2	2	2
2	2	2	2	2	1	2	2	2	1
2	2	2	2	2	2	2	2	2	2
2	2	#NULL!	#NULL!	#NULL!	#NULL!	#NULL!	#NULL!	#NULL!	#NULL!
2	2	#NULL!	#NULL!	#NULL!	#NULL!	#NULL!	#NULL!	#NULL!	#NULL!
#NULL!	2	2	2	2	2	2	2	2	2
2	2	2	2	2	2	2	2	2	2
#NULL!	2	2	1	2	1	2	2	2	2
#NULL!	2	2	2	2	2	2	2	2	2
2	2	2	2	2	2	2	2	1	2
2	2	2	2	2	2	2	2	2	2
2	2	#NULL!	#NULL!	#NULL!	#NULL!	#NULL!	#NULL!	#NULL!	#NULL!
#NULL!	2	2	2	2	2	2	2	2	2
2	2	2	2	2	2	2	2	2	2
2	1	1	2	2	2	2	2	2	2
2	2	2	2	2	2	2	2	2	2
2	2	2	2	2	2	2	2	2	2
2	2	#NULL!	#NULL!	#NULL!	#NULL!	#NULL!	#NULL!	#NULL!	#NULL!
2	2	2	1	1	2	2	1	2	2

1	2	1	2	2	2	2	2	2	2
2	2	2	2	2	2	2	2	2	2
2	2	1	2	2	1	1	2	1	2
2	2	2	2	2	2	2	2	2	2
2	2	2	2	2	2	2	2	2	2
2	2	2	2	2	2	1	1	1	2
2	2	2	2	1	2	2	2	2	1
2	2	2	1	2	2	2	2	2	2
2	2	1	2	2	1	1	2	1	2
2	1	1	1	2	1	1	1	1	2
2	2	2	2	2	2	2	2	2	2
2	2	2	2	2	2	2	2	2	2
2	2	2	2	2	2	2	2	2	2
2	2	1	2	2	1	1	2	1	2
2	2	2	#NULL!	2	2	1	2	#NULL!	1
2	2	2	2	2	2	2	2	2	2
2	2	2	1	2	1	2	1	2	1
#NULL!	2	2	2	2	2	2	2	2	2
2	1	2	2	2	2	1	2	2	1
2	2	2	2	2	2	2	2	2	2
2	2	1	2	2	2	2	2	1	2
1	#NULL!	#NULL!	#NULL!	#NULL!	#NULL!	#NULL!	#NULL!	#NULL!	#NULL!
2	2	2	2	2	2	2	2	2	2
1	2	2	2	2	2	2	2	2	2
2	2	2	2	2	2	2	2	2	2
2	2	1	2	2	2	2	2	2	2
1	2	2	1	2	2	2	2	2	2
1	2	2	2	2	2	2	2	2	2
1	2	1	2	1	1	1	1	1	2
1	2	1	2	2	2	2	2	2	2
1	2	1	1	1	1	1	1	1	2
1	2	1	2	2	2	2	2	2	2
2	2	2	2	2	2	2	2	2	2
#NULL!	#NULL!	#NULL!	#NULL!	#NULL!	#NULL!	#NULL!	#NULL!	#NULL!	#NULL!
2	1	#NULL!	#NULL!	#NULL!	#NULL!	#NULL!	#NULL!	#NULL!	#NULL!
1	2	1	2	1	1	1	2	1	2
#NULL!	2	2	2	2	2	2	2	2	2
1	2	2	2	2	2	2	2	2	2
1	2	2	2	2	2	2	2	2	2
2	2	2	2	2	2	2	2	2	2
1	2	2	1	2	2	2	2	2	2
#NULL!	#NULL!	#NULL!	#NULL!	#NULL!	#NULL!	#NULL!	#NULL!	#NULL!	#NULL!
#NULL!	#NULL!	#NULL!	#NULL!	#NULL!	#NULL!	#NULL!	#NULL!	#NULL!	#NULL!
1	2	2	2	2	2	2	2	2	2
1	2	2	1	2	2	2	2	1	2
#NULL!	#NULL!	#NULL!	#NULL!	#NULL!	#NULL!	#NULL!	#NULL!	#NULL!	#NULL!
#NULL!	#NULL!	#NULL!	#NULL!	#NULL!	#NULL!	#NULL!	#NULL!	#NULL!	#NULL!

#NULL!	2	2	2	2	2	2	2	2	2
1	2	2	2	2	2	2	2	2	1
1	2	2	1	2	2	1	2	2	1
2	2	1	2	2	1	2	2	1	2
2	2	2	2	2	1	1	1	1	2
1	2	2	1	2	2	1	2	2	1
1	2	2	2	1	2	2	2	2	2
2	2	#NULL!	#NULL!	#NULL!	#NULL!	#NULL!	#NULL!	#NULL!	#NULL!
1	2	1	2	2	2	2	2	2	2
#NULL!	#NULL!	#NULL!	#NULL!	#NULL!	#NULL!	#NULL!	#NULL!	#NULL!	#NULL!
1	2	2	2	2	2	2	2	2	2
2	2	2	1	1	1	1	1	2	2
#NULL!	2	2	2	2	2	2	1	1	2
#NULL!	#NULL!	#NULL!	#NULL!	#NULL!	#NULL!	#NULL!	#NULL!	#NULL!	#NULL!
1	2	2	2	2	2	2	2	2	2
#NULL!	#NULL!	#NULL!	#NULL!	#NULL!	#NULL!	#NULL!	#NULL!	#NULL!	#NULL!
#NULL!	#NULL!	#NULL!	#NULL!	#NULL!	#NULL!	#NULL!	#NULL!	#NULL!	#NULL!
1	2	2	2	2	2	2	2	2	2
2	2	2	2	2	2	2	2	2	2
2	2	2	2	2	2	2	2	2	2
2	2	2	2	2	2	2	2	2	2
1	2	2	2	2	2	2	2	2	2
2	2	1	1	1	1	1	1	1	1
1	2	1	2	2	1	2	1	2	2
2	2	2	2	2	#NULL!	2	#NULL!	#NULL!	1
2	2	#NULL!	#NULL!	#NULL!	#NULL!	#NULL!	#NULL!	#NULL!	#NULL!
2	2	2	2	2	2	2	2	2	2
#NULL!	2	1	2	2	2	2	2	2	2
2	2	2	2	2	2	2	2	2	2
2	2	2	1	1	1	1	1	1	2
1	2	2	2	2	2	2	2	2	2
2	2	2	2	2	2	2	2	2	2
#NULL!	2	2	2	2	2	2	2	2	2
2	2	2	2	2	2	2	2	2	2
1	2	2	2	2	2	2	2	2	2
2	2	2	2	2	2	2	2	2	2
2	1	1	1	1	1	1	1	1	1
2	#NULL!	#NULL!	#NULL!	#NULL!	#NULL!	2	#NULL!	#NULL!	#NULL!
2	#NULL!	#NULL!	#NULL!	#NULL!	#NULL!	#NULL!	#NULL!	#NULL!	#NULL!
#NULL!	2	2	2	2	2	2	2	2	2
2	1	1	1	1	1	1	1	1	1
2	2	2	2	2	2	2	2	2	2
#NULL!	2	2	2	2	2	2	2	2	2
2	2	#NULL!	#NULL!	#NULL!	#NULL!	#NULL!	#NULL!	#NULL!	#NULL!
2	2	2	2	2	2	2	2	2	2
#NULL!	#NULL!	#NULL!	#NULL!	#NULL!	#NULL!	#NULL!	#NULL!	#NULL!	#NULL!
2	2	2	2	2	2	2	2	2	2

2	2	2	2	1	2	1	2	2	#NULL!
2	#NULL!	#NULL!	#NULL!	#NULL!	#NULL!	#NULL!	#NULL!	#NULL!	2
#NULL!	2	2	2	2	2	2	2	2	2
2	2	2	2	2	2	2	2	2	2
1	1	1	1	2	2	2	2	2	2
2	2	2	2	2	2	2	2	2	2
2	2	2	2	2	2	2	2	1	2
#NULL!	#NULL!	#NULL!	#NULL!	#NULL!	#NULL!	#NULL!	#NULL!	#NULL!	#NULL!
2	2	2	2	2	2	2	2	2	2
2	2	2	2	2	2	2	2	2	2
2	2	2	2	2	2	2	2	2	2
2	2	2	2	2	2	2	2	2	2
2	2	1	#NULL!	2	1	#NULL!	2	1	1
2	2	1	1	2	2	1	1	1	2
2	2	2	2	2	2	2	2	2	2
2	2	2	2	2	2	2	2	2	2
2	2	2	2	2	2	2	2	2	2
2	2	2	2	2	2	2	2	2	2
#NULL!	#NULL!	#NULL!	#NULL!	#NULL!	#NULL!	#NULL!	#NULL!	#NULL!	#NULL!
#NULL!	#NULL!	#NULL!	#NULL!	#NULL!	#NULL!	#NULL!	#NULL!	#NULL!	#NULL!
2	1	2	2	1	2	1	2	2	2
2	2	2	2	2	2	2	2	2	2
2	#NULL!	#NULL!	#NULL!	#NULL!	#NULL!	#NULL!	#NULL!	#NULL!	#NULL!
#NULL!	#NULL!	#NULL!	#NULL!	#NULL!	#NULL!	#NULL!	#NULL!	#NULL!	#NULL!
2	1	1	1	1	1	1	1	1	1
2	#NULL!	#NULL!	#NULL!	#NULL!	#NULL!	#NULL!	#NULL!	#NULL!	#NULL!
#NULL!	#NULL!	#NULL!	#NULL!	#NULL!	#NULL!	#NULL!	#NULL!	#NULL!	#NULL!
#NULL!	#NULL!	#NULL!	#NULL!	#NULL!	#NULL!	#NULL!	#NULL!	#NULL!	#NULL!
#NULL!	2	2	1	2	2	2	2	1	2
2	2	2	2	2	2	2	2	2	2
2	2	2	2	2	2	2	2	2	2
1	2	2	2	#NULL!	2	2	2	#NULL!	2
2	2	2	2	2	2	1	2	2	2
2	2	2	2	2	2	2	2	2	2
2	2	2	2	2	2	2	2	2	2
2	2	2	2	2	2	2	2	2	2
2	2	2	2	2	2	2	2	1	1
2	2	2	1	2	1	2	1	2	2
2	2	2	1	2	2	1	2	2	2
1	2	1	2	2	2	2	2	2	2
2	2	2	2	2	1	1	2	2	2
1	2	2	2	2	2	2	2	2	2
1	2	#NULL!	#NULL!	#NULL!	#NULL!	#NULL!	#NULL!	#NULL!	#NULL!
2	#NULL!	#NULL!	#NULL!	2	#NULL!	#NULL!	#NULL!	#NULL!	#NULL!
2	2	1	2	2	2	2	1	2	2
2	2	2	2	2	2	2	2	2	2
2	2	2	2	2	2	2	2	2	2

1	2	2	2	2	2	2	2	1	1
#NULL!	#NULL!	#NULL!	#NULL!	#NULL!	#NULL!	#NULL!	#NULL!	#NULL!	#NULL!
#NULL!	#NULL!	#NULL!	#NULL!	#NULL!	#NULL!	#NULL!	#NULL!	#NULL!	#NULL!
#NULL!	#NULL!	#NULL!	#NULL!	#NULL!	#NULL!	#NULL!	#NULL!	#NULL!	#NULL!
#NULL!	#NULL!	#NULL!	#NULL!	#NULL!	#NULL!	#NULL!	#NULL!	#NULL!	#NULL!
1	#NULL!	#NULL!	#NULL!	#NULL!	#NULL!	#NULL!	#NULL!	#NULL!	#NULL!
#NULL!	#NULL!	#NULL!	#NULL!	#NULL!	#NULL!	#NULL!	#NULL!	#NULL!	#NULL!
#NULL!	#NULL!	#NULL!	#NULL!	#NULL!	#NULL!	#NULL!	#NULL!	#NULL!	#NULL!
#NULL!	#NULL!	#NULL!	#NULL!	#NULL!	#NULL!	#NULL!	#NULL!	#NULL!	#NULL!
2	#NULL!	#NULL!	#NULL!	#NULL!	#NULL!	#NULL!	#NULL!	2	#NULL!
2	2	2	2	2	2	2	2	2	2
2	2	2	2	2	2	2	1	2	1
#NULL!	#NULL!	#NULL!	#NULL!	#NULL!	#NULL!	#NULL!	#NULL!	2	2
2	2	2	2	2	2	2	2	2	2
2	2	2	2	2	2	2	2	2	2
#NULL!	#NULL!	#NULL!	#NULL!	#NULL!	#NULL!	#NULL!	#NULL!	#NULL!	#NULL!
1	2	2	2	2	2	2	2	1	1
2	2	2	2	2	2	2	2	2	2
2	2	2	2	2	2	2	2	2	2
2	2	2	2	2	2	2	2	2	2
2	2	1	2	2	2	1	2	2	1
2	1	1	2	1	2	2	2	1	2
1	1	1	1	1	1	1	1	1	1
2	2	2	2	2	2	2	2	2	2
2	2	2	2	2	2	2	2	2	2
1	2	2	2	2	2	2	2	2	2
2	2	2	2	2	2	2	2	2	2
1	2	2	2	2	2	2	2	2	2
2	2	2	2	2	2	2	2	2	2
2	2	2	2	2	2	2	2	2	2
#NULL!	#NULL!	#NULL!	#NULL!	#NULL!	#NULL!	#NULL!	#NULL!	#NULL!	#NULL!
2	2	2	2	2	2	2	2	2	2
1	1	2	2	2	2	2	2	2	2
1	2	#NULL!	#NULL!	#NULL!	#NULL!	#NULL!	#NULL!	#NULL!	#NULL!
#NULL!	1	1	1	1	1	1	1	1	1
2	2	2	2	2	2	2	2	2	2
2	2	2	2	2	2	2	2	2	2
2	2	2	2	2	2	2	2	2	2
2	2	1	1	2	1	2	1	2	1
#NULL!	#NULL!	#NULL!	#NULL!	#NULL!	#NULL!	#NULL!	#NULL!	#NULL!	#NULL!
1	2	2	2	2	1	2	1	2	1
2	2	2	2	2	2	2	2	2	2
2	2	2	2	2	2	2	2	2	2
1	2	1	2	2	2	2	2	1	2
1	2	2	2	2	2	2	2	2	2
#NULL!	#NULL!	#NULL!	#NULL!	#NULL!	#NULL!	#NULL!	#NULL!	#NULL!	#NULL!
#NULL!	#NULL!	#NULL!	#NULL!	#NULL!	#NULL!	#NULL!	#NULL!	#NULL!	#NULL!

#NULL!	#NULL!	#NULL!	#NULL!	#NULL!	#NULL!	#NULL!	#NULL!	#NULL!	#NULL!
2	2	2	2	2	2	2	2	2	2
2	2	#NULL!	#NULL!	#NULL!	#NULL!	#NULL!	#NULL!	#NULL!	#NULL!
#NULL!	#NULL!	#NULL!	#NULL!	#NULL!	#NULL!	#NULL!	#NULL!	#NULL!	#NULL!
#NULL!	#NULL!	#NULL!	#NULL!	#NULL!	#NULL!	#NULL!	#NULL!	#NULL!	#NULL!
#NULL!	#NULL!	#NULL!	#NULL!	#NULL!	#NULL!	#NULL!	#NULL!	#NULL!	#NULL!
#NULL!	#NULL!	#NULL!	#NULL!	#NULL!	#NULL!	#NULL!	#NULL!	#NULL!	#NULL!
#NULL!	#NULL!	#NULL!	#NULL!	#NULL!	#NULL!	#NULL!	#NULL!	#NULL!	#NULL!
#NULL!	#NULL!	#NULL!	#NULL!	#NULL!	#NULL!	#NULL!	#NULL!	#NULL!	#NULL!
#NULL!	#NULL!	#NULL!	#NULL!	#NULL!	#NULL!	#NULL!	#NULL!	#NULL!	#NULL!
#NULL!	#NULL!	#NULL!	#NULL!	#NULL!	#NULL!	#NULL!	#NULL!	#NULL!	#NULL!
#NULL!	#NULL!	#NULL!	#NULL!	#NULL!	#NULL!	#NULL!	#NULL!	#NULL!	#NULL!
#NULL!	#NULL!	#NULL!	#NULL!	#NULL!	#NULL!	#NULL!	#NULL!	#NULL!	#NULL!
#NULL!	#NULL!	#NULL!	#NULL!	#NULL!	#NULL!	#NULL!	#NULL!	#NULL!	#NULL!
#NULL!	#NULL!	#NULL!	#NULL!	#NULL!	#NULL!	#NULL!	#NULL!	#NULL!	#NULL!
#NULL!	#NULL!	#NULL!	#NULL!	#NULL!	#NULL!	#NULL!	#NULL!	#NULL!	#NULL!
#NULL!	#NULL!	#NULL!	#NULL!	#NULL!	#NULL!	#NULL!	#NULL!	#NULL!	#NULL!
#NULL!	#NULL!	#NULL!	#NULL!	#NULL!	#NULL!	#NULL!	#NULL!	#NULL!	#NULL!
#NULL!	#NULL!	#NULL!	#NULL!	#NULL!	#NULL!	#NULL!	#NULL!	#NULL!	#NULL!
#NULL!	#NULL!	#NULL!	#NULL!	#NULL!	#NULL!	#NULL!	#NULL!	#NULL!	#NULL!
#NULL!	#NULL!	#NULL!	#NULL!	#NULL!	#NULL!	#NULL!	#NULL!	#NULL!	#NULL!
#NULL!	#NULL!	#NULL!	#NULL!	#NULL!	#NULL!	#NULL!	#NULL!	#NULL!	#NULL!
#NULL!	#NULL!	#NULL!	#NULL!	#NULL!	#NULL!	#NULL!	#NULL!	#NULL!	#NULL!
2	2	2	1	2	2	1	2	2	1
2	2	2	2	2	2	2	2	2	2
1	2	2	2	2	2	2	2	2	2
1	2	2	2	2	2	2	2	2	2
1	2	2	2	2	2	1	2	2	2
2	2	#NULL!	#NULL!	#NULL!	#NULL!	#NULL!	#NULL!	#NULL!	#NULL!
2	2	2	2	2	2	2	2	2	2
1	2	2	2	2	2	2	2	2	1
2	2	2	2	2	2	2	2	2	2
2	2	2	2	2	2	2	1	1	1
2	#NULL!	#NULL!	#NULL!	#NULL!	#NULL!	#NULL!	#NULL!	#NULL!	#NULL!
2	2	1	1	1	2	2	2	2	2
1	2	1	2	2	1	1	2	1	2
2	#NULL!	#NULL!	#NULL!	#NULL!	#NULL!	#NULL!	#NULL!	#NULL!	#NULL!
1	2	2	2	2	2	2	2	2	2
1	2	1	2	2	2	2	2	1	2
1	2	#NULL!	#NULL!	#NULL!	#NULL!	#NULL!	#NULL!	#NULL!	#NULL!
1	2	2	2	2	2	2	2	2	2
1	2	2	2	2	2	2	2	1	2
1	2	#NULL!	#NULL!	#NULL!	#NULL!	#NULL!	#NULL!	#NULL!	#NULL!
1	2	2	2	2	2	1	2	2	2
1	2	1	1	2	1	1	2	1	2
2	2	2	2	2	2	2	2	2	2
2	2	2	2	1	2	1	2	1	2

1	2	2	2	2	2	2	2	1	2
1	2	2	2	2	2	2	2	2	2
2	2	1	2	2	1	1	2	1	2
1	2	2	2	2	2	2	2	2	2
1	2	2	2	1	2	1	2	1	2
1	2	#NULL!	#NULL!	#NULL!	#NULL!	#NULL!	#NULL!	#NULL!	#NULL!
1	2	2	1	2	2	2	2	2	2
1	2	#NULL!	#NULL!	#NULL!	#NULL!	#NULL!	#NULL!	#NULL!	#NULL!
2	2	2	2	2	2	2	2	2	2
2	2	2	2	2	2	2	2	2	2
1	#NULL!	#NULL!	#NULL!	2	#NULL!	#NULL!	#NULL!	#NULL!	#NULL!
1	2	#NULL!	#NULL!	#NULL!	#NULL!	#NULL!	#NULL!	#NULL!	#NULL!
2	#NULL!	#NULL!	#NULL!	#NULL!	2	#NULL!	#NULL!	#NULL!	#NULL!
#NULL!	#NULL!	#NULL!	#NULL!	2	#NULL!	#NULL!	#NULL!	#NULL!	#NULL!
1	#NULL!	#NULL!	#NULL!	#NULL!	#NULL!	#NULL!	#NULL!	#NULL!	#NULL!
#NULL!	#NULL!	#NULL!	#NULL!	#NULL!	#NULL!	#NULL!	#NULL!	#NULL!	#NULL!
#NULL!	#NULL!	#NULL!	#NULL!	#NULL!	#NULL!	#NULL!	#NULL!	#NULL!	#NULL!
2	2	2	2	2	2	2	2	2	2
1	#NULL!	#NULL!	#NULL!	#NULL!	#NULL!	#NULL!	#NULL!	#NULL!	2
1	2	2	2	1	2	1	2	1	1
1	2	2	2	2	2	2	2	2	2
1	#NULL!	#NULL!	#NULL!	#NULL!	2	#NULL!	#NULL!	2	#NULL!
1	2	#NULL!	#NULL!	#NULL!	#NULL!	#NULL!	#NULL!	#NULL!	#NULL!
1	#NULL!	#NULL!	#NULL!	#NULL!	#NULL!	2	#NULL!	#NULL!	#NULL!
1	2	#NULL!	#NULL!	#NULL!	#NULL!	#NULL!	#NULL!	#NULL!	#NULL!
1	2	1	2	2	1	2	1	2	2
1	2	1	2	2	1	2	2	2	1
1	2	2	1	2	2	2	2	1	1
2	2	2	2	2	2	2	2	2	2
#NULL!	#NULL!	#NULL!	#NULL!	#NULL!	#NULL!	#NULL!	#NULL!	#NULL!	#NULL!
2	2	2	1	1	2	2	2	2	2
1	1	1	2	2	1	2	1	2	2
2	1	1	1	1	1	1	1	1	1
1	2	2	1	2	1	2	2	1	2
2	2	1	2	2	1	1	2	1	2
2	#NULL!	2	#NULL!	#NULL!	#NULL!	#NULL!	#NULL!	#NULL!	#NULL!
2	2	2	2	2	1	2	2	2	2
2	2	1	2	2	1	1	2	2	2
2	2	1	2	1	2	1	2	2	1
2	2	2	2	2	2	1	2	2	2
1	2	1	2	2	1	1	2	1	2
1	2	1	2	2	2	1	2	1	2
1	2	1	2	2	1	1	2	1	2
2	2	1	1	1	1	2	2	2	2
1	2	1	2	#NULL!	2	2	2	1	2
2	2	2	#NULL!	#NULL!	#NULL!	#NULL!	#NULL!	#NULL!	#NULL!
1	2	1	2	2	2	2	2	2	2

1	2	2	2	2	2	2	2	2	2
1	2	2	1	2	1	2	1	1	1
2	#NULL!	#NULL!	#NULL!	#NULL!	#NULL!	#NULL!	#NULL!	#NULL!	#NULL!
1	2	1	1	1	1	1	1	1	2
#NULL!	#NULL!	#NULL!	#NULL!	#NULL!	#NULL!	#NULL!	#NULL!	#NULL!	#NULL!
1	2	1	2	2	2	2	2	1	2
1	2	2	2	2	2	1	2	1	2
#NULL!	#NULL!	#NULL!	#NULL!	#NULL!	#NULL!	#NULL!	#NULL!	#NULL!	#NULL!
2	2	#NULL!	#NULL!	#NULL!	#NULL!	#NULL!	#NULL!	#NULL!	#NULL!
2	2	2	2	2	2	2	2	2	2
2	2	2	2	1	2	1	2	2	2
2	2	1	1	1	1	2	1	1	1
1	2	1	2	2	1	1	2	2	2
#NULL!	#NULL!	#NULL!	#NULL!	#NULL!	#NULL!	#NULL!	#NULL!	#NULL!	#NULL!
#NULL!	#NULL!	#NULL!	#NULL!	#NULL!	#NULL!	#NULL!	#NULL!	#NULL!	#NULL!
1	1	#NULL!	2	2	#NULL!	1	1	1	2
1	2	#NULL!	#NULL!	#NULL!	#NULL!	#NULL!	#NULL!	#NULL!	#NULL!
#NULL!	#NULL!	#NULL!	#NULL!	#NULL!	#NULL!	#NULL!	#NULL!	#NULL!	#NULL!
2	1	2	#NULL!	2	1	2	2	2	1
1	2	1	2	2	1	1	1	2	1
1	2	2	2	2	2	2	2	2	2
1	2	2	2	2	2	2	2	2	2
2	2	2	2	1	1	2	1	2	1
1	2	1	2	1	1	1	2	1	2
2	2	2	2	2	2	2	2	2	2
#NULL!	#NULL!	#NULL!	#NULL!	#NULL!	#NULL!	#NULL!	#NULL!	#NULL!	#NULL!
1	2	#NULL!	#NULL!	#NULL!	#NULL!	#NULL!	#NULL!	#NULL!	#NULL!
1	2	1	2	2	1	2	2	1	1
1	1	1	1	1	1	2	2	2	2
1	2	2	2	1	2	2	1	2	2
#NULL!	#NULL!	#NULL!	#NULL!	#NULL!	#NULL!	#NULL!	#NULL!	#NULL!	#NULL!
#NULL!	#NULL!	#NULL!	#NULL!	#NULL!	#NULL!	#NULL!	#NULL!	#NULL!	#NULL!
#NULL!	#NULL!	#NULL!	#NULL!	#NULL!	#NULL!	#NULL!	#NULL!	#NULL!	#NULL!
#NULL!	#NULL!	#NULL!	#NULL!	#NULL!	#NULL!	#NULL!	#NULL!	#NULL!	#NULL!
#NULL!	#NULL!	#NULL!	#NULL!	#NULL!	#NULL!	#NULL!	#NULL!	#NULL!	#NULL!
#NULL!	#NULL!	#NULL!	#NULL!	#NULL!	#NULL!	#NULL!	#NULL!	#NULL!	#NULL!
#NULL!	#NULL!	#NULL!	#NULL!	#NULL!	#NULL!	#NULL!	#NULL!	#NULL!	#NULL!
#NULL!	#NULL!	#NULL!	#NULL!	#NULL!	#NULL!	#NULL!	#NULL!	#NULL!	#NULL!
#NULL!	#NULL!	#NULL!	#NULL!	#NULL!	#NULL!	#NULL!	#NULL!	#NULL!	#NULL!
#NULL!	#NULL!	#NULL!	#NULL!	#NULL!	#NULL!	#NULL!	#NULL!	#NULL!	#NULL!
#NULL!	#NULL!	#NULL!	#NULL!	#NULL!	#NULL!	#NULL!	#NULL!	#NULL!	#NULL!
#NULL!	#NULL!	#NULL!	#NULL!	#NULL!	#NULL!	#NULL!	#NULL!	#NULL!	#NULL!
#NULL!	#NULL!	#NULL!	#NULL!	#NULL!	#NULL!	#NULL!	#NULL!	#NULL!	#NULL!
#NULL!	#NULL!	#NULL!	#NULL!	#NULL!	#NULL!	#NULL!	#NULL!	#NULL!	#NULL!
#NULL!	#NULL!	#NULL!	#NULL!	#NULL!	#NULL!	#NULL!	#NULL!	#NULL!	#NULL!
#NULL!	#NULL!	#NULL!	#NULL!	#NULL!	#NULL!	#NULL!	#NULL!	#NULL!	#NULL!
#NULL!	#NULL!	#NULL!	#NULL!	#NULL!	#NULL!	#NULL!	#NULL!	#NULL!	#NULL!

#NULL!	#NULL!	#NULL!	#NULL!	#NULL!	#NULL!	#NULL!	#NULL!	#NULL!	#NULL!
#NULL!	#NULL!	#NULL!	#NULL!	#NULL!	#NULL!	#NULL!	#NULL!	#NULL!	#NULL!
#NULL!	#NULL!	#NULL!	#NULL!	#NULL!	#NULL!	#NULL!	#NULL!	#NULL!	#NULL!
#NULL!	#NULL!	#NULL!	#NULL!	#NULL!	#NULL!	#NULL!	#NULL!	#NULL!	#NULL!
#NULL!	#NULL!	#NULL!	#NULL!	#NULL!	#NULL!	#NULL!	#NULL!	#NULL!	#NULL!
#NULL!	#NULL!	#NULL!	#NULL!	#NULL!	#NULL!	#NULL!	#NULL!	#NULL!	#NULL!
#NULL!	#NULL!	#NULL!	#NULL!	#NULL!	#NULL!	#NULL!	#NULL!	#NULL!	#NULL!
#NULL!	#NULL!	#NULL!	#NULL!	#NULL!	#NULL!	#NULL!	#NULL!	#NULL!	#NULL!
#NULL!	#NULL!	#NULL!	#NULL!	#NULL!	#NULL!	#NULL!	#NULL!	#NULL!	#NULL!
#NULL!	#NULL!	#NULL!	#NULL!	#NULL!	#NULL!	#NULL!	#NULL!	#NULL!	#NULL!
#NULL!	#NULL!	#NULL!	#NULL!	#NULL!	#NULL!	#NULL!	#NULL!	#NULL!	#NULL!
#NULL!	#NULL!	#NULL!	#NULL!	#NULL!	#NULL!	#NULL!	#NULL!	#NULL!	#NULL!
#NULL!	#NULL!	#NULL!	#NULL!	#NULL!	#NULL!	#NULL!	#NULL!	#NULL!	#NULL!
#NULL!	#NULL!	#NULL!	#NULL!	#NULL!	#NULL!	#NULL!	#NULL!	#NULL!	#NULL!
#NULL!	#NULL!	#NULL!	#NULL!	#NULL!	#NULL!	#NULL!	#NULL!	#NULL!	#NULL!
#NULL!	#NULL!	#NULL!	#NULL!	#NULL!	#NULL!	#NULL!	#NULL!	#NULL!	#NULL!
#NULL!	#NULL!	#NULL!	#NULL!	#NULL!	#NULL!	#NULL!	#NULL!	#NULL!	#NULL!
#NULL!	#NULL!	#NULL!	#NULL!	#NULL!	#NULL!	#NULL!	#NULL!	#NULL!	#NULL!
#NULL!	#NULL!	#NULL!	#NULL!	#NULL!	#NULL!	#NULL!	#NULL!	#NULL!	#NULL!
1	2	2	2	2	2	2	2	2	2
#NULL!	#NULL!	#NULL!	#NULL!	#NULL!	#NULL!	#NULL!	#NULL!	#NULL!	#NULL!
#NULL!	#NULL!	#NULL!	#NULL!	#NULL!	#NULL!	#NULL!	#NULL!	#NULL!	#NULL!
#NULL!	#NULL!	#NULL!	#NULL!	#NULL!	#NULL!	#NULL!	#NULL!	#NULL!	#NULL!
#NULL!	#NULL!	#NULL!	#NULL!	#NULL!	#NULL!	#NULL!	#NULL!	#NULL!	#NULL!
#NULL!	#NULL!	#NULL!	#NULL!	#NULL!	#NULL!	#NULL!	#NULL!	#NULL!	#NULL!
#NULL!	#NULL!	#NULL!	#NULL!	#NULL!	#NULL!	#NULL!	#NULL!	#NULL!	#NULL!
#NULL!	#NULL!	#NULL!	#NULL!	#NULL!	#NULL!	#NULL!	#NULL!	#NULL!	#NULL!
#NULL!	#NULL!	#NULL!	#NULL!	#NULL!	#NULL!	#NULL!	#NULL!	#NULL!	#NULL!
#NULL!	#NULL!	#NULL!	#NULL!	#NULL!	#NULL!	#NULL!	#NULL!	#NULL!	#NULL!
#NULL!	#NULL!	#NULL!	#NULL!	#NULL!	#NULL!	#NULL!	#NULL!	#NULL!	#NULL!
#NULL!	#NULL!	#NULL!	#NULL!	#NULL!	#NULL!	#NULL!	#NULL!	#NULL!	#NULL!
#NULL!	#NULL!	#NULL!	#NULL!	#NULL!	#NULL!	#NULL!	#NULL!	#NULL!	#NULL!
#NULL!	#NULL!	#NULL!	#NULL!	#NULL!	#NULL!	#NULL!	#NULL!	#NULL!	#NULL!
#NULL!	#NULL!	#NULL!	#NULL!	#NULL!	#NULL!	#NULL!	#NULL!	#NULL!	#NULL!
#NULL!	#NULL!	#NULL!	#NULL!	#NULL!	#NULL!	#NULL!	#NULL!	#NULL!	#NULL!
2	2	2	2	2	2	2	2	2	2
#NULL!	#NULL!	#NULL!	#NULL!	#NULL!	#NULL!	#NULL!	#NULL!	#NULL!	#NULL!
1	2	2	2	2	2	1	2	1	2
1	2	1	2	1	2	2	2	2	2
2	1	1	1	1	1	1	1	1	1
#NULL!	#NULL!	#NULL!	#NULL!	#NULL!	#NULL!	#NULL!	#NULL!	#NULL!	#NULL!
1	#NULL!	#NULL!	#NULL!	#NULL!	#NULL!	#NULL!	#NULL!	#NULL!	#NULL!
1	2	#NULL!	#NULL!	#NULL!	#NULL!	#NULL!	#NULL!	#NULL!	#NULL!
1	#NULL!	#NULL!	#NULL!	#NULL!	#NULL!	#NULL!	#NULL!	#NULL!	#NULL!
2	2	2	2	2	2	2	1	2	2
2	#NULL!	#NULL!	#NULL!	2	#NULL!	#NULL!	#NULL!	#NULL!	#NULL!
1	2	2	2	2	2	2	2	2	2

1	2	1	2	2	2	2	2	1	2
2	2	2	2	2	2	2	2	2	2
1	2	2	2	2	2	2	2	2	2
2	2	2	2	2	2	2	2	1	2
1	2	2	2	2	1	1	2	1	2
2	2	2	2	2	2	1	2	1	1
2	2	2	2	2	2	2	2	2	2
2	2	2	2	2	2	2	2	2	2
1	2	2	2	1	2	2	2	2	1
2	2	2	2	2	1	1	2	2	2
2	2	#NULL!	#NULL!	#NULL!	#NULL!	#NULL!	#NULL!	#NULL!	#NULL!
2	2	2	1	2	2	1	2	2	1
#NULL!	#NULL!	#NULL!	#NULL!	#NULL!	#NULL!	#NULL!	#NULL!	#NULL!	#NULL!
2	2	2	1	2	2	2	2	2	2
2	2	2	1	2	2	2	2	2	2
2	2	1	2	2	2	1	2	2	2
#NULL!	#NULL!	#NULL!	#NULL!	#NULL!	#NULL!	#NULL!	#NULL!	#NULL!	#NULL!
2	2	2	#NULL!	#NULL!	#NULL!	#NULL!	#NULL!	#NULL!	#NULL!
2	2	1	2	2	2	2	2	2	2
1	2	2	2	2	2	2	2	1	2
2	2	2	2	1	1	2	#NULL!	1	2
1	2	1	2	2	1	1	2	1	2
1	2	1	2	2	2	2	2	2	2
1	2	1	2	1	1	1	2	1	2
1	1	1	1	1	1	1	1	1	1
2	2	2	2	2	2	2	2	2	2
2	2	2	2	2	2	2	2	2	2
#NULL!	2	1	2	1	2	2	1	2	1
2	2	2	2	2	2	2	2	2	2
#NULL!	#NULL!	#NULL!	#NULL!	#NULL!	#NULL!	#NULL!	#NULL!	#NULL!	#NULL!
#NULL!	#NULL!	#NULL!	#NULL!	#NULL!	#NULL!	#NULL!	#NULL!	#NULL!	#NULL!
1	2	2	2	2	2	2	2	2	2
1	2	2	2	2	2	2	2	2	2
2	#NULL!	#NULL!	#NULL!	#NULL!	#NULL!	#NULL!	#NULL!	#NULL!	#NULL!
#NULL!	#NULL!	#NULL!	#NULL!	#NULL!	#NULL!	#NULL!	#NULL!	#NULL!	#NULL!
2	#NULL!	#NULL!	#NULL!	#NULL!	#NULL!	#NULL!	#NULL!	#NULL!	#NULL!
1	#NULL!	#NULL!	#NULL!	#NULL!	#NULL!	#NULL!	#NULL!	#NULL!	#NULL!
1	2	2	1	2	2	2	1	2	2
1	#NULL!	#NULL!	#NULL!	#NULL!	#NULL!	#NULL!	#NULL!	#NULL!	#NULL!
#NULL!	#NULL!	#NULL!	#NULL!	#NULL!	#NULL!	#NULL!	#NULL!	#NULL!	#NULL!
1	2	2	2	2	2	2	2	2	2
2	#NULL!	#NULL!	#NULL!	#NULL!	#NULL!	#NULL!	#NULL!	#NULL!	#NULL!
1	2	2	2	1	1	1	2	1	2
1	1	1	1	1	1	1	2	1	1
2	2	2	2	2	2	2	2	1	2
2	1	1	1	1	1	1	1	1	2
2	2	1	2	2	1	2	2	2	1

2	2	1	1	2	1	2	1	2	1
#NULL!	2	2	2	2	2	2	2	2	1
1	2	2	2	2	2	2	2	2	2
1	2	2	2	2	2	2	1	1	2
2	2	2	2	1	1	2	2	1	2
1	2	1	2	2	1	2	2	2	1
1	2	2	2	2	2	2	2	1	1
2	2	1	2	2	1	2	2	1	2
1	2	2	2	2	2	2	2	2	2
#NULL!	2	#NULL!	2	#NULL!	2	#NULL!	2	1	#NULL!
1	2	1	1	1	1	1	1	1	1
2	2	2	2	2	2	2	2	1	2
1	2	2	2	2	2	2	2	2	2
2	2	2	1	2	2	1	2	2	1
2	2	2	2	2	2	2	2	2	2
1	2	1	2	2	1	1	1	1	2
1	2	2	2	1	2	1	2	2	1
2	2	#NULL!	#NULL!	#NULL!	#NULL!	#NULL!	#NULL!	#NULL!	#NULL!
2	2	2	2	2	2	2	2	2	1
2	2	2	2	1	1	1	1	1	1
2	2	2	2	2	1	1	2	2	2
2	2	2	1	2	2	2	2	2	2
1	2	1	2	2	2	1	2	2	2
2	2	2	2	2	2	2	2	2	2
2	2	2	2	2	2	2	2	2	2
2	1	2	2	2	2	1	2	2	2
2	2	2	2	2	1	2	2	1	2
1	2	2	2	2	2	2	1	2	2
2	2	2	2	2	2	2	2	2	1
2	2	1	2	2	1	2	2	2	2
2	2	1	2	2	2	2	2	2	2
1	2	2	1	2	2	1	2	2	1
2	2	2	2	2	2	2	2	2	2
2	2	2	2	1	1	2	1	1	2
1	2	2	1	2	2	1	2	2	1
2	2	1	1	1	1	1	1	1	2
2	2	2	2	2	2	2	1	2	2
#NULL!	#NULL!	#NULL!	#NULL!	#NULL!	#NULL!	#NULL!	#NULL!	#NULL!	#NULL!
1	2	1	2	2	1	2	1	1	2
2	2	1	#NULL!	2	2	2	1	2	2
2	2	1	1	1	2	1	2	1	2
1	2	2	1	2	2	1	2	2	1
2	2	1	2	2	2	1	2	2	2
1	2	1	2	2	1	2	2	1	2
1	2	2	2	2	2	2	2	2	2
2	2	1	1	1	1	1	1	1	1
2	1	1	1	1	1	1	1	1	#NULL!

1	2	2	2	1	2	2	1	2	2
#NULL!	2	2	2	2	2	2	2	2	2
1	2	2	2	2	2	2	1	1	1
2	2	2	2	1	2	2	2	2	1
1	2	2	2	2	2	2	2	2	2
1	2	2	2	2	2	1	1	1	2
2	2	2	2	2	2	2	2	2	2
#NULL!	#NULL!	#NULL!	#NULL!	#NULL!	#NULL!	#NULL!	#NULL!	#NULL!	#NULL!
#NULL!	#NULL!	#NULL!	#NULL!	#NULL!	#NULL!	#NULL!	#NULL!	#NULL!	#NULL!
1	2	1	2	1	2	2	2	1	2
1	2	2	2	2	2	2	2	2	2
2	2	2	2	2	2	2	2	2	2
2	2	2	2	2	2	2	2	2	2
1	2	2	2	2	2	2	2	2	2
2	2	2	2	2	2	2	2	2	2
2	2	2	2	2	2	2	2	2	2
2	2	2	2	2	2	2	2	2	2
2	2	2	2	2	2	2	2	2	2
1	2	2	2	2	1	2	2	2	2
2	2	2	2	2	2	2	2	2	2
2	2	1	1	2	2	2	2	2	2
2	2	2	2	2	2	2	2	2	2
2	2	2	2	2	2	2	2	2	2
#NULL!	2	2	2	2	2	2	2	1	2
2	2	2	2	2	2	2	2	2	2
1	2	2	2	2	2	2	2	2	2
1	2	2	2	2	2	2	2	2	2
2	2	2	#NULL!	2	2	2	2	1	2
1	2	2	2	2	2	2	2	2	2
2	2	2	2	2	2	2	2	2	2
2	2	2	2	2	2	2	2	2	2
1	2	2	2	2	2	2	2	2	2
2	2	2	2	2	2	2	2	2	2
2	2	2	2	2	2	2	2	2	2
1	2	2	2	2	2	2	2	2	2
1	2	2	2	2	2	2	2	2	2
1	2	2	2	2	2	2	2	2	2
1	2	2	2	2	2	2	2	2	2
2	2	2	2	2	2	2	2	2	2
#NULL!	2	1	1	2	2	2	1	1	2
1	2	2	2	2	2	2	2	2	2
2	2	2	2	2	2	2	2	2	2
1	2	1	2	2	2	2	2	1	1
#NULL!	2	2	2	2	2	2	2	2	2
2	2	2	1	1	1	2	2	1	1
1	2	2	2	2	2	2	2	2	2
2	2	2	2	2	2	2	2	2	2

2	2	2	2	2	2	2	2	2	2
2	2	2	2	2	2	2	2	2	2
2	2	2	2	2	2	2	2	2	2
1	2	2	2	2	2	2	2	2	2
1	2	2	2	2	2	2	2	2	2
2	2	2	2	2	2	2	2	2	2
2	#NULL!	#NULL!	#NULL!	#NULL!	#NULL!	#NULL!	#NULL!	#NULL!	#NULL!
1	2	2	2	2	2	2	2	2	2
2	#NULL!	#NULL!	#NULL!	#NULL!	#NULL!	#NULL!	#NULL!	#NULL!	#NULL!
2	2	2	2	2	2	2	2	2	2
1	2	2	2	2	1	2	2	1	2
1	2	2	2	2	2	2	2	2	2
2	2	1	2	2	1	2	1	1	2
2	#NULL!	#NULL!	#NULL!	#NULL!	#NULL!	#NULL!	#NULL!	#NULL!	#NULL!
2	2	2	2	2	2	2	2	2	2
2	2	1	2	2	2	2	2	2	2
1	2	2	2	2	2	2	2	2	2
1	2	2	2	2	2	2	2	2	2
1	2	#NULL!	#NULL!	#NULL!	#NULL!	#NULL!	#NULL!	#NULL!	#NULL!
2	2	2	#NULL!	2	2	2	2	2	2
2	2	2	2	2	2	2	2	2	2
1	#NULL!	#NULL!	#NULL!	#NULL!	#NULL!	#NULL!	#NULL!	#NULL!	#NULL!
#NULL!	#NULL!	#NULL!	2	#NULL!	#NULL!	#NULL!	#NULL!	#NULL!	#NULL!
2	1	1	1	1	1	1	1	1	1
1	2	2	1	2	2	1	2	2	1
2	1	1	1	1	1	2	2	2	1
#NULL!	2	2	2	2	2	2	2	2	2
2	2	2	2	1	1	1	2	#NULL!	2
2	2	2	2	2	2	2	2	2	#NULL!
1	2	2	2	2	2	2	2	2	2
2	2	2	2	2	2	2	2	2	2
2	2	2	2	2	1	1	1	1	1
2	2	2	2	2	2	2	2	2	2
2	2	#NULL!	#NULL!	#NULL!	2	#NULL!	#NULL!	#NULL!	#NULL!
2	2	2	2	2	2	2	2	2	2
1	2	#NULL!	#NULL!	#NULL!	#NULL!	#NULL!	#NULL!	#NULL!	#NULL!
2	1	2	2	1	1	2	2	1	1
2	2	2	2	2	2	2	2	2	2
2	2	2	2	2	2	2	2	2	2
2	2	2	2	2	2	2	2	2	2
2	2	1	#NULL!	1	#NULL!	2	#NULL!	2	2
2	2	2	2	2	2	2	2	2	2
2	#NULL!	#NULL!	#NULL!	#NULL!	2	#NULL!	#NULL!	#NULL!	#NULL!
1	2	2	2	2	2	2	2	1	1
2	2	2	2	2	2	2	2	2	2
2	1	1	1	1	1	1	1	1	1
1	2	2	2	2	2	2	2	2	2

2	#NULL!	#NULL!	#NULL!	#NULL!	#NULL!	#NULL!	#NULL!	2	#NULL!
2	2	2	2	2	2	2	2	2	2
1	2	2	2	2	2	2	2	2	2
2	2	2	2	2	2	2	2	2	2
2	#NULL!	2	#NULL!	#NULL!	#NULL!	#NULL!	#NULL!	#NULL!	#NULL!
1	2	2	2	2	1	1	2	2	2
2	1	1	1	1	1	1	1	1	1
1	2	2	2	2	2	2	2	2	2
1	2	2	2	2	2	2	2	2	2
1	2	2	2	2	2	2	1	1	1
2	#NULL!	#NULL!	#NULL!	#NULL!	#NULL!	#NULL!	#NULL!	#NULL!	#NULL!
1	2	2	2	2	2	2	2	2	2
1	2	2	2	2	2	2	2	2	2
2	2	2	2	2	2	2	2	2	2
2	2	2	2	2	2	2	2	2	2
2	1	1	1	1	1	1	1	1	1
2	2	2	2	2	2	2	2	2	2
1	1	1	1	1	2	2	2	2	2
#NULL!	#NULL!	#NULL!	#NULL!	#NULL!	#NULL!	#NULL!	#NULL!	#NULL!	#NULL!
#NULL!	2	2	2	1	2	1	2	2	2
2	2	2	2	2	2	2	2	2	2
1	2	2	2	2	2	2	2	2	2
1	2	2	2	2	2	2	2	2	2
1	2	2	2	2	2	2	2	2	2
2	1	1	#NULL!	1	1	1	1	1	1
1	2	2	2	2	2	2	2	2	2
1	2	#NULL!	2	2	1	2	2	2	1
1	2	2	2	2	2	2	2	2	2
2	2	2	2	#NULL!	2	2	2	#NULL!	2
2	2	2	2	2	2	2	2	2	2
1	2	2	#NULL!	1	2	2	1	2	1
2	2	2	2	2	1	1	1	2	2
2	2	2	2	2	2	2	2	2	2
1	2	2	2	2	2	2	2	2	2
2	2	2	2	2	2	2	2	2	2
2	2	2	2	2	2	2	2	2	2
1	2	2	2	2	2	2	2	2	2
1	1	1	1	1	2	2	2	2	2
1	2	2	1	2	2	1	2	2	1
2	1	1	1	1	1	1	1	1	1
1	2	2	2	2	2	2	2	1	2
#NULL!	2	2	#NULL!	1	1	1	1	2	2
#NULL!	#NULL!	#NULL!	#NULL!	#NULL!	#NULL!	#NULL!	#NULL!	#NULL!	#NULL!
#NULL!	1	1	1	1	1	1	1	1	1
1	#NULL!	#NULL!	#NULL!	#NULL!	#NULL!	#NULL!	#NULL!	#NULL!	#NULL!
2	2	2	2	2	2	2	2	2	2
1	2	2	2	2	2	2	2	1	2

1	2	2	2	2	2	2	2	2	2
1	2	1	1	1	1	1	1	1	2
#NULL!	#NULL!	#NULL!	#NULL!	#NULL!	#NULL!	#NULL!	#NULL!	#NULL!	#NULL!
1	2	1	1	1	1	1	1	1	2
1	2	2	#NULL!	2	2	#NULL!	2	2	2
2	2	2	2	2	2	2	2	2	2
2	2	2	2	2	2	2	2	2	2
#NULL!	2	2	2	2	2	2	2	2	2
2	1	2	2	2	2	2	2	1	1
1	2	1	2	2	2	2	2	2	2
2	2	2	2	2	2	2	2	2	2
2	2	2	2	1	2	1	2	2	1
1	2	2	2	2	2	2	2	2	2
1	2	1	2	1	2	1	2	1	2
2	2	2	2	2	2	2	2	2	2
2	2	2	2	2	2	2	2	2	2
2	2	2	2	2	2	2	2	2	2
2	2	2	2	2	2	2	2	2	2
2	2	2	2	2	2	2	2	2	2
2	2	2	2	1	1	2	2	#NULL!	2
2	2	2	2	2	2	2	2	2	2
2	1	1	1	1	1	1	1	1	1
1	2	2	2	2	2	2	2	2	2
2	2	2	2	2	2	2	2	2	2
1	2	2	2	2	2	2	2	2	2
2	2	2	2	2	2	2	2	2	2
2	2	2	1	2	2	2	2	2	2
1	2	2	2	2	2	2	2	2	2
2	2	2	1	2	2	2	2	2	1
2	2	2	2	2	2	2	2	2	2
2	2	2	2	1	2	2	2	2	2
2	2	2	1	2	2	1	1	2	1
2	2	2	2	2	2	2	2	2	2
#NULL!	2	2	2	2	2	2	2	2	2
2	2	2	2	2	2	2	2	1	1
1	2	2	1	1	2	2	1	2	1
2	#NULL!	#NULL!	#NULL!	#NULL!	#NULL!	#NULL!	#NULL!	#NULL!	#NULL!
#NULL!	#NULL!	#NULL!	#NULL!	#NULL!	#NULL!	#NULL!	#NULL!	#NULL!	#NULL!
2	2	2	2	2	2	2	2	2	2
2	2	2	2	2	2	2	2	2	2
#NULL!	2	2	1	2	2	2	2	2	2
2	2	2	2	2	2	2	2	2	2
2	2	2	2	2	2	2	2	2	2
#NULL!	2	2	2	2	2	2	2	2	2
#NULL!	#NULL!	#NULL!	#NULL!	#NULL!	#NULL!	#NULL!	#NULL!	#NULL!	#NULL!
1	2	2	#NULL!	2	2	2	2	2	2
2	2	2	2	2	2	2	2	2	2

#NULL!	#NULL!	#NULL!	#NULL!	#NULL!	#NULL!	#NULL!	#NULL!	#NULL!	#NULL!
1	2	2	2	1	2	2	2	1	2
2	2	2	1	2	1	2	2	1	2
#NULL!	2	1	2	2	1	2	2	1	2
2	2	1	2	2	1	1	2	#NULL!	2
2	2	2	1	2	2	2	2	2	2
#NULL!	2	2	1	2	2	2	2	1	2
#NULL!	#NULL!	#NULL!	#NULL!	#NULL!	#NULL!	#NULL!	#NULL!	#NULL!	#NULL!
2	2	2	2	2	2	2	2	2	2
2	1	2	2	2	1	1	1	2	2
2	2	2	2	2	2	2	2	2	2
1	2	2	2	2	2	1	#NULL!	1	1
2	2	2	2	2	2	2	1	1	2
2	2	2	2	2	2	2	2	2	2
1	2	2	1	2	2	1	2	2	2
2	2	2	2	2	2	2	2	2	2
2	2	2	2	2	2	2	2	2	2
2	2	2	2	2	2	2	2	2	2
#NULL!	#NULL!	#NULL!	#NULL!	#NULL!	#NULL!	#NULL!	#NULL!	#NULL!	#NULL!
#NULL!	#NULL!	#NULL!	#NULL!	#NULL!	#NULL!	#NULL!	#NULL!	#NULL!	#NULL!
#NULL!	#NULL!	#NULL!	#NULL!	#NULL!	#NULL!	#NULL!	#NULL!	#NULL!	#NULL!
2	2	2	1	2	2	1	2	2	2
2	2	2	2	1	1	2	2	2	1
#NULL!	2	2	2	2	2	1	1	1	2
#NULL!	2	2	2	2	1	1	1	2	2
2	1	1	1	2	2	2	2	2	2
2	2	1	2	2	2	2	2	1	2
2	2	2	2	1	1	2	2	2	2
2	2	2	1	1	2	2	2	2	2
2	2	2	2	2	1	2	2	1	2
2	#NULL!	#NULL!	#NULL!	#NULL!	#NULL!	#NULL!	#NULL!	#NULL!	#NULL!
2	2	2	2	2	2	2	2	2	2
1	2	1	1	1	1	1	1	1	1
2	2	2	2	2	2	2	2	2	2
1	1	1	1	1	1	1	1	1	1
2	2	2	2	2	2	2	2	2	2
2	1	1	1	1	1	1	1	1	1
2	2	2	2	2	2	2	2	2	2
#NULL!	#NULL!	#NULL!	#NULL!	#NULL!	#NULL!	#NULL!	#NULL!	#NULL!	#NULL!
#NULL!	#NULL!	#NULL!	#NULL!	#NULL!	#NULL!	#NULL!	#NULL!	#NULL!	#NULL!
2	2	2	1	2	2	2	2	2	1
2	2	2	2	2	2	2	2	2	2
1	2	1	2	2	2	2	2	1	2
2	2	2	1	2	1	2	2	1	2
2	#NULL!	#NULL!	#NULL!	#NULL!	#NULL!	#NULL!	#NULL!	#NULL!	#NULL!
1	1	1	1	1	1	1	1	1	1
2	2	2	2	2	1	2	2	1	2

#NULL!	2	2	2	2	2	2	2	2	2
2	2	2	2	2	2	2	2	2	2
1	2	2	1	2	2	2	1	2	2
2	2	#NULL!	#NULL!	1	#NULL!	#NULL!	2	#NULL!	1
2	2	1	2	1	2	1	2	#NULL!	1
2	1	1	1	1	1	1	1	1	1
1	2	2	2	2	2	2	2	2	2
1	1	1	1	1	1	1	1	1	1
2	2	1	1	1	2	2	2	2	1
#NULL!	2	2	2	1	2	2	1	2	2
2	2	2	2	2	2	2	2	2	2
2	2	2	2	2	2	1	2	2	1
#NULL!	2	2	2	2	2	1	2	1	2
2	2	2	2	1	2	2	1	2	2
2	2	2	2	2	2	2	2	2	2
2	1	1	1	1	2	2	2	1	1
2	1	1	2	2	2	2	2	2	2
2	2	2	1	2	2	1	2	2	1
2	#NULL!	#NULL!	#NULL!	#NULL!	#NULL!	#NULL!	#NULL!	#NULL!	#NULL!
2	2	1	2	2	2	2	2	1	2
#NULL!	2	2	1	2	1	2	2	2	2
2	1	1	1	1	1	1	2	1	2
1	2	2	2	2	2	2	2	2	2
2	2	2	2	1	1	2	2	2	2
2	2	2	1	2	2	1	2	2	1
2	2	2	2	2	2	2	1	2	1
2	2	2	2	2	2	2	2	2	2
2	2	2	1	2	2	1	2	2	1
2	2	2	2	1	2	2	2	2	1
2	2	2	2	2	2	2	2	2	2
2	2	2	1	1	2	2	1	2	2
2	2	2	2	2	2	2	2	2	2
2	2	2	2	2	2	2	2	2	2
1	2	2	1	1	1	2	2	2	1
2	2	2	1	2	2	1	2	2	1
1	2	2	2	2	2	2	2	2	2
2	2	2	1	2	1	2	2	2	1
2	1	1	1	1	1	1	1	1	1
2	2	2	2	2	2	2	2	2	2
2	2	2	2	2	1	2	1	2	2
2	2	2	2	2	2	2	2	2	2
1	2	2	1	2	2	1	2	2	1
2	2	2	2	2	2	2	2	1	2
1	2	2	2	2	2	2	2	2	2
2	2	2	2	1	1	2	1	1	1
2	2	2	2	2	2	2	1	2	2
2	1	2	1	1	2	1	2	2	1

2	2	2	2	2	2	2	2	2	2
2	2	1	1	1	2	2	1	2	2
2	2	2	2	2	2	2	2	2	2
1	2	2	2	2	2	2	2	2	2
2	2	2	1	2	2	1	2	2	1
1	2	2	1	2	2	1	2	2	1
2	2	2	1	2	2	2	2	2	2
2	2	2	2	2	2	2	2	2	2
2	2	2	2	2	2	2	2	2	1
2	1	1	1	1	1	1	1	1	1
1	2	2	1	2	1	2	1	2	2
2	2	2	1	2	2	2	2	2	1
1	2	2	2	2	2	2	2	2	2
2	2	1	2	2	2	1	2	2	2
2	2	2	1	2	1	2	2	2	2
1	2	1	2	2	2	2	2	1	2
2	2	2	2	2	2	2	2	1	2
2	2	2	1	1	1	1	1	1	1
2	2	2	1	2	2	1	2	2	1
1	2	1	2	2	2	2	1	2	2
2	1	1	1	1	1	1	1	1	1
2	1	1	1	1	1	1	1	1	1
2	1	2	1	2	2	1	2	2	2
1	2	1	2	2	2	2	2	2	2
2	2	2	2	2	2	2	2	2	2
#NULL!	2	2	2	2	2	2	2	2	2
1	2	1	1	1	2	2	2	2	2
1	2	2	1	2	2	1	2	2	1
#NULL!	2	2	2	2	2	2	2	2	2
#NULL!	#NULL!	#NULL!	#NULL!	#NULL!	#NULL!	#NULL!	#NULL!	#NULL!	#NULL!
#NULL!	#NULL!	#NULL!	#NULL!	#NULL!	#NULL!	#NULL!	#NULL!	#NULL!	#NULL!
#NULL!	#NULL!	#NULL!	#NULL!	#NULL!	#NULL!	#NULL!	#NULL!	#NULL!	#NULL!
1	2	2	2	2	1	1	1	1	1
#NULL!	#NULL!	#NULL!	#NULL!	#NULL!	#NULL!	#NULL!	#NULL!	#NULL!	#NULL!
#NULL!	1	1	1	1	1	2	2	2	2
#NULL!	#NULL!	#NULL!	#NULL!	#NULL!	#NULL!	#NULL!	#NULL!	#NULL!	#NULL!
2	2	2	2	2	2	2	2	2	2
2	#NULL!	#NULL!	#NULL!	#NULL!	#NULL!	#NULL!	#NULL!	#NULL!	#NULL!
#NULL!	2	2	2	#NULL!	#NULL!	#NULL!	2	2	2
1	2	1	2	1	2	1	2	1	2
#NULL!	#NULL!	#NULL!	#NULL!	#NULL!	#NULL!	#NULL!	#NULL!	#NULL!	#NULL!
2	2	2	2	2	2	2	2	2	2
1	2	#NULL!	2	1	2	1	2	1	2
#NULL!	2	1	1	2	1	2	2	2	2
1	2	1	2	2	2	2	2	2	1
1	2	2	2	2	1	2	2	2	2
1	2	1	1	1	2	2	2	2	2

1	2	2	2	2	2	2	2	2	1
#NULL!	2	2	2	2	2	2	2	2	2
#NULL!	#NULL!	#NULL!	#NULL!	#NULL!	#NULL!	#NULL!	#NULL!	#NULL!	#NULL!
1	2	2	1	2	2	2	1	1	2
1	2	2	1	#NULL!	2	2	1	2	2
1	2	2	2	2	1	2	2	2	2
1	#NULL!	#NULL!	2	#NULL!	#NULL!	#NULL!	#NULL!	2	#NULL!
2	2	2	1	1	2	2	2	1	2
2	2	2	2	2	1	1	2	2	2
1	1	1	1	1	1	1	1	1	1
2	2	1	2	2	1	2	2	1	2
1	2	2	1	2	2	2	2	2	1
2	2	1	2	2	2	1	2	2	2
2	2	2	2	2	2	2	2	2	2
2	2	2	2	2	2	2	2	2	2
#NULL!	#NULL!	#NULL!	#NULL!	#NULL!	#NULL!	#NULL!	#NULL!	#NULL!	#NULL!
2	2	2	2	2	2	2	2	2	2
2	2	2	2	2	2	2	2	2	2
2	2	2	2	2	2	2	2	2	2
2	2	2	2	2	2	2	2	2	2
1	2	1	2	2	2	2	2	#NULL!	2
1	2	1	2	2	2	2	2	#NULL!	2
1	2	1	2	2	2	2	2	#NULL!	2
1	2	2	2	2	2	1	2	1	2
1	2	1	2	2	1	1	2	1	2
1	2	1	2	2	1	1	2	1	2
1	2	1	2	2	2	2	2	2	2
1	2	1	1	2	1	2	2	2	1
1	2	2	2	2	2	2	2	2	2
1	2	1	2	2	2	2	2	1	2
1	2	1	2	2	#NULL!	2	2	#NULL!	2
2	#NULL!	2	#NULL!	#NULL!	#NULL!	#NULL!	#NULL!	#NULL!	#NULL!
1	2	2	2	2	2	2	2	2	2
1	2	1	2	1	1	1	2	1	2
1	2	2	2	2	2	2	2	2	2
1	2	2	2	1	1	2	2	2	2
1	2	2	2	1	1	2	2	2	2
1	2	1	2	2	2	1	#NULL!	2	2
1	#NULL!	#NULL!	2	1	2	#NULL!	2	#NULL!	2
1	2	1	2	2	2	2	2	2	2
1	2	2	2	2	1	1	2	1	2
1	2	#NULL!	#NULL!	#NULL!	2	2	2	#NULL!	2
1	2	2	2	2	2	2	2	2	#NULL!
1	2	1	2	2	1	1	2	2	2
1	2	2	2	2	2	2	2	2	2
1	2	1	2	2	2	2	2	2	#NULL!
1	2	2	2	2	2	2	2	1	2

1	2	2	2	1	1	2	2	2	2
1	2	2	2	2	2	2	2	2	2
1	2	1	2	2	1	1	2	1	2
1	2	2	2	2	2	2	2	2	2
1	2	1	2	2	1	2	1	#NULL!	2
1	2	1	2	2	1	2	1	1	2
2	2	2	2	2	2	2	2	2	2
1	2	2	2	2	2	2	2	1	2
1	#NULL!	#NULL!	#NULL!	#NULL!	#NULL!	#NULL!	#NULL!	#NULL!	#NULL!
1	2	1	2	2	2	2	2	2	2
2	#NULL!	1	2	2	1	2	2	2	2
1	2	2	2	2	2	2	2	2	2
1	2	1	2	2	1	2	1	1	2
1	2	1	2	2	1	1	2	1	2
1	2	1	2	2	1	1	2	1	2
1	2	1	2	2	1	1	2	1	2
1	2	1	2	2	1	1	2	1	2
2	2	#NULL!	2	1	1	2	2	2	#NULL!
1	2	1	2	2	1	1	2	1	2
1	2	#NULL!	2	2	2	2	1	2	2
2	1	2	1	2	2	2	1	2	2
2	2	1	1	1	1	1	1	2	2
1	2	2	2	2	2	2	2	1	2
1	2	1	2	1	1	2	2	1	2
2	1	2	2	2	2	2	2	2	2
1	2	1	1	1	1	2	2	1	2
1	2	2	2	1	1	1	1	1	1
1	2	1	2	1	1	1	1	1	2
2	2	1	2	2	2	1	2	1	2
1	2	1	2	2	2	1	2	2	2
2	2	2	2	2	2	2	2	2	2
1	2	1	2	1	1	1	1	1	2
1	#NULL!	#NULL!	#NULL!	#NULL!	#NULL!	#NULL!	#NULL!	#NULL!	#NULL!
1	2	1	2	2	2	#NULL!	#NULL!	1	2
1	2	1	2	1	1	1	2	1	2
1	2	1	2	1	1	1	2	1	2
1	2	1	2	2	2	#NULL!	2	1	2
1	2	1	2	1	#NULL!	1	2	1	2
1	2	1	2	1	1	1	2	1	#NULL!
1	2	1	2	2	1	1	2	1	2
1	2	1	2	2	1	1	2	1	2
1	2	1	2	1	1	1	1	2	2
1	2	1	2	#NULL!	2	2	#NULL!	#NULL!	#NULL!
1	2	1	2	1	1	1	2	1	2
1	2	1	2	1	2	2	2	2	2
1	2	2	2	2	2	1	2	1	2
1	2	1	#NULL!	#NULL!	#NULL!	2	2	1	2

1	2	1	2	2	2	1	2	1	2
1	2	1	2	1	1	1	2	#NULL!	2
1	2	1	2	2	2	1	2	1	2
1	2	2	2	2	2	2	2	2	1
1	2	1	2	2	1	1	2	1	2
1	2	2	2	2	2	2	2	2	2
1	1	2	2	1	2	2	2	2	2
#NULL!	2	#NULL!	2	1	1	#NULL!	2	2	2
#NULL!	2	#NULL!	2	#NULL!	1	1	2	2	2
1	2	1	2	2	1	1	2	1	2
1	2	1	2	1	1	1	2	1	2
1	#NULL!	#NULL!	#NULL!	#NULL!	#NULL!	#NULL!	#NULL!	#NULL!	2
1	2	1	2	2	2	1	2	1	2
1	2	1	1	1	2	2	2	1	2
2	2	#NULL!	2	1	2	2	2	2	2
2	2	2	2	2	2	1	2	2	2
1	#NULL!	#NULL!	#NULL!	#NULL!	#NULL!	#NULL!	#NULL!	#NULL!	2
1	#NULL!	#NULL!	#NULL!	#NULL!	#NULL!	#NULL!	#NULL!	#NULL!	2
1	2	2	2	1	2	2	2	2	2
1	2	2	2	2	2	2	2	2	2
1	2	1	2	2	1	1	2	1	2
1	2	1	2	2	2	2	1	1	2
1	2	1	2	1	1	1	2	1	2
1	2	1	2	#NULL!	2	2	2	#NULL!	2
2	2	1	1	1	2	1	2	2	2
2	2	1	1	1	2	1	2	2	2
2	2	1	2	1	2	2	2	1	2
1	2	2	2	2	2	2	2	2	2
1	2	2	2	2	2	2	2	2	2
1	2	2	2	2	2	2	2	1	2
1	2	2	2	2	2	2	2	2	2
2	2	1	2	2	1	2	1	2	2
1	2	2	2	2	2	2	2	1	2
1	2	1	2	2	1	1	2	1	2
1	2	2	2	2	2	1	1	2	2
1	2	2	2	2	2	2	2	2	1
#NULL!	#NULL!	#NULL!	#NULL!	#NULL!	#NULL!	#NULL!	#NULL!	#NULL!	#NULL!
1	2	2	2	2	2	2	2	2	2
1	2	2	2	2	2	2	2	2	2
1	2	1	2	2	2	1	2	2	2
1	2	1	2	2	1	1	2	1	2
1	2	1	2	2	1	2	1	1	2
2	2	2	2	2	2	2	2	1	2
#NULL!	2	2	2	2	2	2	2	2	2
2	2	2	2	2	2	2	2	1	2
1	2	1	2	2	2	1	1	1	2
1	2	1	2	2	2	2	2	2	2

1	2	1	2	2	2	2	2	1	2
1	2	2	2	2	1	2	2	2	2
1	2	1	2	2	2	2	2	2	2
1	2	2	2	2	2	2	2	2	2
1	2	2	2	2	2	2	2	2	2
2	2	1	2	1	2	1	1	2	2
1	2	2	2	2	2	2	2	2	2
1	#NULL!	#NULL!	#NULL!	#NULL!	#NULL!	#NULL!	#NULL!	#NULL!	#NULL!
1	#NULL!	#NULL!	#NULL!	#NULL!	#NULL!	#NULL!	#NULL!	#NULL!	#NULL!
1	2	2	2	2	2	1	2	1	2
1	2	1	2	1	1	2	2	1	2
1	2	1	2	1	1	1	1	1	2
2	2	2	2	2	2	2	2	2	2
1	2	2	2	1	1	2	2	2	2
1	2	2	2	2	2	2	2	2	2
1	#NULL!	#NULL!	#NULL!	#NULL!	#NULL!	#NULL!	#NULL!	#NULL!	2
1	2	2	2	2	1	2	2	1	2
1	2	2	2	2	2	2	2	2	2
1	2	1	2	2	1	1	2	1	2
1	2	1	2	2	2	2	2	1	2
1	#NULL!	#NULL!	#NULL!	#NULL!	#NULL!	#NULL!	#NULL!	#NULL!	2
1	2	2	2	2	1	1	2	1	2
1	#NULL!	#NULL!	#NULL!	#NULL!	#NULL!	#NULL!	#NULL!	#NULL!	#NULL!
1	2	2	2	2	2	1	2	2	2
1	2	1	2	2	2	1	2	1	2
1	2	1	2	2	1	2	2	2	2
1	2	2	2	2	2	2	2	#NULL!	2
#NULL!	#NULL!	#NULL!	#NULL!	#NULL!	#NULL!	#NULL!	#NULL!	#NULL!	#NULL!
#NULL!	#NULL!	#NULL!	#NULL!	#NULL!	#NULL!	#NULL!	#NULL!	#NULL!	#NULL!
1	2	1	1	2	2	1	2	1	2
1	2	2	2	2	2	1	2	1	2
1	2	2	2	2	1	1	2	1	2
1	#NULL!	#NULL!	2	1	2	#NULL!	#NULL!	#NULL!	#NULL!
1	#NULL!	#NULL!	#NULL!	#NULL!	#NULL!	#NULL!	#NULL!	#NULL!	#NULL!
1	2	1	2	2	1	1	2	1	2
1	2	2	2	1	1	2	2	1	2
1	2	1	2	2	2	1	2	1	2
1	2	1	2	2	2	2	2	2	2
1	2	1	2	1	1	1	2	1	2
1	2	1	2	2	2	1	2	1	2
1	2	2	2	2	2	1	2	1	2
1	2	1	2	2	2	1	2	1	2
1	2	1	2	2	1	2	2	1	2
1	1	2	2	1	#NULL!	#NULL!	#NULL!	#NULL!	#NULL!
1	2	2	2	1	1	2	2	2	2
2	1	1	1	1	1	1	1	1	1
2	2	1	2	1	1	1	2	2	2

1	2	1	2	2	2	2	2	2	2
2	2	1	2	1	#NULL!	#NULL!	#NULL!	#NULL!	#NULL!
#NULL!	#NULL!	#NULL!	#NULL!	#NULL!	#NULL!	#NULL!	#NULL!	#NULL!	#NULL!
1	2	1	2	1	1	1	2	1	2
1	2	1	2	2	1	#NULL!	2	2	2
1	2	2	2	2	2	2	2	2	2
1	2	2	2	2	2	2	2	1	2
1	2	1	2	2	1	2	2	1	2
1	2	#NULL!	#NULL!	#NULL!	2	#NULL!	#NULL!	#NULL!	#NULL!
1	2	1	2	1	1	1	2	2	1
#NULL!	2	1	1	1	2	2	2	1	2
1	2	2	2	2	2	2	2	2	2
1	2	2	2	2	2	1	2	2	2
1	#NULL!	#NULL!	2	#NULL!	2	#NULL!	#NULL!	#NULL!	#NULL!
1	2	2	2	2	2	2	2	2	2
1	2	1	2	2	1	1	2	1	2
1	#NULL!	#NULL!	#NULL!	#NULL!	#NULL!	#NULL!	#NULL!	#NULL!	#NULL!
1	#NULL!	#NULL!	#NULL!	#NULL!	#NULL!	#NULL!	#NULL!	#NULL!	2
1	2	2	2	2	1	1	2	1	2
1	2	1	1	2	2	2	2	1	2
1	2	1	2	2	1	1	2	1	2
1	2	1	1	1	1	1	1	1	2
1	2	1	1	1	1	1	1	1	2
1	2	1	2	2	1	1	2	2	2
1	2	#NULL!	2	2	2	2	2	1	2
1	2	2	2	1	2	2	2	2	2
1	2	1	2	2	2	1	2	2	2
1	2	1	2	2	2	1	2	2	2
1	2	2	2	1	2	2	2	2	2
1	2	2	2	2	2	2	2	2	2
1	2	2	1	2	2	2	1	1	2
1	2	2	2	2	2	2	2	2	1
1	2	1	2	2	1	1	2	#NULL!	2
1	2	2	2	2	2	2	2	2	2
1	2	1	2	2	2	1	2	1	2
1	#NULL!	#NULL!	#NULL!	#NULL!	#NULL!	#NULL!	#NULL!	#NULL!	2
1	2	1	2	1	1	1	2	#NULL!	2
1	2	#NULL!	2	#NULL!	2	#NULL!	#NULL!	#NULL!	2
1	2	2	2	2	2	2	2	2	2
1	2	1	2	2	2	2	2	2	2
2	2	1	2	2	1	2	2	1	1
1	2	1	2	1	1	1	2	2	1
1	2	#NULL!	#NULL!	#NULL!	#NULL!	#NULL!	#NULL!	#NULL!	#NULL!
1	#NULL!	#NULL!	2	#NULL!	#NULL!	#NULL!	#NULL!	#NULL!	#NULL!
1	2	2	2	2	1	1	2	1	2
1	2	1	2	2	1	1	1	1	2
1	2	#NULL!	#NULL!	#NULL!	#NULL!	#NULL!	#NULL!	#NULL!	1

1	2	1	2	2	1	1	1	1	2
1	2	2	2	2	2	2	2	1	2
1	2	2	2	2	2	2	2	#NULL!	2
1	2	1	2	2	2	1	2	2	2
1	2	2	2	2	2	1	1	1	2
1	2	2	2	2	2	1	2	1	2
1	2	2	2	2	2	2	1	2	2
1	2	2	2	2	2	2	2	2	2
2	2	2	2	2	2	2	2	2	2
1	2	2	2	2	2	2	2	2	2
1	2	2	2	2	2	2	2	1	2
1	2	#NULL!	#NULL!	#NULL!	#NULL!	#NULL!	#NULL!	#NULL!	#NULL!
1	2	#NULL!	#NULL!	#NULL!	#NULL!	#NULL!	#NULL!	#NULL!	#NULL!
1	2	#NULL!	#NULL!	#NULL!	#NULL!	#NULL!	#NULL!	#NULL!	#NULL!
1	2	#NULL!	#NULL!	#NULL!	#NULL!	#NULL!	#NULL!	#NULL!	#NULL!
1	2	#NULL!	#NULL!	#NULL!	#NULL!	#NULL!	#NULL!	#NULL!	#NULL!
1	2	1	2	1	2	1	2	1	2
2	2	2	2	1	1	1	2	2	1
1	#NULL!	#NULL!	2	#NULL!	#NULL!	#NULL!	#NULL!	#NULL!	#NULL!
1	2	1	2	2	2	1	2	1	2
1	2	1	2	2	1	1	2	2	2
1	#NULL!	#NULL!	#NULL!	#NULL!	#NULL!	#NULL!	#NULL!	#NULL!	#NULL!
1	2	1	2	1	2	2	2	2	2
1	2	1	2	1	1	2	2	1	2
1	2	2	2	1	2	2	2	1	2
1	2	2	2	2	1	1	2	1	2
1	2	2	2	2	2	#NULL!	1	1	2
1	2	1	1	1	1	1	2	1	2
1	2	2	2	2	2	2	2	1	2
1	2	1	#NULL!	2	1	1	2	1	2
1	2	2	2	2	2	2	2	2	2
1	2	#NULL!	2	#NULL!	2	#NULL!	2	#NULL!	2
1	2	1	2	1	1	1	2	1	2
1	2	1	2	2	1	2	2	2	2
1	2	2	2	2	2	1	2	1	2
1	2	2	2	2	2	2	1	1	2
1	2	2	2	2	2	2	2	2	2
1	2	2	1	2	1	1	2	1	2
1	#NULL!	#NULL!	#NULL!	#NULL!	#NULL!	#NULL!	#NULL!	#NULL!	#NULL!
1	2	1	2	2	1	1	2	1	2
2	2	2	1	2	1	2	1	2	2
2	2	2	1	2	1	2	2	1	2
1	2	1	2	2	2	1	2	1	2
1	2	2	2	2	2	2	2	2	2
1	2	1	2	2	1	1	2	1	2
1	2	#NULL!	2	2	1	1	2	2	2
1	2	1	2	1	1	1	2	2	2

1	2	1	2	1	1	1	1	1	2
1	2	1	2	2	2	2	2	2	2
1	2	2	2	2	2	1	1	1	2
1	2	2	2	2	1	1	2	1	2
1	2	2	2	2	2	1	1	1	2
1	2	1	2	2	2	2	2	1	2
1	2	1	2	1	1	1	2	2	2
1	2	2	2	2	1	1	2	1	2
1	2	2	2	2	2	2	2	2	1
1	2	2	2	2	1	2	2	2	2
1	2	1	2	2	2	1	2	2	2
1	2	2	2	2	2	2	2	2	2
1	2	1	2	2	1	1	2	2	2
1	2	1	2	2	2	2	2	2	2
1	2	2	2	2	2	2	2	2	2
1	2	2	2	2	2	2	2	2	2
1	2	2	2	2	2	2	2	2	2
1	2	2	2	2	2	2	2	2	2
2	2	1	2	2	1	1	2	2	2
1	2	1	2	1	1	1	1	1	2
1	2	1	2	1	1	1	1	1	2
#NULL!	2	1	1	2	1	1	2	1	2
1	2	1	2	2	2	1	2	1	2
1	2	1	1	2	2	2	2	2	2
1	2	2	2	2	2	2	2	2	2
#NULL!	#NULL!	#NULL!	#NULL!	#NULL!	#NULL!	#NULL!	#NULL!	#NULL!	#NULL!
#NULL!	#NULL!	#NULL!	#NULL!	#NULL!	#NULL!	#NULL!	#NULL!	#NULL!	#NULL!
1	2	#NULL!	#NULL!	#NULL!	#NULL!	#NULL!	#NULL!	#NULL!	#NULL!
#NULL!	2	1	2	2	1	1	2	1	2
1	1	#NULL!	2	#NULL!	2	1	#NULL!	2	1
1	2	2	2	2	2	1	2	1	2
1	2	2	2	2	2	1	2	1	2
1	#NULL!	#NULL!	#NULL!	#NULL!	#NULL!	#NULL!	#NULL!	#NULL!	2
#NULL!	#NULL!	#NULL!	#NULL!	#NULL!	#NULL!	#NULL!	#NULL!	#NULL!	#NULL!
1	#NULL!	#NULL!	#NULL!	#NULL!	#NULL!	#NULL!	#NULL!	#NULL!	#NULL!
1	#NULL!	#NULL!	#NULL!	#NULL!	#NULL!	#NULL!	#NULL!	#NULL!	#NULL!
1	#NULL!	#NULL!	#NULL!	#NULL!	#NULL!	#NULL!	#NULL!	#NULL!	2
1	#NULL!	#NULL!	#NULL!	#NULL!	#NULL!	#NULL!	#NULL!	#NULL!	2
1	2	#NULL!	#NULL!	#NULL!	#NULL!	#NULL!	#NULL!	#NULL!	#NULL!
2	1	2	2	1	2	1	2	1	2
1	2	1	2	2	1	1	2	1	2
1	2	#NULL!	#NULL!	#NULL!	#NULL!	#NULL!	#NULL!	#NULL!	#NULL!
1	2	#NULL!	#NULL!	#NULL!	#NULL!	#NULL!	#NULL!	#NULL!	#NULL!
1	2	2	2	2	1	2	2	2	2
1	#NULL!	#NULL!	#NULL!	#NULL!	#NULL!	#NULL!	#NULL!	#NULL!	#NULL!
1	2	#NULL!	#NULL!	#NULL!	#NULL!	#NULL!	#NULL!	#NULL!	#NULL!
1	2	#NULL!	#NULL!	#NULL!	#NULL!	#NULL!	#NULL!	#NULL!	#NULL!

1	2	1	2	2	2	1	2	1	2
1	#NULL!	#NULL!	#NULL!	#NULL!	#NULL!	#NULL!	#NULL!	#NULL!	#NULL!
1	#NULL!	2	#NULL!	#NULL!	#NULL!	#NULL!	#NULL!	#NULL!	#NULL!
1	2	#NULL!	2	1	1	1	2	2	2
1	2	#NULL!	2	1	1	1	2	2	2
1	2	#NULL!	#NULL!	#NULL!	#NULL!	#NULL!	#NULL!	#NULL!	#NULL!
1	2	#NULL!	#NULL!	#NULL!	#NULL!	#NULL!	#NULL!	#NULL!	#NULL!
1	2	#NULL!	#NULL!	#NULL!	#NULL!	#NULL!	#NULL!	#NULL!	#NULL!
1	#NULL!	#NULL!	2	#NULL!	#NULL!	#NULL!	#NULL!	#NULL!	#NULL!
1	2	#NULL!	#NULL!	#NULL!	#NULL!	#NULL!	#NULL!	#NULL!	#NULL!
1	2	1	2	2	1	1	1	1	2
1	2	1	1	1	1	2	2	2	2
1	2	1	2	2	1	2	2	1	2
1	2	2	2	2	2	2	2	2	2
1	2	2	#NULL!	1	1	1	1	1	2
1	#NULL!	#NULL!	#NULL!	#NULL!	#NULL!	#NULL!	#NULL!	#NULL!	#NULL!
1	2	2	2	2	2	2	1	1	2
1	2	2	2	2	2	2	1	1	2
1	2	1	1	1	1	2	2	2	2
1	2	1	2	1	1	1	2	2	2
1	2	1	2	1	1	1	2	2	2
1	2	2	2	2	2	2	2	2	2
1	2	#NULL!	#NULL!	#NULL!	#NULL!	#NULL!	#NULL!	#NULL!	#NULL!
1	2	#NULL!	2	2	2	2	2	2	2
1	1	2	2	1	2	2	1	1	1
1	2	1	2	2	2	1	2	2	2
1	2	1	1	1	2	1	1	1	1
1	2	1	2	2	1	1	2	1	2
1	2	1	2	2	2	2	1	1	2
2	2	2	2	2	2	2	2	1	2
1	2	2	2	2	2	1	2	1	2
1	2	2	2	2	1	2	2	1	2
1	2	2	2	2	2	2	1	1	2
1	2	2	2	2	2	1	2	1	2
1	2	2	2	2	2	2	2	2	2
1	2	1	2	2	1	1	1	2	2
1	2	1	2	1	1	1	1	1	2
1	2	1	2	2	1	1	2	1	2
1	2	1	2	1	1	1	2	1	2
1	2	1	1	1	1	1	2	1	2
1	2	1	2	2	2	#NULL!	#NULL!	#NULL!	#NULL!
1	2	2	2	2	2	2	2	1	2
1	2	2	2	1	1	1	2	2	2
1	2	1	2	2	2	2	2	1	2
1	2	#NULL!	2	2	2	2	2	1	2
1	2	1	2	2	2	1	2	2	2
1	2	1	2	2	2	2	2	1	2

1	2	1	2	2	1	1	2	1	2
1	2	1	2	2	1	2	2	1	2
1	2	2	2	2	2	1	1	1	2
1	2	1	2	2	2	2	2	1	2
1	2	1	2	2	2	2	2	1	2
1	2	1	2	2	2	2	2	1	2
1	2	1	2	2	2	2	2	1	2
1	2	1	2	2	1	1	2	1	2
1	2	2	2	2	2	1	2	1	2
1	2	1	2	2	1	1	2	2	2
1	2	2	2	2	2	2	2	2	2
1	2	1	1	2	#NULL!	1	1	2	2
1	2	1	2	2	2	1	2	1	2
1	2	1	2	2	2	1	2	1	2
1	2	1	2	2	1	1	1	1	2
1	2	1	1	2	1	1	1	2	2
2	2	2	2	2	2	2	2	2	2
1	2	2	2	2	2	2	2	2	2
2	1	2	1	2	1	1	2	1	2
1	2	2	2	2	2	2	2	1	2
1	2	1	2	2	1	2	2	1	2
1	2	1	2	2	1	1	2	1	2
1	2	1	2	2	2	2	1	1	2
1	2	2	2	2	2	2	2	2	2
1	2	2	2	2	2	2	2	2	2
1	2	1	2	2	1	1	2	1	2
2	2	2	2	2	1	1	1	1	2
2	2	2	2	2	2	2	2	2	2
1	2	2	2	2	2	2	2	1	2
1	2	2	2	2	2	2	2	2	2
2	2	2	1	1	2	1	2	2	1
1	2	2	2	2	1	2	1	1	2
1	2	1	2	2	1	1	2	2	2
1	2	2	2	2	2	2	2	2	2
1	2	1	2	2	2	2	1	2	2
1	2	1	2	2	2	2	1	1	2
1	1	1	1	1	2	2	2	2	2
1	1	1	1	1	2	2	2	2	2
1	2	2	1	2	1	2	2	2	2
1	2	1	2	2	1	2	1	1	2
1	2	1	2	2	1	2	2	2	2
1	2	2	2	2	1	1	2	1	2
1	2	2	2	2	1	1	2	1	2
1	2	2	2	2	2	2	2	2	2
1	2	1	1	1	1	2	2	1	2
1	2	2	2	2	2	2	2	1	2
1	2	1	2	1	1	1	1	1	#NULL!

1	2	2	2	2	2	2	2	2	2
1	2	1	2	2	2	2	2	2	2
1	2	1	2	1	1	1	1	1	2
1	1	1	2	1	1	2	2	2	2
1	2	1	2	2	1	2	2	1	2
1	2	2	2	2	1	2	2	1	2
1	2	1	2	1	1	2	2	1	2
1	2	2	2	2	2	2	2	2	2
1	1	2	1	1	2	2	2	2	2
1	2	1	2	2	1	1	2	1	2
1	2	2	2	2	2	2	2	2	2
1	2	1	1	1	1	1	2	1	2
#NULL!	2	#NULL!	#NULL!	#NULL!	#NULL!	#NULL!	#NULL!	#NULL!	#NULL!
1	2	1	1	1	2	2	2	2	2
1	2	1	2	1	1	1	1	1	2
2	2	2	2	2	2	2	2	2	2
1	2	2	2	2	1	1	2	1	2
1	2	1	2	2	1	1	2	1	2
1	2	1	2	2	1	2	2	1	2
1	2	2	2	2	2	2	2	1	2
1	2	1	2	1	2	2	1	1	2
1	2	1	2	1	1	1	2	2	2
1	2	2	2	2	1	2	2	1	2
1	2	1	2	2	1	1	2	1	2
1	2	2	2	2	2	2	2	2	2
1	2	1	2	2	1	1	2	1	2
1	2	1	2	2	1	1	2	1	2
2	1	2	2	1	2	1	2	1	2
1	2	2	2	2	1	2	2	2	2
1	2	#NULL!	#NULL!	#NULL!	#NULL!	#NULL!	#NULL!	#NULL!	#NULL!
1	2	2	2	2	1	2	2	2	2
1	#NULL!	#NULL!	#NULL!	#NULL!	#NULL!	#NULL!	#NULL!	#NULL!	#NULL!
1	2	2	2	1	2	2	2	2	2
1	2	2	2	2	2	2	2	1	2
1	2	1	1	2	1	1	2	2	2
1	2	2	2	2	2	2	2	2	2
2	2	2	2	2	2	2	2	2	2
1	#NULL!	#NULL!	#NULL!	#NULL!	#NULL!	#NULL!	2	#NULL!	#NULL!
1	2	2	1	2	2	2	1	2	1
2	2	2	2	2	2	2	2	2	2
1	2	2	2	2	2	2	2	2	2
1	1	1	2	1	1	1	2	1	1
1	1	1	2	2	1	1	2	2	2
1	2	#NULL!	2	2	#NULL!	1	#NULL!	#NULL!	2
2	2	2	2	2	2	2	2	2	2
1	2	2	2	2	2	2	2	2	2
1	2	1	2	2	2	1	1	2	2

1	2	1	2	1	1	2	2	2	2
1	2	2	2	2	2	2	2	2	2
1	2	2	2	2	2	2	2	2	2
1	2	2	2	2	2	2	2	2	2
2	2	2	2	2	2	2	2	2	2
1	2	1	2	2	1	1	2	1	2
1	2	1	2	2	1	2	2	2	2
1	2	#NULL!	#NULL!	#NULL!	#NULL!	#NULL!	#NULL!	#NULL!	#NULL!
1	2	1	2	1	1	2	2	2	2
1	2	2	2	2	2	2	2	2	2
1	2	1	2	2	2	2	2	2	1
1	2	2	2	2	2	1	2	1	2
1	2	2	2	2	2	2	2	2	2
1	2	2	2	2	2	2	2	2	2
1	2	1	1	1	1	1	2	1	1
1	2	1	1	1	1	1	2	1	1
1	2	2	2	2	2	2	2	2	2
1	2	1	2	2	2	2	1	2	2
1	2	1	1	2	1	2	2	1	2
1	2	2	2	2	2	2	2	#NULL!	2
1	2	2	2	2	2	1	2	1	1
1	2	2	2	2	2	2	2	2	2
1	2	2	2	2	2	2	2	2	2
1	2	1	2	2	2	2	2	1	2
1	2	2	2	2	1	1	2	1	2
1	2	2	2	2	2	2	2	2	2
1	2	2	2	2	2	2	2	2	2
1	2	2	2	2	2	2	2	2	2
1	2	2	2	2	2	2	2	2	2
1	#NULL!	#NULL!	#NULL!	#NULL!	2	#NULL!	#NULL!	#NULL!	#NULL!
1	2	2	2	2	2	2	2	2	2
1	2	1	1	2	2	2	2	2	2
1	2	2	2	2	2	2	2	2	2
1	2	2	2	2	2	2	2	1	2
1	#NULL!	#NULL!	#NULL!	#NULL!	#NULL!	#NULL!	#NULL!	#NULL!	#NULL!
1	2	2	2	2	2	2	2	2	2
1	2	2	2	2	2	2	2	2	2
#NULL!	2	#NULL!	#NULL!	2	#NULL!	#NULL!	#NULL!	2	2
1	2	1	1	1	2	1	1	2	2
1	2	2	2	2	2	2	2	1	2
1	2	1	2	2	2	2	2	1	2
1	2	1	2	2	2	2	2	1	2
2	2	2	2	2	2	2	2	2	2
1	2	1	2	2	1	1	1	2	1
1	2	1	2	1	1	1	2	1	2
1	2	1	2	2	2	2	2	2	2
1	2	1	1	2	1	1	2	1	2

1	2	1	2	1	1	2	2	1	2
1	2	1	2	1	1	2	2	1	2
1	2	1	2	2	1	2	2	1	2
1	2	1	2	2	2	2	2	2	2
1	2	1	1	2	1	1	2	2	2
1	2	1	1	2	1	1	1	2	2
1	2	1	2	2	2	2	1	1	2
1	2	2	2	2	2	2	2	2	2
1	2	1	1	1	1	2	#NULL!	2	2
1	2	1	2	2	2	2	1	2	2
1	2	1	2	2	1	1	1	1	2
1	2	1	2	2	2	2	2	1	1
1	2	1	2	2	2	2	2	1	1
1	2	2	1	1	1	2	2	2	2
1	2	1	2	2	2	1	2	1	2
1	2	1	2	2	1	1	2	1	2
2	2	2	2	2	2	2	2	1	2
1	2	2	2	2	2	1	2	2	2
1	2	1	2	2	1	1	2	1	2
1	2	1	2	2	1	1	1	1	2
1	2	2	2	2	2	2	2	2	2
1	2	1	1	2	1	1	2	1	2
1	2	#NULL!	#NULL!	1	1	1	2	2	2
1	#NULL!	2	#NULL!	1	1	1	2	2	2
1	2	#NULL!	#NULL!	#NULL!	#NULL!	#NULL!	#NULL!	#NULL!	#NULL!
1	#NULL!	#NULL!	2	#NULL!	#NULL!	#NULL!	#NULL!	#NULL!	#NULL!
1	2	1	1	2	1	1	2	1	2
1	#NULL!	#NULL!	#NULL!	#NULL!	#NULL!	#NULL!	#NULL!	#NULL!	#NULL!
1	2	#NULL!	#NULL!	#NULL!	#NULL!	#NULL!	#NULL!	#NULL!	#NULL!
2	#NULL!	#NULL!	2	#NULL!	#NULL!	#NULL!	#NULL!	#NULL!	#NULL!
1	#NULL!	#NULL!	2	#NULL!	#NULL!	#NULL!	#NULL!	#NULL!	#NULL!
1	#NULL!	#NULL!	2	#NULL!	#NULL!	#NULL!	#NULL!	#NULL!	#NULL!
1	2	#NULL!	#NULL!	#NULL!	#NULL!	#NULL!	#NULL!	#NULL!	#NULL!
1	2	#NULL!	#NULL!	#NULL!	#NULL!	#NULL!	#NULL!	#NULL!	#NULL!
1	2	2	2	2	2	2	2	1	1
1	2	1	2	2	1	1	2	1	2
1	2	2	2	2	2	2	2	2	2
1	2	1	2	2	2	2	1	2	2
1	2	2	2	2	2	2	2	2	2
1	2	2	2	2	2	2	2	2	2
1	2	1	1	2	#NULL!	1	1	2	2
1	2	2	2	1	2	2	2	2	2
1	2	2	2	2	1	2	2	2	2
1	2	1	2	2	2	2	2	2	2
1	2	2	2	2	2	1	2	2	2
1	2	1	2	2	1	2	2	1	2
1	2	2	2	2	#NULL!	#NULL!	2	2	#NULL!

1	2	2	2	2	2	2	2	2	2
1	2	2	2	2	2	2	2	2	2
1	2	2	1	1	1	2	2	2	2
1	1	2	2	2	2	2	1	2	2
2	2	2	2	2	2	2	2	2	2
1	2	1	2	2	1	1	2	1	2
#NULL!	#NULL!	#NULL!	#NULL!	#NULL!	#NULL!	#NULL!	#NULL!	#NULL!	#NULL!
1	2	2	2	2	2	2	2	2	2
1	2	2	2	2	2	2	2	2	2
1	2	2	2	2	2	2	2	1	1
1	2	2	2	2	2	2	2	2	2
1	2	2	2	2	1	1	2	1	2
1	2	2	2	2	1	2	1	2	2
2	1	1	1	1	1	1	1	1	1
1	2	2	2	2	2	2	2	2	2
1	2	2	2	2	1	2	2	2	2
#NULL!	#NULL!	#NULL!	#NULL!	#NULL!	#NULL!	#NULL!	#NULL!	#NULL!	#NULL!
1	2	2	2	1	2	2	2	1	2
1	2	2	2	2	2	2	2	2	2
1	2	2	2	2	2	2	2	2	2
1	2	2	2	2	2	2	2	2	2
1	2	2	2	2	2	2	2	2	2
1	2	2	2	2	2	2	2	2	2
2	2	2	2	2	2	2	2	2	2
1	2	2	2	2	2	2	2	2	2
2	2	1	2	2	1	2	2	1	2
1	2	2	2	2	1	2	2	1	2
1	2	2	2	2	2	2	2	2	2
2	2	2	2	2	2	2	2	2	2
1	2	2	2	2	2	2	2	2	2
1	2	2	2	1	2	2	2	2	2
2	2	2	2	2	2	2	2	2	2
1	2	1	2	2	2	2	2	2	2
1	2	2	2	2	2	2	2	2	2
1	#NULL!	#NULL!	#NULL!	#NULL!	#NULL!	#NULL!	#NULL!	#NULL!	2
#NULL!	2	2	2	2	2	2	2	2	2
1	2	2	2	2	2	2	2	2	2
1	2	2	2	2	2	2	2	2	2
1	2	2	2	2	1	1	2	2	2
1	2	1	2	2	2	1	2	1	#NULL!
1	2	2	2	2	2	2	2	2	2
1	2	2	2	2	2	2	2	2	2

vacb4sex2	earlysex2	multipartn	smoke2	alcohol2	over4baby	highfat2	birthpill2	attack2	familyhx2
2	2	2	2	2	2	2	2	2	2
1	2	1	2	1	2	1	2	1	2
2	2	2	2	2	2	2	2	2	2
1	2	2	2	2	2	2	2	2	2
2	2	2	2	2	2	2	2	2	2
2	2	2	2	2	2	2	2	2	2
2	2	2	1	2	2	2	1	2	2
2	2	2	2	1	1	2	2	2	2
1	2	2	2	#NULL!	2	2	2	#NULL!	2
2	1	2	2	#NULL!	2	1	2	2	1
2	2	2	2	2	2	2	2	2	2
2	2	2	2	2	2	2	1	2	2
2	2	2	2	1	1	1	2	2	2
2	1	2	2	2	1	1	1	1	2
2	2	2	2	2	2	2	2	2	2
2	2	2	2	2	2	2	2	2	2
#NULL!	#NULL!	#NULL!	#NULL!	#NULL!	#NULL!	#NULL!	#NULL!	#NULL!	#NULL!
2	2	2	2	2	2	1	2	2	2
#NULL!	#NULL!	#NULL!	#NULL!	#NULL!	#NULL!	#NULL!	#NULL!	#NULL!	#NULL!
#NULL!	#NULL!	#NULL!	#NULL!	#NULL!	#NULL!	#NULL!	#NULL!	#NULL!	#NULL!
2	2	2	2	2	2	2	2	2	2
2	2	2	2	2	2	2	2	2	2
2	2	2	2	2	2	2	2	2	1
2	2	2	2	2	2	2	2	2	2
#NULL!	2	#NULL!	#NULL!	#NULL!	#NULL!	#NULL!	#NULL!	#NULL!	#NULL!
2	2	2	2	2	2	2	2	2	2
2	2	1	1	2	1	1	2	1	2
2	2	2	2	2	2	2	2	2	2
1	2	2	2	2	2	2	1	2	2
#NULL!	2	2	1	2	2	2	2	2	1
1	1	2	2	2	2	1	1	1	2
2	2	2	2	2	1	2	2	2	2
1	1	1	1	1	1	1	1	1	1
2	2	#NULL!	2	2	2	2	2	2	2
2	2	2	2	2	2	2	2	2	2
2	1	1	2	2	2	1	1	1	1
2	2	2	2	2	2	2	2	2	2
2	2	#NULL!	2	2	2	2	1	1	1
2	2	2	2	2	2	2	2	1	1
2	1	2	2	2	2	2	2	2	1
2	2	2	2	2	2	2	2	2	2
#NULL!	2	#NULL!	#NULL!	#NULL!	#NULL!	2	#NULL!	2	#NULL!
#NULL!	1	#NULL!	#NULL!	#NULL!	#NULL!	#NULL!	#NULL!	#NULL!	#NULL!
2	2	2	2	2	2	2	2	2	2
2	2	2	2	2	2	2	2	2	1
#NULL!	#NULL!	#NULL!	#NULL!	#NULL!	#NULL!	#NULL!	#NULL!	#NULL!	#NULL!

2	2	2	2	2	2	2	2	2	1
2	2	2	2	2	2	2	2	2	2
2	2	2	2	2	2	2	2	2	2
2	2	2	2	2	2	2	2	2	2
1	2	#NULL!	2	1	1	1	1	1	1
1	2	#NULL!	#NULL!	1	1	1	1	1	1
2	2	1	1	2	2	2	1	2	2
2	2	2	2	2	2	2	2	2	2
1	2	2	2	2	2	2	2	2	2
2	2	1	2	2	1	2	2	1	2
#NULL!	#NULL!	2	#NULL!	#NULL!	#NULL!	#NULL!	#NULL!	#NULL!	#NULL!
2	2	2	2	2	2	2	2	2	2
1	2	2	2	2	2	2	2	2	2
2	2	2	2	2	2	1	1	2	2
2	2	2	2	2	2	2	2	2	2
1	2	2	2	1	2	2	2	2	2
1	2	2	2	2	2	2	1	1	1
2	2	2	1	1	2	1	2	#NULL!	2
2	2	2	2	2	2	2	2	2	2
2	2	2	2	2	2	1	#NULL!	2	2
2	2	2	2	2	2	2	2	2	2
2	2	2	2	2	2	1	1	1	2
2	2	2	2	2	2	2	2	1	1
2	2	2	2	2	2	2	2	2	2
2	2	2	2	2	2	2	2	2	2
2	2	2	2	2	2	2	2	2	2
1	1	1	1	1	1	1	2	1	1
2	2	2	2	1	1	2	2	2	2
2	2	2	2	2	1	2	2	2	2
1	1	1	2	1	1	1	2	1	1
#NULL!	#NULL!	#NULL!	#NULL!	#NULL!	#NULL!	#NULL!	#NULL!	#NULL!	#NULL!
2	2	2	2	2	1	1	2	2	1
2	2	1	#NULL!	#NULL!	#NULL!	#NULL!	#NULL!	#NULL!	2
2	2	2	2	2	2	2	2	2	2
2	2	2	2	1	1	1	#NULL!	#NULL!	1
2	2	2	2	2	2	2	2	2	2
#NULL!	2	2	2	2	2	2	2	#NULL!	2
2	2	2	2	2	1	2	2	2	2
2	2	1	1	2	2	2	2	1	2
1	2	2	1	2	2	2	2	2	2
2	2	2	2	2	2	2	2	2	2
#NULL!	#NULL!	#NULL!	#NULL!	#NULL!	#NULL!	#NULL!	#NULL!	#NULL!	#NULL!
2	2	2	2	1	#NULL!	#NULL!	#NULL!	2	2
2	1	2	2	2	2	2	1	2	2
2	2	2	2	2	2	2	2	2	2
1	1	2	2	2	2	1	2	1	2
2	2	1	2	1	1	2	2	2	2

2	2	2	1	2	2	1	2	2	2
1	1	1	1	1	1	1	1	1	1
2	2	2	2	2	2	2	2	2	2
1	2	2	2	2	2	2	2	2	2
2	2	2	2	2	2	2	2	2	2
1	2	2	2	2	2	2	2	2	1
2	2	2	2	2	2	2	2	2	2
#NULL!	#NULL!	#NULL!	#NULL!	#NULL!	#NULL!	#NULL!	#NULL!	#NULL!	#NULL!
2	#NULL!	2	2	2	2	2	2	2	2
2	2	2	2	2	1	2	2	1	2
2	2	2	2	2	2	1	2	2	1
2	2	2	2	2	2	2	2	1	2
2	2	2	1	2	2	2	2	2	2
2	2	2	2	2	2	2	2	2	2
2	1	2	2	2	2	1	1	2	2
2	1	#NULL!	2	2	2	2	2	1	2
2	2	2	2	2	2	2	2	2	2
2	2	2	2	2	1	1	2	1	2
2	2	2	2	2	2	#NULL!	2	2	#NULL!
2	2	2	2	2	2	2	2	2	2
1	2	1	2	1	2	1	2	1	2
2	2	2	2	2	2	2	2	2	2
2	2	2	1	2	2	2	2	2	2
2	2	2	2	2	2	2	2	2	2
1	2	2	2	2	2	2	2	2	2
2	2	2	2	2	2	2	1	1	1
2	2	2	2	2	1	2	1	2	1
2	2	2	2	2	2	2	2	2	2
1	2	2	2	2	2	2	2	2	2
2	2	1	2	2	1	2	2	2	1
1	2	2	2	2	2	1	2	2	2
2	2	2	1	2	2	2	2	2	2
2	1	2	2	2	1	2	2	2	2
2	2	2	2	2	2	2	2	2	2
1	2	1	2	2	2	2	#NULL!	1	2
2	2	2	2	2	2	2	1	2	2
1	2	2	2	2	2	2	2	#NULL!	2
2	2	2	2	2	2	2	2	1	1
#NULL!	#NULL!	#NULL!	#NULL!	#NULL!	#NULL!	#NULL!	#NULL!	#NULL!	#NULL!
1	1	1	1	1	1	1	1	1	1
2	2	2	2	2	1	2	2	2	2
#NULL!	#NULL!	#NULL!	#NULL!	#NULL!	#NULL!	#NULL!	#NULL!	#NULL!	#NULL!
2	2	2	2	2	2	2	2	2	2
2	2	2	2	2	2	1	2	2	2
#NULL!	#NULL!	#NULL!	#NULL!	#NULL!	#NULL!	#NULL!	#NULL!	#NULL!	#NULL!
2	1	2	2	2	2	1	1	1	1
2	2	2	2	#NULL!	2	2	2	2	2

2	1	1	1	1	1	#NULL!	2	2	2
2	2	2	2	2	2	2	2	2	1
1	1	1	1	1	1	1	1	1	1
2	2	2	2	2	2	2	2	2	2
1	2	2	2	2	2	2	2	1	1
1	2	2	#NULL!	2	1	2	1	2	1
1	2	#NULL!	1	2	1	2	1	2	1
1	2	2	1	2	1	2	2	1	2
#NULL!	2	#NULL!	#NULL!	#NULL!	#NULL!	#NULL!	#NULL!	#NULL!	#NULL!
2	2	2	2	2	2	2	2	2	2
1	2	1	2	2	2	2	2	1	1
2	2	2	2	2	2	2	2	2	2
2	2	2	2	2	2	2	2	2	2
2	2	2	2	2	2	2	2	1	2
2	2	2	2	1	2	2	2	2	2
2	2	2	2	2	1	2	2	2	1
2	2	2	2	2	2	2	2	2	2
2	2	2	1	2	2	2	2	2	2
#NULL!	2	#NULL!	#NULL!	#NULL!	#NULL!	#NULL!	#NULL!	#NULL!	#NULL!
2	2	2	2	2	1	2	2	2	2
2	2	2	2	2	2	2	2	2	2
2	2	2	2	2	2	2	2	1	2
#NULL!	1	1	1	1	1	1	1	1	1
2	2	2	2	2	#NULL!	2	2	2	2
#NULL!	1	2	2	2	2	2	1	1	1
1	1	#NULL!	2	2	2	1	2	1	1
1	2	2	2	2	1	1	2	#NULL!	#NULL!
2	2	2	2	2	1	1	1	1	1
2	2	2	2	2	2	2	1	1	2
2	2	2	2	2	1	1	1	1	1
2	2	2	2	2	2	2	2	2	2
1	2	2	2	2	2	2	2	2	2
2	2	2	2	2	2	2	2	2	2
1	1	1	1	1	1	2	2	2	2
2	2	2	2	2	2	2	2	2	1
2	1	1	1	1	1	2	2	2	2
2	1	1	1	1	1	1	1	1	1
2	2	2	1	1	1	1	2	1	1
2	2	2	2	2	1	1	1	2	2
1	2	2	2	2	2	2	2	2	2
#NULL!	#NULL!	#NULL!	#NULL!	#NULL!	#NULL!	#NULL!	#NULL!	#NULL!	#NULL!
2	2	2	2	2	2	2	2	2	2
2	2	2	2	2	2	2	2	2	2
#NULL!	#NULL!	#NULL!	#NULL!	#NULL!	#NULL!	#NULL!	#NULL!	#NULL!	#NULL!
#NULL!	#NULL!	#NULL!	#NULL!	#NULL!	#NULL!	#NULL!	#NULL!	#NULL!	#NULL!
2	2	2	2	1	1	1	1	2	2
1	2	2	2	1	1	1	1	1	2

#NULL!	#NULL!	#NULL!	#NULL!	2	#NULL!	#NULL!	#NULL!	#NULL!	#NULL!
#NULL!	#NULL!	#NULL!	#NULL!	#NULL!	#NULL!	#NULL!	2	#NULL!	#NULL!
#NULL!	#NULL!	2	#NULL!	#NULL!	#NULL!	#NULL!	#NULL!	#NULL!	#NULL!
#NULL!	#NULL!	#NULL!	#NULL!	#NULL!	#NULL!	#NULL!	2	#NULL!	#NULL!
#NULL!	2	#NULL!	#NULL!	#NULL!	#NULL!	#NULL!	#NULL!	#NULL!	#NULL!
#NULL!	#NULL!	#NULL!	#NULL!	#NULL!	#NULL!	#NULL!	#NULL!	#NULL!	#NULL!
2	2	2	2	2	2	2	2	2	2
#NULL!	#NULL!	#NULL!	#NULL!	#NULL!	#NULL!	#NULL!	#NULL!	#NULL!	#NULL!
2	2	2	2	2	1	1	1	2	2
#NULL!	#NULL!	#NULL!	#NULL!	#NULL!	2	#NULL!	#NULL!	#NULL!	#NULL!
#NULL!	#NULL!	#NULL!	#NULL!	#NULL!	#NULL!	2	#NULL!	#NULL!	#NULL!
#NULL!	#NULL!	#NULL!	#NULL!	#NULL!	2	#NULL!	#NULL!	#NULL!	#NULL!
2	2	2	2	2	2	2	2	2	2
2	2	2	1	2	2	1	2	2	1
#NULL!	#NULL!	2	#NULL!	#NULL!	#NULL!	#NULL!	#NULL!	#NULL!	#NULL!
2	2	2	2	2	2	2	1	1	1
1	1	1	1	1	1	1	1	1	1
2	1	2	2	2	2	2	2	2	2
2	2	2	2	2	2	2	2	#NULL!	2
2	2	2	2	2	2	2	2	2	2
2	2	2	2	2	1	2	2	2	2
#NULL!	#NULL!	#NULL!	#NULL!	#NULL!	#NULL!	#NULL!	#NULL!	#NULL!	#NULL!
2	2	2	2	2	1	2	2	2	2
1	2	2	2	2	1	2	2	2	2
2	2	2	2	2	2	2	2	2	2
2	2	2	2	2	2	2	2	2	2
2	2	2	2	2	1	2	2	2	2
2	2	2	2	2	2	2	2	2	2
1	1	1	1	1	1	1	1	1	1
2	2	2	2	2	1	1	1	2	1
2	2	2	2	2	2	2	2	2	2
2	2	2	2	2	2	2	2	2	2
#NULL!	#NULL!	#NULL!	#NULL!	#NULL!	#NULL!	#NULL!	#NULL!	#NULL!	#NULL!
1	2	2	2	2	2	2	2	2	2
2	2	2	2	2	2	1	1	2	2
2	2	2	2	2	2	1	2	2	2
2	2	1	2	2	1	2	2	1	2
2	2	2	2	2	2	2	2	2	2
#NULL!	#NULL!	2	#NULL!	#NULL!	#NULL!	#NULL!	#NULL!	#NULL!	#NULL!
2	2	2	2	2	2	2	2	2	2
2	2	2	2	2	2	1	2	2	2
1	2	2	2	1	2	1	2	2	2
2	2	2	2	2	2	1	1	1	2
2	2	2	2	2	2	2	2	2	2
2	2	2	2	2	2	2	2	2	2
2	2	2	2	2	1	1	2	2	2
#NULL!	#NULL!	#NULL!	#NULL!	#NULL!	#NULL!	#NULL!	#NULL!	#NULL!	#NULL!

2	2	2	2	#NULL!	2	2	2	2	2
2	2	2	2	2	1	1	1	2	2
2	2	2	2	2	2	2	2	2	2
#NULL!	#NULL!	#NULL!	#NULL!	#NULL!	#NULL!	#NULL!	#NULL!	#NULL!	#NULL!
2	2	2	2	2	2	2	2	2	2
2	2	2	2	2	1	2	2	2	2
2	2	1	1	1	1	1	1	2	2
2	2	2	2	2	1	2	2	2	2
2	2	2	2	2	2	1	2	2	2
2	2	2	2	2	2	2	2	2	2
2	2	2	2	2	2	2	2	2	2
#NULL!	1	#NULL!	#NULL!	#NULL!	#NULL!	#NULL!	#NULL!	#NULL!	#NULL!
#NULL!	2	#NULL!	#NULL!	#NULL!	#NULL!	#NULL!	#NULL!	#NULL!	#NULL!
#NULL!	2	#NULL!	#NULL!	#NULL!	#NULL!	#NULL!	#NULL!	#NULL!	#NULL!
2	2	#NULL!	#NULL!	#NULL!	#NULL!	2	#NULL!	#NULL!	2
#NULL!	#NULL!	#NULL!	#NULL!	#NULL!	#NULL!	#NULL!	#NULL!	#NULL!	#NULL!
#NULL!	#NULL!	#NULL!	#NULL!	#NULL!	#NULL!	#NULL!	#NULL!	#NULL!	#NULL!
#NULL!	#NULL!	#NULL!	#NULL!	#NULL!	#NULL!	#NULL!	#NULL!	#NULL!	#NULL!
2	#NULL!	2	#NULL!	2	#NULL!	#NULL!	2	#NULL!	2
#NULL!	#NULL!	#NULL!	#NULL!	#NULL!	#NULL!	#NULL!	#NULL!	#NULL!	#NULL!
#NULL!	#NULL!	#NULL!	#NULL!	#NULL!	#NULL!	#NULL!	#NULL!	#NULL!	#NULL!
2	2	2	2	2	2	2	2	2	2
2	2	2	2	2	2	2	2	2	2
2	2	2	2	2	2	2	2	2	2
2	2	2	2	2	2	2	2	2	2
#NULL!	#NULL!	#NULL!	#NULL!	#NULL!	#NULL!	#NULL!	#NULL!	#NULL!	#NULL!
2	2	2	2	2	2	2	1	2	2
#NULL!	#NULL!	#NULL!	#NULL!	#NULL!	#NULL!	#NULL!	#NULL!	#NULL!	#NULL!
#NULL!	#NULL!	#NULL!	#NULL!	#NULL!	#NULL!	#NULL!	#NULL!	#NULL!	#NULL!
2	2	2	2	2	2	2	2	2	2
#NULL!	#NULL!	#NULL!	#NULL!	#NULL!	#NULL!	#NULL!	#NULL!	#NULL!	#NULL!
#NULL!	#NULL!	#NULL!	#NULL!	#NULL!	#NULL!	#NULL!	#NULL!	#NULL!	#NULL!
#NULL!	#NULL!	#NULL!	#NULL!	#NULL!	#NULL!	#NULL!	#NULL!	#NULL!	#NULL!
#NULL!	#NULL!	#NULL!	#NULL!	#NULL!	#NULL!	#NULL!	#NULL!	#NULL!	#NULL!
2	#NULL!	1	2	#NULL!	2	#NULL!	2	#NULL!	1
2	#NULL!	2	#NULL!	#NULL!	#NULL!	2	#NULL!	#NULL!	#NULL!
#NULL!	#NULL!	#NULL!	#NULL!	#NULL!	#NULL!	#NULL!	#NULL!	#NULL!	#NULL!
#NULL!	#NULL!	#NULL!	#NULL!	#NULL!	#NULL!	#NULL!	#NULL!	#NULL!	#NULL!
#NULL!	#NULL!	#NULL!	#NULL!	#NULL!	#NULL!	#NULL!	#NULL!	#NULL!	#NULL!
#NULL!	1	#NULL!	#NULL!	#NULL!	#NULL!	#NULL!	#NULL!	#NULL!	#NULL!
#NULL!	2	#NULL!	#NULL!	#NULL!	#NULL!	#NULL!	#NULL!	#NULL!	#NULL!
#NULL!	#NULL!	#NULL!	#NULL!	#NULL!	#NULL!	#NULL!	#NULL!	#NULL!	#NULL!
1	1	1	1	1	1	1	1	1	1
2	2	2	1	2	2	1	2	2	2
2	2	2	1	2	2	1	2	2	2
#NULL!	2	#NULL!	#NULL!	#NULL!	#NULL!	#NULL!	2	#NULL!	#NULL!
#NULL!	2	#NULL!	#NULL!	#NULL!	#NULL!	#NULL!	#NULL!	#NULL!	#NULL!

#NULL!	#NULL!	2	#NULL!	#NULL!	#NULL!	#NULL!	#NULL!	#NULL!	#NULL!
#NULL!	1	#NULL!	#NULL!	#NULL!	#NULL!	#NULL!	#NULL!	#NULL!	#NULL!
1	1	1	1	1	1	1	2	2	2
1	1	2	2	1	2	2	1	2	2
2	1	2	2	2	1	2	1	2	1
2	2	2	2	2	2	2	2	1	1
2	2	2	1	1	2	2	2	2	1
2	2	1	2	2	2	2	1	2	2
2	2	2	2	2	2	2	1	1	1
1	1	1	1	1	1	1	1	1	1
#NULL!	#NULL!	#NULL!	#NULL!	#NULL!	#NULL!	#NULL!	#NULL!	#NULL!	#NULL!
#NULL!	#NULL!	#NULL!	#NULL!	#NULL!	#NULL!	#NULL!	#NULL!	#NULL!	#NULL!
#NULL!	#NULL!	#NULL!	#NULL!	#NULL!	#NULL!	#NULL!	#NULL!	#NULL!	#NULL!
#NULL!	#NULL!	2	#NULL!	#NULL!	#NULL!	#NULL!	#NULL!	#NULL!	#NULL!
1	2	2	2	2	2	2	2	2	2
2	2	2	2	2	1	1	1	1	2
#NULL!	#NULL!	#NULL!	#NULL!	#NULL!	#NULL!	#NULL!	#NULL!	#NULL!	#NULL!
2	2	2	2	2	2	2	2	2	2
2	2	2	2	2	2	2	2	2	2
2	2	2	2	2	2	2	2	2	2
2	2	2	2	1	2	2	1	2	2
2	2	2	2	2	2	2	2	2	2
1	1	#NULL!	#NULL!	#NULL!	#NULL!	#NULL!	#NULL!	#NULL!	#NULL!
2	2	2	2	2	2	2	2	2	2
2	2	2	2	2	2	2	2	1	1
2	2	2	2	2	2	2	2	2	2
2	2	2	2	2	2	2	2	2	2
1	1	2	2	2	2	2	1	1	1
1	2	2	2	2	2	2	2	2	2
2	2	2	2	2	2	2	2	2	2
2	2	2	2	2	2	2	2	2	2
2	2	2	2	2	2	2	2	2	2
1	1	1	1	1	1	1	1	1	1
1	1	2	#NULL!	2	2	2	2	2	2
2	1	1	2	2	2	2	1	1	1
2	2	2	2	1	#NULL!	2	2	1	2
#NULL!	#NULL!	#NULL!	#NULL!	#NULL!	#NULL!	#NULL!	#NULL!	#NULL!	#NULL!
2	2	2	2	#NULL!	2	2	2	2	2
1	1	1	1	1	1	1	1	1	1
1	1	1	1	1	1	1	1	1	1
#NULL!	2	#NULL!	#NULL!	#NULL!	#NULL!	#NULL!	#NULL!	#NULL!	#NULL!
#NULL!	#NULL!	#NULL!	#NULL!	#NULL!	#NULL!	#NULL!	#NULL!	#NULL!	#NULL!
#NULL!	#NULL!	#NULL!	#NULL!	1	#NULL!	#NULL!	#NULL!	#NULL!	#NULL!
2	2	2	2	2	2	2	2	2	2
2	2	2	2	2	2	2	2	2	2
2	2	2	2	2	2	2	2	2	2
1	2	2	2	2	2	2	2	2	1

#NULL!	#NULL!	#NULL!	#NULL!	#NULL!	#NULL!	#NULL!	2	#NULL!	#NULL!
#NULL!	2	2	#NULL!	#NULL!	#NULL!	#NULL!	#NULL!	#NULL!	#NULL!
#NULL!	#NULL!	#NULL!	#NULL!	#NULL!	#NULL!	2	#NULL!	#NULL!	#NULL!
#NULL!	#NULL!	#NULL!	2	#NULL!	#NULL!	#NULL!	#NULL!	#NULL!	#NULL!
#NULL!	#NULL!	#NULL!	#NULL!	#NULL!	#NULL!	2	#NULL!	#NULL!	#NULL!
2	1	1	2	2	2	2	2	2	2
#NULL!	#NULL!	#NULL!	#NULL!	#NULL!	#NULL!	#NULL!	2	#NULL!	#NULL!
#NULL!	2	#NULL!	#NULL!	#NULL!	#NULL!	#NULL!	#NULL!	#NULL!	#NULL!
#NULL!	2	#NULL!	#NULL!	#NULL!	#NULL!	#NULL!	#NULL!	#NULL!	#NULL!
#NULL!	2	#NULL!	#NULL!	#NULL!	#NULL!	#NULL!	#NULL!	#NULL!	#NULL!
#NULL!	#NULL!	#NULL!	#NULL!	#NULL!	#NULL!	#NULL!	#NULL!	2	#NULL!
#NULL!	#NULL!	#NULL!	#NULL!	#NULL!	#NULL!	#NULL!	#NULL!	#NULL!	1
#NULL!	2	#NULL!	#NULL!	#NULL!	#NULL!	#NULL!	#NULL!	#NULL!	#NULL!
#NULL!	#NULL!	#NULL!	#NULL!	#NULL!	#NULL!	#NULL!	1	#NULL!	#NULL!
#NULL!	#NULL!	#NULL!	2	#NULL!	#NULL!	#NULL!	#NULL!	#NULL!	#NULL!
#NULL!	#NULL!	#NULL!	#NULL!	#NULL!	#NULL!	#NULL!	#NULL!	#NULL!	#NULL!
#NULL!	#NULL!	#NULL!	#NULL!	2	#NULL!	#NULL!	#NULL!	#NULL!	#NULL!
#NULL!	2	2	1	2	1	2	2	2	2
2	2	2	2	2	2	1	2	2	2
2	2	2	2	2	2	2	2	2	2
2	2	2	2	1	1	2	2	1	1
2	2	2	2	2	2	2	2	2	2
2	2	2	2	2	2	2	2	2	2
1	2	1	2	2	2	1	2	1	2
1	2	1	2	2	2	2	2	1	2
#NULL!	#NULL!	#NULL!	#NULL!	#NULL!	#NULL!	#NULL!	#NULL!	#NULL!	#NULL!
2	2	2	2	2	2	1	2	2	1
2	1	1	1	1	1	1	1	1	2
2	2	2	2	2	2	2	2	2	1
2	2	2	2	2	2	2	2	2	2
2	2	2	2	2	2	2	2	#NULL!	1
2	2	2	2	2	2	2	2	2	2
2	2	2	2	2	2	2	2	1	1
2	2	2	2	2	2	2	2	2	2
#NULL!	#NULL!	#NULL!	#NULL!	#NULL!	#NULL!	#NULL!	#NULL!	#NULL!	#NULL!
2	2	2	2	2	2	2	2	2	2
#NULL!	#NULL!	#NULL!	#NULL!	#NULL!	#NULL!	#NULL!	#NULL!	#NULL!	#NULL!
2	2	2	2	2	2	2	2	2	2
#NULL!	#NULL!	#NULL!	#NULL!	#NULL!	#NULL!	#NULL!	#NULL!	#NULL!	#NULL!
2	2	2	2	2	2	2	2	2	2
2	2	2	2	2	2	1	1	2	2
1	1	1	1	1	1	1	1	1	1
1	2	2	2	2	#NULL!	2	2	1	2
2	2	2	2	2	2	2	2	1	1
2	1	1	1	#NULL!	2	2	2	2	2
2	2	1	2	2	2	1	1	2	2
2	2	2	2	2	2	2	2	2	2

2	2	2	2	2	2	1	1	1	2
#NULL!	#NULL!	#NULL!	#NULL!	#NULL!	2	#NULL!	#NULL!	#NULL!	#NULL!
1	#NULL!	#NULL!	#NULL!	2	2	2	2	2	2
2	2	1	2	2	1	2	1	2	1
2	1	1	2	#NULL!	2	2	2	2	2
1	1	1	2	2	2	2	2	2	1
2	2	2	2	2	2	2	2	2	2
2	2	2	2	2	2	2	2	2	2
#NULL!	#NULL!	#NULL!	#NULL!	#NULL!	#NULL!	#NULL!	#NULL!	#NULL!	#NULL!
2	2	2	2	2	2	2	2	2	2
2	2	2	2	2	1	1	2	1	1
#NULL!	2	#NULL!	#NULL!	#NULL!	#NULL!	#NULL!	#NULL!	#NULL!	#NULL!
#NULL!	2	#NULL!	#NULL!	#NULL!	#NULL!	#NULL!	#NULL!	#NULL!	#NULL!
2	2	2	2	2	2	2	2	2	2
#NULL!	#NULL!	#NULL!	#NULL!	#NULL!	#NULL!	#NULL!	#NULL!	#NULL!	#NULL!
2	2	2	2	2	1	1	1	1	1
#NULL!	#NULL!	#NULL!	#NULL!	#NULL!	#NULL!	#NULL!	#NULL!	#NULL!	#NULL!
#NULL!	2	#NULL!	#NULL!	#NULL!	#NULL!	#NULL!	#NULL!	#NULL!	#NULL!
#NULL!	#NULL!	#NULL!	#NULL!	#NULL!	#NULL!	2	#NULL!	#NULL!	#NULL!
1	1	2	1	2	1	2	1	2	1
2	2	2	2	2	2	2	2	2	2
2	2	2	2	2	2	2	2	2	2
2	2	2	2	2	2	2	2	2	2
#NULL!	#NULL!	#NULL!	#NULL!	#NULL!	#NULL!	#NULL!	#NULL!	#NULL!	#NULL!
#NULL!	2	#NULL!	#NULL!	#NULL!	#NULL!	#NULL!	#NULL!	#NULL!	#NULL!
2	2	2	2	2	2	2	2	2	2
2	2	2	1	2	2	2	1	1	1
2	2	2	1	2	2	1	2	2	2
#NULL!	#NULL!	#NULL!	#NULL!	#NULL!	#NULL!	#NULL!	#NULL!	#NULL!	#NULL!
#NULL!	2	2	2	2	1	#NULL!	#NULL!	#NULL!	#NULL!
2	2	2	2	2	2	2	2	2	2
2	2	1	2	2	1	2	2	1	2
2	2	2	2	1	2	2	2	2	2
2	2	2	2	1	2	2	2	2	2
2	2	2	2	2	1	#NULL!	2	1	1
2	2	2	2	2	2	2	2	1	1
#NULL!	#NULL!	#NULL!	#NULL!	#NULL!	#NULL!	#NULL!	#NULL!	#NULL!	#NULL!
#NULL!	#NULL!	#NULL!	#NULL!	#NULL!	#NULL!	#NULL!	#NULL!	#NULL!	#NULL!
1	2	2	1	2	1	#NULL!	2	2	2
2	2	2	2	2	2	2	2	2	2
2	2	2	2	2	2	2	2	2	2
2	2	2	2	2	2	2	2	2	2
2	2	2	2	2	2	2	2	2	2
2	2	2	2	1	2	2	2	2	2
1	2	2	2	2	2	2	2	2	2
2	2	2	2	2	2	2	2	2	2
1	1	1	1	2	2	2	2	2	1

1	2	2	2	2	2	2	2	2	2
2	2	2	1	2	2	2	2	1	2
#NULL!	#NULL!	#NULL!	#NULL!	#NULL!	#NULL!	#NULL!	#NULL!	#NULL!	#NULL!
#NULL!	#NULL!	#NULL!	#NULL!	#NULL!	#NULL!	#NULL!	#NULL!	#NULL!	#NULL!
2	2	#NULL!	#NULL!	#NULL!	#NULL!	#NULL!	#NULL!	#NULL!	#NULL!
1	2	2	#NULL!	2	1	2	2	1	2
#NULL!	2	#NULL!	#NULL!	#NULL!	2	#NULL!	#NULL!	2	#NULL!
1	1	#NULL!	1	#NULL!	#NULL!	1	#NULL!	#NULL!	1
#NULL!	#NULL!	#NULL!	#NULL!	#NULL!	#NULL!	#NULL!	#NULL!	#NULL!	#NULL!
#NULL!	#NULL!	#NULL!	#NULL!	#NULL!	#NULL!	#NULL!	#NULL!	#NULL!	#NULL!
#NULL!	#NULL!	#NULL!	#NULL!	#NULL!	#NULL!	#NULL!	#NULL!	#NULL!	#NULL!
#NULL!	#NULL!	#NULL!	#NULL!	#NULL!	#NULL!	#NULL!	#NULL!	#NULL!	#NULL!
#NULL!	#NULL!	#NULL!	#NULL!	#NULL!	#NULL!	#NULL!	#NULL!	#NULL!	#NULL!
#NULL!	#NULL!	#NULL!	#NULL!	#NULL!	#NULL!	#NULL!	#NULL!	#NULL!	#NULL!
#NULL!	#NULL!	#NULL!	#NULL!	#NULL!	#NULL!	#NULL!	#NULL!	#NULL!	#NULL!
#NULL!	2	#NULL!	#NULL!	#NULL!	#NULL!	#NULL!	#NULL!	#NULL!	#NULL!
1	1	1	1	1	1	1	1	1	1
#NULL!	2	#NULL!	#NULL!	#NULL!	#NULL!	#NULL!	#NULL!	#NULL!	#NULL!
#NULL!	2	#NULL!	#NULL!	#NULL!	#NULL!	#NULL!	#NULL!	#NULL!	#NULL!
2	2	#NULL!	#NULL!	2	#NULL!	#NULL!	2	#NULL!	2
#NULL!	#NULL!	#NULL!	#NULL!	#NULL!	#NULL!	#NULL!	#NULL!	#NULL!	#NULL!
#NULL!	2	#NULL!	#NULL!	#NULL!	#NULL!	#NULL!	#NULL!	#NULL!	#NULL!
2	2	2	2	2	2	2	2	2	2
#NULL!	#NULL!	#NULL!	#NULL!	#NULL!	#NULL!	#NULL!	#NULL!	#NULL!	#NULL!
#NULL!	#NULL!	#NULL!	#NULL!	#NULL!	#NULL!	#NULL!	#NULL!	#NULL!	#NULL!
#NULL!	2	#NULL!	#NULL!	#NULL!	#NULL!	#NULL!	#NULL!	#NULL!	#NULL!
#NULL!	#NULL!	#NULL!	#NULL!	#NULL!	#NULL!	#NULL!	#NULL!	#NULL!	#NULL!
2	2	#NULL!	2	#NULL!	#NULL!	2	#NULL!	#NULL!	2
2	2	2	2	2	2	#NULL!	2	#NULL!	2
#NULL!	#NULL!	#NULL!	#NULL!	#NULL!	#NULL!	#NULL!	#NULL!	#NULL!	#NULL!
#NULL!	#NULL!	#NULL!	#NULL!	#NULL!	#NULL!	#NULL!	#NULL!	#NULL!	#NULL!
2	2	2	2	#NULL!	2	#NULL!	#NULL!	2	#NULL!
#NULL!	#NULL!	#NULL!	#NULL!	#NULL!	#NULL!	#NULL!	#NULL!	#NULL!	#NULL!
#NULL!	#NULL!	#NULL!	#NULL!	#NULL!	#NULL!	#NULL!	#NULL!	#NULL!	#NULL!
#NULL!	#NULL!	#NULL!	#NULL!	#NULL!	#NULL!	#NULL!	#NULL!	#NULL!	#NULL!
#NULL!	#NULL!	#NULL!	#NULL!	#NULL!	#NULL!	#NULL!	#NULL!	#NULL!	#NULL!
#NULL!	#NULL!	#NULL!	#NULL!	#NULL!	#NULL!	#NULL!	#NULL!	#NULL!	#NULL!
#NULL!	#NULL!	#NULL!	#NULL!	#NULL!	#NULL!	#NULL!	#NULL!	#NULL!	#NULL!
2	2	2	1	2	2	2	1	2	2
2	2	2	1	2	2	1	2	2	1
#NULL!	2	1	1	2	2	#NULL!	#NULL!	2	#NULL!
#NULL!	#NULL!	#NULL!	#NULL!	#NULL!	#NULL!	#NULL!	#NULL!	#NULL!	#NULL!
2	2	2	1	2	2	1	2	2	2
2	2	2	1	2	1	2	2	2	2
2	1	1	1	1	1	1	1	2	2
2	2	2	2	2	1	2	2	2	2
#NULL!	2	2	2	2	2	2	2	2	1

#NULL!	#NULL!	#NULL!	#NULL!	#NULL!	#NULL!	#NULL!	#NULL!	#NULL!	#NULL!
2	2	1	2	2	2	2	2	2	2
2	2	2	2	2	2	2	2	2	2
2	2	2	2	2	2	2	2	2	2
2	2	2	2	2	2	2	1	1	1
1	2	2	1	2	2	2	1	2	2
#NULL!	#NULL!	#NULL!	#NULL!	#NULL!	#NULL!	#NULL!	#NULL!	#NULL!	#NULL!
2	2	1	2	2	2	2	1	2	2
2	2	2	2	2	2	2	2	2	2
2	2	2	2	2	2	2	2	2	2
2	2	2	#NULL!	2	#NULL!	2	2	2	2
#NULL!	#NULL!	#NULL!	#NULL!	#NULL!	#NULL!	#NULL!	#NULL!	#NULL!	#NULL!
#NULL!	#NULL!	#NULL!	#NULL!	#NULL!	#NULL!	#NULL!	#NULL!	#NULL!	#NULL!
#NULL!	#NULL!	#NULL!	2	#NULL!	#NULL!	#NULL!	#NULL!	#NULL!	#NULL!
#NULL!	#NULL!	#NULL!	2	#NULL!	#NULL!	#NULL!	#NULL!	#NULL!	#NULL!
2	2	2	2	2	2	2	2	2	2
2	2	2	2	2	2	2	2	2	#NULL!
2	2	2	2	1	1	2	2	1	1
2	1	1	2	1	1	1	1	1	1
2	2	2	2	2	2	2	2	2	2
2	2	2	2	2	2	2	2	2	2
#NULL!	#NULL!	#NULL!	#NULL!	#NULL!	#NULL!	#NULL!	#NULL!	#NULL!	#NULL!
2	2	1	2	1	2	2	1	2	2
#NULL!	#NULL!	#NULL!	#NULL!	2	#NULL!	#NULL!	#NULL!	#NULL!	#NULL!
#NULL!	#NULL!	#NULL!	#NULL!	#NULL!	#NULL!	#NULL!	#NULL!	#NULL!	#NULL!
2	2	2	1	1	1	2	2	2	2
#NULL!	#NULL!	#NULL!	#NULL!	2	#NULL!	#NULL!	#NULL!	#NULL!	#NULL!
2	2	2	2	2	1	1	1	2	2
#NULL!	#NULL!	#NULL!	#NULL!	#NULL!	#NULL!	#NULL!	#NULL!	#NULL!	#NULL!
#NULL!	#NULL!	#NULL!	#NULL!	#NULL!	#NULL!	#NULL!	#NULL!	#NULL!	#NULL!
1	2	2	2	1	2	1	2	1	2
1	2	2	1	2	1	2	1	2	1
#NULL!	#NULL!	#NULL!	#NULL!	#NULL!	#NULL!	#NULL!	#NULL!	#NULL!	#NULL!
#NULL!	2	#NULL!	#NULL!	#NULL!	#NULL!	#NULL!	#NULL!	#NULL!	#NULL!
2	2	2	2	1	2	1	2	2	2
#NULL!	#NULL!	#NULL!	#NULL!	#NULL!	#NULL!	#NULL!	#NULL!	#NULL!	#NULL!
2	2	2	1	2	2	1	2	1	2
2	2	2	2	#NULL!	#NULL!	#NULL!	#NULL!	#NULL!	#NULL!
2	2	2	2	2	2	2	2	2	2
#NULL!	#NULL!	#NULL!	#NULL!	#NULL!	#NULL!	#NULL!	#NULL!	#NULL!	#NULL!
2	2	2	2	2	2	2	2	2	2
2	2	2	2	2	2	2	2	2	2
2	2	2	2	2	2	2	2	2	2
2	2	2	2	2	2	2	2	2	2
1	2	1	1	1	2	1	2	1	1
2	2	2	2	2	2	2	2	2	2
2	2	2	2	2	1	1	2	1	2

#NULL!	#NULL!	2	#NULL!	#NULL!	#NULL!	#NULL!	#NULL!	#NULL!	#NULL!
2	2	2	2	2	2	2	2	2	2
2	2	2	2	2	2	2	2	2	2
#NULL!	2	#NULL!	#NULL!	#NULL!	#NULL!	#NULL!	#NULL!	#NULL!	#NULL!
#NULL!	2	#NULL!	#NULL!	#NULL!	#NULL!	#NULL!	#NULL!	#NULL!	#NULL!
#NULL!	#NULL!	#NULL!	#NULL!	#NULL!	#NULL!	#NULL!	#NULL!	#NULL!	#NULL!
#NULL!	#NULL!	#NULL!	#NULL!	#NULL!	#NULL!	#NULL!	#NULL!	#NULL!	#NULL!
2	2	2	2	2	2	2	2	2	2
2	2	2	2	2	2	2	2	2	2
#NULL!	#NULL!	#NULL!	2	2	#NULL!	#NULL!	#NULL!	#NULL!	#NULL!
2	2	2	2	2	2	2	2	2	2
1	2	2	2	2	2	2	2	1	1
2	2	2	2	2	2	2	2	2	2
2	2	2	2	2	2	2	2	2	2
#NULL!	#NULL!	#NULL!	#NULL!	#NULL!	#NULL!	#NULL!	#NULL!	#NULL!	#NULL!
#NULL!	#NULL!	#NULL!	#NULL!	#NULL!	#NULL!	#NULL!	#NULL!	#NULL!	#NULL!
#NULL!	#NULL!	#NULL!	#NULL!	#NULL!	#NULL!	#NULL!	#NULL!	#NULL!	#NULL!
#NULL!	#NULL!	#NULL!	#NULL!	#NULL!	#NULL!	#NULL!	#NULL!	#NULL!	#NULL!
#NULL!	2	#NULL!	#NULL!	#NULL!	#NULL!	#NULL!	#NULL!	#NULL!	#NULL!
#NULL!	2	#NULL!	#NULL!	#NULL!	#NULL!	#NULL!	#NULL!	#NULL!	#NULL!
#NULL!	#NULL!	#NULL!	#NULL!	#NULL!	#NULL!	#NULL!	#NULL!	#NULL!	#NULL!
#NULL!	#NULL!	#NULL!	#NULL!	#NULL!	#NULL!	#NULL!	#NULL!	#NULL!	#NULL!
2	2	#NULL!	2	2	2	2	#NULL!	2	2
#NULL!	2	#NULL!	#NULL!	#NULL!	#NULL!	#NULL!	#NULL!	#NULL!	#NULL!
#NULL!	#NULL!	#NULL!	#NULL!	#NULL!	#NULL!	#NULL!	#NULL!	#NULL!	#NULL!
#NULL!	1	#NULL!	#NULL!	#NULL!	#NULL!	#NULL!	#NULL!	#NULL!	#NULL!
#NULL!	#NULL!	#NULL!	#NULL!	#NULL!	#NULL!	#NULL!	#NULL!	#NULL!	#NULL!
#NULL!	#NULL!	#NULL!	#NULL!	#NULL!	#NULL!	#NULL!	#NULL!	#NULL!	#NULL!
2	2	#NULL!	#NULL!	#NULL!	#NULL!	#NULL!	#NULL!	#NULL!	2
#NULL!	1	#NULL!	#NULL!	#NULL!	#NULL!	#NULL!	#NULL!	#NULL!	#NULL!
#NULL!	#NULL!	#NULL!	#NULL!	#NULL!	#NULL!	#NULL!	#NULL!	#NULL!	#NULL!
2	2	2	2	2	2	2	2	2	2
#NULL!	#NULL!	#NULL!	#NULL!	#NULL!	#NULL!	#NULL!	#NULL!	#NULL!	#NULL!
#NULL!	#NULL!	#NULL!	#NULL!	#NULL!	#NULL!	#NULL!	#NULL!	#NULL!	#NULL!
#NULL!	#NULL!	#NULL!	#NULL!	#NULL!	#NULL!	#NULL!	#NULL!	#NULL!	#NULL!
#NULL!	#NULL!	#NULL!	#NULL!	#NULL!	#NULL!	#NULL!	#NULL!	#NULL!	#NULL!
#NULL!	#NULL!	#NULL!	#NULL!	#NULL!	#NULL!	#NULL!	#NULL!	#NULL!	#NULL!
#NULL!	#NULL!	#NULL!	#NULL!	#NULL!	#NULL!	#NULL!	#NULL!	#NULL!	#NULL!
#NULL!	#NULL!	#NULL!	#NULL!	#NULL!	#NULL!	#NULL!	#NULL!	#NULL!	#NULL!
#NULL!	#NULL!	#NULL!	#NULL!	#NULL!	#NULL!	#NULL!	#NULL!	#NULL!	#NULL!
2	2	#NULL!	2	#NULL!	2	2	2	2	2
#NULL!	2	#NULL!	#NULL!	#NULL!	#NULL!	#NULL!	#NULL!	#NULL!	#NULL!
#NULL!	#NULL!	#NULL!	#NULL!	#NULL!	#NULL!	#NULL!	#NULL!	#NULL!	#NULL!
#NULL!	#NULL!	#NULL!	#NULL!	#NULL!	#NULL!	#NULL!	#NULL!	#NULL!	#NULL!
#NULL!	#NULL!	#NULL!	#NULL!	#NULL!	#NULL!	#NULL!	#NULL!	#NULL!	#NULL!
#NULL!	#NULL!	#NULL!	#NULL!	#NULL!	#NULL!	#NULL!	#NULL!	#NULL!	#NULL!
#NULL!	#NULL!	#NULL!	#NULL!	#NULL!	#NULL!	#NULL!	#NULL!	#NULL!	#NULL!

#NULL!	#NULL!	#NULL!	#NULL!	#NULL!	#NULL!	#NULL!	#NULL!	#NULL!	#NULL!
1	1	1	1	1	1	1	1	1	1
#NULL!	#NULL!	#NULL!	#NULL!	#NULL!	#NULL!	#NULL!	#NULL!	#NULL!	#NULL!
#NULL!	#NULL!	#NULL!	#NULL!	#NULL!	#NULL!	#NULL!	#NULL!	#NULL!	#NULL!
#NULL!	#NULL!	#NULL!	#NULL!	#NULL!	#NULL!	#NULL!	#NULL!	#NULL!	#NULL!
#NULL!	#NULL!	#NULL!	#NULL!	#NULL!	#NULL!	#NULL!	#NULL!	#NULL!	#NULL!
#NULL!	#NULL!	#NULL!	#NULL!	#NULL!	#NULL!	#NULL!	#NULL!	#NULL!	#NULL!
#NULL!	#NULL!	#NULL!	#NULL!	#NULL!	#NULL!	#NULL!	#NULL!	#NULL!	#NULL!
#NULL!	#NULL!	#NULL!	#NULL!	#NULL!	#NULL!	#NULL!	#NULL!	#NULL!	#NULL!
#NULL!	#NULL!	#NULL!	#NULL!	#NULL!	#NULL!	#NULL!	#NULL!	#NULL!	#NULL!
#NULL!	#NULL!	#NULL!	#NULL!	#NULL!	#NULL!	#NULL!	#NULL!	#NULL!	#NULL!
#NULL!	#NULL!	#NULL!	#NULL!	#NULL!	#NULL!	#NULL!	#NULL!	#NULL!	#NULL!
#NULL!	#NULL!	#NULL!	#NULL!	#NULL!	#NULL!	#NULL!	#NULL!	#NULL!	#NULL!
#NULL!	#NULL!	#NULL!	#NULL!	#NULL!	#NULL!	#NULL!	#NULL!	#NULL!	#NULL!
#NULL!	#NULL!	#NULL!	#NULL!	#NULL!	#NULL!	#NULL!	#NULL!	#NULL!	#NULL!
#NULL!	#NULL!	#NULL!	#NULL!	#NULL!	#NULL!	#NULL!	#NULL!	#NULL!	#NULL!
#NULL!	#NULL!	#NULL!	#NULL!	#NULL!	#NULL!	#NULL!	#NULL!	#NULL!	#NULL!
2	2	2	2	2	2	2	2	2	2
#NULL!	#NULL!	#NULL!	#NULL!	#NULL!	#NULL!	#NULL!	#NULL!	#NULL!	#NULL!
#NULL!	#NULL!	#NULL!	#NULL!	#NULL!	#NULL!	#NULL!	#NULL!	#NULL!	#NULL!
#NULL!	#NULL!	#NULL!	#NULL!	#NULL!	#NULL!	#NULL!	#NULL!	#NULL!	#NULL!
#NULL!	#NULL!	#NULL!	#NULL!	#NULL!	#NULL!	#NULL!	#NULL!	#NULL!	#NULL!
#NULL!	#NULL!	#NULL!	#NULL!	#NULL!	#NULL!	#NULL!	#NULL!	#NULL!	#NULL!
#NULL!	#NULL!	#NULL!	#NULL!	#NULL!	#NULL!	#NULL!	#NULL!	#NULL!	#NULL!
2	2	2	2	2	2	2	2	2	2
#NULL!	#NULL!	#NULL!	#NULL!	#NULL!	#NULL!	#NULL!	#NULL!	#NULL!	#NULL!
#NULL!	#NULL!	#NULL!	#NULL!	#NULL!	#NULL!	#NULL!	#NULL!	#NULL!	#NULL!
2	2	#NULL!	#NULL!	#NULL!	2	#NULL!	#NULL!	#NULL!	2
#NULL!	#NULL!	#NULL!	#NULL!	#NULL!	#NULL!	#NULL!	#NULL!	#NULL!	#NULL!
#NULL!	#NULL!	#NULL!	#NULL!	#NULL!	#NULL!	#NULL!	#NULL!	#NULL!	#NULL!
2	2	2	2	2	2	2	2	2	2
2	2	2	2	2	2	2	2	2	2
#NULL!	#NULL!	#NULL!	#NULL!	#NULL!	#NULL!	#NULL!	#NULL!	#NULL!	#NULL!
1	1	1	2	2	2	2	2	2	2
#NULL!	#NULL!	#NULL!	#NULL!	#NULL!	#NULL!	#NULL!	#NULL!	#NULL!	#NULL!
2	2	2	2	2	2	2	2	2	2
#NULL!	2	#NULL!	#NULL!	#NULL!	#NULL!	#NULL!	#NULL!	#NULL!	#NULL!
#NULL!	#NULL!	#NULL!	#NULL!	#NULL!	#NULL!	#NULL!	#NULL!	#NULL!	#NULL!
2	2	2	2	2	2	2	2	2	2
1	1	#NULL!	#NULL!	#NULL!	#NULL!	#NULL!	#NULL!	#NULL!	#NULL!
#NULL!	#NULL!	#NULL!	#NULL!	#NULL!	#NULL!	#NULL!	#NULL!	#NULL!	#NULL!
#NULL!	#NULL!	#NULL!	#NULL!	#NULL!	#NULL!	#NULL!	#NULL!	#NULL!	#NULL!
2	2	2	2	2	2	2	2	2	2
2	2	2	2	2	2	2	2	2	2
2	#NULL!	2	#NULL!	1	2	#NULL!	1	2	#NULL!
#NULL!	#NULL!	#NULL!	#NULL!	#NULL!	#NULL!	#NULL!	#NULL!	#NULL!	#NULL!
#NULL!	#NULL!	#NULL!	#NULL!	#NULL!	#NULL!	#NULL!	#NULL!	#NULL!	#NULL!

2	2	#NULL!	#NULL!	#NULL!	#NULL!	#NULL!	2	#NULL!	#NULL!
#NULL!	#NULL!	#NULL!	#NULL!	#NULL!	#NULL!	#NULL!	#NULL!	#NULL!	#NULL!
1	2	#NULL!	#NULL!	2	#NULL!	2	1	2	#NULL!
#NULL!	#NULL!	#NULL!	#NULL!	#NULL!	#NULL!	#NULL!	#NULL!	#NULL!	#NULL!
#NULL!	#NULL!	#NULL!	#NULL!	#NULL!	#NULL!	#NULL!	#NULL!	#NULL!	#NULL!
#NULL!	#NULL!	#NULL!	#NULL!	#NULL!	#NULL!	#NULL!	#NULL!	#NULL!	#NULL!
#NULL!	2	#NULL!	#NULL!	#NULL!	#NULL!	#NULL!	#NULL!	#NULL!	#NULL!
#NULL!	2	#NULL!	#NULL!	#NULL!	#NULL!	#NULL!	#NULL!	#NULL!	#NULL!
2	2	#NULL!	#NULL!	#NULL!	#NULL!	2	#NULL!	#NULL!	#NULL!
#NULL!	#NULL!	#NULL!	#NULL!	#NULL!	#NULL!	#NULL!	#NULL!	#NULL!	#NULL!
#NULL!	#NULL!	#NULL!	#NULL!	#NULL!	#NULL!	#NULL!	#NULL!	#NULL!	#NULL!
#NULL!	#NULL!	#NULL!	#NULL!	#NULL!	#NULL!	#NULL!	#NULL!	#NULL!	#NULL!
#NULL!	#NULL!	#NULL!	#NULL!	#NULL!	#NULL!	#NULL!	#NULL!	#NULL!	#NULL!
#NULL!	#NULL!	#NULL!	#NULL!	#NULL!	#NULL!	#NULL!	#NULL!	#NULL!	#NULL!
2	2	2	2	2	1	2	2	2	2
2	2	2	1	2	2	1	2	1	2
#NULL!	#NULL!	#NULL!	2	#NULL!	#NULL!	#NULL!	#NULL!	#NULL!	#NULL!
2	2	2	2	2	2	2	2	2	2
2	2	2	2	1	2	2	2	2	1
#NULL!	2	#NULL!	#NULL!	#NULL!	#NULL!	#NULL!	#NULL!	#NULL!	#NULL!
#NULL!	#NULL!	#NULL!	#NULL!	2	#NULL!	#NULL!	#NULL!	#NULL!	#NULL!
#NULL!	2	#NULL!	#NULL!	#NULL!	#NULL!	#NULL!	#NULL!	#NULL!	#NULL!
2	1	1	1	1	1	1	1	1	1
2	2	1	2	2	1	2	2	1	2
2	2	2	1	1	1	1	2	1	2
2	2	2	2	2	2	2	2	2	2
1	2	2	2	2	2	2	2	1	1
2	2	2	2	2	2	2	2	1	#NULL!
1	2	2	2	1	1	2	2	2	1
2	2	2	2	2	2	2	2	2	2
2	2	2	2	2	2	2	2	2	2
#NULL!	2	#NULL!	#NULL!	#NULL!	#NULL!	#NULL!	#NULL!	#NULL!	#NULL!
#NULL!	2	#NULL!	#NULL!	#NULL!	#NULL!	#NULL!	#NULL!	#NULL!	#NULL!
#NULL!	2	#NULL!	#NULL!	#NULL!	#NULL!	#NULL!	#NULL!	#NULL!	#NULL!
#NULL!	2	#NULL!	#NULL!	#NULL!	#NULL!	#NULL!	#NULL!	#NULL!	#NULL!
#NULL!	#NULL!	#NULL!	#NULL!	#NULL!	#NULL!	#NULL!	#NULL!	#NULL!	#NULL!
#NULL!	#NULL!	#NULL!	#NULL!	#NULL!	#NULL!	#NULL!	#NULL!	#NULL!	#NULL!
2	#NULL!	#NULL!	2	#NULL!	#NULL!	#NULL!	#NULL!	2	#NULL!
#NULL!	#NULL!	#NULL!	#NULL!	#NULL!	#NULL!	#NULL!	#NULL!	#NULL!	#NULL!
2	2	#NULL!	#NULL!	2	#NULL!	2	#NULL!	#NULL!	2
#NULL!	#NULL!	#NULL!	#NULL!	#NULL!	#NULL!	#NULL!	#NULL!	#NULL!	#NULL!
2	2	2	2	2	2	2	2	2	2
#NULL!	#NULL!	#NULL!	#NULL!	#NULL!	#NULL!	#NULL!	#NULL!	#NULL!	#NULL!
#NULL!	#NULL!	#NULL!	#NULL!	#NULL!	#NULL!	#NULL!	#NULL!	#NULL!	#NULL!
#NULL!	#NULL!	#NULL!	#NULL!	#NULL!	#NULL!	#NULL!	#NULL!	#NULL!	#NULL!
2	2	2	2	2	2	2	2	2	2
2	2	1	2	1	2	1	2	1	1

2	2	2	2	2	2	2	2	2	2
2	2	2	2	2	2	2	2	2	2
2	2	2	2	2	2	2	2	2	2
1	2	2	1	2	1	2	2	2	2
1	1	1	1	1	1	1	1	1	1
2	2	2	2	2	2	2	2	2	2
2	2	2	2	2	2	2	2	2	2
1	1	1	1	1	1	1	1	1	1
#NULL!	#NULL!	#NULL!	#NULL!	#NULL!	#NULL!	#NULL!	#NULL!	#NULL!	#NULL!
2	2	2	2	2	2	2	2	2	2
2	#NULL!	2	#NULL!	#NULL!	#NULL!	2	#NULL!	#NULL!	#NULL!
2	#NULL!	2	2	#NULL!	2	#NULL!	1	2	#NULL!
2	2	2	2	2	2	2	2	2	2
1	2	2	2	2	2	2	2	1	2
1	1	2	2	2	2	2	2	2	2
2	2	2	2	2	2	2	2	2	2
2	2	1	2	2	2	2	2	1	1
1	2	1	2	2	1	2	2	1	1
#NULL!	#NULL!	#NULL!	#NULL!	#NULL!	#NULL!	#NULL!	#NULL!	#NULL!	#NULL!
2	2	2	2	1	2	2	2	2	2
2	2	1	1	1	1	2	2	2	2
2	2	2	2	2	2	2	1	2	2
2	2	2	2	2	2	2	2	2	1
2	2	2	2	2	1	2	2	1	2
2	2	2	2	2	1	2	2	2	2
2	2	2	2	2	2	2	2	2	2
2	2	2	2	2	2	2	2	2	2
2	2	1	2	1	2	2	2	1	2
2	2	2	2	2	2	2	2	2	2
2	2	2	2	2	2	2	2	2	1
1	1	1	1	1	1	1	1	1	1
#NULL!	#NULL!	#NULL!	#NULL!	#NULL!	#NULL!	#NULL!	#NULL!	#NULL!	#NULL!
1	1	1	1	1	1	1	1	1	1
2	2	2	2	2	2	2	2	2	2
#NULL!	#NULL!	2	#NULL!	#NULL!	#NULL!	#NULL!	#NULL!	#NULL!	#NULL!
2	1	2	2	1	2	1	2	2	2
2	2	2	2	2	2	2	2	2	2
2	2	2	2	2	2	2	2	2	2
2	2	2	2	2	2	2	2	2	2
#NULL!	#NULL!	#NULL!	#NULL!	#NULL!	#NULL!	#NULL!	#NULL!	#NULL!	#NULL!
2	2	2	2	2	2	2	2	2	2
#NULL!	#NULL!	#NULL!	#NULL!	#NULL!	#NULL!	#NULL!	#NULL!	#NULL!	#NULL!
2	2	2	1	2	2	2	1	1	2
2	2	2	2	2	2	2	2	2	2
1	2	1	2	1	2	1	2	1	2
#NULL!	#NULL!	#NULL!	#NULL!	#NULL!	#NULL!	#NULL!	#NULL!	#NULL!	#NULL!
2	1	2	2	1	1	1	2	1	2

#NULL!	#NULL!	#NULL!	#NULL!	#NULL!	#NULL!	#NULL!	#NULL!	#NULL!	#NULL!
1	1	1	1	1	1	1	1	1	1
1	2	2	2	2	2	1	1	2	1
1	2	1	1	1	1	1	2	1	2
1	2	2	2	2	2	2	2	2	1
#NULL!	#NULL!	#NULL!	#NULL!	#NULL!	#NULL!	#NULL!	#NULL!	#NULL!	#NULL!
2	2	2	2	2	2	2	2	2	2
1	1	1	1	1	1	1	1	1	2
2	2	2	2	2	1	2	2	2	2
2	2	2	2	2	2	2	2	2	2
2	2	2	2	1	1	2	2	2	2
2	2	2	2	2	2	2	2	2	2
#NULL!	#NULL!	#NULL!	#NULL!	#NULL!	#NULL!	#NULL!	#NULL!	#NULL!	#NULL!
2	2	2	1	1	2	2	2	2	1
2	2	2	2	2	1	2	2	2	1
2	2	2	2	2	2	2	2	2	2
2	2	2	2	1	1	1	2	1	1
2	1	2	2	1	#NULL!	2	1	2	1
2	2	2	2	2	2	2	2	2	2
#NULL!	#NULL!	2	#NULL!	#NULL!	#NULL!	#NULL!	#NULL!	#NULL!	#NULL!
#NULL!	#NULL!	#NULL!	#NULL!	#NULL!	#NULL!	#NULL!	#NULL!	#NULL!	#NULL!
2	2	2	2	2	2	2	2	2	2
1	2	1	2	2	2	2	2	2	1
1	2	1	2	1	2	1	2	1	2
#NULL!	#NULL!	#NULL!	#NULL!	#NULL!	#NULL!	#NULL!	#NULL!	#NULL!	#NULL!
2	2	2	2	2	1	2	2	2	2
#NULL!	#NULL!	#NULL!	2	#NULL!	#NULL!	#NULL!	#NULL!	#NULL!	#NULL!
2	2	2	2	2	2	2	2	2	2
2	2	2	2	2	2	2	2	2	2
2	2	2	2	2	2	2	2	2	2
2	1	1	1	2	2	2	1	2	2
#NULL!	#NULL!	#NULL!	#NULL!	#NULL!	#NULL!	#NULL!	#NULL!	#NULL!	#NULL!
#NULL!	#NULL!	#NULL!	#NULL!	#NULL!	#NULL!	#NULL!	#NULL!	#NULL!	#NULL!
2	2	2	2	2	2	1	2	2	2
2	2	2	2	1	1	2	2	1	2
#NULL!	#NULL!	#NULL!	#NULL!	#NULL!	#NULL!	#NULL!	#NULL!	#NULL!	#NULL!
#NULL!	#NULL!	#NULL!	#NULL!	#NULL!	#NULL!	#NULL!	#NULL!	#NULL!	#NULL!
2	2	1	2	2	2	2	1	2	1
2	2	2	2	2	2	2	2	1	1
2	2	2	2	2	2	2	2	2	2
2	2	2	2	2	2	2	2	2	2
2	2	2	2	2	2	2	2	2	2
2	2	2	2	2	2	2	2	2	2
2	2	2	2	2	2	2	2	2	2
2	2	2	2	2	2	1	2	1	1
2	2	2	2	2	1	1	2	1	2
2	2	2	2	2	2	2	2	2	2

1	1	1	2	1	2	2	2	2	1
2	2	2	2	2	1	2	2	2	2
2	2	2	2	2	2	2	2	2	2
2	2	2	2	2	2	2	1	2	2
2	2	2	2	2	2	2	2	2	2
2	2	2	2	2	1	1	2	1	1
2	2	2	2	2	1	1	2	1	2
2	2	2	2	2	2	2	2	2	2
2	2	2	2	2	2	2	2	2	2
#NULL!	#NULL!	#NULL!	#NULL!	#NULL!	#NULL!	#NULL!	#NULL!	#NULL!	#NULL!
2	1	1	1	1	1	1	1	1	1
2	2	1	2	2	2	2	2	2	1
2	2	2	2	2	2	1	2	2	2
2	2	2	2	2	2	2	2	2	2
1	1	1	1	1	2	1	1	1	2
#NULL!	#NULL!	#NULL!	#NULL!	#NULL!	#NULL!	#NULL!	#NULL!	#NULL!	#NULL!
#NULL!	#NULL!	#NULL!	#NULL!	#NULL!	#NULL!	#NULL!	#NULL!	#NULL!	#NULL!
#NULL!	#NULL!	#NULL!	#NULL!	#NULL!	#NULL!	#NULL!	2	#NULL!	#NULL!
#NULL!	#NULL!	#NULL!	#NULL!	#NULL!	#NULL!	#NULL!	#NULL!	#NULL!	#NULL!
2	2	2	2	#NULL!	#NULL!	#NULL!	#NULL!	#NULL!	#NULL!
2	2	2	1	2	2	#NULL!	1	2	2
#NULL!	#NULL!	#NULL!	#NULL!	#NULL!	#NULL!	#NULL!	#NULL!	#NULL!	#NULL!
#NULL!	#NULL!	#NULL!	#NULL!	#NULL!	#NULL!	#NULL!	#NULL!	#NULL!	#NULL!
2	2	2	2	2	2	2	2	2	2
2	2	2	2	#NULL!	2	2	2	2	2
2	2	2	2	2	2	2	2	2	2
2	2	2	1	1	2	2	2	1	1
2	2	2	2	2	1	2	1	2	1
2	2	2	2	2	1	2	2	2	2
2	2	2	2	1	2	2	1	2	2
#NULL!	#NULL!	#NULL!	#NULL!	#NULL!	#NULL!	#NULL!	#NULL!	#NULL!	#NULL!
2	2	2	2	1	2	2	2	2	1
2	2	2	2	2	2	2	1	2	1
2	1	1	1	2	2	2	2	2	2
2	2	2	2	2	2	2	2	2	2
2	2	2	2	2	2	2	2	2	2
#NULL!	#NULL!	#NULL!	#NULL!	#NULL!	#NULL!	#NULL!	#NULL!	#NULL!	#NULL!
2	2	2	2	2	2	2	2	2	2
2	2	2	2	2	2	2	2	2	2
2	2	1	2	1	1	1	2	1	2
1	1	1	1	1	1	1	1	1	1
2	2	2	2	1	2	2	2	2	1
2	2	2	2	2	2	2	2	2	2
2	2	2	2	2	2	2	2	2	2
1	2	2	#NULL!	1	2	1	2	1	2
1	2	2	2	2	1	2	2	2	2
2	1	2	2	2	2	2	2	1	1

1	2	2	1	2	2	2	2	2	2
2	2	2	2	2	2	2	2	2	2
1	2	2	2	2	2	2	2	2	1
2	2	2	2	2	2	2	2	2	2
2	1	1	1	1	2	1	1	2	1
#NULL!	#NULL!	#NULL!	#NULL!	#NULL!	#NULL!	#NULL!	#NULL!	#NULL!	#NULL!
#NULL!	2	#NULL!	#NULL!	#NULL!	#NULL!	#NULL!	#NULL!	#NULL!	#NULL!
1	1	1	1	1	1	1	1	1	1
#NULL!	#NULL!	#NULL!	#NULL!	#NULL!	#NULL!	#NULL!	#NULL!	#NULL!	#NULL!
#NULL!	#NULL!	#NULL!	#NULL!	#NULL!	#NULL!	#NULL!	#NULL!	#NULL!	#NULL!
2	2	2	2	2	2	2	2	2	2
2	2	2	2	2	2	2	2	2	2
2	2	2	2	2	2	2	2	2	2
2	#NULL!	#NULL!	#NULL!	2	#NULL!	#NULL!	#NULL!	#NULL!	#NULL!
2	2	2	#NULL!	#NULL!	#NULL!	#NULL!	#NULL!	#NULL!	#NULL!
2	2	2	1	#NULL!	1	1	2	1	1
2	2	2	2	2	2	2	2	2	2
#NULL!	#NULL!	#NULL!	#NULL!	#NULL!	#NULL!	#NULL!	#NULL!	#NULL!	#NULL!
2	2	2	2	2	2	2	2	2	2
2	2	2	2	2	2	2	2	2	2
2	2	1	2	2	1	2	2	1	2
#NULL!	#NULL!	#NULL!	#NULL!	#NULL!	#NULL!	#NULL!	#NULL!	#NULL!	#NULL!
2	2	2	2	2	2	2	2	2	2
2	2	2	2	2	2	2	2	2	2
2	2	2	2	2	2	2	2	2	2
2	2	2	2	2	2	2	2	2	2
2	2	2	2	2	2	2	2	2	2
2	2	2	2	2	2	2	2	2	2
2	2	2	#NULL!	2	#NULL!	2	2	1	2
2	2	2	2	2	2	2	2	2	2
2	2	1	2	2	2	2	1	2	2
2	2	2	2	1	2	1	2	2	1
2	2	2	2	2	2	2	2	2	2
2	2	2	2	2	2	2	2	2	2
2	2	2	2	2	2	2	2	2	2
2	2	2	2	2	2	2	1	1	2
2	2	2	2	2	2	2	2	2	2
1	2	2	2	2	2	2	2	2	2
2	2	2	2	2	#NULL!	2	#NULL!	2	1
2	1	1	#NULL!	2	2	2	2	1	2
2	2	2	1	1	1	2	2	1	1
1	2	2	2	2	2	2	2	2	2
2	2	2	2	2	2	2	2	2	2
2	2	2	2	2	2	2	2	2	2
2	2	2	2	2	2	2	2	2	2
2	2	2	2	2	2	1	2	2	2
2	2	2	2	2	2	2	2	2	2

2	2	1	2	2	2	2	1	2	2
2	2	2	2	2	1	2	1	2	2
1	2	1	2	1	2	1	2	1	2
2	2	2	2	2	2	2	2	2	2
#NULL!	1	#NULL!	#NULL!	#NULL!	#NULL!	#NULL!	#NULL!	#NULL!	#NULL!
#NULL!	#NULL!	#NULL!	#NULL!	#NULL!	2	#NULL!	#NULL!	#NULL!	#NULL!
2	1	2	1	2	1	2	1	2	1
#NULL!	2	#NULL!	#NULL!	#NULL!	#NULL!	#NULL!	#NULL!	#NULL!	#NULL!
#NULL!	2	#NULL!	#NULL!	#NULL!	#NULL!	#NULL!	#NULL!	#NULL!	#NULL!
#NULL!	#NULL!	#NULL!	#NULL!	#NULL!	#NULL!	#NULL!	#NULL!	#NULL!	#NULL!
#NULL!	#NULL!	#NULL!	#NULL!	#NULL!	#NULL!	#NULL!	#NULL!	1	#NULL!
#NULL!	#NULL!	#NULL!	2	#NULL!	#NULL!	#NULL!	#NULL!	#NULL!	#NULL!
#NULL!	2	#NULL!	#NULL!	#NULL!	#NULL!	#NULL!	#NULL!	#NULL!	#NULL!
#NULL!	#NULL!	#NULL!	#NULL!	#NULL!	#NULL!	#NULL!	#NULL!	2	#NULL!
2	2	2	2	2	1	2	2	2	2
#NULL!	2	#NULL!	#NULL!	#NULL!	#NULL!	#NULL!	#NULL!	#NULL!	#NULL!
#NULL!	#NULL!	#NULL!	#NULL!	#NULL!	#NULL!	#NULL!	#NULL!	#NULL!	#NULL!
#NULL!	#NULL!	#NULL!	#NULL!	#NULL!	#NULL!	2	#NULL!	#NULL!	#NULL!
#NULL!	#NULL!	#NULL!	#NULL!	#NULL!	#NULL!	2	#NULL!	#NULL!	#NULL!
#NULL!	#NULL!	#NULL!	#NULL!	2	#NULL!	#NULL!	#NULL!	#NULL!	#NULL!
#NULL!	#NULL!	#NULL!	#NULL!	#NULL!	#NULL!	#NULL!	#NULL!	#NULL!	#NULL!
2	2	2	2	2	2	2	2	2	2
2	2	2	2	2	2	2	2	2	2
2	2	2	1	1	1	1	2	2	2
1	2	2	2	2	2	2	1	2	1
#NULL!	#NULL!	#NULL!	#NULL!	#NULL!	#NULL!	#NULL!	#NULL!	#NULL!	#NULL!
2	2	2	2	2	2	2	2	1	2
#NULL!	#NULL!	#NULL!	#NULL!	#NULL!	#NULL!	2	#NULL!	#NULL!	#NULL!
2	2	2	2	2	2	2	2	2	2
2	2	2	2	2	1	2	2	2	1
1	1	2	#NULL!	2	#NULL!	2	1	2	2
#NULL!	2	#NULL!	#NULL!	#NULL!	#NULL!	#NULL!	#NULL!	#NULL!	#NULL!
2	2	2	2	2	2	2	2	2	2
#NULL!	#NULL!	#NULL!	2	#NULL!	#NULL!	#NULL!	#NULL!	#NULL!	#NULL!
#NULL!	2	#NULL!	#NULL!	#NULL!	#NULL!	#NULL!	#NULL!	#NULL!	#NULL!
2	2	2	1	#NULL!	2	2	2	2	2
2	2	1	1	2	2	2	2	2	2
#NULL!	#NULL!	#NULL!	#NULL!	#NULL!	#NULL!	#NULL!	#NULL!	#NULL!	#NULL!
#NULL!	#NULL!	#NULL!	#NULL!	#NULL!	#NULL!	#NULL!	#NULL!	#NULL!	#NULL!
2	2	2	2	2	2	2	2	2	2
#NULL!	#NULL!	#NULL!	#NULL!	#NULL!	#NULL!	#NULL!	#NULL!	#NULL!	#NULL!
2	2	2	2	2	2	2	2	2	2
1	1	1	1	1	1	1	1	1	1
#NULL!	#NULL!	#NULL!	#NULL!	2	#NULL!	#NULL!	#NULL!	#NULL!	#NULL!
2	2	2	2	2	2	2	2	2	2
2	2	2	2	2	2	2	2	2	2
#NULL!	#NULL!	#NULL!	#NULL!	#NULL!	#NULL!	#NULL!	#NULL!	#NULL!	#NULL!

#NULL!	#NULL!	#NULL!	#NULL!	#NULL!	#NULL!	#NULL!	#NULL!	#NULL!	#NULL!
2	#NULL!	2	#NULL!	#NULL!	2	#NULL!	#NULL!	2	#NULL!
#NULL!	#NULL!	#NULL!	#NULL!	#NULL!	#NULL!	#NULL!	#NULL!	#NULL!	#NULL!
2	2	2	2	2	2	2	2	2	2
2	#NULL!	2	#NULL!	#NULL!	#NULL!	2	2	#NULL!	#NULL!
2	1	2	1	2	2	1	2	1	2
#NULL!	#NULL!	#NULL!	#NULL!	#NULL!	#NULL!	#NULL!	#NULL!	#NULL!	#NULL!
#NULL!	#NULL!	#NULL!	#NULL!	#NULL!	#NULL!	#NULL!	#NULL!	#NULL!	#NULL!
2	#NULL!	2	#NULL!	#NULL!	2	#NULL!	#NULL!	#NULL!	2
2	2	2	2	2	2	2	2	2	2
#NULL!	#NULL!	#NULL!	#NULL!	#NULL!	#NULL!	#NULL!	#NULL!	#NULL!	#NULL!
2	2	#NULL!	#NULL!	2	#NULL!	#NULL!	2	#NULL!	#NULL!
2	2	2	2	2	2	2	2	2	2
#NULL!	#NULL!	#NULL!	#NULL!	#NULL!	#NULL!	#NULL!	#NULL!	#NULL!	#NULL!
#NULL!	2	2	2	2	2	2	2	2	2
#NULL!	#NULL!	#NULL!	#NULL!	#NULL!	#NULL!	#NULL!	#NULL!	#NULL!	#NULL!
#NULL!	#NULL!	#NULL!	#NULL!	#NULL!	#NULL!	#NULL!	#NULL!	#NULL!	#NULL!
#NULL!	#NULL!	#NULL!	#NULL!	#NULL!	#NULL!	#NULL!	#NULL!	#NULL!	#NULL!
#NULL!	#NULL!	#NULL!	#NULL!	#NULL!	#NULL!	#NULL!	#NULL!	#NULL!	#NULL!
#NULL!	#NULL!	#NULL!	#NULL!	#NULL!	#NULL!	#NULL!	#NULL!	#NULL!	#NULL!
2	2	2	2	2	2	2	2	2	2
2	2	2	2	2	2	2	2	2	2
1	2	1	2	1	2	2	2	2	1
2	2	2	2	2	1	1	2	2	2
1	2	2	2	2	2	2	1	1	2
2	2	2	2	2	2	2	2	2	2
2	2	2	2	2	2	2	2	2	2
2	2	2	2	2	2	2	2	2	2
1	2	2	1	2	2	2	2	2	1
2	2	2	2	2	2	2	2	2	2
2	2	2	2	2	2	1	1	1	1
2	2	2	2	1	1	2	2	2	2
2	2	2	2	2	2	2	2	2	2
#NULL!	#NULL!	#NULL!	#NULL!	#NULL!	#NULL!	#NULL!	#NULL!	#NULL!	#NULL!
2	2	2	1	2	2	2	2	2	1
2	2	2	2	2	2	2	2	2	2
2	2	2	2	2	2	2	2	2	2
2	2	2	2	2	2	2	2	2	2
1	2	2	1	2	2	1	2	1	2
2	2	2	2	1	2	1	2	2	2
2	2	2	2	2	2	2	2	2	2
#NULL!	#NULL!	#NULL!	#NULL!	#NULL!	#NULL!	#NULL!	#NULL!	#NULL!	#NULL!
#NULL!	2	2	2	2	1	#NULL!	#NULL!	#NULL!	#NULL!
#NULL!	#NULL!	#NULL!	#NULL!	#NULL!	#NULL!	#NULL!	#NULL!	#NULL!	#NULL!
#NULL!	2	2	2	2	2	2	2	2	2
2	2	2	2	2	2	2	2	2	2
#NULL!	#NULL!	#NULL!	#NULL!	#NULL!	#NULL!	#NULL!	#NULL!	#NULL!	#NULL!

1	2	2	2	2	2	2	2	2	2
#NULL!	2	2	2	2	2	2	2	2	2
2	2	2	2	2	2	2	#NULL!	1	#NULL!
2	2	2	2	2	2	2	2	2	2
#NULL!	2	2	2	#NULL!	2	#NULL!	2	#NULL!	#NULL!
2	2	2	2	2	2	2	2	2	2
2	2	2	2	2	2	2	2	2	2
2	2	#NULL!	2	2	2	2	2	1	1
1	1	1	1	1	1	1	1	1	1
1	2	2	2	2	2	2	2	2	1
#NULL!	#NULL!	#NULL!	#NULL!	#NULL!	#NULL!	#NULL!	#NULL!	#NULL!	#NULL!
2	2	2	2	2	2	2	2	2	2
2	2	2	2	2	2	2	2	2	2
2	2	2	2	2	2	2	2	2	2
2	2	2	2	2	2	2	2	2	2
2	2	2	2	2	2	2	2	2	2
2	2	2	2	2	2	2	2	2	2
2	2	2	2	2	2	2	2	2	2
2	2	2	2	2	2	2	2	2	2
#NULL!	#NULL!	#NULL!	#NULL!	#NULL!	#NULL!	#NULL!	#NULL!	#NULL!	#NULL!
2	2	2	2	2	2	2	2	2	2
2	2	2	2	2	2	#NULL!	#NULL!	2	2
#NULL!	#NULL!	#NULL!	#NULL!	#NULL!	#NULL!	#NULL!	#NULL!	#NULL!	#NULL!
2	2	2	2	2	2	2	2	2	2
#NULL!	#NULL!	#NULL!	#NULL!	#NULL!	#NULL!	#NULL!	#NULL!	#NULL!	#NULL!
2	2	2	2	2	2	2	2	2	2
2	2	2	2	2	2	2	2	2	2
2	2	2	2	2	2	2	2	2	2
#NULL!	#NULL!	#NULL!	#NULL!	#NULL!	#NULL!	#NULL!	#NULL!	#NULL!	#NULL!
2	2	#NULL!	2	2	#NULL!	2	#NULL!	2	2
#NULL!	#NULL!	#NULL!	#NULL!	#NULL!	#NULL!	#NULL!	#NULL!	#NULL!	#NULL!
#NULL!	#NULL!	#NULL!	#NULL!	#NULL!	#NULL!	#NULL!	#NULL!	#NULL!	#NULL!
2	2	2	2	2	2	2	2	2	2
2	2	2	2	2	#NULL!	2	2	2	2
2	2	2	2	2	2	2	2	2	2
#NULL!	#NULL!	#NULL!	#NULL!	#NULL!	#NULL!	#NULL!	#NULL!	#NULL!	#NULL!
2	2	2	2	2	2	2	2	2	2
2	2	2	2	2	2	2	2	2	2
#NULL!	#NULL!	#NULL!	#NULL!	#NULL!	#NULL!	#NULL!	#NULL!	#NULL!	#NULL!
#NULL!	#NULL!	#NULL!	#NULL!	#NULL!	#NULL!	#NULL!	#NULL!	#NULL!	#NULL!
2	2	2	#NULL!	2	#NULL!	2	2	#NULL!	2
#NULL!	#NULL!	#NULL!	#NULL!	#NULL!	#NULL!	#NULL!	#NULL!	#NULL!	#NULL!
#NULL!	2	2	2	#NULL!	2	2	2	2	2
2	#NULL!	#NULL!	#NULL!	#NULL!	#NULL!	#NULL!	#NULL!	#NULL!	#NULL!
2	2	2	2	2	2	2	2	2	2
2	2	2	2	2	#NULL!	#NULL!	2	#NULL!	2
2	2	2	#NULL!	2	2	#NULL!	#NULL!	2	#NULL!

2	2	2	2	2	2	2	2	2	2
#NULL!	2	#NULL!	2	2	#NULL!	2	2	2	#NULL!
1	2	2	2	2	2	2	2	2	2
2	2	2	2	2	2	2	2	2	2
2	2	2	2	2	2	2	2	2	2
#NULL!	#NULL!	#NULL!	#NULL!	#NULL!	#NULL!	#NULL!	#NULL!	#NULL!	#NULL!
2	2	2	2	2	2	2	2	2	2
2	2	2	2	2	2	2	2	2	2
2	#NULL!	#NULL!	2	#NULL!	2	#NULL!	2	#NULL!	#NULL!
2	#NULL!	2	2	2	#NULL!	2	2	#NULL!	2
#NULL!	#NULL!	#NULL!	#NULL!	#NULL!	#NULL!	#NULL!	#NULL!	#NULL!	#NULL!
#NULL!	#NULL!	#NULL!	#NULL!	#NULL!	#NULL!	#NULL!	#NULL!	#NULL!	#NULL!
#NULL!	#NULL!	#NULL!	#NULL!	#NULL!	#NULL!	#NULL!	#NULL!	#NULL!	#NULL!
#NULL!	#NULL!	#NULL!	#NULL!	#NULL!	#NULL!	#NULL!	#NULL!	#NULL!	#NULL!
#NULL!	#NULL!	#NULL!	#NULL!	#NULL!	#NULL!	#NULL!	#NULL!	#NULL!	#NULL!
#NULL!	#NULL!	#NULL!	#NULL!	#NULL!	#NULL!	#NULL!	#NULL!	#NULL!	#NULL!
2	2	2	2	2	2	2	2	2	2
#NULL!	#NULL!	#NULL!	#NULL!	#NULL!	#NULL!	#NULL!	#NULL!	#NULL!	#NULL!
2	2	2	2	2	2	2	2	2	2
2	2	2	2	2	2	2	2	1	1
2	2	2	2	2	2	2	2	1	1
2	2	2	2	2	2	2	2	1	1
2	2	2	2	2	2	2	2	1	1
2	2	2	2	2	2	2	2	1	1
2	2	2	2	2	2	2	2	1	1
2	2	2	2	2	2	2	2	1	1
2	2	2	2	2	2	2	2	1	1
2	2	2	2	2	2	2	1	1	1
2	2	2	2	2	2	2	2	1	1
2	2	2	2	2	2	2	2	1	1
2	2	2	2	2	2	2	2	1	1
2	2	2	2	2	2	2	2	1	1
2	2	2	2	2	2	2	2	1	1
2	2	2	2	2	2	2	2	1	1
2	2	2	2	2	2	2	2	1	1
2	2	2	2	2	2	2	2	1	1
2	2	2	2	2	2	2	2	1	1
2	2	2	2	2	2	2	2	1	1
2	2	2	2	2	2	2	2	1	1
2	2	2	2	2	2	2	2	1	1
2	2	2	2	2	2	2	2	1	1
2	2	2	2	2	2	2	2	1	1
2	2	2	2	2	2	2	2	1	1
2	2	2	2	2	2	2	2	1	1
#NULL!	#NULL!	#NULL!	#NULL!	#NULL!	#NULL!	#NULL!	#NULL!	#NULL!	#NULL!
#NULL!	#NULL!	#NULL!	#NULL!	#NULL!	#NULL!	#NULL!	#NULL!	#NULL!	#NULL!
#NULL!	#NULL!	#NULL!	#NULL!	#NULL!	#NULL!	#NULL!	#NULL!	#NULL!	#NULL!

#NULL!	#NULL!	#NULL!	#NULL!	#NULL!	#NULL!	#NULL!	#NULL!	#NULL!	#NULL!
#NULL!	#NULL!	#NULL!	#NULL!	#NULL!	#NULL!	#NULL!	#NULL!	#NULL!	#NULL!
#NULL!	#NULL!	#NULL!	#NULL!	#NULL!	#NULL!	#NULL!	#NULL!	#NULL!	#NULL!
#NULL!	#NULL!	#NULL!	#NULL!	#NULL!	#NULL!	#NULL!	#NULL!	#NULL!	#NULL!
#NULL!	#NULL!	#NULL!	#NULL!	#NULL!	#NULL!	#NULL!	#NULL!	#NULL!	#NULL!
#NULL!	#NULL!	#NULL!	#NULL!	#NULL!	#NULL!	#NULL!	#NULL!	#NULL!	#NULL!
#NULL!	#NULL!	#NULL!	#NULL!	#NULL!	#NULL!	#NULL!	#NULL!	#NULL!	#NULL!
#NULL!	#NULL!	#NULL!	#NULL!	#NULL!	#NULL!	#NULL!	#NULL!	#NULL!	#NULL!
#NULL!	#NULL!	#NULL!	#NULL!	#NULL!	#NULL!	#NULL!	#NULL!	#NULL!	#NULL!
#NULL!	#NULL!	#NULL!	#NULL!	#NULL!	#NULL!	#NULL!	#NULL!	#NULL!	#NULL!
#NULL!	#NULL!	#NULL!	#NULL!	#NULL!	#NULL!	#NULL!	#NULL!	#NULL!	#NULL!
#NULL!	#NULL!	#NULL!	#NULL!	#NULL!	#NULL!	#NULL!	#NULL!	#NULL!	#NULL!
#NULL!	#NULL!	#NULL!	#NULL!	#NULL!	#NULL!	#NULL!	#NULL!	#NULL!	#NULL!
#NULL!	#NULL!	#NULL!	#NULL!	#NULL!	#NULL!	#NULL!	#NULL!	#NULL!	#NULL!
#NULL!	#NULL!	#NULL!	#NULL!	#NULL!	#NULL!	#NULL!	#NULL!	#NULL!	#NULL!
#NULL!	#NULL!	#NULL!	#NULL!	#NULL!	#NULL!	#NULL!	#NULL!	#NULL!	#NULL!
#NULL!	#NULL!	#NULL!	#NULL!	#NULL!	#NULL!	#NULL!	#NULL!	#NULL!	#NULL!
#NULL!	#NULL!	#NULL!	#NULL!	#NULL!	#NULL!	#NULL!	#NULL!	#NULL!	#NULL!
#NULL!	#NULL!	#NULL!	#NULL!	#NULL!	#NULL!	#NULL!	#NULL!	#NULL!	#NULL!
2	2	2	2	2	2	2	2	1	1
2	2	2	2	2	2	2	2	1	1
2	2	2	2	2	2	2	2	1	1
2	2	2	2	2	2	2	2	1	1
2	2	2	2	2	2	2	2	1	1
2	2	2	2	2	2	2	2	1	1
2	2	2	2	2	2	2	2	1	1
2	2	2	2	2	2	2	2	1	1
2	2	2	2	2	2	2	2	1	1
2	2	2	2	2	2	2	2	1	1
2	2	2	2	2	2	1	1	#NULL!	2
2	2	2	2	2	2	2	2	1	1
2	2	2	2	2	2	2	2	1	1
2	2	2	2	2	2	2	2	1	1
2	2	2	2	2	2	2	2	1	1
2	2	2	2	2	2	2	2	1	1
#NULL!	#NULL!	2	#NULL!	#NULL!	1	#NULL!	#NULL!	1	2
2	2	2	2	2	2	2	2	1	1
2	2	2	2	2	2	2	2	1	1
2	2	2	2	2	2	2	2	1	1
2	2	2	2	2	2	2	2	1	1
2	2	2	2	2	2	2	2	1	1
2	2	2	2	2	2	2	2	1	1
2	2	2	2	2	2	2	2	1	1
2	2	2	2	2	#NULL!	2	2	1	1
2	2	2	2	2	2	2	2	1	1
2	2	2	2	2	2	2	2	1	1
2	2	2	2	2	2	2	2	1	1

2	2	2	2	2	2	2	2	1	1
2	2	2	2	2	2	2	2	1	2
2	2	2	2	2	2	2	2	1	1
2	2	2	2	2	2	2	#NULL!	1	1
2	2	2	2	2	2	2	2	1	1
2	2	2	2	2	2	2	2	1	1
2	2	2	2	2	2	2	2	1	1
2	2	2	2	2	2	2	2	1	1
2	2	2	2	2	2	2	2	1	1
2	2	2	2	2	2	2	2	1	1
2	2	2	2	2	2	2	2	1	1
2	2	2	2	2	2	2	2	1	1
2	2	2	2	2	2	2	2	1	1
2	2	2	2	2	2	2	1	1	2
2	2	2	2	2	2	2	2	1	1
2	2	2	2	2	2	2	2	1	1
2	2	2	2	2	2	2	#NULL!	2	1
2	2	2	2	2	2	2	2	1	1
2	2	2	2	2	2	2	2	1	1
2	2	2	2	2	2	2	2	1	1
2	2	2	2	2	2	2	2	1	1
2	2	2	2	2	2	2	1	1	2
2	2	2	2	2	2	2	1	1	2
2	2	2	2	2	2	2	2	1	1
2	2	2	2	2	2	2	2	1	1
2	2	2	2	2	2	2	2	1	1
2	2	2	2	2	2	2	2	2	1
2	2	2	2	2	2	2	2	1	1
2	2	2	2	2	2	2	2	1	1
2	2	2	2	2	#NULL!	2	2	1	1
2	2	2	2	2	2	2	2	1	1
2	2	2	2	2	2	2	2	1	1
#NULL!	#NULL!	#NULL!	#NULL!	#NULL!	#NULL!	#NULL!	#NULL!	1	1
2	2	2	2	2	2	2	2	1	1
#NULL!	#NULL!	#NULL!	#NULL!	#NULL!	#NULL!	#NULL!	#NULL!	#NULL!	#NULL!
2	2	2	2	2	2	2	2	1	1
2	2	2	2	2	2	2	2	1	1
2	2	2	2	2	2	2	2	1	1
2	2	2	2	2	2	2	2	1	1
2	2	2	2	2	#NULL!	#NULL!	#NULL!	#NULL!	#NULL!
2	2	2	2	2	2	2	2	1	1
2	2	2	2	2	2	2	2	1	2
2	2	2	2	2	2	2	2	1	1
2	2	2	2	2	2	2	2	1	1
2	2	2	2	2	2	2	2	1	1
2	2	2	2	2	2	2	2	1	1
2	2	2	2	2	2	2	2	1	1

2	2	2	2	2	2	2	2	1	1
2	2	2	2	2	2	2	2	1	1
2	2	2	2	2	2	2	2	1	1
2	2	2	2	2	2	2	2	1	1
2	2	2	2	2	2	2	2	1	1
2	2	2	2	2	2	2	2	1	1
2	2	2	2	2	2	2	2	1	1
2	2	2	2	2	2	2	2	1	1
2	2	2	2	2	2	2	2	1	1
2	2	2	2	2	2	2	2	1	1
2	2	2	2	2	2	2	2	1	1
2	2	2	2	2	2	2	2	1	2
2	2	2	2	2	2	2	2	1	1
2	2	2	2	2	2	2	2	1	1
2	2	2	2	2	2	2	2	1	1
2	2	2	2	2	2	2	2	1	1
2	2	2	2	2	2	2	2	1	1
2	2	2	2	2	2	2	2	1	1
2	2	2	2	2	2	2	2	1	1
2	2	2	2	2	2	2	1	1	#NULL!
2	2	2	2	2	2	2	2	1	1
2	2	2	2	2	2	2	2	1	1
2	2	2	2	2	2	2	2	1	1
2	2	2	2	2	2	1	2	1	1
2	2	2	2	2	2	2	2	1	1
2	2	2	2	#NULL!	2	2	2	2	#NULL!
2	2	2	2	2	2	2	2	1	1
2	2	2	2	2	2	2	2	1	1
2	2	2	2	2	2	2	2	1	1
2	2	2	2	2	2	2	2	1	1
2	2	2	2	2	2	2	2	1	1
2	2	2	2	2	2	2	2	1	1
2	2	2	2	2	2	2	2	1	1
2	2	2	2	2	2	2	2	1	1
2	2	2	2	2	2	2	2	1	1
2	2	2	2	2	2	2	1	1	#NULL!
2	2	2	2	2	2	2	2	1	1
#NULL!	2	2	2	2	2	2	2	1	1
2	2	2	2	2	2	2	2	1	1
2	2	2	2	2	2	2	2	1	1
2	2	2	2	2	2	2	2	1	1
#NULL!	#NULL!	#NULL!	#NULL!	#NULL!	#NULL!	#NULL!	#NULL!	#NULL!	#NULL!
2	2	2	2	2	2	2	2	2	1
2	2	2	2	2	2	2	2	1	1
2	2	2	2	2	2	2	2	1	1
2	2	2	2	2	2	2	1	1	2
2	2	2	2	2	2	2	1	1	2

2	2	2	2	2	2	1	2	1	1
2	2	2	2	2	2	2	2	1	1
2	2	2	2	2	2	2	2	1	1
2	2	2	2	2	2	2	2	1	1
2	2	2	2	2	2	2	2	1	1
2	2	2	2	2	2	2	2	1	1
2	2	2	2	2	2	2	1	1	1
2	2	2	2	2	2	2	2	1	1
2	2	2	2	2	2	2	2	1	1
#NULL!	#NULL!	#NULL!	#NULL!	#NULL!	#NULL!	#NULL!	#NULL!	#NULL!	#NULL!
2	2	2	2	2	2	2	2	1	1
2	2	2	2	2	2	2	2	1	1
2	2	2	2	2	2	2	2	1	1
2	2	2	2	2	2	2	2	1	1
2	2	2	2	2	2	2	2	1	1
2	2	2	2	2	2	2	2	1	1
2	2	2	2	2	2	2	2	1	1
2	2	2	2	2	2	2	2	1	1
2	2	2	2	2	2	2	2	1	1
2	2	2	2	2	2	2	2	1	1
2	2	2	2	2	2	2	#NULL!	1	2
2	2	2	2	2	2	2	2	1	1
2	2	2	2	2	2	2	2	1	1
2	2	2	2	2	2	2	2	1	1
2	2	2	2	2	2	2	2	1	1
2	2	2	2	2	2	2	2	1	1
2	2	2	2	2	2	2	2	1	1
#NULL!	#NULL!	#NULL!	#NULL!	#NULL!	#NULL!	#NULL!	#NULL!	#NULL!	#NULL!
#NULL!	#NULL!	#NULL!	#NULL!	#NULL!	#NULL!	#NULL!	#NULL!	#NULL!	#NULL!
#NULL!	#NULL!	#NULL!	#NULL!	#NULL!	#NULL!	#NULL!	#NULL!	#NULL!	#NULL!
#NULL!	#NULL!	#NULL!	#NULL!	#NULL!	#NULL!	#NULL!	#NULL!	#NULL!	#NULL!
#NULL!	#NULL!	#NULL!	#NULL!	#NULL!	#NULL!	#NULL!	#NULL!	#NULL!	#NULL!
#NULL!	#NULL!	#NULL!	#NULL!	#NULL!	#NULL!	#NULL!	#NULL!	#NULL!	#NULL!
#NULL!	#NULL!	#NULL!	#NULL!	#NULL!	#NULL!	#NULL!	#NULL!	#NULL!	#NULL!
#NULL!	#NULL!	#NULL!	#NULL!	#NULL!	#NULL!	#NULL!	#NULL!	#NULL!	#NULL!
#NULL!	#NULL!	#NULL!	#NULL!	#NULL!	#NULL!	#NULL!	#NULL!	#NULL!	#NULL!
#NULL!	#NULL!	#NULL!	#NULL!	#NULL!	#NULL!	#NULL!	#NULL!	#NULL!	#NULL!
#NULL!	#NULL!	#NULL!	#NULL!	#NULL!	#NULL!	#NULL!	#NULL!	#NULL!	#NULL!
#NULL!	#NULL!	#NULL!	#NULL!	#NULL!	#NULL!	#NULL!	#NULL!	#NULL!	#NULL!
#NULL!	#NULL!	#NULL!	#NULL!	#NULL!	#NULL!	#NULL!	#NULL!	#NULL!	#NULL!
#NULL!	#NULL!	#NULL!	#NULL!	#NULL!	#NULL!	#NULL!	#NULL!	#NULL!	#NULL!
#NULL!	#NULL!	#NULL!	#NULL!	#NULL!	#NULL!	#NULL!	#NULL!	#NULL!	#NULL!
#NULL!	#NULL!	#NULL!	#NULL!	#NULL!	#NULL!	#NULL!	#NULL!	#NULL!	#NULL!
#NULL!	#NULL!	#NULL!	#NULL!	#NULL!	#NULL!	#NULL!	#NULL!	#NULL!	#NULL!
#NULL!	#NULL!	#NULL!	#NULL!	#NULL!	#NULL!	#NULL!	#NULL!	#NULL!	#NULL!
#NULL!	#NULL!	#NULL!	#NULL!	#NULL!	#NULL!	#NULL!	#NULL!	#NULL!	#NULL!
#NULL!	#NULL!	#NULL!	#NULL!	#NULL!	#NULL!	#NULL!	#NULL!	#NULL!	#NULL!

#NULL!	#NULL!	#NULL!	#NULL!	#NULL!	#NULL!	#NULL!	#NULL!	#NULL!	#NULL!
#NULL!	#NULL!	#NULL!	#NULL!	#NULL!	#NULL!	#NULL!	#NULL!	#NULL!	#NULL!
#NULL!	#NULL!	#NULL!	#NULL!	#NULL!	#NULL!	#NULL!	#NULL!	#NULL!	#NULL!
#NULL!	#NULL!	#NULL!	#NULL!	#NULL!	#NULL!	#NULL!	#NULL!	#NULL!	#NULL!
#NULL!	#NULL!	#NULL!	#NULL!	#NULL!	#NULL!	#NULL!	#NULL!	#NULL!	#NULL!
#NULL!	#NULL!	#NULL!	#NULL!	#NULL!	#NULL!	#NULL!	#NULL!	#NULL!	#NULL!
#NULL!	#NULL!	#NULL!	#NULL!	#NULL!	#NULL!	#NULL!	#NULL!	#NULL!	#NULL!
#NULL!	#NULL!	#NULL!	#NULL!	#NULL!	#NULL!	#NULL!	#NULL!	#NULL!	#NULL!
#NULL!	#NULL!	#NULL!	#NULL!	#NULL!	#NULL!	#NULL!	#NULL!	#NULL!	#NULL!
#NULL!	#NULL!	#NULL!	#NULL!	#NULL!	#NULL!	#NULL!	#NULL!	#NULL!	#NULL!
#NULL!	#NULL!	#NULL!	#NULL!	#NULL!	#NULL!	#NULL!	#NULL!	#NULL!	#NULL!
#NULL!	#NULL!	#NULL!	#NULL!	#NULL!	#NULL!	#NULL!	#NULL!	#NULL!	#NULL!
#NULL!	#NULL!	#NULL!	#NULL!	#NULL!	#NULL!	#NULL!	#NULL!	#NULL!	#NULL!
2	2	2	2	2	2	2	2	2	2
2	2	2	2	2	2	2	2	2	2
2	2	2	2	2	2	2	2	2	2
2	2	1	2	#NULL!	2	2	2	2	2
2	2	2	2	2	2	2	2	2	2
2	2	2	2	2	2	2	2	2	2
1	2	2	2	1	2	2	2	2	2
2	2	2	2	2	2	2	2	2	2
2	1	2	1	2	1	1	1	1	1
#NULL!	#NULL!	#NULL!	#NULL!	#NULL!	#NULL!	#NULL!	#NULL!	#NULL!	#NULL!
1	2	1	2	2	1	2	1	1	1
2	#NULL!	#NULL!	#NULL!	#NULL!	#NULL!	2	#NULL!	#NULL!	#NULL!
2	2	2	2	2	2	2	1	1	1
#NULL!	#NULL!	#NULL!	#NULL!	#NULL!	#NULL!	#NULL!	#NULL!	#NULL!	#NULL!
2	2	2	2	2	2	2	2	2	2
2	2	2	2	2	2	1	2	2	2
2	2	2	2	2	2	2	2	2	2
2	2	2	2	2	2	2	1	1	1
2	2	1	2	2	1	2	2	2	2
#NULL!	#NULL!	2	#NULL!	#NULL!	#NULL!	#NULL!	#NULL!	#NULL!	#NULL!
2	2	2	2	2	2	2	2	2	2
2	2	2	1	#NULL!	2	2	2	2	#NULL!
#NULL!	2	#NULL!	#NULL!	#NULL!	#NULL!	#NULL!	#NULL!	#NULL!	#NULL!
2	2	2	2	2	2	2	2	2	2
2	2	2	2	1	1	1	2	2	2
2	1	2	2	2	2	2	2	2	2
2	2	1	2	2	1	2	2	2	2
#NULL!	#NULL!	#NULL!	#NULL!	#NULL!	#NULL!	#NULL!	#NULL!	#NULL!	#NULL!
#NULL!	#NULL!	#NULL!	#NULL!	#NULL!	#NULL!	#NULL!	#NULL!	#NULL!	#NULL!
2	2	2	2	2	2	2	2	2	2
2	2	2	2	2	2	2	2	2	2
#NULL!	#NULL!	#NULL!	#NULL!	#NULL!	#NULL!	#NULL!	#NULL!	#NULL!	#NULL!
#NULL!	#NULL!	#NULL!	#NULL!	#NULL!	#NULL!	#NULL!	#NULL!	#NULL!	#NULL!
#NULL!	#NULL!	#NULL!	#NULL!	#NULL!	#NULL!	#NULL!	#NULL!	#NULL!	#NULL!

2	#NULL!	#NULL!	#NULL!	#NULL!	#NULL!	2	#NULL!	#NULL!	#NULL!
#NULL!	#NULL!	#NULL!	#NULL!	#NULL!	#NULL!	#NULL!	#NULL!	#NULL!	#NULL!
1	2	2	1	2	2	1	2	1	2
2	2	2	2	2	2	2	2	2	2
2	2	2	1	1	1	1	2	2	2
#NULL!	#NULL!	#NULL!	#NULL!	#NULL!	#NULL!	#NULL!	2	#NULL!	#NULL!
2	2	2	2	2	2	2	2	2	2
2	2	2	2	2	2	2	2	2	2
2	2	2	2	2	2	2	2	2	2
2	2	2	1	1	1	1	2	2	2
1	1	#NULL!	2	1	2	1	1	1	1
#NULL!	#NULL!	#NULL!	#NULL!	#NULL!	#NULL!	#NULL!	#NULL!	#NULL!	#NULL!
#NULL!	#NULL!	1	#NULL!	#NULL!	#NULL!	#NULL!	#NULL!	#NULL!	#NULL!
2	2	2	2	2	2	2	2	2	2
2	2	2	2	2	2	2	2	2	2
2	2	2	2	2	2	2	2	2	2
2	2	2	1	2	2	1	2	2	1
2	2	2	2	2	2	2	2	2	2
2	2	2	#NULL!	2	#NULL!	2	2	2	2
#NULL!	#NULL!	#NULL!	#NULL!	#NULL!	#NULL!	#NULL!	#NULL!	#NULL!	#NULL!
2	2	2	2	2	2	2	2	2	2
2	2	2	2	2	2	2	2	2	2
2	2	2	2	2	2	2	2	2	2
2	2	2	2	2	2	2	2	2	2
2	2	2	2	2	2	2	2	2	2
2	2	2	2	2	2	2	2	1	1
2	2	2	2	2	2	2	2	2	2
2	2	2	2	2	2	2	2	2	2
2	2	#NULL!	#NULL!	#NULL!	#NULL!	#NULL!	#NULL!	#NULL!	#NULL!
2	2	2	2	2	2	2	2	2	1
2	1	1	2	2	1	1	2	1	1
2	2	2	2	2	2	2	2	2	2
2	2	2	2	2	2	2	2	2	2
2	2	2	2	2	2	2	2	2	2
1	2	2	2	2	2	2	2	2	2
2	2	2	2	2	2	2	2	1	1
2	1	1	1	2	2	2	2	2	2
#NULL!	2	2	2	2	2	2	2	#NULL!	#NULL!
#NULL!	2	#NULL!	#NULL!	#NULL!	#NULL!	#NULL!	#NULL!	#NULL!	#NULL!
2	2	2	2	2	2	2	2	2	2
2	2	2	2	2	2	2	2	1	1
2	2	2	2	2	2	2	1	1	2
2	2	2	1	2	2	1	2	2	1
#NULL!	#NULL!	#NULL!	#NULL!	#NULL!	#NULL!	#NULL!	#NULL!	#NULL!	#NULL!
2	2	2	2	2	2	2	2	2	2
2	2	1	1	1	1	1	1	1	#NULL!
2	2	2	1	1	1	2	2	1	1

2	2	2	2	2	2	2	2	2	2
2	2	2	2	2	2	1	2	1	2
2	1	1	1	1	1	1	1	1	1
2	2	1	2	1	2	1	1	1	2
2	2	2	2	2	2	2	2	2	2
2	2	2	2	2	2	2	2	2	2
2	2	2	2	2	2	2	2	2	2
1	2	2	1	1	1	1	2	2	2
2	2	2	2	2	2	2	2	2	2
1	1	2	2	1	2	1	2	2	2
2	2	2	2	2	2	2	2	2	2
2	2	2	2	2	2	2	2	2	2
2	2	2	2	2	2	2	2	2	2
1	1	1	1	1	1	1	1	2	1
2	2	2	2	2	2	2	2	2	2
2	2	2	2	2	1	2	1	2	1
2	2	2	2	2	2	2	2	2	2
2	2	2	2	2	2	2	2	2	2
2	2	2	2	2	2	2	2	2	2
2	2	2	1	2	2	1	2	2	1
2	2	2	2	2	2	2	2	2	2
1	2	2	2	2	2	2	2	2	2
2	2	2	2	2	2	2	2	2	2
1	2	2	2	2	2	1	1	1	1
2	2	2	2	2	2	2	1	1	#NULL!
#NULL!	#NULL!	#NULL!	#NULL!	#NULL!	#NULL!	#NULL!	#NULL!	#NULL!	#NULL!
#NULL!	#NULL!	#NULL!	#NULL!	#NULL!	#NULL!	#NULL!	#NULL!	#NULL!	#NULL!
2	2	2	2	2	2	2	2	2	2
#NULL!	#NULL!	#NULL!	#NULL!	#NULL!	#NULL!	#NULL!	#NULL!	#NULL!	#NULL!
2	1	1	2	1	2	2	2	1	1
2	2	2	2	2	2	2	2	1	1
2	2	1	2	2	2	1	2	2	1
1	#NULL!	#NULL!	#NULL!	#NULL!	#NULL!	#NULL!	#NULL!	#NULL!	#NULL!
2	2	2	2	2	2	2	2	2	2
#NULL!	#NULL!	2	2	2	2	#NULL!	2	2	2
2	2	2	2	2	2	2	2	2	2
#NULL!	#NULL!	#NULL!	#NULL!	#NULL!	#NULL!	#NULL!	#NULL!	#NULL!	#NULL!
2	1	1	2	2	2	2	2	2	2
#NULL!	#NULL!	#NULL!	#NULL!	#NULL!	#NULL!	#NULL!	#NULL!	#NULL!	#NULL!
2	2	2	2	2	2	2	2	2	2
2	2	1	2	1	2	1	2	1	2
2	2	2	2	2	2	2	2	2	2
2	2	1	2	2	1	2	2	2	2
#NULL!	#NULL!	#NULL!	#NULL!	#NULL!	#NULL!	#NULL!	#NULL!	#NULL!	#NULL!
#NULL!	#NULL!	#NULL!	#NULL!	#NULL!	#NULL!	#NULL!	#NULL!	#NULL!	#NULL!
#NULL!	#NULL!	#NULL!	#NULL!	#NULL!	#NULL!	#NULL!	#NULL!	#NULL!	#NULL!
2	2	2	2	2	2	2	2	2	2

#NULL!	#NULL!	#NULL!	#NULL!	#NULL!	#NULL!	#NULL!	#NULL!	#NULL!	#NULL!
#NULL!	#NULL!	#NULL!	#NULL!	#NULL!	#NULL!	#NULL!	#NULL!	#NULL!	#NULL!
2	1	2	2	1	2	2	2	2	2
#NULL!	#NULL!	#NULL!	#NULL!	#NULL!	#NULL!	#NULL!	#NULL!	#NULL!	#NULL!
#NULL!	#NULL!	#NULL!	#NULL!	#NULL!	#NULL!	#NULL!	#NULL!	#NULL!	#NULL!
2	1	1	1	1	1	1	1	1	1
1	2	2	2	1	1	2	2	2	2
#NULL!	#NULL!	#NULL!	#NULL!	#NULL!	#NULL!	#NULL!	#NULL!	#NULL!	#NULL!
2	2	2	2	2	2	2	2	2	2
2	2	2	1	2	2	1	2	2	1
2	2	2	1	2	2	1	2	2	1
2	2	2	2	2	2	2	2	2	2
2	2	2	2	2	2	2	2	2	2
#NULL!	#NULL!	#NULL!	#NULL!	#NULL!	#NULL!	#NULL!	#NULL!	#NULL!	#NULL!
2	2	2	2	2	2	2	2	2	2
2	2	2	2	2	2	2	2	2	2
2	2	2	2	2	2	2	2	2	2
2	2	1	2	2	2	2	1	1	2
2	2	2	2	1	1	2	2	2	2
#NULL!	#NULL!	#NULL!	#NULL!	#NULL!	#NULL!	#NULL!	#NULL!	#NULL!	#NULL!
2	2	1	2	2	2	2	1	1	2
2	1	1	1	1	2	#NULL!	2	#NULL!	2
2	2	2	2	2	2	2	2	2	2
2	2	1	2	2	2	2	2	1	1
1	2	1	1	1	2	1	1	1	2
#NULL!	#NULL!	#NULL!	#NULL!	#NULL!	#NULL!	#NULL!	#NULL!	#NULL!	#NULL!
2	2	2	1	1	1	2	1	1	2
#NULL!	#NULL!	#NULL!	#NULL!	#NULL!	#NULL!	#NULL!	#NULL!	#NULL!	#NULL!
2	1	1	1	1	1	1	1	1	1
2	2	2	2	2	2	2	2	2	2
2	2	2	2	2	2	2	2	2	2
#NULL!	#NULL!	#NULL!	#NULL!	#NULL!	#NULL!	#NULL!	#NULL!	#NULL!	2
#NULL!	#NULL!	#NULL!	#NULL!	#NULL!	#NULL!	#NULL!	#NULL!	#NULL!	2
2	2	2	2	2	2	2	2	2	2
2	2	2	2	2	2	2	2	1	1
1	2	2	1	2	2	2	1	2	2
#NULL!	2	2	2	2	2	#NULL!	#NULL!	#NULL!	#NULL!
2	2	2	2	2	1	1	1	2	2
2	2	2	2	2	2	2	2	2	2
#NULL!	2	#NULL!	#NULL!	#NULL!	#NULL!	#NULL!	#NULL!	#NULL!	#NULL!
2	2	2	2	2	2	2	2	2	2
2	2	2	2	2	2	2	2	2	2
2	2	2	2	2	2	2	2	2	2
2	2	2	2	2	2	2	2	2	2
2	2	2	2	2	2	2	2	2	2
#NULL!	2	#NULL!	#NULL!	#NULL!	#NULL!	#NULL!	#NULL!	#NULL!	#NULL!
1	2	1	1	2	2	2	2	2	2

2	2	2	2	2	1	2	2	2	2
2	2	2	2	2	2	2	2	2	2
2	2	2	1	1	1	1	1	1	1
2	2	2	2	2	2	2	2	2	2
2	2	2	2	2	2	2	2	2	2
2	2	2	2	2	2	2	2	2	1
2	2	2	1	1	1	2	2	2	2
2	2	2	2	1	2	2	2	1	2
2	2	2	2	2	2	2	2	2	2
2	2	2	2	2	1	1	1	1	2
2	2	2	2	2	1	2	1	2	2
2	2	2	2	2	2	2	2	2	2
2	2	2	2	2	2	2	2	2	2
2	2	2	2	2	2	2	2	2	1
2	1	2	2	#NULL!	#NULL!	2	#NULL!	2	2
2	2	2	2	2	2	2	2	2	2
2	2	#NULL!	2	#NULL!	2	#NULL!	2	2	2
2	2	2	2	2	2	2	2	#NULL!	2
2	2	2	2	1	2	2	2	1	2
2	2	2	2	2	2	2	2	2	2
2	2	2	2	2	2	2	2	2	2
#NULL!	#NULL!	#NULL!	#NULL!	#NULL!	#NULL!	#NULL!	#NULL!	#NULL!	#NULL!
2	2	2	2	2	2	2	2	2	2
2	2	2	2	2	2	2	2	2	2
2	2	2	2	2	#NULL!	2	2	#NULL!	2
2	2	2	2	2	2	2	2	2	2
2	2	2	2	2	2	2	2	2	2
2	2	2	2	2	2	2	2	2	2
2	2	2	2	2	2	#NULL!	1	1	1
2	2	2	2	2	2	2	2	1	2
2	2	2	2	2	2	1	2	1	1
2	2	2	2	2	2	2	2	2	2
2	1	2	2	2	2	2	2	2	2
#NULL!	#NULL!	#NULL!	#NULL!	#NULL!	#NULL!	#NULL!	#NULL!	#NULL!	#NULL!
#NULL!	#NULL!	#NULL!	#NULL!	#NULL!	#NULL!	2	#NULL!	#NULL!	#NULL!
2	2	2	2	2	2	1	2	1	1
2	2	2	2	2	2	2	2	2	2
2	2	2	2	2	2	2	1	2	2
2	2	2	2	2	2	2	2	#NULL!	2
2	2	2	2	2	2	2	2	2	2
2	2	2	2	2	1	2	1	2	1
#NULL!	#NULL!	#NULL!	#NULL!	#NULL!	#NULL!	#NULL!	#NULL!	#NULL!	#NULL!
#NULL!	#NULL!	#NULL!	#NULL!	#NULL!	#NULL!	#NULL!	#NULL!	#NULL!	#NULL!
2	2	2	2	2	2	2	2	2	2
1	2	2	2	1	1	1	2	2	2
#NULL!	#NULL!	#NULL!	#NULL!	#NULL!	#NULL!	#NULL!	#NULL!	#NULL!	#NULL!
#NULL!	#NULL!	#NULL!	#NULL!	#NULL!	#NULL!	#NULL!	#NULL!	#NULL!	#NULL!

2	2	2	2	2	2	2	2	2	2
1	1	1	1	1	1	2	2	2	2
2	2	2	1	2	2	1	2	2	1
2	2	2	2	2	2	2	2	2	2
2	2	2	2	2	2	2	2	2	2
2	2	2	1	2	2	1	2	2	1
2	2	2	2	2	2	2	2	2	2
#NULL!	#NULL!	#NULL!	#NULL!	#NULL!	#NULL!	#NULL!	2	#NULL!	#NULL!
2	2	2	2	2	1	1	1	2	2
#NULL!	#NULL!	#NULL!	#NULL!	#NULL!	#NULL!	#NULL!	#NULL!	#NULL!	#NULL!
2	2	2	2	2	2	2	2	2	2
2	2	2	2	2	2	2	2	2	2
2	2	2	2	2	2	2	2	2	2
#NULL!	#NULL!	#NULL!	#NULL!	#NULL!	#NULL!	#NULL!	#NULL!	#NULL!	#NULL!
2	2	2	2	2	2	2	2	2	2
#NULL!	#NULL!	#NULL!	#NULL!	#NULL!	#NULL!	#NULL!	#NULL!	#NULL!	#NULL!
#NULL!	#NULL!	#NULL!	#NULL!	#NULL!	#NULL!	#NULL!	#NULL!	#NULL!	#NULL!
2	2	2	2	2	2	2	2	2	2
2	2	2	2	2	2	2	2	2	2
2	2	2	2	2	2	2	2	2	2
2	2	2	2	2	2	2	2	2	2
2	2	2	2	2	2	2	2	2	2
1	2	2	2	2	2	2	2	2	2
2	2	2	2	1	2	2	2	1	2
#NULL!	1	1	1	#NULL!	1	1	1	1	#NULL!
#NULL!	2	#NULL!	#NULL!	#NULL!	#NULL!	#NULL!	#NULL!	#NULL!	#NULL!
2	2	2	2	2	2	2	2	2	2
2	2	2	2	2	2	#NULL!	2	2	2
2	2	2	2	2	2	2	2	2	2
2	1	2	2	2	1	2	2	2	2
2	2	2	2	2	2	2	2	2	2
2	2	2	2	2	2	2	2	2	2
2	2	2	2	2	2	2	2	2	2
2	2	2	2	2	2	2	2	2	2
2	2	2	2	2	2	2	2	2	2
2	2	2	2	2	2	2	2	2	2
1	2	1	2	2	1	2	1	2	2
#NULL!	#NULL!	#NULL!	#NULL!	#NULL!	#NULL!	#NULL!	2	#NULL!	#NULL!
2	#NULL!	#NULL!	#NULL!	#NULL!	#NULL!	#NULL!	2	#NULL!	#NULL!
2	2	2	2	2	2	2	2	2	2
1	1	1	1	1	1	1	1	1	1
2	#NULL!	2	#NULL!	#NULL!	#NULL!	#NULL!	#NULL!	#NULL!	#NULL!
2	2	2	2	2	2	2	2	2	2
#NULL!	2	#NULL!	#NULL!	#NULL!	#NULL!	#NULL!	#NULL!	#NULL!	#NULL!
2	1	1	1	1	1	1	1	1	1
#NULL!	#NULL!	#NULL!	#NULL!	#NULL!	#NULL!	#NULL!	#NULL!	#NULL!	#NULL!
2	2	2	2	2	2	2	2	2	2

1	1	2	2	2	2	2	1	2	1
#NULL!	#NULL!	#NULL!	#NULL!	#NULL!	#NULL!	#NULL!	#NULL!	#NULL!	#NULL!
2	2	2	2	2	2	2	2	2	2
2	2	2	2	2	2	2	2	2	2
2	2	2	2	2	2	2	2	2	2
2	2	2	2	1	2	2	2	2	2
2	2	2	2	2	2	2	2	2	2
#NULL!	#NULL!	#NULL!	#NULL!	#NULL!	#NULL!	#NULL!	#NULL!	#NULL!	#NULL!
2	2	2	2	2	2	2	2	2	2
2	2	2	2	2	2	2	2	2	2
2	2	2	2	2	2	2	2	2	2
2	2	2	2	2	2	2	2	2	2
2	2	2	2	2	2	2	2	2	2
2	2	2	1	2	2	2	1	1	1
1	2	1	2	1	2	2	2	1	2
2	2	1	2	#NULL!	1	2	2	2	2
2	2	2	2	2	2	2	2	2	2
2	2	2	2	2	2	2	2	2	2
#NULL!	#NULL!	#NULL!	#NULL!	#NULL!	#NULL!	#NULL!	#NULL!	#NULL!	#NULL!
#NULL!	#NULL!	#NULL!	#NULL!	#NULL!	#NULL!	#NULL!	#NULL!	#NULL!	#NULL!
1	2	2	2	2	2	2	2	2	2
2	2	2	2	2	2	2	2	1	2
#NULL!	#NULL!	#NULL!	#NULL!	#NULL!	#NULL!	#NULL!	#NULL!	#NULL!	#NULL!
#NULL!	#NULL!	#NULL!	#NULL!	#NULL!	#NULL!	#NULL!	#NULL!	#NULL!	#NULL!
1	1	1	1	1	1	1	1	1	1
#NULL!	#NULL!	#NULL!	#NULL!	#NULL!	#NULL!	#NULL!	#NULL!	#NULL!	#NULL!
#NULL!	#NULL!	#NULL!	#NULL!	#NULL!	#NULL!	#NULL!	#NULL!	#NULL!	#NULL!
#NULL!	#NULL!	#NULL!	#NULL!	#NULL!	#NULL!	#NULL!	#NULL!	#NULL!	#NULL!
2	2	2	2	2	2	1	1	2	2
2	2	2	2	2	2	2	2	2	2
2	2	2	2	2	2	2	2	2	2
2	2	2	2	#NULL!	2	2	2	2	2
1	1	2	2	2	2	1	2	2	1
2	2	2	2	2	2	2	2	2	2
2	2	2	2	2	2	2	2	2	2
2	2	2	2	2	2	2	2	2	2
2	2	2	2	2	2	2	2	2	1
1	2	2	1	#NULL!	2	1	#NULL!	2	1
2	2	2	2	2	2	1	2	2	2
2	2	2	2	2	2	2	2	1	1
2	2	2	2	2	2	2	1	1	2
2	2	2	2	2	2	2	2	2	2
#NULL!	2	#NULL!	#NULL!	#NULL!	#NULL!	#NULL!	#NULL!	#NULL!	#NULL!
#NULL!	#NULL!	#NULL!	#NULL!	#NULL!	2	#NULL!	#NULL!	#NULL!	#NULL!
1	2	2	2	2	1	2	2	2	2
2	2	2	2	2	2	2	2	2	2
2	2	2	2	2	2	2	2	2	2

1	2	2	2	2	#NULL!	2	2	2	2
#NULL!	#NULL!	#NULL!	#NULL!	#NULL!	#NULL!	#NULL!	#NULL!	#NULL!	#NULL!
#NULL!	#NULL!	#NULL!	#NULL!	#NULL!	#NULL!	#NULL!	#NULL!	#NULL!	#NULL!
#NULL!	#NULL!	#NULL!	#NULL!	#NULL!	#NULL!	#NULL!	#NULL!	#NULL!	#NULL!
#NULL!	#NULL!	#NULL!	#NULL!	#NULL!	#NULL!	#NULL!	#NULL!	#NULL!	#NULL!
2	#NULL!	#NULL!	#NULL!	#NULL!	#NULL!	2	#NULL!	#NULL!	#NULL!
#NULL!	#NULL!	#NULL!	#NULL!	#NULL!	#NULL!	#NULL!	#NULL!	#NULL!	#NULL!
#NULL!	#NULL!	#NULL!	#NULL!	#NULL!	#NULL!	#NULL!	#NULL!	#NULL!	#NULL!
#NULL!	#NULL!	#NULL!	#NULL!	#NULL!	#NULL!	#NULL!	#NULL!	#NULL!	#NULL!
#NULL!	2	#NULL!	#NULL!	#NULL!	#NULL!	#NULL!	#NULL!	#NULL!	#NULL!
2	2	2	2	2	2	2	2	2	2
2	2	2	1	2	2	2	1	1	2
#NULL!	2	2	2	2	2	2	2	2	2
2	2	1	2	1	2	2	2	2	2
2	2	2	2	2	2	2	2	2	2
#NULL!	#NULL!	#NULL!	#NULL!	#NULL!	#NULL!	#NULL!	#NULL!	#NULL!	#NULL!
1	2	2	2	2	2	2	2	2	2
2	2	2	2	2	2	2	2	2	2
#NULL!	2	2	2	2	2	2	2	2	2
2	2	2	2	2	2	2	1	2	1
2	2	2	1	2	2	1	2	1	2
1	2	1	2	1	2	2	1	2	2
1	2	2	2	2	2	2	2	2	2
2	2	2	2	2	2	2	2	2	2
2	2	2	2	2	2	2	2	2	2
2	2	2	2	2	2	2	2	2	2
2	1	1	1	1	1	1	1	1	1
2	2	2	2	2	2	2	2	2	2
2	2	2	2	2	2	2	2	2	2
2	2	2	2	2	2	2	2	2	2
#NULL!	#NULL!	#NULL!	#NULL!	#NULL!	#NULL!	#NULL!	#NULL!	#NULL!	#NULL!
2	2	2	2	2	2	2	2	2	2
2	2	2	2	2	2	2	2	2	2
#NULL!	2	#NULL!	#NULL!	#NULL!	#NULL!	#NULL!	#NULL!	#NULL!	#NULL!
1	#NULL!	#NULL!	#NULL!	#NULL!	#NULL!	#NULL!	#NULL!	#NULL!	#NULL!
2	2	2	2	2	2	2	2	2	2
2	2	2	2	2	2	2	2	2	2
2	2	2	2	2	2	2	2	2	2
2	2	1	1	1	1	2	1	1	1
#NULL!	#NULL!	#NULL!	#NULL!	#NULL!	#NULL!	#NULL!	#NULL!	#NULL!	#NULL!
1	2	2	2	2	2	2	2	2	2
2	2	2	2	2	2	2	2	2	2
2	2	2	2	2	2	2	2	2	2
2	2	2	2	2	2	2	2	2	2
2	2	2	2	2	2	2	2	2	2
#NULL!	#NULL!	#NULL!	#NULL!	#NULL!	#NULL!	#NULL!	#NULL!	#NULL!	#NULL!
#NULL!	#NULL!	#NULL!	#NULL!	#NULL!	#NULL!	#NULL!	#NULL!	#NULL!	#NULL!

#NULL!	#NULL!	#NULL!	#NULL!	#NULL!	#NULL!	#NULL!	#NULL!	#NULL!	#NULL!
2	2	2	2	2	2	2	2	1	1
#NULL!	2	#NULL!	#NULL!	#NULL!	#NULL!	#NULL!	#NULL!	#NULL!	#NULL!
#NULL!	#NULL!	#NULL!	#NULL!	#NULL!	#NULL!	#NULL!	#NULL!	#NULL!	#NULL!
#NULL!	#NULL!	#NULL!	#NULL!	#NULL!	#NULL!	#NULL!	#NULL!	#NULL!	#NULL!
#NULL!	#NULL!	#NULL!	#NULL!	#NULL!	#NULL!	#NULL!	#NULL!	#NULL!	#NULL!
#NULL!	#NULL!	#NULL!	#NULL!	#NULL!	#NULL!	#NULL!	#NULL!	#NULL!	#NULL!
#NULL!	#NULL!	#NULL!	#NULL!	#NULL!	#NULL!	#NULL!	#NULL!	#NULL!	#NULL!
#NULL!	#NULL!	#NULL!	#NULL!	#NULL!	#NULL!	#NULL!	#NULL!	#NULL!	#NULL!
#NULL!	#NULL!	#NULL!	#NULL!	#NULL!	#NULL!	#NULL!	#NULL!	#NULL!	#NULL!
#NULL!	#NULL!	#NULL!	#NULL!	#NULL!	#NULL!	#NULL!	#NULL!	#NULL!	#NULL!
#NULL!	#NULL!	#NULL!	#NULL!	#NULL!	#NULL!	#NULL!	#NULL!	#NULL!	#NULL!
#NULL!	#NULL!	#NULL!	#NULL!	#NULL!	#NULL!	#NULL!	#NULL!	#NULL!	#NULL!
#NULL!	#NULL!	#NULL!	#NULL!	#NULL!	#NULL!	#NULL!	#NULL!	#NULL!	#NULL!
#NULL!	#NULL!	#NULL!	#NULL!	#NULL!	#NULL!	#NULL!	#NULL!	#NULL!	#NULL!
#NULL!	#NULL!	#NULL!	#NULL!	#NULL!	#NULL!	#NULL!	#NULL!	#NULL!	#NULL!
#NULL!	#NULL!	#NULL!	#NULL!	#NULL!	#NULL!	#NULL!	#NULL!	#NULL!	#NULL!
#NULL!	#NULL!	#NULL!	#NULL!	#NULL!	#NULL!	#NULL!	#NULL!	#NULL!	#NULL!
#NULL!	#NULL!	#NULL!	#NULL!	#NULL!	#NULL!	#NULL!	#NULL!	#NULL!	#NULL!
#NULL!	#NULL!	#NULL!	#NULL!	#NULL!	#NULL!	#NULL!	#NULL!	#NULL!	#NULL!
#NULL!	#NULL!	#NULL!	#NULL!	#NULL!	#NULL!	#NULL!	#NULL!	#NULL!	#NULL!
#NULL!	#NULL!	#NULL!	#NULL!	#NULL!	#NULL!	#NULL!	#NULL!	#NULL!	#NULL!
#NULL!	#NULL!	#NULL!	#NULL!	#NULL!	#NULL!	#NULL!	#NULL!	#NULL!	#NULL!
2	2	2	2	2	2	2	2	2	2
2	2	2	2	2	2	2	2	2	2
2	2	2	2	2	2	2	2	2	2
2	2	2	2	2	2	2	2	2	2
2	2	2	1	1	2	2	1	2	2
#NULL!	2	2	2	2	2	2	2	1	1
2	2	2	2	2	2	2	2	2	2
1	2	2	2	2	2	2	1	#NULL!	2
2	1	2	2	2	2	2	2	2	2
1	2	2	2	2	2	2	1	1	1
#NULL!	2	2	2	2	2	2	2	1	2
2	2	2	2	2	2	2	2	1	2
2	2	2	2	2	2	2	2	2	2
#NULL!	2	2	2	2	2	2	2	2	2
2	2	2	2	2	2	2	2	2	2
2	2	2	2	2	2	2	2	1	2
#NULL!	2	#NULL!	#NULL!	#NULL!	#NULL!	#NULL!	#NULL!	#NULL!	#NULL!
2	2	2	2	2	2	1	2	2	2
2	2	2	2	2	2	2	2	2	1
#NULL!	2	#NULL!	#NULL!	#NULL!	#NULL!	2	#NULL!	#NULL!	#NULL!
2	2	2	2	2	2	2	2	2	2
2	2	2	2	2	2	2	2	1	2
2	2	2	2	2	2	2	2	2	2
1	1	2	2	2	2	2	2	#NULL!	2

2	2	2	2	2	2	2	2	2	2
2	2	2	2	2	2	2	2	1	2
2	2	2	2	2	2	2	2	2	2
2	2	2	2	2	2	2	2	2	2
2	1	2	2	1	2	2	2	2	2
#NULL!	1	#NULL!	#NULL!	#NULL!	#NULL!	#NULL!	#NULL!	#NULL!	#NULL!
2	2	2	2	2	2	2	1	1	2
#NULL!	2	#NULL!	#NULL!	#NULL!	#NULL!	#NULL!	#NULL!	#NULL!	#NULL!
2	2	2	2	2	2	2	2	2	2
2	2	2	2	2	2	2	2	2	2
#NULL!	#NULL!	#NULL!	#NULL!	2	#NULL!	#NULL!	#NULL!	#NULL!	#NULL!
#NULL!	2	#NULL!	#NULL!	#NULL!	#NULL!	#NULL!	#NULL!	#NULL!	#NULL!
#NULL!	2	#NULL!	#NULL!	#NULL!	#NULL!	#NULL!	#NULL!	#NULL!	#NULL!
#NULL!	2	#NULL!	#NULL!	#NULL!	#NULL!	#NULL!	#NULL!	#NULL!	#NULL!
#NULL!	#NULL!	2	#NULL!	#NULL!	#NULL!	#NULL!	#NULL!	#NULL!	#NULL!
#NULL!	#NULL!	#NULL!	#NULL!	#NULL!	#NULL!	#NULL!	#NULL!	#NULL!	#NULL!
#NULL!	#NULL!	#NULL!	#NULL!	#NULL!	#NULL!	#NULL!	#NULL!	#NULL!	#NULL!
2	#NULL!	#NULL!	#NULL!	#NULL!	#NULL!	#NULL!	#NULL!	#NULL!	#NULL!
#NULL!	#NULL!	#NULL!	2	#NULL!	#NULL!	#NULL!	#NULL!	#NULL!	#NULL!
2	2	2	2	1	2	2	1	1	1
2	#NULL!	2	2	2	2	2	2	2	2
#NULL!	#NULL!	2	#NULL!	#NULL!	#NULL!	#NULL!	#NULL!	#NULL!	#NULL!
#NULL!	#NULL!	2	#NULL!	#NULL!	#NULL!	#NULL!	#NULL!	#NULL!	#NULL!
#NULL!	#NULL!	2	#NULL!	#NULL!	#NULL!	#NULL!	#NULL!	#NULL!	#NULL!
#NULL!	2	#NULL!	#NULL!	#NULL!	#NULL!	#NULL!	#NULL!	#NULL!	#NULL!
1	2	2	1	2	2	2	1	2	2
2	1	2	1	2	2	2	1	1	1
2	2	1	2	2	2	2	2	1	1
2	2	2	2	2	2	2	2	2	2
#NULL!	#NULL!	#NULL!	#NULL!	#NULL!	#NULL!	#NULL!	#NULL!	#NULL!	#NULL!
2	2	2	2	2	2	1	2	2	2
2	2	2	1	1	2	1	1	1	1
1	1	1	1	1	1	1	1	1	1
2	2	1	2	1	2	2	2	1	2
2	2	2	1	1	1	1	1	1	2
#NULL!	1	#NULL!	#NULL!	#NULL!	#NULL!	#NULL!	#NULL!	#NULL!	#NULL!
2	2	2	2	2	2	2	2	2	2
2	2	2	2	2	2	2	2	1	2
2	2	2	2	2	2	2	1	1	1
2	2	2	2	2	2	1	2	1	1
2	2	2	2	2	2	2	2	2	2
2	2	2	2	2	2	2	2	2	2
2	2	1	2	2	2	2	2	1	1
2	2	2	2	2	1	1	1	1	2
2	2	2	2	2	2	2	1	1	2
#NULL!	2	2	2	2	1	2	2	1	2
2	2	2	2	2	2	2	2	1	2

2	2	2	2	2	2	2	2	1	2
1	2	2	2	#NULL!	2	2	1	#NULL!	2
#NULL!	#NULL!	#NULL!	#NULL!	#NULL!	#NULL!	#NULL!	#NULL!	#NULL!	#NULL!
2	#NULL!	2	2	2	2	2	2	1	#NULL!
#NULL!	#NULL!	#NULL!	#NULL!	#NULL!	#NULL!	#NULL!	#NULL!	#NULL!	#NULL!
2	2	2	2	2	2	2	2	1	2
2	2	2	2	2	2	2	2	2	1
#NULL!	#NULL!	#NULL!	#NULL!	#NULL!	#NULL!	#NULL!	#NULL!	#NULL!	#NULL!
#NULL!	2	#NULL!	#NULL!	#NULL!	#NULL!	#NULL!	#NULL!	#NULL!	#NULL!
2	2	2	2	2	2	2	2	2	2
2	2	1	2	2	2	2	2	1	2
2	#NULL!	2	2	2	2	2	1	1	2
2	2	2	2	2	2	2	#NULL!	2	2
#NULL!	#NULL!	#NULL!	#NULL!	#NULL!	#NULL!	#NULL!	#NULL!	#NULL!	#NULL!
#NULL!	#NULL!	#NULL!	#NULL!	#NULL!	#NULL!	#NULL!	#NULL!	#NULL!	#NULL!
2	2	2	2	2	2	2	2	2	2
#NULL!	2	#NULL!	#NULL!	#NULL!	#NULL!	#NULL!	#NULL!	#NULL!	#NULL!
#NULL!	#NULL!	#NULL!	#NULL!	#NULL!	#NULL!	#NULL!	#NULL!	#NULL!	#NULL!
1	1	1	1	1	1	1	1	1	1
2	2	2	2	2	2	2	2	2	2
2	2	2	2	2	2	2	2	2	2
2	2	2	2	2	2	2	2	2	2
1	2	2	1	1	1	2	2	2	2
2	2	2	2	2	2	2	2	1	2
2	2	2	2	2	2	2	2	2	2
#NULL!	#NULL!	#NULL!	#NULL!	#NULL!	#NULL!	#NULL!	#NULL!	#NULL!	#NULL!
#NULL!	2	#NULL!	#NULL!	#NULL!	#NULL!	#NULL!	#NULL!	#NULL!	#NULL!
2	2	2	2	2	1	2	1	1	2
2	1	1	2	2	2	2	2	2	1
1	2	1	2	1	2	1	1	#NULL!	2
#NULL!	#NULL!	#NULL!	#NULL!	#NULL!	#NULL!	#NULL!	#NULL!	#NULL!	#NULL!
#NULL!	#NULL!	#NULL!	#NULL!	#NULL!	#NULL!	#NULL!	#NULL!	#NULL!	#NULL!
#NULL!	#NULL!	#NULL!	#NULL!	#NULL!	#NULL!	#NULL!	#NULL!	#NULL!	#NULL!
#NULL!	#NULL!	#NULL!	#NULL!	#NULL!	#NULL!	#NULL!	#NULL!	#NULL!	#NULL!
#NULL!	#NULL!	#NULL!	#NULL!	#NULL!	#NULL!	#NULL!	#NULL!	#NULL!	#NULL!
#NULL!	#NULL!	#NULL!	#NULL!	#NULL!	#NULL!	#NULL!	#NULL!	#NULL!	#NULL!
#NULL!	#NULL!	#NULL!	#NULL!	#NULL!	#NULL!	#NULL!	#NULL!	#NULL!	#NULL!
#NULL!	#NULL!	#NULL!	#NULL!	#NULL!	#NULL!	#NULL!	#NULL!	#NULL!	#NULL!
#NULL!	#NULL!	#NULL!	#NULL!	#NULL!	#NULL!	#NULL!	#NULL!	#NULL!	#NULL!
#NULL!	#NULL!	#NULL!	#NULL!	#NULL!	#NULL!	#NULL!	#NULL!	#NULL!	#NULL!
#NULL!	#NULL!	#NULL!	#NULL!	#NULL!	#NULL!	#NULL!	#NULL!	#NULL!	#NULL!
#NULL!	#NULL!	#NULL!	#NULL!	#NULL!	#NULL!	#NULL!	#NULL!	#NULL!	#NULL!
#NULL!	#NULL!	#NULL!	#NULL!	#NULL!	#NULL!	#NULL!	#NULL!	#NULL!	#NULL!
#NULL!	#NULL!	#NULL!	#NULL!	#NULL!	#NULL!	#NULL!	#NULL!	#NULL!	#NULL!
#NULL!	#NULL!	#NULL!	#NULL!	#NULL!	#NULL!	#NULL!	#NULL!	#NULL!	#NULL!
#NULL!	#NULL!	#NULL!	#NULL!	#NULL!	#NULL!	#NULL!	#NULL!	#NULL!	#NULL!
#NULL!	#NULL!	#NULL!	#NULL!	#NULL!	#NULL!	#NULL!	#NULL!	#NULL!	#NULL!

#NULL!	#NULL!	#NULL!	#NULL!	#NULL!	#NULL!	#NULL!	#NULL!	#NULL!	#NULL!
#NULL!	#NULL!	#NULL!	#NULL!	#NULL!	#NULL!	#NULL!	#NULL!	#NULL!	#NULL!
#NULL!	#NULL!	#NULL!	#NULL!	#NULL!	#NULL!	#NULL!	#NULL!	#NULL!	#NULL!
#NULL!	#NULL!	#NULL!	#NULL!	#NULL!	#NULL!	#NULL!	#NULL!	#NULL!	#NULL!
#NULL!	#NULL!	#NULL!	#NULL!	#NULL!	#NULL!	#NULL!	#NULL!	#NULL!	#NULL!
#NULL!	#NULL!	#NULL!	#NULL!	#NULL!	#NULL!	#NULL!	#NULL!	#NULL!	#NULL!
#NULL!	#NULL!	#NULL!	#NULL!	#NULL!	#NULL!	#NULL!	#NULL!	#NULL!	#NULL!
#NULL!	#NULL!	#NULL!	#NULL!	#NULL!	#NULL!	#NULL!	#NULL!	#NULL!	#NULL!
#NULL!	#NULL!	#NULL!	#NULL!	#NULL!	#NULL!	#NULL!	#NULL!	#NULL!	#NULL!
#NULL!	#NULL!	#NULL!	#NULL!	#NULL!	#NULL!	#NULL!	#NULL!	#NULL!	#NULL!
#NULL!	#NULL!	#NULL!	#NULL!	#NULL!	#NULL!	#NULL!	#NULL!	#NULL!	#NULL!
#NULL!	#NULL!	#NULL!	#NULL!	#NULL!	#NULL!	#NULL!	#NULL!	#NULL!	#NULL!
#NULL!	#NULL!	#NULL!	#NULL!	#NULL!	#NULL!	#NULL!	#NULL!	#NULL!	#NULL!
#NULL!	#NULL!	#NULL!	#NULL!	#NULL!	#NULL!	#NULL!	#NULL!	#NULL!	#NULL!
#NULL!	#NULL!	#NULL!	#NULL!	#NULL!	#NULL!	#NULL!	#NULL!	#NULL!	#NULL!
#NULL!	#NULL!	#NULL!	#NULL!	#NULL!	#NULL!	#NULL!	#NULL!	#NULL!	#NULL!
#NULL!	#NULL!	#NULL!	#NULL!	#NULL!	#NULL!	#NULL!	#NULL!	#NULL!	#NULL!
#NULL!	#NULL!	#NULL!	#NULL!	#NULL!	#NULL!	#NULL!	#NULL!	#NULL!	#NULL!
#NULL!	#NULL!	#NULL!	#NULL!	#NULL!	#NULL!	#NULL!	#NULL!	#NULL!	#NULL!
2	#NULL!	#NULL!	#NULL!	#NULL!	#NULL!	#NULL!	#NULL!	#NULL!	#NULL!
#NULL!	#NULL!	#NULL!	#NULL!	#NULL!	#NULL!	#NULL!	#NULL!	#NULL!	#NULL!
#NULL!	#NULL!	#NULL!	#NULL!	#NULL!	#NULL!	#NULL!	#NULL!	#NULL!	#NULL!
#NULL!	#NULL!	#NULL!	#NULL!	#NULL!	#NULL!	#NULL!	#NULL!	#NULL!	#NULL!
#NULL!	#NULL!	#NULL!	#NULL!	#NULL!	#NULL!	#NULL!	#NULL!	#NULL!	#NULL!
#NULL!	#NULL!	#NULL!	#NULL!	#NULL!	#NULL!	#NULL!	#NULL!	#NULL!	#NULL!
#NULL!	#NULL!	#NULL!	#NULL!	#NULL!	#NULL!	#NULL!	#NULL!	#NULL!	#NULL!
#NULL!	#NULL!	#NULL!	#NULL!	#NULL!	#NULL!	#NULL!	#NULL!	#NULL!	#NULL!
#NULL!	#NULL!	#NULL!	#NULL!	#NULL!	#NULL!	#NULL!	#NULL!	#NULL!	#NULL!
#NULL!	#NULL!	#NULL!	#NULL!	#NULL!	#NULL!	#NULL!	#NULL!	#NULL!	#NULL!
#NULL!	#NULL!	#NULL!	#NULL!	#NULL!	#NULL!	#NULL!	#NULL!	#NULL!	#NULL!
#NULL!	#NULL!	#NULL!	#NULL!	#NULL!	#NULL!	#NULL!	#NULL!	#NULL!	#NULL!
#NULL!	#NULL!	#NULL!	#NULL!	#NULL!	#NULL!	#NULL!	#NULL!	#NULL!	#NULL!
#NULL!	#NULL!	#NULL!	#NULL!	#NULL!	#NULL!	#NULL!	#NULL!	#NULL!	#NULL!
#NULL!	#NULL!	#NULL!	#NULL!	#NULL!	#NULL!	#NULL!	#NULL!	#NULL!	#NULL!
#NULL!	#NULL!	#NULL!	#NULL!	#NULL!	#NULL!	#NULL!	#NULL!	#NULL!	#NULL!
2	2	2	2	2	2	2	2	2	2
#NULL!	#NULL!	#NULL!	#NULL!	#NULL!	#NULL!	#NULL!	#NULL!	#NULL!	#NULL!
2	1	1	2	2	1	2	1	1	1
2	2	2	2	2	2	2	2	2	1
1	1	1	1	1	1	1	1	1	1
#NULL!	#NULL!	#NULL!	#NULL!	#NULL!	#NULL!	#NULL!	#NULL!	#NULL!	#NULL!
#NULL!	2	2	2	2	2	1	2	1	1
#NULL!	#NULL!	2	#NULL!	#NULL!	#NULL!	#NULL!	#NULL!	#NULL!	#NULL!
#NULL!	2	2	2	2	2	1	2	1	1
2	2	2	2	2	2	1	2	2	2
#NULL!	#NULL!	2	#NULL!	#NULL!	2	2	#NULL!	#NULL!	#NULL!
2	2	2	2	2	2	2	2	2	2

2	2	2	2	2	2	2	2	1	2
2	2	2	2	2	2	2	2	2	1
2	2	2	2	2	2	2	2	2	2
2	2	2	1	1	2	#NULL!	2	2	2
2	2	2	2	2	2	2	2	1	2
2	2	2	2	2	2	2	1	1	2
2	2	2	2	2	2	2	2	2	1
2	2	2	2	2	2	2	2	2	1
2	2	1	2	2	1	#NULL!	2	1	2
2	2	2	2	2	1	2	1	#NULL!	2
#NULL!	#NULL!	2	#NULL!	#NULL!	#NULL!	#NULL!	#NULL!	#NULL!	#NULL!
1	2	#NULL!	2	1	2	2	#NULL!	2	2
#NULL!	#NULL!	#NULL!	#NULL!	#NULL!	#NULL!	#NULL!	#NULL!	#NULL!	#NULL!
2	2	2	2	2	#NULL!	#NULL!	2	2	2
2	2	2	2	2	1	1	2	2	2
2	2	2	2	2	2	2	2	2	2
#NULL!	#NULL!	#NULL!	#NULL!	#NULL!	#NULL!	#NULL!	#NULL!	#NULL!	#NULL!
#NULL!	2	2	#NULL!	#NULL!	#NULL!	#NULL!	#NULL!	#NULL!	#NULL!
2	2	2	2	2	2	2	2	2	2
2	1	2	2	2	2	2	2	2	2
2	2	2	2	2	2	2	2	1	2
2	2	2	2	2	2	2	2	2	2
2	2	2	2	2	2	1	2	2	2
2	2	2	2	2	2	1	1	1	2
1	1	1	1	1	1	2	1	2	1
2	2	1	2	2	2	1	2	2	2
2	2	2	2	2	2	2	2	2	2
2	2	2	2	1	1	1	2	2	1
2	2	2	2	2	2	2	2	2	2
#NULL!	#NULL!	#NULL!	#NULL!	#NULL!	#NULL!	#NULL!	#NULL!	#NULL!	#NULL!
#NULL!	#NULL!	#NULL!	#NULL!	#NULL!	#NULL!	#NULL!	#NULL!	#NULL!	#NULL!
2	2	2	2	2	2	2	2	2	2
2	2	2	2	2	2	2	2	2	2
#NULL!	#NULL!	#NULL!	#NULL!	#NULL!	#NULL!	#NULL!	#NULL!	#NULL!	#NULL!
#NULL!	#NULL!	#NULL!	#NULL!	#NULL!	#NULL!	#NULL!	#NULL!	#NULL!	#NULL!
#NULL!	#NULL!	#NULL!	#NULL!	#NULL!	#NULL!	#NULL!	#NULL!	#NULL!	#NULL!
#NULL!	2	2	2	2	1	2	2	1	2
2	2	2	2	2	2	2	2	2	2
#NULL!	#NULL!	#NULL!	#NULL!	#NULL!	#NULL!	#NULL!	#NULL!	#NULL!	#NULL!
#NULL!	#NULL!	#NULL!	#NULL!	#NULL!	#NULL!	#NULL!	#NULL!	#NULL!	#NULL!
2	2	2	2	2	2	2	1	1	1
#NULL!	#NULL!	#NULL!	#NULL!	#NULL!	#NULL!	#NULL!	#NULL!	#NULL!	#NULL!
2	2	2	2	2	2	2	2	2	1
2	1	1	2	2	1	2	1	2	2
1	2	2	2	1	1	2	2	2	2
1	1	1	1	1	2	1	1	1	1
2	2	2	1	2	1	1	2	2	1

2	2	1	2	1	2	1	2	1	2
2	2	2	2	2	2	2	1	1	2
1	2	2	1	2	1	2	2	2	2
2	2	2	2	2	2	2	2	2	2
2	2	2	1	2	2	2	2	2	2
2	2	2	1	2	2	1	2	2	1
1	2	2	1	2	2	2	2	2	2
2	2	1	2	2	1	2	2	2	1
2	2	2	2	2	2	2	1	2	2
2	2	2	#NULL!	#NULL!	2	#NULL!	2	#NULL!	#NULL!
1	1	1	1	1	1	1	2	2	1
2	2	2	2	1	2	2	2	2	2
2	1	2	1	#NULL!	2	2	2	1	2
2	2	2	1	1	2	2	2	1	1
2	2	2	1	1	2	1	2	1	2
2	2	2	2	2	1	2	2	2	2
2	2	2	2	2	2	1	2	2	2
#NULL!	#NULL!	2	#NULL!	#NULL!	#NULL!	#NULL!	#NULL!	#NULL!	#NULL!
2	2	2	1	2	2	2	1	2	2
2	2	2	2	2	1	1	2	2	2
2	2	2	1	2	2	1	2	2	2
2	2	2	2	2	2	2	2	2	2
2	2	2	2	2	2	1	1	1	2
2	2	2	2	2	2	2	2	2	2
2	2	1	1	2	1	2	2	1	2
2	2	2	1	1	1	2	1	1	2
2	2	2	2	2	2	2	2	2	2
2	2	1	2	2	2	2	1	1	1
2	2	2	2	2	2	2	2	2	2
2	2	2	2	2	1	2	2	1	2
1	1	1	2	2	2	2	1	2	2
2	2	2	2	1	2	2	2	1	2
2	2	2	2	2	1	1	2	2	2
1	2	2	2	1	#NULL!	#NULL!	#NULL!	2	2
2	2	2	1	2	2	1	2	2	1
2	2	2	2	2	1	1	2	2	1
2	2	2	1	1	2	2	2	1	2
#NULL!	#NULL!	#NULL!	#NULL!	#NULL!	#NULL!	#NULL!	#NULL!	#NULL!	#NULL!
#NULL!	2	2	2	2	1	2	2	1	1
1	2	2	2	2	1	2	2	2	2
2	2	1	2	2	1	1	1	1	1
2	2	2	1	2	2	1	2	2	1
2	2	2	1	1	1	1	2	2	2
2	2	2	1	2	1	2	1	2	2
2	2	2	2	2	2	2	2	2	2
1	1	2	1	1	1	1	1	1	1
#NULL!	#NULL!	#NULL!	#NULL!	#NULL!	#NULL!	#NULL!	#NULL!	#NULL!	#NULL!

2	2	2	#NULL!	2	2	2	2	2	2
2	2	2	2	2	2	2	2	2	2
1	2	2	2	2	2	2	2	2	1
2	2	2	2	2	2	2	1	1	2
2	2	2	2	2	2	2	2	2	2
2	2	2	2	2	1	2	2	2	1
2	2	2	2	2	2	2	2	2	2
#NULL!	#NULL!	#NULL!	#NULL!	#NULL!	#NULL!	#NULL!	#NULL!	#NULL!	#NULL!
#NULL!	#NULL!	#NULL!	#NULL!	#NULL!	#NULL!	#NULL!	#NULL!	#NULL!	#NULL!
2	2	2	1	2	1	2	2	2	2
2	2	2	2	2	2	2	2	1	2
2	2	2	2	2	2	2	2	2	2
2	2	2	2	2	2	2	2	2	2
2	2	2	2	2	2	2	2	2	2
2	2	2	2	2	2	2	2	2	2
2	2	2	2	2	2	2	2	2	2
2	2	2	2	2	2	2	2	2	2
2	1	2	2	1	2	1	2	2	2
1	2	2	2	2	2	2	2	2	2
2	2	2	2	2	2	2	2	2	2
2	2	2	2	2	2	2	2	2	2
2	2	2	2	2	2	2	2	2	2
2	2	2	2	2	2	2	2	2	2
2	1	2	2	2	2	1	2	2	#NULL!
2	2	2	2	2	2	2	2	2	2
2	2	2	2	2	2	2	2	2	2
2	2	2	2	2	2	2	2	1	2
2	2	2	2	#NULL!	2	#NULL!	2	#NULL!	2
2	2	1	1	1	1	2	2	2	2
2	2	2	2	2	2	2	2	2	2
2	2	2	2	2	2	2	2	2	2
2	2	2	2	2	2	2	2	1	2
2	2	2	2	2	2	2	2	2	2
2	2	2	2	2	2	2	2	2	2
2	2	2	2	2	2	2	2	2	2
2	2	2	2	2	2	2	2	2	2
2	2	2	2	2	2	2	2	2	2
2	2	2	2	2	2	2	2	2	2
2	2	2	2	2	2	2	2	2	2
2	2	2	2	2	1	2	2	2	1
2	2	2	2	2	2	2	2	2	2
2	2	2	2	2	2	2	2	2	2
#NULL!	2	2	2	1	1	1	2	2	2
2	2	2	2	2	2	2	2	2	2
2	2	2	2	2	2	2	2	1	2
2	2	2	2	2	2	2	2	2	2
2	2	2	2	2	2	2	2	2	2

2	2	2	2	2	2	2	2	2	2
2	2	2	2	2	2	2	2	2	2
2	2	2	2	2	1	2	2	1	2
2	2	2	2	2	2	2	2	2	2
2	2	2	2	2	2	2	2	2	2
2	2	2	2	2	2	2	2	2	2
#NULL!	#NULL!	#NULL!	#NULL!	#NULL!	#NULL!	#NULL!	#NULL!	#NULL!	#NULL!
2	2	1	2	1	2	2	2	2	2
2	#NULL!	2	#NULL!	#NULL!	#NULL!	#NULL!	#NULL!	#NULL!	#NULL!
2	1	1	1	1	1	1	1	1	1
2	2	2	2	2	2	2	1	1	1
2	2	2	2	2	2	2	2	2	2
2	1	2	2	2	1	1	2	2	2
#NULL!	1	1	1	1	1	1	1	1	1
2	#NULL!	#NULL!	#NULL!	#NULL!	#NULL!	#NULL!	#NULL!	#NULL!	#NULL!
2	2	2	2	2	2	2	2	2	2
2	2	2	2	2	2	2	2	2	2
2	2	2	2	2	2	2	2	2	2
#NULL!	2	#NULL!	#NULL!	#NULL!	#NULL!	#NULL!	#NULL!	#NULL!	#NULL!
2	1	1	1	2	2	2	2	2	2
2	2	1	2	2	2	2	2	2	2
2	#NULL!	#NULL!	#NULL!	#NULL!	#NULL!	2	#NULL!	#NULL!	#NULL!
#NULL!	#NULL!	2	#NULL!	#NULL!	#NULL!	#NULL!	#NULL!	#NULL!	#NULL!
1	#NULL!	2	2	1	1	1	1	1	#NULL!
2	2	2	1	1	2	2	2	2	2
2	2	2	2	#NULL!	2	2	2	2	2
1	2	1	1	1	1	1	1	1	1
2	2	2	2	#NULL!	2	1	#NULL!	2	2
2	2	2	2	2	1	2	1	#NULL!	2
2	2	#NULL!	2	2	2	2	1	1	1
2	2	2	2	2	2	2	2	2	2
1	1	2	2	2	2	2	2	2	2
2	2	2	2	2	2	2	2	2	2
#NULL!	2	#NULL!	#NULL!	#NULL!	2	#NULL!	#NULL!	#NULL!	2
2	2	2	2	2	2	2	2	2	2
#NULL!	2	#NULL!	#NULL!	#NULL!	#NULL!	#NULL!	#NULL!	#NULL!	#NULL!
1	2	1	2	2	2	2	2	#NULL!	2
2	1	1	1	1	1	1	1	1	1
2	2	2	2	2	2	2	2	2	2
2	2	2	2	#NULL!	2	2	2	2	2
#NULL!	2	#NULL!	2	2	2	2	2	2	2
2	2	2	2	2	2	2	2	2	2
#NULL!	#NULL!	#NULL!	#NULL!	2	#NULL!	#NULL!	#NULL!	#NULL!	#NULL!
1	1	1	1	1	1	2	2	2	2
2	2	2	2	2	2	2	2	2	2
1	2	2	2	2	2	2	2	2	2
2	2	2	2	2	2	2	2	2	2

#NULL!	#NULL!	#NULL!	2	#NULL!	#NULL!	#NULL!	#NULL!	#NULL!	#NULL!
2	2	2	2	2	2	2	2	2	2
2	2	2	2	2	2	2	2	2	2
2	2	2	2	2	2	2	2	2	2
#NULL!	2	#NULL!	#NULL!	#NULL!	#NULL!	#NULL!	#NULL!	#NULL!	#NULL!
2	2	2	2	2	2	1	1	1	2
1	1	1	1	1	1	1	1	1	1
2	2	2	2	2	2	2	2	2	2
2	2	2	2	2	2	2	2	2	2
1	2	2	2	2	2	1	1	1	1
#NULL!	#NULL!	#NULL!	#NULL!	#NULL!	#NULL!	#NULL!	#NULL!	#NULL!	#NULL!
2	2	2	2	2	2	2	2	2	2
2	2	2	2	2	2	2	2	2	2
2	2	2	2	2	2	2	2	2	2
2	2	2	2	2	2	2	2	2	2
1	2	2	2	2	2	2	2	2	2
2	2	2	2	2	2	2	2	2	2
2	2	2	2	2	2	2	2	2	2
#NULL!	#NULL!	#NULL!	#NULL!	#NULL!	#NULL!	#NULL!	#NULL!	#NULL!	#NULL!
2	2	2	2	2	1	2	2	2	1
2	2	2	2	2	2	2	2	2	2
2	2	2	1	1	1	1	1	2	2
2	2	2	2	2	2	2	2	2	2
2	2	2	2	2	2	2	2	2	2
1	1	1	#NULL!	1	#NULL!	1	#NULL!	1	1
2	2	2	2	2	2	2	2	2	2
2	2	2	2	2	1	2	2	2	2
2	2	2	2	2	2	2	2	2	2
2	2	2	2	2	2	#NULL!	2	1	2
2	2	2	2	2	2	2	2	2	2
2	2	1	2	#NULL!	2	2	1	2	2
2	2	2	2	2	2	2	1	1	1
2	2	2	2	1	1	1	1	1	1
1	2	#NULL!	2	1	2	2	2	#NULL!	1
2	2	2	2	2	2	2	2	2	2
2	2	2	2	2	2	2	2	2	2
2	2	2	2	2	2	2	2	2	2
2	2	2	2	2	2	2	2	2	2
2	2	2	1	2	2	1	2	2	2
1	1	1	1	1	1	1	1	1	1
2	2	2	1	2	2	2	1	2	1
2	2	2	2	2	2	2	2	2	2
#NULL!	#NULL!	#NULL!	#NULL!	#NULL!	#NULL!	#NULL!	#NULL!	#NULL!	#NULL!
1	1	1	1	1	1	1	1	1	#NULL!
#NULL!	#NULL!	#NULL!	#NULL!	#NULL!	#NULL!	#NULL!	#NULL!	#NULL!	#NULL!
2	2	2	2	2	2	2	2	2	2
2	2	2	2	2	2	2	2	1	1

#NULL!	2	2	2	2	2	2	2	2	2
2	1	1	1	1	1	2	1	2	1
#NULL!	#NULL!	#NULL!	#NULL!	#NULL!	#NULL!	#NULL!	#NULL!	#NULL!	#NULL!
2	1	1	1	1	1	2	1	2	1
#NULL!	2	2	1	2	2	2	2	1	2
2	2	2	2	2	2	2	2	2	2
2	2	2	2	2	2	2	2	2	2
2	2	2	2	2	2	2	2	2	2
1	2	2	2	2	2	2	2	2	2
2	2	2	2	1	2	1	2	2	2
2	2	2	2	2	2	1	2	2	2
2	2	2	1	2	1	#NULL!	2	1	2
2	2	2	2	2	2	2	2	2	2
2	2	1	2	1	2	2	2	1	2
2	2	2	2	2	2	2	2	2	2
2	2	2	2	2	2	2	2	2	2
2	2	2	2	2	2	2	2	2	2
2	2	2	2	2	2	2	2	2	2
2	2	2	2	2	2	2	2	2	2
1	1	2	2	2	2	2	2	2	2
2	2	2	2	2	2	2	2	2	2
1	2	2	2	2	2	2	2	2	2
2	2	2	2	2	2	2	2	2	2
2	2	2	2	2	2	2	2	2	2
2	2	2	2	2	2	2	2	2	2
2	2	2	2	2	2	2	2	2	2
2	2	1	1	2	2	2	2	2	2
2	2	2	2	2	2	2	2	2	2
2	2	2	2	2	1	1	2	1	2
2	2	2	2	1	1	2	2	2	2
2	2	2	2	2	1	#NULL!	2	2	2
2	2	2	2	1	1	1	1	1	2
2	2	2	2	2	2	2	2	2	2
2	1	2	1	2	1	1	2	1	2
1	1	2	1	1	1	1	2	2	2
2	2	2	2	2	2	1	1	2	1
#NULL!	#NULL!	#NULL!	#NULL!	#NULL!	#NULL!	#NULL!	#NULL!	#NULL!	#NULL!
#NULL!	#NULL!	#NULL!	#NULL!	#NULL!	#NULL!	#NULL!	#NULL!	#NULL!	#NULL!
2	1	1	1	1	1	1	1	1	1
2	2	2	2	2	2	2	2	2	2
1	#NULL!	2	2	2	2	2	2	2	2
2	2	2	2	2	2	2	2	2	2
2	2	2	2	2	2	2	2	2	2
2	2	2	2	2	2	2	2	2	2
#NULL!	#NULL!	#NULL!	#NULL!	#NULL!	#NULL!	#NULL!	#NULL!	#NULL!	#NULL!
1	2	2	1	2	2	2	2	2	2
2	2	2	2	2	2	2	2	2	2

#NULL!	#NULL!	#NULL!	#NULL!	#NULL!	#NULL!	#NULL!	#NULL!	#NULL!	#NULL!
2	2	2	2	2	2	2	2	1	2
1	1	2	2	1	2	2	2	1	2
2	2	2	1	1	2	2	2	1	2
2	2	2	1	1	1	1	1	1	1
1	2	2	2	1	2	2	2	2	1
1	2	2	2	2	2	1	1	1	1
#NULL!	#NULL!	#NULL!	#NULL!	#NULL!	#NULL!	#NULL!	#NULL!	#NULL!	#NULL!
2	2	2	2	2	2	2	2	2	2
1	1	2	2	1	2	2	1	1	2
2	2	1	2	2	2	2	2	2	2
1	1	2	2	2	1	2	2	2	1
2	2	2	2	2	1	2	2	2	2
2	2	2	2	2	2	2	2	2	2
1	2	2	1	2	2	1	2	2	1
2	2	2	2	2	2	2	2	2	2
2	1	2	2	2	2	2	2	1	2
2	2	2	2	2	2	2	2	2	2
#NULL!	#NULL!	#NULL!	#NULL!	#NULL!	#NULL!	#NULL!	#NULL!	#NULL!	#NULL!
#NULL!	#NULL!	#NULL!	#NULL!	#NULL!	#NULL!	#NULL!	#NULL!	#NULL!	#NULL!
#NULL!	#NULL!	#NULL!	#NULL!	#NULL!	#NULL!	#NULL!	#NULL!	#NULL!	#NULL!
2	2	2	1	2	2	1	2	2	2
2	2	2	2	1	1	1	2	2	2
2	1	1	1	1	2	2	2	2	2
2	2	2	2	2	2	2	2	1	1
1	1	1	1	1	1	1	1	1	1
2	2	1	2	2	2	2	2	1	2
2	2	2	2	2	#NULL!	2	1	1	1
1	2	2	1	2	1	2	1	2	2
2	2	2	2	2	2	2	2	2	2
#NULL!	2	2	2	1	2	2	2	2	2
2	2	2	2	2	2	2	2	2	1
2	1	1	2	2	1	1	#NULL!	2	1
2	1	1	1	1	1	1	1	1	1
1	1	1	1	1	1	1	1	1	1
2	2	2	2	2	2	2	2	2	2
1	1	1	1	1	1	1	1	1	1
2	2	2	2	2	2	2	2	2	2
#NULL!	#NULL!	#NULL!	#NULL!	#NULL!	#NULL!	#NULL!	#NULL!	#NULL!	#NULL!
#NULL!	#NULL!	#NULL!	#NULL!	#NULL!	#NULL!	#NULL!	#NULL!	#NULL!	#NULL!
1	2	2	2	2	2	2	2	2	2
2	2	2	2	2	2	2	2	2	2
2	2	2	2	2	2	1	1	2	2
2	1	2	1	2	2	1	2	1	2
#NULL!	#NULL!	#NULL!	2	2	#NULL!	#NULL!	#NULL!	#NULL!	#NULL!
1	1	1	1	1	1	1	1	1	1
2	2	2	2	2	2	2	2	2	2

2	2	2	2	2	2	2	2	2	2
2	2	2	2	1	2	2	1	2	2
1	2	2	1	2	2	2	1	2	2
2	2	2	2	1	#NULL!	2	2	2	2
2	2	2	2	1	2	1	2	2	2
1	2	1	#NULL!	2	2	2	1	1	1
2	2	2	2	2	2	2	2	2	2
1	1	1	1	1	1	1	1	1	1
2	2	2	2	1	2	2	1	2	2
2	2	1	2	2	2	2	2	1	2
2	2	2	2	2	2	2	2	2	2
2	2	2	1	1	2	1	1	1	1
1	2	#NULL!	#NULL!	2	2	2	1	2	1
1	2	2	2	2	2	2	1	2	2
2	2	2	2	2	2	2	2	2	2
1	1	1	1	1	1	1	1	1	2
2	2	2	2	2	2	2	2	2	1
2	2	2	2	2	2	2	2	2	2
#NULL!	#NULL!	#NULL!	#NULL!	#NULL!	#NULL!	#NULL!	#NULL!	#NULL!	#NULL!
2	1	2	2	1	2	2	2	2	2
2	2	1	2	1	2	2	1	2	2
1	2	1	2	2	1	1	2	1	2
2	2	2	2	2	2	2	2	2	1
1	2	2	2	2	2	1	2	2	2
2	2	2	1	2	2	1	2	2	1
1	2	2	2	2	1	2	2	1	2
2	2	2	2	2	2	2	2	2	2
2	2	2	1	2	2	2	2	2	1
2	2	2	1	2	2	2	1	2	2
2	2	2	2	2	2	2	2	2	2
2	1	1	1	2	2	2	2	2	2
2	2	2	2	2	2	2	2	2	2
2	2	2	2	2	2	2	2	2	2
1	2	2	2	1	1	2	2	1	1
2	2	2	1	2	2	1	2	2	1
2	2	2	1	2	2	2	2	2	2
2	2	2	2	2	1	1	2	2	1
1	1	1	1	1	1	1	1	1	1
#NULL!	2	2	2	2	2	1	1	1	1
2	2	2	1	2	1	2	2	2	1
2	2	2	2	2	2	2	2	2	2
2	2	2	1	2	2	1	2	2	2
1	2	2	1	2	1	2	1	2	#NULL!
2	2	2	2	2	2	2	2	2	2
2	2	1	1	1	1	1	1	1	2
1	2	2	2	1	2	2	1	2	1
2	2	2	2	2	2	2	2	2	2

2	2	2	2	2	1	1	1	1	2
2	2	2	1	2	2	2	2	1	2
2	2	2	1	2	1	2	2	2	2
2	2	2	2	2	2	2	2	2	2
2	2	2	2	2	1	2	2	1	2
2	2	2	2	2	2	2	2	1	1
2	2	2	2	2	2	2	2	1	2
2	2	2	1	2	2	2	2	1	2
1	2	2	2	2	2	2	1	2	2
1	1	1	2	1	1	2	2	1	2
1	2	1	2	1	2	1	2	1	2
2	2	2	2	2	2	2	2	1	2
2	2	2	2	2	2	2	2	2	2
1	2	1	2	1	1	1	2	2	2
1	2	2	1	2	2	1	2	2	1
2	2	2	1	1	1	1	1	1	2
1	2	2	2	2	2	2	2	2	2
1	2	2	2	2	1	1	1	2	2
2	2	2	1	2	2	1	2	2	2
1	2	1	2	2	2	1	2	2	2
1	2	2	2	1	2	1	2	1	2
1	1	1	1	1	1	1	1	1	1
1	1	1	2	2	1	1	2	2	1
2	2	2	2	2	2	2	2	2	2
2	2	2	2	2	2	2	2	2	2
2	2	2	2	2	2	2	2	2	2
1	2	2	2	2	1	1	1	2	1
2	2	2	2	2	2	1	2	1	2
2	2	2	2	2	2	2	2	2	2
#NULL!	#NULL!	#NULL!	#NULL!	#NULL!	#NULL!	#NULL!	#NULL!	#NULL!	#NULL!
#NULL!	#NULL!	#NULL!	#NULL!	#NULL!	#NULL!	#NULL!	#NULL!	#NULL!	#NULL!
#NULL!	#NULL!	#NULL!	#NULL!	#NULL!	#NULL!	#NULL!	#NULL!	#NULL!	#NULL!
1	2	2	1	1	1	1	1	1	1
#NULL!	#NULL!	#NULL!	#NULL!	#NULL!	#NULL!	#NULL!	#NULL!	#NULL!	#NULL!
2	2	2	2	2	2	2	2	2	2
#NULL!	#NULL!	#NULL!	#NULL!	#NULL!	#NULL!	#NULL!	#NULL!	#NULL!	#NULL!
2	2	2	2	2	2	2	2	2	2
2	#NULL!	2	#NULL!	#NULL!	#NULL!	#NULL!	#NULL!	#NULL!	#NULL!
2	#NULL!	#NULL!	2	2	2	2	2	2	2
2	2	2	2	2	2	1	2	1	2
#NULL!	#NULL!	#NULL!	#NULL!	#NULL!	#NULL!	#NULL!	#NULL!	#NULL!	#NULL!
2	2	2	2	2	2	2	2	2	1
2	1	1	1	1	1	1	1	1	1
2	1	1	1	1	1	1	1	1	1
2	2	2	2	1	2	2	2	2	2
2	1	2	2	2	2	1	2	1	2
1	2	2	2	2	1	1	1	2	1

2	2	2	2	2	1	1	1	2	1
2	2	2	2	2	2	2	2	2	2
#NULL!	#NULL!	#NULL!	#NULL!	#NULL!	#NULL!	#NULL!	#NULL!	#NULL!	#NULL!
2	2	2	2	2	2	2	2	1	2
#NULL!	2	2	2	2	2	2	1	#NULL!	#NULL!
2	2	2	2	2	2	1	2	2	2
#NULL!	#NULL!	#NULL!	2	#NULL!	#NULL!	#NULL!	#NULL!	#NULL!	#NULL!
2	2	2	1	2	2	2	2	1	2
2	2	2	2	2	2	1	2	2	1
1	1	1	1	1	1	1	1	1	1
2	2	1	2	2	1	2	2	1	2
2	1	2	2	2	1	1	2	2	2
1	2	1	2	2	2	2	#NULL!	2	2
2	2	2	2	2	2	2	2	2	2
2	2	2	2	2	2	2	2	2	2
2	1	#NULL!	#NULL!	#NULL!	2	#NULL!	#NULL!	#NULL!	#NULL!
2	2	2	2	2	2	2	2	2	2
2	2	2	2	2	2	2	2	2	2
2	2	2	2	2	2	2	2	2	2
2	2	2	2	2	2	2	2	2	2
2	2	2	2	2	#NULL!	#NULL!	#NULL!	2	2
2	2	2	2	2	#NULL!	#NULL!	#NULL!	2	2
2	2	2	2	2	2	#NULL!	#NULL!	#NULL!	2
2	2	2	2	2	2	2	2	2	2
2	2	2	2	2	2	2	2	2	2
2	2	2	2	#NULL!	#NULL!	#NULL!	#NULL!	#NULL!	#NULL!
2	2	2	2	2	2	1	1	2	1
2	2	2	1	2	2	2	1	1	2
2	2	2	2	2	2	2	1	2	2
2	2	2	2	2	2	2	2	1	2
2	2	2	2	2	2	2	1	1	1
#NULL!	#NULL!	#NULL!	2	2	#NULL!	#NULL!	#NULL!	#NULL!	2
2	2	2	2	2	2	2	2	2	2
2	2	2	2	2	2	2	1	2	2
2	2	2	2	2	1	2	2	2	2
2	1	1	2	2	1	2	2	1	2
2	1	1	2	2	1	2	2	1	2
#NULL!	#NULL!	2	2	2	2	#NULL!	#NULL!	#NULL!	2
2	2	2	2	2	2	1	1	1	2
1	2	2	2	2	2	2	2	2	2
2	2	2	2	2	2	1	1	1	2
#NULL!	2	2	2	2	2	#NULL!	#NULL!	#NULL!	#NULL!
2	2	2	2	2	2	2	2	#NULL!	2
2	2	2	2	2	2	2	2	2	2
2	2	2	2	2	2	1	1	1	2
2	2	2	2	2	2	1	2	1	2
2	2	2	2	2	2	1	1	1	2

2	1	1	2	2	1	2	2	1	2
2	2	#NULL!	1	1	#NULL!	#NULL!	#NULL!	#NULL!	#NULL!
2	2	2	2	2	2	2	2	2	2
2	2	2	2	2	2	2	2	2	2
2	2	2	2	#NULL!	2	2	2	1	#NULL!
2	2	2	2	2	2	2	2	1	#NULL!
2	2	2	2	2	2	2	2	2	2
2	2	2	2	2	2	2	2	2	2
#NULL!	#NULL!	#NULL!	#NULL!	#NULL!	#NULL!	#NULL!	#NULL!	#NULL!	#NULL!
2	2	2	2	2	2	2	2	1	2
2	2	2	2	2	2	1	2	2	2
2	2	2	2	2	2	2	2	2	2
2	2	2	2	#NULL!	2	2	2	1	#NULL!
2	2	2	2	#NULL!	2	2	2	1	#NULL!
2	2	2	2	#NULL!	2	2	2	#NULL!	1
2	2	2	2	2	2	2	2	#NULL!	#NULL!
2	2	2	2	2	2	2	2	1	1
1	2	1	2	2	2	2	2	1	1
2	2	2	2	2	2	2	2	1	#NULL!
2	2	2	2	2	1	2	1	2	2
1	2	2	1	2	2	1	2	2	2
2	2	2	#NULL!	2	#NULL!	2	2	2	2
2	2	2	2	2	2	2	2	1	2
2	1	1	2	2	2	2	1	1	2
2	1	2	2	2	2	2	2	2	2
2	2	2	2	2	1	2	2	2	2
1	1	1	1	1	2	1	1	1	1
2	2	2	2	2	2	2	2	1	2
2	2	2	2	2	2	1	2	1	1
2	2	2	2	2	2	2	2	2	2
2	2	2	2	2	1	2	2	2	2
1	2	2	1	1	2	1	1	1	2
#NULL!	2	2	#NULL!	#NULL!	2	2	#NULL!	#NULL!	2
2	2	2	2	#NULL!	2	2	#NULL!	#NULL!	#NULL!
2	2	2	2	2	2	2	#NULL!	1	2
2	2	2	2	2	2	2	2	1	2
#NULL!	#NULL!	2	2	2	2	2	2	#NULL!	2
2	2	2	2	2	2	2	1	1	2
2	2	2	2	2	2	2	1	#NULL!	2
2	2	2	2	2	2	2	1	1	2
2	2	2	2	2	2	2	2	1	2
2	2	1	2	2	1	2	2	1	1
#NULL!	1	1	2	2	#NULL!	2	2	1	2
2	2	2	2	2	2	2	2	1	2
2	2	2	2	2	2	2	2	1	2
2	2	2	2	2	2	2	1	1	2
2	2	2	2	#NULL!	2	1	#NULL!	#NULL!	#NULL!

2	2	2	1	1	2	1	2	1	2
2	2	2	2	2	2	2	1	1	2
2	2	2	2	2	2	1	2	1	2
1	2	2	2	1	2	1	1	1	2
2	2	2	2	2	2	2	2	1	2
2	2	2	2	2	2	2	1	1	2
2	#NULL!	#NULL!	#NULL!	2	#NULL!	#NULL!	#NULL!	#NULL!	#NULL!
#NULL!	2	1	#NULL!	2	2	2	#NULL!	2	2
1	2	1	2	2	2	2	1	2	2
2	2	2	2	2	2	2	2	1	2
2	2	2	2	2	2	2	2	2	2
#NULL!	2	2	2	2	2	2	#NULL!	#NULL!	#NULL!
2	2	2	2	2	2	2	2	1	1
2	2	2	2	2	2	1	2	1	1
2	2	2	2	2	2	2	2	2	2
2	2	2	2	2	2	2	2	2	2
#NULL!	#NULL!	2	2	2	2	2	#NULL!	#NULL!	#NULL!
#NULL!	#NULL!	2	2	2	2	2	#NULL!	#NULL!	#NULL!
2	2	2	2	2	2	2	2	2	1
2	2	2	2	2	2	2	2	2	2
2	2	2	2	2	1	1	1	2	2
2	2	2	2	2	2	2	2	1	1
1	1	2	2	2	2	2	2	1	2
2	2	2	2	2	2	2	2	2	2
2	1	2	2	2	1	2	2	2	2
2	1	2	2	2	1	2	1	1	1
2	2	2	1	1	1	1	2	2	2
2	2	2	2	2	2	2	2	2	2
2	2	2	2	2	2	2	2	2	2
2	2	2	2	2	2	2	1	1	2
2	2	2	2	2	2	2	2	2	2
2	2	2	#NULL!	2	2	2	1	2	2
2	2	2	2	2	2	2	2	1	2
2	2	2	2	1	2	2	2	2	1
2	1	1	1	1	2	2	2	2	2
2	2	2	2	2	2	2	2	2	2
#NULL!	2	2	2	2	2	2	2	2	1
2	2	2	2	2	2	2	2	1	1
2	2	2	2	2	2	#NULL!	2	1	1
2	2	2	2	2	2	2	1	1	1
2	2	2	2	2	2	2	1	1	2
2	2	2	2	2	2	2	1	1	1
2	2	2	2	2	1	2	2	1	2
2	#NULL!	#NULL!	#NULL!	#NULL!	#NULL!	#NULL!	#NULL!	#NULL!	#NULL!
2	#NULL!	2	2	2	2	1	2	2	2
2	2	2	2	2	2	2	1	1	1
2	2	2	2	2	2	2	2	2	2

2	2	2	2	2	2	2	2	1	2
2	2	2	2	2	2	2	2	1	2
2	2	2	2	2	2	2	2	2	2
2	2	2	2	2	2	2	2	2	2
#NULL!	2	2	2	2	2	2	2	2	2
2	2	2	1	1	2	1	2	2	1
2	#NULL!	2	2	2	#NULL!	#NULL!	2	#NULL!	#NULL!
#NULL!	2	2	2	2	2	2	2	2	2
#NULL!	#NULL!	#NULL!	#NULL!	#NULL!	#NULL!	#NULL!	#NULL!	#NULL!	#NULL!
2	2	2	2	2	2	2	2	2	2
2	2	2	2	2	2	2	2	2	2
2	2	2	2	2	2	2	2	1	2
2	2	2	2	2	2	2	2	2	2
2	2	2	2	2	2	2	2	1	1
2	#NULL!	2	2	2	2	2	#NULL!	2	2
#NULL!	2	#NULL!	#NULL!	#NULL!	#NULL!	#NULL!	#NULL!	#NULL!	#NULL!
2	2	2	2	2	2	2	2	2	2
2	2	2	2	2	2	2	2	2	2
2	2	2	2	2	2	2	2	2	2
2	2	2	2	2	2	2	2	2	2
#NULL!	2	#NULL!	#NULL!	#NULL!	#NULL!	#NULL!	#NULL!	#NULL!	#NULL!
2	2	2	1	1	2	1	2	1	1
#NULL!	2	2	2	2	2	2	2	1	1
2	2	2	2	2	2	2	2	1	2
2	2	2	2	2	2	2	2	2	2
2	2	2	2	2	1	2	2	2	2
2	2	2	2	2	2	2	2	2	2
#NULL!	#NULL!	#NULL!	#NULL!	#NULL!	#NULL!	#NULL!	#NULL!	#NULL!	#NULL!
#NULL!	#NULL!	#NULL!	#NULL!	#NULL!	#NULL!	#NULL!	#NULL!	#NULL!	#NULL!
2	2	2	2	1	2	2	2	1	2
2	2	2	2	2	2	2	2	1	1
1	2	2	2	2	2	2	2	2	2
#NULL!	2	2	2	2	2	2	1	2	1
#NULL!	2	2	2	2	2	2	1	1	2
2	2	2	2	2	2	1	1	1	2
2	2	2	2	2	2	2	2	1	2
2	2	2	2	2	2	2	1	2	2
2	2	2	2	2	2	1	1	2	2
2	2	2	2	2	2	2	1	1	2
2	2	2	2	2	2	2	1	2	2
2	2	2	2	2	2	1	2	2	2
2	2	2	2	2	2	2	2	1	2
2	2	2	2	2	2	2	1	1	2
#NULL!	#NULL!	#NULL!	#NULL!	#NULL!	#NULL!	#NULL!	#NULL!	#NULL!	#NULL!
2	2	2	2	2	2	2	2	1	2
1	2	2	2	2	2	2	2	2	2
2	#NULL!	1	2	#NULL!	2	2	2	2	2

2	2	2	2	2	2	1	1	1	2
#NULL!	2	2	2	2	2	2	2	2	2
#NULL!	#NULL!	#NULL!	#NULL!	#NULL!	#NULL!	#NULL!	#NULL!	#NULL!	#NULL!
2	2	2	2	2	2	2	2	1	1
2	#NULL!	#NULL!	2	2	2	#NULL!	#NULL!	#NULL!	1
2	2	2	2	2	2	2	2	2	2
2	#NULL!	2	#NULL!	#NULL!	2	2	2	1	2
2	2	2	1	1	2	1	2	1	2
#NULL!	#NULL!	2	#NULL!	#NULL!	#NULL!	#NULL!	2	#NULL!	#NULL!
2	2	2	2	2	2	2	2	2	2
2	2	2	1	2	2	1	1	2	1
2	2	2	2	2	2	2	2	2	2
2	2	2	2	2	2	2	2	1	1
2	#NULL!	2	2	2	2	#NULL!	#NULL!	#NULL!	2
2	2	2	2	2	2	2	2	2	2
2	2	2	2	2	2	2	1	1	1
#NULL!	2	2	#NULL!	#NULL!	2	#NULL!	#NULL!	#NULL!	#NULL!
#NULL!	2	2	2	#NULL!	2	#NULL!	#NULL!	#NULL!	#NULL!
2	2	2	2	2	2	2	2	2	2
2	2	2	2	2	2	1	1	2	1
2	2	2	2	1	1	1	2	#NULL!	2
1	2	2	2	2	2	2	2	1	1
1	2	2	2	2	2	2	2	1	1
2	2	2	2	2	2	2	2	1	2
2	2	2	#NULL!	2	2	2	2	2	2
2	2	2	1	1	2	2	2	1	1
2	2	2	2	2	2	2	2	#NULL!	1
2	2	2	1	1	2	2	2	1	1
2	2	2	1	1	2	2	2	2	1
2	2	2	1	1	2	2	2	2	1
2	2	2	2	2	2	2	1	2	1
2	2	2	2	2	2	2	2	2	2
2	2	2	1	1	2	1	1	2	2
2	2	2	2	2	2	2	2	2	2
2	2	2	2	2	2	2	2	1	1
#NULL!	#NULL!	2	#NULL!	#NULL!	2	#NULL!	#NULL!	#NULL!	2
2	2	2	1	1	2	1	2	1	1
#NULL!	2	2	#NULL!	#NULL!	2	#NULL!	#NULL!	#NULL!	#NULL!
2	2	2	2	2	2	2	2	2	#NULL!
2	2	2	2	2	2	2	2	2	2
2	2	1	2	1	2	1	2	2	1
2	2	2	1	2	1	1	2	2	1
#NULL!	#NULL!	2	#NULL!	#NULL!	#NULL!	#NULL!	#NULL!	#NULL!	#NULL!
#NULL!	#NULL!	2	#NULL!	#NULL!	#NULL!	#NULL!	#NULL!	#NULL!	#NULL!
2	2	2	2	2	2	2	2	2	1
2	2	2	2	2	2	2	2	1	1
2	#NULL!	2	2	#NULL!	2	#NULL!	#NULL!	#NULL!	#NULL!

2	2	2	2	1	2	1	1	1	1
2	2	2	2	2	2	2	2	2	2
2	2	2	#NULL!	2	2	2	2	#NULL!	#NULL!
2	2	2	2	2	2	2	1	1	1
2	2	2	2	2	2	2	2	2	2
2	2	2	2	2	2	2	2	2	1
2	2	2	2	2	2	2	2	2	2
2	2	2	2	2	2	2	2	2	2
2	2	2	2	2	2	2	2	2	2
2	2	2	2	2	2	2	2	2	1
2	2	2	2	2	2	2	2	2	2
#NULL!	#NULL!	2	#NULL!	#NULL!	#NULL!	#NULL!	#NULL!	#NULL!	#NULL!
#NULL!	2	#NULL!	#NULL!	#NULL!	#NULL!	#NULL!	#NULL!	#NULL!	#NULL!
#NULL!	#NULL!	#NULL!	#NULL!	2	#NULL!	#NULL!	#NULL!	#NULL!	#NULL!
#NULL!	#NULL!	#NULL!	#NULL!	#NULL!	#NULL!	#NULL!	#NULL!	#NULL!	#NULL!
#NULL!	#NULL!	#NULL!	#NULL!	#NULL!	2	#NULL!	#NULL!	#NULL!	#NULL!
1	#NULL!	1	2	2	1	1	2	2	1
2	2	1	2	1	2	2	2	2	2
#NULL!	#NULL!	#NULL!	2	#NULL!	#NULL!	#NULL!	#NULL!	#NULL!	#NULL!
2	2	2	2	2	2	2	2	2	2
2	2	2	2	2	2	2	2	1	1
#NULL!	2	2	2	2	#NULL!	2	2	1	2
2	2	2	2	2	2	2	2	1	2
1	2	2	2	2	2	2	2	1	1
2	2	2	2	2	2	2	2	2	1
2	2	2	2	2	2	2	2	1	2
2	2	2	2	#NULL!	2	1	1	1	1
2	1	2	2	2	2	2	1	1	1
2	2	2	2	2	2	#NULL!	#NULL!	#NULL!	#NULL!
2	2	2	2	2	2	#NULL!	#NULL!	#NULL!	2
2	2	2	2	2	2	2	2	2	2
2	#NULL!	2	2	2	#NULL!	2	#NULL!	#NULL!	#NULL!
2	2	2	2	2	2	2	2	2	1
2	2	2	2	2	2	1	1	1	2
2	2	2	1	1	2	2	2	1	1
2	2	2	2	2	2	2	2	2	2
2	2	2	2	2	2	2	2	2	2
2	2	2	2	1	2	1	1	1	2
#NULL!	#NULL!	#NULL!	#NULL!	#NULL!	#NULL!	#NULL!	#NULL!	#NULL!	#NULL!
2	#NULL!	2	2	2	2	2	1	1	2
1	2	1	2	2	2	2	1	1	1
2	2	1	2	2	2	2	2	#NULL!	2
2	2	2	2	2	2	2	2	1	2
2	2	2	2	2	2	2	2	2	2
2	2	2	2	2	2	2	2	2	2
2	2	2	2	2	2	2	2	2	2
2	2	2	2	2	2	1	2	2	1

2	2	2	2	1	2	1	1	1	1
2	2	2	2	2	2	2	2	2	2
2	2	2	2	2	1	2	1	2	2
2	2	2	2	2	2	2	2	2	1
2	#NULL!	#NULL!	#NULL!	#NULL!	#NULL!	#NULL!	#NULL!	#NULL!	#NULL!
2	2	2	2	2	2	1	1	1	1
2	2	2	1	1	2	1	1	1	1
2	2	2	2	2	2	2	2	2	1
1	2	2	2	2	2	2	2	2	#NULL!
2	2	2	2	2	2	2	2	2	2
2	2	2	2	2	2	2	2	2	1
2	2	2	2	2	2	2	2	2	2
2	2	2	2	2	2	2	1	2	1
2	2	2	2	2	2	1	1	1	1
2	2	2	2	1	2	2	2	1	1
2	2	2	2	2	2	2	2	2	2
2	2	2	2	2	2	2	2	2	2
2	2	2	#NULL!	2	2	2	2	2	2
2	2	2	2	2	2	1	1	1	1
2	2	2	2	2	2	2	2	2	2
2	2	2	2	2	2	2	2	2	2
2	2	2	2	2	2	2	2	1	#NULL!
2	2	2	2	2	2	2	2	1	2
2	1	1	2	2	2	2	2	1	2
2	2	2	2	2	2	2	2	2	2
#NULL!	#NULL!	#NULL!	#NULL!	#NULL!	#NULL!	#NULL!	#NULL!	#NULL!	#NULL!
#NULL!	#NULL!	#NULL!	#NULL!	#NULL!	#NULL!	#NULL!	#NULL!	#NULL!	#NULL!
#NULL!	2	#NULL!	#NULL!	#NULL!	#NULL!	#NULL!	#NULL!	#NULL!	#NULL!
2	2	2	2	2	2	2	2	1	2
1	2	2	2	2	2	2	2	1	1
2	2	2	2	2	2	2	2	2	2
2	2	2	2	2	2	2	2	2	#NULL!
#NULL!	#NULL!	2	#NULL!	#NULL!	#NULL!	#NULL!	#NULL!	#NULL!	#NULL!
2	#NULL!	2	#NULL!	#NULL!	#NULL!	#NULL!	#NULL!	#NULL!	#NULL!
2	#NULL!	2	#NULL!	#NULL!	#NULL!	#NULL!	#NULL!	#NULL!	#NULL!
2	#NULL!	2	#NULL!	#NULL!	#NULL!	#NULL!	#NULL!	#NULL!	#NULL!
#NULL!	#NULL!	2	#NULL!	#NULL!	#NULL!	#NULL!	#NULL!	#NULL!	#NULL!
#NULL!	#NULL!	2	#NULL!	#NULL!	#NULL!	#NULL!	#NULL!	#NULL!	#NULL!
#NULL!	2	#NULL!	#NULL!	#NULL!	#NULL!	#NULL!	#NULL!	#NULL!	#NULL!
2	2	2	2	2	2	2	1	2	2
2	2	2	2	2	2	2	2	1	2
#NULL!	#NULL!	2	#NULL!	#NULL!	#NULL!	#NULL!	#NULL!	#NULL!	#NULL!
#NULL!	#NULL!	2	#NULL!	#NULL!	#NULL!	#NULL!	#NULL!	#NULL!	#NULL!
2	2	2	2	2	2	2	2	1	1
#NULL!	#NULL!	#NULL!	#NULL!	#NULL!	#NULL!	#NULL!	#NULL!	#NULL!	#NULL!
#NULL!	#NULL!	#NULL!	#NULL!	#NULL!	2	#NULL!	#NULL!	#NULL!	#NULL!
#NULL!	#NULL!	#NULL!	#NULL!	#NULL!	2	#NULL!	#NULL!	#NULL!	#NULL!

2	2	2	2	2	2	2	1	1	1
#NULL!	#NULL!	#NULL!	#NULL!	#NULL!	#NULL!	#NULL!	#NULL!	#NULL!	#NULL!
#NULL!	#NULL!	#NULL!	#NULL!	2	#NULL!	#NULL!	#NULL!	#NULL!	#NULL!
2	#NULL!	2	1	#NULL!	2	1	2	2	1
2	#NULL!	2	1	#NULL!	2	1	2	2	1
#NULL!	#NULL!	#NULL!	#NULL!	2	#NULL!	#NULL!	#NULL!	#NULL!	#NULL!
#NULL!	#NULL!	2	#NULL!	#NULL!	#NULL!	#NULL!	#NULL!	#NULL!	#NULL!
#NULL!	#NULL!	2	#NULL!	#NULL!	#NULL!	#NULL!	#NULL!	#NULL!	#NULL!
#NULL!	#NULL!	2	#NULL!	#NULL!	#NULL!	#NULL!	#NULL!	#NULL!	#NULL!
#NULL!	#NULL!	2	#NULL!	#NULL!	#NULL!	#NULL!	#NULL!	#NULL!	#NULL!
2	2	2	2	2	2	2	2	1	1
1	2	2	2	2	2	2	2	1	2
2	2	2	2	1	2	2	2	1	2
2	2	2	2	2	2	2	2	2	2
2	2	2	2	2	2	1	2	1	1
#NULL!	2	2	1	1	2	1	2	2	1
2	2	2	1	1	1	1	1	2	2
2	2	2	1	1	2	2	2	2	2
1	2	2	2	2	2	2	2	1	2
2	2	2	2	2	2	2	2	2	2
2	2	2	2	2	2	2	2	2	2
2	2	2	2	2	2	2	2	2	2
#NULL!	2	#NULL!	#NULL!	#NULL!	#NULL!	#NULL!	#NULL!	#NULL!	#NULL!
2	2	2	2	2	2	2	2	2	2
1	1	2	#NULL!	2	2	2	1	1	1
2	2	2	2	2	2	2	2	1	1
#NULL!	2	2	2	2	2	1	1	1	1
2	2	2	2	2	2	1	2	2	1
2	2	2	2	2	1	2	2	2	1
2	2	2	1	2	2	1	2	1	2
2	2	2	2	2	2	2	2	1	1
2	2	2	1	1	2	2	2	1	1
2	2	2	1	1	2	2	2	2	1
2	2	2	2	2	2	2	2	2	1
2	2	2	2	2	2	2	2	2	1
2	2	2	2	2	2	2	2	2	1
2	2	2	1	1	2	#NULL!	#NULL!	#NULL!	#NULL!
2	2	2	1	1	1	1	2	1	1
2	2	2	1	1	1	1	1	1	1
2	2	2	2	2	1	2	2	2	1
#NULL!	#NULL!	#NULL!	#NULL!	#NULL!	#NULL!	#NULL!	#NULL!	#NULL!	#NULL!
2	2	2	2	2	1	1	2	2	2
2	2	2	2	1	1	1	1	2	1
2	2	2	2	2	2	2	2	2	2
2	2	2	2	2	2	2	2	2	2
2	2	2	2	2	2	2	2	2	2
2	2	2	2	2	2	2	2	1	1

2	2	2	2	2	2	1	2	1	1
2	2	2	2	2	2	2	2	2	2
2	2	2	2	2	2	2	2	2	2
2	2	2	2	2	2	2	2	2	2
2	2	2	2	2	2	2	2	2	2
2	2	2	2	2	2	2	2	2	2
2	2	2	2	2	2	2	2	1	1
2	2	2	2	2	2	2	2	2	1
2	2	2	2	2	2	2	2	2	2
2	2	2	1	1	1	1	2	1	1
2	2	2	2	2	2	2	2	2	2
2	2	2	2	2	1	1	1	#NULL!	2
2	#NULL!	2	2	2	2	2	1	2	2
2	2	2	2	2	2	1	2	2	2
1	2	2	1	1	1	2	2	2	2
2	2	2	2	2	1	1	1	2	2
2	2	2	2	2	2	2	2	2	2
2	2	2	2	2	2	2	2	2	2
1	2	1	1	2	2	2	1	2	1
2	2	2	2	2	2	2	2	1	2
2	2	2	2	2	2	2	1	1	2
2	2	2	2	2	2	1	1	1	2
2	2	2	2	2	2	2	2	2	2
2	2	2	2	2	2	2	2	2	2
2	2	2	2	2	2	2	2	2	2
2	2	2	2	2	2	2	2	1	1
2	2	1	1	2	2	1	1	2	2
2	1	2	1	2	2	2	2	2	1
2	2	2	2	2	2	2	2	2	2
2	2	2	2	2	2	2	1	1	2
2	2	2	1	1	1	1	1	2	2
2	2	2	2	2	2	1	1	1	1
2	2	2	2	2	2	2	1	1	1
2	2	2	1	1	2	2	1	1	1
2	2	2	2	2	2	2	2	2	2
2	2	2	2	2	2	2	2	2	2
2	2	2	2	1	2	1	1	1	1
2	2	2	2	1	2	1	1	1	1
2	2	2	2	2	2	2	1	1	1
2	2	2	2	2	2	1	2	1	1
2	2	2	2	2	2	1	2	1	1
2	2	2	1	1	2	2	1	2	1
2	2	2	1	1	2	2	1	#NULL!	1
2	2	2	2	2	2	2	2	2	2
2	2	2	2	2	2	1	1	1	2
2	2	2	2	2	2	2	2	2	2
2	2	2	2	2	2	2	2	1	2

2	1	2	2	2	1	2	2	2	1
2	2	2	#NULL!	#NULL!	2	#NULL!	#NULL!	#NULL!	#NULL!
2	2	2	2	2	2	2	2	2	1
2	2	2	2	2	2	2	2	1	2
2	2	2	2	2	2	2	2	2	2
2	2	2	2	2	2	2	2	2	2
2	2	2	1	1	2	2	2	1	1
2	2	2	2	2	2	2	2	2	2
2	2	2	2	1	1	1	1	1	2
2	2	2	1	2	2	1	2	1	2
2	2	2	2	2	2	2	2	2	2
2	2	2	1	1	2	1	1	1	1
#NULL!	2	#NULL!	#NULL!	#NULL!	#NULL!	#NULL!	#NULL!	#NULL!	#NULL!
2	2	2	1	1	1	1	2	1	2
2	2	2	2	2	2	2	1	1	1
2	2	2	2	2	2	2	2	2	2
2	2	2	1	1	2	1	2	2	2
2	2	2	2	2	2	2	2	2	2
2	2	2	2	2	2	2	2	2	2
2	2	2	2	2	2	2	2	2	2
2	2	2	2	2	2	2	2	2	2
2	2	2	2	2	2	2	2	2	2
2	2	2	2	2	2	2	1	1	1
2	2	2	2	2	2	2	2	2	1
2	2	2	2	#NULL!	2	2	2	2	2
2	2	2	2	1	2	1	2	1	1
2	2	2	1	1	2	1	2	2	1
2	2	2	2	2	2	2	1	2	2
2	2	2	2	2	2	2	1	1	#NULL!
#NULL!	#NULL!	2	#NULL!	#NULL!	#NULL!	#NULL!	#NULL!	#NULL!	#NULL!
2	2	2	2	2	2	1	2	2	1
#NULL!	2	2	2	2	2	1	2	2	2
2	2	2	2	2	2	2	2	2	2
2	2	2	2	2	2	2	2	2	2
2	2	2	2	2	2	2	2	2	2
2	2	2	2	2	2	2	2	2	2
2	#NULL!	#NULL!	#NULL!	#NULL!	#NULL!	#NULL!	#NULL!	#NULL!	#NULL!
#NULL!	#NULL!	#NULL!	#NULL!	2	#NULL!	#NULL!	#NULL!	#NULL!	#NULL!
2	2	1	2	2	2	2	2	2	2
2	2	2	2	2	2	2	2	2	2
2	2	2	2	2	2	1	2	2	2
2	2	2	2	2	2	2	2	1	2
2	2	2	2	2	2	2	2	2	2
2	2	2	2	#NULL!	2	2	1	#NULL!	2
1	2	2	2	2	2	2	2	2	2
2	2	2	2	2	2	2	2	2	2
2	2	2	2	2	2	2	1	1	2

2	2	2	2	2	2	2	2	1	2
2	2	2	2	2	2	2	2	2	2
2	2	2	2	2	2	2	2	2	2
2	2	2	2	2	2	2	2	2	2
2	2	2	2	2	2	2	2	2	2
2	2	2	2	2	2	2	1	#NULL!	1
2	2	2	2	2	2	2	2	1	2
#NULL!	#NULL!	#NULL!	#NULL!	#NULL!	#NULL!	2	#NULL!	#NULL!	#NULL!
2	2	2	2	2	2	2	2	1	2
2	2	2	2	2	2	2	2	2	2
2	2	2	2	2	2	2	2	1	#NULL!
1	2	1	2	1	2	2	1	2	2
2	2	2	2	2	2	2	1	2	2
2	2	2	2	2	2	2	2	2	2
2	2	2	2	1	2	1	2	1	1
2	2	2	2	1	2	1	2	1	1
2	2	2	2	2	2	2	2	2	2
2	2	2	2	2	2	2	2	2	2
1	#NULL!	#NULL!	#NULL!	2	#NULL!	#NULL!	#NULL!	#NULL!	#NULL!
2	2	2	2	2	1	1	1	2	2
2	2	2	2	2	2	2	2	1	2
2	2	2	2	2	2	2	2	2	2
2	2	2	2	2	2	2	2	2	2
2	2	2	2	2	2	2	2	1	2
2	2	2	2	2	2	2	2	1	1
2	2	2	2	2	#NULL!	2	2	#NULL!	2
2	2	2	2	2	2	2	2	1	2
2	2	2	1	2	2	2	2	1	2
2	2	2	1	2	2	2	2	1	2
#NULL!	#NULL!	#NULL!	2	#NULL!	#NULL!	#NULL!	#NULL!	#NULL!	#NULL!
2	2	2	1	2	2	2	2	1	2
2	2	2	2	2	2	#NULL!	2	2	2
2	2	2	2	2	2	2	2	1	2
2	2	2	2	2	2	2	2	2	2
#NULL!	#NULL!	#NULL!	#NULL!	#NULL!	#NULL!	#NULL!	#NULL!	#NULL!	#NULL!
2	2	2	2	2	2	2	2	2	2
2	2	2	2	2	2	2	2	2	2
2	2	2	2	2	1	1	1	#NULL!	2
2	2	2	2	2	1	1	1	1	2
2	2	2	2	2	2	2	2	1	1
2	2	2	2	2	2	2	2	1	2
2	2	2	2	2	2	2	2	1	2
2	2	2	2	2	2	2	2	2	2
2	2	2	2	2	2	2	1	1	2
2	2	2	1	1	1	1	1	1	1
2	2	2	2	2	2	2	1	1	2
2	2	2	2	2	2	2	2	1	2

2	2	2	2	1	1	1	1	1	2
2	2	2	2	1	1	1	1	1	2
2	2	2	1	1	1	1	1	1	1
2	2	2	2	2	2	2	2	1	2
2	2	2	2	2	1	1	1	1	2
2	2	2	2	2	1	1	1	1	2
2	#NULL!	2	2	2	2	1	2	1	2
2	2	2	2	2	2	2	2	2	2
#NULL!	2	2	2	2	2	2	#NULL!	2	1
2	2	2	2	1	2	2	2	1	2
2	2	2	2	2	2	2	2	1	1
2	2	2	2	2	2	2	2	2	2
2	2	2	2	2	2	2	2	2	2
1	2	2	2	1	1	1	2	2	2
2	2	2	2	2	2	2	2	1	1
2	2	2	2	2	2	2	1	1	1
2	2	2	2	2	2	2	2	2	2
2	2	2	2	2	2	2	2	2	2
2	2	2	2	2	2	1	2	1	1
1	#NULL!	#NULL!	#NULL!	#NULL!	#NULL!	#NULL!	#NULL!	#NULL!	#NULL!
2	2	2	2	2	1	2	2	2	2
2	2	2	2	2	2	2	2	1	2
2	#NULL!	2	1	#NULL!	2	1	#NULL!	#NULL!	1
2	#NULL!	2	1	#NULL!	2	1	2	2	1
#NULL!	#NULL!	#NULL!	#NULL!	#NULL!	#NULL!	2	#NULL!	#NULL!	#NULL!
#NULL!	#NULL!	2	#NULL!	#NULL!	#NULL!	#NULL!	#NULL!	#NULL!	#NULL!
2	2	2	2	2	2	2	2	1	1
2	#NULL!	2	#NULL!	#NULL!	#NULL!	#NULL!	#NULL!	#NULL!	#NULL!
#NULL!	2	#NULL!	#NULL!	#NULL!	#NULL!	#NULL!	#NULL!	#NULL!	#NULL!
#NULL!	#NULL!	#NULL!	2	#NULL!	#NULL!	#NULL!	#NULL!	#NULL!	#NULL!
#NULL!	2	#NULL!	#NULL!	#NULL!	#NULL!	#NULL!	#NULL!	#NULL!	#NULL!
#NULL!	2	#NULL!	#NULL!	#NULL!	#NULL!	#NULL!	#NULL!	#NULL!	#NULL!
#NULL!	1	#NULL!	#NULL!	#NULL!	#NULL!	#NULL!	#NULL!	#NULL!	#NULL!
#NULL!	2	#NULL!	#NULL!	#NULL!	#NULL!	#NULL!	#NULL!	#NULL!	#NULL!
2	2	2	2	2	2	2	2	1	2
2	2	2	2	2	2	2	2	2	2
2	2	2	2	2	2	2	2	2	2
2	2	2	2	2	2	2	2	2	2
2	2	2	2	2	2	2	2	1	2
2	2	2	2	2	2	2	2	1	2
2	#NULL!	#NULL!	#NULL!	#NULL!	#NULL!	#NULL!	#NULL!	#NULL!	#NULL!
2	#NULL!	#NULL!	2	2	1	2	2	2	2
2	2	2	2	2	2	2	2	2	2
2	2	2	2	2	2	2	2	1	2
2	2	2	2	2	2	2	2	1	1
2	2	2	2	2	2	1	2	1	2
2	2	2	2	2	2	2	2	2	2

2	2	2	2	2	2	2	2	2	2
2	2	2	2	2	2	2	2	2	2
2	2	2	1	2	2	2	#NULL!	1	2
2	2	2	1	2	1	2	#NULL!	2	2
2	2	2	2	2	2	2	2	2	2
2	2	2	2	2	2	1	1	2	1
#NULL!	#NULL!	#NULL!	#NULL!	#NULL!	#NULL!	#NULL!	#NULL!	#NULL!	#NULL!
2	2	2	2	2	2	2	2	2	1
1	2	2	2	2	2	2	2	2	2
2	2	2	2	2	2	2	2	2	2
2	2	2	2	2	2	2	2	2	2
2	2	2	2	2	2	2	2	2	2
2	2	1	2	1	2	2	1	2	2
1	1	2	2	1	1	1	2	1	1
2	2	2	2	#NULL!	2	1	2	1	1
2	2	2	2	2	2	2	1	1	1
#NULL!	#NULL!	#NULL!	#NULL!	#NULL!	#NULL!	#NULL!	#NULL!	#NULL!	#NULL!
2	2	2	2	2	2	1	2	2	2
2	2	2	2	2	2	2	2	2	2
2	2	2	2	2	2	2	2	2	2
2	2	2	2	2	2	2	2	2	2
2	2	2	2	2	1	1	1	2	2
1	2	2	2	1	2	2	2	1	2
2	2	2	2	2	2	2	2	2	2
2	2	2	2	2	2	2	2	2	2
2	2	2	1	2	2	2	2	2	2
2	2	2	2	2	2	2	2	1	2
2	2	2	2	2	2	2	2	2	2
2	2	2	2	2	2	2	2	2	2
2	2	2	2	2	2	2	2	2	2
2	2	2	2	2	2	2	2	1	2
2	2	2	2	2	2	2	2	2	2
2	2	2	2	2	2	1	2	1	2
2	2	2	2	2	2	1	2	2	2
2	#NULL!	#NULL!	#NULL!	#NULL!	2	#NULL!	#NULL!	#NULL!	#NULL!
2	2	2	2	2	2	2	2	2	2
2	2	2	2	2	2	2	2	2	2
2	2	2	2	2	2	2	2	2	2
2	2	2	2	2	2	2	2	2	1
2	2	2	2	2	2	2	2	2	2
2	2	2	2	2	2	2	2	2	2
2	2	2	2	2	2	2	2	2	2

warts2	uti2	std2	poison2	inherited2	discharge2	postsexble	dysmenorr	menorrhag	Asymptom
2	2	2	2	2	2	2	2	2	2
1	2	1	2	1	2	2	2	2	2
2	2	2	2	2	2	2	2	2	2
2	2	2	2	2	2	2	2	#NULL!	1
1	2	2	1	1	2	2	2	2	1
2	2	2	2	1	1	1	1	1	1
1	2	2	2	2	2	2	1	2	1
1	2	2	2	2	2	2	2	2	1
2	2	2	1	2	2	2	2	2	2
2	1	2	2	2	2	2	2	2	1
2	2	2	2	2	2	2	2	2	2
2	2	2	2	2	2	2	2	2	2
2	2	2	2	2	2	2	2	2	1
2	2	2	2	2	2	2	2	2	2
2	2	2	2	2	2	2	2	2	2
2	2	2	2	2	2	2	2	2	2
#NULL!	#NULL!	#NULL!	#NULL!	#NULL!	#NULL!	#NULL!	#NULL!	#NULL!	#NULL!
2	2	2	2	2	2	2	2	2	2
#NULL!	#NULL!	#NULL!	#NULL!	#NULL!	#NULL!	#NULL!	#NULL!	#NULL!	#NULL!
#NULL!	#NULL!	#NULL!	#NULL!	#NULL!	#NULL!	#NULL!	#NULL!	#NULL!	#NULL!
2	2	2	2	2	2	2	2	2	2
2	2	2	2	2	2	2	2	2	2
2	2	2	2	2	2	2	2	2	2
2	2	2	2	2	2	2	2	2	2
#NULL!	#NULL!	#NULL!	#NULL!	#NULL!	2	#NULL!	#NULL!	#NULL!	#NULL!
2	2	2	1	1	2	2	2	2	2
1	1	2	1	1	2	1	2	2	2
2	2	2	2	2	2	2	2	2	2
1	2	2	1	2	2	2	1	1	2
2	2	1	2	1	2	1	1	2	2
2	2	1	1	2	2	2	1	1	2
2	2	2	2	2	2	2	2	2	2
1	1	1	2	1	1	1	1	2	1
2	2	2	2	2	2	2	2	2	2
2	2	2	2	#NULL!	2	2	2	2	2
2	2	2	2	1	1	1	2	1	2
2	2	2	2	2	2	2	2	2	2
2	2	2	1	2	2	2	2	2	2
2	2	2	1	1	2	2	2	2	2
1	#NULL!	2	1	2	2	1	1	2	2
2	2	2	1	2	2	2	1	2	2
#NULL!	#NULL!	#NULL!	#NULL!	1	2	2	2	1	2
#NULL!	#NULL!	#NULL!	#NULL!	#NULL!	#NULL!	#NULL!	#NULL!	#NULL!	#NULL!
2	2	2	2	2	2	2	2	2	2
2	2	2	2	2	2	2	2	2	2
#NULL!	#NULL!	#NULL!	#NULL!	#NULL!	#NULL!	#NULL!	#NULL!	#NULL!	#NULL!

1	2	2	1	1	2	2	2	2	1
1	2	1	2	2	2	2	2	2	2
2	2	2	2	2	2	2	2	2	1
2	2	2	2	2	2	2	2	2	2
2	2	2	1	2	1	1	1	1	1
2	2	2	1	2	1	1	1	1	1
1	2	2	1	1	2	2	1	2	1
2	2	2	2	2	2	2	1	2	2
2	2	2	2	2	2	2	2	2	1
2	1	2	2	2	2	#NULL!	#NULL!	#NULL!	#NULL!
2	#NULL!	#NULL!	#NULL!	#NULL!	2	#NULL!	#NULL!	#NULL!	#NULL!
1	2	2	2	2	2	2	2	2	2
2	2	2	2	2	2	2	2	2	2
2	2	2	2	2	2	2	2	2	2
1	2	1	2	2	1	2	2	2	2
2	1	2	2	2	2	2	2	2	1
1	2	2	2	1	#NULL!	#NULL!	#NULL!	#NULL!	#NULL!
2	2	1	2	2	2	1	1	1	2
2	2	2	2	2	2	2	2	2	2
1	1	#NULL!	2	1	#NULL!	#NULL!	#NULL!	#NULL!	#NULL!
2	2	2	2	2	2	1	1	1	1
1	2	2	1	1	2	2	2	2	2
2	2	2	2	2	2	2	2	2	1
2	2	2	2	2	2	2	2	2	2
1	1	2	2	2	2	2	2	2	2
2	2	2	2	2	2	2	2	2	2
2	2	2	1	1	1	1	1	1	1
2	2	2	2	2	2	2	1	1	2
2	2	2	2	2	2	2	2	2	1
1	2	2	1	1	1	1	1	1	1
#NULL!	#NULL!	#NULL!	#NULL!	#NULL!	#NULL!	#NULL!	#NULL!	#NULL!	#NULL!
2	2	2	1	2	2	2	2	2	2
2	#NULL!	2	#NULL!	2	1	2	2	#NULL!	#NULL!
2	2	2	2	2	2	2	2	2	2
2	2	2	#NULL!	2	2	2	2	#NULL!	2
2	2	2	2	2	2	2	2	2	2
2	2	2	2	2	1	#NULL!	#NULL!	#NULL!	#NULL!
2	1	2	2	2	2	2	2	2	2
2	2	1	1	2	2	2	2	2	2
2	2	2	2	2	2	2	2	2	2
2	2	2	2	2	2	2	2	2	1
#NULL!	#NULL!	#NULL!	#NULL!	#NULL!	#NULL!	#NULL!	#NULL!	#NULL!	#NULL!
2	2	2	2	1	2	2	2	1	2
2	2	2	2	2	2	2	2	2	2
2	2	2	2	2	2	2	2	2	2
1	2	2	2	2	2	2	2	2	1
2	2	1	1	2	2	2	1	2	2

2	2	1	2	2	2	2	2	2	2
1	1	1	1	1	1	1	1	1	1
2	2	2	2	2	2	2	2	2	2
1	2	2	2	2	2	2	2	2	2
2	2	2	2	2	2	2	2	2	2
2	2	2	2	2	2	2	2	2	2
2	2	2	1	1	2	2	2	2	2
#NULL!	#NULL!	#NULL!	#NULL!	#NULL!	#NULL!	#NULL!	#NULL!	#NULL!	#NULL!
2	2	2	2	2	2	2	2	2	2
2	2	2	1	2	2	2	1	1	2
2	2	2	2	2	2	2	2	1	2
2	2	2	2	1	2	2	2	2	2
2	2	2	2	2	2	2	2	1	1
2	2	2	2	2	2	2	2	2	2
2	2	2	2	2	2	1	1	2	2
2	2	#NULL!	2	2	1	2	2	2	2
2	2	2	2	2	2	2	2	1	2
2	2	2	1	1	2	2	2	2	2
2	2	2	2	#NULL!	2	2	2	2	2
2	2	2	2	2	2	2	2	2	2
1	2	1	2	1	2	2	2	2	2
2	2	2	2	2	2	1	1	2	2
2	2	2	2	2	2	2	1	2	2
2	2	2	2	2	2	2	2	2	2
2	2	2	2	2	2	2	2	2	2
2	2	2	1	1	2	2	2	2	2
2	2	2	2	1	2	2	1	1	1
2	2	2	2	2	2	2	2	2	2
2	2	2	2	2	2	2	2	2	2
2	2	2	1	2	2	1	2	2	2
2	1	1	1	1	2	2	2	1	2
1	1	1	1	2	2	2	1	1	2
#NULL!	2	2	2	2	2	2	2	2	#NULL!
2	2	2	2	2	2	2	2	2	2
2	#NULL!	1	2	1	2	2	1	2	2
2	1	2	1	2	2	2	2	1	2
2	2	#NULL!	1	1	2	2	2	2	2
2	2	2	2	2	2	2	2	2	2
#NULL!	#NULL!	#NULL!	#NULL!	#NULL!	#NULL!	#NULL!	#NULL!	#NULL!	#NULL!
1	1	1	1	1	1	1	1	1	1
2	2	2	2	2	2	2	2	2	2
#NULL!	#NULL!	#NULL!	#NULL!	#NULL!	#NULL!	#NULL!	#NULL!	#NULL!	#NULL!
2	2	2	2	2	2	2	2	2	2
2	2	2	2	2	2	2	2	1	2
#NULL!	#NULL!	#NULL!	#NULL!	#NULL!	#NULL!	#NULL!	#NULL!	#NULL!	#NULL!
2	2	2	2	1	2	1	2	2	1
2	2	2	2	2	2	2	2	2	2

2	1	2	1	2	2	1	1	2	2
2	2	2	2	2	2	2	2	2	2
1	1	1	1	1	1	1	1	1	1
2	2	2	2	2	2	2	2	2	2
2	2	2	1	1	2	2	2	2	2
2	1	2	2	2	2	1	2	1	2
1	2	#NULL!	2	2	2	2	#NULL!	2	#NULL!
1	2	#NULL!	2	2	2	2	#NULL!	2	2
#NULL!	#NULL!	#NULL!	#NULL!	#NULL!	2	#NULL!	#NULL!	#NULL!	#NULL!
2	2	2	2	2	2	2	2	2	2
1	1	2	2	2	2	2	2	1	2
2	2	2	2	2	2	2	2	2	2
2	2	2	2	2	2	2	2	2	2
2	2	2	2	2	2	2	2	2	1
2	2	2	2	2	2	2	2	2	2
2	2	2	2	2	2	2	2	2	2
2	2	2	2	2	2	2	2	2	2
2	2	2	2	2	2	2	2	2	2
#NULL!	#NULL!	#NULL!	#NULL!	#NULL!	2	#NULL!	#NULL!	#NULL!	#NULL!
2	1	2	2	2	2	2	2	1	2
2	2	2	2	2	2	2	2	2	1
2	2	2	2	2	2	2	2	2	2
1	1	1	1	1	1	1	1	1	1
2	2	2	2	2	2	2	2	2	2
2	2	2	2	1	2	2	2	2	1
1	1	2	2	1	2	1	1	2	2
#NULL!	#NULL!	#NULL!	#NULL!	#NULL!	#NULL!	#NULL!	#NULL!	#NULL!	#NULL!
1	1	1	1	1	2	2	2	2	2
2	2	2	2	2	2	1	2	2	1
1	1	1	2	2	2	2	2	2	2
2	2	2	2	2	2	2	2	2	2
2	2	2	2	2	2	2	2	2	2
2	2	2	2	2	2	2	2	2	1
1	1	1	1	1	1	1	1	1	1
1	2	2	2	2	2	2	2	2	2
2	2	2	2	2	1	1	1	2	2
1	1	1	1	1	2	2	2	2	2
2	2	2	1	1	2	2	2	2	1
2	1	2	2	2	2	2	2	2	2
2	2	2	2	2	2	2	2	2	2
#NULL!	#NULL!	#NULL!	#NULL!	#NULL!	#NULL!	#NULL!	#NULL!	#NULL!	#NULL!
2	2	2	2	2	2	2	2	2	2
2	2	2	2	2	2	2	2	2	2
#NULL!	#NULL!	#NULL!	#NULL!	#NULL!	#NULL!	#NULL!	#NULL!	#NULL!	#NULL!
#NULL!	#NULL!	#NULL!	#NULL!	#NULL!	#NULL!	#NULL!	#NULL!	#NULL!	#NULL!
2	2	2	2	2	2	2	2	2	2
2	2	2	1	1	2	2	2	2	2

#NULL!	#NULL!	#NULL!	#NULL!	#NULL!	2	#NULL!	#NULL!	#NULL!	#NULL!
#NULL!	#NULL!	#NULL!	#NULL!	2	#NULL!	#NULL!	2	#NULL!	#NULL!
#NULL!	#NULL!	#NULL!	#NULL!	2	2	2	2	1	2
#NULL!	#NULL!	#NULL!	#NULL!	#NULL!	2	#NULL!	#NULL!	#NULL!	#NULL!
#NULL!	#NULL!	#NULL!	#NULL!	#NULL!	1	1	1	1	1
#NULL!	#NULL!	#NULL!	#NULL!	#NULL!	#NULL!	#NULL!	#NULL!	#NULL!	#NULL!
2	2	2	2	2	2	2	2	2	2
#NULL!	#NULL!	#NULL!	#NULL!	#NULL!	#NULL!	#NULL!	#NULL!	#NULL!	#NULL!
2	1	2	2	2	1	2	2	2	2
#NULL!	#NULL!	#NULL!	#NULL!	#NULL!	#NULL!	#NULL!	#NULL!	#NULL!	2
#NULL!	#NULL!	#NULL!	#NULL!	#NULL!	2	#NULL!	#NULL!	#NULL!	#NULL!
#NULL!	#NULL!	#NULL!	#NULL!	#NULL!	#NULL!	#NULL!	#NULL!	#NULL!	#NULL!
2	2	2	2	2	2	2	2	2	2
2	2	2	1	2	2	2	2	1	1
#NULL!	#NULL!	#NULL!	#NULL!	#NULL!	#NULL!	#NULL!	2	#NULL!	#NULL!
1	1	1	1	1	2	1	2	2	1
1	1	1	1	1	2	2	2	2	2
2	2	2	2	2	2	2	2	2	2
2	2	2	2	2	2	2	2	2	2
2	2	2	2	2	2	2	2	2	2
2	2	2	2	2	2	2	2	1	2
#NULL!	#NULL!	#NULL!	#NULL!	#NULL!	#NULL!	#NULL!	#NULL!	#NULL!	#NULL!
2	2	2	2	2	2	2	2	2	2
2	2	2	2	2	2	2	2	2	2
2	2	2	2	2	2	2	2	2	2
2	2	2	2	2	2	2	2	2	2
2	2	2	2	2	2	2	2	2	2
2	2	2	2	2	2	2	2	1	2
1	1	1	1	1	1	1	1	1	1
1	2	2	2	2	2	2	1	2	1
2	2	2	2	2	2	2	2	2	2
2	2	2	2	2	2	2	2	2	2
#NULL!	#NULL!	#NULL!	#NULL!	#NULL!	#NULL!	#NULL!	#NULL!	#NULL!	#NULL!
2	2	2	2	2	2	2	2	2	2
2	2	2	2	2	2	1	2	2	2
1	2	2	2	2	2	2	1	2	2
2	2	2	2	1	2	2	1	#NULL!	#NULL!
2	2	2	2	2	2	2	2	2	2
#NULL!	#NULL!	#NULL!	#NULL!	#NULL!	2	#NULL!	#NULL!	#NULL!	#NULL!
2	2	2	2	2	2	2	2	2	2
2	2	2	2	2	2	2	2	2	2
2	2	2	1	2	2	1	2	2	2
2	2	2	2	2	2	2	2	2	2
2	2	2	2	2	2	2	2	2	2
2	2	2	2	2	1	1	1	1	1
2	1	2	2	2	2	2	2	2	2
#NULL!	#NULL!	#NULL!	#NULL!	#NULL!	#NULL!	#NULL!	#NULL!	#NULL!	#NULL!

2	2	#NULL!	2	2	2	2	2	2	2
2	2	#NULL!	2	2	2	2	2	2	2
2	2	2	2	2	2	2	2	2	2
#NULL!	#NULL!	#NULL!	#NULL!	#NULL!	#NULL!	#NULL!	#NULL!	#NULL!	#NULL!
2	2	2	2	2	2	2	2	2	2
1	2	1	2	1	2	2	2	2	2
2	2	2	2	1	2	2	1	2	2
2	2	2	2	2	2	1	2	2	2
2	2	2	2	2	2	2	2	2	2
2	2	1	2	2	2	2	2	2	2
2	2	2	2	2	2	2	2	1	2
#NULL!	#NULL!	#NULL!	#NULL!	#NULL!	2	#NULL!	#NULL!	#NULL!	#NULL!
#NULL!	#NULL!	#NULL!	#NULL!	#NULL!	2	#NULL!	#NULL!	#NULL!	#NULL!
#NULL!	#NULL!	#NULL!	#NULL!	#NULL!	2	#NULL!	#NULL!	#NULL!	#NULL!
#NULL!	#NULL!	2	#NULL!	2	2	#NULL!	#NULL!	#NULL!	2
#NULL!	#NULL!	#NULL!	#NULL!	#NULL!	#NULL!	#NULL!	#NULL!	#NULL!	#NULL!
#NULL!	#NULL!	#NULL!	#NULL!	#NULL!	#NULL!	#NULL!	#NULL!	#NULL!	#NULL!
#NULL!	#NULL!	#NULL!	#NULL!	#NULL!	#NULL!	#NULL!	#NULL!	#NULL!	#NULL!
#NULL!	#NULL!	2	#NULL!	2	#NULL!	2	2	#NULL!	#NULL!
#NULL!	#NULL!	#NULL!	#NULL!	#NULL!	#NULL!	#NULL!	#NULL!	#NULL!	#NULL!
#NULL!	#NULL!	#NULL!	#NULL!	#NULL!	#NULL!	#NULL!	#NULL!	#NULL!	#NULL!
2	2	2	2	2	2	2	2	2	2
2	2	2	2	2	2	2	2	2	2
2	2	2	2	2	2	2	2	2	2
2	2	2	2	2	2	2	2	2	2
#NULL!	#NULL!	#NULL!	#NULL!	#NULL!	#NULL!	#NULL!	#NULL!	#NULL!	#NULL!
2	2	2	2	2	2	2	2	2	2
#NULL!	#NULL!	#NULL!	#NULL!	#NULL!	#NULL!	#NULL!	#NULL!	#NULL!	#NULL!
#NULL!	#NULL!	#NULL!	#NULL!	#NULL!	#NULL!	#NULL!	#NULL!	#NULL!	#NULL!
2	2	2	2	2	2	2	2	2	2
#NULL!	#NULL!	#NULL!	#NULL!	#NULL!	#NULL!	#NULL!	#NULL!	#NULL!	#NULL!
#NULL!	#NULL!	#NULL!	#NULL!	#NULL!	#NULL!	#NULL!	#NULL!	#NULL!	#NULL!
#NULL!	#NULL!	#NULL!	#NULL!	#NULL!	#NULL!	#NULL!	#NULL!	#NULL!	#NULL!
#NULL!	#NULL!	#NULL!	#NULL!	#NULL!	#NULL!	#NULL!	#NULL!	#NULL!	#NULL!
2	2	#NULL!	#NULL!	#NULL!	#NULL!	#NULL!	#NULL!	#NULL!	#NULL!
#NULL!	2	#NULL!	#NULL!	#NULL!	#NULL!	2	2	#NULL!	1
#NULL!	#NULL!	#NULL!	#NULL!	#NULL!	#NULL!	#NULL!	#NULL!	#NULL!	#NULL!
#NULL!	#NULL!	#NULL!	#NULL!	#NULL!	#NULL!	#NULL!	#NULL!	#NULL!	#NULL!
#NULL!	#NULL!	#NULL!	#NULL!	#NULL!	#NULL!	#NULL!	#NULL!	#NULL!	#NULL!
#NULL!	#NULL!	#NULL!	#NULL!	#NULL!	1	#NULL!	#NULL!	#NULL!	#NULL!
#NULL!	#NULL!	#NULL!	#NULL!	#NULL!	2	#NULL!	#NULL!	#NULL!	#NULL!
#NULL!	#NULL!	#NULL!	#NULL!	#NULL!	#NULL!	#NULL!	#NULL!	#NULL!	#NULL!
1	1	1	1	1	1	1	1	1	1
2	1	2	2	#NULL!	2	2	2	2	2
2	1	2	1	2	2	2	2	2	2
#NULL!	#NULL!	2	#NULL!	2	2	2	2	2	#NULL!
#NULL!	#NULL!	#NULL!	#NULL!	#NULL!	2	#NULL!	#NULL!	#NULL!	#NULL!

#NULL!	#NULL!	#NULL!	#NULL!	#NULL!	#NULL!	2	#NULL!	#NULL!	#NULL!
#NULL!	#NULL!	#NULL!	#NULL!	#NULL!	#NULL!	#NULL!	1	#NULL!	#NULL!
2	2	2	2	2	1	1	2	2	2
1	2	2	1	2	2	2	1	2	2
2	2	1	2	1	2	2	2	2	2
1	1	1	2	2	2	2	2	1	1
2	2	2	1	2	2	2	2	2	2
2	2	2	2	2	2	2	2	2	1
1	2	2	2	2	2	2	2	2	2
1	1	1	1	1	1	1	1	1	1
#NULL!	#NULL!	#NULL!	#NULL!	#NULL!	#NULL!	#NULL!	#NULL!	#NULL!	#NULL!
#NULL!	#NULL!	#NULL!	#NULL!	#NULL!	#NULL!	#NULL!	#NULL!	#NULL!	#NULL!
#NULL!	#NULL!	#NULL!	#NULL!	#NULL!	#NULL!	#NULL!	#NULL!	#NULL!	#NULL!
#NULL!	#NULL!	#NULL!	#NULL!	#NULL!	2	#NULL!	#NULL!	#NULL!	#NULL!
2	2	2	2	2	2	2	2	2	2
1	2	2	2	2	2	2	2	2	1
#NULL!	#NULL!	#NULL!	#NULL!	#NULL!	#NULL!	#NULL!	#NULL!	#NULL!	#NULL!
2	2	2	2	2	2	2	2	2	2
2	2	2	2	2	2	2	2	2	2
2	2	2	2	2	2	2	2	2	2
2	2	1	2	2	2	2	2	2	2
2	2	2	2	2	2	2	2	2	2
#NULL!	#NULL!	#NULL!	#NULL!	#NULL!	2	#NULL!	#NULL!	#NULL!	#NULL!
2	2	2	2	2	2	2	2	2	2
1	2	2	1	1	2	2	2	2	2
2	2	2	2	2	2	2	2	2	2
2	2	2	2	2	2	2	2	2	2
2	2	2	2	2	2	2	2	2	2
#NULL!	1	1	1	1	2	2	2	2	2
2	2	2	2	2	2	2	2	2	2
2	2	2	2	2	2	2	2	2	2
2	2	2	2	2	2	2	2	2	2
2	1	2	1	2	2	2	1	2	2
2	2	2	2	2	2	2	2	2	1
2	2	1	2	2	2	2	2	2	2
1	2	2	2	1	1	1	2	2	2
#NULL!	#NULL!	#NULL!	#NULL!	#NULL!	#NULL!	#NULL!	#NULL!	#NULL!	#NULL!
2	2	2	2	2	2	2	2	2	2
1	1	1	1	1	1	1	1	1	1
1	1	1	1	1	1	1	1	1	1
#NULL!	#NULL!	#NULL!	#NULL!	#NULL!	2	#NULL!	#NULL!	#NULL!	#NULL!
#NULL!	#NULL!	#NULL!	#NULL!	#NULL!	#NULL!	#NULL!	#NULL!	#NULL!	#NULL!
#NULL!	#NULL!	#NULL!	#NULL!	#NULL!	#NULL!	#NULL!	#NULL!	2	#NULL!
2	2	2	2	2	2	2	2	2	2
2	2	2	2	2	2	2	2	2	2
2	2	2	2	2	2	2	2	2	2
1	1	1	1	1	1	1	2	2	2

#NULL!	#NULL!	#NULL!	#NULL!	#NULL!	2	#NULL!	#NULL!	#NULL!	#NULL!
#NULL!	#NULL!	#NULL!	#NULL!	#NULL!	2	#NULL!	#NULL!	#NULL!	#NULL!
#NULL!	#NULL!	#NULL!	#NULL!	#NULL!	#NULL!	#NULL!	2	#NULL!	#NULL!
#NULL!	#NULL!	#NULL!	#NULL!	#NULL!	#NULL!	#NULL!	#NULL!	2	#NULL!
#NULL!	2	#NULL!	#NULL!	1	#NULL!	2	#NULL!	#NULL!	#NULL!
2	2	2	2	2	1	1	2	2	2
#NULL!	#NULL!	#NULL!	#NULL!	#NULL!	2	#NULL!	#NULL!	#NULL!	#NULL!
#NULL!	#NULL!	#NULL!	#NULL!	#NULL!	2	#NULL!	#NULL!	#NULL!	#NULL!
#NULL!	#NULL!	#NULL!	#NULL!	#NULL!	2	#NULL!	#NULL!	#NULL!	#NULL!
#NULL!	#NULL!	#NULL!	#NULL!	#NULL!	2	#NULL!	#NULL!	#NULL!	#NULL!
#NULL!	#NULL!	#NULL!	#NULL!	#NULL!	2	#NULL!	#NULL!	#NULL!	#NULL!
#NULL!	#NULL!	#NULL!	#NULL!	#NULL!	#NULL!	#NULL!	2	#NULL!	#NULL!
#NULL!	#NULL!	#NULL!	#NULL!	#NULL!	2	#NULL!	#NULL!	#NULL!	#NULL!
#NULL!	#NULL!	#NULL!	#NULL!	#NULL!	#NULL!	#NULL!	2	#NULL!	#NULL!
#NULL!	#NULL!	#NULL!	#NULL!	#NULL!	2	#NULL!	#NULL!	#NULL!	#NULL!
#NULL!	#NULL!	#NULL!	#NULL!	#NULL!	#NULL!	#NULL!	#NULL!	#NULL!	#NULL!
2	#NULL!	#NULL!	#NULL!	#NULL!	2	#NULL!	#NULL!	2	#NULL!
2	#NULL!	2	2	1	1	2	2	1	#NULL!
2	2	2	2	2	2	2	1	2	2
2	2	2	2	2	2	2	2	2	2
2	2	2	1	1	2	2	2	2	1
2	1	2	1	2	2	2	2	2	2
2	2	2	2	2	2	2	2	2	2
1	2	2	2	1	2	1	2	#NULL!	2
2	2	2	2	2	2	1	2	1	2
#NULL!	#NULL!	#NULL!	#NULL!	#NULL!	#NULL!	#NULL!	#NULL!	#NULL!	#NULL!
2	2	2	2	1	2	2	2	2	2
2	2	2	2	2	2	2	2	2	2
2	2	2	1	2	2	2	1	2	2
2	2	2	2	2	2	2	2	2	2
2	2	2	2	1	2	2	2	1	2
2	2	2	2	2	2	2	2	2	2
1	1	1	1	1	2	2	2	2	2
2	2	2	2	2	2	2	2	2	2
#NULL!	#NULL!	#NULL!	#NULL!	#NULL!	#NULL!	#NULL!	#NULL!	#NULL!	#NULL!
2	2	2	2	2	2	2	2	2	2
#NULL!	#NULL!	#NULL!	#NULL!	#NULL!	#NULL!	#NULL!	#NULL!	#NULL!	#NULL!
2	2	2	2	2	2	2	2	2	2
#NULL!	#NULL!	#NULL!	#NULL!	#NULL!	#NULL!	#NULL!	#NULL!	#NULL!	#NULL!
2	2	2	2	2	2	2	2	2	2
2	2	1	2	2	2	2	2	2	2
1	1	1	1	1	1	1	1	1	1
2	2	2	1	1	2	1	#NULL!	1	2
2	2	2	2	2	2	2	1	2	1
1	#NULL!	2	2	2	2	2	2	2	2
2	1	2	1	2	2	2	2	2	2
2	2	2	2	2	2	2	2	2	2

2	2	2	2	2	2	2	2	2	2
#NULL!	#NULL!	#NULL!	#NULL!	#NULL!	1	#NULL!	#NULL!	#NULL!	#NULL!
2	2	1	1	2	2	2	2	2	2
2	1	2	1	2	2	#NULL!	2	1	2
2	2	2	2	2	2	2	1	2	2
1	1	1	1	2	2	2	2	1	1
2	2	2	2	2	2	2	2	2	2
2	2	2	2	2	2	2	2	2	2
#NULL!	#NULL!	#NULL!	#NULL!	#NULL!	#NULL!	#NULL!	#NULL!	#NULL!	#NULL!
2	2	2	2	2	2	2	2	2	2
2	2	1	2	1	2	2	2	2	2
#NULL!	#NULL!	#NULL!	#NULL!	#NULL!	2	#NULL!	#NULL!	#NULL!	#NULL!
#NULL!	#NULL!	#NULL!	#NULL!	#NULL!	2	#NULL!	#NULL!	#NULL!	#NULL!
2	1	1	1	1	1	1	1	2	2
#NULL!	#NULL!	#NULL!	#NULL!	2	#NULL!	#NULL!	#NULL!	#NULL!	#NULL!
2	2	2	2	2	2	2	2	2	2
#NULL!	#NULL!	#NULL!	#NULL!	#NULL!	#NULL!	#NULL!	#NULL!	#NULL!	#NULL!
#NULL!	#NULL!	#NULL!	#NULL!	#NULL!	2	#NULL!	#NULL!	#NULL!	#NULL!
#NULL!	#NULL!	#NULL!	#NULL!	#NULL!	#NULL!	#NULL!	#NULL!	2	#NULL!
2	1	2	1	2	1	2	1	2	1
2	2	2	2	2	2	2	2	2	2
2	2	2	2	2	2	2	1	1	2
2	2	2	2	2	2	2	2	2	2
2	#NULL!	#NULL!	#NULL!	#NULL!	#NULL!	#NULL!	#NULL!	#NULL!	#NULL!
#NULL!	#NULL!	#NULL!	#NULL!	#NULL!	2	#NULL!	#NULL!	#NULL!	#NULL!
2	2	2	2	2	2	2	2	2	2
1	1	1	1	1	2	2	2	2	2
2	2	1	1	2	2	2	2	2	1
#NULL!	#NULL!	#NULL!	#NULL!	#NULL!	#NULL!	#NULL!	#NULL!	#NULL!	#NULL!
#NULL!	#NULL!	#NULL!	#NULL!	#NULL!	2	#NULL!	#NULL!	#NULL!	#NULL!
2	2	2	2	2	2	2	2	2	2
2	1	2	2	1	2	2	1	2	2
2	2	2	2	2	2	2	2	2	2
2	2	2	2	2	2	2	2	2	2
2	2	2	1	2	2	2	1	1	1
1	1	1	2	2	2	2	1	2	2
#NULL!	#NULL!	#NULL!	#NULL!	#NULL!	#NULL!	#NULL!	#NULL!	#NULL!	#NULL!
#NULL!	#NULL!	#NULL!	#NULL!	#NULL!	#NULL!	#NULL!	#NULL!	#NULL!	#NULL!
2	1	2	2	2	1	2	2	1	2
2	2	2	2	2	2	2	2	2	2
2	2	2	2	2	2	2	2	2	2
2	2	2	2	2	2	2	2	2	2
2	2	2	2	2	2	2	2	2	2
2	2	1	2	2	2	2	1	2	2
2	2	2	2	2	2	2	2	2	2
2	2	2	2	2	2	2	2	2	2
1	1	1	1	1	1	1	1	1	2

2	2	2	2	2	2	2	2	2	2
2	1	2	1	2	2	2	1	#NULL!	2
#NULL!	2	#NULL!	#NULL!	#NULL!	#NULL!	#NULL!	#NULL!	2	#NULL!
#NULL!	2	#NULL!	#NULL!	#NULL!	#NULL!	#NULL!	#NULL!	#NULL!	#NULL!
#NULL!	#NULL!	#NULL!	#NULL!	#NULL!	2	#NULL!	#NULL!	#NULL!	#NULL!
2	1	2	2	1	1	2	#NULL!	2	1
#NULL!	2	#NULL!	#NULL!	2	2	#NULL!	2	#NULL!	#NULL!
#NULL!	1	#NULL!	1	#NULL!	2	#NULL!	#NULL!	2	#NULL!
#NULL!	#NULL!	#NULL!	#NULL!	#NULL!	#NULL!	#NULL!	#NULL!	#NULL!	#NULL!
#NULL!	#NULL!	#NULL!	#NULL!	#NULL!	#NULL!	#NULL!	#NULL!	#NULL!	#NULL!
#NULL!	#NULL!	#NULL!	#NULL!	#NULL!	#NULL!	#NULL!	#NULL!	#NULL!	#NULL!
#NULL!	#NULL!	#NULL!	#NULL!	#NULL!	#NULL!	#NULL!	#NULL!	#NULL!	#NULL!
#NULL!	#NULL!	#NULL!	#NULL!	#NULL!	#NULL!	#NULL!	#NULL!	#NULL!	#NULL!
#NULL!	#NULL!	#NULL!	#NULL!	#NULL!	#NULL!	#NULL!	#NULL!	#NULL!	#NULL!
#NULL!	#NULL!	#NULL!	#NULL!	#NULL!	#NULL!	#NULL!	#NULL!	#NULL!	#NULL!
#NULL!	#NULL!	#NULL!	#NULL!	#NULL!	2	#NULL!	#NULL!	#NULL!	#NULL!
1	1	1	1	1	1	1	1	1	1
#NULL!	#NULL!	#NULL!	#NULL!	#NULL!	2	#NULL!	#NULL!	#NULL!	#NULL!
#NULL!	#NULL!	#NULL!	#NULL!	#NULL!	2	#NULL!	#NULL!	#NULL!	#NULL!
#NULL!	2	#NULL!	#NULL!	2	2	#NULL!	#NULL!	2	#NULL!
#NULL!	#NULL!	#NULL!	#NULL!	#NULL!	#NULL!	#NULL!	#NULL!	#NULL!	#NULL!
#NULL!	#NULL!	#NULL!	#NULL!	#NULL!	2	#NULL!	#NULL!	#NULL!	#NULL!
2	2	2	2	2	2	2	2	2	2
#NULL!	#NULL!	#NULL!	#NULL!	#NULL!	#NULL!	#NULL!	#NULL!	#NULL!	#NULL!
#NULL!	#NULL!	#NULL!	#NULL!	#NULL!	#NULL!	#NULL!	#NULL!	#NULL!	#NULL!
#NULL!	#NULL!	#NULL!	#NULL!	#NULL!	2	#NULL!	#NULL!	#NULL!	#NULL!
#NULL!	#NULL!	#NULL!	#NULL!	#NULL!	#NULL!	#NULL!	#NULL!	#NULL!	#NULL!
#NULL!	2	#NULL!	#NULL!	2	2	#NULL!	2	#NULL!	2
#NULL!	2	#NULL!	2	#NULL!	2	2	1	2	1
#NULL!	#NULL!	#NULL!	#NULL!	#NULL!	#NULL!	#NULL!	#NULL!	#NULL!	#NULL!
#NULL!	#NULL!	#NULL!	#NULL!	#NULL!	#NULL!	#NULL!	#NULL!	#NULL!	#NULL!
2	2	2	#NULL!	2	2	#NULL!	2	#NULL!	2
#NULL!	#NULL!	#NULL!	#NULL!	#NULL!	#NULL!	#NULL!	#NULL!	#NULL!	#NULL!
#NULL!	#NULL!	#NULL!	#NULL!	#NULL!	#NULL!	#NULL!	#NULL!	#NULL!	#NULL!
#NULL!	#NULL!	#NULL!	#NULL!	#NULL!	#NULL!	#NULL!	#NULL!	#NULL!	#NULL!
#NULL!	#NULL!	#NULL!	#NULL!	#NULL!	#NULL!	#NULL!	#NULL!	#NULL!	#NULL!
#NULL!	#NULL!	#NULL!	#NULL!	#NULL!	#NULL!	#NULL!	#NULL!	#NULL!	#NULL!
#NULL!	#NULL!	#NULL!	#NULL!	#NULL!	#NULL!	#NULL!	#NULL!	#NULL!	#NULL!
2	2	1	2	2	2	2	1	2	2
2	1	2	2	1	2	1	2	2	1
#NULL!	2	#NULL!	2	2	2	#NULL!	#NULL!	#NULL!	#NULL!
#NULL!	#NULL!	#NULL!	#NULL!	#NULL!	#NULL!	#NULL!	#NULL!	#NULL!	#NULL!
2	2	1	2	2	2	2	1	2	2
1	2	2	1	2	2	2	2	2	2
2	2	2	2	2	2	2	2	1	1
2	2	2	1	2	2	2	2	2	1
2	2	1	2	2	2	1	1	1	1

2	#NULL!	#NULL!	#NULL!	#NULL!	#NULL!	#NULL!	#NULL!	#NULL!	#NULL!
1	1	1	2	1	2	2	2	2	1
2	2	2	2	2	2	2	2	2	2
2	2	2	2	2	2	2	2	2	2
1	1	1	1	1	2	1	2	1	2
2	2	2	2	2	2	2	2	2	2
#NULL!	#NULL!	#NULL!	#NULL!	#NULL!	#NULL!	#NULL!	#NULL!	#NULL!	#NULL!
2	2	2	2	2	2	2	1	2	2
2	2	2	2	2	2	2	2	2	2
2	2	2	2	2	2	2	2	2	2
2	2	2	2	2	2	1	1	2	1
#NULL!	#NULL!	2	#NULL!	#NULL!	#NULL!	#NULL!	2	#NULL!	#NULL!
2	#NULL!	#NULL!	#NULL!	#NULL!	#NULL!	#NULL!	#NULL!	#NULL!	#NULL!
#NULL!	#NULL!	#NULL!	#NULL!	#NULL!	2	#NULL!	#NULL!	#NULL!	#NULL!
#NULL!	#NULL!	#NULL!	#NULL!	#NULL!	#NULL!	#NULL!	#NULL!	#NULL!	#NULL!
2	2	2	2	2	2	2	2	2	2
#NULL!	#NULL!	#NULL!	#NULL!	#NULL!	1	2	2	2	2
2	2	2	1	2	2	2	2	2	2
1	1	2	1	1	2	1	1	1	1
2	2	2	2	2	2	2	2	2	2
2	2	2	2	2	2	2	2	2	2
#NULL!	#NULL!	#NULL!	#NULL!	#NULL!	#NULL!	#NULL!	#NULL!	#NULL!	#NULL!
2	2	2	2	2	1	2	2	1	1
#NULL!	#NULL!	#NULL!	#NULL!	#NULL!	#NULL!	2	#NULL!	#NULL!	#NULL!
2	#NULL!	#NULL!	#NULL!	#NULL!	#NULL!	#NULL!	#NULL!	#NULL!	#NULL!
2	2	1	1	1	2	2	2	2	2
#NULL!	#NULL!	#NULL!	#NULL!	#NULL!	#NULL!	#NULL!	2	#NULL!	#NULL!
2	2	2	2	2	2	2	2	2	2
#NULL!	#NULL!	#NULL!	#NULL!	#NULL!	#NULL!	#NULL!	#NULL!	#NULL!	#NULL!
2	#NULL!	#NULL!	#NULL!	#NULL!	#NULL!	#NULL!	#NULL!	#NULL!	#NULL!
1	2	2	2	1	1	2	1	2	2
2	1	2	1	2	1	2	1	2	1
#NULL!	#NULL!	#NULL!	#NULL!	#NULL!	#NULL!	#NULL!	#NULL!	#NULL!	#NULL!
#NULL!	#NULL!	#NULL!	#NULL!	#NULL!	2	#NULL!	#NULL!	#NULL!	#NULL!
1	2	2	1	2	2	2	1	2	2
#NULL!	#NULL!	#NULL!	#NULL!	#NULL!	#NULL!	#NULL!	#NULL!	#NULL!	#NULL!
2	1	2	2	1	#NULL!	#NULL!	#NULL!	#NULL!	#NULL!
#NULL!	#NULL!	#NULL!	#NULL!	#NULL!	#NULL!	2	#NULL!	#NULL!	#NULL!
2	2	2	2	2	2	2	2	2	2
#NULL!	#NULL!	#NULL!	#NULL!	#NULL!	#NULL!	#NULL!	#NULL!	#NULL!	#NULL!
2	2	2	2	2	2	2	2	2	2
2	2	2	2	2	2	2	2	2	2
2	2	2	2	2	2	2	1	1	1
2	2	2	2	2	2	2	2	2	2
1	2	1	1	2	1	2	1	#NULL!	2
2	2	2	2	2	2	2	2	2	2
1	2	2	2	2	2	2	2	2	2

#NULL!	#NULL!	#NULL!	#NULL!	#NULL!	2	#NULL!	#NULL!	#NULL!	#NULL!
2	2	2	2	2	2	2	2	2	2
2	2	2	2	2	2	2	2	2	2
#NULL!	#NULL!	#NULL!	#NULL!	#NULL!	#NULL!	#NULL!	#NULL!	#NULL!	#NULL!
#NULL!	#NULL!	#NULL!	#NULL!	#NULL!	2	#NULL!	#NULL!	#NULL!	#NULL!
#NULL!	#NULL!	#NULL!	#NULL!	#NULL!	#NULL!	#NULL!	#NULL!	#NULL!	#NULL!
#NULL!	#NULL!	#NULL!	#NULL!	#NULL!	#NULL!	#NULL!	#NULL!	#NULL!	#NULL!
2	2	2	2	2	2	2	2	2	2
2	2	2	2	2	2	2	2	2	2
#NULL!	#NULL!	#NULL!	#NULL!	#NULL!	#NULL!	#NULL!	#NULL!	#NULL!	#NULL!
2	2	2	2	2	2	2	2	2	2
2	2	2	2	2	2	2	2	2	2
2	2	2	2	2	2	2	2	2	2
2	2	2	2	2	2	2	2	2	2
#NULL!	#NULL!	#NULL!	#NULL!	#NULL!	#NULL!	#NULL!	#NULL!	#NULL!	#NULL!
#NULL!	#NULL!	#NULL!	#NULL!	#NULL!	#NULL!	#NULL!	#NULL!	#NULL!	#NULL!
#NULL!	#NULL!	#NULL!	#NULL!	#NULL!	#NULL!	#NULL!	#NULL!	#NULL!	#NULL!
#NULL!	#NULL!	#NULL!	#NULL!	#NULL!	#NULL!	#NULL!	#NULL!	#NULL!	#NULL!
#NULL!	#NULL!	#NULL!	#NULL!	#NULL!	2	#NULL!	#NULL!	#NULL!	#NULL!
#NULL!	#NULL!	#NULL!	#NULL!	#NULL!	2	#NULL!	#NULL!	#NULL!	#NULL!
#NULL!	#NULL!	#NULL!	#NULL!	#NULL!	#NULL!	#NULL!	#NULL!	#NULL!	#NULL!
#NULL!	#NULL!	#NULL!	#NULL!	#NULL!	#NULL!	#NULL!	#NULL!	#NULL!	#NULL!
2	2	2	2	2	2	2	2	2	2
#NULL!	#NULL!	#NULL!	#NULL!	#NULL!	2	#NULL!	#NULL!	#NULL!	#NULL!
#NULL!	#NULL!	#NULL!	#NULL!	#NULL!	#NULL!	#NULL!	#NULL!	#NULL!	#NULL!
#NULL!	#NULL!	#NULL!	#NULL!	#NULL!	#NULL!	#NULL!	#NULL!	#NULL!	#NULL!
#NULL!	#NULL!	#NULL!	#NULL!	#NULL!	#NULL!	#NULL!	#NULL!	#NULL!	#NULL!
#NULL!	#NULL!	#NULL!	#NULL!	#NULL!	#NULL!	#NULL!	#NULL!	#NULL!	#NULL!
#NULL!	#NULL!	#NULL!	#NULL!	2	#NULL!	#NULL!	#NULL!	#NULL!	#NULL!
#NULL!	#NULL!	#NULL!	#NULL!	#NULL!	1	#NULL!	#NULL!	#NULL!	#NULL!
#NULL!	#NULL!	#NULL!	#NULL!	#NULL!	#NULL!	#NULL!	#NULL!	#NULL!	#NULL!
2	2	2	2	2	#NULL!	#NULL!	#NULL!	#NULL!	#NULL!
#NULL!	#NULL!	#NULL!	#NULL!	#NULL!	#NULL!	#NULL!	#NULL!	#NULL!	#NULL!
#NULL!	#NULL!	#NULL!	#NULL!	#NULL!	#NULL!	#NULL!	#NULL!	#NULL!	#NULL!
#NULL!	#NULL!	#NULL!	#NULL!	#NULL!	#NULL!	#NULL!	#NULL!	#NULL!	#NULL!
#NULL!	#NULL!	#NULL!	#NULL!	#NULL!	#NULL!	#NULL!	#NULL!	#NULL!	#NULL!
#NULL!	#NULL!	#NULL!	#NULL!	#NULL!	#NULL!	#NULL!	#NULL!	#NULL!	#NULL!
#NULL!	#NULL!	#NULL!	#NULL!	#NULL!	#NULL!	#NULL!	#NULL!	#NULL!	#NULL!
#NULL!	#NULL!	#NULL!	#NULL!	#NULL!	#NULL!	#NULL!	#NULL!	#NULL!	#NULL!
#NULL!	#NULL!	#NULL!	#NULL!	#NULL!	#NULL!	#NULL!	#NULL!	#NULL!	#NULL!
2	2	2	2	2	2	2	2	2	2
#NULL!	#NULL!	#NULL!	#NULL!	#NULL!	2	#NULL!	#NULL!	#NULL!	#NULL!
#NULL!	#NULL!	#NULL!	#NULL!	#NULL!	#NULL!	#NULL!	#NULL!	#NULL!	#NULL!
#NULL!	#NULL!	#NULL!	#NULL!	#NULL!	#NULL!	#NULL!	#NULL!	#NULL!	#NULL!
#NULL!	#NULL!	#NULL!	#NULL!	#NULL!	#NULL!	#NULL!	#NULL!	#NULL!	#NULL!
#NULL!	#NULL!	#NULL!	#NULL!	#NULL!	#NULL!	#NULL!	#NULL!	#NULL!	#NULL!
#NULL!	#NULL!	#NULL!	#NULL!	#NULL!	#NULL!	#NULL!	#NULL!	#NULL!	#NULL!

#NULL!	#NULL!	#NULL!	#NULL!	#NULL!	#NULL!	#NULL!	#NULL!	#NULL!	#NULL!
1	1	1	1	1	2	2	2	2	2
#NULL!	#NULL!	#NULL!	#NULL!	#NULL!	#NULL!	#NULL!	#NULL!	#NULL!	#NULL!
#NULL!	#NULL!	#NULL!	#NULL!	#NULL!	#NULL!	#NULL!	#NULL!	#NULL!	#NULL!
#NULL!	#NULL!	#NULL!	#NULL!	#NULL!	#NULL!	#NULL!	#NULL!	#NULL!	#NULL!
#NULL!	#NULL!	#NULL!	#NULL!	#NULL!	#NULL!	#NULL!	#NULL!	#NULL!	#NULL!
#NULL!	#NULL!	#NULL!	#NULL!	#NULL!	#NULL!	#NULL!	#NULL!	#NULL!	#NULL!
#NULL!	#NULL!	#NULL!	#NULL!	#NULL!	#NULL!	#NULL!	#NULL!	#NULL!	#NULL!
#NULL!	#NULL!	#NULL!	#NULL!	#NULL!	#NULL!	#NULL!	#NULL!	#NULL!	#NULL!
#NULL!	#NULL!	#NULL!	#NULL!	#NULL!	#NULL!	#NULL!	#NULL!	#NULL!	#NULL!
#NULL!	#NULL!	#NULL!	#NULL!	#NULL!	#NULL!	#NULL!	#NULL!	#NULL!	#NULL!
#NULL!	#NULL!	#NULL!	#NULL!	#NULL!	#NULL!	#NULL!	#NULL!	#NULL!	#NULL!
#NULL!	#NULL!	#NULL!	#NULL!	#NULL!	#NULL!	#NULL!	#NULL!	#NULL!	#NULL!
#NULL!	#NULL!	#NULL!	#NULL!	#NULL!	#NULL!	#NULL!	#NULL!	#NULL!	#NULL!
#NULL!	#NULL!	#NULL!	#NULL!	#NULL!	#NULL!	#NULL!	#NULL!	#NULL!	#NULL!
#NULL!	#NULL!	#NULL!	#NULL!	#NULL!	#NULL!	#NULL!	#NULL!	#NULL!	#NULL!
#NULL!	#NULL!	#NULL!	#NULL!	#NULL!	#NULL!	#NULL!	#NULL!	#NULL!	#NULL!
#NULL!	#NULL!	#NULL!	#NULL!	#NULL!	#NULL!	#NULL!	#NULL!	#NULL!	#NULL!
2	2	2	2	2	2	2	2	2	2
#NULL!	#NULL!	#NULL!	#NULL!	#NULL!	#NULL!	#NULL!	#NULL!	#NULL!	#NULL!
#NULL!	#NULL!	#NULL!	#NULL!	#NULL!	#NULL!	#NULL!	#NULL!	#NULL!	#NULL!
#NULL!	#NULL!	#NULL!	#NULL!	#NULL!	#NULL!	#NULL!	#NULL!	#NULL!	#NULL!
#NULL!	#NULL!	#NULL!	#NULL!	#NULL!	#NULL!	#NULL!	#NULL!	#NULL!	#NULL!
#NULL!	#NULL!	#NULL!	#NULL!	#NULL!	#NULL!	#NULL!	#NULL!	#NULL!	#NULL!
#NULL!	#NULL!	#NULL!	#NULL!	#NULL!	#NULL!	#NULL!	#NULL!	#NULL!	#NULL!
2	2	2	2	2	2	2	2	2	2
#NULL!	#NULL!	#NULL!	#NULL!	#NULL!	#NULL!	#NULL!	#NULL!	#NULL!	#NULL!
#NULL!	#NULL!	#NULL!	#NULL!	#NULL!	#NULL!	#NULL!	#NULL!	#NULL!	#NULL!
#NULL!	2	2	2	2	2	2	2	2	2
#NULL!	#NULL!	#NULL!	#NULL!	#NULL!	#NULL!	#NULL!	#NULL!	#NULL!	#NULL!
#NULL!	#NULL!	#NULL!	#NULL!	#NULL!	#NULL!	#NULL!	#NULL!	#NULL!	#NULL!
2	2	2	2	2	2	2	2	2	2
2	2	2	2	2	2	2	1	2	2
#NULL!	#NULL!	#NULL!	#NULL!	#NULL!	#NULL!	#NULL!	#NULL!	#NULL!	#NULL!
2	2	2	2	2	2	2	2	2	2
#NULL!	#NULL!	#NULL!	#NULL!	#NULL!	#NULL!	#NULL!	#NULL!	#NULL!	#NULL!
2	2	2	2	2	2	2	2	2	2
#NULL!	#NULL!	#NULL!	#NULL!	#NULL!	#NULL!	#NULL!	#NULL!	#NULL!	#NULL!
#NULL!	#NULL!	#NULL!	#NULL!	#NULL!	#NULL!	#NULL!	#NULL!	#NULL!	#NULL!
2	2	2	2	2	2	2	2	2	2
#NULL!	#NULL!	#NULL!	#NULL!	1	1	#NULL!	#NULL!	#NULL!	#NULL!
#NULL!	#NULL!	#NULL!	#NULL!	#NULL!	#NULL!	#NULL!	#NULL!	#NULL!	#NULL!
#NULL!	#NULL!	#NULL!	#NULL!	#NULL!	#NULL!	#NULL!	#NULL!	#NULL!	#NULL!
2	2	2	2	2	2	2	2	2	2
2	2	2	2	2	2	2	2	2	2
2	#NULL!	2	#NULL!	2	2	#NULL!	2	#NULL!	1
#NULL!	#NULL!	#NULL!	#NULL!	#NULL!	#NULL!	#NULL!	#NULL!	#NULL!	#NULL!
#NULL!	#NULL!	#NULL!	#NULL!	#NULL!	#NULL!	#NULL!	#NULL!	#NULL!	#NULL!

#NULL!	#NULL!	#NULL!	#NULL!	2	2	#NULL!	#NULL!	2	#NULL!
#NULL!	#NULL!	#NULL!	#NULL!	#NULL!	#NULL!	#NULL!	#NULL!	#NULL!	#NULL!
2	1	2	2	1	#NULL!	#NULL!	#NULL!	#NULL!	#NULL!
#NULL!	#NULL!	#NULL!	#NULL!	#NULL!	#NULL!	#NULL!	#NULL!	#NULL!	#NULL!
#NULL!	#NULL!	#NULL!	#NULL!	#NULL!	#NULL!	#NULL!	#NULL!	#NULL!	#NULL!
#NULL!	#NULL!	#NULL!	#NULL!	#NULL!	#NULL!	#NULL!	#NULL!	#NULL!	#NULL!
#NULL!	#NULL!	#NULL!	#NULL!	#NULL!	2	#NULL!	#NULL!	#NULL!	#NULL!
#NULL!	#NULL!	#NULL!	#NULL!	#NULL!	2	#NULL!	#NULL!	#NULL!	#NULL!
2	#NULL!	#NULL!	2	#NULL!	2	#NULL!	2	#NULL!	2
#NULL!	#NULL!	#NULL!	#NULL!	#NULL!	#NULL!	#NULL!	#NULL!	#NULL!	#NULL!
#NULL!	#NULL!	#NULL!	#NULL!	#NULL!	#NULL!	#NULL!	#NULL!	#NULL!	#NULL!
#NULL!	#NULL!	#NULL!	#NULL!	#NULL!	#NULL!	#NULL!	#NULL!	#NULL!	#NULL!
#NULL!	#NULL!	#NULL!	#NULL!	#NULL!	#NULL!	#NULL!	#NULL!	#NULL!	#NULL!
#NULL!	#NULL!	#NULL!	#NULL!	#NULL!	#NULL!	#NULL!	#NULL!	#NULL!	#NULL!
2	2	1	2	1	2	1	1	2	2
1	2	2	1	2	2	2	1	2	2
#NULL!	#NULL!	#NULL!	#NULL!	#NULL!	#NULL!	#NULL!	#NULL!	#NULL!	2
2	2	2	2	2	2	2	2	2	2
1	1	1	2	2	2	2	2	1	1
#NULL!	#NULL!	#NULL!	#NULL!	#NULL!	2	#NULL!	#NULL!	#NULL!	#NULL!
#NULL!	#NULL!	#NULL!	#NULL!	#NULL!	2	#NULL!	#NULL!	#NULL!	#NULL!
#NULL!	#NULL!	#NULL!	#NULL!	#NULL!	2	#NULL!	#NULL!	#NULL!	#NULL!
1	1	1	1	1	2	2	2	2	2
2	1	2	2	1	2	2	1	2	2
2	2	2	1	1	2	2	2	2	2
2	2	2	2	2	2	2	2	2	2
1	1	1	1	1	#NULL!	#NULL!	#NULL!	#NULL!	#NULL!
2	2	2	2	1	2	2	2	2	2
1	2	2	#NULL!	2	2	2	2	2	2
2	2	2	2	2	2	2	2	2	2
2	2	2	2	2	2	2	2	2	2
#NULL!	#NULL!	#NULL!	#NULL!	#NULL!	#NULL!	#NULL!	#NULL!	#NULL!	#NULL!
#NULL!	#NULL!	#NULL!	#NULL!	#NULL!	2	#NULL!	#NULL!	#NULL!	#NULL!
#NULL!	#NULL!	#NULL!	#NULL!	#NULL!	#NULL!	#NULL!	#NULL!	#NULL!	#NULL!
#NULL!	#NULL!	#NULL!	#NULL!	#NULL!	2	#NULL!	#NULL!	#NULL!	#NULL!
#NULL!	#NULL!	#NULL!	#NULL!	#NULL!	#NULL!	#NULL!	#NULL!	#NULL!	#NULL!
#NULL!	#NULL!	#NULL!	#NULL!	#NULL!	#NULL!	#NULL!	#NULL!	#NULL!	#NULL!
#NULL!	#NULL!	#NULL!	2	#NULL!	#NULL!	2	#NULL!	#NULL!	2
#NULL!	#NULL!	#NULL!	#NULL!	#NULL!	#NULL!	#NULL!	#NULL!	#NULL!	#NULL!
2	#NULL!	2	#NULL!	2	2	#NULL!	#NULL!	2	#NULL!
#NULL!	#NULL!	#NULL!	#NULL!	#NULL!	#NULL!	#NULL!	#NULL!	#NULL!	#NULL!
2	2	2	2	2	2	2	2	2	2
#NULL!	#NULL!	#NULL!	#NULL!	#NULL!	#NULL!	#NULL!	#NULL!	#NULL!	#NULL!
#NULL!	#NULL!	#NULL!	#NULL!	#NULL!	#NULL!	#NULL!	#NULL!	#NULL!	#NULL!
#NULL!	#NULL!	#NULL!	#NULL!	#NULL!	#NULL!	#NULL!	#NULL!	#NULL!	#NULL!
2	2	2	2	2	2	2	2	2	2
2	2	#NULL!	2	1	2	2	2	2	2

2	2	2	2	2	2	#NULL!	2	2	2
2	2	2	2	2	2	2	2	2	1
2	2	2	2	2	2	2	2	2	2
2	2	2	1	1	2	2	2	2	2
1	1	1	1	1	1	1	1	1	1
2	2	2	2	2	2	2	2	2	2
2	2	2	2	2	2	2	2	2	2
1	1	1	1	1	1	1	1	1	1
#NULL!	#NULL!	#NULL!	#NULL!	#NULL!	#NULL!	#NULL!	#NULL!	#NULL!	#NULL!
2	2	2	2	2	2	2	2	2	2
#NULL!	2	#NULL!	#NULL!	2	2	#NULL!	#NULL!	2	#NULL!
1	2	#NULL!	1	2	1	2	2	2	2
2	2	2	2	2	2	2	2	2	2
2	1	2	2	2	2	2	2	2	1
2	1	1	1	1	1	1	1	1	1
2	2	2	2	2	2	2	2	2	2
1	2	2	2	2	2	2	2	1	2
1	2	1	1	1	2	1	1	1	1
#NULL!	#NULL!	#NULL!	#NULL!	#NULL!	#NULL!	#NULL!	#NULL!	#NULL!	#NULL!
2	1	2	2	2	2	2	2	2	2
2	2	2	2	2	2	2	1	2	2
2	2	2	2	1	2	2	2	2	1
2	1	2	2	2	2	2	2	2	1
2	2	1	2	1	2	2	2	2	2
2	2	2	2	2	2	2	2	2	2
2	2	2	2	2	2	2	2	2	2
2	2	2	2	2	2	2	2	2	2
2	1	2	2	2	2	2	2	2	2
2	2	2	2	2	2	2	2	2	2
2	2	2	1	1	2	2	2	2	1
1	1	1	1	1	1	1	1	1	1
#NULL!	#NULL!	#NULL!	#NULL!	#NULL!	#NULL!	#NULL!	2	2	#NULL!
1	1	1	1	1	1	1	1	1	1
2	2	2	2	2	2	2	2	2	2
#NULL!	#NULL!	#NULL!	#NULL!	#NULL!	#NULL!	2	#NULL!	#NULL!	#NULL!
2	2	1	1	1	1	2	2	1	1
2	2	2	2	2	1	1	1	1	1
2	2	2	2	2	2	2	2	2	2
2	2	2	2	2	2	2	2	2	2
#NULL!	#NULL!	#NULL!	#NULL!	#NULL!	#NULL!	#NULL!	#NULL!	#NULL!	#NULL!
2	2	2	2	2	2	2	2	2	2
#NULL!	#NULL!	#NULL!	#NULL!	#NULL!	#NULL!	#NULL!	#NULL!	#NULL!	#NULL!
2	1	2	2	2	2	1	2	2	1
2	2	2	2	2	2	2	2	2	2
1	2	1	2	1	2	2	1	2	2
#NULL!	#NULL!	#NULL!	#NULL!	#NULL!	#NULL!	#NULL!	2	#NULL!	#NULL!
1	1	2	2	2	2	2	1	2	1

#NULL!	#NULL!	#NULL!	#NULL!	#NULL!	#NULL!	#NULL!	#NULL!	#NULL!	#NULL!
1	1	1	1	1	1	1	1	1	1
2	2	2	2	2	#NULL!	1	1	2	1
1	2	2	1	1	1	1	2	2	2
1	1	2	2	2	2	2	2	2	2
#NULL!	#NULL!	#NULL!	#NULL!	#NULL!	#NULL!	#NULL!	#NULL!	#NULL!	#NULL!
2	2	2	2	2	1	1	1	1	1
2	2	2	1	1	2	1	2	1	2
2	2	2	1	2	2	2	2	2	2
2	2	2	2	2	2	2	2	2	2
2	2	2	2	2	2	2	2	2	2
2	2	2	2	2	2	2	2	2	2
#NULL!	#NULL!	#NULL!	#NULL!	#NULL!	#NULL!	#NULL!	#NULL!	#NULL!	#NULL!
2	2	1	1	1	1	1	2	1	2
2	2	2	1	2	2	2	2	2	2
2	2	2	2	2	2	2	2	2	2
1	1	1	1	1	2	2	2	2	2
2	1	2	1	2	2	2	2	2	2
2	2	2	2	2	2	2	2	2	2
#NULL!	#NULL!	#NULL!	#NULL!	#NULL!	#NULL!	#NULL!	#NULL!	#NULL!	2
#NULL!	#NULL!	#NULL!	#NULL!	#NULL!	#NULL!	#NULL!	#NULL!	#NULL!	#NULL!
2	2	2	2	2	2	2	2	2	2
2	1	1	1	2	2	2	2	2	2
1	2	1	2	1	2	1	2	2	1
#NULL!	#NULL!	#NULL!	#NULL!	#NULL!	#NULL!	#NULL!	#NULL!	#NULL!	#NULL!
1	2	1	2	2	2	1	2	1	2
#NULL!	#NULL!	#NULL!	#NULL!	#NULL!	2	#NULL!	#NULL!	#NULL!	#NULL!
2	2	2	2	2	2	2	2	2	2
2	2	2	2	2	2	2	2	2	2
2	2	2	2	2	2	2	2	2	2
2	2	1	2	2	2	2	1	2	1
#NULL!	#NULL!	#NULL!	#NULL!	#NULL!	#NULL!	#NULL!	#NULL!	#NULL!	#NULL!
#NULL!	#NULL!	#NULL!	#NULL!	#NULL!	#NULL!	#NULL!	#NULL!	#NULL!	#NULL!
2	2	2	2	2	2	2	2	2	2
2	2	2	1	2	2	2	2	1	1
#NULL!	#NULL!	#NULL!	#NULL!	#NULL!	#NULL!	#NULL!	#NULL!	#NULL!	#NULL!
#NULL!	#NULL!	#NULL!	#NULL!	#NULL!	#NULL!	#NULL!	#NULL!	#NULL!	#NULL!
2	1	2	1	2	2	2	2	2	2
2	2	1	1	1	2	2	2	2	1
2	2	2	2	2	2	2	2	2	2
2	2	2	2	2	2	2	2	2	2
2	2	2	2	2	2	2	2	2	2
2	2	2	2	2	2	2	2	2	2
2	2	2	2	2	2	2	2	2	2
2	2	2	1	2	2	2	2	1	2
2	1	2	1	2	2	2	2	2	2
2	2	2	2	2	2	2	2	2	2

1	1	1	1	1	1	1	2	2	2
2	2	2	1	2	2	2	2	1	2
2	2	2	2	2	2	2	2	2	2
1	2	2	2	2	2	2	2	2	2
2	2	2	2	2	2	2	2	2	2
2	2	2	1	2	2	2	2	1	2
2	2	2	1	2	2	2	2	2	2
2	2	2	2	2	2	2	2	2	2
2	2	2	2	2	2	2	2	2	1
#NULL!	#NULL!	#NULL!	#NULL!	#NULL!	#NULL!	#NULL!	#NULL!	#NULL!	#NULL!
1	1	1	1	1	2	2	2	2	2
2	2	2	2	1	2	2	2	2	2
2	2	2	2	2	2	2	2	2	2
2	2	2	2	2	2	2	2	2	2
2	1	#NULL!	2	1	1	1	1	1	1
#NULL!	#NULL!	#NULL!	#NULL!	2	#NULL!	#NULL!	2	#NULL!	2
#NULL!	#NULL!	#NULL!	#NULL!	#NULL!	#NULL!	#NULL!	#NULL!	#NULL!	#NULL!
#NULL!	1	#NULL!	#NULL!	#NULL!	#NULL!	#NULL!	#NULL!	#NULL!	2
#NULL!	#NULL!	#NULL!	#NULL!	#NULL!	#NULL!	#NULL!	#NULL!	#NULL!	#NULL!
#NULL!	#NULL!	#NULL!	#NULL!	#NULL!	#NULL!	#NULL!	#NULL!	#NULL!	#NULL!
2	2	2	2	2	2	2	1	1	2
#NULL!	#NULL!	#NULL!	#NULL!	#NULL!	#NULL!	#NULL!	#NULL!	#NULL!	#NULL!
#NULL!	#NULL!	#NULL!	#NULL!	#NULL!	#NULL!	#NULL!	#NULL!	#NULL!	#NULL!
2	2	2	2	2	2	2	2	2	2
2	2	2	2	2	2	2	2	2	2
2	2	2	2	2	2	2	2	2	2
1	2	2	2	1	2	2	1	1	2
2	#NULL!	#NULL!	#NULL!	#NULL!	#NULL!	#NULL!	#NULL!	#NULL!	#NULL!
2	2	2	2	2	2	2	2	2	2
2	2	1	2	1	2	1	2	2	2
#NULL!	#NULL!	#NULL!	#NULL!	#NULL!	#NULL!	#NULL!	#NULL!	#NULL!	#NULL!
2	2	2	1	2	2	2	2	2	2
2	2	1	2	2	2	2	1	2	2
2	2	2	#NULL!	2	2	2	2	1	1
2	1	1	2	2	2	2	2	2	2
2	2	2	2	2	2	2	2	2	2
#NULL!	#NULL!	#NULL!	#NULL!	#NULL!	#NULL!	#NULL!	#NULL!	#NULL!	#NULL!
2	2	2	2	2	2	2	2	2	2
2	2	2	2	2	2	2	2	2	2
2	2	2	2	2	2	2	1	2	2
1	1	#NULL!	2	2	2	2	2	2	2
2	2	2	1	2	2	2	2	2	2
2	2	2	2	2	2	2	2	2	2
2	1	1	1	1	2	2	2	2	2
2	2	1	2	1	2	2	1	2	1
2	2	2	2	2	2	2	2	2	2
1	1	1	1	1	1	1	1	2	2

2	2	2	2	2	2	2	1	1	1
2	2	2	2	2	2	2	2	2	2
1	1	1	1	1	2	2	1	2	#NULL!
2	2	2	2	2	2	2	2	2	2
1	2	1	2	1	1	1	1	2	1
#NULL!	#NULL!	#NULL!	#NULL!	#NULL!	#NULL!	#NULL!	#NULL!	#NULL!	#NULL!
#NULL!	#NULL!	#NULL!	#NULL!	#NULL!	2	#NULL!	#NULL!	#NULL!	#NULL!
1	1	1	1	1	2	2	2	2	2
#NULL!	#NULL!	#NULL!	#NULL!	#NULL!	#NULL!	#NULL!	#NULL!	#NULL!	#NULL!
#NULL!	#NULL!	#NULL!	#NULL!	#NULL!	#NULL!	#NULL!	#NULL!	#NULL!	#NULL!
2	2	2	2	2	2	1	2	2	2
2	2	2	2	2	2	2	2	2	2
2	2	2	2	2	2	2	2	2	1
#NULL!	#NULL!	#NULL!	#NULL!	#NULL!	2	#NULL!	2	#NULL!	#NULL!
#NULL!	#NULL!	#NULL!	#NULL!	#NULL!	2	2	2	2	2
1	2	2	1	2	1	1	1	1	2
2	2	2	2	2	2	2	2	2	2
#NULL!	#NULL!	#NULL!	#NULL!	#NULL!	#NULL!	#NULL!	#NULL!	#NULL!	#NULL!
2	2	2	2	2	2	2	2	2	2
2	2	2	2	2	2	2	1	1	2
1	2	2	2	1	2	2	2	2	2
#NULL!	#NULL!	#NULL!	#NULL!	#NULL!	#NULL!	#NULL!	#NULL!	#NULL!	#NULL!
2	2	2	2	2	2	2	2	2	2
2	2	2	2	2	2	2	2	2	2
2	2	2	2	2	2	2	2	2	2
2	2	2	2	2	2	2	2	2	2
2	2	2	2	2	2	2	2	2	2
2	2	2	2	2	2	2	2	2	2
1	2	1	2	2	2	2	2	2	2
2	2	2	2	2	2	2	2	2	2
2	2	2	2	1	2	2	2	2	2
1	2	2	2	2	2	2	2	1	1
2	1	1	2	2	2	1	1	#NULL!	2
2	2	2	2	2	2	2	2	2	2
2	2	2	2	2	2	2	2	2	2
2	2	2	2	2	2	2	1	2	2
2	2	2	2	2	2	2	2	2	2
2	2	2	2	2	2	2	2	2	2
2	1	1	2	2	2	2	2	2	2
2	2	2	1	2	2	2	2	2	1
2	2	2	1	2	2	2	2	2	2
1	2	2	2	2	2	1	2	2	2
2	2	2	2	2	2	2	2	2	2
2	2	2	2	2	2	2	2	2	2
2	2	2	2	2	2	2	2	2	2
2	2	2	2	2	2	2	2	2	2
2	2	2	2	2	2	2	2	2	2

1	2	1	2	1	2	2	1	2	1
1	2	2	1	2	2	2	2	2	1
1	2	1	2	1	2	1	2	1	2
2	2	2	2	2	2	2	2	2	2
#NULL!	#NULL!	#NULL!	#NULL!	#NULL!	2	#NULL!	#NULL!	#NULL!	#NULL!
#NULL!	#NULL!	#NULL!	#NULL!	#NULL!	#NULL!	#NULL!	2	#NULL!	#NULL!
2	2	1	2	1	2	1	2	1	2
#NULL!	#NULL!	#NULL!	#NULL!	#NULL!	2	#NULL!	#NULL!	#NULL!	#NULL!
#NULL!	#NULL!	#NULL!	#NULL!	#NULL!	2	#NULL!	#NULL!	#NULL!	#NULL!
#NULL!	#NULL!	#NULL!	#NULL!	#NULL!	#NULL!	#NULL!	#NULL!	#NULL!	#NULL!
#NULL!	#NULL!	#NULL!	#NULL!	#NULL!	#NULL!	#NULL!	#NULL!	#NULL!	1
#NULL!	#NULL!	#NULL!	#NULL!	#NULL!	#NULL!	#NULL!	#NULL!	#NULL!	2
#NULL!	#NULL!	#NULL!	#NULL!	#NULL!	#NULL!	#NULL!	#NULL!	#NULL!	#NULL!
#NULL!	#NULL!	#NULL!	#NULL!	#NULL!	#NULL!	2	#NULL!	#NULL!	#NULL!
2	2	2	2	1	1	1	1	1	2
#NULL!	#NULL!	#NULL!	#NULL!	#NULL!	2	#NULL!	#NULL!	#NULL!	#NULL!
#NULL!	#NULL!	#NULL!	#NULL!	#NULL!	#NULL!	#NULL!	#NULL!	#NULL!	#NULL!
#NULL!	#NULL!	#NULL!	#NULL!	#NULL!	#NULL!	#NULL!	#NULL!	2	#NULL!
#NULL!	#NULL!	#NULL!	#NULL!	#NULL!	#NULL!	#NULL!	#NULL!	2	#NULL!
#NULL!	#NULL!	#NULL!	#NULL!	#NULL!	#NULL!	#NULL!	#NULL!	#NULL!	2
#NULL!	#NULL!	#NULL!	#NULL!	#NULL!	#NULL!	#NULL!	#NULL!	#NULL!	#NULL!
2	2	2	2	2	2	2	2	2	2
2	2	2	2	2	2	1	2	2	2
2	2	2	2	2	2	2	2	2	2
2	2	2	1	2	2	2	2	2	2
#NULL!	#NULL!	#NULL!	#NULL!	#NULL!	#NULL!	#NULL!	#NULL!	#NULL!	#NULL!
2	2	2	2	2	2	2	2	2	1
#NULL!	#NULL!	#NULL!	#NULL!	#NULL!	#NULL!	#NULL!	#NULL!	#NULL!	#NULL!
1	1	1	2	1	2	2	2	2	2
2	2	2	2	2	2	2	2	2	2
1	2	2	1	2	2	1	1	1	1
#NULL!	#NULL!	#NULL!	#NULL!	#NULL!	2	#NULL!	#NULL!	#NULL!	#NULL!
2	2	2	2	2	2	2	2	2	2
#NULL!	#NULL!	#NULL!	#NULL!	#NULL!	2	#NULL!	#NULL!	#NULL!	#NULL!
#NULL!	#NULL!	#NULL!	#NULL!	#NULL!	1	#NULL!	#NULL!	#NULL!	#NULL!
2	2	2	2	2	2	2	2	2	2
2	2	2	2	#NULL!	2	2	2	2	2
#NULL!	#NULL!	#NULL!	#NULL!	#NULL!	#NULL!	#NULL!	#NULL!	#NULL!	#NULL!
#NULL!	#NULL!	#NULL!	#NULL!	#NULL!	#NULL!	#NULL!	#NULL!	#NULL!	#NULL!
2	2	2	2	2	2	2	2	2	2
#NULL!	#NULL!	#NULL!	#NULL!	#NULL!	#NULL!	#NULL!	#NULL!	#NULL!	#NULL!
2	2	2	2	2	2	2	2	2	2
1	1	1	1	1	1	1	1	1	1
#NULL!	2	#NULL!	#NULL!	#NULL!	#NULL!	#NULL!	2	#NULL!	#NULL!
2	2	2	2	2	2	2	2	2	2
2	2	2	2	2	2	2	2	2	2
#NULL!	#NULL!	#NULL!	#NULL!	#NULL!	#NULL!	#NULL!	#NULL!	#NULL!	#NULL!

#NULL!	#NULL!	#NULL!	#NULL!	#NULL!	#NULL!	#NULL!	#NULL!	#NULL!	#NULL!
2	#NULL!	#NULL!	2	#NULL!	2	#NULL!	2	#NULL!	#NULL!
#NULL!	#NULL!	#NULL!	#NULL!	#NULL!	#NULL!	#NULL!	#NULL!	#NULL!	#NULL!
2	2	2	2	2	2	2	2	2	2
#NULL!	#NULL!	#NULL!	#NULL!	#NULL!	#NULL!	#NULL!	#NULL!	#NULL!	#NULL!
#NULL!	1	2	1	2	1	2	1	2	2
#NULL!	#NULL!	#NULL!	#NULL!	#NULL!	#NULL!	#NULL!	#NULL!	#NULL!	#NULL!
#NULL!	#NULL!	#NULL!	#NULL!	#NULL!	#NULL!	#NULL!	#NULL!	#NULL!	#NULL!
#NULL!	#NULL!	2	#NULL!	2	#NULL!	2	#NULL!	#NULL!	2
2	2	2	2	2	#NULL!	#NULL!	#NULL!	#NULL!	#NULL!
#NULL!	#NULL!	#NULL!	#NULL!	#NULL!	#NULL!	#NULL!	#NULL!	#NULL!	#NULL!
2	#NULL!	#NULL!	2	#NULL!	#NULL!	2	#NULL!	#NULL!	2
2	2	2	2	2	2	2	2	2	2
#NULL!	#NULL!	#NULL!	#NULL!	#NULL!	#NULL!	#NULL!	#NULL!	#NULL!	#NULL!
2	2	2	2	2	2	2	2	2	2
#NULL!	#NULL!	#NULL!	#NULL!	#NULL!	#NULL!	#NULL!	#NULL!	#NULL!	#NULL!
#NULL!	#NULL!	#NULL!	#NULL!	#NULL!	#NULL!	#NULL!	#NULL!	#NULL!	#NULL!
#NULL!	#NULL!	#NULL!	#NULL!	#NULL!	#NULL!	#NULL!	#NULL!	#NULL!	#NULL!
#NULL!	#NULL!	#NULL!	#NULL!	#NULL!	#NULL!	#NULL!	#NULL!	#NULL!	#NULL!
#NULL!	#NULL!	#NULL!	#NULL!	#NULL!	#NULL!	#NULL!	#NULL!	#NULL!	#NULL!
2	2	2	2	2	2	2	2	2	2
2	2	2	2	2	2	2	2	2	2
2	2	2	1	2	1	1	1	1	1
1	2	1	2	2	2	2	2	2	2
2	1	1	1	1	2	2	2	2	2
2	2	2	2	2	2	2	2	2	2
2	2	2	2	2	2	2	2	2	2
2	2	2	2	2	2	2	2	2	2
2	2	2	2	2	2	2	2	2	1
2	2	2	2	2	2	2	2	2	2
1	2	2	2	1	2	2	2	2	2
2	1	2	2	1	2	2	2	2	1
2	2	2	2	2	2	2	2	2	2
#NULL!	#NULL!	#NULL!	#NULL!	#NULL!	#NULL!	#NULL!	#NULL!	#NULL!	#NULL!
2	2	2	1	2	2	2	2	2	2
2	2	2	2	2	2	2	2	2	2
2	2	2	2	2	2	2	2	2	2
2	2	2	2	2	2	2	2	2	2
1	2	1	2	1	2	2	1	2	2
1	2	2	1	2	2	2	2	#NULL!	2
2	2	2	2	2	#NULL!	#NULL!	#NULL!	#NULL!	#NULL!
#NULL!	#NULL!	#NULL!	#NULL!	#NULL!	#NULL!	#NULL!	#NULL!	#NULL!	#NULL!
#NULL!	#NULL!	#NULL!	#NULL!	#NULL!	1	2	2	2	2
#NULL!	#NULL!	#NULL!	#NULL!	#NULL!	#NULL!	#NULL!	#NULL!	#NULL!	#NULL!
2	2	2	2	2	#NULL!	#NULL!	2	#NULL!	2
2	2	2	2	2	2	2	2	2	2
#NULL!	#NULL!	#NULL!	#NULL!	#NULL!	#NULL!	#NULL!	#NULL!	#NULL!	#NULL!

2	2	2	1	1	2	2	1	1	1
2	2	2	2	1	2	2	2	2	2
2	2	2	2	2	#NULL!	#NULL!	#NULL!	#NULL!	#NULL!
2	2	2	2	2	2	2	2	2	2
#NULL!	#NULL!	2	2	2	#NULL!	#NULL!	#NULL!	#NULL!	#NULL!
2	2	2	2	2	2	2	2	2	2
2	1	1	1	1	2	2	2	2	2
1	2	2	2	2	2	2	#NULL!	1	2
1	1	1	1	1	1	1	1	1	1
2	2	2	1	2	2	2	2	2	2
#NULL!	#NULL!	#NULL!	#NULL!	#NULL!	#NULL!	#NULL!	#NULL!	#NULL!	#NULL!
2	2	2	2	2	2	2	2	2	2
2	2	2	2	2	2	2	2	2	2
2	2	2	2	2	2	2	2	2	2
2	2	2	2	2	#NULL!	#NULL!	#NULL!	#NULL!	#NULL!
2	2	2	2	2	2	2	2	#NULL!	2
2	2	2	2	2	2	2	2	2	2
2	2	2	2	2	2	2	2	2	2
2	2	2	2	2	2	2	2	2	2
#NULL!	#NULL!	#NULL!	#NULL!	#NULL!	#NULL!	#NULL!	#NULL!	#NULL!	#NULL!
2	2	2	2	2	2	2	2	2	2
2	#NULL!	#NULL!	2	2	2	2	2	2	2
#NULL!	#NULL!	#NULL!	#NULL!	#NULL!	#NULL!	#NULL!	#NULL!	#NULL!	#NULL!
2	2	2	2	2	2	2	2	2	2
#NULL!	#NULL!	#NULL!	#NULL!	#NULL!	#NULL!	#NULL!	#NULL!	#NULL!	#NULL!
2	2	2	2	2	2	2	2	2	2
2	2	#NULL!	2	2	2	2	2	2	2
2	2	2	2	#NULL!	2	2	#NULL!	2	2
#NULL!	#NULL!	#NULL!	#NULL!	#NULL!	#NULL!	#NULL!	#NULL!	#NULL!	#NULL!
2	2	2	#NULL!	2	#NULL!	#NULL!	#NULL!	#NULL!	#NULL!
#NULL!	#NULL!	#NULL!	#NULL!	#NULL!	#NULL!	#NULL!	#NULL!	#NULL!	#NULL!
#NULL!	#NULL!	#NULL!	#NULL!	#NULL!	#NULL!	#NULL!	#NULL!	#NULL!	#NULL!
2	2	2	2	2	2	#NULL!	2	2	2
2	2	2	2	2	2	2	2	2	2
2	2	2	2	2	2	2	2	2	2
#NULL!	#NULL!	#NULL!	#NULL!	#NULL!	#NULL!	#NULL!	#NULL!	#NULL!	#NULL!
2	2	2	2	2	2	2	2	2	2
2	2	2	2	2	2	2	2	2	2
#NULL!	#NULL!	#NULL!	#NULL!	#NULL!	#NULL!	#NULL!	#NULL!	#NULL!	#NULL!
#NULL!	#NULL!	#NULL!	#NULL!	#NULL!	#NULL!	#NULL!	#NULL!	#NULL!	#NULL!
2	2	#NULL!	#NULL!	#NULL!	#NULL!	#NULL!	#NULL!	#NULL!	#NULL!
#NULL!	#NULL!	#NULL!	#NULL!	#NULL!	#NULL!	#NULL!	#NULL!	#NULL!	#NULL!
#NULL!	2	2	2	#NULL!	2	2	2	2	2
#NULL!	#NULL!	#NULL!	#NULL!	#NULL!	#NULL!	#NULL!	#NULL!	#NULL!	#NULL!
2	2	2	2	2	2	2	2	2	2
#NULL!	2	#NULL!	2	2	2	2	2	2	2
2	2	2	2	2	2	2	2	2	2

2	2	2	2	2	2	2	2	2	2
2	#NULL!	2	2	#NULL!	2	2	#NULL!	2	2
2	2	2	2	2	2	2	2	2	2
2	2	2	2	2	2	2	2	2	2
2	2	2	2	2	2	2	2	2	2
#NULL!	#NULL!	#NULL!	#NULL!	#NULL!	#NULL!	#NULL!	#NULL!	#NULL!	#NULL!
2	2	2	1	2	1	2	2	2	2
2	2	2	2	2	2	2	2	2	2
2	#NULL!	#NULL!	2	#NULL!	#NULL!	#NULL!	#NULL!	#NULL!	#NULL!
2	2	2	2	2	#NULL!	#NULL!	#NULL!	#NULL!	#NULL!
#NULL!	#NULL!	#NULL!	#NULL!	#NULL!	#NULL!	#NULL!	#NULL!	#NULL!	#NULL!
#NULL!	#NULL!	#NULL!	#NULL!	#NULL!	#NULL!	#NULL!	#NULL!	#NULL!	#NULL!
#NULL!	#NULL!	#NULL!	#NULL!	#NULL!	#NULL!	#NULL!	#NULL!	#NULL!	#NULL!
#NULL!	#NULL!	#NULL!	#NULL!	#NULL!	#NULL!	#NULL!	#NULL!	#NULL!	#NULL!
#NULL!	#NULL!	#NULL!	#NULL!	#NULL!	#NULL!	#NULL!	#NULL!	#NULL!	#NULL!
#NULL!	#NULL!	#NULL!	#NULL!	#NULL!	#NULL!	#NULL!	#NULL!	#NULL!	#NULL!
2	2	2	2	2	#NULL!	#NULL!	#NULL!	#NULL!	#NULL!
#NULL!	#NULL!	#NULL!	#NULL!	#NULL!	#NULL!	#NULL!	#NULL!	#NULL!	#NULL!
2	2	2	2	2	2	2	2	2	2
2	2	2	1	1	2	2	1	1	2
2	2	2	1	1	2	2	1	1	2
2	2	2	1	1	2	2	1	1	2
2	2	2	1	1	2	2	1	1	2
2	2	2	1	1	2	2	1	1	2
2	2	2	1	1	2	2	1	1	2
2	2	2	1	1	2	2	2	1	2
2	2	2	1	1	2	2	1	1	2
2	2	2	1	1	2	2	1	1	2
2	2	2	1	1	2	2	1	1	2
2	2	1	1	1	2	2	1	1	2
2	2	2	1	1	2	2	1	1	2
2	2	2	1	1	2	2	1	1	2
2	2	2	1	1	2	2	1	1	2
2	2	2	1	1	2	2	1	#NULL!	2
2	2	2	1	1	2	2	1	1	2
2	2	2	1	1	2	2	1	1	2
2	2	2	1	1	2	2	1	1	2
2	2	2	1	1	2	2	1	1	2
2	2	2	1	1	2	2	1	1	2
2	2	2	1	1	2	2	1	1	2
2	2	2	1	1	2	2	1	1	2
2	2	2	1	1	2	2	1	1	2
2	2	2	1	1	2	2	1	1	2
2	2	2	1	1	2	2	2	1	1
#NULL!	#NULL!	#NULL!	#NULL!	#NULL!	#NULL!	#NULL!	#NULL!	#NULL!	#NULL!
#NULL!	#NULL!	#NULL!	#NULL!	#NULL!	#NULL!	#NULL!	#NULL!	#NULL!	#NULL!
#NULL!	#NULL!	#NULL!	#NULL!	#NULL!	#NULL!	#NULL!	#NULL!	#NULL!	#NULL!

#NULL!	#NULL!	#NULL!	#NULL!	#NULL!	#NULL!	#NULL!	#NULL!	#NULL!	#NULL!
#NULL!	#NULL!	#NULL!	#NULL!	#NULL!	#NULL!	#NULL!	#NULL!	#NULL!	#NULL!
#NULL!	#NULL!	#NULL!	#NULL!	#NULL!	#NULL!	#NULL!	#NULL!	#NULL!	#NULL!
#NULL!	#NULL!	#NULL!	#NULL!	#NULL!	#NULL!	#NULL!	#NULL!	#NULL!	#NULL!
#NULL!	#NULL!	#NULL!	#NULL!	#NULL!	#NULL!	#NULL!	#NULL!	#NULL!	#NULL!
#NULL!	#NULL!	#NULL!	#NULL!	#NULL!	#NULL!	#NULL!	#NULL!	#NULL!	#NULL!
#NULL!	#NULL!	#NULL!	#NULL!	#NULL!	#NULL!	#NULL!	#NULL!	#NULL!	#NULL!
#NULL!	#NULL!	#NULL!	#NULL!	#NULL!	#NULL!	#NULL!	#NULL!	#NULL!	#NULL!
#NULL!	#NULL!	#NULL!	#NULL!	#NULL!	#NULL!	#NULL!	#NULL!	#NULL!	#NULL!
#NULL!	#NULL!	#NULL!	#NULL!	#NULL!	#NULL!	#NULL!	#NULL!	#NULL!	#NULL!
#NULL!	#NULL!	#NULL!	#NULL!	#NULL!	#NULL!	#NULL!	#NULL!	#NULL!	#NULL!
#NULL!	#NULL!	#NULL!	#NULL!	#NULL!	#NULL!	#NULL!	#NULL!	#NULL!	#NULL!
#NULL!	#NULL!	#NULL!	#NULL!	#NULL!	#NULL!	#NULL!	#NULL!	#NULL!	#NULL!
#NULL!	#NULL!	#NULL!	#NULL!	#NULL!	#NULL!	#NULL!	#NULL!	#NULL!	#NULL!
#NULL!	#NULL!	#NULL!	#NULL!	#NULL!	#NULL!	#NULL!	#NULL!	#NULL!	#NULL!
#NULL!	#NULL!	#NULL!	#NULL!	#NULL!	#NULL!	#NULL!	#NULL!	#NULL!	#NULL!
#NULL!	#NULL!	#NULL!	#NULL!	#NULL!	#NULL!	#NULL!	#NULL!	#NULL!	#NULL!
#NULL!	#NULL!	#NULL!	#NULL!	#NULL!	#NULL!	#NULL!	#NULL!	#NULL!	#NULL!
#NULL!	#NULL!	#NULL!	#NULL!	#NULL!	#NULL!	#NULL!	#NULL!	#NULL!	#NULL!
2	2	2	1	1	2	2	1	1	2
2	2	2	1	1	2	2	1	1	2
2	2	2	1	1	2	2	1	1	2
2	2	2	1	1	2	2	1	1	2
2	2	2	1	1	2	2	1	1	2
2	2	2	1	1	2	2	1	1	2
2	2	2	1	1	2	2	1	1	2
2	2	2	1	1	2	2	1	1	2
2	2	2	1	1	2	2	1	1	2
2	2	2	1	1	2	2	1	1	2
2	2	2	1	1	2	2	1	1	1
2	2	2	1	1	2	2	1	1	2
2	2	2	1	1	2	2	1	1	2
2	2	2	1	1	2	2	1	1	2
2	2	2	1	1	2	2	1	1	2
2	2	2	1	1	2	2	1	1	2
#NULL!	2	2	#NULL!	2	2	#NULL!	1	1	#NULL!
2	2	2	1	1	2	2	1	1	2
2	2	2	1	1	2	2	1	1	2
2	2	2	1	1	2	2	1	1	2
2	2	1	1	1	2	2	1	1	2
2	2	2	1	1	2	2	1	2	#NULL!
2	2	2	1	1	2	2	1	1	2
2	2	2	1	1	2	2	1	1	2
2	2	2	1	1	2	2	1	1	2
2	2	2	1	1	2	2	1	1	2
2	2	2	1	1	2	2	1	1	2
2	2	2	1	1	2	2	1	1	2

2	2	2	1	1	2	2	1	1	2
#NULL!	#NULL!	#NULL!	#NULL!	#NULL!	2	2	1	1	2
2	2	2	1	1	2	2	1	1	1
2	2	2	1	1	2	2	1	1	2
2	2	2	1	1	2	2	1	1	2
2	2	2	1	1	2	2	1	1	2
2	2	2	1	1	2	2	1	1	2
2	2	2	1	1	2	2	1	1	2
2	2	2	1	1	2	2	1	1	#NULL!
2	2	2	1	1	2	2	1	1	2
2	2	2	1	1	2	2	1	1	2
2	2	2	1	1	2	2	1	1	2
2	2	2	1	1	2	2	1	1	2
2	2	2	1	1	2	2	2	1	2
2	2	2	1	1	2	2	1	1	2
2	2	2	1	1	2	2	1	1	2
2	1	2	1	1	2	#NULL!	1	1	2
2	2	2	1	1	2	2	1	1	2
2	2	2	1	1	2	2	1	1	2
2	2	2	1	1	2	2	1	1	2
2	2	2	1	1	2	2	1	1	2
2	1	2	1	1	2	2	1	1	2
2	2	2	1	1	2	2	2	2	2
2	2	2	1	1	2	2	1	1	2
2	2	2	1	1	2	2	1	1	2
2	2	2	1	1	2	2	1	1	2
2	2	2	1	1	2	2	1	1	2
2	2	2	1	1	2	2	1	1	2
2	2	2	1	1	2	2	2	1	2
2	2	2	1	1	2	2	2	1	#NULL!
2	2	2	1	1	2	2	1	1	2
2	2	2	1	1	2	2	1	1	2
2	#NULL!	2	#NULL!	#NULL!	2	1	1	1	2
2	2	2	1	1	2	2	1	1	2
#NULL!	#NULL!	#NULL!	#NULL!	#NULL!	#NULL!	#NULL!	#NULL!	#NULL!	#NULL!
2	2	2	1	1	2	2	1	1	2
2	2	2	1	1	2	2	1	1	2
2	2	2	1	1	2	2	1	1	2
2	2	2	1	1	2	2	1	1	2
#NULL!	#NULL!	#NULL!	#NULL!	#NULL!	2	2	1	1	2
2	2	2	1	1	2	2	1	1	2
2	2	2	2	1	2	2	1	1	2
2	2	2	1	1	2	2	1	1	2
2	2	2	1	1	2	2	1	1	2
2	2	2	1	1	2	2	1	1	2
2	2	2	1	1	2	2	1	1	2
2	2	2	1	1	2	2	1	1	2

2	2	2	1	1	2	2	2	1	1
2	2	2	1	1	2	2	1	1	2
2	2	2	1	1	2	2	1	1	2
2	2	2	1	1	2	2	1	1	2
2	2	2	1	1	2	2	1	1	2
2	2	2	1	1	2	2	1	1	2
2	2	2	1	1	2	2	1	1	2
2	2	2	1	1	2	2	1	1	2
2	2	2	1	1	2	2	1	1	2
2	2	2	1	1	2	2	1	1	2
2	2	2	1	1	2	2	1	1	2
2	2	2	1	2	2	2	1	1	2
2	2	2	1	1	2	2	1	1	2
2	2	2	1	1	2	2	1	1	2
2	2	2	1	1	2	2	1	1	#NULL!
2	2	2	1	1	2	2	1	1	2
2	2	2	1	1	2	2	1	1	2
2	2	2	1	1	2	2	1	1	2
2	2	2	1	1	2	2	1	1	2
2	2	2	1	1	2	1	1	#NULL!	1
2	2	2	1	1	2	2	1	1	2
2	2	2	1	1	2	2	1	1	2
2	2	2	1	1	2	2	1	1	2
2	2	2	1	1	2	2	1	1	2
2	2	2	1	1	2	2	1	1	2
2	2	2	1	1	2	2	1	1	2
2	2	2	1	1	2	2	1	1	2
2	2	2	1	1	2	2	1	1	2
2	2	2	1	1	2	2	1	1	2
2	2	2	1	1	2	2	1	1	2
2	2	2	1	1	2	2	1	1	2
2	2	2	1	1	2	2	1	1	2
2	2	2	1	1	2	2	2	1	1
2	2	2	1	1	2	2	2	1	1
2	2	2	1	1	2	2	1	1	2
2	2	2	1	1	2	2	1	1	2
2	2	2	1	1	2	2	1	1	2
2	2	2	1	1	2	2	1	1	2
2	2	2	1	1	2	2	1	1	2
2	2	2	1	1	2	2	1	1	2
2	2	2	1	1	2	2	1	1	2
#NULL!	#NULL!	#NULL!	#NULL!	#NULL!	#NULL!	#NULL!	#NULL!	#NULL!	#NULL!
1	2	#NULL!	2	1	2	2	1	1	2
2	2	2	1	1	2	2	1	1	2
2	2	2	1	1	2	2	1	1	2
2	2	2	1	1	2	2	1	1	2
2	1	1	1	1	2	2	1	1	2

2	2	2	1	1	2	2	1	1	2
2	2	2	1	1	2	2	1	1	2
2	2	2	1	1	2	2	1	2	2
2	2	2	1	1	2	2	1	1	2
2	2	2	1	1	2	2	1	1	2
2	2	2	1	1	2	2	1	1	2
2	2	2	1	1	2	2	1	1	2
2	2	2	1	1	2	2	1	1	2
2	2	2	1	1	2	2	1	1	2
#NULL!	#NULL!	#NULL!	#NULL!	#NULL!	#NULL!	#NULL!	#NULL!	#NULL!	#NULL!
2	2	2	1	1	2	2	1	1	2
2	2	2	1	1	2	2	1	1	2
2	2	2	1	1	2	2	1	1	2
2	2	2	1	1	2	2	1	1	2
2	2	2	1	1	2	2	1	1	2
2	2	2	1	1	2	2	1	1	2
2	2	2	1	1	2	2	1	1	2
2	2	2	1	1	2	2	1	1	2
2	2	2	1	1	2	2	1	1	2
2	2	2	1	1	2	2	1	1	2
2	2	2	1	1	2	2	1	1	2
2	2	2	1	1	2	2	1	1	2
2	2	2	1	1	2	2	1	1	2
2	2	2	1	1	2	2	1	1	2
2	2	2	1	1	2	2	1	1	2
2	2	2	1	1	2	2	1	1	2
2	2	2	1	1	2	2	1	1	2
#NULL!	#NULL!	#NULL!	#NULL!	#NULL!	#NULL!	#NULL!	#NULL!	#NULL!	#NULL!
#NULL!	#NULL!	#NULL!	#NULL!	#NULL!	#NULL!	#NULL!	#NULL!	#NULL!	#NULL!
#NULL!	#NULL!	#NULL!	#NULL!	#NULL!	#NULL!	#NULL!	#NULL!	#NULL!	#NULL!
#NULL!	#NULL!	#NULL!	#NULL!	#NULL!	#NULL!	#NULL!	#NULL!	#NULL!	#NULL!
#NULL!	#NULL!	#NULL!	#NULL!	#NULL!	#NULL!	#NULL!	#NULL!	#NULL!	#NULL!
#NULL!	#NULL!	#NULL!	#NULL!	#NULL!	#NULL!	#NULL!	#NULL!	#NULL!	#NULL!
#NULL!	#NULL!	#NULL!	#NULL!	#NULL!	#NULL!	#NULL!	#NULL!	#NULL!	#NULL!
#NULL!	#NULL!	#NULL!	#NULL!	#NULL!	#NULL!	#NULL!	#NULL!	#NULL!	#NULL!
#NULL!	#NULL!	#NULL!	#NULL!	#NULL!	#NULL!	#NULL!	#NULL!	#NULL!	#NULL!
#NULL!	#NULL!	#NULL!	#NULL!	#NULL!	#NULL!	#NULL!	#NULL!	#NULL!	#NULL!
#NULL!	#NULL!	#NULL!	#NULL!	#NULL!	#NULL!	#NULL!	#NULL!	#NULL!	#NULL!
#NULL!	#NULL!	#NULL!	#NULL!	#NULL!	#NULL!	#NULL!	#NULL!	#NULL!	#NULL!
#NULL!	#NULL!	#NULL!	#NULL!	#NULL!	#NULL!	#NULL!	#NULL!	#NULL!	#NULL!
#NULL!	#NULL!	#NULL!	#NULL!	#NULL!	#NULL!	#NULL!	#NULL!	#NULL!	#NULL!
#NULL!	#NULL!	#NULL!	#NULL!	#NULL!	#NULL!	#NULL!	#NULL!	#NULL!	#NULL!
#NULL!	#NULL!	#NULL!	#NULL!	#NULL!	#NULL!	#NULL!	#NULL!	#NULL!	#NULL!
#NULL!	#NULL!	#NULL!	#NULL!	#NULL!	#NULL!	#NULL!	#NULL!	#NULL!	#NULL!
#NULL!	#NULL!	#NULL!	#NULL!	#NULL!	#NULL!	#NULL!	#NULL!	#NULL!	#NULL!
#NULL!	#NULL!	#NULL!	#NULL!	#NULL!	#NULL!	#NULL!	#NULL!	#NULL!	#NULL!
#NULL!	#NULL!	#NULL!	#NULL!	#NULL!	#NULL!	#NULL!	#NULL!	#NULL!	#NULL!

#NULL!	#NULL!	#NULL!	#NULL!	#NULL!	#NULL!	#NULL!	#NULL!	#NULL!	#NULL!
#NULL!	#NULL!	#NULL!	#NULL!	#NULL!	#NULL!	#NULL!	#NULL!	#NULL!	#NULL!
#NULL!	#NULL!	#NULL!	#NULL!	#NULL!	#NULL!	#NULL!	#NULL!	#NULL!	#NULL!
#NULL!	#NULL!	#NULL!	#NULL!	#NULL!	#NULL!	#NULL!	#NULL!	#NULL!	#NULL!
#NULL!	#NULL!	#NULL!	#NULL!	#NULL!	#NULL!	#NULL!	#NULL!	#NULL!	#NULL!
#NULL!	#NULL!	#NULL!	#NULL!	#NULL!	#NULL!	#NULL!	#NULL!	#NULL!	#NULL!
#NULL!	#NULL!	#NULL!	#NULL!	#NULL!	#NULL!	#NULL!	#NULL!	#NULL!	#NULL!
#NULL!	#NULL!	#NULL!	#NULL!	#NULL!	#NULL!	#NULL!	#NULL!	#NULL!	#NULL!
#NULL!	#NULL!	#NULL!	#NULL!	#NULL!	#NULL!	#NULL!	#NULL!	#NULL!	#NULL!
#NULL!	#NULL!	#NULL!	#NULL!	#NULL!	#NULL!	#NULL!	#NULL!	#NULL!	#NULL!
#NULL!	#NULL!	#NULL!	#NULL!	#NULL!	#NULL!	#NULL!	#NULL!	#NULL!	#NULL!
#NULL!	#NULL!	#NULL!	#NULL!	#NULL!	#NULL!	#NULL!	#NULL!	#NULL!	#NULL!
[truncated: 122,539 more chars]
