# Supplementary material for: Outcomes from integrating anti-cervical cancer teachings into the curriculum of high schools in a South-Eastern Nigerian State
Source: BMC Public Health. 2022 Oct 14;22:1914. doi: 10.1186/s12889-022-14231-4 (PMC9562070; doi:10.1186/s12889-022-14231-4)
Supplement: Supplementary file 7 — Additional file 7. [file 12889_2022_14231_MOESM7_ESM.pdf]

Comparing Phases 1 and 2 interventions for anti-cervical cancer teachings for high schools in South East Nigeria

Phase	Phase2Onl	ID	Gender	Age	Age2	StateofSch	ClassofStu	SchoolGen	HeardofBre
1	9	1	2	16	2	1	3	1	2
1	9	2	2	15	2	1	1	1	1
1	9	3	2	16	2	1	3	1	1
1	9	4	2	17	2	1	1	1	1
1	9	5	2	15	2	1	1	1	1
1	9	6	2	0	#NULL!	1	1	1	1
1	9	7	2	16	2	1	3	1	1
1	9	8	2	18	2	1	3	1	1
1	9	9	2	18	2	1	3	1	1
1	9	10	2	15	2	1	3	1	1
1	9	11	2	17	2	1	3	1	1
1	9	12	2	17	2	1	3	1	1
1	9	13	2	16	2	1	3	1	1
1	9	14	2	15	2	1	3	1	1
1	9	15	2	16	2	1	3	1	1
1	9	16	2	16	2	1	3	1	1
1	9	17	2	15	2	1	3	1	1
1	9	18	2	16	2	1	3	1	1
1	9	19	2	16	2	1	3	1	1
1	9	20	2	17	2	9	9	1	1
1	9	21	2	17	2	1	3	1	1
1	9	22	2	18	2	1	3	1	1
1	9	23	2	17	2	1	3	1	1
1	9	24	2	16	2	1	3	1	1
1	9	25	2	18	2	1	3	1	1
1	9	26	2	17	2	1	3	1	1
1	9	27	2	18	2	1	3	1	1
1	9	28	2	16	2	1	3	1	1
1	9	29	2	17	2	1	3	1	1
1	9	30	2	18	2	1	3	1	1
1	9	31	2	16	2	1	3	1	1
1	9	32	2	18	2	1	3	1	1
1	9	33	2	16	2	1	3	1	2
1	9	34	2	14	1	1	3	1	1
1	9	35	2	16	2	1	3	1	1
1	9	36	2	15	2	1	3	1	1
1	9	37	2	17	2	1	3	1	1
1	9	38	2	16	2	1	3	1	1
1	9	39	2	17	2	1	3	1	1
1	9	40	2	16	2	1	3	1	1
1	9	41	2	17	2	1	3	1	1
1	9	42	2	0	#NULL!	1	3	1	2
1	9	43	2	17	2	1	3	1	1
1	9	44	2	16	2	1	3	1	1
1	9	45	2	19	2	1	3	1	1
1	9	46	2	18	2	1	3	1	1

1	9	47	2	17	2	1	3	1	1
1	9	48	2	18	2	1	3	1	1
1	9	49	2	17	2	1	3	1	1
1	9	50	2	15	2	1	3	1	1
1	9	51	2	17	2	1	3	1	1
1	9	52	2	16	2	1	3	1	1
1	9	53	2	17	2	1	3	1	2
1	9	54	2	15	2	1	3	1	1
1	9	55	2	17	2	1	3	1	1
1	9	56	2	15	2	1	3	1	1
1	9	57	2	17	2	1	3	1	1
1	9	58	2	19	2	1	3	1	1
1	9	59	2	15	2	1	3	1	1
1	9	60	2	16	2	1	3	1	1
1	9	61	2	15	2	1	3	1	1
1	9	62	2	16	2	1	3	1	1
1	9	63	2	16	2	1	3	1	1
1	9	64	2	0	#NULL!	1	3	1	1
1	9	65	2	16	2	1	3	1	1
1	9	66	2	17	2	1	3	1	1
1	9	67	2	17	2	1	3	1	1
1	9	68	2	17	2	1	3	1	1
1	9	69	2	18	2	1	3	1	1
1	9	70	2	17	2	1	3	1	1
1	9	71	2	17	2	1	3	1	1
1	9	72	2	15	2	1	3	1	1
1	9	73	2	17	2	1	3	1	1
1	9	74	2	16	2	1	3	1	1
1	9	75	2	18	2	1	3	1	1
1	9	76	2	17	2	1	3	1	1
1	9	77	2	17	2	1	3	1	1
1	9	78	2	17	2	1	3	1	1
1	9	79	2	19	2	1	3	1	1
1	9	80	2	0	#NULL!	1	3	1	1
1	9	81	2	18	2	1	3	1	1
1	9	82	9	19	2	1	3	1	1
1	9	83	9	16	2	1	3	1	1
1	9	84	2	15	2	1	3	1	1
1	9	85	2	17	2	1	3	1	1
1	9	86	2	17	2	1	3	1	1
1	9	87	2	17	2	1	3	1	1
1	9	88	2	17	2	1	3	1	1
1	9	89	2	17	2	1	3	1	1
1	9	90	2	16	2	1	3	1	1
1	9	91	2	16	2	1	3	1	1
1	9	92	2	16	2	1	3	1	2
1	9	93	2	16	2	1	3	1	2

1	9	94	2	16	2	1	3	1	1
1	9	95	2	17	2	1	3	1	1
1	9	96	2	15	2	1	3	1	1
1	9	97	2	16	2	1	3	1	1
1	9	98	2	16	2	1	3	1	2
1	9	99	2	16	2	1	3	1	1
1	9	100	2	17	2	1	3	1	1
1	9	101	2	17	2	1	3	1	1
1	9	102	2	17	2	1	3	1	1
1	9	103	2	16	2	1	3	1	1
1	9	104	2	15	2	1	3	1	1
1	9	105	2	17	2	1	3	1	1
1	9	106	2	17	2	1	3	1	1
1	9	107	2	16	2	1	3	1	1
1	9	108	2	17	2	1	3	1	1
1	9	109	2	16	2	1	3	1	1
1	9	110	2	16	2	1	3	1	1
1	9	111	2	15	2	1	3	1	1
1	9	112	2	17	2	1	3	1	1
1	9	113	2	16	2	1	3	1	1
1	9	114	2	20	3	1	3	1	1
1	9	115	2	16	2	1	3	1	1
1	9	116	2	18	2	1	3	1	2
1	9	117	2	15	2	1	3	1	2
1	9	118	2	17	2	1	3	1	1
1	9	119	2	16	2	1	3	1	1
1	9	120	2	16	2	1	3	1	1
1	9	121	2	16	2	1	3	1	2
1	9	122	2	18	2	1	3	1	1
1	9	123	2	18	2	1	3	1	1
1	9	124	2	17	2	1	3	1	1
1	9	125	2	16	2	1	3	1	2
1	9	126	2	19	2	1	3	1	2
1	9	127	2	17	2	1	3	1	1
1	9	128	2	17	2	1	3	1	1
1	9	129	2	16	2	1	3	1	1
1	9	130	2	17	2	1	3	1	1
1	9	131	2	15	2	1	3	1	1
1	9	132	2	18	2	1	3	1	1
1	9	133	2	17	2	1	3	1	1
1	9	134	2	19	2	1	3	1	1
1	9	135	2	18	2	1	3	1	1
1	9	136	2	18	2	1	3	1	2
1	9	137	2	15	2	1	3	1	1
1	9	138	2	17	2	1	3	1	2
1	9	139	2	17	2	1	3	1	1
1	9	140	2	16	2	1	3	1	2

1	9	141	2	19	2	1	3	1	1
1	9	142	2	17	2	1	3	1	1
1	9	143	2	18	2	1	3	1	1
1	9	144	2	18	2	1	3	1	1
1	9	145	2	18	2	1	3	1	1
1	9	146	2	17	2	1	3	1	1
1	9	147	2	21	3	1	9	1	1
1	9	148	2	15	2	1	3	1	1
1	9	149	2	15	2	1	3	1	1
1	9	150	2	18	2	1	3	1	1
1	9	151	2	18	2	1	3	1	1
1	9	152	2	19	2	1	3	1	1
1	9	153	2	20	3	1	3	1	1
1	9	154	2	16	2	1	3	1	1
1	9	155	2	16	2	1	3	1	1
1	9	156	2	17	2	1	3	1	1
1	9	157	2	18	2	1	3	1	1
1	9	158	2	16	2	1	3	1	1
1	9	159	2	19	2	1	3	1	1
1	9	160	2	15	2	1	2	1	2
1	9	161	2	16	2	1	2	1	1
1	9	162	2	17	2	1	2	1	1
1	9	163	2	17	2	1	2	1	1
1	9	164	2	16	2	1	2	1	2
1	9	165	2	16	2	1	2	1	2
1	9	166	2	17	2	1	2	1	1
1	9	167	2	15	2	1	2	1	2
1	9	168	2	15	2	1	2	1	1
1	9	169	2	15	2	1	2	1	1
1	9	170	2	16	2	1	2	1	1
1	9	171	2	17	2	1	3	1	1
1	9	172	2	18	2	1	3	1	1
1	9	173	2	16	2	1	2	1	2
1	9	174	2	17	2	1	2	1	2
1	9	175	2	16	2	1	2	1	2
1	9	176	2	16	2	1	2	1	1
1	9	177	2	16	2	1	2	1	1
1	9	178	2	16	2	1	2	1	1
1	9	179	2	16	2	1	2	1	1
1	9	180	2	17	2	1	2	1	1
1	9	181	2	16	2	1	2	1	1
1	9	182	2	16	2	1	2	1	2
1	9	183	2	15	2	1	2	1	1
1	9	184	2	20	3	1	2	1	1
1	9	185	2	15	2	1	2	1	1
1	9	186	2	20	3	1	2	1	1
1	9	187	2	16	2	1	2	1	1

1	9	188	2	17	2	1	2	1	2
1	9	189	2	15	2	1	2	1	1
1	9	190	9	15	2	1	2	1	2
1	9	191	2	16	2	1	2	1	1
1	9	192	2	15	2	1	2	1	1
1	9	193	2	15	2	1	2	1	1
1	9	194	2	16	2	1	2	1	1
1	9	195	2	15	2	1	3	1	1
1	9	196	2	16	2	1	3	1	1
1	9	197	2	16	2	1	3	1	1
1	9	198	9	0	#NULL!	1	3	1	1
1	9	199	2	17	2	1	3	1	1
1	9	200	2	18	2	1	3	1	1
1	9	201	2	17	2	1	2	1	1
1	9	202	2	17	2	1	2	1	1
1	9	203	2	15	2	1	2	1	1
1	9	204	2	16	2	1	2	1	1
1	9	205	2	17	2	1	2	1	1
1	9	206	2	15	2	1	2	1	1
1	9	207	2	16	2	1	2	1	1
1	9	208	2	16	2	1	2	1	1
1	9	209	2	15	2	1	2	1	1
1	9	210	2	16	2	1	2	1	1
1	9	211	2	15	2	1	2	1	1
1	9	212	2	16	2	1	2	1	1
1	9	213	2	16	2	1	2	1	1
1	9	214	2	16	2	1	2	1	2
1	9	215	2	16	2	1	2	1	2
1	9	216	2	16	2	1	2	1	1
1	9	217	2	16	2	1	2	1	1
1	9	218	2	15	2	1	2	1	1
1	9	219	2	19	2	1	2	1	1
1	9	220	2	16	2	1	2	1	1
1	9	221	2	16	2	1	2	1	2
1	9	222	2	16	2	1	2	1	1
1	9	223	2	16	2	1	2	1	1
1	9	224	2	15	2	1	2	1	1
1	9	225	2	17	2	1	2	1	3
1	9	226	2	15	2	1	2	1	1
1	9	227	2	17	2	1	2	1	1
1	9	228	2	17	2	1	2	1	1
1	9	229	2	15	2	1	2	1	1
1	9	230	2	16	2	1	2	1	1
1	9	231	2	15	2	1	2	1	1
1	9	232	2	15	2	1	2	1	1
1	9	233	2	15	2	1	2	1	1
1	9	234	2	16	2	1	2	1	1

1	9	235	2	15	2	1	2	1	1
1	9	236	2	16	2	1	2	1	1
1	9	237	2	15	2	1	2	1	1
1	9	238	2	18	2	1	2	1	1
1	9	239	2	16	2	1	2	1	1
1	9	240	2	16	2	1	3	9	1
1	9	241	2	17	2	1	3	1	1
1	9	242	2	17	2	1	3	1	1
1	9	243	2	16	2	1	3	1	1
1	9	244	2	17	2	1	3	1	1
1	9	245	2	16	2	1	3	1	1
1	9	246	2	17	2	1	3	1	2
1	9	247	2	17	2	1	3	1	1
1	9	248	2	16	2	1	3	1	1
1	9	249	2	16	2	1	3	1	1
1	9	250	2	16	2	1	3	1	2
1	9	251	2	17	2	1	3	1	1
1	9	252	2	17	2	1	3	1	1
1	9	253	2	17	2	1	3	1	2
1	9	254	2	16	2	1	3	1	1
1	9	255	2	17	2	1	3	1	1
1	9	256	2	17	2	1	3	1	1
1	9	257	2	16	2	1	3	1	1
1	9	258	2	17	2	1	3	1	1
1	9	259	2	15	2	1	3	1	1
1	9	260	2	18	2	1	3	1	1
1	9	261	2	16	2	1	3	1	1
1	9	262	2	15	2	1	3	1	1
1	9	263	2	0	#NULL!	1	3	1	1
1	9	264	2	16	2	1	3	1	1
1	9	265	2	18	2	1	3	1	1
1	9	266	2	17	2	1	3	1	1
1	9	267	2	18	2	1	3	1	1
1	9	268	2	17	2	1	3	1	1
1	9	269	9	17	2	1	3	1	1
1	9	270	2	17	2	1	3	1	2
1	9	271	2	16	2	1	3	1	1
1	9	272	2	16	2	1	3	1	1
1	9	273	2	18	2	1	3	1	1
1	9	274	2	17	2	1	3	1	1
1	9	275	2	15	2	1	2	1	1
1	9	276	2	14	1	1	2	1	1
1	9	277	2	0	#NULL!	1	2	1	1
1	9	278	2	15	2	1	2	1	1
1	9	279	2	16	2	1	2	1	1
1	9	280	2	16	2	1	2	1	1
1	9	281	2	17	2	1	2	1	1

1	9	282	2	15	2	1	2	1	1
1	9	283	2	16	2	1	2	1	1
1	9	284	2	16	2	1	2	1	1
1	9	285	2	13	1	1	2	1	1
1	9	286	2	15	2	1	2	1	1
1	9	287	2	15	2	1	2	1	1
1	9	288	2	16	2	1	2	1	1
1	9	289	2	16	2	1	2	1	1
1	9	290	2	16	2	1	2	1	1
1	9	291	2	16	2	1	2	1	1
1	9	292	2	17	2	1	2	1	1
1	9	293	2	14	1	1	2	1	1
1	9	294	2	15	2	1	2	1	2
1	9	295	2	16	2	1	2	1	2
1	9	296	2	17	2	1	2	1	1
1	9	297	2	20	3	1	2	1	1
1	9	298	2	15	2	1	2	1	1
1	9	299	2	15	2	1	2	1	1
1	9	300	2	16	2	1	2	1	1
1	9	301	2	14	1	1	2	1	1
1	9	302	2	16	2	1	2	1	1
1	9	303	2	0	#NULL!	1	2	1	1
1	9	304	2	17	2	1	2	1	1
1	9	305	2	17	2	1	2	1	1
1	9	306	2	16	2	1	2	1	1
1	9	307	2	18	2	1	3	1	1
1	9	308	2	18	2	1	3	1	1
1	9	309	2	16	2	1	3	1	1
1	9	310	2	17	2	1	3	1	1
1	9	311	2	18	2	1	3	1	1
1	9	312	2	17	2	1	3	1	1
1	9	313	2	17	2	1	3	1	1
1	9	314	2	17	2	9	9	9	9
1	9	315	2	17	2	9	3	1	1
1	9	316	2	18	2	1	3	1	1
1	9	317	2	17	2	1	3	1	1
1	9	318	2	17	2	1	3	1	1
1	9	319	2	16	2	1	3	1	1
1	9	320	2	17	2	1	3	1	1
1	9	321	2	17	2	1	3	1	1
1	9	322	2	19	2	1	3	1	1
1	9	323	2	17	2	1	3	1	1
1	9	324	9	17	2	1	3	1	1
1	9	325	2	16	2	1	3	1	1
1	9	326	2	17	2	1	3	1	1
1	9	327	2	17	2	1	3	1	1
1	9	328	2	16	2	1	3	1	1

1	9	329	2	15	2	1	3	1	1
1	9	330	2	16	2	1	3	1	1
1	9	331	2	16	2	1	3	1	1
1	9	332	2	15	2	1	3	1	1
1	9	333	2	17	2	1	3	1	1
1	9	334	2	17	2	1	3	1	1
1	9	335	2	17	2	1	3	1	1
1	9	336	2	18	2	1	3	1	1
1	9	337	2	16	2	1	2	1	1
1	9	338	2	16	2	1	2	1	1
1	9	339	2	16	2	1	2	1	1
1	9	340	2	15	2	1	2	1	1
1	9	341	2	15	2	1	2	1	1
1	9	342	2	16	2	1	1	1	1
1	9	343	2	16	2	1	2	1	1
1	9	344	2	16	2	1	2	1	1
1	9	345	2	17	2	1	2	1	1
1	9	346	2	17	2	1	2	1	1
1	9	347	2	16	2	1	2	1	1
1	9	348	2	14	1	1	2	1	1
1	9	349	2	16	2	1	2	1	1
1	9	350	2	16	2	1	2	1	1
1	9	351	2	16	2	1	2	1	1
1	9	352	2	14	1	1	2	1	1
1	9	353	2	14	1	1	2	1	1
1	9	354	2	16	2	1	2	1	1
1	9	355	2	15	2	1	2	1	1
1	9	356	2	15	2	1	2	1	1
1	9	357	2	16	2	1	2	1	1
1	9	358	2	16	2	1	2	1	1
1	9	359	2	15	2	1	2	1	1
1	9	360	2	15	2	1	2	1	1
1	9	361	2	16	2	1	2	1	1
1	9	362	2	15	2	1	2	1	1
1	9	363	2	15	2	1	2	1	1
1	9	364	2	15	2	1	2	1	1
1	9	365	2	15	2	1	2	1	1
1	9	366	2	15	2	1	2	1	1
1	9	367	2	15	2	1	2	1	1
1	9	368	2	14	1	1	2	1	3
1	9	369	2	16	2	1	2	1	1
1	9	370	2	15	2	1	2	1	1
1	9	371	2	15	2	1	2	1	1
1	9	372	2	13	1	1	2	1	1
1	9	373	2	14	1	1	2	1	1
1	9	374	2	15	2	1	2	1	1
1	9	375	2	16	2	1	2	1	1

1	9	376	2	16	2	1	2	1	1
1	9	377	2	14	1	1	2	1	1
1	9	378	2	16	2	1	2	1	2
1	9	379	2	16	2	1	2	1	1
1	9	380	2	14	1	1	2	1	1
1	9	381	2	15	2	1	2	1	1
1	9	382	2	0	#NULL!	1	2	1	1
1	9	383	2	15	2	1	2	1	1
1	9	384	2	15	2	1	2	1	1
1	9	385	2	14	1	1	2	1	1
1	9	386	2	14	1	1	2	1	1
1	9	387	2	16	2	1	2	1	1
1	9	388	2	17	2	1	2	1	1
1	9	389	2	16	2	1	2	1	1
1	9	390	2	17	2	1	2	1	1
1	9	391	2	16	2	1	2	1	1
1	9	392	2	16	2	1	2	1	1
1	9	393	2	14	1	1	2	1	1
1	9	394	2	16	2	1	2	1	2
1	9	395	2	16	2	1	2	1	1
1	9	396	2	15	2	1	2	1	1
1	9	397	2	15	2	1	2	1	1
1	9	398	2	16	2	1	2	1	1
1	9	399	2	15	2	1	2	1	1
1	9	400	2	16	2	1	2	1	1
1	9	401	2	19	2	1	2	1	1
1	9	402	2	15	2	1	2	1	1
1	9	403	2	15	2	1	2	1	1
1	9	404	2	12	1	1	2	1	1
1	9	405	2	17	2	1	2	1	1
1	9	406	2	16	2	1	2	1	1
1	9	407	2	17	2	1	2	1	1
1	9	408	2	16	2	1	2	1	1
1	9	409	2	15	2	1	2	1	2
1	9	410	2	16	2	1	2	1	2
1	9	411	2	0	#NULL!	1	2	1	1
1	9	412	2	14	1	1	2	1	1
1	9	413	2	16	2	1	2	1	1
1	9	414	2	18	2	1	2	1	1
1	9	415	2	15	2	9	2	1	1
1	9	416	2	0	#NULL!	1	2	1	1
1	9	417	2	15	2	1	1	1	1
1	9	418	2	15	2	1	2	1	1
1	9	419	2	17	2	1	2	1	1
1	9	420	2	16	2	1	2	1	1
1	9	421	2	16	2	1	2	1	1
1	9	422	2	17	2	1	2	1	1

1	9	423	2	16	2	1	2	1	1
1	9	424	2	15	2	1	2	1	1
1	9	425	2	16	2	1	2	1	1
1	9	426	2	18	2	1	2	1	2
1	9	427	2	16	2	9	9	9	9
1	9	428	2	15	2	1	2	1	2
1	9	429	2	17	2	1	2	1	1
1	9	430	2	16	2	1	2	1	1
1	9	431	2	19	2	1	2	1	1
1	9	432	2	16	2	1	2	1	2
1	9	433	2	16	2	1	2	1	1
1	9	434	2	14	1	1	2	1	1
1	9	435	2	15	2	1	2	1	1
1	9	436	2	15	2	1	2	1	1
1	9	437	2	15	2	1	2	1	1
1	9	438	2	15	2	1	2	1	1
1	9	439	2	16	2	1	2	1	1
1	9	440	2	15	2	1	2	1	2
1	9	441	2	16	2	1	2	1	1
1	9	442	2	14	1	1	2	1	1
1	9	443	2	15	2	1	2	1	1
1	9	444	2	16	2	1	2	1	2
1	9	445	2	15	2	1	2	1	1
1	9	446	2	15	2	1	2	1	1
1	9	447	2	14	1	1	2	1	1
1	9	448	2	15	2	1	2	1	1
1	9	449	2	17	2	1	2	1	1
1	9	450	2	16	2	1	2	1	1
1	9	451	2	16	2	9	2	1	1
1	9	452	2	15	2	1	2	1	1
1	9	453	2	15	2	1	2	1	1
1	9	454	2	15	2	1	2	1	1
1	9	455	2	16	2	1	2	1	1
1	9	456	2	16	2	1	2	1	1
1	9	457	2	14	1	9	2	1	1
1	9	458	2	16	2	1	2	1	1
1	9	459	2	15	2	1	2	1	1
1	9	460	2	16	2	1	2	1	1
1	9	461	2	15	2	1	2	1	1
1	9	462	2	15	2	1	2	1	1
1	9	463	2	16	2	1	2	1	1
1	9	464	2	19	2	1	3	1	1
1	9	465	2	16	2	1	3	1	1
1	9	466	2	19	2	1	3	1	1
1	9	467	2	16	2	1	3	1	1
1	9	468	2	17	2	1	3	1	1
1	9	469	9	17	2	1	3	1	1

1	9	470	2	16	2	1	2	1	2
1	9	471	2	19	2	1	3	1	1
1	9	472	2	19	2	1	3	1	1
1	9	473	2	17	2	1	3	1	2
1	9	474	2	18	2	1	3	1	1
1	9	475	2	0	#NULL!	1	3	1	1
1	9	476	2	19	2	1	3	1	1
1	9	477	2	18	2	1	3	1	1
1	9	478	2	15	2	1	3	1	1
1	9	479	2	17	2	1	3	1	1
1	9	480	2	0	#NULL!	1	3	1	1
1	9	481	2	17	2	1	2	1	1
1	9	482	2	16	2	1	3	1	2
1	9	483	2	17	2	1	3	1	1
1	9	484	2	17	2	1	2	1	2
1	9	485	2	16	2	1	2	1	2
1	9	486	2	17	2	1	2	1	1
1	9	487	2	18	2	1	2	1	1
1	9	488	2	15	2	1	2	1	1
1	9	489	2	14	1	1	2	1	1
1	9	490	9	0	#NULL!	9	9	9	9
1	9	491	2	17	2	1	2	1	1
1	9	492	2	15	2	1	2	1	1
1	9	493	2	17	2	1	2	1	1
1	9	494	2	16	2	1	2	1	1
1	9	495	2	16	2	1	2	1	1
1	9	496	2	16	2	1	2	1	1
1	9	497	2	16	2	1	2	1	1
1	9	498	2	16	2	1	2	1	1
1	9	499	2	16	2	1	2	1	1
1	9	500	2	16	2	1	2	1	1
1	9	501	2	15	2	1	2	1	1
1	9	502	2	16	2	1	2	1	1
1	9	503	2	16	2	1	2	1	2
1	9	504	2	14	1	1	2	1	1
1	9	505	2	15	2	1	2	1	1
1	9	506	2	15	2	1	2	1	1
1	9	507	2	14	1	1	2	1	1
1	9	508	2	17	2	1	2	1	2
1	9	509	2	15	2	1	2	1	1
1	9	510	2	14	1	1	2	1	1
1	9	511	2	15	2	1	2	1	1
1	9	512	2	17	2	1	2	1	1
1	9	513	2	16	2	1	2	1	1
1	9	514	2	15	2	1	2	1	1
1	9	515	2	16	2	1	2	1	1
1	9	516	2	16	2	1	2	1	1

1	9	517	2	16	2	1	2	1	1
1	9	518	2	15	2	1	2	1	1
1	9	519	2	15	2	1	2	1	1
1	9	520	2	15	2	1	2	1	1
1	9	521	2	15	2	1	2	1	1
1	9	522	2	17	2	1	2	1	1
1	9	523	2	14	1	1	2	1	1
1	9	524	2	16	2	1	2	1	1
1	9	525	2	15	2	1	2	1	1
1	9	526	2	16	2	1	2	1	1
1	9	527	2	15	2	1	2	1	1
1	9	528	2	15	2	1	2	1	1
1	9	529	2	16	2	1	2	1	1
1	9	530	2	16	2	1	2	1	1
1	9	531	2	17	2	1	2	1	1
1	9	532	2	14	1	1	2	1	1
1	9	533	2	16	2	1	2	1	1
1	9	534	2	17	2	1	2	1	1
1	9	535	2	14	1	1	2	1	2
1	9	536	2	16	2	1	2	1	1
1	9	537	2	15	2	1	2	1	1
1	9	538	2	15	2	1	2	1	1
1	9	539	2	17	2	1	2	1	1
1	9	540	2	17	2	1	2	1	1
1	9	541	2	15	2	1	2	1	1
1	9	542	2	14	1	1	2	1	1
1	9	543	2	15	2	1	2	1	1
1	9	544	2	15	2	1	2	1	1
1	9	545	2	15	2	1	2	3	1
1	9	546	2	16	2	1	3	1	1
1	9	547	2	16	2	1	3	1	1
1	9	548	2	17	2	1	3	1	1
1	9	549	2	16	2	1	3	1	1
1	9	550	2	18	2	1	3	1	1
1	9	551	2	17	2	1	3	1	2
1	9	552	2	16	2	1	3	1	1
1	9	553	2	17	2	1	3	1	1
1	9	554	2	15	2	1	3	1	1
1	9	555	2	17	2	1	3	1	1
1	9	556	2	18	2	1	3	1	1
1	9	557	2	0	#NULL!	1	3	1	1
1	9	558	2	15	2	1	3	1	1
1	9	559	2	17	2	1	3	1	1
1	9	560	2	18	2	1	3	1	1
1	9	561	2	16	2	1	2	1	1
1	9	562	2	16	2	1	2	1	1
1	9	563	2	15	2	1	2	1	1

1	9	564	2	16	2	1	2	1	1
1	9	565	2	17	2	1	2	1	1
1	9	566	2	15	2	1	2	1	1
1	9	567	2	17	2	1	2	1	1
1	9	568	2	18	2	1	2	1	1
1	9	569	2	15	2	1	2	1	1
1	9	570	2	17	2	1	2	1	1
1	9	571	2	16	2	1	2	1	1
1	9	572	2	16	2	1	2	1	1
1	9	573	2	16	2	1	2	1	1
1	9	574	2	16	2	1	2	1	1
1	9	575	2	16	2	1	2	1	1
1	9	576	2	16	2	1	2	1	1
1	9	577	2	19	2	1	2	1	1
1	9	578	2	15	2	1	2	1	1
1	9	579	2	16	2	1	2	1	1
1	9	580	2	17	2	1	2	1	1
1	9	581	2	15	2	1	2	1	1
1	9	582	1	0	#NULL!	1	2	2	3
1	9	583	1	15	2	1	2	2	3
1	9	584	1	15	2	1	2	2	2
1	9	585	1	17	2	1	2	2	1
1	9	586	1	15	2	1	2	2	1
1	9	587	1	16	2	1	2	2	1
1	9	588	2	18	2	1	3	3	1
1	9	589	1	0	#NULL!	1	3	3	1
1	9	590	2	18	2	1	3	3	1
1	9	591	1	16	2	1	3	3	1
1	9	592	1	0	#NULL!	1	3	3	1
1	9	593	1	17	2	1	3	3	1
1	9	594	1	19	2	1	3	3	1
1	9	595	2	16	2	1	3	3	1
1	9	596	2	17	2	1	3	3	1
1	9	597	1	17	2	1	3	3	1
1	9	598	1	18	2	1	3	3	1
1	9	599	1	15	2	1	3	3	1
1	9	600	2	13	1	1	9	3	1
1	9	601	2	13	1	1	9	3	1
1	9	602	2	13	1	1	9	3	1
1	9	603	2	15	2	1	9	3	1
1	9	604	1	18	2	9	9	9	9
1	9	605	1	14	1	1	9	3	1
1	9	606	2	15	2	1	9	3	1
1	9	607	2	15	2	1	9	3	1
1	9	608	1	14	1	1	9	3	2
1	9	609	2	13	1	1	9	3	1
1	9	610	1	14	1	1	9	3	1

1	9	611	2	14	1	1	3	3	1
1	9	612	2	14	1	1	9	3	3
1	9	613	9	14	1	1	9	3	9
1	9	614	2	17	2	1	9	2	1
1	9	615	1	15	2	1	9	3	1
1	9	616	1	0	#NULL!	1	9	3	1
1	9	617	1	17	2	1	9	3	1
1	9	618	9	13	1	1	9	3	1
1	9	619	2	14	1	1	9	3	3
1	9	620	9	0	#NULL!	1	9	9	9
1	9	621	2	15	2	9	9	9	9
1	9	622	2	14	1	1	9	3	1
1	9	623	1	0	#NULL!	1	9	3	2
1	9	624	2	13	1	1	9	3	3
1	9	625	2	14	1	1	9	3	1
1	9	626	2	18	2	1	3	3	1
1	9	627	2	13	1	1	9	3	1
1	9	628	2	13	1	1	9	3	1
1	9	629	2	13	1	1	9	3	1
1	9	630	1	12	1	1	9	3	1
1	9	631	1	12	1	1	9	3	1
1	9	632	9	0	#NULL!	1	9	3	2
1	9	633	9	0	#NULL!	1	1	3	2
1	9	634	2	14	1	1	9	3	1
1	9	635	2	13	1	1	9	3	1
1	9	636	2	14	1	1	9	3	2
1	9	637	2	14	1	1	9	3	1
1	9	638	2	14	1	1	9	3	2
1	9	639	1	12	1	1	9	3	1
1	9	640	2	15	2	1	9	3	1
1	9	641	2	15	2	1	9	3	2
1	9	642	2	15	2	1	9	3	1
1	9	643	2	15	2	1	9	3	1
1	9	644	2	16	2	1	9	3	1
1	9	645	2	16	2	1	9	3	1
1	9	646	2	13	1	1	9	3	1
1	9	647	2	15	2	1	9	3	1
1	9	648	2	13	1	1	3	3	2
1	9	649	2	12	1	1	9	3	2
1	9	650	2	18	2	1	9	1	1
1	9	651	2	12	1	1	9	3	1
1	9	652	2	13	1	1	9	3	1
1	9	653	1	14	1	1	9	3	1
1	9	654	2	13	1	1	9	3	1
1	9	655	1	14	1	1	9	3	1
1	9	656	1	14	1	1	9	3	1
1	9	657	1	16	2	1	3	3	1

1	9	658	2	15	2	1	3	3	1
1	9	659	2	15	2	1	3	3	1
1	9	660	1	19	2	1	3	3	1
1	9	661	2	18	2	1	3	3	1
1	9	662	2	16	2	1	3	3	1
1	9	663	1	16	2	1	3	3	1
1	9	664	1	20	3	1	3	3	3
1	9	665	2	0	#NULL!	1	3	3	1
1	9	666	2	14	1	1	9	3	2
1	9	667	2	15	2	1	3	3	1
1	9	668	1	14	1	1	9	3	3
1	9	669	2	15	2	1	9	3	1
1	9	670	2	13	1	1	9	3	1
1	9	671	2	12	1	1	9	3	1
1	9	672	1	13	1	1	9	3	1
1	9	673	2	13	1	1	9	3	1
1	9	674	2	13	1	1	3	3	1
1	9	675	2	13	1	1	9	3	1
1	9	676	2	14	1	1	9	3	1
1	9	677	1	13	1	1	9	3	1
1	9	678	2	14	1	1	9	3	1
1	9	679	1	14	1	1	9	3	1
1	9	680	2	15	2	1	9	3	1
1	9	681	1	13	1	1	9	3	1
1	9	682	1	18	2	1	3	3	2
1	9	683	1	13	1	1	9	3	2
1	9	684	2	14	1	1	9	3	1
1	9	685	2	13	1	1	9	3	1
1	9	686	2	14	1	1	9	3	1
1	9	687	1	15	2	1	9	3	3
1	9	688	1	15	2	1	9	3	1
1	9	689	2	16	2	1	9	3	2
1	9	690	1	14	1	1	9	3	1
1	9	691	2	14	1	1	9	3	1
1	9	692	2	14	1	1	9	3	2
1	9	693	2	13	1	1	9	3	2
1	9	694	2	14	1	1	9	3	1
1	9	695	2	13	1	1	9	3	1
1	9	696	2	14	1	1	9	3	2
1	9	697	2	13	1	1	9	3	1
1	9	698	1	14	1	1	9	2	2
1	9	699	2	15	2	1	9	3	1
1	9	700	1	14	1	1	9	3	1
1	9	701	2	13	1	1	9	3	1
1	9	702	2	13	1	9	9	9	9
1	9	703	1	14	1	1	9	3	2
1	9	704	2	14	1	1	9	3	1

1	9	705	1	12	1	1	9	3	1
1	9	706	2	13	1	1	9	3	1
1	9	707	1	14	1	1	3	3	1
1	9	708	2	14	1	1	9	3	2
1	9	709	1	16	2	1	3	3	2
1	9	710	1	15	2	1	3	3	2
1	9	711	1	14	1	1	3	3	3
1	9	712	1	16	2	1	3	3	2
1	9	713	1	16	2	1	3	3	2
1	9	714	9	0	#NULL!	9	9	9	9
1	9	715	2	14	1	1	9	3	1
1	9	716	2	15	2	1	9	3	1
1	9	717	2	11	1	1	9	3	1
1	9	718	2	15	2	1	9	3	3
1	9	719	2	13	1	1	9	3	1
1	9	720	1	13	1	1	9	3	1
1	9	721	1	13	1	1	9	3	1
1	9	722	1	15	2	1	9	3	1
1	9	723	1	15	2	1	3	3	1
1	9	724	1	14	1	1	9	3	1
1	9	725	2	16	2	9	9	3	1
1	9	726	1	11	1	1	9	3	1
1	9	727	1	15	2	2	9	3	2
1	9	728	1	14	1	1	9	3	1
1	9	729	1	14	1	1	9	3	1
1	9	730	1	13	1	1	9	3	1
1	9	731	1	12	1	1	9	3	1
1	9	732	1	11	1	1	9	3	1
1	9	733	1	14	1	2	9	3	1
1	9	734	1	15	2	1	9	3	3
1	9	735	1	15	2	2	9	3	1
1	9	736	1	14	1	1	9	3	1
1	9	737	2	14	1	1	3	3	1
1	9	738	1	13	1	1	9	3	1
1	9	739	1	14	1	1	9	3	1
1	9	740	1	15	2	1	9	3	2
1	9	741	1	13	1	1	1	2	1
1	9	742	2	15	2	1	9	3	1
1	9	743	1	14	1	1	9	3	1
1	9	744	1	15	2	1	2	2	1
1	9	745	1	16	2	1	2	2	1
1	9	746	1	17	2	2	2	2	1
1	9	747	1	17	2	1	2	2	1
1	9	748	1	17	2	1	2	2	2
1	9	749	1	17	2	1	2	2	1
1	9	750	1	14	1	1	2	2	1
1	9	751	1	15	2	1	2	2	1

1	9	752	1	16	2	1	2	2	1
1	9	753	1	15	2	1	1	2	1
1	9	754	1	16	2	1	2	2	2
1	9	755	1	16	2	1	2	2	1
1	9	756	1	15	2	1	2	2	2
1	9	757	1	16	2	1	2	2	1
1	9	758	1	16	2	1	2	2	2
1	9	759	1	15	2	1	2	2	1
1	9	760	9	0	#NULL!	9	3	2	1
1	9	761	1	15	2	1	2	2	1
1	9	762	1	17	2	1	2	2	2
1	9	763	1	16	2	1	2	2	1
1	9	764	1	15	2	1	2	2	1
1	9	765	1	16	2	1	2	2	1
1	9	766	1	17	2	1	2	2	1
1	9	767	1	17	2	1	2	2	1
1	9	768	1	13	1	1	2	2	1
1	9	769	1	21	3	1	2	2	1
1	9	770	1	16	2	1	2	2	1
1	9	771	1	15	2	1	2	2	1
1	9	772	1	17	2	1	2	2	2
1	9	773	1	15	2	1	2	2	1
1	9	774	1	16	2	1	2	2	1
1	9	775	1	16	2	1	2	2	1
1	9	776	1	17	2	1	2	2	2
1	9	777	1	18	2	1	2	3	1
1	9	778	1	15	2	1	2	2	1
1	9	779	1	17	2	1	2	2	2
1	9	780	1	16	2	1	2	2	1
1	9	781	1	15	2	1	2	2	1
1	9	782	1	16	2	1	2	2	1
1	9	783	1	15	2	1	2	2	1
1	9	784	1	18	2	1	2	2	1
1	9	785	1	14	1	1	2	2	2
1	9	786	1	16	2	1	2	2	2
1	9	787	1	18	2	1	2	2	1
1	9	788	1	15	2	1	2	2	1
1	9	789	1	15	2	1	2	2	1
1	9	790	1	15	2	1	2	2	1
1	9	791	1	16	2	1	2	2	1
1	9	792	1	15	2	1	2	2	1
1	9	793	1	16	2	1	2	2	1
1	9	794	1	15	2	1	2	2	2
1	9	795	1	16	2	1	2	2	2
1	9	796	1	16	2	1	2	2	1
1	9	797	1	15	2	1	2	2	2
1	9	798	1	16	2	1	2	2	2

1	9	799	1	15	2	1	2	2	1
1	9	800	1	16	2	1	2	2	2
1	9	801	1	18	2	1	2	2	1
1	9	802	1	16	2	1	2	2	2
1	9	803	1	13	1	1	2	2	1
1	9	804	1	15	2	1	2	2	2
1	9	805	1	15	2	1	2	2	2
1	9	806	1	16	2	1	2	2	1
1	9	807	1	20	3	1	2	2	1
1	9	808	1	15	2	1	2	2	2
1	9	809	1	18	2	1	2	2	1
1	9	810	9	0	#NULL!	1	2	2	2
1	9	811	1	16	2	1	2	2	1
1	9	812	1	15	2	1	2	2	1
1	9	813	1	16	2	1	2	2	1
1	9	814	1	15	2	1	2	2	2
1	9	815	1	16	2	1	2	2	2
1	9	816	1	16	2	1	2	2	1
1	9	817	1	16	2	1	2	2	1
1	9	818	1	0	#NULL!	1	2	2	1
1	9	819	1	17	2	1	2	2	1
1	9	820	1	16	2	1	2	2	1
1	9	821	1	16	2	1	2	2	1
1	9	822	1	17	2	1	2	2	1
1	9	823	1	15	2	1	2	2	1
1	9	824	1	16	2	1	2	2	1
1	9	825	1	15	2	1	2	2	1
1	9	826	1	14	1	1	2	2	1
1	9	827	1	16	2	1	2	2	1
1	9	828	1	17	2	1	2	2	1
1	9	829	9	15	2	1	2	9	1
1	9	830	1	16	2	1	2	2	1
1	9	831	1	17	2	1	2	2	1
1	9	832	1	16	2	1	2	2	1
1	9	833	1	0	#NULL!	1	2	2	1
1	9	834	1	16	2	1	2	2	1
1	9	835	9	15	2	1	2	2	1
1	9	836	1	15	2	1	2	2	1
1	9	837	1	16	2	9	9	2	2
1	9	838	1	15	2	9	9	2	2
1	9	839	1	16	2	1	2	2	1
1	9	840	1	17	2	1	2	2	1
1	9	841	1	18	2	1	2	2	1
1	9	842	1	17	2	1	2	2	1
1	9	843	1	16	2	1	2	2	2
1	9	844	1	15	2	1	2	2	1
1	9	845	1	19	2	1	2	2	1

1	9	846	1	18	2	1	2	2	1
1	9	847	1	16	2	1	2	2	2
1	9	848	1	17	2	1	2	2	2
1	9	849	1	17	2	1	2	2	1
1	9	850	1	18	2	1	2	2	1
1	9	851	1	14	1	1	2	2	2
1	9	852	1	17	2	1	2	2	2
1	9	853	1	16	2	1	9	9	9
1	9	854	1	17	2	1	2	2	1
1	9	855	1	16	2	1	2	2	1
1	9	856	1	16	2	1	2	2	2
1	9	857	1	17	2	1	2	2	1
1	9	858	1	18	2	1	2	2	1
1	9	859	1	18	2	1	2	2	1
1	9	860	1	17	2	1	2	2	2
1	9	861	1	16	2	1	2	2	2
1	9	862	1	19	2	1	2	2	2
1	9	863	1	16	2	1	2	2	1
1	9	864	1	16	2	1	2	2	1
1	9	865	1	17	2	1	2	2	2
1	9	866	1	16	2	1	2	2	1
1	9	867	1	15	2	1	2	2	1
1	9	868	1	0	#NULL!	1	2	2	1
1	9	869	1	17	2	1	2	2	1
1	9	870	1	17	2	1	2	2	2
1	9	871	1	16	2	1	2	2	2
1	9	872	1	17	2	1	2	2	1
1	9	873	1	16	2	1	2	2	1
1	9	874	1	15	2	1	2	2	1
1	9	875	1	16	2	1	2	2	1
1	9	876	1	16	2	1	2	2	1
1	9	877	1	0	#NULL!	1	2	2	1
1	9	878	1	16	2	1	2	2	1
1	9	879	1	17	2	1	2	2	2
1	9	880	1	19	2	1	2	2	1
1	9	881	1	15	2	1	2	2	1
1	9	882	1	17	2	1	2	2	2
1	9	883	1	18	2	1	2	2	2
1	9	884	1	18	2	1	2	2	1
1	9	885	1	16	2	1	2	2	2
1	9	886	1	16	2	1	2	2	1
1	9	887	1	16	2	1	2	2	2
1	9	888	1	18	2	1	2	2	1
1	9	889	1	17	2	1	2	2	1
1	9	890	1	16	2	1	2	2	1
1	9	891	1	16	2	1	2	2	2
1	9	892	1	15	2	1	2	2	1

1	9	893	1	15	2	1	2	2	2
1	9	894	1	15	2	1	2	2	1
1	9	895	1	18	2	1	2	2	2
1	9	896	1	18	2	1	2	2	2
1	9	897	1	15	2	1	2	2	1
1	9	898	1	16	2	1	2	2	1
1	9	899	1	15	2	1	2	2	2
1	9	900	1	15	2	1	2	2	1
1	9	901	1	15	2	1	2	2	1
1	9	902	1	16	2	1	2	2	1
1	9	903	1	0	#NULL!	1	2	2	1
1	9	904	1	18	2	1	2	2	1
1	9	905	1	14	1	1	2	2	1
1	9	906	1	16	2	1	2	2	2
1	9	907	1	16	2	1	2	2	1
1	9	908	1	15	2	1	2	2	2
1	9	909	1	16	2	1	2	2	1
1	9	910	1	15	2	1	2	2	1
1	9	911	1	15	2	1	2	2	1
1	9	912	1	18	2	1	2	2	1
1	9	913	1	16	2	1	2	2	2
1	9	914	1	17	2	1	2	2	2
1	9	915	1	19	2	1	2	2	1
1	9	916	1	17	2	1	2	2	1
1	9	917	1	16	2	1	2	2	2
1	9	918	1	15	2	1	2	2	2
1	9	919	1	16	2	1	2	2	1
1	9	920	1	16	2	1	2	2	1
1	9	921	1	16	2	1	2	2	1
1	9	922	2	14	1	1	9	3	3
1	9	923	2	17	2	1	3	3	1
1	9	924	2	15	2	1	9	3	1
1	9	925	1	15	2	1	9	3	2
1	9	926	2	14	1	1	9	3	1
1	9	927	2	0	#NULL!	1	3	3	3
1	9	928	1	18	2	1	3	3	1
1	9	929	2	19	2	1	3	3	1
1	9	930	1	17	2	1	3	3	2
1	9	931	2	17	2	1	3	3	1
1	9	932	2	16	2	1	3	3	2
1	9	933	1	0	#NULL!	1	3	3	1
1	9	934	1	17	2	1	3	3	1
1	9	935	1	17	2	1	3	3	2
1	9	936	2	17	2	1	3	3	3
1	9	937	1	18	2	1	3	3	2
1	9	938	2	19	2	1	3	3	2
1	9	939	2	19	2	1	3	3	1

1	9	940	2	17	2	1	3	3	1
1	9	941	2	19	2	1	3	3	3
1	9	942	1	20	3	1	3	3	1
1	9	943	2	17	2	1	3	3	1
1	9	944	1	18	2	1	3	3	1
1	9	945	9	0	#NULL!	9	9	9	9
1	9	946	2	16	2	1	3	3	1
1	9	947	2	18	2	1	3	3	1
1	9	948	2	16	2	1	3	3	1
1	9	949	2	17	2	1	3	3	1
1	9	950	2	17	2	1	3	3	1
1	9	951	2	17	2	1	3	3	1
1	9	952	2	17	2	1	3	3	9
1	9	953	2	15	2	1	3	3	2
1	9	954	2	17	2	1	3	3	1
1	9	955	9	16	2	1	3	3	1
1	9	956	1	19	2	1	3	3	2
1	9	957	9	18	2	1	3	2	2
1	9	958	2	16	2	1	3	3	1
1	9	959	2	15	2	1	3	3	1
1	9	960	2	18	2	1	3	3	1
1	9	961	2	17	2	1	3	3	1
1	9	962	2	17	2	2	3	3	1
1	9	963	2	15	2	1	3	3	2
1	9	964	2	17	2	1	3	3	1
1	9	965	2	17	2	1	3	3	1
1	9	966	2	17	2	1	3	3	1
1	9	967	2	17	2	1	3	3	1
1	9	968	2	17	2	1	3	3	1
1	9	969	9	16	2	1	3	3	1
1	9	970	2	15	2	1	3	3	1
1	9	971	2	17	2	1	3	3	9
1	9	972	2	16	2	1	3	3	1
1	9	973	2	16	2	1	3	3	1
1	9	974	1	17	2	1	3	3	1
1	9	975	2	14	1	1	3	3	1
1	9	976	2	14	1	1	3	3	1
1	9	977	2	15	2	1	3	3	1
1	9	978	2	15	2	1	3	3	1
1	9	979	2	18	2	1	3	3	1
1	9	980	2	19	2	1	3	3	1
1	9	981	1	17	2	1	3	3	2
1	9	982	2	16	2	1	3	3	1
1	9	983	1	23	3	1	3	3	2
1	9	984	1	18	2	1	3	3	1
1	9	985	1	18	2	1	3	3	1
1	9	986	2	16	2	1	3	3	1

1	9	987	2	17	2	1	3	3	1
1	9	988	2	16	2	1	3	3	2
1	9	989	1	16	2	1	3	3	1
1	9	990	2	16	2	1	3	3	1
1	9	991	2	16	2	1	3	3	1
1	9	992	1	16	2	1	3	3	1
1	9	993	1	16	2	1	3	3	2
1	9	994	1	15	2	1	3	3	1
1	9	995	1	17	2	1	3	3	1
1	9	996	1	17	2	1	3	3	2
1	9	997	2	0	#NULL!	1	3	3	1
1	9	998	1	19	2	1	3	3	1
1	9	999	2	17	2	1	3	3	1
1	9	1000	2	18	2	1	3	3	1
1	9	1001	2	16	2	1	2	3	1
1	9	1002	2	16	2	1	3	3	1
1	9	1003	1	18	2	1	3	3	1
1	9	1004	2	16	2	1	3	3	1
1	9	1005	2	17	2	1	3	3	1
1	9	1006	2	15	2	1	3	3	1
1	9	1007	2	16	2	1	3	3	1
1	9	1008	1	17	2	1	3	3	1
1	9	1009	2	17	2	1	3	3	1
1	9	1010	2	16	2	1	3	3	1
1	9	1011	2	15	2	1	3	3	1
1	9	1012	2	15	2	1	3	3	1
1	9	1013	2	18	2	1	3	3	1
1	9	1014	2	17	2	2	3	3	2
1	9	1015	2	17	2	1	3	3	1
1	9	1016	1	19	2	1	3	3	1
1	9	1017	2	18	2	1	3	2	1
1	9	1018	1	17	2	1	3	3	3
1	9	1019	1	17	2	1	3	3	1
1	9	1020	2	17	2	1	3	3	1
1	9	1021	1	19	2	1	3	3	2
1	9	1022	2	17	2	1	3	3	1
1	9	1023	1	19	2	1	3	3	1
1	9	1024	1	18	2	1	3	3	1
1	9	1025	2	14	1	1	3	3	1
1	9	1026	1	17	2	1	3	3	1
1	9	1027	1	17	2	1	3	3	1
1	9	1028	1	18	2	1	3	3	1
1	9	1029	1	18	2	1	3	3	2
1	9	1030	1	19	2	1	3	3	2
1	9	1031	1	20	3	1	3	3	1
1	9	1032	1	0	#NULL!	1	3	3	1
1	9	1033	1	17	2	1	3	3	1

1	9	1034	1	18	2	1	3	3	2
1	9	1035	1	21	3	1	3	3	1
1	9	1036	1	17	2	1	3	3	2
1	9	1037	2	17	2	1	3	3	1
1	9	1038	1	17	2	1	3	3	1
1	9	1039	1	18	2	1	3	3	2
1	9	1040	2	18	2	1	3	3	1
1	9	1041	2	16	2	1	3	3	1
1	9	1042	2	16	2	1	3	3	1
1	9	1043	1	14	1	1	9	3	1
1	9	1044	1	17	2	1	2	3	1
1	9	1045	1	15	2	1	2	3	3
1	9	1046	1	17	2	1	2	3	1
1	9	1047	2	19	2	1	2	3	1
1	9	1048	2	16	2	1	2	3	3
1	9	1049	2	15	2	1	2	3	1
1	9	1050	2	16	2	1	2	3	1
1	9	1051	2	16	2	1	2	3	1
1	9	1052	2	14	1	1	2	3	1
1	9	1053	2	17	2	1	2	3	1
1	9	1054	2	16	2	1	2	3	1
1	9	1055	1	16	2	1	2	3	1
1	9	1056	1	16	2	1	2	3	1
1	9	1057	1	19	2	1	2	3	2
1	9	1058	2	16	2	1	2	3	1
1	9	1059	2	15	2	2	2	3	1
1	9	1060	2	14	1	1	2	3	1
1	9	1061	2	17	2	1	2	3	1
1	9	1062	2	13	1	1	2	3	1
1	9	1063	2	17	2	1	2	3	1
1	9	1064	2	16	2	1	2	3	1
1	9	1065	1	19	2	1	2	3	2
1	9	1066	1	18	2	1	2	3	2
1	9	1067	1	16	2	1	2	3	2
1	9	1068	2	15	2	2	2	3	1
1	9	1069	2	16	2	2	2	3	1
1	9	1070	2	13	1	1	2	3	1
1	9	1071	2	18	2	1	2	3	1
1	9	1072	2	18	2	9	2	3	1
1	9	1073	1	16	2	1	2	3	1
1	9	1074	1	16	2	1	2	3	1
1	9	1075	2	16	2	1	2	3	2
1	9	1076	2	16	2	1	2	3	1
1	9	1077	2	16	2	1	2	3	1
1	9	1078	2	16	2	1	2	2	2
1	9	1079	2	14	1	1	2	3	1
1	9	1080	2	16	2	1	2	3	1

1	9	1081	2	17	2	1	3	3	2
1	9	1082	2	17	2	1	3	3	1
1	9	1083	2	17	2	1	3	3	1
1	9	1084	1	15	2	1	9	3	1
1	9	1085	2	14	1	1	9	3	1
1	9	1086	2	15	2	1	2	3	2
1	9	1087	2	15	2	1	2	3	2
1	9	1088	2	0	#NULL!	1	2	3	1
1	9	1089	2	16	2	1	2	3	1
1	9	1090	2	14	1	1	2	3	1
1	9	1091	2	15	2	1	2	3	2
1	9	1092	1	14	1	1	2	3	1
1	9	1093	1	18	2	1	2	3	1
1	9	1094	2	16	2	1	2	3	2
1	9	1095	2	16	2	1	2	3	1
1	9	1096	2	15	2	1	2	3	1
1	9	1097	2	15	2	1	2	3	1
1	9	1098	2	15	2	1	2	3	1
1	9	1099	2	14	1	2	2	3	1
1	9	1100	1	17	2	1	2	3	1
1	9	1101	1	18	2	1	2	3	2
1	9	1102	1	0	#NULL!	1	2	3	2
1	9	1103	1	15	2	1	2	3	1
1	9	1104	2	18	2	1	2	3	1
1	9	1105	2	17	2	1	2	3	1
1	9	1106	1	16	2	1	2	3	3
1	9	1107	1	16	2	1	2	3	1
1	9	1108	1	16	2	1	2	3	1
1	9	1109	1	17	2	1	2	3	1
1	9	1110	2	17	2	1	2	3	1
1	9	1111	2	17	2	1	2	3	1
1	9	1112	2	16	2	1	2	3	1
1	9	1113	2	16	2	1	2	3	1
1	9	1114	2	15	2	1	2	3	1
1	9	1115	2	15	2	1	2	3	1
1	9	1116	1	17	2	1	2	3	1
1	9	1117	1	15	2	1	2	3	2
1	9	1118	2	17	2	1	2	3	1
1	9	1119	2	16	2	1	2	3	1
1	9	1120	2	15	2	1	2	3	1
1	9	1121	1	18	2	2	2	3	1
1	9	1122	2	0	#NULL!	1	2	3	1
1	9	1123	2	15	2	1	2	3	2
1	9	1124	2	16	2	1	2	3	2
1	9	1125	2	14	1	1	2	3	1
1	9	1126	2	15	2	1	2	3	1
1	9	1127	2	16	2	1	2	3	1

1	9	1128	2	17	2	1	2	3	1
1	9	1129	1	17	2	1	2	3	1
1	9	1130	2	17	2	1	2	3	1
1	9	1131	2	15	2	1	2	3	1
1	9	1132	2	15	2	1	2	3	1
1	9	1133	1	16	2	1	2	3	1
1	9	1134	2	16	2	1	2	3	1
1	9	1135	1	0	#NULL!	1	2	2	2
1	9	1136	1	17	2	1	2	3	1
1	9	1137	1	16	2	1	2	2	2
1	9	1138	1	16	2	1	2	3	1
1	9	1139	2	17	2	2	2	3	1
1	9	1140	1	17	2	1	2	3	1
1	9	1141	1	17	2	1	2	3	1
1	9	1142	2	16	2	1	2	3	1
1	9	1143	2	15	2	1	2	3	1
1	9	1144	2	16	2	1	2	3	1
1	9	1145	1	17	2	1	2	3	1
1	9	1146	1	16	2	1	2	3	1
1	9	1147	1	16	2	1	2	3	1
1	9	1148	1	17	2	1	2	3	1
1	9	1149	2	15	2	1	2	3	1
1	9	1150	2	16	2	1	2	3	1
1	9	1151	2	17	2	1	2	3	1
1	9	1152	2	16	2	1	2	3	1
1	9	1153	2	17	2	1	2	3	9
1	9	1154	2	15	2	1	2	3	1
1	9	1155	1	18	2	1	2	3	2
1	9	1156	2	17	2	1	2	3	1
1	9	1157	2	17	2	1	2	3	1
1	9	1158	2	16	2	1	2	3	1
1	9	1159	2	16	2	1	2	1	1
1	9	1160	2	16	2	1	2	3	1
1	9	1161	2	17	2	1	2	3	1
1	9	1162	2	15	2	1	2	3	1
1	9	1163	2	15	2	1	2	3	1
1	9	1164	2	16	2	1	2	3	1
1	9	1165	1	17	2	1	2	3	1
1	9	1166	1	16	2	1	2	3	1
1	9	1167	1	16	2	1	2	3	1
1	9	1168	1	15	2	1	2	3	1
1	9	1169	2	15	2	1	2	3	1
1	9	1170	2	15	2	1	2	3	1
1	9	1171	2	15	2	1	2	3	1
1	9	1172	2	17	2	1	2	3	1
1	9	1173	2	14	1	1	2	3	1
1	9	1174	2	16	2	1	2	3	1

1	9	1175	1	15	2	1	2	3	1
1	9	1176	2	16	2	1	2	3	1
1	9	1177	1	17	2	1	2	3	2
1	9	1178	1	18	2	1	2	3	1
1	9	1179	1	19	2	1	2	3	1
1	9	1180	9	9	1	1	2	3	1
1	9	1181	1	15	2	1	2	3	1
1	9	1182	9	9	1	1	2	3	1
1	9	1183	1	17	2	1	2	3	1
1	9	1184	1	16	2	1	2	3	1
1	9	1185	1	16	2	1	2	3	1
1	9	1186	1	16	2	1	2	3	1
1	9	1187	1	16	2	1	2	3	2
1	9	1188	1	18	2	1	2	3	1
1	9	1189	1	16	2	1	2	3	1
1	9	1190	9	0	#NULL!	9	9	9	9
1	9	1191	1	19	2	2	2	3	1
1	9	1192	1	17	2	1	2	3	1
1	9	1193	1	16	2	1	2	3	2
1	9	1194	2	17	2	1	2	1	1
1	9	1195	2	14	1	1	2	3	1
1	9	1196	1	16	2	1	2	3	1
1	9	1197	2	19	2	1	2	3	1
1	9	1198	2	17	2	1	2	3	1
1	9	1199	2	15	2	1	2	3	2
1	9	1200	1	18	2	1	2	3	1
1	9	1201	2	16	2	1	2	3	1
1	9	1202	1	17	2	1	2	2	2
1	9	1203	1	15	2	1	2	3	2
1	9	1204	2	15	2	1	2	3	1
1	9	1205	9	0	#NULL!	9	9	9	9
1	9	1206	1	17	2	1	2	3	3
1	9	1207	1	16	2	1	2	2	2
1	9	1208	1	15	2	1	2	3	1
1	9	1209	1	15	2	1	2	3	1
1	9	1210	1	16	2	1	2	3	1
1	9	1211	2	15	2	1	2	3	1
1	9	1212	2	15	2	1	2	3	1
1	9	1213	2	14	1	1	2	3	1
1	9	1214	2	14	1	1	2	3	1
1	9	1215	2	16	2	1	2	3	1
1	9	1216	2	16	2	1	2	3	1
1	9	1217	1	16	2	1	2	3	1
1	9	1218	1	16	2	1	2	3	1
1	9	1219	2	14	1	1	2	3	1
1	9	1220	1	17	2	1	2	3	1
1	9	1221	2	15	2	1	2	3	1

1	9	1222	2	15	2	1	2	3	1
1	9	1223	2	17	2	1	2	3	1
1	9	1224	2	18	2	1	2	3	1
1	9	1225	1	16	2	1	2	3	2
1	9	1226	2	16	2	2	2	3	9
1	9	1227	2	16	2	1	2	3	1
1	9	1228	2	16	2	1	2	3	1
1	9	1229	2	14	1	1	2	3	1
1	9	1230	2	16	2	1	2	3	1
1	9	1231	1	15	2	1	2	3	1
1	9	1232	2	15	2	1	2	3	1
1	9	1233	2	16	2	1	2	3	1
1	9	1234	1	17	2	1	2	3	1
1	9	1235	2	16	2	1	2	3	1
1	9	1236	1	15	2	2	1	2	1
1	9	1237	1	16	2	1	1	2	1
1	9	1238	1	15	2	1	1	2	1
1	9	1239	1	15	2	1	1	2	1
1	9	1240	1	16	2	1	1	2	2
1	9	1241	1	14	1	1	1	2	1
1	9	1242	1	13	1	1	1	2	2
1	9	1243	1	15	2	1	1	2	1
1	9	1244	1	14	1	1	1	2	1
1	9	1245	1	14	1	1	1	2	1
1	9	1246	1	15	2	1	1	2	1
1	9	1247	1	16	2	1	1	2	2
1	9	1248	1	17	2	1	1	2	1
1	9	1249	1	16	2	1	1	2	1
1	9	1250	1	15	2	1	1	9	9
1	9	1251	1	16	2	1	1	2	1
1	9	1252	1	16	2	1	1	2	1
1	9	1253	1	16	2	1	1	2	1
1	9	1254	1	15	2	1	1	2	1
1	9	1255	1	14	1	1	1	2	2
1	9	1256	1	14	1	1	1	2	1
1	9	1257	1	14	1	1	1	2	1
1	9	1258	1	15	2	1	1	2	1
1	9	1259	1	14	1	1	1	2	1
1	9	1260	1	15	2	1	1	2	3
1	9	1261	1	14	1	1	1	2	9
1	9	1262	1	15	2	2	1	2	1
1	9	1263	1	15	2	1	1	2	1
1	9	1264	1	16	2	1	1	2	1
1	9	1265	1	15	2	1	1	2	1
1	9	1266	1	14	1	1	1	2	3
1	9	1267	1	16	2	2	1	2	1
1	9	1268	1	14	1	1	1	2	3

1	9	1269	1	16	2	1	1	2	3
1	9	1270	1	16	2	2	1	2	2
1	9	1271	1	15	2	1	1	2	1
1	9	1272	1	15	2	1	1	2	1
1	9	1273	1	14	1	1	1	2	1
1	9	1274	1	16	2	1	1	2	1
1	9	1275	1	14	1	1	1	2	1
1	9	1276	1	16	2	1	1	2	1
1	9	1277	1	18	2	1	1	2	1
1	9	1278	1	16	2	9	9	9	9
1	9	1279	1	16	2	9	9	9	1
1	9	1280	1	15	2	1	1	2	2
1	9	1281	1	15	2	1	1	2	1
1	9	1282	1	15	2	1	1	2	1
1	9	1283	1	15	2	1	1	2	1
1	9	1284	1	14	1	1	1	2	1
1	9	1285	1	15	2	1	1	2	1
1	9	1286	1	17	2	1	1	2	1
1	9	1287	1	15	2	1	1	3	1
1	9	1288	1	14	1	1	1	2	1
1	9	1289	1	14	1	1	1	2	1
1	9	1290	1	14	1	1	1	2	1
1	9	1291	1	17	2	1	1	2	1
1	9	1292	1	0	#NULL!	1	1	2	1
1	9	1293	1	14	1	1	1	2	1
1	9	1294	1	15	2	1	1	2	1
1	9	1295	1	15	2	1	1	2	1
1	9	1296	2	17	2	1	1	9	9
1	9	1297	1	15	2	1	1	2	2
1	9	1298	1	14	1	1	1	2	1
1	9	1299	1	0	#NULL!	1	1	2	1
1	9	1300	1	15	2	1	1	2	1
1	9	1301	1	16	2	2	1	2	1
1	9	1302	1	15	2	1	1	2	1
1	9	1303	1	18	2	2	1	2	1
1	9	1304	9	19	2	1	1	2	2
1	9	1305	1	16	2	1	1	2	1
1	9	1306	9	18	2	1	1	2	1
1	9	1307	1	16	2	1	1	2	1
1	9	1308	1	16	2	1	1	2	1
1	9	1309	1	15	2	1	1	2	1
1	9	1310	1	17	2	1	1	2	1
1	9	1311	1	15	2	1	1	2	1
1	9	1312	1	17	2	1	1	2	1
1	9	1313	1	15	2	1	1	2	1
1	9	1314	1	18	2	1	1	2	1
1	9	1315	9	0	#NULL!	9	9	9	9

1	9	1316	1	15	2	1	1	2	2
1	9	1317	1	15	2	1	1	2	1
1	9	1318	1	14	1	1	1	2	1
1	9	1319	1	14	1	1	1	2	1
1	9	1320	1	16	2	1	1	2	2
1	9	1321	1	15	2	1	1	2	1
1	9	1322	1	18	2	1	1	2	1
1	9	1323	1	15	2	1	1	2	2
1	9	1324	1	16	2	1	1	2	1
1	9	1325	1	15	2	1	1	2	1
1	9	1326	1	15	2	1	1	2	1
1	9	1327	1	15	2	1	1	2	1
1	9	1328	1	16	2	1	1	2	1
1	9	1329	1	14	1	1	1	2	2
1	9	1330	1	16	2	1	1	2	1
1	9	1331	1	15	2	1	1	2	1
1	9	1332	1	18	2	1	1	2	1
1	9	1333	1	16	2	1	1	2	1
1	9	1334	1	14	1	1	1	2	1
1	9	1335	1	15	2	1	1	2	1
1	9	1336	1	13	1	1	1	2	1
1	9	1337	1	15	2	1	1	2	1
1	9	1338	1	13	1	1	1	2	1
1	9	1339	1	13	1	1	1	2	1
1	9	1340	1	15	2	1	1	2	1
1	9	1341	1	15	2	1	1	2	1
1	9	1342	1	15	2	1	1	2	1
1	9	1343	1	15	2	1	1	2	2
1	9	1344	1	16	2	9	9	9	9
1	9	1345	1	15	2	1	1	2	1
1	9	1346	1	0	#NULL!	1	1	2	1
1	9	1347	1	15	2	1	1	2	1
1	9	1348	1	13	1	1	1	2	1
1	9	1349	1	14	1	1	1	2	1
1	9	1350	1	14	1	1	1	2	1
1	9	1351	1	16	2	1	1	2	1
1	9	1352	1	15	2	1	1	2	1
1	9	1353	1	16	2	1	1	2	1
1	9	1354	1	18	2	1	1	2	2
1	9	1355	1	13	1	1	1	2	1
1	9	1356	1	15	2	1	1	2	2
1	9	1357	1	15	2	1	1	2	1
1	9	1358	1	15	2	1	1	2	1
1	9	1359	1	15	2	1	1	2	1
1	9	1360	1	15	2	1	1	2	1
1	9	1361	1	15	2	1	1	2	1
1	9	1362	1	15	2	1	1	2	1

1	9	1363	1	15	2	1	1	2	1
1	9	1364	1	14	1	1	1	2	1
1	9	1365	1	15	2	1	1	2	3
1	9	1366	1	13	1	1	1	2	1
1	9	1367	1	14	1	1	1	2	1
1	9	1368	1	15	2	1	1	2	1
1	9	1369	1	19	2	1	1	2	2
1	9	1370	1	16	2	1	1	2	1
1	9	1371	1	14	1	1	1	2	1
1	9	1372	1	15	2	1	1	2	1
1	9	1373	1	14	1	1	1	2	1
1	9	1374	1	16	2	1	1	2	1
1	9	1375	1	14	1	1	1	2	2
1	9	1376	1	15	2	1	1	2	2
1	9	1377	1	15	2	1	1	2	1
1	9	1378	1	14	1	1	1	2	1
1	9	1379	1	16	2	1	1	2	1
1	9	1380	1	14	1	1	1	2	1
1	9	1381	1	16	2	1	1	2	2
1	9	1382	1	14	1	1	1	2	2
1	9	1383	1	14	1	1	1	2	1
1	9	1384	1	15	2	1	1	2	1
1	9	1385	1	16	2	1	1	2	2
1	9	1386	1	14	1	1	1	2	2
1	9	1387	1	14	1	1	1	2	1
1	9	1388	1	14	1	1	1	2	1
1	9	1389	1	14	1	1	1	2	1
1	9	1390	1	14	1	1	1	2	2
1	9	1391	1	15	2	1	1	2	1
1	9	1392	1	15	2	1	1	2	1
1	9	1393	1	15	2	1	1	2	1
1	9	1394	1	15	2	1	1	2	2
1	9	1395	1	15	2	1	3	2	1
1	9	1396	1	14	1	1	1	2	1
1	9	1397	1	15	2	1	1	2	2
1	9	1398	1	15	2	1	1	2	3
1	9	1399	1	13	1	1	1	2	3
1	9	1400	1	14	1	1	1	2	1
1	9	1401	1	13	1	1	1	2	3
1	9	1402	1	16	2	1	1	2	1
1	9	1403	1	15	2	1	1	2	1
1	9	1404	1	15	2	1	1	2	1
1	9	1405	1	14	1	1	1	2	1
1	9	1406	1	17	2	1	3	2	1
1	9	1407	1	16	2	1	3	2	2
1	9	1408	1	18	2	1	3	2	1
1	9	1409	1	16	2	1	3	2	1

1	9	1410	1	16	2	1	3	2	1
1	9	1411	1	18	2	1	3	2	2
1	9	1412	1	17	2	1	3	2	1
1	9	1413	1	18	2	1	3	2	1
1	9	1414	1	0	#NULL!	1	3	2	1
1	9	1415	1	16	2	1	3	2	1
1	9	1416	1	17	2	1	3	2	1
1	9	1417	1	19	2	1	3	2	2
1	9	1418	1	16	2	1	3	2	3
1	9	1419	1	19	2	1	3	2	1
1	9	1420	1	17	2	1	3	2	1
1	9	1421	1	17	2	1	3	2	1
1	9	1422	1	17	2	1	3	2	2
1	9	1423	1	17	2	1	3	2	1
1	9	1424	1	0	#NULL!	1	3	2	1
1	9	1425	1	16	2	1	3	2	2
1	9	1426	1	17	2	1	3	2	1
1	9	1427	1	17	2	1	3	2	1
1	9	1428	1	16	2	1	3	2	1
1	9	1429	1	19	2	1	3	2	1
1	9	1430	1	19	2	1	3	2	2
1	9	1431	1	17	2	1	3	2	3
1	9	1432	1	17	2	1	3	2	1
1	9	1433	1	18	2	1	3	2	1
1	9	1434	1	17	2	1	3	2	1
1	9	1435	1	18	2	1	3	2	3
1	9	1436	1	21	3	1	3	2	1
1	9	1437	1	17	2	1	3	1	1
1	9	1438	1	19	2	1	3	2	1
1	9	1439	1	0	#NULL!	1	3	2	1
1	9	1440	1	18	2	1	3	2	1
1	9	1441	1	18	2	1	3	2	1
1	9	1442	1	18	2	1	3	2	2
1	9	1443	1	18	2	1	3	2	1
1	9	1444	1	17	2	1	3	2	1
1	9	1445	1	15	2	1	3	2	1
1	9	1446	1	17	2	1	3	2	3
1	9	1447	1	17	2	1	3	2	1
1	9	1448	1	15	2	1	3	2	1
1	9	1449	1	16	2	1	3	2	1
1	9	1450	1	15	2	1	3	2	1
1	9	1451	1	17	2	1	3	2	2
1	9	1452	1	19	2	1	3	2	1
1	9	1453	1	18	2	1	3	2	1
1	9	1454	1	18	2	1	3	2	1
1	9	1455	1	17	2	1	3	2	3
1	9	1456	1	16	2	1	3	2	1

1	9	1457	1	17	2	1	3	2	1
1	9	1458	1	16	2	1	3	2	1
1	9	1459	1	17	2	1	3	2	1
1	9	1460	1	18	2	1	3	2	2
1	9	1461	1	18	2	1	3	2	2
1	9	1462	1	17	2	1	3	2	1
1	9	1463	1	18	2	1	3	2	1
1	9	1464	1	17	2	1	3	2	2
1	9	1465	1	17	2	1	3	2	1
1	9	1466	1	18	2	1	3	2	1
1	9	1467	1	18	2	1	3	2	1
1	9	1468	1	18	2	1	3	2	2
1	9	1469	1	16	2	1	3	2	1
1	9	1470	1	17	2	1	3	2	1
1	9	1471	1	17	2	1	3	2	1
1	9	1472	1	18	2	1	3	2	1
1	9	1473	1	18	2	1	3	2	1
1	9	1474	1	18	2	1	3	2	1
1	9	1475	1	17	2	1	3	2	1
1	9	1476	1	0	#NULL!	1	3	2	1
1	9	1477	1	17	2	1	3	2	1
1	9	1478	1	16	2	1	3	2	2
1	9	1479	1	17	2	1	3	2	2
1	9	1480	1	17	2	1	3	2	2
1	9	1481	1	20	3	1	3	2	2
1	9	1482	1	18	2	1	3	2	1
1	9	1483	1	17	2	1	3	2	1
1	9	1484	1	19	2	1	3	2	1
1	9	1485	1	15	2	1	3	2	1
1	9	1486	1	18	2	1	3	2	3
1	9	1487	1	17	2	1	3	2	2
1	9	1488	1	17	2	1	3	2	2
1	9	1489	1	17	2	1	3	2	1
1	9	1490	1	15	2	1	3	2	1
1	9	1491	1	0	#NULL!	1	3	2	1
1	9	1492	1	16	2	1	3	2	1
1	9	1493	1	17	2	1	3	2	1
1	9	1494	1	16	2	1	3	2	1
1	9	1495	1	18	2	1	3	3	1
1	9	1496	1	17	2	1	3	2	1
1	9	1497	1	0	#NULL!	1	3	2	1
1	9	1498	1	17	2	1	3	2	1
1	9	1499	1	17	2	1	3	2	1
1	9	1500	1	18	2	1	3	2	3
1	9	1501	1	17	2	1	3	2	1
1	9	1502	1	0	#NULL!	1	3	2	1
1	9	1503	1	18	2	1	3	2	1

1	9	1504	1	17	2	1	3	2	1
1	9	1505	1	15	2	1	3	2	1
1	9	1506	1	17	2	1	3	2	2
1	9	1507	1	17	2	1	3	2	1
1	9	1508	1	18	2	1	3	2	1
1	9	1509	1	17	2	1	3	2	1
1	9	1510	1	17	2	1	3	2	1
1	9	1511	1	18	2	1	3	2	1
1	9	1512	1	17	2	1	3	2	1
1	9	1513	1	0	#NULL!	1	3	2	1
1	9	1514	1	19	2	1	3	2	1
1	9	1515	1	19	2	1	3	2	1
1	9	1516	1	17	2	1	3	3	1
1	9	1517	1	18	2	1	3	2	1
1	9	1518	1	16	2	1	3	2	1
1	9	1519	1	17	2	1	3	2	1
1	9	1520	1	17	2	1	3	2	1
1	9	1521	1	15	2	1	3	2	1
1	9	1522	9	0	#NULL!	9	9	9	9
1	9	1523	1	15	2	1	3	2	1
1	9	1524	1	18	2	1	3	2	1
1	9	1525	1	18	2	1	3	2	1
1	9	1526	1	16	2	1	3	2	1
1	9	1527	1	18	2	1	3	2	1
1	9	1528	1	16	2	1	3	2	1
1	9	1529	1	17	2	1	3	2	1
1	9	1530	1	20	3	1	3	2	1
1	9	1531	1	17	2	1	3	2	1
1	9	1532	1	0	#NULL!	1	3	2	1
1	9	1533	1	17	2	1	3	2	1
1	9	1534	1	17	2	1	3	2	1
1	9	1535	1	17	2	1	3	2	1
1	9	1536	1	17	2	1	3	2	1
1	9	1537	1	17	2	1	3	2	1
1	9	1538	1	17	2	1	3	9	1
1	9	1539	1	16	2	1	3	2	1
1	9	1540	1	17	2	1	3	2	1
1	9	1541	1	16	2	1	3	2	1
1	9	1542	1	17	2	1	3	2	1
1	9	1543	1	16	2	1	3	2	1
1	9	1544	1	14	1	1	3	2	1
1	9	1545	1	17	2	1	3	2	1
1	9	1546	1	16	2	1	3	2	1
1	9	1547	1	18	2	1	3	2	1
1	9	1548	1	19	2	1	3	2	1
1	9	1549	1	17	2	1	3	2	1
1	9	1550	1	18	2	1	3	2	2

1	9	1551	1	16	2	1	3	2	1
1	9	1552	1	17	2	1	3	2	1
1	9	1553	1	16	2	1	3	2	1
1	9	1554	1	18	2	1	3	2	1
1	9	1555	1	17	2	1	3	2	1
1	9	1556	1	17	2	1	3	2	1
1	9	1557	1	18	2	1	3	2	2
1	9	1558	1	30	3	1	3	2	3
1	9	1559	1	19	2	1	3	2	2
1	9	1560	1	18	2	1	3	2	1
1	9	1561	2	14	1	1	1	3	1
1	9	1562	1	17	2	1	1	3	1
1	9	1563	1	16	2	1	1	3	3
1	9	1564	2	15	2	1	1	3	2
1	9	1565	1	17	2	1	1	3	1
1	9	1566	1	12	1	1	1	3	1
1	9	1567	1	15	2	1	1	3	1
1	9	1568	9	0	#NULL!	2	1	3	2
1	9	1569	1	16	2	1	1	3	1
1	9	1570	1	17	2	1	1	2	1
1	9	1571	1	18	2	1	1	3	1
1	9	1572	1	17	2	1	1	3	2
1	9	1573	2	14	1	1	1	3	1
1	9	1574	2	16	2	1	1	3	1
1	9	1575	9	15	2	1	1	3	1
1	9	1576	2	15	2	1	1	3	1
1	9	1577	1	14	1	1	1	3	1
1	9	1578	2	14	1	1	1	3	1
1	9	1579	2	15	2	1	1	3	1
1	9	1580	2	19	2	1	1	3	3
1	9	1581	1	15	2	1	1	3	1
1	9	1582	1	15	2	1	1	3	2
1	9	1583	1	16	2	1	1	2	1
1	9	1584	1	16	2	1	1	3	2
1	9	1585	2	18	2	1	1	3	1
1	9	1586	2	14	1	1	1	3	2
1	9	1587	1	16	2	1	1	3	1
1	9	1588	9	17	2	1	1	3	1
1	9	1589	9	0	#NULL!	1	1	3	1
1	9	1590	2	17	2	1	1	3	1
1	9	1591	9	14	1	1	1	3	1
1	9	1592	1	14	1	1	1	3	2
1	9	1593	2	16	2	1	1	3	1
1	9	1594	2	15	2	1	1	3	1
1	9	1595	2	14	1	1	1	3	1
1	9	1596	1	14	1	1	1	3	1
1	9	1597	1	16	2	1	1	3	2

1	9	1598	1	16	2	1	1	3	1
1	9	1599	2	14	1	1	1	3	1
1	9	1600	2	15	2	1	1	3	1
1	9	1601	2	15	2	1	1	3	1
1	9	1602	2	13	1	1	1	3	1
1	9	1603	2	16	2	1	1	3	1
1	9	1604	2	15	2	1	1	3	1
1	9	1605	2	14	1	1	1	3	1
1	9	1606	1	14	1	1	1	3	1
1	9	1607	2	15	2	1	1	3	1
1	9	1608	1	16	2	2	1	3	1
1	9	1609	2	14	1	1	1	3	1
1	9	1610	2	14	1	1	1	3	1
1	9	1611	2	15	2	1	1	3	1
1	9	1612	2	14	1	1	1	3	2
1	9	1613	2	17	2	1	1	3	1
1	9	1614	1	15	2	1	1	3	1
1	9	1615	1	15	2	1	1	3	2
1	9	1616	2	15	2	1	1	3	1
1	9	1617	1	14	1	1	1	3	1
1	9	1618	2	15	2	1	1	3	1
1	9	1619	2	14	1	1	1	3	1
1	9	1620	2	15	2	1	1	3	1
1	9	1621	2	14	1	1	1	3	1
1	9	1622	2	16	2	1	1	3	1
1	9	1623	2	17	2	1	1	3	1
1	9	1624	1	16	2	1	1	3	2
1	9	1625	1	17	2	1	1	3	1
1	9	1626	2	16	2	1	1	1	1
1	9	1627	2	15	2	1	1	3	1
1	9	1628	2	16	2	1	1	3	1
1	9	1629	2	13	1	1	1	3	1
1	9	1630	2	16	2	1	1	3	3
1	9	1631	2	14	1	1	1	3	1
1	9	1632	2	14	1	1	1	3	1
1	9	1633	2	15	2	1	1	3	1
1	9	1634	2	14	1	1	1	3	1
1	9	1635	1	15	2	1	1	3	1
1	9	1636	2	15	2	1	1	3	2
1	9	1637	2	14	1	1	1	3	1
1	9	1638	1	18	2	1	1	3	2
1	9	1639	1	14	1	1	1	3	2
1	9	1640	2	15	2	1	1	3	3
1	9	1641	2	16	2	1	1	3	1
1	9	1642	2	15	2	1	1	3	1
1	9	1643	2	14	1	1	1	3	1
1	9	1644	2	16	2	1	1	3	2

1	9	1645	1	20	3	1	1	2	2
1	9	1646	2	15	2	1	1	3	1
1	9	1647	1	16	2	1	1	3	1
1	9	1648	2	16	2	1	1	3	1
1	9	1649	2	18	2	1	1	3	1
1	9	1650	9	16	2	1	1	3	1
1	9	1651	2	14	1	1	1	3	2
1	9	1652	2	16	2	1	1	3	1
1	9	1653	1	17	2	1	1	3	1
1	9	1654	2	16	2	1	1	3	1
1	9	1655	2	15	2	1	1	3	1
1	9	1656	2	15	2	1	1	3	1
1	9	1657	2	14	1	1	1	3	1
1	9	1658	2	15	2	1	1	3	1
1	9	1659	2	14	1	1	1	3	2
1	9	1660	2	14	1	1	1	3	1
1	9	1661	2	16	2	1	1	3	1
1	9	1662	2	14	1	1	1	3	2
1	9	1663	2	16	2	1	1	3	1
1	9	1664	2	15	2	1	1	3	1
1	9	1665	2	15	2	1	1	3	2
1	9	1666	2	16	2	1	1	3	1
1	9	1667	9	16	2	1	1	3	1
1	9	1668	2	14	1	1	1	3	2
1	9	1669	2	13	1	1	1	3	1
1	9	1670	2	15	2	1	1	3	1
1	9	1671	2	15	2	1	1	3	1
1	9	1672	2	15	2	1	1	3	1
1	9	1673	1	13	1	1	1	3	1
1	9	1674	2	15	2	1	1	3	1
1	9	1675	2	14	1	1	1	3	1
1	9	1676	2	15	2	1	1	3	1
1	9	1677	2	14	1	1	1	3	1
1	9	1678	2	15	2	1	1	3	1
1	9	1679	1	14	1	1	1	3	1
1	9	1680	2	16	2	1	1	3	1
1	9	1681	2	13	1	1	1	3	1
1	9	1682	1	15	2	1	1	3	2
1	9	1683	1	15	2	1	1	3	1
1	9	1684	1	15	2	1	1	3	1
1	9	1685	1	14	1	1	1	3	3
1	9	1686	1	15	2	1	1	3	1
1	9	1687	1	18	2	1	1	3	1
1	9	1688	1	16	2	1	3	3	2
1	9	1689	1	16	2	1	1	3	2
1	9	1690	1	16	2	1	1	3	1
1	9	1691	1	15	2	1	1	3	1

1	9	1692	1	14	1	1	1	3	1
1	9	1693	1	15	2	1	1	3	3
1	9	1694	1	0	#NULL!	1	1	3	1
1	9	1695	1	15	2	1	1	3	1
1	9	1696	1	15	2	1	1	3	1
1	9	1697	1	16	2	1	1	3	1
1	9	1698	1	15	2	1	1	3	1
1	9	1699	1	14	1	1	1	3	1
1	9	1700	2	15	2	2	3	3	1
1	9	1701	2	15	2	2	3	3	1
1	9	1702	2	18	2	2	3	3	1
1	9	1703	2	17	2	2	3	3	1
1	9	1704	2	15	2	2	3	3	2
1	9	1705	2	18	2	2	3	3	1
1	9	1706	2	18	2	2	3	3	1
1	9	1707	1	17	2	2	3	3	1
1	9	1708	2	19	2	2	3	3	1
1	9	1709	1	18	2	2	3	3	1
1	9	1710	1	17	2	2	3	3	1
1	9	1711	1	16	2	2	3	3	1
1	9	1712	2	16	2	2	3	3	1
1	9	1713	2	15	2	2	3	3	1
1	9	1714	2	15	2	2	3	3	1
1	9	1715	1	17	2	2	3	3	3
1	9	1716	1	18	2	2	3	3	1
1	9	1717	9	19	2	2	3	3	1
1	9	1718	1	17	2	2	3	3	1
1	9	1719	2	16	2	2	1	3	2
1	9	1720	2	14	1	2	1	3	1
1	9	1721	2	13	1	2	1	3	1
1	9	1722	2	15	2	2	3	3	1
1	9	1723	2	15	2	2	1	3	1
1	9	1724	1	13	1	2	1	3	3
1	9	1725	1	16	2	9	1	3	1
1	9	1726	1	15	2	2	1	3	1
1	9	1727	2	17	2	2	1	3	1
1	9	1728	1	15	2	2	2	3	1
1	9	1729	1	16	2	2	2	2	1
1	9	1730	1	15	2	2	2	3	1
1	9	1731	1	16	2	2	2	3	2
1	9	1732	2	14	1	2	1	3	1
1	9	1733	1	15	2	2	1	3	1
1	9	1734	2	13	1	2	1	3	1
1	9	1735	1	15	2	2	1	3	1
1	9	1736	1	9	1	2	1	2	2
1	9	1737	1	17	2	2	2	3	3
1	9	1738	1	14	1	2	1	3	2

1	9	1739	1	14	1	2	1	3	2
1	9	1740	1	14	1	2	1	3	2
1	9	1741	1	14	1	2	1	3	1
1	9	1742	1	14	1	2	1	3	1
1	9	1743	1	15	2	2	1	3	2
1	9	1744	1	14	1	2	1	3	1
1	9	1745	1	15	2	2	1	3	1
1	9	1746	1	14	1	2	1	3	2
1	9	1747	2	15	2	2	1	3	2
1	9	1748	2	14	1	2	1	3	1
1	9	1749	2	15	2	2	1	3	1
1	9	1750	1	17	2	2	3	3	3
1	9	1751	1	15	2	2	1	3	2
1	9	1752	1	14	1	2	1	3	1
1	9	1753	1	15	2	2	1	3	2
1	9	1754	1	15	2	2	1	3	2
1	9	1755	1	15	2	2	1	3	1
1	9	1756	1	14	1	2	1	3	2
1	9	1757	1	16	2	2	2	3	2
1	9	1758	1	17	2	2	2	3	2
1	9	1759	1	17	2	2	2	3	3
1	9	1760	1	16	2	2	2	3	3
1	9	1761	1	14	1	2	1	3	1
1	9	1762	1	14	1	2	1	3	2
1	9	1763	1	15	2	2	1	3	2
1	9	1764	1	14	1	2	1	3	2
1	9	1765	1	15	2	2	1	3	2
1	9	1766	1	16	2	2	1	3	1
1	9	1767	1	15	2	2	1	3	2
1	9	1768	1	14	1	2	1	3	2
1	9	1769	1	15	2	2	1	3	2
1	9	1770	1	14	1	2	1	3	1
1	9	1771	1	15	2	2	1	3	2
1	9	1772	1	14	1	2	1	3	2
1	9	1773	2	14	1	2	1	3	2
1	9	1774	2	14	1	2	1	3	2
1	9	1775	2	13	1	2	1	3	2
1	9	1776	2	14	1	2	1	3	2
1	9	1777	2	14	1	2	1	3	2
1	9	1778	2	15	2	2	1	3	2
1	9	1779	2	14	1	2	1	3	3
1	9	1780	2	14	1	2	1	3	2
1	9	1781	2	14	1	2	1	3	2
1	9	1782	2	13	1	2	1	3	1
1	9	1783	2	13	1	2	1	3	2
1	9	1784	2	14	1	2	1	3	2
1	9	1785	2	14	1	2	1	3	2

1	9	1786	2	15	2	2	1	3	2
1	9	1787	2	15	2	2	1	3	2
1	9	1788	2	15	2	2	1	3	2
1	9	1789	2	14	1	2	1	3	2
1	9	1790	2	14	1	2	1	3	2
1	9	1791	2	15	2	2	1	3	2
1	9	1792	2	15	2	2	1	3	2
1	9	1793	2	14	1	2	1	3	1
1	9	1794	2	17	2	2	2	3	1
1	9	1795	2	16	2	2	2	3	3
1	9	1796	2	14	1	2	1	3	1
1	9	1797	2	15	2	2	1	3	1
1	9	1798	2	15	2	2	1	3	1
1	9	1799	2	15	2	2	1	3	1
1	9	1800	2	14	1	2	1	3	1
1	9	1801	2	15	2	2	1	3	1
1	9	1802	2	15	2	2	1	3	1
1	9	1803	2	16	2	2	1	3	3
1	9	1804	1	15	2	2	1	3	1
1	9	1805	1	15	2	2	1	3	2
1	9	1806	1	14	1	2	1	3	1
1	9	1807	1	14	1	2	1	3	1
1	9	1808	1	15	2	2	1	3	1
1	9	1809	1	16	2	2	2	3	3
1	9	1810	1	17	2	2	2	3	2
1	9	1811	1	17	2	2	2	3	3
1	9	1812	1	16	2	2	2	3	3
1	9	1813	1	17	2	2	2	3	2
1	9	1814	1	16	2	2	2	3	2
1	9	1815	1	16	2	2	2	3	2
1	9	1816	1	15	2	2	2	3	1
1	9	1817	1	16	2	2	2	3	3
1	9	1818	1	16	2	2	2	3	3
1	9	1819	1	16	2	2	2	3	2
1	9	1820	1	15	2	2	2	3	2
1	9	1821	1	16	2	2	2	3	2
1	9	1822	1	16	2	2	2	3	2
1	9	1823	1	17	2	2	2	3	1
1	9	1824	1	15	2	2	1	3	1
1	9	1825	1	16	2	2	1	3	1
1	9	1826	1	14	1	2	1	3	2
1	9	1827	1	13	1	2	1	3	2
1	9	1828	2	14	1	2	1	3	1
1	9	1829	2	15	2	2	1	3	1
1	9	1830	1	14	1	2	1	3	1
1	9	1831	1	14	1	2	1	3	2
1	9	1832	1	14	1	2	1	3	2

1	9	1833	1	15	2	2	1	3	2
1	9	1834	1	14	1	2	1	3	2
1	9	1835	1	14	1	2	1	3	2
1	9	1836	1	15	2	2	1	3	2
1	9	1837	1	17	2	2	3	3	1
1	9	1838	2	17	2	2	3	3	1
1	9	1839	9	18	2	2	3	3	1
1	9	1840	2	17	2	2	3	3	1
1	9	1841	2	16	2	2	3	3	1
1	9	1842	1	17	2	2	3	3	1
1	9	1843	2	16	2	2	3	3	1
1	9	1844	2	17	2	2	3	3	1
1	9	1845	1	17	2	2	3	3	1
1	9	1846	1	17	2	2	3	3	1
1	9	1847	1	17	2	2	3	3	1
1	9	1848	1	15	2	2	2	3	1
1	9	1849	1	17	2	2	2	3	1
1	9	1850	1	15	2	2	2	3	1
1	9	1851	1	20	3	9	2	3	1
1	9	1852	1	16	2	2	2	3	1
1	9	1853	1	16	2	2	2	3	1
1	9	1854	1	15	2	2	2	3	1
1	9	1855	2	15	2	2	2	3	1
1	9	1856	1	16	2	2	2	3	1
1	9	1857	2	18	2	2	3	3	1
1	9	1858	1	16	2	2	3	3	1
1	9	1859	1	16	2	2	3	3	1
1	9	1860	2	16	2	2	1	3	1
1	9	1861	2	20	3	2	1	3	1
1	9	1862	2	16	2	2	1	3	1
1	9	1863	1	16	2	2	1	3	1
1	9	1864	1	0	#NULL!	2	1	3	1
1	9	1865	1	15	2	2	1	3	1
1	9	1866	1	16	2	2	2	3	1
1	9	1867	1	16	2	2	2	3	1
1	9	1868	2	15	2	2	1	3	1
1	9	1869	2	12	1	2	1	3	1
1	9	1870	2	14	1	2	1	3	1
1	9	1871	2	13	1	2	1	3	1
1	9	1872	2	14	1	2	1	3	1
1	9	1873	1	17	2	2	1	3	1
1	9	1874	1	15	2	2	1	3	1
1	9	1875	2	18	2	2	3	3	1
1	9	1876	1	17	2	2	3	3	1
1	9	1877	1	16	2	2	1	3	1
1	9	1878	2	15	2	2	1	3	1
1	9	1879	2	15	2	2	1	3	1

1	9	1880	2	15	2	2	1	3	1
1	9	1881	2	15	2	2	1	3	1
1	9	1882	1	16	2	2	1	3	1
1	9	1883	2	16	2	2	1	3	1
1	9	1884	2	14	1	2	1	3	1
1	9	1885	1	15	2	2	1	3	1
1	9	1886	1	15	2	2	1	3	1
1	9	1887	2	14	1	2	1	3	2
1	9	1888	1	15	2	2	1	3	1
1	9	1889	1	16	2	2	1	3	1
1	9	1890	2	15	2	2	1	3	2
1	9	1891	2	15	2	2	1	2	1
1	9	1892	1	15	2	2	1	3	2
1	9	1893	2	14	1	2	1	3	1
1	9	1894	1	15	2	2	1	3	1
1	9	1895	2	14	1	2	1	3	3
1	9	1896	2	15	2	2	1	3	1
1	9	1897	2	15	2	2	1	3	1
1	9	1898	2	15	2	2	1	3	1
1	9	1899	2	15	2	2	1	3	1
1	9	1900	9	0	#NULL!	2	1	3	1
1	9	1901	2	11	1	9	1	3	2
1	9	1902	2	14	1	2	1	3	1
1	9	1903	9	0	#NULL!	2	1	2	2
1	9	1904	1	16	2	2	1	3	1
1	9	1905	1	16	2	2	1	3	2
1	9	1906	1	15	2	2	1	3	1
1	9	1907	2	14	1	2	1	3	1
1	9	1908	1	16	2	2	1	3	2
1	9	1909	1	16	2	2	1	3	2
1	9	1910	1	16	2	2	1	3	2
1	9	1911	2	15	2	2	1	3	2
1	9	1912	1	15	2	2	1	3	1
1	9	1913	9	0	#NULL!	9	9	9	9
1	9	1914	9	0	#NULL!	2	1	3	1
1	9	1915	1	19	2	2	1	3	1
1	9	1916	1	19	2	2	1	3	1
1	9	1917	1	14	1	2	1	3	1
1	9	1918	1	16	2	2	2	3	1
1	9	1919	1	17	2	2	2	3	2
1	9	1920	1	15	2	2	2	3	1
1	9	1921	2	17	2	2	2	3	1
1	9	1922	1	17	2	2	2	3	1
1	9	1923	2	0	#NULL!	2	2	3	1
1	9	1924	1	17	2	2	2	3	1
1	9	1925	9	15	2	2	2	3	1
1	9	1926	1	15	2	2	2	3	1

1	9	1927	1	19	2	2	2	3	1
1	9	1928	1	16	2	2	2	9	2
1	9	1929	1	17	2	2	2	3	2
1	9	1930	1	17	2	2	2	1	1
1	9	1931	1	16	2	2	2	3	2
1	9	1932	1	16	2	2	2	9	1
1	9	1933	1	15	2	2	2	2	2
1	9	1934	1	15	2	2	2	3	1
1	9	1935	2	15	2	2	2	3	1
1	9	1936	1	17	2	2	2	9	2
1	9	1937	2	14	1	2	1	3	2
1	9	1938	2	14	1	2	1	3	1
1	9	1939	2	19	2	2	2	1	1
1	9	1940	2	15	2	2	2	1	1
1	9	1941	2	17	2	2	2	1	1
1	9	1942	2	16	2	2	2	1	1
1	9	1943	2	18	2	2	2	1	1
1	9	1944	2	15	2	2	2	1	1
1	9	1945	2	16	2	2	2	1	1
1	9	1946	2	15	2	2	2	1	1
1	9	1947	2	15	2	2	2	1	3
1	9	1948	2	15	2	2	2	1	1
1	9	1949	2	15	2	2	2	1	1
1	9	1950	2	15	2	2	2	1	1
1	9	1951	2	14	1	2	2	1	1
1	9	1952	2	16	2	2	2	1	1
1	9	1953	2	14	1	2	2	1	1
1	9	1954	2	0	#NULL!	2	2	1	1
1	9	1955	2	17	2	2	1	1	1
1	9	1956	2	14	1	2	1	1	1
1	9	1957	2	14	1	2	1	1	3
1	9	1958	2	15	2	2	1	1	1
1	9	1959	2	14	1	2	1	1	1
1	9	1960	2	15	2	2	1	1	1
1	9	1961	2	15	2	2	1	1	1
1	9	1962	2	14	1	2	1	1	1
1	9	1963	2	15	2	2	1	1	1
1	9	1964	2	14	1	2	1	1	1
1	9	1965	2	15	2	2	1	1	1
1	9	1966	2	15	2	2	1	1	1
1	9	1967	2	16	2	2	1	1	1
1	9	1968	2	13	1	2	1	1	1
1	9	1969	2	15	2	2	1	1	1
1	9	1970	2	14	1	2	1	1	1
1	9	1971	2	14	1	2	1	1	1
1	9	1972	2	14	1	2	1	1	1
1	9	1973	2	13	1	9	1	9	1

1	9	1974	2	15	2	2	1	1	1
1	9	1975	2	15	2	2	1	1	2
1	9	1976	2	13	1	2	3	1	2
1	9	1977	2	13	1	2	1	1	1
1	9	1978	2	17	2	2	1	1	1
1	9	1979	2	15	2	2	1	1	1
1	9	1980	2	15	2	2	1	1	2
1	9	1981	2	15	2	2	1	1	1
1	9	1982	2	14	1	2	1	1	1
1	9	1983	2	15	2	2	1	1	1
1	9	1984	2	14	1	2	1	1	1
1	9	1985	1	15	2	2	1	1	1
1	9	1986	2	16	2	2	1	1	1
1	9	1987	2	17	2	2	1	1	1
1	9	1988	2	15	2	2	1	1	1
1	9	1989	2	15	2	2	1	1	1
1	9	1990	2	0	#NULL!	2	2	1	1
1	9	1991	2	15	2	2	1	1	1
1	9	1992	2	15	2	2	1	1	1
1	9	1993	2	16	2	2	1	1	1
1	9	1994	2	17	2	2	2	1	1
1	9	1995	2	0	#NULL!	2	2	1	1
1	9	1996	2	16	2	2	1	1	1
1	9	1997	2	14	1	9	1	1	1
1	9	1998	2	14	1	2	1	1	1
1	9	1999	2	16	2	2	1	1	1
1	9	2000	2	15	2	2	1	1	1
1	9	2001	2	15	2	2	1	1	1
1	9	2002	2	16	2	2	1	1	1
1	9	2003	2	16	2	2	1	1	1
1	9	2004	2	15	2	2	1	1	1
1	9	2005	2	16	2	2	1	1	1
1	9	2006	2	15	2	2	1	1	1
1	9	2007	2	16	2	2	1	1	2
1	9	2008	2	15	2	2	1	1	1
1	9	2009	2	15	2	2	1	1	1
1	9	2010	2	16	2	2	1	1	1
1	9	2011	2	15	2	2	1	1	1
1	9	2012	2	14	1	2	1	1	1
1	9	2013	2	15	2	9	1	1	2
1	9	2014	2	15	2	2	1	1	1
1	9	2015	2	13	1	2	1	1	1
1	9	2016	2	14	1	2	1	1	1
1	9	2017	2	15	2	2	1	1	1
1	9	2018	2	15	2	2	1	1	1
1	9	2019	2	15	2	2	1	1	1
1	9	2020	2	15	2	2	1	1	1

1	9	2021	2	15	2	2	1	1	1
1	9	2022	2	15	2	2	1	1	1
1	9	2023	2	17	2	2	3	1	1
1	9	2024	2	17	2	2	3	1	2
1	9	2025	2	16	2	2	3	1	1
1	9	2026	2	18	2	2	3	1	1
1	9	2027	2	17	2	2	3	1	1
1	9	2028	2	15	2	2	3	1	1
1	9	2029	2	17	2	2	3	1	1
1	9	2030	2	16	2	2	3	1	1
1	9	2031	2	0	#NULL!	2	3	1	1
1	9	2032	2	17	2	2	3	1	1
1	9	2033	2	18	2	2	3	1	1
1	9	2034	2	19	2	2	3	1	1
1	9	2035	2	15	2	2	3	1	3
1	9	2036	2	0	#NULL!	2	3	3	1
1	9	2037	2	17	2	2	3	1	1
1	9	2038	2	17	2	2	3	1	1
1	9	2039	2	17	2	2	3	1	1
1	9	2040	2	15	2	2	3	1	1
1	9	2041	2	17	2	2	3	1	1
1	9	2042	2	17	2	2	3	1	1
1	9	2043	2	17	2	2	3	1	1
1	9	2044	2	18	2	2	3	1	1
1	9	2045	2	18	2	2	3	1	1
1	9	2046	2	17	2	1	3	1	2
1	9	2047	2	17	2	2	3	1	1
1	9	2048	2	17	2	2	3	1	1
1	9	2049	2	17	2	2	3	1	1
1	9	2050	2	15	2	2	3	1	1
1	9	2051	2	16	2	2	3	1	2
1	9	2052	2	10	1	2	3	1	1
1	9	2053	9	0	#NULL!	2	3	1	1
1	9	2054	2	16	2	2	3	1	1
1	9	2055	2	17	2	2	3	1	1
1	9	2056	2	17	2	2	3	1	1
1	9	2057	2	17	2	2	3	1	1
1	9	2058	2	17	2	2	3	1	1
1	9	2059	2	17	2	2	3	1	1
1	9	2060	2	18	2	2	3	1	1
1	9	2061	2	17	2	2	3	1	1
1	9	2062	2	15	2	2	2	1	2
1	9	2063	2	16	2	2	2	1	1
1	9	2064	2	16	2	9	2	1	1
1	9	2065	2	16	2	2	2	1	1
1	9	2066	2	14	1	2	2	1	1
1	9	2067	2	15	2	2	2	1	1

1	9	2068	2	15	2	2	2	1	1
1	9	2069	2	16	2	2	2	1	1
1	9	2070	2	17	2	2	2	1	1
1	9	2071	2	15	2	2	2	1	3
1	9	2072	2	0	#NULL!	2	2	1	1
1	9	2073	2	17	2	2	3	1	1
1	9	2074	2	16	2	2	2	3	1
1	9	2075	1	19	2	2	2	2	1
1	9	2076	1	17	2	2	2	2	2
1	9	2077	1	16	2	2	1	2	1
1	9	2078	1	16	2	2	1	2	1
1	9	2079	1	15	2	1	1	2	2
1	9	2080	1	20	3	2	1	2	1
1	9	2081	1	15	2	2	1	2	3
1	9	2082	1	15	2	2	1	2	1
1	9	2083	9	15	2	2	1	2	1
1	9	2084	1	14	1	2	1	2	1
1	9	2085	1	13	1	2	1	2	3
1	9	2086	1	17	2	2	1	2	1
1	9	2087	1	0	#NULL!	2	1	1	1
1	9	2088	1	17	2	2	1	2	2
1	9	2089	1	16	2	2	1	2	3
1	9	2090	1	19	2	2	1	2	1
1	9	2091	1	15	2	2	2	2	1
1	9	2092	1	10	1	2	2	2	2
1	9	2093	1	0	#NULL!	2	2	2	1
1	9	2094	1	17	2	2	2	2	1
1	9	2095	1	10	1	2	2	2	2
1	9	2096	1	13	1	2	2	2	1
1	9	2097	1	17	2	2	2	2	1
1	9	2098	1	14	1	2	2	2	1
1	9	2099	1	17	2	2	2	2	1
1	9	2100	1	19	2	2	2	2	2
1	9	2101	1	18	2	2	2	2	3
1	9	2102	9	0	#NULL!	2	2	1	1
1	9	2103	1	16	2	2	2	2	1
1	9	2104	1	18	2	2	2	2	1
1	9	2105	1	18	2	2	2	2	1
1	9	2106	1	18	2	2	2	2	2
1	9	2107	1	19	2	9	2	9	9
1	9	2108	1	16	2	9	9	9	9
1	9	2109	1	0	#NULL!	2	2	2	1
1	9	2110	1	17	2	2	2	2	1
1	9	2111	1	17	2	2	2	2	1
1	9	2112	1	17	2	2	3	3	1
1	9	2113	2	17	2	2	3	3	1
1	9	2114	1	17	2	2	2	2	1

1	9	2115	1	0	#NULL!	2	2	2	1
1	9	2116	1	16	2	2	2	2	1
1	9	2117	1	16	2	2	2	2	2
1	9	2118	1	17	2	2	2	2	1
1	9	2119	1	14	1	2	2	2	1
1	9	2120	1	16	2	2	2	2	1
1	9	2121	1	18	2	2	1	2	1
1	9	2122	1	16	2	2	2	2	1
1	9	2123	1	17	2	2	3	2	1
1	9	2124	1	17	2	2	3	2	2
1	9	2125	1	20	3	2	3	2	1
1	9	2126	1	18	2	2	3	2	1
1	9	2127	1	19	2	2	3	2	1
1	9	2128	1	16	2	2	3	2	1
1	9	2129	1	18	2	2	3	2	1
1	9	2130	1	17	2	2	3	2	2
1	9	2131	1	18	2	2	3	2	1
1	9	2132	1	20	3	2	3	2	1
1	9	2133	1	18	2	2	3	2	1
1	9	2134	1	17	2	2	1	2	1
1	9	2135	1	18	2	2	1	1	1
1	9	2136	1	18	2	2	3	2	2
1	9	2137	1	18	2	2	3	3	3
1	9	2138	2	18	2	2	3	3	1
1	9	2139	2	16	2	2	3	3	1
1	9	2140	2	18	2	2	3	3	1
1	9	2141	2	16	2	2	3	3	1
1	9	2142	1	15	2	2	3	3	1
1	9	2143	2	15	2	2	3	3	2
1	9	2144	2	17	2	2	3	3	1
1	9	2145	1	15	2	2	3	3	1
1	9	2146	2	16	2	2	3	3	1
1	9	2147	1	17	2	2	3	3	1
1	9	2148	2	16	2	2	3	3	1
1	9	2149	2	15	2	2	3	3	1
1	9	2150	1	19	2	2	3	3	1
1	9	2151	1	17	2	2	3	3	1
1	9	2152	2	18	2	2	3	3	1
1	9	2153	2	19	2	2	3	3	1
1	9	2154	1	15	2	2	2	2	1
1	9	2155	1	17	2	2	3	3	1
1	9	2156	2	14	1	2	1	3	1
1	9	2157	2	15	2	2	1	3	1
1	9	2158	2	15	2	2	1	3	1
1	9	2159	2	16	2	2	1	2	1
1	9	2160	9	18	2	2	2	2	1
1	9	2161	1	0	#NULL!	2	1	2	1

1	9	2162	1	13	1	2	1	2	1
1	9	2163	9	0	#NULL!	2	1	3	3
1	9	2164	1	15	2	2	1	2	2
1	9	2165	9	0	#NULL!	9	9	9	9
1	9	2166	1	17	2	2	1	2	1
1	9	2167	1	17	2	2	1	2	1
1	9	2168	1	19	2	2	1	2	1
1	9	2169	1	19	2	9	9	2	2
1	9	2170	9	25	3	2	3	2	2
1	9	2171	1	15	2	2	1	2	1
1	9	2172	1	0	#NULL!	2	1	2	2
1	9	2173	1	16	2	2	1	2	2
1	9	2174	1	19	2	2	1	1	2
1	9	2175	1	16	2	2	1	2	1
1	9	2176	1	15	2	2	1	2	1
1	9	2177	9	0	#NULL!	2	1	3	1
1	9	2178	1	14	1	2	1	2	1
1	9	2179	1	0	#NULL!	2	1	2	1
1	9	2180	1	0	#NULL!	2	1	2	1
1	9	2181	1	0	#NULL!	2	1	2	2
1	9	2182	1	18	2	2	1	2	1
1	9	2183	9	0	#NULL!	2	1	2	1
1	9	2184	1	16	2	2	1	2	2
1	9	2185	1	14	1	1	1	1	1
1	9	2186	9	0	#NULL!	9	9	9	9
1	9	2187	2	16	2	2	1	3	1
1	9	2188	2	18	2	2	1	3	1
1	9	2189	2	16	2	2	1	3	1
1	9	2190	9	0	#NULL!	9	9	9	9
1	9	2191	9	0	#NULL!	9	9	9	9
1	9	2192	9	0	#NULL!	9	9	9	9
1	9	2193	9	0	#NULL!	9	9	9	9
1	9	2194	9	0	#NULL!	9	9	9	9
1	9	2195	2	20	3	2	2	3	1
1	9	2196	1	17	2	2	2	3	1
1	9	2197	9	0	#NULL!	2	2	3	1
1	9	2198	1	16	2	2	2	3	2
1	9	2199	2	15	2	2	2	3	1
1	9	2200	9	15	2	2	1	3	1
1	9	2201	1	17	2	2	2	3	1
1	9	2202	2	12	1	2	3	3	1
1	9	2203	2	17	2	2	3	3	1
1	9	2204	2	17	2	2	3	3	1
1	9	2205	2	18	2	2	3	3	1
1	9	2206	2	16	2	2	3	3	1
1	9	2207	2	15	2	2	3	3	1
1	9	2208	1	16	2	2	3	3	1

1	9	2209	1	19	2	2	3	3	1
1	9	2210	2	12	1	9	3	3	1
1	9	2211	2	16	2	2	3	3	1
1	9	2212	1	16	2	2	1	3	2
1	9	2213	1	20	3	2	1	3	2
1	9	2214	1	14	1	2	1	3	1
1	9	2215	2	17	2	2	1	3	2
1	9	2216	1	13	1	2	1	3	2
1	9	2217	2	18	2	2	1	3	1
1	9	2218	2	12	1	2	1	3	2
1	9	2219	2	17	2	2	1	3	1
1	9	2220	1	13	1	2	1	3	2
1	9	2221	2	16	2	9	2	3	1
1	9	2222	1	18	2	2	2	2	2
1	9	2223	1	15	2	2	2	2	1
1	9	2224	1	17	2	9	9	9	9
1	9	2225	1	16	2	2	2	2	1
1	9	2226	1	17	2	2	2	2	1
1	9	2227	1	17	2	2	2	2	1
1	9	2228	1	17	2	2	2	2	1
1	9	2229	1	17	2	2	2	2	1
1	9	2230	1	18	2	2	1	2	1
1	9	2231	1	18	2	2	2	2	1
1	9	2232	1	16	2	2	2	2	1
1	9	2233	1	16	2	2	2	2	1
1	9	2234	1	17	2	2	2	2	1
1	9	2235	1	15	2	2	2	2	1
1	9	2236	1	15	2	2	2	2	1
1	9	2237	1	17	2	2	2	2	1
1	9	2238	1	18	2	2	2	2	2
1	9	2239	1	16	2	2	2	2	1
1	9	2240	1	19	2	2	3	2	2
1	9	2241	1	18	2	2	3	2	1
1	9	2242	1	18	2	2	3	3	1
1	9	2243	1	0	#NULL!	2	3	2	1
1	9	2244	1	0	#NULL!	2	3	2	1
1	9	2245	1	25	3	2	3	2	1
1	9	2246	1	19	2	2	3	2	1
1	9	2247	1	18	2	2	3	2	1
1	9	2248	1	18	2	2	3	2	1
1	9	2249	1	30	3	2	3	2	1
1	9	2250	2	14	1	2	1	3	1
1	9	2251	9	16	2	2	1	3	1
1	9	2252	2	15	2	2	1	3	1
1	9	2253	2	15	2	2	1	3	1
1	9	2254	9	0	#NULL!	9	1	3	1
1	9	2255	2	14	1	2	1	3	1

1	9	2256	1	18	2	2	1	3	1
1	9	2257	1	16	2	2	1	3	2
1	9	2258	2	0	#NULL!	2	1	3	1
1	9	2259	2	14	1	2	1	3	1
1	9	2260	1	15	2	2	1	3	1
1	9	2261	1	15	2	2	1	3	1
1	9	2262	2	16	2	2	2	3	1
1	9	2263	2	15	2	2	2	3	1
1	9	2264	2	15	2	2	2	3	1
1	9	2265	2	16	2	2	2	3	1
1	9	2266	2	16	2	2	2	3	1
1	9	2267	2	13	1	2	2	3	1
1	9	2268	9	16	2	2	2	2	2
1	9	2269	2	15	2	2	2	3	1
1	9	2270	2	16	2	2	2	3	1
1	9	2271	1	18	2	2	2	3	1
1	9	2272	2	13	1	2	2	3	1
1	9	2273	2	15	2	2	2	3	1
1	9	2274	2	17	2	2	2	3	1
1	9	2275	2	15	2	2	2	3	1
1	9	2276	1	16	2	2	2	3	1
1	9	2277	2	15	2	2	2	3	1
1	9	2278	1	15	2	2	2	3	1
1	9	2279	2	15	2	2	2	3	1
1	9	2280	1	17	2	2	2	3	1
1	9	2281	1	15	2	2	2	3	1
1	9	2282	1	18	2	2	2	2	1
1	9	2283	9	0	#NULL!	2	2	2	2
1	9	2284	1	17	2	2	2	2	2
1	9	2285	1	16	2	2	2	2	1
1	9	2286	1	15	2	2	2	2	1
1	9	2287	1	16	2	2	2	2	1
1	9	2288	1	18	2	2	3	2	1
1	9	2289	1	20	3	2	3	2	1
1	9	2290	1	17	2	2	3	2	2
1	9	2291	1	19	2	2	3	2	2
1	9	2292	1	17	2	2	3	2	1
1	9	2293	1	18	2	2	3	2	1
1	9	2294	1	18	2	2	3	2	1
1	9	2295	1	18	2	2	3	2	1
1	9	2296	1	19	2	2	3	2	2
1	9	2297	9	19	2	2	3	2	1
1	9	2298	1	15	2	2	1	2	1
1	9	2299	1	17	2	2	1	2	2
1	9	2300	1	14	1	2	1	2	1
1	9	2301	1	14	1	2	1	2	1
1	9	2302	1	14	1	2	1	2	1

1	9	2303	1	16	2	2	1	2	1
1	9	2304	2	14	1	2	1	3	1
1	9	2305	2	17	2	2	1	3	1
1	9	2306	2	0	#NULL!	2	3	3	2
1	9	2307	1	14	1	2	1	3	1
1	9	2308	2	17	2	2	3	3	1
1	9	2309	2	16	2	2	3	3	1
1	9	2310	2	0	#NULL!	2	3	1	1
1	9	2311	1	17	2	2	3	3	1
1	9	2312	2	16	2	2	3	3	1
1	9	2313	1	17	2	2	3	3	1
1	9	2314	2	16	2	2	3	3	1
1	9	2315	2	14	1	2	3	3	1
1	9	2316	2	17	2	2	3	3	1
1	9	2317	1	17	2	2	3	3	1
1	9	2318	1	13	1	2	2	3	1
1	9	2319	1	16	2	2	3	3	1
1	9	2320	1	16	2	2	3	3	1
1	9	2321	2	16	2	2	3	3	1
1	9	2322	1	17	2	2	3	3	1
1	9	2323	2	18	2	2	3	3	1
1	9	2324	1	18	2	2	3	3	1
1	9	2325	1	17	2	2	3	3	2
1	9	2326	1	15	2	2	3	3	1
1	9	2327	2	17	2	2	3	3	1
1	9	2328	1	18	2	2	3	3	1
1	9	2329	2	15	2	2	3	3	1
1	9	2330	2	0	#NULL!	2	3	3	1
1	9	2331	1	15	2	2	3	3	1
1	9	2332	2	16	2	2	3	3	1
1	9	2333	1	16	2	2	3	3	1
1	9	2334	2	14	1	2	3	3	1
1	9	2335	1	17	2	2	3	3	1
1	9	2336	1	16	2	2	3	3	1
1	9	2337	1	17	2	2	3	3	1
1	9	2338	2	17	2	2	3	3	1
1	9	2339	2	14	1	2	3	1	2
1	9	2340	1	14	1	2	3	3	2
1	9	2341	1	17	2	2	3	3	1
1	9	2342	2	0	#NULL!	2	2	3	1
1	9	2343	2	15	2	2	2	3	1
1	9	2344	9	15	2	2	2	3	1
1	9	2345	1	15	2	2	1	3	1
1	9	2346	1	17	2	2	1	3	2
1	9	2347	2	16	2	2	1	3	2
1	9	2348	1	16	2	2	1	3	1
1	9	2349	2	16	2	2	1	3	1

1	9	2350	1	16	2	2	1	3	1
1	9	2351	2	17	2	2	1	3	3
1	9	2352	2	15	2	2	1	3	1
1	9	2353	2	15	2	2	1	3	1
1	9	2354	1	13	1	2	1	3	1
1	9	2355	2	14	1	2	1	3	1
1	9	2356	2	15	2	2	1	3	1
1	9	2357	2	14	1	2	1	3	1
1	9	2358	2	14	1	2	1	3	1
1	9	2359	2	14	1	2	1	3	1
1	9	2360	2	15	2	2	1	3	1
1	9	2361	2	15	2	2	1	3	1
1	9	2362	2	14	1	2	1	3	1
1	9	2363	1	17	2	2	1	3	2
1	9	2364	2	16	2	2	3	3	1
1	9	2365	2	16	2	2	3	3	1
1	9	2366	2	17	2	2	3	3	1
1	9	2367	2	0	#NULL!	2	3	3	2
1	9	2368	2	0	#NULL!	2	3	3	1
1	9	2369	1	17	2	2	3	3	1
1	9	2370	1	15	2	2	3	3	1
1	9	2371	2	16	2	2	3	3	1
1	9	2372	2	16	2	2	3	3	1
1	9	2373	1	17	2	2	3	3	1
1	9	2374	2	16	2	2	3	3	1
1	9	2375	1	17	2	2	3	3	1
1	9	2376	2	17	2	2	3	3	1
1	9	2377	2	16	2	2	3	3	1
1	9	2378	1	17	2	2	3	3	1
1	9	2379	2	17	2	2	3	3	1
1	9	2380	2	17	2	2	3	3	1
1	9	2381	2	16	2	2	3	3	1
1	9	2382	1	16	2	2	1	3	1
1	9	2383	1	16	2	2	1	3	1
1	9	2384	2	17	2	2	1	3	1
1	9	2385	9	0	#NULL!	2	1	1	1
1	9	2386	2	15	2	2	1	3	1
1	9	2387	2	14	1	2	1	3	1
1	9	2388	2	15	2	2	1	3	1
1	9	2389	1	15	2	2	1	3	2
1	9	2390	2	13	1	2	1	3	2
1	9	2391	2	12	1	2	1	3	1
1	9	2392	9	0	#NULL!	9	9	9	9
1	9	2393	2	0	#NULL!	2	1	3	1
1	9	2394	1	14	1	2	1	3	1
1	9	2395	2	17	2	2	1	3	1
1	9	2396	2	16	2	2	1	3	1

1	9	2397	2	14	1	2	1	3	1
1	9	2398	2	16	2	2	2	3	1
1	9	2399	2	15	2	2	2	3	1
1	9	2400	2	15	2	2	2	3	1
1	9	2401	1	17	2	2	2	3	2
1	9	2402	1	14	1	2	2	3	1
1	9	2403	1	16	2	2	1	3	2
1	9	2404	1	16	2	2	1	3	1
1	9	2405	1	18	2	2	1	3	2
1	9	2406	2	15	2	2	2	3	1
1	9	2407	1	16	2	2	2	3	1
1	9	2408	2	16	2	2	2	3	1
1	9	2409	2	15	2	2	2	3	1
1	9	2410	2	17	2	2	3	3	1
1	9	2411	1	16	2	2	2	3	1
1	9	2412	2	15	2	2	2	3	3
1	9	2413	2	14	1	2	1	3	1
1	9	2414	2	15	2	2	2	3	1
1	9	2415	2	14	1	2	2	3	1
1	9	2416	1	16	2	2	2	3	1
1	9	2417	1	17	2	2	2	3	1
1	9	2418	2	16	2	2	2	3	2
1	9	2419	2	15	2	2	2	3	1
1	9	2420	2	15	2	2	2	3	3
1	9	2421	9	0	#NULL!	9	9	9	9
1	9	2422	2	15	2	2	2	3	1
1	9	2423	1	15	2	2	1	3	1
1	9	2424	2	15	2	2	2	1	1
1	9	2425	2	0	#NULL!	2	2	3	2
1	9	2426	2	18	2	2	2	3	2
1	9	2427	2	16	2	2	2	3	1
1	9	2428	2	14	1	2	2	3	1
1	9	2429	2	16	2	2	2	3	2
1	9	2430	2	17	2	2	2	3	1
1	9	2431	2	15	2	2	2	3	1
1	9	2432	2	15	2	2	2	3	1
1	9	2433	2	17	2	2	2	3	1
1	9	2434	2	15	2	2	2	3	2
1	9	2435	2	16	2	2	2	3	1
1	9	2436	2	16	2	2	2	3	1
1	9	2437	2	17	2	2	2	3	1
1	9	2438	2	16	2	2	2	3	1
1	9	2439	2	16	2	2	2	3	1
1	9	2440	1	16	2	2	2	3	1
1	9	2441	2	16	2	2	2	3	1
1	9	2442	9	0	#NULL!	9	9	9	9
1	9	2443	2	15	2	2	2	3	1

1	9	2444	2	17	2	2	2	3	1
1	9	2445	1	18	2	2	2	3	1
1	9	2446	2	15	2	2	2	3	1
1	9	2447	2	16	2	2	2	3	2
1	9	2448	2	17	2	2	2	3	1
1	9	2449	2	15	2	2	2	3	1
1	9	2450	1	20	3	2	2	3	2
1	9	2451	2	16	2	2	2	3	3
1	9	2452	2	17	2	2	2	3	1
1	9	2453	2	15	2	2	3	3	2
1	9	2454	2	16	2	2	2	3	2
1	9	2455	1	18	2	2	2	2	1
1	9	2456	1	20	3	2	2	2	1
1	9	2457	1	17	2	2	2	2	1
1	9	2458	1	15	2	2	2	2	1
1	9	2459	1	18	2	2	2	2	1
1	9	2460	1	20	3	9	2	2	2
1	9	2461	2	16	2	2	3	3	1
1	9	2462	2	13	1	2	1	3	1
1	9	2463	2	13	1	2	2	3	1
1	9	2464	9	0	#NULL!	9	9	9	9
1	9	2465	2	15	2	2	1	3	1
1	9	2466	1	15	2	2	2	3	1
1	9	2467	2	16	2	2	3	3	2
1	9	2468	9	17	2	2	3	3	2
1	9	2469	2	16	2	2	3	3	1
1	9	2470	2	16	2	2	3	3	2
1	9	2471	1	15	2	2	2	3	2
1	9	2472	2	16	2	2	3	3	2
1	9	2473	2	16	2	2	3	3	2
1	9	2474	9	0	#NULL!	9	9	9	9
1	9	2475	9	0	#NULL!	9	9	9	9
1	9	2476	9	0	#NULL!	9	9	9	9
1	9	2477	2	15	2	2	1	3	1
1	9	2478	2	15	2	2	1	3	2
1	9	2479	2	15	2	2	1	3	1
1	9	2480	2	15	2	2	1	3	1
1	9	2481	2	15	2	2	2	3	1
1	9	2482	2	16	2	2	2	3	1
1	9	2483	2	16	2	2	2	3	1
1	9	2484	2	15	2	2	2	3	1
1	9	2485	2	16	2	2	2	3	1
1	9	2486	1	16	2	2	2	3	1
1	9	2487	2	15	2	2	2	3	1
1	9	2488	2	15	2	2	2	3	1
1	9	2489	1	9	1	2	2	3	1
1	9	2490	9	0	#NULL!	9	9	9	9

1	9	2491	2	16	2	2	2	3	2
1	9	2492	2	16	2	2	2	3	1
1	9	2493	2	15	2	2	2	3	2
1	9	2494	1	0	#NULL!	2	2	3	1
1	9	2495	2	16	2	2	2	3	1
1	9	2496	9	0	#NULL!	9	9	9	9
1	9	2497	2	16	2	2	2	3	1
1	9	2498	1	18	2	2	3	3	1
1	9	2499	1	17	2	2	3	3	1
1	9	2500	1	19	2	2	3	3	1
1	9	2501	1	19	2	2	3	3	1
1	9	2502	1	15	2	2	1	3	1
1	9	2503	1	17	2	2	3	3	1
1	9	2504	2	17	2	2	3	3	1
1	9	2505	1	17	2	2	3	3	1
1	9	2506	1	18	2	2	3	3	1
1	9	2507	1	18	2	2	3	3	1
1	9	2508	1	14	1	2	3	3	1
1	9	2509	2	17	2	2	3	3	1
1	9	2510	1	15	2	2	3	3	1
1	9	2511	2	16	2	2	3	3	1
1	9	2512	2	16	2	2	3	3	1
1	9	2513	2	16	2	2	3	3	1
1	9	2514	1	19	2	2	3	3	1
1	9	2515	1	19	2	2	3	3	1
1	9	2516	2	16	2	2	3	3	1
1	9	2517	2	16	2	2	3	3	1
1	9	2518	2	16	2	2	3	3	1
1	9	2519	2	14	1	2	3	3	1
1	9	2520	2	16	2	2	3	3	1
1	9	2521	1	17	2	2	3	3	1
1	9	2522	2	16	2	2	3	3	1
1	9	2523	2	16	2	2	3	3	2
1	9	2524	2	16	2	2	3	3	2
1	9	2525	2	17	2	2	3	3	1
1	9	2526	2	16	2	2	3	3	1
1	9	2527	2	17	2	2	3	3	1
1	9	2528	2	16	2	2	3	3	1
1	9	2529	2	16	2	2	3	3	1
1	9	2530	2	17	2	2	3	3	1
1	9	2531	2	16	2	2	3	3	1
1	9	2532	2	15	2	2	3	3	1
1	9	2533	2	15	2	2	3	3	1
1	9	2534	2	17	2	2	3	3	1
1	9	2535	2	16	2	2	3	3	1
1	9	2536	2	17	2	2	3	3	1
1	9	2537	2	17	2	2	3	3	1

1	9	2538	2	20	3	2	3	3	1
1	9	2539	2	15	2	2	3	3	1
1	9	2540	2	19	2	2	3	3	1
1	9	2541	2	17	2	2	3	3	1
1	9	2542	2	16	2	2	3	3	1
1	9	2543	2	16	2	2	2	3	1
1	9	2544	2	16	2	2	1	3	1
1	9	2545	1	15	2	2	1	3	1
1	9	2546	2	14	1	1	1	3	1
1	9	2547	1	15	2	2	1	3	2
1	9	2548	2	19	2	2	2	3	1
1	9	2549	1	18	2	2	3	2	1
1	9	2550	2	18	2	2	3	3	1
1	9	2551	2	15	2	2	3	3	1
1	9	2552	1	19	2	2	3	2	1
1	9	2553	1	18	2	2	3	2	1
1	9	2554	1	15	2	2	3	2	1
1	9	2555	1	17	2	2	3	2	1
1	9	2556	1	16	2	2	3	2	2
1	9	2557	2	17	2	2	3	3	1
1	9	2558	2	16	2	2	3	3	1
1	9	2559	2	17	2	2	3	3	1
1	9	2560	2	17	2	2	3	3	1
1	9	2561	2	18	2	2	3	3	1
1	9	2562	1	16	2	2	3	3	1
1	9	2563	2	16	2	2	3	3	1
1	9	2564	2	16	2	2	3	3	1
1	9	2565	1	17	2	2	3	3	1
1	9	2566	1	17	2	2	2	3	1
1	9	2567	2	15	2	2	2	3	2
1	9	2568	2	16	2	2	2	3	1
1	9	2569	1	0	#NULL!	2	2	3	1
1	9	2570	2	19	2	2	2	3	1
1	9	2571	1	14	1	2	1	3	1
1	9	2572	1	15	2	2	1	3	1
1	9	2573	2	15	2	2	1	3	1
1	9	2574	2	15	2	2	2	3	1
1	9	2575	2	16	2	2	2	3	1
1	9	2576	2	15	2	2	2	3	1
1	9	2577	2	16	2	2	2	3	1
1	9	2578	1	16	2	2	2	2	1
1	9	2579	2	16	2	2	2	3	1
1	9	2580	2	16	2	2	2	3	1
1	9	2581	1	16	2	2	2	2	1
1	9	2582	1	16	2	2	2	3	1
1	9	2583	2	18	2	2	2	3	2
1	9	2584	2	15	2	2	2	3	1

1	9	2585	2	15	2	2	2	3	1
1	9	2586	2	16	2	2	2	3	1
1	9	2587	2	0	#NULL!	2	2	3	1
1	9	2588	2	16	2	2	2	3	1
1	9	2589	2	17	2	2	2	3	1
1	9	2590	1	0	#NULL!	2	1	3	1
1	9	2591	2	15	2	2	2	2	2
1	9	2592	2	16	2	2	2	3	1
1	9	2593	2	15	2	2	2	3	3
1	9	2594	2	16	2	2	1	3	1
1	9	2595	2	17	2	2	1	3	1
1	9	2596	2	17	2	2	3	3	1
1	9	2597	1	20	3	2	2	3	1
1	9	2598	1	16	2	2	2	3	1
1	9	2599	2	16	2	2	2	3	1
1	9	2600	2	14	1	2	1	3	1
1	9	2601	1	0	#NULL!	2	1	3	1
1	9	2602	2	0	#NULL!	2	1	3	1
1	9	2603	2	15	2	2	1	3	1
1	9	2604	2	15	2	2	1	3	1
1	9	2605	2	15	2	2	1	3	1
1	9	2606	2	18	2	2	2	3	1
1	9	2607	1	16	2	2	2	3	1
1	9	2608	2	16	2	2	2	3	1
1	9	2609	2	19	2	2	2	1	1
1	9	2610	2	16	2	2	2	3	1
1	9	2611	1	19	2	2	2	2	1
1	9	2612	1	16	2	2	2	3	1
1	9	2613	2	14	1	2	2	3	1
1	9	2614	9	16	2	2	3	3	1
1	9	2615	2	17	2	2	1	3	1
1	9	2616	2	15	2	2	1	3	1
1	9	2617	1	15	2	2	1	3	1
1	9	2618	1	15	2	2	1	3	2
1	9	2619	1	15	2	2	1	3	2
1	9	2620	1	16	2	2	1	3	1
1	9	2621	1	16	2	2	1	3	1
1	9	2622	1	16	2	2	1	3	1
1	9	2623	1	18	2	2	1	3	1
1	9	2624	2	14	1	2	1	3	1
1	9	2625	2	17	2	2	1	3	1
1	9	2626	2	14	1	2	1	3	1
1	9	2627	2	15	2	2	1	3	1
1	9	2628	2	15	2	2	1	3	1
1	9	2629	2	15	2	2	1	3	1
1	9	2630	1	14	1	2	1	3	1
1	9	2631	1	15	2	2	1	3	1

1	9	2632	1	15	2	2	1	3	1
1	9	2633	1	16	2	2	1	3	1
1	9	2634	2	14	1	2	1	3	1
1	9	2635	2	15	2	2	1	3	1
1	9	2636	2	15	2	2	1	3	1
1	9	2637	2	20	3	2	3	3	1
1	9	2638	2	19	2	2	3	3	1
1	9	2639	1	16	2	2	3	3	1
1	9	2640	2	17	2	2	1	3	1
1	9	2641	1	13	1	2	1	3	2
1	9	2642	2	14	1	2	1	3	1
1	9	2643	2	14	1	2	1	3	1
1	9	2644	1	14	1	2	1	3	1
1	9	2645	2	0	#NULL!	2	1	3	9
1	9	2646	1	15	2	2	1	3	2
1	9	2647	2	15	2	2	3	3	1
1	9	2648	2	17	2	2	1	3	1
1	9	2649	1	22	3	2	3	2	1
1	9	2650	1	18	2	2	3	2	1
1	9	2651	1	19	2	2	3	2	1
1	9	2652	2	17	2	2	3	3	1
1	9	2653	2	17	2	2	3	3	1
1	9	2654	2	18	2	2	3	3	1
1	9	2655	2	18	2	2	3	2	2
1	9	2656	2	18	2	2	3	2	2
1	9	2657	1	13	1	2	1	3	1
1	9	2658	2	13	1	2	1	3	1
1	9	2659	1	13	1	2	1	3	2
1	9	2660	1	18	2	2	3	3	1
1	9	2661	1	19	2	2	3	2	1
1	9	2662	1	16	2	2	2	3	1
1	9	2663	9	0	#NULL!	9	9	9	9
1	9	2664	9	0	#NULL!	2	3	1	1
1	9	2665	2	17	2	2	1	3	1
1	9	2666	1	15	2	2	1	3	1
1	9	2667	2	14	1	2	1	3	1
1	9	2668	1	15	2	2	1	3	1
1	9	2669	1	14	1	2	1	3	1
1	9	2670	2	14	1	2	1	3	1
1	9	2671	1	16	2	2	1	3	1
1	9	2672	2	16	2	2	1	3	1
1	9	2673	2	16	2	2	1	3	1
1	9	2674	2	15	2	2	1	3	1
1	9	2675	2	14	1	2	1	3	1
1	9	2676	1	17	2	2	3	3	1
1	9	2677	1	14	1	2	1	3	1
1	9	2678	2	15	2	2	1	3	1

1	9	2679	2	18	2	2	1	3	1
1	9	2680	2	13	1	2	1	3	1
1	9	2681	2	15	2	2	1	3	1
1	9	2682	1	14	1	2	1	3	1
1	9	2683	2	15	2	2	1	3	1
1	9	2684	2	14	1	2	3	3	1
1	9	2685	2	15	2	2	3	3	1
2	2	1	1	16	2	2	2	3	2
2	2	2	1	16	2	1	2	3	2
2	2	3	1	15	2	2	1	3	2
2	2	4	2	15	2	9	2	3	2
2	2	5	2	18	2	2	3	3	9
2	2	6	2	16	2	2	2	3	1
2	2	7	2	16	2	2	2	1	2
2	2	8	2	15	2	2	2	1	1
2	2	9	2	15	2	2	2	1	1
2	2	10	2	15	2	2	2	1	1
2	2	11	2	14	1	2	2	1	1
2	2	12	2	15	2	2	2	1	2
2	2	13	2	14	1	2	2	1	1
2	2	14	2	17	2	2	2	1	1
2	2	15	2	15	2	2	2	1	1
2	2	16	2	16	2	2	2	1	2
2	2	17	2	0	#NULL!	2	2	1	1
2	2	18	2	16	2	2	2	1	1
2	2	19	2	17	2	2	2	1	1
2	2	20	2	15	2	2	2	1	1
2	2	21	2	17	2	2	3	1	1
2	2	22	2	17	2	2	3	1	1
2	2	23	2	17	2	2	3	1	1
2	2	24	2	17	2	2	3	1	2
2	2	25	2	14	1	2	1	1	3
2	2	26	2	14	1	2	1	1	2
2	2	27	2	13	1	2	1	1	1
2	2	28	2	15	2	2	1	1	1
2	2	29	2	14	1	2	1	1	1
2	2	30	2	15	2	2	1	1	1
2	2	31	2	13	1	2	1	1	1
2	2	32	2	15	2	2	1	1	1
2	2	33	2	14	1	2	1	3	1
2	2	34	2	16	2	2	1	1	1
2	2	35	2	13	1	2	1	1	1
2	2	36	2	15	2	2	1	1	1
2	2	37	2	15	2	2	1	1	1
2	2	38	2	15	2	2	1	1	1
2	2	39	2	15	2	2	1	1	1
2	2	40	2	15	2	2	2	1	1

2	2	41	2	15	2	2	3	3	1
2	2	42	2	16	2	2	3	3	1
2	2	43	2	15	2	2	3	3	1
2	2	44	2	17	2	2	3	3	1
2	2	45	2	13	1	2	1	3	1
2	2	46	9	14	1	2	1	3	1
2	2	47	2	14	1	2	1	3	1
2	2	48	2	10	1	2	1	9	1
2	2	49	2	14	1	2	1	1	1
2	2	50	2	13	1	2	1	3	1
2	2	51	2	16	2	2	1	3	1
2	2	52	2	14	1	2	1	3	1
2	2	53	1	16	2	2	1	3	1
2	2	54	2	18	2	2	1	3	2
2	2	55	2	29	3	2	1	3	1
2	2	56	1	15	2	2	1	3	1
2	2	57	2	13	1	2	1	3	1
2	2	58	2	14	1	2	1	3	2
2	2	59	1	16	2	2	2	2	2
2	2	60	1	15	2	2	1	3	1
2	2	61	2	15	2	2	2	3	1
2	2	62	2	14	1	2	2	3	2
2	2	63	1	15	2	2	1	3	1
2	2	64	2	16	2	2	1	3	1
2	2	65	2	14	1	2	1	3	1
2	2	66	1	0	#NULL!	2	1	3	3
2	2	67	2	16	2	2	1	3	3
2	2	68	2	16	2	2	1	3	1
2	2	69	2	16	2	2	2	3	1
2	2	70	1	16	2	2	2	3	1
2	2	71	1	15	2	2	2	3	2
2	2	72	9	0	#NULL!	2	1	3	2
2	2	73	2	15	2	2	1	3	1
2	2	74	1	15	2	2	1	3	1
2	2	75	2	15	2	2	1	3	1
2	2	76	2	15	2	2	1	3	1
2	2	77	2	13	1	2	1	3	1
2	2	78	2	14	1	2	1	3	1
2	2	79	2	15	2	2	1	3	1
2	2	80	1	13	1	2	1	3	1
2	2	81	2	0	#NULL!	2	1	3	1
2	2	82	1	16	2	2	1	3	1
2	2	83	2	14	1	2	1	3	1
2	2	84	2	14	1	2	1	3	1
2	2	85	2	15	2	2	1	3	1
2	2	86	1	15	2	2	1	3	1
2	2	87	2	16	2	2	1	3	1

2	2	88	9	15	2	2	1	3	1
2	2	89	1	16	2	2	1	3	1
2	2	90	2	13	1	2	1	3	1
2	2	91	1	16	2	2	1	3	1
2	2	92	1	18	2	2	1	3	1
2	2	93	2	16	2	2	1	3	1
2	2	94	2	19	2	2	1	3	1
2	2	95	2	15	2	2	1	3	1
2	2	96	1	16	2	2	1	3	1
2	2	97	2	11	1	2	1	3	1
2	2	98	1	19	2	2	1	3	1
2	2	99	2	16	2	2	1	3	1
2	2	100	2	15	2	2	1	3	1
2	2	101	1	19	2	2	1	3	1
2	2	102	2	15	2	2	1	3	1
2	2	103	2	16	2	2	1	3	1
2	2	104	2	14	1	2	1	3	1
2	2	105	2	15	2	2	2	3	1
2	2	106	2	15	2	2	2	3	2
2	2	107	2	16	2	2	1	3	1
2	2	108	2	16	2	2	1	3	2
2	2	109	2	15	2	2	1	3	2
2	2	110	1	17	2	2	2	3	1
2	2	111	2	16	2	2	1	3	1
2	2	112	2	17	2	2	1	3	1
2	2	113	1	14	1	2	1	3	1
2	2	114	2	14	1	2	1	3	1
2	2	115	2	15	2	2	1	3	1
2	2	116	1	15	2	2	2	3	1
2	2	117	9	17	2	2	1	3	3
2	2	118	2	15	2	2	1	3	2
2	2	119	2	16	2	2	1	3	1
2	2	120	1	15	2	2	2	3	1
2	2	121	2	16	2	2	1	3	1
2	2	122	2	15	2	2	1	3	1
2	2	123	1	14	1	2	1	3	1
2	2	124	2	15	2	2	2	3	2
2	2	125	2	16	2	2	2	3	2
2	2	126	2	16	2	2	2	3	2
2	2	127	2	17	2	2	2	1	1
2	2	128	2	16	2	2	2	3	1
2	2	129	2	15	2	2	2	3	1
2	2	130	1	16	2	2	2	3	1
2	2	131	2	15	2	2	1	3	1
2	2	132	2	14	1	2	1	3	1
2	2	133	2	13	1	2	1	3	3
2	2	134	2	16	2	2	2	3	2

2	2	135	9	0	#NULL!	9	9	9	9
2	2	136	1	15	2	2	2	3	2
2	2	137	1	15	2	2	2	3	2
2	2	138	9	14	1	2	1	3	1
2	2	139	2	14	1	2	1	3	1
2	2	140	2	16	2	2	1	3	1
2	2	141	2	19	2	2	1	3	1
2	2	142	1	13	1	2	1	3	1
2	2	143	1	15	2	2	2	3	2
2	2	144	1	15	2	2	1	3	1
2	2	145	1	17	2	2	1	3	1
2	2	146	1	17	2	2	1	3	1
2	2	147	2	16	2	2	1	9	1
2	2	148	2	19	2	2	1	3	2
2	2	149	1	15	2	2	1	3	1
2	2	150	1	16	2	2	1	3	1
2	2	151	1	16	2	2	1	3	1
2	2	152	2	14	1	2	1	3	1
2	2	153	2	0	#NULL!	2	1	3	1
2	2	154	1	20	3	2	1	3	2
2	2	155	1	16	2	2	2	3	2
2	2	156	1	16	2	2	2	3	2
2	2	157	1	16	2	2	2	3	2
2	2	158	1	16	2	2	2	3	2
2	2	159	1	17	2	2	1	3	1
2	2	160	2	15	2	2	1	3	1
2	2	161	2	16	2	2	1	3	2
2	2	162	1	17	2	2	3	2	1
2	2	163	1	19	2	2	3	2	2
2	2	164	1	19	2	2	3	2	3
2	2	165	1	18	2	2	3	2	1
2	2	166	1	15	2	2	1	3	1
2	2	167	1	19	2	2	3	2	1
2	2	168	1	15	2	2	1	3	1
2	2	169	1	16	2	2	1	3	1
2	2	170	1	16	2	2	2	3	1
2	2	171	9	0	#NULL!	9	9	9	9
2	2	172	1	14	1	2	1	3	2
2	2	173	1	18	2	2	1	3	1
2	2	174	1	19	2	9	2	9	9
2	2	175	1	13	1	2	1	3	1
2	2	176	2	18	2	2	1	3	1
2	2	177	1	40	3	2	1	2	1
2	2	178	2	14	1	2	1	3	1
2	2	179	2	15	2	2	2	3	1
2	2	180	2	17	2	1	3	1	2
2	2	181	1	17	2	1	1	1	1

2	2	182	2	14	1	2	1	3	1
2	2	183	1	19	2	2	1	3	1
2	2	184	9	0	#NULL!	9	9	9	9
2	2	185	9	0	#NULL!	9	9	9	9
2	2	186	2	15	2	2	2	3	2
2	2	187	1	17	2	2	1	2	1
2	2	188	1	17	2	2	1	2	3
2	2	189	1	0	#NULL!	2	3	2	1
2	2	190	1	18	2	2	3	2	1
2	2	191	1	17	2	2	3	2	1
2	2	192	1	17	2	2	3	2	1
2	2	193	1	18	2	2	3	2	1
2	2	194	1	17	2	2	3	2	1
2	2	195	1	18	2	2	3	2	2
2	2	196	9	0	#NULL!	2	3	2	1
2	2	197	1	18	2	2	1	2	2
2	2	198	1	14	1	2	1	2	1
2	2	199	9	0	#NULL!	2	1	1	1
2	2	200	1	17	2	2	1	2	1
2	2	201	1	18	2	9	9	9	9
2	2	202	1	20	3	2	2	2	3
2	2	203	1	16	2	2	1	2	1
2	2	204	9	0	#NULL!	2	2	9	1
2	2	205	1	18	2	9	1	2	1
2	2	206	1	18	2	2	3	2	3
2	2	207	1	18	2	2	3	3	1
2	2	208	1	17	2	2	3	2	1
2	2	209	1	17	2	2	3	2	1
2	2	210	1	17	2	2	3	2	1
2	2	211	9	19	2	2	3	2	1
2	2	212	1	19	2	2	3	2	1
2	2	213	1	17	2	2	1	2	1
2	2	214	9	9	1	2	1	2	1
2	2	215	1	17	2	2	1	2	1
2	2	216	9	0	#NULL!	9	9	9	9
2	2	217	1	16	2	2	1	2	1
2	2	218	1	18	2	2	1	2	1
2	2	219	1	18	2	2	3	2	1
2	2	220	1	18	2	2	3	2	1
2	2	221	1	19	2	2	3	2	2
2	2	222	1	18	2	2	3	3	2
2	2	223	1	14	1	2	1	2	2
2	2	224	1	14	1	2	1	2	1
2	2	225	1	15	2	2	1	2	1
2	2	226	1	14	1	2	1	2	1
2	2	227	1	19	2	2	1	1	1
2	2	228	1	17	2	2	1	2	1

2	2	229	1	13	1	2	1	2	1
2	2	230	1	15	2	2	1	2	1
2	2	231	1	15	2	2	1	2	2
2	2	232	1	17	2	2	1	2	1
2	2	233	9	0	#NULL!	9	1	2	2
2	2	234	1	0	#NULL!	2	1	2	2
2	2	235	9	0	#NULL!	2	3	2	1
2	2	236	1	19	2	2	3	2	1
2	2	237	1	22	3	2	3	2	1
2	2	238	1	17	2	2	3	2	1
2	2	239	1	14	1	2	3	2	1
2	2	240	1	17	2	2	3	2	1
2	2	241	1	17	2	2	3	2	9
2	2	242	1	40	3	2	1	2	2
2	2	243	1	16	2	2	1	2	1
2	2	244	1	14	1	2	1	2	1
2	2	245	1	17	2	2	1	2	1
2	2	246	1	15	2	2	1	2	1
2	2	247	1	14	1	2	1	2	1
2	2	248	1	18	2	2	3	2	1
2	2	249	1	16	2	2	2	3	2
2	2	250	9	0	#NULL!	9	9	9	9
2	2	251	1	14	1	2	1	2	2
2	2	252	1	14	1	2	1	2	2
2	2	253	1	16	2	2	2	2	2
2	2	254	9	0	#NULL!	9	9	9	9
2	2	255	9	0	#NULL!	9	9	9	9
2	2	256	1	14	1	2	2	2	2
2	2	257	1	16	2	2	2	2	2
2	2	258	1	17	2	2	3	2	3
2	2	259	1	16	2	2	2	2	2
2	2	260	1	16	2	2	2	2	2
2	2	261	1	16	2	2	2	2	2
2	2	262	9	0	#NULL!	9	9	9	9
2	2	263	9	0	#NULL!	9	9	9	9
2	2	264	1	16	2	2	2	2	1
2	2	265	9	0	#NULL!	9	9	9	9
2	2	266	1	18	2	2	3	2	2
2	2	267	1	16	2	2	2	3	2
2	2	268	1	19	2	2	3	2	2
2	2	269	1	15	2	2	1	2	2
2	2	270	1	17	2	2	2	2	2
2	2	271	1	18	2	2	3	2	2
2	2	272	1	19	2	2	3	2	2
2	2	273	1	17	2	2	3	2	2
2	2	274	1	0	#NULL!	2	2	9	1
2	2	275	9	0	#NULL!	9	9	9	9

2	2	276	9	0	#NULL!	2	2	2	1
2	2	277	1	10	1	1	1	1	1
2	2	278	1	17	2	2	2	2	1
2	2	279	1	17	2	1	2	2	1
2	2	280	1	17	2	2	2	2	1
2	2	281	9	0	#NULL!	2	2	2	1
2	2	282	1	18	2	2	2	2	1
2	2	283	1	18	2	2	2	2	1
2	2	284	1	17	2	2	2	2	1
2	2	285	1	19	2	9	2	2	1
2	2	286	1	0	#NULL!	1	3	1	1
2	2	287	1	13	1	2	1	2	1
2	2	288	1	15	2	2	1	2	1
2	2	289	1	15	2	2	1	2	1
2	2	290	9	0	#NULL!	9	9	9	9
2	2	291	1	20	3	1	1	2	2
2	2	292	9	0	#NULL!	9	9	9	9
2	2	293	9	0	#NULL!	9	9	9	9
2	2	294	2	16	2	2	3	1	1
2	2	295	1	19	2	2	2	2	1
2	2	296	2	16	2	2	2	1	1
2	2	297	1	18	2	2	2	2	2
2	2	298	2	15	2	2	3	1	1
2	2	299	1	18	2	2	3	2	1
2	2	300	1	17	2	2	3	2	1
2	2	301	1	19	2	2	3	2	2
2	2	302	1	27	3	2	3	2	1
2	2	303	1	18	2	2	3	2	1
2	2	304	1	20	3	2	3	2	1
2	2	305	1	18	2	2	3	2	1
2	2	306	1	15	2	2	3	2	1
2	2	307	1	20	3	2	3	2	1
2	2	308	1	12	1	2	3	2	1
2	2	309	1	18	2	2	3	2	1
2	2	310	1	19	2	2	3	2	3
2	2	311	1	18	2	2	3	2	1
2	2	312	1	17	2	2	3	2	1
2	2	313	1	18	2	2	3	3	1
2	2	314	1	19	2	2	3	2	3
2	2	315	1	20	3	2	3	2	3
2	2	316	1	20	3	2	3	2	2
2	2	317	1	20	3	2	3	2	1
2	2	318	1	16	2	2	3	2	3
2	2	319	1	18	2	2	2	2	1
2	2	320	9	19	2	2	2	2	2
2	2	321	9	18	2	2	2	2	2
2	2	322	1	18	2	2	2	2	2

2	2	323	9	0	#NULL!	2	2	2	1
2	2	324	1	19	2	2	2	2	2
2	2	325	1	18	2	2	2	2	1
2	2	326	1	17	2	2	2	2	1
2	2	327	2	13	1	2	2	2	2
2	2	328	1	15	2	2	2	2	1
2	2	329	1	13	1	2	2	2	1
2	2	330	1	18	2	2	1	2	1
2	2	331	9	16	2	2	2	2	1
2	2	332	1	18	2	2	2	2	1
2	2	333	1	21	3	2	2	2	1
2	2	334	1	18	2	2	2	2	2
2	2	335	1	18	2	2	1	2	1
2	2	336	1	17	2	2	1	2	1
2	2	337	1	15	2	2	1	2	2
2	2	338	9	28	3	2	1	2	1
2	2	339	1	16	2	2	2	2	1
2	2	340	1	17	2	2	2	2	1
2	2	341	1	16	2	2	2	2	2
2	2	342	1	17	2	2	2	2	2
2	2	343	1	19	2	2	2	2	1
2	2	344	1	17	2	2	2	2	1
2	2	345	1	16	2	2	2	2	2
2	2	346	1	18	2	2	1	3	2
2	2	347	1	16	2	2	1	3	1
2	2	348	1	15	2	2	1	3	1
2	2	349	2	14	1	2	1	3	1
2	2	350	2	14	1	2	1	3	1
2	2	351	1	14	1	2	1	3	2
2	2	352	2	14	1	2	1	3	2
2	2	353	2	15	2	2	1	3	1
2	2	354	2	16	2	2	1	1	1
2	2	355	2	17	2	2	1	3	9
2	2	356	1	16	2	2	1	3	1
2	2	357	1	14	1	2	1	3	1
2	2	358	1	17	2	2	1	3	1
2	2	359	1	14	1	2	1	3	1
2	2	360	1	15	2	2	1	3	1
2	2	361	9	17	2	2	1	3	1
2	2	362	2	16	2	2	3	3	2
2	2	363	1	19	2	2	3	3	2
2	2	364	2	16	2	2	3	3	1
2	2	365	1	0	#NULL!	2	3	3	1
2	2	366	2	18	2	2	3	3	3
2	2	367	2	16	2	2	3	3	1
2	2	368	1	12	1	2	3	3	1
2	2	369	2	22	3	2	3	3	1

2	2	370	2	16	2	2	2	3	9
2	2	371	2	16	2	2	2	3	1
2	2	372	2	17	2	2	2	3	1
2	2	373	1	16	2	9	2	3	1
2	2	374	2	17	2	2	2	3	1
2	2	375	1	16	2	2	2	3	1
2	2	376	1	16	2	2	2	3	1
2	2	377	1	15	2	2	2	3	1
2	2	378	2	14	1	1	1	3	1
2	2	379	2	14	1	2	2	3	2
2	2	380	1	20	3	1	3	2	2
2	2	381	2	18	2	2	3	3	1
2	2	382	1	0	#NULL!	2	2	3	1
2	2	383	2	0	#NULL!	2	2	3	1
2	2	384	1	18	2	2	2	3	2
2	2	385	2	15	2	2	2	3	1
2	2	386	2	16	2	2	2	3	1
2	2	387	2	16	2	2	2	3	1
2	2	388	2	16	2	2	2	3	1
2	2	389	2	15	2	2	2	3	1
2	2	390	2	15	2	2	2	3	1
2	2	391	2	16	2	2	2	3	3
2	2	392	9	0	#NULL!	9	9	9	9
2	2	393	2	15	2	2	2	1	2
2	2	394	2	15	2	2	1	1	1
2	2	395	2	14	1	2	2	3	1
2	2	396	2	16	2	2	2	3	1
2	2	397	2	14	1	2	2	3	2
2	2	398	2	15	2	2	1	1	1
2	2	399	2	15	2	2	1	1	1
2	2	400	2	14	1	2	1	1	1
2	2	401	2	15	2	2	1	1	1
2	2	402	2	15	2	2	1	1	1
2	2	403	2	19	2	2	1	1	1
2	2	404	2	15	2	2	1	1	2
2	2	405	2	15	2	2	1	1	1
2	2	406	9	0	#NULL!	9	9	9	9
2	2	407	2	14	1	2	1	1	1
2	2	408	2	15	2	2	1	1	1
2	2	409	2	17	2	2	2	1	1
2	2	410	2	16	2	2	2	1	1
2	2	411	2	16	2	2	2	1	2
2	2	412	2	17	2	2	2	1	1
2	2	413	2	16	2	2	2	1	1
2	2	414	2	17	2	2	2	1	1
2	2	415	2	16	2	2	1	1	1
2	2	416	2	15	2	2	1	1	1

2	2	417	2	15	2	2	1	1	1
2	2	418	2	15	2	2	1	1	1
2	2	419	2	15	2	2	1	1	1
2	2	420	9	0	#NULL!	9	9	9	9
2	2	421	2	15	2	9	1	1	1
2	2	422	2	15	2	2	1	1	1
2	2	423	2	14	1	2	1	1	1
2	2	424	2	14	1	2	1	1	1
2	2	425	2	15	2	2	1	1	1
2	2	426	2	15	2	2	1	1	1
2	2	427	2	18	2	2	3	1	2
2	2	428	2	18	2	2	3	1	1
2	2	429	1	16	2	2	2	3	2
2	2	430	1	16	2	2	2	3	2
2	2	431	1	17	2	2	2	3	3
2	2	432	1	14	1	2	1	3	9
2	2	433	1	14	1	2	1	3	1
2	2	434	1	14	1	2	1	3	2
2	2	435	1	15	2	2	1	3	1
2	2	436	1	14	1	2	1	3	2
2	2	437	1	15	2	2	1	3	1
2	2	438	1	20	3	2	3	3	2
2	2	439	1	19	2	2	3	3	3
2	2	440	1	19	2	2	3	3	1
2	2	441	2	18	2	2	3	3	1
2	2	442	1	18	2	2	3	3	1
2	2	443	1	18	2	2	3	3	9
2	2	444	1	18	2	2	3	3	2
2	2	445	2	19	2	2	3	3	2
2	2	446	1	19	2	2	3	3	2
2	2	447	9	0	#NULL!	9	9	9	9
2	2	448	2	18	2	2	2	3	1
2	2	449	2	17	2	2	2	3	1
2	2	450	2	18	2	2	2	3	1
2	2	451	2	18	2	2	2	3	1
2	2	452	2	17	2	2	2	3	2
2	2	453	2	17	2	2	2	3	3
2	2	454	2	17	2	2	2	3	1
2	2	455	2	16	2	2	2	3	2
2	2	456	2	17	2	2	2	3	3
2	2	457	2	18	2	2	2	3	2
2	2	458	9	0	#NULL!	9	9	9	9
2	2	459	2	17	2	2	2	3	3
2	2	460	1	14	1	2	1	3	2
2	2	461	2	15	2	2	1	1	1
2	2	462	2	15	2	2	1	1	1
2	2	463	2	13	1	2	1	1	1

2	2	464	2	17	2	2	1	1	1
2	2	465	2	15	2	2	1	1	1
2	2	466	2	15	2	2	1	1	1
2	2	467	9	0	#NULL!	9	1	1	1
2	2	468	2	15	2	2	1	1	1
2	2	469	2	15	2	2	1	1	1
2	2	470	2	16	2	2	1	1	1
2	2	471	2	14	1	2	1	1	1
2	2	472	2	14	1	2	1	1	1
2	2	473	2	13	1	2	1	1	1
2	2	474	2	14	1	2	1	1	1
2	2	475	2	15	2	2	1	1	1
2	2	476	9	0	#NULL!	9	9	9	9
2	2	477	2	0	#NULL!	2	1	1	1
2	2	478	2	15	2	2	1	1	1
2	2	479	2	16	2	2	1	1	1
2	2	480	2	14	1	9	1	1	1
2	2	481	2	14	1	2	1	1	1
2	2	482	2	13	1	2	1	1	1
2	2	483	9	14	1	2	1	1	2
2	2	484	9	18	2	2	1	1	1
2	2	485	2	17	2	2	3	1	1
2	2	486	2	15	2	2	1	1	1
2	2	487	2	16	2	2	1	1	1
2	2	488	2	16	2	2	1	1	1
2	2	489	2	14	1	2	1	1	1
2	2	490	2	15	2	2	1	1	2
2	2	491	2	14	1	2	1	1	1
2	2	492	2	15	2	2	1	1	1
2	2	493	2	12	1	2	1	1	1
2	2	494	2	16	2	2	1	1	1
2	2	495	2	16	2	2	1	1	1
2	2	496	2	14	1	2	1	1	1
2	2	497	2	14	1	2	1	1	1
2	2	498	2	15	2	1	1	1	1
2	2	499	2	17	2	2	1	1	1
2	2	500	2	18	2	2	1	1	1
2	2	501	9	0	#NULL!	2	1	1	2
2	2	502	2	17	2	2	1	1	1
2	2	503	2	16	2	2	1	1	1
2	2	504	2	16	2	2	1	1	1
2	2	505	2	16	2	2	1	1	1
2	2	506	2	15	2	2	1	1	9
2	2	507	2	15	2	2	1	1	1
2	2	508	2	15	2	2	1	1	1
2	2	509	2	13	1	2	1	1	1
2	2	510	2	15	2	2	1	1	1

2	2	511	2	15	2	2	1	3	1
2	2	512	2	14	1	2	1	1	1
2	2	513	2	15	2	2	1	1	1
2	2	514	2	16	2	2	1	1	1
2	2	515	2	16	2	2	1	1	2
2	2	516	2	16	2	2	1	1	1
2	2	517	2	14	1	2	1	1	1
2	2	518	2	14	1	2	1	1	1
2	2	519	2	15	2	2	1	1	1
2	2	520	2	16	2	2	1	1	1
2	2	521	2	14	1	2	1	1	1
2	2	522	2	0	#NULL!	9	2	9	1
2	2	523	9	0	#NULL!	9	2	9	1
2	2	524	2	16	2	2	3	1	1
2	2	525	2	18	2	2	3	1	1
2	2	526	2	0	#NULL!	2	3	1	1
2	2	527	2	15	2	2	3	1	1
2	2	528	2	12	1	2	3	1	1
2	2	529	2	17	2	2	3	1	1
2	2	530	2	17	2	2	3	1	2
2	2	531	2	17	2	2	3	1	2
2	2	532	1	16	2	2	2	3	2
2	2	533	1	17	2	2	2	3	3
2	2	534	1	0	#NULL!	2	3	3	9
2	2	535	1	18	2	2	3	3	2
2	2	536	1	18	2	2	3	3	3
2	2	537	1	19	2	2	3	3	2
2	2	538	1	18	2	2	3	3	9
2	2	539	1	19	2	2	3	3	2
2	2	540	1	0	#NULL!	2	2	3	2
2	2	541	1	16	2	2	2	3	2
2	2	542	1	16	2	2	2	3	2
2	2	543	1	17	2	2	2	3	9
2	2	544	2	16	2	2	2	3	1
2	2	545	1	17	2	2	2	3	2
2	2	546	1	16	2	2	2	3	2
2	2	547	1	17	2	2	2	3	2
2	2	548	1	17	2	2	2	3	2
2	2	549	1	16	2	2	2	3	2
2	2	550	1	17	2	2	2	3	2
2	2	551	1	19	2	2	3	3	3
2	2	552	1	20	3	2	3	3	2
2	2	553	1	19	2	2	3	3	2
2	2	554	2	18	2	2	3	3	9
2	2	555	2	17	2	2	3	3	2
2	2	556	2	18	2	2	3	3	3
2	2	557	2	18	2	2	3	3	1

2	2	558	2	20	3	2	3	3	1
2	2	559	2	19	2	2	3	3	2
2	2	560	2	18	2	2	3	3	3
2	2	561	1	14	1	2	1	3	2
2	2	562	1	15	2	2	1	3	2
2	2	563	9	0	#NULL!	9	9	9	9
2	2	564	2	18	2	2	2	3	2
2	2	565	2	17	2	2	2	3	3
2	2	566	2	17	2	2	2	3	2
2	2	567	2	16	2	2	2	3	1
2	2	568	2	13	1	2	1	3	2
2	2	569	2	14	1	2	1	3	2
2	2	570	2	15	2	2	1	3	2
2	2	571	2	15	2	2	1	3	1
2	2	572	9	0	#NULL!	9	9	9	9
2	2	573	9	0	#NULL!	9	9	9	9
2	2	574	9	0	#NULL!	9	9	9	9
2	2	575	9	0	#NULL!	9	9	9	9
2	2	576	9	0	#NULL!	9	9	9	9
2	2	577	9	0	#NULL!	9	9	9	9
2	2	578	1	14	1	2	1	3	2
2	2	579	1	15	2	2	1	3	2
2	2	580	1	15	2	2	1	3	1
2	2	581	2	16	2	2	2	3	2
2	2	582	1	14	1	2	1	3	2
2	2	583	1	15	2	2	1	3	2
2	2	584	1	15	2	2	1	3	2
2	2	585	2	14	1	2	1	3	1
2	2	586	2	15	2	2	1	3	1
2	2	587	1	15	2	2	1	3	2
2	2	588	1	14	1	2	1	3	2
2	2	589	1	15	2	2	1	3	1
2	2	590	2	18	2	2	3	3	3
2	2	591	2	19	2	2	2	3	2
2	2	592	2	0	#NULL!	2	2	3	2
2	2	593	2	18	2	2	2	3	1
2	2	594	2	16	2	2	2	3	1
2	2	595	2	17	2	2	2	3	2
2	2	596	2	17	2	2	2	3	3
2	2	597	2	17	2	2	2	3	1
2	2	598	2	18	2	2	2	3	1
2	2	599	1	14	1	2	1	3	1
2	2	600	9	18	2	2	3	3	2
2	2	601	2	0	#NULL!	2	3	3	2
2	2	602	2	19	2	2	3	3	3
2	2	603	2	19	2	2	3	3	2
2	2	604	2	18	2	2	3	3	2

2	2	605	2	18	2	2	3	3	2
2	2	606	2	18	2	2	3	3	2
2	2	607	2	18	2	2	3	3	1
2	2	608	1	19	2	2	3	3	2
2	2	609	2	17	2	2	3	3	2
2	2	610	1	18	2	2	3	3	2
2	2	611	2	18	2	2	3	3	3
2	2	612	1	19	2	2	3	3	9
2	2	613	1	17	2	2	3	3	2
2	2	614	1	20	3	2	3	3	2
2	2	615	1	18	2	2	3	3	9
2	2	616	1	19	2	2	3	3	2
2	2	617	2	18	2	2	3	3	1
2	2	618	2	18	2	2	3	3	2
2	2	619	2	18	2	2	3	3	1
2	2	620	1	19	2	2	3	3	1
2	2	621	1	18	2	2	3	3	2
2	2	622	1	19	2	2	3	3	2
2	2	623	2	17	2	2	3	1	1
2	2	624	2	16	2	2	3	1	2
2	2	625	2	15	2	2	1	1	1
2	2	626	2	15	2	2	1	1	1
2	2	627	2	16	2	2	1	1	2
2	2	628	2	15	2	2	1	1	1
2	2	629	2	14	1	2	1	1	1
2	2	630	2	16	2	2	3	1	2
2	2	631	2	17	2	2	2	1	1
2	2	632	2	15	2	2	2	1	1
2	2	633	2	14	1	2	2	1	1
2	2	634	2	16	2	2	2	1	1
2	2	635	2	15	2	2	3	1	1
2	2	636	2	17	2	2	3	1	1
2	2	637	2	17	2	2	3	1	1
2	2	638	2	16	2	2	3	1	1
2	2	639	2	16	2	2	3	1	2
2	2	640	2	16	2	2	2	1	1
2	2	641	2	16	2	2	2	1	1
2	2	642	2	16	2	2	2	1	2
2	2	643	2	15	2	2	2	1	1
2	2	644	2	14	1	2	1	1	1
2	2	645	2	14	1	2	2	1	1
2	2	646	9	0	#NULL!	9	9	9	9
2	2	647	9	0	#NULL!	9	9	9	9
2	2	648	1	19	2	2	3	3	1
2	2	649	1	15	2	2	1	3	2
2	2	650	1	18	2	2	3	3	2
2	2	651	2	16	2	2	2	3	1

2	2	652	2	18	2	2	3	3	1
2	2	653	1	18	2	2	3	3	1
2	2	654	1	16	2	2	2	3	2
2	2	655	2	14	1	2	1	3	2
2	2	656	1	16	2	2	2	3	2
2	2	657	2	16	2	2	2	3	1
2	2	658	2	15	2	2	2	3	1
2	2	659	2	16	2	2	2	3	1
2	2	660	2	15	2	2	2	3	1
2	2	661	2	16	2	2	2	3	1
2	2	662	2	16	2	9	9	9	9
2	2	663	2	15	2	2	2	3	1
2	2	664	2	15	2	2	2	3	1
2	2	665	1	18	2	2	3	3	2
2	2	666	1	17	2	2	2	3	2
2	2	667	1	18	2	2	3	3	1
2	2	668	2	18	2	2	3	3	1
2	2	669	2	16	2	2	2	3	1
2	2	670	1	14	1	2	2	3	1
2	2	671	1	17	2	2	2	3	1
2	2	672	9	0	#NULL!	9	9	9	9
2	2	673	1	16	2	2	2	3	1
2	2	674	1	16	2	2	2	3	1
2	2	675	2	17	2	2	2	3	1
2	2	676	2	14	1	2	2	3	1
2	2	677	2	16	2	2	2	3	1
2	2	678	2	16	2	2	2	3	1
2	2	679	2	15	2	9	9	9	9
2	2	680	9	16	2	2	2	3	1
2	2	681	2	16	2	2	2	3	1
2	2	682	1	15	2	2	2	3	1
2	2	683	2	16	2	2	2	3	1
2	2	684	2	16	2	2	2	3	1
2	2	685	2	15	2	2	2	3	1
2	2	686	2	15	2	2	2	3	1
2	2	687	2	17	2	2	2	3	1
2	2	688	2	15	2	2	2	3	1
2	2	689	2	16	2	9	9	9	9
2	2	690	1	16	2	2	2	3	1
2	2	691	9	0	#NULL!	9	9	9	9
2	2	692	1	16	2	2	2	3	1
2	2	693	2	15	2	2	2	3	1
2	2	694	1	14	1	2	2	3	1
2	2	695	1	18	2	2	3	3	1
2	2	696	1	16	2	2	2	3	1
2	2	697	1	17	2	2	2	3	2
2	2	698	2	16	2	2	1	3	2

2	2	699	2	14	1	2	1	3	1
2	2	700	1	16	2	2	2	3	1
2	2	701	2	16	2	2	2	3	1
2	2	702	1	15	2	2	2	3	1
2	2	703	1	16	2	2	2	3	1
2	2	704	1	18	2	2	2	3	1
2	2	705	1	15	2	2	2	3	2
2	2	706	1	20	3	2	2	2	1
2	2	707	9	0	#NULL!	2	2	3	2
2	2	708	2	16	2	9	9	9	9
2	2	709	9	16	2	2	2	3	1
2	2	710	1	17	2	2	2	3	2
2	2	711	1	17	2	2	2	2	1
2	2	712	1	19	2	2	1	3	2
2	2	713	2	16	2	9	9	9	9
2	2	714	2	14	1	2	1	3	1
2	2	715	2	17	2	2	1	3	1
2	2	716	2	15	2	2	1	3	1
2	2	717	1	17	2	2	1	3	1
2	2	718	2	15	2	2	2	3	1
2	2	719	2	16	2	2	2	3	1
2	2	720	2	16	2	2	1	3	2
2	2	721	2	16	2	2	1	3	2
2	2	722	1	16	2	2	1	2	1
2	2	723	1	15	2	2	1	1	2
2	2	724	2	15	2	2	1	3	1
2	2	725	2	16	2	9	9	9	9
2	2	726	1	17	2	2	2	3	1
2	2	727	1	17	2	2	2	3	1
2	2	728	1	15	2	9	9	9	9
2	2	729	1	17	2	2	2	3	1
2	2	730	1	15	2	2	2	3	1
2	2	731	2	15	2	2	1	3	1
2	2	732	1	15	2	2	1	3	1
2	2	733	1	15	2	2	1	3	1
2	2	734	1	15	2	9	1	3	3
2	2	735	1	12	1	2	1	3	1
2	2	736	1	16	2	2	1	3	2
2	2	737	1	0	#NULL!	9	9	9	9
2	2	738	1	15	2	2	1	3	1
2	2	739	2	15	2	2	1	3	1
2	2	740	9	16	2	9	9	9	9
2	2	741	1	14	1	2	1	3	1
2	2	742	1	10	1	9	9	9	9
2	2	743	2	0	#NULL!	9	9	9	9
2	2	744	2	0	#NULL!	2	1	3	1
2	2	745	2	14	1	2	1	3	1

2	2	746	2	10	1	2	1	3	1
2	2	747	2	14	1	2	1	3	1
2	2	748	1	15	2	2	1	3	1
2	2	749	2	15	2	9	9	9	9
2	2	750	2	15	2	2	1	3	1
2	2	751	2	15	2	2	1	3	1
2	2	752	2	16	2	2	1	3	1
2	2	753	2	15	2	2	1	3	1
2	2	754	2	15	2	2	1	3	1
2	2	755	1	16	2	2	1	3	1
2	2	756	2	14	1	2	1	3	1
2	2	757	2	15	2	2	1	3	1
2	2	758	2	15	2	2	1	3	1
2	2	759	1	15	2	2	1	3	1
2	2	760	1	15	2	2	1	3	1
2	2	761	9	0	#NULL!	9	9	9	9
2	2	762	1	16	2	2	1	2	1
2	2	763	1	17	2	2	1	2	1
2	2	764	1	15	2	2	1	2	3
2	2	765	1	16	2	2	1	2	3
2	2	766	1	10	1	2	1	2	1
2	2	767	1	12	1	2	1	2	2
2	2	768	1	0	#NULL!	2	1	2	1
2	2	769	1	17	2	2	1	2	1
2	2	770	9	0	#NULL!	9	9	9	9
2	2	771	1	16	2	2	1	2	1
2	2	772	1	17	2	2	1	2	1
2	2	773	1	17	2	2	1	2	1
2	2	774	1	16	2	2	1	2	2
2	2	775	1	17	2	9	9	9	9
2	2	776	1	18	2	2	1	2	1
2	2	777	1	17	2	2	1	2	1
2	2	778	9	0	#NULL!	2	1	2	1
2	2	779	1	16	2	2	1	2	1
2	2	780	1	18	2	2	1	2	1
2	2	781	1	18	2	2	1	2	1
2	2	782	1	18	2	2	1	2	1
2	2	783	1	17	2	2	1	2	1
2	2	784	1	18	2	2	1	2	1
2	2	785	1	18	2	2	1	2	1
2	2	786	1	16	2	2	1	2	1
2	2	787	1	15	2	2	1	2	1
2	2	788	1	17	2	2	2	2	2
2	2	789	1	17	2	2	2	2	1
2	2	790	1	0	#NULL!	2	2	2	1
2	2	791	1	14	1	2	1	2	2
2	2	792	1	0	#NULL!	2	1	1	1

2	2	793	1	15	2	2	1	2	2
2	2	794	1	15	2	2	1	2	3
2	2	795	1	16	2	2	1	2	1
2	2	796	1	15	2	2	1	2	3
2	2	797	1	16	2	2	1	2	1
2	2	798	1	17	2	2	3	2	1
2	2	799	1	15	2	9	3	2	2
2	2	800	1	18	2	2	3	2	1
2	2	801	1	19	2	2	3	2	3
2	2	802	1	16	2	2	3	2	1
2	2	803	1	18	2	2	3	2	2
2	2	804	1	18	2	2	3	2	1
2	2	805	1	18	2	2	3	2	1
2	2	806	1	20	3	2	3	2	3
2	2	807	1	15	2	2	2	2	1
2	2	808	1	16	2	2	2	2	2
2	2	809	2	18	2	2	1	3	1
2	2	810	2	15	2	9	1	3	1
2	2	811	2	14	1	2	3	3	1
2	2	812	2	17	2	2	3	3	1
2	2	813	2	17	2	2	3	3	1
2	2	814	2	16	2	2	3	3	1
2	2	815	2	16	2	2	3	3	1
2	2	816	2	17	2	2	3	3	1
2	2	817	2	17	2	2	3	3	1
2	2	818	2	16	2	2	3	3	1
2	2	819	2	17	2	2	3	3	3
2	2	820	2	17	2	2	3	3	1
2	2	821	2	0	#NULL!	2	3	3	1
2	2	822	2	17	2	2	3	3	1
2	2	823	2	16	2	2	3	3	1
2	2	824	2	19	2	2	3	3	1
2	2	825	2	16	2	2	3	3	1
2	2	826	2	17	2	2	3	3	1
2	2	827	2	15	2	2	1	3	1
2	2	828	9	0	#NULL!	2	1	3	1
2	2	829	9	0	#NULL!	9	9	9	9
2	2	830	2	15	2	2	1	3	9
2	2	831	2	11	1	2	1	3	1
2	2	832	2	15	2	2	1	3	1
2	2	833	2	15	2	2	1	3	1
2	2	834	1	14	1	2	1	3	1
2	2	835	1	14	1	2	1	3	1
2	2	836	1	14	1	2	1	3	1
2	2	837	1	0	#NULL!	2	1	3	1
2	2	838	2	15	2	2	1	3	1
2	2	839	1	14	1	2	1	3	1

2	2	840	2	16	2	2	1	3	1
2	2	841	1	16	2	2	1	3	2
2	2	842	2	15	2	2	1	3	1
2	2	843	2	14	1	2	1	3	1
2	2	844	2	14	1	2	1	3	1
2	2	845	2	13	1	2	1	3	1
2	2	846	2	14	1	2	1	3	1
2	2	847	2	15	2	2	1	3	1
2	2	848	2	14	1	2	1	3	1
2	2	849	1	0	#NULL!	2	1	2	1
2	2	850	2	16	2	2	1	1	1
2	2	851	2	15	2	2	2	1	1
2	2	852	2	14	1	2	2	1	1
2	2	853	2	15	2	2	2	1	1
2	2	854	2	16	2	2	2	1	1
2	2	855	2	14	1	2	2	1	1
2	2	856	2	15	2	2	2	1	1
2	2	857	2	16	2	2	2	1	1
2	2	858	2	15	2	2	2	1	1
2	2	859	2	15	2	2	2	1	2
2	2	860	2	16	2	2	2	1	1
2	2	861	2	16	2	2	2	1	1
2	2	862	2	16	2	9	9	9	9
2	2	863	2	16	2	2	2	1	2
2	2	864	2	15	2	2	2	1	2
2	2	865	2	15	2	2	2	1	2
2	2	866	2	0	#NULL!	2	3	1	1
2	2	867	2	15	2	2	2	3	2
2	2	868	1	19	2	2	3	2	1
2	2	869	1	23	3	9	3	9	9
2	2	870	1	15	2	2	2	2	1
2	2	871	1	15	2	2	1	2	1
2	2	872	1	15	2	2	1	2	2
2	2	873	1	16	2	2	1	2	1
2	2	874	1	16	2	2	1	2	1
2	2	875	1	16	2	2	1	2	2
2	2	876	1	15	2	2	1	2	2
2	2	877	1	0	#NULL!	9	9	9	9
2	2	878	1	19	2	2	2	2	1
2	2	879	1	17	2	2	2	2	1
2	2	880	1	16	2	1	1	3	1
2	2	881	1	18	2	2	2	2	1
2	2	882	1	17	2	9	2	2	1
2	2	883	1	19	2	2	2	9	9
2	2	884	1	16	2	2	2	2	1
2	2	885	1	16	2	2	1	3	1
2	2	886	1	15	2	2	1	3	1

2	2	887	1	17	2	2	2	3	3
2	2	888	1	16	2	2	2	3	2
2	2	889	2	15	2	2	1	3	1
2	2	890	2	16	2	2	2	3	1
2	2	891	1	15	2	2	1	3	1
2	2	892	1	16	2	2	2	3	2
2	2	893	1	15	2	2	1	3	2
2	2	894	2	18	2	2	3	3	1
2	2	895	2	17	2	2	3	3	1
2	2	896	1	15	2	2	9	3	9
2	2	897	2	14	1	2	1	3	2
2	2	898	1	16	2	2	2	3	2
2	2	899	2	14	1	2	1	3	2
2	2	900	2	15	2	2	1	3	2
2	2	901	1	18	2	2	3	3	2
2	2	902	2	15	2	2	2	3	1
2	2	903	2	16	2	2	2	3	2
2	2	904	2	18	2	2	3	3	1
2	2	905	2	18	2	2	3	3	1
2	2	906	1	17	2	2	3	3	2
2	2	907	2	14	1	2	1	3	1
2	2	908	1	17	2	2	3	3	2
2	2	909	2	15	2	2	2	3	1
2	2	910	1	16	2	2	2	3	2
2	2	911	2	13	1	2	1	3	2
2	2	912	2	16	2	2	2	3	1
2	2	913	2	16	2	2	1	3	1
2	2	914	2	15	2	2	1	3	1
2	2	915	2	15	2	2	1	3	1
2	2	916	1	16	2	2	2	3	1
2	2	917	9	0	#NULL!	2	1	3	1
2	2	918	9	0	#NULL!	9	9	9	9
2	2	919	1	15	2	2	1	3	2
2	2	920	2	14	1	2	1	3	1
2	2	921	2	15	2	2	1	2	1
2	2	922	2	15	2	2	1	3	1
2	2	923	1	16	2	2	1	3	1
2	2	924	1	15	2	2	1	3	3
2	2	925	1	25	3	2	3	2	1
2	2	926	9	17	2	2	3	2	1
2	2	927	1	15	2	2	1	2	1
2	2	928	9	0	#NULL!	2	1	2	1
2	2	929	1	15	2	2	1	2	3
2	2	930	1	16	2	2	1	2	1
2	2	931	1	14	1	2	1	2	1
2	2	932	1	15	2	2	1	2	1
2	2	933	1	14	1	2	1	2	1

2	2	934	1	0	#NULL!	9	1	2	1
2	2	935	1	18	2	2	1	2	1
2	2	936	1	16	2	2	1	2	2
2	2	937	1	18	2	2	1	2	1
2	2	938	1	19	2	9	1	1	9
2	2	939	1	16	2	2	1	2	1
2	2	940	1	17	2	2	1	2	3
2	2	941	1	17	2	2	1	2	1
2	2	942	2	15	2	2	1	3	1
2	2	943	1	16	2	2	1	3	1
2	2	944	1	17	2	2	3	3	1
2	2	945	1	15	2	2	1	3	1
2	2	946	2	16	2	2	1	3	1
2	2	947	1	16	2	2	2	3	1
2	2	948	1	14	1	2	1	3	2
2	2	949	2	15	2	2	1	3	1
2	2	950	1	15	2	2	1	3	1
2	2	951	2	16	2	2	2	3	1
2	2	952	2	16	2	2	2	3	1
2	2	953	2	18	2	2	3	3	1
2	2	954	1	17	2	2	3	3	1
2	2	955	2	16	2	2	2	3	1
2	2	956	2	14	1	2	1	3	1
2	2	957	2	16	2	2	1	3	1
2	2	958	2	16	2	2	2	3	1
2	2	959	1	16	2	2	1	3	2
2	2	960	2	15	2	2	1	3	1
2	2	961	1	17	2	2	2	3	2
2	2	962	9	0	#NULL!	9	9	9	9
2	2	963	1	18	2	2	3	3	1
2	2	964	2	16	2	9	2	3	1
2	2	965	2	14	1	2	1	3	1
2	2	966	2	15	2	2	1	3	1
2	2	967	2	15	2	2	1	3	1
2	2	968	2	15	2	2	1	3	3
2	2	969	2	16	2	2	3	3	1
2	2	970	1	16	2	2	3	3	3
2	2	971	1	18	2	2	3	3	3
2	2	972	2	17	2	2	3	3	1
2	2	973	2	16	2	2	2	3	1
2	2	974	1	15	2	2	1	3	1
2	2	975	1	16	2	2	2	3	3
2	2	976	2	15	2	2	1	3	1
2	2	977	2	14	1	2	1	3	1
2	2	978	1	16	2	2	9	3	9
2	2	979	1	16	2	2	3	3	2
2	2	980	1	16	2	2	2	3	1

2	2	981	1	17	2	2	2	3	2
2	2	982	2	17	2	2	3	3	1
2	2	983	1	17	2	2	2	3	2
2	2	984	2	15	2	2	1	3	1
2	2	985	1	16	2	2	2	3	2
2	2	986	2	18	2	2	3	3	1
2	2	987	2	15	2	1	1	3	1
2	2	988	1	15	2	2	1	3	2
2	2	989	2	16	2	2	2	3	1
2	2	990	2	15	2	2	2	1	1
2	2	991	2	19	2	2	3	3	1
2	2	992	1	17	2	2	2	3	2
2	2	993	2	17	2	2	3	3	1
2	2	994	2	18	2	2	3	3	1
2	2	995	1	16	2	2	3	3	1
2	2	996	2	16	2	2	2	3	1
2	2	997	1	17	2	2	3	3	3
2	2	998	1	16	2	2	2	3	1
2	2	999	1	15	2	2	1	3	3
2	2	1000	2	15	2	2	1	1	3
2	2	1001	2	16	2	2	2	3	1
2	2	1002	1	16	2	2	2	3	3
2	2	1003	2	15	2	2	1	3	1
2	2	1004	9	0	#NULL!	9	9	9	9
2	2	1005	1	16	2	2	2	2	1
2	2	1006	2	15	2	1	1	1	1
2	2	1007	2	16	2	1	1	1	1
2	2	1008	2	16	2	1	1	1	1
2	2	1009	2	15	2	1	1	1	1
2	2	1010	2	15	2	1	1	1	1
2	2	1011	2	15	2	1	1	1	1
2	2	1012	2	19	2	1	1	1	1
2	2	1013	2	14	1	1	1	1	1
2	2	1014	2	15	2	1	1	1	1
2	2	1015	2	15	2	1	1	1	1
2	2	1016	2	14	1	1	1	1	1
2	2	1017	2	17	2	1	1	1	1
2	2	1018	2	15	2	1	1	1	1
2	2	1019	2	13	1	1	1	1	1
2	2	1020	2	15	2	1	1	1	1
2	2	1021	2	0	#NULL!	1	1	1	1
2	2	1022	2	17	2	1	1	1	1
2	2	1023	2	14	1	1	1	1	1
2	2	1024	2	14	1	1	1	1	1
2	2	1025	2	14	1	1	1	1	1
2	2	1026	2	13	1	1	1	1	1
2	2	1027	2	13	1	1	1	1	1

2	2	1028	2	15	2	1	1	1	1
2	2	1029	2	15	2	1	1	1	1
2	2	1030	2	15	2	1	1	1	1
2	2	1031	2	0	#NULL!	9	9	9	9
2	2	1032	2	17	2	1	1	1	1
2	2	1033	2	15	2	2	1	1	1
2	2	1034	2	15	2	1	1	1	3
2	2	1035	2	14	1	1	1	1	1
2	2	1036	9	0	#NULL!	9	9	9	9
2	2	1037	2	15	2	1	1	1	1
2	2	1038	2	16	2	1	1	1	2
2	2	1039	2	13	1	1	1	1	1
2	2	1040	2	14	1	1	1	1	1
2	2	1041	2	14	1	1	1	1	1
2	2	1042	9	0	#NULL!	9	9	9	9
2	2	1043	2	15	2	2	1	1	1
2	2	1044	2	15	2	1	1	1	1
2	2	1045	2	16	2	1	1	1	1
2	2	1046	2	14	1	1	1	1	1
2	2	1047	2	14	1	1	1	1	1
2	2	1048	2	12	1	1	1	1	1
2	2	1049	2	16	2	1	1	1	1
2	2	1050	2	15	2	1	1	1	2
2	2	1051	2	13	1	2	1	1	1
2	2	1052	2	16	2	2	1	1	9
2	2	1053	2	16	2	1	1	1	1
2	2	1054	2	17	2	1	1	1	1
2	2	1055	2	15	2	1	1	1	1
2	2	1056	2	14	1	1	1	1	1
2	2	1057	2	16	2	1	1	1	1
2	2	1058	2	15	2	1	1	1	1
2	2	1059	2	13	1	1	1	1	1
2	2	1060	2	14	1	1	1	1	1
2	2	1061	2	13	1	1	1	1	1
2	2	1062	2	14	1	1	1	1	1
2	2	1063	2	15	2	1	1	1	1
2	2	1064	2	16	2	1	1	1	1
2	2	1065	2	13	1	1	1	1	1
2	2	1066	2	15	2	1	1	1	1
2	2	1067	2	15	2	1	1	1	1
2	2	1068	2	15	2	1	1	1	1
2	2	1069	2	16	2	1	1	1	1
2	2	1070	2	15	2	1	1	1	1
2	2	1071	2	13	1	9	1	1	1
2	2	1072	2	13	1	1	1	1	1
2	2	1073	2	15	2	1	1	1	1
2	2	1074	2	15	2	1	1	1	1

2	2	1075	2	14	1	1	1	1	1
2	2	1076	2	15	2	1	1	1	1
2	2	1077	2	14	1	1	1	1	1
2	2	1078	2	15	2	2	1	1	1
2	2	1079	2	15	2	1	1	1	1
2	2	1080	2	15	2	1	1	1	1
2	2	1081	2	15	2	1	1	1	1
2	2	1082	2	15	2	1	1	1	1
2	2	1083	2	16	2	1	1	1	1
2	2	1084	2	14	1	1	1	1	1
2	2	1085	2	15	2	1	1	1	1
2	2	1086	2	14	1	1	1	1	1
2	2	1087	2	15	2	1	1	1	1
2	2	1088	9	0	#NULL!	9	9	9	9
2	2	1089	2	14	1	1	1	1	1
2	2	1090	2	15	2	1	1	1	1
2	2	1091	2	14	1	1	1	1	1
2	2	1092	2	14	1	1	1	1	1
2	2	1093	2	15	2	1	1	1	1
2	2	1094	2	15	2	1	1	1	1
2	2	1095	2	15	2	1	1	1	1
2	2	1096	2	13	1	1	1	1	1
2	2	1097	2	16	2	1	1	1	1
2	2	1098	2	15	2	1	1	1	1
2	2	1099	2	15	2	1	1	1	1
2	2	1100	2	14	1	1	1	1	1
2	2	1101	2	14	1	1	1	1	1
2	2	1102	9	0	#NULL!	9	9	9	9
2	2	1103	2	15	2	1	1	1	1
2	2	1104	2	14	1	1	1	1	1
2	2	1105	2	14	1	1	1	1	1
2	2	1106	2	15	2	1	1	1	1
2	2	1107	2	16	2	1	1	1	1
2	2	1108	2	15	2	1	1	1	1
2	2	1109	2	16	2	1	1	1	1
2	2	1110	2	15	2	1	1	1	1
2	2	1111	2	14	1	1	1	1	1
2	2	1112	2	15	2	1	1	1	1
2	2	1113	2	16	2	1	1	1	1
2	2	1114	2	16	2	1	1	1	1
2	2	1115	2	15	2	1	1	1	1
2	2	1116	2	0	#NULL!	1	1	1	1
2	2	1117	2	15	2	1	1	1	1
2	2	1118	2	15	2	1	1	1	1
2	2	1119	2	16	2	1	1	1	1
2	2	1120	2	15	2	1	1	1	1
2	2	1121	2	16	2	1	1	1	1

2	2	1122	2	16	2	1	1	1	1
2	2	1123	2	14	1	1	1	1	1
2	2	1124	2	14	1	1	1	1	1
2	2	1125	2	15	2	1	1	1	1
2	2	1126	2	0	#NULL!	1	1	1	1
2	2	1127	2	14	1	1	1	1	1
2	2	1128	2	15	2	1	1	1	1
2	2	1129	2	13	1	1	1	1	1
2	2	1130	2	14	1	1	1	1	1
2	2	1131	2	15	2	1	1	1	1
2	2	1132	2	15	2	1	1	1	1
2	2	1133	2	15	2	1	1	1	1
2	2	1134	2	13	1	1	1	1	1
2	2	1135	2	18	2	1	1	1	1
2	2	1136	2	14	1	1	1	1	1
2	2	1137	2	14	1	1	1	1	1
2	2	1138	2	13	1	1	1	1	1
2	2	1139	2	17	2	1	1	1	1
2	2	1140	2	16	2	1	1	1	1
2	2	1141	2	17	2	1	1	1	1
2	2	1142	2	15	2	1	1	1	1
2	2	1143	2	0	#NULL!	1	1	1	1
2	2	1144	2	14	1	1	1	1	1
2	2	1145	2	17	2	1	1	1	1
2	2	1146	2	17	2	1	1	1	1
2	2	1147	9	0	#NULL!	9	9	9	9
2	2	1148	2	14	1	1	1	1	1
2	2	1149	2	15	2	1	1	1	1
2	2	1150	2	15	2	1	1	1	1
2	2	1151	2	13	1	1	1	1	1
2	2	1152	2	14	1	1	1	1	1
2	2	1153	2	16	2	1	1	1	1
2	2	1154	2	14	1	1	1	1	1
2	2	1155	2	15	2	1	1	1	1
2	2	1156	2	13	1	1	1	1	1
2	2	1157	2	16	2	1	1	1	1
2	2	1158	2	15	2	1	1	1	1
2	2	1159	2	14	1	1	1	1	1
2	2	1160	2	15	2	1	1	1	1
2	2	1161	2	14	1	1	1	1	1
2	2	1162	2	14	1	1	1	1	1
2	2	1163	2	14	1	1	1	1	1
2	2	1164	2	13	1	1	1	1	1
2	2	1165	2	13	1	1	1	1	1
2	2	1166	2	14	1	1	1	1	1
2	2	1167	2	16	2	1	1	1	1
2	2	1168	2	16	2	1	1	1	1

2	2	1169	9	0	#NULL!	9	9	9	9
2	2	1170	2	15	2	1	1	1	1
2	2	1171	2	16	2	1	1	1	1
2	2	1172	2	15	2	1	1	1	1
2	2	1173	2	17	2	1	1	1	1
2	2	1174	2	17	2	1	1	1	1
2	2	1175	2	18	2	1	1	1	1
2	2	1176	2	15	2	1	1	1	1
2	2	1177	2	14	1	1	1	1	1
2	2	1178	2	15	2	1	1	1	1
2	2	1179	2	15	2	1	1	1	1
2	2	1180	2	15	2	1	1	1	1
2	2	1181	2	14	1	1	1	1	1
2	2	1182	2	19	2	1	1	1	1
2	2	1183	2	15	2	1	1	1	1
2	2	1184	2	13	1	1	1	1	1
2	2	1185	2	15	2	1	1	1	1
2	2	1186	2	13	1	1	1	1	1
2	2	1187	2	15	2	1	1	1	1
2	2	1188	2	17	2	1	1	1	1
2	2	1189	2	16	2	1	1	1	1
2	2	1190	2	14	1	1	1	1	1
2	2	1191	2	14	1	1	1	1	1
2	2	1192	2	16	2	1	1	1	1
2	2	1193	2	14	1	1	1	1	1
2	2	1194	2	14	1	1	1	1	1
2	2	1195	2	15	2	1	1	1	1
2	2	1196	2	14	1	1	1	1	1
2	2	1197	2	16	2	2	1	1	1
2	2	1198	2	13	1	1	1	1	1
2	2	1199	2	14	1	1	1	1	1
2	2	1200	2	14	1	1	1	1	1
2	2	1201	2	14	1	1	1	1	1
2	2	1202	2	15	2	1	1	1	1
2	2	1203	9	0	#NULL!	1	1	1	1
2	2	1204	2	14	1	1	1	1	1
2	2	1205	2	14	1	1	1	1	1
2	2	1206	2	15	2	9	1	1	1
2	2	1207	2	16	2	1	1	1	1
2	2	1208	2	17	2	9	1	1	1
2	2	1209	2	15	2	1	1	1	1
2	2	1210	2	16	2	1	1	1	1
2	2	1211	2	14	1	1	1	1	1
2	2	1212	2	15	2	1	1	1	1
2	2	1213	2	14	1	9	1	1	9
2	2	1214	2	16	2	1	1	1	1
2	2	1215	2	15	2	2	1	1	1

2	2	1216	2	14	1	1	1	1	1
2	2	1217	2	14	1	1	1	1	1
2	2	1218	2	17	2	1	1	1	1
2	2	1219	2	14	1	1	1	1	1
2	2	1220	2	15	2	1	1	1	1
2	2	1221	9	16	2	1	1	1	2
2	2	1222	2	15	2	1	1	1	1
2	2	1223	2	14	1	1	1	1	1
2	2	1224	2	15	2	1	1	1	1
2	2	1225	9	14	1	2	1	1	1
2	2	1226	2	15	2	1	1	1	1
2	2	1227	2	15	2	1	1	1	1
2	2	1228	2	13	1	1	1	1	1
2	2	1229	2	16	2	1	1	1	1
2	2	1230	2	14	1	1	1	1	1
2	2	1231	2	0	#NULL!	1	1	1	1
2	2	1232	9	9	1	9	9	9	9
2	2	1233	2	16	2	1	1	3	9
2	2	1234	2	15	2	1	1	1	1
2	2	1235	2	17	2	1	1	3	1
2	2	1236	1	15	2	1	1	3	1
2	2	1237	2	16	2	1	1	3	1
2	2	1238	2	22	3	1	1	3	1
2	2	1239	2	16	2	1	1	3	1
2	2	1240	2	15	2	1	1	3	2
2	2	1241	1	15	2	1	1	3	1
2	2	1242	2	15	2	1	1	3	1
2	2	1243	2	15	2	1	1	3	2
2	2	1244	2	14	1	1	1	3	1
2	2	1245	2	14	1	1	1	3	1
2	2	1246	2	15	2	1	1	3	1
2	2	1247	2	15	2	1	1	3	1
2	2	1248	2	17	2	1	1	3	1
2	2	1249	2	13	1	1	1	3	1
2	2	1250	2	14	1	2	1	3	1
2	2	1251	2	16	2	1	1	3	1
2	2	1252	1	16	2	1	1	3	2
2	2	1253	2	15	2	1	1	3	1
2	2	1254	2	15	2	1	1	3	1
2	2	1255	2	15	2	1	1	3	1
2	2	1256	1	14	1	1	1	3	1
2	2	1257	2	15	2	9	1	3	1
2	2	1258	2	14	1	2	1	3	1
2	2	1259	2	15	2	1	1	3	1
2	2	1260	2	14	1	1	1	3	1
2	2	1261	2	16	2	1	1	3	1
2	2	1262	9	0	#NULL!	1	1	3	2

2	2	1263	1	17	2	1	1	3	2
2	2	1264	2	15	2	2	1	3	2
2	2	1265	9	16	2	2	1	3	1
2	2	1266	2	15	2	1	1	1	1
2	2	1267	2	15	2	1	1	1	1
2	2	1268	2	14	1	1	1	3	2
2	2	1269	2	17	2	2	1	3	1
2	2	1270	1	15	2	1	1	3	2
2	2	1271	2	20	3	1	1	3	1
2	2	1272	2	15	2	1	1	3	1
2	2	1273	1	15	2	1	1	3	1
2	2	1274	2	13	1	1	1	3	2
2	2	1275	2	14	1	1	1	3	1
2	2	1276	2	14	1	1	1	3	2
2	2	1277	2	14	1	1	1	3	2
2	2	1278	2	15	2	1	1	3	1
2	2	1279	2	14	1	1	1	3	1
2	2	1280	1	15	2	1	1	3	2
2	2	1281	1	14	1	1	1	3	1
2	2	1282	2	14	1	1	1	3	2
2	2	1283	2	19	2	1	1	3	1
2	2	1284	2	16	2	1	1	3	1
2	2	1285	2	14	1	1	1	3	2
2	2	1286	1	15	2	2	1	3	2
2	2	1287	1	18	2	1	1	3	3
2	2	1288	1	14	1	1	1	3	1
2	2	1289	1	17	2	1	1	3	1
2	2	1290	2	14	1	1	1	3	2
2	2	1291	1	13	1	1	1	3	1
2	2	1292	2	14	1	1	1	3	1
2	2	1293	1	14	1	1	1	3	1
2	2	1294	2	14	1	1	1	3	1
2	2	1295	2	17	2	1	1	3	2
2	2	1296	2	15	2	2	1	3	2
2	2	1297	2	19	2	1	1	3	1
2	2	1298	2	15	2	1	1	3	1
2	2	1299	2	14	1	1	1	3	1
2	2	1300	9	0	#NULL!	9	9	9	9
2	2	1301	2	15	2	1	1	3	2
2	2	1302	2	14	1	1	1	3	1
2	2	1303	2	14	1	1	1	3	1
2	2	1304	9	0	#NULL!	9	9	9	9
2	2	1305	2	16	2	1	1	3	1
2	2	1306	2	15	2	1	1	1	1
2	2	1307	9	0	#NULL!	9	9	9	9
2	2	1308	2	15	2	2	1	3	1
2	2	1309	1	15	2	1	1	3	2

2	2	1310	2	16	2	1	1	3	1
2	2	1311	1	17	2	1	1	3	1
2	2	1312	2	15	2	1	1	3	1
2	2	1313	1	15	2	1	1	3	1
2	2	1314	2	14	1	1	1	1	1
2	2	1315	2	12	1	1	1	1	1
2	2	1316	2	13	1	1	1	3	1
2	2	1317	1	14	1	1	1	3	1
2	2	1318	2	16	2	1	1	3	1
2	2	1319	9	0	#NULL!	9	9	9	9
2	2	1320	2	15	2	1	1	3	1
2	2	1321	2	16	2	1	1	3	1
2	2	1322	2	14	1	1	1	3	1
2	2	1323	1	15	2	1	1	3	3
2	2	1324	1	14	1	1	1	3	1
2	2	1325	2	15	2	1	1	3	3
2	2	1326	9	0	#NULL!	9	9	9	9
2	2	1327	1	19	2	1	1	3	3
2	2	1328	1	14	1	1	1	3	2
2	2	1329	1	17	2	1	1	3	1
2	2	1330	9	0	#NULL!	9	9	9	9
2	2	1331	2	15	2	1	1	3	3
2	2	1332	1	17	2	1	1	3	1
2	2	1333	2	14	1	1	1	3	1
2	2	1334	2	14	1	1	1	3	2
2	2	1335	1	18	2	1	1	3	1
2	2	1336	2	14	1	1	1	3	1
2	2	1337	2	16	2	1	1	3	1
2	2	1338	9	0	#NULL!	9	9	9	9
2	2	1339	9	0	#NULL!	9	9	9	9
2	2	1340	2	16	2	1	1	3	1
2	2	1341	2	15	2	1	1	3	1
2	2	1342	1	14	1	1	1	3	1
2	2	1343	2	13	1	1	1	3	1
2	2	1344	2	15	2	1	1	3	1
2	2	1345	2	14	1	1	1	3	1
2	2	1346	2	12	1	1	1	1	1
2	2	1347	1	15	2	1	1	3	2
2	2	1348	1	14	1	1	1	3	2
2	2	1349	1	19	2	1	1	9	2
2	2	1350	1	15	2	1	1	3	2
2	2	1351	2	15	2	1	1	3	9
2	2	1352	1	15	2	1	1	3	1
2	2	1353	1	14	1	1	1	3	1
2	2	1354	2	15	2	1	1	3	1
2	2	1355	2	15	2	1	1	3	1
2	2	1356	2	17	2	1	1	3	1

2	2	1357	2	14	1	1	1	3	1
2	2	1358	2	16	2	1	1	3	1
2	2	1359	1	14	1	1	1	3	1
2	2	1360	1	15	2	1	1	3	2
2	2	1361	1	14	1	1	1	2	2
2	2	1362	1	14	1	1	1	3	2
2	2	1363	2	15	2	1	1	3	3
2	2	1364	2	16	2	1	1	3	1
2	2	1365	2	15	2	1	1	3	1
2	2	1366	1	17	2	1	1	3	1
2	2	1367	2	18	2	1	1	3	2
2	2	1368	2	14	1	1	1	3	1
2	2	1369	2	19	2	1	1	3	1
2	2	1370	1	16	2	1	1	3	1
2	2	1371	2	15	2	1	1	3	1
2	2	1372	2	16	2	1	1	3	2
2	2	1373	1	18	2	1	1	3	1
2	2	1374	1	17	2	1	1	3	1
2	2	1375	1	19	2	2	1	3	2
2	2	1376	1	16	2	1	1	3	3
2	2	1377	1	18	2	1	1	3	1
2	2	1378	2	14	1	1	1	3	1
2	2	1379	2	15	2	1	1	3	2
2	2	1380	1	15	2	1	1	3	1
2	2	1381	2	16	2	1	1	3	2
2	2	1382	2	15	2	1	1	3	1
2	2	1383	1	12	1	1	1	3	1
2	2	1384	2	14	1	1	1	3	2
2	2	1385	2	15	2	1	1	3	2
2	2	1386	1	15	2	1	1	3	1
2	2	1387	2	17	2	1	1	3	1
2	2	1388	9	0	#NULL!	9	9	9	9
2	2	1389	2	14	1	1	1	3	1
2	2	1390	1	16	2	1	1	3	2
2	2	1391	1	19	2	1	1	3	1
2	2	1392	2	16	2	1	1	3	1
2	2	1393	2	14	1	1	1	3	1
2	2	1394	2	14	1	1	1	3	1
2	2	1395	2	15	2	1	1	3	1
2	2	1396	1	16	2	1	1	3	1
2	2	1397	1	17	2	1	1	3	1
2	2	1398	2	50	3	9	9	9	9
2	2	1399	1	15	2	1	1	3	2
2	2	1400	2	14	1	1	1	3	1
2	2	1401	2	0	#NULL!	1	1	3	1
2	2	1402	2	14	1	1	1	3	1
2	2	1403	1	16	2	1	1	3	1

2	2	1404	1	15	2	1	1	3	3
2	2	1405	2	14	1	1	1	3	1
2	2	1406	1	12	1	1	1	3	1
2	2	1407	1	14	1	1	1	3	1
2	2	1408	2	14	1	1	1	1	1
2	2	1409	1	16	2	2	1	3	2
2	2	1410	2	14	1	1	1	3	1
2	2	1411	1	15	2	1	1	3	1
2	2	1412	2	14	1	1	1	3	1
2	2	1413	2	14	1	1	1	3	1
2	2	1414	1	17	2	1	1	3	1
2	2	1415	1	2	1	1	1	3	1
2	2	1416	1	14	1	1	1	3	1
2	2	1417	1	14	1	1	1	3	1
2	2	1418	2	13	1	1	1	3	1
2	2	1419	2	14	1	1	1	3	1
2	2	1420	2	15	2	1	1	3	1
2	2	1421	1	14	1	1	1	3	3
2	2	1422	2	15	2	1	1	3	1
2	2	1423	2	16	2	1	1	3	1
2	2	1424	1	17	2	1	1	3	1
2	2	1425	2	14	1	1	1	3	1
2	2	1426	2	15	2	1	1	3	3
2	2	1427	2	14	1	1	1	3	1
2	2	1428	2	15	2	1	1	3	1
2	2	1429	2	16	2	1	1	3	1
2	2	1430	2	15	2	1	1	3	1
2	2	1431	1	17	2	1	3	3	1
2	2	1432	2	17	2	1	3	3	1
2	2	1433	1	16	2	1	3	3	1
2	2	1434	1	18	2	1	3	3	3
2	2	1435	2	17	2	1	3	3	1
2	2	1436	2	16	2	1	3	3	1
2	2	1437	2	16	2	1	3	3	1
2	2	1438	2	16	2	1	3	3	1
2	2	1439	2	16	2	1	3	3	1
2	2	1440	2	15	2	1	3	3	1
2	2	1441	2	16	2	1	3	3	1
2	2	1442	2	17	2	1	3	3	1
2	2	1443	2	16	2	1	3	3	1
2	2	1444	1	14	1	2	3	3	1
2	2	1445	2	17	2	1	3	3	1
2	2	1446	2	17	2	1	3	3	1
2	2	1447	2	16	2	1	3	3	1
2	2	1448	1	35	3	1	1	3	1
2	2	1449	1	10	1	1	2	3	3
2	2	1450	1	20	3	1	1	3	1

2	2	1451	9	0	#NULL!	9	9	9	9
2	2	1452	9	0	#NULL!	9	9	9	9
2	2	1453	2	25	3	2	1	1	1
2	2	1454	1	30	3	2	1	2	1
2	2	1455	9	0	#NULL!	9	9	9	9
2	2	1456	1	28	3	1	1	3	1
2	2	1457	9	0	#NULL!	9	9	9	9
2	2	1458	1	0	#NULL!	1	3	3	1
2	2	1459	1	25	3	1	3	3	2
2	2	1460	1	30	3	1	3	3	2
2	2	1461	2	17	2	1	3	3	1
2	2	1462	2	17	2	1	3	3	1
2	2	1463	1	16	2	2	3	3	1
2	2	1464	1	29	3	1	3	3	1
2	2	1465	1	13	1	1	3	3	1
2	2	1466	9	0	#NULL!	9	9	9	9
2	2	1467	2	20	3	1	1	1	1
2	2	1468	2	15	2	1	3	3	2
2	2	1469	2	14	1	1	3	1	9
2	2	1470	1	20	3	1	3	3	1
2	2	1471	1	18	2	1	3	3	1
2	2	1472	9	0	#NULL!	9	9	9	9
2	2	1473	9	0	#NULL!	9	9	9	9
2	2	1474	2	13	1	1	3	3	1
2	2	1475	2	13	1	2	3	3	1
2	2	1476	2	17	2	1	3	3	1
2	2	1477	1	8	1	2	1	1	2
2	2	1478	2	19	2	1	3	3	1
2	2	1479	2	16	2	1	3	3	1
2	2	1480	2	18	2	1	3	3	3
2	2	1481	1	17	2	1	3	3	2
2	2	1482	2	18	2	1	3	1	1
2	2	1483	2	16	2	1	3	3	1
2	2	1484	2	17	2	1	3	3	9
2	2	1485	9	0	#NULL!	9	9	9	9
2	2	1486	2	17	2	1	3	3	2
2	2	1487	1	19	2	1	3	3	1
2	2	1488	2	16	2	1	3	3	1
2	2	1489	2	18	2	1	3	3	3
2	2	1490	1	16	2	2	3	3	1
2	2	1491	2	14	1	1	3	3	1
2	2	1492	2	13	1	1	3	3	1
2	2	1493	2	13	1	1	3	1	2
2	2	1494	2	17	2	9	3	3	1
2	2	1495	1	40	3	1	3	3	2
2	2	1496	2	15	2	1	3	3	1
2	2	1497	1	20	3	1	3	1	3

2	2	1498	2	14	1	1	3	3	1
2	2	1499	1	12	1	1	3	3	2
2	2	1500	2	14	1	2	3	3	1
2	2	1501	2	12	1	1	3	3	3
2	2	1502	9	0	#NULL!	1	2	2	1
2	2	1503	2	15	2	1	3	3	1
2	2	1504	2	16	2	1	3	3	2
2	2	1505	2	16	2	1	3	3	1
2	2	1506	1	17	2	1	3	3	3
2	2	1507	2	17	2	1	3	3	1
2	2	1508	9	0	#NULL!	2	1	3	1
2	2	1509	2	18	2	1	3	3	2
2	2	1510	2	17	2	1	3	3	2
2	2	1511	2	17	2	1	3	3	1
2	2	1512	2	19	2	9	3	3	1
2	2	1513	2	16	2	1	3	3	1
2	2	1514	2	16	2	1	3	3	1
2	2	1515	2	14	1	1	3	3	1
2	2	1516	9	0	#NULL!	1	3	3	1
2	2	1517	2	15	2	1	3	3	2
2	2	1518	2	18	2	1	3	3	1
2	2	1519	2	17	2	1	3	3	1
2	2	1520	2	16	2	1	3	3	1
2	2	1521	2	17	2	1	3	3	1
2	2	1522	2	16	2	2	1	3	1
2	2	1523	2	17	2	1	3	3	1
2	2	1524	2	19	2	1	3	3	3
2	2	1525	2	16	2	1	3	3	1
2	2	1526	1	15	2	1	3	3	1
2	2	1527	9	0	#NULL!	9	9	9	9
2	2	1528	1	17	2	1	3	3	9
2	2	1529	1	18	2	1	3	3	1
2	2	1530	1	18	2	1	3	3	1
2	2	1531	1	19	2	1	3	3	1
2	2	1532	1	20	3	1	3	3	1
2	2	1533	2	19	2	1	3	3	1
2	2	1534	2	17	2	1	3	3	2
2	2	1535	2	16	2	1	3	3	1
2	2	1536	1	20	3	1	3	3	2
2	2	1537	2	18	2	1	3	3	3
2	2	1538	2	17	2	1	3	3	1
2	2	1539	2	16	2	1	3	3	1
2	2	1540	1	15	2	1	3	3	1
2	2	1541	9	18	2	1	3	3	1
2	2	1542	1	13	1	1	3	3	1
2	2	1543	1	18	2	1	3	3	1
2	2	1544	1	16	2	2	3	3	1

2	2	1545	1	16	2	1	3	3	2
2	2	1546	2	19	2	1	3	3	1
2	2	1547	2	16	2	1	3	3	1
2	2	1548	2	17	2	1	3	3	2
2	2	1549	2	18	2	1	3	3	1
2	2	1550	1	0	#NULL!	1	3	3	1
2	2	1551	1	19	2	1	3	3	1
2	2	1552	1	17	2	1	3	3	3
2	2	1553	1	18	2	1	3	3	1
2	2	1554	9	0	#NULL!	9	9	9	9
2	2	1555	1	17	2	1	3	3	3
2	2	1556	2	0	#NULL!	1	3	3	1
2	2	1557	9	0	#NULL!	9	9	9	9
2	2	1558	9	0	#NULL!	9	9	9	9
2	2	1559	9	0	#NULL!	9	9	9	9
2	2	1560	2	14	1	1	3	3	1
2	2	1561	2	18	2	1	3	3	1
2	2	1562	2	17	2	1	3	3	1
2	2	1563	2	17	2	1	3	3	1
2	2	1564	1	17	2	1	3	3	3
2	2	1565	2	13	1	1	3	3	1
2	2	1566	1	17	2	1	3	3	1
2	2	1567	2	17	2	1	3	3	1
2	2	1568	2	16	2	2	3	3	1
2	2	1569	2	16	2	1	3	3	2
2	2	1570	2	16	2	1	3	3	1
2	2	1571	2	15	2	1	3	3	1
2	2	1572	2	17	2	1	3	3	1
2	2	1573	2	15	2	2	3	3	1
2	2	1574	2	16	2	1	3	3	1
2	2	1575	1	17	2	1	3	3	1
2	2	1576	2	18	2	2	3	3	2
2	2	1577	2	16	2	2	3	3	2
2	2	1578	2	15	2	1	3	3	1
2	2	1579	9	0	#NULL!	9	9	9	9
2	2	1580	1	17	2	1	3	3	2
2	2	1581	9	0	#NULL!	9	9	9	9
2	2	1582	1	17	2	1	3	3	1
2	2	1583	1	17	2	1	3	3	3
2	2	1584	1	19	2	1	2	3	1
2	2	1585	2	17	2	1	2	1	2
2	2	1586	1	16	2	1	2	3	2
2	2	1587	2	16	2	1	2	3	1
2	2	1588	2	16	2	2	2	3	1
2	2	1589	9	16	2	1	2	3	1
2	2	1590	9	0	#NULL!	9	9	9	9
2	2	1591	2	16	2	1	2	3	1

2	2	1592	1	18	2	1	2	3	1
2	2	1593	2	0	#NULL!	1	2	3	2
2	2	1594	2	17	2	1	2	3	1
2	2	1595	2	16	2	1	2	3	1
2	2	1596	2	16	2	1	2	3	2
2	2	1597	2	14	1	1	2	3	1
2	2	1598	9	0	#NULL!	9	9	9	9
2	2	1599	2	0	#NULL!	1	2	3	1
2	2	1600	2	17	2	1	2	3	1
2	2	1601	9	0	#NULL!	9	9	9	9
2	2	1602	9	0	#NULL!	9	9	9	9
2	2	1603	9	0	#NULL!	9	9	9	9
2	2	1604	9	0	#NULL!	9	9	9	9
2	2	1605	9	0	#NULL!	9	9	9	9
2	2	1606	9	0	#NULL!	9	9	9	9
2	2	1607	9	0	#NULL!	9	9	9	9
2	2	1608	9	0	#NULL!	9	9	9	9
2	2	1609	9	0	#NULL!	9	9	9	9
2	2	1610	9	0	#NULL!	9	9	9	9
2	2	1611	9	0	#NULL!	9	9	9	9
2	2	1612	9	0	#NULL!	9	9	9	9
2	2	1613	9	0	#NULL!	9	9	9	9
2	2	1614	9	0	#NULL!	9	9	9	9
2	2	1615	9	0	#NULL!	9	9	9	9
2	2	1616	9	0	#NULL!	9	9	9	9
2	2	1617	9	0	#NULL!	9	9	9	9
2	2	1618	9	0	#NULL!	9	9	9	9
2	2	1619	9	0	#NULL!	9	9	9	9
2	2	1620	9	0	#NULL!	9	9	9	9
2	2	1621	1	16	2	1	2	3	1
2	2	1622	2	15	2	1	2	3	1
2	2	1623	2	15	2	1	2	3	1
2	2	1624	2	15	2	1	2	3	1
2	2	1625	2	14	1	1	2	3	1
2	2	1626	2	15	2	1	2	3	1
2	2	1627	1	15	2	1	2	3	1
2	2	1628	2	17	2	1	2	3	1
2	2	1629	2	18	2	1	2	3	1
2	2	1630	1	16	2	1	2	3	2
2	2	1631	2	15	2	1	2	3	1
2	2	1632	2	16	2	1	2	3	1
2	2	1633	2	14	1	1	2	3	1
2	2	1634	2	15	2	1	2	3	1
2	2	1635	2	16	2	1	2	3	1
2	2	1636	2	16	2	1	2	3	1
2	2	1637	1	16	2	1	2	3	1
2	2	1638	2	19	2	1	2	3	1

2	2	1639	1	18	2	1	2	3	1
2	2	1640	2	14	1	1	2	3	1
2	2	1641	2	17	2	1	2	3	1
2	2	1642	2	15	2	1	2	3	1
2	2	1643	1	17	2	1	2	2	1
2	2	1644	1	16	2	1	2	3	1
2	2	1645	2	14	1	1	2	3	1
2	2	1646	2	17	2	1	2	3	1
2	2	1647	2	16	2	1	2	3	1
2	2	1648	2	14	1	1	2	3	1
2	2	1649	2	15	2	2	2	2	1
2	2	1650	2	15	2	1	2	3	1
2	2	1651	2	15	2	1	2	3	1
2	2	1652	2	17	2	1	2	3	1
2	2	1653	2	16	2	1	2	3	1
2	2	1654	2	18	2	1	2	3	1
2	2	1655	2	16	2	1	2	3	1
2	2	1656	2	16	2	1	2	3	1
2	2	1657	2	16	2	1	2	3	1
2	2	1658	1	16	2	1	2	3	1
2	2	1659	2	14	1	1	2	3	1
2	2	1660	2	15	2	1	2	3	1
2	2	1661	2	16	2	1	2	3	1
2	2	1662	1	16	2	1	2	3	1
2	2	1663	2	16	2	1	2	3	1
2	2	1664	1	14	1	1	2	3	1
2	2	1665	2	17	2	1	2	3	1
2	2	1666	1	17	2	1	2	3	1
2	2	1667	2	15	2	1	2	3	1
2	2	1668	2	17	2	1	2	3	1
2	2	1669	1	17	2	1	2	3	1
2	2	1670	2	19	2	2	2	1	1
2	2	1671	1	0	#NULL!	1	2	3	1
2	2	1672	2	15	2	1	2	3	1
2	2	1673	2	16	2	1	2	3	1
2	2	1674	1	16	2	1	2	3	1
2	2	1675	2	16	2	1	2	1	1
2	2	1676	2	16	2	2	2	3	1
2	2	1677	2	15	2	1	2	3	1
2	2	1678	2	17	2	1	2	3	1
2	2	1679	2	16	2	1	2	3	1
2	2	1680	1	17	2	1	2	3	1
2	2	1681	2	18	2	1	2	3	1
2	2	1682	2	17	2	1	2	3	3
2	2	1683	9	16	2	1	2	2	2
2	2	1684	1	16	2	1	2	3	1
2	2	1685	2	14	1	1	2	3	1

2	2	1686	2	15	2	1	2	3	1
2	2	1687	2	16	2	1	2	3	1
2	2	1688	2	17	2	1	2	3	1
2	2	1689	2	17	2	1	2	3	1
2	2	1690	2	15	2	1	2	3	1
2	2	1691	2	16	2	1	2	3	1
2	2	1692	2	15	2	1	2	3	1
2	2	1693	2	17	2	1	3	3	1
2	2	1694	2	15	2	1	2	3	1
2	2	1695	2	16	2	1	2	3	1
2	2	1696	1	15	2	1	2	3	1
2	2	1697	2	9	1	1	2	3	1
2	2	1698	2	18	2	1	2	3	1
2	2	1699	2	16	2	1	2	3	1
2	2	1700	2	15	2	1	2	3	3
2	2	1701	1	18	2	1	2	3	1
2	2	1702	1	16	2	1	2	3	1
2	2	1703	2	16	2	1	2	3	1
2	2	1704	9	15	2	1	2	3	1
2	2	1705	2	16	2	1	2	3	1
2	2	1706	2	19	2	1	2	3	1
2	2	1707	1	14	1	1	2	3	1
2	2	1708	2	17	2	1	2	3	1
2	2	1709	2	17	2	1	2	3	1
2	2	1710	1	18	2	1	2	3	1
2	2	1711	2	15	2	1	2	3	1
2	2	1712	2	14	1	1	2	3	1
2	2	1713	2	14	1	1	2	3	1
2	2	1714	1	16	2	1	2	3	2
2	2	1715	2	17	2	1	2	3	1
2	2	1716	2	15	2	1	2	3	1
2	2	1717	2	16	2	1	2	3	3
2	2	1718	2	0	#NULL!	2	2	3	1
2	2	1719	2	16	2	1	2	3	1
2	2	1720	2	17	2	1	2	3	1
2	2	1721	2	14	1	1	2	3	1
2	2	1722	9	0	#NULL!	9	9	9	9
2	2	1723	9	0	#NULL!	9	9	9	9
2	2	1724	9	0	#NULL!	9	9	9	9
2	2	1725	9	0	#NULL!	9	9	9	9
2	2	1726	9	0	#NULL!	9	9	9	9
2	2	1727	9	0	#NULL!	9	9	9	9
2	2	1728	9	0	#NULL!	9	9	9	9
2	2	1729	9	0	#NULL!	9	9	9	9
2	2	1730	9	0	#NULL!	9	9	9	9
2	2	1731	9	0	#NULL!	9	9	9	9
2	2	1732	9	0	#NULL!	9	9	9	9

2	2	1733	9	0	#NULL!	9	9	9	9
2	2	1734	9	0	#NULL!	9	9	9	9
2	2	1735	9	0	#NULL!	9	9	9	9
2	2	1736	9	0	#NULL!	9	9	9	9
2	2	1737	9	0	#NULL!	9	9	9	9
2	2	1738	9	0	#NULL!	9	9	9	9
2	2	1739	9	0	#NULL!	9	9	9	9
2	2	1740	9	0	#NULL!	9	9	9	9
2	2	1741	9	0	#NULL!	9	9	9	9
2	2	1742	9	0	#NULL!	9	9	9	9
2	2	1743	9	0	#NULL!	9	9	9	9
2	2	1744	9	0	#NULL!	9	9	9	9
2	2	1745	9	0	#NULL!	9	9	9	9
2	2	1746	9	0	#NULL!	9	9	9	9
2	2	1747	9	0	#NULL!	1	2	3	1
2	2	1748	9	0	#NULL!	9	9	9	9
2	2	1749	9	0	#NULL!	9	9	9	9
2	2	1750	9	0	#NULL!	9	9	9	9
2	2	1751	9	0	#NULL!	9	9	9	9
2	2	1752	9	0	#NULL!	9	9	9	9
2	2	1753	9	0	#NULL!	9	9	9	9
2	2	1754	9	0	#NULL!	9	9	9	9
2	2	1755	9	0	#NULL!	9	9	9	9
2	2	1756	9	0	#NULL!	9	9	9	9
2	2	1757	9	0	#NULL!	9	9	9	9
2	2	1758	2	0	#NULL!	1	2	3	1
2	2	1759	9	0	#NULL!	9	9	9	9
2	2	1760	9	0	#NULL!	9	9	9	9
2	2	1761	9	0	#NULL!	9	9	9	9
2	2	1762	9	0	#NULL!	9	9	9	9
2	2	1763	9	0	#NULL!	9	9	9	9
2	2	1764	9	0	#NULL!	9	9	9	9
2	2	1765	9	0	#NULL!	9	9	9	9
2	2	1766	9	0	#NULL!	9	9	9	9
2	2	1767	9	0	#NULL!	9	9	9	9
2	2	1768	9	0	#NULL!	9	9	9	9
2	2	1769	9	0	#NULL!	9	9	9	9
2	2	1770	9	0	#NULL!	9	9	9	9
2	2	1771	9	0	#NULL!	9	9	9	9
2	2	1772	9	0	#NULL!	9	9	9	9
2	2	1773	9	0	#NULL!	9	9	9	9
2	2	1774	1	16	2	1	2	3	1
2	2	1775	2	17	2	1	2	3	1
2	2	1776	2	16	2	1	2	3	1
2	2	1777	2	17	2	1	2	3	1
2	2	1778	1	17	2	1	2	3	3
2	2	1779	2	17	2	1	2	3	1

2	2	1780	1	15	2	1	2	3	1
2	2	1781	2	17	2	1	2	3	1
2	2	1782	1	15	2	1	2	3	2
2	2	1783	1	15	2	1	2	3	1
2	2	1784	1	16	2	1	2	2	1
2	2	1785	2	16	2	1	2	3	1
2	2	1786	2	15	2	1	2	3	1
2	2	1787	2	0	#NULL!	1	2	3	1
2	2	1788	2	16	2	1	2	3	1
2	2	1789	2	14	1	1	2	3	1
2	2	1790	2	15	2	1	2	3	1
2	2	1791	2	14	1	1	2	3	1
2	2	1792	1	14	1	1	2	3	1
2	2	1793	2	16	2	1	2	3	1
2	2	1794	1	17	2	1	2	3	2
2	2	1795	1	16	2	1	2	3	1
2	2	1796	2	14	1	1	2	3	1
2	2	1797	2	17	2	1	2	3	1
2	2	1798	1	19	2	1	2	3	1
2	2	1799	2	16	2	1	2	3	1
2	2	1800	2	16	2	1	2	3	1
2	2	1801	1	18	2	1	2	3	1
2	2	1802	1	0	#NULL!	1	2	3	1
2	2	1803	1	16	2	2	2	3	1
2	2	1804	2	14	1	1	2	3	1
2	2	1805	2	15	2	1	2	3	1
2	2	1806	2	17	2	1	2	3	1
2	2	1807	2	15	2	2	2	3	1
2	2	1808	2	16	2	1	2	3	1
2	2	1809	2	15	2	1	2	3	1
2	2	1810	1	17	2	1	2	3	2
2	2	1811	1	16	2	1	3	2	1
2	2	1812	2	16	2	1	3	2	2
2	2	1813	1	15	2	1	3	2	2
2	2	1814	1	15	2	1	3	2	1
2	2	1815	1	0	#NULL!	1	3	2	1
2	2	1816	1	17	2	1	3	2	1
2	2	1817	1	0	#NULL!	1	3	2	1
2	2	1818	1	0	#NULL!	1	3	2	1
2	2	1819	1	18	2	1	3	2	1
2	2	1820	1	18	2	1	3	2	1
2	2	1821	1	18	2	1	3	2	1
2	2	1822	1	18	2	1	3	2	1
2	2	1823	1	18	2	1	3	2	1
2	2	1824	1	18	2	1	3	2	1
2	2	1825	1	18	2	1	3	2	1
2	2	1826	1	18	2	1	3	2	1

2	2	1827	2	18	2	2	3	1	1
2	2	1828	1	16	2	1	3	2	1
2	2	1829	1	17	2	1	2	2	1
2	2	1830	1	14	1	2	3	2	2
2	2	1831	1	14	1	1	3	2	2
2	2	1832	1	12	1	1	3	2	2
2	2	1833	1	16	2	1	3	2	2
2	2	1834	1	16	2	1	3	2	2
2	2	1835	1	30	3	1	3	2	2
2	2	1836	1	15	2	1	3	2	2
2	2	1837	1	17	2	1	3	2	1
2	2	1838	1	16	2	1	3	2	2
2	2	1839	1	14	1	1	3	2	1
2	2	1840	1	18	2	1	3	2	1
2	2	1841	1	14	1	1	3	2	2
2	2	1842	1	16	2	1	3	2	1
2	2	1843	1	18	2	1	3	2	1
2	2	1844	1	17	2	1	3	2	2
2	2	1845	1	14	1	1	3	2	2
2	2	1846	1	14	1	1	3	2	1
2	2	1847	1	18	2	1	3	2	2
2	2	1848	1	18	2	1	3	2	2
2	2	1849	1	18	2	1	3	2	1
2	2	1850	1	19	2	1	3	2	1
2	2	1851	1	18	2	1	3	2	1
2	2	1852	1	18	2	1	3	2	3
2	2	1853	1	18	2	1	3	2	2
2	2	1854	1	18	2	1	3	2	2
2	2	1855	1	16	2	1	3	2	1
2	2	1856	1	19	2	1	3	2	1
2	2	1857	1	14	1	1	3	2	2
2	2	1858	1	18	2	1	3	2	2
2	2	1859	1	18	2	1	3	2	1
2	2	1860	1	19	2	1	3	2	1
2	2	1861	1	17	2	1	3	2	2
2	2	1862	1	18	2	1	3	2	1
2	2	1863	1	18	2	1	3	2	1
2	2	1864	1	16	2	1	3	2	2
2	2	1865	1	16	2	1	3	2	2
2	2	1866	1	18	2	1	3	2	2
2	2	1867	1	16	2	1	3	2	1
2	2	1868	1	13	1	1	3	2	2
2	2	1869	1	20	3	1	3	2	1
2	2	1870	1	14	1	1	3	2	1
2	2	1871	1	14	1	1	3	2	1
2	2	1872	1	14	1	1	3	2	1
2	2	1873	1	15	2	1	3	2	2

2	2	1874	1	14	1	1	3	2	2
2	2	1875	1	12	1	1	3	2	2
2	2	1876	1	14	1	1	3	2	2
2	2	1877	1	13	1	1	3	2	3
2	2	1878	1	18	2	1	3	2	2
2	2	1879	2	18	2	1	3	1	2
2	2	1880	2	18	2	1	3	1	1
2	2	1881	1	15	2	1	3	2	2
2	2	1882	1	17	2	1	3	2	1
2	2	1883	1	23	3	1	3	2	1
2	2	1884	1	20	3	1	3	2	1
2	2	1885	1	29	3	9	3	2	2
2	2	1886	1	19	2	1	3	2	1
2	2	1887	1	12	1	1	3	1	3
2	2	1888	9	0	#NULL!	1	3	2	9
2	2	1889	1	19	2	1	3	2	2
2	2	1890	1	17	2	1	3	2	1
2	2	1891	1	18	2	1	3	2	1
2	2	1892	1	15	2	1	3	2	2
2	2	1893	1	20	3	1	3	2	1
2	2	1894	2	19	2	1	3	1	2
2	2	1895	2	19	2	1	3	1	2
2	2	1896	1	19	2	1	3	9	9
2	2	1897	1	20	3	1	3	2	1
2	2	1898	1	19	2	1	3	2	1
2	2	1899	1	18	2	1	3	2	1
2	2	1900	1	14	1	1	3	2	2
2	2	1901	1	17	2	1	3	2	1
2	2	1902	1	19	2	1	3	2	1
2	2	1903	1	17	2	1	3	2	2
2	2	1904	1	18	2	1	3	2	1
2	2	1905	1	18	2	1	3	2	1
2	2	1906	1	17	2	1	3	2	1
2	2	1907	1	19	2	2	3	2	1
2	2	1908	2	23	3	2	3	2	2
2	2	1909	1	18	2	1	3	2	2
2	2	1910	1	18	2	9	9	9	9
2	2	1911	1	17	2	1	3	2	1
2	2	1912	1	19	2	9	9	9	9
2	2	1913	1	17	2	1	3	2	2
2	2	1914	1	17	2	1	3	2	1
2	2	1915	1	17	2	1	3	2	1
2	2	1916	1	19	2	1	3	2	1
2	2	1917	1	16	2	1	3	2	1
2	2	1918	1	17	2	9	9	9	9
2	2	1919	1	16	2	1	3	2	1
2	2	1920	1	17	2	1	3	2	1

2	2	1921	1	17	2	1	3	2	1
2	2	1922	1	20	3	9	2	2	1
2	2	1923	1	14	1	1	2	2	1
2	2	1924	1	20	3	1	2	2	1
2	2	1925	1	15	2	1	2	9	9
2	2	1926	1	18	2	1	3	2	1
2	2	1927	1	14	1	1	2	2	1
2	2	1928	1	19	2	1	2	2	1
2	2	1929	1	16	2	1	3	2	2
2	2	1930	1	18	2	1	2	2	1
2	2	1931	1	21	3	1	2	2	1
2	2	1932	1	16	2	9	9	9	9
2	2	1933	1	15	2	1	2	2	2
2	2	1934	1	18	2	1	2	2	1
2	2	1935	1	18	2	1	2	2	1
2	2	1936	9	0	#NULL!	1	2	2	2
2	2	1937	1	19	2	1	2	2	2
2	2	1938	1	19	2	2	2	1	2
2	2	1939	1	17	2	1	2	2	2
2	2	1940	1	18	2	1	2	2	1
2	2	1941	1	17	2	2	2	2	1
2	2	1942	1	17	2	1	2	2	1
2	2	1943	1	17	2	1	2	2	1
2	2	1944	1	18	2	1	2	2	1
2	2	1945	1	16	2	1	2	2	1
2	2	1946	1	15	2	1	2	2	1
2	2	1947	1	16	2	1	2	2	1
2	2	1948	1	16	2	1	2	3	1
2	2	1949	1	16	2	1	2	2	1
2	2	1950	1	17	2	1	2	2	1
2	2	1951	1	30	3	1	2	3	1
2	2	1952	1	17	2	1	2	2	2
2	2	1953	1	14	1	1	2	2	2
2	2	1954	1	15	2	1	2	2	1
2	2	1955	1	18	2	1	2	2	2
2	2	1956	1	18	2	1	3	3	1
2	2	1957	9	0	#NULL!	9	9	9	9
2	2	1958	1	25	3	1	3	3	2
2	2	1959	1	18	2	1	2	2	2
2	2	1960	1	16	2	1	2	2	1
2	2	1961	1	17	2	1	2	2	1
2	2	1962	1	16	2	1	2	3	1
2	2	1963	1	16	2	1	2	2	2
2	2	1964	1	19	2	1	2	2	1
2	2	1965	1	18	2	1	2	2	3
2	2	1966	1	29	3	1	2	2	3
2	2	1967	1	18	2	1	3	2	1

2	2	1968	1	22	3	1	2	2	1
2	2	1969	1	18	2	1	2	2	1
2	2	1970	1	16	2	1	2	2	2
2	2	1971	1	17	2	1	2	2	1
2	2	1972	1	16	2	1	2	2	1
2	2	1973	1	18	2	1	2	2	1
2	2	1974	1	15	2	1	2	3	1
2	2	1975	1	16	2	1	2	2	1
2	2	1976	1	15	2	1	2	2	1
2	2	1977	1	27	3	1	3	2	1
2	2	1978	1	16	2	1	2	2	1
2	2	1979	1	16	2	1	2	2	1
2	2	1980	1	18	2	1	2	2	1
2	2	1981	1	16	2	1	1	1	1
2	2	1982	1	16	2	1	2	2	1
2	2	1983	1	17	2	1	2	2	1
2	2	1984	1	12	1	1	2	2	1
2	2	1985	1	25	3	2	3	3	3
2	2	1986	1	15	2	1	2	2	1
2	2	1987	1	15	2	1	2	2	1
2	2	1988	1	50	3	2	3	1	2
2	2	1989	1	20	3	2	3	1	2
2	2	1990	1	18	2	1	2	2	2
2	2	1991	1	16	2	1	2	2	1
2	2	1992	1	5	1	2	3	1	9
2	2	1993	9	17	2	1	2	2	1
2	2	1994	1	15	2	1	2	2	1
2	2	1995	1	17	2	1	2	2	1
2	2	1996	1	13	1	1	2	2	2
2	2	1997	1	16	2	1	2	2	1
2	2	1998	1	13	1	1	2	2	2
2	2	1999	1	17	2	1	2	2	1
2	2	2000	1	16	2	1	2	2	1
2	2	2001	1	16	2	1	2	2	1
2	2	2002	1	18	2	1	2	2	1
2	2	2003	1	15	2	1	2	2	2
2	2	2004	9	0	#NULL!	9	9	9	9
2	2	2005	1	16	2	1	2	2	1
2	2	2006	1	39	3	1	2	2	3
2	2	2007	1	25	3	1	2	2	1
2	2	2008	1	15	2	1	2	2	1
2	2	2009	9	0	#NULL!	9	9	9	9
2	2	2010	1	16	2	1	2	2	3
2	2	2011	1	18	2	1	2	3	3
2	2	2012	1	16	2	1	2	2	1
2	2	2013	1	19	2	1	2	2	1
2	2	2014	1	16	2	1	2	2	1

2	2	2015	1	12	1	1	2	2	2
2	2	2016	9	0	#NULL!	9	9	9	9
2	2	2017	1	13	1	1	2	2	2
2	2	2018	1	14	1	1	2	2	1
2	2	2019	1	15	2	1	2	2	1
2	2	2020	1	15	2	1	2	2	2
2	2	2021	1	17	2	1	2	2	1
2	2	2022	1	16	2	1	2	2	1
2	2	2023	1	12	1	1	2	2	2
2	2	2024	1	16	2	1	2	2	1
2	2	2025	1	14	1	2	1	2	1
2	2	2026	1	13	1	9	1	2	2
2	2	2027	1	14	1	1	1	2	1
2	2	2028	1	13	1	1	1	2	1
2	2	2029	9	0	#NULL!	9	9	9	9
2	2	2030	1	13	1	1	1	2	1
2	2	2031	1	15	2	1	1	2	1
2	2	2032	1	14	1	1	1	2	2
2	2	2033	1	14	1	1	1	2	1
2	2	2034	1	13	1	1	1	2	1
2	2	2035	1	16	2	1	1	2	2
2	2	2036	1	12	1	1	1	2	1
2	2	2037	1	14	1	1	1	2	2
2	2	2038	1	13	1	1	1	2	1
2	2	2039	1	14	1	1	1	2	1
2	2	2040	1	14	1	1	1	2	1
2	2	2041	1	14	1	1	1	2	1
2	2	2042	1	15	2	1	1	2	2
2	2	2043	1	12	1	1	1	2	2
2	2	2044	1	15	2	2	1	2	1
2	2	2045	1	14	1	1	1	1	1
2	2	2046	9	0	#NULL!	9	9	9	9
2	2	2047	1	13	1	1	1	2	1
2	2	2048	1	14	1	1	1	2	1
2	2	2049	1	14	1	1	1	2	1
2	2	2050	1	14	1	1	1	2	1
2	2	2051	1	13	1	1	1	2	1
2	2	2052	9	0	#NULL!	9	9	9	9
2	2	2053	1	14	1	9	1	2	1
2	2	2054	1	14	1	1	1	2	1
2	2	2055	1	11	1	1	1	2	1
2	2	2056	1	13	1	1	1	2	1
2	2	2057	9	0	#NULL!	1	1	2	2
2	2	2058	1	15	2	1	1	2	2
2	2	2059	1	13	1	1	3	2	3
2	2	2060	1	15	2	1	1	2	1
2	2	2061	1	14	1	1	1	2	1

2	2	2062	1	15	2	1	1	2	2
2	2	2063	1	15	2	1	1	2	1
2	2	2064	1	14	1	1	9	2	2
2	2	2065	1	14	1	1	9	2	2
2	2	2066	1	14	1	1	1	3	1
2	2	2067	1	15	2	2	3	3	2
2	2	2068	1	14	1	9	9	9	9
2	2	2069	1	15	2	1	1	2	1
2	2	2070	1	14	1	1	1	2	1
2	2	2071	1	15	2	1	1	2	1
2	2	2072	1	16	2	1	1	2	1
2	2	2073	1	16	2	1	1	2	1
2	2	2074	1	16	2	1	1	2	1
2	2	2075	1	0	#NULL!	2	1	2	1
2	2	2076	1	16	2	1	1	2	1
2	2	2077	1	14	1	1	1	2	1
2	2	2078	1	16	2	1	1	2	1
2	2	2079	1	14	1	2	1	2	2
2	2	2080	1	14	1	1	1	2	1
2	2	2081	1	15	2	1	1	2	1
2	2	2082	9	0	#NULL!	1	1	1	1
2	2	2083	1	15	2	1	1	2	3
2	2	2084	1	15	2	2	1	2	1
2	2	2085	1	14	1	1	1	2	1
2	2	2086	1	16	2	1	1	2	1
2	2	2087	1	15	2	1	1	2	1
2	2	2088	9	0	#NULL!	9	9	9	9
2	2	2089	9	0	#NULL!	1	1	2	1
2	2	2090	1	14	1	1	1	2	1
2	2	2091	9	0	#NULL!	9	9	9	9
2	2	2092	1	16	2	1	1	2	2
2	2	2093	1	14	1	1	1	2	2
2	2	2094	1	15	2	1	1	2	2
2	2	2095	1	16	2	1	1	2	1
2	2	2096	1	15	2	1	1	2	1
2	2	2097	1	15	2	1	1	2	1
2	2	2098	1	15	2	1	1	2	1
2	2	2099	1	15	2	1	1	2	1
2	2	2100	9	18	2	1	1	2	1
2	2	2101	1	15	2	1	1	2	2
2	2	2102	1	13	1	1	1	2	3
2	2	2103	9	0	#NULL!	1	1	2	2
2	2	2104	9	0	#NULL!	1	1	2	1
2	2	2105	1	15	2	1	1	2	1
2	2	2106	1	16	2	1	1	2	1
2	2	2107	1	16	2	1	1	2	1
2	2	2108	1	16	2	1	1	2	1

2	2	2109	1	15	2	1	1	2	1
2	2	2110	1	15	2	1	1	2	1
2	2	2111	1	15	2	1	1	2	1
2	2	2112	1	16	2	1	1	2	1
2	2	2113	9	17	2	1	2	2	1
2	2	2114	1	18	2	1	1	2	1
2	2	2115	9	13	1	1	1	2	1
2	2	2116	1	14	1	1	1	2	1
2	2	2117	1	15	2	1	1	2	1
2	2	2118	9	0	#NULL!	1	1	2	2
2	2	2119	1	15	2	1	1	2	2
2	2	2120	1	15	2	1	1	2	2
2	2	2121	1	15	2	1	1	1	1
2	2	2122	1	15	2	1	1	2	2
2	2	2123	1	13	1	1	1	2	1
2	2	2124	1	13	1	1	1	2	1
2	2	2125	1	13	1	1	1	2	2
2	2	2126	1	13	1	1	1	2	1
2	2	2127	1	18	2	1	1	2	2
2	2	2128	1	18	2	2	1	2	1
2	2	2129	1	19	2	2	1	1	2
2	2	2130	1	15	2	1	1	2	2
2	2	2131	1	15	2	1	1	2	1
2	2	2132	1	13	1	1	1	2	1
2	2	2133	1	14	1	1	1	2	3
2	2	2134	1	15	2	1	1	2	1
2	2	2135	1	14	1	1	1	2	1
2	2	2136	1	12	1	1	1	2	2
2	2	2137	1	17	2	1	1	2	2
2	2	2138	1	14	1	1	1	2	1
2	2	2139	1	14	1	1	1	2	1
2	2	2140	1	14	1	1	1	2	2
2	2	2141	1	14	1	2	1	2	1
2	2	2142	1	15	2	1	1	2	1
2	2	2143	1	14	1	1	1	2	1
2	2	2144	1	29	3	2	1	2	1
2	2	2145	1	15	2	1	1	2	1
2	2	2146	1	0	#NULL!	1	1	2	3
2	2	2147	1	14	1	1	1	1	1
2	2	2148	1	19	2	2	1	2	1
2	2	2149	1	13	1	1	1	2	1
2	2	2150	1	15	2	1	1	2	1
2	2	2151	1	14	1	1	1	2	1
2	2	2152	1	14	1	1	1	2	1
2	2	2153	1	15	2	1	1	2	1
2	2	2154	1	15	2	1	1	2	1
2	2	2155	1	15	2	1	1	2	2

2	2	2156	1	14	1	1	1	2	1
2	2	2157	1	14	1	1	1	2	1
2	2	2158	1	15	2	1	1	2	1
2	2	2159	9	0	#NULL!	9	9	9	9
2	2	2160	1	16	2	1	9	9	1
2	2	2161	1	19	2	2	1	2	1
2	2	2162	1	15	2	1	1	2	1
2	2	2163	1	16	2	1	1	2	1
2	2	2164	1	15	2	1	1	2	1
2	2	2165	1	14	1	1	1	2	1
2	2	2166	1	14	1	1	1	2	1
2	2	2167	1	13	1	1	1	2	1
2	2	2168	1	13	1	1	1	2	2
2	2	2169	1	14	1	1	1	2	1
2	2	2170	1	13	1	1	1	2	1
2	2	2171	1	14	1	1	1	2	1
2	2	2172	1	14	1	1	1	2	1
2	2	2173	1	17	2	1	1	2	1
2	2	2174	1	14	1	1	1	2	1
2	2	2175	1	14	1	1	1	2	1
2	2	2176	1	14	1	9	1	2	1
2	2	2177	1	13	1	1	1	2	1
2	2	2178	1	12	1	1	1	2	3
2	2	2179	1	14	1	1	1	2	1
2	2	2180	1	14	1	1	1	2	1
2	2	2181	1	13	1	1	1	2	1
2	2	2182	1	14	1	1	1	2	1
2	2	2183	1	14	1	1	1	2	1
2	2	2184	1	0	#NULL!	1	1	2	1
2	2	2185	1	15	2	1	1	2	1
2	2	2186	1	15	2	1	1	2	2
2	2	2187	1	13	1	1	1	2	2
2	2	2188	1	15	2	1	1	2	2
2	2	2189	1	14	1	1	1	2	1
2	2	2190	1	14	1	1	1	2	1
2	2	2191	1	14	1	1	1	2	1
2	2	2192	1	15	2	1	1	2	1
2	2	2193	1	15	2	1	1	2	1
2	2	2194	1	30	3	1	3	2	1
2	2	2195	1	14	1	1	1	2	2
2	2	2196	9	0	#NULL!	1	1	2	1
2	2	2197	9	0	#NULL!	1	1	2	1
2	2	2198	1	14	1	1	1	2	2
2	2	2199	1	15	2	1	1	2	2
2	2	2200	1	14	1	1	1	2	1
2	2	2201	1	14	1	1	1	2	1
2	2	2202	1	17	2	1	1	2	1

2	2	2203	1	15	2	1	1	2	1
2	2	2204	1	14	1	1	1	2	1
2	2	2205	1	15	2	1	1	2	1
2	2	2206	1	13	1	1	1	2	1
2	2	2207	1	16	2	1	1	2	2
2	2	2208	1	17	2	1	1	2	2
2	2	2209	1	2	1	1	1	2	2
2	2	2210	1	13	1	1	1	2	1
2	2	2211	1	16	2	1	1	2	1
2	2	2212	1	16	2	1	1	2	1
2	2	2213	1	15	2	1	1	2	1
2	2	2214	1	17	2	1	1	2	1
2	2	2215	1	14	1	1	1	2	1
2	2	2216	1	14	1	1	1	2	1
2	2	2217	1	13	1	1	1	2	1
2	2	2218	1	14	1	1	1	2	1
2	2	2219	1	12	1	1	1	2	2
2	2	2220	1	13	1	2	1	2	1
2	2	2221	1	13	1	1	1	2	1
2	2	2222	1	15	2	1	1	2	2
2	2	2223	1	15	2	1	1	2	2
2	2	2224	1	0	#NULL!	1	1	2	1
2	2	2225	1	14	1	1	1	2	2
2	2	2226	1	13	1	1	1	2	2
2	2	2227	1	15	2	1	1	2	2
2	2	2228	1	14	1	1	1	2	1
2	2	2229	2	0	#NULL!	1	3	1	1
2	2	2230	2	19	2	1	3	1	1
2	2	2231	2	18	2	1	3	1	1
2	2	2232	2	16	2	1	3	1	1
2	2	2233	2	16	2	1	3	1	1
2	2	2234	2	0	#NULL!	1	3	1	1
2	2	2235	2	16	2	1	3	1	1
2	2	2236	2	16	2	1	3	1	1
2	2	2237	2	16	2	1	3	1	1
2	2	2238	9	0	#NULL!	9	9	9	9
2	2	2239	2	17	2	1	3	1	1
2	2	2240	9	0	#NULL!	1	3	1	1
2	2	2241	2	15	2	1	3	1	1
2	2	2242	2	16	2	1	3	1	1
2	2	2243	2	16	2	1	3	1	1
2	2	2244	2	16	2	1	3	1	1
2	2	2245	2	0	#NULL!	1	3	1	1
2	2	2246	2	16	2	1	3	1	1
2	2	2247	2	18	2	1	3	1	1
2	2	2248	2	18	2	1	3	1	1
2	2	2249	2	17	2	1	3	1	1

2	2	2250	2	16	2	1	3	1	1
2	2	2251	2	17	2	1	3	1	1
2	2	2252	2	20	3	1	3	1	1
2	2	2253	2	17	2	1	3	1	1
2	2	2254	2	17	2	1	3	1	1
2	2	2255	2	17	2	1	3	1	1
2	2	2256	2	17	2	2	3	1	1
2	2	2257	2	17	2	1	3	1	1
2	2	2258	2	19	2	1	3	1	1
2	2	2259	2	16	2	1	3	1	1
2	2	2260	2	17	2	1	3	1	1
2	2	2261	2	16	2	1	3	1	1
2	2	2262	2	18	2	1	3	1	1
2	2	2263	2	16	2	1	3	1	1
2	2	2264	2	16	2	1	3	1	1
2	2	2265	2	16	2	1	3	1	1
2	2	2266	2	17	2	1	3	9	1
2	2	2267	2	18	2	1	3	1	1
2	2	2268	2	17	2	1	3	1	1
2	2	2269	9	0	#NULL!	9	9	9	1
2	2	2270	9	0	#NULL!	1	9	9	1
2	2	2271	2	16	2	1	3	1	1
2	2	2272	2	18	2	1	3	1	1
2	2	2273	2	18	2	1	3	1	1
2	2	2274	2	15	2	1	9	9	1
2	2	2275	2	15	2	1	3	1	1
2	2	2276	2	16	2	1	3	1	1
2	2	2277	2	17	2	1	3	1	1
2	2	2278	2	17	2	1	3	1	1
2	2	2279	2	18	2	1	3	1	1
2	2	2280	9	0	#NULL!	1	3	1	1
2	2	2281	2	16	2	1	3	1	1
2	2	2282	2	16	2	1	3	1	1
2	2	2283	2	17	2	1	3	1	1
2	2	2284	2	17	2	1	3	1	1
2	2	2285	2	17	2	1	3	1	1
2	2	2286	9	0	#NULL!	1	3	9	1
2	2	2287	2	17	2	1	3	1	1
2	2	2288	2	17	2	1	3	1	1
2	2	2289	2	16	2	1	3	1	1
2	2	2290	2	17	2	1	3	1	1
2	2	2291	2	16	2	1	3	1	1
2	2	2292	2	18	2	1	3	1	1
2	2	2293	2	18	2	1	3	1	1
2	2	2294	2	17	2	1	3	1	1
2	2	2295	2	17	2	1	3	1	1
2	2	2296	2	16	2	1	3	1	1

2	2	2297	2	17	2	1	3	1	1
2	2	2298	2	16	2	1	3	1	1
2	2	2299	2	15	2	1	3	1	1
2	2	2300	9	0	#NULL!	9	9	9	9
2	2	2301	2	15	2	1	3	1	1
2	2	2302	2	16	2	1	3	1	1
2	2	2303	2	17	2	1	3	1	1
2	2	2304	2	16	2	1	3	1	1
2	2	2305	2	16	2	1	3	1	1
2	2	2306	2	17	2	1	3	1	1
2	2	2307	2	16	2	1	3	1	1
2	2	2308	2	17	2	1	3	1	1
2	2	2309	2	17	2	1	3	1	1
2	2	2310	2	19	2	1	3	1	1
2	2	2311	2	18	2	1	3	1	1
2	2	2312	2	19	2	1	3	1	1
2	2	2313	2	17	2	1	3	1	1
2	2	2314	2	20	3	1	3	1	1
2	2	2315	2	17	2	1	3	1	2
2	2	2316	2	17	2	1	3	1	1
2	2	2317	2	18	2	1	3	1	1
2	2	2318	2	17	2	1	3	1	1
2	2	2319	2	16	2	1	3	1	1
2	2	2320	2	16	2	1	3	1	1
2	2	2321	2	17	2	1	3	1	1
2	2	2322	2	16	2	1	3	1	1
2	2	2323	2	17	2	1	3	1	1
2	2	2324	2	17	2	1	3	1	1
2	2	2325	2	15	2	2	3	1	1
2	2	2326	2	18	2	1	3	1	1
2	2	2327	2	16	2	1	3	1	1
2	2	2328	2	16	2	1	3	1	1
2	2	2329	2	17	2	1	3	1	1
2	2	2330	2	17	2	1	3	1	9
2	2	2331	9	18	2	1	3	1	1
2	2	2332	2	0	#NULL!	1	3	1	1
2	2	2333	2	17	2	1	3	1	1
2	2	2334	2	18	2	1	3	1	1
2	2	2335	2	17	2	1	3	1	2
2	2	2336	2	17	2	1	3	1	1
2	2	2337	2	17	2	1	3	1	1
2	2	2338	2	17	2	1	3	1	1
2	2	2339	9	16	2	2	3	1	1
2	2	2340	9	17	2	1	3	1	1
2	2	2341	9	17	2	1	3	1	1
2	2	2342	2	17	2	1	3	1	2
2	2	2343	2	16	2	1	3	1	1

2	2	2344	2	16	2	1	3	1	1
2	2	2345	2	17	2	1	3	1	1
2	2	2346	2	16	2	9	3	1	1
2	2	2347	2	15	2	1	3	1	2
2	2	2348	2	16	2	1	3	1	1
2	2	2349	2	18	2	1	3	1	1
2	2	2350	2	16	2	1	3	1	1
2	2	2351	2	18	2	1	3	1	1
2	2	2352	2	17	2	1	3	1	1
2	2	2353	2	16	2	1	3	1	1
2	2	2354	2	15	2	1	3	1	1
2	2	2355	2	0	#NULL!	2	3	1	1
2	2	2356	2	16	2	1	3	1	9
2	2	2357	2	16	2	1	3	1	1
2	2	2358	2	17	2	1	3	1	1
2	2	2359	2	16	2	1	3	1	1
2	2	2360	2	15	2	1	3	1	1
2	2	2361	2	15	2	1	3	1	1
2	2	2362	2	17	2	1	3	1	1
2	2	2363	2	15	2	1	3	1	1
2	2	2364	2	16	2	1	3	1	1
2	2	2365	2	16	2	1	3	1	1
2	2	2366	2	16	2	1	3	1	1
2	2	2367	2	16	2	1	3	1	1
2	2	2368	2	17	2	1	3	1	1
2	2	2369	2	16	2	1	3	1	1
2	2	2370	2	16	2	1	3	1	1
2	2	2371	2	17	2	1	3	1	1
2	2	2372	9	0	#NULL!	1	3	1	1
2	2	2373	9	19	2	1	3	1	1
2	2	2374	2	16	2	1	3	1	1
2	2	2375	2	16	2	1	3	1	1
2	2	2376	2	15	2	1	3	1	1
2	2	2377	2	16	2	1	3	1	1
2	2	2378	2	15	2	1	3	1	1
2	2	2379	2	17	2	1	3	1	1
2	2	2380	2	15	2	2	3	1	1
2	2	2381	2	18	2	1	3	1	1
2	2	2382	2	15	2	1	3	1	1
2	2	2383	2	16	2	1	3	1	1
2	2	2384	2	16	2	1	3	1	1
2	2	2385	2	0	#NULL!	1	3	1	1
2	2	2386	2	17	2	1	3	1	1
2	2	2387	9	17	2	1	3	1	1
2	2	2388	2	17	2	1	3	1	1
2	2	2389	2	15	2	1	3	1	1
2	2	2390	2	17	2	2	3	1	1

2	2	2391	2	16	2	1	3	1	1
2	2	2392	2	16	2	1	3	1	1
2	2	2393	2	16	2	1	3	1	1
2	2	2394	2	16	2	1	3	1	1
2	2	2395	2	18	2	9	3	1	1
2	2	2396	2	16	2	1	3	1	1
2	2	2397	2	0	#NULL!	1	3	1	1
2	2	2398	2	17	2	1	3	1	1
2	2	2399	9	0	#NULL!	9	9	9	9
2	2	2400	2	18	2	1	3	1	1
2	2	2401	2	18	2	1	3	1	1
2	2	2402	2	16	2	1	3	1	1
2	2	2403	2	18	2	1	3	1	1
2	2	2404	2	16	2	1	3	1	1
2	2	2405	2	18	2	1	3	1	1
2	2	2406	2	17	2	1	3	1	1
2	2	2407	2	17	2	2	3	1	1
2	2	2408	2	17	2	1	3	1	1
2	2	2409	2	19	2	1	3	1	1
2	2	2410	2	17	2	1	3	1	1
2	2	2411	2	18	2	9	3	1	1
2	2	2412	2	17	2	1	3	1	1
2	2	2413	2	18	2	1	3	1	1
2	2	2414	2	16	2	1	3	1	1
2	2	2415	2	17	2	1	3	1	1
2	2	2416	2	17	2	1	3	1	1
2	2	2417	2	17	2	2	3	1	1
2	2	2418	2	15	2	1	3	1	1
2	2	2419	2	16	2	1	3	1	1
2	2	2420	2	19	2	1	3	1	1
2	2	2421	2	17	2	1	3	1	1
2	2	2422	2	17	2	1	3	1	1
2	2	2423	2	16	2	1	3	1	1
2	2	2424	2	15	2	1	3	1	1
2	2	2425	2	18	2	1	3	1	1
2	2	2426	2	16	2	1	3	1	1
2	2	2427	2	15	2	2	3	1	1
2	2	2428	2	17	2	1	3	1	1
2	2	2429	2	0	#NULL!	9	3	1	1
2	2	2430	2	17	2	1	3	1	1
2	2	2431	2	17	2	1	3	1	2
2	2	2432	2	16	2	1	3	1	1
2	2	2433	2	17	2	1	3	1	2
2	2	2434	2	17	2	1	3	1	1
2	2	2435	2	17	2	1	3	1	1
2	2	2436	2	17	2	1	3	1	1
2	2	2437	2	17	2	1	3	9	1

2	2	2438	2	16	2	1	3	1	1
2	2	2439	2	17	2	1	3	1	1
2	2	2440	2	17	2	1	3	1	1
2	2	2441	2	16	2	1	3	1	1
2	2	2442	2	18	2	1	3	1	1
2	2	2443	2	15	2	1	3	1	1
2	2	2444	2	17	2	1	3	1	1
2	2	2445	2	16	2	1	3	1	1
2	2	2446	2	16	2	1	3	1	1
2	2	2447	2	18	2	1	3	1	1
2	2	2448	2	17	2	1	3	1	1
2	2	2449	2	16	2	1	3	1	1
2	2	2450	2	15	2	1	3	1	1
2	2	2451	2	17	2	2	3	1	1
2	2	2452	2	16	2	1	3	1	2
2	2	2453	2	18	2	1	3	1	1
2	2	2454	2	15	2	1	3	1	1
2	2	2455	2	17	2	1	3	1	1
2	2	2456	2	17	2	1	3	1	1
2	2	2457	2	16	2	1	3	1	1
2	2	2458	2	17	2	1	3	1	1
2	2	2459	2	18	2	1	3	1	1
2	2	2460	2	16	2	1	3	1	1
2	2	2461	2	18	2	1	3	1	1
2	2	2462	2	18	2	1	3	1	1
2	2	2463	2	18	2	1	3	1	1
2	2	2464	2	15	2	1	3	1	1
2	2	2465	2	15	2	1	3	1	1
2	2	2466	2	16	2	1	3	1	1
2	2	2467	2	19	2	1	3	1	1
2	2	2468	2	17	2	1	3	1	1
2	2	2469	2	17	2	1	3	1	1
2	2	2470	2	15	2	1	3	1	1
2	2	2471	2	17	2	1	3	1	1
2	2	2472	2	18	2	1	3	1	1
2	2	2473	2	15	2	1	3	1	1
2	2	2474	2	17	2	1	3	1	1
2	2	2475	2	16	2	1	3	1	1
2	2	2476	2	15	2	1	3	1	1
2	2	2477	2	14	1	1	3	1	1
2	2	2478	2	17	2	1	3	1	1
2	2	2479	2	17	2	1	3	1	1
2	2	2480	2	15	2	1	3	1	1
2	2	2481	2	15	2	1	3	1	1
2	2	2482	9	0	#NULL!	1	3	1	1
2	2	2483	2	18	2	1	3	1	1
2	2	2484	2	17	2	1	3	1	1

2	2	2485	9	18	2	1	3	1	1
2	2	2486	2	16	2	1	3	1	2
2	2	2487	2	16	2	1	3	1	1
2	2	2488	2	19	2	1	3	1	1
2	2	2489	2	17	2	1	3	1	1
2	2	2490	2	14	1	1	2	1	1
2	2	2491	2	16	2	1	2	1	1
2	2	2492	2	15	2	1	2	1	1
2	2	2493	2	17	2	1	2	1	1
2	2	2494	2	14	1	1	2	1	1
2	2	2495	2	15	2	1	2	1	1
2	2	2496	2	12	1	1	2	1	1
2	2	2497	2	15	2	1	2	1	1
2	2	2498	2	14	1	1	2	1	1
2	2	2499	2	16	2	1	2	1	1
2	2	2500	2	15	2	1	2	1	1
2	2	2501	2	15	2	1	2	1	1
2	2	2502	2	17	2	1	2	1	1
2	2	2503	2	17	2	1	2	1	1
2	2	2504	2	15	2	1	2	1	2
2	2	2505	2	14	1	1	2	1	1
2	2	2506	2	17	2	1	2	1	1
2	2	2507	2	16	2	1	2	1	1
2	2	2508	2	15	2	1	2	1	1
2	2	2509	2	16	2	1	2	1	1
2	2	2510	2	15	2	1	2	1	1
2	2	2511	2	15	2	1	2	1	1
2	2	2512	2	17	2	1	2	1	1
2	2	2513	2	16	2	1	2	1	1
2	2	2514	2	16	2	1	2	1	1
2	2	2515	2	15	2	1	2	1	1
2	2	2516	2	16	2	1	2	1	1
2	2	2517	2	15	2	1	2	1	2
2	2	2518	2	15	2	1	2	1	1
2	2	2519	2	16	2	1	2	1	1
2	2	2520	2	18	2	2	2	1	2
2	2	2521	2	15	2	1	2	1	1
2	2	2522	2	14	1	1	2	1	1
2	2	2523	2	16	2	1	2	1	1
2	2	2524	2	16	2	1	2	1	1
2	2	2525	2	16	2	1	2	1	2
2	2	2526	2	17	2	1	2	1	1
2	2	2527	2	16	2	1	2	1	2
2	2	2528	2	17	2	1	2	1	2
2	2	2529	2	17	2	1	2	1	1
2	2	2530	2	16	2	1	2	1	1
2	2	2531	2	16	2	1	2	1	1

2	2	2532	2	15	2	1	2	1	1
2	2	2533	2	15	2	1	2	1	1
2	2	2534	2	16	2	1	2	1	1
2	2	2535	2	17	2	2	2	1	1
2	2	2536	2	17	2	1	2	1	2
2	2	2537	2	18	2	1	2	1	1
2	2	2538	2	16	2	1	2	1	1
2	2	2539	2	15	2	1	2	1	1
2	2	2540	2	19	2	1	2	1	1
2	2	2541	2	16	2	1	2	1	1
2	2	2542	2	16	2	1	2	1	1
2	2	2543	2	18	2	1	2	1	1
2	2	2544	2	18	2	9	9	9	9
2	2	2545	2	17	2	1	2	1	1
2	2	2546	2	15	2	1	2	1	1
2	2	2547	2	16	2	1	2	1	1
2	2	2548	2	17	2	1	2	1	1
2	2	2549	2	15	2	1	2	1	1
2	2	2550	2	16	2	1	2	1	1
2	2	2551	2	15	2	1	2	1	1
2	2	2552	2	16	2	1	2	1	1
2	2	2553	2	15	2	1	2	1	1
2	2	2554	2	15	2	1	2	1	1
2	2	2555	2	16	2	2	2	1	1
2	2	2556	2	15	2	1	2	1	1
2	2	2557	2	17	2	1	2	1	2
2	2	2558	2	15	2	1	2	1	1
2	2	2559	2	17	2	1	2	1	1
2	2	2560	2	16	2	1	2	1	1
2	2	2561	2	14	1	1	2	1	1
2	2	2562	2	15	2	1	2	1	1
2	2	2563	2	15	2	1	2	1	1
2	2	2564	2	14	1	2	2	3	2
2	2	2565	2	15	2	1	2	1	1
2	2	2566	2	15	2	1	2	1	1
2	2	2567	2	16	2	1	2	1	1
2	2	2568	2	15	2	1	2	1	1
2	2	2569	2	15	2	1	2	1	1
2	2	2570	2	17	2	1	2	1	1
2	2	2571	2	16	2	1	2	1	1
2	2	2572	2	16	2	1	2	1	1
2	2	2573	2	16	2	1	2	1	1
2	2	2574	2	14	1	1	2	1	1
2	2	2575	2	15	2	1	2	1	1
2	2	2576	2	15	2	1	2	1	1
2	2	2577	2	15	2	1	2	1	1
2	2	2578	2	16	2	1	2	1	1

2	2	2579	2	17	2	1		2	1	1
2	2	2580	2	20	3	1		2	1	1
2	2	2581	2	17	2	1		2	1	3
2	2	2582	2	15	2	1		2	1	3
2	2	2583	2	17	2	1		2	1	1
2	2	2584	2	16	2	1		2	1	1
2	2	2585	2	16	2	2		2	1	1
2	2	2586	2	15	2	1		2	1	1
2	2	2587	2	18	2	1		2	1	1
2	2	2588	2	14	1	1		2	1	3
2	2	2589	2	15	2	1		2	1	1
2	2	2590	2	17	2	1		2	1	3
2	2	2591	2	16	2	1		2	1	1
2	2	2592	2	15	2	1		2	1	1
2	2	2593	2	16	2	1		2	1	1
2	2	2594	2	16	2	1		2	1	1
2	2	2595	2	16	2	1		2	1	1
2	2	2596	2	21	3	1		2	1	1
2	2	2597	2	15	2	1		2	1	1
2	2	2598	2	16	2	1		2	1	1
2	2	2599	2	16	2	1		2	1	1
2	2	2600	2	16	2	1		2	1	1
2	2	2601	2	15	2	1		2	1	1
2	2	2602	2	13	1	1		2	1	1
2	2	2603	2	15	2	1		2	1	1
2	2	2604	2	15	2	1		2	1	1
2	2	2605	2	15	2	1		2	1	1
2	2	2606	2	16	2	1		2	1	1
2	2	2607	2	16	2	1		2	1	1
2	2	2608	2	15	2	1		2	1	1
2	2	2609	2	15	2	1		2	9	1
2	2	2610	2	14	1	1		2	1	1
2	2	2611	2	14	1	1		2	1	1
2	2	2612	2	15	2	1		2	1	1
2	2	2613	2	15	2	1		2	1	1
2	2	2614	2	16	2	1		2	1	1
2	2	2615	2	14	1	2		2	1	1
2	2	2616	2	15	2	1		2	1	1
2	2	2617	2	0	#NULL!	1		2	1	1
2	2	2618	2	16	2	1		2	1	1
2	2	2619	2	16	2	1		2	1	1
2	2	2620	2	17	2	1		2	1	1
2	2	2621	2	15	2	1		2	1	1
2	2	2622	2	14	1	2		2	1	1
2	2	2623	2	15	2	1		2	1	1
2	2	2624	2	17	2	1		2	1	1
2	2	2625	2	15	2	1		2	1	1

2	2	2626	2	14	1	2	2	1	1
2	2	2627	2	17	2	1	2	1	1
2	2	2628	2	15	2	2	2	1	1
2	2	2629	2	16	2	1	2	1	1
2	2	2630	2	15	2	1	2	1	1
2	2	2631	2	0	#NULL!	2	2	1	1
2	2	2632	2	15	2	1	2	1	1
2	2	2633	2	14	1	1	2	1	1
2	2	2634	2	15	2	1	2	1	1
2	2	2635	2	15	2	1	2	1	1
2	2	2636	2	15	2	1	2	1	1
2	2	2637	2	16	2	1	2	1	1
2	2	2638	2	14	1	1	2	1	1
2	2	2639	2	13	1	1	2	1	1
2	2	2640	2	16	2	1	2	1	1
2	2	2641	2	15	2	1	2	1	1
2	2	2642	2	14	1	1	2	1	1
2	2	2643	2	14	1	1	2	1	1
2	2	2644	2	15	2	1	2	1	1
2	2	2645	2	16	2	1	2	1	1
2	2	2646	2	14	1	1	2	1	1
2	2	2647	2	17	2	1	2	1	1
2	2	2648	2	15	2	1	2	1	1
2	2	2649	2	14	1	1	2	1	1
2	2	2650	2	16	2	1	2	1	1
2	2	2651	2	15	2	1	2	1	1
2	2	2652	2	16	2	2	2	1	1
2	2	2653	2	15	2	1	2	1	1
2	2	2654	2	16	2	1	2	1	1
2	2	2655	2	16	2	2	2	1	1
2	2	2656	2	14	1	1	2	1	1
2	2	2657	2	16	2	1	2	1	1
2	2	2658	2	15	2	1	2	1	1
2	2	2659	2	17	2	1	2	1	1
2	2	2660	2	16	2	2	2	1	1
2	2	2661	2	16	2	1	2	1	1
2	2	2662	2	15	2	1	2	1	1
2	2	2663	2	17	2	1	2	1	1
2	2	2664	2	15	2	1	2	1	1
2	2	2665	2	15	2	1	2	1	1
2	2	2666	2	16	2	1	2	1	1
2	2	2667	2	15	2	1	2	1	1
2	2	2668	2	16	2	1	2	1	1
2	2	2669	2	20	3	1	2	1	1
2	2	2670	2	12	1	1	2	1	1
2	2	2671	2	18	2	1	2	1	1
2	2	2672	2	16	2	1	2	1	1

2	2	2673	2	16	2	1	2	1	1
2	2	2674	2	15	2	1	2	1	1
2	2	2675	2	18	2	2	2	1	1
2	2	2676	2	17	2	1	1	1	2
2	2	2677	2	15	2	1	2	1	1
2	2	2678	2	16	2	1	2	1	1
2	2	2679	2	14	1	1	2	1	1
2	2	2680	2	14	1	1	2	1	1
2	2	2681	2	16	2	1	2	1	1
2	2	2682	2	14	1	1	2	1	1
2	2	2683	2	15	2	1	2	1	1
2	2	2684	2	15	2	1	2	1	1
2	2	2685	2	16	2	1	2	1	1
2	2	2686	2	15	2	1	2	1	1
2	2	2687	2	15	2	1	2	1	1
2	2	2688	2	16	2	1	2	1	1
2	2	2689	2	0	#NULL!	1	2	1	1
2	2	2690	2	17	2	1	2	1	1
2	2	2691	2	18	2	1	2	1	1
2	2	2692	9	15	2	1	2	1	1
2	2	2693	2	16	2	1	2	1	1
2	2	2694	2	15	2	1	2	1	1
2	2	2695	2	17	2	1	2	1	1
2	2	2696	2	19	2	1	2	1	1
2	2	2697	2	16	2	1	2	1	1
2	2	2698	2	16	2	1	2	1	1
2	2	2699	2	16	2	1	2	1	1
2	2	2700	2	15	2	1	2	1	1
2	2	2701	2	16	2	1	2	1	1
2	2	2702	2	16	2	1	2	1	1
2	2	2703	2	15	2	1	2	1	1
2	2	2704	9	18	2	1	2	1	1
2	2	2705	2	16	2	1	2	1	1
2	2	2706	2	19	2	1	2	1	1
2	2	2707	2	16	2	1	2	1	1
2	2	2708	2	15	2	1	2	1	1
2	2	2709	2	15	2	1	2	1	1
2	2	2710	2	17	2	1	2	1	1
2	2	2711	2	15	2	1	2	1	1
2	2	2712	2	15	2	1	2	1	1
2	2	2713	9	16	2	1	2	1	1
2	2	2714	2	17	2	1	2	1	1
2	2	2715	2	16	2	2	2	1	1
2	2	2716	2	17	2	1	2	1	1
2	2	2717	2	15	2	1	2	1	1
2	2	2718	2	15	2	1	2	1	1
2	2	2719	2	15	2	1	2	1	1

2	2	2720	2	15	2	1	2	1	1
2	2	2721	2	15	2	1	2	1	1
2	2	2722	2	17	2	1	2	1	2
2	2	2723	2	16	2	1	2	1	1
2	2	2724	2	15	2	1	2	1	1
2	2	2725	2	15	2	1	2	1	1
2	2	2726	2	16	2	1	2	1	1
2	2	2727	2	15	2	1	2	1	1
2	2	2728	2	16	2	1	2	1	1
2	2	2729	2	16	2	1	2	1	1
2	2	2730	2	16	2	1	2	1	1
2	2	2731	2	17	2	1	2	1	1
2	2	2732	2	16	2	1	2	1	1
2	2	2733	2	16	2	1	2	1	1
2	2	2734	2	16	2	1	2	1	1
2	2	2735	2	15	2	1	2	1	9
2	2	2736	2	15	2	1	2	1	1
2	2	2737	2	16	2	1	2	1	1
2	2	2738	2	15	2	1	2	1	1
2	2	2739	2	16	2	1	2	1	1
2	2	2740	2	16	2	1	2	1	1
2	2	2741	2	16	2	1	2	1	1
2	2	2742	2	14	1	1	2	1	1
2	2	2743	2	15	2	1	1	1	1
2	2	2744	2	16	2	1	2	1	1
2	2	2745	2	14	1	1	2	1	1
2	2	2746	2	18	2	1	2	3	3
2	2	2747	2	15	2	2	2	1	1
2	2	2748	2	15	2	1	2	1	1
2	2	2749	2	15	2	1	2	1	1
2	2	2750	2	15	2	2	2	1	1
2	2	2751	2	17	2	1	2	1	1
2	2	2752	2	16	2	1	2	1	1
2	2	2753	2	18	2	1	2	1	2
2	2	2754	2	17	2	1	2	1	1
2	2	2755	1	18	2	9	2	1	1
2	2	2756	2	16	2	1	2	1	1
2	2	2757	2	16	2	2	2	1	1
2	2	2758	2	16	2	1	2	1	1
2	2	2759	2	15	2	1	2	1	1
2	2	2760	2	16	2	1	2	1	1
2	2	2761	2	15	2	1	2	1	1
2	2	2762	2	15	2	1	2	1	1
2	2	2763	2	18	2	1	2	1	1
2	2	2764	2	16	2	2	2	1	1
2	2	2765	2	15	2	1	2	1	1
2	2	2766	2	17	2	1	2	1	1

2	2	2767	2	20	3	1	2	1	1
2	2	2768	2	16	2	1	2	1	1
2	2	2769	2	16	2	1	2	1	1
2	2	2770	2	18	2	1	2	1	1
2	2	2771	2	18	2	1	2	1	1
2	2	2772	2	16	2	1	2	1	1
2	2	2773	2	15	2	1	2	1	1
2	2	2774	2	16	2	1	2	1	1
2	2	2775	2	15	2	1	2	1	1
2	2	2776	2	16	2	1	2	1	1
2	2	2777	2	16	2	2	2	1	1
2	2	2778	2	16	2	1	2	1	1
2	2	2779	2	16	2	1	2	1	1
2	2	2780	2	15	2	1	2	1	1
2	2	2781	2	15	2	1	2	1	1
2	2	2782	2	19	2	1	2	1	2
2	2	2783	2	17	2	1	2	1	1
2	2	2784	2	13	1	1	2	1	1
2	2	2785	2	17	2	1	2	1	1
2	2	2786	2	14	1	1	2	1	1
2	2	2787	2	17	2	1	2	1	1
2	2	2788	2	15	2	1	2	1	1
2	2	2789	2	16	2	1	2	1	1
2	2	2790	2	15	2	1	2	1	1
2	2	2791	2	15	2	1	2	1	1
2	2	2792	2	15	2	1	2	1	1
2	2	2793	2	17	2	2	2	1	1
2	2	2794	2	16	2	1	2	1	1
2	2	2795	2	18	2	1	2	1	1
2	2	2796	2	16	2	1	2	1	1
2	2	2797	2	14	1	1	2	1	1
2	2	2798	2	15	2	1	2	1	1
2	2	2799	2	15	2	1	2	1	1
2	2	2800	2	16	2	1	2	1	1
2	2	2801	2	16	2	1	2	1	1
2	2	2802	2	16	2	1	2	1	1
2	2	2803	2	16	2	2	2	1	1
2	2	2804	2	17	2	1	2	1	1
2	2	2805	2	17	2	1	2	1	1
2	2	2806	2	15	2	1	2	1	1
2	2	2807	2	14	1	1	2	1	1
2	2	2808	2	18	2	1	2	1	1
2	2	2809	2	16	2	1	2	1	1
2	2	2810	2	16	2	1	2	1	1
2	2	2811	2	15	2	1	2	1	1
2	2	2812	9	0	#NULL!	9	9	9	9
2	2	2813	2	14	1	1	2	1	1

2	2	2814	2	15	2	1	2	1	1	

HeardofBS	PainBreast	ChestPain	Headache	BreastLum	ArmpitLum	DischargeC	DischargeP	DischargeS	Women20
2	1	3	2	3	2	2	2	2	9
2	3	3	1	2	1	2	3	2	3
1	1	3	3	1	1	2	2	2	1
2	1	3	1	1	2	2	2	2	3
2	3	3	1	9	9	9	9	9	9
2	1	3	1	1	2	2	2	2	3
1	2	2	2	1	2	2	2	2	1
1	1	2	1	2	1	1	3	3	1
1	1	9	9	9	9	9	9	9	2
2	2	2	9	9	9	9	9	9	2
2	1	2	2	2	2	2	2	2	1
2	1	2	1	2	2	2	2	2	1
1	1	2	2	2	2	2	2	2	2
1	1	2	3	2	2	3	3	3	2
2	1	2	2	1	2	2	2	2	2
1	1	2	2	1	1	2	2	2	2
2	1	2	3	2	2	1	2	3	1
1	1	2	2	1	2	2	2	2	1
1	1	2	2	1	3	3	1	2	3
9	2	1	1	2	2	2	2	2	2
1	2	2	2	1	2	1	2	2	1
1	1	1	1	1	1	2	2	2	2
2	2	3	2	2	2	2	2	1	1
1	2	2	2	1	2	1	2	2	1
1	1	2	2	1	1	2	2	2	1
1	1	2	2	1	2	2	2	2	2
1	2	2	2	1	2	2	2	2	1
2	1	2	1	2	2	2	1	2	1
2	1	2	1	1	1	2	3	3	1
2	1	1	9	9	9	2	2	2	2
2	1	2	1	2	2	2	2	2	2
2	2	1	1	1	2	2	2	2	2
1	1	1	1	1	1	1	2	2	1
1	2	1	1	1	2	2	2	2	2
2	2	1	1	2	2	2	2	2	2
1	2	1	1	1	2	2	2	2	2
2	2	1	1	2	2	2	2	2	2
1	1	1	3	1	1	1	3	2	3
2	1	2	1	1	1	2	3	3	1
2	1	2	1	1	1	2	3	3	1
2	1	9	1	1	1	2	3	3	1
1	1	9	9	9	9	9	9	9	2
1	1	3	1	1	1	1	3	2	9
1	1	3	9	1	3	9	9	9	1
1	2	2	2	1	2	2	2	2	1
1	1	1	3	1	1	1	3	2	1

2	1	1	9	1	1	1	3	1	1
1	1	9	9	1	9	9	9	9	1
1	1	1	3	1	2	1	9	2	3
1	1	1	1	1	2	1	2	1	3
1	9	9	9	1	9	9	9	9	1
1	1	1	3	1	1	1	3	3	3
1	1	1	1	1	3	3	3	2	1
1	1	9	9	9	9	9	9	9	1
1	1	3	3	1	9	9	9	9	1
1	1	1	1	1	1	1	2	1	3
1	9	9	9	1	9	9	9	2	1
1	9	9	9	9	1	9	9	9	1
1	1	2	2	1	1	2	3	3	1
9	2	2	2	1	1	1	1	1	1
2	9	9	9	9	1	9	9	9	9
1	1	2	2	1	3	2	2	2	1
1	1	2	3	1	1	1	2	2	1
1	3	9	1	1	1	1	2	1	1
2	1	3	2	3	2	1	3	3	1
1	1	1	3	1	1	2	2	1	1
2	1	1	3	1	3	1	1	3	1
2	1	1	3	1	3	9	1	3	9
1	1	1	3	1	9	1	1	3	1
1	1	3	3	1	3	1	3	9	1
1	1	1	3	1	1	1	9	2	1
1	1	9	9	1	1	1	9	9	9
2	1	3	3	1	1	3	3	1	9
2	1	3	3	1	2	1	3	1	2
2	1	1	3	1	2	2	3	2	2
1	9	9	9	1	9	9	9	9	1
1	1	9	9	9	1	1	9	9	9
1	1	1	2	2	1	2	2	2	3
1	1	1	1	1	9	9	2	1	9
1	9	9	9	9	1	1	3	2	1
1	1	2	2	1	1	2	2	2	2
1	1	1	1	1	1	2	2	2	1
1	1	1	1	1	2	2	2	2	1
2	1	2	2	1	1	2	3	2	2
1	1	9	9	9	1	9	9	9	1
1	1	1	1	1	1	1	1	1	1
1	1	1	1	1	1	1	2	1	1
1	1	2	1	1	9	9	2	1	3
2	1	3	3	1	3	3	3	3	1
1	1	1	2	1	2	1	2	1	1
2	1	3	1	1	3	1	3	2	1
2	3	3	3	3	3	3	3	3	9
2	1	1	1	9	1	3	3	3	1

1	1	2	3	1	2	2	3	2	2
1	1	2	2	1	2	2	3	2	3
1	1	1	2	1	3	1	2	1	1
1	1	2	2	1	2	2	3	2	3
1	1	2	2	1	1	2	2	2	1
2	1	1	2	1	1	2	2	2	9
1	1	1	1	1	1	2	2	2	3
1	1	1	2	1	3	1	2	1	1
1	1	1	2	1	3	1	2	1	1
1	1	2	9	1	2	1	3	1	3
3	2	1	1	2	1	1	2	1	3
9	1	2	3	1	2	2	2	2	3
1	1	2	3	1	2	2	2	1	1
1	1	2	2	1	2	2	2	2	1
3	2	3	2	9	1	3	3	1	1
2	1	3	2	1	2	1	2	3	1
1	1	3	3	1	9	2	2	2	1
1	1	2	3	1	2	2	2	1	2
1	1	1	9	1	1	9	9	9	1
2	1	1	9	1	9	9	9	9	1
2	1	1	9	1	2	2	2	2	2
1	1	3	3	1	3	2	3	2	1
1	3	3	1	2	1	1	1	2	2
2	1	3	3	1	3	1	3	3	3
1	2	2	1	2	9	1	3	1	2
1	2	2	2	1	2	2	2	2	2
2	1	9	9	9	9	9	9	9	1
2	1	2	2	1	2	2	2	2	1
2	2	2	3	2	2	2	2	2	2
2	9	9	9	1	9	9	9	9	9
2	1	2	2	1	2	2	3	2	2
3	2	2	9	9	9	9	9	9	1
2	1	3	1	2	3	3	3	3	2
2	2	2	3	2	2	2	3	2	3
2	9	9	9	1	9	9	9	9	9
2	9	9	9	9	9	9	9	9	1
1	2	2	1	1	2	2	2	3	1
1	1	3	3	2	1	2	3	2	1
1	1	9	9	9	9	9	9	9	1
1	1	2	2	3	3	1	2	3	1
1	3	3	3	1	3	3	3	3	2
1	3	1	3	3	3	3	3	3	2
3	9	9	9	9	9	9	9	9	1
2	1	2	2	1	2	2	2	2	2
3	1	3	2	1	3	2	3	2	2
2	1	1	3	1	1	2	1	1	1
2	1	3	2	1	3	2	3	2	1

2	2	2	2	2	2	2	2	2	9
1	1	9	9	9	9	9	9	9	1
2	2	2	3	2	3	2	2	2	1
1	3	1	1	3	3	3	3	3	2
3	3	1	1	3	3	3	3	3	2
1	3	3	9	1	9	9	9	9	1
9	1	2	9	3	2	2	9	9	2
1	3	3	3	1	3	3	3	3	2
2	2	3	2	2	2	2	2	2	3
2	2	2	2	2	2	2	2	2	2
2	1	2	3	2	2	3	2	1	1
2	1	2	2	2	2	2	2	1	1
9	2	2	2	9	9	9	9	9	2
2	2	2	2	2	2	2	2	2	2
2	2	2	3	2	3	2	2	2	1
2	2	2	3	2	2	2	3	2	2
2	1	1	1	2	2	2	2	2	2
2	1	3	9	3	3	9	9	9	3
1	9	9	9	1	9	9	9	9	9
2	1	1	1	1	1	2	2	2	1
1	1	9	9	9	9	9	9	9	1
2	1	1	1	1	1	1	2	2	1
2	1	1	1	1	1	1	1	1	2
2	1	3	1	3	3	3	3	3	1
2	1	3	1	3	3	3	3	3	1
1	1	1	1	1	1	1	1	1	1
2	1	1	1	1	1	2	2	2	1
1	1	2	9	9	9	9	9	9	1
2	3	3	3	1	3	3	3	3	1
2	2	2	2	2	1	1	3	2	1
1	1	3	1	3	3	1	3	3	1
3	1	2	2	1	2	1	2	1	1
2	1	1	1	1	9	2	2	2	1
2	3	3	1	3	3	3	3	3	1
2	1	1	2	2	2	2	2	2	2
3	1	2	2	1	1	1	3	1	1
1	2	3	3	3	3	3	3	3	1
1	1	3	3	3	1	1	1	1	1
2	1	2	2	1	1	2	2	2	1
2	2	3	3	1	2	3	1	1	3
1	1	3	3	1	1	3	3	1	1
2	1	3	1	3	3	3	3	3	1
9	1	1	1	1	1	1	1	1	1
3	1	1	1	1	1	1	1	1	1
1	3	3	3	3	3	3	3	3	1
3	1	1	1	1	1	1	1	1	1
3	1	1	1	1	1	1	1	1	1

1	1	1	1	1	1	1	1	1	1
1	1	3	1	1	1	1	1	3	1
2	3	3	3	3	3	3	3	3	1
1	1	1	1	1	1	1	1	1	1
3	3	3	1	3	3	3	3	3	1
1	1	1	1	1	1	1	1	1	1
1	1	1	1	1	1	1	1	1	1
1	1	1	2	1	2	2	2	2	1
1	1	1	1	1	1	1	1	1	1
1	1	2	2	1	9	2	3	1	1
1	1	1	3	2	2	2	2	2	1
1	9	9	9	1	1	9	9	1	9
1	1	1	1	1	1	1	1	1	1
1	1	2	2	1	1	1	3	9	1
1	1	3	3	1	3	2	2	1	2
1	1	2	2	1	1	1	2	2	3
2	1	2	2	2	2	1	2	2	3
2	1	1	1	1	1	1	1	1	1
1	1	3	3	1	3	1	1	1	2
2	1	2	2	1	2	2	2	2	2
1	2	2	3	1	1	3	3	2	1
2	1	3	1	3	1	3	3	3	1
2	9	9	9	9	9	9	9	1	1
3	1	2	2	1	2	1	2	2	2
2	1	3	1	3	1	3	3	3	1
2	1	3	3	1	3	1	1	1	9
2	1	2	2	1	2	2	2	2	2
2	1	2	3	1	3	1	1	1	1
2	2	2	2	2	2	2	2	2	1
1	1	2	3	3	3	1	1	1	3
3	1	3	3	1	3	1	1	1	3
3	1	3	3	1	3	1	1	1	3
1	1	3	3	1	3	1	1	1	3
2	1	1	2	1	3	1	2	1	1
3	1	2	2	1	1	1	2	2	2
2	2	2	1	1	1	1	2	1	2
3	1	1	3	1	1	1	2	2	2
1	1	2	2	1	1	1	2	2	1
1	1	2	1	1	2	2	2	2	3
2	2	2	1	1	3	1	3	1	1
1	2	2	2	2	2	2	2	2	3
2	1	3	1	9	3	2	2	2	2
2	1	1	1	1	2	3	2	2	9
2	1	1	1	1	2	2	2	3	2
2	1	1	1	2	2	1	2	2	1
2	1	1	2	1	1	2	2	2	1
2	1	2	2	1	2	1	2	1	9

2	1	2	2	2	2	1	2	2	2
3	1	2	2	2	2	1	2	2	2
1	3	2	3	1	2	2	2	2	2
2	1	1	1	2	2	2	2	2	2
2	2	1	3	2	3	1	3	2	2
1	1	2	2	1	1	2	3	2	1
1	1	1	3	1	1	3	2	2	9
1	2	2	3	1	1	2	3	2	1
1	1	2	3	2	2	1	3	1	1
2	2	3	3	1	3	3	3	3	1
2	1	3	3	1	3	2	2	1	2
2	1	2	1	3	2	2	2	2	1
2	1	2	3	2	2	2	2	2	1
1	1	1	2	2	3	2	2	2	1
2	1	1	2	1	2	2	3	3	2
2	1	3	2	2	1	1	2	2	1
2	1	1	2	2	3	2	2	2	1
2	1	1	3	2	3	2	3	3	2
2	1	3	2	2	2	1	3	2	1
2	1	3	3	1	3	1	2	2	1
1	1	3	1	1	1	1	2	1	1
2	1	1	2	1	2	2	2	2	2
2	1	1	2	2	3	2	2	2	1
1	2	3	9	1	2	1	3	2	2
2	2	9	9	9	9	9	9	9	9
2	3	1	2	2	2	2	2	2	2
2	3	1	2	2	2	2	2	2	2
2	3	2	9	1	3	1	3	1	1
2	2	2	1	2	2	2	2	2	2
3	1	2	3	1	3	2	2	2	1
3	1	2	2	2	2	2	2	2	1
2	1	1	2	1	1	2	2	1	2
2	1	1	9	1	1	9	9	9	1
1	2	3	1	2	2	2	3	2	2
3	2	2	2	2	2	2	2	2	2
1	9	9	9	9	9	9	9	9	1
2	1	1	9	1	1	9	9	9	3
1	1	9	3	1	1	1	3	2	3
3	1	1	2	1	1	1	1	2	1
9	1	2	3	1	1	2	3	3	1
2	3	3	2	3	2	3	3	3	1
1	1	2	2	1	3	2	2	2	2
2	1	1	3	1	2	2	3	2	2
2	1	2	2	1	2	1	1	2	1
2	1	2	3	1	2	2	2	2	1
2	1	1	3	2	3	3	3	3	2
2	1	2	2	1	1	2	2	3	1

1	1	2	1	1	2	2	2	2	2
1	1	1	2	1	2	3	3	2	1
3	1	1	1	1	2	1	1	1	2
2	1	2	3	1	2	1	2	1	1
2	1	3	3	1	2	2	3	2	3
2	1	1	2	1	2	1	3	3	1
2	1	2	1	1	2	2	2	2	1
1	9	9	9	1	9	9	9	9	2
1	1	2	1	1	1	1	2	2	1
2	1	2	1	2	2	2	2	2	2
2	1	2	2	2	2	1	2	2	1
1	1	2	2	1	1	1	2	2	1
2	2	2	2	1	1	1	2	3	1
2	1	3	3	1	3	3	3	3	1
2	2	1	3	1	3	1	3	3	3
2	1	1	1	1	2	2	1	2	1
1	3	1	3	2	2	1	1	2	2
3	1	1	1	1	1	1	2	3	1
2	1	1	1	1	1	1	2	3	3
2	1	2	2	2	2	2	2	2	2
2	1	2	3	1	2	1	2	2	3
2	1	1	2	1	2	1	3	3	1
1	1	2	3	1	2	2	3	2	1
2	1	2	2	1	2	1	2	1	2
2	1	2	2	1	2	1	1	2	1
1	1	9	9	9	9	9	9	9	9
1	1	3	3	1	3	9	9	1	1
1	1	3	2	1	1	2	3	3	1
1	1	1	2	1	1	2	2	2	1
1	3	2	2	1	2	2	2	2	1
1	1	2	3	1	2	2	3	1	1
1	3	3	3	2	1	2	3	3	9
9	1	9	9	9	9	9	9	9	1
1	3	3	3	2	1	3	3	3	1
1	2	9	2	1	1	2	2	2	1
3	1	3	3	1	1	9	1	1	1
1	1	2	2	1	2	2	3	2	1
1	1	3	3	1	1	1	1	1	1
1	1	3	3	1	1	1	2	1	1
2	3	3	3	3	2	1	3	2	9
1	9	9	9	9	9	1	9	9	9
1	1	3	3	1	1	1	1	1	1
1	2	2	2	2	2	2	2	1	1
1	1	3	3	1	2	2	3	3	3
1	1	1	2	1	3	2	2	2	1
2	1	3	1	1	3	2	2	2	1
1	1	1	2	1	1	1	2	2	1

1	1	2	2	1	1	2	3	3	1
1	1	2	2	1	1	2	3	3	1
1	9	9	9	9	1	9	9	9	1
1	1	1	9	1	1	1	9	2	3
3	9	9	9	1	9	9	9	9	9
1	1	2	1	1	1	2	2	2	2
1	1	2	1	1	1	2	2	2	1
1	2	9	1	9	2	9	9	9	2
3	1	2	2	1	9	1	9	1	3
1	1	2	2	1	3	2	1	1	1
1	1	3	3	1	9	2	2	1	2
1	1	1	1	3	3	2	2	3	3
1	2	3	3	1	2	2	3	3	1
1	3	2	3	3	3	3	3	3	1
1	1	3	3	1	2	1	2	1	2
2	2	3	9	1	3	1	2	2	2
1	1	1	1	1	2	1	2	1	3
1	1	1	1	1	1	1	2	1	2
1	1	1	1	1	1	1	9	1	3
2	3	2	3	1	2	3	2	2	2
2	1	1	3	1	1	3	2	2	3
2	1	3	3	1	3	1	3	2	3
2	1	3	3	1	3	1	3	2	3
1	2	3	3	1	2	3	2	1	2
2	1	2	2	1	2	2	2	2	2
2	2	3	2	1	1	2	2	2	1
2	2	2	1	1	2	2	2	2	2
1	2	3	3	1	3	2	3	3	1
2	2	3	3	1	2	1	2	2	2
2	2	3	2	1	1	1	3	3	1
2	2	2	1	2	2	3	2	3	2
2	2	1	3	1	1	1	2	3	2
2	1	1	2	2	1	2	2	1	1
2	3	1	2	1	3	9	3	3	2
2	3	1	9	2	2	2	2	2	3
2	3	2	3	1	1	9	2	1	1
2	1	1	3	1	2	3	2	1	1
2	1	2	2	1	2	2	2	2	2
2	2	2	1	1	2	2	2	2	2
2	2	3	3	3	3	2	2	3	3
1	1	2	1	2	1	2	2	2	2
2	2	3	1	2	2	1	2	2	1
2	1	2	3	1	2	2	2	1	1
2	2	3	2	1	2	2	2	2	1
1	2	3	3	1	3	2	3	3	1
3	1	3	1	1	1	1	3	3	1
2	3	2	3	1	1	1	2	1	1

1	1	1	9	1	3	9	2	3	2
1	1	2	2	1	3	1	2	2	1
2	9	9	1	9	9	9	9	9	2
2	3	9	3	9	1	9	1	9	2
2	1	2	1	1	2	3	3	3	1
1	1	3	3	1	2	2	2	2	1
2	2	2	2	1	3	2	2	2	2
1	1	2	2	1	1	1	2	2	1
1	1	2	2	1	1	2	9	2	1
2	2	2	2	1	1	1	1	2	3
3	2	3	2	1	2	2	1	2	1
1	2	2	2	1	2	1	1	1	2
1	1	2	2	2	1	2	2	2	1
2	1	1	1	1	3	1	2	2	2
2	1	1	1	1	3	1	2	2	2
1	1	1	2	1	1	1	2	1	1
2	1	3	3	2	3	2	3	3	1
1	1	2	3	1	2	1	2	2	1
1	1	2	3	1	2	1	2	2	1
1	9	9	9	9	9	9	9	9	9
3	1	3	1	1	3	3	3	3	1
1	1	2	2	1	2	1	3	2	2
1	1	2	3	1	2	1	3	2	2
2	1	1	1	1	1	1	2	1	1
1	1	2	2	2	2	3	1	1	1
1	3	9	3	1	3	9	2	2	2
2	1	3	3	1	3	3	3	3	9
2	1	1	3	1	1	2	2	2	2
2	1	2	3	1	2	1	3	2	3
2	9	9	9	9	1	9	9	9	9
1	1	2	2	1	2	2	2	1	2
2	1	2	1	2	2	3	2	2	2
1	1	2	2	2	2	2	2	2	2
2	1	3	3	9	3	3	3	3	2
2	1	9	9	1	2	1	3	1	3
2	1	2	3	1	1	1	2	2	2
2	1	2	2	1	2	2	2	1	9
2	2	1	1	3	3	2	1	2	1
2	1	3	3	1	2	2	2	2	2
1	1	2	3	1	2	1	2	1	1
2	3	3	2	1	1	2	3	2	1
2	3	3	1	2	9	2	3	2	3
2	1	2	3	1	1	1	2	2	3
2	1	2	9	1	2	1	3	2	2
3	2	3	3	1	2	1	2	2	2
2	1	1	1	1	1	1	1	1	1
2	1	1	2	1	1	1	1	1	1

2	1	1	1	1	1	1	1	1	1
1	1	1	3	1	1	1	3	1	1
2	1	1	2	1	1	1	1	1	1
2	1	1	1	1	1	2	2	2	1
9	1	1	3	1	1	1	1	1	2
1	1	1	2	1	1	1	1	1	1
2	1	1	1	1	1	1	1	1	3
2	1	1	1	1	1	1	1	1	1
2	1	2	2	1	2	1	1	1	3
2	1	1	1	1	1	1	1	1	1
2	1	1	3	1	1	1	1	1	2
1	1	1	1	1	1	1	1	1	1
2	1	1	2	1	1	2	2	1	1
1	1	1	2	1	2	1	1	2	1
2	1	1	2	1	1	1	1	1	1
1	1	1	2	1	2	1	1	1	1
9	1	1	2	1	1	1	1	1	1
1	1	1	2	1	2	3	2	2	1
1	1	1	2	1	2	3	2	2	1
1	1	1	2	1	2	3	2	2	1
2	1	1	2	1	1	1	1	1	1
3	1	1	2	1	1	1	1	1	1
1	1	1	2	1	1	1	1	1	1
1	1	1	1	1	1	1	1	1	1
1	1	3	1	1	1	1	1	1	1
1	1	1	1	1	1	1	1	1	1
2	1	1	1	1	1	1	1	1	1
3	1	1	3	1	1	1	1	1	1
2	1	1	9	1	1	1	1	1	1
3	1	1	1	1	1	1	1	1	1
1	1	1	1	1	1	1	1	1	2
2	1	1	2	1	2	1	2	2	1
1	1	1	1	1	1	1	1	1	1
1	1	1	1	1	1	1	1	1	1
1	1	1	1	1	1	1	1	1	2
1	1	1	1	1	1	1	1	1	1
3	1	1	2	1	1	3	1	1	3
3	1	1	1	1	1	1	1	1	1
3	1	1	1	1	1	1	1	1	1
1	1	1	1	1	1	1	1	1	1
2	1	3	3	1	1	1	3	1	1
2	1	2	2	1	1	2	2	2	1
2	1	9	9	9	9	9	9	9	1
2	1	2	2	1	2	2	2	2	2
2	2	9	9	9	9	9	9	9	2
2	1	2	2	2	2	3	2	2	2
2	9	3	3	1	1	2	2	2	9

2	1	2	2	1	2	2	2	2	2
1	1	1	3	1	1	2	3	3	1
1	1	1	3	1	1	2	3	3	1
1	1	9	3	1	2	1	2	2	3
2	1	3	9	1	2	2	3	1	1
1	1	2	2	1	1	1	2	1	3
2	1	2	2	1	2	1	2	2	2
9	1	2	2	1	2	3	2	2	1
1	1	3	1	1	2	2	2	2	2
1	2	2	2	2	2	2	2	2	2
1	1	1	2	2	9	9	9	9	9
2	9	9	9	1	9	9	9	9	9
2	1	3	3	3	1	3	3	3	1
2	1	2	2	1	1	2	3	2	2
2	9	9	9	9	1	9	9	9	9
2	9	9	9	9	1	9	9	9	9
2	1	9	9	9	9	9	9	9	1
2	9	9	9	1	9	9	9	9	9
1	9	9	9	1	9	9	9	9	9
1	3	3	2	3	3	3	3	3	1
9	9	9	9	9	9	9	9	9	9
1	1	3	3	1	1	3	3	1	3
1	2	3	9	1	2	2	3	2	2
1	1	9	9	9	9	9	9	9	1
2	9	9	9	9	9	9	9	9	9
2	9	9	9	9	1	9	9	9	9
1	9	9	9	1	9	9	9	9	2
2	1	3	3	1	2	2	2	3	2
2	1	3	3	1	2	1	2	3	1
1	9	9	9	1	9	9	9	9	9
2	9	9	9	1	9	9	9	9	9
2	1	2	3	1	2	1	3	2	1
2	3	9	3	2	2	2	9	9	1
2	1	3	3	1	3	3	3	1	1
2	2	2	2	2	2	2	2	2	2
1	2	3	3	1	2	3	3	3	1
1	1	2	1	1	2	1	2	2	1
1	2	3	3	2	3	3	3	3	1
2	3	3	2	2	3	2	2	2	2
2	2	3	2	3	2	2	3	2	1
2	2	3	3	1	2	1	2	1	3
1	1	3	3	1	3	3	3	3	1
2	3	2	1	1	2	2	2	2	1
2	2	2	3	1	2	1	3	2	1
2	3	3	3	1	2	3	2	3	1
1	3	3	3	1	2	1	2	2	3
3	1	2	1	1	2	2	3	3	1

3	9	3	3	1	3	2	2	2	2
2	1	2	3	1	1	2	2	2	1
2	3	3	3	1	2	3	2	3	1
2	1	2	3	1	3	2	2	2	2
3	2	3	3	1	2	3	3	2	2
2	2	3	3	1	3	1	3	2	3
2	2	2	2	2	3	3	3	3	1
1	1	3	3	1	1	2	1	2	1
1	1	1	2	1	3	2	1	2	1
1	3	3	3	2	2	2	2	2	1
9	3	3	3	3	2	2	2	2	1
1	1	2	2	1	2	3	3	1	2
2	2	2	3	2	2	3	2	3	2
2	2	2	3	2	2	3	2	3	2
2	2	3	3	1	2	1	2	1	1
2	2	3	3	3	1	3	3	3	2
3	9	1	3	1	2	2	3	3	1
1	3	3	3	3	3	3	3	3	1
2	3	3	3	1	1	2	3	3	1
1	1	3	3	1	2	1	2	2	1
2	1	3	3	1	1	2	2	2	1
2	1	3	3	1	3	1	3	2	3
2	1	2	3	1	1	2	2	2	1
1	1	2	3	1	1	2	2	2	1
1	2	3	3	1	2	2	3	3	1
2	1	2	3	1	3	2	2	2	2
1	1	3	3	9	3	3	3	3	1
1	1	2	3	2	1	9	2	2	1
1	1	2	2	3	3	2	2	2	2
2	1	3	3	1	3	3	2	3	3
1	1	9	9	1	2	1	3	2	9
2	1	1	2	1	2	2	2	2	2
2	1	3	3	2	2	2	2	2	2
2	1	3	1	2	1	1	2	2	2
3	1	3	2	1	1	3	1	2	3
2	9	9	9	1	9	9	9	9	1
2	1	2	2	1	2	2	2	2	1
1	1	1	1	1	3	1	3	1	1
3	1	3	3	2	2	2	2	2	2
2	1	1	3	1	3	1	3	2	1
1	1	9	9	1	1	9	9	9	1
1	1	1	3	1	2	2	2	2	1
1	9	9	9	1	9	9	9	9	1
1	1	2	3	1	2	2	2	2	3
3	1	3	1	1	3	1	2	1	1
1	1	2	2	2	2	2	2	2	2
2	1	9	9	9	9	9	9	9	9

2	9	9	9	1	9	9	9	9	9
2	9	9	9	1	9	9	9	9	9
2	9	9	9	9	1	9	9	9	9
2	9	9	9	9	9	9	9	9	9
2	9	9	9	1	9	9	9	9	1
2	9	9	9	1	9	9	9	9	1
2	2	9	1	2	2	2	2	2	9
3	3	3	3	1	1	1	9	9	9
2	9	9	9	9	1	9	9	9	1
3	2	9	9	9	9	9	9	9	2
3	1	3	3	1	2	2	9	2	9
3	2	9	9	9	9	9	9	9	2
2	1	3	3	1	1	9	9	9	9
2	9	9	9	1	9	9	9	9	9
2	9	9	9	1	9	9	9	9	9
2	9	9	9	1	9	9	9	9	9
2	9	9	9	9	1	9	9	9	9
2	9	9	9	9	9	1	9	9	1
3	1	1	2	2	2	2	2	2	2
1	3	1	1	2	1	1	2	1	1
2	1	1	1	1	1	1	1	1	1
3	1	1	2	1	1	1	1	2	3
2	2	2	9	2	2	3	2	1	3
1	1	1	1	1	3	1	3	2	1
3	1	1	2	1	2	2	9	3	2
1	1	9	9	9	9	9	9	9	1
1	1	2	1	1	2	2	2	2	1
1	1	2	1	1	3	2	3	1	2
1	1	9	9	9	9	9	9	9	1
2	1	3	3	1	2	2	2	2	2
2	1	1	1	1	1	1	1	2	1
2	1	2	2	1	3	2	2	2	3
1	1	2	2	1	1	2	1	2	1
1	1	2	3	1	3	2	3	1	1
1	1	1	2	1	3	2	2	2	1
3	1	3	3	2	3	3	3	3	3
2	1	2	3	1	2	1	1	2	2
2	1	3	3	1	3	1	1	1	2
2	1	2	3	1	2	3	1	1	9
2	1	2	2	1	2	1	2	1	2
9	1	1	2	1	1	1	9	1	1
3	3	1	3	9	1	1	9	3	1
1	1	2	3	1	2	1	2	2	3
1	1	1	1	1	1	1	1	1	1
2	1	3	3	1	3	1	3	3	2
1	1	2	3	1	2	1	2	2	9
3	1	1	1	1	2	2	2	2	2

2	1	2	3	2	3	3	3	3	1
1	1	1	1	1	3	1	1	3	1
2	1	3	3	1	3	1	1	1	3
1	1	2	1	1	2	1	2	1	1
1	1	1	2	1	2	2	2	2	2
1	1	1	2	3	2	3	1	3	2
3	1	1	1	2	1	2	1	2	1
2	1	2	3	2	3	3	3	3	1
1	1	1	1	9	9	1	1	1	1
9	9	9	9	9	9	9	9	9	9
9	9	1	9	9	9	9	9	9	9
2	1	1	2	1	1	2	2	1	1
2	1	3	3	3	3	3	3	3	3
3	2	2	1	1	2	2	2	2	1
2	2	1	1	3	9	1	1	2	1
1	1	9	9	9	9	9	9	9	1
1	9	9	9	1	9	9	9	9	1
2	1	3	3	1	3	1	1	1	2
2	1	2	2	2	1	1	2	1	1
2	1	2	2	1	2	1	2	2	1
3	1	3	2	1	1	2	1	2	2
2	1	3	3	3	3	3	3	3	3
2	1	2	3	1	2	3	3	3	3
2	9	9	9	9	9	9	9	9	9
2	1	2	2	2	2	2	9	9	9
2	3	3	3	3	3	3	3	2	3
2	1	2	2	1	2	1	2	2	1
1	3	3	1	3	9	9	1	3	3
3	1	1	1	1	2	2	2	2	2
1	1	9	9	9	1	9	9	9	9
2	1	1	3	1	3	2	1	2	1
2	1	1	3	1	1	1	1	1	9
3	1	1	2	2	2	2	2	2	1
2	1	2	2	1	2	1	1	2	2
2	9	9	9	9	9	1	9	9	9
1	9	9	9	1	9	9	9	9	9
1	9	9	9	1	9	9	9	9	9
2	1	1	2	1	2	1	2	2	1
2	1	1	2	1	2	3	3	2	1
1	1	1	2	1	2	1	9	2	1
1	1	1	2	1	2	1	2	2	1
3	1	1	1	1	1	1	1	1	1
2	2	2	3	2	3	1	1	2	1
2	1	1	2	1	2	1	2	2	1
2	1	1	3	2	3	1	1	2	1
1	2	2	1	3	3	3	3	3	3
2	1	3	3	1	3	1	1	1	3

2	3	3	3	1	3	3	3	3	3
1	1	1	1	1	1	1	1	2	1
2	1	2	2	1	3	1	1	1	1
2	1	2	2	1	2	1	2	1	1
2	2	2	1	1	2	2	2	2	2
1	1	1	1	1	1	1	1	1	1
2	1	1	3	2	3	2	3	2	1
1	9	9	9	1	9	1	9	9	1
2	1	1	1	2	3	2	9	3	3
1	3	3	1	1	2	2	2	2	1
3	2	2	2	2	2	2	2	2	2
9	9	9	9	9	9	9	9	9	9
3	1	2	1	3	3	3	3	3	1
1	3	2	1	1	2	2	3	9	9
3	1	2	1	1	2	1	3	3	2
3	3	2	3	1	2	2	3	2	3
2	2	2	1	3	3	3	3	3	1
1	2	2	1	3	3	3	3	3	1
1	2	2	1	3	3	3	3	3	1
2	1	3	3	1	3	3	3	2	1
1	1	2	3	1	2	3	2	1	9
2	1	3	3	1	3	1	1	1	2
2	9	9	9	1	9	9	9	9	9
2	1	3	3	1	3	1	3	2	1
2	3	2	1	9	1	3	1	3	1
2	2	2	1	3	3	3	3	3	3
2	1	2	2	1	2	1	2	2	1
2	1	1	3	1	3	1	3	1	1
2	9	1	9	9	9	9	9	9	1
3	2	2	2	2	2	2	2	2	2
2	1	1	1	1	1	2	3	3	1
3	2	3	2	2	3	2	3	2	2
1	1	2	1	2	3	1	2	1	3
2	3	3	1	3	2	2	2	3	2
2	3	3	3	3	3	3	3	3	3
2	1	2	3	1	3	2	3	3	2
3	1	2	3	3	3	3	3	3	1
3	1	2	1	3	3	3	3	3	1
2	2	2	1	3	3	3	3	3	1
2	1	1	3	1	1	1	3	2	1
3	1	1	2	1	2	2	1	3	2
2	1	3	1	1	1	2	3	1	2
2	1	3	3	3	3	3	3	3	1
2	3	3	1	3	9	1	3	1	1
9	9	9	9	9	9	9	9	9	9
2	3	3	3	3	3	3	3	3	3
2	2	2	1	2	2	2	2	2	2

2	1	2	1	1	1	1	3	3	2
1	1	3	3	3	3	3	3	3	9
1	1	1	3	1	3	1	1	2	2
2	2	1	2	1	3	1	1	1	3
2	1	1	3	1	2	2	2	2	2
2	1	1	2	1	2	2	2	2	2
3	1	1	1	3	3	1	1	9	2
2	1	1	3	1	2	2	2	2	2
2	1	1	2	1	2	2	2	2	2
9	9	9	9	9	9	9	9	9	9
2	3	2	1	9	2	2	2	2	1
1	1	3	3	1	2	3	3	2	3
2	2	2	1	1	2	2	2	2	1
1	1	1	1	1	3	1	1	3	1
1	1	3	1	1	2	1	2	2	1
3	3	3	3	1	1	2	1	1	1
2	1	1	1	2	2	2	2	2	1
2	1	1	9	2	2	2	2	2	1
2	1	1	1	2	2	2	2	2	1
3	1	1	3	2	2	2	2	2	1
2	1	1	1	1	1	1	3	9	1
2	1	2	1	3	2	1	2	2	1
2	3	3	1	3	3	3	3	3	1
1	1	2	2	1	2	2	2	2	2
1	2	2	2	2	1	2	2	2	2
1	9	9	9	9	9	9	9	1	9
1	9	9	9	9	9	9	9	1	9
2	1	3	1	1	3	1	2	1	1
3	3	3	1	3	3	3	3	3	1
3	2	3	3	2	2	2	2	3	2
3	2	2	1	1	1	9	3	1	1
2	1	1	2	1	2	1	9	2	2
2	1	1	3	2	1	2	2	1	1
1	9	9	9	9	9	9	9	1	9
2	1	3	1	1	2	2	3	1	2
2	3	3	3	3	3	3	3	3	3
2	1	2	2	1	2	2	2	2	1
1	1	1	1	3	3	3	3	3	9
1	1	9	9	9	9	9	9	9	9
2	1	2	1	1	1	2	3	2	1
2	9	9	9	1	9	9	9	9	1
2	3	3	1	3	3	3	3	3	2
1	3	3	1	3	3	3	3	3	2
2	2	2	2	2	2	2	3	3	2
2	3	3	1	3	3	3	3	3	2
1	1	1	2	1	2	1	2	1	3
2	1	1	2	1	2	2	2	2	1

2	2	2	2	2	2	2	2	2	2
1	2	2	3	1	2	2	2	2	1
1	3	1	1	1	1	1	1	1	1
1	1	2	3	9	9	9	9	9	1
2	1	9	9	9	9	9	9	9	1
2	2	1	3	1	1	1	1	2	1
1	1	1	2	1	3	2	3	2	1
2	1	9	9	9	9	9	9	9	9
3	1	2	1	3	2	3	1	2	1
2	2	9	9	9	9	9	9	9	1
2	2	2	2	2	2	2	2	2	2
1	1	3	3	2	3	3	2	2	1
1	1	3	3	1	2	1	2	2	1
2	2	2	2	2	2	2	2	2	1
1	1	1	3	1	3	1	2	1	1
2	2	2	2	2	2	2	2	2	2
3	2	2	2	2	2	2	2	2	2
3	1	3	3	1	2	1	3	1	9
2	1	1	2	2	2	3	1	3	1
2	1	3	2	2	3	1	2	1	9
2	1	1	3	2	2	3	1	1	1
1	1	3	3	2	3	3	2	2	1
2	1	2	2	1	2	2	2	2	2
2	1	2	3	2	2	2	2	2	1
2	1	1	2	1	1	2	2	2	1
2	2	3	2	3	2	3	2	3	1
2	1	1	2	3	1	2	3	1	2
2	3	3	1	3	1	3	3	3	1
2	1	1	2	1	2	1	1	1	2
1	1	3	3	2	3	3	2	2	1
2	1	9	9	9	9	9	9	9	3
2	1	1	1	1	1	2	2	2	1
1	3	3	3	1	3	1	1	1	3
2	1	2	2	1	2	1	1	2	2
2	1	2	3	9	9	9	9	9	1
3	1	1	1	1	1	1	1	1	1
2	2	2	2	2	2	2	2	2	2
2	1	2	2	9	1	1	2	3	1
2	3	1	1	3	1	3	3	2	1
2	1	2	1	1	2	1	2	1	1
3	1	1	2	2	2	2	1	2	1
1	2	2	2	2	2	2	2	2	2
2	2	1	3	1	3	1	1	1	1
1	1	1	3	1	3	3	3	3	1
2	1	2	2	1	3	3	3	3	2
2	3	3	3	3	3	3	3	3	3
2	1	2	3	1	2	2	2	2	1

2	1	2	3	9	2	9	9	9	1
2	3	3	3	3	3	3	3	3	3
2	3	3	3	3	3	3	3	3	3
2	3	2	2	2	2	2	2	2	2
1	1	2	2	1	2	2	2	1	1
3	1	3	3	1	3	2	2	1	1
2	3	3	3	3	3	3	3	3	2
1	1	1	3	3	3	2	2	3	1
2	2	3	9	1	2	3	1	2	2
2	3	3	3	3	3	3	3	3	2
1	1	3	2	1	3	1	2	1	1
2	3	3	1	3	3	3	3	3	2
2	9	1	9	9	9	9	9	9	1
2	1	3	2	1	2	1	2	2	2
1	1	2	2	2	2	2	2	2	1
2	3	1	3	1	3	3	9	1	9
2	3	1	3	3	3	3	9	1	1
2	3	3	1	1	1	1	1	1	1
2	1	1	1	2	1	2	2	2	1
1	1	3	2	1	2	1	2	2	2
2	1	1	1	3	3	1	3	3	1
2	1	3	2	1	2	2	2	2	1
2	1	1	2	1	2	2	2	2	2
2	1	1	1	1	1	1	1	1	1
2	1	3	2	1	3	3	3	3	1
1	1	1	9	1	9	1	9	1	1
3	2	2	2	2	2	2	2	2	2
1	1	2	2	1	1	9	3	1	1
2	1	1	3	1	3	1	1	2	1
2	3	2	3	3	3	2	2	9	3
1	1	1	1	1	1	1	1	1	1
2	1	1	1	1	1	1	3	3	1
1	1	2	1	1	1	1	1	1	1
1	1	1	1	2	1	1	1	1	2
2	1	1	3	2	9	3	1	1	3
1	3	3	9	2	3	3	1	1	3
1	9	9	9	1	9	9	9	9	9
2	1	1	1	1	1	1	1	1	1
3	1	2	2	2	2	2	2	2	2
3	2	2	2	2	2	2	2	2	2
2	1	1	1	2	1	1	9	2	3
3	1	1	2	1	2	1	1	1	1
2	1	2	3	2	9	9	9	9	3
2	1	2	2	1	1	1	3	2	1
3	2	2	2	2	2	2	2	2	2
1	1	1	1	3	3	3	3	3	3
2	1	2	2	2	2	2	2	2	3

2	1	1	2	2	3	3	9	1	1
2	3	3	1	3	3	3	3	3	1
2	1	2	3	1	1	2	3	2	3
2	1	9	9	9	9	9	9	9	1
2	1	1	3	2	3	2	3	3	2
2	1	2	3	1	1	2	3	2	3
1	3	3	1	3	3	3	3	3	9
9	9	9	9	9	9	9	9	9	9
2	3	3	3	1	1	1	3	3	1
3	2	2	2	2	2	2	2	2	2
2	1	1	1	1	1	1	1	1	1
1	3	1	3	3	3	3	9	9	1
2	1	9	2	2	9	9	9	9	3
2	1	3	3	2	2	2	2	2	2
2	1	1	1	1	1	1	1	1	1
2	1	1	9	2	3	3	2	3	2
2	1	1	2	2	2	2	2	2	2
1	1	1	1	1	1	1	1	1	1
1	1	2	2	2	1	2	1	2	3
2	1	3	3	2	2	1	2	2	1
2	3	3	9	1	2	2	3	2	1
2	1	3	3	1	3	2	2	2	1
2	3	2	1	3	3	3	2	2	2
2	1	2	9	1	1	1	2	9	1
2	3	2	1	2	3	2	2	9	2
2	1	2	2	9	1	1	3	3	1
1	3	3	3	3	2	3	3	3	2
1	1	3	3	1	2	1	3	3	1
1	1	2	3	2	2	1	2	2	1
2	1	2	3	1	1	2	9	9	1
2	1	2	1	1	2	2	1	1	1
9	1	3	3	3	9	1	3	2	1
1	1	2	1	1	3	3	2	1	1
2	3	3	3	1	3	3	3	3	1
1	1	9	9	1	9	1	9	9	1
3	1	2	1	3	1	1	3	2	1
2	3	3	3	9	3	3	3	3	2
2	3	3	3	3	3	3	3	3	1
2	2	3	1	9	2	1	1	1	9
2	3	3	3	3	3	3	3	3	3
1	1	1	3	3	3	9	9	9	3
2	9	9	3	9	9	9	9	9	1
2	3	3	3	3	3	3	3	3	1
9	3	3	3	3	3	3	1	1	1
1	1	2	2	1	2	1	2	2	1
1	1	1	1	3	3	3	3	3	2
2	1	1	2	1	1	1	3	1	1

2	3	1	3	3	3	3	3	3	3
2	3	3	3	1	3	3	3	3	1
2	1	2	3	1	3	1	2	1	1
2	1	1	1	1	1	1	1	1	3
2	3	3	3	3	3	3	3	3	2
2	1	3	2	9	9	9	3	9	1
2	2	2	2	2	2	2	2	2	2
2	3	3	3	3	3	3	3	3	2
2	3	3	3	3	3	3	3	3	2
2	2	2	2	2	1	2	1	2	2
2	1	1	2	1	2	3	3	2	1
3	1	1	2	2	1	1	2	2	3
2	1	1	3	1	1	3	1	3	3
2	3	3	1	3	3	3	3	3	2
1	1	1	2	1	1	1	2	2	1
2	2	2	2	2	2	2	2	2	2
1	1	1	3	1	3	2	2	1	1
1	1	2	2	1	3	1	2	2	1
2	1	3	3	1	2	2	2	1	1
1	9	9	9	9	1	9	9	9	9
2	3	3	3	3	3	3	3	3	1
2	3	3	3	3	3	3	3	3	3
1	1	1	3	3	3	3	3	3	3
3	1	3	3	1	1	1	2	1	1
2	3	3	3	3	3	3	3	3	1
2	3	1	3	3	3	3	3	3	3
1	3	3	3	3	3	3	3	3	2
2	1	2	3	1	3	1	2	2	1
2	1	1	3	1	2	3	3	2	3
2	2	2	3	1	2	1	1	2	2
2	1	1	1	1	2	3	3	3	1
1	1	1	1	1	1	1	3	3	1
2	2	2	3	2	2	1	2	2	1
2	1	2	1	1	1	1	2	2	1
2	1	9	9	1	9	1	2	1	1
2	1	1	3	1	2	9	9	9	1
2	1	9	9	9	9	9	9	9	1
2	1	2	2	2	2	2	2	2	9
1	1	1	2	1	1	1	1	2	3
1	3	3	3	3	3	3	3	3	2
2	1	1	2	1	1	2	1	1	1
1	1	3	3	3	3	1	3	3	1
2	2	2	2	1	2	2	2	2	2
2	2	9	9	9	9	9	9	9	9
2	9	9	9	9	9	9	9	9	9
2	1	1	1	1	1	1	1	1	1
2	1	3	2	1	1	1	2	1	1

1	1	2	2	3	9	1	1	2	9
2	1	1	1	9	1	2	2	2	1
1	1	3	3	1	3	1	3	3	1
1	9	9	9	1	9	9	9	9	9
2	1	2	3	2	2	2	2	3	3
9	9	9	9	9	9	9	9	9	9
2	2	2	1	2	2	2	2	2	3
2	1	2	1	1	1	2	2	3	1
2	3	3	1	3	3	3	3	3	3
2	1	2	2	2	3	1	2	2	1
2	1	3	3	2	1	1	2	2	1
2	1	1	1	1	1	2	3	2	1
9	1	9	9	9	9	9	9	9	9
2	1	9	9	9	9	9	9	9	9
2	1	2	1	2	1	3	2	1	9
2	3	3	9	1	2	2	3	3	1
2	1	1	2	3	1	2	3	1	1
1	3	3	2	1	3	3	3	3	3
2	1	3	3	2	2	1	2	1	3
1	1	2	3	1	1	1	2	2	3
2	1	2	1	1	2	2	2	2	1
2	1	3	3	1	3	1	2	2	1
2	1	1	2	2	2	2	2	2	2
2	1	2	1	2	2	2	3	3	2
3	3	3	3	3	3	3	3	2	1
1	1	2	2	1	1	1	1	2	3
1	1	9	9	9	9	9	9	9	1
3	1	1	1	2	2	1	1	1	2
1	1	2	2	1	1	1	1	2	2
2	3	3	9	1	2	2	3	3	1
1	1	2	3	1	1	1	2	2	3
9	1	9	9	9	9	9	9	9	9
1	1	2	1	3	2	1	1	2	2
2	1	1	2	1	2	2	2	2	1
2	1	2	2	2	2	2	2	2	2
1	1	3	3	1	1	1	2	2	9
1	1	9	9	9	9	9	9	9	2
1	1	9	9	9	9	9	9	9	2
2	1	2	3	1	2	1	2	2	1
2	1	1	2	2	2	2	2	2	1
1	9	9	9	1	9	9	9	9	1
2	1	2	1	1	1	1	1	2	1
2	1	1	1	1	2	3	3	3	1
2	1	1	1	1	2	3	3	3	1
1	1	1	1	1	1	1	1	1	1
3	1	2	1	1	1	2	1	2	1
3	1	9	9	9	9	9	9	9	1

1	9	9	9	9	9	9	9	9	1
2	1	3	2	1	2	1	2	2	1
1	1	1	1	9	1	2	9	2	3
2	9	9	9	9	9	9	1	9	1
1	1	9	9	9	9	9	9	9	1
1	1	1	2	1	2	1	3	1	3
2	1	1	3	1	1	2	2	2	2
1	1	3	3	1	3	1	3	2	1
3	3	2	1	3	3	3	3	3	1
2	1	1	3	1	1	2	2	2	2
2	1	2	3	1	1	1	3	2	1
2	9	9	9	1	9	9	9	9	2
1	1	2	2	1	1	1	2	1	1
2	1	2	2	2	3	1	2	3	2
2	1	2	2	1	1	2	2	1	2
2	3	3	1	3	1	3	3	3	3
1	1	1	3	1	3	2	2	2	1
1	1	1	2	1	2	1	2	1	1
2	1	2	1	2	2	2	2	1	2
1	1	3	1	1	3	3	3	2	1
2	1	1	3	1	2	1	1	2	3
2	9	9	1	9	9	9	9	9	9
2	1	1	3	1	2	1	1	2	2
3	1	2	2	2	2	2	2	2	3
2	1	1	2	1	2	1	2	2	2
2	1	1	2	2	2	2	2	2	1
1	2	2	2	1	2	2	2	2	3
2	1	2	1	1	3	1	1	2	1
2	1	2	2	1	3	2	3	2	1
2	1	3	3	3	3	3	3	3	1
2	9	9	1	9	9	9	9	9	1
3	2	1	2	2	1	1	9	3	2
1	1	1	1	1	2	1	2	2	1
3	2	3	2	1	2	3	2	2	1
2	1	1	3	9	2	3	1	3	1
1	1	9	9	9	9	9	9	9	9
2	9	9	9	9	9	9	9	9	9
2	2	2	2	2	2	2	2	2	2
2	1	3	1	1	3	2	2	2	3
2	1	2	1	3	2	1	1	2	1
1	3	1	2	3	2	1	2	1	1
2	1	1	1	1	9	1	1	1	1
2	1	1	9	2	1	3	1	1	1
2	1	2	2	3	1	2	1	3	1
9	3	2	3	1	9	3	1	2	3
2	1	9	9	9	9	9	9	9	1
1	1	3	3	2	2	9	3	3	2

2	2	1	2	1	2	1	2	2	1
2	1	2	2	1	3	1	2	2	1
2	3	3	3	3	3	3	3	3	3
2	1	3	2	1	1	9	3	9	1
1	1	2	3	1	2	1	1	2	1
2	2	1	2	1	2	1	2	2	1
1	1	2	1	1	3	1	3	2	2
2	1	1	3	1	2	1	1	2	2
1	1	2	1	1	2	2	2	2	3
1	3	3	1	3	3	3	1	1	1
2	1	3	3	2	2	2	2	2	1
3	1	3	2	2	2	2	2	2	2
3	9	9	9	9	1	9	9	9	1
2	1	3	3	1	1	2	1	2	2
2	1	3	3	2	3	1	2	2	2
1	1	3	3	1	2	1	2	1	1
1	1	3	2	1	2	1	2	1	1
1	1	3	3	1	2	1	2	1	1
2	1	2	3	1	2	1	1	3	1
2	2	2	3	1	3	2	3	2	3
2	1	1	1	1	1	1	3	1	1
2	1	3	3	1	1	3	3	1	1
2	3	1	2	3	2	3	3	2	2
2	3	3	1	2	2	2	2	9	1
2	1	2	3	1	2	2	2	1	2
2	2	3	3	2	3	3	2	2	2
2	2	2	1	2	2	2	2	2	2
2	2	2	2	1	2	2	2	2	2
2	1	3	1	1	3	1	3	1	3
1	1	3	2	3	1	1	3	1	3
2	1	2	1	2	3	3	3	3	1
3	3	3	1	2	2	2	2	9	1
1	3	3	2	2	2	2	2	9	1
2	1	2	2	9	9	9	9	9	1
2	1	1	1	2	1	1	1	2	2
1	1	1	1	1	2	1	2	3	1
2	1	3	3	1	9	9	9	9	1
2	1	3	3	1	2	1	3	3	1
2	1	3	3	1	2	3	2	3	1
1	3	1	1	3	3	3	3	3	1
2	3	3	2	2	2	3	1	2	3
2	1	3	1	3	3	3	3	3	3
1	1	3	3	3	3	3	3	3	1
2	1	1	3	1	3	1	1	3	1
2	1	9	9	9	9	9	9	9	1
1	1	9	9	9	9	9	9	9	1
2	1	9	9	9	9	9	9	9	1

2	3	3	3	2	2	3	3	2	3
3	2	1	2	3	1	1	3	2	2
1	1	1	9	1	1	1	1	1	1
2	1	3	3	1	2	1	2	2	2
1	1	1	1	1	1	1	3	1	1
2	1	9	9	9	9	9	9	9	1
2	1	9	9	9	9	9	9	9	1
9	9	9	9	1	9	9	9	9	9
2	1	9	9	9	9	9	9	9	1
1	1	9	9	9	9	9	9	9	9
1	1	9	9	9	9	9	9	9	9
9	1	1	1	3	1	1	1	1	1
1	1	9	9	9	9	9	9	9	1
2	1	3	3	1	1	1	3	1	1
2	1	3	3	1	3	2	1	2	1
1	1	2	2	1	2	2	2	2	2
2	2	2	2	1	2	2	2	2	1
3	9	9	9	1	9	9	9	9	9
1	9	9	9	1	9	9	9	9	1
2	1	9	9	9	9	9	9	9	1
1	1	3	2	2	3	3	9	3	1
2	1	9	9	9	9	9	9	9	1
2	1	1	1	2	2	2	2	2	1
2	1	1	2	2	2	3	2	2	1
1	1	1	1	1	2	2	2	2	1
1	1	1	1	1	1	1	1	1	1
2	1	2	3	1	3	1	2	2	1
1	1	1	1	2	2	2	2	2	1
2	1	1	1	1	2	2	2	2	1
2	1	1	3	2	2	2	2	2	1
1	1	1	1	1	3	3	3	3	1
2	1	2	2	1	2	2	2	2	1
2	2	2	2	2	1	1	1	1	1
1	1	9	9	9	9	9	9	9	1
2	1	1	1	2	1	1	1	1	1
2	1	2	2	1	1	2	2	2	1
3	1	1	2	2	2	2	2	2	1
1	1	9	9	9	9	9	9	9	1
2	1	2	2	1	2	1	3	3	1
2	3	3	3	3	3	1	3	3	1
2	2	2	1	3	9	3	3	3	2
9	3	3	3	3	3	3	2	1	3
2	3	3	3	1	3	1	3	3	3
2	1	1	2	1	1	1	1	1	1
1	1	3	3	1	3	3	1	1	1
1	1	9	9	1	3	2	3	2	3
2	1	2	1	1	2	2	2	2	1

2	1	3	3	1	3	3	2	1	1
2	1	1	1	2	3	2	1	3	2
2	9	3	3	9	2	3	3	2	1
2	1	2	2	1	1	2	2	2	1
2	1	3	2	1	1	1	3	1	2
2	3	1	1	1	9	2	2	9	9
2	2	3	3	3	3	2	1	1	1
2	3	3	3	3	3	3	3	3	1
2	3	1	1	1	2	2	9	3	9
2	3	1	1	1	1	2	2	3	1
2	9	9	9	2	9	9	9	9	9
3	2	2	2	2	3	1	3	2	1
1	1	1	1	2	2	2	2	2	1
3	3	1	1	2	2	1	2	2	2
2	1	1	1	2	2	2	2	2	1
1	1	1	3	1	2	2	3	2	1
3	2	1	1	1	1	2	2	2	1
1	1	2	3	1	2	2	2	2	2
1	1	1	1	1	1	1	1	1	1
1	9	9	9	9	9	1	9	9	9
1	1	1	1	1	1	1	1	1	1
2	1	3	2	1	2	1	1	3	1
3	1	2	2	1	2	1	1	2	2
3	1	1	2	1	2	1	2	3	1
1	1	1	2	1	1	1	2	2	1
2	3	3	1	3	3	3	3	3	3
2	1	1	3	1	3	1	1	1	3
2	1	1	1	1	2	9	2	3	3
1	1	2	2	1	2	2	2	2	1
2	1	2	2	1	1	2	9	1	3
1	1	2	3	1	2	3	2	3	1
1	1	1	1	2	2	2	2	2	2
2	1	1	2	1	1	2	2	2	1
2	1	1	2	1	3	2	2	2	1
2	1	1	1	1	2	2	2	2	1
1	1	2	1	2	1	1	1	2	3
3	3	2	2	1	2	1	2	2	2
2	1	2	2	2	1	1	1	1	1
2	1	2	2	1	1	1	1	2	1
9	2	2	2	2	2	2	1	2	2
1	1	3	3	1	1	1	3	3	1
2	3	3	3	3	3	3	3	3	2
2	1	3	2	1	3	1	3	3	1
3	1	2	1	2	2	2	2	2	1
2	1	1	1	1	2	1	1	1	3
2	3	3	3	3	3	9	3	3	3
1	1	2	2	1	2	2	2	2	1

1	2	2	1	3	2	1	3	2	1
2	1	1	1	1	1	1	1	3	1
2	9	9	9	9	9	1	9	9	9
3	3	1	3	3	3	3	3	3	3
1	9	9	9	9	9	1	9	9	9
2	3	3	1	1	2	1	9	3	3
2	1	9	9	9	9	9	9	9	1
2	1	2	1	3	3	2	3	2	1
1	2	3	9	2	3	2	3	3	2
2	1	1	1	2	3	1	2	2	3
2	1	2	2	1	1	2	9	2	3
2	1	1	3	1	2	1	1	2	1
2	1	1	1	2	2	1	1	2	2
1	1	1	1	2	1	1	1	2	1
1	1	1	2	2	3	3	2	2	1
9	9	9	9	9	9	9	9	9	9
3	2	1	1	1	3	2	2	2	3
2	3	3	3	3	3	3	3	3	3
2	3	1	1	3	2	3	1	3	3
1	3	9	1	3	9	2	2	2	1
2	1	2	2	1	1	1	1	2	2
1	3	1	1	3	3	3	3	3	1
2	1	1	1	1	1	1	1	1	1
3	9	3	3	3	1	2	2	2	1
2	2	2	1	2	2	1	2	2	2
2	1	1	2	1	2	1	2	2	1
1	3	9	9	9	9	9	9	9	9
2	1	1	1	1	1	1	2	2	2
2	1	2	3	1	2	2	3	1	1
2	2	2	2	1	2	2	2	2	1
9	9	9	9	9	9	9	9	9	9
3	2	2	2	2	2	2	1	1	1
3	1	3	1	2	3	1	1	2	1
1	2	2	2	2	2	2	2	2	2
2	2	2	2	2	2	2	3	3	2
3	1	2	3	1	2	1	3	2	1
1	3	3	3	3	3	3	3	3	3
2	1	2	3	1	3	1	1	2	1
2	1	2	3	1	3	1	1	2	1
2	1	2	3	1	2	2	2	2	3
2	1	1	2	2	2	2	2	2	1
2	1	1	1	1	1	2	2	2	1
2	1	3	2	1	9	2	3	2	3
1	1	9	9	9	9	9	9	9	1
1	9	9	9	1	9	9	9	9	1
2	1	3	2	1	9	2	3	2	3
2	3	1	3	3	3	3	3	1	3

1	3	3	1	3	3	3	3	3	3
2	1	2	2	1	2	2	2	1	2
2	1	9	9	9	9	9	9	9	1
2	3	3	1	3	3	3	3	3	3
2	1	3	1	3	2	1	1	3	3
3	1	1	1	1	2	1	2	3	2
3	1	2	1	2	1	2	2	2	2
2	2	3	3	3	3	1	3	2	1
2	1	2	2	1	1	2	2	1	3
3	1	2	2	1	2	1	3	3	1
2	9	9	9	9	9	9	9	9	1
2	9	9	9	9	9	9	9	9	1
1	2	2	2	2	2	2	2	2	2
1	1	1	1	1	2	1	2	2	1
2	9	9	9	9	1	9	9	9	9
2	1	9	1	1	1	1	2	2	2
3	1	2	3	1	1	3	1	3	3
2	1	1	3	1	1	3	3	1	2
2	1	3	1	1	2	1	3	2	1
2	1	3	1	1	3	3	2	1	3
2	1	2	3	1	3	2	2	1	2
2	1	3	1	1	3	1	1	1	1
1	3	1	3	1	3	1	2	3	1
1	1	1	3	1	3	1	3	3	1
9	1	1	3	2	3	1	2	2	1
2	1	3	3	3	3	3	3	3	1
1	9	9	9	1	9	9	9	9	1
2	1	3	1	1	3	2	2	1	3
9	1	1	1	1	2	9	9	1	1
1	1	1	2	1	2	3	1	2	1
2	1	1	1	1	9	3	3	1	1
1	1	2	3	1	2	1	1	2	1
2	1	1	1	2	3	2	1	3	1
2	3	3	1	3	3	3	3	3	1
2	1	2	2	1	3	2	1	1	1
3	2	2	2	1	2	1	2	2	2
2	1	1	3	1	2	2	9	9	9
2	2	3	3	1	3	2	3	1	1
2	1	9	9	9	9	9	9	9	9
9	3	3	1	3	3	3	3	3	1
2	1	1	3	1	1	2	1	2	1
2	1	1	3	1	2	1	3	3	9
2	1	1	3	9	1	9	3	3	1
2	1	1	3	1	2	3	3	1	1
3	1	1	2	1	3	1	3	3	1
2	1	1	1	1	2	3	3	1	1
3	3	1	1	3	3	3	3	3	3

3	3	3	1	3	3	3	3	3	3
2	1	1	1	9	9	9	9	9	9
2	3	3	3	3	1	3	3	3	1
1	3	1	1	3	1	3	1	1	3
2	1	3	3	1	3	1	3	1	1
1	1	3	3	1	3	1	1	3	1
2	1	1	3	1	1	2	1	2	3
9	1	1	1	3	2	1	3	3	2
2	3	3	3	3	3	3	3	3	3
9	1	1	1	1	2	3	3	1	1
2	1	1	1	1	2	3	3	1	1
2	1	3	2	2	1	3	2	2	1
1	1	1	1	1	3	3	2	2	2
2	1	1	1	3	2	1	3	3	2
1	2	2	2	2	2	2	3	2	3
1	1	3	1	1	3	1	2	3	1
2	3	3	2	1	3	1	3	2	2
1	3	3	2	2	3	1	2	1	2
2	1	1	3	1	3	3	3	3	1
2	2	1	1	2	2	2	2	2	2
1	1	1	1	1	2	2	2	1	1
1	1	1	2	3	2	2	3	2	1
3	1	1	1	2	1	2	2	2	2
3	1	1	1	2	2	1	1	1	3
3	1	1	2	2	1	2	2	2	2
1	1	2	1	2	2	1	2	3	2
1	1	2	2	3	1	2	2	1	1
9	1	3	2	1	3	3	3	3	3
2	2	2	2	3	3	3	3	3	3
2	1	9	9	9	9	9	9	9	1
2	2	1	2	1	1	2	3	2	1
1	1	1	1	1	1	1	2	3	1
3	1	1	2	2	3	2	3	2	2
1	1	1	2	1	3	1	2	2	2
2	1	2	1	3	1	1	2	1	2
2	2	2	2	2	2	2	2	2	2
2	1	1	3	1	1	1	3	3	3
1	1	1	2	1	3	1	1	3	1
1	1	2	3	1	1	3	3	2	1
1	1	2	3	1	3	2	1	2	1
1	1	1	2	1	2	1	2	1	2
1	1	1	2	2	3	2	3	3	1
1	1	1	1	2	2	2	3	1	1
1	1	1	2	1	2	1	2	1	1
3	3	1	1	1	3	2	2	2	2
2	1	1	3	1	3	2	2	2	2
9	1	1	3	1	2	1	2	3	1

2	2	1	1	1	2	2	3	2	3
1	1	3	1	1	2	1	2	2	2
2	1	1	1	2	1	1	2	1	1
1	1	2	1	3	9	1	1	2	2
2	2	3	2	2	3	3	2	3	3
1	1	1	1	1	1	2	2	1	3
2	2	1	3	2	3	2	3	2	2
2	3	3	2	3	3	3	3	3	3
1	1	1	1	1	1	1	2	1	3
2	1	2	3	2	2	2	2	2	2
2	1	9	9	9	9	9	9	9	1
2	1	1	2	1	3	3	3	3	2
2	1	1	2	9	9	9	9	9	2
2	2	3	2	2	3	3	2	3	3
2	1	2	1	3	2	2	3	3	2
1	1	3	1	3	3	3	3	3	3
1	2	1	1	2	2	2	2	3	2
2	1	1	1	2	2	9	1	2	2
1	1	1	1	2	2	2	3	3	3
1	1	1	2	2	2	2	2	2	3
2	3	3	3	3	3	3	3	3	3
2	9	9	9	9	9	9	9	9	2
2	1	9	9	9	9	9	9	9	1
2	1	1	3	2	2	3	1	2	2
2	1	1	3	1	2	2	1	2	2
1	1	1	1	3	1	2	2	2	2
2	9	9	9	1	9	9	9	9	9
2	2	1	1	2	2	2	3	2	3
9	2	3	2	1	2	2	2	2	3
1	1	9	9	9	9	9	9	9	1
2	1	1	2	1	1	1	1	3	1
2	1	1	9	1	2	1	2	2	1
2	1	3	9	2	1	3	1	1	2
2	1	1	1	1	1	2	9	3	2
2	1	1	1	1	1	1	1	1	3
2	1	2	1	2	1	2	2	3	3
2	1	1	2	1	1	2	1	1	1
1	1	3	3	1	3	2	2	3	1
9	1	2	3	1	1	1	3	2	1
2	1	2	2	1	1	2	2	3	3
2	1	3	9	1	2	2	2	1	1
3	1	3	3	1	2	1	2	2	3
1	1	1	1	1	3	3	3	3	1
3	1	9	3	9	2	1	2	2	1
2	1	3	3	1	3	2	2	2	1
2	2	2	2	1	2	3	3	1	1
2	3	2	3	1	1	2	2	2	3

2	1	3	3	1	1	3	3	2	2
2	1	1	2	1	2	2	2	2	1
2	1	1	1	3	3	3	3	3	9
2	1	9	9	9	9	9	9	9	9
3	1	2	1	3	1	1	2	1	1
1	1	3	1	3	3	2	3	1	1
2	3	3	2	3	1	3	3	3	3
1	1	3	2	3	2	3	3	3	1
2	1	2	1	1	3	2	2	2	1
2	1	2	2	1	2	2	2	2	2
2	1	3	3	3	2	2	1	2	2
2	1	1	2	1	2	1	1	2	1
1	2	2	2	2	2	2	2	2	2
2	3	3	3	3	3	3	3	3	3
1	1	3	3	2	1	3	3	3	1
3	2	3	3	1	2	1	1	1	2
2	2	2	1	3	3	2	3	3	2
2	1	3	3	1	3	1	3	3	1
2	1	1	1	3	3	1	3	1	1
2	3	3	3	3	1	3	1	1	3
2	1	2	3	2	2	1	1	2	2
2	1	2	2	3	2	2	2	2	2
2	1	3	2	9	2	2	2	2	2
2	1	1	2	1	2	1	1	1	1
1	1	1	1	1	2	1	2	2	1
2	1	2	2	1	2	2	2	3	3
2	1	1	3	1	3	2	1	3	1
2	3	3	1	3	3	3	3	3	1
1	1	1	3	3	3	3	3	3	1
2	1	1	1	1	1	1	1	1	2
2	1	1	3	2	3	3	3	3	2
2	3	3	3	3	3	3	3	2	2
2	1	3	3	2	3	1	3	3	3
2	9	9	9	9	9	1	9	9	9
2	3	3	3	3	1	3	2	3	1
3	3	1	3	3	3	3	3	3	3
3	3	3	1	3	3	3	3	3	3
3	1	3	2	1	3	2	1	1	1
3	1	1	2	1	2	1	2	2	2
3	2	1	1	2	2	2	2	2	2
2	3	1	1	3	3	3	3	3	2
1	1	3	9	1	3	3	3	3	3
1	1	1	1	1	1	1	1	1	2
2	1	2	2	2	2	2	2	2	2
2	1	1	1	1	2	2	2	2	3
1	1	3	3	3	2	1	3	1	1
2	1	3	3	1	3	1	3	2	1

2	1	2	3	9	2	2	2	2	2
3	2	3	3	2	2	2	2	2	2
2	2	2	2	2	2	2	2	2	2
2	2	2	2	2	2	2	2	2	2
2	2	2	2	2	2	2	2	2	2
2	2	2	2	3	2	2	2	2	2
2	1	2	3	1	2	1	2	2	3
2	2	2	2	2	2	2	2	2	1
2	2	3	3	2	3	1	2	2	2
2	1	2	2	3	3	2	1	3	2
2	2	2	2	2	2	2	2	2	2
2	3	2	2	2	2	2	2	2	2
2	2	3	3	1	2	1	2	3	2
2	2	2	2	2	2	2	2	2	2
2	2	2	2	2	2	2	2	2	3
2	2	2	3	1	2	2	2	2	2
2	2	2	2	2	2	2	2	2	1
2	2	1	2	2	2	2	2	2	2
2	2	3	3	1	1	1	3	3	1
3	1	2	3	1	2	1	1	2	1
2	2	2	2	2	2	2	2	2	2
3	2	3	3	2	2	2	2	2	2
3	2	2	3	2	2	2	2	2	3
2	2	2	2	2	2	2	2	2	2
2	2	2	2	1	2	2	2	2	2
2	1	1	2	9	1	1	1	2	1
2	2	2	2	1	1	2	2	2	2
2	2	2	2	2	2	2	2	2	2
2	2	2	2	1	2	2	2	2	2
3	2	3	3	3	2	1	2	2	3
2	3	3	3	3	3	3	3	3	3
3	3	2	2	2	2	2	2	2	2
2	2	2	2	2	2	2	2	2	2
2	3	2	2	2	9	3	2	2	3
2	2	2	2	2	2	2	2	2	2
2	1	2	3	1	2	2	3	2	1
2	1	2	3	1	2	2	2	2	1
2	2	2	2	2	2	2	2	2	2
2	1	3	2	2	2	2	2	2	2
2	2	2	2	2	2	2	2	2	2
2	1	3	3	1	2	1	2	2	1
2	2	2	2	2	2	2	2	2	2
2	2	2	2	2	2	2	2	2	2
2	2	2	2	2	2	2	2	2	2
2	2	2	2	2	2	2	2	2	2
3	2	3	3	2	2	2	2	2	2
2	2	2	2	2	2	2	2	2	2

2	2	2	2	2	2	2	2	2	2
2	2	2	2	2	2	2	2	2	2
1	2	1	3	3	1	1	3	2	3
9	2	2	2	1	2	2	2	2	2
2	2	2	2	2	2	2	2	2	2
2	2	2	2	2	2	2	2	2	2
3	1	1	1	1	2	2	2	2	1
2	9	9	9	1	9	9	9	9	1
1	1	1	2	1	2	1	1	2	1
1	1	2	2	1	2	1	2	2	1
1	1	2	2	1	1	2	2	3	2
2	2	2	2	2	2	2	2	2	2
2	2	2	3	1	2	2	2	2	2
1	1	1	1	1	1	1	1	1	9
2	1	3	3	2	3	1	3	3	3
2	2	3	2	2	2	2	2	2	1
2	2	2	2	2	2	2	2	2	1
3	2	3	3	2	2	2	2	2	2
2	1	1	1	1	1	1	1	1	1
1	1	1	3	1	9	2	2	2	2
1	1	9	9	9	9	9	9	9	1
2	2	1	2	2	2	2	2	3	2
2	2	2	2	2	2	2	2	2	2
2	2	2	2	2	2	2	2	2	2
2	2	2	2	2	2	2	2	2	2
2	1	1	3	1	3	2	2	2	2
2	9	9	9	1	9	9	9	9	2
2	2	2	3	2	2	2	2	2	1
2	2	2	2	2	2	2	2	2	2
2	1	1	9	9	1	1	1	2	2
2	2	2	2	2	2	2	2	2	2
2	3	3	3	3	3	3	3	3	1
2	1	1	2	1	3	2	2	1	3
1	3	3	3	3	3	3	3	3	1
2	3	3	2	1	1	2	1	1	3
1	1	2	3	1	1	3	9	9	1
1	3	2	2	1	1	1	2	1	1
1	1	2	3	1	1	1	2	1	1
1	1	1	2	1	2	2	2	2	3
1	1	2	2	1	2	3	3	2	1
1	2	3	2	1	2	1	2	3	2
2	1	2	3	1	2	2	2	2	1
1	1	1	2	1	1	1	2	1	9
3	2	2	2	2	2	2	2	2	2
2	1	1	2	1	1	1	2	1	2
1	1	1	1	1	1	2	2	2	1
2	1	2	3	1	2	1	2	1	1

2	1	2	3	1	2	1	1	2	2
1	1	2	9	2	3	9	1	1	1
1	1	1	2	1	1	1	2	1	3
1	2	1	2	1	2	1	2	2	1
1	1	3	3	1	3	2	3	2	1
1	1	3	2	3	2	2	3	1	1
1	1	2	1	1	3	1	2	2	1
3	9	9	9	1	9	9	9	9	9
2	1	1	2	1	2	2	2	2	2
2	1	1	3	3	1	2	2	2	2
1	1	1	2	1	1	1	1	1	2
1	1	1	2	1	1	1	1	1	2
1	3	3	3	3	3	3	3	3	1
2	1	3	3	1	2	2	1	2	2
1	1	1	2	1	2	1	1	1	3
1	1	1	2	1	2	2	2	2	2
2	1	1	2	1	2	2	2	2	2
2	3	3	3	3	3	3	3	3	2
9	1	1	2	1	2	1	2	2	1
2	1	3	1	1	3	3	2	2	1
2	1	2	3	2	2	3	2	2	2
2	2	2	3	1	2	1	2	2	1
2	1	2	3	1	3	2	2	2	3
2	2	2	3	9	9	9	9	9	9
2	1	2	3	1	2	1	2	1	2
2	1	2	1	1	2	1	1	3	1
3	1	3	9	1	1	1	2	2	3
2	2	9	9	9	9	9	9	9	2
1	1	2	2	1	2	2	2	2	2
1	1	2	2	3	3	3	3	3	1
1	1	2	2	1	2	1	2	1	1
2	1	2	3	3	3	2	2	2	3
2	2	2	2	1	2	2	2	2	1
2	2	2	2	1	2	2	2	2	1
2	2	2	2	1	2	2	2	2	1
2	2	2	2	2	2	2	2	2	2
1	1	1	9	1	2	9	1	1	3
2	2	2	2	2	2	2	2	2	1
2	2	2	2	1	2	2	2	2	1
2	2	2	2	1	2	2	2	2	1
3	1	1	1	1	1	3	3	3	2
2	2	2	2	2	2	2	2	2	3
2	1	2	2	1	2	2	2	2	2
2	1	1	2	1	2	3	3	1	3
2	9	9	9	9	9	9	9	9	9
2	1	1	1	2	2	2	1	1	1
2	2	3	3	3	3	3	3	3	3

1	1	2	2	1	1	2	3	2	1
2	2	1	2	1	3	3	1	2	1
2	2	2	2	2	2	2	2	2	1
2	1	1	2	1	2	1	2	2	1
2	2	2	2	2	2	2	2	2	1
2	1	9	2	2	2	2	2	2	2
2	1	2	2	2	2	2	2	2	2
2	3	3	3	3	3	3	3	3	3
2	1	3	3	1	1	1	3	3	1
3	2	2	2	2	2	2	2	2	2
2	1	2	1	1	1	1	1	1	1
2	1	9	1	2	1	2	1	2	1
2	3	1	1	3	1	3	3	2	2
2	9	2	2	2	2	2	1	2	1
2	3	3	3	3	3	3	3	3	1
2	3	2	3	3	1	1	2	1	2
2	1	1	1	3	3	2	2	2	3
3	2	2	1	2	3	1	3	1	3
1	9	9	9	1	9	9	9	9	1
2	1	3	1	3	1	9	2	1	1
2	1	3	2	1	3	2	2	1	1
2	1	1	3	1	2	3	1	1	3
2	1	1	1	1	1	3	2	1	9
2	1	3	3	1	3	1	2	2	1
9	1	3	2	2	3	1	2	1	1
2	3	3	1	3	3	3	3	3	2
2	1	1	2	1	3	1	3	3	2
2	3	3	3	1	2	1	2	1	1
1	2	3	1	2	2	3	2	3	2
1	3	3	2	1	3	3	3	2	9
2	1	1	1	3	3	2	2	2	1
2	2	2	2	1	2	2	2	2	2
3	1	1	2	1	3	1	3	1	1
2	3	3	3	3	3	3	3	2	3
3	2	2	2	1	2	2	2	2	1
2	1	2	9	1	3	2	3	3	3
2	1	1	3	3	3	3	3	3	3
2	2	2	2	2	2	2	2	2	2
2	1	2	1	1	2	1	3	1	1
1	1	9	9	9	9	9	9	9	9
1	1	1	2	2	2	1	2	1	2
1	3	3	1	3	2	2	1	3	1
2	1	1	3	2	3	2	2	2	1
2	1	2	1	1	3	2	3	2	1
2	1	1	2	1	1	2	1	1	1
2	1	3	3	3	3	3	3	3	1
2	3	3	3	3	3	3	3	3	3

2	3	3	3	2	2	3	1	2	2
1	1	1	3	1	3	1	1	1	3
2	1	3	3	2	2	2	2	3	1
2	1	1	1	1	3	3	3	3	1
2	1	3	3	1	1	1	1	1	1
2	1	2	2	1	2	1	3	1	2
2	1	1	1	1	1	1	1	1	1
2	9	9	9	9	9	1	9	9	2
2	1	3	3	1	2	1	3	2	1
2	3	1	3	2	3	3	3	3	3
2	1	1	2	1	2	1	2	2	1
2	1	1	2	1	2	1	2	2	2
2	1	3	2	2	3	2	2	2	2
2	1	1	3	1	1	1	1	3	1
2	1	2	1	1	1	1	2	2	3
2	1	2	2	2	2	2	2	2	2
2	3	3	3	3	3	2	1	2	2
2	2	3	3	3	3	2	2	2	2
2	1	1	3	2	3	2	2	2	2
2	1	1	1	3	2	1	1	1	1
2	1	1	2	1	9	2	2	2	2
2	9	9	9	9	1	9	9	9	9
2	1	2	2	1	3	2	1	1	3
2	2	3	3	2	2	2	3	2	3
2	1	2	3	2	3	2	1	3	2
2	1	9	1	1	2	2	2	2	2
2	2	2	2	2	2	2	2	2	9
2	2	2	2	2	3	1	1	2	1
2	2	1	3	1	2	3	3	3	3
1	1	1	1	1	1	1	1	1	1
1	1	1	1	3	1	3	3	3	3
2	1	2	3	1	1	2	2	3	9
3	1	3	2	2	3	2	2	3	2
1	3	3	3	1	3	1	3	3	1
1	1	2	3	1	2	1	3	2	1
2	2	2	2	1	2	1	3	2	1
1	1	1	3	1	2	1	3	3	3
2	1	1	9	1	2	2	2	2	1
2	1	2	3	2	3	2	2	2	2
2	1	1	9	9	9	9	9	9	2
2	1	1	2	1	2	1	2	1	1
2	1	1	2	1	2	1	2	2	1
3	1	1	1	2	1	1	2	2	2
2	1	2	3	1	2	1	2	3	1
1	2	2	3	1	2	1	2	1	1
2	1	3	3	1	3	2	3	3	3
2	1	2	3	1	2	3	2	1	1

2	1	2	3	1	2	1	2	1	1
2	2	2	2	2	2	2	2	2	1
1	1	3	2	9	3	2	9	1	2
1	3	1	2	1	2	1	3	2	3
2	1	2	2	1	2	1	1	1	1
2	9	9	9	2	2	9	9	9	2
2	1	3	1	1	3	2	2	2	1
2	1	9	9	9	9	9	9	9	1
2	1	2	3	2	2	2	3	2	1
1	3	1	2	1	2	1	1	2	3
3	2	3	3	1	2	1	3	2	2
2	2	2	3	1	3	3	2	2	2
3	1	2	3	1	3	2	3	2	1
1	1	1	3	1	2	1	2	1	1
2	9	2	2	2	2	9	9	9	9
1	1	2	1	2	3	2	2	2	3
1	1	1	1	1	1	1	1	1	1
2	1	2	2	2	1	2	2	3	2
2	3	1	2	1	2	1	1	2	3
1	1	2	2	3	3	1	2	2	1
2	1	2	1	1	3	1	1	3	1
1	2	1	1	3	3	3	3	3	2
1	1	1	1	1	2	1	1	1	2
1	3	1	1	3	3	3	3	3	1
1	1	2	2	1	1	1	2	1	1
1	1	1	1	1	1	1	1	2	2
2	1	2	3	1	2	2	3	2	1
2	1	2	2	1	2	1	2	2	1
2	1	1	2	1	2	2	3	2	3
2	1	1	2	1	1	2	2	1	1
3	1	1	2	1	1	1	2	2	1
1	1	1	2	2	2	2	1	2	1
3	1	1	2	2	1	2	1	1	1
3	1	1	2	1	2	2	2	2	1
3	1	2	3	1	2	3	1	3	1
1	1	3	3	1	3	2	3	1	1
2	1	1	1	2	2	1	1	1	1
2	1	3	3	1	3	1	1	3	1
2	1	1	2	2	2	1	2	2	1
1	1	1	2	2	2	1	1	1	1
1	3	3	3	3	3	3	3	3	3
2	1	1	3	1	2	1	1	2	2
1	3	1	1	2	2	1	3	2	1
1	2	2	2	2	2	2	2	2	3
2	1	2	3	2	2	2	3	2	3
1	2	2	2	2	2	2	2	2	2
1	1	2	2	3	2	3	2	2	2

2	1	2	2	1	3	2	3	2	1
1	3	1	1	2	1	3	2	3	2
2	2	1	1	2	2	2	2	2	2
1	1	2	3	2	1	3	3	1	2
2	2	2	2	2	2	2	2	2	1
3	2	1	2	3	1	1	2	1	3
3	2	1	2	1	3	2	2	1	3
2	3	3	3	3	3	3	3	3	1
1	1	2	2	1	2	2	2	2	1
1	1	2	2	1	2	2	2	2	1
1	1	1	2	1	1	2	2	2	2
1	2	2	2	2	2	2	2	2	2
3	3	3	3	3	3	3	3	3	2
9	1	1	1	2	2	1	2	2	1
1	1	2	2	1	2	2	2	2	1
1	1	3	2	1	3	1	2	3	3
2	1	2	1	1	1	1	9	2	1
1	1	9	1	3	2	2	9	2	3
2	3	3	3	3	3	3	3	3	2
2	1	3	3	2	2	2	2	2	2
2	1	2	1	1	2	2	1	2	2
1	1	2	2	1	2	2	2	2	1
1	3	3	3	3	3	3	3	3	1
3	3	2	2	3	3	2	3	3	3
2	1	2	9	2	1	2	1	2	1
2	2	2	2	2	2	2	2	2	2
1	1	2	9	1	2	2	3	1	2
3	3	3	2	2	2	3	3	2	2
1	1	3	3	1	3	1	1	1	3
2	1	3	3	1	3	1	2	2	3
1	1	3	3	1	3	1	1	1	1
1	1	1	1	1	1	1	1	1	2
1	1	3	3	1	3	3	3	3	1
1	1	1	1	1	2	2	2	1	1
1	1	3	3	1	3	1	1	1	1
2	2	2	2	2	2	2	2	2	1
3	2	2	1	3	3	2	3	1	3
1	2	3	3	1	2	2	1	3	3
1	3	1	2	1	3	3	1	3	3
2	3	2	2	3	3	3	3	3	3
3	2	2	2	2	2	2	2	2	2
3	2	2	1	3	3	2	2	2	2
2	2	2	2	2	2	2	2	2	2
3	2	2	1	2	2	2	2	2	3
2	2	2	1	3	3	2	2	2	3
3	2	9	2	9	9	9	2	9	2
2	1	1	1	1	1	2	2	2	2

2	2	1	2	2	9	1	2	2	1
2	1	2	2	1	1	1	2	2	2
2	2	2	1	1	2	2	1	1	1
2	1	1	1	2	3	3	3	1	2
2	2	2	2	3	3	1	1	2	2
2	2	2	1	1	1	1	2	2	2
1	2	2	2	2	1	1	2	2	2
2	2	2	2	1	1	1	1	1	2
2	1	1	1	2	2	1	1	1	1
2	2	2	1	1	1	2	2	2	2
1	1	2	2	1	2	1	1	1	1
3	2	2	2	2	1	2	2	2	2
2	2	2	2	2	2	2	2	2	2
2	2	2	2	2	2	2	2	2	2
2	2	2	2	2	2	2	2	2	2
2	2	2	2	2	2	2	2	2	2
2	1	2	2	1	2	2	2	2	1
2	2	2	2	2	2	2	2	2	2
2	2	2	9	9	2	9	2	2	2
2	2	2	2	2	2	2	2	2	2
2	2	2	2	2	2	2	2	2	2
3	2	2	2	2	2	2	2	2	2
2	1	2	2	1	2	2	2	2	1
2	2	2	2	2	2	2	2	2	2
2	2	2	2	2	2	2	2	2	2
2	2	2	2	2	2	2	2	2	2
2	2	2	2	2	2	2	2	2	2
2	2	2	2	2	2	2	2	2	2
2	2	2	2	2	2	2	2	2	2
2	3	3	3	3	3	3	3	3	3
2	2	2	2	2	2	2	2	2	2
2	2	2	2	2	2	2	2	2	2
2	2	2	2	2	2	2	2	2	2
2	1	2	2	1	2	2	2	2	1
2	2	1	2	1	9	1	9	1	9
2	1	9	1	2	9	9	1	9	9
2	9	2	9	1	2	9	9	2	9
2	2	9	2	9	9	2	9	2	2
2	1	2	9	1	9	1	9	1	9
2	1	1	1	1	1	1	1	9	1
3	9	3	9	2	9	1	9	9	3
2	2	2	2	2	1	1	1	2	2
2	3	1	1	1	2	2	2	2	1
1	2	2	2	2	2	2	2	2	2
2	2	9	9	1	3	2	9	1	1
2	2	9	9	2	9	9	2	9	2
2	2	2	2	2	1	1	2	3	1

2	2	2	2	2	2	2	1	2	2
2	2	2	2	2	2	2	2	2	2
2	2	2	2	2	2	1	1	2	2
2	1	1	2	2	1	2	3	1	1
2	1	1	1	1	2	2	1	2	2
2	1	2	3	2	1	2	2	3	1
2	1	2	1	2	1	2	1	2	1
2	1	9	9	1	9	9	3	3	2
1	2	2	2	2	2	2	1	1	2
3	2	1	2	1	2	1	2	1	2
1	1	1	1	1	2	2	2	2	1
1	1	2	2	1	2	2	2	2	1
1	1	1	1	1	2	2	2	3	1
1	1	1	1	1	2	2	2	2	1
2	1	1	1	1	2	2	1	1	1
2	1	2	2	1	3	3	2	2	1
2	1	2	2	1	3	2	2	2	1
2	1	2	2	1	1	2	2	1	2
1	1	1	1	1	2	2	1	1	2
2	2	2	2	3	2	2	1	1	2
2	1	2	2	1	1	2	2	1	2
2	2	2	1	1	2	1	1	2	2
2	1	3	2	1	1	2	2	2	1
3	1	1	2	2	1	1	2	2	2
2	3	2	2	3	2	2	2	1	1
3	3	3	3	3	3	2	2	3	3
3	1	1	1	1	2	2	2	2	3
2	3	2	2	2	2	3	2	2	2
2	3	3	2	2	2	2	2	3	3
2	2	2	2	2	2	2	2	2	2
1	2	2	2	2	2	2	2	2	2
3	1	1	2	2	2	2	2	2	2
3	2	2	2	1	2	2	2	1	1
2	3	2	2	2	1	2	2	2	2
2	2	2	2	1	1	2	2	2	2
2	2	2	2	2	2	2	2	2	2
2	2	2	2	1	2	3	2	2	2
1	1	2	2	1	2	2	2	2	1
2	1	2	2	1	2	1	1	1	1
2	2	2	1	1	2	2	2	1	1
2	2	2	1	1	2	1	1	2	2
2	3	3	3	3	3	3	3	3	3
2	1	3	2	2	1	1	2	2	1
1	1	3	2	1	2	2	2	2	1
2	2	1	1	2	2	1	1	1	2
2	1	2	1	1	2	1	2	3	1
2	2	1	1	2	2	1	1	9	1

2	2	2	2	9	2	9	2	2	2
2	1	1	2	2	2	1	1	1	1
2	2	2	2	2	2	2	2	1	2
2	1	2	2	1	1	2	2	9	2
3	1	2	3	1	3	2	2	2	1
2	1	1	1	1	2	2	2	1	1
1	1	2	2	1	2	1	2	1	1
2	1	2	2	1	1	3	2	3	1
2	1	2	2	1	3	2	2	2	1
2	1	2	3	1	3	3	3	1	3
3	2	2	2	2	2	2	2	2	1
2	1	1	2	1	9	1	1	1	1
1	1	2	1	2	3	2	1	2	2
2	1	1	2	1	1	2	1	1	1
3	1	2	3	1	3	2	2	2	1
2	2	2	1	1	1	2	2	2	2
2	1	2	1	1	1	2	2	2	2
2	1	1	2	1	1	2	2	2	1
3	1	1	1	1	1	1	1	1	3
2	1	2	1	1	3	2	2	2	2
2	1	1	3	1	2	1	1	1	1
2	1	2	1	1	3	2	2	2	1
1	1	3	3	1	2	1	2	1	2
2	3	3	3	3	3	3	3	3	3
1	1	1	3	1	2	2	2	2	1
3	2	2	2	3	3	3	3	3	2
2	1	1	1	2	1	2	1	1	1
1	1	2	1	2	2	2	2	2	2
1	1	1	2	2	1	1	1	1	1
3	1	1	1	2	1	2	9	3	2
2	1	2	2	1	3	2	3	3	1
2	1	1	2	2	2	1	3	2	3
1	1	2	2	2	2	2	2	2	2
1	1	2	2	1	1	1	2	2	2
2	2	1	3	1	3	1	1	2	1
2	1	1	3	1	1	2	2	1	1
1	1	2	3	9	1	2	3	1	3
2	1	2	1	1	1	2	2	1	2
2	1	2	3	1	1	1	3	3	1
1	1	3	3	1	3	2	2	2	3
2	1	3	3	1	3	2	1	3	1
2	1	1	2	2	2	3	3	3	2
3	1	2	2	1	2	2	2	2	3
2	3	2	2	3	3	3	3	2	2
1	1	1	2	1	2	2	2	2	1
2	1	1	1	2	2	1	3	3	2
2	1	1	1	2	2	1	2	2	2

2	1	3	3	1	3	1	3	3	1
2	1	3	3	1	3	1	1	1	1
2	1	2	2	2	1	2	2	2	1
1	1	1	2	1	2	1	2	3	2
1	1	1	2	1	3	2	2	2	1
2	1	1	1	1	3	1	3	2	3
1	1	9	2	1	2	1	2	2	2
3	1	3	3	3	2	9	9	2	3
2	1	2	3	1	2	2	2	2	1
1	1	9	1	2	9	9	9	9	1
1	1	3	3	2	2	1	1	2	3
1	1	2	3	2	1	2	1	3	2
1	2	3	2	1	3	2	1	3	2
2	1	2	3	9	2	9	3	3	3
3	1	1	2	1	2	2	2	2	1
3	1	1	2	2	2	2	2	2	1
1	1	2	2	1	2	1	2	2	2
1	1	2	2	1	2	1	2	2	2
2	1	1	9	3	3	3	3	3	3
2	1	3	3	1	3	3	3	1	1
9	3	3	3	3	3	3	3	3	3
2	3	3	3	3	3	3	3	3	3
2	9	2	3	2	3	2	3	1	2
2	1	1	2	2	1	1	1	2	1
3	2	2	2	2	2	2	2	2	2
1	3	3	3	1	1	1	3	3	1
1	2	2	3	1	2	2	2	2	2
2	2	2	2	2	2	2	2	2	2
2	2	1	1	3	2	2	3	2	1
1	3	1	2	3	3	1	2	2	2
1	2	3	1	1	2	3	3	1	3
2	1	2	3	1	2	1	2	1	1
1	1	1	2	3	2	3	3	3	3
9	3	2	1	3	3	3	3	3	3
3	1	1	2	2	2	2	2	2	2
1	2	1	1	2	3	3	2	1	1
2	2	2	3	1	1	2	2	1	2
2	2	1	1	2	3	3	2	1	1
1	2	2	2	2	2	2	2	2	2
1	1	2	3	2	2	3	2	2	2
2	1	2	2	1	2	1	3	1	1
1	1	2	1	1	1	1	3	1	3
3	1	2	3	1	2	1	2	2	2
1	1	1	2	1	1	1	3	1	1
2	3	3	3	3	3	3	3	3	3
1	2	2	3	1	1	2	2	3	1
1	1	1	2	2	1	1	1	2	1

3	3	2	1	2	2	1	1	2	1
1	1	1	3	1	3	1	3	3	1
2	1	1	3	1	1	1	3	3	1
1	2	2	2	2	2	2	2	2	2
2	2	1	2	2	1	3	2	2	1
2	9	9	9	9	9	9	9	1	9
1	2	2	2	2	2	2	2	2	2
1	2	2	2	1	2	2	2	2	2
3	1	3	1	1	2	1	2	2	1
2	1	2	2	2	2	2	2	2	3
2	2	2	1	1	2	1	1	2	1
1	1	2	3	2	9	1	2	3	3
2	3	9	9	9	9	9	9	9	3
3	1	9	9	9	9	9	9	9	9
2	1	9	9	9	9	9	9	9	9
1	2	2	2	2	2	2	2	2	9
1	2	2	2	2	1	2	2	2	1
2	2	2	2	1	2	2	2	2	2
1	1	1	2	2	2	3	2	2	1
3	1	1	2	1	2	2	2	2	1
3	1	9	9	2	2	2	9	3	1
2	1	3	3	1	3	1	1	1	2
2	1	3	3	1	3	2	3	3	2
1	2	2	2	2	1	2	2	2	2
2	1	1	1	2	3	3	3	2	3
2	1	1	1	3	2	1	2	3	3
2	1	1	1	2	3	3	3	2	3
2	1	9	9	9	9	9	9	9	9
2	2	2	2	1	2	2	2	2	1
2	3	3	1	3	1	3	1	1	3
2	2	3	3	1	2	1	3	1	2
2	1	1	3	1	1	3	3	3	1
1	9	9	3	1	9	1	3	2	3
1	1	2	2	1	2	3	3	1	3
1	9	3	3	1	3	1	3	2	2
3	2	3	2	2	2	1	1	1	2
1	1	2	2	3	3	2	2	2	3
2	1	1	2	2	2	2	3	3	2
1	1	3	3	2	3	2	3	2	3
1	1	2	3	1	3	2	2	2	1
2	3	2	2	1	1	1	1	1	2
2	2	3	2	1	2	2	2	2	2
2	1	2	2	2	2	3	2	3	1
3	2	1	2	2	1	3	1	2	1
1	2	2	2	1	1	1	1	1	2
1	2	2	2	1	2	2	2	2	2
3	2	2	2	2	2	1	2	2	1

3	1	1	1	1	1	1	2	2	1
2	1	2	2	1	2	2	2	2	3
2	1	1	1	2	1	1	1	9	1
2	1	3	1	1	1	3	2	2	3
3	1	3	1	1	2	3	1	1	1
2	1	1	1	2	2	2	2	2	3
2	3	3	1	3	1	3	3	3	3
2	1	3	3	1	2	1	2	3	2
2	3	3	1	3	3	3	3	3	3
2	1	2	2	2	1	1	1	1	1
3	1	2	2	1	2	2	2	2	2
2	1	9	9	9	9	9	9	9	1
3	1	2	3	1	3	1	3	2	3
1	1	1	1	2	2	2	2	1	1
1	1	9	1	9	2	1	3	2	9
2	1	3	1	9	3	1	1	1	9
2	1	3	3	1	3	3	9	9	1
1	1	3	3	1	3	1	2	1	1
1	1	2	1	1	3	3	1	2	1
1	1	1	1	3	3	1	1	1	3
2	1	2	1	1	3	2	2	2	2
2	1	1	1	2	2	3	1	2	1
2	3	3	3	3	3	3	3	3	1
2	1	3	2	1	1	2	2	3	1
2	2	2	2	2	2	2	2	2	2
2	3	2	3	9	1	2	3	1	1
2	1	9	9	1	9	2	9	9	1
2	2	3	3	2	1	2	1	3	3
2	1	2	1	1	2	2	2	1	3
2	1	1	1	1	1	1	1	1	1
1	1	2	2	1	1	2	2	2	2
2	1	2	2	1	2	1	2	1	1
1	1	3	3	1	1	3	3	1	2
2	1	3	3	1	3	1	1	1	1
1	1	3	2	2	3	2	3	1	1
3	1	1	3	2	3	1	1	3	3
2	1	3	3	3	2	2	2	2	2
2	1	1	1	3	1	9	9	9	2
1	2	2	2	2	1	2	2	9	1
2	2	2	2	1	1	1	1	1	2
1	1	3	3	2	9	9	9	2	9
2	1	3	3	1	1	3	3	1	3
2	1	2	3	2	3	2	3	1	2
2	2	3	3	1	3	2	1	3	2
2	2	3	3	1	3	2	1	9	2
2	2	2	3	1	3	2	3	2	2
2	3	2	3	1	2	1	2	2	2

1	1	9	3	1	2	2	2	2	1
3	1	2	1	3	2	2	1	3	1
1	9	9	9	1	9	9	9	9	1
2	9	9	9	9	3	9	9	9	9
1	9	9	9	1	9	9	9	9	1
1	9	9	9	1	9	9	9	9	1
2	1	3	3	1	2	1	2	3	1
2	1	2	3	1	2	2	3	2	1
2	1	1	2	1	2	2	1	1	1
1	1	3	3	1	1	2	3	1	1
1	1	2	1	1	1	2	2	2	1
1	1	3	2	1	3	1	3	2	1
2	1	1	1	1	2	1	1	2	1
2	1	2	2	1	2	2	2	2	1
2	3	3	3	3	3	3	3	3	2
2	1	9	9	9	9	9	9	9	9
9	1	3	3	3	9	1	2	2	3
1	1	2	3	1	2	1	2	1	1
1	1	2	3	1	2	1	1	3	1
2	1	9	9	9	9	9	9	9	9
9	1	9	9	9	9	9	9	9	9
2	3	3	3	3	3	3	3	9	1
2	1	2	3	3	2	1	2	3	1
2	1	1	3	3	2	1	2	1	1
2	9	9	9	1	9	9	9	9	9
3	2	9	9	9	9	9	9	9	2
1	1	3	3	1	2	3	2	3	1
1	1	2	3	1	3	2	3	2	3
1	1	1	2	2	3	2	2	3	1
2	1	1	2	2	1	2	3	2	3
1	2	1	2	1	2	2	3	2	3
2	1	3	3	1	1	3	3	3	2
2	1	9	9	1	9	1	9	9	2
2	1	1	2	1	2	2	2	2	3
1	2	1	2	1	2	2	3	2	3
1	1	1	3	1	3	2	1	1	1
2	1	3	2	1	2	1	3	2	2
1	1	2	2	1	2	2	2	2	2
1	1	1	2	1	2	1	2	2	1
2	1	3	2	1	1	2	2	2	3
1	1	3	1	1	2	1	2	2	2
2	1	1	1	2	2	2	2	2	1
1	1	2	2	3	3	3	3	3	2
2	2	9	9	9	9	9	9	9	9
1	1	9	9	9	9	9	9	9	9
2	1	2	2	2	2	3	3	1	1
2	1	2	2	2	2	2	1	1	3

2	1	1	1	1	1	1	2	3	1
2	2	1	2	2	2	2	2	2	3
2	1	1	1	1	2	2	2	3	1
2	2	1	1	2	1	2	2	2	2
2	1	2	2	2	2	2	2	3	2
1	2	1	2	1	2	1	1	1	2
1	1	2	9	1	2	1	2	2	2
1	1	1	3	1	1	2	2	1	1
2	3	3	3	3	3	3	3	3	9
2	1	1	2	1	1	2	2	2	1
2	2	2	2	2	2	2	2	2	2
2	1	2	3	1	3	1	3	1	2
1	1	2	3	1	2	2	1	2	1
3	2	2	1	2	2	3	1	9	1
2	2	2	2	2	2	2	2	2	2
2	1	2	2	2	2	2	2	3	2
1	2	3	1	2	3	1	2	3	1
3	2	2	2	2	3	3	3	2	2
3	2	2	1	1	3	1	2	2	1
1	3	2	3	3	2	2	2	2	1
2	1	2	2	2	3	3	2	2	1
2	3	3	3	3	3	3	3	3	2
2	2	1	3	2	2	2	2	2	2
2	1	2	9	2	1	2	2	2	3
1	2	2	2	1	1	2	2	3	1
2	3	1	3	2	3	3	3	2	2
2	1	1	2	2	3	2	1	1	2
1	1	1	3	1	2	2	2	2	1
3	1	1	3	2	2	3	1	2	2
1	1	2	3	1	3	1	2	2	1
1	1	1	2	2	2	2	1	2	2
2	3	3	3	3	3	3	3	3	3
2	3	3	3	3	3	3	3	3	3
2	1	2	1	1	3	1	2	2	1
1	2	1	1	1	2	1	1	3	1
2	1	1	1	1	2	3	1	3	1
1	1	2	3	1	3	1	2	1	1
1	1	2	3	2	3	1	2	3	2
2	1	2	2	2	2	2	2	2	2
9	1	1	1	2	2	2	1	1	1
9	1	2	3	1	2	2	1	2	2
2	9	9	9	1	9	9	9	9	1
2	1	2	2	2	2	2	2	2	1
2	3	2	3	1	2	2	2	1	3
1	1	2	2	1	2	2	2	2	2
1	2	2	2	2	2	3	3	1	1
2	1	2	1	1	2	2	2	3	1

2	3	3	1	1	1	1	1	1	3
2	1	2	2	1	2	1	2	2	1
1	1	1	1	2	1	2	2	2	3
2	1	1	1	2	3	1	1	1	3
2	1	1	1	1	2	2	3	3	2
3	1	1	1	1	9	9	3	9	2
2	1	1	1	1	1	1	1	1	2
2	1	1	1	9	2	3	3	2	3
2	9	9	3	9	9	1	9	2	9
1	1	1	2	2	2	1	1	1	1
1	3	3	3	3	3	2	2	2	3
2	2	3	3	2	3	2	2	2	1
2	1	1	2	1	3	2	1	2	1
2	2	2	2	1	2	1	1	2	1
1	1	1	1	1	1	2	2	9	2
1	1	1	2	3	1	3	2	3	1
1	1	1	1	1	1	3	1	1	1
2	1	3	1	3	1	1	2	2	3
1	1	2	2	1	2	1	2	2	2
1	2	2	2	1	2	2	2	2	2
1	1	2	9	1	2	1	2	1	1
2	3	3	3	3	3	3	3	3	3
2	1	3	3	1	2	1	2	2	1
1	1	9	2	2	2	2	3	1	3
1	2	2	3	1	3	2	1	1	1
1	2	2	2	2	2	2	2	2	1
1	2	2	2	2	2	2	2	2	1
3	1	3	2	1	2	3	9	2	3
2	1	3	1	2	3	2	1	1	1
1	1	2	2	1	2	1	2	2	1
1	1	1	2	2	1	1	9	2	1
1	1	2	1	3	9	2	2	2	2
2	1	2	1	2	9	1	2	2	1
3	1	1	2	2	3	3	3	2	1
2	1	2	3	1	3	2	2	3	3
3	1	2	2	1	2	1	2	3	1
2	2	2	2	2	2	2	3	2	2
2	1	3	3	2	1	2	2	3	3
1	1	9	9	9	9	9	9	9	9
1	1	1	2	2	2	1	2	2	2
2	2	2	2	2	2	2	3	2	2
1	1	3	1	3	3	3	3	3	1
2	9	1	1	9	2	2	2	2	2
2	1	1	1	1	2	2	2	2	1
2	2	2	9	1	9	1	2	1	1
1	3	2	3	2	1	2	2	1	1
2	3	3	3	3	3	3	3	3	9

1	1	1	3	1	1	9	9	9	1
3	2	2	2	2	2	2	2	2	2
2	3	3	3	3	3	3	3	3	3
9	9	9	9	9	9	9	9	9	9
2	2	2	2	2	2	1	1	1	1
1	1	2	2	1	1	1	2	2	3
2	3	2	2	2	2	2	2	2	2
9	2	2	2	2	2	2	2	2	1
2	3	3	3	3	3	3	3	3	3
1	1	2	3	2	2	1	2	3	3
2	3	3	2	3	3	3	3	3	1
2	1	3	1	2	3	3	1	3	1
2	3	3	3	3	3	3	3	3	2
2	2	3	2	2	2	3	2	3	2
2	2	3	2	2	2	3	2	3	2
2	2	2	2	2	2	2	2	2	9
2	1	9	9	9	9	9	9	9	9
3	3	3	3	3	3	3	3	3	2
2	3	3	3	3	3	3	3	3	9
2	1	9	9	9	9	9	9	9	3
2	2	3	2	2	2	3	2	3	2
2	2	3	3	3	1	2	3	2	2
2	1	9	9	9	9	9	9	9	2
1	3	3	3	3	3	3	3	3	3
9	1	1	1	1	1	1	1	1	1
2	1	1	3	1	3	3	3	3	1
2	1	1	1	9	9	9	9	9	9
1	2	2	2	2	1	1	2	2	2
9	9	9	9	9	9	9	9	9	9
9	9	9	9	9	9	9	9	9	9
9	9	9	9	9	9	9	9	9	9
9	9	9	9	9	9	9	9	9	9
9	9	9	9	9	9	9	9	9	9
2	1	3	2	1	2	2	2	2	2
2	1	3	2	9	2	9	1	3	1
9	3	1	1	3	3	3	9	3	1
2	2	2	2	2	2	2	2	2	1
3	1	2	3	9	3	2	2	2	1
1	1	1	1	9	1	1	9	1	9
2	3	3	1	3	3	3	3	3	2
1	2	2	2	2	2	2	2	2	2
3	1	2	1	1	2	1	2	2	1
1	1	1	2	1	1	1	2	2	2
2	2	2	2	2	2	2	2	2	2
1	2	2	2	2	2	1	1	1	1
1	1	2	3	2	2	3	2	1	1
1	1	2	1	2	2	3	2	2	2

3	2	2	2	2	2	2	2	2	2
1	1	1	1	1	1	1	1	1	2
1	2	2	3	1	1	2	2	2	1
2	1	2	3	1	2	2	1	2	1
2	1	2	3	1	2	3	1	2	1
1	1	1	1	1	1	1	1	1	1
2	3	9	2	9	1	3	3	1	2
3	2	2	2	2	2	2	2	2	1
3	2	1	2	2	2	2	2	2	2
9	2	2	1	1	9	1	2	3	2
2	1	2	1	2	1	2	3	2	1
2	1	1	2	2	9	1	1	1	1
1	1	2	2	1	1	2	1	2	1
2	3	3	1	3	3	3	3	3	3
2	1	9	9	9	1	2	9	9	3
9	1	1	3	1	3	1	3	3	1
2	1	1	2	2	3	3	1	2	2
2	1	2	2	1	1	3	2	2	3
2	2	2	9	3	2	2	3	2	1
2	1	2	3	1	3	2	2	1	1
2	9	1	9	9	9	9	9	9	1
2	1	1	1	3	1	3	3	3	3
2	1	2	2	1	1	2	2	2	1
2	9	9	9	2	9	9	9	9	2
1	9	9	1	9	9	9	9	9	9
2	9	9	9	1	9	9	9	9	1
3	1	1	3	2	3	3	2	3	3
1	2	2	1	1	2	2	2	2	2
2	9	9	9	2	9	9	9	9	2
2	1	1	1	1	1	1	1	2	2
2	3	1	1	1	1	1	1	1	1
2	1	1	1	1	1	1	1	1	1
2	1	1	2	1	2	1	1	1	2
3	1	1	1	1	1	2	1	2	1
2	2	2	2	2	2	2	2	2	1
2	1	2	2	2	2	2	2	2	2
1	1	1	1	1	3	1	1	1	1
3	1	1	1	1	1	1	1	1	2
2	2	1	3	2	2	2	1	2	2
2	1	1	9	1	2	1	2	2	1
1	3	9	9	9	9	9	9	9	3
3	1	3	3	1	1	2	2	2	1
3	3	3	1	3	3	3	3	3	2
3	3	1	2	1	2	2	9	1	1
1	9	9	9	1	9	9	9	9	9
1	1	1	1	1	1	1	1	1	1
2	1	3	3	1	3	1	3	3	3

2	1	2	3	9	1	2	2	2	3
2	1	1	2	2	2	1	1	2	1
1	1	2	2	1	3	1	2	1	3
1	2	3	3	1	1	3	2	2	3
3	3	3	2	3	3	3	3	3	2
3	3	3	2	3	3	3	3	3	9
2	1	1	1	2	9	2	2	2	1
2	3	3	1	3	3	3	3	3	3
2	2	1	2	1	2	1	2	1	1
2	1	1	3	1	3	1	3	1	1
2	1	9	3	1	3	9	9	9	3
2	1	1	3	1	2	2	2	2	1
2	3	3	3	3	9	2	2	2	2
2	2	1	1	2	2	1	2	1	3
1	1	1	1	1	1	1	1	1	1
2	1	1	2	1	2	2	2	1	1
1	1	3	3	1	3	3	3	3	1
2	1	1	1	1	1	1	1	1	2
2	3	2	3	1	9	3	3	2	3
1	1	2	2	1	2	2	2	2	1
1	1	1	1	1	1	9	9	1	1
2	1	1	2	1	2	2	2	2	3
2	1	1	1	1	1	1	1	1	1
2	1	3	3	1	2	2	2	2	1
2	1	1	1	1	1	1	1	1	1
2	3	3	1	3	3	3	3	3	3
2	1	2	3	1	2	2	2	2	1
2	1	3	3	3	3	3	3	3	3
2	3	3	3	3	3	3	3	3	3
2	1	3	2	1	2	3	2	1	2
3	1	1	2	2	3	3	2	3	3
2	1	9	9	9	9	9	9	9	2
2	3	2	2	2	2	2	2	2	2
2	1	2	9	3	2	9	1	3	1
3	1	2	1	3	1	1	2	3	2
1	1	3	1	1	1	2	1	3	1
2	1	1	3	2	2	2	1	1	1
2	1	3	3	1	1	3	2	3	3
1	1	9	9	9	9	9	9	9	1
2	2	3	3	1	3	2	2	2	2
2	3	3	3	2	2	2	2	2	1
1	2	3	2	3	2	2	2	2	2
2	1	1	2	1	1	2	2	9	2
2	9	9	9	9	9	9	9	9	2
1	2	3	3	1	3	1	2	1	1
1	1	2	2	2	2	2	9	1	1
1	1	1	1	2	2	3	3	3	1

2	2	2	2	2	2	2	2	2	1
1	1	1	1	9	9	9	9	1	9
2	1	1	1	9	9	9	9	1	9
2	1	2	1	3	3	3	3	2	2
2	1	2	2	1	2	1	3	1	1
1	1	9	1	9	9	9	9	9	9
3	9	9	9	1	9	9	9	9	1
1	1	1	1	2	2	1	1	2	1
1	1	2	2	1	2	1	3	9	1
2	1	2	2	2	1	2	3	1	1
1	1	2	3	1	2	2	2	2	2
2	1	2	1	1	2	2	1	2	1
1	3	2	2	1	2	2	2	2	1
1	3	2	2	1	2	2	2	2	1
1	1	9	3	1	3	2	2	2	1
1	1	1	1	1	1	1	1	2	1
3	1	2	1	2	2	2	2	2	3
3	1	2	1	2	2	2	2	2	3
2	1	2	2	1	2	2	2	3	1
2	1	1	3	2	2	2	3	1	1
1	2	2	2	1	1	2	2	2	2
3	1	1	2	1	1	2	1	1	1
2	1	1	1	1	1	1	1	1	9
1	1	1	2	1	3	1	1	2	1
1	1	3	3	3	3	3	3	3	1
2	2	2	2	1	2	2	2	2	1
1	3	3	3	3	3	3	3	3	3
2	1	1	1	3	3	3	2	2	2
2	1	2	3	1	2	1	1	1	1
1	1	1	1	1	1	1	1	2	1
1	2	2	2	2	2	2	2	2	2
1	1	1	1	1	1	3	9	2	2
1	1	2	9	1	3	1	2	1	1
1	1	2	3	2	3	3	2	2	1
1	1	1	2	9	1	1	1	1	3
2	2	2	2	2	2	2	2	2	2
3	1	2	1	3	1	2	1	3	2
2	2	2	2	2	2	2	2	2	2
1	1	2	2	1	2	2	2	2	1
1	2	3	9	1	2	2	2	2	2
2	2	2	2	1	3	2	2	2	2
1	1	2	3	2	1	2	3	1	1
2	1	1	3	1	1	1	1	2	2
2	1	1	2	1	2	2	1	1	1
1	1	1	3	1	2	1	1	1	1
2	1	2	3	2	1	1	1	2	1
3	2	2	1	2	2	2	2	2	2

2	1	1	2	1	2	2	1	1	1
2	3	9	9	9	1	9	9	2	3
2	1	2	2	1	2	2	2	2	1
3	1	9	9	1	9	1	9	9	1
3	1	2	2	2	2	2	2	2	3
2	2	2	2	2	2	2	3	2	1
1	1	3	2	1	2	3	3	2	2
2	2	2	3	3	3	3	3	3	2
1	1	9	2	1	2	2	1	3	1
2	2	2	2	2	2	2	2	2	3
2	1	1	1	1	2	1	2	2	1
2	1	1	1	1	2	2	2	2	1
2	2	2	2	2	2	2	2	2	3
1	1	1	3	3	1	3	1	2	1
2	1	2	2	1	2	1	3	2	2
1	1	1	2	1	3	2	1	2	1
2	2	2	1	1	1	1	1	1	1
1	9	9	9	1	9	9	9	9	1
2	3	3	1	3	3	2	2	2	2
1	1	2	2	9	1	2	2	1	1
1	1	2	3	1	1	2	1	2	1
1	1	2	3	1	1	2	2	2	1
1	1	2	3	1	3	2	2	3	2
1	1	2	3	2	1	3	2	1	2
2	1	3	3	1	3	1	3	2	1
2	1	2	3	1	2	2	3	3	1
1	1	2	3	1	2	2	2	2	2
2	2	3	3	1	2	2	3	3	1
1	2	3	2	1	2	1	2	1	1
2	2	2	3	1	3	2	3	3	1
1	1	2	1	1	3	2	2	2	3
1	1	1	1	2	1	1	1	1	2
2	1	3	1	2	3	3	2	1	3
2	1	1	1	1	1	1	1	1	2
1	1	3	9	1	2	1	2	1	3
2	1	2	1	3	1	2	2	2	3
2	1	3	3	1	3	2	2	2	1
3	2	3	2	1	9	2	1	3	2
1	1	3	3	1	2	1	3	3	1
2	2	2	3	1	3	2	2	2	2
3	1	2	3	9	1	2	1	3	1
2	1	2	1	2	3	2	3	1	1
9	9	9	9	9	9	9	9	9	9
1	1	3	3	3	2	1	3	1	1
2	1	2	1	1	1	1	2	1	1
1	1	1	1	1	2	1	2	1	1
3	1	2	2	1	1	3	2	2	1

2	3	3	1	3	3	3	1	2	3
1	3	3	3	1	3	3	3	3	1
2	1	1	1	2	2	2	1	2	2
1	1	2	3	1	2	1	3	3	1
2	3	1	2	2	3	3	3	1	3
2	1	1	2	1	2	2	1	2	2
2	1	3	3	1	2	2	2	1	3
1	1	1	1	1	1	1	1	1	1
1	1	1	1	1	1	1	2	2	2
2	1	2	3	1	2	1	2	2	1
2	2	2	2	1	2	2	1	9	2
2	1	1	1	1	2	1	1	2	1
2	1	1	1	1	2	2	1	1	1
3	1	1	1	1	2	2	1	1	1
2	2	3	3	1	3	1	1	2	1
2	2	2	3	1	2	3	2	3	1
2	1	1	3	1	2	3	3	3	2
2	1	3	3	1	2	1	2	2	2
3	1	1	1	1	2	3	3	3	3
2	1	2	1	1	1	2	2	2	1
2	3	1	3	3	3	3	3	3	1
2	9	9	9	9	9	9	2	9	9
2	2	1	1	2	3	3	3	3	2
1	2	1	2	1	3	2	2	3	3
9	9	9	9	9	9	9	9	9	9
1	9	9	9	9	9	1	9	9	9
2	1	3	3	1	3	1	3	3	3
1	9	9	9	9	9	9	9	9	9
2	3	3	3	1	2	1	9	1	2
2	3	3	1	3	3	3	3	2	1
3	1	2	1	1	2	2	2	3	1
3	1	1	1	1	2	2	2	3	9
1	1	3	2	3	2	9	9	9	9
2	2	1	2	1	3	2	2	3	3
2	1	3	2	1	3	1	3	3	3
2	1	3	3	1	3	1	3	3	2
2	1	1	1	1	2	1	9	1	1
2	1	1	9	9	9	2	2	2	9
2	1	2	2	1	2	1	2	3	1
2	1	1	1	1	2	1	3	2	1
2	1	2	3	1	3	1	2	1	1
1	1	1	1	1	1	1	1	1	3
2	1	3	3	2	9	1	9	9	1
2	1	2	3	1	1	2	3	2	1
1	1	9	9	9	9	9	9	9	9
9	9	9	9	9	9	9	9	9	9
1	9	9	9	1	9	9	9	9	9

2	1	2	9	1	2	3	3	3	1
2	9	9	9	1	9	9	9	2	9
2	1	3	2	3	3	3	3	3	2
2	2	1	3	2	3	2	3	2	1
2	1	2	2	1	2	3	1	2	2
2	3	3	3	2	3	1	3	2	1
2	3	3	3	1	2	2	2	2	3
1	3	3	3	3	3	3	3	3	2
2	1	1	1	1	1	1	1	1	2
2	2	2	2	2	2	2	2	3	1
1	2	3	2	1	2	1	3	2	1
2	1	1	1	2	1	1	1	1	2
2	1	2	1	1	2	2	1	3	1
2	9	1	1	1	1	2	2	2	1
1	1	2	2	1	2	1	2	2	2
3	1	1	1	1	1	1	1	9	1
2	1	9	1	1	9	1	1	1	1
2	1	2	3	2	2	2	3	1	1
2	2	2	2	1	2	2	2	2	2
2	1	3	3	1	1	2	1	1	3
9	9	9	9	9	9	9	9	9	9
2	3	3	1	2	2	2	2	2	2
1	1	1	1	2	2	2	2	2	1
1	1	1	1	2	2	2	1	1	1
1	1	1	2	2	2	1	1	1	1
2	2	2	2	2	2	2	2	2	1
2	2	2	2	3	3	3	3	3	2
1	1	1	2	2	3	3	3	3	1
1	1	2	2	2	2	2	2	2	1
1	2	2	2	1	2	2	2	2	1
9	9	9	9	9	9	9	9	9	9
9	9	9	9	9	9	9	9	9	9
9	9	9	9	9	9	9	9	9	9
2	3	3	1	1	2	1	3	2	2
2	1	1	1	1	2	1	1	1	3
2	1	2	2	3	2	2	2	2	2
1	1	2	2	1	2	2	2	1	1
2	1	1	3	9	3	1	3	1	1
1	1	1	1	1	1	1	1	1	2
2	2	2	2	3	2	3	2	3	3
2	3	3	3	1	3	3	2	2	9
3	2	1	9	1	1	1	2	3	1
2	1	2	2	1	1	2	2	1	1
1	1	2	3	1	2	2	2	2	2
2	1	2	2	3	2	2	2	2	3
2	1	1	1	1	1	1	1	1	1
9	9	9	9	9	9	9	9	9	9

1	1	1	1	1	1	1	1	1	1
2	2	2	2	1	2	2	1	2	2
1	1	1	1	1	1	1	1	1	2
2	1	3	3	1	3	1	3	2	1
2	1	9	9	1	9	9	9	9	3
9	9	9	9	9	9	9	9	9	9
1	1	2	2	1	9	1	1	2	2
1	1	9	9	9	9	9	9	9	1
3	1	9	9	9	9	9	9	9	9
1	1	1	1	1	1	1	3	3	1
1	9	9	9	1	9	9	9	9	1
2	1	9	9	9	9	9	9	9	9
3	1	1	2	1	1	1	2	1	1
2	1	2	3	1	1	1	2	3	1
2	1	1	1	3	3	2	1	2	1
1	1	2	2	1	2	2	3	2	1
3	1	2	1	1	1	1	1	1	1
1	1	2	2	1	1	1	1	1	1
1	1	2	2	1	2	2	3	3	1
2	1	2	1	3	3	2	2	2	1
2	1	2	1	1	2	2	2	2	1
3	1	2	3	1	2	2	2	2	1
1	2	2	2	1	1	1	2	2	1
2	1	2	3	1	3	2	3	3	3
1	1	3	2	3	1	3	2	3	2
1	1	3	1	1	1	1	1	2	3
2	2	2	2	2	2	2	2	2	3
2	1	2	3	1	2	2	2	2	1
3	1	2	1	2	2	2	2	2	1
1	1	2	2	3	3	2	2	3	1
1	1	2	1	2	2	2	2	2	1
1	1	2	1	1	3	2	2	2	3
2	2	2	3	1	3	1	2	2	1
2	1	1	2	1	1	2	3	3	1
3	1	2	2	9	9	1	9	9	9
2	2	2	3	2	3	3	3	1	3
2	1	2	3	1	3	2	3	2	1
3	1	2	3	1	2	1	2	1	1
1	1	2	2	1	2	2	2	2	3
3	3	3	1	2	9	1	3	2	2
2	9	9	9	1	9	9	9	9	1
1	9	9	9	1	9	9	9	9	1
2	1	9	9	9	1	9	9	9	1
1	1	2	3	1	3	2	3	2	2
1	9	9	9	1	9	9	9	9	1
2	1	3	3	1	1	1	2	3	9
2	1	2	1	1	2	3	3	2	2

2	1	2	2	2	2	2	1	2	1
1	1	1	2	3	2	2	2	2	2
2	1	3	2	1	3	1	1	3	1
3	1	2	2	1	1	1	2	3	2
2	1	1	3	2	2	2	2	2	3
1	1	2	2	1	2	1	2	1	2
2	1	3	3	1	2	2	3	1	1
2	1	2	3	1	2	1	3	2	2
1	1	3	3	1	3	2	2	2	1
1	3	1	2	2	1	2	1	9	1
2	1	2	2	1	2	2	1	1	3
2	2	2	3	3	1	2	2	2	2
1	1	3	2	1	1	2	3	1	1
2	2	2	3	3	3	3	1	1	1
2	1	3	3	1	2	1	2	1	1
2	1	1	3	1	3	2	2	1	9
2	1	2	2	1	2	2	2	2	1
1	1	3	1	3	1	3	2	2	3
1	1	1	2	2	2	2	2	2	2
2	2	2	2	2	2	2	2	2	1
1	1	3	3	3	3	2	2	2	1
3	1	2	2	1	2	1	2	1	3
2	1	2	2	1	2	2	2	2	2
2	3	3	3	1	2	2	3	2	1
1	1	1	1	1	1	1	1	1	1
1	1	3	1	2	3	2	2	2	3
1	2	2	3	1	2	1	3	2	1
1	1	1	1	1	1	1	1	1	1
3	2	2	2	1	2	1	2	3	1
2	1	2	1	2	2	2	2	2	1
9	1	2	3	1	2	2	2	2	1
2	9	3	3	1	1	1	3	2	1
2	1	2	1	1	1	1	2	2	2
1	1	3	2	1	3	2	3	1	1
1	1	1	2	1	2	2	1	2	1
9	1	1	1	2	2	2	2	3	1
1	1	1	1	1	1	1	2	1	2
2	1	2	2	2	2	2	2	2	2
2	1	3	3	1	3	1	3	1	1
3	1	1	1	1	1	1	1	1	1
2	1	1	3	1	2	3	2	1	1
2	1	3	3	1	2	1	3	2	1
2	1	2	3	2	3	2	2	2	1
1	1	1	2	1	1	1	2	1	1
9	1	3	3	1	3	1	2	1	1
1	3	9	9	1	1	2	9	2	2
2	2	3	3	1	3	1	3	3	3

2	1	3	2	1	3	1	3	3	3
3	1	2	2	2	2	3	3	3	3
1	2	2	2	2	2	2	2	2	3
3	1	2	3	2	2	1	2	3	1
2	1	1	2	2	2	2	2	2	1
2	1	1	3	1	1	1	2	2	2
1	1	2	2	2	3	3	3	2	2
2	1	2	3	1	1	1	2	2	3
3	1	2	3	2	3	1	2	3	1
2	1	2	3	1	3	1	2	2	1
1	1	3	9	1	2	1	2	1	3
1	1	1	2	1	3	2	2	1	1
9	2	2	2	2	2	2	2	9	1
2	1	2	2	1	2	1	2	1	1
2	3	3	3	1	3	3	3	3	1
2	1	2	2	1	2	2	2	2	2
2	1	1	3	1	1	1	2	2	2
1	1	1	2	1	2	2	2	2	2
1	1	1	2	2	2	2	2	2	2
2	1	1	2	1	3	2	2	2	1
2	1	3	1	1	2	1	2	3	1
2	1	1	1	1	1	1	1	1	3
3	2	2	3	1	3	2	3	2	1
1	1	2	3	1	3	2	2	2	3
1	1	1	3	1	2	3	1	2	1
2	1	3	2	1	1	1	3	2	1
1	3	3	3	3	3	3	3	3	1
1	1	2	2	1	2	2	2	1	1
2	2	2	2	1	2	2	2	2	1
2	1	2	1	1	2	2	2	1	3
2	1	2	1	2	2	2	2	2	2
2	2	2	2	2	2	2	2	2	2
2	1	2	2	1	2	2	2	3	2
2	1	2	3	1	1	1	2	2	1
2	1	3	1	2	2	1	3	2	3
2	3	1	2	1	1	1	2	2	1
2	1	2	3	1	2	2	2	2	1
1	1	1	1	1	2	1	2	1	1
3	1	3	1	2	3	1	2	3	3
1	2	3	3	2	2	2	2	2	2
1	2	3	3	1	2	2	3	3	1
1	1	3	3	1	9	2	2	2	1
1	2	3	3	1	2	2	2	2	1
1	1	3	3	1	1	1	9	2	1
1	1	2	2	1	2	2	2	2	3
1	3	2	1	2	2	1	2	2	2
2	1	2	3	1	2	1	3	2	2

2	1	2	3	2	2	1	3	2	2
2	2	2	3	2	2	1	3	2	2
2	1	2	3	1	3	2	3	3	1
1	2	3	3	1	2	2	3	3	1
2	1	3	3	1	1	1	1	2	1
2	3	3	1	9	3	2	2	3	3
2	1	2	3	2	3	2	3	2	2
2	2	1	3	2	2	1	3	3	1
3	1	2	3	1	2	2	3	1	1
2	1	2	2	2	2	2	2	2	3
1	1	3	1	1	2	3	2	1	3
2	1	1	1	2	2	3	3	3	1
2	1	1	1	2	1	3	3	1	3
1	2	2	2	1	2	1	2	2	3
2	1	1	1	1	1	1	1	1	1
1	1	2	2	1	2	2	3	3	1
3	2	2	1	2	2	2	2	2	2
1	3	1	1	1	1	1	3	2	1
1	3	3	9	3	2	9	1	3	2
1	1	1	2	1	2	1	1	2	1
2	1	1	1	2	2	2	2	2	1
2	1	1	2	2	2	2	3	3	1
1	1	1	2	2	1	1	1	1	1
1	2	2	1	2	2	2	2	2	1
1	2	1	2	2	1	2	2	2	2
2	1	2	2	1	2	2	3	3	1
3	1	1	1	2	2	3	3	3	1
2	1	2	2	2	2	2	2	2	3
3	1	3	1	2	3	1	2	3	3
1	1	1	2	1	2	1	1	2	1
2	1	2	3	1	2	2	2	2	1
9	9	9	9	9	9	9	9	9	9
1	1	1	1	1	2	2	2	1	3
1	1	1	1	3	3	3	3	1	2
2	1	1	1	1	2	1	2	2	1
1	2	1	3	1	3	1	1	1	1
2	1	1	1	1	1	1	3	3	1
2	1	1	2	1	2	2	2	2	2
3	2	2	2	2	2	2	2	2	1
2	1	1	1	1	2	2	2	2	2
1	1	2	3	1	2	1	2	1	1
1	1	2	3	1	2	1	2	1	1
1	1	3	3	1	2	2	2	3	3
1	1	3	9	1	1	1	3	2	1
1	2	1	2	1	2	3	1	2	1
2	2	2	3	9	1	2	3	3	2
3	1	2	1	3	3	3	3	2	1

1	1	1	2	2	2	2	2	3	2
2	1	1	1	1	3	3	3	2	1
3	1	9	1	1	1	1	1	1	1
2	1	2	2	1	2	2	2	2	1
1	3	2	2	2	2	2	2	2	3
1	1	3	3	1	1	1	1	1	3
1	1	9	3	1	3	2	1	2	1
2	2	2	2	2	2	2	2	2	2
2	2	2	2	2	2	2	2	2	2
2	2	2	2	2	2	2	2	2	2
2	3	3	3	3	3	3	3	3	2
1	1	1	2	2	2	3	3	3	1
1	2	2	2	2	2	2	2	2	2
2	1	2	2	3	2	3	1	2	3
1	2	1	1	1	2	2	2	2	1
1	2	1	1	2	9	1	2	2	1
1	1	1	1	1	2	1	1	2	2
1	2	2	2	2	2	2	2	2	3
1	1	1	2	2	2	3	2	1	1
2	1	2	1	1	2	1	2	2	2
2	3	2	1	1	2	2	1	1	3
2	2	2	2	2	2	2	2	2	2
1	2	2	1	2	2	2	2	2	2
1	1	2	1	3	2	2	2	2	2
2	1	3	3	3	3	3	3	3	3
2	1	2	2	1	3	2	2	2	2
1	1	2	2	1	2	3	2	1	2
1	1	2	2	1	1	1	3	1	1
1	1	1	1	1	1	1	2	2	1
1	1	1	2	1	1	1	1	1	1
1	1	1	1	1	2	2	2	1	1
3	2	2	2	2	2	2	2	2	2
2	1	3	3	1	3	1	3	3	3
1	1	9	9	9	9	9	9	9	9
1	1	1	2	1	1	1	1	3	1
1	1	2	3	1	2	2	2	2	1
2	1	1	3	3	3	1	1	1	1
3	3	3	3	1	2	2	2	3	3
1	1	3	3	1	3	2	2	2	3
2	1	1	2	1	2	1	9	2	3
2	1	1	1	1	2	1	3	3	1
2	1	2	1	1	1	9	9	9	1
2	3	1	1	2	2	9	9	2	3
1	1	1	3	2	3	2	1	2	1
2	2	3	3	1	3	2	2	1	2
1	1	2	2	1	2	2	2	2	1
2	9	1	3	2	2	1	3	2	2

1	1	2	3	1	2	2	2	2	3
2	1	9	9	9	9	9	9	9	3
1	9	9	9	9	9	9	9	9	1
1	1	2	2	1	1	2	2	2	1
1	1	2	2	1	2	2	3	2	3
1	3	2	1	3	3	3	3	3	9
1	1	1	2	2	2	1	1	2	1
1	1	2	2	2	2	1	1	2	1
1	1	2	2	2	2	2	2	2	1
1	2	2	2	1	2	2	1	2	2
1	1	3	3	1	3	1	1	9	3
2	1	3	3	1	3	1	1	3	1
2	2	2	2	3	2	2	2	3	2
2	1	2	3	1	2	3	3	2	2
1	1	1	1	1	3	3	3	1	1
1	3	2	1	3	3	3	2	1	3
2	9	9	9	1	9	9	9	9	9
1	1	1	2	2	2	2	3	2	1
2	2	2	2	1	1	1	1	1	1
2	1	1	1	1	1	1	1	1	1
1	1	1	1	2	2	1	1	2	1
1	1	1	2	2	1	1	1	2	1
2	2	2	2	2	9	1	2	2	3
2	3	1	3	1	9	3	3	1	1
2	1	3	2	1	3	2	2	2	2
3	2	3	3	3	3	3	3	3	2
3	3	1	3	3	3	3	3	9	3
1	1	2	1	1	2	2	1	2	2
1	1	1	2	2	2	2	1	1	1
2	1	1	2	2	3	3	3	3	2
2	1	2	2	3	1	2	2	2	1
2	2	2	2	1	1	1	2	1	2
2	1	1	2	9	2	2	1	1	3
2	1	1	1	1	2	2	1	2	2
2	1	1	2	1	2	2	1	1	1
1	1	3	3	1	2	9	1	9	1
1	2	2	2	2	2	2	2	2	9
2	3	3	3	2	1	3	3	3	1
2	1	3	3	1	3	1	1	1	3
1	1	2	2	2	1	1	1	1	1
2	1	2	2	1	1	9	9	9	2
2	1	1	3	1	2	1	1	1	1
1	2	3	3	1	3	3	2	1	3
2	1	2	2	1	1	2	2	2	1
2	1	3	3	1	3	1	3	3	1
1	2	2	2	1	1	2	2	2	1
1	2	3	3	1	3	2	2	3	1

1	1	9	1	1	1	1	1	1	3
1	9	9	9	9	9	9	9	9	1
2	2	2	2	1	2	2	2	2	1
2	1	1	2	1	2	2	3	3	1
1	1	1	1	1	1	1	1	1	1
1	1	2	2	1	2	2	2	2	1
2	2	1	3	2	1	2	1	3	1
3	3	1	1	3	3	3	1	3	3
3	1	1	2	1	1	2	2	2	1
2	2	1	1	1	2	3	3	2	1
1	2	2	2	3	2	1	1	1	1
1	1	2	2	1	2	2	3	2	1
2	2	2	1	2	2	2	2	2	1
1	3	3	3	3	3	3	3	3	1
1	1	2	3	2	2	2	2	2	2
3	1	2	2	1	9	2	3	1	1
2	1	2	2	1	3	1	2	3	3
1	1	3	3	1	2	2	1	1	3
1	1	2	2	1	2	2	2	2	1
1	1	2	2	1	2	2	2	2	2
2	1	2	2	1	2	1	1	1	2
9	1	2	2	1	2	1	1	1	2
3	3	3	3	1	3	2	2	1	1
2	2	3	3	3	3	2	2	2	2
1	1	1	3	1	3	1	9	2	2
1	2	2	2	1	2	2	1	1	1
2	1	1	3	3	3	3	3	3	3
1	1	1	2	1	1	1	2	2	1
1	1	1	1	1	1	2	1	1	1
3	1	2	1	2	1	2	9	2	1
2	3	3	1	3	3	3	3	3	3
2	2	1	3	2	2	1	3	2	3
2	3	2	1	1	2	2	1	1	1
2	1	2	1	2	1	2	2	2	1
1	1	2	1	2	2	2	2	2	1
1	1	2	3	1	2	2	2	3	1
1	1	1	1	2	2	1	1	1	1
2	3	3	3	3	3	3	3	3	1
1	2	1	2	3	1	2	2	9	3
1	1	2	1	1	2	2	2	2	1
1	1	2	1	3	3	3	3	1	1
1	2	2	2	2	2	2	2	2	1
1	1	1	2	2	1	2	9	2	1
2	1	1	9	1	9	1	9	1	1
2	1	1	1	1	3	2	1	3	1
3	3	3	3	3	3	3	3	3	1
1	1	1	2	2	2	2	1	2	1

9	9	9	9	9	9	9	9	9	9
1	1	2	2	1	1	2	2	2	1
1	2	2	2	1	1	2	1	1	2
1	1	3	3	1	3	2	3	1	1
1	1	2	2	1	2	2	2	1	2
2	1	1	9	1	2	2	2	1	1
2	1	3	3	1	1	3	1	3	1
2	3	3	3	3	3	3	3	3	3
1	3	3	3	3	3	3	3	3	3
1	1	2	2	2	2	2	2	2	1
2	1	1	2	1	2	1	2	2	1
2	3	3	3	3	3	3	3	3	3
2	1	3	3	1	3	1	9	2	1
2	1	1	3	2	2	3	2	1	3
2	9	9	9	9	9	9	9	9	2
2	1	2	3	2	2	2	2	2	2
9	1	1	1	1	1	1	1	1	2
1	1	3	3	1	3	1	3	2	2
2	2	2	2	9	2	2	2	2	2
2	9	1	3	2	2	2	2	2	3
1	1	1	2	3	2	2	2	2	1
1	2	1	1	1	1	1	1	1	1
1	1	1	1	1	2	2	1	1	1
1	1	1	2	2	2	2	1	1	1
2	1	1	2	1	2	2	2	2	2
2	1	1	3	2	2	1	2	2	3
2	1	9	9	9	9	9	9	9	2
1	1	2	3	9	1	2	2	2	2
2	3	3	2	2	2	2	3	2	2
3	1	1	2	1	1	2	9	2	2
3	1	2	2	1	2	3	3	3	1
2	3	3	1	3	3	3	3	3	3
2	1	2	3	1	2	1	2	2	1
2	1	1	3	1	1	2	2	2	3
2	1	1	2	1	1	2	2	2	2
2	1	2	2	2	1	1	1	1	1
9	9	9	9	9	9	9	9	9	9
2	2	2	2	2	2	2	2	2	2
3	1	2	2	2	1	1	1	2	1
9	1	1	1	1	1	1	1	1	1
1	1	1	2	1	2	2	2	2	1
2	1	1	1	1	1	1	1	1	1
1	1	1	1	1	1	1	1	1	1
1	1	2	3	1	1	2	2	1	2
1	1	3	3	3	3	1	3	1	1
1	1	1	1	1	2	2	2	2	2
1	2	1	2	1	2	1	2	1	3

1	2	2	2	2	2	2	2	2	2
2	1	2	2	3	1	1	1	1	1
9	9	9	9	9	9	9	9	9	9
9	9	9	9	9	9	9	9	9	9
1	1	1	2	2	1	1	2	2	1
3	2	2	1	1	1	2	2	2	1
2	9	9	1	9	9	9	9	9	9
1	9	9	9	1	9	9	9	9	1
1	1	1	2	1	2	1	1	1	1
3	9	9	9	1	9	9	9	9	1
2	2	3	3	1	1	2	3	2	3
3	1	1	1	2	2	2	2	2	1
1	2	2	9	1	2	2	2	2	1
2	1	2	1	3	3	1	3	1	3
3	1	1	3	1	2	1	1	1	2
2	9	9	9	9	9	1	9	9	9
2	1	9	9	9	9	9	9	9	9
2	1	9	9	9	9	9	9	9	9
2	1	1	1	2	2	1	1	1	1
9	2	3	1	1	2	2	3	3	2
1	9	2	9	9	9	9	9	9	9
1	1	1	2	1	1	2	2	2	1
9	3	3	3	3	3	3	3	3	3
1	2	2	2	1	1	1	1	1	2
2	1	1	2	1	2	1	1	1	1
2	2	3	2	1	2	2	2	3	1
2	2	2	2	1	2	2	2	1	1
2	1	2	3	1	3	1	2	2	3
2	3	1	1	3	9	3	3	3	2
2	1	1	3	1	2	2	1	1	1
2	2	3	3	2	1	2	2	2	2
2	2	3	1	2	2	2	2	2	2
1	1	1	1	2	2	2	2	2	2
1	1	2	2	2	2	3	3	3	2
9	9	9	9	9	9	9	9	9	9
1	1	1	2	1	1	1	2	2	1
2	1	2	2	2	2	2	2	2	2
1	1	2	1	2	1	1	2	1	1
2	3	3	3	2	3	3	9	2	1
2	1	1	1	2	2	2	2	1	1
2	1	2	9	1	3	1	3	1	1
1	1	1	1	2	1	1	1	1	2
2	1	3	1	2	3	2	1	1	3
3	1	2	2	1	1	1	1	1	2
2	1	3	1	2	3	2	1	1	3
2	1	1	1	1	1	1	2	2	2
1	1	1	1	2	2	1	1	2	2

2	1	2	2	2	1	1	3	3	1
2	3	3	3	3	3	3	3	3	1
2	1	1	1	2	2	1	1	1	1
3	2	2	2	2	2	2	2	2	1
1	1	1	3	1	3	2	1	2	1
2	3	3	3	3	3	3	3	3	9
1	1	1	1	1	2	1	3	1	1
1	1	1	1	1	2	1	1	2	3
1	1	1	1	1	2	2	3	3	1
1	1	3	2	1	3	1	2	1	1
3	1	1	2	2	2	2	2	2	3
2	1	2	3	2	2	2	2	2	2
1	1	1	3	2	2	1	1	1	1
1	1	1	2	2	2	2	2	2	3
2	1	2	2	1	1	1	1	1	2
2	1	2	1	1	1	1	1	1	1
3	1	1	1	1	1	1	1	1	1
3	1	1	1	1	1	1	1	1	2
2	2	9	9	9	9	9	9	9	2
2	2	2	2	9	2	2	2	2	2
2	9	9	3	1	9	9	2	1	2
9	9	9	9	9	9	9	9	9	9
2	9	1	9	9	1	9	9	9	2
2	9	2	1	9	9	9	9	9	9
2	2	9	2	9	9	2	9	2	9
9	9	9	9	9	9	9	9	9	9
9	9	9	9	9	9	9	9	9	9
2	2	2	2	2	2	2	2	2	2
2	2	2	2	2	2	2	2	2	2
3	3	3	3	3	3	3	3	3	2
2	2	2	1	1	2	2	2	2	2
2	9	2	9	9	2	9	2	2	9
2	2	2	2	2	2	2	2	2	2
9	9	9	9	9	9	9	9	9	9
9	9	9	9	9	9	9	9	9	9
2	1	1	2	2	2	2	2	2	2
9	9	9	2	9	9	9	9	9	9
2	9	9	9	9	9	9	9	9	9
2	9	9	9	9	9	9	9	9	9
2	9	2	9	9	2	9	9	2	2
2	9	9	2	1	9	3	1	9	9
2	9	9	1	9	9	3	1	9	9
2	9	9	9	9	9	9	9	9	9
2	9	2	9	9	9	2	9	9	9
2	9	9	3	1	3	9	2	1	9
1	3	9	9	9	9	9	9	9	3
9	9	9	9	9	2	9	9	9	9

2	1	9	9	9	9	9	9	9	1
1	3	3	3	3	3	3	3	3	3
2	3	1	3	1	1	1	1	1	2
1	1	2	3	1	2	3	1	1	2
2	1	1	1	1	1	1	1	1	1
2	9	1	9	9	9	9	9	9	9
2	9	9	9	1	9	9	9	9	1
2	1	9	9	9	9	9	9	9	1
3	1	1	2	1	3	2	3	2	3
1	1	3	9	3	2	9	2	3	3
1	2	2	2	2	2	2	2	2	1
1	1	2	2	1	2	2	2	2	2
2	1	1	2	1	2	1	3	2	1
2	1	3	1	1	1	1	1	1	3
9	1	2	1	1	1	1	1	1	9
2	1	1	2	1	2	2	2	2	1
9	9	9	9	9	9	9	9	9	9
9	9	9	9	9	9	9	9	9	9
1	1	9	9	9	9	9	9	9	9
1	1	1	2	2	1	2	2	1	1
1	1	1	1	1	1	1	1	1	1
2	1	1	1	2	2	1	2	1	2
1	9	9	9	9	9	9	9	9	9
2	1	1	2	1	2	2	2	2	2
3	1	1	2	3	1	1	1	1	1
2	1	1	1	1	1	1	1	1	1
1	1	3	3	1	3	1	1	3	3
2	2	2	3	2	2	2	2	2	1
1	3	3	3	2	2	1	1	2	1
1	1	1	1	1	1	1	1	1	2
1	2	3	1	1	1	1	1	1	2
1	2	2	2	2	2	2	2	2	2
2	2	2	2	2	2	2	2	2	2
1	1	2	3	1	2	3	1	2	1
2	2	2	2	2	2	2	2	2	3
2	1	1	1	1	1	1	1	1	2
2	2	2	2	2	2	2	2	2	3
1	2	2	2	2	2	2	2	2	2
2	2	3	2	1	2	3	2	3	2
3	2	2	2	2	2	2	2	2	2
2	3	3	9	3	3	3	3	3	3
1	1	1	2	2	2	2	2	2	1
2	3	2	2	2	2	1	1	1	9
2	2	1	2	3	3	2	3	2	3
2	3	3	3	3	3	3	3	3	3
2	3	3	3	3	3	3	3	3	3
1	2	2	2	2	2	2	2	9	2

2	2	1	2	1	3	1	2	3	1
2	1	1	1	1	1	1	1	2	1
2	2	1	1	3	1	2	3	3	3
2	2	2	2	2	2	2	2	2	1
2	2	2	2	2	2	2	2	2	2
1	2	2	2	2	2	2	2	2	1
2	1	1	2	1	3	2	1	2	2
2	1	1	3	1	1	2	2	2	9
2	9	9	9	1	9	9	9	9	9
2	1	3	2	1	2	3	2	3	1
1	1	9	9	9	9	9	9	9	9
9	2	2	1	2	3	2	1	2	2
2	1	9	9	9	9	9	9	9	9
2	2	9	9	9	9	9	9	9	3
1	2	2	2	2	2	2	2	2	2
1	2	9	9	9	9	9	9	9	1
2	1	9	9	9	9	9	9	9	9
2	1	9	9	9	9	9	9	9	9
2	2	9	9	9	9	9	9	9	9
2	9	9	2	9	9	9	9	9	9
1	1	9	9	9	9	9	9	9	1
2	9	9	2	9	9	9	9	9	9
2	2	2	1	2	1	3	2	1	2
2	1	9	2	1	3	2	1	3	2
1	1	2	1	1	2	3	1	2	1
2	1	2	2	2	1	1	2	2	2
1	1	2	3	1	2	1	2	3	3
1	1	2	2	1	2	1	2	1	1
2	1	1	3	1	3	1	1	2	1
2	2	1	2	1	2	3	2	2	2
1	2	9	2	1	2	3	3	3	1
2	3	3	3	3	3	3	3	3	3
2	2	2	2	2	2	2	2	2	9
2	1	2	2	2	3	2	3	2	1
3	1	1	2	1	2	2	2	2	2
1	1	2	2	1	1	1	1	1	1
3	1	1	2	1	1	2	1	1	1
2	2	2	2	2	2	2	2	2	2
1	1	2	2	1	1	1	1	2	1
2	1	1	1	1	1	1	1	1	1
2	9	9	9	9	9	9	9	9	2
3	2	2	3	1	2	1	2	2	1
3	3	3	3	3	3	3	3	3	9
2	1	1	1	2	2	2	2	2	1
1	1	1	9	2	2	2	2	2	2
1	2	2	2	2	2	2	2	2	2
2	1	9	9	9	9	9	9	9	1

1	3	3	3	2	3	2	3	3	3
1	9	9	9	1	9	9	9	9	1
3	1	3	2	1	2	1	3	1	2
1	1	1	2	2	2	2	2	2	2
1	1	1	2	9	2	2	1	3	1
1	1	1	1	1	1	1	1	1	2
1	1	1	1	2	2	3	2	1	3
1	2	9	9	1	9	9	9	9	9
1	1	9	9	2	2	9	9	9	2
2	1	2	9	3	2	3	2	3	1
2	2	2	2	2	2	2	2	2	3
2	1	9	2	2	2	2	2	2	2
3	1	3	3	1	3	2	2	2	1
1	1	1	2	1	2	2	2	1	2
1	9	9	9	9	9	9	9	9	9
1	1	1	3	1	2	2	3	2	2
1	1	1	3	1	2	1	2	1	1
1	1	9	9	9	9	9	9	9	1
1	1	1	1	1	1	2	2	1	3
1	1	9	9	9	9	9	9	9	1
1	1	2	2	1	2	1	2	2	1
2	1	1	2	1	2	1	2	2	2
9	9	9	9	9	9	9	9	9	9
2	2	9	9	9	9	9	9	9	2
2	9	9	9	1	9	9	9	9	1
1	1	3	3	1	3	2	3	3	3
1	2	2	2	2	2	2	2	2	2
2	1	3	1	3	3	3	2	2	2
2	1	2	1	2	2	2	2	2	2
2	9	9	9	9	1	9	9	9	1
1	1	2	1	2	2	1	2	2	1
2	2	2	2	2	1	2	2	2	1
1	2	2	2	1	2	2	1	1	1
2	1	1	1	2	1	2	1	2	2
2	2	2	1	2	3	3	3	3	3
2	1	9	9	9	9	9	9	9	1
9	9	9	9	9	9	9	9	9	9
1	1	2	1	1	1	1	1	1	1
2	1	1	1	1	1	1	2	2	1
2	1	1	1	1	1	1	9	9	9
2	1	2	1	1	1	1	2	2	1
1	1	2	2	1	1	1	1	1	2
2	1	1	2	1	1	1	9	9	1
3	1	2	2	1	1	1	1	1	2
1	3	2	9	3	1	2	2	9	2
2	2	2	9	1	1	2	2	1	2
1	2	2	2	1	2	2	2	2	1

1	1	3	3	1	1	2	3	2	2
1	3	3	3	3	3	3	2	2	2
2	2	2	1	1	2	1	2	1	2
9	1	1	1	1	1	1	1	1	2
1	1	1	1	1	1	1	1	1	2
2	3	3	3	3	3	3	3	3	2
1	1	2	2	2	2	2	2	2	2
1	2	9	9	1	9	9	9	9	1
1	3	1	9	9	9	9	9	9	9
1	9	9	9	1	9	9	9	9	1
1	1	1	2	2	3	1	2	9	1
1	1	1	1	2	1	1	1	1	3
2	2	2	2	9	9	2	9	2	2
2	2	9	2	2	9	2	9	2	2
3	9	9	9	9	9	9	9	9	9
9	2	9	9	9	2	9	9	9	9
2	1	2	2	1	2	2	2	2	1
2	2	2	2	2	2	2	2	2	2
2	1	2	2	2	2	2	2	2	2
2	2	2	2	2	2	2	2	2	2
2	1	9	9	1	9	9	9	9	2
2	2	9	9	9	9	9	9	9	2
3	3	3	3	3	3	3	3	3	3
1	1	9	9	9	9	9	9	9	1
1	2	9	9	9	9	9	9	9	2
1	2	9	9	9	9	9	9	9	2
9	9	9	9	9	9	9	9	9	9
2	2	9	9	9	9	9	9	9	2
2	2	2	2	2	2	2	2	2	2
2	2	2	2	2	2	2	2	2	2
9	9	9	9	9	9	9	9	9	9
1	1	9	9	9	9	9	9	9	1
1	2	2	1	1	1	1	1	1	1
1	1	1	9	1	1	9	9	9	1
1	1	3	3	1	1	3	3	3	1
2	2	2	2	2	2	2	2	2	2
3	2	2	2	2	9	9	9	9	1
1	1	2	3	1	1	3	3	3	1
2	1	9	1	9	1	9	1	9	1
3	2	9	2	9	9	9	1	9	1
2	2	2	2	2	2	2	2	2	2
9	3	3	3	3	2	2	2	2	1
3	2	2	2	2	2	2	2	2	2
2	2	2	2	2	2	2	2	2	2
1	1	1	1	1	1	2	2	2	1
2	2	1	1	2	2	2	9	2	2
3	9	9	9	9	9	1	9	9	1

1	1	3	3	1	1	2	2	1	2
1	1	3	3	1	2	1	2	3	2
2	1	1	1	2	2	3	9	3	3
2	3	3	3	1	1	1	1	1	3
1	1	2	3	1	2	2	1	2	2
1	1	2	3	1	2	2	3	2	1
1	1	9	9	9	9	9	9	9	1
2	1	3	3	9	1	1	2	1	3
1	1	2	2	2	2	2	2	2	2
1	1	9	9	1	9	1	9	1	1
2	1	2	2	2	2	2	2	2	2
3	1	1	3	3	2	1	3	2	1
9	1	2	1	2	2	1	1	1	3
3	1	1	3	3	2	1	3	2	1
1	1	3	3	1	3	1	1	1	3
2	1	2	2	1	1	1	9	1	2
2	1	2	2	2	2	2	2	2	2
2	9	9	9	9	9	9	9	9	1
2	2	9	9	1	3	9	9	9	9
2	1	2	3	1	2	2	1	1	1
2	9	9	9	2	9	2	9	9	9
1	1	1	2	2	2	2	1	1	2
2	2	3	3	1	1	2	2	3	1
2	2	2	2	1	2	2	2	2	2
2	1	1	3	1	1	3	9	2	2
3	1	1	2	2	2	1	1	2	1
2	2	9	9	9	9	9	9	9	2
3	2	3	1	3	3	2	3	1	1
2	3	2	3	1	3	3	3	3	3
1	1	9	9	9	9	9	9	9	1
3	1	9	9	1	2	1	9	9	9
1	1	1	1	1	1	1	1	1	1
1	9	9	9	9	1	9	9	9	1
2	1	2	2	1	1	1	1	1	2
1	9	9	9	9	9	9	9	9	9
1	1	9	9	9	9	9	9	9	9
2	1	1	2	2	2	2	2	2	2
2	3	2	1	2	1	3	2	3	2
1	1	2	1	1	1	1	2	3	2
1	1	1	2	1	2	1	1	1	1
1	1	1	2	2	2	1	3	1	1
1	2	2	3	2	3	2	9	9	2
1	2	3	3	2	2	2	2	3	3
2	1	1	1	1	1	1	1	1	1
3	1	2	2	1	2	2	2	2	3
1	2	2	2	2	2	2	2	2	2
3	1	9	3	1	2	2	2	2	1

3	1	9	9	1	2	2	2	2	1
2	1	2	1	3	1	1	2	3	3
2	1	2	3	2	1	1	2	2	3
1	1	9	1	3	3	3	3	3	3
2	1	1	1	3	2	1	2	2	2
1	1	1	3	3	3	3	1	1	3
3	1	9	9	9	9	9	9	9	9
1	1	2	3	1	2	2	2	2	2
2	1	3	3	1	2	2	3	2	1
1	1	2	3	1	1	1	2	1	3
2	1	2	2	2	2	2	2	2	1
9	1	1	3	9	9	9	9	9	9
9	1	9	1	9	9	9	9	9	9
1	1	2	2	2	1	2	2	2	2
1	1	1	2	1	1	2	2	2	2
1	1	2	2	1	2	2	2	2	2
3	2	2	3	2	2	2	2	2	2
1	1	2	3	2	2	2	2	2	3
1	1	2	2	1	2	1	2	2	2
2	2	2	2	2	2	2	2	2	2
1	1	3	3	3	3	2	3	2	3
2	2	9	9	2	9	2	9	2	2
3	9	9	9	9	9	2	9	9	2
9	9	9	9	9	9	9	9	9	9
2	1	9	9	9	9	9	9	9	1
3	2	2	2	2	2	2	2	2	2
2	2	9	9	9	9	9	9	9	2
9	9	9	9	9	9	9	9	9	9
2	2	2	2	2	2	2	2	2	2
2	2	9	9	9	9	9	9	9	2
2	9	9	9	9	9	9	9	9	9
2	3	9	9	9	9	9	9	9	3
9	9	9	9	9	9	9	9	9	9
1	1	1	1	1	1	1	1	1	1
2	3	9	9	9	9	9	9	2	2
2	3	9	9	9	9	9	9	3	3
2	3	9	9	9	9	9	9	9	3
2	2	2	2	2	2	2	2	2	2
2	1	1	1	1	1	1	1	1	1
2	3	9	9	9	9	9	9	9	3
3	3	3	3	3	3	3	3	3	2
2	9	9	9	9	9	9	9	9	9
2	2	9	9	9	9	9	9	9	2
9	9	9	9	9	9	9	9	9	9
2	2	9	1	9	9	2	9	9	1
3	1	9	9	9	9	9	9	9	1
1	2	2	2	2	2	2	2	2	2

1	2	9	9	9	9	9	9	9	2
2	2	9	9	9	9	9	9	9	2
3	2	9	9	9	9	9	9	9	9
2	2	2	2	2	2	2	2	2	2
2	2	2	2	2	2	2	2	2	2
9	9	9	9	9	9	9	9	9	9
2	1	2	1	1	9	1	1	1	1
3	2	1	9	1	2	1	2	2	2
2	2	9	9	9	2	9	9	9	1
1	1	3	3	1	2	2	2	2	1
2	2	9	9	9	9	9	9	9	2
2	2	9	9	9	9	9	9	9	2
2	2	9	9	9	9	9	9	9	2
2	3	9	9	9	9	9	9	9	2
9	9	9	9	9	9	9	9	9	9
9	9	9	9	9	9	9	9	9	9
9	9	9	9	9	9	9	9	9	9
9	9	9	9	9	9	9	9	9	9
9	9	9	9	9	9	9	9	9	9
9	9	9	9	9	9	9	9	9	9
2	2	2	2	2	2	2	2	2	2
2	2	2	2	2	2	2	2	2	2
2	1	2	2	1	2	2	2	2	1
2	2	2	2	2	2	2	2	2	2
2	2	2	2	2	2	2	2	2	2
2	2	2	2	2	2	2	2	2	2
2	2	2	2	2	2	2	2	2	2
2	2	9	9	9	9	9	9	9	2
2	1	9	9	9	9	9	9	9	1
2	2	2	2	2	2	2	2	2	2
2	2	2	2	2	2	2	2	2	2
2	1	9	9	1	2	2	2	2	1
3	9	9	9	9	9	9	9	9	9
2	2	2	2	2	2	2	2	2	3
2	9	9	9	9	9	9	9	9	9
1	1	1	1	1	1	1	1	1	2
1	1	1	1	1	1	1	1	1	1
2	2	2	3	2	1	2	3	2	1
3	2	1	1	2	2	2	2	2	1
1	2	3	3	1	9	2	2	2	1
1	2	2	2	2	2	2	2	2	2
2	1	9	9	1	9	9	9	9	1
2	3	9	9	9	9	9	9	3	3
2	2	9	9	9	9	9	9	9	2
3	1	1	1	1	1	1	1	1	1
2	3	9	9	9	9	9	9	3	3
2	2	9	9	9	9	9	9	9	2

2	9	9	9	9	9	9	9	9	9
2	2	2	2	2	2	2	2	2	2
1	1	9	9	1	1	9	9	9	1
2	1	9	1	9	1	9	1	2	9
2	2	9	9	9	9	9	9	2	2
2	2	9	9	9	2	9	9	2	2
3	3	9	9	9	9	9	9	9	2
9	9	9	9	9	9	9	9	9	9
2	3	9	9	9	9	9	9	3	3
2	3	9	9	9	9	9	9	9	3
9	9	9	9	9	9	9	9	9	9
2	2	9	9	9	9	9	9	9	2
1	1	9	9	1	1	9	9	9	1
2	2	9	9	9	9	9	9	9	2
1	2	2	2	9	2	2	2	2	2
1	1	9	9	9	9	9	9	9	1
2	2	9	9	9	9	9	9	9	2
2	9	9	9	9	9	9	9	9	9
2	1	3	3	1	1	1	3	3	3
2	9	9	9	9	9	9	9	9	9
2	1	1	2	1	2	3	1	1	3
1	3	2	3	1	2	1	2	2	2
2	9	9	9	1	9	9	9	9	9
2	9	9	9	1	9	9	9	9	9
2	1	3	2	2	2	2	9	1	1
2	2	2	2	1	2	2	9	9	2
1	1	1	3	2	2	2	3	2	1
2	9	9	1	9	9	9	9	9	2
3	1	2	2	3	2	3	1	1	3
3	1	2	2	3	2	3	1	2	2
1	1	2	9	1	2	2	2	2	2
1	1	2	2	1	2	2	2	2	2
1	1	2	2	1	1	2	2	2	3
1	2	2	1	2	2	1	2	2	3
1	2	3	3	9	9	9	9	9	1
2	1	2	1	1	1	2	9	1	2
3	2	9	9	9	9	9	9	9	2
2	2	9	9	9	9	9	9	9	1
1	1	2	1	1	3	1	1	1	1
2	9	9	9	9	9	9	2	9	9
9	1	3	3	1	2	9	9	9	1
9	9	9	9	9	9	9	9	9	9
9	9	9	9	9	9	9	9	9	9
1	9	2	9	2	9	9	2	9	9
2	9	9	9	9	9	9	9	9	9
2	2	2	9	2	9	2	9	2	2
1	9	2	9	9	2	9	9	2	2

1	2	2	2	2	2	2	2	2	2
1	2	2	2	2	2	2	2	2	2
2	2	2	2	2	2	2	2	2	2
2	2	2	2	2	2	2	2	2	2
2	2	2	2	2	2	2	2	2	2
3	3	1	1	2	3	2	1	2	2
1	1	2	1	1	3	1	2	3	2
2	2	3	2	1	2	2	2	2	2
1	1	2	1	1	1	1	2	2	2
2	1	2	1	3	2	1	2	1	3
9	3	3	1	3	3	3	3	3	3
1	1	1	2	3	2	2	2	1	1
2	1	2	2	1	2	2	2	3	1
2	3	1	1	3	3	3	3	3	3
2	2	2	2	2	2	2	2	2	2
1	2	2	2	2	2	2	2	2	2
1	2	2	2	2	2	2	2	2	2
1	2	2	2	2	2	2	2	2	2
1	1	2	1	2	3	3	2	1	1
2	2	1	3	1	3	2	1	2	1
9	1	2	2	2	2	2	3	3	3
3	2	2	2	2	2	2	2	2	2
2	3	1	3	3	3	3	3	3	2
3	1	2	3	1	3	2	3	2	1
1	1	2	2	9	1	2	2	3	1
1	1	3	2	1	2	1	3	2	3
3	2	1	1	2	1	2	1	2	2
9	1	3	2	1	1	1	2	2	2
1	2	1	1	2	1	3	2	1	2
2	1	2	2	1	2	1	2	2	3
3	2	2	1	1	2	2	1	1	1
2	1	2	3	2	2	2	3	3	2
1	2	2	3	2	3	3	3	3	2
3	3	2	3	2	3	2	2	3	2
3	1	3	3	1	3	2	2	3	1
1	1	1	2	1	3	1	3	3	1
2	1	1	2	2	2	1	3	3	3
9	2	1	1	2	2	3	2	2	1
2	3	2	3	3	3	3	3	3	3
9	1	1	3	1	1	1	2	3	3
3	9	9	9	1	9	9	9	9	9
2	2	1	3	3	3	2	3	3	2
1	1	2	2	1	3	3	2	2	1
1	1	1	1	1	1	1	1	1	1
2	1	2	2	1	3	3	2	2	1
2	3	2	1	3	2	9	9	9	9
2	3	2	9	2	2	2	2	2	2

3	3	3	1	3	3	3	3	3	1
1	1	1	2	2	3	2	1	3	1
2	1	2	2	2	3	2	1	3	1
3	3	3	3	2	2	3	2	3	2
3	3	3	1	3	3	3	3	3	2
3	3	2	3	2	2	3	2	3	2
2	3	3	3	3	3	3	3	3	2
1	3	3	3	3	3	3	3	3	3
2	1	1	2	2	2	2	3	3	1
9	1	2	1	3	3	2	3	2	3
1	1	9	2	9	1	2	2	3	2
1	1	1	3	1	2	1	1	2	1
1	2	3	2	1	1	1	2	2	1
2	2	3	2	3	9	2	3	1	3
9	1	3	3	1	3	1	1	1	1
1	1	2	2	2	2	1	3	3	1
2	2	2	2	1	2	1	2	2	2
9	3	2	3	2	2	2	2	3	3
3	1	2	9	2	1	9	1	2	2
1	2	2	1	1	3	2	2	2	1
9	1	3	2	2	1	1	2	3	2
2	2	2	2	2	2	2	2	2	2
2	2	2	2	2	9	1	2	2	2
2	2	2	2	2	2	2	2	2	3
2	3	2	2	3	3	3	3	3	2
2	3	3	1	3	3	3	3	3	2
9	1	2	1	1	2	1	2	2	1
1	1	1	2	2	1	1	2	2	2
1	3	3	2	3	3	3	3	2	2
9	1	2	1	3	2	3	2	1	1
1	3	2	3	3	3	3	3	3	3
1	3	3	1	2	1	2	3	2	1
1	2	3	1	2	3	2	2	1	2
2	3	2	2	2	1	2	2	2	2
2	2	3	9	2	3	2	2	2	3
3	2	2	2	2	2	2	2	2	2
1	3	3	3	3	3	3	3	3	2
2	3	9	9	9	9	9	9	9	3
9	1	2	2	2	1	2	1	2	2
3	1	2	1	1	2	2	2	1	3
2	1	3	2	1	3	1	2	3	1
9	3	1	3	3	2	3	2	2	3
1	1	2	1	1	1	1	2	1	3
9	3	1	2	3	1	2	2	1	2
9	1	3	9	1	1	1	3	3	3
3	1	2	2	1	2	2	1	2	2
2	1	2	1	1	1	2	2	2	1

2	2	2	2	2	2	2	2	2	2
2	2	2	2	2	2	2	2	2	2
3	2	2	2	2	2	2	2	2	2
9	1	2	2	2	1	2	1	2	2
2	1	1	1	2	2	2	2	1	1
3	2	2	2	2	3	2	2	2	3
1	2	3	2	2	2	2	3	2	2
2	1	1	2	2	2	1	2	2	1
3	2	2	2	2	2	2	2	2	3
2	1	1	1	2	1	1	3	1	3
2	1	1	1	9	2	1	2	2	9
2	1	1	2	1	2	1	2	2	1
2	1	1	2	2	2	1	2	2	1
3	1	2	1	1	2	1	2	9	1
3	3	3	2	1	2	2	3	2	3
9	2	9	9	9	9	9	9	9	9
2	1	1	3	1	2	2	1	2	3
2	1	1	3	1	2	2	1	2	3
2	9	9	9	1	9	9	9	9	9
1	9	9	9	1	9	9	9	9	2
2	1	1	2	2	2	3	1	2	3
1	9	9	9	9	9	9	9	9	9
1	9	3	1	3	3	3	3	3	1
1	1	9	9	9	9	9	9	9	1
9	1	9	9	9	9	9	9	9	9
2	3	3	3	1	3	2	3	2	3
3	2	9	3	2	2	2	2	2	1
2	2	2	9	9	9	1	9	9	1
2	1	2	1	1	2	3	1	2	1
9	1	1	1	2	2	2	2	3	2
1	2	2	2	2	2	2	2	2	1
1	1	1	1	2	2	2	2	3	3
2	1	1	2	1	1	2	2	3	2
1	3	9	9	9	9	9	9	9	1
9	2	2	2	2	2	2	2	2	2
2	2	2	3	1	2	1	2	2	2
3	3	3	3	1	1	9	9	9	9
1	1	1	2	3	3	2	2	2	1
2	1	2	3	1	2	1	3	2	1
3	2	2	2	2	2	2	2	2	3
2	2	2	2	2	2	2	2	2	2
1	1	1	1	2	9	2	9	3	3
2	9	1	9	9	9	9	9	9	1
2	3	3	1	2	2	2	2	2	1
1	1	1	2	2	1	2	2	2	2
3	3	3	3	3	3	3	3	3	1
1	1	2	2	1	1	1	2	2	2

2	1	2	9	1	2	1	2	3	2
2	2	1	1	2	2	2	2	2	2
2	2	1	1	1	2	2	2	2	2
3	3	2	3	1	3	3	3	2	1
2	9	9	9	9	9	9	9	9	9
2	2	2	1	2	2	2	3	3	3
2	2	3	1	2	2	1	1	2	3
2	1	1	1	3	3	3	3	3	2
3	2	3	3	1	1	2	2	3	3
9	2	2	3	1	2	1	2	1	1
2	2	3	9	3	3	3	3	3	3
3	9	9	9	9	1	1	1	1	1
2	9	9	9	1	9	9	9	9	1
3	3	3	3	3	3	3	3	3	3
2	2	3	3	3	3	3	3	3	3
2	1	2	2	2	2	2	2	2	3
1	9	1	1	2	2	2	2	2	2
2	1	3	3	1	1	3	3	3	1
1	2	3	3	2	2	2	1	1	1
1	9	9	9	9	9	1	9	9	3
2	9	9	9	2	9	9	9	9	1
2	9	9	9	1	9	9	9	9	9
2	2	2	2	1	1	1	1	1	3
1	1	1	1	1	3	9	1	1	9
3	1	2	3	1	2	2	2	2	2
1	2	2	2	2	2	2	2	2	2
2	1	1	1	2	2	3	3	2	2
1	1	1	1	1	1	1	1	9	1
2	1	2	2	1	1	1	2	1	2
2	1	2	2	1	2	2	2	2	2
1	1	1	3	1	1	1	3	1	1
1	3	2	2	2	2	2	2	2	2
2	2	2	2	2	2	2	2	2	2
2	2	9	9	9	9	9	9	9	2
2	2	9	2	3	2	3	2	1	1
2	3	3	3	3	3	3	3	3	2
9	1	1	1	1	1	1	1	1	1
9	1	1	2	1	2	2	1	2	3
1	1	1	2	1	2	1	1	9	1
1	1	2	2	1	1	1	2	2	2
1	1	1	2	2	1	1	1	1	2
2	2	2	2	2	2	2	2	2	1
1	2	2	2	2	2	2	2	2	1
1	1	3	2	1	3	3	3	1	1
1	1	2	3	1	3	2	3	2	3
3	1	1	1	1	2	2	3	3	3
1	1	9	9	9	9	9	9	9	9

1	2	1	2	1	2	1	2	1	2
2	1	2	2	1	2	2	2	2	1
1	1	2	2	1	2	2	2	2	3
1	2	1	1	2	2	2	2	2	2
1	1	2	1	1	2	2	2	2	1
2	3	9	9	9	9	9	9	9	3
2	1	2	1	2	3	2	1	2	3
1	1	2	1	1	2	9	1	2	3
2	3	3	1	3	3	3	3	3	1
2	1	2	2	1	2	2	2	2	1
1	2	2	9	1	3	1	2	1	3
2	1	3	3	1	2	1	3	2	1
1	1	2	3	1	1	1	2	3	1
2	1	9	9	9	9	9	9	9	1
2	1	9	9	9	9	9	9	9	1
1	9	9	9	1	9	9	9	9	9
2	1	2	9	1	2	2	2	1	2
1	1	1	1	1	1	1	1	1	3
1	1	1	1	1	1	1	2	2	1
2	1	9	9	9	9	9	9	9	2
2	1	1	1	3	2	2	2	2	2
1	2	2	2	1	1	1	9	9	1
9	1	2	9	2	2	2	9	1	3
2	9	9	9	1	9	9	9	9	9
2	9	9	9	1	9	9	9	9	9
2	1	9	9	9	9	9	9	9	9
1	1	1	1	1	1	1	1	1	1
2	1	1	2	2	2	1	1	1	3
9	3	2	2	3	2	3	2	2	1
9	3	3	3	3	3	3	3	3	2
2	1	1	9	9	9	9	1	1	2
3	1	1	3	3	3	1	1	2	1
2	1	1	1	3	3	3	3	3	1
2	2	9	9	9	9	9	9	9	2
3	2	2	2	2	2	2	2	3	1
2	1	3	9	1	3	1	3	1	1
2	3	2	1	3	3	3	3	3	3
9	2	9	9	9	9	9	9	9	1
3	1	1	1	1	1	2	2	3	3
2	1	9	9	9	9	9	9	9	3
1	1	9	9	9	9	9	9	9	1
1	1	1	2	2	1	2	2	2	2
1	1	9	9	9	9	9	9	9	1
9	1	1	1	1	1	1	1	9	1
2	1	2	2	1	2	3	2	2	3
3	2	2	2	2	2	2	2	2	2
1	2	2	2	2	2	2	2	2	2

3	2	2	2	2	2	2	2	2	2
2	3	3	3	3	3	3	3	3	3
1	2	2	2	2	2	2	2	2	2
1	1	1	1	1	1	1	1	1	2
1	1	1	1	1	1	1	1	9	1
2	2	2	2	2	2	2	2	2	1
2	9	3	1	3	9	1	3	1	9
1	2	9	2	2	9	2	2	2	9
1	2	9	2	8	2	2	2	9	9
9	9	9	9	9	9	9	9	9	9
2	9	1	3	1	9	1	9	1	1
2	1	1	3	2	1	1	9	1	1
2	9	3	1	9	9	3	1	9	9
2	9	2	9	2	9	9	2	9	2
2	9	2	9	9	9	2	9	2	2
1	1	1	1	1	1	1	1	1	1
2	2	2	2	2	2	2	2	9	2
1	2	9	9	2	9	9	2	9	2
1	2	2	2	2	2	2	2	2	2
2	3	3	3	3	3	3	3	3	3
1	2	2	2	2	2	2	2	2	2
2	9	9	9	9	9	9	9	9	9
1	2	9	2	9	9	2	9	2	2
2	2	2	2	2	2	2	2	2	2
2	9	2	1	9	2	1	2	9	9
1	2	2	2	2	2	2	2	2	2
2	2	2	2	2	2	2	2	2	2
1	2	2	2	2	2	2	2	2	2
1	1	2	1	3	1	1	2	3	1
2	1	1	9	2	1	1	1	1	1
2	3	3	3	3	3	3	3	3	2
9	3	3	3	3	3	3	3	3	3
2	2	3	1	3	1	2	1	2	3
2	1	2	2	1	2	2	3	2	2
1	1	2	1	3	2	1	2	3	3
2	1	2	2	1	2	2	2	2	2
2	3	3	3	3	3	3	3	3	2
3	1	2	2	2	2	2	2	2	3
2	2	3	3	2	2	2	2	1	2
1	1	1	1	2	2	1	2	2	3
2	1	2	2	1	2	1	1	1	1
1	9	1	9	9	9	9	9	9	1
3	3	2	3	1	3	3	3	2	2
2	1	3	3	1	3	1	2	2	1
2	2	2	2	2	2	2	2	3	2
2	2	2	3	2	2	2	2	2	2
1	1	1	1	3	3	3	3	3	1

2	1	1	3	2	2	3	2	3	3
2	1	1	3	1	2	3	1	2	3
1	9	9	9	9	9	9	9	9	9
2	1	3	3	2	1	2	1	1	1
9	1	1	1	2	2	9	9	3	3
1	1	3	1	3	3	3	3	3	1
2	3	1	2	1	2	2	2	2	2
3	3	9	3	3	2	2	2	2	2
1	1	1	1	1	1	1	1	1	1
1	2	2	2	2	2	2	2	2	2
1	1	1	1	1	2	2	2	2	1
1	2	2	2	2	2	2	2	2	2
1	1	1	1	1	1	1	2	1	1
1	1	1	9	1	1	3	3	1	1
2	3	3	3	3	3	3	3	3	3
1	1	2	1	1	1	1	1	2	1
1	1	1	1	1	1	1	9	9	1
1	1	1	1	1	1	1	1	1	1
1	1	1	1	1	1	1	1	1	1
1	1	1	1	1	1	1	1	1	1
1	1	1	1	1	2	2	2	2	1
1	1	1	1	1	1	1	1	1	2
1	1	1	1	2	2	2	1	1	1
1	1	1	2	2	1	2	2	2	1
1	1	1	1	1	1	1	1	1	1
2	2	2	2	2	2	2	2	2	2
1	1	9	9	9	9	9	9	9	1
2	1	1	1	2	2	2	2	2	2
9	9	9	9	9	9	9	9	9	9
1	1	1	1	1	1	1	1	1	1
1	1	1	9	1	1	1	1	1	1
1	1	2	1	1	1	1	1	1	2
1	1	2	1	1	1	1	2	1	1
1	1	1	1	2	1	1	1	1	1
3	2	2	9	2	2	2	2	2	2
1	1	1	1	1	9	1	1	1	1
3	2	2	2	2	2	2	2	2	2
3	2	2	2	2	2	2	2	2	2
1	1	1	1	1	1	1	9	1	1
1	1	1	1	1	1	1	1	1	1
1	1	1	1	1	1	2	2	2	1
3	2	2	2	2	2	2	2	2	2
1	1	2	1	2	1	2	1	2	1
1	1	1	1	1	2	2	1	1	1
9	9	9	9	9	9	9	9	9	9
2	2	2	2	2	2	2	2	2	2
1	2	2	2	2	2	2	2	2	2

2	2	2	2	2	2	2	2	2	2
1	1	1	1	1	1	1	1	1	1
2	2	2	2	2	2	2	2	2	2
1	1	1	1	1	2	1	2	1	1
2	2	2	2	2	2	2	2	2	2
1	1	1	1	1	1	1	1	1	1
1	1	2	1	1	2	1	1	1	1
2	2	2	2	2	2	2	2	2	2
1	1	1	1	1	1	1	1	1	1
1	1	1	1	9	1	1	1	9	1
1	1	1	1	1	1	1	1	1	1
2	2	2	2	2	2	2	2	2	2
1	1	1	1	1	1	1	1	1	1
1	1	1	1	1	1	1	1	1	1
1	2	9	2	2	9	2	2	2	2
1	1	1	1	1	1	1	1	1	1
3	2	2	9	2	2	2	2	2	9
1	1	9	1	1	1	1	9	1	1
3	2	2	2	2	2	2	2	2	2
1	1	1	1	1	1	1	1	1	1
1	1	1	1	1	1	1	1	1	1
3	2	2	2	2	2	2	2	2	2
1	1	1	1	1	1	1	9	9	1
9	9	9	9	9	9	9	9	9	9
2	2	2	2	2	2	2	2	2	2
1	1	3	1	1	1	1	3	3	1
1	1	3	2	1	1	1	1	3	1
1	1	3	2	1	1	1	1	3	1
1	1	3	2	1	1	1	3	1	1
1	1	3	2	1	1	1	3	3	1
1	1	3	2	1	1	1	3	3	1
1	1	3	2	1	1	1	3	3	1
1	1	3	2	1	1	1	9	3	1
1	1	3	1	1	1	1	3	3	1
1	1	3	2	1	1	1	3	3	1
1	1	3	1	1	1	1	3	3	1
1	1	3	2	1	1	1	3	1	1
1	1	3	2	1	1	1	3	3	1
1	1	3	2	1	1	1	3	3	1
1	1	3	2	1	1	1	3	3	1
1	1	3	1	9	9	1	9	3	1
1	3	3	1	2	1	1	2	3	1
1	1	3	2	1	1	1	3	3	1
1	1	1	2	1	1	1	3	3	1
1	1	3	2	1	1	1	3	3	1
1	1	3	2	1	1	1	3	3	1
1	3	3	2	1	1	1	3	3	1

1	1	3	2	1	1	1	3	3	1
1	1	3	2	1	1	1	3	3	1
1	9	9	1	1	1	3	3	3	1
9	9	9	9	9	9	9	9	9	9
3	9	9	9	9	9	9	9	9	9
1	9	9	9	9	9	9	9	9	9
3	9	9	9	9	9	9	9	9	9
1	9	9	9	9	9	9	9	9	3
9	9	9	9	9	9	9	9	9	9
2	9	9	9	9	9	9	9	9	9
2	9	9	9	9	9	9	9	9	9
1	9	9	9	9	9	9	9	9	9
2	9	9	9	9	9	9	9	9	9
1	9	9	9	9	9	9	9	9	9
9	9	9	9	9	9	9	9	9	9
1	9	9	9	9	9	9	9	9	1
2	9	9	9	9	9	9	9	9	1
1	9	9	9	9	9	9	9	9	9
1	9	9	9	9	9	9	9	9	1
1	9	9	9	9	9	9	9	9	1
1	9	9	9	9	9	9	9	9	9
9	9	9	9	9	9	9	9	9	9
2	9	9	9	9	9	9	9	9	1
9	9	9	9	9	9	9	9	9	1
9	9	9	9	9	9	9	9	9	9
1	1	3	2	1	1	1	3	3	1
1	1	3	2	1	1	1	3	3	1
1	1	3	2	1	1	1	3	1	1
1	1	3	2	1	9	1	3	3	1
1	1	3	1	1	1	1	3	3	1
1	1	3	2	1	1	1	3	3	1
1	1	3	2	1	1	1	3	3	1
1	1	3	1	1	1	1	3	3	1
1	1	3	2	1	1	1	3	3	1
1	3	2	1	1	9	9	3	3	1
1	3	3	9	9	1	1	1	1	1
1	1	3	1	1	1	1	3	3	1
1	1	3	3	9	9	1	3	3	1
1	1	3	2	1	1	1	9	3	1
1	1	3	3	1	1	1	3	3	1
1	1	3	1	1	1	1	3	3	1
2	2	9	1	1	9	2	9	3	3
1	3	2	2	1	1	1	3	9	1
1	1	3	3	3	9	1	3	3	1
1	1	3	3	9	9	1	3	3	1
1	9	9	9	9	9	1	9	9	1
1	3	3	1	1	1	1	3	3	1

1	1	3	2	1	1	1	3	3	1
1	3	3	2	1	9	1	3	1	1
1	1	3	2	1	1	1	3	3	1
2	3	3	2	1	1	1	3	3	1
1	1	3	1	1	1	1	3	3	1
1	1	3	1	1	1	1	3	3	1
1	1	3	2	1	1	1	1	1	1
1	1	3	1	1	1	1	3	3	1
3	1	3	1	1	1	1	3	3	1
1	1	3	2	1	1	1	3	3	1
1	1	3	1	1	1	1	3	3	1
1	1	3	2	1	1	1	3	3	1
3	1	3	1	1	1	1	3	3	1
9	1	3	3	1	1	1	1	3	1
1	1	3	3	1	1	1	1	3	1
1	3	2	1	9	9	1	3	9	1
1	1	3	2	1	1	1	3	1	1
1	3	2	1	3	9	9	9	9	1
1	1	3	2	1	1	1	3	3	1
1	9	3	2	1	1	3	3	3	1
1	3	3	2	1	9	9	9	9	1
1	1	3	2	3	2	3	3	3	1
1	1	3	2	1	1	1	3	3	1
1	1	3	2	1	1	1	3	3	1
1	1	2	1	1	1	2	3	3	1
1	1	3	2	1	1	1	3	3	1
1	1	3	2	1	1	1	3	3	3
9	1	3	2	1	1	1	9	3	1
1	1	2	1	1	1	1	3	3	1
1	3	3	2	1	1	1	3	3	1
1	1	3	2	1	1	1	3	3	1
1	1	3	2	1	1	1	3	3	1
1	3	1	1	1	3	3	3	1	1
1	1	3	2	1	1	1	3	3	3
1	1	3	3	1	1	1	1	3	1
1	1	3	3	9	1	1	1	3	1
1	3	2	1	1	9	9	3	3	1
1	3	3	2	1	1	1	9	3	1
2	3	3	2	1	1	1	9	3	1
1	1	3	1	1	1	1	3	3	1
1	9	9	9	9	9	9	9	9	1
1	3	2	3	1	1	1	3	3	1
1	1	3	2	1	1	1	3	3	1
1	3	3	1	1	1	3	3	3	1
1	9	9	1	9	9	9	9	9	1
1	9	9	9	9	9	9	9	9	1
1	3	2	1	1	1	3	3	3	1

1	1	3	2	1	1	1	1	1	1
1	3	3	2	1	1	1	1	1	1
1	1	3	1	1	1	1	3	3	1
1	1	3	1	1	1	1	3	3	1
1	1	3	2	1	1	1	3	3	1
1	1	3	1	1	1	1	3	3	1
1	1	3	1	1	2	3	9	3	1
1	1	3	2	1	1	1	3	3	1
1	1	3	1	1	1	1	3	3	1
1	1	3	2	1	1	1	3	3	1
1	1	3	2	1	1	1	3	3	1
1	1	3	2	1	1	1	3	3	1
1	1	3	2	1	1	1	3	3	1
1	1	3	2	1	1	1	3	3	1
1	1	2	1	1	1	1	3	3	1
1	1	3	2	1	1	1	3	3	1
1	1	3	1	1	1	1	3	3	1
1	1	3	1	1	1	1	3	3	1
1	1	3	2	1	1	1	3	3	1
1	1	3	2	1	1	1	3	3	1
1	1	9	3	1	1	1	1	1	3
1	1	3	2	1	1	1	1	1	3
1	1	3	2	1	1	1	3	3	1
1	1	3	1	1	1	1	3	3	3
1	1	3	2	1	1	1	3	3	1
9	9	9	9	9	9	9	9	9	9
1	1	3	2	1	1	3	3	3	1
1	1	3	2	9	1	1	1	1	1
1	1	3	2	1	1	1	3	1	1
1	1	3	1	1	1	1	3	3	1
2	9	9	9	9	9	9	9	9	3
1	1	3	2	1	1	1	3	3	3
1	1	3	2	1	1	1	3	3	1
1	1	3	3	1	1	1	3	3	1
1	1	3	2	1	1	1	3	3	1
1	1	3	2	1	1	1	3	3	1
1	1	3	2	1	1	1	3	3	1
1	1	3	1	1	1	1	3	1	1
1	1	3	2	1	1	3	3	3	1
1	1	3	2	1	1	3	3	3	1
1	1	3	1	1	1	1	3	3	1
1	1	3	1	1	1	3	3	3	1
1	1	3	1	1	1	3	3	3	1
1	1	3	2	1	1	1	3	3	1
1	1	3	1	1	1	1	3	3	1
1	1	3	1	1	1	1	3	3	1
1	1	3	2	1	1	1	3	3	1

9	9	9	9	9	9	9	9	9	9
1	1	3	1	1	1	1	3	3	1
1	1	3	3	1	1	1	3	3	1
1	1	3	2	1	1	1	3	3	1
1	1	3	1	1	1	1	3	3	1
1	1	3	1	1	1	1	3	3	1
1	1	3	2	1	1	1	3	3	1
1	1	3	2	1	1	1	3	3	1
1	1	3	1	1	1	1	3	3	1
1	1	3	2	1	1	1	3	3	1
1	1	3	1	1	1	1	3	3	1
1	1	3	3	1	1	1	3	3	1
1	1	3	2	1	1	1	3	3	1
1	1	3	2	1	9	1	9	3	1
1	1	3	2	1	1	1	3	3	1
1	1	3	1	1	1	3	3	3	1
1	1	9	9	9	9	1	3	3	1
1	1	3	2	1	1	1	3	3	1
1	1	3	1	1	1	1	3	3	1
1	1	3	2	1	1	2	3	3	1
1	3	2	1	1	1	1	3	3	1
1	1	3	1	1	1	1	3	3	1
1	1	3	2	1	1	1	3	3	1
1	1	3	2	1	1	1	3	3	1
1	1	3	2	1	1	1	3	3	1
1	1	3	2	1	1	1	3	3	1
1	1	3	2	1	1	1	3	3	1
1	1	3	2	1	1	1	1	3	1
1	3	1	9	1	1	1	3	3	1
1	3	1	2	1	1	1	3	3	1
1	1	3	2	1	1	1	1	3	1
1	1	3	2	1	1	1	1	1	1
1	1	2	3	1	1	1	1	1	1
2	9	9	9	9	9	9	9	9	9
9	9	9	9	9	9	9	9	9	9
3	9	9	9	9	9	9	9	9	9
1	9	9	9	9	9	9	9	9	9
1	9	9	9	9	9	9	9	9	9
2	9	9	9	9	9	9	9	9	2
1	9	9	9	9	9	9	9	9	9
2	9	9	9	9	9	9	9	9	2
1	9	9	9	9	9	9	9	9	9
3	9	9	9	9	9	9	9	9	9
3	9	9	9	9	9	9	9	9	9
9	9	9	9	9	9	9	9	9	9
2	9	9	9	9	9	9	9	9	9
2	9	9	9	9	9	9	9	9	9

2	9	9	9	9	9	9	9	9	1
1	1	2	2	1	1	2	2	2	2
1	9	9	9	9	9	9	9	9	1
1	9	9	9	9	9	9	9	9	9
1	9	9	9	9	9	9	9	9	9
2	9	9	9	9	9	9	9	9	9
1	9	9	9	9	9	9	9	9	9
1	9	9	9	9	9	9	9	9	1
2	9	9	9	9	9	9	9	9	9
1	9	9	9	9	9	9	9	9	9
1	9	9	9	9	9	9	9	9	9
2	9	9	9	9	9	9	9	9	9
1	9	9	9	9	9	9	9	9	1
1	3	2	2	2	2	2	2	2	2
1	9	9	9	9	9	9	9	9	9
1	9	9	9	9	9	9	9	9	9
9	9	9	9	9	9	9	9	9	9
9	9	9	9	9	9	9	9	9	9
1	9	9	9	9	9	9	9	9	9
1	1	1	1	3	2	1	2	2	1
2	1	2	9	1	2	2	2	2	2
2	1	3	3	1	3	2	3	3	2
2	1	1	1	1	3	3	2	3	3
1	2	2	2	1	1	1	2	2	1
3	1	1	1	1	3	1	3	3	2
2	2	2	1	2	1	3	2	1	2
1	1	2	2	1	1	1	2	2	1
2	9	9	9	9	9	9	9	9	3
2	1	2	2	1	1	1	3	3	3
3	1	2	2	9	2	2	2	2	2
2	9	9	9	1	9	9	9	9	1
1	1	2	2	2	2	2	2	2	2
2	9	9	9	9	9	9	9	9	2
2	1	1	2	1	1	1	2	3	2
1	1	3	1	1	1	3	9	2	1
1	3	1	3	1	1	1	2	2	2
2	2	3	2	3	2	3	2	2	3
2	1	2	1	2	1	3	2	1	1
2	9	9	9	1	9	9	9	9	9
2	1	2	1	3	2	2	3	1	1
1	1	9	9	9	9	9	9	9	1
2	1	9	9	9	9	9	9	9	2
3	1	2	2	1	2	1	1	1	1
1	1	2	1	1	1	3	2	2	1
1	1	9	9	1	1	1	9	9	1
2	3	1	1	3	3	3	3	3	9
3	9	9	9	9	9	9	9	9	9

2	1	1	3	1	1	2	1	1	1
1	1	2	1	1	1	1	1	1	1
1	9	9	9	9	9	9	9	9	3
2	9	9	9	9	9	9	9	9	9
2	9	9	9	9	9	9	9	9	2
3	1	2	2	2	2	2	2	2	2
2	9	9	9	1	9	9	9	9	1
2	9	9	9	9	9	9	9	9	9
2	2	3	2	3	2	1	3	3	3
1	1	1	1	1	1	1	1	1	2
2	1	9	9	9	9	9	9	9	1
2	9	2	9	9	9	9	9	9	2
1	1	1	1	1	2	2	2	2	1
2	2	2	2	2	2	2	2	2	2
2	2	2	2	2	2	2	2	2	2
2	2	2	2	2	1	1	1	1	1
1	1	2	2	1	1	1	1	1	2
2	9	9	9	9	9	9	9	9	9
1	9	9	9	9	1	9	9	1	1
2	2	2	2	2	2	2	2	2	2
2	1	2	2	1	2	2	2	2	3
1	1	2	3	1	2	3	2	3	2
2	9	9	9	9	9	9	9	9	3
2	2	1	2	3	3	1	2	1	1
2	1	9	9	1	3	1	2	3	1
3	1	2	2	3	2	1	2	3	9
2	1	1	1	1	1	1	1	1	2
2	2	2	2	2	2	2	2	2	1
1	2	2	2	2	2	2	2	2	2
2	2	2	2	2	2	2	2	2	2
3	2	2	2	2	2	2	2	2	2
2	1	3	3	1	1	2	2	1	2
2	2	2	2	2	2	2	2	2	3
1	1	1	1	1	1	1	1	1	3
3	1	2	2	1	2	1	2	2	2
1	1	1	2	1	1	1	1	1	1
1	3	3	2	2	2	3	3	3	3
9	1	1	2	1	1	1	1	2	2
2	1	1	1	1	1	1	1	1	2
1	1	2	2	1	2	1	1	1	1
2	1	2	9	1	1	2	3	1	2
9	1	3	2	3	1	1	2	3	9
1	1	1	9	1	3	3	2	2	2
1	9	9	1	1	1	2	9	9	9
9	2	9	9	9	9	9	9	9	9
1	1	1	2	1	1	2	2	2	1
2	1	2	1	2	2	2	2	2	2

1	1	1	2	2	2	2	2	2	2
1	1	2	3	1	2	3	1	2	1
1	1	2	1	2	2	1	2	2	1
1	1	1	1	1	1	1	1	1	1
1	1	1	2	2	2	3	2	2	1
3	1	3	3	1	1	2	2	2	1
1	1	1	2	1	1	2	1	1	3
1	1	1	1	1	2	2	3	1	2
3	2	3	2	1	2	3	2	3	2
9	1	2	2	1	2	2	2	2	9
1	1	2	1	2	2	1	2	2	1
1	1	2	2	1	2	1	2	2	1
1	1	2	3	1	3	1	3	3	1
3	2	3	2	9	1	2	2	2	1
3	2	2	2	2	2	2	2	2	2
2	1	1	2	3	3	2	1	2	2
9	2	2	2	2	2	2	2	2	2
3	2	2	2	2	2	2	2	2	2
2	2	2	2	2	2	2	2	2	1
2	2	2	2	2	2	2	2	2	2
9	2	2	2	2	2	2	2	2	9
1	2	3	2	2	2	2	2	2	2
1	3	3	3	3	3	3	3	3	3
1	2	2	2	2	2	2	2	2	2
2	2	2	2	2	2	2	2	2	1
2	1	2	3	1	2	3	1	2	1
1	1	2	1	2	2	2	2	2	1
2	1	1	1	1	1	1	1	1	1
9	2	2	2	2	2	2	2	2	9
9	1	1	1	1	1	1	1	1	9
1	1	1	3	1	3	1	3	1	1
3	9	9	9	9	9	9	9	9	1
2	1	1	1	1	9	3	3	1	2
3	1	2	2	1	1	2	2	2	3
1	3	2	3	1	3	1	3	2	1
1	1	2	1	1	1	2	3	2	3
1	1	2	3	1	1	1	2	3	1
2	1	2	9	1	2	3	1	2	9
2	2	2	2	1	1	1	1	2	1
1	3	2	2	3	3	3	3	3	2
1	1	9	2	3	9	2	1	2	1
9	9	9	9	9	9	9	9	9	9
1	1	1	2	1	1	1	1	1	1
1	2	2	1	1	1	2	1	1	2
1	1	3	3	1	3	2	2	9	2
2	1	2	2	2	2	2	2	2	2
1	1	2	3	1	2	3	2	3	2

1	1	1	2	1	1	2	1	2	1
3	1	2	3	1	1	1	2	3	1
2	9	9	9	9	9	9	9	9	1
2	9	9	9	9	9	9	9	9	9
2	9	9	9	9	9	9	9	9	9
1	1	1	1	1	1	1	1	1	1
2	1	1	2	1	2	3	2	2	3
2	1	2	2	1	2	1	3	1	1
2	1	2	2	1	2	1	2	3	1
2	1	3	1	3	3	1	3	1	1
2	9	9	9	9	9	9	9	9	1
1	1	2	2	1	1	1	3	2	2
3	3	1	3	3	2	3	3	3	3
1	1	2	2	1	2	2	2	3	1
1	1	1	2	1	1	1	2	1	1
3	1	2	3	1	2	3	1	2	3
2	1	2	3	2	1	2	3	1	1
1	2	2	2	2	2	2	2	2	2
2	2	2	2	2	2	2	2	2	2
3	1	1	3	1	1	3	3	3	3
2	2	2	2	2	2	2	2	2	2
1	1	2	9	1	1	2	2	2	3
2	1	3	3	1	1	1	1	1	2
2	1	3	3	2	3	2	2	3	3
2	2	1	3	2	1	1	1	1	1
2	1	2	3	1	1	2	3	1	1
2	1	3	3	2	3	1	2	3	2
3	1	3	3	3	2	1	1	1	1
9	1	3	3	2	2	2	2	2	1
1	1	3	3	2	3	2	2	3	2
3	3	3	3	3	3	3	3	3	1
9	9	9	9	9	9	9	9	9	9
2	1	2	3	1	2	3	1	2	2
2	1	1	2	2	2	2	2	2	3
1	1	2	3	1	2	1	1	1	2
3	1	2	1	1	3	1	1	2	1
2	2	2	2	2	2	2	2	2	2
2	1	9	9	9	9	9	9	9	9
2	1	2	1	1	2	2	2	2	1
1	1	2	3	2	3	1	3	2	3
1	1	1	1	1	2	1	3	2	3
9	2	1	2	1	3	2	1	3	1
2	3	3	3	3	3	3	3	3	1
9	1	3	3	1	3	1	1	2	1
1	1	1	2	1	2	2	2	2	3
2	1	9	9	9	9	9	9	9	2
1	2	2	1	1	2	1	3	2	1

3	2	2	2	2	2	2	2	2	2
1	3	3	3	1	1	1	3	3	3
3	2	2	2	2	2	2	2	2	2
3	2	2	2	2	2	2	2	2	2
1	1	3	2	2	2	2	2	2	1
3	2	2	2	3	3	3	2	2	2
1	1	1	1	1	1	1	1	1	1
2	2	2	2	2	2	2	2	2	2
1	1	3	3	1	3	2	3	2	2
2	1	2	2	1	2	1	2	2	2
3	2	2	2	2	2	2	2	2	1
3	3	3	2	2	2	2	2	2	2
1	1	1	3	1	1	1	1	3	1
1	1	1	3	1	2	1	1	1	1
1	1	1	3	1	1	1	9	1	1
1	1	3	3	1	1	1	3	3	3
2	2	1	3	1	3	1	3	2	3
3	2	2	2	2	2	2	2	2	2
2	2	1	3	1	3	1	3	2	3
1	1	2	3	1	3	2	1	1	1
2	2	2	2	2	2	2	2	2	2
1	1	3	3	1	1	1	2	2	1
2	2	1	3	2	3	2	2	2	9
1	1	2	2	2	2	1	2	2	2
3	3	3	2	1	2	2	2	2	1
2	1	2	3	2	1	1	1	1	2
1	1	3	3	1	1	1	1	1	1
1	1	2	3	1	3	3	3	1	2
1	9	9	9	9	9	9	9	1	9
1	2	2	2	2	2	2	2	2	2
2	2	2	2	2	2	2	2	2	2
1	1	1	2	1	1	1	1	1	3
1	2	2	3	2	2	2	2	2	3
1	2	2	2	2	2	2	2	2	2
1	3	3	3	1	1	1	3	1	1
1	1	1	2	1	1	1	2	2	3
1	1	2	2	2	3	1	3	1	1
2	1	1	2	1	2	2	2	1	1
2	1	2	1	2	1	1	1	1	3
1	9	9	9	9	9	9	9	9	1
1	2	9	9	9	9	9	9	9	2
1	1	1	2	1	1	1	3	1	3
3	2	2	2	2	2	2	2	2	2
1	1	1	1	3	3	3	2	2	1
1	2	2	2	1	1	1	1	1	1
1	2	2	2	2	2	2	2	2	1
1	1	2	3	2	1	3	1	3	1

9	9	9	9	9	9	9	9	9	9
9	9	9	9	9	9	9	9	9	9
1	1	1	1	1	1	1	1	1	1
1	1	2	2	3	2	3	2	3	1
9	9	9	9	9	9	9	9	9	9
1	2	2	2	2	2	2	9	2	1
9	2	2	2	2	2	2	2	2	2
1	1	3	3	3	3	2	2	2	1
2	1	2	3	1	2	3	1	2	1
2	1	9	1	3	1	9	1	3	1
1	1	2	3	3	3	2	2	1	1
1	1	2	3	1	2	3	1	2	1
3	2	2	2	1	2	2	1	2	2
1	2	9	9	9	9	9	9	9	2
1	1	1	1	1	1	2	3	3	2
9	9	9	9	9	9	9	9	9	9
1	1	1	1	1	1	1	1	1	2
1	1	3	3	2	1	1	3	1	3
9	1	2	1	3	1	1	2	2	1
1	1	2	2	3	2	3	2	2	1
1	1	1	1	1	1	1	1	1	1
9	9	9	9	9	9	9	9	9	9
9	9	9	9	9	9	9	9	9	9
1	1	2	2	1	2	1	2	2	2
2	2	2	2	2	2	2	2	2	2
1	2	2	2	1	2	3	3	3	1
3	2	2	2	2	2	2	2	2	2
1	1	9	1	1	1	1	1	1	2
3	3	3	3	2	2	2	2	2	1
1	2	3	2	1	2	3	1	3	2
2	2	2	2	2	9	2	2	2	1
1	9	9	9	1	9	9	9	9	9
1	1	1	1	1	1	1	2	2	1
1	2	1	2	1	1	1	1	1	2
9	2	2	2	2	2	2	2	2	9
2	2	2	3	2	2	2	2	2	3
1	2	1	2	3	1	1	2	2	1
2	2	2	2	2	2	2	2	2	2
3	2	2	2	2	2	2	2	2	2
1	2	2	2	2	2	2	2	2	2
1	1	2	2	1	2	2	2	2	1
2	2	2	2	2	2	2	2	2	2
2	3	3	3	3	3	3	3	3	2
2	9	9	9	1	9	9	9	9	1
2	9	9	9	9	9	9	2	9	9
1	2	2	2	2	2	2	2	2	2
3	2	2	2	2	2	2	2	2	2

1	9	9	9	1	9	9	9	9	9
3	1	1	1	1	1	2	2	2	1
1	2	9	9	9	9	9	9	9	2
3	2	2	2	2	2	2	2	2	2
1	2	1	2	2	1	2	2	1	1
1	2	2	2	2	2	2	2	2	2
3	2	3	1	2	3	1	3	2	2
2	1	9	9	9	9	9	9	9	1
3	2	2	2	2	2	2	2	2	2
2	1	1	2	1	2	3	2	2	2
2	2	2	2	2	2	2	2	3	2
2	2	2	1	2	1	1	2	2	1
1	1	1	2	1	2	1	1	1	2
2	1	2	2	3	1	2	2	2	1
2	2	2	2	2	2	2	2	2	2
2	9	9	9	9	9	9	9	9	1
2	9	9	9	1	9	9	9	9	3
1	2	2	2	2	2	2	2	2	2
1	1	1	9	1	9	1	2	3	2
2	1	2	2	1	3	2	3	1	2
2	1	2	1	1	2	1	1	1	1
2	1	1	1	1	1	1	1	1	2
2	2	2	2	2	2	2	2	2	2
2	2	2	2	2	2	2	2	2	2
1	3	3	1	1	1	1	2	3	3
2	2	2	2	1	2	2	2	1	1
1	1	1	2	1	1	1	2	1	2
1	1	2	2	1	2	2	2	2	2
2	1	1	3	3	3	2	1	2	1
9	9	9	9	9	9	9	9	9	9
9	3	3	3	3	3	3	3	3	3
2	9	9	9	1	9	9	9	9	1
2	9	9	9	9	9	9	9	9	9
2	9	9	9	9	9	9	9	9	1
1	1	1	1	2	2	2	2	3	3
1	1	1	2	1	1	1	1	1	1
2	2	2	2	2	2	2	2	2	3
1	1	2	9	9	1	9	2	1	1
2	1	2	1	1	2	2	2	2	1
3	2	2	2	2	2	2	2	2	2
1	1	1	2	1	1	1	1	1	2
1	1	1	1	1	1	1	2	2	1
2	1	3	1	2	1	2	1	3	2
2	2	3	2	1	2	9	3	2	2
1	1	2	2	1	1	1	2	1	2
1	1	1	2	1	2	2	2	3	3
3	3	3	2	2	3	3	2	2	1

2	2	1	9	2	3	9	2	1	3
1	9	9	9	9	9	9	9	1	1
1	9	9	2	9	9	9	9	9	3
2	1	2	1	3	2	3	1	2	2
2	2	2	2	2	2	2	2	2	2
2	2	2	2	2	2	2	2	2	2
1	3	3	3	1	1	1	2	2	1
9	9	9	9	9	9	9	9	9	9
1	9	9	9	9	9	9	9	9	9
9	9	9	9	9	9	9	9	9	9
2	9	9	9	9	9	9	9	9	2
2	9	9	1	9	9	9	9	9	1
9	9	9	9	9	9	9	9	9	9
9	9	9	9	9	9	9	9	9	9
9	9	9	9	9	9	9	9	9	9
1	9	9	9	1	9	9	9	9	9
1	2	2	2	2	2	2	2	2	2
1	1	2	2	2	2	3	3	3	1
1	1	2	2	1	1	1	1	2	1
3	3	2	2	3	3	2	3	2	2
1	2	2	2	2	2	2	2	2	1
1	1	2	3	2	3	2	3	2	1
1	2	3	2	3	2	2	2	2	1
2	1	2	2	2	2	2	2	2	2
1	2	1	1	2	1	1	1	1	2
2	2	3	3	2	2	1	2	2	3
2	1	2	2	3	2	1	3	2	3
2	3	3	2	1	2	9	1	1	3
2	1	1	1	1	1	1	1	1	1
1	2	2	2	2	2	2	2	2	2
1	2	2	2	2	2	2	2	2	2
2	3	3	3	3	3	3	3	3	2
1	3	3	3	3	3	3	3	3	2
1	1	1	1	1	1	1	2	2	1
9	2	2	2	2	2	2	2	2	9
1	2	2	2	2	2	2	2	2	3
9	9	9	9	9	9	9	9	9	9
1	2	2	2	2	2	2	2	2	2
3	1	2	1	1	1	1	1	1	1
1	9	9	9	1	9	9	9	9	1
2	3	3	3	3	3	3	3	3	3
2	1	1	1	1	1	1	1	1	1
2	2	2	2	2	2	2	2	2	2
1	2	2	2	2	2	2	2	2	2
1	1	1	3	1	2	3	1	3	1
9	9	9	9	9	9	9	9	9	9
1	1	2	3	1	2	2	3	2	1

2	2	2	2	2	2	2	2	2	2
2	2	2	2	2	2	2	2	2	2
1	1	2	2	1	1	1	2	1	3
1	1	2	2	1	1	1	1	2	1
2	9	9	9	9	9	9	9	9	9
2	2	2	2	1	2	2	2	2	2
9	9	9	9	9	9	9	9	9	9
2	3	3	3	1	1	2	2	2	3
1	2	3	3	1	1	1	1	1	3
9	9	9	9	9	9	9	9	9	9
9	9	9	9	9	9	9	9	9	9
9	9	9	9	9	9	9	9	9	9
9	9	9	9	9	9	9	9	9	9
9	9	9	9	9	9	9	9	9	9
9	9	9	9	9	9	9	9	9	9
9	9	9	9	9	9	9	9	9	9
9	9	9	9	9	9	9	9	9	9
9	9	9	9	9	9	9	9	9	9
9	9	9	9	9	9	9	9	9	9
9	9	9	9	9	9	9	9	9	9
9	9	9	9	9	9	9	9	9	9
9	9	9	9	9	9	9	9	9	9
9	9	9	9	9	9	9	9	9	9
9	9	9	9	9	9	9	9	9	9
9	9	9	9	9	9	9	9	9	9
9	9	9	9	9	9	9	9	9	9
9	9	9	9	9	9	9	9	9	9
9	9	9	9	9	9	9	9	9	9
9	9	9	9	9	9	9	9	9	9
1	2	1	1	2	1	3	2	1	1
2	2	2	1	2	2	2	2	2	2
1	1	1	2	1	1	2	2	2	2
1	1	2	2	1	2	2	2	2	1
2	1	3	3	2	2	2	3	2	1
1	1	9	9	9	9	9	9	9	1
1	2	2	2	2	2	2	2	2	2
1	1	2	2	1	1	1	9	2	1
3	1	2	2	2	2	2	2	2	1
2	2	2	1	3	1	2	1	3	1
1	1	1	1	1	1	1	1	1	2
1	1	2	2	1	2	2	2	2	1
1	1	2	1	1	1	1	2	1	1
1	1	2	2	1	1	1	3	1	1
1	1	1	1	1	1	2	2	2	1
1	1	9	2	1	1	1	2	1	1
1	1	9	9	9	9	9	9	9	1
1	1	2	2	1	2	1	2	2	1

1	1	1	3	1	1	1	3	1	1
1	1	3	9	9	3	9	3	9	1
1	1	2	2	1	1	2	2	2	1
2	1	2	2	1	1	3	3	1	1
2	1	2	2	2	2	2	2	2	2
1	1	3	3	2	3	2	3	2	2
1	1	1	2	1	1	1	2	1	1
1	1	1	1	1	1	1	2	1	1
1	3	3	3	1	1	1	3	1	1
1	1	2	2	1	1	2	2	2	3
1	1	2	1	2	2	1	2	2	2
2	1	9	9	9	9	9	9	9	1
1	1	2	1	1	1	1	2	1	1
1	9	9	9	1	9	9	9	9	1
2	1	9	9	9	9	9	9	9	1
2	1	9	9	9	9	9	9	9	1
1	9	9	9	1	9	9	9	9	1
1	9	9	9	1	9	9	9	9	1
1	9	9	9	1	9	9	9	9	9
1	9	9	9	1	9	9	9	9	1
1	1	9	9	9	9	9	9	9	9
2	1	1	3	1	3	1	3	3	3
1	1	3	3	3	3	1	1	3	3
2	1	3	1	2	2	2	2	1	1
1	1	9	9	9	9	9	9	9	9
1	1	3	3	1	3	1	3	1	3
1	9	9	9	1	9	9	9	9	1
3	1	9	9	9	9	9	9	9	3
1	9	9	9	1	9	9	9	9	1
1	9	9	9	1	9	9	9	9	3
1	9	9	9	1	9	9	9	9	1
1	1	2	3	2	1	2	3	2	1
1	1	1	3	3	1	1	3	3	1
2	1	2	3	1	2	1	2	3	2
1	1	1	2	1	1	1	2	1	1
1	2	2	2	2	2	2	2	2	2
2	1	2	3	1	1	1	1	1	1
1	1	1	3	1	9	1	3	3	1
2	3	3	3	3	3	3	3	3	1
2	1	3	1	1	3	1	3	1	9
1	1	2	3	1	2	1	2	1	2
2	1	9	9	9	9	9	9	9	1
1	2	2	2	2	2	2	2	2	2
2	2	2	2	2	2	2	2	2	3
1	2	3	3	2	1	2	3	2	3
1	2	1	2	1	1	1	2	3	2
1	1	2	2	1	1	1	2	2	1

1	1	1	2	9	1	2	2	2	9
2	1	1	1	1	1	3	3	1	1
2	3	3	3	2	2	2	1	1	2
1	1	1	2	1	1	2	2	2	2
1	9	9	9	1	9	9	9	9	2
1	9	9	9	9	1	9	9	9	1
1	2	1	1	1	1	1	2	2	3
1	2	2	2	1	9	3	2	3	1
2	1	1	2	1	1	1	1	1	1
1	9	9	9	1	9	9	9	1	1
1	2	9	9	1	2	9	9	3	2
2	1	1	3	1	1	1	2	1	1
1	1	1	2	1	1	1	3	2	1
1	9	9	9	9	9	9	9	9	1
3	2	9	9	9	9	9	9	9	2
1	2	2	2	2	2	3	3	3	1
1	2	2	2	2	2	2	2	2	2
1	9	9	9	9	9	9	9	9	1
1	1	1	2	1	1	1	2	2	1
1	2	3	9	1	9	9	1	3	3
1	9	9	9	9	9	1	9	9	9
1	3	3	3	3	3	3	3	3	1
1	9	9	9	1	9	9	9	9	9
1	9	9	9	9	9	9	9	9	1
1	2	2	2	2	2	2	2	2	3
1	1	2	1	1	1	1	2	2	1
1	1	3	3	1	1	1	3	2	3
1	1	1	1	1	1	1	1	1	1
3	3	1	3	3	3	3	3	3	3
1	1	1	2	1	1	1	2	1	1
1	1	2	2	1	2	1	2	2	2
9	9	9	9	9	9	9	9	9	9
2	9	9	9	1	9	9	9	9	1
1	1	3	3	1	1	1	3	1	1
2	3	3	3	3	3	3	3	3	1
1	1	3	3	1	1	1	1	1	3
9	9	9	9	9	9	9	9	9	9
9	9	9	9	9	9	9	9	9	9
9	9	9	9	9	9	9	9	9	9
9	9	9	9	9	9	9	9	9	9
9	9	9	9	9	9	9	9	9	9
9	9	9	9	9	9	9	9	9	9
9	9	9	9	9	9	9	9	9	9
9	9	9	9	9	9	9	9	9	9
9	9	9	9	9	9	9	9	9	9
9	9	9	9	9	9	9	9	9	9
9	9	9	9	9	9	9	9	9	9

9	9	9	9	9	9	9	9	9	9
9	9	9	9	9	9	9	9	9	9
9	9	9	9	9	9	9	9	9	9
9	9	9	9	9	9	9	9	9	9
9	9	9	9	9	9	9	9	9	9
9	9	9	9	9	9	9	9	9	9
9	9	9	9	9	9	9	9	9	9
9	9	9	9	9	9	9	9	9	9
9	9	9	9	9	9	9	9	9	9
9	9	9	9	9	9	9	9	9	9
9	9	9	9	9	9	9	9	9	9
9	9	9	9	9	9	9	9	9	9
9	9	9	9	9	9	9	9	9	9
9	9	9	9	9	9	9	9	9	9
1	9	9	9	9	9	9	9	9	9
9	9	9	9	9	9	9	9	9	9
9	9	9	9	9	9	9	9	9	9
9	9	9	9	9	9	9	9	9	9
9	9	9	9	9	9	9	9	9	9
9	9	9	9	9	9	9	9	9	9
9	9	9	9	9	9	9	9	9	9
9	9	9	9	9	9	9	9	9	9
9	9	9	9	9	9	9	9	9	9
9	9	9	9	9	9	9	9	9	9
9	9	9	9	9	9	9	9	9	9
1	1	2	1	1	2	1	2	2	1
9	9	9	9	9	9	9	9	9	9
9	9	9	9	9	9	9	9	9	9
9	9	9	9	9	9	9	9	9	9
9	9	9	9	9	9	9	9	9	9
9	9	9	9	9	9	9	9	9	9
9	9	9	9	9	9	9	9	9	9
9	9	9	9	9	9	9	9	9	9
9	9	9	9	9	9	9	9	9	9
9	9	9	9	9	9	9	9	9	9
9	9	9	9	9	9	9	9	9	9
9	9	9	9	9	9	9	9	9	9
9	9	9	9	9	9	9	9	9	9
9	9	9	9	9	9	9	9	9	9
9	9	9	9	9	9	9	9	9	9
1	2	2	2	2	2	2	2	2	2
1	1	3	3	3	3	3	3	3	1
1	1	3	3	1	1	2	3	3	2
1	1	1	1	1	1	3	3	1	3
3	3	3	3	3	3	3	3	3	3
1	1	1	1	1	1	1	1	1	3

1	9	9	9	9	9	9	9	9	1
1	1	9	9	9	9	9	9	9	3
2	9	9	9	9	9	9	9	9	1
3	2	2	2	2	1	3	1	2	2
2	1	1	2	1	1	1	1	1	1
1	1	2	3	1	2	2	2	2	1
1	1	9	2	1	1	1	2	1	1
1	1	1	2	1	1	1	2	2	1
1	1	1	2	1	1	9	2	2	9
1	1	3	3	1	2	1	2	2	1
1	2	2	2	1	1	1	2	1	2
1	1	2	1	1	1	1	1	1	1
1	1	1	2	1	1	1	2	1	1
1	1	1	1	1	1	2	2	2	1
2	2	1	2	9	3	2	2	1	2
2	1	3	3	1	1	2	1	1	3
1	9	9	9	1	1	9	9	9	1
1	1	3	3	1	3	1	1	1	2
1	1	1	2	1	1	1	2	2	3
1	1	1	2	1	2	1	2	2	2
1	2	1	3	1	1	1	2	1	2
1	1	3	1	1	1	1	2	1	3
1	1	1	2	1	1	2	2	1	2
3	1	3	3	1	3	3	3	3	2
3	1	1	1	1	1	1	2	1	2
1	1	1	1	1	1	1	1	1	1
2	1	2	1	1	9	2	3	2	2
1	1	2	2	1	1	1	2	2	1
1	1	1	2	1	1	1	2	2	1
1	9	9	9	9	9	1	9	9	1
2	3	3	3	3	3	3	3	3	2
2	1	1	1	2	1	1	2	1	2
2	1	2	1	1	9	1	9	1	2
2	3	3	1	1	1	2	2	2	3
1	2	2	2	2	2	2	2	2	2
1	1	2	1	1	1	1	2	1	2
1	2	2	3	2	3	3	3	3	1
1	1	2	1	1	1	1	2	2	2
1	1	2	1	1	1	1	2	1	2
2	9	9	9	9	9	9	9	9	9
1	9	9	9	9	9	9	9	9	9
2	9	9	9	9	9	9	9	9	2
1	9	9	9	9	9	9	9	9	2
1	9	9	9	9	9	9	9	9	3
1	9	9	9	9	9	9	9	9	2
2	9	9	9	9	9	9	9	9	9
1	1	1	2	3	1	2	3	2	1

1	1	2	2	1	2	2	2	2	1
1	1	1	1	1	2	9	1	1	1
2	1	3	3	1	3	1	3	1	3
2	1	1	1	1	1	1	1	1	1
1	3	1	3	3	3	3	3	3	1
2	1	3	3	1	3	1	3	1	1
2	2	2	2	2	2	2	2	2	1
2	2	3	3	1	2	3	2	1	2
2	2	2	2	2	2	2	2	2	1
2	1	1	3	1	3	3	9	1	1
1	2	2	2	2	3	1	1	1	1
2	1	2	2	1	2	3	9	2	3
2	1	9	3	1	9	1	9	1	1
3	1	1	2	3	2	1	2	2	3
2	1	1	3	1	2	1	1	1	2
2	9	9	9	1	9	9	9	9	1
1	2	2	2	1	2	2	2	2	1
2	2	1	1	3	2	1	1	3	1
2	1	1	3	1	2	3	2	2	1
2	1	3	3	1	3	1	1	1	1
2	1	1	1	3	1	2	2	2	3
2	1	1	1	1	2	1	1	2	1
1	3	2	2	2	2	2	3	2	1
2	1	9	9	9	9	9	9	9	9
2	1	2	3	1	1	2	3	1	1
2	9	9	9	1	9	9	9	9	9
1	3	2	1	1	1	2	3	2	3
2	2	2	2	1	2	1	2	1	2
2	1	3	3	1	9	1	1	1	1
2	1	3	1	1	3	1	2	2	1
2	1	2	1	1	2	2	1	1	1
2	1	3	3	1	3	1	3	3	1
3	1	2	1	1	3	1	2	3	1
2	3	3	3	3	3	3	3	3	1
2	1	1	2	2	2	2	2	2	1
2	1	1	3	2	3	1	2	3	3
2	1	1	3	1	3	2	2	2	1
2	1	2	3	2	1	2	3	1	3
2	1	1	1	1	2	3	1	1	1
2	1	3	3	1	3	1	3	3	1
2	1	1	1	1	1	1	1	1	1
2	1	1	1	1	3	3	1	3	3
9	1	2	3	1	2	3	2	1	1
2	9	9	9	9	9	9	9	9	9
2	1	9	1	1	1	3	1	1	1
2	1	3	3	1	1	3	1	2	1
2	1	1	1	3	3	1	1	1	1

2	1	2	3	1	2	3	1	2	2
2	1	3	1	1	3	1	1	3	1
2	2	2	2	1	2	3	2	2	3
3	2	2	2	2	2	2	2	2	2
2	1	3	9	2	2	1	1	2	2
2	3	3	3	3	3	3	3	3	2
1	1	3	3	3	3	3	2	2	1
2	1	1	1	2	9	1	1	2	9
1	1	2	2	2	2	2	2	2	1
2	2	1	3	3	2	2	2	1	2
2	1	1	2	2	2	2	2	1	2
2	2	2	2	3	3	3	2	2	1
2	2	2	2	2	2	2	2	2	2
3	9	9	9	9	9	9	9	9	3
9	9	9	9	9	9	9	9	9	9
3	1	2	3	2	1	2	1	3	1
1	2	2	2	2	2	2	2	2	2
2	2	2	2	2	2	2	2	2	2
2	2	2	2	2	2	2	2	2	2
1	2	2	1	1	1	1	1	1	2
2	2	2	2	2	2	2	2	2	2
2	2	2	2	2	2	2	2	2	2
9	2	2	2	2	2	2	2	2	2
1	1	2	2	2	2	2	2	2	2
1	2	2	3	2	2	2	2	3	2
1	3	2	2	2	2	2	2	2	2
2	2	2	2	1	1	2	2	2	2
2	2	2	2	2	2	2	2	2	2
2	2	2	2	2	2	2	2	2	2
2	2	2	1	2	3	3	2	2	2
2	2	2	2	2	2	2	2	2	2
2	2	2	2	2	2	2	2	2	2
1	2	2	2	2	2	2	2	2	2
3	1	2	3	1	1	2	2	3	1
2	1	2	3	2	2	3	1	1	2
1	2	2	2	2	2	2	2	2	2
9	2	2	2	2	2	2	2	2	2
1	2	2	2	2	2	2	2	2	2
9	2	2	2	2	2	2	2	2	2
2	2	2	2	2	2	2	2	2	2
1	2	2	2	2	2	2	2	2	2
1	2	2	2	2	2	2	2	2	2
1	2	2	2	2	2	2	9	2	1
2	2	2	2	2	2	2	2	2	2
9	2	2	2	2	2	2	2	2	2
2	3	3	1	1	1	1	3	3	1
2	2	2	2	2	2	2	2	2	2

2	2	2	2	2	2	2	2	2	2
1	1	2	2	1	3	3	3	3	2
1	2	2	2	2	2	2	2	2	2
1	2	1	1	2	2	3	2	2	1
9	2	2	2	2	2	2	2	2	2
2	2	2	2	2	2	2	2	2	2
1	2	2	2	2	2	2	2	2	1
1	1	2	2	1	1	2	2	2	1
2	1	1	2	1	1	2	2	2	2
2	2	2	2	2	2	2	2	2	2
1	1	2	3	2	1	2	2	3	1
9	2	2	2	2	2	2	2	2	2
1	9	9	9	9	9	9	9	9	1
1	1	1	1	3	1	3	1	1	1
1	1	9	9	9	9	9	9	9	1
2	2	2	2	2	2	2	2	2	2
2	3	3	3	3	3	3	3	3	2
1	2	2	2	2	2	2	1	1	1
2	1	1	1	1	1	1	1	1	3
1	3	9	9	9	9	9	9	9	3
1	2	2	2	2	2	2	2	2	2
2	1	2	2	1	2	1	2	1	1
1	1	1	2	2	2	2	2	2	2
1	1	1	1	1	1	1	1	1	1
1	1	1	9	9	9	9	9	9	1
1	2	2	2	2	2	2	2	2	2
1	2	3	1	1	1	1	1	2	1
1	9	9	9	9	9	9	9	1	1
1	1	9	9	9	9	9	9	9	1
2	2	2	9	3	1	2	3	2	2
1	1	2	3	2	3	2	3	1	1
2	2	1	1	1	1	2	2	2	2
2	1	2	3	1	1	2	2	1	2
1	1	1	3	3	9	1	1	1	3
1	1	2	1	2	1	9	1	3	2
1	1	1	1	1	1	1	1	1	1
9	1	1	1	1	1	1	1	1	9
2	2	2	3	3	3	3	3	3	2
2	2	2	2	2	2	2	2	2	2
2	1	9	9	9	9	9	2	9	2
2	1	2	2	2	2	2	2	2	1
1	1	9	9	9	9	9	9	9	9
1	1	1	1	1	3	1	1	3	1
1	2	2	2	2	2	2	2	2	2
3	2	2	2	2	2	2	2	2	2
3	2	2	2	2	2	2	2	2	2
1	2	3	9	1	1	1	2	9	1

1	1	1	2	1	2	1	2	1	2
2	9	2	9	9	9	9	9	9	9
2	2	2	1	9	2	1	1	1	1
1	1	2	2	2	2	2	2	2	1
1	1	1	1	1	1	1	1	1	2
1	1	9	9	9	9	9	9	9	2
1	1	1	1	1	1	1	1	1	1
1	2	2	2	2	2	2	2	2	1
1	1	1	1	1	1	1	1	1	1
1	2	2	2	2	2	2	2	2	2
1	2	9	9	9	9	9	9	9	2
1	1	2	1	3	2	2	3	1	2
1	3	3	3	3	3	3	3	3	1
1	1	1	1	1	1	1	1	1	1
2	1	1	1	1	1	1	1	1	2
1	1	1	1	3	3	3	3	3	1
2	3	3	3	3	3	3	3	3	2
1	2	2	2	2	2	2	2	2	1
2	2	2	2	2	2	2	2	2	2
2	2	2	2	2	2	2	2	2	2
2	2	2	2	2	2	2	2	2	2
2	1	1	2	1	2	1	2	1	2
2	1	1	1	1	1	1	1	1	1
1	2	1	1	1	1	1	1	1	3
9	9	9	9	9	9	9	9	9	9
2	1	2	1	2	1	2	1	2	1
1	1	1	1	1	1	1	2	2	1
1	1	1	3	1	1	2	2	2	1
1	1	1	1	1	1	1	1	1	1
2	1	2	9	1	2	1	2	2	3
2	9	9	9	9	9	9	9	9	3
1	2	1	1	1	1	1	1	1	2
3	2	1	1	2	3	2	1	2	1
2	3	2	2	2	1	2	1	2	2
1	2	1	1	1	1	2	9	3	1
2	2	2	2	2	2	2	2	2	2
9	3	3	3	3	3	3	3	3	3
2	2	2	2	2	2	2	2	2	1
3	3	3	3	3	3	3	3	3	3
1	1	2	1	3	2	1	2	3	1
1	2	2	2	2	2	2	2	2	2
9	3	3	3	3	3	3	3	3	2
2	2	3	2	3	2	1	2	1	2
3	3	3	3	3	3	3	2	2	2
3	1	3	2	1	2	3	1	2	3
1	3	3	3	3	3	3	3	3	3
1	1	1	1	1	1	1	1	1	1

2	2	1	2	1	3	2	1	2	2
9	9	9	9	9	9	9	9	9	9
2	3	3	3	3	3	3	3	3	3
3	2	2	1	1	1	1	1	1	1
1	2	2	2	2	2	2	2	9	2
1	1	2	2	1	1	1	2	3	1
1	2	2	2	2	2	2	2	2	1
2	1	1	3	3	3	1	3	3	3
3	3	3	3	3	3	3	3	3	3
1	1	1	3	3	3	1	3	3	3
2	1	1	2	1	3	1	1	9	2
2	2	2	2	2	9	9	2	2	2
1	2	2	2	2	2	2	2	2	1
2	2	2	2	2	2	2	2	2	2
9	2	2	2	2	2	2	2	2	9
2	3	3	2	1	3	2	1	2	3
2	2	2	2	2	2	2	2	2	2
2	1	3	2	3	2	3	2	3	3
2	2	2	2	2	2	2	2	2	2
2	1	2	2	1	3	1	1	1	3
2	2	2	2	2	2	2	2	2	2
2	1	2	2	1	1	2	2	2	2
3	2	2	2	2	2	2	2	2	2
2	1	1	3	1	3	2	3	2	2
2	1	1	2	9	2	2	2	2	2
1	1	2	2	3	2	2	2	2	1
1	2	1	2	1	2	1	2	2	2
2	2	2	2	2	2	2	2	2	3
2	1	1	2	1	1	2	2	2	3
1	2	2	2	2	2	2	2	2	1
1	1	1	1	1	1	1	1	1	1
9	1	3	3	1	1	3	2	1	9
2	1	1	3	3	2	1	1	1	1
1	3	3	3	1	3	1	3	1	1
2	1	3	2	2	3	1	2	3	1
2	1	2	2	1	2	1	2	3	2
2	1	3	3	1	1	2	2	3	2
9	2	2	2	2	2	2	2	2	9
2	2	2	2	2	2	2	2	2	1
2	1	9	2	3	1	3	2	3	3
2	1	3	3	1	3	1	3	3	1
2	1	2	2	1	2	1	2	1	1
2	2	2	2	2	2	2	2	2	1
2	9	9	9	9	9	9	9	9	2
3	2	2	2	2	2	2	2	1	2
2	1	3	2	2	1	2	3	2	2
2	2	2	1	2	1	2	3	2	2

2	2	2	2	2	2	2	2	2	2
2	2	2	2	2	2	2	2	2	2
2	2	2	2	2	2	2	2	2	2
2	2	1	1	1	1	2	3	1	3
2	1	1	2	1	3	1	1	9	1
2	2	2	2	2	2	2	2	2	1
9	9	9	9	9	9	9	9	9	9
1	1	2	1	2	1	2	3	1	1
2	1	1	2	9	3	1	1	2	2
2	1	1	1	1	3	1	3	3	2
2	1	2	2	1	1	2	3	1	2
2	1	1	1	1	2	2	2	2	2
2	1	3	2	2	1	2	1	3	2
2	9	9	9	9	9	9	9	9	1
2	1	1	2	2	2	2	2	2	1
1	1	1	2	1	3	3	3	1	1
1	1	1	2	1	1	1	2	2	2
2	1	1	2	2	2	3	2	3	3
2	1	2	2	2	2	2	2	2	2
1	1	1	1	1	1	1	1	1	1
1	2	2	2	3	1	2	2	1	1
3	2	2	2	2	2	2	2	2	2
1	2	1	2	3	1	2	1	2	3
2	1	1	9	1	2	3	3	3	3
2	9	9	9	9	9	9	9	1	1
1	2	2	2	2	2	2	2	2	3
9	9	9	9	9	9	9	9	9	9
1	1	2	1	3	1	2	3	2	1
3	2	2	2	1	1	3	3	3	2
9	3	3	2	2	1	1	2	3	1
2	1	1	2	2	2	2	3	3	1
2	2	2	2	2	3	3	2	3	2
1	1	2	2	2	3	2	3	2	3
2	1	2	1	3	2	1	1	2	2
1	1	3	3	1	3	1	3	3	2
1	1	2	3	1	1	1	2	2	1
2	3	3	2	2	2	1	1	1	1
2	2	3	1	2	3	2	1	3	1
1	1	1	3	3	3	1	3	3	1
2	3	3	3	3	3	3	3	3	2
3	2	2	2	2	2	2	2	2	2
2	2	2	2	2	2	2	2	2	2
2	9	9	9	9	9	9	9	9	3
2	2	2	2	2	2	2	2	2	2
1	2	2	2	1	1	1	1	1	2
1	2	2	2	1	1	1	1	1	2
1	2	2	2	2	2	2	2	2	1

2	1	2	3	2	1	2	3	2	2
1	1	1	1	1	1	1	2	3	2
2	2	3	3	2	3	2	2	2	2
2	9	9	9	9	9	9	9	9	2
1	3	3	3	3	3	3	3	3	1
2	1	1	3	1	2	1	2	2	2
2	3	3	3	3	3	3	3	3	3
1	1	1	1	1	3	3	9	2	3
1	1	2	3	2	3	2	1	1	2
2	9	9	9	9	9	9	9	9	9
2	1	1	1	1	2	2	3	2	2
2	3	3	3	3	3	3	3	3	3
1	1	1	1	1	1	1	1	1	1
2	3	3	3	3	3	3	3	3	2
3	2	2	3	2	1	2	2	3	3
1	1	9	1	1	2	2	1	2	1
2	2	2	2	2	2	2	2	2	2
2	1	1	2	1	2	2	2	2	1
2	1	1	1	2	1	2	1	2	1
2	9	9	9	9	1	9	9	9	1
2	2	2	2	2	2	2	2	2	2
3	3	3	3	2	2	3	2	3	2
2	1	1	2	1	1	1	1	2	3
2	2	2	2	2	2	2	2	2	2
3	2	2	2	2	2	2	2	2	2
2	1	1	1	1	1	1	2	2	2
2	1	2	2	1	2	1	3	2	3
2	3	3	3	3	3	2	3	2	2
2	1	2	2	1	2	2	2	1	2
3	1	1	2	1	3	2	2	2	1
2	1	2	3	1	2	3	1	2	1
2	1	2	3	3	3	3	3	3	1
2	1	2	3	2	2	1	2	1	3
2	1	1	2	3	2	1	2	3	2
2	2	3	2	1	3	2	1	2	3
2	1	1	1	1	1	1	1	1	2
1	3	3	3	3	3	3	3	3	3
3	1	1	2	2	2	2	2	2	3
1	1	1	1	1	1	1	1	1	1
1	1	1	1	1	1	2	2	2	1
2	1	3	3	3	1	1	2	2	3
2	1	3	1	3	1	2	9	2	1
1	1	3	1	3	2	3	2	3	1
2	1	2	3	1	3	1	1	1	3
2	1	1	3	2	2	2	1	2	3
2	1	1	1	3	1	1	3	2	1
2	3	3	3	3	3	3	3	3	2

2	1	3	3	1	1	1	1	1	1
1	3	2	3	1	2	1	2	3	2
2	1	1	2	1	9	1	9	3	3
9	2	3	9	2	3	2	9	2	9
1	2	1	3	2	3	2	1	2	2
1	1	3	3	1	3	1	3	1	1
2	1	2	2	1	2	2	2	2	1
3	1	3	2	1	2	1	2	3	2
3	2	1	1	2	1	2	2	2	1
2	1	1	1	1	1	1	1	1	1
2	1	2	3	1	2	3	1	3	1
2	3	2	2	2	2	2	2	2	1
3	1	2	3	1	2	2	1	3	1
1	1	2	3	1	2	3	3	2	1
2	1	3	3	1	3	1	3	1	1
2	1	3	3	1	3	1	3	2	2
3	1	2	3	2	3	2	3	1	1
2	1	2	3	2	1	2	2	1	3
1	1	1	1	1	1	1	1	1	1
2	1	3	9	3	3	1	1	1	3
2	1	1	3	1	1	3	3	2	3
2	1	1	3	3	1	1	3	3	1
3	1	1	2	2	3	3	2	2	1
1	1	1	1	1	1	1	1	1	3
2	3	2	1	3	2	1	1	1	3
2	1	1	2	1	1	1	1	1	1
2	1	2	1	2	2	1	2	2	2
1	1	1	3	3	3	3	1	3	3
2	1	1	1	1	1	1	3	1	3
2	1	2	2	1	2	1	1	2	1
2	1	1	1	2	2	2	2	2	1
2	2	2	2	2	2	2	2	2	2
1	1	1	3	2	1	2	2	1	1
1	1	2	3	1	2	1	2	3	1
1	2	2	2	2	2	2	2	2	3
2	9	9	9	9	9	9	9	9	9
2	9	9	9	9	9	9	9	9	1
2	9	9	9	9	9	9	9	9	2
1	2	2	2	2	2	1	1	1	2
2	9	9	9	1	9	9	9	9	1
2	2	2	2	2	2	2	2	2	2
1	2	2	2	2	2	2	2	2	1
3	2	2	3	2	2	2	3	2	3
2	1	2	2	1	2	2	2	2	9
2	1	9	9	9	9	9	9	9	9
1	2	1	2	2	2	2	3	2	3
1	2	3	3	1	2	2	2	3	2

2	1	1	3	1	1	2	1	2	2
2	3	3	3	3	3	3	3	3	1
2	3	3	3	3	3	3	3	3	1
1	1	1	3	2	3	2	1	1	1
1	2	2	2	2	2	2	2	2	2
1	1	1	3	2	1	2	2	1	1
2	1	2	2	2	1	1	3	3	1
2	1	2	2	2	2	2	1	1	2
1	2	2	2	2	2	2	2	2	3
2	1	2	3	1	3	2	2	1	1
1	2	2	3	2	2	2	3	2	3
2	2	2	3	2	2	2	3	2	3
2	9	9	9	1	9	2	9	2	3
2	1	1	2	1	3	2	2	2	3
2	1	3	3	1	2	1	2	3	1
1	3	3	3	3	3	3	3	3	2
2	2	3	2	1	3	2	2	1	2
1	1	3	2	1	3	2	3	2	3
1	1	3	1	2	9	1	3	2	1
2	2	2	2	2	2	2	2	2	2
2	2	2	2	2	2	2	2	2	2
2	1	1	2	2	2	2	2	2	1
2	2	2	2	2	2	2	2	2	2
2	2	2	2	2	2	2	2	2	2
2	2	2	2	2	2	2	2	2	2
2	9	2	9	9	9	9	9	9	1
1	1	3	1	1	1	1	1	1	1
1	1	3	1	1	1	1	1	1	1
1	1	9	9	1	1	1	1	1	1
1	1	2	2	1	1	1	2	2	1
1	1	3	3	1	1	1	1	1	1
1	1	9	9	1	9	1	1	1	1
1	1	3	3	1	1	1	1	1	1
1	1	3	3	1	1	1	1	1	1
1	1	2	2	1	1	1	1	1	1
9	9	9	9	1	9	1	1	9	9
1	1	9	9	9	1	1	1	1	1
1	1	9	9	9	9	9	9	9	1
1	1	3	3	1	1	1	1	1	1
1	1	3	3	1	1	1	1	1	1
1	1	9	9	1	1	1	1	1	1
1	1	3	3	1	1	1	1	1	1
1	1	9	9	1	1	1	1	1	1
1	1	3	9	1	1	1	1	1	1
1	1	3	3	1	1	1	1	1	1
1	1	3	3	1	1	1	1	1	1
1	1	3	2	1	1	1	1	1	1

1	1	9	9	1	9	9	9	9	9
1	1	3	3	1	1	1	1	1	1
1	9	9	9	1	9	9	9	9	3
1	1	9	9	1	9	1	1	1	1
1	1	3	3	1	1	1	1	1	1
1	1	3	9	1	1	1	1	1	1
1	1	3	1	1	9	1	1	1	1
1	1	3	1	1	1	1	1	1	1
1	1	3	3	1	1	1	1	1	1
1	1	1	1	1	1	1	1	1	1
1	1	3	3	1	1	1	1	1	1
1	1	3	3	1	1	1	1	1	1
1	1	1	1	1	1	1	1	1	1
1	1	1	2	1	2	1	2	2	1
1	1	3	3	9	1	1	1	1	1
1	1	9	9	1	1	1	1	1	1
1	1	3	2	3	1	1	1	1	1
1	1	3	1	1	1	1	2	2	1
1	1	3	3	1	1	1	1	1	1
1	1	3	1	1	1	1	1	1	1
1	1	9	1	1	1	1	1	1	1
1	1	3	3	1	1	1	1	1	1
1	1	3	1	1	1	1	9	9	1
1	1	3	1	1	1	1	9	9	1
1	1	3	3	1	1	1	1	1	1
1	1	1	1	1	1	9	9	9	1
1	9	9	9	9	9	9	2	9	1
1	9	9	9	9	9	9	9	9	1
1	1	2	3	1	1	1	1	1	1
1	1	3	3	1	1	1	1	1	1
1	1	3	3	1	1	1	1	1	1
1	1	3	3	1	3	1	1	1	1
1	1	3	1	1	1	1	3	3	1
1	1	3	1	3	3	3	3	3	1
1	1	3	3	1	1	1	1	1	1
1	1	9	9	9	1	1	1	1	1
2	1	1	3	1	1	1	1	1	1
1	1	3	3	1	3	1	1	3	1
1	1	9	9	1	1	1	1	1	1
1	1	9	9	1	1	1	1	1	1
1	1	3	3	1	1	1	1	1	1
1	1	3	3	1	3	1	1	1	1
1	9	9	9	1	9	1	1	9	1
1	1	3	3	1	1	1	1	1	1
1	1	3	3	1	3	1	3	1	1
1	1	3	3	1	3	1	1	1	1
1	1	3	3	1	1	1	1	1	1

1	1	3	1	1	1	1	1	1	1
1	1	9	9	1	9	1	1	1	1
1	1	9	1	1	1	1	1	1	1
9	1	3	3	1	1	1	1	1	9
1	1	3	3	1	1	1	1	1	3
1	1	9	9	1	9	1	1	9	1
1	1	3	3	1	1	1	1	1	1
1	1	3	3	1	1	1	1	1	1
1	1	3	3	1	2	1	1	1	1
1	1	3	3	1	1	1	1	1	1
1	1	9	9	1	1	1	9	9	1
2	9	9	9	1	9	1	1	9	1
1	1	3	3	1	2	3	3	3	1
1	1	3	1	1	1	1	9	9	1
1	1	3	1	1	1	1	9	9	1
1	1	3	3	1	1	1	3	2	1
2	2	2	3	1	2	1	2	2	2
1	1	9	9	1	9	9	9	9	1
1	3	3	3	2	1	1	3	9	1
2	1	2	2	1	2	2	2	2	3
1	2	2	2	2	2	2	2	2	2
1	1	1	9	1	1	1	2	2	1
1	1	9	9	9	9	9	9	9	1
1	9	9	9	1	9	9	9	9	1
1	1	1	1	1	1	1	1	1	1
1	1	2	1	1	1	1	1	3	1
1	1	1	3	1	1	1	2	2	1
1	2	2	3	1	1	2	3	2	1
1	1	2	3	1	2	2	3	2	1
1	1	1	2	1	1	2	2	2	1
1	1	9	9	9	9	9	9	9	1
1	1	2	3	1	2	1	3	2	1
2	9	9	9	9	9	9	9	9	1
1	1	1	3	2	1	2	1	3	3
1	1	2	2	1	1	2	2	2	1
1	2	2	3	1	1	1	2	2	1
1	2	2	2	1	2	2	2	2	1
2	1	2	2	1	1	2	3	2	1
1	2	2	3	1	2	2	2	2	2
1	9	9	9	1	9	9	9	9	1
1	1	1	3	1	2	2	3	2	1
1	1	1	1	1	2	2	2	2	1
1	1	1	1	1	9	1	2	2	1
1	1	1	1	1	2	9	1	2	1
1	1	1	1	1	2	1	1	2	1
1	1	3	3	1	3	1	3	2	3
1	1	1	3	1	1	1	3	1	1

1	1	2	3	1	1	1	2	1	1
1	1	1	2	1	2	2	2	2	1
1	3	3	3	3	3	3	3	3	2
2	1	1	1	2	9	2	2	2	1
1	1	2	3	1	1	1	2	1	1
1	9	9	9	1	9	9	9	9	2
1	1	3	3	1	1	1	1	1	1
1	2	2	2	1	1	2	2	2	1
1	1	2	2	1	2	1	2	1	1
1	2	2	2	1	2	2	2	2	1
1	1	9	1	1	1	1	9	1	1
1	2	1	1	1	2	2	2	2	1
1	2	2	2	2	2	2	2	2	9
1	1	2	2	1	2	2	2	2	2
1	1	2	3	1	1	1	3	2	1
1	1	2	3	1	1	2	2	2	3
1	2	2	3	1	3	1	1	1	2
1	3	3	3	1	3	3	3	3	1
1	1	3	2	1	1	2	2	2	3
1	1	1	2	1	1	1	2	2	1
1	1	9	1	2	9	1	2	2	1
1	9	9	9	9	1	9	9	9	1
1	1	1	2	1	1	1	3	1	1
1	1	2	2	1	1	2	2	1	1
1	1	1	1	1	1	1	3	3	1
1	1	1	2	1	1	1	2	1	1
1	3	9	9	9	9	9	9	9	1
1	1	1	2	9	1	1	2	1	1
1	1	2	2	2	2	2	2	2	1
1	1	3	3	3	3	3	3	3	1
1	1	2	1	1	3	2	1	3	1
1	1	2	1	1	3	2	1	3	1
1	9	9	9	1	1	9	9	1	1
1	9	9	9	9	9	9	9	9	9
1	9	9	9	9	9	9	9	9	1
1	1	2	3	1	2	3	1	2	1
1	1	9	9	9	1	1	9	9	1
1	1	1	2	1	1	2	2	1	1
1	1	9	3	1	1	1	1	1	1
1	1	9	9	9	1	1	1	1	1
1	9	3	3	1	1	1	1	1	1
1	1	9	9	1	1	1	1	1	1
1	1	1	3	1	9	1	1	1	1
1	3	3	3	1	1	1	3	1	1
1	1	3	3	1	1	1	1	1	1
1	1	2	3	1	1	1	1	1	1
1	1	3	1	1	1	1	1	2	1

1	1	9	9	1	9	1	1	1	1
1	1	3	3	1	2	1	1	1	1
1	1	9	9	1	9	1	1	9	1
1	1	3	3	1	1	1	1	1	1
1	1	3	3	1	1	1	1	1	3
1	1	9	9	1	9	9	9	9	1
1	1	3	3	1	1	1	3	1	1
1	1	9	9	1	9	1	1	9	1
9	9	9	9	9	9	9	9	9	9
1	9	9	9	9	9	9	9	9	1
1	1	1	1	9	2	9	2	9	1
1	1	3	3	1	1	1	3	3	1
1	9	9	9	1	9	9	9	9	1
1	1	9	9	9	9	9	9	9	1
1	1	9	9	9	9	9	9	9	9
1	9	9	9	1	9	9	9	9	1
1	1	3	1	1	3	3	3	3	1
1	1	2	2	2	2	2	2	2	1
1	1	2	2	1	1	2	2	1	1
1	9	9	9	1	9	9	9	9	1
1	1	1	1	1	2	1	2	2	1
1	1	1	2	1	1	2	2	2	1
1	9	9	9	1	9	9	9	1	1
1	9	9	9	1	9	9	9	9	1
1	9	9	9	1	9	9	9	9	1
1	3	3	3	3	1	3	3	3	1
1	1	1	3	1	2	1	2	1	1
1	9	9	9	1	9	9	9	9	1
1	9	9	9	1	9	9	9	9	1
1	1	1	2	1	1	1	1	1	1
1	1	9	9	1	9	1	9	1	1
1	1	1	2	1	1	1	2	1	1
1	3	2	9	1	9	9	9	1	1
1	1	2	1	1	2	3	3	3	2
1	1	1	2	2	3	1	2	2	1
1	1	1	2	2	3	1	2	2	1
1	1	1	2	1	1	2	3	2	1
1	1	2	1	1	1	1	2	3	1
1	1	1	9	1	1	1	2	2	1
1	1	3	3	1	1	3	3	3	1
1	1	1	2	1	1	2	2	2	2
1	9	9	9	1	9	9	9	9	1
1	9	9	9	1	9	9	9	9	1
1	1	9	9	1	1	9	9	9	1
1	1	1	1	1	1	1	2	3	1
1	1	2	2	1	1	1	1	1	1
1	2	3	2	1	2	3	2	3	2

1	1	2	3	1	2	3	2	2	1
1	9	9	9	1	9	9	9	9	1
1	9	9	9	1	9	9	9	9	1
1	9	9	9	1	1	9	9	9	1
1	9	9	9	1	9	9	9	9	1
1	2	9	9	1	1	1	9	1	1
1	1	3	1	1	1	1	3	1	1
1	1	1	2	1	1	3	3	2	1
1	9	9	9	1	9	9	9	9	1
1	2	2	1	1	2	2	2	2	1
1	1	9	1	1	3	1	3	3	1
1	1	9	9	9	9	9	9	9	1
1	2	3	3	2	2	2	2	2	1
1	1	2	2	1	2	1	2	2	1
1	1	2	2	1	1	1	2	2	1
1	1	1	1	1	2	2	1	2	1
1	2	1	2	1	2	1	2	1	1
1	1	9	9	9	9	9	9	9	1
1	1	9	9	9	9	9	9	9	1
1	9	9	9	1	9	9	9	9	1
1	9	9	9	1	9	9	9	9	1
1	9	9	9	1	9	9	9	9	1
1	3	9	3	1	3	3	3	3	1
1	1	2	2	1	2	2	2	2	1
1	1	9	9	9	9	9	9	9	1
1	1	1	2	1	1	1	2	2	1
1	1	1	1	1	1	2	2	1	1
1	1	2	2	1	1	1	2	2	1
1	2	2	2	1	1	1	2	2	1
1	2	3	3	1	2	1	2	3	1
1	2	3	3	2	2	1	3	3	1
1	9	9	9	1	9	9	9	9	1
1	1	2	2	1	1	1	9	3	1
1	1	2	9	1	2	2	2	2	1
1	1	9	9	1	1	1	9	9	1
1	9	9	9	1	9	9	9	9	1
1	1	2	2	1	1	2	3	2	1
1	1	9	9	9	9	9	9	9	1
1	9	9	9	9	9	1	9	9	1
2	1	2	3	1	1	2	2	2	1
1	1	1	2	1	2	1	2	1	1
1	3	3	3	1	3	3	3	3	1
1	1	2	2	1	2	1	2	2	2
1	1	3	3	1	1	1	1	1	1
1	9	9	9	1	1	1	1	1	9
2	9	3	3	1	9	1	1	9	1
1	1	3	3	3	2	2	3	3	1

1	1	2	2	2	2	9	1	2	1
1	1	2	2	1	1	2	2	2	1
1	1	1	2	1	1	1	2	2	1
1	1	1	2	2	2	2	1	2	2
1	9	9	9	1	9	9	9	9	1
1	1	1	3	1	1	1	9	1	2
1	1	1	3	1	1	1	3	1	1
1	2	2	3	1	1	1	2	2	2
1	1	1	2	1	2	1	2	2	1
3	1	2	2	1	1	1	2	2	1
3	1	1	2	1	2	1	3	2	1
1	1	2	3	1	1	3	3	3	1
1	1	3	2	1	3	1	3	1	1
1	1	2	3	1	1	1	1	2	1
1	2	3	3	1	3	1	2	1	1
1	1	2	2	1	1	1	3	3	3
1	1	2	2	1	1	2	2	2	1
1	1	2	2	1	2	2	2	2	1
1	1	2	2	1	1	1	1	1	1
1	1	1	3	1	1	1	2	2	2
2	1	1	1	1	1	2	2	2	2
1	1	2	3	1	1	1	1	1	1
1	1	2	1	2	2	2	2	2	1
1	2	2	2	2	2	2	2	2	2
1	2	2	2	2	2	2	2	2	1
1	1	1	1	1	1	1	2	2	1
1	1	2	3	1	2	1	2	3	1
1	2	2	3	2	2	3	2	2	2
1	2	2	2	1	1	1	2	2	1
1	1	2	2	1	2	1	2	2	3
1	1	2	9	1	2	1	2	2	2
1	2	2	3	1	1	1	2	2	9
2	1	1	2	1	1	1	1	1	1
1	1	2	9	1	2	1	2	2	2
1	1	2	2	1	2	1	2	2	2
9	1	1	1	1	2	3	3	1	1
1	1	2	2	1	2	1	2	2	1
1	1	1	2	1	2	1	2	1	1
2	1	9	9	9	9	9	9	9	1
1	1	9	9	9	9	9	9	9	1
1	9	9	9	1	9	9	9	9	1
1	9	9	9	1	9	9	9	9	9
1	1	9	9	9	9	9	9	9	2
1	1	9	9	9	9	9	9	9	9
1	9	9	9	1	9	9	9	9	9
1	9	9	9	1	9	3	9	9	2
1	1	3	1	1	2	1	2	2	2

1	1	9	9	9	9	9	9	9	1
1	1	9	9	9	9	9	9	9	1
1	2	2	3	1	2	2	2	2	2
1	9	9	9	9	9	1	9	9	9
1	1	9	9	9	9	9	9	9	1
1	1	9	9	9	9	9	9	9	1
1	1	1	1	1	1	1	2	1	1
9	2	1	1	3	2	3	2	3	3
1	1	9	9	9	9	9	9	9	1
1	9	9	9	1	9	9	9	9	3
1	9	9	9	1	9	9	9	9	1
1	1	9	9	9	9	9	9	9	1
9	9	9	9	1	9	9	9	9	1
1	9	9	9	1	9	9	9	9	1
1	9	9	9	9	9	1	9	9	9
1	1	9	9	9	9	9	9	9	1
1	1	1	1	1	1	3	9	1	1
1	1	3	3	1	1	1	3	2	1
1	1	3	3	1	1	1	3	2	1
1	1	1	1	1	1	1	3	3	1
1	1	1	1	1	1	1	1	1	1
1	1	9	9	1	1	1	1	3	1
1	1	9	9	1	1	1	1	3	1
3	1	1	2	1	1	1	9	3	1
1	1	3	3	1	1	1	3	2	1
2	1	2	2	1	2	2	2	2	2
1	1	2	2	1	1	2	2	2	1
2	1	3	3	1	1	1	1	1	2
1	9	9	9	1	9	9	9	9	1
1	1	1	3	2	3	1	2	9	2
1	1	1	3	9	1	2	1	1	1
1	1	1	2	1	2	2	1	1	1
1	3	3	3	3	3	3	3	3	3
1	1	1	1	1	1	1	1	2	3
1	1	1	3	1	1	1	1	1	1
1	1	1	1	2	2	2	3	2	2
1	1	1	1	1	1	1	1	2	1
1	1	1	3	1	1	2	2	2	1
3	1	2	2	1	1	1	3	3	1
1	1	2	2	1	2	1	2	2	1
1	1	1	1	1	1	1	1	1	3
1	1	1	1	1	1	1	1	1	2
1	1	1	3	1	1	1	3	1	3
1	1	1	3	1	1	1	3	1	3
1	1	1	3	1	3	1	3	3	3
1	1	3	3	1	2	1	2	2	3
1	1	1	1	1	2	1	1	2	1

2	1	1	3	1	1	1	1	1	1
2	1	1	1	1	1	2	2	1	3
3	1	3	3	1	1	1	1	1	3
1	1	3	3	1	1	1	3	1	3
1	1	3	3	1	1	1	3	1	3
1	1	2	2	1	1	1	2	1	3
1	1	1	1	1	1	2	2	2	1
1	1	1	1	1	1	1	1	1	1
1	1	1	1	1	1	1	2	2	3
1	1	3	3	1	1	1	3	1	3
1	1	3	3	1	1	1	3	1	3
3	1	3	3	1	1	1	3	1	3
1	1	2	2	1	1	1	2	1	3
1	1	2	2	1	1	1	2	1	1
1	1	2	2	1	1	1	3	1	2
1	1	1	9	1	1	1	9	1	3
1	1	1	3	1	1	1	2	2	1
1	1	9	9	1	1	1	1	1	1
1	1	2	2	1	1	2	2	1	1
1	1	2	3	1	1	1	2	1	1
1	1	3	3	1	3	1	3	1	1
1	2	2	2	2	2	2	2	2	2
1	1	1	9	1	2	1	9	1	1
1	2	2	2	1	2	2	2	2	1
2	1	3	1	1	3	3	1	3	1
1	1	1	2	1	1	1	2	1	1
1	1	3	3	1	3	2	3	3	1
1	1	1	3	1	3	1	1	1	1
2	1	1	2	1	1	1	1	1	2
2	1	1	2	1	1	1	1	1	2
2	1	1	2	1	1	1	1	1	2
1	1	1	2	1	2	1	2	2	3
2	1	2	2	1	2	1	2	3	1
2	1	1	2	1	2	1	1	2	2
1	1	2	2	1	1	1	2	2	2
1	1	2	3	1	2	2	3	2	1
2	3	3	2	2	2	3	3	3	2
1	2	3	3	2	2	3	2	3	2
1	1	2	1	1	1	1	1	1	1
1	1	1	3	1	1	1	1	1	1
2	1	1	2	1	1	1	1	1	9
1	1	3	3	1	1	1	3	1	3
3	2	3	3	1	3	3	2	2	1
1	2	2	2	2	2	2	2	2	1
1	1	3	3	1	1	1	1	1	1
1	1	1	3	1	1	1	2	2	1
1	1	2	1	2	3	2	2	2	1

1	1	1	1	1	3	1	1	1	9
1	1	1	1	1	3	1	1	1	1
1	1	1	1	1	1	1	3	3	3
1	1	1	1	1	1	3	9	2	1
1	1	1	2	1	2	1	2	2	1
1	1	1	2	1	1	2	2	2	3
1	1	1	2	2	3	1	2	1	1
2	2	2	2	2	2	2	2	2	2
1	1	1	1	1	1	1	3	3	3
1	1	2	2	1	2	1	2	1	1
1	1	2	1	1	1	1	2	2	1
1	1	2	2	1	2	1	2	2	3
1	1	2	3	1	2	3	3	3	1
1	1	2	2	1	2	2	2	2	2
1	1	1	2	1	3	3	2	2	2
1	1	1	1	1	1	1	3	3	1
1	1	1	2	1	2	2	2	2	3
1	2	2	2	2	2	3	2	3	1
1	9	9	9	1	9	9	9	9	1
1	1	2	1	2	1	2	2	2	1
1	1	1	3	1	3	3	3	3	1
2	1	2	2	1	2	1	1	1	2
1	1	2	1	1	3	1	2	3	1
1	1	2	2	1	1	1	2	2	2
1	1	2	9	1	2	1	3	2	2
1	1	1	1	1	1	2	2	2	1
1	1	1	1	1	1	2	2	1	1
1	1	1	2	1	1	2	3	2	1
1	1	3	3	1	1	1	1	1	1
1	1	1	1	1	2	2	2	1	1
1	1	3	3	1	1	1	1	1	2
1	1	1	3	1	1	1	3	1	3
1	1	3	3	1	1	1	1	3	1
1	2	1	2	2	1	3	2	3	2
1	1	9	9	9	9	9	9	9	2
1	9	2	9	1	9	9	9	9	9
1	2	2	3	2	2	3	2	2	2
1	1	1	2	1	1	1	2	2	1
2	2	2	2	1	2	2	3	1	1
2	1	1	2	1	1	1	2	1	3
1	9	9	9	1	9	9	9	1	2
2	1	1	1	1	1	1	2	2	1
2	2	2	2	1	1	2	2	2	2
2	1	9	9	9	9	9	9	9	9
1	1	3	1	1	2	1	3	2	1
1	1	2	1	1	1	3	3	3	2
1	9	9	9	1	9	9	9	9	2

1	1	1	2	1	1	1	1	2	2
1	1	2	1	1	1	1	2	1	1
1	9	9	9	1	9	9	9	9	2
2	1	2	2	1	1	1	2	2	2
3	1	3	3	1	2	3	2	2	2
1	1	2	2	1	1	1	2	1	2
1	1	2	2	1	2	1	2	2	2
1	1	1	1	1	1	2	2	2	1
1	1	1	2	1	1	2	2	3	1
1	2	3	3	1	1	2	2	2	2
2	1	2	1	2	2	3	2	3	1
1	1	1	2	1	2	1	2	2	1
1	1	2	1	1	2	1	2	1	1
1	1	9	9	1	9	9	9	9	9
2	1	2	2	2	2	2	2	2	1
2	1	2	2	1	2	1	1	2	3
1	1	2	2	1	1	2	2	2	1
1	1	9	9	9	9	9	9	9	2
1	1	1	1	2	1	2	2	2	2
1	1	2	2	2	2	2	2	2	1
1	2	3	3	1	3	1	1	1	1
1	2	3	3	1	3	3	2	2	1
1	1	1	1	1	1	1	1	1	1
1	1	1	3	1	1	1	3	1	1
1	1	2	2	1	2	2	1	2	1
1	1	9	9	1	1	2	3	1	3
1	1	3	3	1	1	2	3	1	3
1	1	1	2	1	1	1	1	1	1
1	1	2	2	1	2	2	2	2	1
1	1	2	3	2	1	1	3	2	1
1	1	3	2	1	2	1	3	3	3
1	1	1	1	1	9	1	1	1	1
1	1	2	2	1	1	2	3	2	2
1	1	2	3	1	1	1	1	1	2
1	1	1	1	1	1	1	1	1	2
1	1	9	9	9	9	9	9	9	1
1	1	2	3	1	1	1	1	1	2
1	1	2	1	3	2	2	2	1	1
1	1	2	2	1	1	1	1	1	1
1	1	2	2	1	2	1	2	2	1
1	1	1	2	1	2	1	3	3	1
1	1	2	1	1	1	2	1	2	1
1	1	1	2	1	1	1	2	2	1
1	9	9	9	9	9	9	9	9	1
1	1	2	3	1	1	1	3	1	3
1	1	3	3	1	1	1	3	2	1
1	1	2	2	1	1	1	2	2	1

1	1	1	3	1	1	1	3	1	1
2	1	2	2	1	2	2	2	2	2
2	1	3	3	1	3	1	2	3	1
1	1	3	3	1	3	1	3	1	1
1	1	3	3	1	3	1	3	1	1
1	1	2	3	1	1	1	1	1	1
1	1	3	3	1	3	1	3	1	1
1	1	3	3	1	3	1	3	1	1
1	1	3	3	1	1	1	3	2	1
1	1	2	2	1	1	1	1	1	1
1	1	3	3	1	3	1	3	1	1
1	1	3	3	1	3	1	3	1	1
1	1	1	3	1	1	1	3	1	1
1	1	1	3	1	2	1	2	3	1
1	1	3	3	1	1	1	1	2	1
9	1	3	3	1	1	1	3	2	1
1	1	3	3	1	9	1	3	9	1
1	1	1	3	1	1	1	3	2	1
1	1	1	1	1	1	1	2	1	1
1	1	1	1	1	2	3	2	1	3
1	1	1	1	1	1	2	2	1	3
1	1	1	1	1	1	1	2	3	2
2	1	1	3	1	2	1	1	2	1
1	2	2	3	1	1	1	2	2	1
1	1	1	2	1	2	1	1	1	1
9	1	1	1	9	3	9	9	9	1
1	1	1	1	2	2	1	1	2	2
1	3	3	3	1	1	1	3	3	1
1	9	9	9	9	1	9	9	9	1
1	9	9	9	1	9	9	9	9	3
1	1	9	9	9	9	9	9	9	2
1	9	9	9	9	9	1	9	9	9
2	3	3	3	1	1	2	3	3	1
2	9	9	9	1	9	9	1	9	9
1	2	9	9	9	9	9	9	9	3
2	9	9	9	9	9	1	9	9	9
2	9	9	9	1	9	9	9	9	9
1	9	9	9	1	9	9	9	9	9
3	1	9	9	9	9	9	9	9	2
1	9	9	9	1	9	9	9	9	1
1	2	2	3	1	1	9	9	9	9
1	1	1	3	1	1	1	2	2	1
1	1	1	2	1	1	1	1	1	2
1	1	1	3	1	1	1	3	1	3
1	1	2	2	1	1	2	9	2	3
1	1	2	2	1	1	2	3	2	2
1	1	9	1	1	1	1	1	1	1

1	1	3	3	1	1	1	3	2	2
2	1	2	1	1	2	3	3	2	1
1	1	2	2	1	1	1	1	2	2
2	2	2	3	2	3	2	2	2	1
1	1	3	1	3	1	1	1	3	1
1	1	1	2	1	1	2	2	1	1
1	1	1	1	2	2	1	1	2	3
1	1	1	2	1	2	1	2	2	2
1	2	2	3	1	2	2	2	2	1
2	1	1	3	1	1	1	1	1	2
2	1	1	2	2	2	3	2	2	1
1	1	3	1	1	1	1	1	1	1
2	2	2	2	2	2	2	2	9	9
1	1	3	1	1	3	2	2	2	2
1	1	2	1	1	2	2	2	2	1
2	2	2	2	2	2	2	2	2	1
1	1	2	1	1	2	2	2	2	3
2	1	2	1	1	1	1	2	2	2
2	1	1	1	1	2	3	3	3	2
2	3	3	3	3	3	3	3	3	9
3	1	2	2	2	2	2	2	2	2
2	1	2	2	1	2	1	1	2	3
1	2	1	9	2	1	2	2	2	2
1	1	2	1	2	9	1	2	1	3
1	1	1	1	1	1	1	1	1	2
1	1	1	1	1	1	1	1	1	2
1	1	1	1	1	1	1	1	1	3
1	1	1	1	3	3	2	2	2	1
1	1	2	1	1	2	2	2	2	1
2	1	1	2	1	1	1	1	1	2
1	1	1	2	1	1	1	2	2	3
1	2	2	1	3	1	2	3	1	1
1	1	1	2	1	2	2	2	1	1
1	1	1	1	1	1	1	1	1	1
2	2	2	2	2	2	2	2	2	1
1	2	2	2	2	2	2	2	2	2
1	9	9	9	1	9	9	9	9	9
2	2	9	1	3	2	9	1	3	1
1	2	2	2	2	2	2	2	2	1
2	2	2	3	1	9	2	2	2	1
1	9	9	9	1	9	9	9	9	9
2	9	2	2	2	2	2	2	2	1
2	2	2	2	2	2	2	2	2	2
1	2	2	3	2	2	1	2	2	1
3	1	2	3	1	2	2	2	2	1
9	1	2	2	1	1	1	2	1	1
1	1	2	2	1	2	1	2	2	1

1	1	2	2	1	2	1	2	2	1

WomenMa	WomenInt	WomenPre	WomenMe	CABreastH	Daily	Weekly	Monthly	SixMonthly	Yearly
9	9	9	9	1	9	9	9	9	9
3	1	2	3	3	2	3	1	3	3
3	3	3	2	2	1	1	2	2	2
9	3	1	9	3	2	9	1	1	2
9	9	9	9	9	9	9	9	9	9
3	1	2	3	3	2	3	1	1	9
2	2	2	2	2	2	2	1	2	2
1	3	3	3	2	3	2	1	1	2
9	9	9	9	9	2	9	9	9	9
9	9	9	9	9	2	9	9	9	9
2	2	2	2	2	2	9	9	1	9
2	3	3	3	3	1	2	1	3	2
2	2	2	2	2	2	2	2	2	2
1	2	1	2	1	2	2	1	1	3
2	3	3	2	2	2	2	2	2	2
2	2	2	9	1	1	2	2	2	3
2	2	1	3	1	2	1	1	1	2
3	3	3	3	3	1	3	3	3	3
3	3	3	3	3	2	2	2	2	2
2	2	2	2	2	2	2	2	2	2
3	3	3	3	2	3	3	3	2	3
2	2	3	3	3	2	1	2	2	2
1	3	2	2	2	9	1	9	9	9
3	3	3	3	2	2	1	2	2	2
2	2	2	2	2	1	2	3	3	3
2	2	2	2	2	1	2	2	2	2
2	2	2	2	2	2	2	1	2	2
3	3	3	3	1	1	2	1	2	2
3	3	2	2	2	2	2	1	3	3
2	2	2	2	2	2	2	2	2	2
2	2	2	2	2	2	2	2	2	2
2	2	2	2	2	2	2	2	2	2
3	3	3	3	2	1	2	2	3	3
2	2	2	2	2	2	2	2	2	2
2	2	2	2	2	2	2	2	2	2
2	2	2	2	2	2	2	2	2	2
2	2	2	2	2	2	2	2	2	2
3	3	3	3	3	1	3	3	3	3
3	3	2	2	2	2	2	1	3	3
3	3	2	2	2	2	2	1	3	3
3	3	2	2	2	2	2	1	3	3
9	9	9	9	9	2	9	9	9	9
9	9	9	9	9	2	1	1	3	3
3	3	3	3	3	1	1	1	3	3
2	2	2	2	2	2	2	2	1	2
1	1	3	1	3	3	3	3	3	3

1	1	3	3	1	3	3	9	1	1
9	9	9	9	9	9	1	9	9	9
9	9	9	3	9	9	9	9	9	9
3	1	2	3	2	1	3	3	3	3
9	9	9	9	9	9	1	9	9	9
3	3	3	3	3	3	3	1	3	3
3	3	3	3	1	1	3	3	3	3
9	9	9	9	9	9	9	1	9	9
9	9	9	9	9	9	9	9	1	9
3	1	3	2	2	1	3	3	3	3
9	2	9	9	9	9	9	1	2	9
9	9	9	9	9	1	9	9	9	9
3	2	2	2	3	1	9	9	1	9
3	3	3	3	2	2	1	9	1	9
9	9	9	9	9	9	9	1	9	9
3	3	3	3	3	3	3	3	1	3
3	2	3	2	3	9	9	1	9	9
9	3	3	2	3	1	1	1	3	3
3	3	2	2	1	1	1	3	2	3
3	3	1	2	3	1	2	2	1	1
3	3	3	3	3	3	3	1	3	3
9	9	9	9	9	3	3	1	3	3
3	3	3	3	3	3	3	1	3	3
3	3	1	9	1	1	1	1	2	3
1	1	2	1	2	3	1	3	1	3
9	9	9	9	9	1	9	9	9	9
9	9	9	9	9	9	9	9	9	9
3	3	3	3	1	3	1	3	2	2
3	3	3	3	3	1	3	3	3	3
9	9	9	9	9	9	9	1	9	9
9	9	9	9	9	1	9	9	9	9
3	3	1	3	3	1	3	3	3	3
9	1	9	9	9	9	9	9	9	9
3	3	3	3	9	3	1	9	9	9
1	2	2	2	1	1	1	3	3	3
9	2	1	2	2	1	2	1	2	1
2	1	2	2	1	1	2	1	2	2
2	2	2	2	2	1	9	9	9	9
9	9	9	9	9	9	9	1	9	9
3	3	3	3	3	1	3	3	3	3
3	3	3	2	3	1	1	9	9	3
3	3	3	3	1	2	1	9	3	3
3	3	3	3	3	3	1	3	3	3
3	2	2	3	3	1	2	1	3	3
3	9	9	9	9	9	9	1	9	9
9	9	9	9	9	9	9	1	9	9
9	9	9	9	9	9	9	1	9	9

2	2	3	2	1	3	1	2	2	2
2	2	3	3	1	2	2	2	2	2
2	3	3	3	3	1	2	2	3	3
2	2	3	2	1	2	2	2	1	2
3	2	3	3	2	2	2	2	1	2
9	3	9	9	1	2	3	3	3	3
3	3	3	3	3	1	3	3	3	3
3	3	3	3	2	3	1	1	1	3
2	3	3	3	3	1	2	2	3	3
2	2	2	2	1	1	1	1	2	2
1	3	2	2	1	3	2	1	1	2
2	2	2	2	1	1	2	2	2	2
2	3	3	3	3	1	2	1	3	3
1	9	9	9	9	1	2	3	3	3
3	2	1	2	3	2	1	1	2	2
2	3	1	2	2	2	1	3	2	1
2	3	3	3	1	3	2	1	1	1
2	2	2	2	2	2	2	2	2	2
3	3	3	3	3	1	2	2	2	2
3	3	3	3	3	1	3	9	9	9
3	3	3	3	3	1	3	9	9	9
9	9	9	9	9	9	9	9	1	9
2	2	2	1	2	3	2	2	1	3
3	3	3	1	1	9	9	1	9	9
1	1	2	1	2	3	2	3	3	3
2	2	2	2	2	2	2	2	2	2
9	9	9	9	9	9	9	9	1	9
2	2	2	2	2	9	9	9	1	9
2	2	3	3	2	2	2	2	2	2
9	9	9	9	1	9	9	1	9	9
2	2	2	2	2	2	2	2	2	2
2	2	2	2	2	2	2	2	2	2
2	2	2	2	1	2	2	2	2	3
3	3	3	2	2	2	2	2	2	2
9	9	9	9	1	9	9	1	9	9
1	1	3	3	3	3	1	3	3	3
3	3	3	3	2	1	3	2	3	3
2	3	3	3	2	3	3	2	1	3
9	9	9	9	9	9	1	9	9	9
3	3	3	3	3	2	2	2	1	2
2	2	2	2	2	3	3	1	3	3
2	2	2	2	2	3	3	1	3	3
2	2	2	2	2	2	2	2	2	2
1	1	2	2	2	3	2	1	1	2
3	2	1	3	1	2	1	2	2	3
3	3	3	3	3	3	1	1	2	3
3	1	2	3	3	1	2	3	3	3

2	2	2	2	2	3	3	3	3	3
9	9	9	9	9	9	9	9	1	9
3	2	3	2	1	2	2	1	3	3
9	9	9	9	9	2	2	1	2	2
9	9	9	9	9	2	2	1	2	2
9	9	9	9	9	9	1	9	9	9
1	2	3	2	1	1	2	2	2	3
3	3	3	3	2	2	2	2	3	3
1	3	3	3	2	2	2	2	2	2
2	2	2	2	2	2	2	2	2	2
3	3	2	3	2	2	2	2	2	3
3	3	2	3	2	2	2	2	2	2
9	9	9	9	9	1	9	9	9	9
3	3	3	3	3	2	2	2	2	2
3	2	3	2	1	2	1	2	3	3
2	2	2	2	2	2	2	2	2	2
2	2	2	2	2	2	2	2	2	2
3	3	3	2	2	3	1	9	9	9
9	9	1	9	9	9	9	1	9	9
3	3	3	3	1	3	3	1	3	3
3	3	3	3	3	3	3	1	3	3
3	3	3	3	3	3	3	1	3	3
3	1	3	3	3	2	3	3	2	3
3	3	3	3	3	3	3	1	3	3
3	3	3	3	3	3	3	1	3	3
3	3	3	3	1	9	9	1	9	9
3	3	3	3	1	3	3	1	3	3
3	3	3	3	3	9	9	1	9	9
3	3	3	3	3	3	3	1	3	3
3	3	3	3	3	3	3	1	3	3
2	2	1	1	1	1	1	1	2	3
3	2	2	3	1	1	2	1	2	3
3	1	3	3	1	3	3	1	3	3
3	3	3	3	3	3	3	1	3	3
3	3	3	3	3	3	3	1	3	3
3	3	3	3	2	3	2	1	3	3
3	3	3	3	2	3	3	1	3	3
3	3	3	3	1	9	9	1	9	9
3	3	3	3	3	3	3	1	3	3
3	3	3	3	3	3	3	1	3	3
3	3	3	3	3	3	3	1	3	3
3	3	3	3	3	3	3	1	3	3
3	3	3	3	1	9	9	1	9	9
3	3	3	3	1	3	3	1	3	3
3	3	3	3	3	3	3	1	3	3
3	3	3	3	1	3	3	1	3	3
3	3	3	3	1	3	3	1	3	3

3	3	3	3	3	3	3	1	3	3
3	3	3	3	3	3	3	1	3	3
3	3	3	3	3	3	3	1	3	3
3	3	3	3	3	3	3	3	1	3
2	3	3	3	3	3	3	1	3	3
3	3	3	3	3	3	3	1	3	3
3	3	3	3	3	3	3	1	3	3
3	3	3	3	3	1	2	3	3	3
3	3	3	3	3	1	3	3	3	3
3	3	2	3	1	3	1	2	3	3
3	3	3	3	3	1	3	3	3	3
9	9	9	9	9	9	9	9	9	9
3	3	3	3	3	1	9	9	9	9
3	3	3	3	3	3	3	1	3	3
1	3	2	2	3	1	2	1	9	3
1	2	1	2	1	2	1	1	1	2
3	2	1	1	2	9	1	9	1	1
1	1	1	1	1	1	1	1	1	1
2	2	2	2	2	2	2	1	2	2
2	2	2	2	1	2	2	2	2	2
3	1	2	1	1	1	1	1	1	2
2	2	2	2	2	2	2	2	2	2
9	9	9	9	9	2	9	1	2	3
3	1	1	1	1	1	2	2	3	3
2	2	2	2	2	2	2	2	2	2
2	2	9	9	9	2	2	2	2	2
2	2	2	2	1	2	2	2	2	2
2	2	2	2	2	2	2	2	2	2
2	3	2	2	2	2	2	2	2	2
2	3	2	2	2	1	2	2	2	3
3	3	3	3	3	2	2	1	2	2
3	3	3	3	3	2	2	1	2	2
3	3	3	3	3	1	2	1	2	2
2	1	1	2	2	1	2	2	2	2
1	2	1	2	1	2	2	2	2	2
3	1	2	2	3	1	2	2	2	2
1	2	2	2	1	3	3	2	1	1
1	2	1	2	1	2	2	2	2	2
3	3	3	3	3	2	2	2	1	2
3	3	3	3	3	2	3	3	1	3
2	2	3	3	3	2	2	2	2	2
2	2	2	2	1	2	2	2	2	2
1	9	9	9	9	1	9	9	9	9
2	3	2	2	3	1	9	9	9	9
3	3	3	2	2	2	3	1	1	1
2	3	3	3	2	2	2	1	1	3
3	2	2	3	1	3	2	3	2	1

3	3	1	1	2	1	2	2	2	2
3	3	1	1	2	1	2	2	2	2
2	3	1	2	2	2	2	2	1	2
2	2	1	1	2	3	2	2	1	2
3	3	3	3	2	2	2	1	1	2
3	3	2	3	1	2	1	3	3	3
9	9	9	9	9	9	9	9	9	9
2	3	2	3	1	2	3	3	1	2
1	3	3	3	1	2	3	1	1	2
3	3	3	3	3	3	9	9	9	9
2	2	2	2	2	2	2	2	2	2
1	3	3	1	2	2	2	1	3	1
1	3	3	3	1	2	3	1	1	2
2	2	2	2	3	2	1	2	3	3
1	2	3	2	3	1	2	3	3	3
2	3	3	3	1	1	1	1	2	3
2	2	2	2	3	2	1	3	3	3
2	2	2	3	3	2	2	1	2	2
3	2	2	3	1	2	1	2	3	3
2	3	3	3	2	3	3	2	1	2
2	3	2	2	1	2	1	2	1	3
1	2	1	2	2	2	1	1	2	2
2	2	2	2	3	2	1	2	3	3
3	2	3	2	2	3	3	1	2	2
9	9	9	9	9	2	9	9	9	9
2	2	2	2	2	2	2	2	2	2
2	2	2	2	2	2	2	2	2	2
3	3	2	3	2	3	9	1	1	3
2	1	1	1	2	2	2	1	1	3
9	9	9	9	9	2	2	2	2	2
2	2	2	2	2	2	2	1	2	2
3	3	2	2	2	2	2	2	2	2
3	3	3	3	3	1	9	9	9	9
3	3	3	3	3	2	2	3	3	3
2	2	2	2	2	2	2	2	2	2
2	2	2	2	2	2	2	2	2	2
3	3	3	2	2	3	2	1	1	2
3	1	9	9	9	9	9	9	9	9
3	2	3	3	3	3	3	2	1	3
3	9	3	2	2	3	9	2	2	1
2	2	2	2	2	1	3	3	3	3
3	3	3	3	2	1	1	1	2	2
3	2	3	2	3	3	3	1	2	3
3	3	3	3	3	2	2	2	2	2
3	1	3	3	2	2	1	2	1	1
1	3	3	3	1	1	2	3	3	3
3	2	3	2	1	3	1	2	2	3

2	2	2	2	2	2	1	2	2	2
3	2	2	3	1	3	2	1	3	3
2	2	2	2	1	2	2	1	1	2
3	2	3	3	1	2	3	3	1	3
3	3	3	3	2	1	2	3	3	3
3	3	2	3	3	3	3	1	1	3
2	2	1	2	1	2	1	1	1	2
9	9	9	9	9	9	9	9	1	9
3	9	2	3	2	2	2	1	2	2
2	2	2	2	2	2	2	2	2	2
1	1	1	1	3	2	2	2	2	2
2	2	2	2	2	1	3	3	3	3
2	3	3	3	3	3	1	1	1	1
9	9	9	9	9	9	1	9	9	9
3	1	3	1	3	1	3	3	1	3
3	3	3	3	3	2	2	2	2	2
3	3	3	3	3	1	3	3	3	3
2	3	3	2	2	2	1	1	1	1
2	2	2	1	1	1	2	3	1	1
1	2	2	2	1	2	1	2	1	2
3	2	3	2	1	2	2	2	2	2
3	3	2	3	3	2	1	1	3	3
2	2	3	3	3	3	1	2	3	3
2	2	2	2	2	2	2	2	2	2
3	3	3	3	3	2	2	2	2	2
9	9	9	9	1	9	9	9	9	9
3	3	3	3	1	2	2	2	2	2
3	3	3	3	2	2	2	1	2	3
3	3	3	3	3	2	2	2	2	2
2	2	2	2	2	2	1	1	3	2
3	3	3	3	2	2	1	2	2	2
9	9	9	9	9	9	9	1	3	2
9	9	9	9	9	9	1	9	9	9
3	3	3	3	3	3	3	1	2	3
3	2	2	2	2	2	2	1	2	2
2	1	9	3	1	3	1	9	1	2
3	3	3	3	3	1	1	2	2	3
1	1	3	3	1	3	1	1	1	2
3	1	3	3	1	3	1	1	3	3
9	9	9	9	9	3	3	2	2	1
9	9	9	9	9	1	9	9	9	9
3	1	3	3	1	3	1	3	3	3
2	2	2	2	2	2	2	1	2	2
3	3	3	3	2	3	3	1	2	3
3	3	3	3	3	2	3	1	1	3
3	2	3	3	3	3	2	2	1	2
3	3	3	3	1	2	1	2	2	2

3	3	2	2	1	2	1	1	2	3
3	3	2	2	1	2	1	1	2	3
9	9	9	9	9	9	9	1	9	9
3	3	3	2	1	3	1	1	3	3
9	9	9	9	9	9	9	9	1	9
3	3	1	2	3	3	1	3	2	2
3	3	3	3	1	1	2	2	2	2
2	2	1	2	9	2	9	9	9	9
2	3	3	3	1	3	3	1	2	3
1	3	1	2	1	3	3	1	2	2
3	3	3	3	3	3	3	3	1	3
2	2	3	3	3	2	2	2	2	2
2	3	3	3	1	2	1	3	2	3
3	3	3	3	3	3	1	3	3	3
3	3	3	3	3	3	3	3	1	3
2	3	2	2	2	3	3	2	3	3
3	3	3	3	3	3	3	3	1	3
3	2	2	2	2	2	2	2	1	2
3	3	3	3	3	2	3	3	1	3
2	1	2	3	2	2	1	2	2	3
3	3	9	2	1	3	3	3	1	1
3	3	3	3	2	3	2	1	3	3
3	3	3	2	3	3	2	1	3	3
3	2	2	3	1	3	1	1	3	2
2	2	2	2	2	2	2	2	2	1
3	2	3	3	3	3	2	2	3	2
2	3	2	3	3	3	2	1	2	3
2	3	3	3	3	3	3	2	1	3
1	1	2	3	2	2	1	1	2	3
3	2	3	3	3	3	1	1	3	3
3	3	2	3	3	1	3	2	3	3
3	3	2	3	3	1	3	2	3	3
1	1	1	2	2	1	1	1	2	2
2	2	2	2	2	2	2	2	2	2
3	2	2	2	2	2	2	2	2	2
3	3	3	3	2	3	2	1	2	3
3	3	3	3	3	2	2	2	2	2
1	2	2	2	1	3	2	1	2	2
3	1	2	2	2	3	1	3	2	2
3	3	3	2	3	3	2	1	3	3
3	2	1	2	2	2	2	2	2	2
3	3	2	2	1	1	3	2	1	2
3	1	2	3	1	2	1	2	1	1
3	3	2	2	3	3	2	2	1	3
2	3	3	3	3	3	3	2	1	2
3	2	3	3	3	2	1	1	3	3
3	3	3	3	3	2	2	1	2	2

2	3	3	9	1	2	2	1	2	3
2	3	1	3	1	2	2	1	1	2
9	9	9	9	9	2	9	9	9	9
3	3	2	2	1	3	2	2	2	3
3	2	1	3	1	3	3	1	1	2
3	2	2	2	3	3	1	3	2	3
2	2	2	2	2	3	1	1	2	1
2	2	2	2	2	1	3	3	2	3
2	2	2	2	2	1	3	3	3	3
3	3	3	3	3	1	2	2	2	2
3	2	1	2	1	3	2	1	1	3
3	3	3	3	1	3	1	1	1	3
1	1	3	3	3	3	2	1	2	3
3	2	2	2	2	3	2	2	2	2
3	2	2	2	2	3	2	2	2	2
3	3	3	3	3	2	2	1	2	3
3	3	3	3	3	2	3	2	2	2
3	3	3	3	3	3	2	1	3	3
3	3	3	3	3	2	1	1	3	3
9	9	9	9	9	9	9	9	9	9
2	3	2	3	1	1	2	2	3	3
3	3	3	3	3	3	3	3	2	3
3	3	3	3	3	3	3	3	2	3
2	2	2	2	1	2	2	2	2	2
3	3	3	3	1	3	2	1	2	3
3	2	3	2	1	2	1	2	2	2
9	9	9	9	9	9	1	9	9	9
2	2	2	2	2	3	2	1	2	3
3	2	3	2	1	2	2	2	2	2
9	9	9	9	1	9	1	9	9	9
1	2	2	2	1	2	2	2	1	2
1	1	2	2	2	1	2	3	3	2
1	2	2	2	1	2	2	2	1	2
9	9	9	9	9	9	9	9	1	9
1	3	2	3	2	1	2	3	2	2
3	2	3	2	3	3	3	1	2	3
9	9	9	9	9	9	9	9	9	9
3	3	2	3	1	3	2	1	1	3
2	3	2	2	2	3	2	1	2	2
2	2	2	1	1	3	1	2	3	3
2	3	3	2	3	3	2	2	1	3
3	1	2	3	3	2	3	1	3	3
2	3	2	1	1	2	3	3	1	2
9	3	3	3	3	3	2	1	3	3
3	3	3	3	3	2	2	2	2	2
3	3	3	3	2	9	9	1	9	9
3	3	3	3	3	3	3	3	3	3

3	3	3	3	2	9	9	1	9	9
3	3	3	3	1	3	3	1	3	3
3	3	3	3	2	3	3	1	3	3
3	3	3	3	1	3	3	1	3	3
3	3	3	3	3	3	1	9	9	9
3	3	3	3	3	3	3	1	3	3
3	3	3	3	3	2	2	1	2	2
3	9	9	3	3	3	3	1	1	1
2	3	3	3	3	2	2	2	9	2
1	3	3	3	3	2	2	2	2	2
3	3	3	3	1	3	1	9	9	9
3	3	3	3	9	3	3	1	3	3
3	3	3	3	1	3	3	1	3	3
3	3	3	3	3	3	3	1	3	3
3	3	3	3	2	3	3	1	3	3
3	3	3	3	1	3	3	1	3	3
3	9	3	3	1	3	3	1	3	3
3	3	3	3	3	3	3	1	3	3
3	3	3	3	1	3	3	1	3	3
3	3	3	3	1	3	3	1	3	3
3	3	3	3	1	9	9	1	9	9
3	3	3	3	1	3	3	1	3	3
3	3	3	3	1	3	3	1	3	3
3	3	3	3	3	3	3	1	3	3
3	3	3	3	3	3	3	1	3	3
3	3	3	3	3	3	3	1	3	3
3	3	3	3	3	3	3	1	3	3
3	3	3	3	1	3	3	1	3	3
3	3	3	3	9	3	3	1	3	3
3	3	3	3	3	3	3	1	3	3
3	3	3	3	3	3	3	1	3	3
3	3	3	2	2	2	2	1	2	2
3	3	3	3	1	3	3	1	3	3
3	3	3	3	1	3	3	1	3	3
1	2	2	2	1	9	9	1	9	9
3	3	3	3	3	3	9	1	2	3
3	2	2	3	2	2	1	1	2	2
3	3	3	3	3	2	2	1	3	3
3	3	3	3	3	3	3	1	3	3
3	3	3	3	3	3	3	1	3	3
3	3	3	3	3	3	3	1	3	3
2	2	2	2	2	2	2	1	1	2
9	9	9	9	9	9	1	9	9	9
2	2	2	2	2	2	3	3	3	3
9	9	9	9	9	2	9	9	9	9
2	2	2	2	2	2	2	1	2	2
9	9	9	9	9	9	1	1	2	9

2	2	2	2	2	2	2	2	2	2
3	1	3	2	1	3	3	1	3	3
3	1	3	2	1	3	3	1	3	3
2	2	9	2	9	3	1	2	2	3
3	2	3	1	3	3	2	1	1	3
3	2	2	2	1	9	9	9	9	1
3	3	2	1	2	1	2	3	3	3
1	2	3	2	2	2	3	1	2	1
1	2	1	2	1	1	2	2	3	3
2	2	2	2	2	2	2	2	2	2
9	9	9	9	9	9	9	9	9	9
9	9	9	1	9	9	9	9	9	1
3	3	3	3	3	9	1	2	3	9
2	2	2	2	2	2	2	1	1	2
9	1	9	9	9	9	9	1	9	9
9	1	9	9	9	9	9	1	9	9
9	9	9	9	9	9	9	1	9	9
9	1	9	9	9	9	1	9	9	9
9	1	9	9	9	9	1	9	9	9
3	3	9	9	9	9	9	9	9	9
9	9	9	9	9	9	9	9	9	9
3	3	3	3	1	1	3	3	3	3
1	3	2	3	3	3	2	1	1	3
9	9	9	9	9	9	1	9	9	9
9	9	9	9	9	1	9	9	9	9
9	1	9	9	9	9	9	1	9	9
9	9	9	9	9	9	1	9	9	9
1	2	2	2	1	2	2	3	3	2
3	3	2	1	2	2	1	1	3	3
9	9	9	1	9	9	1	9	9	9
9	9	9	9	1	9	1	9	9	9
2	2	3	2	2	3	2	2	1	2
2	3	2	3	3	3	2	3	3	3
3	3	3	3	3	3	3	3	1	1
2	2	2	2	2	2	2	2	2	2
3	3	3	2	3	3	3	2	1	3
3	3	2	2	3	3	3	1	1	2
3	3	3	3	3	3	3	1	2	3
2	2	2	3	2	2	2	3	3	2
2	3	2	3	2	1	2	2	3	3
2	3	2	3	3	2	1	2	2	2
3	3	3	3	1	3	1	3	1	3
3	3	1	1	1	3	2	1	1	3
3	3	2	3	1	2	1	3	3	3
1	3	2	2	1	2	1	3	3	2
3	3	3	3	3	3	3	2	1	2
3	3	3	2	1	2	2	1	1	3

1	2	2	3	3	1	2	2	3	3
3	3	3	2	1	9	9	9	9	9
2	3	2	3	3	2	1	2	3	3
1	3	1	1	2	3	1	2	3	3
1	2	3	3	3	3	3	2	1	2
3	3	3	3	3	3	1	3	3	3
2	2	2	3	3	2	2	1	9	9
2	3	1	2	1	3	1	2	1	2
3	3	2	3	2	2	1	1	2	3
2	3	3	3	1	2	2	1	2	2
2	3	2	3	2	2	2	2	3	3
3	2	2	2	1	3	2	2	1	1
2	3	3	3	3	3	2	2	3	3
2	3	3	3	3	3	2	2	3	3
3	3	3	3	3	3	2	1	1	2
2	2	2	1	1	2	2	2	2	2
3	3	3	3	3	2	2	1	2	2
3	3	3	2	3	3	3	2	2	2
3	3	3	3	1	3	3	2	1	3
2	3	2	3	1	3	2	1	3	3
3	3	1	3	1	3	3	1	2	2
3	3	3	3	3	3	2	1	3	3
2	3	3	3	2	3	2	1	2	1
2	2	3	3	2	3	2	1	2	1
3	3	3	2	2	2	2	3	1	2
3	2	3	3	2	1	2	3	3	3
3	3	3	3	1	3	3	1	1	3
3	3	3	3	2	3	3	1	1	3
3	2	2	2	2	3	2	1	1	3
2	3	2	1	1	1	2	3	3	1
9	3	3	2	3	2	3	1	2	2
1	2	2	2	3	1	2	3	3	1
2	2	2	2	2	2	2	2	2	2
2	2	2	2	1	2	2	1	1	2
3	2	3	3	3	3	2	2	2	3
9	9	9	9	9	9	9	9	9	2
2	2	2	3	2	3	2	2	1	1
3	3	3	2	1	3	1	1	2	1
2	2	2	2	2	2	2	2	2	2
3	2	3	2	2	3	2	1	2	2
3	2	3	2	1	1	1	1	3	3
2	1	2	1	2	3	2	2	1	3
9	9	9	9	9	1	9	9	9	9
2	2	2	2	1	1	2	2	2	2
3	1	2	3	3	3	3	3	3	3
2	2	2	2	2	1	2	1	2	2
9	1	9	9	9	9	2	9	9	9

9	9	9	9	1	9	9	9	1	9
9	9	9	9	1	9	9	1	9	9
9	9	9	1	9	9	9	1	9	9
9	9	9	1	9	9	9	9	1	9
9	9	9	9	9	9	9	9	1	9
9	9	9	9	9	1	9	9	9	9
9	9	9	9	2	9	9	1	9	9
9	9	9	9	9	9	9	9	1	9
9	9	9	9	9	9	9	9	1	9
9	9	9	9	9	2	9	9	9	9
3	9	9	9	9	9	9	9	9	9
9	9	9	9	9	2	9	9	9	9
3	9	9	9	9	1	9	9	9	9
9	1	9	9	9	9	9	1	9	9
9	9	9	9	1	9	9	1	9	9
9	9	9	9	1	1	9	9	9	9
9	9	9	9	9	9	9	1	9	9
9	9	9	9	9	9	9	1	9	9
2	2	2	2	2	2	2	2	2	2
1	1	1	1	1	1	1	2	1	3
1	1	1	1	1	1	1	1	1	1
3	3	3	3	3	3	3	1	1	2
1	2	2	2	1	3	3	1	3	3
3	1	1	1	3	3	3	1	1	1
1	2	3	2	3	2	2	1	3	3
9	3	3	3	3	3	3	1	3	3
3	3	3	3	3	3	1	1	3	3
3	1	2	2	2	3	2	1	2	1
3	3	3	3	3	3	3	1	3	3
1	9	1	2	3	2	2	2	2	2
1	1	2	2	3	1	1	2	2	1
3	3	3	3	3	3	1	3	3	3
3	2	2	2	2	2	2	2	1	2
3	3	2	3	3	3	1	2	3	3
2	1	2	2	1	1	1	3	3	3
3	3	3	3	3	2	1	3	3	3
3	2	2	2	1	3	1	2	3	3
3	3	2	2	2	2	9	9	9	9
3	9	9	9	9	2	9	3	9	1
3	3	3	3	2	2	3	3	3	3
1	9	1	9	9	9	9	9	9	9
3	1	3	3	3	1	3	3	3	3
9	9	9	9	9	9	9	9	9	9
1	1	3	1	1	1	1	1	3	1
3	3	1	2	2	3	2	2	1	2
9	9	9	9	9	2	2	1	1	1
2	2	2	1	1	2	1	1	2	2

2	1	2	1	1	1	2	3	1	3
3	3	1	1	3	3	3	2	2	3
3	1	3	1	1	3	3	3	1	3
2	1	2	1	1	1	2	1	1	2
2	2	2	2	2	2	1	1	2	2
2	2	2	2	2	2	2	2	1	3
2	1	2	2	2	2	2	2	2	2
2	1	2	1	1	1	2	3	1	3
1	2	1	1	1	1	1	1	1	3
9	9	9	9	9	9	9	9	9	9
9	9	9	9	9	9	9	9	9	1
3	3	2	3	2	3	2	1	3	2
3	3	3	3	3	3	3	3	3	3
1	2	3	2	1	3	2	1	2	2
1	9	3	3	2	9	9	1	3	3
9	9	9	9	9	1	9	9	9	9
9	9	9	9	9	9	1	9	9	9
3	2	2	2	9	2	2	2	2	2
3	3	2	3	1	3	3	1	3	3
2	3	2	2	3	3	3	3	1	2
1	2	1	2	1	3	1	1	3	3
3	3	3	3	3	3	3	3	3	3
3	3	3	3	3	3	3	3	3	3
9	9	9	9	9	9	9	9	9	9
9	9	9	9	9	9	9	9	9	9
3	3	9	2	1	3	3	3	1	3
2	1	2	2	3	3	3	3	2	3
3	1	9	3	1	9	9	9	9	9
2	2	9	1	1	2	1	1	2	2
9	9	9	9	1	9	9	9	9	9
3	3	3	3	3	3	1	3	3	3
3	3	3	1	1	3	3	1	3	3
1	3	1	3	1	3	1	2	1	1
1	1	1	1	1	3	1	2	2	3
9	1	9	9	1	9	1	9	9	9
9	9	9	9	1	9	9	9	1	9
9	9	9	9	1	9	9	9	1	9
2	2	2	2	1	1	3	3	2	2
2	2	2	2	1	1	3	3	2	2
2	2	2	2	1	1	3	3	2	2
2	2	2	2	1	1	3	3	2	2
2	3	2	3	2	2	3	1	2	3
2	3	2	3	3	1	2	3	1	2
2	1	2	2	1	1	3	3	2	2
2	3	2	1	1	1	2	3	1	2
2	3	2	2	3	3	2	3	2	2
1	1	1	3	3	3	3	3	3	1

3	3	3	3	1	3	2	1	3	1
3	2	2	2	3	1	3	3	3	3
3	3	3	3	3	1	3	3	3	3
2	3	3	3	3	2	2	1	1	2
2	2	2	2	2	3	3	1	2	3
1	1	1	1	1	1	1	1	1	1
3	2	2	3	2	2	9	1	2	2
9	9	9	9	9	9	1	9	9	9
3	3	3	1	1	2	2	2	1	3
3	3	3	2	2	2	2	1	2	2
2	2	2	2	2	2	2	2	2	2
1	1	1	3	1	9	1	9	9	9
2	3	2	2	1	2	2	1	2	2
1	2	2	2	2	2	2	1	2	2
3	1	3	3	3	3	2	3	2	2
1	2	2	2	2	2	2	3	1	3
2	2	3	3	1	2	3	2	2	1
2	3	2	3	1	2	3	2	2	1
2	3	2	3	1	2	3	2	2	1
1	3	3	1	3	3	3	1	3	3
9	9	9	9	1	2	1	3	1	2
3	3	2	2	2	2	9	9	9	9
9	9	9	9	1	3	9	9	9	1
2	1	2	3	3	3	3	3	1	3
9	2	1	3	9	1	2	3	2	3
2	3	2	2	3	1	2	1	3	2
2	2	2	1	2	2	3	2	3	1
3	1	3	3	3	1	1	1	1	3
9	9	9	9	9	9	9	9	9	1
2	2	2	2	2	9	1	1	2	3
3	2	3	3	1	3	1	3	3	3
9	9	9	9	9	3	9	9	9	9
2	1	2	3	1	1	2	3	2	1
2	1	1	1	3	2	1	1	2	2
3	3	3	3	3	3	3	3	3	3
3	3	3	3	1	3	3	2	1	2
2	3	2	3	1	2	3	2	2	1
2	3	2	3	1	2	3	2	2	1
2	3	2	3	1	2	3	2	2	1
3	1	1	3	1	1	3	3	3	3
1	3	2	1	1	2	1	3	1	1
2	9	1	2	3	1	2	2	1	3
2	1	3	3	3	3	1	3	3	3
3	3	9	3	1	1	3	3	3	3
9	9	9	9	9	9	9	9	9	9
3	3	3	3	3	3	3	3	3	3
2	2	1	2	2	2	1	2	2	2

2	2	2	2	2	1	1	1	1	1
9	9	1	1	9	1	9	9	9	9
1	3	1	1	1	9	9	9	9	9
3	1	3	3	1	3	3	3	1	3
2	2	2	2	2	9	9	1	1	2
2	2	2	2	2	2	1	1	2	2
1	1	1	2	3	1	2	1	2	2
2	2	2	2	2	2	1	1	2	2
2	2	2	2	2	2	1	1	2	2
9	9	9	9	9	9	9	9	9	9
2	3	3	2	3	2	3	1	2	2
3	3	1	3	1	1	2	3	3	3
1	3	3	2	3	3	3	1	2	3
3	3	1	1	3	3	3	2	2	3
3	3	3	3	3	3	3	1	1	3
3	3	3	1	2	1	1	1	1	1
2	3	3	3	2	3	3	3	1	3
2	3	3	3	2	1	9	9	9	9
2	3	3	3	2	1	9	9	9	9
2	1	3	3	1	1	3	3	3	3
2	2	1	3	3	1	2	1	1	1
3	3	2	2	3	9	1	1	2	3
3	3	3	3	3	9	9	9	1	9
2	2	2	2	2	2	1	2	2	2
2	2	2	2	2	2	3	3	3	3
9	9	9	9	1	9	9	1	9	9
9	9	9	9	1	9	9	1	9	9
3	1	1	3	3	3	3	3	1	3
3	3	3	3	3	9	9	9	1	9
2	2	2	2	2	2	2	2	2	2
3	2	3	3	1	9	9	1	9	9
1	1	2	3	1	3	2	1	1	3
2	1	2	2	1	2	2	1	1	3
9	9	9	9	1	9	9	1	9	9
1	2	3	2	3	3	1	3	2	3
3	3	3	3	3	3	3	3	3	3
3	2	2	2	2	1	2	2	2	2
3	9	9	9	9	9	9	9	9	9
9	9	9	9	1	9	9	9	9	1
3	3	2	1	3	2	2	2	2	2
3	3	3	3	3	3	1	3	3	3
2	2	2	2	2	2	2	1	2	2
2	2	2	2	2	3	1	3	3	3
2	2	2	2	2	2	2	2	2	2
2	2	2	2	2	2	2	2	2	2
3	3	3	3	3	3	3	1	3	3
3	3	3	3	3	3	1	3	3	3

2	2	2	2	2	3	3	2	1	2
3	3	3	3	3	2	2	2	2	2
3	3	3	3	3	3	3	1	3	3
3	3	2	3	3	3	2	1	1	2
9	9	9	9	9	1	9	9	9	9
1	2	1	2	2	3	1	2	2	1
2	2	2	2	3	2	2	2	2	2
9	9	9	9	1	1	9	9	9	9
2	2	1	2	3	1	2	1	2	3
9	9	9	9	9	9	9	2	9	9
2	2	2	2	2	2	2	2	2	2
3	2	3	3	9	9	1	9	9	9
3	2	2	2	2	9	9	1	9	9
2	2	2	2	2	2	2	2	2	2
3	3	3	1	1	1	2	1	2	3
2	2	2	2	2	2	2	2	2	2
2	2	2	2	2	2	2	2	2	2
9	9	9	9	9	1	3	2	2	3
2	1	3	2	1	9	2	1	1	2
9	9	9	9	9	9	9	9	9	9
2	2	9	9	9	9	9	1	9	9
3	3	3	3	2	3	3	1	2	2
2	2	2	2	2	2	2	2	2	2
1	2	1	1	1	1	1	1	1	1
3	3	2	3	1	2	2	2	2	2
2	1	3	3	3	1	2	2	2	2
1	3	2	9	2	3	3	1	3	3
3	3	3	3	1	3	3	1	3	3
2	2	2	2	2	2	2	2	2	2
3	3	3	3	3	3	3	1	2	2
3	3	3	3	3	9	9	1	9	9
3	3	2	2	2	3	2	1	2	3
3	1	3	3	1	3	3	1	3	3
2	2	2	2	2	2	1	2	2	2
1	2	2	3	1	3	2	2	1	2
3	3	3	3	2	3	3	3	3	1
2	2	2	2	2	2	2	1	2	2
1	3	2	1	2	2	2	1	1	1
2	1	3	2	3	1	9	9	9	9
3	1	3	1	1	2	1	2	2	3
3	3	2	3	3	3	3	3	1	2
2	2	2	2	2	2	2	2	1	2
3	1	3	3	3	3	3	3	1	3
1	9	3	3	1	3	3	3	1	1
2	2	2	2	2	2	2	2	2	2
3	3	3	3	3	3	3	3	3	3
2	2	2	3	2	3	2	2	1	3

3	3	2	1	1	3	3	2	1	2
1	3	3	3	3	9	9	9	9	9
3	3	3	3	3	3	3	3	3	3
2	2	2	2	2	2	2	2	2	2
3	3	3	3	2	2	2	2	1	2
2	1	1	3	1	1	1	1	2	3
1	3	1	3	1	2	2	2	2	2
3	3	3	2	3	3	1	1	2	3
1	2	3	1	2	1	2	3	1	2
2	2	2	2	2	2	2	2	2	2
3	3	3	3	1	3	3	1	2	3
2	2	2	2	2	2	2	2	2	2
9	9	9	9	9	9	9	9	1	9
2	1	1	1	2	1	2	2	2	2
2	2	3	2	3	1	2	2	2	2
9	9	9	9	9	3	3	3	1	3
9	9	9	9	9	3	3	3	9	1
3	3	3	1	3	3	3	1	2	3
3	2	2	2	3	1	1	1	1	2
2	1	1	1	3	1	2	2	2	2
3	3	3	3	1	1	1	1	3	3
1	1	3	1	2	1	1	1	1	1
3	3	3	3	1	3	3	1	2	2
2	1	2	3	1	3	2	1	2	3
3	3	3	3	3	3	3	3	3	1
9	1	2	3	9	9	2	1	2	3
2	2	2	2	2	2	2	2	2	2
3	2	3	3	3	1	1	2	2	2
3	2	2	2	2	1	9	9	9	9
2	3	2	2	2	3	2	2	2	1
1	1	1	1	1	2	1	2	1	2
1	1	1	1	1	1	1	1	1	1
1	1	1	1	1	1	1	1	1	1
1	1	2	2	2	1	1	1	1	1
1	1	2	3	1	3	9	2	1	2
9	1	9	1	1	3	3	1	1	1
9	1	9	9	9	9	9	9	1	9
1	1	1	1	1	1	1	1	1	1
2	2	2	2	2	2	2	2	2	2
2	2	2	2	2	2	2	2	2	2
3	1	1	3	1	3	1	2	2	3
2	1	3	1	1	1	1	1	2	3
3	9	9	9	1	9	9	1	9	9
1	3	2	1	9	2	2	2	2	1
2	2	2	2	2	2	2	2	2	2
3	3	3	1	3	3	3	3	1	1
3	3	2	3	3	2	2	2	2	2

3	2	9	3	2	3	3	1	3	3
3	3	3	3	3	3	1	2	2	3
3	1	3	3	2	1	2	3	3	3
9	9	9	9	9	9	9	9	1	9
3	1	2	3	1	2	9	1	2	3
2	1	3	2	1	2	2	1	2	3
9	9	9	9	9	9	9	9	3	9
9	9	9	9	9	9	9	9	9	9
3	3	3	3	3	3	3	3	1	1
2	2	2	2	2	2	2	1	2	2
9	1	9	9	1	9	9	1	9	9
3	3	3	3	3	1	3	3	2	2
3	9	9	9	1	2	2	1	2	2
1	2	3	3	1	2	1	2	2	3
1	1	2	2	2	2	2	2	2	2
1	2	9	3	2	2	1	2	3	3
1	1	2	2	2	3	2	1	1	2
9	1	9	9	1	9	9	1	9	9
2	1	2	3	3	3	2	1	2	3
2	1	2	1	1	2	2	2	2	2
3	3	9	2	2	2	1	2	3	2
2	2	2	3	2	3	3	1	2	2
2	1	2	1	2	2	3	1	2	3
1	1	2	2	2	2	2	2	2	2
3	1	1	3	9	2	3	2	1	3
3	1	3	9	9	3	3	1	1	3
3	3	9	1	3	3	3	2	2	1
3	3	3	3	1	3	3	1	1	3
2	2	2	2	2	3	9	1	2	2
2	3	2	1	1	2	2	1	3	2
2	3	2	1	3	3	1	2	2	3
1	3	2	2	9	3	2	1	1	2
3	3	3	2	2	2	2	2	3	3
3	1	3	1	3	1	1	1	3	3
9	9	9	9	9	9	9	9	1	9
3	2	3	3	3	3	1	2	3	3
1	1	1	9	1	1	2	1	1	1
3	1	3	1	3	1	1	1	3	3
2	9	9	3	9	1	1	9	1	2
1	3	3	3	3	1	3	3	3	3
1	3	3	3	1	3	9	1	1	3
9	9	9	9	9	1	9	9	9	9
1	1	1	1	1	3	3	1	1	3
3	3	1	3	3	3	3	1	1	3
3	2	2	2	2	3	2	1	1	2
1	2	1	9	9	9	9	9	9	9
3	3	1	2	1	1	2	2	1	2

1	1	1	3	2	9	9	9	9	1
3	3	3	2	2	2	9	1	3	2
2	1	2	1	3	1	2	1	2	1
3	3	3	3	3	3	1	3	3	2
2	2	2	2	2	2	2	2	2	2
3	2	3	3	2	3	3	2	1	2
2	2	2	2	1	3	3	2	1	3
2	1	2	2	2	2	2	2	2	2
9	1	2	2	2	2	2	2	2	2
1	2	1	3	2	2	2	2	2	2
3	3	3	2	2	3	3	2	1	2
3	3	2	2	3	3	3	3	1	2
1	1	1	1	3	3	1	1	1	3
2	3	3	2	3	2	3	2	3	2
1	9	9	9	9	9	9	9	9	9
3	2	2	2	1	3	3	2	1	3
3	3	3	3	3	3	1	3	3	3
2	3	3	2	1	2	2	1	2	2
2	3	2	2	1	2	2	1	1	3
9	1	9	9	9	9	9	9	1	9
9	9	9	9	9	9	9	1	9	9
3	3	3	3	3	3	3	3	3	3
3	3	3	3	1	1	1	3	3	3
3	3	3	1	1	3	3	1	3	3
3	3	3	1	1	1	3	3	3	3
1	1	1	3	2	9	9	1	9	9
1	2	1	3	2	3	2	1	1	9
3	3	3	2	2	3	2	1	1	3
1	2	1	1	1	3	3	1	2	2
3	2	2	9	1	3	2	1	2	3
1	3	3	3	3	2	1	3	2	3
1	1	3	1	1	1	1	1	9	1
1	2	1	1	3	1	2	2	2	1
3	3	3	3	1	1	3	3	3	3
1	3	3	3	1	3	2	2	3	2
3	3	3	3	3	3	1	3	3	3
9	9	9	9	9	9	9	1	9	9
9	9	9	9	9	2	2	2	2	2
3	3	3	3	3	3	3	3	3	3
2	2	2	2	2	3	1	2	2	2
3	3	3	2	1	1	2	1	1	3
3	3	3	3	1	1	3	3	1	3
2	2	2	2	2	1	2	2	2	2
9	9	1	9	9	9	9	1	9	9
9	9	9	9	9	9	9	9	9	9
3	3	3	1	3	1	2	3	3	3
2	3	3	3	3	2	2	1	3	1

9	9	9	9	9	1	2	9	1	3
2	1	1	1	1	3	3	2	3	3
3	3	3	3	3	1	3	3	3	3
9	9	9	9	1	9	9	9	2	9
3	3	3	3	2	3	3	3	1	2
9	9	9	9	9	9	9	9	9	9
2	1	1	2	2	1	3	1	1	1
2	3	3	3	3	2	9	1	1	2
3	3	3	3	3	1	3	3	3	3
3	3	3	3	3	3	1	2	3	3
3	3	3	3	3	3	1	2	3	3
3	3	3	3	3	3	1	2	1	3
9	1	9	9	9	1	9	9	9	9
9	1	9	9	9	1	9	9	9	9
9	9	9	9	9	2	2	1	1	1
3	3	3	2	2	3	2	1	1	1
3	1	3	3	1	3	1	3	3	3
3	3	3	3	3	2	2	3	1	3
1	2	3	2	1	2	2	1	2	3
3	3	3	3	2	2	1	2	2	2
3	2	2	2	2	3	1	1	1	1
2	2	1	2	2	3	1	1	1	1
2	2	2	2	2	2	2	2	2	2
3	2	2	3	3	3	3	2	1	3
3	2	3	2	3	2	1	2	2	2
3	2	3	3	3	9	9	9	9	9
1	1	3	3	1	1	9	9	9	9
2	2	2	2	2	2	2	2	2	2
3	3	2	2	2	9	9	9	9	9
3	3	3	2	2	3	2	1	1	1
3	3	3	3	2	2	1	2	2	2
9	1	9	9	9	1	9	9	9	9
3	3	3	3	3	1	9	9	9	9
3	3	3	3	2	3	2	1	1	2
1	1	1	2	2	2	2	1	1	1
9	9	9	9	1	2	1	2	2	3
9	9	9	9	9	9	9	1	9	9
9	9	9	9	9	9	1	9	9	9
3	3	2	3	3	1	2	1	3	1
1	1	1	3	1	2	2	2	2	2
9	9	9	9	9	9	9	9	1	9
2	1	1	2	1	1	3	3	3	3
1	3	3	3	3	1	3	3	3	3
3	3	3	3	3	3	1	3	3	3
1	1	1	1	1	3	2	1	2	1
3	1	2	2	1	2	1	3	2	3
9	9	9	9	9	9	9	1	9	9

1	1	3	3	1	9	9	9	9	9
3	3	3	3	3	2	2	1	1	1
3	1	2	1	1	2	2	2	1	1
9	9	9	9	9	9	9	1	9	9
9	9	9	9	9	9	1	9	9	9
3	3	3	2	1	3	2	3	3	3
3	1	2	1	3	1	1	1	2	3
3	2	3	2	2	1	2	3	3	3
1	3	1	3	2	1	2	2	3	3
3	1	2	1	3	1	1	2	2	2
3	3	3	3	1	2	1	1	3	3
9	9	9	9	9	2	2	2	2	2
3	3	3	3	3	1	2	3	2	2
3	3	3	3	1	2	2	2	2	2
3	2	2	3	1	3	2	1	2	2
3	3	1	2	1	3	3	3	3	3
3	3	3	3	3	1	3	3	3	3
3	3	3	3	2	1	2	3	3	3
2	3	9	2	3	2	3	3	2	2
3	3	3	3	3	3	1	3	3	3
2	2	2	3	3	3	1	3	3	3
1	9	9	9	9	9	9	9	9	1
2	2	1	2	1	2	2	2	2	2
2	3	2	2	2	3	3	3	3	3
2	1	2	2	1	2	2	2	2	2
1	1	2	2	1	2	2	2	1	2
3	2	2	3	2	3	1	2	2	2
3	3	3	3	3	3	3	1	3	3
3	3	3	2	3	1	1	1	1	1
3	3	3	3	3	3	3	3	1	3
9	9	9	9	9	9	9	1	9	9
3	1	2	9	1	2	1	3	2	2
9	9	9	9	9	9	9	1	9	9
3	3	1	2	1	3	3	2	1	3
1	1	1	2	3	1	2	3	2	2
9	9	1	9	9	1	9	9	9	9
9	9	9	9	9	9	9	9	9	9
2	2	2	2	2	2	2	2	2	2
3	3	3	3	1	2	2	1	2	2
2	1	3	1	2	1	2	1	1	2
2	9	2	1	3	2	3	1	1	3
1	1	1	1	1	1	1	9	9	9
1	2	1	1	1	1	1	1	1	2
2	3	9	1	3	1	2	3	1	3
9	3	1	2	3	3	1	2	2	3
9	9	9	9	9	9	9	9	2	9
3	3	3	3	2	1	1	3	3	3

2	1	1	2	1	2	1	2	3	2
3	3	3	3	2	9	9	1	9	9
3	3	3	3	3	3	3	3	3	3
2	2	3	3	3	3	2	1	1	2
3	3	2	2	2	3	2	2	1	2
2	1	1	2	1	2	1	2	3	2
1	3	3	2	1	1	1	2	3	3
2	2	1	2	1	2	2	2	2	2
1	3	3	3	3	2	2	1	2	2
3	1	1	3	3	3	1	3	3	3
2	2	2	2	2	2	2	2	2	2
2	2	2	2	2	2	2	2	2	2
9	9	9	9	9	9	9	1	9	9
2	2	2	2	2	2	2	2	2	2
3	3	3	3	2	3	2	2	1	2
1	2	2	3	1	3	2	1	2	2
3	3	3	3	3	1	3	3	3	3
3	3	3	3	3	1	3	3	3	3
3	3	3	3	3	2	2	2	1	3
3	2	3	3	1	3	3	1	3	3
3	3	1	2	2	2	2	1	1	1
1	3	3	3	3	3	3	1	3	3
3	1	3	3	2	3	3	3	2	2
3	3	2	2	2	3	1	3	3	3
2	2	2	2	2	2	2	2	2	2
1	2	1	2	2	3	3	1	2	3
2	2	2	2	2	2	2	2	2	2
2	2	2	2	2	1	3	3	3	3
1	1	1	2	1	3	1	1	3	3
1	2	1	1	2	1	1	2	3	3
3	3	3	3	3	1	1	3	3	3
3	3	2	2	2	3	1	3	3	3
3	3	3	2	2	1	3	9	2	1
3	1	2	2	1	3	3	1	1	2
1	2	2	9	3	1	2	1	3	1
3	3	2	3	1	2	2	1	2	2
3	1	1	1	1	3	1	1	1	2
3	3	3	3	2	2	2	2	9	2
3	3	3	3	2	2	3	3	2	3
1	3	2	2	2	3	3	2	1	2
1	1	3	1	3	3	1	3	3	3
3	3	2	2	1	1	3	3	3	3
3	3	3	3	3	9	1	9	9	9
3	3	3	1	1	3	1	1	9	3
9	9	9	9	9	9	1	9	9	9
9	9	9	9	9	9	1	9	9	9
9	9	9	9	9	9	1	9	9	9

3	1	3	3	1	3	1	2	1	2
3	2	2	1	2	2	3	2	2	1
3	3	3	3	3	3	3	1	3	3
3	1	2	3	3	3	3	2	1	2
1	1	3	1	1	1	1	1	3	1
9	9	9	9	9	9	1	9	9	9
9	9	9	9	9	9	1	9	9	9
9	9	9	1	9	9	1	9	9	9
9	9	9	9	9	9	1	9	9	9
1	9	9	9	9	9	9	1	9	9
1	9	9	9	1	9	9	1	9	9
1	1	1	1	1	1	1	1	9	9
9	9	9	9	9	9	1	9	9	9
3	1	3	3	1	3	3	3	1	3
3	3	3	2	3	3	3	2	1	1
2	2	3	3	3	2	1	2	2	2
1	1	1	1	1	2	2	1	2	2
9	1	9	9	9	9	9	9	1	9
9	9	9	9	9	1	9	9	9	9
9	9	9	9	9	9	1	9	9	9
3	3	1	2	2	9	9	9	9	9
9	9	9	9	9	9	1	9	9	9
1	1	1	1	1	2	2	1	1	1
9	9	9	9	9	9	9	1	9	9
3	3	3	3	3	2	2	1	2	2
2	2	2	2	2	2	1	2	2	2
2	3	2	2	1	3	3	1	2	1
2	1	1	1	1	2	1	2	2	2
2	2	2	1	1	1	1	1	2	2
3	3	3	3	3	2	2	1	2	2
1	1	3	3	3	1	1	1	1	1
2	2	2	2	1	3	1	3	3	3
1	1	1	2	2	2	2	2	1	2
9	9	9	9	9	9	9	9	9	9
2	2	2	2	1	1	1	2	1	1
2	2	2	2	1	2	1	2	2	2
2	2	2	2	1	2	1	2	2	2
9	9	9	9	9	9	9	1	9	9
1	3	2	3	1	1	2	2	3	3
3	3	1	3	1	9	9	9	9	9
1	3	9	3	3	2	2	2	2	2
3	1	3	3	1	2	3	3	1	3
3	3	3	3	1	3	3	1	3	3
1	1	1	9	2	2	2	2	2	2
2	2	2	3	3	1	3	3	3	3
3	3	3	3	1	3	2	2	3	1
3	3	3	3	3	3	2	2	1	2

3	2	3	3	3	3	3	3	2	1
1	1	2	3	1	2	3	3	2	2
9	9	9	9	9	3	3	3	3	3
3	3	3	2	1	2	1	2	2	2
3	3	3	3	3	3	3	1	2	3
9	2	9	9	9	9	1	9	9	9
3	3	3	1	1	3	2	1	1	3
3	3	3	3	3	3	1	2	1	3
9	2	9	9	9	9	1	9	9	9
3	2	2	2	3	2	1	2	3	3
9	2	9	9	9	9	1	9	9	9
3	3	3	2	2	3	3	1	2	1
1	1	1	2	1	2	1	2	2	2
1	1	2	9	1	2	1	1	2	3
3	1	9	2	1	3	1	2	2	2
2	2	2	3	3	3	1	3	1	3
3	2	2	2	2	2	2	2	1	1
3	3	3	2	3	2	1	2	3	3
3	3	3	3	3	3	3	3	1	2
9	1	9	9	9	9	9	1	9	9
3	3	3	3	3	3	3	3	1	2
3	3	2	3	1	3	3	3	1	2
3	3	3	3	3	2	2	2	2	2
3	2	3	1	2	1	1	1	1	1
3	3	3	3	3	2	2	1	2	2
3	3	3	3	3	1	1	1	1	1
1	1	3	3	3	3	3	1	3	3
3	3	9	2	1	1	1	3	1	1
2	3	3	3	1	2	1	2	2	2
3	1	1	3	3	3	3	3	1	1
3	1	2	3	1	3	1	2	1	3
2	1	2	2	2	1	2	2	2	2
2	2	1	1	1	2	1	1	1	1
1	1	1	1	1	2	2	2	1	2
2	2	2	2	2	2	2	1	2	2
1	2	9	2	2	2	2	1	2	2
2	2	2	2	1	2	1	2	2	2
2	2	2	1	1	2	2	1	2	2
2	2	2	2	2	2	2	1	2	2
2	9	1	2	2	2	2	2	2	2
3	3	9	3	3	3	3	3	1	3
3	3	3	9	1	2	2	2	2	2
2	3	3	3	3	3	3	2	1	2
1	1	2	2	2	2	1	1	1	2
1	1	3	3	1	3	3	3	3	3
3	3	3	3	3	3	3	3	3	3
3	3	3	3	3	2	2	2	1	3

2	2	2	2	1	1	2	2	3	3
3	3	1	3	1	2	2	1	1	1
9	9	9	1	9	9	9	1	9	9
3	3	3	1	9	9	9	9	3	9
9	9	9	1	9	9	1	9	9	9
3	1	3	3	3	9	9	1	1	9
9	9	9	9	9	9	9	1	9	9
3	3	2	2	3	2	3	2	1	1
2	2	2	2	2	2	2	2	2	2
3	3	3	3	1	3	1	1	3	1
3	1	3	3	1	1	1	1	9	3
3	1	1	1	1	3	3	1	1	1
1	1	1	2	2	2	2	1	1	1
1	1	3	2	2	2	2	2	2	2
1	1	1	1	1	1	1	2	1	1
9	9	9	9	9	9	9	9	9	9
3	3	3	3	3	2	2	2	2	2
3	3	3	3	3	9	9	9	9	3
2	2	3	1	2	9	9	9	9	9
1	1	1	1	1	2	2	2	2	2
2	1	1	1	1	2	1	1	1	1
1	3	2	2	2	3	3	2	1	2
2	2	2	2	2	1	2	2	2	2
2	2	2	3	1	2	2	2	2	2
2	1	2	3	2	2	1	1	1	2
3	3	3	3	3	3	3	1	1	3
9	1	9	9	9	9	9	1	9	9
2	1	2	2	2	2	2	1	2	2
3	3	3	2	3	3	1	2	3	3
3	1	1	3	3	2	2	1	1	1
9	9	9	9	9	9	9	9	9	9
2	2	1	3	2	1	1	2	3	1
3	1	2	1	2	1	1	1	2	2
2	2	2	2	2	2	2	2	2	2
2	2	2	2	2	3	3	3	2	2
3	2	2	2	1	1	2	2	3	3
1	1	1	3	3	3	3	1	3	3
3	3	1	2	3	3	2	1	2	3
3	3	2	2	3	1	1	2	3	3
3	3	3	3	1	1	1	2	3	3
1	1	2	2	2	2	2	1	2	2
3	3	3	3	2	1	1	3	2	3
3	2	3	2	2	3	2	1	1	3
9	9	9	9	9	9	9	9	1	9
9	9	9	9	9	9	9	1	9	9
3	2	3	2	2	3	2	1	1	3
3	3	3	3	3	3	3	1	3	3

3	3	3	3	3	1	3	3	2	3
2	2	2	1	1	2	1	2	2	2
9	9	9	9	9	9	9	1	9	9
3	3	3	3	3	1	9	9	9	9
3	9	9	9	9	9	9	9	9	9
3	2	3	2	1	2	2	2	1	2
3	3	3	3	3	2	2	1	3	3
3	3	3	3	3	3	3	3	1	3
3	2	9	3	2	3	2	1	1	3
3	1	1	2	3	1	3	3	3	3
9	9	9	9	9	9	9	1	9	9
9	9	9	9	9	1	9	9	9	9
2	2	2	2	2	2	2	2	2	2
2	3	2	2	2	1	9	3	9	9
1	9	9	9	9	1	9	9	9	9
3	1	3	3	1	3	3	1	1	1
3	1	1	3	3	3	1	2	3	1
3	3	3	1	2	1	1	1	3	3
1	3	2	3	3	1	3	1	2	3
3	2	2	3	1	3	2	2	1	3
1	2	1	3	3	1	1	2	3	3
3	3	3	1	1	3	3	3	1	3
1	2	1	2	3	1	1	3	3	3
9	9	9	9	9	2	2	2	1	2
3	3	3	3	3	1	2	2	1	1
1	1	3	3	1	3	2	2	2	1
9	9	9	9	9	9	9	9	1	9
3	2	2	3	1	1	3	2	9	1
3	3	3	2	3	2	3	9	9	9
3	2	3	3	2	1	1	1	2	2
3	3	3	3	3	2	2	1	1	3
1	1	1	1	1	2	3	3	3	3
2	1	3	2	1	2	2	1	3	2
3	2	3	3	3	3	1	1	2	2
3	2	3	2	3	3	1	3	2	3
2	1	1	2	1	2	2	2	2	2
9	9	9	9	9	2	2	2	2	2
3	3	3	3	2	2	2	2	2	2
9	9	9	9	1	9	9	1	9	9
3	2	3	2	3	3	1	1	2	3
3	3	3	3	1	2	2	3	2	1
1	1	3	2	1	2	2	2	2	2
3	2	1	1	3	1	2	9	1	3
3	1	3	2	1	1	2	1	2	3
3	3	3	2	3	2	2	2	1	2
3	3	3	3	3	2	2	1	1	3
3	3	3	3	3	2	2	2	1	3

3	3	3	3	3	3	3	3	1	3
9	9	9	9	9	9	9	9	9	9
1	3	3	3	3	9	1	9	9	9
3	3	3	1	1	3	3	1	1	3
3	3	3	1	3	3	1	1	3	3
3	3	3	1	1	3	3	1	3	3
3	3	3	9	9	2	3	9	2	1
2	1	1	1	2	2	2	1	1	1
3	3	3	3	1	3	3	1	3	3
3	3	3	3	3	2	2	1	1	3
3	3	3	3	3	2	2	1	1	3
3	1	2	2	3	1	2	3	2	1
3	3	3	3	2	3	2	3	2	3
2	1	1	1	2	2	2	1	1	1
1	1	2	2	1	2	1	2	1	3
1	2	1	2	3	2	2	2	2	2
3	3	2	2	1	2	2	2	2	2
1	1	1	3	2	3	2	1	1	2
3	1	3	3	3	3	3	1	1	1
2	2	2	2	2	1	2	2	2	2
3	3	3	3	3	1	1	1	3	3
3	1	2	1	1	3	2	1	3	1
3	3	3	3	3	2	2	2	2	2
3	3	3	3	3	2	2	2	2	2
1	2	2	1	3	2	2	1	2	2
1	2	1	2	3	1	2	3	3	2
3	3	3	2	3	3	3	2	1	2
3	3	3	3	3	3	3	3	3	3
3	3	3	3	3	3	3	1	1	3
9	9	9	9	9	9	1	9	9	9
3	2	3	3	3	3	2	1	2	3
1	2	1	3	2	1	1	1	1	1
3	2	2	2	3	2	3	1	2	2
3	3	3	3	1	2	2	2	2	2
1	1	1	1	1	1	1	1	1	1
2	2	2	2	2	2	2	2	2	2
1	3	3	3	1	3	1	3	3	3
3	2	3	2	3	2	1	1	2	2
3	3	3	1	1	3	2	1	2	9
2	1	1	1	2	1	2	2	2	2
2	2	2	2	1	9	9	1	9	9
2	3	3	3	3	3	3	2	3	1
3	3	1	2	3	2	2	1	2	1
3	3	3	3	3	1	3	3	2	3
2	2	2	2	2	2	2	2	2	2
2	3	3	3	3	2	2	2	2	2
3	3	3	3	3	3	3	1	1	2

3	2	3	2	3	2	3	3	1	2
2	3	1	1	3	2	2	1	3	2
2	2	2	2	1	2	2	2	2	2
1	2	2	1	1	1	1	1	1	1
1	2	3	2	2	3	1	1	3	1
3	3	3	3	3	2	2	2	2	2
3	3	3	3	2	3	1	2	2	2
3	3	3	3	2	2	2	2	2	2
3	1	3	3	2	2	2	2	2	2
2	2	2	2	2	2	2	2	2	2
9	9	9	9	9	9	2	9	9	9
2	3	2	3	1	9	1	2	3	3
9	9	9	9	9	9	9	9	9	9
1	2	3	2	2	3	1	1	3	1
2	2	3	3	2	2	2	2	2	2
3	3	3	3	3	9	9	9	9	9
2	2	2	2	3	2	2	1	2	2
3	3	1	1	3	2	1	3	2	3
3	3	3	1	1	3	1	1	3	1
3	3	3	3	3	2	2	2	2	2
3	3	3	9	9	1	1	1	1	2
1	2	3	3	3	3	2	1	2	3
9	9	9	9	9	9	9	1	9	9
2	1	3	3	1	2	2	2	2	2
2	2	3	1	1	3	3	1	1	1
3	3	3	2	1	2	2	2	2	2
9	9	9	1	9	9	1	9	9	9
3	2	2	2	3	2	3	3	3	2
2	3	3	2	1	3	2	2	2	3
9	9	9	9	9	1	9	9	9	9
3	3	3	1	1	2	2	1	1	2
2	2	2	2	2	1	1	1	1	1
3	3	3	1	1	9	9	1	9	9
1	3	2	3	3	1	1	3	3	3
3	3	3	3	3	1	3	3	3	3
3	1	9	9	9	3	3	2	1	2
3	3	3	9	1	3	1	3	3	3
3	3	2	9	3	3	2	1	1	3
1	1	1	1	1	9	9	1	9	9
3	3	3	3	3	3	2	1	2	2
2	3	2	3	1	3	3	2	1	2
1	3	3	2	1	3	2	1	2	3
3	3	3	3	2	2	2	2	2	2
2	2	2	3	1	3	1	2	2	2
3	2	2	2	3	1	3	3	2	3
2	2	3	3	3	2	2	1	2	2
3	2	3	3	3	3	3	1	3	3

3	3	3	3	3	3	3	3	1	2
2	2	2	2	2	2	2	2	2	2
9	9	9	9	9	9	9	1	9	9
9	9	9	1	9	9	9	1	9	9
3	1	2	2	1	1	1	2	2	2
3	3	3	3	3	3	3	3	3	1
3	3	3	3	2	2	2	2	2	2
2	2	3	2	3	3	3	1	2	3
3	3	1	2	1	3	1	2	1	3
3	3	3	3	3	3	1	3	3	3
3	2	2	3	1	3	2	2	1	1
1	1	2	2	1	2	1	3	1	3
2	2	2	2	2	2	2	2	2	2
3	3	3	3	3	3	3	3	3	3
2	2	3	2	3	2	2	1	3	3
1	2	1	2	1	3	3	2	1	1
2	3	9	2	3	3	2	1	3	3
3	3	3	3	1	1	1	3	3	3
1	1	3	3	3	2	2	2	2	2
1	3	1	2	1	2	2	1	1	2
3	2	2	3	2	3	3	1	3	3
1	2	2	2	2	3	1	2	2	2
1	2	2	2	2	3	1	2	2	2
3	1	1	3	1	3	1	3	3	3
3	3	3	3	1	3	3	1	1	3
3	3	3	3	3	3	3	3	1	3
3	3	1	1	3	3	1	2	3	3
3	3	1	1	3	3	1	2	3	3
3	2	2	3	3	2	2	2	2	2
2	2	2	2	2	3	3	3	1	3
2	3	3	2	2	1	2	3	2	2
1	2	2	1	2	3	1	3	2	2
3	3	3	3	3	3	3	3	1	3
9	1	3	3	3	1	2	2	2	2
3	3	3	3	3	2	1	2	2	2
3	3	3	3	3	3	3	3	3	3
3	3	3	3	3	3	3	3	3	3
2	3	1	2	1	3	2	2	1	3
1	2	1	2	2	2	2	2	2	2
2	2	1	2	3	2	2	2	2	2
1	3	1	3	3	3	3	1	2	2
3	3	3	3	1	3	3	3	3	1
1	1	2	1	1	1	1	1	1	1
2	2	2	2	2	1	2	2	2	2
2	2	2	3	2	2	2	2	2	3
3	3	2	2	1	1	3	3	3	3
3	1	3	3	3	2	2	1	2	3

3	1	2	2	2	2	1	2	3	2
2	2	2	2	2	2	9	2	9	2
2	1	1	2	2	2	2	2	2	2
2	2	2	2	2	2	2	2	2	2
2	2	2	2	2	2	2	2	2	2
2	2	2	2	2	1	2	2	2	2
2	1	1	2	1	2	1	3	2	3
3	3	1	3	2	1	2	1	3	2
1	3	2	2	2	2	2	2	2	2
2	3	2	3	3	2	2	2	2	2
2	2	2	2	2	2	2	2	2	2
2	2	2	2	2	2	2	2	2	2
2	2	2	2	2	2	2	2	2	2
2	2	2	2	2	2	2	2	2	2
1	3	3	1	2	2	2	2	2	2
2	2	2	2	2	2	2	1	2	2
3	3	1	2	1	1	2	2	2	2
2	2	2	2	2	2	2	2	2	2
3	1	2	3	1	1	1	1	3	3
2	2	3	2	3	3	1	2	3	2
2	2	2	2	2	2	2	2	2	2
2	2	2	2	2	2	2	2	2	2
2	2	3	3	1	3	3	3	2	3
2	2	2	2	2	2	2	2	2	2
2	2	2	2	2	2	2	2	2	2
2	2	2	2	2	9	9	9	1	9
2	2	2	2	2	2	2	2	2	2
2	2	2	2	2	2	2	2	2	2
2	2	2	2	2	2	2	2	2	2
3	3	3	3	3	3	3	3	1	3
3	3	3	3	3	2	2	2	2	2
2	2	2	2	2	2	2	2	2	2
2	2	2	2	2	2	2	2	2	2
3	3	3	2	2	2	2	2	2	2
2	2	2	2	2	2	2	2	2	2
3	3	2	3	2	3	3	2	1	1
3	2	2	2	2	2	2	2	2	2
2	2	2	2	2	2	2	2	2	2
2	2	2	2	2	3	3	1	2	2
2	2	2	2	2	2	1	2	2	2
3	3	2	1	2	1	1	2	3	3
2	2	2	2	2	2	2	2	2	2
2	2	2	2	2	2	2	2	2	2
2	2	2	2	2	2	2	2	2	2
2	2	2	2	2	2	2	2	2	2
2	2	2	2	2	2	3	2	3	2
2	2	2	2	2	2	2	2	2	2

2	2	2	2	2	2	2	2	2	2
2	2	2	2	2	2	2	2	2	2
3	3	2	2	2	3	2	1	2	3
2	2	2	2	2	2	2	2	2	2
2	2	2	2	2	2	2	2	2	2
2	2	2	2	2	3	3	3	1	2
3	3	3	3	2	2	2	2	2	1
9	9	9	9	9	9	9	1	9	9
3	1	3	3	1	3	1	1	3	3
3	3	3	3	2	2	2	1	3	3
3	2	2	3	1	2	2	1	2	2
2	2	2	2	2	2	2	2	2	2
2	1	2	2	1	2	2	2	2	2
9	1	9	9	9	9	1	9	9	9
3	3	3	3	3	3	3	3	1	3
2	2	2	2	2	2	1	1	2	2
2	2	2	2	2	2	1	2	2	2
2	2	2	2	2	2	2	2	2	2
3	1	3	3	1	1	3	3	3	3
2	2	2	2	2	2	1	2	2	2
9	9	9	9	9	9	1	9	9	9
2	2	2	2	2	2	2	2	2	2
2	2	2	2	2	2	2	2	2	2
2	2	2	2	2	2	2	2	2	2
2	2	2	2	2	2	2	2	2	2
2	2	2	2	2	2	2	2	2	2
9	9	9	9	9	2	9	9	9	9
2	2	2	2	2	2	2	2	2	2
2	2	2	2	2	2	2	2	2	2
2	2	2	2	2	2	2	2	2	2
2	2	2	2	2	2	2	2	2	2
3	3	2	3	2	3	3	2	3	2
3	1	3	3	3	3	3	1	3	3
3	3	3	3	1	3	3	1	3	3
3	2	2	3	1	3	1	1	3	3
3	3	3	3	3	3	2	1	1	3
3	3	3	2	1	3	3	2	1	1
3	3	3	3	3	1	1	1	2	3
3	2	2	3	2	3	1	1	2	3
3	2	3	2	2	3	2	2	2	2
3	3	2	3	2	3	3	1	1	2
1	1	1	2	1	3	3	1	3	3
1	1	1	2	2	2	2	1	1	3
2	2	2	2	2	2	2	2	2	2
2	1	1	1	2	3	3	1	9	9
2	1	2	2	1	2	2	2	1	1
2	3	2	3	3	2	1	2	2	1

3	3	2	2	1	2	1	3	2	1
1	2	9	1	2	1	2	1	3	1
1	1	1	2	2	1	1	1	2	2
2	1	2	1	2	3	2	3	3	3
2	2	3	1	2	2	1	2	3	1
3	1	2	3	1	2	1	3	2	3
3	1	2	3	1	3	1	2	1	2
9	1	9	9	9	9	9	9	2	9
2	2	2	2	2	2	2	2	2	2
1	2	2	2	2	2	2	2	2	2
2	2	2	2	2	2	2	1	2	2
2	2	2	2	2	2	2	1	2	2
3	3	3	3	3	3	3	3	3	3
2	2	2	2	2	3	3	1	2	9
3	3	3	3	3	1	3	3	3	3
2	2	2	2	2	2	1	2	2	2
2	2	2	2	2	1	2	2	2	2
3	3	3	3	3	2	9	1	3	3
3	3	3	3	3	3	2	1	2	3
3	2	3	3	2	3	9	1	1	2
3	3	3	3	3	3	1	2	3	3
9	9	9	9	9	9	9	1	9	9
3	1	3	3	1	3	3	1	1	2
9	9	9	9	9	9	9	9	9	9
2	3	1	2	1	1	2	2	1	3
3	2	3	1	1	1	3	3	1	2
3	3	3	3	3	3	3	1	3	3
9	9	9	9	9	2	9	9	9	9
2	2	2	2	2	1	2	2	2	2
1	1	2	2	1	9	1	9	9	9
1	1	2	2	1	9	1	9	9	9
2	2	2	3	3	3	1	2	3	3
2	2	2	2	1	3	1	1	3	3
2	2	2	2	1	3	1	1	3	3
2	2	2	2	1	3	1	1	3	3
2	2	2	2	2	2	2	2	2	2
3	3	3	3	3	2	1	3	2	3
2	2	2	2	1	3	1	1	3	3
2	2	2	2	1	2	1	1	3	3
2	2	2	2	1	3	1	1	3	3
2	2	1	2	2	2	2	2	2	2
3	3	3	3	2	3	3	3	1	3
2	2	2	2	2	2	2	2	2	2
2	1	2	3	3	2	2	3	1	3
9	1	9	9	9	1	9	9	9	9
1	2	2	2	1	1	1	1	2	2
3	2	2	2	2	2	2	2	1	9

3	2	2	1	2	2	1	2	2	3
3	3	1	2	1	1	2	1	3	2
3	3	3	3	3	3	3	3	1	2
3	3	3	3	3	2	1	2	2	2
2	2	2	2	2	2	2	1	1	1
2	2	2	2	2	2	2	2	2	2
2	2	2	2	2	2	2	2	2	2
3	3	3	3	2	3	3	3	3	3
3	3	3	1	1	3	1	3	3	3
2	2	2	2	2	2	2	2	2	2
1	1	2	2	2	2	2	2	1	2
1	2	1	1	1	1	1	2	1	1
3	3	1	1	3	1	3	3	2	3
2	1	2	3	3	2	2	1	3	2
3	1	3	2	2	3	3	1	3	2
3	1	3	2	3	3	1	2	1	3
3	3	1	1	1	3	1	3	3	3
1	1	3	2	2	1	3	3	2	3
2	2	2	2	2	2	1	2	2	2
3	3	1	1	2	1	3	2	1	3
3	2	3	3	1	2	1	1	2	3
1	1	3	1	3	1	3	3	2	3
9	9	9	9	1	9	1	9	9	9
3	3	3	3	3	3	3	3	1	3
1	2	3	1	1	2	1	1	2	2
2	2	3	3	2	2	2	2	2	2
3	2	2	3	1	3	3	1	3	2
3	2	2	3	3	3	2	1	1	1
9	1	3	2	3	1	2	3	1	2
3	3	3	3	3	1	9	9	9	9
3	2	2	2	2	2	2	2	2	2
2	2	1	2	2	2	1	2	2	2
3	3	1	1	3	1	1	1	1	3
3	3	3	3	3	3	3	3	3	3
2	2	2	2	2	2	2	1	2	2
3	2	2	1	1	2	1	3	3	2
3	3	9	3	1	1	3	3	3	3
2	2	2	2	2	2	2	2	2	2
1	1	2	1	1	2	2	1	1	1
9	9	9	9	1	9	1	9	9	9
1	3	3	3	1	2	2	2	2	2
3	2	3	2	1	1	2	1	3	1
3	3	3	3	3	3	2	1	1	3
3	2	2	3	1	1	2	2	3	3
3	1	1	1	1	3	2	1	1	2
3	3	1	3	1	2	2	2	2	2
3	3	3	3	3	9	9	9	9	9

3	1	2	2	3	2	1	2	2	1
2	3	2	3	1	2	1	1	3	3
3	3	3	3	3	1	3	3	3	3
3	3	3	3	1	3	1	3	3	3
3	3	3	3	3	9	9	9	9	9
2	2	2	2	2	2	3	3	3	1
3	3	2	1	2	1	1	2	2	3
2	2	2	2	1	2	2	9	2	2
3	3	3	3	3	3	2	3	3	3
3	3	9	2	1	2	2	3	2	3
3	3	3	3	1	1	1	2	3	3
2	3	2	2	2	2	2	2	1	2
2	2	2	1	1	3	2	2	1	2
3	1	1	1	1	3	3	1	1	1
2	2	2	2	1	3	3	2	1	3
2	2	2	2	2	2	2	2	2	2
1	1	2	2	2	3	2	2	2	1
1	3	1	2	2	2	2	2	2	2
3	3	3	3	3	3	2	1	1	3
1	2	1	9	1	3	2	1	1	3
2	2	2	2	2	1	2	1	2	2
9	9	1	9	9	9	9	2	9	9
3	2	3	3	3	2	2	2	2	1
3	3	3	3	3	3	3	3	3	3
3	3	2	3	3	3	3	9	9	9
2	2	2	2	2	2	1	2	2	2
9	9	9	9	9	3	1	3	2	2
3	3	3	3	1	2	2	2	2	2
3	3	3	3	3	2	3	3	3	3
2	2	2	3	2	2	1	2	2	3
1	1	1	1	1	3	3	1	1	3
9	9	9	9	9	3	1	3	3	3
2	2	2	3	3	2	3	2	2	3
3	3	2	2	1	3	3	1	1	1
2	1	2	2	1	2	3	2	1	2
3	3	3	2	2	2	2	1	1	1
2	3	1	2	1	3	9	9	1	9
3	1	1	1	1	3	1	2	2	3
2	2	3	3	3	3	2	1	2	3
9	9	9	9	9	9	9	2	9	9
2	2	2	2	1	1	2	2	2	2
3	2	2	1	2	3	3	2	1	1
2	1	1	2	2	3	3	1	2	2
3	1	3	1	1	3	2	1	2	1
2	1	3	3	1	2	1	3	2	3
3	3	3	3	1	2	1	1	3	3
3	2	2	3	1	3	2	1	2	3

3	2	2	3	1	3	2	1	2	3
3	2	2	2	2	2	2	2	2	2
2	2	1	2	3	2	2	3	3	3
2	1	2	9	9	1	2	1	2	1
3	3	3	3	3	2	2	2	1	2
1	1	3	3	3	3	1	1	3	1
2	3	3	3	1	1	9	9	9	9
9	9	9	9	9	9	9	1	9	9
3	1	3	3	1	2	1	2	2	2
3	1	3	1	3	1	9	1	1	1
2	2	2	2	2	2	2	2	2	2
3	3	3	3	2	3	3	1	2	2
3	2	3	3	3	9	1	9	9	9
3	2	2	3	1	1	2	3	3	3
9	2	9	2	9	9	2	2	9	9
3	2	2	1	2	2	2	3	3	3
1	1	1	1	1	1	1	1	1	1
2	3	3	1	2	2	2	1	2	2
3	1	3	3	3	2	3	1	1	9
3	1	3	1	2	3	2	1	2	3
3	1	3	1	1	2	3	1	1	2
3	3	3	3	3	1	3	3	3	3
2	1	1	2	2	2	2	2	1	2
3	1	3	3	3	3	3	2	1	2
3	3	3	3	3	1	3	3	3	3
1	1	1	1	2	2	2	2	1	2
2	3	3	2	3	2	1	2	1	3
3	3	3	3	1	3	3	3	1	3
3	3	3	3	3	3	1	3	3	3
3	3	3	3	1	1	1	3	1	3
1	2	1	2	1	2	1	2	1	1
2	1	1	1	1	1	2	1	1	2
1	1	1	1	2	1	1	1	2	1
1	2	2	2	1	2	2	2	1	2
3	1	1	1	3	3	3	1	2	3
3	3	2	3	1	3	2	1	1	3
2	2	9	1	1	2	2	1	2	2
3	3	3	3	3	3	3	3	1	3
2	1	1	1	2	1	2	2	2	1
2	2	2	1	1	1	2	2	2	1
3	3	3	3	3	3	3	3	3	3
3	1	3	3	3	3	1	3	1	3
3	3	3	3	3	3	3	1	3	3
3	3	3	3	3	3	3	3	3	3
2	3	3	3	2	2	3	1	3	3
1	1	1	1	1	2	2	2	2	2
3	2	2	2	2	2	2	2	2	2

3	2	2	2	2	2	1	3	3	3
1	1	1	3	3	3	3	2	1	3
2	2	2	2	2	2	2	2	2	2
2	1	2	3	2	2	1	1	2	3
3	3	3	3	3	3	3	1	3	3
1	1	1	1	2	2	1	1	3	1
3	3	3	9	2	3	3	1	3	3
1	2	1	2	2	2	2	2	1	2
3	3	3	3	3	2	2	2	2	2
3	3	3	3	3	2	2	2	2	2
2	2	2	2	2	2	2	2	2	2
2	2	2	2	2	2	2	2	2	2
2	2	2	2	9	2	2	2	2	2
3	3	3	3	3	3	3	3	1	3
3	3	3	3	3	2	2	2	2	2
3	3	3	3	1	3	3	1	3	3
3	3	3	3	3	3	3	3	1	3
3	3	1	3	2	2	2	1	2	2
2	2	2	1	1	2	2	2	2	2
1	1	2	3	2	3	3	3	1	1
2	2	2	2	2	2	2	2	1	2
3	3	3	3	3	2	2	2	2	2
3	3	3	3	3	3	3	3	3	3
2	2	3	3	3	3	3	2	3	3
1	1	2	1	2	2	2	1	2	3
2	2	2	2	2	2	2	2	2	2
1	3	2	1	3	1	2	3	1	3
2	1	1	1	2	3	3	3	2	2
3	3	3	3	3	9	9	9	9	9
3	3	3	3	2	3	3	2	1	1
3	3	3	3	3	2	1	2	2	2
2	2	2	2	2	2	2	2	2	2
2	1	3	1	3	1	3	1	1	2
2	2	1	1	2	2	1	2	2	2
3	3	3	3	1	3	3	1	2	3
2	2	1	1	3	2	1	1	2	2
3	3	3	3	1	1	3	3	3	3
3	1	2	2	3	2	2	3	3	2
3	3	3	1	3	3	1	1	3	3
3	3	3	3	3	3	3	3	3	3
2	2	2	2	2	2	2	2	2	2
2	2	2	2	2	2	2	2	2	2
2	2	2	2	2	2	2	2	2	2
3	3	3	2	3	3	3	3	3	3
3	3	3	2	3	3	3	3	3	3
9	9	2	9	2	2	9	2	9	2
2	2	1	1	1	1	1	1	2	2

1	2	2	2	1	1	1	2	2	2
2	2	1	1	1	1	2	2	1	1
2	2	3	2	2	2	2	1	1	2
2	2	2	2	2	2	2	2	2	2
2	2	2	2	1	2	2	2	2	2
2	2	1	1	1	2	2	2	2	1
2	3	3	2	2	2	2	1	1	1
2	1	1	1	1	2	1	1	1	1
1	2	2	1	1	1	1	1	2	2
2	1	1	1	1	2	2	2	1	1
2	2	3	3	2	3	2	1	3	3
2	1	2	2	2	1	3	2	3	3
2	2	2	2	2	2	2	2	2	2
2	2	2	2	2	2	2	2	2	2
2	2	2	2	2	2	2	2	2	2
2	2	2	2	2	2	2	2	2	2
2	2	2	2	2	2	2	2	2	2
2	2	2	2	2	2	2	2	2	2
2	2	2	2	2	2	2	2	2	2
2	2	2	2	2	2	2	2	2	2
2	2	2	2	2	2	9	2	2	2
2	2	2	2	2	2	2	2	2	2
2	2	2	2	2	2	2	1	2	2
2	2	2	2	2	2	2	2	2	2
2	2	2	2	2	2	2	2	2	2
2	2	2	2	2	2	2	2	2	2
2	2	2	2	2	2	2	2	2	2
2	2	2	2	2	2	2	2	2	2
2	2	2	2	2	2	2	2	2	2
3	3	3	3	3	3	3	3	3	3
2	2	2	2	2	2	2	2	2	2
2	2	2	2	2	2	2	2	2	2
2	2	2	2	2	2	2	2	2	2
2	2	2	2	2	2	2	2	2	2
1	9	1	9	1	1	2	1	2	9
3	1	9	3	1	9	3	1	3	1
9	1	9	1	9	1	9	2	9	1
9	2	9	2	2	2	9	2	9	2
9	9	9	9	9	9	9	9	9	9
1	1	1	1	1	1	1	1	1	1
9	1	9	3	1	9	1	9	9	1
2	1	1	1	2	2	2	2	1	1
1	2	1	1	2	1	2	3	2	1
2	2	2	2	2	2	2	2	2	2
9	2	9	1	1	1	9	2	1	1
9	9	9	2	2	9	2	9	2	9
3	2	1	1	2	2	2	2	2	2

2	2	2	1	2	2	2	2	2	2
1	2	2	2	2	2	2	3	2	1
2	2	1	2	2	2	2	2	2	1
1	2	1	1	2	1	1	2	1	3
1	2	2	2	1	1	2	1	2	3
2	2	3	3	2	2	2	2	3	3
1	1	1	1	1	1	1	1	1	1
2	2	2	2	2	2	2	2	2	2
1	2	1	1	2	2	1	2	1	2
1	2	1	1	2	2	1	2	1	2
2	2	1	1	1	2	1	1	2	2
3	2	2	1	1	2	2	1	2	2
2	3	2	2	1	2	2	1	3	3
2	2	2	1	1	1	1	1	9	9
2	2	2	2	3	2	2	1	3	3
3	2	2	2	3	2	2	1	3	3
3	2	2	2	3	2	2	1	3	3
2	1	2	2	2	1	2	2	2	1
1	2	1	1	2	2	2	1	1	2
2	1	1	2	2	2	2	1	1	2
2	2	1	1	2	2	2	2	1	1
2	3	2	2	1	2	2	2	1	1
2	2	2	2	3	2	2	2	2	2
2	2	2	1	2	2	2	3	3	2
2	2	3	2	2	2	2	3	2	2
3	3	2	1	1	3	2	1	1	2
3	3	2	2	2	3	2	2	1	2
3	2	2	2	2	2	3	3	3	3
2	2	2	2	2	3	3	2	2	3
2	2	2	2	2	2	2	2	2	2
2	2	2	2	2	2	2	9	2	2
2	2	2	2	2	2	2	2	2	2
2	2	1	2	1	2	2	2	2	2
2	2	2	2	2	2	2	2	2	2
2	2	2	2	2	2	1	2	2	2
2	2	2	2	2	3	3	3	3	3
3	2	2	3	2	2	3	2	1	2
1	2	2	2	2	2	2	2	2	2
2	3	2	1	2	2	2	1	2	2
2	2	3	2	1	3	2	1	2	3
1	1	2	2	2	2	2	1	2	2
3	3	3	3	3	3	3	3	3	3
2	1	1	2	2	1	1	1	2	2
3	3	2	2	2	2	1	1	2	2
2	2	2	2	2	1	2	2	1	2
2	1	1	2	2	1	1	2	2	1
2	2	2	2	1	2	2	1	1	2

2	2	2	2	2	2	2	2	2	2
1	2	2	2	2	1	2	2	2	2
2	1	2	2	2	2	2	1	2	2
1	2	2	2	2	2	2	2	2	3
2	3	2	3	2	2	3	1	2	3
3	1	2	3	3	3	2	2	2	2
3	3	3	3	3	2	2	1	2	3
3	3	3	2	3	3	2	1	3	3
1	1	2	2	1	1	1	2	3	3
3	3	3	3	2	2	2	2	2	2
3	2	3	3	3	3	3	2	3	2
3	1	3	2	9	2	1	3	3	3
1	2	3	2	1	3	2	1	2	1
3	1	2	3	2	2	2	2	2	2
2	3	2	3	2	2	3	1	2	3
2	2	2	2	1	2	9	1	2	3
2	2	2	2	1	2	2	2	2	2
2	1	1	2	1	2	2	2	1	2
3	3	3	3	3	3	3	3	3	9
2	2	2	2	2	2	2	2	2	2
3	2	3	2	3	3	3	3	1	2
3	2	2	2	2	2	2	2	2	2
3	1	2	2	2	2	3	9	1	3
2	2	2	3	3	3	2	1	1	3
3	3	3	3	3	3	2	2	2	2
3	2	3	3	3	2	2	1	3	3
3	1	2	3	2	2	1	3	2	3
3	3	3	3	1	3	3	3	3	1
1	1	1	1	2	2	2	2	2	1
3	2	3	1	1	2	3	2	3	2
3	2	1	3	1	3	2	1	1	2
2	2	3	3	2	2	1	3	3	3
2	2	2	2	2	2	2	2	2	2
2	2	2	1	1	2	2	2	2	2
3	2	2	3	3	2	1	2	1	3
3	3	3	3	1	1	3	1	3	3
2	1	2	3	1	3	3	2	1	3
3	3	3	3	2	2	2	2	1	1
3	3	3	2	1	3	3	1	2	3
3	3	3	2	1	3	3	3	1	3
1	3	2	2	1	1	1	1	1	3
2	2	2	2	2	2	2	1	1	2
3	3	3	2	3	2	2	2	2	2
2	2	2	1	1	2	2	1	2	3
2	2	1	1	2	2	2	1	2	2
2	1	2	2	3	2	2	3	2	3
2	1	9	3	3	2	2	3	2	3

3	3	3	3	1	3	3	2	1	1
2	1	3	2	1	3	1	1	2	3
2	3	3	2	1	3	2	2	1	1
3	2	2	1	1	2	1	2	2	2
3	3	3	1	1	3	1	2	2	2
3	1	3	2	1	3	2	1	2	3
2	2	2	2	1	2	2	1	2	2
1	1	2	1	3	2	3	2	2	1
3	3	3	3	1	2	2	2	1	3
9	9	9	9	9	2	9	9	9	9
3	1	1	2	3	1	2	3	1	3
1	1	2	2	3	2	3	2	3	2
1	3	2	1	3	2	2	1	1	3
3	2	2	3	3	1	2	3	3	3
3	3	2	2	2	1	1	1	2	2
2	2	2	2	2	2	2	2	2	2
3	1	2	2	3	2	2	2	2	2
3	1	2	2	3	2	3	2	3	2
3	3	3	3	3	3	3	3	3	3
3	3	3	3	3	3	3	3	3	1
3	3	3	3	3	3	3	3	3	3
3	3	3	3	3	3	3	3	3	3
2	1	3	2	3	2	2	3	2	1
2	1	9	2	1	2	2	3	3	3
2	2	2	2	2	2	2	2	2	2
3	1	3	3	1	1	3	2	3	3
2	3	3	2	2	1	2	2	3	3
2	2	2	2	2	2	2	2	2	2
2	3	2	3	2	2	3	3	1	2
1	1	3	2	2	2	3	1	2	3
3	3	3	3	3	3	2	1	3	3
3	3	2	2	1	3	2	1	2	3
3	3	3	2	2	2	2	2	1	3
3	3	3	3	3	3	3	3	3	3
3	3	3	2	1	1	3	2	2	2
3	1	2	2	2	3	2	1	2	3
1	2	2	3	1	3	1	1	2	3
3	1	2	2	2	3	2	1	2	3
2	2	2	2	2	2	2	2	2	2
2	3	2	2	1	2	2	2	1	2
2	1	3	2	1	1	1	2	3	2
3	3	3	3	2	3	3	3	3	3
2	2	2	2	2	2	2	2	2	2
3	3	3	3	3	1	1	3	3	3
3	3	3	3	3	3	3	3	3	3
3	3	3	3	1	2	1	3	3	3
1	2	2	2	1	2	2	1	1	2

2	3	2	1	2	2	2	1	2	9
3	3	9	3	1	3	1	3	1	3
3	2	2	3	3	3	3	2	1	1
2	2	2	2	2	2	2	2	2	2
1	3	3	2	2	3	2	1	2	2
9	9	9	9	2	9	9	2	9	9
2	2	2	2	2	2	2	2	2	2
2	2	2	2	2	3	3	2	2	3
3	3	3	3	3	2	2	1	1	3
3	2	2	3	2	3	2	2	2	3
1	2	2	1	1	2	2	1	2	2
3	2	2	1	1	3	2	2	1	3
9	9	9	9	9	9	9	1	9	9
9	9	9	1	9	9	9	1	9	9
9	9	9	1	9	9	2	9	9	9
2	2	2	1	1	2	1	2	2	2
2	2	2	2	2	1	2	2	2	2
3	1	2	3	1	1	3	3	3	3
3	3	3	3	3	2	1	2	3	3
3	2	2	2	2	3	2	2	2	3
3	2	1	1	3	2	3	9	1	2
3	3	3	9	3	3	3	1	2	3
3	2	2	2	2	2	2	1	3	3
2	2	2	2	2	2	2	2	2	2
2	3	1	3	1	3	3	1	1	1
3	2	1	1	2	1	1	1	2	1
2	3	1	3	1	3	3	1	1	1
9	2	9	9	9	9	9	9	1	9
1	2	2	2	2	2	2	2	2	2
3	3	3	3	1	3	3	1	3	3
3	3	3	3	1	2	1	3	1	2
3	3	3	2	2	3	3	2	1	2
3	3	3	3	3	3	3	3	3	3
3	3	3	3	2	2	1	2	2	2
3	3	3	3	3	3	3	1	2	2
2	2	2	2	3	2	2	2	1	3
3	2	2	2	3	2	1	2	3	3
2	2	2	2	2	3	3	3	1	3
1	2	3	2	1	3	1	3	2	3
3	3	3	3	3	3	3	2	1	1
2	3	2	2	1	2	2	2	2	1
2	3	2	2	1	3	3	1	2	9
2	2	2	3	2	3	2	2	1	3
3	2	3	3	1	1	1	1	1	2
1	1	2	2	2	2	2	1	2	2
1	2	2	2	2	2	2	1	2	2
2	2	2	2	2	2	2	1	9	9

1	1	1	2	1	2	2	2	1	1
2	2	2	2	1	3	2	2	2	2
9	2	9	3	1	3	2	9	1	9
3	3	3	3	1	2	1	3	2	2
3	3	2	3	1	2	1	2	2	2
1	3	2	9	1	3	3	2	2	2
3	3	3	3	3	1	2	3	3	3
1	3	2	3	1	3	2	1	1	3
3	3	3	3	1	3	3	3	1	3
2	2	2	2	2	2	2	2	1	9
1	1	2	2	1	2	2	2	1	3
9	9	9	9	9	9	1	9	9	9
3	2	2	2	1	3	3	2	1	1
2	2	2	2	1	2	2	2	2	3
9	9	9	9	9	9	1	9	9	9
9	9	1	9	9	9	9	9	1	9
3	3	3	9	3	1	2	3	3	3
2	3	2	3	2	2	1	2	1	3
3	3	3	9	1	3	3	3	1	1
3	3	3	3	3	3	3	1	3	3
2	3	2	3	1	3	3	2	1	1
3	1	3	3	3	1	9	9	9	9
3	3	3	3	3	2	3	3	3	3
3	3	3	2	3	3	2	1	1	2
2	2	2	2	2	3	3	2	1	3
1	3	2	3	2	2	2	2	2	2
9	9	9	9	9	9	9	9	1	9
1	2	3	2	1	3	2	1	2	3
3	3	3	2	3	3	3	1	2	3
2	3	9	9	9	9	9	9	1	9
2	2	1	1	1	9	9	2	9	9
3	3	3	3	3	3	1	2	3	3
3	3	3	3	3	1	1	3	3	3
3	3	3	3	1	3	3	3	1	1
9	9	9	9	9	1	9	9	9	9
2	1	3	1	2	2	3	1	3	3
3	3	3	3	3	3	1	2	2	2
2	2	2	2	1	2	2	2	2	1
2	2	2	2	3	3	2	1	2	2
2	2	1	1	1	1	2	2	2	2
9	9	9	9	1	1	2	2	2	2
3	3	3	3	1	3	1	3	3	3
3	2	2	1	3	2	2	1	2	2
3	2	1	3	3	2	1	2	3	3
3	2	2	3	1	2	2	2	3	3
3	3	2	3	1	2	2	3	1	1
3	2	2	3	1	2	1	2	3	3

3	3	3	3	3	1	1	3	3	3
2	2	1	1	3	1	1	1	3	1
9	9	9	9	9	1	9	9	9	9
9	9	9	9	1	9	9	9	9	1
9	9	9	9	9	9	9	1	9	9
9	9	9	9	9	1	9	9	9	9
3	3	3	3	1	3	1	3	3	3
3	3	3	3	3	2	2	2	2	2
2	3	2	2	1	2	2	1	2	2
3	3	3	3	2	1	3	3	3	3
3	3	2	3	2	3	3	1	1	1
3	3	3	3	3	3	3	3	1	3
2	1	1	1	1	2	2	2	2	2
2	1	2	2	2	3	3	2	1	3
3	2	2	2	2	3	3	3	3	3
9	9	9	9	9	9	1	9	9	9
3	3	3	3	1	3	3	3	1	3
3	3	3	3	1	3	1	2	3	3
3	3	3	3	3	3	1	2	2	2
9	9	9	9	1	9	2	9	9	9
1	9	9	9	9	9	9	1	9	9
3	3	3	3	3	9	9	9	9	9
3	3	3	2	3	2	1	1	1	1
3	2	3	2	3	3	2	2	1	1
1	9	9	9	9	9	9	1	9	9
9	9	9	9	9	9	9	1	9	9
3	3	3	3	3	1	1	2	3	3
3	3	3	3	3	1	2	3	3	3
3	3	3	3	3	2	1	3	3	3
1	3	1	2	1	1	1	1	1	1
3	3	3	3	2	3	1	2	2	2
2	1	3	2	2	2	2	2	2	2
2	2	2	2	2	2	2	2	2	2
3	3	3	3	3	3	3	3	3	3
3	3	3	3	2	3	3	3	2	2
3	3	3	3	3	3	1	3	3	3
3	3	3	3	3	2	2	2	1	2
2	2	2	2	2	1	1	1	1	1
2	2	2	2	2	2	1	2	2	2
2	2	2	2	2	2	3	1	1	3
1	2	1	2	1	1	1	2	3	3
1	1	1	2	2	1	2	2	2	2
2	2	2	1	2	2	2	1	2	2
9	9	9	9	1	9	2	9	9	9
9	9	9	9	1	2	9	9	9	9
3	2	3	3	3	3	3	1	1	2
2	2	2	1	3	2	1	2	2	2

1	1	2	1	1	1	2	1	2	1
3	3	3	2	2	2	2	2	2	1
2	2	2	2	2	2	2	1	2	3
2	3	3	2	2	2	2	2	2	2
2	1	1	2	3	3	3	2	2	2
3	3	1	2	1	2	3	1	2	1
3	3	2	2	1	2	1	2	2	2
2	3	1	2	3	1	2	3	1	2
9	9	9	9	9	9	9	9	9	9
2	9	1	2	1	2	2	1	2	1
2	2	2	2	2	2	2	2	2	2
3	1	2	2	1	2	1	1	2	3
2	3	1	2	3	1	3	2	3	2
2	2	2	2	2	2	1	2	2	2
2	2	2	2	2	2	2	2	2	2
2	2	2	2	2	3	1	2	2	2
2	3	1	3	2	1	2	3	1	2
2	2	2	2	2	3	1	2	3	2
3	2	2	1	3	3	3	2	2	2
1	1	1	3	2	3	3	3	3	1
1	1	1	2	3	1	1	1	1	1
2	2	2	2	2	3	3	3	3	3
2	1	1	1	1	2	2	2	3	3
3	2	2	1	2	1	3	3	3	3
2	2	2	2	2	2	2	2	2	2
3	1	2	3	2	3	2	3	2	3
1	1	2	3	1	2	2	2	2	3
3	2	2	3	2	3	2	1	2	1
3	3	3	2	2	2	1	1	3	3
2	3	3	2	3	3	2	1	1	2
9	2	9	1	2	2	1	2	3	2
3	1	3	3	9	9	9	9	9	9
3	3	3	3	3	3	3	3	3	3
1	3	1	1	2	2	1	1	1	2
1	1	1	1	1	1	1	2	2	2
3	3	2	1	2	2	1	1	2	2
3	2	1	1	1	3	2	2	1	2
3	1	2	1	2	1	2	2	3	1
2	2	2	2	2	2	2	2	2	2
1	1	1	1	1	2	2	2	3	3
3	1	1	3	1	2	3	1	3	3
9	9	9	9	9	9	9	2	9	9
2	1	2	2	2	1	2	2	2	2
3	2	2	3	3	3	2	1	3	3
2	2	2	2	2	2	2	2	2	2
3	3	3	3	3	3	3	1	3	3
3	3	3	3	3	1	2	3	3	3

3	3	2	3	9	3	1	9	9	9
3	3	3	2	2	1	2	2	2	1
9	2	1	1	1	3	1	3	3	3
3	1	3	1	3	3	3	3	1	3
2	1	3	3	3	3	2	3	3	3
2	2	2	2	2	2	2	2	2	2
2	3	2	3	2	3	3	3	3	3
1	1	1	3	2	2	2	2	2	2
9	1	3	2	9	9	9	1	2	3
3	3	3	3	3	2	2	2	2	2
3	3	3	3	3	2	2	2	2	2
2	3	1	2	1	3	2	1	2	3
3	2	3	2	2	9	9	9	9	9
3	3	3	3	1	3	3	1	2	3
2	2	2	2	2	3	3	3	1	3
1	3	3	1	1	3	3	1	1	1
3	3	1	3	3	9	9	1	9	9
2	3	3	2	2	1	2	3	1	2
2	3	2	3	3	2	2	2	1	3
1	2	2	2	2	2	2	1	2	2
2	1	2	1	2	1	2	1	2	1
3	3	3	3	3	3	3	3	3	3
3	2	3	1	1	2	2	1	3	3
3	2	2	1	3	3	2	2	2	1
2	3	2	2	3	3	2	3	1	2
3	3	3	3	3	3	3	1	3	3
3	3	3	3	3	3	3	3	3	3
2	2	2	3	1	2	3	1	1	2
2	3	1	3	1	3	2	1	1	3
3	3	3	3	3	2	2	1	1	2
3	3	3	3	2	2	2	2	2	2
9	2	3	2	2	2	2	1	2	2
2	1	2	2	1	1	2	2	2	2
3	3	3	3	3	2	2	2	2	2
3	3	3	3	3	3	1	2	1	3
9	9	9	9	9	9	9	1	9	9
3	3	3	3	2	3	2	2	2	3
3	2	2	2	1	1	2	3	3	3
9	9	9	9	1	1	9	9	9	9
3	2	2	3	3	2	2	1	2	2
3	3	3	3	2	3	2	2	2	3
2	2	1	1	2	3	2	3	1	2
2	2	2	2	2	2	2	2	2	2
1	1	1	1	1	2	2	2	2	2
3	3	3	3	3	9	9	9	9	9
1	3	1	2	3	1	2	3	2	1
9	9	9	9	9	3	3	3	3	3

1	1	1	1	1	2	2	1	2	2
2	2	2	2	2	2	2	2	2	2
3	3	3	3	3	3	3	3	3	3
9	9	9	9	9	9	9	9	9	9
2	2	1	1	1	2	2	1	3	3
1	2	1	3	2	1	3	2	9	1
2	2	2	2	2	2	2	2	2	2
9	9	9	9	9	2	3	1	3	2
3	3	3	3	3	3	3	3	3	3
3	1	3	3	3	3	1	3	3	3
2	1	1	3	3	3	3	3	3	3
3	3	9	9	3	3	1	3	1	2
1	3	3	3	2	3	1	3	9	2
3	1	1	1	1	2	2	2	2	2
2	2	2	2	2	2	2	2	2	2
3	3	3	3	3	2	2	2	2	2
9	9	9	9	1	9	9	9	2	9
9	9	9	9	9	1	9	9	9	9
9	9	9	9	9	9	9	9	9	9
9	9	9	9	9	2	9	9	9	9
2	2	2	2	2	2	2	2	2	2
2	2	2	2	2	3	3	3	3	3
3	2	3	2	2	2	2	1	1	1
3	3	3	3	9	3	3	3	3	3
1	1	1	1	1	1	2	3	2	2
2	2	2	2	1	9	9	1	9	9
9	9	9	9	9	9	9	9	9	9
1	2	2	2	2	2	2	2	3	3
9	9	9	9	9	9	9	9	9	9
9	9	9	9	9	9	9	9	9	9
9	9	9	9	9	9	9	9	9	9
9	9	9	9	9	9	9	9	9	9
9	9	9	9	9	9	9	9	9	9
3	3	3	2	2	3	3	3	1	3
9	2	3	2	1	2	1	3	2	1
3	1	3	3	2	3	2	1	2	2
2	9	3	1	3	2	2	2	2	1
3	3	3	3	3	9	9	9	9	9
9	9	9	9	9	1	9	9	9	9
2	1	1	2	2	1	1	1	3	3
2	2	2	2	2	2	2	2	2	2
2	2	2	2	2	2	1	1	2	2
2	2	2	2	2	1	2	2	2	2
2	2	2	2	2	2	2	2	2	2
2	2	2	1	1	2	1	2	1	2
2	3	2	2	1	1	2	1	2	2
3	1	2	2	1	1	1	2	3	3

2	2	2	2	2	2	2	2	2	2
2	2	2	2	2	2	2	2	2	2
3	3	2	3	3	1	1	9	1	3
1	1	2	2	3	1	2	3	1	2
2	3	1	2	3	1	2	3	1	2
1	1	1	1	1	1	1	1	1	1
9	9	3	1	2	1	2	9	1	2
2	2	2	2	2	2	2	2	1	2
2	2	2	2	2	2	2	2	2	2
2	2	2	2	2	2	2	2	2	2
3	3	3	3	3	3	1	3	3	3
9	2	2	2	1	2	1	1	2	2
2	2	1	2	1	3	1	2	1	2
3	3	2	3	3	2	2	3	3	3
1	2	3	3	3	1	3	3	3	3
3	3	1	3	2	3	3	3	3	1
2	3	1	2	3	1	2	2	3	3
3	3	3	3	3	3	2	2	3	3
3	9	2	1	2	2	9	2	9	1
3	1	3	3	1	3	2	1	2	3
9	9	9	9	9	9	9	9	1	9
3	9	2	9	1	3	3	3	3	3
2	3	1	2	2	2	9	1	3	2
9	9	9	9	9	9	9	9	9	9
9	2	9	9	9	9	2	9	9	9
9	9	9	9	9	1	9	9	9	9
3	1	1	2	1	3	2	1	3	3
3	2	3	3	3	2	3	3	2	2
9	9	9	9	9	9	9	9	2	9
2	3	3	2	1	2	2	3	3	2
3	1	3	1	1	1	3	1	3	1
3	3	3	3	1	3	1	1	2	3
3	3	2	2	1	1	2	2	3	3
2	2	2	2	2	1	1	1	1	1
2	1	1	2	2	3	2	1	1	2
2	2	1	2	2	2	2	2	2	2
3	3	1	3	1	3	3	3	1	3
2	2	2	2	2	2	2	2	2	2
3	2	2	2	2	3	2	1	2	3
3	1	2	3	2	3	3	3	2	1
9	9	9	9	9	2	9	9	9	9
3	2	3	3	3	1	1	3	3	3
2	2	2	2	2	3	3	1	1	3
3	3	3	3	1	1	3	2	3	3
9	1	9	9	9	9	1	9	9	9
1	1	1	1	1	1	1	1	1	1
9	9	9	9	9	1	2	2	2	2

3	2	3	3	2	1	9	3	9	1
2	3	2	3	3	2	2	2	2	3
3	3	3	3	3	3	3	1	1	3
2	3	2	2	1	3	2	1	1	1
2	2	2	2	2	2	2	2	2	2
9	9	9	9	9	9	9	9	9	9
2	2	3	2	3	3	1	2	2	3
3	3	3	3	3	2	2	2	2	2
2	3	3	2	3	3	2	1	2	3
3	3	3	3	1	2	2	1	2	2
3	3	3	3	3	1	3	1	3	3
2	1	1	3	2	1	1	3	3	2
2	2	2	2	2	9	9	2	9	9
3	3	3	3	3	2	2	1	1	2
1	1	1	1	1	3	3	3	1	3
3	3	3	3	3	2	1	1	3	3
3	3	3	3	3	3	3	3	3	3
2	2	2	2	2	2	1	2	2	2
2	3	2	3	2	3	2	2	3	3
2	2	2	2	2	2	2	2	2	2
1	2	2	1	1	2	2	1	2	2
3	3	3	3	2	2	2	2	2	2
3	3	3	3	3	1	3	1	1	1
2	2	2	3	1	2	1	2	1	1
1	1	1	1	1	1	1	1	1	1
3	9	9	9	9	1	9	9	9	9
3	3	3	2	3	2	3	1	3	3
2	3	3	3	2	2	2	3	2	3
9	9	9	9	9	3	1	3	3	3
2	2	2	3	3	3	2	2	2	2
3	3	2	3	3	3	1	2	2	2
9	9	9	9	9	1	9	9	9	9
2	2	2	2	2	2	2	2	2	2
3	2	1	3	2	1	2	3	9	1
1	3	1	2	1	1	3	1	2	2
3	3	1	2	3	3	3	1	1	3
2	2	2	2	2	2	2	1	2	2
3	1	3	3	3	3	3	3	3	3
3	3	2	2	3	2	9	9	9	9
2	1	1	2	2	2	2	2	2	2
3	1	9	3	2	3	3	1	3	2
3	3	2	2	3	2	2	3	2	3
2	2	2	2	1	3	2	3	1	1
1	1	1	9	9	9	9	9	9	9
3	3	3	3	3	3	3	1	3	3
3	3	3	2	2	1	2	3	3	3
1	2	2	2	2	2	1	2	2	2

2	3	3	1	2	3	3	9	2	2
9	9	9	9	1	9	9	1	9	9
9	9	9	1	2	9	9	9	9	9
2	1	2	2	2	2	2	2	1	3
3	3	3	3	3	1	3	3	3	1
9	9	9	9	9	9	9	9	9	9
9	9	9	9	9	9	9	9	1	9
3	3	1	1	2	3	3	3	2	1
3	3	3	3	3	1	2	3	3	3
3	3	3	3	2	2	2	2	2	2
3	2	3	3	1	3	2	1	2	3
3	2	2	2	2	2	2	1	3	3
2	2	2	2	2	1	1	1	1	1
2	2	2	2	2	1	1	1	1	1
1	2	3	2	2	2	1	2	2	2
2	2	2	3	2	2	2	2	3	2
3	3	3	3	3	2	2	2	3	3
3	3	3	2	1	3	3	1	2	2
3	3	3	3	3	1	3	2	3	2
3	2	2	2	1	1	2	3	3	3
2	2	2	1	2	2	2	1	2	2
1	1	1	1	1	1	1	1	1	1
9	1	9	9	9	9	9	1	9	9
2	2	2	2	2	2	2	2	2	2
3	3	3	3	3	9	9	9	1	9
1	1	1	1	1	2	2	1	2	2
3	3	1	3	1	3	1	3	3	3
2	1	2	2	1	1	1	1	2	2
2	2	3	1	3	3	2	1	2	3
3	3	3	2	3	1	2	2	2	3
2	1	2	2	2	2	2	1	2	1
2	1	3	9	1	2	2	2	2	2
3	2	3	2	1	2	1	1	3	3
2	1	1	3	3	2	3	2	2	3
3	9	2	3	3	1	2	2	3	3
2	2	2	2	2	2	2	2	2	2
2	3	2	1	3	1	2	1	3	2
2	2	2	2	2	1	2	2	2	2
2	2	2	3	3	3	2	2	1	2
3	3	3	3	3	9	9	1	9	9
2	2	2	2	1	9	9	9	1	9
2	3	2	1	2	2	3	1	2	3
3	3	2	3	1	3	3	3	1	2
3	3	3	2	2	2	2	2	1	1
2	1	1	1	1	1	2	2	1	1
2	2	2	2	2	2	2	2	2	2
3	2	2	3	2	2	2	2	2	2

3	3	3	2	2	2	1	2	2	2
1	9	9	9	2	3	1	2	9	1
9	9	9	9	9	9	9	9	1	9
3	3	3	3	3	3	3	1	1	1
3	3	3	3	1	2	1	2	2	2
1	2	3	2	1	2	2	2	2	2
3	3	3	3	3	3	3	3	3	3
2	1	1	3	1	3	1	2	3	3
1	2	9	2	3	2	1	2	3	9
3	3	3	3	3	3	3	3	2	3
3	3	2	1	1	3	1	2	2	3
3	3	3	3	3	3	1	1	2	2
3	3	3	3	3	3	3	3	2	9
1	1	1	1	3	3	3	1	3	2
3	3	3	3	3	1	2	3	3	3
3	3	3	3	3	1	2	2	2	2
1	1	1	1	2	2	2	1	2	2
9	9	9	9	9	9	1	9	9	9
3	2	2	2	2	2	2	2	2	2
2	1	2	1	2	1	1	2	1	2
2	1	2	3	2	1	2	1	1	1
3	3	2	3	3	1	1	1	2	3
2	2	2	2	2	3	3	1	2	3
1	3	2	3	1	1	2	1	2	1
3	3	2	3	1	3	2	1	1	1
9	9	9	9	9	2	2	1	3	3
3	2	2	3	2	2	1	2	2	2
3	3	3	2	3	3	3	3	1	2
3	3	2	3	3	3	2	1	3	3
2	2	1	1	1	3	3	2	1	1
3	3	3	3	3	3	2	3	3	3
3	3	3	3	3	3	2	3	2	2
2	1	1	2	1	1	1	1	2	2
3	1	2	3	1	1	2	3	1	2
3	3	3	3	1	1	3	3	3	3
2	1	9	9	9	1	3	2	2	1
2	2	3	2	3	2	2	1	1	2
2	2	2	2	2	2	2	2	2	2
3	1	1	1	2	3	1	1	2	3
2	2	2	2	2	2	2	2	2	2
3	3	3	3	1	3	2	1	3	3
1	2	3	3	2	3	1	2	3	1
9	9	9	9	9	9	9	9	9	9
3	3	3	3	1	1	3	3	3	3
2	2	2	2	1	3	3	1	1	2
3	2	3	3	1	3	3	1	3	3
3	3	3	3	3	1	2	3	3	3

1	1	1	1	1	3	3	1	3	2
3	3	3	3	3	2	2	1	1	2
2	2	2	2	2	2	2	2	2	2
3	3	2	2	1	3	1	1	2	3
3	3	3	3	3	3	3	3	3	3
2	2	2	2	2	2	2	2	2	2
1	1	3	1	1	3	3	3	1	3
1	1	1	1	1	1	1	1	1	1
2	2	1	1	2	3	3	1	1	1
1	2	1	3	1	1	3	3	3	3
2	2	2	2	1	9	9	1	9	9
3	3	3	3	3	2	2	2	1	3
3	1	3	3	3	1	3	3	3	3
3	1	3	3	3	1	3	3	3	3
3	3	3	2	3	3	3	1	2	3
2	3	2	3	2	2	1	2	2	3
2	1	2	2	1	2	1	1	2	3
3	3	3	3	3	2	3	3	2	3
3	3	3	3	1	1	1	1	2	3
1	1	1	1	1	1	9	1	1	1
3	3	3	3	3	1	3	3	3	3
9	9	9	9	3	9	9	9	9	3
9	9	9	9	1	9	9	1	9	9
3	2	1	1	1	3	1	2	3	1
9	9	9	9	9	9	9	9	9	9
9	9	9	9	1	9	1	9	9	9
9	9	9	9	9	1	2	2	2	2
9	1	9	9	9	2	9	9	9	9
3	3	3	2	1	3	3	1	1	2
3	1	2	2	1	3	1	3	1	3
3	2	3	1	1	3	2	1	3	3
9	1	9	9	9	9	9	1	9	9
9	9	9	9	9	9	9	9	9	9
2	2	1	2	3	2	3	2	2	2
2	3	3	3	1	1	2	3	2	3
3	3	2	2	1	1	2	3	2	3
3	2	2	1	2	2	2	1	1	2
9	1	9	9	1	9	1	1	9	9
3	3	2	2	3	1	1	1	2	3
3	3	3	2	2	3	3	3	1	2
3	3	3	3	2	2	2	3	1	1
3	3	2	3	1	3	2	1	1	1
9	9	9	9	9	9	9	9	1	9
3	2	3	1	3	9	1	9	9	9
1	9	9	9	9	9	9	1	9	9
9	9	9	9	9	9	9	9	9	9
9	9	9	9	1	9	9	1	9	9

1	1	2	3	1	2	1	1	3	3
9	9	9	9	1	1	9	9	9	9
9	9	9	9	1	9	9	9	1	9
3	3	2	3	3	2	1	3	3	3
2	3	1	2	1	1	1	2	3	3
3	3	3	3	3	2	1	1	3	3
3	3	3	3	3	3	3	2	1	2
2	2	2	2	2	2	2	2	2	2
2	2	2	2	1	3	3	3	3	3
3	3	3	2	3	3	2	1	3	3
1	1	2	1	1	2	1	2	3	3
2	2	2	1	2	1	2	1	2	1
3	1	3	1	3	1	3	1	3	1
1	1	2	1	1	1	2	3	3	3
2	2	2	2	2	2	2	2	1	2
1	1	1	2	1	9	9	1	9	9
3	1	1	1	1	1	2	1	1	1
3	3	3	3	3	2	2	2	2	1
1	1	2	2	2	2	2	2	2	3
2	2	2	2	1	2	2	1	2	2
9	9	9	9	9	9	9	9	9	9
2	2	2	2	2	1	2	2	2	3
1	2	2	2	3	2	2	2	2	2
1	2	2	2	2	2	2	2	2	3
1	1	2	2	2	1	2	2	2	2
1	2	2	2	2	2	2	2	2	2
2	2	3	3	3	2	2	2	2	2
2	2	3	3	3	2	2	3	3	3
2	2	2	2	3	2	2	2	2	2
1	2	2	2	1	1	1	2	2	2
9	9	9	9	9	9	9	9	9	9
9	9	9	9	9	9	9	9	9	9
9	9	9	9	9	9	9	9	9	9
3	3	3	2	1	1	2	3	3	3
3	3	3	9	1	3	2	1	2	3
2	2	2	2	2	2	2	2	2	2
1	1	2	1	2	2	2	2	1	1
3	3	3	3	1	3	3	1	1	1
2	1	1	1	1	1	1	9	1	3
3	3	3	3	3	2	2	2	2	2
9	9	9	9	1	3	2	2	1	3
3	2	2	1	2	3	3	2	1	2
1	1	1	2	1	1	2	1	2	2
2	3	2	2	3	3	3	1	3	3
3	3	3	3	2	3	2	3	1	3
3	3	3	3	3	3	3	3	1	2
9	9	9	9	9	9	9	9	9	9

2	2	2	3	2	3	2	2	2	2
2	2	2	2	2	2	2	2	2	2
3	2	2	3	3	3	2	1	1	3
3	3	3	1	1	2	1	1	1	3
3	3	3	3	3	1	3	3	3	3
9	9	9	9	9	9	9	9	9	9
2	3	1	1	1	2	1	1	2	2
9	9	9	1	9	9	9	1	9	9
9	9	9	2	9	9	9	9	9	9
9	9	9	9	1	9	1	9	9	9
9	9	9	9	9	9	9	1	9	9
9	1	9	9	9	9	9	3	1	9
3	1	1	3	2	2	1	2	2	2
2	2	2	2	3	2	1	2	2	3
3	3	3	3	1	3	2	2	1	2
3	3	3	3	3	1	2	3	3	3
1	1	1	1	1	2	2	1	1	1
1	1	1	1	1	2	2	1	1	1
3	3	3	3	2	3	3	1	2	3
3	3	3	3	3	3	2	3	3	3
3	3	3	2	2	2	2	2	2	2
3	2	3	3	3	3	2	1	1	2
3	2	1	1	3	3	1	3	3	3
3	3	3	3	3	9	9	9	9	9
3	2	2	1	1	1	1	1	1	1
3	3	3	1	3	3	3	1	2	1
3	3	2	2	2	2	2	2	2	2
3	3	2	3	3	3	3	2	1	2
3	2	2	2	1	2	2	2	2	2
2	9	3	2	3	1	2	3	2	2
2	2	2	2	2	1	1	1	1	1
3	3	3	3	3	3	2	3	3	3
2	3	2	2	1	3	2	1	1	3
3	3	2	1	2	2	2	2	2	1
1	9	9	2	9	9	9	9	9	9
3	3	3	3	3	3	3	1	1	1
3	3	2	3	2	3	3	1	1	1
3	1	3	3	3	3	3	1	3	3
3	3	3	3	3	3	1	2	2	3
2	2	2	2	1	1	2	2	2	2
9	9	9	9	9	9	9	9	1	9
9	9	9	9	9	9	9	2	9	9
9	9	9	9	9	9	9	2	9	9
3	3	3	3	1	3	2	2	2	2
9	9	9	9	9	9	1	9	9	9
9	9	9	1	9	9	9	9	9	9
2	3	2	3	1	3	2	1	2	2

2	2	2	2	3	2	2	1	3	3
3	3	3	3	3	1	2	2	2	2
9	1	9	3	1	1	3	2	1	2
3	3	2	3	3	2	3	1	3	3
3	3	3	3	3	1	2	2	2	2
3	2	2	2	3	2	2	2	2	2
1	3	2	3	1	3	3	1	1	2
3	1	2	2	2	3	1	2	3	3
3	3	3	3	3	3	2	1	3	3
2	1	1	2	1	1	2	2	3	1
3	3	3	3	3	3	1	1	1	1
3	2	1	1	1	1	3	3	3	3
3	3	3	3	3	3	2	1	3	3
2	3	2	2	2	2	1	2	2	2
3	3	3	3	2	2	1	3	2	2
9	9	2	1	1	2	2	2	2	2
3	3	3	3	3	3	3	1	2	2
1	3	3	3	3	2	2	1	3	3
2	2	1	2	2	2	2	2	3	3
1	2	2	2	3	2	2	1	1	1
2	2	2	3	2	2	2	2	1	1
2	2	2	2	1	2	2	2	2	2
2	2	2	2	2	2	2	2	2	2
2	3	3	3	2	3	2	1	2	3
3	3	3	3	3	3	2	1	3	3
3	3	3	3	3	2	2	2	2	2
3	3	3	3	3	1	3	3	3	3
3	3	3	3	1	1	1	1	1	3
2	3	2	2	3	9	9	2	1	9
3	3	3	3	3	2	2	1	3	3
2	3	2	2	3	1	2	2	3	3
3	3	3	1	3	3	3	2	1	3
2	3	3	3	2	1	3	3	3	3
3	2	3	3	3	1	3	3	3	3
1	1	2	1	2	1	2	3	2	2
1	1	1	2	2	2	2	2	2	2
2	2	2	2	2	2	2	1	2	2
3	3	3	3	1	2	2	1	9	2
1	3	3	1	2	2	1	1	2	1
2	2	2	2	2	3	3	3	3	1
3	3	3	3	3	1	3	3	3	3
3	3	2	3	3	3	3	2	1	1
2	3	3	3	3	1	1	1	2	3
2	1	2	3	3	3	2	1	2	2
3	1	3	1	1	9	1	9	9	9
2	2	2	2	2	2	2	2	2	2
3	2	2	3	3	1	1	2	3	3

2	3	1	3	1	1	2	3	2	3
3	3	3	3	1	2	2	2	2	2
2	2	2	2	2	3	2	2	3	3
2	3	3	2	2	3	2	1	1	2
2	2	2	2	2	2	2	2	2	2
3	3	3	3	3	2	2	2	1	2
1	2	2	2	2	2	2	2	2	2
3	2	2	2	2	2	2	2	2	2
3	3	3	3	1	3	2	2	3	3
3	2	1	1	1	3	2	1	1	1
3	3	3	3	1	1	3	3	3	3
3	2	3	2	2	3	2	2	2	1
1	1	1	1	1	3	3	3	3	3
2	2	2	2	3	2	1	2	2	2
3	3	3	3	3	9	9	3	9	9
2	2	2	2	2	2	2	1	2	2
3	3	3	3	3	2	2	2	1	2
1	3	3	3	1	2	2	2	2	2
3	3	3	2	2	1	2	2	2	2
2	2	2	3	3	1	2	3	3	3
3	3	1	3	1	1	2	3	1	2
3	3	3	3	3	3	3	3	3	3
3	2	2	3	1	1	1	2	3	3
3	3	3	2	1	1	2	2	1	3
3	3	2	2	3	1	2	2	3	2
9	1	1	3	1	1	3	2	2	3
2	1	2	1	2	1	2	2	1	3
2	3	3	3	1	3	2	1	1	3
2	2	2	3	3	3	2	3	1	3
2	9	2	2	1	3	2	2	2	2
2	2	2	2	2	3	3	3	3	3
3	3	3	2	2	2	2	2	2	2
3	2	3	2	3	2	3	3	2	3
2	1	2	2	1	1	1	1	1	1
2	1	3	2	3	2	2	2	2	2
2	2	2	2	1	2	2	1	2	2
3	3	2	3	1	3	3	1	2	2
3	2	3	3	2	3	3	1	3	3
9	1	1	1	2	3	1	2	3	3
3	3	2	3	3	3	1	2	3	3
3	3	3	3	3	3	3	1	3	3
3	3	3	3	3	3	3	1	2	3
3	2	2	2	2	3	1	1	3	3
3	3	2	2	1	3	3	1	1	3
3	3	3	3	3	2	2	2	2	2
2	2	2	2	2	3	3	2	2	3
3	2	2	2	1	2	1	2	2	2

2	1	2	3	1	3	1	1	2	2
3	1	3	2	1	1	2	2	2	2
3	3	3	3	3	2	1	3	3	3
3	3	3	3	3	3	3	1	3	3
3	3	3	3	1	3	1	2	1	3
1	3	3	1	9	2	3	9	2	3
1	2	3	2	2	2	3	2	2	3
3	3	2	1	2	3	3	3	1	2
3	2	2	3	1	3	2	1	2	3
3	3	3	3	1	2	2	2	2	2
3	2	9	1	3	3	2	1	1	2
3	3	3	3	3	2	1	1	3	3
1	1	1	1	3	3	3	2	1	1
3	3	2	3	2	3	1	1	3	2
1	1	1	1	2	3	2	2	2	9
3	3	3	3	2	3	3	1	2	3
3	2	2	3	2	2	2	2	2	2
3	1	3	3	1	1	1	1	1	1
1	2	2	9	9	1	2	3	2	1
9	1	2	2	3	2	2	2	2	2
1	1	2	3	2	1	1	2	2	3
2	2	2	3	3	1	2	2	3	3
1	2	2	2	2	1	2	2	2	3
1	2	2	2	3	2	2	2	2	2
1	2	2	2	3	2	2	2	2	2
3	3	3	3	2	3	3	1	2	3
3	3	3	3	3	2	1	1	3	3
3	3	3	3	1	2	2	2	2	2
9	1	1	1	2	3	1	2	3	3
9	1	2	2	3	2	2	2	2	2
3	3	2	3	1	3	3	1	2	2
9	9	9	9	9	9	9	9	9	9
3	2	2	3	3	2	2	1	2	2
2	2	2	1	1	2	2	1	2	2
2	2	2	3	1	2	2	1	3	3
3	3	3	3	1	3	3	2	1	1
3	3	3	3	3	3	1	1	1	1
2	2	2	2	2	2	1	2	9	2
3	3	3	3	3	3	2	2	2	3
2	2	2	2	1	2	2	2	2	2
2	1	2	1	1	1	1	2	1	2
2	1	2	1	1	1	1	2	1	2
3	2	1	3	1	3	2	1	2	3
3	3	3	3	1	3	1	9	9	9
2	1	3	2	2	2	3	2	3	1
2	2	2	2	1	3	3	2	2	3
3	3	3	3	3	9	1	3	3	3

2	3	2	2	1	2	9	2	1	1
1	1	1	1	1	1	1	2	3	3
1	1	1	1	1	1	1	1	1	1
3	3	2	2	2	2	2	1	3	3
3	3	3	3	2	3	3	3	1	2
3	3	3	3	3	2	3	1	2	3
3	1	3	2	1	3	3	2	1	1
2	2	2	2	2	3	3	3	3	3
2	2	2	2	2	2	2	2	2	2
2	2	2	2	2	2	2	2	2	2
2	2	2	2	2	2	2	2	2	2
2	2	2	1	2	1	2	2	2	3
2	2	2	2	2	2	2	2	2	2
2	2	2	2	3	3	3	3	1	2
3	3	2	3	2	3	2	1	1	3
2	1	2	2	2	1	1	2	2	2
1	3	2	1	2	2	2	2	2	2
3	3	3	3	1	3	3	1	3	3
1	2	3	2	1	1	1	2	2	3
2	2	2	2	2	1	1	1	1	1
3	2	2	2	1	2	1	2	2	2
2	2	2	2	2	2	2	2	2	2
2	2	2	2	2	3	3	3	2	2
2	2	2	2	2	2	2	2	2	2
3	3	3	3	3	1	3	3	1	3
2	2	2	2	2	2	2	2	2	2
3	3	3	3	2	2	1	1	2	1
3	1	1	3	3	3	3	1	3	3
3	2	3	2	3	2	3	1	3	3
3	3	3	3	3	2	3	1	3	3
2	2	2	2	2	2	2	1	2	3
2	1	2	2	2	2	2	2	2	2
3	3	3	3	1	3	3	3	1	3
9	9	9	9	1	9	9	1	9	9
3	3	3	3	3	3	3	1	1	1
3	3	3	3	3	3	2	1	1	2
2	3	2	1	3	3	3	1	2	1
3	3	3	3	1	3	3	1	2	2
3	3	3	3	1	2	1	2	2	2
3	3	3	3	3	1	3	3	3	3
2	9	1	3	1	3	1	1	1	2
2	1	9	1	9	1	2	2	1	1
3	2	2	3	2	3	3	2	3	3
1	2	3	3	2	1	1	1	2	3
3	1	2	3	3	2	1	2	1	1
3	3	3	3	3	1	1	1	2	3
2	2	2	3	2	1	1	1	2	2

3	2	2	1	3	2	1	1	2	3
9	9	9	9	9	9	1	9	9	9
9	9	9	9	9	9	1	9	9	9
3	3	3	2	1	3	1	9	2	3
3	3	3	3	3	3	3	3	1	3
9	9	9	9	9	9	9	9	9	9
1	2	2	2	2	1	1	2	3	3
2	2	2	3	2	1	1	2	2	3
2	2	2	1	2	2	2	1	2	2
1	1	2	2	2	2	2	1	2	2
3	3	3	3	3	3	3	3	3	1
3	3	1	3	1	3	1	1	3	3
2	2	2	2	1	2	2	2	2	2
1	3	3	3	1	3	3	2	1	1
3	3	3	9	9	3	3	3	1	2
1	2	3	3	1	3	3	3	3	1
2	9	9	9	9	9	9	9	1	9
1	2	2	2	3	1	2	2	2	3
1	2	2	2	2	1	1	2	2	2
1	1	1	2	2	1	1	2	2	2
1	2	2	2	1	1	1	2	2	2
1	2	2	2	2	1	1	1	1	2
2	3	2	2	3	3	1	2	2	2
2	3	3	2	3	3	3	1	1	1
2	2	2	2	1	2	2	2	2	2
2	2	2	2	2	2	2	2	2	2
3	3	3	3	3	3	3	3	3	3
3	1	2	3	2	2	2	1	2	3
2	2	2	2	2	1	2	2	2	2
1	1	1	2	3	1	2	2	3	3
1	2	2	2	1	1	2	2	3	3
2	2	2	2	2	1	2	2	2	2
1	3	3	3	1	2	1	1	2	3
3	3	2	2	1	1	2	2	3	2
1	3	3	3	3	2	1	1	3	3
3	3	2	1	1	3	3	3	3	1
9	9	9	9	9	9	9	9	9	9
3	3	3	3	3	3	3	3	1	3
3	9	3	2	2	3	1	1	2	3
2	2	2	2	2	2	2	2	2	2
3	3	9	1	2	3	1	3	3	3
3	3	2	1	1	3	3	3	2	1
2	3	3	3	2	2	1	2	1	2
2	2	2	2	3	2	2	2	1	3
3	3	3	3	3	3	3	1	1	3
2	2	2	1	1	2	2	2	1	2
2	3	3	3	2	3	2	1	1	2

3	3	3	3	3	2	2	2	2	2
3	1	1	3	1	1	2	1	1	1
2	2	3	2	2	3	2	2	1	1
2	2	3	3	3	3	3	3	2	1
3	1	2	3	2	1	2	2	2	2
3	9	9	9	9	2	2	1	2	2
2	1	3	3	1	2	2	3	1	1
3	3	3	3	1	3	3	1	3	3
2	2	3	3	2	3	3	3	2	1
2	3	1	2	3	1	2	2	1	3
2	2	1	2	2	1	1	2	2	2
2	2	3	2	1	2	2	1	3	2
2	3	3	2	3	1	3	3	2	2
3	3	2	3	3	1	3	9	2	2
3	1	2	3	1	1	3	3	3	3
2	3	3	1	2	2	9	1	9	9
1	2	1	1	1	9	9	9	9	9
3	3	3	3	1	3	3	1	1	2
1	2	2	3	2	1	2	2	3	3
2	2	2	2	2	2	2	2	2	2
1	1	1	3	3	3	3	1	3	3
1	1	1	3	3	3	3	1	3	3
3	3	3	2	1	3	1	2	1	3
2	2	2	2	2	3	2	1	9	1
2	2	2	9	1	9	1	9	9	9
2	2	3	2	2	1	2	2	2	3
1	1	3	3	3	3	3	3	3	3
3	3	3	3	1	3	3	3	1	2
1	2	2	3	2	1	1	2	2	3
2	2	2	2	2	1	1	1	1	1
3	3	3	3	3	2	2	2	2	2
3	3	3	3	3	1	2	2	3	3
1	1	2	2	1	1	1	2	2	2
1	2	1	2	1	1	2	1	1	1
1	1	1	2	1	2	2	2	1	3
3	3	2	3	3	1	1	2	2	3
1	1	2	1	1	1	1	2	2	2
2	1	2	2	1	2	1	3	3	3
3	3	3	3	3	3	2	1	2	1
2	3	1	2	1	1	1	1	1	2
3	2	3	3	3	2	1	3	1	1
1	1	1	1	1	2	2	1	2	2
9	9	9	9	9	9	9	1	9	9
3	3	2	2	1	3	2	2	1	2
3	3	3	3	3	3	3	1	3	3
3	3	3	3	1	3	2	3	9	2
1	2	2	2	2	1	2	1	1	1

9	9	9	9	9	9	9	9	9	9
1	2	2	2	2	1	2	2	1	1
1	1	9	2	2	2	2	2	2	3
3	3	3	3	3	1	2	3	3	3
3	3	3	3	2	2	3	2	1	3
3	9	3	2	1	1	1	2	2	3
1	1	2	2	3	1	9	3	2	3
3	3	3	3	3	3	3	3	3	3
3	3	3	3	3	3	3	3	3	3
3	3	3	3	1	1	3	3	1	3
2	1	2	3	1	3	3	1	2	2
3	1	2	2	1	3	1	2	3	2
9	9	9	9	1	9	2	9	1	2
3	3	2	1	2	2	1	2	1	3
2	2	2	2	2	2	2	2	2	2
2	2	2	2	1	2	2	2	2	2
3	1	1	2	1	2	3	1	3	1
2	2	2	3	1	1	9	9	3	3
2	2	9	2	1	2	2	2	2	2
3	3	3	3	3	3	3	3	3	3
1	2	1	1	2	2	2	1	1	1
1	2	1	1	1	2	2	1	1	1
1	2	1	1	1	1	1	2	2	2
1	2	2	2	1	2	2	1	1	1
2	2	2	2	1	1	2	2	3	3
3	3	3	2	1	2	2	3	2	3
9	9	9	9	9	9	9	2	9	9
3	3	3	2	2	3	1	2	2	2
3	3	3	3	3	2	1	2	2	2
1	1	1	1	1	3	3	2	1	1
2	3	3	3	3	2	2	1	2	2
3	1	1	2	3	3	3	2	3	3
3	3	2	3	3	3	1	1	3	3
3	1	2	2	3	2	1	1	1	2
3	3	3	3	1	2	2	2	1	1
1	1	2	2	1	2	1	1	1	2
9	9	9	9	9	9	9	9	9	9
2	2	2	2	2	2	2	2	2	2
2	2	1	1	2	2	2	1	2	2
1	1	1	1	1	1	1	1	1	1
2	2	2	2	2	3	3	3	3	3
1	1	1	1	1	2	1	1	1	1
1	1	1	1	1	2	2	2	2	2
3	2	3	3	2	3	1	1	2	3
3	3	3	3	1	9	1	1	9	9
3	3	3	9	3	3	3	3	3	3
9	3	9	3	9	1	3	1	1	1

2	2	2	2	2	2	2	2	2	2
1	2	2	3	2	3	1	1	2	3
9	9	9	9	9	9	9	9	9	9
9	9	9	9	9	9	9	9	9	9
1	2	2	1	1	1	2	2	1	1
2	2	2	3	2	2	1	2	2	2
9	9	2	9	9	9	9	9	1	9
9	9	9	9	9	2	9	9	9	9
3	3	3	3	3	9	9	1	9	9
9	9	9	9	9	1	9	9	9	9
3	3	3	3	3	3	3	2	1	3
3	3	3	3	3	2	2	1	2	3
3	3	3	3	3	3	2	2	1	3
1	2	1	3	2	9	9	9	9	9
1	2	1	1	1	3	3	1	3	3
9	1	9	9	9	9	9	2	9	9
9	9	9	2	9	9	9	1	9	9
2	9	9	9	9	9	9	9	9	1
1	1	1	1	2	2	2	2	2	2
2	3	3	1	1	2	2	3	3	1
9	2	9	9	9	9	1	9	9	9
3	3	1	2	2	3	3	3	2	3
3	3	3	3	3	3	3	3	3	3
1	1	1	1	2	2	2	1	1	2
2	2	2	2	3	2	2	1	1	1
2	2	2	2	2	9	9	2	9	9
3	1	2	3	2	1	2	3	3	3
2	2	2	3	2	3	2	2	2	2
2	2	2	2	2	2	2	1	1	2
3	3	3	3	3	2	3	3	3	3
3	2	1	2	2	2	2	2	2	2
1	2	2	2	2	3	2	2	1	2
2	1	1	1	3	1	1	2	2	3
2	1	2	1	1	2	2	2	1	1
9	9	9	9	9	9	9	9	9	9
2	1	2	2	2	1	2	1	2	2
2	2	2	1	2	2	2	2	1	2
1	1	1	1	1	1	1	1	1	1
2	3	2	3	2	3	2	1	3	3
2	3	2	2	3	1	3	3	3	3
3	3	3	3	1	3	3	3	3	1
1	2	1	1	2	2	2	2	2	2
1	3	3	2	2	1	1	2	2	2
1	1	2	2	2	2	2	2	1	2
1	1	2	3	2	1	2	3	2	2
2	1	2	2	2	2	1	2	2	2
1	2	2	2	1	1	2	2	2	2

3	2	1	3	2	3	1	1	2	1
2	1	3	3	3	3	2	1	1	1
1	2	2	2	2	2	2	2	2	2
2	1	3	1	3	2	2	1	2	2
1	1	2	3	3	3	3	2	1	2
9	9	9	9	9	9	9	9	9	9
3	3	3	3	3	1	2	3	9	3
3	3	3	2	1	1	3	2	2	3
1	1	1	2	1	2	2	2	2	2
3	3	3	3	3	3	3	1	3	3
3	3	3	3	3	2	2	2	2	2
3	3	2	2	3	3	9	2	1	3
2	3	1	1	3	1	1	1	3	1
2	1	1	1	1	1	2	2	2	2
2	2	2	2	2	2	2	2	9	9
1	1	1	2	2	1	1	1	1	1
1	1	2	2	1	1	1	1	2	2
3	2	2	1	1	1	1	1	1	1
9	9	9	9	9	2	9	9	9	9
2	2	2	2	2	2	2	2	2	2
9	2	2	2	9	9	2	9	2	2
9	9	9	9	9	9	9	9	9	9
9	9	2	9	2	2	9	9	2	9
2	9	9	2	9	9	2	1	2	9
1	9	2	9	2	2	2	9	2	2
9	9	9	9	9	9	9	9	9	9
9	9	9	9	9	9	9	9	9	9
2	2	2	2	2	2	2	2	2	2
2	2	2	2	2	2	2	2	2	2
3	3	3	3	3	2	2	2	2	3
2	2	2	2	2	2	2	2	2	2
9	2	9	9	2	9	2	9	2	2
2	2	2	2	2	2	2	2	2	2
9	9	9	9	9	9	9	9	9	9
9	9	9	9	9	9	9	9	9	9
2	2	2	2	2	2	2	2	2	2
9	9	9	9	9	9	9	9	9	9
9	9	9	9	9	9	9	9	9	9
9	9	9	9	9	9	9	9	9	9
9	2	9	2	9	2	9	2	9	2
2	9	9	3	9	9	9	2	1	9
3	1	9	3	1	3	1	9	1	2
9	9	9	9	9	9	9	9	9	9
2	9	9	9	9	9	9	2	9	9
2	9	9	2	9	9	2	9	9	2
9	9	9	9	9	3	9	9	9	9
9	1	9	9	9	1	9	9	9	9

9	9	9	9	9	9	9	1	9	9
3	3	3	3	3	3	3	3	3	3
2	2	1	1	2	2	1	1	2	1
1	2	1	2	1	1	2	1	3	2
1	9	2	1	9	9	1	2	1	1
9	1	9	9	9	1	9	9	9	9
9	9	9	9	9	9	9	1	9	9
9	9	9	9	9	9	9	1	9	9
2	1	1	1	2	2	9	9	9	9
2	9	1	2	3	3	2	1	2	3
1	1	1	1	1	1	2	1	2	1
2	1	2	1	1	2	2	2	2	2
3	3	3	3	3	2	1	1	3	3
3	1	1	1	1	3	1	2	2	3
9	9	9	9	9	9	9	9	9	9
2	3	3	3	3	3	3	3	3	3
9	9	9	9	9	9	9	9	9	9
9	9	9	9	9	9	9	9	9	9
1	9	9	9	9	9	9	9	1	9
1	1	1	1	9	1	1	1	1	2
1	1	1	1	1	1	1	1	1	1
1	2	1	1	1	1	2	2	2	2
9	9	9	9	9	9	9	9	9	9
2	1	2	2	1	1	2	2	2	2
1	1	1	1	1	1	1	1	1	1
3	1	3	3	1	3	3	1	2	3
3	1	3	2	2	9	9	1	9	9
3	3	3	3	1	2	3	1	2	3
3	3	3	2	3	3	2	3	1	1
2	2	2	2	2	2	2	2	2	2
2	2	2	2	2	2	2	2	1	2
2	2	2	2	2	2	2	2	2	2
2	2	2	2	2	2	2	2	2	2
2	3	1	2	3	1	1	1	1	1
3	3	2	2	2	2	2	3	3	3
2	2	2	2	2	1	2	2	1	2
2	2	2	2	2	2	2	2	2	2
2	2	2	2	2	2	2	2	2	2
2	2	2	2	3	3	3	2	2	3
2	2	9	3	3	2	2	2	2	2
3	3	3	3	3	2	2	2	2	3
3	3	3	3	2	2	2	3	1	2
9	9	9	9	9	9	9	9	9	9
1	2	1	1	2	3	2	1	2	2
3	3	3	3	3	3	3	3	3	3
3	3	3	3	3	3	3	3	3	3
2	2	2	2	2	2	2	1	1	1

1	2	3	1	2	3	2	1	3	1
3	2	3	3	3	1	3	3	3	1
1	1	2	2	2	1	2	2	2	3
1	1	1	1	1	3	3	3	2	3
2	2	2	2	2	2	2	2	2	2
1	1	1	9	9	2	2	2	9	9
2	1	2	2	2	2	3	1	2	2
9	1	9	9	9	1	9	9	9	9
1	9	9	9	9	9	1	9	9	9
2	3	1	2	3	1	2	3	1	2
9	2	9	9	9	2	9	9	9	9
2	1	1	2	3	3	3	2	9	1
9	1	9	9	9	9	2	9	9	9
9	9	9	9	9	9	9	9	1	9
2	2	2	2	9	2	2	2	2	2
9	9	9	9	9	2	9	9	9	9
1	9	9	9	9	9	2	9	9	9
2	9	9	9	9	9	9	2	9	9
9	2	9	9	9	9	2	9	9	9
1	9	9	9	9	9	9	3	9	9
9	9	9	9	9	9	1	9	9	9
9	2	9	9	9	2	9	9	9	9
3	2	1	2	3	2	3	2	1	3
1	3	2	1	1	3	2	1	3	2
3	2	1	1	2	1	2	1	1	2
2	2	2	3	2	2	2	2	2	2
3	3	3	2	3	3	2	1	2	3
3	2	2	3	3	1	1	1	2	3
2	3	2	1	1	1	1	1	1	1
3	1	2	2	2	3	1	2	2	1
3	3	3	9	3	2	2	1	3	3
3	3	3	3	3	1	3	3	3	3
3	1	3	3	2	2	2	2	2	2
3	3	1	3	2	1	3	3	3	3
2	1	2	3	2	9	2	1	1	3
2	1	3	3	3	3	1	2	2	2
3	2	1	2	1	1	1	3	1	1
2	2	2	2	2	2	2	2	2	2
1	1	2	2	3	1	1	1	1	1
9	9	9	9	9	2	9	9	9	9
3	1	3	3	3	9	9	9	9	9
3	3	3	3	3	2	2	2	2	2
9	9	9	9	9	9	9	9	9	9
2	1	2	2	1	2	1	1	1	1
9	1	1	2	2	2	2	2	2	2
2	2	2	2	2	2	2	2	2	2
9	9	9	9	9	9	2	9	9	9

3	3	3	3	3	1	2	3	3	3
9	9	9	9	9	9	9	9	1	9
3	2	3	2	1	3	3	1	1	2
2	2	2	2	2	2	2	2	2	2
2	3	1	2	3	3	3	3	1	2
2	2	2	2	2	2	2	2	9	2
2	1	2	3	3	3	2	2	1	2
9	9	9	9	1	9	9	1	9	9
9	9	9	9	9	9	9	1	9	9
2	3	1	2	3	1	2	1	3	2
3	3	3	3	3	3	3	3	3	3
2	2	2	2	2	9	9	1	9	9
2	1	2	2	2	3	2	2	1	2
2	1	2	2	2	2	2	2	1	2
9	9	9	9	9	9	9	9	9	9
3	3	3	3	2	3	3	2	1	2
3	3	3	3	1	3	3	3	1	3
9	9	9	9	9	9	9	2	9	9
2	2	2	2	1	2	1	1	2	3
9	9	9	9	9	1	9	9	9	9
3	2	3	3	2	9	9	9	1	9
3	3	3	2	2	3	3	3	1	3
9	9	9	9	9	9	9	9	9	9
9	9	9	9	9	3	9	9	9	9
9	9	9	9	9	9	9	1	9	9
3	2	9	2	3	2	2	2	3	2
2	2	2	2	2	2	2	2	2	2
2	3	3	2	1	3	2	2	2	1
1	2	3	2	1	2	2	2	2	2
9	9	9	9	9	9	9	9	1	9
3	9	9	9	9	9	9	9	1	9
1	1	2	2	1	1	1	1	1	1
1	1	1	1	1	1	1	1	1	1
1	2	3	1	2	1	2	3	1	1
2	2	3	3	3	3	2	2	2	2
9	9	9	9	9	3	9	9	9	9
9	9	9	9	9	9	9	9	9	9
1	2	2	1	1	1	2	1	1	1
3	2	2	2	2	2	2	1	2	2
3	3	3	3	3	1	1	2	2	2
3	3	3	3	3	3	1	3	1	1
2	1	2	1	2	1	2	2	1	2
3	2	3	3	3	1	2	2	1	3
1	2	1	1	2	1	1	2	2	2
3	2	1	2	1	2	3	2	3	1
1	2	2	1	2	1	1	1	1	1
3	3	2	2	3	2	2	2	2	2

2	2	2	3	3	2	2	2	2	2
2	3	3	2	2	3	3	3	2	2
3	3	2	3	1	3	2	1	1	1
2	2	2	2	2	9	3	1	9	9
2	2	2	2	2	2	2	2	2	2
2	2	2	2	2	2	2	1	2	2
2	2	2	2	2	1	2	2	2	2
9	9	9	9	9	1	9	9	9	9
9	9	9	1	9	9	9	9	9	1
9	9	9	9	9	9	1	9	9	9
2	2	2	2	2	3	2	1	1	1
3	3	3	2	2	2	3	3	3	2
2	2	2	2	2	2	2	2	2	2
9	2	2	2	2	2	9	2	2	2
9	9	9	9	9	9	9	9	9	9
9	2	9	9	9	9	9	2	9	9
2	2	2	2	2	2	2	2	2	2
2	2	2	2	2	2	2	2	2	2
2	2	9	9	9	2	2	2	9	9
2	2	2	2	2	2	2	2	2	2
2	2	2	2	2	2	2	2	2	2
9	9	9	9	9	2	9	9	9	9
3	3	3	3	3	3	3	3	3	3
9	9	9	9	9	1	9	9	9	9
9	9	9	9	9	2	9	9	9	9
9	9	9	9	9	2	9	9	9	9
9	9	9	9	9	9	9	9	9	9
9	9	9	9	9	2	9	9	9	9
2	2	2	2	2	3	3	3	3	3
2	2	2	2	2	2	2	2	2	2
9	9	9	9	9	9	9	9	9	9
9	9	9	9	9	9	9	1	9	9
9	1	9	9	9	1	9	1	9	9
9	9	9	9	9	9	1	1	9	9
3	3	3	3	3	3	3	2	3	3
9	2	9	2	2	2	9	2	2	2
9	2	1	2	2	2	9	2	9	2
3	3	3	3	3	3	3	1	3	3
9	1	9	1	9	3	1	3	2	1
9	1	9	9	9	9	9	9	9	1
2	2	2	2	2	2	2	2	2	2
9	9	9	1	9	9	1	9	2	1
2	9	2	2	2	2	9	9	2	2
2	2	2	2	2	2	2	2	2	2
2	3	2	3	3	2	1	3	1	3
2	2	2	2	2	2	2	2	2	2
9	9	9	9	9	9	1	9	9	9

3	3	2	3	3	1	2	3	3	3
1	1	2	3	2	1	1	2	9	3
3	3	2	2	2	2	3	2	3	1
3	3	3	3	1	3	3	1	3	3
1	2	3	2	1	1	2	1	2	1
3	3	3	2	2	1	2	3	3	3
9	9	9	9	9	9	9	1	9	9
1	3	2	1	3	2	1	3	2	1
2	2	2	2	2	2	2	2	2	2
9	9	9	9	9	1	9	9	9	9
2	2	2	1	2	2	2	2	2	2
3	2	3	3	2	3	2	2	1	1
3	3	3	3	3	3	3	3	3	3
3	2	3	3	2	3	2	2	1	1
3	2	3	3	1	3	2	2	3	3
2	2	2	2	1	1	9	9	1	9
2	3	2	3	3	2	2	9	2	2
2	3	3	2	2	2	2	2	2	3
9	9	9	9	9	9	1	9	9	9
2	3	2	2	2	1	3	2	1	3
2	9	9	9	9	2	9	9	9	9
2	2	2	2	2	2	1	1	1	1
2	3	2	3	2	2	2	1	3	3
3	3	3	3	3	2	3	3	3	3
3	3	3	3	3	2	1	2	3	2
3	3	3	3	2	2	2	1	2	3
9	9	9	9	9	9	9	1	9	9
3	3	3	3	3	3	3	9	1	3
3	9	3	2	2	3	2	2	3	2
9	9	9	9	9	9	9	9	1	9
9	9	9	9	9	9	9	1	9	9
1	1	1	1	1	1	2	2	2	2
9	9	9	9	9	9	9	2	9	9
2	2	1	2	9	1	1	1	2	1
9	9	9	9	9	9	9	9	9	9
1	9	9	9	9	9	9	1	9	9
2	3	3	3	3	3	3	3	1	3
3	3	2	3	2	2	3	2	1	3
2	2	3	1	2	9	9	9	9	9
1	1	2	9	9	9	9	1	9	9
3	2	1	1	1	2	1	1	2	2
3	2	3	2	3	9	9	1	9	9
1	1	3	1	2	2	1	9	9	9
1	1	1	1	1	1	1	1	1	1
3	3	3	3	3	3	3	3	1	3
2	2	2	2	2	2	2	2	2	2
9	9	9	9	9	9	9	1	9	9

3	3	3	3	3	9	9	9	9	3
3	2	3	1	3	9	9	9	1	9
3	3	3	3	3	3	3	2	2	2
2	2	3	3	1	2	3	1	2	2
1	3	1	2	3	2	2	3	2	2
3	1	1	1	3	3	3	3	3	3
9	9	9	1	9	9	9	9	2	9
2	2	2	2	2	2	2	2	2	2
2	2	1	1	2	3	1	1	1	1
3	3	3	3	3	2	2	2	2	2
2	1	2	1	1	2	2	2	2	2
9	9	9	9	9	9	9	9	9	9
9	9	9	9	9	9	9	9	9	9
2	2	2	2	2	9	9	1	9	9
2	2	2	2	2	1	2	1	2	2
2	2	2	2	2	9	9	2	9	9
2	2	2	2	2	2	2	2	2	2
2	2	2	2	2	2	2	2	2	2
3	3	3	2	3	2	1	1	3	3
2	2	2	2	2	2	2	2	2	2
2	2	2	2	2	3	3	1	2	3
9	2	9	2	2	2	9	2	9	2
9	9	9	2	9	2	9	9	2	9
9	9	9	9	9	9	9	9	9	9
9	9	9	9	9	1	9	9	9	9
9	2	9	2	2	2	9	2	9	9
9	9	9	9	9	2	9	9	9	9
9	9	9	9	9	9	9	9	9	9
2	2	2	2	2	2	2	2	2	2
9	9	9	9	9	2	9	9	9	9
9	9	9	9	9	9	9	9	9	9
9	9	9	9	9	3	9	9	9	9
9	9	9	9	9	9	9	9	9	9
1	1	1	1	1	1	1	1	1	1
9	9	9	3	3	2	9	9	2	3
9	9	9	9	3	3	9	9	9	3
9	9	9	9	9	3	9	9	9	9
2	2	2	2	2	2	2	2	2	2
1	1	1	1	1	1	1	1	1	1
9	9	9	9	9	3	9	9	9	9
2	2	2	2	2	2	2	2	2	2
9	9	9	9	9	9	9	9	9	9
9	9	9	9	9	2	9	9	9	9
9	9	9	9	9	9	9	9	9	9
9	9	2	9	3	1	9	2	9	3
9	9	9	9	9	1	9	9	9	9
2	2	2	2	2	2	2	2	2	2

9	9	9	9	9	2	9	9	9	9
9	9	9	9	9	2	9	9	9	9
9	9	2	9	9	9	1	9	9	2
2	2	2	2	2	2	2	2	2	2
2	2	2	2	2	2	2	2	2	2
9	9	9	9	9	9	9	9	9	9
1	1	1	1	1	1	1	1	1	1
2	2	2	2	2	2	2	2	2	2
9	9	9	9	1	2	9	9	1	2
2	3	1	2	2	2	2	1	3	3
9	9	9	9	9	2	9	9	9	9
9	9	9	9	9	2	9	9	9	9
9	9	9	9	9	2	9	9	9	9
9	9	9	9	9	1	9	9	9	9
9	9	9	9	9	9	9	9	9	9
9	9	9	9	9	9	9	9	9	9
9	9	9	9	9	9	9	9	9	9
9	9	9	9	9	9	9	9	9	9
9	9	9	9	9	9	9	9	9	9
9	9	9	9	9	9	9	9	9	9
2	2	2	2	2	2	2	2	2	2
2	2	2	2	2	2	2	2	2	2
2	2	2	2	2	2	2	2	2	2
2	2	2	2	2	2	2	2	2	2
2	2	2	2	2	2	2	2	2	2
2	2	2	2	2	2	2	2	2	2
2	2	2	2	2	2	2	2	2	2
9	9	9	9	9	2	9	9	9	9
9	9	9	9	9	1	9	9	9	9
2	2	2	2	2	2	2	2	2	2
2	2	2	2	2	2	2	2	2	2
2	2	2	2	2	2	2	1	2	2
9	9	9	9	9	9	9	9	9	9
3	3	3	3	3	2	1	9	2	2
9	9	9	9	9	9	9	9	9	9
2	2	2	2	2	3	3	3	3	3
1	1	1	1	1	1	1	1	1	1
2	1	1	2	1	2	1	2	3	2
1	1	1	1	1	1	1	1	1	1
1	1	1	1	1	1	1	3	3	3
1	1	1	1	1	1	1	1	1	1
9	9	9	9	9	9	9	1	9	9
9	9	9	9	3	3	9	9	9	3
9	2	2	2	2	2	2	2	2	2
1	1	1	1	1	1	1	1	1	1
9	9	9	9	3	3	9	9	9	3
9	9	9	9	9	2	9	9	9	9

9	9	9	9	9	9	9	9	9	9
2	2	2	2	2	2	2	2	2	2
9	9	9	9	9	9	9	1	9	9
1	9	1	9	1	1	9	1	9	1
9	9	9	9	2	2	9	9	9	2
9	9	2	9	2	2	9	2	9	2
9	9	9	9	9	2	9	1	9	9
9	9	9	9	9	9	9	9	9	9
9	9	3	9	1	2	9	9	1	3
9	9	9	9	9	3	9	9	9	9
9	9	9	9	9	9	9	9	9	9
9	9	9	9	9	2	9	9	9	9
9	9	9	9	9	9	1	1	9	9
9	9	9	9	9	2	9	9	9	9
9	9	2	9	2	1	1	9	9	1
1	1	9	9	9	2	1	1	1	2
9	9	9	9	9	2	9	9	9	9
9	9	9	9	9	9	9	9	9	9
3	1	3	3	3	3	3	3	1	3
9	9	9	9	9	9	9	9	9	9
3	3	3	3	3	2	1	1	9	3
3	1	9	2	2	2	2	3	1	1
9	9	1	9	9	9	9	9	9	1
9	9	9	9	1	1	2	2	2	2
3	2	3	3	2	2	2	2	2	2
2	2	2	2	2	2	2	9	2	9
3	3	3	3	2	1	2	2	2	2
9	9	9	9	9	9	1	9	9	9
3	3	3	3	3	3	3	3	1	2
2	2	3	2	1	2	1	2	1	2
3	3	3	3	3	2	2	1	2	2
3	3	3	2	3	2	2	1	2	2
3	3	3	3	3	3	1	3	3	3
3	3	3	3	3	2	1	3	3	3
1	2	3	2	2	2	1	1	2	3
2	2	2	2	2	2	2	2	2	2
9	9	9	9	9	2	9	9	9	9
9	9	9	9	9	1	9	9	9	9
3	3	3	3	3	2	3	2	2	3
9	9	9	2	9	9	9	3	9	9
9	9	9	9	9	2	9	9	9	9
9	9	9	9	9	9	9	9	9	9
9	9	9	9	9	9	9	9	9	9
2	9	9	2	2	9	2	9	9	2
9	9	9	9	9	9	9	9	9	9
9	2	9	2	2	2	9	2	9	2
9	9	2	9	2	2	9	9	2	2

2	2	2	2	2	2	2	2	2	2
2	2	2	2	2	2	2	2	2	2
2	2	2	2	2	2	2	2	2	2
2	2	2	2	2	2	2	2	2	2
2	2	2	2	2	2	2	2	2	2
2	1	2	1	2	2	2	2	2	1
2	2	3	2	2	3	2	1	1	3
2	2	2	2	1	2	2	2	1	3
3	2	2	2	1	9	9	9	1	9
3	3	3	3	3	3	1	3	1	2
3	3	3	3	3	3	3	3	3	3
3	3	3	9	1	2	2	2	1	1
2	2	2	2	1	2	2	1	1	2
3	3	3	3	3	3	3	3	3	3
2	2	2	2	2	2	2	2	2	2
2	2	2	2	2	2	2	2	2	2
2	2	2	2	2	2	2	2	2	2
2	2	2	2	2	2	2	2	2	2
3	3	3	3	3	3	1	3	3	3
3	2	2	3	3	1	1	1	2	3
2	1	1	2	3	2	2	1	2	2
2	2	2	2	2	2	2	2	2	2
3	2	3	2	2	2	3	2	3	2
2	2	2	3	3	2	2	1	2	3
3	3	3	3	2	1	2	2	3	3
2	2	3	2	1	2	2	1	3	3
1	3	3	2	2	2	1	1	1	2
3	1	2	3	2	3	2	2	1	3
3	1	2	2	2	2	1	3	1	2
1	2	1	3	1	3	3	2	1	2
3	3	3	3	3	3	3	1	3	3
3	2	2	2	2	2	3	2	2	2
3	3	3	3	2	1	2	2	2	3
3	3	3	2	2	1	2	3	3	2
1	3	3	2	1	3	1	1	2	3
3	3	3	3	3	1	1	1	1	1
3	3	3	3	1	3	3	3	1	2
2	2	1	1	2	2	1	2	2	2
3	3	3	3	3	3	3	3	3	3
3	3	3	3	2	2	2	2	2	2
9	1	9	9	9	9	1	9	9	9
3	3	2	3	3	3	2	1	3	2
3	3	2	2	3	1	1	1	1	1
1	1	1	1	1	1	1	1	1	1
3	3	2	2	3	1	1	1	1	1
9	9	9	9	9	9	9	9	9	9
2	2	2	3	1	3	2	2	2	3

9	9	9	9	9	9	1	9	9	9
3	2	2	3	3	3	3	1	2	3
3	2	2	3	3	3	3	1	2	3
3	2	3	2	2	2	3	2	3	2
3	2	3	2	3	3	3	2	2	2
1	2	2	3	2	2	3	2	3	3
1	1	1	2	2	1	2	2	2	2
3	3	3	3	3	3	3	3	3	3
1	3	1	3	1	2	3	9	1	1
3	3	3	3	3	3	2	2	2	3
1	3	1	2	2	1	2	1	2	3
3	3	3	3	3	3	3	3	3	3
2	2	2	1	9	1	2	2	2	2
1	2	3	1	2	1	2	3	2	1
3	3	3	3	1	3	2	1	1	2
3	3	3	3	3	3	3	3	1	3
3	3	2	3	2	3	2	2	2	2
2	3	3	3	1	3	3	2	2	3
2	2	9	2	2	1	9	1	2	3
1	2	1	2	3	2	2	1	1	1
1	3	2	2	1	2	1	1	1	3
3	2	3	2	2	2	2	2	2	2
3	3	3	2	3	3	3	3	3	3
3	3	3	3	3	2	2	2	2	2
9	2	2	2	2	3	3	3	3	3
3	2	2	2	3	2	9	9	9	9
1	2	1	1	2	2	2	2	1	2
1	1	1	2	1	2	1	2	2	9
3	3	2	3	3	3	2	3	2	3
1	2	2	2	1	1	1	1	2	3
3	3	3	3	3	3	3	3	3	3
2	3	2	1	2	2	1	3	2	1
2	2	2	2	2	9	9	9	9	1
2	2	2	2	2	3	3	3	3	3
3	2	2	2	2	2	3	2	2	2
2	2	2	2	2	2	2	2	2	2
3	1	3	2	3	2	1	2	2	1
9	9	9	9	9	3	9	9	9	9
3	3	2	3	2	3	1	2	2	2
3	3	3	3	3	2	2	2	2	2
3	3	3	2	1	1	1	2	2	3
1	3	3	2	1	3	1	2	3	2
3	3	3	3	3	1	1	3	3	3
3	2	3	1	1	2	3	2	2	3
3	3	3	3	3	3	3	1	2	2
3	3	2	3	2	2	1	2	2	2
3	1	3	3	1	3	1	3	3	3

2	2	2	2	2	2	2	2	2	2
2	2	2	2	2	2	2	2	2	2
2	2	2	2	2	2	2	2	2	2
3	3	2	3	2	3	1	2	2	2
2	3	3	3	2	2	1	1	1	2
3	3	3	3	2	2	2	2	2	2
2	3	2	2	2	2	2	2	2	2
2	3	3	2	3	9	1	2	2	2
3	3	3	3	2	2	2	2	2	2
3	3	3	3	3	2	2	1	3	3
9	9	9	9	9	9	9	9	9	9
2	3	3	2	3	3	1	2	2	2
2	3	3	2	3	3	1	2	2	2
3	2	3	2	1	2	2	2	2	2
3	3	2	2	1	2	2	2	2	2
9	9	9	9	9	9	9	9	9	9
2	3	3	2	2	3	2	1	3	2
2	3	3	2	2	3	2	1	3	2
9	1	9	9	9	9	9	9	1	9
9	1	9	9	9	9	9	9	9	9
2	1	2	3	2	3	3	3	2	2
9	9	9	9	1	9	9	1	9	9
9	1	3	1	1	9	9	1	9	9
9	9	9	9	9	9	1	9	9	9
9	9	9	9	9	9	9	9	9	9
3	2	3	2	1	2	2	2	2	1
3	3	2	3	1	1	3	1	3	3
9	9	9	9	9	9	9	9	9	1
1	2	1	1	1	2	1	2	3	1
2	2	2	2	1	1	2	2	2	2
2	2	2	2	2	3	3	1	3	3
2	2	2	2	2	2	2	2	2	2
2	2	2	9	1	3	2	3	1	1
3	2	3	2	3	9	1	9	9	9
2	2	2	2	2	2	2	2	2	2
3	2	9	1	2	2	1	3	2	1
9	9	9	9	9	9	9	9	9	9
1	3	9	2	1	1	2	1	2	2
3	3	3	3	3	3	3	2	2	2
2	2	2	2	9	3	3	1	3	3
2	1	2	2	1	2	2	2	2	2
2	2	2	2	2	2	2	2	2	2
9	9	9	9	9	9	9	9	1	9
3	2	3	2	2	2	2	2	2	2
3	3	3	2	2	2	2	2	2	2
1	1	1	1	1	1	1	1	1	1
2	1	2	2	2	3	3	2	1	1

3	2	3	3	3	2	2	2	2	2
2	1	1	1	1	1	1	1	2	3
2	9	9	9	9	1	9	9	9	9
3	3	3	3	3	3	2	3	3	2
9	9	9	9	9	9	9	9	9	9
3	3	2	3	3	3	2	2	2	3
1	2	2	1	3	2	2	1	2	3
2	2	2	2	2	2	2	2	2	2
9	3	2	1	2	3	3	3	3	3
2	1	2	3	2	2	2	3	2	3
2	3	2	3	2	2	3	1	3	2
1	1	1	9	1	3	3	3	3	3
9	9	9	9	9	9	9	1	9	9
3	3	3	3	3	3	3	3	3	3
3	3	3	3	9	2	3	2	3	9
3	2	2	1	3	2	2	3	1	2
2	2	2	2	2	2	2	2	2	2
2	3	3	3	3	3	3	3	1	3
2	2	1	1	2	2	2	1	1	3
3	3	3	3	3	1	9	9	9	9
9	9	9	9	9	9	9	1	9	9
9	9	9	1	9	1	9	9	9	9
3	2	2	3	3	2	1	1	3	3
3	3	1	1	1	3	3	1	3	3
3	3	3	3	2	3	2	1	2	3
3	3	3	3	3	1	2	3	3	3
2	2	2	2	2	2	2	2	2	2
3	3	3	3	1	1	2	3	2	3
2	2	2	2	2	2	2	2	2	2
2	2	2	2	2	2	2	2	2	2
2	2	2	2	2	1	3	3	3	3
2	2	2	2	2	2	2	2	2	2
3	3	3	3	3	2	2	2	2	2
9	9	9	9	9	2	9	9	9	9
3	3	3	3	3	2	2	2	2	2
2	2	2	2	2	2	2	2	2	2
1	1	1	1	1	1	1	1	1	1
3	9	9	9	9	1	2	1	2	3
2	2	1	2	1	2	2	2	9	1
2	2	2	2	2	2	2	2	2	2
2	3	2	2	2	3	3	2	3	3
2	3	2	2	2	2	3	1	2	2
1	1	1	1	1	2	2	2	2	2
3	3	3	9	3	1	3	3	3	3
3	3	2	2	3	1	2	3	2	3
3	3	3	3	2	1	2	3	3	3
9	9	9	9	1	9	9	1	9	9

2	2	2	2	2	1	2	1	2	2
3	2	2	2	2	3	2	1	2	2
3	3	3	3	3	2	2	3	3	3
2	3	3	3	3	3	3	3	3	1
1	1	2	3	3	1	3	2	2	3
9	9	9	9	9	3	9	9	9	9
3	3	3	9	2	3	3	1	3	2
9	3	2	2	1	2	1	3	2	3
2	3	2	3	2	2	3	2	3	2
3	3	3	3	2	1	2	2	2	3
2	3	2	1	2	1	2	2	2	1
3	2	1	2	3	3	2	1	1	1
3	2	3	3	1	3	2	1	3	2
9	9	9	9	9	1	9	9	9	9
9	9	9	9	9	1	9	9	9	9
1	9	9	9	9	1	9	9	9	9
2	2	2	3	1	2	2	2	1	2
3	3	3	9	9	3	9	9	9	9
2	3	3	3	1	3	2	2	1	2
9	9	9	9	9	1	9	9	9	9
2	2	2	2	2	1	2	2	2	2
9	9	9	9	9	9	9	9	1	9
2	1	2	1	2	3	1	1	3	3
9	1	9	9	9	9	9	1	9	9
9	1	9	9	9	9	1	9	9	9
9	2	9	9	9	9	9	2	9	9
1	1	1	1	1	1	1	1	1	1
3	3	3	3	3	2	2	1	1	3
2	2	2	2	2	2	3	2	2	2
2	3	2	3	3	3	2	2	3	2
1	2	3	3	3	1	3	3	3	3
3	3	3	3	2	2	1	3	3	3
3	3	1	3	3	3	3	3	3	1
9	9	9	9	9	9	9	9	1	9
2	2	1	2	2	2	2	2	2	2
3	3	9	3	3	9	9	9	9	9
1	1	1	3	1	3	3	3	3	1
9	9	9	9	9	1	9	9	9	9
3	3	3	3	3	3	3	3	3	3
9	9	9	9	1	2	9	9	9	9
9	9	9	9	9	1	9	9	9	9
2	2	2	2	2	1	1	2	1	2
9	9	9	9	9	9	9	9	9	9
2	3	1	1	9	1	1	1	1	1
3	3	3	3	3	3	2	2	3	3
9	2	2	2	2	2	2	2	2	2
2	2	2	2	2	2	2	2	2	2

2	2	2	2	2	2	2	2	2	2
3	3	3	3	3	3	3	3	3	3
2	2	2	2	2	9	2	9	9	2
2	1	1	1	1	2	2	2	2	2
1	1	1	1	1	2	2	2	2	2
3	1	3	1	1	1	3	1	3	1
1	3	2	1	1	1	9	3	1	9
2	9	2	2	2	2	2	2	2	2
2	9	2	9	2	9	2	2	2	9
9	9	9	9	9	9	9	9	9	9
9	3	2	1	1	1	9	2	1	9
2	1	2	1	1	1	3	1	3	1
2	9	2	2	9	9	2	9	2	2
3	1	9	2	9	9	1	3	2	1
9	2	9	2	2	9	2	2	2	2
1	1	1	1	1	2	2	2	2	2
2	2	2	2	2	2	2	2	2	2
9	9	9	2	2	2	9	9	2	9
2	2	2	2	2	2	2	2	2	2
3	3	3	3	3	3	3	3	3	3
2	2	2	2	2	2	2	2	2	2
9	9	9	9	9	9	9	9	9	9
9	9	2	9	2	2	9	2	9	2
2	2	2	2	2	2	2	2	2	2
1	2	9	2	1	3	1	9	3	1
2	2	2	2	2	2	2	2	2	2
2	2	2	2	2	2	2	2	2	2
2	2	2	2	2	2	2	2	2	2
2	9	2	1	3	2	2	1	9	1
2	9	1	2	9	2	1	1	3	1
2	2	2	3	2	3	2	2	2	3
3	3	3	3	3	3	3	3	3	3
3	3	2	1	1	1	2	3	2	1
3	3	2	2	1	2	2	1	2	2
3	3	3	1	2	1	3	1	2	1
3	2	3	2	2	2	2	1	2	3
2	3	2	2	2	2	2	3	2	3
2	2	2	3	2	9	9	9	9	9
2	1	2	2	2	3	3	1	1	1
3	9	3	3	3	3	1	9	9	9
3	3	3	3	3	1	1	1	3	3
1	1	1	1	2	1	9	2	1	2
3	3	3	3	3	3	1	3	3	3
3	2	2	2	1	2	2	1	2	2
1	2	2	3	2	1	2	2	2	3
3	3	2	3	3	3	3	2	2	3
3	1	3	1	1	3	3	3	3	1

3	2	3	2	3	3	9	1	2	3
2	3	3	2	2	3	2	2	1	2
9	9	9	9	9	9	9	9	9	9
9	9	9	9	9	1	2	2	1	2
2	2	2	9	2	2	2	2	2	2
3	1	3	1	1	9	9	1	9	9
1	1	1	2	2	3	2	3	1	1
2	2	2	2	2	9	9	9	9	9
1	1	1	1	1	2	2	2	2	2
2	2	2	2	2	2	2	2	2	2
1	1	1	2	2	1	1	1	1	1
2	2	2	2	2	2	2	2	2	2
1	1	2	2	2	1	1	1	9	9
3	3	3	3	3	3	1	3	3	3
3	3	3	3	3	3	3	3	3	3
2	1	1	2	2	1	1	1	1	1
1	1	1	1	2	1	1	1	1	2
1	1	1	1	1	9	1	9	2	2
1	1	1	1	1	9	1	2	2	2
1	2	2	2	1	9	1	2	2	2
1	1	1	2	2	2	2	1	1	9
2	2	1	1	1	9	2	2	2	2
2	1	1	1	1	1	2	1	1	2
1	2	2	1	1	1	1	1	2	2
1	1	1	1	1	9	1	2	2	2
2	2	2	2	2	2	2	2	2	2
2	1	2	1	2	1	1	1	1	1
2	2	2	2	2	2	2	2	2	2
9	9	9	9	9	9	9	9	9	9
1	1	1	1	1	9	1	2	2	2
9	1	1	1	1	1	1	1	1	1
1	2	1	1	1	1	2	1	1	2
1	1	1	1	1	1	2	1	2	1
1	1	1	1	2	1	1	1	2	2
9	2	9	2	2	2	9	2	2	2
9	1	1	1	1	1	1	1	1	1
2	2	2	2	2	2	2	2	2	2
2	2	2	2	2	2	2	2	2	2
1	1	1	1	1	1	1	1	1	1
1	1	1	1	1	1	1	1	1	1
1	1	1	1	1	2	2	2	2	2
2	2	2	2	2	2	2	2	2	2
2	2	2	1	1	1	1	1	1	1
1	1	1	1	1	1	1	2	2	1
9	9	9	9	9	9	9	9	9	9
2	2	2	2	2	2	2	9	2	2
2	2	2	2	2	2	2	2	2	2

2	2	2	2	2	2	2	2	2	2
1	1	1	1	1	9	1	2	2	2
2	2	2	2	2	2	2	2	2	2
1	1	1	2	1	1	1	1	2	1
2	2	2	2	2	2	2	2	2	2
1	1	1	1	1	9	1	9	9	9
2	1	1	1	2	2	2	2	2	2
9	2	2	2	2	2	2	2	2	2
1	1	1	1	1	9	1	2	2	2
9	1	1	1	9	1	1	2	2	1
1	1	1	1	1	1	1	1	1	1
2	2	2	2	2	2	2	2	2	2
1	1	1	1	1	1	1	1	1	1
1	1	2	2	1	9	1	2	2	2
9	2	9	2	2	2	9	2	2	2
1	1	1	1	1	1	1	1	1	1
2	2	9	2	2	2	2	2	9	2
9	1	9	1	1	9	1	1	1	1
9	2	2	2	2	2	9	2	2	2
1	1	1	1	1	1	1	1	1	1
1	1	1	1	1	9	1	9	9	9
2	2	2	2	2	2	2	2	2	2
1	1	1	1	1	2	1	2	2	2
9	9	9	9	9	9	9	9	9	9
2	2	2	2	2	2	2	2	2	2
3	3	3	3	3	3	2	1	3	3
3	3	3	3	3	3	2	1	3	3
3	3	3	3	3	3	2	1	3	3
3	3	3	3	3	3	2	1	3	3
3	3	3	3	3	3	2	1	3	3
3	3	3	3	3	3	2	1	3	3
9	3	3	3	3	2	2	1	3	3
3	3	3	3	3	3	2	1	3	3
9	9	9	9	9	3	2	1	9	3
3	3	3	3	3	3	2	1	3	3
3	3	3	3	3	3	2	1	3	3
3	3	9	3	3	3	2	1	3	3
3	3	9	3	3	3	2	1	3	3
3	3	3	3	3	3	3	2	1	3
3	3	9	3	3	3	2	1	3	3
3	3	3	3	3	3	2	1	3	3
3	3	3	3	3	3	2	1	3	3
3	3	3	3	3	3	2	1	3	3
3	3	3	3	3	3	2	1	3	3
3	3	3	3	3	3	2	1	3	3
3	3	3	3	3	3	2	1	3	3
3	3	3	3	3	3	2	1	3	3

3	3	3	3	3	3	2	1	3	3
3	3	3	3	3	3	2	1	3	3
3	3	3	3	3	3	2	1	3	3
9	9	9	9	9	9	9	9	9	9
9	9	9	9	9	9	9	9	9	9
9	9	9	9	9	9	9	9	9	9
9	9	9	9	9	9	9	9	9	9
3	2	2	3	3	3	2	1	2	2
9	9	9	9	9	9	9	9	9	9
9	9	9	9	9	9	9	9	9	9
9	9	9	9	9	9	9	9	9	9
9	9	9	9	9	9	9	9	9	9
9	9	9	9	9	9	9	9	9	9
9	9	9	9	9	9	9	9	9	9
9	9	9	9	9	9	9	9	9	9
9	9	9	9	9	9	9	9	9	9
9	9	9	9	9	9	9	9	9	9
9	9	9	9	9	9	9	9	9	9
1	2	2	2	1	1	2	2	2	3
9	9	9	9	9	9	9	9	9	9
9	9	9	9	9	9	9	9	9	9
9	9	9	9	9	9	9	9	9	9
1	1	1	1	1	1	2	1	1	3
9	9	9	9	9	9	9	9	9	9
9	9	9	9	9	9	9	9	9	9
3	3	3	3	3	3	2	1	3	3
3	3	3	3	3	3	2	1	3	3
3	1	3	3	3	3	2	1	3	3
3	3	3	3	3	3	2	1	3	3
3	3	3	3	3	3	2	1	3	3
3	3	3	3	3	3	2	1	3	3
3	3	3	3	3	3	2	1	3	3
3	3	3	3	3	3	2	1	3	3
3	3	3	3	3	3	2	1	3	3
3	3	3	3	3	3	2	1	3	3
3	3	3	3	3	3	1	3	3	3
3	3	3	3	3	3	2	1	3	3
3	3	3	3	3	3	3	1	3	3
3	3	3	3	3	3	2	1	3	3
3	3	3	3	9	3	2	1	3	3
3	3	3	3	3	3	3	1	3	3
9	9	9	9	9	3	9	9	9	9
3	3	3	3	3	3	3	1	3	3
3	3	3	3	3	3	3	1	3	3
3	3	3	3	3	3	3	1	3	3
3	3	3	3	3	3	2	1	3	3
3	3	3	3	9	3	9	1	3	3

3	3	3	3	3	3	2	1	3	3
3	3	3	3	3	3	2	1	3	3
3	3	3	3	3	3	2	1	3	3
3	3	3	3	3	3	3	1	3	3
3	3	3	3	3	3	2	1	3	3
3	3	3	3	3	3	2	1	3	3
3	3	3	3	3	3	2	1	3	3
3	3	3	3	3	3	2	1	3	3
3	3	3	3	3	3	2	1	3	3
3	3	3	3	3	3	2	1	3	3
3	3	3	3	3	3	2	1	3	3
3	3	3	3	3	3	2	1	3	3
3	3	3	3	3	3	2	1	3	3
3	3	3	3	3	3	3	2	3	3
3	3	3	3	3	3	3	2	3	3
3	3	3	3	3	3	2	1	3	3
3	3	3	3	3	3	2	1	3	3
3	3	3	3	3	3	2	1	3	3
3	3	3	3	3	3	2	1	3	3
3	3	3	3	3	3	3	1	3	3
3	3	3	3	3	3	3	1	3	3
3	3	3	3	3	3	2	1	3	3
3	3	3	3	3	3	3	1	3	3
3	3	3	3	3	3	2	1	3	3
3	3	3	3	3	9	2	1	3	3
3	3	3	3	3	3	2	1	3	3
3	3	3	3	3	3	2	1	3	3
3	3	3	3	3	3	2	1	3	3
3	3	3	3	3	3	3	1	3	3
3	3	3	3	3	3	3	1	3	3
3	3	3	3	3	3	2	1	3	3
3	3	3	3	3	3	3	1	3	3
3	3	3	3	3	3	2	1	3	3
3	3	3	3	3	1	3	1	3	3
3	3	3	3	3	3	3	1	3	3
3	3	3	3	3	3	3	1	3	3
3	3	3	3	3	3	2	1	3	3
3	3	3	3	3	3	2	1	3	3
3	3	3	3	3	9	9	9	9	9
3	3	3	3	3	3	2	1	3	3
3	3	3	3	3	2	3	1	3	3
3	3	3	3	3	3	2	1	3	3
3	3	3	3	3	3	3	1	3	3
3	3	3	3	3	3	3	1	3	3
3	3	3	3	3	3	3	3	3	3
3	3	3	3	3	3	2	1	3	3
3	3	3	3	3	1	2	1	3	3

3	3	3	3	3	3	2	1	3	3
3	3	3	3	3	3	3	1	3	3
3	3	3	3	3	3	3	1	3	3
3	3	3	3	3	3	2	1	3	3
3	3	3	3	3	3	2	1	3	3
3	3	3	3	3	3	2	1	3	3
3	3	3	3	3	3	2	1	3	3
3	3	3	3	3	3	2	1	3	3
3	3	3	3	3	3	2	1	3	3
3	3	3	3	3	3	2	1	3	3
3	3	3	3	3	3	2	1	3	3
3	3	3	3	3	3	2	1	3	3
3	3	3	3	3	3	2	1	3	3
3	3	3	3	3	3	2	1	3	3
3	3	3	3	3	3	2	1	3	3
3	3	3	3	3	3	2	1	3	3
3	3	3	3	3	3	2	1	3	3
3	3	3	3	3	3	2	1	3	3
3	3	3	3	3	3	2	1	3	3
3	1	3	3	3	3	2	3	3	3
3	3	3	3	3	3	3	1	3	3
3	3	3	3	3	3	2	1	3	3
3	3	3	3	3	3	2	1	3	3
3	3	3	3	3	3	2	1	3	3
3	3	3	3	3	3	2	1	3	3
9	9	9	9	9	9	9	9	9	9
3	3	3	3	3	3	3	1	3	3
3	3	3	3	3	3	2	1	3	3
3	3	3	3	3	3	2	1	3	3
3	3	3	3	3	3	2	1	3	3
3	3	3	1	9	9	9	9	9	9
3	3	3	3	3	3	2	1	3	3
3	3	3	3	3	3	2	1	3	3
3	3	9	3	3	3	2	1	3	3
3	3	3	3	3	3	2	1	3	3
3	3	3	3	3	3	2	1	3	3
3	3	3	3	3	3	2	1	3	3
3	3	3	3	3	3	2	1	3	3
3	3	3	3	3	3	3	1	3	3
3	3	3	3	3	3	3	1	3	3
3	3	3	3	3	3	2	1	3	3
3	3	3	3	3	3	2	1	3	3
3	3	3	3	3	1	2	1	3	3
3	3	3	3	3	3	2	1	3	3
3	3	3	3	3	3	2	1	3	3
3	3	3	3	3	3	2	1	3	3
3	3	3	3	3	3	2	1	3	3

9	9	9	9	9	9	9	9	9	9
3	3	3	3	3	3	2	1	3	3
3	3	3	3	3	3	2	1	3	3
3	3	3	3	3	3	2	1	3	3
3	3	3	3	3	3	2	1	3	3
3	3	3	3	3	3	2	1	3	3
3	3	3	3	3	3	2	1	3	3
3	3	3	3	3	3	2	1	3	3
3	3	3	3	3	3	2	1	3	3
3	3	3	3	3	3	2	1	3	3
3	3	3	3	3	3	2	1	3	3
3	3	3	3	3	3	2	1	3	3
3	3	3	3	3	3	2	1	3	3
3	3	3	3	3	3	9	1	3	3
3	3	3	3	3	3	3	1	3	3
3	3	3	3	3	3	2	1	3	3
3	3	3	3	3	3	2	1	3	3
3	3	3	3	3	3	2	1	3	3
3	3	3	3	3	3	2	1	3	3
3	3	3	3	3	3	2	1	3	3
3	3	3	3	3	3	2	1	3	3
3	3	3	3	3	3	2	1	3	3
3	3	3	3	3	3	2	1	3	3
3	3	3	3	3	3	2	1	3	3
3	3	3	3	3	3	2	1	3	3
3	3	3	3	3	3	2	1	3	3
3	3	3	3	3	3	2	1	3	3
3	3	3	3	3	3	2	1	3	3
3	3	3	3	3	3	2	1	3	3
3	3	3	3	3	3	2	1	3	3
3	3	3	3	3	3	2	1	3	3
3	3	3	3	3	3	2	1	3	3
3	3	3	3	3	3	2	1	3	3
9	2	9	9	9	9	9	9	9	9
9	2	9	9	9	9	9	9	9	9
9	2	9	9	9	9	9	9	9	9
9	2	9	9	9	9	9	9	9	9
9	9	9	9	9	9	9	9	9	9
2	2	2	2	2	2	2	9	9	9
9	9	9	9	9	9	9	9	9	9
9	9	9	9	9	9	9	9	9	9
9	9	9	9	9	9	9	9	9	9
9	9	9	9	9	9	9	9	9	9
9	9	9	9	9	9	9	9	9	9
9	9	9	9	9	9	9	9	9	9
9	9	9	9	9	9	9	9	9	9
9	9	9	9	9	9	9	9	9	9

3	3	3	9	9	9	9	9	9	9
2	1	3	3	1	3	2	1	2	3
1	1	1	1	1	1	1	1	1	1
9	9	9	9	9	9	9	9	9	9
9	9	9	9	9	9	9	9	9	9
9	9	9	9	9	9	9	9	9	9
9	9	9	9	9	9	9	9	9	9
3	3	3	3	9	9	9	9	9	9
9	9	9	9	9	9	9	9	9	9
9	9	1	9	9	9	9	9	1	9
9	9	9	9	9	9	9	9	9	9
9	1	9	9	9	9	2	9	9	9
3	3	3	1	2	1	1	1	1	3
2	2	2	2	1	3	3	1	3	3
9	9	9	9	9	9	9	9	9	9
9	9	9	9	9	9	9	9	9	9
9	9	9	9	9	9	9	9	9	9
9	9	9	9	9	9	9	9	9	9
9	9	9	9	9	9	9	9	9	9
3	3	1	1	3	3	1	1	2	1
2	2	2	2	2	2	2	2	2	2
3	3	3	3	3	2	2	1	2	2
3	3	1	3	1	3	3	2	1	1
2	3	3	3	3	1	3	2	2	3
3	3	3	9	1	2	3	2	3	3
1	3	1	3	1	1	3	1	1	3
3	1	3	3	1	2	1	2	1	3
3	3	3	3	3	1	1	1	1	1
3	2	3	2	2	3	2	3	2	3
3	3	3	3	3	3	3	3	3	3
9	9	9	9	9	9	9	1	9	9
2	2	2	2	2	2	2	2	2	2
3	2	2	2	2	2	2	2	2	2
1	1	1	1	3	3	1	3	3	3
3	3	3	9	1	2	3	1	3	2
3	2	2	2	2	2	2	2	3	3
3	3	3	3	3	3	3	3	3	3
2	3	1	2	1	1	2	3	1	2
1	9	9	9	9	9	9	1	9	9
2	2	3	2	2	3	2	2	2	2
9	9	9	9	9	1	9	9	9	9
9	9	9	9	9	9	9	9	1	9
2	1	2	2	1	2	2	2	1	2
2	9	3	3	1	3	3	2	1	2
9	9	9	9	1	9	1	9	9	9
9	9	9	9	9	1	2	2	2	2
9	9	9	9	9	9	9	9	9	9

3	1	3	3	1	1	9	1	2	3
2	1	2	2	1	3	3	1	3	3
3	3	3	3	3	9	9	9	9	9
9	9	9	9	1	1	1	1	1	9
2	2	2	2	1	9	9	9	1	9
2	2	2	2	2	2	2	2	2	2
9	9	9	9	9	2	9	9	9	9
9	9	9	9	9	9	9	9	9	9
3	3	3	3	3	2	3	1	2	2
1	2	2	2	1	1	1	1	3	3
9	9	9	9	9	1	9	9	9	9
9	9	9	9	9	9	9	2	9	9
2	2	2	2	2	2	2	1	2	2
2	2	2	2	2	2	2	2	2	2
2	2	2	2	2	2	2	2	2	2
1	1	1	1	1	1	2	2	2	2
2	1	1	2	2	2	2	2	2	2
9	9	9	9	9	9	9	9	9	9
9	9	9	9	9	9	9	1	9	9
2	2	2	2	2	2	2	2	2	2
2	3	2	2	2	2	2	2	3	3
3	3	3	3	3	2	2	2	3	2
3	3	3	3	3	1	1	1	1	1
2	2	3	2	1	3	9	1	2	1
1	9	1	2	1	1	2	1	1	1
9	9	9	9	9	2	1	3	2	3
3	3	3	2	2	2	2	2	2	2
2	2	2	2	2	2	2	2	2	2
2	2	2	2	2	2	2	2	2	2
3	2	2	2	2	3	2	1	2	2
2	2	2	2	2	2	2	2	2	2
2	1	1	2	3	3	2	2	2	9
2	2	3	2	2	3	3	2	2	2
3	3	3	2	2	2	2	2	2	2
2	3	2	2	2	2	2	2	2	2
2	1	1	1	2	1	1	1	2	2
3	3	3	3	3	1	3	3	3	3
3	3	3	3	3	3	1	2	3	3
2	2	2	2	1	2	1	1	1	1
2	2	2	2	2	3	2	2	1	1
1	3	2	1	1	2	2	2	2	2
9	9	9	9	9	9	9	9	9	9
2	2	2	2	2	2	3	2	2	2
2	2	2	9	9	3	3	3	3	3
9	9	9	9	9	9	9	9	9	9
3	3	3	3	3	1	3	3	3	3
3	3	2	2	3	3	2	2	2	3

2	2	3	3	2	2	2	2	2	2
2	3	1	2	3	1	2	3	1	2
3	3	3	9	3	2	3	3	3	3
3	1	3	3	1	1	1	3	3	3
2	2	2	2	2	1	1	1	1	1
2	2	2	2	2	1	2	2	2	2
3	2	2	2	2	2	2	2	2	3
1	2	3	2	3	3	2	3	2	3
2	2	2	2	2	1	1	1	1	1
9	9	9	9	9	9	9	9	9	9
3	3	3	3	3	2	3	3	3	3
2	2	2	2	2	1	2	2	1	2
2	2	2	3	2	1	2	2	3	2
2	2	2	2	2	2	1	2	2	2
2	2	2	2	2	2	2	2	2	2
2	1	3	2	3	1	3	2	3	2
2	2	2	2	2	2	2	2	2	2
2	2	2	2	2	2	2	2	2	2
1	1	1	1	1	2	2	2	2	2
2	2	2	2	2	2	2	2	2	2
9	9	9	9	9	9	9	9	9	9
1	2	2	2	2	1	2	2	2	2
3	3	3	3	1	3	3	3	3	3
2	2	2	2	2	2	2	2	2	2
2	2	2	2	2	2	2	2	2	2
2	3	1	2	1	1	2	3	1	2
1	1	3	3	3	2	2	2	2	2
1	1	1	2	1	2	2	2	1	1
9	9	9	9	9	9	9	9	9	9
9	9	9	9	9	9	9	9	9	9
3	3	3	3	3	3	3	3	3	3
3	1	1	1	1	2	1	1	2	1
1	2	3	1	1	1	1	2	3	3
3	3	3	3	3	1	3	3	3	3
3	3	1	1	1	1	3	1	3	2
3	3	3	2	2	1	2	2	2	2
3	3	2	2	2	1	2	2	2	3
9	9	9	1	9	9	9	9	9	2
1	2	3	3	3	3	2	2	2	3
1	3	2	1	2	2	2	2	2	2
1	1	1	1	1	9	9	9	1	9
9	9	9	9	9	9	9	9	9	9
2	1	2	3	1	1	1	3	3	3
2	2	1	2	3	2	2	2	2	2
3	2	3	2	3	3	1	2	1	1
2	2	2	2	2	2	2	2	2	2
3	2	3	3	2	2	1	3	3	2

3	1	3	1	1	3	2	1	2	3
3	3	2	2	2	1	2	2	2	3
3	2	3	9	9	9	9	9	9	9
9	9	9	9	9	9	9	9	9	9
2	9	9	9	9	9	9	9	9	9
1	1	1	1	1	9	9	9	1	9
3	2	3	2	2	1	2	2	3	3
3	2	3	3	2	2	1	1	3	3
3	3	3	1	2	3	2	2	1	2
3	3	3	3	1	1	3	3	3	3
2	2	2	2	2	2	2	2	1	2
3	3	2	3	2	2	1	3	3	3
3	3	3	3	1	3	3	3	3	3
3	3	2	2	3	3	3	3	3	3
3	1	1	1	1	1	2	1	1	2
2	2	2	2	2	2	3	1	2	1
2	1	3	1	2	1	2	3	1	2
2	2	2	2	2	1	1	3	3	3
2	2	2	2	2	2	2	2	2	2
3	3	3	3	1	1	1	2	3	3
2	2	2	2	2	2	2	2	2	2
3	3	3	3	3	1	2	2	2	2
3	1	1	3	1	3	3	3	1	1
3	1	3	3	1	2	1	3	3	3
3	3	3	3	3	1	1	1	3	3
3	3	2	3	1	9	9	9	9	9
1	3	1	2	3	3	3	2	1	1
3	3	1	2	1	3	1	2	3	3
3	3	3	3	3	1	3	3	3	3
3	3	3	3	1	1	3	3	3	3
3	2	2	3	3	1	3	2	1	3
9	9	9	9	9	9	9	9	9	9
1	2	1	2	1	1	2	2	1	2
3	3	3	3	1	2	2	1	3	3
2	2	2	2	2	2	2	3	3	2
2	1	2	2	3	3	3	1	2	2
2	2	2	2	2	3	2	2	2	2
9	9	9	9	1	9	9	1	9	9
3	3	3	1	2	3	2	1	3	3
1	2	2	3	2	3	3	3	1	1
2	3	1	2	2	1	2	3	3	3
9	1	2	3	2	2	1	2	3	2
3	9	3	9	9	1	2	2	2	2
2	2	2	2	2	3	2	1	1	2
3	2	3	3	3	1	3	3	3	3
9	9	9	9	9	2	9	9	9	9
2	3	1	2	1	1	2	3	2	1

2	2	2	2	2	2	2	2	2	2
3	3	3	3	3	2	2	2	2	2
2	2	2	2	2	2	2	2	2	2
2	2	2	2	2	2	2	2	2	2
2	2	3	9	1	2	2	1	1	1
2	2	3	2	3	2	2	2	1	1
3	3	3	2	3	1	1	2	2	3
2	2	2	2	2	2	2	2	2	2
3	3	3	2	3	3	1	2	3	3
2	3	3	3	2	2	2	1	2	3
3	3	1	3	3	2	2	2	2	2
2	2	3	3	3	2	2	2	2	2
3	3	3	2	3	1	1	3	3	3
3	3	3	2	3	2	1	2	2	1
3	3	3	3	1	1	1	3	3	3
3	3	3	3	3	3	3	3	3	3
3	3	3	3	3	1	3	3	3	2
2	2	2	2	2	3	3	3	3	3
3	3	3	3	3	3	3	3	3	3
3	3	3	3	1	3	2	1	1	3
2	2	2	2	2	1	1	1	1	1
3	2	3	2	2	2	2	2	2	2
9	9	9	9	3	9	9	9	1	9
3	2	3	2	2	2	2	2	2	2
2	3	2	2	1	2	2	2	2	2
3	3	2	1	1	2	1	1	1	3
2	2	2	1	1	2	2	1	1	1
3	1	3	2	2	3	3	1	3	3
9	9	9	9	1	9	2	9	9	9
2	2	2	2	2	3	3	1	3	3
2	2	2	2	2	9	9	9	1	9
1	2	2	2	1	2	2	2	2	2
3	2	2	2	3	1	1	3	3	3
3	2	2	2	2	2	2	2	2	2
3	3	3	3	3	3	3	1	3	3
3	1	2	2	1	3	2	1	2	2
3	3	3	3	3	3	3	1	3	3
2	2	2	2	2	1	2	2	2	2
9	2	1	1	1	3	2	2	2	2
2	2	2	2	2	1	2	2	2	2
9	9	9	9	9	2	9	9	9	9
3	3	9	1	3	3	3	1	3	3
2	1	2	2	2	2	2	2	2	2
2	1	1	2	1	1	1	1	1	1
1	1	1	1	1	1	1	1	1	1
1	1	2	2	2	2	2	2	2	2
2	2	3	2	1	1	2	3	2	1

9	9	9	9	9	9	9	9	9	9
9	9	9	9	9	9	9	9	9	9
1	1	1	1	1	1	1	1	1	1
2	3	2	2	3	1	2	3	2	3
9	9	9	9	9	9	9	9	9	9
2	2	2	2	2	1	1	1	1	1
2	2	2	2	2	2	2	2	2	2
2	1	2	2	1	1	1	1	1	1
3	2	3	2	2	1	2	3	1	2
2	3	2	1	2	1	2	3	2	1
3	3	3	3	3	3	3	1	3	3
2	3	1	2	3	1	2	3	1	2
2	1	2	2	2	1	2	2	2	2
9	9	9	9	9	1	9	9	9	9
1	3	3	2	1	1	1	1	2	3
9	9	9	9	9	9	9	9	9	9
2	2	2	2	2	1	1	1	1	1
3	3	2	2	3	2	2	1	3	3
2	2	2	2	2	2	2	1	3	1
2	2	2	2	2	2	2	2	2	2
1	1	1	1	1	1	1	1	1	1
9	9	9	9	9	9	9	9	9	9
9	9	9	9	9	9	9	9	9	9
2	1	1	1	2	2	3	3	3	3
2	2	2	2	2	2	2	2	2	2
1	1	2	2	2	2	9	9	9	9
2	2	2	2	2	2	2	2	2	2
2	2	2	2	2	9	9	9	9	9
3	3	3	3	2	3	3	3	3	1
1	3	2	1	2	2	1	2	3	2
2	9	2	3	2	2	9	1	3	3
1	9	9	9	9	9	9	1	9	9
9	1	9	2	2	2	2	1	2	2
2	2	2	2	2	9	1	9	9	9
9	9	9	9	9	9	9	9	9	9
3	3	3	3	2	3	2	1	2	2
1	2	1	2	1	1	2	1	2	1
2	2	2	2	2	2	2	2	2	2
2	2	2	2	2	2	2	2	2	2
2	2	2	2	2	2	2	2	2	2
1	2	1	2	1	1	2	2	2	2
2	2	2	2	2	2	2	2	2	2
2	2	2	2	2	3	3	3	3	3
9	9	9	9	9	9	9	1	9	9
9	9	9	9	2	9	9	9	9	2
2	2	2	2	2	2	2	2	2	2
2	2	2	2	2	2	2	2	2	2

9	1	9	9	9	9	1	9	9	9
2	1	2	1	2	2	2	3	1	2
9	9	9	9	9	2	9	9	9	9
2	2	2	2	2	2	2	2	2	2
2	1	2	2	1	2	1	2	1	2
2	2	2	2	2	2	2	2	2	2
1	2	1	2	3	3	9	9	9	9
9	9	9	9	9	9	9	1	9	9
2	2	2	2	2	2	2	2	2	2
3	3	2	2	1	3	3	1	2	3
2	2	2	2	2	3	3	3	3	3
3	2	3	3	3	3	3	2	2	1
1	1	2	3	1	2	2	1	1	2
2	3	1	2	3	2	1	2	3	1
2	2	2	2	2	2	2	2	2	2
1	1	1	1	1	9	9	9	9	1
3	3	3	3	1	3	3	3	3	1
2	2	2	2	2	2	2	2	2	2
2	3	2	3	2	3	2	2	2	2
3	2	3	3	3	3	2	2	3	3
3	1	2	2	2	1	2	3	2	2
1	2	3	2	2	1	2	3	2	2
2	2	2	2	2	2	1	2	2	2
2	2	2	2	2	2	2	2	2	2
3	3	3	2	1	3	2	3	1	1
3	3	2	2	2	1	2	3	1	3
2	2	2	2	2	2	1	2	3	3
3	3	3	2	3	2	2	1	2	2
2	3	1	3	2	1	2	1	3	2
9	9	9	9	9	9	9	9	9	9
2	3	3	3	1	2	2	2	2	2
9	9	9	9	9	9	9	9	1	9
9	9	9	9	9	9	9	9	9	1
9	9	9	9	9	9	9	9	1	9
1	2	3	3	3	2	2	2	3	3
3	3	3	3	3	2	2	2	2	2
3	3	3	3	3	2	2	2	2	2
3	1	1	3	9	1	9	9	9	9
2	1	1	3	1	2	2	2	2	2
2	2	2	2	2	2	2	2	2	2
2	2	9	9	9	9	9	1	9	9
3	1	3	2	3	2	2	1	1	2
3	3	3	2	1	1	2	9	3	1
9	1	2	3	3	1	2	1	2	1
3	1	2	2	1	2	1	3	3	3
3	3	3	3	3	3	3	1	3	3
2	3	2	2	2	1	2	3	2	1

2	3	1	2	1	9	9	9	9	1
9	9	9	9	9	9	9	1	9	9
9	9	9	9	9	9	9	1	9	9
3	1	2	1	3	1	3	2	3	1
2	2	2	2	2	2	2	2	2	2
2	2	2	2	2	2	2	2	2	2
3	2	1	3	2	1	2	3	1	2
9	9	9	9	9	9	9	9	9	9
9	9	9	9	9	9	9	9	9	9
9	9	9	9	9	9	9	9	9	9
2	2	2	2	2	9	9	1	9	9
9	9	9	9	1	9	9	1	9	9
9	9	9	9	9	9	9	9	9	9
9	9	9	9	9	9	9	9	9	9
9	9	9	9	9	9	9	9	9	9
9	1	9	9	9	1	9	9	9	9
2	2	1	2	1	1	2	1	2	1
1	2	1	1	2	1	1	1	1	1
2	2	1	2	1	1	1	2	3	3
2	2	3	2	2	2	2	2	2	2
1	2	1	2	1	2	2	2	2	2
2	2	2	2	2	1	2	2	2	2
1	1	1	1	2	1	2	1	9	9
9	2	2	2	2	1	2	2	2	2
3	2	2	3	2	1	1	2	3	3
2	1	2	2	1	3	3	2	9	1
3	2	2	2	3	3	2	1	3	3
3	2	2	2	3	3	2	3	1	3
1	1	1	1	1	3	9	3	3	3
2	2	2	2	2	3	3	3	3	3
2	2	2	2	2	2	2	2	2	2
2	2	2	2	2	2	2	2	2	2
2	2	2	2	2	2	2	2	2	2
1	1	1	2	2	2	1	1	2	2
9	9	9	9	9	9	9	9	9	9
2	2	2	2	2	2	2	2	2	2
9	9	9	9	9	9	9	9	9	9
2	2	2	2	2	2	2	2	2	2
1	1	1	1	1	1	1	1	1	1
9	9	9	9	9	1	9	9	9	9
3	3	3	3	3	3	3	3	3	3
3	1	2	1	1	1	1	1	3	3
2	2	2	2	2	2	2	2	2	2
2	2	2	2	2	2	2	2	2	2
3	2	1	1	3	3	1	3	3	3
9	9	9	9	9	9	9	9	9	9
3	2	3	1	1	2	1	1	2	1

2	2	2	2	2	3	1	3	3	3
2	2	2	2	2	2	2	2	2	2
3	3	3	3	3	2	1	3	3	3
3	3	3	2	2	2	2	1	2	2
9	9	9	9	9	9	9	9	9	9
2	2	2	2	2	2	2	2	2	2
9	9	9	9	9	9	9	9	9	9
3	1	2	1	3	2	2	2	2	2
3	3	3	3	3	1	1	1	9	1
9	9	9	9	9	9	9	9	9	9
9	9	9	9	9	9	9	9	9	9
9	9	9	9	9	9	9	9	9	9
9	9	9	9	9	9	9	9	9	9
9	9	9	9	9	9	9	9	9	9
9	9	9	9	9	9	9	9	9	9
9	9	9	9	9	9	9	9	9	9
9	9	9	9	9	9	9	9	9	9
9	9	9	9	9	9	9	9	9	9
9	9	9	9	9	9	9	9	9	9
9	9	9	9	9	9	9	9	9	9
9	9	9	9	9	9	9	9	9	9
9	9	9	9	9	9	9	9	9	9
9	9	9	9	9	9	9	9	9	9
9	9	9	9	9	9	9	9	9	9
9	9	9	9	9	9	9	9	9	9
9	9	9	9	9	9	9	9	9	9
9	9	9	9	9	9	9	9	9	9
9	9	9	9	9	9	9	9	9	9
9	9	9	9	9	9	9	9	9	9
2	3	2	1	3	1	2	3	1	2
2	2	2	2	2	2	2	2	2	2
2	2	2	2	2	1	2	2	2	2
2	3	2	3	3	2	1	1	2	2
3	3	3	3	3	3	3	1	3	3
9	9	9	9	9	9	9	1	9	9
2	2	2	2	2	2	2	2	2	2
3	9	3	3	3	3	3	1	3	3
1	1	1	1	3	9	9	1	9	9
2	2	2	2	2	3	3	1	2	2
3	3	3	3	3	1	3	3	3	3
9	9	9	9	9	9	9	1	9	9
3	2	3	2	3	1	1	3	3	3
3	1	1	1	1	3	3	1	3	3
3	3	2	2	2	1	1	2	2	2
3	2	3	2	3	1	1	3	3	3
9	9	9	9	9	9	9	1	9	9
3	3	3	3	3	1	2	3	2	3

2	3	3	1	3	3	3	1	3	3
9	9	2	9	9	3	9	1	9	9
3	1	3	3	3	3	3	1	3	1
3	3	3	3	3	3	3	1	3	3
2	2	2	2	2	1	2	3	2	3
2	2	2	2	2	2	2	1	2	2
2	1	1	1	1	1	2	2	1	3
3	3	3	3	1	3	1	1	1	1
3	3	3	3	3	9	9	1	9	9
3	3	3	3	1	3	2	2	3	1
1	2	1	2	3	2	1	1	9	2
9	9	9	9	9	1	9	9	9	9
3	2	9	2	3	1	1	2	3	3
9	9	9	9	9	9	9	9	9	1
9	9	9	9	9	2	9	9	9	9
9	9	9	9	9	2	9	9	9	9
9	9	9	9	9	9	9	1	9	9
9	9	9	9	9	9	9	1	9	9
1	9	9	9	9	9	9	9	1	9
9	9	9	9	9	9	9	9	1	9
1	9	9	9	9	9	1	9	9	9
3	3	3	1	3	3	3	3	1	3
3	3	3	3	3	3	3	3	3	3
3	2	2	3	3	2	2	2	2	2
9	1	9	9	9	9	9	9	1	9
3	1	3	3	3	1	3	3	3	3
9	9	9	9	9	9	9	1	9	9
3	3	3	1	2	3	3	1	3	3
9	9	9	9	9	9	9	1	9	9
9	9	9	9	9	9	1	9	9	9
9	9	9	9	9	9	9	1	9	9
2	1	2	3	3	1	2	3	2	1
9	1	1	3	3	3	3	1	1	3
3	3	3	3	2	1	2	3	2	3
3	3	3	3	2	3	1	2	2	3
2	2	2	2	2	2	2	2	2	2
3	3	3	1	1	2	2	9	2	1
3	3	3	3	1	1	3	1	9	3
3	3	3	3	3	3	3	1	3	3
9	9	9	9	9	1	3	1	1	3
3	2	3	3	2	2	3	1	1	3
9	9	9	9	9	9	1	9	9	9
2	2	2	2	2	2	2	2	2	2
3	3	3	3	2	2	2	2	2	2
2	2	2	2	1	2	2	2	2	2
3	2	2	2	1	2	1	1	2	3
2	2	2	2	3	2	2	1	2	3

3	2	2	2	1	2	1	2	2	2
9	9	9	9	9	3	1	3	3	3
2	2	2	2	2	3	3	3	3	1
1	3	2	2	3	3	1	2	1	2
9	9	9	9	9	9	1	9	9	9
9	9	9	9	9	9	9	1	9	9
3	1	1	1	2	2	2	2	1	3
3	3	3	3	3	3	3	1	3	3
3	3	3	3	3	3	3	1	3	3
9	9	9	9	9	1	9	9	9	9
9	1	9	1	3	1	9	9	9	2
3	1	3	2	3	3	3	1	1	1
3	3	3	3	2	3	2	1	1	3
3	3	2	2	3	2	9	9	9	9
9	9	9	9	9	2	9	9	9	9
3	3	2	2	2	1	9	9	9	9
2	2	2	2	2	2	2	2	2	2
1	3	1	1	1	1	9	9	9	9
3	1	3	3	2	3	1	2	3	3
9	1	3	1	9	3	9	1	9	2
9	9	9	9	1	9	9	9	1	9
3	2	2	2	1	3	2	1	3	3
9	9	9	1	9	9	1	9	9	9
3	3	9	1	9	3	1	2	9	3
3	3	3	3	1	3	3	3	3	3
2	1	2	1	2	2	2	2	2	2
3	3	3	3	1	3	3	2	2	1
3	3	3	3	3	1	3	3	2	3
3	1	3	3	2	3	3	3	3	3
3	3	3	3	3	2	2	2	2	2
2	2	2	2	2	1	2	2	2	2
9	9	9	9	9	9	9	9	9	9
3	1	3	1	1	1	9	9	9	9
3	1	2	1	1	3	3	1	3	3
3	3	3	3	3	3	2	2	2	2
1	2	2	2	1	9	9	1	9	9
9	9	9	9	9	9	9	9	9	9
9	9	9	9	9	9	9	9	9	9
9	9	9	9	9	9	9	9	9	9
9	9	9	9	9	9	9	9	9	9
9	9	9	9	9	9	9	9	9	9
9	9	9	9	9	9	9	9	9	9
9	9	9	9	9	9	9	9	9	9
9	9	9	9	9	9	9	9	9	9
9	9	9	9	9	9	9	9	9	9
9	9	9	9	9	9	9	9	9	9
9	9	9	9	9	9	9	9	9	9

9	9	9	9	9	9	9	9	9	9
9	9	9	9	9	9	9	9	9	9
9	9	9	9	9	9	9	9	9	9
9	9	9	9	9	9	9	9	9	9
9	9	9	9	9	9	9	9	9	9
9	9	9	9	9	9	9	9	9	9
9	9	9	9	9	9	9	9	9	9
9	9	9	9	9	9	9	9	9	9
9	9	9	9	9	9	9	9	9	9
9	9	9	9	9	9	9	9	9	9
9	9	9	9	9	9	9	9	9	9
9	9	9	9	9	9	9	9	9	9
9	9	9	9	9	9	9	9	9	9
9	9	9	9	9	9	9	9	9	9
9	9	9	9	9	9	9	9	9	9
9	9	9	9	9	9	9	9	9	9
9	9	9	9	9	9	9	9	9	9
9	9	9	9	9	9	9	9	9	9
9	9	9	9	9	9	9	9	9	9
9	9	9	9	9	9	9	9	9	9
9	9	9	9	9	9	9	9	9	9
9	9	9	9	9	9	9	9	9	9
9	9	9	9	9	9	9	9	9	9
9	9	9	9	9	9	9	9	9	9
9	9	9	9	9	9	9	9	9	9
2	2	2	2	2	2	2	1	2	2
9	9	9	9	9	9	9	9	9	9
9	9	9	9	9	9	9	9	9	9
9	9	9	9	9	9	9	9	9	9
9	9	9	9	9	9	9	9	9	9
9	9	9	9	9	9	9	9	9	9
9	9	9	9	9	9	9	9	9	9
9	9	9	9	9	9	9	9	9	9
9	9	9	9	9	9	9	9	9	9
9	9	9	9	9	9	9	9	9	9
9	9	9	9	9	9	9	9	9	9
9	9	9	9	9	9	9	9	9	9
9	9	9	9	9	9	9	9	9	9
9	9	9	9	9	9	9	9	9	9
9	9	9	9	9	9	9	9	9	9
9	9	9	9	9	9	9	9	9	9
9	9	9	9	9	2	2	9	9	9
1	1	1	1	1	1	3	3	3	3
3	3	3	3	3	1	1	2	1	3
3	1	3	3	2	3	1	2	3	3
3	3	3	3	3	3	3	3	3	3
3	3	3	3	3	3	3	3	3	3

3	3	3	1	1	2	1	3	3	3
9	9	9	9	9	2	9	9	9	9
3	3	3	1	1	2	1	3	3	3
2	2	2	1	3	2	2	1	2	3
2	1	1	3	1	1	2	3	1	2
3	3	3	3	3	3	2	2	2	2
3	2	3	2	3	1	1	3	3	3
1	1	1	1	1	3	3	1	3	3
9	9	9	9	9	1	1	2	3	3
3	3	3	3	3	3	1	3	3	3
3	3	3	3	3	2	2	2	1	2
2	9	1	1	3	2	1	1	3	3
2	2	2	1	3	2	2	1	2	2
3	2	2	2	2	3	2	1	3	3
3	3	2	2	2	3	2	1	2	2
2	1	1	2	3	3	2	1	2	1
9	9	9	9	9	9	9	1	9	9
3	3	3	2	2	2	2	2	2	3
3	3	3	3	3	1	2	3	2	3
2	3	2	2	2	2	2	2	2	1
2	3	2	2	2	2	2	2	1	3
3	3	3	3	3	3	3	1	3	3
2	2	2	2	3	3	3	2	1	2
2	2	2	2	2	1	2	3	1	2
1	1	1	3	1	1	1	1	3	3
2	3	1	1	1	1	1	1	1	1
9	9	9	9	9	9	2	9	9	9
3	2	2	2	1	3	2	1	2	3
3	3	3	2	3	3	3	2	1	3
9	9	9	9	9	9	9	1	9	9
2	2	2	2	2	3	1	3	3	3
1	2	1	2	1	3	3	3	1	2
2	2	2	1	1	2	2	2	2	2
3	1	3	2	1	1	3	1	3	1
2	2	2	2	2	2	2	2	2	2
2	2	2	2	2	2	2	2	2	2
2	1	2	3	1	2	9	9	9	9
2	2	2	2	2	1	2	2	2	2
2	2	2	2	2	2	2	2	2	2
9	9	9	9	9	9	9	1	9	9
9	9	9	9	9	9	9	1	9	9
2	2	2	2	2	9	9	1	9	9
1	2	1	2	2	9	9	9	9	9
1	2	2	2	2	2	2	2	2	2
1	1	2	2	1	2	2	2	2	2
9	9	9	9	9	9	9	9	9	9
1	1	1	3	2	3	3	1	1	2

3	1	2	3	2	2	2	1	1	3
3	3	3	3	3	1	1	1	1	3
3	3	3	3	1	3	3	1	1	3
3	1	1	1	1	1	1	1	1	1
1	1	3	3	1	3	1	3	3	3
3	3	1	1	1	3	1	1	1	3
2	2	2	2	2	3	2	1	1	1
3	2	2	3	2	2	3	1	3	2
2	2	2	2	1	1	2	3	1	2
3	1	3	1	1	1	1	9	9	9
2	1	2	2	2	1	2	3	2	1
3	2	3	3	2	3	2	3	3	3
1	3	3	1	1	1	1	1	3	3
2	1	3	2	1	1	2	3	2	3
3	3	3	3	3	2	1	2	2	3
1	1	1	1	1	1	9	9	9	9
3	3	3	3	3	2	3	1	3	3
3	1	1	3	1	1	1	3	3	2
1	1	1	1	1	2	2	2	2	2
3	1	1	3	1	1	1	3	3	2
1	1	1	1	1	1	1	1	1	1
3	3	3	3	3	3	1	1	1	1
2	2	3	2	3	2	2	2	3	3
9	9	1	9	9	9	1	9	9	9
3	3	2	2	1	1	2	2	3	1
1	9	9	9	9	9	1	9	9	9
3	2	1	3	2	2	3	3	2	2
2	3	2	3	1	9	9	1	9	9
1	1	1	3	1	1	3	2	1	1
3	1	2	3	1	3	3	1	2	2
2	2	3	3	1	1	3	1	1	2
3	3	3	3	3	1	3	3	3	3
2	3	1	1	1	1	1	1	1	3
3	1	3	1	3	1	3	3	1	3
1	1	3	1	1	1	1	1	3	3
3	2	3	2	1	3	2	2	1	2
3	3	3	3	3	2	3	3	1	3
2	1	2	3	1	1	2	3	3	2
1	1	3	3	1	1	3	3	3	3
3	3	3	3	1	1	3	3	3	3
1	3	3	1	1	1	1	1	9	3
3	3	3	3	1	3	3	1	3	3
2	1	3	2	1	1	2	3	3	2
9	9	9	9	9	9	9	9	9	9
3	3	3	3	9	3	3	3	1	3
3	1	2	3	1	3	1	1	2	3
3	1	3	3	1	1	1	1	1	3

2	2	2	2	2	1	1	1	1	1
3	3	3	3	1	3	1	1	1	3
1	3	3	1	1	2	3	3	3	3
2	2	2	2	2	2	2	2	2	2
3	2	3	2	3	1	3	3	2	2
2	3	3	9	9	3	3	2	9	3
2	2	1	2	3	3	2	1	1	2
9	9	9	9	9	9	9	9	9	9
3	3	3	3	3	2	2	1	3	3
2	2	2	2	2	2	2	2	2	2
2	2	2	2	2	1	1	2	2	2
1	1	2	2	3	2	2	2	1	1
2	2	2	2	2	2	2	2	2	2
3	2	2	2	2	3	1	2	2	2
9	9	9	9	9	9	9	9	9	9
9	2	2	3	1	1	2	2	2	3
2	2	2	2	2	2	2	2	2	2
2	2	2	2	2	2	2	2	2	2
2	2	2	2	2	2	2	2	2	2
2	2	2	2	2	1	1	2	2	2
2	2	2	2	2	2	2	2	2	2
2	2	2	2	2	2	2	2	2	2
2	2	2	2	2	2	2	2	2	2
2	2	2	2	2	2	2	2	2	2
2	2	2	2	2	1	2	2	2	2
2	2	2	2	2	2	1	2	2	2
3	2	2	2	3	2	2	2	1	1
2	2	2	2	2	2	2	2	2	2
2	2	2	1	2	2	2	2	2	1
2	2	2	2	2	2	2	2	2	2
2	2	2	2	2	2	2	2	2	2
2	2	2	2	2	2	2	2	2	2
2	2	2	2	2	2	2	2	2	2
1	1	1	1	1	2	2	1	2	2
2	2	2	9	1	1	2	2	3	1
2	2	2	2	2	2	2	2	2	2
2	2	2	2	2	2	2	2	2	2
2	2	2	2	2	2	2	2	2	2
2	2	2	2	2	2	2	2	2	2
2	2	2	2	2	2	2	2	2	2
2	2	2	2	2	2	2	2	2	2
2	2	2	2	2	2	2	2	2	2
9	2	2	2	2	2	2	2	2	2
2	2	2	2	2	2	2	2	2	2
2	2	2	2	2	2	2	2	2	2
1	1	1	1	1	3	3	1	3	1
2	2	2	2	2	2	2	2	2	2

2	2	2	2	2	9	9	9	9	9
1	1	3	2	2	1	2	3	3	3
2	2	2	2	2	2	2	2	2	2
1	2	2	3	2	1	2	9	9	9
2	2	2	2	2	2	2	2	2	2
2	2	2	2	2	2	2	2	2	2
2	2	3	2	2	3	3	2	1	2
2	1	2	2	3	3	3	1	2	1
2	2	2	2	2	2	2	2	2	2
2	2	2	2	2	2	2	2	2	2
2	3	2	1	2	1	2	3	2	1
2	2	2	2	2	2	2	2	2	2
9	9	9	9	9	9	9	9	9	9
1	1	2	3	2	1	2	3	1	2
9	9	9	9	9	9	9	1	9	9
2	2	2	2	2	2	2	2	2	2
2	2	2	2	2	2	2	2	2	2
2	2	2	2	2	2	2	1	2	2
2	3	3	2	3	3	3	1	2	3
9	9	9	9	9	3	9	9	9	9
2	9	9	9	1	2	2	2	2	1
2	1	1	2	2	2	1	2	2	2
2	2	2	2	2	2	2	1	2	2
9	1	1	1	1	1	1	9	2	1
2	1	9	9	9	9	9	1	9	9
2	2	2	2	2	2	2	1	2	2
9	2	3	1	1	2	1	9	2	1
9	9	9	9	9	9	9	1	9	9
1	9	9	9	9	9	9	1	9	9
9	9	9	9	9	3	3	9	2	3
2	3	1	2	1	3	1	3	1	2
1	2	2	1	2	3	2	2	2	3
3	3	1	1	3	3	3	2	1	2
2	2	2	1	1	3	1	3	9	1
2	2	3	2	3	3	3	3	1	1
9	9	9	9	9	1	9	9	1	9
9	9	9	9	9	9	9	9	9	9
2	2	2	2	2	2	2	2	2	2
2	2	2	2	2	2	2	2	2	2
9	2	9	9	9	2	9	2	9	9
1	1	1	1	1	9	9	9	9	9
9	1	9	9	9	9	9	1	9	9
3	1	1	1	1	3	1	3	1	3
2	2	2	2	2	2	2	2	2	2
2	2	2	2	2	2	2	2	2	2
2	2	2	2	2	2	2	2	2	2
1	9	2	3	1	2	3	9	1	2

2	1	2	2	2	2	2	2	2	2
9	9	9	9	2	9	1	9	9	9
1	1	1	1	1	1	1	1	1	1
1	1	1	1	1	3	3	1	3	3
2	3	2	3	2	2	2	2	2	2
9	9	9	9	9	9	9	9	9	1
1	1	1	1	1	1	1	1	1	1
2	2	2	2	2	2	2	1	2	2
1	1	1	1	1	1	1	1	1	1
2	2	2	2	2	2	2	2	2	2
9	9	9	9	9	9	9	2	9	9
3	2	1	2	3	2	1	2	1	3
3	3	3	3	3	3	3	3	3	3
1	1	1	1	1	1	1	1	1	1
2	2	2	2	2	2	2	1	1	1
1	1	1	1	1	3	1	1	1	1
2	2	2	2	2	2	2	2	2	2
1	1	1	1	1	1	1	1	1	1
2	2	2	2	2	2	2	2	2	2
2	2	2	2	2	2	2	2	2	2
2	2	2	2	2	2	2	2	2	2
2	2	2	2	2	2	2	2	2	2
1	1	1	1	1	1	1	1	1	1
1	3	3	2	3	3	1	2	2	2
9	9	9	9	9	9	9	9	9	9
2	9	1	2	1	1	2	1	2	1
1	2	2	1	1	1	1	2	2	3
3	3	3	3	3	3	3	1	3	3
3	3	3	3	3	3	3	3	3	3
3	3	3	3	1	2	2	1	1	3
3	3	3	3	3	3	3	3	3	3
2	1	2	1	2	1	2	2	1	2
2	1	1	1	1	2	1	1	1	1
2	2	2	2	2	2	2	2	2	2
9	9	9	9	9	2	9	9	9	9
2	3	2	1	1	2	2	2	2	2
3	3	3	3	3	2	2	2	2	2
3	3	3	3	3	1	2	3	2	2
3	3	3	3	3	3	3	3	3	3
2	9	1	2	3	2	1	3	2	1
2	2	2	2	2	2	2	2	2	2
2	2	3	3	2	2	2	2	2	2
3	9	1	2	3	1	2	3	2	1
2	2	2	2	2	2	2	2	2	2
2	2	3	3	2	1	2	3	1	2
3	3	3	3	3	3	3	3	3	3
2	1	3	3	1	1	2	3	1	2

1	3	2	1	2	1	2	2	3	1
9	9	9	9	9	9	9	9	9	9
3	3	3	3	3	3	3	3	3	3
1	1	1	1	2	2	2	3	1	1
2	3	2	2	2	2	2	2	2	3
3	3	3	2	2	1	2	3	3	3
1	1	1	1	1	2	2	2	2	2
3	3	3	3	3	3	3	1	3	3
3	3	3	3	3	2	2	2	2	2
3	3	3	3	3	3	3	1	3	3
1	3	2	1	2	3	1	2	2	3
2	2	2	2	2	2	2	2	2	2
2	1	2	3	2	2	2	2	2	2
2	2	2	2	2	2	2	2	2	2
9	9	9	9	9	9	9	9	9	9
3	9	1	3	3	1	3	2	1	3
2	2	2	2	2	2	2	2	2	2
3	1	3	2	1	1	3	1	2	3
2	2	2	2	2	2	2	2	2	2
3	3	1	3	3	3	3	1	1	3
2	2	2	2	2	2	2	2	2	2
2	2	2	2	2	2	2	2	2	2
2	2	2	2	2	2	2	2	2	2
1	3	3	3	3	2	1	1	2	3
1	2	3	1	2	2	2	2	2	2
9	9	9	9	9	3	2	2	2	1
2	2	2	2	1	3	3	1	2	3
3	1	2	1	2	2	1	3	2	1
3	1	2	1	2	2	1	3	2	1
2	3	2	2	1	2	2	2	2	2
1	1	1	1	1	1	1	1	1	1
9	9	9	9	9	9	9	9	9	9
2	3	1	3	3	3	2	1	1	3
2	3	3	3	3	3	2	3	1	1
1	3	9	2	3	9	9	1	9	9
3	3	2	3	3	2	1	2	3	3
3	2	1	3	1	3	1	1	1	1
9	9	9	9	9	9	9	9	9	9
3	1	2	2	2	2	2	2	2	2
3	2	2	3	3	2	3	2	3	2
1	1	2	1	3	2	2	1	3	1
2	1	1	2	9	2	2	2	1	2
1	1	1	1	1	2	2	2	2	2
2	2	2	2	2	9	9	9	9	9
2	2	2	2	1	1	2	2	2	2
3	3	3	3	2	3	2	2	3	3
3	3	3	3	2	3	2	9	3	3

2	2	2	2	2	2	2	2	2	2
2	2	2	2	2	2	2	2	2	2
2	2	2	2	2	2	2	2	2	2
1	9	9	1	3	3	2	9	3	2
3	1	2	1	2	9	9	9	9	9
3	1	2	3	3	2	2	2	2	2
9	9	9	9	9	9	9	9	9	9
2	2	3	1	3	1	2	2	9	2
1	3	2	1	2	3	1	2	2	3
1	3	3	3	3	3	3	1	3	3
1	3	2	2	3	2	3	9	1	3
2	3	3	3	2	2	3	2	2	2
3	2	3	1	2	3	3	3	1	1
1	1	1	1	2	2	2	2	2	2
3	2	2	2	1	2	1	2	2	2
3	2	3	3	3	3	2	1	3	3
3	3	3	3	2	2	2	3	3	3
2	3	3	1	2	3	3	3	1	3
2	2	2	2	3	2	2	2	2	2
1	1	1	1	1	9	9	1	9	9
2	3	9	1	2	3	2	2	1	3
2	2	2	2	2	2	2	2	2	2
1	3	1	2	1	1	2	3	2	3
1	1	3	3	1	3	3	3	3	3
3	9	3	3	3	3	2	9	1	9
3	3	3	3	3	2	2	2	2	2
9	9	9	9	9	9	9	9	9	9
2	9	2	1	3	3	2	1	3	1
3	1	1	1	2	1	2	2	2	2
2	1	2	2	2	1	1	2	2	3
1	2	2	3	3	3	3	1	2	1
2	3	3	3	3	3	2	2	2	2
2	1	3	2	3	9	9	9	9	1
2	1	1	3	2	1	1	2	1	1
2	2	1	3	2	2	2	1	3	3
1	1	1	2	1	1	1	2	2	2
2	3	2	1	2	1	2	3	2	2
2	1	3	2	2	3	2	2	1	1
3	3	3	3	3	3	2	3	3	3
2	2	2	2	2	2	2	2	2	2
2	2	2	2	2	2	2	2	2	2
2	2	2	2	2	2	2	2	2	2
3	3	3	3	3	3	3	3	3	3
2	2	2	2	2	3	3	1	2	2
2	1	2	1	1	2	3	2	1	2
3	1	2	1	1	2	3	2	1	2
1	1	1	1	1	2	2	2	2	2

3	1	3	2	1	2	2	2	2	2
3	2	2	1	3	2	3	1	1	2
2	2	2	2	2	2	2	2	2	2
9	9	9	9	9	3	3	3	1	2
3	3	3	3	3	3	3	3	3	3
3	2	3	3	3	2	2	2	3	1
3	1	2	3	2	2	1	1	3	2
3	1	3	3	3	3	3	3	1	3
3	2	1	2	2	2	2	3	1	2
9	9	9	9	9	9	9	9	9	9
3	2	3	2	3	2	3	2	3	2
3	3	3	3	3	3	3	3	3	3
1	1	3	1	1	1	1	1	1	1
2	2	2	2	2	3	3	3	3	3
2	2	3	1	2	2	3	3	2	1
3	2	3	1	2	2	3	1	9	3
2	2	2	2	2	2	2	2	2	2
3	3	3	3	3	3	3	3	1	2
1	1	1	1	1	1	1	1	1	1
9	9	9	9	9	9	9	1	9	9
2	2	9	1	1	1	1	1	1	1
2	2	2	2	2	3	3	3	3	3
3	3	3	1	1	1	1	1	1	1
2	2	2	2	2	1	9	1	9	1
2	2	9	2	2	1	2	3	2	3
2	1	1	3	1	2	2	3	2	3
3	1	2	3	9	3	1	2	3	2
2	1	1	1	2	2	2	2	3	3
2	3	3	3	3	2	2	2	2	2
2	3	1	2	1	2	2	1	3	3
2	3	1	2	3	1	2	3	1	2
2	1	3	3	3	2	2	1	2	1
2	3	1	2	1	3	3	3	1	3
3	3	3	3	3	3	3	2	1	2
1	2	3	2	1	1	2	3	1	2
2	2	2	2	2	2	2	2	2	2
1	2	1	1	2	3	3	2	2	3
2	2	2	2	2	1	1	1	1	1
2	3	2	3	2	1	3	3	3	3
1	1	3	1	2	1	3	3	1	1
3	1	1	3	2	3	1	1	3	3
3	3	3	3	1	1	3	3	3	3
3	3	3	2	3	1	1	1	3	3
1	1	3	3	1	3	3	3	3	3
3	1	3	1	1	1	3	3	3	3
3	3	3	2	3	1	1	2	2	2
2	2	2	2	2	2	2	2	2	2

1	2	3	1	3	3	1	3	3	3
1	2	2	3	2	3	2	1	3	2
2	3	2	2	1	1	1	2	3	2
9	9	9	9	9	9	9	9	9	9
1	1	2	3	2	3	3	3	3	3
1	3	1	9	3	1	3	1	3	1
2	2	2	3	2	2	1	2	2	2
3	1	2	3	1	1	2	3	2	3
2	2	2	2	2	2	2	2	2	2
1	1	3	1	1	1	1	1	1	1
3	1	2	1	3	1	2	3	1	2
1	1	3	2	2	3	1	2	2	2
3	3	2	3	1	1	1	2	3	3
3	3	3	3	2	3	2	1	2	1
2	3	1	2	1	1	2	1	2	1
3	3	1	3	1	3	2	1	3	3
2	3	1	2	3	1	2	3	2	3
1	1	3	3	1	3	3	9	1	2
1	1	1	1	1	3	1	1	1	1
1	1	3	1	1	3	1	3	1	3
1	2	2	3	2	3	2	2	2	3
1	3	1	1	1	3	3	1	1	1
3	1	2	3	1	2	2	2	1	3
3	3	3	3	3	3	3	3	3	3
2	1	3	3	3	3	1	2	3	3
3	2	9	1	2	3	3	3	1	2
2	3	2	3	2	2	2	2	2	2
3	3	2	3	1	3	1	1	1	3
3	3	9	3	3	3	1	3	1	3
2	1	2	2	1	2	2	2	2	2
2	2	1	1	2	2	1	1	3	3
2	2	2	2	2	2	2	2	2	2
1	3	3	3	3	1	3	1	3	2
2	3	1	9	9	1	3	1	2	2
2	2	2	2	2	3	2	2	2	3
9	9	9	9	9	9	3	9	9	9
2	1	3	3	1	9	9	9	9	9
1	9	9	9	9	9	9	9	9	9
2	2	2	2	2	1	1	2	2	2
9	9	9	9	9	9	9	1	9	9
2	2	2	2	2	3	3	3	3	3
1	1	1	1	1	2	2	2	2	2
2	2	3	3	2	3	3	2	2	2
1	9	9	9	9	1	9	9	9	9
9	9	9	9	9	3	3	3	2	2
1	3	2	3	3	2	2	2	2	2
3	2	3	2	1	2	1	3	2	3

2	3	1	2	1	1	2	1	1	3
3	3	3	3	1	3	2	2	1	1
3	3	3	2	2	1	1	2	1	1
2	3	1	2	2	1	2	1	2	3
2	2	2	2	2	1	2	1	2	1
1	3	3	3	3	1	3	3	3	2
9	9	9	9	9	9	9	1	9	9
2	1	2	1	1	1	1	1	1	1
2	2	3	2	3	3	2	2	2	3
2	3	1	2	1	2	1	1	3	3
2	2	2	2	2	3	3	2	2	3
2	2	3	3	2	3	2	3	3	3
1	3	3	3	3	9	9	9	1	9
2	3	2	3	3	3	2	3	1	1
3	3	3	2	1	3	2	2	1	2
3	1	1	3	3	3	2	3	1	3
3	2	1	2	3	2	3	2	1	3
2	9	2	1	1	3	2	1	1	3
1	1	1	3	2	1	1	1	1	1
2	2	2	2	2	2	2	2	2	2
2	2	2	2	2	2	2	2	2	2
3	3	3	2	2	9	9	9	9	9
2	2	1	2	1	3	2	2	2	2
2	2	2	2	2	2	2	2	2	2
2	2	2	2	2	2	2	2	2	2
3	2	3	3	1	3	2	3	1	2
9	9	9	9	9	1	2	3	9	9
9	9	9	9	9	1	9	9	9	9
9	9	9	9	9	1	9	9	9	9
2	2	2	2	1	1	1	2	2	2
3	3	3	3	3	1	3	3	3	3
9	9	9	9	9	1	9	9	9	9
2	1	2	2	1	1	9	9	9	9
2	1	2	2	2	1	2	2	2	2
2	1	2	2	2	2	2	2	2	2
9	9	9	9	9	9	9	9	9	9
9	9	9	9	9	1	9	9	9	9
9	9	9	9	9	9	1	9	9	9
2	2	2	1	1	1	3	3	3	3
3	3	3	3	3	1	3	3	3	3
9	9	9	9	9	1	9	9	9	9
3	2	2	3	3	1	9	9	9	9
9	9	9	9	9	1	1	9	9	9
9	9	9	9	9	1	9	9	9	9
9	9	9	9	9	1	9	9	9	9
3	1	3	3	9	1	3	3	3	3
3	3	3	3	1	1	3	3	3	3

9	9	9	1	9	1	9	9	9	9
9	9	9	9	9	1	9	9	9	9
1	2	3	9	9	1	9	9	1	9
9	9	9	9	9	1	9	9	9	9
3	1	1	2	1	1	1	1	3	3
2	2	3	2	2	1	2	2	2	2
9	9	9	9	1	1	9	9	9	9
2	2	3	3	2	1	2	2	2	2
3	3	3	3	3	1	2	2	2	2
1	1	1	1	1	1	1	1	1	1
9	9	9	9	9	1	9	9	9	9
9	9	9	9	9	1	9	9	9	9
1	1	1	1	1	1	9	9	9	9
1	2	2	2	2	2	2	1	2	2
9	9	9	9	9	1	9	9	9	9
9	9	9	9	9	1	9	9	9	9
2	2	2	2	2	1	9	9	9	9
2	2	2	2	2	1	9	9	9	9
9	9	9	9	9	1	9	9	9	9
9	9	9	9	9	1	9	9	9	9
9	9	9	9	9	1	9	9	9	9
9	9	9	9	9	1	9	9	9	9
9	9	9	9	9	1	9	9	9	9
9	9	9	9	9	1	9	9	9	9
9	9	9	9	9	1	9	9	9	9
2	2	3	3	3	1	9	9	9	9
9	9	9	9	9	1	9	9	9	9
9	9	9	9	9	1	9	9	9	9
3	3	3	3	3	3	3	3	3	3
9	9	9	9	9	1	9	9	9	9
2	1	2	2	1	1	9	9	9	9
3	3	3	3	9	3	3	9	9	1
3	3	3	3	3	1	3	3	3	3
9	9	9	9	9	1	9	9	9	9
3	3	3	3	3	1	3	3	3	3
3	3	3	3	1	1	9	9	9	9
2	2	2	1	1	1	2	2	2	2
1	3	3	3	1	1	3	3	3	3
9	9	9	9	9	1	9	9	9	9
9	9	9	9	9	9	9	9	9	9
9	9	9	9	9	1	9	9	9	9
3	3	3	3	3	1	3	3	3	3
9	9	9	9	9	1	9	9	9	9
9	9	9	9	9	1	9	9	9	9
3	3	3	3	3	1	3	3	3	3
3	3	3	3	3	1	2	2	1	2
3	3	3	3	3	3	1	1	1	3

2	3	3	3	3	1	1	3	3	3
9	9	9	9	9	1	9	9	9	9
9	9	9	9	9	1	9	9	9	9
9	9	9	9	9	9	9	9	9	9
3	3	3	2	3	1	9	9	9	9
9	9	3	9	9	1	9	9	9	9
3	1	3	1	3	1	9	9	9	9
3	3	3	3	1	3	3	3	3	1
3	3	3	2	3	1	9	9	9	9
3	1	3	1	1	1	9	9	9	9
9	9	9	9	9	1	9	9	9	9
9	9	9	9	9	1	9	9	9	9
3	3	3	1	1	3	1	1	1	3
3	3	3	3	3	3	3	1	3	3
3	3	3	3	3	3	3	1	3	3
3	3	3	3	3	3	3	1	3	3
3	3	3	3	3	2	2	1	1	1
3	3	3	3	3	9	9	9	1	9
9	9	9	9	9	9	9	9	9	1
3	2	2	3	3	3	2	2	1	2
2	2	2	2	2	2	2	2	2	2
2	1	2	2	2	3	3	1	1	2
3	3	9	9	9	9	9	9	1	9
9	9	9	9	9	9	9	9	1	9
3	3	2	9	3	1	1	1	1	2
3	3	2	3	2	1	1	1	1	2
3	1	3	3	2	3	1	1	3	3
3	2	2	2	2	2	1	3	2	3
3	3	3	3	3	3	3	3	2	1
3	3	3	3	3	3	3	3	1	3
3	3	3	3	3	3	3	3	2	1
3	3	3	3	3	9	9	1	9	9
3	2	3	2	3	2	3	1	1	2
3	2	9	1	2	3	2	2	3	3
3	3	3	3	3	3	2	3	3	3
2	2	2	1	1	2	2	2	1	1
2	2	2	2	2	2	2	1	2	2
3	1	2	2	3	2	1	1	1	3
1	3	2	1	3	2	2	1	2	1
9	9	9	9	9	9	9	9	1	9
3	3	3	3	3	3	3	3	2	1
3	3	3	2	1	3	3	2	2	1
9	9	9	9	9	9	1	9	9	9
9	9	9	9	9	9	1	9	9	9
9	9	9	9	9	9	1	9	9	9
3	2	9	1	3	1	1	3	2	3
3	3	3	3	3	3	3	3	3	1

3	3	3	3	3	1	1	3	3	3
3	3	3	3	2	2	2	2	3	3
2	2	2	2	2	2	2	2	2	2
3	3	3	3	3	2	2	2	3	3
9	9	9	9	9	9	9	3	9	9
3	3	3	3	3	9	9	9	1	9
9	9	9	9	9	1	9	9	9	9
1	2	2	3	2	3	2	2	1	2
3	3	3	3	1	2	2	2	2	1
2	2	2	2	2	2	2	2	2	1
2	3	2	9	3	3	3	2	1	1
2	2	3	1	9	3	3	3	3	3
9	9	9	9	9	2	1	2	2	2
3	3	3	3	1	3	3	1	3	3
9	9	9	9	9	9	9	9	9	1
3	3	3	3	3	9	9	1	9	9
3	3	3	3	3	2	2	2	2	3
3	3	3	3	3	3	1	3	3	3
3	3	3	3	3	3	1	2	3	3
3	3	2	3	3	9	1	9	9	9
9	9	9	9	9	9	1	9	9	9
9	9	9	9	9	9	1	9	9	9
2	1	2	3	3	1	1	3	3	3
2	2	2	2	2	2	1	2	2	2
3	3	3	3	3	3	1	3	3	3
3	2	3	1	3	9	1	9	9	9
9	9	9	9	9	9	1	9	9	9
9	9	9	9	9	1	9	9	9	9
2	2	2	2	3	9	1	9	9	9
3	3	3	3	1	1	3	3	3	3
3	3	2	9	3	2	1	3	3	3
3	3	2	9	3	2	1	3	3	3
9	9	9	9	9	9	1	9	9	9
9	9	9	9	9	9	9	9	9	9
9	9	9	9	9	1	9	9	9	9
3	2	2	2	9	1	1	1	3	2
9	9	9	9	9	3	1	3	3	3
3	3	9	2	3	2	1	3	3	3
9	9	9	9	9	1	9	9	9	9
3	3	3	3	3	1	9	3	9	9
3	3	3	3	3	1	2	2	2	2
9	9	9	9	9	1	9	9	9	9
2	3	3	3	3	1	1	1	1	3
3	3	3	3	3	1	3	3	3	3
3	3	3	3	3	1	1	3	3	3
2	3	3	3	3	1	1	1	1	3
1	1	1	3	1	1	9	9	9	9

9	9	9	9	9	1	9	9	1	9
3	3	3	3	3	1	3	3	3	3
9	9	9	9	9	1	9	9	1	9
3	2	3	3	3	1	3	3	3	3
9	9	9	9	9	9	1	9	9	9
9	9	9	9	9	1	9	9	9	9
3	3	3	3	3	1	3	3	3	3
9	9	9	9	9	1	9	9	9	9
9	9	9	9	9	9	9	9	9	9
9	9	9	9	9	9	9	9	9	1
3	9	9	9	9	9	1	9	9	9
3	3	3	3	1	3	3	1	1	3
2	1	2	1	2	2	1	2	9	2
9	9	9	9	9	9	1	9	9	9
9	9	9	9	9	9	9	9	9	9
9	9	9	9	9	9	1	9	9	9
3	3	3	3	3	1	3	3	3	3
1	9	9	9	9	1	9	9	9	9
3	3	3	3	3	1	2	2	2	3
9	9	9	9	9	9	1	9	9	9
1	3	1	1	1	9	1	2	1	1
3	3	3	3	3	3	1	2	3	3
9	9	9	9	9	9	1	9	9	9
9	9	9	9	9	9	1	9	9	9
9	9	9	9	9	9	1	9	9	9
3	3	3	3	3	3	1	3	3	3
3	3	3	3	1	1	3	3	9	3
9	9	9	9	9	9	1	9	9	9
9	9	9	9	9	9	1	9	9	9
3	3	3	3	3	1	9	9	9	9
9	9	9	9	9	9	1	9	9	9
2	2	3	1	1	2	1	1	3	1
3	3	3	3	3	9	1	9	9	9
3	2	3	1	3	2	1	2	3	3
3	3	1	1	2	9	1	9	9	9
3	3	3	3	3	1	1	3	3	3
3	3	3	3	3	2	3	3	3	3
2	2	1	1	2	3	1	3	3	3
3	3	3	2	2	1	1	1	1	1
3	3	3	3	3	3	1	3	3	3
3	2	3	3	3	1	2	1	1	3
9	9	9	9	9	9	1	9	9	9
9	9	9	9	9	9	1	9	9	9
9	1	9	9	9	9	1	9	9	9
3	3	3	3	1	3	1	2	3	3
3	3	3	1	1	3	1	3	3	3
2	2	3	2	2	2	1	2	3	3

3	3	2	3	3	2	1	3	3	3
9	9	9	9	9	9	9	1	9	9
9	9	9	9	9	9	1	1	9	9
9	9	9	9	9	1	9	9	9	9
3	3	3	3	3	1	9	9	9	9
3	2	3	2	1	9	1	9	9	9
3	3	3	3	1	3	1	3	3	3
3	1	1	2	1	9	1	9	9	9
9	9	9	9	9	9	1	9	9	9
3	3	3	3	2	2	1	1	2	2
3	3	3	3	3	9	1	9	9	9
9	9	9	9	9	9	1	9	9	9
2	2	2	2	2	2	1	2	2	3
3	2	2	2	1	2	1	2	2	2
3	3	3	3	3	3	1	3	2	3
3	3	2	3	2	3	1	2	2	3
3	2	2	2	3	3	1	2	2	3
3	3	3	3	3	9	1	9	9	9
9	9	9	9	9	9	1	9	9	9
9	9	9	9	9	9	1	9	9	9
9	9	9	9	9	9	1	9	9	9
9	9	9	9	9	9	1	9	9	9
3	3	3	3	3	3	1	3	3	3
3	2	2	1	3	2	1	2	2	2
3	3	3	3	3	9	9	9	9	9
9	9	9	9	9	9	1	9	9	9
3	3	3	3	3	2	1	3	3	3
3	3	2	2	2	3	1	2	9	9
3	3	3	3	2	2	1	1	2	2
2	3	3	3	3	1	1	2	2	3
2	2	2	2	2	3	1	3	3	3
3	3	9	9	9	9	1	9	9	9
3	2	3	3	3	3	1	1	1	3
3	3	3	3	3	2	1	2	2	2
3	3	3	3	3	1	1	9	9	9
9	9	9	9	9	9	1	9	9	9
3	3	3	3	3	3	1	3	3	3
9	9	9	9	9	9	1	9	9	9
9	9	9	9	9	9	1	9	9	9
2	3	3	2	1	2	1	2	2	3
2	3	3	3	3	2	1	2	2	1
3	3	3	1	1	2	1	2	2	2
3	3	3	3	2	2	1	3	3	3
3	3	3	3	3	3	3	1	2	2
9	9	9	9	3	9	9	9	1	9
9	9	9	9	9	1	9	9	9	9
3	2	3	3	3	3	2	2	3	2

3	3	3	2	3	2	2	2	2	2
9	9	9	9	9	9	9	9	1	9
2	1	1	2	1	2	2	2	1	1
2	3	3	2	2	1	1	2	3	2
9	9	9	9	9	9	1	9	9	9
3	3	3	3	1	3	3	1	1	3
3	1	3	3	3	3	3	1	3	3
1	1	2	2	2	3	2	1	2	2
3	2	2	2	2	3	3	2	1	2
2	2	2	2	2	2	2	1	3	3
2	2	2	2	1	3	2	1	2	3
2	2	1	2	2	3	3	1	3	3
2	1	3	2	1	1	1	3	1	2
2	2	2	2	2	2	2	1	2	3
2	3	2	3	3	2	1	1	2	2
3	3	3	3	3	3	3	2	1	2
2	2	2	2	3	2	2	1	2	2
3	2	2	2	2	9	2	9	9	9
3	3	3	3	3	3	2	1	2	2
2	2	2	2	2	2	2	2	2	2
2	2	2	2	2	2	2	2	2	2
3	1	2	2	1	3	2	2	1	1
2	2	2	2	2	2	2	2	2	2
2	2	2	2	2	2	2	2	2	2
3	3	3	3	3	2	2	2	2	2
3	2	2	3	2	3	1	2	2	3
2	3	2	3	3	2	3	1	2	1
2	3	3	3	2	2	1	2	2	1
9	9	9	9	9	9	9	1	9	9
3	3	2	2	3	1	1	2	3	3
2	1	1	1	2	2	2	1	1	1
9	9	9	9	9	9	9	9	9	9
3	3	1	1	1	2	3	3	3	3
2	1	1	1	2	9	1	9	9	9
2	1	1	1	2	9	9	1	9	9
2	2	2	9	3	1	9	2	9	3
2	3	2	2	3	2	3	1	1	2
3	2	2	2	1	2	1	2	2	2
2	2	2	3	1	2	2	1	2	1
2	2	2	3	1	2	2	1	2	1
9	9	9	9	9	9	9	9	1	9
9	9	9	1	9	9	9	9	9	1
2	1	1	9	2	1	9	9	9	9
9	1	9	9	9	1	9	9	9	9
9	9	9	9	9	9	1	9	9	9
2	1	1	1	9	9	9	1	9	9
3	2	2	3	2	1	2	2	3	3

9	9	9	9	9	9	9	9	9	1
9	9	9	9	9	9	9	9	9	1
2	3	3	3	2	2	1	2	2	2
9	1	9	9	9	1	9	9	9	9
9	9	9	9	9	9	1	9	9	9
9	9	9	9	9	9	1	9	9	9
3	1	3	3	3	1	1	1	3	3
1	2	3	1	2	1	3	2	1	2
9	9	9	9	9	9	1	9	9	9
2	1	3	2	3	3	2	1	2	3
3	2	2	2	1	2	2	1	2	3
9	9	9	9	9	9	9	1	9	9
9	9	9	9	9	9	9	1	9	9
9	9	9	9	9	9	9	1	9	9
9	9	9	9	9	9	9	1	9	9
9	9	9	9	9	1	9	9	9	9
3	3	3	3	3	2	1	2	3	3
2	1	2	3	3	3	1	3	3	3
3	3	3	3	3	2	1	3	3	3
2	3	3	3	3	1	2	2	2	2
3	3	3	3	3	3	3	1	3	3
3	3	3	3	3	3	3	3	1	3
3	3	3	3	3	3	3	3	1	3
3	2	3	3	3	3	3	3	1	3
2	1	2	3	3	3	1	3	3	3
2	1	2	3	1	2	1	1	2	1
2	1	2	2	2	1	1	2	2	2
2	2	2	2	1	2	2	2	2	2
9	9	9	9	9	9	9	1	9	9
2	1	1	2	1	1	2	3	2	1
3	1	3	3	3	3	2	3	3	3
3	1	3	2	3	3	1	1	1	2
3	3	3	3	3	9	9	9	9	9
3	3	3	3	3	2	2	2	2	2
3	3	3	3	3	3	3	1	3	3
2	1	2	2	2	9	9	9	9	9
3	1	3	3	3	2	2	1	1	3
3	2	2	2	2	3	3	1	2	2
3	1	3	3	3	3	1	1	3	3
2	1	2	3	3	2	2	1	2	2
3	3	3	3	3	2	3	1	3	3
1	3	3	2	3	3	1	1	1	2
3	1	3	3	3	1	3	3	3	3
3	1	3	3	3	3	1	3	3	3
3	1	3	3	3	3	3	1	3	3
3	3	3	2	3	2	1	1	2	3
3	1	3	3	3	1	9	9	9	9

3	1	3	3	3	3	1	1	1	3
3	1	2	2	3	3	2	1	2	3
3	1	3	3	3	3	9	1	3	3
3	1	3	3	3	3	9	1	3	3
3	1	3	3	3	3	3	1	3	3
3	3	2	3	3	1	2	1	1	2
2	3	2	3	2	1	2	2	3	2
2	3	1	2	1	1	1	1	2	3
3	1	2	2	1	2	2	2	2	2
3	1	3	3	3	3	3	1	3	3
3	1	3	3	3	3	3	1	3	3
3	1	3	3	3	3	3	1	3	3
3	3	2	3	3	1	3	1	3	3
3	3	3	3	3	3	2	1	3	3
3	3	3	2	2	2	1	1	3	3
9	9	9	9	9	9	9	9	1	9
3	2	2	1	9	1	1	3	3	2
9	9	9	9	9	9	9	9	1	9
1	2	2	1	1	2	2	1	2	2
2	3	2	2	2	3	3	1	2	2
3	3	3	1	1	3	2	1	1	3
2	2	2	2	2	2	2	2	2	2
1	9	1	2	2	2	1	9	1	9
1	2	2	2	2	3	3	1	2	2
3	3	1	1	3	1	3	2	1	3
2	1	1	3	1	1	1	3	2	2
3	3	3	3	1	1	3	3	3	3
3	3	3	3	3	1	3	1	1	3
9	1	2	2	9	2	1	2	1	3
2	1	2	3	1	2	1	2	2	3
2	1	2	3	1	3	1	2	2	3
3	3	2	2	3	2	1	1	3	2
3	3	3	2	1	3	2	3	3	3
2	2	2	2	2	1	2	2	2	2
2	2	3	2	2	1	2	2	3	3
3	2	2	2	2	2	2	1	2	2
3	3	2	2	3	2	2	2	2	2
3	3	2	2	3	3	3	1	1	2
3	3	3	3	3	2	2	9	1	2
1	1	9	3	2	1	3	1	1	3
9	1	2	2	9	2	1	2	1	3
3	1	3	3	3	3	3	1	3	3
3	3	3	3	3	3	3	1	2	3
9	3	3	3	3	2	1	1	2	3
9	9	9	9	9	9	9	9	1	9
3	3	3	2	2	3	3	1	3	2
3	3	3	3	3	1	2	2	2	2

9	9	9	9	9	9	9	9	9	9
9	3	3	1	1	1	3	2	1	3
3	3	3	3	2	3	3	1	3	3
3	3	3	3	3	3	3	2	2	2
3	2	3	3	3	3	3	3	3	2
3	1	2	1	3	3	3	1	3	3
3	2	2	1	3	3	1	1	1	2
2	2	2	2	2	2	2	2	2	2
3	3	3	3	2	3	3	1	3	3
3	2	2	3	3	2	2	2	2	1
3	3	3	2	3	3	3	1	1	2
3	2	3	3	2	2	3	3	3	3
3	3	3	2	9	9	9	9	1	9
3	3	3	3	3	3	3	2	2	2
1	3	2	2	2	1	1	2	2	2
3	3	3	3	2	3	3	1	2	2
3	1	3	3	3	2	3	3	2	1
3	2	2	3	3	3	1	3	3	3
3	2	2	3	3	9	9	1	9	9
3	3	2	2	2	1	2	3	3	3
3	3	3	3	3	3	1	2	3	3
2	2	2	2	2	2	2	2	2	2
2	1	1	3	1	3	1	2	1	2
3	3	3	3	3	1	2	2	3	3
3	3	3	3	3	1	2	1	2	2
3	1	3	3	3	3	3	1	3	3
2	3	2	3	1	2	2	1	1	3
2	2	2	2	3	9	1	9	9	9
3	3	3	3	3	3	3	1	3	3
9	9	9	9	9	3	3	1	1	3
3	3	3	3	3	3	3	2	3	3
3	2	1	2	3	3	1	1	3	3
3	1	3	3	3	3	3	3	1	3
2	1	1	1	2	2	9	1	2	2
9	9	9	9	9	1	9	9	9	9
2	9	2	9	9	3	1	9	9	9
2	3	3	3	2	2	1	2	2	1
1	2	2	3	2	1	2	3	3	3
3	2	2	3	2	3	2	1	1	3
3	3	3	3	3	9	9	1	9	9
3	2	3	3	2	9	9	9	9	1
2	2	2	3	2	2	2	1	2	2
2	2	2	2	2	2	2	2	2	2
9	1	9	9	9	9	3	9	9	9
2	3	2	3	2	2	1	1	1	1
3	3	2	3	2	2	1	2	3	3
3	3	3	3	3	9	9	1	9	9

3	2	3	2	3	3	2	1	1	3
1	2	1	2	1	2	1	1	2	2
3	2	3	2	2	2	1	2	2	3
3	2	2	2	1	3	2	3	3	3
2	3	3	3	2	2	2	2	2	2
3	3	3	3	3	1	1	2	2	3
3	2	2	2	2	1	3	3	3	3
3	3	3	3	2	1	1	1	1	1
3	3	3	3	3	2	1	1	2	3
2	1	2	2	1	3	2	1	2	3
2	3	2	2	3	1	2	3	1	3
3	3	3	2	3	1	3	3	2	3
3	3	3	3	1	2	2	1	3	3
9	9	9	9	1	9	9	9	1	9
3	3	3	3	1	2	2	2	2	2
3	2	2	2	2	3	2	2	2	2
2	2	2	2	2	2	2	1	3	3
3	2	2	3	2	3	2	9	9	9
2	2	2	2	2	2	2	2	2	2
2	1	2	2	2	1	2	2	2	2
3	3	3	3	3	3	3	1	2	3
3	3	3	3	3	3	3	1	2	3
2	2	2	3	1	9	9	9	1	9
1	2	1	2	1	3	1	1	1	1
9	9	9	9	9	9	9	1	9	9
3	3	3	3	3	3	3	1	3	3
3	3	3	3	3	3	3	1	3	3
3	1	2	1	1	3	2	1	1	2
2	2	2	2	2	2	2	1	2	1
3	3	3	3	2	3	3	1	3	3
3	3	3	3	3	3	2	1	3	3
9	1	2	2	1	1	1	1	1	2
1	2	3	3	1	3	1	1	1	2
1	2	3	3	1	2	2	2	2	2
1	2	3	3	1	3	3	1	1	1
9	9	9	9	9	9	9	1	9	9
1	2	1	1	1	3	3	1	1	2
2	3	1	1	2	1	1	2	1	2
9	9	9	9	1	1	1	1	2	2
3	3	3	2	1	2	1	1	2	2
3	3	3	2	1	3	3	3	1	2
2	2	2	2	2	1	1	1	2	2
2	2	2	2	2	1	1	1	2	2
9	9	9	9	9	9	9	9	1	9
3	3	3	3	3	3	3	1	3	3
1	2	2	1	1	2	1	1	2	3
3	2	3	3	3	1	1	1	1	1

3	1	1	1	1	3	1	1	1	1
2	2	2	2	2	2	2	2	2	2
3	3	3	2	3	3	3	1	2	3
1	3	1	1	1	3	1	1	1	3
1	2	3	1	1	2	2	1	1	1
3	3	2	1	1	3	1	3	3	3
1	3	2	1	1	2	1	1	2	2
1	3	2	1	1	2	1	1	2	2
3	2	2	3	3	3	3	1	3	1
2	3	2	2	1	2	2	1	1	1
3	3	3	1	1	2	1	1	2	2
2	1	2	1	3	2	1	1	1	2
1	1	3	3	1	3	2	1	3	1
3	3	2	3	3	1	1	3	2	3
3	1	3	3	3	3	3	1	3	3
2	1	2	3	3	3	1	3	3	3
3	1	3	3	3	3	3	1	3	3
3	1	3	3	3	3	3	1	3	3
3	1	2	3	3	3	3	1	2	2
3	3	3	2	3	3	2	3	2	3
1	3	3	3	3	3	3	3	1	3
3	3	3	9	2	1	2	3	3	3
1	2	2	2	1	1	2	2	2	2
3	2	2	3	2	3	1	1	2	1
9	9	9	9	9	9	9	1	9	9
3	1	3	3	3	9	9	9	9	9
1	2	1	1	3	2	1	3	2	1
2	2	2	3	2	1	1	2	3	3
3	2	2	2	1	2	1	2	2	2
2	1	3	2	3	3	2	1	2	3
9	9	9	9	9	9	1	9	9	9
9	9	9	9	9	9	9	9	1	9
9	9	9	9	9	9	9	1	9	9
9	9	9	9	1	9	9	9	9	1
9	9	9	9	9	3	9	9	9	9
9	9	9	9	1	9	9	1	9	9
9	9	9	9	1	9	9	9	1	9
9	9	9	9	1	9	9	9	9	1
9	9	9	9	9	1	9	9	9	9
9	9	9	9	9	9	1	9	9	9
9	9	9	9	9	2	1	1	2	2
3	1	3	3	3	3	3	1	3	3
2	2	2	1	1	2	1	1	2	2
1	2	1	2	1	3	1	1	1	1
3	3	3	3	2	1	1	1	2	2
1	2	3	3	1	3	1	1	1	2
2	2	1	1	1	9	9	9	1	9

3	3	3	3	3	1	9	9	9	9
2	1	2	2	3	2	2	9	1	1
3	3	3	3	3	2	2	1	2	2
2	2	1	3	2	2	2	2	2	2
3	1	3	3	3	3	3	3	1	3
2	3	2	1	1	3	3	1	3	3
2	1	2	2	3	2	2	1	2	3
3	2	2	3	2	2	3	3	2	2
2	2	2	2	2	1	2	2	2	2
2	3	3	3	2	1	1	2	1	2
2	2	3	2	2	2	2	1	1	1
9	9	9	9	9	9	9	1	9	9
9	9	9	9	9	9	9	9	9	9
2	1	3	3	2	2	2	2	2	2
2	3	3	3	2	3	3	2	2	2
2	2	2	2	2	2	2	3	2	2
3	3	3	3	3	2	2	2	2	2
3	3	3	3	3	3	2	1	1	2
1	2	2	2	2	2	2	2	2	1
9	9	1	9	9	9	9	1	9	9
2	2	2	2	2	2	2	2	2	2
1	1	2	3	1	2	2	2	2	2
1	2	1	9	1	2	2	2	2	2
3	1	3	3	2	3	3	1	1	3
1	1	1	2	3	3	3	1	3	3
2	2	2	2	2	2	2	2	2	2
3	3	3	3	1	3	3	3	3	3
2	2	2	2	2	2	2	2	2	2
2	3	3	3	2	3	3	2	2	2
2	2	2	2	2	3	3	1	3	3
3	3	3	3	1	3	3	1	3	3
2	3	3	3	3	1	9	9	9	9
3	2	3	2	3	2	1	1	3	3
1	1	1	1	1	2	1	2	3	3
1	1	1	1	1	2	2	2	2	2
2	2	2	2	2	2	2	2	2	2
1	9	9	9	9	9	9	9	1	9
2	2	1	1	1	1	1	1	1	1
9	9	9	9	9	3	3	1	3	3
3	3	3	2	2	1	3	3	3	3
9	1	9	9	9	1	9	9	9	9
3	2	2	2	2	2	1	1	2	2
2	2	2	2	2	2	2	2	2	2
3	3	2	2	3	3	2	1	2	1
1	2	3	3	3	2	9	1	2	3
3	3	3	3	3	3	3	3	2	3
3	3	2	2	3	2	1	1	2	2

3	3	2	2	3	2	1	1	2	3

Anytime	DuringPeri	SeventoTe	MidCycle	SeventoTe	EarlyPerio	LatePeriod	NoBabies	ManyBabi	HighFat
1	2	3	9	2	3	2	2	3	2
2	1	3	1	2	2	3	3	1	2
2	2	2	2	2	3	3	3	2	2
2	1	3	1	2	2	3	9	2	3
9	9	9	9	9	9	9	9	9	9
2	1	3	1	2	2	3	3	1	2
1	2	2	2	2	2	2	2	2	2
3	2	1	1	2	1	1	1	1	1
2	9	9	9	9	2	9	9	9	9
9	2	9	9	9	1	9	9	9	9
2	2	2	2	2	9	2	9	9	9
1	1	2	3	3	9	9	9	9	9
2	1	2	2	2	2	2	2	2	2
3	3	1	3	2	2	3	3	3	2
2	2	2	2	2	3	2	3	3	2
2	1	2	2	2	3	3	1	3	2
1	2	9	1	2	2	3	2	9	1
1	2	2	2	2	3	3	1	3	2
1	3	3	2	2	3	3	1	3	2
2	2	2	2	2	3	3	3	3	2
2	2	2	2	2	2	3	3	3	3
2	2	2	2	3	2	3	3	3	1
1	3	3	3	3	9	9	2	9	9
2	2	2	2	3	2	2	2	2	3
1	1	2	2	2	2	2	2	2	2
2	2	2	2	2	2	2	2	2	2
1	2	2	2	2	2	2	2	2	2
2	1	2	2	2	2	2	2	2	2
1	1	2	2	2	1	1	2	2	2
2	2	2	2	2	2	2	2	2	2
2	2	2	2	2	2	2	2	2	2
2	2	2	2	2	3	3	3	3	2
2	2	1	2	2	3	2	2	2	1
2	2	2	2	2	3	3	3	3	2
2	2	2	2	2	3	3	3	3	2
2	2	2	2	1	3	3	3	3	2
2	2	2	2	2	3	3	3	3	2
9	9	1	2	1	1	3	3	1	3
1	1	2	2	2	1	1	9	9	9
1	1	2	2	2	9	9	9	9	9
9	9	1	2	2	2	2	2	2	2
2	9	9	9	9	2	9	9	9	9
1	3	3	3	2	3	3	3	3	3
1	3	3	1	3	3	3	3	3	3
1	2	2	2	2	2	3	3	9	9
9	9	9	9	9	3	1	1	2	3

1	2	1	1	3	3	3	3	3	3
9	9	9	9	9	9	9	9	9	9
9	9	9	9	9	2	9	3	3	9
1	1	2	2	2	2	2	3	3	2
1	9	9	9	9	9	9	9	9	1
3	3	1	3	3	3	3	3	3	1
1	1	1	1	1	3	3	1	3	3
9	1	1	9	9	9	9	9	9	9
1	9	1	9	9	9	9	9	9	9
1	1	2	3	2	1	2	2	3	1
9	9	1	9	9	9	9	9	9	1
9	9	1	9	9	9	9	9	9	9
9	9	9	9	9	2	2	2	3	2
2	2	2	2	2	9	9	9	9	9
9	9	9	9	9	2	2	3	3	2
3	3	1	3	3	3	3	3	3	2
1	2	2	2	2	2	2	2	2	1
1	1	2	1	2	9	9	9	9	1
1	2	3	1	2	3	1	3	3	3
1	2	2	1	2	1	3	3	2	2
3	1	3	1	3	3	3	1	3	3
3	1	3	1	3	3	3	1	3	3
3	1	3	1	3	3	3	1	3	3
2	1	1	1	2	9	9	9	9	3
3	9	3	2	1	3	1	1	2	3
9	9	9	9	9	9	9	9	9	1
9	9	9	9	9	9	9	9	9	9
2	1	2	1	2	3	2	3	2	2
2	3	1	2	1	1	3	3	1	3
9	9	1	9	9	3	3	3	2	3
9	9	9	9	9	2	3	3	3	3
9	1	2	2	2	3	3	3	3	1
9	9	9	9	9	2	2	1	2	2
9	9	1	9	9	3	3	3	9	2
1	1	2	1	1	3	3	3	2	1
9	1	2	1	2	2	2	3	3	3
9	9	9	9	9	2	3	3	3	3
1	2	2	2	2	3	3	3	3	2
9	9	9	9	9	9	9	9	9	9
1	3	3	1	1	3	3	3	3	1
1	9	9	9	9	3	2	1	2	1
3	3	2	3	1	3	3	3	3	1
9	9	9	9	9	3	3	3	3	1
1	1	1	1	1	2	2	3	3	1
9	9	9	9	1	3	9	3	3	2
9	9	9	9	9	9	9	9	9	9
9	1	9	9	9	9	9	9	9	1

1	2	2	2	2	3	3	2	3	2
1	2	2	2	2	3	3	2	2	2
1	2	2	2	2	9	9	9	9	9
2	2	2	1	2	3	3	3	3	2
1	2	2	2	2	9	2	2	2	1
2	1	2	1	2	1	1	2	1	2
3	3	3	3	3	2	2	3	3	2
1	2	1	2	2	3	3	3	1	1
1	2	2	2	2	2	2	3	3	1
1	2	1	1	1	2	1	2	3	1
1	1	2	2	2	3	2	1	2	1
1	2	2	2	2	3	3	3	3	2
1	2	3	3	2	3	3	3	3	1
9	9	1	9	9	9	9	9	9	9
1	2	2	3	1	2	3	3	2	1
2	1	3	2	1	9	9	9	2	9
1	2	1	2	2	2	2	3	3	2
2	2	1	2	2	2	2	2	2	2
2	2	2	2	2	2	2	2	2	2
9	9	9	9	9	2	2	2	2	2
2	1	3	2	2	2	2	2	2	2
9	9	9	9	1	9	9	9	9	9
1	3	1	2	3	2	2	2	2	2
1	3	3	3	1	1	3	3	3	3
1	1	2	2	1	3	2	2	2	1
2	2	2	2	2	2	2	2	2	2
9	9	9	9	1	9	9	9	9	9
9	9	9	9	1	9	9	9	9	9
2	2	2	2	2	2	2	3	3	2
9	9	1	9	9	9	9	9	9	1
2	2	2	2	2	2	2	2	2	2
1	3	2	2	2	2	2	2	2	2
2	1	2	2	2	2	2	2	2	1
2	2	2	2	2	3	3	3	3	3
9	9	1	9	9	9	9	9	9	1
3	1	3	1	3	1	3	3	3	3
1	2	2	9	2	2	2	2	2	2
2	2	1	2	2	2	3	3	1	2
1	9	9	9	9	1	9	9	9	9
1	2	1	2	1	3	3	3	3	3
3	3	1	3	3	2	3	3	3	1
3	3	1	3	3	2	3	3	3	1
1	3	2	2	2	2	2	2	2	2
3	2	2	2	2	2	2	2	2	2
1	2	1	2	1	2	2	2	2	2
2	1	1	2	2	2	2	2	2	1
3	1	3	3	3	2	2	2	2	2

9	1	2	2	2	9	9	9	9	9
9	9	9	9	1	3	3	3	2	2
1	2	1	2	2	3	3	2	2	2
3	3	1	3	3	2	3	3	1	3
3	3	1	3	3	2	3	2	9	3
1	9	9	9	9	3	2	2	3	1
2	1	2	1	2	2	2	1	2	1
2	2	2	2	2	2	2	3	3	2
2	3	3	3	3	2	2	2	2	2
1	2	2	2	2	2	2	2	2	2
2	1	3	2	2	3	3	2	2	3
2	1	3	2	2	3	3	2	2	3
9	9	1	9	9	2	9	9	9	9
1	2	2	2	2	2	2	2	2	2
1	2	1	2	2	3	3	2	2	2
2	2	2	2	2	3	3	2	2	2
2	2	2	2	2	2	2	2	2	2
9	9	1	9	9	3	3	3	2	2
9	9	9	9	1	9	9	9	9	9
3	1	1	2	3	1	2	1	3	2
3	3	1	3	3	9	9	9	9	9
3	3	1	3	1	3	3	3	3	3
2	9	1	2	2	2	1	2	2	2
1	3	3	3	3	3	3	3	3	3
1	3	1	3	3	3	3	3	3	3
9	9	1	9	9	9	9	9	9	9
3	3	1	2	3	3	2	2	3	3
9	9	1	9	9	9	9	9	9	9
3	3	1	3	3	3	3	3	3	3
3	3	1	3	3	3	3	3	3	3
1	1	2	2	2	3	2	2	2	1
2	3	2	2	1	2	2	3	3	1
3	3	1	2	3	9	9	9	9	3
3	3	1	3	3	3	2	3	3	9
3	3	1	3	3	3	2	3	3	2
2	3	1	9	3	3	2	3	3	2
1	3	3	3	3	2	3	2	3	3
1	3	1	3	3	2	3	2	3	3
3	3	1	3	3	3	2	3	3	2
3	3	1	3	3	3	3	3	3	2
3	3	1	3	3	3	3	3	3	3
1	3	3	3	3	3	3	3	3	3
9	9	1	9	9	9	9	9	9	9
3	3	1	3	3	3	3	3	3	3
3	3	1	3	3	1	3	3	3	3
3	3	1	3	3	3	3	3	3	3
3	3	1	3	3	3	3	3	3	3

3	3	1	3	3	3	3	3	3	3
3	3	1	3	3	9	9	9	9	9
3	3	1	3	3	3	9	9	9	9
3	3	3	3	1	9	9	9	9	9
3	3	1	3	3	3	3	3	3	3
1	1	1	1	1	3	1	3	3	3
3	3	1	3	3	3	3	3	3	3
2	2	1	2	2	2	2	3	9	1
1	3	3	1	1	3	3	3	3	1
9	9	9	9	9	9	9	3	9	9
9	9	9	9	9	2	2	3	2	1
1	9	9	9	9	9	9	9	9	9
9	9	9	9	1	3	3	3	3	1
3	3	1	2	3	3	3	3	3	2
1	1	2	1	3	2	2	1	2	2
1	1	1	1	1	2	2	2	2	2
3	3	1	9	2	3	2	2	2	2
1	1	1	1	1	3	3	1	1	1
1	2	2	2	2	2	2	2	2	2
2	1	1	2	1	2	2	2	2	2
1	1	1	2	2	2	1	2	2	1
1	2	2	2	2	2	2	2	2	2
2	3	2	1	2	1	2	2	1	2
1	2	2	2	2	9	9	9	9	9
1	2	2	2	2	2	2	2	2	2
9	9	9	9	9	2	2	2	2	2
2	2	2	2	2	2	2	2	2	2
1	2	2	2	2	2	2	2	2	2
2	2	2	2	2	2	3	3	3	3
1	2	2	2	2	2	2	2	2	2
2	1	2	2	2	2	2	2	2	2
2	1	2	2	2	2	2	2	2	2
1	2	2	2	2	2	2	2	3	2
1	2	2	2	2	2	3	1	1	2
2	2	2	1	2	2	2	2	2	2
2	1	1	1	1	1	2	2	2	1
2	2	1	1	1	2	1	3	3	1
2	3	3	3	1	3	3	2	2	2
2	2	1	2	2	2	2	2	3	2
2	1	2	9	2	2	3	2	3	3
2	2	2	2	2	2	3	2	3	2
1	2	2	2	2	2	2	2	2	2
9	9	9	9	1	1	9	9	9	9
9	9	9	9	1	9	9	2	9	9
3	3	2	2	2	3	3	2	2	3
3	2	1	2	2	2	2	2	3	1
2	2	2	2	2	2	2	2	2	1

1	2	2	2	2	3	3	3	3	2
1	2	2	2	2	3	3	3	3	2
2	2	1	2	2	1	2	2	2	2
2	2	1	1	2	2	2	2	2	1
1	1	2	3	2	3	2	2	3	1
1	3	1	2	2	3	1	2	3	1
1	3	9	9	9	3	3	1	3	3
3	2	2	2	2	2	3	2	3	1
1	2	3	3	2	1	2	3	3	1
1	1	3	3	3	3	3	3	3	3
2	2	2	2	2	3	3	2	9	1
2	3	1	1	2	3	2	3	3	1
1	2	3	3	2	1	2	3	3	1
3	1	2	2	2	1	2	2	2	2
3	2	2	2	2	3	3	2	2	2
1	3	2	2	1	2	3	1	3	3
3	1	2	1	3	3	2	1	2	3
2	2	2	2	2	2	2	3	3	2
1	2	2	2	2	3	3	1	3	3
2	2	1	2	2	3	3	2	1	1
2	2	1	1	2	2	2	1	3	1
2	1	1	2	1	2	2	1	2	1
3	1	2	2	2	3	2	1	2	2
2	2	1	3	2	3	2	2	3	3
9	1	9	9	9	9	9	2	9	9
2	2	2	2	2	2	2	2	2	2
2	2	2	2	2	2	2	2	2	2
3	2	1	3	1	3	2	2	9	1
3	1	2	2	2	2	2	3	3	2
1	1	1	2	2	2	2	2	2	2
2	2	2	2	2	9	1	9	9	9
2	1	2	2	1	2	2	2	2	2
9	1	9	9	9	9	9	9	9	9
1	3	2	2	2	3	3	3	3	2
2	2	2	2	2	1	9	9	9	9
1	3	2	2	2	2	2	2	2	2
3	2	2	1	2	3	3	3	2	2
1	1	2	1	2	2	1	2	2	2
3	1	1	2	1	2	2	2	3	2
2	2	2	1	1	3	3	3	2	3
1	2	3	2	2	2	2	2	2	2
1	1	2	2	2	3	2	2	2	2
1	3	1	2	2	3	3	3	3	2
2	2	2	2	2	2	2	2	2	2
9	9	1	9	9	3	2	2	3	2
1	2	2	2	3	2	3	2	3	2
1	2	1	2	3	3	3	1	3	2

1	1	2	2	1	2	2	3	2	2
2	1	2	2	1	3	3	3	3	2
2	2	1	2	1	2	2	2	2	1
3	2	2	2	1	3	3	2	2	2
2	1	2	1	2	3	3	3	3	2
1	1	2	1	2	3	3	2	2	1
1	2	1	2	2	2	2	3	3	1
9	2	9	9	9	9	9	9	9	1
2	1	2	1	2	3	2	3	3	2
2	2	2	2	2	3	2	2	2	2
1	2	2	2	2	2	2	2	2	2
1	2	2	2	2	2	2	3	2	2
1	1	1	1	1	1	1	1	1	1
9	9	1	9	9	3	3	3	3	2
3	1	1	1	3	3	1	1	3	1
2	2	2	2	2	3	3	3	3	3
1	3	3	3	3	3	3	3	3	2
1	2	3	1	2	1	1	3	2	2
2	1	1	1	2	2	1	3	1	2
1	1	2	1	2	2	1	2	2	1
2	2	2	2	2	2	1	1	2	2
2	1	1	2	3	3	3	3	3	1
3	2	1	2	3	3	3	3	2	1
1	2	2	2	2	2	2	2	2	2
2	2	2	2	2	2	2	2	2	2
9	9	9	9	9	9	9	9	9	9
9	9	9	9	9	1	1	2	3	1
2	2	1	2	3	3	3	3	3	3
2	2	2	2	2	2	2	3	3	1
2	1	1	2	2	2	2	3	3	1
1	2	2	2	2	2	2	2	2	2
2	1	3	2	1	9	9	9	9	9
1	9	9	9	9	9	9	9	9	9
3	2	3	3	3	3	3	3	3	2
2	2	1	2	2	3	3	3	3	1
1	3	3	3	3	3	3	3	2	9
1	1	2	1	2	2	2	3	3	1
1	3	3	3	3	3	3	3	3	3
3	1	1	3	3	3	3	2	2	1
3	2	1	2	2	9	9	9	9	9
9	9	1	9	9	1	2	3	1	2
1	3	3	3	3	1	3	3	9	1
1	2	2	2	2	2	2	3	2	2
2	9	1	3	3	3	3	3	3	2
2	3	3	1	3	2	3	3	3	3
2	1	9	1	3	3	3	1	3	1
1	2	1	2	2	1	2	2	3	2

1	3	2	2	3	2	2	3	2	9
1	3	2	2	3	2	2	3	2	9
9	9	9	9	1	9	9	9	9	1
3	3	1	3	3	1	3	3	3	3
9	9	9	9	9	1	9	9	9	9
2	1	2	1	2	2	3	3	3	1
1	2	2	2	2	2	2	2	2	2
2	9	2	9	9	3	3	3	3	1
3	3	1	3	3	2	2	3	9	2
3	2	1	2	1	3	2	2	2	1
2	2	2	2	2	2	3	3	3	2
2	2	2	2	2	2	2	2	2	2
3	1	2	1	3	2	3	3	3	1
1	2	3	3	3	1	2	3	2	3
2	2	2	2	2	3	2	3	3	2
3	2	3	2	2	2	2	3	3	3
2	3	1	2	2	2	2	2	3	1
2	2	2	2	2	3	2	3	2	2
9	9	9	9	9	3	3	9	3	1
1	3	2	3	2	3	2	3	2	3
3	2	1	2	3	3	2	3	1	3
1	2	3	2	2	1	3	3	3	1
1	2	3	2	2	2	3	3	3	1
3	2	2	1	2	2	1	3	2	2
2	1	2	2	2	2	2	2	2	2
2	2	2	3	3	2	3	9	2	1
2	2	2	2	2	3	2	3	3	2
3	2	1	2	2	1	2	2	2	2
2	1	2	3	1	2	3	3	3	2
1	2	2	1	1	3	3	3	3	1
2	1	3	2	2	3	2	3	3	2
2	1	3	1	2	3	2	3	3	2
1	1	3	2	3	1	3	1	3	3
1	2	2	2	2	2	2	2	2	2
1	2	2	2	2	2	2	2	2	2
2	3	2	1	2	3	3	2	3	2
3	2	3	2	2	2	2	2	3	2
1	2	1	2	2	1	2	2	3	1
2	2	2	2	2	2	2	2	2	2
1	1	1	1	2	3	1	1	2	2
3	1	2	2	3	2	2	2	2	2
1	2	2	2	2	2	2	2	2	2
2	1	1	9	1	2	3	2	1	1
3	3	2	2	2	2	2	2	2	2
2	3	2	3	1	2	2	3	3	3
3	1	3	2	1	3	2	3	3	1
2	2	2	2	2	3	3	2	2	2

1	3	1	2	2	2	2	2	2	2
1	2	2	1	2	3	3	2	2	1
9	2	9	9	9	2	9	9	9	9
1	2	1	2	1	2	2	2	2	2
1	2	2	3	2	3	2	2	3	1
1	2	2	3	1	3	2	3	3	3
2	3	1	2	1	3	3	2	3	2
1	2	2	2	3	2	2	3	3	2
1	2	2	2	3	2	2	3	3	2
1	1	2	1	2	2	2	3	2	1
1	2	1	2	2	2	2	3	3	2
1	2	1	2	2	3	2	3	3	1
2	2	2	2	2	3	3	2	2	2
2	3	2	2	2	3	3	2	2	3
2	3	2	2	2	3	3	2	2	3
1	1	1	2	2	2	2	2	2	2
2	3	1	1	3	3	2	2	3	1
1	2	2	2	2	2	2	1	2	2
1	2	2	2	2	2	2	1	2	3
9	9	9	9	9	9	9	9	9	9
3	3	2	2	2	2	3	1	2	3
1	9	9	9	9	2	2	2	2	2
1	9	9	9	9	3	3	2	2	3
1	3	2	1	2	2	2	3	3	1
2	2	1	2	2	3	3	3	2	2
1	2	2	2	3	2	1	2	2	3
9	9	1	9	9	3	3	3	3	2
1	2	2	2	2	2	2	2	2	2
2	2	2	2	2	2	1	2	1	2
9	9	9	9	9	9	9	1	9	9
3	2	2	2	2	2	2	2	2	2
1	2	2	2	2	2	2	2	1	1
3	2	2	2	2	2	2	2	2	2
9	9	9	9	9	3	3	3	3	1
2	1	1	2	1	3	2	2	3	1
1	3	1	2	2	2	2	2	2	2
9	9	9	9	9	3	2	3	3	2
2	1	1	3	2	3	2	3	2	3
2	2	2	2	1	1	2	3	3	2
1	2	2	1	2	2	1	3	3	1
1	2	2	3	2	2	3	2	3	3
2	1	3	1	2	2	3	3	1	2
3	2	1	2	2	2	2	2	2	3
1	2	2	2	2	3	3	2	3	3
2	3	2	2	2	1	2	3	2	3
9	9	1	9	9	3	3	3	2	3
3	3	3	3	3	3	3	3	2	3

9	9	1	9	9	3	3	3	2	3
3	3	3	3	1	1	3	3	3	3
3	3	1	3	3	3	3	3	3	3
3	1	1	2	2	1	2	1	9	2
1	1	1	3	3	3	3	1	3	9
3	3	1	3	3	3	3	3	3	3
2	2	2	2	2	2	2	2	2	2
3	1	1	3	1	1	3	3	3	3
2	2	1	2	2	2	2	2	2	2
3	3	3	3	1	3	3	3	3	3
9	9	9	9	9	3	2	3	3	2
9	9	1	9	9	3	3	3	2	3
3	3	3	3	1	3	3	3	3	3
3	3	1	3	3	3	2	3	3	3
3	3	1	3	3	3	3	3	3	3
3	1	1	3	3	3	3	3	3	3
2	2	2	2	2	2	2	2	2	2
2	2	2	2	2	2	2	2	2	2
3	1	1	3	3	3	3	3	3	3
3	1	1	3	3	3	3	3	3	9
3	3	1	3	3	3	3	3	3	3
3	3	1	3	3	1	3	3	3	3
3	1	1	3	3	3	3	3	3	3
1	3	1	3	3	3	3	3	3	1
3	3	3	3	1	3	3	3	3	3
3	3	1	3	3	3	3	3	3	9
3	3	1	3	3	3	3	3	3	3
3	3	1	3	3	3	3	3	3	3
3	3	3	3	1	3	3	3	3	3
3	3	1	3	3	3	3	3	3	3
9	9	9	9	9	1	3	3	3	3
2	2	1	3	3	3	3	3	2	3
3	3	1	3	3	3	3	3	3	3
3	3	1	3	3	3	3	3	3	3
9	9	1	9	9	9	9	9	9	9
3	3	1	3	3	3	9	9	9	9
1	2	2	2	2	2	2	1	2	1
1	2	1	1	3	3	1	3	3	1
3	3	1	3	3	3	3	3	3	3
3	3	1	3	3	3	1	3	3	3
3	3	1	3	3	3	9	9	9	9
2	1	1	1	2	1	2	2	2	2
9	9	9	9	9	9	9	9	9	9
2	2	2	2	2	2	2	2	2	2
2	9	9	9	9	2	9	9	9	9
1	2	2	2	1	2	2	2	2	2
3	9	3	2	1	9	2	3	3	1

2	2	2	2	2	2	2	2	2	2
1	1	2	3	2	1	2	2	3	1
1	1	2	3	2	1	2	2	3	1
1	2	2	2	2	3	2	9	2	3
3	2	2	2	2	2	2	1	3	2
9	9	9	1	9	9	9	9	9	9
1	2	2	1	2	1	2	2	2	2
2	1	2	3	1	2	3	2	2	1
2	1	2	2	2	2	2	3	2	2
2	2	2	2	2	2	2	2	2	2
9	9	9	9	9	1	2	1	2	1
9	1	9	9	9	9	9	9	9	9
3	3	1	3	3	9	9	9	9	9
1	1	2	2	2	1	2	2	2	2
9	9	1	9	9	9	1	9	9	9
9	9	1	9	9	9	1	9	9	9
9	1	9	9	9	2	9	9	9	9
9	1	9	9	9	9	9	9	9	1
9	1	9	9	9	9	9	9	9	1
9	9	9	9	9	9	9	9	9	9
9	9	9	9	9	3	1	3	9	1
1	3	3	3	3	3	3	3	3	3
9	1	9	9	9	3	2	3	3	1
1	9	9	9	9	9	9	9	9	9
1	9	9	9	9	2	9	9	9	9
9	9	2	9	9	9	9	9	9	9
9	1	9	9	9	1	9	9	9	9
1	2	2	1	2	1	2	2	2	3
9	9	9	9	9	2	1	3	2	3
9	1	9	9	9	9	9	9	9	9
9	1	9	9	9	1	9	9	9	9
1	2	2	2	2	3	3	2	2	2
3	3	2	2	2	3	3	2	3	2
1	1	1	1	1	3	3	3	3	3
1	2	2	2	2	2	2	2	1	2
1	1	2	2	2	2	2	3	3	2
3	1	2	2	1	3	2	1	3	2
3	3	3	3	1	3	3	3	3	2
3	2	2	2	2	3	3	3	2	2
2	2	3	2	1	2	2	3	1	2
2	1	2	2	3	2	3	2	2	1
1	1	3	2	3	3	3	1	3	2
1	2	3	2	2	3	2	1	2	1
2	2	2	2	1	2	2	3	2	1
1	2	2	2	2	3	2	2	2	2
3	1	2	2	2	3	3	3	3	2
3	1	1	2	1	2	2	2	1	1

2	1	2	2	3	2	2	3	3	2
1	2	2	3	2	9	9	9	9	9
1	2	2	3	3	3	3	3	3	1
2	1	1	3	2	3	1	2	3	1
3	2	3	1	2	3	3	3	3	2
2	2	2	2	2	3	3	3	3	3
1	2	2	2	2	3	3	3	2	3
1	2	3	1	2	3	2	3	3	2
1	2	3	2	2	2	2	3	2	1
2	2	2	2	2	3	3	2	3	3
2	2	2	2	2	3	3	2	3	3
2	1	2	3	2	3	3	3	3	2
3	2	2	2	2	3	2	2	2	2
3	2	2	2	2	3	2	2	2	2
2	1	2	2	2	2	3	3	2	2
2	2	2	2	2	3	3	3	3	2
1	2	2	2	2	3	3	3	2	1
2	1	3	1	3	2	2	2	2	2
3	3	1	3	1	3	3	3	1	3
3	2	2	1	2	3	3	2	3	1
1	3	1	1	3	2	2	2	2	1
1	2	3	2	2	2	3	3	3	1
1	3	2	2	2	2	3	3	3	2
1	3	2	2	2	2	3	3	3	2
1	2	3	3	2	3	2	3	3	2
2	1	2	2	2	2	2	2	2	2
1	1	1	9	3	1	1	3	3	3
1	1	1	1	3	1	1	3	3	1
2	1	3	2	1	2	2	2	2	1
2	1	1	3	2	2	2	3	2	3
3	1	2	3	3	2	3	3	2	2
2	2	2	3	2	2	2	3	3	2
3	2	2	2	2	2	2	2	2	2
2	1	2	2	3	2	3	2	3	2
1	2	1	2	1	2	1	3	1	1
9	9	9	9	2	9	9	9	9	9
2	2	2	2	2	2	2	2	2	2
1	3	1	2	1	3	1	3	2	1
3	3	2	2	2	3	3	3	3	2
1	9	2	2	1	3	9	3	2	1
1	1	9	9	9	2	2	3	3	1
1	3	1	3	3	1	2	3	3	1
9	9	1	9	9	9	9	9	9	1
1	2	2	2	2	3	3	3	3	2
1	3	3	3	3	3	3	3	3	3
1	2	2	2	2	2	2	2	2	3
9	1	9	9	9	9	9	9	9	1

1	9	9	9	9	9	9	9	9	9
9	1	9	9	9	9	9	9	9	9
9	1	9	9	9	2	9	9	9	9
9	1	9	9	9	2	9	9	9	9
9	1	9	9	9	9	9	9	9	9
9	1	9	9	9	9	9	9	9	9
9	1	9	9	9	9	9	9	9	9
1	9	9	9	9	9	9	9	9	9
1	9	9	9	9	9	9	9	9	9
2	9	9	9	9	2	9	9	9	9
9	9	9	9	9	9	9	9	9	9
2	9	9	9	9	2	9	9	9	9
9	9	1	9	9	9	9	9	9	9
9	9	1	9	9	9	1	9	9	9
1	9	9	9	9	9	9	2	9	9
1	9	9	9	9	1	9	9	9	9
1	9	9	9	9	9	9	9	9	9
9	9	9	9	1	9	9	9	9	9
2	2	2	2	2	2	2	2	2	2
1	1	1	1	1	1	1	1	1	2
1	1	1	1	1	9	9	9	9	9
3	1	1	2	3	1	2	3	3	2
2	1	2	1	2	2	2	3	2	2
1	1	1	1	3	1	3	1	3	1
2	1	3	9	2	2	2	2	1	3
2	2	1	3	3	2	3	2	1	2
2	2	2	2	2	2	3	3	3	2
3	1	2	3	2	3	2	3	3	3
3	3	3	1	3	2	2	2	2	1
3	1	2	1	2	2	2	2	2	2
1	1	1	2	1	1	1	2	2	1
3	1	2	2	2	2	2	3	3	3
1	3	2	2	2	2	2	2	2	2
1	3	2	3	3	3	3	1	2	2
1	1	2	2	1	2	2	2	2	1
2	1	2	2	3	1	2	2	2	2
2	3	2	2	2	2	3	2	2	2
2	1	3	2	1	2	2	2	2	1
9	9	9	9	9	1	3	3	1	1
1	1	2	2	2	2	1	2	2	1
9	9	9	9	9	1	1	1	9	9
1	1	3	1	3	3	3	3	1	9
9	9	9	9	9	2	2	1	2	2
1	1	1	1	1	3	1	3	1	1
1	2	3	1	2	3	3	3	2	2
9	9	9	9	9	2	2	1	2	2
1	2	2	2	2	1	2	2	3	1

2	3	2	3	3	1	2	2	2	2
1	1	1	1	3	2	1	3	3	3
3	3	3	1	3	3	1	1	3	1
2	1	1	2	1	1	2	1	2	1
2	2	2	2	2	1	2	2	2	2
2	1	1	2	2	1	2	2	2	2
2	1	2	2	2	2	1	2	2	2
2	3	2	1	1	1	2	2	2	2
1	1	1	1	3	1	1	1	2	1
9	9	9	9	9	2	9	9	9	9
1	9	9	9	9	1	3	2	9	1
1	2	3	9	2	1	1	3	3	3
3	1	1	2	2	1	1	1	2	1
2	3	1	2	2	3	3	2	2	1
1	2	9	3	1	1	1	2	2	2
9	9	9	9	9	3	3	1	1	2
9	9	9	1	9	1	1	1	1	1
2	2	2	2	2	2	2	2	2	2
1	2	2	3	3	2	3	3	1	2
3	3	3	1	3	1	3	3	3	3
3	3	1	2	2	1	3	2	2	3
3	1	1	2	2	1	1	1	2	1
3	3	3	3	3	1	2	2	2	2
9	9	9	9	9	1	3	1	3	2
9	9	9	9	9	1	3	3	2	3
3	3	3	1	3	1	3	1	3	1
3	3	3	2	3	1	3	3	3	3
9	9	9	9	9	3	3	3	3	1
1	2	2	2	2	1	2	2	9	1
1	9	9	9	9	9	9	9	9	9
1	3	1	2	2	2	2	2	2	2
3	1	2	2	2	3	3	1	9	9
2	1	1	1	1	1	2	2	2	3
1	2	1	1	2	2	3	3	3	3
9	9	1	9	9	9	9	9	9	9
9	9	9	9	1	9	9	9	9	9
1	9	9	9	9	9	9	9	9	9
1	2	2	2	2	2	2	2	3	2
1	2	2	2	2	2	1	2	3	2
1	2	2	2	2	2	1	2	3	2
1	2	2	2	2	2	2	2	3	2
2	1	3	2	3	2	2	1	2	2
1	2	3	2	1	1	2	3	1	2
1	2	2	2	2	2	2	2	3	2
1	2	3	2	1	1	2	3	1	2
3	2	2	3	3	1	2	3	2	1
1	3	3	3	3	3	3	3	2	3

3	3	1	3	3	3	3	3	3	3
1	1	1	2	2	2	2	3	3	2
1	3	3	3	3	1	1	1	3	3
1	1	1	2	2	2	2	2	2	2
1	2	2	2	2	2	2	2	2	2
1	1	1	1	1	2	1	2	1	2
1	2	9	1	3	2	2	2	3	2
9	9	9	9	9	9	9	9	9	1
3	9	3	3	3	1	2	3	3	3
2	1	1	2	1	3	3	3	3	2
2	2	2	2	2	3	3	3	3	3
1	9	9	9	9	1	1	1	9	1
1	3	2	2	3	1	2	3	3	2
1	3	2	2	3	1	2	3	2	1
3	2	2	3	3	1	2	3	2	1
2	1	2	3	2	1	2	3	2	1
1	3	2	2	3	1	2	3	2	1
1	3	2	2	3	1	2	3	2	1
1	3	2	2	3	1	2	3	2	1
3	3	1	1	3	1	3	3	3	3
1	2	1	3	2	2	1	3	1	2
2	2	2	3	1	2	2	2	2	1
1	9	9	9	9	3	3	3	1	3
3	3	2	2	1	2	1	2	2	3
1	2	1	3	2	1	3	3	1	1
3	3	1	2	3	2	1	3	3	3
3	3	3	2	3	1	1	1	2	1
1	1	2	2	2	1	2	1	3	1
1	9	9	9	9	9	9	9	9	9
3	2	2	2	2	3	3	3	3	3
1	9	1	3	2	3	3	1	3	2
3	9	9	9	9	2	2	3	2	3
3	2	3	1	2	2	1	2	1	3
3	1	1	2	1	1	1	2	2	1
3	3	3	3	3	3	3	3	3	3
1	3	2	3	3	3	3	3	3	2
1	3	2	2	3	2	2	1	2	2
1	3	2	2	3	2	2	1	2	2
1	3	2	2	3	2	2	1	2	2
3	3	3	2	3	1	3	1	3	3
1	3	1	1	1	2	1	3	2	2
1	1	2	2	9	3	2	1	9	3
1	2	1	3	2	1	2	1	3	2
3	3	1	3	3	3	3	3	3	1
9	9	9	9	9	9	9	1	9	9
3	1	1	2	2	1	1	1	1	1
2	1	2	2	2	1	2	2	2	2

1	2	3	2	3	2	9	9	9	9
1	9	9	9	9	1	9	9	9	9
9	1	9	9	9	1	9	9	9	9
3	3	3	1	3	1	3	1	3	1
3	1	2	2	2	1	2	2	2	2
2	2	2	2	2	1	2	2	2	2
1	3	1	1	2	1	2	1	2	3
2	2	2	2	2	1	2	2	2	2
2	2	2	2	2	1	2	2	2	2
9	9	9	9	9	1	2	2	2	2
2	2	3	3	2	3	3	3	2	2
3	1	2	1	2	3	3	3	3	2
3	2	2	2	2	1	3	2	1	1
1	1	1	1	3	2	1	1	3	3
1	1	2	1	2	2	1	1	3	3
1	3	3	1	2	2	3	3	3	1
3	3	3	3	1	1	2	3	3	3
9	9	9	9	9	3	9	9	9	9
3	9	9	9	9	3	9	9	9	9
1	2	1	3	3	1	2	1	3	1
1	1	1	2	3	1	3	1	2	1
3	2	2	2	2	2	2	3	2	2
1	9	9	9	9	3	3	3	1	3
2	2	2	2	2	2	2	2	2	2
3	3	3	3	3	3	3	3	3	3
2	9	9	9	9	2	9	9	9	9
2	9	9	9	9	2	9	9	9	9
1	1	3	3	3	3	1	3	3	3
1	9	9	9	9	1	3	3	1	3
3	3	3	3	3	2	2	2	2	2
9	9	9	9	9	2	2	2	3	2
3	2	3	2	1	1	1	1	1	9
3	3	3	1	1	1	3	1	1	1
2	9	9	9	9	2	9	9	9	9
1	2	1	3	2	3	2	2	1	1
3	3	3	3	3	3	1	3	3	3
1	9	2	1	1	2	2	2	2	1
9	9	9	9	9	9	9	9	9	9
9	9	9	9	1	9	9	2	9	9
2	2	2	2	2	2	2	2	2	2
9	9	9	9	9	3	9	9	9	9
2	1	2	2	2	2	2	2	2	2
2	2	2	2	2	2	2	2	2	2
2	2	3	2	3	2	3	3	3	2
2	2	2	2	2	2	2	2	2	2
2	2	2	2	2	2	2	2	2	2
1	1	2	2	2	3	3	3	2	2

2	2	2	2	2	2	2	2	2	2
1	2	2	2	2	3	3	3	2	2
9	9	9	9	9	2	2	2	2	1
9	9	9	9	9	3	3	2	2	1
1	9	9	9	9	9	9	9	9	9
1	1	2	3	3	3	1	1	2	3
2	2	2	2	2	2	2	2	2	2
9	1	9	9	9	9	9	9	9	1
1	1	1	1	1	1	2	3	1	2
9	2	9	9	9	2	9	9	9	9
2	2	2	2	2	2	2	2	2	2
9	1	9	9	9	2	2	3	2	2
9	1	9	9	9	9	9	9	9	9
2	2	2	2	2	2	2	2	2	2
3	1	2	1	1	1	3	2	3	1
2	2	2	2	2	2	2	2	2	2
2	2	2	2	2	2	2	2	2	2
3	3	3	3	3	3	9	2	2	1
1	3	3	1	2	9	9	9	9	9
9	9	9	9	9	3	1	9	3	1
1	9	9	9	9	9	9	9	9	2
3	1	3	1	2	3	3	2	2	3
2	2	2	2	2	2	2	2	2	2
2	2	2	2	2	2	2	2	2	2
2	2	2	2	2	2	2	2	2	2
1	3	2	1	1	2	3	2	3	2
3	3	3	1	3	3	3	3	3	3
3	2	2	2	2	2	2	2	2	2
2	2	2	2	2	2	2	2	2	2
1	3	2	2	2	3	3	3	3	3
1	9	9	9	9	9	9	9	9	9
3	2	2	2	2	3	2	3	2	2
1	3	3	3	3	3	3	3	3	3
2	2	2	2	2	2	2	2	2	2
1	2	2	2	3	3	2	3	3	1
3	3	3	1	3	3	2	3	1	1
2	2	2	2	2	2	2	2	2	2
1	2	2	2	3	1	2	2	2	3
1	2	2	1	2	2	2	2	2	2
1	1	2	2	1	1	2	1	3	1
3	1	1	2	1	3	1	2	2	1
2	2	2	2	2	2	2	2	2	2
1	2	3	3	3	3	3	3	3	3
1	1	1	3	2	2	1	2	3	3
2	2	2	2	2	2	2	2	2	2
3	3	3	3	3	3	3	3	3	3
3	2	2	3	2	2	2	2	3	2

9	9	9	9	9	3	3	2	2	3
3	1	1	3	3	3	3	2	2	2
3	3	3	3	3	1	3	3	3	1
2	2	2	2	2	2	2	2	2	2
2	2	2	2	2	1	2	3	3	2
1	3	3	1	3	3	2	1	2	3
2	2	2	2	2	2	2	2	2	2
2	1	1	2	2	2	1	3	1	3
9	1	9	9	9	1	2	3	1	2
2	2	2	2	2	9	9	9	9	9
1	3	1	2	3	2	1	2	3	1
2	2	2	2	2	2	9	9	9	9
9	1	9	9	9	9	9	9	9	9
9	9	1	1	1	2	2	2	3	2
1	1	3	3	3	1	2	2	2	2
3	1	3	3	2	3	3	3	3	3
3	3	3	3	1	2	2	2	2	2
3	1	1	1	1	3	3	1	3	1
2	2	1	3	2	2	1	3	2	2
1	1	2	2	2	2	2	2	3	2
1	3	3	1	3	9	9	9	9	9
2	1	2	2	1	1	1	1	1	9
2	2	2	2	2	2	2	2	2	2
2	1	2	1	1	3	1	2	3	1
3	1	1	3	3	1	3	1	9	3
2	1	2	9	1	9	9	9	9	1
2	2	2	2	2	2	2	2	2	2
1	9	9	9	9	9	9	9	9	1
1	9	9	9	9	2	2	2	2	2
2	2	3	2	2	3	2	2	2	2
1	1	1	1	1	1	1	1	1	1
1	1	1	1	1	1	2	1	3	1
1	1	1	1	1	1	1	1	2	2
1	1	1	1	1	1	1	1	1	1
2	1	2	1	3	9	9	9	9	9
3	9	1	1	1	3	1	3	1	1
9	9	9	1	9	9	9	9	9	9
1	1	1	1	1	1	1	1	1	1
2	2	2	2	2	2	2	2	2	2
2	2	2	2	2	2	2	2	2	2
2	1	2	3	1	1	1	3	3	3
2	1	2	1	2	1	1	1	1	1
2	1	3	1	2	2	1	1	2	3
2	2	2	2	2	3	1	2	2	1
2	2	2	2	2	2	2	2	2	2
3	3	3	1	1	1	2	3	2	2
1	2	2	2	2	2	3	3	2	3

3	1	1	1	2	1	3	3	1	1
2	2	2	3	3	1	3	3	3	3
1	1	2	1	3	2	2	2	2	3
9	2	9	9	9	9	9	9	9	9
9	1	9	9	9	9	9	9	9	9
2	1	2	3	2	1	2	3	1	3
9	9	9	9	9	9	9	9	9	9
9	9	9	9	9	3	3	3	3	3
3	3	3	3	1	1	1	1	3	3
2	2	2	2	2	2	2	2	2	2
9	1	9	9	9	9	9	9	9	9
2	2	2	3	1	3	1	2	9	3
1	2	2	2	2	2	1	1	2	2
3	3	3	2	2	2	2	1	2	2
2	2	2	2	2	1	2	2	2	2
2	3	1	2	3	2	1	2	3	3
1	2	3	2	2	3	1	2	1	2
9	1	9	9	9	9	9	9	9	9
2	1	3	1	2	2	9	9	9	9
3	2	2	1	1	2	2	1	2	1
9	9	9	9	9	2	2	2	2	2
1	1	2	2	2	2	2	2	9	3
1	1	2	9	2	9	9	9	9	9
2	2	2	2	2	3	3	3	1	1
1	3	2	2	3	1	2	3	9	1
9	9	9	9	9	3	3	3	9	3
3	1	2	3	2	2	3	2	3	2
1	3	1	1	3	3	3	3	3	1
2	1	2	2	2	2	1	3	3	3
3	1	3	2	1	3	2	1	2	1
1	2	1	3	2	2	2	3	3	2
1	3	2	2	3	2	3	2	3	2
1	2	2	1	3	3	3	2	9	1
3	3	3	3	3	3	3	3	3	3
9	9	1	9	9	9	1	9	9	9
1	2	3	3	2	1	2	3	3	3
1	1	9	1	1	1	9	1	9	1
3	3	3	3	3	3	3	3	3	3
2	1	3	9	2	2	9	9	1	9
1	3	3	3	3	3	1	3	3	3
3	1	1	3	1	3	1	3	3	3
1	9	9	9	9	9	9	9	9	9
1	1	1	1	1	1	1	1	1	1
1	1	3	9	3	3	3	3	3	1
1	2	2	2	3	9	9	9	9	9
9	9	9	9	9	2	2	2	2	2
2	1	1	2	2	2	1	3	2	1

9	1	9	9	9	3	1	3	3	3
2	3	3	3	3	2	3	3	3	3
1	2	1	2	1	9	9	9	9	9
3	3	3	3	3	1	3	3	3	3
2	2	2	2	2	2	2	2	2	2
2	1	2	3	2	1	1	3	3	3
2	2	2	2	2	3	3	3	3	3
2	2	2	2	2	2	2	2	2	2
2	2	2	2	2	2	2	2	2	2
2	2	2	2	3	2	2	2	2	2
2	1	2	3	2	2	3	2	2	1
3	3	2	2	2	2	2	2	3	3
3	1	3	3	1	3	1	3	1	3
3	1	2	2	3	2	3	2	3	2
9	9	9	9	9	2	2	2	2	2
2	1	2	3	1	3	3	3	3	3
1	2	3	2	3	3	3	3	3	1
2	2	2	2	2	2	2	2	2	2
9	9	9	9	9	3	2	2	2	3
1	9	9	9	9	9	9	9	9	1
9	9	1	9	9	9	9	9	9	9
3	3	3	3	3	3	3	3	3	3
1	3	3	3	3	3	3	3	3	1
1	1	3	1	1	1	2	3	3	1
1	3	3	3	3	1	3	3	9	1
9	1	9	9	9	3	1	3	3	3
9	9	9	9	9	3	3	3	3	2
2	1	3	1	2	1	2	3	2	3
3	2	3	2	1	2	3	2	3	1
1	3	1	1	3	2	2	1	2	2
1	2	2	2	2	3	3	2	3	1
1	1	1	1	1	3	1	3	1	1
1	2	1	2	3	1	2	1	2	1
1	1	2	2	2	3	1	2	2	3
2	9	9	9	9	9	9	9	9	9
1	3	3	3	3	2	2	2	2	2
1	9	9	9	9	2	9	2	2	2
3	2	2	2	2	2	2	2	2	2
9	9	9	9	1	2	3	2	3	1
1	2	2	2	2	3	3	3	3	1
1	2	2	3	1	1	2	3	2	1
1	2	1	2	1	2	2	2	2	2
1	1	1	1	1	1	1	1	1	1
9	9	2	9	9	2	9	9	9	9
3	9	9	9	9	3	9	9	9	9
1	3	2	9	3	3	3	3	3	3
2	1	2	1	2	3	2	2	3	2

1	3	3	3	3	3	3	3	3	1
2	2	2	1	2	3	3	3	3	3
1	3	3	3	3	3	3	3	3	1
9	2	9	9	9	2	2	1	1	1
2	2	2	2	2	3	3	3	3	3
9	9	9	9	9	3	3	3	3	2
3	2	2	2	2	2	2	2	2	3
3	1	2	3	2	3	3	3	3	3
1	1	3	3	3	3	3	3	3	3
3	3	3	3	3	3	2	1	9	9
3	3	1	2	3	9	9	9	9	9
3	3	3	3	3	9	9	9	9	9
9	9	9	9	9	2	9	9	9	9
1	9	9	9	9	3	9	9	9	9
3	1	1	2	1	9	9	9	9	9
3	1	2	2	2	3	3	3	3	2
1	3	3	2	2	1	1	2	1	1
2	2	1	1	2	2	1	2	1	1
3	1	3	3	3	3	3	3	2	2
2	2	2	2	2	3	3	3	3	1
1	1	2	2	2	2	2	2	2	1
1	2	2	2	3	2	2	1	2	2
2	2	2	2	2	2	2	2	2	2
3	2	2	2	1	3	2	2	3	3
2	2	2	2	1	2	2	2	2	1
1	3	2	1	2	1	1	3	3	2
9	9	9	9	9	3	1	9	1	3
2	2	2	2	2	2	2	2	2	2
1	3	2	1	2	1	1	3	3	2
3	1	2	2	2	3	3	3	3	2
2	2	2	2	2	3	3	3	3	1
9	9	9	9	9	2	9	9	9	9
1	1	3	3	2	3	3	3	3	3
2	2	2	2	2	2	2	2	2	2
2	1	1	3	3	1	1	2	1	1
9	9	9	9	9	3	3	3	3	2
9	9	2	9	9	9	9	9	9	9
9	9	2	9	9	9	9	9	9	9
1	2	1	2	2	3	2	2	3	2
1	1	1	1	1	1	2	2	2	2
1	9	9	9	9	9	9	9	9	9
1	3	2	1	1	1	1	1	1	1
1	1	1	1	1	3	3	3	3	1
1	3	3	3	3	3	3	3	3	2
1	2	3	2	3	3	1	1	3	1
2	1	2	2	2	2	2	2	2	2
1	9	9	9	9	9	9	9	9	9

9	9	9	9	9	3	1	1	1	3
2	2	1	1	1	2	2	2	2	2
1	1	1	2	2	1	9	1	3	2
9	1	9	9	9	9	9	9	9	9
9	1	9	9	9	9	9	9	9	9
1	1	2	1	2	1	9	9	9	9
1	1	2	1	2	1	2	1	2	3
1	2	2	2	2	2	2	2	2	2
2	3	2	3	1	1	2	1	3	1
1	3	3	2	2	1	2	1	2	3
2	1	2	1	2	3	2	3	3	1
2	1	1	2	3	1	3	2	2	1
2	3	9	1	1	3	3	1	3	1
1	2	1	2	2	2	2	2	9	2
3	1	2	2	1	3	2	3	2	1
3	3	3	3	3	3	3	3	3	3
3	3	1	3	3	3	3	3	3	1
3	2	1	2	1	2	2	1	2	2
1	1	2	3	1	1	2	3	3	3
3	2	1	1	1	2	3	3	3	3
2	2	3	3	2	2	2	3	3	2
9	9	1	9	9	2	9	9	9	9
2	1	2	1	2	3	3	3	2	2
3	2	3	3	3	2	2	2	3	2
2	1	2	2	2	2	3	2	2	2
2	1	1	2	1	2	2	2	2	2
2	2	2	2	2	3	2	2	2	2
1	2	2	9	1	3	1	3	3	1
1	1	2	2	2	2	2	2	2	1
3	3	1	3	3	3	3	3	3	1
1	9	9	9	9	9	9	9	9	9
1	2	2	2	2	2	2	2	2	1
9	9	1	9	1	2	2	2	2	1
2	1	1	9	3	2	2	1	3	2
1	2	3	2	3	1	2	3	2	1
1	9	9	9	9	9	9	9	9	9
9	9	9	9	9	9	9	9	9	9
2	2	2	2	2	2	2	2	2	2
2	2	2	1	2	3	3	3	3	1
1	1	2	2	1	1	2	2	1	2
1	2	3	1	2	1	1	1	1	1
9	9	1	9	9	1	9	1	9	1
1	1	1	2	1	1	2	1	1	2
1	2	1	2	3	1	2	1	2	1
1	3	2	3	1	2	1	1	2	3
1	9	9	9	9	2	2	3	1	2
2	2	2	2	2	1	2	3	2	1

1	2	2	1	1	1	2	1	2	2
9	9	9	9	9	9	9	9	9	9
1	2	3	3	3	3	3	3	3	3
3	1	2	2	3	2	2	3	3	2
2	1	2	2	1	1	1	3	1	1
1	2	2	1	1	1	2	1	2	2
1	2	3	2	2	3	2	1	1	2
2	2	2	2	2	3	3	3	2	2
1	2	2	2	2	2	2	2	2	2
3	1	1	3	3	3	1	3	1	2
2	1	3	2	2	9	9	9	9	9
2	2	2	2	2	2	2	2	2	2
1	9	9	9	9	9	9	9	9	9
1	1	2	2	2	2	2	2	1	1
2	3	2	2	2	3	3	2	2	3
9	9	9	9	9	9	9	9	9	9
3	2	3	3	3	3	3	2	3	3
1	9	9	9	9	9	9	9	9	9
2	1	1	2	1	2	2	1	1	2
1	2	2	2	2	3	3	3	3	2
1	1	2	2	2	1	1	3	1	2
3	3	1	3	1	2	1	1	3	3
1	3	2	9	3	3	2	1	3	1
9	9	9	9	9	1	2	2	2	2
1	2	2	2	2	2	2	2	2	2
3	2	2	3	2	3	3	2	2	3
2	2	2	2	2	9	9	9	9	9
1	2	2	2	2	2	2	2	2	2
2	3	1	2	1	3	3	3	3	3
1	1	1	1	1	1	2	3	1	1
3	3	3	1	3	3	2	1	2	1
1	3	2	2	1	1	2	2	2	2
3	9	1	2	3	1	3	3	1	2
2	2	2	2	2	1	2	3	2	1
1	1	2	2	2	1	2	2	2	2
1	2	2	2	2	1	2	3	3	1
2	1	1	2	2	2	1	1	2	1
2	3	3	2	2	3	2	2	2	2
3	1	2	3	1	3	2	2	3	1
2	2	1	2	3	2	2	2	2	1
3	2	3	1	3	3	3	2	1	3
2	3	3	3	3	1	3	9	3	3
9	9	9	9	9	3	3	9	3	3
3	1	9	9	9	9	9	9	9	9
9	1	9	9	9	9	9	9	9	9
9	1	9	9	9	9	9	9	9	9
9	1	9	9	9	9	9	9	9	1

3	1	2	2	2	3	3	3	3	2
2	3	2	2	2	2	3	2	3	2
3	9	9	1	9	9	9	9	9	9
1	2	3	3	3	3	2	3	3	3
1	1	1	1	1	3	1	3	1	1
9	1	9	9	9	1	9	9	9	9
9	1	9	9	9	9	9	9	9	9
1	9	9	9	9	9	9	9	9	9
1	9	9	9	9	1	3	3	9	9
9	1	9	9	9	9	9	9	9	9
9	1	9	9	9	9	9	9	9	9
9	9	9	9	9	1	1	2	3	3
1	9	9	9	9	9	9	9	9	9
3	3	3	1	3	3	3	3	3	3
3	1	1	9	3	3	3	1	3	9
2	1	1	1	1	3	2	2	2	1
1	2	2	2	2	2	2	2	2	1
9	9	9	1	9	9	9	9	9	9
9	1	9	9	9	1	9	9	9	9
9	1	9	9	9	1	9	9	9	9
9	9	9	9	9	1	2	3	1	2
9	9	9	9	9	9	9	9	9	9
1	1	1	1	1	1	2	1	1	1
1	9	9	9	9	9	9	9	9	9
1	2	2	1	1	1	1	1	2	2
2	1	2	2	2	1	1	1	2	2
3	1	2	3	1	3	1	2	2	2
2	1	2	2	2	1	1	1	1	1
2	1	2	2	2	1	1	1	2	2
1	2	2	2	2	2	2	1	1	1
1	1	1	2	2	1	1	2	2	1
3	1	3	3	3	2	2	2	2	1
1	1	2	2	2	3	3	3	3	3
9	1	9	9	9	9	9	9	9	9
2	1	2	2	2	3	1	1	1	1
2	1	2	2	2	2	1	1	1	1
2	1	2	2	2	1	1	1	2	1
9	1	9	9	9	9	9	9	9	9
1	2	3	2	3	3	9	3	2	2
9	1	1	3	1	1	3	3	3	1
3	3	3	3	3	3	3	3	3	3
2	1	2	3	1	1	2	3	2	1
3	3	9	1	3	3	3	3	3	3
2	2	2	2	2	2	2	2	2	2
9	1	3	3	3	1	3	1	1	2
3	1	3	2	3	2	2	2	2	2
2	2	2	2	3	3	2	2	2	1

1	3	2	1	3	3	2	3	3	1
3	2	3	1	3	2	1	3	2	2
3	9	9	9	9	3	3	3	3	3
2	1	2	2	3	2	2	3	3	1
1	3	3	3	3	3	3	3	3	1
9	1	9	9	9	9	2	9	9	9
1	3	2	3	2	3	3	3	3	1
1	2	2	2	1	2	1	2	2	1
9	1	9	9	9	9	2	9	9	9
2	1	2	2	3	2	3	2	1	2
9	1	9	9	9	9	2	9	9	9
2	2	1	1	3	3	3	3	3	1
2	2	2	2	2	1	1	1	1	1
1	2	2	2	1	3	3	3	3	3
2	1	3	1	1	3	3	3	3	3
1	1	2	3	3	1	2	1	3	1
1	2	1	2	2	2	2	2	2	1
2	1	2	2	2	2	2	2	2	2
2	1	1	3	1	3	1	1	3	3
9	9	1	9	9	9	9	9	9	9
3	3	3	1	2	3	1	1	3	2
2	2	2	2	2	2	2	1	3	2
2	2	2	2	2	2	2	2	2	2
2	2	2	2	2	3	2	1	1	1
2	2	1	2	2	3	2	2	2	2
1	3	3	3	3	1	1	1	2	2
3	1	3	3	3	1	1	1	1	1
1	1	1	1	1	2	3	3	2	3
2	2	2	2	2	2	2	2	2	2
1	3	1	1	3	3	3	3	3	9
3	1	2	2	1	1	2	2	3	3
2	1	2	2	2	1	2	1	1	1
2	1	1	1	2	2	2	1	1	1
1	1	1	2	1	2	2	2	2	2
1	2	2	2	2	2	2	1	1	2
1	2	2	2	2	3	3	3	3	3
1	2	2	2	2	2	2	2	2	2
2	1	1	1	1	2	2	2	2	1
2	2	2	2	2	2	2	2	2	1
2	2	2	2	2	2	2	2	2	3
3	3	1	3	3	2	3	2	2	2
2	2	2	2	2	3	3	3	3	2
3	2	1	1	2	3	3	1	2	1
1	1	1	1	1	1	2	2	1	2
3	2	3	3	3	9	2	1	2	2
3	3	3	3	3	3	3	3	3	3
1	2	2	2	2	2	2	2	2	2

2	1	2	2	2	3	2	2	2	3
2	1	1	2	1	3	3	3	3	3
9	9	9	1	9	1	9	9	9	9
3	9	9	9	9	9	9	9	9	9
9	9	1	9	9	9	9	9	9	9
1	1	1	9	9	1	1	2	2	2
1	9	9	9	9	9	9	9	9	9
1	2	2	2	2	3	2	2	2	1
2	2	2	2	2	2	2	2	2	2
3	1	1	3	1	2	2	2	2	1
9	9	9	9	9	2	2	2	2	2
1	1	3	1	2	2	2	2	2	1
1	1	1	1	2	1	1	1	1	2
2	2	2	2	2	2	2	2	2	2
1	1	3	1	1	1	1	1	2	2
9	9	9	9	9	2	2	2	2	2
2	2	2	2	2	2	2	2	2	2
3	1	3	9	3	3	3	3	3	3
9	9	9	9	9	9	2	9	2	1
2	2	2	2	2	2	2	2	2	2
2	1	1	1	1	2	2	2	2	2
2	2	1	2	3	2	2	2	2	1
1	2	2	2	2	2	2	2	2	2
2	2	2	2	2	3	3	3	9	2
2	1	2	2	2	2	1	1	1	1
2	2	2	2	2	2	2	2	2	2
1	9	9	9	9	9	9	9	9	9
2	2	1	2	2	3	3	3	3	3
2	1	3	2	2	2	3	2	2	3
2	2	2	2	2	1	1	2	2	2
9	9	9	9	9	9	9	9	9	9
2	1	1	2	3	2	1	2	1	3
1	1	3	1	2	1	3	1	2	1
3	2	2	2	2	3	2	3	3	2
2	2	2	3	3	2	2	2	2	2
2	2	3	1	1	3	2	2	1	1
3	3	3	3	3	1	1	3	3	3
1	1	2	1	3	3	2	3	2	1
3	2	1	2	1	3	2	2	3	2
1	1	1	2	2	1	3	1	3	2
2	2	1	2	2	2	2	2	2	2
1	2	2	9	2	2	2	2	2	3
3	2	2	3	1	2	2	2	2	2
9	9	9	9	1	9	9	9	9	9
9	9	9	9	1	1	9	9	9	9
3	2	2	3	1	2	9	9	9	9
1	2	2	2	2	9	9	9	9	3

1	3	2	3	2	3	2	3	3	1
2	1	2	2	1	2	2	2	2	2
9	1	9	9	9	9	9	9	9	9
9	9	9	9	9	1	3	1	2	3
9	9	9	9	9	3	3	1	2	3
1	2	3	2	1	3	2	2	2	2
2	1	2	2	2	2	2	2	2	2
2	2	2	2	2	2	2	2	2	2
3	3	1	2	1	3	2	3	3	3
1	9	2	3	1	3	3	3	1	3
9	9	1	9	9	9	9	9	9	1
1	9	9	9	9	9	9	9	9	9
2	2	2	2	2	2	2	2	2	2
1	9	9	9	9	9	9	9	9	1
9	1	9	9	9	9	9	9	9	9
1	3	3	1	1	3	2	1	3	3
1	2	1	2	3	2	3	2	1	1
1	2	1	1	3	3	3	1	3	3
1	2	3	1	3	1	1	1	1	1
3	2	1	2	2	2	3	2	2	2
1	2	2	3	1	1	1	3	3	2
3	3	1	1	3	3	3	1	3	1
1	1	2	3	1	1	3	3	1	3
3	2	1	2	2	2	3	3	3	1
1	2	2	2	2	2	2	2	2	2
3	1	2	9	2	1	1	9	9	9
9	9	9	1	9	2	3	3	3	1
3	1	3	1	1	2	1	3	3	2
9	9	9	9	9	1	2	3	3	3
1	1	2	2	3	1	2	2	2	3
3	1	2	3	2	1	3	3	3	1
3	1	1	1	3	2	2	3	2	2
2	1	3	1	1	1	2	2	3	1
3	2	2	1	1	3	2	3	2	3
1	1	2	2	2	2	2	2	2	2
2	1	2	2	2	2	2	2	2	2
3	1	2	1	2	2	1	1	3	3
1	2	2	3	3	2	2	2	2	2
1	9	9	9	9	9	1	9	9	9
2	3	3	2	3	3	2	3	2	3
9	9	2	9	9	9	9	9	1	9
2	2	1	2	2	1	2	2	2	1
9	2	9	9	9	1	9	9	9	9
9	2	9	9	9	1	9	9	9	9
2	2	2	2	2	3	2	3	1	2
1	1	3	1	3	1	3	3	1	1
1	3	3	3	3	3	3	3	3	3

3	3	3	3	3	3	3	3	3	3
9	9	9	9	9	9	9	9	9	9
9	9	9	1	9	3	3	3	3	3
3	3	1	3	3	1	1	1	3	1
1	1	3	1	3	1	3	3	1	3
3	3	1	1	3	1	3	1	3	1
2	3	2	9	2	2	2	2	3	2
3	1	2	1	1	2	1	1	2	2
3	3	1	3	3	1	3	1	3	3
1	1	9	1	3	1	1	3	3	3
1	1	3	1	3	1	3	3	3	1
1	2	3	2	1	1	3	1	2	3
3	2	3	2	2	2	3	2	3	1
3	3	1	3	3	1	3	1	2	3
1	2	1	1	2	1	2	2	2	2
2	2	2	2	2	2	3	1	3	2
2	3	3	2	2	3	2	3	2	3
3	1	2	2	2	2	3	3	2	1
3	1	3	3	3	1	3	3	3	3
2	2	2	2	2	2	2	2	2	2
1	3	3	2	1	2	2	2	2	2
2	1	2	3	2	3	2	2	2	2
1	1	2	2	2	3	3	3	3	3
2	2	2	2	2	3	2	2	2	2
2	2	2	1	2	1	1	1	2	2
1	2	1	2	2	2	1	2	1	2
2	2	2	2	2	3	2	3	9	1
2	2	1	1	3	3	3	2	2	3
1	1	1	1	3	2	3	1	3	1
1	9	9	9	9	1	9	9	9	9
2	3	2	3	3	3	2	2	3	2
3	3	3	3	3	1	3	3	3	2
2	2	3	3	3	1	1	2	3	1
1	3	3	3	3	2	1	2	2	1
1	1	1	1	1	1	1	3	3	1
2	2	2	2	2	2	2	2	2	2
3	3	3	3	3	3	2	2	2	3
1	2	2	3	2	1	3	2	3	2
1	2	2	1	3	2	1	2	3	1
2	2	2	2	2	2	2	2	2	2
9	1	9	9	9	9	9	9	9	9
1	3	3	3	3	9	9	9	9	9
1	3	1	2	3	1	2	3	1	2
1	3	3	3	3	1	3	2	2	1
2	2	2	2	2	1	2	2	2	2
3	3	2	2	2	3	3	2	9	1
1	1	2	2	3	9	9	9	9	9

2	2	3	2	3	1	2	3	3	1
2	1	2	3	2	3	3	3	3	3
1	3	2	2	3	2	2	1	2	3
1	1	2	3	1	2	1	2	1	1
3	2	1	1	2	9	9	9	9	9
3	1	3	3	3	1	1	1	1	1
1	2	2	2	2	2	2	3	2	2
2	2	2	2	2	2	2	2	2	2
1	2	2	2	2	1	2	3	2	2
1	2	1	2	2	2	1	2	2	2
2	9	9	9	9	3	9	9	9	9
1	2	2	3	2	1	2	3	3	3
9	9	9	9	9	1	2	3	3	2
3	9	1	3	1	9	9	9	9	9
2	2	2	2	2	2	2	2	2	3
9	9	9	9	9	9	9	9	9	9
2	2	2	3	3	3	2	2	2	2
3	2	2	1	1	1	1	3	3	3
3	3	1	3	1	3	3	3	2	1
1	3	3	3	3	2	2	2	2	2
1	2	1	1	3	3	3	3	3	1
2	2	2	2	2	2	2	2	2	2
1	9	9	9	9	9	9	9	9	9
1	2	2	2	2	1	3	2	2	2
2	3	2	1	3	2	2	2	2	1
2	2	3	2	2	2	2	2	2	2
1	9	9	9	9	1	9	9	9	9
2	2	3	2	3	2	2	3	2	2
3	2	2	1	2	2	2	3	3	3
1	9	9	9	9	9	9	9	9	1
3	1	1	2	3	2	2	3	3	3
2	2	2	2	1	2	2	2	2	2
1	9	9	9	9	1	3	3	2	3
2	2	2	2	2	2	2	3	3	1
1	3	3	2	2	2	2	3	3	2
2	2	2	2	2	1	2	3	1	1
1	2	2	1	2	2	2	2	2	2
1	3	3	3	2	3	3	3	3	3
9	9	1	9	9	9	9	9	9	9
1	3	2	3	3	3	3	3	3	3
3	1	2	3	2	2	1	2	3	2
3	3	2	1	2	3	2	3	3	2
2	2	2	2	2	2	2	3	3	2
3	3	2	2	1	3	2	3	2	1
1	2	2	2	3	2	2	1	3	1
9	9	9	1	9	2	2	1	1	2
1	2	2	2	2	2	2	3	3	2

1	3	3	2	3	3	3	3	3	3
2	2	2	2	2	2	2	2	2	1
2	9	9	9	9	2	2	2	2	1
9	9	1	9	9	9	9	9	9	9
2	2	2	2	2	2	2	2	2	2
1	3	3	3	3	9	9	9	9	9
2	2	2	2	2	2	2	2	2	2
3	3	1	1	3	3	3	3	3	2
1	3	2	3	2	2	2	3	3	3
9	9	9	9	9	2	2	3	2	3
2	9	1	1	2	3	3	2	2	2
2	1	2	3	1	2	2	1	2	3
2	2	2	2	2	2	2	2	2	2
3	3	3	3	3	3	3	3	3	3
2	1	2	1	3	2	1	2	1	3
2	2	2	1	3	2	2	3	3	2
2	3	2	3	3	3	3	1	3	3
3	3	1	1	3	2	2	2	2	2
2	2	2	2	2	2	2	2	2	2
1	3	2	3	2	2	2	2	2	2
3	2	2	2	2	3	2	2	2	2
3	2	2	1	2	2	2	3	3	2
3	2	2	1	2	2	2	3	3	2
1	3	3	1	3	2	2	2	2	2
2	2	2	2	2	3	3	3	3	1
3	3	3	1	2	1	2	3	2	3
3	1	1	2	1	1	2	1	3	2
3	1	1	2	3	1	2	1	3	2
1	1	2	3	2	3	3	2	2	2
2	2	2	2	2	2	1	2	2	1
2	3	3	3	3	3	2	3	2	2
3	3	3	2	2	2	2	3	3	2
3	3	2	2	2	3	2	3	3	2
1	2	2	2	2	2	2	2	2	2
2	2	2	2	2	3	3	3	3	3
3	3	3	3	3	2	2	2	2	2
2	2	2	2	2	2	2	2	2	2
2	1	3	2	1	1	2	2	3	2
2	3	3	3	3	2	2	3	2	2
2	2	2	3	2	2	2	2	3	2
1	3	3	3	3	2	2	3	3	1
1	3	3	2	3	2	3	3	2	3
1	1	1	2	3	1	1	1	1	1
1	2	2	2	2	1	1	2	2	1
2	2	2	2	3	2	2	3	3	2
1	2	3	3	3	3	3	3	3	9
9	9	9	9	9	9	9	9	9	9

2	1	3	2	3	3	2	1	2	3
1	2	3	2	2	2	2	1	1	2
1	2	2	2	2	9	9	9	9	9
2	2	2	2	2	2	2	1	1	1
2	2	2	2	2	2	2	2	2	2
2	2	2	2	2	2	2	2	2	2
1	1	1	1	2	2	2	3	3	2
9	9	9	9	9	1	2	2	1	1
9	9	9	9	9	2	2	2	2	2
2	2	2	2	2	2	2	2	2	2
2	2	2	2	2	2	2	2	2	2
2	2	2	2	2	2	2	2	2	2
2	2	2	2	2	2	2	2	2	2
2	2	2	2	2	2	2	2	2	2
3	3	3	3	3	2	2	2	2	2
9	9	9	9	9	2	2	2	2	2
2	2	9	9	9	2	2	2	2	2
9	9	9	9	9	2	2	2	2	2
9	9	9	9	9	2	2	3	3	3
2	1	1	2	1	2	2	3	2	3
2	2	2	2	2	2	2	2	2	2
1	2	2	2	2	2	2	2	2	2
2	2	2	2	2	3	3	3	3	2
2	2	2	2	2	2	2	2	2	2
9	9	9	9	9	2	2	2	2	2
9	9	9	9	9	9	9	9	9	9
2	2	2	2	2	2	2	2	2	2
2	2	2	2	2	2	2	2	2	2
2	2	2	2	2	2	2	2	2	2
1	1	1	2	1	3	2	3	3	3
2	2	3	2	2	3	2	2	2	2
2	2	2	2	2	2	2	2	2	2
2	2	2	2	2	2	2	2	2	2
2	2	2	2	2	2	2	2	2	2
2	2	2	2	2	9	9	9	9	9
2	2	2	2	2	1	2	2	2	2
2	2	2	2	2	2	2	2	2	2
2	2	2	2	2	2	2	2	2	2
3	2	2	2	2	2	2	2	2	2
9	9	9	9	9	2	2	2	2	2
9	9	9	9	9	3	3	2	2	2
2	2	2	2	2	2	2	2	2	2
9	9	9	9	9	2	2	2	2	2
2	2	2	2	2	2	2	2	2	2
2	2	2	2	2	2	2	2	2	2
1	2	3	2	2	2	2	2	2	2
2	2	2	2	2	2	2	2	2	2

2	2	2	2	2	2	2	2	2	2
9	9	9	9	9	2	2	2	2	2
1	2	2	2	2	2	3	3	2	1
2	2	2	2	2	2	2	2	2	2
2	2	2	2	2	2	2	2	2	2
9	9	9	9	9	2	2	3	3	3
1	1	2	2	3	9	9	9	9	9
9	2	9	9	9	9	9	9	9	9
1	2	2	2	3	3	1	3	3	1
3	2	2	1	2	3	2	2	2	3
1	2	3	2	3	2	2	2	2	2
2	2	2	2	2	2	2	2	2	2
9	9	9	9	9	2	2	2	2	2
9	1	9	9	9	9	9	1	9	9
3	3	3	3	3	3	3	3	3	3
2	2	2	2	2	2	2	2	2	2
2	2	2	2	2	2	2	2	2	2
2	2	2	2	2	2	2	3	9	9
1	2	2	2	2	9	9	9	9	9
1	2	2	2	2	2	2	2	1	2
1	9	9	9	9	2	9	9	9	9
2	2	2	2	2	2	2	2	2	2
9	9	9	9	9	2	2	2	2	2
2	9	2	2	2	2	2	2	2	2
2	2	2	2	2	2	2	2	2	2
2	2	2	2	2	2	2	2	2	2
2	9	9	9	9	9	9	9	9	9
2	2	2	2	2	2	2	2	2	2
2	2	2	2	2	2	2	2	2	2
9	9	9	9	9	2	1	2	9	9
2	2	2	2	2	2	2	2	2	2
1	2	1	3	3	2	3	1	2	2
2	2	2	2	1	3	3	1	2	3
1	1	1	1	1	2	2	2	2	2
1	2	3	3	2	3	3	3	3	1
1	3	1	3	1	3	3	1	1	2
3	2	2	3	3	3	3	2	3	1
1	2	2	2	2	1	2	1	2	1
1	2	2	1	1	1	1	3	3	1
1	2	2	1	2	2	2	2	2	2
1	2	2	2	2	3	3	3	3	1
9	9	9	1	9	2	2	2	1	2
2	2	1	2	1	1	1	3	3	2
2	2	2	2	2	2	2	2	2	2
1	1	2	1	3	1	1	1	9	1
2	2	2	2	2	2	2	2	2	2
1	2	2	2	2	1	2	2	1	1

2	3	1	2	3	2	3	1	2	1
1	2	1	2	1	1	9	3	1	2
1	1	1	1	1	3	1	2	3	1
3	1	2	1	2	1	3	3	3	2
3	3	2	1	2	9	9	9	9	9
2	1	2	1	3	1	2	3	3	1
1	2	3	1	2	2	1	3	2	3
1	9	9	9	9	2	9	9	9	9
2	2	2	2	2	2	2	2	2	2
1	2	2	2	2	2	2	2	2	2
2	2	2	2	2	3	3	3	3	3
2	2	2	2	2	3	3	3	3	3
3	3	3	3	3	2	2	2	2	2
2	2	2	2	2	2	2	2	2	2
2	1	2	1	1	3	3	3	3	2
2	2	2	2	2	2	2	2	2	2
2	2	2	2	2	2	2	2	2	2
1	3	3	3	3	3	3	3	3	3
9	9	9	9	9	3	3	3	3	2
3	2	1	2	1	1	1	3	2	2
9	9	9	9	9	3	3	2	3	2
1	9	9	9	9	9	9	9	9	9
3	1	1	1	2	2	2	1	3	3
9	9	9	9	9	9	9	9	9	9
1	2	3	1	2	1	2	2	1	3
3	1	2	2	2	2	2	1	1	1
2	2	2	2	2	1	3	3	3	2
2	9	9	9	9	2	9	9	9	9
9	9	9	9	9	2	2	2	2	2
9	9	9	9	9	9	9	9	9	9
2	9	9	9	9	3	3	3	2	3
1	1	2	2	2	9	9	9	9	9
3	1	1	2	1	9	9	9	9	9
3	1	1	2	1	1	2	2	2	1
3	1	1	2	1	1	2	2	2	1
2	2	2	2	2	2	9	9	9	9
1	2	3	1	2	9	9	9	9	9
3	1	1	2	1	1	2	2	2	1
2	1	2	2	1	2	2	2	2	2
9	9	9	9	9	2	2	2	2	2
2	2	2	2	2	2	9	9	9	9
2	2	2	2	2	2	2	2	2	2
2	2	2	2	2	2	2	2	2	2
9	9	9	9	9	9	9	9	9	9
9	2	9	9	9	3	9	9	9	9
1	1	2	2	1	1	1	1	1	1
1	2	2	2	2	2	2	2	2	2

1	2	2	1	1	9	9	9	9	9
9	9	9	9	9	1	2	2	1	1
2	1	3	3	3	3	3	3	3	3
9	2	9	9	9	2	3	1	3	2
2	2	2	2	2	2	2	2	2	2
9	9	9	9	9	3	3	3	3	3
2	2	2	2	2	2	2	2	2	2
2	2	2	2	1	9	9	9	9	9
9	1	9	9	9	9	9	9	9	9
2	2	2	2	2	9	9	9	9	9
2	2	2	1	1	1	1	1	1	1
1	2	2	2	1	2	3	3	2	2
1	2	3	3	3	3	2	2	2	2
3	3	3	3	3	3	3	3	3	3
2	1	3	1	3	3	3	2	2	1
3	2	1	2	2	3	2	3	3	1
1	1	1	2	1	1	2	3	3	3
2	3	1	2	1	3	3	2	3	1
2	2	2	2	1	2	2	2	1	2
3	2	1	3	1	1	1	3	1	3
2	3	1	2	1	1	3	2	1	2
3	1	1	3	3	1	3	3	3	3
1	9	9	9	9	3	2	3	2	1
1	3	3	3	3	3	3	3	3	2
1	1	2	2	1	3	1	2	1	3
2	2	2	2	2	3	2	2	2	2
1	2	2	2	2	2	2	3	3	2
3	9	1	1	2	2	2	3	3	1
2	3	2	3	3	2	1	3	2	3
9	1	3	3	3	3	1	3	3	3
2	1	2	2	2	1	2	3	2	2
1	1	2	1	1	3	3	3	3	3
3	2	2	2	1	1	3	1	3	1
3	3	3	3	3	3	3	3	3	3
2	2	2	2	1	2	3	3	3	3
1	2	3	2	1	2	2	3	1	2
3	3	3	1	3	3	2	1	3	3
2	2	2	2	2	2	2	2	2	2
2	2	1	1	2	1	2	2	3	1
9	9	1	9	9	9	9	9	9	9
3	1	3	3	2	3	3	3	3	3
1	2	3	3	1	2	1	2	3	1
2	3	1	2	2	2	2	3	3	2
2	1	1	2	3	3	3	2	2	1
1	2	1	1	2	2	1	3	3	2
3	3	3	3	3	3	2	3	2	3
9	9	9	9	9	3	2	3	3	3

1	9	9	9	9	1	2	1	2	2
9	9	9	9	9	3	3	2	3	2
3	3	3	3	3	3	3	3	3	3
1	3	3	3	3	3	3	3	3	3
1	1	2	1	2	3	3	3	3	1
3	1	2	2	2	3	3	3	3	1
9	9	9	9	9	1	1	1	1	1
2	2	2	2	2	3	2	2	2	2
3	3	3	1	3	1	3	1	1	3
2	1	2	1	2	2	2	3	3	1
1	3	3	2	3	3	1	3	9	1
2	2	2	2	2	2	2	2	2	2
1	2	2	2	2	2	2	3	2	2
3	1	1	3	1	1	1	1	3	3
1	1	2	2	1	2	2	2	3	2
2	2	2	2	2	2	2	2	2	2
1	2	3	2	2	3	2	2	2	2
2	2	2	2	2	2	2	2	2	1
1	1	2	3	3	3	2	1	3	2
1	2	1	1	2	1	2	3	2	9
1	2	2	3	3	3	3	3	3	2
9	1	9	9	9	9	9	9	9	9
1	3	2	2	2	2	3	2	2	2
3	2	2	2	2	3	3	2	3	3
3	3	3	2	3	3	3	3	3	2
1	2	2	2	1	1	2	2	2	2
1	3	2	2	1	2	2	2	2	2
2	2	2	2	2	2	2	2	2	2
3	3	3	3	3	3	3	3	3	3
2	1	1	1	1	2	2	3	3	1
3	1	1	2	1	1	1	3	3	3
2	1	2	3	1	3	3	2	9	1
1	2	3	2	3	3	2	3	1	3
2	2	3	1	3	2	2	2	2	2
1	1	2	3	1	1	2	3	2	1
2	2	2	2	2	2	2	2	2	2
1	2	2	1	2	3	2	1	3	1
3	1	2	1	1	3	2	3	2	1
3	2	2	1	2	1	2	2	3	9
2	9	9	9	9	2	9	9	9	9
2	2	1	2	2	2	2	2	1	3
2	1	2	2	1	2	3	3	1	1
2	2	2	2	2	2	1	2	2	2
2	1	2	1	2	3	2	2	2	9
3	1	2	3	2	2	3	2	3	3
2	1	3	2	3	9	9	9	1	9
3	1	2	2	1	2	1	3	3	3

3	1	2	2	1	2	1	3	3	2
2	2	2	2	2	2	2	2	2	2
1	1	3	3	3	1	3	3	2	3
1	2	1	3	2	2	1	3	2	1
2	2	2	2	2	3	3	3	3	2
1	3	3	1	1	3	1	3	3	9
1	2	2	1	2	2	2	2	2	1
9	9	1	9	9	1	9	9	9	9
2	2	2	2	2	1	3	2	3	2
1	2	3	1	2	2	1	3	2	3
2	2	2	2	2	2	2	2	2	2
3	3	2	2	1	2	2	2	3	3
9	9	9	9	1	9	9	9	9	9
2	1	1	9	2	2	3	3	2	1
9	9	1	9	9	2	9	2	2	9
1	1	2	2	3	2	1	3	2	2
1	1	1	1	1	9	1	1	1	1
2	2	1	2	2	2	2	1	1	1
2	1	3	2	1	1	3	3	2	1
2	2	3	1	2	2	3	2	3	3
1	1	2	1	1	3	1	3	3	3
2	2	2	2	2	3	2	2	2	2
1	2	2	2	2	2	2	1	2	2
3	2	2	3	2	3	3	3	2	2
1	3	3	1	3	9	9	9	9	1
2	2	1	3	3	1	2	2	2	1
2	9	3	2	1	1	2	2	1	3
2	2	1	2	1	2	2	1	2	1
1	3	3	3	3	2	2	3	2	2
1	2	2	2	2	3	2	2	2	1
1	2	2	1	1	1	2	9	1	2
1	1	1	2	2	2	2	2	2	2
1	1	1	2	1	1	1	1	1	1
2	2	1	2	2	1	1	1	1	1
3	1	3	1	2	1	1	2	3	1
1	1	2	3	2	9	9	9	9	1
1	2	2	2	2	1	1	1	1	2
3	3	3	1	3	3	3	3	3	3
1	1	1	2	2	1	2	1	1	1
1	2	2	1	1	1	1	2	2	2
2	2	3	3	3	3	3	3	3	3
3	3	3	3	3	1	1	2	2	1
1	1	3	2	1	1	3	1	1	1
2	2	2	2	2	2	3	3	3	2
1	2	2	2	3	2	3	2	3	3
3	3	3	3	3	3	3	3	3	3
2	2	2	2	2	2	2	2	2	2

1	2	1	3	3	2	3	1	2	2
3	1	2	3	3	3	1	3	3	3
2	2	2	2	2	2	2	2	2	2
2	2	1	2	2	1	2	3	3	2
1	3	3	3	3	1	3	3	3	3
1	2	3	1	2	1	2	1	2	3
1	3	3	3	3	9	1	9	9	9
1	3	3	2	2	2	2	1	1	1
2	2	2	2	2	2	2	2	2	2
2	2	2	2	2	2	2	2	2	2
2	2	2	2	2	2	2	2	2	2
2	2	2	2	2	2	2	2	2	2
1	1	1	1	1	1	1	1	1	1
3	3	3	3	3	2	2	2	2	2
1	2	2	2	2	2	2	3	3	3
2	2	1	1	2	3	3	2	2	2
1	2	2	2	9	2	2	2	2	2
9	1	1	2	2	3	1	2	2	1
2	2	2	2	2	2	2	3	3	2
1	2	2	2	3	2	2	2	1	1
2	1	2	2	2	2	2	2	2	2
2	2	2	2	2	2	2	2	2	2
3	3	3	3	3	2	2	2	9	2
3	3	2	2	3	3	3	3	3	3
2	1	2	1	2	1	2	3	2	1
2	2	2	2	2	2	2	2	2	2
2	1	2	1	2	2	1	3	1	2
3	2	2	2	3	2	2	3	3	2
2	2	2	2	2	2	2	2	2	2
1	2	1	2	2	2	2	2	3	2
1	2	2	2	3	3	2	2	1	2
2	2	2	2	2	1	2	3	3	2
1	1	2	2	2	1	2	2	2	2
1	2	2	2	3	1	1	1	3	3
1	2	2	1	2	2	3	2	3	2
1	1	2	2	1	2	3	3	3	3
2	2	1	9	2	1	1	1	1	3
2	3	2	2	1	2	2	1	3	2
1	3	3	3	3	3	3	3	3	3
3	3	3	3	3	3	3	3	3	3
2	2	2	2	2	2	2	2	2	2
2	2	2	2	2	2	2	2	2	2
2	2	2	2	2	2	2	2	2	2
3	3	3	2	2	3	3	3	3	3
3	3	3	2	2	3	3	3	3	3
2	9	2	9	2	2	9	2	9	9
2	2	2	1	1	1	2	1	1	2

2	2	1	1	1	2	2	1	1	1
1	2	2	2	1	1	2	2	1	1
1	2	2	2	2	1	2	2	2	1
2	2	1	1	1	1	2	2	1	1
2	2	3	3	2	2	2	2	1	1
2	1	2	2	2	2	2	2	1	1
2	2	1	1	1	2	3	1	2	2
2	2	2	1	1	2	1	2	2	2
1	1	2	3	3	1	2	1	1	2
2	2	3	3	3	2	3	1	1	2
1	1	2	3	2	1	2	2	1	1
1	2	2	2	3	1	2	2	2	3
2	2	2	2	2	2	2	2	2	2
2	2	2	2	2	2	2	2	2	2
2	2	2	2	2	2	2	2	2	2
2	2	2	2	2	2	2	2	2	2
1	2	2	2	2	2	2	2	2	2
2	2	2	2	2	2	2	2	2	2
2	2	2	2	2	2	2	2	9	2
2	2	2	2	2	2	2	3	3	2
2	2	2	2	2	2	2	2	2	2
2	2	2	2	2	2	2	2	2	2
2	2	2	2	2	1	2	2	2	1
2	2	2	2	2	1	1	2	2	1
2	2	2	2	2	2	2	2	2	2
2	2	2	2	2	2	2	2	2	2
2	2	2	2	2	2	2	2	2	2
2	2	2	2	2	2	2	2	2	2
2	2	2	2	2	2	1	1	2	2
3	3	3	3	3	2	2	2	2	2
2	2	2	2	2	2	2	2	2	2
2	2	2	2	2	2	2	2	2	2
2	2	2	2	2	2	2	2	2	2
2	2	2	2	2	2	2	2	2	2
9	1	9	1	9	1	9	1	9	1
9	1	3	1	2	1	3	1	9	3
9	1	9	9	9	1	9	1	9	1
2	9	2	9	2	2	9	9	2	9
9	9	9	9	9	9	9	9	9	9
1	1	1	1	1	1	1	1	1	9
1	9	2	1	9	1	9	2	1	9
2	1	1	1	1	2	1	2	2	1
1	2	2	2	1	1	2	2	2	2
2	2	2	2	2	2	2	2	2	2
1	9	1	9	1	1	9	2	9	9
9	2	9	2	9	2	9	9	9	2
2	1	2	1	2	2	2	2	2	2

2	2	2	2	2	2	1	1	2	1
2	1	1	2	2	2	2	2	9	2
2	2	2	2	2	2	2	2	2	2
1	1	1	1	3	1	1	2	2	1
2	1	1	2	2	1	1	2	2	2
2	2	2	3	3	2	3	2	3	2
1	2	1	1	1	1	1	1	2	2
2	2	2	2	2	2	2	2	2	2
2	2	2	3	2	2	1	1	2	2
2	1	2	2	1	2	1	2	2	3
1	2	2	2	2	1	2	2	2	1
1	2	2	2	2	2	3	3	2	2
1	2	2	3	3	1	1	2	2	1
1	2	2	1	1	1	2	2	2	1
1	2	2	2	2	2	2	2	2	2
1	2	2	3	3	1	2	2	2	2
3	2	1	3	3	2	2	2	2	2
1	2	3	2	2	2	2	2	1	1
2	2	2	1	1	1	2	1	1	2
2	1	2	3	2	2	3	2	1	2
2	1	1	2	2	2	3	2	1	2
2	2	2	1	1	2	2	1	2	2
2	2	2	2	2	1	1	2	1	1
2	2	3	3	2	3	1	1	2	2
1	2	3	2	1	2	2	3	2	2
3	2	1	1	2	3	2	2	3	2
2	2	2	2	2	3	3	2	2	2
3	2	2	3	3	2	3	3	3	2
3	2	1	1	3	2	2	2	2	3
2	2	2	2	2	2	2	2	2	2
2	9	2	2	2	2	2	2	2	2
2	2	2	2	2	3	3	3	3	3
1	2	1	2	1	2	2	2	2	2
1	2	2	1	2	2	2	2	2	3
3	2	2	1	3	2	3	2	1	3
3	3	3	3	3	3	3	3	3	3
2	2	2	3	2	2	2	2	2	2
2	2	2	2	2	2	2	2	2	2
1	2	2	2	3	2	2	2	2	2
1	2	3	2	1	1	2	1	2	1
2	1	1	3	2	2	3	2	1	2
3	3	3	3	3	3	3	3	3	3
1	2	3	2	1	1	2	2	1	1
1	2	3	2	1	2	2	2	2	2
1	2	2	3	3	2	1	2	1	2
1	2	2	2	1	1	2	1	1	1
1	2	3	2	1	2	1	2	1	2

2	2	2	2	2	9	1	9	1	9
1	2	2	2	2	1	1	2	2	2
1	2	2	2	1	1	1	2	2	2
2	2	2	3	3	1	1	2	2	1
1	2	2	3	1	3	2	1	2	2
1	2	2	2	1	2	1	2	2	2
1	1	1	2	2	2	3	3	3	2
1	1	1	2	2	2	1	3	1	3
1	2	1	2	1	3	2	2	3	1
1	2	3	2	2	2	2	2	2	2
1	2	1	2	2	3	3	2	3	1
2	1	2	2	2	2	2	2	1	2
1	2	1	2	2	1	2	3	1	2
2	1	2	2	2	2	3	2	2	3
1	2	3	2	1	3	2	1	2	2
2	2	2	3	2	3	2	2	2	2
1	2	2	2	2	3	2	2	2	2
1	2	2	1	2	2	2	2	2	1
1	1	3	3	3	1	3	3	3	1
1	3	3	2	3	3	3	3	3	2
1	2	2	3	3	2	2	3	3	3
1	3	2	2	3	3	3	3	3	2
2	2	1	1	3	2	2	2	2	1
3	3	9	2	2	2	3	3	3	2
1	2	2	2	2	2	2	2	2	1
2	2	3	2	3	3	3	2	3	3
3	2	1	2	3	2	3	3	2	1
3	2	2	2	2	2	1	2	3	2
1	2	1	1	1	1	1	1	1	1
1	2	2	3	2	2	2	2	3	3
3	3	1	2	1	2	2	3	1	2
1	2	2	1	1	2	2	2	3	2
2	2	2	2	2	2	2	2	2	2
1	2	2	2	2	2	2	2	2	2
2	1	3	9	2	1	2	2	3	1
2	3	2	2	2	2	2	2	2	2
1	2	3	2	3	2	2	3	3	3
3	3	1	2	1	9	2	2	2	1
3	3	2	3	2	3	3	2	2	2
3	2	1	2	2	2	2	3	3	2
1	1	2	2	1	3	3	2	9	1
1	2	2	3	1	2	1	2	2	2
2	2	2	2	2	3	3	2	2	2
2	2	3	2	3	2	2	3	3	2
2	2	2	2	2	2	2	2	2	2
2	2	3	2	2	2	2	2	2	1
2	2	3	2	3	3	2	3	2	2

3	3	2	1	2	3	3	2	2	2
2	3	1	2	1	3	2	2	3	1
1	2	2	2	1	2	2	3	2	2
1	2	2	2	2	2	2	2	2	2
1	2	2	2	2	3	3	2	3	2
1	1	1	1	1	1	2	2	1	1
2	2	2	2	1	2	2	2	2	2
1	1	2	2	1	2	1	2	3	2
3	1	1	2	1	1	1	3	3	1
2	9	9	9	9	9	9	9	9	9
2	1	1	3	1	2	1	1	3	2
2	1	3	2	3	1	2	2	3	2
1	2	3	2	1	1	9	2	9	3
1	2	2	2	2	1	3	2	3	2
1	2	2	2	2	2	2	2	2	2
1	1	1	2	2	1	2	1	1	2
2	2	2	2	2	2	2	2	2	9
2	2	2	2	3	2	2	2	2	2
3	3	3	3	3	3	3	3	3	3
1	9	3	1	1	1	1	1	3	3
3	3	3	3	3	3	3	3	3	3
3	3	3	3	3	3	3	3	3	3
1	2	2	2	2	2	2	2	2	2
1	1	2	9	3	1	1	3	3	2
2	2	2	2	2	2	2	2	2	2
3	3	1	3	2	9	9	3	9	1
3	2	2	2	3	3	2	2	2	2
2	2	2	2	2	2	2	2	2	2
2	2	2	3	2	2	3	1	2	1
1	9	3	2	1	1	3	2	1	3
3	3	1	2	3	3	3	3	3	2
1	2	3	2	2	1	2	2	2	2
2	1	2	2	2	2	2	3	2	3
3	3	3	3	3	2	3	3	3	3
1	1	2	2	2	2	2	3	2	1
2	2	1	2	3	2	1	1	3	2
2	1	3	3	2	2	1	1	3	2
2	2	1	2	3	2	1	1	9	2
2	2	2	2	2	2	2	2	2	2
2	3	3	2	2	2	2	3	2	2
1	1	2	2	1	2	2	2	2	2
1	2	3	3	3	3	3	1	3	3
2	2	2	2	2	3	3	3	3	2
1	1	1	1	1	3	3	3	3	9
3	3	3	3	3	3	3	3	3	3
1	1	1	3	1	3	3	3	2	1
1	1	2	2	2	2	1	2	1	1

2	2	1	2	3	2	2	2	1	1
1	3	3	2	1	3	1	3	1	1
2	2	2	1	1	2	2	3	2	9
2	2	2	2	2	2	2	2	2	2
2	2	1	3	3	3	2	3	9	3
9	9	9	2	9	9	9	9	9	9
2	2	2	2	2	2	2	2	2	2
2	2	2	2	2	2	2	2	2	2
1	1	1	2	1	2	3	2	2	2
2	2	2	2	2	2	2	2	3	2
2	2	1	1	1	2	1	2	2	1
1	2	2	2	2	2	2	1	3	3
1	9	9	9	9	9	9	9	9	9
1	9	9	9	9	9	9	9	9	1
9	9	9	9	1	9	9	9	9	9
2	2	2	2	1	2	2	2	2	2
2	1	2	2	2	1	1	1	1	1
1	2	2	2	3	1	2	2	3	3
2	2	2	2	1	3	3	3	3	2
1	2	2	2	2	2	2	2	2	3
3	3	3	2	2	3	2	3	3	3
3	9	1	2	3	3	3	2	2	2
2	2	1	2	2	3	3	2	2	2
2	2	2	2	2	3	2	2	2	2
3	3	3	2	1	1	3	1	3	3
2	9	1	2	9	2	2	1	9	2
3	3	3	2	1	1	3	1	3	3
9	9	1	9	9	9	9	9	9	9
2	2	2	2	2	9	9	9	9	9
1	3	1	9	2	1	3	3	3	3
1	2	2	3	2	2	2	3	3	2
3	2	2	3	1	3	3	2	2	1
1	3	3	3	3	3	3	3	3	3
2	1	1	3	1	3	2	2	2	2
1	2	2	2	2	3	3	3	3	3
2	3	2	1	2	2	3	3	2	2
3	2	1	3	3	3	3	3	3	2
3	2	3	2	2	3	3	3	3	3
3	3	3	1	1	3	3	3	3	9
1	3	3	3	3	3	3	3	2	1
2	2	1	2	3	1	1	2	2	2
1	2	2	2	3	2	2	3	2	2
1	2	2	3	3	2	3	2	2	2
1	2	1	2	1	2	2	1	2	2
2	2	2	1	2	2	2	2	2	2
1	2	2	2	2	2	2	2	2	1
1	2	2	3	3	2	1	1	1	2

2	2	2	2	2	1	1	1	1	1
1	2	2	2	2	1	3	3	3	1
9	3	1	9	2	9	9	9	9	9
1	9	3	2	2	3	1	2	3	2
2	2	3	2	1	2	2	3	9	2
2	2	1	3	2	3	1	2	2	1
1	3	2	3	3	3	3	3	2	2
1	2	3	1	2	3	2	1	2	3
3	1	3	3	3	3	3	3	3	3
1	2	2	2	2	2	2	2	2	1
2	2	2	2	2	2	2	2	2	3
1	9	9	9	9	9	9	9	9	9
3	1	2	3	1	1	2	1	3	3
1	1	2	2	2	2	2	2	2	2
9	9	9	1	9	3	2	1	2	2
3	2	1	9	1	3	3	3	9	2
1	3	3	3	3	3	3	3	3	3
2	1	3	2	1	2	2	1	2	3
1	2	3	1	2	3	1	2	2	1
3	3	1	3	3	3	3	3	3	9
3	2	2	2	2	2	2	2	2	2
1	3	3	3	3	3	3	3	3	3
1	1	1	2	2	3	1	1	3	3
3	2	1	2	3	1	3	2	2	2
2	2	2	2	2	2	2	2	2	2
3	2	1	9	2	3	2	2	2	3
9	9	2	9	9	2	9	9	9	9
1	2	3	1	1	2	3	1	3	1
2	3	2	2	2	2	2	9	9	9
9	9	1	9	9	9	9	9	9	9
9	2	9	9	9	9	9	2	9	9
2	1	1	2	1	2	1	2	2	2
1	1	1	1	1	1	1	3	3	3
3	3	3	2	2	3	2	3	3	2
3	9	9	9	9	3	1	2	3	1
1	2	3	1	2	2	2	1	1	1
3	3	2	2	2	1	2	1	1	1
1	2	1	2	2	1	2	2	3	3
1	2	2	2	2	2	1	2	1	1
1	2	2	2	2	2	2	2	3	1
1	2	9	2	9	9	9	9	9	9
3	3	3	1	3	3	3	3	3	3
2	2	2	2	1	9	2	2	2	3
2	1	3	2	3	3	3	3	2	1
2	1	3	2	3	3	3	3	3	1
1	3	2	2	3	3	3	3	3	1
2	1	2	9	2	2	3	3	3	2

1	3	2	2	2	2	2	3	2	2
1	1	3	2	3	1	3	1	1	2
9	1	9	9	9	9	9	9	9	9
9	9	1	9	9	9	9	9	9	9
9	9	1	9	9	9	9	9	9	9
2	9	9	9	9	3	3	3	9	1
2	2	1	1	3	2	2	3	2	1
2	2	2	2	2	2	2	2	2	2
1	2	2	2	2	2	2	2	2	1
1	2	1	2	3	3	2	3	3	1
2	1	1	1	1	2	2	2	9	1
1	2	2	2	2	3	3	2	2	2
2	2	2	2	2	2	2	1	2	2
1	2	3	2	2	2	2	2	2	2
3	2	2	3	3	3	3	3	3	3
9	9	9	9	9	9	9	9	9	9
2	2	9	9	3	2	9	2	2	2
1	2	1	1	3	3	2	3	3	1
1	2	3	2	2	3	2	3	3	1
9	9	9	9	9	9	9	9	9	9
9	9	9	9	1	1	9	9	9	9
9	9	9	9	9	1	3	1	3	3
1	1	1	2	1	1	2	1	2	3
3	1	2	2	1	9	9	9	9	9
9	9	2	9	9	1	1	3	9	3
9	1	9	9	9	9	9	9	9	9
1	1	2	1	3	3	3	3	3	2
1	1	2	3	3	3	3	3	3	2
1	2	2	1	1	2	3	2	3	3
2	2	2	2	2	2	2	2	3	1
1	2	2	3	3	2	2	2	2	2
2	2	1	2	2	3	2	2	2	1
2	2	2	2	2	2	2	2	2	2
1	1	3	3	3	3	3	3	3	2
2	3	3	2	3	2	2	2	2	2
3	1	3	3	3	1	3	3	3	1
1	2	2	2	2	2	2	2	2	1
1	2	1	1	1	1	1	3	3	3
2	2	2	2	2	1	3	3	3	3
1	3	3	2	2	3	2	2	2	2
1	1	2	2	2	2	1	1	2	2
1	2	2	2	2	3	2	2	2	2
2	2	2	2	2	1	1	2	2	1
9	2	9	9	9	9	9	9	9	9
2	9	9	9	9	9	9	9	2	9
1	3	3	3	1	3	3	3	3	3
2	2	2	2	2	1	1	1	1	1

1	1	1	1	1	2	2	2	2	2
3	3	2	2	2	2	1	3	3	1
2	2	1	2	3	2	1	2	2	2
2	2	2	2	2	2	2	2	2	2
1	1	2	2	3	1	1	1	2	2
2	3	1	2	1	2	3	3	2	3
3	2	2	2	2	3	2	3	9	1
1	2	3	1	2	1	2	1	2	3
9	9	9	9	9	9	9	9	9	9
1	2	1	2	1	3	2	1	1	9
2	2	2	2	2	2	2	2	2	2
1	1	2	1	1	2	2	2	3	3
1	2	3	1	2	1	2	3	1	2
2	2	2	2	2	2	2	2	2	2
2	2	2	2	2	2	2	2	2	2
1	2	3	2	3	2	1	3	1	2
3	1	2	3	1	1	2	3	1	2
2	2	1	2	3	3	2	3	1	2
1	2	1	1	1	2	1	2	2	2
2	2	2	2	2	2	2	2	2	2
2	2	2	2	2	1	1	2	1	2
3	3	3	3	3	3	3	3	3	3
2	2	2	3	3	2	2	2	3	3
1	3	3	2	3	1	2	2	3	2
2	2	2	2	2	2	2	2	2	2
1	3	3	3	3	3	3	3	3	2
1	2	2	2	2	2	2	2	2	2
1	2	1	3	1	1	1	2	3	9
2	2	2	2	2	2	2	2	1	3
3	1	1	2	2	9	9	9	9	9
2	3	1	2	2	1	1	2	1	2
9	9	9	9	9	9	9	9	9	9
3	3	3	3	3	3	3	3	3	3
2	2	1	2	1	1	1	1	1	1
3	2	1	2	3	1	2	2	2	3
1	1	2	3	2	2	1	3	1	2
2	2	1	1	3	3	2	1	2	3
2	2	2	2	2	2	3	3	2	1
2	2	2	2	2	2	2	2	2	2
1	1	2	3	1	1	2	3	3	3
2	1	3	9	2	2	3	2	3	1
9	9	1	9	9	9	9	9	9	2
1	1	2	2	2	2	2	2	2	2
1	3	2	2	2	3	2	2	2	2
2	2	2	2	2	2	2	2	2	2
3	2	2	2	2	2	2	3	3	2
1	1	2	2	2	1	2	2	1	2

9	9	9	9	9	1	1	1	3	3
1	2	2	3	3	2	1	3	3	2
2	2	2	1	2	2	1	3	3	1
3	9	1	3	3	2	3	3	3	3
3	1	2	3	3	3	2	3	3	1
2	2	2	2	2	2	2	2	2	2
1	3	3	3	1	1	3	3	3	3
2	2	3	2	3	2	2	2	2	3
1	9	2	9	3	9	2	9	3	9
1	3	3	1	3	1	2	2	2	2
1	3	3	2	3	2	3	3	3	2
2	2	1	9	3	2	3	3	2	3
2	1	3	3	2	9	9	9	9	9
2	3	3	3	2	2	3	1	3	2
3	2	2	1	3	2	3	2	3	2
1	1	3	3	3	3	1	3	3	2
1	9	9	9	9	9	9	9	9	9
1	3	2	3	2	1	3	2	1	3
3	1	2	2	3	3	1	2	2	2
2	2	2	2	2	2	2	2	2	1
1	2	1	2	1	1	2	1	2	1
3	3	3	3	3	3	3	3	3	3
2	2	2	2	3	2	2	2	2	2
2	2	2	3	3	3	3	3	3	2
1	2	2	3	1	2	2	3	2	2
2	2	2	2	2	2	2	2	2	2
3	2	2	2	2	2	2	2	2	2
1	3	3	2	2	3	2	3	3	3
1	2	3	2	2	3	1	1	2	1
1	3	2	2	1	2	2	2	2	3
1	1	1	2	2	1	2	1	1	1
1	2	2	2	2	3	3	3	3	2
1	2	2	2	2	1	2	1	2	1
1	2	2	2	2	2	2	2	2	2
1	1	2	1	2	2	2	3	2	2
9	9	9	2	9	9	9	9	9	9
2	3	3	3	3	2	3	3	3	3
3	3	2	1	2	3	3	2	3	2
9	9	9	1	9	1	3	9	2	1
1	2	2	2	2	2	2	2	2	2
2	3	3	3	3	2	3	3	3	3
2	2	2	1	3	2	2	3	3	1
2	2	2	2	2	2	2	2	2	2
2	2	2	2	2	2	2	2	2	2
9	9	9	9	9	1	1	1	2	1
3	2	1	2	3	3	2	2	3	2
3	3	3	3	3	3	3	3	9	9

1	2	2	2	2	9	9	9	9	9
2	2	2	2	2	3	3	3	3	2
3	3	3	3	3	3	3	3	3	3
9	9	9	9	9	1	3	3	3	3
1	2	2	3	3	3	2	2	2	2
1	2	1	2	2	2	1	2	3	2
2	2	2	2	2	2	2	2	2	2
1	3	1	3	1	2	2	2	2	2
3	3	3	3	3	3	3	3	3	3
1	3	3	3	3	1	2	3	3	3
1	1	3	2	2	3	3	3	3	3
3	2	3	9	3	3	1	2	3	1
3	1	2	3	3	3	1	2	2	2
2	2	1	3	2	2	3	3	9	2
2	2	2	2	2	3	3	3	3	2
1	1	1	1	1	1	2	2	2	1
9	9	9	2	9	9	9	9	9	1
1	9	9	9	9	2	9	9	9	9
9	9	9	9	9	3	3	3	3	3
2	9	9	9	9	2	9	9	9	9
2	2	2	2	2	3	3	3	9	2
1	1	1	1	1	1	2	2	1	1
2	2	3	3	2	3	2	3	2	3
3	3	3	3	3	3	3	3	3	3
1	2	3	2	2	1	2	2	1	2
9	9	9	9	1	9	9	9	9	9
9	9	9	9	9	9	9	9	9	9
2	2	2	3	3	2	2	2	2	2
9	9	9	9	9	9	9	9	9	9
9	9	9	9	9	9	9	9	9	9
9	9	9	9	9	9	9	9	9	9
9	9	9	9	9	9	9	9	9	9
9	9	9	9	9	9	9	9	9	9
1	1	2	2	2	1	2	2	2	3
1	3	2	3	1	2	1	3	2	3
2	1	2	2	2	2	9	9	9	9
9	9	9	9	9	9	9	9	9	9
9	9	9	9	9	3	3	3	3	3
9	1	1	9	1	1	9	1	9	1
1	1	1	1	1	2	2	2	2	2
2	2	2	2	2	2	2	2	2	2
2	1	2	1	2	3	2	2	2	3
1	1	2	2	2	2	2	2	2	1
2	2	2	2	2	2	2	2	2	2
1	2	1	2	1	2	1	1	2	2
1	2	3	2	2	1	2	1	3	2
2	1	2	3	2	1	2	2	3	1

2	2	2	2	2	2	2	2	2	2
2	2	2	2	2	2	2	2	2	2
1	2	2	2	2	2	2	3	3	2
1	2	2	3	1	1	2	3	1	2
1	2	3	1	1	1	1	1	2	2
2	1	3	1	1	1	1	2	3	9
9	2	9	1	3	3	1	2	9	2
1	2	2	2	2	2	2	2	2	2
2	2	2	2	2	2	2	2	2	2
2	2	2	2	2	9	9	9	9	1
3	3	1	2	1	3	2	2	2	2
2	3	3	2	2	1	2	1	2	2
3	1	2	3	1	1	2	3	3	9
1	1	2	2	2	2	1	3	9	2
1	3	3	3	3	1	2	2	2	1
3	1	1	1	1	3	3	3	3	3
1	1	2	2	3	2	2	3	3	1
1	3	2	2	2	9	9	9	9	9
1	2	2	2	2	2	3	2	9	2
1	2	3	2	2	2	2	2	3	3
9	9	9	9	2	9	9	9	9	9
3	3	3	3	3	3	2	3	3	9
1	1	1	1	1	1	3	3	3	3
9	9	9	9	9	3	3	1	1	1
9	2	9	9	9	1	9	9	9	9
9	2	9	9	9	9	2	9	9	9
3	1	2	2	3	1	2	2	1	3
2	3	2	2	1	3	2	2	3	3
9	9	9	9	9	9	9	9	9	9
1	2	2	3	2	1	2	2	2	3
1	3	1	3	1	1	3	1	3	9
1	1	3	3	3	3	3	3	3	3
2	3	1	2	1	1	2	2	2	1
2	1	2	2	2	2	3	3	3	2
1	1	2	2	2	1	3	3	2	3
2	2	2	2	2	2	2	2	2	2
1	1	1	1	3	3	3	1	3	2
2	2	2	2	1	2	2	3	3	2
1	2	3	1	2	3	2	2	2	3
1	1	2	1	3	3	2	3	9	9
1	9	9	9	9	2	9	1	3	9
1	2	2	3	2	2	2	1	2	3
3	3	3	3	3	2	2	2	2	2
1	2	2	2	1	2	3	3	3	2
9	9	1	9	9	3	2	1	3	1
1	1	1	1	1	1	1	1	1	1
2	2	2	2	2	3	3	2	2	3

1	2	3	9	1	3	1	3	3	3
1	2	3	2	3	3	2	3	2	2
1	1	2	2	2	2	2	3	3	2
3	1	1	2	2	3	2	2	2	2
2	2	2	2	2	2	2	2	3	3
9	9	9	9	9	2	2	2	3	3
1	1	2	3	2	2	3	2	1	9
2	2	2	2	2	2	2	3	9	1
1	2	2	2	2	3	2	2	3	1
2	2	2	1	1	2	2	3	2	2
3	1	1	2	3	3	3	3	3	1
3	1	3	1	1	1	3	2	1	2
9	9	9	9	9	9	9	9	9	1
3	2	2	3	1	1	3	2	1	2
3	1	1	2	1	3	2	2	3	3
1	1	3	3	3	1	3	3	3	3
3	3	1	3	3	3	3	3	1	3
2	2	2	2	1	2	2	2	2	2
3	2	3	2	3	3	2	3	9	2
9	1	1	1	1	1	2	2	2	2
2	2	2	1	2	2	2	2	2	1
3	3	2	2	2	2	2	2	2	2
1	1	1	1	1	1	3	1	1	1
2	1	1	2	1	2	2	2	1	2
1	1	1	1	1	1	1	1	1	1
9	9	9	9	9	1	1	1	1	1
2	3	3	3	2	2	2	3	3	2
2	2	3	3	3	3	3	3	3	3
1	3	3	3	3	3	3	3	3	3
2	2	2	2	2	2	2	3	2	3
3	1	2	1	2	1	2	3	3	3
2	9	9	9	9	9	9	9	9	9
2	2	2	2	2	2	2	2	2	2
1	2	1	3	2	1	3	2	9	1
2	1	2	1	2	2	1	2	1	1
1	3	3	1	3	1	3	3	1	3
2	1	2	2	2	1	2	2	2	2
2	3	3	3	3	2	3	2	2	3
9	1	1	2	3	1	3	3	1	1
2	2	2	2	2	2	2	2	2	2
2	9	9	1	2	3	2	2	2	3
2	3	2	2	2	2	2	2	2	3
1	3	1	2	2	2	2	3	2	3
9	9	9	9	9	1	3	3	1	2
2	3	3	1	3	3	3	3	3	3
1	2	2	2	2	1	2	2	2	1
2	2	2	2	2	1	2	2	2	2

1	2	1	3	3	3	2	2	1	2
9	9	9	1	9	9	9	9	9	9
9	9	9	9	9	9	9	9	9	9
1	2	2	3	3	3	2	2	2	3
1	3	3	1	3	9	9	9	1	9
1	9	9	9	1	9	9	9	9	1
9	9	9	1	9	9	9	9	9	1
1	1	1	3	1	1	9	2	2	2
3	1	2	2	3	1	3	3	1	1
1	2	2	2	2	3	3	2	3	2
1	2	2	1	2	2	2	2	2	2
2	1	2	3	1	2	2	2	2	2
1	2	2	2	2	1	2	2	2	2
1	2	2	2	2	1	2	2	2	2
1	2	2	2	2	1	2	2	2	2
1	2	2	2	2	2	2	2	2	2
2	2	3	3	3	2	2	2	2	2
2	2	2	2	2	2	2	2	2	2
2	2	2	2	2	2	2	2	2	2
2	2	3	1	2	2	2	2	3	3
2	2	1	2	2	2	2	2	2	2
1	1	1	1	1	1	2	1	1	1
9	1	9	9	9	9	9	9	1	9
2	2	2	2	2	2	2	2	2	2
2	2	1	2	2	3	9	2	1	2
3	2	2	1	3	2	1	1	1	1
3	3	2	2	2	1	2	2	2	3
1	1	2	3	9	2	2	1	3	3
1	2	3	3	2	2	2	3	3	2
1	2	2	2	2	2	2	3	1	1
2	2	2	2	2	2	2	2	2	2
1	1	2	9	2	2	2	2	3	2
2	2	2	2	2	1	2	2	3	1
2	1	1	2	3	1	3	2	3	1
1	2	3	3	3	3	3	3	3	3
2	2	9	9	9	2	2	2	3	3
1	2	1	3	2	2	2	2	2	2
2	2	2	2	2	2	2	2	2	2
2	2	2	2	2	2	2	2	2	2
9	9	9	1	9	2	3	3	2	9
9	9	1	9	9	2	2	2	2	2
1	2	3	2	1	1	2	3	2	2
1	1	2	2	2	2	1	3	3	3
2	1	2	3	2	1	1	2	2	1
1	1	2	1	1	3	2	2	3	2
2	2	2	2	2	2	2	2	2	2
2	2	2	2	2	2	2	2	2	2

1	2	2	2	2	1	1	2	2	1
9	9	9	9	1	1	9	3	9	2
9	1	9	9	9	2	2	2	2	2
3	1	3	2	2	2	2	2	2	3
2	2	1	1	1	3	1	3	3	3
2	2	2	2	2	3	2	3	2	1
3	3	3	3	3	3	3	3	3	3
2	2	2	1	1	2	2	3	3	2
2	3	1	3	2	2	9	2	9	2
3	2	2	2	2	2	2	2	2	2
2	2	1	3	3	2	2	2	2	2
1	1	2	2	3	2	2	2	2	2
3	2	2	2	2	2	2	2	2	2
3	2	2	1	2	1	3	9	1	1
1	3	3	3	3	3	3	2	3	2
1	1	2	2	2	2	2	2	2	2
2	2	1	3	3	2	2	2	2	2
1	9	9	9	9	9	9	9	9	9
2	1	2	2	2	2	2	2	2	2
1	1	1	1	1	1	1	1	2	1
1	2	2	1	1	1	3	3	3	1
1	2	2	2	2	2	2	3	3	2
2	1	3	2	3	2	3	3	1	2
2	2	2	2	2	2	2	2	1	2
2	2	1	2	1	3	3	1	1	1
1	2	2	3	3	1	2	2	3	2
1	2	2	2	2	2	2	2	2	2
1	3	1	2	2	3	2	3	3	3
3	2	1	2	3	3	2	2	2	1
2	2	3	1	1	3	2	3	3	3
2	1	3	2	1	2	3	2	2	2
2	2	2	2	2	1	3	3	3	1
1	1	2	3	2	3	3	2	1	2
2	1	3	2	1	9	9	9	9	9
9	3	1	3	3	1	3	3	3	1
2	3	1	2	1	3	2	1	2	3
1	1	3	2	3	1	2	2	3	3
2	2	2	2	2	2	2	2	3	1
2	1	1	2	9	2	2	2	2	2
3	1	2	2	2	1	2	3	3	3
1	3	2	1	2	9	9	9	9	9
3	1	3	3	3	3	2	1	3	2
9	9	9	9	9	9	9	9	9	9
3	3	1	2	3	1	3	3	3	3
2	2	2	2	2	2	1	1	2	2
1	1	2	3	2	3	3	3	3	2
1	9	2	2	2	3	2	2	2	3

3	2	3	3	3	3	3	3	3	1
3	3	1	3	3	9	9	9	9	9
2	2	2	2	2	3	3	3	2	2
1	2	2	2	2	2	2	2	3	1
3	3	3	3	3	2	2	2	2	2
2	1	2	1	2	2	1	2	1	2
2	2	3	1	2	2	1	1	3	2
1	1	1	1	1	1	1	1	1	1
1	1	1	1	1	2	2	2	2	2
1	2	1	1	2	2	9	9	9	9
2	2	2	2	2	2	2	2	2	2
2	2	2	2	2	2	2	2	2	2
9	9	9	9	9	3	3	3	1	3
9	9	9	9	9	3	3	3	1	2
1	2	3	1	3	2	1	3	3	3
2	2	3	1	2	3	2	2	2	1
3	2	2	2	2	3	3	3	3	3
1	3	2	3	3	3	3	3	3	2
2	1	1	1	2	3	3	3	3	9
1	1	1	1	1	1	1	1	1	1
2	1	2	2	2	2	2	2	2	2
9	9	3	9	9	9	9	3	9	9
1	9	9	9	9	3	2	3	3	3
1	2	3	2	1	3	1	2	3	1
9	9	9	9	9	1	2	3	1	2
9	9	1	9	9	9	9	9	9	1
2	2	2	2	2	2	2	2	2	2
9	9	1	9	9	1	1	9	9	9
1	2	2	2	3	3	1	3	3	3
3	3	1	3	3	1	2	1	3	1
2	3	1	2	1	2	2	2	3	3
9	1	9	9	9	9	9	9	9	9
9	9	9	9	9	2	3	3	3	1
1	3	2	2	1	3	2	3	2	2
1	3	2	2	3	3	3	1	3	3
1	3	2	2	3	3	3	1	3	2
2	2	2	2	1	2	2	2	2	1
9	9	9	9	1	9	9	9	9	9
1	3	2	2	2	2	3	3	3	3
3	2	3	1	2	2	2	2	9	1
2	2	2	2	2	2	3	2	2	1
1	2	1	2	2	2	2	1	9	2
9	9	1	9	9	3	3	1	9	2
1	9	9	9	9	9	9	9	9	9
1	9	9	9	9	1	2	2	2	2
9	9	9	9	9	1	1	2	2	1
1	9	9	9	9	9	9	9	9	9

1	1	3	2	2	2	3	3	3	3
1	9	9	9	9	9	1	9	9	9
9	2	1	2	3	2	2	1	3	1
2	2	3	2	1	2	3	2	3	2
2	2	1	1	2	3	3	3	3	1
1	2	2	2	2	2	2	2	2	2
3	1	2	3	1	1	2	1	3	2
1	1	1	1	1	2	2	2	2	2
3	3	3	3	3	2	2	2	2	2
9	9	9	9	9	9	9	9	9	9
1	1	1	1	1	3	3	2	3	1
1	1	2	2	1	1	2	2	1	2
1	3	1	3	1	1	3	1	3	1
1	1	2	2	1	1	2	1	2	1
1	2	2	2	2	2	2	2	2	2
1	1	2	1	1	1	2	1	1	1
1	9	1	1	1	1	1	1	1	1
2	3	1	2	2	2	1	2	2	3
1	2	2	2	3	1	2	2	2	1
1	1	1	2	2	1	2	2	2	1
9	9	9	9	9	9	9	9	9	9
2	2	2	3	3	2	2	1	1	2
1	2	2	2	2	1	1	2	2	2
2	2	2	3	2	1	2	2	2	2
1	1	2	2	2	1	1	2	2	2
2	2	2	2	2	1	1	2	2	2
2	2	2	2	2	2	2	2	2	2
2	3	3	3	3	2	2	2	3	3
1	2	2	2	2	1	2	2	3	2
1	2	2	2	2	1	2	2	2	2
9	9	9	9	9	9	9	9	9	9
9	9	9	9	9	9	9	9	9	9
9	9	9	9	9	9	9	9	9	9
3	2	1	1	1	3	3	3	3	2
2	3	1	3	3	3	3	3	3	3
2	1	2	2	2	2	2	2	2	2
2	2	2	1	1	1	2	2	2	1
2	3	1	1	1	3	2	3	3	2
1	2	1	1	1	3	3	2	2	1
2	2	2	2	2	2	3	3	3	2
1	3	2	3	3	3	3	3	3	3
2	3	1	2	2	2	3	3	2	2
1	2	2	2	2	2	2	2	2	2
3	1	3	3	3	3	3	1	3	2
2	2	3	1	1	2	2	2	2	2
3	3	9	1	1	3	3	3	3	3
9	9	9	9	9	3	3	3	3	2

1	2	2	2	2	3	3	3	3	2
2	2	2	2	2	2	2	2	9	2
3	2	3	2	3	3	3	3	3	2
3	1	2	1	3	1	3	3	1	1
3	3	3	3	3	3	3	3	3	3
9	9	9	9	9	3	2	2	2	1
2	2	2	3	1	2	2	2	2	1
9	9	9	9	1	1	9	9	9	9
1	9	9	9	9	9	9	9	9	1
1	9	9	9	9	9	9	9	9	9
9	9	9	1	9	1	9	9	9	9
9	1	9	9	9	9	1	9	9	9
2	1	2	2	2	1	2	2	2	2
1	3	1	9	2	9	9	9	9	9
1	2	2	3	2	2	2	3	3	2
1	2	3	1	1	2	2	2	2	2
2	1	2	2	1	1	1	1	2	2
2	1	2	2	1	1	1	1	2	2
1	2	2	2	2	3	2	3	3	1
2	1	3	2	1	2	3	3	3	3
2	2	2	2	2	2	2	2	2	1
1	2	3	2	2	2	3	2	2	3
1	3	1	2	1	9	9	9	9	1
9	9	9	9	9	3	3	3	3	3
1	1	1	1	1	1	3	2	3	2
3	1	1	1	1	1	1	3	2	1
2	2	2	2	2	3	3	2	3	2
2	2	2	2	2	3	3	2	3	2
2	2	2	2	2	2	2	2	2	2
2	2	1	2	2	1	2	2	2	2
1	2	2	2	2	1	2	2	2	2
2	1	3	2	1	2	3	3	3	3
2	2	2	1	2	3	3	3	3	2
3	1	1	1	2	1	1	2	1	1
9	9	9	9	9	2	9	2	9	2
2	2	3	3	1	3	3	3	3	2
2	2	3	3	1	2	3	3	3	3
2	1	2	3	1	3	3	3	3	3
1	1	2	2	2	2	2	2	3	2
1	2	2	2	1	2	2	2	2	3
9	9	1	9	9	1	9	9	9	9
9	9	9	9	2	9	9	9	9	9
9	9	9	9	1	9	9	9	9	9
1	2	2	2	2	3	3	2	2	2
1	9	9	9	9	9	9	9	9	9
9	1	9	9	9	2	2	2	2	2
1	2	1	2	2	3	2	1	3	2

1	2	2	2	3	2	2	1	1	2
1	1	2	2	2	2	2	2	2	3
3	2	1	9	3	3	1	2	3	1
2	2	3	2	1	3	2	3	2	3
2	3	2	3	3	2	3	2	2	3
3	3	1	3	3	2	2	2	2	2
3	3	3	1	3	1	3	1	3	2
3	3	3	2	1	3	2	2	2	3
2	3	3	1	3	3	3	3	3	3
2	1	2	1	1	1	2	3	1	2
2	2	2	2	2	2	3	3	3	3
1	2	1	2	1	2	2	1	2	2
2	3	2	1	3	3	3	1	1	2
1	2	2	9	2	3	3	3	3	2
3	2	1	2	1	1	2	3	1	1
3	1	2	2	1	2	2	2	2	2
1	2	3	2	2	3	1	3	1	1
2	2	2	2	2	2	2	2	2	2
2	2	2	2	2	1	2	2	2	1
1	1	2	3	2	3	3	2	3	2
1	2	2	2	3	1	1	2	1	1
1	1	2	2	2	2	3	3	3	2
2	2	2	2	2	1	2	2	2	1
1	2	1	2	3	2	2	3	3	2
1	1	1	2	2	3	3	3	3	3
2	2	2	2	2	3	3	3	3	2
1	2	2	2	2	2	2	2	2	2
1	1	1	1	1	1	3	3	3	1
1	1	2	2	1	2	2	2	2	2
1	1	2	2	2	2	2	1	1	2
1	1	2	2	2	2	1	1	2	2
3	1	2	3	3	1	2	3	3	3
1	9	9	9	9	1	1	2	2	2
1	1	2	2	2	3	3	2	2	3
3	1	2	3	1	1	1	2	3	2
2	2	2	2	2	2	2	2	2	2
2	1	2	2	2	1	1	2	2	2
1	2	2	2	2	2	2	2	2	2
1	3	1	2	9	1	3	9	3	1
1	1	1	1	1	2	2	2	2	2
3	2	1	1	1	2	1	2	1	2
3	2	1	1	1	3	3	9	2	1
1	2	2	2	2	3	3	3	3	1
2	2	1	2	3	3	3	3	3	1
9	1	9	9	9	2	1	2	1	3
3	3	1	2	1	2	2	2	2	2
1	3	2	2	3	3	3	1	3	3

1	3	2	2	3	3	3	1	3	3
2	2	2	2	2	1	1	2	2	2
2	2	2	2	2	2	1	2	2	2
2	2	2	2	2	1	2	2	1	2
1	1	2	2	2	1	1	2	2	2
1	2	2	2	3	1	2	2	1	3
1	2	2	3	3	1	2	2	3	2
2	2	2	2	2	2	2	2	2	2
3	1	3	3	3	3	3	3	2	1
1	1	2	1	2	1	1	2	1	3
9	3	1	3	3	1	3	3	3	1
3	2	2	2	3	3	2	2	2	3
1	1	1	1	1	2	2	2	2	2
2	1	2	2	2	1	2	2	2	2
9	9	9	9	9	3	3	3	3	3
2	2	1	2	2	9	9	9	2	1
1	1	2	2	2	3	1	3	3	3
1	2	2	2	2	2	2	2	3	2
3	1	1	2	2	2	2	2	2	9
1	2	2	2	2	3	3	3	3	3
1	2	2	1	2	2	2	1	3	1
3	3	3	3	3	1	1	3	1	3
1	3	2	2	3	3	2	2	2	2
2	2	2	3	3	3	2	2	3	2
3	2	9	2	3	1	2	1	1	2
2	1	1	2	1	1	3	3	3	3
1	1	2	1	1	2	1	2	1	1
3	2	2	2	1	2	3	2	3	2
1	2	3	3	3	2	1	1	2	2
2	2	2	2	2	3	2	3	3	3
3	3	3	3	3	1	2	2	2	3
1	2	2	2	2	1	3	3	3	3
1	2	3	2	3	2	2	3	1	2
1	1	2	2	1	1	2	1	3	3
2	2	1	2	2	2	2	2	2	1
2	2	2	2	2	3	1	3	3	2
2	1	2	1	1	1	3	2	2	1
1	1	2	3	2	1	1	2	1	3
3	1	1	1	2	1	2	1	2	3
1	2	2	1	2	3	3	3	2	1
3	1	2	2	2	3	3	3	3	3
1	1	2	2	3	3	3	3	3	2
1	2	3	2	2	2	2	2	2	1
1	1	2	2	1	1	2	3	1	2
2	2	2	2	2	3	3	2	3	3
3	3	2	2	3	2	1	2	1	2
2	2	2	2	2	3	2	2	2	2

1	2	2	3	2	3	2	2	2	3
1	1	2	2	2	2	2	2	3	3
1	1	3	3	1	1	2	2	2	2
3	1	3	3	3	3	3	3	3	3
3	1	1	2	3	3	3	2	3	2
2	2	2	2	2	2	2	2	2	2
2	3	2	3	2	2	3	2	2	2
2	2	1	1	1	2	1	1	1	3
2	1	1	9	2	2	3	2	9	1
1	2	2	2	2	3	1	3	3	3
1	3	1	2	3	3	2	3	3	2
1	1	2	2	2	3	3	3	3	3
1	1	1	2	2	3	2	3	1	3
3	3	3	3	3	3	2	2	2	3
2	2	2	2	2	3	3	3	3	3
1	2	2	2	2	3	2	3	3	1
2	2	2	2	2	2	2	2	2	2
1	3	3	1	3	1	3	3	3	3
1	2	3	2	1	1	2	3	2	1
3	3	2	2	2	2	2	2	2	2
1	2	2	2	2	1	2	2	2	1
1	2	2	2	3	1	2	2	3	1
1	2	2	3	3	1	2	2	2	1
2	2	2	2	2	2	2	2	2	3
2	2	2	2	2	2	2	2	2	3
1	2	2	2	2	3	2	3	3	1
1	1	2	2	2	3	3	3	3	3
1	2	2	2	2	3	1	3	3	3
3	1	1	1	2	1	2	1	2	3
3	3	2	2	2	2	2	2	2	2
2	1	2	1	1	1	3	2	2	1
9	9	9	9	9	9	9	9	9	9
1	2	2	2	2	1	1	2	2	1
3	2	2	1	2	2	2	2	2	2
1	1	1	2	2	1	1	3	3	3
1	2	1	2	3	3	1	3	3	2
3	1	1	1	1	1	1	3	3	3
1	2	3	1	2	1	2	3	1	2
2	2	2	2	2	2	2	2	2	2
1	2	2	2	2	2	1	3	2	1
1	1	1	1	1	2	2	2	3	3
1	1	1	1	1	2	2	2	3	3
1	3	2	1	1	3	2	3	3	9
1	3	9	9	3	3	3	9	1	2
2	1	2	3	2	2	1	1	2	3
3	2	2	2	2	3	3	1	9	3
3	3	1	2	2	3	2	2	2	3

2	3	1	2	2	2	1	9	3	1
1	3	3	1	1	3	3	3	3	1
1	1	2	1	1	1	1	1	1	9
1	2	2	2	2	3	3	3	3	2
3	3	2	2	2	3	3	3	2	2
3	1	2	2	2	2	2	2	2	2
1	1	2	2	3	2	2	9	1	1
3	3	3	3	3	2	2	2	2	2
2	2	2	2	2	2	2	2	2	2
2	2	2	2	2	2	2	2	2	2
2	2	2	2	2	2	2	2	2	2
1	2	2	3	3	1	2	3	2	2
2	2	2	2	2	2	2	2	2	2
2	2	2	2	2	2	2	2	2	2
2	2	2	2	2	2	2	2	2	2
1	1	1	1	2	1	2	2	2	2
3	2	2	3	2	2	1	2	3	1
3	2	1	3	1	3	3	3	3	1
1	2	2	3	3	1	1	2	2	3
2	2	2	2	2	3	2	1	2	2
3	1	1	2	2	1	2	3	2	2
2	2	2	2	2	2	2	2	2	2
3	2	3	3	3	2	2	2	2	2
1	2	2	2	2	2	3	3	3	2
3	3	1	3	3	2	2	2	2	2
1	2	2	2	3	2	3	3	3	1
1	2	2	1	1	2	2	2	2	2
3	2	1	2	2	2	2	2	3	2
3	2	1	3	3	2	2	2	2	2
3	3	1	2	3	2	2	3	3	2
2	2	1	3	1	1	2	2	2	2
2	2	2	2	2	2	2	2	2	3
3	3	1	3	3	3	1	3	1	3
1	9	9	9	9	9	9	9	9	9
1	3	3	3	3	3	3	3	1	1
2	2	2	2	2	2	2	3	2	2
3	2	1	1	3	2	1	2	3	2
1	2	2	2	2	3	3	3	3	3
2	2	2	2	2	2	2	2	2	2
1	3	2	3	3	9	9	9	9	9
3	1	1	2	1	9	1	3	1	2
2	1	2	1	1	2	9	1	2	9
3	3	3	2	3	1	1	1	2	3
1	1	2	3	2	2	2	2	2	1
1	2	3	3	2	3	3	3	3	2
3	2	2	2	2	3	3	2	3	2
1	2	2	3	3	1	2	1	2	2

2	1	2	2	2	2	2	2	3	2
9	2	9	9	9	3	9	2	9	9
1	9	9	9	9	9	9	9	9	9
1	2	3	2	2	3	3	3	3	2
2	2	2	1	3	2	2	1	1	1
9	9	9	9	9	9	9	9	9	9
1	2	2	3	3	1	2	2	3	2
1	2	2	3	3	1	2	2	1	2
2	1	2	2	2	2	1	2	2	2
2	1	1	2	2	2	2	1	2	2
1	1	3	9	3	3	3	3	3	3
3	1	3	2	3	3	3	3	3	1
3	2	3	2	3	3	2	2	2	2
3	2	9	9	3	1	3	3	3	3
9	1	1	1	1	9	9	1	1	1
2	2	1	2	1	2	2	1	2	3
9	9	9	2	9	2	9	2	9	9
1	1	2	2	2	1	1	2	2	2
2	2	1	1	1	1	1	1	1	2
2	1	1	1	1	1	1	3	3	2
1	2	2	2	2	1	1	2	2	2
1	1	1	2	2	1	1	1	2	2
3	2	2	2	1	2	3	3	1	1
2	3	2	1	1	9	9	2	9	1
2	2	2	2	2	2	2	2	2	2
3	3	3	3	3	2	3	3	2	9
3	3	3	3	3	3	3	3	3	3
2	1	1	2	2	1	2	3	3	2
2	2	2	1	1	1	1	1	1	2
1	2	2	3	2	1	2	2	3	2
1	2	2	3	2	1	2	2	1	1
2	1	3	2	2	2	2	3	2	1
1	1	3	2	1	3	1	3	3	1
2	1	1	2	2	1	3	2	3	1
3	1	2	1	2	3	3	3	3	1
1	1	3	3	3	3	3	3	3	9
9	9	9	9	9	9	9	9	9	9
2	2	2	2	2	2	2	2	2	2
1	1	2	3	3	3	1	2	9	9
2	1	2	2	2	2	1	2	2	2
3	3	1	3	2	3	3	1	9	1
2	2	2	2	1	2	2	2	2	2
2	3	1	1	1	1	3	3	3	2
1	2	2	3	2	2	1	1	2	2
3	1	1	1	3	1	3	3	2	2
1	2	2	2	2	1	2	2	1	1
2	3	1	3	2	2	1	3	2	1

2	2	2	2	2	9	9	9	9	9
1	1	3	2	3	1	2	3	3	1
1	2	2	2	2	1	2	2	3	3
1	2	2	3	2	2	2	3	3	2
3	2	3	1	2	2	2	2	2	2
2	2	2	2	2	2	2	3	3	2
2	3	1	1	2	1	2	1	2	3
3	2	2	3	2	1	3	9	1	9
2	2	2	2	3	2	2	2	2	2
1	2	2	3	2	1	1	1	1	2
2	2	1	1	3	1	1	2	3	2
3	2	2	2	1	2	1	1	1	1
1	2	2	2	2	2	2	3	2	2
3	2	3	2	1	3	2	3	1	2
1	2	2	2	2	2	2	2	2	2
3	1	3	2	3	3	3	3	3	2
9	9	9	9	9	9	9	9	9	9
1	1	2	2	1	1	2	2	1	1
1	2	1	1	1	1	1	1	2	3
1	2	2	2	2	2	2	2	2	2
2	2	2	2	2	2	2	3	1	2
2	2	2	2	2	2	2	3	1	9
1	1	2	3	2	1	2	1	3	1
1	3	3	3	3	3	3	2	2	2
9	9	1	2	1	3	3	3	3	3
2	1	2	2	1	1	1	1	2	2
3	3	3	3	3	1	3	3	3	2
3	3	2	1	2	2	2	3	3	3
2	2	2	1	1	1	1	1	1	1
2	2	2	2	2	2	2	2	2	2
2	2	2	2	2	2	2	2	2	2
1	2	3	2	2	3	3	3	3	3
1	2	2	2	2	1	1	2	2	3
2	1	2	1	2	2	1	2	1	2
2	2	1	2	1	1	2	1	3	3
2	1	1	1	2	3	2	3	3	3
1	1	2	3	2	1	2	1	2	2
1	2	2	1	2	2	2	2	2	2
2	2	3	2	1	1	2	3	2	2
1	2	9	1	2	1	2	1	2	1
1	2	3	3	1	3	2	2	1	2
2	9	1	3	1	1	2	3	2	9
2	9	9	9	9	2	9	9	9	9
3	2	1	2	3	2	3	2	2	1
1	3	3	2	2	3	1	9	9	9
3	2	9	2	2	1	3	9	1	3
1	2	2	3	2	1	1	2	1	1

9	9	9	9	9	9	9	9	9	9
1	2	1	1	1	1	2	2	1	2
1	2	2	3	3	1	2	2	3	2
3	3	2	2	1	2	2	2	2	1
1	3	2	2	3	2	2	2	2	2
1	2	2	1	1	2	1	2	3	1
3	3	9	1	2	3	2	3	9	2
3	3	3	3	3	3	3	3	3	3
3	3	3	3	3	3	3	3	2	9
1	1	2	2	3	2	2	2	2	2
1	2	2	3	1	2	1	1	3	3
3	3	3	3	3	3	3	1	2	3
2	9	1	9	9	2	3	1	2	1
1	2	3	1	2	1	2	3	1	2
2	2	2	2	2	1	9	9	9	9
2	2	2	2	2	3	1	2	2	2
1	1	1	1	1	2	1	2	3	2
9	9	9	9	9	2	2	2	2	2
2	2	2	2	2	2	2	2	2	2
2	2	2	2	2	1	1	2	2	2
1	2	2	1	1	1	1	1	1	1
1	1	2	2	2	1	2	1	2	2
1	2	2	1	1	2	2	2	2	2
1	1	2	2	2	1	2	2	3	2
1	2	2	3	2	2	2	3	3	2
1	2	3	1	3	2	1	1	3	1
9	2	9	9	9	9	9	9	9	9
3	3	3	3	3	3	3	2	3	2
2	2	2	2	2	3	3	3	3	3
3	2	1	1	1	2	3	2	2	2
3	1	2	2	2	3	3	2	3	1
1	3	9	2	3	3	3	1	2	3
1	3	3	2	2	2	3	3	3	2
1	2	2	2	3	9	9	9	9	9
1	2	3	2	2	3	3	2	3	2
1	2	2	1	1	1	1	2	2	1
9	9	9	9	9	2	2	2	3	1
2	2	2	2	2	2	2	2	2	2
1	1	2	2	2	1	2	2	1	1
1	1	1	1	1	1	3	3	3	3
2	3	2	2	2	2	2	2	2	2
2	2	2	1	1	2	1	1	1	1
3	3	3	3	3	1	2	2	2	2
3	1	2	3	3	2	2	3	3	3
1	9	9	9	9	9	9	9	9	9
3	3	3	3	3	3	3	3	3	3
2	1	1	2	1	3	2	2	1	2

2	2	2	2	2	2	2	2	2	2
2	1	1	1	3	1	3	2	3	1
9	9	9	9	9	9	9	9	9	9
9	9	9	9	9	9	9	9	9	9
2	1	1	1	1	1	2	2	1	1
1	2	2	3	3	1	1	1	1	1
9	9	9	1	9	9	9	9	9	9
1	9	9	9	9	9	9	9	9	9
1	3	2	2	2	1	1	3	2	1
1	9	9	9	9	1	9	9	9	9
2	2	2	2	2	3	2	2	3	3
2	1	2	2	2	9	9	9	9	9
2	2	2	2	2	3	3	3	2	2
9	9	9	9	9	3	1	2	3	1
2	1	3	2	1	1	2	1	3	2
9	9	9	1	9	9	2	9	9	9
9	2	9	9	9	9	9	9	9	1
3	9	9	9	9	9	2	9	9	9
2	2	2	2	2	2	2	1	2	2
3	2	3	1	2	2	2	3	2	1
9	9	3	9	9	2	9	9	9	9
2	2	3	3	3	3	3	3	3	3
3	3	3	3	3	3	3	3	3	3
2	1	1	1	1	2	1	1	1	2
1	2	1	2	2	1	2	2	2	1
2	2	2	2	3	3	3	3	3	3
1	2	2	3	2	2	2	2	2	1
1	2	3	1	2	3	2	2	2	3
2	2	2	2	2	3	3	2	2	2
1	2	1	9	2	1	3	3	3	2
2	2	2	2	2	2	2	2	2	2
2	1	1	1	1	2	1	2	2	3
2	1	1	1	3	2	2	1	2	2
2	2	1	3	2	2	2	2	1	2
9	9	9	9	9	3	3	3	3	3
1	1	2	2	1	1	1	2	2	2
2	2	2	2	1	2	2	2	2	2
2	2	2	2	2	1	1	2	2	2
2	3	3	2	1	3	9	9	9	9
3	1	2	2	2	2	2	2	3	3
3	1	2	9	1	3	1	3	3	3
1	1	2	2	2	2	2	2	2	2
1	2	2	2	3	2	2	3	1	1
9	2	9	9	9	2	1	1	1	1
1	2	2	3	3	2	2	3	2	2
2	2	1	2	3	1	1	1	1	2
1	1	2	2	1	2	1	2	2	1

1	1	1	1	1	1	1	3	9	2
1	2	3	1	2	1	1	3	3	3
2	2	2	2	2	9	9	9	9	9
2	2	2	2	2	2	2	2	2	1
3	1	2	9	3	1	1	3	3	1
9	9	9	9	9	9	9	9	9	9
1	1	3	3	3	3	3	2	3	1
1	1	3	3	2	1	3	3	3	1
1	1	1	1	2	1	1	1	1	1
1	2	2	1	3	1	3	3	3	1
2	2	2	2	2	3	3	3	3	2
3	3	2	3	2	3	2	3	3	3
1	2	1	2	1	3	3	3	3	3
2	3	2	2	2	1	2	2	2	1
1	2	2	1	2	1	1	1	2	1
3	2	2	1	1	2	2	1	1	1
2	1	9	2	2	2	2	2	2	3
1	2	2	2	2	2	9	9	9	9
2	9	9	9	9	2	9	9	9	9
2	2	2	2	2	2	2	2	2	2
9	1	9	9	2	9	3	1	9	9
2	9	9	9	2	2	2	9	2	9
2	9	9	9	1	9	1	1	9	2
9	3	1	9	9	9	1	9	9	9
2	9	2	9	2	2	9	2	9	2
9	9	9	9	9	9	9	9	9	9
9	9	9	9	9	9	9	9	9	9
2	2	2	2	2	2	2	2	2	2
2	2	2	2	2	2	2	2	2	2
2	2	3	3	3	3	3	3	3	3
2	2	2	2	2	1	1	2	2	2
2	9	2	9	2	2	9	2	9	9
2	2	2	2	2	2	2	2	2	2
9	9	9	9	9	9	9	9	9	9
9	9	9	9	9	9	9	9	9	9
1	2	1	1	1	2	2	2	2	2
2	9	9	9	9	9	2	9	9	9
9	9	9	9	9	9	9	9	9	9
9	9	9	9	9	9	9	9	9	9
9	2	2	9	2	2	9	2	9	9
9	9	3	1	9	9	3	1	9	9
9	1	9	3	1	9	9	1	9	9
9	9	9	9	9	9	9	9	9	9
9	2	9	9	9	9	2	9	9	9
9	9	2	1	9	9	9	1	9	3
3	9	9	9	9	3	9	9	9	9
9	9	1	9	9	2	9	9	9	9

1	9	9	9	9	9	9	9	9	9
3	3	3	3	3	3	3	3	3	3
1	1	2	1	1	2	1	1	1	1
1	2	3	1	2	1	2	1	3	1
9	1	2	9	9	1	1	1	9	1
9	9	1	9	9	3	9	9	9	9
9	9	2	9	9	2	9	9	9	9
2	9	9	9	9	9	9	9	9	2
3	9	9	9	9	9	2	9	3	3
3	1	2	1	3	3	2	1	3	2
2	1	3	3	2	2	3	3	2	2
2	2	1	1	2	2	2	2	2	2
2	3	3	3	2	2	2	3	3	3
1	2	3	1	1	2	2	1	1	2
9	9	9	9	9	9	9	9	9	9
3	3	3	3	3	3	3	3	3	3
9	9	9	9	9	9	9	9	9	9
9	9	9	9	9	9	9	9	9	9
9	1	9	9	9	1	9	9	9	9
1	2	1	3	1	1	1	1	1	2
1	1	1	1	1	1	1	1	1	1
1	1	2	1	1	1	2	1	1	1
9	9	9	9	9	9	9	9	9	9
1	2	2	2	2	1	2	2	2	1
1	1	1	1	1	1	1	1	1	1
1	2	2	2	2	3	3	3	9	2
3	1	2	1	3	3	1	2	1	2
1	1	2	2	3	2	2	3	3	3
1	2	1	3	2	2	3	3	2	3
2	2	2	2	2	2	2	2	2	2
2	2	2	2	2	2	2	2	2	2
2	2	2	2	2	2	2	2	2	2
2	2	2	2	2	2	2	2	2	2
1	2	3	1	2	1	2	3	1	2
2	2	2	2	2	2	2	2	2	2
1	2	3	2	3	1	1	1	2	2
2	2	2	2	2	2	2	2	2	2
2	2	2	2	2	2	2	2	2	2
2	2	1	2	1	2	2	3	2	3
2	3	3	3	2	2	2	2	2	2
2	3	2	3	1	3	2	1	3	3
1	2	9	3	3	1	2	3	2	3
9	9	9	9	9	9	9	9	9	9
2	2	2	2	2	2	2	2	2	2
3	3	3	3	3	3	3	3	3	3
3	3	3	3	3	3	3	3	3	3
2	1	1	2	2	2	9	9	9	9

2	3	1	2	3	2	3	1	3	2
2	2	1	1	1	3	3	1	1	1
2	2	1	1	1	1	1	1	1	1
2	3	3	3	3	1	1	1	2	2
2	2	2	2	2	2	2	2	2	2
2	2	2	2	2	2	2	2	2	2
2	3	1	2	3	2	3	1	2	3
1	9	9	9	9	1	9	9	9	9
9	1	9	9	9	9	9	9	9	9
1	9	9	9	9	1	9	9	9	9
9	9	9	9	1	2	9	9	9	9
2	2	2	1	1	1	1	1	1	1
3	9	9	9	9	2	9	9	9	9
9	9	2	9	9	9	9	9	9	2
2	2	2	2	2	2	2	2	2	2
9	2	9	9	9	9	9	9	9	9
9	9	2	9	9	9	9	9	9	2
1	9	9	9	9	9	9	9	9	9
9	2	9	9	9	2	9	9	9	9
1	9	9	9	9	9	9	2	9	9
1	9	9	9	9	1	9	9	9	9
9	9	9	9	2	9	9	2	9	9
3	2	9	9	9	9	2	9	9	9
1	2	3	1	3	2	1	3	1	2
1	2	2	1	2	1	2	1	3	1
2	2	2	2	2	2	2	2	2	2
1	1	2	2	3	3	2	2	2	1
1	2	1	1	2	3	2	3	3	1
1	1	1	1	1	1	2	1	3	1
1	2	3	2	1	2	3	2	9	2
2	1	3	2	2	3	3	3	2	3
3	3	3	3	3	3	3	3	3	3
2	2	2	2	2	2	2	2	1	2
1	2	2	3	2	3	2	3	3	3
9	1	2	1	2	1	1	2	2	1
2	2	2	1	1	1	1	1	1	2
1	3	2	1	3	1	2	2	2	2
2	2	2	2	2	3	3	1	1	2
2	2	3	3	2	2	2	1	1	2
1	1	1	1	1	1	1	2	2	1
9	9	9	9	9	9	9	9	9	9
1	2	2	2	2	3	3	3	3	2
9	9	9	9	9	9	9	9	9	9
1	1	1	1	2	2	2	1	1	2
2	2	1	2	2	1	1	2	2	2
2	2	2	2	2	2	2	2	2	2
1	9	9	1	9	9	9	9	9	9

3	3	3	3	3	3	3	3	3	3
1	9	9	9	9	3	3	3	3	1
3	2	2	1	1	3	3	2	2	3
2	2	2	2	2	2	2	2	2	2
1	2	3	1	2	1	2	2	3	2
2	2	2	2	2	2	2	2	2	2
3	1	2	2	3	1	2	2	2	2
9	9	2	9	9	9	9	9	9	9
9	9	2	9	9	2	2	2	2	2
1	2	3	1	2	1	2	2	9	1
1	1	1	1	2	2	2	2	2	2
2	9	9	9	9	2	9	9	9	9
2	1	1	2	2	1	1	2	2	2
1	1	2	2	2	1	2	2	9	3
9	9	9	9	9	9	9	9	9	9
1	2	2	2	2	3	3	2	3	2
3	3	1	3	3	3	3	3	3	3
1	9	9	9	9	9	9	9	9	9
9	9	9	9	9	2	9	9	9	9
2	9	9	9	9	2	1	2	2	3
9	9	2	9	9	2	9	9	9	9
3	2	2	1	2	2	3	1	9	3
9	9	9	9	9	9	9	9	9	9
1	9	9	9	9	2	9	9	9	9
9	9	1	9	9	9	9	9	2	9
3	2	2	2	3	3	3	3	3	3
2	2	2	2	2	2	2	2	2	2
2	2	2	2	2	3	3	3	3	2
2	1	2	2	2	2	2	2	1	2
9	9	9	1	9	9	9	9	9	1
9	9	1	9	9	9	9	9	9	9
2	1	1	1	1	1	2	2	2	2
1	1	1	1	1	1	1	1	1	1
2	1	1	2	1	2	3	1	2	3
2	2	2	2	2	2	2	2	3	3
2	9	9	9	9	9	9	9	9	9
9	9	9	9	9	2	1	1	2	2
1	2	1	2	1	2	1	2	3	2
3	1	1	2	2	2	2	3	2	2
1	1	2	3	3	2	2	2	2	2
1	3	3	2	2	3	3	3	1	1
1	2	1	2	3	1	2	1	2	1
9	9	9	9	9	9	9	9	9	9
3	3	2	2	1	1	1	2	2	2
3	1	2	2	2	2	3	3	2	3
2	2	2	2	2	1	1	1	1	1
1	2	2	1	3	2	2	2	2	2

2	2	2	2	2	2	2	2	2	2
2	2	2	2	2	2	2	2	2	2
1	2	3	2	1	2	1	2	1	2
3	2	3	2	3	3	3	3	3	3
2	2	2	2	2	2	2	2	2	2
3	3	3	3	3	3	3	3	3	3
2	2	2	2	2	2	9	9	9	9
9	1	9	9	9	9	9	9	9	9
9	9	1	9	9	9	9	9	1	9
9	1	9	9	9	1	9	9	9	9
2	2	2	1	2	9	9	9	9	9
3	2	2	3	2	3	2	2	9	1
2	2	2	2	2	2	2	2	2	2
2	9	2	2	2	2	2	9	2	2
9	9	9	9	9	9	9	9	9	9
9	9	1	9	9	9	9	9	9	9
2	2	2	2	2	2	2	2	2	2
2	2	2	2	2	2	2	2	2	2
2	2	2	2	9	2	2	2	2	2
2	2	2	2	2	2	2	2	2	2
2	2	2	2	2	2	2	2	2	2
2	9	9	9	9	2	9	9	9	9
3	3	3	3	3	3	3	3	3	3
1	9	9	9	9	1	9	9	9	9
2	9	9	9	9	2	9	9	9	9
2	9	9	9	9	2	9	9	9	9
9	9	9	9	9	9	9	9	9	9
2	9	9	9	9	2	9	9	9	9
2	2	2	2	2	2	2	2	2	2
2	2	2	2	2	2	2	2	2	2
9	9	9	9	9	9	9	9	9	9
9	9	1	9	9	2	9	9	9	9
1	9	2	9	9	9	9	9	9	9
9	9	1	9	9	2	2	2	9	2
3	3	1	3	3	2	2	2	2	2
2	9	2	9	2	9	9	9	9	9
2	9	2	9	2	2	9	2	9	2
3	3	1	9	3	2	2	2	2	2
1	9	3	1	9	1	9	1	9	1
1	9	9	9	2	9	9	9	9	9
3	3	3	3	3	3	3	3	3	3
2	9	1	9	1	9	9	9	9	9
2	2	2	2	2	1	1	9	2	9
1	2	2	2	2	9	9	9	9	9
1	1	3	2	1	2	1	3	3	1
2	2	2	2	2	2	2	2	2	2
1	9	9	9	9	9	9	9	9	2

1	2	2	2	2	2	1	3	3	1
2	3	2	3	2	2	3	2	2	1
1	2	3	2	3	2	3	2	3	2
3	1	3	3	3	3	3	3	3	3
1	2	2	9	1	1	1	1	2	2
2	2	2	2	2	9	9	9	9	9
9	9	9	1	9	9	9	9	9	1
1	3	3	2	1	1	2	2	3	3
9	9	9	9	9	2	2	2	2	2
1	9	9	9	9	1	1	1	1	1
2	2	2	2	2	2	2	2	2	2
2	3	2	1	3	2	3	2	2	3
3	3	3	3	3	3	3	3	3	3
2	3	2	1	3	2	3	2	2	3
1	3	2	3	3	2	3	3	3	1
2	2	2	2	2	2	2	2	2	2
1	2	2	2	2	2	3	3	3	3
1	2	9	2	3	2	1	3	2	2
9	9	1	9	9	3	3	3	3	3
9	9	1	2	2	2	2	2	2	2
9	9	9	9	2	9	9	9	2	9
1	1	1	2	2	2	2	1	1	1
2	2	2	2	2	2	2	3	3	2
1	2	2	2	2	2	2	2	2	1
3	1	2	3	1	3	3	3	3	3
1	2	3	2	3	2	2	2	2	2
1	9	9	9	9	2	2	2	2	2
3	3	1	3	3	1	1	1	1	2
1	3	2	9	1	1	2	1	9	3
1	9	9	9	9	9	9	1	9	9
1	9	9	9	9	9	9	9	9	9
2	2	2	2	2	1	1	1	1	1
9	2	9	9	9	9	2	2	2	2
2	2	1	1	1	3	3	3	3	3
9	9	9	9	9	9	9	9	9	9
9	9	9	1	9	9	9	9	9	1
3	2	3	2	1	2	2	2	2	1
2	3	1	3	2	3	2	3	2	3
9	9	9	9	9	9	9	9	9	9
9	1	9	9	9	9	9	9	9	9
2	2	1	1	2	1	2	2	2	1
9	9	9	9	9	9	9	9	9	9
1	1	2	2	1	1	3	3	2	2
1	1	1	1	1	3	3	3	3	3
2	1	2	2	2	3	1	2	2	3
2	2	2	2	2	2	2	2	2	2
9	9	9	1	9	2	1	2	3	2

1	3	3	3	3	3	3	3	3	3
1	9	9	9	9	2	9	9	9	9
1	3	3	2	3	2	2	2	3	3
2	1	2	9	1	2	1	1	2	3
2	2	2	2	2	2	2	2	2	2
1	3	3	3	2	2	3	3	3	2
9	9	9	9	2	9	9	9	9	9
1	2	2	2	2	2	2	2	2	2
2	1	1	2	2	3	3	2	3	1
1	2	2	2	2	2	2	2	2	2
2	2	2	2	2	2	3	3	3	2
9	9	9	9	9	9	9	9	9	9
9	9	9	9	9	1	1	2	1	1
1	9	9	9	9	2	2	2	2	2
2	1	2	1	1	2	2	2	1	1
9	9	9	9	1	2	2	2	2	2
3	3	3	3	3	2	2	2	2	2
9	2	9	9	9	3	1	3	2	2
2	2	1	1	2	3	3	3	3	2
2	2	2	2	2	2	2	2	2	2
3	1	3	1	1	3	1	3	3	3
2	9	2	9	2	3	3	3	3	3
2	9	2	2	2	9	9	9	9	9
9	9	9	9	9	9	9	9	9	9
1	9	9	9	9	1	9	9	9	9
2	9	2	9	2	9	9	9	2	9
2	9	9	9	9	2	9	9	9	9
9	9	9	9	9	9	9	9	9	9
2	2	2	2	2	2	2	2	2	2
2	9	9	9	9	2	9	9	9	9
9	9	9	9	9	9	9	9	9	9
3	9	9	9	9	3	9	9	9	9
9	9	9	9	9	9	9	9	9	9
1	1	1	1	1	1	9	1	1	1
2	9	3	9	3	3	3	3	9	9
3	9	9	9	3	9	9	9	9	9
3	9	9	9	9	3	9	9	3	9
2	2	2	2	2	2	2	2	2	2
1	1	1	1	1	1	1	1	1	1
3	9	9	9	9	3	9	9	9	9
2	2	9	2	2	2	1	9	2	1
9	9	9	9	9	9	9	9	9	9
2	9	9	9	9	2	9	9	9	9
9	9	9	9	9	9	9	9	9	9
3	9	2	9	1	1	9	9	1	9
1	9	9	9	9	1	9	9	9	9
2	2	2	2	2	2	2	2	2	2

2	9	9	9	9	2	9	9	9	9
2	9	9	9	9	2	9	9	9	9
2	9	9	9	9	2	9	9	9	9
2	2	2	2	2	2	2	2	2	2
2	2	2	2	2	9	9	9	9	9
9	9	9	9	9	9	9	9	9	9
1	1	1	3	2	9	9	9	9	9
2	2	2	2	2	1	2	2	2	2
1	9	2	9	1	9	2	9	9	9
2	2	2	2	2	2	9	2	9	9
2	9	9	9	9	9	9	9	9	9
2	9	9	9	9	9	9	9	9	9
2	9	9	9	9	9	9	9	9	9
2	9	9	9	9	2	9	9	9	9
9	9	9	9	9	9	9	9	9	9
9	9	9	9	9	9	9	9	9	9
9	9	9	9	9	9	9	9	9	9
9	9	9	9	9	9	9	9	9	9
9	9	9	9	9	9	9	9	9	9
9	9	9	9	9	9	9	9	9	9
2	2	2	2	2	2	2	2	2	2
2	2	2	2	2	2	2	2	2	2
2	2	2	2	2	2	2	2	2	2
2	2	2	2	2	2	2	2	2	2
2	2	2	2	2	9	9	9	9	9
2	2	2	2	2	2	2	2	2	2
2	2	2	2	2	9	9	9	9	9
2	9	9	9	9	2	2	9	9	9
9	9	9	9	9	9	9	9	9	9
2	2	2	2	2	9	9	9	9	9
2	2	2	2	2	2	2	2	2	2
1	2	2	2	2	2	2	2	2	2
9	9	9	9	9	9	9	9	9	9
1	2	2	2	2	1	2	1	2	1
9	9	9	9	9	9	9	9	9	9
2	2	2	2	2	2	2	2	2	2
1	1	1	1	1	1	1	1	1	1
2	1	3	1	2	2	2	2	2	2
1	2	2	2	2	9	9	9	9	9
1	2	1	2	1	2	1	2	3	2
1	1	1	1	1	9	9	9	9	9
1	9	9	9	9	2	2	2	2	2
3	9	9	9	3	2	9	9	9	9
2	2	2	2	2	2	2	9	2	9
1	1	1	1	1	1	1	1	1	1
3	9	9	9	3	3	9	9	9	9
2	9	9	9	9	9	9	9	9	9

9	9	9	9	9	9	9	9	9	9
2	2	2	2	2	2	2	2	2	2
9	9	1	9	9	9	2	9	2	9
1	9	1	9	1	1	9	3	1	9
2	9	9	9	2	2	9	9	9	9
2	9	2	9	2	2	9	9	2	9
2	9	9	1	9	2	9	9	9	9
9	9	9	9	9	9	9	9	9	9
2	9	1	9	3	2	9	9	9	1
3	9	9	9	9	3	9	9	9	9
9	9	9	9	9	9	9	9	9	9
2	9	9	9	9	9	9	9	9	9
9	1	1	1	9	2	9	9	9	9
2	9	9	9	9	2	9	9	9	9
9	3	1	9	9	1	2	9	2	9
1	1	2	2	2	1	1	1	9	1
9	9	9	9	9	9	9	9	9	9
9	9	9	9	9	9	9	9	9	9
3	3	3	1	3	9	9	9	9	9
9	9	9	9	9	9	9	9	9	9
2	3	2	9	2	3	3	3	2	2
3	2	3	1	2	3	3	2	1	3
2	9	9	9	9	9	9	9	9	9
2	2	1	9	9	2	2	2	2	2
2	2	2	2	2	2	2	2	2	2
9	1	2	9	9	2	2	9	2	9
1	9	9	9	9	2	9	9	9	9
9	9	2	9	9	1	9	9	9	9
1	2	2	2	2	3	3	3	3	1
1	2	2	3	3	1	2	3	1	2
3	3	1	2	2	2	2	3	2	2
2	2	1	2	2	2	3	3	3	2
1	1	3	3	2	3	3	3	3	2
1	3	2	2	2	2	2	2	2	2
3	1	1	3	2	3	2	3	3	2
1	2	2	2	2	2	2	2	2	2
1	2	2	2	2	2	9	9	9	9
1	9	9	9	9	2	9	9	9	9
1	2	2	9	3	3	2	3	3	1
9	9	9	2	9	9	9	9	9	9
1	9	9	9	9	1	9	9	9	9
9	9	9	9	9	9	9	9	9	9
9	9	9	9	9	9	9	9	9	9
9	9	1	9	1	9	2	9	9	2
9	9	9	9	9	9	9	9	9	9
2	9	2	9	2	2	9	2	9	9
2	9	9	2	9	2	9	9	2	9

2	2	2	2	2	2	2	2	2	2
2	2	2	2	2	2	2	2	2	2
2	2	2	2	2	2	2	2	2	2
2	2	2	2	2	9	9	9	9	9
2	2	2	2	2	2	2	2	2	2
2	1	3	2	2	2	3	2	2	3
2	2	1	2	2	2	1	3	3	2
2	2	2	3	2	2	2	3	3	3
9	9	9	9	1	9	9	9	9	1
1	2	1	2	2	2	2	3	2	3
3	3	3	3	3	3	3	3	3	3
1	2	2	2	2	2	3	3	2	2
2	2	1	2	2	3	3	2	2	2
3	3	3	3	3	3	3	3	3	3
2	2	2	2	2	2	2	2	2	2
2	2	2	2	2	2	2	2	2	2
2	2	2	2	2	2	9	9	2	9
2	2	2	2	2	2	2	2	2	2
1	1	2	2	1	2	2	2	2	2
2	1	2	3	2	3	2	1	2	3
1	2	2	3	3	2	1	2	3	3
2	2	2	2	2	2	2	2	2	2
2	1	2	3	1	2	2	2	1	2
2	2	2	3	2	2	2	3	2	2
2	2	2	2	2	2	2	3	3	3
1	1	1	1	3	3	3	1	2	3
1	1	2	2	2	2	1	2	2	3
3	2	1	3	2	2	2	1	3	1
1	2	3	1	2	2	2	3	2	2
3	2	2	2	1	2	3	3	3	2
1	3	3	3	3	2	2	2	2	1
9	1	2	2	2	3	3	3	3	2
1	2	2	2	2	2	2	2	2	2
2	9	2	2	3	2	3	2	2	2
3	2	2	2	1	3	3	9	2	9
1	2	1	1	1	1	1	2	3	1
1	3	3	3	3	3	3	3	3	3
2	2	1	2	2	3	3	3	3	3
3	3	3	3	3	3	3	3	3	3
2	2	2	2	2	2	2	2	2	2
9	1	9	9	9	9	1	9	9	9
2	3	2	1	2	3	2	3	2	1
2	2	2	2	2	3	3	2	2	2
1	1	1	1	1	2	2	2	2	2
2	2	2	2	2	3	3	2	2	2
9	9	9	9	9	9	9	9	9	9
1	3	2	2	2	2	2	2	3	2

9	9	9	1	9	9	9	9	9	9
3	2	2	2	3	2	1	2	1	2
3	2	1	2	3	2	9	9	1	9
2	3	3	2	2	2	1	2	1	2
2	2	2	3	2	2	2	3	2	3
2	3	2	3	3	2	3	1	2	1
2	1	1	1	2	2	1	2	2	1
3	3	3	3	3	3	3	3	3	3
1	1	1	3	1	3	1	3	1	1
1	3	3	2	3	2	2	1	3	3
1	1	2	1	3	1	1	2	3	3
1	2	1	1	3	2	3	3	3	3
3	3	3	3	3	2	2	2	2	2
1	2	1	3	3	2	1	2	2	1
3	3	2	1	2	3	3	3	3	1
2	2	2	2	2	2	2	2	2	2
2	1	2	1	2	3	2	2	3	3
1	3	2	2	2	2	2	2	3	2
1	9	2	1	2	2	1	2	1	2
3	9	3	2	2	1	1	3	2	1
1	2	2	2	2	3	2	3	3	2
1	2	2	2	2	3	2	2	3	2
3	2	2	3	2	1	2	3	3	3
2	3	2	3	3	2	3	2	3	2
2	2	2	2	2	2	2	2	2	2
2	9	9	9	9	9	2	9	9	9
2	1	2	2	2	2	1	2	1	1
1	2	1	1	2	2	1	2	2	1
3	3	2	2	3	2	3	3	2	3
3	2	1	2	1	2	2	1	3	2
3	3	3	3	3	3	3	3	3	3
1	2	1	3	2	1	1	2	3	1
1	9	9	9	9	9	9	9	9	9
2	2	2	2	2	2	2	2	2	2
2	2	2	2	2	2	2	2	2	2
2	2	2	2	2	2	2	2	2	2
1	3	2	2	1	2	1	3	9	2
2	9	9	9	9	2	3	2	9	1
1	2	2	2	2	3	3	3	3	2
1	3	2	2	2	3	3	3	3	3
1	3	2	3	2	3	2	3	3	2
1	2	2	3	2	2	2	3	2	9
3	1	1	1	1	3	2	2	2	2
2	3	2	3	2	2	3	2	3	2
3	3	3	3	1	3	3	3	3	2
1	1	2	2	2	3	3	3	2	1
1	2	2	2	2	2	2	2	2	2

2	2	2	2	2	2	2	2	2	2
2	2	2	2	2	2	2	2	2	2
2	2	2	2	2	2	2	2	2	2
1	2	2	2	2	3	3	3	3	2
2	2	1	1	2	3	2	3	3	2
1	2	2	2	2	2	2	2	2	2
3	1	2	2	2	3	2	2	1	2
1	2	2	2	2	3	3	3	3	2
1	3	3	3	3	2	2	2	2	2
2	2	2	2	2	2	2	3	3	1
9	9	9	9	9	2	2	2	2	2
1	2	2	2	2	3	3	3	3	2
1	2	2	2	2	3	3	3	3	2
2	2	2	2	2	2	2	2	2	2
2	3	2	2	2	2	2	3	2	2
9	9	9	9	9	9	9	9	9	9
1	1	3	2	2	1	2	3	2	2
1	1	3	2	2	1	2	3	2	1
9	9	9	2	9	9	9	9	9	9
9	9	9	9	9	9	9	9	9	9
2	3	2	2	2	3	1	3	2	2
9	9	9	9	1	9	9	9	9	9
9	1	9	9	9	9	9	9	9	9
1	9	9	9	9	9	9	9	1	9
9	9	9	9	9	9	9	9	9	9
2	2	2	2	2	2	2	2	2	2
1	2	3	2	3	3	3	3	9	1
9	1	9	9	9	9	9	9	9	9
1	2	1	2	1	9	9	9	9	9
1	2	2	2	2	2	2	2	2	2
3	3	3	1	3	2	2	2	1	3
1	2	2	2	2	2	2	2	2	2
3	1	2	9	3	2	3	3	1	9
9	9	9	9	9	9	9	9	9	9
2	2	2	2	2	2	2	2	2	2
2	2	2	1	2	2	2	2	2	3
9	9	9	9	9	9	9	9	9	9
1	2	2	1	3	1	2	3	1	2
1	2	2	2	2	1	1	2	3	2
2	2	2	2	2	2	1	2	2	2
2	2	2	2	1	2	1	2	2	2
1	2	2	2	2	2	2	2	2	2
9	1	9	9	9	9	1	9	9	9
2	2	2	2	2	1	3	3	2	1
2	2	2	2	2	2	2	2	2	2
2	2	2	2	2	3	2	1	1	2
1	1	1	1	1	2	2	1	1	1

2	1	3	2	3	2	3	3	3	1
1	2	2	3	2	2	2	2	3	1
9	9	9	9	9	3	3	3	2	2
2	3	1	2	3	3	2	3	2	3
9	9	9	9	9	2	1	9	3	1
3	3	3	3	3	3	3	3	3	3
1	1	3	3	2	3	3	1	2	2
2	2	2	2	2	2	2	2	2	2
2	3	2	3	1	2	2	9	3	3
1	2	2	2	2	2	1	2	2	2
1	3	2	2	3	3	2	3	2	3
2	2	2	2	2	2	1	2	2	2
9	9	1	9	9	9	9	9	9	9
3	3	3	3	3	3	3	3	3	3
9	3	2	3	9	3	3	3	3	3
2	1	2	2	2	2	1	2	2	3
2	2	2	2	2	2	1	3	9	3
9	9	9	9	9	9	9	9	9	9
2	3	1	1	1	1	1	2	2	2
1	9	9	9	9	9	9	9	9	1
1	9	9	9	9	9	9	9	9	9
9	9	9	9	9	9	9	9	9	9
1	2	1	2	1	2	3	1	1	2
1	3	9	2	3	1	3	3	3	1
1	3	3	2	3	3	3	3	3	3
1	2	2	2	2	2	2	3	3	2
2	9	3	2	1	2	3	9	2	2
1	1	1	1	1	1	1	1	3	1
2	2	2	2	2	2	2	2	2	2
2	2	2	2	2	2	2	2	2	2
1	1	1	1	1	3	3	3	3	1
2	2	2	2	2	2	2	2	2	2
2	2	2	2	2	2	2	2	2	2
2	9	9	9	9	2	9	9	9	9
2	2	2	2	2	2	2	2	2	2
2	2	2	2	2	2	2	2	2	9
1	1	1	1	1	1	1	1	1	1
3	2	1	3	2	2	3	2	1	3
1	2	2	2	1	2	2	1	1	1
1	2	2	2	2	2	2	2	2	2
2	1	2	2	2	2	1	3	9	2
2	1	2	3	1	2	9	1	3	2
1	1	1	1	1	1	1	1	1	1
1	3	3	3	3	2	2	3	3	2
3	9	3	2	1	1	3	2	2	2
1	2	2	3	2	3	2	3	2	2
1	9	9	9	9	9	9	9	9	9

1	2	2	1	1	2	2	2	9	1
1	2	2	2	2	2	2	2	2	2
1	1	2	2	2	2	2	2	2	2
3	3	3	3	3	3	3	3	3	3
1	1	2	1	2	3	3	1	3	3
3	9	9	9	9	3	3	3	3	3
2	3	9	1	2	3	3	3	3	3
3	2	1	2	2	3	2	3	2	3
3	2	3	2	3	3	3	2	2	3
1	2	2	2	2	2	2	2	2	1
1	2	3	1	2	1	2	9	9	1
1	9	9	9	9	9	9	1	9	9
3	2	1	2	3	3	3	3	3	2
1	9	9	9	9	9	9	9	9	9
1	9	9	9	9	9	9	9	9	9
9	9	9	9	9	9	9	9	9	9
2	2	1	9	2	2	1	2	2	1
3	9	9	9	9	9	9	9	2	9
1	2	3	2	2	3	3	3	3	2
2	9	9	9	9	2	9	9	9	9
2	2	2	2	2	2	2	2	2	1
9	9	9	9	9	9	9	2	9	9
3	1	3	2	1	2	3	3	2	2
9	9	1	9	9	9	9	1	9	9
9	9	1	9	9	9	9	1	9	9
9	9	2	9	9	9	9	2	9	9
1	1	1	1	1	1	1	1	1	1
2	2	1	2	3	2	2	1	1	2
2	2	2	2	2	2	2	2	2	2
1	2	1	1	2	3	3	3	2	3
2	3	3	3	3	1	1	1	1	2
1	3	2	2	2	3	3	3	2	3
3	3	3	3	1	3	1	1	1	3
9	9	1	9	9	9	9	9	9	1
2	2	2	2	2	2	2	2	2	2
1	1	3	3	3	9	1	3	3	3
3	1	3	1	3	1	1	3	1	1
1	9	9	9	9	3	9	9	9	9
3	3	3	3	3	3	3	3	3	3
2	9	9	9	9	3	9	9	9	9
1	9	9	9	9	1	9	9	9	9
1	2	9	9	9	1	1	2	1	1
1	9	9	9	9	1	1	1	1	1
2	3	1	1	1	9	9	9	9	9
1	3	2	2	2	9	9	9	9	9
2	2	2	2	2	2	2	2	2	2
2	2	2	2	2	2	2	2	2	2

2	2	2	2	2	2	2	2	2	2
3	3	3	3	3	3	3	3	3	3
9	2	9	2	9	2	9	9	9	2
1	2	2	2	2	2	2	2	2	2
2	2	2	2	2	2	2	2	2	2
1	9	1	9	1	1	9	1	3	1
3	1	3	1	9	9	1	9	9	9
1	1	1	1	1	2	2	9	9	2
9	2	2	2	2	2	2	2	9	2
9	9	9	9	9	1	1	1	1	1
1	9	9	1	9	1	2	9	3	1
1	9	1	3	1	1	9	1	9	3
3	1	9	2	9	9	1	9	9	9
3	1	3	2	1	2	1	3	9	1
1	9	1	1	1	1	9	2	9	2
1	1	1	1	1	2	2	2	2	2
2	2	2	2	2	2	2	2	2	2
2	9	9	2	9	2	9	2	9	9
2	2	2	2	2	2	2	2	2	2
3	3	3	3	3	9	9	9	9	9
2	2	2	2	2	2	2	2	2	2
9	9	9	9	9	9	9	9	9	9
9	2	9	2	2	2	9	9	9	2
2	2	2	2	2	9	9	9	9	9
9	1	9	3	1	3	1	3	2	1
2	2	2	2	2	2	2	2	2	2
2	2	2	2	2	2	2	2	2	2
2	2	2	2	2	2	2	2	2	2
2	1	1	3	2	1	2	2	1	3
1	1	2	9	1	1	2	1	1	1
3	2	3	2	3	2	3	2	3	2
3	3	3	3	3	3	3	3	3	3
2	9	1	1	2	3	1	2	1	2
1	2	2	2	2	2	2	2	2	1
1	1	3	1	3	1	3	2	1	1
1	2	2	2	2	2	2	3	3	1
2	9	2	3	2	2	2	2	2	2
2	2	3	3	3	3	2	1	2	3
1	1	2	2	2	2	2	2	2	2
2	9	1	1	1	1	2	1	2	1
2	1	1	3	3	1	2	1	2	2
1	1	3	2	3	1	1	1	1	1
1	3	3	3	3	3	1	2	3	1
2	1	2	2	2	3	3	9	1	1
2	2	2	1	3	1	2	3	3	2
3	2	2	9	2	2	2	3	3	9
1	2	3	1	1	3	1	1	1	3

1	3	2	1	2	3	2	2	3	2
3	2	2	2	2	2	3	2	2	2
9	9	9	9	9	9	9	9	9	9
1	2	1	1	2	9	9	9	9	9
1	2	2	2	2	2	2	2	2	2
9	1	9	9	9	3	1	1	3	2
3	9	9	9	9	1	3	3	1	2
9	9	9	9	9	1	3	3	2	2
1	2	2	2	2	2	2	2	2	2
2	2	2	2	2	2	2	2	2	2
1	1	1	2	2	2	2	2	2	2
2	2	2	2	2	2	2	2	2	2
2	2	2	2	2	1	1	1	1	1
1	1	1	1	1	1	2	1	1	1
3	3	3	3	3	3	3	3	3	3
1	2	1	2	1	1	1	2	1	2
1	1	1	1	1	1	1	1	1	1
1	9	9	9	9	2	2	2	2	2
2	2	2	2	2	2	2	2	2	2
1	2	2	2	2	2	2	2	2	2
2	2	2	2	2	2	2	2	2	2
1	9	9	9	9	2	2	2	2	2
1	2	1	2	1	1	1	2	1	1
1	1	2	2	2	1	1	1	2	1
1	1	1	1	1	2	2	2	2	2
2	2	2	2	2	2	2	2	2	2
2	1	2	1	2	2	3	2	1	9
2	2	2	2	2	2	2	2	2	2
9	9	9	9	9	9	9	9	9	9
1	9	9	9	9	2	2	2	2	2
1	1	1	1	1	1	1	1	9	1
2	1	1	1	1	1	2	1	2	1
1	1	1	2	1	1	1	2	2	1
1	2	1	1	2	1	2	1	1	2
1	2	2	9	2	2	2	2	9	2
1	1	1	1	1	1	1	1	1	1
2	2	2	2	2	2	2	2	2	2
2	2	2	2	2	2	2	2	2	2
1	1	1	1	1	2	2	2	2	2
2	2	2	2	2	2	2	2	2	2
2	2	2	2	2	2	2	2	2	2
2	2	2	9	2	9	9	9	9	9
1	1	1	1	2	1	1	1	1	1
1	2	2	9	1	1	2	1	1	1
9	9	9	9	9	2	2	2	2	9
2	2	2	9	2	2	2	2	2	2
2	2	2	2	2	2	2	2	2	2

2	2	2	2	2	2	2	2	2	2
2	2	2	9	2	2	2	2	2	2
2	2	2	2	2	2	2	2	2	2
1	1	1	2	1	1	1	2	1	1
2	2	2	2	2	2	2	2	2	2
2	1	1	9	2	9	1	2	2	2
2	2	2	2	9	1	2	1	1	1
2	2	2	2	2	2	2	2	2	9
1	1	2	2	2	2	2	2	2	2
1	1	1	2	9	1	1	1	2	1
1	1	1	1	1	1	1	1	1	1
2	2	2	2	2	2	2	2	2	2
1	2	2	2	2	2	2	2	2	2
1	2	2	2	2	1	1	1	1	2
9	2	2	9	2	9	9	9	9	9
1	1	9	1	1	2	2	2	9	2
9	9	9	9	9	2	2	9	2	2
1	9	1	9	1	1	1	9	1	1
9	9	9	9	9	9	9	9	9	9
1	1	1	1	1	1	1	1	1	1
1	9	9	9	9	1	1	1	1	9
2	2	2	2	2	2	2	2	2	2
1	1	1	1	1	9	9	9	9	9
9	9	9	9	9	9	9	9	9	9
2	2	2	2	2	2	2	2	2	2
1	2	3	2	3	1	3	1	3	1
1	2	3	2	3	1	3	1	3	1
1	2	3	2	3	1	3	1	3	1
1	2	3	2	3	1	3	1	3	1
1	2	2	2	3	1	3	1	9	1
1	2	3	2	3	1	3	1	9	1
1	2	3	2	3	2	3	1	3	1
1	2	3	2	3	1	3	1	3	1
1	2	3	2	3	1	3	1	1	1
1	2	3	2	3	1	3	1	3	1
1	3	3	2	3	1	3	2	3	1
1	2	3	2	3	1	3	1	3	1
1	2	2	2	3	1	3	1	3	1
1	2	3	2	3	1	3	1	3	1
1	2	3	2	3	1	3	1	3	1
1	2	3	2	3	1	3	1	3	1
1	2	3	2	3	1	3	1	3	1
1	2	3	2	3	1	3	1	3	1
1	2	3	2	3	1	3	1	1	1
1	2	3	2	3	1	3	1	1	1
1	2	3	2	3	1	3	1	1	1
1	2	3	2	3	1	3	1	3	1

1	2	3	2	3	1	3	1	3	1
1	2	3	2	3	1	3	1	3	1
1	2	3	2	3	1	3	3	3	1
9	9	9	9	9	9	9	9	9	9
9	9	9	9	9	9	9	9	9	9
9	9	9	9	9	9	9	9	9	9
9	9	9	9	9	9	9	9	9	9
9	9	9	9	9	9	9	9	9	9
9	9	9	9	9	9	9	9	9	9
9	9	9	9	9	9	9	9	9	9
9	9	9	9	9	9	9	9	9	9
9	9	9	9	9	9	9	9	9	9
9	9	9	9	9	9	9	9	9	9
9	9	9	9	9	9	9	9	9	9
9	9	9	9	9	9	9	9	9	9
9	9	9	9	9	9	9	9	9	9
9	9	9	9	9	9	9	9	9	9
9	9	9	9	9	9	9	9	9	9
1	2	9	2	2	9	9	9	9	9
9	9	9	9	9	9	9	9	9	9
9	9	9	9	9	9	9	9	9	9
9	9	9	9	9	9	9	9	9	9
9	9	9	9	9	9	9	9	9	9
9	9	9	9	9	9	9	9	9	9
9	9	9	9	9	9	9	9	9	9
1	2	3	2	3	1	3	1	3	1
1	2	3	2	3	1	3	1	3	1
1	2	3	2	3	1	3	1	3	1
1	2	3	2	3	1	3	1	3	1
1	2	2	2	2	1	3	1	3	1
1	2	3	2	3	1	3	1	3	1
1	2	3	2	3	1	3	1	3	1
1	2	3	2	3	1	3	1	3	1
1	2	3	2	3	1	3	1	3	1
1	3	2	2	3	1	3	1	3	1
1	3	2	2	3	1	3	1	3	1
1	2	3	2	3	1	3	1	3	1
1	2	3	2	3	1	3	1	3	1
1	2	3	2	3	1	3	1	3	1
1	2	2	2	3	1	3	1	3	1
1	2	2	2	2	1	3	1	3	1
1	2	9	2	3	2	9	9	3	3
1	2	3	2	3	1	3	1	3	1
1	2	3	2	3	1	3	1	3	1
1	2	3	2	3	1	3	1	3	1
1	2	3	2	3	3	9	1	3	1
1	2	3	2	3	1	3	1	3	1

1	2	3	2	3	1	3	1	3	1
1	2	3	2	3	1	3	3	9	1
1	2	3	2	3	1	1	3	3	1
1	3	3	2	3	1	3	1	9	3
1	2	3	2	3	1	3	1	3	1
1	2	3	2	3	1	3	1	3	1
1	2	3	2	3	1	3	1	3	1
1	2	3	2	3	1	1	1	9	9
1	2	3	2	3	1	3	1	3	1
1	2	3	2	3	1	3	1	3	1
3	3	2	9	3	1	3	1	3	1
1	2	9	2	3	1	1	3	3	1
1	2	3	2	3	1	3	1	1	1
1	9	3	2	9	1	3	1	3	1
1	2	3	2	3	1	3	1	3	1
1	2	3	2	3	1	3	1	3	1
1	2	3	2	3	1	3	1	3	1
1	3	2	3	2	1	3	1	3	1
1	2	3	2	3	1	3	1	3	1
1	2	3	2	3	1	3	1	3	1
1	2	3	2	3	1	3	1	3	1
1	2	3	2	3	1	3	1	3	1
1	2	3	2	3	1	3	1	1	1
1	2	3	2	3	1	3	1	3	1
1	2	3	2	3	3	1	3	1	1
1	2	3	2	3	1	3	1	3	1
1	2	3	2	3	1	3	1	3	1
1	2	3	2	3	1	1	3	1	1
1	2	3	2	3	1	3	1	3	1
1	2	3	2	3	1	3	1	3	1
1	2	2	2	3	1	3	2	3	1
1	2	3	2	3	1	3	1	3	1
1	2	3	9	2	1	1	3	1	1
1	2	3	2	3	1	3	1	3	1
1	2	3	3	3	1	3	1	3	1
1	9	3	9	3	9	3	1	3	1
1	2	3	2	3	1	3	1	3	1
1	2	9	2	3	1	3	1	3	1
1	2	9	2	3	1	3	1	3	1
1	2	3	2	3	1	3	1	3	1
9	9	9	9	9	9	9	9	9	9
1	2	2	2	3	1	9	1	3	1
1	3	3	2	3	1	3	3	3	1
1	3	3	3	3	1	3	1	3	1
1	3	3	9	3	9	3	1	1	1
1	2	9	2	3	1	9	9	9	9
1	3	2	3	2	1	3	1	3	1

1	2	3	9	3	1	3	1	3	1
1	3	3	3	3	3	3	1	3	1
1	2	3	2	3	1	3	1	3	1
1	2	3	2	3	1	3	1	3	1
1	2	3	2	3	1	3	1	3	1
1	2	3	2	3	1	3	1	3	1
1	2	3	2	3	1	3	1	3	1
1	2	3	2	3	1	3	1	3	1
1	2	3	2	3	1	3	1	3	1
1	2	3	2	3	1	3	1	3	1
1	2	3	2	3	1	3	1	3	1
1	2	3	2	3	1	3	1	3	1
1	2	3	2	3	1	3	1	3	1
1	2	3	2	3	1	3	1	9	1
1	2	3	2	3	1	3	1	3	1
1	2	3	2	3	1	3	1	3	1
1	2	3	2	3	1	3	1	3	1
1	2	3	2	3	1	3	1	3	1
1	2	3	2	3	1	3	1	3	1
1	2	3	2	3	1	3	1	3	1
1	2	3	2	3	1	3	1	9	1
1	2	3	2	3	1	3	1	3	1
1	2	3	2	3	1	3	1	3	1
1	2	3	2	3	1	3	1	3	1
1	2	3	2	3	1	3	1	3	1
9	9	9	9	9	9	9	9	9	9
1	2	3	2	3	1	3	1	3	1
1	2	3	2	3	1	3	1	3	1
1	2	3	2	3	1	3	1	3	1
1	2	3	2	3	1	3	1	3	1
1	2	3	1	3	1	3	2	9	9
1	2	3	2	3	1	3	1	3	1
1	2	3	2	3	1	3	1	1	1
1	2	3	2	3	1	3	1	3	1
1	2	3	3	3	1	3	1	3	1
1	2	3	2	3	1	3	1	3	1
1	2	3	2	3	1	3	1	3	1
1	2	3	2	3	1	3	1	3	1
1	2	3	2	3	1	1	3	3	1
1	2	3	2	3	1	1	3	3	1
1	2	2	2	3	1	3	1	3	1
1	2	2	2	3	1	3	1	3	1
1	2	3	2	3	1	3	3	1	3
1	2	3	2	3	1	3	1	1	1
1	2	3	2	3	1	3	1	3	1
1	2	3	2	3	1	3	1	3	1
1	2	3	2	3	1	3	1	3	1

9	9	9	9	9	9	9	9	9	9
1	2	3	2	3	1	3	1	3	1
1	2	3	2	3	1	3	1	3	1
1	2	3	2	3	1	3	9	3	1
1	2	3	2	3	1	3	1	3	1
1	2	3	2	3	1	3	1	3	1
1	2	3	2	3	1	3	1	3	1
1	2	3	2	3	1	3	1	3	1
1	2	3	2	3	1	3	1	3	1
1	2	3	2	3	1	3	1	3	1
1	2	3	2	3	1	3	1	3	1
1	2	3	2	3	1	3	1	3	1
1	2	3	2	3	1	3	1	3	1
1	3	2	2	3	1	3	9	3	3
1	2	3	2	3	1	3	1	3	1
1	2	3	2	3	1	3	1	3	1
1	2	3	2	3	1	3	1	3	1
1	2	3	2	3	1	3	1	3	1
1	2	3	2	3	1	3	1	3	1
1	2	2	3	3	1	3	1	3	1
1	2	3	2	3	1	3	1	3	3
1	2	3	2	3	1	3	1	3	1
1	2	3	2	3	1	3	1	3	1
1	2	3	2	3	1	3	1	3	1
1	3	2	2	3	1	3	1	3	1
1	2	3	2	3	1	3	1	3	1
1	2	3	2	3	1	3	1	3	1
1	2	3	2	3	1	3	1	3	1
1	2	3	2	3	1	3	1	3	1
1	2	3	2	3	1	3	1	3	1
1	2	3	2	3	1	3	1	3	1
1	2	3	2	3	1	3	1	3	1
3	2	3	2	3	1	3	1	3	1
9	9	9	9	9	9	9	9	9	9
9	9	9	9	9	9	9	9	9	9
9	9	9	9	9	9	9	9	9	9
9	9	9	9	9	9	9	9	9	9
9	9	9	9	9	9	9	9	9	9
9	9	9	9	9	9	9	9	9	9
9	9	9	9	9	9	9	9	9	9
9	9	9	9	9	9	9	9	9	9
9	9	9	9	9	9	9	9	9	9
9	9	9	9	9	9	9	9	9	9
9	9	9	9	9	9	9	9	9	9
9	9	9	9	9	9	9	9	9	9
9	9	9	9	9	9	9	9	9	9
9	9	9	9	9	9	9	9	9	9

9	9	9	9	9	9	9	9	9	9
1	2	2	3	1	1	2	1	2	1
1	1	1	1	1	1	9	1	1	1
9	9	9	9	9	9	9	9	9	9
9	9	9	9	9	9	9	9	9	9
9	9	9	9	9	9	9	9	9	9
9	9	9	9	9	9	9	9	9	9
9	9	9	9	9	9	9	9	9	9
9	9	9	9	9	9	9	9	9	9
1	9	9	9	9	9	9	9	9	9
9	9	9	9	9	9	9	9	9	9
9	9	9	9	9	9	9	9	9	9
1	9	9	9	9	9	9	9	9	9
2	2	2	2	2	2	3	3	3	1
9	9	9	9	9	9	9	9	9	9
9	9	9	9	9	9	9	9	9	9
9	9	9	9	9	9	9	9	9	9
9	9	9	9	9	9	9	9	9	9
9	9	9	9	9	9	9	9	9	9
9	9	9	9	9	1	2	3	1	1
2	2	2	2	2	2	2	2	2	2
2	1	2	2	2	2	2	2	2	2
3	3	9	2	1	3	1	1	3	3
1	2	2	3	2	2	2	2	2	2
1	2	2	2	2	3	1	1	3	3
1	2	1	2	3	2	1	1	2	3
2	2	1	2	1	1	1	2	1	1
1	3	1	3	1	1	3	1	3	1
1	3	3	2	3	1	3	2	2	3
3	2	3	3	3	2	2	3	3	9
2	9	9	9	9	9	9	9	1	9
2	2	2	2	2	2	2	2	2	2
3	2	2	2	2	2	9	9	9	9
1	1	1	2	2	3	2	3	3	1
1	3	2	3	1	3	3	3	3	2
1	3	3	3	3	3	3	3	3	2
2	2	3	3	2	2	2	3	2	2
1	2	1	3	2	1	2	1	2	1
9	9	1	9	9	9	9	9	9	9
3	2	2	2	2	3	3	2	3	1
9	1	9	9	9	9	9	1	9	9
2	9	9	9	9	2	9	9	9	9
1	2	2	1	1	1	2	2	2	1
3	3	2	1	1	3	1	2	3	1
1	9	1	9	9	9	9	9	9	1
1	1	1	9	3	1	1	2	2	3
9	9	9	9	9	9	9	9	9	9

1	9	2	1	3	1	3	3	1	1
2	1	2	2	2	2	2	2	3	9
1	1	1	1	1	1	1	1	1	1
9	9	9	9	9	9	9	9	9	9
2	1	2	2	2	9	9	9	9	9
2	2	2	2	2	2	2	2	3	2
9	9	9	2	9	9	9	9	9	9
9	9	9	9	9	9	9	9	9	9
1	2	2	1	2	2	2	1	2	2
1	3	3	2	2	2	2	2	2	2
2	9	9	9	9	9	9	9	9	9
9	2	9	9	9	9	9	9	9	2
2	2	2	2	2	2	2	2	2	2
2	2	2	2	2	2	2	2	2	2
2	2	2	2	2	2	2	2	2	2
2	2	3	1	1	2	2	2	1	1
2	3	1	2	3	1	2	3	2	3
9	9	9	9	9	9	9	9	9	9
1	9	9	9	9	9	9	9	9	9
2	2	2	2	2	2	2	2	2	2
1	2	2	2	2	2	2	2	2	2
3	1	2	2	2	2	1	3	2	3
1	3	1	3	1	1	3	1	3	1
1	1	1	1	1	2	2	2	2	3
1	2	2	1	1	2	1	1	1	2
1	2	1	2	3	9	9	2	2	9
2	2	2	2	2	2	2	2	2	2
2	2	1	2	2	2	2	2	2	2
2	2	2	2	2	2	2	2	2	2
2	2	2	2	2	2	2	2	2	2
3	3	3	2	2	3	2	2	2	2
1	1	1	2	2	2	2	2	1	3
3	3	2	2	2	2	2	2	2	2
3	2	2	2	2	2	2	2	3	3
1	2	2	2	2	2	2	3	2	2
2	2	1	1	1	2	1	3	2	1
3	3	2	1	2	3	3	3	3	3
2	3	2	2	2	1	1	1	3	2
1	2	1	2	1	1	1	1	1	1
1	2	1	2	1	2	2	1	1	1
2	1	2	1	2	2	2	1	2	1
3	3	3	3	3	2	2	2	3	2
3	2	2	2	2	3	2	2	2	2
3	1	3	3	3	2	2	2	9	1
2	9	9	9	9	2	9	9	9	9
1	3	3	3	3	3	3	3	3	3
3	3	3	2	2	2	2	2	2	2

1	2	2	3	2	2	2	2	2	2
1	2	3	1	2	1	2	3	1	2
3	2	2	2	3	2	1	1	3	3
1	1	1	1	1	1	1	1	1	1
1	3	2	2	2	1	1	3	9	2
1	2	2	2	2	2	2	2	2	2
2	2	2	2	2	2	2	2	2	2
1	2	3	1	3	2	2	2	3	1
2	3	2	2	2	2	2	2	2	9
2	2	3	1	2	2	3	3	3	2
2	1	2	2	2	3	3	2	3	3
1	2	2	2	2	2	2	2	2	2
2	2	3	2	3	2	2	1	3	3
2	1	2	3	1	3	2	1	3	2
2	2	2	2	2	2	2	2	2	2
1	2	3	2	2	3	2	1	3	2
2	2	2	2	2	2	2	2	2	2
2	2	2	2	2	2	2	2	2	2
1	1	1	1	1	2	2	2	2	2
2	2	2	2	2	2	2	2	2	2
2	2	2	2	2	2	2	2	2	2
2	1	1	2	1	2	1	2	1	2
3	3	3	3	3	3	3	3	3	3
2	2	2	2	2	2	2	2	2	2
2	2	2	2	2	2	2	2	2	2
1	2	3	1	2	1	2	3	1	2
2	2	2	1	2	1	2	3	1	1
1	1	1	3	3	1	1	1	1	1
2	2	2	2	2	2	2	2	2	2
1	3	1	1	1	1	1	1	1	1
3	3	3	3	3	3	3	3	3	1
9	9	9	9	9	9	9	9	9	9
2	1	3	2	1	1	2	3	3	2
3	1	3	3	2	2	2	2	2	3
1	2	1	2	1	1	1	1	2	2
1	1	2	2	3	3	2	1	3	2
1	2	2	2	2	2	2	3	3	2
2	9	2	9	1	1	3	2	9	1
2	2	2	3	3	2	2	2	3	3
1	1	1	1	1	1	2	2	1	2
3	2	1	2	2	1	1	2	3	1
9	9	9	9	9	9	9	9	9	9
1	1	3	2	3	1	3	3	1	1
1	2	1	2	3	2	2	2	2	2
2	3	3	3	2	2	2	3	3	3
2	1	1	2	1	2	2	1	2	1
1	2	3	3	2	2	2	3	2	3

1	3	2	1	2	2	1	2	1	2
1	2	2	2	2	2	2	3	3	2
9	9	9	9	9	9	9	9	9	9
9	9	9	9	9	9	9	9	9	9
9	9	9	9	9	9	9	9	9	9
3	2	1	2	2	1	2	3	2	2
1	2	3	2	2	3	2	3	2	2
3	3	2	9	2	3	9	3	9	2
3	2	1	2	2	3	2	3	3	2
1	3	3	2	3	3	3	3	3	1
2	2	2	1	2	9	9	9	9	9
1	3	3	3	2	2	2	1	1	3
3	3	3	3	3	2	3	3	2	1
1	1	2	2	2	2	2	1	2	1
1	1	1	1	1	2	1	2	3	1
1	2	3	1	2	1	2	3	1	2
1	3	1	2	1	2	2	2	1	1
2	2	2	2	2	2	2	2	2	2
2	2	2	2	2	2	2	2	2	2
1	3	1	3	1	1	1	3	3	1
2	2	2	2	2	2	2	2	2	2
1	2	2	2	2	2	2	2	2	1
1	2	1	1	3	3	3	1	1	3
3	2	3	2	2	3	2	3	2	1
3	3	1	2	2	3	1	1	2	2
1	3	3	2	3	3	1	1	2	3
3	2	3	1	2	3	2	3	2	1
1	3	3	2	3	3	1	1	2	3
2	2	2	2	2	2	2	2	2	2
3	2	3	2	2	1	3	3	3	2
1	3	2	1	2	3	3	3	3	3
9	9	9	9	9	9	9	9	9	9
2	2	3	1	2	2	2	1	3	2
2	3	3	2	1	3	3	1	3	3
3	3	3	3	3	2	2	2	2	2
2	3	1	2	3	1	2	1	2	1
2	2	2	2	2	2	2	2	2	2
1	9	9	9	9	9	2	9	9	9
3	2	1	2	2	3	2	2	1	2
1	1	3	2	2	2	2	2	2	1
3	2	3	3	2	2	1	3	3	2
2	3	1	2	1	2	1	2	1	3
2	2	2	2	2	2	2	2	2	2
2	2	1	3	1	2	2	1	1	1
1	2	2	2	2	3	2	1	2	3
9	9	2	9	9	2	9	9	9	9
3	2	1	2	3	1	2	2	1	2

2	2	2	2	2	2	2	2	2	2
2	2	2	2	2	2	2	2	2	2
2	2	2	2	2	2	2	2	2	2
2	2	2	2	2	2	2	2	2	2
2	3	2	2	2	1	2	3	1	1
2	2	2	2	2	2	2	2	2	2
1	3	2	1	2	1	3	1	2	2
2	2	2	2	2	2	2	2	2	2
1	3	2	2	3	3	3	3	3	3
2	2	2	2	2	2	2	1	3	2
2	2	2	2	2	2	2	2	2	2
3	3	3	3	3	2	2	2	2	2
1	3	2	2	3	1	1	3	3	3
1	2	1	1	2	1	1	3	3	3
1	1	1	1	1	3	3	3	3	1
1	3	3	3	3	3	3	3	3	3
2	3	3	2	1	3	3	2	3	1
1	2	2	2	2	2	2	2	2	2
2	3	3	2	1	3	3	2	3	1
1	1	2	2	2	3	3	3	2	1
2	2	2	2	2	2	2	2	2	2
2	2	2	2	2	2	2	2	2	2
1	2	2	2	2	2	2	2	9	2
1	1	2	2	2	2	2	2	2	2
2	2	2	2	2	2	2	2	2	2
2	2	2	1	2	2	2	2	2	2
1	1	1	2	1	1	1	1	3	1
3	3	1	3	3	1	3	1	1	3
2	9	9	9	9	9	9	9	9	2
3	3	1	3	3	1	2	2	2	2
1	2	2	2	2	2	2	2	2	2
1	2	2	2	2	2	2	3	2	2
1	2	2	2	2	3	3	3	2	2
1	2	2	2	2	3	3	3	2	2
3	3	1	3	3	1	3	1	1	1
2	2	1	2	1	1	1	1	1	1
3	3	1	3	3	1	3	1	1	3
1	1	1	2	2	3	3	3	3	1
1	2	2	3	2	1	2	2	2	3
2	2	1	2	2	1	2	2	2	2
2	9	9	9	9	2	9	9	9	9
3	3	1	3	3	1	3	1	1	3
2	2	2	2	2	2	2	2	2	2
2	3	1	1	1	1	1	1	1	3
1	1	2	2	2	1	1	2	2	2
2	2	2	2	2	2	2	2	2	2
1	2	3	2	2	1	2	2	3	2

9	9	9	9	9	9	9	9	9	9
9	9	9	9	9	9	9	9	9	9
1	1	1	1	1	1	1	1	1	1
1	2	2	3	3	1	2	2	3	3
9	9	9	9	9	9	9	9	9	9
1	2	2	2	2	1	1	1	1	1
2	2	2	2	2	2	2	2	2	2
1	3	3	3	2	1	1	1	1	1
1	2	3	1	2	1	2	3	1	2
1	2	3	2	1	1	2	3	2	1
2	2	1	3	3	1	2	3	1	2
1	2	3	1	2	1	2	3	1	2
1	2	3	2	2	2	2	3	2	2
1	9	9	9	9	1	9	9	9	9
1	1	1	1	1	2	1	3	1	1
9	9	9	9	9	9	9	9	9	9
1	1	1	1	1	2	2	2	2	2
3	3	1	2	2	2	3	3	3	2
1	1	1	1	1	2	1	1	2	3
1	2	2	3	2	2	2	2	2	2
1	1	1	1	1	1	1	1	1	1
9	9	9	9	9	9	9	9	9	9
9	9	9	9	9	9	9	9	9	9
3	3	3	3	3	2	2	2	2	2
2	2	2	2	2	2	2	2	2	2
2	2	2	1	3	1	2	2	1	2
2	2	2	2	2	2	2	2	2	2
2	2	2	2	2	2	2	2	9	9
2	2	9	1	2	2	2	1	2	3
2	1	2	3	2	1	2	3	2	1
1	9	2	9	2	2	2	2	2	9
1	9	9	9	9	9	9	9	9	9
2	1	2	2	2	2	2	2	2	1
2	2	2	2	2	2	2	2	9	1
2	2	2	2	2	1	1	2	2	1
3	2	1	2	2	3	2	3	3	2
1	2	1	2	3	1	1	2	2	3
2	2	2	2	2	2	2	2	2	2
2	2	2	2	2	2	2	2	2	2
2	2	2	2	2	2	2	2	2	2
1	2	2	2	2	2	2	2	2	2
2	2	2	2	2	2	2	2	2	2
3	3	3	3	3	3	3	3	3	3
1	9	9	9	9	9	9	9	9	9
9	9	9	9	2	9	9	9	9	9
2	2	2	2	2	2	2	2	2	2
2	2	2	2	2	2	2	2	2	2

1	9	9	9	9	9	9	9	9	1
1	2	2	2	1	1	2	1	2	1
2	9	9	9	9	2	9	9	9	9
2	2	2	2	2	2	2	2	2	2
1	2	1	2	2	2	1	2	1	1
2	2	2	2	2	2	2	2	2	2
3	2	9	2	3	1	2	1	2	1
1	9	9	9	9	9	9	9	9	1
2	2	2	2	2	1	1	1	1	1
2	1	2	1	2	2	1	3	2	2
2	3	2	3	2	2	2	3	3	3
2	2	2	1	2	2	1	1	2	2
2	1	1	2	2	2	2	1	1	1
1	2	3	2	2	2	2	1	1	3
2	2	2	2	2	2	2	2	2	2
3	3	3	3	1	9	9	9	1	9
9	9	9	9	1	9	9	9	9	9
2	2	2	2	2	2	2	2	2	2
1	2	2	3	3	9	3	2	2	2
2	1	2	3	3	2	2	2	2	2
2	2	2	1	2	2	2	1	2	2
2	2	9	1	2	2	2	1	2	2
2	2	2	2	2	2	2	2	2	2
2	2	2	2	2	2	2	2	2	2
3	2	2	2	3	2	3	2	3	2
1	2	1	3	1	1	3	1	2	2
2	1	2	1	2	2	1	1	2	1
1	2	2	2	2	2	2	1	3	3
2	1	3	2	2	1	2	1	3	3
9	9	9	9	9	9	9	9	9	9
2	2	1	2	2	3	3	3	3	3
1	9	9	9	9	9	9	9	9	1
9	9	9	9	9	9	9	9	9	9
9	9	1	9	9	9	9	9	9	9
1	2	2	2	1	1	2	2	2	3
2	2	2	2	2	3	3	3	3	3
2	2	2	2	2	2	2	2	2	2
1	9	9	9	9	1	9	9	9	9
1	2	3	3	2	2	2	2	3	1
2	2	2	2	2	2	2	2	2	2
9	9	1	9	9	9	9	9	9	1
1	1	1	2	2	2	2	2	2	1
1	2	2	3	1	1	3	1	2	3
1	2	9	2	1	1	2	9	9	3
2	3	1	2	3	1	2	3	1	2
3	3	1	3	3	1	3	1	1	1
1	2	3	2	2	2	2	2	2	2

1	2	3	9	1	1	2	2	2	2
9	9	1	9	9	1	9	9	9	9
9	9	9	9	1	9	9	9	1	9
2	3	1	2	3	1	2	1	3	1
2	2	2	2	2	2	2	2	2	2
2	2	2	2	2	2	2	2	2	2
2	1	3	1	2	2	2	9	1	1
9	9	9	9	9	9	9	9	9	9
9	9	9	9	9	9	9	9	9	9
9	9	9	9	9	9	9	9	9	9
1	9	9	9	9	9	9	9	9	9
9	9	1	9	9	9	9	9	9	9
9	9	9	9	9	9	9	9	9	9
9	9	9	9	9	9	9	9	9	9
9	9	9	9	9	9	9	9	9	9
9	9	1	9	9	9	9	9	9	1
2	2	2	1	2	2	2	1	2	2
1	2	1	1	1	1	1	1	2	9
1	3	1	9	3	2	1	2	2	1
1	2	2	2	2	2	2	2	2	2
1	2	2	2	2	2	2	2	2	2
1	2	2	2	2	1	2	2	2	2
1	3	3	3	3	1	1	1	1	2
1	2	2	2	2	2	2	2	2	1
1	9	1	2	2	1	1	2	9	1
2	2	2	3	3	2	3	2	3	2
2	1	2	1	3	1	3	2	3	2
2	1	2	1	3	1	3	2	3	1
2	2	2	2	2	3	3	3	3	3
2	2	2	2	2	2	2	2	2	2
2	2	2	2	2	3	3	3	3	3
3	3	3	3	3	3	3	3	3	3
2	2	2	2	2	2	2	2	2	2
2	3	1	2	3	1	2	3	1	2
2	2	2	2	2	2	2	2	2	2
2	2	2	2	2	2	2	2	2	2
9	9	9	9	9	9	9	9	9	9
2	1	2	2	2	2	2	2	1	2
1	1	1	1	1	2	1	1	1	1
9	9	1	9	9	1	9	9	9	9
3	3	3	3	3	3	3	3	3	3
1	1	1	1	1	2	2	2	2	2
2	2	2	2	2	2	2	2	2	2
2	2	2	2	2	2	2	2	2	2
1	2	1	2	3	1	1	3	1	3
9	9	9	9	9	9	9	9	9	9
2	2	1	1	1	1	1	2	3	2

1	1	2	2	2	2	2	2	2	2
2	2	2	2	2	2	2	2	2	2
2	2	1	2	1	2	2	2	2	2
2	2	1	2	2	3	3	2	1	2
9	9	9	9	9	9	9	9	9	9
2	2	2	2	2	2	2	2	1	2
9	9	9	9	9	9	9	9	9	9
2	2	2	2	2	3	2	2	2	2
9	1	9	9	9	1	9	9	9	9
9	9	9	9	9	9	9	9	9	9
9	9	9	9	9	9	9	9	9	9
9	9	9	9	9	9	9	9	9	9
9	9	9	9	9	9	9	9	9	9
9	9	9	9	9	9	9	9	9	9
9	9	9	9	9	9	9	9	9	9
9	9	9	9	9	9	9	9	9	9
9	9	9	9	9	9	9	9	9	9
9	9	9	9	9	9	9	9	9	9
9	9	9	9	9	9	9	9	9	9
9	9	9	9	9	9	9	9	9	9
9	9	9	9	9	9	9	9	9	9
9	9	9	9	9	9	9	9	9	9
9	9	9	9	9	9	9	9	9	9
9	9	9	9	9	9	9	9	9	9
9	9	9	9	9	9	9	9	9	9
9	9	9	9	9	9	9	9	9	9
9	9	9	9	9	9	9	9	9	9
9	9	9	9	9	9	9	9	9	9
9	9	9	9	9	9	9	9	9	9
1	2	3	2	2	1	2	1	3	1
2	2	2	2	2	2	2	2	2	2
1	2	3	3	3	2	1	1	2	1
2	2	1	1	2	1	1	1	2	1
3	3	3	1	3	1	1	3	2	2
2	3	1	2	2	1	2	2	1	2
2	2	2	2	2	2	2	2	2	2
3	3	1	9	3	1	3	3	2	3
1	3	2	2	2	1	2	2	2	2
1	2	2	2	2	2	2	2	2	2
1	3	2	2	2	3	3	1	2	1
9	9	1	9	9	1	3	1	3	2
3	3	1	3	2	1	2	1	1	2
3	3	1	3	3	1	3	1	1	1
2	3	2	2	1	2	2	2	1	1
3	3	1	3	2	1	2	1	1	1
9	1	9	9	9	1	9	9	9	9
1	2	1	2	2	1	2	2	1	3

1	3	1	3	3	1	3	1	3	1
1	9	9	9	9	9	9	9	9	1
3	3	1	3	3	1	1	1	1	3
3	3	1	3	1	1	3	1	3	2
2	2	2	2	2	1	2	2	2	2
3	3	2	2	1	2	2	2	2	2
1	1	1	2	2	1	1	1	2	1
3	3	1	2	2	1	2	1	2	2
3	1	1	3	1	1	1	1	1	1
3	3	1	2	2	1	1	1	2	1
3	2	3	2	2	2	1	2	1	2
1	9	9	9	9	1	9	9	9	9
3	1	2	2	3	1	2	1	1	1
1	9	9	9	9	1	9	9	9	9
1	9	9	9	9	2	9	9	9	9
1	9	9	9	9	2	9	9	9	9
1	9	9	9	9	9	9	1	9	9
9	9	1	9	9	1	9	9	9	9
9	9	1	9	9	9	9	1	9	9
9	9	1	9	9	9	9	9	1	9
9	9	1	9	9	9	9	9	9	9
3	3	3	3	1	1	1	3	3	3
3	3	3	3	3	3	3	3	3	3
2	2	2	2	2	2	2	2	2	2
9	9	1	9	9	9	1	9	9	9
3	3	3	3	3	1	3	3	1	1
9	9	1	9	9	1	9	9	9	9
1	9	9	9	9	9	9	9	1	9
9	9	1	9	9	1	9	9	9	9
1	9	9	9	9	9	9	9	9	9
9	9	1	9	9	1	9	9	9	9
1	2	3	2	1	2	2	3	2	1
1	3	3	3	1	1	1	3	1	1
1	2	3	2	2	2	2	3	2	2
1	3	1	3	3	2	2	2	2	3
2	2	2	2	2	2	2	2	2	2
3	3	2	9	9	3	3	2	2	3
3	3	1	3	1	1	1	9	9	9
1	3	3	3	3	1	3	3	3	1
1	3	1	2	1	3	1	2	2	1
2	2	2	2	3	3	3	2	3	3
9	9	1	9	9	9	1	9	9	9
2	2	2	2	2	2	2	2	2	2
2	2	2	2	2	2	2	2	2	2
2	2	2	2	2	3	3	1	2	3
2	2	2	2	2	2	1	3	2	2
2	2	1	2	1	1	2	1	1	1

2	2	1	2	1	1	2	1	1	1
3	3	1	1	3	1	1	3	3	3
2	2	3	2	1	2	2	2	2	2
2	3	1	1	1	1	3	3	1	3
1	9	9	9	9	9	9	9	9	1
9	9	1	9	9	9	9	1	9	9
2	1	2	9	3	1	1	1	1	1
3	2	1	3	3	2	3	1	3	3
1	3	1	3	3	1	3	3	3	1
1	9	9	9	9	9	9	9	1	9
2	9	1	9	1	3	2	1	9	9
2	1	1	2	1	3	1	2	3	1
3	1	1	3	2	1	2	1	1	1
9	9	9	9	9	9	9	9	9	9
2	9	9	9	9	2	9	9	9	9
1	2	1	1	2	3	3	3	3	3
2	2	2	2	2	2	2	2	2	2
3	3	1	3	2	3	3	3	1	1
9	1	1	1	2	1	1	2	1	1
3	9	1	9	2	1	3	1	2	9
1	9	9	9	9	1	9	9	9	9
1	1	2	2	2	2	1	2	2	2
9	9	1	9	9	9	9	9	9	9
3	1	1	9	1	9	9	9	9	9
2	3	2	3	2	3	3	3	3	3
2	1	1	2	2	1	2	1	1	1
3	3	1	3	2	3	3	3	3	2
3	2	3	3	2	3	1	3	2	3
3	3	2	3	3	3	2	3	3	3
3	3	1	2	2	1	1	1	1	1
2	2	1	2	2	2	2	2	2	2
9	9	9	9	9	9	9	9	9	9
1	3	1	2	2	1	2	3	1	3
1	3	3	1	3	3	3	3	3	1
3	3	3	3	3	3	3	3	3	3
2	3	1	2	3	1	2	3	2	2
9	9	9	9	9	9	9	9	9	9
9	9	9	9	9	9	9	9	9	9
9	9	9	9	9	9	9	9	9	9
9	9	9	9	9	9	9	9	9	9
9	9	9	9	9	9	9	9	9	9
9	9	9	9	9	9	9	9	9	9
9	9	9	9	9	9	9	9	9	9
9	9	9	9	9	9	9	9	9	9
9	9	9	9	9	9	9	9	9	9
9	9	9	9	9	9	9	9	9	9
9	9	9	9	9	9	9	9	9	9

9	9	9	9	9	9	9	9	9	9
9	9	9	9	9	9	9	9	9	9
9	9	9	9	9	9	9	9	9	9
9	9	9	9	9	9	9	9	9	9
9	9	9	9	9	9	9	9	9	9
9	9	9	9	9	9	9	9	9	9
9	9	9	9	9	9	9	9	9	9
9	9	9	9	9	9	9	9	9	9
9	9	9	9	9	9	9	9	9	9
9	9	9	9	9	9	9	9	9	9
9	9	9	9	9	9	9	9	9	9
9	9	9	9	9	9	9	9	9	9
9	9	9	9	9	9	9	9	9	9
9	9	9	9	9	9	9	9	9	9
9	9	9	9	9	9	9	9	9	9
9	9	9	9	9	9	9	9	9	9
9	9	9	9	9	9	9	9	9	9
9	9	9	9	9	9	9	9	9	9
9	9	9	9	9	9	9	9	9	9
9	9	9	9	9	9	9	9	9	9
9	9	9	9	9	9	9	9	9	9
9	9	9	9	9	9	9	9	9	9
9	9	9	9	9	9	9	9	9	9
9	9	9	9	9	9	9	9	9	9
9	9	9	9	9	9	9	9	9	9
2	2	1	2	2	1	2	2	2	2
9	9	9	9	9	9	9	9	9	9
9	9	9	9	9	9	9	9	9	9
9	9	9	9	9	9	9	9	9	9
9	9	9	9	9	9	9	9	9	9
9	9	9	9	9	9	9	9	9	9
9	9	9	9	9	9	9	9	9	9
9	9	9	9	9	9	9	9	9	9
9	9	9	9	9	9	9	9	9	9
9	9	9	9	9	9	9	9	9	9
9	9	9	9	9	9	9	9	9	9
9	9	9	9	9	9	9	9	9	9
9	9	9	9	9	9	9	9	9	9
9	9	9	9	9	9	9	9	9	9
9	9	9	9	9	9	9	9	9	9
9	9	9	9	9	9	9	9	9	9
2	2	2	2	2	2	2	2	2	2
1	3	3	3	3	3	3	3	3	3
1	2	9	9	3	3	2	3	3	2
1	3	1	3	1	1	1	1	1	1
3	3	3	3	3	3	3	3	3	3
1	3	3	3	3	1	1	1	1	1

1	2	2	3	2	9	9	1	9	9
2	9	9	9	9	1	9	9	9	9
1	2	2	3	2	9	9	1	9	9
2	2	2	2	3	2	2	2	3	1
1	2	1	1	1	1	2	1	1	1
2	2	2	2	2	3	2	3	2	2
3	3	1	3	2	1	2	1	1	1
1	2	2	2	1	1	2	3	2	1
1	2	1	1	3	1	2	1	1	1
2	2	1	2	2	1	3	1	3	2
2	2	2	2	2	1	2	2	1	2
3	3	1	2	1	1	2	1	9	3
3	2	1	2	2	1	1	1	1	1
3	3	1	3	3	1	2	2	2	2
2	3	2	1	2	2	1	3	2	2
2	1	1	3	2	1	2	1	3	2
9	9	1	9	9	1	9	9	9	9
1	2	3	3	3	2	2	3	2	2
1	2	1	2	2	1	2	1	1	3
2	1	2	2	2	1	3	3	2	2
2	1	2	1	2	2	2	3	3	2
2	2	2	9	1	3	3	2	2	2
2	2	1	2	2	1	2	1	2	1
1	2	3	2	1	2	1	1	2	3
9	1	1	1	1	1	1	3	3	1
1	2	1	2	1	2	3	3	3	2
1	2	3	9	1	2	1	2	2	2
2	2	1	2	1	1	2	1	1	1
3	1	1	9	3	1	1	2	2	2
9	9	1	9	9	9	9	9	9	9
3	3	3	3	3	3	3	3	3	3
3	3	3	1	1	1	2	3	1	2
1	1	1	1	1	1	1	1	2	9
3	2	1	3	1	3	2	1	2	2
1	2	2	2	2	2	2	2	2	2
2	2	2	2	2	2	2	2	2	2
9	9	9	9	9	2	2	3	2	2
2	2	2	2	2	2	2	2	2	2
2	2	2	2	2	2	2	2	2	2
9	9	9	9	9	9	9	9	9	9
9	9	9	9	9	9	9	9	9	9
9	9	9	9	9	9	9	9	9	9
9	9	9	9	9	9	9	9	9	9
2	1	2	2	2	9	9	9	9	9
9	9	9	9	9	9	9	9	9	9
9	9	9	9	9	9	9	9	9	9
3	2	2	2	2	2	2	1	3	2

2	2	2	1	1	2	2	2	2	2
1	1	1	1	1	1	2	2	2	1
1	3	3	1	3	3	3	3	3	1
1	1	1	2	3	1	3	1	3	1
1	3	3	3	3	3	1	3	3	3
3	1	3	1	3	1	3	3	1	3
1	1	2	1	2	2	2	2	2	2
1	2	3	2	1	1	2	3	1	2
1	2	2	2	2	2	2	2	2	2
1	1	1	1	3	1	3	1	9	1
1	1	1	2	2	1	3	3	1	2
1	2	3	1	2	3	2	1	3	2
1	1	1	1	1	1	1	9	1	1
3	1	2	3	1	2	1	3	2	1
1	1	1	3	2	3	1	2	2	2
1	9	9	9	9	1	9	9	9	9
1	3	3	3	3	2	2	2	3	3
1	3	1	1	2	1	2	3	1	1
1	1	1	1	1	3	1	3	1	1
1	3	1	1	2	1	3	3	3	2
3	1	3	2	1	1	2	1	3	1
1	3	1	1	1	3	1	2	3	2
1	2	2	1	1	2	2	2	2	2
9	9	9	9	1	9	9	1	9	9
1	2	3	2	1	1	2	1	2	1
9	9	1	9	9	9	9	9	9	1
1	3	2	3	2	1	1	2	3	2
2	2	1	2	2	2	2	1	2	2
9	3	1	1	1	1	9	9	3	2
1	3	1	2	2	1	1	1	2	2
1	2	3	1	2	1	3	2	3	3
3	3	3	3	1	3	1	3	3	3
1	1	2	3	1	1	2	1	2	2
1	1	3	1	1	1	3	3	1	1
3	1	2	2	1	1	1	2	2	1
1	2	2	3	2	1	2	3	3	2
1	1	3	3	3	2	1	3	1	3
1	3	2	3	1	2	1	2	3	1
3	3	1	1	1	1	3	2	2	2
3	3	3	3	1	3	3	1	3	3
1	1	1	1	3	1	1	3	1	1
1	3	9	1	1	1	3	1	3	3
1	2	3	1	3	1	2	1	3	2
9	9	9	9	9	9	9	9	9	9
1	3	3	3	3	1	3	3	3	1
1	2	1	2	3	3	1	3	1	3
1	3	1	3	1	1	3	3	3	1

1	1	2	2	3	1	2	2	1	3
1	3	1	3	1	3	1	3	3	1
3	2	2	1	1	1	2	2	3	1
2	2	2	2	2	2	2	2	2	2
1	1	2	2	3	9	9	2	9	9
3	3	2	2	2	3	3	3	3	3
1	1	2	3	1	2	1	2	2	2
1	3	1	1	1	3	1	2	3	2
2	3	3	1	1	2	2	9	2	9
1	1	1	2	3	2	2	2	2	1
2	2	2	2	2	1	1	1	1	1
2	2	2	3	3	2	2	3	3	2
2	2	2	2	2	2	2	2	2	2
9	9	9	9	9	9	9	9	9	9
9	9	9	9	9	9	9	9	9	9
1	1	2	1	3	1	2	3	2	1
2	2	2	2	2	2	2	2	2	2
2	2	2	2	2	2	2	2	2	2
2	2	2	2	2	2	2	2	2	2
1	1	1	1	2	2	2	2	2	2
2	2	2	2	2	2	2	2	2	2
2	2	2	2	2	2	2	2	2	2
2	2	2	2	2	2	2	2	2	2
2	2	2	2	2	2	3	2	3	2
2	2	2	2	2	2	2	2	2	2
1	2	2	2	2	2	2	2	2	2
2	2	2	3	1	2	2	2	2	2
2	2	2	2	2	2	2	2	2	2
2	2	2	2	2	2	2	2	2	2
2	2	2	2	2	2	2	2	2	2
2	2	2	2	2	2	2	2	2	2
2	2	2	2	2	2	2	2	2	2
2	2	2	2	2	2	2	2	2	2
1	2	3	1	2	1	2	1	2	9
1	1	2	2	2	2	2	1	1	3
2	2	2	2	9	2	2	2	2	2
2	2	2	2	2	2	2	2	2	2
2	2	2	2	2	2	2	2	2	2
2	2	2	2	2	2	2	2	2	2
2	2	2	2	2	2	2	2	2	2
1	2	2	2	2	2	2	2	2	2
2	2	2	2	2	2	2	2	2	2
2	2	2	2	2	2	2	2	2	2
2	2	2	2	2	2	2	2	2	2
2	2	2	2	2	2	2	2	2	2
1	3	3	1	1	3	3	3	3	1
2	2	2	2	2	2	2	2	2	2

2	2	2	2	2	2	2	2	2	2
2	1	1	2	3	2	2	1	1	3
2	2	2	2	2	2	2	2	2	2
1	9	9	9	9	2	1	2	1	2
3	3	3	3	3	3	3	3	3	3
2	2	2	2	2	2	9	2	2	2
2	2	2	2	2	2	2	2	2	2
2	2	2	2	2	2	2	2	2	2
2	2	2	2	2	2	2	2	2	2
2	2	2	2	2	2	2	2	2	2
1	2	3	2	1	2	3	2	1	2
2	2	2	2	2	2	2	2	2	2
3	3	1	1	3	1	9	9	9	9
1	1	1	1	1	1	2	3	1	3
1	9	9	9	9	9	9	9	9	9
2	2	2	2	2	2	2	2	2	2
3	3	3	3	3	3	3	3	3	3
1	1	1	1	1	1	1	1	1	1
3	3	2	1	2	2	2	3	3	3
3	9	9	9	9	3	9	9	9	9
2	2	2	2	2	2	2	2	2	2
2	1	1	2	2	2	1	2	2	2
1	2	2	2	2	2	2	2	2	2
1	1	1	2	1	1	1	1	1	1
1	9	9	9	9	9	9	9	9	9
1	2	3	3	3	2	2	2	2	2
1	2	1	1	2	1	2	1	3	2
9	1	9	9	9	9	9	9	9	9
1	9	9	9	9	1	9	9	9	9
2	2	9	2	2	2	2	2	2	2
1	2	3	1	2	1	3	2	3	1
2	1	3	2	2	2	2	3	3	3
2	1	2	2	2	1	1	3	1	2
1	1	3	9	1	1	1	1	3	9
1	2	9	1	3	3	1	2	9	1
1	1	1	1	1	1	1	1	1	1
3	3	3	3	3	3	3	3	3	3
2	2	2	2	2	2	2	2	2	2
2	2	2	2	2	2	2	2	2	2
1	9	1	9	9	9	3	2	9	9
1	3	3	2	2	2	2	2	2	2
9	9	1	9	9	9	9	9	9	9
1	3	1	1	3	1	3	1	1	1
3	3	3	3	3	2	2	2	2	2
2	2	2	2	2	2	2	2	2	2
2	2	2	2	2	2	2	2	2	2
2	9	3	1	2	2	2	2	2	2

1	2	2	2	2	2	2	2	2	2
9	9	9	9	2	9	9	3	9	9
3	3	3	1	1	1	1	1	1	1
1	3	3	2	2	2	2	2	2	2
3	2	2	3	3	2	2	2	2	2
2	2	2	2	2	2	2	2	2	2
1	1	1	1	1	1	1	1	1	1
2	2	2	2	2	2	2	2	2	2
1	1	1	1	1	1	1	1	1	1
2	2	2	2	2	2	2	2	2	2
2	9	9	9	9	9	9	9	9	9
1	2	2	1	3	2	3	2	1	2
3	9	9	9	9	3	3	3	3	3
1	1	1	1	1	1	1	1	1	1
1	2	3	9	1	1	1	2	2	1
1	1	1	1	1	3	3	3	3	3
2	2	2	2	2	1	1	1	1	1
2	2	2	2	2	2	2	2	2	2
2	2	2	2	2	2	2	2	2	2
2	2	2	2	2	2	2	2	2	2
2	2	2	2	2	2	2	2	2	2
2	2	2	2	2	2	2	2	2	2
1	1	1	1	1	1	1	1	1	1
2	3	3	3	3	1	1	3	3	2
9	9	9	9	9	9	9	9	9	9
1	2	1	2	1	1	2	1	2	3
1	1	1	2	2	1	1	1	1	1
1	3	3	3	3	3	3	3	2	2
3	3	3	3	3	2	2	3	2	9
2	2	1	2	2	1	1	1	3	3
3	3	3	3	3	3	3	3	3	3
2	2	2	1	2	2	2	1	1	1
1	2	3	2	1	1	1	1	1	2
2	2	2	2	2	2	1	2	2	2
3	2	2	2	3	1	2	1	2	1
2	2	2	2	2	2	2	2	2	2
1	1	1	1	1	2	2	2	2	2
2	2	2	2	2	2	2	2	2	2
3	3	3	3	3	3	3	3	3	3
1	1	1	3	3	2	1	3	1	2
2	2	2	2	2	2	2	2	2	2
2	2	2	2	2	2	2	2	2	2
2	3	1	2	3	2	1	3	2	2
3	3	3	3	3	2	2	2	2	2
1	2	3	1	2	1	2	3	1	2
3	3	3	3	3	3	3	3	3	3
1	1	1	1	1	1	2	1	2	1

1	3	2	3	2	1	1	1	1	1
9	9	9	9	9	9	9	9	9	9
3	3	3	3	3	3	3	9	9	9
1	1	1	1	1	1	1	1	1	1
2	2	2	2	2	2	2	2	2	2
1	2	1	3	3	3	3	3	2	1
1	1	1	1	9	1	1	1	1	1
1	3	3	3	3	3	3	3	3	1
3	3	3	3	3	2	2	2	2	9
1	3	3	3	3	3	3	3	3	1
3	1	2	2	2	1	1	2	1	1
2	2	2	2	2	2	2	2	2	2
2	2	2	2	2	2	2	2	2	2
2	2	2	2	2	2	2	2	2	2
2	2	2	2	2	1	1	1	1	1
1	1	1	1	1	2	3	1	1	2
2	2	2	2	2	1	1	1	1	1
3	2	1	2	1	1	2	3	3	2
2	2	2	2	2	2	2	2	2	2
9	9	9	9	9	1	3	2	1	3
2	2	2	2	2	2	2	2	2	2
2	2	2	2	2	2	2	2	2	2
2	2	2	2	2	2	2	2	2	2
2	2	2	2	2	3	2	3	2	2
2	2	2	2	2	2	2	2	2	2
2	1	3	3	2	3	3	1	2	1
1	2	2	2	1	2	2	2	2	2
2	1	1	2	3	2	2	1	2	1
2	1	1	2	3	1	3	3	2	2
2	2	2	2	2	2	2	2	2	2
1	1	1	1	1	1	1	1	1	1
1	1	1	3	1	3	1	3	1	3
1	1	3	1	1	1	1	2	1	1
3	3	3	3	2	1	2	3	2	1
1	3	3	1	2	1	3	2	1	2
1	2	3	2	3	3	3	3	2	2
9	1	9	2	2	2	2	2	2	2
2	2	2	2	2	2	2	2	2	2
1	2	2	2	1	2	2	2	2	2
3	3	3	3	2	1	2	3	2	1
1	1	2	1	3	1	2	3	1	3
2	2	2	2	2	2	2	2	2	2
2	2	2	2	2	2	2	2	2	2
9	9	9	9	9	9	9	9	9	9
2	2	2	2	1	1	2	2	1	2
3	2	3	1	2	2	3	2	2	2
3	2	3	2	1	2	2	1	2	3

2	2	2	2	2	2	2	2	2	2
2	2	2	2	2	2	2	2	2	2
2	2	2	2	2	2	2	2	2	2
2	9	1	2	3	2	3	1	2	3
3	1	2	2	2	1	1	2	1	1
2	1	2	2	2	2	2	2	2	2
9	9	9	9	9	9	9	9	9	9
1	2	9	3	9	1	2	3	1	2
1	1	2	2	2	1	1	2	1	1
1	3	1	9	1	1	3	3	3	3
1	3	1	1	3	3	1	2	2	3
3	2	1	2	2	3	1	3	2	2
1	2	3	2	1	1	2	2	1	1
2	1	2	2	2	9	9	9	9	9
3	3	3	3	3	3	3	3	3	1
3	3	3	3	3	3	3	3	3	1
1	2	2	2	2	2	2	2	2	1
1	3	2	2	3	1	2	2	2	2
2	2	2	2	2	2	2	2	2	2
1	2	2	1	1	1	1	1	1	1
1	2	3	2	3	1	2	3	2	1
2	2	2	2	2	2	2	2	2	2
1	2	1	1	2	1	2	2	1	1
1	1	3	1	9	3	1	2	2	1
9	9	9	9	1	9	9	9	9	9
2	2	2	2	2	2	2	2	2	2
9	9	9	9	9	9	9	9	9	9
1	2	3	1	2	1	2	3	3	2
1	3	3	2	2	3	2	1	2	1
1	1	3	3	3	1	1	3	3	3
2	2	1	1	3	3	3	2	2	2
3	3	3	2	2	2	2	3	3	3
3	1	2	2	2	1	2	2	3	2
1	1	2	3	2	1	1	2	2	2
3	1	2	2	1	2	2	3	1	3
1	2	2	2	3	2	2	3	3	1
2	2	2	2	2	1	1	1	2	2
2	2	1	2	2	2	1	3	1	3
1	3	3	3	3	1	3	3	3	1
2	2	2	2	2	2	2	2	2	2
2	2	2	2	2	2	2	2	2	2
2	2	2	2	2	2	2	2	2	3
3	3	3	3	3	3	3	3	3	3
1	2	1	3	2	2	2	2	3	1
3	2	1	2	2	2	1	1	1	1
3	2	1	1	2	2	1	1	1	1
3	3	3	3	3	1	1	1	1	1

2	2	3	3	3	1	1	1	2	3
1	2	1	3	2	2	3	2	1	1
2	2	2	3	3	2	2	2	2	2
1	9	9	9	9	9	9	9	9	9
3	3	3	3	3	3	3	3	3	3
2	2	2	3	3	2	2	2	2	2
2	3	3	3	3	1	1	2	3	3
1	1	3	2	3	3	3	3	3	3
1	2	3	1	2	2	3	2	2	2
9	9	9	9	9	9	9	9	9	9
2	3	2	3	2	2	2	3	2	2
3	3	3	3	3	3	3	3	3	3
1	1	9	2	2	2	2	2	2	2
3	3	3	3	3	3	3	3	3	3
1	2	2	2	3	3	3	3	3	2
1	2	3	2	1	1	2	3	3	3
2	2	2	2	2	2	2	2	2	2
1	3	2	3	2	2	2	3	1	3
1	1	1	1	1	1	2	1	2	1
1	9	9	9	9	1	9	9	9	9
2	1	1	1	2	2	2	1	1	1
2	3	3	3	3	2	3	3	2	2
3	3	3	1	3	1	1	1	1	1
2	2	2	2	2	2	2	2	2	2
1	1	2	2	2	3	3	2	2	3
1	1	2	9	1	3	2	3	2	1
1	3	2	3	2	3	1	2	3	2
3	3	9	3	3	3	3	3	3	3
2	2	2	2	2	2	2	2	2	2
2	2	2	2	2	2	1	3	2	2
1	2	3	1	2	1	2	3	1	2
3	2	2	3	1	1	3	3	3	3
2	1	2	2	3	3	1	2	3	3
1	3	3	2	2	2	2	3	9	1
1	3	1	2	3	2	3	9	3	1
2	2	2	2	2	2	2	2	2	2
1	1	1	2	3	1	2	1	2	1
2	2	2	2	2	2	2	2	2	2
1	2	3	2	1	1	2	3	1	2
1	2	3	3	3	1	1	2	3	3
2	3	1	2	3	1	2	3	1	2
3	3	3	1	1	1	3	2	3	2
1	3	3	9	1	1	2	2	2	3
3	3	3	3	3	2	2	2	1	2
3	1	3	1	1	1	1	1	2	2
1	3	3	1	2	1	1	2	2	3
2	1	2	2	2	2	1	2	2	1

3	1	2	3	2	1	3	1	3	1
1	2	2	2	1	1	1	2	3	2
3	2	3	1	3	3	2	1	3	9
1	2	3	2	2	2	2	3	2	2
2	1	2	1	2	1	3	2	2	1
1	3	3	3	3	1	3	9	1	3
1	1	2	2	2	3	3	3	3	2
1	2	3	1	3	1	3	2	1	3
2	2	2	2	2	2	2	2	2	2
1	1	3	1	3	1	1	3	1	1
1	2	3	1	2	1	2	3	1	2
1	1	3	1	2	2	2	2	2	2
1	3	2	3	1	2	1	2	3	2
2	3	2	1	3	2	1	2	3	1
1	2	2	1	3	1	1	2	1	2
3	1	3	3	3	3	3	3	3	1
1	2	3	2	3	1	2	3	2	3
1	3	2	1	3	1	2	3	1	2
1	1	1	1	1	1	1	1	1	1
3	3	1	1	3	1	3	1	1	1
1	2	2	3	2	1	2	3	2	2
9	1	3	1	3	3	1	1	1	3
1	1	2	2	3	1	1	3	3	2
1	3	2	9	9	3	3	3	3	3
1	1	3	2	3	1	2	3	1	2
1	3	1	2	1	3	1	1	1	2
1	2	2	2	2	2	2	2	2	2
3	1	3	3	3	2	1	3	3	3
3	1	3	2	1	1	3	2	1	1
1	2	2	2	3	2	2	3	3	1
2	2	2	2	2	2	2	3	1	3
2	2	2	2	2	2	2	2	2	2
1	1	1	3	3	1	1	3	2	1
2	1	3	2	3	2	1	2	1	2
2	2	2	2	2	2	2	2	2	2
9	9	9	9	9	9	9	9	9	9
9	9	9	9	9	9	9	9	9	9
9	9	9	9	9	9	9	9	9	9
1	2	2	2	2	2	2	2	2	2
9	1	9	9	9	1	9	9	9	9
3	3	3	3	3	2	2	2	2	2
2	2	2	2	2	2	2	2	2	2
3	3	3	1	2	2	2	3	2	2
2	2	2	2	2	2	2	2	2	2
3	2	9	2	3	9	2	9	2	9
3	2	3	2	2	2	2	3	2	3
1	3	2	1	2	3	2	3	2	3

2	1	1	2	2	3	1	1	2	2
1	3	3	2	3	3	3	2	2	2
1	3	3	2	3	3	3	2	2	2
1	2	3	2	1	1	2	3	3	3
1	1	1	1	1	2	2	2	2	2
1	1	1	3	3	1	1	3	2	1
2	2	2	2	2	9	9	9	9	9
1	2	1	2	2	1	2	3	2	2
2	2	2	2	2	2	2	2	2	2
2	1	2	1	1	1	3	3	2	1
2	2	2	2	2	3	2	2	2	2
3	3	3	1	2	2	2	3	2	2
1	2	1	3	2	1	2	1	1	2
1	3	2	2	3	2	3	3	1	2
1	3	1	2	1	1	2	1	3	3
1	3	3	3	2	3	3	3	3	2
3	1	2	3	2	2	3	2	1	2
3	1	2	3	2	3	2	1	2	3
1	1	2	1	1	1	1	3	1	1
2	2	2	2	2	2	2	2	2	2
2	2	2	2	2	2	2	2	2	2
2	3	3	3	3	3	3	2	2	2
2	2	2	2	2	3	1	2	2	2
2	2	2	2	2	2	2	2	2	2
2	2	2	2	2	2	2	2	2	2
1	2	3	1	2	3	2	3	1	1
9	9	2	9	9	9	1	1	9	9
9	9	1	9	9	9	1	1	9	9
9	9	1	9	9	9	1	1	9	9
1	1	2	2	2	1	1	1	1	1
3	3	1	3	3	3	1	1	3	3
9	9	1	9	9	9	1	1	9	9
9	9	1	9	9	2	1	1	2	2
1	2	1	2	1	1	1	1	9	9
1	2	1	9	2	1	2	1	2	1
9	9	9	9	9	9	9	1	9	9
9	9	1	9	9	1	9	1	9	9
1	9	9	9	9	9	9	1	9	9
2	2	1	3	3	3	3	1	3	3
1	3	3	3	3	3	3	1	3	3
9	9	1	9	9	9	1	1	9	9
1	3	1	2	2	2	1	1	3	3
9	9	1	9	9	9	1	1	9	9
9	9	1	9	9	9	1	1	9	9
9	9	1	9	9	9	1	1	9	9
9	9	1	9	9	9	1	1	9	9
1	3	1	3	3	1	3	1	3	3

1	9	9	9	9	1	9	1	1	9
9	9	1	9	9	9	1	1	9	9
9	9	9	9	9	1	9	9	9	9
9	9	1	9	9	9	1	1	9	9
1	3	1	3	2	3	1	1	3	3
3	2	1	2	2	9	1	1	9	9
9	9	1	9	9	9	1	1	9	9
2	2	1	2	9	3	1	1	1	3
1	2	2	2	1	1	1	1	1	1
1	1	1	1	1	1	1	1	1	1
9	9	1	9	9	9	9	1	9	9
9	9	1	9	9	9	9	1	9	9
1	1	1	1	1	1	1	1	1	1
2	2	2	2	2	1	3	1	1	1
9	9	1	9	9	9	1	1	9	9
9	9	1	9	9	3	1	1	3	9
2	2	1	2	2	2	1	1	2	2
2	2	1	2	2	2	1	1	2	2
9	9	1	9	9	1	9	1	9	9
9	9	1	9	9	9	1	1	9	9
1	9	1	9	9	9	1	1	9	9
9	9	1	9	9	9	1	1	9	9
9	9	1	9	9	9	9	1	9	9
9	9	1	9	9	9	9	1	9	9
9	9	1	9	9	9	1	1	9	9
1	9	9	9	9	1	1	1	1	9
1	9	9	9	9	9	1	9	9	9
9	9	1	9	9	9	9	1	9	9
1	1	1	1	1	3	3	1	1	1
9	9	9	9	1	9	9	1	9	9
1	1	1	1	1	3	3	1	3	3
3	3	1	3	3	3	3	1	3	3
3	3	1	3	3	3	3	1	3	3
9	9	1	9	9	3	3	1	9	3
9	9	1	9	9	3	1	1	3	3
9	9	1	9	9	9	9	1	9	9
2	2	1	2	2	9	1	1	1	1
3	3	1	3	3	3	1	1	3	3
9	9	1	9	9	9	1	1	9	9
9	9	1	9	9	9	1	1	9	9
9	9	1	9	9	9	1	1	9	9
3	3	1	3	3	3	3	1	1	1
9	9	1	9	9	9	9	1	9	9
9	9	1	9	9	9	9	1	9	9
9	9	1	9	9	3	1	1	3	3
1	3	2	3	2	3	1	1	1	1
1	1	1	3	2	3	1	1	3	3

1	2	2	2	2	1	3	1	3	1
9	9	1	9	9	9	1	1	9	9
9	9	1	9	9	9	9	1	3	1
9	9	1	9	9	3	1	1	3	3
9	9	1	9	9	1	3	1	3	3
9	9	1	9	9	9	1	1	9	9
9	9	1	9	9	9	1	1	9	9
3	3	1	3	3	1	1	1	9	9
9	9	1	9	9	9	1	1	9	9
1	3	1	3	1	1	1	1	1	3
9	9	1	9	9	9	1	1	1	9
9	1	9	9	9	9	1	9	9	9
3	3	1	3	1	1	3	9	9	9
9	9	1	9	9	9	9	1	9	9
9	9	1	9	9	9	9	1	9	9
3	3	1	3	3	1	3	1	1	1
1	1	2	2	2	1	3	1	1	1
9	1	9	9	9	9	9	9	9	1
9	9	9	1	9	9	9	1	9	1
2	1	2	2	2	3	2	1	2	2
1	1	2	2	2	1	3	1	2	2
2	1	2	1	2	1	2	1	1	1
9	9	9	1	9	9	9	9	9	1
9	9	9	1	9	1	9	9	9	9
1	2	2	1	2	2	2	2	1	1
1	1	2	1	1	1	1	1	1	1
1	3	3	3	3	3	3	3	3	2
2	2	2	2	1	3	3	2	9	1
3	2	2	9	2	1	3	1	1	1
1	3	3	3	3	1	2	1	1	1
9	9	9	9	9	9	3	9	9	9
2	2	1	2	2	1	1	1	1	1
2	3	2	1	3	1	2	1	3	2
3	3	2	2	2	2	3	1	1	1
2	1	1	2	1	2	2	2	1	1
1	2	2	2	2	1	3	1	1	1
1	2	2	2	2	2	2	1	2	2
1	2	2	1	3	1	2	2	1	2
3	2	2	9	2	1	1	1	1	1
9	9	1	9	9	9	9	9	9	9
3	2	2	2	2	1	3	1	1	1
1	2	2	2	1	1	1	1	2	1
2	1	1	2	2	1	9	1	1	2
2	1	1	2	2	1	2	1	1	2
2	1	1	2	2	1	9	1	1	2
1	1	2	3	2	1	3	1	1	1
3	3	1	2	3	1	3	1	1	1

3	3	1	3	1	1	3	1	1	1
2	1	2	2	2	1	2	3	3	2
1	1	2	2	2	1	1	2	9	2
2	9	2	2	2	1	2	3	3	9
9	9	9	1	9	1	1	1	1	1
2	9	9	9	9	9	9	9	9	1
9	9	1	9	9	3	1	1	3	1
1	2	1	2	1	1	2	1	1	1
2	2	2	2	2	1	1	1	9	1
1	2	2	2	2	2	2	2	2	2
1	1	2	3	2	9	9	9	1	9
1	2	2	2	2	1	2	3	1	2
1	2	2	2	2	2	2	2	2	2
1	2	3	3	3	1	2	1	1	2
9	2	9	9	9	3	1	1	3	1
1	9	9	9	9	1	3	3	1	1
3	2	3	9	3	1	3	1	1	1
3	3	3	3	1	3	3	3	3	3
3	9	1	2	2	2	2	3	2	2
3	3	1	3	3	9	9	9	9	9
3	2	1	2	2	2	9	9	1	2
9	9	1	9	9	9	9	9	9	9
1	2	1	2	2	2	2	3	1	1
2	9	1	2	1	9	9	9	9	9
3	3	1	3	3	3	2	3	1	1
9	9	1	9	9	1	2	3	3	2
3	9	9	9	9	2	9	9	9	9
1	9	9	9	9	9	9	9	9	9
2	2	2	2	1	2	2	2	1	2
3	3	3	3	3	1	3	3	3	3
9	3	1	2	3	2	2	3	1	2
9	3	1	2	2	1	2	3	1	2
9	9	1	9	9	9	9	9	1	1
9	9	1	9	9	9	9	9	9	9
9	9	1	9	9	9	9	9	9	9
2	3	1	2	2	3	2	3	2	1
9	9	1	9	9	3	3	3	1	9
3	2	1	3	1	2	3	3	1	1
9	9	1	9	9	9	1	1	9	9
3	9	1	3	3	1	9	1	3	9
3	2	2	2	1	1	3	1	3	3
1	9	9	9	9	9	9	9	9	9
2	2	1	2	2	1	1	1	3	1
1	3	1	3	2	2	2	3	3	3
3	3	1	3	3	3	1	1	3	3
2	2	1	2	2	1	1	1	3	1
1	2	1	2	1	2	1	1	3	2

9	9	1	9	9	9	9	1	9	9
3	1	1	3	1	1	1	1	3	3
9	9	1	9	9	9	9	1	9	9
3	3	1	3	3	3	1	1	3	3
9	9	1	9	9	9	1	1	9	9
9	9	1	9	9	1	9	1	9	9
3	3	1	3	3	3	3	1	3	3
9	9	1	9	1	9	9	1	9	9
9	9	9	9	9	9	9	9	9	9
1	9	9	9	9	9	9	9	9	9
1	2	9	9	9	9	1	9	3	3
2	2	1	2	2	2	2	3	1	1
9	9	1	9	9	9	9	9	1	9
9	9	1	9	9	9	1	9	9	9
9	9	1	9	9	9	9	9	9	1
9	9	1	9	9	9	9	9	9	9
1	3	3	3	1	3	3	3	3	3
2	2	1	2	2	2	2	2	1	2
3	9	1	3	1	3	3	3	1	1
9	9	1	9	9	9	9	9	9	9
1	3	1	1	1	1	1	3	1	1
3	2	1	2	1	3	2	2	1	1
9	9	1	9	9	9	9	9	9	9
9	1	9	9	9	2	2	3	1	3
9	9	1	9	9	9	9	9	1	9
3	3	1	3	3	3	3	3	1	2
3	3	1	3	3	1	2	1	3	3
9	9	1	9	9	1	9	9	9	9
9	9	1	9	9	1	9	9	9	9
1	3	1	1	3	2	2	2	2	2
9	1	9	9	9	9	9	1	1	1
2	9	1	1	2	2	1	2	1	1
9	9	1	3	9	9	9	9	1	9
1	2	1	3	2	3	2	3	1	2
1	1	2	2	1	1	3	3	2	2
1	2	2	2	1	1	2	3	2	2
9	1	2	1	1	2	3	3	3	1
2	9	1	1	2	2	2	2	1	2
1	1	1	9	9	3	3	3	2	1
3	3	1	3	3	3	3	3	1	1
2	2	1	3	1	3	3	3	2	2
9	9	1	9	9	9	1	9	9	9
9	9	1	9	9	3	3	3	3	9
9	9	1	9	9	9	9	9	9	9
3	3	3	2	1	2	2	2	9	1
1	3	1	3	2	3	3	3	3	9
2	3	1	3	2	2	3	3	1	2

1	3	1	3	3	3	3	3	3	3
9	9	1	9	9	9	1	9	9	9
9	9	1	9	9	9	1	9	9	9
1	9	9	9	9	9	9	9	1	9
1	9	9	9	9	9	9	9	9	9
9	9	1	9	9	1	9	9	1	9
3	3	3	3	1	3	3	1	1	3
1	9	1	9	9	2	2	3	1	1
9	9	1	9	9	9	9	9	9	9
2	1	1	2	2	3	2	1	1	3
3	3	1	3	3	3	3	1	1	1
1	9	9	9	9	2	9	9	9	9
3	2	1	2	2	2	2	2	1	3
1	2	2	2	2	2	2	2	2	2
2	2	1	2	2	3	3	3	3	3
2	1	1	2	1	2	2	2	1	1
3	2	1	3	2	3	3	3	1	1
9	1	9	9	9	9	9	9	9	9
9	9	1	9	9	9	9	9	9	9
9	9	1	9	1	9	9	9	1	9
9	9	1	9	9	9	9	9	9	9
9	9	1	9	9	9	9	9	1	9
3	3	1	3	3	9	9	9	9	9
3	3	1	3	2	2	2	2	2	2
9	1	9	9	9	9	9	9	9	9
3	3	1	2	2	2	1	2	2	2
2	1	2	3	1	3	3	3	1	1
3	2	1	2	2	2	2	3	2	3
2	2	1	2	2	2	2	3	2	2
2	3	1	2	3	3	2	3	1	2
3	3	1	3	3	3	3	3	2	3
9	9	1	9	9	9	9	9	1	9
3	3	1	3	3	2	2	3	3	3
3	3	1	3	3	2	2	2	1	2
9	9	1	9	9	9	9	9	1	9
9	9	1	9	9	9	9	9	9	1
2	2	1	2	1	3	2	3	1	2
9	9	1	1	9	9	9	9	9	9
9	9	1	9	9	9	9	9	9	1
1	2	9	2	1	3	1	1	3	1
9	9	1	9	9	3	9	1	9	9
3	3	1	3	3	2	3	3	2	2
2	2	2	2	2	1	3	1	2	1
2	2	1	2	2	3	1	1	1	1
3	9	9	9	9	9	9	3	1	9
9	9	1	9	9	9	1	1	9	9
3	2	1	2	3	3	3	1	2	2

2	3	3	2	2	1	2	3	3	3
9	9	9	1	9	1	2	1	1	1
1	1	1	1	1	1	2	1	2	2
2	1	2	2	1	2	1	2	2	1
9	9	1	9	9	9	9	9	9	9
3	2	1	1	1	3	3	3	2	3
3	3	1	3	3	3	3	3	1	2
3	3	1	3	3	2	3	3	3	2
1	2	2	1	2	3	2	1	2	2
2	2	2	2	1	3	3	3	1	1
2	2	2	1	2	3	3	2	1	2
3	1	2	1	2	2	2	2	1	1
1	3	2	2	3	3	3	3	1	3
2	2	2	2	2	9	2	2	1	2
2	3	2	1	3	3	3	3	1	1
1	2	2	2	2	3	2	1	1	2
1	1	2	2	2	2	2	3	3	1
2	2	2	2	2	2	2	2	2	2
3	1	1	2	2	2	3	3	1	3
1	1	2	2	2	3	1	1	2	2
1	2	2	2	2	2	2	2	1	2
2	2	2	2	2	3	3	2	2	2
1	2	2	2	2	2	2	2	2	2
2	2	2	2	2	2	2	2	2	2
2	2	2	2	2	2	2	2	2	2
1	1	1	1	1	2	2	3	1	1
2	3	1	2	3	3	2	3	1	2
1	2	2	3	2	3	3	3	1	2
1	2	2	2	2	9	1	3	2	2
1	3	2	2	2	2	2	2	1	2
2	2	1	2	1	2	2	2	1	2
1	2	2	3	2	3	3	3	1	2
1	2	2	3	2	2	2	1	1	2
2	2	1	9	1	2	2	2	1	2
2	2	1	2	1	2	2	2	1	2
1	1	3	9	3	3	2	9	1	2
2	1	2	2	2	3	3	3	1	1
2	2	1	2	1	3	3	2	2	3
2	1	2	2	1	9	9	9	9	9
2	1	2	2	1	1	9	9	9	9
9	9	1	9	9	9	9	9	9	9
9	1	9	9	9	1	9	9	9	9
1	9	9	9	9	9	9	9	9	9
1	9	9	9	9	9	9	9	9	9
9	1	9	9	9	9	9	9	1	9
1	2	2	3	1	3	3	3	1	2
1	2	3	2	1	3	2	3	1	2

1	9	9	9	9	9	9	9	9	1
1	9	9	9	9	9	9	9	9	1
1	2	2	3	2	3	3	3	1	2
1	9	9	9	9	9	9	9	9	9
1	9	9	9	9	1	9	9	9	9
1	9	9	9	9	1	9	9	9	9
1	2	2	2	2	3	3	3	2	2
1	3	2	2	3	3	1	3	2	3
1	9	9	9	9	1	9	9	9	9
1	9	9	9	9	9	9	9	9	9
1	9	9	9	9	9	9	9	9	9
2	9	9	9	9	9	9	9	9	9
9	9	1	9	9	9	9	9	1	9
9	9	1	9	9	9	9	9	1	9
9	9	9	9	1	9	9	9	1	9
1	9	9	9	9	9	9	9	9	9
3	3	1	3	1	3	3	3	3	1
1	3	3	3	3	3	3	3	3	3
1	3	1	3	3	3	3	3	3	1
3	3	1	3	3	3	3	3	2	3
1	1	1	2	1	3	3	3	3	3
3	3	1	3	3	3	3	3	1	3
3	3	1	3	3	3	3	3	1	3
3	3	1	3	3	3	3	3	1	3
1	3	3	3	3	3	3	3	3	3
2	2	2	2	1	2	2	2	2	2
1	1	1	2	2	1	2	3	1	2
1	3	1	2	3	1	1	3	3	1
9	9	9	9	1	9	9	9	9	1
1	1	1	2	1	2	3	1	2	2
3	3	3	3	1	3	3	3	3	3
3	3	1	1	3	3	3	2	1	1
9	1	9	9	9	9	1	9	1	3
2	1	1	2	3	3	3	3	1	3
2	3	1	1	3	3	3	1	3	3
9	1	2	2	3	2	1	2	1	3
3	2	1	3	1	3	2	2	3	2
9	9	9	9	9	2	2	1	9	1
1	3	1	1	3	2	2	3	3	3
3	3	1	3	3	2	2	3	3	3
1	1	2	2	3	2	2	2	3	1
1	3	1	1	1	1	3	3	3	3
3	3	1	3	3	3	3	3	1	3
3	3	1	3	1	3	3	3	3	3
3	3	3	3	1	3	3	3	3	9
3	1	1	1	1	3	3	3	1	2
3	2	1	3	3	1	1	2	1	3

1	1	1	9	1	3	3	1	3	3
3	3	1	2	3	2	3	2	3	3
1	3	3	3	3	3	3	3	1	1
1	3	3	3	3	3	3	3	1	1
1	3	3	3	3	3	3	1	3	3
1	1	1	2	2	2	3	3	2	2
2	3	1	2	3	3	2	1	1	9
1	1	1	1	1	3	3	1	1	1
1	1	1	2	1	1	2	3	2	9
1	3	3	3	3	3	3	3	1	3
1	3	3	3	3	3	3	3	1	1
1	3	3	3	3	3	3	3	1	3
1	1	1	2	2	2	3	3	2	2
1	1	1	9	1	3	2	2	2	2
3	2	1	2	2	2	2	2	2	2
1	1	1	1	1	1	1	9	9	9
3	1	2	2	2	1	2	2	2	2
1	9	2	2	2	1	1	1	1	9
1	2	9	9	9	9	1	1	2	2
1	2	2	3	2	1	3	1	3	2
3	1	1	1	1	1	1	1	3	1
2	2	2	2	2	2	2	2	2	2
1	1	1	9	1	2	2	9	1	9
1	2	2	2	2	2	2	3	3	2
1	3	1	3	1	1	3	2	1	1
1	1	1	2	2	2	3	3	2	2
1	2	2	2	2	3	3	1	1	2
1	1	1	1	1	3	3	3	3	1
2	3	1	2	3	2	2	2	2	1
2	3	1	2	3	2	2	2	2	1
3	2	1	2	3	2	2	2	2	1
2	2	1	1	2	1	2	2	1	2
1	2	2	2	2	3	2	3	2	2
2	2	2	2	2	3	3	1	1	2
1	2	2	2	2	2	2	2	1	2
2	2	2	1	2	2	2	2	2	1
2	3	2	2	2	2	3	2	2	3
2	2	1	2	2	2	3	3	3	2
9	9	9	9	9	2	2	1	1	1
3	1	2	3	3	3	3	3	1	1
2	3	1	2	3	2	2	2	2	1
1	3	3	3	3	3	3	1	3	3
3	2	1	2	9	3	3	2	2	3
3	2	2	2	2	3	3	2	2	3
1	9	9	9	9	3	3	3	1	3
2	2	1	1	2	1	3	3	1	3
1	2	2	2	2	1	2	3	1	2

1	1	3	3	3	3	9	1	1	2
1	1	3	3	3	3	1	1	1	2
3	1	2	2	1	1	1	2	1	1
2	2	2	2	2	3	3	3	2	1
2	2	2	2	2	3	3	3	3	2
2	1	2	2	2	3	1	3	1	3
2	1	2	9	1	1	1	2	2	2
2	2	2	2	2	2	2	2	2	2
3	1	2	2	1	1	1	2	1	1
2	2	2	2	1	2	2	2	1	3
1	2	2	1	3	1	2	1	1	1
1	1	2	2	3	2	2	1	1	2
1	9	9	9	9	3	3	3	9	2
2	2	2	2	2	3	3	3	3	2
1	2	2	2	2	1	2	2	2	2
2	2	1	1	1	3	3	3	1	3
1	1	2	3	3	1	3	3	3	1
2	1	2	1	2	3	1	1	1	3
2	9	9	9	9	9	9	9	9	9
1	2	2	2	2	2	2	3	2	2
1	3	3	3	3	1	3	3	1	3
2	2	2	2	2	2	2	2	2	2
2	2	1	2	1	3	3	2	1	3
1	2	2	2	2	2	2	1	1	2
1	1	2	2	2	2	2	2	1	2
1	2	9	1	3	2	2	2	1	1
2	1	1	3	1	3	3	3	1	1
1	1	1	2	2	1	2	3	2	1
3	3	1	3	3	3	3	3	1	2
3	3	1	3	3	3	2	3	2	3
3	3	1	3	3	3	3	3	3	3
3	3	1	3	3	3	3	3	3	3
3	3	1	3	3	3	3	3	1	3
1	2	1	3	2	3	3	3	1	2
2	2	1	1	1	1	1	1	1	1
9	2	1	9	9	9	9	2	9	9
1	2	2	3	2	3	3	3	1	2
1	1	3	2	3	3	3	3	1	2
2	1	2	2	1	2	2	3	1	1
2	2	2	2	2	2	2	3	1	1
9	9	1	9	9	9	9	9	1	9
1	2	2	2	2	3	3	3	1	3
2	2	2	2	2	2	2	2	2	2
9	1	9	9	9	9	9	2	9	9
2	3	1	1	1	2	1	1	2	3
2	1	2	2	1	3	3	3	3	1
3	9	9	9	9	9	9	9	9	9

1	2	2	2	2	1	3	3	1	2
2	1	1	2	1	1	2	2	1	1
2	1	2	3	3	2	3	2	9	2
3	2	3	2	2	3	3	3	3	1
2	2	2	1	3	3	3	3	2	2
1	3	2	2	2	3	2	3	2	2
1	2	3	3	3	1	2	3	1	2
1	2	2	2	2	2	2	2	1	2
2	2	2	2	2	2	2	2	2	2
1	2	1	2	2	2	2	2	2	2
1	2	2	1	2	1	2	2	1	2
2	2	3	2	2	2	3	3	3	2
2	2	2	2	2	3	3	3	1	1
3	9	9	9	9	9	9	9	9	9
2	2	2	2	2	2	2	2	1	2
2	2	3	9	2	3	3	3	2	2
2	1	1	2	1	2	2	2	1	2
9	9	1	9	9	9	9	9	9	9
2	1	1	2	1	1	1	3	3	3
1	2	2	2	2	2	2	2	2	2
3	9	9	3	9	3	3	2	2	3
3	2	1	2	1	3	3	2	2	3
1	2	1	1	1	1	2	2	2	2
3	3	1	1	1	2	1	1	9	1
9	1	9	9	9	9	9	9	9	9
1	1	1	3	1	1	1	1	1	1
3	1	1	3	1	1	1	1	1	1
2	1	2	2	2	1	1	2	2	1
2	1	2	2	2	1	2	2	2	2
2	3	2	2	2	1	2	2	2	2
1	2	3	3	3	1	1	2	3	1
2	1	2	2	2	1	1	1	9	1
1	1	2	2	2	1	2	1	1	1
1	1	1	2	2	1	2	1	1	1
1	1	1	1	2	1	2	2	1	1
9	9	9	9	1	3	9	9	9	9
1	1	1	2	2	1	2	1	1	1
2	3	3	2	1	2	2	1	1	1
1	1	1	1	1	1	2	1	2	1
1	2	2	2	2	3	3	1	1	2
3	1	1	2	1	1	2	3	1	2
1	1	1	2	1	1	2	1	2	2
1	1	1	2	2	1	1	1	2	2
1	2	2	2	2	1	1	1	1	2
3	3	1	3	3	1	1	1	3	1
1	1	2	2	2	1	3	1	2	2
1	2	2	2	2	2	2	1	1	2

3	3	1	1	1	1	1	1	1	1
1	2	2	2	2	2	2	1	2	1
1	1	3	2	3	3	3	3	1	1
1	1	2	1	1	1	1	1	3	3
1	1	1	2	2	1	2	1	1	3
1	3	1	2	1	1	1	1	1	1
1	1	2	3	3	1	3	1	3	3
1	1	2	3	3	1	3	1	3	3
3	1	1	2	2	1	3	1	3	1
3	1	1	2	1	1	3	1	1	1
1	1	2	3	3	1	3	1	3	3
1	2	2	2	2	1	1	1	1	2
1	1	3	1	3	3	1	3	1	3
1	1	1	2	2	3	2	1	1	2
1	3	3	3	3	3	3	3	3	3
1	3	1	3	3	3	3	3	3	1
1	1	3	3	3	3	3	3	3	3
1	1	3	3	3	3	3	3	3	3
1	1	1	1	2	2	2	2	1	1
2	3	1	2	3	3	2	3	2	2
3	2	1	3	3	1	2	3	1	1
1	1	2	2	2	2	2	2	1	1
1	2	2	2	2	2	2	3	1	2
1	1	2	2	2	2	1	1	2	1
9	9	1	9	9	3	3	3	3	3
3	3	3	3	1	3	3	3	3	3
2	1	2	2	1	3	2	1	3	1
1	2	2	2	2	9	1	9	9	9
1	9	9	9	9	9	9	9	9	9
1	9	9	9	9	9	9	9	9	9
1	9	9	9	9	9	9	9	9	1
9	9	9	9	1	9	9	9	1	9
1	2	2	2	2	3	3	3	1	2
9	9	1	9	9	9	9	9	9	9
2	9	9	9	9	2	9	9	9	9
9	9	1	9	9	9	9	9	9	9
1	9	9	9	9	9	9	9	9	9
1	9	9	9	9	9	9	9	9	9
2	9	9	9	9	3	9	9	9	9
1	9	9	9	9	3	2	2	3	2
1	2	2	3	2	3	3	3	1	2
3	3	1	3	3	2	3	3	1	2
1	2	2	2	2	1	1	1	1	2
3	3	1	1	1	1	1	1	1	1
1	1	1	2	2	1	2	1	1	1
1	1	1	2	2	1	2	1	1	1
1	2	2	2	2	1	1	1	1	2

1	3	3	3	3	2	2	3	1	3
2	2	1	1	2	1	2	2	1	2
1	2	2	2	2	2	2	3	1	2
3	3	3	2	3	3	3	2	1	3
1	3	3	3	1	1	3	3	1	3
2	1	2	1	1	3	2	2	1	1
1	2	2	1	3	2	2	2	1	2
2	2	2	2	2	2	2	3	2	2
1	2	2	3	2	3	3	3	3	3
3	3	3	3	1	3	1	2	2	1
1	1	1	1	2	1	2	2	2	3
2	1	1	1	1	2	2	2	1	1
2	2	2	2	2	2	2	3	2	2
2	2	2	2	2	2	1	2	2	2
2	2	2	3	2	2	2	3	1	1
2	3	1	2	3	2	2	2	2	2
1	2	2	2	2	3	3	2	2	1
2	2	2	1	2	3	2	3	2	2
1	2	1	2	2	2	1	2	1	1
3	3	3	1	3	3	3	3	3	3
2	2	2	2	2	2	2	2	2	2
2	2	2	2	2	2	2	2	2	2
1	2	2	2	2	3	3	2	1	2
1	2	1	2	2	3	1	3	9	2
2	2	1	3	1	1	1	1	1	1
1	1	1	1	1	1	1	1	1	1
1	1	2	2	2	1	1	1	1	1
2	2	2	2	2	1	1	1	1	2
2	2	2	3	2	2	2	3	1	1
1	3	3	3	3	2	1	2	1	3
1	1	2	2	2	1	1	2	1	2
2	2	1	2	1	2	3	2	1	2
1	2	2	2	2	2	1	2	1	1
1	2	1	1	1	2	2	3	1	1
2	2	2	2	2	2	2	2	1	2
2	2	2	2	2	9	9	9	1	9
9	9	9	9	1	9	9	9	9	9
1	2	1	2	1	1	2	2	1	1
3	3	1	3	3	1	1	1	1	1
1	1	2	2	3	3	3	3	1	3
1	3	3	3	3	3	3	3	3	3
9	1	2	2	2	9	2	2	2	2
1	1	2	3	2	2	2	2	2	2
3	2	1	2	2	3	3	2	2	2
1	2	3	2	3	3	3	3	2	1
1	2	1	2	1	2	2	2	2	2
1	2	2	2	2	2	2	2	1	2

1	2	2	2	2	2	2	2	1	2

BirthContr	FamilyBrea	EarlyInterc	UTI	BSEnoneec	BSEnecessa	BSEshy	BSEpainful	BSEfearofC	BSEtimewa
3	1	1	1	1	1	1	2	3	2
1	3	1	1	1	1	1	1	3	2
2	2	2	2	1	1	3	3	3	3
1	9	1	1	9	1	1	1	3	2
9	9	9	9	1	1	1	9	9	9
1	3	1	1	1	1	1	1	3	2
2	2	1	2	3	1	3	3	3	3
3	1	3	9	1	2	3	1	3	3
9	9	9	9	1	9	3	2	2	3
9	9	9	9	3	9	9	9	9	9
9	9	9	9	2	2	2	2	2	9
9	9	1	9	2	2	1	1	1	1
2	2	2	2	1	1	3	3	3	3
3	3	3	2	2	1	3	2	2	2
2	3	3	2	1	9	3	3	3	3
2	1	3	2	2	1	3	3	3	3
2	2	3	2	3	2	9	1	1	3
2	1	3	2	2	1	3	3	3	3
2	1	3	2	2	1	3	1	3	3
3	3	2	2	3	1	3	3	2	1
3	2	3	3	1	1	3	3	3	3
2	3	3	3	1	3	3	3	3	3
9	3	9	9	2	1	3	3	2	3
3	2	3	3	1	1	3	3	3	3
1	2	2	2	1	1	3	3	2	3
2	2	2	2	1	1	2	1	2	3
2	2	2	2	1	1	3	1	3	3
2	2	1	2	3	1	3	1	3	3
2	3	2	3	3	1	3	3	1	3
2	2	2	2	1	2	1	2	2	2
2	2	2	2	1	2	1	2	2	2
3	3	2	2	3	1	3	3	3	3
2	1	2	1	3	1	3	3	3	3
2	3	2	2	3	1	3	3	3	3
2	3	2	2	3	1	3	3	3	3
2	3	2	2	3	9	3	3	3	3
2	3	2	2	3	1	3	3	3	3
1	1	1	3	3	1	3	3	3	3
9	9	9	9	9	9	9	9	9	9
9	9	9	9	3	1	3	3	1	3
2	1	2	2	2	1	1	3	3	3
9	9	9	9	1	2	3	3	2	3
3	3	3	3	3	1	3	3	9	3
3	3	3	2	9	1	3	9	9	3
9	3	1	3	3	1	3	3	2	3
2	3	1	3	3	1	3	3	3	3

1	3	1	1	3	1	3	2	3	3
1	9	9	9	3	1	1	3	3	3
1	3	3	2	9	1	3	3	3	3
1	3	1	1	2	1	1	2	1	3
9	9	9	9	3	1	3	1	3	3
1	3	3	1	3	1	3	3	3	3
1	3	3	3	3	1	3	3	3	3
1	9	9	9	1	1	3	3	9	3
1	9	9	9	9	1	9	9	9	9
2	3	1	2	2	1	1	2	3	3
9	9	9	9	1	9	9	9	9	9
1	9	9	9	2	9	3	3	3	3
1	3	2	1	2	1	3	3	1	3
1	9	9	9	2	1	3	3	2	3
1	2	1	2	1	1	3	3	3	3
1	3	3	3	2	1	3	3	3	3
2	3	2	2	3	1	3	3	9	3
9	9	9	9	3	1	3	3	3	3
1	3	3	3	3	1	3	3	2	3
2	1	3	3	1	1	1	3	1	1
1	3	3	3	3	3	3	1	3	3
1	3	3	3	3	3	3	1	3	3
1	3	3	3	3	3	3	1	3	3
9	9	9	3	3	1	3	3	3	3
2	3	1	3	9	1	9	1	9	9
9	9	1	2	9	1	1	9	1	9
9	9	9	9	3	1	3	3	3	3
1	3	1	1	1	1	3	1	2	3
1	1	1	3	3	1	3	3	3	3
1	2	1	1	3	1	3	3	1	2
3	1	1	2	9	1	1	1	1	1
2	3	3	1	1	1	3	3	3	3
2	2	1	9	9	1	1	2	2	2
9	1	1	1	3	2	3	3	3	3
2	1	2	1	3	1	1	2	3	3
1	9	9	9	2	1	3	3	3	3
1	1	3	3	3	3	3	1	1	1
2	2	1	2	3	1	3	3	3	3
1	9	9	9	3	1	3	3	3	3
1	3	1	1	3	1	3	3	3	3
1	1	1	2	9	1	3	2	3	3
3	3	2	1	1	1	3	3	3	3
3	3	3	1	1	1	3	3	3	3
1	1	2	1	3	1	3	1	3	3
2	3	2	1	3	1	3	3	2	3
9	9	9	9	1	1	3	3	3	3
9	9	9	9	1	1	3	3	1	1

1	3	3	2	3	1	3	3	3	3
1	2	2	2	3	1	3	1	3	3
9	9	9	9	3	1	3	1	1	3
1	3	2	2	3	1	3	1	3	3
9	2	2	2	3	1	3	3	3	3
2	2	2	2	2	1	9	9	9	9
1	3	2	2	3	1	3	3	3	3
1	3	1	2	3	1	3	3	3	1
2	3	2	2	9	1	9	9	9	9
2	3	1	9	2	1	3	3	3	3
1	2	1	1	1	1	1	1	3	2
2	2	3	2	3	1	3	3	2	3
1	2	3	3	9	9	9	9	9	9
9	9	9	9	9	9	9	9	9	9
2	3	1	2	3	1	3	1	2	3
9	9	9	9	1	1	3	3	2	3
2	3	2	2	3	1	3	2	3	3
2	2	2	2	3	1	3	2	3	3
2	2	2	2	3	3	3	3	3	3
2	2	2	2	3	1	3	2	2	2
2	3	1	2	3	1	3	3	3	3
1	9	9	9	1	1	3	3	3	3
2	2	2	2	3	2	2	2	2	2
3	1	9	1	1	1	3	3	3	3
2	2	2	3	1	3	1	9	9	1
2	2	2	2	3	1	2	2	2	2
1	9	9	9	3	1	3	3	3	3
1	9	9	9	3	9	9	9	9	9
3	2	3	2	3	1	3	3	3	3
9	9	9	9	1	1	3	2	2	2
2	2	2	2	2	1	1	2	2	3
2	2	2	2	1	1	2	1	2	2
2	3	2	2	2	2	3	1	3	1
3	2	3	3	3	1	3	3	1	3
9	9	9	9	1	1	3	2	2	2
3	3	3	3	3	1	1	3	3	1
2	1	2	3	3	1	3	3	1	3
3	2	2	2	3	2	3	2	3	3
9	9	9	9	1	9	9	9	9	9
1	1	1	1	1	1	3	3	3	3
3	2	3	2	2	1	3	3	3	3
3	2	3	2	2	1	3	3	3	3
2	2	2	2	1	1	2	1	2	2
2	2	2	2	3	3	3	2	3	1
2	2	2	2	1	1	3	3	1	3
2	3	2	2	3	1	3	1	1	3
2	1	2	2	1	1	3	3	1	3

9	9	9	1	2	2	1	9	9	9
2	3	2	2	3	1	3	3	3	3
2	3	3	2	3	1	3	1	3	3
3	2	3	2	2	1	3	3	3	3
3	2	3	2	2	2	2	1	3	3
3	2	1	2	2	1	9	1	3	3
3	2	3	1	3	1	2	1	1	3
2	2	2	2	3	1	3	3	3	3
2	2	2	2	1	1	9	2	2	3
2	2	2	2	2	1	2	2	2	2
3	3	2	2	3	1	3	3	2	2
3	3	2	9	3	1	3	3	2	2
9	9	9	9	9	1	9	1	9	9
2	2	2	2	3	1	3	3	3	3
2	3	3	2	3	1	3	1	3	3
2	3	3	2	1	2	3	1	1	1
2	2	2	2	1	2	3	1	3	1
3	2	3	2	1	1	3	3	1	1
9	1	9	9	1	9	9	9	9	9
1	2	1	1	2	1	3	3	1	3
9	9	1	9	3	1	3	3	1	3
2	3	1	1	3	1	3	3	1	3
2	2	1	1	1	1	3	3	3	3
3	3	1	3	2	1	3	3	1	3
3	3	1	3	2	1	3	3	3	3
9	9	1	9	3	1	1	3	1	3
9	1	1	1	2	1	3	3	1	3
9	9	1	9	2	1	3	9	3	3
3	3	1	3	3	1	2	3	1	3
3	3	1	1	3	1	3	3	1	3
1	2	3	1	3	1	3	1	1	3
2	3	1	1	1	2	3	2	2	3
9	1	1	9	2	1	3	3	1	3
1	1	1	9	3	1	9	3	3	1
1	1	1	1	3	1	9	3	3	1
1	1	1	2	3	1	3	3	1	3
1	2	1	2	2	3	3	3	1	3
1	2	1	2	3	1	3	9	1	3
1	1	1	2	3	1	3	3	3	3
1	3	2	3	3	1	3	3	1	3
1	3	1	3	3	1	3	3	1	3
3	9	1	3	2	1	3	3	1	3
9	9	1	9	3	1	1	3	1	3
1	3	1	3	2	1	3	3	1	3
3	3	1	3	3	1	3	3	1	3
1	3	1	3	2	1	3	3	1	3
1	3	1	3	2	1	3	3	1	3

1	3	1	3	9	1	3	3	1	3
1	1	9	9	3	1	3	3	1	3
9	9	1	9	3	1	3	3	1	3
9	9	1	9	3	1	3	3	1	3
3	3	1	3	3	1	3	3	1	3
1	1	1	1	3	1	3	3	1	3
1	3	1	9	3	1	3	3	1	3
2	2	3	3	3	1	3	3	3	3
1	3	1	1	3	1	3	3	3	3
9	9	9	9	3	1	3	3	3	3
2	3	3	3	3	1	1	3	3	3
9	9	9	9	3	1	3	3	2	3
1	3	1	1	3	1	3	3	3	3
1	3	1	1	3	1	1	3	1	3
2	1	1	1	1	1	3	3	3	3
2	1	2	2	1	1	3	3	3	3
2	2	2	9	2	1	1	1	1	2
1	1	1	1	2	2	1	1	1	1
1	3	2	2	2	1	3	3	3	3
2	2	2	2	1	1	3	3	3	3
1	1	2	1	2	1	3	2	2	1
2	3	9	2	2	1	3	3	3	3
2	3	1	2	2	1	1	2	2	2
9	9	1	9	2	1	3	2	2	1
2	2	2	2	2	1	3	3	3	3
2	2	3	9	2	1	2	2	2	2
2	2	2	2	1	1	3	3	3	3
2	2	3	9	2	1	2	2	2	2
3	3	3	3	3	9	1	1	2	2
1	2	3	3	2	1	3	3	3	3
2	2	2	2	3	2	2	2	2	2
2	2	2	2	3	2	2	2	2	2
1	2	3	3	2	1	3	3	3	3
2	1	1	2	1	1	2	3	3	3
2	2	2	2	1	1	2	1	2	2
1	1	2	2	1	1	2	2	2	2
1	2	2	1	2	1	1	1	1	2
1	2	3	2	1	1	2	3	2	3
1	2	3	3	1	1	3	3	3	3
3	3	1	2	2	1	3	3	3	3
2	2	2	2	1	2	2	2	3	2
2	2	2	2	1	1	3	3	3	3
9	9	9	9	3	1	3	3	9	9
9	9	9	9	3	1	3	3	9	9
2	1	1	1	1	3	3	3	1	1
1	2	1	2	1	2	1	1	2	3
1	3	2	2	1	1	1	1	1	2

2	2	3	2	2	1	1	1	2	3
2	3	3	2	2	1	1	3	3	3
2	2	2	2	1	1	3	3	3	3
1	2	2	2	2	2	2	1	2	2
2	2	2	1	1	1	3	3	1	3
2	3	2	2	3	1	3	3	3	3
3	3	3	3	3	1	3	3	3	3
1	3	2	2	3	3	2	1	1	2
2	3	3	1	3	1	3	3	3	3
3	3	3	3	9	1	1	1	3	3
2	2	2	2	1	3	1	2	2	2
1	3	3	2	1	1	3	3	3	3
2	3	3	1	1	1	3	3	3	3
2	2	2	2	2	3	2	2	2	3
2	3	1	2	2	1	3	1	2	3
2	3	3	3	3	1	3	3	3	3
2	3	3	2	9	2	3	3	2	1
3	2	3	2	1	2	2	9	2	2
2	3	2	2	3	3	3	3	3	2
3	3	3	2	1	2	1	1	3	9
1	2	2	1	2	2	1	1	1	1
2	2	2	2	2	1	2	1	2	2
2	3	3	2	9	1	3	3	2	3
2	2	2	2	2	1	3	2	1	3
9	9	9	9	9	1	9	9	9	9
2	2	2	2	2	2	2	2	2	2
2	2	2	2	2	2	2	2	2	2
3	2	3	1	1	3	3	3	3	3
2	2	1	2	1	1	3	2	2	1
2	1	2	2	2	1	3	1	3	2
9	9	9	9	9	1	9	9	9	9
2	2	3	3	3	3	3	3	3	1
9	9	9	2	3	3	3	3	1	1
2	3	2	2	2	1	1	1	9	3
9	9	9	9	1	9	9	9	9	9
2	2	2	2	1	1	2	1	2	2
3	2	3	2	2	1	3	2	3	3
9	1	1	2	1	1	3	3	3	3
2	3	3	1	3	1	1	1	1	1
2	2	2	3	3	2	3	1	3	3
2	2	2	2	2	1	3	3	3	3
1	2	2	2	1	1	3	3	3	2
2	3	3	2	2	2	1	3	3	3
2	2	2	2	1	1	3	2	3	2
1	2	2	1	2	1	2	3	3	2
3	2	3	1	1	3	2	3	3	3
2	3	2	1	1	1	3	2	3	3

2	2	1	1	2	1	1	2	2	2
2	2	1	1	2	1	3	3	3	3
2	2	2	1	3	1	3	3	3	3
1	2	1	2	3	3	3	2	2	3
2	1	2	2	3	2	3	1	3	1
2	2	1	1	1	1	3	2	3	1
1	2	2	1	2	1	3	3	3	3
9	9	9	9	3	1	3	3	3	3
2	2	2	1	3	1	3	1	2	3
2	3	3	2	3	1	3	2	2	2
2	2	2	2	2	2	2	2	2	2
3	3	2	2	3	1	3	3	3	3
1	1	1	1	1	1	1	1	1	1
2	3	3	2	1	1	3	3	3	3
1	1	3	1	1	9	3	1	3	1
3	3	3	2	3	3	3	3	3	3
2	2	3	1	2	1	3	1	3	3
2	3	1	1	2	1	1	1	1	2
1	1	1	1	1	2	1	2	3	1
1	1	1	2	1	1	3	9	9	9
3	3	2	3	3	1	3	3	3	2
1	2	2	2	3	1	3	3	3	3
3	3	1	2	3	1	3	2	3	3
2	2	2	2	1	1	3	1	1	2
2	2	2	2	1	1	3	2	3	2
9	9	9	9	3	1	3	2	3	3
1	2	2	1	1	1	3	3	3	9
3	3	3	1	3	1	3	2	2	3
1	3	2	2	1	1	3	3	3	3
2	3	3	2	9	1	3	3	2	3
2	2	1	2	3	1	3	1	1	3
9	9	9	9	1	3	3	3	3	3
9	9	9	1	3	1	3	3	2	3
3	3	3	2	3	3	3	1	3	3
3	3	3	1	3	1	3	3	3	3
1	3	3	3	3	1	3	3	1	3
3	2	2	2	1	1	1	3	3	3
1	3	3	1	1	1	3	3	3	3
2	3	2	2	3	1	3	3	3	1
9	9	9	9	1	3	9	9	3	3
3	1	2	3	1	1	1	3	1	3
3	2	2	2	3	1	3	3	1	3
2	2	2	1	3	1	3	3	2	2
3	3	3	2	1	3	2	3	3	3
3	3	1	2	1	1	3	3	3	3
3	1	2	2	1	1	3	3	3	3
2	1	2	3	3	1	3	3	3	3

9	9	9	9	3	1	3	3	2	3
9	9	9	9	3	1	3	3	2	3
9	9	9	9	9	1	9	9	9	9
3	3	3	2	3	1	3	3	3	3
9	9	9	9	9	9	9	9	9	9
1	2	2	2	1	1	3	3	3	2
1	1	2	1	3	1	3	2	1	3
3	3	3	3	3	9	3	3	3	3
1	3	1	2	2	1	3	3	1	3
2	1	2	2	1	1	1	2	3	3
3	3	2	2	3	2	2	1	1	3
2	3	1	1	3	1	2	3	3	3
2	3	2	1	3	1	3	2	3	3
2	3	2	2	3	2	2	1	1	3
3	3	2	2	3	2	2	1	1	3
3	3	3	3	2	1	3	1	1	3
3	1	3	2	3	2	2	1	1	3
3	3	1	1	3	1	2	3	3	3
2	3	1	1	3	1	3	3	3	3
3	2	1	1	1	3	3	3	2	3
3	3	2	2	1	3	3	1	3	3
2	2	1	2	3	1	3	3	3	3
2	2	1	2	3	1	3	3	3	3
3	1	1	2	2	1	3	3	3	3
2	1	2	2	1	2	3	3	3	1
2	3	3	2	1	1	2	1	2	2
2	3	3	2	3	1	3	2	2	3
2	3	2	2	1	3	1	2	2	2
2	3	2	2	2	2	3	2	2	3
2	3	3	2	2	1	3	2	1	1
3	3	3	2	3	2	3	1	3	2
1	3	2	2	3	2	3	1	3	2
2	1	2	2	1	1	1	3	3	3
2	2	2	2	2	1	2	2	1	2
2	2	2	2	2	1	2	2	1	2
2	2	3	1	1	3	3	2	3	2
2	3	3	2	3	1	3	3	3	3
2	3	1	1	3	1	3	2	2	3
2	3	1	1	3	1	2	2	2	2
2	3	1	2	3	1	2	2	1	3
2	2	2	2	3	2	2	3	3	3
2	1	1	2	1	2	3	2	2	3
2	2	1	1	3	1	3	3	3	3
2	2	1	2	1	2	3	2	2	3
2	2	1	3	1	3	1	2	2	3
1	2	1	1	2	1	3	3	2	3
2	2	2	1	2	1	3	2	3	2

3	1	2	1	3	3	3	2	2	3
1	2	1	3	1	1	3	1	3	1
9	9	9	9	2	1	1	2	3	3
2	2	2	2	1	1	3	9	1	1
2	1	3	2	3	1	3	3	3	3
2	3	2	2	3	1	3	3	3	3
2	2	2	2	1	1	3	1	2	2
3	3	2	2	3	1	3	3	3	3
3	3	2	2	3	1	3	3	3	3
2	1	2	1	3	1	1	1	1	1
2	1	2	2	3	1	2	2	1	3
2	1	3	1	3	1	1	3	1	3
2	1	2	2	2	1	3	3	3	3
2	3	1	2	1	2	3	3	3	3
2	3	1	2	1	2	3	3	3	3
2	1	1	2	1	1	3	3	3	3
3	1	3	3	1	3	1	1	1	3
2	3	3	2	2	1	1	2	3	3
2	3	3	2	2	1	3	2	3	3
9	1	9	9	2	3	2	2	3	3
1	3	3	3	3	1	3	1	3	3
2	2	2	2	1	1	2	2	1	1
2	2	3	2	2	1	2	3	1	3
1	2	2	1	3	1	3	2	1	2
1	1	1	1	3	1	3	1	2	3
1	2	1	1	2	1	3	2	2	3
2	3	3	2	1	1	3	3	3	3
2	1	2	2	3	1	3	3	3	3
1	3	2	3	1	1	1	2	3	2
9	9	9	9	1	1	3	1	1	3
2	2	2	2	1	2	3	3	3	2
1	3	3	3	1	1	3	3	1	3
2	2	2	2	1	2	3	3	3	2
2	3	3	2	3	1	3	3	3	3
1	3	1	1	3	1	3	3	3	3
2	2	3	1	1	1	1	1	2	1
2	3	3	3	2	2	2	3	3	3
3	1	1	3	1	2	1	3	3	3
2	2	2	2	2	2	2	3	3	3
2	1	1	1	3	1	1	1	1	3
2	2	3	2	1	2	1	1	2	1
1	3	1	1	1	1	1	1	3	2
2	2	3	1	1	1	3	3	2	3
3	2	2	2	3	3	3	1	3	1
3	3	3	3	9	9	9	9	9	9
2	2	1	1	3	1	3	3	1	3
3	3	3	3	3	1	3	3	1	3

2	2	1	1	3	1	3	3	1	3
3	1	1	3	2	1	3	1	1	3
3	3	1	3	3	1	3	3	1	3
9	2	1	1	2	1	3	3	1	3
9	9	9	9	3	1	3	3	1	3
1	3	1	2	1	1	3	3	1	3
2	2	2	2	9	1	3	3	1	3
1	1	1	1	3	1	3	3	3	3
2	2	2	2	3	1	3	3	1	3
3	3	1	3	2	1	3	3	1	3
1	3	1	1	2	1	3	3	3	3
2	2	1	1	3	1	9	9	1	3
3	3	1	3	3	1	3	3	1	3
3	3	1	3	2	1	3	3	1	3
3	3	1	3	3	1	3	3	1	3
3	3	1	3	3	1	1	3	1	3
2	2	2	2	3	2	1	3	1	3
2	2	2	2	3	1	3	3	1	3
3	3	1	3	3	1	3	3	1	3
3	3	1	3	3	1	3	3	1	3
3	3	1	1	2	1	3	1	1	3
3	1	1	1	2	1	1	1	1	3
3	3	1	1	3	1	3	3	1	3
1	3	1	1	2	1	1	1	2	3
1	3	1	3	3	1	3	3	1	3
1	3	1	9	3	1	3	3	1	3
3	3	1	3	2	1	1	3	1	3
3	3	1	1	3	1	3	3	1	3
3	3	3	3	3	1	3	3	9	3
1	3	1	2	2	1	3	3	1	3
3	9	3	3	2	1	3	3	1	3
3	2	1	3	2	1	3	3	1	3
3	1	1	3	2	1	3	3	1	3
9	1	1	3	3	1	3	3	1	3
9	9	1	9	3	1	3	3	1	3
9	9	9	9	3	1	3	3	1	3
2	2	1	2	1	1	3	3	3	3
1	3	1	1	3	1	3	3	1	3
3	3	1	3	3	1	3	3	1	3
1	1	1	1	3	1	3	3	1	3
1	9	1	9	2	1	3	3	1	3
2	2	2	1	3	1	3	3	3	3
1	9	9	9	2	1	3	3	2	2
2	2	2	2	3	1	3	2	2	3
9	9	9	9	3	1	3	3	3	3
2	2	2	2	2	1	3	3	2	3
1	3	9	9	3	1	3	9	2	3

2	2	2	2	3	3	3	2	2	2
2	3	1	1	3	1	3	3	3	2
2	3	1	1	3	1	3	3	3	2
3	9	3	1	3	1	3	3	3	2
2	2	3	1	3	1	3	3	3	3
9	1	9	9	1	2	2	3	3	3
3	3	2	2	1	1	3	1	2	3
2	1	1	2	2	3	2	1	2	2
2	1	1	2	3	1	3	3	3	3
2	2	2	2	2	1	1	1	1	1
1	2	2	1	1	1	1	2	3	2
9	1	9	9	9	9	9	9	9	9
1	9	9	9	9	1	9	9	9	9
2	3	2	2	2	1	3	1	2	3
9	9	9	9	9	9	9	9	9	9
9	9	9	9	9	1	9	9	9	9
9	9	9	9	1	9	9	9	9	9
9	9	9	9	9	9	9	9	9	9
9	9	9	9	9	9	9	9	9	9
9	9	9	9	3	1	3	2	3	3
9	9	9	1	1	9	1	9	1	3
3	3	3	3	1	9	1	3	3	3
3	3	3	2	3	1	2	3	3	3
9	9	1	9	9	1	9	9	9	9
9	9	9	9	9	9	9	9	9	9
9	9	1	1	3	1	3	3	3	3
9	9	9	9	9	9	9	9	9	9
3	2	1	1	3	2	3	1	2	2
1	2	1	1	3	1	3	1	9	9
9	1	9	9	9	9	9	9	9	9
9	9	9	9	9	9	9	9	9	9
2	2	1	2	2	1	3	3	1	3
2	2	2	2	2	1	1	2	2	2
3	1	3	3	1	1	3	3	3	3
2	1	2	2	3	2	3	2	2	1
2	2	3	2	3	1	3	3	3	3
3	3	2	1	3	1	3	2	2	2
3	3	1	3	3	1	3	3	3	3
2	3	3	3	2	2	2	2	2	2
3	3	3	2	2	1	3	2	1	3
1	3	1	2	3	1	3	2	3	3
1	1	1	1	3	1	3	3	3	3
2	1	2	1	3	1	3	3	1	3
1	1	1	1	3	1	2	3	3	3
2	2	1	1	3	1	3	3	2	3
2	3	1	2	2	1	3	3	3	3
1	3	2	2	3	1	3	3	2	3

2	3	1	2	1	1	3	2	2	2
9	9	9	9	3	1	3	3	3	3
2	1	3	2	3	1	3	3	3	3
2	3	2	2	3	1	1	1	2	3
1	3	1	1	3	3	3	3	2	3
2	2	2	2	1	2	3	2	2	2
3	3	3	3	2	1	3	3	1	3
1	2	1	1	2	1	3	3	3	2
2	1	1	1	3	1	3	1	1	2
2	3	2	2	2	1	1	2	2	2
3	3	2	2	2	1	1	2	2	2
2	2	2	2	1	1	3	3	2	3
2	3	3	2	2	2	3	2	1	2
2	3	3	2	2	2	3	2	1	2
3	3	3	2	3	2	1	1	3	3
2	2	2	2	1	1	2	2	2	2
2	2	2	1	1	2	3	2	2	2
2	2	2	2	1	1	3	1	2	1
3	2	2	2	2	1	3	1	1	3
2	3	2	2	3	1	3	2	3	3
1	3	3	2	3	1	3	1	3	3
2	2	1	2	3	1	3	3	3	3
2	3	1	1	3	1	1	3	3	3
2	3	1	2	3	1	1	3	3	3
2	3	2	2	3	2	2	1	1	3
3	3	1	2	2	1	3	2	2	2
9	3	3	3	1	1	3	3	3	3
3	1	3	1	1	1	3	3	3	9
1	1	1	2	3	1	1	2	1	1
3	3	1	3	2	1	2	3	2	3
2	1	3	2	1	1	3	1	1	1
2	2	1	2	9	9	9	9	9	9
2	2	3	2	2	2	1	2	1	2
2	3	2	2	2	3	3	3	3	3
1	3	3	1	3	1	1	1	1	3
9	9	9	2	9	2	9	9	9	9
2	2	2	2	2	2	1	2	1	3
3	3	1	1	3	1	3	1	3	3
2	2	2	2	2	1	2	2	3	3
1	2	1	3	3	1	3	3	3	3
2	3	2	3	3	1	3	3	3	3
1	2	1	2	1	2	3	2	3	1
9	9	9	9	1	1	3	3	3	3
2	2	3	2	3	1	3	3	2	3
2	3	1	2	3	1	3	3	3	3
3	3	2	3	2	2	9	9	9	9
9	9	9	9	9	1	9	9	9	9

9	9	2	9	9	1	9	9	9	9
9	9	1	9	9	9	9	9	9	9
9	9	9	9	9	1	9	9	9	9
9	9	9	9	1	1	3	1	3	3
9	9	9	1	3	1	3	3	3	3
9	1	9	1	1	1	1	9	9	9
9	9	9	1	1	1	1	1	1	9
9	9	1	1	9	1	9	9	9	9
9	9	1	1	9	1	1	9	9	9
9	9	9	9	1	9	9	9	9	9
9	9	9	9	1	9	9	9	3	9
9	9	9	9	1	9	9	9	9	9
9	9	9	1	9	1	9	9	9	9
9	9	9	9	9	1	9	9	9	9
9	9	9	9	1	1	1	1	3	3
9	9	9	9	1	9	9	9	9	9
9	9	1	1	1	9	9	9	9	9
9	9	9	1	1	1	3	1	3	3
2	2	2	2	2	2	2	2	2	2
1	3	1	1	3	2	1	1	2	1
1	9	9	9	1	1	1	1	1	1
2	1	2	3	1	1	3	1	2	3
1	1	1	9	3	1	1	2	2	3
1	3	1	1	1	1	3	3	3	3
3	3	1	2	2	2	3	3	3	3
1	1	3	2	1	3	1	2	2	2
1	1	2	2	1	2	3	2	3	3
2	2	2	3	1	3	3	9	2	3
2	2	2	2	1	3	3	2	2	2
2	2	2	2	1	1	2	2	9	3
1	1	1	1	1	1	3	3	3	3
2	3	2	3	2	1	3	3	3	3
2	2	2	2	1	1	3	1	1	3
3	1	3	3	9	9	9	9	9	9
2	2	1	2	1	1	1	2	1	2
2	2	2	2	1	1	1	1	2	1
1	3	2	1	2	2	2	1	2	3
1	2	3	3	3	3	1	2	1	2
9	9	9	9	1	3	1	1	1	1
2	2	1	2	1	1	3	1	2	1
2	1	1	9	1	3	1	2	2	1
3	1	1	1	9	1	3	3	3	1
2	2	2	2	3	3	3	1	2	1
1	3	1	3	1	3	1	1	1	1
2	1	3	3	3	3	3	3	3	3
2	3	2	2	3	3	3	1	2	1
1	1	1	1	9	2	2	2	3	3

2	3	1	3	1	3	1	3	9	9
1	3	3	3	1	1	2	1	2	2
1	3	3	1	1	9	3	3	1	1
1	1	1	2	2	1	1	2	1	2
2	2	2	2	1	2	3	3	2	1
2	2	2	2	1	2	3	3	2	1
2	1	1	1	1	1	3	2	2	1
2	3	1	3	1	3	1	3	9	9
1	1	1	1	1	1	1	2	1	2
9	9	9	9	1	1	3	3	3	3
1	1	2	3	1	2	3	1	2	1
2	3	1	1	1	1	3	3	3	3
2	2	3	2	2	1	3	2	1	2
2	3	1	2	2	1	1	2	1	2
2	2	2	2	1	2	2	1	3	1
3	1	2	3	3	1	3	2	3	3
1	1	1	1	1	1	1	1	1	1
3	3	3	3	3	3	9	9	9	9
9	1	2	2	1	1	3	3	3	3
3	3	1	3	3	1	3	3	1	3
2	1	1	1	1	3	3	3	3	3
2	2	3	2	2	1	2	3	3	3
2	2	1	3	1	1	1	2	3	2
3	2	3	3	2	1	3	1	3	2
3	3	2	2	3	3	2	2	2	2
3	1	3	3	1	3	1	1	3	1
3	3	1	3	3	1	3	3	1	3
9	3	1	1	3	1	3	3	3	3
1	1	1	1	9	2	2	2	9	3
9	9	1	9	1	3	3	3	3	3
1	2	1	2	3	3	3	3	3	3
9	9	9	9	3	3	3	3	3	9
2	3	1	1	3	1	3	3	3	3
2	3	2	9	3	1	3	3	3	3
9	9	2	9	3	1	3	3	3	3
9	9	1	9	1	3	3	2	3	3
9	9	1	9	1	3	3	3	3	3
2	2	2	2	1	1	3	2	3	3
2	2	2	2	1	1	3	2	2	2
2	2	2	2	1	1	3	2	2	2
2	2	1	2	1	1	3	2	3	2
2	2	2	2	3	3	3	1	2	1
3	2	3	1	3	1	2	1	2	1
2	2	1	2	1	1	3	2	3	2
3	2	3	1	3	1	2	1	2	1
1	1	1	2	1	3	3	1	1	1
3	3	3	3	3	1	1	1	1	3

3	3	3	3	3	1	1	1	3	3
2	3	2	2	3	1	3	3	2	3
2	1	1	1	3	1	3	3	3	3
1	2	1	2	1	2	1	2	2	1
2	2	2	2	3	1	3	3	3	3
3	9	3	3	1	2	1	2	1	3
3	2	3	2	1	3	1	2	2	2
9	1	9	9	1	1	3	3	3	3
3	3	3	3	1	1	3	3	3	3
1	2	2	2	2	1	3	2	3	3
3	3	3	3	3	3	3	3	3	3
9	9	1	9	1	3	1	3	3	3
2	1	2	1	1	3	3	1	1	1
1	1	1	2	1	3	3	1	1	1
1	1	1	2	1	3	3	1	1	1
2	1	1	2	1	3	1	3	2	3
1	1	1	2	1	3	3	1	1	1
1	1	1	2	1	3	3	1	1	1
1	1	1	2	1	3	3	1	1	1
3	3	1	1	1	3	1	1	1	3
1	3	2	1	3	3	1	2	3	1
1	2	3	3	2	2	2	2	2	2
3	1	3	2	1	3	3	9	1	3
3	2	2	3	3	1	3	2	1	2
1	1	1	1	3	9	3	3	1	2
2	3	3	1	1	3	3	3	1	3
1	2	2	2	1	2	1	2	1	2
1	1	3	1	2	1	3	3	1	3
9	9	9	9	1	9	9	9	9	9
3	3	3	3	3	3	3	3	3	3
1	1	1	1	1	1	3	3	3	1
2	3	2	3	2	9	1	3	2	1
2	1	2	3	3	1	2	1	2	1
9	2	2	3	1	2	1	1	1	2
3	2	3	3	1	3	3	2	3	3
3	1	3	2	1	1	1	3	3	3
2	2	9	9	3	2	1	1	1	2
2	2	2	2	1	1	2	1	1	1
2	2	3	2	1	3	2	1	1	1
1	1	1	1	1	1	1	3	3	1
3	1	1	2	1	3	3	1	1	2
3	3	1	1	1	1	1	9	1	3
1	3	2	3	1	2	1	3	2	1
1	3	1	1	3	1	3	3	3	3
9	9	9	9	1	1	3	3	2	3
1	1	1	1	1	9	1	2	1	2
2	2	2	2	1	1	1	1	1	1

9	9	9	9	3	1	3	2	3	3
9	9	9	9	1	9	1	9	9	9
9	9	9	9	1	9	9	9	9	9
3	1	3	3	1	3	1	1	3	1
2	2	2	2	1	1	3	3	2	1
2	2	2	2	1	2	3	3	2	1
1	1	3	3	1	1	3	1	2	1
2	2	2	2	1	2	3	3	2	1
2	2	2	2	1	2	3	3	9	9
2	2	2	2	1	2	3	3	2	1
9	2	3	2	2	1	3	1	1	1
3	2	1	2	3	2	3	1	1	3
2	1	1	2	1	1	1	1	2	1
2	2	1	3	1	1	1	1	3	1
1	2	3	1	3	3	1	1	3	3
2	1	3	1	1	1	1	3	3	2
3	3	3	3	3	1	1	2	3	3
9	9	9	9	3	1	9	9	9	9
9	9	9	9	3	1	9	9	9	9
1	1	1	1	2	1	3	3	1	3
3	2	1	3	1	1	3	1	1	1
3	3	2	2	1	3	3	2	2	2
3	3	3	3	1	1	1	2	1	1
2	2	2	2	2	2	2	2	2	2
3	3	3	3	3	3	3	3	3	3
9	9	9	9	3	9	2	2	2	2
9	9	9	9	3	9	2	2	2	2
1	3	1	2	3	1	2	3	1	3
3	3	3	3	1	1	1	2	1	1
2	2	2	2	2	2	2	2	2	2
1	2	3	3	3	1	9	9	9	9
2	2	1	1	1	3	2	2	1	2
9	1	1	1	1	1	3	9	1	1
9	9	9	9	3	9	2	2	2	2
3	3	2	1	1	3	3	1	1	2
3	3	3	3	1	3	3	9	1	2
2	2	1	1	1	3	1	2	2	2
9	9	9	9	1	3	3	1	1	1
9	1	3	9	1	9	1	9	2	9
2	2	2	2	3	1	1	1	1	1
9	9	9	9	1	3	1	1	1	1
2	2	2	2	3	1	1	2	2	2
2	2	2	2	2	1	3	3	2	2
2	2	2	2	2	2	2	2	2	3
2	2	2	2	2	2	2	1	3	1
2	2	2	2	2	2	2	2	2	2
2	3	2	2	1	3	3	3	3	3

2	2	2	2	1	2	3	3	2	2
2	1	2	2	2	2	2	2	2	2
2	1	2	1	1	3	3	3	3	9
3	1	2	2	9	9	9	9	9	9
9	9	9	9	1	9	9	9	9	9
1	2	1	2	2	1	2	2	1	2
2	2	2	2	1	1	3	2	2	2
9	9	9	9	3	3	3	3	3	3
2	2	1	2	1	1	1	2	1	2
9	9	9	9	9	3	9	9	9	9
2	2	2	2	2	2	2	2	2	2
2	1	1	1	1	1	3	3	1	1
9	9	1	9	1	1	3	3	3	1
2	2	2	2	2	2	2	2	2	2
1	1	1	2	1	2	3	1	1	2
2	2	2	2	3	3	2	2	2	2
2	2	2	2	2	2	2	2	2	2
2	2	1	1	1	1	1	3	1	2
1	9	1	1	1	1	9	9	9	9
1	1	1	2	1	3	3	3	3	3
9	9	9	9	2	9	9	9	9	9
3	1	2	2	1	3	1	2	1	1
2	2	2	2	2	2	2	2	2	2
2	2	2	2	1	2	1	2	2	1
2	2	2	2	1	1	3	3	3	2
3	2	3	3	1	2	1	2	3	1
3	3	3	3	2	9	9	9	9	9
2	2	2	2	9	1	9	9	9	9
2	2	2	2	2	2	2	2	2	2
1	3	3	2	3	1	2	2	3	3
1	9	9	9	9	1	9	9	9	9
2	2	3	2	1	1	1	2	3	3
3	3	1	3	3	3	3	3	3	3
2	2	2	2	1	1	2	2	2	2
2	2	1	2	1	2	1	1	1	1
3	1	1	1	1	1	1	1	1	1
2	2	2	2	2	2	2	2	2	2
1	3	1	2	1	2	2	3	3	1
2	1	1	1	2	2	1	1	2	2
2	1	2	3	1	2	3	3	3	3
2	2	3	2	1	1	3	3	2	3
2	2	2	2	2	2	2	2	2	2
1	2	3	1	3	1	1	3	3	3
3	2	1	1	1	1	3	3	3	3
2	2	2	2	1	2	2	2	2	2
3	3	3	3	3	3	3	3	3	3
3	3	2	2	1	2	3	9	1	2

2	3	1	2	1	1	2	2	2	2
2	2	2	2	2	2	2	2	2	2
3	1	3	3	1	2	2	2	2	2
2	2	1	2	2	2	2	2	2	2
3	2	1	1	1	2	3	3	1	3
2	1	1	2	1	1	3	1	2	1
2	2	2	2	3	2	2	2	2	2
1	1	3	3	2	2	2	2	1	2
3	1	2	3	1	1	3	3	3	3
9	9	9	9	1	1	3	1	2	1
1	3	3	1	1	3	3	1	1	3
9	9	9	9	9	2	9	9	9	9
9	9	1	9	9	1	9	9	9	9
2	2	1	2	1	1	1	2	1	2
2	2	2	2	1	2	1	2	2	3
2	3	1	3	1	3	1	1	2	1
2	2	2	2	9	1	2	2	2	2
2	1	3	1	3	1	3	3	1	1
1	2	1	3	1	2	2	1	1	2
2	2	1	2	1	1	1	2	1	2
9	1	9	9	1	1	1	3	3	3
1	1	1	1	1	2	2	1	2	1
2	2	2	2	1	2	3	3	2	3
1	2	1	2	2	1	1	3	3	3
3	9	3	3	1	3	1	3	3	3
1	2	1	2	2	1	1	3	3	3
2	2	2	2	2	2	2	2	2	2
9	9	9	9	9	1	9	9	9	9
2	2	2	2	1	1	2	2	2	2
3	2	1	2	1	2	2	2	2	3
1	1	1	1	1	1	1	1	1	1
1	1	1	1	1	1	1	1	1	1
3	1	2	1	1	1	1	2	1	1
2	1	2	1	1	2	2	1	1	2
9	9	9	9	1	1	1	3	1	1
1	3	1	1	1	3	1	9	1	1
9	9	1	9	3	1	3	3	3	3
1	1	1	1	1	1	1	1	1	1
2	2	2	2	1	1	2	2	2	2
2	2	2	2	1	1	2	2	2	2
2	2	1	2	3	1	2	3	3	1
1	1	1	1	2	3	3	2	2	1
2	2	1	3	1	9	9	9	9	9
2	2	2	2	1	1	2	1	2	1
2	2	2	2	1	1	2	2	2	2
3	1	1	1	1	1	2	3	1	1
3	3	1	3	2	2	2	2	2	3

3	1	1	1	1	1	3	1	1	1
3	3	3	9	3	1	3	3	3	3
2	2	2	2	3	1	3	3	2	3
9	9	1	9	9	9	1	9	9	9
9	1	9	9	3	9	2	9	9	9
3	2	1	2	1	1	2	3	1	2
9	9	1	9	1	1	2	2	2	2
1	3	1	1	3	1	3	3	3	3
3	3	3	1	1	9	9	9	9	2
2	2	2	2	2	2	2	2	2	2
9	9	1	9	1	9	9	9	9	9
3	2	3	3	1	2	2	2	2	2
2	2	1	2	2	2	2	2	2	2
2	2	1	1	2	1	2	2	2	2
2	2	2	2	1	1	2	2	2	2
9	2	1	3	2	3	2	1	2	3
1	2	3	2	1	2	2	1	3	2
9	9	1	1	1	9	9	9	9	9
9	9	9	9	1	9	9	9	9	9
1	2	1	2	2	2	2	2	2	2
2	2	2	2	1	2	2	2	2	2
3	3	1	2	1	3	9	3	3	3
9	9	9	9	1	3	3	9	9	9
1	1	1	1	1	9	2	2	2	2
2	3	1	2	2	1	2	2	3	1
3	1	3	1	3	3	3	3	3	3
2	2	9	2	1	1	3	3	2	3
1	2	1	3	3	3	3	1	1	3
3	3	3	3	3	1	2	9	2	2
2	3	2	1	1	3	3	2	3	3
3	1	1	1	1	1	3	3	3	2
2	2	1	2	1	1	3	3	2	3
1	3	2	2	3	3	2	3	3	3
3	3	3	3	1	3	3	3	3	3
9	9	9	9	1	1	1	2	2	3
2	2	1	3	1	1	3	1	1	1
1	1	2	1	1	1	1	3	1	1
3	3	3	3	1	3	3	1	1	1
2	1	9	3	2	1	1	9	3	2
3	3	3	3	1	3	3	3	3	3
3	1	1	1	1	3	1	1	3	1
9	9	1	9	1	1	1	2	1	3
1	1	1	1	1	1	1	3	1	1
3	3	1	3	1	1	3	3	3	3
9	9	9	1	3	1	3	1	3	3
2	2	2	2	1	1	2	3	3	2
1	1	1	2	1	1	2	2	1	2

3	1	1	3	1	3	3	3	1	1
3	3	3	3	3	3	3	3	3	3
9	9	1	9	1	1	1	2	1	3
1	1	1	1	2	3	3	1	2	1
2	2	2	2	3	3	2	2	1	1
2	3	1	1	2	2	2	2	2	2
3	3	3	3	2	3	3	1	3	2
2	2	2	2	1	1	1	1	1	1
2	2	2	2	1	1	1	1	1	1
2	1	3	2	2	1	3	1	2	1
1	2	1	2	2	3	1	2	2	3
2	3	2	3	1	1	1	1	2	3
1	3	3	3	3	3	1	1	1	1
3	2	2	2	3	1	3	2	3	2
2	2	2	2	2	1	1	2	2	2
3	3	3	3	2	3	3	1	3	2
3	3	3	1	3	1	1	3	3	3
2	1	1	2	1	3	2	2	2	2
2	1	3	2	2	1	1	2	2	3
9	9	9	9	9	1	9	9	9	9
9	9	1	9	1	9	9	9	9	9
3	3	3	3	1	1	3	1	1	1
3	3	3	3	1	3	3	1	3	3
3	3	3	1	3	3	3	3	3	3
1	3	3	3	1	3	3	3	1	3
3	1	1	3	1	3	3	3	1	1
2	3	1	2	1	1	3	1	1	3
2	1	1	2	3	2	1	2	1	1
1	2	1	1	3	1	1	3	3	1
2	3	1	3	1	3	1	3	3	3
2	3	2	1	3	1	3	2	2	2
1	3	1	3	1	1	1	1	1	1
2	3	1	2	2	1	2	2	1	2
2	1	2	2	1	1	1	1	3	3
9	1	1	9	2	1	3	2	2	3
2	3	2	2	1	2	2	1	2	1
9	1	2	2	1	2	9	1	1	1
2	2	2	2	1	1	3	2	2	2
3	2	2	2	9	1	2	2	1	3
2	2	2	1	1	1	3	3	1	3
2	2	1	3	1	2	1	3	1	2
2	2	2	2	2	2	2	2	2	2
1	1	3	3	1	3	3	3	2	1
9	9	9	9	2	1	9	9	9	9
9	9	9	9	3	9	9	9	9	9
3	3	1	2	3	1	3	3	3	3
2	3	1	1	1	1	3	2	3	3

1	1	1	3	1	9	3	3	3	3
3	3	3	1	3	1	3	2	3	2
3	3	3	2	1	1	3	3	3	3
1	3	2	1	1	2	1	1	2	3
3	3	3	3	2	2	3	3	3	3
3	3	3	3	3	3	3	2	3	3
2	2	2	2	3	9	2	2	2	2
3	3	3	3	2	1	3	3	3	3
3	3	3	2	3	1	3	3	1	3
9	9	9	9	1	3	3	1	3	3
9	9	9	9	1	3	3	2	3	3
9	9	9	9	1	3	3	2	3	3
9	9	9	9	1	3	2	9	9	9
9	9	9	9	1	9	9	9	9	9
9	9	9	9	1	3	3	2	3	3
2	2	3	2	3	1	3	3	3	3
2	9	2	1	1	3	3	3	1	1
2	2	2	2	1	2	2	2	2	2
3	3	1	2	1	1	3	9	1	3
2	1	2	1	1	1	3	3	3	3
9	2	3	1	1	3	3	3	3	3
1	3	2	3	2	1	3	2	9	2
2	2	2	2	1	1	2	2	2	1
1	1	1	2	3	1	3	2	3	3
2	2	2	2	1	1	2	9	1	2
2	2	3	1	1	1	1	3	1	3
3	3	3	2	3	1	3	3	3	3
2	1	1	1	1	3	1	3	3	1
2	2	3	1	1	1	1	3	1	3
2	2	3	2	3	1	3	3	3	3
2	1	2	1	1	1	3	3	3	3
9	9	9	9	1	3	2	9	9	9
3	3	3	1	1	1	1	3	3	3
2	2	2	2	1	1	3	2	3	3
2	1	2	2	1	3	1	1	1	1
2	2	2	2	1	1	3	3	3	3
9	9	9	2	1	2	3	2	3	3
9	9	9	2	1	2	3	3	2	3
2	3	2	2	1	2	3	1	2	2
2	2	2	2	2	2	2	2	2	2
1	9	9	9	3	1	3	3	3	3
1	1	1	2	1	1	3	3	3	3
3	3	3	2	3	1	3	3	3	3
2	2	2	2	2	2	1	2	1	2
3	3	1	3	1	3	1	3	1	3
2	1	1	2	3	2	3	2	2	3
9	9	9	1	9	1	9	9	9	9

1	3	3	1	1	1	2	2	2	2
2	2	2	2	1	1	3	2	2	3
1	9	1	1	1	1	1	1	1	1
9	9	1	9	1	2	1	3	2	3
9	1	9	9	3	1	3	3	1	3
9	9	9	9	9	1	9	9	9	9
1	2	1	3	1	1	1	1	2	1
1	2	3	2	1	1	3	3	2	3
1	2	2	3	1	2	3	2	2	3
1	2	1	3	1	1	1	1	2	3
1	3	2	1	1	1	3	2	3	3
1	3	2	1	1	2	1	2	9	1
1	1	2	2	3	1	3	1	2	2
2	2	2	1	1	1	3	3	3	3
1	2	2	2	2	2	1	3	2	2
3	3	3	3	1	1	3	3	3	3
3	3	3	1	3	1	3	2	3	3
1	3	2	2	1	1	3	3	3	3
1	2	1	2	3	1	3	3	3	3
1	3	1	1	1	2	1	2	1	3
2	2	2	2	1	2	3	3	3	3
9	9	9	9	1	2	2	1	1	2
2	3	1	2	1	2	1	1	1	2
2	3	2	2	2	2	2	2	2	2
2	2	2	2	1	2	3	2	2	3
2	2	2	2	9	1	1	9	9	9
2	2	2	2	1	1	2	3	3	2
1	3	2	1	3	1	1	1	1	2
2	1	2	1	1	3	3	3	3	3
3	3	3	3	3	1	3	3	3	3
1	9	9	9	1	9	9	3	9	9
2	2	2	2	2	2	2	2	2	2
2	2	2	1	1	1	1	9	9	9
2	3	3	3	1	1	1	2	1	2
3	2	1	2	1	3	1	2	3	2
9	9	1	9	1	9	1	9	9	9
9	9	9	9	9	9	9	9	9	9
2	2	2	2	2	2	2	2	2	2
1	3	2	2	2	2	1	2	3	3
2	2	1	3	1	2	1	2	3	2
1	1	1	1	1	2	2	3	1	3
9	1	1	1	1	1	1	1	1	1
1	1	2	1	3	1	2	1	1	1
2	1	2	3	1	2	1	2	3	1
2	1	3	2	1	9	3	2	1	3
3	1	2	3	9	9	9	9	9	9
2	3	2	1	3	3	2	1	3	2

1	1	2	1	2	1	1	1	2	1
9	9	9	9	9	9	9	9	9	9
3	3	3	3	3	3	1	3	3	3
2	3	1	2	1	1	1	1	1	2
3	3	2	3	3	1	3	1	1	3
1	1	2	1	2	1	1	1	2	1
2	1	3	2	1	9	9	9	9	9
2	3	9	2	1	1	3	2	3	3
2	2	2	9	1	1	1	1	1	1
1	3	1	2	1	3	3	1	3	1
9	9	9	9	9	9	9	9	9	9
2	2	2	2	1	9	9	9	9	9
1	9	9	9	9	1	9	9	9	9
2	1	1	1	1	3	3	3	1	1
3	3	2	2	2	1	3	1	2	1
9	9	9	9	1	1	3	2	2	3
3	3	3	3	9	1	3	3	3	3
9	9	9	1	9	1	9	9	9	9
1	3	1	2	2	1	3	3	2	3
2	3	3	3	2	3	2	1	2	3
1	3	2	1	2	1	3	3	1	3
3	3	3	3	1	1	2	2	1	1
2	3	3	1	2	3	1	2	3	1
2	2	2	2	2	1	3	3	2	3
2	2	2	2	3	1	3	2	2	2
2	2	1	1	1	1	2	1	2	2
9	9	9	9	9	9	9	9	9	9
2	2	2	2	1	1	3	3	3	3
3	2	3	1	2	1	3	2	3	2
9	3	3	3	2	2	1	3	2	2
2	3	3	2	3	2	3	3	3	3
2	2	2	2	2	1	1	2	1	3
2	1	3	1	1	3	1	2	9	2
2	3	2	2	1	3	3	3	2	2
2	2	2	2	3	1	2	1	1	1
2	3	1	1	3	1	3	1	2	3
2	1	1	2	1	2	1	2	2	1
1	3	3	3	1	3	1	1	1	1
1	3	1	2	1	3	3	2	2	3
1	2	1	1	1	1	3	1	1	2
2	1	3	3	3	9	2	3	2	3
3	3	3	3	3	3	3	3	3	3
3	3	1	3	1	9	1	9	3	3
9	9	1	9	1	1	1	3	3	3
9	9	9	9	1	1	3	3	3	3
9	9	1	9	1	1	3	3	3	3
9	9	9	9	1	1	1	3	3	3

2	2	1	2	1	1	3	3	2	2
1	3	2	2	2	3	1	2	2	2
9	1	1	9	2	1	3	3	3	3
2	1	2	2	3	1	3	3	3	3
1	3	1	3	1	1	1	1	1	1
9	9	9	9	3	1	3	3	3	3
1	9	9	9	3	3	3	3	3	3
1	9	9	9	1	1	3	3	3	3
9	9	9	1	1	1	1	3	3	3
9	9	1	9	1	1	9	3	3	3
9	9	1	9	9	1	9	3	3	9
9	2	1	2	3	3	3	3	3	3
9	9	9	1	1	1	1	3	3	3
3	1	1	3	1	3	3	1	1	1
1	3	1	3	1	3	3	2	3	3
2	2	1	2	3	1	3	2	3	3
2	2	2	2	3	1	3	9	3	3
9	9	1	9	3	1	3	3	3	9
9	9	9	9	3	1	3	3	3	3
9	9	9	9	1	1	1	1	3	3
3	1	3	2	1	3	2	3	1	1
9	9	9	9	1	1	3	3	3	3
1	1	1	2	1	1	3	3	3	3
9	9	9	1	1	1	3	3	3	3
2	2	2	1	1	1	3	3	3	3
1	1	2	2	1	1	3	3	3	3
2	2	2	2	2	3	1	3	1	3
2	1	1	1	1	1	1	3	3	3
2	1	1	1	1	1	3	3	3	3
1	1	1	1	1	1	3	3	3	3
1	1	1	1	1	1	3	3	3	3
2	2	2	2	3	1	3	3	3	3
3	3	3	3	1	1	9	2	2	2
1	9	9	9	1	1	1	3	3	3
1	1	1	1	1	1	1	3	3	3
1	1	1	1	9	1	3	3	3	3
1	3	3	3	1	1	3	3	3	3
1	9	9	9	1	1	9	3	3	3
1	3	3	3	3	1	3	3	3	3
3	3	3	3	1	1	1	3	9	1
3	3	3	3	2	3	2	3	1	2
1	3	1	3	3	1	3	3	1	3
3	1	3	1	1	9	3	2	3	3
2	2	2	2	1	2	1	1	2	2
2	1	1	1	1	1	1	1	1	3
2	3	2	2	1	1	1	1	1	2
1	3	2	3	1	3	3	2	2	3

3	3	2	3	3	3	3	2	3	3
3	3	3	2	3	3	3	2	3	3
2	2	2	3	1	2	3	1	2	9
2	1	2	2	2	1	3	2	2	3
2	1	3	2	9	1	3	2	3	3
9	9	9	9	2	9	9	9	9	9
2	1	2	3	3	1	3	2	2	3
1	2	2	2	1	3	3	2	2	1
9	9	9	9	1	9	9	9	9	9
3	3	2	2	1	1	9	9	9	9
9	9	9	9	1	9	9	9	9	9
3	2	9	9	9	9	9	9	9	9
1	2	2	2	2	1	9	1	1	2
2	1	2	1	3	2	2	9	1	9
1	1	1	1	1	1	3	3	3	9
2	1	3	3	3	1	3	2	1	1
1	1	2	1	3	2	3	1	2	1
2	2	1	2	3	1	3	3	3	3
3	1	3	1	1	3	1	1	1	1
9	9	1	9	1	9	9	9	9	9
1	2	3	1	1	3	1	1	1	1
2	1	2	3	1	1	3	2	3	3
2	2	2	2	9	1	3	2	2	3
1	1	1	1	1	1	1	1	1	1
2	2	1	2	3	1	3	2	2	3
1	1	1	1	3	1	3	3	3	3
1	2	2	2	1	3	1	1	9	9
2	3	2	3	2	3	2	2	2	2
1	2	2	2	2	1	2	2	1	2
9	1	1	3	2	9	9	9	9	1
1	1	3	1	1	1	1	1	3	1
1	1	1	1	1	1	1	1	1	1
2	2	2	1	1	1	3	1	1	1
2	1	2	2	2	1	1	1	1	1
2	1	1	1	1	1	3	3	3	3
3	3	2	1	3	2	2	2	2	3
2	2	2	2	2	2	1	2	2	2
1	1	1	1	1	2	2	2	1	1
1	1	1	1	1	2	2	2	1	1
3	3	3	3	1	2	2	2	2	2
2	1	2	1	1	3	3	1	3	3
3	2	3	2	1	1	3	2	2	2
1	3	2	2	2	1	3	3	1	3
2	2	2	2	1	1	3	3	2	2
2	3	1	2	3	3	3	3	3	3
3	3	3	3	3	3	3	3	3	3
2	2	2	2	2	1	3	1	2	3

2	2	2	2	9	9	9	9	9	9
9	2	3	1	2	1	3	3	3	3
9	9	9	9	9	9	9	3	9	9
9	9	9	1	1	9	9	9	9	9
9	9	1	9	1	9	9	9	9	9
2	2	2	2	1	1	3	2	9	1
9	9	1	9	9	2	9	9	9	9
2	3	1	2	1	1	3	2	1	3
1	2	2	2	2	2	3	3	2	3
2	2	2	2	1	3	2	2	2	2
2	2	2	2	1	1	3	3	3	3
1	1	3	1	1	3	2	1	1	1
1	1	1	1	1	2	1	1	1	1
2	2	2	2	1	3	1	3	3	3
2	2	1	1	1	3	3	1	1	1
2	2	1	2	1	1	1	2	1	1
2	2	2	2	1	1	1	2	2	2
3	3	3	3	3	3	3	3	3	3
3	2	3	2	3	1	1	3	1	2
2	2	2	2	1	1	1	1	1	1
2	2	2	2	2	3	3	2	3	2
1	2	1	1	1	2	1	1	1	1
2	2	2	2	2	2	1	2	2	2
2	2	2	2	3	3	1	1	1	2
1	1	1	1	2	1	3	3	2	2
2	2	2	2	1	2	2	2	2	2
9	9	9	1	1	2	3	1	1	1
3	3	1	3	1	3	3	3	1	3
3	3	2	3	1	2	3	2	3	2
1	1	1	1	1	2	1	1	2	2
9	9	9	9	2	9	9	9	9	9
1	2	1	3	2	3	2	3	2	3
3	1	2	1	1	1	1	2	1	3
3	3	3	2	2	1	3	3	2	3
3	3	3	3	1	3	3	3	2	2
2	1	3	2	1	2	3	3	3	2
3	3	1	1	1	1	1	1	1	3
1	3	1	1	1	9	1	3	3	1
1	2	3	3	1	2	3	1	2	1
1	2	2	2	2	1	3	1	1	2
2	2	2	2	1	1	3	2	9	2
1	2	2	2	1	1	2	2	2	2
2	2	2	2	3	1	1	3	1	9
9	9	1	9	1	9	9	9	9	9
9	9	9	9	9	1	1	9	9	9
9	9	9	9	9	9	9	9	9	9
3	9	9	3	2	1	3	3	2	3

1	3	2	2	2	1	3	3	2	3
2	2	2	2	2	2	1	1	2	1
9	9	1	9	1	3	1	3	3	1
1	2	3	1	1	3	3	3	3	3
3	1	9	3	1	1	3	9	3	9
2	2	3	2	1	2	2	2	9	3
2	2	2	2	2	1	3	3	3	3
2	2	2	2	2	1	3	2	3	3
2	1	2	2	3	2	2	2	2	2
1	3	1	1	3	3	1	1	3	3
9	9	9	1	9	1	9	9	9	9
9	9	9	1	2	9	1	1	2	3
2	2	2	2	1	1	2	1	2	9
9	9	9	9	2	1	2	1	3	2
9	1	9	9	1	9	1	9	9	9
3	3	1	3	3	1	1	1	3	9
2	2	1	2	2	1	3	1	2	1
3	1	3	2	3	1	3	3	3	3
1	9	1	1	2	9	1	1	1	1
3	3	2	1	1	3	2	1	2	2
2	2	2	1	3	2	2	3	2	1
1	3	1	3	1	3	1	3	1	3
3	3	1	1	1	3	3	1	3	1
2	1	3	3	3	2	3	3	3	2
2	3	1	3	2	2	2	2	2	2
1	9	9	9	3	3	3	3	3	3
2	1	3	3	3	2	3	3	3	2
1	3	1	2	1	1	3	3	1	1
2	1	3	3	3	2	3	2	3	2
2	3	1	2	3	3	2	2	2	2
1	3	3	1	3	1	3	3	3	1
2	3	2	2	3	2	2	2	3	1
1	2	1	3	2	1	1	1	2	1
3	9	2	2	1	1	3	2	2	3
2	3	1	1	2	3	1	3	3	2
2	1	1	9	3	2	2	1	2	2
2	2	2	2	2	2	1	2	2	2
2	2	2	3	1	3	3	1	1	3
9	9	9	9	1	9	9	9	9	9
2	3	3	3	1	1	2	2	3	2
9	9	9	9	3	9	9	9	9	9
2	2	1	2	1	3	9	9	9	9
9	9	9	9	1	3	2	3	2	2
9	9	9	9	1	3	2	3	1	2
3	3	3	3	2	3	1	1	3	3
3	3	1	1	1	3	1	3	1	1
3	3	3	3	2	2	3	3	3	3

3	3	3	3	1	3	3	3	3	3
9	3	9	9	2	1	9	3	9	9
3	3	1	3	3	3	3	3	3	3
1	1	1	1	3	1	3	1	3	3
3	3	1	3	3	1	3	3	3	3
3	3	1	3	1	3	1	1	1	1
3	3	2	1	1	9	2	2	1	1
2	1	1	2	1	1	2	2	2	2
3	1	1	3	1	3	3	3	3	3
3	3	3	1	3	2	3	2	9	1
1	3	3	1	1	1	3	3	1	1
2	3	1	2	3	1	3	3	2	2
2	3	2	3	1	3	1	1	2	1
1	1	2	3	9	1	3	3	3	3
1	1	2	1	1	1	1	1	2	1
3	1	2	2	2	3	3	1	1	2
2	1	1	2	1	3	3	1	2	2
1	3	3	2	1	3	1	3	3	2
3	1	3	1	1	1	3	3	3	3
2	2	2	2	2	2	2	2	2	2
2	2	2	2	3	1	3	1	3	3
1	3	3	1	1	1	2	2	3	1
3	3	3	3	3	3	3	3	3	3
3	1	1	1	1	1	1	3	1	3
1	3	2	2	3	1	3	3	1	2
2	1	2	3	1	1	2	3	3	1
2	3	1	2	1	2	2	3	1	2
2	3	3	3	3	1	2	2	2	3
3	1	1	2	2	1	3	1	2	1
9	9	9	9	9	1	3	9	9	9
1	2	1	3	1	2	3	2	2	3
1	3	1	2	1	1	1	1	1	1
2	1	3	2	3	1	3	3	1	2
2	1	2	2	2	2	3	3	3	1
1	1	1	1	3	3	3	1	2	1
2	2	2	2	2	9	3	3	3	3
3	3	2	2	1	1	3	1	3	3
3	3	1	2	1	3	2	2	2	2
1	1	3	2	1	3	1	2	2	2
2	2	2	1	1	1	3	3	3	3
9	1	9	9	9	9	9	9	9	9
9	9	9	9	3	9	3	2	2	3
1	2	3	2	1	2	1	2	3	1
2	3	1	1	3	1	3	2	1	3
2	3	3	2	1	1	2	2	2	2
2	3	3	1	1	3	1	3	3	3
9	9	9	9	9	9	9	9	9	9

2	1	1	2	1	3	1	1	2	1
3	1	3	3	1	1	1	3	3	3
2	1	2	1	3	3	3	3	3	3
2	3	2	1	1	2	1	2	1	2
9	9	9	9	9	9	9	9	9	9
1	1	1	1	1	9	1	1	1	1
2	2	2	2	1	1	3	2	3	3
2	2	2	2	2	2	2	2	2	2
1	2	1	2	2	3	3	1	1	1
2	1	1	1	1	3	3	2	1	3
9	9	9	9	1	3	3	3	3	3
2	3	2	3	1	2	1	2	2	2
2	3	2	3	1	2	3	2	2	3
9	9	9	9	9	9	9	9	9	9
3	2	3	3	2	1	2	2	2	2
9	9	9	9	9	9	9	9	9	9
3	3	2	3	1	2	2	3	3	2
3	1	1	2	1	3	3	3	3	3
1	2	3	3	1	1	3	2	3	1
2	1	2	2	1	1	2	2	2	2
3	3	3	3	1	1	1	3	1	1
2	2	2	2	3	1	2	2	3	3
9	9	1	9	9	1	9	9	9	9
2	2	2	2	1	3	2	1	3	2
2	2	1	2	1	1	3	2	2	2
2	2	2	2	1	1	2	2	2	2
9	9	9	9	1	9	3	9	3	9
2	2	2	2	2	1	2	2	2	1
3	2	2	2	3	3	1	2	2	2
9	9	9	9	9	1	9	9	9	9
2	1	1	3	3	1	3	2	3	3
3	1	1	1	3	3	2	2	3	1
3	1	3	1	3	3	3	1	1	3
2	1	2	2	1	1	3	2	3	3
2	1	1	1	1	3	3	2	3	3
2	3	2	2	3	3	3	3	3	3
2	3	1	1	1	1	1	2	3	3
3	1	1	3	1	1	3	3	3	3
9	1	9	9	1	2	9	9	9	9
3	3	3	3	2	2	3	3	3	3
3	2	1	2	2	1	3	3	2	1
2	3	1	3	1	1	3	3	3	3
2	3	2	2	1	1	3	3	3	3
1	1	2	1	1	1	3	9	2	2
1	2	1	2	3	3	3	3	3	3
2	3	2	2	2	1	2	1	1	3
2	3	2	2	1	3	3	2	3	2

3	1	2	1	2	2	2	2	2	2
2	2	2	2	3	1	3	2	2	2
1	2	2	1	2	2	2	1	1	1
9	9	1	9	1	1	2	2	2	2
2	2	1	2	2	3	2	1	1	2
9	9	1	9	9	1	9	9	9	9
2	2	2	2	2	2	2	2	2	2
2	1	9	1	3	1	1	3	2	3
2	2	2	3	1	1	2	3	3	3
2	2	1	2	3	1	3	2	3	3
3	3	1	2	9	3	3	2	2	2
2	3	1	1	1	2	1	1	2	1
2	2	2	2	2	2	2	2	2	2
3	3	3	3	3	3	3	3	3	3
2	3	3	3	3	1	3	1	1	2
2	2	1	3	3	3	2	2	3	1
2	3	3	3	2	3	2	1	1	1
2	2	2	2	3	1	1	2	9	3
2	2	3	3	1	1	3	3	3	3
2	2	2	2	1	1	2	2	2	1
2	3	2	2	1	1	1	3	3	1
3	1	3	2	3	1	3	2	3	1
2	3	1	2	3	1	3	2	3	1
2	2	2	2	3	3	3	1	3	1
1	1	1	2	1	1	3	3	3	3
3	1	2	2	2	2	1	1	1	2
3	3	1	2	3	1	3	2	3	3
3	3	1	2	3	1	3	2	3	3
2	2	2	2	2	3	3	2	2	2
2	2	2	2	1	1	3	1	1	3
3	2	3	3	1	3	3	2	2	2
3	2	3	2	3	1	3	2	3	1
3	3	3	1	3	1	3	3	3	3
2	2	2	2	1	1	3	1	3	3
3	3	3	3	1	3	1	1	3	1
2	2	2	2	1	9	3	2	2	1
2	2	2	2	1	3	3	2	2	1
2	2	1	3	1	2	2	1	1	2
1	3	1	2	1	3	1	2	1	1
2	2	1	2	2	2	2	2	2	2
1	2	1	3	1	2	3	2	3	1
3	1	2	2	3	1	3	1	3	3
1	1	1	1	1	1	2	1	2	2
2	1	1	2	1	1	1	2	2	2
3	1	3	3	3	1	3	2	1	3
2	1	3	1	1	1	3	3	3	3
9	9	1	9	3	1	3	3	3	2

3	2	2	1	2	1	3	2	2	1
3	3	3	1	3	2	2	2	2	2
9	9	9	9	1	1	2	2	3	3
1	2	2	2	3	3	2	2	2	2
2	2	2	2	1	1	9	3	3	3
2	2	2	2	2	1	3	2	2	2
1	3	2	2	2	1	3	3	3	3
3	2	1	2	3	1	3	3	3	3
3	2	1	2	1	1	3	2	3	2
2	2	3	2	1	2	3	2	2	3
2	2	2	2	1	2	3	2	2	3
2	2	2	2	1	1	3	1	1	1
2	2	2	2	1	3	3	2	2	2
2	2	2	2	3	1	3	3	1	3
2	2	2	2	1	3	1	1	1	1
2	2	2	2	1	1	2	2	3	3
3	2	1	2	2	2	2	3	3	3
2	2	2	2	1	1	3	1	3	2
3	1	1	2	1	1	1	2	3	3
1	1	2	2	2	1	3	2	1	3
2	2	2	2	1	2	2	2	2	2
2	2	2	2	3	2	2	2	2	2
2	3	3	2	1	2	3	2	2	2
2	2	2	2	2	1	3	2	3	2
2	2	2	2	1	1	2	2	1	3
9	9	1	9	2	1	3	2	1	3
2	2	2	2	1	1	2	2	2	2
2	2	2	2	2	2	2	2	2	2
2	2	2	2	2	1	2	2	2	2
2	3	2	1	3	1	3	3	3	3
2	2	2	2	2	2	2	2	2	2
2	2	2	2	2	2	2	2	2	2
2	2	2	2	2	2	2	2	2	2
2	2	2	2	2	1	2	2	2	2
9	9	9	9	1	9	3	3	9	9
2	2	2	2	1	1	2	2	2	2
2	2	2	2	1	1	2	2	2	2
2	2	2	2	1	2	2	2	2	2
2	2	2	2	2	2	2	2	2	2
2	2	2	2	1	1	3	2	3	3
2	2	2	2	2	2	2	2	2	2
2	2	2	2	1	1	3	3	3	3
2	2	2	2	1	1	3	3	3	3
2	2	2	2	2	2	2	9	2	2
2	2	2	2	1	2	2	2	2	2
3	2	3	1	1	1	3	2	3	2
2	2	2	2	1	1	9	3	3	3

2	2	2	2	2	2	2	2	2	2
2	2	1	2	2	2	2	2	2	2
1	1	1	1	1	1	3	3	3	3
2	2	2	2	2	2	1	2	2	2
2	2	2	2	2	2	2	2	2	2
3	3	2	1	2	3	3	1	3	2
9	9	1	9	1	1	3	3	3	3
9	9	2	9	9	2	3	3	3	3
3	1	1	3	1	1	3	3	1	1
2	2	1	2	2	1	3	3	3	3
2	2	1	1	1	1	3	3	3	1
2	2	1	2	1	3	2	2	2	2
2	2	1	2	1	1	3	3	3	3
9	9	1	1	1	3	1	1	3	9
3	1	1	3	1	1	1	1	1	3
2	2	2	2	2	1	3	2	1	3
2	2	2	2	3	2	2	2	2	2
9	9	9	9	1	1	2	3	2	3
9	9	9	9	1	1	1	1	1	1
2	2	2	2	1	1	3	2	2	3
9	9	9	9	1	1	3	3	3	9
2	2	2	2	2	2	2	2	2	2
2	2	2	2	1	2	3	2	3	3
2	2	2	2	2	2	2	2	2	2
2	2	2	2	1	2	2	2	2	2
2	2	2	2	1	2	2	2	2	2
9	9	1	9	9	1	9	9	9	9
2	2	2	2	1	3	2	2	2	2
2	2	2	2	1	2	2	2	2	2
2	9	1	9	2	1	3	2	1	3
2	2	2	2	2	1	3	2	1	3
3	1	2	2	1	3	1	2	1	3
1	1	3	3	3	3	1	2	3	2
2	2	2	2	1	1	1	2	1	3
1	2	1	1	2	1	3	3	3	3
1	3	2	1	2	2	3	1	1	1
3	3	3	2	3	1	3	2	3	3
2	1	2	2	3	1	3	2	3	3
2	1	1	1	3	1	3	3	3	3
2	3	1	2	1	2	2	2	3	2
3	3	3	3	3	1	3	3	3	3
2	2	2	2	3	2	3	2	2	3
2	3	1	1	1	1	3	2	2	3
2	2	2	2	2	2	2	2	2	2
1	1	1	1	2	3	3	1	2	3
2	1	1	1	1	2	1	1	3	2
2	2	1	1	3	1	3	2	1	1

3	2	1	2	2	1	1	2	9	3
1	1	3	2	1	2	1	2	1	3
2	3	2	1	1	1	3	2	2	3
1	3	2	1	1	1	3	1	3	3
9	9	9	9	3	2	1	2	9	1
2	2	1	1	3	2	1	9	1	2
1	1	1	1	1	1	2	1	1	3
9	9	9	9	3	1	2	9	9	9
2	2	2	2	2	1	2	2	2	2
2	2	2	2	2	2	2	2	2	2
3	3	3	1	1	3	3	1	1	1
3	3	3	1	1	3	3	1	1	1
2	2	2	2	1	2	3	3	3	3
2	1	2	2	3	1	1	2	1	2
2	2	3	3	3	2	3	2	3	2
2	2	2	2	1	2	3	2	2	3
2	2	2	2	2	1	1	2	1	2
3	9	1	9	1	1	3	3	2	2
2	2	1	1	2	2	3	2	2	3
3	1	1	2	1	3	3	3	3	9
1	2	2	2	1	3	3	3	3	3
9	9	9	9	2	2	2	2	2	2
1	1	1	2	1	3	3	2	2	2
9	9	2	9	9	1	9	9	9	9
1	3	2	1	1	3	1	2	3	1
2	1	1	2	1	1	1	2	1	1
2	1	1	1	1	2	2	1	3	2
9	9	9	9	1	3	2	2	2	2
2	2	2	2	2	1	2	2	2	2
9	9	9	9	9	1	9	9	9	9
3	3	1	3	1	3	3	3	3	2
9	9	9	9	9	9	9	9	9	9
9	9	9	9	9	9	9	9	9	9
2	2	1	1	3	1	3	2	2	2
2	2	1	1	3	1	3	2	2	2
9	9	9	9	1	1	3	1	1	1
9	9	9	9	3	3	1	2	3	1
2	2	1	1	3	1	3	2	2	2
2	2	2	2	1	1	1	1	3	1
2	2	2	2	1	1	1	1	3	1
9	9	9	9	9	3	9	9	9	9
2	2	2	2	1	1	3	3	3	3
2	2	2	2	1	2	2	2	2	1
9	9	9	9	9	9	9	9	9	9
9	9	9	9	3	9	9	9	9	9
2	2	2	1	1	2	1	2	2	1
2	2	2	2	2	9	2	2	2	2

9	9	9	9	1	1	3	3	3	1
3	2	1	2	3	1	3	3	3	3
3	3	3	3	2	1	3	3	1	3
1	2	1	1	9	9	9	9	9	9
2	2	2	2	2	2	2	2	2	2
3	3	3	3	1	9	3	3	3	3
2	2	2	2	2	2	2	2	2	2
9	9	9	9	1	1	3	3	3	3
9	9	9	9	1	9	9	9	9	9
9	9	9	9	2	2	2	2	3	2
2	2	2	1	2	2	3	1	3	2
2	2	2	2	3	1	1	3	1	2
2	3	3	1	2	3	3	1	2	1
3	3	3	3	3	2	3	3	3	3
1	3	2	3	1	3	3	2	3	2
2	1	1	2	3	1	3	3	1	3
1	3	1	1	3	3	3	3	3	3
2	1	2	1	2	1	1	1	2	3
2	2	2	2	1	2	2	2	2	2
1	1	3	1	3	1	2	3	1	2
1	3	1	2	3	2	2	3	3	2
3	1	1	1	1	3	1	1	1	1
2	2	1	2	1	1	1	1	1	1
1	2	3	3	1	3	3	2	2	2
3	3	3	3	3	1	1	1	3	3
2	2	2	2	2	2	2	1	2	2
2	2	2	3	1	3	3	2	3	3
2	2	1	2	2	1	3	3	3	3
2	2	1	2	1	3	1	2	2	3
3	3	3	3	2	3	1	2	3	1
3	3	2	2	1	1	1	1	1	1
3	3	3	2	1	2	3	3	3	3
1	3	1	1	2	1	3	3	2	3
3	3	3	3	1	9	3	3	3	3
3	3	1	2	1	2	2	1	2	2
1	2	1	2	3	2	3	1	2	3
3	3	3	3	3	1	1	1	3	3
2	2	2	2	1	1	9	1	1	1
2	1	1	1	1	3	1	1	1	1
9	1	9	9	1	3	3	2	1	1
3	3	3	1	1	1	2	9	9	2
2	3	2	1	2	1	1	2	3	1
3	3	3	2	3	3	3	2	3	2
1	2	3	2	1	1	3	3	1	3
1	1	1	1	1	3	3	2	3	3
2	3	2	2	1	3	2	2	2	2
3	3	3	3	2	2	2	2	2	2

2	1	2	2	1	2	1	3	2	1
1	2	2	3	1	1	1	2	3	3
3	3	3	3	2	1	1	1	1	2
1	3	3	3	1	2	3	3	3	3
1	1	1	1	1	1	1	1	3	3
2	1	3	2	3	3	1	1	2	1
1	1	1	1	9	9	9	9	9	9
2	3	2	2	2	1	3	2	1	2
3	3	3	3	3	1	3	1	3	3
2	3	2	1	1	3	1	1	2	3
1	3	3	3	3	2	3	1	3	3
2	2	2	2	1	3	2	3	2	1
2	2	2	2	2	2	2	2	2	2
3	1	1	1	1	1	3	3	1	3
1	1	1	1	3	1	3	3	3	3
2	2	2	2	2	2	3	2	2	1
1	2	1	2	2	2	2	2	3	2
2	3	2	2	1	1	1	2	1	2
1	1	3	2	3	3	3	1	1	3
9	1	2	2	1	1	3	3	3	2
2	2	1	1	3	1	3	1	1	1
9	9	2	9	3	2	3	2	2	2
3	3	3	3	3	2	1	1	2	3
3	2	2	2	2	3	3	2	2	2
2	2	2	2	3	1	3	2	2	3
2	2	2	2	1	1	3	3	3	3
2	2	2	2	3	9	2	2	2	2
2	1	1	2	1	3	2	1	2	1
3	3	3	3	2	3	3	3	2	1
2	3	2	1	3	1	3	2	2	3
3	3	3	3	1	3	3	3	3	1
3	3	1	1	3	1	3	3	2	3
2	2	2	3	2	1	1	2	3	2
2	2	1	1	1	3	3	3	3	3
1	2	1	3	1	1	1	2	3	1
2	2	2	2	3	1	1	3	1	3
3	1	1	1	3	1	3	1	1	3
2	2	1	1	1	3	3	2	2	3
2	1	3	2	1	3	3	3	2	1
9	9	9	9	3	9	1	9	9	9
3	2	3	2	1	1	3	2	3	2
2	3	2	1	1	1	2	1	3	3
2	2	2	2	1	2	2	2	2	2
1	1	1	1	1	1	3	1	1	3
3	3	3	1	3	1	3	1	3	3
9	9	9	9	3	1	3	2	3	3
3	1	2	2	3	2	2	1	1	2

3	2	2	2	3	2	2	1	2	1
2	2	2	2	2	2	2	2	2	2
3	3	3	1	3	3	3	3	3	3
3	3	2	1	2	1	1	2	1	3
2	2	2	2	9	1	9	9	9	9
1	1	3	3	9	9	9	9	9	9
2	1	3	3	1	9	9	9	9	9
1	1	1	1	1	1	3	2	2	2
2	2	2	2	9	9	9	9	9	9
2	3	1	3	1	3	1	1	1	1
2	1	1	2	1	3	2	1	1	1
2	3	3	2	1	3	3	1	1	3
9	9	1	9	9	1	2	3	1	3
1	2	1	1	9	1	3	3	3	3
9	9	9	9	1	9	9	2	2	2
2	2	2	2	3	1	3	3	2	3
1	1	1	9	9	9	3	2	2	1
2	2	2	1	3	3	1	1	1	1
3	3	1	3	3	3	3	3	3	3
2	2	2	1	3	2	3	1	3	2
1	3	2	1	1	1	1	1	1	1
2	1	2	2	2	1	2	3	3	3
2	2	2	2	1	3	3	1	3	1
2	3	3	2	1	3	3	3	2	3
9	9	9	9	3	1	3	2	3	3
1	1	1	2	2	2	3	3	2	1
3	2	3	2	2	1	2	3	2	3
2	3	2	1	2	3	3	2	2	3
2	3	1	3	3	3	3	3	3	3
1	2	2	2	1	1	9	3	1	2
1	2	1	2	9	2	1	2	2	1
2	2	2	2	1	2	2	2	2	2
2	1	1	1	1	3	1	1	1	1
2	2	2	1	1	1	3	3	3	3
2	3	1	2	3	1	1	2	3	2
9	9	9	9	2	3	1	2	3	3
2	2	2	1	2	1	1	1	1	1
1	1	3	1	1	3	3	3	3	3
3	2	2	1	1	1	3	3	3	3
1	1	1	2	1	1	2	2	2	1
3	3	3	3	2	2	3	3	3	3
1	1	2	2	2	1	1	1	2	2
1	3	3	1	1	3	3	2	1	2
3	3	1	3	1	3	2	1	1	2
3	2	1	1	1	3	1	3	3	3
3	3	3	3	2	3	1	3	3	3
2	2	2	2	1	3	3	2	1	1

1	3	1	1	1	3	3	2	1	1
3	3	1	3	1	2	3	2	2	2
2	2	2	2	2	2	2	2	2	3
1	2	2	1	1	2	2	1	1	2
3	3	3	3	1	1	3	3	1	1
1	3	2	1	1	2	3	1	2	1
9	9	9	9	1	9	2	9	9	9
2	3	2	1	1	3	2	2	2	1
2	2	2	2	2	1	3	2	1	3
2	2	2	2	2	1	3	2	1	3
2	2	2	2	3	3	3	3	3	3
2	2	2	2	2	2	2	2	2	2
1	1	1	1	1	2	2	2	2	1
2	2	2	2	1	9	2	2	2	2
3	3	3	3	2	1	3	3	2	3
2	2	3	1	1	1	2	2	2	3
2	2	2	2	3	1	3	2	2	1
2	3	3	2	2	2	2	2	2	3
3	1	2	2	1	3	1	1	2	2
1	2	1	2	1	3	3	3	1	2
2	2	2	2	2	2	1	1	2	2
2	2	2	2	2	1	3	2	1	3
9	2	2	2	2	2	2	2	2	2
3	3	3	3	1	2	1	1	1	1
2	1	2	1	2	1	2	1	2	3
2	2	2	2	2	2	2	2	2	2
1	2	2	1	2	3	2	1	3	2
2	3	3	2	2	2	2	2	3	3
2	2	2	2	1	1	3	1	1	3
2	3	2	2	1	2	3	3	3	2
2	3	1	3	1	1	3	2	2	3
1	2	3	2	1	1	1	2	9	1
2	3	2	2	1	2	1	1	1	2
2	2	1	3	2	2	3	9	2	2
2	1	1	3	3	1	3	3	3	3
3	2	2	2	2	1	1	3	1	9
3	2	1	2	1	1	1	2	2	1
2	2	2	2	3	3	3	2	2	3
1	3	3	3	3	3	3	1	1	1
3	3	3	3	3	3	3	3	3	3
2	2	2	2	2	2	2	2	2	2
2	2	2	2	2	2	2	2	2	2
2	2	2	2	2	2	2	2	2	2
3	3	3	3	3	3	3	3	3	3
3	3	3	3	3	3	3	3	3	3
2	9	9	2	2	9	9	9	2	9
2	1	1	1	1	2	1	1	1	2

2	2	1	1	2	2	1	1	1	9
2	2	2	1	2	2	1	1	1	1
1	1	2	2	2	1	1	2	1	1
1	2	2	2	2	1	1	1	1	1
1	1	1	1	1	2	2	1	1	1
2	3	2	1	1	3	2	2	1	1
1	1	2	1	1	3	2	2	1	1
1	1	2	2	1	3	1	2	1	1
2	2	1	1	1	2	1	1	1	1
2	2	2	1	2	1	2	2	1	1
1	2	2	1	2	3	2	2	2	1
1	2	3	3	2	1	9	2	9	9
2	2	2	2	9	3	2	2	2	2
2	2	2	2	9	3	2	2	2	2
2	2	2	2	9	3	2	2	2	2
2	2	2	2	9	3	2	2	2	2
2	2	2	2	9	3	2	2	2	2
2	2	2	2	9	3	2	2	2	2
2	2	2	2	2	9	9	2	9	9
2	2	2	2	2	2	2	2	1	1
2	2	2	2	2	2	9	2	2	2
2	2	2	2	2	2	2	2	2	2
2	2	2	2	2	2	2	2	2	2
1	1	1	2	2	2	2	2	2	9
2	2	2	2	2	3	2	2	2	2
2	2	2	2	2	3	2	2	2	2
2	2	2	2	2	2	2	2	2	2
2	2	2	2	1	9	2	2	2	2
1	1	1	1	1	3	1	2	3	2
2	2	9	9	2	2	1	2	2	1
2	2	2	2	2	2	2	2	2	2
2	2	2	2	9	3	2	2	2	2
2	2	2	2	2	3	2	2	2	2
2	2	2	2	9	3	2	2	2	2
9	1	9	9	1	9	3	1	3	2
1	9	3	1	9	1	9	1	2	9
9	1	9	1	3	1	9	1	2	9
9	2	9	2	2	9	2	9	2	9
9	9	9	9	9	9	9	9	9	9
1	2	9	1	1	9	1	9	1	9
3	1	9	1	1	9	2	9	3	1
1	2	2	1	2	1	1	3	3	2
2	2	2	1	1	3	1	2	3	1
2	2	2	2	2	2	2	2	2	2
1	9	3	1	1	9	9	2	9	9
9	9	9	2	9	2	9	9	2	9
2	2	1	2	2	1	1	1	2	1

2	2	1	2	2	1	1	2	3	3
1	1	1	2	2	2	1	1	1	2
2	2	2	2	1	2	2	2	2	1
1	1	2	1	1	3	1	2	2	2
3	1	2	2	1	3	1	1	2	1
2	2	3	3	1	3	2	2	2	1
1	2	1	1	3	1	2	3	2	1
2	1	1	1	2	1	1	2	2	2
1	1	1	2	2	3	2	2	2	2
2	1	1	2	1	3	1	2	2	1
1	1	3	3	2	1	1	2	2	2
1	1	2	2	3	1	1	1	2	3
1	2	2	2	3	1	1	2	1	1
1	1	2	2	2	1	1	2	2	2
2	1	2	2	3	1	2	1	1	1
2	2	2	2	3	1	1	2	2	2
2	2	2	2	9	1	3	3	3	3
2	2	1	1	2	3	3	1	2	2
2	1	1	1	1	3	3	2	1	2
2	2	1	2	2	9	2	2	1	2
2	3	2	2	2	3	1	2	2	1
2	1	1	2	2	1	1	2	2	3
2	1	1	2	1	3	2	1	2	1
2	1	1	2	1	3	2	2	1	2
3	2	2	2	3	1	1	3	2	2
2	2	2	3	3	2	2	2	1	1
2	2	2	1	3	1	3	2	1	2
2	1	2	2	1	3	3	1	3	3
2	2	2	2	2	1	9	2	3	2
2	2	2	2	2	2	2	2	2	2
2	2	2	2	2	9	2	2	2	2
3	3	3	3	3	3	9	3	3	3
2	2	2	2	1	3	1	3	1	2
2	2	2	2	3	1	3	3	3	3
2	2	1	2	1	3	2	2	2	2
9	3	3	3	3	3	3	9	3	3
2	2	2	2	2	3	2	2	2	2
2	2	2	2	2	2	2	2	2	2
2	2	2	2	1	3	2	2	2	2
2	1	2	2	1	2	1	1	1	2
2	1	1	2	2	3	2	2	1	1
3	3	3	3	2	2	2	2	2	2
2	2	3	3	2	1	2	1	1	3
2	2	2	3	3	1	2	1	1	2
2	1	2	3	2	2	1	2	2	2
1	1	2	2	1	2	1	2	1	2
1	1	2	1	1	2	1	2	2	1

9	1	9	9	2	9	9	9	9	9
2	2	1	2	1	2	1	1	1	2
2	2	2	2	1	2	1	2	2	3
1	2	2	2	1	2	2	1	1	1
3	3	1	3	1	2	3	2	3	3
2	2	1	1	3	1	3	3	3	3
1	2	1	3	3	1	3	3	3	3
2	3	2	1	2	1	1	3	2	3
1	1	1	1	1	1	3	3	3	3
2	1	3	2	1	1	2	2	2	2
3	2	2	3	1	1	1	2	3	2
2	1	2	2	3	1	1	2	2	3
3	1	3	2	3	2	1	2	3	1
2	1	1	2	2	2	3	2	1	2
3	3	1	3	1	2	3	2	3	2
2	2	2	2	1	1	3	3	1	3
2	2	2	2	1	1	3	3	1	3
2	2	2	2	1	1	2	2	1	2
3	3	1	2	1	3	3	3	1	3
2	3	3	2	1	3	1	2	2	1
1	2	2	3	9	1	9	9	9	9
2	3	3	2	1	3	2	2	2	2
2	1	3	1	1	2	3	1	2	3
2	2	2	3	1	2	3	3	3	3
2	3	2	2	3	3	3	2	3	3
3	2	2	2	3	2	1	2	1	2
2	3	1	2	2	1	3	3	1	2
2	1	2	1	1	3	3	3	1	3
1	1	1	1	1	1	1	1	1	1
2	2	1	1	1	1	2	1	1	3
3	2	1	3	2	1	1	2	2	3
3	1	1	1	1	2	3	3	3	3
2	2	2	2	2	2	2	2	2	2
2	2	1	2	2	1	2	2	2	2
2	3	1	2	1	9	3	3	3	3
2	1	2	1	1	1	2	3	3	3
2	1	1	2	1	2	1	3	3	2
1	2	1	2	3	1	1	2	2	2
1	2	1	2	1	1	1	3	3	9
2	2	2	3	1	2	3	1	3	2
1	2	2	2	1	2	3	2	3	3
2	2	3	3	2	2	3	9	2	2
2	2	2	2	3	1	3	3	2	3
3	1	2	2	3	3	1	2	1	2
2	2	2	2	2	2	2	2	2	2
2	1	1	2	1	1	1	3	1	3
2	3	1	1	1	1	2	1	1	3

2	1	3	3	1	1	3	3	3	3
9	1	1	2	1	1	3	3	3	3
2	1	2	2	3	2	2	2	2	2
2	1	1	2	1	3	3	2	2	3
2	3	1	1	1	3	3	3	1	3
2	3	2	1	1	1	1	1	1	1
2	2	2	2	3	1	3	3	3	3
9	1	3	3	1	9	2	1	2	1
3	1	3	3	1	1	3	3	3	3
9	9	9	9	9	9	9	9	9	9
1	3	1	2	1	9	1	2	1	1
2	2	1	1	1	3	1	2	3	2
2	1	2	3	9	9	1	3	2	2
2	2	2	2	1	1	3	1	1	2
2	2	2	2	1	3	3	1	3	3
2	2	2	2	2	2	2	2	2	1
9	3	1	1	2	2	2	2	3	2
9	3	1	1	1	1	2	2	2	2
3	3	3	3	3	3	3	3	3	3
3	3	3	3	1	1	1	3	3	3
3	3	3	3	3	3	3	3	3	3
3	3	3	3	3	3	3	3	3	3
2	2	2	2	1	2	3	1	3	2
1	3	3	2	2	3	3	2	2	9
2	2	2	2	2	2	2	2	2	2
9	3	9	9	3	9	1	9	9	2
2	2	2	2	1	2	2	1	2	1
2	2	2	2	1	1	2	2	2	2
3	2	2	1	2	3	2	2	2	2
1	2	3	1	1	1	1	2	1	3
9	2	3	1	3	1	3	3	3	1
2	1	1	1	2	2	3	1	2	2
2	1	2	3	2	2	2	3	2	3
3	3	3	3	3	3	3	3	3	3
2	3	1	2	1	1	3	3	3	3
2	3	1	1	1	1	2	2	2	2
2	3	1	1	1	1	2	2	2	2
2	3	1	1	1	1	2	2	2	2
2	2	2	2	1	3	2	2	2	2
3	2	2	2	2	3	2	2	2	2
3	1	1	1	1	2	3	1	1	1
3	3	3	3	3	1	3	1	3	3
3	1	3	2	1	3	2	2	2	2
1	1	3	3	3	1	3	3	3	3
3	3	3	3	1	2	3	3	3	3
2	1	1	1	3	1	3	2	2	3
2	2	1	1	1	1	1	1	2	3

1	2	2	2	1	3	3	3	2	2
9	1	3	2	3	1	1	3	9	1
2	3	3	3	1	1	3	3	3	3
2	2	2	2	2	2	2	2	2	2
2	2	2	2	3	3	3	3	2	2
9	9	9	9	9	2	9	2	9	1
2	2	2	2	2	2	2	2	2	2
2	2	2	2	2	2	2	2	2	2
2	2	1	2	3	1	3	2	1	2
2	2	3	3	1	2	2	3	3	3
1	2	2	1	1	2	1	1	2	3
2	1	1	3	1	2	1	3	2	3
9	9	1	9	1	9	9	9	9	9
9	9	9	9	9	9	9	9	1	9
9	9	2	9	9	9	9	9	9	9
3	3	1	3	3	3	1	3	2	2
2	2	1	2	1	3	2	2	2	3
1	9	2	1	1	1	3	1	1	9
2	3	1	2	1	1	3	3	1	3
3	2	1	2	3	1	3	3	3	3
3	3	3	3	2	3	3	3	3	3
2	1	1	1	1	3	3	3	3	3
2	1	1	1	1	1	3	3	3	3
2	2	1	2	2	2	2	2	2	2
1	3	1	1	3	1	3	3	1	3
1	2	2	2	1	1	3	2	2	2
1	3	1	1	3	1	3	3	1	3
9	9	9	2	3	3	3	2	2	3
9	9	9	9	3	3	3	3	3	3
3	1	1	3	3	1	3	3	3	3
3	2	3	2	1	3	3	2	3	2
2	3	2	1	9	1	3	2	3	3
2	3	3	1	2	1	3	9	2	2
1	1	3	1	2	1	1	2	3	3
3	3	3	3	3	1	3	3	3	3
3	3	3	3	1	9	3	3	3	3
2	1	2	3	3	1	2	2	2	2
3	2	3	2	1	2	1	3	3	3
3	3	1	3	1	1	3	3	2	2
2	3	3	2	1	1	3	3	3	3
3	3	2	2	3	1	1	1	2	2
1	2	2	3	3	1	3	3	3	3
2	2	2	1	1	3	3	1	3	3
2	2	1	2	3	1	3	2	3	2
2	2	2	2	1	1	3	2	3	3
2	2	3	3	1	1	3	3	3	3
2	1	2	2	3	1	3	2	3	2

2	2	2	1	2	2	2	2	2	2
2	1	2	1	3	3	3	3	3	1
9	9	9	1	9	1	9	9	9	9
3	3	2	1	1	2	2	3	2	3
2	2	1	2	3	1	2	2	3	3
3	3	1	2	2	3	3	1	1	3
2	2	2	2	2	1	3	2	3	3
3	3	3	3	1	2	3	2	2	1
3	3	1	3	3	3	1	2	3	2
2	2	2	3	3	3	3	3	3	3
2	2	9	1	3	2	2	2	3	2
9	1	9	9	1	3	1	3	9	3
2	1	2	1	1	1	1	2	3	3
1	1	1	2	2	1	9	3	2	9
2	1	1	2	2	1	3	2	3	3
9	9	9	9	3	1	3	9	2	3
3	3	3	3	3	1	3	3	3	3
1	2	3	3	2	1	3	3	3	1
1	3	2	1	3	1	3	3	3	3
3	1	3	3	3	1	3	1	3	3
2	2	2	2	2	1	3	2	2	3
3	3	3	3	9	9	9	9	9	9
3	3	3	3	3	3	3	3	3	3
2	3	2	1	1	2	1	3	2	2
2	2	2	2	1	1	3	3	3	3
2	1	3	2	3	9	3	9	9	9
9	9	9	9	2	1	3	2	9	1
2	1	2	1	1	1	3	1	2	1
9	9	9	9	9	1	9	9	9	9
9	9	1	9	1	9	2	9	9	9
9	9	9	9	9	1	9	9	9	9
2	3	1	2	2	1	3	2	2	3
3	3	3	3	3	1	1	3	1	3
2	1	3	2	3	1	3	2	3	3
3	2	1	3	3	1	3	1	3	2
1	2	2	1	2	1	3	3	9	3
1	2	2	1	2	1	3	3	9	3
3	3	3	3	1	2	1	2	2	2
2	2	1	3	2	1	3	2	1	2
2	2	2	2	3	1	2	1	2	2
9	9	2	9	1	1	9	3	3	3
3	1	3	1	3	3	3	1	1	3
2	2	2	3	3	1	3	1	3	3
2	3	1	1	1	2	1	2	3	3
2	3	1	1	1	2	1	2	3	3
2	3	2	2	1	2	1	1	2	2
2	2	1	2	1	1	1	2	3	3

2	2	2	1	3	1	3	3	1	3
1	1	2	3	1	1	2	2	1	1
9	9	1	9	3	1	3	3	3	3
9	1	9	9	3	1	3	1	3	3
9	1	9	9	3	1	3	3	3	3
1	3	3	1	3	1	3	3	3	3
1	3	1	1	2	3	1	1	3	3
2	2	2	2	3	2	2	2	2	9
2	1	1	1	9	9	9	9	9	9
1	1	2	2	3	1	3	3	1	2
2	2	2	2	2	2	3	3	2	2
2	3	2	1	3	1	3	1	2	2
1	1	2	1	3	3	9	2	9	3
2	2	1	1	1	1	3	3	2	3
3	3	3	2	2	3	3	2	9	9
9	9	9	9	9	9	9	9	9	9
3	3	1	1	1	3	2	2	1	2
1	1	2	2	2	1	3	3	1	1
3	2	2	1	9	9	9	9	9	9
9	9	9	9	1	2	9	1	9	9
9	9	9	9	9	1	9	1	9	9
3	3	3	3	2	1	3	3	3	3
3	9	9	9	9	9	3	3	3	3
9	9	9	9	9	9	9	9	9	9
3	3	3	1	3	1	3	3	3	3
9	9	9	2	2	1	2	2	1	2
1	3	3	3	2	1	3	3	3	3
1	3	3	3	3	1	3	3	3	3
3	3	3	3	3	1	3	3	3	3
2	3	2	3	3	3	3	3	3	3
2	2	2	2	1	1	2	2	2	2
2	2	1	2	1	3	3	1	3	3
2	2	2	2	2	2	2	3	2	3
2	3	3	3	3	1	3	3	2	3
2	2	2	2	1	1	2	1	2	2
1	3	3	3	1	1	3	3	3	3
2	3	2	2	3	3	3	1	3	3
3	3	3	1	3	1	3	3	3	3
2	2	2	2	3	1	3	2	2	3
2	2	1	2	3	1	3	3	1	9
3	2	2	1	1	1	1	2	2	2
1	1	1	1	1	3	2	2	1	2
1	2	2	2	1	2	2	2	1	2
9	9	9	1	1	9	3	9	9	9
9	9	9	9	2	9	9	9	9	9
3	1	3	3	3	1	3	1	3	3
2	3	3	3	1	1	1	1	1	1

2	2	1	2	2	1	2	2	1	2
2	3	2	2	3	1	3	2	2	2
2	2	2	2	2	1	2	2	2	3
2	2	2	2	2	3	1	1	1	3
2	2	2	2	3	1	2	2	1	1
2	3	3	2	1	1	1	3	1	2
2	1	2	3	2	1	2	3	1	3
1	2	3	2	1	2	3	1	2	3
9	9	9	9	9	9	9	9	9	9
1	1	2	2	1	2	2	1	2	1
2	2	2	2	2	9	2	2	2	2
1	2	1	1	2	1	3	2	3	3
3	1	2	1	1	2	1	2	3	1
2	2	2	2	2	2	2	2	2	2
2	2	2	2	2	9	3	2	2	2
3	1	2	2	2	2	3	2	1	2
3	1	2	3	1	2	3	1	2	3
3	1	2	3	3	2	2	2	2	2
2	2	2	3	2	1	1	1	1	1
2	2	2	2	3	3	3	3	3	3
2	3	1	1	1	3	1	1	2	3
3	3	3	3	3	3	3	3	3	3
2	2	2	2	2	2	2	3	3	2
2	2	1	3	2	2	2	1	2	3
2	3	3	3	2	3	2	2	2	2
2	3	3	2	2	3	3	9	9	9
2	2	2	2	3	1	2	3	3	1
2	9	1	3	1	2	1	1	1	1
9	1	2	1	1	2	2	1	3	2
9	9	9	9	9	9	9	9	9	9
2	2	2	3	1	1	2	2	2	3
9	9	9	9	9	9	9	9	9	9
3	3	3	3	3	3	3	3	3	3
1	1	1	1	2	1	1	9	1	1
2	3	2	3	1	2	3	1	3	1
2	1	2	1	1	2	1	2	1	2
9	2	1	2	1	2	3	2	1	1
2	2	2	2	1	2	1	2	9	1
2	2	2	2	2	2	2	2	2	2
2	1	2	3	1	2	2	2	2	2
2	3	1	2	2	3	2	3	1	3
9	9	9	9	9	1	9	9	9	9
2	2	2	2	2	2	2	1	2	2
2	3	2	2	1	1	3	2	3	2
2	2	2	2	2	1	2	2	9	2
2	3	1	2	3	1	3	3	3	2
1	1	2	3	3	1	3	3	2	1

9	1	9	1	1	3	3	3	3	3
2	2	2	1	3	3	3	2	2	3
3	1	3	2	1	3	1	3	1	3
3	3	1	2	1	2	3	1	1	2
2	3	2	2	1	2	2	1	2	3
2	2	2	2	2	2	2	2	2	2
3	3	1	1	3	3	3	3	1	1
1	2	1	2	3	1	3	3	1	1
9	9	1	9	9	1	9	9	9	9
3	3	2	2	1	1	3	2	2	1
2	3	2	2	3	1	3	1	3	3
2	1	3	2	3	3	3	1	3	3
9	9	9	9	9	9	9	9	9	9
2	2	2	2	1	1	3	2	2	3
2	3	1	1	3	3	1	1	1	1
1	1	3	3	2	1	1	1	1	2
9	9	1	9	3	3	3	1	3	1
1	3	2	1	1	3	2	3	1	2
2	2	3	3	3	1	3	1	1	1
2	2	2	2	2	1	1	1	2	2
2	1	2	1	1	2	1	2	1	2
3	3	3	3	3	3	3	3	3	3
2	2	2	2	1	1	1	2	2	2
2	2	3	2	3	1	3	3	2	2
3	3	2	1	1	1	3	3	2	3
2	3	1	2	3	1	3	3	3	2
2	3	3	2	3	1	3	3	3	2
2	3	3	3	3	2	2	1	2	3
2	1	3	3	2	1	3	1	1	2
1	2	1	2	3	1	3	3	3	3
1	1	2	2	2	2	2	2	2	2
2	3	1	2	2	2	2	2	2	3
2	2	2	2	1	1	2	1	2	2
1	1	1	1	2	1	3	3	3	3
3	3	2	2	2	1	3	2	3	2
2	1	2	9	1	9	9	9	9	9
3	3	3	3	1	2	3	3	3	1
2	3	2	2	1	3	3	2	3	1
2	2	3	1	3	1	3	3	3	9
2	2	2	2	2	2	2	2	2	2
3	3	3	3	1	2	3	3	3	1
3	3	1	2	1	2	3	1	1	3
2	2	2	1	1	1	3	3	3	3
2	2	2	2	1	1	3	9	1	2
2	1	1	2	9	9	9	9	9	9
3	3	1	3	3	1	3	2	3	1
9	9	9	9	1	1	3	1	9	9

9	1	9	9	1	1	3	3	3	3
3	1	9	3	2	3	3	1	2	2
3	1	3	1	1	3	3	1	1	3
3	3	1	1	3	3	3	3	3	3
2	2	2	2	3	2	2	1	1	2
2	1	1	3	2	9	2	1	1	1
2	2	2	2	2	2	2	2	2	2
9	1	1	9	9	9	1	1	1	1
3	3	3	3	2	3	1	3	2	3
3	3	3	3	2	2	2	1	2	1
3	3	3	3	3	3	3	3	3	3
2	3	2	2	1	2	3	2	3	1
2	2	2	2	1	2	3	3	3	3
2	2	2	3	1	2	2	3	1	3
3	1	9	3	2	3	3	1	2	2
3	2	2	2	3	3	2	1	2	3
9	9	9	9	3	1	3	2	3	3
9	9	9	9	9	9	9	9	9	9
3	3	3	3	9	9	9	9	9	9
9	9	9	9	3	9	2	1	3	2
3	1	2	3	1	2	2	2	2	2
2	1	9	3	1	2	2	1	2	1
2	2	2	3	1	1	1	3	2	3
3	3	3	3	2	2	2	2	2	2
1	2	2	3	1	2	1	3	2	1
9	2	9	9	2	9	2	2	2	3
9	9	9	9	2	9	9	9	9	2
3	2	2	2	2	2	2	2	1	2
9	9	9	9	9	9	9	9	9	9
9	9	9	9	9	9	9	9	9	9
9	9	9	9	9	9	9	9	9	9
9	9	9	9	9	9	9	9	9	9
9	9	9	9	9	9	9	9	9	9
1	1	1	2	1	2	2	2	2	1
1	3	1	2	2	3	3	1	2	3
9	9	9	9	2	1	2	3	2	3
9	9	9	9	9	9	9	9	9	9
2	2	2	2	3	1	3	2	1	3
9	1	9	1	9	9	9	9	1	1
2	2	2	2	2	2	2	2	2	2
2	2	2	2	2	2	2	2	2	2
1	2	2	1	2	1	3	2	3	3
2	1	1	2	1	1	2	2	2	2
2	2	2	2	1	2	2	2	2	2
1	1	1	2	1	1	1	1	1	1
2	1	1	2	1	2	1	2	2	2
2	3	2	3	2	3	1	2	3	2

2	2	2	2	2	2	2	2	2	2
2	2	2	2	2	2	2	2	2	2
2	3	2	2	9	1	9	9	9	9
3	1	2	3	1	2	1	1	1	2
2	3	3	3	1	2	1	1	2	2
3	3	3	1	1	1	1	1	1	3
2	2	9	3	1	9	1	9	9	2
2	2	2	2	9	1	2	2	2	2
2	2	2	2	2	2	2	2	2	2
3	2	1	2	2	2	2	2	2	2
2	2	2	2	3	1	3	3	3	3
2	2	2	3	2	9	2	2	2	1
1	2	9	9	9	9	9	9	9	9
1	2	3	2	1	1	1	1	1	1
2	9	3	2	2	3	1	2	1	9
1	1	1	1	1	1	1	1	3	3
1	2	2	3	1	2	2	3	2	2
9	9	9	9	9	9	9	9	9	9
1	2	3	2	3	1	2	3	2	2
1	3	2	2	1	2	2	2	2	2
1	9	9	9	1	9	9	9	9	9
2	1	3	2	3	3	1	2	1	3
3	3	1	2	1	2	3	2	2	2
1	1	1	1	9	1	9	9	9	9
9	9	9	9	3	3	1	9	9	9
9	9	9	9	9	2	9	9	9	9
2	1	1	2	2	2	2	2	2	3
3	2	1	1	2	2	3	3	3	3
9	9	9	9	9	1	9	9	9	9
3	2	2	2	2	2	2	2	2	2
1	3	1	3	1	3	1	3	1	3
3	3	1	1	3	1	3	3	3	3
1	3	1	1	1	1	3	2	3	3
1	3	1	1	1	1	3	1	1	1
2	1	1	2	9	9	9	9	9	9
2	2	2	2	3	3	3	3	3	3
1	3	1	1	1	1	1	1	3	1
3	3	2	2	1	1	3	2	2	2
2	1	1	1	1	2	1	2	3	1
9	9	9	9	1	9	9	1	1	1
2	9	1	3	2	1	2	3	2	1
2	3	2	2	3	1	3	3	3	3
2	2	2	2	2	3	3	2	2	3
2	2	3	2	2	1	3	2	3	3
2	3	1	2	9	1	3	3	3	3
1	1	1	1	1	1	1	1	1	1
3	3	3	3	1	1	1	3	3	3

1	2	3	1	1	2	1	2	3	1
2	2	9	1	1	1	3	3	3	2
2	1	1	3	3	1	1	1	2	3
2	2	1	3	3	1	2	9	3	3
3	3	2	2	1	2	2	1	1	1
3	3	2	2	1	2	2	1	1	1
1	2	3	3	3	1	3	3	3	2
2	2	3	1	1	3	3	3	3	3
2	3	2	3	3	1	3	2	2	3
2	1	3	2	3	3	3	3	1	1
2	3	2	2	3	1	3	3	3	2
2	3	2	2	3	1	1	2	1	1
9	2	9	3	3	3	3	3	3	3
1	3	1	3	3	2	3	3	2	2
2	1	1	1	3	1	3	1	3	3
3	3	3	3	3	1	3	3	1	1
3	3	3	3	1	1	3	3	3	3
2	2	2	2	1	3	3	3	3	3
2	3	2	2	3	2	3	2	2	2
9	1	1	1	3	1	2	2	2	2
2	2	1	2	3	2	1	2	2	1
2	2	9	2	2	1	3	2	2	3
1	1	1	1	1	1	1	1	3	9
1	3	9	3	1	1	1	1	1	1
1	1	1	1	1	1	1	1	1	1
1	1	1	1	1	1	1	1	1	1
2	3	9	2	1	1	2	2	2	2
3	1	1	3	3	3	2	3	1	3
3	1	1	3	2	2	2	2	2	2
2	3	2	2	3	1	3	3	3	3
3	3	3	3	1	3	3	3	2	3
9	9	9	9	1	1	2	9	3	1
2	2	2	2	2	2	2	2	2	2
3	2	1	2	3	3	1	3	1	3
2	2	3	3	2	2	1	2	1	3
1	3	3	3	3	1	1	1	2	3
2	2	2	2	1	3	2	2	2	2
1	3	1	1	3	3	3	3	3	3
2	2	1	2	1	1	1	1	3	3
2	2	2	2	1	3	3	2	2	2
2	2	2	2	3	3	2	2	2	2
3	2	2	3	2	3	2	3	3	2
2	2	3	2	1	1	1	3	3	3
2	2	3	3	3	3	2	1	2	1
2	1	1	1	2	3	3	1	2	2
3	2	2	2	3	3	2	1	2	3
2	1	1	1	3	3	1	1	2	3

2	1	1	1	1	2	3	1	1	1
9	9	9	1	1	1	9	9	9	1
9	9	9	1	1	1	9	9	1	1
2	2	2	2	2	3	3	3	3	3
9	9	9	9	1	9	9	9	9	9
9	9	9	1	1	1	9	9	9	9
9	9	9	9	9	9	9	9	9	9
2	3	3	3	1	2	1	1	1	2
2	1	3	1	2	1	3	3	2	3
2	3	3	2	2	1	2	3	3	3
2	2	2	2	2	3	3	2	2	2
2	2	2	2	2	2	3	3	2	2
2	2	2	3	1	9	3	3	3	3
2	2	2	3	1	9	3	3	3	3
2	2	2	2	2	1	2	2	2	2
2	2	2	2	2	2	2	2	2	2
3	3	3	2	2	3	3	9	3	3
1	1	2	2	1	1	3	3	2	3
2	3	3	2	2	1	3	3	3	3
2	2	1	2	1	1	2	1	2	1
2	2	2	2	3	1	3	3	2	3
1	2	1	1	1	1	1	1	1	1
9	9	9	9	1	2	2	2	2	2
2	2	2	2	2	2	2	2	2	2
2	2	2	2	2	2	2	2	2	2
1	1	2	1	1	3	3	2	9	1
3	3	3	2	1	2	1	1	1	2
2	1	1	2	9	9	9	9	9	9
1	3	2	2	2	3	3	2	1	2
1	2	3	2	1	3	1	1	1	1
2	2	1	2	1	1	3	3	3	2
2	1	1	2	3	1	3	1	1	1
2	3	2	3	2	1	3	3	1	2
1	1	2	2	1	1	3	1	2	3
3	3	3	3	3	1	3	3	2	3
3	3	2	2	2	9	2	9	2	2
2	2	2	2	2	2	2	2	2	2
2	2	2	2	2	1	2	2	2	2
2	2	2	2	1	1	2	2	2	2
2	3	9	2	9	9	9	9	9	9
2	2	2	2	2	2	3	2	1	3
1	1	2	3	1	2	3	2	1	1
3	1	2	1	3	3	1	1	2	1
2	1	1	2	2	1	2	1	1	2
2	1	1	1	1	2	3	1	1	1
2	2	2	2	2	2	2	2	2	2
2	2	2	2	3	1	3	2	2	2

2	1	1	2	2	1	2	1	1	1
9	9	9	9	3	9	9	9	9	9
2	1	2	2	3	1	1	1	2	9
3	3	3	3	3	1	1	1	2	3
3	3	3	3	3	1	2	2	2	1
9	1	2	2	2	1	3	2	2	3
3	3	3	3	3	3	2	3	3	2
2	2	1	1	2	1	3	3	3	3
9	2	3	1	1	1	3	2	9	2
2	2	2	2	1	2	3	3	2	2
2	3	1	2	3	1	3	1	3	2
2	2	2	2	2	3	3	3	3	3
2	2	2	2	1	2	3	3	2	2
1	1	1	2	1	1	1	1	1	1
2	2	2	2	1	2	3	2	2	3
2	2	2	2	1	1	3	3	3	3
2	2	2	1	3	1	3	2	1	1
9	9	1	9	3	1	3	3	3	3
2	2	2	2	3	2	2	2	9	9
1	1	1	1	1	1	1	1	1	2
1	1	2	1	1	1	3	2	3	2
3	3	2	2	3	1	3	3	3	3
2	2	1	2	2	1	3	2	3	2
2	2	2	2	2	2	2	1	2	1
2	1	1	2	2	1	1	1	1	2
1	2	3	2	1	1	2	9	2	3
2	1	1	3	1	1	3	3	3	3
2	3	2	2	3	1	3	9	2	1
2	3	2	3	1	2	1	3	2	1
2	3	3	2	3	1	2	1	3	3
2	3	2	2	2	1	2	2	2	2
2	2	3	3	2	1	1	2	2	3
3	3	2	2	1	3	3	1	1	1
9	9	9	9	1	2	3	1	2	3
1	1	3	1	3	1	3	3	3	3
3	1	2	2	2	3	2	3	1	2
2	3	2	3	1	3	3	3	3	3
1	2	1	1	3	2	2	2	3	1
2	2	2	2	9	1	3	2	2	3
2	2	2	3	3	1	3	2	2	3
9	9	9	9	9	9	9	9	9	9
1	2	3	1	1	2	2	3	3	2
9	9	9	9	1	1	3	3	3	3
2	3	3	3	3	1	3	3	3	3
1	2	2	2	1	1	3	3	3	3
2	1	1	1	1	1	3	1	3	3
2	3	2	2	2	3	3	2	3	3

2	3	1	2	1	3	2	1	3	3
9	9	9	2	9	9	9	9	9	9
2	1	1	1	1	3	1	2	2	2
2	3	2	2	1	1	3	3	3	3
2	2	2	2	1	2	2	2	2	2
1	2	1	2	2	2	2	2	2	2
1	2	3	2	1	2	3	2	3	2
1	1	1	1	1	1	1	1	1	1
2	2	2	2	2	2	2	2	2	2
9	9	9	9	3	3	3	1	1	2
2	2	2	2	1	9	9	9	9	9
2	3	3	3	2	1	3	2	3	3
3	3	1	3	1	1	1	1	1	1
2	2	2	2	2	3	1	1	1	1
2	3	3	3	1	3	3	3	3	3
2	3	1	1	2	1	3	2	3	3
9	1	9	3	2	3	3	1	3	2
2	2	3	2	1	1	1	2	3	9
2	2	1	1	1	1	3	1	3	2
1	1	1	2	1	3	1	2	2	9
2	2	2	2	1	2	2	1	1	1
9	9	9	9	1	3	2	2	3	9
2	3	1	2	3	2	1	1	1	2
2	3	1	3	1	3	2	1	2	1
3	1	2	3	1	2	2	2	2	1
9	9	9	9	3	2	3	3	2	3
2	2	2	2	1	1	3	3	3	3
9	9	9	9	1	1	1	2	1	1
1	1	9	1	2	1	1	2	3	3
2	2	2	2	1	3	3	3	1	3
3	2	1	1	2	1	2	1	1	3
2	3	2	3	1	2	1	1	3	2
2	3	2	3	1	9	2	3	3	2
1	3	2	2	3	1	3	2	3	3
2	3	3	2	1	1	1	3	1	3
3	3	3	3	1	1	1	3	1	3
1	2	2	1	2	1	2	1	1	3
9	9	9	2	9	9	9	1	9	9
2	3	2	1	3	3	2	3	3	3
9	2	2	2	3	1	3	2	3	3
2	3	2	1	1	3	3	2	1	2
9	1	2	2	2	1	1	3	3	2
9	1	3	2	1	1	3	2	2	2
9	9	9	9	9	9	9	9	9	9
2	2	2	2	1	1	1	1	1	1
2	2	2	2	1	2	1	1	1	1
9	1	9	9	1	3	3	3	2	9

3	3	1	3	3	1	3	3	1	3
9	9	9	9	2	9	9	9	9	9
2	1	3	2	2	3	1	2	1	3
1	1	2	2	2	1	3	2	2	3
2	2	2	1	1	1	2	1	2	2
2	2	3	3	2	1	3	3	3	3
1	3	2	1	3	3	2	2	1	1
2	2	2	2	1	3	3	3	3	3
2	2	2	2	1	3	1	3	3	3
9	9	9	9	9	9	9	9	9	9
2	3	1	1	3	1	2	3	3	3
3	2	1	3	1	3	2	1	1	1
3	1	3	1	1	3	1	3	1	3
3	1	2	1	3	2	1	2	1	3
2	2	2	2	2	1	3	3	3	3
1	1	1	1	3	1	2	1	1	1
1	1	1	1	1	1	1	3	1	1
3	3	1	2	1	2	2	1	3	2
2	2	2	2	1	2	1	1	2	2
2	2	2	2	1	2	2	2	1	2
9	9	9	9	9	9	9	9	9	9
2	2	2	2	1	2	1	1	1	2
2	2	2	3	2	2	2	2	2	1
1	1	1	1	1	2	2	1	2	2
2	2	1	1	1	2	2	2	2	2
2	2	2	2	2	2	2	2	2	2
2	2	2	2	2	2	1	1	1	1
3	3	2	2	2	3	3	2	2	3
2	1	2	2	2	2	2	2	2	3
2	2	2	2	3	2	1	1	2	2
9	9	9	9	9	9	9	9	9	9
9	9	9	9	9	9	9	9	9	9
9	9	9	9	9	9	9	9	9	9
9	1	2	2	2	1	3	3	3	3
9	1	3	3	2	1	3	3	2	3
2	2	2	2	9	2	3	2	2	3
2	2	1	1	1	3	1	1	1	1
2	1	1	1	1	2	3	1	1	2
1	1	1	1	1	3	1	1	1	1
2	3	2	2	1	1	3	2	2	3
3	3	3	3	1	3	3	3	1	1
2	1	2	1	3	1	3	2	3	3
2	1	1	2	2	1	3	2	2	2
2	1	3	2	1	1	3	3	3	3
2	1	2	2	2	3	1	2	2	3
3	3	1	1	1	1	2	3	3	3
1	3	1	3	1	1	3	3	3	3

2	3	3	2	1	2	1	1	1	2
2	2	2	2	1	1	3	1	1	2
2	3	3	2	1	2	1	1	2	2
1	1	1	3	3	3	1	1	3	1
3	3	3	3	3	1	3	3	3	9
2	2	3	1	1	1	1	3	3	3
2	2	2	2	3	3	3	1	1	1
9	9	9	9	1	9	9	9	9	1
9	9	9	9	1	3	9	9	9	9
9	1	9	9	1	1	3	1	1	1
9	9	9	9	1	2	2	2	9	9
9	9	9	9	1	9	9	9	1	9
2	2	2	2	1	1	3	1	2	3
9	9	9	9	2	3	1	1	1	2
3	1	3	3	1	1	3	3	3	2
2	2	2	2	3	1	3	3	3	3
2	2	1	2	1	1	1	2	1	2
2	2	1	2	1	1	1	2	1	2
2	2	2	3	1	1	3	3	3	3
1	9	2	1	3	3	1	2	1	2
2	2	2	2	3	2	3	2	2	2
2	2	2	2	1	1	3	2	2	3
9	9	9	9	3	1	3	1	1	1
3	3	3	3	1	3	2	2	2	2
1	2	3	2	3	1	3	2	3	2
2	3	1	1	3	1	3	1	1	2
2	2	2	2	1	1	3	3	2	2
2	2	2	2	1	1	3	2	1	3
2	2	2	2	1	2	2	2	2	2
2	3	3	3	1	2	2	1	2	3
2	2	2	3	1	9	3	3	3	3
1	3	2	1	3	3	1	1	1	1
2	3	3	2	3	1	3	1	2	3
2	2	2	3	1	1	3	3	2	1
9	2	9	9	2	9	2	3	9	9
2	3	3	3	3	2	3	2	3	3
2	2	1	1	1	1	3	2	2	3
2	2	2	2	1	1	3	3	3	2
2	3	2	2	3	1	3	2	1	3
2	2	2	2	2	1	3	2	2	2
9	9	9	9	3	3	3	1	1	1
9	9	9	9	9	1	9	9	9	9
9	9	9	9	9	1	9	9	9	9
2	2	2	2	3	1	3	2	2	3
1	9	9	9	3	1	2	2	3	2
2	2	2	2	3	1	3	1	2	3
1	2	2	2	3	2	2	3	3	3

2	2	1	2	1	1	3	2	2	2
2	1	1	1	3	3	2	2	2	1
2	2	2	3	3	3	3	1	1	1
1	3	3	1	1	3	3	2	3	2
3	3	3	3	2	2	2	2	2	2
2	2	2	2	3	1	3	3	3	3
3	1	2	1	1	1	3	9	2	3
3	3	3	3	1	3	3	2	1	1
1	3	2	2	3	1	3	1	1	3
2	1	1	2	2	1	1	2	3	1
2	2	2	2	3	1	3	3	2	3
3	3	1	2	1	3	3	3	2	1
3	3	1	3	3	1	3	3	3	3
2	3	2	3	1	1	3	3	3	1
2	1	2	2	3	1	3	2	2	3
2	2	2	2	3	1	3	2	2	2
2	2	1	1	3	1	2	1	2	2
2	2	2	2	2	2	2	2	2	2
2	2	2	2	1	2	2	1	2	2
2	2	2	2	2	1	2	2	3	3
2	1	2	2	1	3	3	2	1	1
2	2	1	2	2	1	3	3	3	3
2	3	3	2	1	2	1	1	3	3
2	3	2	2	1	2	2	3	3	3
9	1	1	3	1	3	3	3	3	3
2	3	3	2	1	1	3	3	3	3
2	1	2	2	2	1	2	3	1	2
3	1	3	1	3	1	3	3	3	3
2	2	2	1	2	2	2	2	1	2
2	2	2	2	1	3	1	1	2	2
2	3	2	3	9	9	9	9	9	9
3	3	1	3	1	2	3	3	3	3
2	2	2	2	1	2	1	1	1	1
3	3	3	3	2	1	2	2	1	1
1	2	1	2	1	9	2	2	3	1
2	2	2	2	2	2	1	1	1	1
3	3	2	2	1	2	2	2	2	2
2	2	2	2	1	1	3	2	2	2
3	3	1	1	1	3	1	1	9	1
2	1	2	2	9	1	9	1	3	3
1	1	1	1	1	1	3	3	3	9
2	3	2	2	3	1	3	3	2	3
2	2	2	2	2	2	2	2	2	2
3	1	1	1	3	1	3	2	1	2
3	2	1	2	3	1	3	2	3	1
2	2	2	2	2	1	2	2	2	2
2	3	3	2	1	1	1	3	1	3

2	3	3	2	1	1	1	3	1	3
2	2	2	2	1	2	1	2	2	2
2	2	3	3	1	1	3	3	3	3
2	2	2	1	3	2	1	1	1	2
2	2	3	3	1	2	1	2	2	2
2	2	2	2	2	2	1	1	2	2
2	1	1	1	1	2	1	1	1	2
2	3	2	2	1	1	3	2	3	1
3	2	3	3	1	2	2	3	2	1
3	3	3	1	1	1	3	1	3	2
1	1	3	1	3	1	3	3	3	3
3	2	2	1	2	3	3	1	3	3
2	2	2	2	2	2	1	1	1	1
2	1	1	2	2	1	3	2	2	2
3	3	3	3	1	1	3	3	3	1
1	9	9	9	9	2	3	2	3	3
3	3	3	3	1	2	2	1	1	1
2	2	2	2	1	2	1	1	1	2
2	2	2	2	1	1	1	1	1	1
2	2	2	3	1	1	3	3	3	3
1	1	1	2	2	1	3	3	2	3
2	3	3	3	3	1	1	3	1	3
3	3	3	2	1	3	2	1	1	1
2	2	2	2	1	3	3	1	1	1
1	2	2	1	1	1	2	2	1	1
2	3	2	2	1	3	1	3	2	1
1	2	1	1	3	2	1	2	1	3
2	2	2	2	1	1	3	2	2	3
2	3	3	3	3	3	1	3	3	3
2	3	2	2	3	2	3	3	3	2
3	3	3	3	3	3	3	3	3	3
1	1	1	1	3	1	3	3	3	3
3	2	3	2	1	2	2	2	2	2
2	1	1	3	1	3	3	3	3	3
2	1	3	2	2	9	2	9	1	3
2	3	2	2	3	3	1	1	1	1
2	3	2	2	1	3	3	1	2	1
3	1	2	2	1	3	3	2	1	2
2	1	2	1	1	1	1	3	3	3
2	3	2	2	9	1	3	1	1	3
2	2	1	3	2	1	3	1	1	3
1	3	1	1	3	1	3	1	1	3
2	2	2	2	3	1	1	1	1	3
2	1	1	2	3	1	3	3	3	3
3	3	3	3	3	1	3	2	3	3
9	1	2	2	1	2	1	2	3	2
2	2	2	2	1	2	2	2	3	1

3	3	3	3	1	3	2	1	3	2
2	1	1	2	1	3	2	1	3	9
3	2	2	2	1	2	2	2	1	1
2	2	1	3	2	1	9	1	1	3
1	3	1	3	3	3	3	1	3	1
2	2	2	2	2	2	2	2	2	2
2	3	2	2	1	1	2	2	2	2
3	3	3	1	1	1	1	3	2	3
3	1	1	9	3	3	1	9	1	1
3	3	3	3	3	1	2	2	2	2
3	1	1	1	1	2	2	1	1	1
2	3	3	2	1	1	3	3	3	3
2	1	1	1	3	1	1	2	2	1
2	3	9	2	1	1	3	2	2	2
3	3	2	1	1	1	1	2	2	2
2	2	2	1	1	1	3	3	3	3
2	2	2	2	3	1	3	2	2	2
1	1	1	1	3	1	2	2	2	2
2	3	2	1	1	3	1	2	3	2
2	2	2	2	1	2	3	1	3	1
1	2	2	2	1	1	1	2	2	2
2	2	2	2	1	3	2	2	2	1
2	2	2	2	2	2	2	2	2	2
2	2	2	2	1	2	2	2	2	1
2	2	2	2	1	2	2	2	2	1
2	2	2	1	1	1	3	3	3	3
2	3	3	2	1	1	3	3	3	3
3	3	3	3	3	1	2	2	2	2
2	1	2	1	1	1	1	3	3	3
2	2	2	2	1	2	3	1	3	1
2	3	2	2	1	3	3	1	2	1
9	9	9	9	9	9	9	9	9	9
2	3	3	3	2	1	2	2	2	3
2	2	2	1	1	1	3	3	3	1
3	1	1	1	1	3	1	3	3	3
3	2	1	1	2	1	1	2	3	3
3	1	1	1	3	3	1	3	3	3
3	1	2	3	1	3	1	2	3	1
2	2	2	2	3	1	3	3	3	3
2	3	2	1	1	2	1	2	3	2
1	3	1	2	3	3	3	2	3	1
1	3	1	2	3	3	3	2	3	1
1	3	2	3	1	3	2	1	1	1
9	3	3	2	2	1	2	9	2	2
2	2	1	2	1	2	2	3	1	2
2	1	3	2	9	2	1	1	3	3
2	2	3	1	1	1	3	3	3	3

2	2	2	1	1	1	2	2	1	1
3	3	1	1	1	1	3	3	3	3
1	1	1	1	3	9	9	1	1	1
1	3	2	2	1	2	3	2	3	3
2	2	2	2	2	1	3	2	3	2
2	2	2	2	3	1	3	3	3	3
1	1	1	1	1	1	3	3	1	3
2	2	2	2	2	2	2	2	2	9
2	2	2	2	2	2	2	2	2	2
2	2	2	2	2	2	2	2	2	2
2	2	2	2	2	2	2	3	3	3
2	1	2	2	1	3	2	2	1	1
2	2	2	2	2	2	2	2	2	2
2	2	2	2	2	2	2	2	2	2
2	2	2	2	1	1	3	3	3	3
2	2	2	2	1	2	3	3	3	2
2	3	2	2	3	2	1	2	2	3
1	1	1	2	3	1	3	3	3	3
3	2	2	2	1	3	2	2	2	2
2	2	2	2	1	1	1	2	2	2
1	2	2	2	3	3	3	3	3	2
2	2	2	2	3	2	1	2	2	2
2	2	2	2	3	2	3	3	3	3
9	3	2	2	1	2	1	2	2	2
2	2	1	2	2	1	3	3	1	2
2	2	2	3	1	3	3	2	2	1
2	2	2	2	2	1	1	2	3	2
3	3	2	2	3	1	3	1	3	3
2	2	2	2	3	1	3	2	1	3
2	3	1	1	3	1	3	3	3	3
2	2	2	2	2	1	2	2	3	3
2	2	2	2	2	2	2	2	2	2
3	3	3	3	3	3	3	3	3	9
9	1	9	9	3	1	3	3	3	3
2	3	2	2	1	1	3	3	9	3
2	3	3	2	2	2	1	2	3	3
1	2	3	1	1	9	1	1	1	1
3	1	2	2	2	2	1	1	1	1
1	1	2	2	2	1	3	3	3	3
9	9	1	9	3	3	3	1	3	3
1	3	1	1	1	2	3	1	1	3
3	9	1	1	3	1	3	1	1	1
3	3	3	3	3	3	3	2	3	2
3	2	2	2	3	1	3	3	3	3
1	2	3	1	1	2	3	2	1	2
2	3	2	3	3	1	3	3	3	3
3	1	2	3	2	3	3	2	1	2

2	2	2	2	2	2	1	2	3	3
9	2	1	9	3	3	2	9	9	9
9	9	9	9	9	9	9	9	9	9
1	2	2	2	1	1	2	2	3	2
2	2	2	2	1	1	3	3	3	1
9	9	9	9	9	9	9	9	9	9
2	3	3	2	1	2	1	2	2	2
2	3	2	2	1	3	2	2	1	1
2	2	2	2	2	1	1	1	1	1
2	2	2	1	3	3	3	2	3	1
1	3	3	3	3	1	3	3	3	3
3	1	3	3	3	1	3	1	3	3
2	2	3	2	3	2	1	3	2	9
3	1	1	1	2	1	3	1	1	3
9	9	9	1	1	3	1	1	1	1
9	1	3	2	3	1	2	2	2	2
9	9	9	9	1	9	9	2	9	9
2	2	1	1	1	3	1	2	2	3
2	2	1	1	2	2	2	2	1	1
2	2	1	1	1	2	1	1	2	2
1	1	1	1	1	1	1	1	1	2
2	1	1	1	1	3	1	2	2	1
2	2	2	2	3	2	2	2	2	3
3	1	1	3	3	1	3	3	1	3
2	2	2	2	2	2	2	2	9	2
2	3	3	3	3	3	3	3	3	3
3	3	3	2	1	3	2	3	2	2
2	2	3	2	2	3	3	1	2	2
2	1	1	1	1	2	1	1	1	2
1	2	2	2	1	3	1	2	2	3
2	2	2	2	1	2	1	2	2	3
2	1	1	1	3	9	9	2	2	3
2	1	1	1	3	3	1	3	2	3
2	1	1	1	3	3	3	1	1	1
2	1	1	1	2	1	1	1	1	3
1	3	3	3	3	1	3	3	3	3
9	9	9	9	9	9	9	9	9	9
2	2	2	2	2	2	3	2	3	2
9	9	9	9	9	9	9	9	1	9
2	2	1	1	2	3	1	3	3	3
2	9	1	1	3	3	1	9	1	1
2	1	1	2	1	2	1	2	2	2
1	2	1	1	1	3	3	1	2	1
1	2	1	3	3	1	3	3	3	2
2	2	3	2	3	1	2	3	1	3
2	2	1	2	3	1	2	1	1	1
2	3	2	1	3	3	1	1	1	1

9	9	9	9	9	9	9	9	9	9
1	1	1	1	1	1	2	1	1	3
2	2	1	2	3	1	3	3	2	1
2	1	2	1	1	2	3	9	2	1
2	2	2	2	1	1	1	1	1	1
2	2	2	2	1	1	1	2	3	3
1	1	2	3	1	2	1	2	3	2
3	3	3	3	1	1	3	1	1	1
2	2	2	2	1	3	3	3	2	3
2	2	1	3	3	1	1	1	2	2
1	1	1	1	1	1	1	2	2	1
1	1	2	1	1	2	2	2	1	1
2	2	2	2	3	1	3	2	3	2
3	3	1	1	1	3	1	1	1	3
1	2	1	2	2	1	1	3	3	3
2	3	1	1	1	1	3	2	2	3
9	9	9	9	1	3	3	1	2	1
2	2	2	2	2	1	1	1	2	2
2	2	1	1	1	2	1	1	1	2
2	2	2	1	2	1	2	3	1	3
1	1	1	3	1	1	3	2	3	3
1	1	1	3	1	1	3	2	3	3
1	3	1	1	1	2	1	1	1	3
2	2	2	2	2	9	2	3	3	3
3	3	3	1	3	1	3	3	3	1
1	1	1	2	1	3	2	2	2	1
3	1	1	1	1	1	3	3	3	3
2	1	2	3	1	1	1	3	1	3
2	2	1	1	2	1	1	1	1	2
2	2	2	2	1	1	2	2	2	2
2	2	2	2	2	2	2	2	2	2
2	3	3	3	1	1	3	3	3	3
1	1	1	1	3	2	2	2	2	2
1	2	2	1	2	1	2	9	1	2
2	2	2	2	1	2	2	1	2	2
2	3	1	2	3	3	1	2	3	1
2	2	1	1	1	2	1	1	1	1
1	2	1	1	3	1	3	2	2	2
1	3	2	2	3	1	2	2	1	1
2	1	2	1	1	9	2	1	2	1
1	3	3	2	3	1	3	2	3	9
2	2	2	3	1	3	1	2	2	2
9	9	9	9	2	2	2	2	2	2
1	1	1	3	1	2	1	1	1	1
9	9	9	9	9	9	9	9	9	9
3	3	3	3	1	1	3	1	1	1
1	1	1	1	1	2	1	1	1	2

9	9	9	9	9	9	9	9	9	9
2	2	1	1	1	2	2	2	2	2
2	2	2	2	1	2	1	2	1	1
2	2	2	2	3	1	3	1	3	3
2	2	2	2	3	1	3	1	3	3
2	1	1	2	1	3	3	3	1	2
1	1	3	2	1	1	2	2	2	9
3	3	3	3	9	3	3	3	3	3
3	3	9	3	1	1	1	1	1	1
2	2	2	2	3	2	3	2	2	2
1	1	1	2	1	1	3	3	1	1
3	9	1	3	1	2	2	2	3	2
9	1	9	9	1	9	9	9	9	9
3	1	2	3	1	2	2	2	3	3
9	9	9	9	1	3	1	1	1	2
2	2	2	2	9	9	9	9	9	9
1	2	3	1	1	2	2	1	3	3
3	1	1	1	1	2	3	3	3	3
2	2	2	2	2	2	2	2	2	2
3	3	3	3	1	1	2	2	2	2
1	2	2	1	1	2	1	2	2	1
2	2	1	2	2	1	1	1	1	1
2	2	1	1	1	2	1	2	2	1
2	2	2	2	1	2	2	2	2	2
2	1	2	1	1	2	3	9	2	1
2	2	3	1	1	3	1	9	1	1
9	9	2	9	9	9	9	9	1	9
2	2	3	1	1	1	3	3	3	3
3	2	3	3	1	3	3	3	3	1
1	1	2	1	3	3	1	2	1	2
2	3	3	2	1	1	3	3	3	3
3	2	1	3	1	2	2	9	1	3
2	2	3	2	3	1	3	3	3	3
9	9	1	9	1	3	3	2	3	3
2	2	1	1	9	9	9	9	9	9
1	1	1	1	2	1	1	1	1	1
2	2	2	2	2	2	2	2	2	2
2	2	2	2	1	1	2	2	2	2
2	2	2	2	1	2	3	3	2	2
3	3	1	3	1	1	1	1	1	1
3	2	3	2	3	1	1	1	2	2
1	1	1	1	1	1	1	1	3	1
2	2	2	2	1	1	1	1	1	1
1	3	1	2	3	1	1	1	1	2
9	1	1	9	3	1	3	3	3	3
3	3	3	3	1	2	2	2	2	2
1	2	1	1	2	1	2	9	9	2

2	2	2	2	2	2	2	2	2	2
2	2	2	2	1	3	3	1	3	2
9	9	9	9	9	9	9	9	9	9
9	9	9	9	9	9	9	9	9	9
2	2	1	1	1	2	1	1	1	1
2	2	2	2	2	2	1	1	1	1
2	9	9	9	9	9	9	9	9	9
2	9	9	9	2	1	3	3	3	3
1	1	1	3	3	1	3	3	3	3
9	9	9	9	2	1	3	3	2	3
2	3	1	3	1	1	3	3	9	9
9	9	9	9	9	9	9	9	9	9
2	1	1	1	3	3	3	2	1	2
2	3	2	1	3	3	3	1	1	2
2	2	2	3	1	2	3	2	2	3
9	9	9	9	9	9	9	9	1	9
9	9	9	9	9	1	9	9	9	9
9	9	9	9	9	9	2	9	9	9
1	1	2	3	1	3	1	2	3	2
3	1	3	2	2	3	3	3	1	1
9	9	9	9	9	1	9	9	9	9
3	3	3	3	2	2	2	2	2	2
3	3	3	3	3	3	3	3	3	3
2	2	2	2	2	1	1	1	2	2
2	2	2	2	1	1	3	2	2	3
2	2	3	1	3	3	2	2	2	2
9	2	1	2	1	2	3	3	3	3
2	2	2	1	2	2	9	2	3	1
2	3	2	3	1	1	2	1	1	1
1	2	3	2	1	1	3	2	3	3
2	2	2	2	1	2	2	2	2	2
3	2	2	2	3	1	3	3	3	2
2	2	2	2	1	2	2	2	1	2
2	2	2	2	2	3	1	2	2	2
3	3	3	3	3	1	1	3	3	2
2	1	1	1	3	1	2	3	1	3
2	2	1	2	3	1	1	1	1	2
3	3	1	3	3	1	3	3	3	3
9	2	9	9	1	2	1	1	2	9
2	1	2	1	1	1	1	3	1	2
2	1	3	1	3	1	2	1	9	1
1	2	2	2	3	3	3	3	2	3
1	1	1	9	1	2	2	1	2	3
2	2	2	2	3	1	3	2	1	1
1	1	1	2	1	2	2	1	3	2
2	2	1	1	1	3	1	2	2	1
1	1	1	1	1	2	9	1	1	1

1	1	1	2	1	1	3	1	2	1
2	2	2	1	2	1	3	2	1	2
9	9	9	9	2	1	1	1	1	3
3	1	2	3	9	2	2	2	2	2
1	2	3	3	3	1	3	1	3	2
9	9	9	9	9	9	9	9	9	9
1	3	3	1	3	1	3	3	3	3
3	1	3	1	1	1	3	1	3	3
2	2	1	2	3	3	3	3	1	3
1	3	2	3	1	1	3	2	3	3
2	2	3	3	2	1	3	2	3	3
3	2	2	2	2	3	1	2	2	9
3	3	3	3	1	1	3	3	3	3
1	1	1	1	1	2	2	1	1	2
2	2	2	2	2	1	2	1	2	2
1	1	2	2	1	1	1	1	1	2
2	2	2	2	2	2	2	2	2	2
9	9	9	9	1	9	9	9	9	9
9	9	9	9	2	9	9	9	9	9
2	2	2	2	2	2	2	2	2	2
3	1	9	9	3	1	9	9	3	1
2	9	2	9	2	2	2	9	2	9
3	1	9	9	9	1	1	9	9	1
1	9	9	9	9	1	9	9	1	2
9	2	9	2	9	2	2	9	9	2
9	9	9	9	9	9	9	9	9	9
9	9	9	9	9	9	9	9	9	9
2	2	2	2	2	2	2	2	2	2
2	2	2	2	2	2	2	2	2	2
3	3	3	3	1	2	1	1	1	1
2	1	2	2	2	2	2	2	2	2
2	9	2	9	9	2	9	9	2	9
2	2	2	2	2	2	2	2	2	2
9	9	9	9	9	9	9	9	9	9
9	9	9	9	9	9	9	9	9	9
2	2	2	2	1	1	2	2	1	2
9	9	9	9	9	2	9	9	9	9
9	9	9	9	9	9	9	9	9	9
9	9	9	9	9	9	9	9	9	9
2	9	9	2	9	9	9	9	9	9
3	1	9	9	3	1	9	9	1	2
1	9	9	9	1	2	9	1	2	9
9	9	9	9	9	9	9	9	9	9
9	9	2	9	9	2	9	9	9	2
2	9	1	2	1	2	9	9	9	1
9	9	9	9	3	9	9	9	9	9
9	9	9	9	9	3	9	9	9	9

1	9	9	9	1	9	9	9	9	9
3	3	3	3	3	3	3	3	3	3
9	1	3	3	3	3	3	3	3	3
2	1	2	1	1	2	1	3	1	2
1	1	1	1	1	9	9	9	1	9
9	9	9	9	1	9	9	9	9	9
9	9	9	9	9	1	9	9	9	9
9	9	9	9	2	9	9	9	9	9
3	9	2	2	1	3	2	2	2	1
2	1	3	2	2	1	2	3	1	2
2	2	3	3	1	2	2	1	1	2
2	1	2	2	1	2	2	2	2	2
2	2	3	2	1	1	3	1	3	3
1	2	1	1	2	1	1	2	2	3
9	9	9	9	9	9	9	9	9	9
3	3	3	3	3	3	3	3	3	3
9	9	9	9	9	9	9	9	9	9
9	9	9	9	9	9	9	9	9	9
9	9	9	9	9	1	9	9	9	9
2	3	1	2	1	9	9	9	1	9
1	1	1	1	1	1	1	1	1	1
2	2	2	2	3	3	1	1	1	2
9	9	9	9	9	9	9	9	9	9
1	2	1	1	1	2	2	2	3	3
1	1	1	1	1	1	1	1	1	1
3	3	1	2	3	3	1	1	1	1
3	1	2	1	3	1	1	3	1	3
2	1	2	1	1	1	3	1	3	3
2	2	2	3	3	1	2	1	2	3
2	2	2	2	2	2	2	2	2	2
2	2	2	2	1	2	2	2	2	2
2	2	2	2	2	2	2	2	2	2
2	2	2	2	2	2	2	2	2	2
3	1	2	3	1	2	1	2	3	1
2	2	2	2	2	3	2	3	9	2
2	3	3	3	1	1	2	2	2	1
2	2	2	2	1	2	2	2	2	2
2	2	2	2	2	2	2	2	2	2
2	3	1	2	2	1	2	3	2	3
2	2	2	2	3	2	2	2	2	2
3	3	3	3	1	3	1	1	1	1
1	2	3	2	2	2	2	2	2	2
9	9	9	9	9	9	9	9	9	9
2	2	1	2	3	3	3	2	2	2
3	3	3	3	3	3	3	3	3	3
3	3	3	3	3	3	3	3	3	3
9	9	9	9	2	9	2	9	9	9

1	2	1	3	1	1	2	1	2	1
2	2	1	1	9	1	9	9	9	9
1	1	1	1	2	2	2	2	2	2
2	2	1	1	3	3	3	2	2	1
2	2	2	2	2	2	2	2	2	2
2	2	2	2	2	3	2	2	2	1
1	2	3	1	2	1	2	3	1	2
9	9	9	9	1	9	1	9	9	9
1	9	9	9	9	9	9	9	9	1
9	9	9	9	9	2	9	9	9	1
9	9	9	9	2	9	9	9	1	9
1	1	1	1	1	2	1	1	1	2
9	9	9	9	1	9	9	9	9	9
9	9	9	9	9	1	9	9	9	9
1	2	2	2	2	1	1	2	2	1
9	9	1	9	1	9	9	9	9	9
9	9	9	9	3	9	9	9	9	3
2	9	9	9	3	9	9	3	9	9
9	9	9	9	2	9	9	9	9	9
9	9	9	9	9	3	9	9	1	9
9	9	9	9	2	9	9	9	9	9
9	9	9	9	9	9	9	9	9	9
9	9	9	9	3	9	9	9	2	9
3	1	2	3	2	3	2	3	1	2
2	2	1	2	1	2	1	1	3	1
3	3	3	3	1	2	2	2	2	2
1	3	1	2	3	1	1	2	1	2
2	3	1	1	2	1	3	1	3	3
2	1	3	2	1	2	1	2	3	1
2	3	2	2	2	1	3	1	2	2
2	2	1	2	1	1	3	2	2	3
3	3	3	3	2	3	2	9	2	3
1	3	1	2	2	3	2	1	1	2
3	1	1	1	1	1	3	1	2	3
2	2	2	2	3	2	1	2	2	2
9	1	1	1	2	1	1	1	1	1
2	1	2	1	2	3	1	2	1	3
1	3	2	2	1	2	2	2	2	2
1	1	1	1	2	2	1	1	2	2
1	1	1	1	2	2	1	2	2	2
9	9	9	9	9	9	9	9	9	9
2	3	3	2	3	9	3	3	2	3
9	9	9	9	9	9	9	9	9	9
1	1	2	1	1	1	1	1	2	2
1	2	2	2	3	2	2	1	2	2
2	2	2	2	2	2	2	2	2	2
9	9	9	9	1	1	3	3	2	3

3	3	3	3	1	3	3	2	3	3
3	3	3	3	1	3	3	3	1	3
2	3	2	1	3	1	3	3	2	3
2	2	2	2	2	2	2	2	2	2
2	3	3	1	3	1	1	1	2	2
2	2	2	2	2	2	2	2	2	2
2	3	3	2	3	1	2	1	2	3
9	9	1	9	1	1	1	9	9	9
2	9	3	3	9	2	2	2	2	2
2	1	3	2	2	3	1	2	3	1
2	2	2	2	3	3	3	3	3	3
9	9	9	9	2	2	9	9	9	9
2	1	1	1	3	1	3	1	1	3
2	2	1	1	3	2	3	1	1	3
9	9	9	9	9	9	9	9	9	9
3	1	3	2	3	1	3	3	1	3
3	3	1	3	3	1	3	3	1	3
9	9	9	2	9	1	9	9	9	9
9	9	9	9	2	9	9	9	9	9
2	2	2	2	1	2	1	2	2	3
9	9	9	9	9	1	9	9	9	9
2	2	3	1	2	2	3	2	2	1
9	9	9	9	9	9	9	9	9	9
9	9	9	9	9	9	9	9	9	9
9	9	9	9	9	1	9	9	2	9
3	3	3	3	1	3	3	2	1	1
2	2	2	2	2	2	2	2	2	2
2	1	1	1	1	3	1	1	1	3
2	2	3	2	3	2	2	2	9	2
9	9	9	9	9	9	9	9	9	9
9	1	9	9	2	1	3	3	3	3
1	1	1	2	3	1	1	2	2	1
1	1	1	1	2	1	1	1	1	1
2	1	1	1	1	2	1	2	3	1
3	3	3	3	2	2	2	2	2	2
9	1	9	9	9	2	9	9	9	9
2	2	1	2	3	2	2	2	2	2
1	2	2	2	1	1	1	2	3	1
2	2	2	2	1	3	1	2	2	2
2	1	2	2	1	1	2	2	2	2
1	1	1	1	1	1	3	3	1	1
1	2	2	2	1	2	2	2	1	2
9	9	9	9	9	9	9	9	9	9
2	2	2	2	9	9	9	9	9	9
9	2	3	2	2	9	2	3	2	3
1	1	1	1	2	1	1	9	2	1
2	2	2	2	2	1	1	9	1	9

2	2	2	2	3	1	3	3	3	3
2	2	2	1	2	2	2	2	2	3
2	2	1	1	1	2	1	1	2	3
3	3	3	3	2	2	2	2	2	2
2	2	2	2	1	1	3	3	2	3
3	3	3	3	1	1	2	2	2	2
9	9	9	9	3	3	3	3	3	3
9	1	9	9	2	1	3	3	3	2
9	9	9	9	1	9	9	9	1	9
9	9	9	9	9	1	9	9	9	9
9	9	9	1	2	3	1	1	2	2
3	3	3	3	1	1	3	2	2	3
2	2	2	2	2	2	2	2	2	2
2	2	9	2	2	2	2	9	2	9
9	9	9	9	9	9	9	9	9	9
9	9	9	9	9	9	9	9	9	9
2	2	2	2	9	3	2	2	2	2
2	2	2	2	9	3	2	2	2	2
2	2	2	9	9	3	2	2	2	2
2	2	2	2	9	9	9	9	9	9
2	2	2	2	9	3	2	2	2	2
9	9	9	9	2	9	9	9	9	9
3	3	3	3	3	3	3	3	3	3
9	9	9	9	1	9	9	9	9	9
9	9	9	9	2	9	9	9	9	9
9	9	9	2	2	9	9	9	9	9
9	9	9	9	9	9	9	9	9	9
9	9	9	9	2	9	9	9	9	9
2	2	2	2	2	2	2	2	2	2
9	2	2	2	9	9	9	9	9	9
9	9	9	9	9	9	9	9	9	9
9	9	9	9	9	9	9	9	9	9
9	9	9	9	9	9	9	9	9	9
9	9	2	9	3	3	3	3	3	3
2	2	2	2	1	1	3	3	3	3
9	9	9	9	9	9	9	9	9	9
9	2	9	2	2	9	2	9	2	9
2	2	2	2	2	2	2	2	2	2
9	1	9	1	1	9	1	9	1	9
9	9	9	9	9	9	9	9	9	9
3	3	3	3	3	3	3	3	3	3
9	9	9	9	9	9	9	9	9	9
2	9	2	2	1	9	9	9	9	9
9	9	9	9	2	2	2	2	2	2
1	3	2	1	2	1	1	1	3	2
2	2	2	2	2	1	1	2	3	2
9	9	9	9	9	1	1	9	9	9

2	3	2	2	3	1	1	3	2	9
3	1	2	1	1	1	3	2	3	1
3	1	2	3	1	2	1	2	3	1
3	3	3	1	1	1	1	3	1	3
2	1	2	1	1	2	1	2	1	2
1	9	9	9	2	1	1	3	2	3
9	9	9	9	9	1	9	9	9	9
2	3	3	2	1	1	2	3	2	1
2	2	2	2	3	3	9	1	1	1
1	1	1	1	1	1	1	1	1	1
2	2	2	2	2	2	2	2	2	2
1	2	3	2	1	2	2	1	3	3
3	3	3	3	3	3	3	3	3	3
1	3	2	2	3	2	2	2	2	2
3	1	1	2	3	1	3	3	3	3
2	2	1	1	3	3	3	3	9	1
3	3	2	2	2	2	2	2	2	2
2	2	1	2	2	2	2	2	2	2
3	3	3	3	3	3	3	3	1	2
2	2	2	2	2	2	2	2	2	2
9	9	9	9	9	9	2	9	9	9
2	1	1	2	2	3	3	2	2	3
3	2	2	2	2	1	1	2	2	2
2	2	2	2	3	1	3	1	3	1
2	3	3	2	2	3	1	3	3	3
2	2	2	2	2	2	2	2	2	2
2	3	3	2	2	1	3	2	2	9
2	1	3	1	1	1	3	3	3	1
2	1	2	1	2	3	3	1	2	9
9	9	9	9	1	9	9	9	9	9
9	1	9	9	9	1	9	9	9	9
1	1	1	1	1	1	1	1	1	1
2	2	2	2	9	9	1	9	9	9
3	3	3	3	2	1	3	3	3	3
9	9	9	9	9	9	9	9	9	9
9	9	9	9	9	1	9	9	9	9
2	2	2	2	3	1	3	1	1	1
1	2	3	2	2	3	3	2	3	2
9	9	9	9	9	9	9	9	9	9
1	9	9	9	1	9	9	9	9	9
1	1	9	1	1	1	3	2	1	3
9	9	9	9	9	9	9	9	9	9
1	3	3	2	2	2	2	3	2	3
3	3	3	3	1	1	1	1	1	1
2	3	2	2	1	1	1	1	2	2
2	2	2	2	2	1	3	2	2	2
1	1	2	1	1	1	3	3	2	3

3	3	3	3	2	2	2	2	2	2
9	9	9	9	2	9	3	2	1	2
3	3	3	3	3	2	2	2	3	3
3	3	3	3	1	1	2	2	3	3
2	2	3	3	3	2	2	3	2	2
2	2	3	1	1	1	1	3	2	2
9	9	2	9	9	1	2	9	9	9
2	2	2	2	3	1	3	1	3	1
2	2	2	2	1	3	3	2	3	3
2	2	2	2	9	9	9	9	9	9
3	2	1	1	2	1	3	2	1	3
9	9	9	9	9	9	9	9	9	9
2	2	2	2	9	9	9	9	9	9
2	2	2	2	2	2	2	2	2	2
2	2	2	1	1	2	3	3	3	3
2	2	2	2	2	1	3	2	2	3
2	2	2	2	1	1	1	1	1	1
3	2	3	2	2	2	3	3	3	3
2	3	2	3	2	1	3	2	2	3
2	2	2	2	2	2	2	2	2	2
3	3	3	2	1	1	3	1	2	3
3	3	3	3	3	3	3	3	3	3
9	9	9	9	9	9	9	9	9	9
9	9	9	9	9	9	9	9	9	9
9	9	9	9	1	9	9	9	9	9
9	9	9	9	2	9	9	9	9	9
9	9	9	9	9	9	9	9	9	9
9	9	9	9	9	9	9	9	9	9
2	2	2	2	2	2	2	9	2	2
9	9	9	9	2	9	9	9	9	9
9	9	9	9	9	9	9	9	9	9
9	9	9	9	3	9	9	9	9	9
9	9	9	9	9	9	9	9	9	9
9	2	2	2	1	1	1	1	1	1
9	2	2	2	2	9	2	2	3	9
9	9	9	9	3	9	9	9	3	9
9	9	9	3	9	9	9	9	9	9
2	2	2	2	2	2	2	2	2	2
1	1	1	1	9	9	9	9	9	9
9	9	9	9	3	9	9	9	3	9
9	1	9	2	1	9	9	1	2	2
9	9	9	9	9	9	9	9	9	9
9	9	9	9	2	9	9	9	9	9
9	9	9	9	9	9	9	9	9	9
2	9	9	3	1	9	2	9	9	1
9	9	9	9	1	9	9	9	9	9
2	2	2	2	2	2	2	2	2	2

9	9	9	9	2	9	9	9	9	9
9	9	9	9	2	9	9	9	9	9
9	9	9	9	2	9	9	9	9	9
2	2	2	2	9	9	9	9	9	9
9	9	9	9	9	3	2	2	2	2
9	9	9	9	9	9	9	9	9	9
9	9	9	9	9	9	9	9	9	9
2	2	2	2	2	2	2	2	2	2
2	9	9	9	2	9	9	2	9	9
2	9	9	9	2	9	9	2	9	9
9	9	9	9	9	9	9	9	9	9
9	9	9	9	9	9	9	9	9	9
9	9	9	9	9	9	9	9	9	9
9	9	9	9	2	9	9	9	9	9
9	9	9	9	9	9	9	9	9	9
9	9	9	9	9	9	9	9	9	9
9	9	9	9	9	9	9	9	9	9
9	9	9	9	9	9	9	9	9	9
9	9	9	9	9	9	9	9	9	9
9	9	9	9	9	9	9	9	9	9
2	2	2	2	9	3	2	2	2	2
2	2	2	2	2	2	2	2	2	2
2	2	2	2	9	3	2	2	2	2
2	2	2	2	2	2	2	2	2	2
9	9	9	9	2	2	2	2	2	2
2	2	2	2	2	2	2	2	2	2
9	9	9	9	9	9	9	9	9	9
9	9	9	9	1	9	9	9	9	9
9	9	9	9	9	1	2	2	2	9
9	9	9	9	9	2	2	2	9	2
2	2	2	2	2	2	2	2	2	2
2	2	2	2	9	9	9	9	9	9
9	9	9	9	9	9	9	9	9	9
2	1	1	1	1	2	3	1	2	2
9	9	9	9	9	9	9	9	9	9
2	2	2	2	9	9	9	9	9	9
1	1	1	1	1	1	1	1	1	1
2	2	2	2	3	2	1	3	2	9
9	9	9	9	9	9	9	9	9	9
2	1	1	1	1	2	2	2	2	2
9	9	9	9	9	9	9	9	9	9
2	2	2	2	1	9	1	9	9	9
9	9	2	2	2	9	9	9	2	9
2	2	9	2	2	2	2	2	2	2
1	1	1	1	1	1	1	1	1	1
9	9	9	3	3	9	9	9	9	9
9	9	9	9	9	9	9	9	9	9

9	9	9	9	9	9	9	9	9	9
2	2	2	2	2	2	2	2	2	2
9	9	9	9	3	1	3	9	9	9
1	9	3	1	1	9	9	1	9	9
9	9	9	2	2	9	9	9	9	9
9	2	9	2	2	9	9	9	2	9
9	9	9	9	2	9	9	9	9	9
9	9	9	9	9	9	9	9	9	9
9	9	9	3	2	9	9	1	9	3
9	9	9	9	3	9	9	9	9	9
9	9	9	9	9	9	9	9	9	9
9	9	9	9	9	9	9	9	9	9
9	9	9	9	2	9	9	9	9	9
9	9	9	9	2	9	9	9	9	9
2	9	2	9	2	9	9	2	9	9
9	9	9	9	2	2	2	2	2	9
9	9	9	9	9	9	9	9	9	9
9	9	9	9	9	9	9	9	9	9
9	9	9	9	9	9	9	9	9	9
9	9	9	9	9	9	9	9	9	9
9	1	2	1	1	1	3	3	3	2
1	2	1	1	1	1	3	1	1	3
9	9	2	9	1	9	9	9	9	9
2	2	2	2	3	1	1	3	1	3
2	2	2	2	1	1	3	3	3	3
9	9	9	9	2	2	2	2	2	2
9	9	9	9	9	1	9	9	9	9
9	9	9	9	3	9	9	9	9	9
2	3	3	3	2	3	3	2	2	2
3	1	2	3	1	2	3	1	2	3
2	3	2	2	2	1	3	3	3	3
2	2	2	2	2	1	3	3	1	3
2	2	3	3	1	9	3	3	3	3
2	2	2	2	3	1	3	3	3	3
2	3	1	1	2	1	3	3	2	3
2	2	2	2	1	1	3	3	3	3
9	9	9	9	1	9	9	9	9	9
9	9	9	9	3	9	9	9	9	9
3	3	1	1	1	9	9	9	9	9
9	2	9	9	9	9	9	9	9	9
9	9	9	9	2	9	9	9	9	9
9	9	9	9	9	9	9	9	9	9
9	9	9	9	9	9	9	9	9	9
9	9	9	2	9	2	9	2	9	9
9	9	9	9	9	9	9	9	9	9
2	9	9	2	9	2	9	9	2	9
9	2	9	2	2	9	2	9	2	9

2	2	2	2	2	2	2	2	2	2
2	2	2	2	2	2	2	2	2	2
2	2	2	2	2	2	2	2	2	2
9	9	9	9	9	9	9	9	9	9
2	2	2	2	1	3	2	2	2	2
2	2	3	2	1	1	2	3	2	2
2	2	1	1	2	2	3	2	3	3
2	2	1	2	2	1	3	3	2	3
9	9	9	9	3	3	3	3	3	1
2	2	3	2	3	2	3	3	1	2
3	3	3	3	3	1	3	3	3	3
2	1	2	2	3	1	3	1	2	3
1	1	2	1	2	1	3	1	2	1
3	3	3	3	3	3	3	3	3	3
2	2	2	2	2	2	2	2	2	2
2	2	2	2	2	2	2	2	2	2
9	9	2	9	9	2	9	2	9	9
2	2	2	2	2	2	2	2	2	2
2	2	1	2	1	3	3	1	1	2
1	2	1	3	3	1	2	3	1	2
2	3	2	2	2	2	2	3	3	3
2	2	2	2	2	2	2	2	2	2
1	2	1	2	2	2	1	2	1	2
2	3	3	3	1	2	3	3	3	2
1	3	2	2	9	1	3	3	3	3
3	2	2	3	2	1	1	3	1	3
3	3	2	2	1	3	3	2	3	2
2	1	3	1	1	2	3	1	2	3
3	1	1	2	1	3	2	1	1	2
2	3	1	2	3	1	1	2	3	3
2	1	2	1	1	2	3	3	3	2
2	2	2	2	3	1	2	2	2	3
2	1	1	1	3	1	2	1	2	3
3	3	2	2	3	1	3	2	9	2
1	2	3	2	1	1	3	2	2	3
2	3	3	3	2	1	3	3	3	3
3	3	3	2	2	3	3	1	3	3
3	3	1	2	2	2	3	1	3	3
3	3	3	3	3	3	3	3	3	3
2	2	2	2	1	1	3	3	3	3
9	9	9	9	1	9	9	9	9	9
3	3	3	3	3	1	2	3	1	2
2	2	2	2	3	3	1	1	1	2
2	2	2	2	2	2	2	2	2	2
2	2	2	2	3	3	1	1	1	1
9	9	9	9	9	9	9	9	9	9
2	2	2	2	1	1	3	3	3	3

1	9	9	9	1	1	3	3	3	3
1	2	1	2	1	1	2	1	3	2
9	9	1	2	1	1	1	2	3	2
3	2	3	1	2	2	2	3	2	3
3	3	3	3	3	3	3	3	3	3
2	3	2	3	2	2	3	3	2	3
2	1	1	2	1	2	1	3	2	2
3	3	3	3	3	3	3	3	3	3
3	1	3	3	3	1	3	3	9	3
3	3	3	2	1	1	2	3	3	3
1	3	2	2	1	3	3	3	2	2
3	2	3	2	1	1	3	1	3	3
2	2	3	3	3	2	3	2	2	3
2	9	1	3	2	2	2	3	1	3
2	1	1	1	1	3	1	1	2	1
2	2	2	2	2	2	1	2	2	2
3	2	2	3	9	2	2	2	2	3
2	2	2	2	1	1	3	3	3	3
2	2	2	1	1	3	2	1	2	2
3	3	2	3	1	1	1	2	3	2
2	2	1	2	3	1	3	2	3	3
2	2	2	2	1	9	3	3	3	9
3	2	2	2	3	3	1	1	1	2
3	3	2	2	1	2	3	2	1	2
2	2	2	2	1	2	2	2	2	2
9	9	9	9	1	9	9	9	9	9
2	2	2	1	1	1	1	2	2	2
2	2	2	2	1	1	1	1	2	1
2	3	2	2	2	2	2	3	2	3
2	3	1	3	2	3	2	3	1	3
3	3	3	3	3	3	3	3	3	3
2	2	3	3	1	1	2	3	2	1
9	1	9	9	2	2	1	2	2	2
2	2	2	2	2	2	2	2	2	2
2	2	2	2	3	3	2	2	2	2
2	2	2	2	2	2	2	2	2	2
3	2	3	1	3	1	1	3	1	3
2	9	9	1	1	3	2	1	2	2
2	2	1	1	1	1	3	2	2	1
2	1	3	2	1	1	2	3	3	1
3	1	2	2	1	1	3	2	3	3
1	2	3	3	2	3	2	3	9	2
2	1	2	2	1	1	3	3	3	3
3	2	1	2	1	1	2	2	3	1
1	3	3	2	2	1	3	3	3	3
2	2	1	1	2	3	2	3	2	2
2	2	1	2	1	1	3	2	2	3

2	2	2	2	2	2	2	2	2	2
2	2	2	2	1	2	2	2	2	2
2	2	2	2	2	2	2	2	2	2
2	2	1	1	1	1	3	2	2	1
3	3	3	3	3	1	1	1	1	3
2	2	2	2	2	1	2	2	1	3
3	2	1	1	1	2	1	3	1	1
2	2	1	1	1	1	3	2	2	1
2	2	2	2	2	1	2	2	1	3
2	2	3	2	1	3	2	2	1	3
2	1	2	1	2	1	1	2	2	9
2	2	1	1	1	1	3	2	2	1
2	2	1	1	1	1	3	2	2	1
2	2	2	2	2	2	2	2	2	2
2	2	2	2	1	1	2	2	2	2
9	9	9	9	9	9	9	9	9	9
1	3	1	2	1	3	3	3	3	3
2	1	3	1	1	3	3	3	3	3
1	9	9	9	2	1	3	3	1	2
2	9	1	9	1	1	1	2	2	3
2	2	2	2	2	3	3	2	2	2
9	9	9	1	9	1	9	9	9	9
9	9	9	9	9	9	9	9	9	9
9	9	9	9	1	9	9	9	2	9
9	9	9	9	9	9	9	9	9	9
2	2	2	2	3	3	3	3	3	3
1	1	3	3	1	1	1	1	3	3
9	9	1	9	9	9	9	9	9	9
9	9	9	9	9	9	9	9	9	9
2	2	2	2	3	3	2	2	2	2
2	2	3	3	1	1	2	1	2	2
2	2	2	2	3	3	2	2	2	2
2	2	3	3	3	3	2	1	2	1
9	9	9	9	1	3	2	1	9	3
2	2	2	2	1	2	3	2	1	1
2	1	3	1	1	3	2	2	3	1
9	9	9	9	9	9	9	9	9	9
1	2	1	3	1	1	2	1	2	3
3	3	3	3	3	1	3	2	2	1
2	2	1	1	2	2	2	2	2	2
1	2	3	3	2	2	2	2	9	2
2	2	2	2	3	3	2	2	2	2
9	9	9	9	1	1	9	9	9	9
1	2	2	2	1	1	2	2	2	2
2	2	2	2	1	1	3	3	3	2
1	2	3	3	2	2	1	2	1	1
2	3	1	1	2	1	1	1	1	1

1	1	1	1	1	1	3	3	1	3
1	2	3	2	9	1	3	1	2	1
1	1	2	2	1	2	2	1	9	1
1	3	3	3	3	1	2	3	1	2
3	1	1	3	1	1	2	1	1	1
3	3	3	3	3	2	3	3	2	3
3	2	1	9	2	2	1	2	3	2
2	2	2	2	2	2	2	2	2	2
3	3	1	3	3	3	3	3	2	2
2	2	2	2	2	2	1	2	2	2
2	3	1	2	2	3	2	2	1	2
2	2	2	2	2	2	2	2	2	2
9	9	9	1	9	1	3	9	9	9
3	3	3	2	1	1	3	3	1	2
3	3	3	3	3	9	9	9	9	9
2	2	2	2	3	1	2	2	2	1
2	2	2	3	1	1	2	2	2	2
9	9	9	9	1	2	1	1	1	1
1	1	1	2	2	3	1	1	1	2
9	9	9	9	1	9	9	1	9	1
9	9	9	9	2	9	9	9	9	9
9	9	9	9	1	1	1	1	1	9
1	2	2	2	1	1	3	3	3	3
1	3	3	2	1	1	1	3	3	3
2	2	2	2	3	3	3	1	3	2
2	2	3	2	3	1	3	3	3	3
2	2	2	2	1	2	2	2	3	2
3	3	3	9	3	3	1	1	3	3
2	3	1	2	2	1	3	3	1	3
2	2	2	2	2	2	2	2	2	2
3	3	9	1	3	1	3	3	3	3
2	2	2	2	2	2	2	2	2	2
3	2	2	2	1	1	2	2	2	2
9	9	9	9	9	1	9	9	9	9
2	2	3	2	2	2	2	3	2	3
2	3	2	2	2	1	3	3	3	3
1	1	1	1	1	1	1	1	1	1
1	2	1	3	2	1	1	2	3	1
1	2	1	3	1	2	2	2	1	2
2	2	2	2	3	1	3	1	2	2
2	2	2	2	1	3	1	3	9	1
1	2	2	1	1	2	2	3	1	3
1	1	1	1	1	1	1	1	1	1
2	2	3	2	3	1	3	3	3	3
1	3	3	2	1	1	1	2	1	3
3	2	3	2	2	2	3	3	1	1
9	9	1	9	1	3	3	1	1	1

1	2	1	2	1	2	3	3	9	3
2	2	2	2	1	1	3	3	2	9
2	2	2	2	1	1	3	3	2	3
3	3	3	3	9	9	9	9	9	9
2	3	3	2	2	1	3	9	3	3
3	3	3	3	3	3	3	3	3	3
3	3	3	1	3	1	3	1	2	3
2	1	2	1	3	1	3	3	2	3
2	3	2	3	2	2	2	2	2	2
2	2	2	2	2	2	2	2	2	2
3	2	1	9	3	9	1	2	9	3
9	9	9	9	9	1	9	9	9	9
2	3	2	2	1	1	3	3	3	3
9	9	1	9	1	9	9	9	9	9
9	9	1	9	1	9	9	9	9	9
9	9	9	1	1	1	3	3	3	3
2	1	9	9	1	9	3	9	9	9
9	9	9	9	9	1	9	9	9	9
2	3	3	2	1	1	3	3	1	9
9	9	9	9	9	3	9	9	9	9
2	1	1	2	1	1	2	2	2	3
9	9	9	9	9	9	9	9	9	9
2	3	2	3	1	2	9	9	9	9
9	9	9	9	1	9	9	9	9	9
9	9	9	9	1	9	9	9	9	9
9	9	9	9	9	2	9	2	9	9
1	1	1	1	3	1	1	1	1	1
2	2	1	2	2	3	1	1	2	2
2	2	2	2	1	1	2	2	2	3
2	2	3	1	1	2	3	3	3	2
9	2	1	1	3	3	3	9	3	1
3	3	2	1	3	1	3	3	3	3
3	1	1	3	1	3	1	3	1	1
9	9	9	9	2	9	9	9	9	9
2	2	2	2	1	2	3	1	2	1
3	1	1	1	3	1	3	3	3	1
3	1	1	3	3	1	3	3	2	3
9	9	9	9	1	9	9	9	9	9
3	3	3	3	1	1	1	1	1	1
9	9	9	9	9	1	9	9	9	9
9	9	9	9	1	9	9	9	9	9
2	2	2	1	1	2	1	1	1	1
1	1	1	1	1	1	1	1	1	1
9	9	9	9	9	9	9	9	9	9
9	9	9	9	9	9	9	9	9	9
2	2	2	2	2	2	2	2	2	2
2	2	2	2	2	2	2	2	2	2

2	2	2	2	2	2	2	2	2	2
3	3	3	3	3	3	3	3	3	3
9	9	2	9	9	2	9	9	2	9
2	2	2	2	2	2	2	2	2	2
2	2	2	2	2	2	2	2	2	2
3	1	9	1	9	1	3	1	3	2
1	3	1	9	9	1	9	3	1	9
9	2	2	9	1	2	9	9	1	2
9	9	2	9	2	9	9	1	9	9
1	1	1	1	2	2	2	2	2	2
9	3	9	1	1	9	1	3	3	1
1	9	1	9	9	1	9	3	1	9
2	1	3	1	1	2	9	3	1	3
3	9	1	2	9	1	9	9	9	2
9	2	9	2	2	2	9	2	9	2
2	2	2	9	2	2	2	2	2	2
2	2	2	2	2	2	2	2	2	2
2	9	9	2	2	9	9	2	9	9
2	2	2	2	2	2	2	2	2	2
9	9	9	9	9	9	9	9	9	9
2	2	2	2	2	2	2	2	2	2
9	9	9	9	9	9	9	9	9	9
9	9	2	9	9	9	9	9	9	9
9	9	9	9	9	9	9	9	9	9
9	3	1	9	9	1	9	3	1	9
2	2	2	2	2	2	2	2	2	2
2	2	2	2	2	1	3	2	2	2
2	2	2	2	2	2	2	2	2	2
2	1	3	2	1	2	2	9	1	3
9	2	1	2	1	2	1	3	1	2
2	2	3	3	3	3	2	2	2	2
3	3	3	3	2	2	1	1	2	3
3	1	2	3	2	3	3	2	1	3
2	2	2	2	1	2	3	3	3	3
1	3	3	1	1	1	3	3	1	2
1	2	2	2	1	1	3	3	2	3
2	2	2	2	2	2	3	3	9	2
3	2	1	2	3	2	2	3	1	2
2	2	2	2	1	2	2	2	2	2
1	1	1	1	2	1	2	2	2	2
2	2	1	3	1	2	1	1	2	3
1	1	1	1	2	2	2	2	2	2
1	3	3	3	3	3	3	3	3	3
1	1	1	3	1	1	1	1	1	1
2	2	2	3	1	3	3	3	3	3
2	3	2	2	3	3	1	2	2	3
3	1	1	3	1	3	1	3	1	1

3	3	2	3	3	3	9	9	9	9
3	3	2	2	1	3	2	3	3	2
9	9	9	9	2	2	2	1	1	3
9	9	9	9	9	9	9	9	9	9
2	2	2	2	3	3	2	2	2	2
9	1	2	3	3	9	2	3	1	1
2	2	3	3	3	2	2	2	1	2
2	3	2	2	1	1	2	1	3	9
2	2	2	2	2	2	2	2	2	2
2	2	2	2	2	2	2	2	2	2
2	2	2	2	2	2	2	2	2	2
2	2	2	2	2	2	2	2	2	2
1	1	1	1	1	1	1	1	1	1
1	1	2	3	1	1	1	2	3	3
3	3	3	3	3	3	3	3	3	3
1	2	1	2	1	2	1	1	1	1
1	1	9	9	1	1	1	1	1	1
2	2	2	2	2	2	2	2	2	2
2	2	2	2	2	2	2	2	2	2
2	2	2	2	2	2	2	2	2	2
2	2	2	2	2	2	2	2	2	2
2	2	2	2	1	1	1	1	9	1
1	1	9	1	1	1	1	1	1	1
1	1	2	2	1	1	1	2	2	1
2	2	2	2	2	2	2	2	2	2
2	2	2	2	2	2	2	2	2	2
1	1	1	1	1	2	1	2	3	2
2	2	2	2	2	2	2	2	2	2
9	9	9	9	9	9	9	9	9	9
2	2	2	2	2	2	2	2	2	2
9	1	1	1	9	9	9	9	9	9
1	1	1	2	1	2	1	2	1	2
2	1	1	1	1	1	1	1	1	1
9	1	1	1	9	1	1	1	1	1
2	2	2	2	2	2	2	9	2	2
1	1	9	1	1	1	1	9	1	1
2	2	2	2	2	2	2	2	2	2
2	2	2	2	2	2	2	2	2	2
2	2	2	9	2	2	2	2	2	2
2	2	2	2	3	1	2	3	3	2
2	2	2	2	2	2	2	2	2	2
9	9	9	9	9	9	9	9	9	9
1	1	1	2	1	1	1	1	1	2
1	1	1	1	1	1	2	1	1	1
2	2	9	2	9	1	1	1	1	1
2	2	2	2	2	2	2	2	2	2
2	2	2	2	2	2	2	2	2	2

2	2	2	2	1	1	1	1	1	1
2	2	2	2	2	2	2	2	2	2
2	2	2	2	2	2	2	2	2	2
2	1	1	2	1	2	1	1	1	1
2	2	2	2	2	2	2	2	2	2
2	2	2	2	3	1	2	2	2	9
1	1	1	1	1	1	2	1	1	2
2	9	2	2	9	9	1	1	9	1
2	2	2	2	3	1	1	1	1	1
1	1	1	1	9	1	1	2	1	1
1	1	1	1	2	1	1	1	1	2
2	2	2	2	2	2	2	2	2	2
2	2	2	2	3	1	2	2	2	2
2	1	2	2	3	1	2	2	2	2
9	9	9	9	9	9	9	9	9	9
9	2	2	2	1	1	1	9	1	9
2	9	2	2	9	9	2	2	9	2
9	1	1	1	1	1	1	1	1	9
9	9	9	9	9	9	9	9	9	9
2	2	2	2	1	2	1	1	1	1
1	9	9	9	1	1	1	1	1	1
2	2	2	2	2	2	2	2	9	9
9	9	9	9	9	9	9	9	9	9
9	9	9	9	9	9	9	9	9	9
2	2	2	2	2	2	2	2	2	2
1	1	1	1	3	1	3	3	3	3
1	1	1	1	3	1	3	3	3	3
1	1	1	1	3	1	3	3	3	3
1	1	1	1	3	1	3	3	3	3
1	1	1	1	3	1	3	3	3	3
1	1	1	1	3	1	3	3	3	3
1	1	1	1	3	1	3	3	3	3
1	1	1	1	9	1	3	3	3	3
1	1	1	1	3	1	3	3	3	3
1	1	1	1	3	1	3	3	3	3
1	1	1	1	3	1	3	3	3	3
1	1	1	1	3	1	3	3	3	3
1	1	1	1	3	1	3	3	3	3
1	1	1	1	3	1	3	3	3	3
1	1	1	1	3	1	3	3	3	3
1	1	1	1	3	1	3	3	3	3
1	1	1	1	1	3	1	3	3	3
1	1	1	1	3	1	3	3	3	3
1	1	1	1	3	1	3	3	3	3
1	1	1	1	9	1	3	3	3	3
1	1	1	1	3	1	3	3	3	3
1	1	1	1	3	1	3	3	3	3

1	1	1	1	3	1	3	3	3	3
1	1	1	1	3	1	3	3	3	3
1	1	1	1	3	9	1	3	3	3
9	9	9	9	9	9	9	9	9	9
9	9	9	9	9	9	9	9	9	9
9	9	9	9	9	9	9	9	9	9
9	9	9	9	9	9	9	9	9	9
9	9	9	9	9	9	9	9	9	9
9	9	9	9	9	9	9	9	9	9
9	9	9	9	9	9	9	9	9	9
9	9	9	9	9	9	9	9	9	9
9	9	9	9	9	9	9	9	9	9
9	9	9	9	9	9	9	9	9	9
9	9	9	9	9	9	9	9	9	9
9	9	9	9	9	9	9	9	9	9
9	9	9	9	9	9	9	9	9	9
9	9	9	9	9	9	9	9	9	9
9	9	9	9	9	9	9	9	9	9
9	9	9	9	9	9	9	9	9	9
9	9	9	9	9	9	9	9	9	9
9	9	9	9	9	9	9	9	9	9
9	9	9	9	9	9	9	9	9	9
9	9	9	9	9	9	9	9	9	9
9	9	9	9	9	9	9	9	9	9
9	9	9	9	9	9	9	9	9	9
1	1	1	1	3	1	3	3	3	3
1	1	1	1	3	1	3	3	3	3
1	1	1	1	3	1	3	3	3	3
1	1	1	1	3	1	3	3	3	3
1	1	1	1	3	1	3	3	3	3
1	1	1	1	3	1	3	3	3	3
1	1	1	1	3	1	3	3	3	3
1	1	1	1	3	1	3	3	3	3
1	1	1	1	3	1	3	3	3	3
1	1	1	1	3	1	3	3	3	3
1	1	1	1	3	1	3	3	3	3
1	1	1	1	3	1	3	3	3	3
1	1	1	1	3	1	3	3	3	3
1	3	1	1	3	1	3	3	3	3
1	1	1	1	3	1	3	3	3	3
1	1	1	1	3	1	3	3	3	3
9	9	9	9	9	1	9	9	9	9
1	1	1	1	3	1	3	3	3	3
9	1	1	1	3	1	3	3	3	3
1	1	1	1	3	1	3	3	3	3
1	1	9	9	3	1	3	3	3	3
9	9	9	1	3	1	3	3	3	3

1	1	1	1	3	1	3	3	3	3
1	3	1	1	3	3	3	3	3	3
1	1	1	1	3	1	3	3	3	3
1	1	1	1	3	1	3	3	3	3
1	1	1	1	3	1	3	3	3	3
1	1	1	1	3	1	3	3	3	3
1	1	1	1	3	1	3	3	3	3
9	9	9	9	3	1	3	3	3	3
1	1	1	1	3	1	3	3	3	3
1	1	1	1	3	1	3	3	3	3
1	1	1	1	3	1	3	3	3	3
1	1	1	1	3	1	3	3	3	3
1	1	1	1	3	1	3	3	3	3
1	1	1	1	3	1	3	3	3	3
1	1	1	1	3	1	3	3	3	3
1	1	1	1	3	1	3	3	3	3
1	1	1	1	3	1	3	3	3	3
1	1	1	1	3	1	3	3	3	3
1	1	1	1	3	1	3	3	3	3
1	1	1	1	3	1	3	3	3	3
1	3	1	1	3	1	3	3	3	3
1	1	1	1	3	1	3	3	3	3
1	1	1	1	3	1	3	3	3	3
1	1	1	1	3	1	3	3	3	3
1	1	1	1	3	1	3	3	3	3
1	1	1	1	3	1	3	3	3	3
1	1	1	1	3	1	3	3	3	3
1	1	1	1	3	1	3	3	3	3
1	1	1	1	1	3	1	3	3	3
1	1	1	1	3	1	3	3	3	3
1	1	1	1	3	1	3	3	3	3
1	9	1	1	3	1	3	3	3	3
1	1	1	1	3	1	3	3	3	3
1	1	1	1	3	1	3	3	3	3
1	1	1	1	3	1	3	3	3	3
1	1	1	1	3	1	3	3	3	3
1	1	1	1	3	1	3	3	3	3
1	1	1	1	3	1	3	3	3	3
1	1	1	1	3	1	3	3	3	3
1	1	1	1	3	1	3	3	3	3
9	9	9	9	9	9	9	9	9	9
1	1	1	1	3	1	3	3	3	3
1	1	3	1	3	1	3	3	3	3
1	1	1	1	3	1	3	3	3	3
1	1	1	1	3	1	3	3	3	3
9	9	9	9	3	1	3	3	3	3
1	1	1	1	3	1	3	3	3	3

1	1	1	1	3	1	3	3	3	3
1	1	1	1	1	1	3	3	3	3
9	1	1	9	3	1	3	3	3	3
1	1	1	1	3	1	3	3	3	1
1	1	1	1	3	1	3	3	3	3
1	1	1	1	3	1	3	3	3	3
1	1	1	1	1	1	3	3	3	3
1	1	1	1	3	1	3	3	3	3
1	1	1	1	3	1	3	3	3	3
1	1	1	1	3	1	3	3	3	3
1	1	1	1	3	1	3	3	3	3
1	1	1	1	3	1	3	3	3	3
1	1	1	1	3	1	3	3	3	3
1	1	1	1	3	1	3	3	3	3
1	1	1	1	3	1	3	3	3	3
1	1	1	1	3	1	3	3	3	3
1	1	1	1	9	1	3	3	3	3
1	1	1	1	3	1	3	3	3	3
1	1	1	1	3	1	3	3	3	3
1	1	1	1	3	1	3	3	3	3
1	1	1	1	9	9	9	9	9	9
1	1	1	1	3	1	3	3	3	3
1	1	1	1	3	1	3	3	3	3
1	1	1	1	3	1	3	3	3	3
1	1	1	1	3	1	3	3	3	3
9	9	9	9	9	9	9	9	9	9
1	1	1	1	3	1	3	3	3	3
1	1	1	1	3	1	3	3	3	3
1	1	1	1	3	1	3	3	3	3
1	1	1	1	3	1	3	3	3	3
9	9	9	9	9	9	9	9	9	9
1	1	1	1	3	1	3	3	3	3
1	1	1	1	3	1	3	3	3	3
1	1	1	1	3	1	3	3	3	3
1	1	1	1	3	1	3	3	3	3
1	1	1	1	3	1	3	3	3	3
1	1	1	1	3	1	3	3	3	3
1	1	1	1	3	1	3	3	3	3
1	1	1	1	3	9	1	3	3	3
1	1	1	1	3	9	1	3	3	3
1	1	1	1	3	1	3	3	3	3
1	1	1	1	3	1	3	3	3	3
1	1	1	1	3	1	3	3	3	3
1	1	1	1	3	1	3	3	3	3
1	1	1	1	3	1	3	3	3	3
1	1	1	1	3	1	3	3	3	3
1	1	1	1	3	1	3	3	3	3

9	9	9	9	9	9	9	9	9	9
9	1	1	1	3	1	3	3	3	3
1	1	1	1	9	1	3	3	3	3
1	1	1	1	3	1	3	3	3	3
1	1	1	1	3	1	3	3	3	3
1	1	1	1	3	1	3	3	3	3
1	1	1	1	3	1	3	3	3	3
1	1	1	1	3	1	3	3	3	3
1	1	1	1	3	1	3	3	3	3
1	1	1	1	3	1	3	3	3	3
1	1	1	1	3	1	3	3	3	3
1	1	1	1	3	1	3	3	3	3
1	1	1	1	3	1	3	3	3	3
9	2	3	3	3	1	3	3	3	3
1	1	1	1	3	1	3	3	3	3
1	1	1	1	3	1	3	3	3	3
1	1	1	1	3	1	3	3	3	3
1	1	1	1	3	1	3	3	3	3
1	1	1	1	3	1	3	3	3	3
1	1	1	1	3	1	3	3	3	3
1	1	1	1	3	1	3	3	3	3
1	1	1	1	3	1	3	3	3	3
1	1	1	1	3	1	3	3	3	3
1	1	1	1	3	1	3	3	3	3
1	1	1	1	3	1	3	3	3	3
1	1	1	1	3	1	3	3	3	3
1	1	1	1	3	1	3	3	3	3
1	1	1	1	3	1	3	3	3	3
1	1	1	1	3	1	3	3	3	3
3	1	1	1	3	1	3	3	3	3
1	1	1	1	3	1	3	3	3	3
1	1	1	1	3	1	3	3	3	3
1	1	1	1	3	1	3	3	1	3
9	9	9	9	9	9	9	9	9	9
9	9	9	9	9	9	9	9	9	9
9	9	9	9	9	9	9	9	9	9
9	9	9	9	9	9	9	9	9	9
9	9	9	9	9	9	9	9	9	9
9	9	9	9	9	9	9	9	9	9
9	9	9	9	9	9	9	9	9	9
9	9	9	9	9	9	9	9	9	9
9	9	9	9	9	9	9	9	9	9
9	9	9	9	9	9	9	9	9	9
9	9	9	9	9	9	9	9	9	9
9	9	9	9	9	9	9	9	9	9
9	9	9	9	9	9	9	9	9	9
9	9	9	9	9	9	9	9	9	9

9	9	9	9	9	9	9	9	9	9
2	1	1	1	1	1	1	3	3	3
9	9	9	9	1	1	1	1	1	1
9	9	9	9	9	9	9	9	9	9
9	9	9	9	9	9	9	9	9	9
9	9	9	9	9	9	9	9	9	9
9	9	9	9	9	9	9	9	9	9
9	9	9	9	9	9	9	9	9	9
9	9	9	9	9	9	9	9	9	9
9	9	9	9	9	9	9	9	9	9
9	9	9	9	9	9	9	9	9	9
9	9	9	9	9	9	9	9	9	9
9	9	9	9	9	9	9	9	9	9
2	3	3	2	1	1	3	9	9	9
9	9	9	9	9	9	9	9	9	9
9	9	9	9	9	9	9	9	9	9
9	9	9	9	9	9	9	9	9	9
9	9	9	9	9	9	9	9	9	9
9	9	9	9	9	9	9	9	9	9
1	1	1	2	3	3	3	3	1	3
2	2	2	2	1	2	2	2	2	3
2	1	2	1	1	3	3	3	3	1
1	3	3	3	1	2	2	3	3	2
2	3	2	2	1	1	1	2	3	3
3	3	3	3	3	3	1	1	1	3
3	3	3	3	1	3	1	2	1	2
2	2	1	1	2	1	3	3	3	3
3	1	3	1	1	3	1	3	1	3
3	3	2	2	2	1	3	1	9	2
3	3	3	3	2	3	2	2	3	2
9	9	9	9	2	1	3	3	2	3
2	2	2	2	2	2	2	2	2	2
9	9	9	9	9	9	9	9	9	9
3	2	1	1	3	1	3	3	3	3
1	1	1	1	1	1	3	3	1	3
2	2	2	2	3	1	3	3	3	3
3	1	3	1	2	3	2	3	9	2
2	1	2	1	1	2	1	2	1	3
9	9	1	9	9	1	9	9	9	9
1	2	2	2	1	1	3	2	3	3
9	9	9	9	3	1	3	1	3	3
9	9	9	9	2	9	2	9	9	9
2	1	2	1	1	2	2	2	2	2
2	1	1	3	1	1	2	1	1	2
9	9	1	1	9	1	3	3	1	2
2	3	2	1	1	1	3	9	1	9
9	9	9	9	9	9	9	9	9	9

2	3	1	1	1	2	2	1	1	1
2	2	2	2	2	1	2	2	2	2
1	1	1	1	1	1	1	1	1	1
9	9	9	9	9	9	9	9	9	9
9	9	9	9	9	9	9	9	9	9
2	2	2	2	1	3	2	2	1	2
9	9	1	9	1	9	9	1	9	9
9	9	9	9	9	9	9	9	9	9
2	3	3	2	1	3	2	2	3	2
1	3	2	1	1	1	3	2	3	3
9	1	9	9	1	1	3	3	1	1
9	9	9	9	1	9	9	9	9	9
2	2	2	2	1	1	9	9	9	9
2	2	2	2	2	2	2	2	2	2
2	2	2	2	2	2	2	2	2	2
1	3	3	2	3	3	1	1	1	1
2	3	2	3	2	3	3	3	1	2
9	9	9	9	9	9	9	9	9	9
9	9	1	9	1	1	9	9	9	9
2	2	2	2	3	2	2	2	1	2
2	2	1	2	2	2	1	2	2	3
3	1	1	2	1	1	2	3	3	3
3	1	3	1	1	3	1	3	1	3
2	3	1	2	1	1	3	1	1	3
1	1	1	1	1	9	1	1	2	1
2	2	1	1	3	1	1	2	3	9
2	2	2	2	2	2	2	2	2	2
2	2	2	2	2	2	2	2	2	2
2	2	2	2	2	2	2	2	2	2
2	2	2	2	2	2	2	2	2	2
2	3	2	2	2	2	2	2	2	2
3	2	2	1	2	1	3	3	3	3
2	2	2	2	2	2	2	2	2	2
3	3	3	3	3	1	1	2	3	3
2	9	3	2	2	3	1	2	3	3
1	2	1	1	3	1	2	3	3	1
3	3	2	2	3	1	3	3	3	3
1	1	2	2	2	1	3	2	3	3
1	1	1	1	2	1	3	2	3	3
1	3	3	3	3	1	2	1	1	2
2	1	2	2	3	1	3	2	2	2
3	1	2	1	3	2	2	3	3	3
2	2	2	2	1	1	2	3	3	3
2	9	9	9	1	1	1	1	1	1
9	9	9	9	2	9	9	9	9	9
3	3	3	3	3	1	3	3	3	3
3	3	3	3	2	2	2	3	3	3

2	2	2	2	2	1	2	1	2	1
3	1	2	3	1	2	1	2	3	1
2	3	3	3	2	1	3	2	2	3
1	1	1	1	1	1	1	1	1	1
2	2	3	3	1	1	1	3	3	3
2	2	2	2	2	1	2	2	2	2
2	3	2	2	2	2	2	2	2	2
2	3	2	3	2	1	2	2	2	3
1	1	1	1	2	2	1	1	1	1
3	2	3	3	1	2	1	2	3	2
2	2	2	1	3	1	3	3	3	3
1	2	2	2	1	1	3	3	3	3
3	3	2	3	1	1	3	3	3	3
1	1	2	3	1	3	2	3	3	2
2	2	2	2	2	2	2	2	2	2
3	2	3	2	1	1	1	2	2	1
2	2	2	2	2	2	2	2	2	2
2	2	2	2	1	2	2	2	2	2
2	2	2	2	2	3	3	3	3	3
2	2	2	2	2	2	2	2	2	2
2	2	2	2	2	2	2	2	2	2
2	2	2	2	2	2	2	2	2	2
3	3	3	3	3	3	3	3	3	3
2	2	2	2	2	2	2	2	2	2
2	2	2	2	2	2	2	2	2	2
3	1	2	3	1	2	1	2	3	1
2	3	2	3	2	2	2	2	2	2
1	1	1	3	1	1	2	2	2	2
2	2	2	2	9	2	2	2	2	2
1	1	1	1	3	3	1	1	1	1
1	3	3	3	9	1	3	3	3	3
9	9	9	9	9	9	9	9	9	9
1	1	2	3	1	1	3	1	1	2
2	2	1	2	2	2	3	2	2	2
1	2	3	2	2	1	1	2	1	2
1	3	3	2	3	1	2	3	3	3
1	3	2	2	2	1	2	2	2	3
1	2	3	2	1	1	1	2	9	2
3	3	2	2	3	3	3	3	2	2
2	2	2	2	1	1	1	1	1	1
2	1	2	1	1	9	1	2	9	1
9	9	9	9	9	9	9	9	9	9
1	1	1	1	1	1	1	3	2	3
2	1	1	1	3	1	3	2	2	1
2	3	3	9	9	9	9	9	9	9
2	1	2	1	1	1	1	1	1	1
3	2	3	2	1	1	3	3	3	3

1	1	1	2	3	1	3	1	1	3
1	3	2	2	2	1	2	2	2	3
9	9	9	9	9	9	9	9	9	9
9	9	9	9	9	9	9	9	9	9
9	9	9	9	9	9	9	9	9	9
3	2	1	1	9	9	9	9	9	9
3	3	2	1	3	1	1	3	1	3
1	2	3	2	9	9	9	9	9	9
3	2	2	2	2	1	3	3	2	3
1	3	1	3	1	3	1	1	3	9
9	9	9	9	9	9	9	9	9	9
3	3	2	2	3	1	3	3	3	3
3	2	2	3	3	1	3	2	3	3
1	2	2	2	3	1	3	3	3	2
1	1	1	1	3	1	3	3	2	1
3	1	2	3	2	2	2	2	2	2
3	2	1	2	9	2	2	2	2	1
2	2	2	2	2	2	2	2	2	2
2	2	2	2	1	2	2	2	2	2
1	1	3	1	1	1	1	1	3	1
2	2	2	2	2	2	2	2	2	2
2	2	1	1	3	1	3	3	3	3
3	1	1	3	1	3	2	3	1	1
2	1	1	2	3	1	3	3	1	3
1	2	3	9	9	1	3	9	1	9
1	3	2	1	1	2	1	3	1	3
2	1	1	2	3	1	3	3	1	3
1	2	3	1	1	1	2	1	3	3
2	2	2	2	3	1	3	3	3	3
3	2	2	3	3	1	9	9	9	9
3	3	3	3	1	1	3	2	3	3
9	9	9	9	9	9	9	9	9	9
2	1	1	1	1	2	1	2	1	3
3	3	3	3	1	1	1	1	1	1
2	2	2	2	1	3	3	3	3	3
2	3	1	1	1	3	2	1	3	2
2	2	2	2	3	2	3	2	3	1
9	1	9	9	9	1	9	9	9	9
2	2	2	2	2	1	3	3	2	3
3	3	3	1	2	1	1	3	2	2
1	1	3	1	3	1	2	9	1	1
2	1	2	3	1	2	3	1	2	1
2	2	2	2	2	2	2	2	2	2
1	3	3	2	3	1	3	1	2	1
2	3	2	2	3	1	3	3	3	3
9	9	9	9	3	9	1	9	9	9
2	3	3	2	9	9	9	9	9	9

2	2	2	2	1	2	1	2	2	2
2	2	2	2	3	2	3	3	3	3
2	2	2	2	1	2	2	2	2	2
2	2	2	2	1	2	1	2	2	2
1	2	2	9	1	1	3	1	1	3
2	2	2	2	2	2	2	2	2	2
1	1	3	1	3	2	1	1	1	2
2	2	2	2	1	2	2	2	2	2
3	3	1	3	1	1	3	2	2	1
2	2	1	1	3	1	3	3	3	3
2	2	2	2	2	2	2	2	2	2
2	2	2	2	2	2	2	2	2	3
3	3	3	1	1	3	3	2	2	2
2	1	1	3	1	1	3	3	3	3
1	1	1	1	1	1	1	1	3	1
2	3	3	3	3	1	3	3	3	3
1	2	1	2	2	2	3	3	3	3
2	2	2	2	3	3	3	3	3	3
1	2	1	2	2	2	3	3	3	3
1	3	3	2	1	2	3	3	3	3
2	2	2	2	1	1	3	3	3	3
2	3	2	2	2	1	2	2	2	2
3	2	3	1	2	1	9	2	3	2
2	2	2	2	2	2	2	3	2	2
2	2	2	2	1	2	2	2	2	3
2	2	2	1	1	1	3	1	3	3
1	1	1	1	3	1	3	3	1	3
3	3	1	3	3	1	1	2	2	3
9	9	9	9	3	1	3	3	2	3
2	2	2	2	2	2	2	2	2	2
2	2	2	2	2	2	2	2	2	2
2	2	1	1	3	1	3	3	3	3
2	3	2	2	1	1	3	3	3	3
2	2	2	2	1	1	3	3	3	3
3	1	1	3	3	1	3	3	3	3
1	1	1	1	2	1	3	2	2	3
3	3	1	2	3	1	3	1	3	3
1	1	1	1	2	2	2	2	2	2
2	2	2	1	1	3	1	2	1	2
2	2	2	2	1	2	2	2	2	2
9	9	9	9	2	2	2	2	2	2
1	1	1	1	2	1	3	3	9	3
2	2	2	2	2	2	2	2	2	2
3	3	3	3	1	2	1	2	2	2
2	2	3	2	1	2	2	2	2	2
2	2	2	2	2	2	2	2	2	2
2	1	2	3	1	3	2	2	1	3

9	9	9	9	9	9	9	9	9	9
9	9	9	9	9	9	9	9	9	9
1	1	1	1	1	1	1	1	1	1
2	2	3	2	1	2	3	2	3	1
9	9	9	9	9	9	9	9	9	9
2	2	2	2	9	9	9	9	9	9
2	2	2	2	2	2	2	2	2	2
1	1	1	1	3	1	1	3	2	3
3	1	2	3	1	2	1	2	3	1
2	3	2	1	1	2	2	3	1	2
3	2	3	3	1	2	2	2	1	1
3	1	2	3	1	2	1	2	3	1
2	2	2	2	1	2	1	2	3	2
9	9	9	9	1	9	1	9	9	9
1	2	2	2	1	1	1	2	2	1
9	9	9	9	9	9	9	9	9	9
2	2	2	2	1	1	1	1	1	1
2	2	3	3	3	3	3	3	3	3
1	3	2	1	1	1	1	2	3	2
2	2	3	2	1	2	2	2	2	2
1	1	1	1	1	1	1	1	1	1
9	9	9	9	9	9	9	9	9	9
9	9	9	9	9	9	9	9	9	9
2	2	2	2	1	1	2	3	3	3
2	2	2	2	2	2	2	2	2	2
1	2	1	3	2	2	1	1	1	2
2	2	2	2	2	2	2	2	2	2
2	2	2	2	2	2	2	2	2	2
2	1	2	2	3	1	1	2	2	1
2	3	1	2	1	2	2	1	3	2
2	9	2	9	1	2	2	2	9	9
1	9	9	9	9	1	9	9	9	9
1	1	1	1	3	1	3	3	3	3
2	2	2	1	1	1	3	3	9	2
1	1	1	1	1	1	1	1	1	1
2	2	2	2	3	1	9	9	9	9
3	2	1	1	1	1	1	2	1	2
2	2	2	2	2	2	2	2	2	2
2	2	2	2	2	2	2	2	2	2
2	2	2	2	2	2	2	2	2	2
2	2	2	2	3	1	1	1	1	1
2	2	2	2	2	2	2	2	2	2
3	3	3	3	3	3	3	3	3	2
1	9	9	9	1	9	9	9	9	9
9	9	2	9	9	9	9	9	9	9
2	2	2	2	2	2	2	2	2	2
2	2	2	2	2	2	2	2	2	2

9	9	9	9	9	1	9	9	9	9
2	2	2	1	1	2	1	2	1	2
9	9	9	9	1	1	2	2	9	9
2	2	2	2	2	2	2	2	2	2
9	1	2	2	2	2	1	2	2	1
2	2	2	2	2	2	2	2	2	2
2	1	3	2	2	1	2	3	1	3
9	9	9	9	1	9	9	9	9	9
1	1	1	1	2	2	2	2	2	2
2	2	2	2	3	3	3	3	3	3
3	3	3	3	2	2	2	2	2	2
2	1	2	9	3	3	2	2	2	1
1	2	1	1	2	1	3	2	2	2
3	2	1	2	2	1	1	2	2	2
2	2	2	2	2	2	2	2	2	2
9	9	9	9	1	1	3	3	3	3
9	1	9	9	1	1	3	3	3	3
2	2	2	2	2	2	2	2	2	2
2	2	3	1	2	2	9	3	1	1
3	3	2	3	3	1	2	2	1	3
1	2	1	2	1	2	1	1	1	2
9	1	1	2	1	2	1	1	1	2
2	2	2	2	1	1	2	2	2	2
2	2	2	2	2	2	2	2	2	2
3	2	3	2	1	1	1	2	1	1
1	2	1	2	2	1	2	2	1	2
2	1	1	1	1	1	1	2	2	2
3	2	2	2	1	1	2	2	2	2
2	3	1	2	1	2	2	1	1	2
9	9	9	9	9	9	9	9	9	9
3	3	3	3	2	9	2	2	2	2
9	9	9	9	9	1	9	9	9	9
9	9	9	9	9	9	9	9	9	9
9	9	9	9	9	9	9	9	9	9
3	3	3	3	1	2	2	2	2	2
3	2	3	3	3	1	1	3	1	3
2	2	2	2	2	2	2	2	2	2
9	9	9	9	1	9	9	9	1	3
1	3	2	1	1	1	2	2	2	2
2	2	2	2	2	2	2	2	2	2
9	9	9	9	1	1	2	3	3	3
1	1	1	1	2	1	3	3	3	3
1	1	2	1	1	2	1	2	1	3
1	2	3	1	1	2	2	1	2	1
3	2	1	1	1	1	2	2	3	2
2	1	1	1	3	1	3	3	3	3
2	2	2	2	1	2	2	2	3	3

3	1	2	1	2	2	2	2	2	2
9	9	9	9	1	9	9	9	9	9
9	9	9	9	2	9	9	9	9	9
2	3	1	2	1	2	1	2	1	3
2	2	2	2	2	2	2	2	2	2
2	2	2	2	2	2	2	2	2	2
1	3	3	3	1	1	1	1	1	3
9	9	9	9	9	9	9	9	9	9
9	9	9	9	9	9	9	9	9	9
9	9	9	9	9	9	9	9	9	9
9	9	9	9	9	9	9	9	9	9
1	9	9	9	1	9	9	9	9	9
9	9	9	9	9	9	9	9	9	9
9	9	9	9	9	9	9	9	9	9
9	9	9	9	9	9	9	9	9	9
9	9	9	9	3	1	3	3	3	3
2	2	2	2	2	2	2	2	2	2
1	2	1	2	1	2	1	2	1	3
1	1	1	1	3	1	3	3	3	3
2	2	2	2	1	2	2	2	2	2
2	2	2	2	2	1	3	3	3	2
2	1	2	1	1	2	2	3	1	3
3	1	3	2	2	2	1	1	2	2
2	2	2	2	3	1	3	3	2	3
3	2	2	3	2	1	1	1	1	1
1	2	1	2	3	2	1	2	1	1
9	1	2	1	1	2	1	2	9	2
2	1	2	3	1	2	1	2	3	2
3	3	3	3	1	1	1	1	1	1
2	2	2	2	2	2	2	2	2	2
3	3	3	3	2	2	2	2	2	2
3	3	3	3	2	2	2	2	2	2
2	2	2	2	3	3	3	3	3	3
1	3	2	1	2	1	1	2	1	2
2	2	2	2	2	2	2	2	2	2
2	2	2	2	2	2	2	2	2	2
9	9	9	9	9	9	9	9	9	9
2	2	2	2	2	2	2	2	2	2
1	1	1	1	1	1	1	1	1	1
9	9	9	9	9	1	1	9	9	9
3	3	3	3	3	3	2	2	2	2
2	2	2	2	3	2	2	2	2	2
2	2	2	2	2	2	2	2	2	2
2	2	2	2	2	2	2	2	2	2
3	2	2	3	3	1	1	1	3	1
9	9	9	9	9	9	9	9	9	9
3	3	1	2	2	1	3	1	1	3

2	2	2	2	2	2	2	2	2	2
2	2	2	2	2	2	2	2	2	2
2	2	2	2	2	1	3	3	3	3
2	3	1	1	1	1	3	3	3	3
9	9	9	9	9	9	9	9	9	9
2	2	2	2	2	2	2	2	2	2
9	9	9	9	9	9	9	9	9	9
2	2	2	2	2	2	3	3	3	3
9	9	9	9	9	1	9	9	9	9
9	9	9	9	9	9	9	9	9	9
9	9	9	9	9	9	9	9	9	9
9	9	9	9	9	9	9	9	9	9
9	9	9	9	9	9	9	9	9	9
9	9	9	9	9	9	9	9	9	9
9	9	9	9	9	9	9	9	9	9
9	9	9	9	9	9	9	9	9	9
9	9	9	9	9	9	9	9	9	9
9	9	9	9	9	9	9	9	9	9
9	9	9	9	9	9	9	9	9	9
9	9	9	9	9	9	9	9	9	9
9	9	9	9	9	9	9	9	9	9
9	9	9	9	9	9	9	9	9	9
9	9	9	9	9	9	9	9	9	9
9	9	9	9	9	9	9	9	9	9
9	9	9	9	9	9	9	9	9	9
9	9	9	9	9	9	9	9	9	9
9	9	9	9	9	9	9	9	9	9
9	9	9	9	9	9	9	9	9	9
9	9	9	9	9	9	9	9	9	9
2	1	3	2	1	2	1	2	3	2
2	2	2	2	1	2	2	2	2	2
3	3	3	3	3	1	3	3	3	3
2	2	2	2	3	1	3	3	3	3
2	3	2	1	3	1	3	3	3	3
2	2	1	1	9	1	3	3	3	3
2	2	2	2	1	1	3	2	2	2
2	2	2	2	3	1	3	3	3	3
2	2	2	2	2	1	1	1	1	1
2	2	2	2	1	2	2	3	2	3
1	3	3	3	3	1	3	3	3	3
2	1	1	2	3	1	3	3	3	3
3	1	1	1	3	1	3	2	3	3
3	2	1	3	3	1	3	2	2	3
2	2	2	2	3	1	3	2	2	3
1	2	9	1	1	1	1	3	3	3
9	9	9	9	1	9	9	9	9	9
2	2	1	2	1	1	3	1	3	3

3	3	1	3	1	1	3	3	3	3
9	9	9	9	1	9	1	9	9	2
1	1	1	3	3	1	3	3	3	1
1	2	1	3	3	1	3	3	1	3
2	2	2	2	3	3	3	2	2	2
2	2	2	2	3	1	3	3	3	3
2	2	1	2	2	1	3	3	3	3
2	1	2	1	2	1	3	3	3	3
1	1	1	1	1	1	3	3	3	3
2	2	2	2	3	1	3	3	2	3
3	2	1	3	2	2	3	2	2	2
9	9	9	3	1	9	9	9	9	9
2	2	1	2	3	1	3	3	3	3
9	9	9	9	9	1	9	9	9	9
9	9	9	9	3	1	3	3	2	3
9	9	9	9	3	1	3	3	2	3
9	9	9	9	2	1	9	9	9	9
9	9	9	9	2	1	9	9	9	9
9	9	9	9	1	9	9	1	9	9
9	9	9	9	1	9	9	9	9	9
9	9	1	9	1	1	3	3	3	3
3	3	3	3	1	1	3	3	3	3
1	9	1	1	1	1	3	1	3	3
2	2	2	2	1	2	2	2	2	2
9	9	9	9	9	1	9	9	9	9
3	1	1	3	3	1	3	3	3	3
9	9	9	9	2	1	1	2	3	3
9	9	9	9	1	9	9	9	9	9
9	9	9	9	1	1	1	3	1	3
9	9	1	9	9	1	9	9	9	9
9	9	9	9	9	1	9	9	9	9
1	2	3	3	1	2	2	2	1	2
3	3	1	3	3	1	1	3	3	1
2	3	3	1	3	1	3	3	3	3
1	1	3	3	2	1	3	3	2	3
2	2	2	2	2	2	2	2	2	2
1	1	3	1	3	1	1	3	3	3
9	9	9	9	3	1	3	3	3	3
3	3	3	3	2	1	1	2	3	1
1	1	1	1	3	1	1	1	1	3
3	2	3	1	3	1	3	1	1	3
9	9	9	9	1	9	9	9	9	9
2	2	2	2	2	2	2	2	2	2
2	2	2	2	3	9	3	3	3	3
1	2	2	2	2	3	2	2	3	2
2	1	3	2	2	2	2	2	2	2
2	2	1	2	2	1	2	3	2	2

2	2	1	2	2	1	3	1	2	2
1	3	1	1	3	3	3	3	3	3
2	2	2	3	2	2	2	1	1	3
3	3	1	1	3	1	3	3	3	3
9	9	9	9	3	1	3	3	2	3
9	9	9	9	3	1	3	3	3	3
1	1	1	1	1	1	1	1	3	3
3	3	3	3	2	1	2	2	3	2
3	3	1	3	1	1	3	3	3	3
9	9	9	9	3	1	3	1	1	1
2	9	1	9	2	9	9	2	9	9
2	3	3	2	2	3	1	1	2	3
1	1	1	1	3	1	3	3	3	3
9	9	9	9	9	9	9	9	9	9
9	9	9	9	2	9	2	9	9	9
3	3	3	3	1	1	1	1	1	1
2	2	2	2	2	2	2	2	2	2
3	1	3	3	3	1	3	3	3	3
2	2	1	1	2	1	2	3	2	3
1	9	9	9	9	9	9	9	9	9
9	9	9	9	9	9	9	9	9	9
2	2	1	3	3	3	3	3	3	3
9	1	9	9	9	1	9	9	9	1
9	9	9	9	9	9	9	9	9	9
3	3	3	3	2	3	3	3	3	2
2	1	2	2	2	1	3	3	1	2
2	1	3	1	1	1	3	3	3	3
1	1	1	1	1	1	1	1	1	1
3	1	3	1	3	2	3	3	3	1
2	1	1	1	3	1	3	3	3	3
2	2	2	2	3	1	1	1	2	2
9	9	9	9	9	9	9	9	9	9
1	9	9	9	2	9	9	9	9	9
3	1	1	1	3	1	3	3	3	3
3	3	3	3	3	3	3	3	3	3
3	2	3	2	2	3	3	3	3	1
9	9	9	9	9	9	9	9	9	9
9	9	9	9	9	9	9	9	9	9
9	9	9	9	9	9	9	9	9	9
9	9	9	9	9	9	9	9	9	9
9	9	9	9	9	9	9	9	9	9
9	9	9	9	9	9	9	9	9	9
9	9	9	9	9	9	9	9	9	9
9	9	9	9	9	9	9	9	9	9
9	9	9	9	9	9	9	9	9	9
9	9	9	9	9	9	9	9	9	9
9	9	9	9	9	9	9	9	9	9

9	9	9	9	9	9	9	9	9	9
9	9	9	9	9	9	9	9	9	9
9	9	9	9	9	9	9	9	9	9
9	9	9	9	9	9	9	9	9	9
9	9	9	9	9	9	9	9	9	9
9	9	9	9	9	9	9	9	9	9
9	9	9	9	9	9	9	9	9	9
9	9	9	9	9	9	9	9	9	9
9	9	9	9	9	9	9	9	9	9
9	9	9	9	9	9	9	9	9	9
9	9	9	9	9	9	9	9	9	9
9	9	9	9	9	9	9	9	9	9
9	9	9	9	9	9	9	9	9	9
9	9	9	9	9	9	9	9	9	9
9	9	9	9	9	9	9	9	9	9
9	9	9	9	9	9	9	9	9	9
9	9	9	9	9	9	9	9	9	9
9	9	9	9	9	9	9	9	9	9
9	9	9	9	9	9	9	9	9	9
9	9	9	9	9	9	9	9	9	9
9	9	9	9	9	9	9	9	9	9
9	9	9	9	9	9	9	9	9	9
9	9	9	9	9	9	9	9	9	9
9	9	9	9	9	9	9	9	9	9
9	9	9	9	9	9	9	9	9	9
2	2	1	2	2	1	2	2	2	2
9	9	9	9	9	9	9	9	9	9
9	9	9	9	9	9	9	9	9	9
9	9	9	9	9	9	9	9	9	9
9	9	9	9	9	9	9	9	9	9
9	9	9	9	9	9	9	9	9	9
9	9	9	9	9	9	9	9	9	9
9	9	9	9	9	9	9	9	9	9
9	9	9	9	9	9	9	9	9	9
9	9	9	9	9	9	9	9	9	9
9	9	9	9	9	9	9	9	9	9
9	9	9	9	9	9	9	9	9	9
9	9	9	9	9	9	9	9	9	9
9	9	9	9	9	9	9	9	9	9
9	9	9	9	9	9	9	9	9	9
9	9	9	9	9	9	9	9	9	9
2	2	2	2	2	9	2	9	9	9
3	3	3	3	1	1	3	1	3	3
3	3	2	3	3	1	3	9	1	3
1	3	1	1	3	1	3	3	3	3
3	3	3	3	2	2	2	2	2	2
1	1	1	1	1	1	1	1	1	1

9	9	9	9	1	1	3	2	3	3
9	9	9	9	9	1	9	9	9	9
9	9	9	9	1	1	3	2	3	3
2	3	2	1	2	2	2	2	2	2
2	3	1	1	1	1	1	3	3	3
2	2	2	2	3	1	3	3	3	3
1	2	9	1	1	1	1	3	3	3
2	3	1	1	3	1	3	3	3	3
2	1	1	2	1	1	3	1	2	3
2	1	1	3	2	1	3	2	2	3
2	1	1	2	3	1	3	1	3	3
9	1	1	1	2	2	1	2	3	3
2	3	2	2	3	1	3	3	3	3
2	2	2	2	2	1	3	3	3	3
2	1	3	2	2	2	1	2	3	2
1	1	2	1	1	3	3	3	3	1
9	9	9	9	9	1	9	9	1	9
9	2	3	3	3	1	3	2	1	3
2	2	1	1	1	1	3	3	3	3
2	1	1	2	2	1	3	1	2	3
2	2	2	1	2	1	3	1	2	3
2	2	2	1	3	1	3	3	3	3
2	1	2	2	9	9	9	9	9	9
2	1	3	3	3	1	3	1	1	3
2	1	1	2	1	1	1	2	1	2
1	1	1	1	2	9	1	1	2	1
2	3	1	9	3	1	3	3	1	3
2	2	1	2	2	1	3	3	3	2
1	1	1	1	3	1	1	3	3	3
9	9	1	9	3	1	3	3	3	3
3	3	3	3	3	3	3	3	3	3
2	1	2	1	1	3	1	1	1	1
1	1	1	2	1	1	1	1	1	1
1	3	1	2	1	2	3	1	3	2
2	2	2	2	2	2	2	2	2	2
2	2	2	2	3	1	2	2	2	2
2	3	3	2	2	2	2	3	2	3
2	2	2	2	2	1	3	2	2	2
2	2	2	2	3	1	2	2	2	2
9	9	9	9	9	9	9	9	9	9
9	9	9	1	9	9	9	9	9	9
9	9	9	9	9	9	9	9	9	9
9	9	9	9	1	1	2	3	1	2
9	9	9	9	1	1	2	2	1	2
9	9	9	9	1	1	9	9	9	9
9	9	9	9	9	9	9	9	9	9
1	2	2	2	1	2	3	1	2	2

2	2	2	2	3	2	1	2	1	3
1	2	1	2	3	1	3	3	3	3
1	1	1	3	1	3	3	1	3	3
3	1	1	1	3	1	1	1	3	3
3	3	3	3	3	1	3	1	3	3
1	3	1	3	3	1	3	3	3	9
2	2	2	2	2	2	2	2	2	2
1	2	3	1	1	3	2	1	1	3
2	2	2	2	1	2	2	1	2	2
1	1	1	3	1	3	3	1	1	1
2	2	3	2	1	1	2	3	3	3
1	2	1	2	1	3	1	2	3	1
1	3	3	3	1	1	3	1	9	3
9	2	3	1	3	2	2	3	1	2
2	1	1	1	3	1	3	3	3	3
9	9	9	9	1	9	9	9	9	9
1	3	3	3	3	1	3	1	3	3
3	3	1	1	1	3	1	3	1	2
1	1	3	3	3	1	1	3	1	3
3	3	1	1	1	1	3	3	3	3
1	2	1	3	3	1	1	1	1	1
3	2	3	2	1	9	3	1	1	1
1	2	1	2	2	2	2	1	3	2
9	9	9	9	9	1	9	9	9	9
3	3	2	1	1	1	3	3	1	1
9	9	9	9	1	1	3	3	3	3
1	2	3	1	1	2	1	1	2	2
3	1	1	2	1	3	2	1	1	1
3	3	1	1	1	1	3	3	3	3
1	1	1	3	1	3	3	1	1	3
1	2	1	2	1	2	2	3	1	1
3	3	1	3	1	1	3	3	3	3
2	1	1	1	1	1	3	3	3	3
1	3	3	3	1	1	1	2	1	3
2	2	2	2	1	1	1	3	3	3
3	1	2	1	1	3	1	2	3	1
3	3	3	3	2	3	1	2	1	1
2	1	2	3	1	1	1	2	3	1
2	1	1	1	1	1	2	1	3	3
3	3	1	3	1	1	3	3	3	3
1	3	3	3	1	1	1	1	1	1
3	3	1	1	3	1	3	3	3	3
3	1	2	1	1	2	1	2	3	1
9	9	9	9	9	9	9	9	9	9
3	3	3	3	3	1	3	3	1	3
1	1	3	2	3	1	1	9	3	3
3	1	1	1	1	1	1	1	3	3

3	2	3	2	1	2	1	2	3	1
3	3	1	1	1	1	1	1	1	3
2	2	2	2	2	2	2	1	2	3
2	2	2	2	2	2	2	2	2	2
9	9	9	9	3	3	2	1	2	3
3	3	3	3	3	3	3	3	3	3
1	2	3	1	1	1	2	3	1	2
3	2	3	2	1	3	1	1	1	1
1	2	2	2	3	1	2	2	2	2
1	3	2	3	1	2	1	2	2	3
2	2	2	2	1	1	2	2	2	2
1	1	1	2	1	1	2	2	2	1
2	2	2	2	2	2	2	2	2	2
9	9	9	9	9	9	9	9	9	9
9	9	9	9	9	9	9	9	9	9
2	3	2	1	1	2	1	2	1	2
2	2	2	2	1	1	2	2	2	2
2	2	2	2	2	2	2	2	2	2
2	2	2	2	1	3	2	2	2	2
2	2	2	2	2	2	1	1	1	1
2	2	2	2	2	2	3	2	2	2
2	2	2	2	2	2	2	2	2	2
2	2	2	2	2	2	2	2	2	3
1	2	2	2	2	2	2	2	2	2
2	2	2	2	1	2	1	3	2	2
2	2	2	2	3	2	2	2	2	2
2	2	3	2	1	1	2	2	2	2
2	2	2	2	2	2	2	2	2	2
2	2	2	2	2	2	2	2	2	2
2	2	2	2	1	3	2	2	1	2
2	2	2	2	2	2	2	2	2	2
2	2	2	2	1	2	2	1	2	2
2	2	2	2	1	1	2	2	2	2
1	2	3	2	1	2	1	2	3	1
3	3	3	3	2	2	2	2	2	2
2	2	2	2	2	2	2	2	2	2
2	2	2	2	2	2	2	2	2	2
2	2	2	2	1	1	2	2	2	2
2	2	2	2	2	2	2	2	2	2
2	2	2	2	2	2	2	2	2	2
2	2	2	2	1	2	2	2	2	2
2	2	2	2	1	1	2	2	2	2
2	2	2	2	2	2	2	2	2	2
2	2	2	2	2	2	2	2	2	2
2	2	2	2	2	2	2	2	2	2
1	3	1	1	1	1	3	3	3	1
2	2	2	2	1	1	2	2	2	2

2	2	2	2	2	2	2	2	2	2
3	3	1	2	2	2	1	1	3	1
2	2	2	2	2	2	2	2	2	2
1	1	2	2	1	2	2	2	1	1
3	3	3	3	2	2	2	2	2	2
2	2	2	2	3	3	3	3	3	3
2	2	2	2	2	2	2	2	2	2
2	2	2	2	1	3	2	2	2	2
2	2	2	2	1	2	2	2	2	2
2	2	2	2	2	2	2	2	2	2
3	2	1	2	1	2	3	2	1	2
2	2	2	2	2	2	2	2	2	2
9	9	9	9	3	1	1	1	1	1
3	3	9	1	1	1	1	1	1	1
9	9	1	9	1	9	9	9	9	9
2	2	2	2	2	2	2	2	2	2
3	2	2	2	2	2	2	2	3	3
1	1	1	1	1	1	1	1	1	1
3	3	3	3	2	2	2	3	3	2
9	9	9	9	1	1	1	3	9	9
2	2	2	2	1	3	2	2	2	2
1	2	1	2	1	9	2	2	2	1
2	2	2	2	1	1	3	2	2	2
9	3	2	1	1	1	1	1	1	1
9	9	1	9	1	9	9	9	9	9
2	1	3	3	1	1	2	2	1	1
2	1	1	2	1	1	1	2	1	2
9	9	9	1	1	9	9	9	9	9
9	9	9	9	9	1	9	9	9	9
2	2	2	2	1	3	2	3	3	1
3	2	2	3	1	2	1	3	2	9
3	3	3	3	2	2	1	1	1	1
9	2	2	1	3	1	2	2	1	2
1	9	2	1	1	1	1	3	3	3
2	1	2	3	1	2	1	2	9	1
1	1	1	1	1	1	1	1	1	1
3	3	3	3	3	3	1	2	1	1
2	2	2	2	2	3	3	2	1	3
2	2	2	2	2	2	2	2	2	2
9	9	9	9	1	2	9	2	9	9
2	2	2	1	1	1	3	3	3	3
9	9	9	1	1	9	9	9	9	9
1	3	3	3	1	1	1	1	3	3
2	2	2	2	1	1	1	1	1	1
2	2	2	2	2	2	2	2	2	2
2	2	2	2	2	2	2	2	2	2
3	3	1	1	9	9	9	9	9	9

2	2	2	2	1	1	2	2	2	2
9	9	9	9	9	1	9	9	9	9
1	1	1	1	1	1	1	1	1	1
2	2	2	1	1	1	2	3	2	2
2	2	2	2	2	1	2	2	2	2
2	2	2	2	2	1	2	1	2	2
1	1	1	1	1	1	1	1	1	1
2	2	2	2	1	1	2	2	2	2
1	1	1	1	1	1	1	1	1	1
2	2	2	2	2	2	2	2	2	2
9	9	2	9	9	1	9	9	1	9
3	1	2	3	3	3	1	2	2	3
3	3	3	3	3	3	3	3	3	3
1	1	1	1	1	2	2	2	2	2
3	1	2	3	1	1	2	2	2	2
3	3	3	3	1	1	1	1	1	1
1	1	1	1	1	2	1	2	3	2
2	2	2	2	3	3	3	3	3	3
2	2	2	2	1	3	1	1	1	1
2	2	2	2	1	3	1	1	1	1
2	2	2	2	2	2	2	2	2	2
3	3	2	2	3	2	3	2	2	2
1	1	1	1	1	1	1	1	1	1
3	3	3	3	1	1	3	3	3	3
9	9	9	9	9	9	9	9	9	9
1	2	1	2	1	2	2	1	2	1
2	2	2	1	3	3	1	1	1	1
2	3	1	2	1	3	2	2	2	2
2	3	3	3	1	1	1	1	1	1
3	3	2	2	2	1	1	2	9	1
3	3	3	3	9	3	9	3	9	3
1	2	2	2	1	2	2	1	1	1
2	2	3	3	1	1	1	2	9	1
1	2	1	2	2	2	2	2	1	2
2	2	2	3	1	9	2	9	9	9
2	2	2	2	2	3	1	2	1	2
2	2	2	2	1	2	1	1	9	2
2	2	2	2	2	2	2	2	2	2
3	3	3	3	3	3	3	3	3	3
1	3	1	2	1	1	2	2	3	1
2	2	2	2	2	2	2	2	2	2
2	2	2	2	3	2	3	2	3	2
2	3	1	3	1	3	2	3	9	2
2	2	2	2	2	2	3	3	9	2
3	1	2	3	1	2	3	1	2	1
3	3	3	3	3	3	3	3	3	3
2	3	1	1	1	1	1	2	1	2

1	1	1	1	2	2	3	2	3	2
9	9	9	9	9	9	9	9	9	9
3	3	3	3	3	3	3	3	3	3
1	1	1	1	1	2	1	1	2	2
2	2	2	2	2	2	2	2	2	2
2	2	2	1	2	1	3	3	3	3
1	1	1	1	1	1	1	1	1	1
3	1	3	3	1	1	3	3	3	3
1	1	1	1	1	1	1	1	1	1
3	1	3	3	1	1	3	3	3	3
1	1	1	1	1	3	1	1	2	3
2	2	2	2	2	2	2	2	2	2
2	2	2	2	2	2	2	2	2	2
2	2	2	2	2	2	2	2	2	2
1	1	1	1	1	1	2	2	2	2
9	1	3	2	1	3	1	2	1	1
1	1	1	1	2	2	2	2	2	2
1	2	3	1	1	3	2	3	2	1
2	2	2	2	2	3	2	3	2	2
2	1	3	1	2	1	2	3	2	2
2	2	2	2	2	2	2	2	2	2
2	2	2	2	1	1	3	3	3	3
2	2	2	2	2	2	2	2	2	2
1	3	2	2	2	3	2	1	3	1
2	2	1	2	1	2	2	1	2	2
2	2	3	3	3	1	3	3	2	3
2	2	2	2	1	1	1	1	1	1
1	2	3	2	1	2	3	1	1	1
1	1	1	1	1	2	3	1	1	1
2	2	2	2	2	2	2	2	2	2
1	1	1	1	1	1	1	1	1	1
3	3	3	3	2	3	2	2	2	2
2	3	3	3	1	2	1	1	1	1
3	2	3	1	3	1	1	3	3	3
2	1	2	2	1	9	2	3	2	2
1	2	2	3	2	1	2	3	2	3
2	2	2	2	1	1	3	1	2	3
2	1	2	2	1	3	3	2	1	2
1	2	2	2	1	1	1	1	1	1
3	2	2	3	2	2	2	2	2	2
1	3	3	3	1	2	1	1	1	3
2	1	2	2	1	2	1	2	3	2
2	2	2	2	1	2	2	2	2	2
9	9	9	9	9	9	9	9	9	9
1	2	2	2	1	2	2	2	2	2
9	1	3	3	1	3	2	3	3	1
2	3	3	2	1	3	2	3	3	1

2	2	2	2	2	2	2	2	2	2
2	2	2	2	2	2	2	2	2	2
2	2	2	2	2	2	2	2	2	2
3	9	3	2	9	9	9	9	9	9
1	1	1	1	1	3	1	1	2	3
2	2	2	2	2	2	2	2	2	2
9	9	9	9	9	9	9	9	9	9
3	1	3	1	1	2	2	3	1	3
1	1	1	1	1	1	2	3	1	2
3	3	1	3	2	1	3	3	1	1
1	1	9	1	3	3	3	3	3	3
2	3	3	3	1	1	3	2	1	3
3	2	3	1	1	1	3	3	3	3
9	9	9	9	9	9	9	9	9	9
3	2	1	2	1	2	2	1	1	2
1	3	3	1	1	1	3	1	3	3
2	2	3	2	2	2	2	2	2	1
2	2	2	3	1	3	1	2	3	2
2	2	2	2	2	1	9	9	9	9
1	1	1	1	1	1	3	1	2	3
3	1	2	2	1	2	3	1	3	2
2	2	2	2	2	2	2	2	2	2
2	3	2	3	1	2	2	1	2	3
2	2	3	2	1	9	3	3	3	2
9	9	1	9	1	9	9	9	9	9
2	2	2	2	2	2	2	2	2	2
9	9	9	9	9	9	9	9	9	9
1	2	3	2	1	2	1	2	2	1
3	3	3	2	1	2	2	2	1	1
2	2	2	1	1	2	2	3	3	2
1	1	3	3	1	1	2	2	2	1
3	3	3	3	3	3	3	3	3	2
1	2	2	3	1	2	2	3	2	1
1	2	1	2	1	1	2	2	2	2
2	2	1	2	1	1	3	2	2	3
2	1	1	2	1	1	3	3	3	3
2	3	3	3	1	1	2	2	3	3
2	9	2	1	1	2	2	1	2	3
3	3	3	3	3	3	3	1	3	3
2	2	2	2	2	2	2	2	2	2
2	2	2	2	2	2	2	2	2	2
3	3	3	3	2	2	2	9	1	2
3	3	3	3	1	3	3	3	3	3
2	3	2	1	2	2	2	3	3	3
1	1	1	1	1	2	1	2	3	1
1	1	1	1	1	2	2	1	3	2
1	1	1	1	1	3	1	1	2	1

3	3	2	1	2	2	2	2	2	2
2	3	2	1	3	1	3	3	2	2
2	3	3	3	1	2	3	3	2	2
9	9	9	9	9	9	9	9	9	9
3	3	3	3	3	3	3	3	3	3
2	3	3	2	1	1	3	3	3	3
3	3	3	3	3	3	3	3	3	2
3	1	9	1	1	1	1	1	3	3
3	2	1	2	3	1	2	1	2	3
9	9	9	9	1	2	2	2	2	2
2	3	3	3	1	3	2	2	3	2
3	3	3	3	3	3	3	3	3	3
2	2	2	2	1	1	1	1	1	1
3	3	3	3	3	3	3	3	3	3
2	2	2	1	2	3	1	2	1	2
1	2	3	1	1	1	3	2	3	1
2	2	2	2	2	2	2	2	2	2
2	3	2	3	2	2	2	2	2	2
2	1	2	1	1	2	2	2	2	1
9	9	9	9	9	1	9	9	9	9
1	1	1	1	1	1	9	1	9	2
3	3	3	3	3	3	3	3	3	3
2	1	1	1	1	1	1	1	1	1
2	2	2	2	2	2	2	2	2	2
2	2	2	2	2	2	2	2	2	2
3	1	1	1	1	1	1	2	2	2
3	1	2	1	1	3	1	3	1	1
3	3	3	3	9	9	9	9	9	9
2	2	2	2	3	3	3	1	3	3
2	3	3	1	2	1	2	3	1	3
3	1	2	3	1	2	3	1	2	3
3	3	1	1	1	3	3	1	1	3
3	1	2	2	1	1	1	1	1	1
2	3	2	1	1	3	1	2	3	1
2	1	3	2	2	1	2	1	3	1
2	2	2	2	1	1	3	3	3	3
2	3	3	2	1	1	2	3	2	3
2	2	2	2	3	3	3	3	3	3
3	1	2	3	1	2	2	2	1	2
3	3	3	3	1	3	2	2	3	3
3	1	2	3	1	1	1	3	1	1
3	1	1	2	1	1	1	2	1	3
2	3	2	3	1	1	1	2	2	1
1	1	1	1	1	1	3	3	9	2
2	2	1	1	1	3	1	1	1	1
9	1	1	2	3	1	1	2	1	2
2	2	2	2	2	2	2	2	2	2

3	1	1	3	1	1	1	1	1	1
3	2	1	2	1	2	1	2	1	2
2	2	2	1	1	1	3	9	2	9
3	2	2	3	1	2	2	1	2	1
2	1	3	2	2	2	1	2	1	3
1	2	3	3	3	1	1	3	3	3
2	3	1	3	2	3	3	3	1	3
1	2	1	2	1	3	1	2	3	2
2	2	2	9	1	2	2	2	2	2
1	3	3	1	3	1	1	1	3	1
3	1	2	3	1	2	1	2	3	1
2	2	2	2	1	1	1	2	2	3
1	2	2	1	1	3	2	1	2	3
2	3	1	2	1	2	1	3	9	1
1	2	1	3	3	3	1	1	1	3
1	2	2	2	2	3	1	2	2	2
2	3	2	2	1	2	1	2	1	2
3	2	1	2	1	1	2	3	1	1
1	1	1	1	3	1	1	1	1	3
3	3	1	3	3	1	3	1	1	3
1	1	2	2	3	3	1	1	9	9
3	3	3	3	3	3	3	3	3	1
1	1	1	1	1	3	1	3	1	1
3	3	3	3	1	1	1	3	9	2
1	1	1	1	1	1	3	2	2	2
1	1	1	1	1	1	1	1	1	2
2	1	2	2	1	9	1	1	1	2
1	3	1	1	3	3	3	3	3	3
3	1	1	2	1	1	3	3	3	3
2	1	1	1	1	1	1	1	1	1
2	3	2	2	1	3	2	2	2	2
2	2	2	2	2	2	2	2	2	2
1	3	3	3	1	3	3	1	1	2
1	3	2	1	1	9	2	1	3	2
2	2	2	2	2	2	2	2	2	2
9	9	9	9	9	9	9	9	9	9
9	9	9	9	9	9	9	9	9	9
9	9	9	9	9	9	9	9	9	9
2	2	2	2	1	1	2	2	2	2
9	9	9	9	1	1	9	9	9	9
2	2	2	2	2	2	2	2	2	2
2	2	2	2	1	1	1	3	3	3
2	3	2	3	3	2	2	1	1	2
2	2	2	2	1	3	3	3	3	3
9	2	2	2	3	1	2	9	9	9
2	3	3	2	1	1	2	2	1	1
2	3	2	3	3	1	3	3	3	3

2	3	3	1	1	1	1	1	3	1
1	3	3	3	3	1	3	3	2	3
1	3	3	3	3	1	3	3	2	3
2	1	3	2	1	1	3	1	2	1
2	2	2	2	1	3	1	2	1	2
1	3	3	3	1	3	3	1	1	2
9	9	2	9	3	3	3	2	1	1
2	1	1	2	1	2	1	2	2	2
2	2	2	2	3	3	2	2	3	2
1	1	2	1	3	3	3	1	1	1
2	2	2	2	3	1	3	3	9	3
2	3	2	3	3	2	2	1	2	1
3	9	9	9	1	9	1	9	2	9
3	1	2	1	1	3	2	3	1	1
3	3	1	1	3	1	3	3	2	3
2	3	2	1	3	3	3	3	2	3
3	2	2	1	1	2	3	2	1	2
2	3	2	3	1	3	3	3	3	3
9	3	1	2	1	1	1	2	9	2
2	2	2	2	2	2	2	2	2	2
2	2	2	2	2	2	2	2	2	2
3	9	9	9	1	9	9	2	2	2
2	2	2	2	1	1	3	3	3	3
2	2	2	2	2	2	2	2	2	2
2	2	2	2	2	2	2	2	2	2
9	9	9	9	2	2	2	2	2	2
9	1	3	9	1	1	1	2	1	3
9	1	3	9	1	1	3	9	9	9
9	1	3	9	9	1	9	9	9	9
1	1	1	2	3	1	3	3	3	3
3	1	3	3	3	1	3	3	3	3
9	1	9	9	9	1	9	9	9	9
2	2	2	2	3	1	3	3	3	3
9	1	3	3	2	1	3	9	9	9
2	1	1	2	2	1	3	2	2	2
9	1	3	3	3	1	3	3	3	3
9	1	3	3	3	1	3	9	9	9
9	9	9	9	9	1	3	9	9	9
3	1	3	3	3	1	3	3	3	3
3	1	3	3	1	1	3	3	3	3
9	1	9	9	3	1	3	9	9	9
3	1	3	3	3	1	3	3	3	3
9	1	9	9	3	1	3	9	9	9
9	1	9	9	9	1	3	9	9	9
9	1	3	3	3	1	3	3	3	9
9	1	3	9	1	1	3	9	9	9
3	1	3	3	3	1	3	3	3	3

9	2	1	9	9	1	9	9	9	9
9	1	3	3	2	1	3	3	3	3
9	9	9	9	1	1	3	9	3	3
9	1	3	3	3	1	3	9	9	9
2	1	3	3	3	1	3	3	3	3
9	1	3	3	3	1	3	9	9	9
9	1	9	9	9	1	9	9	9	9
2	1	9	2	3	1	3	2	2	2
2	1	3	3	2	1	3	2	3	2
1	1	1	1	1	1	1	1	1	1
9	1	3	3	3	1	3	9	9	9
9	1	3	3	3	1	3	9	9	9
1	1	1	1	2	1	3	1	1	1
1	1	1	2	2	1	2	2	2	2
9	1	3	3	2	1	3	9	9	9
9	1	9	9	3	1	3	3	3	3
2	1	3	3	3	1	3	3	3	3
1	2	1	2	3	1	3	3	2	3
9	1	3	3	3	1	3	9	9	9
9	1	3	3	3	1	3	9	9	9
9	1	3	3	3	1	3	9	9	9
9	1	3	9	3	1	3	9	9	9
9	1	9	9	3	1	3	3	2	9
9	1	9	9	9	1	3	9	9	9
9	1	3	3	3	1	3	9	9	9
9	9	9	9	3	1	9	9	9	9
9	9	9	9	9	1	3	1	3	2
9	1	1	9	2	1	3	9	9	9
1	1	1	2	3	1	3	2	2	3
9	1	3	3	2	1	3	9	9	9
1	1	2	1	1	1	3	3	3	3
3	3	3	3	1	1	1	1	1	1
3	3	3	3	1	1	3	3	1	3
3	1	3	3	1	1	3	3	2	3
3	1	3	3	2	1	3	2	3	3
9	9	9	9	3	1	3	9	9	9
1	1	3	3	3	1	3	1	1	1
3	1	3	3	3	1	3	2	3	3
9	1	3	3	3	1	3	9	9	9
9	1	9	9	2	1	3	9	9	9
9	1	9	9	3	1	3	9	9	3
2	1	1	1	3	1	3	3	2	3
9	1	9	9	2	1	3	2	2	2
9	1	3	1	2	1	3	3	3	3
3	1	9	9	3	1	3	3	3	3
1	1	1	3	3	1	3	3	3	3
1	1	1	3	3	1	3	3	3	3

1	1	3	3	2	1	3	3	3	3
9	1	1	9	2	1	3	9	9	9
1	1	1	1	3	1	3	3	3	9
3	1	3	3	3	1	3	2	3	3
3	1	3	3	3	1	1	3	3	3
9	1	9	9	3	1	3	3	3	3
9	1	3	3	9	1	3	3	1	3
9	1	1	3	3	1	3	3	3	3
9	1	3	3	2	1	3	9	3	3
1	1	1	1	9	1	3	3	3	3
9	1	1	9	3	1	3	3	3	3
9	1	9	9	3	1	3	3	3	3
9	9	9	9	3	1	1	3	3	3
9	1	9	9	1	9	3	9	9	9
9	1	9	9	1	9	3	9	9	9
3	3	3	3	2	1	3	3	3	3
2	2	1	2	3	1	3	1	2	3
9	9	9	9	9	1	9	9	9	9
9	2	9	9	3	1	3	3	3	3
2	2	2	2	2	1	1	2	3	3
2	2	2	2	3	3	3	3	3	2
2	2	1	1	3	1	3	3	3	3
9	9	9	9	9	9	9	9	9	9
9	9	9	9	1	1	3	9	3	3
2	1	2	3	3	1	3	3	3	3
1	1	1	1	1	1	3	3	3	1
2	3	1	3	2	1	3	3	3	3
3	2	2	3	3	1	3	3	2	1
2	1	2	2	2	1	3	2	2	3
1	1	1	1	3	1	3	3	3	3
9	9	9	9	1	1	3	3	3	3
2	2	3	1	1	1	3	3	3	3
1	2	2	1	3	2	2	3	3	2
2	3	1	2	3	1	1	1	1	3
2	1	2	2	3	1	3	3	3	3
3	1	1	2	2	1	3	3	2	9
2	2	2	2	3	1	3	3	1	2
1	1	3	2	1	1	9	9	9	9
1	1	1	2	3	1	2	3	3	2
9	9	1	9	1	1	3	3	3	3
2	1	2	2	2	1	3	2	2	3
1	1	2	2	1	1	3	3	1	3
2	1	9	9	1	1	3	1	3	1
2	2	1	2	1	1	3	1	3	3
2	1	9	9	1	1	3	1	3	1
3	3	1	2	1	1	3	3	3	3
2	1	1	1	3	1	3	3	3	3

2	1	1	2	3	1	3	3	3	3
2	2	2	2	3	1	3	3	2	2
2	2	2	2	1	1	2	2	2	2
2	2	2	2	3	1	3	3	2	2
1	1	1	2	3	1	3	3	3	3
9	9	9	9	1	1	9	9	1	3
2	1	3	3	3	1	3	3	9	3
1	1	1	2	1	1	3	2	2	2
2	1	1	2	3	1	3	3	3	3
2	2	2	2	3	1	3	2	2	3
9	9	1	1	3	1	3	1	3	3
2	3	3	3	1	3	1	1	1	1
2	2	2	2	2	1	3	3	2	3
2	1	1	2	3	2	2	3	3	2
3	1	1	2	1	1	3	3	3	3
2	1	1	3	3	1	3	3	3	3
2	1	1	2	3	1	2	3	3	3
3	1	3	3	3	1	3	3	3	3
9	1	1	2	3	1	3	3	3	3
9	9	9	9	2	1	3	3	2	3
2	2	1	2	2	1	3	3	3	3
9	9	9	9	9	1	9	9	9	9
2	2	1	2	3	1	3	3	3	3
9	9	9	9	2	1	1	2	2	2
1	1	3	3	3	1	3	3	3	3
2	1	1	2	3	1	3	3	2	3
9	9	9	9	1	9	9	9	9	9
9	9	9	9	9	1	3	3	3	3
2	2	2	2	3	1	3	3	9	9
3	1	1	3	1	1	1	1	3	3
1	3	2	3	3	1	3	3	2	3
9	3	2	3	9	1	3	3	9	3
9	9	1	9	3	1	3	1	1	3
9	9	9	9	9	9	9	9	9	9
9	9	9	9	9	9	9	9	9	9
2	2	3	1	3	1	3	2	2	3
9	9	9	1	3	1	3	3	3	3
1	3	1	3	3	1	3	3	3	3
9	1	9	9	3	1	3	3	3	3
3	1	1	9	9	1	3	3	3	3
3	1	3	3	2	1	3	9	9	9
9	1	9	1	2	1	3	3	3	3
3	1	1	1	3	1	3	3	3	3
3	2	3	3	2	1	3	2	3	3
3	1	3	3	3	1	3	3	3	3
3	1	1	1	3	1	3	3	3	3
2	1	3	2	3	1	3	1	3	3

9	1	9	9	3	1	3	2	3	3
3	1	1	3	3	1	3	3	3	3
9	9	3	9	2	9	3	9	9	9
3	1	3	3	3	1	3	3	3	3
9	1	3	3	3	1	3	3	3	3
9	1	9	9	3	1	3	3	3	3
3	1	3	3	3	1	3	3	3	3
9	1	1	3	2	1	3	9	3	9
9	9	9	9	9	9	9	9	9	9
9	9	1	9	3	1	3	2	3	3
3	3	2	9	1	9	1	1	3	3
1	3	1	1	3	1	3	3	3	3
9	9	9	9	9	1	1	1	2	2
9	9	9	9	3	1	3	3	2	3
9	9	9	9	1	9	9	9	9	9
9	9	9	9	9	1	9	9	9	9
3	1	1	1	1	1	3	3	3	3
2	1	1	1	3	1	3	2	3	2
2	3	1	1	3	1	3	2	3	3
9	9	9	9	9	1	9	9	9	9
3	1	1	1	3	1	1	1	3	1
2	3	1	1	3	1	3	3	3	3
9	9	9	9	9	1	9	9	9	9
9	3	2	9	3	1	3	3	3	3
9	9	9	9	3	1	3	3	3	3
3	3	3	2	3	1	3	3	1	3
1	1	1	1	3	1	3	3	3	3
9	9	9	9	3	1	3	3	3	3
9	9	9	9	3	1	3	3	3	3
1	1	1	1	3	1	3	3	3	1
9	1	9	9	1	1	9	9	3	9
2	2	1	2	1	1	1	1	1	1
9	9	9	9	9	1	3	2	3	3
3	3	1	1	2	1	1	1	2	3
2	9	1	1	1	1	1	1	1	1
2	3	1	1	1	1	1	1	1	1
2	3	1	2	2	1	3	3	2	3
1	2	1	1	3	1	3	3	2	1
3	2	2	3	3	1	3	2	3	3
3	3	1	3	3	1	3	3	3	3
3	3	1	1	3	1	3	3	3	3
9	9	9	9	9	1	9	9	9	9
9	9	1	9	9	1	3	3	3	3
1	9	1	9	9	1	9	9	9	9
1	1	2	2	2	1	3	9	1	3
2	3	3	2	1	1	3	3	3	3
3	3	1	2	3	1	3	3	3	2

2	2	2	2	9	9	3	3	3	3
9	9	9	9	9	1	9	9	9	9
9	9	9	9	9	1	9	9	9	9
1	9	1	9	3	1	3	2	3	3
9	9	1	9	9	1	3	9	1	9
9	9	1	9	3	1	3	9	9	9
3	3	3	3	3	1	3	3	3	3
1	3	1	1	9	1	9	9	9	9
9	9	1	9	1	1	1	1	3	3
3	3	1	1	2	1	3	2	1	3
1	1	1	3	3	1	3	3	3	3
9	9	9	9	2	1	3	2	3	3
3	2	1	2	3	1	3	3	3	3
2	2	2	2	1	1	3	3	1	3
3	3	3	3	3	1	3	9	3	3
2	3	1	1	2	1	3	2	2	2
2	3	3	3	3	1	3	1	3	3
1	9	9	9	3	1	9	9	9	9
1	9	9	9	3	1	3	3	3	3
9	9	9	9	9	1	9	9	9	9
9	9	1	9	9	1	9	9	9	9
9	9	9	9	9	1	9	9	9	9
9	9	3	3	3	1	3	3	3	3
2	2	1	2	1	1	3	2	2	2
1	9	9	9	3	1	3	3	3	3
1	1	2	2	3	1	3	3	3	3
1	3	1	1	3	1	1	2	1	3
2	3	2	2	3	1	3	3	2	3
3	3	2	2	3	1	3	3	2	3
2	2	3	3	2	1	3	2	2	3
3	3	1	2	1	1	3	2	3	3
9	9	1	9	9	1	9	9	9	9
3	3	9	3	3	1	3	3	3	3
2	2	2	2	1	1	3	3	3	3
9	9	1	1	3	1	3	3	3	3
9	9	9	9	3	1	3	3	3	3
2	3	2	3	3	1	3	2	3	3
9	9	1	9	9	1	9	9	9	9
9	9	9	9	3	1	3	3	3	3
1	2	2	3	1	1	1	3	3	2
9	9	9	9	2	1	3	3	3	3
2	1	3	3	2	1	3	3	3	3
2	1	2	2	3	1	3	3	3	3
9	1	3	3	2	1	3	2	2	2
9	1	9	9	3	1	3	3	9	9
9	1	3	3	3	1	3	3	3	3
2	2	3	2	2	1	2	3	2	3

3	3	1	2	2	1	2	3	2	9
2	1	1	1	3	1	3	3	2	3
2	1	1	2	3	1	3	3	3	3
2	1	1	1	3	1	3	3	2	3
1	9	9	9	9	1	9	9	9	9
9	1	1	1	3	1	3	3	1	3
3	3	1	3	3	1	3	3	3	3
1	2	1	2	3	1	2	3	3	3
1	1	1	1	3	1	3	3	3	3
1	3	1	1	3	1	3	3	3	3
2	2	2	2	1	1	1	2	3	3
3	3	1	2	2	2	1	2	2	3
2	3	1	1	1	1	3	3	3	2
2	2	1	2	3	1	3	1	2	3
1	3	1	2	3	1	3	2	3	3
1	2	1	2	1	1	1	1	1	2
2	2	1	2	3	1	3	3	3	3
2	2	1	2	1	2	2	2	2	2
3	3	1	1	3	1	3	1	1	3
1	3	1	2	3	1	3	3	3	3
1	2	1	2	1	1	2	2	2	2
3	3	1	1	1	1	3	3	3	3
2	2	2	2	1	2	2	2	3	3
2	2	2	2	2	2	2	2	2	2
2	2	2	2	2	2	2	2	2	2
2	2	1	1	3	1	3	3	3	1
1	2	1	2	2	1	1	3	3	1
3	2	3	2	3	1	3	3	3	1
3	3	2	1	3	1	3	3	2	3
1	1	1	1	3	1	3	3	3	3
2	2	1	1	2	1	3	2	3	3
3	1	3	2	3	1	1	1	2	2
2	1	2	1	2	2	2	2	2	2
2	2	1	1	9	1	3	2	3	3
2	2	1	1	3	1	3	2	3	3
9	1	1	1	3	1	1	3	3	3
2	2	1	3	2	1	2	2	3	3
3	1	1	1	2	1	3	3	3	3
9	9	1	9	9	1	9	9	9	9
9	9	9	9	9	1	9	9	9	9
9	9	1	9	9	9	9	9	9	9
9	9	9	9	9	1	9	9	9	9
9	9	1	9	9	1	9	9	9	9
9	9	1	9	9	1	9	9	9	9
9	9	9	9	9	1	9	9	9	9
2	3	1	3	3	9	9	9	9	9
1	2	1	1	2	1	3	3	2	3

9	9	9	9	9	1	9	9	9	9
9	9	9	9	9	1	9	9	9	9
3	3	1	2	3	1	1	3	3	3
9	9	1	9	9	1	9	9	9	9
9	9	9	9	9	1	9	9	9	9
9	9	9	9	9	1	9	9	9	9
2	3	1	1	3	1	3	3	3	3
3	1	2	3	1	3	2	1	1	3
9	9	9	9	9	1	9	9	9	9
9	9	1	9	9	1	9	9	9	9
9	9	1	9	9	1	9	9	9	9
9	9	1	9	1	9	9	9	9	9
9	9	9	9	9	1	9	9	9	9
9	9	9	9	9	1	9	9	9	9
9	9	9	9	3	1	3	3	3	3
1	9	9	9	9	1	9	9	9	9
3	3	1	1	3	1	3	3	1	3
3	3	1	1	3	1	3	3	3	3
1	2	1	1	2	1	3	3	3	3
1	3	1	2	3	1	3	3	3	3
1	3	1	1	3	1	3	3	1	3
1	3	1	1	3	1	3	3	3	3
1	3	1	1	3	1	3	3	3	3
1	1	1	1	3	3	1	1	1	2
1	1	1	1	3	1	3	3	3	3
1	1	1	1	3	1	3	3	9	3
2	1	1	1	1	1	3	1	3	3
1	1	1	1	1	1	3	1	3	3
9	9	9	9	9	1	9	9	9	9
1	3	1	2	3	2	2	9	1	2
3	3	1	1	1	2	3	1	2	9
1	2	1	1	3	1	3	3	3	3
3	3	3	3	9	9	9	9	9	9
3	3	1	2	3	1	1	3	3	3
3	3	1	1	2	1	3	3	2	3
2	3	1	2	3	1	3	2	3	3
3	3	1	1	3	1	3	2	3	3
1	3	1	3	1	1	3	3	2	3
2	3	1	1	3	1	3	1	3	3
1	3	1	2	1	1	3	3	2	3
1	2	1	1	1	1	1	3	3	3
3	3	1	1	3	1	3	3	3	3
1	3	1	1	3	1	3	3	3	3
1	3	1	3	3	1	3	3	3	3
3	3	1	1	3	1	3	3	1	3
1	3	1	2	3	1	1	1	2	3
3	2	1	3	1	1	3	3	3	3

1	1	1	1	2	1	3	1	3	3
2	2	1	1	3	1	2	3	1	3
3	3	3	3	3	1	3	3	3	3
3	3	3	3	3	1	3	3	3	3
3	3	1	3	3	1	3	3	3	3
1	2	1	2	2	1	3	3	3	3
9	9	9	9	3	1	3	3	3	3
1	2	1	1	3	1	3	1	1	3
1	3	1	1	3	1	1	1	1	3
3	3	1	3	3	1	3	3	3	3
3	3	3	3	3	1	3	3	3	3
3	3	1	3	3	1	3	3	3	3
1	2	1	2	2	1	3	3	3	3
9	9	1	1	3	1	3	3	3	3
2	3	1	2	3	1	3	1	1	3
1	9	1	9	3	1	3	3	1	3
2	1	1	2	3	1	3	3	3	3
1	1	9	9	3	1	3	9	3	3
2	1	1	2	3	1	1	1	3	3
2	1	1	2	3	1	1	1	3	3
3	1	1	3	3	1	3	3	3	3
2	2	2	2	2	2	3	3	3	3
9	1	1	1	1	2	2	3	3	3
2	2	1	2	1	2	1	1	2	2
2	3	2	1	1	3	1	1	3	2
2	2	1	2	3	1	3	3	3	3
3	1	1	1	3	1	3	1	3	3
3	3	1	1	3	1	3	3	3	3
2	1	1	1	3	1	3	1	3	3
2	1	1	1	3	1	3	1	3	3
3	1	1	1	3	1	3	1	3	3
3	3	1	1	3	1	3	2	3	3
2	3	3	2	3	1	3	3	3	3
2	3	9	2	3	1	2	1	3	2
2	2	2	1	3	1	3	3	3	3
2	2	1	2	2	1	3	2	3	3
2	3	2	2	2	2	3	3	2	3
2	2	2	2	3	1	3	1	3	3
3	3	1	1	3	1	1	3	1	2
3	3	1	2	3	1	1	1	3	3
2	1	1	1	3	1	3	1	3	3
3	3	1	3	3	1	3	3	3	3
3	3	1	2	3	1	3	3	3	3
1	3	1	2	2	2	1	3	1	3
3	3	1	1	1	1	3	1	3	3
1	3	1	1	1	1	3	3	3	3
2	3	1	1	3	2	3	2	2	2

3	3	1	3	3	1	3	3	3	3
3	3	1	3	3	1	3	3	3	3
2	3	1	1	3	2	1	2	2	3
3	3	1	1	3	1	3	3	2	3
2	3	3	3	2	1	3	2	2	3
1	3	1	3	1	1	1	3	3	3
3	3	2	2	1	1	3	3	3	3
2	1	2	2	2	2	2	2	2	2
2	3	1	1	3	2	1	2	3	2
1	3	1	1	1	1	3	3	3	3
1	3	1	1	1	1	3	3	2	3
2	2	2	2	3	1	3	3	3	3
2	2	1	2	3	3	3	3	3	9
2	3	1	2	2	1	3	2	3	2
2	2	2	2	2	1	3	3	3	3
3	3	1	1	2	1	3	3	2	3
1	3	1	2	3	1	3	3	3	3
1	2	1	1	3	1	3	3	3	3
9	9	1	9	1	9	9	9	9	9
1	1	2	2	1	1	3	3	1	3
3	3	1	1	1	1	1	3	3	3
2	2	2	2	3	1	3	1	2	2
1	2	1	2	3	1	3	2	1	3
1	1	1	1	3	1	3	3	3	3
1	1	1	2	3	1	3	3	3	3
1	2	1	1	1	3	3	3	3	3
1	3	1	1	2	1	3	3	3	3
2	3	1	1	3	1	1	1	1	3
3	1	1	1	3	1	3	3	3	3
1	2	1	1	3	1	3	3	2	3
3	3	1	3	2	1	1	9	3	9
1	3	1	1	3	1	3	3	3	3
1	3	1	1	3	1	3	3	3	3
3	1	2	1	9	9	9	9	9	9
2	9	2	9	1	9	2	9	9	9
9	9	2	1	2	1	2	9	2	9
3	2	3	2	3	1	3	3	3	1
1	1	1	1	3	1	3	3	3	3
2	3	1	1	3	1	3	3	3	3
3	2	1	1	1	1	3	2	3	3
9	9	1	9	9	1	9	1	9	9
1	3	1	1	2	1	1	1	1	3
2	2	2	2	2	1	1	2	2	2
9	9	9	9	9	2	9	1	9	9
2	1	2	1	1	1	1	2	2	1
2	1	2	2	3	1	3	3	3	3
9	9	1	9	3	1	3	3	3	3

3	2	1	1	3	1	3	3	3	3
2	2	1	1	3	1	1	3	3	3
1	2	9	9	9	1	9	9	9	9
1	3	1	1	1	2	1	2	1	3
3	3	1	2	3	1	3	2	2	2
3	3	1	1	3	1	1	1	1	3
2	3	1	1	3	1	3	3	3	3
2	2	1	1	2	1	3	3	2	2
2	2	2	2	1	1	3	2	3	3
2	1	1	2	3	1	3	2	1	2
2	3	2	2	1	2	2	2	1	2
3	9	1	2	3	1	3	3	3	3
1	1	1	2	1	1	3	1	1	3
9	1	9	9	9	9	9	9	9	9
2	3	1	1	1	2	1	2	2	2
2	2	1	1	3	1	3	3	2	3
2	2	2	3	2	1	1	2	2	2
9	9	1	1	3	1	3	2	2	3
9	1	1	1	3	3	3	3	3	3
2	2	2	2	2	1	2	2	2	2
3	3	2	3	3	1	3	1	3	2
3	3	1	3	3	1	3	3	3	3
2	2	1	1	2	1	3	3	3	3
9	9	9	9	1	1	1	9	1	1
9	9	1	9	3	1	3	3	3	3
1	1	1	3	2	1	3	1	2	9
2	1	1	3	2	1	3	1	2	3
1	1	1	1	1	1	3	1	1	3
2	2	2	2	2	1	3	3	3	3
2	1	1	2	3	1	3	3	3	3
2	2	1	3	3	1	3	3	3	3
9	1	9	2	1	1	3	1	1	1
2	1	2	2	1	1	3	3	3	3
2	1	2	2	1	1	3	2	3	3
2	1	2	2	1	1	3	2	3	3
9	9	9	9	1	9	2	9	9	1
2	1	2	2	1	1	3	2	3	3
2	3	2	1	1	1	2	1	2	3
2	1	1	1	3	1	3	3	2	3
2	1	2	3	3	1	3	3	3	3
2	1	1	2	1	1	9	3	3	3
2	1	1	1	2	1	2	2	2	2
1	1	1	2	2	1	3	3	3	3
2	1	1	2	9	9	9	9	9	9
3	1	1	1	3	1	3	3	3	3
2	1	1	3	2	1	3	3	1	1
2	1	2	2	3	1	3	3	3	3

3	1	1	1	3	1	1	3	3	3
2	2	2	2	3	1	1	2	2	2
3	1	1	2	3	1	3	3	3	3
3	1	2	3	3	1	3	3	1	3
3	1	1	3	3	1	1	3	3	3
2	1	1	1	3	3	1	1	1	1
3	1	1	3	3	1	1	1	1	3
3	1	1	3	3	1	1	1	1	3
3	3	3	3	1	1	3	3	1	3
2	1	1	1	3	1	3	3	2	3
3	1	1	3	3	1	1	1	1	3
2	1	1	2	3	1	1	1	1	3
3	1	1	1	9	1	3	3	3	3
2	3	2	2	2	1	3	3	3	3
3	3	1	1	3	1	3	3	3	3
1	2	1	1	2	1	3	3	3	3
3	3	1	1	3	1	3	3	3	3
3	9	1	1	3	1	3	3	3	3
2	1	1	1	3	1	3	3	2	3
3	2	1	2	3	1	3	3	2	3
2	3	1	1	3	1	3	3	3	3
2	3	1	1	3	1	3	3	3	3
3	2	1	1	3	1	2	3	2	1
1	2	1	2	2	1	1	1	2	3
1	3	1	3	3	1	3	3	3	3
3	3	3	1	3	1	1	3	3	3
2	1	1	1	2	1	3	1	2	1
9	9	1	9	3	1	3	3	2	3
9	9	1	9	9	1	9	9	9	9
9	9	1	9	9	1	9	9	9	9
9	9	9	9	9	1	9	9	9	9
9	9	9	9	3	1	3	3	3	3
2	3	1	1	9	1	3	3	3	3
9	9	1	9	9	9	9	9	9	9
9	9	9	9	1	9	9	9	9	9
9	9	1	9	9	9	9	9	1	9
9	9	1	9	9	9	9	9	9	9
9	9	1	9	9	9	9	9	9	9
9	9	9	9	9	9	9	9	9	9
1	1	1	1	2	3	3	3	3	3
3	1	3	2	3	1	1	1	2	2
2	2	1	1	2	1	1	3	1	3
2	1	1	2	3	1	3	3	3	3
3	1	1	1	3	1	1	3	3	3
2	1	2	2	1	1	3	3	3	9
2	1	2	2	1	1	3	3	3	3
2	1	1	2	3	1	3	9	3	3

3	3	1	1	3	1	3	3	3	3
2	2	1	1	2	1	3	1	9	3
2	2	1	1	1	1	3	2	3	3
2	2	1	1	1	2	3	2	2	2
3	1	1	1	3	3	1	1	1	1
2	2	1	1	3	1	3	3	3	3
2	1	1	2	1	1	1	2	3	3
2	2	2	3	3	3	1	1	1	3
3	3	3	3	3	1	3	3	3	3
2	1	3	2	2	3	3	3	3	3
2	1	1	1	1	1	3	1	1	3
1	2	2	1	2	1	3	1	2	3
2	2	2	2	9	9	9	9	9	9
2	2	2	2	2	1	1	2	2	3
2	2	1	1	2	1	3	3	1	2
2	2	2	2	3	3	1	2	3	1
2	2	2	2	1	2	1	3	3	3
1	2	1	1	3	1	3	3	3	3
1	3	3	3	1	1	1	1	2	1
3	3	3	2	2	2	3	3	1	2
2	2	2	2	3	1	2	1	3	3
2	2	1	1	2	3	3	3	3	3
1	1	1	1	3	2	2	3	3	3
3	1	2	1	1	1	1	2	1	2
1	1	1	1	2	2	3	2	2	1
1	1	1	1	1	1	1	1	1	1
1	3	3	1	1	1	1	1	1	1
3	3	2	2	2	2	2	2	2	2
2	2	1	1	9	9	9	9	9	9
1	2	1	1	3	1	2	3	9	1
2	1	1	1	3	1	1	1	1	3
3	1	3	2	2	2	3	3	2	2
1	2	1	1	3	1	3	2	3	2
1	1	1	1	1	1	3	1	3	3
2	1	2	2	1	2	2	2	2	2
9	9	1	9	1	1	3	3	1	3
9	9	1	9	3	1	3	9	3	3
1	1	3	2	1	1	1	1	1	1
1	2	3	2	3	2	2	9	1	2
2	2	1	1	3	1	3	3	3	3
3	3	3	3	9	1	1	1	3	2
2	2	2	2	9	9	9	9	9	9
2	2	2	2	2	2	2	2	2	2
2	3	1	2	1	1	3	1	2	1
1	3	3	1	1	2	2	3	2	2
1	2	1	1	3	1	3	3	3	3
2	2	2	2	1	1	3	2	2	3

2	2	2	2	1	1	3	2	2	3

BSEdirty	BSEdisliket	BSElumpse	CaBreastCo	CaBreastfr	CaBreastTr	BSEdetects	EverDoneB	StandMirro	Liedown
1	1	1	1	1	1	1	1	1	2
1	1	3	1	1	3	1	1	3	3
3	3	1	1	1	2	1	2	3	1
1	1	3	1	1	3	1	1	3	3
9	9	9	9	9	9	1	9	1	9
1	1	3	1	1	3	1	1	1	3
3	3	1	2	1	2	1	1	9	1
3	3	1	1	2	2	1	1	1	2
3	3	2	1	2	3	1	3	1	9
9	9	9	1	1	3	9	9	2	2
9	9	9	2	2	2	2	1	1	9
1	3	3	1	1	2	2	1	2	1
3	2	1	2	2	2	2	1	3	1
3	2	2	1	2	2	1	1	2	1
3	1	1	1	2	2	1	1	1	1
3	3	1	1	1	3	1	2	1	1
2	1	1	1	1	2	2	2	1	2
3	3	1	1	1	3	1	2	1	1
3	3	1	3	1	3	1	2	1	1
1	3	2	1	2	2	2	1	1	2
3	3	1	1	2	3	1	2	1	1
3	3	1	1	2	3	1	2	1	1
3	3	9	2	2	2	1	4	1	1
3	9	1	1	3	3	1	4	9	1
3	3	1	1	1	2	2	3	1	1
2	2	1	2	2	3	1	2	2	1
3	2	1	1	3	3	1	1	3	1
3	3	1	1	1	3	1	2	1	1
3	3	1	1	1	3	1	4	3	1
2	1	1	1	1	3	1	1	3	1
2	1	1	1	1	3	1	1	3	1
3	3	1	1	1	2	1	1	1	2
3	3	1	1	1	1	1	3	2	1
3	3	1	1	1	2	1	3	1	2
3	3	1	1	1	3	1	1	1	3
3	3	1	1	1	2	1	3	1	2
3	3	1	1	1	3	1	1	1	3
3	3	1	1	1	3	1	4	1	1
9	9	9	9	9	9	9	9	3	1
3	3	1	1	1	3	1	1	3	1
3	3	1	1	1	2	1	5	3	1
2	1	1	1	1	2	1	1	1	2
3	3	9	1	1	3	2	2	2	3
3	3	1	1	1	3	1	4	1	3
3	3	1	1	1	3	1	2	3	3
3	3	1	2	1	2	2	2	1	9

3	3	1	1	1	9	1	1	1	1
3	3	1	1	1	3	1	4	3	3
3	3	1	1	1	3	1	4	9	1
3	3	1	1	3	1	1	2	2	1
3	3	1	1	1	3	1	2	1	1
3	3	1	1	1	3	1	5	1	1
3	3	1	1	3	3	1	5	3	1
3	3	1	1	1	3	1	5	1	3
9	9	1	1	1	9	1	4	1	9
2	3	1	2	3	3	1	4	1	3
9	9	9	1	9	9	9	1	9	9
3	3	1	1	1	1	1	3	1	1
3	1	1	2	1	1	1	5	1	3
3	2	1	1	2	1	1	6	1	9
3	1	1	1	1	3	1	5	1	3
3	3	1	1	1	9	1	4	1	2
3	3	1	1	1	3	1	5	1	2
3	3	1	1	1	3	1	4	1	1
3	3	1	1	1	3	2	2	2	3
2	3	3	1	1	3	1	1	1	1
1	3	1	1	1	3	1	5	1	9
1	1	1	1	1	3	1	5	3	1
1	3	1	1	1	3	1	4	9	9
3	3	1	1	1	3	1	2	1	3
9	9	1	9	1	1	1	4	9	1
9	1	1	1	1	9	1	3	1	1
3	3	1	1	1	9	1	6	1	3
3	3	1	1	1	3	1	4	3	3
3	3	1	1	2	3	1	3	1	1
3	3	1	1	1	3	1	3	1	3
9	1	1	3	3	3	3	1	1	1
3	3	1	3	3	3	3	3	1	1
3	1	1	1	2	2	2	9	1	1
3	3	1	1	3	3	1	4	2	3
3	3	1	1	1	1	1	2	1	1
3	3	1	1	1	1	1	3	1	1
1	1	1	1	1	1	1	1	1	1
3	3	1	1	1	3	1	1	2	3
3	3	1	1	1	3	1	3	9	9
3	3	1	1	1	3	1	2	1	3
3	3	1	1	1	2	1	1	1	9
3	3	1	1	1	3	1	5	1	2
3	3	1	1	1	3	1	1	3	3
3	3	1	1	2	3	1	4	1	1
3	3	1	9	9	9	1	4	1	3
3	9	1	9	9	9	9	3	1	1
1	1	1	9	1	9	1	3	1	9

3	3	1	1	2	2	1	1	2	1
3	3	1	1	2	2	1	1	2	1
1	1	1	1	1	3	1	5	1	1
3	3	1	1	2	2	1	1	2	1
3	3	1	1	3	3	1	2	9	9
9	1	1	9	9	9	9	2	1	1
3	3	1	1	1	3	1	3	1	1
3	3	1	1	3	3	1	2	1	3
9	9	1	1	1	3	1	3	1	1
3	3	1	1	1	3	1	4	1	3
3	3	1	1	1	2	1	2	1	2
3	3	1	2	2	2	1	9	2	1
9	9	1	9	9	9	1	3	1	3
9	2	1	1	1	1	1	1	1	3
1	3	1	2	1	1	1	1	1	2
3	1	1	1	2	3	1	4	2	1
3	3	1	1	1	2	2	2	9	1
2	2	1	2	2	3	2	2	1	1
3	3	1	1	1	3	1	2	1	1
1	1	1	1	1	3	1	2	9	9
9	3	1	1	1	3	1	2	3	1
3	3	1	1	1	3	1	5	1	9
2	2	2	2	2	2	2	4	2	2
1	1	1	1	1	3	1	1	3	3
2	1	1	1	2	1	1	9	1	1
2	2	1	1	2	2	1	1	3	1
3	3	1	1	1	3	1	2	1	3
9	9	1	1	9	9	9	2	1	3
3	3	1	2	2	3	2	3	3	2
2	1	1	1	1	1	1	1	2	2
2	2	1	2	2	2	1	1	1	2
1	1	2	2	2	3	2	2	3	2
2	2	1	1	2	9	1	1	2	2
3	2	1	2	3	2	2	3	2	3
2	1	1	1	1	1	1	1	2	2
3	3	1	1	3	3	1	2	3	3
2	1	1	1	1	2	1	1	1	3
1	2	1	2	2	3	1	1	3	1
9	9	1	1	1	9	9	2	9	1
3	3	1	1	1	3	1	6	1	1
3	3	1	1	1	3	1	5	1	1
3	3	1	1	1	3	1	5	1	1
1	1	2	2	2	3	2	2	3	2
1	1	1	1	3	3	1	1	1	2
2	2	2	2	2	2	1	3	2	2
3	3	1	1	1	2	1	4	3	1
1	2	1	1	2	2	1	3	2	2

9	9	1	1	9	9	9	1	2	3
3	3	1	1	1	3	1	1	1	3
3	3	1	1	1	2	1	1	1	2
3	3	9	1	1	3	1	5	1	1
3	3	3	3	2	2	2	5	1	1
3	1	1	1	1	3	1	2	9	1
1	2	3	1	3	2	1	2	3	3
3	3	1	1	2	2	1	4	2	3
2	2	2	2	1	2	2	1	2	2
2	2	1	2	2	2	2	1	2	2
3	2	1	2	1	3	1	1	3	2
3	2	1	2	1	3	1	1	3	2
9	9	9	9	1	3	2	4	1	9
3	3	1	1	1	1	1	2	1	3
3	3	1	1	1	2	1	1	1	2
1	1	1	1	1	2	2	1	1	2
2	3	1	1	1	3	1	1	9	9
3	1	1	1	1	3	1	4	1	3
9	9	9	9	9	1	9	1	9	1
3	3	1	1	1	3	1	1	1	1
3	3	1	1	1	3	1	1	1	9
3	3	1	1	1	3	1	2	1	1
3	3	1	1	1	3	1	1	1	2
3	3	1	1	1	3	1	1	1	1
3	3	1	1	1	3	1	1	1	1
3	3	1	1	1	3	1	1	1	1
3	3	1	1	1	3	1	1	1	1
3	3	1	1	1	3	1	1	1	1
3	3	1	1	1	3	1	1	1	1
3	3	1	1	1	3	1	1	1	1
3	1	1	1	1	3	1	5	1	1
3	1	1	1	1	2	1	1	1	2
3	3	1	1	1	3	1	1	9	9
9	9	1	1	1	3	1	1	1	1
9	9	1	1	1	3	1	1	1	1
3	3	1	1	1	3	1	2	1	1
3	3	3	1	1	3	1	1	1	1
3	3	1	1	1	3	1	1	1	1
3	3	1	1	1	3	1	1	1	9
3	3	1	1	1	3	1	1	1	1
3	3	1	1	1	3	1	1	1	1
3	3	1	1	1	3	1	5	1	1
3	3	1	1	1	3	1	1	9	9
3	3	1	1	1	3	1	1	1	1
3	3	1	1	1	3	1	1	1	1
3	3	1	1	1	3	1	1	1	1
3	3	1	1	1	3	1	1	1	1

3	3	1	1	1	3	1	1	1	1
3	3	1	1	1	3	1	1	1	1
3	3	1	1	1	3	1	1	1	1
3	3	1	1	1	3	1	1	1	1
3	3	1	1	1	3	1	1	1	1
3	3	1	1	1	3	1	2	1	1
3	3	1	1	1	3	1	1	1	1
3	3	1	1	1	9	1	5	1	3
3	3	1	1	1	3	1	2	1	3
3	3	9	1	1	3	1	9	3	1
3	1	1	3	3	3	1	2	1	3
3	3	1	1	1	1	1	3	1	1
3	3	1	1	1	3	1	2	1	3
3	3	1	1	1	3	1	1	9	9
3	2	1	1	1	2	1	5	3	3
3	2	1	1	2	3	1	4	1	2
1	1	1	2	2	2	1	1	1	1
1	1	1	1	1	1	1	1	1	1
3	3	1	1	1	3	1	3	2	1
3	3	1	1	2	2	1	4	2	2
1	1	1	1	1	3	2	1	1	2
3	3	3	1	1	2	1	1	2	2
1	3	1	1	1	2	1	1	1	2
2	2	1	1	1	2	2	3	1	2
3	3	2	1	1	2	1	1	2	2
2	2	2	1	1	2	1	1	2	2
3	3	1	1	2	2	1	4	2	2
2	2	2	1	1	2	1	1	2	2
2	2	2	2	2	2	2	1	2	2
3	3	1	1	1	3	1	2	1	1
2	2	1	1	1	3	2	1	2	2
2	2	1	1	1	3	2	1	2	2
3	3	1	1	1	3	1	2	1	1
1	1	1	1	1	3	1	1	1	3
2	2	1	1	2	2	1	2	1	2
1	1	1	1	1	1	1	1	2	2
1	1	1	2	2	2	1	1	1	1
3	1	1	1	1	2	1	5	1	2
3	3	1	1	1	3	1	2	3	2
2	1	1	3	1	2	1	3	2	2
1	2	3	1	2	2	2	1	1	2
3	3	1	1	2	2	1	1	2	2
9	9	9	1	9	9	9	3	9	9
9	9	9	1	9	9	9	3	9	9
1	1	1	1	1	1	1	1	1	2
1	2	1	1	1	2	1	1	3	2
1	1	1	1	1	3	1	1	2	9

2	3	1	1	1	2	1	1	1	1
2	3	1	1	1	2	1	1	1	1
3	3	1	1	1	2	1	1	2	2
1	1	1	1	3	2	1	1	2	2
3	3	1	3	1	3	1	1	2	1
3	2	1	1	2	3	1	4	1	1
2	2	1	1	1	3	3	3	1	3
2	2	1	1	3	3	2	7	2	3
3	3	1	1	1	3	1	5	1	3
3	3	1	1	3	3	3	3	3	3
2	2	1	1	1	2	2	1	1	2
3	3	1	3	3	2	1	3	3	3
3	3	1	3	3	2	1	3	3	3
2	3	2	2	2	2	2	2	2	3
3	3	1	1	2	2	1	3	1	3
3	3	1	1	1	3	1	2	2	3
2	3	1	1	2	3	2	3	3	3
2	2	2	1	2	3	2	1	2	2
3	2	1	1	1	3	3	1	3	1
1	1	1	1	1	3	2	1	1	2
1	1	1	1	2	1	1	1	1	1
2	2	1	1	1	2	1	2	1	2
2	3	1	1	2	3	1	4	2	3
3	2	1	2	1	3	1	3	1	1
9	9	1	2	1	9	9	1	1	9
2	2	2	2	2	2	2	1	2	2
2	2	2	2	2	2	2	1	2	2
3	3	3	1	9	2	1	1	1	1
2	2	2	2	2	2	2	2	9	9
2	3	1	2	2	2	2	1	9	1
1	9	9	1	9	9	9	2	1	9
3	3	1	2	2	2	1	2	1	1
1	1	1	3	1	1	1	1	2	1
3	3	1	3	2	2	1	2	1	1
1	9	9	9	9	1	9	1	1	9
1	1	2	2	2	3	2	2	3	2
3	3	1	1	1	3	1	1	2	1
2	1	2	1	1	3	1	4	1	3
3	3	1	1	2	2	1	1	2	1
3	3	1	1	3	3	1	2	2	1
3	3	3	1	3	2	1	3	3	2
2	2	1	1	1	2	1	4	1	3
3	3	1	1	2	2	1	5	1	3
2	2	1	1	1	3	1	3	2	2
3	2	1	1	1	2	1	1	1	9
3	9	1	1	1	2	1	3	2	2
2	3	1	2	1	1	2	5	3	2

9	9	9	9	9	9	9	3	9	9
3	3	1	1	1	2	1	2	1	3
3	3	1	1	1	3	1	5	2	2
3	3	1	1	1	2	1	3	1	2
3	3	1	1	3	2	1	1	2	3
3	2	1	1	1	3	1	1	1	2
3	3	1	1	1	1	1	1	1	2
3	3	1	1	1	3	3	3	1	1
3	3	1	1	3	3	1	4	1	9
2	3	1	2	2	2	2	2	2	1
2	2	2	2	2	2	2	1	2	2
3	3	1	1	2	3	1	3	1	2
1	1	1	1	1	1	1	2	9	9
3	1	3	1	1	3	1	2	3	1
1	1	1	3	1	1	1	2	1	1
3	3	1	1	1	3	1	1	1	3
3	3	1	1	1	1	1	1	2	2
1	2	1	1	1	1	2	6	1	1
2	1	2	1	2	1	1	1	1	3
9	9	9	9	9	9	9	9	9	9
3	1	1	2	2	2	3	1	2	3
2	3	1	1	2	2	1	4	1	2
3	3	1	1	1	3	1	4	1	3
3	1	1	2	2	2	2	1	2	2
2	2	1	1	1	3	1	3	2	2
1	2	1	1	3	3	1	4	3	3
2	3	1	1	1	2	1	3	3	2
3	3	1	1	1	3	1	1	2	2
2	3	1	1	1	3	1	3	3	1
2	1	2	1	1	3	1	1	3	2
3	3	1	1	1	3	1	2	1	2
1	3	1	2	3	3	1	1	1	3
3	3	1	1	1	3	1	4	9	9
3	3	1	1	1	3	1	1	3	3
3	3	1	2	1	3	1	1	1	2
1	1	1	1	3	3	1	1	3	1
3	3	1	1	1	3	1	5	3	3
3	3	1	1	1	3	1	4	3	1
3	3	1	1	3	3	1	4	3	1
9	3	1	2	2	2	1	1	2	2
3	1	1	1	3	3	1	2	2	3
1	9	1	1	1	2	1	4	3	1
9	9	9	1	2	2	2	5	9	9
3	3	1	1	2	3	1	1	2	2
3	3	1	3	3	3	1	2	1	3
3	3	1	1	1	2	1	2	2	1
3	3	1	1	2	3	1	5	1	1

1	3	1	1	1	2	1	2	3	2
1	3	1	1	1	2	1	2	3	2
9	9	1	9	9	9	1	2	9	9
3	3	1	1	3	3	1	3	1	1
9	9	1	1	9	9	9	1	9	9
3	3	1	1	2	2	1	4	2	2
3	3	1	1	1	2	1	4	1	2
3	3	9	3	9	3	9	1	9	9
3	3	1	1	1	3	1	1	1	1
3	3	1	1	2	1	1	2	1	2
2	2	1	1	1	3	1	1	1	3
3	1	1	2	2	3	2	1	3	3
3	3	1	2	1	3	1	4	1	3
2	2	1	1	1	3	1	2	1	2
2	2	1	1	1	3	1	1	1	3
1	1	1	1	3	3	1	2	3	2
2	2	1	1	1	3	1	1	1	3
1	2	1	1	2	3	1	2	3	3
1	1	1	1	1	3	1	6	3	3
3	3	1	1	2	2	1	1	2	2
3	1	1	1	3	3	1	1	3	2
3	3	1	1	1	2	1	2	3	3
3	3	1	1	1	3	1	5	3	3
3	3	1	1	2	3	1	4	1	2
2	1	1	1	1	1	2	1	2	2
1	2	1	1	2	3	2	1	2	1
3	3	1	2	1	2	1	1	1	1
2	1	2	2	2	2	2	1	1	1
1	3	1	1	2	2	1	1	2	2
2	2	1	1	3	3	1	2	2	1
2	3	1	1	1	2	1	3	2	3
1	3	1	1	1	2	1	3	2	3
3	3	2	2	3	2	3	1	3	3
1	2	1	2	2	2	2	1	2	2
1	2	1	2	2	2	2	1	2	2
3	3	1	1	1	1	1	1	1	3
3	1	1	2	1	2	1	3	1	3
2	2	1	1	1	1	1	2	9	1
2	2	1	2	2	2	1	1	2	2
3	2	1	1	3	3	3	4	2	3
3	3	1	1	3	1	2	2	2	1
3	3	1	2	2	2	1	2	1	1
3	3	1	1	1	1	1	1	1	2
2	3	1	2	2	2	1	1	1	2
3	3	1	1	1	2	1	1	1	1
3	3	1	1	1	3	1	5	2	1
3	2	1	1	1	3	1	1	2	2

3	3	1	1	1	2	1	1	3	3
3	3	1	1	1	2	1	3	1	2
2	2	1	1	2	3	2	2	1	2
1	1	2	1	1	2	1	1	1	3
3	3	1	1	1	2	1	1	3	1
1	2	1	1	2	2	1	5	3	3
2	2	1	1	2	2	2	1	1	3
3	3	1	1	2	3	1	3	1	2
3	3	1	1	2	3	1	3	3	3
1	1	1	1	1	2	2	1	2	2
1	2	1	1	2	2	1	4	1	1
3	1	1	1	2	2	1	2	1	3
3	3	1	1	1	1	1	2	3	3
2	2	2	2	1	3	2	1	2	2
2	2	2	2	1	3	2	1	2	2
3	1	1	1	1	2	1	1	2	1
1	3	1	3	1	2	1	1	1	2
3	3	1	1	1	2	1	1	1	2
3	3	1	1	1	2	1	1	1	2
2	3	1	1	1	3	1	1	9	1
3	3	1	1	1	1	1	2	1	1
3	3	1	1	1	3	1	1	2	1
3	3	1	1	1	3	1	3	9	9
1	1	1	1	2	1	1	1	2	2
3	3	1	1	1	1	1	2	1	2
2	3	1	2	1	3	1	4	3	3
3	1	2	1	2	3	1	2	3	3
3	1	9	1	3	2	3	9	9	9
1	1	1	2	2	1	1	1	3	3
9	1	1	1	1	2	2	1	2	1
2	2	1	2	2	2	2	1	1	2
3	3	1	2	2	2	1	3	1	2
2	2	1	2	2	2	2	6	2	2
3	3	1	1	1	3	3	9	1	1
3	3	1	1	1	1	1	1	1	1
1	1	1	2	2	1	2	2	1	9
2	3	2	3	3	3	3	1	2	3
3	1	1	1	1	3	1	1	3	2
3	2	2	2	2	2	2	1	3	2
1	1	1	1	1	2	1	4	1	3
2	1	1	2	3	2	3	1	1	2
1	1	3	1	1	3	1	9	1	3
1	1	1	1	1	2	1	3	1	1
1	3	1	1	1	2	3	1	3	1
9	9	9	9	9	9	9	9	9	9
2	2	1	1	1	3	1	5	1	1
3	3	3	1	1	3	1	1	9	9

2	1	9	1	1	3	1	5	1	1
3	3	1	1	1	3	1	2	1	1
3	3	1	1	1	3	1	1	1	1
3	3	1	1	1	3	1	1	1	1
3	3	1	1	1	3	1	1	1	1
3	3	1	1	1	3	1	2	1	1
3	3	1	1	1	3	1	1	1	3
3	3	1	1	1	1	1	1	1	3
3	3	1	1	1	3	1	1	1	3
3	3	1	1	1	3	1	1	1	1
3	9	3	1	3	2	2	1	1	3
3	3	1	9	1	3	1	1	1	1
3	3	1	1	1	3	1	1	1	3
3	3	1	1	1	3	1	1	1	3
3	3	1	1	1	3	1	1	1	1
3	3	1	1	1	3	1	1	1	3
3	3	1	1	1	3	1	1	1	3
3	3	1	1	1	3	1	1	1	3
3	3	1	1	1	3	1	1	1	3
3	3	1	1	1	3	1	1	1	9
1	3	1	1	1	3	1	1	1	3
3	3	1	1	1	3	1	1	1	1
3	3	1	1	1	3	1	1	1	1
3	3	1	1	1	3	1	1	1	1
3	3	1	1	1	3	1	1	1	1
3	3	1	1	1	3	1	1	1	1
3	3	1	1	1	3	1	1	1	1
1	3	1	1	1	3	1	1	1	1
3	3	1	1	1	3	1	1	1	1
3	3	1	1	1	3	1	1	1	1
3	3	1	1	1	3	1	2	1	3
3	3	1	1	1	3	1	1	1	3
3	3	1	1	1	3	1	3	1	1
3	3	1	1	1	3	1	3	1	1
3	3	1	1	1	3	1	1	1	1
3	3	1	1	1	3	1	2	1	1
2	2	1	1	1	3	1	5	1	2
3	3	1	1	1	3	1	1	1	1
3	3	1	1	1	3	1	1	1	1
3	3	1	1	1	3	1	1	1	1
3	3	1	1	1	3	1	2	1	1
3	3	1	2	1	2	1	5	2	2
9	1	1	1	1	2	1	3	1	1
3	3	1	1	1	2	1	1	2	2
3	2	1	1	2	2	2	1	2	9
2	2	1	2	2	2	2	2	1	3
3	3	1	1	1	3	1	4	3	3

2	2	2	1	2	2	2	1	2	2
1	1	1	1	1	2	1	4	1	3
1	1	1	1	1	2	1	4	1	3
3	3	1	1	1	2	1	2	2	1
3	2	1	1	1	2	1	3	1	2
3	1	1	1	2	2	3	1	2	1
3	2	1	1	1	2	3	3	1	3
2	2	2	2	2	1	2	1	2	2
3	3	1	1	3	2	1	3	2	2
1	1	1	1	2	2	2	1	2	2
1	1	1	1	1	2	1	9	1	3
1	9	9	9	9	9	1	1	1	9
9	9	1	1	1	9	9	4	9	9
3	2	1	2	2	2	2	1	2	2
9	1	9	9	9	9	1	1	9	9
9	1	9	9	9	9	1	3	1	9
9	9	9	9	9	9	1	1	1	9
9	9	1	1	9	9	9	1	1	9
9	9	1	1	9	9	9	1	9	9
3	3	1	9	1	3	1	2	1	3
3	3	3	3	3	3	1	3	1	3
3	9	3	9	3	3	1	3	1	3
2	2	1	1	1	3	1	5	1	3
9	9	9	1	9	9	9	3	1	9
9	9	1	9	9	9	1	3	9	9
1	2	1	1	1	3	1	2	1	1
9	9	1	9	9	9	1	3	9	9
3	2	1	1	2	3	1	2	1	3
1	2	1	1	1	3	1	4	1	3
1	9	9	9	9	9	1	1	9	9
9	9	1	1	9	9	1	3	1	9
3	3	2	1	1	3	1	5	1	2
2	2	1	2	3	2	2	1	2	2
3	3	1	1	1	2	1	3	1	3
1	2	1	1	1	1	2	3	1	2
3	2	1	2	1	3	1	2	1	3
2	3	1	1	1	3	1	1	1	2
3	3	1	3	2	3	1	1	3	3
2	2	2	2	2	2	2	1	2	2
3	3	1	1	1	2	1	4	2	3
3	2	3	1	2	3	1	3	9	9
3	3	1	1	1	1	1	3	3	2
2	1	1	1	1	1	1	1	2	1
3	1	1	1	1	3	1	4	2	2
3	2	1	1	1	2	1	3	1	3
3	9	1	1	2	2	2	3	1	3
3	3	1	1	1	3	1	3	1	3

3	1	1	1	2	2	1	2	9	9
3	3	1	1	1	2	1	1	3	3
3	2	1	1	1	1	1	1	3	3
2	3	1	1	1	2	2	1	1	1
2	3	1	1	2	3	2	1	1	3
3	2	1	1	2	2	2	1	2	2
3	3	1	1	3	3	1	1	1	3
1	2	1	1	1	3	1	1	1	1
1	2	1	1	1	1	1	4	1	2
2	2	1	1	2	3	2	1	2	2
2	2	1	1	2	3	2	2	2	3
3	3	1	2	1	2	1	4	1	3
2	2	2	2	3	2	2	1	2	2
2	2	2	2	3	2	2	1	2	2
1	1	2	1	2	3	1	1	1	1
2	2	2	1	2	3	1	1	1	2
3	3	1	2	1	3	1	5	1	2
1	1	1	1	1	1	1	1	2	2
3	3	1	2	1	2	2	3	2	3
2	1	1	1	1	3	1	1	1	3
1	3	1	1	1	3	1	4	3	3
3	3	1	1	1	3	1	2	3	3
3	3	1	1	1	3	1	2	1	3
3	3	1	1	1	3	1	2	1	3
2	2	1	1	1	3	1	2	1	2
3	3	1	1	1	2	1	5	2	2
3	1	1	1	3	3	1	2	1	1
1	2	1	1	3	3	1	2	1	1
1	3	1	1	1	2	1	1	1	2
2	3	1	1	3	3	2	1	2	2
1	1	1	1	1	2	1	9	1	3
9	9	1	1	1	2	1	9	2	9
2	1	2	1	2	3	2	1	3	3
3	1	1	2	3	3	1	4	3	9
2	2	1	1	1	3	3	2	1	2
9	9	9	2	9	9	9	1	2	9
3	2	1	1	3	3	1	1	2	1
3	3	1	1	1	3	1	3	1	1
3	2	2	3	2	2	2	1	2	2
3	3	1	1	1	3	1	3	1	2
2	2	1	1	2	3	1	3	1	2
1	2	1	1	1	2	1	1	1	2
3	3	1	3	1	3	1	4	3	3
3	3	1	2	2	2	1	9	2	1
3	3	1	1	1	1	1	3	1	3
9	9	9	1	2	3	1	3	1	3
9	9	1	1	9	9	9	3	9	9

9	9	1	9	9	9	1	1	1	9
9	9	1	9	9	9	1	1	1	9
9	9	9	1	9	9	9	1	9	9
3	3	1	1	1	3	1	7	1	9
1	3	1	1	1	3	1	3	1	9
9	9	9	9	9	1	9	2	1	9
9	9	9	2	2	2	2	1	1	9
9	9	1	9	9	9	1	1	1	9
9	9	9	9	9	9	1	2	1	9
9	9	9	9	9	9	1	1	1	9
3	9	1	1	3	3	1	1	1	3
9	9	9	9	9	9	1	1	1	9
9	9	9	9	9	9	1	1	1	9
9	9	9	1	9	9	9	3	1	9
3	2	2	1	9	1	1	1	1	9
9	9	9	1	9	9	9	1	1	9
9	9	9	9	9	9	9	1	1	9
3	3	1	1	1	3	1	1	1	9
2	2	2	2	2	2	2	1	2	2
1	2	1	1	1	1	1	1	1	1
1	1	1	1	1	1	1	1	1	1
1	1	1	1	2	2	1	1	3	3
1	1	1	2	1	1	1	1	1	2
3	1	1	1	1	1	1	2	1	3
3	3	1	2	2	3	1	1	2	2
3	2	2	2	1	2	3	5	1	3
2	2	1	1	1	2	1	1	2	2
3	2	2	1	2	2	1	1	1	3
1	9	1	2	2	2	2	1	2	2
3	2	2	3	9	2	2	1	2	2
3	3	3	1	1	1	1	1	3	3
3	3	1	2	2	3	1	4	1	3
3	3	1	1	2	3	1	1	2	2
9	1	9	1	3	3	1	1	1	3
3	3	1	1	1	2	1	1	1	2
2	2	2	1	2	2	2	1	1	2
2	2	2	1	2	3	2	1	2	3
1	1	1	2	3	1	3	2	2	3
1	1	1	1	1	2	1	1	2	1
3	3	1	1	1	2	1	1	1	3
2	1	2	1	2	3	2	9	2	2
3	3	1	1	1	1	1	3	1	3
1	2	2	1	1	3	1	3	3	1
1	3	1	1	1	3	1	2	1	2
3	3	1	1	1	1	1	1	1	3
1	2	2	1	1	3	3	1	9	9
1	2	3	1	2	2	2	1	1	2

1	1	2	1	1	3	2	1	1	3
1	1	1	1	1	2	2	2	1	3
3	1	1	3	1	3	1	1	1	3
1	1	1	1	1	1	1	1	1	2
3	3	1	2	1	3	1	1	3	1
3	3	1	2	1	3	1	1	3	1
2	2	1	1	1	1	2	4	2	1
1	1	2	1	1	3	2	1	1	3
1	1	1	1	2	1	3	1	1	9
3	3	1	1	1	1	1	1	1	3
3	2	1	1	2	1	3	1	2	3
2	2	1	1	1	1	1	3	1	3
2	2	2	2	2	2	2	9	2	2
1	1	1	2	3	1	3	2	1	2
2	2	1	1	3	1	3	1	2	2
2	1	1	1	2	3	1	2	1	2
1	1	1	1	1	1	1	4	1	1
9	9	9	1	9	9	9	1	9	9
3	1	1	1	2	1	1	3	1	3
3	3	1	1	1	3	1	1	3	3
3	3	3	1	2	9	1	1	1	1
3	2	2	2	3	3	3	9	2	2
2	2	3	1	2	3	3	2	1	1
3	3	2	1	3	2	1	2	1	3
2	2	1	1	1	3	1	1	2	2
3	3	1	1	1	3	3	1	1	3
3	3	1	1	1	3	1	1	1	3
3	3	3	1	1	3	1	3	3	1
1	2	3	1	2	2	2	1	1	1
3	1	1	1	1	3	3	3	1	3
3	3	1	1	1	3	1	4	3	1
1	3	1	1	1	3	3	1	3	3
3	2	1	1	1	2	1	1	1	1
3	1	1	1	1	3	3	1	1	3
9	1	1	1	1	3	3	1	1	3
3	1	1	1	1	2	1	1	1	3
3	1	1	1	1	3	2	4	1	3
1	9	9	9	9	9	9	9	9	9
2	1	1	1	1	1	1	1	2	2
2	1	1	1	1	1	1	1	9	9
1	1	1	9	9	9	9	9	9	9
1	2	2	2	9	2	3	9	3	9
2	3	1	1	1	2	3	1	1	3
1	1	1	9	9	9	9	9	9	9
2	3	1	1	1	2	3	1	1	3
2	2	2	1	2	2	2	9	2	3
1	1	1	1	1	1	1	1	1	3

3	3	1	1	1	3	1	1	1	3
2	2	1	1	1	2	1	3	1	1
3	1	1	1	1	1	1	1	1	3
1	1	1	1	1	2	2	1	1	2
3	3	1	3	2	2	1	4	2	2
1	3	2	1	3	2	3	2	3	9
1	2	2	1	2	3	2	1	9	9
3	3	1	1	3	3	1	2	1	3
3	3	3	3	3	2	1	1	3	3
3	3	1	1	1	1	1	5	3	3
3	3	3	3	3	3	3	1	3	3
3	3	1	1	1	3	1	1	1	3
2	2	2	1	2	2	1	1	2	3
2	2	2	1	2	2	2	1	2	3
2	2	2	1	2	2	2	1	2	3
2	3	1	1	2	2	2	1	2	3
2	2	2	1	2	2	2	1	2	3
2	2	2	1	2	2	2	1	2	3
2	2	2	1	2	2	2	1	2	3
1	3	1	1	1	1	1	1	1	3
1	2	3	2	1	3	2	4	1	3
3	3	3	1	2	3	3	9	1	3
1	1	1	1	2	2	2	3	2	2
2	1	1	1	1	2	1	1	1	2
3	2	3	3	3	3	3	1	1	3
3	3	2	1	2	2	2	1	2	1
1	2	1	1	2	2	2	1	1	9
3	3	1	1	1	1	1	1	1	1
9	9	9	9	9	9	1	3	9	9
3	3	3	3	3	3	3	7	1	3
1	1	1	1	2	1	3	1	3	3
3	1	2	2	3	2	3	7	2	9
2	1	3	2	1	3	2	3	2	1
2	3	3	1	2	1	2	1	2	3
3	3	3	3	3	3	3	1	3	3
3	3	1	2	1	1	1	1	3	3
3	1	1	2	1	1	1	1	1	9
2	2	1	1	1	2	1	1	1	3
2	2	1	1	1	2	1	1	2	1
1	1	1	1	1	1	1	1	1	1
1	1	2	1	1	1	2	1	1	2
3	1	1	1	1	2	1	1	1	2
2	1	3	3	1	2	1	1	1	2
3	3	1	1	1	1	1	3	3	1
3	2	1	2	3	3	1	2	1	1
1	2	1	1	2	1	2	1	1	1
2	9	3	1	1	1	1	1	1	1

3	3	1	1	2	3	1	3	1	3
9	9	9	9	9	1	9	1	1	9
9	9	9	1	9	9	9	1	1	9
3	3	1	1	1	3	3	1	3	1
3	3	1	2	1	3	1	1	1	3
3	3	1	2	1	3	1	1	3	1
1	3	1	1	3	1	1	2	2	3
3	3	1	2	1	3	1	1	3	1
3	3	1	2	1	3	1	1	9	1
3	3	1	2	1	3	2	1	1	3
2	2	1	1	1	1	2	1	1	2
3	3	1	2	3	3	1	3	1	1
2	1	1	1	1	2	1	3	1	2
3	3	2	1	1	3	1	2	1	3
1	3	1	1	1	3	1	1	3	3
3	1	1	1	1	3	1	1	2	1
3	3	3	1	2	3	1	1	1	2
9	9	9	9	1	9	9	9	1	9
9	9	9	9	1	9	9	1	1	9
3	3	1	1	1	1	9	1	1	1
1	2	2	3	1	1	1	1	1	1
2	2	2	1	1	1	2	1	2	1
1	1	1	1	1	1	1	9	1	1
2	2	2	2	2	2	2	9	2	2
3	3	3	3	3	3	3	9	3	3
2	2	1	2	1	3	1	1	2	2
2	2	1	2	1	3	1	1	2	2
3	2	1	1	1	3	1	1	3	1
1	1	1	1	1	1	1	1	1	1
2	2	2	2	2	2	2	1	2	2
9	9	9	2	1	2	2	1	2	3
1	1	1	1	1	2	1	1	1	2
2	1	3	1	1	1	1	1	9	1
2	2	1	2	1	3	1	1	2	2
1	1	1	1	1	3	1	4	1	3
2	2	2	2	2	2	2	9	2	2
1	1	2	1	1	2	1	1	1	2
1	3	1	1	1	1	1	1	9	9
9	3	9	9	2	9	1	1	1	9
1	1	1	1	1	3	1	1	1	3
1	1	1	1	1	1	3	1	3	3
2	2	2	2	2	2	2	9	2	2
2	2	2	2	2	2	2	1	1	2
2	2	3	2	3	2	2	1	2	2
1	1	2	1	2	2	2	1	2	2
2	2	2	2	2	2	2	9	2	2
1	2	1	3	1	2	3	9	9	9

2	2	2	2	2	2	2	1	9	9
2	2	2	2	2	3	2	1	2	2
9	9	9	1	1	1	1	9	9	9
9	9	9	1	1	3	1	9	9	9
9	9	9	9	9	9	9	1	1	9
1	2	3	1	2	1	3	1	3	2
2	2	1	1	2	2	2	1	2	2
9	3	1	1	1	3	1	1	3	3
3	1	2	1	2	1	2	1	1	1
9	9	9	9	2	9	9	1	3	9
2	2	2	2	2	2	2	9	2	2
1	2	1	1	1	2	1	4	1	2
1	1	1	1	1	2	1	9	1	2
2	2	2	1	1	2	1	1	2	2
1	1	1	1	1	3	1	1	1	3
2	2	2	2	2	2	2	1	2	2
2	2	2	1	1	2	1	1	2	2
3	2	3	3	3	1	1	1	2	2
9	9	9	1	1	3	1	1	2	2
3	3	2	1	1	1	1	1	3	3
9	9	9	1	9	9	9	1	9	1
1	1	1	1	1	2	3	1	1	3
2	2	2	2	2	2	2	1	2	2
1	1	3	1	2	2	1	1	3	3
2	2	2	1	1	3	1	1	2	2
1	2	2	1	2	3	2	2	2	3
9	9	9	9	9	9	1	1	9	9
9	9	9	9	9	9	9	1	9	1
2	2	2	1	2	2	2	1	3	3
3	3	1	1	2	1	1	1	2	2
9	9	9	1	9	9	1	1	9	1
3	1	1	1	1	2	1	1	1	3
3	3	1	3	3	3	1	2	1	3
3	3	1	2	2	2	2	1	1	2
2	3	1	2	2	2	2	1	1	2
3	1	3	1	1	1	1	2	1	3
2	2	2	2	2	2	2	1	2	2
2	1	1	1	2	2	1	4	1	1
2	2	2	1	1	2	1	1	2	2
3	2	1	1	1	2	1	4	1	2
3	2	1	1	2	2	2	1	2	2
2	2	2	1	2	2	2	1	2	2
3	3	1	1	1	3	2	1	2	3
3	1	1	1	1	3	1	1	1	2
2	2	1	2	2	2	1	1	2	2
3	3	3	3	3	3	3	9	3	3
2	1	2	2	1	2	2	1	2	2

2	2	2	1	2	2	2	1	2	2
2	2	2	2	2	2	2	5	2	2
2	2	2	2	2	1	2	1	2	2
2	2	2	2	2	2	2	1	2	2
3	3	1	1	2	2	2	2	2	2
3	3	1	1	1	1	2	4	3	3
2	2	2	2	2	1	2	1	2	2
3	1	1	1	1	3	1	1	1	2
2	3	1	1	1	1	1	1	2	2
3	3	1	2	2	2	2	1	3	3
2	1	1	1	1	3	1	6	1	3
9	9	9	2	9	9	9	9	9	2
9	9	9	9	1	9	9	1	9	9
2	2	2	2	1	2	1	1	2	2
3	3	3	1	2	2	2	1	1	2
1	1	1	1	3	1	1	1	2	1
2	2	1	2	2	2	1	1	2	1
3	3	1	1	1	1	1	1	1	3
1	2	1	1	2	2	3	1	1	2
2	2	1	2	2	2	3	3	2	3
3	3	2	3	3	1	1	1	3	3
3	3	3	1	1	1	1	1	1	2
2	2	1	1	2	2	1	1	2	2
9	3	1	1	2	2	1	1	1	1
3	3	1	3	3	3	1	1	3	3
9	3	1	1	2	2	1	1	1	9
2	2	2	1	1	2	1	1	2	2
9	9	1	2	1	2	1	1	2	1
2	2	2	1	3	2	1	1	2	2
1	2	1	1	2	3	1	1	2	3
1	1	1	1	1	1	1	2	1	2
1	1	1	1	3	1	1	1	1	1
1	1	1	1	1	1	1	2	3	3
1	1	2	1	1	1	1	1	2	2
2	1	2	1	1	3	2	1	3	3
1	1	1	1	1	3	1	1	3	3
3	3	1	1	1	3	1	2	1	9
1	1	1	1	1	1	1	1	1	1
2	2	2	2	2	2	2	1	2	2
2	2	2	2	2	2	2	1	2	2
2	1	1	2	1	3	1	1	1	3
1	1	1	1	1	1	1	1	1	1
9	9	2	1	1	1	1	1	1	2
1	1	1	1	2	2	1	1	1	2
2	2	2	2	2	2	2	1	2	2
2	3	1	1	2	1	3	1	1	2
2	2	2	2	1	3	2	1	3	3

9	1	9	1	3	1	1	1	3	3
3	3	1	3	3	2	2	1	3	3
3	3	1	1	1	2	1	1	1	3
9	9	9	9	1	9	9	5	9	9
9	9	9	2	9	9	1	2	1	2
3	2	1	3	1	2	3	1	2	2
2	2	1	2	1	2	1	1	2	2
3	3	1	1	1	3	1	1	3	3
2	9	9	2	1	1	1	9	2	3
2	2	2	2	2	2	2	1	2	2
9	9	9	9	9	1	9	1	9	9
3	3	2	1	1	2	3	1	1	2
2	2	2	2	2	2	2	9	2	2
2	2	2	2	2	2	1	1	2	2
2	2	2	2	2	2	2	1	2	2
2	2	3	2	3	1	3	1	2	3
1	2	3	1	2	2	3	4	2	1
9	9	9	9	9	9	1	1	1	9
9	9	9	9	2	9	9	4	9	9
2	2	2	2	2	2	2	9	2	2
2	2	2	2	2	2	2	1	3	2
1	1	2	2	2	2	2	9	2	2
9	9	9	9	9	9	1	1	9	9
2	2	1	1	2	1	1	1	2	9
2	2	3	1	2	3	1	2	1	2
3	3	1	3	1	3	1	1	1	3
3	3	1	3	2	3	1	3	1	3
1	3	1	1	1	3	3	1	1	1
2	2	2	1	3	3	3	1	1	3
3	9	2	1	1	3	2	1	1	2
2	3	1	1	1	3	1	4	1	2
3	3	1	1	1	3	1	3	1	3
3	3	1	1	1	3	1	3	1	3
3	3	3	3	3	9	3	1	3	3
2	3	1	1	1	3	1	1	1	9
2	1	1	1	1	2	1	1	3	2
2	1	1	1	1	1	1	2	1	1
3	3	3	3	3	3	3	1	3	3
9	1	9	2	9	1	9	1	9	1
3	3	3	3	1	3	3	1	3	3
1	3	1	1	3	3	3	6	1	3
1	3	1	1	1	3	1	2	1	9
3	1	1	1	1	1	1	1	1	3
3	1	1	3	1	3	1	1	2	2
1	1	1	1	1	2	1	1	1	2
1	1	1	2	3	2	1	1	3	3
2	2	1	2	1	1	1	1	1	2

1	1	3	2	2	2	2	1	2	2
3	2	2	9	1	3	2	1	1	2
1	3	1	1	1	9	1	2	1	9
1	3	1	1	3	3	1	7	1	1
1	2	1	1	1	2	1	1	3	2
2	2	2	1	3	3	1	1	2	2
1	1	1	2	2	2	2	1	2	2
1	1	1	2	2	2	2	1	9	9
1	1	1	2	2	2	2	1	9	9
1	2	1	1	2	3	2	1	2	2
1	1	1	1	1	2	2	1	1	9
3	1	2	1	1	3	1	1	1	2
1	1	1	1	1	3	3	1	1	3
3	2	3	1	2	3	3	9	2	1
2	2	1	1	2	3	2	2	1	3
1	1	1	2	2	2	2	1	2	2
3	1	1	1	1	3	1	1	1	1
2	2	1	1	2	1	1	1	2	2
2	3	1	1	3	2	1	1	1	2
9	9	1	9	1	9	9	1	9	9
9	9	9	1	9	9	9	1	1	9
1	1	1	1	1	1	1	2	1	3
1	3	1	1	3	3	1	1	3	3
3	3	3	3	3	3	3	1	1	1
3	3	3	3	3	3	3	1	3	3
1	1	3	1	3	3	1	1	2	2
3	1	1	1	1	3	1	1	1	3
2	3	1	1	1	2	1	1	1	2
1	3	1	1	1	1	1	1	1	1
3	2	3	1	1	1	1	1	1	2
2	3	1	1	2	2	2	4	1	2
1	1	1	1	1	3	1	3	3	1
2	1	1	1	1	2	1	3	1	2
3	9	9	1	1	2	2	3	2	1
1	2	1	1	1	2	2	2	1	3
2	1	1	1	1	2	9	2	2	2
2	1	1	1	1	2	1	1	2	2
2	2	2	1	2	3	2	1	2	2
2	2	3	1	3	3	1	1	2	2
3	3	1	1	3	2	1	1	3	3
1	2	1	1	2	1	2	1	1	2
2	2	2	2	2	2	2	1	2	2
1	1	1	1	1	1	1	1	1	1
9	9	9	2	9	9	9	1	2	9
9	9	9	3	9	9	9	1	3	2
3	3	1	3	1	3	1	3	1	3
3	1	1	1	1	2	1	1	1	2

3	3	1	1	1	2	1	4	1	3
3	3	1	2	1	2	2	2	1	3
3	1	1	2	1	3	1	7	1	3
3	3	1	1	1	3	1	5	2	1
3	3	3	1	3	2	2	1	2	2
3	3	2	2	3	2	3	1	3	2
3	2	2	2	9	9	9	1	2	2
3	3	1	1	2	2	1	1	3	3
3	3	3	1	3	3	3	3	1	3
3	3	1	1	1	1	1	1	1	3
3	3	1	1	1	1	1	1	1	3
3	3	1	1	1	1	1	1	1	3
9	9	9	2	9	9	9	1	9	9
9	9	9	1	9	9	9	2	9	9
3	3	1	1	1	1	1	1	1	3
3	1	1	2	3	2	1	1	2	2
2	1	1	1	1	1	1	2	1	1
2	2	2	2	2	2	2	1	2	2
3	3	1	1	1	1	1	5	2	3
3	1	1	1	3	3	1	1	2	2
3	3	9	1	1	2	2	1	2	2
1	2	3	2	2	2	3	2	1	3
2	2	1	9	9	9	9	1	2	2
3	3	1	1	2	3	2	2	2	3
2	2	1	2	1	2	2	1	2	2
3	1	1	2	2	2	1	2	3	3
3	3	1	1	2	3	1	2	1	2
1	1	2	3	3	1	1	4	1	1
3	1	1	2	2	2	1	2	3	3
3	1	1	2	3	2	1	1	2	2
3	1	1	1	3	3	1	1	2	2
9	9	9	2	9	9	9	1	9	9
2	1	1	1	2	3	2	3	1	3
3	3	1	1	1	2	2	1	2	2
1	2	2	1	1	2	1	1	1	3
3	3	1	1	1	2	1	1	1	1
2	2	1	3	2	2	2	2	1	1
3	2	2	1	9	9	9	3	1	9
1	1	1	1	1	3	2	1	1	2
2	2	2	1	2	2	2	2	2	2
1	1	1	1	1	3	1	2	1	3
3	1	1	2	3	1	1	1	1	3
3	3	1	1	1	3	1	3	1	3
2	2	2	1	1	2	2	1	2	2
1	1	1	1	1	1	1	3	1	1
2	2	2	2	2	2	2	1	2	2
9	9	9	9	1	9	9	1	9	9

2	2	2	2	2	2	2	2	3	1
3	1	1	1	1	2	1	1	2	2
2	1	1	1	1	1	1	1	9	9
3	3	1	1	1	2	1	2	1	9
3	3	1	1	1	2	1	2	1	2
9	9	9	1	9	9	9	1	9	9
3	1	2	1	2	1	1	1	1	1
3	3	1	2	1	3	1	3	1	3
2	3	2	1	1	2	3	9	1	2
3	1	2	1	2	3	2	1	1	1
3	2	1	1	1	3	1	4	3	3
2	2	1	1	2	1	2	1	1	3
2	2	1	1	1	3	1	2	2	2
3	3	1	1	1	2	2	1	1	3
3	2	1	1	1	2	1	1	2	2
3	1	9	3	3	3	2	3	2	2
2	3	1	1	1	3	1	1	1	3
3	3	1	1	1	3	1	5	2	2
3	1	1	1	1	2	3	4	1	2
3	3	1	1	1	3	1	2	1	1
3	3	1	1	1	2	1	3	1	3
1	2	1	2	2	2	2	1	2	2
1	1	1	1	1	3	2	3	1	2
2	2	2	3	3	3	3	1	2	2
2	1	1	1	1	1	1	2	2	2
9	9	9	1	1	2	1	1	1	2
2	3	1	1	2	2	1	1	2	2
3	3	1	1	3	1	2	1	2	2
3	3	1	1	2	2	1	3	1	3
1	3	3	1	3	3	3	1	9	9
9	9	9	1	1	1	3	3	9	9
1	3	2	1	1	2	2	1	2	2
9	9	9	1	1	2	1	1	9	1
1	1	1	2	1	2	1	1	1	2
2	3	2	1	2	3	2	1	1	2
9	9	9	9	9	9	9	2	9	9
9	9	9	9	9	9	9	9	9	9
2	2	2	2	2	2	2	1	2	2
1	1	1	1	1	3	1	1	2	2
2	3	1	1	2	1	1	1	1	2
3	3	3	1	1	1	1	1	1	1
1	1	1	2	2	2	2	9	1	1
2	1	1	1	1	1	2	1	3	1
2	1	2	1	2	1	3	2	1	2
2	1	2	1	2	3	1	1	1	3
9	9	9	9	9	9	9	9	9	9
1	1	3	3	3	2	1	1	3	2

2	1	2	1	2	1	2	9	1	2
9	9	9	9	9	9	9	9	9	9
3	3	9	1	1	1	1	2	3	3
1	9	1	2	2	2	2	1	2	2
1	1	1	1	1	3	1	2	2	2
2	1	2	1	2	1	2	9	1	2
9	1	1	1	1	2	2	1	3	1
3	3	1	1	1	2	1	1	1	3
1	1	1	1	2	2	2	2	2	2
2	3	1	1	1	3	1	4	1	3
9	9	9	9	9	9	9	9	9	9
9	9	9	9	9	9	9	9	9	9
9	9	1	9	9	9	1	1	9	9
1	1	1	1	1	2	3	1	1	2
3	1	1	1	1	3	1	2	1	2
3	3	1	1	1	3	2	5	2	3
3	3	3	9	9	9	9	9	9	9
9	9	9	1	1	3	1	2	1	9
3	2	1	1	1	2	1	5	1	3
2	2	1	1	1	3	3	1	1	3
3	1	1	2	2	1	1	4	2	2
1	2	3	1	3	1	1	1	1	1
2	2	2	2	2	2	3	4	3	3
3	1	2	2	2	2	2	1	2	2
2	2	2	1	1	2	1	1	2	2
3	1	1	1	1	3	1	1	3	2
9	9	9	1	2	2	2	1	9	9
3	3	1	2	2	2	1	3	1	3
3	3	1	1	1	2	2	1	1	2
2	2	2	3	3	2	2	2	2	2
3	3	3	3	1	1	1	1	1	3
3	3	1	1	2	3	2	5	1	3
3	9	9	9	9	9	9	9	9	9
2	2	1	3	2	1	3	1	2	2
1	1	1	1	1	2	2	1	1	1
2	3	2	1	1	1	1	1	2	1
1	1	1	1	1	1	1	1	1	1
1	1	1	1	1	3	2	1	1	2
3	3	1	3	1	3	1	1	3	3
1	2	3	1	2	1	3	1	1	2
1	1	3	3	1	2	2	1	1	3
3	3	3	3	3	3	3	9	3	3
3	3	1	1	1	3	1	1	9	9
3	3	1	1	1	3	1	1	9	9
3	3	1	1	1	3	1	2	1	3
3	3	1	1	1	3	1	1	3	3
3	3	1	1	1	3	1	1	9	9

2	2	2	2	2	2	2	1	2	2
3	3	2	2	2	2	2	1	2	2
3	9	1	1	1	1	3	5	1	1
3	3	1	1	1	1	1	1	1	3
1	1	1	1	1	3	1	3	3	1
3	3	1	1	1	3	1	2	1	3
1	3	1	1	1	3	1	2	9	9
3	3	1	1	1	3	1	1	1	9
3	3	1	1	1	3	1	2	9	9
3	3	1	1	1	3	1	3	9	9
9	9	1	1	1	3	1	3	9	9
3	3	1	1	1	3	1	2	9	9
3	3	1	1	1	3	1	1	1	9
1	1	1	1	3	3	1	1	3	3
3	3	1	1	1	3	2	1	1	2
3	3	1	1	1	3	1	7	1	3
3	3	1	1	1	2	1	1	1	1
1	1	1	1	1	1	2	5	9	1
3	3	1	1	1	3	1	5	9	9
3	1	1	1	1	3	1	1	9	9
2	1	2	1	2	1	3	9	1	2
3	3	1	1	1	3	1	3	9	9
3	3	1	1	1	3	1	4	1	1
3	3	1	1	1	3	1	2	9	9
3	3	1	1	1	3	1	2	1	1
3	3	1	1	1	3	1	1	1	1
3	1	1	2	3	2	1	1	3	2
3	3	1	1	1	3	1	4	1	1
3	3	1	1	1	3	1	2	3	2
3	3	1	1	1	3	1	2	1	1
3	3	1	1	1	3	1	1	1	1
3	3	1	1	1	3	1	1	2	1
2	2	1	1	1	3	1	1	3	1
3	3	1	1	1	3	1	1	1	9
3	3	1	1	1	3	1	1	1	1
3	3	1	1	1	3	3	4	1	1
3	3	1	1	1	3	1	4	1	3
3	3	1	1	1	3	1	1	1	9
3	3	1	2	3	3	3	1	1	3
3	1	3	1	3	3	1	2	3	3
2	2	2	2	2	2	2	1	2	3
1	1	1	3	3	2	1	4	1	2
3	2	3	2	1	1	1	1	1	2
2	1	2	2	2	2	2	1	9	9
1	1	1	1	1	3	2	3	1	1
3	1	3	1	3	3	1	1	1	1
3	3	1	1	1	2	1	1	1	3

3	2	1	1	1	3	1	9	9	9
2	3	3	3	1	2	3	2	9	9
2	2	1	1	9	9	9	9	9	9
3	3	1	1	1	2	1	1	9	9
3	3	1	3	1	2	1	1	2	2
9	9	1	1	9	9	9	9	9	9
3	3	1	1	1	2	3	1	2	2
3	1	1	2	2	2	2	2	1	2
9	9	1	1	9	9	9	1	2	9
9	9	9	1	2	9	9	9	9	9
9	9	1	1	9	9	9	1	2	9
9	9	9	9	9	9	9	9	9	9
2	2	1	1	1	1	1	1	2	2
2	9	2	1	2	1	3	4	1	3
3	2	2	1	2	3	1	3	1	3
1	1	2	1	3	3	1	3	1	1
1	1	1	1	1	2	1	3	1	2
3	2	1	1	1	3	1	1	1	2
1	1	1	1	1	1	1	1	1	1
9	9	9	1	9	9	9	1	1	9
1	1	1	1	1	1	1	1	1	1
3	3	1	1	1	2	1	1	2	2
3	3	1	1	1	2	2	1	2	2
1	1	1	1	2	2	1	1	1	1
3	3	1	1	1	3	1	1	1	1
3	3	1	1	1	3	1	3	1	3
3	1	1	1	3	2	2	4	1	9
3	3	3	1	2	1	2	1	2	2
2	2	2	2	2	2	1	1	1	9
9	9	9	2	3	1	9	1	3	9
3	1	1	1	1	1	1	1	1	3
1	1	1	1	1	1	1	9	3	3
1	1	1	1	2	2	2	1	2	3
1	1	1	1	2	2	2	1	2	2
3	3	1	1	1	3	1	1	1	2
3	3	1	1	2	2	1	1	1	3
2	2	1	1	1	2	2	1	1	2
1	1	1	2	2	2	1	1	1	1
1	2	2	2	2	2	1	1	1	1
2	2	2	2	1	1	1	1	1	2
1	3	1	1	2	2	2	1	1	3
2	2	2	1	2	1	2	1	2	2
3	3	1	1	3	3	1	4	9	3
1	2	1	1	1	2	1	1	1	2
2	3	3	1	1	3	3	1	3	3
3	3	3	1	1	1	1	1	1	3
3	3	1	1	1	2	1	1	2	1

9	9	9	9	9	9	9	9	9	9
3	3	1	1	1	2	1	4	1	1
9	9	9	9	2	9	9	1	9	9
9	9	9	9	1	9	9	1	9	9
9	9	9	9	1	9	9	1	9	9
1	1	1	1	1	2	1	1	2	1
9	9	9	9	9	9	1	5	9	9
3	3	1	1	1	2	1	1	1	2
1	2	1	2	2	2	2	1	2	9
2	1	2	2	1	2	2	1	2	3
3	3	3	1	1	2	2	1	9	9
1	3	1	1	2	1	1	1	2	1
1	2	1	1	2	1	1	1	1	1
3	3	3	1	3	1	3	1	2	2
1	1	3	1	1	2	2	1	1	1
2	2	3	2	2	2	1	1	1	9
2	2	2	2	2	2	2	1	2	2
3	3	3	3	3	3	3	2	3	3
2	2	3	2	3	2	1	9	3	1
1	1	1	2	2	2	2	6	3	3
1	2	1	1	1	2	2	1	2	2
2	2	2	1	2	2	1	1	1	1
2	2	2	2	2	2	2	1	2	2
2	2	2	1	2	3	2	1	2	2
2	2	1	1	2	2	2	1	2	2
2	2	2	1	1	1	1	1	2	2
1	1	1	1	1	3	1	1	1	1
3	3	3	1	1	2	1	1	1	3
2	3	2	2	1	2	2	1	1	1
2	2	1	1	1	1	1	1	2	2
9	9	9	9	9	9	9	9	9	9
2	2	1	3	2	3	3	1	2	3
1	2	3	1	2	2	3	9	2	1
2	2	1	1	2	2	1	1	2	2
1	1	1	1	1	1	2	1	2	2
3	3	3	1	1	2	1	1	1	1
1	1	1	1	1	3	1	2	3	1
1	1	1	1	1	3	2	1	1	1
1	1	1	1	1	2	1	1	1	1
3	2	1	1	1	2	1	1	1	3
2	2	2	2	2	2	2	1	2	2
2	2	2	2	2	2	2	1	1	1
9	9	9	9	9	9	9	9	9	9
9	9	9	1	9	9	9	1	9	9
9	9	9	1	9	9	9	1	1	9
9	9	9	9	9	9	9	9	2	2
3	3	1	1	1	3	1	4	2	2

3	3	1	1	3	3	1	3	3	1
1	2	1	1	2	1	1	1	1	2
1	1	1	1	3	2	1	1	1	9
3	3	3	1	2	2	3	3	1	3
9	1	1	1	1	9	1	1	9	9
2	2	1	1	1	3	1	1	2	2
3	3	1	1	2	3	1	5	9	9
3	3	1	1	1	1	1	1	1	9
2	2	2	3	2	3	2	1	2	2
3	1	3	1	1	3	1	1	1	3
9	9	1	9	9	9	1	2	9	9
3	2	2	9	9	9	9	1	2	2
1	2	1	1	2	1	2	1	1	3
3	3	1	1	1	2	1	3	2	2
9	9	9	1	9	9	9	1	1	9
3	1	1	1	1	1	1	5	3	3
3	2	2	2	3	2	2	1	2	3
3	3	1	2	1	2	1	4	2	3
1	1	1	9	1	9	9	2	1	2
2	2	1	1	1	2	1	1	2	2
2	1	2	1	2	2	1	9	1	1
1	1	1	1	1	3	3	1	3	3
1	3	1	2	1	3	2	1	2	2
2	3	1	3	1	3	1	1	1	2
2	2	1	1	1	3	1	1	3	3
3	3	3	1	3	1	1	1	1	2
2	3	1	3	1	3	1	1	1	2
2	1	3	1	2	1	2	1	1	2
3	2	3	9	9	9	9	9	2	3
1	1	2	1	2	3	1	1	1	1
1	1	1	3	2	3	1	3	1	3
1	3	1	1	1	3	1	1	1	3
2	3	1	1	1	1	1	3	2	3
3	2	3	1	1	2	2	4	1	3
1	1	2	1	1	3	1	1	1	3
1	1	1	1	1	2	1	1	2	2
2	2	2	1	1	2	1	1	1	3
3	1	3	1	1	2	1	1	3	3
9	9	9	9	9	1	9	1	9	1
2	3	2	2	2	2	3	1	2	2
9	9	9	9	9	1	9	1	9	9
9	9	9	2	9	9	9	1	9	1
1	3	1	1	2	3	1	1	1	2
2	3	1	1	2	3	1	1	1	2
3	1	1	1	1	3	1	1	1	3
1	1	1	1	3	1	1	9	1	3
3	3	3	1	3	3	3	1	1	1

3	3	2	3	3	3	3	1	3	3
9	9	9	1	9	9	9	1	9	9
3	3	1	1	3	3	1	1	1	3
1	1	3	1	1	3	1	1	1	3
3	3	1	1	1	3	1	1	1	3
1	1	1	1	1	3	1	1	1	3
1	3	1	2	1	2	1	2	2	1
2	2	1	3	2	2	3	1	2	1
3	3	1	1	3	3	1	1	1	3
3	2	1	1	2	1	3	1	3	9
1	3	1	1	3	1	1	1	1	3
1	1	1	1	2	2	3	4	1	2
2	2	1	2	1	1	1	1	2	2
3	3	2	1	3	3	1	2	1	3
2	2	1	2	1	1	1	1	2	1
3	2	1	1	1	2	1	1	1	3
2	3	1	1	1	3	1	1	1	3
3	2	1	1	1	3	2	1	2	3
3	1	1	1	3	3	1	1	1	3
2	2	2	2	2	2	2	1	2	2
3	3	1	3	2	2	2	1	1	2
2	3	2	1	2	1	2	9	2	2
3	3	3	3	3	3	3	1	3	3
3	1	3	1	3	2	1	1	9	9
3	3	1	1	1	1	1	1	2	2
1	2	2	1	2	2	1	9	1	2
3	1	2	1	1	2	1	1	2	1
3	3	1	1	2	1	3	9	2	2
3	3	2	1	1	3	1	1	1	2
9	9	9	9	9	9	1	1	9	1
1	2	1	2	1	9	2	1	2	3
1	1	1	3	1	2	2	2	1	3
3	2	1	2	3	1	2	1	2	2
3	2	1	1	2	1	1	1	2	3
1	3	1	3	1	2	1	1	1	1
3	3	3	3	3	3	3	9	2	2
3	3	1	1	1	2	1	1	2	2
2	2	2	2	3	1	2	1	9	9
1	2	1	1	2	1	3	1	1	2
2	3	3	1	1	3	1	4	1	3
9	9	1	9	9	9	1	1	9	9
9	9	9	9	9	9	9	9	9	9
2	1	3	3	1	3	1	1	2	1
3	3	1	1	1	3	1	1	3	3
2	2	2	1	2	2	2	1	3	3
3	3	1	1	1	3	1	1	1	3
9	9	9	9	9	9	9	9	9	9

1	2	1	1	2	2	2	1	1	3
3	3	1	1	1	1	1	9	9	9
3	1	1	2	1	2	2	1	2	2
3	1	1	1	2	1	9	9	9	9
9	9	9	9	9	9	9	9	9	9
1	1	1	1	1	1	1	1	3	3
3	3	1	2	2	2	2	1	2	2
2	2	2	2	2	2	2	9	2	2
2	1	1	1	2	1	3	1	3	2
2	1	1	2	1	1	2	1	1	2
3	3	1	9	9	9	9	9	9	9
1	9	1	1	2	3	1	1	9	9
1	3	2	2	3	3	3	9	9	9
9	9	9	9	9	9	9	9	1	1
2	3	1	2	2	2	2	1	2	3
9	9	9	9	9	9	9	9	1	1
2	2	3	1	2	2	3	1	1	9
3	3	1	3	3	3	1	2	9	9
1	2	3	3	2	2	1	1	3	3
2	2	2	2	2	2	1	1	2	2
3	1	1	2	3	3	1	2	3	3
3	3	1	3	3	2	1	1	2	2
9	9	1	1	9	9	9	1	1	9
1	3	2	1	3	1	2	1	1	3
2	1	1	1	1	2	1	1	2	3
2	2	2	2	2	2	2	1	2	2
3	9	1	1	9	3	9	1	1	9
2	2	1	1	3	1	3	1	1	2
1	1	2	2	2	2	1	1	2	2
9	9	9	9	9	9	1	3	9	9
1	3	1	1	1	1	1	1	1	2
2	1	1	2	1	2	2	1	2	2
1	1	3	1	1	3	3	1	1	3
2	2	1	1	1	1	1	1	1	3
3	3	1	1	1	1	2	1	1	3
3	3	1	3	1	2	1	2	3	3
1	1	1	1	1	3	1	1	1	3
1	1	1	3	3	3	1	5	3	3
9	9	1	9	9	1	9	1	1	9
3	3	3	3	3	3	3	1	2	2
3	3	1	1	1	3	1	1	2	3
2	3	1	1	1	2	1	1	3	3
3	3	2	2	2	2	2	4	1	3
3	2	1	2	2	2	1	1	3	3
3	3	1	3	1	3	1	3	1	2
2	3	1	1	2	3	2	2	1	3
1	2	1	1	1	3	1	1	9	9

2	2	2	2	2	2	2	1	2	2
3	2	1	2	2	3	2	1	1	3
1	1	1	9	2	2	2	1	2	1
3	2	1	2	1	3	1	1	2	2
1	1	2	2	2	3	2	1	3	3
9	9	9	1	9	9	9	1	9	9
2	2	2	2	2	2	2	2	1	2
3	3	1	9	1	2	1	1	1	3
3	3	1	1	3	3	1	1	1	2
2	2	1	1	2	2	2	1	2	2
1	2	1	1	1	3	1	1	2	2
3	3	1	1	2	2	1	1	1	2
2	2	2	2	2	2	2	1	2	2
3	3	3	3	3	3	3	1	3	3
1	3	2	1	2	1	2	1	2	2
1	2	1	2	2	3	1	1	1	2
1	3	1	1	1	3	2	1	3	3
3	3	1	1	1	2	1	1	2	2
3	3	3	1	1	3	1	1	3	2
2	2	2	1	1	2	2	1	2	3
1	1	1	1	1	3	1	1	1	3
2	2	1	1	1	3	2	1	1	3
2	2	1	1	1	3	2	1	1	3
3	1	1	1	2	2	2	1	1	1
3	3	1	1	1	3	1	2	1	2
3	2	3	2	1	2	1	1	3	3
3	3	1	3	1	3	1	1	1	2
3	3	1	3	1	3	1	1	1	2
2	2	2	1	2	2	2	1	2	2
3	1	1	1	1	2	1	1	1	1
2	2	2	2	2	2	2	1	3	3
2	2	1	1	1	3	2	1	1	3
3	3	1	1	1	2	1	1	1	3
3	3	1	2	2	3	1	4	1	3
1	3	1	1	1	3	1	1	1	1
1	1	1	1	2	2	1	1	2	2
1	1	1	1	2	2	1	1	2	2
3	1	2	1	2	2	2	1	2	2
1	1	1	1	1	3	2	1	2	2
2	2	2	2	2	2	2	1	2	2
3	3	1	2	1	1	1	1	2	3
3	3	1	1	1	2	1	2	2	2
3	2	1	1	1	1	1	1	2	2
1	2	2	1	2	1	1	2	1	2
1	3	2	3	1	1	1	5	1	3
3	3	1	1	3	3	9	1	1	3
2	2	1	1	1	1	1	1	1	3

2	3	1	2	2	2	2	1	2	2
2	2	2	1	1	3	1	1	2	2
3	2	1	1	2	1	2	1	1	3
2	2	2	1	2	1	9	1	2	1
9	1	9	2	2	2	2	9	2	2
2	2	2	2	2	2	2	1	2	2
3	2	1	1	1	2	1	1	1	9
3	1	1	1	1	3	1	1	1	3
1	1	1	2	2	3	1	1	2	2
3	3	1	3	2	3	2	1	2	2
3	3	1	3	2	3	2	1	2	2
2	1	1	2	2	3	2	2	2	2
2	1	2	9	9	9	9	2	2	2
3	3	1	1	1	1	1	1	9	9
1	1	3	1	1	1	2	1	2	2
2	2	1	1	1	1	1	1	1	2
3	1	1	1	1	3	1	1	1	3
2	2	1	1	1	1	1	1	1	2
2	3	1	1	1	1	1	1	1	3
3	2	1	1	1	1	1	1	1	2
2	2	2	2	2	2	2	1	2	2
2	2	2	1	2	2	1	1	2	2
1	2	2	2	3	2	2	1	2	2
2	1	2	2	2	2	2	1	2	2
2	2	1	1	1	1	1	1	1	2
1	1	2	1	1	2	1	1	1	3
2	2	2	1	1	3	1	1	3	3
2	2	2	2	2	2	2	1	2	2
2	2	1	2	2	2	2	1	2	2
3	3	1	1	2	1	1	1	1	2
2	2	2	3	2	2	2	1	3	3
2	2	2	2	2	2	2	1	2	2
2	2	2	2	2	2	2	9	2	2
2	2	2	2	2	2	2	1	2	2
9	9	9	9	9	9	1	1	2	2
2	2	2	2	2	2	2	1	2	2
2	2	2	2	2	2	2	1	2	3
2	2	2	2	2	2	2	1	2	2
2	2	2	2	2	2	2	1	9	9
3	2	1	1	1	2	1	1	1	2
2	2	2	2	2	2	2	9	1	2
1	1	1	2	2	2	2	1	1	2
3	3	1	1	1	2	1	1	2	2
2	2	2	2	2	2	2	1	2	2
2	2	2	2	2	2	2	1	1	2
1	1	1	2	2	3	3	1	2	2
9	1	1	2	2	2	2	1	1	2

2	2	2	2	2	2	2	9	2	2
2	2	2	1	2	2	2	1	1	2
1	2	2	2	3	1	1	5	1	3
2	2	1	1	1	1	2	1	1	2
2	2	2	2	2	2	2	1	2	2
2	2	2	2	2	1	3	1	1	2
3	3	1	3	1	1	1	1	1	9
3	3	1	1	1	2	1	1	1	1
3	1	1	1	1	1	1	2	1	3
3	3	1	1	1	2	1	7	1	3
3	3	1	1	1	2	1	2	1	3
2	2	2	2	1	2	2	1	2	2
2	2	1	2	3	3	1	1	1	3
9	9	9	1	1	3	1	1	2	2
1	1	1	1	1	1	1	1	1	9
3	2	1	3	1	1	1	5	1	2
2	2	1	1	1	1	1	1	1	2
3	2	1	1	1	1	1	1	1	2
1	1	9	1	1	1	1	1	1	1
2	2	1	1	1	2	1	1	1	3
2	2	1	3	1	3	1	1	1	2
2	2	2	2	2	2	2	9	1	2
3	2	1	2	2	2	2	1	1	3
2	2	2	2	2	2	2	1	2	2
2	2	2	2	2	2	2	1	2	2
2	2	2	2	2	2	2	1	2	2
9	9	9	9	9	9	1	2	1	9
2	2	1	1	1	2	2	1	2	2
2	2	2	1	1	1	2	1	1	2
9	9	9	9	9	9	9	9	2	1
3	2	1	2	2	2	1	1	1	2
2	3	2	1	2	3	1	1	1	2
1	2	9	3	2	3	1	1	9	9
2	2	2	1	1	1	1	2	1	2
2	2	1	1	3	1	1	1	9	9
3	3	1	1	3	2	1	1	1	3
3	3	3	1	1	3	1	3	1	3
3	3	1	1	1	3	1	1	2	2
3	3	2	1	2	3	1	1	1	9
1	2	1	3	1	1	2	1	2	2
3	3	1	1	1	3	1	1	1	3
3	1	1	2	1	1	2	1	1	1
3	3	1	1	2	3	1	1	1	3
2	2	2	2	2	2	2	1	2	2
3	3	1	1	2	3	1	4	1	3
1	1	1	1	1	2	1	1	2	2
2	3	1	1	1	1	1	1	1	2

1	1	1	2	1	2	2	1	2	3
3	2	1	1	2	1	3	9	1	2
3	3	1	3	2	3	9	1	1	3
3	3	1	1	1	3	1	1	3	1
3	2	3	3	2	3	1	2	3	2
1	1	1	2	2	3	2	1	1	2
1	1	1	1	3	1	1	1	1	3
9	9	9	2	9	9	9	1	2	9
1	2	1	1	2	2	1	2	2	2
2	2	2	2	2	2	2	2	2	2
1	1	1	1	1	3	2	9	3	2
1	1	1	1	1	3	2	1	3	2
3	3	3	1	1	1	2	1	2	2
2	2	1	2	2	2	2	1	2	2
2	2	2	1	1	3	1	1	2	2
3	3	1	2	1	2	1	1	2	2
1	2	1	2	2	2	2	1	2	2
3	1	1	1	1	2	1	1	3	2
3	3	1	1	1	2	1	1	2	2
3	2	1	1	1	2	1	1	1	3
2	3	3	2	2	1	1	1	9	9
1	2	1	1	1	2	1	1	9	9
3	2	1	1	1	2	2	1	1	3
9	9	1	1	9	9	9	1	1	9
1	2	3	1	2	1	3	3	1	3
3	3	1	1	1	1	1	1	1	2
1	2	1	2	3	1	1	1	1	1
2	2	2	1	1	2	3	1	1	2
2	2	2	1	1	2	1	3	2	2
9	9	9	9	9	9	1	1	1	9
3	2	2	3	3	3	1	1	1	3
9	9	9	9	9	9	9	9	9	9
9	9	9	9	9	9	9	9	9	9
2	2	1	1	1	2	1	1	2	2
2	2	1	1	1	2	1	1	2	2
1	1	1	1	1	2	1	1	1	2
1	1	1	1	3	3	1	1	3	1
2	2	1	1	1	2	1	1	2	2
3	2	1	1	1	1	1	1	1	3
3	2	1	1	1	1	1	1	1	3
9	9	9	9	9	9	9	9	9	9
3	3	3	2	1	3	1	1	2	2
1	2	1	1	2	2	3	2	2	2
9	9	9	9	9	9	9	9	9	9
9	3	9	3	9	9	9	9	3	9
1	1	1	1	1	1	2	1	1	1
2	2	2	1	1	1	1	1	2	1

1	3	1	9	9	9	9	9	9	9
3	1	1	1	1	3	1	1	1	3
3	3	1	1	1	3	1	9	1	3
9	9	9	1	1	2	2	1	1	2
2	2	1	1	1	2	2	1	9	9
3	3	3	3	3	3	3	9	3	3
2	2	2	2	2	2	2	1	2	2
3	3	3	2	1	3	1	1	3	2
9	9	9	9	9	9	1	1	9	9
2	9	1	1	2	2	2	1	2	2
3	3	1	1	2	3	1	3	3	3
2	2	2	2	2	2	2	1	2	2
2	2	1	1	1	3	3	1	2	2
3	3	3	3	3	3	3	1	3	3
2	2	1	2	2	3	2	1	3	3
3	2	1	3	1	1	2	1	3	1
3	1	1	1	1	1	2	1	1	2
2	3	1	3	1	1	2	6	1	1
2	2	2	2	2	2	2	1	9	9
2	1	1	3	1	2	1	1	1	2
2	3	1	1	1	2	1	3	1	3
1	1	1	1	3	1	2	1	2	2
3	2	1	1	1	2	1	1	9	9
2	2	2	1	1	3	3	1	2	2
3	2	3	1	3	1	1	5	1	3
2	2	1	1	2	2	2	1	2	2
3	3	1	1	1	2	2	1	1	2
1	9	3	1	1	2	1	1	1	1
1	3	2	1	3	1	2	2	1	2
1	1	1	1	3	3	3	1	9	9
1	1	1	2	2	2	2	1	2	2
3	3	3	1	2	1	1	1	3	1
3	3	1	1	1	3	1	1	1	3
3	3	1	1	2	2	3	1	3	3
2	3	1	1	1	3	1	1	1	1
2	1	1	1	1	3	2	2	1	2
3	3	1	1	1	1	1	4	1	3
9	3	9	2	3	2	2	1	2	2
1	1	1	1	1	1	1	1	2	2
1	3	1	1	1	1	3	1	9	9
2	2	3	2	3	3	3	1	9	9
2	3	1	1	2	3	1	1	2	1
3	2	1	2	1	3	3	1	1	3
1	1	1	1	3	2	1	1	2	2
1	1	1	1	1	2	3	1	1	3
2	2	2	1	1	2	2	1	2	3
9	9	9	3	3	3	1	1	3	2

2	1	2	1	2	1	2	9	1	3
3	2	1	1	1	2	1	1	1	3
3	3	3	1	1	3	1	1	3	3
2	3	2	1	1	3	3	1	2	3
3	3	1	1	1	3	1	1	1	3
1	2	1	3	1	3	2	1	2	2
9	9	9	9	9	9	9	9	2	2
3	3	1	1	2	3	1	1	2	2
3	3	1	1	1	3	1	1	1	3
1	1	1	1	1	3	1	1	2	1
2	2	1	9	9	9	9	1	1	1
2	2	2	2	2	2	1	1	1	2
2	2	2	2	2	2	2	1	2	2
1	1	1	1	1	1	1	2	1	3
3	3	1	1	2	3	1	1	9	9
2	2	1	1	2	9	1	1	2	2
2	3	1	3	3	3	2	1	2	2
3	3	1	2	1	3	1	1	1	3
1	1	1	1	1	1	1	1	3	1
3	2	1	1	1	2	2	1	1	3
1	3	1	1	1	3	1	2	1	2
3	3	1	1	2	2	1	5	9	9
3	1	1	1	3	3	2	1	2	3
3	1	1	1	1	2	2	1	9	1
2	2	1	3	3	3	3	1	2	2
3	1	1	1	1	2	1	1	2	2
2	2	2	2	2	2	2	1	2	2
2	2	2	1	1	2	2	1	2	2
3	3	3	3	3	3	3	1	3	9
3	3	1	1	1	1	1	1	1	2
1	1	1	3	1	3	1	1	3	3
3	3	1	1	2	3	3	3	3	1
2	2	2	2	1	2	2	1	2	2
3	3	1	1	2	3	1	1	3	3
2	1	1	1	2	2	1	1	1	2
3	3	1	2	1	3	1	1	2	2
3	3	1	1	1	3	1	4	3	1
1	1	1	1	1	3	3	1	2	1
1	1	1	1	1	3	1	1	1	1
9	9	9	9	9	9	1	1	2	9
3	3	1	1	1	3	1	1	2	2
1	2	3	1	1	3	3	1	2	1
3	2	1	2	2	2	2	1	1	3
3	2	1	1	1	3	1	3	1	3
3	3	1	1	3	3	3	5	1	3
3	3	1	1	3	3	1	1	9	9
3	1	1	1	1	2	2	1	2	2

2	2	1	1	2	2	1	1	1	2
2	2	2	2	2	2	2	1	2	3
3	9	9	3	3	3	3	1	3	3
2	3	1	1	1	2	3	1	2	3
9	9	1	1	2	3	1	1	1	3
9	9	9	9	9	9	9	9	2	1
9	9	9	1	1	2	1	1	3	1
2	3	1	1	1	1	1	1	9	9
9	9	9	9	9	9	9	9	1	2
2	1	1	3	1	1	1	2	1	2
2	2	1	1	1	2	2	1	2	3
1	1	1	1	1	3	1	1	1	2
1	1	1	1	1	3	1	4	2	2
3	3	1	1	3	2	1	2	3	1
2	9	9	2	2	9	2	1	9	9
2	2	1	1	2	3	2	1	1	1
1	3	9	9	9	9	9	9	1	1
1	1	1	1	1	2	3	1	3	1
3	3	1	1	1	3	1	1	1	1
3	3	1	1	1	3	1	2	2	3
1	1	1	1	3	3	1	1	1	2
3	3	3	1	1	3	1	2	3	3
2	1	1	1	2	1	3	1	2	2
3	3	1	2	3	2	1	1	1	3
2	2	1	1	1	3	1	3	1	1
1	1	1	1	1	2	1	1	1	2
1	2	3	1	2	3	2	3	3	2
1	1	1	1	3	3	1	1	1	2
3	3	2	2	2	2	2	1	2	2
3	3	1	1	1	2	1	1	1	2
2	1	1	1	2	2	1	1	2	2
2	1	2	2	2	2	2	1	2	2
1	2	1	1	3	3	2	1	2	2
3	3	1	1	1	3	1	1	1	1
1	1	3	1	2	3	2	2	1	2
3	3	1	1	1	3	1	1	1	2
1	1	1	1	1	2	2	4	2	2
3	3	3	1	3	1	3	1	3	3
3	1	3	1	1	2	1	1	1	2
1	1	1	2	2	2	1	1	1	1
3	3	3	2	2	2	2	1	1	1
2	1	1	1	2	2	1	1	1	1
3	3	1	1	1	2	2	1	3	1
2	3	3	1	2	3	3	2	2	2
2	2	2	1	1	2	2	1	2	3
3	3	3	1	1	3	3	1	2	2
2	2	2	1	3	2	2	1	1	1

1	3	1	1	1	3	2	1	1	2
2	3	2	1	1	1	1	1	1	2
1	2	2	1	1	3	2	1	1	1
3	2	1	1	2	3	3	1	1	1
1	1	1	1	1	1	1	4	2	1
2	2	3	1	1	1	2	1	1	2
9	9	9	1	2	1	2	1	1	2
2	3	1	1	2	3	2	2	2	2
3	2	1	1	2	2	1	1	2	1
3	2	1	1	2	2	1	1	2	2
3	3	1	1	2	2	2	1	2	2
2	2	2	2	2	2	2	2	2	2
1	1	1	2	2	2	2	1	1	1
3	2	2	1	1	3	1	1	3	3
3	1	1	1	1	2	1	7	1	2
3	3	1	1	1	2	1	5	1	1
3	2	1	1	1	3	1	1	2	2
2	2	1	9	2	2	2	3	1	2
2	2	1	1	2	2	2	1	2	2
1	2	2	1	1	1	2	1	3	3
2	2	1	1	1	2	2	1	2	2
3	2	1	1	2	2	1	1	2	1
2	2	9	2	2	2	2	1	3	3
3	3	2	3	3	3	3	9	3	1
1	2	1	2	1	1	2	2	2	9
2	2	2	2	2	2	2	9	2	2
1	2	2	2	1	3	1	1	2	1
2	2	3	3	2	2	3	1	2	2
3	2	1	1	1	2	1	1	2	2
1	3	1	1	2	3	2	1	2	2
1	2	1	3	1	2	1	1	2	2
3	3	1	1	2	3	3	3	2	1
2	2	2	1	2	2	1	1	1	1
2	2	2	2	1	3	2	1	1	2
3	2	1	1	1	2	2	1	1	3
1	1	1	1	1	3	2	1	1	1
1	2	1	1	2	2	3	2	3	2
2	3	3	1	3	2	3	4	2	3
3	3	3	1	2	2	1	1	2	1
1	2	2	1	3	2	2	1	2	1
2	2	2	2	2	2	2	1	2	2
2	2	2	2	2	2	2	9	2	2
2	2	2	2	2	2	2	1	2	2
3	3	3	3	3	3	3	1	3	3
3	3	3	3	3	3	3	1	3	3
9	9	2	2	9	9	2	1	2	2
2	1	1	3	2	1	1	1	2	1

2	1	1	1	1	2	2	1	1	2
1	1	2	2	1	1	1	1	2	2
2	1	1	2	2	1	1	1	1	1
2	1	1	1	2	2	1	1	1	2
2	1	1	2	2	2	1	1	1	2
1	2	2	1	1	2	2	1	2	2
2	1	1	1	2	2	2	1	2	2
1	2	1	2	1	1	2	1	3	3
1	2	1	1	2	1	1	1	1	1
1	1	1	2	2	1	1	1	2	2
2	1	2	2	2	1	1	1	1	1
2	2	2	1	1	1	1	1	1	1
2	2	2	2	2	2	2	1	1	1
2	2	2	2	2	2	2	1	1	1
2	2	2	2	2	2	2	1	1	1
2	2	2	2	2	2	2	1	2	2
2	2	2	2	2	2	2	1	2	2
2	2	2	2	2	2	2	1	2	2
2	2	2	2	2	2	2	1	2	2
2	1	1	2	2	2	2	1	2	1
2	2	2	2	2	2	2	1	2	9
2	2	2	2	2	2	2	1	3	3
2	2	2	2	2	2	2	1	1	1
1	1	1	2	2	2	1	1	1	2
2	2	2	2	2	2	2	1	2	2
2	2	2	2	2	2	2	1	2	2
2	2	2	2	2	2	2	1	1	1
2	2	2	2	2	1	1	2	1	2
2	1	1	1	2	3	2	1	1	2
2	2	2	3	3	2	2	1	3	3
2	2	2	2	2	2	2	1	2	2
2	2	2	2	2	2	2	1	2	2
2	2	2	1	2	2	3	1	2	2
2	2	2	2	2	2	2	7	2	2
1	1	1	1	3	1	1	1	9	1
1	9	9	9	1	9	1	1	1	3
3	1	9	3	1	9	1	1	1	2
2	9	2	2	9	2	2	1	2	9
9	9	9	9	9	9	9	9	9	9
1	9	1	1	9	1	1	1	1	9
3	1	1	1	2	1	1	1	1	9
2	1	1	2	1	1	1	1	2	1
1	1	1	1	2	3	1	1	1	9
2	2	2	2	2	2	2	1	2	2
1	9	1	1	1	1	2	1	1	9
9	2	9	2	9	2	2	1	2	9
1	2	1	2	1	2	2	1	2	9

2	1	2	2	1	2	2	1		2	9
1	2	2	1	2	2	2	1		2	9
1	1	2	2	2	2	2	1		2	9
1	1	2	1	2	3	2	1		9	9
1	2	1	1	1	2	1	1		9	9
1	1	1	1	2	3	3	1		9	2
1	2	2	1	2	2	1	1		9	9
3	2	2	2	2	2	1	1		2	9
2	1	2	2	1	2	2	1		2	1
2	2	2	2	2	2	2	1		2	9
2	1	1	1	2	2	2	1		2	1
2	2	2	1	2	2	3	1		3	2
1	1	1	1	3	2	1	1		2	2
1	1	1	1	2	2	2	2		3	2
2	2	1	2	1	2	3	1		3	2
2	3	2	3	2	2	1	1		1	2
3	3	1	1	2	2	2	1		2	2
1	2	1	1	2	1	2	1		2	3
2	1	1	1	9	9	9	1		2	2
2	3	2	2	2	1	1	1		2	2
2	1	2	1	2	2	2	1		2	2
2	2	1	2	2	1	1	1		1	2
2	2	3	1	2	2	1	1		1	1
3	2	1	1	2	3	2	1		1	2
2	1	2	1	2	3	2	1		3	2
2	2	1	3	2	1	2	1		3	3
2	2	3	2	2	2	2	1		9	2
1	3	1	2	2	2	2	1		3	2
2	2	2	2	2	3	2	1		1	1
2	2	2	2	2	2	2	1		2	2
2	1	1	2	2	1	1	1		2	9
3	3	3	2	2	2	2	1		3	3
1	1	1	1	1	1	1	1		2	1
3	3	3	2	2	2	2	1		2	2
2	2	2	2	2	2	2	1		3	2
3	3	3	3	3	3	3	1		3	3
2	2	2	2	2	2	2	1		2	2
2	2	2	2	2	2	2	1		9	9
2	2	2	1	2	2	1	1		1	1
2	2	2	1	2	2	2	1		2	2
2	3	2	2	1	3	2	1		2	1
2	3	3	2	2	2	2	9		2	2
2	1	2	1	2	2	3	1		3	2
3	2	2	1	2	2	2	1		2	2
2	2	2	1	2	2	2	1		1	1
1	2	3	2	1	2	3	2		1	2
1	2	2	3	2	2	1	2		1	2

9	2	9	2	9	9	9	1	1	1
2	1	1	1	1	1	2	1	2	2
2	2	1	1	2	3	2	2	1	2
2	2	2	2	2	2	1	1	2	2
2	3	2	2	1	2	3	7	3	2
3	3	1	1	1	2	1	1	2	2
3	3	1	2	1	3	3	3	3	2
3	2	1	1	2	2	1	5	1	1
3	1	1	1	1	2	1	1	1	1
2	2	1	3	3	3	2	1	2	2
3	3	2	2	1	1	1	1	2	2
3	1	1	1	1	2	2	1	1	2
2	3	1	1	2	1	2	1	2	1
2	3	2	2	3	1	2	3	2	3
1	3	2	1	1	3	2	1	3	2
3	1	1	1	1	3	1	1	1	1
3	1	1	1	1	3	1	1	1	1
1	1	1	1	1	2	1	1	2	2
1	3	3	3	2	2	3	2	2	3
2	2	2	2	2	2	2	9	2	2
9	9	9	1	1	2	1	9	1	1
2	3	2	2	2	2	2	1	2	2
2	3	1	1	2	1	1	5	1	2
3	2	1	1	1	3	1	5	1	3
3	3	1	1	1	3	1	3	1	2
1	2	1	1	1	2	1	1	2	3
3	3	1	3	1	1	2	1	2	1
2	1	1	1	1	1	1	2	2	1
1	1	1	1	1	1	1	1	1	1
2	2	1	2	1	2	2	1	2	2
3	3	1	1	1	3	1	1	1	3
3	2	1	1	1	9	2	1	1	3
2	2	2	2	2	3	2	9	2	2
2	2	2	1	1	2	2	1	2	2
3	3	1	1	1	2	1	1	2	2
3	3	2	1	1	1	1	1	2	2
2	3	1	2	1	1	1	1	2	1
2	2	1	1	3	2	1	1	2	2
3	1	1	1	2	1	1	2	3	1
3	3	1	1	1	2	1	1	3	2
3	3	1	1	1	1	1	1	2	2
2	2	2	2	9	2	2	1	1	2
2	2	1	1	1	2	2	2	3	3
1	2	1	2	3	3	2	1	2	2
1	1	1	2	2	2	2	1	2	2
1	3	1	1	1	1	1	1	2	2
1	3	1	1	1	1	1	1	2	2

3	3	1	1	1	2	1	1	1	3
3	3	1	1	1	1	1	1	1	3
2	2	2	2	2	2	2	1	2	2
2	2	3	1	1	2	2	1	2	2
3	2	2	2	2	2	2	1	2	2
1	1	1	1	1	1	1	5	2	2
3	3	1	1	1	2	1	2	1	1
2	1	3	1	3	3	1	1	2	3
3	3	3	1	1	1	1	1	2	2
9	9	9	9	9	9	9	9	9	9
2	3	3	1	3	1	2	1	1	3
1	1	1	1	1	2	2	1	1	1
1	3	1	1	2	2	2	2	2	2
2	2	1	2	2	2	2	2	2	2
1	1	1	1	2	2	3	1	2	2
1	2	2	2	1	2	2	9	2	2
2	3	2	2	1	2	2	1	2	2
2	2	2	2	1	3	2	1	2	2
1	3	3	9	9	9	9	1	3	3
3	3	3	1	1	3	1	1	3	3
3	3	3	3	3	3	3	9	3	3
3	3	3	3	3	3	3	9	3	3
3	3	1	2	3	3	2	2	1	9
3	2	3	1	3	2	3	1	3	2
2	2	2	2	2	2	2	1	2	2
9	2	9	3	1	2	1	9	1	1
1	2	1	1	2	2	1	1	3	3
2	2	2	2	2	2	2	1	1	2
2	2	2	2	2	2	2	9	2	2
2	1	3	2	1	3	1	5	3	2
3	3	1	3	3	2	9	4	1	1
2	2	2	2	1	1	2	1	2	2
1	2	2	1	2	1	2	1	1	3
3	3	9	3	3	3	3	1	3	3
3	2	1	2	1	3	1	2	1	2
2	2	2	1	2	3	1	1	3	2
2	2	2	1	2	3	1	1	3	2
2	2	2	1	2	3	1	1	3	2
2	2	2	2	2	2	2	1	2	2
2	2	2	3	3	2	3	1	2	2
2	2	1	1	1	1	1	1	1	2
3	2	2	1	2	3	1	5	1	1
2	2	2	2	2	2	2	1	1	2
3	3	1	1	1	1	1	3	3	1
3	3	1	1	1	3	1	1	1	3
3	3	1	1	1	2	1	3	1	1
2	2	1	1	2	1	1	1	1	1

2	2	2	2	2	2	2	2	2	2
1	2	1	1	1	1	3	1	1	3
3	2	1	3	2	3	1	3	1	3
2	2	2	2	2	2	2	9	2	2
3	2	2	2	2	2	3	1	3	3
3	3	1	1	1	1	9	1	2	2
2	2	2	2	2	2	2	1	2	2
2	2	2	2	2	3	1	6	2	2
3	1	1	1	1	2	3	4	1	2
2	2	2	2	3	2	2	1	1	2
2	2	1	1	2	2	1	1	1	2
3	3	1	3	2	1	1	1	2	1
1	9	9	9	9	9	1	1	9	9
9	9	9	1	9	9	9	1	9	1
9	9	1	1	9	9	9	1	9	9
3	2	2	2	2	2	1	1	3	1
2	2	1	2	2	2	1	4	2	2
2	1	1	1	3	2	1	3	1	9
3	3	1	1	1	1	1	1	1	1
3	3	3	1	3	1	1	1	1	2
1	1	1	1	3	3	1	1	1	1
3	3	1	2	1	1	1	1	1	1
3	3	1	2	1	1	1	1	1	1
2	2	2	2	2	2	2	1	2	2
3	2	1	3	1	1	1	1	1	1
2	2	1	1	1	1	1	1	3	1
3	2	1	3	1	1	1	1	1	1
9	9	1	1	1	2	1	4	1	9
3	3	1	1	1	3	1	1	9	9
3	3	1	1	1	3	1	1	1	3
3	3	1	1	3	3	1	5	2	3
2	3	1	3	1	2	1	1	1	3
2	1	1	3	3	3	1	4	1	1
3	3	1	2	1	1	1	2	1	1
3	3	1	1	3	3	1	2	1	1
3	3	1	1	2	9	1	4	1	9
2	2	1	2	2	2	1	2	2	3
3	3	1	1	1	2	2	1	2	2
2	2	1	1	1	1	2	5	3	3
3	3	1	1	1	3	1	7	3	1
1	2	2	2	3	2	1	1	1	2
3	3	1	1	2	2	1	1	2	2
3	3	1	1	1	1	1	1	2	1
2	1	1	2	2	2	1	2	2	1
2	3	1	2	2	1	1	2	1	1
3	3	1	1	1	3	1	2	1	3
2	2	1	1	1	2	1	1	1	2

1	2	1	1	2	2	1	2	1	1
2	2	1	1	2	2	1	1	3	3
9	9	9	9	9	9	1	1	9	9
3	2	1	1	1	1	1	1	1	1
3	3	1	1	1	2	1	4	3	3
1	1	1	1	1	3	1	2	1	1
3	3	1	2	1	2	2	1	1	2
1	3	1	1	2	3	2	1	1	2
2	2	1	1	2	3	3	1	3	3
3	3	1	1	1	1	3	1	1	2
3	2	1	1	2	3	1	2	2	3
1	1	1	1	2	2	1	1	3	3
3	2	1	3	2	3	1	1	2	2
3	3	1	1	1	3	1	2	3	1
3	9	1	1	1	2	1	2	3	1
1	3	1	1	1	3	1	1	3	2
3	3	1	1	1	3	1	2	1	3
2	3	1	1	1	2	1	2	1	2
3	3	1	1	1	3	1	2	1	1
3	1	1	1	3	3	1	1	1	3
1	2	1	2	1	3	1	1	3	3
9	9	9	9	9	9	9	9	9	9
3	3	3	3	3	3	3	2	3	3
3	1	2	1	1	1	1	1	2	2
3	3	1	1	3	2	2	3	2	2
9	9	9	2	9	9	9	6	3	9
2	3	1	2	1	2	1	2	9	1
3	1	2	2	1	3	2	1	1	2
9	9	1	1	9	9	9	3	9	9
9	9	9	9	9	9	1	7	9	9
9	9	9	9	9	2	9	1	9	9
3	3	1	1	2	2	2	2	1	2
1	1	1	1	1	1	1	2	9	1
1	3	1	1	1	2	1	1	1	1
3	1	2	3	2	1	2	4	3	2
3	3	3	1	1	1	1	3	1	3
3	3	3	1	1	1	1	3	1	3
2	2	2	3	2	2	2	3	3	3
1	3	1	1	2	1	2	2	2	1
2	2	2	2	3	2	1	2	1	2
3	3	1	1	1	3	1	1	9	1
3	3	3	1	1	3	1	1	1	3
3	1	2	2	2	3	1	1	2	3
2	1	1	1	1	3	1	1	3	1
2	1	1	1	1	3	1	1	3	1
3	2	1	1	1	3	1	1	3	1
2	1	1	1	1	3	1	1	3	1

3	3	1	1	2	3	1	4	9	1
3	1	1	1	1	1	1	4	1	2
3	3	1	1	1	1	1	4	1	1
3	3	1	3	3	3	1	2	9	9
3	3	1	1	1	3	1	3	9	9
3	3	1	1	2	2	1	5	1	2
1	2	1	1	1	3	1	1	1	3
2	2	2	1	1	2	1	1	1	3
9	9	9	9	9	9	9	9	9	9
3	3	1	1	3	3	1	1	1	3
2	2	1	3	2	9	3	4	2	2
3	1	1	2	1	2	1	1	2	3
9	2	9	1	1	2	2	1	1	3
3	3	1	1	1	2	2	2	1	3
9	9	9	9	9	9	9	9	9	9
9	9	9	9	9	9	9	9	9	9
3	3	1	1	2	3	2	1	2	2
1	3	1	1	1	3	1	1	3	1
9	9	9	9	9	9	9	9	1	1
9	9	9	9	9	9	9	1	9	9
9	9	9	1	9	9	9	1	9	9
3	3	1	1	1	1	2	1	1	2
3	3	2	9	9	9	9	9	9	9
9	9	9	9	9	9	9	9	9	9
3	3	1	1	1	3	1	1	1	1
1	2	1	1	3	2	1	1	2	1
3	3	1	1	1	3	1	2	3	1
3	3	1	1	2	3	1	4	3	2
3	3	1	1	1	3	1	2	1	2
3	3	2	1	1	3	1	2	1	3
2	3	2	2	1	2	3	1	3	3
3	3	1	1	1	2	1	1	1	3
3	2	1	2	3	2	2	1	1	2
3	3	1	1	1	2	1	2	1	2
2	1	1	1	2	2	2	1	2	2
3	3	1	1	1	3	1	1	1	1
3	2	1	1	3	2	1	4	2	3
3	3	1	1	2	3	1	1	1	1
3	3	1	1	1	3	1	1	1	1
3	3	1	1	1	1	1	1	1	3
3	2	1	1	2	2	1	2	2	1
2	1	1	3	2	2	1	1	3	1
2	2	2	1	2	2	3	1	1	3
9	9	9	1	9	9	9	3	2	9
9	9	9	9	2	9	9	1	9	9
1	1	1	3	2	2	1	1	1	1
3	3	2	1	1	1	2	1	2	1

2	2	2	1	2	2	2	1	1	2
2	2	1	1	1	2	1	1	1	2
2	2	1	2	2	2	1	1	3	1
1	1	1	2	2	2	2	1	2	2
2	2	3	1	2	2	3	1	1	2
3	1	2	1	1	3	1	3	1	2
3	2	1	1	2	2	1	2	3	1
1	2	3	1	2	1	3	2	2	2
9	9	9	9	9	9	9	9	9	9
2	1	2	2	2	1	1	4	1	2
2	2	2	2	2	2	2	1	2	2
3	3	3	1	1	3	1	1	2	2
2	3	2	1	2	3	1	1	1	2
2	2	2	2	2	2	2	2	2	2
2	2	2	2	2	3	2	1	2	3
2	2	2	2	1	3	2	1	2	1
1	2	3	1	2	3	1	2	2	2
2	2	2	2	2	2	2	1	2	3
3	2	2	1	1	1	2	4	1	1
3	3	3	3	3	3	2	1	3	1
3	2	1	1	2	2	1	1	1	1
3	3	3	3	3	3	3	1	2	2
2	1	1	2	2	1	1	2	3	3
2	2	3	2	3	2	1	2	2	2
2	2	2	2	2	2	1	1	2	2
9	9	9	9	9	9	9	9	3	2
2	1	1	1	1	1	3	1	2	2
2	2	9	2	2	2	1	1	1	1
1	3	2	1	3	2	1	1	1	2
9	9	9	9	9	9	9	9	9	9
3	2	2	1	2	3	1	1	2	2
9	9	9	9	9	9	9	9	9	9
3	3	3	9	9	9	9	9	3	3
2	1	1	1	3	3	1	1	1	1
2	1	2	1	2	1	1	2	3	2
1	2	1	2	3	2	2	2	1	2
2	3	2	1	2	1	1	1	1	2
2	1	2	2	2	3	3	2	2	3
2	2	2	2	2	2	2	1	2	2
2	1	2	2	2	2	1	1	1	2
2	3	1	2	2	2	2	9	2	2
9	9	1	1	9	9	9	1	3	1
2	2	2	1	2	2	1	1	1	2
3	3	3	2	1	1	1	1	3	2
3	2	1	3	2	2	2	9	2	2
3	3	1	3	1	1	1	3	3	2
3	3	2	1	1	1	1	1	1	2

9	3	1	1	1	1	1	1	3	1
3	3	3	2	1	3	1	1	3	3
1	2	2	1	2	2	2	4	2	2
2	2	2	2	1	3	1	1	3	1
3	3	3	2	1	1	9	1	2	1
2	2	2	2	2	2	2	9	2	2
1	1	1	3	1	3	3	1	1	3
1	1	1	3	1	3	3	1	2	3
9	9	1	9	1	9	9	2	9	9
1	1	1	1	3	3	1	1	3	1
3	3	1	1	1	2	1	1	3	3
2	3	1	2	1	3	1	1	2	3
9	9	9	9	9	9	9	9	9	9
1	1	1	3	1	3	3	1	2	2
1	1	1	3	3	3	3	2	1	3
3	1	1	1	1	1	2	1	1	1
1	1	1	1	1	3	3	1	9	9
3	2	1	1	3	2	1	9	1	3
1	1	1	1	1	2	1	1	1	3
1	1	1	3	1	2	1	1	1	3
1	2	1	1	2	1	2	9	1	9
3	3	3	3	3	3	3	1	3	3
3	1	3	2	2	2	2	1	2	2
3	1	3	1	2	2	2	3	3	2
3	2	1	1	1	3	1	2	2	1
3	3	1	3	1	1	2	4	2	3
3	1	1	3	1	1	2	5	1	1
1	3	1	1	2	3	2	1	2	1
1	1	2	1	1	2	1	1	1	3
3	3	1	2	1	2	1	4	2	1
2	2	2	2	2	2	2	1	2	2
1	1	1	2	2	2	2	9	2	2
2	1	2	2	2	1	2	3	1	2
3	3	1	1	2	2	1	4	2	2
2	2	1	2	2	3	1	2	3	3
9	9	9	9	9	9	9	9	9	9
3	2	2	3	2	3	9	1	2	2
2	1	1	1	3	3	2	1	3	3
9	9	9	1	2	3	1	2	1	2
2	2	2	2	2	2	2	1	2	2
3	2	2	3	2	3	9	1	2	2
3	3	1	1	1	2	1	1	1	1
3	3	1	1	2	2	1	1	2	2
2	1	2	2	2	2	2	9	2	2
9	9	9	9	9	9	9	9	9	9
2	3	1	1	1	3	1	1	1	3
9	9	9	9	9	9	9	9	9	9

3	3	3	1	2	1	1	3	1	1
2	2	1	9	1	9	1	1	1	3
1	1	1	9	9	9	9	1	9	9
1	1	1	3	1	3	1	5	1	3
2	3	2	2	1	2	1	1	1	2
2	1	2	2	2	1	3	9	1	2
2	2	2	2	2	2	2	9	2	2
1	1	1	1	1	1	1	9	9	3
1	3	2	2	3	2	1	1	2	3
1	2	2	1	2	2	2	1	1	3
3	3	3	3	3	3	3	9	9	9
3	2	3	3	1	3	1	9	9	1
2	2	1	3	3	3	2	1	3	3
3	2	1	1	2	3	1	1	3	3
2	2	9	2	2	2	2	9	3	3
9	9	9	9	9	9	9	9	3	3
3	2	1	1	1	3	1	1	1	3
9	9	9	9	9	9	9	9	9	9
9	9	9	9	9	9	9	9	9	9
1	1	3	1	1	1	2	1	1	2
2	2	2	2	2	2	2	1	3	2
1	3	3	3	2	2	2	9	2	2
3	3	2	2	2	2	2	9	3	2
2	2	2	3	3	3	3	1	3	3
2	3	2	1	2	3	2	2	1	2
2	9	1	1	1	2	1	1	3	2
9	9	9	1	1	2	9	4	3	9
2	3	3	2	2	2	3	1	1	2
9	9	9	9	9	9	9	9	9	9
9	9	9	9	9	9	9	9	9	9
9	9	9	9	9	9	9	9	9	9
9	9	9	9	9	9	9	9	9	9
9	9	9	9	9	9	9	9	9	9
2	1	2	2	2	2	2	1	1	1
1	2	1	1	3	1	1	1	2	1
2	3	1	1	1	2	2	1	1	3
9	9	9	9	9	9	9	9	9	9
3	3	1	1	1	2	1	1	1	2
1	1	1	9	1	9	1	1	1	1
2	2	2	2	2	2	2	1	2	9
2	2	2	2	2	2	2	1	2	2
3	2	1	1	2	3	1	1	1	2
2	2	2	2	2	2	2	2	2	2
2	2	2	1	2	2	2	9	2	2
2	2	2	2	2	1	1	2	2	1
1	1	2	1	2	2	2	4	1	1
1	2	3	1	2	2	3	3	1	2

2	2	2	2	2	2	2	1	2	2
2	2	2	2	2	2	2	9	2	2
9	9	9	9	9	9	9	9	9	9
2	3	3	1	2	3	2	7	1	2
3	3	2	1	2	3	2	1	1	3
3	3	1	1	1	1	3	1	1	1
9	1	3	2	9	3	1	1	1	9
2	2	2	2	2	2	2	1	2	2
2	2	2	2	2	2	2	2	2	2
2	2	2	2	2	3	3	1	2	2
3	3	3	1	1	1	1	1	3	2
1	2	2	1	2	3	2	9	2	3
9	9	9	9	9	9	9	9	9	9
1	1	1	1	1	1	1	1	1	1
3	1	2	2	2	2	1	2	1	2
1	1	1	1	1	1	1	1	1	1
1	1	2	1	2	3	2	1	2	2
9	9	9	9	9	9	9	9	9	9
2	1	2	1	2	2	2	4	1	3
2	2	2	3	2	2	2	1	2	2
9	9	9	1	9	9	9	1	9	9
9	9	9	3	1	3	3	1	3	2
2	1	2	2	2	2	1	9	9	9
9	9	9	9	9	9	9	9	9	9
9	9	9	9	1	9	9	1	1	9
9	9	9	9	2	9	9	1	9	9
3	3	3	1	1	3	2	1	1	1
2	2	3	3	3	1	1	9	3	2
9	9	9	9	9	9	9	1	9	9
2	1	1	2	2	2	1	1	1	1
1	3	1	1	3	1	3	1	1	3
3	3	1	1	1	1	1	1	1	1
3	3	1	1	1	1	1	1	1	2
1	1	1	1	3	3	1	1	3	3
9	9	9	2	2	2	2	9	2	2
3	3	3	1	2	2	2	1	2	2
3	2	1	1	1	1	1	1	1	3
3	3	1	2	1	2	2	1	2	2
2	2	2	1	1	1	1	1	2	2
1	1	1	1	1	3	2	4	1	3
2	1	2	3	1	2	1	9	3	2
3	3	1	2	1	2	1	3	1	3
2	3	2	1	1	1	2	2	2	3
3	3	1	2	3	2	1	1	2	2
3	1	1	1	1	3	1	3	1	3
1	1	1	1	1	1	1	1	1	1
1	1	1	3	3	1	1	1	3	3

2	1	2	1	2	3	1	2	3	3
2	1	1	1	1	2	1	3	1	3
3	3	1	1	1	3	1	3	1	3
3	3	1	1	1	2	1	1	2	1
9	1	1	1	1	2	1	1	2	2
9	1	1	1	1	2	1	9	2	2
3	2	1	1	1	1	1	1	3	3
3	3	1	1	2	2	1	1	3	3
3	2	1	2	2	2	1	1	2	2
1	1	1	1	1	3	1	2	1	1
3	3	1	1	1	1	1	1	1	2
1	1	3	1	2	1	3	1	1	2
3	3	3	3	3	3	3	1	3	3
2	1	1	1	1	2	2	1	1	2
1	2	1	3	1	3	1	1	2	1
3	1	9	1	1	3	1	2	9	9
3	3	1	1	1	1	1	1	1	1
3	3	1	1	1	2	1	1	2	1
3	2	2	3	2	3	2	1	1	2
2	2	2	1	1	9	1	1	1	1
1	2	2	1	1	1	1	1	2	2
2	2	2	2	2	2	2	1	2	2
1	1	1	1	1	1	1	9	1	1
1	1	1	1	2	2	3	1	3	2
1	1	1	1	1	1	1	1	1	1
1	1	1	1	1	1	1	1	3	3
3	3	3	2	1	1	1	1	3	3
2	1	2	3	3	2	1	1	2	3
2	2	2	3	3	3	3	1	1	2
2	2	2	2	2	2	2	1	2	2
1	3	1	1	3	3	3	1	3	3
2	9	9	1	9	9	9	1	9	9
2	2	2	2	2	2	2	1	2	2
2	3	1	3	2	3	1	1	3	3
1	3	1	2	1	1	3	4	3	2
1	2	3	1	3	1	3	1	1	3
2	2	2	2	2	2	2	2	2	2
3	3	3	3	3	3	3	1	3	3
2	2	3	1	1	2	1	1	2	2
2	2	2	1	2	2	2	9	2	2
3	2	3	1	3	3	2	1	2	2
2	3	3	3	3	3	3	2	2	3
3	1	1	1	1	3	1	1	3	3
9	1	1	2	1	2	1	1	2	2
2	2	1	1	1	1	3	1	9	9
2	1	1	1	1	3	2	1	1	2
1	3	1	1	1	2	1	3	1	2

2	2	1	1	1	2	3	1	2	2
9	9	1	1	1	3	1	1	9	9
9	9	1	1	1	9	1	9	9	9
2	2	2	2	1	2	2	1	2	2
9	9	9	9	9	9	1	1	1	9
9	9	9	1	1	1	9	3	1	9
9	9	1	9	9	9	1	7	9	1
1	2	1	1	3	2	1	1	1	1
2	3	1	1	1	1	1	1	1	2
2	2	1	2	1	2	2	1	1	2
2	2	1	1	2	2	1	1	2	2
1	1	2	1	3	2	2	1	2	2
3	3	3	1	2	2	2	1	2	2
3	3	3	1	2	2	2	1	2	2
2	2	2	1	2	1	1	1	2	2
2	2	2	2	2	2	2	1	1	2
3	2	2	1	1	2	2	1	3	3
1	1	1	1	2	2	1	1	2	2
3	3	1	2	2	2	2	2	1	1
2	2	2	1	1	3	2	1	3	1
3	3	1	1	2	2	1	2	2	1
1	1	1	1	1	2	1	1	1	1
2	2	2	2	2	2	2	1	9	9
2	2	2	2	2	2	2	1	2	2
2	2	2	1	1	1	1	1	1	3
1	9	1	1	1	1	1	1	2	2
2	2	2	2	2	2	3	1	3	3
9	9	9	9	9	9	9	9	9	9
3	2	1	1	1	2	1	3	1	2
1	1	2	1	2	2	2	1	1	3
2	2	2	2	2	2	1	2	1	1
1	1	1	1	1	2	1	3	1	1
3	3	1	1	1	3	1	5	1	2
2	2	1	1	3	1	1	1	1	3
3	3	1	1	1	2	1	4	1	3
2	2	1	2	1	3	1	9	9	2
2	2	2	2	2	2	2	1	2	2
2	2	2	1	2	2	2	1	2	2
3	2	1	1	1	2	1	1	2	2
9	9	9	9	9	9	9	9	9	9
3	1	1	2	2	2	1	1	1	3
2	2	3	2	1	2	3	5	1	2
1	2	2	1	2	1	3	1	3	1
1	2	2	1	1	1	1	1	2	2
1	2	1	1	1	2	1	1	1	1
2	2	2	2	2	2	2	1	2	2
2	2	2	1	2	2	2	1	3	1

2	2	1	1	1	1	1	1	2	2
9	9	9	2	9	1	9	1	3	9
2	3	1	1	2	1	1	9	1	9
9	2	1	1	2	3	2	1	1	2
3	2	1	2	2	2	2	1	2	2
3	3	1	2	1	1	1	1	1	3
2	3	2	3	3	3	3	1	3	2
3	1	3	1	1	2	1	1	1	3
3	9	2	9	9	1	9	1	9	3
1	2	1	1	2	2	2	1	1	2
3	2	1	1	2	3	9	9	9	9
3	3	1	1	1	1	1	1	1	3
1	2	9	1	2	2	2	1	1	2
1	1	1	1	1	1	1	1	1	1
2	2	1	1	1	3	2	1	2	2
3	3	1	2	1	2	1	4	1	3
3	1	1	1	1	1	1	1	2	2
3	9	1	1	1	3	1	3	1	1
9	9	9	9	9	9	9	9	9	9
1	2	1	1	1	1	2	1	1	1
1	1	1	1	1	3	1	1	1	3
3	3	1	1	1	3	1	2	1	2
2	2	2	2	3	3	2	1	3	1
1	2	1	2	1	3	1	3	2	1
2	1	1	1	1	1	1	1	3	3
2	1	1	2	1	2	1	1	2	2
3	3	1	1	2	2	1	2	2	2
3	2	1	1	1	3	1	1	1	2
3	1	2	1	2	3	2	1	1	2
2	3	2	1	3	3	2	1	3	1
2	2	2	2	2	2	2	1	2	2
2	1	1	1	2	3	1	4	2	2
3	3	1	1	1	3	1	1	1	3
1	2	3	1	2	3	1	5	1	1
3	3	1	1	1	3	1	4	1	3
3	3	3	1	2	3	1	3	1	1
3	3	3	1	1	3	1	4	2	1
1	1	1	1	1	2	1	1	1	2
3	3	1	1	2	2	1	1	2	2
3	3	1	1	1	2	1	1	2	2
9	9	9	9	9	9	9	9	9	9
1	1	1	1	2	1	2	3	3	2
3	3	1	1	1	3	1	4	1	9
3	3	1	1	1	3	1	1	1	3
3	3	1	1	1	3	1	1	1	2
1	3	1	3	3	1	1	1	3	1
3	3	2	1	1	2	1	3	1	3

3	3	2	1	1	3	1	1	3	3
9	9	1	9	9	9	1	2	1	9
1	2	2	1	1	1	2	1	2	2
3	3	1	1	2	3	3	4	1	3
2	2	2	2	2	2	2	1	2	2
2	2	2	1	1	3	3	1	2	2
3	2	1	2	1	1	1	3	1	2
1	1	1	1	1	1	1	9	1	1
2	2	2	2	2	2	2	9	9	9
1	1	3	1	1	2	2	9	1	2
9	9	9	1	2	2	1	9	9	9
3	1	1	2	2	2	2	1	1	3
1	1	1	1	1	3	1	1	1	3
1	1	1	1	1	3	1	1	2	3
1	2	1	1	1	3	1	1	2	2
3	3	1	1	1	3	1	3	2	3
1	1	1	1	2	9	1	1	3	1
1	1	1	1	1	2	1	1	3	3
3	3	1	1	1	3	1	1	1	1
1	1	1	1	2	2	9	1	1	1
1	1	1	1	1	1	1	2	2	2
1	1	2	3	3	3	3	1	1	3
3	2	1	1	9	3	1	1	9	9
1	2	2	1	1	2	3	1	1	1
1	3	3	2	2	3	3	1	1	2
3	3	1	1	1	2	1	2	9	1
3	3	1	1	1	3	1	1	1	3
1	9	3	3	2	1	3	1	2	1
1	9	1	1	1	2	3	1	1	3
3	3	1	1	1	3	1	1	3	3
2	2	1	2	1	2	1	9	3	3
2	3	2	2	3	2	3	3	1	2
1	3	2	3	2	1	2	3	3	2
2	3	1	1	1	2	1	2	1	3
2	1	1	1	1	3	1	1	1	3
2	1	1	1	1	3	1	1	1	3
2	2	1	1	2	2	1	1	1	2
9	9	1	9	1	1	9	1	1	1
3	3	1	1	1	1	1	1	2	3
3	3	1	1	1	1	1	1	3	2
3	1	1	3	1	2	1	1	1	1
2	2	2	2	3	2	3	4	1	2
2	2	1	1	3	2	1	1	1	2
9	9	9	9	9	9	9	9	1	2
1	1	1	1	2	3	1	3	2	9
2	2	1	2	1	2	2	1	1	1
1	2	1	1	1	3	3	1	2	2

3	3	1	1	1	3	1	1	1	9
9	9	9	9	9	9	9	9	3	3
2	3	1	1	2	1	1	2	1	3
3	3	1	2	1	2	1	1	3	3
2	2	2	2	1	2	2	1	2	2
3	3	1	1	1	3	1	1	1	3
1	1	2	1	2	2	1	9	3	3
3	3	3	3	3	3	3	9	2	2
3	3	3	1	3	3	3	1	2	2
9	9	9	9	9	9	9	9	9	9
2	1	1	3	2	2	3	3	1	3
1	1	1	2	1	3	2	1	1	2
1	3	1	1	3	1	3	4	1	3
1	3	1	1	2	1	2	1	1	2
2	2	1	1	1	3	1	1	2	2
1	1	1	2	1	1	1	1	9	9
2	1	1	1	1	1	1	1	1	9
2	2	2	3	1	2	2	1	9	1
2	1	1	3	2	2	1	1	1	2
2	2	2	2	2	2	1	1	1	2
9	9	9	9	9	9	9	9	9	9
2	2	1	1	2	2	1	1	1	2
2	1	1	2	2	2	1	1	1	1
2	1	1	2	2	1	1	2	2	2
2	1	1	2	2	2	1	1	1	1
2	1	1	2	2	1	1	1	1	1
2	1	1	2	2	2	1	1	1	1
3	3	3	3	2	2	3	1	2	2
2	2	2	3	2	2	1	2	2	2
2	1	1	2	2	2	1	1	1	1
9	9	9	9	9	9	9	9	9	9
9	9	9	9	9	9	9	9	9	9
9	9	9	9	9	9	9	9	9	9
3	3	1	1	1	3	1	1	1	2
3	3	1	1	2	3	1	1	1	2
3	2	1	1	2	2	1	1	2	2
1	1	1	1	1	1	2	1	2	2
3	3	3	1	1	2	1	1	1	2
2	2	1	1	2	2	1	4	2	2
3	1	1	1	2	1	1	1	1	9
1	1	1	1	3	3	1	1	1	3
3	3	1	1	1	1	1	1	1	3
2	2	2	2	2	2	2	1	1	2
3	3	1	1	1	3	1	5	3	1
3	2	1	2	3	1	3	1	2	2
3	3	9	3	3	3	1	1	1	1
1	1	1	1	3	3	3	1	1	3

1	1	1	1	3	2	2	1	2	3
1	1	2	1	1	1	1	1	2	2
3	2	1	2	1	2	2	1	1	3
3	1	3	1	1	1	3	1	3	1
9	9	9	9	9	9	9	1	1	2
2	1	2	2	3	2	2	1	1	2
1	1	1	1	1	2	2	1	1	3
9	9	9	1	9	9	9	1	1	9
9	9	9	1	1	2	2	1	9	9
9	3	1	9	9	9	9	9	1	9
1	9	2	1	2	9	1	1	1	9
9	9	9	1	9	9	9	1	9	1
2	1	1	1	1	2	1	2	1	1
2	2	2	2	2	2	2	2	1	2
3	3	1	1	3	2	1	1	1	3
3	2	1	1	1	2	1	1	1	2
2	1	1	1	1	2	2	1	1	2
2	1	1	1	1	2	2	1	1	2
3	3	3	2	1	2	1	3	1	1
3	2	1	2	3	2	1	1	3	1
3	3	1	2	1	2	1	2	1	3
2	2	1	1	1	2	1	1	2	2
3	3	1	1	1	2	1	5	2	1
2	2	2	1	2	2	1	1	9	9
3	2	3	1	2	1	2	1	1	2
3	1	1	1	1	1	3	5	3	1
2	2	2	2	2	2	2	1	2	2
3	2	1	1	1	2	1	1	2	1
2	2	2	2	1	2	2	1	2	2
3	3	2	1	2	2	2	9	2	3
3	3	3	1	2	2	2	1	2	2
2	1	1	1	1	2	1	1	9	2
2	3	1	1	2	3	1	1	3	2
2	3	1	1	1	3	2	2	1	3
9	3	9	9	9	2	3	1	9	2
3	3	2	1	3	2	2	1	2	2
3	3	1	1	1	2	1	1	2	2
2	3	2	1	1	2	1	1	2	2
3	3	1	2	1	3	1	2	2	1
2	2	2	1	3	1	1	1	1	3
1	3	3	3	3	1	1	1	1	1
9	9	1	9	9	9	1	2	9	9
9	9	1	9	9	9	1	2	9	9
3	3	1	1	2	2	2	1	2	2
1	3	2	1	1	3	1	1	3	1
3	1	1	2	1	2	1	2	3	9
3	2	1	3	3	2	1	1	2	2

2	3	1	2	2	3	1	1	1	3
2	1	1	1	3	2	1	2	3	3
2	1	1	2	1	1	1	1	1	3
1	1	1	1	2	2	2	1	1	2
2	2	2	2	2	2	2	1	1	2
3	3	1	1	1	2	1	2	2	2
2	3	1	1	3	3	2	3	9	1
1	1	1	1	1	2	1	1	1	3
3	3	1	1	1	3	1	5	1	1
1	2	3	2	2	1	2	1	1	3
3	3	1	1	2	3	1	1	1	1
3	1	2	2	1	2	2	1	1	2
3	3	1	1	1	2	1	5	3	1
3	9	1	2	1	3	2	1	9	1
2	2	1	1	1	1	1	1	2	2
2	2	1	3	2	2	2	1	2	2
2	2	2	3	3	2	2	3	1	2
2	2	2	2	2	2	2	1	2	2
1	2	2	2	2	1	1	2	1	1
2	1	1	2	1	1	1	1	2	2
2	1	1	2	1	2	3	1	1	1
2	2	1	2	1	1	1	5	1	2
1	1	1	1	2	3	2	4	3	3
3	3	1	1	1	2	1	1	2	1
3	1	1	1	1	2	2	1	1	1
3	3	1	1	2	3	1	3	2	2
3	3	1	1	1	3	1	2	2	2
3	3	1	1	1	1	1	2	1	1
3	2	1	2	2	2	1	1	2	2
3	3	3	2	2	2	1	3	1	1
9	9	9	9	9	9	9	9	9	9
1	9	9	9	9	9	9	9	1	1
2	1	1	2	2	2	1	2	1	1
2	1	1	2	1	2	1	2	1	2
2	3	2	1	2	2	1	1	1	2
2	1	1	2	2	2	1	2	1	1
2	1	1	2	2	2	1	2	1	1
2	2	2	1	2	1	2	4	2	1
1	3	1	1	1	3	1	1	2	1
3	3	3	1	1	1	1	7	1	1
3	3	1	1	1	1	1	2	1	3
3	3	1	1	3	3	1	1	2	1
1	2	1	1	1	2	1	1	1	2
3	3	1	3	1	1	1	1	1	3
2	3	1	1	1	1	1	5	1	2
2	2	2	2	2	2	1	1	2	2
2	1	1	1	1	3	1	1	1	3

2	1	1	1	1	3	1	1	1	3
2	1	2	1	2	2	2	2	1	1
3	3	1	2	1	2	2	7	1	1
2	1	1	2	2	1	1	2	1	1
2	1	1	2	2	2	1	2	1	2
2	1	1	1	2	2	2	1	1	1
2	1	1	2	2	2	1	2	1	1
2	2	2	2	2	2	2	1	1	2
1	1	1	1	2	1	2	3	3	2
1	1	1	1	1	3	1	2	1	3
3	3	1	1	1	3	1	2	1	3
2	1	1	1	1	2	3	1	1	1
1	1	1	1	1	1	1	7	3	3
2	2	2	1	1	2	1	1	1	2
3	1	1	1	3	3	1	1	1	3
3	3	1	2	2	2	1	1	3	2
3	3	2	2	2	1	1	1	3	3
2	1	1	2	2	2	1	1	1	1
1	1	1	1	1	1	1	1	3	2
3	3	1	1	1	2	1	3	1	3
3	1	1	1	1	2	1	3	1	1
1	1	1	3	1	3	1	2	1	3
2	3	1	1	1	3	2	1	2	3
1	1	1	1	1	2	2	1	1	3
2	2	1	1	1	1	2	2	1	2
1	3	1	3	1	3	1	2	1	3
3	3	2	2	1	2	1	1	1	3
9	2	1	1	1	1	1	1	3	3
3	3	1	3	3	3	1	1	3	3
3	3	1	1	1	3	1	2	3	1
3	3	3	3	3	3	3	1	3	3
3	3	1	1	1	3	1	4	1	3
2	2	2	2	2	2	2	1	1	2
3	3	3	1	2	1	1	1	3	3
2	1	2	1	2	3	1	1	1	2
2	2	2	2	1	2	2	1	2	2
2	2	3	1	1	3	1	1	1	3
3	3	2	1	3	3	1	2	1	2
3	3	2	1	3	2	1	4	3	2
3	3	1	1	1	2	1	4	1	1
3	2	1	1	1	3	1	2	1	3
3	3	1	1	1	2	1	2	2	1
3	3	1	2	1	3	1	2	2	2
3	3	1	1	3	2	1	4	2	2
3	3	1	1	2	2	1	1	2	2
3	2	1	1	2	1	3	1	1	2
3	2	2	2	2	3	1	1	2	2

2	3	1	1	1	3	1	1	3	2
1	1	1	1	1	2	1	1	1	2
2	1	2	3	2	1	1	2	2	3
3	2	1	1	2	9	1	2	1	3
3	3	1	1	1	3	3	1	3	3
2	2	2	2	2	2	2	1	1	1
1	2	1	1	1	2	1	1	1	3
1	2	1	1	2	3	2	1	1	1
3	3	1	1	2	3	3	2	9	9
2	2	2	2	2	2	2	1	2	2
1	1	1	1	1	3	1	1	2	2
1	1	1	1	2	3	1	2	1	3
3	1	1	1	1	1	1	1	1	1
2	2	1	2	2	2	2	1	2	2
2	2	3	1	1	1	9	2	1	1
3	3	1	1	1	2	1	3	1	1
2	2	2	1	2	2	2	1	3	1
2	2	2	1	3	1	1	1	2	2
1	2	3	1	2	3	2	2	1	2
3	3	1	2	2	2	2	1	2	2
3	2	1	1	2	2	2	2	1	2
2	2	1	1	2	2	1	2	2	2
2	1	1	2	2	1	1	2	1	2
1	2	1	2	2	1	2	1	1	2
1	2	1	2	2	1	2	1	1	2
3	3	1	1	1	2	1	3	1	1
1	1	1	1	2	3	1	2	1	3
2	2	2	2	2	2	2	1	2	2
3	3	2	1	3	2	1	4	3	2
3	3	1	2	2	2	2	1	2	2
2	2	3	1	1	3	1	1	1	3
9	9	9	9	9	9	9	9	9	9
2	2	2	1	2	2	1	1	1	2
3	3	1	1	1	3	1	1	3	3
3	1	1	1	1	1	1	1	1	1
1	1	1	1	3	3	2	1	3	3
3	3	1	1	1	1	1	1	3	1
2	3	1	1	2	3	1	9	1	2
3	3	1	2	1	2	1	3	2	3
1	2	3	1	2	3	1	1	1	2
1	1	1	1	2	1	1	1	9	9
1	1	1	9	2	1	1	1	1	3
1	1	1	1	1	3	1	1	3	1
2	2	1	2	1	3	1	1	2	2
2	1	2	2	3	2	2	1	2	3
3	3	1	1	1	2	1	1	1	9
1	3	1	2	3	3	1	1	3	3

1	1	2	1	2	2	1	1	2	1
3	3	1	2	1	3	1	1	1	3
1	1	1	1	1	1	1	1	1	1
1	3	1	3	1	2	1	1	2	2
3	3	1	2	2	2	1	1	2	2
3	3	1	1	2	2	1	5	1	3
3	1	1	1	1	1	1	1	1	2
3	3	3	1	1	2	2	2	2	2
2	2	2	2	2	2	2	3	2	3
2	2	2	3	3	3	3	2	2	2
3	3	3	2	2	2	2	2	2	2
2	2	3	3	2	2	1	1	2	2
2	2	2	2	2	2	2	2	2	2
2	2	2	1	2	2	2	2	2	2
3	1	3	3	3	3	3	1	2	2
2	1	1	3	2	1	3	1	2	3
1	2	3	1	2	2	3	2	1	2
1	2	1	3	3	1	3	2	2	2
1	2	1	3	2	2	1	1	1	2
1	2	1	1	2	1	3	1	1	1
2	1	1	1	2	2	2	2	2	1
2	2	1	2	2	2	2	2	2	2
3	3	3	2	2	2	2	2	2	2
1	1	1	1	1	1	1	9	9	9
1	1	3	1	3	2	1	2	1	1
3	1	1	1	1	1	1	2	3	2
1	3	1	2	1	3	1	1	2	1
3	3	1	1	1	3	1	1	1	1
3	3	1	1	1	2	1	1	1	1
3	3	1	1	1	3	1	1	1	1
3	1	1	2	1	2	1	1	1	1
2	2	2	3	3	2	2	2	2	2
1	9	1	1	2	3	2	2	1	3
3	3	1	1	1	1	1	9	9	9
3	3	1	1	1	3	1	3	1	1
2	1	2	1	2	3	2	1	2	3
1	1	1	2	1	3	1	1	2	1
2	2	2	1	2	2	1	2	3	2
3	3	1	1	2	2	1	3	1	1
3	3	1	1	2	2	1	9	9	9
3	1	1	1	1	2	1	1	9	1
9	1	2	1	2	1	1	1	2	1
3	3	2	3	3	3	3	3	2	3
2	1	1	1	1	3	1	1	3	1
3	2	1	1	1	3	1	1	2	1
3	3	1	1	1	2	1	3	1	1
3	1	3	1	2	2	3	1	2	2

2	2	1	2	2	2	2	9	9	9
9	9	9	9	9	9	2	9	9	9
9	9	9	9	9	9	9	9	9	9
2	2	2	1	2	2	1	2	2	2
2	2	1	1	1	2	1	2	1	2
9	9	9	9	9	9	9	9	9	9
2	1	1	1	2	2	1	1	1	2
1	1	1	1	1	2	1	1	1	2
1	1	1	2	2	1	1	1	1	2
3	2	1	1	1	2	1	1	1	2
3	3	1	3	1	9	1	3	1	1
3	3	1	3	1	9	1	3	1	1
1	2	1	3	2	1	2	3	2	3
3	3	1	1	1	1	1	2	2	2
1	1	3	1	1	1	1	1	9	1
3	1	2	1	3	1	1	2	1	3
9	9	9	2	9	9	9	9	1	9
2	2	1	1	2	2	2	1	2	2
1	2	2	2	2	1	1	3	3	3
1	1	2	1	1	2	2	1	1	2
2	1	1	1	2	2	2	1	1	2
2	2	2	1	2	2	2	1	2	2
3	3	2	2	2	3	1	1	2	2
2	3	1	3	1	2	1	2	3	2
2	2	1	1	2	2	1	2	2	2
3	3	1	3	3	3	3	9	9	9
3	2	2	2	2	2	2	2	2	2
2	1	1	2	2	2	3	2	2	1
2	1	1	2	2	1	1	1	1	2
2	2	2	1	2	2	2	1	2	2
2	1	2	1	2	3	2	1	2	3
3	3	1	2	2	2	2	1	3	2
1	3	1	1	1	1	3	3	1	1
1	2	1	1	2	3	2	1	2	2
1	1	1	1	1	1	1	2	2	1
3	3	1	3	1	1	1	3	3	1
9	9	9	9	9	9	9	9	9	9
2	3	1	1	3	3	1	3	1	1
9	9	1	9	9	9	1	1	2	1
2	3	1	2	1	3	3	2	2	2
1	1	9	1	3	3	3	3	3	1
2	2	1	1	1	1	2	2	2	2
1	1	1	1	2	2	3	1	3	2
2	2	2	2	2	1	2	1	1	2
2	2	1	1	1	2	1	3	3	3
2	2	1	2	2	2	1	1	1	2
1	1	1	1	1	3	3	2	3	1

9	9	9	9	9	9	9	9	9	9
3	3	1	1	1	1	1	1	2	1
1	2	2	2	2	1	2	2	2	2
3	1	1	2	1	3	1	1	3	1
1	2	3	3	3	2	2	3	3	2
3	3	2	1	1	2	1	2	2	2
1	1	3	1	2	3	1	2	1	3
1	1	1	1	1	1	1	3	1	1
2	2	1	1	1	1	2	1	2	2
1	1	2	2	2	2	1	2	1	1
1	2	3	1	2	2	2	1	2	2
1	1	1	1	1	2	1	1	2	1
2	2	1	2	2	2	1	1	2	2
3	3	3	1	3	3	1	1	3	3
3	2	1	1	1	2	1	2	3	1
3	2	1	1	1	2	1	3	3	1
1	1	1	1	2	2	3	1	3	2
2	1	1	1	2	2	1	1	1	2
2	1	1	1	2	1	1	1	1	1
3	3	1	1	1	2	1	1	1	1
3	3	1	1	2	1	1	1	3	2
3	3	1	1	1	1	1	1	3	2
2	1	1	1	2	1	1	1	2	2
3	3	3	1	1	3	1	1	1	2
3	3	1	1	9	9	9	1	3	1
1	1	1	1	2	2	1	3	1	2
3	1	1	1	1	1	1	3	3	1
3	3	1	2	1	2	1	1	2	1
2	1	1	2	2	1	1	1	1	1
2	2	2	2	2	2	2	1	2	2
2	2	2	2	2	2	2	2	2	2
3	3	1	1	1	3	1	1	3	1
2	1	1	1	1	3	1	1	3	1
2	1	1	1	2	2	1	1	1	1
2	1	2	1	1	2	2	1	2	1
1	3	1	1	1	3	2	1	3	1
2	1	1	1	2	1	1	2	2	1
1	2	1	1	1	2	1	3	3	2
2	2	3	1	2	1	3	3	3	2
1	1	1	2	2	1	2	1	9	1
3	2	1	1	1	3	1	1	2	1
2	3	3	1	2	3	2	1	2	2
2	2	2	3	3	3	3	2	2	2
2	1	1	1	1	2	1	1	1	2
9	9	9	9	9	9	9	3	3	9
1	1	1	1	1	1	1	3	1	1
1	1	1	1	1	2	1	2	2	1

9	9	9	9	9	9	9	9	9	9
2	1	1	1	2	2	3	1	2	3
2	1	1	2	2	1	1	1	2	2
3	3	1	1	1	3	1	2	1	1
3	1	1	1	1	2	2	1	3	3
1	1	1	1	1	2	1	1	3	1
1	3	1	1	1	1	2	1	3	1
3	3	3	3	3	3	3	3	3	3
1	1	1	3	3	3	3	3	3	3
2	2	2	1	2	1	1	3	2	2
1	3	3	1	2	1	2	2	3	1
1	2	3	1	3	1	2	2	3	2
9	9	9	1	2	3	1	2	9	2
1	3	2	1	2	3	1	1	9	2
1	1	1	1	1	3	1	1	9	9
9	9	9	1	3	3	2	2	2	2
2	1	1	2	1	1	3	1	2	1
3	3	1	1	1	2	1	1	3	1
2	2	1	1	2	2	1	2	2	2
2	2	2	3	3	3	3	1	1	1
2	1	1	2	2	1	2	1	1	2
2	2	1	1	2	1	1	1	1	2
1	2	1	1	2	2	1	1	1	2
2	1	1	1	2	1	1	1	2	2
3	1	1	2	1	3	1	1	3	9
1	1	1	1	3	1	1	2	3	3
9	9	9	9	1	9	9	2	2	2
3	3	2	1	2	2	1	1	3	1
1	1	1	2	1	3	3	3	3	3
2	2	1	2	2	1	2	1	3	2
3	3	3	1	1	1	1	3	3	1
3	1	1	2	1	3	2	1	3	1
3	3	1	2	1	3	1	1	3	1
3	2	1	2	1	1	1	3	3	3
9	9	9	9	9	9	9	1	2	1
1	2	1	1	1	2	1	1	1	2
2	2	2	2	2	2	2	1	2	1
2	2	2	2	1	1	1	1	1	1
2	2	2	2	2	2	2	1	1	2
1	1	1	1	1	1	1	1	1	1
2	2	2	1	2	2	2	1	2	2
1	1	1	3	3	3	1	3	3	3
1	1	1	3	3	3	3	2	2	2
2	3	1	1	1	3	1	1	1	1
3	3	3	1	1	1	1	1	3	1
2	2	2	3	3	3	3	2	2	2
2	1	1	2	2	2	2	2	2	1

2	2	2	2	2	2	2	1	3	2
2	2	2	3	1	1	2	1	2	2
9	9	9	9	9	9	9	9	9	9
9	9	9	9	9	9	9	9	9	9
2	1	1	1	1	2	1	1	2	1
2	1	1	2	2	1	1	1	1	2
1	9	9	9	3	9	9	9	9	9
3	3	1	1	1	3	1	3	1	2
3	3	1	1	1	1	1	1	1	1
3	3	1	1	1	2	1	1	9	9
9	9	9	9	9	9	1	9	9	9
9	9	9	9	9	9	9	9	9	9
1	1	1	1	1	1	2	2	2	2
3	1	1	9	9	9	9	9	9	9
2	3	1	2	1	2	2	1	3	2
9	9	9	9	1	9	9	9	9	9
9	9	9	9	2	9	9	1	9	9
9	9	9	9	2	9	9	1	9	9
1	2	2	1	2	2	2	2	2	2
2	2	1	2	2	1	1	2	1	2
9	9	9	9	3	9	9	1	9	9
2	2	2	3	3	3	3	2	2	2
3	3	3	3	3	3	3	3	3	3
1	2	2	1	2	2	2	2	1	1
3	3	1	2	1	2	2	1	2	1
2	2	2	3	3	2	2	2	2	2
3	1	1	2	1	2	3	1	3	3
2	2	9	1	2	2	2	9	9	9
3	1	1	2	1	2	2	1	2	1
1	3	2	1	1	2	1	3	1	1
2	2	2	2	2	2	2	2	2	2
3	3	3	2	3	2	2	2	1	1
2	2	2	2	2	2	1	3	2	1
2	2	2	1	2	2	3	1	1	1
3	3	3	3	3	3	3	3	3	3
1	3	1	1	1	1	1	1	3	1
3	2	3	1	2	2	2	1	2	2
3	3	1	1	1	3	1	2	2	2
9	9	9	1	9	9	9	9	9	9
1	1	1	1	1	1	3	2	2	1
1	1	3	3	1	3	3	3	1	3
2	2	2	1	1	2	3	3	1	1
2	1	3	2	1	2	2	2	3	1
1	1	1	1	1	2	1	1	2	2
1	2	3	2	1	3	2	2	1	2
1	2	2	2	1	2	2	1	1	1
1	2	2	2	3	1	1	1	2	2

1	3	1	2	3	2	1	1	3	2
1	1	1	1	2	3	1	1	2	1
1	1	3	9	9	2	9	2	2	2
2	2	2	3	2	2	2	1	2	3
1	1	2	1	1	3	1	1	3	1
9	9	9	9	9	9	9	9	9	9
3	3	1	1	1	3	1	1	2	1
3	1	1	1	1	3	1	3	3	3
3	3	3	1	1	1	1	1	3	1
3	3	1	1	1	3	1	1	1	2
3	3	1	3	1	2	1	1	1	2
1	2	3	2	3	2	1	2	2	2
2	3	3	1	1	3	1	1	3	3
1	2	2	2	3	2	2	2	2	2
1	2	2	1	2	3	2	2	2	2
3	2	2	2	2	2	2	2	1	2
2	1	2	1	1	1	1	2	2	2
9	9	9	1	9	9	9	2	9	9
9	9	9	2	9	9	9	2	9	9
2	2	2	2	2	2	2	2	2	2
9	3	1	9	1	3	1	2	9	2
9	2	2	2	2	2	2	2	9	9
9	1	1	1	9	1	1	9	9	9
9	1	9	9	2	1	9	9	9	9
9	2	2	2	2	2	2	9	2	9
9	9	9	9	9	9	9	9	9	9
9	9	9	9	9	9	9	9	9	9
2	2	2	2	2	2	2	2	2	2
2	2	2	2	2	2	2	2	2	2
2	1	1	1	1	1	2	1	1	1
2	2	2	2	2	2	2	1	1	2
2	2	2	2	9	2	2	9	9	9
2	2	2	2	2	2	2	2	2	2
9	9	9	9	9	9	9	9	9	9
9	9	9	9	9	9	9	9	9	9
2	2	1	1	1	2	2	1	2	2
9	9	9	9	2	9	9	9	2	9
9	9	9	9	9	9	9	9	9	9
9	9	9	9	9	9	9	9	9	9
9	9	9	9	9	9	9	9	9	9
9	1	2	1	3	2	1	9	9	1
9	1	9	9	9	1	9	9	1	9
9	9	9	9	9	9	9	9	9	9
9	9	2	2	9	9	2	9	2	9
2	9	9	9	1	9	9	9	9	9
9	9	9	3	9	9	9	3	9	9
9	9	1	2	9	9	1	1	9	9

9	9	9	1	2	2	2	9	9	9
3	3	3	3	3	3	3	3	3	3
1	2	3	1	2	2	1	1	2	3
1	2	3	2	1	2	1	2	1	2
3	9	3	1	1	1	2	1	1	2
9	9	9	1	9	9	9	1	9	9
9	9	9	1	9	9	9	2	9	9
9	9	9	9	1	9	9	2	9	9
1	3	3	2	2	3	1	3	2	1
3	1	2	3	1	2	3	2	3	1
1	1	2	2	3	3	2	2	2	2
2	2	2	1	1	1	2	2	2	2
2	3	2	2	1	1	1	2	3	1
1	1	2	1	2	3	1	1	3	1
9	9	9	9	9	9	9	2	3	2
3	3	3	3	3	3	3	3	3	3
9	9	9	9	9	9	9	9	9	9
9	9	9	9	9	9	9	9	9	9
9	9	9	1	9	9	9	9	1	9
9	9	9	9	2	9	9	9	2	9
1	1	1	1	1	1	1	1	1	1
2	3	3	1	1	1	1	1	2	1
9	9	9	9	9	9	9	9	9	9
2	3	2	1	1	1	1	1	2	2
1	1	1	1	1	1	1	1	1	1
1	3	3	1	1	1	2	1	2	2
1	1	3	1	3	1	2	3	3	3
3	2	1	1	1	2	1	2	2	2
2	2	3	1	1	2	3	3	3	2
2	2	2	2	2	2	2	2	2	2
2	2	2	1	2	1	2	2	2	2
2	2	2	1	1	1	1	2	2	2
2	2	2	2	2	2	2	2	2	2
2	3	1	1	2	3	1	1	2	1
3	2	3	3	3	3	3	2	2	2
1	2	1	2	1	2	2	2	3	2
2	2	2	2	2	2	2	2	2	2
2	2	2	2	2	2	2	2	2	2
3	3	2	2	2	3	1	2	3	2
2	2	2	2	3	3	3	2	2	3
1	3	2	2	3	1	3	1	1	1
2	2	2	1	2	3	9	1	2	3
9	9	9	9	9	9	9	9	9	9
2	2	2	9	1	3	2	2	2	2
3	3	3	3	3	3	3	3	3	3
3	3	3	3	3	3	3	3	3	3
9	9	9	2	9	9	9	2	9	9

2	2	1	2	2	1	1	2	1	2
2	9	9	9	2	9	9	9	9	9
2	2	2	1	1	1	1	1	1	1
1	2	2	1	2	2	2	1	2	2
2	2	2	2	2	2	2	2	2	2
1	2	1	2	1	2	2	2	2	2
3	1	2	2	3	1	3	9	9	9
9	9	9	2	9	9	9	1	9	9
9	9	9	9	1	9	9	9	9	1
9	9	9	9	2	9	9	3	9	9
9	9	9	9	9	2	9	9	9	1
1	1	1	1	1	1	1	1	1	1
9	9	9	3	9	9	9	2	9	9
9	9	9	1	9	9	9	9	9	9
1	2	2	1	1	2	2	2	2	2
9	9	9	1	9	9	9	2	9	9
9	9	9	9	2	9	9	9	2	9
9	9	9	9	9	2	9	9	9	9
9	9	9	2	9	9	9	2	9	9
9	9	9	9	2	9	9	9	9	9
9	9	9	1	9	9	9	1	9	9
9	9	9	9	9	9	9	9	9	9
9	9	9	9	2	9	9	9	9	9
3	1	2	3	1	2	1	2	1	2
2	1	1	1	1	2	2	1	1	2
2	2	2	2	2	2	2	2	2	2
3	3	1	1	2	3	1	2	1	1
3	2	1	1	1	3	1	1	2	3
2	3	1	1	1	1	1	1	2	1
2	1	2	2	2	3	1	2	3	1
3	3	1	2	2	3	3	2	2	2
3	3	3	3	3	3	3	2	2	2
1	1	1	1	3	3	2	2	2	2
3	3	1	1	1	1	1	1	3	1
1	3	2	1	1	2	3	1	2	2
1	2	1	1	1	2	1	1	1	1
1	2	2	1	2	3	1	1	2	2
2	2	2	1	1	2	1	1	2	1
2	2	3	2	2	2	2	1	1	1
2	1	1	1	1	1	1	1	1	1
9	9	9	9	9	9	9	9	9	9
3	3	2	1	2	3	2	2	2	2
9	9	9	9	9	9	9	9	9	9
2	2	2	2	2	2	1	1	1	1
2	1	2	3	3	3	3	3	3	3
2	2	2	2	2	2	2	2	2	2
3	3	1	1	1	2	1	2	2	1

3	3	1	1	3	3	1	1	1	1
3	3	1	1	3	3	1	1	1	2
3	3	1	2	1	3	1	1	2	1
2	2	2	2	2	2	2	2	2	2
2	2	1	1	1	2	1	1	2	3
2	2	2	2	2	2	2	2	2	2
2	2	3	2	3	2	3	2	1	3
9	9	9	9	1	9	9	2	2	2
2	2	2	1	9	9	9	3	3	2
2	3	2	1	2	9	3	2	2	1
3	3	3	3	3	3	3	3	3	2
9	9	9	2	9	9	9	3	3	3
3	3	1	1	1	1	1	1	1	1
3	3	1	1	1	2	1	1	1	1
9	9	9	9	9	9	9	9	9	9
3	3	1	1	1	2	2	2	2	1
2	3	1	1	1	3	1	1	1	1
9	9	9	1	9	9	9	9	9	9
9	9	9	1	9	9	9	9	9	9
3	3	3	3	3	3	3	1	9	1
9	9	1	1	1	2	1	9	9	9
2	2	2	2	1	2	2	2	3	2
9	9	9	9	9	9	9	9	9	9
9	9	9	9	9	2	9	9	9	2
9	9	9	9	1	9	9	9	9	9
1	2	2	3	2	3	2	2	2	3
2	2	2	2	2	2	2	2	2	2
1	1	1	1	3	1	2	2	2	2
2	2	1	2	1	3	2	2	1	2
9	9	1	1	9	9	9	9	9	9
3	3	1	1	1	2	2	1	9	9
1	1	1	1	2	2	1	2	2	1
1	1	1	1	1	1	1	1	1	1
2	3	1	1	2	2	3	1	2	3
2	2	2	3	3	3	3	1	2	2
1	9	9	9	2	9	9	2	9	9
2	2	2	2	2	2	2	2	2	2
2	3	1	2	3	1	2	1	2	3
2	1	1	2	3	1	1	3	1	2
2	1	1	1	2	2	2	1	1	1
1	1	1	1	1	3	1	1	3	1
1	2	3	2	1	3	2	9	9	9
9	9	9	9	9	9	9	9	9	9
9	9	9	9	9	9	9	9	9	9
1	2	2	2	3	1	3	3	2	1
2	1	2	1	2	1	2	2	1	2
1	9	1	1	2	1	2	9	1	1

3	3	3	1	2	3	2	1	1	1
3	3	1	1	3	2	2	3	3	3
2	1	2	1	2	3	1	1	3	2
2	2	2	3	3	3	3	2	2	2
2	1	2	1	1	1	2	1	2	2
2	2	2	2	2	2	2	2	2	2
3	3	3	1	2	2	2	1	1	1
2	2	1	2	1	2	1	1	2	1
9	9	9	9	2	9	9	9	2	9
9	9	9	9	9	9	1	9	9	9
1	1	9	1	2	2	1	9	9	9
3	3	3	1	3	2	2	3	2	2
2	2	2	2	2	2	2	2	9	2
2	2	2	2	2	2	2	2	2	9
9	9	9	9	9	9	9	9	9	9
9	9	9	9	9	9	9	9	9	9
2	2	2	2	2	2	2	2	2	2
2	2	2	2	2	2	2	9	9	9
2	2	2	2	2	2	2	2	2	2
9	9	9	9	9	9	9	9	9	9
2	2	2	2	9	9	9	2	2	2
9	9	9	2	9	9	9	2	9	9
3	3	3	3	3	3	3	3	3	3
9	9	9	1	9	9	9	1	9	9
9	9	2	2	9	9	2	2	9	9
9	9	2	2	2	2	2	2	9	9
9	9	9	9	9	9	9	9	9	9
9	9	9	2	9	9	9	2	9	9
2	2	2	2	2	2	2	2	2	2
9	9	9	9	9	9	9	9	9	9
9	9	9	9	9	9	9	9	9	9
9	9	9	9	9	9	9	9	9	9
9	9	9	9	9	9	9	9	9	9
3	2	2	2	2	2	2	3	3	2
3	3	3	1	1	3	1	1	3	1
9	9	9	9	9	9	9	9	9	9
2	9	2	2	2	2	2	2	9	9
2	2	2	2	2	2	2	2	9	2
9	1	9	1	9	1	1	1	9	9
9	9	9	9	9	9	9	9	9	9
3	3	3	3	3	3	3	2	2	2
9	9	9	9	9	9	9	9	9	9
9	9	9	1	9	9	9	1	9	9
2	2	2	2	2	2	2	9	9	9
3	3	1	1	2	2	3	1	1	1
2	3	1	1	3	2	3	2	1	2
9	9	9	1	9	9	9	2	9	9

1	2	9	9	9	9	9	9	9	9
2	3	1	1	3	3	2	2	1	3
2	3	1	1	2	3	2	1	2	1
2	3	1	3	3	3	3	3	3	3
1	3	3	1	2	1	2	1	2	2
3	1	1	1	1	2	2	2	1	1
9	9	9	9	9	9	1	9	1	9
2	1	1	1	2	2	3	2	1	2
1	1	1	1	1	2	2	2	2	2
1	1	1	1	1	3	1	1	1	1
2	2	2	2	1	2	2	2	2	3
2	1	2	2	2	1	3	2	3	1
3	3	3	1	1	1	1	3	3	3
1	1	1	2	2	1	2	2	3	3
3	3	1	3	2	2	1	2	2	2
1	1	1	1	1	2	1	2	2	1
2	2	2	2	2	2	2	2	2	2
2	2	2	1	2	3	9	3	2	9
3	3	1	1	2	3	1	1	3	2
2	2	2	2	2	2	2	2	2	2
9	9	9	9	2	9	9	1	9	9
2	2	1	1	2	1	1	1	2	1
3	2	1	1	1	2	1	3	1	1
1	3	1	1	1	2	3	1	2	2
1	9	9	1	1	3	1	3	1	1
2	2	2	2	2	2	2	2	2	2
3	1	1	1	2	3	3	1	1	2
1	1	1	1	1	1	1	1	9	1
3	3	3	1	1	3	3	1	3	3
9	9	9	1	9	9	9	9	1	9
9	9	1	9	9	9	1	9	9	1
1	1	1	1	1	1	1	1	1	1
9	1	9	9	1	9	9	9	9	2
3	1	1	1	1	3	2	1	1	1
9	9	9	9	9	9	9	9	9	9
9	9	9	9	9	9	1	9	9	9
3	1	2	1	3	2	2	2	3	2
3	2	3	2	3	2	1	3	2	3
9	9	9	9	9	9	9	9	9	9
9	9	9	1	9	9	9	1	9	9
2	2	1	1	2	1	1	1	1	2
9	9	9	9	9	9	9	9	9	9
2	3	2	3	3	2	2	2	3	2
2	1	1	1	1	1	1	1	1	1
2	2	2	2	2	2	2	2	2	2
2	2	1	2	2	2	2	1	1	1
3	3	1	2	2	2	1	2	1	2

2	2	2	2	2	2	2	2	2	2
3	2	3	2	3	2	2	2	2	2
3	3	3	3	3	3	3	2	2	2
2	2	2	1	2	3	3	2	2	2
2	2	2	1	2	2	2	2	2	9
3	2	2	1	9	2	2	2	3	1
9	9	9	9	9	9	1	9	9	9
1	1	1	1	2	2	1	2	2	2
3	1	1	1	1	2	1	1	3	2
9	9	1	9	9	9	1	9	1	9
3	3	1	1	3	2	2	1	9	9
9	9	9	9	9	9	9	9	9	9
9	9	9	9	9	9	9	9	9	9
2	2	2	2	2	2	2	1	9	1
3	3	3	2	2	2	1	2	2	2
2	2	1	1	1	2	1	3	1	1
1	1	1	1	2	2	2	1	2	1
2	2	1	1	1	2	1	9	1	1
3	3	1	1	2	2	1	1	3	1
2	2	2	2	2	2	2	2	2	2
2	1	1	1	3	3	3	2	2	1
3	3	3	3	3	3	3	9	9	9
9	9	9	9	9	9	9	9	9	9
9	9	9	9	9	9	9	9	9	9
9	9	9	1	9	9	9	1	9	9
9	9	9	2	9	9	9	2	9	9
9	9	9	9	9	9	9	9	9	9
9	9	9	9	9	9	9	9	9	9
2	2	2	2	2	2	2	2	2	2
9	9	9	2	9	9	9	2	9	9
9	9	9	9	9	9	9	9	9	9
9	9	9	3	9	9	9	3	9	9
9	9	9	9	9	9	9	9	9	9
1	1	1	1	1	1	1	1	9	1
9	2	2	2	9	2	9	2	9	9
9	9	3	3	9	9	3	3	9	9
9	9	9	9	9	9	9	9	9	9
2	2	2	2	2	2	2	2	2	2
9	9	9	9	9	9	9	9	9	9
9	9	3	3	3	3	3	9	9	9
1	1	1	1	1	9	1	9	9	9
9	9	9	9	9	9	9	9	9	9
9	9	9	2	9	9	9	2	9	9
9	9	9	9	9	9	9	9	9	9
9	2	3	1	2	3	2	9	9	9
9	9	9	1	9	9	9	1	9	9
2	2	2	2	2	2	2	2	2	2

9	9	9	2	9	9	9	2	9	9
9	9	9	2	9	9	9	9	9	9
9	9	9	2	9	9	9	2	9	9
9	9	9	9	9	9	9	9	9	9
2	2	2	2	2	2	2	9	9	9
9	9	9	9	9	9	9	9	9	9
9	9	9	9	9	9	9	9	9	9
2	2	2	9	9	9	9	9	9	9
9	2	9	2	9	9	9	3	9	9
2	9	9	2	9	9	9	9	9	9
9	9	9	9	9	9	9	9	9	9
9	9	9	9	9	9	9	9	9	9
9	9	9	9	9	9	9	9	9	9
9	9	9	1	9	9	9	9	9	9
9	9	9	9	9	9	9	9	9	9
9	9	9	9	9	9	9	9	9	9
9	9	9	9	9	9	9	9	9	9
9	9	9	9	9	9	9	9	9	9
9	9	9	9	9	9	9	9	9	9
9	9	9	9	9	9	9	9	9	9
2	2	2	2	2	2	2	9	9	9
2	2	2	2	2	2	2	9	9	9
2	2	9	2	2	2	2	9	9	9
2	2	2	2	2	2	2	2	2	2
2	2	9	2	9	9	9	9	9	9
2	2	2	2	2	2	2	2	2	2
9	9	9	9	9	9	9	9	9	9
9	9	9	1	9	9	9	2	9	9
9	9	9	2	9	9	9	9	9	9
2	2	9	2	2	2	2	9	9	9
2	2	2	2	2	2	2	9	9	9
9	9	1	9	9	9	1	9	9	9
9	9	9	9	9	9	9	9	9	9
9	3	2	1	2	2	2	2	2	9
9	9	9	9	9	9	9	9	9	9
9	9	9	9	9	9	9	9	9	9
1	1	1	1	1	1	1	1	1	1
1	2	2	1	2	3	2	1	3	3
9	9	9	9	9	9	9	9	9	9
2	2	2	2	1	1	1	2	2	2
9	9	9	9	9	9	9	9	9	9
1	9	9	1	9	9	9	9	9	1
9	9	2	2	9	2	2	9	9	9
2	2	2	2	2	2	2	2	9	9
1	1	1	1	1	1	1	1	1	1
9	9	3	3	9	9	3	3	9	9
9	9	9	9	9	9	9	9	9	9

9	9	9	9	9	9	9	9	9	9
2	2	2	2	2	2	2	2	2	2
9	9	9	1	1	9	9	2	9	9
1	9	1	1	9	1	9	1	9	1
9	9	2	2	9	9	2	2	9	9
9	9	2	2	9	2	2	9	9	9
9	9	9	2	9	9	9	2	9	9
9	9	9	9	9	9	9	9	9	9
2	1	3	1	3	9	2	2	9	9
9	9	9	3	9	9	9	9	9	9
9	9	9	9	9	9	9	9	9	9
9	9	9	9	9	9	9	9	9	9
9	9	9	2	9	9	9	2	9	9
9	9	9	2	9	9	9	2	9	9
2	9	9	2	9	2	9	2	9	9
9	9	9	2	2	2	2	2	2	2
9	9	9	9	9	9	9	9	9	9
9	9	9	9	9	9	9	9	9	9
9	9	9	9	9	9	9	9	9	9
9	9	9	9	9	9	9	9	9	9
3	3	1	3	2	1	1	1	2	2
2	1	1	1	2	2	1	1	1	1
9	9	9	2	9	9	9	2	9	9
2	2	1	2	2	2	2	2	2	2
2	2	2	2	2	2	2	2	2	2
2	2	2	2	2	2	2	2	9	9
9	9	9	1	9	9	9	9	1	9
3	9	9	9	9	1	9	9	9	9
3	2	3	1	1	3	1	1	2	1
1	2	3	1	2	3	1	1	2	3
3	1	1	1	2	3	2	1	1	1
3	3	1	2	2	3	1	1	3	1
3	3	1	1	1	3	1	3	3	3
3	2	3	1	1	3	9	1	2	2
3	2	1	1	1	2	2	9	1	2
3	1	9	1	1	2	1	1	1	1
9	9	9	9	9	9	9	1	1	1
9	9	9	1	9	9	9	1	9	9
9	9	9	1	9	9	9	9	1	9
9	1	9	9	1	9	9	3	9	9
9	9	9	1	9	9	9	2	9	9
9	9	9	9	9	9	9	9	9	9
9	9	9	9	9	9	9	9	9	9
2	9	2	2	9	2	9	9	2	9
9	9	9	9	9	9	9	9	9	9
2	9	2	2	9	2	2	2	9	9
2	9	2	2	9	2	2	9	9	9

2	2	2	2	2	2	2	2	2	2
2	2	2	2	2	2	2	9	9	9
2	2	2	2	2	2	2	9	9	9
9	9	9	9	9	9	9	9	9	9
2	2	2	2	2	2	2	2	2	2
1	2	1	2	2	2	2	3	3	2
2	2	1	1	2	2	1	1	3	1
2	2	1	1	1	3	1	1	2	2
3	3	1	1	2	2	1	1	1	1
1	2	3	2	2	2	2	3	1	1
3	3	3	3	3	3	3	1	1	2
3	3	1	1	1	2	1	1	2	1
1	2	1	1	1	2	1	1	2	2
3	3	3	3	3	3	3	3	3	3
2	2	2	2	2	2	2	2	2	2
2	2	2	2	2	2	2	2	2	2
2	9	2	2	9	9	2	2	9	9
2	2	2	2	2	2	2	2	9	2
2	2	2	2	2	2	2	2	2	2
2	1	1	1	1	1	2	1	3	2
3	3	3	3	3	1	2	1	1	1
2	2	2	2	2	2	2	2	2	2
3	1	2	2	2	1	1	1	1	2
3	3	1	2	1	2	1	2	2	2
3	3	1	1	1	3	1	9	9	9
2	2	1	1	2	2	1	1	3	3
1	2	3	3	2	2	3	2	1	2
1	2	1	1	1	1	1	3	3	1
2	1	1	3	2	2	1	3	1	1
3	2	1	1	1	3	1	1	2	1
3	3	1	1	1	2	2	1	2	2
3	3	1	1	2	3	1	2	2	2
3	3	2	1	1	2	1	1	2	1
2	3	1	2	1	3	1	2	3	1
2	2	1	1	2	2	1	1	3	2
3	3	1	3	1	3	1	3	1	1
3	2	1	1	3	3	1	2	2	3
3	2	3	2	1	3	1	3	3	1
3	3	1	3	3	3	3	3	3	3
3	2	1	2	2	3	3	1	3	2
9	9	9	1	9	9	9	9	9	9
3	3	3	3	3	3	3	3	3	2
1	1	1	9	9	9	9	1	2	1
2	2	2	2	2	2	2	2	2	2
1	1	2	1	1	1	1	3	1	2
9	9	9	9	9	9	9	9	9	9
3	3	1	2	2	2	2	2	2	2

3	3	1	1	1	9	1	1	3	9
2	1	2	1	3	1	2	2	2	1
1	1	2	2	2	3	2	2	2	2
2	2	3	2	3	2	2	2	3	2
3	3	3	2	2	3	2	3	2	3
2	2	3	1	2	3	2	2	3	2
2	2	2	1	1	2	1	9	9	9
3	3	2	3	3	3	3	3	3	3
1	1	1	1	1	1	3	1	1	1
3	3	1	1	1	3	3	3	1	2
3	1	1	2	3	2	1	1	2	1
3	3	1	9	9	9	9	1	3	1
3	3	2	3	3	3	3	2	2	3
1	3	1	2	2	3	3	2	2	2
1	2	1	1	1	1	1	3	1	3
2	2	2	1	1	2	1	2	2	2
3	2	2	3	3	3	3	3	2	2
3	3	1	2	2	2	2	2	2	2
2	2	2	2	3	2	2	9	9	9
2	2	2	2	3	2	2	2	3	2
3	2	1	1	2	2	1	2	1	2
1	3	3	1	1	3	1	2	2	2
2	2	2	2	3	3	3	3	3	2
1	2	2	1	2	2	2	2	2	2
2	2	2	2	2	2	2	2	2	2
9	9	9	1	9	9	9	9	1	9
2	2	1	1	1	1	1	1	9	1
2	1	2	2	1	2	1	1	3	1
2	2	3	2	3	2	3	2	3	3
2	3	2	2	2	2	1	2	2	3
3	3	3	1	1	1	1	3	3	3
3	1	3	1	2	3	2	1	2	3
1	2	2	1	3	2	1	1	9	9
2	2	2	2	2	2	2	2	2	2
2	2	2	2	2	2	2	2	2	2
2	2	2	2	2	2	2	2	2	2
2	2	3	1	3	1	2	2	3	2
2	2	2	2	9	2	2	9	9	9
1	1	2	1	1	2	2	9	9	9
3	3	1	1	2	1	1	1	3	2
3	3	1	1	1	2	1	1	3	3
9	1	2	2	3	2	1	1	9	9
3	3	1	1	1	3	1	1	1	1
2	2	1	1	2	1	2	2	2	2
3	3	1	1	1	3	3	3	2	1
1	1	1	1	1	3	1	1	2	1
3	3	1	1	1	2	1	1	9	9

2	2	2	2	2	2	2	2	2	2
2	2	2	2	2	2	2	2	2	2
2	2	2	2	2	2	2	2	2	2
1	1	2	1	1	2	2	2	2	2
3	3	1	1	1	3	1	1	3	2
3	3	1	1	1	2	1	2	2	2
2	2	2	2	2	3	3	3	2	3
1	1	2	1	1	2	1	2	2	2
3	3	1	1	1	2	1	2	2	2
1	1	3	2	1	3	1	1	2	2
2	2	2	2	1	2	2	2	2	2
1	1	2	1	1	2	1	2	2	2
1	1	2	1	1	2	1	2	2	2
2	2	2	2	2	2	2	2	2	2
2	2	2	2	2	2	2	2	2	2
9	9	9	9	9	9	9	9	9	9
3	3	3	2	1	1	1	2	2	3
3	3	3	1	2	2	1	2	2	1
3	3	1	1	1	3	1	1	2	1
2	2	1	1	3	3	1	1	9	2
1	1	1	3	2	1	3	3	3	3
9	9	9	9	9	9	1	1	9	1
9	9	9	9	9	9	9	9	9	9
9	9	9	9	1	9	9	1	9	9
9	9	9	9	9	9	9	9	9	9
3	3	1	1	2	2	2	2	2	2
2	1	1	1	1	1	1	1	3	1
9	9	9	1	1	3	9	9	9	9
9	9	9	9	9	9	9	9	9	9
2	2	2	1	2	2	1	2	2	2
2	1	1	3	2	1	2	1	1	1
2	2	2	1	2	2	1	2	2	2
3	1	2	1	3	2	1	2	1	3
1	1	1	1	1	1	1	3	1	3
3	1	1	1	3	3	1	1	2	1
2	2	2	2	2	2	2	2	1	3
9	9	9	9	9	9	9	9	9	9
1	1	1	1	2	1	3	1	2	1
2	3	2	1	2	2	3	1	2	3
2	2	3	3	3	1	1	2	2	2
9	2	2	1	2	2	2	2	2	2
2	2	2	1	2	2	1	2	2	2
9	9	9	1	1	2	1	2	2	1
2	2	2	2	2	2	2	2	2	2
3	2	2	2	2	2	2	1	1	2
1	1	1	3	3	3	3	3	3	3
3	2	2	1	2	2	2	1	1	1

1	3	1	1	1	1	1	1	3	1
1	1	1	1	1	2	1	2	2	1
1	1	9	1	1	2	1	2	2	1
3	2	2	3	3	3	3	3	3	2
9	2	1	1	1	1	3	2	1	1
2	3	3	3	2	2	3	3	3	3
3	2	2	2	1	1	3	2	2	1
2	2	2	2	2	2	2	2	2	2
2	2	1	2	2	2	3	2	2	2
2	2	2	2	2	2	2	2	2	2
3	1	3	2	2	1	3	3	3	3
2	2	2	2	2	2	2	2	2	2
9	9	9	9	9	9	1	9	9	9
3	2	1	3	3	3	3	3	3	3
9	9	1	1	2	2	1	9	9	9
3	2	2	1	2	2	2	2	2	1
2	2	2	2	2	2	2	2	2	2
1	1	1	1	1	1	1	1	2	1
2	2	1	2	1	1	2	1	2	2
9	9	9	1	9	9	9	9	9	9
9	9	1	9	9	9	1	9	9	9
3	1	1	1	1	1	1	9	9	1
3	3	1	1	1	3	1	1	2	1
3	2	1	2	3	1	1	3	3	1
1	1	1	1	1	2	1	3	3	3
3	3	1	2	1	2	1	1	2	1
2	2	2	2	1	2	2	1	1	9
3	3	1	1	1	3	1	3	3	9
3	3	1	1	1	2	1	1	3	1
2	2	2	1	2	2	2	2	2	2
3	3	1	1	1	3	1	1	1	1
2	2	2	2	2	2	2	2	2	2
2	2	2	2	2	2	2	2	2	2
1	9	9	2	9	9	9	2	2	2
2	1	2	2	3	2	2	2	2	3
3	3	3	2	2	2	2	3	3	3
1	1	1	1	1	1	1	2	2	2
2	1	3	1	2	2	3	1	3	2
1	1	1	1	1	2	2	2	2	2
2	2	2	2	2	2	2	2	2	2
1	1	1	1	1	2	1	2	2	2
1	2	1	2	3	1	2	2	1	3
1	1	1	1	1	1	1	1	1	1
3	3	1	1	1	2	1	1	3	1
2	3	2	2	3	2	3	1	2	3
2	1	2	1	1	2	1	1	3	2
1	1	1	1	1	1	1	9	1	9

3	3	3	3	3	3	1	1	2	2
3	2	2	2	2	2	2	2	2	2
3	3	1	1	2	2	2	1	2	1
9	9	9	9	9	9	9	2	2	2
3	1	9	2	2	2	1	1	3	2
3	3	3	2	2	2	2	2	2	2
2	2	1	1	2	2	3	1	1	1
1	2	1	1	1	3	1	1	3	1
2	2	2	2	2	3	2	2	3	2
2	2	2	2	2	2	2	2	2	2
2	9	9	2	3	2	3	2	1	2
9	9	9	9	1	9	9	9	1	9
2	3	3	3	2	3	2	2	2	3
9	9	9	9	9	9	1	9	1	9
9	9	9	9	9	9	1	9	1	9
3	3	1	3	3	3	3	9	1	1
9	9	1	9	9	9	2	9	1	9
9	9	9	9	9	9	2	9	9	9
9	9	9	9	9	9	9	9	9	2
9	9	9	9	1	9	9	3	9	9
3	2	1	1	1	2	1	1	3	2
9	9	2	9	9	9	2	2	9	9
9	9	9	9	9	9	9	9	9	9
9	9	9	9	1	9	9	1	9	9
9	9	9	9	1	9	9	1	9	9
9	9	9	9	2	9	9	9	9	9
1	1	1	1	1	1	1	9	9	9
2	1	1	1	1	2	2	2	2	2
3	3	1	1	1	3	1	1	2	2
3	3	2	3	2	2	2	3	3	3
2	3	9	1	1	3	2	1	1	1
3	3	2	1	1	3	1	1	2	1
1	1	1	3	1	1	1	3	1	3
9	9	9	9	9	9	1	2	9	9
2	2	2	2	1	2	2	2	3	2
1	1	1	1	1	1	1	1	3	1
3	1	3	3	1	1	3	3	3	3
9	9	9	1	9	9	9	2	9	9
1	1	1	2	3	2	1	3	3	2
9	9	9	1	9	9	9	9	9	2
9	9	9	1	9	9	9	1	9	9
2	2	2	2	3	2	3	1	1	2
1	1	1	1	1	1	1	1	1	1
9	9	9	9	9	9	9	9	9	9
9	9	9	9	9	9	9	9	9	9
2	2	2	2	2	2	2	2	2	2
2	2	2	2	2	2	2	2	9	2

2	2	2	2	2	2	2	2	2	2
3	3	3	3	3	3	3	3	3	3
9	2	9	2	9	9	2	9	2	9
2	2	2	2	2	2	2	1	1	1
2	2	2	2	2	2	2	2	2	2
1	1	1	1	3	1	1	9	1	3
9	1	9	1	9	1	9	9	9	9
9	1	9	1	2	2	9	9	1	9
1	2	9	9	1	9	1	9	1	9
2	2	2	2	2	2	2	2	2	2
9	1	9	1	9	3	1	9	3	1
9	1	1	2	9	2	2	9	1	9
9	1	2	2	1	3	1	9	9	9
1	2	9	1	9	3	1	9	9	9
9	2	2	2	9	2	2	2	9	9
2	2	2	2	2	2	2	2	2	2
2	2	2	2	2	2	2	9	9	9
2	9	2	9	2	9	2	9	2	9
2	2	2	2	2	2	2	2	2	2
9	9	9	9	9	9	9	9	9	9
2	2	2	2	2	2	2	2	2	2
9	9	9	9	9	9	9	9	9	9
9	9	9	9	9	9	9	9	9	9
9	9	9	9	9	9	9	9	9	9
9	3	1	3	1	3	1	9	9	9
2	2	2	2	2	2	2	9	9	9
2	2	2	2	2	2	2	2	2	2
2	2	2	2	2	2	2	2	2	2
2	2	1	2	2	3	1	1	3	2
9	2	3	1	1	2	3	1	2	2
3	3	3	2	2	2	3	2	2	2
3	2	1	2	2	2	2	1	3	2
1	3	1	2	3	2	1	1	1	1
3	3	1	2	1	2	1	3	3	3
1	1	3	1	1	3	2	1	3	1
3	2	1	2	1	2	1	3	3	2
2	3	3	3	2	2	3	2	2	3
3	2	2	2	1	3	2	2	3	1
2	2	2	1	1	2	2	2	2	2
2	2	2	2	2	2	2	9	9	9
1	1	3	1	1	3	2	1	3	1
2	2	2	3	3	3	3	3	3	3
3	2	3	2	1	2	1	1	3	2
1	1	1	3	3	3	3	1	1	3
3	1	1	1	2	2	2	2	2	2
2	3	2	3	2	3	2	2	2	3
1	1	1	3	1	1	1	3	1	3

9	9	9	9	9	9	9	9	9	9
3	3	2	2	1	3	2	2	2	2
3	1	1	9	9	9	9	9	9	9
9	9	9	9	9	9	9	1	2	2
2	2	2	1	2	2	1	2	2	2
3	1	2	2	3	1	3	3	3	2
2	2	1	1	1	2	2	2	2	1
1	1	2	1	3	2	1	1	1	3
2	2	9	1	1	1	1	2	2	2
2	2	2	2	2	2	2	2	2	2
2	2	2	2	2	2	2	1	1	1
2	2	2	2	2	2	2	1	1	1
1	1	1	1	1	1	1	1	1	1
3	1	2	2	1	1	1	1	2	1
3	3	3	3	3	3	3	3	3	3
1	1	2	2	3	2	1	1	3	1
9	9	9	1	1	1	1	2	2	2
2	2	2	1	1	1	1	2	2	2
2	2	2	2	2	2	2	2	2	2
2	2	2	2	2	2	2	2	2	2
2	2	2	2	2	2	2	9	9	9
1	1	1	1	1	1	1	1	1	1
1	1	2	2	1	1	1	1	1	1
2	1	2	2	1	1	1	1	1	1
2	2	2	2	2	2	2	9	9	9
2	2	2	2	2	2	2	2	2	2
1	2	1	1	2	3	2	1	2	3
2	2	2	2	2	2	2	1	1	1
9	9	9	9	9	9	9	9	9	9
2	2	2	2	2	2	2	2	2	2
9	9	9	9	9	9	9	9	9	9
1	1	1	1	1	2	1	1	1	1
1	2	1	2	1	1	1	1	2	1
1	1	1	1	9	1	1	1	1	1
2	2	2	2	2	2	2	2	2	9
1	1	1	1	1	1	1	2	2	2
2	2	2	2	2	2	2	2	2	2
2	2	2	2	2	2	2	2	2	2
2	2	9	2	2	2	2	2	2	2
3	2	1	1	1	1	1	1	1	1
2	2	2	2	2	2	2	2	2	2
9	9	9	9	9	9	9	9	9	9
2	2	1	1	2	2	2	1	1	1
1	1	2	1	1	1	1	1	2	1
1	1	1	1	1	1	1	9	9	9
2	2	2	2	2	2	2	2	2	2
2	2	2	2	2	2	2	2	2	2

2	2	2	1	1	1	1	9	9	9
2	2	2	2	2	2	2	2	2	2
2	2	2	2	2	2	2	2	2	2
1	1	1	1	2	1	1	1	1	1
2	2	2	2	2	2	2	2	2	2
9	1	1	1	1	3	1	1	1	9
1	1	2	1	2	1	1	1	2	1
1	1	9	1	1	1	9	2	2	9
1	1	1	1	1	1	1	1	1	1
1	2	1	1	2	1	2	1	1	1
2	1	1	1	1	1	1	2	2	2
2	2	2	2	2	2	2	2	2	2
2	2	2	1	1	1	1	1	1	1
2	2	1	2	2	2	2	2	2	2
9	9	9	9	9	9	9	9	9	9
1	1	1	1	1	1	1	9	1	1
2	2	2	2	2	2	2	2	2	9
1	1	1	1	1	1	1	2	2	9
9	9	9	9	9	9	9	9	9	9
1	1	1	1	2	2	2	9	9	9
1	1	9	1	1	1	1	9	9	9
2	2	2	3	3	3	9	9	9	9
9	9	9	9	9	9	9	2	2	2
9	9	9	9	9	9	9	9	9	9
2	2	2	2	2	2	2	2	2	2
3	1	1	1	1	3	1	1	1	1
3	1	1	1	1	3	1	1	1	1
3	1	1	1	1	3	1	1	1	1
3	1	1	1	1	3	1	1	1	1
3	1	1	1	1	3	1	1	1	1
3	1	1	1	1	3	1	1	1	1
3	1	1	1	1	3	1	1	1	1
3	1	1	1	1	3	1	1	1	1
3	3	1	1	1	3	1	1	1	9
3	1	1	1	1	3	1	1	1	1
3	1	1	1	1	3	1	1	1	1
3	1	1	1	1	3	1	1	1	1
3	1	1	1	1	3	1	1	1	1
3	1	1	1	1	3	1	9	1	1
3	1	1	1	1	3	1	1	1	1
3	1	1	1	1	3	1	1	1	1
3	1	1	1	1	3	1	1	1	1
3	1	1	1	1	3	1	1	1	1
3	1	1	1	1	3	1	1	1	1
3	1	1	1	1	3	1	1	1	1
3	1	1	1	1	3	1	1	1	1
3	1	1	1	1	3	1	1	1	1

3	1	1	1	1	3	1	1	1	1
3	1	1	1	1	3	1	1	1	1
3	1	1	1	1	3	1	1	1	1
9	9	9	9	9	9	9	9	9	9
9	9	9	9	9	9	9	9	9	9
9	9	9	9	9	9	9	9	9	9
9	9	9	9	9	9	9	9	9	9
9	9	9	9	9	9	9	9	9	9
9	9	9	9	9	9	9	9	9	9
9	9	9	9	9	9	9	9	9	9
9	9	9	9	9	9	9	9	9	9
9	9	9	9	9	9	9	9	9	9
9	9	9	9	9	9	9	9	9	9
9	9	9	9	9	9	9	9	9	9
9	9	9	9	9	9	9	9	9	9
9	9	9	9	9	9	9	9	9	9
9	9	9	9	9	9	9	9	9	9
9	9	9	9	9	9	9	9	9	9
9	9	9	9	9	9	9	9	9	9
9	9	9	9	9	9	9	9	9	9
9	9	9	9	9	9	9	9	9	9
9	9	9	9	9	9	9	9	9	9
9	9	9	9	9	9	9	9	9	9
9	9	9	9	9	9	9	9	9	9
9	9	9	9	9	9	9	9	9	9
3	1	1	1	1	3	1	1	1	1
3	1	1	1	1	3	1	1	1	1
3	1	1	1	1	3	1	1	1	1
3	1	1	1	1	3	1	1	1	1
3	1	1	1	1	3	1	1	1	1
3	1	1	1	1	3	1	1	1	1
3	1	1	1	1	3	1	1	1	1
3	1	1	1	1	3	1	1	1	1
3	1	1	1	1	3	1	1	1	1
3	1	1	1	1	3	1	1	1	1
3	1	1	1	1	3	1	1	1	1
3	1	1	1	1	3	9	1	1	1
3	1	1	1	1	3	1	1	1	1
3	1	1	1	1	3	1	1	1	1
3	1	1	1	1	3	1	1	1	1
3	1	1	1	1	3	1	1	1	1
9	9	1	9	1	9	1	1	9	1
3	1	1	1	1	3	1	1	1	1
3	1	1	1	1	3	1	1	1	1
3	1	1	1	1	3	1	1	1	1
3	1	1	1	1	3	1	1	1	1
3	3	1	1	1	3	1	1	1	1

3	1	1	1	1	3	1	1	1	1
3	1	1	1	1	3	1	1	1	1
3	1	1	1	1	3	1	1	1	1
3	3	1	1	1	3	1	1	1	1
3	1	1	1	1	3	1	1	1	1
3	1	1	1	1	3	1	1	1	1
3	1	1	1	1	3	1	1	1	1
3	1	1	1	1	3	1	1	1	1
1	1	1	1	3	3	1	1	1	1
3	1	1	1	1	3	1	1	1	1
3	1	1	1	1	3	3	1	1	1
3	1	1	1	1	3	1	1	1	1
1	1	1	1	1	3	1	1	1	1
3	1	1	1	1	3	1	1	1	1
3	1	1	1	1	3	1	1	1	1
3	1	1	1	1	3	1	1	1	1
3	1	1	1	1	3	1	1	1	1
3	1	1	1	1	3	1	1	1	1
3	1	1	1	1	3	1	1	1	1
3	1	1	1	1	3	1	1	1	1
3	1	1	1	1	3	1	1	1	1
3	1	1	1	1	3	1	1	1	1
3	1	1	1	1	3	1	1	1	1
3	1	1	1	1	3	1	1	1	1
3	1	1	1	1	3	1	1	1	1
3	1	1	1	1	3	1	1	1	1
3	1	1	1	1	3	1	1	1	1
3	1	1	1	3	3	1	1	1	1
3	1	1	1	1	3	1	1	1	1
3	1	1	1	1	3	1	1	1	1
3	1	1	1	1	3	1	1	1	1
3	1	1	1	1	3	1	1	1	1
3	1	1	1	1	3	1	1	1	1
3	1	1	1	1	3	1	1	1	1
3	1	1	1	1	3	1	1	1	1
3	1	1	1	1	3	1	1	1	1
3	1	1	1	1	3	1	1	1	1
3	1	1	1	1	3	1	1	1	1
3	1	1	1	1	3	1	1	9	9
3	1	1	1	1	3	1	1	1	1
9	9	9	9	9	9	9	9	9	9
3	1	1	1	1	3	1	1	1	1
3	1	1	1	1	3	1	1	1	1
3	1	1	9	1	3	1	1	1	1
3	1	1	1	1	3	1	1	1	1
3	1	1	1	1	3	1	1	1	1
3	1	1	1	1	3	1	1	1	1

3	1	1	1	1	3	1	1	1	1
3	3	1	1	1	3	1	1	1	1
3	1	1	1	1	3	1	1	1	1
3	1	1	1	1	3	1	1	1	1
3	1	1	1	1	3	1	1	1	1
3	1	1	1	1	3	1	1	1	1
3	1	1	1	1	3	1	1	1	1
3	1	1	1	1	3	1	1	1	1
3	1	1	1	1	3	1	1	1	1
3	1	1	1	1	3	1	1	1	1
3	1	1	1	1	3	1	1	1	1
3	1	1	1	1	3	1	1	1	1
3	1	1	1	1	3	1	1	1	1
3	1	1	1	1	3	1	1	1	1
3	1	1	1	1	3	1	1	1	1
3	1	1	1	1	3	1	1	1	1
3	1	1	1	1	3	1	1	1	1
3	1	1	1	1	3	1	1	1	1
3	1	1	1	1	3	1	1	1	1
3	1	1	1	1	3	1	1	1	1
9	9	9	9	9	9	9	1	1	1
3	1	1	1	1	3	1	1	1	1
3	1	1	1	1	3	1	1	1	1
3	1	1	1	1	3	1	1	1	1
3	1	1	1	1	3	1	1	1	1
9	9	9	9	9	9	9	9	9	9
3	1	1	1	1	3	1	1	1	1
3	1	1	1	1	3	1	1	1	1
3	1	1	1	1	3	1	1	1	1
3	1	1	1	1	3	1	1	1	1
9	9	9	9	9	9	9	1	1	1
3	1	1	1	1	3	1	1	1	1
3	1	1	1	1	3	1	1	1	1
3	1	1	1	1	3	1	1	1	1
3	1	1	1	1	3	1	1	1	1
3	1	1	1	1	3	1	1	1	1
3	1	1	1	1	3	1	1	1	1
3	1	1	1	1	3	1	1	1	1
3	1	1	1	1	3	1	1	1	1
3	1	1	1	1	3	1	1	1	1
3	1	1	1	1	3	1	1	1	1
3	1	1	1	1	3	1	1	1	1
3	1	1	1	1	3	1	1	1	1
3	1	1	1	1	3	1	1	1	1
3	1	1	1	1	3	1	1	1	1
3	1	1	1	1	1	3	1	1	1
3	1	1	1	1	3	1	1	1	1

9	9	9	9	9	9	9	9	9	9
3	1	1	1	1	3	1	1	1	1
3	1	1	1	1	3	1	1	1	1
3	1	1	1	1	3	1	1	1	1
3	1	1	1	1	3	1	1	1	1
3	1	1	1	1	3	1	1	1	1
3	1	1	1	1	3	1	1	1	1
3	1	1	1	1	3	1	1	1	1
3	1	1	1	1	3	1	1	1	1
3	1	1	1	1	3	1	1	1	1
3	1	1	1	1	3	1	1	1	1
3	1	1	1	1	3	1	1	1	1
3	1	1	1	1	3	1	1	1	1
3	1	1	1	1	3	1	1	1	1
3	1	1	1	1	3	1	1	1	1
3	1	1	1	1	9	1	1	1	1
3	1	1	1	1	3	1	1	1	1
3	1	1	1	1	3	1	1	1	1
3	1	1	1	1	3	1	1	1	1
3	1	1	1	1	3	1	1	1	1
3	1	1	1	1	3	1	1	1	1
3	1	1	1	1	3	1	1	1	1
3	1	1	1	1	3	1	1	1	1
3	1	1	1	1	3	1	1	1	1
3	1	1	1	1	3	1	1	1	1
3	1	1	1	1	3	1	1	1	1
3	1	1	1	1	3	1	1	1	1
3	1	1	1	1	3	1	1	1	1
3	1	1	1	1	3	1	1	1	1
3	1	1	1	1	3	1	1	1	1
3	1	1	1	1	3	1	1	1	1
3	1	1	1	1	3	1	1	1	1
3	1	1	1	1	3	1	1	1	1
9	9	9	9	9	9	9	9	9	9
9	9	9	9	9	9	9	9	9	9
9	9	9	9	9	9	9	9	9	9
9	9	9	9	9	9	9	9	9	9
9	9	9	9	9	9	9	9	9	9
9	9	9	9	9	9	9	9	9	9
9	9	9	9	9	9	9	9	9	9
9	9	9	9	9	9	9	9	9	9
9	9	9	9	9	9	9	9	9	9
9	9	9	9	9	9	9	9	9	9
9	9	9	9	9	9	9	9	9	9
9	9	9	9	9	9	9	9	9	9
9	9	9	9	9	9	9	9	9	9
9	9	9	9	9	9	9	9	9	9

9	9	9	9	9	9	9	9	9	9
2	2	1	9	9	9	9	9	9	9
1	1	1	1	1	1	1	9	9	9
9	9	9	9	9	9	9	9	9	9
9	9	9	9	9	9	9	9	9	9
9	9	9	9	9	9	9	9	9	9
9	9	9	9	9	9	9	9	9	9
9	9	9	9	9	9	9	9	9	9
9	9	9	9	9	9	9	9	9	9
9	9	9	9	9	9	9	9	9	9
9	9	9	9	9	9	9	9	9	9
9	9	9	9	9	9	9	9	9	9
9	9	9	9	9	9	9	9	9	9
9	9	9	9	9	9	9	9	9	9
9	9	9	9	9	9	9	9	9	9
9	9	9	9	9	9	9	9	9	9
9	9	9	9	9	9	9	9	9	9
9	9	9	9	9	9	9	9	9	9
9	9	9	9	9	9	9	9	9	9
3	1	1	1	1	3	2	1	2	1
2	2	2	2	2	2	2	2	2	2
3	3	1	1	1	2	1	1	3	1
1	1	3	2	3	1	1	1	1	1
3	3	1	1	1	3	1	1	3	1
3	1	1	1	1	3	1	1	1	3
3	3	2	1	2	3	2	1	2	1
3	2	1	2	1	2	1	1	1	1
1	3	1	1	3	1	3	1	3	1
2	3	1	1	1	3	1	3	3	1
3	3	2	2	3	2	3	2	3	2
3	3	1	1	1	2	1	9	9	9
2	2	2	2	2	2	2	2	2	2
9	9	9	9	9	3	3	9	9	9
3	3	1	1	1	3	3	3	1	3
1	1	1	2	1	3	2	2	2	2
3	3	1	3	2	2	1	2	2	1
2	3	2	2	2	2	3	1	3	3
1	2	1	3	2	1	2	2	1	3
9	9	9	1	9	9	9	1	9	9
3	2	1	1	2	3	3	2	1	2
3	3	1	1	1	3	3	1	3	1
9	9	9	2	9	9	9	1	9	9
2	2	1	1	1	2	1	2	2	1
2	3	3	1	2	2	1	2	1	1
3	3	1	1	3	3	1	3	1	1
1	1	1	1	1	1	1	3	1	1
9	9	9	9	9	9	9	9	9	9

1	1	1	1	1	1	1	9	9	9
2	2	2	1	1	1	1	2	1	1
1	1	1	9	9	9	9	1	1	1
9	9	9	9	9	9	9	9	9	9
9	9	9	9	9	9	9	9	9	9
2	2	1	1	2	2	2	3	3	2
9	9	9	9	9	9	1	9	9	9
9	9	9	9	9	9	9	9	9	9
1	2	3	3	2	2	2	2	1	2
3	3	1	1	1	3	1	3	2	2
3	3	1	1	1	1	1	1	2	2
9	9	9	9	9	9	2	2	9	9
9	9	9	2	2	2	2	2	2	2
2	2	2	2	2	2	2	2	2	2
2	2	2	2	2	2	2	2	2	2
1	2	2	2	2	1	1	1	2	2
1	2	3	1	1	3	3	3	2	1
9	9	9	9	9	9	9	9	9	9
9	9	9	9	9	1	9	1	9	2
2	2	2	2	2	2	2	2	2	2
1	1	1	2	3	2	2	3	1	2
1	3	1	1	2	1	1	2	1	1
1	3	1	1	3	1	3	1	3	1
3	3	1	2	2	2	2	2	2	2
3	1	1	1	2	1	1	2	9	1
2	2	1	1	2	1	3	2	1	9
2	2	2	2	2	2	2	2	2	2
2	2	2	2	2	2	2	2	2	2
2	2	2	2	2	2	2	2	2	2
2	2	2	2	2	2	2	2	2	2
2	2	2	2	2	2	2	2	2	2
3	1	3	2	2	3	1	3	3	1
2	2	2	2	2	2	2	2	2	2
2	3	1	2	2	2	2	2	2	2
1	1	1	2	2	2	1	3	1	2
3	3	1	1	1	2	1	1	1	1
3	3	1	1	1	1	1	3	1	2
1	2	1	1	1	2	1	3	1	1
3	3	1	1	1	1	1	2	1	1
3	2	1	1	1	3	1	1	1	1
2	2	1	1	2	2	1	1	1	2
3	2	2	3	3	3	2	3	2	3
3	2	1	1	2	2	2	2	1	1
2	9	9	2	2	2	2	2	2	2
9	9	9	2	9	9	9	2	9	9
3	3	3	1	1	2	1	3	1	1
3	3	3	2	2	2	2	1	1	1

2	2	2	2	2	2	2	1	1	1
2	3	1	1	2	3	1	1	2	3
2	3	1	3	2	3	1	3	1	2
1	1	1	1	1	1	1	1	1	1
3	2	1	3	1	3	2	1	1	1
2	2	2	1	1	3	1	3	1	1
2	2	2	2	2	2	2	2	1	1
3	1	1	2	3	2	2	2	2	2
1	2	2	2	2	2	1	2	9	1
2	3	2	2	3	2	3	2	3	3
3	3	1	1	1	3	1	2	1	1
2	3	1	1	1	2	1	3	1	1
3	3	1	1	1	2	1	2	1	1
1	3	3	1	1	2	3	2	3	1
2	2	2	2	2	2	2	2	2	2
2	2	1	2	1	2	3	1	2	1
2	2	2	2	2	2	2	2	2	2
2	2	2	2	2	2	2	2	2	2
3	3	3	2	2	2	2	1	1	1
2	2	2	2	2	2	2	2	2	2
2	2	2	2	2	2	2	2	2	2
2	2	2	2	3	2	3	2	3	2
3	3	3	3	3	3	3	3	3	3
2	2	2	2	2	2	2	2	2	2
2	2	2	2	2	2	2	2	2	2
2	3	1	1	2	3	1	1	2	3
2	2	2	1	2	1	2	1	2	1
1	1	1	1	1	1	2	1	1	1
2	2	2	2	2	2	2	2	2	2
1	1	1	1	1	1	1	1	1	1
3	3	1	1	1	3	1	1	1	1
9	9	9	9	9	9	9	9	9	9
3	1	2	2	3	1	3	3	1	2
2	2	1	1	2	2	2	2	2	1
1	1	3	1	2	1	1	1	2	2
3	3	1	1	2	9	1	2	1	1
3	2	1	1	1	3	1	2	1	1
1	3	2	2	2	1	2	1	2	2
3	3	3	2	2	2	2	2	2	2
1	1	1	2	1	2	1	3	1	1
9	9	2	1	2	1	1	1	1	1
9	9	9	9	9	9	9	2	2	2
3	3	1	9	9	9	9	9	9	9
2	2	1	1	2	2	2	1	2	1
9	9	9	9	9	9	9	9	9	9
1	1	1	1	1	2	1	1	1	1
3	2	1	2	1	2	1	1	1	1

1	1	1	3	1	2	1	1	1	1
3	2	1	1	1	3	1	2	1	1
9	9	9	9	9	9	9	9	9	9
9	9	9	9	9	9	9	9	9	9
9	9	9	9	9	9	9	9	9	9
9	9	9	9	2	9	9	1	2	3
1	2	2	1	2	2	3	1	1	1
9	9	9	9	9	9	9	9	9	9
3	3	1	1	2	3	1	2	2	1
3	1	3	1	1	3	3	1	1	1
9	9	9	9	9	9	9	9	9	9
3	3	1	1	1	3	1	3	1	1
3	3	2	2	3	3	3	3	3	2
2	3	1	2	2	2	1	1	2	2
3	3	1	1	1	2	1	1	1	1
2	2	2	1	2	3	2	1	2	3
3	1	2	1	2	1	3	1	2	1
2	2	2	2	2	2	2	2	2	2
2	2	2	2	2	2	2	2	2	2
1	1	3	1	1	3	1	9	9	9
2	2	2	2	2	2	2	2	2	2
3	3	1	1	1	2	1	2	1	1
3	3	3	3	3	3	3	3	3	1
3	2	3	1	3	2	3	3	1	1
1	1	1	1	2	3	2	1	2	1
1	1	2	1	2	1	3	9	9	9
3	2	3	3	1	2	3	3	1	1
3	2	2	2	1	3	2	1	1	1
3	3	1	1	1	3	1	1	1	1
9	9	9	9	9	9	9	3	1	2
3	3	2	2	2	3	2	2	3	3
9	9	9	9	9	9	9	9	9	9
1	3	1	1	1	3	1	1	1	1
1	1	1	2	1	1	1	2	2	2
3	3	1	2	2	3	1	2	2	2
2	1	1	1	2	2	3	1	3	1
2	2	1	2	1	2	2	1	1	2
9	9	9	1	9	9	9	1	9	9
3	3	1	1	2	2	1	3	1	1
2	3	1	1	1	3	2	3	1	1
1	2	1	3	1	2	1	1	3	1
3	2	1	2	1	3	1	1	2	1
2	2	2	2	2	2	2	9	9	9
3	3	1	1	1	3	1	1	2	1
1	3	1	1	1	2	1	3	1	1
9	9	9	9	9	1	9	2	9	9
9	9	9	9	9	9	9	1	3	2

2	2	2	2	2	2	2	2	2	2
3	1	1	1	1	3	1	3	1	2
2	2	2	2	2	2	2	2	2	2
2	2	2	2	2	2	2	2	2	2
3	9	9	1	9	9	9	1	9	2
2	2	2	2	2	2	2	2	2	2
3	3	1	1	1	3	1	1	1	1
2	2	2	2	2	2	2	2	2	2
3	1	1	1	2	3	1	1	3	2
3	3	1	1	1	2	1	3	3	1
2	2	2	2	2	2	2	1	3	1
3	2	2	2	2	3	3	2	2	2
2	2	2	1	1	3	1	1	1	1
3	2	1	1	1	3	1	1	2	1
3	1	1	1	1	3	1	1	1	1
3	3	1	1	1	3	1	2	3	1
3	3	1	3	1	3	3	2	3	1
3	2	2	2	3	2	3	3	3	3
3	3	1	1	1	3	1	2	2	2
3	9	1	1	1	1	1	1	2	1
3	3	1	9	9	9	9	9	9	9
2	2	1	2	2	2	1	1	1	1
3	2	3	2	2	2	3	9	2	2
2	1	1	1	2	2	1	1	2	2
2	2	2	2	2	2	2	2	2	3
3	2	1	1	1	3	1	1	2	2
3	3	1	1	1	2	1	1	3	1
3	2	1	1	1	3	1	1	1	3
3	2	1	1	2	3	2	3	3	3
2	2	2	3	2	3	2	1	1	2
2	2	2	2	2	2	2	2	2	2
3	1	1	1	1	3	1	1	3	2
2	2	1	1	1	3	1	1	3	2
3	3	1	1	1	2	1	1	2	2
3	1	1	1	1	3	1	1	1	1
3	2	1	1	1	3	1	1	1	1
3	3	1	1	1	3	1	1	1	1
2	1	1	1	1	1	1	3	1	1
1	2	1	2	1	1	2	1	2	2
2	2	2	1	2	2	2	9	9	9
2	2	2	2	2	2	2	2	9	9
3	3	1	1	1	3	1	1	1	1
2	2	2	2	2	2	2	2	2	2
1	2	3	1	2	1	2	1	2	1
2	1	1	1	1	1	1	1	1	1
2	2	2	2	2	2	2	2	2	2
2	1	2	1	2	3	2	1	2	3

9	9	9	9	9	9	9	9	9	9
9	9	9	9	9	9	9	9	9	9
1	1	1	1	1	1	1	1	1	1
2	3	2	1	2	3	2	1	1	3
9	9	9	9	9	9	9	9	9	9
9	9	9	9	9	9	9	9	9	9
2	2	2	2	2	2	2	2	2	2
1	1	1	2	3	2	1	2	3	2
2	3	1	1	2	3	1	1	2	3
2	2	3	1	2	3	2	1	2	3
1	3	3	1	2	3	3	1	2	2
2	3	1	1	2	3	1	1	2	3
2	2	2	1	2	3	2	1	2	2
9	9	9	2	9	9	9	1	9	9
2	1	2	2	2	2	2	1	1	2
9	9	9	9	9	9	9	9	9	9
1	1	1	1	1	1	1	1	1	1
3	3	3	1	1	2	3	1	3	2
3	3	1	1	2	2	2	9	9	9
3	2	2	2	3	2	2	9	9	9
1	1	1	1	1	1	1	2	2	2
9	9	9	9	9	9	9	9	9	9
9	9	9	9	9	9	9	9	9	9
3	3	3	1	1	2	1	2	2	2
2	2	2	2	2	2	2	2	2	2
2	1	2	2	2	2	2	3	3	1
2	2	2	2	2	2	2	2	2	2
2	2	2	2	2	2	2	2	9	2
3	1	3	1	1	2	2	1	1	3
3	1	2	2	3	1	2	2	3	1
1	9	2	1	2	2	2	1	1	9
9	9	1	9	9	9	1	9	9	9
3	3	1	1	1	1	1	1	1	1
9	3	3	1	2	2	1	1	1	1
1	1	1	3	3	3	3	1	2	1
9	9	9	1	1	2	2	1	2	1
2	3	1	1	1	1	1	1	1	2
2	2	2	9	9	9	9	2	2	2
2	2	2	2	2	2	2	2	2	2
2	2	2	2	2	2	2	2	2	2
1	1	1	1	1	3	1	1	1	1
2	2	2	2	2	2	2	2	2	2
2	2	2	2	2	2	2	2	2	2
9	9	9	1	9	9	9	1	9	9
9	9	2	9	9	9	2	9	9	9
2	2	2	2	2	2	2	2	2	2
2	2	2	2	2	2	2	2	2	2

9	9	9	1	2	3	1	9	9	9
1	2	2	1	2	3	2	1	2	2
1	1	1	2	9	9	9	1	9	9
2	2	2	2	2	2	2	3	3	3
2	2	9	1	2	2	1	9	9	9
2	2	2	2	2	2	2	2	2	2
1	2	3	3	2	3	1	1	3	2
9	9	9	9	1	9	9	1	9	9
2	2	2	2	2	2	2	2	2	2
3	3	1	1	1	2	2	2	3	3
2	2	2	3	3	3	3	1	1	1
1	1	2	3	3	2	1	2	2	2
3	2	1	1	1	3	1	1	1	1
2	3	2	2	2	1	2	1	2	3
2	2	2	2	2	2	2	2	2	2
3	3	3	9	3	3	3	1	1	1
3	1	1	1	1	2	1	1	3	1
2	2	2	2	2	2	2	2	2	2
1	1	2	2	2	2	2	2	2	2
3	2	3	1	2	3	1	3	3	1
1	1	1	1	1	2	1	1	1	1
1	1	1	1	1	2	1	1	1	1
2	2	2	1	2	2	2	2	2	2
2	2	2	2	2	2	2	2	2	2
1	2	3	2	3	2	2	1	2	2
1	3	2	2	2	1	1	2	2	9
2	2	2	1	2	3	1	1	2	1
3	3	1	1	2	2	1	1	2	1
1	3	3	2	1	2	2	1	1	2
9	9	9	9	9	9	9	9	9	9
2	2	2	2	2	2	2	3	3	3
9	9	9	9	1	9	9	9	1	9
9	9	9	9	9	9	9	9	9	9
9	9	9	9	9	9	9	9	9	9
1	1	1	3	3	3	1	1	2	2
3	3	3	1	1	1	1	1	1	1
2	2	2	2	2	2	2	2	2	2
1	1	3	1	2	9	1	1	3	2
2	2	2	2	2	2	2	2	2	2
2	2	2	2	2	2	2	2	2	2
3	2	1	1	1	1	1	1	9	1
3	3	1	1	1	1	1	1	1	1
3	2	1	1	2	2	1	1	2	1
2	2	3	3	1	2	1	1	9	3
1	3	3	2	3	1	2	1	2	3
3	3	1	1	1	2	1	1	1	1
2	2	1	1	1	1	1	1	1	1

2	2	2	2	2	2	3	2	2	2
9	1	9	9	9	9	1	1	9	9
1	9	9	9	1	9	9	9	9	9
1	2	3	1	3	2	1	1	2	2
2	2	2	2	2	2	2	2	2	2
2	2	2	2	2	2	2	2	2	2
3	2	2	1	1	2	3	1	2	3
9	9	9	9	9	9	9	9	9	9
9	9	9	9	9	9	9	9	9	9
9	9	9	9	9	9	9	9	9	9
9	9	9	9	9	9	9	9	9	9
9	9	1	1	9	9	9	1	9	9
9	9	9	9	9	9	9	9	9	9
9	9	9	9	9	9	9	9	9	9
9	9	9	9	9	9	9	9	9	9
2	2	1	1	2	3	1	9	9	9
2	2	9	2	2	2	2	1	2	2
1	2	1	2	2	2	2	1	2	2
3	3	1	1	1	1	1	1	1	1
2	2	2	2	2	2	2	2	1	2
2	2	2	1	1	2	1	2	2	2
1	2	2	2	1	3	2	1	3	2
1	1	2	1	1	2	1	2	1	1
3	3	1	2	1	3	1	2	2	2
2	1	2	1	1	3	1	1	3	1
2	1	1	1	1	2	1	1	1	1
3	2	1	1	2	1	3	1	2	1
1	2	1	1	2	1	3	1	2	1
1	1	1	2	2	2	2	3	3	3
2	2	2	2	2	2	2	2	2	2
2	2	2	2	2	2	2	2	2	2
2	2	2	2	2	2	2	2	2	2
3	3	3	3	3	3	3	2	2	2
1	2	1	1	2	1	2	1	1	1
2	2	2	2	2	2	2	2	2	2
2	2	2	2	2	2	2	2	2	2
9	9	9	9	9	9	9	9	9	9
2	2	2	2	2	2	2	2	2	2
1	1	1	2	2	2	2	1	1	1
9	9	9	1	9	9	9	1	9	9
2	2	2	3	3	3	3	9	9	9
2	2	2	2	2	2	2	2	2	2
2	2	2	2	2	2	2	2	2	2
2	2	2	2	2	2	2	2	2	2
2	2	1	1	1	1	3	3	3	1
9	9	9	9	9	9	9	9	9	9
3	2	2	1	1	1	1	2	2	1

2	2	2	2	2	2	2	2	2	2
2	2	2	2	2	2	2	2	2	2
3	3	1	1	1	3	1	1	1	1
3	3	1	1	1	3	1	1	1	1
9	9	9	9	9	9	9	9	9	9
2	2	2	1	2	2	1	2	1	2
9	9	9	9	9	9	9	9	9	9
2	2	2	1	2	3	2	3	1	1
9	9	9	9	9	1	9	1	9	9
9	9	9	9	9	9	9	9	9	9
9	9	9	9	9	9	9	9	9	9
9	9	9	9	9	9	9	9	9	9
9	9	9	9	9	9	9	9	9	9
9	9	9	9	9	9	9	9	9	9
9	9	9	9	9	9	9	9	9	9
9	9	9	9	9	9	9	9	9	9
9	9	9	9	9	9	9	9	9	9
9	9	9	9	9	9	9	9	9	9
9	9	9	9	9	9	9	9	9	9
9	9	9	9	9	9	9	9	9	9
9	9	9	9	9	9	9	9	9	9
9	9	9	9	9	9	9	9	9	9
9	9	9	9	9	9	9	9	9	9
9	9	9	9	9	9	9	9	9	9
9	9	9	9	9	9	9	9	9	9
9	9	9	9	9	9	9	9	9	9
9	9	9	9	9	9	9	9	9	9
9	9	9	9	9	9	9	9	9	9
9	9	9	9	9	9	9	9	9	9
1	2	3	1	2	3	2	1	2	3
2	2	2	1	1	2	1	2	2	2
3	3	1	3	1	1	1	1	1	1
3	3	1	1	2	2	1	1	1	1
3	3	1	1	1	3	1	3	1	1
3	3	1	1	9	9	1	1	1	1
2	2	2	1	2	2	1	1	1	1
3	2	1	1	1	2	1	1	1	1
1	2	1	1	2	2	1	1	1	2
1	1	1	2	1	2	1	1	1	2
3	3	1	1	9	1	1	2	1	1
3	3	1	1	2	2	1	1	1	1
3	3	1	1	1	2	1	1	3	1
3	3	1	1	1	3	1	1	1	1
3	3	1	1	1	2	1	1	1	1
1	3	1	1	1	2	1	1	1	1
9	9	9	1	9	9	9	9	9	9
3	3	1	1	1	2	1	1	1	1

3	3	3	1	1	3	1	1	1	1
9	9	9	1	2	9	9	1	9	9
3	3	1	1	1	2	1	1	1	1
3	3	1	1	1	1	1	1	1	1
2	2	2	1	2	2	2	1	2	2
9	2	1	3	3	2	1	1	1	1
3	3	1	1	1	2	1	1	1	1
3	3	1	1	1	1	1	3	1	1
3	3	1	1	1	3	1	1	1	1
3	3	1	2	1	2	1	1	1	1
3	2	3	2	1	2	3	2	1	2
9	9	9	1	9	9	9	1	9	9
3	3	1	1	1	2	1	1	1	1
9	9	9	1	9	9	9	1	9	9
3	3	1	1	1	2	1	1	2	1
3	3	1	1	1	2	1	1	2	1
9	9	1	1	9	9	9	1	9	9
9	9	1	9	9	9	1	9	9	9
9	9	9	1	9	9	9	1	9	9
9	9	9	9	9	9	1	1	9	9
3	3	1	1	3	1	1	1	9	9
3	3	1	1	3	1	3	9	9	9
3	1	3	1	1	1	1	1	1	1
2	2	2	1	1	2	1	1	2	1
9	9	9	9	9	9	1	1	9	9
3	3	1	1	1	3	1	3	1	3
3	1	1	1	1	3	1	1	1	9
9	9	1	1	9	9	1	3	1	9
3	3	1	1	1	3	1	1	9	9
9	9	9	1	9	9	9	1	9	9
9	9	9	1	9	9	9	1	9	9
3	2	1	1	2	3	2	1	2	2
3	3	1	3	1	3	1	1	1	1
3	3	1	2	2	2	1	1	1	1
3	3	1	1	2	1	1	1	1	1
2	2	2	2	2	2	2	2	2	2
3	3	1	1	1	1	1	1	1	1
3	3	1	3	9	9	1	1	1	1
2	1	3	1	3	3	3	1	2	1
3	1	1	1	3	3	1	1	3	1
3	1	1	1	1	3	1	1	2	1
9	9	9	1	9	9	9	1	9	9
2	2	2	1	2	2	2	1	2	9
3	3	3	2	1	3	2	2	2	3
3	2	2	2	3	2	3	2	3	2
2	2	2	2	2	2	2	2	2	2
3	3	3	1	1	3	3	1	1	1

2	3	1	1	1	3	1	1	1	1
3	9	1	1	1	3	1	1	1	1
3	3	2	2	2	1	3	2	2	2
3	3	1	1	2	3	1	1	1	1
3	3	1	1	1	2	1	9	1	1
3	3	1	1	1	3	1	1	1	1
3	3	1	1	1	2	1	1	1	1
2	2	3	2	1	3	3	1	2	3
3	3	1	1	3	2	1	1	1	1
1	1	3	1	1	3	1	1	9	3
9	9	9	2	3	1	2	1	9	9
1	3	1	1	1	2	1	1	3	2
3	3	1	1	1	3	1	1	1	1
9	9	9	9	9	9	9	9	9	9
9	9	9	2	9	9	9	2	9	9
1	1	1	2	2	2	2	2	2	3
2	2	2	2	2	2	2	2	1	2
3	3	3	1	1	3	1	1	3	3
2	1	1	1	1	1	1	1	1	1
9	9	9	9	9	9	9	9	9	9
9	9	9	9	9	9	9	9	9	9
3	3	3	3	3	3	3	2	2	3
9	9	9	1	9	9	9	1	9	9
9	9	9	9	9	9	9	9	9	9
2	2	1	3	3	3	3	1	1	1
3	3	1	1	1	2	1	1	1	1
3	3	1	2	3	3	2	1	1	1
1	1	1	1	1	1	1	1	1	1
1	1	3	3	3	3	3	3	1	2
3	3	1	1	1	2	1	1	1	1
1	1	1	1	1	1	1	1	1	1
9	9	9	9	9	9	9	9	9	9
9	9	9	9	9	9	9	1	9	9
3	1	1	1	3	3	1	1	1	3
3	3	3	3	3	3	3	3	3	2
3	3	1	1	2	3	2	1	1	1
9	9	9	9	9	9	9	9	9	9
9	9	9	9	9	9	9	9	9	9
9	9	9	9	9	9	9	9	9	9
9	9	9	9	9	9	9	9	9	9
9	9	9	9	9	9	9	9	9	9
9	9	9	9	9	9	9	9	9	9
9	9	9	9	9	9	9	9	9	9
9	9	9	9	9	9	9	9	9	9
9	9	9	9	9	9	9	9	9	9
9	9	9	9	9	9	9	9	9	9
9	9	9	9	9	9	9	9	9	9

9	9	9	9	9	9	9	9	9	9
9	9	9	9	9	9	9	9	9	9
9	9	9	9	9	9	9	9	9	9
9	9	9	9	9	9	9	9	9	9
9	9	9	9	9	9	9	9	9	9
9	9	9	9	9	9	9	9	9	9
9	9	9	9	9	9	9	9	9	9
9	9	9	9	9	9	9	9	9	9
9	9	9	9	9	9	9	9	9	9
9	9	9	9	9	9	9	9	9	9
9	9	9	9	9	9	9	9	9	9
9	9	9	9	9	9	9	9	9	9
9	9	9	9	9	9	9	9	9	9
9	9	9	9	9	9	9	9	9	9
9	9	9	9	9	9	9	9	9	9
9	9	9	9	9	9	9	9	9	9
9	9	9	9	9	9	9	9	9	9
9	9	9	9	9	9	9	9	9	9
9	9	9	9	9	9	9	9	9	9
9	9	9	9	9	9	9	9	9	9
9	9	9	9	9	9	9	9	9	9
9	9	9	9	9	9	9	9	9	9
9	9	9	9	9	9	9	9	9	9
9	9	9	9	9	9	9	9	9	9
9	9	9	9	9	9	9	9	9	9
2	2	2	2	2	2	2	1	1	1
9	9	9	9	9	9	9	9	9	9
9	9	9	9	9	9	9	9	9	9
9	9	9	9	9	9	9	9	9	9
9	9	9	9	9	9	9	9	9	9
9	9	9	9	9	9	9	9	9	9
9	9	9	9	9	9	9	9	9	9
9	9	9	9	9	9	9	9	9	9
9	9	9	9	9	9	9	9	9	9
9	9	9	9	9	9	9	9	9	9
9	9	9	9	9	9	9	9	9	9
9	9	9	9	9	9	9	9	9	9
9	9	9	9	9	9	9	9	9	9
9	9	9	9	9	9	9	9	9	9
9	9	9	9	9	9	9	9	9	9
9	9	9	9	9	9	9	9	9	9
9	9	9	2	9	9	9	2	2	2
1	3	1	1	1	1	1	3	1	1
3	3	1	1	1	3	1	1	1	1
3	3	1	1	1	3	1	1	3	1
2	2	2	3	3	3	3	2	3	3
1	1	1	1	1	1	1	3	1	1

2	3	1	1	2	3	1	1	3	1
9	9	1	1	9	9	9	1	9	9
2	3	1	1	2	3	1	1	3	1
2	1	2	2	2	1	2	2	2	2
3	1	1	1	1	2	2	1	1	1
3	3	1	1	1	2	1	3	2	3
1	3	1	1	1	2	1	1	1	1
3	3	1	1	1	3	1	1	1	1
3	2	1	1	3	3	1	1	1	1
3	3	1	1	1	1	1	1	1	1
3	3	1	1	1	3	1	1	1	1
3	3	1	1	1	2	1	1	1	1
3	3	1	1	1	3	1	1	9	1
3	2	1	1	1	3	1	1	1	1
1	3	2	3	2	1	2	3	2	2
3	3	1	1	1	1	1	1	1	1
9	9	9	9	9	9	9	1	1	1
2	2	1	2	2	3	1	1	2	2
3	3	3	1	1	2	1	1	1	1
3	3	1	2	2	2	1	1	1	1
3	2	1	2	2	2	1	1	1	1
3	1	1	1	1	2	1	1	1	1
9	9	9	1	1	2	1	1	1	1
3	9	1	1	1	3	1	1	1	1
2	1	1	1	1	3	1	1	1	1
3	1	3	1	2	1	1	3	1	2
3	3	1	1	1	3	1	1	1	1
3	3	1	1	1	3	1	1	1	1
3	3	1	1	1	3	1	1	1	1
3	3	1	3	1	1	1	1	1	3
3	3	3	3	3	3	3	3	3	3
2	1	2	1	2	1	2	1	2	1
1	1	1	2	2	2	2	1	1	1
1	2	1	3	2	1	1	1	2	3
2	2	2	2	2	2	2	2	2	2
2	2	2	2	2	2	2	2	2	2
2	2	1	2	2	3	2	2	2	2
2	2	2	1	1	2	1	2	2	2
2	2	2	1	2	2	1	2	2	2
9	9	9	9	9	9	9	9	9	9
9	9	9	9	9	9	9	9	9	9
9	9	9	9	9	9	9	9	9	9
1	2	3	2	1	2	1	9	9	9
1	1	3	9	9	9	9	9	9	9
9	9	9	2	1	3	1	9	9	9
9	9	9	9	9	9	9	9	9	9
2	3	1	1	1	2	1	1	2	3

1	2	1	1	1	2	1	9	9	9
3	3	1	1	1	3	1	1	2	1
3	3	1	1	1	3	1	3	3	3
3	3	3	3	1	1	1	1	1	1
3	3	9	3	3	3	1	3	3	3
3	1	1	1	1	1	3	1	3	1
2	2	2	3	2	3	2	2	3	2
2	2	3	2	3	2	2	1	1	1
1	2	1	1	2	2	2	2	1	3
1	1	3	1	1	1	1	1	3	1
2	2	2	2	1	2	1	2	2	1
2	1	2	1	2	1	3	1	2	3
9	1	1	1	1	3	1	3	3	1
1	2	1	2	3	2	1	3	2	3
3	3	1	1	1	1	1	1	1	1
9	9	9	1	9	9	9	1	9	9
3	3	1	1	1	3	1	3	3	1
1	3	1	1	2	1	2	1	2	1
2	2	1	1	1	2	1	1	2	1
3	3	1	1	1	3	1	3	1	1
1	1	1	1	1	1	1	3	1	1
3	1	9	2	2	2	3	1	1	2
2	2	2	2	2	3	2	2	2	2
9	9	1	9	9	9	1	9	9	9
1	2	2	1	1	1	2	2	3	1
3	3	1	3	1	3	1	3	3	2
3	3	1	3	2	3	2	3	2	1
1	2	1	3	1	1	2	3	3	3
3	3	1	1	1	3	9	3	1	1
1	3	1	1	1	2	1	3	3	1
2	2	1	1	2	3	1	1	2	1
3	1	3	1	1	1	1	1	3	1
3	3	1	1	1	3	1	1	2	1
2	3	3	1	2	3	1	1	1	2
1	3	1	1	3	1	1	1	1	3
3	2	1	1	1	2	1	1	3	2
1	1	1	1	1	2	1	1	2	1
2	2	1	1	2	3	1	1	2	3
3	9	1	1	1	1	3	2	3	2
3	1	3	1	1	1	1	1	3	1
1	1	1	1	3	1	1	1	1	1
1	1	1	1	3	3	1	1	1	1
2	1	3	1	2	1	3	1	2	3
9	9	9	9	9	9	9	9	9	9
3	1	1	1	3	3	1	1	3	3
3	1	1	1	3	3	1	1	3	1
3	3	1	1	1	1	1	1	3	1

2	3	1	1	3	2	3	1	2	3
1	1	1	9	1	1	1	1	3	1
2	2	2	3	2	1	1	2	3	3
2	2	2	2	2	2	2	2	2	2
3	2	1	2	3	2	1	1	1	1
3	3	3	3	3	3	9	3	3	3
2	1	2	1	1	2	1	1	2	1
1	1	1	1	1	1	1	9	9	9
2	3	1	2	2	2	2	2	2	3
2	2	1	2	2	3	3	1	1	1
2	2	2	1	1	1	2	3	3	3
1	1	3	1	2	3	1	1	1	2
2	2	2	2	2	2	2	2	2	2
9	9	9	9	9	9	9	9	9	9
9	9	9	9	9	9	9	9	9	9
1	2	2	1	2	2	1	1	2	2
2	2	2	2	2	2	2	1	2	2
2	2	2	2	2	2	2	2	2	2
2	2	3	2	3	3	3	2	2	2
1	1	2	2	2	2	2	2	1	1
2	2	1	2	2	2	2	1	1	1
2	2	2	2	2	2	2	2	2	1
3	2	3	2	2	2	2	2	2	2
2	2	2	2	2	2	2	1	2	2
2	2	2	2	2	2	2	1	2	1
2	2	2	2	2	2	2	2	2	2
2	2	2	1	1	2	2	2	2	2
2	2	2	2	2	2	2	2	2	2
2	2	2	2	2	2	2	2	2	2
2	2	2	2	2	2	2	1	1	2
2	2	2	2	2	2	2	2	2	2
2	2	2	2	2	2	2	2	2	2
2	2	2	2	2	2	2	1	2	2
2	1	2	1	2	3	2	1	2	2
2	2	2	2	2	2	2	1	1	1
2	2	2	2	2	2	2	2	2	2
2	2	2	2	2	2	2	2	2	2
2	2	2	2	2	2	2	1	2	2
2	2	2	2	2	2	2	2	2	2
2	2	2	2	2	2	2	2	2	2
2	2	1	2	2	2	2	1	2	2
2	2	1	2	2	2	2	1	2	2
2	2	2	2	2	2	2	2	2	2
2	2	2	2	2	2	2	2	2	2
2	2	2	2	2	2	2	2	2	2
3	2	1	3	3	3	1	1	3	1
3	3	1	2	2	2	2	1	3	2

2	2	2	2	2	2	2	2	2	2
3	2	2	2	1	2	2	2	2	1
2	2	2	2	2	2	2	2	2	2
1	1	3	1	2	3	3	2	1	1
2	2	2	2	2	2	2	3	3	3
3	3	3	9	3	3	3	3	3	3
2	2	2	2	2	2	2	2	2	2
2	2	2	1	1	2	2	2	2	2
2	2	2	2	2	2	2	1	2	2
2	2	2	2	2	2	2	2	2	2
3	2	1	2	3	3	2	1	2	3
2	2	2	2	2	2	2	2	2	2
1	1	3	1	1	3	3	9	9	9
1	1	1	1	2	3	1	1	1	1
9	9	9	1	1	3	1	1	9	9
2	2	2	2	2	2	2	2	2	2
3	3	2	2	2	2	2	2	3	2
1	1	1	2	2	1	1	3	3	3
3	3	2	2	2	2	2	2	2	3
9	9	9	1	9	9	9	3	9	9
2	2	2	1	1	1	1	2	2	2
2	2	1	2	1	2	2	1	1	1
2	2	2	1	1	1	1	1	1	2
1	1	1	1	2	1	1	1	1	1
9	9	9	9	9	9	1	9	1	9
3	3	2	3	1	1	1	1	1	1
2	2	2	2	2	2	2	1	2	2
9	9	9	1	9	9	9	1	9	9
9	9	9	1	9	9	9	1	9	9
1	2	2	1	1	2	2	1	3	1
1	1	2	1	2	3	1	2	3	9
2	3	3	1	2	1	1	1	9	3
3	1	1	1	3	1	1	1	2	3
1	1	1	1	1	3	1	1	9	3
3	1	3	1	2	3	1	1	2	3
1	1	1	1	1	1	1	1	1	1
2	2	2	2	2	2	2	3	3	3
2	2	3	2	2	2	2	2	2	2
2	2	2	2	2	2	2	2	2	2
9	2	9	1	2	2	9	1	9	9
3	3	1	1	1	1	1	1	2	2
9	9	9	1	9	9	9	9	9	9
3	3	1	1	1	3	3	1	3	1
1	1	1	3	3	3	3	2	2	2
2	2	2	2	2	2	2	2	2	2
2	2	2	2	2	2	2	2	2	2
9	9	9	9	9	9	9	1	3	3

2	2	2	2	2	2	2	2	2	2
9	9	9	9	1	9	9	9	9	9
1	1	1	2	2	3	1	2	2	2
2	2	2	1	2	2	2	1	1	1
2	2	2	1	1	3	1	3	9	2
2	2	2	1	1	1	1	2	2	2
1	1	1	1	1	1	2	1	1	1
2	2	2	2	2	2	2	2	2	2
1	1	1	1	1	1	1	1	1	1
2	2	2	2	2	2	2	2	2	2
9	9	9	1	9	9	9	9	9	1
2	1	1	3	2	2	1	1	1	2
3	3	3	3	9	9	9	3	3	3
2	2	2	1	1	1	1	1	1	1
2	2	2	2	2	2	2	2	2	2
1	1	1	1	1	3	3	1	1	1
1	2	3	2	2	2	2	3	3	3
3	3	3	3	3	3	3	1	1	1
1	1	2	2	2	2	2	2	2	2
1	1	2	2	2	2	2	2	2	2
2	2	2	2	2	2	2	2	2	2
2	2	2	2	2	2	2	1	1	1
1	1	1	1	1	1	1	1	1	1
3	3	3	1	2	2	3	1	3	3
9	9	9	9	9	9	9	9	9	9
3	1	1	1	2	1	3	9	9	9
1	2	2	1	1	2	2	1	1	2
2	2	2	2	2	2	2	2	2	2
1	1	1	1	1	1	1	1	1	1
1	1	1	1	2	2	2	1	2	2
9	9	3	9	3	9	3	3	3	3
2	2	1	1	2	2	2	1	1	1
2	1	2	1	1	2	3	1	2	2
1	2	1	2	2	2	2	2	1	2
9	9	9	2	9	2	9	1	2	1
2	1	1	2	2	2	2	2	2	3
2	2	1	1	2	1	2	2	9	1
2	2	2	2	2	2	2	2	2	2
3	3	3	3	3	3	3	1	9	1
2	3	1	2	3	1	2	1	2	3
2	2	2	2	2	2	2	2	2	2
3	2	3	2	2	3	2	2	2	2
3	3	3	2	2	2	2	1	2	3
2	2	2	3	2	2	2	2	2	2
2	1	2	1	2	3	1	1	2	3
3	3	3	3	3	3	3	3	3	3
3	1	2	1	2	3	1	1	2	1

3	2	1	2	1	3	1	2	2	2
9	9	9	9	9	9	9	9	9	9
3	3	3	3	3	3	3	3	9	9
1	1	2	1	1	2	2	1	1	1
2	2	2	2	2	2	2	2	2	2
3	3	1	1	1	2	1	1	1	2
1	1	1	2	2	2	9	1	1	1
1	1	1	3	3	3	1	1	3	3
1	1	1	2	1	1	1	2	2	2
1	1	1	3	3	3	1	1	3	3
1	1	1	1	1	1	3	1	1	2
2	2	2	2	2	2	2	2	2	2
2	2	2	2	2	2	2	2	2	2
2	2	2	2	2	2	2	2	2	2
1	1	1	1	1	1	1	2	2	2
2	1	2	2	1	2	2	2	1	2
2	1	2	2	2	2	2	2	2	2
2	3	1	1	2	2	3	1	2	3
2	2	2	1	2	3	1	1	2	2
3	2	1	3	3	3	3	1	1	1
2	2	2	2	2	2	2	2	2	2
2	2	1	1	2	3	2	3	2	1
2	2	2	1	2	2	2	1	2	2
1	2	1	1	1	1	2	2	2	2
2	2	1	1	2	2	2	2	2	2
1	1	1	1	1	3	1	2	2	1
1	2	1	2	2	2	1	1	2	2
1	1	1	2	2	2	2	2	2	2
3	1	1	1	2	1	1	3	1	1
2	2	2	2	2	2	2	2	2	2
1	1	1	1	1	1	1	1	1	1
2	2	2	2	2	2	2	2	2	2
1	1	3	1	1	9	3	1	1	3
3	1	3	1	1	1	1	2	3	3
2	3	2	2	2	3	1	1	2	2
3	3	1	1	2	3	1	1	3	1
1	1	1	1	3	2	2	1	3	3
3	2	2	2	1	3	2	1	2	2
1	2	1	2	2	2	2	2	2	2
2	2	2	2	2	2	2	2	2	2
1	1	1	1	1	3	3	1	2	2
1	2	3	1	2	2	1	3	3	3
2	2	2	2	2	2	2	1	9	2
9	9	9	9	9	9	9	9	9	9
1	1	9	2	2	1	2	2	2	2
1	2	1	1	1	2	1	2	2	1
1	2	1	1	1	2	1	2	2	1

2	2	2	2	2	2	2	2	2	2
2	2	2	2	2	2	2	2	2	2
2	2	2	2	2	2	2	2	2	2
9	9	9	1	1	1	3	9	9	9
1	1	1	1	1	1	3	1	1	2
2	2	2	9	1	3	2	2	2	2
9	9	9	9	9	9	9	9	9	9
1	3	1	1	1	2	3	1	2	2
3	1	2	1	1	1	2	3	3	1
1	1	1	1	1	3	2	1	3	1
3	3	1	1	1	3	1	1	3	3
3	3	1	1	1	3	1	1	3	9
3	3	1	1	1	3	1	1	3	1
9	9	9	9	9	9	9	9	9	9
1	1	2	2	2	3	2	3	3	3
3	3	3	3	2	3	2	3	3	3
2	2	1	1	2	2	2	2	2	2
3	3	2	1	2	2	3	1	3	2
9	9	9	9	9	9	9	1	2	2
2	3	1	1	1	3	1	1	1	1
2	2	3	1	2	3	2	1	2	3
2	2	2	2	2	2	2	2	2	2
2	2	1	2	3	2	2	1	2	2
2	1	2	2	2	2	2	2	2	1
9	9	9	9	1	9	9	1	9	9
2	2	2	2	2	2	2	9	9	9
9	9	9	9	9	9	9	9	9	9
2	3	3	1	2	2	3	3	2	3
2	3	2	1	2	3	2	2	1	1
1	1	2	3	2	1	2	1	1	2
1	2	3	1	2	3	1	1	1	2
3	2	2	3	3	3	3	2	3	3
3	1	2	2	1	2	2	2	2	2
2	2	2	1	1	1	2	2	2	2
1	3	2	1	1	2	3	2	3	2
3	3	1	2	1	1	1	1	3	1
3	2	2	2	2	3	1	3	3	3
2	1	2	2	3	2	1	1	3	2
3	3	1	1	1	3	3	3	3	2
2	2	2	3	3	3	3	2	2	2
2	2	2	2	2	2	2	2	2	2
2	2	2	2	2	2	2	2	2	2
3	3	3	3	3	3	3	3	3	3
1	2	3	9	9	9	9	2	2	2
2	3	1	1	2	3	3	9	9	9
2	3	2	1	1	2	3	9	9	9
1	1	1	3	3	3	3	1	2	3

2	2	2	2	2	2	2	2	2	2
1	2	3	1	2	3	1	3	2	1
2	3	2	1	3	3	2	2	1	2
9	9	9	9	9	9	9	9	9	9
3	3	3	3	3	3	3	3	3	3
3	2	3	1	2	3	2	1	3	1
2	2	2	1	1	1	1	1	1	1
1	2	1	1	1	3	1	2	2	1
1	2	3	2	1	2	1	1	2	3
2	2	2	1	1	2	1	2	2	2
3	2	3	2	3	2	3	1	2	1
3	3	3	3	3	3	3	1	3	3
1	1	1	1	1	1	1	1	1	1
3	3	3	3	3	3	3	3	3	3
1	1	1	2	2	1	2	2	2	3
3	3	1	2	1	3	1	1	1	1
2	2	2	2	2	2	2	2	2	2
2	2	2	2	2	2	2	1	1	1
9	1	2	1	2	1	2	9	9	2
9	9	9	9	1	9	9	1	2	3
2	2	1	2	2	3	3	2	2	2
3	3	3	2	2	2	2	3	3	3
1	1	1	1	1	1	1	3	1	1
2	2	2	1	2	3	1	1	2	3
2	2	2	1	2	2	2	2	2	2
3	1	2	1	1	2	2	2	3	2
1	3	1	1	1	3	3	1	2	3
9	9	9	9	9	9	9	3	3	3
1	2	1	1	3	3	2	3	3	3
2	2	1	1	1	2	2	2	2	3
1	2	3	1	2	3	2	1	2	3
1	1	1	1	1	1	3	2	2	2
1	1	1	1	1	2	3	2	2	3
2	1	2	1	2	3	2	1	2	3
2	1	1	2	1	3	1	2	3	1
3	3	3	1	1	2	2	1	1	1
2	1	3	1	2	3	1	1	2	3
3	3	3	2	2	2	2	1	1	1
2	2	2	2	2	2	2	2	2	2
3	1	1	1	1	3	3	1	1	1
3	1	2	1	2	3	1	1	2	3
2	3	2	1	1	3	1	3	2	3
2	2	1	1	3	2	3	2	1	3
3	1	1	1	3	3	3	3	2	1
1	1	1	1	1	3	1	1	1	1
1	2	3	1	1	1	3	1	2	1
2	3	3	3	3	2	2	1	2	2

1	1	1	1	1	3	1	3	3	1
1	2	3	1	2	3	2	1	2	1
3	1	1	2	1	2	2	1	2	1
2	2	1	1	2	3	1	1	2	3
2	1	2	3	2	1	2	1	2	3
1	1	3	3	3	1	1	1	1	3
1	1	2	1	1	1	1	2	3	3
1	2	3	1	2	3	1	1	3	1
2	2	2	3	3	1	2	2	2	2
3	3	3	1	1	1	1	1	1	1
2	3	1	1	2	3	1	1	2	3
3	2	2	1	1	2	2	1	3	2
1	2	1	1	2	1	3	1	1	2
2	2	1	1	3	2	1	1	2	1
1	1	3	3	1	1	1	1	3	1
1	1	1	3	1	1	1	3	3	1
3	1	3	1	2	3	1	1	2	3
2	1	3	2	2	3	1	1	2	3
3	3	3	1	1	1	1	1	1	1
1	3	1	1	1	3	3	1	1	3
2	1	2	3	3	2	2	1	1	1
1	1	1	1	1	1	1	1	3	1
1	1	1	1	1	1	1	1	3	1
3	1	1	1	1	1	3	3	3	3
2	1	1	1	1	2	3	1	2	1
1	1	1	1	2	2	1	2	1	1
1	1	1	1	2	2	1	1	1	2
3	3	1	1	1	3	3	1	1	1
3	1	1	1	1	1	1	1	3	1
3	1	1	1	1	1	1	1	9	1
2	2	2	1	1	2	2	2	2	2
2	2	2	2	2	2	2	2	2	2
1	1	1	1	3	3	1	3	3	2
1	2	1	3	2	1	1	1	2	3
2	2	2	2	2	2	2	2	2	2
9	9	9	9	9	9	9	9	9	9
9	9	9	9	9	9	9	9	9	9
9	9	9	9	9	9	9	9	9	9
2	2	2	1	1	2	2	2	2	2
9	9	9	9	9	9	1	9	1	9
2	2	2	3	3	3	3	2	2	2
3	3	3	2	2	2	2	9	9	9
1	1	2	1	2	2	1	1	2	1
9	9	9	9	9	9	2	1	9	9
9	1	1	9	1	1	1	1	2	1
1	2	2	1	3	3	2	2	2	3
3	2	1	1	2	3	1	9	9	9

1	1	1	3	1	3	1	1	3	3
2	2	2	3	1	2	1	3	1	2
2	2	2	3	3	2	1	3	1	2
3	1	3	1	2	3	1	1	3	1
1	3	2	1	2	3	2	1	2	3
1	1	1	1	3	3	1	3	3	2
1	9	9	1	1	2	2	1	2	2
2	2	2	1	1	2	2	2	2	2
2	2	3	3	2	3	2	2	2	2
3	3	1	1	3	1	1	1	2	1
3	1	1	1	1	9	1	2	2	2
2	1	2	2	1	3	1	1	2	1
9	9	1	9	9	9	1	9	9	9
1	1	2	2	2	1	3	2	2	1
3	3	1	1	1	3	1	3	1	2
3	3	3	3	3	3	3	3	3	3
3	3	1	1	1	2	3	2	3	1
1	3	1	1	3	2	3	3	2	2
3	1	2	1	2	2	2	1	3	2
2	2	2	2	2	2	2	2	2	2
2	2	2	2	2	2	2	2	2	2
2	9	9	1	2	9	2	9	1	2
3	3	3	2	2	2	2	2	2	2
2	2	2	2	2	2	2	2	2	2
2	2	2	2	2	2	2	2	2	2
2	2	2	2	2	2	2	9	2	2
1	1	1	1	1	1	1	1	1	1
9	9	1	1	1	1	1	1	1	1
9	9	1	1	1	1	1	1	1	1
3	3	1	1	1	1	1	1	1	1
3	3	1	1	1	1	1	1	1	1
9	9	9	1	9	9	9	1	1	1
3	3	9	1	1	2	2	3	3	1
9	9	1	1	1	1	1	1	1	1
2	1	1	1	1	1	1	1	2	1
3	3	1	1	9	1	1	1	1	1
9	9	9	1	1	1	1	1	1	1
9	9	9	9	1	9	2	1	9	9
3	3	1	1	1	1	1	1	1	1
3	3	1	1	1	1	1	1	1	1
9	9	9	1	1	1	1	1	1	1
3	3	1	1	1	1	1	1	1	1
9	9	1	1	1	1	1	1	1	1
9	9	9	1	1	1	1	9	1	1
9	9	9	1	9	1	1	1	1	1
9	9	9	1	1	1	1	1	1	1
3	3	1	1	1	1	1	1	1	1

9	9	1	1	1	1	1	1	9	1
3	3	3	1	1	1	1	1	1	1
3	9	9	9	9	9	9	1	1	1
9	9	9	1	1	1	1	9	1	1
3	3	1	1	3	1	1	1	1	1
9	9	9	1	9	1	1	3	1	1
9	9	1	1	1	1	1	1	1	1
2	2	1	1	1	1	1	3	3	2
3	3	1	1	1	1	1	1	1	1
1	1	1	1	1	1	1	1	1	1
9	9	9	1	1	1	1	1	1	1
9	9	9	1	1	1	1	1	1	1
1	1	1	1	1	1	1	1	1	1
2	2	1	1	1	1	1	1	3	1
9	9	9	1	1	1	1	1	1	1
3	3	1	1	1	1	1	1	1	1
3	3	1	1	1	1	1	1	1	1
3	3	1	1	1	1	1	1	1	1
9	9	9	1	1	1	1	1	1	1
9	9	9	1	1	1	1	1	1	1
9	9	9	1	1	1	1	1	1	1
9	9	9	1	1	1	1	9	1	1
9	9	9	1	1	1	1	1	1	1
9	9	9	1	1	1	1	1	1	1
9	9	9	1	1	1	1	1	1	1
9	9	9	1	9	9	1	1	3	1
1	1	1	1	9	9	9	1	1	9
9	9	9	1	9	9	1	1	1	1
3	3	1	1	1	1	1	1	1	1
9	9	1	1	1	1	1	1	1	1
3	3	1	1	1	1	1	3	1	1
1	1	1	1	1	1	1	1	3	1
3	3	3	1	3	1	1	1	1	1
3	3	2	1	1	1	1	1	3	1
3	3	1	1	1	1	1	1	1	1
9	9	9	1	1	1	1	9	1	9
2	1	1	1	1	1	1	1	1	1
3	3	1	1	1	1	1	1	1	1
9	9	9	1	1	1	1	9	1	1
9	9	9	1	9	1	1	1	1	1
9	9	1	1	1	1	1	1	1	1
3	3	1	1	1	1	1	1	1	1
2	2	1	1	9	1	9	1	1	1
3	3	1	1	1	1	1	1	1	1
3	3	1	1	1	1	1	1	1	1
3	3	1	1	1	1	1	1	1	1
3	3	1	1	1	1	1	1	1	1

3	3	1	1	1	2	1	1	1	1
9	9	9	1	9	9	1	1	1	1
9	9	1	1	1	1	1	1	1	1
3	3	1	1	1	1	1	1	1	1
3	3	1	1	1	1	1	1	1	1
3	3	1	1	1	1	1	1	1	1
3	3	1	1	1	1	1	1	1	1
3	3	1	1	1	1	1	1	1	1
3	3	1	1	1	1	1	1	1	1
3	3	1	1	1	1	1	1	1	1
3	3	1	1	1	1	1	1	1	1
3	9	1	1	1	1	1	1	1	1
3	3	3	1	1	1	2	9	9	1
9	9	9	1	1	1	1	1	1	1
9	9	9	1	1	1	1	1	1	1
3	1	1	1	1	1	1	1	1	1
3	3	1	3	1	2	1	1	3	1
9	9	9	9	1	9	9	1	9	9
3	3	1	1	1	3	1	1	1	1
3	2	1	2	2	3	1	1	2	2
3	2	2	1	1	1	2	2	2	2
3	3	1	1	1	2	1	1	2	1
9	9	1	9	9	9	1	9	9	9
3	3	1	1	1	1	1	9	9	9
2	1	1	1	1	3	1	1	1	1
1	3	1	1	1	1	1	1	1	1
3	3	1	1	1	3	1	1	3	1
2	2	1	1	1	3	2	2	2	1
3	3	2	1	2	3	2	3	3	3
3	3	1	1	1	1	1	1	3	1
3	1	1	1	1	3	1	1	1	1
3	1	1	1	1	2	1	1	3	9
2	2	1	2	1	1	1	1	3	1
3	2	2	1	1	3	2	3	1	1
3	3	1	1	1	2	1	1	1	1
3	2	1	1	1	1	1	1	1	1
3	3	1	1	1	2	1	2	1	1
9	9	1	9	1	9	9	1	9	9
2	3	1	1	1	3	1	1	2	1
3	1	1	1	1	2	1	1	3	1
3	3	2	1	2	3	2	3	3	3
3	3	1	1	1	2	1	1	2	1
3	1	1	1	1	3	1	2	1	9
3	1	1	1	1	3	1	2	1	1
3	1	1	1	1	3	1	2	1	1
3	3	1	1	1	3	1	1	1	1
3	3	1	1	1	1	1	1	1	1

3	3	1	1	1	3	1	1	1	1
3	1	1	1	1	2	1	1	2	1
2	2	2	3	3	3	3	2	2	2
1	1	1	1	9	1	1	1	2	1
3	3	3	1	1	1	1	1	3	1
1	2	1	1	1	3	9	1	9	9
3	3	1	1	1	1	1	1	1	1
3	3	1	1	1	3	1	1	2	1
3	3	1	1	1	2	1	1	3	1
3	3	1	1	2	2	1	9	1	2
3	1	1	1	1	3	1	9	9	9
2	1	3	3	1	1	1	1	1	2
3	3	3	1	2	2	2	1	1	1
2	2	1	1	1	1	1	1	2	1
3	3	1	1	1	3	1	1	3	1
3	3	1	1	1	3	1	1	1	1
3	3	1	1	1	1	1	1	1	1
3	3	3	1	1	3	1	1	3	1
3	3	1	1	2	2	1	1	1	1
3	3	3	1	1	3	2	1	3	1
3	2	1	1	1	2	2	2	1	1
9	9	9	1	9	9	9	9	9	9
3	3	1	1	1	3	1	1	1	2
2	1	1	1	1	2	1	1	2	1
3	3	1	1	1	1	1	1	1	1
3	3	1	1	1	3	1	1	1	1
9	9	9	1	9	9	9	9	9	9
3	1	1	1	1	3	1	1	1	1
9	9	9	9	9	9	9	9	1	2
3	3	3	3	3	3	3	3	3	1
3	3	1	1	1	3	1	1	3	1
3	3	1	1	1	3	1	1	3	1
3	1	1	1	1	9	1	9	9	1
9	9	9	9	9	9	9	9	9	9
9	9	9	9	9	9	9	9	9	9
2	2	1	1	1	2	1	1	3	1
3	3	1	1	1	3	1	1	3	1
3	3	1	1	1	3	1	1	2	1
3	3	1	1	1	1	1	1	1	1
3	3	3	1	9	1	1	1	1	1
9	9	9	1	1	1	1	1	1	1
2	3	1	1	1	1	1	1	1	1
3	3	1	1	1	1	1	1	1	1
2	3	1	1	1	1	2	1	1	1
3	3	1	1	3	1	1	1	1	1
3	3	1	1	1	1	1	1	1	1
3	1	1	1	1	1	1	1	1	1

2	3	1	1	1	1	1	1	1	1
3	3	1	1	1	1	1	1	1	1
9	9	9	1	1	1	1	1	1	1
3	3	1	1	1	1	1	1	1	1
9	9	1	1	1	1	1	1	1	1
3	3	1	1	9	9	9	1	1	1
3	3	1	1	1	1	1	1	1	1
3	3	3	1	1	1	1	1	1	1
9	9	9	9	9	9	9	9	9	9
1	3	1	1	1	3	1	3	3	1
3	3	1	1	1	3	1	1	1	1
1	3	1	1	1	3	1	1	1	1
2	1	1	1	1	2	1	9	9	1
3	2	1	9	9	9	1	1	3	1
9	9	9	1	9	9	9	1	9	9
9	9	1	1	1	3	1	9	9	9
3	1	3	1	1	3	1	1	1	1
3	9	9	1	1	2	1	1	1	1
3	3	1	1	1	2	1	2	3	2
9	9	1	9	9	9	1	9	9	9
1	3	1	1	1	3	1	1	2	1
3	3	1	1	1	3	1	2	1	1
9	9	9	1	9	9	1	1	1	1
3	3	1	1	1	3	1	3	1	1
3	3	1	1	1	2	1	1	1	1
3	3	1	1	1	3	1	2	2	1
3	3	1	1	1	3	1	1	3	1
3	3	1	1	1	3	1	1	3	2
3	3	1	1	1	3	1	1	3	2
3	3	1	1	1	1	1	1	1	1
9	9	1	1	1	2	1	1	9	1
1	1	1	1	2	2	1	1	1	1
3	3	1	1	1	3	1	9	9	1
2	3	1	1	1	3	1	1	2	1
1	1	1	1	2	2	1	1	1	1
1	1	1	1	2	3	1	1	1	1
2	3	1	2	3	3	1	1	1	1
2	3	1	1	1	2	1	1	2	1
2	2	1	1	1	2	1	1	9	1
3	3	1	1	1	3	1	1	3	1
3	3	1	1	1	3	1	1	1	1
9	9	9	1	9	9	1	1	3	1
3	3	1	1	3	3	1	3	1	3
9	9	1	1	9	9	1	1	9	9
3	3	1	1	1	2	1	1	2	1
3	3	1	1	1	3	1	1	1	1
3	3	1	1	2	2	2	1	3	1

3	3	3	1	1	2	1	2	1	1
9	9	9	1	9	9	9	9	9	9
9	9	9	1	9	9	9	9	9	9
3	3	1	1	1	3	1	1	3	1
9	1	1	1	1	3	1	1	1	1
9	9	1	1	1	3	1	9	9	9
3	3	1	1	1	3	1	1	3	1
9	9	1	1	1	3	1	2	1	1
3	9	1	1	1	1	1	2	2	1
3	2	1	1	1	3	1	3	1	1
3	3	1	1	1	3	1	3	3	1
3	3	1	1	1	2	1	1	1	2
3	2	2	1	2	2	1	2	1	2
3	2	1	1	1	3	1	1	1	1
3	3	1	1	1	3	1	1	1	1
3	3	1	1	1	2	1	1	2	2
3	3	1	1	1	2	1	1	1	1
9	9	9	1	9	9	9	1	9	9
9	9	9	1	9	9	9	1	9	9
9	9	9	1	9	9	9	9	9	9
9	9	9	1	9	9	9	1	9	9
9	9	9	1	9	9	9	9	9	9
3	3	3	3	1	3	1	3	1	1
3	2	1	1	1	3	1	3	9	1
3	3	1	1	1	3	1	9	9	9
3	1	1	1	1	3	1	3	1	1
3	3	1	1	1	1	1	1	2	9
2	2	1	1	1	2	1	3	3	1
1	2	1	1	1	2	1	3	2	1
3	3	1	1	2	3	2	1	2	3
3	3	1	1	1	2	1	3	3	1
9	9	1	1	1	9	1	2	9	1
3	1	1	1	1	3	1	1	2	1
3	1	1	1	1	3	1	1	3	1
3	3	1	1	1	2	1	9	9	1
3	3	1	1	1	3	1	3	3	1
3	3	1	1	1	2	1	1	2	1
9	9	1	9	1	9	9	1	1	1
3	3	1	1	9	9	9	1	1	1
1	3	1	1	1	2	1	1	2	1
3	3	1	1	1	3	1	1	1	1
3	3	3	2	2	2	1	1	2	1
3	3	1	1	2	1	1	1	3	1
3	2	1	1	1	1	1	1	1	1
9	9	9	1	1	1	1	1	1	3
3	9	1	1	1	1	1	1	1	1
2	3	2	1	2	1	2	1	3	1

2	2	2	1	2	1	2	1	3	1
3	2	1	1	1	2	1	1	3	1
3	1	1	1	1	1	1	1	2	2
3	1	1	1	1	1	1	1	1	1
9	9	1	1	1	2	1	1	1	1
3	3	3	1	1	3	1	1	1	1
3	3	1	1	1	3	1	1	1	1
3	3	1	1	1	2	1	1	9	1
3	3	1	1	1	2	1	1	2	1
3	3	1	1	1	2	1	1	2	2
2	2	1	2	1	1	1	1	3	1
3	3	1	1	2	3	1	2	2	2
3	2	1	1	1	2	2	1	3	1
3	2	1	1	1	3	1	1	2	1
3	2	1	1	1	2	2	1	2	1
2	3	1	1	1	3	1	1	1	1
3	2	1	1	1	2	1	1	3	1
2	2	2	2	2	2	2	2	2	2
3	1	2	1	1	3	1	2	3	2
3	3	1	1	1	3	1	1	3	1
2	2	2	1	1	2	2	2	2	2
3	3	1	1	1	2	1	1	3	1
3	3	2	1	1	2	1	2	2	2
2	2	2	2	2	2	2	2	2	2
2	2	2	2	2	2	2	1	2	1
3	3	1	1	1	3	1	1	2	1
3	3	1	1	1	3	1	1	2	1
2	3	1	1	1	3	2	1	9	9
3	3	1	1	2	2	1	1	3	1
3	3	1	1	2	1	1	1	3	1
3	3	1	1	2	2	1	1	1	1
2	3	1	1	1	2	1	1	1	1
2	2	2	1	1	1	1	1	1	2
1	9	9	1	2	9	1	1	1	1
1	9	9	1	1	3	1	1	3	3
3	3	1	3	1	3	1	3	3	1
2	2	1	1	1	2	1	1	2	1
3	3	1	2	1	2	1	1	2	1
9	9	9	9	9	9	1	1	9	9
9	9	1	9	9	9	1	9	9	9
9	9	1	9	9	9	1	9	9	9
9	9	1	9	9	9	1	9	9	9
9	9	9	9	9	9	1	1	9	9
9	9	9	9	9	9	1	1	9	9
9	9	9	1	9	9	9	1	9	9
9	9	9	1	1	1	1	9	9	9
3	3	1	1	1	2	1	1	3	1

9	9	9	9	9	9	1	1	9	9
9	9	9	9	9	9	1	1	9	9
2	3	1	1	2	3	1	1	1	1
9	9	9	1	9	9	9	1	9	9
9	9	9	1	9	9	9	1	9	9
9	9	9	1	9	9	9	1	9	9
3	3	1	1	1	3	1	1	2	1
2	3	2	2	1	2	3	2	3	1
9	9	9	1	9	9	9	1	9	9
9	9	9	9	9	9	1	1	9	9
9	9	9	9	9	9	1	1	9	9
9	9	1	9	9	9	1	1	9	9
9	9	9	1	9	9	9	9	9	1
9	9	9	1	9	9	9	1	9	1
3	3	1	1	1	3	1	1	9	9
9	9	9	1	9	9	9	1	9	9
3	3	1	1	1	3	1	1	3	1
3	3	1	1	1	1	1	1	3	1
3	3	1	1	1	1	1	1	3	1
3	3	1	1	1	3	1	1	1	1
3	3	1	1	1	3	1	1	1	1
3	3	1	1	1	3	1	1	1	1
1	3	1	1	1	3	1	1	1	1
1	1	1	1	1	3	2	1	1	1
3	3	1	1	1	1	1	1	3	1
3	3	1	1	1	1	1	1	1	1
3	3	1	1	1	3	1	1	1	1
3	3	2	1	3	1	1	1	3	1
9	9	9	9	9	9	1	9	9	1
2	3	1	1	2	3	1	2	1	2
1	3	1	3	1	2	2	3	2	1
3	1	1	1	1	2	2	1	3	1
9	9	9	9	1	1	9	3	3	3
3	3	1	1	1	3	1	1	1	1
3	3	1	1	1	3	1	1	3	1
2	2	2	1	1	3	1	1	3	1
3	3	3	1	1	3	1	1	3	1
2	3	1	1	2	3	1	1	3	2
3	3	1	1	1	2	1	1	3	1
3	3	1	1	2	3	1	1	3	1
3	1	2	1	1	1	1	1	3	1
3	3	1	1	1	3	1	1	1	1
3	1	1	1	1	3	1	1	3	1
3	3	1	1	1	3	1	1	3	1
3	3	1	1	1	3	1	1	3	1
3	3	1	1	3	3	1	1	2	1
3	3	1	1	1	3	1	1	3	1

3	3	1	3	1	3	1	1	1	1
3	1	1	1	1	2	3	1	2	2
3	3	1	1	3	3	1	1	3	1
3	3	1	1	3	3	1	1	3	1
3	3	1	9	3	3	1	1	3	1
3	3	1	1	2	3	1	1	2	1
3	3	1	2	2	3	1	1	1	1
3	3	1	1	1	3	1	1	1	1
3	1	1	1	1	2	1	1	2	1
3	3	1	1	3	3	1	1	3	1
3	3	1	1	3	3	1	1	3	1
3	3	1	1	3	3	1	1	3	1
3	3	1	1	2	3	1	1	2	1
3	3	1	1	2	3	1	1	1	1
3	3	1	1	1	3	1	1	2	1
3	3	3	1	1	3	1	1	1	1
3	3	1	1	1	2	1	1	1	1
3	1	1	1	1	2	1	1	1	1
1	1	1	1	1	3	1	1	1	1
1	1	1	1	1	3	1	1	1	1
3	3	1	3	1	3	1	1	1	1
3	3	1	1	1	1	1	2	1	1
3	2	1	1	1	3	2	1	1	1
2	2	2	1	2	2	2	1	2	1
3	1	1	3	3	3	1	3	1	3
3	3	1	1	1	3	1	1	2	1
3	3	1	1	1	2	1	1	3	1
3	3	1	1	1	1	1	1	1	1
3	3	1	1	1	1	1	1	3	1
3	3	1	1	1	1	1	2	3	1
3	3	1	1	1	1	1	9	1	1
3	3	1	1	1	2	1	2	2	1
3	1	1	1	2	3	1	2	2	2
2	2	1	2	1	2	2	2	2	2
3	3	1	1	2	2	1	1	1	1
3	2	1	1	1	2	1	2	2	2
3	3	2	1	2	2	2	2	2	2
3	3	1	1	2	3	1	1	2	1
9	1	9	1	1	3	1	1	3	1
3	3	1	1	1	3	1	1	2	1
3	3	1	1	1	1	1	1	3	1
3	3	1	1	3	3	1	1	3	1
1	2	1	1	1	3	1	1	3	3
3	1	1	3	3	3	2	1	3	2
1	3	1	1	1	3	1	3	3	1
3	1	1	1	1	3	1	1	3	1
2	2	1	1	1	3	1	2	2	2

3	3	1	1	1	3	1	1	2	1
3	3	1	1	1	3	1	1	2	1
2	2	1	1	1	2	2	1	3	1
3	3	1	1	1	1	1	1	2	1
3	3	1	1	2	2	1	1	2	1
3	1	2	1	1	3	1	3	3	2
3	1	1	1	1	3	1	2	3	1
2	1	2	1	1	1	1	2	2	2
1	1	1	1	1	3	1	1	2	1
3	1	1	1	1	3	1	1	3	1
3	3	1	1	1	2	1	1	1	1
3	3	1	1	1	1	2	2	1	1
2	3	1	1	1	2	1	3	3	3
3	3	1	1	1	3	2	2	2	2
2	3	3	3	3	3	1	1	2	2
3	3	1	1	1	3	1	2	1	1
3	3	1	1	2	2	1	2	1	1
3	3	1	1	1	3	1	1	2	1
9	9	9	1	9	9	9	9	9	9
3	1	1	1	1	2	1	1	3	2
3	3	1	1	1	3	3	1	1	3
2	2	2	1	1	1	1	3	3	1
3	3	1	1	1	2	1	1	3	1
3	3	1	1	1	1	1	1	1	1
3	3	1	1	1	1	1	1	1	1
3	3	1	1	1	2	2	1	2	1
3	1	1	1	3	3	1	1	3	1
3	3	1	1	2	2	1	2	1	1
3	3	3	1	3	3	1	1	1	1
3	3	1	1	1	3	1	1	3	1
3	2	2	1	1	3	3	1	1	1
3	3	1	1	1	3	1	1	1	1
3	3	1	1	1	3	1	1	1	1
9	9	9	9	9	9	9	1	1	1
9	9	9	1	9	9	9	1	1	1
2	9	2	1	2	2	1	1	9	9
2	3	1	1	1	3	2	2	3	2
3	3	1	1	1	3	1	1	2	1
3	1	1	1	1	2	1	1	2	2
3	3	1	1	1	2	1	1	2	1
9	9	1	1	3	3	1	1	9	9
1	1	1	1	1	3	1	1	2	1
2	2	2	1	2	2	2	2	2	2
9	9	9	3	9	9	9	9	9	9
1	1	1	1	1	2	1	1	1	1
3	3	1	1	1	2	1	1	2	1
3	2	1	1	1	1	1	9	9	9

3	3	1	3	1	3	1	1	2	1
2	2	1	1	1	2	1	1	2	1
9	9	1	9	9	9	1	9	9	9
3	3	2	1	1	1	1	2	2	2
2	2	2	2	2	2	2	2	3	1
3	1	1	1	2	3	1	1	1	1
3	3	1	1	1	3	1	3	2	2
2	2	1	1	1	2	1	1	1	1
3	3	1	1	2	2	1	1	2	1
2	1	1	1	2	2	1	1	2	2
3	2	2	1	2	2	2	1	2	1
3	3	1	1	1	3	1	3	2	2
3	3	1	2	1	3	3	1	1	1
9	9	1	1	9	9	9	9	1	9
2	2	2	2	1	2	2	2	2	2
3	2	1	1	3	1	1	1	2	3
3	3	1	1	1	2	1	1	1	1
3	2	1	2	1	2	2	2	2	1
3	3	1	1	1	1	1	1	3	1
2	2	2	2	2	2	2	2	2	2
2	1	1	1	1	3	1	1	3	3
1	1	1	1	1	3	1	1	3	3
3	3	1	1	1	3	1	1	2	1
2	1	1	1	1	1	1	1	1	1
3	3	1	1	1	2	1	1	1	2
1	1	1	1	1	1	1	1	1	1
1	1	1	1	1	1	1	1	1	1
3	1	1	1	1	2	1	1	1	1
3	3	1	2	1	2	2	1	1	2
3	2	1	3	1	3	1	3	1	1
3	2	1	1	1	2	1	2	1	2
1	1	2	1	1	1	2	1	1	1
3	3	1	1	1	3	1	1	1	1
3	2	1	1	1	2	1	1	1	1
3	2	1	1	1	2	1	1	1	1
9	9	9	1	9	2	9	9	9	9
3	2	1	1	1	2	1	1	1	1
3	3	1	1	1	1	1	1	1	1
3	2	1	1	1	1	1	1	1	1
3	3	1	1	1	1	1	1	1	2
1	2	1	1	2	3	1	1	2	1
2	1	1	1	1	2	1	1	1	1
3	1	1	1	1	2	1	1	1	1
9	9	9	9	9	9	9	1	1	1
3	3	1	1	1	3	1	1	1	1
3	3	1	1	1	3	1	1	1	1
3	3	1	1	1	1	1	2	1	1

3	3	1	1	1	3	1	1	1	1
2	1	1	1	1	3	1	1	1	1
3	3	1	1	1	1	1	1	1	1
3	1	1	1	1	1	1	1	1	1
3	3	1	1	1	3	1	3	1	1
1	1	1	1	1	1	1	2	1	1
1	1	1	1	1	1	1	1	1	1
1	1	1	1	1	1	1	1	1	1
3	3	1	1	1	3	1	1	1	1
3	2	1	1	1	2	1	1	1	1
1	1	1	1	1	1	1	1	1	1
1	1	1	1	1	1	1	1	1	1
3	9	1	1	1	3	1	3	1	1
3	1	1	3	1	3	1	1	1	1
3	3	1	1	1	1	1	1	3	1
3	3	1	1	1	1	1	1	3	1
3	3	1	1	1	3	1	1	3	1
3	3	1	1	1	3	1	1	3	1
3	3	1	1	1	2	1	3	3	1
3	3	1	3	1	1	3	1	3	1
3	3	1	1	1	3	1	1	3	1
3	3	1	1	1	3	1	1	3	1
1	2	1	1	1	2	2	1	2	2
1	1	1	1	1	3	1	1	3	1
3	3	1	1	1	3	1	1	3	1
3	3	1	1	1	1	1	1	3	3
2	1	1	1	3	1	1	1	1	1
3	3	1	1	2	2	1	1	3	3
9	9	9	9	9	9	1	1	9	9
9	9	9	9	9	9	1	1	9	9
9	9	9	9	9	1	9	9	9	1
3	3	1	1	1	3	1	1	9	9
3	1	1	1	1	3	1	1	3	3
9	9	1	9	9	9	1	9	9	9
9	9	9	2	9	9	9	2	9	9
9	9	9	1	9	9	9	9	9	9
9	9	1	9	1	9	9	1	9	9
9	9	1	9	1	9	9	1	9	9
9	9	1	9	9	2	9	1	9	9
2	1	2	1	2	1	1	1	9	9
2	3	1	1	1	3	1	1	1	1
3	3	1	1	1	3	1	1	1	1
3	3	1	1	1	1	1	2	1	1
3	3	1	1	1	3	1	1	3	1
3	3	1	1	1	3	1	1	1	1
3	3	1	1	1	3	1	1	1	1
3	1	1	1	1	2	1	1	1	1

3	3	1	1	1	3	1	1	3	1
2	3	1	1	1	2	2	2	3	1
3	3	1	2	1	2	1	2	3	1
3	3	1	1	1	2	2	1	2	3
1	1	1	1	1	1	3	1	1	3
3	3	1	1	1	1	1	1	3	2
1	1	1	2	2	1	2	1	2	2
3	3	1	1	1	2	2	2	1	1
3	9	1	1	3	2	1	2	3	1
3	3	1	1	1	3	1	3	3	1
1	1	1	1	1	1	1	1	1	1
3	3	1	1	1	3	1	1	1	1
9	9	9	9	9	9	9	9	9	9
3	2	3	1	1	3	2	2	2	1
2	3	1	1	1	1	1	1	3	2
2	3	1	2	2	2	2	3	3	3
3	3	3	1	1	1	1	1	1	2
3	3	1	1	1	2	1	1	1	1
1	1	1	2	2	2	1	1	3	1
3	2	2	3	3	3	3	2	2	3
3	3	1	1	1	3	1	1	3	1
1	1	1	1	9	3	1	2	2	2
3	3	1	1	1	1	1	1	1	1
1	2	1	9	9	9	9	1	2	3
2	1	2	1	2	3	1	1	2	3
1	1	1	1	1	1	1	1	1	1
2	1	2	1	1	1	1	1	2	1
2	2	2	3	3	2	2	2	2	2
9	9	9	9	9	9	9	1	3	2
2	2	1	1	1	1	1	2	9	1
1	1	1	1	1	1	1	1	1	1
1	1	3	2	3	2	1	1	2	3
3	3	1	2	1	2	3	1	2	1
3	3	1	1	1	1	1	1	1	1
2	2	2	2	2	2	2	2	2	2
3	3	1	1	1	3	2	2	2	2
3	3	1	1	1	3	1	1	1	1
1	1	1	1	1	1	1	1	1	1
3	1	2	2	2	1	3	2	2	2
3	3	1	1	2	3	1	2	1	1
3	2	1	1	1	3	2	2	2	1
9	9	9	9	9	9	9	1	1	2
2	2	2	2	2	2	2	2	2	2
3	1	1	1	1	3	1	1	3	1
3	2	1	1	1	3	1	3	3	2
3	3	1	1	1	1	1	2	1	1
3	2	1	1	1	2	1	1	2	1

3	2	1	1	1	2	1	1	2	1

RhandtoLb	Fingerpads	Fingernails	Palms	Onefinger	Threemidd	FingersinCi	FingersVer	FingersWe	PressNippl
3	2	3	2	2	2	2	3	2	2
1	3	2	1	2	1	1	1	3	2
1	2	1	2	2	2	3	2	2	2
1	3	2	1	2	1	1	1	3	2
9	9	9	9	9	9	9	9	9	9
1	3	2	1	2	1	1	1	3	2
1	2	2	2	2	2	2	2	2	1
1	1	2	3	2	2	1	2	2	1
9	2	9	2	9	9	2	9	9	9
9	1	9	9	9	9	9	9	9	9
1	9	2	9	2	2	1	2	2	2
1	3	3	3	1	2	2	2	2	1
1	1	2	2	1	2	2	2	2	2
1	2	3	2	3	2	1	3	2	1
1	2	2	2	2	2	2	2	2	2
2	2	3	1	3	3	2	2	2	1
1	2	3	2	2	1	1	2	1	1
2	2	3	1	3	3	2	2	2	1
2	2	3	1	3	3	2	2	2	1
1	1	3	2	3	3	2	1	2	3
1	1	2	1	1	3	1	1	1	1
1	1	2	1	1	3	1	1	1	1
1	2	1	9	2	2	9	9	9	1
1	1	3	1	1	2	1	1	2	1
2	2	2	2	2	2	2	2	2	2
1	2	3	2	2	2	2	2	2	2
2	2	2	2	2	2	2	2	2	2
1	2	3	2	3	2	2	1	2	1
1	3	3	1	3	9	1	1	1	1
1	3	3	1	3	2	2	2	2	2
1	3	3	1	3	2	2	2	2	2
1	1	3	1	3	2	9	1	2	1
1	1	3	3	2	1	1	2	2	1
1	1	3	1	3	2	1	2	1	1
1	1	3	3	3	2	9	1	2	1
1	1	3	1	3	2	1	2	1	1
1	1	3	3	3	2	9	1	2	1
1	1	3	3	3	9	1	1	1	1
1	3	3	1	3	1	1	1	1	1
1	3	3	1	3	9	1	1	1	1
1	3	2	1	2	1	1	1	1	1
1	2	2	2	3	9	2	2	2	2
2	1	3	1	3	2	2	1	2	1
1	1	3	1	3	3	1	1	3	1
1	3	3	1	3	1	3	2	1	1
9	9	9	9	9	9	9	9	9	9

2	2	3	3	3	2	3	2	1	1
1	2	1	2	2	1	1	1	1	2
1	9	9	9	9	1	1	1	1	1
1	1	3	2	2	2	1	1	2	1
1	2	3	2	3	2	1	9	1	1
1	1	3	1	3	3	1	1	1	1
1	3	3	3	3	1	1	3	3	1
1	1	3	3	9	1	1	1	1	1
1	9	9	9	9	9	1	9	9	9
1	3	2	3	1	2	1	1	3	2
9	9	9	9	9	9	9	9	9	9
1	3	3	3	1	1	1	1	1	1
1	1	3	2	3	1	1	1	1	2
1	2	9	1	9	3	2	1	9	2
2	3	2	1	2	3	1	1	1	2
1	1	3	3	3	2	1	1	1	3
1	2	2	2	3	1	1	2	2	1
1	2	9	1	1	9	1	1	1	1
2	1	3	1	3	2	2	1	2	1
1	3	2	2	3	1	2	1	2	1
9	9	9	9	9	9	1	9	9	9
9	9	9	9	9	3	1	3	3	1
9	9	9	9	9	9	1	9	9	9
1	2	1	2	3	3	1	2	9	1
9	9	1	9	9	9	9	1	9	9
1	1	3	1	2	3	1	1	1	1
1	1	3	1	3	3	1	1	1	1
1	1	2	1	3	2	1	1	1	2
1	3	3	3	3	1	1	1	1	1
1	1	2	1	2	2	1	1	9	1
1	1	2	1	2	3	1	1	1	1
1	2	1	1	3	3	1	3	1	3
1	1	3	1	2	3	1	1	1	1
1	3	3	1	3	2	1	1	2	3
1	2	2	3	3	2	1	2	2	1
1	3	3	3	2	1	1	1	1	9
1	1	1	1	1	1	1	1	1	1
1	2	2	3	3	1	1	1	1	3
1	1	3	1	3	3	1	1	1	1
1	1	3	1	3	3	1	1	1	1
1	1	2	3	3	1	2	1	9	1
1	3	3	3	3	3	9	9	9	9
1	1	3	1	1	1	1	1	3	3
1	1	1	1	1	2	1	1	1	1
1	3	3	1	3	3	1	1	2	1
1	1	3	3	9	9	1	1	2	2
1	1	9	9	9	9	9	9	9	9

1	2	3	2	2	1	1	1	1	1
1	2	2	2	2	2	1	1	1	2
1	2	3	3	3	1	1	1	1	1
1	2	2	2	2	1	1	1	1	2
1	1	3	3	1	1	1	1	3	2
1	1	2	2	2	2	1	1	2	1
1	1	1	2	3	1	1	1	1	1
1	3	1	3	3	1	1	2	2	1
1	2	3	2	1	1	1	2	2	1
1	1	2	3	3	1	1	1	1	1
1	1	1	1	3	3	1	2	2	2
1	2	3	2	2	2	1	1	1	1
1	1	1	3	3	1	1	1	1	1
1	1	3	3	3	1	1	1	1	1
1	2	3	3	1	2	1	1	2	1
1	2	3	1	2	3	1	2	1	2
1	2	2	1	2	2	2	2	1	2
1	1	3	2	1	1	1	3	3	1
1	2	1	2	1	1	1	1	1	1
9	9	9	9	9	9	9	9	9	9
1	2	2	2	3	2	1	1	1	1
1	1	3	1	3	3	1	1	2	1
2	2	2	3	1	2	2	2	2	2
1	3	3	3	3	1	1	9	9	9
1	1	1	3	3	1	1	1	1	3
1	1	3	2	2	2	1	2	2	2
1	1	9	3	3	2	2	1	2	1
1	3	2	1	3	2	1	2	1	1
2	2	2	2	2	2	2	2	2	2
2	2	2	3	2	2	2	2	2	2
2	2	2	2	2	2	2	2	2	2
2	2	3	2	1	2	2	2	2	2
2	1	3	1	3	2	1	1	3	1
2	2	2	2	2	2	3	2	3	2
2	2	2	2	2	2	2	2	2	2
3	3	2	1	3	9	2	2	1	2
2	1	2	2	2	1	9	1	2	2
1	1	3	1	3	3	3	2	2	2
9	9	1	2	9	9	9	9	9	9
1	3	3	1	3	2	3	2	2	1
1	2	2	3	3	1	1	2	1	3
1	2	2	3	3	1	1	1	2	1
2	2	3	2	2	2	2	2	2	2
1	3	3	2	2	2	1	1	1	1
1	2	2	3	2	2	1	2	2	2
1	1	2	3	2	2	1	1	2	1
2	2	2	1	2	2	1	2	2	1

3	3	3	3	3	3	3	3	3	3
1	1	3	1	2	3	2	1	2	1
1	1	3	2	2	2	2	1	1	1
1	2	2	3	3	1	1	1	2	1
1	1	1	1	1	1	1	1	1	1
1	1	3	3	2	1	1	1	2	1
2	2	3	3	3	3	3	2	2	3
1	2	3	1	3	3	2	2	2	1
2	2	2	2	2	2	2	2	2	2
2	2	2	2	2	2	2	2	2	2
2	3	2	2	9	3	2	2	2	3
2	3	3	2	2	3	2	2	2	3
9	9	9	9	9	9	9	1	9	9
1	1	1	2	2	9	1	1	2	2
1	1	3	2	2	2	2	1	1	1
1	1	3	2	2	2	2	1	1	1
1	9	9	9	9	9	9	9	9	9
1	1	3	3	1	1	1	1	1	3
9	9	9	9	9	9	9	9	9	9
1	1	1	1	3	3	1	1	1	1
9	9	9	9	9	9	9	1	1	1
1	1	1	1	3	3	1	1	1	1
1	1	1	1	3	3	1	1	1	1
1	1	1	1	3	3	1	1	1	1
1	1	1	1	3	3	1	1	1	1
1	1	1	1	3	3	1	1	1	1
1	1	1	1	3	3	1	1	1	1
1	1	1	3	3	9	1	1	1	1
1	1	1	1	3	3	1	1	1	1
1	1	1	1	3	3	1	1	1	1
9	2	3	3	2	2	3	1	3	2
3	2	2	2	2	2	1	1	2	2
9	9	9	9	9	9	9	9	9	9
1	1	1	1	3	3	1	1	1	1
1	1	1	1	3	3	1	1	1	1
1	1	1	1	3	3	1	1	1	1
1	1	1	1	3	3	1	1	1	1
1	1	1	1	3	3	1	1	1	1
1	1	1	3	3	1	1	1	1	1
1	9	1	1	1	3	1	1	1	1
1	1	1	1	3	3	1	1	1	1
1	1	1	1	3	3	1	1	1	1
9	9	9	9	9	9	9	9	9	9
1	1	1	1	1	3	3	1	1	1
1	1	1	1	3	3	1	1	1	1
1	1	1	3	1	1	1	1	1	1
1	1	1	1	3	3	1	1	1	1

1	1	1	1	3	3	1	1	1	1
1	1	1	1	1	3	1	1	1	1
1	1	1	1	1	3	1	1	1	1
1	1	1	1	3	3	1	1	1	1
1	1	1	1	1	1	1	1	1	1
1	1	1	1	2	2	1	1	1	1
1	1	1	1	3	3	1	1	1	1
1	1	3	3	3	1	1	1	1	1
1	1	3	1	3	1	1	1	1	1
1	1	3	2	3	1	1	1	1	1
1	1	3	3	3	1	1	1	1	1
1	1	3	3	3	1	1	3	1	3
1	1	3	1	3	3	1	1	1	1
9	9	9	9	3	3	1	1	1	1
1	2	2	1	1	3	1	2	2	2
1	2	2	1	3	3	2	1	1	1
2	2	2	2	1	1	1	1	1	1
1	1	1	1	1	1	1	1	1	1
1	2	2	1	2	2	1	2	2	1
2	2	2	2	2	2	2	2	2	2
1	1	2	2	3	1	1	1	1	2
2	2	2	2	2	2	2	2	2	2
1	3	2	1	2	2	1	2	1	3
1	1	3	1	2	2	2	1	2	1
2	2	2	2	2	2	2	2	2	2
2	2	2	2	2	2	2	2	2	2
2	2	2	2	2	2	2	2	2	2
2	2	2	2	2	2	2	2	2	2
2	2	2	2	2	2	2	2	2	2
1	2	2	1	2	2	2	2	2	1
2	2	2	2	2	2	2	1	1	2
2	2	2	2	2	2	2	1	1	2
1	2	2	2	2	2	1	2	2	1
1	2	3	3	2	2	2	1	1	3
2	2	2	1	2	2	2	2	1	1
2	2	2	2	2	2	2	2	2	2
2	2	2	2	1	1	1	1	2	2
1	1	2	3	3	3	1	1	1	2
2	2	2	2	2	2	2	1	2	2
3	3	3	1	3	3	2	3	1	1
3	2	1	2	2	2	2	2	2	2
2	2	2	2	2	2	2	2	2	2
9	9	9	9	9	9	9	9	9	9
9	9	9	9	9	9	9	9	9	9
2	3	3	3	2	2	2	3	2	2
1	2	1	3	2	1	3	2	2	1
9	9	9	9	9	9	9	9	9	9

1	2	2	2	2	2	1	2	2	1
1	2	2	2	2	2	1	2	2	1
2	2	2	2	2	2	2	1	2	2
2	2	3	2	2	2	2	2	1	1
2	3	2	1	2	1	2	2	1	2
1	1	2	1	2	3	1	1	2	1
1	1	3	1	3	3	2	1	2	1
2	2	3	2	2	2	2	2	2	1
2	2	2	3	3	3	1	1	2	1
3	3	3	3	3	3	1	1	3	3
1	1	2	2	2	2	2	3	2	2
1	2	3	3	3	1	1	2	1	1
1	3	2	3	3	3	1	1	2	1
1	3	3	3	2	2	2	2	2	1
1	3	3	1	3	2	2	2	2	1
1	3	2	3	3	3	2	3	2	2
1	2	3	3	2	2	2	2	1	2
2	2	2	2	2	2	2	2	2	2
1	2	3	3	3	2	2	3	2	2
1	1	2	2	2	2	2	2	1	1
2	2	2	1	3	3	2	2	2	1
1	2	2	2	2	2	1	1	2	1
1	3	3	3	2	2	2	2	2	1
2	2	2	2	2	2	2	3	3	3
1	9	9	1	9	9	9	9	9	1
2	2	2	2	2	2	2	2	2	2
2	2	2	2	2	2	2	2	2	2
1	2	2	3	9	2	1	9	1	2
9	9	9	9	9	9	9	9	9	9
1	1	2	2	2	2	2	1	2	2
9	9	9	9	9	9	9	1	9	9
1	1	2	2	2	9	9	9	1	1
1	1	3	3	1	3	1	3	2	3
1	2	1	2	2	2	1	2	1	2
9	9	9	9	9	9	9	9	9	9
2	2	3	2	1	2	2	2	2	2
1	2	1	2	2	2	1	2	2	1
1	1	3	2	3	2	3	2	1	2
1	1	2	2	3	1	1	1	1	1
1	1	3	1	3	2	1	1	2	3
1	3	3	1	3	3	2	2	2	1
2	2	3	2	2	2	1	1	1	1
1	2	3	1	2	1	1	1	1	1
2	2	2	2	2	2	2	2	2	1
9	9	9	9	9	9	9	9	9	9
2	2	2	9	1	2	2	2	2	1
1	2	3	2	3	1	1	2	2	1

9	9	9	9	9	9	9	9	9	9
2	1	1	2	3	1	9	9	9	9
1	1	2	1	2	1	1	1	1	1
1	1	2	1	3	9	1	1	1	1
2	2	2	2	2	2	1	3	3	3
1	3	1	2	3	2	1	1	3	1
2	1	1	2	2	2	2	2	1	1
1	1	3	1	2	3	1	1	1	1
1	1	2	2	2	2	2	3	1	1
2	2	1	2	2	2	2	2	2	2
2	2	2	2	2	2	2	2	2	2
2	2	3	1	3	2	1	1	1	2
9	9	9	9	9	9	9	9	9	9
1	1	3	3	1	1	1	1	1	3
3	1	1	3	1	3	3	1	3	3
2	1	2	2	2	9	1	1	2	2
1	1	3	3	1	1	1	1	1	2
1	2	2	1	2	1	1	1	2	1
1	2	2	2	1	1	2	3	1	2
9	9	9	9	9	9	9	9	9	9
2	3	1	2	3	2	1	2	1	3
9	2	3	2	3	1	1	1	1	1
1	3	2	3	3	1	1	2	2	1
2	2	2	2	2	2	2	2	2	2
2	2	2	2	2	2	2	2	2	1
1	9	9	9	9	1	1	1	2	1
1	3	3	3	2	1	2	1	1	1
3	1	3	9	3	9	1	1	2	2
1	3	3	1	3	3	2	1	2	2
1	1	3	3	1	1	1	1	1	2
1	1	1	3	3	1	1	1	1	2
1	2	3	3	3	3	2	3	3	3
9	9	9	9	9	9	1	9	9	9
1	3	2	3	1	1	1	3	3	3
1	3	3	1	2	1	1	1	2	2
1	1	3	3	3	1	3	2	2	1
1	1	3	1	3	3	1	1	1	1
1	1	3	3	3	3	1	1	1	1
1	1	3	3	3	1	1	1	1	1
1	1	3	3	3	1	3	1	2	1
2	3	3	3	2	2	1	3	3	3
1	1	3	3	3	1	1	1	1	1
1	2	2	2	3	2	3	2	2	3
2	2	2	2	2	2	2	2	2	2
1	1	3	3	3	3	3	3	2	1
1	2	2	2	2	2	2	1	2	1
1	3	3	3	3	1	1	1	3	1

1	1	3	3	2	2	1	1	2	2
1	1	3	3	2	2	1	1	2	2
9	9	9	9	9	9	9	1	9	9
1	3	3	3	3	3	1	1	2	1
9	9	9	9	9	9	9	9	9	9
1	2	3	3	3	2	1	2	2	1
1	1	1	2	1	1	1	1	1	1
1	1	3	9	9	9	9	1	9	2
1	9	1	1	3	3	1	1	1	1
1	2	3	1	3	2	3	2	2	1
2	2	2	2	3	3	1	2	2	3
2	3	3	3	2	2	2	2	2	2
1	1	3	2	3	3	2	2	2	1
2	3	2	2	2	2	1	1	2	2
1	3	2	2	3	3	1	2	2	3
2	1	2	2	3	1	2	2	1	1
1	3	2	2	3	3	1	2	2	2
2	1	1	1	1	2	2	2	2	1
1	1	1	1	1	3	1	1	1	1
1	1	2	2	2	2	2	2	2	2
1	2	2	3	9	2	1	1	3	2
2	2	3	2	3	1	1	1	2	1
2	2	3	1	3	3	1	1	2	2
3	3	3	3	3	3	1	1	1	1
2	2	2	2	2	2	2	2	2	2
1	2	2	2	2	2	2	2	2	2
1	2	3	1	2	2	2	2	2	1
2	2	3	2	3	2	2	2	2	2
2	2	3	2	2	3	2	2	1	1
1	1	1	1	1	2	1	1	2	1
1	2	3	2	2	2	2	2	2	2
1	2	3	2	2	2	2	2	2	2
2	3	3	2	2	1	2	3	2	3
2	2	2	2	2	2	2	2	2	2
2	2	2	2	2	2	2	2	2	2
2	3	3	2	2	2	1	1	2	3
1	3	3	2	3	3	2	2	3	1
9	9	9	9	9	9	1	1	9	1
2	2	2	2	2	2	2	2	2	2
1	2	2	3	2	2	2	2	3	2
2	2	2	1	2	2	1	2	2	2
2	2	2	1	2	2	2	1	2	1
1	2	2	2	2	3	1	1	1	1
2	2	2	2	2	2	2	2	2	1
2	2	2	1	2	2	1	2	2	1
1	1	3	1	2	2	1	1	2	1
2	2	2	2	2	2	2	2	2	2

2	1	3	3	3	3	2	2	1	2
1	1	2	3	2	1	1	1	1	2
2	1	2	2	2	2	3	3	2	3
1	2	2	1	2	2	1	2	2	2
3	2	3	2	2	2	1	2	2	1
2	2	3	2	3	3	1	1	2	2
2	2	2	1	2	2	2	1	9	2
2	2	3	1	3	3	1	1	1	1
2	2	3	2	2	1	1	1	1	2
1	2	3	1	2	2	1	1	2	1
1	2	1	2	1	2	1	2	2	2
1	1	1	1	2	2	1	1	1	1
1	3	3	1	3	3	1	1	2	2
2	2	2	2	2	2	2	2	2	2
2	2	2	2	2	2	2	2	2	2
1	1	1	1	1	2	1	1	1	2
1	3	3	1	3	3	1	2	2	1
2	2	2	2	2	2	1	1	1	1
2	2	2	2	2	2	1	1	1	1
9	9	9	9	9	9	9	9	9	9
1	2	2	1	2	2	1	1	2	1
2	2	2	2	2	2	2	2	2	2
9	9	9	9	9	9	9	9	9	9
2	2	2	2	2	2	2	2	2	2
1	1	3	3	2	2	1	1	1	1
1	3	3	9	1	2	9	2	3	1
1	2	2	2	2	2	2	2	2	3
9	9	9	9	9	9	9	9	9	9
2	2	2	2	2	1	2	2	2	2
3	1	2	1	3	2	1	3	1	2
1	2	1	2	2	2	2	2	2	2
2	2	1	3	3	2	3	1	1	2
2	2	1	2	2	2	2	2	2	2
1	1	3	1	1	2	1	1	1	1
1	1	1	2	1	1	1	1	1	1
1	3	2	1	2	1	2	1	1	1
3	3	3	3	3	3	3	3	3	3
2	1	2	1	2	3	2	1	3	2
2	1	2	2	2	2	2	2	1	3
1	1	3	1	2	2	1	1	2	2
2	1	1	2	3	2	2	3	2	1
1	3	2	1	2	1	1	1	3	2
2	1	3	2	3	3	1	1	1	1
3	2	3	3	3	2	3	3	3	3
9	9	9	9	9	9	9	9	9	9
1	1	1	3	3	1	1	1	1	1
9	9	9	9	9	9	1	1	1	9

1	1	1	1	1	1	1	1	1	1
1	1	1	1	3	3	1	1	1	1
1	1	1	1	3	3	1	1	1	1
1	1	1	1	3	3	1	1	1	1
1	1	1	1	1	1	1	1	1	1
1	1	1	1	3	3	1	1	1	1
1	1	1	1	3	3	1	1	1	1
1	1	1	1	3	3	1	1	1	1
1	1	1	1	1	3	3	1	1	1
1	1	1	1	1	3	1	1	1	1
1	2	1	1	2	2	9	1	2	1
1	1	1	1	3	3	1	1	1	1
1	1	1	1	3	3	1	1	1	1
1	1	1	1	3	3	1	1	1	1
1	1	1	1	3	3	1	1	1	1
1	1	1	1	3	3	1	1	1	1
1	1	2	2	2	2	1	2	2	1
1	1	1	3	3	1	1	1	1	1
1	1	1	1	3	3	1	1	1	1
1	1	1	1	3	3	1	1	1	1
1	1	1	1	3	3	1	1	1	1
1	1	1	1	3	3	1	1	1	1
1	1	1	1	3	3	1	1	1	1
1	1	1	1	3	3	1	1	1	1
1	1	1	1	3	3	1	1	1	1
1	1	1	1	3	3	1	1	1	1
1	1	1	1	3	3	1	1	1	1
1	1	1	1	3	3	1	1	1	1
1	1	1	3	1	3	3	1	1	1
1	1	1	1	3	3	1	1	1	1
1	1	1	1	3	3	1	1	1	1
1	1	1	1	3	3	1	1	1	1
1	1	1	1	3	3	1	1	1	1
1	1	1	1	3	3	1	1	1	1
1	1	1	1	3	3	1	1	1	1
1	1	1	1	3	3	1	1	1	1
1	1	3	1	3	2	1	1	1	1
1	1	1	1	1	3	1	1	1	1
1	1	1	1	3	3	1	1	1	1
1	1	1	1	3	3	1	1	1	1
1	9	1	1	3	3	1	1	1	1
1	2	2	1	2	2	2	2	2	1
2	1	2	2	3	2	2	3	1	1
1	2	2	2	2	2	2	2	2	2
9	9	9	9	9	9	9	9	9	9
1	2	2	2	2	2	1	1	2	2
1	1	3	1	3	3	1	1	1	2

2	2	2	2	2	2	2	2	2	2
1	1	2	1	2	2	1	2	1	1
1	1	2	1	2	2	1	2	1	1
1	2	2	3	3	1	1	1	1	1
2	1	1	1	3	1	1	1	1	1
2	2	3	2	2	2	1	2	1	1
1	2	2	2	2	2	2	2	2	2
1	2	2	2	2	2	2	2	2	3
1	2	3	3	2	2	1	1	1	2
2	2	2	2	2	2	1	2	2	2
1	1	2	1	2	2	1	2	1	1
9	9	9	9	9	9	9	9	9	9
1	9	1	2	9	2	1	9	9	9
2	2	2	2	2	2	2	1	2	2
9	9	1	9	9	9	9	9	9	9
9	9	9	9	9	9	9	1	9	9
9	9	9	9	9	9	9	9	9	9
9	9	9	9	9	9	9	9	9	9
1	9	9	9	9	9	9	9	9	9
1	1	3	3	3	2	2	2	1	2
3	3	3	3	3	3	3	3	3	3
3	3	3	3	3	3	3	3	3	3
1	2	3	2	1	3	1	2	1	2
9	9	9	9	9	9	9	9	9	9
9	9	9	9	1	9	9	9	9	9
1	9	9	9	9	1	9	9	9	1
1	9	9	9	9	9	9	9	9	9
1	1	2	3	1	3	3	2	2	1
1	1	3	2	2	1	2	2	2	1
1	9	9	9	9	9	9	9	9	9
9	9	9	9	9	9	9	9	9	9
1	2	2	1	2	2	2	2	2	2
2	2	2	2	2	2	2	2	2	2
2	3	1	1	3	3	1	1	1	1
1	1	2	1	2	2	2	2	1	1
1	2	2	3	3	3	2	2	2	1
2	1	2	1	3	3	2	2	2	9
3	3	3	2	3	3	3	3	2	2
2	2	2	2	2	2	2	2	2	2
1	1	3	1	3	2	1	2	1	1
9	9	9	9	9	9	9	9	9	1
1	1	3	3	3	3	1	1	3	2
1	1	2	1	1	1	1	1	2	2
1	2	1	1	3	2	1	2	1	2
1	3	3	1	3	3	3	2	2	3
2	2	2	2	2	2	1	1	1	2
1	1	3	2	3	3	1	1	1	1

9	9	9	9	9	9	9	9	9	9
1	2	2	2	2	2	2	2	2	2
3	3	3	3	3	3	2	2	2	2
2	3	3	2	2	2	1	1	2	1
2	2	3	1	2	2	1	1	2	1
2	2	2	2	2	2	2	2	2	2
2	2	2	2	2	2	2	2	2	2
1	1	1	1	2	1	1	1	2	1
2	3	3	1	3	3	2	1	1	1
2	2	2	2	2	2	2	2	2	2
1	1	3	2	2	2	2	2	2	2
1	2	3	1	3	2	2	2	1	1
2	2	2	2	2	2	2	2	2	2
2	2	2	2	2	2	2	2	2	2
2	2	2	2	2	2	2	2	2	2
2	2	2	2	2	2	2	2	2	2
2	2	2	2	2	2	1	1	2	2
2	2	2	2	2	2	2	2	2	2
1	2	3	3	3	3	2	2	2	1
1	1	3	2	3	2	1	1	1	1
1	2	3	3	1	1	1	1	1	2
2	2	3	1	3	3	2	2	2	2
1	1	3	1	3	3	1	1	1	1
1	1	3	1	3	3	1	1	1	1
1	2	2	2	2	2	2	1	1	2
1	2	2	2	2	2	1	1	2	2
1	3	3	3	1	1	3	3	1	1
1	3	3	3	2	1	2	3	1	2
2	2	3	1	1	2	1	1	1	1
1	3	2	3	3	3	2	2	2	2
1	1	1	3	1	1	1	2	2	2
2	2	9	9	9	9	9	9	9	9
3	2	3	3	3	2	2	2	2	2
1	3	3	1	3	3	3	2	2	2
1	1	3	1	3	3	2	1	2	1
9	9	9	9	9	9	9	9	9	9
1	2	2	2	2	2	2	2	2	2
1	1	3	3	3	1	3	3	1	1
2	2	2	2	2	2	2	2	2	2
1	2	1	3	3	1	1	1	2	2
9	9	9	9	9	9	1	1	2	9
2	1	2	3	3	2	1	1	1	1
3	1	3	3	3	3	1	1	1	1
1	2	3	2	2	2	1	1	1	1
1	1	3	1	3	3	1	1	1	1
3	2	1	2	1	2	2	2	2	2
1	9	9	9	9	9	9	9	9	9

9	9	9	9	9	9	9	9	9	9
9	9	9	9	9	9	9	9	9	9
1	9	9	9	9	9	9	9	9	9
9	9	9	9	9	9	9	9	9	9
9	9	9	9	9	9	9	9	9	9
9	1	9	9	9	9	9	9	9	9
9	9	9	9	9	9	9	9	9	9
9	9	9	9	9	9	9	9	9	9
9	9	9	9	9	9	9	9	9	9
9	9	9	9	9	9	9	9	9	9
3	1	3	3	1	3	9	9	1	9
9	9	9	9	9	9	9	9	9	9
9	9	9	9	9	9	9	9	9	9
9	9	9	9	9	9	9	9	9	9
9	9	9	9	9	9	9	9	9	9
9	9	9	9	9	9	9	9	9	9
9	9	9	9	9	9	9	9	9	9
9	9	9	9	9	9	9	9	9	9
2	2	2	2	2	2	2	2	2	2
1	1	2	1	1	1	1	2	1	1
1	1	1	1	1	1	1	1	1	1
3	1	2	2	2	2	3	2	1	2
2	9	2	1	1	2	1	2	2	2
1	1	1	1	3	1	3	1	1	1
2	2	2	2	3	3	3	2	3	2
2	2	1	1	2	3	3	2	1	3
2	2	2	2	2	2	2	2	2	2
2	2	2	2	2	3	3	2	2	2
2	2	2	2	2	2	2	2	2	2
2	1	9	2	2	3	2	1	2	2
3	1	2	1	1	1	1	1	1	2
2	2	1	1	3	3	2	2	2	1
2	2	3	3	3	2	2	2	2	2
3	2	3	3	3	3	1	1	3	1
1	2	2	2	2	2	2	2	2	1
2	2	2	1	3	3	3	3	3	2
9	1	2	2	2	2	2	2	2	2
1	2	1	2	2	3	1	3	2	1
2	1	1	1	2	2	2	2	1	1
1	2	1	2	3	3	1	1	1	1
9	2	1	2	2	1	2	1	2	1
1	1	1	3	1	3	1	1	3	1
1	1	1	1	3	1	1	1	1	9
1	1	1	2	3	1	1	1	1	3
1	1	3	2	2	2	2	2	2	2
9	9	9	9	9	9	9	9	9	9
2	2	3	1	1	2	2	3	1	2

1	2	3	3	3	3	1	2	2	1
1	1	1	9	2	9	9	9	9	9
1	3	1	3	1	1	3	1	1	3
1	2	2	9	9	9	9	9	9	9
1	2	1	3	2	3	2	2	2	2
1	2	3	2	2	2	2	2	2	2
2	2	2	9	9	9	9	9	9	9
1	2	3	3	3	1	2	2	1	2
1	3	1	2	1	1	1	3	1	1
1	1	3	3	3	3	1	1	2	1
1	2	9	1	1	2	1	1	1	1
1	1	2	2	1	2	1	1	1	2
3	3	2	2	2	2	2	2	2	2
3	1	2	3	1	2	3	1	2	3
2	2	2	2	2	2	2	2	2	2
1	3	1	1	1	3	2	2	2	1
1	1	1	2	2	1	1	1	1	2
9	9	9	9	9	9	9	9	9	9
1	1	3	9	2	3	1	2	1	2
3	3	3	3	3	3	1	3	3	3
3	2	2	2	3	3	3	3	3	3
3	3	2	2	2	2	2	2	2	2
2	3	3	3	3	3	2	2	2	2
1	3	2	2	9	3	1	3	3	3
2	2	2	2	2	2	2	2	2	2
3	1	1	3	1	3	3	1	1	3
3	3	3	3	3	3	2	3	3	3
1	3	1	3	3	3	3	3	1	1
2	2	1	3	2	1	9	1	1	1
1	2	3	1	3	3	1	1	2	1
1	1	3	1	3	3	1	1	1	1
3	3	3	3	3	3	3	9	9	9
2	1	1	2	2	2	1	3	2	1
1	3	3	2	3	3	1	2	1	2
1	2	3	3	9	3	1	9	9	1
1	2	3	1	3	3	1	2	2	1
1	2	3	1	3	3	1	2	2	1
9	9	9	9	9	9	9	9	9	9
2	2	2	2	2	2	9	9	9	9
9	9	9	9	9	9	9	9	9	9
9	9	9	9	9	9	9	9	9	9
3	2	3	2	1	2	2	3	2	2
3	2	3	1	2	3	1	2	3	1
9	9	9	9	9	9	9	9	9	9
3	2	3	1	2	3	1	2	3	1
1	2	3	1	3	2	1	1	1	2
1	1	3	3	3	1	1	3	3	1

3	3	3	3	3	3	3	3	3	3
1	1	2	1	2	2	1	1	2	1
2	2	2	3	3	3	3	3	2	2
2	2	3	2	2	3	1	2	2	1
2	2	2	2	2	2	2	2	2	2
1	9	1	9	9	1	9	1	9	1
9	9	9	9	9	9	9	9	9	9
1	3	3	1	3	2	2	2	1	1
3	3	3	3	3	3	3	3	3	3
1	1	3	3	3	1	1	1	2	2
3	3	3	3	3	3	3	3	3	3
1	1	3	3	1	3	1	3	1	1
1	2	2	1	2	9	2	2	3	2
1	2	3	1	2	2	1	1	1	1
3	2	3	1	2	2	1	1	1	1
2	2	3	2	2	9	2	2	3	2
1	2	3	1	2	2	1	1	1	1
1	2	3	1	2	2	1	1	1	1
1	2	3	1	2	2	1	1	1	1
1	3	3	3	3	3	3	3	3	1
2	1	1	2	9	1	3	2	1	3
2	3	1	2	3	3	3	3	3	3
1	3	3	1	3	3	1	1	1	1
2	2	2	2	3	2	2	2	3	3
3	3	3	3	3	3	3	3	3	3
3	2	3	1	2	2	1	1	1	1
9	9	9	1	9	9	9	9	9	9
1	2	2	3	3	3	1	1	1	1
1	9	9	9	9	9	9	9	9	9
3	3	3	3	3	3	3	3	3	3
1	2	1	1	2	2	1	1	2	1
3	2	2	3	2	3	2	3	2	2
3	1	2	3	2	1	3	1	3	2
3	3	3	3	3	3	3	3	3	3
3	3	3	3	3	3	3	3	3	3
3	3	3	3	3	9	9	9	9	9
9	9	9	9	9	9	9	9	9	9
1	2	1	1	1	1	1	2	1	1
2	1	2	2	1	1	1	1	2	2
1	1	3	3	3	1	2	2	2	1
2	2	2	3	2	2	9	3	9	1
1	3	3	1	2	2	1	2	1	3
3	9	9	1	9	2	1	1	1	9
1	1	3	1	3	3	3	3	1	1
1	2	1	1	3	3	2	2	1	1
1	1	1	1	1	1	1	1	1	1
1	2	1	2	2	2	2	2	2	2

2	1	3	3	3	3	2	2	1	1
9	9	9	9	9	9	9	9	9	9
9	9	9	9	9	9	9	9	9	9
3	1	3	3	1	3	1	3	1	3
2	2	2	2	2	2	2	2	2	2
1	2	1	3	2	3	2	2	2	2
2	9	3	2	9	2	2	2	2	2
1	2	3	1	3	2	2	2	2	2
1	2	3	1	3	2	1	2	2	2
2	1	3	2	1	2	1	3	2	1
3	2	2	1	3	9	2	3	2	2
1	2	3	3	2	1	1	2	1	1
2	2	3	1	3	2	2	2	1	1
1	1	1	1	3	1	2	3	3	2
1	3	3	3	3	3	1	1	1	1
2	3	2	2	1	2	3	2	1	2
3	1	2	3	1	2	3	1	2	3
9	9	9	9	9	9	9	9	9	9
9	9	9	9	9	9	9	9	9	9
2	2	3	2	3	2	3	1	2	3
1	1	1	1	9	2	2	2	1	1
2	2	2	2	2	2	2	2	2	1
1	1	1	1	1	1	1	1	1	1
2	2	2	2	2	2	2	2	2	2
3	3	3	3	3	3	3	3	3	3
3	2	9	2	2	2	3	2	3	3
3	2	3	2	2	3	2	3	3	9
1	1	3	3	3	1	1	1	1	1
1	1	1	1	1	1	1	1	1	1
2	2	2	2	2	2	2	2	2	2
2	3	1	2	3	2	2	3	3	2
1	1	2	3	3	2	1	2	1	1
9	9	9	9	9	9	9	9	9	9
3	2	9	2	2	3	2	3	3	9
1	2	2	2	2	2	1	2	1	2
2	2	2	2	2	2	2	2	2	2
2	2	2	2	2	2	2	2	1	1
9	9	9	9	9	9	9	9	9	9
9	9	9	9	9	1	3	9	9	2
1	1	1	1	2	2	1	2	2	1
3	3	3	3	3	3	3	3	3	3
2	2	2	2	2	2	2	2	2	2
2	2	2	2	2	2	2	2	2	2
2	2	2	2	2	2	2	2	2	2
2	2	2	2	2	2	2	2	2	2
2	2	2	2	2	2	2	2	2	2
9	9	9	9	9	9	9	9	9	9

9	9	9	9	9	9	9	9	9	9
2	2	2	2	2	2	2	2	2	2
9	9	9	9	9	9	9	9	9	9
9	9	9	9	9	9	9	9	9	9
9	9	9	9	9	9	9	9	9	9
1	1	1	3	3	1	2	1	2	3
2	2	2	2	2	2	2	2	2	2
3	3	3	3	3	3	3	3	3	3
1	1	1	1	1	1	2	3	2	3
9	9	9	9	9	9	9	9	9	9
2	2	2	2	2	2	2	2	2	2
1	2	2	1	2	2	2	2	2	1
1	1	2	2	2	2	2	2	2	1
2	2	2	2	2	2	2	2	2	2
3	1	1	1	3	3	3	1	1	1
2	2	2	2	2	2	2	2	2	2
2	2	2	2	2	2	2	2	2	2
2	2	2	2	2	2	2	2	2	2
2	1	2	1	2	2	1	2	2	1
1	1	1	2	2	3	3	3	3	3
9	9	9	9	9	9	9	9	9	9
1	2	2	2	3	3	3	3	2	2
2	2	2	2	2	2	2	2	2	2
1	1	3	1	2	2	1	1	1	1
2	2	2	2	2	2	2	2	2	2
2	3	3	2	3	3	2	3	2	3
9	9	9	9	9	9	9	9	9	1
9	9	9	9	9	9	9	9	9	9
3	3	3	3	3	3	3	3	3	3
2	2	2	2	2	2	2	2	2	2
9	9	9	9	9	9	9	9	9	9
1	3	3	1	2	2	2	2	2	1
1	1	3	3	3	3	1	1	3	1
2	2	1	2	2	2	2	2	2	2
1	1	1	1	1	1	1	1	1	1
1	1	3	3	3	3	3	3	3	3
2	2	2	2	2	2	2	2	2	2
2	2	1	2	1	2	1	3	2	3
2	2	2	2	2	2	2	2	2	2
9	9	9	1	9	9	9	9	9	9
2	2	2	2	2	2	2	2	2	2
2	2	2	2	2	2	2	2	2	2
2	3	3	3	3	3	3	3	1	1
1	1	3	1	3	2	2	2	2	2
2	2	2	2	2	2	2	2	2	2
3	3	3	3	3	3	3	3	3	3
2	2	2	2	2	2	2	2	2	2

2	2	2	2	2	2	2	2	2	2
2	2	2	2	2	2	2	2	2	2
2	2	2	2	2	2	2	2	2	2
2	2	2	2	2	2	2	2	2	2
1	3	3	3	2	2	2	2	2	2
1	1	3	2	2	2	1	2	3	1
2	2	2	2	2	2	2	2	2	2
1	3	2	2	1	2	1	3	2	1
2	2	2	2	2	2	2	2	2	3
1	1	2	2	2	2	1	2	2	2
2	3	3	3	1	3	3	1	1	1
9	9	9	9	9	9	9	9	9	9
9	9	9	9	9	1	9	9	9	9
2	2	2	2	2	2	2	2	2	2
2	2	2	2	2	2	2	2	2	2
3	2	2	2	3	3	3	3	3	2
2	2	3	3	2	3	2	3	3	2
1	1	1	9	3	3	1	2	2	1
2	1	2	3	2	1	2	3	2	2
2	3	1	2	2	3	2	2	2	3
3	3	3	3	3	3	3	1	1	1
2	2	2	2	2	2	2	2	2	2
2	2	2	2	2	2	2	2	2	2
1	2	2	3	3	3	3	3	3	1
3	3	3	3	1	1	1	1	1	3
1	9	9	9	9	9	9	9	9	1
2	2	2	2	2	2	2	2	2	2
1	1	2	1	2	2	1	1	2	1
2	2	2	2	2	2	2	9	9	9
2	2	2	3	3	3	2	3	3	1
1	9	1	2	2	1	1	1	1	1
1	3	2	1	1	1	2	2	3	3
3	3	3	3	3	3	3	3	1	1
1	1	2	2	2	1	1	1	1	1
3	3	3	3	3	3	3	3	3	3
1	1	1	1	1	1	3	3	1	1
9	9	9	9	9	9	9	9	9	9
1	1	1	1	1	1	1	1	1	1
2	2	2	2	2	2	2	2	2	2
2	2	2	2	2	2	2	2	2	2
2	2	1	3	3	3	1	2	3	1
1	1	2	2	2	2	1	1	2	1
3	2	3	2	2	3	3	2	2	1
2	2	1	2	2	2	2	2	2	2
2	2	2	2	2	2	2	2	2	2
3	2	1	3	2	1	3	2	3	2
2	3	2	2	3	2	3	2	2	2

1	1	2	9	1	3	2	3	2	3
3	3	3	3	3	3	3	3	3	3
2	2	3	1	2	2	3	2	2	3
9	1	9	9	9	9	9	9	9	9
9	2	9	9	9	9	9	9	9	9
2	2	2	3	3	3	2	2	3	2
2	2	2	2	2	2	2	2	2	2
3	3	3	3	3	3	3	3	3	3
2	3	1	2	2	2	2	2	2	2
2	2	2	2	2	2	2	2	2	2
1	9	9	9	9	9	9	9	9	9
3	2	1	2	2	3	1	3	2	3
2	2	2	2	2	2	2	2	2	2
2	2	2	2	2	2	2	1	9	1
2	2	2	2	2	2	2	2	2	2
3	3	3	3	3	9	2	2	2	2
2	3	2	2	1	2	3	2	2	1
9	9	9	9	9	9	9	9	9	9
9	9	9	2	9	9	9	9	9	9
2	2	2	2	2	2	2	1	2	2
2	2	2	2	2	2	2	2	2	2
2	2	2	2	2	2	2	2	2	2
9	9	9	9	9	9	9	9	9	9
9	9	9	9	9	9	9	9	9	9
2	2	3	1	2	3	3	1	2	3
1	3	3	3	1	3	3	3	3	3
2	2	3	3	3	3	2	3	2	3
1	1	3	3	3	2	1	2	1	1
1	3	3	3	3	3	3	3	3	3
1	2	2	3	3	3	1	2	2	1
1	1	1	1	2	3	1	1	1	1
1	2	3	3	3	3	3	3	1	2
1	1	3	2	3	2	1	2	9	2
3	3	3	3	3	3	3	3	3	3
1	1	1	1	1	1	1	1	1	1
1	1	2	3	3	2	1	2	3	1
1	1	1	1	1	1	1	1	1	3
3	3	3	3	3	3	3	3	3	3
9	9	1	9	9	1	9	9	1	9
3	3	3	3	3	3	3	3	3	3
3	3	3	3	3	3	1	1	1	3
3	1	1	3	3	1	1	3	1	1
3	3	3	3	3	3	1	1	1	3
2	2	2	2	2	2	2	2	2	2
1	1	1	2	2	2	1	1	1	1
1	1	1	1	2	1	1	1	1	3
1	1	2	2	2	2	1	1	2	1

2	2	2	2	2	2	2	2	2	2
1	2	1	2	2	2	2	2	2	2
9	1	1	3	1	1	9	9	1	1
1	3	3	1	3	2	1	1	1	2
2	3	3	3	3	3	3	3	3	3
2	2	2	2	2	2	2	2	2	2
2	2	2	2	2	2	2	2	2	2
1	9	9	9	9	9	9	9	9	9
1	9	9	9	9	9	9	9	9	9
1	3	1	2	2	2	2	2	2	2
9	9	9	9	9	9	9	9	9	9
1	1	3	3	3	3	1	2	2	2
1	3	3	1	3	3	9	1	1	1
3	1	3	3	1	3	2	2	3	2
1	2	2	1	2	2	3	2	2	2
2	2	2	2	2	2	2	2	2	2
1	1	3	1	3	3	1	1	1	1
2	2	2	2	2	2	2	2	2	2
1	3	3	2	3	2	2	2	2	3
9	9	1	9	9	9	9	9	9	9
9	9	9	9	9	9	9	9	9	9
1	1	1	1	1	1	3	3	3	3
1	1	1	3	3	3	3	1	1	1
3	2	2	2	2	2	1	1	1	1
3	3	3	3	3	3	3	3	3	3
2	2	2	2	2	2	2	2	2	2
1	1	2	1	3	9	1	2	1	1
3	2	3	3	3	2	2	1	1	1
3	3	2	1	1	1	1	1	3	2
2	1	2	1	3	3	1	2	2	1
1	2	2	2	2	2	2	2	2	2
1	1	1	1	3	1	1	1	1	3
2	2	1	2	2	3	1	2	2	2
1	3	2	1	2	2	2	1	3	2
2	2	1	2	2	2	2	2	1	2
2	2	2	2	2	2	2	2	2	2
2	2	2	2	2	2	2	2	2	2
2	2	2	2	2	2	2	2	2	2
3	2	2	2	3	3	3	9	2	1
3	2	3	1	2	3	1	2	3	1
1	2	3	2	1	3	2	1	2	1
2	2	2	2	2	2	2	2	2	2
1	1	1	1	1	1	1	1	1	1
9	9	9	9	9	9	9	9	9	9
2	2	2	2	2	2	2	2	2	2
3	3	3	1	3	2	2	2	2	2
1	1	3	1	2	3	2	1	1	1

2	3	3	2	2	3	1	1	2	1
3	3	3	3	2	2	2	2	3	1
2	3	3	1	2	3	1	1	3	3
1	2	3	1	3	3	1	1	1	1
2	2	2	2	2	2	2	2	2	2
3	2	2	2	2	2	2	2	3	2
2	3	3	3	2	2	2	2	2	2
3	2	2	2	2	2	2	2	9	1
1	1	1	1	1	1	1	1	1	1
2	2	2	2	3	2	2	2	1	1
2	2	2	2	2	2	3	2	2	2
2	3	3	2	2	2	3	2	2	2
1	9	9	9	9	9	9	9	9	9
9	1	9	9	9	9	9	9	9	9
2	2	3	2	3	3	3	2	2	2
2	3	3	3	3	3	2	2	2	2
9	9	9	9	9	9	9	9	9	9
2	2	2	2	2	2	2	2	1	1
1	2	1	3	1	1	3	2	2	3
2	2	2	2	2	2	2	2	2	2
1	2	1	3	3	3	2	1	1	2
2	1	9	2	1	2	3	2	3	3
2	2	2	2	2	2	2	2	2	2
2	1	3	2	3	1	1	1	1	2
2	2	2	2	2	2	2	2	2	2
1	3	3	2	2	3	2	1	2	2
3	3	3	3	3	3	3	3	3	3
1	1	2	2	2	2	9	2	2	2
1	3	3	2	2	3	2	1	2	2
2	3	3	3	3	2	2	2	2	3
2	2	2	2	2	2	2	2	2	2
1	9	9	9	9	9	9	9	9	9
1	3	1	1	3	3	1	1	3	2
2	2	2	2	2	2	2	2	2	2
1	1	2	3	1	3	3	2	2	2
1	1	2	2	2	2	1	1	1	2
1	1	2	2	1	2	1	1	1	2
9	9	9	9	9	9	9	9	9	9
2	2	2	2	2	2	2	2	2	2
2	2	2	2	2	2	2	2	2	2
1	1	3	3	3	3	1	3	2	1
2	3	2	2	2	3	1	2	3	2
2	2	2	1	2	2	1	1	1	1
2	2	2	2	2	2	2	2	2	2
3	1	1	9	1	1	3	9	2	2
2	2	2	2	2	2	2	2	2	2
9	9	9	9	9	9	9	1	9	9

2	2	2	2	2	2	2	2	2	2
2	2	2	2	2	2	2	1	1	1
9	9	9	9	9	9	1	9	9	9
9	9	9	9	9	9	9	9	9	9
1	2	2	2	2	1	1	1	1	1
9	9	9	9	9	9	9	9	9	9
1	2	2	2	3	3	3	1	1	1
1	1	1	1	2	3	2	1	1	3
3	2	2	2	3	2	2	1	3	1
2	2	3	3	2	2	1	1	2	2
2	2	2	9	1	2	2	1	1	2
2	1	2	1	2	1	2	2	1	2
1	2	2	1	2	2	1	1	1	1
1	1	2	2	2	2	1	1	2	2
2	1	2	1	3	2	1	2	1	1
3	3	3	3	3	3	3	3	3	3
1	1	3	1	3	3	3	3	2	2
1	1	3	2	3	1	1	1	1	1
3	3	3	1	2	2	3	1	1	1
1	1	3	3	3	1	1	1	3	2
2	1	2	2	3	3	2	3	2	2
2	2	2	2	1	2	1	1	1	2
3	3	3	3	3	3	3	2	1	1
2	2	2	2	2	2	2	2	2	2
2	2	2	2	2	2	2	1	2	1
2	1	2	2	2	2	2	2	2	1
2	2	2	2	2	2	2	2	2	2
2	2	2	2	2	2	2	2	2	2
2	1	1	1	3	1	2	3	2	2
9	9	9	9	9	9	9	9	9	9
1	9	9	9	9	9	9	9	9	9
2	1	2	1	2	2	2	2	2	2
1	9	9	9	9	9	9	9	9	9
3	1	2	3	2	1	3	1	3	1
3	2	2	1	2	1	2	3	2	1
9	9	9	9	9	9	9	9	9	9
9	9	9	9	9	9	9	9	9	9
2	2	2	2	2	2	2	2	2	2
2	2	2	2	9	9	9	9	9	9
1	1	2	2	2	1	1	1	3	2
1	1	1	1	1	1	1	1	1	1
2	1	3	1	3	1	2	1	2	1
2	1	1	1	2	1	2	1	1	1
1	2	1	2	2	3	2	1	2	2
2	1	2	3	1	2	2	3	1	2
9	9	9	9	9	9	9	9	9	9
2	1	3	2	1	3	2	2	1	3

1	2	1	2	2	1	2	1	1	2
9	9	9	9	9	9	9	9	9	9
9	9	9	9	9	9	9	9	9	9
2	2	2	2	2	2	2	2	2	2
1	2	3	1	3	2	1	1	1	1
1	2	1	2	2	1	2	1	1	2
3	1	2	2	2	2	1	1	1	1
1	1	2	2	2	2	2	2	2	2
2	2	2	2	2	2	2	2	2	2
1	1	3	9	3	3	1	1	3	1
9	9	9	9	9	9	9	9	9	9
9	9	9	9	9	9	9	9	9	9
9	1	9	9	9	9	9	9	9	9
2	2	2	2	2	2	1	1	3	2
2	1	2	1	2	2	2	1	2	3
1	2	2	2	2	2	9	9	9	9
9	9	9	9	9	9	9	9	9	9
9	9	9	9	9	9	1	9	9	9
2	2	2	9	3	2	1	1	1	1
3	1	1	9	3	3	3	3	2	1
1	1	1	2	1	1	1	1	2	1
2	2	2	3	3	2	2	1	1	1
3	3	3	3	3	3	3	3	3	3
2	2	2	2	2	2	2	2	2	2
2	2	2	2	2	2	2	2	2	2
2	2	2	3	3	2	1	1	2	2
9	9	9	9	9	9	9	9	9	9
1	3	3	2	3	2	2	1	2	1
3	1	2	3	1	2	3	2	1	2
2	2	2	2	2	2	2	2	2	2
3	1	3	3	3	1	1	1	3	1
1	2	2	1	2	2	1	1	1	1
9	9	9	9	9	9	9	9	9	9
3	3	1	1	1	3	2	1	2	1
2	2	2	2	2	2	2	2	2	2
2	2	2	2	2	2	2	2	2	2
1	1	2	2	2	2	1	1	2	2
2	2	2	2	2	2	2	2	2	2
2	2	1	2	1	1	1	1	1	1
2	1	2	3	1	2	1	2	2	1
2	3	2	2	3	1	1	3	2	1
3	3	3	3	3	3	3	3	3	3
9	9	9	9	9	9	9	9	9	9
9	9	9	9	9	9	1	9	9	9
2	2	9	2	2	2	1	2	2	2
1	1	3	1	3	9	9	9	9	9
9	9	9	9	9	9	9	9	9	9

2	2	2	2	2	2	2	2	2	2
3	2	3	3	2	2	2	2	1	2
1	9	3	1	3	3	2	1	2	1
1	1	3	1	2	2	2	2	2	2
1	1	1	1	1	3	1	1	1	3
2	3	3	3	3	3	3	3	9	9
1	9	9	9	9	9	9	9	9	9
1	3	3	3	3	3	1	3	1	1
9	9	9	9	9	9	9	9	9	9
9	9	9	9	9	9	9	9	9	9
9	9	9	9	9	9	9	9	9	9
9	9	9	9	9	9	9	9	9	9
9	9	9	9	9	9	9	9	9	9
1	3	1	3	3	1	3	3	3	3
1	1	2	3	3	1	1	1	1	1
3	2	2	2	2	2	2	1	2	1
1	1	1	1	1	1	1	1	2	2
1	1	3	9	9	9	1	9	9	9
9	9	9	9	9	9	9	9	9	9
9	9	9	9	1	9	9	9	9	9
1	1	1	9	1	9	3	2	9	1
9	9	9	9	9	9	9	9	9	9
1	1	1	1	1	1	1	1	1	1
9	9	9	9	9	9	9	9	9	9
1	1	1	1	1	2	1	1	2	2
1	2	2	1	1	1	1	1	2	2
1	3	2	1	2	2	3	3	3	2
1	1	1	1	1	1	1	2	2	2
1	1	1	1	1	1	1	1	1	1
1	2	2	1	1	1	1	2	2	2
1	1	1	1	1	1	1	1	1	1
1	2	1	1	1	1	2	2	1	1
1	1	1	2	2	2	2	1	1	1
9	9	9	9	9	9	9	9	9	9
1	2	2	2	2	1	1	1	1	1
1	1	1	1	2	2	2	1	1	1
1	3	3	1	2	2	2	3	3	3
9	9	9	9	9	9	9	9	9	9
1	1	1	1	2	2	1	1	2	1
1	3	3	3	3	3	3	1	1	3
2	3	2	2	2	2	2	2	2	2
1	2	3	1	3	2	1	2	1	1
3	1	1	3	9	9	9	9	9	9
9	9	9	9	9	9	9	9	9	9
1	1	1	1	1	3	1	3	3	1
3	1	2	9	1	3	2	9	3	3
1	2	3	2	3	2	3	1	1	1

9	9	9	9	9	9	9	9	9	9
9	9	9	9	9	9	9	9	9	9
9	9	9	9	9	9	9	9	9	9
9	9	9	9	9	9	9	9	9	9
1	2	3	2	3	2	1	1	3	1
9	9	9	9	9	9	9	9	9	9
2	2	2	2	2	2	2	2	2	2
1	1	1	1	2	1	9	9	9	9
9	9	9	9	9	9	9	9	9	9
9	9	9	9	9	9	9	9	9	9
9	9	9	9	9	9	9	9	9	9
9	9	9	9	9	9	9	9	9	9
2	2	2	2	2	2	1	1	1	1
2	2	2	1	3	1	3	2	2	3
3	3	3	3	3	3	1	1	9	2
1	1	1	1	3	2	1	1	2	1
2	1	1	1	1	1	2	2	2	1
1	2	2	1	2	2	1	1	1	1
1	1	1	2	2	2	2	1	1	1
9	9	9	9	9	9	9	9	9	9
1	1	1	1	1	2	2	2	2	2
2	1	2	1	3	3	1	1	3	3
1	2	2	2	2	2	2	2	2	2
1	3	9	1	9	2	1	3	1	1
2	1	2	2	2	1	1	2	1	1
1	1	1	3	3	3	3	3	3	3
3	2	9	9	1	9	9	9	2	9
2	1	1	1	3	3	3	2	2	2
9	9	9	9	9	9	9	9	9	9
9	9	9	9	9	9	9	9	9	9
3	3	3	3	3	1	1	3	1	1
3	3	3	3	3	3	3	3	3	3
3	3	3	3	3	3	3	3	3	3
1	1	1	1	1	1	2	2	1	2
2	2	2	1	1	1	1	2	2	1
1	2	2	2	2	2	1	1	2	2
1	2	2	2	2	2	2	2	2	2
1	1	2	2	2	2	1	1	1	1
1	1	2	2	2	1	1	1	1	1
2	2	2	2	2	2	2	2	2	2
1	1	3	3	3	3	1	1	1	1
2	2	2	2	2	2	2	2	2	2
3	3	3	3	3	3	3	3	1	1
2	2	2	1	2	2	1	2	2	1
1	2	3	1	2	2	3	2	1	1
3	3	3	3	3	3	3	3	3	3
2	2	2	2	2	2	2	2	2	1

9	9	9	9	9	9	9	9	9	9
1	1	1	1	2	2	3	3	2	2
9	9	2	9	9	9	9	9	9	9
9	9	9	9	9	9	9	9	9	9
1	9	9	9	9	9	9	9	9	9
1	1	2	2	1	1	1	1	2	1
9	9	9	9	9	9	9	9	9	9
1	2	3	1	2	3	2	2	2	1
9	9	9	9	9	9	9	9	9	9
2	2	2	3	2	2	2	2	2	2
9	9	9	9	9	9	9	9	9	9
2	2	2	2	2	2	2	2	2	2
2	1	1	1	2	2	2	1	1	1
2	2	2	2	2	2	2	2	2	2
1	1	1	2	2	1	1	2	2	1
9	9	9	9	9	9	9	9	9	9
2	2	2	2	2	2	2	2	2	2
3	3	3	3	3	3	3	3	3	3
2	2	3	1	9	9	9	9	9	9
3	3	3	3	3	3	3	3	3	3
2	2	2	2	2	2	2	2	2	2
3	3	3	1	1	2	2	2	2	2
2	2	2	2	2	2	2	2	2	2
2	2	2	2	2	2	2	2	2	2
2	1	1	1	1	1	1	1	1	1
2	2	2	2	2	2	2	2	2	2
3	1	3	3	1	3	1	3	2	2
3	3	3	1	2	3	1	3	3	3
1	1	2	2	2	2	2	1	1	1
2	2	1	1	1	1	1	2	2	2
9	9	9	9	9	9	9	9	9	9
1	2	3	1	2	2	3	1	2	1
3	1	2	3	2	1	2	2	3	1
2	2	2	2	2	2	2	2	2	2
2	3	3	2	2	2	2	2	2	2
1	2	2	2	1	1	1	2	1	1
1	1	3	3	3	3	3	1	1	1
1	1	2	2	2	2	2	1	1	1
1	2	1	1	1	1	1	1	2	2
1	2	2	2	2	2	1	1	2	2
2	2	2	2	2	2	2	2	2	2
1	1	1	1	1	1	1	1	1	1
9	9	9	9	9	9	9	9	9	9
9	9	9	9	9	9	9	9	9	9
9	9	9	9	9	9	9	9	9	9
2	2	9	1	2	9	2	2	2	1
2	2	2	2	2	2	2	2	2	1

1	1	3	3	3	1	1	1	1	1
2	2	2	2	2	2	2	2	1	2
9	9	9	9	9	9	9	9	9	9
3	2	3	1	2	3	1	2	1	3
9	9	9	9	9	9	9	9	9	9
2	2	2	2	2	2	3	2	3	2
9	1	9	9	9	9	9	9	9	9
9	9	9	9	9	9	9	9	9	9
2	2	2	2	2	2	1	2	2	2
3	3	3	1	1	1	1	1	1	1
9	9	9	9	9	9	9	9	9	9
9	9	9	9	9	9	9	9	9	9
2	1	2	1	1	2	2	2	2	1
1	2	3	1	3	3	2	2	2	1
9	9	9	9	9	9	9	9	9	9
1	1	1	1	1	1	1	3	1	2
2	1	2	3	2	2	1	3	1	2
1	1	3	1	3	2	2	2	3	1
2	2	2	2	2	2	2	2	2	2
3	1	2	3	3	3	2	2	1	1
2	3	3	2	2	1	1	2	2	1
1	1	3	1	3	1	1	1	3	1
1	2	3	3	3	3	1	3	1	1
1	2	2	2	3	3	2	1	1	1
2	2	2	2	2	2	2	2	2	2
2	2	2	9	1	1	1	3	2	2
1	2	2	2	3	3	2	1	1	1
1	1	3	2	2	3	2	1	1	2
1	3	3	9	9	3	2	3	2	2
1	2	2	2	1	2	2	2	1	2
1	3	3	9	1	3	3	1	2	3
2	3	3	3	2	3	2	2	2	3
1	2	9	9	9	9	9	9	9	9
1	3	3	1	3	9	3	3	2	2
1	1	3	2	3	2	3	1	2	1
2	2	2	2	2	2	2	2	2	2
1	3	3	1	3	3	3	2	2	2
3	2	3	3	3	3	1	2	2	1
9	9	9	9	9	9	9	9	9	9
2	2	2	3	2	3	2	3	2	9
9	9	9	9	9	2	9	9	9	9
9	9	9	9	9	9	9	9	9	9
1	2	2	2	2	2	2	3	2	2
1	2	2	1	2	2	1	9	9	9
1	3	3	1	3	3	2	2	3	3
1	1	3	1	1	1	3	1	3	1
3	3	3	3	3	1	1	3	3	9

3	3	3	3	3	3	3	3	3	3
9	9	9	9	9	9	9	9	9	9
1	1	3	3	3	3	1	3	3	3
3	1	3	1	3	3	1	3	1	1
3	3	3	1	3	3	3	3	3	1
1	1	1	3	1	3	3	1	2	2
3	1	3	9	1	2	2	1	3	1
2	2	2	2	2	2	2	2	2	2
3	3	1	1	3	3	3	1	1	3
2	1	3	1	3	2	3	1	2	3
1	3	1	3	1	1	3	1	1	1
1	3	2	3	1	2	3	2	1	3
3	2	2	3	3	3	2	2	3	2
3	3	3	3	2	1	2	1	2	3
1	2	2	2	2	1	1	1	3	1
1	1	3	3	3	3	2	1	1	1
1	1	3	3	2	3	1	1	2	2
2	3	3	2	1	3	2	1	1	1
3	3	3	1	3	3	3	3	1	1
2	2	2	2	2	2	2	2	2	2
1	1	2	1	1	2	2	2	2	2
2	2	2	2	2	2	2	3	3	3
3	3	3	3	3	3	3	3	3	3
9	9	9	9	9	9	9	9	9	9
2	3	3	2	2	2	1	1	1	1
1	2	1	2	2	1	2	1	1	1
3	2	1	3	2	1	3	2	1	3
3	3	3	2	3	3	2	2	3	3
1	1	1	1	3	3	1	1	1	1
9	9	9	9	9	9	9	9	9	9
1	2	3	2	2	2	2	2	2	2
2	1	2	3	1	2	3	2	3	1
2	3	3	3	9	9	1	2	3	1
2	2	3	3	2	3	2	2	2	1
1	3	1	1	1	9	2	9	1	3
2	2	2	2	2	2	2	2	2	2
2	2	2	2	2	9	3	3	3	3
9	9	9	9	9	9	9	9	9	9
2	3	2	2	2	1	2	3	2	2
3	2	3	3	1	2	3	1	2	3
9	9	9	9	9	9	9	9	9	9
9	9	9	9	9	9	9	9	9	9
2	2	3	1	2	1	2	2	3	2
3	3	3	1	3	3	3	1	3	3
2	2	2	2	2	2	2	2	2	2
3	1	9	3	3	3	2	2	2	2
9	9	9	9	9	9	9	9	9	9

2	2	3	2	1	2	2	3	3	3
9	9	9	9	9	9	9	9	9	9
2	3	3	2	3	3	2	2	2	3
9	9	9	9	9	9	9	9	9	9
9	9	9	9	9	9	9	9	9	9
3	3	3	3	3	3	3	3	3	3
2	2	2	2	2	2	2	2	2	2
2	2	2	2	2	2	2	2	2	2
3	3	3	3	3	1	1	1	1	1
1	3	3	3	3	3	2	3	2	3
9	9	9	9	9	9	9	9	9	9
9	9	9	9	9	9	9	9	9	9
9	9	9	9	9	9	9	9	9	9
1	1	2	2	2	2	1	1	2	2
2	2	2	2	2	2	2	2	2	2
1	2	2	2	2	2	2	2	2	2
9	9	9	9	9	9	9	9	9	9
9	9	9	9	9	9	9	9	9	9
3	3	3	3	3	3	3	3	3	3
2	2	2	2	2	2	2	2	2	2
3	3	3	3	3	3	3	3	3	3
2	2	2	2	2	2	2	2	2	2
9	9	9	9	9	9	9	9	9	9
2	1	2	2	1	3	2	1	2	3
2	3	1	2	3	2	2	1	1	2
1	2	1	2	3	3	3	2	2	3
9	9	9	9	9	9	9	9	9	9
2	2	2	2	2	2	2	2	2	2
2	2	2	2	2	2	2	2	2	2
9	9	9	9	9	9	9	9	1	9
1	1	3	3	3	2	2	3	1	1
2	2	2	2	2	2	2	2	2	2
1	3	1	3	1	3	1	3	1	3
1	1	3	2	2	3	1	2	2	2
1	1	3	3	3	3	3	1	1	1
2	3	2	2	1	2	2	2	1	3
3	3	3	3	3	3	3	3	1	1
1	1	3	3	3	3	3	3	3	1
9	9	9	9	9	9	9	9	9	9
2	2	2	2	2	2	2	2	2	2
1	2	3	1	2	2	3	2	3	1
2	2	2	1	2	2	3	3	1	2
3	3	3	3	3	3	3	3	3	3
3	3	3	3	3	3	2	2	2	1
2	2	2	1	2	2	2	2	1	3
2	2	2	2	3	2	3	2	1	2
9	9	9	9	9	9	9	9	9	9

2	2	2	2	2	2	2	2	2	3
9	2	3	2	2	2	2	2	2	2
1	1	2	1	2	2	1	1	1	1
1	3	3	9	2	2	2	2	2	2
3	3	3	3	3	2	3	9	2	1
1	9	9	9	9	9	9	9	9	9
2	2	2	2	2	2	2	2	2	2
1	2	2	1	2	9	2	2	2	2
1	2	2	2	3	1	2	1	2	1
2	2	2	2	2	2	2	2	2	2
3	2	2	3	2	2	2	2	2	2
1	3	1	3	9	3	3	2	2	1
2	2	2	2	2	2	2	2	2	2
3	3	3	3	3	3	3	3	3	3
1	3	3	2	2	3	3	3	3	2
2	3	2	1	2	3	2	2	1	3
3	3	3	2	2	3	3	3	3	3
3	1	2	3	2	3	2	3	3	2
2	2	2	2	2	2	2	2	2	2
1	3	3	3	2	2	2	2	2	2
3	3	3	3	3	3	1	1	2	1
1	2	3	3	3	2	2	3	2	2
1	2	3	3	3	2	2	3	2	2
1	1	2	2	2	1	1	1	1	1
2	1	2	2	2	2	2	2	2	1
2	3	2	3	3	2	2	2	2	2
2	2	2	2	2	1	9	1	2	2
2	2	2	2	2	1	9	1	2	2
2	2	2	2	2	2	2	2	2	2
1	1	2	2	2	2	1	1	1	1
3	2	2	2	2	3	2	3	2	3
2	3	3	2	9	3	2	2	3	2
3	3	3	3	3	3	3	1	1	1
1	2	3	1	2	2	2	2	3	3
1	1	3	3	2	2	2	3	3	2
2	2	2	2	2	2	2	2	2	2
2	2	2	2	2	2	2	2	2	2
2	2	2	2	2	3	1	1	2	3
2	2	2	2	2	2	2	2	2	2
2	2	2	2	2	2	2	2	2	2
1	2	2	3	3	2	1	1	2	1
1	1	3	3	3	3	2	2	3	1
2	2	2	2	2	2	2	2	2	2
2	2	2	2	1	2	2	2	2	2
1	2	3	2	2	2	2	3	2	1
1	1	3	1	1	3	2	1	1	1
1	2	3	1	2	2	2	1	1	1

1	3	2	3	2	2	1	2	3	2
2	2	2	2	2	2	2	2	2	2
1	2	3	2	2	2	2	2	2	1
2	1	2	2	2	2	2	2	2	2
2	2	2	2	2	2	2	2	2	2
2	2	2	2	2	2	2	2	2	2
1	2	3	1	2	2	1	1	1	1
1	1	2	2	3	2	2	1	2	1
2	2	2	2	2	2	2	2	2	2
2	2	3	3	3	2	1	2	2	2
2	2	3	3	3	2	1	2	2	2
2	2	2	2	2	2	2	2	2	2
2	2	2	2	2	2	2	2	2	2
9	9	9	9	9	9	9	9	9	9
2	2	2	2	2	2	2	2	2	2
2	2	1	2	2	2	1	2	2	1
1	1	2	2	3	2	2	1	2	2
2	2	2	2	2	2	1	1	2	2
1	1	1	1	3	3	1	1	1	1
2	1	2	1	3	2	1	2	1	2
2	2	2	2	2	2	2	2	2	2
2	2	2	2	2	2	2	2	2	2
2	2	2	2	3	2	3	2	3	2
2	2	2	2	2	2	2	2	2	2
2	2	2	1	2	2	1	2	2	2
1	1	3	2	3	3	9	2	2	2
3	3	3	3	3	2	2	2	2	2
2	2	2	2	2	2	2	2	2	2
2	2	2	2	2	2	2	2	2	2
2	1	2	2	2	2	2	2	1	1
3	3	3	3	3	3	3	3	3	3
2	2	2	2	2	2	2	2	2	2
2	2	2	2	2	2	2	2	2	2
2	2	2	2	2	2	2	2	2	2
2	2	2	2	2	2	2	2	2	2
2	2	2	2	2	2	2	2	2	2
2	2	2	2	2	2	2	2	2	2
2	2	2	2	2	2	2	2	2	2
9	9	9	9	9	9	9	9	9	9
2	2	1	2	2	2	1	2	2	2
1	2	2	1	2	2	1	1	1	1
2	2	2	1	2	2	2	2	2	2
2	2	2	2	2	2	2	2	2	2
2	2	2	2	2	2	2	2	2	2
2	2	2	2	2	2	2	2	2	2
2	2	2	2	2	2	2	2	2	2
2	2	2	2	2	2	2	2	2	2

2	2	2	2	2	2	2	2	2	2
2	2	2	2	2	2	2	2	2	2
3	1	1	2	3	3	1	1	3	1
2	2	2	2	2	2	2	2	2	2
2	2	2	2	2	2	2	2	2	2
3	2	2	1	2	2	1	2	2	2
9	9	9	9	9	9	9	9	9	9
1	1	3	3	3	3	1	2	1	1
1	1	3	3	3	3	3	1	1	1
1	2	3	3	3	1	2	2	1	1
1	1	2	1	2	2	1	1	1	1
2	3	3	2	3	3	3	2	2	2
1	2	2	2	3	2	1	1	2	1
2	2	2	2	2	2	2	2	2	2
1	1	3	1	3	1	2	2	2	1
2	2	2	2	2	2	2	2	2	2
2	2	2	2	2	2	2	2	2	2
1	1	2	2	2	1	1	1	1	1
1	1	1	1	2	2	1	1	1	1
2	2	2	2	3	1	2	2	2	2
1	1	3	1	3	1	1	1	1	1
3	1	2	2	2	2	3	1	2	2
3	2	2	2	2	2	2	2	2	2
2	2	2	2	2	2	2	2	2	2
2	2	2	2	2	2	2	2	2	2
2	2	2	2	2	2	2	2	2	2
9	9	9	9	9	9	9	9	9	9
2	2	2	2	2	2	2	2	2	2
2	2	2	2	2	2	2	2	2	2
1	2	2	2	2	2	2	2	2	2
2	2	2	2	2	2	2	2	2	2
3	2	2	2	2	2	2	2	2	2
9	9	9	9	9	9	9	9	9	9
1	1	2	1	1	2	3	3	3	3
9	9	9	9	9	9	9	9	9	9
1	1	1	3	3	2	1	1	1	1
3	2	3	3	3	3	3	3	2	2
1	1	2	1	1	2	1	2	1	1
3	2	2	2	2	2	2	2	2	2
3	3	3	3	3	3	3	3	3	3
1	2	2	2	2	1	1	1	1	1
1	2	2	2	1	2	2	2	2	2
1	1	2	1	2	3	1	1	2	1
2	2	2	2	2	2	2	2	2	2
1	2	1	1	1	2	1	2	1	1
9	2	2	2	2	2	2	2	2	2
1	1	1	1	1	2	1	2	1	2

3	2	2	1	2	3	2	2	3	2
3	1	2	1	1	2	3	1	1	2
1	2	2	3	1	9	2	3	3	3
1	3	2	3	3	2	2	1	2	1
1	2	2	3	2	3	2	2	2	2
3	1	2	3	1	2	3	2	2	2
3	2	2	2	1	1	1	1	1	3
9	9	9	9	9	9	9	9	9	9
2	2	2	2	2	2	2	2	2	2
2	2	2	2	2	2	2	2	2	2
2	2	2	2	2	2	2	9	1	2
2	2	2	2	2	2	2	2	1	2
2	2	2	2	9	9	9	9	9	9
2	2	2	2	2	2	2	2	2	2
2	2	2	2	2	2	2	2	2	2
2	2	2	2	2	2	2	2	2	2
2	2	2	1	2	2	1	1	2	1
1	2	3	2	1	2	1	3	3	3
2	2	2	2	2	2	2	2	2	2
1	2	3	1	3	9	2	2	2	2
9	9	9	9	9	9	9	9	9	9
9	9	9	9	9	9	9	9	9	9
2	2	3	3	3	3	3	2	3	1
9	9	9	9	9	9	9	9	9	9
1	2	2	1	3	1	3	1	2	1
1	1	3	1	2	2	1	1	3	1
1	1	1	1	1	1	1	1	1	1
2	2	2	2	2	2	2	2	1	1
2	2	2	2	2	2	2	2	2	2
9	9	9	9	9	9	9	9	9	9
3	2	3	3	3	3	2	2	3	3
9	9	9	9	9	9	9	9	9	9
9	9	9	9	9	9	9	9	9	9
2	2	2	2	2	2	2	2	2	2
2	2	2	2	2	2	2	2	2	2
2	2	2	2	2	2	2	2	2	2
2	2	2	9	1	9	2	3	2	2
2	2	2	2	2	2	2	2	2	2
2	2	2	1	2	2	2	2	2	2
2	2	2	1	2	2	2	2	2	2
9	9	9	9	9	9	9	9	9	9
2	2	2	2	2	2	2	2	2	2
2	2	2	2	2	2	2	2	2	2
9	9	9	9	9	9	9	9	9	9
9	9	9	9	9	9	3	9	3	9
2	2	1	1	1	1	2	1	1	1
1	1	2	2	1	1	1	1	1	2

9	9	9	9	9	9	9	9	9	9
1	1	2	2	3	2	2	1	2	1
3	3	3	3	3	3	3	3	3	1
2	2	2	2	2	2	2	2	2	2
9	9	9	9	9	9	9	9	9	9
3	3	3	3	3	3	3	3	3	3
2	2	2	2	2	2	2	2	2	2
3	3	9	2	3	2	2	2	2	3
9	9	9	9	9	9	9	9	9	9
2	2	2	2	2	2	2	2	2	2
1	3	3	3	3	3	1	1	1	1
2	2	2	2	2	2	2	2	2	2
1	2	2	2	2	2	2	1	2	3
3	3	3	3	3	3	3	3	3	3
1	9	9	9	9	9	9	9	9	9
1	2	3	3	3	3	1	2	1	1
2	2	2	2	2	2	1	1	1	1
3	2	3	2	1	3	1	2	1	3
9	9	1	9	9	9	9	9	9	9
1	3	2	1	3	3	1	1	3	2
2	1	3	1	2	1	3	1	2	1
2	2	2	2	2	2	2	2	2	2
9	9	9	9	9	9	9	9	9	9
2	2	2	2	2	2	2	2	2	2
3	1	1	1	1	1	1	1	3	3
2	2	2	2	2	2	2	2	2	2
1	2	2	2	3	2	2	3	1	1
1	1	3	3	3	3	1	1	1	1
3	2	3	2	1	3	1	2	2	2
9	9	9	9	9	9	9	9	9	9
2	2	2	2	2	2	2	2	2	2
3	1	2	2	1	2	2	2	2	1
1	3	3	3	3	3	3	1	3	1
3	3	3	3	3	3	3	3	3	3
1	1	1	1	1	1	2	1	1	1
2	2	3	2	2	1	1	1	1	2
3	3	3	1	9	3	3	3	3	3
2	2	2	2	2	2	2	2	2	2
2	2	1	2	3	1	1	2	2	1
9	9	9	9	9	9	9	9	9	9
9	9	9	9	9	9	9	9	9	9
3	1	3	1	2	3	1	3	1	2
1	2	3	3	3	2	2	1	1	1
1	2	2	3	2	1	2	1	2	1
1	2	3	2	1	3	1	2	2	2
2	2	2	2	1	1	2	2	2	1
2	2	2	2	3	2	2	3	3	2

1	1	3	3	3	1	1	3	1	1
1	2	1	9	3	2	2	1	3	1
1	2	3	2	2	2	2	2	2	1
2	2	2	2	2	3	3	3	3	3
1	1	3	1	3	3	1	1	3	1
2	2	2	2	2	2	2	2	2	2
2	2	2	2	2	2	2	2	2	2
3	3	2	3	3	3	3	2	2	2
1	1	3	1	3	3	1	1	1	1
2	2	2	2	2	2	2	2	2	2
2	2	1	2	9	1	9	1	2	1
2	2	2	2	1	2	2	1	1	2
2	2	2	2	2	3	2	2	3	2
1	1	3	1	3	3	1	1	1	1
9	9	9	9	9	9	9	9	9	9
2	2	2	2	2	2	2	2	2	2
3	2	3	3	2	3	2	3	3	2
1	3	2	2	2	2	2	2	2	2
1	1	3	3	3	2	2	1	1	3
2	3	2	1	3	1	1	1	2	1
1	2	3	1	3	3	1	2	1	1
9	9	9	9	9	9	9	9	9	9
2	2	2	9	2	2	1	2	2	3
9	9	9	9	9	9	9	9	9	9
2	2	2	2	2	2	2	2	2	2
2	2	2	2	2	9	3	3	3	9
3	2	1	2	3	2	1	2	3	2
2	2	2	2	2	2	2	2	2	2
3	3	3	3	3	3	3	3	3	3
1	2	2	1	2	2	2	2	2	1
3	3	3	3	3	3	3	3	3	1
2	2	3	1	2	3	3	3	2	1
2	3	3	2	3	2	3	3	2	3
9	3	3	3	3	3	2	1	1	2
1	1	2	2	3	3	1	2	1	2
2	2	2	2	2	2	2	2	2	2
1	3	3	3	3	3	1	1	1	1
1	3	3	3	3	3	2	2	2	2
2	2	2	2	2	2	2	2	2	2
9	9	9	9	9	9	9	9	9	9
2	2	2	3	2	2	2	3	3	3
1	1	1	2	3	1	2	1	3	1
2	3	3	3	3	2	3	2	3	2
1	1	3	2	3	2	1	1	1	1
1	3	3	3	3	2	3	3	1	1
2	9	9	9	9	9	9	9	9	9
2	2	2	2	3	3	2	2	3	2

1	2	1	2	1	1	2	1	2	1
2	3	2	2	2	3	2	2	2	2
3	3	3	3	3	3	3	1	1	1
2	1	3	2	2	3	1	2	3	1
2	1	2	2	2	2	2	2	2	1
2	2	1	9	2	1	1	2	2	2
1	2	3	3	3	9	2	2	2	1
9	9	9	9	9	9	9	9	9	9
3	9	9	9	9	9	9	9	9	9
1	3	2	9	1	2	1	2	1	2
2	2	2	2	3	3	2	2	2	2
2	2	3	2	2	2	2	2	2	3
3	2	3	3	3	3	2	1	1	3
1	3	3	1	3	3	3	1	3	1
9	2	9	9	9	9	9	9	9	9
1	3	3	1	2	2	3	3	2	2
1	1	2	1	1	1	2	2	2	2
1	3	2	3	3	3	3	3	2	2
3	3	3	1	3	3	1	1	3	1
2	3	1	3	3	1	2	2	2	3
1	2	3	2	3	2	1	1	1	1
1	1	3	3	3	3	1	1	1	2
2	2	1	2	1	1	1	2	2	1
3	1	3	3	3	3	1	2	3	3
1	2	3	1	2	3	1	1	1	2
2	2	2	2	2	2	1	2	1	1
1	2	3	1	2	3	2	2	3	2
2	1	1	1	2	2	1	1	1	2
2	2	2	2	2	2	2	2	2	2
1	2	2	2	2	2	2	2	2	1
1	2	1	1	1	2	2	2	1	2
2	2	2	2	2	2	2	2	2	2
2	2	2	2	2	2	2	2	2	2
1	1	1	2	2	2	2	1	2	2
3	1	1	1	2	2	2	3	3	3
1	1	3	1	3	2	1	1	2	1
2	2	1	2	1	1	1	2	2	2
3	3	3	3	3	3	3	3	3	3
2	1	1	1	1	2	1	1	2	1
2	2	2	1	1	1	1	1	1	2
2	2	3	3	3	3	2	2	2	2
2	1	1	1	1	2	2	3	1	1
2	1	2	3	3	3	1	3	3	3
1	2	2	3	3	2	2	2	2	2
3	3	3	3	2	2	1	1	2	2
2	2	2	2	2	2	2	2	1	1
1	1	2	2	1	1	1	3	2	2

1	3	3	2	3	3	1	1	2	1
2	2	1	2	2	2	1	1	2	2
1	2	2	2	2	2	3	2	1	2
2	1	1	1	1	2	2	2	1	1
2	3	3	3	3	1	2	2	1	1
2	1	1	2	3	3	2	2	1	1
2	2	3	2	2	1	1	1	1	2
2	1	1	2	2	1	2	2	1	2
1	1	2	2	2	2	2	2	1	2
1	1	1	2	2	2	2	2	2	1
2	2	2	2	2	2	2	2	2	2
2	2	2	2	2	2	2	2	2	2
1	1	1	1	1	1	1	1	1	1
3	3	3	3	3	3	3	3	3	3
2	2	2	2	2	2	2	2	2	1
1	2	1	2	2	2	2	2	2	2
2	2	2	2	9	9	9	9	9	1
2	2	2	2	9	1	1	2	2	2
2	2	2	2	2	2	2	2	2	2
2	2	2	1	1	1	2	3	2	3
2	2	2	2	2	2	2	2	2	2
1	1	2	2	2	2	2	2	1	2
3	3	3	3	3	3	3	3	3	3
3	3	3	3	2	9	2	3	2	3
1	2	9	1	2	1	2	1	1	2
2	2	2	2	2	2	2	2	2	2
3	2	2	3	1	1	1	1	2	3
2	2	3	3	3	3	2	2	2	2
2	2	2	2	2	2	2	2	2	2
2	2	2	2	2	2	2	2	1	1
2	2	2	3	2	2	2	2	1	1
2	1	2	3	3	2	1	1	3	2
2	2	2	1	1	1	1	1	2	2
2	2	2	2	2	3	3	3	3	2
3	3	3	3	3	3	2	2	3	1
3	2	3	3	1	3	2	2	2	2
1	1	2	2	3	3	1	1	1	1
1	2	2	2	3	3	2	2	2	2
1	1	1	1	1	1	1	1	3	3
1	1	2	1	2	1	1	1	1	1
2	2	2	2	2	2	2	2	2	2
2	2	2	2	2	2	2	2	2	2
2	2	2	2	2	2	2	2	2	2
3	3	3	3	3	3	3	3	3	3
3	3	3	3	3	3	3	3	3	3
2	2	2	1	1	2	2	2	2	3
1	1	1	1	1	1	1	1	1	1

2	1	1	2	2	2	2	3	3	2
2	1	1	2	2	2	1	1	2	2
2	2	2	2	1	1	1	2	2	1
2	3	3	2	2	1	1	2	3	2
2	2	1	1	1	2	1	1	2	2
2	1	1	1	2	2	2	1	1	1
2	1	1	1	1	1	1	1	2	2
3	3	3	2	2	1	1	1	2	2
2	2	2	2	1	1	1	2	2	2
1	1	2	2	1	2	2	1	1	2
2	2	2	1	1	1	2	2	2	2
2	2	2	1	1	2	2	1	1	1
2	2	2	2	2	1	1	1	1	1
1	1	2	1	1	2	1	1	2	1
1	1	1	1	2	2	2	1	1	1
1	1	2	2	1	1	1	2	2	1
2	2	2	2	2	2	2	2	2	2
2	2	2	2	2	2	2	2	2	2
2	2	2	2	2	2	2	2	2	9
2	2	3	3	3	2	1	1	1	2
9	9	2	9	9	9	9	9	2	9
3	3	3	3	3	3	3	3	3	3
2	2	2	1	1	1	1	1	1	2
1	1	2	1	1	2	1	1	1	1
2	2	1	1	1	2	2	1	1	1
2	2	2	2	2	2	2	2	2	2
2	2	2	2	1	1	1	1	2	1
1	1	1	2	1	1	2	2	1	1
2	2	1	1	1	2	1	1	2	2
3	2	1	2	2	1	2	2	2	2
2	1	1	1	1	1	1	2	2	1
2	2	2	2	2	2	2	2	2	2
2	2	2	2	2	2	2	2	2	2
1	1	1	2	2	2	1	1	2	2
9	1	9	3	1	9	1	9	1	9
2	3	3	3	9	1	2	3	9	1
9	1	9	2	1	9	1	9	1	9
2	9	2	9	2	9	2	9	2	9
9	9	9	9	9	9	9	9	9	9
1	9	1	9	1	9	1	1	1	9
9	9	2	9	9	9	9	9	9	9
1	1	2	2	1	1	1	1	1	2
9	9	1	9	9	9	1	9	9	9
2	2	2	2	2	2	2	2	2	2
1	9	1	9	1	9	1	1	9	1
9	2	9	9	2	9	9	2	9	9
9	9	9	2	9	9	9	9	2	9

9	9	2	9	9	9	2	9	9	9
9	9	9	9	2	9	9	9	2	9
9	2	9	9	2	9	9	2	9	9
9	9	9	9	9	9	9	9	9	9
9	1	2	9	9	9	9	9	1	3
9	9	9	9	9	9	2	9	9	9
9	9	9	9	9	9	9	9	9	9
9	9	9	9	9	9	9	9	9	9
2	1	2	1	2	1	2	1	2	1
9	9	9	9	9	2	9	9	9	9
1	2	1	1	1	2	1	1	2	1
2	1	1	2	2	2	1	1	3	2
2	2	1	1	2	2	1	1	1	1
2	1	1	1	1	2	2	1	1	2
2	2	1	1	2	2	1	1	1	2
2	2	1	2	2	1	1	2	2	1
2	2	2	2	2	2	2	2	2	2
1	1	1	1	2	1	1	2	1	1
2	1	1	2	3	2	1	2	2	3
1	1	2	1	1	1	2	1	2	1
2	1	2	2	1	1	2	2	1	2
2	1	2	2	1	2	2	1	2	2
2	2	1	2	2	1	2	1	2	2
3	3	2	2	1	2	2	3	2	2
2	2	1	2	2	3	2	2	1	2
3	3	3	2	2	1	2	3	2	2
9	9	9	9	9	9	9	9	9	2
1	2	2	2	3	1	2	2	2	2
2	3	2	1	2	2	3	2	1	2
2	2	2	2	2	2	2	2	3	3
9	9	9	9	9	9	9	9	9	9
3	3	3	3	3	3	3	1	3	2
2	2	3	2	2	1	2	2	1	2
2	2	2	2	2	2	2	2	2	2
1	2	2	2	2	3	2	2	1	2
3	3	9	3	3	3	3	3	3	3
2	2	2	2	2	2	2	2	2	2
9	9	9	9	9	9	9	9	9	9
1	2	2	1	1	2	1	2	2	2
2	2	2	2	2	2	2	2	3	2
2	1	1	2	2	1	2	1	1	2
2	2	2	2	2	2	2	2	2	2
2	2	1	2	2	2	2	1	1	1
2	2	2	2	3	3	2	2	2	2
1	1	2	1	2	1	2	2	1	2
1	1	2	1	2	1	2	1	2	2
1	1	2	1	2	1	1	1	2	1

2	2	2	3	2	2	1	1	2	3
2	2	2	2	1	1	1	2	1	2
2	2	1	1	2	2	2	1	2	2
2	2	2	2	2	2	2	2	2	2
2	2	3	2	3	2	3	2	1	3
2	2	1	2	2	2	1	1	1	1
1	2	1	3	3	2	2	1	2	2
1	2	3	1	3	1	2	1	1	1
3	2	2	2	2	2	1	9	1	1
2	2	3	2	2	2	2	2	2	2
2	3	3	3	2	2	2	2	3	2
2	2	2	2	2	2	2	2	2	2
2	3	1	3	2	1	1	2	3	1
2	2	3	2	1	2	3	2	2	3
2	2	3	2	3	2	3	2	2	1
3	3	3	9	2	3	2	1	1	1
3	3	3	2	2	3	2	3	1	1
2	2	2	2	2	2	2	2	2	2
1	3	3	3	2	3	3	2	3	2
2	2	2	2	2	2	2	2	2	2
1	2	2	2	2	2	1	1	2	2
2	2	2	2	2	2	2	2	2	2
1	1	3	2	3	2	1	1	1	1
3	3	2	2	2	9	2	2	2	2
2	2	2	2	2	2	2	2	1	1
2	2	3	3	3	3	3	3	3	3
3	2	2	3	3	1	2	2	1	2
2	2	2	2	2	2	1	1	2	2
1	1	1	1	1	1	1	1	1	1
2	2	2	2	2	2	2	2	2	2
2	2	2	2	2	2	3	2	1	1
1	2	2	2	2	2	3	2	3	2
2	2	2	3	2	2	2	9	1	2
2	2	2	2	2	2	2	2	2	2
2	2	2	2	2	9	1	2	1	2
2	2	2	2	2	2	1	1	2	2
1	2	2	2	3	3	3	2	2	2
2	2	2	2	2	2	2	2	2	1
1	2	2	3	3	2	2	3	2	1
1	2	1	3	3	3	1	2	1	3
1	2	1	2	1	2	1	2	1	2
2	2	2	2	2	3	3	3	2	3
1	2	3	2	2	3	2	1	1	1
3	2	3	3	3	2	2	3	2	3
2	2	2	2	2	2	2	2	2	2
2	2	2	2	2	2	2	2	2	2
2	2	2	2	2	2	1	2	3	2

1	1	3	2	2	3	1	1	3	1
1	3	3	1	3	2	1	2	1	1
2	2	2	2	2	2	2	2	2	2
2	2	2	2	2	2	2	2	2	2
2	2	2	2	2	2	2	2	2	2
2	2	2	3	2	2	2	2	2	2
2	2	2	2	2	2	1	1	2	2
1	2	2	1	3	1	2	3	2	1
2	2	2	2	2	2	2	2	2	2
9	9	9	9	9	9	9	9	9	9
1	2	2	9	1	2	2	3	1	2
2	2	2	1	1	1	1	1	2	2
1	1	3	2	2	2	1	2	1	3
2	2	2	2	2	2	1	1	2	2
2	2	2	2	2	2	2	2	2	2
2	2	2	1	2	2	1	2	2	2
2	2	2	2	2	2	2	2	2	2
2	2	2	2	2	2	2	2	2	2
3	3	3	3	3	3	3	3	3	3
3	3	1	1	1	1	1	1	3	3
3	3	3	3	3	3	3	3	3	3
3	3	3	3	3	3	3	3	3	3
1	2	2	2	2	2	2	3	3	3
2	3	2	1	3	2	3	2	1	1
2	2	2	2	2	2	2	2	2	2
2	2	1	1	2	2	2	1	1	2
2	3	2	1	1	1	1	2	2	2
2	2	1	1	1	1	2	2	2	2
2	2	2	2	2	2	2	2	2	2
1	3	2	1	2	2	1	2	1	2
2	2	2	3	1	1	1	1	2	1
2	2	3	2	9	2	2	2	2	2
2	3	2	3	1	2	2	2	1	2
3	3	3	3	3	3	3	3	3	3
1	2	3	2	2	2	2	2	1	1
2	2	2	2	2	3	3	3	1	2
2	2	2	3	3	2	1	1	1	2
3	2	3	3	3	2	1	2	2	9
2	2	2	2	2	2	2	2	2	2
2	2	2	2	2	2	2	2	2	3
2	2	2	2	2	2	2	2	2	1
1	2	1	1	3	1	1	1	3	1
2	2	2	2	2	2	2	2	2	2
1	1	3	3	3	1	1	1	1	1
3	3	3	3	1	3	3	3	3	3
1	2	2	1	3	3	1	1	2	1
1	1	1	1	2	3	3	2	2	1

2	3	2	2	2	2	2	2	3	3
1	3	3	3	3	1	3	3	1	1
1	2	3	3	3	3	3	2	2	2
2	2	2	3	2	2	2	2	2	2
2	3	2	2	1	3	3	3	3	2
9	9	9	9	9	9	9	9	9	9
2	2	2	2	2	2	2	2	2	2
2	2	2	2	2	2	2	2	2	2
1	3	3	1	2	2	2	2	2	1
3	2	3	3	3	3	3	3	3	3
3	2	1	2	2	1	2	3	1	1
1	2	2	2	3	1	2	2	2	2
1	9	9	9	9	9	9	9	9	9
9	9	9	9	9	9	9	9	9	9
9	9	9	9	9	2	9	9	9	9
3	3	2	2	2	2	3	2	2	3
2	2	2	2	3	3	1	2	2	2
2	2	2	9	3	9	3	9	2	9
1	3	2	2	9	2	1	1	1	1
2	2	3	2	3	2	2	1	2	1
1	2	2	2	3	3	3	3	3	3
1	1	2	1	2	1	1	1	1	1
1	2	2	2	2	3	1	1	1	1
2	2	2	2	2	2	2	2	2	2
1	2	3	3	3	3	1	2	3	2
1	2	1	2	2	2	1	1	2	2
1	2	3	3	3	3	1	2	3	2
9	9	9	9	9	9	9	9	9	9
9	9	9	9	9	9	9	1	9	9
1	1	1	3	1	3	3	3	3	1
1	2	2	2	3	2	1	2	1	1
2	3	2	1	2	3	2	2	3	1
2	3	3	1	3	3	1	2	1	1
1	1	2	9	3	3	1	3	2	1
1	1	3	2	3	3	2	2	2	1
1	2	3	3	2	2	2	2	2	2
2	2	2	2	3	3	1	3	3	3
1	2	2	2	2	2	2	2	2	2
1	1	1	3	3	2	2	1	2	2
1	3	3	3	3	3	1	1	3	1
2	2	1	1	2	2	2	3	2	2
2	2	2	2	2	3	2	2	1	2
2	2	2	2	2	3	2	2	2	1
2	2	3	2	2	2	2	2	2	2
1	1	3	3	3	2	2	2	2	2
1	1	1	2	2	2	2	2	2	2
1	2	2	1	2	2	1	2	2	3

2	1	1	1	1	1	1	1	1	1
3	3	3	3	2	2	3	2	2	2
1	9	9	9	9	9	9	9	9	9
1	2	3	1	3	3	1	2	1	1
1	3	2	1	3	2	1	1	2	1
1	3	3	1	3	2	2	2	2	2
1	2	3	2	2	1	1	2	2	1
1	3	3	1	2	1	2	3	2	1
3	3	3	3	3	3	3	3	3	3
2	2	2	2	2	3	2	2	1	2
2	3	3	2	3	2	2	2	2	2
2	2	2	2	2	2	2	2	1	2
3	3	3	1	2	2	3	2	3	3
1	2	9	1	3	3	2	1	1	1
1	2	2	2	2	2	2	2	2	1
1	2	1	3	3	3	3	2	3	2
1	3	3	9	9	3	1	3	3	3
2	2	2	3	2	3	1	2	2	1
9	1	2	2	2	9	1	2	2	2
1	3	3	3	3	3	1	1	1	1
3	3	3	3	3	3	3	3	3	1
9	9	9	9	9	9	9	9	9	9
3	3	3	3	3	3	3	3	3	2
2	1	3	2	1	3	2	1	3	2
1	2	2	2	2	1	2	2	2	2
9	9	9	9	9	9	9	9	9	9
9	9	9	9	9	9	1	9	9	9
1	3	2	2	3	2	1	3	1	2
1	9	9	9	9	9	9	9	9	9
1	9	9	9	9	9	9	9	9	9
9	9	9	9	9	9	2	9	9	9
1	1	2	2	2	2	2	1	2	2
9	9	9	9	9	9	9	9	9	9
3	2	1	2	3	1	2	3	1	2
3	1	3	1	3	1	2	3	2	1
3	3	3	3	3	3	3	3	3	3
3	3	3	3	3	3	3	3	3	3
3	2	2	1	1	1	1	1	1	2
2	2	2	2	2	2	2	2	2	2
2	3	2	2	1	1	2	2	1	1
1	9	9	9	9	9	1	1	9	1
1	3	2	2	2	2	2	2	2	2
1	2	3	2	2	2	2	2	2	2
1	1	3	2	9	1	1	1	1	2
1	1	3	2	9	1	1	1	1	2
1	1	2	3	2	1	1	1	1	2
1	1	3	2	1	2	1	1	1	1

1	1	3	1	3	1	1	3	2	2
2	3	2	1	2	9	2	1	2	3
1	1	3	1	3	1	1	3	3	3
9	9	9	9	9	9	9	9	9	9
9	9	9	9	9	9	1	9	9	9
2	1	3	1	2	9	1	2	1	1
1	1	3	1	3	1	2	3	1	1
1	1	2	9	9	9	9	9	9	9
9	9	9	9	9	9	9	9	9	9
1	2	1	3	3	1	1	1	3	1
2	2	2	2	2	2	2	2	2	2
1	2	2	2	2	2	2	2	2	2
3	1	3	3	3	3	3	3	2	2
2	2	2	2	2	9	9	9	9	9
9	9	9	9	9	9	9	9	9	9
9	9	9	9	9	9	9	9	9	9
2	2	2	2	2	2	3	2	2	2
1	2	3	3	3	1	1	1	3	1
1	2	2	2	2	2	1	1	2	1
9	9	9	9	9	9	9	9	9	9
1	9	9	9	9	9	9	9	9	9
3	1	2	3	1	2	2	2	1	1
9	9	9	9	9	9	9	9	9	9
9	9	9	9	9	9	9	9	9	9
1	1	1	3	3	1	1	2	1	1
3	3	3	3	3	3	1	2	2	2
1	1	1	2	1	1	1	1	2	2
1	1	1	3	3	2	1	1	1	3
2	2	2	2	2	2	1	1	1	1
1	2	2	2	2	9	1	1	2	2
3	3	3	3	3	3	3	3	3	3
1	1	2	2	2	2	2	2	2	1
2	1	2	2	2	2	1	2	2	1
1	2	2	3	3	3	9	1	1	1
2	2	2	2	2	2	2	2	2	2
1	3	3	3	1	3	1	1	1	1
1	2	2	2	2	1	2	2	1	1
1	2	2	3	2	2	1	1	1	2
1	2	3	3	2	2	1	2	2	3
1	1	1	1	3	3	2	2	1	1
1	1	2	2	2	2	2	2	2	1
3	1	1	1	1	1	2	2	2	1
2	2	1	1	1	1	2	2	2	1
9	9	9	9	9	9	9	9	9	9
9	9	9	9	2	9	9	9	9	9
1	1	1	1	2	3	1	2	2	2
2	2	2	2	2	3	2	2	2	1

2	2	1	2	2	2	2	2	2	3
2	3	3	1	2	2	2	2	2	2
2	2	2	2	2	2	2	2	3	3
2	2	2	2	2	2	2	2	2	2
2	2	2	2	2	2	2	2	1	1
1	2	3	1	1	3	2	2	1	1
1	1	3	2	2	3	3	2	3	1
2	1	2	1	2	3	1	2	1	2
9	9	9	9	9	9	9	9	9	9
2	1	1	1	1	1	1	2	2	1
2	2	2	2	2	2	2	2	2	2
2	2	2	2	2	2	2	2	2	2
2	2	2	2	2	2	2	1	2	3
2	2	2	2	2	2	2	2	2	2
9	2	3	3	3	3	3	3	3	2
2	1	2	3	2	2	3	2	2	3
1	2	3	1	2	3	2	3	1	2
1	2	3	1	2	3	1	2	3	1
1	2	2	2	2	2	2	2	3	3
2	2	1	2	2	3	3	1	2	3
3	3	2	2	2	1	2	1	3	2
2	2	2	2	2	2	2	2	2	2
2	3	2	3	2	2	3	2	2	2
1	1	1	3	2	1	1	3	2	2
2	2	2	2	2	2	2	2	2	2
3	1	9	9	3	3	3	2	2	2
2	2	2	2	2	2	2	2	2	2
2	2	3	2	9	2	1	1	2	9
3	1	2	3	1	2	3	1	2	3
9	9	9	9	9	9	9	9	9	9
2	1	3	2	2	1	1	1	2	1
9	9	9	9	9	9	9	9	9	9
3	3	3	3	9	9	9	9	9	9
1	1	3	3	3	3	1	1	1	1
1	2	1	2	1	3	1	3	2	1
1	3	2	1	3	2	1	2	3	2
3	3	2	1	1	2	3	3	2	1
1	2	2	2	2	3	3	3	3	2
2	2	2	2	2	2	2	2	2	2
2	2	2	2	2	1	1	2	2	1
2	2	1	1	1	3	3	3	2	2
1	1	3	3	2	2	1	1	1	2
2	2	2	2	2	2	2	2	2	2
2	2	2	2	2	2	2	2	2	2
2	1	3	2	9	9	1	2	2	9
2	2	2	2	2	2	2	2	2	1
2	2	2	2	3	2	3	2	3	2

9	3	3	3	3	3	2	2	2	1
1	2	2	2	2	2	2	2	2	2
1	2	2	3	1	2	2	2	1	2
9	9	9	9	2	9	9	9	9	9
2	1	3	2	2	3	3	1	2	1
2	2	2	2	2	2	2	2	2	2
1	3	1	3	3	1	3	3	1	1
2	2	3	3	2	2	1	2	2	1
9	9	9	9	9	9	1	9	9	9
3	2	2	2	2	2	2	2	2	2
1	3	3	3	3	3	1	1	1	2
2	1	2	1	3	1	1	1	2	1
9	9	9	9	9	9	9	9	9	9
2	2	2	2	2	2	2	2	2	2
3	3	3	9	1	3	2	1	2	3
1	3	3	3	1	3	2	1	1	2
1	9	9	9	9	9	9	9	9	9
9	9	9	9	9	9	9	9	9	9
2	1	2	2	1	3	3	1	3	1
3	3	3	3	3	3	2	2	2	1
2	1	2	1	2	1	2	1	1	2
3	3	3	3	3	3	3	3	3	3
2	2	2	2	2	2	2	2	2	2
1	3	2	3	2	1	2	2	2	2
1	2	1	3	1	9	1	2	3	1
2	2	3	2	3	3	1	1	2	2
9	2	3	2	3	3	2	2	2	2
1	2	3	2	3	2	3	1	1	1
1	2	2	3	3	1	1	1	2	1
1	3	3	1	3	3	2	2	2	1
2	2	2	2	2	2	2	2	2	2
2	2	2	2	2	2	2	2	3	3
2	9	1	2	2	1	2	2	1	2
1	2	2	2	2	2	2	1	2	2
2	2	2	1	2	2	2	2	2	1
9	9	9	9	9	9	9	9	9	9
2	1	2	2	2	2	2	3	3	2
2	3	3	3	3	3	2	2	2	2
3	3	3	1	1	1	2	2	1	2
2	1	2	2	2	2	2	2	2	2
2	1	2	2	2	2	2	3	3	2
1	2	2	2	2	2	1	2	2	1
2	2	2	2	2	2	2	2	2	2
2	2	2	2	2	2	2	2	2	2
9	9	9	9	9	9	9	9	9	9
2	1	3	1	2	3	2	2	1	2
9	9	9	9	9	9	9	9	9	9

1	2	2	2	2	2	1	1	2	2
1	1	9	3	2	1	2	1	1	1
9	9	9	9	9	9	9	9	9	9
1	1	3	3	3	3	1	1	1	1
2	1	1	1	2	2	1	1	2	2
2	1	2	3	2	2	1	1	2	2
2	2	2	2	2	2	2	2	2	2
2	9	9	9	1	9	9	9	2	3
2	1	2	3	2	3	1	2	1	2
1	2	2	1	2	2	1	1	1	1
9	9	9	9	9	9	9	9	9	9
3	1	3	1	3	3	1	3	1	3
3	3	3	2	1	2	9	2	9	2
2	9	3	2	2	2	3	2	3	3
2	2	2	3	9	3	2	3	2	2
3	9	3	3	9	3	9	2	2	2
1	2	2	2	1	2	2	1	3	3
9	9	9	9	9	9	9	9	9	9
9	9	9	9	9	9	9	9	9	9
3	2	1	3	2	2	2	1	3	2
2	3	9	1	2	1	2	3	2	1
1	3	3	1	1	1	2	2	3	3
3	2	9	2	2	3	2	3	2	3
3	3	3	3	3	3	3	3	3	3
3	2	1	2	3	2	1	2	3	2
2	2	2	2	2	2	2	2	2	1
9	9	9	9	9	9	9	9	9	9
2	1	2	2	3	3	2	2	3	3
9	9	9	9	9	9	9	9	9	9
9	9	9	9	9	9	9	9	9	9
9	9	9	9	9	9	9	9	9	9
9	9	9	9	9	9	9	9	9	9
9	9	9	9	9	9	9	9	9	9
1	1	2	1	1	1	1	1	1	2
1	2	1	2	1	2	3	2	2	3
2	2	3	2	2	2	2	3	2	2
9	9	9	9	9	9	9	9	9	9
3	1	3	2	3	3	2	2	2	2
1	1	1	9	1	1	1	9	1	1
9	9	9	9	9	9	9	9	9	9
2	2	2	2	2	2	2	2	2	2
2	2	2	1	2	2	2	2	2	2
2	2	2	2	1	2	1	1	2	2
2	2	2	2	2	2	2	2	2	2
3	1	2	3	1	2	3	1	2	3
2	1	3	2	2	1	2	2	3	2
3	2	3	2	3	2	1	1	2	2

2	2	2	2	2	2	2	2	2	2
2	2	2	2	2	2	2	2	2	2
9	9	9	9	9	9	9	9	9	9
3	1	2	3	1	2	3	1	2	3
2	3	1	1	1	1	2	2	2	2
1	1	1	2	2	1	1	3	3	3
9	3	1	9	2	9	2	9	9	2
2	2	2	2	2	2	2	2	2	2
2	2	2	2	2	2	2	2	2	2
2	2	2	2	2	2	2	2	2	2
2	2	2	2	2	2	2	2	2	2
1	1	2	2	3	2	2	1	2	2
9	9	9	9	9	9	9	9	9	9
1	1	1	1	1	1	1	1	1	1
2	2	3	2	3	9	3	2	1	2
1	1	3	3	3	3	1	1	3	1
2	3	3	3	1	1	1	2	2	2
9	9	9	9	9	9	9	9	9	9
2	2	1	3	1	2	2	3	2	3
2	2	3	3	2	3	2	9	9	9
9	9	9	9	9	9	9	9	9	9
3	1	2	2	3	3	1	3	1	1
9	9	9	9	9	9	9	9	9	9
9	9	9	9	9	9	9	9	9	9
9	9	9	9	9	9	9	9	9	9
9	9	9	9	9	9	9	9	9	9
1	2	2	1	2	2	1	2	2	1
3	3	3	3	2	2	3	2	2	1
9	9	9	9	9	9	9	9	9	9
1	1	1	1	2	2	1	1	2	2
1	3	1	3	1	3	1	3	1	3
1	3	3	3	1	3	3	3	1	1
1	1	2	2	3	2	2	2	2	1
2	2	1	2	2	2	2	2	2	2
2	2	2	2	2	2	2	2	2	2
2	2	2	2	2	2	2	2	2	2
1	3	1	1	1	3	1	3	1	3
2	2	2	2	2	2	2	2	2	2
3	2	2	2	2	3	2	3	2	9
3	1	1	3	3	1	1	2	2	1
1	9	2	9	1	2	1	2	9	2
3	2	3	2	3	3	2	2	2	1
2	2	2	2	2	2	2	2	2	2
1	1	3	1	3	2	1	1	2	9
1	1	1	2	1	3	2	1	1	1
1	1	1	1	1	1	1	1	1	1
3	3	3	3	3	3	3	3	2	2

3	3	3	1	3	3	1	1	2	2
3	2	2	3	2	3	3	3	2	2
1	3	3	1	3	3	1	3	2	2
1	1	3	2	2	1	1	1	2	1
2	2	2	2	2	2	2	2	2	2
2	2	2	2	2	2	2	2	2	2
2	2	2	2	2	2	2	2	2	2
2	2	3	3	3	3	3	3	3	3
2	2	2	2	2	2	2	2	2	2
1	1	3	3	3	3	3	1	2	1
9	9	3	1	1	2	2	2	2	1
1	1	1	3	2	1	3	1	2	1
3	3	3	3	3	3	3	3	3	3
2	2	3	2	2	3	1	2	3	1
2	1	3	2	1	1	2	2	2	1
9	9	9	9	9	9	9	9	9	9
3	3	1	3	1	3	1	1	1	3
2	2	2	2	2	2	2	2	2	2
2	3	2	2	3	9	2	3	2	2
3	3	3	3	3	3	3	3	3	3
2	2	2	2	2	2	2	2	2	2
2	2	2	2	2	2	2	2	2	2
1	1	1	1	1	1	1	1	1	1
2	3	3	3	2	3	2	2	3	2
1	1	1	1	1	1	1	1	1	1
3	3	3	3	3	3	3	3	3	3
2	2	3	2	2	2	3	2	2	3
3	2	3	3	2	3	2	3	2	3
3	1	3	2	2	2	1	2	2	3
2	2	2	2	2	2	2	3	3	3
3	3	3	3	3	3	3	3	3	3
9	9	9	9	9	9	9	9	9	9
2	2	2	2	2	2	2	2	2	2
1	3	2	9	1	9	1	2	3	1
1	2	1	3	2	1	3	1	2	3
1	2	3	2	1	3	1	3	1	3
2	2	2	2	2	2	2	2	2	2
3	3	3	3	3	3	3	3	3	3
1	1	1	3	3	3	2	2	1	2
2	2	2	2	1	2	2	2	2	2
2	2	2	2	2	2	2	2	2	2
2	2	3	2	3	2	2	3	1	1
1	2	2	3	2	2	1	1	1	2
2	3	2	2	1	1	2	2	1	1
9	9	9	9	9	9	9	9	9	9
3	1	2	3	1	2	3	1	2	1
2	2	2	2	2	2	2	2	2	2

1	1	2	2	2	2	2	1	1	1
9	9	9	9	1	9	1	1	1	1
1	9	9	9	1	9	1	1	1	1
1	1	2	2	2	2	1	2	2	2
9	9	9	9	9	9	9	9	9	9
1	9	1	9	9	9	9	9	9	1
9	9	9	9	9	9	9	9	9	9
2	2	2	2	2	2	2	2	2	2
2	1	2	1	2	2	2	1	3	1
3	2	2	2	3	2	3	3	2	1
2	2	2	2	2	2	2	2	2	2
2	2	2	2	2	2	2	2	2	2
2	2	2	2	2	2	2	2	2	2
2	2	2	2	2	2	2	2	2	2
2	2	2	2	2	2	2	2	2	2
2	2	2	2	2	2	2	2	2	2
1	1	1	2	3	3	1	1	3	3
2	2	2	2	2	2	2	2	2	2
1	2	3	2	2	2	2	2	2	2
2	2	2	2	3	9	2	3	1	2
1	2	2	2	1	2	1	2	2	2
1	1	1	1	1	1	1	1	1	1
1	9	2	9	1	9	9	9	9	9
2	2	2	2	2	2	2	2	2	2
1	1	3	2	1	2	1	1	1	1
2	2	2	2	2	1	1	1	1	1
3	2	2	2	1	2	2	3	2	2
9	9	9	9	9	9	9	9	9	9
2	2	2	1	2	3	2	1	1	1
2	2	2	2	2	2	2	2	2	2
2	2	2	3	2	2	2	2	2	2
1	1	2	1	1	1	1	1	1	1
1	2	3	2	1	3	2	1	1	1
1	1	2	2	3	1	1	2	2	2
1	2	2	3	3	1	1	1	1	1
2	2	2	2	2	3	3	3	3	3
2	2	2	2	2	2	2	2	2	2
2	2	2	2	2	2	2	2	2	2
2	2	2	2	2	2	2	2	2	1
9	9	9	9	9	9	9	9	9	9
1	1	3	2	9	1	2	1	2	2
3	3	2	2	1	2	2	2	1	2
2	1	1	1	2	2	3	2	1	1
1	1	2	2	2	2	1	1	1	1
2	2	2	1	3	3	3	3	3	2
2	2	2	2	2	2	2	2	2	2
2	2	2	2	2	2	2	2	2	2

1	1	2	2	2	2	1	1	1	1
9	9	2	9	9	9	1	9	9	9
2	2	2	1	2	2	2	2	2	2
2	3	2	3	3	1	2	3	1	2
2	2	2	2	2	2	2	2	2	2
1	3	3	1	2	3	3	2	2	1
2	3	2	3	2	3	2	3	2	3
1	1	2	2	3	3	1	1	1	1
1	3	9	9	2	3	9	9	9	1
2	2	2	3	3	3	1	2	2	2
9	9	9	9	9	9	9	9	1	1
3	3	2	1	3	3	3	3	1	1
2	2	2	2	2	2	2	2	2	2
1	1	1	1	1	1	2	1	3	1
2	2	2	2	2	2	2	2	2	1
1	2	2	2	3	1	1	1	1	1
2	2	2	2	2	2	2	2	1	1
1	1	3	1	3	9	1	1	1	1
9	9	9	9	9	9	9	9	9	9
1	1	2	1	2	1	1	1	1	1
3	2	2	1	1	2	2	1	2	1
2	2	2	3	3	1	1	2	2	2
2	2	2	2	2	2	2	3	2	2
3	1	2	1	1	2	3	1	2	1
2	2	2	2	2	2	2	2	2	2
2	1	2	1	1	2	2	3	2	2
2	2	2	2	2	2	1	1	1	1
1	2	2	2	2	2	2	2	1	1
3	2	1	2	3	2	2	1	2	3
1	1	1	3	1	3	3	3	3	3
2	2	2	2	2	2	2	2	2	2
2	3	3	2	2	2	2	1	2	3
1	2	3	2	1	2	1	3	3	1
2	3	1	2	3	1	2	3	1	3
1	1	3	3	3	3	1	3	1	1
2	3	3	2	1	1	1	3	1	1
1	3	2	2	1	1	1	1	2	2
1	1	2	2	2	2	2	1	1	2
2	2	2	2	2	2	2	2	2	2
1	3	3	3	3	2	1	1	1	1
9	9	9	9	9	9	9	9	9	9
1	2	3	1	3	2	2	1	2	3
9	9	9	3	3	3	1	3	1	1
3	1	1	2	3	1	1	3	1	1
1	3	3	1	3	3	1	1	1	1
1	1	1	3	3	1	1	1	3	3
2	2	2	3	2	3	1	2	2	3

1	3	2	2	2	3	2	3	2	3
9	9	9	9	9	9	9	9	9	9
2	2	2	2	2	2	2	2	2	2
1	1	9	3	2	3	9	9	9	9
2	2	2	2	2	2	2	2	2	2
2	2	2	2	2	2	2	2	2	2
1	1	2	1	3	1	1	1	2	1
1	1	1	1	1	1	1	1	1	1
9	9	9	9	9	9	9	9	9	9
1	2	3	1	2	1	2	1	2	1
9	9	9	9	9	9	9	9	9	9
2	2	3	2	3	3	2	2	2	2
1	1	2	1	2	2	2	2	1	1
1	3	3	3	2	2	2	2	2	1
3	3	3	3	3	3	2	2	2	2
2	3	2	1	2	3	2	2	3	1
2	2	3	3	3	3	3	3	2	1
1	1	3	3	3	3	1	1	1	1
1	1	3	3	3	2	3	3	1	2
1	1	1	1	1	1	1	1	1	1
2	2	2	2	2	1	1	1	1	1
3	3	9	1	2	2	3	2	3	1
9	9	9	9	9	9	9	9	9	9
2	3	1	3	1	3	1	3	1	1
3	1	2	3	1	2	3	1	2	3
1	2	3	3	3	1	1	1	1	1
2	1	3	3	3	2	1	1	1	1
1	3	3	2	3	2	2	1	1	1
1	2	2	2	2	2	2	2	2	1
1	2	3	2	3	2	1	1	1	1
3	2	1	1	1	2	1	1	2	2
3	1	2	3	2	9	2	9	2	2
3	2	1	3	2	3	2	2	2	3
1	2	2	1	3	2	1	2	1	1
1	1	3	3	3	2	3	1	2	3
1	3	3	3	3	2	3	2	2	3
1	1	1	2	2	2	1	2	1	1
9	9	9	9	9	9	9	9	9	9
1	2	2	2	2	2	2	2	2	2
2	2	2	2	3	2	2	3	2	9
2	2	2	2	2	1	1	1	1	2
1	1	1	2	2	2	2	1	2	1
1	2	2	2	2	3	2	3	2	1
1	2	1	2	2	3	1	2	2	3
1	1	1	1	1	2	2	1	2	1
1	3	3	1	3	1	1	1	1	1
1	1	2	1	2	3	1	2	1	1

3	1	3	1	3	1	1	2	1	1
1	9	9	9	9	9	9	9	9	9
1	2	3	2	1	3	2	1	3	2
1	2	3	2	1	1	3	2	2	1
2	2	2	2	2	2	2	2	2	2
1	2	3	3	3	2	2	1	2	1
1	2	3	2	3	1	2	9	2	9
2	2	2	2	2	2	2	2	2	2
2	2	2	2	2	2	2	2	2	2
9	9	9	9	9	9	9	9	9	9
2	3	3	3	2	3	3	1	1	1
2	1	1	2	2	3	3	2	3	1
1	3	1	3	1	3	1	3	1	3
1	3	2	1	2	1	2	1	3	1
2	2	2	2	2	2	2	2	2	2
9	9	9	9	9	9	9	9	9	9
9	9	9	9	9	9	9	9	9	9
2	2	1	1	1	1	2	2	2	2
2	2	1	2	2	2	2	2	2	2
2	1	2	1	1	2	2	2	2	2
9	9	9	9	9	9	9	9	9	9
2	2	1	2	2	2	1	1	1	1
2	2	2	2	1	1	1	1	2	2
2	1	1	2	2	2	1	1	1	2
2	2	2	2	2	1	1	2	2	2
1	2	2	2	2	3	3	3	3	2
2	2	1	1	1	1	2	2	2	1
3	3	3	2	3	3	3	3	3	3
2	1	1	2	2	2	2	3	2	2
2	2	2	3	3	3	3	3	3	3
9	9	9	9	9	9	9	9	9	9
9	9	9	9	9	9	9	9	9	9
9	9	9	9	9	9	9	9	9	9
2	1	3	1	9	3	1	2	1	1
9	9	1	2	2	2	1	2	1	1
2	2	2	2	2	2	2	2	2	1
2	2	2	2	2	2	2	2	2	2
2	2	9	2	2	9	1	1	1	1
1	1	2	2	1	2	2	2	2	1
2	2	2	2	2	2	2	2	2	2
3	3	3	3	3	3	2	1	3	1
2	2	1	3	3	1	1	1	1	1
2	2	2	2	2	2	2	2	2	1
1	1	3	3	9	1	1	1	3	3
2	2	2	2	2	2	2	2	2	1
1	1	1	1	1	1	1	1	1	1
1	3	3	1	3	3	3	1	3	1

3	3	3	3	3	3	3	3	3	3
1	2	2	2	2	2	2	2	2	1
2	2	2	2	3	2	1	3	2	3
1	1	1	3	3	1	1	1	1	1
3	3	3	3	3	3	2	9	9	9
1	1	2	2	2	3	1	3	2	1
2	3	3	3	3	3	1	2	2	1
9	9	9	9	9	9	9	9	9	9
9	9	9	9	9	9	9	2	9	9
1	9	9	9	9	9	9	9	9	9
9	9	9	9	1	9	9	9	9	9
9	9	9	9	9	9	9	9	9	9
1	1	1	1	1	1	1	1	1	1
3	2	2	2	2	1	1	2	2	1
3	3	3	3	3	3	2	3	2	1
1	2	3	2	3	1	1	1	1	2
1	1	2	1	2	2	1	1	1	1
1	1	2	1	2	2	1	1	1	1
1	2	2	1	3	2	2	2	2	2
2	2	3	2	3	1	3	2	3	2
2	2	2	2	2	2	2	2	2	1
2	2	3	2	2	3	2	2	2	2
1	2	3	1	3	3	1	1	2	1
9	9	9	9	9	9	9	9	9	9
1	1	2	3	2	1	2	3	2	1
3	3	3	3	3	3	3	1	1	2
2	2	2	2	2	2	2	2	2	2
2	2	2	1	2	3	2	2	2	2
2	2	2	2	2	2	2	2	2	2
1	2	3	2	2	3	3	2	2	2
2	2	2	2	2	2	2	2	2	2
9	9	9	9	9	9	9	9	9	9
1	2	3	1	3	3	2	2	2	1
1	1	2	1	3	2	1	2	2	3
9	9	9	9	9	9	9	9	9	2
2	2	2	2	2	2	2	2	2	2
2	2	2	2	2	2	2	2	2	2
2	2	2	2	2	2	2	2	2	2
1	2	2	2	2	2	2	2	2	1
3	3	3	1	3	3	3	3	2	2
1	1	1	1	3	3	3	3	3	3
9	9	9	9	9	9	9	9	9	9
1	9	9	9	9	9	9	9	9	9
2	2	2	2	2	2	2	2	2	2
9	1	9	9	1	9	2	9	1	9
1	3	3	1	3	3	3	3	2	1
1	1	3	3	2	2	2	2	2	2

1	1	3	1	3	2	1	2	1	2
3	2	2	2	3	3	2	2	2	2
1	2	3	3	3	3	1	1	1	2
2	3	2	3	2	2	3	2	2	2
2	2	2	2	2	2	2	2	2	2
1	1	3	3	3	9	1	1	1	1
3	2	3	1	3	3	2	3	2	2
2	2	2	2	1	2	3	2	1	2
1	2	3	1	3	3	3	3	3	1
1	2	1	2	3	1	2	2	1	3
1	1	2	2	2	2	1	2	3	2
1	2	2	1	2	2	2	2	2	1
9	1	1	3	3	3	3	3	1	1
2	2	2	2	2	2	2	1	2	2
2	2	2	2	2	2	2	2	2	2
2	2	2	2	2	2	2	2	2	2
2	1	1	3	3	3	2	2	2	2
2	2	2	2	2	2	2	2	2	2
2	2	2	1	1	1	2	2	2	2
2	2	2	1	1	1	1	3	3	2
2	2	1	2	2	3	2	2	1	2
1	1	3	1	3	3	3	2	2	1
1	2	2	2	2	2	2	2	2	1
2	2	3	2	3	2	2	2	2	2
1	2	2	2	1	3	3	2	2	2
2	2	2	2	2	2	2	2	2	2
2	2	2	2	2	2	2	2	2	2
1	1	1	1	1	1	1	1	1	1
2	2	2	2	2	2	2	2	2	2
1	2	2	3	2	2	3	2	2	2
9	9	9	9	9	9	9	9	9	9
2	2	3	2	2	1	1	2	1	1
2	2	2	2	2	1	2	2	2	2
2	2	1	2	2	2	2	3	3	2
2	1	3	9	2	1	2	2	1	2
2	2	2	2	2	2	1	1	2	2
2	2	2	1	2	3	2	2	1	2
2	2	2	2	2	2	2	3	2	1
2	2	3	2	1	2	2	1	9	1
1	1	1	1	1	1	1	1	1	2
1	1	3	3	3	3	1	1	1	1
2	2	2	2	2	2	1	2	2	1
2	2	2	2	2	2	2	2	1	1
1	3	3	3	2	1	3	2	1	2
1	1	1	2	9	2	1	2	1	2
2	2	2	2	2	2	2	2	2	2
1	1	3	3	3	2	3	1	2	3

1	1	3	3	3	2	3	1	2	3
2	2	2	1	2	2	2	2	2	2
2	2	1	1	2	2	1	2	2	1
1	2	2	2	1	1	2	3	2	1
2	3	2	2	1	2	2	2	1	2
2	2	2	1	1	2	2	2	2	2
1	1	1	1	2	2	1	2	2	1
1	2	2	2	2	2	2	2	2	2
1	3	2	1	3	2	1	3	2	1
1	1	3	1	2	2	2	1	2	2
1	1	3	3	3	9	1	9	1	1
1	2	2	2	2	2	2	2	2	2
3	3	3	3	3	3	3	3	3	3
2	2	2	2	2	2	2	2	2	1
3	3	3	3	3	3	3	1	3	1
2	2	2	2	2	2	3	3	2	1
3	3	3	3	3	2	2	3	1	1
2	2	2	3	3	2	2	1	2	2
2	2	2	3	3	9	3	2	9	2
1	2	3	1	3	1	1	1	2	1
2	2	2	3	3	3	2	2	3	2
1	3	3	3	3	3	3	3	3	3
1	2	2	2	3	2	2	2	2	1
1	2	1	2	2	2	1	2	2	2
1	2	1	2	1	3	2	1	2	2
3	2	2	3	1	3	1	2	3	2
3	1	2	2	2	1	1	1	2	2
2	2	2	2	2	2	1	2	2	2
2	2	2	2	2	1	1	3	2	1
2	2	2	2	2	2	1	2	2	2
3	3	3	3	3	3	3	3	3	3
1	1	3	3	3	3	1	9	1	1
2	2	2	2	3	2	3	2	3	2
2	1	2	3	2	2	1	2	2	1
1	3	2	1	2	3	1	2	1	2
2	2	3	2	3	3	2	2	2	2
1	2	3	2	1	2	1	1	1	2
2	1	2	1	2	3	2	3	3	2
3	1	1	1	2	3	1	1	2	1
1	2	3	1	3	2	2	2	1	2
1	1	3	1	3	3	3	3	1	2
1	2	3	1	3	3	2	2	1	3
1	1	1	2	2	2	2	2	2	2
2	2	3	1	2	1	2	2	1	1
2	2	2	2	2	2	2	2	2	2
2	1	2	1	1	2	2	2	1	3
2	2	2	2	2	2	2	2	2	2

1	2	1	1	3	3	3	2	1	2
1	1	2	3	9	3	3	2	1	2
2	1	2	2	2	2	2	2	2	2
1	1	2	1	3	3	3	3	1	2
1	2	3	3	3	3	2	2	3	1
2	2	2	1	2	2	2	3	3	2
2	2	2	2	2	2	2	2	2	2
2	2	2	1	1	1	1	1	1	1
9	9	9	9	9	9	9	9	9	9
2	2	2	2	2	2	2	2	2	2
2	2	2	2	2	2	2	2	1	1
1	2	2	2	3	3	1	1	1	1
1	1	1	1	2	2	3	2	2	2
2	2	2	2	2	2	2	2	2	2
1	1	1	1	1	1	1	1	1	1
1	2	2	1	3	2	1	2	1	1
2	2	2	2	2	2	2	2	2	2
1	2	2	1	1	2	2	1	2	1
3	2	1	2	3	2	1	2	3	2
2	2	2	2	2	2	2	2	2	2
2	1	2	2	2	2	1	2	2	2
1	1	2	2	2	2	2	2	2	2
2	2	3	2	2	2	2	2	3	2
2	3	2	2	2	3	2	2	2	2
2	3	2	2	2	3	1	1	1	1
1	2	2	1	3	2	1	2	1	1
1	2	2	2	3	3	1	1	1	1
2	2	2	2	2	2	2	2	2	2
3	1	1	1	2	3	1	1	2	1
2	2	2	2	2	2	2	2	2	2
1	2	3	2	1	2	1	1	1	2
9	9	9	9	9	9	9	9	9	9
2	2	2	2	3	2	2	2	1	2
1	3	3	3	1	3	1	1	1	1
1	1	3	3	3	3	9	2	2	2
2	2	2	2	3	3	2	3	3	1
1	1	3	3	3	1	1	1	1	1
3	3	2	1	2	3	3	3	1	1
1	2	1	2	1	2	2	2	2	1
3	2	1	2	1	2	3	1	2	3
9	1	2	1	2	2	1	1	1	2
1	2	9	1	2	1	1	1	1	2
2	1	1	1	3	2	1	2	1	1
2	2	2	2	2	2	1	1	1	1
2	1	2	1	2	2	3	2	2	3
9	9	9	9	9	9	9	9	9	9
3	3	3	3	2	2	2	2	2	2

2	3	2	2	2	3	2	2	2	1
1	3	3	1	3	3	3	3	3	3
1	1	1	1	1	1	1	1	1	1
2	2	2	2	2	2	2	2	2	2
2	2	2	2	2	2	2	2	2	2
2	2	2	1	2	2	2	2	2	2
2	1	2	1	2	2	1	1	2	2
3	3	3	1	1	1	2	2	2	2
2	2	3	9	3	9	2	3	2	2
2	2	2	2	2	2	2	2	2	2
2	2	2	2	2	2	2	2	2	2
2	1	1	2	2	2	2	2	2	2
2	2	3	3	3	9	3	3	3	3
2	2	2	2	2	2	2	2	2	2
3	2	2	1	2	2	2	2	2	1
2	2	2	9	1	1	3	3	3	3
1	2	3	2	2	1	3	2	1	3
2	2	2	2	2	2	2	2	2	2
2	1	2	2	2	2	2	2	1	2
1	1	1	1	1	2	2	2	2	2
2	2	2	1	2	2	2	1	2	2
2	2	2	2	2	2	2	2	2	2
2	2	2	2	2	2	2	2	2	2
9	9	9	9	9	9	9	9	9	9
1	3	2	2	2	1	1	1	1	1
2	2	2	2	2	2	2	2	2	3
2	2	1	2	3	3	2	2	2	3
1	2	2	2	1	1	1	1	1	1
1	3	3	2	2	1	2	2	9	1
1	2	3	1	2	1	1	3	1	1
1	2	1	2	2	2	2	1	1	1
2	2	2	2	2	2	2	2	2	2
9	3	2	2	2	2	3	2	2	2
9	9	9	9	9	9	9	1	9	9
2	3	2	2	9	1	1	1	1	1
2	3	2	9	1	2	1	1	1	2
1	3	9	2	3	1	2	1	3	1
2	2	2	2	2	2	2	2	2	2
1	1	2	3	3	2	2	2	1	3
9	9	9	9	9	9	9	1	9	9
3	3	2	3	3	1	1	9	1	1
2	3	1	2	9	1	2	3	1	9
1	3	2	2	3	2	3	2	3	1
2	2	2	2	2	2	2	2	2	2
2	1	2	2	1	1	1	1	1	2
1	3	2	2	1	1	3	1	2	1
2	3	3	2	3	2	9	1	3	1

9	9	9	9	9	9	9	9	9	9
9	9	9	9	9	9	9	9	9	9
9	9	9	9	9	9	9	9	9	9
2	2	1	2	2	2	1	2	2	2
2	2	2	2	1	2	1	2	2	2
9	9	9	9	9	9	9	9	9	9
2	3	2	2	2	2	3	2	2	3
2	1	1	2	2	3	2	2	2	2
2	2	1	1	2	2	2	1	2	1
1	3	2	2	1	1	2	2	1	1
3	3	1	1	3	1	1	1	1	1
3	3	1	1	3	1	1	1	1	1
3	2	3	3	9	9	3	3	3	2
2	2	2	2	2	2	2	2	2	2
9	1	9	9	1	1	1	1	1	1
3	1	3	2	1	2	2	3	1	3
9	9	9	9	9	9	1	9	9	9
2	1	1	2	2	2	1	1	1	2
3	3	3	3	3	3	2	2	1	1
2	1	1	2	2	2	1	1	2	2
2	2	2	3	2	1	2	2	2	1
1	2	2	2	3	2	2	2	3	2
3	3	2	3	3	2	3	3	3	2
3	2	1	3	2	1	1	1	1	2
2	2	2	2	2	2	2	2	2	2
9	9	9	9	9	9	9	9	9	9
2	2	2	2	2	2	2	2	2	2
2	3	2	3	9	2	3	1	2	9
2	1	1	2	2	2	2	2	2	2
3	2	2	2	3	2	1	2	2	3
1	2	2	1	2	1	2	1	2	2
2	1	3	3	9	2	2	3	2	3
1	3	3	2	1	1	2	9	3	1
1	2	3	2	1	2	3	3	3	2
1	2	2	3	2	3	2	1	1	1
3	3	1	3	3	3	1	1	1	3
9	9	9	9	9	9	9	9	9	9
1	3	3	3	1	1	2	2	1	1
9	9	1	3	3	9	2	2	1	1
2	2	2	2	2	2	2	2	2	2
3	3	3	3	3	3	3	3	3	1
2	2	2	2	2	2	2	2	2	2
1	3	2	2	2	3	3	1	1	3
2	1	1	1	1	1	2	3	2	1
3	3	1	2	3	2	3	2	1	1
2	1	2	1	2	2	2	1	1	2
2	3	2	2	3	1	2	1	1	1

9	9	9	9	9	9	9	9	9	9
3	1	1	9	9	9	9	9	9	9
2	2	2	1	2	2	1	1	2	2
2	3	2	1	1	1	1	2	2	2
2	2	2	3	3	3	3	3	2	2
2	2	2	2	2	2	2	1	2	2
1	2	3	1	2	3	1	2	3	2
1	1	1	3	9	2	1	2	3	3
2	2	2	2	2	2	2	2	2	1
1	1	2	1	1	1	2	1	1	2
1	2	2	1	2	2	3	2	1	2
2	2	1	1	2	2	2	1	1	2
2	2	2	2	2	2	2	2	2	2
1	3	1	3	3	3	1	9	1	2
2	2	3	2	2	1	1	2	2	1
2	9	2	9	3	1	2	2	1	2
1	3	1	2	1	3	3	9	9	9
2	3	2	1	2	2	3	2	1	2
1	2	1	1	1	2	1	1	1	1
2	2	2	2	2	2	2	2	1	1
1	1	2	3	3	1	2	1	1	1
1	1	2	3	3	1	2	3	1	2
1	2	2	1	2	2	1	2	2	3
2	3	3	3	3	3	3	3	1	1
1	1	3	9	9	1	1	1	1	1
2	2	2	2	3	2	2	2	2	3
1	1	1	1	1	3	1	1	1	3
2	2	1	3	3	2	2	2	1	2
1	1	1	2	1	1	1	2	1	1
1	3	2	2	2	3	2	1	2	2
2	2	2	2	2	2	2	2	2	2
2	2	3	3	3	9	2	1	1	2
1	3	3	1	9	1	1	1	1	1
2	1	2	1	2	1	2	1	2	1
1	1	2	2	2	2	2	1	2	2
2	3	1	2	3	2	3	2	1	1
2	1	1	2	2	1	1	2	1	1
1	1	1	1	2	3	2	1	2	1
3	1	2	9	1	1	3	1	1	2
2	2	1	2	2	1	1	2	1	2
1	1	1	2	3	2	1	2	1	2
2	1	3	2	1	1	2	2	3	1
2	2	2	2	2	2	2	2	2	2
1	1	1	2	2	1	1	2	2	2
9	9	9	9	9	9	9	9	9	9
1	1	2	3	9	2	1	2	3	3
2	2	2	2	1	1	3	2	2	2

9	9	9	9	9	9	9	9	9	9
2	2	2	1	2	2	1	2	2	1
1	2	3	2	1	2	2	2	2	2
2	3	3	3	9	1	1	1	1	1
3	2	1	2	1	3	2	1	1	1
1	2	1	3	2	1	1	1	1	2
2	9	1	2	9	3	1	2	2	3
3	3	3	3	3	3	3	3	3	3
3	3	3	3	3	3	3	3	3	3
1	1	1	1	2	2	2	2	2	2
2	2	1	1	2	3	3	2	1	3
3	2	2	3	2	2	2	2	1	2
1	2	1	3	1	9	3	1	9	1
1	2	9	1	1	2	2	1	1	9
9	9	9	9	9	9	9	9	9	9
2	2	2	2	2	2	2	2	2	2
3	1	2	2	2	2	2	2	1	1
2	2	2	2	2	2	2	1	1	3
2	2	2	2	9	2	2	2	2	2
2	2	2	3	3	3	3	3	3	2
2	3	3	2	2	2	2	2	2	1
2	1	1	2	2	2	2	1	2	2
2	2	2	1	2	2	3	2	2	1
1	1	1	2	2	1	1	2	2	1
9	9	9	9	9	9	9	9	9	9
3	3	3	3	2	3	3	3	1	1
2	3	2	3	2	1	2	1	2	3
1	2	2	2	2	2	1	2	2	1
3	3	3	3	3	3	3	3	3	3
2	2	2	2	2	1	2	2	1	2
2	3	1	1	1	1	1	2	3	3
3	2	3	3	1	2	1	1	1	3
2	2	3	2	2	1	2	2	1	2
3	3	3	3	3	3	2	2	2	2
1	1	2	3	3	2	2	2	2	2
2	2	2	2	2	2	1	2	3	2
2	1	2	1	1	1	2	2	1	2
1	1	1	2	2	2	2	1	1	1
2	2	2	2	2	2	2	2	2	2
1	1	1	3	3	3	1	1	3	3
2	3	2	2	2	2	1	2	2	1
1	1	2	2	1	1	1	1	2	1
2	2	2	2	2	2	2	2	2	2
2	3	1	3	3	1	2	3	1	1
3	1	1	3	3	3	1	1	1	1
2	2	2	2	2	2	2	2	2	2
1	2	2	1	1	2	2	2	2	2

2	2	2	2	2	2	2	2	2	2
2	2	2	2	2	2	2	2	2	3
9	9	9	9	9	9	9	9	9	9
9	9	9	9	9	9	9	9	9	9
1	1	2	2	1	1	1	2	1	1
2	1	1	1	2	2	2	2	2	2
9	9	9	9	9	9	1	9	9	9
2	1	2	3	3	2	2	2	3	3
1	1	1	2	1	1	1	1	1	1
9	9	9	9	9	9	9	9	9	9
9	9	9	9	9	9	9	9	1	9
9	9	9	9	9	9	9	9	9	9
3	3	3	3	2	2	2	3	1	1
9	9	9	9	9	9	9	9	9	9
1	2	2	3	2	2	3	2	1	2
1	9	9	9	9	9	9	9	9	9
9	9	9	9	9	9	9	9	9	9
9	9	9	9	9	9	9	9	9	9
1	2	2	1	1	2	2	1	2	2
3	2	1	2	3	1	2	2	2	2
9	9	9	9	9	9	9	9	9	9
2	3	3	3	3	3	2	3	3	3
3	3	3	3	3	3	3	3	3	3
2	2	1	1	2	2	2	1	2	2
1	1	2	1	2	1	1	2	1	2
2	2	2	3	2	2	3	2	2	2
1	3	1	3	3	3	2	2	2	2
9	9	9	9	9	9	9	9	9	9
1	1	1	1	1	1	1	1	1	1
1	1	1	2	2	1	1	1	1	1
2	2	2	2	2	2	2	2	2	2
2	2	1	1	1	1	1	1	1	1
1	2	1	1	1	1	1	1	1	1
2	2	2	1	1	1	1	1	1	1
3	3	3	2	2	2	2	2	2	3
1	1	3	3	3	1	3	3	3	3
2	2	2	2	2	2	2	2	2	2
2	1	2	2	2	2	1	2	2	2
9	9	9	9	9	9	9	9	9	9
3	3	2	2	2	3	3	2	3	2
3	3	1	1	1	1	1	2	1	1
1	1	1	1	1	1	1	1	1	2
2	3	9	2	1	2	2	1	2	1
1	1	2	1	1	1	2	1	1	2
2	1	1	1	3	3	3	1	2	3
1	1	1	2	3	2	1	1	2	2
1	1	1	2	2	2	2	2	2	2

2	1	3	2	1	3	1	3	2	1
1	2	3	3	2	2	1	2	1	2
2	2	2	2	2	2	2	2	2	2
2	1	2	2	1	3	3	3	3	3
1	1	2	9	1	3	2	1	2	3
9	9	9	9	9	9	9	9	9	9
2	2	1	3	1	1	1	2	3	3
3	3	3	3	3	3	3	1	2	1
3	2	3	3	3	1	1	1	1	3
3	2	2	2	1	2	3	3	2	2
3	3	2	2	2	2	2	2	2	2
1	2	2	2	2	2	3	2	3	2
1	1	3	1	3	3	3	3	3	1
2	2	2	2	1	1	2	2	2	2
2	1	2	1	1	1	1	1	1	1
1	1	2	1	1	1	1	2	2	2
2	2	2	2	2	2	2	1	1	1
9	9	9	9	9	9	9	9	9	9
9	9	9	9	9	9	9	9	9	9
2	2	2	2	2	2	2	2	2	2
9	9	2	9	2	9	9	2	9	9
2	9	9	9	9	9	2	9	9	9
9	9	9	9	9	9	9	9	9	9
9	9	9	9	9	9	9	9	9	9
2	9	9	9	9	9	2	9	9	9
9	9	9	9	9	9	9	9	9	9
9	9	9	9	9	9	9	9	9	9
2	2	2	2	2	2	2	2	2	2
2	2	2	2	2	2	2	2	2	2
2	2	2	2	2	2	2	2	1	1
2	1	1	1	1	1	2	2	2	2
9	9	9	9	9	9	9	9	9	9
2	2	2	2	2	2	2	2	2	2
9	9	9	9	9	9	9	9	9	9
9	9	9	9	9	9	9	9	9	9
1	2	2	1	2	2	1	2	2	1
9	9	2	9	9	9	9	2	9	9
9	9	9	9	9	9	9	9	9	9
9	9	9	9	9	9	9	9	9	9
9	9	9	9	9	9	9	9	9	9
9	9	3	1	9	9	3	1	9	3
9	2	1	9	2	1	9	2	1	9
9	9	9	9	9	9	9	9	9	9
9	9	9	9	2	9	9	9	9	9
9	9	9	9	9	9	9	9	9	9
9	9	9	9	9	9	9	9	9	9
9	9	9	9	9	2	9	9	9	9

9	9	9	9	9	9	9	9	9	9
3	3	3	3	3	3	3	3	3	3
1	2	1	2	1	1	3	1	2	1
3	1	2	1	3	1	2	2	1	3
1	1	1	1	9	1	9	9	2	9
9	9	9	9	9	9	9	9	9	9
9	9	9	9	9	9	9	9	9	9
9	9	9	9	9	9	9	9	9	9
3	3	1	2	1	1	1	1	1	2
2	3	1	2	3	1	2	3	1	2
2	2	3	3	3	3	3	3	3	3
2	2	2	3	3	3	3	3	3	2
2	3	2	2	3	2	2	2	1	2
1	1	3	2	1	3	3	2	1	1
3	2	3	2	3	2	3	2	3	2
3	3	3	3	3	3	3	3	3	3
9	9	9	9	9	9	9	9	9	9
9	9	9	9	9	9	9	9	9	9
9	9	9	9	9	9	9	9	9	9
9	9	9	9	9	9	9	2	9	9
1	1	1	1	1	1	1	1	1	1
1	3	3	3	3	3	2	2	1	1
9	9	9	9	9	9	9	9	9	9
2	2	2	2	2	2	1	2	1	2
1	1	1	1	1	1	1	1	1	1
2	2	2	2	2	2	2	2	2	2
3	3	3	3	3	3	1	2	2	3
2	2	2	2	2	2	2	2	2	2
1	2	3	1	2	3	2	1	3	1
2	2	2	2	2	2	2	2	2	2
2	2	2	2	2	2	2	2	2	2
2	2	2	2	2	2	2	2	2	2
2	2	2	2	2	2	2	2	2	2
3	3	3	1	2	1	2	3	1	2
3	2	2	2	3	3	3	9	2	2
1	2	1	2	1	1	1	2	1	1
2	2	2	2	2	2	2	2	2	2
2	2	2	2	2	2	2	2	2	2
2	2	2	2	3	2	1	2	2	3
3	9	2	2	2	2	2	3	3	3
1	3	3	9	3	1	3	1	2	9
1	2	3	1	2	3	2	2	2	2
9	9	9	9	9	9	9	9	9	9
2	2	2	2	2	2	2	2	2	2
3	3	3	3	3	3	3	3	3	3
3	3	3	3	3	3	3	3	3	3
9	9	9	9	9	9	2	9	9	9

1	2	3	1	2	3	2	3	9	9
9	9	9	1	9	9	9	9	9	9
1	1	1	1	1	1	1	1	1	1
1	1	2	2	2	2	2	2	2	2
2	2	2	2	2	2	2	2	2	2
2	2	3	3	3	3	3	2	3	3
9	9	9	9	2	9	9	9	9	9
9	9	9	9	9	9	9	9	9	9
9	9	9	9	9	9	9	9	9	9
9	9	9	9	9	1	9	9	9	9
9	9	9	9	9	9	9	9	9	9
1	1	1	1	1	1	1	1	1	1
9	9	9	9	9	9	9	9	9	9
9	1	9	9	9	2	9	9	9	9
2	1	1	2	2	1	2	2	1	1
9	9	9	9	9	9	9	9	9	9
9	9	9	9	9	9	9	9	9	9
9	2	9	9	9	9	9	9	9	9
9	9	9	9	9	9	9	9	9	9
2	9	9	9	9	9	9	9	9	9
9	9	9	9	9	9	9	9	9	9
9	9	9	9	9	9	9	9	9	9
9	9	2	9	9	9	9	9	2	9
3	2	1	2	3	1	2	3	1	2
2	2	3	2	2	2	1	1	1	2
2	2	2	2	2	2	2	2	2	2
2	3	2	3	2	1	2	2	1	1
2	3	3	3	2	1	1	2	1	2
2	3	2	1	2	2	2	2	2	2
2	3	2	2	2	3	2	2	2	1
2	2	2	2	2	2	2	2	2	3
9	2	2	2	9	2	2	2	2	2
2	2	2	2	2	2	2	2	2	2
3	3	3	3	3	2	2	2	2	2
2	2	3	1	2	1	2	3	1	2
1	1	1	1	1	1	1	1	1	1
2	3	2	1	2	1	1	1	1	2
1	1	3	3	2	1	1	3	3	3
1	1	2	2	2	2	2	2	3	3
2	2	2	2	2	2	2	1	1	1
9	9	9	9	9	9	9	9	9	9
2	2	2	2	2	2	2	2	2	2
9	9	9	9	9	9	9	9	9	9
1	2	2	2	1	2	2	2	2	2
2	3	3	2	3	2	2	3	2	2
2	2	2	2	2	2	2	2	2	2
2	2	2	2	9	1	2	2	2	1

3	1	2	2	1	1	3	3	2	3
1	3	3	3	2	2	3	1	1	3
1	2	2	3	2	1	1	2	1	1
2	2	2	2	2	2	2	2	2	2
2	2	1	2	2	3	1	2	2	2
2	2	2	2	2	2	2	2	2	2
2	9	1	2	2	1	2	3	1	2
2	2	2	2	2	2	2	2	2	2
2	2	9	1	1	1	1	2	2	1
2	3	1	2	3	1	2	3	1	2
2	2	2	2	2	2	2	2	2	2
2	2	2	2	2	2	2	2	3	3
2	2	2	1	1	1	2	2	3	3
1	1	2	3	1	1	1	1	1	1
9	9	9	9	9	9	9	9	9	9
2	2	2	2	2	2	2	1	3	3
1	2	1	3	1	2	1	1	1	1
9	9	9	9	9	9	9	9	9	9
9	9	9	9	9	9	9	9	9	9
9	1	9	1	1	1	9	1	9	1
9	9	9	9	2	9	9	9	9	9
1	1	2	2	2	3	3	1	1	1
9	9	9	9	9	9	9	9	9	9
9	9	9	9	9	9	9	9	9	9
9	9	9	9	9	9	1	9	9	9
3	2	3	2	2	3	2	3	2	3
2	2	2	2	2	2	2	2	2	2
2	2	2	2	2	2	2	2	2	2
2	2	2	1	2	2	2	2	2	2
9	9	9	9	9	9	9	9	9	9
9	9	9	9	9	9	9	9	9	9
1	2	2	1	1	1	1	1	1	2
1	2	3	1	1	2	1	1	1	1
1	1	1	2	2	2	3	3	3	2
2	2	3	3	2	3	2	3	3	2
9	9	9	9	9	9	9	9	9	9
2	2	2	2	2	2	2	2	2	2
1	2	3	1	2	3	1	2	3	1
2	1	1	1	2	1	1	1	1	1
1	3	3	3	3	3	3	3	3	3
1	3	1	1	3	1	1	1	1	1
9	9	9	9	9	9	9	9	9	9
9	9	9	9	9	9	9	9	9	9
9	9	9	9	9	9	9	9	9	9
2	3	1	9	3	2	1	2	3	2
1	2	1	2	1	2	1	2	1	2
2	3	2	3	2	1	1	1	1	3

1	3	3	3	3	1	2	3	2	1
3	2	2	2	2	2	2	2	2	2
1	2	1	1	2	3	2	3	1	2
2	2	2	2	2	2	3	3	3	3
2	2	2	2	2	2	2	2	2	2
3	3	2	2	2	2	2	2	3	3
1	1	1	1	2	2	2	2	2	2
2	3	9	2	3	2	1	2	3	1
9	9	9	9	9	9	9	9	9	9
9	9	9	9	9	9	9	1	9	9
9	9	9	9	9	1	9	9	9	9
2	3	2	3	2	2	3	1	2	2
2	9	2	9	9	2	9	2	9	2
2	2	9	9	2	2	9	2	9	2
9	9	9	9	9	9	9	9	9	9
9	9	9	9	9	9	9	9	9	9
2	2	2	2	2	2	2	2	2	2
9	9	9	9	9	9	9	9	9	9
2	2	2	2	9	9	9	9	9	9
9	9	9	9	9	9	9	9	9	9
2	2	2	9	9	9	9	9	9	9
9	9	9	9	9	9	9	9	9	9
3	3	3	3	3	3	3	3	3	3
9	9	9	9	9	9	9	9	9	9
9	9	9	9	9	9	9	9	9	9
2	9	9	2	9	2	9	2	9	2
9	9	9	9	9	9	9	9	9	9
9	9	9	9	9	9	9	9	9	9
2	2	2	2	2	2	2	2	2	2
9	9	9	9	9	9	9	9	9	9
9	9	9	9	9	9	9	9	9	9
9	9	9	9	9	9	9	9	9	9
9	9	9	9	9	9	9	9	9	9
9	2	9	9	9	2	9	9	9	9
1	3	3	3	1	1	9	9	9	9
9	9	9	9	9	9	9	9	9	9
9	9	9	2	9	9	9	9	9	9
9	2	9	2	2	2	9	2	9	2
2	1	9	1	9	9	1	9	1	9
9	9	9	9	9	9	9	9	9	9
9	2	9	2	2	2	2	2	2	2
9	9	9	9	9	9	9	9	9	9
9	9	2	9	9	9	9	9	9	9
9	9	9	9	9	9	9	9	9	9
1	1	2	3	1	1	1	3	1	1
3	1	3	2	2	2	1	2	1	3
9	9	9	9	9	9	9	9	9	9

9	9	9	9	9	9	9	9	9	9
2	2	1	3	2	1	2	3	2	1
2	2	3	2	3	2	1	2	3	1
3	3	3	3	3	3	3	3	3	3
1	2	2	1	2	2	1	2	1	2
9	9	9	9	9	9	9	9	9	9
9	9	9	9	9	9	9	9	9	9
3	3	2	1	1	3	2	1	1	2
2	2	2	2	2	2	2	2	2	2
1	1	1	1	1	1	1	1	1	1
2	2	2	2	2	2	2	2	2	2
2	2	2	3	2	1	2	1	1	3
3	3	3	3	3	3	3	3	3	3
2	2	1	1	2	3	2	3	2	2
2	2	2	2	2	3	3	3	2	2
2	2	2	2	2	2	2	2	1	2
3	3	3	3	3	3	3	2	2	2
9	9	9	9	9	9	9	9	1	9
2	2	1	3	3	9	9	9	9	9
2	2	2	2	2	2	2	2	2	2
9	9	9	9	9	9	9	9	9	9
1	3	2	3	3	1	1	2	2	2
2	3	2	2	2	2	1	2	1	1
1	2	2	3	3	1	1	1	1	1
3	2	1	3	2	1	2	3	1	1
2	2	2	2	2	2	2	2	2	2
2	2	2	2	2	2	2	2	2	2
3	3	3	3	3	3	2	2	1	3
2	3	3	3	3	2	3	2	1	1
9	9	9	9	9	9	9	9	9	9
9	9	9	9	9	9	9	9	9	9
1	1	2	2	2	2	2	2	2	2
1	9	9	3	9	9	9	9	3	9
1	2	2	2	2	2	1	2	2	2
9	9	9	9	9	9	9	9	9	9
9	9	9	9	9	9	9	9	2	9
3	2	9	2	9	2	2	3	9	2
2	3	1	3	1	3	1	2	3	2
9	9	9	9	9	9	9	9	9	9
9	9	9	9	9	9	9	9	9	9
2	3	3	3	2	1	2	2	3	2
9	9	9	9	9	9	9	9	9	9
3	2	1	1	2	3	2	1	2	3
1	1	1	1	1	1	1	1	1	1
2	2	2	2	2	2	2	2	2	2
2	2	2	2	2	2	2	2	2	2
2	2	2	2	2	2	2	2	2	2

2	2	2	2	2	2	2	2	2	2
2	2	2	2	2	2	2	2	2	2
2	2	2	2	2	2	2	2	2	2
2	2	2	2	2	2	2	2	2	2
2	9	2	9	9	9	2	9	2	9
2	3	2	2	2	2	2	2	3	3
9	9	2	9	9	9	9	9	9	9
2	2	2	2	2	2	2	2	2	2
2	3	1	3	3	3	2	1	1	3
9	9	9	9	9	9	9	9	9	9
9	9	9	9	9	9	9	9	9	9
9	9	9	9	9	9	9	9	9	9
9	9	9	9	9	9	9	9	9	9
1	2	2	2	2	1	1	2	2	2
2	2	2	2	2	2	2	2	2	2
9	9	9	9	9	9	9	9	9	9
3	3	3	3	3	2	2	2	2	2
2	2	1	2	1	2	1	2	2	2
2	2	2	2	3	1	2	2	1	1
2	2	2	2	2	2	2	2	2	2
3	2	1	2	3	1	3	3	2	3
9	9	9	9	9	9	9	9	9	9
9	9	9	9	9	9	9	9	9	9
9	9	9	9	9	9	9	9	9	9
9	9	9	9	9	9	9	9	9	9
9	9	9	9	9	9	9	9	9	9
9	9	9	9	9	9	9	9	9	9
9	9	9	9	9	9	9	9	9	9
2	2	2	2	2	2	2	2	2	2
9	9	9	9	9	9	9	9	9	9
9	9	9	9	9	9	9	9	9	9
9	9	9	9	9	9	9	9	9	9
9	9	9	9	9	9	9	9	9	9
9	2	9	9	1	9	9	1	9	9
9	9	2	9	9	9	9	2	9	9
9	9	9	9	9	3	9	9	9	9
9	9	9	9	9	9	9	9	9	9
2	2	2	9	2	9	9	9	9	2
9	9	9	9	9	9	9	9	9	9
9	9	9	9	9	9	9	9	9	9
9	9	9	9	9	9	9	9	9	9
9	9	9	9	9	9	9	9	9	9
9	9	9	9	9	9	9	9	9	9
9	9	9	9	9	9	9	9	9	9
9	9	9	9	9	9	9	9	9	9
9	9	9	9	9	9	9	9	9	9
2	2	2	2	2	2	2	2	2	2

9	9	9	9	9	9	9	9	9	9
9	9	9	9	9	9	9	9	9	9
9	9	9	9	9	9	9	9	9	9
9	9	9	9	9	9	9	9	9	9
9	9	9	9	9	9	9	9	9	9
9	9	9	9	9	9	9	9	9	9
9	9	9	9	9	9	9	9	9	9
9	9	9	9	9	9	9	9	9	9
9	9	9	9	9	9	9	9	9	9
9	9	9	9	9	9	9	9	9	9
9	9	9	9	9	9	9	9	9	9
9	9	9	9	9	9	9	9	9	9
9	9	9	9	9	9	9	9	9	9
9	9	9	9	9	9	9	9	9	9
9	9	9	9	9	9	9	9	9	9
9	9	9	9	9	9	9	9	9	9
9	9	9	9	9	9	9	9	9	9
9	9	9	9	9	9	9	9	9	9
9	9	9	9	9	9	9	9	9	9
9	9	9	9	9	9	9	9	9	9
9	9	9	9	9	9	9	9	9	9
9	9	9	9	9	9	9	9	9	9
9	9	9	9	9	9	9	9	9	9
2	2	2	2	2	2	2	2	2	2
9	9	9	9	9	9	9	9	9	9
2	2	2	2	2	2	2	2	2	2
9	9	9	9	9	9	9	9	9	9
9	9	9	9	9	9	9	9	9	9
9	9	9	9	9	9	9	9	9	9
9	9	9	9	9	9	9	9	9	9
9	9	9	9	9	9	9	9	9	9
9	9	9	9	9	9	9	9	9	9
9	9	9	9	9	9	9	9	9	9
2	9	3	2	9	9	2	9	3	9
9	9	9	9	9	9	9	9	9	9
9	9	9	9	9	9	9	9	9	9
1	1	1	1	1	1	1	1	1	1
2	2	1	1	2	2	3	2	9	1
9	9	9	9	9	9	9	9	9	9
2	2	2	2	2	2	2	1	1	1
9	9	9	9	9	9	9	9	9	9
9	9	9	9	9	9	9	9	9	9
9	9	9	9	9	9	9	9	9	9
9	9	9	9	9	9	9	9	9	9
1	1	1	1	1	1	1	1	1	1
9	9	9	9	9	9	9	9	9	9
9	9	9	9	9	9	9	9	9	9

9	9	9	9	9	9	9	9	9	9
2	2	2	2	2	2	2	2	3	3
2	9	9	9	9	9	9	9	9	9
9	9	9	1	9	1	2	9	1	9
9	9	9	9	9	9	9	9	9	9
9	9	9	9	9	9	9	9	9	9
9	9	9	9	9	2	9	9	9	9
9	9	9	9	9	9	9	9	9	9
1	9	3	9	9	1	9	2	3	9
9	9	9	9	9	9	9	9	9	9
9	9	9	9	9	9	9	9	9	9
9	9	9	9	9	9	9	9	9	9
9	9	9	9	9	9	9	9	9	9
9	9	9	9	9	9	9	9	9	9
9	9	2	9	9	9	2	9	9	9
2	2	2	2	2	9	9	9	9	9
9	9	9	9	9	9	9	9	9	9
9	9	9	9	9	9	9	9	9	9
9	9	9	9	9	9	9	9	9	9
9	9	9	9	9	9	9	9	9	9
2	2	2	2	2	2	2	2	2	2
2	1	3	1	1	1	1	1	2	1
9	9	9	9	9	9	9	9	9	9
2	2	2	2	2	2	2	2	2	2
2	2	2	2	2	2	2	2	2	2
3	9	9	9	9	2	9	9	3	9
9	9	9	9	9	9	9	9	9	9
9	9	1	9	9	9	9	9	9	9
3	3	2	1	2	3	1	2	1	3
1	2	3	1	2	3	1	2	3	1
1	1	2	3	2	1	1	1	1	2
2	3	2	2	2	2	2	2	1	2
1	1	1	1	1	1	1	3	3	1
1	2	1	3	2	2	2	2	2	2
3	2	2	2	2	2	2	2	2	2
1	1	1	1	1	1	1	1	1	1
1	1	1	1	1	9	9	9	9	9
9	9	9	9	9	9	9	9	9	9
9	9	9	9	9	9	9	9	9	9
9	9	9	9	9	9	9	9	9	9
9	9	9	9	9	9	9	9	9	9
9	9	9	9	9	9	9	9	9	9
9	9	9	9	9	9	9	9	9	9
9	2	9	9	9	2	9	9	2	9
9	9	9	9	9	9	9	9	9	9
2	9	9	9	2	9	2	9	2	9
9	9	9	9	9	9	9	9	9	9

2	2	2	2	2	2	2	2	2	2
9	9	9	9	9	9	9	9	9	9
9	9	9	9	9	9	9	9	9	9
9	9	9	9	9	9	9	9	9	9
2	2	2	2	2	2	2	2	2	2
2	3	2	2	2	3	2	3	2	3
1	2	1	9	2	1	1	1	1	2
2	2	2	2	2	2	2	2	2	1
1	1	1	2	2	1	1	1	1	1
1	2	1	3	2	2	3	2	3	2
2	3	3	3	3	3	3	3	3	3
2	2	1	2	1	2	1	1	1	2
2	2	1	3	3	2	1	1	1	1
3	3	3	3	3	3	3	3	3	3
2	2	2	2	2	2	2	2	2	2
2	2	2	2	2	2	2	2	2	2
9	2	9	9	9	2	9	9	2	9
9	9	9	9	1	9	2	9	1	9
2	2	2	2	2	2	2	2	2	2
2	1	3	2	2	2	2	2	3	1
1	1	2	2	1	3	2	3	3	2
2	2	2	2	2	2	2	2	2	2
2	1	3	2	3	2	3	1	2	1
2	2	2	2	2	2	2	2	2	2
9	9	9	9	9	9	9	9	9	9
2	2	2	2	2	2	2	2	1	3
3	2	2	2	3	3	2	3	3	3
1	2	3	2	1	1	2	3	1	1
2	3	1	2	3	2	2	3	1	2
2	3	2	1	2	2	2	2	1	2
2	2	1	2	2	1	1	1	1	1
2	2	2	2	2	2	2	2	2	1
1	2	2	3	1	1	1	2	1	1
2	2	3	2	2	2	2	2	2	2
2	2	2	2	2	1	1	2	1	3
1	3	2	3	1	1	3	2	1	3
3	3	3	3	3	2	2	3	3	2
2	2	3	2	2	2	2	2	2	2
3	3	3	3	3	3	3	3	3	3
2	2	2	2	2	2	2	2	2	2
9	1	9	9	9	9	9	9	9	9
2	3	2	2	3	3	3	3	2	2
2	3	2	1	2	1	1	2	2	2
2	2	2	2	2	2	2	2	2	2
2	2	2	2	1	1	2	1	1	2
9	9	9	9	9	9	9	9	9	9
2	2	2	2	2	2	2	2	2	2

9	9	9	9	9	9	9	9	9	9
3	3	3	3	2	1	3	3	2	2
2	2	2	2	2	2	2	2	2	2
3	2	3	1	2	1	1	1	2	3
2	3	2	3	2	3	2	3	2	3
3	3	1	2	3	2	3	2	2	2
9	9	9	9	9	9	9	9	9	9
3	3	3	3	3	3	3	3	3	3
3	3	3	1	1	1	1	1	3	3
2	2	2	3	2	1	1	9	1	2
2	1	2	3	3	3	2	1	1	2
1	3	3	3	2	1	1	1	1	1
2	2	2	2	2	2	2	2	2	2
2	2	2	2	2	2	2	3	3	9
3	3	3	3	2	2	2	1	1	1
2	2	2	2	2	2	2	2	2	1
2	2	2	2	2	3	3	3	2	2
2	2	9	2	9	2	2	2	2	2
9	9	9	9	9	9	9	9	9	9
2	2	2	2	2	2	3	3	2	3
1	2	2	2	2	1	1	1	2	2
2	2	2	2	2	2	2	2	2	2
2	2	2	2	2	2	2	3	3	3
2	2	2	2	2	2	2	2	2	2
2	2	2	2	2	2	2	2	2	2
9	9	9	9	9	9	9	9	9	9
2	9	1	2	2	2	2	2	1	2
2	1	2	1	2	2	1	1	1	1
3	3	3	3	2	3	2	3	2	2
2	3	1	2	3	2	3	2	2	2
3	3	3	3	3	3	3	3	3	3
2	1	2	3	3	2	2	1	1	2
9	9	9	9	9	9	9	9	9	9
2	2	2	2	2	2	2	2	2	2
2	2	2	2	2	2	2	2	2	2
2	2	2	2	2	2	2	2	2	2
2	1	3	2	3	2	3	1	1	2
9	9	9	9	9	9	9	9	9	9
9	9	9	9	9	9	9	9	9	9
2	2	2	2	2	2	1	1	1	1
2	3	1	3	3	1	2	2	1	2
9	9	9	9	9	9	9	9	9	9
2	2	2	2	2	3	3	3	3	3
2	2	2	2	2	2	2	1	1	1
3	3	3	3	3	1	2	1	2	1
2	1	1	2	2	2	2	1	1	2
9	9	9	9	9	9	9	9	9	9

2	2	2	2	2	2	2	2	2	2
2	2	2	2	2	2	2	2	2	2
2	2	2	2	2	2	2	2	2	2
2	2	2	2	2	2	2	2	2	2
2	2	2	2	2	2	2	1	1	2
2	2	2	2	2	2	2	2	2	2
3	3	3	2	1	3	2	2	1	1
2	2	2	2	2	2	2	2	2	2
2	2	2	2	2	2	2	2	2	2
2	2	2	2	2	2	2	2	2	2
2	2	2	2	2	2	2	1	2	2
2	2	2	2	2	2	2	2	2	2
2	2	2	2	2	2	2	2	2	2
2	2	2	2	2	2	2	2	2	2
2	2	2	2	2	2	2	2	2	2
9	9	9	9	9	9	9	9	9	9
3	3	3	3	3	3	3	2	1	1
3	2	3	1	2	2	1	3	1	1
2	2	2	2	2	1	2	2	2	2
1	2	2	1	2	1	2	2	2	1
3	3	3	3	3	3	3	3	2	2
1	1	2	1	2	1	9	1	2	1
9	9	9	9	9	9	9	9	9	9
9	9	9	2	9	9	9	9	2	9
9	9	9	9	9	9	9	9	9	9
2	2	2	2	2	2	2	2	2	2
3	2	2	2	2	2	2	2	3	9
9	9	9	9	9	9	9	9	9	9
9	9	9	9	9	9	9	9	9	9
2	2	2	2	2	2	2	2	2	2
1	1	1	2	2	1	1	2	2	2
2	2	2	2	2	2	2	2	2	2
2	1	2	2	1	3	3	1	1	2
2	3	3	3	2	3	3	3	3	2
2	1	1	2	1	1	2	1	3	1
2	2	2	2	3	2	1	2	3	1
9	9	9	9	9	9	9	9	9	9
3	1	1	1	3	1	2	1	3	1
1	2	3	2	1	3	2	1	3	1
1	2	2	2	2	2	2	3	3	3
2	2	2	2	2	2	2	2	2	2
2	2	2	2	2	2	2	2	2	2
1	1	2	9	9	9	9	9	9	9
2	2	2	2	2	2	2	2	2	2
2	2	2	2	2	2	2	2	2	2
2	2	2	2	3	3	3	3	3	3
1	1	3	1	1	3	1	1	1	1

2	3	2	2	2	3	1	1	1	1
2	2	2	2	2	2	2	2	2	2
2	2	2	2	2	2	2	2	2	2
3	2	3	1	2	2	2	3	2	3
2	3	1	3	1	3	1	1	2	1
2	3	3	2	3	2	2	2	3	3
3	3	2	3	1	1	2	2	2	1
2	2	2	2	2	2	2	2	2	2
2	3	9	1	9	1	3	2	9	2
2	2	2	2	2	2	2	2	2	2
3	2	3	2	3	1	2	3	3	3
2	2	2	2	2	2	2	2	2	2
9	9	9	9	9	9	1	9	9	9
3	3	3	3	3	3	3	3	3	3
9	9	9	9	9	9	9	9	9	9
2	2	2	3	3	3	3	2	2	2
2	2	2	2	2	2	2	2	2	2
1	3	3	2	9	1	1	2	1	1
1	1	2	2	1	1	2	3	3	2
9	9	9	9	9	9	9	9	9	9
9	9	9	9	1	9	1	9	9	9
9	9	9	9	9	9	9	9	9	9
2	3	1	2	2	1	2	2	1	1
1	1	1	3	1	1	1	1	1	1
3	3	1	3	9	2	2	2	1	2
1	2	2	3	2	1	1	2	1	1
2	9	2	2	2	2	2	2	2	2
1	3	1	3	1	3	1	3	1	3
1	3	2	2	1	9	2	2	1	2
2	2	2	2	2	2	2	2	2	2
1	1	1	3	1	1	1	1	1	1
2	2	2	2	2	2	2	2	2	2
2	2	2	2	2	2	2	2	2	2
2	2	2	2	2	2	2	2	2	2
2	3	2	3	2	2	1	2	3	2
3	3	3	3	3	3	3	3	3	3
2	2	2	1	2	1	2	2	1	2
1	3	2	1	2	3	2	2	3	1
2	2	2	2	2	2	2	2	2	3
2	2	2	2	2	2	2	2	2	2
2	1	1	1	2	2	2	2	2	1
2	3	2	2	2	3	2	2	2	3
1	1	1	1	1	1	1	1	1	1
2	2	2	2	2	2	2	2	2	2
2	3	2	3	1	3	1	3	2	3
2	2	2	2	2	2	2	2	2	2
9	9	9	9	9	1	3	1	3	1

2	1	1	2	1	2	2	2	2	2
2	2	2	2	2	2	2	2	2	2
2	3	1	3	2	1	1	1	1	2
2	2	2	2	2	2	2	2	2	2
2	3	2	3	1	2	2	2	3	2
2	2	2	2	2	2	2	2	2	2
1	1	1	2	3	2	1	3	1	2
1	3	3	3	1	1	1	2	1	2
3	2	3	2	3	2	3	2	3	2
2	2	2	2	2	2	2	2	2	2
1	2	3	1	3	3	1	9	2	1
9	9	9	9	9	9	9	9	9	9
2	2	2	2	3	2	2	2	3	2
9	9	9	9	9	9	9	9	9	9
9	9	9	9	9	9	9	9	9	9
1	1	1	3	3	3	3	3	3	3
9	9	9	9	9	9	9	9	9	9
9	9	2	9	9	9	9	9	9	9
9	9	9	9	9	9	9	9	9	9
9	9	9	9	9	9	9	9	9	9
2	2	1	2	3	2	2	2	2	1
9	9	9	9	9	9	9	9	9	9
9	9	9	9	9	9	9	9	9	9
9	9	9	9	9	9	9	9	9	9
9	9	9	9	9	9	9	9	9	9
9	2	9	9	9	9	9	9	9	9
9	9	9	9	9	9	9	9	9	9
2	2	2	2	2	2	2	2	2	2
2	2	2	2	2	2	2	2	2	2
3	3	3	3	2	2	2	3	1	3
3	2	2	2	1	2	1	1	3	3
2	2	3	2	2	2	3	2	2	1
1	1	3	1	1	1	3	3	3	1
9	9	9	9	9	9	9	9	9	9
2	2	2	2	2	2	2	2	2	3
1	1	3	9	1	1	1	1	3	3
3	3	1	3	1	3	3	2	1	3
9	9	9	9	9	9	9	9	9	9
1	2	2	2	2	2	2	2	2	2
9	9	9	9	9	9	9	9	9	9
9	9	9	9	9	9	9	9	9	9
1	2	1	2	2	1	1	2	3	2
1	1	1	1	1	1	1	1	1	1
9	9	9	9	9	9	9	9	9	9
9	9	9	9	9	9	9	9	9	9
2	2	2	2	2	2	2	2	2	2
9	2	9	9	2	9	2	9	2	2

2	2	2	2	2	2	2	2	2	2
3	3	3	3	3	3	3	3	3	3
9	9	9	2	9	9	9	9	9	2
1	1	1	1	1	1	1	1	1	2
2	2	2	2	2	2	2	2	2	2
1	9	3	1	9	3	1	9	3	1
9	9	9	9	9	9	9	9	9	9
9	3	1	9	9	9	3	1	9	3
9	9	3	1	9	9	9	1	9	9
2	2	2	2	2	2	2	2	2	2
3	9	2	1	2	1	9	3	2	1
9	3	1	3	1	3	2	1	9	9
9	9	9	9	9	9	9	9	9	9
9	9	9	9	9	9	9	9	9	9
2	9	2	9	9	2	9	2	9	2
2	2	2	2	2	2	2	2	2	2
9	9	9	9	9	9	9	9	9	9
9	2	9	9	2	9	9	2	9	9
2	2	2	2	2	2	2	2	2	2
9	9	9	9	9	9	9	9	9	9
2	2	2	2	2	2	2	2	2	2
9	9	9	9	9	9	9	9	9	9
9	9	9	9	9	9	9	9	9	9
9	9	9	9	9	9	9	9	9	9
9	9	9	9	9	9	9	9	9	9
9	9	9	9	9	9	9	9	9	9
2	2	2	2	2	2	2	2	2	2
2	2	2	2	2	2	2	2	2	2
1	3	2	2	3	2	1	2	1	3
1	3	1	1	1	2	3	1	1	2
2	2	2	2	2	2	3	3	3	3
2	2	2	2	2	2	2	2	2	2
1	1	1	1	1	1	1	1	1	1
9	2	3	2	2	3	3	1	1	1
1	3	3	1	1	1	1	2	3	1
2	2	2	2	2	2	2	2	2	2
2	3	2	2	2	2	2	3	3	3
2	1	2	3	3	2	2	3	3	2
2	2	2	2	2	2	2	2	2	2
9	9	9	9	9	9	9	9	9	9
2	2	1	2	1	1	2	1	1	2
3	3	3	3	3	3	3	3	3	3
2	2	2	2	2	2	2	2	2	2
1	3	1	2	1	2	1	2	2	2
2	2	2	2	2	2	2	2	2	2
2	2	3	3	2	3	3	2	2	2
1	1	9	3	2	1	1	1	2	2

9	9	9	9	9	9	9	9	9	9
2	2	2	2	2	2	2	2	2	2
9	9	9	9	9	9	9	9	9	9
1	2	1	2	1	2	2	1	2	1
2	2	2	2	2	2	2	2	2	2
3	3	2	3	2	2	2	2	2	2
2	3	2	2	2	1	3	2	1	1
3	3	3	2	9	1	1	2	1	1
2	2	2	2	2	2	2	2	2	2
9	9	2	2	2	2	2	2	2	2
1	1	1	1	2	2	2	2	2	2
1	1	1	1	9	2	2	2	2	2
1	1	1	1	1	1	1	1	1	1
3	3	3	1	1	2	1	2	1	2
3	3	3	3	3	3	3	3	3	3
1	3	3	3	1	1	1	2	1	1
2	2	2	2	2	2	2	2	2	2
2	2	2	2	2	2	2	2	2	1
2	2	2	2	2	2	2	2	1	1
2	2	2	1	1	1	1	1	1	1
9	9	9	9	9	9	9	9	9	9
1	9	1	9	9	1	1	1	2	2
1	1	1	1	1	1	2	2	2	2
2	2	2	2	1	1	1	1	2	1
9	9	9	9	9	9	9	9	9	9
2	2	2	2	2	2	2	2	2	2
1	1	2	2	1	1	2	1	2	1
1	1	2	2	2	2	2	2	2	2
9	9	9	9	9	9	9	9	9	9
2	2	2	2	2	2	2	2	2	2
9	9	9	9	9	9	9	9	9	9
1	1	1	1	1	1	2	2	1	1
1	1	1	1	2	2	1	2	1	1
1	1	1	9	1	1	1	2	2	2
2	9	2	2	9	2	2	2	9	2
2	2	2	2	2	2	2	9	2	2
2	2	2	2	2	2	2	9	2	2
2	2	2	2	2	2	2	2	2	2
2	2	2	2	2	2	2	2	2	2
1	1	1	1	1	1	1	1	1	1
2	2	2	2	2	2	2	2	2	2
9	9	9	9	9	9	9	9	9	9
2	2	1	1	1	1	2	2	2	2
1	1	1	1	1	2	2	1	1	1
9	9	9	9	9	9	9	9	9	9
2	2	2	2	2	2	2	2	2	2
2	2	2	2	2	2	9	2	9	9

9	9	9	9	9	9	9	9	9	9
2	2	2	2	2	2	2	2	2	2
2	2	2	2	2	2	2	2	2	2
1	1	1	1	1	1	2	1	1	2
2	2	2	2	2	2	9	2	2	9
9	9	1	9	9	9	9	9	9	9
1	2	1	2	1	2	2	1	1	1
2	9	2	9	9	2	2	9	9	2
1	1	1	1	1	1	1	1	1	1
1	1	1	1	1	2	1	2	1	1
2	2	2	2	2	2	2	2	2	2
2	2	2	2	2	2	2	2	2	2
1	1	1	1	1	1	1	1	1	1
2	2	2	2	2	2	2	2	2	2
9	9	9	9	9	9	9	9	9	9
1	1	9	1	9	1	9	9	2	2
9	2	9	2	9	2	9	2	2	9
2	2	9	2	9	2	2	2	2	9
9	9	9	9	9	9	9	9	9	9
9	9	9	9	9	9	9	9	9	9
9	9	9	9	9	9	9	9	9	9
9	9	9	9	9	9	9	9	9	9
2	2	9	2	2	9	9	9	9	9
9	9	9	9	9	9	9	9	9	9
2	2	2	2	2	2	2	2	2	2
1	3	3	3	1	1	1	1	3	1
1	3	3	3	1	1	1	1	3	1
1	3	3	3	1	1	1	1	3	1
1	9	3	3	1	1	1	1	1	1
1	3	3	1	3	1	1	1	3	1
1	3	3	3	1	1	1	1	3	1
1	3	3	3	1	1	1	1	1	1
9	3	3	3	1	1	1	1	3	1
9	3	3	1	3	1	1	1	3	1
1	3	9	3	1	1	1	1	3	1
1	3	3	3	1	1	1	1	3	1
1	3	3	3	1	1	1	1	3	1
1	3	3	3	1	1	1	3	1	1
1	3	3	3	9	1	1	1	3	1
1	3	3	3	1	1	1	1	3	1
1	3	3	3	1	1	1	1	3	1
1	3	3	3	3	1	1	3	3	1
1	3	3	3	3	1	1	1	3	1
1	3	3	3	1	1	1	1	3	1
1	9	3	3	1	1	9	1	3	1
9	3	3	3	1	1	1	1	3	1
1	3	3	3	1	1	1	1	3	1

1	3	3	3	1	1	1	1	3	1
1	3	3	3	1	1	1	1	3	1
1	3	3	3	1	1	1	3	1	1
9	9	9	9	9	9	9	9	9	9
9	9	9	9	9	9	9	9	9	9
9	9	9	9	9	9	9	9	9	9
9	9	9	9	9	9	9	9	9	9
9	9	9	9	9	9	9	9	9	9
9	9	9	9	9	9	9	9	9	9
9	9	9	9	9	9	9	9	9	9
9	9	9	9	9	9	9	9	9	9
9	9	9	9	9	9	9	9	9	9
9	9	9	9	9	9	9	9	9	9
9	9	9	9	9	9	9	9	9	9
9	9	9	9	9	9	9	9	9	9
9	9	9	9	9	9	9	9	9	9
9	9	9	9	9	9	9	9	9	9
9	9	9	9	9	9	9	9	9	9
9	9	9	9	9	9	9	9	9	9
9	9	9	9	9	9	9	9	9	9
9	9	9	9	9	9	9	9	9	9
9	9	9	9	9	9	9	9	9	9
9	9	9	9	9	9	9	9	9	9
9	9	9	9	9	9	9	9	9	9
9	9	9	9	9	9	9	9	9	9
3	3	3	3	1	1	1	1	3	1
1	3	3	3	1	1	1	1	3	1
1	3	3	3	1	1	1	1	3	1
1	3	3	3	1	1	1	1	3	1
1	3	3	3	1	1	3	1	3	1
9	3	3	3	3	1	3	1	3	1
1	2	2	3	1	1	1	1	3	1
1	3	3	3	1	1	1	1	3	1
1	3	3	3	1	1	1	1	1	3
1	3	3	3	1	1	1	1	3	1
1	3	3	1	1	1	1	1	3	1
1	3	3	3	1	1	1	3	1	1
1	3	3	3	1	1	1	1	1	1
1	3	3	3	1	1	1	1	3	1
1	3	3	3	1	1	1	3	3	1
1	3	3	9	1	1	1	1	3	1
9	9	9	9	9	9	9	9	9	9
1	3	3	3	1	1	1	1	3	1
1	3	3	3	1	1	1	1	1	1
1	3	3	3	1	1	1	1	1	1
1	3	3	3	1	1	1	9	3	1
1	3	3	1	3	1	1	1	3	1

1	3	3	3	1	1	1	1	3	1
1	3	3	9	1	1	1	1	3	1
9	3	3	3	1	1	1	1	3	1
1	1	9	3	1	1	1	3	1	1
3	3	9	9	1	1	1	1	1	1
1	3	3	3	1	1	1	1	1	1
1	3	3	1	3	1	1	3	1	1
3	3	3	3	1	1	1	1	1	1
1	3	3	3	1	1	1	1	3	1
1	3	3	3	1	1	1	1	3	1
1	3	3	3	1	1	1	1	3	1
1	3	3	3	1	1	1	1	3	1
1	1	3	3	3	1	1	1	1	1
1	3	3	3	1	1	1	3	3	1
1	3	3	3	1	1	1	1	3	1
1	3	3	3	1	1	1	1	3	1
1	3	3	3	1	1	1	3	3	1
1	3	9	3	1	1	1	1	3	1
3	3	3	3	1	1	1	1	3	1
1	3	3	3	1	1	1	1	3	1
1	3	3	9	1	1	1	1	3	1
1	3	3	3	1	1	1	1	3	1
1	3	3	3	1	1	1	1	3	1
1	3	3	3	1	1	1	1	3	1
1	3	3	3	1	1	1	1	3	2
1	3	3	3	1	1	1	1	3	1
1	3	3	3	1	1	1	1	3	1
3	3	3	1	1	1	1	1	3	1
3	3	3	3	3	1	1	3	1	1
1	1	3	3	1	1	1	1	3	1
1	3	3	3	1	1	1	1	3	1
3	3	3	9	1	1	1	1	3	1
1	3	3	3	1	1	1	1	3	1
1	1	3	3	3	1	1	1	9	1
3	3	3	3	1	1	3	1	3	1
1	3	3	3	1	1	1	1	3	1
1	3	3	3	1	1	1	1	3	1
1	3	3	3	3	1	1	1	1	3
9	9	9	9	9	9	9	9	9	9
1	3	3	9	1	1	1	1	3	1
9	9	9	9	9	9	9	9	9	9
1	3	3	3	1	1	3	1	3	1
1	1	3	3	1	1	1	1	3	9
1	3	3	3	1	1	1	1	3	1
1	1	3	3	1	1	1	1	3	1
1	3	3	3	1	1	1	3	1	3
3	3	3	1	1	1	3	3	1	1

3	3	3	3	1	1	1	1	1	1
1	3	3	3	1	1	1	1	3	1
1	3	3	3	1	1	3	1	3	1
1	3	3	3	1	1	1	1	3	1
1	3	3	3	1	1	1	1	3	1
1	3	3	3	1	1	1	1	3	1
1	3	3	3	1	1	1	1	3	1
1	3	3	3	1	1	1	1	3	1
1	3	3	3	1	1	1	1	3	1
1	3	3	3	1	1	1	1	3	1
1	3	3	3	1	1	1	3	1	1
3	1	3	3	1	1	1	1	3	1
1	3	3	3	1	1	1	1	3	1
1	3	3	3	1	1	3	3	3	1
1	1	3	3	1	1	1	1	3	1
1	3	3	3	1	1	1	1	3	1
1	3	3	3	1	1	1	1	3	1
1	3	3	3	1	1	1	1	3	1
3	3	3	3	1	1	1	1	3	1
1	3	3	3	1	1	1	1	3	1
3	3	3	3	9	9	9	9	9	9
1	3	3	3	1	1	1	1	3	1
1	3	3	3	1	1	1	1	1	1
3	3	3	3	1	1	1	1	1	1
3	3	3	3	1	1	1	1	1	1
9	9	9	9	9	9	9	9	9	9
3	3	3	3	1	1	1	1	3	1
1	3	3	3	1	1	1	3	1	1
1	3	3	3	1	1	1	1	3	1
3	3	9	3	1	1	1	1	3	1
2	1	2	2	1	9	9	9	9	1
1	3	3	9	3	1	1	1	3	1
1	3	3	3	1	1	1	1	3	1
1	1	3	3	1	1	1	1	3	1
1	3	3	3	1	1	1	1	3	1
1	3	1	3	1	1	1	1	1	1
1	3	3	3	1	1	1	1	3	1
1	3	3	3	1	1	1	1	3	1
3	3	3	1	1	1	1	3	1	1
1	3	3	3	1	1	1	1	3	1
1	3	3	3	1	1	1	1	3	1
1	3	3	3	1	1	1	9	3	1
1	3	3	3	1	1	1	1	3	1
1	3	3	3	1	1	1	1	3	1
1	3	3	3	1	1	1	1	3	1
1	3	3	3	1	1	1	1	3	1
1	3	3	3	1	1	1	1	3	1

9	9	9	9	9	9	9	9	9	9
1	3	3	3	1	1	1	1	3	1
1	3	3	3	1	1	1	1	3	1
1	3	3	1	1	1	1	1	3	1
1	3	3	3	1	1	1	1	3	1
1	3	3	3	1	1	1	1	3	1
1	3	3	3	1	1	1	1	3	1
1	3	3	3	1	1	1	1	3	1
9	3	3	3	1	1	1	1	3	1
1	3	3	1	1	1	3	1	3	1
1	3	3	3	1	1	1	1	3	1
1	3	3	3	1	1	3	1	3	1
1	3	3	3	1	1	3	3	3	1
1	3	3	3	1	1	1	1	3	1
1	3	3	3	1	1	3	3	3	1
3	3	1	1	1	1	1	3	1	1
1	3	3	3	1	1	1	1	3	1
1	3	3	3	1	1	1	1	3	1
1	3	3	3	1	1	1	1	3	1
1	3	3	3	1	1	1	1	3	1
1	3	1	3	9	1	1	1	3	1
1	3	3	3	1	1	1	1	3	1
1	3	3	3	1	1	1	1	3	1
1	3	3	3	1	1	1	1	3	1
1	3	3	3	1	1	1	1	3	1
1	3	3	3	1	1	1	1	1	1
1	3	3	3	1	1	1	1	3	1
1	1	3	3	3	1	1	1	3	1
1	3	3	3	1	1	1	1	3	1
1	3	3	3	1	1	1	1	3	1
1	3	3	3	1	1	1	1	1	1
1	1	3	3	1	1	1	1	3	1
1	1	3	3	1	1	1	1	1	1
9	9	9	9	9	9	9	9	9	9
9	9	9	9	9	9	9	9	9	9
9	9	9	9	9	9	9	9	9	9
9	9	9	9	9	9	9	9	9	9
9	9	9	9	9	9	9	9	9	9
9	9	9	9	9	9	9	9	9	9
9	9	9	9	9	9	9	9	9	9
9	9	9	9	9	9	9	9	9	9
9	9	9	9	9	9	9	9	9	9
9	9	9	9	9	9	9	9	9	9
9	9	9	9	9	9	9	9	9	9
9	9	9	9	9	9	9	9	9	9
9	9	9	9	9	9	9	9	9	9
9	9	9	9	9	9	9	9	9	9

9	9	9	9	9	9	9	9	9	9
9	9	9	9	9	9	9	9	9	9
9	9	9	9	9	9	9	9	9	9
9	9	9	9	9	9	9	9	9	9
9	9	9	9	9	9	9	9	9	9
9	9	9	9	9	9	9	9	9	9
9	9	9	9	9	9	9	9	9	9
9	9	9	9	9	9	9	9	9	9
9	9	9	9	9	9	9	9	9	9
9	9	9	9	9	9	9	9	9	9
9	9	9	9	9	9	9	9	9	9
9	9	9	9	9	9	9	9	9	9
9	9	9	9	9	9	9	9	9	9
9	9	9	9	9	9	9	9	9	9
9	9	9	9	9	9	9	9	9	9
9	9	9	9	9	9	9	9	9	9
9	9	9	9	9	9	9	9	9	9
9	9	9	9	9	9	9	9	9	9
9	9	9	9	9	9	9	9	9	9
2	2	1	1	2	1	1	2	3	1
2	2	2	2	2	2	2	2	2	2
1	2	2	2	2	2	2	2	3	2
2	3	1	2	3	2	1	3	1	1
2	3	1	2	1	3	2	1	1	1
3	3	3	3	3	1	1	1	1	1
2	3	1	2	2	1	3	2	1	1
1	1	3	3	1	1	1	1	1	1
1	1	1	1	3	2	1	2	3	1
2	3	9	9	9	9	9	9	9	9
2	3	3	2	2	2	3	3	3	2
9	9	1	9	9	9	9	9	9	9
2	3	3	3	3	3	3	2	2	2
9	9	9	9	9	9	9	9	9	9
1	1	1	3	3	3	3	3	3	1
1	3	2	3	1	2	1	1	1	1
1	3	3	3	1	1	1	2	2	1
3	3	3	3	3	3	3	3	3	3
1	2	3	1	2	3	1	2	3	2
9	9	9	9	9	9	9	9	9	9
1	2	3	1	2	1	2	3	2	3
1	3	1	3	1	1	1	1	1	1
9	9	9	9	9	9	9	9	9	9
1	2	2	2	2	2	2	2	2	2
2	3	2	1	1	1	3	3	2	2
3	3	1	3	3	3	2	1	1	1
3	1	1	1	1	1	1	3	2	1
9	9	9	9	9	9	9	9	9	9

9	9	9	9	9	9	9	9	9	9
2	2	2	1	2	2	2	2	2	2
1	1	9	1	9	1	1	1	9	1
9	9	9	9	9	9	9	9	9	9
9	9	9	9	9	9	9	9	9	9
2	2	2	3	3	2	2	2	2	2
9	9	1	9	9	9	9	9	9	9
9	9	9	9	9	9	9	9	9	9
2	3	2	2	3	2	3	2	3	2
1	2	2	2	2	2	2	1	2	1
2	1	1	2	2	1	1	1	1	1
9	9	9	9	9	9	9	9	9	9
2	2	2	2	2	2	2	2	2	2
2	2	2	2	2	2	2	2	2	2
2	2	2	2	2	2	2	2	2	2
2	3	3	3	1	1	1	1	2	2
2	9	2	9	2	1	2	2	1	3
9	9	9	9	9	9	9	9	9	9
9	9	9	9	9	9	9	9	9	9
2	2	2	2	2	2	2	2	2	2
2	2	2	2	2	2	2	2	2	2
3	2	1	2	3	1	1	2	1	3
1	1	1	1	3	2	1	2	3	1
2	2	2	2	2	2	2	2	2	2
1	1	1	3	2	2	1	1	1	3
3	9	3	9	9	9	9	9	9	9
2	2	3	2	2	2	2	2	3	2
2	2	2	2	2	2	2	2	2	2
2	2	2	2	2	2	2	2	2	2
2	2	2	2	2	2	2	2	2	2
2	2	2	2	2	2	2	2	2	2
1	2	2	1	1	3	3	2	2	1
2	2	2	2	2	2	2	2	2	2
2	2	2	2	2	2	2	2	2	2
2	2	1	2	2	2	2	2	2	2
1	2	1	2	2	1	1	1	1	1
3	3	3	3	3	1	1	2	2	2
2	3	2	3	2	1	2	3	1	1
1	1	1	2	1	1	1	2	1	1
1	2	1	3	3	1	1	1	1	2
2	2	2	2	1	1	2	1	2	1
2	3	2	3	1	3	2	3	2	3
1	1	1	2	2	2	2	2	2	2
2	2	2	2	2	2	2	2	2	2
9	9	9	9	9	9	9	9	9	9
2	2	1	3	3	1	1	1	2	1
1	1	1	2	2	2	2	2	2	2

1	2	1	2	2	2	2	2	2	1
1	2	3	1	2	3	1	2	3	1
1	3	2	3	2	2	2	2	1	2
1	3	1	2	1	1	1	1	1	1
1	1	1	2	2	2	3	3	3	2
2	3	3	3	2	1	2	2	1	1
2	2	2	2	2	2	2	2	2	1
3	3	2	2	2	3	2	2	3	2
1	2	2	2	2	2	2	2	2	2
2	3	2	3	2	3	2	2	3	2
3	1	1	1	1	2	3	2	2	2
1	1	1	1	1	1	1	2	2	2
3	3	1	3	3	1	3	3	1	3
2	2	3	2	2	1	2	2	2	1
2	2	2	2	2	2	2	2	2	2
3	1	2	1	2	3	1	2	3	2
2	2	2	2	2	2	2	2	2	2
2	2	2	2	2	2	2	2	2	2
1	1	1	1	1	1	1	1	1	1
2	2	3	2	2	2	2	2	2	2
2	2	2	2	2	2	2	2	2	2
3	2	3	2	2	3	2	2	3	2
3	3	3	3	3	3	3	3	3	3
2	2	2	2	2	2	2	2	2	2
2	2	2	2	2	2	2	2	2	2
1	2	3	1	2	3	1	2	3	1
2	1	2	1	2	1	1	2	1	1
1	1	2	2	2	2	2	2	1	1
2	2	2	2	2	2	2	2	2	2
1	3	3	3	3	3	3	1	1	1
3	1	3	3	1	1	1	1	1	1
9	9	9	9	9	9	9	9	9	9
2	3	2	3	1	2	1	2	1	2
2	2	2	2	2	2	2	2	2	2
1	3	1	2	3	2	1	1	1	1
2	2	1	2	1	1	2	1	2	1
2	3	2	3	2	1	2	2	1	1
3	1	2	1	2	2	1	2	2	3
2	2	2	3	3	3	3	3	3	3
1	1	1	1	1	2	2	1	2	1
1	1	2	2	9	9	1	1	1	3
2	2	2	2	2	2	9	9	9	9
9	9	9	9	9	9	9	9	9	9
2	2	2	2	2	2	1	1	2	2
9	9	9	9	9	9	9	9	9	9
1	2	1	2	2	2	2	2	1	2
2	2	2	3	3	9	2	2	2	2

2	2	1	1	2	1	2	1	1	2
2	3	2	1	2	2	1	1	1	2
9	9	9	9	9	9	9	9	9	9
9	9	9	9	9	9	9	9	9	9
9	9	9	9	9	9	9	9	9	9
2	2	2	1	1	1	1	1	3	3
2	1	3	3	9	9	9	9	9	9
9	9	9	9	9	9	9	9	9	9
3	2	2	3	3	2	3	2	1	2
1	1	1	1	1	3	3	3	3	3
9	9	9	9	9	9	9	9	9	9
2	3	1	3	3	3	2	2	3	3
3	3	2	2	2	3	2	3	3	3
3	2	2	3	3	3	2	2	2	2
1	2	1	3	2	1	1	1	1	1
1	2	3	1	2	3	1	2	3	1
2	1	2	3	1	2	1	3	2	1
2	2	2	2	2	2	2	2	2	2
2	2	2	2	2	2	2	2	2	2
9	9	9	9	9	9	9	9	9	9
2	2	2	2	2	2	2	2	2	2
1	2	2	2	2	1	9	1	1	1
1	1	1	1	1	1	3	1	1	3
2	3	3	2	3	3	1	1	2	2
3	1	3	1	2	2	2	1	2	3
9	9	9	9	9	9	9	9	9	9
2	3	3	3	3	1	1	2	2	1
2	2	3	3	1	3	2	1	2	3
1	3	1	2	3	3	3	3	3	2
3	3	2	1	3	1	2	3	2	2
3	2	3	2	9	3	2	3	3	2
9	9	9	9	9	9	9	9	9	9
1	1	1	1	2	1	3	2	3	2
2	2	2	2	2	2	2	2	2	2
2	2	2	2	3	3	3	3	3	3
2	2	1	2	2	3	1	2	1	2
2	2	2	2	2	2	2	2	2	2
9	9	9	9	9	9	9	9	9	9
2	3	3	1	3	1	1	3	3	2
2	2	3	3	2	2	2	2	3	2
2	3	1	9	3	1	2	2	2	3
2	3	1	2	1	2	1	3	1	2
9	9	9	9	9	9	9	9	9	9
1	3	1	3	2	1	1	1	1	2
2	3	1	3	3	1	1	1	2	1
9	9	9	9	9	9	9	9	9	9
2	2	1	1	2	3	2	1	2	3

2	2	2	2	2	2	2	2	2	2
2	2	2	2	2	2	2	2	2	2
2	2	2	2	2	2	2	2	2	2
2	2	2	2	2	2	2	2	2	2
9	9	9	9	9	9	9	9	9	9
2	2	2	2	2	2	2	2	2	2
3	3	2	3	3	1	1	1	1	1
2	2	2	2	2	2	2	2	2	2
2	2	2	2	2	2	2	2	2	2
2	2	1	2	3	3	3	2	2	2
1	3	3	3	3	3	3	1	1	3
2	2	2	2	3	3	2	3	3	3
1	1	1	3	1	1	1	1	1	1
2	3	1	3	3	1	2	1	2	2
1	1	1	3	1	1	1	1	1	1
3	3	1	3	3	1	1	2	3	3
2	2	3	3	2	2	2	2	2	2
3	3	3	3	3	3	3	3	2	2
2	2	2	2	2	2	2	2	2	2
1	2	1	3	2	2	1	1	1	2
9	9	9	9	9	9	9	9	9	9
1	2	2	2	2	1	2	1	1	1
2	2	3	1	3	3	2	2	1	2
2	2	2	2	2	2	2	1	2	2
3	2	2	2	2	2	3	2	2	3
2	2	2	2	2	2	1	1	1	1
1	3	1	3	3	1	1	1	1	1
1	3	3	3	1	9	9	9	1	3
3	3	3	3	3	1	2	2	3	3
2	2	2	2	2	2	2	2	2	2
2	2	2	2	2	2	9	3	2	9
1	1	2	2	3	2	2	1	1	1
2	2	1	3	2	2	3	1	1	2
2	2	2	3	3	2	2	2	2	2
1	3	3	3	1	1	3	3	1	1
1	3	3	3	1	1	2	2	1	1
1	2	3	3	1	1	1	1	1	1
1	1	2	2	2	2	2	1	1	1
1	1	2	2	2	2	2	1	1	1
9	9	9	9	9	9	9	9	9	9
9	9	9	9	9	9	9	9	9	9
1	3	3	3	1	1	3	1	1	1
2	2	2	2	2	2	2	2	2	2
1	1	1	1	1	2	2	3	3	3
1	1	1	1	1	2	2	2	2	2
2	2	2	2	2	2	2	2	2	2
2	3	1	2	3	1	1	2	2	3

9	9	9	9	9	9	9	9	9	9
9	9	9	9	9	9	9	9	9	9
1	1	1	1	1	1	1	1	1	1
2	3	1	2	2	3	2	3	2	2
9	9	9	9	9	9	9	9	9	9
9	9	9	9	9	9	9	9	9	9
2	2	2	2	2	2	2	2	2	2
3	1	3	1	1	1	1	1	1	1
1	2	3	1	2	3	1	2	3	1
2	1	2	3	1	2	3	1	3	2
2	3	3	3	1	1	2	2	2	2
1	2	3	1	2	3	1	2	3	1
2	2	3	2	1	2	3	2	1	2
9	9	9	9	9	9	9	9	9	9
1	1	2	3	2	2	1	2	2	2
9	9	9	9	9	9	9	9	9	9
1	1	1	1	1	1	1	1	1	1
1	1	1	3	1	1	1	1	1	1
9	9	9	9	9	9	9	9	9	9
9	9	9	9	9	9	9	9	9	9
2	2	1	2	2	2	2	2	2	2
9	9	9	9	9	9	9	9	9	9
9	9	9	9	9	9	9	9	9	9
2	2	2	2	2	2	2	2	2	2
2	2	2	2	2	2	2	2	2	2
2	2	2	2	2	2	9	1	1	1
2	2	2	2	2	2	2	2	2	2
2	2	2	2	2	2	2	2	2	2
3	3	3	2	3	3	2	2	3	3
2	3	2	1	2	1	2	3	2	1
2	9	2	2	2	9	9	3	3	9
9	1	9	9	9	9	9	9	9	9
1	2	2	2	1	1	1	1	1	1
1	1	1	1	1	1	1	1	1	1
2	1	1	1	1	1	1	1	1	1
1	1	1	3	1	1	1	1	1	1
1	2	2	2	1	1	2	1	2	1
2	2	2	2	2	2	2	2	2	2
2	2	2	2	2	2	2	2	2	2
2	2	2	2	2	2	2	2	2	2
1	1	1	1	1	1	1	1	1	1
2	2	2	2	2	2	2	2	2	2
2	2	2	2	2	2	2	2	2	2
9	9	9	9	9	9	9	9	9	9
9	9	9	9	9	9	9	9	9	9
2	2	2	2	2	2	2	2	2	2
2	2	2	2	2	2	2	2	2	2

9	9	9	9	9	9	9	9	9	9
2	2	2	2	2	2	2	2	2	2
9	9	9	9	9	9	9	9	9	9
3	3	3	3	3	3	3	3	3	3
9	9	9	9	9	9	9	9	9	9
2	2	2	2	2	2	2	2	2	2
1	3	1	2	1	2	1	3	1	2
9	9	9	9	9	9	9	9	9	9
2	2	2	2	2	2	2	2	2	2
3	3	3	3	2	2	2	3	2	2
2	2	2	2	2	2	2	2	2	3
2	2	2	1	1	3	1	2	2	2
1	1	1	2	2	2	2	2	1	1
1	2	2	2	3	2	2	2	2	3
2	2	2	2	2	2	2	2	2	2
3	3	3	3	3	3	2	1	1	1
1	3	3	3	3	1	1	3	1	1
2	2	2	2	2	2	2	2	2	2
2	2	3	3	2	2	2	2	2	2
1	2	3	2	2	3	2	3	2	2
2	2	1	1	1	2	1	1	1	1
2	2	1	1	1	2	1	1	1	1
2	2	2	2	2	2	1	2	2	2
2	2	2	2	2	2	2	2	2	2
3	2	3	2	3	2	2	2	3	3
1	9	2	9	2	3	2	1	2	2
1	1	2	3	2	1	2	1	1	1
2	2	1	2	2	2	2	2	1	1
3	3	2	1	2	3	2	2	1	1
9	9	9	9	9	9	9	9	9	9
3	3	3	3	3	3	3	3	3	3
9	9	9	9	9	9	9	9	9	9
9	9	9	9	9	9	9	9	9	9
9	9	9	9	9	9	9	9	9	9
2	2	2	2	2	2	3	3	3	3
1	1	1	1	1	1	1	1	1	1
2	2	2	2	2	2	2	2	2	2
1	1	1	9	1	3	1	2	1	1
2	2	2	2	2	2	2	2	2	2
2	2	2	2	2	2	2	2	2	2
1	1	1	1	1	1	1	1	1	1
1	3	3	3	1	1	1	1	1	1
1	3	1	1	3	2	2	1	1	2
2	9	2	1	2	1	3	2	1	2
2	3	1	2	1	1	1	3	2	2
1	3	3	3	1	1	2	1	1	1
1	1	1	1	1	1	1	1	1	1

2	2	2	2	2	2	2	2	2	2
9	9	9	9	9	9	9	9	9	9
2	9	9	9	9	9	9	9	9	9
3	1	2	3	2	3	1	2	1	2
2	2	2	2	2	2	2	2	2	2
2	2	2	2	2	2	2	2	2	2
2	1	2	2	2	3	3	1	1	1
9	9	9	9	9	9	9	9	9	9
9	9	9	9	9	9	9	9	9	9
9	9	9	9	9	9	9	9	9	9
9	9	9	9	9	9	9	9	9	9
9	9	9	9	9	9	9	9	9	9
9	9	9	9	9	9	9	9	9	9
9	9	9	9	9	9	9	9	9	9
9	9	9	9	9	9	9	9	9	9
9	9	9	9	9	9	9	9	9	9
2	2	2	2	2	2	2	2	2	2
2	2	2	3	2	2	3	2	2	2
1	1	3	3	3	1	1	2	1	1
2	2	2	2	2	2	3	2	3	2
2	2	2	2	2	2	2	2	2	2
3	2	2	2	3	2	3	2	3	2
1	1	1	2	2	1	9	2	1	2
2	2	2	2	2	2	2	2	2	2
1	1	1	3	2	1	1	2	2	2
2	3	3	3	3	3	2	2	1	1
3	2	2	2	3	2	1	2	3	1
3	2	2	3	2	1	2	3	1	2
3	9	3	3	3	3	3	3	3	3
2	2	2	2	2	2	2	2	2	2
2	2	2	2	2	2	2	2	2	2
2	2	2	2	2	2	2	2	2	2
2	2	2	2	2	2	9	3	3	3
2	2	3	2	1	1	2	1	1	2
2	2	2	2	2	2	2	2	2	2
2	2	2	2	2	2	2	2	2	2
9	9	9	9	9	9	9	9	9	9
2	2	2	2	2	2	2	2	2	2
1	1	1	1	1	1	1	1	1	1
9	9	9	9	9	9	9	9	9	9
9	9	9	9	9	9	9	9	9	9
2	2	2	2	2	2	2	2	2	2
2	2	2	2	2	2	2	2	2	2
2	2	2	2	2	2	2	2	2	2
1	1	1	3	2	1	3	2	1	1
9	9	9	9	9	9	9	9	9	9
2	2	1	2	1	1	1	2	1	2

2	2	2	2	2	2	2	2	2	2
2	2	2	2	3	3	3	3	2	2
1	3	3	3	1	1	1	1	1	1
1	3	3	9	2	2	2	1	1	1
9	9	9	9	9	9	9	9	9	9
2	2	2	1	2	1	2	2	2	1
9	9	9	9	9	9	9	9	9	9
1	1	2	1	1	1	1	1	2	2
9	9	9	9	9	9	9	9	9	9
9	9	9	9	9	9	9	9	9	9
9	9	9	9	9	9	9	9	9	9
9	9	9	9	9	9	9	9	9	9
9	9	9	9	9	9	9	9	9	9
9	9	9	9	9	9	9	9	9	9
9	9	9	9	9	9	9	9	9	9
9	9	9	9	9	9	9	9	9	9
9	9	9	9	9	9	9	9	9	9
9	9	9	9	9	9	9	9	9	9
9	9	9	9	9	9	9	9	9	9
9	9	9	9	9	9	9	9	9	9
9	9	9	9	9	9	9	9	9	9
9	9	9	9	9	9	9	9	9	9
9	9	9	9	9	9	9	9	9	9
9	9	9	9	9	9	9	9	9	9
9	9	9	9	9	9	9	9	9	9
9	9	9	9	9	9	9	9	9	9
9	9	9	9	9	9	9	9	9	9
9	9	9	9	9	9	9	9	9	9
9	9	9	9	9	9	9	9	9	9
2	1	2	3	2	1	2	3	2	1
2	2	2	2	2	2	2	2	2	2
1	3	3	3	3	1	1	1	1	2
1	3	3	3	2	2	1	1	1	1
1	3	9	3	3	2	1	2	1	1
1	3	2	2	1	1	2	3	2	2
1	1	1	2	1	1	1	2	1	2
1	3	1	3	3	3	1	1	2	2
2	1	2	2	2	1	2	2	1	1
2	2	2	2	9	2	1	1	1	1
1	3	3	1	1	1	1	1	1	1
1	2	1	2	3	1	2	3	1	2
1	3	2	3	3	1	1	1	1	1
1	3	3	3	2	1	2	2	1	1
1	3	3	3	1	1	1	1	1	1
1	3	1	2	1	1	1	2	1	1
9	9	9	9	9	9	9	9	9	9
1	2	2	3	3	1	1	1	1	2

2	2	3	3	1	1	1	2	1	1
9	9	9	9	9	9	1	9	9	9
1	3	1	3	3	1	1	2	2	1
1	3	1	3	1	1	3	3	1	1
2	2	2	2	2	2	3	2	3	3
1	2	3	1	9	1	2	2	3	2
1	2	2	2	2	1	1	1	1	1
1	1	1	1	1	1	1	1	1	1
2	1	3	3	2	1	1	2	1	1
1	3	3	2	2	1	2	2	1	1
3	2	1	2	3	2	3	2	1	2
9	9	9	9	9	9	9	9	9	9
1	1	1	1	2	1	1	1	1	1
9	9	9	9	9	9	9	9	9	9
1	1	1	2	3	1	1	1	1	1
1	1	1	2	9	1	1	1	1	1
9	9	9	9	9	9	9	9	9	9
9	1	9	9	9	9	9	9	9	9
9	9	9	9	9	9	9	9	9	9
9	9	9	9	9	9	9	9	9	9
9	9	9	9	9	9	9	9	9	9
9	9	9	9	9	9	9	9	9	9
1	3	3	3	3	3	3	1	1	1
2	2	2	2	2	2	2	2	2	2
9	9	9	9	9	9	9	9	9	9
3	3	1	3	1	1	1	3	3	3
9	9	9	9	9	9	9	9	9	9
9	9	9	9	9	9	9	9	9	9
9	9	9	9	9	9	9	9	9	9
9	9	9	9	9	9	9	9	9	9
9	9	9	9	9	9	9	9	9	9
1	2	3	3	2	1	2	2	3	3
1	3	3	3	1	3	1	3	1	1
2	3	2	2	2	1	9	2	2	3
1	2	1	2	1	1	1	2	1	1
2	2	2	2	2	2	2	2	2	2
1	1	1	1	2	1	2	1	1	1
1	3	3	3	1	1	1	3	1	1
1	3	3	3	2	1	3	3	3	3
3	1	1	3	1	2	3	1	1	1
2	2	2	3	2	2	2	3	2	9
9	9	9	9	9	9	9	9	9	9
1	1	2	1	2	2	2	2	2	1
2	2	2	2	2	2	2	2	2	2
1	2	1	2	3	1	2	3	3	1
2	2	2	2	2	2	2	2	2	2
1	3	3	3	3	3	3	1	1	1

1	1	3	1	2	3	3	1	1	1
1	1	1	3	3	1	1	1	1	1
1	1	1	3	3	3	2	2	1	1
1	3	3	3	1	1	1	1	1	1
9	9	9	9	9	9	1	9	1	1
1	3	2	3	1	1	1	1	3	1
1	3	3	3	1	1	1	2	3	1
2	3	2	3	9	3	3	3	3	3
1	3	3	3	1	1	1	2	2	1
3	3	3	3	1	9	3	3	3	1
9	9	9	9	1	9	9	2	9	9
2	3	1	3	2	2	2	2	1	1
1	3	1	3	1	1	1	1	1	1
9	9	9	9	9	9	9	9	9	9
9	9	9	9	9	9	9	9	9	9
3	2	2	2	2	2	2	2	2	2
2	2	2	2	2	2	2	2	2	2
3	3	3	3	3	1	2	3	3	3
1	2	2	2	1	1	1	1	1	1
9	9	9	9	9	9	9	9	9	9
9	9	9	9	9	9	9	9	9	9
3	3	3	9	9	1	1	1	1	1
9	9	9	9	9	9	9	9	9	9
9	9	9	9	9	9	9	9	9	9
1	1	1	1	1	1	1	1	1	1
1	3	3	3	3	3	3	1	1	1
1	3	1	2	1	1	1	2	1	1
1	1	1	1	1	1	1	1	1	1
2	2	2	3	2	2	2	2	1	3
1	3	2	3	1	2	1	2	3	1
1	1	1	1	1	1	1	1	1	1
9	9	9	9	9	9	9	9	9	9
9	9	9	9	9	9	9	9	9	9
1	3	3	1	1	1	1	1	1	1
2	2	2	2	2	2	1	1	1	1
1	3	2	3	1	1	3	3	1	1
9	9	9	9	9	9	9	9	9	9
9	9	9	9	9	9	9	9	9	9
9	9	9	9	9	9	9	9	9	9
9	9	9	9	9	9	9	9	9	9
9	9	9	9	9	9	9	9	9	9
9	9	9	9	9	9	9	9	9	9
9	9	9	9	9	9	9	9	9	9
9	9	9	9	9	9	9	9	9	9
9	9	9	9	9	9	9	9	9	9
9	9	9	9	9	9	9	9	9	9
9	9	9	9	9	9	9	9	9	9

9	9	9	9	9	9	9	9	9	9
9	9	9	9	9	9	9	9	9	9
9	9	9	9	9	9	9	9	9	9
9	9	9	9	9	9	9	9	9	9
9	9	9	9	9	9	9	9	9	9
9	9	9	9	9	9	9	9	9	9
9	9	9	9	9	9	9	9	9	9
9	9	9	9	9	9	9	9	9	9
9	9	9	9	9	9	9	9	9	9
9	9	9	9	9	9	9	9	9	9
9	9	9	9	9	9	9	9	9	9
9	9	9	9	9	9	9	9	9	9
9	9	9	9	9	9	9	9	9	9
9	9	9	9	9	9	9	9	9	9
9	9	9	9	9	9	9	9	9	9
9	9	9	9	9	9	9	9	9	9
9	9	9	9	9	9	9	9	9	9
9	9	9	9	9	9	9	9	9	9
9	9	9	9	9	9	9	9	9	9
9	9	9	9	9	9	9	9	9	9
9	9	9	9	9	9	9	9	9	9
9	9	9	9	9	9	9	9	9	9
9	9	9	9	9	9	9	9	9	9
9	9	9	9	9	9	9	9	9	9
9	9	9	9	9	9	9	9	9	9
2	2	2	2	2	2	2	2	2	2
9	9	9	9	9	9	9	9	9	9
9	9	9	9	9	9	9	9	9	9
9	9	9	9	9	9	9	9	9	9
9	9	9	9	9	9	9	9	9	9
9	9	9	9	9	9	9	9	9	9
9	9	9	9	9	9	9	9	9	9
9	9	9	9	9	9	9	9	9	9
9	9	9	9	9	9	9	9	9	9
9	9	9	9	9	9	9	9	9	9
9	9	9	9	9	9	9	9	9	9
9	9	9	9	9	9	9	9	9	9
9	9	9	9	9	9	9	9	9	9
9	9	9	9	9	9	9	9	9	9
9	9	9	9	9	9	9	9	9	9
9	9	9	9	9	9	9	9	9	9
2	2	2	2	2	2	2	2	2	2
3	3	3	3	3	3	3	3	3	3
2	2	3	2	2	1	3	2	1	1
1	3	1	3	1	1	1	3	1	1
3	3	3	3	3	3	3	3	3	3
1	1	1	1	1	1	1	1	1	1

1	3	3	2	1	3	1	3	1	1
9	9	9	9	9	9	9	9	9	9
1	3	3	2	1	3	1	3	1	1
1	3	1	2	2	2	2	1	3	2
1	2	2	2	2	2	2	1	1	1
2	2	2	2	2	2	2	2	2	2
1	3	1	2	1	1	1	2	1	1
1	2	3	3	1	1	1	3	1	1
1	1	3	3	1	1	1	2	2	1
1	3	2	3	9	1	2	2	1	1
2	2	2	2	2	1	2	2	1	1
1	2	2	2	1	1	1	1	1	1
1	3	3	3	3	1	1	1	1	1
1	3	1	3	3	2	1	1	1	1
2	1	9	2	1	2	3	1	1	3
1	1	2	3	1	1	2	2	1	2
1	9	9	9	1	1	9	9	9	1
2	3	2	2	2	3	1	2	2	3
1	3	3	3	1	2	1	2	2	1
1	2	1	2	2	1	2	2	1	1
2	2	1	2	3	2	2	2	1	1
1	2	2	2	2	1	1	1	1	1
1	2	2	2	2	2	2	2	2	9
2	2	2	2	2	1	1	3	9	1
1	2	1	2	2	1	1	1	1	1
1	2	1	1	1	2	2	1	1	3
9	2	2	2	2	2	9	2	1	1
1	3	3	3	3	3	3	1	1	1
1	1	1	3	3	1	1	1	2	2
3	3	9	9	1	1	1	1	1	1
3	3	3	3	3	3	3	3	3	3
2	1	2	1	2	1	2	1	2	1
1	1	1	1	1	1	2	2	2	2
1	3	1	2	3	1	1	2	3	3
2	2	2	2	2	2	2	2	2	2
2	2	2	2	2	2	9	9	9	9
1	1	2	2	2	2	1	2	3	2
2	2	2	2	2	2	2	2	2	2
2	2	2	2	2	2	2	2	2	2
9	9	9	9	9	9	9	9	9	9
9	9	9	9	9	9	9	9	9	9
9	9	9	9	9	9	9	9	9	9
9	9	9	9	9	9	9	9	9	9
9	9	9	9	9	9	9	9	9	9
9	9	9	9	9	9	9	9	9	9
9	9	9	9	9	9	9	9	9	9
2	1	2	2	2	2	1	1	1	2

9	9	9	9	9	9	9	9	9	9
1	1	2	1	2	1	1	1	1	1
3	3	3	3	3	1	1	1	3	3
1	3	1	1	1	1	1	1	1	1
3	3	3	3	3	1	3	3	3	3
1	3	1	3	1	1	1	3	1	1
3	2	3	2	3	2	3	2	3	2
1	1	1	1	1	2	1	2	2	3
1	2	3	2	2	2	2	2	3	2
1	1	1	3	3	1	3	1	3	9
2	1	9	3	3	2	2	2	1	2
1	2	3	2	1	2	3	2	1	2
1	1	3	3	1	3	1	3	3	1
1	2	1	2	1	2	3	3	2	1
1	1	3	3	3	1	1	2	2	1
2	9	9	9	9	2	9	1	9	2
3	3	3	3	3	3	3	3	3	3
2	1	1	3	9	1	3	1	1	2
2	1	1	1	2	2	2	1	2	2
1	3	3	3	3	1	1	1	1	3
1	1	1	1	1	1	1	1	1	1
3	1	9	1	9	1	3	2	3	2
3	2	2	2	2	2	9	2	3	2
9	9	9	9	1	9	9	9	9	9
2	2	1	2	3	2	1	2	1	2
2	1	1	2	1	1	1	3	1	2
1	1	2	3	2	2	1	2	3	2
3	3	3	3	3	3	3	3	3	3
1	3	3	3	1	1	1	1	9	9
2	3	3	1	2	1	1	1	1	3
1	1	2	3	1	1	2	2	1	1
1	1	3	1	3	3	3	1	3	1
1	2	3	1	9	1	3	1	1	2
3	1	1	3	1	1	1	1	2	2
3	3	2	2	2	2	2	2	2	1
3	3	3	2	2	1	2	2	1	3
1	2	2	2	2	2	2	2	2	1
1	2	3	1	2	3	1	2	3	1
2	2	2	2	2	2	2	2	2	2
1	3	1	3	3	3	3	3	1	3
1	1	3	1	1	1	1	1	1	1
3	3	1	3	3	3	3	3	3	1
1	2	3	1	1	2	2	1	2	3
9	9	9	9	9	9	9	9	9	9
3	9	1	3	3	3	3	1	1	1
1	3	9	2	1	1	3	1	1	1
1	1	3	1	3	9	1	1	1	3

1	2	3	9	1	2	3	1	2	3
1	1	3	1	1	1	9	1	1	3
2	1	2	3	3	1	1	1	1	1
2	2	2	2	2	2	2	2	2	2
3	3	3	3	3	3	3	3	3	3
3	3	3	3	3	3	3	3	3	3
2	2	3	3	3	2	1	2	3	2
9	9	9	9	9	9	9	9	9	9
1	3	2	2	2	2	2	2	2	2
2	2	2	2	3	3	3	3	2	2
3	3	2	2	2	2	2	1	1	3
2	3	3	2	2	1	2	3	2	1
2	2	2	2	2	2	2	2	2	2
9	9	9	9	9	9	9	9	9	9
9	9	9	9	9	9	9	9	9	9
1	2	3	1	2	3	2	1	2	3
2	2	2	2	2	2	2	2	2	2
2	2	2	2	2	2	2	2	2	2
2	2	2	1	1	1	2	2	1	1
1	1	2	2	2	2	2	2	2	2
1	1	1	1	1	1	1	1	1	1
1	1	1	1	1	1	1	1	1	1
2	2	2	2	2	3	3	3	2	2
2	2	2	2	2	2	2	2	2	2
2	2	2	2	2	2	1	2	3	3
2	2	2	2	2	2	3	2	2	2
2	2	2	1	1	1	3	2	2	2
2	2	2	2	2	2	2	2	2	2
2	2	2	2	2	2	2	2	2	2
2	2	2	2	2	2	2	2	2	1
2	2	2	2	2	2	2	2	2	2
2	2	2	2	2	2	2	2	2	2
2	2	2	2	2	2	2	2	2	2
1	2	2	1	2	1	3	1	2	3
2	2	2	1	2	1	1	1	1	1
2	2	2	2	2	2	2	2	2	2
2	2	2	2	2	2	2	2	2	2
2	2	2	2	2	2	2	2	2	2
2	2	2	2	2	2	2	2	2	2
2	2	2	2	2	2	2	2	2	2
2	2	2	2	2	2	2	2	2	2
2	2	2	2	2	2	2	2	2	2
2	2	2	2	2	2	2	2	2	2
2	2	2	2	2	2	2	2	2	2
2	2	2	2	2	2	2	2	2	2
1	1	1	3	3	1	1	1	1	1
2	2	2	2	2	2	2	2	2	2

2	2	2	2	2	2	2	2	2	2
1	1	2	3	2	2	1	1	3	2
2	2	2	2	2	2	2	2	2	2
2	2	1	1	2	3	3	3	2	2
3	3	3	3	3	3	3	3	3	3
3	3	3	3	3	3	3	3	3	3
2	2	2	2	2	2	2	2	2	2
2	2	2	2	2	2	2	2	2	2
2	2	2	2	2	2	2	2	2	2
2	2	2	2	2	2	2	2	2	2
3	3	3	2	2	2	1	1	1	1
2	2	2	2	2	2	2	2	2	2
9	9	9	9	9	9	9	9	9	9
1	1	1	1	1	1	1	1	3	1
9	9	9	9	9	9	9	1	9	9
2	2	2	2	2	2	2	2	2	2
3	2	3	2	3	2	2	1	2	2
2	2	2	1	1	1	3	3	3	2
2	2	2	3	2	3	3	3	3	2
9	9	9	9	9	9	9	9	9	9
2	2	2	2	2	2	2	2	2	2
1	2	2	9	9	9	9	9	9	9
2	2	2	2	2	2	2	2	2	2
1	1	1	1	1	1	1	1	1	1
9	9	9	9	9	9	9	9	9	9
2	2	2	2	2	2	2	2	2	2
2	1	1	2	2	2	2	2	1	9
9	9	9	9	9	9	9	9	9	9
9	9	9	9	9	9	9	9	9	9
3	3	3	3	3	3	3	2	3	3
1	2	3	1	2	3	1	2	1	2
3	3	3	3	3	2	3	3	3	3
3	3	2	3	3	2	3	3	3	3
9	1	3	1	1	1	1	1	1	1
1	2	3	9	1	3	1	2	9	1
1	1	1	1	1	1	1	1	1	1
3	2	2	2	2	2	2	2	2	2
2	2	3	3	3	3	2	2	2	3
2	2	2	2	2	2	2	2	2	2
9	1	9	2	9	2	9	9	9	9
2	2	2	2	2	2	2	2	2	2
9	9	9	9	9	9	9	9	9	9
1	3	3	3	1	1	1	1	1	1
2	2	2	2	2	2	2	2	2	2
2	2	2	2	2	2	2	2	2	2
2	2	2	2	2	2	2	2	2	2
2	2	1	2	3	1	3	2	1	2

2	2	2	2	2	2	2	2	2	2
9	9	9	9	2	9	9	9	9	9
2	3	3	2	1	1	1	3	2	2
1	1	3	2	2	1	1	2	2	2
3	3	3	3	9	2	3	3	3	2
2	2	2	2	2	1	2	1	2	1
1	1	1	1	1	1	1	1	1	1
2	2	2	2	2	2	2	2	2	2
1	1	1	1	1	1	1	1	1	1
2	2	2	2	2	2	2	2	2	2
9	9	9	9	9	9	9	9	9	9
3	2	1	2	2	1	1	2	3	2
3	3	3	3	3	3	3	3	3	3
1	1	1	1	1	1	1	2	9	2
2	2	2	2	2	2	2	2	2	2
1	1	1	1	1	1	1	1	1	1
3	3	3	3	3	3	3	3	3	3
1	1	1	1	1	1	1	1	1	1
2	2	2	2	2	2	2	2	2	2
2	2	2	2	2	2	2	2	2	2
2	2	2	2	2	2	2	2	2	2
1	1	1	1	1	1	1	1	1	1
1	1	1	1	1	1	1	1	1	1
3	3	3	1	1	1	1	1	3	3
9	9	9	9	9	9	9	9	9	9
9	9	9	9	9	9	9	9	9	9
2	1	1	1	2	1	1	1	9	2
2	2	2	2	2	2	2	2	2	2
1	1	1	1	1	1	1	1	1	1
2	2	2	2	2	2	2	2	2	2
3	3	3	3	3	3	3	3	3	3
1	1	2	1	1	1	1	1	1	1
1	1	3	1	2	2	1	1	1	2
1	2	2	2	2	2	2	1	2	1
9	9	9	9	2	9	9	2	9	9
3	3	3	3	3	3	3	3	3	3
9	2	9	1	2	1	2	9	2	1
2	2	2	2	2	2	3	3	2	2
1	1	1	1	1	1	1	1	1	1
1	3	2	1	2	2	2	2	2	2
2	2	2	2	2	2	2	2	2	2
2	3	9	2	3	3	2	2	2	2
3	3	3	3	3	2	2	2	2	2
2	2	2	2	2	2	2	2	2	2
1	2	3	1	2	3	1	2	3	1
3	3	3	3	3	3	3	3	3	3
2	9	1	2	9	1	1	2	1	1

2	2	2	2	2	2	3	3	3	3
9	9	9	9	9	9	9	9	9	9
9	9	9	9	9	9	9	9	9	9
1	2	2	2	2	2	1	1	1	1
2	2	2	2	2	2	2	2	2	2
2	2	2	2	1	2	2	2	1	1
1	1	1	1	1	1	1	1	1	1
1	1	3	3	3	1	3	1	1	1
2	2	2	1	1	1	1	1	3	3
1	1	3	3	3	1	3	1	1	1
1	2	3	9	9	1	2	1	2	1
2	2	2	2	2	2	2	2	2	2
2	2	2	2	2	2	2	2	2	2
2	2	2	2	2	2	2	2	2	2
2	2	2	2	2	2	1	1	1	1
2	1	9	2	2	1	1	1	2	3
2	2	2	2	2	2	1	2	3	2
1	3	3	2	3	1	3	1	3	1
1	2	2	2	2	2	2	2	2	2
1	1	3	3	2	2	2	3	2	1
2	2	2	2	2	2	2	2	2	2
2	2	2	2	2	2	2	2	2	2
2	2	2	2	2	2	2	2	2	2
2	2	2	2	2	2	2	2	2	2
2	2	2	9	2	2	2	2	2	2
1	2	2	2	2	2	2	2	3	2
2	2	2	2	2	2	2	2	2	2
2	2	2	2	2	2	2	2	2	2
1	9	2	3	3	1	2	1	3	1
2	2	2	2	2	2	2	2	2	2
1	1	1	1	1	1	1	1	1	1
2	2	2	2	2	2	2	2	2	2
3	2	2	2	3	1	1	1	2	3
3	3	3	3	3	3	2	2	2	2
2	3	2	3	2	2	3	1	3	2
2	2	9	3	3	2	1	2	1	2
1	2	2	2	2	2	1	1	2	2
3	1	1	2	3	3	2	2	2	3
2	2	2	2	2	2	2	2	2	2
2	2	2	2	2	2	2	2	2	2
1	2	2	1	2	2	2	1	1	1
3	3	3	3	3	3	3	3	3	3
2	2	2	2	2	2	2	2	2	2
9	9	9	9	9	9	9	9	9	9
2	2	2	2	2	2	2	2	2	2
2	3	3	2	3	3	2	1	1	2
2	3	3	2	3	2	1	1	2	1

2	2	2	2	2	2	2	2	2	2
2	2	2	2	2	2	2	2	2	2
2	2	2	2	2	2	2	2	2	2
9	9	9	9	9	9	9	9	9	9
1	2	3	9	9	1	2	1	2	1
2	2	2	2	2	2	2	2	2	2
9	9	9	9	9	9	9	9	9	9
3	1	2	1	1	2	1	3	1	3
2	3	2	1	2	3	1	1	3	2
3	3	2	2	3	1	1	1	1	3
1	3	1	3	3	1	1	3	1	1
3	3	2	2	2	1	2	2	1	2
3	3	3	3	3	3	3	3	3	3
9	9	9	9	9	9	9	9	9	9
3	2	2	2	2	2	3	3	3	3
2	1	2	3	2	3	2	3	2	3
2	2	2	1	2	2	2	2	1	1
2	3	2	3	2	3	1	1	2	2
2	2	2	2	2	2	2	2	1	2
1	1	1	1	1	1	1	1	1	1
2	2	1	2	3	2	1	2	2	1
2	2	2	2	2	2	2	2	2	2
1	3	2	1	1	2	1	2	3	3
3	2	3	2	2	2	2	2	2	2
9	9	9	9	9	9	9	9	9	9
9	9	9	9	9	9	9	9	9	9
9	9	9	9	9	9	9	9	9	9
1	3	2	3	1	3	2	2	2	1
1	2	3	2	1	2	2	2	1	2
2	1	1	3	2	2	2	1	1	1
2	2	1	1	1	2	3	2	1	1
3	3	3	3	3	3	3	3	3	3
3	1	3	2	2	1	2	3	1	2
2	1	2	2	2	1	2	1	2	3
1	2	3	1	3	2	2	2	2	3
2	1	3	2	2	1	2	1	1	1
2	2	2	2	1	1	1	1	3	3
2	2	2	2	2	1	1	1	1	3
2	3	3	3	3	2	2	2	2	3
2	2	2	2	2	2	2	2	2	2
2	2	2	2	2	2	2	2	2	2
2	2	2	2	2	2	2	2	2	2
3	3	3	3	3	3	3	3	3	3
2	2	2	2	2	2	2	2	3	3
9	9	9	9	9	9	9	9	9	9
9	9	9	9	9	9	9	9	9	9
1	2	3	1	2	3	1	2	3	1

2	2	2	2	2	2	2	2	2	2
2	3	2	1	2	3	2	1	2	1
3	2	2	2	3	3	3	2	1	1
9	9	9	9	9	9	9	9	9	9
3	3	3	3	3	3	3	3	3	3
1	3	3	3	3	3	3	3	3	2
1	1	1	1	1	1	1	1	1	1
1	3	1	9	2	1	2	3	1	1
1	2	3	1	2	3	1	2	3	3
2	2	1	2	2	2	2	2	2	2
3	2	2	2	2	3	2	3	2	3
3	3	3	3	3	3	3	3	3	3
1	1	1	1	1	1	1	1	1	1
3	3	3	3	3	3	3	3	3	3
3	3	2	2	3	2	2	2	2	2
2	3	2	3	2	3	2	1	1	1
2	2	2	2	2	2	2	2	2	2
1	1	1	3	3	3	2	1	2	3
3	2	2	2	2	2	2	2	2	3
3	3	9	1	2	1	1	1	2	1
2	2	2	2	2	2	2	2	2	2
3	3	3	3	3	3	3	3	3	2
1	1	2	3	1	1	1	3	3	1
1	2	1	2	3	1	2	3	1	2
2	2	2	2	2	2	2	2	2	2
1	2	1	1	2	2	3	1	2	2
2	1	3	2	2	1	3	2	1	3
3	3	3	3	3	3	3	3	3	3
3	3	3	3	3	3	3	3	3	3
3	2	1	1	1	1	1	2	1	3
1	2	3	1	2	3	1	2	3	1
3	3	3	3	3	2	3	3	2	3
3	3	3	3	3	3	3	2	3	2
1	2	3	2	1	2	3	2	1	2
2	9	3	1	2	1	2	1	3	1
1	1	1	2	2	2	2	2	2	2
1	2	3	1	2	3	2	2	1	2
1	1	1	1	1	1	2	2	2	2
2	2	2	2	2	2	2	2	2	2
1	3	3	3	2	2	1	1	1	1
1	2	3	1	2	1	2	3	1	2
2	1	1	1	1	2	3	2	2	1
2	2	1	1	2	3	3	3	1	2
2	3	2	3	3	3	3	3	3	3
1	2	2	1	1	2	2	1	1	2
2	1	2	3	3	2	1	2	2	2
2	2	2	2	2	2	2	2	2	2

1	1	1	1	1	1	1	1	1	1
2	3	2	1	2	1	2	1	2	1
1	2	2	2	2	1	2	1	2	2
2	2	2	2	2	1	2	2	2	2
1	2	3	1	2	2	3	2	1	2
3	3	3	1	1	1	2	1	2	1
2	2	2	1	2	1	2	2	1	1
2	3	1	2	3	2	3	2	2	1
2	2	1	1	1	2	2	2	2	2
1	1	3	3	3	1	1	1	1	1
1	2	3	1	2	3	1	2	3	1
1	3	1	3	2	1	1	1	2	3
3	2	1	2	3	2	2	1	3	2
2	1	3	1	2	3	2	3	2	3
3	1	3	3	2	1	1	1	1	1
1	3	3	3	3	3	3	2	1	2
1	3	2	3	2	2	1	2	3	2
3	1	2	2	3	2	1	2	3	1
1	1	1	1	1	1	1	1	1	1
3	1	3	1	1	1	3	3	1	1
2	2	3	2	3	2	3	1	2	3
1	1	1	3	3	1	1	1	1	3
2	3	1	2	2	1	2	1	1	1
2	2	2	3	2	2	2	1	2	9
1	1	1	3	2	2	2	2	1	1
1	1	2	2	9	1	1	1	1	1
2	3	3	3	3	2	2	3	3	9
1	2	1	2	2	3	3	3	1	3
1	3	1	3	3	3	1	1	1	1
1	3	3	1	2	1	1	2	1	2
2	2	2	2	2	2	2	2	2	2
2	2	2	2	2	2	2	2	2	2
3	3	2	2	2	1	2	1	1	3
1	2	1	2	1	3	2	1	3	1
2	2	2	2	2	2	2	2	2	2
9	9	9	9	9	9	9	9	9	9
9	9	9	9	9	9	9	9	9	9
9	9	9	9	9	9	9	9	9	9
1	1	1	1	1	2	2	2	2	1
9	9	9	9	9	9	9	9	9	9
2	2	2	2	2	2	2	2	2	2
9	9	9	9	9	9	9	9	9	9
2	2	2	2	2	1	2	3	2	2
9	9	9	9	9	9	9	9	9	9
1	1	9	9	9	1	2	2	1	2
2	3	2	1	2	3	2	3	3	3
9	9	9	9	9	9	9	9	9	9

1	3	1	2	2	1	1	1	2	3
2	2	2	2	3	2	3	2	1	3
2	2	2	2	2	2	3	2	3	1
2	3	3	2	2	1	2	3	2	3
2	1	2	3	2	2	2	1	2	3
3	3	2	2	2	1	2	1	1	3
2	1	2	2	3	2	1	2	1	9
1	2	2	2	1	2	1	2	2	2
2	2	2	2	2	2	2	2	2	2
1	2	2	1	2	1	1	2	1	2
2	2	2	2	2	2	2	2	2	2
2	3	1	3	1	2	2	1	2	3
9	9	9	9	9	9	9	9	1	9
1	3	1	2	3	2	1	2	3	2
2	2	2	3	2	2	1	1	1	2
3	3	3	3	3	3	3	3	2	2
2	3	1	2	3	1	2	3	1	2
1	2	3	1	2	3	1	2	3	2
1	1	3	9	2	1	2	1	3	1
2	2	2	2	2	2	2	2	2	2
2	2	2	2	2	2	2	2	2	2
2	9	9	9	9	9	3	9	2	9
2	2	2	2	2	2	2	2	2	2
2	2	2	2	2	2	2	2	2	2
2	2	2	2	2	2	2	2	2	2
2	2	2	2	2	2	2	2	2	2
1	1	1	1	9	1	1	1	1	1
1	1	1	9	9	1	1	1	1	1
1	1	1	9	9	1	1	1	1	1
1	2	2	2	2	1	1	1	1	1
1	3	1	3	3	1	1	1	1	1
1	3	1	3	3	1	1	1	1	1
3	3	3	3	1	1	1	1	1	1
1	2	2	2	2	1	1	2	1	1
2	2	2	2	1	1	1	2	1	1
1	3	1	3	3	1	1	1	1	1
1	3	1	3	3	1	1	1	1	1
9	9	9	9	9	9	9	9	9	9
1	3	1	3	3	1	1	1	1	1
1	3	1	3	3	1	1	1	1	1
1	3	1	3	3	1	1	1	1	1
1	3	1	3	3	1	1	1	1	1
1	3	1	3	3	1	1	1	1	1
1	3	1	3	3	1	1	1	1	1
1	3	1	3	3	1	1	1	1	1
1	3	1	3	3	1	1	1	1	1
1	1	3	3	9	1	1	1	1	1

9	9	9	9	9	9	9	9	9	9
1	1	1	3	3	1	1	1	1	1
1	3	3	9	9	9	1	1	1	1
1	3	1	3	3	1	1	1	1	1
1	3	1	3	3	1	1	1	1	1
3	1	1	3	1	1	9	9	1	1
1	3	1	3	3	1	1	1	1	1
2	2	2	9	3	1	1	1	1	1
1	3	1	3	3	1	1	1	1	1
1	1	1	1	1	1	1	1	1	1
1	3	1	3	3	1	1	1	1	1
1	3	1	3	3	1	1	1	1	1
1	1	1	2	2	1	1	1	1	1
1	1	2	2	2	1	1	1	1	1
1	3	1	3	3	1	1	1	1	1
1	3	1	3	3	1	1	1	1	1
1	3	1	3	3	1	1	1	1	1
3	1	3	3	2	1	1	1	1	1
1	3	3	3	3	1	1	1	1	1
1	3	1	3	3	1	1	1	1	1
3	1	3	3	1	1	1	1	1	1
1	3	1	3	3	1	1	1	1	1
1	1	1	1	3	1	1	1	1	1
1	1	3	1	1	1	1	1	1	1
1	3	1	3	3	1	1	1	1	1
1	1	1	1	1	1	1	1	1	1
1	9	3	2	2	2	2	3	3	1
3	9	1	1	2	1	1	3	3	3
1	3	1	1	3	1	1	1	1	1
1	3	1	3	3	1	1	1	1	1
1	3	3	3	1	1	1	1	1	1
1	3	1	3	3	1	1	1	1	1
1	1	1	3	3	1	1	1	1	1
1	3	9	1	3	2	2	1	3	3
1	3	1	3	3	1	1	1	1	1
9	9	9	1	3	1	9	9	1	1
3	3	1	3	3	1	1	1	1	1
1	3	1	3	3	1	1	1	1	1
1	3	1	3	3	1	1	1	1	1
1	3	1	3	3	1	1	1	1	1
1	3	1	3	3	1	1	1	1	1
1	3	1	3	3	1	1	1	1	1
9	9	9	9	9	9	9	9	9	1
1	3	1	3	3	1	1	1	1	1
1	3	1	3	3	1	1	1	1	1
1	1	1	1	1	1	1	1	1	2
1	3	1	3	3	1	1	1	1	1

1	1	1	3	3	1	1	1	1	1
3	9	1	3	3	1	1	1	1	1
1	3	1	3	3	1	1	1	1	1
1	3	1	3	3	1	1	1	1	1
1	3	1	3	3	1	1	1	1	1
1	2	1	2	2	1	1	1	1	1
1	3	1	3	3	1	1	1	1	1
1	3	1	3	3	1	1	1	1	1
1	3	1	1	3	1	1	1	1	1
1	3	1	3	3	1	1	1	1	1
1	3	3	3	1	1	1	1	1	1
1	1	1	1	1	1	1	1	1	1
3	3	1	3	3	2	1	1	1	1
1	1	1	1	1	1	1	1	1	1
1	1	3	1	1	1	1	1	1	1
1	3	3	3	1	1	1	3	1	1
2	3	1	2	2	1	1	3	2	2
9	9	9	9	9	9	9	9	9	9
1	1	1	3	3	1	1	1	1	1
1	1	3	2	2	2	2	2	2	2
2	9	3	1	3	1	2	2	2	2
1	3	2	1	1	1	1	1	1	1
9	9	9	9	9	9	9	9	9	9
9	9	9	9	9	9	9	9	9	9
1	1	1	1	1	1	1	1	1	1
1	1	1	1	1	1	1	1	1	1
1	3	1	3	3	1	1	1	1	1
1	2	2	3	9	2	1	2	2	1
3	3	3	3	3	2	3	2	2	2
3	1	3	1	3	2	1	2	1	3
1	3	1	1	3	3	3	3	3	1
1	2	2	2	2	1	1	2	1	1
2	1	2	3	2	1	3	2	1	2
2	1	1	1	1	2	1	1	1	1
1	2	2	2	1	1	1	2	2	1
1	2	1	3	1	1	1	2	2	1
1	2	2	1	1	2	2	2	2	2
9	9	1	9	9	9	1	9	9	1
1	3	2	1	2	2	1	2	2	2
1	3	1	3	3	2	1	2	1	2
3	3	3	3	3	2	3	1	1	1
1	2	2	3	1	1	1	1	1	2
3	3	3	2	1	1	1	1	2	2
3	3	3	2	2	2	2	2	2	2
3	3	3	2	2	2	1	2	3	1
1	1	2	2	1	1	2	1	2	3
1	3	9	3	1	1	1	1	1	1

1	3	3	3	1	1	1	1	1	1
2	1	1	2	2	2	1	2	1	1
2	1	2	2	2	1	1	1	1	2
2	1	1	2	2	2	1	2	1	1
1	3	3	3	3	3	1	1	1	1
9	9	9	9	9	9	2	1	1	1
1	3	1	3	3	1	1	1	1	1
1	1	1	1	1	1	2	1	1	1
1	2	1	2	2	1	1	1	1	2
2	2	2	2	2	2	2	2	2	2
9	9	9	9	9	9	9	9	9	9
2	2	3	3	3	2	1	1	2	2
1	1	1	1	1	1	1	1	9	1
2	2	2	2	2	2	9	9	9	1
3	3	1	3	3	2	2	1	2	2
1	3	2	3	2	1	2	1	1	1
1	2	1	2	3	1	3	1	1	3
1	3	3	3	1	1	1	1	1	1
2	1	2	2	2	2	1	1	1	1
1	3	3	3	1	1	3	2	1	1
1	2	2	2	1	2	1	1	2	2
9	9	9	9	9	9	1	9	9	9
2	2	2	3	1	1	1	1	1	1
2	1	2	2	2	1	2	2	1	2
1	3	1	1	1	1	1	1	1	1
2	2	2	3	1	1	1	1	1	1
1	9	9	9	9	9	9	9	9	9
1	1	2	3	9	1	1	2	1	1
2	2	2	2	2	2	2	2	2	2
3	3	3	3	3	3	1	1	1	1
1	2	3	3	2	1	1	1	1	2
1	2	3	3	2	2	1	1	1	1
1	2	2	2	2	2	1	1	1	1
9	9	9	9	9	9	9	9	9	9
9	9	9	9	9	9	9	9	9	9
2	3	1	3	2	1	1	3	2	3
3	3	3	3	1	1	3	3	1	1
1	2	1	2	1	1	1	1	2	1
1	1	1	1	9	1	1	1	1	1
9	9	9	9	9	1	1	1	1	1
1	3	1	3	3	1	1	1	1	1
1	1	1	1	3	2	1	1	1	1
1	3	1	3	3	1	1	1	1	1
1	1	1	3	1	1	1	1	1	1
3	3	1	3	3	1	1	1	1	1
1	1	1	3	1	1	1	1	1	1
2	1	3	2	3	1	1	1	1	1

1	3	1	3	3	1	1	1	1	1
1	3	1	3	3	1	1	1	1	1
1	3	9	3	3	1	1	1	1	1
1	3	1	3	3	1	1	1	1	1
1	1	1	1	3	3	1	1	1	1
1	3	3	9	9	1	1	1	1	1
1	1	1	3	9	1	1	1	1	1
3	1	3	3	3	3	1	1	1	1
9	9	9	9	9	9	9	9	9	9
1	3	3	9	1	1	1	1	1	9
1	2	9	9	1	9	1	9	9	9
1	3	3	3	3	1	1	1	1	1
1	1	2	1	2	1	2	3	1	2
1	3	3	3	1	1	1	2	1	1
9	9	9	9	9	9	9	9	9	9
9	9	9	9	9	9	9	9	9	9
1	1	3	1	3	1	3	3	3	3
1	3	1	9	2	1	1	1	1	2
2	3	3	1	1	2	2	2	1	1
9	9	9	9	9	9	9	9	9	9
1	2	1	2	2	2	1	2	1	1
1	3	1	3	3	2	1	2	1	1
1	9	9	9	1	1	9	1	1	1
1	2	2	9	1	2	1	1	1	1
1	1	3	3	1	1	1	1	1	1
1	2	3	2	2	1	1	2	3	1
3	3	1	3	3	1	1	3	3	3
1	1	1	3	1	1	1	1	1	1
1	1	1	1	3	1	1	1	1	1
1	3	3	1	1	1	1	1	1	1
1	9	9	9	9	1	9	1	1	9
1	1	3	1	2	1	1	1	1	1
1	9	2	2	1	1	9	9	1	1
2	1	1	3	3	1	1	1	3	2
1	1	1	3	1	1	1	1	1	1
1	1	3	1	1	1	1	1	1	1
3	3	3	2	2	1	1	1	2	3
1	2	1	2	3	1	1	1	1	1
2	2	2	2	2	1	1	1	3	1
1	1	1	3	3	1	1	1	3	1
1	3	3	3	2	1	3	2	3	1
2	3	1	3	2	1	1	1	1	3
1	3	3	3	1	1	1	3	1	1
1	9	9	9	9	9	9	9	9	1
1	1	2	2	2	2	2	2	1	1
1	1	1	3	2	1	1	1	1	1
2	1	3	3	2	2	1	2	2	1

2	3	1	3	3	1	1	3	2	3
9	9	9	9	9	9	9	9	9	9
9	9	9	9	9	9	9	9	9	9
1	3	2	3	2	1	1	1	1	1
1	3	1	3	9	3	3	1	1	1
9	9	9	9	9	9	9	9	9	9
3	3	1	3	1	1	1	1	1	1
1	1	3	3	1	1	1	1	1	2
2	2	2	2	2	1	2	1	1	1
1	2	2	2	2	9	1	1	2	1
1	1	1	3	1	1	1	3	1	1
2	1	2	2	2	2	2	1	1	1
2	2	1	2	2	1	2	2	3	2
1	1	2	1	1	1	1	2	1	2
1	2	2	2	1	1	1	1	1	1
3	2	2	2	2	1	2	2	2	3
3	1	1	2	2	1	1	2	1	1
9	9	9	9	9	9	9	9	9	9
9	9	9	9	9	9	9	9	9	9
9	9	9	9	9	9	9	9	9	1
9	9	9	9	9	9	9	9	9	9
9	9	9	9	9	9	9	9	9	1
9	1	3	3	1	1	3	3	3	1
3	3	3	3	3	1	1	3	1	1
9	9	9	9	9	1	9	9	9	9
1	2	3	3	1	1	1	2	2	2
1	3	2	3	3	1	1	2	1	1
1	3	3	1	2	3	2	2	1	1
1	3	3	1	1	1	1	1	1	1
1	2	1	3	1	1	2	2	1	2
3	2	3	3	1	2	1	2	1	1
1	3	3	3	2	1	1	3	1	1
1	3	2	2	2	1	3	1	1	1
2	3	2	3	3	1	1	2	3	3
9	9	9	9	1	1	9	9	9	1
1	3	9	9	9	1	1	1	2	1
1	2	2	2	2	1	1	2	1	1
1	1	9	9	9	1	9	9	9	1
1	3	3	3	3	1	1	2	1	3
1	2	3	3	2	1	1	1	1	1
2	2	2	2	2	1	1	2	1	2
1	3	3	3	1	1	2	2	1	3
2	3	1	3	3	1	2	2	1	2
1	3	1	3	3	1	1	1	1	1
1	3	3	9	3	9	9	1	1	1
1	3	1	3	3	1	1	1	1	1
3	3	2	3	2	3	2	3	2	3

2	2	2	2	2	2	1	2	2	2
2	3	3	2	1	1	2	1	1	1
1	2	2	2	2	2	2	2	1	1
1	1	1	2	2	1	1	1	1	1
1	9	9	9	1	1	9	1	1	1
1	1	1	1	1	1	1	1	1	1
1	3	1	3	3	1	1	1	1	1
1	1	1	2	1	1	1	1	1	1
1	1	2	2	2	2	2	2	2	1
2	2	1	2	3	1	2	3	1	1
3	3	1	2	3	2	2	2	2	2
2	2	2	2	3	2	2	1	2	2
1	2	2	2	2	2	2	9	1	2
2	2	2	2	2	1	2	2	1	1
1	2	1	1	1	2	2	2	2	2
2	2	1	2	2	1	1	1	1	1
2	3	1	2	2	2	3	2	2	1
2	2	2	2	2	2	2	2	2	2
2	2	2	2	2	1	2	2	1	1
1	3	1	2	2	1	1	2	1	1
2	2	2	2	2	2	2	2	2	2
1	3	1	2	2	1	1	1	1	1
2	3	2	2	2	2	2	2	2	2
2	2	2	2	2	2	2	2	2	2
1	2	1	2	2	2	2	2	2	2
1	2	2	2	2	2	2	2	2	2
2	2	2	2	2	2	2	2	2	2
9	9	9	9	9	9	9	9	9	9
1	3	3	1	3	1	2	1	1	1
3	3	3	1	3	2	2	2	3	3
1	1	2	1	1	2	1	2	1	1
3	3	3	3	1	2	2	1	1	2
2	1	1	1	2	2	1	1	1	1
2	1	2	1	1	1	9	1	9	9
3	3	3	3	9	9	9	2	2	1
1	2	3	2	3	1	2	2	2	3
1	2	2	2	2	1	1	1	1	1
1	2	2	2	2	1	1	1	1	1
9	9	9	9	9	9	9	9	9	9
9	9	9	9	9	9	9	9	9	9
9	9	9	9	9	9	9	9	9	9
9	9	9	9	9	9	9	9	9	9
9	9	9	9	9	9	9	9	9	9
9	9	9	9	9	9	9	9	9	9
9	9	9	9	9	9	9	9	9	9
9	9	9	9	9	9	9	9	9	9
3	3	3	3	2	2	3	2	1	2

9	9	9	9	9	9	9	9	9	9
9	9	9	9	9	9	9	9	9	9
1	2	2	3	3	2	2	2	2	2
9	9	9	9	9	9	9	9	9	9
9	9	9	9	9	9	9	9	9	9
9	9	9	9	9	9	9	9	9	9
1	1	1	1	1	1	1	1	2	1
3	2	1	3	2	3	3	3	3	3
9	9	9	9	9	9	9	9	9	9
9	9	9	9	9	9	9	9	9	9
9	9	9	9	9	9	9	9	9	9
9	9	9	9	9	9	9	9	9	9
9	9	9	9	9	9	9	9	9	9
9	9	9	9	9	9	9	9	9	9
9	9	9	9	9	9	9	9	9	9
9	9	9	9	9	9	9	9	9	9
1	1	1	3	3	1	1	3	1	1
1	3	2	3	3	1	1	1	1	1
1	3	2	3	3	1	1	1	1	1
1	2	1	2	2	1	1	1	1	1
1	1	1	1	1	1	1	1	1	1
1	1	1	3	1	1	1	1	1	1
1	1	1	3	1	1	1	1	1	1
1	1	1	1	1	1	1	1	1	1
1	3	2	3	3	1	1	1	1	1
1	1	1	2	1	1	1	2	1	1
1	1	1	2	1	1	1	2	1	1
3	3	3	2	2	2	3	2	1	1
9	9	9	9	9	9	9	9	9	9
2	2	1	1	3	1	1	1	1	2
1	2	3	9	1	3	1	2	2	9
1	3	3	3	1	1	1	1	1	2
3	3	3	3	3	3	1	2	1	2
1	3	1	3	3	1	1	2	1	1
1	1	1	1	1	1	1	1	1	1
1	2	1	2	2	1	1	2	2	2
1	1	1	2	2	1	1	9	1	1
2	3	3	2	2	1	2	2	1	1
1	3	3	1	3	1	1	1	1	2
1	1	1	3	2	1	1	1	1	1
3	3	1	2	2	2	2	2	1	1
1	1	1	3	1	1	1	1	1	1
1	1	1	3	9	1	1	1	1	1
1	1	1	3	3	1	1	1	1	1
1	1	1	3	3	1	1	1	1	1
1	3	1	2	2	1	1	1	1	2
1	3	3	9	1	1	1	2	1	1

1	3	1	3	1	1	1	1	1	1
2	1	3	1	1	1	2	3	3	3
1	1	3	2	9	2	3	1	1	2
1	1	1	2	3	1	1	2	1	1
1	1	3	1	3	1	3	1	1	1
1	1	1	1	1	1	1	1	1	1
1	1	1	1	1	1	1	1	1	1
1	1	1	1	1	1	1	1	1	1
1	3	1	1	1	1	1	1	1	1
1	1	9	3	2	1	3	2	1	2
1	1	1	2	3	1	1	2	1	1
1	1	1	3	9	2	1	9	2	1
1	1	1	1	1	1	1	1	1	1
3	1	9	2	3	1	1	2	1	1
1	2	1	3	2	1	1	2	1	1
1	1	1	1	1	1	1	1	1	1
1	1	2	2	2	1	1	1	1	1
3	3	3	1	1	1	1	1	1	1
1	2	2	2	2	1	1	1	1	1
1	1	2	2	2	1	1	1	1	1
1	3	3	3	1	1	1	1	1	1
1	1	1	2	1	1	1	1	1	1
3	3	1	2	2	2	2	2	1	1
2	2	2	2	2	2	2	2	2	2
1	2	1	3	9	1	3	1	1	2
1	1	1	3	3	3	3	1	1	1
1	3	3	3	3	1	1	2	3	3
1	1	1	1	3	1	1	1	1	1
1	1	1	1	2	1	2	2	1	2
1	1	1	2	2	1	1	2	1	1
1	1	1	1	1	1	1	1	1	1
2	3	3	2	2	2	2	2	1	1
2	2	1	1	2	1	1	1	1	1
2	2	2	2	2	2	2	2	2	2
3	2	2	2	2	2	1	1	1	1
2	1	1	2	2	1	1	1	2	2
3	2	2	2	3	3	3	3	2	2
3	3	2	2	1	2	2	2	1	1
2	2	2	2	2	2	2	2	1	1
1	9	3	2	1	1	1	2	1	1
1	1	1	9	9	9	9	9	9	9
1	1	1	1	2	1	2	2	1	2
3	3	1	3	3	2	2	2	1	3
2	2	2	2	2	2	2	2	2	2
1	1	3	3	3	1	1	1	1	1
3	3	1	3	3	2	1	1	1	1
2	2	2	2	2	2	2	2	2	2

1	1	3	3	1	1	1	1	1	3
1	1	3	3	1	1	1	1	1	3
2	2	2	2	2	1	2	2	1	2
1	3	3	1	2	1	1	2	1	1
2	2	2	1	2	2	2	1	1	2
2	2	2	2	2	2	2	2	2	2
3	3	3	3	2	3	2	2	3	3
2	2	2	2	2	2	2	2	2	2
1	2	1	3	3	1	2	2	3	1
3	3	1	3	3	3	3	1	1	3
2	2	1	3	2	2	2	2	1	1
2	2	1	3	3	3	2	2	2	2
3	3	3	3	3	1	2	2	3	3
2	2	2	2	2	2	2	2	2	2
2	2	1	2	2	2	2	3	2	3
1	3	2	2	1	1	1	1	1	1
2	1	1	2	2	2	2	1	2	1
1	1	2	3	2	1	1	1	1	3
9	9	9	9	9	9	9	9	9	9
2	3	3	3	3	2	2	2	1	2
1	3	3	3	3	1	3	3	3	3
2	2	2	2	2	2	2	2	2	2
2	3	3	1	3	2	2	1	1	1
3	2	2	2	2	1	1	1	1	1
1	2	2	3	1	1	1	1	1	2
2	3	1	1	2	1	2	1	1	1
2	1	1	3	3	1	3	1	1	1
1	1	1	3	2	2	1	1	1	1
1	3	1	3	1	1	1	1	1	1
1	1	3	3	1	1	1	2	2	1
2	2	1	2	2	3	2	2	1	1
3	3	1	2	3	1	1	2	1	1
1	1	1	3	1	1	1	1	1	1
3	3	3	3	1	2	2	1	1	2
1	2	2	3	3	2	2	2	2	2
9	2	9	1	9	9	9	9	9	9
2	2	2	2	1	3	1	1	1	2
3	3	2	2	3	3	3	3	3	1
2	3	2	2	2	2	3	3	2	1
1	2	1	2	2	2	2	2	1	1
9	9	9	9	9	1	9	1	1	1
1	2	1	1	2	1	1	2	2	1
2	2	2	2	9	1	3	2	2	2
2	9	9	9	9	9	9	9	9	9
2	3	1	3	3	3	2	1	1	1
1	3	3	3	1	1	2	2	1	1
9	9	1	9	9	9	9	1	9	9

2	2	1	3	1	1	2	1	1	1
1	2	1	3	3	1	1	1	1	1
9	9	9	9	9	1	9	9	9	9
2	2	3	2	2	2	2	1	1	3
2	2	2	3	3	2	2	2	2	2
1	1	1	2	2	1	1	1	1	1
2	3	1	3	2	1	3	1	1	1
2	2	2	2	2	1	1	1	1	1
2	2	2	2	1	1	1	1	1	1
2	2	1	2	2	1	1	2	1	1
1	2	2	2	3	3	3	2	2	2
2	3	1	3	2	2	1	2	1	1
1	2	2	2	1	1	1	1	1	1
9	9	9	9	9	9	9	9	9	9
2	2	2	2	2	2	2	2	2	2
2	3	2	2	2	2	2	2	2	2
2	2	1	2	3	2	2	2	2	1
1	2	2	2	2	2	1	2	1	2
2	3	3	1	1	2	2	1	1	1
2	2	2	2	2	2	2	2	2	2
2	3	1	3	2	2	2	2	2	2
3	3	1	3	3	2	2	2	1	3
1	1	2	2	1	1	1	1	1	1
1	3	3	1	9	1	1	1	1	1
2	2	1	3	3	2	1	1	1	1
9	3	2	2	2	2	9	1	1	1
9	3	2	2	2	1	2	1	1	1
1	2	1	1	1	1	1	1	1	1
1	2	2	2	2	1	1	1	1	2
3	3	3	3	3	1	1	1	1	2
1	3	3	3	3	2	1	1	1	1
1	2	1	1	1	1	1	1	1	1
2	2	2	3	2	1	2	2	1	1
1	3	1	1	1	1	1	1	1	1
1	3	1	1	1	1	1	1	1	1
9	9	9	9	9	9	9	9	9	9
1	3	1	3	1	1	1	1	1	1
1	2	1	2	2	1	1	1	1	1
1	2	1	2	2	1	1	1	1	1
1	3	2	3	3	1	1	2	1	2
3	2	1	2	1	9	9	9	9	9
1	2	1	2	1	1	1	1	1	1
1	2	1	2	1	1	1	1	1	1
3	3	3	1	1	1	1	1	1	1
1	3	3	3	1	1	1	1	1	1
2	3	3	3	2	1	1	1	1	1
1	2	1	2	2	1	2	2	1	1

1	3	3	1	9	1	1	1	1	1
2	2	2	2	2	3	3	3	2	2
1	3	3	3	3	1	3	3	1	3
3	3	1	3	3	1	1	1	1	3
3	3	3	3	3	1	1	1	3	3
1	3	1	3	1	1	1	1	1	1
3	3	1	3	3	1	1	1	1	3
3	3	1	3	3	1	1	1	1	3
1	3	3	3	3	1	1	1	1	1
1	3	1	3	2	1	1	1	1	1
1	3	3	3	1	1	1	1	1	1
1	3	3	3	1	1	1	1	1	1
3	9	3	3	3	1	1	1	1	1
1	2	1	1	1	1	1	2	1	2
1	3	2	3	3	1	1	1	1	1
1	3	2	3	3	1	1	1	1	1
1	1	1	3	3	1	1	1	1	1
1	1	1	3	3	1	1	1	1	1
1	3	1	3	3	1	1	1	1	1
3	3	1	3	1	3	2	1	1	3
1	3	1	3	3	1	1	2	1	9
1	3	1	3	3	1	1	1	1	1
1	2	1	1	2	2	3	2	1	2
1	3	2	2	2	1	1	1	1	1
1	1	1	1	1	1	1	1	1	1
1	1	1	1	1	1	1	1	1	1
2	2	2	2	1	1	1	1	1	2
3	3	3	3	3	1	1	3	3	1
9	9	9	9	9	9	9	9	9	9
9	9	9	9	9	9	9	9	9	9
9	9	9	9	9	9	9	9	9	9
9	9	9	9	9	9	9	9	9	9
3	3	3	3	3	1	1	1	3	1
9	9	9	9	9	9	9	9	9	9
9	9	9	9	9	9	9	9	9	9
9	9	9	9	9	9	9	9	1	9
9	9	9	9	9	9	9	9	9	9
9	9	9	9	9	9	9	9	9	9
9	9	9	9	9	9	9	9	9	9
9	9	9	9	9	9	9	9	9	9
3	3	3	3	1	2	2	1	1	2
1	1	1	3	3	1	1	1	1	1
1	1	1	2	2	1	1	1	1	1
1	1	1	1	3	1	1	1	1	1
2	2	3	2	1	2	2	2	1	1
2	2	2	3	2	1	2	2	1	1
3	3	3	1	1	1	1	1	1	1

1	1	3	2	2	1	2	1	2	2
1	2	1	3	1	1	1	2	2	1
1	3	3	3	1	1	3	2	2	2
3	3	1	3	2	1	2	2	3	3
3	3	3	3	1	1	1	1	1	3
1	3	2	1	2	1	1	1	2	1
2	2	1	1	1	2	2	2	2	1
2	2	2	2	2	2	2	2	2	2
1	3	1	3	3	1	1	1	1	1
3	1	1	1	1	3	2	2	3	2
1	1	1	2	2	1	1	1	1	1
1	1	2	2	2	1	1	1	2	2
9	9	9	9	9	9	9	9	9	9
2	2	2	2	2	2	2	2	2	2
2	2	1	2	3	3	2	1	1	1
3	3	1	2	3	1	1	1	1	1
2	2	2	2	2	2	2	2	2	2
1	2	2	2	2	1	1	1	1	1
2	2	2	3	1	2	2	2	2	1
3	3	3	3	3	3	2	1	2	3
1	1	2	2	2	2	2	2	2	2
2	2	2	2	2	2	2	2	2	2
1	1	1	1	2	2	2	2	2	2
1	2	1	2	2	1	2	1	2	1
2	2	1	2	2	1	1	1	1	1
1	1	1	1	1	1	1	1	1	1
1	1	1	2	2	3	3	1	2	3
2	2	2	2	2	3	3	2	2	2
2	2	1	2	3	3	2	1	1	1
1	1	1	1	1	1	1	2	1	1
1	1	1	1	1	1	1	1	1	1
2	2	3	2	3	1	2	3	2	1
2	3	1	2	1	2	1	2	1	2
1	1	1	2	2	1	1	1	1	1
2	2	2	2	2	2	2	2	2	2
2	2	2	2	2	2	2	2	2	2
1	1	1	1	1	1	1	1	1	1
1	1	1	1	1	1	1	1	1	1
2	2	2	2	2	2	2	2	2	2
2	1	2	3	1	1	1	1	1	1
2	2	2	2	2	1	1	3	3	2
2	3	3	3	3	3	3	2	2	2
2	2	2	2	2	2	2	2	2	2
2	3	1	3	2	2	2	2	2	1
3	2	2	2	2	2	2	2	2	2
1	1	1	3	1	1	1	1	1	1
2	1	1	2	2	1	1	2	1	1

2	1	1	2	2	1	1	2	1	1

ExamineAr	BreastSize	Nippledevi	Raisopposi	Undressto	HeardCerv	CervicalCA	PapSmear	HPVandVa	Papdetect
2	2	2	2	3	2	2	2	2	2
1	2	2	1	1	2	2	2	2	2
1	1	2	1	2	2	2	1	2	2
9	1	1	1	1	1	1	2	1	2
9	9	9	9	9	2	2	2	2	2
1	2	2	1	1	1	1	2	2	2
2	2	2	2	2	2	2	2	2	9
1	1	1	1	1	1	2	2	2	1
1	9	9	1	9	2	2	2	2	2
9	9	9	9	9	2	2	2	2	2
2	1	2	2	2	2	2	2	9	2
2	1	2	2	3	2	2	2	3	3
2	2	2	2	2	1	2	2	1	1
1	2	2	2	1	1	3	1	3	1
2	2	2	1	1	2	2	2	2	2
1	1	2	1	2	1	2	2	2	1
1	1	2	1	3	1	1	1	3	2
1	1	2	1	2	1	2	2	2	1
2	1	2	1	2	1	2	2	2	1
9	2	1	1	1	2	2	1	1	2
1	1	1	1	1	1	1	2	2	1
1	1	1	1	1	1	1	2	2	1
9	9	9	1	2	2	2	2	2	3
1	1	2	1	2	1	1	3	3	1
2	2	2	2	2	2	2	2	2	2
2	1	1	1	2	2	2	2	2	2
2	2	2	2	2	3	3	3	3	2
1	2	2	2	3	3	3	1	2	1
1	2	2	1	1	2	2	2	2	2
2	2	2	1	1	2	2	9	2	2
2	2	2	1	1	2	2	2	2	2
1	1	1	1	1	1	1	2	2	1
2	1	1	1	1	2	2	2	2	1
1	1	1	1	1	1	1	1	1	1
1	1	1	1	1	1	1	2	2	1
1	1	1	1	1	1	1	2	1	1
1	1	1	1	1	1	1	2	2	1
1	2	2	1	1	1	1	1	1	1
1	2	2	1	1	9	9	9	9	2
1	1	9	1	1	2	2	2	2	2
1	2	2	1	1	2	2	2	2	2
2	2	1	1	2	1	2	2	3	1
3	1	2	2	3	9	9	9	9	1
9	1	1	1	3	1	1	1	3	1
1	1	1	3	3	2	2	2	2	9
9	9	9	9	9	1	2	2	1	1

3	1	2	1	1	1	1	2	2	1
2	1	2	1	1	1	3	2	1	2
1	9	9	1	9	1	1	1	1	1
1	1	1	1	1	1	1	3	1	1
1	9	1	1	1	1	1	1	1	1
1	1	1	1	3	1	1	1	1	1
3	1	3	1	1	1	1	1	1	1
1	1	1	1	1	1	1	1	2	1
1	1	9	1	9	1	1	2	2	1
3	1	2	1	1	1	1	1	2	1
9	9	9	9	9	2	2	2	2	2
1	1	1	1	1	1	1	2	1	2
2	1	2	1	2	1	1	3	2	1
9	1	9	9	1	2	2	2	2	2
1	2	1	2	2	2	2	2	2	2
2	1	2	1	3	1	1	1	2	1
1	1	2	1	1	1	1	2	1	1
1	1	1	1	1	1	1	1	1	1
3	1	1	2	3	1	2	2	2	1
2	2	2	2	2	1	1	1	2	1
9	9	9	9	9	1	1	2	1	1
3	1	3	3	3	1	1	2	2	1
9	9	9	9	9	1	1	2	1	1
1	1	1	1	1	1	2	2	1	1
1	1	9	1	9	1	1	2	3	9
1	1	1	1	1	1	1	2	1	1
1	2	3	1	1	9	9	9	9	9
2	2	1	2	3	1	2	2	2	1
2	1	2	1	1	2	3	3	3	2
1	1	1	1	3	1	1	1	1	1
1	1	1	1	1	1	1	1	1	1
1	3	1	3	3	1	1	1	2	1
1	1	1	1	1	1	1	9	9	1
1	1	1	1	2	1	1	2	1	9
1	1	2	1	1	1	1	1	1	1
1	2	1	1	1	2	2	2	1	9
1	2	2	2	2	9	9	9	9	9
1	2	2	1	1	9	9	9	9	1
1	1	1	1	3	3	1	1	1	1
1	1	1	1	1	1	1	1	1	1
1	1	1	1	1	1	1	1	1	1
1	1	1	1	3	2	2	3	2	1
3	1	3	1	1	1	1	2	2	1
1	1	1	1	1	1	1	1	1	1
3	2	2	1	1	2	2	2	2	1
3	2	2	3	3	1	1	2	1	9
9	9	9	9	9	2	2	2	9	9

1	2	2	1	1	1	1	2	1	1
1	2	2	1	1	1	1	1	1	1
1	1	1	1	1	1	1	1	1	2
1	2	2	1	1	1	9	1	1	1
1	2	2	1	1	2	2	2	2	9
1	2	2	1	2	2	1	2	2	1
1	1	1	1	1	1	1	1	1	1
1	1	1	1	2	1	1	1	2	1
1	2	2	1	1	1	1	1	1	1
1	1	1	1	1	1	1	1	1	1
1	1	1	1	2	2	2	3	1	1
2	1	2	1	2	1	9	1	3	1
2	2	2	1	1	1	1	3	3	1
1	1	1	1	1	3	3	3	3	1
2	3	2	3	2	2	2	1	3	2
1	2	2	3	3	1	2	2	2	3
2	2	2	1	1	2	2	2	1	2
3	1	2	1	1	2	2	2	2	2
1	2	2	1	2	3	2	2	2	1
9	9	9	9	9	2	2	2	2	2
2	2	1	1	2	2	2	2	2	1
3	1	3	1	3	2	2	2	2	1
2	2	2	2	2	2	2	2	2	2
9	9	9	9	9	2	2	2	2	3
3	2	2	1	1	2	3	3	1	3
2	2	2	1	2	2	2	2	2	2
2	2	1	1	3	1	1	1	2	1
2	2	2	2	2	1	1	1	1	1
2	2	2	2	2	2	2	2	2	2
3	2	2	3	3	2	2	2	2	2
2	2	2	2	2	2	2	2	2	2
2	2	3	2	2	3	3	2	3	2
3	2	2	2	2	2	2	2	2	1
2	2	2	2	2	3	2	2	2	2
2	2	2	2	2	2	2	2	2	2
2	1	1	1	1	2	2	2	2	2
2	1	2	2	2	1	1	2	2	2
1	2	2	1	3	2	2	2	2	2
9	1	9	1	9	1	1	2	2	9
2	1	2	1	2	1	1	3	2	1
3	1	2	1	2	2	2	2	2	2
3	3	2	1	2	3	2	2	2	2
2	2	2	2	2	3	3	2	3	2
2	1	1	2	1	1	1	2	2	1
2	2	2	2	2	3	3	3	3	2
1	1	2	1	1	1	2	2	2	1
2	1	2	3	3	2	2	2	2	2

3	3	3	3	3	2	2	2	2	2
2	2	1	2	1	2	2	2	3	1
1	2	2	1	2	1	1	2	2	9
3	3	2	1	2	3	2	2	2	2
1	1	1	1	1	3	2	2	2	9
2	2	2	1	1	2	2	2	2	2
3	2	2	3	3	2	2	2	2	2
2	1	2	1	1	2	3	2	2	2
2	2	2	2	2	2	2	2	2	2
2	2	2	2	2	2	2	2	2	2
2	3	1	2	3	2	2	2	3	2
2	3	1	2	3	2	2	2	9	2
9	9	9	9	9	2	2	2	2	9
2	2	1	3	2	2	2	2	2	1
1	2	2	1	2	1	1	2	2	2
1	2	2	1	2	1	1	2	2	2
9	9	9	9	9	1	1	3	1	9
1	1	3	1	3	2	2	2	2	2
9	9	9	9	9	1	2	2	2	9
1	1	1	1	1	1	2	2	2	1
1	1	1	1	1	9	1	2	2	1
1	1	1	1	1	2	2	2	2	1
1	1	1	1	1	2	2	2	2	1
1	1	1	1	1	2	2	2	2	1
1	1	1	1	1	2	2	2	2	1
1	1	1	1	1	2	9	2	2	1
1	1	1	1	1	1	2	2	2	1
1	1	1	1	1	1	1	2	2	1
1	1	1	1	1	2	3	2	2	1
1	1	1	1	3	1	2	2	2	1
9	2	3	2	2	1	1	2	2	3
3	1	1	2	2	2	2	2	2	2
9	9	9	9	9	9	9	9	9	1
1	1	1	1	3	2	2	2	2	1
1	1	1	1	3	1	2	2	2	1
1	1	1	1	2	2	2	2	2	1
1	1	1	1	1	1	1	2	2	1
1	1	1	1	1	1	2	2	2	1
1	1	1	1	2	2	2	2	2	1
1	1	1	1	1	2	1	2	2	1
1	1	1	1	1	2	2	2	2	1
1	1	1	1	1	2	2	2	2	1
9	9	9	9	9	9	9	9	9	1
1	1	1	1	3	1	2	2	2	1
1	1	1	1	3	1	2	2	2	1
1	1	1	1	3	1	2	2	2	1
1	1	1	1	3	1	2	2	2	1

1	1	1	1	3	1	2	2	2	1
1	1	1	1	1	2	2	2	2	1
1	1	1	1	1	2	2	2	2	1
1	1	1	1	3	1	1	2	2	1
1	1	1	1	1	2	2	2	2	1
1	1	1	1	1	1	1	1	2	1
1	1	1	1	1	2	1	2	2	1
1	1	1	1	1	1	1	1	1	1
1	1	1	1	1	1	1	1	1	1
2	1	1	1	3	1	1	2	1	1
1	1	1	1	1	9	9	9	9	9
1	1	1	1	1	1	1	1	2	1
1	1	1	1	1	1	1	1	1	1
1	1	1	1	1	1	2	2	2	1
2	1	1	2	2	3	3	3	3	1
1	1	2	1	1	2	2	2	2	1
1	1	1	1	1	1	2	2	2	2
1	1	1	1	1	1	2	9	1	1
2	1	1	1	2	1	2	2	2	2
2	1	2	2	1	1	2	2	2	1
1	1	2	3	3	1	1	3	3	2
2	2	2	2	2	1	2	2	2	2
3	2	1	2	1	2	2	2	2	1
2	1	2	2	2	1	3	3	2	1
2	2	2	2	2	2	2	2	2	2
2	2	2	2	2	2	2	2	2	2
2	2	2	2	1	1	2	2	2	1
2	2	2	2	2	2	2	2	2	2
2	2	2	2	2	2	2	2	2	2
2	2	2	2	2	1	2	2	2	2
1	2	1	2	2	1	1	2	3	1
1	2	1	2	2	1	1	2	3	1
2	1	1	1	2	1	2	2	2	2
1	1	1	1	1	1	1	1	1	1
1	1	2	2	2	1	1	3	1	1
2	2	2	2	2	1	1	1	2	2
1	1	3	2	1	2	2	2	2	2
1	3	1	1	2	1	1	2	2	3
2	2	2	2	2	2	2	2	2	1
1	2	1	2	2	1	2	2	2	1
2	2	2	2	2	1	1	1	1	1
2	2	2	2	1	1	2	2	2	1
9	1	9	9	9	2	2	3	2	1
9	1	9	9	9	2	2	3	2	1
1	1	1	1	1	1	1	2	1	1
1	1	2	3	3	1	1	2	2	1
9	9	9	9	9	3	1	2	2	2

2	1	1	2	3	2	2	2	2	2
2	1	1	2	3	2	2	2	2	2
2	2	2	2	2	2	2	2	2	1
2	2	2	2	2	2	2	1	2	1
2	2	2	2	2	2	2	2	2	2
1	1	1	1	2	1	1	1	1	2
1	1	2	1	1	2	2	2	2	1
1	2	1	2	2	2	2	2	2	2
1	1	1	1	2	1	1	2	2	1
3	3	3	3	3	2	2	2	2	3
2	2	2	2	2	2	2	2	2	2
1	1	1	1	2	1	2	3	1	1
1	1	1	1	2	1	2	3	1	1
2	1	1	1	2	1	3	3	3	2
1	2	2	2	3	2	2	2	2	2
2	1	2	2	3	2	3	3	3	1
1	2	1	3	2	1	3	3	2	2
2	2	2	2	2	2	2	1	1	2
2	3	2	1	3	2	2	2	2	2
3	1	1	3	2	2	1	1	1	1
1	1	1	2	3	2	2	2	1	1
1	1	2	1	2	2	3	2	3	1
2	1	1	1	2	1	3	3	2	2
2	2	2	2	2	2	2	2	2	1
9	1	9	9	9	3	3	3	2	9
2	2	2	2	2	2	2	2	2	2
2	2	2	2	2	2	2	2	9	2
9	1	3	2	3	1	2	3	3	1
9	9	9	9	9	2	2	3	3	2
2	2	2	2	1	2	3	3	3	2
9	9	9	9	9	3	1	1	3	9
9	9	9	1	9	2	2	2	2	2
3	1	1	3	3	2	2	2	2	3
1	1	2	1	2	1	1	2	2	2
9	9	9	9	9	1	3	3	3	1
2	2	3	2	2	3	3	3	3	2
1	1	2	1	2	2	2	2	2	1
2	1	1	3	2	1	2	2	2	1
1	1	1	1	1	1	3	3	3	1
1	1	3	2	3	2	1	2	2	2
1	1	1	3	3	1	1	2	2	1
2	2	1	1	3	1	1	2	2	1
1	1	1	1	1	1	1	1	1	1
2	2	2	2	2	1	2	2	2	2
9	9	9	9	9	1	2	2	1	2
1	2	3	2	3	1	2	2	2	1
1	2	3	1	2	2	2	2	2	2

9	9	9	9	9	9	9	9	9	9
9	9	9	9	9	9	9	9	9	9
2	1	1	1	1	1	1	1	2	1
1	1	1	2	1	1	1	2	1	2
2	1	2	2	2	2	2	2	2	1
3	1	2	1	3	2	2	2	1	3
1	1	1	1	1	1	1	1	1	1
1	3	1	1	3	1	1	1	1	1
1	1	1	1	1	1	2	2	2	2
2	2	2	2	2	1	2	1	2	2
2	2	2	2	2	3	3	3	3	2
1	1	2	1	2	1	1	1	2	1
9	9	9	9	9	9	9	9	9	9
3	1	1	1	3	2	2	1	1	1
3	1	1	1	1	1	1	2	1	1
1	2	2	1	3	9	9	9	9	9
2	2	1	1	1	1	1	1	2	2
2	3	2	2	2	2	2	1	2	1
1	1	1	1	3	1	2	3	1	2
9	9	9	9	9	9	9	9	9	9
1	3	1	2	2	2	2	2	2	9
1	1	2	1	2	1	2	2	2	2
1	2	1	2	2	1	1	2	3	2
2	2	2	2	2	1	2	2	2	2
2	2	2	2	2	1	2	2	2	2
1	1	3	1	1	9	2	3	3	1
2	1	2	1	1	1	1	1	1	1
1	1	1	1	3	3	2	3	2	1
1	1	1	1	1	1	2	1	1	1
1	1	2	1	9	1	2	2	2	1
2	1	2	1	2	1	3	3	1	2
2	1	1	3	3	2	2	2	2	3
9	9	9	9	9	1	1	1	1	9
1	3	3	1	3	2	2	2	2	9
2	1	1	1	1	1	2	2	2	1
1	1	1	1	3	1	1	3	3	2
1	1	2	1	3	1	1	1	2	1
1	1	1	3	3	3	1	1	1	1
1	1	1	1	1	1	1	1	2	1
1	2	2	1	1	1	2	2	2	1
3	3	3	3	3	1	1	1	2	1
1	1	1	1	1	1	1	1	2	1
3	3	3	2	2	1	1	1	1	1
2	2	2	2	2	2	2	2	2	2
3	1	2	2	1	1	1	1	1	2
1	1	1	1	1	1	1	2	2	1
1	1	2	1	3	1	2	2	2	2

1	1	1	1	2	3	2	2	2	2
1	1	1	1	2	3	2	2	2	2
9	9	9	9	9	1	1	1	1	1
1	1	1	1	2	1	2	2	2	2
9	9	9	1	9	1	1	1	3	9
1	1	1	2	2	1	2	2	2	9
1	1	1	1	2	1	1	2	2	2
2	1	9	9	9	1	2	2	1	1
1	1	1	1	1	1	2	2	2	1
3	1	2	1	1	1	2	2	2	1
2	2	2	2	3	2	2	2	2	1
2	2	1	2	2	1	1	1	2	2
1	1	1	1	2	1	1	2	1	1
3	3	2	2	3	2	2	2	2	2
2	2	2	2	1	2	2	2	2	1
1	1	2	1	2	2	9	2	2	2
2	2	2	2	1	2	2	2	2	2
3	2	1	2	3	1	2	2	2	2
1	1	1	1	1	1	1	1	2	2
2	2	1	2	2	2	2	2	2	2
1	1	2	1	2	1	2	1	1	1
3	3	2	1	1	2	2	2	2	1
2	1	1	2	1	1	1	2	2	1
2	1	1	2	2	1	1	2	1	1
2	2	2	2	2	2	2	2	2	2
2	2	2	2	2	2	2	2	2	2
1	1	1	1	2	1	2	2	2	1
2	3	2	2	3	2	3	3	3	2
2	1	1	2	2	1	1	3	3	2
1	1	2	1	2	2	2	2	2	1
1	2	1	2	1	3	3	1	1	1
1	2	1	2	1	2	2	2	2	1
1	1	3	3	3	2	2	2	3	1
2	2	2	2	2	2	2	2	2	2
2	2	2	2	2	2	2	2	2	2
2	1	2	9	1	1	1	2	2	1
1	1	1	1	2	3	2	2	2	2
9	1	9	1	9	3	1	2	3	2
2	2	2	2	2	2	2	2	2	2
1	1	1	3	3	2	2	2	2	2
2	2	2	2	2	3	3	3	2	2
2	1	1	2	1	2	2	2	2	2
2	1	1	1	2	1	2	2	1	1
2	2	2	2	2	2	2	2	2	2
2	1	1	2	1	2	2	2	1	1
1	1	2	1	2	1	1	2	3	1
2	2	2	2	2	1	3	2	2	2

3	1	1	2	3	1	2	2	2	1
2	1	1	2	3	1	1	2	1	1
3	3	3	3	3	1	1	2	1	2
2	1	2	2	2	3	2	3	3	2
2	1	2	2	2	3	2	2	1	2
2	1	2	2	3	1	3	2	1	1
9	2	2	9	9	9	9	9	9	9
1	1	2	1	2	1	1	1	1	1
1	1	2	1	2	1	1	1	3	1
3	1	2	2	1	1	2	2	2	2
2	1	2	2	1	2	2	2	2	3
2	1	1	1	1	2	2	2	2	1
1	1	2	1	2	2	2	2	2	2
2	2	2	2	2	2	2	2	2	1
2	2	2	2	2	2	2	2	2	2
1	1	9	2	2	9	9	9	9	1
2	1	1	2	2	2	2	2	2	1
1	1	2	2	1	3	2	2	2	2
1	1	2	2	1	3	2	2	2	2
9	9	9	9	9	1	2	3	2	2
2	1	1	2	2	1	1	1	2	1
2	2	2	2	2	1	3	2	2	2
9	9	9	9	9	9	9	9	9	9
2	2	2	2	2	2	1	1	1	1
1	1	1	1	1	1	1	2	2	1
2	9	2	9	2	9	9	9	9	9
1	2	1	1	3	3	3	1	1	1
9	9	9	9	9	9	9	9	9	9
2	2	2	2	2	1	1	1	1	2
3	1	1	2	2	1	1	2	2	2
2	2	2	2	2	2	2	2	2	9
3	2	2	3	2	9	9	9	9	9
2	2	2	2	2	2	1	1	2	2
1	1	2	1	3	2	2	2	2	2
1	1	1	1	2	1	2	2	2	2
1	1	1	1	1	1	1	1	1	2
2	3	2	3	2	1	2	3	3	3
1	2	1	2	1	1	2	3	2	2
2	2	2	2	2	9	9	9	9	9
1	1	1	1	2	1	2	2	2	1
2	2	2	1	2	2	3	3	2	2
1	2	2	1	1	2	2	2	2	2
1	1	2	3	1	1	3	3	3	1
3	3	3	3	3	1	2	2	2	3
9	9	9	9	9	9	9	9	9	9
1	1	1	1	9	3	2	2	2	1
1	1	1	1	1	2	2	2	2	1

1	1	1	1	1	3	2	2	2	9
1	1	1	1	1	2	2	2	2	1
1	1	1	1	1	2	2	2	2	1
1	1	1	1	1	1	2	2	2	1
1	1	1	1	1	2	2	2	2	1
1	1	1	1	3	1	1	2	2	1
1	1	1	1	1	2	2	2	2	1
1	1	1	1	1	2	2	2	2	1
1	1	1	1	1	2	2	2	2	1
1	1	1	1	3	1	2	2	2	1
1	1	1	1	1	3	2	2	2	9
1	1	1	1	3	2	2	2	2	1
1	1	1	1	1	1	1	2	2	1
1	1	1	1	1	2	2	2	2	1
1	1	1	1	1	9	2	2	2	1
1	1	1	1	1	1	1	2	2	1
2	1	1	1	2	2	2	2	2	1
1	1	1	1	1	1	2	2	2	1
1	1	1	1	1	1	2	2	2	1
1	1	1	1	1	1	2	2	2	1
1	1	1	1	1	1	2	2	2	1
1	1	1	1	1	1	2	2	2	1
1	1	1	1	1	2	2	2	2	1
1	1	1	1	1	1	1	2	1	1
1	1	1	1	3	2	2	2	2	1
1	1	1	1	3	2	2	2	2	1
1	1	1	1	1	2	2	2	2	1
1	1	1	1	1	9	2	2	2	1
1	1	1	3	1	2	1	2	2	1
1	1	1	1	3	2	2	2	2	1
1	1	1	1	3	2	1	2	2	1
1	1	1	1	1	2	2	2	2	1
1	1	1	1	3	2	1	2	2	1
1	1	1	1	1	2	2	2	2	1
1	1	1	1	3	1	1	2	2	1
1	1	1	1	3	1	1	2	2	1
1	1	1	3	3	1	1	2	2	1
1	1	1	1	1	2	1	2	2	1
1	1	1	1	1	2	2	2	2	1
1	1	1	1	9	1	1	1	2	1
1	1	1	1	1	1	2	2	2	1
1	2	2	2	2	3	3	3	3	2
1	1	2	1	1	1	2	2	1	1
2	2	2	2	2	3	3	3	3	2
9	9	9	9	9	2	2	2	2	2
2	2	2	1	2	2	2	2	2	2
1	1	2	2	2	1	2	1	2	9

2	2	2	2	2	3	2	2	2	2
2	1	1	1	2	1	1	2	1	1
2	1	1	1	2	1	1	2	1	1
1	1	1	1	3	1	1	2	2	1
1	2	2	1	3	3	2	3	3	2
1	1	2	1	2	2	2	2	2	2
2	2	2	2	2	3	1	1	3	2
2	2	2	2	2	3	2	9	3	2
1	2	2	1	1	1	1	2	2	1
2	2	2	2	2	1	2	2	2	2
2	1	1	1	2	1	1	1	1	1
9	9	9	9	9	1	2	1	3	9
1	9	9	1	1	1	1	1	1	1
2	1	2	2	2	2	2	2	2	2
9	9	9	9	9	1	2	2	2	9
9	9	9	9	9	1	1	1	2	9
9	9	9	9	9	2	2	2	2	9
9	9	9	9	9	2	2	2	2	1
9	9	9	9	9	2	2	2	2	9
3	2	2	1	2	1	3	2	2	1
9	3	3	3	3	1	3	1	1	2
1	3	3	1	3	1	2	1	2	9
2	1	2	1	1	1	1	2	2	1
9	9	9	9	9	2	3	2	2	1
9	9	9	9	9	2	2	2	2	9
9	9	9	9	9	9	9	9	9	9
9	9	9	9	9	1	1	1	1	1
2	2	1	1	1	2	2	3	2	9
2	3	1	1	1	2	2	3	1	1
9	9	9	9	9	1	2	1	3	9
9	9	9	9	9	1	1	2	2	1
2	2	2	2	2	2	2	2	2	2
2	2	2	2	2	2	2	2	2	2
3	3	1	1	1	1	1	1	1	1
2	2	1	3	1	3	2	2	2	2
1	1	2	2	3	1	1	2	3	2
1	1	2	2	2	1	1	2	2	1
9	1	1	3	3	1	3	2	2	1
2	2	2	2	2	2	2	3	2	2
2	1	1	3	1	1	3	2	3	1
9	9	9	9	9	3	2	2	2	1
3	1	1	1	1	1	1	1	1	1
2	1	2	1	2	2	2	2	2	2
1	1	1	1	2	3	3	3	3	1
3	1	2	3	3	2	2	2	2	2
2	1	1	3	2	1	1	2	2	2
2	1	1	2	1	2	2	2	2	1

9	1	9	9	9	1	1	1	1	1
1	2	3	3	3	1	1	3	1	1
2	1	1	2	3	2	2	2	2	1
1	1	2	1	1	2	2	2	2	1
1	2	2	3	1	1	3	1	2	2
2	2	2	2	2	3	3	2	2	2
2	2	2	2	2	2	2	2	2	2
1	2	2	2	2	2	1	2	2	2
1	2	1	2	2	2	2	2	2	2
2	2	2	2	2	3	2	2	2	2
2	2	2	2	2	3	3	3	2	2
2	2	2	2	2	1	1	1	2	1
2	2	2	2	2	2	2	2	2	2
2	2	2	2	2	2	2	2	2	2
1	1	2	2	2	1	1	2	2	1
2	2	2	2	2	2	2	2	2	2
2	1	2	1	3	3	3	3	3	2
2	2	2	2	2	2	2	2	2	2
1	2	3	3	1	1	2	2	2	2
2	1	2	2	1	1	1	1	2	1
2	1	2	1	2	2	2	2	1	2
2	1	1	2	1	1	1	2	2	1
1	1	1	1	2	1	1	2	2	1
1	1	1	1	2	2	2	2	2	2
2	2	1	2	3	2	2	2	2	2
2	1	2	2	2	2	2	2	2	2
1	1	1	1	3	1	1	2	1	1
1	1	3	1	3	1	2	1	2	1
1	2	2	2	2	1	1	2	2	1
3	3	3	2	2	1	2	2	2	1
2	1	1	2	2	9	9	9	9	2
2	9	9	2	3	2	2	2	2	2
2	2	2	2	3	2	2	2	2	2
2	2	2	3	2	2	2	2	2	1
1	1	1	1	1	2	2	2	2	2
9	9	9	9	9	3	2	1	2	2
2	2	2	1	2	2	3	2	2	2
1	1	1	1	1	1	1	1	1	1
2	2	2	2	2	2	2	2	2	2
1	1	1	1	2	1	1	2	2	1
1	1	2	1	1	9	9	2	2	1
2	1	1	1	1	2	2	3	1	1
1	3	1	1	1	2	2	2	2	1
2	1	2	1	2	1	9	1	3	1
2	1	1	1	1	1	1	1	2	1
2	2	2	2	2	2	2	2	2	2
9	9	9	9	9	2	2	2	1	1

9	9	9	9	9	1	1	2	2	2
9	9	9	9	9	2	2	2	2	9
9	9	9	9	9	2	2	2	2	1
9	9	9	9	9	9	9	9	9	9
9	9	9	9	9	2	1	2	2	1
9	9	9	9	9	1	2	2	1	1
9	9	9	9	9	2	2	2	2	9
9	9	9	9	9	2	2	2	2	1
9	9	9	9	9	2	2	2	2	1
9	9	9	9	9	1	1	1	1	1
9	9	9	9	9	2	2	2	2	9
9	9	9	9	9	1	1	1	1	1
9	9	9	9	9	2	2	2	2	1
9	9	9	9	9	1	1	1	1	9
9	9	9	9	9	2	1	2	3	9
9	9	9	9	9	2	2	2	2	9
9	9	9	9	9	1	2	2	1	9
9	9	9	9	9	1	2	2	2	1
2	2	2	2	2	3	3	3	3	2
1	1	9	1	2	1	1	1	2	1
1	1	1	1	1	1	1	1	1	1
2	1	3	2	2	2	2	2	2	2
3	3	2	2	2	2	2	9	2	2
1	1	1	1	1	3	1	1	1	1
2	3	2	3	2	2	2	9	2	3
1	1	2	3	3	2	1	2	1	1
2	2	2	2	2	1	1	1	2	2
3	2	2	1	1	3	2	2	2	2
2	2	2	2	2	2	2	2	2	2
3	1	2	1	2	1	3	3	3	2
3	2	2	3	1	9	9	9	9	1
3	1	1	3	1	2	2	2	2	2
9	9	9	9	9	2	2	2	2	1
3	3	1	3	3	1	1	2	2	2
1	1	2	1	1	2	2	2	1	1
2	2	2	2	3	2	2	2	2	1
2	2	2	2	2	2	2	2	2	2
2	3	2	1	2	9	9	9	9	2
1	1	2	1	2	1	1	1	1	1
1	1	1	1	2	1	2	1	9	9
2	2	2	2	2	1	2	1	2	2
3	1	1	3	1	1	1	1	1	1
1	1	1	1	1	9	9	9	9	9
1	2	3	1	1	1	3	1	1	1
2	2	2	2	2	2	2	2	2	2
9	9	9	9	9	1	2	2	2	2
3	1	2	2	2	2	3	3	2	2

2	1	1	1	3	2	2	2	2	2
9	9	9	9	9	9	9	9	9	9
1	3	1	1	1	2	2	2	2	1
9	9	9	9	9	9	9	9	9	9
2	2	2	2	2	1	2	1	1	1
2	2	2	2	2	1	2	1	2	1
9	9	9	9	9	9	9	9	9	9
2	1	1	1	3	2	2	2	2	2
1	1	2	2	3	1	1	1	1	2
1	1	1	1	1	2	2	2	2	1
1	1	1	1	1	1	1	1	1	1
2	1	1	2	1	1	1	2	1	2
3	3	3	2	2	2	3	3	3	3
1	2	3	1	2	9	9	9	9	9
2	2	2	2	2	9	9	9	9	9
1	3	2	3	1	1	3	1	2	9
1	1	1	1	1	1	9	2	2	1
9	9	9	9	9	9	9	9	9	9
2	1	1	2	3	1	2	2	1	1
3	3	3	3	3	1	2	2	2	1
3	3	3	3	3	1	2	2	2	3
3	3	3	2	2	2	3	3	3	3
2	1	2	2	3	1	1	1	1	1
3	1	2	2	3	1	2	2	1	3
2	2	2	2	2	1	2	2	2	2
1	3	1	1	1	2	2	2	2	1
3	3	3	3	3	1	1	1	1	1
3	1	1	1	1	2	2	1	1	3
1	2	2	3	1	2	3	3	3	2
1	1	1	1	1	1	2	2	2	1
1	2	1	3	1	2	2	2	1	9
9	9	9	9	9	1	1	1	2	9
1	2	2	1	1	1	3	3	3	1
9	1	1	2	9	3	2	2	2	2
9	9	1	1	9	3	2	2	9	2
1	1	1	1	1	1	2	2	1	1
1	1	1	1	1	2	2	2	2	2
9	9	9	9	9	9	9	9	9	9
9	9	9	9	9	9	9	9	9	9
9	9	9	9	9	9	9	9	9	9
9	9	9	9	9	9	9	9	9	9
3	2	2	3	2	1	1	1	1	3
2	3	2	1	3	2	2	2	1	1
9	9	9	9	9	9	9	9	9	9
2	1	3	2	3	2	2	2	1	1
2	2	2	2	2	2	3	2	2	2
1	9	1	3	3	2	2	2	2	1

3	3	3	3	3	2	2	2	2	3
1	1	1	1	1	1	3	3	3	2
3	1	3	2	3	1	2	1	1	3
1	1	1	2	2	1	3	3	3	1
2	2	2	2	2	2	2	2	2	2
9	9	1	9	1	1	2	1	1	1
9	9	9	9	9	9	9	9	9	9
2	1	2	1	1	1	1	2	3	1
3	3	3	3	3	2	2	2	2	3
2	1	2	1	2	1	3	2	2	2
3	3	3	3	3	2	2	2	2	3
1	1	1	1	1	1	3	2	2	1
2	2	2	3	3	2	3	2	2	2
2	2	1	2	3	2	3	2	2	2
2	2	2	2	3	2	3	2	2	2
2	2	2	2	3	2	3	2	2	2
2	2	2	2	3	2	3	2	2	2
2	2	1	2	3	2	3	2	2	2
2	2	2	2	3	2	3	2	2	2
3	1	1	3	3	2	2	2	2	1
1	2	3	2	3	2	2	2	2	1
3	3	3	3	3	9	9	9	9	2
3	1	1	1	2	2	2	2	2	2
3	2	2	2	2	2	2	2	2	2
3	3	3	3	3	2	2	2	2	3
2	2	2	2	3	2	3	2	2	2
9	9	9	9	9	2	2	2	2	9
3	3	3	1	1	2	2	2	2	1
9	9	9	9	9	1	1	1	1	1
3	3	3	3	3	2	2	2	2	2
1	2	1	3	1	2	2	2	2	1
3	3	2	3	2	3	3	2	2	3
3	1	2	2	1	1	2	3	2	1
3	3	3	3	3	2	3	2	1	9
3	3	3	3	3	2	2	2	2	3
9	1	1	9	2	2	2	2	2	3
9	9	9	9	9	1	2	2	3	1
1	1	1	2	2	3	3	3	1	2
2	2	2	2	3	2	3	2	2	2
2	1	2	1	2	2	2	2	2	1
2	2	1	2	3	2	2	3	3	1
1	3	1	3	3	2	2	2	2	1
2	1	9	2	2	1	2	1	1	1
1	1	3	1	3	1	1	2	1	3
1	2	3	1	3	2	2	1	2	1
1	1	1	1	1	1	1	1	1	1
2	1	2	1	2	2	2	2	2	2

1	1	1	2	3	2	2	2	2	2
9	9	9	9	9	1	1	1	1	3
9	9	9	9	9	2	2	2	1	1
1	3	1	3	1	2	2	2	2	1
2	2	2	2	2	3	2	2	2	2
2	2	2	2	2	1	2	1	1	1
2	2	2	2	3	3	2	2	2	2
2	2	2	2	2	1	2	1	2	1
2	2	2	2	2	1	2	1	1	1
3	2	3	1	2	1	2	1	2	2
2	3	2	2	3	2	2	2	2	2
1	1	1	2	1	2	2	2	2	1
2	1	2	3	1	2	2	2	2	9
1	1	3	2	3	2	3	3	2	3
2	2	1	2	2	1	2	2	2	1
1	2	2	1	1	1	1	3	1	9
1	2	3	1	2	1	2	3	1	3
9	9	9	9	9	1	1	1	2	9
9	9	9	9	9	1	1	1	2	9
1	3	2	3	2	2	2	2	2	1
3	3	3	1	1	1	2	1	2	1
2	2	2	2	2	1	2	2	2	2
1	1	1	1	1	9	9	9	9	9
2	2	2	2	2	2	2	2	2	2
3	3	3	3	3	9	9	9	9	3
9	2	1	3	2	1	1	1	2	2
2	2	1	3	2	1	1	1	2	2
1	1	1	1	1	1	2	2	2	1
1	1	1	1	1	2	2	9	1	1
2	2	2	2	2	2	2	2	2	2
1	1	1	2	3	1	1	2	1	2
1	1	1	1	1	2	2	2	2	1
9	9	9	9	9	1	2	2	2	1
2	2	1	3	2	1	1	1	2	2
1	2	3	3	2	9	9	9	9	2
2	2	2	2	2	3	3	3	3	2
2	1	2	2	2	3	3	3	1	1
9	9	9	9	9	1	1	1	1	9
9	9	9	9	9	2	2	2	2	9
1	1	1	1	1	1	1	2	2	1
3	3	3	3	3	2	2	2	2	2
2	2	2	2	2	3	3	3	3	2
2	2	2	2	2	2	2	2	2	2
2	2	2	2	2	1	1	1	1	2
2	2	2	2	2	3	3	3	3	2
2	2	2	2	2	2	2	2	2	2
9	9	9	9	9	9	9	9	9	9

9	9	9	9	9	9	9	9	9	9
2	2	2	2	2	3	3	2	3	2
9	9	9	9	9	1	1	2	1	1
9	9	9	9	9	1	1	1	1	1
9	9	9	9	9	1	1	1	1	2
1	2	1	2	2	2	2	2	2	3
2	2	2	2	2	9	9	9	9	9
3	3	3	3	3	2	2	2	2	3
2	3	2	2	3	1	1	1	1	1
9	9	9	9	9	2	2	2	2	2
2	2	2	2	2	2	2	2	2	2
2	2	3	2	1	1	2	2	1	2
1	1	1	1	3	1	2	2	1	2
2	2	2	2	2	2	2	2	2	2
2	1	1	1	2	1	2	2	2	1
2	2	2	2	2	2	2	2	2	2
2	2	2	2	2	2	2	2	2	2
2	2	2	2	2	1	1	1	1	2
1	9	1	2	1	1	2	2	2	1
3	3	3	2	2	2	2	2	2	2
9	9	9	9	9	2	2	2	2	1
2	2	2	3	3	2	1	2	2	3
2	2	2	2	2	3	3	3	3	2
2	2	2	1	3	1	3	2	2	2
2	2	2	2	2	1	1	1	1	2
2	3	2	3	3	2	2	2	2	3
9	9	9	9	9	2	2	2	2	9
9	9	9	9	9	2	2	2	2	2
3	3	3	3	3	2	2	2	2	2
2	2	2	2	2	1	1	1	1	2
9	9	9	9	9	2	9	2	1	2
1	1	2	2	3	2	2	2	2	2
3	1	1	3	1	1	2	1	1	1
2	2	2	2	2	1	1	1	1	2
1	1	1	1	1	1	2	2	2	2
3	2	2	2	2	9	9	9	9	2
2	2	2	2	2	2	2	2	2	2
1	2	3	1	3	1	3	3	2	1
2	2	2	2	2	2	2	2	2	2
9	9	1	9	9	2	2	2	2	2
2	2	2	2	2	3	3	3	3	2
2	2	2	2	2	3	2	2	2	2
1	1	1	3	1	2	2	2	2	1
2	2	2	2	2	9	9	9	9	2
2	2	2	2	2	2	2	2	2	2
3	3	3	3	3	2	2	2	2	3
2	2	2	2	2	2	2	2	2	2

2	2	2	2	2	2	2	2	2	2
2	2	2	2	2	2	2	2	2	2
2	2	2	2	2	2	1	2	2	2
2	2	2	2	2	2	2	2	2	2
2	2	2	2	3	3	3	3	3	3
3	1	1	2	1	2	2	2	2	1
2	2	2	2	2	2	2	2	2	2
2	9	1	3	1	2	2	2	2	2
2	9	1	3	1	2	1	2	2	2
2	2	2	2	2	2	2	2	2	2
2	1	1	2	1	1	1	1	1	1
9	9	9	9	9	1	9	2	2	2
9	9	9	9	9	1	1	3	2	9
2	2	2	2	2	9	9	9	9	2
2	2	2	2	2	1	1	1	1	1
2	2	3	3	3	2	2	2	1	3
3	2	2	2	3	1	2	2	2	2
1	1	1	1	1	2	2	2	2	1
1	2	3	2	1	1	9	9	9	9
3	2	2	2	2	2	2	1	2	2
3	1	1	1	3	1	1	2	2	9
2	2	1	1	1	1	1	1	1	1
2	2	2	2	2	3	1	2	3	2
1	1	3	2	3	1	1	1	1	1
3	3	3	3	3	2	2	2	2	3
1	1	9	9	9	1	1	1	1	1
2	2	2	2	2	2	2	2	2	2
1	1	1	1	1	1	1	1	1	1
9	9	9	9	9	2	2	2	2	2
2	2	1	2	3	2	2	2	2	3
1	1	1	1	1	1	1	1	1	1
1	1	1	1	1	1	3	1	1	1
2	2	1	2	1	9	9	9	9	3
1	1	1	1	1	2	2	1	1	1
3	3	3	3	3	2	2	1	1	3
1	1	3	3	2	2	1	3	1	1
9	9	9	9	9	2	2	2	2	1
1	1	1	1	1	2	2	1	1	1
2	2	2	2	2	3	3	3	3	2
2	2	2	2	2	3	3	3	3	2
2	3	1	3	2	1	1	2	2	1
1	1	1	1	1	2	2	2	2	9
2	3	2	1	3	2	2	2	2	1
2	2	2	2	2	2	2	2	2	2
2	2	2	2	2	3	3	3	3	2
3	1	2	3	1	1	1	1	1	1
2	2	2	2	2	2	2	2	1	2

2	1	9	1	2	1	2	2	2	1
3	3	3	3	3	2	2	2	2	3
3	2	2	3	2	2	1	2	2	1
9	9	9	9	9	3	3	2	3	9
9	9	9	9	9	9	9	9	9	1
3	1	2	2	1	2	2	2	2	1
2	1	2	2	3	3	2	2	2	2
3	3	3	3	3	3	2	3	2	3
2	2	2	2	2	3	1	1	3	3
2	2	2	2	2	2	2	2	2	2
9	9	9	9	9	2	2	2	2	9
2	9	2	9	1	1	1	1	3	1
2	2	2	2	2	9	3	3	3	2
2	2	2	1	2	1	3	3	3	2
2	2	2	2	2	2	3	3	3	2
2	2	1	1	2	3	3	3	3	3
2	3	2	2	3	2	1	3	2	3
9	9	9	9	9	1	1	2	2	9
9	9	9	9	9	1	2	3	2	9
1	2	2	2	2	2	2	2	2	2
2	2	2	2	2	1	1	2	2	2
2	2	2	2	2	2	2	2	2	2
9	9	9	9	9	9	9	9	9	9
9	9	9	9	9	9	9	9	9	9
2	3	2	1	2	2	1	3	1	1
3	1	3	1	3	1	1	2	2	1
3	3	3	3	3	2	1	1	2	1
1	1	1	1	1	1	1	1	2	1
3	3	3	3	3	1	2	2	2	3
2	3	1	2	3	2	2	2	2	2
1	1	1	1	3	1	1	1	2	1
2	2	3	3	2	2	2	2	2	1
3	1	2	2	1	1	1	1	1	1
3	3	3	3	3	2	2	2	2	3
1	1	1	1	9	3	3	3	3	3
1	1	1	1	1	2	1	1	2	2
3	2	2	1	1	2	1	1	1	1
3	3	3	3	3	2	2	2	2	3
9	9	1	2	2	1	1	2	1	1
3	3	3	3	3	2	2	2	2	3
1	3	1	3	1	2	2	2	1	1
1	3	1	2	1	2	1	1	2	1
3	3	3	3	3	2	2	2	2	1
2	2	2	2	2	3	3	3	3	2
2	2	2	1	2	1	2	1	1	1
3	3	3	1	3	9	9	9	9	1
2	1	1	2	3	1	2	2	2	1

2	2	2	2	2	2	2	2	2	2
2	2	2	2	2	2	2	2	2	9
1	3	9	1	1	1	1	2	1	1
1	1	1	1	1	1	1	2	1	1
3	3	3	2	2	2	2	2	2	2
2	2	2	2	2	2	1	2	2	1
2	2	2	3	3	2	3	9	2	3
9	9	9	9	9	2	1	1	2	2
9	9	9	9	9	2	1	1	2	2
2	2	3	2	2	2	2	2	2	3
9	9	9	9	9	3	1	9	2	9
3	1	1	2	1	2	2	2	2	2
1	1	1	3	3	2	2	2	2	1
2	3	1	2	1	1	1	1	2	2
1	1	2	2	2	2	1	1	1	1
2	2	2	3	3	2	3	1	2	3
1	1	1	1	1	2	2	2	2	1
2	1	1	2	2	1	1	2	2	1
2	1	2	2	1	2	3	2	3	2
9	9	9	9	9	1	1	2	2	1
9	9	9	9	9	1	2	1	2	1
3	3	3	3	3	1	1	1	1	1
3	1	1	3	3	1	1	1	1	1
1	1	1	1	1	1	2	1	3	1
3	3	3	3	3	2	2	2	2	3
2	2	2	2	2	3	3	3	3	2
1	1	1	1	1	2	2	2	2	1
2	1	2	1	3	2	2	2	2	2
2	1	1	2	1	2	2	2	1	3
3	1	2	1	1	1	1	1	2	1
2	2	2	2	2	2	2	2	2	2
1	1	1	1	1	1	1	1	1	9
1	2	2	2	1	2	3	1	2	1
1	2	2	2	2	3	3	3	3	9
2	2	1	3	2	1	1	2	2	1
2	2	2	2	2	1	2	2	2	2
2	2	2	2	2	2	2	2	2	2
2	2	2	2	2	3	2	3	2	2
1	1	1	1	1	2	2	2	2	1
2	3	3	3	2	2	2	2	2	2
2	3	2	1	2	1	2	2	2	1
2	2	2	2	2	1	1	1	1	2
1	1	1	1	1	1	2	2	2	2
9	9	9	9	9	2	2	2	2	2
2	2	2	2	2	2	2	2	2	3
2	2	2	2	2	1	1	2	2	2
1	1	2	1	1	2	2	2	2	1

2	1	9	2	2	1	2	2	2	9
2	1	2	3	3	9	9	9	9	2
2	1	1	2	3	2	2	2	2	1
2	1	1	2	2	3	3	3	2	1
2	2	2	2	2	3	1	2	2	3
2	3	3	3	3	1	2	2	2	3
2	2	2	2	2	2	2	2	2	2
2	3	9	1	3	2	1	2	3	2
1	1	1	1	1	1	3	3	2	3
2	2	2	1	1	9	9	9	9	9
1	1	2	2	1	9	9	9	9	9
1	1	2	2	1	9	9	9	9	9
9	9	9	9	9	2	3	2	1	1
9	9	9	9	9	3	2	2	1	3
1	1	2	2	1	9	9	9	9	9
3	2	2	2	3	2	2	2	2	1
9	9	9	9	9	9	9	9	9	1
3	3	1	1	2	1	1	1	1	2
1	1	2	1	3	1	2	2	2	2
2	2	2	2	2	1	1	2	2	1
1	2	1	2	1	2	2	2	2	2
2	1	2	2	2	1	2	2	2	2
1	9	1	1	2	1	2	2	2	2
2	2	3	1	2	2	2	2	2	2
2	2	2	2	2	2	2	2	1	2
1	2	2	1	3	1	2	2	2	2
3	3	2	1	2	1	3	1	3	1
9	2	2	2	2	1	1	1	1	1
1	2	2	1	3	1	2	2	2	2
2	2	2	3	2	2	2	2	2	1
2	2	2	2	2	1	1	2	2	1
9	9	9	9	9	2	3	2	1	1
3	1	1	3	2	1	1	1	1	1
2	2	2	2	2	1	1	2	2	2
1	2	1	1	2	2	2	2	2	2
1	1	1	1	1	2	2	2	2	2
1	1	1	1	1	2	2	2	2	2
9	9	9	9	9	1	3	2	1	1
2	2	2	2	2	2	2	2	2	2
2	2	2	2	2	1	3	3	3	2
1	2	1	1	3	2	2	2	2	2
3	1	2	2	2	1	1	2	1	2
1	1	1	2	1	2	2	2	2	2
2	2	2	2	2	2	2	2	1	2
1	2	1	1	1	1	2	1	1	2
2	2	2	2	2	1	1	3	1	2
9	9	9	9	9	2	2	2	2	1

2	2	2	2	2	9	9	9	9	2
2	1	1	2	2	2	2	2	2	1
9	9	9	9	9	1	2	2	3	9
9	9	9	9	9	2	2	2	2	1
1	1	1	1	3	2	2	2	2	1
1	9	9	9	9	2	2	2	2	1
2	2	2	3	3	2	2	1	2	1
2	1	2	1	3	1	1	1	3	1
2	3	3	2	1	1	2	2	3	1
3	3	2	2	1	2	2	1	2	1
2	1	2	1	3	3	3	2	3	9
1	2	3	3	2	1	1	2	2	1
1	1	1	9	1	2	2	2	2	2
2	2	1	2	2	2	2	2	2	2
1	1	1	3	2	2	2	2	3	1
3	3	3	3	1	2	2	2	2	3
2	2	2	2	2	3	3	3	3	2
2	2	1	2	1	1	1	2	2	1
1	1	1	1	1	2	2	2	2	1
3	1	2	1	1	2	2	2	2	1
2	2	2	3	1	3	2	3	2	2
2	1	1	1	1	3	2	2	2	2
1	1	1	1	1	2	2	2	2	2
2	2	2	2	2	3	3	3	3	2
2	1	2	1	9	3	2	2	2	2
1	1	1	2	1	1	2	2	2	2
2	2	2	2	2	2	2	2	2	2
2	2	2	2	2	2	2	2	2	2
2	9	2	2	2	2	2	2	2	2
9	9	9	9	9	9	9	9	9	9
9	9	9	9	9	1	1	2	2	1
2	2	2	2	2	3	3	3	1	2
9	9	9	9	9	2	2	2	2	1
2	3	2	2	3	9	9	9	9	9
2	3	1	2	1	1	1	1	2	1
9	9	9	9	9	2	2	2	2	9
9	9	9	9	9	9	9	9	9	9
2	2	2	2	2	2	2	2	2	2
3	9	9	9	9	1	1	3	3	1
2	1	2	1	2	2	1	2	1	1
1	1	1	1	1	2	1	1	1	1
1	1	2	3	2	1	2	3	2	2
1	1	1	2	2	2	1	2	3	2
3	1	2	3	2	2	1	2	2	1
1	2	2	2	3	1	1	2	1	3
9	9	9	9	9	9	9	9	9	9
2	2	3	3	3	2	2	2	2	3

2	1	2	2	1	2	2	2	2	1
9	9	9	9	9	9	9	9	9	9
9	9	9	9	9	9	9	9	9	9
2	2	2	2	2	3	3	3	3	2
1	1	1	2	2	1	3	2	2	1
2	1	2	2	1	2	2	2	2	1
3	1	1	1	2	1	1	1	1	2
1	1	2	1	1	2	2	2	2	2
2	2	2	2	2	1	1	1	1	2
1	1	1	3	2	1	1	1	1	1
9	9	9	9	9	9	9	9	9	9
9	9	9	9	9	2	2	2	2	9
9	9	9	9	9	1	2	2	2	1
2	2	2	2	2	1	2	1	1	2
3	1	2	2	2	2	2	2	2	2
9	9	9	9	9	9	9	9	9	9
9	9	9	9	9	9	9	9	9	9
9	9	9	9	9	9	9	9	9	9
1	1	2	2	1	1	2	2	2	1
1	1	1	2	1	1	2	2	2	1
2	3	3	1	2	1	1	9	9	9
2	3	3	2	1	2	1	1	2	1
3	3	3	3	3	2	1	1	3	3
2	2	2	2	2	9	9	9	9	9
2	2	2	2	2	1	1	1	1	2
2	1	2	2	2	2	2	2	2	2
9	9	9	9	9	3	3	3	3	9
1	1	1	2	3	3	2	2	3	9
1	2	1	1	1	2	2	2	2	1
2	2	2	2	2	9	9	9	9	2
1	1	1	1	3	1	2	2	2	2
2	2	2	2	2	3	3	3	3	9
9	9	9	9	9	9	9	9	9	9
3	1	2	3	2	1	2	2	2	1
2	2	2	2	1	2	2	1	1	2
2	2	2	2	2	2	2	2	2	2
1	1	2	1	1	3	3	2	2	1
2	2	2	2	2	2	2	2	2	2
2	1	1	3	2	1	2	2	2	1
2	2	1	2	1	2	2	2	2	1
3	1	1	3	2	2	1	2	1	1
3	3	3	3	3	2	9	2	2	9
9	9	9	9	9	9	9	9	9	9
9	9	9	9	9	2	2	2	2	9
2	2	2	2	2	9	9	9	9	9
9	9	9	9	9	9	9	9	9	9
9	9	9	9	9	9	9	9	9	9

2	2	2	2	2	1	1	2	2	2
1	2	2	2	2	3	2	1	2	2
1	1	1	1	1	1	1	2	2	2
1	1	2	2	2	2	2	2	2	2
1	1	1	1	1	1	1	1	1	9
9	9	9	9	9	9	9	9	9	9
9	9	9	9	9	9	9	9	9	9
3	1	1	3	3	9	9	9	9	9
9	9	9	9	9	9	9	9	9	9
9	9	9	9	9	9	9	9	9	9
9	9	9	9	9	9	9	9	9	9
9	9	9	9	9	9	9	9	9	9
9	9	9	9	9	9	9	9	9	9
3	1	3	3	1	2	2	2	2	1
1	1	1	1	3	2	2	2	2	2
1	1	2	2	1	3	1	2	2	2
2	1	2	2	2	2	2	2	2	2
9	9	9	9	9	9	9	9	9	9
9	9	9	9	9	9	9	9	9	9
9	9	9	9	9	9	9	9	9	9
3	9	2	9	1	1	2	2	2	1
9	9	9	9	9	9	9	9	9	9
1	1	1	1	1	1	1	2	2	1
9	9	9	9	9	9	9	9	9	9
2	2	1	1	1	3	1	1	3	1
2	1	1	1	1	1	1	1	1	1
3	1	2	2	1	1	1	1	1	1
2	1	1	1	1	1	1	1	1	2
1	1	1	1	1	1	1	1	1	1
2	2	1	1	1	1	1	1	1	1
2	2	2	2	2	1	1	1	1	1
1	1	2	2	2	3	3	3	3	1
1	1	1	1	1	1	1	1	1	1
9	9	9	9	9	9	9	9	9	9
1	1	1	1	1	1	1	1	1	1
1	1	1	1	1	1	1	1	1	1
3	3	2	2	2	3	3	3	3	2
9	9	9	9	9	9	9	9	9	9
3	1	2	1	3	2	2	1	1	1
3	1	3	3	3	1	2	2	2	3
2	2	2	2	2	2	1	2	2	2
9	9	9	9	9	9	9	9	9	9
9	9	9	9	9	9	9	9	9	9
9	9	9	9	9	9	9	9	9	9
1	1	1	2	3	3	1	2	1	1
3	2	2	3	2	3	1	1	1	2
2	1	1	2	2	1	2	2	2	9

9	9	9	9	9	9	9	9	9	9
9	9	9	9	9	9	9	9	9	9
9	9	9	9	9	9	9	9	9	9
9	9	9	9	9	9	9	9	9	9
2	1	1	9	1	2	2	2	2	9
9	9	9	9	9	9	9	9	9	9
2	2	2	2	2	9	9	9	9	9
9	9	9	9	9	9	9	9	9	9
9	9	9	9	9	2	2	2	2	9
9	9	9	9	9	9	9	9	9	9
9	9	9	9	9	2	2	2	2	9
9	9	9	9	9	9	9	9	9	9
1	1	3	3	3	1	1	2	1	1
1	2	3	1	2	1	1	1	1	1
2	1	1	1	3	2	2	2	2	2
3	1	1	1	1	1	1	1	2	2
1	1	1	1	2	1	1	3	3	2
2	1	1	1	1	1	1	2	2	1
1	1	1	1	1	1	1	2	2	1
9	9	9	9	9	1	1	1	1	1
2	2	2	2	2	1	1	1	1	1
3	1	2	3	2	1	2	2	2	1
2	2	2	2	2	1	1	3	3	2
2	3	1	1	2	2	2	3	2	2
1	1	1	1	2	1	1	1	1	1
3	3	1	1	3	2	2	2	2	1
9	2	9	9	9	2	2	2	2	1
2	2	2	2	2	2	2	1	1	1
9	9	9	9	9	2	2	2	2	1
9	9	9	9	9	9	2	2	1	9
9	1	1	1	3	9	9	9	9	9
3	3	3	3	3	1	1	9	9	1
3	3	3	3	3	1	2	2	2	2
2	2	2	2	2	2	2	2	2	2
1	1	1	2	2	1	2	2	2	1
2	2	2	2	2	2	3	2	3	2
2	2	2	2	2	1	2	2	2	2
1	1	2	2	2	2	2	2	2	2
2	2	1	1	2	2	2	2	2	2
2	2	2	2	2	2	2	2	2	1
1	1	1	3	1	2	2	2	2	2
2	2	2	2	2	2	2	2	2	1
3	1	1	3	1	1	1	1	2	1
1	2	2	1	1	2	3	2	2	1
2	1	2	2	1	1	1	1	1	1
3	3	3	3	3	1	1	1	1	1
2	1	2	1	2	1	2	2	2	2

9	9	9	9	9	9	9	9	9	9
2	1	1	1	1	1	1	1	1	1
9	9	9	9	9	2	2	2	2	9
9	3	9	9	9	1	2	2	2	9
9	9	9	9	9	1	1	2	3	1
1	1	1	1	2	1	2	2	2	9
9	9	9	9	9	3	3	2	2	9
3	3	3	3	3	2	2	2	2	1
9	9	9	9	9	3	3	3	3	2
2	2	3	1	2	2	2	2	3	2
9	9	9	9	9	9	9	9	9	9
2	1	1	2	2	2	2	2	2	2
1	1	2	2	1	2	2	2	2	1
2	2	2	2	2	1	1	1	1	2
1	1	1	1	1	1	1	1	1	1
9	9	9	9	9	9	9	9	9	9
2	2	2	2	2	2	2	2	2	2
3	3	3	3	3	2	1	2	1	3
9	9	9	9	9	2	2	2	2	2
3	3	3	3	3	1	3	1	2	2
2	2	2	2	2	3	3	3	3	2
1	1	1	1	3	1	1	2	3	3
2	2	2	2	2	9	9	9	9	2
2	2	2	2	2	2	2	2	2	2
1	1	1	1	1	2	2	9	3	2
2	2	2	2	2	2	2	2	2	2
3	1	3	1	2	2	2	2	2	1
3	1	1	1	1	2	2	2	1	1
1	1	1	1	1	1	1	1	1	1
2	2	2	1	1	2	2	2	2	2
9	9	9	9	9	9	9	9	9	9
3	2	3	2	1	3	3	3	3	2
2	3	3	2	3	9	9	9	9	9
2	2	2	2	2	2	2	2	2	2
2	2	2	2	2	2	2	2	2	2
1	1	1	1	1	1	1	1	1	1
1	1	1	2	2	1	2	2	1	3
2	2	2	1	1	1	1	1	2	1
2	2	1	1	1	1	1	1	1	1
1	3	1	3	1	1	1	1	1	1
2	2	2	2	2	1	1	2	2	2
1	1	1	1	1	1	1	1	1	1
9	9	9	9	9	9	9	9	9	9
9	9	1	9	9	2	1	1	3	3
9	9	9	9	9	1	1	1	1	1
9	1	2	2	1	9	9	9	9	9
1	1	1	2	2	1	2	9	2	9

1	1	1	1	1	1	1	1	1	9
2	1	2	2	2	3	1	3	3	2
9	9	9	9	9	2	2	2	2	2
2	1	2	3	2	3	1	2	2	2
9	9	9	9	9	1	1	2	2	9
3	3	2	1	3	2	2	2	2	3
9	9	9	9	9	3	3	3	3	2
9	9	9	9	9	9	9	9	9	9
2	1	2	2	2	9	9	9	9	2
2	2	2	2	1	2	9	9	9	9
9	9	1	9	9	2	2	2	2	1
9	9	9	9	9	3	3	3	3	2
2	1	2	2	2	1	3	1	2	2
9	9	9	9	9	9	9	9	9	9
9	9	9	9	9	2	2	2	2	2
9	2	1	2	3	1	3	3	2	1
3	2	1	2	2	3	2	3	2	2
1	1	2	2	1	2	2	2	2	1
3	3	3	3	3	1	1	2	2	1
1	1	2	2	2	2	1	2	3	1
3	1	2	1	2	2	2	2	2	1
1	3	1	3	1	3	3	3	3	1
2	2	1	3	3	1	2	2	2	2
2	2	2	9	3	2	2	2	2	2
2	2	2	2	2	2	2	2	2	2
2	2	1	1	1	1	1	1	1	1
3	2	2	2	3	2	2	2	2	2
2	1	3	1	1	1	1	1	1	1
3	9	2	2	3	1	9	9	9	2
1	2	1	2	2	1	2	1	3	1
1	3	1	1	3	2	2	1	1	1
1	2	2	2	3	1	2	2	2	2
9	9	9	9	9	9	9	9	9	9
3	3	3	3	2	2	1	1	2	1
1	2	2	2	3	2	2	2	2	1
2	2	2	2	2	2	2	2	2	2
2	2	3	2	3	2	2	2	2	1
3	1	1	2	2	1	2	9	2	2
9	9	9	9	9	2	2	2	2	9
2	2	2	2	2	2	2	2	2	2
9	9	9	9	9	2	2	2	2	9
9	9	9	9	9	2	2	2	2	2
2	2	2	3	2	2	2	2	2	1
9	9	9	9	9	2	2	2	2	1
1	2	2	3	1	1	1	1	1	1
1	3	1	3	1	1	1	2	1	1
1	3	1	1	3	1	1	3	3	1

3	3	3	3	3	2	2	2	2	3
9	3	9	9	9	2	2	2	2	9
1	3	2	2	1	2	2	2	2	3
1	1	1	1	1	1	1	1	2	1
3	1	1	3	1	2	2	2	2	1
2	1	3	1	1	2	2	2	2	1
3	1	2	1	2	1	1	1	1	1
2	2	2	2	2	2	1	1	1	2
2	1	3	3	3	2	2	2	2	2
2	3	1	2	3	1	3	2	3	1
2	2	1	3	3	2	2	2	2	1
2	2	1	2	3	2	2	2	1	1
3	2	3	2	3	3	2	2	1	2
1	1	1	1	2	2	2	2	2	2
1	9	1	2	3	1	1	3	3	1
2	1	1	3	1	2	2	2	3	1
3	2	2	2	1	2	2	2	2	2
1	3	1	2	1	2	2	2	2	1
1	3	3	3	1	2	2	2	2	2
2	2	2	2	2	2	2	2	2	2
2	2	2	2	2	2	2	1	1	2
3	1	2	2	2	2	1	3	1	2
3	3	3	3	3	2	9	2	2	2
9	9	9	9	9	9	9	9	9	9
1	1	1	2	2	3	2	3	2	2
2	3	2	3	1	9	9	9	9	1
2	1	3	2	1	2	2	2	2	1
3	2	3	1	1	9	9	9	9	1
1	1	1	1	2	1	2	2	1	1
9	9	9	9	9	2	2	2	2	1
1	3	2	3	2	1	2	1	2	1
2	1	2	1	3	1	1	2	3	3
2	3	3	2	2	3	2	2	2	2
1	1	1	2	2	3	3	3	3	2
3	9	1	1	1	1	1	1	1	1
2	2	2	2	2	2	2	2	2	2
1	2	2	2	2	2	2	2	2	2
9	9	9	9	9	9	9	9	9	9
2	3	2	1	1	2	2	2	2	2
2	1	3	1	3	1	1	2	2	2
9	9	9	9	9	9	9	9	9	9
9	9	9	9	9	9	9	9	9	9
1	2	3	1	2	3	1	2	3	2
3	3	3	3	1	1	2	2	1	9
2	2	2	2	2	2	2	2	2	2
3	1	1	3	3	2	2	2	2	2
9	9	9	9	9	9	9	9	9	9

2	2	2	2	2	2	2	2	2	2
9	9	9	9	9	9	9	9	9	9
1	1	2	1	1	2	2	2	2	1
9	9	9	9	9	9	9	9	9	9
9	9	9	9	9	9	9	9	9	9
3	3	3	3	3	1	1	1	1	3
2	2	2	2	2	2	2	2	2	2
2	2	2	2	2	3	3	3	3	2
1	2	1	1	2	1	3	1	1	3
3	1	2	2	3	2	2	2	2	2
9	9	9	9	9	9	9	9	9	9
9	9	9	9	9	2	2	2	2	9
9	9	9	9	9	9	9	9	9	9
2	1	1	1	1	1	1	1	1	1
2	2	2	2	2	2	2	2	2	2
2	2	2	2	2	2	2	2	2	1
9	9	9	9	9	2	2	2	2	1
9	9	9	9	9	9	9	9	9	9
3	3	3	3	3	3	3	3	3	3
2	2	2	2	2	2	2	2	2	2
3	3	3	3	3	3	3	3	3	1
2	2	2	2	2	2	2	2	2	2
9	9	9	9	9	2	2	3	2	1
1	2	1	2	3	2	2	3	3	2
2	3	3	3	3	1	2	2	2	2
2	3	2	2	2	3	3	3	3	2
9	9	9	9	9	2	1	2	2	1
2	2	2	2	2	1	1	1	1	1
2	2	2	2	2	2	2	2	2	2
9	9	9	9	9	1	3	3	2	1
9	1	1	2	3	2	2	2	2	2
2	2	2	2	2	3	3	3	2	2
1	3	2	2	2	1	2	2	2	1
1	2	1	2	3	2	2	2	2	2
1	1	1	1	1	2	2	2	2	2
2	2	1	2	3	2	1	1	1	2
1	1	1	1	1	1	1	1	1	1
3	1	1	1	3	2	2	2	2	3
9	9	9	9	9	2	2	2	2	2
2	2	2	2	2	2	2	2	2	3
3	1	1	2	2	2	2	2	2	1
2	3	3	3	3	3	2	2	2	1
2	3	3	3	3	2	1	3	3	2
1	1	2	1	3	2	2	2	2	1
1	3	3	3	1	2	2	2	2	1
2	1	1	2	2	2	2	2	2	2
9	9	9	9	9	9	9	9	9	1

3	1	2	2	2	2	2	2	2	2
2	1	2	2	2	2	2	2	3	1
2	2	2	2	1	1	1	1	1	2
2	2	2	2	2	2	2	2	2	1
2	1	2	3	2	2	2	2	2	2
9	9	9	9	9	2	2	1	1	1
2	2	2	2	2	3	3	3	3	2
2	2	2	2	2	1	1	1	1	1
2	2	2	2	2	2	2	2	2	2
2	2	2	2	2	2	2	2	2	2
2	1	1	2	2	2	2	2	2	2
2	3	2	1	3	2	2	2	2	2
2	2	2	2	2	2	2	2	2	2
3	3	3	3	3	2	2	2	2	3
2	3	2	3	2	1	1	2	2	1
2	1	2	3	1	2	2	2	2	2
3	2	2	2	2	2	2	2	2	2
1	1	1	2	3	2	2	2	2	2
2	2	2	2	2	3	3	3	3	2
2	2	2	2	2	2	1	2	2	1
1	1	1	2	1	1	1	1	2	2
1	1	2	3	1	3	2	3	2	1
1	1	2	3	1	3	2	3	2	1
3	3	3	3	2	9	1	2	2	9
1	1	1	2	2	2	2	2	2	2
3	2	2	2	2	2	2	2	2	2
2	1	2	2	1	2	2	2	2	2
2	2	1	2	1	2	2	2	2	2
2	2	2	2	2	2	2	2	2	2
1	1	1	1	1	1	1	1	1	1
2	2	2	2	2	2	2	2	2	3
3	9	2	2	3	3	2	2	3	1
1	1	1	1	2	2	2	2	2	1
3	3	3	3	3	2	9	2	2	1
2	2	2	2	2	1	2	1	1	1
2	2	2	2	2	2	2	2	2	2
2	2	2	2	2	2	2	2	2	2
2	2	2	2	2	3	3	3	3	2
2	2	2	2	2	2	2	2	2	2
2	2	2	2	2	3	3	3	3	2
1	1	1	2	3	2	2	2	2	1
1	1	3	1	2	1	1	2	2	1
2	1	2	2	2	1	1	1	1	1
2	1	2	2	2	1	2	3	3	2
2	2	3	3	3	2	2	2	2	1
1	1	2	1	1	1	1	3	2	1
3	1	1	3	1	1	2	2	2	1

1	2	2	1	3	2	2	3	3	2
2	2	2	2	2	3	3	1	1	2
2	1	1	1	2	1	2	2	2	1
2	2	2	2	2	2	2	2	2	2
2	2	2	2	2	2	2	2	2	2
2	2	2	2	2	3	2	3	1	2
2	1	1	1	2	2	2	2	2	2
1	1	1	1	2	1	2	2	2	1
2	2	2	2	2	2	3	2	2	2
2	2	2	2	2	2	2	2	2	2
2	2	2	2	2	2	2	2	2	2
2	2	2	2	2	3	2	2	2	2
2	2	2	2	2	9	9	9	9	9
9	1	9	9	9	1	2	2	2	9
2	2	2	2	2	1	2	2	2	2
2	1	1	1	2	1	1	2	2	2
2	2	2	2	2	2	2	2	1	2
2	2	1	1	1	1	1	2	2	2
1	1	1	1	1	1	3	2	2	1
1	1	1	2	2	1	2	1	1	1
2	2	2	2	2	1	1	2	2	2
2	2	2	2	2	3	3	3	3	2
2	1	1	2	1	3	1	2	1	2
2	2	2	2	2	2	2	2	2	2
1	1	1	1	2	1	1	2	2	2
2	2	2	1	1	2	2	2	1	2
2	2	2	2	2	2	2	2	2	2
2	2	2	2	2	2	2	2	2	2
2	2	2	2	2	1	2	2	1	2
1	1	1	1	1	9	9	9	9	9
3	3	3	3	3	3	3	3	3	2
2	2	2	2	2	1	3	3	3	2
2	2	2	2	2	3	1	1	3	2
2	2	2	2	2	3	3	2	2	2
2	2	2	2	2	2	2	2	2	2
2	2	2	2	2	3	3	3	3	2
2	2	2	2	2	2	2	2	2	2
2	2	2	2	2	2	2	2	2	2
9	9	9	9	9	1	2	3	3	2
2	2	2	1	1	1	2	2	1	2
2	2	2	2	2	2	2	2	2	2
2	2	2	2	2	3	3	3	3	2
2	2	2	2	2	1	1	2	2	1
2	2	2	2	2	2	2	2	2	2
2	2	2	2	2	2	2	2	2	2
2	2	2	2	2	3	3	3	2	2
2	2	2	2	2	2	2	2	2	2

2	2	2	2	2	1	1	1	2	2
2	2	2	2	2	1	2	2	2	2
1	1	3	1	1	2	2	2	2	2
2	2	2	2	2	1	2	2	1	2
2	2	2	2	2	2	2	2	2	2
2	2	2	2	2	2	2	1	2	2
9	9	9	9	9	2	2	2	2	9
3	1	2	1	1	1	1	3	2	1
1	1	1	2	3	1	1	1	1	1
1	1	2	2	2	1	2	2	2	2
1	1	1	1	1	1	2	2	2	2
2	2	2	2	3	2	2	2	1	2
2	2	1	3	3	2	2	2	2	2
2	2	2	2	2	1	1	2	2	1
1	1	1	1	1	1	1	2	1	2
2	2	2	2	2	1	2	2	1	1
2	2	2	2	2	1	2	2	1	1
2	2	1	1	1	1	3	2	2	1
1	1	1	1	1	2	2	2	2	1
2	2	1	2	2	1	2	1	1	1
1	1	1	1	1	1	1	1	1	1
2	2	1	3	3	1	1	1	1	1
2	2	2	2	2	3	3	2	2	2
2	2	2	2	2	2	2	2	2	2
2	2	2	2	2	9	9	9	9	2
2	2	2	2	2	2	2	2	2	2
9	9	9	9	9	9	9	9	9	9
2	2	2	2	2	2	2	2	2	2
2	2	2	2	2	3	3	3	2	2
2	2	2	2	2	3	3	3	3	2
2	2	2	2	2	2	2	2	2	2
3	2	1	1	1	1	1	1	1	1
9	9	9	9	9	9	9	9	9	9
3	3	3	3	3	1	1	1	1	9
9	9	9	9	9	9	9	9	9	9
1	1	1	1	1	9	9	9	9	9
2	3	3	2	2	3	1	2	2	2
1	1	1	1	1	1	1	2	1	1
2	1	1	1	9	2	2	2	2	1
3	3	2	2	2	2	2	1	3	2
2	1	1	1	2	1	2	2	2	1
2	2	2	2	2	1	2	2	2	2
2	2	2	2	2	2	2	2	2	1
2	2	2	2	2	3	3	3	3	2
1	1	1	1	2	1	1	2	1	2
2	2	2	2	3	1	1	2	2	2
1	2	1	1	1	2	2	2	2	1

1	2	1	1	2	1	2	2	1	2
1	1	1	2	3	9	9	9	9	3
2	9	1	1	2	3	3	3	2	3
2	1	2	1	2	1	1	2	2	1
2	9	2	1	3	1	1	1	2	1
2	2	2	2	2	2	2	2	3	2
2	9	1	2	3	2	2	2	2	2
9	9	9	9	9	2	2	2	2	2
2	2	2	2	2	2	2	2	2	2
2	2	2	2	2	1	2	2	2	2
2	2	2	2	2	1	2	2	2	2
2	2	2	2	2	1	2	2	2	2
9	9	9	9	9	9	9	9	9	2
2	2	2	2	2	1	2	2	9	1
2	2	2	2	2	1	1	3	1	2
2	2	2	2	2	2	2	2	2	2
1	1	2	2	2	1	1	2	2	2
2	3	2	2	3	2	2	2	1	1
2	2	2	2	2	1	1	1	1	1
1	1	1	3	2	1	1	1	2	1
9	9	9	9	9	1	1	2	2	2
9	9	9	9	9	1	1	2	2	2
2	2	2	2	2	2	2	2	2	2
9	9	9	9	9	2	2	2	2	9
2	1	3	1	3	1	2	1	1	2
1	1	1	1	1	1	1	2	2	1
1	1	1	1	1	2	2	2	2	2
1	1	2	2	1	1	2	2	1	1
2	2	2	2	2	1	1	2	2	2
9	9	9	9	9	1	1	1	1	1
3	3	2	3	3	1	1	1	1	1
9	9	9	9	9	2	2	2	2	9
9	9	9	9	9	9	9	9	9	9
2	2	2	2	2	9	9	9	9	9
2	2	2	2	2	9	9	9	9	9
2	1	2	2	1	2	2	2	2	2
3	2	2	2	2	1	1	1	1	1
2	2	2	2	2	9	9	9	9	9
2	2	2	2	2	2	2	2	2	2
2	2	2	2	2	2	2	2	2	2
9	9	9	9	9	1	1	1	1	1
2	2	2	2	2	2	2	2	2	2
2	2	2	2	2	2	2	2	1	2
9	9	9	9	9	3	3	2	2	3
2	9	9	2	2	9	9	9	9	3
2	1	1	1	1	1	1	1	1	1
2	1	1	1	1	2	2	2	2	3

9	9	9	9	9	2	2	2	2	1
1	1	1	1	2	2	2	2	2	1
3	2	3	3	3	1	2	3	2	1
2	2	2	2	2	2	2	2	1	2
9	9	9	9	9	9	9	9	9	9
3	3	3	3	3	9	9	9	9	9
2	2	2	2	2	2	2	2	2	2
3	3	3	3	3	2	2	2	2	2
9	9	9	9	9	1	2	2	1	1
2	2	2	2	2	2	2	2	2	9
1	1	1	3	3	1	1	1	1	1
2	2	2	2	2	1	2	2	2	2
3	3	2	2	1	3	2	2	2	2
3	3	3	3	3	2	2	2	2	2
9	9	9	9	9	9	9	9	9	9
3	2	3	2	2	2	2	2	2	2
1	3	2	2	2	2	2	2	2	1
2	3	3	1	2	2	3	2	1	1
9	9	9	9	9	3	3	3	2	9
2	1	2	1	3	3	2	1	2	1
3	1	2	1	1	3	1	2	3	1
2	2	2	2	2	1	2	2	1	1
9	9	9	9	9	9	9	9	9	1
2	2	2	2	2	1	2	2	2	2
1	1	3	3	3	2	2	2	2	3
2	2	2	2	2	3	2	2	2	2
2	1	1	2	3	2	2	2	1	2
1	1	1	1	3	2	2	2	2	3
3	2	1	9	3	2	1	3	2	1
9	9	9	9	9	2	2	2	2	1
2	2	2	2	2	2	2	2	2	2
2	2	2	2	2	3	3	2	2	1
1	1	1	3	1	3	3	2	2	1
3	3	3	3	3	1	1	1	1	1
1	1	1	1	1	2	2	2	2	9
2	1	1	1	2	3	2	2	2	1
3	3	3	3	3	2	2	2	2	9
9	1	2	1	1	2	2	2	2	2
1	1	2	1	2	3	2	1	2	2
9	9	9	9	9	9	9	9	9	2
9	9	9	9	9	9	9	9	9	9
3	1	3	1	2	2	1	1	3	2
2	1	1	1	2	2	2	2	2	2
2	1	1	2	1	1	1	2	2	1
1	2	2	2	2	1	1	2	2	1
3	2	3	3	3	9	9	9	9	2
2	3	2	3	2	2	2	3	1	3

1	2	1	2	2	1	2	2	2	1
2	1	3	1	2	2	2	2	2	3
1	1	1	2	1	2	2	2	2	2
3	3	3	3	9	1	2	2	2	3
1	3	1	1	3	2	2	2	2	1
2	2	2	2	2	9	9	9	9	9
2	2	2	2	2	1	9	9	9	9
2	2	2	2	2	2	2	2	2	1
1	1	1	1	1	1	1	1	1	1
2	2	2	2	2	1	2	3	2	1
2	1	2	2	1	1	1	2	3	2
1	2	1	2	1	2	2	2	2	2
2	3	2	3	2	2	2	2	2	2
1	1	1	1	1	3	3	3	3	1
9	9	9	9	9	9	9	9	9	9
2	2	2	2	2	3	1	2	2	2
2	1	2	2	2	2	2	1	1	2
2	2	1	2	2	1	2	2	2	2
1	2	2	3	2	2	2	1	1	1
2	1	1	1	2	1	2	1	2	1
2	9	1	1	2	9	9	9	9	9
1	9	9	9	9	9	9	9	9	9
3	2	2	2	2	2	2	2	2	2
9	9	9	9	9	2	2	2	2	2
2	2	2	2	2	9	9	9	9	9
2	9	1	2	2	2	2	2	2	3
2	1	2	2	2	2	2	2	2	2
2	2	2	2	2	2	2	2	2	1
3	3	3	3	3	2	2	2	2	3
1	1	1	1	2	2	2	2	2	1
3	3	3	3	1	2	2	2	2	3
1	3	3	3	3	2	2	2	2	2
2	2	9	9	9	9	9	9	9	9
1	1	1	1	2	2	2	2	2	9
1	3	1	3	2	9	9	9	9	9
2	2	2	2	2	1	2	2	2	2
1	1	1	1	3	1	1	1	1	1
1	2	3	1	2	2	2	2	2	3
2	1	1	2	2	2	2	2	2	1
9	9	9	9	9	2	2	1	2	2
2	2	2	2	2	3	3	3	3	2
2	2	1	2	3	1	2	2	2	2
3	2	2	2	3	1	2	2	2	3
2	1	1	1	3	2	2	2	2	9
3	3	3	2	2	1	1	1	1	3
9	9	9	9	9	2	2	2	2	9
3	1	2	1	2	2	2	2	2	3

2	3	2	1	2	2	2	1	2	1
2	2	3	2	2	2	2	2	2	2
3	9	3	3	3	2	2	2	2	3
2	3	1	2	3	3	2	1	3	2
2	2	1	2	2	2	2	2	2	2
1	2	2	2	2	1	3	2	1	1
3	1	1	3	1	2	2	2	1	1
9	1	9	9	9	2	2	2	2	1
9	9	9	9	9	9	9	9	9	9
1	2	2	1	2	1	1	1	1	1
2	2	2	3	3	2	3	2	2	2
1	2	2	2	2	2	1	2	2	2
2	1	1	3	3	1	3	2	1	2
1	1	1	1	1	1	1	2	1	1
9	9	9	9	9	2	2	1	2	9
1	1	2	1	2	2	1	2	2	2
2	2	2	2	2	1	1	2	2	2
3	3	3	2	3	2	2	2	2	1
3	3	1	3	3	2	2	2	3	2
2	3	2	2	3	2	2	2	2	2
2	1	1	1	2	2	2	1	2	1
1	3	3	1	3	1	1	2	2	1
1	1	1	2	1	3	3	3	3	2
3	1	3	2	2	2	2	2	2	1
1	1	1	9	9	2	1	2	3	1
1	1	2	2	1	1	1	2	2	2
3	1	2	1	2	1	1	2	2	1
1	2	2	2	2	1	2	2	2	1
2	2	2	2	2	1	2	2	2	2
1	1	1	2	2	3	2	2	2	2
2	2	1	2	2	3	1	3	2	2
2	2	9	1	2	3	1	2	1	2
2	2	2	2	2	2	2	2	1	2
2	1	1	1	1	2	2	2	2	1
1	1	2	2	3	1	2	3	1	1
2	1	1	1	3	2	2	2	2	2
1	1	1	1	2	3	1	2	2	1
9	1	3	2	2	2	9	9	9	1
2	2	2	2	2	1	1	2	2	1
2	1	1	1	1	1	1	1	1	1
2	2	3	3	3	1	2	2	2	2
1	1	1	1	1	1	1	1	2	1
2	2	1	1	1	2	2	2	2	1
2	2	2	2	2	2	2	2	2	1
2	2	1	3	3	2	1	1	2	1
2	2	2	2	2	2	1	1	1	2
1	1	2	1	1	1	1	2	1	1

3	1	2	2	1	1	1	2	2	1
2	1	1	1	2	2	1	2	1	1
3	3	2	1	2	1	2	1	2	1
1	1	2	3	1	2	1	1	2	1
3	2	2	1	1	1	1	1	2	1
1	2	2	2	3	1	1	2	1	3
1	1	1	2	3	2	1	1	2	1
2	1	2	2	3	1	2	1	1	1
1	1	2	1	1	2	2	2	2	2
2	1	1	2	1	2	2	2	2	2
2	2	2	2	2	1	3	3	3	2
2	2	2	2	2	2	1	1	2	9
1	1	1	1	1	2	2	1	2	1
3	3	3	3	3	3	1	2	2	2
1	1	2	2	2	2	2	2	2	2
1	1	1	1	2	1	2	2	2	1
1	1	1	9	1	2	3	2	2	1
2	2	1	2	2	2	2	2	2	2
2	2	2	2	2	2	2	2	2	2
1	1	3	2	1	2	2	2	3	1
2	2	2	2	2	1	1	2	2	1
1	1	2	1	1	2	2	2	2	2
3	3	3	3	3	2	2	2	2	3
3	3	3	3	3	9	9	9	9	9
1	2	1	3	3	3	2	2	3	2
2	2	2	2	2	3	3	3	3	3
2	2	3	2	2	2	1	1	1	2
3	3	1	1	1	2	3	9	3	3
1	1	2	1	2	2	2	2	1	2
2	2	1	1	3	2	2	2	2	3
3	1	2	1	2	1	1	2	1	2
2	3	1	1	3	1	1	1	1	1
2	2	1	1	2	2	1	1	2	2
2	2	2	2	2	1	2	2	3	2
2	3	2	3	1	1	1	1	2	1
2	2	2	2	2	3	2	3	3	2
2	2	3	2	2	2	2	1	2	2
3	3	3	2	3	3	3	3	3	3
1	1	1	1	1	2	1	1	1	1
1	2	3	3	2	2	1	2	1	1
2	2	2	2	2	2	2	2	2	2
2	2	2	2	2	2	2	2	2	1
2	2	2	2	2	2	2	2	2	2
3	3	3	3	3	2	2	2	2	3
3	3	3	3	3	2	2	2	2	3
3	3	2	2	2	2	2	2	2	2
1	1	1	1	1	2	1	1	2	1

1	1	2	3	2	1	2	1	1	1
1	1	1	1	1	1	2	2	1	1
1	1	1	2	2	1	2	1	1	1
2	1	1	2	2	1	1	2	2	1
1	1	2	2	1	2	1	1	2	1
1	2	2	2	1	2	2	2	2	1
2	1	1	1	1	2	2	2	2	3
2	1	1	1	1	2	2	2	2	1
1	1	1	2	2	2	2	1	1	1
2	2	2	1	1	2	1	1	2	1
2	2	2	2	2	2	1	1	2	1
1	1	1	1	1	1	2	2	2	1
1	1	1	2	2	2	2	2	2	1
1	1	2	1	1	2	2	2	2	1
1	2	2	1	1	2	2	2	2	1
1	2	2	1	1	2	2	2	2	1
2	2	2	2	2	2	2	2	2	2
2	2	2	2	2	2	9	2	2	1
2	9	2	2	2	2	2	2	2	2
2	1	1	1	2	2	2	2	2	2
9	2	9	9	2	2	2	2	2	2
3	3	3	3	3	2	2	2	2	3
2	2	1	1	1	2	2	2	2	1
2	2	1	1	1	2	2	2	2	1
1	2	2	1	1	2	2	2	2	1
2	2	2	2	2	2	2	2	2	2
1	1	1	1	1	1	2	2	1	1
2	2	1	1	2	2	2	2	2	1
3	2	2	3	1	2	2	1	1	1
2	2	1	1	1	1	1	1	1	1
1	2	1	1	1	2	2	2	2	2
2	2	2	2	2	2	2	2	2	2
2	2	2	2	2	2	2	2	2	2
1	1	2	2	2	2	2	2	2	2
3	1	3	2	1	2	2	2	2	1
3	3	9	1	3	2	2	2	2	1
3	1	9	1	1	2	2	2	2	2
9	2	9	2	2	2	2	2	2	2
9	9	9	9	9	9	9	9	9	9
1	9	1	1	1	2	2	2	2	1
9	9	9	9	9	2	2	2	2	9
2	1	1	1	1	1	1	1	2	1
1	9	9	1	1	2	2	2	2	1
2	2	2	2	2	2	2	2	2	2
9	1	9	1	1	2	2	2	2	1
2	9	9	9	2	2	2	2	2	2
9	9	2	9	9	2	2	2	2	2

2	9	9	2	9	2	2	2	2	2
9	9	2	9	9	2	2	2	2	9
2	9	9	9	2	2	2	2	2	2
9	9	9	9	9	9	9	9	9	9
9	2	9	3	1	2	2	2	2	1
9	9	2	9	9	2	2	2	2	9
9	9	9	9	9	9	9	9	9	9
9	9	9	9	9	2	9	2	2	2
2	1	2	1	2	2	2	2	2	2
2	9	9	2	9	2	2	2	2	2
1	1	2	1	1	2	2	2	2	1
2	1	1	2	2	2	2	2	2	2
2	2	3	2	3	2	2	2	2	1
1	1	1	1	2	1	2	2	2	2
2	1	1	1	2	1	2	2	1	1
1	1	1	1	1	1	2	2	2	1
2	2	2	2	2	2	2	2	2	2
2	1	3	1	3	2	2	2	2	3
2	2	1	1	2	2	2	2	2	1
2	2	1	2	2	1	2	1	2	1
2	1	2	2	3	2	2	2	9	1
1	2	2	2	3	2	1	1	2	1
1	1	2	2	1	1	2	1	2	2
1	2	2	2	3	1	1	1	2	1
2	3	2	2	2	1	2	2	1	1
1	2	2	3	3	2	2	2	2	3
9	9	9	9	9	2	2	2	2	2
2	2	2	2	2	2	2	2	2	3
3	2	2	1	2	1	2	2	1	1
3	3	3	3	3	3	2	2	2	2
9	9	9	9	9	2	2	2	2	2
2	2	2	2	2	3	3	3	3	3
2	2	2	3	1	2	3	3	1	1
2	2	2	2	2	2	2	2	2	2
2	1	2	2	1	2	1	1	2	1
2	2	2	2	3	2	2	2	2	3
2	2	2	2	2	2	2	2	2	2
9	9	9	9	9	2	2	2	2	2
2	2	9	1	2	2	2	2	2	3
2	2	2	2	2	2	2	2	2	2
3	2	2	1	2	2	2	2	2	2
2	2	2	2	2	2	2	2	2	3
3	2	1	2	3	1	1	1	2	3
2	3	3	2	2	2	2	2	2	3
2	1	2	2	3	2	1	1	2	1
1	1	1	2	1	2	2	1	2	1
2	1	2	1	1	2	1	1	1	2

2	2	2	2	1	1	2	2	1	2
2	3	2	2	2	2	1	1	2	1
2	1	2	2	2	2	1	1	2	1
1	2	2	2	2	2	2	2	2	1
2	1	2	3	2	1	2	3	2	2
1	1	1	3	2	2	2	2	2	2
2	1	2	3	2	1	2	1	2	2
1	1	1	1	1	3	1	1	1	1
1	1	1	1	2	1	1	2	2	1
2	2	2	2	2	2	2	2	2	2
2	3	3	3	3	2	2	1	3	3
2	2	2	2	2	2	2	2	2	1
2	2	3	1	3	2	1	3	1	3
1	2	3	1	2	2	3	2	3	3
2	1	1	2	3	1	2	3	2	2
3	1	3	2	3	2	2	2	2	2
9	1	3	2	3	2	2	2	2	2
2	2	2	2	2	2	2	2	2	2
3	2	3	1	3	3	1	2	2	3
2	2	2	2	2	3	2	2	2	2
2	2	2	2	9	2	1	1	1	1
2	2	2	2	2	2	2	2	2	2
2	2	1	3	2	2	2	2	2	2
9	3	2	3	2	2	2	2	2	2
1	1	2	2	2	1	1	1	1	2
3	3	3	3	3	2	2	2	2	2
3	2	2	2	1	2	2	2	3	2
1	1	2	3	3	1	1	1	1	1
1	9	9	1	1	1	1	1	1	1
2	2	2	2	2	2	2	2	2	2
1	1	1	2	3	3	2	2	2	2
2	1	1	2	1	1	1	1	2	1
2	2	2	2	2	1	2	1	2	2
2	2	2	2	2	1	1	2	3	2
9	1	1	3	2	1	1	3	3	2
2	1	2	1	2	2	2	2	2	2
2	1	3	3	3	1	1	3	3	1
1	1	1	1	1	3	2	2	2	2
3	2	2	1	2	2	2	2	2	2
3	2	2	2	3	1	1	2	2	3
1	2	3	2	2	9	1	1	2	1
2	2	2	2	2	2	2	2	2	2
1	1	1	2	3	2	2	2	2	2
3	2	3	3	2	3	3	3	3	2
2	2	2	2	2	2	3	2	2	1
2	2	2	2	2	2	2	2	2	2
2	2	2	2	2	2	2	2	2	2

3	3	1	2	2	2	2	2	2	2
2	1	1	2	1	1	3	3	1	2
2	2	2	2	2	2	2	2	2	2
2	2	2	2	2	1	1	2	2	2
2	2	2	2	2	3	3	3	3	2
2	2	2	2	2	1	2	2	1	2
1	1	2	1	3	2	2	2	2	1
2	3	1	2	3	9	9	9	9	2
2	2	2	2	2	1	1	1	1	2
9	9	9	9	9	9	9	9	9	9
3	1	2	3	1	2	2	2	2	1
2	1	1	1	2	2	1	2	1	1
2	1	3	2	3	3	1	2	3	2
2	1	2	2	2	2	2	2	2	2
2	2	2	2	2	3	3	2	2	1
2	2	2	2	2	3	2	3	2	2
2	2	2	2	2	1	2	2	1	2
2	2	2	2	2	1	2	2	1	2
3	3	3	3	3	2	2	2	2	3
1	1	1	1	1	1	1	2	1	3
3	3	3	3	3	9	9	9	9	3
3	3	3	3	3	2	2	2	2	3
3	3	3	3	3	2	2	2	2	2
1	3	2	3	1	1	1	1	2	2
2	2	2	2	2	3	3	1	3	2
2	2	2	2	2	2	1	2	1	1
3	2	1	1	1	2	1	2	1	3
2	2	2	2	2	1	2	1	2	2
2	2	2	2	2	1	1	1	2	1
2	2	2	1	1	1	2	2	3	2
1	2	2	2	2	2	1	2	1	2
3	2	3	2	3	2	3	2	3	2
3	2	3	3	3	2	2	2	2	2
3	3	3	3	3	2	2	2	2	3
1	1	1	2	2	3	2	2	2	1
2	1	1	9	2	1	2	3	3	2
9	1	1	2	2	1	2	3	3	2
1	1	2	2	2	2	2	2	2	3
2	2	2	2	2	2	2	2	2	2
2	2	2	3	3	2	2	3	1	2
2	2	2	2	2	2	3	2	2	2
1	1	1	2	1	3	2	1	3	3
2	2	2	2	2	3	2	2	2	2
1	1	1	1	1	1	2	2	2	3
3	1	3	3	3	2	2	2	2	3
3	1	1	1	2	1	1	2	1	1
1	2	1	1	1	1	1	2	1	1

3	3	2	2	2	1	3	3	3	1
1	1	1	1	3	2	2	2	2	2
2	2	2	2	2	1	2	2	3	2
2	2	2	2	2	3	3	3	3	2
3	3	2	3	1	3	3	3	3	3
9	9	9	9	9	2	2	1	1	9
2	2	2	2	2	2	2	2	2	2
2	2	2	2	2	3	3	3	3	2
1	1	1	1	1	1	2	2	2	1
3	3	3	3	3	2	2	2	2	2
1	1	1	2	1	1	1	2	1	1
3	1	2	3	2	1	1	3	3	2
9	9	9	9	9	2	2	2	2	1
9	9	9	9	9	1	2	2	2	9
9	9	9	9	9	3	2	2	2	9
2	2	2	2	3	2	2	2	2	2
2	1	1	1	1	1	1	1	1	1
3	9	2	3	3	3	2	2	2	2
2	1	1	1	1	1	1	1	1	2
2	3	2	1	2	1	3	2	2	1
3	3	2	2	2	3	3	3	3	3
1	1	2	1	1	1	1	1	1	2
1	1	1	1	1	1	1	1	1	2
2	2	2	2	2	2	2	2	2	2
1	3	2	1	1	2	2	2	2	2
2	1	2	2	2	1	1	2	2	2
1	3	2	1	1	2	2	2	2	2
9	9	9	9	9	2	2	2	2	2
9	9	9	9	9	2	2	2	2	9
1	1	1	1	3	2	2	1	1	1
1	1	1	2	3	2	2	2	2	1
1	2	1	3	2	2	2	1	2	2
1	1	1	1	1	2	1	1	2	1
1	1	9	1	1	3	3	3	2	3
1	1	1	1	1	2	2	2	2	1
2	1	1	2	2	1	1	1	1	1
2	2	2	3	3	2	2	2	2	2
2	2	2	2	2	2	2	2	2	2
2	2	2	2	2	3	3	3	3	2
1	2	3	1	1	2	2	2	2	2
1	1	1	1	1	2	2	2	2	1
2	2	3	3	3	2	2	2	2	1
1	1	1	2	2	2	2	2	2	1
2	2	2	2	2	3	2	2	2	2
1	1	1	1	1	2	2	2	2	2
1	1	1	1	1	2	2	2	2	1
2	2	2	2	1	2	2	2	3	1

1	1	1	2	2	1	2	1	1	2
2	2	2	2	2	2	2	1	2	3
9	9	9	9	9	1	1	3	3	9
1	1	1	1	2	2	2	1	3	2
1	1	2	1	2	2	3	3	1	2
2	1	2	2	2	2	2	2	2	2
2	1	2	2	2	2	2	2	2	2
2	1	1	1	1	3	2	2	2	1
3	1	3	1	3	2	2	2	1	1
2	2	2	2	2	2	2	2	2	3
2	3	2	2	3	2	2	2	2	2
9	1	1	1	2	3	3	3	3	3
3	2	2	2	2	2	2	2	2	2
1	1	1	2	2	1	1	2	3	1
1	3	2	1	1	1	2	2	2	1
1	1	9	1	1	1	3	1	2	1
3	1	1	1	1	9	2	9	9	9
3	2	1	1	3	2	2	2	2	9
3	1	1	1	3	1	1	2	2	1
3	1	1	1	1	1	2	1	2	1
2	2	2	3	3	1	2	2	2	2
9	9	9	9	9	9	9	9	9	9
1	2	1	1	1	2	2	1	1	3
1	2	3	1	3	2	2	2	1	3
2	2	2	2	2	2	2	2	2	2
9	9	9	9	9	2	2	2	2	2
9	9	9	9	9	2	2	2	2	1
3	1	2	1	3	2	2	2	2	3
9	9	9	9	9	3	3	9	9	9
9	9	9	9	9	1	2	1	1	9
9	9	9	9	9	9	9	9	9	9
1	2	2	2	2	9	9	9	9	9
9	9	9	9	9	2	2	2	2	1
3	1	2	3	1	1	1	1	1	2
3	1	2	3	1	1	2	1	1	3
3	3	3	3	3	1	1	1	1	2
3	3	3	3	3	1	1	1	1	2
2	2	2	2	2	1	1	1	1	2
1	1	1	1	1	1	1	2	9	1
2	2	2	2	2	2	2	2	2	2
9	1	1	1	1	1	2	1	2	2
1	2	2	2	2	3	2	1	1	1
2	2	2	2	2	2	2	2	2	2
1	2	1	1	2	2	2	2	2	2
1	2	1	1	2	2	2	2	2	2
1	2	1	2	1	2	2	2	2	2
2	2	1	1	2	1	2	2	2	2

2	2	2	1	1	1	1	1	3	1
3	2	3	2	3	3	1	3	1	2
1	1	1	1	3	2	2	2	2	1
9	9	9	9	9	9	9	9	9	9
9	9	9	9	9	2	2	2	2	9
9	9	9	9	9	1	1	3	2	2
1	1	1	1	1	1	2	3	3	2
9	9	9	9	9	9	9	9	9	9
9	9	9	9	9	9	9	9	9	9
1	1	1	1	1	1	2	2	1	1
2	2	2	2	2	2	2	2	2	2
2	2	2	2	2	2	2	2	2	3
2	2	3	3	3	1	2	2	1	2
9	9	9	9	9	9	9	9	9	9
9	9	9	9	9	9	9	9	9	9
9	9	9	9	9	2	2	1	2	9
3	9	2	3	3	2	2	2	2	1
2	3	1	3	1	1	1	2	2	1
1	1	1	1	2	1	1	2	1	9
9	9	9	9	9	9	9	9	9	9
9	9	9	9	9	2	2	2	2	9
1	1	1	1	1	9	9	9	9	9
9	9	9	9	9	9	9	9	9	9
9	9	9	9	9	9	9	9	9	9
2	1	1	1	2	1	2	2	2	2
3	3	9	3	2	2	2	2	2	2
1	3	2	1	1	2	2	2	2	1
3	1	2	1	1	1	1	2	2	2
2	3	1	2	2	2	3	3	1	1
2	1	2	1	3	2	2	2	2	2
3	3	3	3	3	3	3	3	2	3
1	1	2	2	2	1	1	2	2	3
2	2	2	2	2	1	1	2	1	2
2	1	2	2	3	2	2	2	2	1
2	2	2	2	2	1	1	3	3	2
3	1	1	1	1	1	1	2	2	1
1	1	1	3	2	1	1	1	2	1
2	1	2	1	1	2	2	2	2	2
2	1	2	1	1	1	2	2	2	1
2	1	2	2	2	2	2	1	1	1
2	2	1	1	2	1	1	1	1	1
1	1	1	1	1	3	2	2	2	1
1	1	1	1	1	2	2	2	2	2
9	9	9	9	9	1	1	3	2	2
9	9	9	9	9	1	1	1	2	9
2	1	1	1	2	2	2	2	2	2
1	2	2	2	2	1	2	2	2	2

3	2	2	2	2	1	2	2	2	2
2	2	2	2	2	2	2	2	2	1
2	2	3	3	3	2	2	2	2	2
2	2	2	2	2	2	2	2	2	2
1	1	1	1	1	2	2	2	2	1
3	1	1	2	1	2	2	2	2	2
2	1	2	1	2	2	1	2	3	1
1	2	3	3	1	1	2	1	2	1
9	9	9	9	9	9	9	9	9	9
1	1	1	2	2	2	2	3	3	3
2	2	2	2	2	2	2	2	2	2
2	2	2	2	2	2	2	2	2	2
3	3	1	1	1	1	2	3	1	2
2	2	2	2	2	3	3	3	3	2
2	2	2	2	2	2	2	2	2	2
2	3	1	1	1	2	2	2	3	2
3	2	3	1	2	1	1	1	1	1
2	3	1	2	3	2	3	1	2	1
3	1	1	1	1	2	3	3	3	2
2	2	3	2	1	2	3	1	2	2
3	1	3	1	2	2	2	2	2	1
2	2	2	2	2	2	1	2	1	2
1	1	2	2	1	2	1	2	1	1
1	2	3	2	1	1	2	1	1	1
2	2	2	2	2	2	2	1	1	2
2	2	3	3	3	2	1	3	3	2
2	2	2	2	2	3	2	2	2	2
2	9	1	2	3	2	1	2	1	1
1	3	2	1	2	2	1	1	2	1
9	9	9	9	9	9	9	9	9	9
1	1	1	1	2	1	2	2	2	1
9	9	9	9	9	9	9	9	9	9
9	9	9	9	9	9	9	9	9	9
1	1	1	1	1	1	1	1	3	1
3	2	1	2	3	1	1	1	1	1
1	2	3	2	1	1	2	2	2	3
1	2	3	2	1	1	2	3	1	3
2	1	1	1	1	2	1	1	2	3
2	2	2	2	2	2	2	2	2	2
1	2	2	3	3	1	1	1	1	1
2	2	1	1	1	9	9	9	9	2
1	2	1	1	2	2	2	2	2	1
2	2	2	1	2	2	2	2	2	2
3	1	2	1	2	2	2	2	3	2
1	2	3	3	3	1	3	3	3	2
2	2	3	2	2	2	2	2	2	2
2	3	2	9	1	1	2	2	2	2

1	1	1	1	1	3	1	2	2	1
2	2	2	2	2	2	3	2	3	2
2	2	1	2	2	2	1	1	3	1
9	9	9	9	9	3	1	1	9	3
2	3	1	2	2	1	2	2	2	2
2	2	2	2	2	9	9	9	9	2
1	1	1	2	1	1	1	1	2	1
3	9	2	2	3	1	2	2	2	1
9	9	9	9	9	2	2	2	2	9
2	2	2	2	2	3	3	3	1	2
2	2	2	3	1	2	2	2	2	2
2	1	1	2	3	2	1	2	2	2
9	9	9	9	9	9	9	9	9	9
2	2	2	2	2	2	2	2	2	2
2	1	1	2	3	1	2	3	2	1
3	3	1	1	2	1	1	1	1	1
9	9	9	9	9	2	2	2	2	1
9	9	9	9	9	9	9	9	9	1
1	1	2	1	1	1	1	2	2	1
3	2	1	1	3	2	2	2	2	2
1	2	1	2	1	1	1	2	2	1
3	3	3	3	3	3	3	3	2	3
2	2	2	2	2	3	3	3	3	2
1	3	3	3	2	2	2	2	2	2
1	1	1	2	1	3	3	2	2	2
2	1	2	2	3	2	2	2	2	2
2	1	1	2	2	3	1	3	1	2
3	1	1	1	2	1	1	3	2	2
1	1	1	2	1	2	2	2	2	2
2	2	2	2	2	1	1	1	2	1
2	2	2	2	2	2	2	2	2	2
3	2	2	2	2	1	3	3	3	2
3	2	3	2	3	1	1	1	1	1
2	1	1	2	2	1	3	2	2	2
1	1	2	2	2	1	3	3	3	1
9	9	9	9	9	9	9	9	9	9
2	2	2	2	2	2	2	2	2	2
2	2	3	2	3	2	9	9	9	9
3	3	2	1	1	2	1	1	1	1
2	2	2	1	2	2	2	2	2	2
2	2	2	2	2	2	2	2	2	2
1	1	1	1	1	1	2	2	2	2
2	2	2	2	2	1	2	2	2	2
2	2	2	2	2	9	9	9	9	2
9	9	9	9	9	9	9	9	9	9
1	3	3	2	2	2	2	2	3	9
9	9	9	9	9	9	9	9	9	9

2	1	2	1	2	2	2	1	3	3
1	1	1	1	1	9	9	9	9	1
9	9	9	9	9	9	9	9	9	9
3	1	1	1	1	1	1	2	3	1
1	1	1	1	1	2	2	2	2	1
1	3	1	2	3	9	9	9	9	3
2	2	2	2	2	2	2	2	2	2
9	1	9	9	9	9	9	9	9	2
3	2	2	1	3	2	3	3	2	3
2	2	2	2	1	9	9	9	9	9
9	9	9	9	9	9	9	9	9	3
1	3	1	3	1	1	3	2	1	3
3	9	2	3	2	2	3	2	2	2
2	2	2	2	1	1	2	1	2	2
2	9	1	2	2	2	2	2	2	2
2	2	2	2	2	1	3	3	3	9
3	3	3	2	1	2	2	2	2	1
9	9	9	9	9	9	9	9	9	9
9	9	9	9	9	2	2	9	9	9
1	3	1	2	3	2	2	2	1	2
2	3	2	1	2	3	3	3	3	2
3	2	1	1	1	1	1	3	3	2
2	3	2	3	2	9	9	9	9	2
3	3	3	3	3	1	1	1	1	3
1	2	3	2	1	1	1	2	3	1
3	1	1	2	2	2	2	2	2	2
9	9	9	9	9	2	2	2	2	2
3	3	3	3	3	2	1	2	1	2
9	9	9	9	9	9	9	9	9	9
9	9	9	9	9	9	9	9	9	9
9	9	9	9	9	9	9	9	9	9
9	9	9	9	9	9	9	9	9	9
9	9	9	9	9	9	9	9	9	9
2	1	1	1	1	2	2	2	2	2
1	3	1	2	3	3	2	2	1	1
2	2	2	2	1	2	2	2	2	2
9	9	9	9	9	9	9	9	9	9
2	1	2	1	1	2	2	2	2	1
1	1	1	1	1	1	1	2	2	1
9	9	9	9	9	2	2	2	2	9
2	2	2	2	2	1	1	1	1	2
1	2	1	1	1	1	1	3	2	2
2	2	2	2	2	1	1	1	1	2
2	2	2	2	2	2	2	2	2	2
2	2	1	2	2	1	1	2	1	1
1	2	2	2	3	1	1	3	3	1
1	1	2	2	1	1	1	2	3	1

2	2	2	2	2	2	2	2	2	2
2	2	2	2	2	1	1	1	1	2
9	9	9	9	9	9	9	9	9	9
1	2	3	1	2	2	1	3	1	1
3	3	3	3	2	2	2	2	2	1
3	3	1	1	1	1	1	1	1	1
9	1	2	9	3	2	2	2	2	3
2	2	2	2	2	2	2	2	2	2
2	2	2	2	2	1	1	1	1	2
2	2	2	2	2	2	2	2	2	3
2	2	2	9	9	1	1	2	3	2
1	2	1	2	2	3	2	2	2	1
9	9	9	9	9	9	9	9	9	9
1	1	1	1	1	1	1	1	1	1
1	1	2	1	2	1	2	1	2	1
3	1	1	1	3	2	2	1	1	1
3	3	3	1	1	2	2	1	2	1
9	9	9	9	9	2	2	2	2	9
2	9	1	2	2	3	2	1	2	1
9	9	9	9	9	9	9	9	9	9
9	1	9	9	9	1	2	2	2	2
2	3	3	3	3	2	1	1	3	3
9	9	9	9	9	9	9	9	9	9
9	9	9	9	9	9	9	9	9	9
9	9	9	9	9	1	2	1	1	1
9	9	9	9	9	9	9	9	9	9
1	1	1	1	2	2	2	2	2	2
2	3	2	3	2	1	1	1	3	2
9	9	9	9	9	2	2	2	2	9
1	1	1	2	2	2	1	2	1	2
1	3	1	3	1	2	2	2	2	1
1	1	1	3	3	2	2	9	9	9
1	1	1	1	1	2	2	2	2	2
2	2	2	2	2	2	1	2	2	2
2	2	2	2	2	2	2	2	2	2
2	2	2	2	2	2	2	2	2	2
1	2	3	1	2	1	1	1	1	2
2	2	2	2	2	1	2	2	2	2
2	2	2	2	2	1	2	2	2	2
1	1	1	1	1	2	1	2	2	9
1	1	2	1	2	9	9	9	9	3
1	1	2	1	3	2	2	2	2	3
2	2	2	2	2	3	2	2	2	1
9	2	9	3	3	1	3	2	3	1
1	1	2	1	1	1	1	1	1	1
1	1	1	1	1	1	1	1	1	1
2	3	2	3	2	2	2	2	2	2

2	1	3	2	3	3	2	2	2	3
2	2	1	2	2	1	2	2	3	2
2	3	2	3	2	1	1	1	1	2
1	1	1	2	1	2	2	2	2	2
2	2	2	2	2	3	3	3	2	2
2	2	2	2	2	3	3	3	2	2
2	2	2	2	1	2	2	2	2	2
3	2	3	3	9	9	9	9	9	3
2	2	2	2	2	2	2	2	2	2
1	1	1	1	3	9	9	9	9	9
2	1	1	1	1	2	1	2	2	1
3	1	2	1	2	2	2	2	2	1
3	3	3	3	3	2	2	2	2	9
2	3	2	2	3	2	2	2	2	3
1	1	1	1	1	9	9	9	9	9
9	9	9	9	9	2	2	2	1	9
1	3	1	3	1	1	2	3	1	3
2	2	2	2	2	2	2	1	2	2
2	3	3	1	2	2	2	2	2	1
3	3	3	3	3	1	1	1	1	3
2	2	2	2	2	1	2	2	2	2
2	2	2	2	2	3	2	3	2	2
1	1	1	1	1	9	9	9	9	9
3	2	9	1	1	1	1	1	1	2
1	1	1	1	1	9	9	9	9	1
3	3	3	3	3	2	2	2	2	3
2	3	2	2	2	2	2	2	2	1
2	3	3	2	2	2	2	2	2	3
3	3	3	2	3	1	1	1	2	3
2	2	2	2	2	2	2	2	2	2
3	3	3	3	3	1	3	3	2	2
9	1	9	9	9	2	1	2	2	9
2	2	2	2	2	2	2	2	2	2
2	1	3	1	2	2	2	2	2	3
3	3	2	1	1	1	2	3	2	1
1	2	3	1	3	1	1	2	2	1
2	2	2	2	2	1	2	2	2	2
3	3	3	3	3	2	2	2	2	2
1	1	2	3	2	1	2	2	2	2
2	2	2	2	2	3	3	3	3	2
3	3	3	2	3	2	2	2	2	2
1	1	2	1	2	1	1	1	1	2
2	1	2	1	2	2	2	2	2	2
1	1	2	2	9	9	9	9	9	3
9	9	9	9	9	9	9	9	9	9
1	9	1	2	1	1	1	3	2	1
2	2	2	2	2	1	3	2	1	2

1	2	2	2	2	2	2	1	1	2
3	1	1	1	3	1	2	2	2	1
3	1	1	1	3	9	9	9	9	1
2	1	2	2	2	2	1	2	1	1
9	9	9	9	9	2	2	2	2	1
1	1	9	9	1	1	1	2	2	9
9	9	9	9	9	1	1	1	1	1
2	2	2	2	2	2	2	3	3	2
2	1	1	1	3	1	3	1	2	2
2	2	2	3	1	3	1	2	2	1
2	2	2	2	2	2	3	2	3	2
2	2	2	2	2	2	3	2	2	1
2	2	2	2	2	1	1	3	3	2
2	2	2	2	2	1	1	1	3	2
2	2	2	2	2	2	3	3	3	2
2	2	2	2	2	1	1	1	1	2
3	3	3	3	3	3	3	3	3	3
2	2	2	2	2	2	2	2	2	2
2	2	2	2	2	2	2	2	2	2
1	2	2	2	2	1	2	3	2	2
1	1	2	1	1	1	2	2	2	1
2	1	1	1	1	1	1	1	1	1
9	9	9	9	1	2	1	2	1	1
2	2	2	2	2	3	3	3	3	2
1	2	2	2	2	2	2	2	2	1
1	2	2	2	2	2	1	2	1	3
1	2	2	1	1	1	2	2	1	2
9	9	9	9	9	9	9	9	9	9
1	1	1	2	2	2	2	2	2	1
2	1	1	2	2	3	3	1	1	2
2	2	2	2	1	2	3	3	3	1
2	1	1	1	3	1	1	2	3	1
2	3	2	1	1	1	1	2	2	2
2	1	2	1	1	3	1	1	2	1
1	1	1	1	3	1	1	1	1	2
2	3	3	3	3	1	2	1	9	1
2	2	2	2	2	3	3	3	3	2
2	2	2	2	2	2	2	2	2	2
1	2	2	2	2	1	2	2	2	2
9	9	9	9	9	9	9	9	9	9
2	1	2	1	1	2	2	2	2	2
3	3	1	1	2	1	1	1	1	2
2	1	2	1	2	2	1	3	1	2
1	1	1	1	1	2	2	2	2	2
2	2	1	1	1	2	1	3	2	1
2	2	2	2	2	3	3	3	3	2
2	2	2	2	2	3	2	3	3	2

1	1	2	2	2	2	2	2	2	2
9	9	9	9	9	3	2	3	2	2
2	2	2	2	2	1	2	2	2	2
3	1	2	3	1	1	1	1	1	1
2	2	2	2	2	2	2	2	2	3
2	2	2	1	1	2	2	2	2	2
3	2	3	2	3	3	3	3	3	3
1	1	1	1	1	2	2	2	2	1
9	2	9	9	9	1	2	2	1	9
2	2	2	1	3	2	2	2	2	3
2	1	1	9	9	3	2	2	2	9
1	1	1	1	2	2	2	2	2	1
2	2	2	2	2	2	1	1	2	2
2	3	1	1	2	2	2	2	2	1
2	2	2	2	2	2	2	2	2	1
2	1	1	1	1	1	1	1	3	1
2	2	2	1	1	1	2	1	2	1
1	9	1	3	3	1	1	1	1	1
9	9	9	9	9	9	9	9	9	9
1	1	1	2	1	1	1	1	3	1
1	1	2	1	2	2	2	2	2	1
1	1	2	1	1	1	1	3	3	1
2	2	2	2	2	1	1	1	1	1
3	1	2	3	1	2	2	1	1	2
2	2	2	2	2	9	9	9	9	9
3	1	1	2	1	3	1	3	2	1
2	2	2	3	3	1	1	2	2	1
2	1	1	1	1	1	2	2	2	2
2	1	2	3	2	1	2	1	2	1
3	1	3	3	2	1	2	2	2	3
2	2	2	2	2	3	3	3	3	2
1	2	2	3	2	1	2	2	1	2
2	1	1	3	1	2	1	3	1	1
2	1	3	2	1	1	1	2	1	1
1	1	1	3	3	1	2	1	2	1
1	2	3	3	3	1	3	2	1	1
2	2	2	2	3	2	2	2	2	1
2	3	2	2	3	2	2	2	2	2
2	2	2	2	2	2	2	2	2	1
1	1	1	2	2	2	2	2	2	1
9	9	9	9	9	9	9	9	9	9
2	2	1	1	1	3	2	2	2	3
9	1	9	3	3	9	9	9	9	1
2	1	1	1	3	2	2	2	1	1
1	1	1	1	3	2	2	2	2	1
3	1	1	1	3	3	2	2	2	1
2	1	1	2	3	1	2	3	2	1

1	3	2	2	3	2	2	2	2	3
9	9	9	9	9	1	2	2	9	9
2	2	2	2	2	1	9	9	9	9
9	9	9	9	9	9	9	9	9	9
2	2	2	2	2	1	2	2	1	2
2	2	2	2	2	2	2	2	2	2
1	2	1	1	2	2	2	2	2	1
1	1	1	1	1	1	1	1	1	1
9	9	9	9	9	9	9	9	9	9
3	1	2	2	3	9	9	9	9	9
9	9	9	9	9	2	2	2	2	2
2	1	2	1	2	2	2	2	3	1
1	1	1	1	1	2	2	2	2	2
1	1	1	1	1	2	2	2	2	2
2	2	2	2	3	1	1	2	2	2
2	1	1	2	2	1	3	2	2	1
1	1	1	1	2	2	2	2	2	1
1	1	1	1	1	2	2	2	2	1
2	1	2	3	3	2	2	1	3	1
1	9	1	2	1	1	1	1	1	9
1	1	1	1	1	1	1	1	1	2
3	3	2	3	3	1	1	3	3	1
9	9	9	9	9	2	2	2	2	1
1	2	2	3	3	1	2	2	2	3
1	2	3	1	2	2	2	2	2	1
1	1	1	1	2	1	1	1	1	1
1	1	2	2	2	1	2	2	2	1
2	1	2	2	3	1	1	2	2	2
2	2	9	3	1	2	2	2	2	1
2	2	2	2	3	2	2	2	1	3
2	3	3	2	1	2	2	2	2	2
3	3	2	2	3	3	1	1	1	2
2	3	1	2	2	2	2	2	2	3
1	1	2	1	1	2	2	2	2	1
3	1	1	3	1	1	3	2	2	2
3	1	1	3	1	1	3	2	2	2
1	1	1	1	2	1	2	1	1	1
9	9	9	9	9	3	2	3	2	2
2	2	2	2	2	3	2	1	2	2
1	2	1	2	1	9	9	9	9	2
2	1	2	3	2	2	2	2	3	1
1	1	1	2	2	2	1	2	1	1
2	1	1	2	1	9	9	9	9	9
9	2	1	3	3	2	2	2	2	1
2	1	2	1	1	2	2	2	1	1
2	1	2	2	2	1	1	1	1	1
1	1	1	1	1	2	2	1	1	1

3	1	1	1	1	1	1	1	1	1
9	9	9	9	9	9	9	9	9	2
3	1	2	3	1	1	1	1	3	1
3	1	2	1	2	2	2	2	2	1
2	2	2	2	2	2	2	2	2	2
3	3	3	3	3	1	1	2	1	1
2	9	2	2	1	1	1	3	1	1
2	2	2	2	2	2	2	2	2	1
2	2	2	2	2	2	2	2	2	1
9	9	9	9	9	9	9	9	9	9
3	2	3	1	1	1	1	1	2	1
2	1	1	1	2	1	1	1	1	1
1	3	1	3	1	1	2	1	1	1
3	2	1	3	1	1	2	2	1	1
2	2	2	2	2	2	2	2	2	2
9	9	9	9	9	9	9	9	9	9
9	9	9	9	9	1	1	1	1	1
1	1	1	1	1	3	2	2	1	1
2	2	2	2	2	1	2	2	2	1
2	2	2	2	2	2	1	1	2	1
9	9	9	9	9	9	9	9	9	9
1	1	1	1	1	1	2	1	2	1
2	2	2	2	2	1	2	2	1	1
3	3	2	2	1	1	2	2	2	1
1	1	1	2	2	2	1	1	2	1
2	2	2	2	2	1	2	2	1	1
1	1	1	1	1	1	2	2	1	1
3	3	3	3	3	2	1	2	1	1
2	1	2	2	1	3	2	2	2	1
3	3	3	3	3	2	1	1	1	3
9	9	9	9	9	9	9	9	9	9
9	9	9	9	9	9	9	9	9	9
9	9	9	9	9	9	9	9	9	9
1	2	2	2	1	2	2	2	3	1
2	1	1	2	3	2	2	2	1	1
2	1	9	2	3	1	3	2	1	1
2	2	2	2	2	1	1	1	2	1
2	1	2	1	1	2	2	2	2	2
2	1	2	2	2	1	3	9	1	1
2	2	2	2	2	2	2	2	2	2
1	1	1	1	3	2	2	2	2	3
1	2	2	1	1	2	1	1	2	1
1	1	2	2	1	2	2	2	2	2
3	1	1	1	1	1	1	2	2	3
1	3	2	1	3	2	2	2	2	2
1	1	1	1	1	1	1	1	1	9
3	1	1	1	1	2	2	2	2	3

3	1	1	3	3	2	2	2	1	2
1	1	2	2	2	2	2	2	2	2
3	3	3	2	2	2	2	2	2	2
1	1	1	3	1	2	2	2	1	1
2	1	1	1	1	2	2	2	2	3
2	3	1	3	3	1	2	2	2	3
2	1	1	2	3	2	2	2	2	2
9	9	9	9	9	9	9	9	9	1
9	9	9	9	9	2	2	3	1	2
9	9	9	9	9	1	1	1	1	1
9	9	9	9	9	2	1	3	3	9
9	9	9	9	9	2	2	2	2	9
1	1	1	1	1	1	3	2	2	2
2	3	2	2	2	2	2	2	9	9
3	1	1	3	3	2	2	2	3	1
2	1	1	2	2	1	1	1	1	1
1	1	1	1	2	1	1	2	2	1
2	1	1	1	2	1	1	2	1	1
2	2	1	3	3	2	2	2	2	2
1	2	3	2	3	2	1	1	3	1
2	2	1	2	1	2	2	2	2	2
2	2	2	2	1	1	3	2	2	2
1	2	2	2	2	1	1	2	2	2
9	9	9	9	9	2	2	2	2	2
2	1	2	3	2	1	1	1	1	1
1	1	1	3	3	2	1	1	1	2
2	2	2	2	2	2	2	2	2	2
3	2	2	2	1	1	2	2	2	1
2	2	2	2	2	3	2	2	2	1
2	2	2	2	2	1	1	2	3	1
2	2	2	2	2	1	1	3	9	2
9	9	9	9	9	1	1	2	2	2
2	1	2	3	2	2	2	2	2	1
1	2	1	1	2	1	1	2	3	2
9	9	9	9	2	3	3	3	9	9
2	2	2	2	2	3	1	2	2	2
2	2	2	2	2	2	2	2	2	2
2	2	2	2	2	2	2	2	2	2
1	1	2	1	1	3	2	2	2	1
2	1	2	2	2	2	2	2	2	2
3	1	1	1	1	3	2	2	1	1
9	9	9	1	9	3	2	2	2	2
9	9	9	9	9	3	2	2	2	2
2	2	2	2	2	2	2	2	2	2
2	9	1	1	3	1	1	3	1	9
1	1	2	3	1	2	2	2	2	2
2	1	2	2	1	2	2	3	3	1

1	1	1	1	2	1	2	2	1	2
2	2	2	2	2	1	2	2	1	2
2	1	2	2	2	2	3	3	1	2
2	2	2	3	3	1	3	2	2	1
2	2	2	2	2	2	3	2	2	2
2	1	1	1	2	3	2	2	2	2
2	3	3	2	2	1	2	2	3	2
3	2	1	2	3	2	2	2	2	2
1	1	1	1	1	1	1	1	1	1
2	1	2	3	1	1	2	1	1	2
1	1	1	1	1	3	2	2	2	1
3	1	2	2	1	2	2	3	2	2
9	1	2	1	1	1	1	1	2	1
1	1	1	1	1	1	2	2	2	2
2	2	2	2	2	2	2	2	2	1
2	2	2	2	2	2	2	2	2	2
3	3	3	3	3	1	1	2	1	1
2	2	2	2	2	2	2	3	3	2
2	2	2	3	2	2	1	2	1	2
2	2	2	1	2	1	3	2	2	1
2	2	1	2	2	1	2	2	1	2
2	1	2	1	3	3	2	2	1	2
2	1	2	2	1	2	2	2	3	1
1	1	2	2	2	2	2	2	2	2
1	1	1	2	1	1	1	1	1	1
2	2	2	2	2	3	3	3	3	2
2	2	2	2	2	1	1	2	1	2
1	1	1	1	1	1	1	1	1	1
2	2	2	2	2	1	1	3	1	2
3	3	2	2	2	2	1	2	1	1
9	9	9	9	9	9	9	9	9	9
2	2	2	2	2	1	2	2	1	1
1	2	2	2	2	2	2	2	1	1
2	2	2	2	2	2	1	2	1	1
2	3	2	2	2	1	2	1	2	2
2	2	2	2	2	2	1	1	1	1
2	2	2	1	2	1	2	2	1	1
3	2	2	2	2	2	2	2	2	2
2	2	1	2	3	1	1	1	1	1
2	2	2	2	2	1	2	1	1	2
1	1	1	2	1	2	2	2	2	2
2	2	1	2	2	2	2	3	1	3
2	2	2	2	2	1	2	2	2	1
2	1	1	2	3	2	2	2	2	1
2	1	1	2	3	1	1	1	2	2
2	2	2	2	2	3	2	2	2	3
3	1	1	3	1	1	3	2	2	2

3	1	1	3	1	1	3	2	2	2
2	2	2	2	2	1	2	1	1	1
1	2	2	2	2	1	2	1	1	1
2	2	2	2	2	2	1	1	1	1
2	2	2	2	3	2	1	1	2	2
2	2	2	1	1	2	1	1	2	1
1	2	2	1	1	1	2	1	1	2
1	2	2	1	2	2	2	2	2	2
3	2	1	3	2	2	2	2	2	3
3	1	1	3	2	2	1	1	1	1
1	1	1	3	3	1	2	1	2	1
2	1	2	1	1	2	2	2	2	1
3	3	3	3	3	3	3	1	1	2
2	1	1	2	2	2	2	2	2	2
1	1	1	1	3	2	2	2	2	3
1	1	2	2	3	3	2	2	2	2
2	2	1	2	1	1	2	2	1	3
3	3	3	2	2	2	1	1	1	1
2	2	2	3	2	3	3	3	3	2
2	1	1	2	1	1	1	2	2	2
3	2	3	3	2	2	1	1	1	1
3	3	3	3	3	2	2	2	2	1
3	1	1	2	2	1	2	2	2	1
2	2	2	2	3	2	2	2	2	2
3	1	2	2	3	1	1	2	1	1
2	1	1	2	1	2	2	1	2	2
1	2	2	1	2	1	1	3	1	1
2	2	2	2	2	2	2	2	2	2
2	1	2	2	2	3	3	3	3	2
1	1	1	2	2	1	2	3	1	2
3	3	3	3	3	2	1	2	2	1
1	1	1	3	3	1	2	1	2	1
3	2	3	1	1	1	2	2	2	1
1	1	1	2	1	2	2	2	2	1
3	1	3	1	2	2	2	2	2	2
2	2	2	2	2	2	2	2	1	1
2	2	1	1	2	1	1	2	2	1
2	1	1	2	3	1	2	3	1	1
2	3	2	1	2	1	2	3	2	1
2	1	9	1	1	1	1	1	1	1
3	1	2	3	1	1	1	1	1	1
2	9	2	1	1	1	1	1	3	1
3	1	1	1	2	1	1	2	2	1
1	9	1	1	1	1	1	1	2	1
2	2	2	2	2	1	2	2	2	1
2	2	3	2	3	2	2	2	2	1
2	2	2	2	2	1	2	3	2	2

1	1	2	2	3	2	2	2	1	2
1	1	2	2	3	2	2	2	1	2
1	1	2	1	1	2	1	1	2	1
3	2	1	2	1	1	1	1	1	1
2	1	1	3	3	1	2	2	2	3
2	2	2	2	2	2	1	1	2	1
2	2	2	2	2	3	3	2	2	2
1	2	2	2	2	2	1	2	1	1
9	9	9	9	9	9	9	9	9	9
2	2	2	2	2	2	2	2	2	2
1	1	1	2	2	1	1	1	1	1
3	3	2	2	1	2	2	2	2	1
3	2	2	2	2	1	1	1	1	2
3	2	3	2	3	3	3	3	3	1
1	1	1	1	1	2	2	2	2	1
2	3	1	3	3	2	2	2	2	2
2	2	2	2	2	3	3	3	1	2
1	2	1	1	1	1	1	1	1	3
1	2	3	2	1	2	2	2	2	1
2	2	2	2	2	2	2	2	3	2
3	2	2	2	2	2	1	1	2	1
1	2	1	2	1	2	1	2	1	1
2	2	2	2	2	2	1	2	2	1
2	2	2	2	2	1	2	1	2	1
1	1	1	1	1	2	2	2	2	1
2	3	1	3	3	2	2	2	2	2
3	3	2	2	1	2	2	2	2	1
2	2	2	2	2	2	2	2	2	2
2	3	2	1	2	1	2	3	2	1
2	2	2	2	2	2	2	2	3	2
2	2	1	1	2	1	1	2	2	1
9	9	9	9	9	9	9	9	9	9
2	2	2	1	2	3	2	2	2	2
3	1	1	1	3	1	1	2	2	1
2	2	2	2	2	2	2	2	2	1
2	2	3	2	3	1	1	1	1	1
1	1	1	3	3	1	1	1	1	1
2	3	2	1	2	1	2	2	2	1
1	1	1	1	1	3	2	2	2	2
1	2	3	1	2	1	1	2	3	1
1	2	1	1	2	2	1	2	1	1
1	2	1	1	2	2	1	2	2	1
3	3	1	1	3	1	1	2	2	1
1	1	2	1	1	1	1	1	1	1
2	2	1	2	3	1	2	3	2	3
9	9	9	9	9	2	2	2	2	2
2	2	2	2	2	1	1	3	1	2

2	1	2	1	2	3	2	2	2	2
3	3	3	3	3	3	2	2	2	2
1	1	1	1	1	1	2	3	2	1
2	2	2	2	2	2	2	2	2	1
2	2	2	2	2	3	2	3	2	2
3	1	1	1	3	1	1	2	2	1
1	1	1	1	2	1	1	2	2	2
2	2	2	3	1	2	2	2	2	2
3	2	3	3	1	2	2	2	2	2
2	2	2	2	1	2	2	2	2	2
2	2	2	2	1	2	2	2	2	3
2	2	2	1	2	2	1	1	2	1
3	3	3	2	1	2	2	2	2	2
2	2	2	2	1	2	2	2	2	2
1	2	2	2	1	1	2	2	1	1
3	3	3	3	1	2	2	1	1	1
2	1	3	2	2	3	2	1	3	2
2	2	2	2	1	2	2	2	1	2
2	2	2	3	2	2	1	2	1	1
2	2	2	2	1	2	1	1	1	1
1	2	2	2	2	2	2	2	2	1
2	2	2	2	1	3	3	2	2	2
2	2	2	2	1	3	1	3	3	2
9	9	9	9	1	9	9	9	9	9
1	1	1	1	2	3	1	3	2	2
9	9	9	9	1	9	9	9	9	9
9	2	3	3	2	1	2	2	3	9
1	9	1	1	4	1	1	1	2	1
1	1	1	1	1	1	1	1	1	1
1	1	1	1	5	1	1	1	1	1
1	1	1	1	5	1	1	1	1	1
2	2	2	2	2	3	2	2	2	2
2	2	2	2	1	9	9	9	9	2
9	9	9	9	1	1	1	1	1	1
3	2	1	1	7	1	1	1	1	2
3	2	2	1	1	2	2	2	3	1
2	1	1	3	4	1	2	2	2	9
2	2	2	2	1	3	1	1	1	1
3	2	1	1	2	1	1	2	2	1
9	9	9	9	3	2	2	2	1	1
2	1	1	2	2	1	1	3	2	1
1	2	3	1	9	2	2	1	1	1
3	2	2	2	1	1	1	2	1	3
2	2	2	2	5	2	1	1	2	2
2	1	2	1	1	2	2	2	2	3
3	2	1	1	4	1	1	1	3	1
1	2	9	2	9	9	9	9	9	1

9	9	9	9	9	9	9	9	9	1
9	2	9	9	2	1	2	2	1	9
9	9	9	9	9	2	2	2	2	9
2	2	2	2	1	1	2	2	2	2
1	2	2	2	2	2	2	3	2	2
9	9	9	9	9	9	9	9	9	9
2	2	2	2	2	1	1	1	1	1
1	2	2	2	2	2	1	1	1	1
2	2	2	2	1	1	2	1	2	2
2	2	1	2	3	1	1	2	2	2
1	3	3	3	5	3	2	1	1	2
9	3	3	3	5	3	2	1	1	2
3	2	3	2	1	2	2	2	3	2
2	2	2	2	1	2	2	2	2	2
2	1	1	1	2	2	1	1	1	1
1	2	1	3	1	2	2	1	2	3
9	9	1	9	1	9	9	9	9	1
2	2	1	1	1	1	2	1	1	1
2	2	1	1	1	2	2	2	2	1
1	1	2	2	1	1	2	1	1	1
2	2	2	2	1	2	1	1	2	1
2	2	2	2	1	2	1	1	1	1
2	2	3	3	9	2	2	2	2	2
3	1	3	2	1	2	2	2	2	1
2	2	2	2	1	2	2	2	2	2
9	9	9	9	9	9	9	9	9	2
2	2	2	2	1	1	2	2	2	3
2	2	2	2	1	3	2	2	1	2
2	2	2	2	9	2	1	1	1	1
1	2	2	2	1	1	1	2	1	1
1	2	2	2	9	1	2	2	1	1
2	2	3	2	1	2	2	2	2	1
1	3	1	3	1	3	3	1	1	1
1	1	2	3	9	2	2	1	2	1
1	1	1	2	1	2	2	2	2	1
1	1	1	3	5	3	1	3	3	3
9	9	9	9	9	9	9	9	9	9
1	3	1	3	1	2	2	2	2	2
1	1	1	9	2	2	2	2	2	1
2	2	2	2	1	2	2	2	2	2
1	3	3	3	1	3	2	2	2	2
2	2	2	2	1	2	2	2	2	2
1	1	3	3	1	1	1	3	1	1
1	2	2	1	1	1	2	2	2	1
2	2	3	3	7	2	2	2	2	3
2	2	2	1	1	1	2	2	2	1
1	1	1	2	1	2	3	1	2	1

9	9	9	9	9	9	9	9	9	9
9	9	9	9	1	2	2	3	2	1
3	2	2	1	1	1	2	2	2	1
1	1	2	2	1	3	2	3	3	2
2	3	2	3	9	1	1	1	1	3
2	1	3	3	1	3	2	2	2	1
1	2	3	1	3	1	2	3	1	1
3	3	3	3	1	3	3	3	3	3
2	2	2	2	1	2	3	2	2	2
3	2	1	1	1	1	2	2	2	1
2	2	2	2	1	1	2	1	1	1
2	3	3	2	1	2	2	2	2	3
2	2	2	2	2	1	2	1	2	1
9	1	3	3	9	9	9	9	9	9
2	2	2	2	1	2	2	2	2	2
1	1	1	2	4	1	3	1	2	1
9	9	9	2	9	1	1	9	2	1
2	2	3	3	1	1	2	1	1	1
1	2	1	1	1	1	1	2	1	1
1	1	2	2	2	3	3	2	2	2
1	1	2	2	1	1	2	2	1	2
2	1	2	2	1	1	9	2	2	2
2	2	2	1	1	2	1	1	1	1
3	1	1	3	1	2	2	2	2	1
1	1	3	3	3	2	9	2	2	2
2	2	1	1	1	2	2	2	2	3
3	1	1	3	9	1	2	2	1	1
1	2	1	3	1	2	2	1	3	1
1	2	1	1	1	2	1	1	2	2
2	2	2	2	1	1	1	1	1	2
2	2	2	2	2	2	3	3	2	1
2	2	2	2	1	2	2	2	2	1
1	1	1	3	2	1	1	2	2	1
1	2	1	2	2	1	1	1	2	1
1	2	2	1	1	2	2	2	2	1
1	2	1	3	1	1	1	3	2	2
3	2	1	1	1	1	2	1	2	1
1	1	1	2	1	2	2	2	2	1
1	2	2	1	1	3	2	2	3	3
1	2	1	1	7	1	1	2	2	1
1	1	1	2	1	2	2	2	2	1
2	2	1	3	9	1	1	2	2	1
2	3	3	3	1	2	2	2	9	2
3	2	2	2	1	2	1	1	1	1
9	9	9	9	9	9	9	9	9	9
3	3	3	3	1	2	2	2	2	3
3	2	1	1	1	2	1	1	1	1

9	9	9	9	9	9	9	9	9	9
2	2	2	2	1	2	1	1	1	1
2	2	1	2	2	2	1	1	1	1
1	3	3	3	3	2	2	2	2	9
1	2	1	1	3	1	1	1	3	1
1	1	1	1	1	2	2	2	2	1
1	2	3	1	1	2	2	2	2	2
3	3	3	3	9	2	2	2	2	1
3	3	3	3	1	1	1	1	1	3
2	2	2	2	1	3	2	2	2	1
1	3	2	1	1	2	2	1	2	1
3	2	3	2	1	2	3	2	2	2
9	9	9	2	9	2	1	2	1	2
1	2	9	3	9	1	1	2	2	3
9	9	9	9	1	3	2	2	2	2
2	2	2	2	9	2	2	2	2	2
1	1	3	3	2	1	1	2	3	1
3	3	3	3	1	2	2	2	1	1
2	2	2	2	9	1	2	2	2	2
2	2	2	2	1	2	2	2	1	2
1	2	2	3	1	2	1	1	1	1
3	3	3	3	1	1	1	1	1	1
1	1	1	1	1	2	1	1	1	1
1	1	2	2	1	2	1	1	1	1
9	9	9	9	1	3	2	2	2	2
1	1	3	2	1	2	2	2	2	3
1	2	1	2	1	1	2	3	3	2
1	1	2	2	1	3	2	2	3	2
2	3	3	2	1	3	3	2	2	2
1	2	2	2	1	3	2	2	2	1
3	3	2	2	5	1	1	2	2	2
3	1	3	1	1	2	2	2	2	3
1	1	2	2	1	2	2	2	2	2
1	2	2	3	1	3	1	1	1	2
2	9	2	2	9	2	2	2	2	2
1	2	1	1	1	1	1	2	2	1
1	2	1	2	7	1	1	1	2	1
2	2	3	3	7	2	2	2	2	1
1	2	1	1	2	1	1	1	1	1
3	3	2	3	2	1	3	1	2	3
2	3	3	3	1	1	1	1	1	2
1	1	1	1	7	1	1	1	1	3
2	2	2	2	1	1	1	1	1	1
2	1	1	1	2	1	1	1	1	1
1	1	1	3	2	2	2	2	2	1
2	2	2	2	9	1	2	1	1	1
1	1	1	1	9	9	2	2	1	9

2	2	2	2	4	1	1	1	2	2
3	1	2	2	1	1	2	2	2	2
9	9	9	9	9	9	9	9	9	9
9	9	9	9	9	9	9	9	9	9
1	1	1	2	1	2	1	1	2	1
2	2	1	1	2	1	2	1	1	1
9	9	9	9	2	1	1	3	2	1
3	3	3	3	1	2	2	2	2	2
1	1	1	1	5	1	2	2	2	2
9	9	9	9	3	1	2	2	2	1
9	9	9	9	1	2	2	2	2	9
9	9	9	9	9	9	9	9	9	9
1	1	2	2	1	1	1	2	2	2
9	9	9	9	9	9	9	9	9	9
3	3	2	3	1	1	3	2	2	1
9	9	9	9	4	2	3	1	1	9
9	9	9	9	1	2	2	2	2	9
9	9	9	9	4	2	2	3	3	9
2	2	2	1	2	1	2	3	3	1
1	1	2	3	9	9	9	9	9	1
9	9	9	9	9	2	1	1	1	3
3	2	2	3	1	2	1	2	1	3
3	3	3	3	9	1	1	1	1	3
2	2	2	2	2	1	2	1	1	3
1	1	1	1	1	3	3	3	3	2
2	2	3	2	1	2	2	2	2	2
9	9	9	9	1	2	1	2	2	1
9	9	9	9	9	9	9	9	9	9
1	1	1	1	1	3	2	2	2	3
1	1	1	2	1	2	2	2	2	1
2	2	2	2	1	3	2	2	2	2
1	1	1	1	1	2	2	2	9	1
2	2	1	1	5	1	2	2	2	2
1	1	1	1	1	1	2	2	3	3
3	3	3	3	1	1	1	1	1	1
3	3	3	3	2	1	1	1	1	1
2	2	2	2	1	3	2	2	2	2
1	1	2	2	1	2	2	1	2	1
9	9	9	9	9	9	9	9	9	9
2	2	2	2	1	2	2	2	2	3
1	1	1	3	2	2	2	2	1	2
2	2	2	2	2	1	2	2	1	1
2	1	3	3	9	2	1	2	1	2
2	2	2	2	1	1	2	2	3	2
2	3	1	9	1	3	2	1	2	2
2	1	1	3	1	2	2	2	2	1
2	2	2	2	1	1	2	1	3	1

3	2	1	3	1	1	1	1	1	3
3	3	2	2	3	1	2	1	3	1
2	2	2	2	1	1	1	2	2	2
3	2	1	1	1	3	3	3	3	2
2	1	2	3	2	1	1	1	2	3
9	9	9	9	9	9	9	9	9	9
2	2	2	2	3	2	2	2	1	1
1	1	1	1	1	1	2	2	2	1
1	1	1	1	1	2	2	2	2	2
9	2	3	2	3	2	9	9	9	9
2	2	2	2	2	1	2	2	2	2
3	2	2	2	1	2	2	3	3	2
2	3	3	3	5	1	1	1	2	1
2	2	2	2	1	3	2	1	3	3
1	1	1	1	1	2	1	2	2	1
1	1	1	1	1	1	2	2	2	3
1	1	1	1	9	2	2	2	3	2
9	9	9	9	9	1	1	2	3	3
9	9	9	9	1	2	2	2	2	2
2	2	2	2	1	1	1	1	1	1
2	9	2	2	9	2	2	2	2	2
9	2	9	2	9	2	2	2	2	9
9	9	9	9	9	9	9	9	9	9
9	9	9	9	9	9	9	9	9	9
2	9	2	2	1	2	2	2	2	2
9	9	9	9	9	9	9	9	9	9
9	9	9	9	9	9	9	9	9	9
2	2	2	2	1	1	2	1	1	1
2	2	2	2	1	2	1	2	1	1
1	2	1	1	1	2	1	1	1	1
1	1	2	2	1	2	2	1	1	1
9	9	9	9	9	9	9	9	9	9
2	2	2	2	1	1	2	1	2	1
9	9	9	9	9	9	9	9	9	9
9	9	9	9	9	9	9	9	9	9
1	2	2	2	1	1	1	2	1	1
9	9	9	9	1	2	2	2	2	9
9	9	9	9	9	9	9	9	9	9
9	9	9	9	9	9	9	9	9	9
9	9	9	9	9	9	9	9	9	9
1	9	3	1	1	2	2	2	2	9
1	9	3	1	1	2	2	2	2	9
9	9	9	9	9	9	9	9	9	9
2	9	9	9	1	9	9	9	9	9
9	9	9	9	9	9	9	9	9	9
9	9	9	9	1	9	9	9	9	3
9	9	9	2	9	9	9	9	9	3

1	9	9	9	1	2	2	2	2	9
3	3	3	3	1	1	1	1	1	3
2	3	2	2	9	2	2	2	1	1
2	1	3	1	1	1	1	1	1	3
9	1	2	1	2	1	1	9	9	1
9	9	9	9	1	1	2	2	1	1
9	9	9	9	1	3	2	2	2	1
9	9	9	9	1	1	2	2	2	9
2	2	3	3	4	1	1	2	2	2
3	1	3	2	1	1	2	1	1	3
3	3	3	2	9	9	9	9	9	2
2	2	2	2	1	1	2	3	9	2
1	2	1	2	1	1	1	2	1	2
1	1	1	1	1	2	2	2	2	2
2	2	2	2	9	3	3	3	3	2
3	3	3	3	1	1	1	1	1	3
9	9	9	9	9	9	9	9	9	9
9	9	9	9	9	9	9	9	9	9
9	9	9	9	2	2	2	2	2	9
9	9	9	9	9	1	1	1	1	9
1	1	1	1	1	1	2	1	1	2
2	3	2	2	9	1	1	1	2	1
9	9	9	9	9	9	9	9	9	9
2	2	1	2	1	1	2	2	2	2
1	1	1	1	9	1	1	1	1	1
2	2	2	2	1	2	2	2	2	1
1	2	3	3	4	1	2	2	2	3
2	2	2	2	1	1	2	2	2	2
2	3	3	3	1	2	2	2	1	2
2	2	2	2	1	1	2	1	1	2
2	2	2	2	1	2	1	2	2	2
2	2	2	2	1	1	1	1	1	2
2	2	2	2	1	2	2	2	2	2
3	2	3	1	2	1	1	1	1	1
2	2	2	2	9	2	3	3	3	2
2	1	2	1	1	1	2	2	1	1
2	2	2	2	1	3	3	3	3	2
2	2	2	2	1	3	3	3	3	2
2	1	2	3	4	2	2	2	2	2
3	9	2	2	9	2	2	2	3	3
1	3	3	3	9	1	1	1	2	1
2	2	2	2	9	2	1	3	3	1
9	9	9	9	9	9	9	9	9	9
2	2	2	2	1	2	2	2	2	2
3	3	3	3	1	2	2	2	2	3
3	3	3	3	9	2	2	2	2	3
9	9	9	9	9	3	3	3	3	2

9	9	9	9	9	9	9	9	9	9
9	9	9	9	9	2	2	2	9	9
1	1	1	1	7	1	1	2	3	2
2	3	2	2	2	2	2	1	3	2
2	2	2	2	9	2	2	2	2	2
3	3	3	3	4	1	1	1	1	2
9	9	9	9	9	1	2	3	2	9
9	9	9	9	1	2	2	1	1	2
9	9	9	9	1	2	2	9	2	9
9	9	9	9	3	2	3	1	1	9
9	9	9	9	1	1	3	3	3	9
1	1	1	1	1	1	1	1	1	3
9	9	9	9	1	3	2	3	3	9
9	9	9	9	1	2	2	2	1	2
1	1	1	2	1	2	2	2	2	2
9	9	9	9	1	2	2	2	2	2
9	9	9	9	9	2	3	2	2	9
2	9	9	9	9	1	9	2	2	9
9	9	9	9	9	2	2	2	2	2
9	9	9	9	9	3	2	3	1	9
9	9	9	9	2	2	2	2	3	2
9	9	9	9	9	9	9	9	9	9
3	9	9	2	3	2	2	2	2	9
3	2	1	2	5	2	2	1	1	2
3	1	3	3	1	1	1	1	1	1
2	2	2	2	1	2	2	2	2	2
2	1	1	1	2	1	1	1	2	1
1	2	2	3	5	9	9	9	9	1
2	2	2	2	1	2	2	2	2	2
2	2	3	2	7	3	3	1	3	2
9	1	2	3	7	2	3	3	3	2
2	2	2	2	1	9	9	9	9	9
2	2	2	2	1	3	2	2	2	2
2	2	2	2	1	2	2	2	2	2
1	3	2	2	1	1	3	1	3	1
1	1	1	1	1	1	1	1	1	1
3	2	2	1	1	1	1	1	1	1
3	3	3	3	1	2	2	2	2	2
3	3	3	3	2	2	1	1	2	2
2	2	1	1	1	2	2	2	2	1
9	9	9	9	9	9	9	9	9	9
2	2	2	2	1	1	2	2	2	2
9	9	9	9	9	9	9	9	9	9
2	2	2	2	3	2	2	1	2	1
3	3	2	3	2	2	2	2	2	9
2	2	2	2	9	1	1	1	1	2
1	2	1	2	1	2	2	2	2	1

1	1	2	3	2	2	2	2	2	3
1	1	1	3	1	2	2	2	1	1
1	1	1	2	1	2	3	2	2	2
2	2	2	2	1	2	2	2	2	3
2	2	2	2	1	1	1	2	2	1
2	2	2	2	1	1	1	1	1	2
2	1	1	3	1	1	1	1	1	1
2	2	2	2	1	2	9	2	2	2
1	1	2	2	1	1	1	1	1	1
3	1	2	3	1	2	2	2	3	2
2	2	2	2	1	2	2	2	2	2
2	2	3	2	1	2	2	2	2	2
9	2	2	2	9	1	1	2	2	1
1	2	2	2	1	1	1	3	2	1
9	9	9	9	9	9	9	9	9	9
3	1	2	2	1	2	2	2	2	2
1	1	1	2	2	1	2	2	2	2
9	9	9	2	5	2	2	2	2	1
9	9	9	9	1	9	9	9	9	1
1	9	1	9	9	2	2	2	1	1
9	9	9	2	1	2	1	2	2	1
2	2	2	2	1	2	2	2	2	2
9	9	9	9	9	9	9	9	9	9
9	9	9	9	1	9	3	2	1	2
9	9	9	9	1	2	3	3	3	9
2	3	2	3	1	3	2	2	2	2
2	2	2	2	9	3	3	3	3	2
3	3	2	3	1	2	2	2	2	2
2	2	2	2	1	2	2	2	2	2
9	9	9	1	1	2	2	2	2	9
9	9	9	9	1	2	2	2	2	2
2	2	2	2	3	2	2	2	2	1
1	1	1	1	3	1	1	1	1	1
1	3	1	1	2	1	1	1	1	2
2	2	2	2	1	9	9	9	9	9
9	9	9	9	4	2	3	1	1	3
2	2	2	2	9	2	2	2	2	2
2	3	1	2	1	2	2	2	2	1
1	1	1	1	1	2	2	2	2	2
3	3	3	3	2	1	2	2	2	3
1	1	1	3	1	1	2	2	2	1
9	9	9	9	9	9	9	9	9	1
9	9	9	9	9	9	9	9	9	9
9	9	9	9	9	9	9	9	9	9
2	1	1	3	2	1	1	1	3	3
1	2	1	2	1	1	1	1	2	1
1	1	1	1	1	1	2	1	2	2

3	3	3	3	6	2	2	2	2	2
2	2	2	2	1	2	2	2	2	2
2	1	1	2	1	2	2	2	2	1
3	3	3	3	9	9	9	9	9	3
2	2	2	2	2	1	2	3	3	2
3	3	3	3	6	1	2	3	3	3
2	2	2	2	2	2	2	2	3	3
2	3	1	2	4	1	1	1	1	1
9	9	9	9	2	3	1	2	1	9
9	9	9	9	2	2	2	2	2	1
9	9	9	9	1	2	2	3	3	2
1	2	3	2	1	1	1	1	1	3
9	9	2	9	1	2	2	2	2	2
9	2	2	2	1	2	2	2	2	3
9	9	9	9	9	9	9	9	9	9
9	9	9	9	9	9	9	9	9	9
2	2	2	2	1	2	2	2	2	9
9	9	9	9	1	2	2	2	2	9
9	9	9	9	1	2	2	2	2	9
9	9	9	9	9	9	9	9	9	9
9	9	9	9	1	2	2	2	2	9
9	9	9	9	1	2	2	2	2	2
3	3	3	3	1	3	3	3	3	3
9	9	9	9	1	1	1	1	1	1
9	9	9	9	1	1	1	1	1	2
9	2	9	2	1	1	1	1	1	2
9	9	9	9	9	9	9	9	9	9
9	9	9	9	1	2	2	2	2	2
2	2	2	2	9	2	2	2	2	2
9	9	9	9	9	9	9	9	9	9
9	9	9	9	9	9	9	9	9	9
9	9	9	9	9	1	1	1	1	2
9	9	9	9	9	9	9	9	9	9
9	9	9	9	1	1	1	1	1	1
9	9	9	9	5	1	1	1	1	1
9	9	9	9	9	9	9	9	9	9
9	9	9	2	1	9	9	9	9	9
9	2	2	2	1	1	1	1	1	2
1	9	1	1	1	9	9	9	9	9
9	9	9	9	9	9	9	9	9	9
2	2	2	1	9	9	9	9	9	9
9	9	9	9	9	9	9	9	9	9
9	2	1	9	1	2	2	2	2	9
9	9	9	9	1	9	9	9	9	9
1	1	2	2	1	2	2	2	2	2
2	1	2	1	4	1	2	3	2	3
9	9	9	9	1	2	2	2	2	9

9	9	9	9	9	9	9	9	9	9
3	2	1	2	1	2	2	2	2	2
2	3	1	2	1	2	2	2	2	1
3	3	3	3	1	1	2	2	2	3
1	3	2	1	9	1	2	1	1	1
9	9	9	9	2	9	9	9	9	2
9	9	9	9	9	1	1	2	2	1
2	3	3	1	2	1	1	3	2	2
2	2	2	2	1	2	2	2	2	2
1	1	1	1	4	1	1	1	1	1
2	2	2	2	1	2	2	2	2	2
3	2	2	2	5	3	3	3	1	2
3	3	3	3	2	2	1	1	1	9
2	2	2	2	1	3	3	2	2	3
2	2	2	2	1	3	3	3	3	2
1	1	1	2	3	1	2	2	2	1
2	3	2	2	4	2	2	9	2	2
9	9	9	9	1	3	3	2	2	9
9	9	9	9	1	2	2	2	2	9
2	2	2	2	1	1	2	1	1	9
9	9	9	9	2	1	2	2	2	2
2	2	2	2	5	3	3	1	2	2
1	2	1	3	1	2	2	2	2	3
1	1	1	2	3	2	2	2	2	1
1	1	1	1	1	2	2	2	2	1
2	2	2	2	1	1	1	1	3	2
2	2	2	2	1	2	2	2	2	2
2	3	2	3	1	9	9	9	9	9
1	1	1	1	1	2	2	2	1	3
9	9	9	9	2	1	2	2	2	2
9	9	9	9	2	2	3	2	2	9
2	2	2	2	1	1	1	1	1	1
9	9	9	9	9	1	9	2	2	9
2	2	2	1	3	2	2	2	2	2
9	9	9	9	9	9	9	9	9	9
9	9	9	9	1	1	1	1	1	9
2	2	2	2	1	1	1	1	2	2
3	2	1	3	1	1	2	2	2	2
9	9	9	9	9	9	9	9	9	9
9	9	9	9	4	1	1	1	1	1
1	2	2	1	1	1	2	2	1	1
9	9	9	9	9	9	9	9	9	9
2	3	2	1	1	3	3	2	9	2
1	1	1	1	3	1	1	1	1	1
2	2	2	2	1	3	3	3	3	2
2	2	2	2	2	1	1	2	2	9
2	2	2	2	1	2	2	2	2	2

2	2	2	2	9	2	2	2	2	1
2	2	2	2	1	2	2	2	2	2
2	2	2	2	1	2	2	2	2	2
2	2	2	2	1	2	2	2	2	2
2	2	2	2	1	2	2	2	2	3
2	3	2	2	2	3	3	3	3	2
9	9	9	9	1	2	3	3	2	2
2	2	2	2	5	3	1	3	3	2
1	1	3	3	1	2	2	2	2	2
9	9	9	9	5	1	1	2	1	9
9	9	9	9	1	2	2	2	2	3
9	9	9	9	9	9	9	9	9	9
9	9	9	9	9	9	9	9	9	9
2	2	2	2	9	2	2	2	2	2
2	2	2	2	1	1	1	1	1	2
9	9	9	9	1	2	2	2	2	9
2	2	2	2	1	1	1	1	1	2
3	2	3	3	1	1	1	1	2	2
2	1	2	2	4	1	1	1	1	1
2	2	2	2	1	2	2	2	2	2
1	2	2	3	1	2	2	3	1	9
9	9	9	9	1	9	9	9	9	9
9	9	9	9	9	9	9	9	9	9
9	9	9	9	9	9	9	9	9	9
9	9	9	9	1	2	2	2	2	1
9	9	9	9	1	2	2	2	2	2
9	9	9	9	9	9	9	9	9	9
9	9	9	9	9	9	9	9	9	9
2	2	2	2	1	2	2	2	2	2
9	9	9	9	1	2	2	2	2	2
9	9	9	9	9	9	9	9	9	9
9	9	9	9	9	2	2	2	2	3
9	9	9	9	9	9	9	9	9	9
9	9	9	1	1	2	2	2	2	9
9	2	9	2	1	2	2	2	2	2
9	9	9	3	1	2	9	2	2	3
9	9	9	9	9	9	9	9	9	9
9	2	2	2	1	2	2	2	2	2
9	9	9	9	9	9	9	9	9	9
9	9	9	9	1	9	9	9	9	9
9	9	9	9	1	2	2	2	2	9
9	9	9	9	9	9	9	9	9	9
9	9	9	9	1	2	2	2	2	9
9	9	9	9	9	9	9	9	9	9
9	9	9	9	1	9	9	9	9	9
9	9	9	9	1	9	9	9	9	9
2	2	2	2	1	1	1	1	1	2

9	9	9	9	1	1	1	1	1	2
9	9	9	9	1	9	9	9	9	9
9	9	9	9	1	2	2	2	2	9
9	9	9	9	9	9	9	9	9	9
9	9	9	9	1	2	2	2	2	9
9	9	9	9	9	9	9	9	9	9
9	9	9	9	9	9	9	9	9	9
9	9	9	9	9	9	9	9	9	3
9	9	9	9	1	2	2	2	2	2
9	9	9	9	1	9	9	9	9	9
9	9	9	9	9	9	9	9	9	9
9	9	9	9	9	9	9	9	9	9
9	9	9	9	9	9	9	9	9	9
9	9	9	9	1	9	9	9	9	9
9	9	9	9	9	9	9	9	9	9
9	9	9	9	9	9	9	9	9	9
9	9	9	9	9	9	9	9	9	9
9	9	9	9	9	9	9	9	9	9
9	9	9	9	9	9	9	9	9	9
9	9	9	9	9	9	9	9	9	9
9	9	9	9	1	2	2	2	2	9
9	9	9	9	1	2	2	2	2	9
9	9	9	9	1	9	9	9	9	9
2	2	2	2	1	2	2	2	2	2
9	9	9	9	1	9	9	9	9	9
2	2	2	2	1	2	2	2	2	9
9	9	9	9	9	9	9	9	9	9
9	9	9	9	1	2	2	9	2	9
9	9	9	9	1	9	9	9	9	9
9	9	9	9	1	2	2	2	2	9
9	9	9	9	1	2	2	2	2	2
9	9	9	9	1	2	2	2	2	9
9	9	9	9	9	9	9	9	9	9
9	2	2	1	1	2	2	3	2	1
9	9	9	9	9	9	9	9	9	9
9	9	9	9	9	9	9	9	9	9
1	1	1	1	1	1	1	1	1	1
1	3	2	1	1	2	2	2	2	2
9	9	9	9	9	9	9	9	9	9
1	2	1	2	1	2	2	2	2	3
9	9	9	9	9	9	9	9	9	9
1	9	1	9	1	2	2	2	2	2
9	9	9	9	1	1	1	1	1	2
9	9	9	9	1	9	9	9	9	9
1	1	1	1	1	2	2	2	2	1
9	9	9	3	1	2	2	2	2	3
9	9	9	9	9	9	9	9	9	9

9	9	9	9	9	9	9	9	9	9
3	3	3	3	2	2	2	2	2	2
9	9	9	9	1	1	1	1	1	1
9	9	9	1	1	2	2	2	2	9
9	9	9	2	1	9	9	9	9	9
9	9	9	9	1	9	9	9	9	9
9	9	9	2	1	3	3	3	3	2
9	9	9	9	9	9	9	9	9	9
1	9	2	3	1	2	2	2	2	2
9	9	9	9	1	9	9	9	9	9
9	9	9	9	9	9	9	9	9	9
9	9	9	9	9	9	9	9	9	9
9	9	9	9	1	1	1	1	1	2
9	9	9	9	9	2	2	2	2	2
9	9	9	2	1	1	1	1	1	2
9	9	9	9	1	2	2	2	2	9
9	9	9	9	9	2	2	2	2	9
9	9	9	9	9	9	9	9	9	9
9	9	9	9	9	9	9	9	9	9
9	9	9	9	9	9	9	9	9	9
2	2	2	2	1	2	2	2	2	1
1	2	1	1	1	2	2	2	2	3
9	9	9	9	1	2	2	2	2	9
2	2	2	2	1	2	2	2	2	2
2	2	2	2	1	2	2	2	2	2
9	9	9	9	3	1	1	1	1	1
9	9	9	9	3	1	1	2	2	1
9	9	9	9	2	1	1	9	1	1
3	2	2	1	1	2	2	2	2	3
2	3	1	2	1	2	2	2	2	1
1	2	1	2	2	2	2	2	2	2
2	2	1	2	1	1	1	2	2	2
1	3	1	1	2	2	2	2	2	1
2	2	2	2	2	3	3	3	1	2
2	2	2	2	3	2	2	3	1	2
1	1	1	1	1	2	2	2	2	1
9	9	9	9	1	2	2	2	2	1
9	9	9	9	1	1	1	9	3	2
9	9	9	9	4	1	2	1	1	9
9	9	9	9	5	2	2	3	2	2
9	9	9	9	1	1	9	2	1	2
9	9	9	9	9	9	9	9	9	9
9	9	9	9	9	9	9	9	9	9
9	9	2	9	1	1	1	1	1	9
9	9	9	9	9	9	9	9	9	9
9	2	9	2	9	2	2	2	2	2
9	9	9	9	1	9	9	9	9	9

2	2	2	2	1	1	1	1	1	2
9	9	9	9	1	9	9	9	9	9
9	9	9	9	1	9	9	9	9	9
9	9	9	9	9	9	9	9	9	9
2	2	2	2	1	2	2	2	2	2
3	2	3	3	1	3	3	2	2	1
1	1	2	2	2	1	3	3	3	1
1	1	2	2	1	1	1	2	2	2
1	1	1	1	2	1	1	3	3	2
1	3	2	1	1	9	9	9	9	3
3	3	3	3	4	3	2	2	2	3
2	2	2	1	1	3	1	2	2	1
1	1	9	1	1	2	2	2	2	2
3	3	3	3	1	2	2	2	2	3
2	2	2	2	1	2	2	2	2	9
2	2	2	2	1	1	1	1	1	2
9	9	2	9	1	2	2	2	2	2
1	9	9	1	1	2	2	2	2	9
2	2	2	2	9	1	2	2	1	2
3	9	1	2	1	1	2	2	1	1
2	1	2	3	1	2	2	3	3	3
2	2	2	2	1	2	2	2	2	2
3	2	1	2	9	2	2	2	2	2
2	2	2	2	9	3	3	3	3	2
9	9	9	9	4	9	9	9	9	9
2	2	3	2	2	1	1	2	2	2
3	2	2	2	1	1	1	2	2	3
2	3	2	3	2	2	1	2	2	1
1	2	3	3	5	1	3	2	2	2
1	1	1	2	1	1	3	1	2	1
1	2	2	2	1	2	2	2	2	1
1	1	1	1	2	2	2	2	2	2
1	2	1	1	2	1	3	2	2	2
2	3	2	2	1	2	2	2	2	2
1	2	2	3	1	2	1	2	2	2
1	2	1	1	2	2	2	2	2	1
2	3	3	3	1	3	2	2	2	3
2	2	2	2	3	2	3	3	3	9
3	3	3	3	1	2	2	2	2	3
2	3	3	2	1	3	3	2	2	2
9	9	9	9	1	3	3	3	3	9
2	3	3	2	1	3	3	3	3	3
1	1	1	2	1	2	2	2	2	2
2	2	2	2	3	1	1	1	1	1
3	3	3	3	1	2	2	1	2	2
9	9	9	9	9	9	9	9	9	9
2	2	2	2	3	1	2	2	2	2

1	9	9	9	5	3	1	2	1	9
1	2	3	1	1	2	1	2	3	1
2	2	2	2	1	9	2	2	2	2
1	3	2	3	1	2	3	3	3	2
3	2	3	2	1	3	2	2	1	9
2	3	3	1	1	2	3	3	2	3
9	9	9	9	2	9	9	9	9	9
3	3	3	3	3	2	2	2	2	3
1	3	1	3	1	1	2	2	1	1
2	3	3	3	3	2	2	2	2	2
1	1	2	2	2	3	1	3	1	1
1	9	9	9	1	9	9	9	9	9
2	2	2	2	4	1	2	2	2	2
1	3	1	2	3	3	3	3	3	1
3	1	2	1	1	2	2	2	1	1
1	2	2	2	1	2	3	3	3	1
3	3	2	3	9	2	3	3	3	1
2	2	2	2	3	1	2	2	2	2
9	9	9	9	1	9	9	9	9	9
2	2	2	3	1	2	2	2	2	3
1	2	3	3	2	1	1	3	1	1
2	2	2	2	1	2	2	2	2	2
1	1	1	1	1	2	2	2	2	2
2	2	2	2	1	1	3	2	2	2
2	2	2	2	1	9	9	9	9	2
9	9	9	9	3	1	3	3	1	2
1	2	1	1	2	9	9	9	9	9
2	2	1	2	9	9	9	9	9	1
2	2	3	3	1	2	2	2	2	2
9	1	3	2	1	2	2	2	3	2
3	3	3	3	9	9	9	9	9	9
3	2	1	2	1	2	3	3	2	1
9	9	9	9	1	2	2	2	2	2
2	2	2	2	9	2	2	3	2	2
2	2	2	2	1	3	3	3	3	2
2	2	2	2	7	3	3	3	3	2
2	1	3	1	2	2	3	1	2	3
9	9	9	9	1	9	9	9	9	9
9	9	9	9	9	9	9	9	9	9
1	1	1	2	1	2	1	1	2	1
3	3	3	2	3	1	1	2	2	1
9	9	9	9	9	9	9	9	9	9
3	3	3	3	1	9	9	9	9	9
1	2	2	2	1	2	2	2	2	2
3	2	1	1	5	1	1	1	2	1
2	1	1	2	3	2	2	2	2	1
9	9	9	9	2	2	2	2	2	2

2	2	2	2	1	2	2	2	2	2
2	2	2	2	1	2	2	2	2	2
2	2	2	2	1	3	3	3	3	2
2	2	2	2	1	2	2	2	2	2
3	2	3	3	1	3	2	2	2	2
2	2	2	2	1	2	2	1	2	2
3	3	3	3	5	1	2	1	2	3
2	2	2	2	1	2	2	2	2	2
2	2	2	2	1	2	2	1	2	2
2	2	2	2	1	1	1	1	1	1
2	2	2	2	4	3	2	2	2	2
2	2	2	2	1	2	2	2	2	2
2	2	2	2	1	2	2	2	2	2
2	2	2	2	9	2	2	2	2	2
2	2	2	2	1	2	2	2	2	2
9	9	9	9	9	9	9	9	9	9
1	1	2	2	1	1	1	2	1	1
2	3	3	3	1	2	2	3	2	2
1	1	2	2	1	2	2	2	2	1
2	1	1	2	1	1	3	1	2	3
2	2	2	2	1	2	2	2	2	3
1	2	1	2	1	1	1	2	1	9
9	9	9	9	9	9	9	9	9	9
9	9	9	2	3	2	3	3	3	9
9	9	9	9	9	9	9	9	9	9
2	2	2	2	9	9	9	9	9	2
2	2	1	1	1	2	2	3	2	1
9	9	9	9	9	9	9	9	9	9
9	9	9	9	9	9	9	9	9	9
2	2	2	2	9	3	3	3	3	2
1	1	1	1	2	1	2	2	1	2
2	2	2	2	1	3	3	3	3	2
3	1	2	3	9	1	2	1	2	1
2	2	3	2	1	1	3	3	2	3
2	1	2	3	1	2	2	2	2	2
2	3	2	3	2	2	1	3	1	2
9	9	9	9	9	9	9	9	9	9
2	1	3	1	3	9	9	9	9	1
3	2	1	2	2	1	2	2	1	2
2	2	2	1	2	1	9	1	1	2
2	2	2	2	1	1	2	2	2	2
2	2	2	2	1	3	3	3	3	2
9	9	9	9	1	9	9	9	9	9
2	2	2	2	9	2	2	2	2	2
2	2	2	2	1	3	3	3	3	2
3	3	3	3	1	1	2	3	2	3
1	1	1	1	1	1	2	2	1	1

1	1	1	1	1	1	2	2	1	2
2	2	2	2	1	2	2	2	2	1
2	2	2	2	1	1	3	3	3	1
1	2	2	3	1	2	2	2	2	2
2	2	2	1	1	1	2	2	2	1
3	3	2	3	1	1	2	2	2	3
2	1	3	3	9	9	9	9	9	2
2	2	2	2	1	2	3	3	3	2
2	9	3	3	1	2	2	1	2	2
2	2	2	2	1	2	3	3	3	2
3	2	3	2	1	1	3	3	3	3
2	2	2	2	1	9	9	9	9	9
9	9	9	9	1	9	9	9	2	9
3	3	3	3	2	2	2	2	2	2
9	9	9	9	9	9	9	9	9	9
2	2	3	1	9	9	9	9	9	9
2	2	2	2	1	2	2	2	2	2
1	1	1	2	9	3	1	2	2	1
2	1	1	2	2	1	1	1	1	2
1	9	9	9	1	3	3	1	1	9
9	9	9	9	1	2	2	2	2	9
9	9	9	9	1	2	2	2	2	1
1	1	2	1	3	1	1	2	3	1
1	1	1	1	4	9	9	9	9	9
1	1	2	2	1	1	2	3	3	1
1	1	1	1	3	1	1	1	3	2
2	9	1	2	3	1	2	3	9	2
1	1	3	1	1	2	2	2	2	9
1	1	2	2	1	2	2	2	2	2
2	2	2	2	1	3	2	2	2	2
1	1	1	1	3	2	2	2	2	2
2	2	2	2	1	3	3	3	3	2
2	2	2	2	1	3	2	2	2	2
2	2	2	2	1	2	2	2	2	2
3	9	1	2	1	3	3	3	3	2
3	3	3	3	1	2	2	2	2	3
3	1	2	1	2	2	2	1	3	2
2	1	2	2	3	1	1	1	1	1
3	3	3	3	1	2	2	2	2	2
2	2	2	2	1	2	2	3	3	2
2	2	2	2	1	1	2	9	2	2
2	1	2	1	1	2	3	1	2	2
1	1	1	1	3	1	1	2	1	1
2	2	2	2	1	2	2	2	2	2
2	3	1	1	1	1	1	2	1	2
2	2	2	2	3	2	2	2	2	2
1	1	1	9	1	1	1	1	1	1

2	2	2	2	2	1	2	2	2	2
2	2	2	2	9	2	2	2	2	2
1	2	1	1	3	1	2	2	2	2
2	2	2	2	9	1	2	2	2	2
2	2	9	2	4	2	2	2	1	1
2	2	2	2	9	2	2	2	2	2
2	2	3	2	1	3	1	2	3	2
1	2	1	3	5	1	2	2	1	1
3	2	3	2	1	1	1	2	2	2
2	2	2	2	1	1	1	1	1	2
2	1	2	2	4	2	3	3	1	2
9	9	9	9	2	2	1	2	2	9
2	2	2	2	1	3	3	3	3	3
9	9	9	9	1	1	1	1	1	1
9	9	9	9	1	1	1	1	1	1
3	3	3	3	4	9	9	9	9	9
9	9	9	9	2	2	2	2	2	9
9	9	9	9	1	2	1	9	3	9
9	9	9	9	9	2	1	2	1	2
9	9	9	9	1	2	3	2	1	9
1	1	2	2	1	2	2	2	2	2
9	9	9	9	1	2	2	9	9	2
9	9	9	9	9	9	9	9	9	9
9	9	9	9	1	2	2	2	2	9
9	9	9	9	1	2	2	2	2	9
9	9	9	9	3	9	9	9	9	9
9	9	9	9	1	9	9	9	9	9
2	2	2	2	1	2	3	3	3	2
2	2	3	3	1	2	2	2	2	2
2	1	2	3	1	2	2	2	2	2
3	2	2	1	2	2	2	2	1	1
1	1	2	2	1	1	2	2	9	9
2	2	2	2	1	2	2	2	2	1
9	9	9	9	1	2	2	2	2	1
2	2	2	2	1	2	2	2	2	3
1	1	3	3	2	1	2	1	2	1
3	3	3	3	2	1	2	2	2	3
9	9	9	9	1	2	2	2	2	3
2	2	2	2	7	2	2	1	1	2
9	9	9	9	1	2	2	2	2	2
9	9	9	9	9	1	1	1	1	1
1	1	3	2	9	9	9	9	9	3
1	1	1	1	9	1	1	1	1	1
9	9	9	9	9	9	9	9	9	9
9	9	9	9	9	2	2	2	2	9
2	2	2	2	9	3	3	3	3	2
2	2	2	2	9	3	3	3	3	9

2	2	2	2	9	1	1	1	3	2
3	3	3	3	1	2	2	2	2	3
9	9	2	9	1	2	2	2	2	9
2	2	9	1	3	1	1	1	1	2
2	2	2	2	3	1	1	1	1	2
3	1	9	1	9	2	2	2	2	9
9	9	9	9	1	9	9	9	9	9
1	9	3	1	1	2	2	2	2	2
9	1	9	9	1	9	9	9	9	9
2	2	2	2	3	1	1	1	1	2
9	3	1	9	1	2	2	2	2	9
2	1	9	2	1	2	2	2	2	9
9	9	9	9	1	2	2	2	2	9
9	9	9	9	1	9	9	9	9	9
9	2	9	2	1	2	2	2	2	2
2	2	2	2	3	1	1	1	1	2
9	9	9	9	1	9	9	9	9	9
2	9	2	9	1	2	2	2	2	2
2	2	2	2	1	2	2	2	2	2
9	9	9	9	9	9	9	9	9	9
2	2	2	2	1	1	1	1	1	2
9	9	9	9	9	9	9	9	9	9
9	9	9	9	9	9	9	9	9	9
9	9	9	9	9	9	9	9	9	9
9	9	9	9	1	9	9	9	9	9
9	9	9	9	1	9	9	9	9	9
2	2	2	2	1	2	2	2	1	2
2	2	2	2	1	2	2	2	2	2
9	2	1	2	2	2	2	2	2	1
1	2	1	1	1	1	1	2	1	1
3	3	3	3	1	2	1	2	2	2
2	2	2	2	1	3	2	3	3	2
1	1	1	1	1	1	1	1	1	1
2	2	2	3	1	2	2	2	2	2
2	3	1	3	2	2	3	3	1	1
2	2	2	2	9	2	2	2	2	2
2	2	3	2	9	1	2	2	2	2
2	2	2	3	1	3	3	2	2	2
2	1	2	2	1	3	3	3	3	2
9	9	9	9	2	9	9	9	9	9
1	1	1	1	1	2	2	2	2	3
3	3	3	3	2	1	1	1	1	3
2	2	2	2	1	2	2	2	2	2
1	1	1	3	1	1	2	1	1	2
2	2	2	2	1	2	2	2	2	2
2	2	2	2	1	2	2	2	2	2
1	1	1	1	1	1	1	2	2	2

9	9	9	9	9	9	9	9	9	9
2	2	2	2	1	1	2	2	2	2
9	9	9	9	9	9	9	9	9	9
2	1	2	1	9	2	2	1	1	9
2	2	2	2	1	3	3	3	3	2
2	2	3	3	5	1	3	2	2	9
1	1	2	1	2	2	3	3	3	2
3	1	2	2	9	9	9	9	9	1
2	2	2	2	5	1	1	1	1	1
2	2	2	2	3	1	1	1	1	1
2	2	2	2	3	1	1	1	1	2
2	2	2	2	9	1	1	1	1	1
1	1	1	1	3	1	1	1	1	1
1	2	1	2	3	1	1	1	1	1
3	3	3	3	1	2	2	2	2	3
1	1	1	1	5	1	1	1	1	1
2	2	2	2	4	3	3	3	3	9
1	1	1	1	4	1	1	1	1	2
1	1	9	1	5	1	1	1	1	2
1	1	1	1	4	1	1	1	1	2
9	9	9	9	9	1	1	1	1	2
2	2	2	1	3	1	1	1	1	1
9	1	1	1	4	1	1	1	1	1
1	1	2	2	4	1	1	1	1	1
9	9	9	9	9	1	1	1	1	1
2	2	2	2	9	3	3	3	3	9
1	1	2	1	9	1	1	1	1	1
2	2	2	2	1	2	2	2	3	2
9	9	9	9	9	9	9	9	9	9
2	2	2	2	1	1	1	1	1	1
9	9	9	9	9	9	9	9	9	9
1	1	1	2	9	1	1	1	1	1
1	1	1	2	1	1	1	1	1	1
2	1	1	1	1	1	1	3	1	1
9	2	2	2	9	1	1	1	1	9
9	2	9	2	4	1	1	1	1	2
2	2	2	2	2	3	3	3	3	9
2	2	2	2	1	1	1	2	2	9
2	2	2	2	2	1	1	1	1	2
1	1	1	1	4	1	1	1	1	1
2	2	2	2	1	1	1	2	3	2
9	9	9	9	9	9	9	9	9	9
1	1	1	1	9	1	1	1	1	1
1	1	1	1	9	1	1	1	1	1
9	9	9	9	2	9	9	9	9	9
2	2	2	2	9	3	3	3	3	9
9	9	9	9	9	1	1	1	1	2

9	9	9	9	9	9	9	9	9	9
2	2	2	2	9	1	1	1	1	2
2	2	2	2	9	1	1	1	1	2
3	2	1	1	1	1	1	1	1	1
2	9	2	2	1	2	2	2	2	2
9	9	9	9	2	1	1	1	1	1
1	1	1	1	1	1	1	1	1	1
9	2	2	2	1	2	2	2	2	2
1	1	1	1	2	1	1	1	1	3
1	1	1	2	9	1	1	1	1	1
2	2	2	2	3	1	1	1	1	2
2	2	2	2	1	3	9	9	9	9
1	1	1	1	3	1	1	1	1	2
2	2	2	2	1	1	1	1	1	2
9	9	9	9	9	3	3	3	3	2
9	2	2	9	9	1	1	1	1	1
2	2	2	9	4	3	3	3	3	9
2	9	2	2	2	1	1	1	1	9
9	9	9	9	9	9	9	9	9	9
9	9	9	9	9	1	1	1	1	9
9	9	9	9	9	3	3	3	3	9
9	9	9	9	9	9	9	9	9	9
9	9	9	9	9	1	1	1	1	1
9	9	9	9	9	9	9	9	9	9
2	2	2	2	9	3	3	3	3	2
1	2	1	1	4	1	1	1	1	1
1	2	1	1	2	1	1	1	1	1
1	2	1	1	4	1	1	1	1	1
1	2	1	1	4	1	1	1	1	1
1	2	1	1	4	1	1	1	1	1
1	2	1	1	4	1	1	1	1	1
1	2	1	1	2	1	1	1	1	1
1	2	1	1	4	2	1	1	1	1
1	2	1	1	1	1	1	1	1	1
1	2	1	1	4	1	1	1	1	1
1	2	1	1	1	2	1	1	1	1
1	1	9	1	9	2	1	1	1	1
1	2	1	1	9	2	1	1	1	1
1	2	1	1	4	1	1	1	1	1
1	2	1	1	4	2	1	1	1	1
1	2	1	1	4	1	1	1	1	1
1	2	1	1	4	2	1	1	1	1
1	2	1	1	4	1	1	1	1	1
1	2	1	1	4	9	9	9	9	1
1	2	1	1	4	1	1	1	1	1
1	2	1	1	4	1	1	1	1	1
1	2	1	1	1	1	1	1	1	1

1	2	1	1	4	1	1	1	1	1
1	2	1	1	4	1	1	1	1	1
1	2	1	1	4	1	1	1	1	3
9	9	9	9	9	9	9	9	9	9
9	9	9	9	9	9	9	9	9	9
9	9	9	9	9	9	9	9	9	9
9	9	9	9	9	9	9	9	9	9
9	9	9	9	9	9	9	9	9	9
9	9	9	9	9	9	9	9	9	9
9	9	9	9	9	9	9	9	9	9
9	9	9	9	9	9	9	9	9	9
9	9	9	9	9	9	9	9	9	9
9	9	9	9	9	9	9	9	9	9
9	9	9	9	9	9	9	9	9	9
9	9	9	9	9	9	9	9	9	9
9	9	9	9	9	9	9	9	9	9
9	9	9	9	9	9	9	9	9	9
9	9	9	9	9	9	9	9	9	9
9	9	9	9	9	9	9	9	9	9
9	9	9	9	9	9	9	9	9	9
9	9	9	9	9	9	9	9	9	9
9	9	9	9	9	9	9	9	9	9
9	9	9	9	9	9	9	9	9	9
9	9	9	9	9	9	9	9	9	9
1	2	1	1	4	1	9	1	1	1
1	2	1	1	4	1	1	1	1	1
1	2	1	1	1	1	1	1	1	1
1	2	1	1	1	1	1	1	1	1
1	2	1	1	1	1	1	1	1	1
1	2	1	1	1	1	1	2	1	1
1	2	1	1	1	1	2	1	1	1
1	2	1	1	1	1	1	1	1	1
1	2	1	1	4	1	2	1	1	1
1	2	1	1	2	1	1	1	1	1
1	2	1	1	1	1	1	1	1	1
2	1	1	1	1	1	1	1	1	1
1	2	1	1	4	1	1	1	1	1
1	2	1	1	4	1	1	1	1	1
1	2	1	1	5	1	1	1	1	1
1	2	1	1	1	1	1	1	1	1
9	9	1	9	1	2	2	2	2	1
1	2	1	1	1	1	1	1	1	1
1	2	1	1	4	1	1	1	1	1
1	2	1	1	4	1	1	1	1	1
1	2	1	1	1	2	1	1	1	1
1	2	1	1	4	1	1	1	1	1

1	2	1	1	4	1	2	1	1	1
1	2	1	1	4	1	1	1	1	1
1	2	1	1	9	1	1	1	1	1
1	2	1	1	1	1	1	1	3	1
1	2	1	1	1	1	1	1	1	1
1	2	1	1	1	9	1	1	1	1
1	2	1	1	1	1	1	1	1	1
2	2	1	1	1	9	1	1	1	1
1	2	1	1	4	3	1	1	1	1
1	2	1	1	1	1	1	1	1	1
1	2	1	1	1	1	1	1	1	1
1	2	1	1	4	1	1	1	1	1
1	2	1	1	1	1	1	1	1	1
1	2	1	1	1	1	1	1	1	1
1	2	1	1	1	1	1	1	1	1
1	2	1	1	4	1	1	1	1	1
1	2	1	1	1	1	1	1	1	1
1	2	1	1	1	1	1	1	1	1
1	2	1	1	4	1	1	1	1	1
1	2	1	1	3	1	1	1	1	1
1	2	1	1	9	2	1	1	1	1
1	2	1	1	4	1	1	1	1	1
1	1	1	1	9	2	1	1	1	1
1	2	1	1	4	1	1	1	1	1
1	2	1	1	4	1	1	1	1	1
1	2	1	1	4	1	1	1	1	1
1	2	1	1	4	1	1	1	1	1
1	2	1	3	4	2	1	1	1	1
1	2	1	1	1	1	1	1	1	1
1	2	1	1	1	1	1	1	1	1
1	2	1	1	1	1	1	1	1	1
1	2	1	1	4	1	1	1	1	1
1	2	1	1	1	1	1	1	1	1
1	2	1	1	4	1	1	1	1	1
1	2	1	1	1	1	1	1	1	1
1	9	1	1	1	1	1	1	1	1
1	2	1	1	2	1	1	1	1	1
1	2	1	1	1	1	9	9	9	1
9	9	9	9	9	9	9	9	9	1
1	2	1	1	4	1	1	1	1	1
9	9	9	9	9	9	9	9	9	9
1	2	1	1	1	2	1	1	1	1
3	1	2	1	1	1	9	1	1	1
1	2	1	1	4	9	9	1	1	1
1	2	1	1	4	1	9	1	1	1
1	2	1	1	9	2	1	1	1	1
1	2	1	1	4	1	1	1	1	1

1	2	1	1	1	1	1	1	1	1
1	2	1	1	1	2	2	1	1	1
1	2	1	1	4	1	1	1	1	1
1	2	1	1	4	1	1	1	1	1
1	2	1	1	4	1	1	1	1	1
1	2	1	1	4	1	1	1	1	1
1	2	1	1	4	1	1	1	1	1
1	2	1	1	4	1	1	1	1	1
1	2	1	1	4	1	1	1	1	1
1	2	1	1	4	1	1	1	1	1
2	1	1	1	4	1	1	1	1	1
1	2	1	1	4	1	1	1	9	1
1	2	1	1	4	1	1	1	1	1
2	2	1	1	9	1	1	1	1	1
1	2	1	1	4	1	1	1	1	1
1	2	1	1	4	1	1	1	1	1
1	2	1	1	4	1	1	1	1	1
1	2	1	1	4	1	1	1	1	1
1	2	1	1	4	1	1	1	1	1
1	2	1	1	4	1	1	1	1	1
9	9	9	9	4	1	1	1	1	1
1	2	1	1	4	1	1	1	1	1
1	2	1	1	4	1	1	1	1	1
1	2	1	1	4	1	1	1	1	1
1	2	1	1	4	1	1	1	1	1
9	9	9	9	9	9	9	9	9	1
1	2	1	1	4	2	1	1	1	1
1	2	1	1	4	1	1	1	1	1
1	2	1	1	4	1	1	1	1	1
1	2	1	1	4	1	1	1	1	1
1	1	1	1	4	1	1	1	1	1
9	1	2	1	1	1	1	1	1	1
1	2	1	1	9	1	1	1	1	1
1	2	1	1	4	1	1	1	1	1
2	1	1	1	1	1	1	1	1	1
1	3	1	1	1	1	1	1	1	1
1	2	1	1	1	1	1	1	1	1
1	2	1	1	1	1	1	1	1	1
2	1	1	2	1	1	1	1	1	3
1	2	1	1	1	1	1	1	1	3
1	2	1	1	1	1	1	1	1	1
1	2	1	1	2	1	1	1	1	1
1	2	1	1	1	1	1	1	1	1
1	2	1	1	4	1	1	1	1	1
1	2	1	1	4	1	1	1	1	1
1	2	1	1	4	1	1	1	1	1
1	2	1	1	3	1	1	1	1	1

9	9	9	9	9	9	9	9	9	9
1	2	1	1	4	1	1	1	1	1
1	2	1	1	3	1	1	1	1	1
1	2	1	1	4	1	1	1	1	1
1	2	1	1	4	1	1	1	1	1
1	2	1	1	4	1	1	1	1	1
1	2	1	1	4	1	1	1	1	1
1	2	1	1	4	1	1	1	1	1
1	2	1	1	1	1	1	1	1	1
1	2	1	1	4	1	1	1	1	1
1	2	1	1	4	1	1	1	1	1
1	2	1	1	4	1	1	1	1	1
1	2	1	1	1	1	1	1	1	1
1	2	1	1	1	9	1	2	1	1
1	2	1	1	1	1	1	1	1	1
1	1	1	1	9	9	9	9	9	9
1	2	1	1	4	1	1	1	1	1
1	2	1	1	4	1	1	1	1	1
1	2	1	1	4	1	1	1	1	1
1	2	1	1	4	1	1	1	1	1
1	2	1	1	4	1	1	1	1	1
1	2	1	1	4	1	1	1	1	1
1	2	1	1	4	1	1	1	1	1
1	2	1	1	4	1	1	1	1	1
1	2	1	1	4	1	1	1	1	1
1	2	1	1	4	1	1	1	1	1
1	2	1	1	4	1	1	1	1	1
1	2	1	1	4	1	1	1	1	1
1	2	1	1	4	1	1	1	1	1
1	2	1	1	1	1	1	1	1	1
1	2	1	1	4	1	1	1	1	1
1	2	1	1	4	1	1	1	1	1
1	2	1	1	4	9	9	9	9	1
9	9	9	9	9	9	9	9	9	9
9	9	9	9	9	9	9	9	9	9
9	9	9	9	9	9	9	9	9	9
9	9	9	9	9	9	9	9	9	9
9	9	9	9	9	9	9	9	9	9
9	9	9	9	9	9	9	9	9	9
9	9	9	9	9	9	9	9	9	9
9	9	9	9	9	9	9	9	9	9
9	9	9	9	9	9	9	9	9	9
9	9	9	9	9	9	9	9	9	9
9	9	9	9	9	9	9	9	9	9
9	9	9	9	9	9	9	9	9	9
9	9	9	9	9	9	9	9	9	9
9	9	9	9	9	9	9	9	9	9

9	9	9	9	9	9	9	9	9	9
9	9	9	9	9	9	9	9	9	9
9	9	9	9	9	9	9	9	9	9
9	9	9	9	9	9	9	9	9	9
9	9	9	9	9	9	9	9	9	9
9	9	9	9	9	9	9	9	9	9
9	9	9	9	9	9	9	9	9	9
9	9	9	9	9	9	9	9	9	9
9	9	9	9	9	9	9	9	9	9
9	9	9	9	9	9	9	9	9	9
9	9	9	9	9	9	9	9	9	9
9	9	9	9	9	9	9	9	9	9
9	9	9	9	9	9	9	9	9	9
9	9	9	9	9	9	9	9	9	9
9	9	9	9	9	9	9	9	9	9
9	9	9	9	9	9	9	9	9	9
9	9	9	9	9	9	9	9	9	9
9	9	9	9	9	9	9	9	9	9
9	9	9	9	9	9	9	9	9	9
2	1	3	3	1	1	1	1	1	3
2	2	2	2	9	3	3	3	3	2
2	2	2	2	2	2	2	2	2	2
3	3	2	2	9	9	9	9	9	1
1	1	1	1	5	1	1	2	1	1
1	3	3	3	9	9	9	9	9	1
1	3	2	2	9	1	1	1	1	1
1	2	1	1	4	1	1	2	1	1
2	3	1	3	5	2	2	3	3	2
9	9	9	9	9	9	9	9	9	9
2	2	2	3	1	3	3	3	3	2
9	9	9	9	1	2	2	2	2	9
3	3	3	3	9	1	1	1	1	2
9	9	9	9	9	9	9	9	9	9
1	1	3	3	2	2	2	2	2	1
2	3	3	2	9	2	3	1	2	3
2	2	2	3	2	1	1	2	2	2
3	3	3	3	1	2	2	2	2	3
3	1	2	3	1	2	2	2	3	2
9	9	9	9	3	1	1	2	2	1
1	3	3	3	9	1	1	2	2	1
1	1	1	1	2	1	1	1	1	1
9	9	9	9	1	1	1	2	1	2
2	2	2	2	1	1	3	1	2	2
2	1	1	1	2	3	2	1	1	1
1	1	1	1	3	1	1	2	1	2
1	1	1	1	9	1	1	1	1	1
9	9	9	9	9	9	9	9	9	9

9	9	9	9	9	9	9	9	9	9
2	2	1	1	2	1	1	1	1	1
1	1	1	1	9	1	1	3	2	1
9	9	9	9	9	9	9	9	9	9
9	9	9	9	9	9	9	9	9	9
2	2	2	2	1	3	3	9	9	9
9	9	9	9	5	1	2	1	2	9
9	9	9	9	9	9	9	9	9	9
1	2	3	2	1	3	3	2	1	2
2	1	2	2	1	1	1	2	2	2
1	1	2	3	2	1	1	2	2	1
9	9	9	9	3	1	1	2	1	9
2	2	2	2	9	2	2	1	1	2
2	2	2	2	9	2	1	1	1	2
2	2	2	2	9	2	2	2	2	2
3	3	3	2	9	1	2	2	2	2
3	3	3	3	1	1	2	3	3	3
9	9	9	9	9	9	9	9	9	9
9	9	9	9	4	2	2	2	2	1
2	2	2	2	1	3	3	3	3	2
2	2	2	2	1	3	3	2	2	2
1	2	3	3	1	2	2	2	2	2
2	3	1	3	1	2	2	3	3	2
2	2	2	2	9	1	1	1	1	2
3	2	1	9	9	1	1	1	1	1
9	9	9	2	9	9	9	9	9	9
2	2	2	2	3	1	2	2	2	2
2	2	2	2	9	9	9	9	9	2
2	2	2	2	9	2	3	3	3	2
2	2	2	2	9	2	2	1	3	2
2	2	2	2	9	9	9	9	9	2
1	3	1	1	1	2	1	1	2	2
2	2	2	2	9	2	2	2	2	2
2	2	2	2	5	2	2	2	3	2
2	2	2	2	1	2	2	2	2	2
1	2	1	2	1	1	1	1	1	1
1	1	2	3	2	1	1	3	1	1
1	1	2	3	1	1	2	2	2	1
1	1	1	1	1	1	2	3	3	1
1	1	1	1	4	1	1	1	1	1
2	1	2	2	9	1	1	2	2	2
2	2	3	1	1	2	2	1	2	1
2	2	2	2	1	2	2	2	2	2
2	2	2	2	9	1	3	3	1	1
9	9	9	9	1	3	9	3	2	2
3	2	1	1	2	2	2	2	2	2
2	1	1	1	2	1	2	2	2	3

2	2	2	2	1	1	2	2	2	2
2	3	1	2	1	1	1	2	2	1
3	2	1	1	1	1	1	2	2	1
1	1	1	1	1	1	1	1	1	1
9	3	2	2	2	1	1	1	1	1
1	2	1	2	2	2	2	2	9	2
1	1	2	1	1	2	2	2	2	2
3	9	1	1	2	2	2	2	2	1
2	2	2	2	9	1	2	2	2	2
9	3	3	2	9	3	1	2	2	2
2	2	2	2	2	1	1	1	1	2
2	2	2	2	2	1	1	1	1	2
2	2	1	3	1	2	2	2	2	1
3	3	1	3	9	3	3	3	3	2
2	2	2	2	1	3	3	3	3	2
2	3	1	3	1	1	2	2	3	1
2	2	2	2	2	2	2	2	2	2
2	2	2	2	1	3	3	3	3	2
1	1	1	1	9	2	2	2	1	2
3	2	2	2	9	9	9	9	9	2
2	2	2	2	9	1	1	1	1	2
3	2	2	2	1	2	2	2	3	2
3	3	3	3	4	1	1	1	1	2
2	2	2	2	1	1	1	2	2	2
2	2	2	2	9	9	9	9	9	2
2	1	2	1	2	2	1	1	3	1
2	1	2	2	2	2	2	2	2	2
1	1	1	2	1	2	2	2	2	1
2	2	2	2	3	2	2	2	2	2
1	1	1	1	9	1	1	1	1	1
1	1	1	1	2	1	1	1	2	1
9	9	9	9	9	9	9	9	9	9
3	2	1	2	2	2	2	2	9	9
2	2	2	1	1	2	2	2	2	2
1	3	1	3	9	1	1	1	1	9
1	2	1	1	2	3	2	2	1	2
1	2	1	1	2	2	2	2	2	1
2	2	1	2	9	1	2	1	2	1
3	3	3	3	9	2	2	2	1	2
3	2	2	2	9	1	1	1	1	2
3	9	2	1	9	1	1	1	1	9
9	9	9	9	9	9	9	9	9	2
9	9	9	9	9	9	9	9	9	9
1	2	2	2	9	9	9	9	9	1
9	9	9	9	9	9	9	9	9	9
2	2	2	2	1	1	1	2	2	2
2	3	3	2	1	2	2	2	2	2

1	1	1	3	3	1	1	1	2	1
1	2	1	2	2	2	2	2	2	1
9	9	9	9	9	9	9	9	9	9
9	9	9	9	9	9	9	9	9	9
9	9	9	9	9	9	9	9	9	9
3	3	3	3	3	1	1	1	1	1
9	9	9	9	9	9	9	9	9	9
9	9	9	9	9	9	9	9	9	9
2	2	2	2	3	1	2	2	1	1
3	3	3	3	9	9	9	9	9	9
9	9	9	9	9	9	9	9	9	9
3	3	3	1	1	1	1	3	3	2
2	3	2	3	1	2	1	3	2	3
2	2	2	2	9	9	9	9	9	9
1	2	1	2	1	1	1	1	1	1
2	3	1	2	2	3	3	3	3	1
2	3	1	2	2	1	2	3	2	2
2	2	2	2	9	3	3	3	3	2
2	2	2	2	1	3	3	3	3	2
9	9	9	9	9	9	9	9	9	9
2	2	2	2	9	3	3	3	3	2
1	1	9	3	1	2	2	2	3	2
1	1	1	3	5	1	3	1	2	3
2	1	2	3	1	2	2	3	2	2
1	2	3	3	9	1	1	1	1	1
9	9	9	9	9	9	9	9	9	9
3	1	2	3	1	2	2	3	2	2
1	2	3	1	2	1	3	1	1	2
2	2	2	2	1	2	2	2	2	2
3	1	2	3	1	2	2	3	2	9
2	3	2	3	9	9	9	9	9	3
9	9	9	9	9	9	9	9	9	9
2	2	2	2	1	2	2	2	2	3
2	2	2	2	9	2	2	2	2	9
3	2	2	2	4	2	2	2	1	2
3	1	1	2	4	2	2	2	3	1
2	2	2	2	1	2	2	2	2	2
9	9	9	9	2	2	2	2	2	1
2	2	2	2	1	2	2	2	2	1
2	2	2	2	5	9	9	9	9	2
1	2	1	2	2	2	1	2	2	2
1	3	1	2	9	9	9	9	9	2
9	9	9	9	9	9	9	9	9	2
1	1	1	1	3	1	1	2	3	1
1	2	1	1	2	2	2	2	2	2
9	9	9	9	1	3	2	2	2	2
1	3	2	2	9	9	9	9	9	1

2	2	2	2	1	1	3	3	3	2
2	2	2	2	1	2	2	2	2	3
2	2	2	2	1	2	2	3	2	2
2	2	2	2	1	1	3	3	3	2
9	9	9	9	9	2	2	1	2	1
9	3	3	3	1	3	3	3	3	2
1	1	1	3	2	1	1	1	1	1
2	2	2	2	9	3	3	3	3	2
2	3	1	1	1	1	2	2	2	2
2	2	1	2	1	2	2	2	2	1
1	1	1	2	1	2	2	2	2	2
2	2	2	2	9	2	2	3	3	2
1	1	1	1	1	2	2	2	2	1
2	2	1	2	1	1	1	2	2	2
1	1	1	3	1	1	1	2	2	1
2	1	3	3	2	2	2	2	3	3
2	2	2	2	1	1	1	2	2	2
2	2	2	2	2	3	2	2	2	2
2	2	2	2	1	1	1	2	2	2
1	1	1	3	1	1	1	2	2	1
9	9	9	9	9	2	2	2	2	2
1	1	1	2	3	2	2	2	2	2
3	1	2	3	1	3	2	2	2	2
1	1	2	2	9	9	9	9	9	1
2	3	2	2	1	2	2	2	3	3
1	1	1	2	1	2	2	2	2	1
1	1	1	1	1	2	2	2	2	1
1	1	3	3	9	1	1	1	1	9
2	2	3	3	1	2	2	2	2	2
2	2	2	2	9	1	1	1	1	2
2	2	9	2	1	1	1	2	2	2
1	1	1	1	2	2	2	2	2	1
1	1	2	1	2	3	3	3	1	2
2	1	2	1	2	1	3	3	1	2
3	1	1	1	4	1	1	1	1	1
1	1	1	2	1	1	1	1	1	1
1	2	3	1	1	1	1	1	1	1
2	1	1	1	3	2	2	2	1	2
1	2	1	1	2	1	1	1	2	1
9	9	9	9	9	9	9	9	9	9
9	9	9	9	2	3	3	3	3	3
1	3	1	3	1	1	1	1	1	1
2	2	2	2	9	9	9	9	9	2
3	3	1	1	2	1	1	1	1	1
1	1	1	1	9	1	1	1	1	1
2	2	2	2	4	1	1	1	3	2
2	3	2	2	2	1	1	1	1	1

9	9	9	9	9	9	9	9	9	9
9	9	9	9	9	9	9	9	9	9
1	1	1	1	9	1	1	1	1	1
2	2	2	2	2	1	1	1	1	1
9	9	9	9	9	9	9	9	9	9
9	9	9	9	9	9	9	9	9	9
2	2	2	2	9	9	9	9	9	2
1	1	1	1	1	1	1	1	1	1
2	3	1	2	3	1	1	1	1	1
1	2	1	2	2	2	2	2	2	2
2	2	1	1	1	2	2	2	2	1
2	3	1	2	1	1	2	3	1	1
2	1	2	2	9	2	2	1	1	1
9	9	9	9	9	2	9	1	3	1
2	1	2	1	1	1	1	1	1	2
9	9	9	9	9	9	9	9	9	9
1	1	1	1	9	1	1	1	1	2
1	1	1	1	4	3	3	3	3	2
9	9	9	9	9	9	9	9	9	1
9	9	9	9	9	9	9	9	9	9
2	2	2	2	9	1	1	1	1	2
9	9	9	9	9	9	9	9	9	9
9	9	9	9	9	9	9	9	9	9
2	2	2	2	2	2	1	1	1	2
2	2	2	2	9	2	3	3	3	2
2	3	3	2	3	1	1	2	2	2
2	2	2	2	1	2	2	2	2	2
2	2	2	2	9	1	1	1	1	2
2	1	2	3	5	1	3	3	3	2
2	3	2	3	4	1	3	2	1	2
9	9	9	9	9	3	2	2	2	1
9	9	9	9	3	1	2	2	2	1
1	1	1	1	4	2	9	2	2	2
1	1	1	1	2	9	9	9	9	2
1	1	1	1	1	1	1	1	2	1
1	1	1	1	5	3	3	3	3	1
1	1	1	9	3	2	1	1	1	1
2	2	2	2	1	2	3	3	3	2
2	2	2	2	9	9	9	9	9	2
2	2	2	2	9	3	3	3	3	2
1	1	1	1	2	1	1	1	1	2
2	2	2	2	9	3	3	3	3	2
2	2	2	2	9	2	2	2	2	3
9	9	9	9	2	1	2	2	2	9
9	2	9	9	9	2	2	2	2	9
2	2	2	2	2	9	9	9	9	2
2	2	2	3	9	3	2	2	2	3

9	9	1	9	3	1	3	3	3	2
2	2	2	2	9	9	9	9	9	1
9	9	9	9	2	2	2	1	3	2
3	3	3	3	1	1	1	2	2	2
9	9	9	9	9	9	9	9	9	9
2	2	2	2	2	2	2	2	2	2
3	1	2	1	1	3	3	3	3	1
9	9	9	9	1	2	2	2	2	9
2	2	2	2	9	9	9	9	9	2
1	2	2	2	1	2	2	2	2	2
3	3	3	3	9	1	2	1	1	3
2	2	2	2	9	1	2	3	3	2
2	1	2	1	4	2	2	2	2	1
1	1	3	2	1	9	9	9	9	9
2	2	2	2	9	1	2	2	2	2
1	1	1	1	9	2	2	2	2	1
1	1	3	3	2	2	2	2	2	2
2	2	2	2	9	3	3	3	3	2
2	2	2	2	2	1	2	1	2	2
2	3	2	3	1	2	2	2	2	2
2	1	1	1	1	1	2	1	2	2
2	1	1	1	1	1	2	1	2	2
2	2	2	2	9	2	2	2	2	2
2	2	2	2	9	1	2	2	2	2
3	2	2	3	5	9	9	9	9	9
2	9	3	1	1	9	9	9	9	9
2	1	2	2	1	2	2	2	2	3
1	2	2	2	1	1	1	2	2	1
2	2	1	1	1	1	2	1	2	9
9	9	9	9	9	9	9	9	9	9
3	3	3	3	9	3	3	3	3	3
9	9	9	9	1	3	3	3	3	9
9	9	9	9	9	9	9	9	9	9
9	9	9	9	9	9	9	9	9	9
3	1	1	1	1	9	9	9	9	1
1	1	1	1	4	2	2	2	2	1
2	2	2	2	4	2	2	2	2	2
1	1	1	1	4	2	2	2	1	1
2	2	2	2	1	2	2	2	2	1
2	2	2	2	9	3	3	3	3	2
1	1	1	1	3	2	2	2	2	1
1	1	1	1	4	1	2	2	2	2
1	9	3	3	9	1	2	1	3	1
3	2	1	2	2	2	2	1	2	1
3	1	1	1	1	3	3	1	3	2
1	1	1	1	4	1	1	1	1	1
1	1	1	1	9	1	1	2	2	1

2	2	2	2	2	2	2	2	1	2
9	9	9	9	1	1	1	2	1	1
9	9	9	9	2	1	1	1	3	9
3	2	2	1	3	1	2	1	3	1
2	2	2	2	9	3	3	3	3	2
2	2	2	2	1	3	3	2	2	2
2	3	1	1	2	1	2	3	1	1
9	9	9	9	9	9	9	9	9	9
9	9	9	9	9	9	9	9	9	9
9	9	9	9	9	9	9	9	9	9
9	9	9	9	9	9	9	9	9	9
9	9	9	9	3	1	1	2	1	9
9	9	9	9	9	9	9	9	9	9
9	9	9	9	9	9	9	9	9	9
9	9	9	9	9	9	9	9	9	9
9	9	9	1	3	3	3	3	3	9
2	2	2	2	4	1	2	3	3	1
1	2	1	2	4	1	2	1	2	1
1	1	1	1	1	9	9	9	9	9
2	3	2	9	4	1	2	2	2	1
2	2	2	2	1	1	1	2	2	2
2	1	1	3	2	9	9	9	9	9
3	3	2	3	4	1	1	1	1	1
2	2	2	2	3	2	2	2	2	2
1	1	1	2	1	2	1	1	2	2
1	2	2	2	1	3	2	2	2	2
3	2	1	3	3	1	2	3	2	1
3	1	2	3	1	1	2	2	2	3
3	3	3	3	9	1	2	2	1	3
2	2	2	2	9	1	1	1	2	2
2	2	2	2	1	2	2	2	2	2
2	2	2	2	9	1	2	2	1	2
3	3	3	3	1	1	1	2	2	2
2	1	1	1	3	1	1	1	1	1
2	2	2	2	1	2	2	2	2	2
2	2	2	2	9	3	3	3	3	2
9	9	9	9	9	9	9	9	9	9
2	2	2	2	9	3	3	3	3	2
1	1	1	1	9	1	1	1	1	3
9	9	9	9	1	1	1	1	1	1
9	9	9	9	9	9	9	9	9	3
2	2	2	2	9	3	3	3	3	2
2	2	2	2	9	3	9	3	3	2
2	2	2	2	1	1	2	1	2	2
3	2	1	3	4	1	1	1	2	1
9	9	9	9	9	9	9	9	9	9
1	2	1	2	3	3	2	2	1	1

2	2	2	2	3	3	3	3	3	2
2	2	2	2	1	2	2	3	2	2
1	1	1	3	2	1	1	1	1	1
1	1	2	2	4	1	1	1	1	1
9	9	9	9	9	9	9	9	9	9
2	2	2	2	9	1	2	2	9	9
9	9	9	9	9	9	9	9	9	9
2	2	2	2	1	1	2	2	3	2
9	9	9	9	5	1	1	1	2	1
9	9	9	9	9	9	9	9	9	9
9	9	9	9	9	9	9	9	9	9
9	9	9	9	9	9	9	9	9	9
9	9	9	9	9	9	9	9	9	9
9	9	9	9	9	9	9	9	9	9
9	9	9	9	9	9	9	9	9	9
9	9	9	9	9	9	9	9	9	9
9	9	9	9	9	9	9	9	9	9
9	9	9	9	9	9	9	9	9	9
9	9	9	9	9	9	9	9	9	9
9	9	9	9	9	9	9	9	9	9
9	9	9	9	9	9	9	9	9	9
9	9	9	9	9	9	9	9	9	9
9	9	9	9	9	9	9	9	9	9
9	9	9	9	9	9	9	9	9	9
9	9	9	9	9	9	9	9	9	9
9	9	9	9	9	9	9	9	9	9
9	9	9	9	9	9	9	9	9	9
9	9	9	9	9	9	9	9	9	9
9	9	9	9	9	9	9	9	9	9
2	3	2	1	3	3	2	1	2	1
2	2	2	2	1	1	2	2	2	2
2	2	2	2	2	1	1	1	1	1
1	2	1	1	3	1	1	2	1	1
9	2	3	2	4	1	3	2	1	2
1	1	1	1	3	1	1	3	3	1
1	2	1	2	3	1	1	2	2	2
3	1	1	1	4	1	1	1	1	1
1	1	2	3	1	3	2	3	3	2
1	1	2	1	1	1	3	2	2	2
1	1	1	1	4	1	1	2	2	9
1	2	2	1	4	1	1	3	3	1
2	2	1	2	1	1	1	1	1	1
1	2	2	1	4	1	1	2	2	9
2	2	1	2	1	1	1	1	1	2
1	1	1	1	1	1	1	1	1	1
9	9	1	9	1	1	9	2	1	1
1	2	1	2	3	1	1	1	1	1

1	1	1	1	1	1	1	2	1	1
9	9	9	9	1	1	2	1	1	1
1	2	1	1	1	1	1	2	1	2
1	1	1	1	1	1	1	1	1	1
3	3	3	3	1	2	2	2	2	2
3	2	2	1	3	1	1	1	2	2
1	2	1	1	3	1	1	1	1	1
1	1	1	1	3	1	1	1	1	1
2	2	1	1	4	1	1	2	2	1
1	2	1	1	5	1	1	1	1	1
1	2	3	1	2	2	1	2	1	2
9	9	9	9	1	2	1	2	1	1
1	1	1	2	3	1	1	2	1	2
9	9	9	9	2	1	1	1	1	1
1	2	2	2	1	2	2	2	2	2
1	2	2	2	9	2	2	2	2	2
9	9	9	9	1	1	1	2	1	9
9	9	9	9	1	1	1	1	1	1
9	9	9	9	5	1	1	1	2	9
9	9	9	9	1	9	9	9	9	9
9	9	9	9	4	1	1	1	1	9
9	9	9	9	9	9	9	9	9	9
1	1	3	3	2	9	9	9	9	9
2	2	2	2	9	2	1	2	2	2
9	9	9	9	9	1	1	2	1	9
3	3	3	3	3	1	1	1	1	1
9	9	9	9	4	1	1	1	1	1
9	9	9	9	4	1	3	2	1	9
9	9	9	9	3	1	1	1	1	1
9	9	9	9	3	1	2	2	1	9
9	9	9	9	4	1	1	1	1	1
2	3	1	2	9	1	1	1	1	2
1	3	3	1	4	1	1	1	1	1
2	2	1	2	2	2	2	1	1	2
1	1	1	1	1	2	1	2	2	1
2	2	2	2	1	9	9	9	9	9
1	2	1	2	1	1	1	2	2	2
1	2	1	3	3	1	1	1	1	3
1	3	2	1	4	3	2	2	2	3
1	1	1	3	1	2	2	2	1	1
2	3	1	1	3	1	1	2	2	1
9	9	9	9	3	1	2	2	2	9
2	2	1	2	1	1	1	2	2	2
2	2	2	2	1	1	2	1	2	1
2	2	3	2	1	2	3	2	3	2
2	2	2	2	1	2	2	2	2	2
1	1	1	2	1	1	1	1	1	1

1	1	1	1	1	1	1	1	1	1
1	1	1	3	1	1	1	2	1	1
2	3	2	1	1	3	3	3	3	2
2	2	1	1	2	9	1	1	1	1
1	9	1	1	3	1	1	2	2	1
1	1	1	1	4	1	1	1	1	1
1	2	1	1	5	1	1	1	1	1
3	3	3	3	1	1	1	1	1	1
1	2	1	1	3	1	1	2	2	9
1	1	1	1	2	1	1	2	1	1
9	9	9	2	1	9	9	9	9	9
1	1	1	1	4	1	1	1	1	1
1	1	1	1	1	1	1	1	1	2
9	9	9	9	9	9	9	9	9	9
9	9	9	9	2	1	1	2	2	2
2	2	2	2	2	3	3	3	3	2
2	2	2	2	1	1	1	2	2	2
3	3	3	3	3	1	1	2	2	1
1	2	1	3	3	1	1	1	1	1
9	9	9	9	1	9	9	9	9	9
9	9	9	9	9	9	9	9	9	9
1	2	2	2	1	1	2	1	1	3
9	9	9	9	1	1	1	1	1	1
9	9	9	9	9	9	9	9	9	9
1	1	1	1	1	3	3	3	3	3
1	1	1	3	1	1	1	1	1	1
1	1	1	1	1	1	1	1	1	2
1	1	1	1	2	1	1	1	1	1
1	2	2	3	3	3	3	1	3	2
3	1	1	1	1	1	1	1	1	1
1	1	1	1	1	1	3	3	3	2
9	9	9	9	9	9	9	9	9	9
9	9	9	9	9	1	1	1	1	1
1	1	1	1	1	1	1	1	1	1
2	2	2	2	1	2	2	1	1	3
1	1	1	1	1	1	1	2	1	2
9	9	9	9	9	9	9	9	9	9
9	9	9	9	9	9	9	9	9	9
9	9	9	9	9	9	9	9	9	9
9	9	9	9	9	9	9	9	9	9
9	9	9	9	9	9	9	9	9	9
9	9	9	9	9	9	9	9	9	9
9	9	9	9	9	9	9	9	9	9
9	9	9	9	9	9	9	9	9	9
9	9	9	9	9	9	9	9	9	9
9	9	9	9	9	9	9	9	9	9
9	9	9	9	9	9	9	9	9	9

9	9	9	9	9	9	9	9	9	9
9	9	9	9	9	9	9	9	9	9
9	9	9	9	9	9	9	9	9	9
9	9	9	9	9	9	9	9	9	9
9	9	9	9	9	9	9	9	9	9
9	9	9	9	9	9	9	9	9	9
9	9	9	9	9	9	9	9	9	9
9	9	9	9	9	9	9	9	9	9
9	9	9	9	9	9	9	9	9	9
9	9	9	9	9	9	9	9	9	9
9	9	9	9	9	9	9	9	9	9
9	9	9	9	9	9	9	9	9	9
9	9	9	9	9	9	9	9	9	9
9	9	9	9	9	9	9	9	9	9
9	9	9	9	9	9	9	9	9	9
9	9	9	9	9	9	9	9	9	9
9	9	9	9	9	9	9	9	9	9
9	9	9	9	9	9	9	9	9	9
9	9	9	9	9	9	9	9	9	9
9	9	9	9	9	9	9	9	9	9
9	9	9	9	9	9	9	9	9	9
9	9	9	9	9	9	9	9	9	9
9	9	9	9	9	9	9	9	9	9
9	9	9	9	9	9	9	9	9	9
9	9	9	9	9	9	9	9	9	9
2	2	2	2	5	1	1	1	1	2
9	9	9	9	9	9	9	9	9	9
9	9	9	9	9	9	9	9	9	9
9	9	9	9	9	9	9	9	9	9
9	9	9	9	9	9	9	9	9	9
9	9	9	9	9	9	9	9	9	9
9	9	9	9	9	9	9	9	9	9
9	9	9	9	9	9	9	9	9	9
9	9	9	9	9	9	9	9	9	9
9	9	9	9	9	9	9	9	9	9
9	9	9	9	9	9	9	9	9	9
9	9	9	9	9	9	9	9	9	9
9	9	9	9	9	9	9	9	9	9
9	9	9	9	9	9	9	9	9	9
9	9	9	9	9	9	9	9	9	9
9	9	9	9	9	9	9	9	9	9
2	2	2	2	2	3	3	3	3	2
3	3	3	3	2	9	9	9	9	9
1	2	2	3	1	1	1	3	1	1
1	1	1	1	3	1	1	1	1	1
3	2	2	3	9	3	3	3	3	3
1	1	1	1	2	9	9	9	9	9

1	1	1	3	9	1	2	3	1	9
9	9	9	9	3	1	1	1	1	1
1	1	1	3	3	1	2	3	1	9
2	2	3	1	1	2	2	3	3	2
2	3	2	2	1	2	2	2	2	9
2	2	2	2	3	1	1	2	1	2
1	1	1	1	2	1	1	2	1	1
1	1	1	1	9	1	1	2	2	2
1	2	1	1	3	1	1	1	1	1
1	1	1	1	5	1	1	2	2	1
1	2	1	2	1	1	1	1	1	1
1	1	1	1	3	1	1	1	2	1
2	2	1	1	1	1	1	1	2	1
2	2	1	3	4	1	1	2	2	1
2	1	2	1	2	2	2	2	1	2
2	2	1	2	1	1	1	3	3	2
9	9	1	9	4	1	1	2	2	1
3	2	3	2	1	1	2	3	2	2
1	1	1	1	1	9	9	9	9	9
2	2	2	2	3	1	3	2	2	2
1	1	2	2	3	1	3	2	2	2
1	1	1	1	4	1	1	2	2	1
9	9	9	9	9	9	9	9	9	9
1	2	1	2	1	1	1	2	2	1
1	1	2	1	3	3	1	1	2	1
3	2	2	3	9	1	2	3	1	1
1	2	1	2	2	1	1	1	2	1
1	1	1	3	1	1	1	1	1	1
1	1	1	3	1	1	1	1	1	1
1	1	1	1	4	1	1	1	1	1
3	3	3	3	9	1	2	1	1	3
2	1	2	1	2	2	2	2	2	1
2	2	2	2	9	2	2	2	2	1
2	2	3	2	9	9	9	9	9	1
2	2	2	2	9	2	1	1	2	2
9	9	9	9	9	9	9	9	9	9
3	3	2	3	1	1	9	9	9	9
2	2	2	2	2	1	1	3	1	2
2	2	2	2	1	1	1	1	1	2
9	9	9	9	9	2	2	2	2	9
9	9	9	9	9	9	9	9	9	9
9	9	9	9	9	2	2	2	2	9
9	9	9	9	9	1	1	1	1	9
9	9	9	9	9	1	1	1	1	2
9	9	9	9	9	1	1	1	1	9
9	9	9	9	9	9	9	9	9	9
3	2	1	3	1	1	1	1	1	1

9	9	9	9	4	1	1	2	2	9
1	1	1	1	1	1	1	1	1	1
3	3	3	1	1	1	1	2	1	3
1	1	1	3	1	1	2	3	2	1
3	3	3	3	3	1	2	1	2	3
1	3	1	3	1	2	2	2	2	1
3	2	3	2	9	1	1	2	2	2
3	2	2	2	9	9	9	9	9	1
2	3	2	2	2	2	2	2	1	1
1	1	1	1	2	1	3	1	1	1
2	1	2	3	5	1	3	3	3	2
3	1	2	2	1	1	2	2	1	2
1	2	2	3	1	1	9	1	1	1
2	3	1	2	3	2	1	1	3	2
1	1	1	1	4	1	2	2	1	1
9	3	2	9	9	9	9	9	9	1
3	3	3	3	2	1	1	1	1	1
1	1	3	1	9	1	2	2	2	1
2	2	1	2	1	1	2	1	1	2
2	1	3	2	3	2	2	2	2	1
1	1	1	1	9	2	2	2	2	1
3	3	3	3	1	1	1	3	1	1
2	2	3	2	1	1	3	1	1	2
9	9	9	9	2	1	2	2	2	1
3	2	1	2	9	1	3	2	3	1
1	1	1	1	2	3	3	3	2	1
3	2	2	1	9	1	3	3	3	1
3	3	3	3	4	1	1	2	2	1
1	2	1	2	9	1	1	1	1	1
1	1	3	1	1	2	2	2	2	2
2	3	1	2	1	2	1	3	3	1
1	1	3	3	1	2	1	1	2	3
1	1	1	1	1	2	2	2	2	1
1	3	1	1	2	1	1	1	1	1
1	2	2	1	2	2	1	2	2	2
1	1	2	3	2	2	2	2	2	2
1	2	1	2	4	3	2	2	2	1
2	3	1	2	2	1	1	1	1	1
2	2	2	2	1	1	2	2	2	2
1	1	3	3	1	2	2	2	2	2
9	1	3	1	5	1	1	1	1	1
1	3	1	3	4	2	2	2	2	1
3	2	1	2	1	2	3	1	2	1
9	9	9	9	9	9	9	9	9	9
1	1	3	3	5	1	1	2	1	1
1	3	3	3	1	1	2	1	2	1
1	1	1	3	1	1	2	2	2	1

1	2	3	1	1	1	1	1	1	1
1	1	1	3	1	1	2	2	2	1
1	3	1	2	2	1	1	1	1	1
2	2	2	2	9	1	1	1	1	2
3	3	3	3	1	2	2	2	2	2
3	3	3	3	1	2	2	2	2	3
3	3	2	2	2	1	1	1	1	1
9	9	9	9	1	9	9	9	9	1
3	2	3	3	1	1	1	2	1	1
1	1	1	1	2	1	2	1	3	1
2	2	2	2	3	1	1	1	1	1
2	3	2	1	2	2	2	1	1	1
2	2	2	2	9	2	2	2	2	2
9	9	9	9	9	9	9	9	9	9
9	9	9	9	9	9	9	9	9	9
2	1	2	3	1	2	2	1	1	1
2	2	2	1	1	1	1	1	1	2
2	2	2	2	9	2	2	2	2	2
2	2	2	2	1	2	2	2	2	2
2	2	2	2	3	1	1	1	1	1
1	1	1	1	1	2	2	2	2	2
1	1	1	1	1	2	2	2	2	2
2	2	2	2	1	2	2	2	2	2
2	2	2	2	1	2	3	3	3	2
3	3	2	2	2	1	1	1	1	2
3	3	3	3	1	2	2	2	2	2
3	3	2	2	9	2	2	2	2	2
2	2	2	2	1	1	1	2	2	2
2	2	2	2	1	1	1	2	2	2
2	2	2	2	9	9	9	9	9	2
2	2	2	2	9	3	3	3	3	2
2	2	2	2	1	2	2	3	1	2
2	2	2	1	1	1	1	1	1	1
1	2	1	2	2	1	1	1	2	1
2	2	2	2	3	1	1	1	1	1
2	2	2	2	9	2	9	2	2	2
2	2	2	2	9	2	2	2	2	2
2	2	2	1	1	1	1	1	1	1
2	2	2	2	9	2	2	2	2	2
2	2	2	2	1	2	2	2	2	2
2	2	2	1	1	1	1	1	1	2
2	2	2	1	1	1	1	1	1	2
2	2	2	2	9	2	1	2	1	2
2	2	2	2	1	2	2	1	1	2
2	2	2	2	9	2	2	2	2	2
1	1	3	1	2	9	9	9	9	1
2	2	2	1	1	1	2	1	1	2

2	2	2	2	1	1	1	2	2	2
2	3	2	1	1	1	1	1	1	2
2	2	2	2	9	9	9	9	9	2
3	3	3	3	1	1	1	1	2	1
3	3	3	3	9	2	2	1	1	2
3	3	3	3	1	3	3	2	2	2
2	2	2	2	9	1	2	2	2	2
2	2	2	2	1	2	1	2	2	2
2	2	2	2	1	1	2	2	2	1
2	2	2	2	1	2	1	1	1	2
2	2	3	3	1	1	1	2	1	2
2	2	2	2	9	2	2	2	2	2
9	9	9	9	1	1	2	2	2	9
3	1	3	1	2	1	1	1	1	1
9	9	9	9	3	1	1	2	2	9
2	2	2	2	3	2	2	2	2	2
2	2	1	2	9	1	1	1	1	1
2	2	1	1	9	1	1	1	1	1
3	2	1	2	1	3	3	3	3	2
9	9	9	9	1	1	2	2	2	9
2	2	2	2	1	2	2	3	2	2
9	9	9	9	1	2	2	2	2	2
2	2	2	2	1	1	1	1	1	1
1	1	1	1	9	1	1	1	1	1
9	9	9	9	1	1	1	1	1	2
2	2	2	2	9	2	2	1	3	1
2	2	2	1	1	1	1	2	2	1
9	9	9	9	1	1	1	1	1	9
9	9	9	9	4	3	3	1	9	9
2	3	3	3	1	1	2	2	2	3
3	1	2	3	9	1	1	1	1	1
3	3	2	2	9	1	2	2	2	3
3	3	2	2	1	1	2	9	9	2
1	9	3	3	1	1	1	1	2	1
2	9	1	3	2	1	1	2	2	1
1	1	1	1	1	1	1	1	1	1
2	2	2	2	9	2	3	3	3	2
2	2	2	2	1	2	2	2	2	2
2	2	2	2	1	3	3	3	3	2
2	9	9	9	1	1	3	2	2	1
2	2	2	2	9	1	3	2	2	2
9	1	9	9	1	1	1	1	1	1
3	3	3	3	9	1	2	2	2	3
2	2	2	2	9	1	1	2	3	1
2	2	2	2	9	3	3	3	3	2
2	2	2	2	9	3	3	3	3	2
3	1	2	3	1	9	3	2	3	2

2	2	2	2	9	1	2	2	2	2
9	9	9	9	3	3	9	2	3	9
1	1	1	1	1	1	1	1	1	1
2	2	2	2	1	1	1	2	2	1
3	3	3	3	9	2	3	3	3	3
2	2	1	2	1	2	3	3	1	1
1	1	1	1	1	2	1	1	2	9
2	2	2	2	1	3	3	3	3	2
1	1	1	1	9	1	1	1	1	1
2	2	2	2	1	3	3	3	3	2
9	9	9	9	4	3	3	3	3	9
3	2	3	2	4	1	1	1	1	1
3	3	3	3	1	2	2	2	2	3
2	3	9	2	9	1	2	1	1	2
2	2	2	2	9	1	2	2	1	2
3	3	3	3	1	1	1	1	1	1
3	3	3	3	3	2	1	3	3	9
1	1	1	1	3	1	1	1	1	1
2	2	2	2	1	1	2	2	1	2
2	2	2	2	9	1	2	2	2	2
2	2	2	2	9	2	2	2	2	2
1	1	1	1	1	2	2	2	2	3
1	1	1	1	9	1	2	2	2	1
3	3	1	3	3	1	1	1	1	3
9	9	9	9	9	9	9	9	9	9
9	9	9	9	9	9	9	9	9	1
2	2	2	2	3	1	1	3	3	2
2	2	2	2	1	1	2	2	1	2
1	1	1	1	1	1	1	1	1	1
2	2	2	2	9	2	2	2	1	2
9	3	9	3	9	2	2	2	2	3
1	1	1	1	3	1	2	2	1	1
3	2	2	1	9	1	1	1	1	1
2	1	2	3	1	3	1	1	1	2
9	2	9	1	2	1	1	2	2	1
3	3	3	3	1	2	2	2	2	2
2	9	2	1	9	1	2	2	1	1
2	2	2	3	2	2	2	2	2	2
1	1	1	1	9	1	3	3	3	1
2	2	2	2	1	2	2	1	1	1
2	2	2	2	3	3	3	3	3	2
3	3	3	3	9	3	3	3	3	2
2	2	2	2	1	1	1	2	1	1
2	2	2	2	2	2	1	1	1	3
2	3	1	2	9	1	2	3	1	1
3	3	3	3	9	2	2	2	2	3
3	1	1	1	3	1	1	1	1	1

1	1	1	1	9	9	9	9	9	2
9	9	9	9	9	9	9	9	9	9
3	3	3	3	9	2	2	2	9	3
1	1	2	2	1	1	2	1	1	9
2	2	2	2	9	3	3	3	3	2
9	2	2	1	5	1	1	1	1	1
1	1	1	1	1	1	1	1	1	1
3	3	3	3	4	1	1	1	1	1
3	3	3	3	3	9	9	9	9	9
3	3	3	3	4	1	1	1	1	1
1	1	1	1	9	1	2	1	1	1
2	2	2	2	5	3	3	3	3	2
2	2	2	2	1	3	3	3	3	2
2	2	2	2	5	9	9	9	9	2
1	1	1	1	5	2	1	1	3	3
1	2	2	3	1	1	2	1	1	1
2	1	2	2	5	2	2	3	2	2
2	2	3	2	1	1	2	2	2	2
2	2	2	2	3	1	1	1	1	2
1	2	2	2	5	1	1	1	1	1
2	2	2	2	5	3	3	3	3	2
2	2	2	2	1	1	2	2	2	2
2	2	2	2	4	2	3	2	2	2
2	2	2	2	1	3	3	3	3	2
2	2	2	2	1	3	3	3	3	2
2	2	2	1	4	1	1	3	3	2
2	2	2	2	1	1	1	3	3	2
2	2	2	2	1	2	2	2	2	3
1	3	2	3	1	1	2	2	1	2
2	2	2	2	1	2	2	2	2	2
1	1	1	1	1	1	1	1	1	1
2	2	2	2	5	1	3	3	3	2
1	1	2	2	2	1	2	1	2	1
2	2	2	2	1	1	2	3	1	2
1	2	2	2	3	2	1	3	3	2
1	2	3	2	1	2	2	2	2	2
2	3	3	1	3	3	3	2	2	2
3	2	1	1	5	1	1	1	2	1
2	2	2	2	1	2	2	2	3	2
2	2	2	2	5	9	9	9	9	2
1	1	1	2	5	1	1	1	2	1
3	3	3	3	5	2	1	2	1	1
2	2	2	2	1	2	2	2	2	9
9	9	9	9	9	9	9	9	9	9
2	2	2	2	1	1	3	1	2	2
1	2	3	1	2	2	2	2	2	2
2	2	3	1	1	9	9	9	9	2

2	2	2	2	1	2	1	2	2	2
2	2	2	2	1	2	2	2	2	2
2	2	2	2	5	9	9	9	9	2
9	9	9	9	9	9	9	9	9	9
1	1	1	1	1	1	2	1	1	1
2	2	2	2	1	3	3	3	3	2
9	9	9	9	9	9	9	9	9	9
1	2	2	2	1	1	1	2	1	1
1	2	3	2	1	3	2	1	2	2
2	1	1	3	1	9	9	9	9	1
1	1	3	3	1	2	2	2	2	1
1	1	2	1	1	1	1	2	2	1
3	3	3	3	9	9	9	9	9	2
9	9	9	9	9	9	9	9	9	9
3	3	3	3	3	2	2	2	2	2
2	2	2	3	1	2	2	3	2	3
1	1	1	1	1	1	2	3	3	2
1	3	2	1	9	1	1	1	1	1
1	1	1	1	1	1	1	2	2	2
1	1	1	1	4	2	2	2	2	1
2	3	2	1	9	1	3	3	1	1
2	2	2	2	9	3	3	3	3	2
3	2	2	1	9	3	3	2	2	1
2	2	2	2	1	2	2	2	2	2
9	9	9	9	1	9	9	9	9	9
9	9	9	9	9	9	9	9	9	9
9	9	9	9	9	9	9	9	9	9
2	3	2	1	1	1	2	3	3	1
3	2	1	2	1	3	2	2	2	2
1	2	2	1	9	9	9	9	9	1
2	2	3	1	9	9	9	9	9	1
3	3	3	2	9	2	2	2	2	3
2	1	3	2	4	3	3	1	2	1
3	3	2	2	1	1	2	2	3	2
3	1	2	1	4	2	1	3	2	1
1	1	2	3	1	3	2	2	2	1
3	2	2	2	9	2	2	2	3	9
3	3	2	1	1	2	2	2	3	2
3	3	3	3	1	1	1	2	1	1
2	2	2	2	9	2	2	2	3	2
2	2	2	2	9	1	1	1	1	3
2	2	2	2	2	2	2	2	2	2
3	3	3	3	9	2	2	1	2	3
1	2	2	3	9	1	1	1	2	2
9	9	9	9	9	9	9	9	9	9
9	9	9	9	9	9	9	9	9	9
2	3	1	2	9	1	2	1	3	1

2	2	2	2	9	2	2	2	2	2
3	1	2	2	2	1	2	1	1	2
2	3	2	1	1	2	2	2	2	1
9	9	9	9	9	2	2	2	2	9
3	3	3	3	9	1	2	2	1	3
1	2	3	1	9	2	2	2	2	2
1	1	1	1	9	9	9	9	9	1
1	1	2	2	1	2	2	2	2	2
2	1	2	3	1	2	1	3	1	2
2	2	2	2	9	2	2	2	2	1
2	2	2	3	1	2	2	2	2	2
3	3	3	3	9	1	2	1	2	3
1	1	1	1	9	1	3	3	1	1
3	3	3	3	9	1	1	1	1	3
2	3	2	2	1	2	2	2	2	2
2	1	1	1	9	9	9	9	9	2
2	2	2	2	1	3	3	3	3	2
1	2	2	2	5	3	3	2	2	1
2	2	2	2	9	9	9	9	9	1
9	1	2	1	3	1	1	2	2	1
2	2	2	2	9	1	1	2	2	1
2	2	2	2	9	2	3	2	2	3
1	1	1	3	1	1	1	2	2	3
3	1	2	1	1	3	3	3	3	1
2	2	2	2	1	3	3	3	3	9
2	3	2	1	9	1	2	2	2	2
3	2	2	1	9	9	9	9	9	1
3	3	3	3	3	2	2	1	2	3
3	3	3	3	1	2	2	2	1	2
3	3	3	3	1	2	1	2	3	1
2	3	1	2	9	2	2	2	2	1
3	3	3	2	1	2	2	2	2	1
3	3	3	2	1	3	2	2	2	2
2	1	2	3	1	2	2	2	2	1
2	3	1	2	5	1	1	1	3	1
2	2	2	2	1	2	2	2	2	2
3	2	2	2	5	2	3	1	2	1
2	2	2	2	9	2	2	2	2	2
2	2	2	2	2	3	3	3	3	2
1	1	1	1	9	1	1	2	1	1
3	1	2	3	9	1	2	2	2	1
1	2	3	1	1	1	1	1	1	1
1	3	1	2	4	1	1	1	2	1
3	3	3	3	9	2	2	2	2	3
2	1	1	2	1	1	2	2	2	1
3	2	1	2	1	1	2	1	2	2
2	2	2	2	9	2	2	2	2	2

1	1	1	1	1	1	2	1	1	1
2	1	2	1	3	1	2	2	3	1
1	2	1	2	2	1	1	1	1	1
2	2	3	3	9	1	2	3	3	1
2	1	2	2	1	3	3	2	2	2
3	3	1	3	9	1	1	2	2	3
1	1	1	3	1	1	2	2	2	2
2	3	1	2	3	1	1	3	3	1
2	2	2	2	9	1	2	2	2	2
1	1	1	1	2	1	1	1	1	1
2	3	1	2	3	2	2	2	2	1
3	3	3	2	1	1	1	1	1	1
1	2	3	2	4	1	1	2	2	1
3	2	2	1	4	1	1	1	2	2
1	1	3	2	3	1	1	1	2	1
1	1	2	3	1	2	2	2	2	3
3	1	2	3	9	1	3	2	1	1
2	3	2	1	1	2	2	2	2	2
1	1	1	1	5	1	1	1	1	1
1	1	3	3	1	1	2	1	2	1
2	3	2	3	1	2	2	2	2	2
1	1	1	3	1	1	2	2	1	1
1	3	1	3	1	2	2	2	2	1
1	1	3	3	1	1	2	2	2	1
1	2	1	1	4	1	1	2	3	1
1	3	3	2	5	2	1	2	1	2
2	2	3	3	1	1	2	3	3	3
3	3	1	3	1	2	2	2	2	3
3	9	3	1	3	1	2	2	2	3
1	2	1	1	4	1	1	2	1	1
2	2	2	2	2	2	2	2	2	2
2	2	2	2	9	9	9	9	9	2
2	1	3	1	4	1	1	1	1	1
2	1	3	2	1	1	2	3	1	2
2	2	2	2	9	9	9	9	9	2
9	9	9	9	9	9	9	9	9	9
9	9	9	9	9	9	9	9	9	9
9	9	9	9	9	9	9	9	9	9
1	2	2	2	3	1	1	1	1	2
9	9	9	9	1	9	9	9	9	9
2	2	2	2	9	9	9	9	9	3
9	9	9	9	9	9	9	9	9	9
1	3	2	3	1	1	2	2	2	2
9	9	9	9	1	2	2	2	2	9
9	1	9	1	9	9	9	9	9	1
2	1	3	2	3	1	1	1	1	2
9	9	9	9	9	9	9	9	9	9

1	2	2	2	2	1	2	2	2	2
3	2	3	1	1	1	2	1	1	1
3	2	3	1	9	9	9	9	9	1
1	2	1	1	9	1	3	2	1	1
2	3	1	2	1	1	2	1	1	1
2	1	3	1	4	1	1	1	1	1
2	1	2	1	9	1	2	2	1	1
2	2	2	1	3	2	2	2	9	1
2	2	2	2	9	9	9	9	9	9
1	1	1	1	3	2	2	2	1	2
2	2	2	2	1	2	9	2	1	2
1	2	2	1	1	1	2	1	1	1
9	9	9	9	1	2	1	3	1	9
1	1	2	3	1	1	2	3	3	1
3	1	2	3	4	1	2	1	2	1
2	3	2	3	1	1	1	1	1	3
3	1	2	3	1	2	2	1	2	2
3	2	2	2	9	3	2	2	1	2
3	2	3	1	9	1	1	1	3	1
2	2	2	2	1	2	2	2	2	2
2	2	2	2	1	2	2	2	2	2
9	1	1	1	9	9	9	9	9	9
2	2	2	2	3	2	3	3	3	2
2	2	2	2	1	3	3	3	3	2
2	2	2	2	1	2	2	2	2	2
2	2	2	2	9	2	2	2	2	2
1	1	1	9	1	1	1	1	1	1
1	1	1	9	1	1	1	1	1	1
1	1	1	9	1	1	1	1	1	1
1	1	1	1	1	1	1	1	1	1
1	1	1	1	3	1	1	1	1	1
1	1	1	3	4	1	1	1	1	1
1	1	1	3	2	1	1	1	1	1
1	2	1	2	1	1	1	1	1	1
1	2	1	2	1	1	1	1	1	1
1	1	1	9	1	1	1	2	1	1
1	1	1	9	2	1	1	1	1	1
9	9	9	9	3	3	1	2	3	9
1	1	1	1	3	1	1	1	1	1
1	1	1	3	1	1	1	1	1	1
1	1	1	9	1	1	2	2	1	2
1	1	1	9	2	1	1	1	1	1
1	1	1	1	9	1	1	3	1	1
1	1	1	9	1	1	1	1	1	1
1	1	1	1	1	1	1	1	1	9
1	1	1	3	1	1	1	2	1	1
1	1	1	3	3	1	1	1	1	1

1	9	1	1	1	1	1	1	1	1
1	1	1	3	1	1	1	1	1	1
1	1	1	1	1	1	1	1	1	1
1	1	1	1	2	1	1	1	1	1
1	1	1	3	4	1	1	1	1	1
1	1	1	9	9	1	1	1	1	1
1	1	1	9	2	1	1	1	1	1
1	1	1	3	1	1	1	1	1	1
1	1	1	1	1	1	1	1	1	1
1	1	1	1	1	1	1	1	1	1
1	1	1	9	1	1	1	1	1	1
1	1	1	9	1	1	1	1	1	1
1	1	1	1	1	1	1	2	2	2
1	2	1	1	1	1	1	1	1	1
1	1	1	3	1	1	1	1	1	9
1	1	1	1	1	1	1	1	1	1
1	1	1	2	1	1	1	2	2	9
1	1	1	2	2	1	3	3	1	2
1	1	1	9	1	1	1	1	1	1
1	1	1	3	1	1	1	1	1	1
1	1	1	3	1	1	1	1	1	1
1	1	1	9	1	1	1	1	1	1
1	1	1	9	1	1	1	3	1	1
1	1	1	1	1	1	1	2	2	1
1	1	1	9	1	1	1	1	1	1
1	1	1	1	1	1	1	3	1	2
9	2	1	2	2	1	2	2	2	3
3	3	3	3	9	1	2	2	2	1
1	1	1	3	1	1	1	1	1	1
1	1	1	9	1	1	1	1	1	1
1	1	1	1	1	1	2	2	2	3
1	1	1	1	1	1	1	1	1	1
1	1	1	9	1	1	3	2	1	1
3	3	3	2	1	1	1	1	1	1
1	1	1	3	1	1	1	1	2	1
1	1	1	9	3	1	1	2	1	1
1	1	1	2	1	1	1	2	2	1
1	1	1	3	1	1	1	1	1	1
1	1	1	9	2	1	1	1	1	9
1	1	1	9	4	1	1	1	1	1
1	1	1	1	1	1	1	1	1	1
1	1	1	1	1	1	1	1	1	1
9	1	9	9	2	1	1	1	1	1
1	1	1	9	1	1	1	1	1	1
1	1	1	3	1	1	1	1	1	1
1	1	1	1	1	1	1	1	1	1
1	1	1	3	4	1	1	1	1	1

1	1	1	1	1	1	1	1	1	1
1	1	1	9	1	1	1	1	1	1
1	1	1	1	1	1	1	1	1	1
1	1	1	2	1	1	1	1	1	1
1	1	1	1	1	1	1	1	1	1
1	1	1	9	1	1	1	1	1	1
1	1	1	9	1	1	2	2	1	1
1	1	1	9	1	1	1	1	1	1
1	1	1	2	2	1	1	1	1	1
1	1	1	9	1	1	1	1	1	1
1	1	1	1	1	1	1	1	1	1
1	3	1	1	1	1	2	2	1	1
1	1	1	1	5	1	1	2	1	3
1	1	1	1	9	9	9	9	9	1
1	1	1	1	9	1	1	9	9	1
1	1	1	1	4	1	1	1	1	1
1	1	2	1	4	2	2	1	1	1
9	9	9	9	1	1	1	1	1	9
1	1	3	1	5	1	1	2	1	1
2	2	2	1	1	1	1	2	1	1
2	2	3	1	1	2	2	3	3	2
1	1	1	3	4	1	1	2	2	1
9	9	9	9	1	1	1	1	1	9
9	9	9	9	1	1	1	1	1	9
1	1	1	1	4	1	1	1	1	1
1	1	1	1	3	1	1	2	1	2
1	1	1	1	3	1	1	1	1	1
1	2	2	3	3	1	1	1	1	1
1	2	3	3	9	1	1	1	1	1
2	2	3	3	3	1	1	1	1	1
1	3	3	1	4	1	1	2	2	1
2	1	1	1	1	1	1	2	2	1
1	2	2	3	1	1	1	2	3	2
1	1	1	2	1	1	1	1	1	2
1	1	1	1	1	1	1	1	1	1
2	1	1	1	1	1	1	1	1	1
2	1	1	1	1	1	1	1	1	1
9	9	9	9	2	1	1	1	2	2
2	2	1	1	1	1	1	2	1	2
1	2	1	1	5	1	1	1	1	1
2	2	1	1	5	1	1	1	1	1
1	1	2	1	3	1	1	1	1	2
2	2	1	1	9	1	1	3	9	9
2	2	2	2	9	1	1	3	1	1
1	1	1	2	9	1	1	3	1	1
2	2	1	1	2	1	1	1	1	1
1	1	1	1	4	1	1	1	1	1

1	2	1	1	3	1	1	1	1	1
2	2	1	1	3	1	1	2	2	1
2	2	2	2	9	9	9	9	9	2
2	9	1	1	3	1	1	2	2	1
1	1	1	1	1	1	2	2	1	1
1	1	1	2	5	1	1	1	1	1
1	1	1	1	1	1	1	1	1	1
3	2	1	1	1	1	1	2	1	1
2	2	1	1	1	1	1	3	1	1
2	2	2	2	1	1	1	2	1	1
9	9	9	9	4	1	1	1	1	1
2	2	3	3	2	2	1	2	2	2
1	1	1	1	2	1	1	1	1	1
1	2	1	1	4	1	1	2	1	9
2	2	1	1	5	1	1	1	1	9
2	2	1	1	1	1	1	1	1	1
3	3	1	1	1	1	1	1	1	1
1	1	1	1	4	1	1	1	1	1
1	2	1	1	1	1	1	2	2	2
1	2	2	1	3	1	1	1	1	1
1	2	1	2	3	3	1	2	1	2
9	9	9	9	3	1	1	1	1	9
1	1	1	2	3	1	1	1	1	1
1	2	1	1	1	1	1	1	1	1
1	1	1	1	2	1	1	1	1	1
1	1	1	1	3	1	1	1	1	1
9	9	9	9	3	1	1	2	1	9
1	2	1	2	3	1	1	1	1	1
2	2	3	2	3	1	1	1	1	9
1	1	1	1	2	1	1	1	1	1
1	1	1	1	2	1	1	1	1	1
1	1	1	1	9	1	1	1	1	1
1	9	1	9	9	1	1	1	1	2
9	9	9	9	9	9	9	9	9	9
9	9	9	9	9	9	9	9	9	9
1	3	1	1	2	1	1	2	1	1
1	1	1	1	3	1	1	1	1	1
1	2	1	1	4	1	1	1	1	1
1	1	1	9	1	1	1	1	1	9
1	1	1	9	9	1	1	1	1	9
1	1	1	3	1	1	1	1	1	1
1	1	1	9	1	1	1	1	1	1
1	1	1	1	1	1	1	1	1	1
1	1	1	1	2	1	1	1	1	1
1	1	1	1	1	1	1	1	1	1
1	1	1	2	4	1	1	1	1	1
1	1	1	9	1	1	1	2	1	1

1	1	1	9	1	1	1	2	1	1
1	1	1	3	1	1	1	1	1	1
1	1	1	1	1	1	1	3	1	3
1	1	1	1	1	1	1	1	1	1
1	1	1	1	9	1	1	2	2	3
1	1	1	9	1	1	1	1	3	1
1	1	1	1	2	1	1	1	1	1
1	1	1	9	1	1	1	2	2	1
9	9	9	9	9	9	9	9	9	9
1	1	1	9	3	1	9	1	1	1
1	9	1	9	1	1	1	1	1	1
1	1	3	3	3	1	1	1	1	1
1	3	1	1	1	1	1	3	1	1
1	2	1	1	5	1	1	1	1	1
9	9	9	9	3	1	1	1	1	2
9	9	9	9	9	1	1	1	1	1
3	3	3	3	9	1	1	9	9	1
2	1	1	1	2	1	1	3	1	1
1	1	1	2	1	1	1	1	1	1
9	9	1	9	4	1	1	1	1	9
1	1	2	1	3	1	1	1	1	1
1	2	1	1	3	1	1	1	1	1
1	9	1	1	1	1	1	9	1	9
1	2	1	3	3	1	2	1	1	9
1	1	1	2	3	1	1	1	1	1
2	2	1	2	1	1	1	1	1	1
1	1	1	3	1	1	1	1	1	1
1	1	1	3	3	1	1	1	1	1
1	1	1	3	3	1	1	1	1	1
1	1	1	1	2	1	1	1	1	1
1	1	1	1	9	1	1	9	1	1
1	1	1	3	3	1	1	1	1	1
1	2	1	1	1	1	1	1	1	1
2	3	1	1	3	1	2	1	1	1
1	1	1	3	3	1	1	1	1	1
1	1	1	3	3	1	1	1	1	1
1	2	1	1	3	1	2	2	1	1
1	1	1	2	2	1	1	1	1	1
1	2	1	1	3	1	1	1	1	1
1	1	1	1	3	1	1	1	1	1
1	2	1	2	4	1	1	1	1	1
1	2	1	1	3	1	1	1	1	9
1	1	1	1	4	1	1	1	1	1
9	1	1	9	3	1	1	1	1	1
2	2	2	2	3	1	1	2	1	1
1	1	1	1	2	3	1	3	1	1
3	2	2	3	2	1	1	3	3	2

1	3	3	1	3	1	1	1	1	1
9	9	1	9	1	1	1	1	1	1
9	9	1	9	1	1	1	1	1	9
1	1	1	1	3	1	1	1	1	1
1	1	1	1	2	1	1	1	1	1
9	9	9	9	9	1	1	1	1	1
1	3	1	3	3	1	1	1	1	1
1	2	1	1	3	1	1	1	1	1
2	2	1	2	1	1	1	1	1	2
1	2	1	1	1	1	1	1	1	1
1	3	1	1	1	1	1	1	1	1
1	2	1	3	1	1	1	1	1	1
2	3	2	3	3	1	1	9	1	2
1	2	1	1	1	1	1	1	1	1
1	1	2	2	3	1	1	1	3	1
1	1	2	2	1	1	1	1	1	1
1	3	2	2	3	1	1	1	1	1
9	9	9	9	4	1	1	1	1	1
9	9	9	9	3	1	1	1	1	1
9	9	9	9	1	1	1	1	1	1
9	9	9	9	1	1	1	1	1	1
9	9	9	9	1	1	1	1	1	1
3	3	3	3	3	1	1	9	1	1
1	1	1	1	4	2	2	2	2	1
9	9	9	9	3	1	1	1	1	9
1	2	1	1	3	1	1	1	1	1
1	1	1	1	3	1	1	1	1	1
1	2	1	3	3	1	1	2	1	9
1	2	1	2	4	1	1	2	1	1
3	2	2	3	3	1	1	2	1	2
2	2	1	1	3	1	1	1	1	1
1	1	1	1	3	1	1	1	1	1
1	1	1	1	3	1	1	3	1	1
1	1	1	1	2	1	1	1	1	1
1	9	1	1	4	1	1	1	1	1
1	2	1	1	3	1	1	1	1	1
1	1	1	1	3	1	1	1	1	1
9	1	1	9	9	1	1	1	1	1
1	2	2	3	3	1	1	1	1	1
2	1	1	2	3	1	1	1	1	1
1	2	1	1	3	1	1	1	1	1
3	2	2	1	3	1	1	1	1	1
1	2	1	1	3	1	1	2	1	2
1	1	1	1	1	1	1	1	1	1
1	1	1	1	1	1	2	1	1	9
1	1	1	1	1	1	1	1	1	1
2	3	2	3	3	1	1	3	3	1

2	2	2	2	9	1	1	3	3	1
1	2	1	1	5	1	1	1	1	1
1	1	1	1	5	1	1	1	1	1
2	2	2	2	2	1	1	1	1	1
1	9	1	1	2	1	1	1	1	1
1	1	1	1	1	1	1	1	1	1
1	1	1	1	3	1	1	1	1	1
1	1	1	1	4	1	1	1	1	1
2	1	1	1	5	1	1	1	1	1
1	1	2	2	4	1	1	1	1	1
1	2	3	3	4	1	1	1	1	1
2	2	2	2	3	1	1	1	1	1
1	1	2	2	2	1	1	1	1	1
1	1	2	1	3	1	1	1	1	1
2	2	2	2	4	1	1	1	1	2
1	1	1	2	4	1	1	1	1	1
1	2	1	3	3	1	1	1	1	1
2	2	2	2	9	1	1	1	1	2
1	1	2	2	1	1	1	1	1	1
1	1	2	1	1	1	1	1	1	1
2	2	2	2	1	1	1	1	1	1
1	1	1	1	3	1	1	1	1	1
2	2	2	2	2	1	1	2	1	2
2	2	2	2	1	1	1	2	1	2
2	2	2	2	4	3	3	3	3	2
2	2	3	3	3	1	1	1	1	1
2	2	3	3	4	1	1	1	1	1
9	9	9	9	9	9	9	9	9	1
1	1	2	1	4	1	1	1	1	1
1	2	1	3	3	1	1	1	1	1
1	2	1	2	2	1	1	1	1	1
1	2	1	1	1	1	9	9	9	9
2	1	1	1	1	9	9	9	9	9
9	9	9	9	3	1	1	1	1	1
1	2	3	9	1	9	9	9	9	1
3	3	3	3	2	1	1	1	1	3
1	1	1	1	4	1	1	1	1	1
1	1	1	1	4	1	1	1	1	1
9	9	9	9	9	1	1	1	1	9
9	9	1	9	1	9	9	9	9	9
9	9	1	9	1	1	2	1	1	9
9	9	1	9	1	1	1	1	1	9
9	9	9	9	1	1	1	1	1	9
9	9	9	9	1	1	1	1	1	9
9	9	9	9	3	1	1	1	1	1
9	9	9	9	9	1	1	1	3	3
1	1	1	1	3	1	1	1	1	1

9	9	9	9	3	1	1	1	1	1
9	9	9	9	5	1	1	1	1	1
1	1	2	2	2	1	1	1	1	2
9	9	9	9	1	1	1	1	1	9
9	9	9	9	3	1	1	1	1	1
9	9	9	9	3	1	1	1	1	1
1	1	1	1	2	1	1	1	1	1
2	1	2	1	9	1	1	2	1	9
9	9	9	9	3	1	1	1	1	9
9	9	9	9	1	1	1	1	1	1
9	9	9	9	1	1	1	1	1	1
9	9	9	9	1	1	1	1	1	1
9	9	9	9	9	1	1	1	1	1
9	9	9	9	3	1	1	1	1	1
9	9	9	9	4	1	1	1	1	9
9	9	9	9	1	1	1	1	1	1
1	1	3	3	3	1	1	1	1	1
1	1	1	1	3	1	1	1	1	1
1	1	1	1	3	1	1	1	1	1
1	1	1	1	4	1	1	2	1	1
1	1	1	1	4	1	1	1	1	1
1	1	3	1	1	1	1	2	1	9
1	1	3	1	1	1	1	2	1	1
1	1	1	1	1	1	2	1	1	1
1	1	1	1	3	1	1	1	1	1
1	1	1	1	3	1	9	1	1	1
1	1	1	1	3	1	1	1	1	1
2	2	9	2	1	1	1	2	1	1
9	9	9	9	1	1	1	1	1	1
1	1	1	1	9	1	1	1	1	1
9	9	9	9	9	1	1	1	1	3
1	1	1	1	3	1	1	1	1	1
1	2	1	2	1	2	1	2	1	2
1	1	1	1	2	1	1	1	1	1
1	1	1	2	1	1	1	1	1	1
1	2	1	2	1	1	1	2	2	2
1	1	1	2	3	1	1	1	1	1
1	2	1	1	3	1	1	3	1	1
2	2	2	1	1	1	1	1	1	1
1	1	1	1	4	1	1	1	1	1
2	1	2	2	3	1	1	1	1	1
1	1	1	1	2	1	1	1	1	1
1	1	1	3	2	1	1	1	1	1
1	1	1	3	3	1	1	1	1	1
1	1	1	1	1	1	1	1	1	1
1	1	1	3	1	1	1	1	1	2
1	1	1	2	4	1	1	3	1	1

1	1	1	1	3	1	2	2	1	1
2	2	1	3	1	1	3	2	1	1
1	1	1	1	3	1	1	1	1	1
1	1	1	1	9	1	1	1	1	1
1	1	3	3	3	1	1	1	1	1
1	1	1	1	2	1	1	1	1	1
1	1	1	1	2	1	1	1	1	1
1	1	1	1	2	1	1	1	1	1
1	1	1	1	3	1	1	1	1	1
1	1	1	1	4	1	3	3	1	1
1	1	1	1	3	1	1	1	1	1
2	1	1	1	4	1	3	1	1	1
1	1	1	1	2	1	1	1	1	1
9	1	1	1	4	1	1	1	1	1
1	1	1	3	2	1	1	1	1	1
1	1	1	1	9	1	1	1	1	1
1	1	1	1	2	1	1	1	1	1
1	1	1	1	4	1	1	1	1	1
1	1	1	3	3	1	1	1	1	1
1	1	1	3	4	1	1	1	1	1
3	1	1	1	1	1	1	1	1	1
1	1	1	1	2	1	1	1	1	1
1	1	1	3	3	1	2	2	2	1
1	2	2	2	2	1	1	1	1	1
1	3	1	3	1	2	1	1	2	3
1	1	1	2	3	1	1	1	1	1
2	3	1	1	2	1	1	1	1	1
1	1	1	1	2	1	1	1	1	1
1	1	1	1	4	1	3	3	1	1
1	2	1	1	1	1	1	2	1	1
1	2	1	1	1	1	1	2	1	1
1	1	3	2	3	1	1	1	1	1
1	1	2	2	1	1	1	2	2	2
2	2	2	2	1	1	2	2	2	2
1	1	1	1	3	1	1	1	1	1
1	2	2	3	3	1	1	1	1	1
2	3	3	2	1	1	1	2	2	2
3	2	2	2	1	1	1	1	1	1
1	1	1	2	1	1	1	1	1	1
1	1	1	2	1	1	1	1	1	1
9	9	9	9	9	1	3	3	1	1
1	1	1	1	4	1	3	3	1	1
1	1	2	3	5	1	1	1	1	3
2	2	2	2	1	1	1	1	1	3
1	1	1	3	4	1	1	1	1	1
1	1	2	2	2	1	1	1	1	1
2	2	2	2	1	1	1	1	1	1

1	1	1	3	5	1	1	1	1	1
1	1	1	3	9	1	1	1	1	1
1	2	2	2	3	1	1	1	1	1
1	1	1	1	1	1	1	1	1	1
1	1	2	1	2	1	1	1	1	1
2	2	2	2	1	1	1	1	1	1
2	3	3	3	5	1	1	1	1	2
2	2	2	2	1	1	1	1	1	1
1	2	1	2	1	1	1	1	1	1
1	1	1	1	1	1	1	1	1	3
2	1	1	1	4	1	1	1	1	1
2	2	2	2	1	1	1	1	1	1
3	2	3	3	4	1	1	1	1	1
2	2	2	1	1	1	1	1	1	2
1	1	2	2	3	1	1	1	1	3
1	2	1	1	4	1	1	1	1	1
1	2	2	1	5	1	1	2	1	2
1	1	3	2	3	1	1	1	1	1
1	9	9	9	9	1	1	1	9	1
1	1	2	3	1	1	1	1	1	1
3	3	3	1	1	1	1	1	1	1
2	2	2	2	1	2	2	2	2	1
1	2	2	1	4	1	1	1	1	1
1	1	1	1	3	1	1	1	1	1
3	2	1	1	2	1	1	1	1	1
1	1	2	2	1	1	1	2	1	1
1	1	1	1	4	1	1	1	1	1
1	1	1	1	3	1	1	1	1	1
1	3	1	1	4	1	1	1	1	1
3	2	1	1	4	1	1	1	1	1
1	1	1	2	4	1	1	1	1	1
1	1	1	1	4	1	1	1	1	1
1	1	3	1	1	1	1	2	1	1
1	2	1	1	9	1	1	1	3	3
1	1	2	2	9	1	1	1	1	2
9	9	9	9	9	1	1	1	1	2
3	2	3	2	1	1	1	1	1	1
1	2	3	3	2	1	1	1	1	9
1	2	2	2	1	1	9	2	1	1
1	1	1	1	1	1	1	2	1	1
9	9	9	9	1	1	1	1	1	1
1	2	1	2	1	1	3	3	1	1
1	1	1	3	9	3	2	2	3	2
9	9	9	9	3	1	1	3	1	9
3	2	1	2	5	1	1	1	1	1
2	2	1	2	3	1	1	2	2	2
9	9	1	9	4	1	2	2	1	2

1	1	2	3	3	1	1	1	1	3
1	1	1	1	3	1	1	2	1	3
9	1	9	9	4	1	1	2	1	1
2	1	2	3	1	1	1	2	2	2
2	2	2	2	4	1	1	1	1	2
1	1	1	1	5	1	1	2	1	1
2	2	2	2	2	1	1	2	1	1
1	2	2	3	3	1	1	1	1	1
1	1	2	1	1	1	1	2	1	2
2	2	2	2	2	1	1	2	1	2
2	3	1	1	5	1	1	2	2	1
1	2	2	2	2	1	1	2	1	1
2	1	1	1	5	1	1	2	1	1
9	9	9	9	1	1	1	1	1	1
2	2	2	2	1	1	1	2	1	1
2	2	2	2	1	1	1	2	1	1
1	1	1	2	4	1	1	1	1	1
2	3	2	2	1	1	1	1	1	2
2	1	2	2	2	1	1	2	1	1
2	2	2	2	2	1	1	1	1	2
2	2	2	2	1	1	1	1	1	1
1	1	3	3	4	1	1	1	1	1
1	1	1	1	1	1	1	1	1	2
1	1	1	3	1	1	1	1	1	1
1	1	1	2	4	1	1	1	1	1
1	1	1	2	9	1	1	1	1	1
1	1	1	2	4	1	1	1	1	1
1	1	1	2	3	1	1	1	1	1
2	2	2	2	4	3	1	3	1	1
2	2	2	2	4	1	1	1	1	1
2	3	2	3	4	1	1	1	1	1
1	1	1	2	1	1	1	1	1	1
1	1	1	2	4	1	1	1	1	1
1	1	1	3	4	1	1	1	1	1
1	1	1	3	4	1	1	1	1	1
9	9	9	9	9	1	2	1	1	9
1	1	1	3	4	1	1	1	1	1
1	1	2	1	2	1	1	1	1	1
1	1	2	1	2	1	1	1	1	1
2	2	2	9	3	1	1	1	1	1
9	9	9	9	4	1	9	1	1	9
1	1	1	2	4	1	1	1	1	1
1	1	1	1	4	1	1	1	1	1
1	1	1	1	4	9	9	9	9	1
1	1	1	1	4	1	1	1	1	1
2	2	2	3	1	1	1	1	1	1
1	1	2	1	9	1	1	1	1	1

1	1	1	3	1	1	1	1	1	1
2	2	2	3	3	1	1	2	2	2
1	1	3	3	1	1	1	1	1	1
1	1	1	1	4	1	1	1	1	1
3	1	3	1	4	1	1	1	1	1
1	1	1	1	1	1	1	1	1	1
1	1	1	1	3	1	1	1	1	1
1	1	1	1	3	1	1	1	1	1
1	9	2	1	1	1	1	1	1	1
1	1	1	1	3	1	1	1	1	1
1	1	1	1	9	1	1	1	1	1
1	1	1	1	4	1	1	1	1	1
1	1	3	1	4	1	1	1	1	1
1	3	2	1	3	1	1	1	1	2
1	1	1	1	3	1	1	1	1	1
1	1	1	1	3	1	1	1	1	1
1	1	1	3	1	1	1	1	1	1
1	1	1	3	4	1	1	3	1	1
1	1	1	3	4	1	1	1	1	1
1	1	1	1	4	1	1	1	1	1
1	1	1	1	3	1	1	1	1	1
1	1	1	1	2	1	1	1	1	1
1	1	2	3	1	1	1	1	3	1
2	1	1	2	3	1	1	1	1	1
1	1	1	1	4	1	1	1	1	1
1	1	1	1	2	1	1	3	1	1
2	1	1	2	9	1	1	3	1	1
3	3	3	3	1	1	1	1	1	1
9	9	9	9	1	1	1	1	1	1
9	9	9	9	9	1	1	1	1	9
9	9	9	9	2	1	1	1	1	1
9	9	9	9	4	1	1	1	1	9
9	9	9	9	4	1	1	1	1	1
9	9	1	9	1	1	2	1	1	9
9	9	9	9	1	1	1	1	1	1
9	9	9	9	1	2	2	2	2	9
9	9	9	9	1	1	1	1	1	9
9	9	9	9	1	1	1	1	1	9
9	9	9	9	1	1	1	2	1	2
9	9	9	9	3	1	1	1	1	1
1	2	1	1	3	1	1	1	1	2
1	1	1	2	4	1	1	1	1	1
1	1	1	2	2	1	1	1	1	1
1	1	1	3	1	1	1	1	1	1
1	1	1	3	4	1	1	1	1	1
1	1	1	3	4	1	1	1	1	1
1	1	1	1	9	1	1	1	1	1

1	1	1	2	5	1	1	1	1	2
1	2	1	2	1	1	1	2	1	1
2	2	2	2	1	1	3	1	1	1
2	3	2	2	9	1	1	2	1	2
1	1	1	3	5	1	1	1	1	1
1	1	1	3	4	1	1	1	1	1
1	2	2	2	3	1	1	2	1	2
2	1	2	1	1	1	1	1	1	2
1	2	1	3	1	1	1	1	1	1
3	1	1	3	9	1	1	2	1	3
2	1	2	2	3	2	2	2	2	1
2	2	2	2	3	1	1	1	1	1
9	9	9	9	9	9	9	9	9	9
2	2	2	2	1	1	1	2	1	2
2	2	3	2	4	1	1	2	1	1
2	2	2	2	4	1	1	2	1	2
2	2	1	1	9	1	2	2	1	2
1	1	1	1	3	1	1	1	1	1
1	2	1	2	1	1	2	1	1	1
3	2	2	3	1	1	1	2	2	3
2	2	2	2	5	1	1	1	1	1
2	2	2	2	5	1	1	1	1	1
2	2	2	2	9	9	9	9	9	9
3	1	2	2	1	1	2	1	1	1
1	1	1	1	9	1	1	2	1	2
1	1	1	1	1	1	1	1	1	1
1	1	1	2	9	1	1	1	1	1
2	2	2	2	1	1	1	1	1	2
2	2	2	2	9	1	1	2	1	1
1	1	1	1	1	2	1	2	2	2
1	1	1	1	1	1	1	1	1	1
2	2	1	2	2	1	2	2	2	2
2	2	3	1	3	1	1	1	1	1
1	1	1	1	3	1	1	1	1	1
2	2	2	2	1	2	2	2	2	2
2	2	2	2	1	1	1	1	1	2
1	1	1	1	2	1	1	1	1	1
1	1	1	1	1	1	1	2	2	2
2	3	2	1	9	1	1	2	1	1
2	2	2	2	4	1	1	1	1	1
2	2	2	2	1	1	2	2	1	9
2	2	2	2	2	1	1	3	9	2
2	2	2	2	1	1	1	1	1	2
2	2	2	2	3	1	1	2	1	2
2	2	2	2	1	1	1	2	1	2
1	1	9	2	1	1	1	2	1	1
1	1	2	1	1	1	1	2	1	2

1	1	2	1	1	1	1	2	1	2

PapforMar	PapOverEi	PapTwoYe	PapfromM	PapHistory	PapTwoYe	PapOnceor	HPVVaccin	HPVVaccin	EarlyOnset
2	2	2	2	2	2	2	2	2	2
2	1	3	3	3	2	1	1	1	2
2	2	2	2	2	2	2	2	2	2
2	1	3	3	3	2	1	1	1	2
2	1	3	3	3	2	1	1	1	2
2	9	3	3	3	2	1	1	1	1
9	2	9	9	9	9	9	9	9	9
2	2	2	2	3	1	2	1	1	1
9	2	9	2	9	2	9	2	2	9
9	9	9	9	9	9	9	9	9	9
2	2	2	3	2	2	2	2	2	3
3	3	3	3	3	3	3	3	2	2
2	2	3	2	1	2	2	1	2	2
2	2	2	2	2	2	2	2	2	2
2	2	2	2	1	2	1	1	1	2
3	2	2	3	3	2	3	1	2	2
2	9	1	2	1	2	2	3	2	1
3	2	2	3	3	2	3	1	2	2
3	2	2	3	3	2	3	1	2	2
2	2	2	2	2	2	2	2	2	3
1	1	1	2	2	1	1	1	1	3
1	1	1	2	2	1	1	1	1	3
2	9	2	2	2	2	2	2	2	2
3	3	3	3	3	1	3	1	1	3
2	2	2	2	2	2	2	2	2	2
2	2	2	2	2	2	2	2	2	2
3	2	1	1	1	1	3	1	1	2
1	1	2	2	2	1	3	1	1	1
2	2	2	2	2	2	2	2	2	2
2	2	2	2	2	2	2	2	2	2
2	2	2	2	2	2	2	2	2	2
2	2	1	2	2	2	2	2	1	2
2	1	1	3	3	2	3	1	1	1
2	2	1	1	2	1	2	1	1	2
2	2	1	2	2	2	2	2	2	2
2	2	1	1	2	1	2	1	1	2
2	2	1	2	2	2	2	2	2	2
3	1	3	3	3	1	3	1	1	1
2	2	2	2	2	2	2	2	2	2
2	2	2	2	2	2	2	2	2	2
2	2	2	2	2	2	2	2	2	2
2	2	1	2	2	2	2	2	2	1
1	2	3	3	9	2	1	1	1	1
3	1	3	3	3	3	3	1	1	3
9	1	2	2	3	3	3	2	3	1
1	2	3	3	9	2	1	1	2	1

3	1	1	2	3	2	3	2	1	1
3	1	2	2	3	1	2	1	1	1
2	2	2	2	2	2	2	1	1	1
2	1	2	2	3	1	2	1	2	1
3	3	2	3	3	2	2	1	1	1
3	3	3	3	3	1	3	1	1	1
3	3	3	3	3	1	3	1	1	1
3	1	9	1	1	9	9	1	1	1
9	1	9	1	1	9	9	1	1	1
2	3	2	1	2	3	1	1	3	1
2	2	2	2	2	2	2	2	2	2
2	1	2	2	2	2	2	1	2	1
3	1	2	3	3	1	3	2	2	1
2	2	2	2	2	2	2	2	2	2
2	2	2	2	2	2	2	2	2	1
3	1	2	3	3	1	3	2	2	1
2	1	2	3	2	1	3	1	1	1
2	1	1	2	1	1	3	1	1	1
1	2	3	3	2	1	1	1	2	1
9	9	9	9	9	9	9	9	9	1
3	3	3	3	3	1	3	1	1	1
3	3	3	3	3	1	3	1	1	1
3	3	3	3	3	1	3	1	1	1
3	1	1	2	2	1	2	1	1	1
1	9	9	1	9	9	1	9	1	1
2	1	1	1	1	1	1	9	1	1
9	9	9	9	9	9	9	9	9	2
3	1	2	2	3	1	2	1	1	1
2	2	2	2	2	2	2	2	2	2
3	2	2	2	1	1	3	1	1	1
2	9	9	9	9	9	9	9	9	1
3	3	3	3	1	3	3	3	3	3
2	9	9	9	9	9	9	9	9	1
3	3	3	3	2	3	3	1	1	1
3	2	2	3	3	1	3	1	1	1
9	9	9	9	9	9	9	9	9	9
9	9	9	9	9	9	9	9	9	9
3	3	2	2	2	2	2	1	1	1
3	1	3	1	3	3	1	1	1	3
3	1	1	3	3	1	3	1	1	1
9	1	1	2	2	1	3	1	1	1
3	9	9	9	9	9	9	9	9	1
1	1	3	3	3	1	3	1	3	1
2	1	2	2	2	1	3	1	1	1
2	1	2	2	2	1	3	1	1	1
9	9	9	9	9	9	9	9	9	9
9	9	9	9	9	9	9	9	9	9

2	2	2	3	1	1	2	1	1	3
2	3	2	3	2	2	3	1	1	1
1	2	2	1	1	1	1	1	1	1
3	2	1	2	2	2	2	2	1	2
9	9	9	9	9	9	9	9	9	9
2	2	2	2	2	2	2	1	1	1
3	3	2	3	1	1	3	1	1	1
2	3	1	2	2	2	3	2	2	1
3	1	1	3	3	1	3	1	1	1
3	1	2	3	3	1	3	1	1	9
3	1	1	1	2	9	2	1	1	1
2	2	1	3	3	3	2	3	1	2
2	1	1	1	1	2	3	9	9	3
2	2	2	2	1	2	9	9	9	1
3	1	2	2	2	1	2	1	2	2
2	2	1	3	2	1	2	2	3	2
2	2	2	2	2	2	2	2	1	2
2	2	2	2	2	2	2	2	1	1
2	1	2	2	1	2	2	9	9	2
2	2	2	2	2	2	2	2	2	2
3	3	3	3	3	1	3	1	1	2
3	1	1	2	2	1	3	2	2	1
2	2	2	2	2	2	2	2	2	1
3	3	3	3	3	3	3	3	3	3
1	2	3	2	2	1	2	2	1	1
2	2	2	2	2	2	2	2	2	2
2	1	2	3	1	2	3	1	2	2
2	1	2	3	2	2	3	1	1	1
2	2	2	2	2	2	2	2	2	2
2	2	2	2	2	2	2	2	2	1
2	2	2	2	2	2	2	2	2	2
2	2	2	2	2	2	2	2	2	2
2	1	2	2	2	2	2	1	1	1
2	3	3	2	2	2	2	2	2	2
2	2	2	2	2	2	2	2	2	1
2	2	2	2	2	2	2	1	2	1
2	2	2	2	2	2	2	2	2	2
2	2	2	2	2	2	2	2	2	2
1	9	9	9	2	9	9	1	9	9
3	1	1	3	3	3	3	1	1	1
2	2	2	2	2	2	2	2	2	2
2	2	2	2	2	2	2	2	2	2
2	2	2	2	2	2	2	2	2	2
2	1	2	1	1	3	2	2	1	2
2	1	2	2	2	2	1	2	2	1
2	2	2	2	2	2	2	1	2	2
2	2	2	2	2	2	2	2	2	2

2	2	2	2	1	1	1	1	2	2
2	1	2	1	2	2	2	2	2	2
2	9	2	1	2	2	2	2	2	2
2	2	2	2	2	2	2	2	2	2
9	9	9	9	9	9	9	9	9	1
2	2	2	2	2	2	2	2	2	1
3	3	3	2	2	3	3	3	2	2
2	2	2	2	2	2	2	2	2	2
2	2	2	2	2	2	2	2	2	2
2	2	2	2	2	2	2	2	2	2
1	2	2	3	2	2	2	2	3	3
3	2	3	2	2	2	2	1	1	2
2	9	9	9	9	9	9	9	9	1
2	2	2	2	2	2	2	2	2	2
2	2	2	2	2	2	2	2	2	1
2	2	2	2	2	2	2	2	2	1
9	9	9	9	9	9	9	9	9	9
2	2	2	2	2	2	2	2	2	2
9	9	9	1	9	9	9	9	9	9
3	3	1	3	3	2	3	1	1	1
3	1	1	3	3	1	3	1	1	1
3	1	1	3	3	2	3	2	1	1
3	1	1	3	3	1	3	1	1	1
3	1	3	3	2	1	3	3	1	1
3	1	1	3	3	2	3	3	1	1
3	1	1	3	9	9	9	9	9	1
3	1	1	3	3	2	3	1	1	1
3	1	1	3	3	2	3	1	1	1
3	1	1	3	3	2	3	1	1	9
3	1	1	3	3	2	3	1	1	1
2	1	2	2	1	2	2	2	2	2
2	2	2	2	2	2	2	2	2	2
3	1	1	3	3	2	3	1	1	1
3	1	1	3	3	2	3	1	1	1
3	1	1	3	2	1	3	1	1	1
3	1	1	3	2	1	3	1	1	1
3	1	1	3	3	2	3	1	1	1
3	1	1	3	3	2	3	1	1	1
3	1	1	3	1	2	3	1	1	1
3	1	1	3	3	2	3	1	1	1
3	1	1	3	3	1	3	1	1	1
3	1	1	3	3	2	3	3	1	1
3	1	1	3	3	2	3	1	1	1
3	1	1	3	3	2	3	1	1	1
3	1	1	3	3	2	3	2	1	1
3	1	1	3	3	2	3	1	1	1
3	1	1	3	3	2	3	1	1	1

3	1	1	3	3	2	3	3	1	1
3	1	1	3	3	2	3	3	1	1
3	1	1	3	3	2	3	3	2	1
3	1	1	3	3	3	3	1	1	1
3	3	1	3	1	2	3	3	1	1
3	1	1	3	3	2	3	1	1	1
3	1	1	3	3	2	3	1	1	1
3	1	3	3	3	1	3	1	1	3
3	1	1	3	3	1	3	1	1	1
3	3	3	3	3	3	3	1	1	1
9	9	9	9	9	9	9	9	9	9
3	1	2	3	3	1	3	1	2	1
3	1	1	3	3	1	3	1	1	1
3	1	1	3	3	2	3	1	1	1
2	2	2	2	1	1	1	1	2	2
1	2	2	2	2	1	2	2	1	1
2	1	2	2	2	2	2	1	1	2
1	1	1	1	1	1	1	1	1	1
2	2	2	2	2	2	2	2	2	1
2	2	2	2	2	2	2	1	1	1
2	2	1	2	1	1	2	1	1	1
2	2	2	2	2	2	2	2	2	2
2	1	2	1	2	3	2	1	2	1
2	1	2	2	2	2	2	1	1	1
2	2	2	2	2	2	2	2	2	2
2	2	2	2	2	2	2	2	2	2
2	2	2	2	2	2	2	1	2	1
2	2	2	2	2	2	2	2	2	2
2	2	2	2	2	2	2	2	2	2
2	2	2	2	2	2	2	2	2	1
3	1	1	2	2	3	3	1	1	1
3	1	1	2	2	3	3	1	1	1
2	2	2	2	2	2	2	2	2	1
1	1	2	2	2	2	2	2	2	1
2	1	2	1	1	1	2	2	2	1
2	2	2	2	2	2	2	2	2	2
2	2	2	2	2	2	2	2	2	2
3	3	3	3	1	1	2	1	1	1
2	1	1	2	2	2	2	2	2	1
2	2	2	2	3	3	2	1	2	1
2	1	2	1	3	3	3	3	3	3
2	2	2	2	2	2	9	1	1	1
9	9	9	9	9	9	9	9	9	2
9	9	9	9	9	9	9	9	9	2
3	3	2	2	3	2	2	1	2	2
2	1	1	3	1	1	2	1	1	1
9	9	9	9	9	9	9	9	9	9

2	2	2	2	2	2	2	2	2	2
2	2	2	2	2	2	2	2	2	2
2	1	2	2	2	1	2	1	2	1
2	2	2	2	2	1	2	1	1	2
2	1	2	1	1	3	3	3	2	1
1	1	2	3	2	3	2	1	2	2
2	2	2	2	2	2	2	1	1	2
2	2	2	2	2	2	2	2	1	2
3	3	3	3	1	1	3	1	1	1
3	3	3	3	3	3	3	3	3	2
2	2	2	2	2	2	2	2	2	2
3	3	3	2	2	2	2	2	2	1
3	3	3	2	2	2	2	2	2	1
2	2	2	2	2	2	3	1	2	2
2	2	2	2	2	2	2	2	2	2
2	2	2	2	1	2	3	1	2	1
2	2	2	2	2	3	3	1	1	1
3	1	3	3	2	1	3	1	2	1
9	9	9	9	9	9	9	9	9	9
2	2	2	2	2	1	3	1	3	9
2	2	2	2	2	1	3	1	1	1
2	2	2	2	2	2	2	2	1	2
2	2	2	2	2	2	3	1	1	2
2	2	2	2	3	1	3	1	2	2
2	9	9	9	9	9	9	9	9	9
2	2	2	2	2	2	2	2	2	2
2	2	2	2	2	2	2	2	2	2
9	1	2	2	2	1	3	2	1	1
2	2	2	2	2	2	2	2	2	2
2	2	2	2	2	2	2	2	2	2
9	1	9	9	9	9	9	9	9	9
2	3	3	3	3	3	3	3	3	3
3	3	3	3	3	3	3	3	3	3
2	2	2	2	2	2	2	2	2	2
9	9	9	9	9	9	9	9	9	1
2	2	2	2	2	2	2	2	2	2
2	2	2	3	3	1	3	1	2	1
3	1	1	3	3	2	2	3	1	1
2	2	2	2	2	2	2	2	2	2
2	2	2	2	3	1	3	2	2	2
2	1	2	2	3	2	2	1	1	1
2	2	2	2	3	1	3	1	1	3
1	1	1	2	2	2	2	2	2	3
2	2	2	2	2	2	2	2	2	2
3	1	2	3	3	2	2	1	2	2
2	2	2	1	1	2	2	2	2	2
2	2	2	2	2	2	2	2	2	2

9	9	9	9	9	9	9	9	9	9
9	9	9	9	9	9	9	9	9	9
2	1	9	9	9	9	9	9	9	9
2	1	1	3	1	1	3	1	1	1
3	3	2	3	2	2	3	2	2	2
1	2	1	2	3	1	2	3	1	2
3	2	1	2	2	1	3	1	1	1
1	1	1	1	1	1	1	1	1	1
3	2	9	3	2	1	1	1	1	1
2	2	2	2	2	2	2	2	2	2
2	2	2	2	2	2	2	2	2	2
2	3	2	2	2	2	3	2	2	9
9	9	9	9	9	9	9	9	9	9
1	1	1	1	1	1	1	1	1	1
1	1	3	3	3	3	1	1	1	3
9	9	9	9	9	9	9	9	9	9
1	1	2	2	2	1	1	2	2	2
2	2	2	1	3	2	1	2	2	2
1	1	1	1	1	1	1	1	3	3
9	9	9	9	9	9	9	9	9	9
9	9	9	9	9	9	9	9	9	9
2	2	2	3	2	2	2	2	2	2
2	3	3	2	2	3	2	1	2	2
2	2	2	2	2	2	2	2	2	1
2	2	2	2	2	2	2	2	2	2
2	2	2	2	3	1	3	2	2	2
1	1	2	2	1	1	2	1	1	2
3	2	2	3	3	2	3	2	2	1
3	2	2	2	3	1	3	1	2	2
2	1	2	2	3	1	3	1	1	2
2	2	2	2	2	2	2	2	2	2
3	2	2	3	3	3	2	3	2	3
9	9	9	9	9	9	9	9	9	9
9	9	9	9	9	9	9	9	9	9
2	2	2	3	3	2	3	1	3	1
2	3	2	3	1	1	3	3	2	1
3	1	1	3	3	1	3	2	2	1
2	3	1	2	1	1	2	1	1	1
3	1	1	2	1	1	3	1	1	1
2	2	2	2	1	2	9	1	2	2
2	3	2	3	2	2	1	1	3	3
3	1	1	3	1	1	3	1	1	3
2	2	2	2	2	2	2	2	2	1
2	2	2	2	2	2	2	2	2	2
3	2	3	3	3	3	3	1	1	3
2	1	9	3	3	2	3	2	2	2
2	2	2	2	2	2	2	2	2	2

3	1	2	3	2	2	2	2	1	9
3	1	2	3	2	2	2	2	1	9
9	9	9	9	9	9	9	9	9	1
2	2	2	2	2	2	2	2	2	2
9	9	9	9	9	9	9	9	1	9
9	9	9	9	9	9	9	9	9	9
2	1	1	2	2	1	3	1	1	1
2	2	2	9	3	9	3	1	9	9
3	1	1	3	3	1	3	1	1	1
1	2	2	3	1	1	2	1	2	1
2	1	2	2	2	3	1	2	1	1
2	2	2	2	2	2	2	2	2	2
3	2	2	3	1	1	3	1	1	1
1	2	2	2	2	3	2	2	2	1
2	1	2	3	3	2	3	1	1	1
2	2	2	2	3	2	3	2	1	2
2	2	2	2	2	2	2	2	2	2
2	2	2	2	2	2	2	2	2	2
2	2	2	2	2	2	2	2	2	2
2	2	2	2	2	2	2	2	2	2
1	2	2	3	1	1	3	1	1	2
2	1	2	3	2	2	3	1	1	1
2	1	2	2	3	2	2	1	2	1
3	2	1	2	3	2	2	1	1	1
2	2	2	2	2	2	2	2	2	2
2	3	2	3	2	3	2	3	2	2
2	2	2	2	2	2	2	2	2	2
2	2	2	2	2	2	2	2	2	2
2	2	2	2	2	2	1	1	1	2
2	2	1	1	2	1	2	1	1	1
3	3	3	2	2	2	3	2	2	2
3	3	3	2	2	2	3	2	2	2
1	2	1	1	3	2	2	1	2	2
2	2	2	2	2	2	2	2	2	2
2	2	2	2	2	2	2	2	2	2
3	1	2	2	1	1	3	1	1	2
2	2	2	2	2	2	2	1	1	1
2	2	2	2	2	2	2	2	2	2
2	2	2	2	2	2	2	2	2	2
2	2	2	2	2	2	2	2	2	2
2	2	2	2	2	2	2	2	2	2
2	2	2	2	2	2	2	2	2	1
3	1	2	1	1	1	2	1	1	1
2	2	2	2	2	2	2	2	2	1
2	1	2	2	2	2	2	1	1	2
3	1	2	2	2	1	3	1	2	1
2	2	2	2	2	2	2	2	2	2

3	3	3	3	2	2	2	1	2	1
3	2	3	3	1	2	2	3	1	1
2	2	2	2	2	2	2	2	2	2
2	2	2	2	2	2	2	2	2	2
2	1	2	2	2	2	2	2	2	2
2	3	3	2	2	1	2	1	2	2
9	9	9	9	9	9	9	9	9	9
2	3	2	2	1	2	2	2	2	2
3	2	2	2	2	2	2	2	2	2
3	1	2	1	2	3	2	2	3	1
3	1	2	1	2	1	2	1	2	1
3	3	3	3	3	1	3	3	1	1
2	2	2	2	2	2	2	2	2	2
2	2	2	2	2	2	2	2	2	2
2	2	2	2	2	2	2	2	2	2
2	1	2	1	1	1	1	1	1	1
3	3	3	3	3	1	3	1	1	3
2	2	2	2	2	2	2	2	2	2
2	2	2	2	2	2	2	2	2	2
2	2	2	2	2	2	2	2	2	2
2	1	2	2	1	1	2	1	2	2
2	2	2	2	2	2	2	2	2	1
9	9	9	9	9	9	9	9	9	9
2	2	2	2	2	2	2	2	2	2
3	1	2	3	3	1	3	1	3	2
9	9	9	9	9	9	9	9	9	9
2	2	2	1	2	1	2	1	1	2
9	9	9	9	9	9	9	9	9	9
2	2	2	2	2	2	2	2	2	1
2	1	1	2	2	1	2	1	1	1
9	9	9	9	9	9	9	9	9	9
9	9	9	9	9	9	9	9	9	9
2	2	2	2	2	2	2	2	2	2
1	2	1	1	1	2	2	1	1	1
3	1	9	1	1	2	9	9	9	9
1	2	1	2	1	1	2	1	2	1
3	3	3	3	3	3	3	3	3	3
1	2	2	2	2	1	2	2	2	2
9	9	9	9	9	9	9	9	9	9
3	1	2	2	3	1	3	1	1	1
2	2	3	2	1	2	2	2	2	2
2	1	3	3	3	2	1	1	1	2
2	2	1	3	2	1	2	3	2	1
3	3	3	3	3	3	3	3	3	2
9	9	9	9	9	9	9	9	9	9
3	1	1	3	3	2	3	1	1	1
3	1	1	3	3	2	3	1	1	1

9	9	9	9	9	9	9	9	9	9
3	1	1	3	3	2	3	1	1	1
3	1	1	3	3	2	3	1	1	1
3	1	1	3	3	2	3	1	1	1
3	3	1	3	3	2	3	1	1	1
3	1	1	3	3	2	3	1	1	1
3	1	1	3	3	2	3	1	1	1
3	1	1	3	3	2	3	1	1	1
3	1	1	3	3	2	3	1	1	1
3	1	1	3	3	2	3	1	1	1
1	3	3	2	2	2	2	2	2	1
3	1	2	3	2	1	1	1	1	1
3	1	1	3	3	1	3	1	1	1
3	1	1	3	3	2	3	3	1	1
3	1	1	3	3	2	3	1	1	1
3	3	1	3	3	2	3	1	1	1
3	1	1	2	3	2	3	2	1	1
3	1	3	3	3	3	3	1	1	1
3	1	1	3	3	2	3	1	1	1
3	1	1	3	3	2	3	1	1	1
3	1	1	3	3	1	3	1	1	1
3	1	1	3	3	2	3	1	1	1
3	1	1	3	3	2	3	1	1	1
3	1	2	3	3	1	3	1	1	1
3	1	1	3	3	2	3	1	1	1
3	1	1	3	3	2	3	1	1	1
3	1	1	3	3	2	3	1	1	1
3	1	1	3	3	2	3	2	1	1
3	1	1	3	3	2	1	1	1	1
3	1	1	3	3	1	3	1	1	1
3	1	1	3	3	2	3	2	1	1
3	1	1	3	3	1	3	1	1	1
3	1	1	3	3	2	3	1	1	1
3	3	1	3	3	2	3	1	1	1
3	1	1	3	3	3	3	1	1	1
3	1	1	3	3	2	3	1	1	1
3	1	1	3	3	2	3	1	1	1
3	1	1	3	3	2	3	1	1	1
3	1	1	3	3	2	3	1	1	1
3	1	1	3	3	2	3	1	1	1
3	1	1	3	3	2	3	1	1	1
2	2	2	2	2	2	2	2	2	2
2	1	2	3	2	1	3	1	1	1
2	2	2	2	2	2	2	2	2	2
9	9	9	9	9	9	9	9	9	2
2	2	2	2	2	2	2	2	2	2
2	9	9	9	9	9	9	9	9	2

2	2	2	2	2	2	2	2	2	2
2	1	2	3	2	3	3	1	1	1
2	1	2	3	2	3	3	1	1	1
1	1	1	3	3	1	2	1	1	1
2	2	2	2	2	2	2	2	2	2
2	1	3	2	1	1	3	2	2	2
2	2	2	2	2	2	2	2	2	2
2	3	2	2	2	2	1	2	2	2
2	2	2	2	3	2	3	1	1	1
2	2	2	2	2	2	2	2	2	2
2	1	2	3	2	3	3	1	1	1
9	9	1	9	9	9	9	9	9	9
3	1	1	3	3	9	9	1	9	1
2	2	2	2	2	2	2	2	2	2
9	9	9	1	9	9	9	9	9	1
9	1	9	9	9	9	9	9	9	1
9	9	9	9	9	9	1	9	9	9
9	9	9	9	9	9	9	9	9	9
9	9	9	9	9	9	9	1	9	9
2	3	2	1	2	1	2	1	2	1
9	2	9	1	1	9	1	9	9	1
9	9	1	9	9	9	9	9	9	1
3	2	3	3	3	1	3	2	2	1
9	9	9	9	9	9	9	9	9	9
9	1	9	9	9	9	9	9	9	1
9	9	9	9	9	9	9	9	9	9
9	9	9	9	9	9	9	9	9	9
9	9	9	9	9	9	9	9	9	9
2	1	2	3	1	2	3	1	1	2
9	9	1	9	9	9	9	9	9	9
9	9	9	9	9	9	9	9	9	1
2	2	2	2	2	2	2	2	1	2
2	2	2	2	2	2	2	2	2	2
3	1	1	3	3	1	3	1	1	1
1	1	1	2	1	1	2	2	1	1
2	1	2	2	2	1	2	2	1	2
3	2	2	2	3	1	3	2	1	1
3	3	2	9	3	3	3	1	1	1
2	2	2	2	2	2	2	2	2	2
2	1	2	2	3	1	3	1	1	1
9	9	9	9	9	9	9	9	9	9
3	2	2	3	2	1	3	1	1	1
2	2	2	2	2	2	2	2	2	2
2	1	1	3	3	1	3	1	1	1
2	2	2	2	2	2	2	2	2	2
2	2	2	2	2	2	2	2	2	2
3	1	2	3	2	2	3	1	1	1

3	2	2	3	2	1	3	1	2	1
3	1	1	3	2	2	3	1	1	1
3	3	3	3	3	1	3	1	2	2
2	2	2	1	1	2	3	1	1	1
3	3	1	3	1	2	2	1	2	1
2	2	2	2	2	2	2	2	2	2
2	3	2	2	3	2	2	1	1	1
2	2	2	2	2	2	2	2	2	2
2	2	2	2	2	2	2	2	2	2
2	2	2	2	2	2	2	2	2	2
2	2	2	2	2	2	2	2	3	2
1	1	2	3	2	2	2	1	2	1
2	2	2	2	2	2	2	2	2	2
2	2	2	2	2	2	2	2	2	2
3	1	2	3	3	1	2	1	2	2
2	2	2	2	2	2	2	2	2	2
2	2	2	2	2	2	2	2	2	2
2	2	2	2	2	2	2	2	2	2
2	2	2	2	2	2	2	2	2	1
2	1	2	3	1	1	3	1	2	1
2	1	2	2	2	2	2	2	2	2
2	1	2	2	3	2	2	2	2	1
3	1	3	2	3	1	3	2	2	1
2	2	2	2	3	1	2	1	2	1
2	2	2	2	2	2	2	2	2	2
2	1	2	2	2	2	2	2	1	2
3	1	1	3	1	1	3	9	1	1
3	1	1	3	1	3	1	3	1	1
2	1	2	3	3	1	3	1	1	1
3	3	3	3	3	3	3	3	3	2
2	2	2	2	2	2	2	2	2	2
2	2	2	2	2	2	2	2	2	9
2	2	2	2	2	2	2	2	2	2
2	2	3	1	3	2	2	3	2	2
2	2	2	2	2	2	2	2	2	2
9	9	9	9	9	9	9	9	9	2
2	2	2	2	2	2	2	2	2	2
2	3	1	2	2	1	3	1	2	1
2	2	2	2	2	2	2	2	2	2
3	1	2	3	3	1	3	2	1	1
9	9	9	9	9	9	9	1	9	2
2	2	2	2	3	1	2	1	1	3
2	2	2	2	3	1	2	1	1	3
2	2	1	3	3	3	2	3	1	1
3	1	2	3	3	1	3	1	1	1
2	2	2	2	2	2	2	2	2	2
9	9	9	9	9	9	9	9	9	2

9	9	9	9	9	9	9	9	9	2
9	9	9	9	1	9	9	9	9	9
9	9	9	9	9	9	9	9	9	9
9	9	9	9	9	9	9	9	9	9
9	9	9	9	9	9	9	9	9	9
9	9	9	9	9	9	9	9	9	1
9	9	2	9	9	9	9	9	9	9
9	9	9	9	9	9	9	9	9	9
9	9	9	9	9	9	9	9	9	9
9	9	9	9	9	9	9	9	9	2
9	9	9	9	9	9	9	9	9	1
9	9	9	9	9	9	9	9	9	2
9	9	9	9	9	9	9	9	9	9
9	1	9	9	9	9	9	9	9	9
9	9	9	9	9	9	9	9	1	9
9	9	9	9	9	9	9	9	1	9
2	9	9	9	9	9	9	9	9	1
3	1	3	3	3	3	3	1	1	1
2	2	2	2	2	2	2	2	2	2
9	9	9	9	9	9	9	9	9	1
1	1	1	1	1	1	1	1	1	1
3	1	2	2	1	3	2	2	3	1
2	1	1	2	2	2	1	1	2	2
1	1	3	2	1	1	1	1	1	1
3	3	2	3	2	3	2	3	2	2
3	2	2	2	1	3	3	3	2	3
2	2	2	2	2	2	2	2	2	2
1	2	2	3	1	2	1	2	3	2
2	2	2	2	2	2	2	2	2	2
2	2	2	2	2	2	2	2	2	2
1	1	1	2	2	2	3	3	3	1
2	2	2	2	2	2	2	2	2	1
2	2	2	2	2	2	2	2	2	2
2	1	3	3	3	2	2	2	2	1
1	2	3	1	2	3	1	2	3	1
2	2	2	3	2	3	2	3	2	1
2	2	2	2	2	2	2	2	2	2
3	2	3	2	3	2	1	1	1	9
1	1	1	1	1	1	1	1	1	1
9	9	9	9	9	9	9	9	9	9
2	2	2	2	2	2	2	2	2	2
1	1	1	1	1	1	1	1	1	3
9	9	9	9	9	9	9	9	9	9
1	1	1	1	3	2	1	1	1	1
2	2	2	2	2	2	2	2	2	2
2	2	2	2	2	2	2	2	2	2
2	2	2	2	3	3	2	2	2	2

2	2	2	2	2	2	2	2	2	1
9	9	9	9	9	9	9	9	9	9
1	3	1	1	3	1	3	1	1	1
9	9	9	9	9	9	9	9	9	9
3	1	2	2	2	2	2	2	2	2
3	1	2	2	2	2	2	2	2	2
9	9	9	9	9	9	9	9	9	9
2	2	2	2	2	2	2	2	2	1
1	3	1	2	1	1	1	1	3	1
3	1	1	2	1	1	3	3	1	2
1	1	1	1	1	1	1	1	1	1
3	1	2	2	2	1	1	1	1	2
3	3	2	2	3	3	2	2	2	2
9	9	9	9	9	9	9	9	9	9
9	9	9	9	9	9	9	9	9	9
9	9	9	9	9	9	9	9	9	9
3	1	1	3	3	2	3	1	1	1
9	9	9	9	9	9	9	9	9	9
3	2	9	3	3	1	3	2	2	2
3	2	1	2	3	1	3	1	2	1
3	3	3	3	2	2	2	1	2	1
3	3	2	2	3	3	3	2	2	2
1	1	1	1	1	1	1	1	1	2
1	3	2	1	3	2	3	1	3	1
2	2	2	2	2	2	2	2	2	2
1	3	1	2	1	2	1	2	1	1
2	3	3	1	2	3	3	3	3	3
1	3	3	1	1	1	3	1	1	1
2	2	2	2	2	2	1	1	2	2
2	2	2	2	3	3	3	2	2	1
9	9	9	9	9	9	9	9	9	9
9	9	1	9	9	9	9	9	9	9
2	1	1	2	2	2	2	2	2	2
3	1	1	1	2	2	9	2	2	1
3	1	1	9	9	2	9	2	9	1
2	2	2	2	1	2	2	1	2	1
2	2	3	2	9	1	3	9	1	1
9	9	9	9	9	9	9	9	9	9
9	9	9	9	9	9	9	9	9	9
9	9	9	9	9	9	9	9	9	9
9	9	9	9	9	9	9	9	9	9
2	3	2	2	1	2	1	2	2	2
2	3	1	2	3	1	2	3	3	2
9	9	9	9	9	9	9	9	9	9
2	3	1	2	1	3	1	2	3	2
2	3	2	2	3	1	2	1	2	1
3	3	3	1	1	3	1	3	3	1

3	3	3	3	3	3	3	3	3	3
2	1	2	2	2	2	2	2	2	2
3	3	3	2	2	3	3	2	2	3
2	2	1	3	2	1	2	2	2	1
2	2	2	2	2	2	2	2	2	2
3	2	9	1	2	3	9	3	2	1
9	9	9	9	9	9	9	9	9	9
2	3	3	9	2	2	3	1	2	3
3	3	3	3	3	3	3	3	3	3
2	1	2	3	3	1	3	1	2	1
3	3	3	3	3	3	3	3	3	3
1	1	1	3	1	1	3	1	1	1
2	3	2	2	3	1	2	1	2	1
2	3	2	2	3	1	2	1	2	1
2	3	2	2	3	1	2	1	2	1
2	3	2	2	3	1	2	1	2	2
2	3	2	2	3	1	2	1	2	1
2	3	2	2	3	1	2	1	2	1
2	3	2	2	3	1	2	1	2	1
3	2	2	1	3	1	2	1	1	1
2	3	2	1	2	1	2	3	3	1
3	2	2	3	2	3	2	9	2	2
2	2	2	2	3	2	3	2	3	2
2	2	2	2	2	2	3	3	3	2
3	3	3	3	3	3	3	3	3	3
2	3	2	2	3	3	2	1	2	1
9	9	9	1	9	9	9	9	9	9
3	1	1	3	3	1	3	3	1	9
9	9	9	9	9	9	9	9	9	9
2	2	2	2	2	2	2	2	2	2
3	2	3	3	3	3	1	1	1	2
2	3	2	3	2	3	2	2	3	2
2	1	3	2	1	3	1	2	1	2
9	9	9	1	9	9	9	9	9	9
3	3	3	3	3	3	3	3	3	3
2	2	2	2	3	3	3	1	2	1
9	9	9	2	9	1	3	9	2	2
2	2	1	2	2	2	1	9	1	2
2	3	2	2	3	1	2	1	2	2
1	2	1	2	3	1	1	1	1	1
1	1	1	2	2	3	1	1	2	2
3	2	1	2	2	9	2	3	2	2
1	2	1	1	3	2	3	1	2	2
1	3	3	1	1	1	3	1	1	1
1	1	2	2	1	1	1	1	2	2
1	1	1	1	1	1	1	1	1	1
2	2	2	2	2	2	2	2	3	2

2	2	2	2	2	2	2	2	2	2
9	9	9	9	9	9	9	9	9	2
9	9	9	9	9	9	9	9	9	9
3	1	1	3	1	1	3	1	3	1
2	9	1	2	1	2	3	1	2	2
3	1	2	2	2	2	2	2	2	2
2	9	1	1	1	1	1	1	1	1
3	1	2	2	2	2	2	2	2	2
3	1	2	2	2	2	2	2	2	2
3	1	2	3	2	1	2	3	3	2
2	3	2	2	3	2	9	9	2	9
2	2	2	1	2	1	3	1	1	1
9	9	9	9	9	9	9	9	9	9
2	3	1	1	3	1	3	1	1	1
2	1	1	1	2	1	3	1	1	1
9	9	9	9	9	9	9	9	9	9
3	3	3	3	3	3	3	3	3	3
9	9	9	9	9	9	9	9	9	9
9	9	9	9	9	9	9	9	9	9
2	3	1	2	3	1	2	3	1	1
2	1	1	1	1	1	1	1	3	1
2	2	2	2	2	2	2	2	2	2
9	9	9	9	9	9	9	9	9	9
2	2	2	2	2	2	2	2	2	2
3	3	3	3	3	3	3	3	3	3
2	3	2	1	2	1	3	1	1	1
2	3	2	1	2	1	3	1	1	1
3	1	2	3	3	1	3	1	3	1
1	1	1	1	1	1	1	1	1	1
2	2	2	2	2	2	2	2	2	2
2	3	2	1	1	1	3	2	2	3
2	2	3	2	2	2	2	3	1	1
3	1	3	2	2	1	2	3	3	1
2	3	2	1	2	1	3	1	1	1
1	1	2	3	2	2	1	1	2	1
2	2	2	2	2	2	2	2	2	2
2	2	2	2	1	1	1	2	2	2
9	9	9	9	9	9	9	9	9	9
9	9	9	2	9	3	9	9	1	9
3	9	1	1	1	2	1	2	1	1
2	2	2	2	2	2	2	2	2	2
2	2	2	2	2	2	2	2	2	2
2	2	2	2	2	2	2	2	2	2
2	2	2	2	2	2	2	2	2	2
2	2	2	2	2	2	2	2	2	2
2	2	2	2	2	2	2	2	2	2
9	9	9	9	9	9	9	9	9	9

9	9	9	9	9	9	9	9	9	9
2	2	2	2	2	2	2	2	2	2
3	1	2	2	3	1	3	1	1	1
3	1	1	2	2	2	3	1	1	1
9	9	9	9	9	9	9	9	9	9
3	2	2	3	2	1	2	3	2	1
9	9	9	9	9	9	9	9	9	9
3	3	3	3	3	3	3	3	3	1
2	1	2	1	1	2	2	1	2	1
9	9	9	9	9	9	9	9	9	9
2	2	2	2	2	2	2	2	2	2
2	2	1	2	2	2	3	2	1	1
2	2	1	2	2	2	3	2	1	1
2	2	2	2	2	2	2	2	2	2
1	3	3	3	2	2	2	1	1	1
2	2	2	2	2	2	2	2	2	2
2	2	2	2	2	2	2	2	2	2
2	2	2	2	2	2	2	2	2	3
2	1	3	2	2	1	3	2	1	1
2	1	1	3	3	1	1	2	2	1
9	9	9	9	9	9	9	9	9	9
3	3	3	3	3	3	3	3	3	2
2	2	2	2	2	2	2	2	9	2
2	2	2	2	2	2	2	2	2	1
2	2	2	2	2	2	2	2	2	2
3	3	3	3	3	3	3	3	3	2
9	9	9	9	9	9	9	2	9	9
2	2	2	2	2	2	2	2	2	2
2	2	2	2	2	2	2	2	2	2
3	2	3	2	1	2	3	2	2	3
2	2	2	2	2	2	2	2	2	2
2	2	2	2	2	2	2	2	2	2
3	1	3	1	1	1	3	3	1	1
2	2	2	2	2	2	2	2	2	2
2	2	2	2	2	2	2	2	2	2
2	2	2	3	3	1	3	1	1	1
2	2	2	2	2	2	2	2	2	2
2	3	1	2	2	2	1	2	1	1
2	2	2	2	2	2	2	2	2	2
2	2	2	2	2	2	2	2	2	2
2	2	2	2	2	2	2	2	2	2
2	2	2	2	2	2	2	2	2	2
3	1	3	3	3	3	3	1	1	1
2	2	2	2	2	2	2	2	2	2
2	2	2	2	2	2	2	2	2	2
3	3	3	3	3	3	3	3	3	3
2	2	2	2	2	2	2	2	2	2

2	2	2	2	2	2	2	2	2	2
2	2	2	2	2	2	2	2	2	2
2	2	2	2	2	2	2	2	2	2
2	2	2	2	2	2	2	2	2	2
2	2	2	2	2	2	2	2	2	2
3	3	1	3	1	2	3	1	1	1
2	2	2	2	2	2	2	2	2	2
3	1	3	2	1	3	2	2	2	2
1	1	2	3	3	1	3	1	1	3
2	2	2	2	2	2	2	2	2	2
1	1	1	3	3	1	3	1	1	1
9	9	9	9	9	9	9	9	9	9
9	9	2	9	9	9	9	9	9	2
1	1	2	2	2	1	3	1	2	2
1	1	1	3	3	3	3	3	3	2
2	2	2	2	2	3	3	3	3	2
3	3	2	2	2	2	3	3	2	2
3	1	9	2	2	2	2	2	2	1
9	9	9	9	9	9	9	9	9	9
2	3	1	2	2	2	3	2	3	3
9	9	9	9	9	9	9	9	9	9
3	2	3	2	1	2	3	2	1	1
2	2	2	2	2	2	2	2	2	1
2	2	2	2	2	2	2	2	2	1
3	3	3	3	3	3	3	3	1	1
2	2	2	3	3	1	3	1	1	1
2	2	2	2	2	2	2	2	2	2
3	1	2	2	3	1	3	1	2	2
2	2	2	2	2	2	2	1	1	2
3	3	2	2	3	2	3	2	3	1
1	1	1	1	1	1	1	1	1	1
1	1	1	1	1	1	1	1	1	3
3	3	3	3	3	3	3	3	3	1
1	1	1	1	1	1	1	1	1	1
3	3	3	3	3	3	3	3	3	3
1	3	3	3	1	1	1	1	3	1
9	9	9	9	9	9	9	9	9	9
1	1	1	1	1	1	1	1	1	1
2	2	2	2	2	2	2	2	2	2
2	2	2	2	2	2	2	2	2	2
2	1	2	2	1	1	3	2	1	2
9	9	9	1	9	9	9	9	9	1
2	3	2	1	3	2	2	2	1	1
2	2	2	2	2	2	2	2	2	2
2	2	2	2	2	2	2	2	2	2
2	2	3	1	2	2	1	3	2	3
2	3	2	2	2	2	1	2	2	1

3	3	1	1	2	1	2	3	1	1
3	3	3	3	3	3	3	3	3	3
2	3	2	1	3	1	2	3	1	3
2	9	9	9	9	9	9	9	9	9
9	9	9	9	9	9	9	9	9	9
9	2	1	2	3	1	2	1	3	3
2	2	2	2	2	2	2	2	2	2
3	3	3	3	3	3	3	3	3	3
3	3	3	3	3	3	3	3	3	2
2	2	2	2	2	2	2	2	2	2
9	9	9	9	9	9	9	9	1	1
2	2	2	3	9	2	3	9	2	2
2	2	2	2	2	2	2	2	2	2
2	2	2	2	2	1	3	3	3	2
2	2	2	2	2	2	2	2	2	2
3	3	3	3	2	2	1	1	1	3
2	1	2	3	2	2	3	3	2	2
1	9	9	9	9	9	9	9	9	1
9	9	9	9	9	9	9	9	2	9
2	2	2	2	2	2	2	2	2	2
2	2	2	2	2	2	2	2	2	2
2	2	2	2	2	2	2	2	2	2
9	9	9	9	9	9	9	9	9	9
9	9	9	9	9	9	9	9	9	9
2	2	3	1	2	3	1	2	3	1
1	1	1	1	1	1	3	1	1	1
2	2	2	2	1	1	1	1	1	2
1	1	1	1	1	1	3	1	1	1
1	3	3	3	1	1	3	3	1	3
2	9	1	2	3	1	2	3	1	2
2	1	2	2	1	1	3	2	1	1
2	3	1	2	2	1	2	3	1	2
3	1	2	2	3	1	3	1	2	1
3	3	3	3	3	3	3	3	3	3
3	3	3	3	3	3	3	3	3	3
2	3	3	3	2	2	3	2	3	1
2	1	3	2	2	2	2	1	2	1
3	3	3	3	3	3	3	3	3	3
9	9	3	1	3	1	9	1	9	1
3	3	3	3	3	3	3	3	3	3
3	3	1	3	1	1	3	1	3	9
3	1	3	1	1	1	2	1	3	1
1	1	1	1	1	1	1	1	1	1
2	2	2	2	2	2	2	2	2	2
2	2	2	1	2	2	2	2	2	2
1	1	1	1	1	1	1	1	1	2
2	1	1	2	2	2	2	1	1	2

2	2	2	2	2	2	2	2	2	2
9	3	2	9	2	9	3	2	3	3
2	1	2	3	1	9	1	9	1	1
3	1	1	3	1	1	1	1	1	3
2	2	2	2	2	2	2	2	2	2
2	1	2	2	2	2	3	2	2	1
2	3	3	2	2	2	2	2	2	2
2	2	2	2	2	2	2	2	2	2
2	2	2	2	2	2	2	2	2	2
2	2	2	2	2	2	2	2	2	2
9	9	9	9	3	9	9	9	9	9
2	2	2	2	2	2	2	2	2	2
1	3	1	1	3	1	3	1	3	1
3	1	2	3	1	2	2	3	3	2
1	1	1	1	1	1	1	1	1	2
2	3	3	2	2	2	2	2	2	1
2	1	2	3	3	1	3	1	1	1
2	2	2	2	2	1	2	1	1	1
2	3	2	2	1	1	3	1	1	1
9	9	9	9	9	9	9	9	9	9
3	1	1	1	3	1	1	1	1	1
1	1	1	1	1	3	2	2	3	1
3	3	3	3	3	3	3	1	1	3
3	3	3	3	3	3	3	3	3	1
3	3	3	3	3	3	3	3	3	3
2	2	2	2	2	2	2	2	2	1
2	1	1	2	1	1	2	1	1	1
1	2	3	1	2	1	2	3	1	1
3	2	1	1	2	3	1	2	2	3
1	2	1	2	1	1	1	1	1	1
2	2	2	2	2	2	2	2	2	2
9	9	9	9	9	9	9	9	9	9
1	2	1	2	2	2	2	1	3	2
9	9	9	9	9	9	9	9	9	9
2	2	2	3	2	1	2	2	2	1
2	2	2	2	2	2	2	2	2	2
2	2	2	2	2	2	2	2	2	2
2	2	2	2	2	2	2	2	2	2
2	2	2	2	2	2	3	3	3	2
2	1	1	2	2	3	1	2	3	1
9	2	2	1	2	1	2	3	1	1
2	2	2	2	2	2	2	2	2	2
3	3	3	2	2	1	2	2	3	1
9	9	9	9	9	9	9	9	9	2
3	3	3	3	3	3	3	3	3	3
2	2	2	2	2	2	2	2	2	2
3	2	2	2	2	1	3	1	1	1

9	9	9	9	9	9	9	9	9	9
3	2	2	1	2	2	9	2	3	2
2	2	3	3	3	3	3	3	3	1
3	1	2	3	2	1	3	1	1	1
3	3	3	3	3	3	3	3	3	2
3	2	2	2	2	9	2	3	2	2
2	2	2	2	9	9	9	9	9	9
9	1	1	3	3	2	3	2	2	2
2	2	2	2	2	2	2	2	2	2
9	9	9	9	9	9	9	9	9	9
9	9	9	9	9	9	9	9	9	9
9	9	9	9	9	9	9	9	9	9
9	9	9	9	9	9	9	9	9	9
9	9	9	9	9	9	9	9	9	3
9	9	9	9	9	9	9	9	9	9
2	2	2	2	2	1	3	1	1	1
1	2	1	1	2	2	1	2	2	1
2	2	2	2	2	2	2	2	2	2
1	3	2	2	2	2	1	3	1	1
2	2	2	2	1	1	2	1	1	1
2	2	2	2	2	2	2	2	2	1
2	1	2	2	2	3	2	2	2	2
2	3	2	1	1	1	3	1	2	1
2	3	3	2	3	2	3	2	1	1
2	2	2	2	2	2	2	2	2	2
2	2	1	2	1	2	3	1	3	2
9	3	9	1	3	9	1	3	3	2
1	1	1	1	1	1	1	1	1	1
2	2	1	2	1	2	3	1	3	2
2	2	2	2	2	1	3	1	1	1
2	2	2	2	1	1	2	1	1	1
9	9	9	9	9	9	9	9	9	9
3	1	1	3	1	2	2	1	3	1
2	2	2	2	2	2	2	2	2	2
2	2	2	2	2	2	2	2	2	2
2	2	2	2	2	2	2	2	2	2
2	2	2	2	2	2	2	2	2	2
9	9	9	9	9	9	9	9	9	2
2	2	2	2	2	2	2	2	2	2
2	2	2	2	2	2	2	2	2	2
2	2	2	2	2	2	2	2	2	2
2	2	2	2	2	2	2	2	2	2
2	2	2	2	2	2	2	2	2	2
2	2	2	2	1	3	3	3	3	1
2	2	2	2	2	2	2	2	2	1
2	2	2	2	2	2	2	2	2	2
9	9	9	9	9	9	9	9	9	9

2	2	2	2	2	2	2	2	2	2
3	2	2	2	2	2	2	2	2	2
9	9	9	1	9	9	9	9	9	9
9	9	9	9	9	9	9	9	9	9
2	1	1	2	1	1	1	2	2	1
9	9	9	9	9	9	9	9	9	1
1	2	2	3	3	1	2	3	1	1
1	2	3	2	3	3	2	1	1	3
2	3	3	2	1	1	2	3	2	9
1	2	2	3	3	2	2	1	1	1
9	9	9	9	9	9	9	9	9	9
2	1	2	1	2	2	1	1	3	2
3	3	3	2	2	2	2	2	2	2
2	2	2	2	2	2	2	2	2	2
2	2	2	2	3	2	2	1	1	2
3	3	3	3	2	3	1	2	3	3
2	2	2	2	2	2	2	2	2	2
2	2	2	2	2	2	2	2	2	2
2	1	2	2	1	3	3	1	2	1
3	1	2	3	3	3	3	3	3	1
2	2	2	2	1	3	3	2	2	3
3	2	2	2	2	2	2	2	2	2
2	1	1	1	2	2	2	1	1	1
2	2	2	2	2	2	2	2	2	3
2	2	2	2	2	2	2	2	2	2
2	2	2	2	2	2	2	2	2	1
2	2	2	2	2	2	2	2	2	2
2	2	2	2	2	2	2	2	2	2
2	2	2	2	2	2	2	2	2	2
9	9	9	9	9	9	9	9	9	9
1	1	1	9	1	9	1	1	1	1
2	2	2	2	2	2	2	2	2	2
1	1	1	1	1	1	1	1	1	9
9	9	9	9	9	9	9	9	9	9
2	3	3	2	1	3	2	1	1	1
9	9	9	9	9	9	9	9	9	9
9	9	9	9	9	9	9	9	9	9
2	2	2	2	2	2	2	2	2	2
3	3	3	3	3	3	3	1	3	9
1	2	1	2	1	1	3	3	2	1
1	1	1	1	1	1	1	1	1	1
3	1	2	3	2	1	1	2	3	1
9	9	9	9	9	9	9	9	9	1
2	2	1	2	2	3	1	2	3	1
2	1	2	2	2	1	3	2	1	2
9	9	9	9	9	9	9	9	9	9
2	2	2	3	1	2	2	3	3	3

2	2	2	1	2	2	2	1	2	1
9	9	9	9	9	9	9	9	9	9
9	9	9	9	9	9	9	9	9	9
2	2	2	2	2	2	2	2	2	2
3	1	2	1	1	1	3	2	1	1
2	2	2	1	2	2	2	1	2	1
1	3	1	2	1	2	3	1	2	1
2	2	2	2	2	2	2	2	2	1
2	2	2	2	2	2	2	2	2	2
3	1	1	3	1	2	1	1	1	1
9	9	9	9	9	9	9	9	9	9
9	9	9	9	9	9	9	9	9	9
9	9	9	9	9	9	9	9	9	1
2	2	2	2	2	2	2	2	2	1
2	1	2	3	2	3	2	3	3	2
9	9	9	9	9	9	9	9	9	9
9	9	9	9	9	9	9	9	9	9
9	9	9	9	9	9	9	9	9	9
2	1	1	2	2	1	2	1	1	1
2	2	2	2	2	2	2	2	2	1
9	9	9	9	9	9	9	9	9	9
3	1	1	1	3	3	2	2	1	1
3	3	3	3	3	3	3	3	3	3
9	9	9	9	9	9	9	9	9	9
2	2	2	2	2	2	2	2	2	2
2	2	2	2	2	2	2	2	2	2
9	9	9	9	9	9	9	9	9	9
9	9	9	9	9	9	9	9	9	9
2	2	2	2	2	2	2	2	2	2
2	2	2	2	2	2	2	2	2	2
1	1	1	2	1	1	2	3	1	1
9	9	9	9	9	9	9	9	9	9
9	9	9	9	9	9	9	9	9	9
2	2	2	2	2	3	3	3	3	1
9	1	2	9	2	1	2	1	2	2
2	2	2	2	2	2	2	2	2	2
1	2	2	2	1	1	1	2	2	2
2	2	2	2	2	2	2	2	2	2
9	2	1	2	1	2	1	2	1	3
2	1	3	1	2	3	1	2	3	1
2	2	3	3	1	2	2	2	3	2
9	9	9	9	9	9	9	9	9	9
9	9	9	9	9	9	9	9	9	9
9	9	9	1	9	9	9	9	9	9
9	9	9	9	9	9	9	9	9	9
9	9	9	9	9	9	9	9	9	9
9	9	9	9	9	9	9	9	9	9

2	2	2	2	2	2	2	2	2	2
3	2	3	2	3	2	3	2	3	2
3	2	2	2	2	2	1	9	1	1
2	2	2	2	2	2	2	2	2	2
9	9	9	9	9	9	9	9	9	9
9	9	9	9	9	9	9	9	9	9
9	9	9	9	9	9	9	9	9	9
9	9	9	9	9	9	9	9	9	9
9	9	9	9	9	9	9	9	9	9
9	9	9	9	9	9	9	9	9	9
9	9	9	9	9	9	9	9	9	9
9	9	9	9	9	9	9	9	9	9
9	9	9	9	9	9	9	9	9	9
1	3	3	1	1	1	1	1	1	1
2	1	1	2	2	2	2	1	1	1
2	2	2	2	2	2	2	2	2	2
2	2	2	2	2	2	2	2	2	2
9	9	9	9	9	9	9	9	9	9
9	9	9	9	9	9	9	9	9	9
9	9	9	9	9	9	9	9	9	9
9	9	9	9	9	2	9	9	9	1
9	9	9	9	9	9	9	9	9	9
1	1	2	2	2	2	2	2	2	2
9	9	9	9	9	9	9	9	9	9
1	1	1	1	1	1	1	1	2	2
1	1	1	1	1	2	2	2	2	1
1	1	1	1	1	1	1	1	1	2
2	2	1	1	1	1	1	1	1	1
1	1	2	2	2	1	1	1	1	2
1	2	2	1	1	1	2	2	1	1
1	1	2	2	2	1	1	1	1	1
1	1	2	2	2	1	1	1	1	2
1	1	1	1	1	1	1	1	1	1
9	9	9	9	9	9	9	9	9	9
1	1	2	1	1	1	1	1	1	2
1	1	1	2	2	2	1	1	1	1
2	2	2	2	2	2	2	2	2	1
9	9	9	9	9	9	9	9	9	9
2	2	2	2	2	2	2	2	2	3
3	1	1	3	1	3	1	1	1	3
3	3	3	3	3	3	3	3	3	3
9	9	9	9	9	9	9	9	9	9
9	9	9	9	9	9	9	9	9	9
9	9	9	9	9	9	9	9	9	9
2	1	9	9	9	9	9	9	9	9
2	2	2	2	2	3	2	1	3	2
9	9	9	9	9	9	9	9	9	9

9	9	9	9	9	9	9	9	9	9
9	9	9	9	9	9	9	9	9	9
9	9	9	9	9	9	9	9	9	9
9	9	9	9	9	9	9	9	9	9
9	9	9	9	9	9	9	9	9	9
9	9	9	9	9	9	9	9	9	9
9	9	9	9	9	9	9	9	9	9
9	9	9	9	9	9	9	9	9	9
9	9	9	9	9	9	9	9	9	9
9	9	9	9	9	9	9	9	9	9
9	2	9	9	9	9	9	9	9	9
9	9	9	9	9	9	9	9	9	9
2	2	2	1	2	2	2	1	1	1
3	1	2	3	2	9	1	1	2	1
2	2	1	1	1	2	2	2	2	1
1	3	2	1	2	3	2	2	1	1
2	2	2	2	2	2	2	2	2	2
1	1	2	2	1	1	1	3	3	3
1	1	2	2	1	1	1	1	2	1
9	9	9	9	9	9	9	9	9	1
1	1	1	1	1	2	2	2	1	1
3	2	1	3	1	1	2	2	1	1
2	2	2	2	2	2	2	2	2	2
3	1	1	1	2	1	1	2	3	1
3	1	2	2	2	1	1	1	1	2
3	3	3	3	3	3	3	1	1	3
2	9	9	2	9	3	2	3	2	1
3	2	2	2	2	2	2	2	2	1
9	9	9	9	9	9	9	9	9	9
9	9	9	9	9	9	9	9	9	3
9	9	9	9	9	9	9	9	9	9
1	1	1	1	1	1	1	1	1	2
2	2	2	2	2	2	2	2	2	2
2	2	1	1	1	1	1	1	2	2
2	1	1	1	1	2	2	1	1	1
2	2	2	2	2	2	2	2	2	2
2	2	2	2	2	2	2	2	2	2
2	2	1	1	1	1	1	1	2	2
2	1	1	2	2	1	2	2	2	2
1	1	2	2	2	1	1	1	2	1
2	2	2	2	2	2	2	2	2	2
1	2	2	1	1	1	2	1	1	1
3	3	3	3	3	1	3	1	1	1
1	1	2	2	2	1	1	1	1	2
1	1	1	2	2	2	1	1	1	1
1	2	2	2	1	1	1	1	1	1
2	2	2	2	2	2	2	2	2	2

9	9	9	9	9	9	9	9	9	9
1	1	3	2	2	2	3	1	1	1
9	9	9	2	9	9	9	9	9	9
9	3	9	9	9	9	9	9	9	9
9	9	9	9	9	9	9	9	9	1
9	9	9	9	9	9	9	9	9	9
2	9	9	9	9	9	9	9	9	9
2	2	2	2	2	2	2	2	2	3
9	9	9	9	9	9	9	9	9	2
2	2	2	2	2	2	2	2	2	2
9	9	9	9	9	9	9	9	9	9
2	2	1	2	1	2	1	2	2	9
1	1	2	2	1	1	1	1	1	1
2	2	2	2	2	2	2	2	2	2
1	1	1	2	2	2	2	2	1	1
9	9	9	9	9	9	9	9	9	9
2	2	2	2	2	2	2	2	2	2
3	3	3	3	3	3	3	3	3	3
1	3	1	2	3	1	2	1	9	9
2	2	2	2	2	2	2	2	2	1
2	2	2	2	2	2	2	2	2	2
2	2	2	3	2	3	2	2	3	3
2	2	2	2	2	2	2	2	2	2
2	2	2	2	2	2	2	2	2	2
2	2	2	2	1	2	2	2	2	2
2	2	2	2	2	2	2	2	2	2
2	3	1	2	2	3	3	1	1	3
3	3	1	1	1	3	3	3	3	1
1	1	1	1	1	1	1	1	1	1
2	2	2	2	1	1	1	1	1	2
9	9	9	9	9	9	9	9	9	9
2	2	2	2	2	2	2	2	2	3
9	9	9	9	9	9	9	9	9	9
2	2	2	2	2	2	2	2	2	2
2	2	2	2	2	2	2	2	2	2
1	2	2	2	1	1	2	1	1	2
3	3	3	3	3	3	3	3	3	3
1	1	2	2	1	1	2	1	1	1
1	1	1	1	1	1	1	1	1	1
2	1	1	1	3	2	2	1	1	2
2	2	2	2	2	2	2	2	2	2
2	2	1	1	2	2	2	2	2	1
9	9	9	9	9	9	9	9	9	9
9	9	9	9	9	9	9	9	9	9
1	9	9	9	9	9	9	9	9	1
9	9	9	9	9	9	9	9	9	9
9	9	9	9	9	9	9	9	9	9

9	9	9	9	9	9	9	9	9	9
2	2	2	2	2	2	2	2	2	2
9	9	9	9	9	9	9	9	9	2
3	1	2	1	1	2	1	2	2	2
9	9	9	9	9	9	9	9	9	9
2	3	3	2	2	1	2	1	3	1
3	3	3	3	3	3	9	2	1	2
9	9	9	9	9	9	9	9	9	9
2	9	9	9	9	9	9	9	9	9
9	9	9	9	9	9	9	9	9	9
9	9	9	9	9	9	9	9	9	9
9	9	9	9	9	9	9	9	9	9
2	1	1	1	2	2	1	2	3	1
9	9	9	9	9	9	9	9	9	9
9	9	9	9	9	9	9	9	9	9
2	1	1	2	3	1	2	3	1	2
1	2	3	2	1	2	3	2	2	1
3	2	2	2	2	1	3	1	2	2
1	1	1	2	2	3	3	3	3	2
2	2	2	1	1	1	2	3	2	1
3	2	1	1	2	2	3	1	2	2
3	1	1	3	3	1	3	1	1	1
3	2	2	1	1	3	1	3	2	1
3	3	1	1	2	1	1	2	2	1
2	2	2	2	2	2	2	2	2	2
2	2	2	2	2	2	2	1	1	1
3	3	3	2	2	2	2	2	2	2
2	1	2	2	2	1	3	1	2	1
1	2	1	1	2	1	2	2	2	2
2	2	1	2	1	2	3	3	3	1
1	3	3	3	1	1	3	1	1	3
2	2	2	2	3	2	2	2	2	1
9	9	9	9	9	9	9	9	9	9
2	3	2	1	3	2	1	1	2	1
2	1	2	2	2	1	3	1	2	1
2	2	2	2	2	2	2	2	2	1
1	1	2	1	2	2	2	2	2	2
2	3	1	2	2	3	1	2	2	1
9	9	9	9	9	9	9	9	1	9
2	2	2	2	2	2	2	2	2	1
9	9	9	9	2	9	9	9	9	9
9	9	9	9	9	9	9	9	9	1
2	2	2	2	3	2	1	2	3	2
9	9	9	9	9	9	9	9	9	1
1	2	1	3	2	1	1	2	1	1
1	1	3	3	1	1	3	3	1	1
1	1	1	1	1	1	1	1	1	2

3	3	3	3	3	3	3	3	3	3
9	9	9	9	9	9	1	9	9	9
3	9	3	3	3	3	3	3	3	2
3	1	3	1	3	1	3	1	1	1
3	1	3	3	3	1	3	1	1	1
1	3	1	3	1	1	3	1	1	1
2	3	1	3	2	3	1	2	1	1
2	2	2	2	2	1	3	1	3	1
2	2	2	2	2	2	2	2	2	2
3	2	3	1	2	3	1	2	3	2
3	1	2	3	1	3	2	1	1	1
2	3	3	2	1	1	3	2	2	2
3	1	2	3	1	3	2	3	1	3
2	2	2	2	2	2	2	2	2	2
2	2	1	3	2	1	2	1	2	1
2	1	2	3	2	3	1	2	1	1
2	2	2	2	2	2	2	2	2	2
2	2	2	2	1	1	3	1	3	1
2	2	2	2	2	2	2	2	2	2
2	2	2	2	2	2	2	2	2	2
2	2	2	2	2	2	2	2	2	2
2	2	1	1	1	1	3	3	3	1
2	2	2	2	2	2	2	2	2	1
9	9	9	9	9	9	9	9	9	9
2	2	2	2	2	2	2	2	2	2
2	2	1	1	1	1	1	2	1	1
2	3	3	2	1	2	3	2	1	2
2	2	1	3	2	2	1	1	2	3
2	1	3	2	1	2	1	1	3	1
9	9	9	9	9	9	9	9	9	9
3	3	2	1	3	2	3	1	2	2
1	1	3	1	2	3	2	1	2	2
3	2	2	3	3	2	1	3	2	3
2	2	2	2	2	2	3	2	2	2
1	2	3	1	2	1	3	1	1	3
2	2	2	2	2	2	2	2	2	2
2	2	2	2	2	2	2	2	2	3
9	9	9	9	9	9	9	9	9	9
2	2	3	3	1	2	2	1	3	1
2	2	2	3	2	2	2	2	2	2
9	9	9	9	9	9	9	9	9	9
9	9	9	9	9	9	9	9	9	9
2	2	2	2	2	2	2	2	2	2
9	9	9	9	9	9	9	9	9	9
2	2	2	2	2	2	2	2	2	2
2	2	2	2	2	2	2	2	2	1
9	9	9	9	9	9	9	9	9	9

3	2	1	2	2	1	2	2	3	2
9	9	9	9	9	9	9	9	9	9
2	2	2	2	2	3	2	1	2	1
9	9	9	9	9	9	9	9	9	9
9	9	9	9	9	9	9	9	9	9
3	3	3	3	3	3	3	3	3	3
2	2	2	2	2	2	2	2	2	2
2	2	2	2	2	2	2	2	2	2
3	3	3	2	2	2	2	2	2	1
2	2	2	2	2	2	2	2	2	2
9	9	9	9	9	9	9	9	9	9
9	9	9	9	9	9	9	9	9	9
9	9	9	9	9	9	9	9	9	9
1	1	1	2	2	2	1	1	1	2
2	2	2	2	2	2	2	2	2	2
1	1	2	2	2	1	1	1	1	1
9	9	9	9	9	9	9	9	9	2
9	9	9	9	9	9	9	9	9	9
3	3	3	3	3	3	3	3	3	1
2	2	2	2	2	2	2	2	2	2
1	1	2	2	1	1	1	1	1	1
3	3	3	3	3	3	3	3	3	3
9	9	9	9	9	9	9	9	9	1
3	2	3	3	2	3	2	2	3	2
2	2	2	3	3	3	3	3	3	1
2	2	2	3	3	2	2	3	3	2
3	9	9	1	9	9	9	1	2	9
1	1	1	1	1	1	1	1	1	1
2	2	2	2	2	2	2	2	2	2
9	9	9	9	9	9	9	9	9	9
2	2	2	1	1	2	3	3	3	3
2	2	2	2	2	2	2	2	2	2
3	1	3	1	2	1	2	2	2	1
2	2	2	2	3	3	2	3	1	2
2	2	2	2	2	2	2	2	2	2
1	3	2	2	2	1	2	9	1	2
3	2	2	2	2	1	3	1	1	1
3	3	3	3	3	3	3	1	3	1
2	2	2	2	2	2	2	2	2	2
3	3	3	3	3	3	3	3	3	3
2	1	1	3	2	1	2	1	2	2
3	1	9	2	2	1	3	1	1	1
2	2	2	2	2	2	2	2	2	2
2	2	2	2	2	2	2	2	3	2
2	1	3	1	3	1	3	1	3	1
3	2	1	1	3	2	1	3	2	1
2	2	2	2	2	2	3	1	1	2

2	2	2	2	2	2	2	2	2	2
2	2	2	2	2	2	2	2	2	2
1	2	1	2	2	1	2	1	1	1
3	2	2	2	1	2	2	1	2	1
2	2	2	2	2	2	2	2	2	1
9	9	9	9	9	9	9	9	9	9
2	2	2	2	2	2	2	2	2	2
1	1	1	2	2	2	2	1	1	1
2	2	2	2	2	2	2	2	2	2
2	2	2	2	2	2	2	2	2	1
2	3	2	2	2	2	2	1	2	1
2	2	2	2	2	2	2	2	2	2
2	2	2	2	2	2	2	2	2	2
3	3	3	3	3	3	3	3	3	3
2	2	3	3	3	2	2	3	2	2
1	2	3	2	1	3	2	1	2	2
3	2	3	2	2	3	2	3	2	3
2	2	1	3	2	3	2	1	3	2
2	2	2	2	2	2	2	2	2	2
1	2	2	1	3	1	2	2	1	2
2	2	2	2	2	2	2	2	2	2
1	2	2	3	2	3	3	1	2	2
1	2	2	3	2	3	3	1	2	2
9	9	9	9	9	9	9	9	9	1
2	2	2	2	2	2	9	1	1	1
2	2	2	2	2	2	2	2	2	2
2	2	2	2	2	2	3	1	1	1
2	2	2	3	3	2	3	2	3	1
2	2	2	2	2	2	2	2	2	2
1	1	1	1	1	1	1	1	1	1
3	3	2	2	2	3	3	3	2	2
2	2	3	3	2	3	2	2	3	2
1	1	1	3	3	3	1	1	1	2
2	2	2	3	2	3	3	3	2	3
1	1	1	3	3	3	1	1	1	2
2	2	2	2	2	2	2	2	2	2
2	2	2	2	2	2	2	2	2	2
2	2	2	2	2	1	2	2	2	2
2	2	2	2	2	2	2	2	2	2
2	2	2	2	2	2	2	2	2	2
2	3	2	3	3	1	3	1	3	1
2	1	2	2	3	1	3	1	2	1
2	2	2	2	2	2	2	2	2	2
2	2	2	2	2	2	2	2	2	2
2	2	3	2	2	2	1	2	3	1
3	1	3	3	3	2	3	1	1	1
3	1	2	2	2	1	3	2	2	1

2	2	2	2	2	2	2	2	2	1
2	2	2	2	2	2	2	2	2	9
3	2	2	2	9	1	3	1	2	1
2	2	2	2	2	2	2	2	2	2
2	2	2	2	2	2	2	2	2	1
2	2	2	2	2	2	2	2	2	2
2	2	2	2	2	2	2	1	1	1
2	1	1	2	2	1	2	1	1	1
2	2	2	2	2	2	2	2	2	2
2	2	2	2	2	2	2	2	2	2
2	2	2	2	2	2	2	2	2	2
9	9	9	9	9	9	9	9	9	9
9	9	9	9	9	9	9	9	9	9
9	2	9	9	9	9	9	9	9	9
2	2	2	2	2	2	2	2	2	1
2	2	2	2	2	2	2	2	2	1
3	1	1	2	2	1	3	1	1	1
2	2	2	2	2	2	2	2	2	1
2	1	1	3	2	1	3	1	1	1
2	1	1	2	1	2	1	1	2	1
2	2	2	2	2	2	2	2	3	1
2	2	2	2	2	2	2	2	2	9
2	2	2	2	2	2	2	2	2	2
2	2	2	2	2	2	2	2	2	2
2	2	2	2	2	2	2	2	2	1
2	2	2	2	1	2	1	1	1	9
2	2	2	2	2	2	2	2	2	1
2	2	2	2	2	2	2	2	2	2
2	2	2	2	2	2	2	2	2	1
9	9	9	9	9	9	9	9	9	9
2	2	2	2	2	2	2	2	2	2
2	2	2	2	2	2	2	2	2	2
2	2	2	2	2	2	2	2	2	1
2	2	2	2	2	2	2	2	2	2
2	2	2	2	2	2	2	2	2	1
2	2	2	2	2	2	2	2	2	2
2	2	2	2	2	2	2	2	2	2
2	2	2	2	2	2	2	2	2	2
2	2	2	2	2	2	2	2	2	2
2	2	2	2	2	2	2	2	2	1
2	2	2	2	2	2	2	2	2	2
2	2	2	2	2	2	2	2	2	2
2	2	2	2	2	2	2	2	2	1
2	2	2	2	2	2	2	2	2	2
2	2	2	2	2	2	2	2	2	2
2	2	2	2	3	2	2	3	2	1
2	2	2	2	2	2	2	2	2	2

2	2	2	2	2	2	2	2	2	1
2	2	2	2	2	2	2	2	2	1
2	2	2	2	2	2	2	2	2	1
2	2	2	2	2	2	2	2	2	2
2	2	2	2	2	2	2	2	2	2
2	1	2	2	2	2	2	2	2	2
9	9	9	9	9	9	9	9	9	9
2	1	2	2	1	1	3	1	1	1
3	1	1	2	1	2	2	2	2	1
2	2	2	2	2	2	2	2	2	1
2	2	2	2	2	2	2	2	2	1
2	2	2	2	2	2	2	2	2	2
2	2	2	2	2	2	2	2	2	1
2	1	9	9	9	9	9	9	1	1
2	2	2	2	2	2	2	1	1	1
2	2	2	2	2	2	2	2	2	1
2	1	1	1	2	2	2	1	1	2
2	1	1	2	2	2	3	2	2	1
1	1	1	1	1	1	1	1	1	1
1	1	2	2	2	2	2	2	2	1
1	1	2	2	3	3	2	2	3	2
2	2	2	9	1	2	2	2	2	1
2	2	2	2	2	2	2	2	2	1
2	2	2	2	2	2	2	2	2	2
2	2	2	2	2	2	2	2	2	2
2	2	2	2	2	2	2	2	2	2
9	9	9	9	9	9	9	9	9	9
2	2	2	2	2	2	2	2	2	2
2	2	2	2	2	2	2	2	2	1
2	2	2	2	2	2	2	2	2	2
2	2	2	2	2	2	2	2	2	2
2	2	2	1	1	2	2	2	2	1
9	9	9	9	9	9	9	9	9	9
9	9	9	9	9	9	9	9	9	9
9	9	9	9	9	9	9	9	9	9
9	9	9	9	9	9	9	9	9	9
2	2	2	2	3	3	3	3	3	1
3	2	3	3	3	3	3	1	1	1
2	1	1	2	2	1	3	3	2	2
2	2	2	2	2	2	2	2	2	2
2	2	3	3	3	2	3	1	2	1
2	2	2	2	2	2	2	2	2	1
1	1	2	2	1	2	1	2	1	1
2	2	2	2	2	2	2	2	2	2
2	2	2	2	2	2	2	2	1	1
2	2	2	2	2	2	2	2	2	1
1	1	1	2	1	2	1	2	1	1

1	3	1	2	3	1	2	9	2	1
2	1	1	2	3	1	9	1	2	1
2	2	9	1	3	1	2	1	1	1
2	1	2	1	2	1	2	1	2	1
3	2	3	2	3	2	1	1	1	2
2	2	2	2	2	3	1	2	2	1
1	1	2	3	2	1	3	1	1	1
9	9	9	9	9	9	9	9	9	2
2	2	2	2	2	2	2	2	2	2
2	2	2	2	2	2	2	2	2	2
2	2	2	2	2	2	2	2	2	2
2	2	2	2	2	2	2	2	2	2
2	2	2	2	2	2	2	2	2	2
2	2	1	2	1	2	3	1	1	1
2	2	2	2	2	2	2	2	2	1
2	2	2	2	2	2	2	2	2	2
2	2	2	2	2	2	2	2	2	2
3	3	2	2	1	1	2	1	1	1
2	1	2	3	3	2	3	1	1	1
3	1	2	3	3	2	3	1	1	1
2	3	3	3	2	3	3	2	2	2
2	2	2	2	2	2	2	2	2	1
3	1	2	3	3	1	3	2	1	1
9	9	9	9	9	9	9	9	9	9
1	3	1	3	1	2	1	3	3	3
3	1	2	2	2	1	3	1	1	1
2	2	2	2	2	2	2	2	2	2
2	1	2	2	2	2	3	2	1	2
2	2	2	2	2	2	2	2	2	2
9	9	9	9	9	9	9	9	9	9
2	2	2	3	3	3	3	3	3	3
9	9	9	9	9	9	9	9	9	9
9	9	9	9	9	9	9	9	9	9
9	9	9	9	9	9	9	9	9	9
9	9	9	9	9	9	9	9	9	9
2	2	2	2	2	2	2	2	2	2
3	2	3	3	3	3	3	3	3	1
9	9	9	9	9	9	9	9	9	9
2	2	2	2	2	2	2	2	2	2
2	2	2	2	2	2	2	2	2	2
3	1	2	3	3	2	3	2	2	3
2	2	2	2	2	2	2	2	2	1
2	2	2	2	2	2	2	1	1	1
2	3	1	2	3	1	2	3	1	3
3	9	9	9	9	9	9	9	9	3
1	2	1	1	1	2	1	1	1	1
3	3	3	3	3	3	3	3	3	3

1	1	1	1	1	1	1	2	1	1
2	1	1	2	2	1	2	1	1	1
1	2	1	1	1	1	1	1	1	1
2	2	2	2	2	2	2	2	2	1
9	9	9	9	9	9	9	9	9	9
9	9	9	9	9	9	9	9	9	9
2	2	2	2	2	2	2	2	2	2
2	2	2	2	2	2	2	2	2	3
9	9	9	9	9	9	9	9	9	9
9	9	9	9	9	9	9	9	9	9
1	2	2	1	2	2	2	1	2	9
2	2	1	1	1	1	1	1	1	2
2	2	2	1	1	3	3	2	2	3
2	2	3	2	2	3	2	3	2	3
9	9	9	2	9	9	9	9	9	3
3	2	3	1	2	1	2	3	1	1
3	1	2	2	1	2	3	1	1	1
3	3	1	1	2	1	2	2	3	2
2	9	9	9	9	9	9	9	9	9
2	3	2	2	1	1	2	3	1	2
3	2	1	2	1	3	1	2	1	1
3	1	1	1	1	2	1	2	1	1
3	1	2	2	1	1	2	1	1	1
2	2	2	2	2	2	2	2	2	2
1	1	1	1	3	3	3	3	3	1
2	2	2	2	2	2	2	2	2	2
2	1	2	3	1	2	3	2	2	2
3	2	2	2	2	2	2	2	2	2
2	3	2	2	2	3	1	2	2	1
[truncated: 408,831 more chars]
