# Supplementary material for: Outcomes from integrating anti-cervical cancer teachings into the curriculum of high schools in a South-Eastern Nigerian State
Source: BMC Public Health. 2022 Oct 14;22:1914. doi: 10.1186/s12889-022-14231-4 (PMC9562070; doi:10.1186/s12889-022-14231-4)
Supplement: Supplementary file 8 — Additional file 8. [file 12889_2022_14231_MOESM8_ESM.pdf]

Comparing Phases 1 and 4 interventions for anti-cervical cancer teachings for high schools in Southeast Nigeria

Phase	ID	Gender	Age	Age2	StateofSch	ClassofStu	SchoolGen	HeardofBr	HeardofBS
1	1	2	16	2	1	3	1	2	2
1	2	2	15	2	1	1	1	1	2
1	3	2	16	2	1	3	1	1	1
1	4	2	17	2	1	1	1	1	2
1	5	2	15	2	1	1	1	1	2
1	6	2	0	#NULL!	1	1	1	1	2
1	7	2	16	2	1	3	1	1	1
1	8	2	18	2	1	3	1	1	1
1	9	2	18	2	1	3	1	1	1
1	10	2	15	2	1	3	1	1	2
1	11	2	17	2	1	3	1	1	2
1	12	2	17	2	1	3	1	1	2
1	13	2	16	2	1	3	1	1	1
1	14	2	15	2	1	3	1	1	1
1	15	2	16	2	1	3	1	1	2
1	16	2	16	2	1	3	1	1	1
1	17	2	15	2	1	3	1	1	2
1	18	2	16	2	1	3	1	1	1
1	19	2	16	2	1	3	1	1	1
1	20	2	17	2	9	9	1	1	9
1	21	2	17	2	1	3	1	1	1
1	22	2	18	2	1	3	1	1	1
1	23	2	17	2	1	3	1	1	2
1	24	2	16	2	1	3	1	1	1
1	25	2	18	2	1	3	1	1	1
1	26	2	17	2	1	3	1	1	1
1	27	2	18	2	1	3	1	1	1
1	28	2	16	2	1	3	1	1	2
1	29	2	17	2	1	3	1	1	2
1	30	2	18	2	1	3	1	1	2
1	31	2	16	2	1	3	1	1	2
1	32	2	18	2	1	3	1	1	2
1	33	2	16	2	1	3	1	2	1
1	34	2	14	1	1	3	1	1	1
1	35	2	16	2	1	3	1	1	2
1	36	2	15	2	1	3	1	1	1
1	37	2	17	2	1	3	1	1	2
1	38	2	16	2	1	3	1	1	1
1	39	2	17	2	1	3	1	1	2
1	40	2	16	2	1	3	1	1	2
1	41	2	17	2	1	3	1	1	2
1	42	2	0	#NULL!	1	3	1	2	1
1	43	2	17	2	1	3	1	1	1
1	44	2	16	2	1	3	1	1	1
1	45	2	19	2	1	3	1	1	1
1	46	2	18	2	1	3	1	1	1

1	47	2	17	2	1	3	1	1	2
1	48	2	18	2	1	3	1	1	1
1	49	2	17	2	1	3	1	1	1
1	50	2	15	2	1	3	1	1	1
1	51	2	17	2	1	3	1	1	1
1	52	2	16	2	1	3	1	1	1
1	53	2	17	2	1	3	1	2	1
1	54	2	15	2	1	3	1	1	1
1	55	2	17	2	1	3	1	1	1
1	56	2	15	2	1	3	1	1	1
1	57	2	17	2	1	3	1	1	1
1	58	2	19	2	1	3	1	1	1
1	59	2	15	2	1	3	1	1	1
1	60	2	16	2	1	3	1	1	9
1	61	2	15	2	1	3	1	1	2
1	62	2	16	2	1	3	1	1	1
1	63	2	16	2	1	3	1	1	1
1	64	2	0	#NULL!	1	3	1	1	1
1	65	2	16	2	1	3	1	1	2
1	66	2	17	2	1	3	1	1	1
1	67	2	17	2	1	3	1	1	2
1	68	2	17	2	1	3	1	1	2
1	69	2	18	2	1	3	1	1	1
1	70	2	17	2	1	3	1	1	1
1	71	2	17	2	1	3	1	1	1
1	72	2	15	2	1	3	1	1	1
1	73	2	17	2	1	3	1	1	2
1	74	2	16	2	1	3	1	1	2
1	75	2	18	2	1	3	1	1	2
1	76	2	17	2	1	3	1	1	1
1	77	2	17	2	1	3	1	1	1
1	78	2	17	2	1	3	1	1	1
1	79	2	19	2	1	3	1	1	1
1	80	2	0	#NULL!	1	3	1	1	1
1	81	2	18	2	1	3	1	1	1
1	82	9	19	2	1	3	1	1	1
1	83	9	16	2	1	3	1	1	1
1	84	2	15	2	1	3	1	1	2
1	85	2	17	2	1	3	1	1	1
1	86	2	17	2	1	3	1	1	1
1	87	2	17	2	1	3	1	1	1
1	88	2	17	2	1	3	1	1	1
1	89	2	17	2	1	3	1	1	2
1	90	2	16	2	1	3	1	1	1
1	91	2	16	2	1	3	1	1	2
1	92	2	16	2	1	3	1	2	2
1	93	2	16	2	1	3	1	2	2

1	94	2	16	2	1	3	1	1	1
1	95	2	17	2	1	3	1	1	1
1	96	2	15	2	1	3	1	1	1
1	97	2	16	2	1	3	1	1	1
1	98	2	16	2	1	3	1	2	1
1	99	2	16	2	1	3	1	1	2
1	100	2	17	2	1	3	1	1	1
1	101	2	17	2	1	3	1	1	1
1	102	2	17	2	1	3	1	1	1
1	103	2	16	2	1	3	1	1	1
1	104	2	15	2	1	3	1	1	3
1	105	2	17	2	1	3	1	1	9
1	106	2	17	2	1	3	1	1	1
1	107	2	16	2	1	3	1	1	1
1	108	2	17	2	1	3	1	1	3
1	109	2	16	2	1	3	1	1	2
1	110	2	16	2	1	3	1	1	1
1	111	2	15	2	1	3	1	1	1
1	112	2	17	2	1	3	1	1	1
1	113	2	16	2	1	3	1	1	2
1	114	2	20	3	1	3	1	1	2
1	115	2	16	2	1	3	1	1	1
1	116	2	18	2	1	3	1	2	1
1	117	2	15	2	1	3	1	2	2
1	118	2	17	2	1	3	1	1	1
1	119	2	16	2	1	3	1	1	1
1	120	2	16	2	1	3	1	1	2
1	121	2	16	2	1	3	1	2	2
1	122	2	18	2	1	3	1	1	2
1	123	2	18	2	1	3	1	1	2
1	124	2	17	2	1	3	1	1	2
1	125	2	16	2	1	3	1	2	3
1	126	2	19	2	1	3	1	2	2
1	127	2	17	2	1	3	1	1	2
1	128	2	17	2	1	3	1	1	2
1	129	2	16	2	1	3	1	1	2
1	130	2	17	2	1	3	1	1	1
1	131	2	15	2	1	3	1	1	1
1	132	2	18	2	1	3	1	1	1
1	133	2	17	2	1	3	1	1	1
1	134	2	19	2	1	3	1	1	1
1	135	2	18	2	1	3	1	1	1
1	136	2	18	2	1	3	1	2	3
1	137	2	15	2	1	3	1	1	2
1	138	2	17	2	1	3	1	2	3
1	139	2	17	2	1	3	1	1	2
1	140	2	16	2	1	3	1	2	2

1	141	2	19	2	1	3	1	1	2
1	142	2	17	2	1	3	1	1	1
1	143	2	18	2	1	3	1	1	2
1	144	2	18	2	1	3	1	1	1
1	145	2	18	2	1	3	1	1	3
1	146	2	17	2	1	3	1	1	1
1	147	2	21	3	1	9	1	1	9
1	148	2	15	2	1	3	1	1	1
1	149	2	15	2	1	3	1	1	2
1	150	2	18	2	1	3	1	1	2
1	151	2	18	2	1	3	1	1	2
1	152	2	19	2	1	3	1	1	2
1	153	2	20	3	1	3	1	1	9
1	154	2	16	2	1	3	1	1	2
1	155	2	16	2	1	3	1	1	2
1	156	2	17	2	1	3	1	1	2
1	157	2	18	2	1	3	1	1	2
1	158	2	16	2	1	3	1	1	2
1	159	2	19	2	1	3	1	1	1
1	160	2	15	2	1	2	1	2	2
1	161	2	16	2	1	2	1	1	1
1	162	2	17	2	1	2	1	1	2
1	163	2	17	2	1	2	1	1	2
1	164	2	16	2	1	2	1	2	2
1	165	2	16	2	1	2	1	2	2
1	166	2	17	2	1	2	1	1	1
1	167	2	15	2	1	2	1	2	2
1	168	2	15	2	1	2	1	1	1
1	169	2	15	2	1	2	1	1	2
1	170	2	16	2	1	2	1	1	2
1	171	2	17	2	1	3	1	1	1
1	172	2	18	2	1	3	1	1	3
1	173	2	16	2	1	2	1	2	2
1	174	2	17	2	1	2	1	2	2
1	175	2	16	2	1	2	1	2	2
1	176	2	16	2	1	2	1	1	3
1	177	2	16	2	1	2	1	1	1
1	178	2	16	2	1	2	1	1	1
1	179	2	16	2	1	2	1	1	2
1	180	2	17	2	1	2	1	1	2
1	181	2	16	2	1	2	1	1	1
1	182	2	16	2	1	2	1	2	2
1	183	2	15	2	1	2	1	1	9
1	184	2	20	3	1	2	1	1	3
1	185	2	15	2	1	2	1	1	1
1	186	2	20	3	1	2	1	1	3
1	187	2	16	2	1	2	1	1	3

1	188	2	17	2	1	2	1	2	1
1	189	2	15	2	1	2	1	1	1
1	190	9	15	2	1	2	1	2	2
1	191	2	16	2	1	2	1	1	1
1	192	2	15	2	1	2	1	1	3
1	193	2	15	2	1	2	1	1	1
1	194	2	16	2	1	2	1	1	1
1	195	2	15	2	1	3	1	1	1
1	196	2	16	2	1	3	1	1	1
1	197	2	16	2	1	3	1	1	1
1	198	9	0	#NULL!	1	3	1	1	1
1	199	2	17	2	1	3	1	1	1
1	200	2	18	2	1	3	1	1	1
1	201	2	17	2	1	2	1	1	1
1	202	2	17	2	1	2	1	1	1
1	203	2	15	2	1	2	1	1	1
1	204	2	16	2	1	2	1	1	2
1	205	2	17	2	1	2	1	1	2
1	206	2	15	2	1	2	1	1	1
1	207	2	16	2	1	2	1	1	2
1	208	2	16	2	1	2	1	1	1
1	209	2	15	2	1	2	1	1	2
1	210	2	16	2	1	2	1	1	2
1	211	2	15	2	1	2	1	1	3
1	212	2	16	2	1	2	1	1	2
1	213	2	16	2	1	2	1	1	2
1	214	2	16	2	1	2	1	2	2
1	215	2	16	2	1	2	1	2	2
1	216	2	16	2	1	2	1	1	2
1	217	2	16	2	1	2	1	1	1
1	218	2	15	2	1	2	1	1	3
1	219	2	19	2	1	2	1	1	3
1	220	2	16	2	1	2	1	1	1
1	221	2	16	2	1	2	1	2	2
1	222	2	16	2	1	2	1	1	3
1	223	2	16	2	1	2	1	1	2
1	224	2	15	2	1	2	1	1	3
1	225	2	17	2	1	2	1	3	1
1	226	2	15	2	1	2	1	1	1
1	227	2	17	2	1	2	1	1	2
1	228	2	17	2	1	2	1	1	1
1	229	2	15	2	1	2	1	1	2
1	230	2	16	2	1	2	1	1	2
1	231	2	15	2	1	2	1	1	2
1	232	2	15	2	1	2	1	1	2
1	233	2	15	2	1	2	1	1	2
1	234	2	16	2	1	2	1	1	2

1	235	2	15	2	1	2	1	1	2
1	236	2	16	2	1	2	1	1	3
1	237	2	15	2	1	2	1	1	1
1	238	2	18	2	1	2	1	1	2
1	239	2	16	2	1	2	1	1	2
1	240	2	16	2	1	3	9	1	1
1	241	2	17	2	1	3	1	1	1
1	242	2	17	2	1	3	1	1	1
1	243	2	16	2	1	3	1	1	1
1	244	2	17	2	1	3	1	1	2
1	245	2	16	2	1	3	1	1	2
1	246	2	17	2	1	3	1	2	2
1	247	2	17	2	1	3	1	1	2
1	248	2	16	2	1	3	1	1	1
1	249	2	16	2	1	3	1	1	2
1	250	2	16	2	1	3	1	2	2
1	251	2	17	2	1	3	1	1	2
1	252	2	17	2	1	3	1	1	2
1	253	2	17	2	1	3	1	2	2
1	254	2	16	2	1	3	1	1	2
1	255	2	17	2	1	3	1	1	1
1	256	2	17	2	1	3	1	1	2
1	257	2	16	2	1	3	1	1	2
1	258	2	17	2	1	3	1	1	1
1	259	2	15	2	1	3	1	1	2
1	260	2	18	2	1	3	1	1	2
1	261	2	16	2	1	3	1	1	2
1	262	2	15	2	1	3	1	1	2
1	263	2	0	#NULL!	1	3	1	1	2
1	264	2	16	2	1	3	1	1	3
1	265	2	18	2	1	3	1	1	3
1	266	2	17	2	1	3	1	1	2
1	267	2	18	2	1	3	1	1	2
1	268	2	17	2	1	3	1	1	1
1	269	9	17	2	1	3	1	1	3
1	270	2	17	2	1	3	1	2	1
1	271	2	16	2	1	3	1	1	2
1	272	2	16	2	1	3	1	1	1
1	273	2	18	2	1	3	1	1	3
1	274	2	17	2	1	3	1	1	9
1	275	2	15	2	1	2	1	1	2
1	276	2	14	1	1	2	1	1	1
1	277	2	0	#NULL!	1	2	1	1	2
1	278	2	15	2	1	2	1	1	2
1	279	2	16	2	1	2	1	1	2
1	280	2	16	2	1	2	1	1	2
1	281	2	17	2	1	2	1	1	2

1	282	2	15	2	1	2	1	1	1
1	283	2	16	2	1	2	1	1	1
1	284	2	16	2	1	2	1	1	3
1	285	2	13	1	1	2	1	1	2
1	286	2	15	2	1	2	1	1	2
1	287	2	15	2	1	2	1	1	2
1	288	2	16	2	1	2	1	1	2
1	289	2	16	2	1	2	1	1	1
1	290	2	16	2	1	2	1	1	1
1	291	2	16	2	1	2	1	1	2
1	292	2	17	2	1	2	1	1	2
1	293	2	14	1	1	2	1	1	1
1	294	2	15	2	1	2	1	2	2
1	295	2	16	2	1	2	1	2	2
1	296	2	17	2	1	2	1	1	2
1	297	2	20	3	1	2	1	1	2
1	298	2	15	2	1	2	1	1	1
1	299	2	15	2	1	2	1	1	3
1	300	2	16	2	1	2	1	1	2
1	301	2	14	1	1	2	1	1	2
1	302	2	16	2	1	2	1	1	2
1	303	2	0	#NULL!	1	2	1	1	2
1	304	2	17	2	1	2	1	1	1
1	305	2	17	2	1	2	1	1	2
1	306	2	16	2	1	2	1	1	2
1	307	2	18	2	1	3	1	1	1
1	308	2	18	2	1	3	1	1	1
1	309	2	16	2	1	3	1	1	1
1	310	2	17	2	1	3	1	1	1
1	311	2	18	2	1	3	1	1	1
1	312	2	17	2	1	3	1	1	1
1	313	2	17	2	1	3	1	1	1
1	314	2	17	2	9	9	9	9	9
1	315	2	17	2	9	3	1	1	1
1	316	2	18	2	1	3	1	1	1
1	317	2	17	2	1	3	1	1	3
1	318	2	17	2	1	3	1	1	1
1	319	2	16	2	1	3	1	1	1
1	320	2	17	2	1	3	1	1	1
1	321	2	17	2	1	3	1	1	2
1	322	2	19	2	1	3	1	1	1
1	323	2	17	2	1	3	1	1	1
1	324	9	17	2	1	3	1	1	1
1	325	2	16	2	1	3	1	1	1
1	326	2	17	2	1	3	1	1	1
1	327	2	17	2	1	3	1	1	2
1	328	2	16	2	1	3	1	1	1

1	329	2	15	2	1	3	1	1	1
1	330	2	16	2	1	3	1	1	1
1	331	2	16	2	1	3	1	1	1
1	332	2	15	2	1	3	1	1	1
1	333	2	17	2	1	3	1	1	3
1	334	2	17	2	1	3	1	1	1
1	335	2	17	2	1	3	1	1	1
1	336	2	18	2	1	3	1	1	1
1	337	2	16	2	1	2	1	1	3
1	338	2	16	2	1	2	1	1	1
1	339	2	16	2	1	2	1	1	1
1	340	2	15	2	1	2	1	1	1
1	341	2	15	2	1	2	1	1	1
1	342	2	16	2	1	1	1	1	1
1	343	2	16	2	1	2	1	1	1
1	344	2	16	2	1	2	1	1	2
1	345	2	17	2	1	2	1	1	1
1	346	2	17	2	1	2	1	1	1
1	347	2	16	2	1	2	1	1	1
1	348	2	14	1	1	2	1	1	2
1	349	2	16	2	1	2	1	1	2
1	350	2	16	2	1	2	1	1	2
1	351	2	16	2	1	2	1	1	2
1	352	2	14	1	1	2	1	1	1
1	353	2	14	1	1	2	1	1	2
1	354	2	16	2	1	2	1	1	2
1	355	2	15	2	1	2	1	1	2
1	356	2	15	2	1	2	1	1	1
1	357	2	16	2	1	2	1	1	2
1	358	2	16	2	1	2	1	1	2
1	359	2	15	2	1	2	1	1	2
1	360	2	15	2	1	2	1	1	2
1	361	2	16	2	1	2	1	1	2
1	362	2	15	2	1	2	1	1	2
1	363	2	15	2	1	2	1	1	2
1	364	2	15	2	1	2	1	1	2
1	365	2	15	2	1	2	1	1	2
1	366	2	15	2	1	2	1	1	2
1	367	2	15	2	1	2	1	1	2
1	368	2	14	1	1	2	1	3	2
1	369	2	16	2	1	2	1	1	1
1	370	2	15	2	1	2	1	1	2
1	371	2	15	2	1	2	1	1	2
1	372	2	13	1	1	2	1	1	2
1	373	2	14	1	1	2	1	1	1
1	374	2	15	2	1	2	1	1	3
1	375	2	16	2	1	2	1	1	2

1	376	2	16	2	1	2	1	1	1
1	377	2	14	1	1	2	1	1	1
1	378	2	16	2	1	2	1	2	2
1	379	2	16	2	1	2	1	1	2
1	380	2	14	1	1	2	1	1	2
1	381	2	15	2	1	2	1	1	1
1	382	2	0	#NULL!	1	2	1	1	2
1	383	2	15	2	1	2	1	1	1
1	384	2	15	2	1	2	1	1	1
1	385	2	14	1	1	2	1	1	2
1	386	2	14	1	1	2	1	1	3
1	387	2	16	2	1	2	1	1	1
1	388	2	17	2	1	2	1	1	1
1	389	2	16	2	1	2	1	1	2
1	390	2	17	2	1	2	1	1	2
1	391	2	16	2	1	2	1	1	1
1	392	2	16	2	1	2	1	1	2
1	393	2	14	1	1	2	1	1	1
1	394	2	16	2	1	2	1	2	1
1	395	2	16	2	1	2	1	1	1
1	396	2	15	2	1	2	1	1	3
1	397	2	15	2	1	2	1	1	1
1	398	2	16	2	1	2	1	1	1
1	399	2	15	2	1	2	1	1	2
1	400	2	16	2	1	2	1	1	1
1	401	2	19	2	1	2	1	1	1
1	402	2	15	2	1	2	1	1	2
1	403	2	15	2	1	2	1	1	2
1	404	2	12	1	1	2	1	1	2
1	405	2	17	2	1	2	1	1	2
1	406	2	16	2	1	2	1	1	1
1	407	2	17	2	1	2	1	1	2
1	408	2	16	2	1	2	1	1	1
1	409	2	15	2	1	2	1	2	2
1	410	2	16	2	1	2	1	2	2
1	411	2	0	#NULL!	1	2	1	1	2
1	412	2	14	1	1	2	1	1	2
1	413	2	16	2	1	2	1	1	2
1	414	2	18	2	1	2	1	1	2
1	415	2	15	2	9	2	1	1	1
1	416	2	0	#NULL!	1	2	1	1	2
1	417	2	15	2	1	1	1	1	2
1	418	2	15	2	1	2	1	1	2
1	419	2	17	2	1	2	1	1	2
1	420	2	16	2	1	2	1	1	3
1	421	2	16	2	1	2	1	1	2
1	422	2	17	2	1	2	1	1	2

1	423	2	16	2	1	2	1	1	2
1	424	2	15	2	1	2	1	1	1
1	425	2	16	2	1	2	1	1	2
1	426	2	18	2	1	2	1	2	2
1	427	2	16	2	9	9	9	9	9
1	428	2	15	2	1	2	1	2	1
1	429	2	17	2	1	2	1	1	2
1	430	2	16	2	1	2	1	1	2
1	431	2	19	2	1	2	1	1	2
1	432	2	16	2	1	2	1	2	2
1	433	2	16	2	1	2	1	1	2
1	434	2	14	1	1	2	1	1	1
1	435	2	15	2	1	2	1	1	2
1	436	2	15	2	1	2	1	1	1
1	437	2	15	2	1	2	1	1	2
1	438	2	15	2	1	2	1	1	1
1	439	2	16	2	1	2	1	1	9
1	440	2	15	2	1	2	1	2	1
1	441	2	16	2	1	2	1	1	1
1	442	2	14	1	1	2	1	1	1
1	443	2	15	2	1	2	1	1	2
1	444	2	16	2	1	2	1	2	3
1	445	2	15	2	1	2	1	1	1
1	446	2	15	2	1	2	1	1	1
1	447	2	14	1	1	2	1	1	1
1	448	2	15	2	1	2	1	1	1
1	449	2	17	2	1	2	1	1	2
1	450	2	16	2	1	2	1	1	3
1	451	2	16	2	9	2	1	1	2
1	452	2	15	2	1	2	1	1	3
1	453	2	15	2	1	2	1	1	1
1	454	2	15	2	1	2	1	1	2
1	455	2	16	2	1	2	1	1	1
1	456	2	16	2	1	2	1	1	1
1	457	2	14	1	9	2	1	1	1
1	458	2	16	2	1	2	1	1	1
1	459	2	15	2	1	2	1	1	3
1	460	2	16	2	1	2	1	1	3
1	461	2	15	2	1	2	1	1	3
1	462	2	15	2	1	2	1	1	1
1	463	2	16	2	1	2	1	1	2
1	464	2	19	2	1	3	1	1	2
1	465	2	16	2	1	3	1	1	2
1	466	2	19	2	1	3	1	1	2
1	467	2	16	2	1	3	1	1	2
1	468	2	17	2	1	3	1	1	2
1	469	9	17	2	1	3	1	1	2

1	470	2	16	2	1	2	1	2	2
1	471	2	19	2	1	3	1	1	1
1	472	2	19	2	1	3	1	1	1
1	473	2	17	2	1	3	1	2	1
1	474	2	18	2	1	3	1	1	2
1	475	2	0	#NULL!	1	3	1	1	1
1	476	2	19	2	1	3	1	1	2
1	477	2	18	2	1	3	1	1	9
1	478	2	15	2	1	3	1	1	1
1	479	2	17	2	1	3	1	1	1
1	480	2	0	#NULL!	1	3	1	1	1
1	481	2	17	2	1	2	1	1	2
1	482	2	16	2	1	3	1	2	2
1	483	2	17	2	1	3	1	1	2
1	484	2	17	2	1	2	1	2	2
1	485	2	16	2	1	2	1	2	2
1	486	2	17	2	1	2	1	1	2
1	487	2	18	2	1	2	1	1	2
1	488	2	15	2	1	2	1	1	1
1	489	2	14	1	1	2	1	1	1
1	490	9	0	#NULL!	9	9	9	9	9
1	491	2	17	2	1	2	1	1	1
1	492	2	15	2	1	2	1	1	1
1	493	2	17	2	1	2	1	1	1
1	494	2	16	2	1	2	1	1	2
1	495	2	16	2	1	2	1	1	2
1	496	2	16	2	1	2	1	1	1
1	497	2	16	2	1	2	1	1	2
1	498	2	16	2	1	2	1	1	2
1	499	2	16	2	1	2	1	1	1
1	500	2	16	2	1	2	1	1	2
1	501	2	15	2	1	2	1	1	2
1	502	2	16	2	1	2	1	1	2
1	503	2	16	2	1	2	1	2	2
1	504	2	14	1	1	2	1	1	2
1	505	2	15	2	1	2	1	1	1
1	506	2	15	2	1	2	1	1	1
1	507	2	14	1	1	2	1	1	1
1	508	2	17	2	1	2	1	2	2
1	509	2	15	2	1	2	1	1	2
1	510	2	14	1	1	2	1	1	2
1	511	2	15	2	1	2	1	1	1
1	512	2	17	2	1	2	1	1	2
1	513	2	16	2	1	2	1	1	2
1	514	2	15	2	1	2	1	1	2
1	515	2	16	2	1	2	1	1	1
1	516	2	16	2	1	2	1	1	3

1	517	2	16	2	1	2	1	1	3
1	518	2	15	2	1	2	1	1	2
1	519	2	15	2	1	2	1	1	2
1	520	2	15	2	1	2	1	1	2
1	521	2	15	2	1	2	1	1	3
1	522	2	17	2	1	2	1	1	2
1	523	2	14	1	1	2	1	1	2
1	524	2	16	2	1	2	1	1	1
1	525	2	15	2	1	2	1	1	1
1	526	2	16	2	1	2	1	1	1
1	527	2	15	2	1	2	1	1	9
1	528	2	15	2	1	2	1	1	1
1	529	2	16	2	1	2	1	1	2
1	530	2	16	2	1	2	1	1	2
1	531	2	17	2	1	2	1	1	2
1	532	2	14	1	1	2	1	1	2
1	533	2	16	2	1	2	1	1	3
1	534	2	17	2	1	2	1	1	1
1	535	2	14	1	1	2	1	2	2
1	536	2	16	2	1	2	1	1	1
1	537	2	15	2	1	2	1	1	2
1	538	2	15	2	1	2	1	1	2
1	539	2	17	2	1	2	1	1	2
1	540	2	17	2	1	2	1	1	1
1	541	2	15	2	1	2	1	1	1
1	542	2	14	1	1	2	1	1	2
1	543	2	15	2	1	2	1	1	1
1	544	2	15	2	1	2	1	1	1
1	545	2	15	2	1	2	3	1	1
1	546	2	16	2	1	3	1	1	2
1	547	2	16	2	1	3	1	1	1
1	548	2	17	2	1	3	1	1	2
1	549	2	16	2	1	3	1	1	2
1	550	2	18	2	1	3	1	1	2
1	551	2	17	2	1	3	1	2	3
1	552	2	16	2	1	3	1	1	2
1	553	2	17	2	1	3	1	1	2
1	554	2	15	2	1	3	1	1	1
1	555	2	17	2	1	3	1	1	3
1	556	2	18	2	1	3	1	1	2
1	557	2	0	#NULL!	1	3	1	1	1
1	558	2	15	2	1	3	1	1	1
1	559	2	17	2	1	3	1	1	1
1	560	2	18	2	1	3	1	1	1
1	561	2	16	2	1	2	1	1	3
1	562	2	16	2	1	2	1	1	1
1	563	2	15	2	1	2	1	1	2

1	564	2	16	2	1	2	1	1	2
1	565	2	17	2	1	2	1	1	2
1	566	2	15	2	1	2	1	1	2
1	567	2	17	2	1	2	1	1	2
1	568	2	18	2	1	2	1	1	2
1	569	2	15	2	1	2	1	1	2
1	570	2	17	2	1	2	1	1	2
1	571	2	16	2	1	2	1	1	3
1	572	2	16	2	1	2	1	1	2
1	573	2	16	2	1	2	1	1	3
1	574	2	16	2	1	2	1	1	3
1	575	2	16	2	1	2	1	1	3
1	576	2	16	2	1	2	1	1	2
1	577	2	19	2	1	2	1	1	2
1	578	2	15	2	1	2	1	1	2
1	579	2	16	2	1	2	1	1	2
1	580	2	17	2	1	2	1	1	2
1	581	2	15	2	1	2	1	1	2
1	582	1	0	#NULL!	1	2	2	3	3
1	583	1	15	2	1	2	2	3	1
1	584	1	15	2	1	2	2	2	2
1	585	1	17	2	1	2	2	1	3
1	586	1	15	2	1	2	2	1	2
1	587	1	16	2	1	2	2	1	1
1	588	2	18	2	1	3	3	1	3
1	589	1	0	#NULL!	1	3	3	1	1
1	590	2	18	2	1	3	3	1	1
1	591	1	16	2	1	3	3	1	1
1	592	1	0	#NULL!	1	3	3	1	1
1	593	1	17	2	1	3	3	1	2
1	594	1	19	2	1	3	3	1	2
1	595	2	16	2	1	3	3	1	2
1	596	2	17	2	1	3	3	1	1
1	597	1	17	2	1	3	3	1	1
1	598	1	18	2	1	3	3	1	1
1	599	1	15	2	1	3	3	1	3
1	600	2	13	1	1	9	3	1	2
1	601	2	13	1	1	9	3	1	2
1	602	2	13	1	1	9	3	1	2
1	603	2	15	2	1	9	3	1	2
1	604	1	18	2	9	9	9	9	9
1	605	1	14	1	1	9	3	1	3
1	606	2	15	2	1	9	3	1	1
1	607	2	15	2	1	9	3	1	1
1	608	1	14	1	1	9	3	2	2
1	609	2	13	1	1	9	3	1	1
1	610	1	14	1	1	9	3	1	3

1	611	2	14	1	1	3	3	1	2
1	612	2	14	1	1	9	3	3	1
1	613	9	14	1	1	9	3	9	2
1	614	2	17	2	1	9	2	1	1
1	615	1	15	2	1	9	3	1	1
1	616	1	0	#NULL!	1	9	3	1	1
1	617	1	17	2	1	9	3	1	3
1	618	9	13	1	1	9	3	1	2
1	619	2	14	1	1	9	3	3	1
1	620	9	0	#NULL!	1	9	9	9	9
1	621	2	15	2	9	9	9	9	9
1	622	2	14	1	1	9	3	1	2
1	623	1	0	#NULL!	1	9	3	2	2
1	624	2	13	1	1	9	3	3	3
1	625	2	14	1	1	9	3	1	2
1	626	2	18	2	1	3	3	1	1
1	627	2	13	1	1	9	3	1	1
1	628	2	13	1	1	9	3	1	2
1	629	2	13	1	1	9	3	1	2
1	630	1	12	1	1	9	3	1	2
1	631	1	12	1	1	9	3	1	3
1	632	9	0	#NULL!	1	9	3	2	2
1	633	9	0	#NULL!	1	1	3	2	2
1	634	2	14	1	1	9	3	1	2
1	635	2	13	1	1	9	3	1	2
1	636	2	14	1	1	9	3	2	2
1	637	2	14	1	1	9	3	1	2
1	638	2	14	1	1	9	3	2	1
1	639	1	12	1	1	9	3	1	3
1	640	2	15	2	1	9	3	1	1
1	641	2	15	2	1	9	3	2	2
1	642	2	15	2	1	9	3	1	2
1	643	2	15	2	1	9	3	1	3
1	644	2	16	2	1	9	3	1	2
1	645	2	16	2	1	9	3	1	2
1	646	2	13	1	1	9	3	1	1
1	647	2	15	2	1	9	3	1	1
1	648	2	13	1	1	3	3	2	2
1	649	2	12	1	1	9	3	2	2
1	650	2	18	2	1	9	1	1	1
1	651	2	12	1	1	9	3	1	1
1	652	2	13	1	1	9	3	1	3
1	653	1	14	1	1	9	3	1	2
1	654	2	13	1	1	9	3	1	2
1	655	1	14	1	1	9	3	1	2
1	656	1	14	1	1	9	3	1	1
1	657	1	16	2	1	3	3	1	2

1	658	2	15	2	1	3	3	1	2
1	659	2	15	2	1	3	3	1	1
1	660	1	19	2	1	3	3	1	2
1	661	2	18	2	1	3	3	1	2
1	662	2	16	2	1	3	3	1	2
1	663	1	16	2	1	3	3	1	1
1	664	1	20	3	1	3	3	3	2
1	665	2	0	#NULL!	1	3	3	1	1
1	666	2	14	1	1	9	3	2	2
1	667	2	15	2	1	3	3	1	1
1	668	1	14	1	1	9	3	3	3
1	669	2	15	2	1	9	3	1	9
1	670	2	13	1	1	9	3	1	3
1	671	2	12	1	1	9	3	1	1
1	672	1	13	1	1	9	3	1	3
1	673	2	13	1	1	9	3	1	3
1	674	2	13	1	1	3	3	1	2
1	675	2	13	1	1	9	3	1	1
1	676	2	14	1	1	9	3	1	1
1	677	1	13	1	1	9	3	1	2
1	678	2	14	1	1	9	3	1	1
1	679	1	14	1	1	9	3	1	2
1	680	2	15	2	1	9	3	1	2
1	681	1	13	1	1	9	3	1	2
1	682	1	18	2	1	3	3	2	2
1	683	1	13	1	1	9	3	2	2
1	684	2	14	1	1	9	3	1	2
1	685	2	13	1	1	9	3	1	2
1	686	2	14	1	1	9	3	1	2
1	687	1	15	2	1	9	3	3	3
1	688	1	15	2	1	9	3	1	2
1	689	2	16	2	1	9	3	2	3
1	690	1	14	1	1	9	3	1	1
1	691	2	14	1	1	9	3	1	2
1	692	2	14	1	1	9	3	2	2
1	693	2	13	1	1	9	3	2	2
1	694	2	14	1	1	9	3	1	3
1	695	2	13	1	1	9	3	1	3
1	696	2	14	1	1	9	3	2	2
1	697	2	13	1	1	9	3	1	2
1	698	1	14	1	1	9	2	2	3
1	699	2	15	2	1	9	3	1	2
1	700	1	14	1	1	9	3	1	2
1	701	2	13	1	1	9	3	1	2
1	702	2	13	1	9	9	9	9	9
1	703	1	14	1	1	9	3	2	2
1	704	2	14	1	1	9	3	1	2

1	705	1	12	1	1	9	3	1	2
1	706	2	13	1	1	9	3	1	1
1	707	1	14	1	1	3	3	1	1
1	708	2	14	1	1	9	3	2	2
1	709	1	16	2	1	3	3	2	2
1	710	1	15	2	1	3	3	2	2
1	711	1	14	1	1	3	3	3	3
1	712	1	16	2	1	3	3	2	2
1	713	1	16	2	1	3	3	2	2
1	714	9	0	#NULL!	9	9	9	9	9
1	715	2	14	1	1	9	3	1	2
1	716	2	15	2	1	9	3	1	1
1	717	2	11	1	1	9	3	1	2
1	718	2	15	2	1	9	3	3	1
1	719	2	13	1	1	9	3	1	1
1	720	1	13	1	1	9	3	1	3
1	721	1	13	1	1	9	3	1	2
1	722	1	15	2	1	9	3	1	2
1	723	1	15	2	1	3	3	1	2
1	724	1	14	1	1	9	3	1	3
1	725	2	16	2	9	9	3	1	2
1	726	1	11	1	1	9	3	1	2
1	727	1	15	2	2	9	3	2	2
1	728	1	14	1	1	9	3	1	1
1	729	1	14	1	1	9	3	1	1
1	730	1	13	1	1	9	3	1	1
1	731	1	12	1	1	9	3	1	1
1	732	1	11	1	1	9	3	1	2
1	733	1	14	1	2	9	3	1	3
1	734	1	15	2	1	9	3	3	3
1	735	1	15	2	2	9	3	1	3
1	736	1	14	1	1	9	3	1	2
1	737	2	14	1	1	3	3	1	2
1	738	1	13	1	1	9	3	1	1
1	739	1	14	1	1	9	3	1	2
1	740	1	15	2	1	9	3	2	2
1	741	1	13	1	1	1	2	1	2
1	742	2	15	2	1	9	3	1	1
1	743	1	14	1	1	9	3	1	1
1	744	1	15	2	1	2	2	1	2
1	745	1	16	2	1	2	2	1	2
1	746	1	17	2	2	2	2	1	2
1	747	1	17	2	1	2	2	1	1
1	748	1	17	2	1	2	2	2	2
1	749	1	17	2	1	2	2	1	2
1	750	1	14	1	1	2	2	1	1
1	751	1	15	2	1	2	2	1	2

1	752	1	16	2	1	2	2	1	2
1	753	1	15	2	1	1	2	1	1
1	754	1	16	2	1	2	2	2	1
1	755	1	16	2	1	2	2	1	1
1	756	1	15	2	1	2	2	2	2
1	757	1	16	2	1	2	2	1	2
1	758	1	16	2	1	2	2	2	1
1	759	1	15	2	1	2	2	1	2
1	760	9	0	#NULL!	9	3	2	1	3
1	761	1	15	2	1	2	2	1	2
1	762	1	17	2	1	2	2	2	2
1	763	1	16	2	1	2	2	1	1
1	764	1	15	2	1	2	2	1	1
1	765	1	16	2	1	2	2	1	2
1	766	1	17	2	1	2	2	1	1
1	767	1	17	2	1	2	2	1	2
1	768	1	13	1	1	2	2	1	3
1	769	1	21	3	1	2	2	1	3
1	770	1	16	2	1	2	2	1	2
1	771	1	15	2	1	2	2	1	2
1	772	1	17	2	1	2	2	2	2
1	773	1	15	2	1	2	2	1	1
1	774	1	16	2	1	2	2	1	2
1	775	1	16	2	1	2	2	1	2
1	776	1	17	2	1	2	2	2	2
1	777	1	18	2	1	2	3	1	2
1	778	1	15	2	1	2	2	1	2
1	779	1	17	2	1	2	2	2	2
1	780	1	16	2	1	2	2	1	2
1	781	1	15	2	1	2	2	1	1
1	782	1	16	2	1	2	2	1	2
1	783	1	15	2	1	2	2	1	2
1	784	1	18	2	1	2	2	1	1
1	785	1	14	1	1	2	2	2	2
1	786	1	16	2	1	2	2	2	2
1	787	1	18	2	1	2	2	1	3
1	788	1	15	2	1	2	2	1	2
1	789	1	15	2	1	2	2	1	2
1	790	1	15	2	1	2	2	1	2
1	791	1	16	2	1	2	2	1	2
1	792	1	15	2	1	2	2	1	3
1	793	1	16	2	1	2	2	1	1
1	794	1	15	2	1	2	2	2	2
1	795	1	16	2	1	2	2	2	1
1	796	1	16	2	1	2	2	1	2
1	797	1	15	2	1	2	2	2	2
1	798	1	16	2	1	2	2	2	2

1	799	1	15	2	1	2	2	1	2
1	800	1	16	2	1	2	2	2	2
1	801	1	18	2	1	2	2	1	2
1	802	1	16	2	1	2	2	2	2
1	803	1	13	1	1	2	2	1	1
1	804	1	15	2	1	2	2	2	3
1	805	1	15	2	1	2	2	2	2
1	806	1	16	2	1	2	2	1	1
1	807	1	20	3	1	2	2	1	2
1	808	1	15	2	1	2	2	2	2
1	809	1	18	2	1	2	2	1	1
1	810	9	0	#NULL!	1	2	2	2	2
1	811	1	16	2	1	2	2	1	2
1	812	1	15	2	1	2	2	1	2
1	813	1	16	2	1	2	2	1	1
1	814	1	15	2	1	2	2	2	2
1	815	1	16	2	1	2	2	2	2
1	816	1	16	2	1	2	2	1	2
1	817	1	16	2	1	2	2	1	2
1	818	1	0	#NULL!	1	2	2	1	1
1	819	1	17	2	1	2	2	1	2
1	820	1	16	2	1	2	2	1	2
1	821	1	16	2	1	2	2	1	2
1	822	1	17	2	1	2	2	1	2
1	823	1	15	2	1	2	2	1	2
1	824	1	16	2	1	2	2	1	1
1	825	1	15	2	1	2	2	1	3
1	826	1	14	1	1	2	2	1	1
1	827	1	16	2	1	2	2	1	2
1	828	1	17	2	1	2	2	1	2
1	829	9	15	2	1	2	9	1	1
1	830	1	16	2	1	2	2	1	2
1	831	1	17	2	1	2	2	1	1
1	832	1	16	2	1	2	2	1	1
1	833	1	0	#NULL!	1	2	2	1	2
1	834	1	16	2	1	2	2	1	1
1	835	9	15	2	1	2	2	1	1
1	836	1	15	2	1	2	2	1	2
1	837	1	16	2	9	9	2	2	3
1	838	1	15	2	9	9	2	2	3
1	839	1	16	2	1	2	2	1	2
1	840	1	17	2	1	2	2	1	3
1	841	1	18	2	1	2	2	1	2
1	842	1	17	2	1	2	2	1	2
1	843	1	16	2	1	2	2	2	3
1	844	1	15	2	1	2	2	1	1
1	845	1	19	2	1	2	2	1	2

1	846	1	18	2	1	2	2	1	2
1	847	1	16	2	1	2	2	2	2
1	848	1	17	2	1	2	2	2	2
1	849	1	17	2	1	2	2	1	2
1	850	1	18	2	1	2	2	1	2
1	851	1	14	1	1	2	2	2	2
1	852	1	17	2	1	2	2	2	1
1	853	1	16	2	1	9	9	9	9
1	854	1	17	2	1	2	2	1	2
1	855	1	16	2	1	2	2	1	3
1	856	1	16	2	1	2	2	2	2
1	857	1	17	2	1	2	2	1	1
1	858	1	18	2	1	2	2	1	2
1	859	1	18	2	1	2	2	1	2
1	860	1	17	2	1	2	2	2	2
1	861	1	16	2	1	2	2	2	2
1	862	1	19	2	1	2	2	2	2
1	863	1	16	2	1	2	2	1	1
1	864	1	16	2	1	2	2	1	1
1	865	1	17	2	1	2	2	2	2
1	866	1	16	2	1	2	2	1	2
1	867	1	15	2	1	2	2	1	2
1	868	1	0	#NULL!	1	2	2	1	2
1	869	1	17	2	1	2	2	1	2
1	870	1	17	2	1	2	2	2	2
1	871	1	16	2	1	2	2	2	2
1	872	1	17	2	1	2	2	1	1
1	873	1	16	2	1	2	2	1	1
1	874	1	15	2	1	2	2	1	1
1	875	1	16	2	1	2	2	1	2
1	876	1	16	2	1	2	2	1	2
1	877	1	0	#NULL!	1	2	2	1	9
1	878	1	16	2	1	2	2	1	1
1	879	1	17	2	1	2	2	2	2
1	880	1	19	2	1	2	2	1	1
1	881	1	15	2	1	2	2	1	3
1	882	1	17	2	1	2	2	2	2
1	883	1	18	2	1	2	2	2	2
1	884	1	18	2	1	2	2	1	2
1	885	1	16	2	1	2	2	2	2
1	886	1	16	2	1	2	2	1	1
1	887	1	16	2	1	2	2	2	2
1	888	1	18	2	1	2	2	1	2
1	889	1	17	2	1	2	2	1	9
1	890	1	16	2	1	2	2	1	1
1	891	1	16	2	1	2	2	2	1
1	892	1	15	2	1	2	2	1	2

1	893	1	15	2	1	2	2	2	2
1	894	1	15	2	1	2	2	1	2
1	895	1	18	2	1	2	2	2	2
1	896	1	18	2	1	2	2	2	2
1	897	1	15	2	1	2	2	1	2
1	898	1	16	2	1	2	2	1	2
1	899	1	15	2	1	2	2	2	2
1	900	1	15	2	1	2	2	1	2
1	901	1	15	2	1	2	2	1	2
1	902	1	16	2	1	2	2	1	2
1	903	1	0	#NULL!	1	2	2	1	2
1	904	1	18	2	1	2	2	1	3
1	905	1	14	1	1	2	2	1	2
1	906	1	16	2	1	2	2	2	2
1	907	1	16	2	1	2	2	1	1
1	908	1	15	2	1	2	2	2	2
1	909	1	16	2	1	2	2	1	1
1	910	1	15	2	1	2	2	1	1
1	911	1	15	2	1	2	2	1	2
1	912	1	18	2	1	2	2	1	1
1	913	1	16	2	1	2	2	2	2
1	914	1	17	2	1	2	2	2	2
1	915	1	19	2	1	2	2	1	1
1	916	1	17	2	1	2	2	1	3
1	917	1	16	2	1	2	2	2	2
1	918	1	15	2	1	2	2	2	2
1	919	1	16	2	1	2	2	1	1
1	920	1	16	2	1	2	2	1	2
1	921	1	16	2	1	2	2	1	2
1	922	2	14	1	1	9	3	3	2
1	923	2	17	2	1	3	3	1	2
1	924	2	15	2	1	9	3	1	1
1	925	1	15	2	1	9	3	2	2
1	926	2	14	1	1	9	3	1	2
1	927	2	0	#NULL!	1	3	3	3	2
1	928	1	18	2	1	3	3	1	2
1	929	2	19	2	1	3	3	1	2
1	930	1	17	2	1	3	3	2	2
1	931	2	17	2	1	3	3	1	1
1	932	2	16	2	1	3	3	2	1
1	933	1	0	#NULL!	1	3	3	1	2
1	934	1	17	2	1	3	3	1	1
1	935	1	17	2	1	3	3	2	2
1	936	2	17	2	1	3	3	3	2
1	937	1	18	2	1	3	3	2	2
1	938	2	19	2	1	3	3	2	2
1	939	2	19	2	1	3	3	1	2

1	940	2	17	2	1	3	3	1	1
1	941	2	19	2	1	3	3	3	2
1	942	1	20	3	1	3	3	1	1
1	943	2	17	2	1	3	3	1	1
1	944	1	18	2	1	3	3	1	2
1	945	9	0	#NULL!	9	9	9	9	9
1	946	2	16	2	1	3	3	1	2
1	947	2	18	2	1	3	3	1	2
1	948	2	16	2	1	3	3	1	2
1	949	2	17	2	1	3	3	1	2
1	950	2	17	2	1	3	3	1	2
1	951	2	17	2	1	3	3	1	2
1	952	2	17	2	1	3	3	9	9
1	953	2	15	2	1	3	3	2	2
1	954	2	17	2	1	3	3	1	2
1	955	9	16	2	1	3	3	1	2
1	956	1	19	2	1	3	3	2	2
1	957	9	18	2	1	3	2	2	1
1	958	2	16	2	1	3	3	1	2
1	959	2	15	2	1	3	3	1	1
1	960	2	18	2	1	3	3	1	2
1	961	2	17	2	1	3	3	1	2
1	962	2	17	2	2	3	3	1	2
1	963	2	15	2	1	3	3	2	2
1	964	2	17	2	1	3	3	1	3
1	965	2	17	2	1	3	3	1	1
1	966	2	17	2	1	3	3	1	1
1	967	2	17	2	1	3	3	1	3
1	968	2	17	2	1	3	3	1	1
1	969	9	16	2	1	3	3	1	2
1	970	2	15	2	1	3	3	1	1
1	971	2	17	2	1	3	3	9	9
1	972	2	16	2	1	3	3	1	1
1	973	2	16	2	1	3	3	1	2
1	974	1	17	2	1	3	3	1	2
1	975	2	14	1	1	3	3	1	1
1	976	2	14	1	1	3	3	1	1
1	977	2	15	2	1	3	3	1	1
1	978	2	15	2	1	3	3	1	2
1	979	2	18	2	1	3	3	1	2
1	980	2	19	2	1	3	3	1	1
1	981	1	17	2	1	3	3	2	2
1	982	2	16	2	1	3	3	1	2
1	983	1	23	3	1	3	3	2	2
1	984	1	18	2	1	3	3	1	1
1	985	1	18	2	1	3	3	1	3
1	986	2	16	2	1	3	3	1	3

1	987	2	17	2	1	3	3	1	1
1	988	2	16	2	1	3	3	2	2
1	989	1	16	2	1	3	3	1	1
1	990	2	16	2	1	3	3	1	2
1	991	2	16	2	1	3	3	1	1
1	992	1	16	2	1	3	3	1	1
1	993	1	16	2	1	3	3	2	2
1	994	1	15	2	1	3	3	1	1
1	995	1	17	2	1	3	3	1	3
1	996	1	17	2	1	3	3	2	2
1	997	2	0	#NULL!	1	3	3	1	2
1	998	1	19	2	1	3	3	1	2
1	999	2	17	2	1	3	3	1	1
1	1000	2	18	2	1	3	3	1	2
1	1001	2	16	2	1	2	3	1	2
1	1002	2	16	2	1	3	3	1	2
1	1003	1	18	2	1	3	3	1	1
1	1004	2	16	2	1	3	3	1	1
1	1005	2	17	2	1	3	3	1	2
1	1006	2	15	2	1	3	3	1	1
1	1007	2	16	2	1	3	3	1	2
1	1008	1	17	2	1	3	3	1	2
1	1009	2	17	2	1	3	3	1	2
1	1010	2	16	2	1	3	3	1	3
1	1011	2	15	2	1	3	3	1	2
1	1012	2	15	2	1	3	3	1	2
1	1013	2	18	2	1	3	3	1	1
1	1014	2	17	2	2	3	3	2	2
1	1015	2	17	2	1	3	3	1	2
1	1016	1	19	2	1	3	3	1	2
1	1017	2	18	2	1	3	2	1	2
1	1018	1	17	2	1	3	3	3	3
1	1019	1	17	2	1	3	3	1	1
1	1020	2	17	2	1	3	3	1	3
1	1021	1	19	2	1	3	3	2	2
1	1022	2	17	2	1	3	3	1	1
1	1023	1	19	2	1	3	3	1	2
1	1024	1	18	2	1	3	3	1	2
1	1025	2	14	1	1	3	3	1	2
1	1026	1	17	2	1	3	3	1	2
1	1027	1	17	2	1	3	3	1	1
1	1028	1	18	2	1	3	3	1	2
1	1029	1	18	2	1	3	3	2	2
1	1030	1	19	2	1	3	3	2	2
1	1031	1	20	3	1	3	3	1	9
1	1032	1	0	#NULL!	1	3	3	1	2
1	1033	1	17	2	1	3	3	1	1

1	1034	1	18	2	1	3	3	2	2
1	1035	1	21	3	1	3	3	1	2
1	1036	1	17	2	1	3	3	2	2
1	1037	2	17	2	1	3	3	1	2
1	1038	1	17	2	1	3	3	1	1
1	1039	1	18	2	1	3	3	2	2
1	1040	2	18	2	1	3	3	1	1
1	1041	2	16	2	1	3	3	1	2
1	1042	2	16	2	1	3	3	1	1
1	1043	1	14	1	1	9	3	1	1
1	1044	1	17	2	1	2	3	1	2
1	1045	1	15	2	1	2	3	3	3
1	1046	1	17	2	1	2	3	1	3
1	1047	2	19	2	1	2	3	1	2
1	1048	2	16	2	1	2	3	3	2
1	1049	2	15	2	1	2	3	1	1
1	1050	2	16	2	1	2	3	1	1
1	1051	2	16	2	1	2	3	1	1
1	1052	2	14	1	1	2	3	1	2
1	1053	2	17	2	1	2	3	1	2
1	1054	2	16	2	1	2	3	1	2
1	1055	1	16	2	1	2	3	1	2
1	1056	1	16	2	1	2	3	1	2
1	1057	1	19	2	1	2	3	2	2
1	1058	2	16	2	1	2	3	1	2
1	1059	2	15	2	2	2	3	1	2
1	1060	2	14	1	1	2	3	1	2
1	1061	2	17	2	1	2	3	1	2
1	1062	2	13	1	1	2	3	1	2
1	1063	2	17	2	1	2	3	1	1
1	1064	2	16	2	1	2	3	1	2
1	1065	1	19	2	1	2	3	2	3
1	1066	1	18	2	1	2	3	2	1
1	1067	1	16	2	1	2	3	2	2
1	1068	2	15	2	2	2	3	1	2
1	1069	2	16	2	2	2	3	1	1
1	1070	2	13	1	1	2	3	1	2
1	1071	2	18	2	1	2	3	1	2
1	1072	2	18	2	9	2	3	1	2
1	1073	1	16	2	1	2	3	1	1
1	1074	1	16	2	1	2	3	1	2
1	1075	2	16	2	1	2	3	2	2
1	1076	2	16	2	1	2	3	1	1
1	1077	2	16	2	1	2	3	1	2
1	1078	2	16	2	1	2	2	2	2
1	1079	2	14	1	1	2	3	1	1
1	1080	2	16	2	1	2	3	1	2

1	1081	2	17	2	1	3	3	2	2
1	1082	2	17	2	1	3	3	1	3
1	1083	2	17	2	1	3	3	1	1
1	1084	1	15	2	1	9	3	1	2
1	1085	2	14	1	1	9	3	1	1
1	1086	2	15	2	1	2	3	2	2
1	1087	2	15	2	1	2	3	2	2
1	1088	2	0	#NULL!	1	2	3	1	9
1	1089	2	16	2	1	2	3	1	2
1	1090	2	14	1	1	2	3	1	1
1	1091	2	15	2	1	2	3	2	1
1	1092	1	14	1	1	2	3	1	9
1	1093	1	18	2	1	2	3	1	1
1	1094	2	16	2	1	2	3	2	2
1	1095	2	16	2	1	2	3	1	2
1	1096	2	15	2	1	2	3	1	1
1	1097	2	15	2	1	2	3	1	2
1	1098	2	15	2	1	2	3	1	3
1	1099	2	14	1	2	2	3	1	1
1	1100	1	17	2	1	2	3	1	2
1	1101	1	18	2	1	2	3	2	1
1	1102	1	0	#NULL!	1	2	3	2	2
1	1103	1	15	2	1	2	3	1	2
1	1104	2	18	2	1	2	3	1	2
1	1105	2	17	2	1	2	3	1	1
1	1106	1	16	2	1	2	3	3	1
1	1107	1	16	2	1	2	3	1	2
1	1108	1	16	2	1	2	3	1	1
1	1109	1	17	2	1	2	3	1	2
1	1110	2	17	2	1	2	3	1	2
1	1111	2	17	2	1	2	3	1	1
1	1112	2	16	2	1	2	3	1	2
1	1113	2	16	2	1	2	3	1	2
1	1114	2	15	2	1	2	3	1	1
1	1115	2	15	2	1	2	3	1	2
1	1116	1	17	2	1	2	3	1	2
1	1117	1	15	2	1	2	3	2	3
1	1118	2	17	2	1	2	3	1	1
1	1119	2	16	2	1	2	3	1	2
1	1120	2	15	2	1	2	3	1	2
1	1121	1	18	2	2	2	3	1	2
1	1122	2	0	#NULL!	1	2	3	1	9
1	1123	2	15	2	1	2	3	2	2
1	1124	2	16	2	1	2	3	2	2
1	1125	2	14	1	1	2	3	1	1
1	1126	2	15	2	1	2	3	1	1
1	1127	2	16	2	1	2	3	1	2

1	1128	2	17	2	1	2	3	1	2
1	1129	1	17	2	1	2	3	1	2
1	1130	2	17	2	1	2	3	1	2
1	1131	2	15	2	1	2	3	1	2
1	1132	2	15	2	1	2	3	1	2
1	1133	1	16	2	1	2	3	1	2
1	1134	2	16	2	1	2	3	1	2
1	1135	1	0	#NULL!	1	2	2	2	2
1	1136	1	17	2	1	2	3	1	2
1	1137	1	16	2	1	2	2	2	2
1	1138	1	16	2	1	2	3	1	2
1	1139	2	17	2	2	2	3	1	3
1	1140	1	17	2	1	2	3	1	1
1	1141	1	17	2	1	2	3	1	3
1	1142	2	16	2	1	2	3	1	2
1	1143	2	15	2	1	2	3	1	1
1	1144	2	16	2	1	2	3	1	3
1	1145	1	17	2	1	2	3	1	1
1	1146	1	16	2	1	2	3	1	1
1	1147	1	16	2	1	2	3	1	1
1	1148	1	17	2	1	2	3	1	1
1	1149	2	15	2	1	2	3	1	2
1	1150	2	16	2	1	2	3	1	3
1	1151	2	17	2	1	2	3	1	3
1	1152	2	16	2	1	2	3	1	1
1	1153	2	17	2	1	2	3	9	2
1	1154	2	15	2	1	2	3	1	2
1	1155	1	18	2	1	2	3	2	2
1	1156	2	17	2	1	2	3	1	1
1	1157	2	17	2	1	2	3	1	2
1	1158	2	16	2	1	2	3	1	1
1	1159	2	16	2	1	2	1	1	1
1	1160	2	16	2	1	2	3	1	2
1	1161	2	17	2	1	2	3	1	2
1	1162	2	15	2	1	2	3	1	2
1	1163	2	15	2	1	2	3	1	1
1	1164	2	16	2	1	2	3	1	3
1	1165	1	17	2	1	2	3	1	2
1	1166	1	16	2	1	2	3	1	2
1	1167	1	16	2	1	2	3	1	9
1	1168	1	15	2	1	2	3	1	1
1	1169	2	15	2	1	2	3	1	2
1	1170	2	15	2	1	2	3	1	2
1	1171	2	15	2	1	2	3	1	3
1	1172	2	17	2	1	2	3	1	2
1	1173	2	14	1	1	2	3	1	2
1	1174	2	16	2	1	2	3	1	1

1	1175	1	15	2	1	2	3	1	1
1	1176	2	16	2	1	2	3	1	2
1	1177	1	17	2	1	2	3	2	2
1	1178	1	18	2	1	2	3	1	3
1	1179	1	19	2	1	2	3	1	1
1	1180	9	9	1	1	2	3	1	2
1	1181	1	15	2	1	2	3	1	2
1	1182	9	9	1	1	2	3	1	2
1	1183	1	17	2	1	2	3	1	1
1	1184	1	16	2	1	2	3	1	2
1	1185	1	16	2	1	2	3	1	2
1	1186	1	16	2	1	2	3	1	2
1	1187	1	16	2	1	2	3	2	2
1	1188	1	18	2	1	2	3	1	1
1	1189	1	16	2	1	2	3	1	1
1	1190	9	0	#NULL!	9	9	9	9	9
1	1191	1	19	2	2	2	3	1	3
1	1192	1	17	2	1	2	3	1	2
1	1193	1	16	2	1	2	3	2	2
1	1194	2	17	2	1	2	1	1	1
1	1195	2	14	1	1	2	3	1	2
1	1196	1	16	2	1	2	3	1	1
1	1197	2	19	2	1	2	3	1	2
1	1198	2	17	2	1	2	3	1	3
1	1199	2	15	2	1	2	3	2	2
1	1200	1	18	2	1	2	3	1	2
1	1201	2	16	2	1	2	3	1	1
1	1202	1	17	2	1	2	2	2	2
1	1203	1	15	2	1	2	3	2	2
1	1204	2	15	2	1	2	3	1	2
1	1205	9	0	#NULL!	9	9	9	9	9
1	1206	1	17	2	1	2	3	3	3
1	1207	1	16	2	1	2	2	2	3
1	1208	1	15	2	1	2	3	1	1
1	1209	1	15	2	1	2	3	1	2
1	1210	1	16	2	1	2	3	1	3
1	1211	2	15	2	1	2	3	1	1
1	1212	2	15	2	1	2	3	1	2
1	1213	2	14	1	1	2	3	1	2
1	1214	2	14	1	1	2	3	1	2
1	1215	2	16	2	1	2	3	1	2
1	1216	2	16	2	1	2	3	1	2
1	1217	1	16	2	1	2	3	1	2
1	1218	1	16	2	1	2	3	1	1
1	1219	2	14	1	1	2	3	1	1
1	1220	1	17	2	1	2	3	1	2
1	1221	2	15	2	1	2	3	1	2

1	1222	2	15	2	1	2	3	1	1
1	1223	2	17	2	1	2	3	1	2
1	1224	2	18	2	1	2	3	1	2
1	1225	1	16	2	1	2	3	2	2
1	1226	2	16	2	2	2	3	9	2
1	1227	2	16	2	1	2	3	1	3
1	1228	2	16	2	1	2	3	1	3
1	1229	2	14	1	1	2	3	1	2
1	1230	2	16	2	1	2	3	1	2
1	1231	1	15	2	1	2	3	1	3
1	1232	2	15	2	1	2	3	1	2
1	1233	2	16	2	1	2	3	1	2
1	1234	1	17	2	1	2	3	1	1
1	1235	2	16	2	1	2	3	1	1
1	1236	1	15	2	2	1	2	1	2
1	1237	1	16	2	1	1	2	1	2
1	1238	1	15	2	1	1	2	1	3
1	1239	1	15	2	1	1	2	1	2
1	1240	1	16	2	1	1	2	2	2
1	1241	1	14	1	1	1	2	1	2
1	1242	1	13	1	1	1	2	2	2
1	1243	1	15	2	1	1	2	1	2
1	1244	1	14	1	1	1	2	1	1
1	1245	1	14	1	1	1	2	1	1
1	1246	1	15	2	1	1	2	1	9
1	1247	1	16	2	1	1	2	2	2
1	1248	1	17	2	1	1	2	1	1
1	1249	1	16	2	1	1	2	1	2
1	1250	1	15	2	1	1	9	9	9
1	1251	1	16	2	1	1	2	1	1
1	1252	1	16	2	1	1	2	1	2
1	1253	1	16	2	1	1	2	1	1
1	1254	1	15	2	1	1	2	1	2
1	1255	1	14	1	1	1	2	2	2
1	1256	1	14	1	1	1	2	1	2
1	1257	1	14	1	1	1	2	1	3
1	1258	1	15	2	1	1	2	1	2
1	1259	1	14	1	1	1	2	1	2
1	1260	1	15	2	1	1	2	3	2
1	1261	1	14	1	1	1	2	9	9
1	1262	1	15	2	2	1	2	1	2
1	1263	1	15	2	1	1	2	1	2
1	1264	1	16	2	1	1	2	1	2
1	1265	1	15	2	1	1	2	1	2
1	1266	1	14	1	1	1	2	3	3
1	1267	1	16	2	2	1	2	1	2
1	1268	1	14	1	1	1	2	3	3

1	1269	1	16	2	1	1	2	3	3
1	1270	1	16	2	2	1	2	2	2
1	1271	1	15	2	1	1	2	1	2
1	1272	1	15	2	1	1	2	1	1
1	1273	1	14	1	1	1	2	1	2
1	1274	1	16	2	1	1	2	1	1
1	1275	1	14	1	1	1	2	1	2
1	1276	1	16	2	1	1	2	1	9
1	1277	1	18	2	1	1	2	1	2
1	1278	1	16	2	9	9	9	9	9
1	1279	1	16	2	9	9	9	1	2
1	1280	1	15	2	1	1	2	2	2
1	1281	1	15	2	1	1	2	1	1
1	1282	1	15	2	1	1	2	1	2
1	1283	1	15	2	1	1	2	1	1
1	1284	1	14	1	1	1	2	1	1
1	1285	1	15	2	1	1	2	1	2
1	1286	1	17	2	1	1	2	1	1
1	1287	1	15	2	1	1	3	1	2
1	1288	1	14	1	1	1	2	1	2
1	1289	1	14	1	1	1	2	1	1
1	1290	1	14	1	1	1	2	1	1
1	1291	1	17	2	1	1	2	1	3
1	1292	1	0	#NULL!	1	1	2	1	3
1	1293	1	14	1	1	1	2	1	3
1	1294	1	15	2	1	1	2	1	1
1	1295	1	15	2	1	1	2	1	1
1	1296	2	17	2	1	1	9	9	9
1	1297	1	15	2	1	1	2	2	2
1	1298	1	14	1	1	1	2	1	2
1	1299	1	0	#NULL!	1	1	2	1	2
1	1300	1	15	2	1	1	2	1	1
1	1301	1	16	2	2	1	2	1	3
1	1302	1	15	2	1	1	2	1	1
1	1303	1	18	2	2	1	2	1	2
1	1304	9	19	2	1	1	2	2	2
1	1305	1	16	2	1	1	2	1	2
1	1306	9	18	2	1	1	2	1	1
1	1307	1	16	2	1	1	2	1	1
1	1308	1	16	2	1	1	2	1	1
1	1309	1	15	2	1	1	2	1	1
1	1310	1	17	2	1	1	2	1	1
1	1311	1	15	2	1	1	2	1	1
1	1312	1	17	2	1	1	2	1	1
1	1313	1	15	2	1	1	2	1	3
1	1314	1	18	2	1	1	2	1	2
1	1315	9	0	#NULL!	9	9	9	9	9

1	1316	1	15	2	1	1	2	2	2
1	1317	1	15	2	1	1	2	1	1
1	1318	1	14	1	1	1	2	1	2
1	1319	1	14	1	1	1	2	1	1
1	1320	1	16	2	1	1	2	2	2
1	1321	1	15	2	1	1	2	1	1
1	1322	1	18	2	1	1	2	1	2
1	1323	1	15	2	1	1	2	2	2
1	1324	1	16	2	1	1	2	1	1
1	1325	1	15	2	1	1	2	1	2
1	1326	1	15	2	1	1	2	1	2
1	1327	1	15	2	1	1	2	1	2
1	1328	1	16	2	1	1	2	1	2
1	1329	1	14	1	1	1	2	2	2
1	1330	1	16	2	1	1	2	1	2
1	1331	1	15	2	1	1	2	1	1
1	1332	1	18	2	1	1	2	1	1
1	1333	1	16	2	1	1	2	1	2
1	1334	1	14	1	1	1	2	1	1
1	1335	1	15	2	1	1	2	1	1
1	1336	1	13	1	1	1	2	1	2
1	1337	1	15	2	1	1	2	1	2
1	1338	1	13	1	1	1	2	1	2
1	1339	1	13	1	1	1	2	1	2
1	1340	1	15	2	1	1	2	1	2
1	1341	1	15	2	1	1	2	1	1
1	1342	1	15	2	1	1	2	1	2
1	1343	1	15	2	1	1	2	2	2
1	1344	1	16	2	9	9	9	9	9
1	1345	1	15	2	1	1	2	1	1
1	1346	1	0	#NULL!	1	1	2	1	2
1	1347	1	15	2	1	1	2	1	2
1	1348	1	13	1	1	1	2	1	2
1	1349	1	14	1	1	1	2	1	2
1	1350	1	14	1	1	1	2	1	2
1	1351	1	16	2	1	1	2	1	2
1	1352	1	15	2	1	1	2	1	2
1	1353	1	16	2	1	1	2	1	1
1	1354	1	18	2	1	1	2	2	9
1	1355	1	13	1	1	1	2	1	2
1	1356	1	15	2	1	1	2	2	2
1	1357	1	15	2	1	1	2	1	3
1	1358	1	15	2	1	1	2	1	1
1	1359	1	15	2	1	1	2	1	3
1	1360	1	15	2	1	1	2	1	2
1	1361	1	15	2	1	1	2	1	2
1	1362	1	15	2	1	1	2	1	2

1	1363	1	15	2	1	1	2	1	2
1	1364	1	14	1	1	1	2	1	2
1	1365	1	15	2	1	1	2	3	2
1	1366	1	13	1	1	1	2	1	2
1	1367	1	14	1	1	1	2	1	3
1	1368	1	15	2	1	1	2	1	1
1	1369	1	19	2	1	1	2	2	2
1	1370	1	16	2	1	1	2	1	1
1	1371	1	14	1	1	1	2	1	2
1	1372	1	15	2	1	1	2	1	2
1	1373	1	14	1	1	1	2	1	2
1	1374	1	16	2	1	1	2	1	2
1	1375	1	14	1	1	1	2	2	1
1	1376	1	15	2	1	1	2	2	2
1	1377	1	15	2	1	1	2	1	1
1	1378	1	14	1	1	1	2	1	3
1	1379	1	16	2	1	1	2	1	2
1	1380	1	14	1	1	1	2	1	2
1	1381	1	16	2	1	1	2	2	2
1	1382	1	14	1	1	1	2	2	2
1	1383	1	14	1	1	1	2	1	2
1	1384	1	15	2	1	1	2	1	2
1	1385	1	16	2	1	1	2	2	2
1	1386	1	14	1	1	1	2	2	2
1	1387	1	14	1	1	1	2	1	1
1	1388	1	14	1	1	1	2	1	2
1	1389	1	14	1	1	1	2	1	2
1	1390	1	14	1	1	1	2	2	2
1	1391	1	15	2	1	1	2	1	1
1	1392	1	15	2	1	1	2	1	2
1	1393	1	15	2	1	1	2	1	2
1	1394	1	15	2	1	1	2	2	2
1	1395	1	15	2	1	3	2	1	2
1	1396	1	14	1	1	1	2	1	2
1	1397	1	15	2	1	1	2	2	2
1	1398	1	15	2	1	1	2	3	3
1	1399	1	13	1	1	1	2	3	3
1	1400	1	14	1	1	1	2	1	3
1	1401	1	13	1	1	1	2	3	3
1	1402	1	16	2	1	1	2	1	3
1	1403	1	15	2	1	1	2	1	2
1	1404	1	15	2	1	1	2	1	1
1	1405	1	14	1	1	1	2	1	1
1	1406	1	17	2	1	3	2	1	2
1	1407	1	16	2	1	3	2	2	2
1	1408	1	18	2	1	3	2	1	1
1	1409	1	16	2	1	3	2	1	2

1	1410	1	16	2	1	3	2	1	2
1	1411	1	18	2	1	3	2	2	3
1	1412	1	17	2	1	3	2	1	2
1	1413	1	18	2	1	3	2	1	2
1	1414	1	0	#NULL!	1	3	2	1	2
1	1415	1	16	2	1	3	2	1	2
1	1416	1	17	2	1	3	2	1	2
1	1417	1	19	2	1	3	2	2	2
1	1418	1	16	2	1	3	2	3	2
1	1419	1	19	2	1	3	2	1	2
1	1420	1	17	2	1	3	2	1	2
1	1421	1	17	2	1	3	2	1	2
1	1422	1	17	2	1	3	2	2	2
1	1423	1	17	2	1	3	2	1	2
1	1424	1	0	#NULL!	1	3	2	1	2
1	1425	1	16	2	1	3	2	2	2
1	1426	1	17	2	1	3	2	1	2
1	1427	1	17	2	1	3	2	1	2
1	1428	1	16	2	1	3	2	1	2
1	1429	1	19	2	1	3	2	1	3
1	1430	1	19	2	1	3	2	2	2
1	1431	1	17	2	1	3	2	3	3
1	1432	1	17	2	1	3	2	1	3
1	1433	1	18	2	1	3	2	1	2
1	1434	1	17	2	1	3	2	1	2
1	1435	1	18	2	1	3	2	3	2
1	1436	1	21	3	1	3	2	1	2
1	1437	1	17	2	1	3	1	1	2
1	1438	1	19	2	1	3	2	1	2
1	1439	1	0	#NULL!	1	3	2	1	3
1	1440	1	18	2	1	3	2	1	2
1	1441	1	18	2	1	3	2	1	3
1	1442	1	18	2	1	3	2	2	2
1	1443	1	18	2	1	3	2	1	2
1	1444	1	17	2	1	3	2	1	2
1	1445	1	15	2	1	3	2	1	2
1	1446	1	17	2	1	3	2	3	2
1	1447	1	17	2	1	3	2	1	2
1	1448	1	15	2	1	3	2	1	2
1	1449	1	16	2	1	3	2	1	2
1	1450	1	15	2	1	3	2	1	2
1	1451	1	17	2	1	3	2	2	2
1	1452	1	19	2	1	3	2	1	2
1	1453	1	18	2	1	3	2	1	2
1	1454	1	18	2	1	3	2	1	2
1	1455	1	17	2	1	3	2	3	3
1	1456	1	16	2	1	3	2	1	2

1	1457	1	17	2	1	3	2	1	2
1	1458	1	16	2	1	3	2	1	2
1	1459	1	17	2	1	3	2	1	1
1	1460	1	18	2	1	3	2	2	9
1	1461	1	18	2	1	3	2	2	2
1	1462	1	17	2	1	3	2	1	2
1	1463	1	18	2	1	3	2	1	3
1	1464	1	17	2	1	3	2	2	2
1	1465	1	17	2	1	3	2	1	1
1	1466	1	18	2	1	3	2	1	1
1	1467	1	18	2	1	3	2	1	1
1	1468	1	18	2	1	3	2	2	2
1	1469	1	16	2	1	3	2	1	2
1	1470	1	17	2	1	3	2	1	1
1	1471	1	17	2	1	3	2	1	2
1	1472	1	18	2	1	3	2	1	2
1	1473	1	18	2	1	3	2	1	2
1	1474	1	18	2	1	3	2	1	3
1	1475	1	17	2	1	3	2	1	2
1	1476	1	0	#NULL!	1	3	2	1	1
1	1477	1	17	2	1	3	2	1	1
1	1478	1	16	2	1	3	2	2	2
1	1479	1	17	2	1	3	2	2	2
1	1480	1	17	2	1	3	2	2	2
1	1481	1	20	3	1	3	2	2	2
1	1482	1	18	2	1	3	2	1	2
1	1483	1	17	2	1	3	2	1	2
1	1484	1	19	2	1	3	2	1	2
1	1485	1	15	2	1	3	2	1	2
1	1486	1	18	2	1	3	2	3	2
1	1487	1	17	2	1	3	2	2	2
1	1488	1	17	2	1	3	2	2	2
1	1489	1	17	2	1	3	2	1	2
1	1490	1	15	2	1	3	2	1	1
1	1491	1	0	#NULL!	1	3	2	1	2
1	1492	1	16	2	1	3	2	1	1
1	1493	1	17	2	1	3	2	1	1
1	1494	1	16	2	1	3	2	1	1
1	1495	1	18	2	1	3	3	1	1
1	1496	1	17	2	1	3	2	1	1
1	1497	1	0	#NULL!	1	3	2	1	1
1	1498	1	17	2	1	3	2	1	2
1	1499	1	17	2	1	3	2	1	1
1	1500	1	18	2	1	3	2	3	3
1	1501	1	17	2	1	3	2	1	2
1	1502	1	0	#NULL!	1	3	2	1	1
1	1503	1	18	2	1	3	2	1	2

1	1504	1	17	2	1	3	2	1	2
1	1505	1	15	2	1	3	2	1	1
1	1506	1	17	2	1	3	2	2	1
1	1507	1	17	2	1	3	2	1	1
1	1508	1	18	2	1	3	2	1	1
1	1509	1	17	2	1	3	2	1	1
1	1510	1	17	2	1	3	2	1	1
1	1511	1	18	2	1	3	2	1	3
1	1512	1	17	2	1	3	2	1	2
1	1513	1	0	#NULL!	1	3	2	1	2
1	1514	1	19	2	1	3	2	1	1
1	1515	1	19	2	1	3	2	1	1
1	1516	1	17	2	1	3	3	1	1
1	1517	1	18	2	1	3	2	1	2
1	1518	1	16	2	1	3	2	1	1
1	1519	1	17	2	1	3	2	1	1
1	1520	1	17	2	1	3	2	1	2
1	1521	1	15	2	1	3	2	1	2
1	1522	9	0	#NULL!	9	9	9	9	9
1	1523	1	15	2	1	3	2	1	2
1	1524	1	18	2	1	3	2	1	2
1	1525	1	18	2	1	3	2	1	2
1	1526	1	16	2	1	3	2	1	2
1	1527	1	18	2	1	3	2	1	2
1	1528	1	16	2	1	3	2	1	2
1	1529	1	17	2	1	3	2	1	2
1	1530	1	20	3	1	3	2	1	3
1	1531	1	17	2	1	3	2	1	2
1	1532	1	0	#NULL!	1	3	2	1	1
1	1533	1	17	2	1	3	2	1	1
1	1534	1	17	2	1	3	2	1	1
1	1535	1	17	2	1	3	2	1	2
1	1536	1	17	2	1	3	2	1	2
1	1537	1	17	2	1	3	2	1	2
1	1538	1	17	2	1	3	9	1	2
1	1539	1	16	2	1	3	2	1	2
1	1540	1	17	2	1	3	2	1	1
1	1541	1	16	2	1	3	2	1	2
1	1542	1	17	2	1	3	2	1	2
1	1543	1	16	2	1	3	2	1	2
1	1544	1	14	1	1	3	2	1	3
1	1545	1	17	2	1	3	2	1	2
1	1546	1	16	2	1	3	2	1	2
1	1547	1	18	2	1	3	2	1	2
1	1548	1	19	2	1	3	2	1	2
1	1549	1	17	2	1	3	2	1	2
1	1550	1	18	2	1	3	2	2	2

1	1551	1	16	2	1	3	2	1	1
1	1552	1	17	2	1	3	2	1	2
1	1553	1	16	2	1	3	2	1	2
1	1554	1	18	2	1	3	2	1	2
1	1555	1	17	2	1	3	2	1	2
1	1556	1	17	2	1	3	2	1	2
1	1557	1	18	2	1	3	2	2	2
1	1558	1	30	3	1	3	2	3	2
1	1559	1	19	2	1	3	2	2	2
1	1560	1	18	2	1	3	2	1	3
1	1561	2	14	1	1	1	3	1	2
1	1562	1	17	2	1	1	3	1	2
1	1563	1	16	2	1	1	3	3	2
1	1564	2	15	2	1	1	3	2	2
1	1565	1	17	2	1	1	3	1	2
1	1566	1	12	1	1	1	3	1	2
1	1567	1	15	2	1	1	3	1	2
1	1568	9	0	#NULL!	2	1	3	2	3
1	1569	1	16	2	1	1	3	1	1
1	1570	1	17	2	1	1	2	1	2
1	1571	1	18	2	1	1	3	1	2
1	1572	1	17	2	1	1	3	2	2
1	1573	2	14	1	1	1	3	1	2
1	1574	2	16	2	1	1	3	1	2
1	1575	9	15	2	1	1	3	1	9
1	1576	2	15	2	1	1	3	1	2
1	1577	1	14	1	1	1	3	1	2
1	1578	2	14	1	1	1	3	1	2
1	1579	2	15	2	1	1	3	1	1
1	1580	2	19	2	1	1	3	3	1
1	1581	1	15	2	1	1	3	1	2
1	1582	1	15	2	1	1	3	2	2
1	1583	1	16	2	1	1	2	1	3
1	1584	1	16	2	1	1	3	2	2
1	1585	2	18	2	1	1	3	1	3
1	1586	2	14	1	1	1	3	2	2
1	1587	1	16	2	1	1	3	1	2
1	1588	9	17	2	1	1	3	1	2
1	1589	9	0	#NULL!	1	1	3	1	2
1	1590	2	17	2	1	1	3	1	1
1	1591	9	14	1	1	1	3	1	1
1	1592	1	14	1	1	1	3	2	1
1	1593	2	16	2	1	1	3	1	2
1	1594	2	15	2	1	1	3	1	2
1	1595	2	14	1	1	1	3	1	2
1	1596	1	14	1	1	1	3	1	2
1	1597	1	16	2	1	1	3	2	2

1	1598	1	16	2	1	1	3	1	2
1	1599	2	14	1	1	1	3	1	1
1	1600	2	15	2	1	1	3	1	2
1	1601	2	15	2	1	1	3	1	2
1	1602	2	13	1	1	1	3	1	2
1	1603	2	16	2	1	1	3	1	2
1	1604	2	15	2	1	1	3	1	2
1	1605	2	14	1	1	1	3	1	2
1	1606	1	14	1	1	1	3	1	2
1	1607	2	15	2	1	1	3	1	2
1	1608	1	16	2	2	1	3	1	2
1	1609	2	14	1	1	1	3	1	2
1	1610	2	14	1	1	1	3	1	2
1	1611	2	15	2	1	1	3	1	2
1	1612	2	14	1	1	1	3	2	2
1	1613	2	17	2	1	1	3	1	2
1	1614	1	15	2	1	1	3	1	2
1	1615	1	15	2	1	1	3	2	2
1	1616	2	15	2	1	1	3	1	2
1	1617	1	14	1	1	1	3	1	2
1	1618	2	15	2	1	1	3	1	2
1	1619	2	14	1	1	1	3	1	2
1	1620	2	15	2	1	1	3	1	2
1	1621	2	14	1	1	1	3	1	2
1	1622	2	16	2	1	1	3	1	2
1	1623	2	17	2	1	1	3	1	2
1	1624	1	16	2	1	1	3	2	2
1	1625	1	17	2	1	1	3	1	2
1	1626	2	16	2	1	1	1	1	2
1	1627	2	15	2	1	1	3	1	1
1	1628	2	16	2	1	1	3	1	1
1	1629	2	13	1	1	1	3	1	2
1	1630	2	16	2	1	1	3	3	3
1	1631	2	14	1	1	1	3	1	1
1	1632	2	14	1	1	1	3	1	1
1	1633	2	15	2	1	1	3	1	2
1	1634	2	14	1	1	1	3	1	1
1	1635	1	15	2	1	1	3	1	2
1	1636	2	15	2	1	1	3	2	2
1	1637	2	14	1	1	1	3	1	2
1	1638	1	18	2	1	1	3	2	2
1	1639	1	14	1	1	1	3	2	2
1	1640	2	15	2	1	1	3	3	3
1	1641	2	16	2	1	1	3	1	2
1	1642	2	15	2	1	1	3	1	1
1	1643	2	14	1	1	1	3	1	2
1	1644	2	16	2	1	1	3	2	2

1	1645	1	20	3	1	1	2	2	2
1	1646	2	15	2	1	1	3	1	2
1	1647	1	16	2	1	1	3	1	1
1	1648	2	16	2	1	1	3	1	1
1	1649	2	18	2	1	1	3	1	2
1	1650	9	16	2	1	1	3	1	2
1	1651	2	14	1	1	1	3	2	2
1	1652	2	16	2	1	1	3	1	2
1	1653	1	17	2	1	1	3	1	2
1	1654	2	16	2	1	1	3	1	1
1	1655	2	15	2	1	1	3	1	3
1	1656	2	15	2	1	1	3	1	2
1	1657	2	14	1	1	1	3	1	3
1	1658	2	15	2	1	1	3	1	1
1	1659	2	14	1	1	1	3	2	2
1	1660	2	14	1	1	1	3	1	1
1	1661	2	16	2	1	1	3	1	1
1	1662	2	14	1	1	1	3	2	2
1	1663	2	16	2	1	1	3	1	2
1	1664	2	15	2	1	1	3	1	1
1	1665	2	15	2	1	1	3	2	2
1	1666	2	16	2	1	1	3	1	1
1	1667	9	16	2	1	1	3	1	1
1	1668	2	14	1	1	1	3	2	1
1	1669	2	13	1	1	1	3	1	1
1	1670	2	15	2	1	1	3	1	1
1	1671	2	15	2	1	1	3	1	2
1	1672	2	15	2	1	1	3	1	2
1	1673	1	13	1	1	1	3	1	2
1	1674	2	15	2	1	1	3	1	2
1	1675	2	14	1	1	1	3	1	3
1	1676	2	15	2	1	1	3	1	1
1	1677	2	14	1	1	1	3	1	3
1	1678	2	15	2	1	1	3	1	3
1	1679	1	14	1	1	1	3	1	3
1	1680	2	16	2	1	1	3	1	1
1	1681	2	13	1	1	1	3	1	2
1	1682	1	15	2	1	1	3	2	2
1	1683	1	15	2	1	1	3	1	2
1	1684	1	15	2	1	1	3	1	1
1	1685	1	14	1	1	1	3	3	1
1	1686	1	15	2	1	1	3	1	2
1	1687	1	18	2	1	1	3	1	1
1	1688	1	16	2	1	3	3	2	1
1	1689	1	16	2	1	1	3	2	2
1	1690	1	16	2	1	1	3	1	1
1	1691	1	15	2	1	1	3	1	1

1	1692	1	14	1	1	1	3	1	2
1	1693	1	15	2	1	1	3	3	1
1	1694	1	0	#NULL!	1	1	3	1	2
1	1695	1	15	2	1	1	3	1	1
1	1696	1	15	2	1	1	3	1	2
1	1697	1	16	2	1	1	3	1	3
1	1698	1	15	2	1	1	3	1	3
1	1699	1	14	1	1	1	3	1	2
1	1700	2	15	2	2	3	3	1	1
1	1701	2	15	2	2	3	3	1	1
1	1702	2	18	2	2	3	3	1	1
1	1703	2	17	2	2	3	3	1	1
1	1704	2	15	2	2	3	3	2	3
1	1705	2	18	2	2	3	3	1	9
1	1706	2	18	2	2	3	3	1	1
1	1707	1	17	2	2	3	3	1	1
1	1708	2	19	2	2	3	3	1	2
1	1709	1	18	2	2	3	3	1	1
1	1710	1	17	2	2	3	3	1	2
1	1711	1	16	2	2	3	3	1	2
1	1712	2	16	2	2	3	3	1	2
1	1713	2	15	2	2	3	3	1	1
1	1714	2	15	2	2	3	3	1	1
1	1715	1	17	2	2	3	3	3	3
1	1716	1	18	2	2	3	3	1	2
1	1717	9	19	2	2	3	3	1	2
1	1718	1	17	2	2	3	3	1	1
1	1719	2	16	2	2	1	3	2	3
1	1720	2	14	1	2	1	3	1	1
1	1721	2	13	1	2	1	3	1	2
1	1722	2	15	2	2	3	3	1	1
1	1723	2	15	2	2	1	3	1	1
1	1724	1	13	1	2	1	3	3	1
1	1725	1	16	2	9	1	3	1	1
1	1726	1	15	2	2	1	3	1	1
1	1727	2	17	2	2	1	3	1	2
1	1728	1	15	2	2	2	3	1	3
1	1729	1	16	2	2	2	2	1	1
1	1730	1	15	2	2	2	3	1	1
1	1731	1	16	2	2	2	3	2	2
1	1732	2	14	1	2	1	3	1	3
1	1733	1	15	2	2	1	3	1	3
1	1734	2	13	1	2	1	3	1	2
1	1735	1	15	2	2	1	3	1	3
1	1736	1	9	1	2	1	2	2	2
1	1737	1	17	2	2	2	3	3	3
1	1738	1	14	1	2	1	3	2	2

1	1739	1	14	1	2	1	3	2	2
1	1740	1	14	1	2	1	3	2	2
1	1741	1	14	1	2	1	3	1	2
1	1742	1	14	1	2	1	3	1	2
1	1743	1	15	2	2	1	3	2	2
1	1744	1	14	1	2	1	3	1	2
1	1745	1	15	2	2	1	3	1	1
1	1746	1	14	1	2	1	3	2	2
1	1747	2	15	2	2	1	3	2	2
1	1748	2	14	1	2	1	3	1	2
1	1749	2	15	2	2	1	3	1	1
1	1750	1	17	2	2	3	3	3	3
1	1751	1	15	2	2	1	3	2	2
1	1752	1	14	1	2	1	3	1	2
1	1753	1	15	2	2	1	3	2	2
1	1754	1	15	2	2	1	3	2	2
1	1755	1	15	2	2	1	3	1	2
1	1756	1	14	1	2	1	3	2	2
1	1757	1	16	2	2	2	3	2	2
1	1758	1	17	2	2	2	3	2	2
1	1759	1	17	2	2	2	3	3	2
1	1760	1	16	2	2	2	3	3	3
1	1761	1	14	1	2	1	3	1	2
1	1762	1	14	1	2	1	3	2	2
1	1763	1	15	2	2	1	3	2	2
1	1764	1	14	1	2	1	3	2	2
1	1765	1	15	2	2	1	3	2	2
1	1766	1	16	2	2	1	3	1	2
1	1767	1	15	2	2	1	3	2	2
1	1768	1	14	1	2	1	3	2	2
1	1769	1	15	2	2	1	3	2	2
1	1770	1	14	1	2	1	3	1	2
1	1771	1	15	2	2	1	3	2	2
1	1772	1	14	1	2	1	3	2	2
1	1773	2	14	1	2	1	3	2	2
1	1774	2	14	1	2	1	3	2	2
1	1775	2	13	1	2	1	3	2	2
1	1776	2	14	1	2	1	3	2	2
1	1777	2	14	1	2	1	3	2	2
1	1778	2	15	2	2	1	3	2	2
1	1779	2	14	1	2	1	3	3	3
1	1780	2	14	1	2	1	3	2	2
1	1781	2	14	1	2	1	3	2	2
1	1782	2	13	1	2	1	3	1	1
1	1783	2	13	1	2	1	3	2	2
1	1784	2	14	1	2	1	3	2	2
1	1785	2	14	1	2	1	3	2	2

1	1786	2	15	2	2	1	3	2	2
1	1787	2	15	2	2	1	3	2	2
1	1788	2	15	2	2	1	3	2	2
1	1789	2	14	1	2	1	3	2	2
1	1790	2	14	1	2	1	3	2	2
1	1791	2	15	2	2	1	3	2	2
1	1792	2	15	2	2	1	3	2	2
1	1793	2	14	1	2	1	3	1	2
1	1794	2	17	2	2	2	3	1	1
1	1795	2	16	2	2	2	3	3	3
1	1796	2	14	1	2	1	3	1	1
1	1797	2	15	2	2	1	3	1	1
1	1798	2	15	2	2	1	3	1	1
1	1799	2	15	2	2	1	3	1	1
1	1800	2	14	1	2	1	3	1	2
1	1801	2	15	2	2	1	3	1	2
1	1802	2	15	2	2	1	3	1	2
1	1803	2	16	2	2	1	3	3	2
1	1804	1	15	2	2	1	3	1	1
1	1805	1	15	2	2	1	3	2	2
1	1806	1	14	1	2	1	3	1	2
1	1807	1	14	1	2	1	3	1	2
1	1808	1	15	2	2	1	3	1	2
1	1809	1	16	2	2	2	3	3	3
1	1810	1	17	2	2	2	3	2	2
1	1811	1	17	2	2	2	3	3	3
1	1812	1	16	2	2	2	3	3	3
1	1813	1	17	2	2	2	3	2	2
1	1814	1	16	2	2	2	3	2	2
1	1815	1	16	2	2	2	3	2	2
1	1816	1	15	2	2	2	3	1	1
1	1817	1	16	2	2	2	3	3	3
1	1818	1	16	2	2	2	3	3	3
1	1819	1	16	2	2	2	3	2	2
1	1820	1	15	2	2	2	3	2	2
1	1821	1	16	2	2	2	3	2	2
1	1822	1	16	2	2	2	3	2	2
1	1823	1	17	2	2	2	3	1	1
1	1824	1	15	2	2	1	3	1	2
1	1825	1	16	2	2	1	3	1	2
1	1826	1	14	1	2	1	3	2	2
1	1827	1	13	1	2	1	3	2	2
1	1828	2	14	1	2	1	3	1	2
1	1829	2	15	2	2	1	3	1	1
1	1830	1	14	1	2	1	3	1	2
1	1831	1	14	1	2	1	3	2	2
1	1832	1	14	1	2	1	3	2	2

1	1833	1	15	2	2	1	3	2	2
1	1834	1	14	1	2	1	3	2	2
1	1835	1	14	1	2	1	3	2	2
1	1836	1	15	2	2	1	3	2	2
1	1837	1	17	2	2	3	3	1	3
1	1838	2	17	2	2	3	3	1	2
1	1839	9	18	2	2	3	3	1	1
1	1840	2	17	2	2	3	3	1	2
1	1841	2	16	2	2	3	3	1	2
1	1842	1	17	2	2	3	3	1	2
1	1843	2	16	2	2	3	3	1	3
1	1844	2	17	2	2	3	3	1	2
1	1845	1	17	2	2	3	3	1	1
1	1846	1	17	2	2	3	3	1	2
1	1847	1	17	2	2	3	3	1	3
1	1848	1	15	2	2	2	3	1	2
1	1849	1	17	2	2	2	3	1	2
1	1850	1	15	2	2	2	3	1	2
1	1851	1	20	3	9	2	3	1	3
1	1852	1	16	2	2	2	3	1	2
1	1853	1	16	2	2	2	3	1	2
1	1854	1	15	2	2	2	3	1	2
1	1855	2	15	2	2	2	3	1	1
1	1856	1	16	2	2	2	3	1	2
1	1857	2	18	2	2	3	3	1	1
1	1858	1	16	2	2	3	3	1	3
1	1859	1	16	2	2	3	3	1	2
1	1860	2	16	2	2	1	3	1	1
1	1861	2	20	3	2	1	3	1	1
1	1862	2	16	2	2	1	3	1	3
1	1863	1	16	2	2	1	3	1	2
1	1864	1	0	#NULL!	2	1	3	1	2
1	1865	1	15	2	2	1	3	1	1
1	1866	1	16	2	2	2	3	1	1
1	1867	1	16	2	2	2	3	1	2
1	1868	2	15	2	2	1	3	1	2
1	1869	2	12	1	2	1	3	1	1
1	1870	2	14	1	2	1	3	1	2
1	1871	2	13	1	2	1	3	1	2
1	1872	2	14	1	2	1	3	1	1
1	1873	1	17	2	2	1	3	1	2
1	1874	1	15	2	2	1	3	1	2
1	1875	2	18	2	2	3	3	1	3
1	1876	1	17	2	2	3	3	1	2
1	1877	1	16	2	2	1	3	1	1
1	1878	2	15	2	2	1	3	1	2
1	1879	2	15	2	2	1	3	1	2

1	1880	2	15	2	2	1	3	1	2
1	1881	2	15	2	2	1	3	1	2
1	1882	1	16	2	2	1	3	1	2
1	1883	2	16	2	2	1	3	1	1
1	1884	2	14	1	2	1	3	1	1
1	1885	1	15	2	2	1	3	1	2
1	1886	1	15	2	2	1	3	1	1
1	1887	2	14	1	2	1	3	2	3
1	1888	1	15	2	2	1	3	1	2
1	1889	1	16	2	2	1	3	1	1
1	1890	2	15	2	2	1	3	2	1
1	1891	2	15	2	2	1	2	1	1
1	1892	1	15	2	2	1	3	2	1
1	1893	2	14	1	2	1	3	1	2
1	1894	1	15	2	2	1	3	1	3
1	1895	2	14	1	2	1	3	3	3
1	1896	2	15	2	2	1	3	1	1
1	1897	2	15	2	2	1	3	1	1
1	1898	2	15	2	2	1	3	1	2
1	1899	2	15	2	2	1	3	1	2
1	1900	9	0	#NULL!	2	1	3	1	9
1	1901	2	11	1	9	1	3	2	2
1	1902	2	14	1	2	1	3	1	2
1	1903	9	0	#NULL!	2	1	2	2	2
1	1904	1	16	2	2	1	3	1	3
1	1905	1	16	2	2	1	3	2	1
1	1906	1	15	2	2	1	3	1	1
1	1907	2	14	1	2	1	3	1	2
1	1908	1	16	2	2	1	3	2	2
1	1909	1	16	2	2	1	3	2	1
1	1910	1	16	2	2	1	3	2	1
1	1911	2	15	2	2	1	3	2	2
1	1912	1	15	2	2	1	3	1	1
1	1913	9	0	#NULL!	9	9	9	9	9
1	1914	9	0	#NULL!	2	1	3	1	3
1	1915	1	19	2	2	1	3	1	1
1	1916	1	19	2	2	1	3	1	2
1	1917	1	14	1	2	1	3	1	2
1	1918	1	16	2	2	2	3	1	1
1	1919	1	17	2	2	2	3	2	1
1	1920	1	15	2	2	2	3	1	2
1	1921	2	17	2	2	2	3	1	1
1	1922	1	17	2	2	2	3	1	3
1	1923	2	0	#NULL!	2	2	3	1	1
1	1924	1	17	2	2	2	3	1	2
1	1925	9	15	2	2	2	3	1	1
1	1926	1	15	2	2	2	3	1	1

1	1927	1	19	2	2	2	3	1	3
1	1928	1	16	2	2	2	9	2	1
1	1929	1	17	2	2	2	3	2	2
1	1930	1	17	2	2	2	1	1	1
1	1931	1	16	2	2	2	3	2	2
1	1932	1	16	2	2	2	9	1	2
1	1933	1	15	2	2	2	2	2	1
1	1934	1	15	2	2	2	3	1	1
1	1935	2	15	2	2	2	3	1	3
1	1936	1	17	2	2	2	9	2	2
1	1937	2	14	1	2	1	3	2	2
1	1938	2	14	1	2	1	3	1	1
1	1939	2	19	2	2	2	1	1	2
1	1940	2	15	2	2	2	1	1	3
1	1941	2	17	2	2	2	1	1	2
1	1942	2	16	2	2	2	1	1	1
1	1943	2	18	2	2	2	1	1	1
1	1944	2	15	2	2	2	1	1	2
1	1945	2	16	2	2	2	1	1	1
1	1946	2	15	2	2	2	1	1	3
1	1947	2	15	2	2	2	1	3	3
1	1948	2	15	2	2	2	1	1	2
1	1949	2	15	2	2	2	1	1	2
1	1950	2	15	2	2	2	1	1	1
1	1951	2	14	1	2	2	1	1	2
1	1952	2	16	2	2	2	1	1	2
1	1953	2	14	1	2	2	1	1	2
1	1954	2	0	#NULL!	2	2	1	1	2
1	1955	2	17	2	2	1	1	1	2
1	1956	2	14	1	2	1	1	1	2
1	1957	2	14	1	2	1	1	3	2
1	1958	2	15	2	2	1	1	1	2
1	1959	2	14	1	2	1	1	1	1
1	1960	2	15	2	2	1	1	1	1
1	1961	2	15	2	2	1	1	1	1
1	1962	2	14	1	2	1	1	1	3
1	1963	2	15	2	2	1	1	1	1
1	1964	2	14	1	2	1	1	1	2
1	1965	2	15	2	2	1	1	1	1
1	1966	2	15	2	2	1	1	1	1
1	1967	2	16	2	2	1	1	1	2
1	1968	2	13	1	2	1	1	1	2
1	1969	2	15	2	2	1	1	1	2
1	1970	2	14	1	2	1	1	1	3
1	1971	2	14	1	2	1	1	1	1
1	1972	2	14	1	2	1	1	1	1
1	1973	2	13	1	9	1	9	1	3

1	1974	2	15	2	2	1	1	1	3
1	1975	2	15	2	2	1	1	2	2
1	1976	2	13	1	2	3	1	2	2
1	1977	2	13	1	2	1	1	1	2
1	1978	2	17	2	2	1	1	1	3
1	1979	2	15	2	2	1	1	1	2
1	1980	2	15	2	2	1	1	2	2
1	1981	2	15	2	2	1	1	1	2
1	1982	2	14	1	2	1	1	1	2
1	1983	2	15	2	2	1	1	1	2
1	1984	2	14	1	2	1	1	1	3
1	1985	1	15	2	2	1	1	1	2
1	1986	2	16	2	2	1	1	1	3
1	1987	2	17	2	2	1	1	1	1
1	1988	2	15	2	2	1	1	1	1
1	1989	2	15	2	2	1	1	1	2
1	1990	2	0	#NULL!	2	2	1	1	2
1	1991	2	15	2	2	1	1	1	1
1	1992	2	15	2	2	1	1	1	1
1	1993	2	16	2	2	1	1	1	1
1	1994	2	17	2	2	2	1	1	2
1	1995	2	0	#NULL!	2	2	1	1	2
1	1996	2	16	2	2	1	1	1	2
1	1997	2	14	1	9	1	1	1	2
1	1998	2	14	1	2	1	1	1	2
1	1999	2	16	2	2	1	1	1	2
1	2000	2	15	2	2	1	1	1	2
1	2001	2	15	2	2	1	1	1	2
1	2002	2	16	2	2	1	1	1	2
1	2003	2	16	2	2	1	1	1	2
1	2004	2	15	2	2	1	1	1	1
1	2005	2	16	2	2	1	1	1	2
1	2006	2	15	2	2	1	1	1	1
1	2007	2	16	2	2	1	1	2	2
1	2008	2	15	2	2	1	1	1	1
1	2009	2	15	2	2	1	1	1	3
1	2010	2	16	2	2	1	1	1	2
1	2011	2	15	2	2	1	1	1	2
1	2012	2	14	1	2	1	1	1	1
1	2013	2	15	2	9	1	1	2	2
1	2014	2	15	2	2	1	1	1	1
1	2015	2	13	1	2	1	1	1	2
1	2016	2	14	1	2	1	1	1	2
1	2017	2	15	2	2	1	1	1	2
1	2018	2	15	2	2	1	1	1	2
1	2019	2	15	2	2	1	1	1	2
1	2020	2	15	2	2	1	1	1	2

1	2021	2	15	2	2	1	1	1	1
1	2022	2	15	2	2	1	1	1	3
1	2023	2	17	2	2	3	1	1	1
1	2024	2	17	2	2	3	1	2	2
1	2025	2	16	2	2	3	1	1	1
1	2026	2	18	2	2	3	1	1	1
1	2027	2	17	2	2	3	1	1	2
1	2028	2	15	2	2	3	1	1	2
1	2029	2	17	2	2	3	1	1	2
1	2030	2	16	2	2	3	1	1	1
1	2031	2	0	#NULL!	2	3	1	1	1
1	2032	2	17	2	2	3	1	1	1
1	2033	2	18	2	2	3	1	1	2
1	2034	2	19	2	2	3	1	1	2
1	2035	2	15	2	2	3	1	3	2
1	2036	2	0	#NULL!	2	3	3	1	2
1	2037	2	17	2	2	3	1	1	9
1	2038	2	17	2	2	3	1	1	1
1	2039	2	17	2	2	3	1	1	1
1	2040	2	15	2	2	3	1	1	2
1	2041	2	17	2	2	3	1	1	9
1	2042	2	17	2	2	3	1	1	2
1	2043	2	17	2	2	3	1	1	2
1	2044	2	18	2	2	3	1	1	2
1	2045	2	18	2	2	3	1	1	2
1	2046	2	17	2	1	3	1	2	3
1	2047	2	17	2	2	3	1	1	1
1	2048	2	17	2	2	3	1	1	1
1	2049	2	17	2	2	3	1	1	1
1	2050	2	15	2	2	3	1	1	2
1	2051	2	16	2	2	3	1	2	1
1	2052	2	10	1	2	3	1	1	2
1	2053	9	0	#NULL!	2	3	1	1	2
1	2054	2	16	2	2	3	1	1	2
1	2055	2	17	2	2	3	1	1	1
1	2056	2	17	2	2	3	1	1	1
1	2057	2	17	2	2	3	1	1	2
1	2058	2	17	2	2	3	1	1	1
1	2059	2	17	2	2	3	1	1	1
1	2060	2	18	2	2	3	1	1	2
1	2061	2	17	2	2	3	1	1	1
1	2062	2	15	2	2	2	1	2	2
1	2063	2	16	2	2	2	1	1	1
1	2064	2	16	2	9	2	1	1	2
1	2065	2	16	2	2	2	1	1	1
1	2066	2	14	1	2	2	1	1	2
1	2067	2	15	2	2	2	1	1	2

1	2068	2	15	2	2	2	1	1	2
1	2069	2	16	2	2	2	1	1	2
1	2070	2	17	2	2	2	1	1	2
1	2071	2	15	2	2	2	1	3	2
1	2072	2	0	#NULL!	2	2	1	1	2
1	2073	2	17	2	2	3	1	1	1
1	2074	2	16	2	2	2	3	1	1
1	2075	1	19	2	2	2	2	1	1
1	2076	1	17	2	2	2	2	2	2
1	2077	1	16	2	2	1	2	1	2
1	2078	1	16	2	2	1	2	1	2
1	2079	1	15	2	1	1	2	2	2
1	2080	1	20	3	2	1	2	1	1
1	2081	1	15	2	2	1	2	3	3
1	2082	1	15	2	2	1	2	1	2
1	2083	9	15	2	2	1	2	1	2
1	2084	1	14	1	2	1	2	1	1
1	2085	1	13	1	2	1	2	3	3
1	2086	1	17	2	2	1	2	1	3
1	2087	1	0	#NULL!	2	1	1	1	1
1	2088	1	17	2	2	1	2	2	2
1	2089	1	16	2	2	1	2	3	2
1	2090	1	19	2	2	1	2	1	2
1	2091	1	15	2	2	2	2	1	2
1	2092	1	10	1	2	2	2	2	1
1	2093	1	0	#NULL!	2	2	2	1	2
1	2094	1	17	2	2	2	2	1	2
1	2095	1	10	1	2	2	2	2	1
1	2096	1	13	1	2	2	2	1	3
1	2097	1	17	2	2	2	2	1	1
1	2098	1	14	1	2	2	2	1	1
1	2099	1	17	2	2	2	2	1	2
1	2100	1	19	2	2	2	2	2	2
1	2101	1	18	2	2	2	2	3	2
1	2102	9	0	#NULL!	2	2	1	1	1
1	2103	1	16	2	2	2	2	1	2
1	2104	1	18	2	2	2	2	1	1
1	2105	1	18	2	2	2	2	1	1
1	2106	1	18	2	2	2	2	2	2
1	2107	1	19	2	9	2	9	9	9
1	2108	1	16	2	9	9	9	9	9
1	2109	1	0	#NULL!	2	2	2	1	2
1	2110	1	17	2	2	2	2	1	2
1	2111	1	17	2	2	2	2	1	2
1	2112	1	17	2	2	3	3	1	1
1	2113	2	17	2	2	3	3	1	1
1	2114	1	17	2	2	2	2	1	2

1	2115	1	0	#NULL!	2	2	2	1	2
1	2116	1	16	2	2	2	2	1	2
1	2117	1	16	2	2	2	2	2	1
1	2118	1	17	2	2	2	2	1	2
1	2119	1	14	1	2	2	2	1	2
1	2120	1	16	2	2	2	2	1	3
1	2121	1	18	2	2	1	2	1	2
1	2122	1	16	2	2	2	2	1	2
1	2123	1	17	2	2	3	2	1	2
1	2124	1	17	2	2	3	2	2	1
1	2125	1	20	3	2	3	2	1	1
1	2126	1	18	2	2	3	2	1	2
1	2127	1	19	2	2	3	2	1	2
1	2128	1	16	2	2	3	2	1	2
1	2129	1	18	2	2	3	2	1	1
1	2130	1	17	2	2	3	2	2	1
1	2131	1	18	2	2	3	2	1	1
1	2132	1	20	3	2	3	2	1	2
1	2133	1	18	2	2	3	2	1	1
1	2134	1	17	2	2	1	2	1	1
1	2135	1	18	2	2	1	1	1	1
1	2136	1	18	2	2	3	2	2	2
1	2137	1	18	2	2	3	3	3	2
1	2138	2	18	2	2	3	3	1	1
1	2139	2	16	2	2	3	3	1	1
1	2140	2	18	2	2	3	3	1	1
1	2141	2	16	2	2	3	3	1	1
1	2142	1	15	2	2	3	3	1	3
1	2143	2	15	2	2	3	3	2	2
1	2144	2	17	2	2	3	3	1	1
1	2145	1	15	2	2	3	3	1	1
1	2146	2	16	2	2	3	3	1	1
1	2147	1	17	2	2	3	3	1	2
1	2148	2	16	2	2	3	3	1	3
1	2149	2	15	2	2	3	3	1	2
1	2150	1	19	2	2	3	3	1	3
1	2151	1	17	2	2	3	3	1	2
1	2152	2	18	2	2	3	3	1	2
1	2153	2	19	2	2	3	3	1	1
1	2154	1	15	2	2	2	2	1	1
1	2155	1	17	2	2	3	3	1	2
1	2156	2	14	1	2	1	3	1	1
1	2157	2	15	2	2	1	3	1	2
1	2158	2	15	2	2	1	3	1	2
1	2159	2	16	2	2	1	2	1	2
1	2160	9	18	2	2	2	2	1	1
1	2161	1	0	#NULL!	2	1	2	1	2

1	2162	1	13	1	2	1	2	1	1
1	2163	9	0	#NULL!	2	1	3	3	3
1	2164	1	15	2	2	1	2	2	2
1	2165	9	0	#NULL!	9	9	9	9	9
1	2166	1	17	2	2	1	2	1	2
1	2167	1	17	2	2	1	2	1	1
1	2168	1	19	2	2	1	2	1	2
1	2169	1	19	2	9	9	2	2	9
1	2170	9	25	3	2	3	2	2	2
1	2171	1	15	2	2	1	2	1	1
1	2172	1	0	#NULL!	2	1	2	2	2
1	2173	1	16	2	2	1	2	2	2
1	2174	1	19	2	2	1	1	2	2
1	2175	1	16	2	2	1	2	1	2
1	2176	1	15	2	2	1	2	1	2
1	2177	9	0	#NULL!	2	1	3	1	2
1	2178	1	14	1	2	1	2	1	2
1	2179	1	0	#NULL!	2	1	2	1	3
1	2180	1	0	#NULL!	2	1	2	1	2
1	2181	1	0	#NULL!	2	1	2	2	2
1	2182	1	18	2	2	1	2	1	2
1	2183	9	0	#NULL!	2	1	2	1	2
1	2184	1	16	2	2	1	2	2	2
1	2185	1	14	1	1	1	1	1	1
1	2186	9	0	#NULL!	9	9	9	9	9
1	2187	2	16	2	2	1	3	1	2
1	2188	2	18	2	2	1	3	1	2
1	2189	2	16	2	2	1	3	1	1
1	2190	9	0	#NULL!	9	9	9	9	9
1	2191	9	0	#NULL!	9	9	9	9	9
1	2192	9	0	#NULL!	9	9	9	9	9
1	2193	9	0	#NULL!	9	9	9	9	9
1	2194	9	0	#NULL!	9	9	9	9	9
1	2195	2	20	3	2	2	3	1	2
1	2196	1	17	2	2	2	3	1	2
1	2197	9	0	#NULL!	2	2	3	1	9
1	2198	1	16	2	2	2	3	2	2
1	2199	2	15	2	2	2	3	1	3
1	2200	9	15	2	2	1	3	1	1
1	2201	1	17	2	2	2	3	1	2
1	2202	2	12	1	2	3	3	1	1
1	2203	2	17	2	2	3	3	1	3
1	2204	2	17	2	2	3	3	1	1
1	2205	2	18	2	2	3	3	1	2
1	2206	2	16	2	2	3	3	1	1
1	2207	2	15	2	2	3	3	1	1
1	2208	1	16	2	2	3	3	1	1

1	2209	1	19	2	2	3	3	1	3
1	2210	2	12	1	9	3	3	1	1
1	2211	2	16	2	2	3	3	1	1
1	2212	1	16	2	2	1	3	2	2
1	2213	1	20	3	2	1	3	2	2
1	2214	1	14	1	2	1	3	1	1
1	2215	2	17	2	2	1	3	2	2
1	2216	1	13	1	2	1	3	2	3
1	2217	2	18	2	2	1	3	1	3
1	2218	2	12	1	2	1	3	2	9
1	2219	2	17	2	2	1	3	1	2
1	2220	1	13	1	2	1	3	2	2
1	2221	2	16	2	9	2	3	1	1
1	2222	1	18	2	2	2	2	2	2
1	2223	1	15	2	2	2	2	1	2
1	2224	1	17	2	9	9	9	9	9
1	2225	1	16	2	2	2	2	1	2
1	2226	1	17	2	2	2	2	1	2
1	2227	1	17	2	2	2	2	1	2
1	2228	1	17	2	2	2	2	1	2
1	2229	1	17	2	2	2	2	1	2
1	2230	1	18	2	2	1	2	1	2
1	2231	1	18	2	2	2	2	1	2
1	2232	1	16	2	2	2	2	1	2
1	2233	1	16	2	2	2	2	1	1
1	2234	1	17	2	2	2	2	1	2
1	2235	1	15	2	2	2	2	1	3
1	2236	1	15	2	2	2	2	1	1
1	2237	1	17	2	2	2	2	1	2
1	2238	1	18	2	2	2	2	2	2
1	2239	1	16	2	2	2	2	1	2
1	2240	1	19	2	2	3	2	2	2
1	2241	1	18	2	2	3	2	1	2
1	2242	1	18	2	2	3	3	1	3
1	2243	1	0	#NULL!	2	3	2	1	2
1	2244	1	0	#NULL!	2	3	2	1	2
1	2245	1	25	3	2	3	2	1	1
1	2246	1	19	2	2	3	2	1	3
1	2247	1	18	2	2	3	2	1	2
1	2248	1	18	2	2	3	2	1	2
1	2249	1	30	3	2	3	2	1	1
1	2250	2	14	1	2	1	3	1	3
1	2251	9	16	2	2	1	3	1	3
1	2252	2	15	2	2	1	3	1	3
1	2253	2	15	2	2	1	3	1	1
1	2254	9	0	#NULL!	9	1	3	1	1
1	2255	2	14	1	2	1	3	1	2

1	2256	1	18	2	2	1	3	1	2
1	2257	1	16	2	2	1	3	2	2
1	2258	2	0	#NULL!	2	1	3	1	1
1	2259	2	14	1	2	1	3	1	1
1	2260	1	15	2	2	1	3	1	3
1	2261	1	15	2	2	1	3	1	3
1	2262	2	16	2	2	2	3	1	2
1	2263	2	15	2	2	2	3	1	2
1	2264	2	15	2	2	2	3	1	2
1	2265	2	16	2	2	2	3	1	2
1	2266	2	16	2	2	2	3	1	2
1	2267	2	13	1	2	2	3	1	2
1	2268	9	16	2	2	2	2	2	2
1	2269	2	15	2	2	2	3	1	2
1	2270	2	16	2	2	2	3	1	1
1	2271	1	18	2	2	2	3	1	2
1	2272	2	13	1	2	2	3	1	1
1	2273	2	15	2	2	2	3	1	2
1	2274	2	17	2	2	2	3	1	2
1	2275	2	15	2	2	2	3	1	1
1	2276	1	16	2	2	2	3	1	1
1	2277	2	15	2	2	2	3	1	2
1	2278	1	15	2	2	2	3	1	2
1	2279	2	15	2	2	2	3	1	2
1	2280	1	17	2	2	2	3	1	2
1	2281	1	15	2	2	2	3	1	2
1	2282	1	18	2	2	2	2	1	2
1	2283	9	0	#NULL!	2	2	2	2	2
1	2284	1	17	2	2	2	2	2	2
1	2285	1	16	2	2	2	2	1	2
1	2286	1	15	2	2	2	2	1	3
1	2287	1	16	2	2	2	2	1	2
1	2288	1	18	2	2	3	2	1	2
1	2289	1	20	3	2	3	2	1	2
1	2290	1	17	2	2	3	2	2	3
1	2291	1	19	2	2	3	2	2	1
1	2292	1	17	2	2	3	2	1	2
1	2293	1	18	2	2	3	2	1	2
1	2294	1	18	2	2	3	2	1	1
1	2295	1	18	2	2	3	2	1	2
1	2296	1	19	2	2	3	2	2	2
1	2297	9	19	2	2	3	2	1	1
1	2298	1	15	2	2	1	2	1	2
1	2299	1	17	2	2	1	2	2	2
1	2300	1	14	1	2	1	2	1	1
1	2301	1	14	1	2	1	2	1	1
1	2302	1	14	1	2	1	2	1	1

1	2303	1	16	2	2	1	2	1	2
1	2304	2	14	1	2	1	3	1	1
1	2305	2	17	2	2	1	3	1	2
1	2306	2	0	#NULL!	2	3	3	2	2
1	2307	1	14	1	2	1	3	1	2
1	2308	2	17	2	2	3	3	1	1
1	2309	2	16	2	2	3	3	1	3
1	2310	2	0	#NULL!	2	3	1	1	1
1	2311	1	17	2	2	3	3	1	1
1	2312	2	16	2	2	3	3	1	2
1	2313	1	17	2	2	3	3	1	1
1	2314	2	16	2	2	3	3	1	2
1	2315	2	14	1	2	3	3	1	1
1	2316	2	17	2	2	3	3	1	1
1	2317	1	17	2	2	3	3	1	1
1	2318	1	13	1	2	2	3	1	1
1	2319	1	16	2	2	3	3	1	3
1	2320	1	16	2	2	3	3	1	3
1	2321	2	16	2	2	3	3	1	2
1	2322	1	17	2	2	3	3	1	2
1	2323	2	18	2	2	3	3	1	1
1	2324	1	18	2	2	3	3	1	3
1	2325	1	17	2	2	3	3	2	2
1	2326	1	15	2	2	3	3	1	1
1	2327	2	17	2	2	3	3	1	1
1	2328	1	18	2	2	3	3	1	2
1	2329	2	15	2	2	3	3	1	1
1	2330	2	0	#NULL!	2	3	3	1	2
1	2331	1	15	2	2	3	3	1	2
1	2332	2	16	2	2	3	3	1	1
1	2333	1	16	2	2	3	3	1	1
1	2334	2	14	1	2	3	3	1	1
1	2335	1	17	2	2	3	3	1	1
1	2336	1	16	2	2	3	3	1	1
1	2337	1	17	2	2	3	3	1	1
1	2338	2	17	2	2	3	3	1	2
1	2339	2	14	1	2	3	1	2	3
1	2340	1	14	1	2	3	3	2	2
1	2341	1	17	2	2	3	3	1	1
1	2342	2	0	#NULL!	2	2	3	1	1
1	2343	2	15	2	2	2	3	1	2
1	2344	9	15	2	2	2	3	1	1
1	2345	1	15	2	2	1	3	1	2
1	2346	1	17	2	2	1	3	2	2
1	2347	2	16	2	2	1	3	2	1
1	2348	1	16	2	2	1	3	1	2
1	2349	2	16	2	2	1	3	1	3

1	2350	1	16	2	2	1	3	1	2
1	2351	2	17	2	2	1	3	3	2
1	2352	2	15	2	2	1	3	1	2
1	2353	2	15	2	2	1	3	1	3
1	2354	1	13	1	2	1	3	1	3
1	2355	2	14	1	2	1	3	1	2
1	2356	2	15	2	2	1	3	1	1
1	2357	2	14	1	2	1	3	1	2
1	2358	2	14	1	2	1	3	1	1
1	2359	2	14	1	2	1	3	1	2
1	2360	2	15	2	2	1	3	1	2
1	2361	2	15	2	2	1	3	1	2
1	2362	2	14	1	2	1	3	1	2
1	2363	1	17	2	2	1	3	2	1
1	2364	2	16	2	2	3	3	1	2
1	2365	2	16	2	2	3	3	1	1
1	2366	2	17	2	2	3	3	1	2
1	2367	2	0	#NULL!	2	3	3	2	1
1	2368	2	0	#NULL!	2	3	3	1	2
1	2369	1	17	2	2	3	3	1	1
1	2370	1	15	2	2	3	3	1	1
1	2371	2	16	2	2	3	3	1	1
1	2372	2	16	2	2	3	3	1	1
1	2373	1	17	2	2	3	3	1	1
1	2374	2	16	2	2	3	3	1	2
1	2375	1	17	2	2	3	3	1	2
1	2376	2	17	2	2	3	3	1	1
1	2377	2	16	2	2	3	3	1	2
1	2378	1	17	2	2	3	3	1	1
1	2379	2	17	2	2	3	3	1	2
1	2380	2	17	2	2	3	3	1	1
1	2381	2	16	2	2	3	3	1	1
1	2382	1	16	2	2	1	3	1	2
1	2383	1	16	2	2	1	3	1	2
1	2384	2	17	2	2	1	3	1	1
1	2385	9	0	#NULL!	2	1	1	1	2
1	2386	2	15	2	2	1	3	1	2
1	2387	2	14	1	2	1	3	1	3
1	2388	2	15	2	2	1	3	1	1
1	2389	1	15	2	2	1	3	2	2
1	2390	2	13	1	2	1	3	2	3
1	2391	2	12	1	2	1	3	1	2
1	2392	9	0	#NULL!	9	9	9	9	9
1	2393	2	0	#NULL!	2	1	3	1	1
1	2394	1	14	1	2	1	3	1	2
1	2395	2	17	2	2	1	3	1	1
1	2396	2	16	2	2	1	3	1	3

1	2397	2	14	1	2	1	3	1	2
1	2398	2	16	2	2	2	3	1	1
1	2399	2	15	2	2	2	3	1	2
1	2400	2	15	2	2	2	3	1	1
1	2401	1	17	2	2	2	3	2	2
1	2402	1	14	1	2	2	3	1	2
1	2403	1	16	2	2	1	3	2	2
1	2404	1	16	2	2	1	3	1	1
1	2405	1	18	2	2	1	3	2	1
1	2406	2	15	2	2	2	3	1	2
1	2407	1	16	2	2	2	3	1	2
1	2408	2	16	2	2	2	3	1	2
1	2409	2	15	2	2	2	3	1	2
1	2410	2	17	2	2	3	3	1	3
1	2411	1	16	2	2	2	3	1	2
1	2412	2	15	2	2	2	3	3	2
1	2413	2	14	1	2	1	3	1	2
1	2414	2	15	2	2	2	3	1	2
1	2415	2	14	1	2	2	3	1	3
1	2416	1	16	2	2	2	3	1	2
1	2417	1	17	2	2	2	3	1	2
1	2418	2	16	2	2	2	3	2	2
1	2419	2	15	2	2	2	3	1	2
1	2420	2	15	2	2	2	3	3	1
1	2421	9	0	#NULL!	9	9	9	9	9
1	2422	2	15	2	2	2	3	1	1
1	2423	1	15	2	2	1	3	1	2
1	2424	2	15	2	2	2	1	1	1
1	2425	2	0	#NULL!	2	2	3	2	2
1	2426	2	18	2	2	2	3	2	2
1	2427	2	16	2	2	2	3	1	3
1	2428	2	14	1	2	2	3	1	3
1	2429	2	16	2	2	2	3	2	1
1	2430	2	17	2	2	2	3	1	2
1	2431	2	15	2	2	2	3	1	2
1	2432	2	15	2	2	2	3	1	2
1	2433	2	17	2	2	2	3	1	2
1	2434	2	15	2	2	2	3	2	2
1	2435	2	16	2	2	2	3	1	2
1	2436	2	16	2	2	2	3	1	2
1	2437	2	17	2	2	2	3	1	2
1	2438	2	16	2	2	2	3	1	1
1	2439	2	16	2	2	2	3	1	2
1	2440	1	16	2	2	2	3	1	2
1	2441	2	16	2	2	2	3	1	1
1	2442	9	0	#NULL!	9	9	9	9	9
1	2443	2	15	2	2	2	3	1	1

1	2444	2	17	2	2	2	3	1	2
1	2445	1	18	2	2	2	3	1	2
1	2446	2	15	2	2	2	3	1	2
1	2447	2	16	2	2	2	3	2	2
1	2448	2	17	2	2	2	3	1	2
1	2449	2	15	2	2	2	3	1	2
1	2450	1	20	3	2	2	3	2	2
1	2451	2	16	2	2	2	3	3	1
1	2452	2	17	2	2	2	3	1	2
1	2453	2	15	2	2	3	3	2	2
1	2454	2	16	2	2	2	3	2	1
1	2455	1	18	2	2	2	2	1	2
1	2456	1	20	3	2	2	2	1	2
1	2457	1	17	2	2	2	2	1	2
1	2458	1	15	2	2	2	2	1	1
1	2459	1	18	2	2	2	2	1	3
1	2460	1	20	3	9	2	2	2	2
1	2461	2	16	2	2	3	3	1	2
1	2462	2	13	1	2	1	3	1	2
1	2463	2	13	1	2	2	3	1	2
1	2464	9	0	#NULL!	9	9	9	9	9
1	2465	2	15	2	2	1	3	1	2
1	2466	1	15	2	2	2	3	1	1
1	2467	2	16	2	2	3	3	2	1
1	2468	9	17	2	2	3	3	2	1
1	2469	2	16	2	2	3	3	1	2
1	2470	2	16	2	2	3	3	2	2
1	2471	1	15	2	2	2	3	2	1
1	2472	2	16	2	2	3	3	2	1
1	2473	2	16	2	2	3	3	2	1
1	2474	9	0	#NULL!	9	9	9	9	9
1	2475	9	0	#NULL!	9	9	9	9	9
1	2476	9	0	#NULL!	9	9	9	9	9
1	2477	2	15	2	2	1	3	1	2
1	2478	2	15	2	2	1	3	2	2
1	2479	2	15	2	2	1	3	1	2
1	2480	2	15	2	2	1	3	1	1
1	2481	2	15	2	2	2	3	1	2
1	2482	2	16	2	2	2	3	1	1
1	2483	2	16	2	2	2	3	1	2
1	2484	2	15	2	2	2	3	1	2
1	2485	2	16	2	2	2	3	1	3
1	2486	1	16	2	2	2	3	1	2
1	2487	2	15	2	2	2	3	1	1
1	2488	2	15	2	2	2	3	1	2
1	2489	1	9	1	2	2	3	1	2
1	2490	9	0	#NULL!	9	9	9	9	9

1	2491	2	16	2	2	2	3	2	1
1	2492	2	16	2	2	2	3	1	2
1	2493	2	15	2	2	2	3	2	1
1	2494	1	0	#NULL!	2	2	3	1	2
1	2495	2	16	2	2	2	3	1	2
1	2496	9	0	#NULL!	9	9	9	9	9
1	2497	2	16	2	2	2	3	1	1
1	2498	1	18	2	2	3	3	1	1
1	2499	1	17	2	2	3	3	1	3
1	2500	1	19	2	2	3	3	1	1
1	2501	1	19	2	2	3	3	1	1
1	2502	1	15	2	2	1	3	1	2
1	2503	1	17	2	2	3	3	1	3
1	2504	2	17	2	2	3	3	1	2
1	2505	1	17	2	2	3	3	1	2
1	2506	1	18	2	2	3	3	1	1
1	2507	1	18	2	2	3	3	1	3
1	2508	1	14	1	2	3	3	1	1
1	2509	2	17	2	2	3	3	1	1
1	2510	1	15	2	2	3	3	1	2
1	2511	2	16	2	2	3	3	1	2
1	2512	2	16	2	2	3	3	1	3
1	2513	2	16	2	2	3	3	1	1
1	2514	1	19	2	2	3	3	1	2
1	2515	1	19	2	2	3	3	1	1
1	2516	2	16	2	2	3	3	1	1
1	2517	2	16	2	2	3	3	1	2
1	2518	2	16	2	2	3	3	1	2
1	2519	2	14	1	2	3	3	1	3
1	2520	2	16	2	2	3	3	1	1
1	2521	1	17	2	2	3	3	1	1
1	2522	2	16	2	2	3	3	1	1
1	2523	2	16	2	2	3	3	2	2
1	2524	2	16	2	2	3	3	2	2
1	2525	2	17	2	2	3	3	1	3
1	2526	2	16	2	2	3	3	1	2
1	2527	2	17	2	2	3	3	1	2
1	2528	2	16	2	2	3	3	1	3
1	2529	2	16	2	2	3	3	1	1
1	2530	2	17	2	2	3	3	1	3
1	2531	2	16	2	2	3	3	1	2
1	2532	2	15	2	2	3	3	1	1
1	2533	2	15	2	2	3	3	1	2
1	2534	2	17	2	2	3	3	1	1
1	2535	2	16	2	2	3	3	1	1
1	2536	2	17	2	2	3	3	1	2
1	2537	2	17	2	2	3	3	1	2

1	2538	2	20	3	2	3	3	1	2
1	2539	2	15	2	2	3	3	1	1
1	2540	2	19	2	2	3	3	1	2
1	2541	2	17	2	2	3	3	1	3
1	2542	2	16	2	2	3	3	1	2
1	2543	2	16	2	2	2	3	1	1
1	2544	2	16	2	2	1	3	1	2
1	2545	1	15	2	2	1	3	1	2
1	2546	2	14	1	1	1	3	1	1
1	2547	1	15	2	2	1	3	2	1
1	2548	2	19	2	2	2	3	1	2
1	2549	1	18	2	2	3	2	1	2
1	2550	2	18	2	2	3	3	1	1
1	2551	2	15	2	2	3	3	1	2
1	2552	1	19	2	2	3	2	1	2
1	2553	1	18	2	2	3	2	1	2
1	2554	1	15	2	2	3	2	1	2
1	2555	1	17	2	2	3	2	1	1
1	2556	1	16	2	2	3	2	2	1
1	2557	2	17	2	2	3	3	1	2
1	2558	2	16	2	2	3	3	1	1
1	2559	2	17	2	2	3	3	1	3
1	2560	2	17	2	2	3	3	1	2
1	2561	2	18	2	2	3	3	1	2
1	2562	1	16	2	2	3	3	1	1
1	2563	2	16	2	2	3	3	1	1
1	2564	2	16	2	2	3	3	1	1
1	2565	1	17	2	2	3	3	1	1
1	2566	1	17	2	2	2	3	1	3
1	2567	2	15	2	2	2	3	2	2
1	2568	2	16	2	2	2	3	1	9
1	2569	1	0	#NULL!	2	2	3	1	2
1	2570	2	19	2	2	2	3	1	2
1	2571	1	14	1	2	1	3	1	1
1	2572	1	15	2	2	1	3	1	1
1	2573	2	15	2	2	1	3	1	9
1	2574	2	15	2	2	2	3	1	1
1	2575	2	16	2	2	2	3	1	2
1	2576	2	15	2	2	2	3	1	2
1	2577	2	16	2	2	2	3	1	3
1	2578	1	16	2	2	2	2	1	2
1	2579	2	16	2	2	2	3	1	2
1	2580	2	16	2	2	2	3	1	2
1	2581	1	16	2	2	2	2	1	1
1	2582	1	16	2	2	2	3	1	9
1	2583	2	18	2	2	2	3	2	1
1	2584	2	15	2	2	2	3	1	2

1	2585	2	15	2	2	2	3	1	2
1	2586	2	16	2	2	2	3	1	3
1	2587	2	0	#NULL!	2	2	3	1	1
1	2588	2	16	2	2	2	3	1	3
1	2589	2	17	2	2	2	3	1	2
1	2590	1	0	#NULL!	2	1	3	1	2
1	2591	2	15	2	2	2	2	2	1
1	2592	2	16	2	2	2	3	1	2
1	2593	2	15	2	2	2	3	3	3
1	2594	2	16	2	2	1	3	1	2
1	2595	2	17	2	2	1	3	1	1
1	2596	2	17	2	2	3	3	1	1
1	2597	1	20	3	2	2	3	1	9
1	2598	1	16	2	2	2	3	1	2
1	2599	2	16	2	2	2	3	1	2
1	2600	2	14	1	2	1	3	1	2
1	2601	1	0	#NULL!	2	1	3	1	2
1	2602	2	0	#NULL!	2	1	3	1	1
1	2603	2	15	2	2	1	3	1	1
1	2604	2	15	2	2	1	3	1	2
1	2605	2	15	2	2	1	3	1	2
1	2606	2	18	2	2	2	3	1	2
1	2607	1	16	2	2	2	3	1	3
1	2608	2	16	2	2	2	3	1	1
1	2609	2	19	2	2	2	1	1	1
1	2610	2	16	2	2	2	3	1	2
1	2611	1	19	2	2	2	2	1	1
1	2612	1	16	2	2	2	3	1	1
1	2613	2	14	1	2	2	3	1	2
1	2614	9	16	2	2	3	3	1	2
1	2615	2	17	2	2	1	3	1	2
1	2616	2	15	2	2	1	3	1	2
1	2617	1	15	2	2	1	3	1	2
1	2618	1	15	2	2	1	3	2	2
1	2619	1	15	2	2	1	3	2	2
1	2620	1	16	2	2	1	3	1	2
1	2621	1	16	2	2	1	3	1	2
1	2622	1	16	2	2	1	3	1	1
1	2623	1	18	2	2	1	3	1	3
1	2624	2	14	1	2	1	3	1	1
1	2625	2	17	2	2	1	3	1	1
1	2626	2	14	1	2	1	3	1	1
1	2627	2	15	2	2	1	3	1	1
1	2628	2	15	2	2	1	3	1	1
1	2629	2	15	2	2	1	3	1	1
1	2630	1	14	1	2	1	3	1	1
1	2631	1	15	2	2	1	3	1	2

1	2632	1	15	2	2	1	3	1	2
1	2633	1	16	2	2	1	3	1	2
1	2634	2	14	1	2	1	3	1	2
1	2635	2	15	2	2	1	3	1	1
1	2636	2	15	2	2	1	3	1	2
1	2637	2	20	3	2	3	3	1	2
1	2638	2	19	2	2	3	3	1	2
1	2639	1	16	2	2	3	3	1	2
1	2640	2	17	2	2	1	3	1	3
1	2641	1	13	1	2	1	3	2	2
1	2642	2	14	1	2	1	3	1	1
1	2643	2	14	1	2	1	3	1	2
1	2644	1	14	1	2	1	3	1	2
1	2645	2	0	#NULL!	2	1	3	9	1
1	2646	1	15	2	2	1	3	2	2
1	2647	2	15	2	2	3	3	1	1
1	2648	2	17	2	2	1	3	1	3
1	2649	1	22	3	2	3	2	1	1
1	2650	1	18	2	2	3	2	1	1
1	2651	1	19	2	2	3	2	1	1
1	2652	2	17	2	2	3	3	1	2
1	2653	2	17	2	2	3	3	1	2
1	2654	2	18	2	2	3	3	1	1
1	2655	2	18	2	2	3	2	2	1
1	2656	2	18	2	2	3	2	2	1
1	2657	1	13	1	2	1	3	1	2
1	2658	2	13	1	2	1	3	1	3
1	2659	1	13	1	2	1	3	2	2
1	2660	1	18	2	2	3	3	1	3
1	2661	1	19	2	2	3	2	1	1
1	2662	1	16	2	2	2	3	1	2
1	2663	9	0	#NULL!	9	9	9	9	9
1	2664	9	0	#NULL!	2	3	1	1	1
1	2665	2	17	2	2	1	3	1	1
1	2666	1	15	2	2	1	3	1	2
1	2667	2	14	1	2	1	3	1	1
1	2668	1	15	2	2	1	3	1	2
1	2669	1	14	1	2	1	3	1	2
1	2670	2	14	1	2	1	3	1	3
1	2671	1	16	2	2	1	3	1	2
1	2672	2	16	2	2	1	3	1	1
1	2673	2	16	2	2	1	3	1	1
1	2674	2	15	2	2	1	3	1	1
1	2675	2	14	1	2	1	3	1	1
1	2676	1	17	2	2	3	3	1	1
1	2677	1	14	1	2	1	3	1	2
1	2678	2	15	2	2	1	3	1	3

1	2679	2	18	2	2	1	3	1	1
1	2680	2	13	1	2	1	3	1	2
1	2681	2	15	2	2	1	3	1	3
1	2682	1	14	1	2	1	3	1	2
1	2683	2	15	2	2	1	3	1	1
1	2684	2	14	1	2	3	3	1	1
1	2685	2	15	2	2	3	3	1	1
4	1	2	16	2	1	3	3	1	2
4	2	2	18	2	1	3	3	1	1
4	3	2	18	2	1	3	3	1	1
4	4	2	18	2	1	3	3	1	1
4	5	2	18	2	1	3	3	1	1
4	6	2	18	2	1	3	3	1	1
4	7	2	18	2	1	3	3	1	1
4	8	2	17	2	1	3	3	1	1
4	9	9	9	1	9	9	9	1	1
4	10	2	15	2	1	3	3	1	1
4	11	2	17	2	1	3	3	1	1
4	12	1	17	2	1	3	3	1	1
4	13	2	19	2	1	3	3	1	1
4	14	1	19	2	1	3	3	1	1
4	15	2	18	2	1	3	3	1	1
4	16	2	18	2	1	3	3	1	1
4	17	2	18	2	1	3	3	1	3
4	18	2	18	2	1	3	3	1	1
4	19	2	18	2	1	3	3	1	1
4	20	2	18	2	1	3	3	1	1
4	21	2	18	2	1	3	3	1	1
4	22	2	18	2	1	3	3	1	1
4	23	2	18	2	1	3	3	1	1
4	24	2	17	2	1	3	3	1	1
4	25	2	17	2	1	3	3	1	1
4	26	9	9	1	9	9	9	9	9
4	27	9	9	1	9	9	9	3	3
4	28	9	9	1	9	9	9	3	3
4	29	9	9	1	9	9	9	3	3
4	30	9	9	1	9	9	9	3	3
4	31	9	9	1	9	9	9	1	1
4	32	9	9	1	9	9	9	1	1
4	33	9	9	1	9	9	9	1	1
4	34	1	15	2	1	3	3	3	1
4	35	2	16	2	1	3	3	1	1
4	36	2	16	2	1	3	3	1	1
4	37	2	16	2	1	3	3	1	1
4	38	2	16	2	1	3	3	1	1
4	39	1	18	2	1	3	3	1	3
4	40	1	13	1	1	3	3	1	3

4	41	1	15	2	1	3	3	1	3
4	42	9	9	1	1	3	3	1	1
4	43	9	9	1	1	3	3	1	1
4	44	9	9	1	1	3	3	1	1
4	45	9	9	1	1	3	3	1	1
4	46	2	18	2	1	3	3	2	2
4	47	2	17	2	1	3	3	1	2
4	48	1	15	2	1	3	3	1	2
4	49	2	18	2	1	3	3	1	1
4	50	1	17	2	1	3	3	1	1
4	51	2	20	3	1	3	3	2	2
4	52	2	19	2	1	3	3	1	1
4	53	2	18	2	1	3	3	1	1
4	54	1	20	3	9	9	9	1	1
4	55	1	18	2	1	3	3	1	2
4	56	2	15	2	1	3	3	1	3
4	57	9	9	1	1	3	3	1	1
4	58	1	21	3	1	3	3	1	2
4	59	2	18	2	1	3	3	1	1
4	60	2	17	2	1	3	3	1	1
4	61	2	19	2	1	3	3	1	2
4	62	1	18	2	1	3	3	1	1
4	63	1	19	2	1	3	3	3	3
4	64	1	17	2	1	3	3	1	1
4	65	1	18	2	1	3	3	1	1
4	66	2	18	2	1	3	3	1	1
4	67	2	18	2	1	3	3	1	1
4	68	2	16	2	1	3	3	1	2
4	69	2	17	2	1	3	3	1	1
4	70	1	18	2	1	3	3	1	3
4	71	2	20	3	1	3	3	1	2
4	72	2	18	2	1	3	3	1	1
4	73	2	18	2	1	3	3	1	1
4	74	9	9	1	1	1	1	1	1
4	75	9	9	1	1	1	1	1	1
4	76	9	9	1	9	9	9	9	9
4	77	9	9	1	1	1	1	1	1
4	78	2	19	2	1	3	3	3	3
4	79	9	9	1	1	1	3	1	1
4	80	1	16	2	1	3	3	1	1
4	81	2	18	2	1	3	3	1	1
4	82	2	18	2	1	3	3	1	1
4	83	2	17	2	1	3	3	1	1
4	84	2	18	2	1	3	3	1	1
4	85	9	9	1	9	9	9	9	9
4	86	1	18	2	1	3	3	1	1
4	87	2	18	2	1	3	3	1	1

4	88	2	18	2	1	3	3	1	2
4	89	2	15	2	1	3	3	1	1
4	90	2	17	2	1	3	3	1	1
4	91	2	17	2	1	3	3	1	1
4	92	2	18	2	1	3	3	1	1
4	93	2	18	2	1	3	3	1	1
4	94	2	17	2	1	3	3	1	1
4	95	2	16	2	1	3	3	1	1
4	96	2	18	2	1	3	3	1	1
4	97	1	18	2	1	3	3	1	1
4	98	2	19	2	1	3	3	1	1
4	99	2	18	2	1	3	3	1	1
4	100	2	18	2	1	3	3	1	1
4	101	2	18	2	1	3	3	1	1
4	102	1	18	2	1	3	3	1	2
4	103	2	18	2	1	3	3	1	1
4	104	1	16	2	1	3	3	1	1
4	105	2	18	2	1	3	3	1	1
4	106	9	9	1	1	3	3	1	1
4	107	1	14	1	1	3	3	2	1
4	108	1	15	2	1	3	3	1	1
4	109	1	13	1	1	2	1	2	2
4	110	1	16	2	1	3	3	1	1
4	111	1	16	2	1	3	3	1	1
4	112	1	16	2	1	3	3	1	1
4	113	2	37	3	1	2	2	1	3
4	114	2	16	2	1	3	3	1	1
4	115	2	18	2	1	3	3	1	1
4	116	2	17	2	1	3	3	1	1
4	117	2	16	2	1	3	3	1	1
4	118	1	20	3	1	3	3	1	1
4	119	1	20	3	1	3	3	1	1
4	120	1	18	2	1	2	3	2	2
4	121	2	9	1	1	3	3	1	1
4	122	2	9	1	1	3	3	1	2
4	123	2	9	1	1	3	3	1	1
4	124	2	14	1	1	1	3	3	3
4	125	1	19	2	1	3	3	1	1
4	126	2	15	2	1	3	3	2	2
4	127	2	20	3	1	3	3	1	1
4	128	2	20	3	1	3	3	1	2
4	129	2	20	3	1	3	3	1	2
4	130	2	20	3	1	3	3	1	2
4	131	2	20	3	1	3	3	1	2
4	132	2	20	3	1	3	3	1	1
4	133	2	20	3	1	3	3	1	1
4	134	2	18	2	1	3	3	1	1

4	135	2	17	2	1	3	3	1	1
4	136	1	16	2	1	3	3	1	1
4	137	2	21	3	1	3	3	1	3
4	138	9	9	1	9	9	9	1	1
4	139	2	19	2	1	3	3	1	1
4	140	1	18	2	1	3	3	1	1
4	141	1	17	2	9	3	3	1	1
4	142	1	18	2	1	3	3	1	1
4	143	1	19	2	1	3	3	1	1
4	144	2	16	2	1	3	3	1	2
4	145	2	16	2	1	3	3	1	1
4	146	2	16	2	1	3	3	1	1
4	147	2	16	2	1	3	3	1	2
4	148	2	18	2	1	3	3	1	1
4	149	2	18	2	1	3	3	1	1
4	150	2	18	2	1	3	3	1	1
4	151	2	19	2	1	1	3	2	2
4	152	2	15	2	1	1	1	1	1
4	153	2	18	2	1	3	1	1	1
4	154	1	20	3	1	3	3	1	1
4	155	9	9	1	9	9	9	9	9
4	156	9	9	1	9	9	9	9	9
4	157	2	19	2	1	3	3	3	2
4	158	2	18	2	9	3	3	1	1
4	159	2	15	2	1	3	3	1	1
4	160	1	20	3	1	3	3	2	2
4	161	2	18	2	1	3	3	1	1
4	162	1	18	2	1	3	2	1	3
4	163	1	9	1	1	3	2	2	2
4	164	1	18	2	1	3	2	1	1
4	165	1	18	2	1	3	2	1	1
4	166	1	17	2	1	3	2	1	2
4	167	1	18	2	1	3	2	2	2
4	168	1	18	2	1	3	2	1	1
4	169	1	17	2	1	3	2	1	1
4	170	1	20	3	1	3	2	1	3
4	171	1	18	2	1	3	2	1	2
4	172	1	18	2	1	3	2	1	1
4	173	1	18	2	1	3	2	2	2
4	174	1	18	2	1	3	2	1	1
4	175	1	18	2	1	3	2	1	2
4	176	1	17	2	1	3	2	1	2
4	177	1	9	1	1	3	2	1	2
4	178	1	18	2	1	3	2	1	1
4	179	1	18	2	1	3	2	1	1
4	180	1	20	3	1	3	2	1	2
4	181	1	18	2	1	3	2	1	1

4	182	1	20	3	1	3	2	1	3
4	183	1	17	2	1	3	2	1	2
4	184	1	17	2	1	3	2	1	1
4	185	1	16	2	1	3	2	1	1
4	186	1	18	2	1	3	2	1	2
4	187	1	18	2	1	3	2	1	1
4	188	1	9	1	1	3	2	1	1
4	189	1	18	2	1	3	2	1	2
4	190	1	19	2	1	3	2	1	1
4	191	1	20	3	1	3	2	1	2
4	192	1	18	2	1	3	2	1	3
4	193	1	18	2	1	3	2	1	1
4	194	1	18	2	1	3	2	1	1
4	195	1	17	2	1	3	2	1	1
4	196	1	18	2	1	3	2	1	1
4	197	1	16	2	1	3	2	1	3
4	198	1	18	2	1	3	2	1	2
4	199	1	16	2	1	3	2	1	3
4	200	1	17	2	1	3	2	1	3
4	201	1	18	2	1	3	2	1	2
4	202	1	17	2	1	3	2	1	1
4	203	1	18	2	1	3	2	1	1
4	204	1	17	2	1	3	2	1	1
4	205	1	19	2	1	3	2	1	1
4	206	1	18	2	1	3	2	1	1
4	207	1	17	2	1	3	2	1	1
4	208	1	18	2	1	3	2	1	2
4	209	1	16	2	1	3	2	1	1
4	210	1	18	2	1	3	2	1	1
4	211	1	23	3	1	3	2	1	2
4	212	1	18	2	1	3	2	1	1
4	213	1	18	2	1	3	2	1	2
4	214	1	17	2	1	3	2	1	1
4	215	1	18	2	1	3	2	1	1
4	216	1	17	2	1	3	2	1	2
4	217	1	20	3	1	3	2	1	3
4	218	1	17	2	1	3	2	1	1
4	219	1	17	2	1	3	2	1	2
4	220	1	14	1	1	3	2	1	1
4	221	1	18	2	1	3	2	1	1
4	222	1	18	2	1	3	2	2	2
4	223	1	17	2	1	3	2	1	1
4	224	1	19	2	1	3	2	1	1
4	225	1	18	2	1	3	2	1	1
4	226	1	18	2	1	3	2	1	2
4	227	1	20	3	1	3	2	1	1
4	228	1	17	2	1	3	2	1	3

4	229	1	18	2	1	3	2	1	2
4	230	1	9	1	1	3	2	1	1
4	231	1	18	2	1	3	2	1	1
4	232	1	18	2	1	3	2	1	1
4	233	1	18	2	1	3	2	2	2
4	234	1	18	2	1	3	2	1	1
4	235	1	18	2	1	3	2	1	2
4	236	1	18	2	1	3	2	1	1
4	237	1	18	2	1	3	2	1	3
4	238	1	18	2	1	3	2	1	1
4	239	1	18	2	1	3	2	1	1
4	240	1	17	2	1	3	2	1	1
4	241	1	18	2	1	3	2	1	1
4	242	1	18	2	1	3	2	1	1
4	243	1	16	2	1	3	2	2	2
4	244	1	20	3	1	3	2	1	1
4	245	1	19	2	1	3	2	1	1
4	246	1	18	2	1	3	2	1	2
4	247	1	19	2	1	3	2	1	2
4	248	1	18	2	1	3	2	1	1
4	249	1	20	3	1	3	2	1	1
4	250	1	17	2	1	3	2	1	1
4	251	1	19	2	1	3	2	2	2
4	252	1	18	2	1	3	2	1	1
4	253	1	18	2	1	3	2	1	2
4	254	1	9	1	1	3	2	1	1
4	255	1	19	2	1	3	2	1	2
4	256	1	18	2	1	3	2	1	2
4	257	1	17	2	1	3	2	1	1
4	258	1	18	2	1	3	2	1	2
4	259	1	16	2	1	3	2	1	2
4	260	1	19	2	1	3	2	1	2
4	261	1	17	2	1	3	2	1	2
4	262	1	18	2	1	3	2	1	2
4	263	1	18	2	1	3	2	3	3
4	264	1	18	2	1	3	2	1	1
4	265	1	18	2	1	3	2	1	2
4	266	1	18	2	1	3	2	1	1
4	267	1	18	2	1	3	2	1	1
4	268	1	18	2	1	3	2	2	2
4	269	1	18	2	1	3	2	1	1
4	270	1	19	2	1	3	2	1	1
4	271	1	18	2	1	3	2	1	1
4	272	1	19	2	1	3	2	1	1
4	273	1	20	3	1	3	2	1	1
4	274	1	19	2	1	3	2	2	2
4	275	1	18	2	1	3	2	1	2

4	276	1	18	2	1	3	2	1	3
4	277	1	17	2	1	3	2	1	1
4	278	1	19	2	1	3	2	1	1
4	279	1	19	2	1	3	2	1	1
4	280	1	17	2	1	3	2	1	2
4	281	1	18	2	1	3	2	1	2
4	282	1	17	2	1	3	2	1	2
4	283	1	16	2	1	3	2	1	1
4	284	1	16	2	1	3	2	2	2
4	285	1	17	2	1	3	2	1	1
4	286	1	18	2	1	3	2	2	2
4	287	2	19	2	1	3	1	1	1
4	288	2	9	1	1	3	1	1	1
4	289	2	17	2	1	3	1	1	1
4	290	2	18	2	1	3	1	1	1
4	291	2	18	2	1	3	1	1	1
4	292	2	21	3	1	3	1	1	1
4	293	2	17	2	1	3	1	1	1
4	294	2	17	2	1	3	1	1	1
4	295	2	19	2	1	3	3	1	1
4	296	2	16	2	1	3	1	1	1
4	297	2	19	2	1	3	1	1	1
4	298	2	18	2	1	3	1	2	1
4	299	2	16	2	1	3	1	1	1
4	300	2	16	2	1	3	1	1	1
4	301	2	18	2	1	3	1	2	2
4	302	2	16	2	1	3	1	1	1
4	303	2	17	2	1	3	1	1	2
4	304	2	16	2	1	3	1	1	1
4	305	2	17	2	1	3	1	1	1
4	306	2	18	2	1	3	1	1	1
4	307	2	17	2	1	3	1	1	1
4	308	2	18	2	1	3	1	1	1
4	309	2	19	2	1	3	1	1	1
4	310	2	17	2	1	3	1	1	1
4	311	2	17	2	1	3	3	1	1
4	312	2	17	2	1	3	1	1	1
4	313	2	17	2	1	3	1	1	1
4	314	2	18	2	1	3	1	1	1
4	315	2	19	2	1	3	1	1	2
4	316	2	18	2	1	3	1	1	1
4	317	2	19	2	1	3	1	1	1
4	318	2	19	2	1	3	3	1	1
4	319	2	18	2	1	3	1	1	1
4	320	2	17	2	1	3	1	1	1
4	321	2	17	2	1	3	1	1	1
4	322	2	16	2	1	3	1	1	1

4	323	2	18	2	1	3	1	1	1
4	324	2	16	2	1	3	1	1	1
4	325	2	18	2	1	3	1	1	1
4	326	2	17	2	1	3	1	1	1
4	327	2	16	2	1	3	1	1	1
4	328	2	16	2	1	3	1	1	1
4	329	2	19	2	1	3	1	1	1
4	330	2	18	2	1	3	1	1	1
4	331	2	18	2	1	3	1	1	1
4	332	2	16	2	1	3	1	1	1
4	333	2	19	2	1	3	1	1	1
4	334	2	17	2	1	3	1	1	1
4	335	2	17	2	1	3	1	1	1
4	336	2	16	2	1	3	1	1	1
4	337	2	16	2	1	3	1	1	1
4	338	9	9	1	9	9	9	3	3
4	339	2	18	2	1	3	1	1	1
4	340	2	18	2	1	3	1	1	1
4	341	2	17	2	1	3	1	1	1
4	342	9	9	1	9	9	9	1	1
4	343	2	17	2	1	3	1	1	1
4	344	2	18	2	1	3	1	1	1
4	345	2	18	2	1	3	1	1	1
4	346	2	17	2	1	3	1	1	1
4	347	2	18	2	1	3	1	1	1
4	348	2	16	2	1	3	1	1	1
4	349	2	17	2	1	3	1	1	1
4	350	2	18	2	1	3	1	1	1
4	351	2	17	2	1	3	1	1	1
4	352	2	18	2	1	3	1	1	1
4	353	2	9	1	1	3	1	1	2
4	354	2	17	2	1	3	1	2	2
4	355	2	9	1	1	3	1	1	1
4	356	2	18	2	1	3	1	1	1
4	357	2	19	2	1	3	1	1	1
4	358	2	17	2	1	3	1	1	1
4	359	2	17	2	1	3	1	1	1
4	360	2	9	1	1	3	1	1	1
4	361	2	18	2	1	3	1	1	1
4	362	2	9	1	1	3	1	1	1
4	363	2	18	2	1	3	1	1	1
4	364	2	17	2	1	3	1	1	1
4	365	2	16	2	1	3	1	1	1
4	366	2	18	2	1	3	1	1	2
4	367	2	17	2	9	3	1	2	9
4	368	2	17	2	1	3	1	1	1
4	369	2	18	2	1	3	1	1	1

4	370	2	17	2	1	3	2	1	1
4	371	2	18	2	1	3	1	1	1
4	372	2	18	2	1	3	1	1	1
4	373	2	17	2	1	3	1	1	1
4	374	2	16	2	1	3	1	1	1
4	375	2	17	2	1	3	1	1	1
4	376	2	18	2	1	3	1	1	1
4	377	2	21	3	1	3	1	1	1
4	378	2	17	2	1	3	1	1	2
4	379	2	18	2	1	3	1	1	1
4	380	2	17	2	1	3	1	1	1
4	381	2	21	3	1	3	1	1	1
4	382	2	18	2	1	3	1	1	1
4	383	2	18	2	1	3	1	1	1
4	384	2	17	2	1	3	1	1	1
4	385	2	18	2	1	3	1	2	1
4	386	2	17	2	1	3	1	1	1
4	387	2	17	2	1	3	1	1	1
4	388	2	9	1	1	3	1	1	1
4	389	2	18	2	1	3	1	1	1
4	390	2	16	2	1	3	1	1	1
4	391	2	18	2	1	3	1	1	1
4	392	2	17	2	1	3	1	1	1
4	393	2	17	2	1	3	1	1	1
4	394	2	16	2	1	3	1	2	2
4	395	2	19	2	1	3	1	1	1
4	396	2	18	2	1	3	1	1	1
4	397	2	18	2	1	3	1	1	1
4	398	2	16	2	1	3	1	1	1
4	399	2	18	2	1	3	1	1	2
4	400	2	17	2	1	3	1	1	1
4	401	2	17	2	1	3	1	1	1
4	402	2	18	2	1	3	1	1	1
4	403	2	17	2	1	3	1	1	1
4	404	2	17	2	1	3	1	1	1
4	405	2	18	2	1	3	1	1	1
4	406	2	18	2	1	3	1	1	1
4	407	2	16	2	1	3	1	1	1
4	408	2	16	2	1	3	1	1	1
4	409	2	18	2	1	3	1	1	1
4	410	2	16	2	1	3	1	1	1
4	411	2	18	2	1	3	1	1	1
4	412	2	18	2	1	3	1	1	1
4	413	2	9	1	1	3	1	1	1
4	414	2	17	2	1	3	1	1	1
4	415	2	17	2	1	3	1	1	1
4	416	2	17	2	1	3	1	1	1

4	417	2	16	2	1	3	1	1	1
4	418	2	19	2	1	3	1	1	1
4	419	2	18	2	1	3	1	1	1
4	420	2	9	1	1	3	1	1	1
4	421	2	21	3	1	3	1	1	1
4	422	2	19	2	1	3	1	1	1
4	423	2	18	2	1	3	1	1	1
4	424	2	16	2	1	3	1	1	1
4	425	2	18	2	1	3	1	1	1
4	426	2	20	3	1	3	1	1	1
4	427	2	17	2	1	3	1	1	1
4	428	2	18	2	1	3	1	1	1
4	429	2	20	3	1	1	3	1	1
4	430	2	15	2	1	3	1	1	1
4	431	2	19	2	1	3	1	1	1
4	432	2	17	2	1	3	1	1	1
4	433	2	17	2	1	3	1	1	1
4	434	2	9	1	1	3	1	1	1
4	435	2	20	3	1	3	1	1	1
4	436	2	17	2	1	3	1	1	1
4	437	2	18	2	1	3	1	1	2
4	438	2	18	2	1	3	1	1	1
4	439	2	18	2	1	3	1	1	1
4	440	2	18	2	1	3	1	1	1
4	441	2	19	2	1	3	1	3	3
4	442	2	18	2	1	3	1	1	2
4	443	2	18	2	1	3	1	2	1
4	444	2	17	2	1	3	1	1	1
4	445	2	18	2	1	3	1	1	1
4	446	2	19	2	9	3	1	1	1
4	447	2	9	1	1	3	1	1	1
4	448	2	17	2	1	3	1	1	3
4	449	2	19	2	1	3	1	1	2
4	450	2	17	2	1	3	1	1	2
4	451	2	18	2	1	3	1	1	1
4	452	2	18	2	1	3	1	1	1
4	453	2	19	2	1	3	1	1	1
4	454	2	18	2	1	3	1	1	1
4	455	2	17	2	1	3	1	1	1
4	456	2	18	2	1	3	1	1	1
4	457	2	17	2	1	3	1	1	1
4	458	2	16	2	1	3	1	1	1
4	459	2	18	2	1	3	1	1	1
4	460	2	17	2	1	3	1	1	1
4	461	2	17	2	1	3	1	1	1
4	462	2	19	2	1	3	1	1	1
4	463	2	17	2	1	3	1	1	1

4	464	2	17	2	1	3	1	1	1
4	465	2	17	2	1	3	1	1	1
4	466	2	19	2	1	3	1	1	1
4	467	2	17	2	1	3	1	1	1
4	468	2	16	2	1	3	1	1	1
4	469	2	17	2	1	3	1	1	1
4	470	2	17	2	1	3	1	1	1
4	471	2	9	1	1	3	1	1	1
4	472	2	21	3	1	3	1	1	1
4	473	2	17	2	1	3	1	1	1
4	474	2	18	2	1	3	1	1	1
4	475	2	16	2	1	3	1	1	1
4	476	2	16	2	1	3	1	1	1
4	477	2	19	2	1	3	1	1	1
4	478	2	18	2	1	3	1	1	1
4	479	2	18	2	1	3	1	2	1
4	480	2	17	2	1	3	1	1	1
4	481	2	18	2	1	3	1	1	1
4	482	2	18	2	1	3	1	1	1
4	483	2	18	2	1	3	1	1	1
4	484	2	17	2	1	3	1	1	1
4	485	2	18	2	1	3	1	1	1
4	486	2	17	2	1	3	1	1	1
4	487	2	19	2	1	3	1	1	1
4	488	2	19	2	1	3	1	1	1
4	489	2	17	2	1	3	1	1	1
4	490	2	18	2	1	3	1	1	1
4	491	2	19	2	1	3	1	1	1
4	492	2	17	2	1	3	1	1	1
4	493	2	15	2	1	3	1	1	1
4	494	2	18	2	1	3	1	1	1
4	495	2	18	2	1	3	1	1	1
4	496	2	17	2	1	3	1	1	1
4	497	2	17	2	1	3	1	1	1
4	498	2	18	2	1	3	1	1	1
4	499	2	18	2	1	3	1	1	1
4	500	2	18	2	1	3	1	1	3

PainBreast	ChestPain	Headache	BreastLum	ArmpitLum	DischargeC	DischargeP	DischargeS	Women20	WomenMa
1	3	2	3	2	2	2	2	9	9
3	3	1	2	1	2	3	2	3	3
1	3	3	1	1	2	2	2	1	3
1	3	1	1	2	2	2	2	3	9
3	3	1	9	9	9	9	9	9	9
1	3	1	1	2	2	2	2	3	3
2	2	2	1	2	2	2	2	1	2
1	2	1	2	1	1	3	3	1	1
1	9	9	9	9	9	9	9	2	9
2	2	9	9	9	9	9	9	2	9
1	2	2	2	2	2	2	2	1	2
1	2	1	2	2	2	2	2	1	2
1	2	2	2	2	2	2	2	2	2
1	2	3	2	2	3	3	3	2	1
1	2	2	1	2	2	2	2	2	2
1	2	2	1	1	2	2	2	2	2
1	2	3	2	2	1	2	3	1	2
1	2	2	1	2	2	2	2	1	3
1	2	2	1	3	3	1	2	3	3
2	1	1	2	2	2	2	2	2	2
2	2	2	1	2	1	2	2	1	3
1	1	1	1	1	2	2	2	2	2
2	3	2	2	2	2	2	1	1	1
2	2	2	1	2	1	2	2	1	3
1	2	2	1	1	2	2	2	1	2
1	2	2	1	2	2	2	2	2	2
2	2	2	1	2	2	2	2	1	2
1	2	1	2	2	2	1	2	1	3
1	2	1	1	1	2	3	3	1	3
1	1	9	9	9	2	2	2	2	2
1	2	1	2	2	2	2	2	2	2
2	1	1	1	2	2	2	2	2	2
1	1	1	1	1	1	2	2	1	3
2	1	1	1	2	2	2	2	2	2
2	1	1	2	2	2	2	2	2	2
2	1	1	1	2	2	2	2	2	2
2	1	1	2	2	2	2	2	2	2
1	1	3	1	1	1	3	2	3	3
1	2	1	1	1	2	3	3	1	3
1	2	1	1	1	2	3	3	1	3
1	9	1	1	1	2	3	3	1	3
1	9	9	9	9	9	9	9	2	9
1	3	1	1	1	1	3	2	9	9
1	3	9	1	3	9	9	9	1	3
2	2	2	1	2	2	2	2	1	2
1	1	3	1	1	1	3	2	1	1

1	1	9	1	1	1	3	1	1	1
1	9	9	1	9	9	9	9	1	9
1	1	3	1	2	1	9	2	3	9
1	1	1	1	2	1	2	1	3	3
9	9	9	1	9	9	9	9	1	9
1	1	3	1	1	1	3	3	3	3
1	1	1	1	3	3	3	2	1	3
1	9	9	9	9	9	9	9	1	9
1	3	3	1	9	9	9	9	1	9
1	1	1	1	1	1	2	1	3	3
9	9	9	1	9	9	9	2	1	9
9	9	9	9	1	9	9	9	1	9
1	2	2	1	1	2	3	3	1	3
2	2	2	1	1	1	1	1	1	3
9	9	9	9	1	9	9	9	9	9
1	2	2	1	3	2	2	2	1	3
1	2	3	1	1	1	2	2	1	3
3	9	1	1	1	1	2	1	1	9
1	3	2	3	2	1	3	3	1	3
1	1	3	1	1	2	2	1	1	3
1	1	3	1	3	1	1	3	1	3
1	1	3	1	3	9	1	3	9	9
1	1	3	1	9	1	1	3	1	3
1	3	3	1	3	1	3	9	1	3
1	1	3	1	1	1	9	2	1	1
1	9	9	1	1	1	9	9	9	9
1	3	3	1	1	3	3	1	9	9
1	3	3	1	2	1	3	1	2	3
1	1	3	1	2	2	3	2	2	3
9	9	9	1	9	9	9	9	1	9
1	9	9	9	1	1	9	9	9	9
1	1	2	2	1	2	2	2	3	3
1	1	1	1	9	9	2	1	9	9
9	9	9	9	1	1	3	2	1	3
1	2	2	1	1	2	2	2	2	1
1	1	1	1	1	2	2	2	1	9
1	1	1	1	2	2	2	2	1	2
1	2	2	1	1	2	3	2	2	2
1	9	9	9	1	9	9	9	1	9
1	1	1	1	1	1	1	1	1	3
1	1	1	1	1	1	2	1	1	3
1	2	1	1	9	9	2	1	3	3
1	3	3	1	3	3	3	3	1	3
1	1	2	1	2	1	2	1	1	3
1	3	1	1	3	1	3	2	1	3
3	3	3	3	3	3	3	3	9	9
1	1	1	9	1	3	3	3	1	9

1	2	3	1	2	2	3	2	2	2
1	2	2	1	2	2	3	2	3	2
1	1	2	1	3	1	2	1	1	2
1	2	2	1	2	2	3	2	3	2
1	2	2	1	1	2	2	2	1	3
1	1	2	1	1	2	2	2	9	9
1	1	1	1	1	2	2	2	3	3
1	1	2	1	3	1	2	1	1	3
1	1	2	1	3	1	2	1	1	2
1	2	9	1	2	1	3	1	3	2
2	1	1	2	1	1	2	1	3	1
1	2	3	1	2	2	2	2	3	2
1	2	3	1	2	2	2	1	1	2
1	2	2	1	2	2	2	2	1	1
2	3	2	9	1	3	3	1	1	3
1	3	2	1	2	1	2	3	1	2
1	3	3	1	9	2	2	2	1	2
1	2	3	1	2	2	2	1	2	2
1	1	9	1	1	9	9	9	1	3
1	1	9	1	9	9	9	9	1	3
1	1	9	1	2	2	2	2	2	3
1	3	3	1	3	2	3	2	1	9
3	3	1	2	1	1	1	2	2	2
1	3	3	1	3	1	3	3	3	3
2	2	1	2	9	1	3	1	2	1
2	2	2	1	2	2	2	2	2	2
1	9	9	9	9	9	9	9	1	9
1	2	2	1	2	2	2	2	1	2
2	2	3	2	2	2	2	2	2	2
9	9	9	1	9	9	9	9	9	9
1	2	2	1	2	2	3	2	2	2
2	2	9	9	9	9	9	9	1	2
1	3	1	2	3	3	3	3	2	2
2	2	3	2	2	2	3	2	3	3
9	9	9	1	9	9	9	9	9	9
9	9	9	9	9	9	9	9	1	1
2	2	1	1	2	2	2	3	1	3
1	3	3	2	1	2	3	2	1	2
1	9	9	9	9	9	9	9	1	9
1	2	2	3	3	1	2	3	1	3
3	3	3	1	3	3	3	3	2	2
3	1	3	3	3	3	3	3	2	2
9	9	9	9	9	9	9	9	1	2
1	2	2	1	2	2	2	2	2	1
1	3	2	1	3	2	3	2	2	3
1	1	3	1	1	2	1	1	1	3
1	3	2	1	3	2	3	2	1	3

2	2	2	2	2	2	2	2	9	2
1	9	9	9	9	9	9	9	1	9
2	2	3	2	3	2	2	2	1	3
3	1	1	3	3	3	3	3	2	9
3	1	1	3	3	3	3	3	2	9
3	3	9	1	9	9	9	9	1	9
1	2	9	3	2	2	9	9	2	1
3	3	3	1	3	3	3	3	2	3
2	3	2	2	2	2	2	2	3	1
2	2	2	2	2	2	2	2	2	2
1	2	3	2	2	3	2	1	1	3
1	2	2	2	2	2	2	1	1	3
2	2	2	9	9	9	9	9	2	9
2	2	2	2	2	2	2	2	2	3
2	2	3	2	3	2	2	2	1	3
2	2	3	2	2	2	3	2	2	2
1	1	1	2	2	2	2	2	2	2
1	3	9	3	3	9	9	9	3	3
9	9	9	1	9	9	9	9	9	9
1	1	1	1	1	2	2	2	1	3
1	9	9	9	9	9	9	9	1	3
1	1	1	1	1	1	2	2	1	3
1	1	1	1	1	1	1	1	2	3
1	3	1	3	3	3	3	3	1	3
1	3	1	3	3	3	3	3	1	3
1	1	1	1	1	1	1	1	1	3
1	1	1	1	1	2	2	2	1	3
1	2	9	9	9	9	9	9	1	3
3	3	3	1	3	3	3	3	1	3
2	2	2	2	1	1	3	2	1	3
1	3	1	3	3	1	3	3	1	2
1	2	2	1	2	1	2	1	1	3
1	1	1	1	9	2	2	2	1	3
3	3	1	3	3	3	3	3	1	3
1	1	2	2	2	2	2	2	2	3
1	2	2	1	1	1	3	1	1	3
2	3	3	3	3	3	3	3	1	3
1	3	3	3	1	1	1	1	1	3
1	2	2	1	1	2	2	2	1	3
2	3	3	1	2	3	1	1	3	3
1	3	3	1	1	3	3	1	1	3
1	3	1	3	3	3	3	3	1	3
1	1	1	1	1	1	1	1	1	3
1	1	1	1	1	1	1	1	1	3
3	3	3	3	3	3	3	3	1	3
1	1	1	1	1	1	1	1	1	3
1	1	1	1	1	1	1	1	1	3

1	1	1	1	1	1	1	1	1	3
1	3	1	1	1	1	1	3	1	3
3	3	3	3	3	3	3	3	1	3
1	1	1	1	1	1	1	1	1	3
3	3	1	3	3	3	3	3	1	2
1	1	1	1	1	1	1	1	1	3
1	1	1	1	1	1	1	1	1	3
1	1	2	1	2	2	2	2	1	3
1	1	1	1	1	1	1	1	1	3
1	2	2	1	9	2	3	1	1	3
1	1	3	2	2	2	2	2	1	3
9	9	9	1	1	9	9	1	9	9
1	1	1	1	1	1	1	1	1	3
1	2	2	1	1	1	3	9	1	3
1	3	3	1	3	2	2	1	2	1
1	2	2	1	1	1	2	2	3	1
1	2	2	2	2	1	2	2	3	3
1	1	1	1	1	1	1	1	1	1
1	3	3	1	3	1	1	1	2	2
1	2	2	1	2	2	2	2	2	2
2	2	3	1	1	3	3	2	1	3
1	3	1	3	1	3	3	3	1	2
9	9	9	9	9	9	9	1	1	9
1	2	2	1	2	1	2	2	2	3
1	3	1	3	1	3	3	3	1	2
1	3	3	1	3	1	1	1	9	2
1	2	2	1	2	2	2	2	2	2
1	2	3	1	3	1	1	1	1	2
2	2	2	2	2	2	2	2	1	2
1	2	3	3	3	1	1	1	3	2
1	3	3	1	3	1	1	1	3	3
1	3	3	1	3	1	1	1	3	3
1	3	3	1	3	1	1	1	3	3
1	1	2	1	3	1	2	1	1	2
1	2	2	1	1	1	2	2	2	1
2	2	1	1	1	1	2	1	2	3
1	1	3	1	1	1	2	2	2	1
1	2	2	1	1	1	2	2	1	1
1	2	1	1	2	2	2	2	3	3
2	2	1	1	3	1	3	1	1	3
2	2	2	2	2	2	2	2	3	2
1	3	1	9	3	2	2	2	2	2
1	1	1	1	2	3	2	2	9	1
1	1	1	1	2	2	2	3	2	2
1	1	1	2	2	1	2	2	1	3
1	1	2	1	1	2	2	2	1	2
1	2	2	1	2	1	2	1	9	3

1	2	2	2	2	1	2	2	2	3
1	2	2	2	2	1	2	2	2	3
3	2	3	1	2	2	2	2	2	2
1	1	1	2	2	2	2	2	2	2
2	1	3	2	3	1	3	2	2	3
1	2	2	1	1	2	3	2	1	3
1	1	3	1	1	3	2	2	9	9
2	2	3	1	1	2	3	2	1	2
1	2	3	2	2	1	3	1	1	1
2	3	3	1	3	3	3	3	1	3
1	3	3	1	3	2	2	1	2	2
1	2	1	3	2	2	2	2	1	1
1	2	3	2	2	2	2	2	1	1
1	1	2	2	3	2	2	2	1	2
1	1	2	1	2	2	3	3	2	1
1	3	2	2	1	1	2	2	1	2
1	1	2	2	3	2	2	2	1	2
1	1	3	2	3	2	3	3	2	2
1	3	2	2	2	1	3	2	1	3
1	3	3	1	3	1	2	2	1	2
1	3	1	1	1	1	2	1	1	2
1	1	2	1	2	2	2	2	2	1
1	1	2	2	3	2	2	2	1	2
2	3	9	1	2	1	3	2	2	3
2	9	9	9	9	9	9	9	9	9
3	1	2	2	2	2	2	2	2	2
3	1	2	2	2	2	2	2	2	2
3	2	9	1	3	1	3	1	1	3
2	2	1	2	2	2	2	2	2	2
1	2	3	1	3	2	2	2	1	9
1	2	2	2	2	2	2	2	1	2
1	1	2	1	1	2	2	1	2	3
1	1	9	1	1	9	9	9	1	3
2	3	1	2	2	2	3	2	2	3
2	2	2	2	2	2	2	2	2	2
9	9	9	9	9	9	9	9	1	2
1	1	9	1	1	9	9	9	3	3
1	9	3	1	1	1	3	2	3	3
1	1	2	1	1	1	1	2	1	3
1	2	3	1	1	2	3	3	1	3
3	3	2	3	2	3	3	3	1	2
1	2	2	1	3	2	2	2	2	3
1	1	3	1	2	2	3	2	2	3
1	2	2	1	2	1	1	2	1	3
1	2	3	1	2	2	2	2	1	3
1	1	3	2	3	3	3	3	2	1
1	2	2	1	1	2	2	3	1	3

1	2	1	1	2	2	2	2	2	2
1	1	2	1	2	3	3	2	1	3
1	1	1	1	2	1	1	1	2	2
1	2	3	1	2	1	2	1	1	3
1	3	3	1	2	2	3	2	3	3
1	1	2	1	2	1	3	3	1	3
1	2	1	1	2	2	2	2	1	2
9	9	9	1	9	9	9	9	2	9
1	2	1	1	1	1	2	2	1	3
1	2	1	2	2	2	2	2	2	2
1	2	2	2	2	1	2	2	1	1
1	2	2	1	1	1	2	2	1	2
2	2	2	1	1	1	2	3	1	2
1	3	3	1	3	3	3	3	1	9
2	1	3	1	3	1	3	3	3	3
1	1	1	1	2	2	1	2	1	3
3	1	3	2	2	1	1	2	2	3
1	1	1	1	1	1	2	3	1	2
1	1	1	1	1	1	2	3	3	2
1	2	2	2	2	2	2	2	2	1
1	2	3	1	2	1	2	2	3	3
1	1	2	1	2	1	3	3	1	3
1	2	3	1	2	2	3	2	1	2
1	2	2	1	2	1	2	1	2	2
1	2	2	1	2	1	1	2	1	3
1	9	9	9	9	9	9	9	9	9
1	3	3	1	3	9	9	1	1	3
1	3	2	1	1	2	3	3	1	3
1	1	2	1	1	2	2	2	1	3
3	2	2	1	2	2	2	2	1	2
1	2	3	1	2	2	3	1	1	3
3	3	3	2	1	2	3	3	9	9
1	9	9	9	9	9	9	9	1	9
3	3	3	2	1	3	3	3	1	3
2	9	2	1	1	2	2	2	1	3
1	3	3	1	1	9	1	1	1	2
1	2	2	1	2	2	3	2	1	3
1	3	3	1	1	1	1	1	1	1
1	3	3	1	1	1	2	1	1	3
3	3	3	3	2	1	3	2	9	9
9	9	9	9	9	1	9	9	9	9
1	3	3	1	1	1	1	1	1	3
2	2	2	2	2	2	2	1	1	2
1	3	3	1	2	2	3	3	3	3
1	1	2	1	3	2	2	2	1	3
1	3	1	1	3	2	2	2	1	3
1	1	2	1	1	1	2	2	1	3

1	2	2	1	1	2	3	3	1	3
1	2	2	1	1	2	3	3	1	3
9	9	9	9	1	9	9	9	1	9
1	1	9	1	1	1	9	2	3	3
9	9	9	1	9	9	9	9	9	9
1	2	1	1	1	2	2	2	2	3
1	2	1	1	1	2	2	2	1	3
2	9	1	9	2	9	9	9	2	2
1	2	2	1	9	1	9	1	3	2
1	2	2	1	3	2	1	1	1	1
1	3	3	1	9	2	2	1	2	3
1	1	1	3	3	2	2	3	3	2
2	3	3	1	2	2	3	3	1	2
3	2	3	3	3	3	3	3	1	3
1	3	3	1	2	1	2	1	2	3
2	3	9	1	3	1	2	2	2	2
1	1	1	1	2	1	2	1	3	3
1	1	1	1	1	1	2	1	2	3
1	1	1	1	1	1	9	1	3	3
3	2	3	1	2	3	2	2	2	2
1	1	3	1	1	3	2	2	3	3
1	3	3	1	3	1	3	2	3	3
1	3	3	1	3	1	3	2	3	3
2	3	3	1	2	3	2	1	2	3
1	2	2	1	2	2	2	2	2	2
2	3	2	1	1	2	2	2	1	3
2	2	1	1	2	2	2	2	2	2
2	3	3	1	3	2	3	3	1	2
2	3	3	1	2	1	2	2	2	1
2	3	2	1	1	1	3	3	1	3
2	2	1	2	2	3	2	3	2	3
2	1	3	1	1	1	2	3	2	3
1	1	2	2	1	2	2	1	1	1
3	1	2	1	3	9	3	3	2	2
3	1	9	2	2	2	2	2	3	3
3	2	3	1	1	9	2	1	1	3
1	1	3	1	2	3	2	1	1	3
1	2	2	1	2	2	2	2	2	1
2	2	1	1	2	2	2	2	2	3
2	3	3	3	3	2	2	3	3	3
1	2	1	2	1	2	2	2	2	3
2	3	1	2	2	1	2	2	1	3
1	2	3	1	2	2	2	1	1	3
2	3	2	1	2	2	2	2	1	3
2	3	3	1	3	2	3	3	1	2
1	3	1	1	1	1	3	3	1	3
3	2	3	1	1	1	2	1	1	3

1	1	9	1	3	9	2	3	2	2
1	2	2	1	3	1	2	2	1	2
9	9	1	9	9	9	9	9	2	9
3	9	3	9	1	9	1	9	2	3
1	2	1	1	2	3	3	3	1	3
1	3	3	1	2	2	2	2	1	3
2	2	2	1	3	2	2	2	2	2
1	2	2	1	1	1	2	2	1	2
1	2	2	1	1	2	9	2	1	2
2	2	2	1	1	1	1	2	3	3
2	3	2	1	2	2	1	2	1	3
2	2	2	1	2	1	1	1	2	3
1	2	2	2	1	2	2	2	1	1
1	1	1	1	3	1	2	2	2	3
1	1	1	1	3	1	2	2	2	3
1	1	2	1	1	1	2	1	1	3
1	3	3	2	3	2	3	3	1	3
1	2	3	1	2	1	2	2	1	3
1	2	3	1	2	1	2	2	1	3
9	9	9	9	9	9	9	9	9	9
1	3	1	1	3	3	3	3	1	2
1	2	2	1	2	1	3	2	2	3
1	2	3	1	2	1	3	2	2	3
1	1	1	1	1	1	2	1	1	2
1	2	2	2	2	3	1	1	1	3
3	9	3	1	3	9	2	2	2	3
1	3	3	1	3	3	3	3	9	9
1	1	3	1	1	2	2	2	2	2
1	2	3	1	2	1	3	2	3	3
9	9	9	9	1	9	9	9	9	9
1	2	2	1	2	2	2	1	2	1
1	2	1	2	2	3	2	2	2	1
1	2	2	2	2	2	2	2	2	1
1	3	3	9	3	3	3	3	2	9
1	9	9	1	2	1	3	1	3	1
1	2	3	1	1	1	2	2	2	3
1	2	2	1	2	2	2	1	9	9
2	1	1	3	3	2	1	2	1	3
1	3	3	1	2	2	2	2	2	2
1	2	3	1	2	1	2	1	1	2
3	3	2	1	1	2	3	2	1	2
3	3	1	2	9	2	3	2	3	3
1	2	3	1	1	1	2	2	3	2
1	2	9	1	2	1	3	2	2	9
2	3	3	1	2	1	2	2	2	3
1	1	1	1	1	1	1	1	1	3
1	1	2	1	1	1	1	1	1	3

1	1	1	1	1	1	1	1	1	3
1	1	3	1	1	1	3	1	1	3
1	1	2	1	1	1	1	1	1	3
1	1	1	1	1	2	2	2	1	3
1	1	3	1	1	1	1	1	2	3
1	1	2	1	1	1	1	1	1	3
1	1	1	1	1	1	1	1	3	3
1	1	1	1	1	1	1	1	1	3
1	2	2	1	2	1	1	1	3	2
1	1	1	1	1	1	1	1	1	1
1	1	3	1	1	1	1	1	2	3
1	1	1	1	1	1	1	1	1	3
1	1	2	1	1	2	2	1	1	3
1	1	2	1	2	1	1	2	1	3
1	1	2	1	1	1	1	1	1	3
1	1	2	1	2	1	1	1	1	3
1	1	2	1	1	1	1	1	1	3
1	1	2	1	2	3	2	2	1	3
1	1	2	1	2	3	2	2	1	3
1	1	2	1	2	3	2	2	1	3
1	1	2	1	1	1	1	1	1	3
1	1	2	1	1	1	1	1	1	3
1	1	2	1	1	1	1	1	1	3
1	1	1	1	1	1	1	1	1	3
1	3	1	1	1	1	1	1	1	3
1	1	1	1	1	1	1	1	1	3
1	1	1	1	1	1	1	1	1	3
1	1	3	1	1	1	1	1	1	3
1	1	9	1	1	1	1	1	1	3
1	1	1	1	1	1	1	1	1	3
1	1	1	1	1	1	1	1	2	3
1	1	2	1	2	1	2	2	1	3
1	1	1	1	1	1	1	1	1	3
1	1	1	1	1	1	1	1	1	3
1	1	1	1	1	1	1	1	2	1
1	1	1	1	1	1	1	1	1	3
1	1	2	1	1	3	1	1	3	3
1	1	1	1	1	1	1	1	1	3
1	1	1	1	1	1	1	1	1	3
1	1	1	1	1	1	1	1	1	3
1	3	3	1	1	1	3	1	1	3
1	2	2	1	1	2	2	2	1	2
1	9	9	9	9	9	9	9	1	9
1	2	2	1	2	2	2	2	2	2
2	9	9	9	9	9	9	9	2	9
1	2	2	2	2	3	2	2	2	2
9	3	3	1	1	2	2	2	9	9

1	2	2	1	2	2	2	2	2	2
1	1	3	1	1	2	3	3	1	3
1	1	3	1	1	2	3	3	1	3
1	9	3	1	2	1	2	2	3	2
1	3	9	1	2	2	3	1	1	3
1	2	2	1	1	1	2	1	3	3
1	2	2	1	2	1	2	2	2	3
1	2	2	1	2	3	2	2	1	1
1	3	1	1	2	2	2	2	2	1
2	2	2	2	2	2	2	2	2	2
1	1	2	2	9	9	9	9	9	9
9	9	9	1	9	9	9	9	9	9
1	3	3	3	1	3	3	3	1	3
1	2	2	1	1	2	3	2	2	2
9	9	9	9	1	9	9	9	9	9
9	9	9	9	1	9	9	9	9	9
1	9	9	9	9	9	9	9	1	9
9	9	9	1	9	9	9	9	9	9
9	9	9	1	9	9	9	9	9	9
3	3	2	3	3	3	3	3	1	3
9	9	9	9	9	9	9	9	9	9
1	3	3	1	1	3	3	1	3	3
2	3	9	1	2	2	3	2	2	1
1	9	9	9	9	9	9	9	1	9
9	9	9	9	9	9	9	9	9	9
9	9	9	9	1	9	9	9	9	9
9	9	9	1	9	9	9	9	2	9
1	3	3	1	2	2	2	3	2	1
1	3	3	1	2	1	2	3	1	3
9	9	9	1	9	9	9	9	9	9
9	9	9	1	9	9	9	9	9	9
1	2	3	1	2	1	3	2	1	2
3	9	3	2	2	2	9	9	1	2
1	3	3	1	3	3	3	1	1	3
2	2	2	2	2	2	2	2	2	2
2	3	3	1	2	3	3	3	1	3
1	2	1	1	2	1	2	2	1	3
2	3	3	2	3	3	3	3	1	3
3	3	2	2	3	2	2	2	2	2
2	3	2	3	2	2	3	2	1	2
2	3	3	1	2	1	2	1	3	2
1	3	3	1	3	3	3	3	1	3
3	2	1	1	2	2	2	2	1	3
2	2	3	1	2	1	3	2	1	3
3	3	3	1	2	3	2	3	1	1
3	3	3	1	2	1	2	2	3	3
1	2	1	1	2	2	3	3	1	3

9	3	3	1	3	2	2	2	2	1
1	2	3	1	1	2	2	2	1	3
3	3	3	1	2	3	2	3	1	2
1	2	3	1	3	2	2	2	2	1
2	3	3	1	2	3	3	2	2	1
2	3	3	1	3	1	3	2	3	3
2	2	2	2	3	3	3	3	1	2
1	3	3	1	1	2	1	2	1	2
1	1	2	1	3	2	1	2	1	3
3	3	3	2	2	2	2	2	1	2
3	3	3	3	2	2	2	2	1	2
1	2	2	1	2	3	3	1	2	3
2	2	3	2	2	3	2	3	2	2
2	2	3	2	2	3	2	3	2	2
2	3	3	1	2	1	2	1	1	3
2	3	3	3	1	3	3	3	2	2
9	1	3	1	2	2	3	3	1	3
3	3	3	3	3	3	3	3	1	3
3	3	3	1	1	2	3	3	1	3
1	3	3	1	2	1	2	2	1	2
1	3	3	1	1	2	2	2	1	3
1	3	3	1	3	1	3	2	3	3
1	2	3	1	1	2	2	2	1	2
1	2	3	1	1	2	2	2	1	2
2	3	3	1	2	2	3	3	1	3
1	2	3	1	3	2	2	2	2	3
1	3	3	9	3	3	3	3	1	3
1	2	3	2	1	9	2	2	1	3
1	2	2	3	3	2	2	2	2	3
1	3	3	1	3	3	2	3	3	2
1	9	9	1	2	1	3	2	9	9
1	1	2	1	2	2	2	2	2	1
1	3	3	2	2	2	2	2	2	2
1	3	1	2	1	1	2	2	2	2
1	3	2	1	1	3	1	2	3	3
9	9	9	1	9	9	9	9	1	9
1	2	2	1	2	2	2	2	1	2
1	1	1	1	3	1	3	1	1	3
1	3	3	2	2	2	2	2	2	2
1	1	3	1	3	1	3	2	1	3
1	9	9	1	1	9	9	9	1	3
1	1	3	1	2	2	2	2	1	2
9	9	9	1	9	9	9	9	1	9
1	2	3	1	2	2	2	2	3	2
1	3	1	1	3	1	2	1	1	3
1	2	2	2	2	2	2	2	2	2
1	9	9	9	9	9	9	9	9	9

9	9	9	1	9	9	9	9	9	9
9	9	9	1	9	9	9	9	9	9
9	9	9	9	1	9	9	9	9	9
9	9	9	9	9	9	9	9	9	9
9	9	9	1	9	9	9	9	1	9
9	9	9	1	9	9	9	9	1	9
2	9	1	2	2	2	2	2	9	9
3	3	3	1	1	1	9	9	9	9
9	9	9	9	1	9	9	9	1	9
2	9	9	9	9	9	9	9	2	9
1	3	3	1	2	2	9	2	9	3
2	9	9	9	9	9	9	9	2	9
1	3	3	1	1	9	9	9	9	3
9	9	9	1	9	9	9	9	9	9
9	9	9	1	9	9	9	9	9	9
9	9	9	1	9	9	9	9	9	9
9	9	9	9	1	9	9	9	9	9
9	9	9	9	9	1	9	9	1	9
1	1	2	2	2	2	2	2	2	2
3	1	1	2	1	1	2	1	1	1
1	1	1	1	1	1	1	1	1	1
1	1	2	1	1	1	1	2	3	3
2	2	9	2	2	3	2	1	3	1
1	1	1	1	3	1	3	2	1	3
1	1	2	1	2	2	9	3	2	1
1	9	9	9	9	9	9	9	1	9
1	2	1	1	2	2	2	2	1	3
1	2	1	1	3	2	3	1	2	3
1	9	9	9	9	9	9	9	1	3
1	3	3	1	2	2	2	2	2	1
1	1	1	1	1	1	1	2	1	1
1	2	2	1	3	2	2	2	3	3
1	2	2	1	1	2	1	2	1	3
1	2	3	1	3	2	3	1	1	3
1	1	2	1	3	2	2	2	1	2
1	3	3	2	3	3	3	3	3	3
1	2	3	1	2	1	1	2	2	3
1	3	3	1	3	1	1	1	2	3
1	2	3	1	2	3	1	1	9	3
1	2	2	1	2	1	2	1	2	3
1	1	2	1	1	1	9	1	1	1
3	1	3	9	1	1	9	3	1	3
1	2	3	1	2	1	2	2	3	9
1	1	1	1	1	1	1	1	1	1
1	3	3	1	3	1	3	3	2	3
1	2	3	1	2	1	2	2	9	9
1	1	1	1	2	2	2	2	2	2

1	2	3	2	3	3	3	3	1	2
1	1	1	1	3	1	1	3	1	3
1	3	3	1	3	1	1	1	3	3
1	2	1	1	2	1	2	1	1	2
1	1	2	1	2	2	2	2	2	2
1	1	2	3	2	3	1	3	2	2
1	1	1	2	1	2	1	2	1	2
1	2	3	2	3	3	3	3	1	2
1	1	1	9	9	1	1	1	1	1
9	9	9	9	9	9	9	9	9	9
9	1	9	9	9	9	9	9	9	9
1	1	2	1	1	2	2	1	1	3
1	3	3	3	3	3	3	3	3	3
2	2	1	1	2	2	2	2	1	1
2	1	1	3	9	1	1	2	1	1
1	9	9	9	9	9	9	9	1	9
9	9	9	1	9	9	9	9	1	9
1	3	3	1	3	1	1	1	2	3
1	2	2	2	1	1	2	1	1	3
1	2	2	1	2	1	2	2	1	2
1	3	2	1	1	2	1	2	2	1
1	3	3	3	3	3	3	3	3	3
1	2	3	1	2	3	3	3	3	3
9	9	9	9	9	9	9	9	9	9
1	2	2	2	2	2	9	9	9	9
3	3	3	3	3	3	3	2	3	3
1	2	2	1	2	1	2	2	1	2
3	3	1	3	9	9	1	3	3	3
1	1	1	1	2	2	2	2	2	2
1	9	9	9	1	9	9	9	9	9
1	1	3	1	3	2	1	2	1	3
1	1	3	1	1	1	1	1	9	3
1	1	2	2	2	2	2	2	1	1
1	2	2	1	2	1	1	2	2	1
9	9	9	9	9	1	9	9	9	9
9	9	9	1	9	9	9	9	9	9
9	9	9	1	9	9	9	9	9	9
1	1	2	1	2	1	2	2	1	2
1	1	2	1	2	3	3	2	1	2
1	1	2	1	2	1	9	2	1	2
1	1	2	1	2	1	2	2	1	2
1	1	1	1	1	1	1	1	1	2
2	2	3	2	3	1	1	2	1	2
1	1	2	1	2	1	2	2	1	2
1	1	3	2	3	1	1	2	1	2
2	2	1	3	3	3	3	3	3	2
1	3	3	1	3	1	1	1	3	1

3	3	3	1	3	3	3	3	3	3
1	1	1	1	1	1	1	2	1	3
1	2	2	1	3	1	1	1	1	3
1	2	2	1	2	1	2	1	1	2
2	2	1	1	2	2	2	2	2	2
1	1	1	1	1	1	1	1	1	1
1	1	3	2	3	2	3	2	1	3
9	9	9	1	9	1	9	9	1	9
1	1	1	2	3	2	9	3	3	3
3	3	1	1	2	2	2	2	1	3
2	2	2	2	2	2	2	2	2	2
9	9	9	9	9	9	9	9	9	1
1	2	1	3	3	3	3	3	1	2
3	2	1	1	2	2	3	9	9	1
1	2	1	1	2	1	3	3	2	3
3	2	3	1	2	2	3	2	3	1
2	2	1	3	3	3	3	3	1	2
2	2	1	3	3	3	3	3	1	2
2	2	1	3	3	3	3	3	1	2
1	3	3	1	3	3	3	2	1	1
1	2	3	1	2	3	2	1	9	9
1	3	3	1	3	1	1	1	2	3
9	9	9	1	9	9	9	9	9	9
1	3	3	1	3	1	3	2	1	2
3	2	1	9	1	3	1	3	1	9
2	2	1	3	3	3	3	3	3	2
1	2	2	1	2	1	2	2	1	2
1	1	3	1	3	1	3	1	1	3
9	1	9	9	9	9	9	9	1	9
2	2	2	2	2	2	2	2	2	2
1	1	1	1	1	2	3	3	1	3
2	3	2	2	3	2	3	2	2	9
1	2	1	2	3	1	2	1	3	2
3	3	1	3	2	2	2	3	2	2
3	3	3	3	3	3	3	3	3	3
1	2	3	1	3	2	3	3	2	3
1	2	3	3	3	3	3	3	1	2
1	2	1	3	3	3	3	3	1	2
2	2	1	3	3	3	3	3	1	2
1	1	3	1	1	1	3	2	1	3
1	1	2	1	2	2	1	3	2	1
1	3	1	1	1	2	3	1	2	2
1	3	3	3	3	3	3	3	1	2
3	3	1	3	9	1	3	1	1	3
9	9	9	9	9	9	9	9	9	9
3	3	3	3	3	3	3	3	3	3
2	2	1	2	2	2	2	2	2	2

1	2	1	1	1	1	3	3	2	2
1	3	3	3	3	3	3	3	9	9
1	1	3	1	3	1	1	2	2	1
2	1	2	1	3	1	1	1	3	3
1	1	3	1	2	2	2	2	2	2
1	1	2	1	2	2	2	2	2	2
1	1	1	3	3	1	1	9	2	1
1	1	3	1	2	2	2	2	2	2
1	1	2	1	2	2	2	2	2	2
9	9	9	9	9	9	9	9	9	9
3	2	1	9	2	2	2	2	1	2
1	3	3	1	2	3	3	2	3	3
2	2	1	1	2	2	2	2	1	1
1	1	1	1	3	1	1	3	1	3
1	3	1	1	2	1	2	2	1	3
3	3	3	1	1	2	1	1	1	3
1	1	1	2	2	2	2	2	1	2
1	1	9	2	2	2	2	2	1	2
1	1	1	2	2	2	2	2	1	2
1	1	3	2	2	2	2	2	1	2
1	1	1	1	1	1	3	9	1	2
1	2	1	3	2	1	2	2	1	3
3	3	1	3	3	3	3	3	1	3
1	2	2	1	2	2	2	2	2	2
2	2	2	2	1	2	2	2	2	2
9	9	9	9	9	9	9	1	9	9
9	9	9	9	9	9	9	1	9	9
1	3	1	1	3	1	2	1	1	3
3	3	1	3	3	3	3	3	1	3
2	3	3	2	2	2	2	3	2	2
2	2	1	1	1	9	3	1	1	3
1	1	2	1	2	1	9	2	2	1
1	1	3	2	1	2	2	1	1	2
9	9	9	9	9	9	9	1	9	9
1	3	1	1	2	2	3	1	2	1
3	3	3	3	3	3	3	3	3	3
1	2	2	1	2	2	2	2	1	3
1	1	1	3	3	3	3	3	9	3
1	9	9	9	9	9	9	9	9	9
1	2	1	1	1	2	3	2	1	3
9	9	9	1	9	9	9	9	1	3
3	3	1	3	3	3	3	3	2	2
3	3	1	3	3	3	3	3	2	2
2	2	2	2	2	2	3	3	2	2
3	3	1	3	3	3	3	3	2	2
1	1	2	1	2	1	2	1	3	3
1	1	2	1	2	2	2	2	1	3

2	2	2	2	2	2	2	2	2	2
2	2	3	1	2	2	2	2	1	3
3	1	1	1	1	1	1	1	1	3
1	2	3	9	9	9	9	9	1	3
1	9	9	9	9	9	9	9	1	9
2	1	3	1	1	1	1	2	1	1
1	1	2	1	3	2	3	2	1	2
1	9	9	9	9	9	9	9	9	9
1	2	1	3	2	3	1	2	1	2
2	9	9	9	9	9	9	9	1	9
2	2	2	2	2	2	2	2	2	2
1	3	3	2	3	3	2	2	1	3
1	3	3	1	2	1	2	2	1	3
2	2	2	2	2	2	2	2	1	2
1	1	3	1	3	1	2	1	1	3
2	2	2	2	2	2	2	2	2	2
2	2	2	2	2	2	2	2	2	2
1	3	3	1	2	1	3	1	9	9
1	1	2	2	2	3	1	3	1	2
1	3	2	2	3	1	2	1	9	9
1	1	3	2	2	3	1	1	1	2
1	3	3	2	3	3	2	2	1	3
1	2	2	1	2	2	2	2	2	2
1	2	3	2	2	2	2	2	1	1
1	1	2	1	1	2	2	2	1	3
2	3	2	3	2	3	2	3	1	2
1	1	2	3	1	2	3	1	2	1
3	3	1	3	1	3	3	3	1	3
1	1	2	1	2	1	1	1	2	2
1	3	3	2	3	3	2	2	1	3
1	9	9	9	9	9	9	9	3	3
1	1	1	1	1	2	2	2	1	3
3	3	3	1	3	1	1	1	3	3
1	2	2	1	2	1	1	2	2	2
1	2	3	9	9	9	9	9	1	1
1	1	1	1	1	1	1	1	1	3
2	2	2	2	2	2	2	2	2	2
1	2	2	9	1	1	2	3	1	1
3	1	1	3	1	3	3	2	1	2
1	2	1	1	2	1	2	1	1	3
1	1	2	2	2	2	1	2	1	3
2	2	2	2	2	2	2	2	2	2
2	1	3	1	3	1	1	1	1	3
1	1	3	1	3	3	3	3	1	1
1	2	2	1	3	3	3	3	2	2
3	3	3	3	3	3	3	3	3	3
1	2	3	1	2	2	2	2	1	2

1	2	3	9	2	9	9	9	1	3
3	3	3	3	3	3	3	3	3	1
3	3	3	3	3	3	3	3	3	3
3	2	2	2	2	2	2	2	2	2
1	2	2	1	2	2	2	1	1	3
1	3	3	1	3	2	2	1	1	2
3	3	3	3	3	3	3	3	2	1
1	1	3	3	3	2	2	3	1	3
2	3	9	1	2	3	1	2	2	1
3	3	3	3	3	3	3	3	2	2
1	3	2	1	3	1	2	1	1	3
3	3	1	3	3	3	3	3	2	2
9	1	9	9	9	9	9	9	1	9
1	3	2	1	2	1	2	2	2	2
1	2	2	2	2	2	2	2	1	2
3	1	3	1	3	3	9	1	9	9
3	1	3	3	3	3	9	1	1	9
3	3	1	1	1	1	1	1	1	3
1	1	1	2	1	2	2	2	1	3
1	3	2	1	2	1	2	2	2	2
1	1	1	3	3	1	3	3	1	3
1	3	2	1	2	2	2	2	1	1
1	1	2	1	2	2	2	2	2	3
1	1	1	1	1	1	1	1	1	2
1	3	2	1	3	3	3	3	1	3
1	1	9	1	9	1	9	1	1	9
2	2	2	2	2	2	2	2	2	2
1	2	2	1	1	9	3	1	1	3
1	1	3	1	3	1	1	2	1	3
3	2	3	3	3	2	2	9	3	2
1	1	1	1	1	1	1	1	1	1
1	1	1	1	1	1	3	3	1	1
1	2	1	1	1	1	1	1	1	1
1	1	1	2	1	1	1	1	2	1
1	1	3	2	9	3	1	1	3	1
3	3	9	2	3	3	1	1	3	9
9	9	9	1	9	9	9	9	9	9
1	1	1	1	1	1	1	1	1	1
1	2	2	2	2	2	2	2	2	2
2	2	2	2	2	2	2	2	2	2
1	1	1	2	1	1	9	2	3	3
1	1	2	1	2	1	1	1	1	2
1	2	3	2	9	9	9	9	3	3
1	2	2	1	1	1	3	2	1	1
2	2	2	2	2	2	2	2	2	2
1	1	1	3	3	3	3	3	3	3
1	2	2	2	2	2	2	2	3	3

1	1	2	2	3	3	9	1	1	3
3	3	1	3	3	3	3	3	1	3
1	2	3	1	1	2	3	2	3	3
1	9	9	9	9	9	9	9	1	9
1	1	3	2	3	2	3	3	2	3
1	2	3	1	1	2	3	2	3	2
3	3	1	3	3	3	3	3	9	9
9	9	9	9	9	9	9	9	9	9
3	3	3	1	1	1	3	3	1	3
2	2	2	2	2	2	2	2	2	2
1	1	1	1	1	1	1	1	1	9
3	1	3	3	3	3	9	9	1	3
1	9	2	2	9	9	9	9	3	3
1	3	3	2	2	2	2	2	2	1
1	1	1	1	1	1	1	1	1	1
1	1	9	2	3	3	2	3	2	1
1	1	2	2	2	2	2	2	2	1
1	1	1	1	1	1	1	1	1	9
1	2	2	2	1	2	1	2	3	2
1	3	3	2	2	1	2	2	1	2
3	3	9	1	2	2	3	2	1	3
1	3	3	1	3	2	2	2	1	2
3	2	1	3	3	3	2	2	2	2
1	2	9	1	1	1	2	9	1	1
3	2	1	2	3	2	2	9	2	3
1	2	2	9	1	1	3	3	1	3
3	3	3	3	2	3	3	3	2	3
1	3	3	1	2	1	3	3	1	3
1	2	3	2	2	1	2	2	1	2
1	2	3	1	1	2	9	9	1	2
1	2	1	1	2	2	1	1	1	2
1	3	3	3	9	1	3	2	1	1
1	2	1	1	3	3	2	1	1	3
3	3	3	1	3	3	3	3	1	3
1	9	9	1	9	1	9	9	1	9
1	2	1	3	1	1	3	2	1	3
3	3	3	9	3	3	3	3	2	1
3	3	3	3	3	3	3	3	1	3
2	3	1	9	2	1	1	1	9	2
3	3	3	3	3	3	3	3	3	1
1	1	3	3	3	9	9	9	3	1
9	9	3	9	9	9	9	9	1	9
3	3	3	3	3	3	3	3	1	1
3	3	3	3	3	3	1	1	1	3
1	2	2	1	2	1	2	2	1	3
1	1	1	3	3	3	3	3	2	1
1	1	2	1	1	1	3	1	1	3

3	1	3	3	3	3	3	3	3	1
3	3	3	1	3	3	3	3	1	3
1	2	3	1	3	1	2	1	1	2
1	1	1	1	1	1	1	1	3	3
3	3	3	3	3	3	3	3	2	2
1	3	2	9	9	9	3	9	1	3
2	2	2	2	2	2	2	2	2	2
3	3	3	3	3	3	3	3	2	2
3	3	3	3	3	3	3	3	2	9
2	2	2	2	1	2	1	2	2	1
1	1	2	1	2	3	3	2	1	3
1	1	2	2	1	1	2	2	3	3
1	1	3	1	1	3	1	3	3	1
3	3	1	3	3	3	3	3	2	2
1	1	2	1	1	1	2	2	1	1
2	2	2	2	2	2	2	2	2	3
1	1	3	1	3	2	2	1	1	3
1	2	2	1	3	1	2	2	1	2
1	3	3	1	2	2	2	1	1	2
9	9	9	9	1	9	9	9	9	9
3	3	3	3	3	3	3	3	1	9
3	3	3	3	3	3	3	3	3	3
1	1	3	3	3	3	3	3	3	3
1	3	3	1	1	1	2	1	1	3
3	3	3	3	3	3	3	3	1	3
3	1	3	3	3	3	3	3	3	1
3	3	3	3	3	3	3	3	2	1
1	2	3	1	3	1	2	2	1	3
1	1	3	1	2	3	3	2	3	1
2	2	3	1	2	1	1	2	2	3
1	1	1	1	2	3	3	3	1	1
1	1	1	1	1	1	3	3	1	1
2	2	3	2	2	1	2	2	1	1
1	2	1	1	1	1	2	2	1	3
1	9	9	1	9	1	2	1	1	1
1	1	3	1	2	9	9	9	1	3
1	9	9	9	9	9	9	9	1	9
1	2	2	2	2	2	2	2	9	9
1	1	2	1	1	1	1	2	3	3
3	3	3	3	3	3	3	3	2	2
1	1	2	1	1	2	1	1	1	3
1	3	3	3	3	1	3	3	1	3
2	2	2	1	2	2	2	2	2	2
2	9	9	9	9	9	9	9	9	9
9	9	9	9	9	9	9	9	9	9
1	1	1	1	1	1	1	1	1	3
1	3	2	1	1	1	2	1	1	2

1	2	2	3	9	1	1	2	9	9
1	1	1	9	1	2	2	2	1	2
1	3	3	1	3	1	3	3	1	3
9	9	9	1	9	9	9	9	9	9
1	2	3	2	2	2	2	3	3	3
9	9	9	9	9	9	9	9	9	9
2	2	1	2	2	2	2	2	3	2
1	2	1	1	1	2	2	3	1	2
3	3	1	3	3	3	3	3	3	3
1	2	2	2	3	1	2	2	1	3
1	3	3	2	1	1	2	2	1	3
1	1	1	1	1	2	3	2	1	3
1	9	9	9	9	9	9	9	9	9
1	9	9	9	9	9	9	9	9	9
1	2	1	2	1	3	2	1	9	9
3	3	9	1	2	2	3	3	1	3
1	1	2	3	1	2	3	1	1	3
3	3	2	1	3	3	3	3	3	3
1	3	3	2	2	1	2	1	3	1
1	2	3	1	1	1	2	2	3	3
1	2	1	1	2	2	2	2	1	3
1	3	3	1	3	1	2	2	1	2
1	1	2	2	2	2	2	2	2	2
1	2	1	2	2	2	3	3	2	3
3	3	3	3	3	3	3	2	1	3
1	2	2	1	1	1	1	2	3	3
1	9	9	9	9	9	9	9	1	1
1	1	1	2	2	1	1	1	2	2
1	2	2	1	1	1	1	2	2	3
3	3	9	1	2	2	3	3	1	3
1	2	3	1	1	1	2	2	3	3
1	9	9	9	9	9	9	9	9	9
1	2	1	3	2	1	1	2	2	3
1	1	2	1	2	2	2	2	1	3
1	2	2	2	2	2	2	2	2	1
1	3	3	1	1	1	2	2	9	9
1	9	9	9	9	9	9	9	2	9
1	9	9	9	9	9	9	9	2	9
1	2	3	1	2	1	2	2	1	3
1	1	2	2	2	2	2	2	1	1
9	9	9	1	9	9	9	9	1	9
1	2	1	1	1	1	1	2	1	2
1	1	1	1	2	3	3	3	1	1
1	1	1	1	2	3	3	3	1	3
1	1	1	1	1	1	1	1	1	1
1	2	1	1	1	2	1	2	1	3
1	9	9	9	9	9	9	9	1	9

9	9	9	9	9	9	9	9	1	1
1	3	2	1	2	1	2	2	1	3
1	1	1	9	1	2	9	2	3	3
9	9	9	9	9	9	1	9	1	9
1	9	9	9	9	9	9	9	1	9
1	1	2	1	2	1	3	1	3	3
1	1	3	1	1	2	2	2	2	3
1	3	3	1	3	1	3	2	1	3
3	2	1	3	3	3	3	3	1	1
1	1	3	1	1	2	2	2	2	3
1	2	3	1	1	1	3	2	1	3
9	9	9	1	9	9	9	9	2	9
1	2	2	1	1	1	2	1	1	3
1	2	2	2	3	1	2	3	2	3
1	2	2	1	1	2	2	1	2	3
3	3	1	3	1	3	3	3	3	3
1	1	3	1	3	2	2	2	1	3
1	1	2	1	2	1	2	1	1	3
1	2	1	2	2	2	2	1	2	2
1	3	1	1	3	3	3	2	1	3
1	1	3	1	2	1	1	2	3	2
9	9	1	9	9	9	9	9	9	1
1	1	3	1	2	1	1	2	2	2
1	2	2	2	2	2	2	2	3	2
1	1	2	1	2	1	2	2	2	2
1	1	2	2	2	2	2	2	1	1
2	2	2	1	2	2	2	2	3	3
1	2	1	1	3	1	1	2	1	3
1	2	2	1	3	2	3	2	1	3
1	3	3	3	3	3	3	3	1	3
9	9	1	9	9	9	9	9	1	9
2	1	2	2	1	1	9	3	2	3
1	1	1	1	2	1	2	2	1	9
2	3	2	1	2	3	2	2	1	3
1	1	3	9	2	3	1	3	1	1
1	9	9	9	9	9	9	9	9	9
9	9	9	9	9	9	9	9	9	9
2	2	2	2	2	2	2	2	2	2
1	3	1	1	3	2	2	2	3	3
1	2	1	3	2	1	1	2	1	2
3	1	2	3	2	1	2	1	1	2
1	1	1	1	9	1	1	1	1	1
1	1	9	2	1	3	1	1	1	1
1	2	2	3	1	2	1	3	1	2
3	2	3	1	9	3	1	2	3	9
1	9	9	9	9	9	9	9	1	9
1	3	3	2	2	9	3	3	2	3

2	1	2	1	2	1	2	2	1	2
1	2	2	1	3	1	2	2	1	3
3	3	3	3	3	3	3	3	3	3
1	3	2	1	1	9	3	9	1	2
1	2	3	1	2	1	1	2	1	3
2	1	2	1	2	1	2	2	1	2
1	2	1	1	3	1	3	2	2	1
1	1	3	1	2	1	1	2	2	2
1	2	1	1	2	2	2	2	3	1
3	3	1	3	3	3	1	1	1	3
1	3	3	2	2	2	2	2	1	2
1	3	2	2	2	2	2	2	2	2
9	9	9	9	1	9	9	9	1	9
1	3	3	1	1	2	1	2	2	2
1	3	3	2	3	1	2	2	2	3
1	3	3	1	2	1	2	1	1	1
1	3	2	1	2	1	2	1	1	3
1	3	3	1	2	1	2	1	1	3
1	2	3	1	2	1	1	3	1	3
2	2	3	1	3	2	3	2	3	3
1	1	1	1	1	1	3	1	1	3
1	3	3	1	1	3	3	1	1	1
3	1	2	3	2	3	3	2	2	3
3	3	1	2	2	2	2	9	1	3
1	2	3	1	2	2	2	1	2	2
2	3	3	2	3	3	2	2	2	1
2	2	1	2	2	2	2	2	2	2
2	2	2	1	2	2	2	2	2	2
1	3	1	1	3	1	3	1	3	1
1	3	2	3	1	1	3	1	3	1
1	2	1	2	3	3	3	3	1	3
3	3	1	2	2	2	2	9	1	3
3	3	2	2	2	2	2	9	1	3
1	2	2	9	9	9	9	9	1	3
1	1	1	2	1	1	1	2	2	1
1	1	1	1	2	1	2	3	1	3
1	3	3	1	9	9	9	9	1	3
1	3	3	1	2	1	3	3	1	3
1	3	3	1	2	3	2	3	1	3
3	1	1	3	3	3	3	3	1	1
3	3	2	2	2	3	1	2	3	1
1	3	1	3	3	3	3	3	3	3
1	3	3	3	3	3	3	3	1	3
1	1	3	1	3	1	1	3	1	3
1	9	9	9	9	9	9	9	1	9
1	9	9	9	9	9	9	9	1	9
1	9	9	9	9	9	9	9	1	9

3	3	3	2	2	3	3	2	3	3
2	1	2	3	1	1	3	2	2	3
1	1	9	1	1	1	1	1	1	3
1	3	3	1	2	1	2	2	2	3
1	1	1	1	1	1	3	1	1	1
1	9	9	9	9	9	9	9	1	9
1	9	9	9	9	9	9	9	1	9
9	9	9	1	9	9	9	9	9	9
1	9	9	9	9	9	9	9	1	9
1	9	9	9	9	9	9	9	9	1
1	9	9	9	9	9	9	9	9	1
1	1	1	3	1	1	1	1	1	1
1	9	9	9	9	9	9	9	1	9
1	3	3	1	1	1	3	1	1	3
1	3	3	1	3	2	1	2	1	3
1	2	2	1	2	2	2	2	2	2
2	2	2	1	2	2	2	2	1	1
9	9	9	1	9	9	9	9	9	9
9	9	9	1	9	9	9	9	1	9
1	9	9	9	9	9	9	9	1	9
1	3	2	2	3	3	9	3	1	3
1	9	9	9	9	9	9	9	1	9
1	1	1	2	2	2	2	2	1	1
1	1	2	2	2	3	2	2	1	9
1	1	1	1	2	2	2	2	1	3
1	1	1	1	1	1	1	1	1	2
1	2	3	1	3	1	2	2	1	2
1	1	1	2	2	2	2	2	1	2
1	1	1	1	2	2	2	2	1	2
1	1	3	2	2	2	2	2	1	3
1	1	1	1	3	3	3	3	1	1
1	2	2	1	2	2	2	2	1	2
2	2	2	2	1	1	1	1	1	1
1	9	9	9	9	9	9	9	1	9
1	1	1	2	1	1	1	1	1	2
1	2	2	1	1	2	2	2	1	2
1	1	2	2	2	2	2	2	1	2
1	9	9	9	9	9	9	9	1	9
1	2	2	1	2	1	3	3	1	1
3	3	3	3	3	1	3	3	1	3
2	2	1	3	9	3	3	3	2	1
3	3	3	3	3	3	2	1	3	3
3	3	3	1	3	1	3	3	3	3
1	1	2	1	1	1	1	1	1	1
1	3	3	1	3	3	1	1	1	2
1	9	9	1	3	2	3	2	3	3
1	2	1	1	2	2	2	2	1	3

1	3	3	1	3	3	2	1	1	3
1	1	1	2	3	2	1	3	2	1
9	3	3	9	2	3	3	2	1	9
1	2	2	1	1	2	2	2	1	3
1	3	2	1	1	1	3	1	2	3
3	1	1	1	9	2	2	9	9	9
2	3	3	3	3	2	1	1	1	3
3	3	3	3	3	3	3	3	1	3
3	1	1	1	2	2	9	3	9	9
3	1	1	1	1	2	2	3	1	3
9	9	9	2	9	9	9	9	9	9
2	2	2	2	3	1	3	2	1	3
1	1	1	2	2	2	2	2	1	1
3	1	1	2	2	1	2	2	2	1
1	1	1	2	2	2	2	2	1	3
1	1	3	1	2	2	3	2	1	2
2	1	1	1	1	2	2	2	1	3
1	2	3	1	2	2	2	2	2	3
1	1	1	1	1	1	1	1	1	3
9	9	9	9	9	1	9	9	9	9
1	1	1	1	1	1	1	1	1	3
1	3	2	1	2	1	1	3	1	3
1	2	2	1	2	1	1	2	2	3
1	1	2	1	2	1	2	3	1	3
1	1	2	1	1	1	2	2	1	3
3	3	1	3	3	3	3	3	3	3
1	1	3	1	3	1	1	1	3	1
1	1	1	1	2	9	2	3	3	3
1	2	2	1	2	2	2	2	1	2
1	2	2	1	1	2	9	1	3	3
1	2	3	1	2	3	2	3	1	3
1	1	1	2	2	2	2	2	2	2
1	1	2	1	1	2	2	2	1	2
1	1	2	1	3	2	2	2	1	1
1	1	1	1	2	2	2	2	1	2
1	2	1	2	1	1	1	2	3	1
3	2	2	1	2	1	2	2	2	2
1	2	2	2	1	1	1	1	1	2
1	2	2	1	1	1	1	2	1	2
2	2	2	2	2	2	1	2	2	2
1	3	3	1	1	1	3	3	1	3
3	3	3	3	3	3	3	3	2	3
1	3	2	1	3	1	3	3	1	2
1	2	1	2	2	2	2	2	1	1
1	1	1	1	2	1	1	1	3	1
3	3	3	3	3	9	3	3	3	3
1	2	2	1	2	2	2	2	1	3

2	2	1	3	2	1	3	2	1	2
1	1	1	1	1	1	1	3	1	3
9	9	9	9	9	1	9	9	9	9
3	1	3	3	3	3	3	3	3	3
9	9	9	9	9	1	9	9	9	9
3	3	1	1	2	1	9	3	3	3
1	9	9	9	9	9	9	9	1	9
1	2	1	3	3	2	3	2	1	3
2	3	9	2	3	2	3	3	2	2
1	1	1	2	3	1	2	2	3	3
1	2	2	1	1	2	9	2	3	3
1	1	3	1	2	1	1	2	1	3
1	1	1	2	2	1	1	2	2	1
1	1	1	2	1	1	1	2	1	1
1	1	2	2	3	3	2	2	1	1
9	9	9	9	9	9	9	9	9	9
2	1	1	1	3	2	2	2	3	3
3	3	3	3	3	3	3	3	3	3
3	1	1	3	2	3	1	3	3	2
3	9	1	3	9	2	2	2	1	1
1	2	2	1	1	1	1	2	2	2
3	1	1	3	3	3	3	3	1	1
1	1	1	1	1	1	1	1	1	2
9	3	3	3	1	2	2	2	1	2
2	2	1	2	2	1	2	2	2	2
1	1	2	1	2	1	2	2	1	3
3	9	9	9	9	9	9	9	9	9
1	1	1	1	1	1	2	2	2	2
1	2	3	1	2	2	3	1	1	3
2	2	2	1	2	2	2	2	1	3
9	9	9	9	9	9	9	9	9	9
2	2	2	2	2	2	1	1	1	2
1	3	1	2	3	1	1	2	1	3
2	2	2	2	2	2	2	2	2	2
2	2	2	2	2	2	3	3	2	2
1	2	3	1	2	1	3	2	1	3
3	3	3	3	3	3	3	3	3	1
1	2	3	1	3	1	1	2	1	3
1	2	3	1	3	1	1	2	1	3
1	2	3	1	2	2	2	2	3	3
1	1	2	2	2	2	2	2	1	1
1	1	1	1	1	2	2	2	1	3
1	3	2	1	9	2	3	2	3	3
1	9	9	9	9	9	9	9	1	9
9	9	9	1	9	9	9	9	1	9
1	3	2	1	9	2	3	2	3	3
3	1	3	3	3	3	3	1	3	3

3	3	1	3	3	3	3	3	3	3
1	2	2	1	2	2	2	1	2	2
1	9	9	9	9	9	9	9	1	9
3	3	1	3	3	3	3	3	3	3
1	3	1	3	2	1	1	3	3	3
1	1	1	1	2	1	2	3	2	3
1	2	1	2	1	2	2	2	2	3
2	3	3	3	3	1	3	2	1	3
1	2	2	1	1	2	2	1	3	3
1	2	2	1	2	1	3	3	1	3
9	9	9	9	9	9	9	9	1	9
9	9	9	9	9	9	9	9	1	9
2	2	2	2	2	2	2	2	2	2
1	1	1	1	2	1	2	2	1	2
9	9	9	9	1	9	9	9	9	1
1	9	1	1	1	1	2	2	2	3
1	2	3	1	1	3	1	3	3	3
1	1	3	1	1	3	3	1	2	3
1	3	1	1	2	1	3	2	1	1
1	3	1	1	3	3	2	1	3	3
1	2	3	1	3	2	2	1	2	1
1	3	1	1	3	1	1	1	1	3
3	1	3	1	3	1	2	3	1	1
1	1	3	1	3	1	3	3	1	9
1	1	3	2	3	1	2	2	1	3
1	3	3	3	3	3	3	3	1	1
9	9	9	1	9	9	9	9	1	9
1	3	1	1	3	2	2	1	3	3
1	1	1	1	2	9	9	1	1	3
1	1	2	1	2	3	1	2	1	3
1	1	1	1	9	3	3	1	1	3
1	2	3	1	2	1	1	2	1	1
1	1	1	2	3	2	1	3	1	2
3	3	1	3	3	3	3	3	1	3
1	2	2	1	3	2	1	1	1	3
2	2	2	1	2	1	2	2	2	2
1	1	3	1	2	2	9	9	9	9
2	3	3	1	3	2	3	1	1	3
1	9	9	9	9	9	9	9	9	9
3	3	1	3	3	3	3	3	1	3
1	1	3	1	1	2	1	2	1	3
1	1	3	1	2	1	3	3	9	1
1	1	3	9	1	9	3	3	1	3
1	1	3	1	2	3	3	1	1	3
1	1	2	1	3	1	3	3	1	3
1	1	1	1	2	3	3	1	1	3
3	1	1	3	3	3	3	3	3	3

3	3	1	3	3	3	3	3	3	3
1	1	1	9	9	9	9	9	9	9
3	3	3	3	1	3	3	3	1	1
3	1	1	3	1	3	1	1	3	3
1	3	3	1	3	1	3	1	1	3
1	3	3	1	3	1	1	3	1	3
1	1	3	1	1	2	1	2	3	3
1	1	1	3	2	1	3	3	2	2
3	3	3	3	3	3	3	3	3	3
1	1	1	1	2	3	3	1	1	3
1	1	1	1	2	3	3	1	1	3
1	3	2	2	1	3	2	2	1	3
1	1	1	1	3	3	2	2	2	3
1	1	1	3	2	1	3	3	2	2
2	2	2	2	2	2	3	2	3	1
1	3	1	1	3	1	2	3	1	1
3	3	2	1	3	1	3	2	2	3
3	3	2	2	3	1	2	1	2	1
1	1	3	1	3	3	3	3	1	3
2	1	1	2	2	2	2	2	2	2
1	1	1	1	2	2	2	1	1	3
1	1	2	3	2	2	3	2	1	3
1	1	1	2	1	2	2	2	2	3
1	1	1	2	2	1	1	1	3	3
1	1	2	2	1	2	2	2	2	1
1	2	1	2	2	1	2	3	2	1
1	2	2	3	1	2	2	1	1	3
1	3	2	1	3	3	3	3	3	3
2	2	2	3	3	3	3	3	3	3
1	9	9	9	9	9	9	9	1	9
2	1	2	1	1	2	3	2	1	3
1	1	1	1	1	1	2	3	1	1
1	1	2	2	3	2	3	2	2	3
1	1	2	1	3	1	2	2	2	3
1	2	1	3	1	1	2	1	2	1
2	2	2	2	2	2	2	2	2	2
1	1	3	1	1	1	3	3	3	1
1	1	2	1	3	1	1	3	1	3
1	2	3	1	1	3	3	2	1	3
1	2	3	1	3	2	1	2	1	2
1	1	2	1	2	1	2	1	2	2
1	1	2	2	3	2	3	3	1	2
1	1	1	2	2	2	3	1	1	3
1	1	2	1	2	1	2	1	1	3
3	1	1	1	3	2	2	2	2	2
1	1	3	1	3	2	2	2	2	2
1	1	3	1	2	1	2	3	1	3

2	1	1	1	2	2	3	2	3	3
1	3	1	1	2	1	2	2	2	2
1	1	1	2	1	1	2	1	1	2
1	2	1	3	9	1	1	2	2	1
2	3	2	2	3	3	2	3	3	1
1	1	1	1	1	2	2	1	3	3
2	1	3	2	3	2	3	2	2	3
3	3	2	3	3	3	3	3	3	3
1	1	1	1	1	1	2	1	3	3
1	2	3	2	2	2	2	2	2	2
1	9	9	9	9	9	9	9	1	9
1	1	2	1	3	3	3	3	2	2
1	1	2	9	9	9	9	9	2	9
2	3	2	2	3	3	2	3	3	1
1	2	1	3	2	2	3	3	2	2
1	3	1	3	3	3	3	3	3	3
2	1	1	2	2	2	2	3	2	2
1	1	1	2	2	9	1	2	2	3
1	1	1	2	2	2	3	3	3	3
1	1	2	2	2	2	2	2	3	3
3	3	3	3	3	3	3	3	3	3
9	9	9	9	9	9	9	9	2	1
1	9	9	9	9	9	9	9	1	9
1	1	3	2	2	3	1	2	2	2
1	1	3	1	2	2	1	2	2	2
1	1	1	3	1	2	2	2	2	3
9	9	9	1	9	9	9	9	9	9
2	1	1	2	2	2	3	2	3	3
2	3	2	1	2	2	2	2	3	2
1	9	9	9	9	9	9	9	1	9
1	1	2	1	1	1	1	3	1	3
1	1	9	1	2	1	2	2	1	2
1	3	9	2	1	3	1	1	2	3
1	1	1	1	1	2	9	3	2	1
1	1	1	1	1	1	1	1	3	3
1	2	1	2	1	2	2	3	3	3
1	1	2	1	1	2	1	1	1	3
1	3	3	1	3	2	2	3	1	3
1	2	3	1	1	1	3	2	1	1
1	2	2	1	1	2	2	3	3	3
1	3	9	1	2	2	2	1	1	2
1	3	3	1	2	1	2	2	3	1
1	1	1	1	3	3	3	3	1	3
1	9	3	9	2	1	2	2	1	2
1	3	3	1	3	2	2	2	1	3
2	2	2	1	2	3	3	1	1	2
3	2	3	1	1	2	2	2	3	3

1	3	3	1	1	3	3	2	2	3
1	1	2	1	2	2	2	2	1	2
1	1	1	3	3	3	3	3	9	9
1	9	9	9	9	9	9	9	9	9
1	2	1	3	1	1	2	1	1	3
1	3	1	3	3	2	3	1	1	3
3	3	2	3	1	3	3	3	3	3
1	3	2	3	2	3	3	3	1	2
1	2	1	1	3	2	2	2	1	3
1	2	2	1	2	2	2	2	2	3
1	3	3	3	2	2	1	2	2	3
1	1	2	1	2	1	1	2	1	1
2	2	2	2	2	2	2	2	2	2
3	3	3	3	3	3	3	3	3	3
1	3	3	2	1	3	3	3	1	2
2	3	3	1	2	1	1	1	2	1
2	2	1	3	3	2	3	3	2	2
1	3	3	1	3	1	3	3	1	3
1	1	1	3	3	1	3	1	1	1
3	3	3	3	1	3	1	1	3	1
1	2	3	2	2	1	1	2	2	3
1	2	2	3	2	2	2	2	2	1
1	3	2	9	2	2	2	2	2	1
1	1	2	1	2	1	1	1	1	3
1	1	1	1	2	1	2	2	1	3
1	2	2	1	2	2	2	3	3	3
1	1	3	1	3	2	1	3	1	3
3	3	1	3	3	3	3	3	1	3
1	1	3	3	3	3	3	3	1	3
1	1	1	1	1	1	1	1	2	2
1	1	3	2	3	3	3	3	2	2
3	3	3	3	3	3	3	2	2	1
1	3	3	2	3	1	3	3	3	3
9	9	9	9	9	1	9	9	9	9
3	3	3	3	1	3	2	3	1	3
3	1	3	3	3	3	3	3	3	3
3	3	1	3	3	3	3	3	3	3
1	3	2	1	3	2	1	1	1	2
1	1	2	1	2	1	2	2	2	1
2	1	1	2	2	2	2	2	2	2
3	1	1	3	3	3	3	3	2	1
1	3	9	1	3	3	3	3	3	3
1	1	1	1	1	1	1	1	2	1
1	2	2	2	2	2	2	2	2	2
1	1	1	1	2	2	2	2	3	2
1	3	3	3	2	1	3	1	1	3
1	3	3	1	3	1	3	2	1	3

1	2	3	9	2	2	2	2	2	3
2	3	3	2	2	2	2	2	2	2
2	2	2	2	2	2	2	2	2	2
2	2	2	2	2	2	2	2	2	2
2	2	2	2	2	2	2	2	2	2
2	2	2	3	2	2	2	2	2	2
1	2	3	1	2	1	2	2	3	2
2	2	2	2	2	2	2	2	1	3
2	3	3	2	3	1	2	2	2	1
1	2	2	3	3	2	1	3	2	2
2	2	2	2	2	2	2	2	2	2
3	2	2	2	2	2	2	2	2	2
2	3	3	1	2	1	2	3	2	2
2	2	2	2	2	2	2	2	2	2
2	2	2	2	2	2	2	2	3	1
2	2	3	1	2	2	2	2	2	2
2	2	2	2	2	2	2	2	1	3
2	1	2	2	2	2	2	2	2	2
2	3	3	1	1	1	3	3	1	3
1	2	3	1	2	1	1	2	1	2
2	2	2	2	2	2	2	2	2	2
2	3	3	2	2	2	2	2	2	2
2	2	3	2	2	2	2	2	3	2
2	2	2	2	2	2	2	2	2	2
2	2	2	1	2	2	2	2	2	2
1	1	2	9	1	1	1	2	1	2
2	2	2	1	1	2	2	2	2	2
2	2	2	2	2	2	2	2	2	2
2	2	2	1	2	2	2	2	2	2
2	3	3	3	2	1	2	2	3	3
3	3	3	3	3	3	3	3	3	3
3	2	2	2	2	2	2	2	2	2
2	2	2	2	2	2	2	2	2	2
3	2	2	2	9	3	2	2	3	3
2	2	2	2	2	2	2	2	2	2
1	2	3	1	2	2	3	2	1	3
1	2	3	1	2	2	2	2	1	3
2	2	2	2	2	2	2	2	2	2
1	3	2	2	2	2	2	2	2	2
2	2	2	2	2	2	2	2	2	2
1	3	3	1	2	1	2	2	1	3
2	2	2	2	2	2	2	2	2	2
2	2	2	2	2	2	2	2	2	2
2	2	2	2	2	2	2	2	2	2
2	2	2	2	2	2	2	2	2	2
2	3	3	2	2	2	2	2	2	2
2	2	2	2	2	2	2	2	2	2

2	2	2	2	2	2	2	2	2	2
2	2	2	2	2	2	2	2	2	2
2	1	3	3	1	1	3	2	3	3
2	2	2	1	2	2	2	2	2	2
2	2	2	2	2	2	2	2	2	2
2	2	2	2	2	2	2	2	2	2
1	1	1	1	2	2	2	2	1	3
9	9	9	1	9	9	9	9	1	9
1	1	2	1	2	1	1	2	1	3
1	2	2	1	2	1	2	2	1	3
1	2	2	1	1	2	2	3	2	3
2	2	2	2	2	2	2	2	2	2
2	2	3	1	2	2	2	2	2	2
1	1	1	1	1	1	1	1	9	9
1	3	3	2	3	1	3	3	3	3
2	3	2	2	2	2	2	2	1	2
2	2	2	2	2	2	2	2	1	2
2	3	3	2	2	2	2	2	2	2
1	1	1	1	1	1	1	1	1	3
1	1	3	1	9	2	2	2	2	2
1	9	9	9	9	9	9	9	1	9
2	1	2	2	2	2	2	3	2	2
2	2	2	2	2	2	2	2	2	2
2	2	2	2	2	2	2	2	2	2
2	2	2	2	2	2	2	2	2	2
1	1	3	1	3	2	2	2	2	2
9	9	9	1	9	9	9	9	2	9
2	2	3	2	2	2	2	2	1	2
2	2	2	2	2	2	2	2	2	2
1	1	9	9	1	1	1	2	2	2
2	2	2	2	2	2	2	2	2	2
3	3	3	3	3	3	3	3	1	3
1	1	2	1	3	2	2	1	3	3
3	3	3	3	3	3	3	3	1	3
3	3	2	1	1	2	1	1	3	3
1	2	3	1	1	3	9	9	1	3
3	2	2	1	1	1	2	1	1	3
1	2	3	1	1	1	2	1	1	3
1	1	2	1	2	2	2	2	3	3
1	2	2	1	2	3	3	2	1	3
2	3	2	1	2	1	2	3	2	3
1	2	3	1	2	2	2	2	1	1
1	1	2	1	1	1	2	1	9	1
2	2	2	2	2	2	2	2	2	2
1	1	2	1	1	1	2	1	2	2
1	1	1	1	1	2	2	2	1	2
1	2	3	1	2	1	2	1	1	2

1	2	3	1	2	1	1	2	2	3
1	2	9	2	3	9	1	1	1	1
1	1	2	1	1	1	2	1	3	1
2	1	2	1	2	1	2	2	1	2
1	3	3	1	3	2	3	2	1	2
1	3	2	3	2	2	3	1	1	3
1	2	1	1	3	1	2	2	1	3
9	9	9	1	9	9	9	9	9	9
1	1	2	1	2	2	2	2	2	2
1	1	3	3	1	2	2	2	2	1
1	1	2	1	1	1	1	1	2	2
1	1	2	1	1	1	1	1	2	2
3	3	3	3	3	3	3	3	1	3
1	3	3	1	2	2	1	2	2	2
1	1	2	1	2	1	1	1	3	3
1	1	2	1	2	2	2	2	2	2
1	1	2	1	2	2	2	2	2	2
3	3	3	3	3	3	3	3	2	3
1	1	2	1	2	1	2	2	1	3
1	3	1	1	3	3	2	2	1	3
1	2	3	2	2	3	2	2	2	3
2	2	3	1	2	1	2	2	1	9
1	2	3	1	3	2	2	2	3	3
2	2	3	9	9	9	9	9	9	9
1	2	3	1	2	1	2	1	2	2
1	2	1	1	2	1	1	3	1	3
1	3	9	1	1	1	2	2	3	3
2	9	9	9	9	9	9	9	2	9
1	2	2	1	2	2	2	2	2	2
1	2	2	3	3	3	3	3	1	1
1	2	2	1	2	1	2	1	1	1
1	2	3	3	3	2	2	2	3	2
2	2	2	1	2	2	2	2	1	2
2	2	2	1	2	2	2	2	1	2
2	2	2	1	2	2	2	2	1	2
2	2	2	2	2	2	2	2	2	2
1	1	9	1	2	9	1	1	3	3
2	2	2	2	2	2	2	2	1	2
2	2	2	1	2	2	2	2	1	2
2	2	2	1	2	2	2	2	1	2
1	1	1	1	1	3	3	3	2	2
2	2	2	2	2	2	2	2	3	3
1	2	2	1	2	2	2	2	2	2
1	1	2	1	2	3	3	1	3	2
9	9	9	9	9	9	9	9	9	9
1	1	1	2	2	2	1	1	1	1
2	3	3	3	3	3	3	3	3	3

1	2	2	1	1	2	3	2	1	3
2	1	2	1	3	3	1	2	1	3
2	2	2	2	2	2	2	2	1	3
1	1	2	1	2	1	2	2	1	3
2	2	2	2	2	2	2	2	1	2
1	9	2	2	2	2	2	2	2	2
1	2	2	2	2	2	2	2	2	2
3	3	3	3	3	3	3	3	3	3
1	3	3	1	1	1	3	3	1	3
2	2	2	2	2	2	2	2	2	2
1	2	1	1	1	1	1	1	1	1
1	9	1	2	1	2	1	2	1	1
3	1	1	3	1	3	3	2	2	3
9	2	2	2	2	2	1	2	1	2
3	3	3	3	3	3	3	3	1	3
3	2	3	3	1	1	2	1	2	3
1	1	1	3	3	2	2	2	3	3
2	2	1	2	3	1	3	1	3	1
9	9	9	1	9	9	9	9	1	2
1	3	1	3	1	9	2	1	1	3
1	3	2	1	3	2	2	1	1	3
1	1	3	1	2	3	1	1	3	1
1	1	1	1	1	3	2	1	9	9
1	3	3	1	3	1	2	2	1	3
1	3	2	2	3	1	2	1	1	1
3	3	1	3	3	3	3	3	2	2
1	1	2	1	3	1	3	3	2	3
3	3	3	1	2	1	2	1	1	3
2	3	1	2	2	3	2	3	2	9
3	3	2	1	3	3	3	2	9	3
1	1	1	3	3	2	2	2	1	3
2	2	2	1	2	2	2	2	2	2
1	1	2	1	3	1	3	1	1	3
3	3	3	3	3	3	3	2	3	3
2	2	2	1	2	2	2	2	1	2
1	2	9	1	3	2	3	3	3	3
1	1	3	3	3	3	3	3	3	3
2	2	2	2	2	2	2	2	2	2
1	2	1	1	2	1	3	1	1	1
1	9	9	9	9	9	9	9	9	9
1	1	2	2	2	1	2	1	2	1
3	3	1	3	2	2	1	3	1	3
1	1	3	2	3	2	2	2	1	3
1	2	1	1	3	2	3	2	1	3
1	1	2	1	1	2	1	1	1	3
1	3	3	3	3	3	3	3	1	3
3	3	3	3	3	3	3	3	3	3

3	3	3	2	2	3	1	2	2	3
1	1	3	1	3	1	1	1	3	2
1	3	3	2	2	2	2	3	1	3
1	1	1	1	3	3	3	3	1	3
1	3	3	1	1	1	1	1	1	3
1	2	2	1	2	1	3	1	2	2
1	1	1	1	1	1	1	1	1	3
9	9	9	9	9	1	9	9	2	2
1	3	3	1	2	1	3	2	1	3
3	1	3	2	3	3	3	3	3	3
1	1	2	1	2	1	2	2	1	3
1	1	2	1	2	1	2	2	2	2
1	3	2	2	3	2	2	2	2	2
1	1	3	1	1	1	1	3	1	3
1	2	1	1	1	1	2	2	3	2
1	2	2	2	2	2	2	2	2	2
3	3	3	3	3	2	1	2	2	1
2	3	3	3	3	2	2	2	2	1
1	1	3	2	3	2	2	2	2	3
1	1	1	3	2	1	1	1	1	1
1	1	2	1	9	2	2	2	2	2
9	9	9	9	1	9	9	9	9	9
1	2	2	1	3	2	1	1	3	3
2	3	3	2	2	2	3	2	3	3
1	2	3	2	3	2	1	3	2	3
1	9	1	1	2	2	2	2	2	2
2	2	2	2	2	2	2	2	9	9
2	2	2	2	3	1	1	2	1	3
2	1	3	1	2	3	3	3	3	3
1	1	1	1	1	1	1	1	1	2
1	1	1	3	1	3	3	3	3	1
1	2	3	1	1	2	2	3	9	9
1	3	2	2	3	2	2	3	2	2
3	3	3	1	3	1	3	3	1	3
1	2	3	1	2	1	3	2	1	2
2	2	2	1	2	1	3	2	1	3
1	1	3	1	2	1	3	3	3	2
1	1	9	1	2	2	2	2	1	3
1	2	3	2	3	2	2	2	2	2
1	1	9	9	9	9	9	9	2	9
1	1	2	1	2	1	2	1	1	2
1	1	2	1	2	1	2	2	1	3
1	1	1	2	1	1	2	2	2	2
1	2	3	1	2	1	2	3	1	3
2	2	3	1	2	1	2	1	1	2
1	3	3	1	3	2	3	3	3	3
1	2	3	1	2	3	2	1	1	3

1	2	3	1	2	1	2	1	1	3
2	2	2	2	2	2	2	2	1	3
1	3	2	9	3	2	9	1	2	2
3	1	2	1	2	1	3	2	3	2
1	2	2	1	2	1	1	1	1	3
9	9	9	2	2	9	9	9	2	1
1	3	1	1	3	2	2	2	1	2
1	9	9	9	9	9	9	9	1	9
1	2	3	2	2	2	3	2	1	3
3	1	2	1	2	1	1	2	3	3
2	3	3	1	2	1	3	2	2	2
2	2	3	1	3	3	2	2	2	3
1	2	3	1	3	2	3	2	1	3
1	1	3	1	2	1	2	1	1	3
9	2	2	2	2	9	9	9	9	9
1	2	1	2	3	2	2	2	3	3
1	1	1	1	1	1	1	1	1	1
1	2	2	2	1	2	2	3	2	2
3	1	2	1	2	1	1	2	3	3
1	2	2	3	3	1	2	2	1	3
1	2	1	1	3	1	1	3	1	3
2	1	1	3	3	3	3	3	2	3
1	1	1	1	2	1	1	1	2	2
3	1	1	3	3	3	3	3	1	3
1	2	2	1	1	1	2	1	1	3
1	1	1	1	1	1	1	2	2	1
1	2	3	1	2	2	3	2	1	2
1	2	2	1	2	1	2	2	1	3
1	1	2	1	2	2	3	2	3	3
1	1	2	1	1	2	2	1	1	3
1	1	2	1	1	1	2	2	1	1
1	1	2	2	2	2	1	2	1	2
1	1	2	2	1	2	1	1	1	1
1	1	2	1	2	2	2	2	1	1
1	2	3	1	2	3	1	3	1	3
1	3	3	1	3	2	3	1	1	3
1	1	1	2	2	1	1	1	1	2
1	3	3	1	3	1	1	3	1	3
1	1	2	2	2	1	2	2	1	2
1	1	2	2	2	1	1	1	1	2
3	3	3	3	3	3	3	3	3	3
1	1	3	1	2	1	1	2	2	3
3	1	1	2	2	1	3	2	1	3
2	2	2	2	2	2	2	2	3	3
1	2	3	2	2	2	3	2	3	2
2	2	2	2	2	2	2	2	2	1
1	2	2	3	2	3	2	2	2	3

1	2	2	1	3	2	3	2	1	3
3	1	1	2	1	3	2	3	2	1
2	1	1	2	2	2	2	2	2	2
1	2	3	2	1	3	3	1	2	2
2	2	2	2	2	2	2	2	1	3
2	1	2	3	1	1	2	1	3	1
2	1	2	1	3	2	2	1	3	3
3	3	3	3	3	3	3	3	1	1
1	2	2	1	2	2	2	2	1	3
1	2	2	1	2	2	2	2	1	3
1	1	2	1	1	2	2	2	2	2
2	2	2	2	2	2	2	2	2	2
3	3	3	3	3	3	3	3	2	2
1	1	1	2	2	1	2	2	1	3
1	2	2	1	2	2	2	2	1	3
1	3	2	1	3	1	2	3	3	3
1	2	1	1	1	1	9	2	1	3
1	9	1	3	2	2	9	2	3	3
3	3	3	3	3	3	3	3	2	2
1	3	3	2	2	2	2	2	2	1
1	2	1	1	2	2	1	2	2	2
1	2	2	1	2	2	2	2	1	3
3	3	3	3	3	3	3	3	1	3
3	2	2	3	3	2	3	3	3	2
1	2	9	2	1	2	1	2	1	1
2	2	2	2	2	2	2	2	2	2
1	2	9	1	2	2	3	1	2	1
3	3	2	2	2	3	3	2	2	2
1	3	3	1	3	1	1	1	3	3
1	3	3	1	3	1	2	2	3	3
1	3	3	1	3	1	1	1	1	3
1	1	1	1	1	1	1	1	2	2
1	3	3	1	3	3	3	3	1	2
1	1	1	1	2	2	2	1	1	2
1	3	3	1	3	1	1	1	1	3
2	2	2	2	2	2	2	2	1	2
2	2	1	3	3	2	3	1	3	3
2	3	3	1	2	2	1	3	3	3
3	1	2	1	3	3	1	3	3	3
3	2	2	3	3	3	3	3	3	3
2	2	2	2	2	2	2	2	2	2
2	2	1	3	3	2	2	2	2	2
2	2	2	2	2	2	2	2	2	2
2	2	1	2	2	2	2	2	3	3
2	2	1	3	3	2	2	2	3	3
2	9	2	9	9	9	2	9	2	9
1	1	1	1	1	2	2	2	2	2

2	1	2	2	9	1	2	2	1	1
1	2	2	1	1	1	2	2	2	2
2	2	1	1	2	2	1	1	1	2
1	1	1	2	3	3	3	1	2	2
2	2	2	3	3	1	1	2	2	2
2	2	1	1	1	1	2	2	2	2
2	2	2	2	1	1	2	2	2	2
2	2	2	1	1	1	1	1	2	2
1	1	1	2	2	1	1	1	1	1
2	2	1	1	1	2	2	2	2	2
1	2	2	1	2	1	1	1	1	2
2	2	2	2	1	2	2	2	2	2
2	2	2	2	2	2	2	2	2	2
2	2	2	2	2	2	2	2	2	2
2	2	2	2	2	2	2	2	2	2
2	2	2	2	2	2	2	2	2	2
1	2	2	1	2	2	2	2	1	2
2	2	2	2	2	2	2	2	2	2
2	2	9	9	2	9	2	2	2	2
2	2	2	2	2	2	2	2	2	2
2	2	2	2	2	2	2	2	2	2
2	2	2	2	2	2	2	2	2	2
1	2	2	1	2	2	2	2	1	2
2	2	2	2	2	2	2	2	2	2
2	2	2	2	2	2	2	2	2	2
2	2	2	2	2	2	2	2	2	2
2	2	2	2	2	2	2	2	2	2
2	2	2	2	2	2	2	2	2	2
2	2	2	2	2	2	2	2	2	2
3	3	3	3	3	3	3	3	3	3
2	2	2	2	2	2	2	2	2	2
2	2	2	2	2	2	2	2	2	2
2	2	2	2	2	2	2	2	2	2
1	2	2	1	2	2	2	2	1	2
2	1	2	1	9	1	9	1	9	1
1	9	1	2	9	9	1	9	9	3
9	2	9	1	2	9	9	2	9	9
2	9	2	9	9	2	9	2	2	9
1	2	9	1	9	1	9	1	9	9
1	1	1	1	1	1	1	9	1	1
9	3	9	2	9	1	9	9	3	9
2	2	2	2	1	1	1	2	2	2
3	1	1	1	2	2	2	2	1	1
2	2	2	2	2	2	2	2	2	2
2	9	9	1	3	2	9	1	1	9
2	9	9	2	9	9	2	9	2	9
2	2	2	2	1	1	2	3	1	3

2	2	2	2	2	2	1	2	2	2
2	2	2	2	2	2	2	2	2	1
2	2	2	2	2	1	1	2	2	2
1	1	2	2	1	2	3	1	1	1
1	1	1	1	2	2	1	2	2	1
1	2	3	2	1	2	2	3	1	2
1	2	1	2	1	2	1	2	1	1
1	9	9	1	9	9	3	3	2	2
2	2	2	2	2	2	1	1	2	1
2	1	2	1	2	1	2	1	2	1
1	1	1	1	2	2	2	2	1	2
1	2	2	1	2	2	2	2	1	3
1	1	1	1	2	2	2	3	1	2
1	1	1	1	2	2	2	2	1	2
1	1	1	1	2	2	1	1	1	2
1	2	2	1	3	3	2	2	1	3
1	2	2	1	3	2	2	2	1	3
1	2	2	1	1	2	2	1	2	2
1	1	1	1	2	2	1	1	2	1
2	2	2	3	2	2	1	1	2	2
1	2	2	1	1	2	2	1	2	2
2	2	1	1	2	1	1	2	2	2
1	3	2	1	1	2	2	2	1	2
1	1	2	2	1	1	2	2	2	2
3	2	2	3	2	2	2	1	1	2
3	3	3	3	3	2	2	3	3	3
1	1	1	1	2	2	2	2	3	3
3	2	2	2	2	3	2	2	2	3
3	3	2	2	2	2	2	3	3	2
2	2	2	2	2	2	2	2	2	2
2	2	2	2	2	2	2	2	2	2
1	1	2	2	2	2	2	2	2	2
2	2	2	1	2	2	2	1	1	2
3	2	2	2	1	2	2	2	2	2
2	2	2	1	1	2	2	2	2	2
2	2	2	2	2	2	2	2	2	2
2	2	2	1	2	3	2	2	2	3
1	2	2	1	2	2	2	2	1	1
1	2	2	1	2	1	1	1	1	2
2	2	1	1	2	2	2	1	1	2
2	2	1	1	2	1	1	2	2	1
3	3	3	3	3	3	3	3	3	3
1	3	2	2	1	1	2	2	1	2
1	3	2	1	2	2	2	2	1	3
2	1	1	2	2	1	1	1	2	2
1	2	1	1	2	1	2	3	1	2
2	1	1	2	2	1	1	9	1	2

2	2	2	9	2	9	2	2	2	2
1	1	2	2	2	1	1	1	1	1
2	2	2	2	2	2	2	1	2	2
1	2	2	1	1	2	2	9	2	1
1	2	3	1	3	2	2	2	1	2
1	1	1	1	2	2	2	1	1	3
1	2	2	1	2	1	2	1	1	3
1	2	2	1	1	3	2	3	1	3
1	2	2	1	3	2	2	2	1	1
1	2	3	1	3	3	3	1	3	3
2	2	2	2	2	2	2	2	1	3
1	1	2	1	9	1	1	1	1	3
1	2	1	2	3	2	1	2	2	1
1	1	2	1	1	2	1	1	1	3
1	2	3	1	3	2	2	2	1	2
2	2	1	1	1	2	2	2	2	2
1	2	1	1	1	2	2	2	2	2
1	1	2	1	1	2	2	2	1	2
1	1	1	1	1	1	1	1	3	3
1	2	1	1	3	2	2	2	2	2
1	1	3	1	2	1	1	1	1	3
1	2	1	1	3	2	2	2	1	3
1	3	3	1	2	1	2	1	2	3
3	3	3	3	3	3	3	3	3	2
1	1	3	1	2	2	2	2	1	3
2	2	2	3	3	3	3	3	2	3
1	1	1	2	1	2	1	1	1	3
1	2	1	2	2	2	2	2	2	3
1	1	2	2	1	1	1	1	1	1
1	1	1	2	1	2	9	3	2	3
1	2	2	1	3	2	3	3	1	3
1	1	2	2	2	1	3	2	3	2
1	2	2	2	2	2	2	2	2	2
1	2	2	1	1	1	2	2	2	2
2	1	3	1	3	1	1	2	1	3
1	1	3	1	1	2	2	1	1	3
1	2	3	9	1	2	3	1	3	2
1	2	1	1	1	2	2	1	2	3
1	2	3	1	1	1	3	3	1	3
1	3	3	1	3	2	2	2	3	3
1	3	3	1	3	2	1	3	1	1
1	1	2	2	2	3	3	3	2	2
1	2	2	1	2	2	2	2	3	3
3	2	2	3	3	3	3	2	2	2
1	1	2	1	2	2	2	2	1	2
1	1	1	2	2	1	3	3	2	2
1	1	1	2	2	1	2	2	2	2

1	3	3	1	3	1	3	3	1	3
1	3	3	1	3	1	1	1	1	2
1	2	2	2	1	2	2	2	1	2
1	1	2	1	2	1	2	3	2	3
1	1	2	1	3	2	2	2	1	3
1	1	1	1	3	1	3	2	3	3
1	9	2	1	2	1	2	2	2	2
1	3	3	3	2	9	9	2	3	1
1	2	3	1	2	2	2	2	1	3
1	9	1	2	9	9	9	9	1	9
1	3	3	2	2	1	1	2	3	3
1	2	3	2	1	2	1	3	2	1
2	3	2	1	3	2	1	3	2	1
1	2	3	9	2	9	3	3	3	3
1	1	2	1	2	2	2	2	1	3
1	1	2	2	2	2	2	2	1	2
1	2	2	1	2	1	2	2	2	3
1	2	2	1	2	1	2	2	2	3
1	1	9	3	3	3	3	3	3	3
1	3	3	1	3	3	3	1	1	3
3	3	3	3	3	3	3	3	3	3
3	3	3	3	3	3	3	3	3	3
9	2	3	2	3	2	3	1	2	2
1	1	2	2	1	1	1	2	1	2
2	2	2	2	2	2	2	2	2	2
3	3	3	1	1	1	3	3	1	3
2	2	3	1	2	2	2	2	2	2
2	2	2	2	2	2	2	2	2	2
2	1	1	3	2	2	3	2	1	2
3	1	2	3	3	1	2	2	2	1
2	3	1	1	2	3	3	1	3	3
1	2	3	1	2	1	2	1	1	3
1	1	2	3	2	3	3	3	3	3
3	2	1	3	3	3	3	3	3	3
1	1	2	2	2	2	2	2	2	3
2	1	1	2	3	3	2	1	1	3
2	2	3	1	1	2	2	1	2	1
2	1	1	2	3	3	2	1	1	3
2	2	2	2	2	2	2	2	2	2
1	2	3	2	2	3	2	2	2	2
1	2	2	1	2	1	3	1	1	2
1	2	1	1	1	1	3	1	3	3
1	2	3	1	2	1	2	2	2	2
1	1	2	1	1	1	3	1	1	3
3	3	3	3	3	3	3	3	3	3
2	2	3	1	1	2	2	3	1	3
1	1	2	2	1	1	1	2	1	1

3	2	1	2	2	1	1	2	1	2
1	1	3	1	3	1	3	3	1	3
1	1	3	1	1	1	3	3	1	3
2	2	2	2	2	2	2	2	2	2
2	1	2	2	1	3	2	2	1	1
9	9	9	9	9	9	9	1	9	9
2	2	2	2	2	2	2	2	2	2
2	2	2	1	2	2	2	2	2	2
1	3	1	1	2	1	2	2	1	3
1	2	2	2	2	2	2	2	3	3
2	2	1	1	2	1	1	2	1	1
1	2	3	2	9	1	2	3	3	3
3	9	9	9	9	9	9	9	3	9
1	9	9	9	9	9	9	9	9	9
1	9	9	9	9	9	9	9	9	9
2	2	2	2	2	2	2	2	9	2
2	2	2	2	1	2	2	2	1	2
2	2	2	1	2	2	2	2	2	3
1	1	2	2	2	3	2	2	1	3
1	1	2	1	2	2	2	2	1	3
1	9	9	2	2	2	9	3	1	3
1	3	3	1	3	1	1	1	2	3
1	3	3	1	3	2	3	3	2	3
2	2	2	2	1	2	2	2	2	2
1	1	1	2	3	3	3	2	3	2
1	1	1	3	2	1	2	3	3	3
1	1	1	2	3	3	3	2	3	2
1	9	9	9	9	9	9	9	9	9
2	2	2	1	2	2	2	2	1	1
3	3	1	3	1	3	1	1	3	3
2	3	3	1	2	1	3	1	2	3
1	1	3	1	1	3	3	3	1	3
9	9	3	1	9	1	3	2	3	3
1	2	2	1	2	3	3	1	3	3
9	3	3	1	3	1	3	2	2	3
2	3	2	2	2	1	1	1	2	2
1	2	2	3	3	2	2	2	3	3
1	1	2	2	2	2	3	3	2	2
1	3	3	2	3	2	3	2	3	1
1	2	3	1	3	2	2	2	1	3
3	2	2	1	1	1	1	1	2	2
2	3	2	1	2	2	2	2	2	2
1	2	2	2	2	3	2	3	1	2
2	1	2	2	1	3	1	2	1	3
2	2	2	1	1	1	1	1	2	1
2	2	2	1	2	2	2	2	2	1
2	2	2	2	2	1	2	2	1	2

1	1	1	1	1	1	2	2	1	1
1	2	2	1	2	2	2	2	3	2
1	1	1	2	1	1	1	9	1	9
1	3	1	1	1	3	2	2	3	3
1	3	1	1	2	3	1	1	1	3
1	1	1	2	2	2	2	2	3	1
3	3	1	3	1	3	3	3	3	3
1	3	3	1	2	1	2	3	2	1
3	3	1	3	3	3	3	3	3	3
1	2	2	2	1	1	1	1	1	2
1	2	2	1	2	2	2	2	2	1
1	9	9	9	9	9	9	9	1	9
1	2	3	1	3	1	3	2	3	3
1	1	1	2	2	2	2	1	1	2
1	9	1	9	2	1	3	2	9	9
1	3	1	9	3	1	1	1	9	9
1	3	3	1	3	3	9	9	1	3
1	3	3	1	3	1	2	1	1	2
1	2	1	1	3	3	1	2	1	3
1	1	1	3	3	1	1	1	3	3
1	2	1	1	3	2	2	2	2	2
1	1	1	2	2	3	1	2	1	3
3	3	3	3	3	3	3	3	1	3
1	3	2	1	1	2	2	3	1	3
2	2	2	2	2	2	2	2	2	2
3	2	3	9	1	2	3	1	1	1
1	9	9	1	9	2	9	9	1	9
2	3	3	2	1	2	1	3	3	1
1	2	1	1	2	2	2	1	3	3
1	1	1	1	1	1	1	1	1	2
1	2	2	1	1	2	2	2	2	2
1	2	2	1	2	1	2	1	1	3
1	3	3	1	1	3	3	1	2	3
1	3	3	1	3	1	1	1	1	3
1	3	2	2	3	2	3	1	1	9
1	1	3	2	3	1	1	3	3	2
1	3	3	3	2	2	2	2	2	3
1	1	1	3	1	9	9	9	2	2
2	2	2	2	1	2	2	9	1	2
2	2	2	1	1	1	1	1	2	2
1	3	3	2	9	9	9	2	9	9
1	3	3	1	1	3	3	1	3	3
1	2	3	2	3	2	3	1	2	3
2	3	3	1	3	2	1	3	2	3
2	3	3	1	3	2	1	9	2	3
2	2	3	1	3	2	3	2	2	3
3	2	3	1	2	1	2	2	2	3

1	9	3	1	2	2	2	2	1	3
1	2	1	3	2	2	1	3	1	2
9	9	9	1	9	9	9	9	1	9
9	9	9	9	3	9	9	9	9	9
9	9	9	1	9	9	9	9	1	9
9	9	9	1	9	9	9	9	1	9
1	3	3	1	2	1	2	3	1	3
1	2	3	1	2	2	3	2	1	3
1	1	2	1	2	2	1	1	1	2
1	3	3	1	1	2	3	1	1	3
1	2	1	1	1	2	2	2	1	3
1	3	2	1	3	1	3	2	1	3
1	1	1	1	2	1	1	2	1	2
1	2	2	1	2	2	2	2	1	2
3	3	3	3	3	3	3	3	2	3
1	9	9	9	9	9	9	9	9	9
1	3	3	3	9	1	2	2	3	3
1	2	3	1	2	1	2	1	1	3
1	2	3	1	2	1	1	3	1	3
1	9	9	9	9	9	9	9	9	9
1	9	9	9	9	9	9	9	9	1
3	3	3	3	3	3	3	9	1	3
1	2	3	3	2	1	2	3	1	3
1	1	3	3	2	1	2	1	1	3
9	9	9	1	9	9	9	9	9	1
2	9	9	9	9	9	9	9	2	9
1	3	3	1	2	3	2	3	1	3
1	2	3	1	3	2	3	2	3	3
1	1	2	2	3	2	2	3	1	3
1	1	2	2	1	2	3	2	3	1
2	1	2	1	2	2	3	2	3	3
1	3	3	1	1	3	3	3	2	2
1	9	9	1	9	1	9	9	2	2
1	1	2	1	2	2	2	2	3	3
2	1	2	1	2	2	3	2	3	3
1	1	3	1	3	2	1	1	1	3
1	3	2	1	2	1	3	2	2	3
1	2	2	1	2	2	2	2	2	2
1	1	2	1	2	1	2	2	1	2
1	3	2	1	1	2	2	2	3	2
1	3	1	1	2	1	2	2	2	1
1	1	1	2	2	2	2	2	1	1
1	2	2	3	3	3	3	3	2	2
2	9	9	9	9	9	9	9	9	9
1	9	9	9	9	9	9	9	9	9
1	2	2	2	2	3	3	1	1	3
1	2	2	2	2	2	1	1	3	2

1	1	1	1	1	1	2	3	1	1
2	1	2	2	2	2	2	2	3	3
1	1	1	1	2	2	2	3	1	2
2	1	1	2	1	2	2	2	2	2
1	2	2	2	2	2	2	3	2	2
2	1	2	1	2	1	1	1	2	3
1	2	9	1	2	1	2	2	2	3
1	1	3	1	1	2	2	1	1	2
3	3	3	3	3	3	3	3	9	9
1	1	2	1	1	2	2	2	1	2
2	2	2	2	2	2	2	2	2	2
1	2	3	1	3	1	3	1	2	3
1	2	3	1	2	2	1	2	1	2
2	2	1	2	2	3	1	9	1	2
2	2	2	2	2	2	2	2	2	2
1	2	2	2	2	2	2	3	2	2
2	3	1	2	3	1	2	3	1	2
2	2	2	2	3	3	3	2	2	2
2	2	1	1	3	1	2	2	1	3
3	2	3	3	2	2	2	2	1	1
1	2	2	2	3	3	2	2	1	1
3	3	3	3	3	3	3	3	2	2
2	1	3	2	2	2	2	2	2	2
1	2	9	2	1	2	2	2	3	3
2	2	2	1	1	2	2	3	1	2
3	1	3	2	3	3	3	2	2	3
1	1	2	2	3	2	1	1	2	1
1	1	3	1	2	2	2	2	1	3
1	1	3	2	2	3	1	2	2	3
1	2	3	1	3	1	2	2	1	2
1	1	2	2	2	2	1	2	2	9
3	3	3	3	3	3	3	3	3	3
3	3	3	3	3	3	3	3	3	3
1	2	1	1	3	1	2	2	1	1
2	1	1	1	2	1	1	3	1	1
1	1	1	1	2	3	1	3	1	3
1	2	3	1	3	1	2	1	1	3
1	2	3	2	3	1	2	3	2	3
1	2	2	2	2	2	2	2	2	2
1	1	1	2	2	2	1	1	1	1
1	2	3	1	2	2	1	2	2	3
9	9	9	1	9	9	9	9	1	9
1	2	2	2	2	2	2	2	1	2
3	2	3	1	2	2	2	1	3	3
1	2	2	1	2	2	2	2	2	2
2	2	2	2	2	3	3	1	1	3
1	2	1	1	2	2	2	3	1	3

3	3	1	1	1	1	1	1	3	3
1	2	2	1	2	1	2	2	1	3
1	1	1	2	1	2	2	2	3	9
1	1	1	2	3	1	1	1	3	3
1	1	1	1	2	2	3	3	2	2
1	1	1	1	9	9	3	9	2	2
1	1	1	1	1	1	1	1	2	2
1	1	1	9	2	3	3	2	3	1
9	9	3	9	9	1	9	2	9	9
1	1	2	2	2	1	1	1	1	3
3	3	3	3	3	2	2	2	3	3
2	3	3	2	3	2	2	2	1	2
1	1	2	1	3	2	1	2	1	3
2	2	2	1	2	1	1	2	1	3
1	1	1	1	1	2	2	9	2	2
1	1	2	3	1	3	2	3	1	1
1	1	1	1	1	3	1	1	1	3
1	3	1	3	1	1	2	2	3	2
1	2	2	1	2	1	2	2	2	2
2	2	2	1	2	2	2	2	2	1
1	2	9	1	2	1	2	1	1	2
3	3	3	3	3	3	3	3	3	3
1	3	3	1	2	1	2	2	1	3
1	9	2	2	2	2	3	1	3	3
2	2	3	1	3	2	1	1	1	2
2	2	2	2	2	2	2	2	1	3
2	2	2	2	2	2	2	2	1	3
1	3	2	1	2	3	9	2	3	2
1	3	1	2	3	2	1	1	1	2
1	2	2	1	2	1	2	2	1	3
1	1	2	2	1	1	9	2	1	3
1	2	1	3	9	2	2	2	2	9
1	2	1	2	9	1	2	2	1	2
1	1	2	2	3	3	3	2	1	3
1	2	3	1	3	2	2	3	3	3
1	2	2	1	2	1	2	3	1	9
2	2	2	2	2	2	3	2	2	3
1	3	3	2	1	2	2	3	3	3
1	9	9	9	9	9	9	9	9	9
1	1	2	2	2	1	2	2	2	3
2	2	2	2	2	2	3	2	2	3
1	3	1	3	3	3	3	3	1	2
9	1	1	9	2	2	2	2	2	2
1	1	1	1	2	2	2	2	1	1
2	2	9	1	9	1	2	1	1	3
3	2	3	2	1	2	2	1	1	1
3	3	3	3	3	3	3	3	9	9

1	1	3	1	1	9	9	9	1	1
2	2	2	2	2	2	2	2	2	2
3	3	3	3	3	3	3	3	3	3
9	9	9	9	9	9	9	9	9	9
2	2	2	2	2	1	1	1	1	2
1	2	2	1	1	1	2	2	3	1
3	2	2	2	2	2	2	2	2	2
2	2	2	2	2	2	2	2	1	9
3	3	3	3	3	3	3	3	3	3
1	2	3	2	2	1	2	3	3	3
3	3	2	3	3	3	3	3	1	2
1	3	1	2	3	3	1	3	1	3
3	3	3	3	3	3	3	3	2	1
2	3	2	2	2	3	2	3	2	3
2	3	2	2	2	3	2	3	2	2
2	2	2	2	2	2	2	2	9	3
1	9	9	9	9	9	9	9	9	9
3	3	3	3	3	3	3	3	2	9
3	3	3	3	3	3	3	3	9	9
1	9	9	9	9	9	9	9	3	9
2	3	2	2	2	3	2	3	2	2
2	3	3	3	1	2	3	2	2	2
1	9	9	9	9	9	9	9	2	3
3	3	3	3	3	3	3	3	3	3
1	1	1	1	1	1	1	1	1	1
1	1	3	1	3	3	3	3	1	2
1	1	1	9	9	9	9	9	9	9
2	2	2	2	1	1	2	2	2	1
9	9	9	9	9	9	9	9	9	9
9	9	9	9	9	9	9	9	9	9
9	9	9	9	9	9	9	9	9	9
9	9	9	9	9	9	9	9	9	9
9	9	9	9	9	9	9	9	9	9
1	3	2	1	2	2	2	2	2	3
1	3	2	9	2	9	1	3	1	9
3	1	1	3	3	3	9	3	1	3
2	2	2	2	2	2	2	2	1	2
1	2	3	9	3	2	2	2	1	3
1	1	1	9	1	1	9	1	9	9
3	3	1	3	3	3	3	3	2	2
2	2	2	2	2	2	2	2	2	2
1	2	1	1	2	1	2	2	1	2
1	1	2	1	1	1	2	2	2	2
2	2	2	2	2	2	2	2	2	2
2	2	2	2	2	1	1	1	1	2
1	2	3	2	2	3	2	1	1	2
1	2	1	2	2	3	2	2	2	3

2	2	2	2	2	2	2	2	2	2
1	1	1	1	1	1	1	1	2	2
2	2	3	1	1	2	2	2	1	3
1	2	3	1	2	2	1	2	1	1
1	2	3	1	2	3	1	2	1	2
1	1	1	1	1	1	1	1	1	1
3	9	2	9	1	3	3	1	2	9
2	2	2	2	2	2	2	2	1	2
2	1	2	2	2	2	2	2	2	2
2	2	1	1	9	1	2	3	2	2
1	2	1	2	1	2	3	2	1	3
1	1	2	2	9	1	1	1	1	9
1	2	2	1	1	2	1	2	1	2
3	3	1	3	3	3	3	3	3	3
1	9	9	9	1	2	9	9	3	1
1	1	3	1	3	1	3	3	1	3
1	1	2	2	3	3	1	2	2	2
1	2	2	1	1	3	2	2	3	3
2	2	9	3	2	2	3	2	1	3
1	2	3	1	3	2	2	1	1	3
9	1	9	9	9	9	9	9	1	9
1	1	1	3	1	3	3	3	3	3
1	2	2	1	1	2	2	2	1	2
9	9	9	2	9	9	9	9	2	9
9	9	1	9	9	9	9	9	9	9
9	9	9	1	9	9	9	9	1	9
1	1	3	2	3	3	2	3	3	3
2	2	1	1	2	2	2	2	2	3
9	9	9	2	9	9	9	9	2	9
1	1	1	1	1	1	1	2	2	2
3	1	1	1	1	1	1	1	1	3
1	1	1	1	1	1	1	1	1	3
1	1	2	1	2	1	1	1	2	3
1	1	1	1	1	2	1	2	1	2
2	2	2	2	2	2	2	2	1	2
1	2	2	2	2	2	2	2	2	2
1	1	1	1	3	1	1	1	1	3
1	1	1	1	1	1	1	1	2	2
2	1	3	2	2	2	1	2	2	3
1	1	9	1	2	1	2	2	1	3
3	9	9	9	9	9	9	9	3	9
1	3	3	1	1	2	2	2	1	3
3	3	1	3	3	3	3	3	2	2
3	1	2	1	2	2	9	1	1	3
9	9	9	1	9	9	9	9	9	9
1	1	1	1	1	1	1	1	1	1
1	3	3	1	3	1	3	3	3	9

1	2	3	9	1	2	2	2	3	3
1	1	2	2	2	1	1	2	1	2
1	2	2	1	3	1	2	1	3	3
2	3	3	1	1	3	2	2	3	2
3	3	2	3	3	3	3	3	2	2
3	3	2	3	3	3	3	3	9	9
1	1	1	2	9	2	2	2	1	2
3	3	1	3	3	3	3	3	3	3
2	1	2	1	2	1	2	1	1	2
1	1	3	1	3	1	3	1	1	3
1	9	3	1	3	9	9	9	3	3
1	1	3	1	2	2	2	2	1	2
3	3	3	3	9	2	2	2	2	2
2	1	1	2	2	1	2	1	3	3
1	1	1	1	1	1	1	1	1	1
1	1	2	1	2	2	2	1	1	3
1	3	3	1	3	3	3	3	1	3
1	1	1	1	1	1	1	1	2	2
3	2	3	1	9	3	3	2	3	2
1	2	2	1	2	2	2	2	1	2
1	1	1	1	1	9	9	1	1	1
1	1	2	1	2	2	2	2	3	3
1	1	1	1	1	1	1	1	1	3
1	3	3	1	2	2	2	2	1	2
1	1	1	1	1	1	1	1	1	1
3	3	1	3	3	3	3	3	3	3
1	2	3	1	2	2	2	2	1	3
1	3	3	3	3	3	3	3	3	2
3	3	3	3	3	3	3	3	3	9
1	3	2	1	2	3	2	1	2	2
1	1	2	2	3	3	2	3	3	3
1	9	9	9	9	9	9	9	2	9
3	2	2	2	2	2	2	2	2	2
1	2	9	3	2	9	1	3	1	3
1	2	1	3	1	1	2	3	2	1
1	3	1	1	1	2	1	3	1	3
1	1	3	2	2	2	1	1	1	2
1	3	3	1	1	3	2	3	3	3
1	9	9	9	9	9	9	9	1	3
2	3	3	1	3	2	2	2	2	2
3	3	3	2	2	2	2	2	1	3
2	3	2	3	2	2	2	2	2	3
1	1	2	1	1	2	2	9	2	2
9	9	9	9	9	9	9	9	2	1
2	3	3	1	3	1	2	1	1	3
1	2	2	2	2	2	9	1	1	3
1	1	1	2	2	3	3	3	1	1

2	2	2	2	2	2	2	2	1	2
1	1	1	9	9	9	9	1	9	9
1	1	1	9	9	9	9	1	9	9
1	2	1	3	3	3	3	2	2	2
1	2	2	1	2	1	3	1	1	3
1	9	1	9	9	9	9	9	9	9
9	9	9	1	9	9	9	9	1	9
1	1	1	2	2	1	1	2	1	3
1	2	2	1	2	1	3	9	1	3
1	2	2	2	1	2	3	1	1	3
1	2	3	1	2	2	2	2	2	3
1	2	1	1	2	2	1	2	1	3
3	2	2	1	2	2	2	2	1	2
3	2	2	1	2	2	2	2	1	2
1	9	3	1	3	2	2	2	1	1
1	1	1	1	1	1	1	2	1	2
1	2	1	2	2	2	2	2	3	3
1	2	1	2	2	2	2	2	3	3
1	2	2	1	2	2	2	3	1	3
1	1	3	2	2	2	3	1	1	3
2	2	2	1	1	2	2	2	2	2
1	1	2	1	1	2	1	1	1	1
1	1	1	1	1	1	1	1	9	9
1	1	2	1	3	1	1	2	1	2
1	3	3	3	3	3	3	3	1	3
2	2	2	1	2	2	2	2	1	1
3	3	3	3	3	3	3	3	3	3
1	1	1	3	3	3	2	2	2	2
1	2	3	1	2	1	1	1	1	2
1	1	1	1	1	1	1	2	1	3
2	2	2	2	2	2	2	2	2	2
1	1	1	1	1	3	9	2	2	2
1	2	9	1	3	1	2	1	1	3
1	2	3	2	3	3	2	2	1	2
1	1	2	9	1	1	1	1	3	3
2	2	2	2	2	2	2	2	2	2
1	2	1	3	1	2	1	3	2	2
2	2	2	2	2	2	2	2	2	2
1	2	2	1	2	2	2	2	1	2
2	3	9	1	2	2	2	2	2	3
2	2	2	1	3	2	2	2	2	2
1	2	3	2	1	2	3	1	1	2
1	1	3	1	1	1	1	2	2	3
1	1	2	1	2	2	1	1	1	3
1	1	3	1	2	1	1	1	1	2
1	2	3	2	1	1	1	2	1	2
2	2	1	2	2	2	2	2	2	3

1	1	2	1	2	2	1	1	1	3
3	9	9	9	1	9	9	2	3	1
1	2	2	1	2	2	2	2	1	9
1	9	9	1	9	1	9	9	1	3
1	2	2	2	2	2	2	2	3	3
2	2	2	2	2	2	3	2	1	1
1	3	2	1	2	3	3	2	2	3
2	2	3	3	3	3	3	3	2	2
1	9	2	1	2	2	1	3	1	1
2	2	2	2	2	2	2	2	3	3
1	1	1	1	2	1	2	2	1	3
1	1	1	1	2	2	2	2	1	3
2	2	2	2	2	2	2	2	3	3
1	1	3	3	1	3	1	2	1	1
1	2	2	1	2	1	3	2	2	3
1	1	2	1	3	2	1	2	1	3
2	2	1	1	1	1	1	1	1	1
9	9	9	1	9	9	9	9	1	9
3	3	1	3	3	2	2	2	2	3
1	2	2	9	1	2	2	1	1	2
1	2	3	1	1	2	1	2	1	2
1	2	3	1	1	2	2	2	1	3
1	2	3	1	3	2	2	3	2	2
1	2	3	2	1	3	2	1	2	1
1	3	3	1	3	1	3	2	1	3
1	2	3	1	2	2	3	3	1	9
1	2	3	1	2	2	2	2	2	3
2	3	3	1	2	2	3	3	1	3
2	3	2	1	2	1	2	1	1	3
2	2	3	1	3	2	3	3	1	2
1	2	1	1	3	2	2	2	3	3
1	1	1	2	1	1	1	1	2	3
1	3	1	2	3	3	2	1	3	2
1	1	1	1	1	1	1	1	2	3
1	3	9	1	2	1	2	1	3	3
1	2	1	3	1	2	2	2	3	2
1	3	3	1	3	2	2	2	1	2
2	3	2	1	9	2	1	3	2	2
1	3	3	1	2	1	3	3	1	3
2	2	3	1	3	2	2	2	2	2
1	2	3	9	1	2	1	3	1	3
1	2	1	2	3	2	3	1	1	1
9	9	9	9	9	9	9	9	9	9
1	3	3	3	2	1	3	1	1	3
1	2	1	1	1	1	2	1	1	2
1	1	1	1	2	1	2	1	1	3
1	2	2	1	1	3	2	2	1	3

3	3	1	3	3	3	1	2	3	1
3	3	3	1	3	3	3	3	1	3
1	1	1	2	2	2	1	2	2	2
1	2	3	1	2	1	3	3	1	3
3	1	2	2	3	3	3	1	3	3
1	1	2	1	2	2	1	2	2	2
1	3	3	1	2	2	2	1	3	1
1	1	1	1	1	1	1	1	1	1
1	1	1	1	1	1	2	2	2	2
1	2	3	1	2	1	2	2	1	1
2	2	2	1	2	2	1	9	2	2
1	1	1	1	2	1	1	2	1	3
1	1	1	1	2	2	1	1	1	3
1	1	1	1	2	2	1	1	1	3
2	3	3	1	3	1	1	2	1	3
2	2	3	1	2	3	2	3	1	2
1	1	3	1	2	3	3	3	2	2
1	3	3	1	2	1	2	2	2	3
1	1	1	1	2	3	3	3	3	3
1	2	1	1	1	2	2	2	1	1
3	1	3	3	3	3	3	3	1	3
9	9	9	9	9	9	2	9	9	9
2	1	1	2	3	3	3	3	2	9
2	1	2	1	3	2	2	3	3	3
9	9	9	9	9	9	9	9	9	9
9	9	9	9	9	1	9	9	9	9
1	3	3	1	3	1	3	3	3	9
9	9	9	9	9	9	9	9	9	9
3	3	3	1	2	1	9	1	2	3
3	3	1	3	3	3	3	2	1	3
1	2	1	1	2	2	2	3	1	3
1	1	1	1	2	2	2	3	9	9
1	3	2	3	2	9	9	9	9	9
2	1	2	1	3	2	2	3	3	2
1	3	2	1	3	1	3	3	3	2
1	3	3	1	3	1	3	3	2	3
1	1	1	1	2	1	9	1	1	3
1	1	9	9	9	2	2	2	9	9
1	2	2	1	2	1	2	3	1	3
1	1	1	1	2	1	3	2	1	3
1	2	3	1	3	1	2	1	1	3
1	1	1	1	1	1	1	1	3	3
1	3	3	2	9	1	9	9	1	9
1	2	3	1	1	2	3	2	1	3
1	9	9	9	9	9	9	9	9	1
9	9	9	9	9	9	9	9	9	9
9	9	9	1	9	9	9	9	9	9

1	2	9	1	2	3	3	3	1	1
9	9	9	1	9	9	9	2	9	9
1	3	2	3	3	3	3	3	2	9
2	1	3	2	3	2	3	2	1	3
1	2	2	1	2	3	1	2	2	2
3	3	3	2	3	1	3	2	1	3
3	3	3	1	2	2	2	2	3	3
3	3	3	3	3	3	3	3	2	2
1	1	1	1	1	1	1	1	2	2
2	2	2	2	2	2	2	3	1	3
2	3	2	1	2	1	3	2	1	1
1	1	1	2	1	1	1	1	2	2
1	2	1	1	2	2	1	3	1	3
9	1	1	1	1	2	2	2	1	1
1	2	2	1	2	1	2	2	2	2
1	1	1	1	1	1	1	9	1	1
1	9	1	1	9	1	1	1	1	3
1	2	3	2	2	2	3	1	1	3
2	2	2	1	2	2	2	2	2	1
1	3	3	1	1	2	1	1	3	2
9	9	9	9	9	9	9	9	9	9
3	3	1	2	2	2	2	2	2	2
1	1	1	2	2	2	2	2	1	1
1	1	1	2	2	2	1	1	1	1
1	1	2	2	2	1	1	1	1	1
2	2	2	2	2	2	2	2	1	1
2	2	2	3	3	3	3	3	2	2
1	1	2	2	3	3	3	3	1	2
1	2	2	2	2	2	2	2	1	2
2	2	2	1	2	2	2	2	1	1
9	9	9	9	9	9	9	9	9	9
9	9	9	9	9	9	9	9	9	9
9	9	9	9	9	9	9	9	9	9
3	3	1	1	2	1	3	2	2	3
1	1	1	1	2	1	1	1	3	3
1	2	2	3	2	2	2	2	2	2
1	2	2	1	2	2	2	1	1	1
1	1	3	9	3	1	3	1	1	3
1	1	1	1	1	1	1	1	2	2
2	2	2	3	2	3	2	3	3	3
3	3	3	1	3	3	2	2	9	9
2	1	9	1	1	1	2	3	1	3
1	2	2	1	1	2	2	1	1	1
1	2	3	1	2	2	2	2	2	2
1	2	2	3	2	2	2	2	3	3
1	1	1	1	1	1	1	1	1	3
9	9	9	9	9	9	9	9	9	9

1	1	1	1	1	1	1	1	1	2
2	2	2	1	2	2	1	2	2	2
1	1	1	1	1	1	1	1	2	3
1	3	3	1	3	1	3	2	1	3
1	9	9	1	9	9	9	9	3	3
9	9	9	9	9	9	9	9	9	9
1	2	2	1	9	1	1	2	2	2
1	9	9	9	9	9	9	9	1	9
1	9	9	9	9	9	9	9	9	9
1	1	1	1	1	1	3	3	1	9
9	9	9	1	9	9	9	9	1	9
1	9	9	9	9	9	9	9	9	9
1	1	2	1	1	1	2	1	1	3
1	2	3	1	1	1	2	3	1	2
1	1	1	3	3	2	1	2	1	3
1	2	2	1	2	2	3	2	1	3
1	2	1	1	1	1	1	1	1	1
1	2	2	1	1	1	1	1	1	1
1	2	2	1	2	2	3	3	1	3
1	2	1	3	3	2	2	2	1	3
1	2	1	1	2	2	2	2	1	3
1	2	3	1	2	2	2	2	1	3
2	2	2	1	1	1	2	2	1	3
1	2	3	1	3	2	3	3	3	3
1	3	2	3	1	3	2	3	2	3
1	3	1	1	1	1	1	2	3	3
2	2	2	2	2	2	2	2	3	3
1	2	3	1	2	2	2	2	1	3
1	2	1	2	2	2	2	2	1	3
1	2	2	3	3	2	2	3	1	2
1	2	1	2	2	2	2	2	1	2
1	2	1	1	3	2	2	2	3	3
2	2	3	1	3	1	2	2	1	2
1	1	2	1	1	2	3	3	1	3
1	2	2	9	9	1	9	9	9	1
2	2	3	2	3	3	3	1	3	3
1	2	3	1	3	2	3	2	1	3
1	2	3	1	2	1	2	1	1	3
1	2	2	1	2	2	2	2	3	3
3	3	1	2	9	1	3	2	2	2
9	9	9	1	9	9	9	9	1	9
9	9	9	1	9	9	9	9	1	9
1	9	9	9	1	9	9	9	1	9
1	2	3	1	3	2	3	2	2	3
9	9	9	1	9	9	9	9	1	9
1	3	3	1	1	1	2	3	9	9
1	2	1	1	2	3	3	2	2	2

1	2	2	2	2	2	1	2	1	2
1	1	2	3	2	2	2	2	2	3
1	3	2	1	3	1	1	3	1	9
1	2	2	1	1	1	2	3	2	3
1	1	3	2	2	2	2	2	3	3
1	2	2	1	2	1	2	1	2	3
1	3	3	1	2	2	3	1	1	1
1	2	3	1	2	1	3	2	2	3
1	3	3	1	3	2	2	2	1	3
3	1	2	2	1	2	1	9	1	2
1	2	2	1	2	2	1	1	3	3
2	2	3	3	1	2	2	2	2	3
1	3	2	1	1	2	3	1	1	3
2	2	3	3	3	3	1	1	1	2
1	3	3	1	2	1	2	1	1	3
1	1	3	1	3	2	2	1	9	9
1	2	2	1	2	2	2	2	1	3
1	3	1	3	1	3	2	2	3	1
1	1	2	2	2	2	2	2	2	2
2	2	2	2	2	2	2	2	1	1
1	3	3	3	3	2	2	2	1	2
1	2	2	1	2	1	2	1	3	2
1	2	2	1	2	2	2	2	2	2
3	3	3	1	2	2	3	2	1	2
1	1	1	1	1	1	1	1	1	3
1	3	1	2	3	2	2	2	3	3
2	2	3	1	2	1	3	2	1	3
1	1	1	1	1	1	1	1	1	3
2	2	2	1	2	1	2	3	1	2
1	2	1	2	2	2	2	2	1	3
1	2	3	1	2	2	2	2	1	2
9	3	3	1	1	1	3	2	1	3
1	2	1	1	1	1	2	2	2	2
1	3	2	1	3	2	3	1	1	3
1	1	2	1	2	2	1	2	1	1
1	1	1	2	2	2	2	3	1	1
1	1	1	1	1	1	2	1	2	2
1	2	2	2	2	2	2	2	2	3
1	3	3	1	3	1	3	1	1	1
1	1	1	1	1	1	1	1	1	2
1	1	3	1	2	3	2	1	1	3
1	3	3	1	2	1	3	2	1	3
1	2	3	2	3	2	2	2	1	2
1	1	2	1	1	1	2	1	1	2
1	3	3	1	3	1	2	1	1	3
3	9	9	1	1	2	9	2	2	2
2	3	3	1	3	1	3	3	3	3

1	3	2	1	3	1	3	3	3	2
1	2	2	2	2	3	3	3	3	3
2	2	2	2	2	2	2	2	3	2
1	2	3	2	2	1	2	3	1	2
1	1	2	2	2	2	2	2	1	2
1	1	3	1	1	1	2	2	2	3
1	2	2	2	3	3	3	2	2	1
1	2	3	1	1	1	2	2	3	3
1	2	3	2	3	1	2	3	1	3
1	2	3	1	3	1	2	2	1	3
1	3	9	1	2	1	2	1	3	3
1	1	2	1	3	2	2	1	1	3
2	2	2	2	2	2	2	9	1	1
1	2	2	1	2	1	2	1	1	2
3	3	3	1	3	3	3	3	1	3
1	2	2	1	2	2	2	2	2	2
1	1	3	1	1	1	2	2	2	3
1	1	2	1	2	2	2	2	2	1
1	1	2	2	2	2	2	2	2	3
1	1	2	1	3	2	2	2	1	2
1	3	1	1	2	1	2	3	1	3
1	1	1	1	1	1	1	1	3	3
2	2	3	1	3	2	3	2	1	3
1	2	3	1	3	2	2	2	3	3
1	1	3	1	2	3	1	2	1	3
1	3	2	1	1	1	3	2	1	9
3	3	3	3	3	3	3	3	1	2
1	2	2	1	2	2	2	1	1	2
2	2	2	1	2	2	2	2	1	2
1	2	1	1	2	2	2	1	3	2
1	2	1	2	2	2	2	2	2	2
2	2	2	2	2	2	2	2	2	3
1	2	2	1	2	2	2	3	2	3
1	2	3	1	1	1	2	2	1	2
1	3	1	2	2	1	3	2	3	2
3	1	2	1	1	1	2	2	1	2
1	2	3	1	2	2	2	2	1	3
1	1	1	1	2	1	2	1	1	3
1	3	1	2	3	1	2	3	3	9
2	3	3	2	2	2	2	2	2	3
2	3	3	1	2	2	3	3	1	3
1	3	3	1	9	2	2	2	1	3
2	3	3	1	2	2	2	2	1	3
1	3	3	1	1	1	9	2	1	3
1	2	2	1	2	2	2	2	3	3
3	2	1	2	2	1	2	2	2	2
1	2	3	1	2	1	3	2	2	3

1	2	3	2	2	1	3	2	2	2
2	2	3	2	2	1	3	2	2	3
1	2	3	1	3	2	3	3	1	3
2	3	3	1	2	2	3	3	1	3
1	3	3	1	1	1	1	2	1	3
3	3	1	9	3	2	2	3	3	1
1	2	3	2	3	2	3	2	2	1
2	1	3	2	2	1	3	3	1	3
1	2	3	1	2	2	3	1	1	3
1	2	2	2	2	2	2	2	3	3
1	3	1	1	2	3	2	1	3	3
1	1	1	2	2	3	3	3	1	3
1	1	1	2	1	3	3	1	3	1
2	2	2	1	2	1	2	2	3	3
1	1	1	1	1	1	1	1	1	1
1	2	2	1	2	2	3	3	1	3
2	2	1	2	2	2	2	2	2	3
3	1	1	1	1	1	3	2	1	3
3	3	9	3	2	9	1	3	2	1
1	1	2	1	2	1	1	2	1	9
1	1	1	2	2	2	2	2	1	1
1	1	2	2	2	2	3	3	1	2
1	1	2	2	1	1	1	1	1	1
2	2	1	2	2	2	2	2	1	1
2	1	2	2	1	2	2	2	2	1
1	2	2	1	2	2	3	3	1	3
1	1	1	2	2	3	3	3	1	3
1	2	2	2	2	2	2	2	3	3
1	3	1	2	3	1	2	3	3	9
1	1	2	1	2	1	1	2	1	9
1	2	3	1	2	2	2	2	1	3
9	9	9	9	9	9	9	9	9	9
1	1	1	1	2	2	2	1	3	3
1	1	1	3	3	3	3	1	2	2
1	1	1	1	2	1	2	2	1	2
2	1	3	1	3	1	1	1	1	3
1	1	1	1	1	1	3	3	1	3
1	1	2	1	2	2	2	2	2	2
2	2	2	2	2	2	2	2	1	3
1	1	1	1	2	2	2	2	2	2
1	2	3	1	2	1	2	1	1	2
1	2	3	1	2	1	2	1	1	2
1	3	3	1	2	2	2	3	3	3
1	3	9	1	1	1	3	2	1	3
2	1	2	1	2	3	1	2	1	2
2	2	3	9	1	2	3	3	2	2
1	2	1	3	3	3	3	2	1	3

1	1	2	2	2	2	2	3	2	2
1	1	1	1	3	3	3	2	1	1
1	9	1	1	1	1	1	1	1	1
1	2	2	1	2	2	2	2	1	3
3	2	2	2	2	2	2	2	3	3
1	3	3	1	1	1	1	1	3	3
1	9	3	1	3	2	1	2	1	3
2	9	9	9	9	9	9	9	3	9
1	9	9	9	9	9	9	9	3	9
1	9	9	9	9	9	9	9	3	9
1	1	1	3	3	3	1	2	2	3
2	2	2	2	3	3	1	2	2	2
1	2	1	3	3	1	3	2	2	3
1	2	1	3	3	1	3	2	2	3
1	2	1	3	3	1	3	2	2	3
3	3	3	1	1	1	3	3	3	1
2	2	2	3	3	3	1	1	3	1
3	9	2	3	2	1	1	2	1	1
2	1	3	3	2	3	2	1	3	1
3	2	1	1	3	2	1	3	2	1
1	2	3	2	3	3	2	1	2	3
2	2	1	1	3	9	2	3	2	3
2	2	2	2	2	2	2	2	3	1
3	2	2	2	2	1	3	3	3	2
1	1	1	2	2	2	2	2	2	2
2	2	2	1	1	1	3	1	2	2
1	1	1	2	2	2	3	3	3	1
1	1	1	2	2	1	2	2	2	2
1	1	2	3	3	3	1	1	2	2
1	1	2	3	3	3	1	1	2	2
1	2	1	3	3	1	3	2	2	3
1	1	1	3	3	1	3	2	2	2
3	3	3	1	1	1	3	3	9	9
2	2	2	2	2	2	2	2	2	2
2	2	2	2	2	2	2	2	2	2
3	3	3	1	1	1	3	3	1	3
3	3	3	1	1	1	3	3	1	3
2	2	2	2	2	2	2	2	2	2
2	2	2	2	2	2	2	2	1	3
3	9	9	9	9	9	9	9	9	9
1	1	1	2	2	2	3	3	3	1
1	1	2	3	3	3	1	1	2	2
1	1	2	3	3	3	1	1	2	2
1	1	2	3	3	3	1	1	2	2
1	1	2	3	3	3	1	1	2	2
9	1	2	2	3	9	2	2	3	9
1	2	3	2	1	3	3	2	3	3

1	3	2	1	3	2	1	3	3	2
2	1	1	2	2	3	1	1	3	1
2	2	2	1	2	3	1	1	3	2
2	3	3	2	3	3	1	1	3	2
2	1	2	3	2	3	1	2	3	2
1	1	2	1	3	3	1	1	3	3
1	2	3	2	3	2	1	1	3	2
2	2	2	2	2	2	2	2	9	9
9	9	9	3	9	9	9	9	3	9
9	9	9	9	9	9	9	9	9	3
3	3	3	1	1	1	3	3	2	3
1	2	2	1	9	1	3	3	1	9
1	2	2	3	9	3	1	1	3	9
1	1	1	3	3	3	1	1	3	1
1	3	3	3	3	3	2	2	3	1
2	2	2	2	2	2	2	2	2	2
2	2	2	2	2	2	2	2	9	2
9	9	9	2	9	9	9	9	2	9
2	9	9	9	9	9	9	9	3	9
1	9	9	9	9	9	9	9	3	9
1	1	2	3	3	3	3	3	3	2
1	2	2	3	3	3	2	1	2	2
2	2	2	2	2	1	3	3	3	1
1	9	9	9	9	9	9	9	3	9
9	9	9	9	2	9	9	9	3	9
9	2	9	9	9	9	9	9	2	9
2	2	2	2	2	2	2	2	1	3
2	9	9	9	9	9	9	9	2	9
1	2	2	3	2	3	2	2	3	2
9	9	9	9	9	9	9	9	2	2
1	3	2	3	3	2	1	3	3	3
1	9	9	9	9	9	9	9	3	9
2	1	1	3	3	3	1	1	3	1
2	2	2	2	2	2	2	2	2	1
2	2	2	2	2	2	2	2	3	1
2	2	2	2	2	2	2	2	9	9
1	1	1	3	3	2	2	2	3	1
1	1	1	3	3	3	1	1	3	1
1	1	1	3	3	3	2	2	3	2
1	9	9	9	9	9	9	9	3	9
1	9	9	9	9	9	9	9	3	9
1	9	9	9	9	9	9	9	3	9
1	9	9	9	9	9	9	9	3	9
1	9	9	9	9	9	9	9	3	9
9	2	9	9	9	9	9	9	2	9
1	2	2	3	2	2	3	3	2	1
1	2	3	3	2	1	2	3	3	3

9	1	9	9	9	9	9	9	9	9
1	2	2	3	2	3	2	2	2	2
9	9	9	9	2	9	9	9	3	9
1	2	2	3	1	3	2	1	3	3
1	1	9	3	3	3	1	1	2	2
1	2	2	3	2	3	2	2	3	3
1	9	9	9	9	9	9	9	2	9
3	3	3	1	1	1	3	3	1	3
1	2	1	2	3	3	2	2	2	2
1	9	9	9	9	9	9	9	3	9
2	1	3	1	1	1	3	3	2	2
1	9	9	3	3	9	9	1	9	9
1	9	9	9	9	9	9	9	3	9
1	9	9	9	9	9	9	9	3	9
2	2	2	2	3	3	1	1	9	9
1	9	9	9	9	9	9	9	3	9
1	1	2	3	3	3	3	3	3	2
1	9	9	9	9	9	9	9	3	9
1	3	3	2	2	2	1	2	1	3
1	1	1	2	2	1	3	3	3	1
1	1	1	3	3	3	1	1	3	3
1	2	3	3	2	1	1	2	3	2
1	1	1	3	3	3	1	1	3	1
3	3	3	1	1	1	3	3	3	3
1	1	2	3	3	3	1	1	3	3
1	1	1	3	2	1	3	3	3	2
1	1	1	3	3	3	1	1	3	2
1	1	1	3	3	3	1	1	3	1
1	2	2	3	3	3	1	1	3	2
1	1	2	3	3	3	3	1	3	3
1	2	3	2	3	2	3	2	2	3
1	2	3	1	9	2	9	1	3	2
9	9	9	9	9	9	3	9	9	9
1	9	9	9	9	9	9	9	3	9
9	9	9	3	9	9	9	9	3	9
9	9	1	9	9	9	9	9	3	9
1	9	9	9	9	9	9	9	2	9
9	9	3	9	9	9	9	9	3	9
9	9	9	9	3	9	9	9	3	9
2	9	9	9	9	9	9	9	2	9
2	9	9	9	9	9	9	9	2	9
2	9	9	9	9	9	9	9	2	9
2	9	9	9	9	9	9	9	2	9
2	9	9	9	9	9	9	9	2	9
2	9	9	9	9	9	9	9	3	9
9	9	9	9	9	9	9	9	3	9
1	1	2	3	3	3	2	2	3	9

1	1	2	3	3	3	3	1	3	2
9	9	2	2	9	1	3	3	3	9
1	9	9	9	9	9	9	9	9	1
3	2	2	2	2	9	1	2	3	2
1	9	9	9	9	9	9	9	3	9
1	9	9	9	9	9	9	9	3	9
1	9	9	9	9	9	9	9	3	9
2	9	9	9	9	9	9	9	3	9
2	9	9	9	9	9	9	9	3	9
2	2	2	2	2	2	2	2	3	2
2	1	2	3	2	3	2	1	2	1
1	2	1	2	3	2	1	2	2	1
2	2	2	2	2	2	2	2	3	2
3	1	1	2	3	3	2	2	3	1
1	1	1	2	2	1	3	2	3	1
1	1	1	3	2	3	1	1	3	1
1	9	9	9	9	9	9	9	9	9
1	9	9	9	9	9	9	9	3	9
1	9	9	9	9	9	9	9	3	9
3	9	9	9	9	9	9	9	9	1
9	9	3	9	9	9	9	9	9	9
3	9	9	9	1	9	9	9	9	9
1	9	9	2	1	1	3	3	2	9
2	9	9	1	9	2	9	9	2	9
2	2	2	2	9	2	2	9	3	9
9	2	9	9	9	9	9	9	3	9
1	9	9	9	9	9	9	9	9	1
2	2	2	2	2	2	2	2	2	2
1	2	2	1	1	3	3	2	2	3
1	9	9	9	9	9	9	9	3	9
1	2	2	2	2	2	2	2	3	2
1	2	2	3	1	3	1	1	3	3
1	1	2	3	2	2	2	2	1	3
9	9	9	3	9	9	9	9	3	9
1	2	2	3	1	3	2	1	3	3
1	1	1	2	2	2	2	2	1	3
1	9	9	9	9	9	9	9	2	9
2	2	1	3	3	3	1	1	9	9
9	9	9	3	9	9	9	9	3	9
1	2	2	3	3	3	1	1	3	3
2	2	2	3	3	3	2	2	3	2
1	2	2	3	2	3	1	2	2	3
2	1	3	2	2	2	2	2	2	2
9	9	9	2	9	9	1	9	2	9
9	9	9	3	9	9	9	9	3	3
2	2	2	2	2	2	2	2	2	9
9	9	9	3	9	9	9	9	3	9

1	1	2	3	1	3	2	2	3	3
1	2	2	3	2	3	2	1	2	2
1	2	2	2	2	3	1	1	3	1
1	2	2	3	1	2	2	2	2	1
1	1	2	3	1	3	1	2	3	2
1	2	2	3	2	3	1	2	3	3
1	3	3	3	3	3	1	1	1	3
1	2	1	3	2	3	1	1	3	1
9	9	9	9	9	9	9	1	9	9
9	9	9	9	9	9	9	9	3	2
2	2	2	2	2	2	2	2	1	3
1	1	3	3	1	3	3	3	3	3
1	1	1	3	3	3	1	1	3	3
1	1	2	3	3	3	1	1	1	3
9	9	9	9	9	9	9	9	2	1
1	2	3	3	3	3	1	2	9	9
2	2	2	2	2	2	2	2	2	2
1	2	2	3	2	2	2	2	3	3
1	2	2	3	2	2	1	2	2	2
1	2	3	3	2	1	1	2	2	2
1	2	2	3	2	3	2	2	2	1
1	1	2	3	2	2	2	2	2	2
1	1	2	3	3	2	2	2	3	3
1	2	2	2	2	2	2	2	3	3
1	1	2	2	2	3	1	1	3	2
1	1	1	3	3	3	2	1	3	3
1	2	1	3	2	3	2	2	3	3
1	1	2	3	1	2	2	2	2	1
2	3	3	3	1	2	2	2	2	3
2	2	2	2	2	2	2	2	2	2
2	2	2	2	2	2	2	2	2	2
2	2	2	2	2	2	2	2	2	2
1	2	2	3	2	2	2	2	2	2
2	2	2	2	2	2	2	2	2	2
2	2	2	2	2	2	2	2	2	2
2	2	2	2	2	2	2	2	9	9
1	1	1	3	3	3	1	1	1	3
2	9	9	9	9	9	9	9	2	9
1	1	1	3	2	3	2	1	2	1
9	9	9	3	9	3	1	1	3	3
1	1	1	2	3	1	3	3	3	3
9	9	9	3	9	9	9	9	9	9
1	1	1	3	3	3	1	1	3	3
2	3	3	3	3	3	3	2	3	3
1	2	2	3	1	2	2	1	2	2
1	1	3	9	3	3	1	2	1	3
1	2	3	3	2	2	2	2	3	3

9	9	9	9	9	9	9	9	2	1
1	2	2	3	3	3	2	2	3	2
1	2	1	1	2	3	2	3	3	1
1	2	1	3	3	3	2	1	2	2
1	1	1	3	3	3	1	1	2	2
9	9	9	9	9	9	9	9	3	9
1	1	1	3	3	3	1	2	3	3
1	1	1	3	2	3	2	2	3	3
2	3	9	3	2	3	1	2	9	9
1	2	3	3	3	2	3	2	3	2
1	1	3	3	2	2	2	2	2	3
1	2	2	3	2	3	2	1	1	2
1	1	2	3	3	3	1	1	3	3
1	2	3	2	1	2	3	2	1	3
1	2	1	3	2	2	1	2	3	3
2	2	2	2	2	2	2	2	2	9
1	2	3	3	1	9	3	3	1	3
9	1	9	9	9	9	9	9	9	9
1	2	1	3	2	3	2	2	3	3
1	1	1	2	2	3	1	2	1	2
2	2	2	2	2	2	2	2	2	2
1	2	2	3	2	2	3	3	2	3
2	2	2	2	2	2	2	2	2	2
1	1	9	3	3	1	3	3	3	3
1	9	9	9	9	9	9	9	3	9
1	2	2	3	2	2	2	2	2	2
2	2	3	2	2	2	2	2	2	2
2	2	2	2	2	2	2	2	2	2
1	2	2	3	3	3	2	1	3	2
1	2	3	2	2	2	2	2	1	3
1	2	1	3	2	3	1	1	9	9
1	9	9	9	9	9	9	9	3	9
1	2	2	3	3	2	2	2	2	3
2	3	9	3	2	2	2	2	2	2
1	3	3	3	2	3	2	2	2	2
1	2	2	3	2	2	2	2	3	3
2	2	2	2	2	2	2	2	3	2
1	1	2	2	3	2	2	1	1	3
1	1	1	3	2	2	2	2	2	2
1	1	1	3	3	2	2	2	3	2
2	2	2	3	2	2	2	2	3	3
1	1	2	3	3	3	2	2	3	3
1	2	2	3	3	3	2	2	3	3
9	1	9	9	9	9	9	9	3	9
2	2	2	3	2	3	2	1	3	3
1	1	3	3	2	1	3	2	3	9
9	9	9	3	9	9	9	9	3	2

2	2	2	2	2	2	2	2	2	2
1	1	2	3	2	3	2	2	3	3
1	2	2	2	2	2	2	2	3	2
2	2	2	2	2	2	2	2	3	3
2	2	2	2	2	3	2	2	2	3
1	1	3	3	1	3	1	2	3	3
2	2	2	2	2	2	2	2	2	2
2	2	2	2	2	2	2	2	2	2
2	2	2	2	2	2	2	2	2	2
1	2	2	2	2	3	2	2	3	2
2	1	2	2	2	2	2	2	2	2
3	3	2	3	1	2	1	2	3	2
9	9	9	9	9	9	9	9	9	9
1	1	3	3	1	3	1	3	3	1
1	9	9	3	9	3	9	9	1	9
1	9	9	9	9	9	9	9	2	9
3	1	9	2	9	3	2	2	3	1
2	3	2	1	2	1	2	3	3	2
9	9	9	3	9	9	9	9	9	9
1	1	2	3	3	3	2	3	3	9
1	2	2	3	2	3	2	1	3	3
1	9	9	9	9	9	9	9	3	9
2	2	2	1	2	3	9	3	2	9
1	1	2	3	2	2	2	2	3	3
3	3	3	3	1	1	3	3	3	3
1	9	9	9	9	9	9	9	9	1
1	3	3	3	1	1	3	3	3	3
3	2	1	2	1	1	3	3	1	1
1	3	3	3	1	3	1	1	3	3
1	9	9	9	9	9	9	9	2	9
1	2	3	3	2	3	2	2	2	3
1	1	1	3	2	1	2	2	2	2
1	1	2	3	3	3	1	1	3	2
9	9	9	2	9	9	9	9	2	9
1	3	3	2	2	2	2	1	2	3
1	1	2	3	3	3	3	2	3	3
1	1	3	9	3	3	1	1	2	2
1	2	2	3	3	3	2	2	3	3
1	3	3	3	3	3	1	1	2	9
9	9	9	3	9	9	9	9	2	9
1	1	2	3	2	3	2	2	3	3
1	2	2	3	3	2	9	1	3	3
2	3	3	1	1	1	3	3	3	2
3	3	3	1	1	1	3	3	3	3
1	1	1	2	1	2	1	2	3	2
1	9	9	9	3	9	9	9	3	9
2	2	1	3	1	1	3	3	2	2

1	2	1	3	3	2	2	2	3	9
9	9	9	9	3	9	9	9	3	3
9	9	9	3	9	9	9	9	9	9
9	9	9	9	3	9	9	9	3	9
1	1	1	2	2	1	1	3	2	2
1	9	9	3	3	3	9	9	3	9
1	1	3	3	1	3	1	1	3	3
3	2	2	2	2	2	2	2	3	3
1	3	1	3	3	2	9	3	3	3
9	9	9	9	3	9	9	9	3	9
2	9	9	9	9	9	9	9	3	3
1	9	9	9	3	9	9	9	3	9
1	2	2	3	3	2	3	2	3	9
1	1	2	3	3	1	2	2	1	3
9	9	9	3	9	9	9	9	3	9
2	9	1	1	2	3	2	3	2	2
2	3	3	2	2	2	2	1	2	3
9	9	1	9	9	9	9	9	9	9
9	9	9	3	9	3	9	9	1	9
1	2	2	2	1	1	2	1	3	2
1	2	2	3	3	3	2	2	3	3
1	1	3	3	3	3	1	3	3	3
1	2	2	3	3	3	2	1	1	3
1	1	1	3	3	3	2	2	3	2
1	1	2	3	3	3	3	1	3	3
1	9	9	3	9	3	9	1	3	3
1	1	1	3	3	3	2	1	3	2
1	1	1	3	3	3	2	1	9	9
1	2	2	3	3	3	2	1	3	2
1	2	2	3	3	3	2	1	3	3
1	9	9	9	9	9	9	9	3	9
2	1	1	2	2	3	2	2	3	2
1	2	3	3	3	3	1	2	3	2
1	9	9	9	9	9	9	9	3	9
1	3	3	3	3	3	3	3	3	3
9	9	9	3	9	9	9	9	9	9
1	1	2	3	2	3	2	1	3	2
9	9	9	3	9	9	9	9	3	9
1	2	1	3	3	3	2	2	3	3
1	9	9	9	9	1	3	3	3	3
1	9	9	3	3	3	9	9	3	9
9	9	9	3	9	9	9	9	3	9
1	3	3	3	3	1	3	1	9	9
9	9	9	3	9	9	9	9	3	9
3	3	3	1	1	1	3	3	9	3
1	2	2	3	3	3	1	2	2	3
1	3	3	3	2	2	2	3	2	3

2	3	3	3	3	2	2	2	3	2
1	9	9	9	9	9	9	9	3	9
1	9	9	3	9	3	9	9	3	9
9	2	9	9	9	9	9	9	2	9
9	9	9	9	3	9	9	9	3	9
1	9	1	9	9	9	9	9	9	1
1	9	9	9	9	9	9	9	3	9
3	3	9	3	3	3	3	3	3	3
1	1	2	3	3	3	2	2	2	2
2	2	3	3	3	2	2	2	2	3
1	2	2	3	3	2	2	2	2	3
1	1	2	3	3	3	2	2	1	3
9	9	9	3	9	9	9	9	3	9
1	2	3	3	3	3	3	1	9	3
1	1	3	3	3	3	3	2	1	3
1	1	3	3	2	2	2	2	3	3
1	2	3	3	3	1	2	1	3	3
1	9	9	9	9	9	9	9	9	9
2	1	2	2	3	2	1	2	2	3
9	9	9	3	9	9	9	9	2	9
1	9	9	9	9	9	9	9	2	9
9	9	9	3	9	9	9	9	3	9
1	1	2	3	2	2	2	2	2	2
1	2	2	3	2	1	2	3	3	3
2	2	2	2	2	2	2	2	2	9
1	2	3	3	3	3	2	2	3	3
1	9	9	9	9	9	9	1	3	9
2	3	1	2	1	3	2	3	3	3
2	2	2	3	2	2	3	3	3	3
1	1	2	2	2	2	2	2	3	3
1	3	3	2	2	3	3	2	3	9
1	1	2	3	3	3	2	3	3	3
1	9	9	9	9	9	9	9	3	9
1	3	3	3	3	2	3	3	3	3
3	3	3	3	1	1	3	3	3	3
1	9	9	9	9	9	9	9	3	9
1	2	2	3	3	3	3	3	3	3
1	2	2	3	3	3	2	2	1	3
1	2	3	3	3	2	3	3	3	3
1	3	3	3	1	3	3	3	3	9
1	2	2	3	2	3	2	1	3	2
2	3	3	3	3	3	2	2	3	3
9	3	9	3	9	1	9	2	9	1
1	9	9	9	9	9	9	9	3	9
3	3	3	2	2	2	3	2	1	3
1	2	3	3	3	2	2	2	3	3
1	2	9	3	3	3	2	1	3	3

2	9	9	3	9	9	9	1	2	9
1	1	1	3	3	3	1	1	3	3
1	1	1	3	3	3	1	1	3	3
1	1	2	3	3	3	2	2	3	3
2	9	2	1	2	9	2	3	2	9
1	1	3	2	2	2	2	2	3	1
1	1	9	3	1	3	3	1	3	9
1	2	2	3	3	3	2	2	1	2
2	2	2	3	1	3	1	1	1	3
1	2	3	3	2	3	2	2	3	3
2	2	3	3	2	3	2	2	3	2
1	9	9	9	9	9	9	9	3	9
1	9	1	9	9	3	1	1	3	1
1	2	3	3	3	2	2	2	1	3
1	9	9	3	3	3	9	9	1	3
1	1	2	3	3	3	2	3	3	1
1	9	9	3	9	3	9	9	3	9
1	2	1	2	1	3	1	2	3	2
1	2	1	3	1	3	3	3	9	9
9	9	9	9	3	9	9	9	9	9
2	2	2	2	2	2	2	2	2	2
1	2	2	3	3	3	1	2	3	3
9	9	1	9	9	9	9	9	9	9
1	1	3	3	3	3	1	9	3	3
3	3	3	1	1	1	3	3	2	2
3	3	3	2	2	3	2	2	1	3
1	2	3	3	3	2	3	3	3	3
1	2	2	3	2	3	2	2	1	3
1	1	3	3	3	3	1	1	3	3
2	1	1	2	3	1	2	2	2	1
9	9	9	3	9	9	9	9	9	9
1	1	2	3	3	1	2	3	9	1
2	2	2	3	3	2	2	2	2	2
2	2	2	3	3	2	2	2	2	2
2	2	3	1	2	2	1	2	1	3
1	9	9	9	9	9	9	9	9	1
2	3	2	2	2	2	2	2	3	3
1	1	1	3	3	3	2	1	1	3
1	2	3	3	2	2	2	2	3	3
1	1	1	3	2	3	2	3	1	3
1	2	2	3	3	2	3	2	3	2
1	2	3	3	2	2	2	1	2	2
1	9	9	3	9	9	9	1	3	9
1	9	9	3	9	3	9	9	3	3
1	1	1	3	2	3	2	1	3	1
1	9	9	9	9	9	9	9	3	9
1	1	1	3	3	3	1	1	3	1

9	9	9	3	9	9	9	9	3	9
1	9	9	9	9	9	9	9	3	9
3	3	3	1	1	1	3	3	2	2
9	9	1	9	9	9	9	9	3	9
1	2	2	3	3	2	2	2	3	2
1	1	2	3	3	3	2	2	2	2
1	1	2	3	3	3	2	1	2	2
1	2	3	3	3	3	1	1	2	1
1	1	1	3	3	3	1	1	3	2
9	9	9	3	9	9	9	9	3	9
1	9	9	3	3	3	9	1	3	9
1	1	1	3	3	3	3	1	3	3
1	9	9	3	9	3	9	9	3	9
1	2	3	3	3	3	2	2	2	3
1	1	3	3	3	3	3	1	3	3
1	1	2	2	3	2	2	2	2	2
1	2	2	3	2	9	2	3	2	3
1	1	3	3	1	1	3	3	3	3
1	1	3	3	3	3	3	3	3	9
9	9	9	3	9	9	9	9	3	9
1	1	2	3	2	3	2	2	2	2
1	1	2	3	3	3	3	3	3	3
1	3	3	3	1	3	2	1	3	3
1	2	2	3	3	2	2	2	3	3
1	1	1	3	3	3	2	2	3	3
1	2	3	3	9	3	3	1	3	3
1	3	3	3	3	3	3	3	3	3
1	1	3	3	3	3	1	2	9	3
1	1	2	3	3	3	1	1	1	3
1	9	9	9	9	9	9	9	3	3
2	2	2	9	9	9	9	9	9	9
3	1	1	3	3	3	1	1	3	3
1	2	3	1	1	1	3	3	1	3
1	2	3	3	3	9	2	3	9	1
1	2	2	3	3	3	2	2	3	3
9	9	9	3	9	9	9	9	9	9
1	2	3	3	2	3	2	2	3	9

WomenInt	WomenPre	WomenMe	CABreastH	Daily	Weekly	Monthly	SixMonthly	Yearly	Anytime
9	9	9	1	9	9	9	9	9	1
1	2	3	3	2	3	1	3	3	2
3	3	2	2	1	1	2	2	2	2
3	1	9	3	2	9	1	1	2	2
9	9	9	9	9	9	9	9	9	9
1	2	3	3	2	3	1	1	9	2
2	2	2	2	2	2	1	2	2	1
3	3	3	2	3	2	1	1	2	3
9	9	9	9	2	9	9	9	9	2
9	9	9	9	2	9	9	9	9	9
2	2	2	2	2	9	9	1	9	2
3	3	3	3	1	2	1	3	2	1
2	2	2	2	2	2	2	2	2	2
2	1	2	1	2	2	1	1	3	3
3	3	2	2	2	2	2	2	2	2
2	2	9	1	1	2	2	2	3	2
2	1	3	1	2	1	1	1	2	1
3	3	3	3	1	3	3	3	3	1
3	3	3	3	2	2	2	2	2	1
2	2	2	2	2	2	2	2	2	2
3	3	3	2	3	3	3	2	3	2
2	3	3	3	2	1	2	2	2	2
3	2	2	2	9	1	9	9	9	1
3	3	3	2	2	1	2	2	2	2
2	2	2	2	1	2	3	3	3	1
2	2	2	2	1	2	2	2	2	2
2	2	2	2	2	2	1	2	2	1
3	3	3	1	1	2	1	2	2	2
3	2	2	2	2	2	1	3	3	1
2	2	2	2	2	2	2	2	2	2
2	2	2	2	2	2	2	2	2	2
2	2	2	2	2	2	2	2	2	2
3	3	3	2	1	2	2	3	3	2
2	2	2	2	2	2	2	2	2	2
2	2	2	2	2	2	2	2	2	2
2	2	2	2	2	2	2	2	2	2
2	2	2	2	2	2	2	2	2	2
3	3	3	3	1	3	3	3	3	9
3	2	2	2	2	2	1	3	3	1
3	2	2	2	2	2	1	3	3	1
3	2	2	2	2	2	1	3	3	9
9	9	9	9	2	9	9	9	9	2
9	9	9	9	2	1	1	3	3	1
3	3	3	3	1	1	1	3	3	1
2	2	2	2	2	2	2	1	2	1
1	3	1	3	3	3	3	3	3	9

1	3	3	1	3	3	9	1	1	1
9	9	9	9	9	1	9	9	9	9
9	9	3	9	9	9	9	9	9	9
1	2	3	2	1	3	3	3	3	1
9	9	9	9	9	1	9	9	9	1
3	3	3	3	3	3	1	3	3	3
3	3	3	1	1	3	3	3	3	1
9	9	9	9	9	9	1	9	9	9
9	9	9	9	9	9	9	1	9	1
1	3	2	2	1	3	3	3	3	1
2	9	9	9	9	9	1	2	9	9
9	9	9	9	1	9	9	9	9	9
2	2	2	3	1	9	9	1	9	9
3	3	3	2	2	1	9	1	9	2
9	9	9	9	9	9	1	9	9	9
3	3	3	3	3	3	3	1	3	3
2	3	2	3	9	9	1	9	9	1
3	3	2	3	1	1	1	3	3	1
3	2	2	1	1	1	3	2	3	1
3	1	2	3	1	2	2	1	1	1
3	3	3	3	3	3	1	3	3	3
9	9	9	9	3	3	1	3	3	3
3	3	3	3	3	3	1	3	3	3
3	1	9	1	1	1	1	2	3	2
1	2	1	2	3	1	3	1	3	3
9	9	9	9	1	9	9	9	9	9
9	9	9	9	9	9	9	9	9	9
3	3	3	1	3	1	3	2	2	2
3	3	3	3	1	3	3	3	3	2
9	9	9	9	9	9	1	9	9	9
9	9	9	9	1	9	9	9	9	9
3	1	3	3	1	3	3	3	3	9
1	9	9	9	9	9	9	9	9	9
3	3	3	9	3	1	9	9	9	9
2	2	2	1	1	1	3	3	3	1
2	1	2	2	1	2	1	2	1	9
1	2	2	1	1	2	1	2	2	9
2	2	2	2	1	9	9	9	9	1
9	9	9	9	9	9	1	9	9	9
3	3	3	3	1	3	3	3	3	1
3	3	2	3	1	1	9	9	3	1
3	3	3	1	2	1	9	3	3	3
3	3	3	3	3	1	3	3	3	9
2	2	3	3	1	2	1	3	3	1
9	9	9	9	9	9	1	9	9	9
9	9	9	9	9	9	1	9	9	9
9	9	9	9	9	9	1	9	9	9

2	3	2	1	3	1	2	2	2	1
2	3	3	1	2	2	2	2	2	1
3	3	3	3	1	2	2	3	3	1
2	3	2	1	2	2	2	1	2	2
2	3	3	2	2	2	2	1	2	1
3	9	9	1	2	3	3	3	3	2
3	3	3	3	1	3	3	3	3	3
3	3	3	2	3	1	1	1	3	1
3	3	3	3	1	2	2	3	3	1
2	2	2	1	1	1	1	2	2	1
3	2	2	1	3	2	1	1	2	1
2	2	2	1	1	2	2	2	2	1
3	3	3	3	1	2	1	3	3	1
9	9	9	9	1	2	3	3	3	9
2	1	2	3	2	1	1	2	2	1
3	1	2	2	2	1	3	2	1	2
3	3	3	1	3	2	1	1	1	1
2	2	2	2	2	2	2	2	2	2
3	3	3	3	1	2	2	2	2	2
3	3	3	3	1	3	9	9	9	9
3	3	3	3	1	3	9	9	9	2
9	9	9	9	9	9	9	1	9	9
2	2	1	2	3	2	2	1	3	1
3	3	1	1	9	9	1	9	9	1
1	2	1	2	3	2	3	3	3	1
2	2	2	2	2	2	2	2	2	2
9	9	9	9	9	9	9	1	9	9
2	2	2	2	9	9	9	1	9	9
2	3	3	2	2	2	2	2	2	2
9	9	9	1	9	9	1	9	9	9
2	2	2	2	2	2	2	2	2	2
2	2	2	2	2	2	2	2	2	1
2	2	2	1	2	2	2	2	3	2
3	3	2	2	2	2	2	2	2	2
9	9	9	1	9	9	1	9	9	9
1	3	3	3	3	1	3	3	3	3
3	3	3	2	1	3	2	3	3	1
3	3	3	2	3	3	2	1	3	2
9	9	9	9	9	1	9	9	9	1
3	3	3	3	2	2	2	1	2	1
2	2	2	2	3	3	1	3	3	3
2	2	2	2	3	3	1	3	3	3
2	2	2	2	2	2	2	2	2	1
1	2	2	2	3	2	1	1	2	3
2	1	3	1	2	1	2	2	3	1
3	3	3	3	3	1	1	2	3	2
1	2	3	3	1	2	3	3	3	3

2	2	2	2	3	3	3	3	3	9
9	9	9	9	9	9	9	1	9	9
2	3	2	1	2	2	1	3	3	1
9	9	9	9	2	2	1	2	2	3
9	9	9	9	2	2	1	2	2	3
9	9	9	9	9	1	9	9	9	1
2	3	2	1	1	2	2	2	3	2
3	3	3	2	2	2	2	3	3	2
3	3	3	2	2	2	2	2	2	2
2	2	2	2	2	2	2	2	2	1
3	2	3	2	2	2	2	2	3	2
3	2	3	2	2	2	2	2	2	2
9	9	9	9	1	9	9	9	9	9
3	3	3	3	2	2	2	2	2	1
2	3	2	1	2	1	2	3	3	1
2	2	2	2	2	2	2	2	2	2
2	2	2	2	2	2	2	2	2	2
3	3	2	2	3	1	9	9	9	9
9	1	9	9	9	9	1	9	9	9
3	3	3	1	3	3	1	3	3	3
3	3	3	3	3	3	1	3	3	3
3	3	3	3	3	3	1	3	3	3
1	3	3	3	2	3	3	2	3	2
3	3	3	3	3	3	1	3	3	1
3	3	3	3	3	3	1	3	3	1
3	3	3	1	9	9	1	9	9	9
3	3	3	1	3	3	1	3	3	3
3	3	3	3	9	9	1	9	9	9
3	3	3	3	3	3	1	3	3	3
3	3	3	3	3	3	1	3	3	3
2	1	1	1	1	1	1	2	3	1
2	2	3	1	1	2	1	2	3	2
1	3	3	1	3	3	1	3	3	3
3	3	3	3	3	3	1	3	3	3
3	3	3	3	3	3	1	3	3	3
3	3	3	2	3	2	1	3	3	2
3	3	3	2	3	3	1	3	3	1
3	3	3	1	9	9	1	9	9	1
3	3	3	3	3	3	1	3	3	3
3	3	3	3	3	3	1	3	3	3
3	3	3	3	3	3	1	3	3	3
3	3	3	3	3	3	1	3	3	1
3	3	3	1	9	9	1	9	9	9
3	3	3	1	3	3	1	3	3	3
3	3	3	3	3	3	1	3	3	3
3	3	3	1	3	3	1	3	3	3
3	3	3	1	3	3	1	3	3	3

3	3	3	3	3	3	1	3	3	3
3	3	3	3	3	3	1	3	3	3
3	3	3	3	3	3	1	3	3	3
3	3	3	3	3	3	3	1	3	3
3	3	3	3	3	3	1	3	3	3
3	3	3	3	3	3	1	3	3	1
3	3	3	3	3	3	1	3	3	3
3	3	3	3	1	2	3	3	3	2
3	3	3	3	1	3	3	3	3	1
3	2	3	1	3	1	2	3	3	9
3	3	3	3	1	3	3	3	3	9
9	9	9	9	9	9	9	9	9	1
3	3	3	3	1	9	9	9	9	9
3	3	3	3	3	3	1	3	3	3
3	2	2	3	1	2	1	9	3	1
2	1	2	1	2	1	1	1	2	1
2	1	1	2	9	1	9	1	1	3
1	1	1	1	1	1	1	1	1	1
2	2	2	2	2	2	1	2	2	1
2	2	2	1	2	2	2	2	2	2
1	2	1	1	1	1	1	1	2	1
2	2	2	2	2	2	2	2	2	1
9	9	9	9	2	9	1	2	3	2
1	1	1	1	1	2	2	3	3	1
2	2	2	2	2	2	2	2	2	1
2	9	9	9	2	2	2	2	2	9
2	2	2	1	2	2	2	2	2	2
2	2	2	2	2	2	2	2	2	1
3	2	2	2	2	2	2	2	2	2
3	2	2	2	1	2	2	2	3	1
3	3	3	3	2	2	1	2	2	2
3	3	3	3	2	2	1	2	2	2
3	3	3	3	1	2	1	2	2	1
1	1	2	2	1	2	2	2	2	1
2	1	2	1	2	2	2	2	2	2
1	2	2	3	1	2	2	2	2	2
2	2	2	1	3	3	2	1	1	2
2	1	2	1	2	2	2	2	2	2
3	3	3	3	2	2	2	1	2	2
3	3	3	3	2	3	3	1	3	2
2	3	3	3	2	2	2	2	2	2
2	2	2	1	2	2	2	2	2	1
9	9	9	9	1	9	9	9	9	9
3	2	2	3	1	9	9	9	9	9
3	3	2	2	2	3	1	1	1	3
3	3	3	2	2	2	1	1	3	3
2	2	3	1	3	2	3	2	1	2

3	1	1	2	1	2	2	2	2	1
3	1	1	2	1	2	2	2	2	1
3	1	2	2	2	2	2	1	2	2
2	1	1	2	3	2	2	1	2	2
3	3	3	2	2	2	1	1	2	1
3	2	3	1	2	1	3	3	3	1
9	9	9	9	9	9	9	9	9	1
3	2	3	1	2	3	3	1	2	3
3	3	3	1	2	3	1	1	2	1
3	3	3	3	3	9	9	9	9	1
2	2	2	2	2	2	2	2	2	2
3	3	1	2	2	2	1	3	1	2
3	3	3	1	2	3	1	1	2	1
2	2	2	3	2	1	2	3	3	3
2	3	2	3	1	2	3	3	3	3
3	3	3	1	1	1	1	2	3	1
2	2	2	3	2	1	3	3	3	3
2	2	3	3	2	2	1	2	2	2
2	2	3	1	2	1	2	3	3	1
3	3	3	2	3	3	2	1	2	2
3	2	2	1	2	1	2	1	3	2
2	1	2	2	2	1	1	2	2	2
2	2	2	3	2	1	2	3	3	3
2	3	2	2	3	3	1	2	2	2
9	9	9	9	2	9	9	9	9	9
2	2	2	2	2	2	2	2	2	2
2	2	2	2	2	2	2	2	2	2
3	2	3	2	3	9	1	1	3	3
1	1	1	2	2	2	1	1	3	3
9	9	9	9	2	2	2	2	2	1
2	2	2	2	2	2	1	2	2	2
3	2	2	2	2	2	2	2	2	2
3	3	3	3	1	9	9	9	9	9
3	3	3	3	2	2	3	3	3	1
2	2	2	2	2	2	2	2	2	2
2	2	2	2	2	2	2	2	2	1
3	3	2	2	3	2	1	1	2	3
1	9	9	9	9	9	9	9	9	1
2	3	3	3	3	3	2	1	3	3
9	3	2	2	3	9	2	2	1	2
2	2	2	2	1	3	3	3	3	1
3	3	3	2	1	1	1	2	2	1
2	3	2	3	3	3	1	2	3	1
3	3	3	3	2	2	2	2	2	2
1	3	3	2	2	1	2	1	1	9
3	3	3	1	1	2	3	3	3	1
2	3	2	1	3	1	2	2	3	1

2	2	2	2	2	1	2	2	2	1
2	2	3	1	3	2	1	3	3	2
2	2	2	1	2	2	1	1	2	2
2	3	3	1	2	3	3	1	3	3
3	3	3	2	1	2	3	3	3	2
3	2	3	3	3	3	1	1	3	1
2	1	2	1	2	1	1	1	2	1
9	9	9	9	9	9	9	1	9	9
9	2	3	2	2	2	1	2	2	2
2	2	2	2	2	2	2	2	2	2
1	1	1	3	2	2	2	2	2	1
2	2	2	2	1	3	3	3	3	1
3	3	3	3	3	1	1	1	1	1
9	9	9	9	9	1	9	9	9	9
1	3	1	3	1	3	3	1	3	3
3	3	3	3	2	2	2	2	2	2
3	3	3	3	1	3	3	3	3	1
3	3	2	2	2	1	1	1	1	1
2	2	1	1	1	2	3	1	1	2
2	2	2	1	2	1	2	1	2	1
2	3	2	1	2	2	2	2	2	2
3	2	3	3	2	1	1	3	3	2
2	3	3	3	3	1	2	3	3	3
2	2	2	2	2	2	2	2	2	1
3	3	3	3	2	2	2	2	2	2
9	9	9	1	9	9	9	9	9	9
3	3	3	1	2	2	2	2	2	9
3	3	3	2	2	2	1	2	3	2
3	3	3	3	2	2	2	2	2	2
2	2	2	2	2	1	1	3	2	2
3	3	3	2	2	1	2	2	2	1
9	9	9	9	9	9	1	3	2	2
9	9	9	9	9	1	9	9	9	1
3	3	3	3	3	3	1	2	3	3
2	2	2	2	2	2	1	2	2	2
1	9	3	1	3	1	9	1	2	1
3	3	3	3	1	1	2	2	3	1
1	3	3	1	3	1	1	1	2	1
1	3	3	1	3	1	1	3	3	3
9	9	9	9	3	3	2	2	1	3
9	9	9	9	1	9	9	9	9	9
1	3	3	1	3	1	3	3	3	1
2	2	2	2	2	2	1	2	2	1
3	3	3	2	3	3	1	2	3	2
3	3	3	3	2	3	1	1	3	2
2	3	3	3	3	2	2	1	2	2
3	3	3	1	2	1	2	2	2	1

3	2	2	1	2	1	1	2	3	1
3	2	2	1	2	1	1	2	3	1
9	9	9	9	9	9	1	9	9	9
3	3	2	1	3	1	1	3	3	3
9	9	9	9	9	9	9	1	9	9
3	1	2	3	3	1	3	2	2	2
3	3	3	1	1	2	2	2	2	1
2	1	2	9	2	9	9	9	9	2
3	3	3	1	3	3	1	2	3	3
3	1	2	1	3	3	1	2	2	3
3	3	3	3	3	3	3	1	3	2
2	3	3	3	2	2	2	2	2	2
3	3	3	1	2	1	3	2	3	3
3	3	3	3	3	1	3	3	3	1
3	3	3	3	3	3	3	1	3	2
3	2	2	2	3	3	2	3	3	3
3	3	3	3	3	3	3	1	3	2
2	2	2	2	2	2	2	1	2	2
3	3	3	3	2	3	3	1	3	9
1	2	3	2	2	1	2	2	3	1
3	9	2	1	3	3	3	1	1	3
3	3	3	2	3	2	1	3	3	1
3	3	2	3	3	2	1	3	3	1
2	2	3	1	3	1	1	3	2	3
2	2	2	2	2	2	2	2	1	2
2	3	3	3	3	2	2	3	2	2
3	2	3	3	3	2	1	2	3	2
3	3	3	3	3	3	2	1	3	3
1	2	3	2	2	1	1	2	3	2
2	3	3	3	3	1	1	3	3	1
3	2	3	3	1	3	2	3	3	2
3	2	3	3	1	3	2	3	3	2
1	1	2	2	1	1	1	2	2	1
2	2	2	2	2	2	2	2	2	1
2	2	2	2	2	2	2	2	2	1
3	3	3	2	3	2	1	2	3	2
3	3	3	3	2	2	2	2	2	3
2	2	2	1	3	2	1	2	2	1
1	2	2	2	3	1	3	2	2	2
3	3	2	3	3	2	1	3	3	1
2	1	2	2	2	2	2	2	2	3
3	2	2	1	1	3	2	1	2	1
1	2	3	1	2	1	2	1	1	2
3	2	2	3	3	2	2	1	3	3
3	3	3	3	3	3	2	1	2	2
2	3	3	3	2	1	1	3	3	3
3	3	3	3	2	2	1	2	2	2

3	3	9	1	2	2	1	2	3	1
3	1	3	1	2	2	1	1	2	1
9	9	9	9	2	9	9	9	9	9
3	2	2	1	3	2	2	2	3	1
2	1	3	1	3	3	1	1	2	1
2	2	2	3	3	1	3	2	3	1
2	2	2	2	3	1	1	2	1	2
2	2	2	2	1	3	3	2	3	1
2	2	2	2	1	3	3	3	3	1
3	3	3	3	1	2	2	2	2	1
2	1	2	1	3	2	1	1	3	1
3	3	3	1	3	1	1	1	3	1
1	3	3	3	3	2	1	2	3	2
2	2	2	2	3	2	2	2	2	2
2	2	2	2	3	2	2	2	2	2
3	3	3	3	2	2	1	2	3	1
3	3	3	3	2	3	2	2	2	2
3	3	3	3	3	2	1	3	3	1
3	3	3	3	2	1	1	3	3	1
9	9	9	9	9	9	9	9	9	9
3	2	3	1	1	2	2	3	3	3
3	3	3	3	3	3	3	2	3	1
3	3	3	3	3	3	3	2	3	1
2	2	2	1	2	2	2	2	2	1
3	3	3	1	3	2	1	2	3	2
2	3	2	1	2	1	2	2	2	1
9	9	9	9	9	1	9	9	9	9
2	2	2	2	3	2	1	2	3	1
2	3	2	1	2	2	2	2	2	2
9	9	9	1	9	1	9	9	9	9
2	2	2	1	2	2	2	1	2	3
1	2	2	2	1	2	3	3	2	1
2	2	2	1	2	2	2	1	2	3
9	9	9	9	9	9	9	1	9	9
3	2	3	2	1	2	3	2	2	2
2	3	2	3	3	3	1	2	3	1
9	9	9	9	9	9	9	9	9	9
3	2	3	1	3	2	1	1	3	2
3	2	2	2	3	2	1	2	2	2
2	2	1	1	3	1	2	3	3	1
3	3	2	3	3	2	2	1	3	1
1	2	3	3	2	3	1	3	3	2
3	2	1	1	2	3	3	1	2	3
3	3	3	3	3	2	1	3	3	1
3	3	3	3	2	2	2	2	2	2
3	3	3	2	9	9	1	9	9	9
3	3	3	3	3	3	3	3	3	3

3	3	3	2	9	9	1	9	9	9
3	3	3	1	3	3	1	3	3	3
3	3	3	2	3	3	1	3	3	3
3	3	3	1	3	3	1	3	3	3
3	3	3	3	3	1	9	9	9	1
3	3	3	3	3	3	1	3	3	3
3	3	3	3	2	2	1	2	2	2
9	9	3	3	3	3	1	1	1	3
3	3	3	3	2	2	2	9	2	2
3	3	3	3	2	2	2	2	2	3
3	3	3	1	3	1	9	9	9	9
3	3	3	9	3	3	1	3	3	9
3	3	3	1	3	3	1	3	3	3
3	3	3	3	3	3	1	3	3	3
3	3	3	2	3	3	1	3	3	3
3	3	3	1	3	3	1	3	3	3
9	3	3	1	3	3	1	3	3	2
3	3	3	3	3	3	1	3	3	2
3	3	3	1	3	3	1	3	3	3
3	3	3	1	3	3	1	3	3	3
3	3	3	1	9	9	1	9	9	3
3	3	3	1	3	3	1	3	3	3
3	3	3	1	3	3	1	3	3	3
3	3	3	3	3	3	1	3	3	1
3	3	3	3	3	3	1	3	3	3
3	3	3	3	3	3	1	3	3	3
3	3	3	3	3	3	1	3	3	3
3	3	3	1	3	3	1	3	3	3
3	3	3	9	3	3	1	3	3	3
3	3	3	3	3	3	1	3	3	3
3	3	3	3	3	3	1	3	3	9
3	3	2	2	2	2	1	2	2	2
3	3	3	1	3	3	1	3	3	3
3	3	3	1	3	3	1	3	3	3
2	2	2	1	9	9	1	9	9	9
3	3	3	3	3	9	1	2	3	3
2	2	3	2	2	1	1	2	2	1
3	3	3	3	2	2	1	3	3	1
3	3	3	3	3	3	1	3	3	3
3	3	3	3	3	3	1	3	3	3
3	3	3	3	3	3	1	3	3	3
2	2	2	2	2	2	1	1	2	2
9	9	9	9	9	1	9	9	9	9
2	2	2	2	2	3	3	3	3	2
9	9	9	9	2	9	9	9	9	2
2	2	2	2	2	2	1	2	2	1
9	9	9	9	9	1	1	2	9	3

2	2	2	2	2	2	2	2	2	2
1	3	2	1	3	3	1	3	3	1
1	3	2	1	3	3	1	3	3	1
2	9	2	9	3	1	2	2	3	1
2	3	1	3	3	2	1	1	3	3
2	2	2	1	9	9	9	9	1	9
3	2	1	2	1	2	3	3	3	1
2	3	2	2	2	3	1	2	1	2
2	1	2	1	1	2	2	3	3	2
2	2	2	2	2	2	2	2	2	2
9	9	9	9	9	9	9	9	9	9
9	9	1	9	9	9	9	9	1	9
3	3	3	3	9	1	2	3	9	3
2	2	2	2	2	2	1	1	2	1
1	9	9	9	9	9	1	9	9	9
1	9	9	9	9	9	1	9	9	9
9	9	9	9	9	9	1	9	9	9
1	9	9	9	9	1	9	9	9	9
1	9	9	9	9	1	9	9	9	9
3	9	9	9	9	9	9	9	9	9
9	9	9	9	9	9	9	9	9	9
3	3	3	1	1	3	3	3	3	1
3	2	3	3	3	2	1	1	3	9
9	9	9	9	9	1	9	9	9	1
9	9	9	9	1	9	9	9	9	1
1	9	9	9	9	9	1	9	9	9
9	9	9	9	9	1	9	9	9	9
2	2	2	1	2	2	3	3	2	1
3	2	1	2	2	1	1	3	3	9
9	9	1	9	9	1	9	9	9	9
9	9	9	1	9	1	9	9	9	9
2	3	2	2	3	2	2	1	2	1
3	2	3	3	3	2	3	3	3	3
3	3	3	3	3	3	3	1	1	1
2	2	2	2	2	2	2	2	2	1
3	3	2	3	3	3	2	1	3	1
3	2	2	3	3	3	1	1	2	3
3	3	3	3	3	3	1	2	3	3
2	2	3	2	2	2	3	3	2	3
3	2	3	2	1	2	2	3	3	2
3	2	3	3	2	1	2	2	2	2
3	3	3	1	3	1	3	1	3	1
3	1	1	1	3	2	1	1	3	1
3	2	3	1	2	1	3	3	3	2
3	2	2	1	2	1	3	3	2	1
3	3	3	3	3	3	2	1	2	3
3	3	2	1	2	2	1	1	3	3

2	2	3	3	1	2	2	3	3	2
3	3	2	1	9	9	9	9	9	1
3	2	3	3	2	1	2	3	3	1
3	1	1	2	3	1	2	3	3	2
2	3	3	3	3	3	2	1	2	3
3	3	3	3	3	1	3	3	3	2
2	2	3	3	2	2	1	9	9	1
3	1	2	1	3	1	2	1	2	1
3	2	3	2	2	1	1	2	3	1
3	3	3	1	2	2	1	2	2	2
3	2	3	2	2	2	2	3	3	2
2	2	2	1	3	2	2	1	1	2
3	3	3	3	3	2	2	3	3	3
3	3	3	3	3	2	2	3	3	3
3	3	3	3	3	2	1	1	2	2
2	2	1	1	2	2	2	2	2	2
3	3	3	3	2	2	1	2	2	1
3	3	2	3	3	3	2	2	2	2
3	3	3	1	3	3	2	1	3	3
3	2	3	1	3	2	1	3	3	3
3	1	3	1	3	3	1	2	2	1
3	3	3	3	3	2	1	3	3	1
3	3	3	2	3	2	1	2	1	1
2	3	3	2	3	2	1	2	1	1
3	3	2	2	2	2	3	1	2	1
2	3	3	2	1	2	3	3	3	2
3	3	3	1	3	3	1	1	3	1
3	3	3	2	3	3	1	1	3	1
2	2	2	2	3	2	1	1	3	2
3	2	1	1	1	2	3	3	1	2
3	3	2	3	2	3	1	2	2	3
2	2	2	3	1	2	3	3	1	2
2	2	2	2	2	2	2	2	2	3
2	2	2	1	2	2	1	1	2	2
2	3	3	3	3	2	2	2	3	1
9	9	9	9	9	9	9	9	2	9
2	2	3	2	3	2	2	1	1	2
3	3	2	1	3	1	1	2	1	1
2	2	2	2	2	2	2	2	2	3
2	3	2	2	3	2	1	2	2	1
2	3	2	1	1	1	1	3	3	1
1	2	1	2	3	2	2	1	3	1
9	9	9	9	1	9	9	9	9	9
2	2	2	1	1	2	2	2	2	1
1	2	3	3	3	3	3	3	3	1
2	2	2	2	1	2	1	2	2	1
1	9	9	9	9	2	9	9	9	9

9	9	9	1	9	9	9	1	9	1
9	9	9	1	9	9	1	9	9	9
9	9	1	9	9	9	1	9	9	9
9	9	1	9	9	9	9	1	9	9
9	9	9	9	9	9	9	1	9	9
9	9	9	9	1	9	9	9	9	9
9	9	9	2	9	9	1	9	9	9
9	9	9	9	9	9	9	1	9	1
9	9	9	9	9	9	9	1	9	1
9	9	9	9	2	9	9	9	9	2
9	9	9	9	9	9	9	9	9	9
9	9	9	9	2	9	9	9	9	2
9	9	9	9	1	9	9	9	9	9
1	9	9	9	9	9	1	9	9	9
9	9	9	1	9	9	1	9	9	1
9	9	9	1	1	9	9	9	9	1
9	9	9	9	9	9	1	9	9	1
9	9	9	9	9	9	1	9	9	9
2	2	2	2	2	2	2	2	2	2
1	1	1	1	1	1	2	1	3	1
1	1	1	1	1	1	1	1	1	1
3	3	3	3	3	3	1	1	2	3
2	2	2	1	3	3	1	3	3	2
1	1	1	3	3	3	1	1	1	1
2	3	2	3	2	2	1	3	3	2
3	3	3	3	3	3	1	3	3	2
3	3	3	3	3	1	1	3	3	2
1	2	2	2	3	2	1	2	1	3
3	3	3	3	3	3	1	3	3	3
9	1	2	3	2	2	2	2	2	3
1	2	2	3	1	1	2	2	1	1
3	3	3	3	3	1	3	3	3	3
2	2	2	2	2	2	2	1	2	1
3	2	3	3	3	1	2	3	3	1
1	2	2	1	1	1	3	3	3	1
3	3	3	3	2	1	3	3	3	2
2	2	2	1	3	1	2	3	3	2
3	2	2	2	2	9	9	9	9	2
9	9	9	9	2	9	3	9	1	9
3	3	3	2	2	3	3	3	3	1
9	1	9	9	9	9	9	9	9	9
1	3	3	3	1	3	3	3	3	1
9	9	9	9	9	9	9	9	9	9
1	3	1	1	1	1	1	3	1	1
3	1	2	2	3	2	2	1	2	1
9	9	9	9	2	2	1	1	1	9
2	2	1	1	2	1	1	2	2	1

1	2	1	1	1	2	3	1	3	2
3	1	1	3	3	3	2	2	3	1
1	3	1	1	3	3	3	1	3	3
1	2	1	1	1	2	1	1	2	2
2	2	2	2	2	1	1	2	2	2
2	2	2	2	2	2	2	1	3	2
1	2	2	2	2	2	2	2	2	2
1	2	1	1	1	2	3	1	3	2
2	1	1	1	1	1	1	1	3	1
9	9	9	9	9	9	9	9	9	9
9	9	9	9	9	9	9	9	1	1
3	2	3	2	3	2	1	3	2	1
3	3	3	3	3	3	3	3	3	3
2	3	2	1	3	2	1	2	2	2
9	3	3	2	9	9	1	3	3	1
9	9	9	9	1	9	9	9	9	9
9	9	9	9	9	1	9	9	9	9
2	2	2	9	2	2	2	2	2	2
3	2	3	1	3	3	1	3	3	1
3	2	2	3	3	3	3	1	2	3
2	1	2	1	3	1	1	3	3	3
3	3	3	3	3	3	3	3	3	3
3	3	3	3	3	3	3	3	3	3
9	9	9	9	9	9	9	9	9	9
9	9	9	9	9	9	9	9	9	9
3	9	2	1	3	3	3	1	3	3
1	2	2	3	3	3	3	2	3	3
1	9	3	1	9	9	9	9	9	9
2	9	1	1	2	1	1	2	2	1
9	9	9	1	9	9	9	9	9	1
3	3	3	3	3	1	3	3	3	1
3	3	1	1	3	3	1	3	3	3
3	1	3	1	3	1	2	1	1	2
1	1	1	1	3	1	2	2	3	1
1	9	9	1	9	1	9	9	9	9
9	9	9	1	9	9	9	1	9	9
9	9	9	1	9	9	9	1	9	1
2	2	2	1	1	3	3	2	2	1
2	2	2	1	1	3	3	2	2	1
2	2	2	1	1	3	3	2	2	1
2	2	2	1	1	3	3	2	2	1
3	2	3	2	2	3	1	2	3	2
3	2	3	3	1	2	3	1	2	1
1	2	2	1	1	3	3	2	2	1
3	2	1	1	1	2	3	1	2	1
3	2	2	3	3	2	3	2	2	3
1	1	3	3	3	3	3	3	1	1

3	3	3	1	3	2	1	3	1	3
2	2	2	3	1	3	3	3	3	1
3	3	3	3	1	3	3	3	3	1
3	3	3	3	2	2	1	1	2	1
2	2	2	2	3	3	1	2	3	1
1	1	1	1	1	1	1	1	1	1
2	2	3	2	2	9	1	2	2	1
9	9	9	9	9	1	9	9	9	9
3	3	1	1	2	2	2	1	3	3
3	3	2	2	2	2	1	2	2	2
2	2	2	2	2	2	2	2	2	2
1	1	3	1	9	1	9	9	9	1
3	2	2	1	2	2	1	2	2	1
2	2	2	2	2	2	1	2	2	1
1	3	3	3	3	2	3	2	2	3
2	2	2	2	2	2	3	1	3	2
2	3	3	1	2	3	2	2	1	1
3	2	3	1	2	3	2	2	1	1
3	2	3	1	2	3	2	2	1	1
3	3	1	3	3	3	1	3	3	3
9	9	9	1	2	1	3	1	2	1
3	2	2	2	2	9	9	9	9	2
9	9	9	1	3	9	9	9	1	1
1	2	3	3	3	3	3	1	3	3
2	1	3	9	1	2	3	2	3	1
3	2	2	3	1	2	1	3	2	3
2	2	1	2	2	3	2	3	1	3
1	3	3	3	1	1	1	1	3	1
9	9	9	9	9	9	9	9	1	1
2	2	2	2	9	1	1	2	3	3
2	3	3	1	3	1	3	3	3	1
9	9	9	9	3	9	9	9	9	3
1	2	3	1	1	2	3	2	1	3
1	1	1	3	2	1	1	2	2	3
3	3	3	3	3	3	3	3	3	3
3	3	3	1	3	3	2	1	2	1
3	2	3	1	2	3	2	2	1	1
3	2	3	1	2	3	2	2	1	1
3	2	3	1	2	3	2	2	1	1
1	1	3	1	1	3	3	3	3	3
3	2	1	1	2	1	3	1	1	1
9	1	2	3	1	2	2	1	3	1
1	3	3	3	3	1	3	3	3	1
3	9	3	1	1	3	3	3	3	3
9	9	9	9	9	9	9	9	9	9
3	3	3	3	3	3	3	3	3	3
2	1	2	2	2	1	2	2	2	2

2	2	2	2	1	1	1	1	1	1
9	1	1	9	1	9	9	9	9	1
3	1	1	1	9	9	9	9	9	9
1	3	3	1	3	3	3	1	3	3
2	2	2	2	9	9	1	1	2	3
2	2	2	2	2	1	1	2	2	2
1	1	2	3	1	2	1	2	2	1
2	2	2	2	2	1	1	2	2	2
2	2	2	2	2	1	1	2	2	2
9	9	9	9	9	9	9	9	9	9
3	3	2	3	2	3	1	2	2	2
3	1	3	1	1	2	3	3	3	3
3	3	2	3	3	3	1	2	3	3
3	1	1	3	3	3	2	2	3	1
3	3	3	3	3	3	1	1	3	1
3	3	1	2	1	1	1	1	1	1
3	3	3	2	3	3	3	1	3	3
3	3	3	2	1	9	9	9	9	9
3	3	3	2	1	9	9	9	9	3
1	3	3	1	1	3	3	3	3	1
2	1	3	3	1	2	1	1	1	1
3	2	2	3	9	1	1	2	3	3
3	3	3	3	9	9	9	1	9	1
2	2	2	2	2	1	2	2	2	2
2	2	2	2	2	3	3	3	3	3
9	9	9	1	9	9	1	9	9	2
9	9	9	1	9	9	1	9	9	2
1	1	3	3	3	3	3	1	3	1
3	3	3	3	9	9	9	1	9	1
2	2	2	2	2	2	2	2	2	3
2	3	3	1	9	9	1	9	9	9
1	2	3	1	3	2	1	1	3	3
1	2	2	1	2	2	1	1	3	3
9	9	9	1	9	9	1	9	9	2
2	3	2	3	3	1	3	2	3	1
3	3	3	3	3	3	3	3	3	3
2	2	2	2	1	2	2	2	2	1
9	9	9	9	9	9	9	9	9	9
9	9	9	1	9	9	9	9	1	9
3	2	1	3	2	2	2	2	2	2
3	3	3	3	3	1	3	3	3	9
2	2	2	2	2	2	1	2	2	2
2	2	2	2	3	1	3	3	3	2
2	2	2	2	2	2	2	2	2	2
2	2	2	2	2	2	2	2	2	2
3	3	3	3	3	3	1	3	3	2
3	3	3	3	3	1	3	3	3	1

2	2	2	2	3	3	2	1	2	2
3	3	3	3	2	2	2	2	2	1
3	3	3	3	3	3	1	3	3	9
3	2	3	3	3	2	1	1	2	9
9	9	9	9	1	9	9	9	9	1
2	1	2	2	3	1	2	2	1	1
2	2	2	3	2	2	2	2	2	2
9	9	9	1	1	9	9	9	9	9
2	1	2	3	1	2	1	2	3	1
9	9	9	9	9	9	2	9	9	9
2	2	2	2	2	2	2	2	2	2
2	3	3	9	9	1	9	9	9	9
2	2	2	2	9	9	1	9	9	9
2	2	2	2	2	2	2	2	2	2
3	3	1	1	1	2	1	2	3	3
2	2	2	2	2	2	2	2	2	2
2	2	2	2	2	2	2	2	2	2
9	9	9	9	1	3	2	2	3	3
1	3	2	1	9	2	1	1	2	1
9	9	9	9	9	9	9	9	9	9
2	9	9	9	9	9	1	9	9	1
3	3	3	2	3	3	1	2	2	3
2	2	2	2	2	2	2	2	2	2
2	1	1	1	1	1	1	1	1	2
3	2	3	1	2	2	2	2	2	2
1	3	3	3	1	2	2	2	2	1
3	2	9	2	3	3	1	3	3	3
3	3	3	1	3	3	1	3	3	3
2	2	2	2	2	2	2	2	2	2
3	3	3	3	3	3	1	2	2	1
3	3	3	3	9	9	1	9	9	1
3	2	2	2	3	2	1	2	3	3
1	3	3	1	3	3	1	3	3	1
2	2	2	2	2	1	2	2	2	2
2	2	3	1	3	2	2	1	2	1
3	3	3	2	3	3	3	3	1	3
2	2	2	2	2	2	1	2	2	2
3	2	1	2	2	2	1	1	1	1
1	3	2	3	1	9	9	9	9	1
1	3	1	1	2	1	2	2	3	1
3	2	3	3	3	3	3	1	2	3
2	2	2	2	2	2	2	1	2	2
1	3	3	3	3	3	3	1	3	1
9	3	3	1	3	3	3	1	1	1
2	2	2	2	2	2	2	2	2	2
3	3	3	3	3	3	3	3	3	3
2	2	3	2	3	2	2	1	3	3

3	2	1	1	3	3	2	1	2	9
3	3	3	3	9	9	9	9	9	3
3	3	3	3	3	3	3	3	3	3
2	2	2	2	2	2	2	2	2	2
3	3	3	2	2	2	2	1	2	2
1	1	3	1	1	1	1	2	3	1
3	1	3	1	2	2	2	2	2	2
3	3	2	3	3	1	1	2	3	2
2	3	1	2	1	2	3	1	2	9
2	2	2	2	2	2	2	2	2	2
3	3	3	1	3	3	1	2	3	1
2	2	2	2	2	2	2	2	2	2
9	9	9	9	9	9	9	1	9	9
1	1	1	2	1	2	2	2	2	9
2	3	2	3	1	2	2	2	2	1
9	9	9	9	3	3	3	1	3	3
9	9	9	9	3	3	3	9	1	3
3	3	1	3	3	3	1	2	3	3
2	2	2	3	1	1	1	1	2	2
1	1	1	3	1	2	2	2	2	1
3	3	3	1	1	1	1	3	3	1
1	3	1	2	1	1	1	1	1	2
3	3	3	1	3	3	1	2	2	2
1	2	3	1	3	2	1	2	3	2
3	3	3	3	3	3	3	3	1	3
1	2	3	9	9	2	1	2	3	2
2	2	2	2	2	2	2	2	2	2
2	3	3	3	1	1	2	2	2	1
2	2	2	2	1	9	9	9	9	1
3	2	2	2	3	2	2	2	1	2
1	1	1	1	2	1	2	1	2	1
1	1	1	1	1	1	1	1	1	1
1	1	1	1	1	1	1	1	1	1
1	2	2	2	1	1	1	1	1	1
1	2	3	1	3	9	2	1	2	2
1	9	1	1	3	3	1	1	1	3
1	9	9	9	9	9	9	1	9	9
1	1	1	1	1	1	1	1	1	1
2	2	2	2	2	2	2	2	2	2
2	2	2	2	2	2	2	2	2	2
1	1	3	1	3	1	2	2	3	2
1	3	1	1	1	1	1	2	3	2
9	9	9	1	9	9	1	9	9	2
3	2	1	9	2	2	2	2	1	2
2	2	2	2	2	2	2	2	2	2
3	3	1	3	3	3	3	1	1	3
3	2	3	3	2	2	2	2	2	1

2	9	3	2	3	3	1	3	3	3
3	3	3	3	3	1	2	2	3	2
1	3	3	2	1	2	3	3	3	1
9	9	9	9	9	9	9	1	9	9
1	2	3	1	2	9	1	2	3	9
1	3	2	1	2	2	1	2	3	2
9	9	9	9	9	9	9	3	9	9
9	9	9	9	9	9	9	9	9	9
3	3	3	3	3	3	3	1	1	3
2	2	2	2	2	2	1	2	2	2
1	9	9	1	9	9	1	9	9	9
3	3	3	3	1	3	3	2	2	2
9	9	9	1	2	2	1	2	2	1
2	3	3	1	2	1	2	2	3	3
1	2	2	2	2	2	2	2	2	2
2	9	3	2	2	1	2	3	3	2
1	2	2	2	3	2	1	1	2	1
1	9	9	1	9	9	1	9	9	9
1	2	3	3	3	2	1	2	3	2
1	2	1	1	2	2	2	2	2	3
3	9	2	2	2	1	2	3	2	9
2	2	3	2	3	3	1	2	2	1
1	2	1	2	2	3	1	2	3	1
1	2	2	2	2	2	2	2	2	2
1	1	3	9	2	3	2	1	3	1
1	3	9	9	3	3	1	1	3	9
3	9	1	3	3	3	2	2	1	3
3	3	3	1	3	3	1	1	3	1
2	2	2	2	3	9	1	2	2	2
3	2	1	1	2	2	1	3	2	3
3	2	1	3	3	1	2	2	3	1
3	2	2	9	3	2	1	1	2	1
3	3	2	2	2	2	2	3	3	1
1	3	1	3	1	1	1	3	3	3
9	9	9	9	9	9	9	1	9	9
2	3	3	3	3	1	2	3	3	1
1	1	9	1	1	2	1	1	1	1
1	3	1	3	1	1	1	3	3	3
9	9	3	9	1	1	9	1	2	2
3	3	3	3	1	3	3	3	3	1
3	3	3	1	3	9	1	1	3	3
9	9	9	9	1	9	9	9	9	1
1	1	1	1	3	3	1	1	3	1
3	1	3	3	3	3	1	1	3	1
2	2	2	2	3	2	1	1	2	1
2	1	9	9	9	9	9	9	9	9
3	1	2	1	1	2	2	1	2	2

1	1	3	2	9	9	9	9	1	9
3	3	2	2	2	9	1	3	2	2
1	2	1	3	1	2	1	2	1	1
3	3	3	3	3	1	3	3	2	3
2	2	2	2	2	2	2	2	2	2
2	3	3	2	3	3	2	1	2	2
2	2	2	1	3	3	2	1	3	2
1	2	2	2	2	2	2	2	2	2
1	2	2	2	2	2	2	2	2	2
2	1	3	2	2	2	2	2	2	2
3	3	2	2	3	3	2	1	2	2
3	2	2	3	3	3	3	1	2	3
1	1	1	3	3	1	1	1	3	3
3	3	2	3	2	3	2	3	2	3
9	9	9	9	9	9	9	9	9	9
2	2	2	1	3	3	2	1	3	2
3	3	3	3	3	1	3	3	3	1
3	3	2	1	2	2	1	2	2	2
3	2	2	1	2	2	1	1	3	9
1	9	9	9	9	9	9	1	9	1
9	9	9	9	9	9	1	9	9	9
3	3	3	3	3	3	3	3	3	3
3	3	3	1	1	1	3	3	3	1
3	3	1	1	3	3	1	3	3	1
3	3	1	1	1	3	3	3	3	1
1	1	3	2	9	9	1	9	9	9
2	1	3	2	3	2	1	1	9	9
3	3	2	2	3	2	1	1	3	2
2	1	1	1	3	3	1	2	2	3
2	2	9	1	3	2	1	2	3	1
3	3	3	3	2	1	3	2	3	1
1	3	1	1	1	1	1	9	1	1
2	1	1	3	1	2	2	2	1	1
3	3	3	1	1	3	3	3	3	1
3	3	3	1	3	2	2	3	2	2
3	3	3	3	3	1	3	3	3	1
9	9	9	9	9	9	1	9	9	1
9	9	9	9	2	2	2	2	2	3
3	3	3	3	3	3	3	3	3	9
2	2	2	2	3	1	2	2	2	1
3	3	2	1	1	2	1	1	3	1
3	3	3	1	1	3	3	1	3	1
2	2	2	2	1	2	2	2	2	1
9	1	9	9	9	9	1	9	9	9
9	9	9	9	9	9	9	9	9	3
3	3	1	3	1	2	3	3	3	1
3	3	3	3	2	2	1	3	1	2

9	9	9	9	1	2	9	1	3	1
1	1	1	1	3	3	2	3	3	2
3	3	3	3	1	3	3	3	3	1
9	9	9	1	9	9	9	2	9	9
3	3	3	2	3	3	3	1	2	2
9	9	9	9	9	9	9	9	9	9
1	1	2	2	1	3	1	1	1	3
3	3	3	3	2	9	1	1	2	3
3	3	3	3	1	3	3	3	3	1
3	3	3	3	3	1	2	3	3	3
3	3	3	3	3	1	2	3	3	3
3	3	3	3	3	1	2	1	3	3
1	9	9	9	1	9	9	9	9	9
1	9	9	9	1	9	9	9	9	1
9	9	9	9	2	2	1	1	1	3
3	3	2	2	3	2	1	1	1	3
1	3	3	1	3	1	3	3	3	1
3	3	3	3	2	2	3	1	3	2
2	3	2	1	2	2	1	2	3	3
3	3	3	2	2	1	2	2	2	2
2	2	2	2	3	1	1	1	1	1
2	1	2	2	3	1	1	1	1	1
2	2	2	2	2	2	2	2	2	2
2	2	3	3	3	3	2	1	3	3
2	3	2	3	2	1	2	2	2	2
2	3	3	3	9	9	9	9	9	1
1	3	3	1	1	9	9	9	9	9
2	2	2	2	2	2	2	2	2	2
3	2	2	2	9	9	9	9	9	1
3	3	2	2	3	2	1	1	1	3
3	3	3	2	2	1	2	2	2	2
1	9	9	9	1	9	9	9	9	9
3	3	3	3	1	9	9	9	9	1
3	3	3	2	3	2	1	1	2	2
1	1	2	2	2	2	1	1	1	2
9	9	9	1	2	1	2	2	3	9
9	9	9	9	9	9	1	9	9	9
9	9	9	9	9	1	9	9	9	9
3	2	3	3	1	2	1	3	1	1
1	1	3	1	2	2	2	2	2	1
9	9	9	9	9	9	9	1	9	1
1	1	2	1	1	3	3	3	3	1
3	3	3	3	1	3	3	3	3	1
3	3	3	3	3	1	3	3	3	1
1	1	1	1	3	2	1	2	1	1
1	2	2	1	2	1	3	2	3	2
9	9	9	9	9	9	1	9	9	1

1	3	3	1	9	9	9	9	9	9
3	3	3	3	2	2	1	1	1	2
1	2	1	1	2	2	2	1	1	1
9	9	9	9	9	9	1	9	9	9
9	9	9	9	9	1	9	9	9	9
3	3	2	1	3	2	3	3	3	1
1	2	1	3	1	1	1	2	3	1
2	3	2	2	1	2	3	3	3	1
3	1	3	2	1	2	2	3	3	2
1	2	1	3	1	1	2	2	2	1
3	3	3	1	2	1	1	3	3	2
9	9	9	9	2	2	2	2	2	2
3	3	3	3	1	2	3	2	2	2
3	3	3	1	2	2	2	2	2	1
2	2	3	1	3	2	1	2	2	3
3	1	2	1	3	3	3	3	3	3
3	3	3	3	1	3	3	3	3	3
3	3	3	2	1	2	3	3	3	3
3	9	2	3	2	3	3	2	2	1
3	3	3	3	3	1	3	3	3	3
2	2	3	3	3	1	3	3	3	2
9	9	9	9	9	9	9	9	1	9
2	1	2	1	2	2	2	2	2	2
3	2	2	2	3	3	3	3	3	3
1	2	2	1	2	2	2	2	2	2
1	2	2	1	2	2	2	1	2	2
2	2	3	2	3	1	2	2	2	2
3	3	3	3	3	3	1	3	3	1
3	3	2	3	1	1	1	1	1	1
3	3	3	3	3	3	3	1	3	3
9	9	9	9	9	9	1	9	9	1
1	2	9	1	2	1	3	2	2	1
9	9	9	9	9	9	1	9	9	9
3	1	2	1	3	3	2	1	3	2
1	1	2	3	1	2	3	2	2	1
9	1	9	9	1	9	9	9	9	1
9	9	9	9	9	9	9	9	9	9
2	2	2	2	2	2	2	2	2	2
3	3	3	1	2	2	1	2	2	2
1	3	1	2	1	2	1	1	2	1
9	2	1	3	2	3	1	1	3	1
1	1	1	1	1	1	9	9	9	9
2	1	1	1	1	1	1	1	2	1
3	9	1	3	1	2	3	1	3	1
3	1	2	3	3	1	2	2	3	1
9	9	9	9	9	9	9	2	9	1
3	3	3	2	1	1	3	3	3	2

1	1	2	1	2	1	2	3	2	1
3	3	3	2	9	9	1	9	9	9
3	3	3	3	3	3	3	3	3	1
2	3	3	3	3	2	1	1	2	3
3	2	2	2	3	2	2	1	2	2
1	1	2	1	2	1	2	3	2	1
3	3	2	1	1	1	2	3	3	1
2	1	2	1	2	2	2	2	2	2
3	3	3	3	2	2	1	2	2	1
1	1	3	3	3	1	3	3	3	3
2	2	2	2	2	2	2	2	2	2
2	2	2	2	2	2	2	2	2	2
9	9	9	9	9	9	1	9	9	1
2	2	2	2	2	2	2	2	2	1
3	3	3	2	3	2	2	1	2	2
2	2	3	1	3	2	1	2	2	9
3	3	3	3	1	3	3	3	3	3
3	3	3	3	1	3	3	3	3	1
3	3	3	3	2	2	2	1	3	2
2	3	3	1	3	3	1	3	3	1
3	1	2	2	2	2	1	1	1	1
3	3	3	3	3	3	1	3	3	3
1	3	3	2	3	3	3	2	2	1
3	2	2	2	3	1	3	3	3	9
2	2	2	2	2	2	2	2	2	1
2	1	2	2	3	3	1	2	3	3
2	2	2	2	2	2	2	2	2	2
2	2	2	2	1	3	3	3	3	1
1	1	2	1	3	1	1	3	3	2
2	1	1	2	1	1	2	3	3	1
3	3	3	3	1	1	3	3	3	3
3	2	2	2	3	1	3	3	3	1
3	3	2	2	1	3	9	2	1	3
1	2	2	1	3	3	1	1	2	2
2	2	9	3	1	2	1	3	1	1
3	2	3	1	2	2	1	2	2	1
1	1	1	1	3	1	1	1	2	2
3	3	3	2	2	2	2	9	2	2
3	3	3	2	2	3	3	2	3	3
3	2	2	2	3	3	2	1	2	2
1	3	1	3	3	1	3	3	3	3
3	2	2	1	1	3	3	3	3	2
3	3	3	3	9	1	9	9	9	9
3	3	1	1	3	1	1	9	3	3
9	9	9	9	9	1	9	9	9	9
9	9	9	9	9	1	9	9	9	9
9	9	9	9	9	1	9	9	9	9

1	3	3	1	3	1	2	1	2	3
2	2	1	2	2	3	2	2	1	2
3	3	3	3	3	3	1	3	3	3
1	2	3	3	3	3	2	1	2	1
1	3	1	1	1	1	1	3	1	1
9	9	9	9	9	1	9	9	9	9
9	9	9	9	9	1	9	9	9	9
9	9	1	9	9	1	9	9	9	1
9	9	9	9	9	1	9	9	9	1
9	9	9	9	9	9	1	9	9	9
9	9	9	1	9	9	1	9	9	9
1	1	1	1	1	1	1	9	9	9
9	9	9	9	9	1	9	9	9	1
1	3	3	1	3	3	3	1	3	3
3	3	2	3	3	3	2	1	1	3
2	3	3	3	2	1	2	2	2	2
1	1	1	1	2	2	1	2	2	1
1	9	9	9	9	9	9	1	9	9
9	9	9	9	1	9	9	9	9	9
9	9	9	9	9	1	9	9	9	9
3	1	2	2	9	9	9	9	9	9
9	9	9	9	9	1	9	9	9	9
1	1	1	1	2	2	1	1	1	1
9	9	9	9	9	9	1	9	9	1
3	3	3	3	2	2	1	2	2	1
2	2	2	2	2	1	2	2	2	2
3	2	2	1	3	3	1	2	1	3
1	1	1	1	2	1	2	2	2	2
2	2	1	1	1	1	1	2	2	2
3	3	3	3	2	2	1	2	2	1
1	3	3	3	1	1	1	1	1	1
2	2	2	1	3	1	3	3	3	3
1	1	2	2	2	2	2	1	2	1
9	9	9	9	9	9	9	9	9	9
2	2	2	1	1	1	2	1	1	2
2	2	2	1	2	1	2	2	2	2
2	2	2	1	2	1	2	2	2	2
9	9	9	9	9	9	1	9	9	9
3	2	3	1	1	2	2	3	3	1
3	1	3	1	9	9	9	9	9	9
3	9	3	3	2	2	2	2	2	3
1	3	3	1	2	3	3	1	3	2
3	3	3	1	3	3	1	3	3	3
1	1	9	2	2	2	2	2	2	2
2	2	3	3	1	3	3	3	3	9
3	3	3	1	3	2	2	3	1	3
3	3	3	3	3	2	2	1	2	2

2	3	3	3	3	3	3	2	1	1
1	2	3	1	2	3	3	2	2	3
9	9	9	9	3	3	3	3	3	3
3	3	2	1	2	1	2	2	2	2
3	3	3	3	3	3	1	2	3	1
2	9	9	9	9	1	9	9	9	9
3	3	1	1	3	2	1	1	3	1
3	3	3	3	3	1	2	1	3	1
2	9	9	9	9	1	9	9	9	9
2	2	2	3	2	1	2	3	3	2
2	9	9	9	9	1	9	9	9	9
3	3	2	2	3	3	1	2	1	2
1	1	2	1	2	1	2	2	2	2
1	2	9	1	2	1	1	2	3	1
1	9	2	1	3	1	2	2	2	2
2	2	3	3	3	1	3	1	3	1
2	2	2	2	2	2	2	1	1	1
3	3	2	3	2	1	2	3	3	2
3	3	3	3	3	3	3	1	2	2
1	9	9	9	9	9	1	9	9	9
3	3	3	3	3	3	3	1	2	3
3	2	3	1	3	3	3	1	2	2
3	3	3	3	2	2	2	2	2	2
2	3	1	2	1	1	1	1	1	2
3	3	3	3	2	2	1	2	2	2
3	3	3	3	1	1	1	1	1	1
1	3	3	3	3	3	1	3	3	3
3	9	2	1	1	1	3	1	1	1
3	3	3	1	2	1	2	2	2	2
1	1	3	3	3	3	3	1	1	1
1	2	3	1	3	1	2	1	3	3
1	2	2	2	1	2	2	2	2	2
2	1	1	1	2	1	1	1	1	2
1	1	1	1	2	2	2	1	2	1
2	2	2	2	2	2	1	2	2	1
2	9	2	2	2	2	1	2	2	1
2	2	2	1	2	1	2	2	2	1
2	2	1	1	2	2	1	2	2	2
2	2	2	2	2	2	1	2	2	2
9	1	2	2	2	2	2	2	2	2
3	9	3	3	3	3	3	1	3	3
3	3	9	1	2	2	2	2	2	2
3	3	3	3	3	3	2	1	2	3
1	2	2	2	2	1	1	1	2	1
1	3	3	1	3	3	3	3	3	3
3	3	3	3	3	3	3	3	3	3
3	3	3	3	2	2	2	1	3	1

2	2	2	1	1	2	2	3	3	2
3	1	3	1	2	2	1	1	1	2
9	9	1	9	9	9	1	9	9	9
3	3	1	9	9	9	9	3	9	3
9	9	1	9	9	1	9	9	9	9
1	3	3	3	9	9	1	1	9	1
9	9	9	9	9	9	1	9	9	1
3	2	2	3	2	3	2	1	1	1
2	2	2	2	2	2	2	2	2	2
3	3	3	1	3	1	1	3	1	3
1	3	3	1	1	1	1	9	3	9
1	1	1	1	3	3	1	1	1	1
1	1	2	2	2	2	1	1	1	1
1	3	2	2	2	2	2	2	2	2
1	1	1	1	1	1	2	1	1	1
9	9	9	9	9	9	9	9	9	9
3	3	3	3	2	2	2	2	2	2
3	3	3	3	9	9	9	9	3	3
2	3	1	2	9	9	9	9	9	9
1	1	1	1	2	2	2	2	2	2
1	1	1	1	2	1	1	1	1	2
3	2	2	2	3	3	2	1	2	2
2	2	2	2	1	2	2	2	2	1
2	2	3	1	2	2	2	2	2	2
1	2	3	2	2	1	1	1	2	2
3	3	3	3	3	3	1	1	3	2
1	9	9	9	9	9	1	9	9	1
1	2	2	2	2	2	1	2	2	2
3	3	2	3	3	1	2	3	3	2
1	1	3	3	2	2	1	1	1	2
9	9	9	9	9	9	9	9	9	9
2	1	3	2	1	1	2	3	1	2
1	2	1	2	1	1	1	2	2	1
2	2	2	2	2	2	2	2	2	3
2	2	2	2	3	3	3	2	2	2
2	2	2	1	1	2	2	3	3	2
1	1	3	3	3	3	1	3	3	3
3	1	2	3	3	2	1	2	3	1
3	2	2	3	1	1	2	3	3	3
3	3	3	1	1	1	2	3	3	1
1	2	2	2	2	2	1	2	2	2
3	3	3	2	1	1	3	2	3	1
2	3	2	2	3	2	1	1	3	3
9	9	9	9	9	9	9	1	9	9
9	9	9	9	9	9	1	9	9	9
2	3	2	2	3	2	1	1	3	3
3	3	3	3	3	3	1	3	3	1

3	3	3	3	1	3	3	2	3	1
2	2	1	1	2	1	2	2	2	2
9	9	9	9	9	9	1	9	9	9
3	3	3	3	1	9	9	9	9	9
9	9	9	9	9	9	9	9	9	9
2	3	2	1	2	2	2	1	2	1
3	3	3	3	2	2	1	3	3	2
3	3	3	3	3	3	3	1	3	2
2	9	3	2	3	2	1	1	3	3
1	1	2	3	1	3	3	3	3	1
9	9	9	9	9	9	1	9	9	9
9	9	9	9	1	9	9	9	9	1
2	2	2	2	2	2	2	2	2	2
3	2	2	2	1	9	3	9	9	1
9	9	9	9	1	9	9	9	9	9
1	3	3	1	3	3	1	1	1	1
1	1	3	3	3	1	2	3	1	1
3	3	1	2	1	1	1	3	3	1
3	2	3	3	1	3	1	2	3	1
2	2	3	1	3	2	2	1	3	3
2	1	3	3	1	1	2	3	3	1
3	3	1	1	3	3	3	1	3	3
2	1	2	3	1	1	3	3	3	1
9	9	9	9	2	2	2	1	2	3
3	3	3	3	1	2	2	1	1	1
1	3	3	1	3	2	2	2	1	3
9	9	9	9	9	9	9	1	9	9
2	2	3	1	1	3	2	9	1	3
3	3	2	3	2	3	9	9	9	9
2	3	3	2	1	1	1	2	2	1
3	3	3	3	2	2	1	1	3	3
1	1	1	1	2	3	3	3	3	3
1	3	2	1	2	2	1	3	2	2
2	3	3	3	3	1	1	2	2	3
2	3	2	3	3	1	3	2	3	1
1	1	2	1	2	2	2	2	2	2
9	9	9	9	2	2	2	2	2	3
3	3	3	2	2	2	2	2	2	1
9	9	9	1	9	9	1	9	9	1
2	3	2	3	3	1	1	2	3	2
3	3	3	1	2	2	3	2	1	9
1	3	2	1	2	2	2	2	2	2
2	1	1	3	1	2	9	1	3	9
1	3	2	1	1	2	1	2	3	9
3	3	2	3	2	2	2	1	2	2
3	3	3	3	2	2	1	1	3	1
3	3	3	3	2	2	2	1	3	1

3	3	3	3	3	3	3	1	3	3
9	9	9	9	9	9	9	9	9	9
3	3	3	3	9	1	9	9	9	9
3	3	1	1	3	3	1	1	3	3
3	3	1	3	3	1	1	3	3	1
3	3	1	1	3	3	1	3	3	3
3	3	9	9	2	3	9	2	1	2
1	1	1	2	2	2	1	1	1	3
3	3	3	1	3	3	1	3	3	3
3	3	3	3	2	2	1	1	3	1
3	3	3	3	2	2	1	1	3	1
1	2	2	3	1	2	3	2	1	1
3	3	3	2	3	2	3	2	3	3
1	1	1	2	2	2	1	1	1	3
1	2	2	1	2	1	2	1	3	1
2	1	2	3	2	2	2	2	2	2
3	2	2	1	2	2	2	2	2	2
1	1	3	2	3	2	1	1	2	3
1	3	3	3	3	3	1	1	1	3
2	2	2	2	1	2	2	2	2	2
3	3	3	3	1	1	1	3	3	1
1	2	1	1	3	2	1	3	1	2
3	3	3	3	2	2	2	2	2	1
3	3	3	3	2	2	2	2	2	2
2	2	1	3	2	2	1	2	2	2
2	1	2	3	1	2	3	3	2	1
3	3	2	3	3	3	2	1	2	2
3	3	3	3	3	3	3	3	3	2
3	3	3	3	3	3	1	1	3	1
9	9	9	9	9	1	9	9	9	1
2	3	3	3	3	2	1	2	3	2
2	1	3	2	1	1	1	1	1	3
2	2	2	3	2	3	1	2	2	2
3	3	3	1	2	2	2	2	2	1
1	1	1	1	1	1	1	1	1	1
2	2	2	2	2	2	2	2	2	2
3	3	3	1	3	1	3	3	3	3
2	3	2	3	2	1	1	2	2	1
3	3	1	1	3	2	1	2	9	1
1	1	1	2	1	2	2	2	2	2
2	2	2	1	9	9	1	9	9	9
3	3	3	3	3	3	2	3	1	1
3	1	2	3	2	2	1	2	1	1
3	3	3	3	1	3	3	2	3	1
2	2	2	2	2	2	2	2	2	2
3	3	3	3	2	2	2	2	2	3
3	3	3	3	3	3	1	1	2	1

2	3	2	3	2	3	3	1	2	2
3	1	1	3	2	2	1	3	2	2
2	2	2	1	2	2	2	2	2	1
2	2	1	1	1	1	1	1	1	1
2	3	2	2	3	1	1	3	1	3
3	3	3	3	2	2	2	2	2	3
3	3	3	2	3	1	2	2	2	1
3	3	3	2	2	2	2	2	2	2
1	3	3	2	2	2	2	2	2	1
2	2	2	2	2	2	2	2	2	1
9	9	9	9	9	2	9	9	9	2
3	2	3	1	9	1	2	3	3	1
9	9	9	9	9	9	9	9	9	9
2	3	2	2	3	1	1	3	1	3
2	3	3	2	2	2	2	2	2	2
3	3	3	3	9	9	9	9	9	9
2	2	2	3	2	2	1	2	2	2
3	1	1	3	2	1	3	2	3	3
3	3	1	1	3	1	1	3	1	3
3	3	3	3	2	2	2	2	2	1
3	3	9	9	1	1	1	1	2	1
2	3	3	3	3	2	1	2	3	2
9	9	9	9	9	9	1	9	9	1
1	3	3	1	2	2	2	2	2	1
2	3	1	1	3	3	1	1	1	2
3	3	2	1	2	2	2	2	2	2
9	9	1	9	9	1	9	9	9	1
2	2	2	3	2	3	3	3	2	2
3	3	2	1	3	2	2	2	3	3
9	9	9	9	1	9	9	9	9	1
3	3	1	1	2	2	1	1	2	3
2	2	2	2	1	1	1	1	1	2
3	3	1	1	9	9	1	9	9	1
3	2	3	3	1	1	3	3	3	2
3	3	3	3	1	3	3	3	3	1
1	9	9	9	3	3	2	1	2	2
3	3	9	1	3	1	3	3	3	1
3	2	9	3	3	2	1	1	3	1
1	1	1	1	9	9	1	9	9	9
3	3	3	3	3	2	1	2	2	1
3	2	3	1	3	3	2	1	2	3
3	3	2	1	3	2	1	2	3	3
3	3	3	2	2	2	2	2	2	2
2	2	3	1	3	1	2	2	2	3
2	2	2	3	1	3	3	2	3	1
2	3	3	3	2	2	1	2	2	9
2	3	3	3	3	3	1	3	3	1

3	3	3	3	3	3	3	1	2	1
2	2	2	2	2	2	2	2	2	2
9	9	9	9	9	9	1	9	9	2
9	9	1	9	9	9	1	9	9	9
1	2	2	1	1	1	2	2	2	2
3	3	3	3	3	3	3	3	1	1
3	3	3	2	2	2	2	2	2	2
2	3	2	3	3	3	1	2	3	3
3	1	2	1	3	1	2	1	3	1
3	3	3	3	3	1	3	3	3	9
2	2	3	1	3	2	2	1	1	2
1	2	2	1	2	1	3	1	3	2
2	2	2	2	2	2	2	2	2	2
3	3	3	3	3	3	3	3	3	3
2	3	2	3	2	2	1	3	3	2
2	1	2	1	3	3	2	1	1	2
3	9	2	3	3	2	1	3	3	2
3	3	3	1	1	1	3	3	3	3
1	3	3	3	2	2	2	2	2	2
3	1	2	1	2	2	1	1	2	1
2	2	3	2	3	3	1	3	3	3
2	2	2	2	3	1	2	2	2	3
2	2	2	2	3	1	2	2	2	3
1	1	3	1	3	1	3	3	3	1
3	3	3	1	3	3	1	1	3	2
3	3	3	3	3	3	3	1	3	3
3	1	1	3	3	1	2	3	3	3
3	1	1	3	3	1	2	3	3	3
2	2	3	3	2	2	2	2	2	1
2	2	2	2	3	3	3	1	3	2
3	3	2	2	1	2	3	2	2	2
2	2	1	2	3	1	3	2	2	3
3	3	3	3	3	3	3	1	3	3
1	3	3	3	1	2	2	2	2	1
3	3	3	3	2	1	2	2	2	2
3	3	3	3	3	3	3	3	3	3
3	3	3	3	3	3	3	3	3	2
3	1	2	1	3	2	2	1	3	2
2	1	2	2	2	2	2	2	2	2
2	1	2	3	2	2	2	2	2	2
3	1	3	3	3	3	1	2	2	1
3	3	3	1	3	3	3	3	1	1
1	2	1	1	1	1	1	1	1	1
2	2	2	2	1	2	2	2	2	1
2	2	3	2	2	2	2	2	3	2
3	2	2	1	1	3	3	3	3	1
1	3	3	3	2	2	1	2	3	9

1	2	2	2	2	1	2	3	2	2
2	2	2	2	2	9	2	9	2	1
1	1	2	2	2	2	2	2	2	1
2	2	2	2	2	2	2	2	2	2
2	2	2	2	2	2	2	2	2	2
2	2	2	2	1	2	2	2	2	2
1	1	2	1	2	1	3	2	3	1
3	1	3	2	1	2	1	3	2	9
3	2	2	2	2	2	2	2	2	9
3	2	3	3	2	2	2	2	2	2
2	2	2	2	2	2	2	2	2	2
2	2	2	2	2	2	2	2	2	2
2	2	2	2	2	2	2	2	2	2
2	2	2	2	2	2	2	2	2	2
3	3	1	2	2	2	2	2	2	3
2	2	2	2	2	2	1	2	2	9
3	1	2	1	1	2	2	2	2	2
2	2	2	2	2	2	2	2	2	9
1	2	3	1	1	1	1	3	3	9
2	3	2	3	3	1	2	3	2	2
2	2	2	2	2	2	2	2	2	2
2	2	2	2	2	2	2	2	2	1
2	3	3	1	3	3	3	2	3	2
2	2	2	2	2	2	2	2	2	2
2	2	2	2	2	2	2	2	2	9
2	2	2	2	9	9	9	1	9	9
2	2	2	2	2	2	2	2	2	2
2	2	2	2	2	2	2	2	2	2
2	2	2	2	2	2	2	2	2	2
3	3	3	3	3	3	3	1	3	1
3	3	3	3	2	2	2	2	2	2
2	2	2	2	2	2	2	2	2	2
2	2	2	2	2	2	2	2	2	2
3	3	2	2	2	2	2	2	2	2
2	2	2	2	2	2	2	2	2	2
3	2	3	2	3	3	2	1	1	2
2	2	2	2	2	2	2	2	2	2
2	2	2	2	2	2	2	2	2	2
2	2	2	2	3	3	1	2	2	3
2	2	2	2	2	1	2	2	2	9
3	2	1	2	1	1	2	3	3	9
2	2	2	2	2	2	2	2	2	2
2	2	2	2	2	2	2	2	2	9
2	2	2	2	2	2	2	2	2	2
2	2	2	2	2	2	2	2	2	2
2	2	2	2	2	3	2	3	2	1
2	2	2	2	2	2	2	2	2	2

2	2	2	2	2	2	2	2	2	2
2	2	2	2	2	2	2	2	2	9
3	2	2	2	3	2	1	2	3	1
2	2	2	2	2	2	2	2	2	2
2	2	2	2	2	2	2	2	2	2
2	2	2	2	3	3	3	1	2	9
3	3	3	2	2	2	2	2	1	1
9	9	9	9	9	9	1	9	9	9
1	3	3	1	3	1	1	3	3	1
3	3	3	2	2	2	1	3	3	3
2	2	3	1	2	2	1	2	2	1
2	2	2	2	2	2	2	2	2	2
1	2	2	1	2	2	2	2	2	9
1	9	9	9	9	1	9	9	9	9
3	3	3	3	3	3	3	1	3	3
2	2	2	2	2	1	1	2	2	2
2	2	2	2	2	1	2	2	2	2
2	2	2	2	2	2	2	2	2	2
1	3	3	1	1	3	3	3	3	1
2	2	2	2	2	1	2	2	2	1
9	9	9	9	9	1	9	9	9	1
2	2	2	2	2	2	2	2	2	2
2	2	2	2	2	2	2	2	2	9
2	2	2	2	2	2	2	2	2	2
2	2	2	2	2	2	2	2	2	2
2	2	2	2	2	2	2	2	2	2
9	9	9	9	2	9	9	9	9	2
2	2	2	2	2	2	2	2	2	2
2	2	2	2	2	2	2	2	2	2
2	2	2	2	2	2	2	2	2	9
2	2	2	2	2	2	2	2	2	2
3	2	3	2	3	3	2	3	2	1
1	3	3	3	3	3	1	3	3	2
3	3	3	1	3	3	1	3	3	1
2	2	3	1	3	1	1	3	3	1
3	3	3	3	3	2	1	1	3	1
3	3	2	1	3	3	2	1	1	3
3	3	3	3	1	1	1	2	3	1
2	2	3	2	3	1	1	2	3	1
2	3	2	2	3	2	2	2	2	1
3	2	3	2	3	3	1	1	2	1
1	1	2	1	3	3	1	3	3	9
1	1	2	2	2	2	1	1	3	2
2	2	2	2	2	2	2	2	2	2
1	1	1	2	3	3	1	9	9	1
1	2	2	1	2	2	2	1	1	2
3	2	3	3	2	1	2	2	1	1

3	2	2	1	2	1	3	2	1	2
2	9	1	2	1	2	1	3	1	1
1	1	2	2	1	1	1	2	2	1
1	2	1	2	3	2	3	3	3	3
2	3	1	2	2	1	2	3	1	3
1	2	3	1	2	1	3	2	3	2
1	2	3	1	3	1	2	1	2	1
1	9	9	9	9	9	9	2	9	1
2	2	2	2	2	2	2	2	2	2
2	2	2	2	2	2	2	2	2	1
2	2	2	2	2	2	1	2	2	2
2	2	2	2	2	2	1	2	2	2
3	3	3	3	3	3	3	3	3	3
2	2	2	2	3	3	1	2	9	2
3	3	3	3	1	3	3	3	3	2
2	2	2	2	2	1	2	2	2	2
2	2	2	2	1	2	2	2	2	2
3	3	3	3	2	9	1	3	3	1
3	3	3	3	3	2	1	2	3	9
2	3	3	2	3	9	1	1	2	3
3	3	3	3	3	1	2	3	3	9
9	9	9	9	9	9	1	9	9	1
1	3	3	1	3	3	1	1	2	3
9	9	9	9	9	9	9	9	9	9
3	1	2	1	1	2	2	1	3	1
2	3	1	1	1	3	3	1	2	3
3	3	3	3	3	3	1	3	3	2
9	9	9	9	2	9	9	9	9	2
2	2	2	2	1	2	2	2	2	9
1	2	2	1	9	1	9	9	9	9
1	2	2	1	9	1	9	9	9	2
2	2	3	3	3	1	2	3	3	1
2	2	2	1	3	1	1	3	3	3
2	2	2	1	3	1	1	3	3	3
2	2	2	1	3	1	1	3	3	3
2	2	2	2	2	2	2	2	2	2
3	3	3	3	2	1	3	2	3	1
2	2	2	1	3	1	1	3	3	3
2	2	2	1	2	1	1	3	3	2
2	2	2	1	3	1	1	3	3	9
2	1	2	2	2	2	2	2	2	2
3	3	3	2	3	3	3	1	3	2
2	2	2	2	2	2	2	2	2	2
1	2	3	3	2	2	3	1	3	9
1	9	9	9	1	9	9	9	9	9
2	2	2	1	1	1	1	2	2	1
2	2	2	2	2	2	2	1	9	1

2	2	1	2	2	1	2	2	3	1
3	1	2	1	1	2	1	3	2	9
3	3	3	3	3	3	3	1	2	2
3	3	3	3	2	1	2	2	2	9
2	2	2	2	2	2	1	1	1	2
2	2	2	2	2	2	2	2	2	9
2	2	2	2	2	2	2	2	2	2
3	3	3	2	3	3	3	3	3	2
3	3	1	1	3	1	3	3	3	9
2	2	2	2	2	2	2	2	2	2
1	2	2	2	2	2	2	1	2	2
2	1	1	1	1	1	2	1	1	1
3	1	1	3	1	3	3	2	3	1
1	2	3	3	2	2	1	3	2	3
1	3	2	2	3	3	1	3	2	2
1	3	2	3	3	1	2	1	3	3
3	1	1	1	3	1	3	3	3	1
1	3	2	2	1	3	3	2	3	2
2	2	2	2	2	1	2	2	2	2
3	1	1	2	1	3	2	1	3	3
2	3	3	1	2	1	1	2	3	2
1	3	1	3	1	3	3	2	3	3
9	9	9	1	9	1	9	9	9	1
3	3	3	3	3	3	3	1	3	1
2	3	1	1	2	1	1	2	2	1
2	3	3	2	2	2	2	2	2	2
2	2	3	1	3	3	1	3	2	1
2	2	3	3	3	2	1	1	1	3
1	3	2	3	1	2	3	1	2	2
3	3	3	3	1	9	9	9	9	9
2	2	2	2	2	2	2	2	2	2
2	1	2	2	2	1	2	2	2	1
3	1	1	3	1	1	1	1	3	3
3	3	3	3	3	3	3	3	3	3
2	2	2	2	2	2	1	2	2	2
2	2	1	1	2	1	3	3	2	1
3	9	3	1	1	3	3	3	3	3
2	2	2	2	2	2	2	2	2	2
1	2	1	1	2	2	1	1	1	2
9	9	9	1	9	1	9	9	9	9
3	3	3	1	2	2	2	2	2	3
2	3	2	1	1	2	1	3	1	1
3	3	3	3	3	2	1	1	3	2
2	2	3	1	1	2	2	3	3	2
1	1	1	1	3	2	1	1	2	1
3	1	3	1	2	2	2	2	2	3
3	3	3	3	9	9	9	9	9	9

1	2	2	3	2	1	2	2	1	1
3	2	3	1	2	1	1	3	3	9
3	3	3	3	1	3	3	3	3	3
3	3	3	1	3	1	3	3	3	1
3	3	3	3	9	9	9	9	9	1
2	2	2	2	2	3	3	3	1	3
3	2	1	2	1	1	2	2	3	9
2	2	2	1	2	2	9	2	2	2
3	3	3	3	3	2	3	3	3	3
3	9	2	1	2	2	3	2	3	2
3	3	3	1	1	1	2	3	3	1
3	2	2	2	2	2	2	1	2	2
2	2	1	1	3	2	2	1	2	1
1	1	1	1	3	3	1	1	1	3
2	2	2	1	3	3	2	1	3	1
2	2	2	2	2	2	2	2	2	2
1	2	2	2	3	2	2	2	1	1
3	1	2	2	2	2	2	2	2	2
3	3	3	3	3	2	1	1	3	1
2	1	9	1	3	2	1	1	3	1
2	2	2	2	1	2	1	2	2	1
9	1	9	9	9	9	2	9	9	9
2	3	3	3	2	2	2	2	1	1
3	3	3	3	3	3	3	3	3	3
3	2	3	3	3	3	9	9	9	3
2	2	2	2	2	1	2	2	2	1
9	9	9	9	3	1	3	2	2	1
3	3	3	1	2	2	2	2	2	2
3	3	3	3	2	3	3	3	3	3
2	2	3	2	2	1	2	2	3	2
1	1	1	1	3	3	1	1	3	3
9	9	9	9	3	1	3	3	3	2
2	2	3	3	2	3	2	2	3	1
3	2	2	1	3	3	1	1	1	2
1	2	2	1	2	3	2	1	2	1
3	3	2	2	2	2	1	1	1	2
3	1	2	1	3	9	9	1	9	1
1	1	1	1	3	1	2	2	3	3
2	3	3	3	3	2	1	2	3	3
9	9	9	9	9	9	2	9	9	2
2	2	2	1	1	2	2	2	2	2
2	2	1	2	3	3	2	1	1	2
1	1	2	2	3	3	1	2	2	2
1	3	1	1	3	2	1	2	1	2
1	3	3	1	2	1	3	2	3	3
3	3	3	1	2	1	1	3	3	2
2	2	3	1	3	2	1	2	3	3

2	2	3	1	3	2	1	2	3	3
2	2	2	2	2	2	2	2	2	2
2	1	2	3	2	2	3	3	3	1
1	2	9	9	1	2	1	2	1	1
3	3	3	3	2	2	2	1	2	2
1	3	3	3	3	1	1	3	1	1
3	3	3	1	1	9	9	9	9	1
9	9	9	9	9	9	1	9	9	9
1	3	3	1	2	1	2	2	2	2
1	3	1	3	1	9	1	1	1	1
2	2	2	2	2	2	2	2	2	2
3	3	3	2	3	3	1	2	2	3
2	3	3	3	9	1	9	9	9	9
2	2	3	1	1	2	3	3	3	2
2	9	2	9	9	2	2	9	9	9
2	2	1	2	2	2	3	3	3	1
1	1	1	1	1	1	1	1	1	1
3	3	1	2	2	2	1	2	2	2
1	3	3	3	2	3	1	1	9	2
1	3	1	2	3	2	1	2	3	2
1	3	1	1	2	3	1	1	2	1
3	3	3	3	1	3	3	3	3	2
1	1	2	2	2	2	2	1	2	1
1	3	3	3	3	3	2	1	2	3
3	3	3	3	1	3	3	3	3	1
1	1	1	2	2	2	2	1	2	2
3	3	2	3	2	1	2	1	3	2
3	3	3	1	3	3	3	1	3	2
3	3	3	3	3	1	3	3	3	1
3	3	3	1	1	1	3	1	3	1
2	1	2	1	2	1	2	1	1	1
1	1	1	1	1	2	1	1	2	1
1	1	1	2	1	1	1	2	1	1
2	2	2	1	2	2	2	1	2	2
1	1	1	3	3	3	1	2	3	3
3	2	3	1	3	2	1	1	3	1
2	9	1	1	2	2	1	2	2	1
3	3	3	3	3	3	3	1	3	3
1	1	1	2	1	2	2	2	1	1
2	2	1	1	1	2	2	2	1	1
3	3	3	3	3	3	3	3	3	2
1	3	3	3	3	1	3	1	3	3
3	3	3	3	3	3	1	3	3	1
3	3	3	3	3	3	3	3	3	2
3	3	3	2	2	3	1	3	3	1
1	1	1	1	2	2	2	2	2	3
2	2	2	2	2	2	2	2	2	2

2	2	2	2	2	1	3	3	3	1
1	1	3	3	3	3	2	1	3	3
2	2	2	2	2	2	2	2	2	2
1	2	3	2	2	1	1	2	3	2
3	3	3	3	3	3	1	3	3	1
1	1	1	2	2	1	1	3	1	1
3	3	9	2	3	3	1	3	3	1
2	1	2	2	2	2	2	1	2	1
3	3	3	3	2	2	2	2	2	2
3	3	3	3	2	2	2	2	2	2
2	2	2	2	2	2	2	2	2	2
2	2	2	2	2	2	2	2	2	2
2	2	2	9	2	2	2	2	2	1
3	3	3	3	3	3	3	1	3	3
3	3	3	3	2	2	2	2	2	1
3	3	3	1	3	3	1	3	3	2
3	3	3	3	3	3	3	1	3	1
3	1	3	2	2	2	1	2	2	9
2	2	1	1	2	2	2	2	2	2
1	2	3	2	3	3	3	1	1	1
2	2	2	2	2	2	2	1	2	2
3	3	3	3	2	2	2	2	2	2
3	3	3	3	3	3	3	3	3	3
2	3	3	3	3	3	2	3	3	3
1	2	1	2	2	2	1	2	3	2
2	2	2	2	2	2	2	2	2	2
3	2	1	3	1	2	3	1	3	2
1	1	1	2	3	3	3	2	2	3
3	3	3	3	9	9	9	9	9	2
3	3	3	2	3	3	2	1	1	1
3	3	3	3	2	1	2	2	2	1
2	2	2	2	2	2	2	2	2	2
1	3	1	3	1	3	1	1	2	1
2	1	1	2	2	1	2	2	2	1
3	3	3	1	3	3	1	2	3	1
2	1	1	3	2	1	1	2	2	1
3	3	3	1	1	3	3	3	3	2
1	2	2	3	2	2	3	3	2	2
3	3	1	3	3	1	1	3	3	1
3	3	3	3	3	3	3	3	3	3
2	2	2	2	2	2	2	2	2	2
2	2	2	2	2	2	2	2	2	2
2	2	2	2	2	2	2	2	2	2
3	3	2	3	3	3	3	3	3	3
3	3	2	3	3	3	3	3	3	3
9	2	9	2	2	9	2	9	2	2
2	1	1	1	1	1	1	2	2	2

2	2	2	1	1	1	2	2	2	2
2	1	1	1	1	2	2	1	1	1
2	3	2	2	2	2	1	1	2	1
2	2	2	2	2	2	2	2	2	2
2	2	2	1	2	2	2	2	2	2
2	1	1	1	2	2	2	2	1	2
3	3	2	2	2	2	1	1	1	2
1	1	1	1	2	1	1	1	1	2
2	2	1	1	1	1	1	2	2	1
1	1	1	1	2	2	2	1	1	2
2	3	3	2	3	2	1	3	3	1
1	2	2	2	1	3	2	3	3	1
2	2	2	2	2	2	2	2	2	2
2	2	2	2	2	2	2	2	2	2
2	2	2	2	2	2	2	2	2	2
2	2	2	2	2	2	2	2	2	2
2	2	2	2	2	2	2	2	2	1
2	2	2	2	2	2	2	2	2	2
2	2	2	2	2	2	2	2	2	2
2	2	2	2	2	2	2	2	2	2
2	2	2	2	2	9	2	2	2	2
2	2	2	2	2	2	2	2	2	2
2	2	2	2	2	2	1	2	2	2
2	2	2	2	2	2	2	2	2	2
2	2	2	2	2	2	2	2	2	2
2	2	2	2	2	2	2	2	2	2
2	2	2	2	2	2	2	2	2	2
2	2	2	2	2	2	2	2	2	2
2	2	2	2	2	2	2	2	2	2
3	3	3	3	3	3	3	3	3	3
2	2	2	2	2	2	2	2	2	2
2	2	2	2	2	2	2	2	2	2
2	2	2	2	2	2	2	2	2	2
2	2	2	2	2	2	2	2	2	2
9	1	9	1	1	2	1	2	9	9
1	9	3	1	9	3	1	3	1	9
1	9	1	9	1	9	2	9	1	9
2	9	2	2	2	9	2	9	2	2
9	9	9	9	9	9	9	9	9	9
1	1	1	1	1	1	1	1	1	1
1	9	3	1	9	1	9	9	1	1
1	1	1	2	2	2	2	1	1	2
2	1	1	2	1	2	3	2	1	1
2	2	2	2	2	2	2	2	2	2
2	9	1	1	1	9	2	1	1	1
9	9	2	2	9	2	9	2	9	9
2	1	1	2	2	2	2	2	2	2

2	2	1	2	2	2	2	2	2	2
2	2	2	2	2	2	3	2	1	2
2	1	2	2	2	2	2	2	1	2
2	1	1	2	1	1	2	1	3	1
2	2	2	1	1	2	1	2	3	2
2	3	3	2	2	2	2	3	3	2
1	1	1	1	1	1	1	1	1	1
2	2	2	2	2	2	2	2	2	2
2	1	1	2	2	1	2	1	2	2
2	1	1	2	2	1	2	1	2	2
2	1	1	1	2	1	1	2	2	1
2	2	1	1	2	2	1	2	2	1
3	2	2	1	2	2	1	3	3	1
2	2	1	1	1	1	1	9	9	1
2	2	2	3	2	2	1	3	3	1
2	2	2	3	2	2	1	3	3	1
2	2	2	3	2	2	1	3	3	3
1	2	2	2	1	2	2	2	1	1
2	1	1	2	2	2	1	1	2	2
1	1	2	2	2	2	1	1	2	2
2	1	1	2	2	2	2	1	1	2
3	2	2	1	2	2	2	1	1	2
2	2	2	3	2	2	2	2	2	2
2	2	1	2	2	2	3	3	2	2
2	3	2	2	2	2	3	2	2	1
3	2	1	1	3	2	1	1	2	3
3	2	2	2	3	2	2	1	2	2
2	2	2	2	2	3	3	3	3	3
2	2	2	2	3	3	2	2	3	3
2	2	2	2	2	2	2	2	2	2
2	2	2	2	2	2	9	2	2	2
2	2	2	2	2	2	2	2	2	2
2	1	2	1	2	2	2	2	2	1
2	2	2	2	2	2	2	2	2	1
2	2	2	2	2	1	2	2	2	3
2	2	2	2	3	3	3	3	3	3
2	2	3	2	2	3	2	1	2	2
2	2	2	2	2	2	2	2	2	2
3	2	1	2	2	2	1	2	2	1
2	3	2	1	3	2	1	2	3	1
1	2	2	2	2	2	1	2	2	2
3	3	3	3	3	3	3	3	3	3
1	1	2	2	1	1	1	2	2	1
3	2	2	2	2	1	1	2	2	1
2	2	2	2	1	2	2	1	2	1
1	1	2	2	1	1	2	2	1	1
2	2	2	1	2	2	1	1	2	1

2	2	2	2	2	2	2	2	2	2
2	2	2	2	1	2	2	2	2	1
1	2	2	2	2	2	1	2	2	1
2	2	2	2	2	2	2	2	3	2
3	2	3	2	2	3	1	2	3	1
1	2	3	3	3	2	2	2	2	1
3	3	3	3	2	2	1	2	3	1
3	3	2	3	3	2	1	3	3	1
1	2	2	1	1	1	2	3	3	1
3	3	3	2	2	2	2	2	2	1
2	3	3	3	3	3	2	3	2	1
1	3	2	9	2	1	3	3	3	2
2	3	2	1	3	2	1	2	1	1
1	2	3	2	2	2	2	2	2	2
3	2	3	2	2	3	1	2	3	1
2	2	2	1	2	9	1	2	3	2
2	2	2	1	2	2	2	2	2	1
1	1	2	1	2	2	2	1	2	1
3	3	3	3	3	3	3	3	9	1
2	2	2	2	2	2	2	2	2	1
2	3	2	3	3	3	3	1	2	1
2	2	2	2	2	2	2	2	2	1
1	2	2	2	2	3	9	1	3	2
2	2	3	3	3	2	1	1	3	3
3	3	3	3	3	2	2	2	2	1
2	3	3	3	2	2	1	3	3	2
1	2	3	2	2	1	3	2	3	3
3	3	3	1	3	3	3	3	1	3
1	1	1	2	2	2	2	2	1	1
2	3	1	1	2	3	2	3	2	1
2	1	3	1	3	2	1	1	2	3
2	3	3	2	2	1	3	3	3	1
2	2	2	2	2	2	2	2	2	2
2	2	1	1	2	2	2	2	2	1
2	2	3	3	2	1	2	1	3	2
3	3	3	1	1	3	1	3	3	2
1	2	3	1	3	3	2	1	3	1
3	3	3	2	2	2	2	1	1	3
3	3	2	1	3	3	1	2	3	3
3	3	2	1	3	3	3	1	3	3
3	2	2	1	1	1	1	1	3	1
2	2	2	2	2	2	1	1	2	1
3	3	2	3	2	2	2	2	2	2
2	2	1	1	2	2	1	2	3	2
2	1	1	2	2	2	1	2	2	2
1	2	2	3	2	2	3	2	3	2
1	9	3	3	2	2	3	2	3	2

3	3	3	1	3	3	2	1	1	3
1	3	2	1	3	1	1	2	3	2
3	3	2	1	3	2	2	1	1	1
2	2	1	1	2	1	2	2	2	1
3	3	1	1	3	1	2	2	2	1
1	3	2	1	3	2	1	2	3	1
2	2	2	1	2	2	1	2	2	2
1	2	1	3	2	3	2	2	1	1
3	3	3	1	2	2	2	1	3	3
9	9	9	9	2	9	9	9	9	2
1	1	2	3	1	2	3	1	3	2
1	2	2	3	2	3	2	3	2	2
3	2	1	3	2	2	1	1	3	1
2	2	3	3	1	2	3	3	3	1
3	2	2	2	1	1	1	2	2	1
2	2	2	2	2	2	2	2	2	1
1	2	2	3	2	2	2	2	2	2
1	2	2	3	2	3	2	3	2	2
3	3	3	3	3	3	3	3	3	3
3	3	3	3	3	3	3	3	1	1
3	3	3	3	3	3	3	3	3	3
3	3	3	3	3	3	3	3	3	3
1	3	2	3	2	2	3	2	1	1
1	9	2	1	2	2	3	3	3	1
2	2	2	2	2	2	2	2	2	2
1	3	3	1	1	3	2	3	3	3
3	3	2	2	1	2	2	3	3	3
2	2	2	2	2	2	2	2	2	2
3	2	3	2	2	3	3	1	2	2
1	3	2	2	2	3	1	2	3	1
3	3	3	3	3	2	1	3	3	3
3	2	2	1	3	2	1	2	3	1
3	3	2	2	2	2	2	1	3	2
3	3	3	3	3	3	3	3	3	3
3	3	2	1	1	3	2	2	2	1
1	2	2	2	3	2	1	2	3	2
2	2	3	1	3	1	1	2	3	2
1	2	2	2	3	2	1	2	3	2
2	2	2	2	2	2	2	2	2	2
3	2	2	1	2	2	2	1	2	2
1	3	2	1	1	1	2	3	2	1
3	3	3	2	3	3	3	3	3	1
2	2	2	2	2	2	2	2	2	2
3	3	3	3	1	1	3	3	3	1
3	3	3	3	3	3	3	3	3	3
3	3	3	1	2	1	3	3	3	1
2	2	2	1	2	2	1	1	2	1

3	2	1	2	2	2	1	2	9	2
3	9	3	1	3	1	3	1	3	1
2	2	3	3	3	3	2	1	1	2
2	2	2	2	2	2	2	2	2	2
3	3	2	2	3	2	1	2	2	2
9	9	9	2	9	9	2	9	9	9
2	2	2	2	2	2	2	2	2	2
2	2	2	2	3	3	2	2	3	2
3	3	3	3	2	2	1	1	3	1
2	2	3	2	3	2	2	2	3	2
2	2	1	1	2	2	1	2	2	2
2	2	1	1	3	2	2	1	3	1
9	9	9	9	9	9	1	9	9	1
9	9	1	9	9	9	1	9	9	1
9	9	1	9	9	2	9	9	9	9
2	2	1	1	2	1	2	2	2	2
2	2	2	2	1	2	2	2	2	2
1	2	3	1	1	3	3	3	3	1
3	3	3	3	2	1	2	3	3	2
2	2	2	2	3	2	2	2	3	1
2	1	1	3	2	3	9	1	2	3
3	3	9	3	3	3	1	2	3	3
2	2	2	2	2	2	1	3	3	2
2	2	2	2	2	2	2	2	2	2
3	1	3	1	3	3	1	1	1	3
2	1	1	2	1	1	1	2	1	2
3	1	3	1	3	3	1	1	1	3
2	9	9	9	9	9	9	1	9	9
2	2	2	2	2	2	2	2	2	2
3	3	3	1	3	3	1	3	3	1
3	3	3	1	2	1	3	1	2	1
3	3	2	2	3	3	2	1	2	3
3	3	3	3	3	3	3	3	3	1
3	3	3	2	2	1	2	2	2	2
3	3	3	3	3	3	1	2	2	1
2	2	2	3	2	2	2	1	3	2
2	2	2	3	2	1	2	3	3	3
2	2	2	2	3	3	3	1	3	3
2	3	2	1	3	1	3	2	3	3
3	3	3	3	3	3	2	1	1	1
3	2	2	1	2	2	2	2	1	2
3	2	2	1	3	3	1	2	9	1
2	2	3	2	3	2	2	1	3	1
2	3	3	1	1	1	1	1	2	1
1	2	2	2	2	2	1	2	2	2
2	2	2	2	2	2	1	2	2	1
2	2	2	2	2	2	1	9	9	1

1	1	2	1	2	2	2	1	1	2
2	2	2	1	3	2	2	2	2	1
2	9	3	1	3	2	9	1	9	9
3	3	3	1	2	1	3	2	2	1
3	2	3	1	2	1	2	2	2	2
3	2	9	1	3	3	2	2	2	2
3	3	3	3	1	2	3	3	3	1
3	2	3	1	3	2	1	1	3	1
3	3	3	1	3	3	3	1	3	3
2	2	2	2	2	2	2	1	9	1
1	2	2	1	2	2	2	1	3	2
9	9	9	9	9	1	9	9	9	1
2	2	2	1	3	3	2	1	1	3
2	2	2	1	2	2	2	2	3	1
9	9	9	9	9	1	9	9	9	9
9	1	9	9	9	9	9	1	9	3
3	3	9	3	1	2	3	3	3	1
3	2	3	2	2	1	2	1	3	2
3	3	9	1	3	3	3	1	1	1
3	3	3	3	3	3	1	3	3	3
3	2	3	1	3	3	2	1	1	3
1	3	3	3	1	9	9	9	9	1
3	3	3	3	2	3	3	3	3	1
3	3	2	3	3	2	1	1	2	3
2	2	2	2	3	3	2	1	3	2
3	2	3	2	2	2	2	2	2	3
9	9	9	9	9	9	9	1	9	9
2	3	2	1	3	2	1	2	3	1
3	3	2	3	3	3	1	2	3	2
3	9	9	9	9	9	9	1	9	9
2	1	1	1	9	9	2	9	9	9
3	3	3	3	3	1	2	3	3	2
3	3	3	3	1	1	3	3	3	1
3	3	3	1	3	3	3	1	1	3
9	9	9	9	1	9	9	9	9	3
1	3	1	2	2	3	1	3	3	1
3	3	3	3	3	1	2	2	2	3
2	2	2	1	2	2	2	2	1	1
2	2	2	3	3	2	1	2	2	1
2	1	1	1	1	2	2	2	2	1
9	9	9	1	1	2	2	2	2	1
3	3	3	1	3	1	3	3	3	3
2	2	1	3	2	2	1	2	2	2
2	1	3	3	2	1	2	3	3	2
2	2	3	1	2	2	2	3	3	2
3	2	3	1	2	2	3	1	1	1
2	2	3	1	2	1	2	3	3	2

3	3	3	3	1	1	3	3	3	1
2	1	1	3	1	1	1	3	1	1
9	9	9	9	1	9	9	9	9	9
9	9	9	1	9	9	9	9	1	9
9	9	9	9	9	9	1	9	9	9
9	9	9	9	1	9	9	9	9	2
3	3	3	1	3	1	3	3	3	2
3	3	3	3	2	2	2	2	2	2
3	2	2	1	2	2	1	2	2	1
3	3	3	2	1	3	3	3	3	1
3	2	3	2	3	3	1	1	1	2
3	3	3	3	3	3	3	1	3	1
1	1	1	1	2	2	2	2	2	2
1	2	2	2	3	3	2	1	3	1
2	2	2	2	3	3	3	3	3	3
9	9	9	9	9	1	9	9	9	9
3	3	3	1	3	3	3	1	3	2
3	3	3	1	3	1	2	3	3	1
3	3	3	3	3	1	2	2	2	1
9	9	9	1	9	2	9	9	9	9
9	9	9	9	9	9	1	9	9	9
3	3	3	3	9	9	9	9	9	9
3	3	2	3	2	1	1	1	1	1
2	3	2	3	3	2	2	1	1	3
9	9	9	9	9	9	1	9	9	9
9	9	9	9	9	9	1	9	9	9
3	3	3	3	1	1	2	3	3	1
3	3	3	3	1	2	3	3	3	1
3	3	3	3	2	1	3	3	3	1
3	1	2	1	1	1	1	1	1	2
3	3	3	2	3	1	2	2	2	1
1	3	2	2	2	2	2	2	2	2
2	2	2	2	2	2	2	2	2	2
3	3	3	3	3	3	3	3	3	1
3	3	3	2	3	3	3	2	2	2
3	3	3	3	3	1	3	3	3	3
3	3	3	3	2	2	2	1	2	1
2	2	2	2	1	1	1	1	1	1
2	2	2	2	2	1	2	2	2	2
2	2	2	2	2	3	1	1	3	1
2	1	2	1	1	1	2	3	3	1
1	1	2	2	1	2	2	2	2	1
2	2	1	2	2	2	1	2	2	2
9	9	9	1	9	2	9	9	9	9
9	9	9	1	2	9	9	9	9	2
2	3	3	3	3	3	1	1	2	1
2	2	1	3	2	1	2	2	2	2

1	2	1	1	1	2	1	2	1	1
3	3	2	2	2	2	2	2	1	3
2	2	2	2	2	2	1	2	3	2
3	3	2	2	2	2	2	2	2	2
1	1	2	3	3	3	2	2	2	1
3	1	2	1	2	3	1	2	1	2
3	2	2	1	2	1	2	2	2	3
3	1	2	3	1	2	3	1	2	1
9	9	9	9	9	9	9	9	9	9
9	1	2	1	2	2	1	2	1	1
2	2	2	2	2	2	2	2	2	2
1	2	2	1	2	1	1	2	3	1
3	1	2	3	1	3	2	3	2	1
2	2	2	2	2	1	2	2	2	2
2	2	2	2	2	2	2	2	2	2
2	2	2	2	3	1	2	2	2	1
3	1	3	2	1	2	3	1	2	3
2	2	2	2	3	1	2	3	2	2
2	2	1	3	3	3	2	2	2	1
1	1	3	2	3	3	3	3	1	2
1	1	2	3	1	1	1	1	1	2
2	2	2	2	3	3	3	3	3	3
1	1	1	1	2	2	2	3	3	2
2	2	1	2	1	3	3	3	3	1
2	2	2	2	2	2	2	2	2	2
1	2	3	2	3	2	3	2	3	1
1	2	3	1	2	2	2	2	3	1
2	2	3	2	3	2	1	2	1	1
3	3	2	2	2	1	1	3	3	2
3	3	2	3	3	2	1	1	2	3
2	9	1	2	2	1	2	3	2	2
1	3	3	9	9	9	9	9	9	9
3	3	3	3	3	3	3	3	3	3
3	1	1	2	2	1	1	1	2	2
1	1	1	1	1	1	2	2	2	3
3	2	1	2	2	1	1	2	2	1
2	1	1	1	3	2	2	1	2	2
1	2	1	2	1	2	2	3	1	2
2	2	2	2	2	2	2	2	2	2
1	1	1	1	2	2	2	3	3	1
1	1	3	1	2	3	1	3	3	2
9	9	9	9	9	9	2	9	9	9
1	2	2	2	1	2	2	2	2	1
2	2	3	3	3	2	1	3	3	1
2	2	2	2	2	2	2	2	2	2
3	3	3	3	3	3	1	3	3	3
3	3	3	3	1	2	3	3	3	1

3	2	3	9	3	1	9	9	9	9
3	3	2	2	1	2	2	2	1	1
2	1	1	1	3	1	3	3	3	2
1	3	1	3	3	3	3	1	3	3
1	3	3	3	3	2	3	3	3	3
2	2	2	2	2	2	2	2	2	2
3	2	3	2	3	3	3	3	3	1
1	1	3	2	2	2	2	2	2	2
1	3	2	9	9	9	1	2	3	1
3	3	3	3	2	2	2	2	2	1
3	3	3	3	2	2	2	2	2	1
3	1	2	1	3	2	1	2	3	2
2	3	2	2	9	9	9	9	9	2
3	3	3	1	3	3	1	2	3	2
2	2	2	2	3	3	3	1	3	3
3	3	1	1	3	3	1	1	1	1
3	1	3	3	9	9	1	9	9	1
3	3	2	2	1	2	3	1	2	1
3	2	3	3	2	2	2	1	3	3
2	2	2	2	2	2	1	2	2	2
1	2	1	2	1	2	1	2	1	1
3	3	3	3	3	3	3	3	3	3
2	3	1	1	2	2	1	3	3	2
2	2	1	3	3	2	2	2	1	2
3	2	2	3	3	2	3	1	2	1
3	3	3	3	3	3	1	3	3	2
3	3	3	3	3	3	3	3	3	3
2	2	3	1	2	3	1	1	2	1
3	1	3	1	3	2	1	1	3	1
3	3	3	3	2	2	1	1	2	1
3	3	3	2	2	2	2	2	2	1
2	3	2	2	2	2	1	2	2	1
1	2	2	1	1	2	2	2	2	1
3	3	3	3	2	2	2	2	2	1
3	3	3	3	3	1	2	1	3	1
9	9	9	9	9	9	1	9	9	9
3	3	3	2	3	2	2	2	3	2
2	2	2	1	1	2	3	3	3	3
9	9	9	1	1	9	9	9	9	9
2	2	3	3	2	2	1	2	2	1
3	3	3	2	3	2	2	2	3	2
2	1	1	2	3	2	3	1	2	2
2	2	2	2	2	2	2	2	2	2
1	1	1	1	2	2	2	2	2	2
3	3	3	3	9	9	9	9	9	9
3	1	2	3	1	2	3	2	1	3
9	9	9	9	3	3	3	3	3	3

1	1	1	1	2	2	1	2	2	1
2	2	2	2	2	2	2	2	2	2
3	3	3	3	3	3	3	3	3	3
9	9	9	9	9	9	9	9	9	9
2	1	1	1	2	2	1	3	3	1
2	1	3	2	1	3	2	9	1	1
2	2	2	2	2	2	2	2	2	2
9	9	9	9	2	3	1	3	2	1
3	3	3	3	3	3	3	3	3	3
1	3	3	3	3	1	3	3	3	1
1	1	3	3	3	3	3	3	3	1
3	9	9	3	3	1	3	1	2	3
3	3	3	2	3	1	3	9	2	3
1	1	1	1	2	2	2	2	2	2
2	2	2	2	2	2	2	2	2	2
3	3	3	3	2	2	2	2	2	1
9	9	9	1	9	9	9	2	9	9
9	9	9	9	1	9	9	9	9	1
9	9	9	9	9	9	9	9	9	9
9	9	9	9	2	9	9	9	9	2
2	2	2	2	2	2	2	2	2	2
2	2	2	2	3	3	3	3	3	1
2	3	2	2	2	2	1	1	1	2
3	3	3	9	3	3	3	3	3	3
1	1	1	1	1	2	3	2	2	1
2	2	2	1	9	9	1	9	9	9
9	9	9	9	9	9	9	9	9	9
2	2	2	2	2	2	2	3	3	2
9	9	9	9	9	9	9	9	9	9
9	9	9	9	9	9	9	9	9	9
9	9	9	9	9	9	9	9	9	9
9	9	9	9	9	9	9	9	9	9
9	9	9	9	9	9	9	9	9	9
3	3	2	2	3	3	3	1	3	1
2	3	2	1	2	1	3	2	1	1
1	3	3	2	3	2	1	2	2	2
9	3	1	3	2	2	2	2	1	9
3	3	3	3	9	9	9	9	9	9
9	9	9	9	1	9	9	9	9	9
1	1	2	2	1	1	1	3	3	1
2	2	2	2	2	2	2	2	2	2
2	2	2	2	2	1	1	2	2	2
2	2	2	2	1	2	2	2	2	1
2	2	2	2	2	2	2	2	2	2
2	2	1	1	2	1	2	1	2	1
3	2	2	1	1	2	1	2	2	1
1	2	2	1	1	1	2	3	3	2

2	2	2	2	2	2	2	2	2	2
2	2	2	2	2	2	2	2	2	2
3	2	3	3	1	1	9	1	3	1
1	2	2	3	1	2	3	1	2	1
3	1	2	3	1	2	3	1	2	1
1	1	1	1	1	1	1	1	1	2
9	3	1	2	1	2	9	1	2	9
2	2	2	2	2	2	2	1	2	1
2	2	2	2	2	2	2	2	2	2
2	2	2	2	2	2	2	2	2	2
3	3	3	3	3	1	3	3	3	3
2	2	2	1	2	1	1	2	2	2
2	1	2	1	3	1	2	1	2	3
3	2	3	3	2	2	3	3	3	1
2	3	3	3	1	3	3	3	3	1
3	1	3	2	3	3	3	3	1	3
3	1	2	3	1	2	2	3	3	1
3	3	3	3	3	2	2	3	3	1
9	2	1	2	2	9	2	9	1	1
1	3	3	1	3	2	1	2	3	1
9	9	9	9	9	9	9	1	9	9
9	2	9	1	3	3	3	3	3	3
3	1	2	2	2	9	1	3	2	1
9	9	9	9	9	9	9	9	9	9
2	9	9	9	9	2	9	9	9	9
9	9	9	9	1	9	9	9	9	9
1	1	2	1	3	2	1	3	3	3
2	3	3	3	2	3	3	2	2	2
9	9	9	9	9	9	9	2	9	9
3	3	2	1	2	2	3	3	2	1
1	3	1	1	1	3	1	3	1	1
3	3	3	1	3	1	1	2	3	1
3	2	2	1	1	2	2	3	3	2
2	2	2	2	1	1	1	1	1	2
1	1	2	2	3	2	1	1	2	1
2	1	2	2	2	2	2	2	2	2
3	1	3	1	3	3	3	1	3	1
2	2	2	2	2	2	2	2	2	2
2	2	2	2	3	2	1	2	3	1
1	2	3	2	3	3	3	2	1	1
9	9	9	9	2	9	9	9	9	1
2	3	3	3	1	1	3	3	3	1
2	2	2	2	3	3	1	1	3	3
3	3	3	1	1	3	2	3	3	1
1	9	9	9	9	1	9	9	9	9
1	1	1	1	1	1	1	1	1	1
9	9	9	9	1	2	2	2	2	2

2	3	3	2	1	9	3	9	1	1
3	2	3	3	2	2	2	2	3	1
3	3	3	3	3	3	1	1	3	1
3	2	2	1	3	2	1	1	1	3
2	2	2	2	2	2	2	2	2	2
9	9	9	9	9	9	9	9	9	9
2	3	2	3	3	1	2	2	3	1
3	3	3	3	2	2	2	2	2	2
3	3	2	3	3	2	1	2	3	1
3	3	3	1	2	2	1	2	2	2
3	3	3	3	1	3	1	3	3	3
1	1	3	2	1	1	3	3	2	3
2	2	2	2	9	9	2	9	9	9
3	3	3	3	2	2	1	1	2	3
1	1	1	1	3	3	3	1	3	3
3	3	3	3	2	1	1	3	3	1
3	3	3	3	3	3	3	3	3	3
2	2	2	2	2	1	2	2	2	2
3	2	3	2	3	2	2	3	3	3
2	2	2	2	2	2	2	2	2	9
2	2	1	1	2	2	1	2	2	2
3	3	3	2	2	2	2	2	2	3
3	3	3	3	1	3	1	1	1	1
2	2	3	1	2	1	2	1	1	2
1	1	1	1	1	1	1	1	1	1
9	9	9	9	1	9	9	9	9	9
3	3	2	3	2	3	1	3	3	2
3	3	3	2	2	2	3	2	3	2
9	9	9	9	3	1	3	3	3	1
2	2	3	3	3	2	2	2	2	2
3	2	3	3	3	1	2	2	2	3
9	9	9	9	1	9	9	9	9	2
2	2	2	2	2	2	2	2	2	2
2	1	3	2	1	2	3	9	1	1
3	1	2	1	1	3	1	2	2	2
3	1	2	3	3	3	1	1	3	1
2	2	2	2	2	2	1	2	2	2
1	3	3	3	3	3	3	3	3	2
3	2	2	3	2	9	9	9	9	9
1	1	2	2	2	2	2	2	2	2
1	9	3	2	3	3	1	3	2	2
3	2	2	3	2	2	3	2	3	2
2	2	2	1	3	2	3	1	1	1
1	1	9	9	9	9	9	9	9	9
3	3	3	3	3	3	1	3	3	2
3	3	2	2	1	2	3	3	3	1
2	2	2	2	2	1	2	2	2	2

3	3	1	2	3	3	9	2	2	1
9	9	9	1	9	9	1	9	9	9
9	9	1	2	9	9	9	9	9	9
1	2	2	2	2	2	2	1	3	1
3	3	3	3	1	3	3	3	1	1
9	9	9	9	9	9	9	9	9	1
9	9	9	9	9	9	9	1	9	9
3	1	1	2	3	3	3	2	1	1
3	3	3	3	1	2	3	3	3	3
3	3	3	2	2	2	2	2	2	1
2	3	3	1	3	2	1	2	3	1
2	2	2	2	2	2	1	3	3	2
2	2	2	2	1	1	1	1	1	1
2	2	2	2	1	1	1	1	1	1
2	3	2	2	2	1	2	2	2	1
2	2	3	2	2	2	2	3	2	1
3	3	3	3	2	2	2	3	3	2
3	3	2	1	3	3	1	2	2	2
3	3	3	3	1	3	2	3	2	2
2	2	2	1	1	2	3	3	3	2
2	2	1	2	2	2	1	2	2	2
1	1	1	1	1	1	1	1	1	1
1	9	9	9	9	9	1	9	9	9
2	2	2	2	2	2	2	2	2	2
3	3	3	3	9	9	9	1	9	2
1	1	1	1	2	2	1	2	2	3
3	1	3	1	3	1	3	3	3	3
1	2	2	1	1	1	1	2	2	1
2	3	1	3	3	2	1	2	3	1
3	3	2	3	1	2	2	2	3	1
1	2	2	2	2	2	1	2	1	2
1	3	9	1	2	2	2	2	2	1
2	3	2	1	2	1	1	3	3	2
1	1	3	3	2	3	2	2	3	2
9	2	3	3	1	2	2	3	3	1
2	2	2	2	2	2	2	2	2	2
3	2	1	3	1	2	1	3	2	1
2	2	2	2	1	2	2	2	2	2
2	2	3	3	3	2	2	1	2	2
3	3	3	3	9	9	1	9	9	9
2	2	2	1	9	9	9	1	9	9
3	2	1	2	2	3	1	2	3	1
3	2	3	1	3	3	3	1	2	1
3	3	2	2	2	2	2	1	1	2
1	1	1	1	1	2	2	1	1	1
2	2	2	2	2	2	2	2	2	2
2	2	3	2	2	2	2	2	2	2

3	3	2	2	2	1	2	2	2	1
9	9	9	2	3	1	2	9	1	9
9	9	9	9	9	9	9	1	9	9
3	3	3	3	3	3	1	1	1	3
3	3	3	1	2	1	2	2	2	2
2	3	2	1	2	2	2	2	2	2
3	3	3	3	3	3	3	3	3	3
1	1	3	1	3	1	2	3	3	2
2	9	2	3	2	1	2	3	9	2
3	3	3	3	3	3	3	2	3	3
3	2	1	1	3	1	2	2	3	2
3	3	3	3	3	1	1	2	2	1
3	3	3	3	3	3	3	2	9	3
1	1	1	3	3	3	1	3	2	3
3	3	3	3	1	2	3	3	3	1
3	3	3	3	1	2	2	2	2	1
1	1	1	2	2	2	1	2	2	2
9	9	9	9	9	1	9	9	9	1
2	2	2	2	2	2	2	2	2	2
1	2	1	2	1	1	2	1	2	1
1	2	3	2	1	2	1	1	1	1
3	2	3	3	1	1	1	2	3	1
2	2	2	2	3	3	1	2	3	2
3	2	3	1	1	2	1	2	1	2
3	2	3	1	3	2	1	1	1	2
9	9	9	9	2	2	1	3	3	1
2	2	3	2	2	1	2	2	2	1
3	3	2	3	3	3	3	1	2	1
3	2	3	3	3	2	1	3	3	3
2	1	1	1	3	3	2	1	1	2
3	3	3	3	3	2	3	3	3	2
3	3	3	3	3	2	3	2	2	2
1	1	2	1	1	1	1	2	2	1
1	2	3	1	1	2	3	1	2	2
3	3	3	1	1	3	3	3	3	9
1	9	9	9	1	3	2	2	1	2
2	3	2	3	2	2	1	1	2	1
2	2	2	2	2	2	2	2	2	2
1	1	1	2	3	1	1	2	3	2
2	2	2	2	2	2	2	2	2	3
3	3	3	1	3	2	1	3	3	1
2	3	3	2	3	1	2	3	1	3
9	9	9	9	9	9	9	9	9	9
3	3	3	1	1	3	3	3	3	3
2	2	2	1	3	3	1	1	2	2
2	3	3	1	3	3	1	3	3	1
3	3	3	3	1	2	3	3	3	1

1	1	1	1	3	3	1	3	2	3
3	3	3	3	2	2	1	1	2	3
2	2	2	2	2	2	2	2	2	2
3	2	2	1	3	1	1	2	3	1
3	3	3	3	3	3	3	3	3	3
2	2	2	2	2	2	2	2	2	2
1	3	1	1	3	3	3	1	3	2
1	1	1	1	1	1	1	1	1	1
2	1	1	2	3	3	1	1	1	1
2	1	3	1	1	3	3	3	3	1
2	2	2	1	9	9	1	9	9	2
3	3	3	3	2	2	2	1	3	2
1	3	3	3	1	3	3	3	3	9
1	3	3	3	1	3	3	3	3	9
3	3	2	3	3	3	1	2	3	1
3	2	3	2	2	1	2	2	3	2
1	2	2	1	2	1	1	2	3	3
3	3	3	3	2	3	3	2	3	1
3	3	3	1	1	1	1	2	3	2
1	1	1	1	1	9	1	1	1	1
3	3	3	3	1	3	3	3	3	2
9	9	9	3	9	9	9	9	3	9
9	9	9	1	9	9	1	9	9	1
2	1	1	1	3	1	2	3	1	1
9	9	9	9	9	9	9	9	9	9
9	9	9	1	9	1	9	9	9	9
9	9	9	9	1	2	2	2	2	2
1	9	9	9	2	9	9	9	9	9
3	3	2	1	3	3	1	1	2	1
1	2	2	1	3	1	3	1	3	3
2	3	1	1	3	2	1	3	3	2
1	9	9	9	9	9	1	9	9	9
9	9	9	9	9	9	9	9	9	9
2	1	2	3	2	3	2	2	2	1
3	3	3	1	1	2	3	2	3	1
3	2	2	1	1	2	3	2	3	1
2	2	1	2	2	2	1	1	2	2
1	9	9	1	9	1	1	9	9	9
3	2	2	3	1	1	1	2	3	1
3	3	2	2	3	3	3	1	2	3
3	3	3	2	2	2	3	1	1	2
3	2	3	1	3	2	1	1	1	1
9	9	9	9	9	9	9	1	9	9
2	3	1	3	9	1	9	9	9	1
9	9	9	9	9	9	1	9	9	1
9	9	9	9	9	9	9	9	9	9
9	9	9	1	9	9	1	9	9	1

1	2	3	1	2	1	1	3	3	1
9	9	9	1	1	9	9	9	9	1
9	9	9	1	9	9	9	1	9	9
3	2	3	3	2	1	3	3	3	2
3	1	2	1	1	1	2	3	3	2
3	3	3	3	2	1	1	3	3	1
3	3	3	3	3	3	2	1	2	3
2	2	2	2	2	2	2	2	2	1
2	2	2	1	3	3	3	3	3	3
3	3	2	3	3	2	1	3	3	9
1	2	1	1	2	1	2	3	3	1
2	2	1	2	1	2	1	2	1	1
1	3	1	3	1	3	1	3	1	1
1	2	1	1	1	2	3	3	3	1
2	2	2	2	2	2	2	1	2	1
1	1	2	1	9	9	1	9	9	1
1	1	1	1	1	2	1	1	1	1
3	3	3	3	2	2	2	2	1	2
1	2	2	2	2	2	2	2	3	1
2	2	2	1	2	2	1	2	2	1
9	9	9	9	9	9	9	9	9	9
2	2	2	2	1	2	2	2	3	2
2	2	2	3	2	2	2	2	2	1
2	2	2	2	2	2	2	2	3	2
1	2	2	2	1	2	2	2	2	1
2	2	2	2	2	2	2	2	2	2
2	3	3	3	2	2	2	2	2	2
2	3	3	3	2	2	3	3	3	2
2	2	2	3	2	2	2	2	2	1
2	2	2	1	1	1	2	2	2	1
9	9	9	9	9	9	9	9	9	9
9	9	9	9	9	9	9	9	9	9
9	9	9	9	9	9	9	9	9	9
3	3	2	1	1	2	3	3	3	3
3	3	9	1	3	2	1	2	3	2
2	2	2	2	2	2	2	2	2	2
1	2	1	2	2	2	2	1	1	2
3	3	3	1	3	3	1	1	1	2
1	1	1	1	1	1	9	1	3	1
3	3	3	3	2	2	2	2	2	2
9	9	9	1	3	2	2	1	3	1
2	2	1	2	3	3	2	1	2	2
1	1	2	1	1	2	1	2	2	1
3	2	2	3	3	3	1	3	3	3
3	3	3	2	3	2	3	1	3	2
3	3	3	3	3	3	3	1	2	3
9	9	9	9	9	9	9	9	9	9

2	2	3	2	3	2	2	2	2	1
2	2	2	2	2	2	2	2	2	2
2	2	3	3	3	2	1	1	3	3
3	3	1	1	2	1	1	1	3	3
3	3	3	3	1	3	3	3	3	3
9	9	9	9	9	9	9	9	9	9
3	1	1	1	2	1	1	2	2	2
9	9	1	9	9	9	1	9	9	9
9	9	2	9	9	9	9	9	9	1
9	9	9	1	9	1	9	9	9	1
9	9	9	9	9	9	1	9	9	9
1	9	9	9	9	9	3	1	9	9
1	1	3	2	2	1	2	2	2	2
2	2	2	3	2	1	2	2	3	1
3	3	3	1	3	2	2	1	2	1
3	3	3	3	1	2	3	3	3	1
1	1	1	1	2	2	1	1	1	2
1	1	1	1	2	2	1	1	1	2
3	3	3	2	3	3	1	2	3	1
3	3	3	3	3	2	3	3	3	2
3	3	2	2	2	2	2	2	2	2
2	3	3	3	3	2	1	1	2	1
2	1	1	3	3	1	3	3	3	1
3	3	3	3	9	9	9	9	9	9
2	2	1	1	1	1	1	1	1	1
3	3	1	3	3	3	1	2	1	3
3	2	2	2	2	2	2	2	2	2
3	2	3	3	3	3	2	1	2	2
2	2	2	1	2	2	2	2	2	2
9	3	2	3	1	2	3	2	2	2
2	2	2	2	1	1	1	1	1	1
3	3	3	3	3	2	3	3	3	2
3	2	2	1	3	2	1	1	3	2
3	2	1	2	2	2	2	2	1	3
9	9	2	9	9	9	9	9	9	9
3	3	3	3	3	3	1	1	1	2
3	2	3	2	3	3	1	1	1	2
1	3	3	3	3	3	1	3	3	2
3	3	3	3	3	1	2	2	3	1
2	2	2	1	1	2	2	2	2	1
9	9	9	9	9	9	9	1	9	9
9	9	9	9	9	9	2	9	9	9
9	9	9	9	9	9	2	9	9	9
3	3	3	1	3	2	2	2	2	1
9	9	9	9	9	1	9	9	9	1
9	9	1	9	9	9	9	9	9	9
3	2	3	1	3	2	1	2	2	1

2	2	2	3	2	2	1	3	3	1
3	3	3	3	1	2	2	2	2	1
1	9	3	1	1	3	2	1	2	3
3	2	3	3	2	3	1	3	3	2
3	3	3	3	1	2	2	2	2	2
2	2	2	3	2	2	2	2	2	3
3	2	3	1	3	3	1	1	2	3
1	2	2	2	3	1	2	3	3	3
3	3	3	3	3	2	1	3	3	2
1	1	2	1	1	2	2	3	1	2
3	3	3	3	3	1	1	1	1	2
2	1	1	1	1	3	3	3	3	1
3	3	3	3	3	2	1	3	3	2
3	2	2	2	2	1	2	2	2	1
3	3	3	2	2	1	3	2	2	3
9	2	1	1	2	2	2	2	2	3
3	3	3	3	3	3	1	2	2	1
3	3	3	3	2	2	1	3	3	2
2	1	2	2	2	2	2	3	3	2
2	2	2	3	2	2	1	1	1	1
2	2	3	2	2	2	2	1	1	1
2	2	2	1	2	2	2	2	2	1
2	2	2	2	2	2	2	2	2	2
3	3	3	2	3	2	1	2	3	1
3	3	3	3	3	2	1	3	3	1
3	3	3	3	2	2	2	2	2	2
3	3	3	3	1	3	3	3	3	1
3	3	3	1	1	1	1	1	3	1
3	2	2	3	9	9	2	1	9	1
3	3	3	3	2	2	1	3	3	1
3	2	2	3	1	2	2	3	3	1
3	3	1	3	3	3	2	1	3	3
3	3	3	2	1	3	3	3	3	1
2	3	3	3	1	3	3	3	3	1
1	2	1	2	1	2	3	2	2	3
1	1	2	2	2	2	2	2	2	2
2	2	2	2	2	2	1	2	2	2
3	3	3	1	2	2	1	9	2	1
3	3	1	2	2	1	1	2	1	1
2	2	2	2	3	3	3	3	1	1
3	3	3	3	1	3	3	3	3	3
3	2	3	3	3	3	2	1	1	3
3	3	3	3	1	1	1	2	3	1
1	2	3	3	3	2	1	2	2	2
1	3	1	1	9	1	9	9	9	9
2	2	2	2	2	2	2	2	2	3
2	2	3	3	1	1	2	3	3	1

3	1	3	1	1	2	3	2	3	1
3	3	3	1	2	2	2	2	2	2
2	2	2	2	3	2	2	3	3	2
3	3	2	2	3	2	1	1	2	2
2	2	2	2	2	2	2	2	2	1
3	3	3	3	2	2	2	1	2	1
2	2	2	2	2	2	2	2	2	1
2	2	2	2	2	2	2	2	2	2
3	3	3	1	3	2	2	3	3	3
2	1	1	1	3	2	1	1	1	1
3	3	3	1	1	3	3	3	3	9
2	3	2	2	3	2	2	2	1	3
1	1	1	1	3	3	3	3	3	1
2	2	2	3	2	1	2	2	2	2
3	3	3	3	9	9	3	9	9	9
2	2	2	2	2	2	1	2	2	2
3	3	3	3	2	2	2	1	2	1
3	3	3	1	2	2	2	2	2	1
3	3	2	2	1	2	2	2	2	3
2	2	3	3	1	2	3	3	3	1
3	1	3	1	1	2	3	1	2	1
3	3	3	3	3	3	3	3	3	3
2	2	3	1	1	1	2	3	3	1
3	3	2	1	1	2	2	1	3	2
3	2	2	3	1	2	2	3	2	3
1	1	3	1	1	3	2	2	3	2
1	2	1	2	1	2	2	1	3	1
3	3	3	1	3	2	1	1	3	3
2	2	3	3	3	2	3	1	3	1
9	2	2	1	3	2	2	2	2	2
2	2	2	2	3	3	3	3	3	3
3	3	2	2	2	2	2	2	2	1
2	3	2	3	2	3	3	2	3	1
1	2	2	1	1	1	1	1	1	1
1	3	2	3	2	2	2	2	2	2
2	2	2	1	2	2	1	2	2	2
3	2	3	1	3	3	1	2	2	2
2	3	3	2	3	3	1	3	3	1
1	1	1	2	3	1	2	3	3	3
3	2	3	3	3	1	2	3	3	1
3	3	3	3	3	3	1	3	3	3
3	3	3	3	3	3	1	2	3	1
2	2	2	2	3	1	1	3	3	1
3	2	2	1	3	3	1	1	3	1
3	3	3	3	2	2	2	2	2	2
2	2	2	2	3	3	2	2	3	3
2	2	2	1	2	1	2	2	2	2

1	2	3	1	3	1	1	2	2	1
1	3	2	1	1	2	2	2	2	1
3	3	3	3	2	1	3	3	3	1
3	3	3	3	3	3	1	3	3	3
3	3	3	1	3	1	2	1	3	3
3	3	1	9	2	3	9	2	3	2
2	3	2	2	2	3	2	2	3	2
3	2	1	2	3	3	3	1	2	2
2	2	3	1	3	2	1	2	3	2
3	3	3	1	2	2	2	2	2	1
2	9	1	3	3	2	1	1	2	1
3	3	3	3	2	1	1	3	3	1
1	1	1	3	3	3	2	1	1	1
3	2	3	2	3	1	1	3	2	3
1	1	1	2	3	2	2	2	9	2
3	3	3	2	3	3	1	2	3	1
2	2	3	2	2	2	2	2	2	2
1	3	3	1	1	1	1	1	1	1
2	2	9	9	1	2	3	2	1	1
1	2	2	3	2	2	2	2	2	3
1	2	3	2	1	1	2	2	3	1
2	2	3	3	1	2	2	3	3	1
2	2	2	2	1	2	2	2	3	1
2	2	2	3	2	2	2	2	2	2
2	2	2	3	2	2	2	2	2	2
3	3	3	2	3	3	1	2	3	1
3	3	3	3	2	1	1	3	3	1
3	3	3	1	2	2	2	2	2	1
1	1	1	2	3	1	2	3	3	3
1	2	2	3	2	2	2	2	2	3
3	2	3	1	3	3	1	2	2	2
9	9	9	9	9	9	9	9	9	9
2	2	3	3	2	2	1	2	2	1
2	2	1	1	2	2	1	2	2	3
2	2	3	1	2	2	1	3	3	1
3	3	3	1	3	3	2	1	1	1
3	3	3	3	3	1	1	1	1	3
2	2	2	2	2	1	2	9	2	1
3	3	3	3	3	2	2	2	3	2
2	2	2	1	2	2	2	2	2	1
1	2	1	1	1	1	2	1	2	1
1	2	1	1	1	1	2	1	2	1
2	1	3	1	3	2	1	2	3	1
3	3	3	1	3	1	9	9	9	1
1	3	2	2	2	3	2	3	1	2
2	2	2	1	3	3	2	2	3	3
3	3	3	3	9	1	3	3	3	3

3	2	2	1	2	9	2	1	1	2
1	1	1	1	1	1	2	3	3	1
1	1	1	1	1	1	1	1	1	1
3	2	2	2	2	2	1	3	3	1
3	3	3	2	3	3	3	1	2	3
3	3	3	3	2	3	1	2	3	3
1	3	2	1	3	3	2	1	1	1
9	9	9	9	1	9	9	9	9	1
9	9	9	9	1	9	9	9	9	1
9	9	9	9	1	9	9	9	9	1
2	1	1	2	1	9	9	9	9	1
1	1	1	2	1	2	2	1	2	2
3	3	3	2	3	1	2	1	2	3
3	3	3	2	3	1	2	1	2	3
3	3	3	2	3	1	2	1	2	3
1	2	2	2	2	2	2	3	3	3
1	2	2	2	3	3	1	2	2	2
1	2	3	9	2	3	3	2	1	2
2	3	2	2	2	2	3	3	2	2
2	1	1	2	1	2	1	2	3	1
1	2	1	2	2	1	1	2	1	2
1	2	1	2	1	2	3	2	3	1
1	2	2	2	1	2	2	2	3	2
2	2	2	2	3	3	1	3	2	2
2	2	2	2	2	2	1	3	3	2
2	2	2	3	3	2	2	3	3	2
1	2	2	2	3	3	2	2	2	1
1	3	3	2	2	1	2	3	3	1
3	2	1	1	2	2	3	1	2	2
3	2	1	1	2	2	3	1	2	2
3	3	3	2	3	1	2	1	2	3
3	3	3	2	3	1	2	2	2	3
9	9	9	9	9	9	9	9	9	3
2	2	2	2	3	3	1	3	3	2
2	2	2	2	3	3	1	3	3	3
3	3	3	3	3	3	1	3	3	2
3	3	3	3	2	2	2	2	2	3
2	2	2	2	3	3	1	3	3	2
3	3	3	3	2	2	2	2	2	2
9	9	9	9	3	3	1	3	3	9
1	3	3	3	2	2	1	3	3	1
3	3	2	2	3	2	3	3	3	2
3	3	2	2	3	2	3	3	3	2
3	3	2	2	3	2	3	3	3	2
3	3	2	2	3	2	3	3	3	2
9	9	9	9	9	9	9	9	1	1
2	1	3	2	1	2	1	2	3	1

2	3	2	3	1	2	1	1	2	2
2	2	2	2	2	2	2	2	2	1
2	2	2	2	1	1	3	2	2	2
3	2	1	1	1	2	3	2	2	1
2	1	2	1	1	1	2	3	1	1
1	1	1	1	1	1	2	3	1	1
1	2	3	2	9	2	3	3	2	9
9	9	9	1	9	9	2	9	9	1
9	9	9	9	9	9	3	9	9	9
3	2	2	3	3	3	2	2	2	9
3	3	3	3	3	3	1	3	3	3
9	9	9	9	9	9	3	9	9	1
9	9	9	9	9	9	3	9	9	1
1	1	1	1	1	1	3	1	1	1
1	3	2	3	9	9	3	9	9	3
2	2	2	2	2	2	2	1	2	2
1	2	3	2	2	1	2	9	1	2
9	9	9	9	2	9	9	9	9	2
9	9	9	9	1	9	9	9	9	1
9	9	9	9	9	1	9	9	9	9
2	2	2	1	9	1	3	9	9	2
1	2	2	1	2	1	2	2	3	2
1	1	1	1	2	2	2	2	2	1
9	9	9	9	9	1	9	9	9	2
9	9	9	9	9	1	9	9	9	9
9	9	9	9	2	9	9	9	9	2
3	2	2	1	2	2	2	2	2	2
9	9	9	9	1	9	9	9	9	1
3	3	3	3	1	1	2	2	3	1
9	1	2	1	1	9	9	9	9	1
1	1	1	3	1	3	3	1	1	1
9	9	9	9	1	9	9	9	9	9
1	1	1	1	1	1	3	2	2	1
2	2	2	2	1	1	3	1	1	2
1	3	3	3	2	2	2	2	2	1
9	9	9	9	1	1	3	1	1	2
1	1	1	1	1	2	2	2	2	2
1	1	1	1	1	1	3	1	1	2
1	2	1	2	1	1	3	2	2	1
9	9	9	9	9	1	9	9	9	9
9	9	9	9	1	9	9	9	9	9
9	9	9	9	1	9	9	9	9	9
9	9	9	9	2	9	9	9	9	1
9	9	9	9	1	9	9	9	9	9
9	9	9	9	2	9	9	9	9	2
2	1	2	1	2	3	2	1	2	2
1	2	3	2	1	3	2	1	2	1

1	9	9	9	9	9	9	9	1	1
2	1	2	1	1	2	2	2	3	1
9	9	9	9	9	9	3	9	9	9
2	3	3	3	2	1	1	3	3	1
3	3	3	3	3	3	1	1	2	9
3	2	9	3	1	2	1	2	3	2
9	9	9	9	9	9	3	9	9	1
3	3	3	3	3	3	1	3	3	3
2	2	2	2	2	2	2	2	2	2
9	9	9	9	9	9	3	9	9	9
2	2	2	2	2	2	2	2	2	3
9	9	9	9	9	9	9	1	9	9
9	9	9	9	1	9	9	9	9	9
9	9	9	9	1	9	9	9	9	9
9	9	1	9	1	1	3	1	1	1
9	9	9	9	1	9	9	9	9	9
2	3	3	2	3	3	1	1	2	3
9	9	9	9	1	9	9	9	9	9
3	3	3	3	3	1	9	2	2	2
1	2	2	3	3	1	2	1	3	1
3	3	3	3	2	2	3	2	2	1
1	2	1	1	1	2	3	1	2	1
2	3	3	3	2	2	2	2	2	1
2	2	2	3	2	2	1	2	3	2
3	2	2	3	1	1	2	3	3	1
2	3	2	1	1	2	2	1	2	1
2	2	2	1	2	1	3	1	1	1
2	2	2	2	1	2	2	2	3	1
1	1	1	1	2	2	3	2	2	1
2	1	3	3	2	3	3	3	3	1
2	3	2	3	2	2	2	2	2	1
9	2	1	2	1	2	9	3	2	1
9	2	9	9	9	9	3	9	9	9
9	9	9	9	9	9	3	9	9	1
9	9	9	9	9	9	3	9	9	9
9	9	9	9	9	9	3	9	9	9
9	9	9	9	1	9	9	9	9	9
9	9	9	9	2	9	9	9	9	9
9	9	9	9	1	9	9	9	9	9
9	9	9	9	2	9	9	9	9	2
9	9	9	9	2	9	9	9	9	2
9	9	9	9	2	9	9	9	9	2
9	9	9	9	2	9	9	9	9	2
9	9	9	9	2	9	9	9	9	2
9	9	9	9	1	9	9	9	9	2
9	9	9	9	9	9	3	9	9	9
9	9	9	9	9	9	3	9	9	9

1	1	3	1	1	1	3	2	2	1
1	9	1	1	1	2	1	3	3	1
9	9	9	9	1	9	9	9	9	9
1	2	1	2	2	1	2	1	2	1
9	9	9	9	1	9	9	9	9	1
9	9	9	9	1	9	9	9	9	1
9	9	9	9	1	9	9	9	9	1
9	9	9	9	1	9	9	9	9	1
9	9	9	9	2	9	9	9	9	1
1	2	2	2	2	1	2	2	1	2
2	1	2	2	1	2	3	2	1	1
2	1	2	1	2	1	2	1	2	1
1	2	2	2	2	2	2	2	2	2
2	2	3	3	2	2	3	1	1	1
2	2	2	2	3	3	2	1	1	1
2	2	2	2	3	2	3	2	2	1
1	9	9	9	9	9	9	9	1	1
9	9	9	9	1	9	9	9	9	1
9	9	9	9	1	9	9	9	9	1
9	9	9	9	1	9	9	9	9	2
9	9	9	9	9	9	9	9	9	9
9	9	9	9	9	9	9	9	9	3
9	9	9	9	2	9	3	9	9	9
9	2	9	9	1	9	9	9	3	1
9	9	9	9	1	9	9	9	9	1
9	9	9	9	2	9	9	9	9	9
9	9	9	9	9	9	3	9	9	9
2	1	2	3	1	2	2	2	2	2
2	3	1	2	2	3	1	1	2	2
9	9	9	9	9	9	9	1	9	9
2	2	2	2	2	2	2	1	2	2
1	2	3	2	2	1	3	3	3	1
3	3	3	3	3	3	2	2	2	2
9	9	9	9	9	9	9	1	9	9
3	3	3	3	3	1	2	3	3	2
3	3	3	3	2	2	2	2	2	1
9	9	9	9	9	9	9	9	2	2
1	9	9	9	9	1	1	2	3	1
9	9	9	9	9	9	3	9	9	2
3	2	3	3	3	3	3	2	2	1
1	1	1	1	3	3	1	2	1	1
3	1	2	1	3	3	3	2	3	1
2	2	2	2	3	3	2	2	1	2
9	9	9	9	9	9	9	2	9	9
3	3	3	2	1	9	9	9	9	1
9	9	9	9	2	9	9	9	9	2
9	9	9	9	9	9	3	9	9	2

3	3	2	3	3	1	3	1	2	2
2	2	2	2	2	2	2	2	2	2
1	3	1	1	1	2	2	1	2	1
2	3	2	1	2	3	2	1	2	2
3	3	3	3	2	1	1	3	3	2
2	2	3	1	3	2	2	1	3	3
3	3	3	1	3	3	3	1	3	3
1	3	9	1	3	1	3	3	3	1
9	9	9	1	9	9	9	1	9	9
2	2	2	2	2	2	2	2	2	2
2	2	2	2	2	2	2	2	2	2
3	3	3	3	3	3	3	3	3	1
1	1	1	1	1	1	1	3	3	1
1	2	2	1	3	3	2	2	1	2
1	3	2	2	3	3	9	1	2	1
9	9	9	1	9	9	9	1	9	1
2	2	2	2	2	2	2	2	2	2
3	3	2	3	2	2	2	1	2	2
1	2	2	2	2	2	2	1	3	2
2	2	2	2	2	2	2	2	2	2
2	1	2	2	1	1	1	3	3	1
2	2	3	3	2	2	3	2	2	2
3	3	3	2	2	2	2	2	2	2
3	3	3	3	2	2	3	3	3	2
2	2	2	2	1	2	2	2	2	1
3	3	3	3	1	3	1	3	3	9
1	3	3	1	1	1	1	3	3	1
1	2	2	1	3	3	3	2	2	1
3	3	3	2	3	2	3	2	3	3
2	2	2	2	2	2	2	2	2	2
2	2	2	2	2	2	2	2	2	2
2	2	2	2	2	2	2	2	2	2
2	2	2	2	2	2	3	2	2	2
2	2	2	2	2	2	2	2	2	2
2	2	2	2	2	2	2	2	2	2
9	9	9	9	9	9	9	9	9	2
3	3	3	3	1	3	1	3	3	3
9	9	9	9	9	9	3	9	9	1
1	3	1	2	1	2	3	1	1	1
3	3	3	1	9	9	3	9	9	9
1	2	2	3	1	1	1	1	2	2
1	9	9	9	9	9	3	9	9	9
3	3	3	3	9	9	3	9	9	1
3	3	3	3	3	3	1	1	3	1
3	3	3	2	3	1	3	1	2	2
2	9	1	2	3	2	2	1	3	1
1	3	3	3	2	2	2	1	2	1

2	1	2	1	3	3	1	1	2	9
2	2	2	1	1	1	2	2	2	1
3	3	1	1	1	2	1	2	1	3
2	2	2	2	1	2	2	2	2	1
2	2	2	2	2	2	2	2	2	2
9	9	9	9	9	1	9	9	9	9
2	2	2	2	2	1	2	2	2	1
3	3	3	3	2	1	9	1	3	1
9	9	9	9	2	1	2	3	2	1
3	2	1	2	3	2	1	3	1	1
3	3	3	2	3	3	3	2	3	1
1	2	2	1	2	2	3	2	2	1
2	1	2	2	2	1	1	2	2	2
3	3	3	3	9	9	3	9	9	3
3	2	2	3	2	3	2	1	2	9
2	2	2	2	2	2	2	2	2	2
3	3	3	3	2	2	2	2	3	2
9	9	9	1	9	9	3	9	9	9
2	2	2	2	1	1	2	2	3	1
2	3	3	2	2	2	2	3	3	1
2	2	2	2	2	2	2	2	2	2
3	3	2	3	2	2	1	3	3	2
2	2	2	2	2	2	2	2	2	2
3	1	3	3	3	3	2	1	2	3
9	9	9	9	9	9	2	9	9	9
2	2	2	2	2	2	2	2	2	2
2	2	2	2	2	2	2	2	2	2
2	2	2	2	2	2	2	2	2	2
2	2	2	1	2	1	3	2	3	9
2	3	9	2	2	2	2	2	2	2
1	9	9	9	3	2	3	3	3	2
9	9	9	9	9	9	9	1	9	1
1	2	1	2	2	2	2	2	2	2
2	2	2	1	2	2	2	2	2	2
2	2	3	3	3	1	3	3	3	2
2	3	2	2	2	2	2	2	2	2
1	3	2	2	2	2	2	2	2	1
2	3	3	2	1	1	2	3	3	1
3	3	3	1	3	2	3	1	3	2
1	1	1	1	2	1	2	2	2	1
2	3	2	3	2	1	3	3	3	2
3	3	3	3	9	9	9	9	3	2
3	3	3	3	3	1	1	3	3	2
9	9	9	9	9	9	9	9	1	9
3	1	3	1	3	1	2	2	2	1
9	9	9	9	9	9	3	9	9	3
2	2	2	2	1	3	1	3	3	2

2	2	2	2	2	2	2	2	2	2
1	2	2	1	1	1	2	3	3	2
2	2	2	2	2	1	2	2	2	1
1	3	1	3	2	3	3	2	2	2
9	3	2	2	3	1	2	1	2	3
3	2	2	2	2	2	2	2	2	2
2	2	2	2	2	2	2	2	2	2
2	2	2	2	2	2	2	2	2	2
2	2	2	2	2	2	2	2	2	2
2	3	3	2	3	2	2	2	2	2
2	2	2	2	2	2	2	2	2	1
3	3	3	3	3	2	3	2	1	3
9	9	9	9	9	9	9	9	9	9
3	1	1	3	3	1	3	3	1	1
9	9	9	9	1	9	9	9	2	1
9	9	9	9	1	9	9	9	9	9
9	3	2	1	2	3	3	3	3	3
9	1	2	1	2	2	2	2	2	2
9	9	9	9	9	9	3	9	9	1
9	9	9	9	9	9	3	9	9	2
1	3	9	1	3	9	9	1	9	9
9	9	9	9	9	9	3	9	9	1
2	3	9	1	2	9	9	9	1	9
2	2	3	2	3	1	3	3	3	2
3	3	3	3	3	3	3	3	3	1
9	9	9	9	3	9	9	9	9	9
3	3	3	3	3	1	1	3	3	3
2	3	3	3	3	3	1	3	3	1
3	3	1	1	3	3	1	1	3	1
9	9	9	9	9	1	9	9	9	2
3	2	2	2	1	2	1	2	3	1
2	2	2	2	2	2	2	2	2	2
3	3	2	2	2	2	2	2	2	1
9	9	9	9	9	9	2	9	9	9
3	3	2	2	2	2	3	2	2	1
3	3	3	2	1	3	1	3	3	3
2	2	2	2	2	2	3	1	2	1
3	3	3	2	2	1	2	2	3	3
9	9	9	9	2	9	9	9	9	2
9	9	9	9	2	9	9	9	9	2
3	3	2	3	2	1	2	3	3	1
2	3	2	3	3	1	3	2	3	1
2	3	3	1	2	1	2	3	1	3
3	3	3	3	3	3	1	3	3	1
9	3	2	2	2	1	1	2	2	3
9	9	9	9	9	9	9	1	9	9
3	3	3	2	2	2	2	2	2	2

9	9	9	1	2	1	2	1	2	1
1	3	1	1	3	3	1	1	3	1
9	9	9	9	9	1	9	9	9	9
9	9	9	9	9	1	9	9	9	9
1	2	3	3	2	2	3	2	3	3
9	9	9	9	9	1	9	9	9	9
1	1	3	1	3	2	3	2	3	1
3	3	3	3	3	3	3	3	3	1
3	3	3	3	1	3	1	3	3	2
9	9	9	9	9	1	9	9	9	9
2	3	3	1	9	1	9	9	9	9
9	9	9	9	9	1	9	9	9	1
9	9	9	9	9	9	3	9	9	9
3	3	2	3	3	1	2	1	2	1
9	9	9	9	9	9	9	1	9	9
1	2	3	1	2	2	1	2	3	2
3	3	3	3	2	2	2	2	2	2
9	9	9	1	9	1	9	9	9	1
9	9	9	9	9	9	3	9	9	9
2	2	1	1	1	2	2	2	2	1
2	3	2	3	3	2	3	3	3	1
1	3	3	3	3	1	3	3	3	1
3	3	3	3	1	1	1	3	3	1
2	2	3	3	1	1	1	3	3	1
3	2	2	1	2	2	3	3	3	2
3	3	3	1	9	9	3	9	9	9
1	1	1	1	2	1	3	3	3	9
9	9	9	9	2	1	3	3	3	1
1	3	3	2	1	1	1	3	3	1
3	3	3	3	1	1	1	3	3	1
9	9	9	9	1	9	9	9	9	9
2	1	2	1	1	2	2	1	2	1
2	1	2	1	1	1	1	3	3	2
9	9	9	9	9	9	9	1	9	1
3	3	3	3	3	3	1	3	3	3
9	9	9	1	9	9	9	1	9	9
2	2	2	2	2	2	3	2	2	2
9	9	9	9	9	1	9	9	9	1
3	3	3	3	2	1	1	3	3	9
3	3	3	3	1	3	1	3	3	1
9	9	9	9	9	1	9	9	9	9
9	9	9	9	9	9	3	9	9	1
9	9	1	9	1	9	9	9	9	1
9	9	9	9	2	9	9	9	9	9
9	9	9	9	2	2	2	2	1	3
3	3	3	3	1	2	2	2	2	2
3	3	3	3	1	9	9	9	9	2

3	3	1	3	9	9	3	9	9	1
9	9	9	9	1	9	9	9	9	1
9	9	9	9	9	1	9	9	9	1
2	9	9	9	1	9	3	9	9	2
9	9	9	9	9	9	3	9	9	1
9	9	9	9	9	1	9	9	9	1
9	9	9	9	2	9	9	9	9	9
3	3	3	3	3	3	1	3	3	3
2	2	2	2	2	2	2	2	2	2
3	3	3	2	3	1	3	3	3	1
2	2	2	2	2	2	3	2	2	1
3	3	2	3	2	1	1	3	3	3
9	9	9	9	9	9	3	9	9	9
1	9	3	3	9	1	3	3	3	2
3	3	2	2	2	2	2	2	2	3
9	9	9	9	9	9	9	1	9	2
3	3	3	3	2	2	2	1	3	1
9	9	9	1	9	1	9	9	9	1
3	3	3	2	2	1	2	2	3	3
9	9	9	9	2	9	9	9	9	2
9	9	9	9	1	9	9	9	9	1
9	9	9	9	9	9	3	9	9	9
2	2	2	2	2	2	2	2	2	2
2	2	1	3	2	2	2	1	3	2
9	9	9	9	2	9	9	9	9	2
3	3	3	3	1	2	2	2	2	2
9	9	9	9	1	9	9	9	9	1
3	2	2	1	1	2	1	3	3	3
2	2	3	3	3	2	2	1	2	1
3	2	2	2	2	2	2	2	2	2
9	9	9	9	9	9	3	9	9	3
1	3	3	1	3	1	3	3	1	2
9	9	9	9	1	9	9	9	9	9
2	1	3	1	1	1	2	3	3	1
3	3	3	3	3	1	1	3	3	3
9	9	9	9	9	9	3	9	9	9
3	3	3	3	1	1	1	3	3	3
2	2	3	3	3	3	1	1	3	2
3	3	3	3	3	3	2	1	3	1
9	9	9	9	1	1	1	3	3	1
1	3	1	1	3	1	3	3	2	2
2	2	2	2	1	1	3	3	3	2
1	2	2	1	2	9	9	9	9	2
9	9	9	9	9	1	9	9	9	9
2	2	3	2	3	3	2	3	3	2
2	2	2	2	1	2	2	3	3	2
3	3	2	2	2	2	2	2	2	2

9	9	9	9	9	1	9	9	9	2
3	3	3	3	1	3	1	3	3	3
3	3	3	3	1	3	1	3	3	3
3	3	3	3	2	2	2	2	2	1
2	9	2	2	3	9	1	2	9	3
2	1	1	3	1	3	3	1	1	1
9	9	9	9	9	1	9	9	9	9
3	3	3	3	2	1	3	2	1	1
3	3	3	3	2	2	2	2	2	1
3	2	2	2	3	2	3	2	3	9
2	3	3	1	1	2	2	3	3	2
9	9	9	9	9	1	9	9	9	9
1	1	1	1	1	1	3	1	1	1
1	2	3	1	2	2	2	1	2	3
3	3	3	3	3	3	1	3	3	1
2	1	3	2	3	3	1	1	3	2
9	9	9	9	9	9	3	9	9	9
3	9	2	3	1	3	2	1	2	1
9	9	9	9	3	3	3	3	3	1
9	9	1	9	9	1	9	9	9	1
2	2	2	1	2	2	2	2	2	2
3	3	3	3	2	1	1	3	3	3
9	9	1	9	9	9	3	9	9	1
3	3	3	3	3	3	1	3	3	3
2	3	1	1	2	3	9	3	3	2
3	3	1	3	1	3	1	3	3	1
2	3	2	3	2	1	1	2	3	1
3	2	3	3	9	9	3	9	9	2
3	3	3	3	3	3	3	1	3	3
1	1	2	2	1	2	3	2	1	3
9	9	9	1	9	9	9	1	9	9
3	2	2	3	1	2	2	3	3	1
2	2	2	2	2	2	2	2	2	2
2	2	2	2	2	2	2	2	2	2
2	3	2	1	3	1	3	1	3	3
9	9	9	9	9	1	9	9	9	9
3	3	2	2	3	3	2	1	2	3
3	3	3	3	2	2	3	2	2	2
3	3	2	2	3	3	2	1	2	3
3	3	3	3	1	1	1	3	3	2
2	3	3	1	3	2	3	2	2	1
2	3	3	2	3	1	1	3	3	3
9	9	9	9	9	9	3	9	9	9
9	1	3	9	3	3	1	1	3	9
2	1	2	3	1	1	3	1	2	1
9	9	9	9	9	9	3	9	9	9
1	1	1	1	1	1	3	1	1	1

9	9	9	9	9	9	9	9	1	1
9	9	9	9	9	9	3	9	9	3
2	2	2	2	2	2	2	2	2	2
9	9	9	9	9	9	3	9	9	9
1	2	2	2	1	1	1	3	3	2
2	2	2	2	2	2	2	2	2	2
2	2	2	2	2	2	2	2	2	2
2	3	3	2	3	1	3	3	3	1
1	3	1	2	3	1	1	3	3	2
9	9	9	9	9	9	9	1	9	9
9	1	2	9	1	9	9	9	9	1
1	2	2	3	2	1	3	3	3	2
9	9	9	9	1	9	9	9	9	9
2	3	1	2	1	2	2	3	3	1
3	3	3	2	3	3	3	3	3	1
2	2	2	1	9	9	9	1	9	1
3	3	3	3	3	1	2	3	3	1
3	3	3	3	1	1	1	3	3	1
9	9	9	9	9	1	9	9	9	1
9	9	9	9	1	9	9	9	9	9
1	1	2	3	2	1	2	1	1	2
2	2	3	3	2	1	1	3	3	2
3	3	3	3	3	1	1	3	3	1
3	2	2	3	2	1	1	3	3	2
3	3	3	3	3	9	3	3	3	2
2	1	3	3	3	1	3	1	3	9
3	3	3	3	3	1	1	3	3	9
3	3	3	3	2	1	3	3	3	1
3	3	3	1	1	1	2	3	2	1
3	3	3	3	3	1	1	3	3	1
9	9	1	9	9	9	9	9	2	9
3	3	2	1	2	1	3	3	3	1
2	3	1	2	3	2	2	2	2	3
9	1	9	3	9	1	9	9	9	1
2	2	3	3	1	1	2	1	3	3
9	9	1	9	9	9	3	9	9	9
9	9	9	9	9	9	3	9	9	1

DuringPeri	SeventoTe	MidCycle	SeventoTe	EarlyPerioc	LatePeriod	NoBabies	ManyBabie	HighFat	BirthContr
2	3	9	2	3	2	2	3	2	3
1	3	1	2	2	3	3	1	2	1
2	2	2	2	3	3	3	2	2	2
1	3	1	2	2	3	9	2	3	1
9	9	9	9	9	9	9	9	9	9
1	3	1	2	2	3	3	1	2	1
2	2	2	2	2	2	2	2	2	2
2	1	1	2	1	1	1	1	1	3
9	9	9	9	2	9	9	9	9	9
2	9	9	9	1	9	9	9	9	9
2	2	2	2	9	2	9	9	9	9
1	2	3	3	9	9	9	9	9	9
1	2	2	2	2	2	2	2	2	2
3	1	3	2	2	3	3	3	2	3
2	2	2	2	3	2	3	3	2	2
1	2	2	2	3	3	1	3	2	2
2	9	1	2	2	3	2	9	1	2
2	2	2	2	3	3	1	3	2	2
3	3	2	2	3	3	1	3	2	2
2	2	2	2	3	3	3	3	2	3
2	2	2	2	2	3	3	3	3	3
2	2	2	3	2	3	3	3	1	2
3	3	3	3	9	9	2	9	9	9
2	2	2	3	2	2	2	2	3	3
1	2	2	2	2	2	2	2	2	1
2	2	2	2	2	2	2	2	2	2
2	2	2	2	2	2	2	2	2	2
1	2	2	2	2	2	2	2	2	2
1	2	2	2	1	1	2	2	2	2
2	2	2	2	2	2	2	2	2	2
2	2	2	2	2	2	2	2	2	2
2	2	2	2	3	3	3	3	2	3
2	1	2	2	3	2	2	2	1	2
2	2	2	2	3	3	3	3	2	2
2	2	2	2	3	3	3	3	2	2
2	2	2	1	3	3	3	3	2	2
2	2	2	2	3	3	3	3	2	2
9	1	2	1	1	3	3	1	3	1
1	2	2	2	1	1	9	9	9	9
1	2	2	2	9	9	9	9	9	9
9	1	2	2	2	2	2	2	2	2
9	9	9	9	2	9	9	9	9	9
3	3	3	2	3	3	3	3	3	3
3	3	1	3	3	3	3	3	3	3
2	2	2	2	2	3	3	9	9	9
9	9	9	9	3	1	1	2	3	2

2	1	1	3	3	3	3	3	3	1
9	9	9	9	9	9	9	9	9	1
9	9	9	9	2	9	3	3	9	1
1	2	2	2	2	2	3	3	2	1
9	9	9	9	9	9	9	9	1	9
3	1	3	3	3	3	3	3	1	1
1	1	1	1	3	3	1	3	3	1
1	1	9	9	9	9	9	9	9	1
9	1	9	9	9	9	9	9	9	1
1	2	3	2	1	2	2	3	1	2
9	1	9	9	9	9	9	9	1	9
9	1	9	9	9	9	9	9	9	1
9	9	9	9	2	2	2	3	2	1
2	2	2	2	9	9	9	9	9	1
9	9	9	9	2	2	3	3	2	1
3	1	3	3	3	3	3	3	2	1
2	2	2	2	2	2	2	2	1	2
1	2	1	2	9	9	9	9	1	9
2	3	1	2	3	1	3	3	3	1
2	2	1	2	1	3	3	2	2	2
1	3	1	3	3	3	1	3	3	1
1	3	1	3	3	3	1	3	3	1
1	3	1	3	3	3	1	3	3	1
1	1	1	2	9	9	9	9	3	9
9	3	2	1	3	1	1	2	3	2
9	9	9	9	9	9	9	9	1	9
9	9	9	9	9	9	9	9	9	9
1	2	1	2	3	2	3	2	2	1
3	1	2	1	1	3	3	1	3	1
9	1	9	9	3	3	3	2	3	1
9	9	9	9	2	3	3	3	3	3
1	2	2	2	3	3	3	3	1	2
9	9	9	9	2	2	1	2	2	2
9	1	9	9	3	3	3	9	2	9
1	2	1	1	3	3	3	2	1	2
1	2	1	2	2	2	3	3	3	1
9	9	9	9	2	3	3	3	3	1
2	2	2	2	3	3	3	3	2	2
9	9	9	9	9	9	9	9	9	1
3	3	1	1	3	3	3	3	1	1
9	9	9	9	3	2	1	2	1	1
3	2	3	1	3	3	3	3	1	3
9	9	9	9	3	3	3	3	1	3
1	1	1	1	2	2	3	3	1	1
9	9	9	1	3	9	3	3	2	2
9	9	9	9	9	9	9	9	9	9
1	9	9	9	9	9	9	9	1	9

2	2	2	2	3	3	2	3	2	1
2	2	2	2	3	3	2	2	2	1
2	2	2	2	9	9	9	9	9	9
2	2	1	2	3	3	3	3	2	1
2	2	2	2	9	2	2	2	1	9
1	2	1	2	1	1	2	1	2	2
3	3	3	3	2	2	3	3	2	1
2	1	2	2	3	3	3	1	1	1
2	2	2	2	2	2	3	3	1	2
2	1	1	1	2	1	2	3	1	2
1	2	2	2	3	2	1	2	1	1
2	2	2	2	3	3	3	3	2	2
2	3	3	2	3	3	3	3	1	1
9	1	9	9	9	9	9	9	9	9
2	2	3	1	2	3	3	2	1	2
1	3	2	1	9	9	9	2	9	9
2	1	2	2	2	2	3	3	2	2
2	1	2	2	2	2	2	2	2	2
2	2	2	2	2	2	2	2	2	2
9	9	9	9	2	2	2	2	2	2
1	3	2	2	2	2	2	2	2	2
9	9	9	1	9	9	9	9	9	1
3	1	2	3	2	2	2	2	2	2
3	3	3	1	1	3	3	3	3	3
1	2	2	1	3	2	2	2	1	2
2	2	2	2	2	2	2	2	2	2
9	9	9	1	9	9	9	9	9	1
9	9	9	1	9	9	9	9	9	1
2	2	2	2	2	2	3	3	2	3
9	1	9	9	9	9	9	9	1	9
2	2	2	2	2	2	2	2	2	2
3	2	2	2	2	2	2	2	2	2
1	2	2	2	2	2	2	2	1	2
2	2	2	2	3	3	3	3	3	3
9	1	9	9	9	9	9	9	1	9
1	3	1	3	1	3	3	3	3	3
2	2	9	2	2	2	2	2	2	2
2	1	2	2	2	3	3	1	2	3
9	9	9	9	1	9	9	9	9	9
2	1	2	1	3	3	3	3	3	1
3	1	3	3	2	3	3	3	1	3
3	1	3	3	2	3	3	3	1	3
3	2	2	2	2	2	2	2	2	2
2	2	2	2	2	2	2	2	2	2
2	1	2	1	2	2	2	2	2	2
1	1	2	2	2	2	2	2	1	2
1	3	3	3	2	2	2	2	2	2

1	2	2	2	9	9	9	9	9	9
9	9	9	1	3	3	3	2	2	2
2	1	2	2	3	3	2	2	2	2
3	1	3	3	2	3	3	1	3	3
3	1	3	3	2	3	2	9	3	3
9	9	9	9	3	2	2	3	1	3
1	2	1	2	2	2	1	2	1	3
2	2	2	2	2	2	3	3	2	2
3	3	3	3	2	2	2	2	2	2
2	2	2	2	2	2	2	2	2	2
1	3	2	2	3	3	2	2	3	3
1	3	2	2	3	3	2	2	3	3
9	1	9	9	2	9	9	9	9	9
2	2	2	2	2	2	2	2	2	2
2	1	2	2	3	3	2	2	2	2
2	2	2	2	3	3	2	2	2	2
2	2	2	2	2	2	2	2	2	2
9	1	9	9	3	3	3	2	2	3
9	9	9	1	9	9	9	9	9	9
1	1	2	3	1	2	1	3	2	1
3	1	3	3	9	9	9	9	9	9
3	1	3	1	3	3	3	3	3	2
9	1	2	2	2	1	2	2	2	2
3	3	3	3	3	3	3	3	3	3
3	1	3	3	3	3	3	3	3	3
9	1	9	9	9	9	9	9	9	9
3	1	2	3	3	2	2	3	3	9
9	1	9	9	9	9	9	9	9	9
3	1	3	3	3	3	3	3	3	3
3	1	3	3	3	3	3	3	3	3
1	2	2	2	3	2	2	2	1	1
3	2	2	1	2	2	3	3	1	2
3	1	2	3	9	9	9	9	3	9
3	1	3	3	3	2	3	3	9	1
3	1	3	3	3	2	3	3	2	1
3	1	9	3	3	2	3	3	2	1
3	3	3	3	2	3	2	3	3	1
3	1	3	3	2	3	2	3	3	1
3	1	3	3	3	2	3	3	2	1
3	1	3	3	3	3	3	3	2	1
3	1	3	3	3	3	3	3	3	1
3	3	3	3	3	3	3	3	3	3
9	1	9	9	9	9	9	9	9	9
3	1	3	3	3	3	3	3	3	1
3	1	3	3	1	3	3	3	3	3
3	1	3	3	3	3	3	3	3	1
3	1	3	3	3	3	3	3	3	1

3	1	3	3	3	3	3	3	3	1
3	1	3	3	9	9	9	9	9	1
3	1	3	3	3	9	9	9	9	9
3	3	3	1	9	9	9	9	9	9
3	1	3	3	3	3	3	3	3	3
1	1	1	1	3	1	3	3	3	1
3	1	3	3	3	3	3	3	3	1
2	1	2	2	2	2	3	9	1	2
3	3	1	1	3	3	3	3	1	1
9	9	9	9	9	9	3	9	9	9
9	9	9	9	2	2	3	2	1	2
9	9	9	9	9	9	9	9	9	9
9	9	9	1	3	3	3	3	1	1
3	1	2	3	3	3	3	3	2	1
1	2	1	3	2	2	1	2	2	2
1	1	1	1	2	2	2	2	2	2
3	1	9	2	3	2	2	2	2	2
1	1	1	1	3	3	1	1	1	1
2	2	2	2	2	2	2	2	2	1
1	1	2	1	2	2	2	2	2	2
1	1	2	2	2	1	2	2	1	1
2	2	2	2	2	2	2	2	2	2
3	2	1	2	1	2	2	1	2	2
2	2	2	2	9	9	9	9	9	9
2	2	2	2	2	2	2	2	2	2
9	9	9	9	2	2	2	2	2	2
2	2	2	2	2	2	2	2	2	2
2	2	2	2	2	2	2	2	2	2
2	2	2	2	2	3	3	3	3	3
2	2	2	2	2	2	2	2	2	1
1	2	2	2	2	2	2	2	2	2
1	2	2	2	2	2	2	2	2	2
2	2	2	2	2	2	2	3	2	1
2	2	2	2	2	3	1	1	2	2
2	2	1	2	2	2	2	2	2	2
1	1	1	1	1	2	2	2	1	1
2	1	1	1	2	1	3	3	1	1
3	3	3	1	3	3	2	2	2	1
2	1	2	2	2	2	2	3	2	1
1	2	9	2	2	3	2	3	3	3
2	2	2	2	2	3	2	3	2	2
2	2	2	2	2	2	2	2	2	2
9	9	9	1	1	9	9	9	9	9
9	9	9	1	9	9	2	9	9	9
3	2	2	2	3	3	2	2	3	2
2	1	2	2	2	2	2	3	1	1
2	2	2	2	2	2	2	2	1	1

2	2	2	2	3	3	3	3	2	2
2	2	2	2	3	3	3	3	2	2
2	1	2	2	1	2	2	2	2	2
2	1	1	2	2	2	2	2	1	1
1	2	3	2	3	2	2	3	1	2
3	1	2	2	3	1	2	3	1	2
3	9	9	9	3	3	1	3	3	3
2	2	2	2	2	3	2	3	1	1
2	3	3	2	1	2	3	3	1	2
1	3	3	3	3	3	3	3	3	3
2	2	2	2	3	3	2	9	1	2
3	1	1	2	3	2	3	3	1	1
2	3	3	2	1	2	3	3	1	2
1	2	2	2	1	2	2	2	2	2
2	2	2	2	3	3	2	2	2	2
3	2	2	1	2	3	1	3	3	2
1	2	1	3	3	2	1	2	3	2
2	2	2	2	2	2	3	3	2	3
2	2	2	2	3	3	1	3	3	2
2	1	2	2	3	3	2	1	1	3
2	1	1	2	2	2	1	3	1	1
1	1	2	1	2	2	1	2	1	2
1	2	2	2	3	2	1	2	2	2
2	1	3	2	3	2	2	3	3	2
1	9	9	9	9	9	2	9	9	9
2	2	2	2	2	2	2	2	2	2
2	2	2	2	2	2	2	2	2	2
2	1	3	1	3	2	2	9	1	3
1	2	2	2	2	2	3	3	2	2
1	1	2	2	2	2	2	2	2	2
2	2	2	2	9	1	9	9	9	9
1	2	2	1	2	2	2	2	2	2
1	9	9	9	9	9	9	9	9	9
3	2	2	2	3	3	3	3	2	2
2	2	2	2	1	9	9	9	9	9
3	2	2	2	2	2	2	2	2	2
2	2	1	2	3	3	3	2	2	3
1	2	1	2	2	1	2	2	2	9
1	1	2	1	2	2	2	3	2	2
2	2	1	1	3	3	3	2	3	2
2	3	2	2	2	2	2	2	2	2
1	2	2	2	3	2	2	2	2	1
3	1	2	2	3	3	3	3	2	2
2	2	2	2	2	2	2	2	2	2
9	1	9	9	3	2	2	3	2	1
2	2	2	3	2	3	2	3	2	3
2	1	2	3	3	3	1	3	2	2

1	2	2	1	2	2	3	2	2	2
1	2	2	1	3	3	3	3	2	2
2	1	2	1	2	2	2	2	1	2
2	2	2	1	3	3	2	2	2	1
1	2	1	2	3	3	3	3	2	2
1	2	1	2	3	3	2	2	1	2
2	1	2	2	2	2	3	3	1	1
2	9	9	9	9	9	9	9	1	9
1	2	1	2	3	2	3	3	2	2
2	2	2	2	3	2	2	2	2	2
2	2	2	2	2	2	2	2	2	2
2	2	2	2	2	2	3	2	2	3
1	1	1	1	1	1	1	1	1	1
9	1	9	9	3	3	3	3	2	2
1	1	1	3	3	1	1	3	1	1
2	2	2	2	3	3	3	3	3	3
3	3	3	3	3	3	3	3	2	2
2	3	1	2	1	1	3	2	2	2
1	1	1	2	2	1	3	1	2	1
1	2	1	2	2	1	2	2	1	1
2	2	2	2	2	1	1	2	2	3
1	1	2	3	3	3	3	3	1	1
2	1	2	3	3	3	3	2	1	3
2	2	2	2	2	2	2	2	2	2
2	2	2	2	2	2	2	2	2	2
9	9	9	9	9	9	9	9	9	9
9	9	9	9	1	1	2	3	1	1
2	1	2	3	3	3	3	3	3	3
2	2	2	2	2	2	3	3	1	1
1	1	2	2	2	2	3	3	1	2
2	2	2	2	2	2	2	2	2	2
1	3	2	1	9	9	9	9	9	9
9	9	9	9	9	9	9	9	9	9
2	3	3	3	3	3	3	3	2	3
2	1	2	2	3	3	3	3	1	3
3	3	3	3	3	3	3	2	9	1
1	2	1	2	2	2	3	3	1	3
3	3	3	3	3	3	3	3	3	1
1	1	3	3	3	3	2	2	1	2
2	1	2	2	9	9	9	9	9	9
9	1	9	9	1	2	3	1	2	3
3	3	3	3	1	3	3	9	1	3
2	2	2	2	2	2	3	2	2	2
9	1	3	3	3	3	3	3	2	3
3	3	1	3	2	3	3	3	3	3
1	9	1	3	3	3	1	3	1	3
2	1	2	2	1	2	2	3	2	2

3	2	2	3	2	2	3	2	9	9
3	2	2	3	2	2	3	2	9	9
9	9	9	1	9	9	9	9	1	9
3	1	3	3	1	3	3	3	3	3
9	9	9	9	1	9	9	9	9	9
1	2	1	2	2	3	3	3	1	1
2	2	2	2	2	2	2	2	2	1
9	2	9	9	3	3	3	3	1	3
3	1	3	3	2	2	3	9	2	1
2	1	2	1	3	2	2	2	1	2
2	2	2	2	2	3	3	3	2	3
2	2	2	2	2	2	2	2	2	2
1	2	1	3	2	3	3	3	1	2
2	3	3	3	1	2	3	2	3	2
2	2	2	2	3	2	3	3	2	3
2	3	2	2	2	2	3	3	3	3
3	1	2	2	2	2	2	3	1	3
2	2	2	2	3	2	3	2	2	3
9	9	9	9	3	3	9	3	1	2
3	2	3	2	3	2	3	2	3	3
2	1	2	3	3	2	3	1	3	3
2	3	2	2	1	3	3	3	1	2
2	3	2	2	2	3	3	3	1	2
2	2	1	2	2	1	3	2	2	3
1	2	2	2	2	2	2	2	2	2
2	2	3	3	2	3	9	2	1	2
2	2	2	2	3	2	3	3	2	2
2	1	2	2	1	2	2	2	2	2
1	2	3	1	2	3	3	3	2	2
2	2	1	1	3	3	3	3	1	2
1	3	2	2	3	2	3	3	2	3
1	3	1	2	3	2	3	3	2	1
1	3	2	3	1	3	1	3	3	2
2	2	2	2	2	2	2	2	2	2
2	2	2	2	2	2	2	2	2	2
3	2	1	2	3	3	2	3	2	2
2	3	2	2	2	2	2	3	2	2
2	1	2	2	1	2	2	3	1	2
2	2	2	2	2	2	2	2	2	2
1	1	1	2	3	1	1	2	2	2
1	2	2	3	2	2	2	2	2	2
2	2	2	2	2	2	2	2	2	2
1	1	9	1	2	3	2	1	1	2
3	2	2	2	2	2	2	2	2	2
3	2	3	1	2	2	3	3	3	2
1	3	2	1	3	2	3	3	1	1
2	2	2	2	3	3	2	2	2	2

3	1	2	2	2	2	2	2	2	3
2	2	1	2	3	3	2	2	1	1
2	9	9	9	2	9	9	9	9	9
2	1	2	1	2	2	2	2	2	2
2	2	3	2	3	2	2	3	1	2
2	2	3	1	3	2	3	3	3	2
3	1	2	1	3	3	2	3	2	2
2	2	2	3	2	2	3	3	2	3
2	2	2	3	2	2	3	3	2	3
1	2	1	2	2	2	3	2	1	2
2	1	2	2	2	2	3	3	2	2
2	1	2	2	3	2	3	3	1	2
2	2	2	2	3	3	2	2	2	2
3	2	2	2	3	3	2	2	3	2
3	2	2	2	3	3	2	2	3	2
1	1	2	2	2	2	2	2	2	2
3	1	1	3	3	2	2	3	1	3
2	2	2	2	2	2	1	2	2	2
2	2	2	2	2	2	1	2	3	2
9	9	9	9	9	9	9	9	9	9
3	2	2	2	2	3	1	2	3	1
9	9	9	9	2	2	2	2	2	2
9	9	9	9	3	3	2	2	3	2
3	2	1	2	2	2	3	3	1	1
2	1	2	2	3	3	3	2	2	1
2	2	2	3	2	1	2	2	3	1
9	1	9	9	3	3	3	3	2	2
2	2	2	2	2	2	2	2	2	2
2	2	2	2	2	1	2	1	2	1
9	9	9	9	9	9	1	9	9	9
2	2	2	2	2	2	2	2	2	2
2	2	2	2	2	2	2	1	1	1
2	2	2	2	2	2	2	2	2	2
9	9	9	9	3	3	3	3	1	2
1	1	2	1	3	2	2	3	1	1
3	1	2	2	2	2	2	2	2	2
9	9	9	9	3	2	3	3	2	2
1	1	3	2	3	2	3	2	3	3
2	2	2	1	1	2	3	3	2	2
2	2	1	2	2	1	3	3	1	2
2	2	3	2	2	3	2	3	3	2
1	3	1	2	2	3	3	1	2	1
2	1	2	2	2	2	2	2	3	2
2	2	2	2	3	3	2	3	3	3
3	2	2	2	1	2	3	2	3	3
9	1	9	9	3	3	3	2	3	2
3	3	3	3	3	3	3	2	3	3

9	1	9	9	3	3	3	2	3	2
3	3	3	1	1	3	3	3	3	3
3	1	3	3	3	3	3	3	3	3
1	1	2	2	1	2	1	9	2	9
1	1	3	3	3	3	1	3	9	9
3	1	3	3	3	3	3	3	3	1
2	2	2	2	2	2	2	2	2	2
1	1	3	1	1	3	3	3	3	1
2	1	2	2	2	2	2	2	2	2
3	3	3	1	3	3	3	3	3	3
9	9	9	9	3	2	3	3	2	1
9	1	9	9	3	3	3	2	3	2
3	3	3	1	3	3	3	3	3	3
3	1	3	3	3	2	3	3	3	3
3	1	3	3	3	3	3	3	3	3
1	1	3	3	3	3	3	3	3	3
2	2	2	2	2	2	2	2	2	2
2	2	2	2	2	2	2	2	2	2
1	1	3	3	3	3	3	3	3	3
1	1	3	3	3	3	3	3	9	3
3	1	3	3	3	3	3	3	3	3
3	1	3	3	1	3	3	3	3	3
1	1	3	3	3	3	3	3	3	3
3	1	3	3	3	3	3	3	1	1
3	3	3	1	3	3	3	3	3	1
3	1	3	3	3	3	3	3	9	1
3	1	3	3	3	3	3	3	3	3
3	1	3	3	3	3	3	3	3	3
3	3	3	1	3	3	3	3	3	3
3	1	3	3	3	3	3	3	3	1
9	9	9	9	1	3	3	3	3	3
2	1	3	3	3	3	3	2	3	3
3	1	3	3	3	3	3	3	3	3
3	1	3	3	3	3	3	3	3	9
9	1	9	9	9	9	9	9	9	9
3	1	3	3	3	9	9	9	9	9
2	2	2	2	2	2	1	2	1	2
2	1	1	3	3	1	3	3	1	1
3	1	3	3	3	3	3	3	3	3
3	1	3	3	3	1	3	3	3	1
3	1	3	3	3	9	9	9	9	1
1	1	1	2	1	2	2	2	2	2
9	9	9	9	9	9	9	9	9	1
2	2	2	2	2	2	2	2	2	2
9	9	9	9	2	9	9	9	9	9
2	2	2	1	2	2	2	2	2	2
9	3	2	1	9	2	3	3	1	1

2	2	2	2	2	2	2	2	2	2
1	2	3	2	1	2	2	3	1	2
1	2	3	2	1	2	2	3	1	2
2	2	2	2	3	2	9	2	3	3
2	2	2	2	2	2	1	3	2	2
9	9	1	9	9	9	9	9	9	9
2	2	1	2	1	2	2	2	2	3
1	2	3	1	2	3	2	2	1	2
1	2	2	2	2	2	3	2	2	2
2	2	2	2	2	2	2	2	2	2
9	9	9	9	1	2	1	2	1	1
1	9	9	9	9	9	9	9	9	9
3	1	3	3	9	9	9	9	9	1
1	2	2	2	1	2	2	2	2	2
9	1	9	9	9	1	9	9	9	9
9	1	9	9	9	1	9	9	9	9
1	9	9	9	2	9	9	9	9	9
1	9	9	9	9	9	9	9	1	9
1	9	9	9	9	9	9	9	1	9
9	9	9	9	9	9	9	9	9	9
9	9	9	9	3	1	3	9	1	9
3	3	3	3	3	3	3	3	3	3
1	9	9	9	3	2	3	3	1	3
9	9	9	9	9	9	9	9	9	9
9	9	9	9	2	9	9	9	9	9
9	2	9	9	9	9	9	9	9	9
1	9	9	9	1	9	9	9	9	9
2	2	1	2	1	2	2	2	3	3
9	9	9	9	2	1	3	2	3	1
1	9	9	9	9	9	9	9	9	9
1	9	9	9	1	9	9	9	9	9
2	2	2	2	3	3	2	2	2	2
3	2	2	2	3	3	2	3	2	2
1	1	1	1	3	3	3	3	3	3
2	2	2	2	2	2	2	1	2	2
1	2	2	2	2	2	3	3	2	2
1	2	2	1	3	2	1	3	2	3
3	3	3	1	3	3	3	3	2	3
2	2	2	2	3	3	3	2	2	2
2	3	2	1	2	2	3	1	2	3
1	2	2	3	2	3	2	2	1	1
1	3	2	3	3	3	1	3	2	1
2	3	2	2	3	2	1	2	1	2
2	2	2	1	2	2	3	2	1	1
2	2	2	2	3	2	2	2	2	2
1	2	2	2	3	3	3	3	2	2
1	1	2	1	2	2	2	1	1	1

1	2	2	3	2	2	3	3	2	2
2	2	3	2	9	9	9	9	9	9
2	2	3	3	3	3	3	3	1	2
1	1	3	2	3	1	2	3	1	2
2	3	1	2	3	3	3	3	2	1
2	2	2	2	3	3	3	3	3	2
2	2	2	2	3	3	3	2	3	3
2	3	1	2	3	2	3	3	2	1
2	3	2	2	2	2	3	2	1	2
2	2	2	2	3	3	2	3	3	2
2	2	2	2	3	3	2	3	3	3
1	2	3	2	3	3	3	3	2	2
2	2	2	2	3	2	2	2	2	2
2	2	2	2	3	2	2	2	2	2
1	2	2	2	2	3	3	2	2	3
2	2	2	2	3	3	3	3	2	2
2	2	2	2	3	3	3	2	1	2
1	3	1	3	2	2	2	2	2	2
3	1	3	1	3	3	3	1	3	3
2	2	1	2	3	3	2	3	1	2
3	1	1	3	2	2	2	2	1	1
2	3	2	2	2	3	3	3	1	2
3	2	2	2	2	3	3	3	2	2
3	2	2	2	2	3	3	3	2	2
2	3	3	2	3	2	3	3	2	2
1	2	2	2	2	2	2	2	2	3
1	1	9	3	1	1	3	3	3	9
1	1	1	3	1	1	3	3	1	3
1	3	2	1	2	2	2	2	1	1
1	1	3	2	2	2	3	2	3	3
1	2	3	3	2	3	3	2	2	2
2	2	3	2	2	2	3	3	2	2
2	2	2	2	2	2	2	2	2	2
1	2	2	3	2	3	2	3	2	2
2	1	2	1	2	1	3	1	1	1
9	9	9	2	9	9	9	9	9	9
2	2	2	2	2	2	2	2	2	2
3	1	2	1	3	1	3	2	1	3
3	2	2	2	3	3	3	3	2	2
9	2	2	1	3	9	3	2	1	1
1	9	9	9	2	2	3	3	1	2
3	1	3	3	1	2	3	3	1	1
9	1	9	9	9	9	9	9	1	9
2	2	2	2	3	3	3	3	2	2
3	3	3	3	3	3	3	3	3	2
2	2	2	2	2	2	2	2	3	3
1	9	9	9	9	9	9	9	1	9

9	9	9	9	9	9	9	9	9	9
1	9	9	9	9	9	9	9	9	9
1	9	9	9	2	9	9	9	9	9
1	9	9	9	2	9	9	9	9	9
1	9	9	9	9	9	9	9	9	9
1	9	9	9	9	9	9	9	9	9
1	9	9	9	9	9	9	9	9	9
9	9	9	9	9	9	9	9	9	9
9	9	9	9	9	9	9	9	9	9
9	9	9	9	2	9	9	9	9	9
9	9	9	9	9	9	9	9	9	9
9	9	9	9	2	9	9	9	9	9
9	1	9	9	9	9	9	9	9	9
9	1	9	9	9	1	9	9	9	9
9	9	9	9	9	9	2	9	9	9
9	9	9	9	1	9	9	9	9	9
9	9	9	9	9	9	9	9	9	9
9	9	9	1	9	9	9	9	9	9
2	2	2	2	2	2	2	2	2	2
1	1	1	1	1	1	1	1	2	1
1	1	1	1	9	9	9	9	9	1
1	1	2	3	1	2	3	3	2	2
1	2	1	2	2	2	3	2	2	1
1	1	1	3	1	3	1	3	1	1
1	3	9	2	2	2	2	1	3	3
2	1	3	3	2	3	2	1	2	1
2	2	2	2	2	3	3	3	2	1
1	2	3	2	3	2	3	3	3	2
3	3	1	3	2	2	2	2	1	2
1	2	1	2	2	2	2	2	2	2
1	1	2	1	1	1	2	2	1	1
1	2	2	2	2	2	3	3	3	2
3	2	2	2	2	2	2	2	2	2
3	2	3	3	3	3	1	2	2	3
1	2	2	1	2	2	2	2	1	2
1	2	2	3	1	2	2	2	2	2
3	2	2	2	2	3	2	2	2	1
1	3	2	1	2	2	2	2	1	1
9	9	9	9	1	3	3	1	1	9
1	2	2	2	2	1	2	2	1	2
9	9	9	9	1	1	1	9	9	2
1	3	1	3	3	3	3	1	9	3
9	9	9	9	2	2	1	2	2	2
1	1	1	1	3	1	3	1	1	1
2	3	1	2	3	3	3	2	2	2
9	9	9	9	2	2	1	2	2	2
2	2	2	2	1	2	2	3	1	1

3	2	3	3	1	2	2	2	2	2
1	1	1	3	2	1	3	3	3	1
3	3	1	3	3	1	1	3	1	1
1	1	2	1	1	2	1	2	1	1
2	2	2	2	1	2	2	2	2	2
1	1	2	2	1	2	2	2	2	2
1	2	2	2	2	1	2	2	2	2
3	2	1	1	1	2	2	2	2	2
1	1	1	3	1	1	1	2	1	1
9	9	9	9	2	9	9	9	9	9
9	9	9	9	1	3	2	9	1	1
2	3	9	2	1	1	3	3	3	2
1	1	2	2	1	1	1	2	1	2
3	1	2	2	3	3	2	2	1	2
2	9	3	1	1	1	2	2	2	2
9	9	9	9	3	3	1	1	2	3
9	9	1	9	1	1	1	1	1	1
2	2	2	2	2	2	2	2	2	3
2	2	3	3	2	3	3	1	2	9
3	3	1	3	1	3	3	3	3	3
3	1	2	2	1	3	2	2	3	2
1	1	2	2	1	1	1	2	1	2
3	3	3	3	1	2	2	2	2	2
9	9	9	9	1	3	1	3	2	3
9	9	9	9	1	3	3	2	3	3
3	3	1	3	1	3	1	3	1	3
3	3	2	3	1	3	3	3	3	3
9	9	9	9	3	3	3	3	1	9
2	2	2	2	1	2	2	9	1	1
9	9	9	9	9	9	9	9	9	9
3	1	2	2	2	2	2	2	2	1
1	2	2	2	3	3	1	9	9	9
1	1	1	1	1	2	2	2	3	2
2	1	1	2	2	3	3	3	3	2
9	1	9	9	9	9	9	9	9	9
9	9	9	1	9	9	9	9	9	9
9	9	9	9	9	9	9	9	9	9
2	2	2	2	2	2	2	3	2	2
2	2	2	2	2	1	2	3	2	2
2	2	2	2	2	1	2	3	2	2
2	2	2	2	2	2	2	3	2	2
1	3	2	3	2	2	1	2	2	2
2	3	2	1	1	2	3	1	2	3
2	2	2	2	2	2	2	3	2	2
2	3	2	1	1	2	3	1	2	3
2	2	3	3	1	2	3	2	1	1
3	3	3	3	3	3	3	2	3	3

3	1	3	3	3	3	3	3	3	3
1	1	2	2	2	2	3	3	2	2
3	3	3	3	1	1	1	3	3	2
1	1	2	2	2	2	2	2	2	1
2	2	2	2	2	2	2	2	2	2
1	1	1	1	2	1	2	1	2	3
2	9	1	3	2	2	2	3	2	3
9	9	9	9	9	9	9	9	1	9
9	3	3	3	1	2	3	3	3	3
1	1	2	1	3	3	3	3	2	1
2	2	2	2	3	3	3	3	3	3
9	9	9	9	1	1	1	9	1	9
3	2	2	3	1	2	3	3	2	2
3	2	2	3	1	2	3	2	1	1
2	2	3	3	1	2	3	2	1	1
1	2	3	2	1	2	3	2	1	2
3	2	2	3	1	2	3	2	1	1
3	2	2	3	1	2	3	2	1	1
3	2	2	3	1	2	3	2	1	1
3	1	1	3	1	3	3	3	3	3
2	1	3	2	2	1	3	1	2	1
2	2	3	1	2	2	2	2	1	1
9	9	9	9	3	3	3	1	3	3
3	2	2	1	2	1	2	2	3	3
2	1	3	2	1	3	3	1	1	1
3	1	2	3	2	1	3	3	3	2
3	3	2	3	1	1	1	2	1	1
1	2	2	2	1	2	1	3	1	1
9	9	9	9	9	9	9	9	9	9
2	2	2	2	3	3	3	3	3	3
9	1	3	2	3	3	1	3	2	1
9	9	9	9	2	2	3	2	3	2
2	3	1	2	2	1	2	1	3	2
1	1	2	1	1	1	2	2	1	9
3	3	3	3	3	3	3	3	3	3
3	2	3	3	3	3	3	3	2	3
3	2	2	3	2	2	1	2	2	2
3	2	2	3	2	2	1	2	2	2
3	2	2	3	2	2	1	2	2	2
3	3	2	3	1	3	1	3	3	1
3	1	1	1	2	1	3	2	2	3
1	2	2	9	3	2	1	9	3	3
2	1	3	2	1	2	1	3	2	1
3	1	3	3	3	3	3	3	1	1
9	9	9	9	9	9	1	9	9	9
1	1	2	2	1	1	1	1	1	1
1	2	2	2	1	2	2	2	2	2

2	3	2	3	2	9	9	9	9	9
9	9	9	9	1	9	9	9	9	9
1	9	9	9	1	9	9	9	9	9
3	3	1	3	1	3	1	3	1	3
1	2	2	2	1	2	2	2	2	2
2	2	2	2	1	2	2	2	2	2
3	1	1	2	1	2	1	2	3	1
2	2	2	2	1	2	2	2	2	2
2	2	2	2	1	2	2	2	2	2
9	9	9	9	1	2	2	2	2	2
2	3	3	2	3	3	3	2	2	9
1	2	1	2	3	3	3	3	2	3
2	2	2	2	1	3	2	1	1	2
1	1	1	3	2	1	1	3	3	2
1	2	1	2	2	1	1	3	3	1
3	3	1	2	2	3	3	3	1	2
3	3	3	1	1	2	3	3	3	3
9	9	9	9	3	9	9	9	9	9
9	9	9	9	3	9	9	9	9	9
2	1	3	3	1	2	1	3	1	1
1	1	2	3	1	3	1	2	1	3
2	2	2	2	2	2	3	2	2	3
9	9	9	9	3	3	3	1	3	3
2	2	2	2	2	2	2	2	2	2
3	3	3	3	3	3	3	3	3	3
9	9	9	9	2	9	9	9	9	9
9	9	9	9	2	9	9	9	9	9
1	3	3	3	3	1	3	3	3	1
9	9	9	9	1	3	3	1	3	3
3	3	3	3	2	2	2	2	2	2
9	9	9	9	2	2	2	3	2	1
2	3	2	1	1	1	1	1	9	2
3	3	1	1	1	3	1	1	1	9
9	9	9	9	2	9	9	9	9	9
2	1	3	2	3	2	2	1	1	3
3	3	3	3	3	1	3	3	3	3
9	2	1	1	2	2	2	2	1	2
9	9	9	9	9	9	9	9	9	9
9	9	9	1	9	9	2	9	9	9
2	2	2	2	2	2	2	2	2	2
9	9	9	9	3	9	9	9	9	9
1	2	2	2	2	2	2	2	2	2
2	2	2	2	2	2	2	2	2	2
2	3	2	3	2	3	3	3	2	2
2	2	2	2	2	2	2	2	2	2
2	2	2	2	2	2	2	2	2	2
1	2	2	2	3	3	3	2	2	2

2	2	2	2	2	2	2	2	2	2
2	2	2	2	3	3	3	2	2	2
9	9	9	9	2	2	2	2	1	2
9	9	9	9	3	3	2	2	1	3
9	9	9	9	9	9	9	9	9	9
1	2	3	3	3	1	1	2	3	1
2	2	2	2	2	2	2	2	2	2
1	9	9	9	9	9	9	9	1	9
1	1	1	1	1	2	3	1	2	2
2	9	9	9	2	9	9	9	9	9
2	2	2	2	2	2	2	2	2	2
1	9	9	9	2	2	3	2	2	2
1	9	9	9	9	9	9	9	9	9
2	2	2	2	2	2	2	2	2	2
1	2	1	1	1	3	2	3	1	1
2	2	2	2	2	2	2	2	2	2
2	2	2	2	2	2	2	2	2	2
3	3	3	3	3	9	2	2	1	2
3	3	1	2	9	9	9	9	9	1
9	9	9	9	3	1	9	3	1	1
9	9	9	9	9	9	9	9	2	9
1	3	1	2	3	3	2	2	3	3
2	2	2	2	2	2	2	2	2	2
2	2	2	2	2	2	2	2	2	2
2	2	2	2	2	2	2	2	2	2
3	2	1	1	2	3	2	3	2	3
3	3	1	3	3	3	3	3	3	3
2	2	2	2	2	2	2	2	2	2
2	2	2	2	2	2	2	2	2	2
3	2	2	2	3	3	3	3	3	1
9	9	9	9	9	9	9	9	9	1
2	2	2	2	3	2	3	2	2	2
3	3	3	3	3	3	3	3	3	3
2	2	2	2	2	2	2	2	2	2
2	2	2	3	3	2	3	3	1	2
3	3	1	3	3	2	3	1	1	3
2	2	2	2	2	2	2	2	2	2
2	2	2	3	1	2	2	2	3	1
2	2	1	2	2	2	2	2	2	2
1	2	2	1	1	2	1	3	1	2
1	1	2	1	3	1	2	2	1	2
2	2	2	2	2	2	2	2	2	2
2	3	3	3	3	3	3	3	3	1
1	1	3	2	2	1	2	3	3	3
2	2	2	2	2	2	2	2	2	2
3	3	3	3	3	3	3	3	3	3
2	2	3	2	2	2	2	3	2	3

9	9	9	9	3	3	2	2	3	2
1	1	3	3	3	3	2	2	2	2
3	3	3	3	1	3	3	3	1	3
2	2	2	2	2	2	2	2	2	2
2	2	2	2	1	2	3	3	2	3
3	3	1	3	3	2	1	2	3	2
2	2	2	2	2	2	2	2	2	2
1	1	2	2	2	1	3	1	3	1
1	9	9	9	1	2	3	1	2	3
2	2	2	2	9	9	9	9	9	9
3	1	2	3	2	1	2	3	1	1
2	2	2	2	2	9	9	9	9	9
1	9	9	9	9	9	9	9	9	9
9	1	1	1	2	2	2	3	2	2
1	3	3	3	1	2	2	2	2	2
1	3	3	2	3	3	3	3	3	2
3	3	3	1	2	2	2	2	2	2
1	1	1	1	3	3	1	3	1	2
2	1	3	2	2	1	3	2	2	1
1	2	2	2	2	2	2	3	2	2
3	3	1	3	9	9	9	9	9	9
1	2	2	1	1	1	1	1	9	1
2	2	2	2	2	2	2	2	2	2
1	2	1	1	3	1	2	3	1	1
1	1	3	3	1	3	1	9	3	3
1	2	9	1	9	9	9	9	1	1
2	2	2	2	2	2	2	2	2	2
9	9	9	9	9	9	9	9	1	9
9	9	9	9	2	2	2	2	2	2
2	3	2	2	3	2	2	2	2	3
1	1	1	1	1	1	1	1	1	1
1	1	1	1	1	2	1	3	1	1
1	1	1	1	1	1	1	2	2	3
1	1	1	1	1	1	1	1	1	2
1	2	1	3	9	9	9	9	9	9
9	1	1	1	3	1	3	1	1	1
9	9	1	9	9	9	9	9	9	9
1	1	1	1	1	1	1	1	1	1
2	2	2	2	2	2	2	2	2	2
2	2	2	2	2	2	2	2	2	2
1	2	3	1	1	1	3	3	3	2
1	2	1	2	1	1	1	1	1	1
1	3	1	2	2	1	1	2	3	2
2	2	2	2	3	1	2	2	1	2
2	2	2	2	2	2	2	2	2	2
3	3	1	1	1	2	3	2	2	3
2	2	2	2	2	3	3	2	3	3

1	1	1	2	1	3	3	1	1	3
2	2	3	3	1	3	3	3	3	3
1	2	1	3	2	2	2	2	3	2
2	9	9	9	9	9	9	9	9	9
1	9	9	9	9	9	9	9	9	9
1	2	3	2	1	2	3	1	3	3
9	9	9	9	9	9	9	9	9	9
9	9	9	9	3	3	3	3	3	1
3	3	3	1	1	1	1	3	3	3
2	2	2	2	2	2	2	2	2	2
1	9	9	9	9	9	9	9	9	9
2	2	3	1	3	1	2	9	3	3
2	2	2	2	2	1	1	2	2	2
3	3	2	2	2	2	1	2	2	2
2	2	2	2	1	2	2	2	2	2
3	1	2	3	2	1	2	3	3	9
2	3	2	2	3	1	2	1	2	1
1	9	9	9	9	9	9	9	9	9
1	3	1	2	2	9	9	9	9	9
2	2	1	1	2	2	1	2	1	1
9	9	9	9	2	2	2	2	2	2
1	2	2	2	2	2	2	9	3	3
1	2	9	2	9	9	9	9	9	9
2	2	2	2	3	3	3	1	1	1
3	2	2	3	1	2	3	9	1	2
9	9	9	9	3	3	3	9	3	3
1	2	3	2	2	3	2	3	2	2
3	1	1	3	3	3	3	3	1	1
1	2	2	2	2	1	3	3	3	3
1	3	2	1	3	2	1	2	1	2
2	1	3	2	2	2	3	3	2	3
3	2	2	3	2	3	2	3	2	2
2	2	1	3	3	3	2	9	1	1
3	3	3	3	3	3	3	3	3	3
9	1	9	9	9	1	9	9	9	9
2	3	3	2	1	2	3	3	3	2
1	9	1	1	1	9	1	9	1	1
3	3	3	3	3	3	3	3	3	3
1	3	9	2	2	9	9	1	9	2
3	3	3	3	3	1	3	3	3	3
1	1	3	1	3	1	3	3	3	3
9	9	9	9	9	9	9	9	9	9
1	1	1	1	1	1	1	1	1	1
1	3	9	3	3	3	3	3	1	3
2	2	2	3	9	9	9	9	9	9
9	9	9	9	2	2	2	2	2	2
1	1	2	2	2	1	3	2	1	1

1	9	9	9	3	1	3	3	3	3
3	3	3	3	2	3	3	3	3	3
2	1	2	1	9	9	9	9	9	9
3	3	3	3	1	3	3	3	3	1
2	2	2	2	2	2	2	2	2	2
1	2	3	2	1	1	3	3	3	2
2	2	2	2	3	3	3	3	3	3
2	2	2	2	2	2	2	2	2	2
2	2	2	2	2	2	2	2	2	2
2	2	2	3	2	2	2	2	2	2
1	2	3	2	2	3	2	2	1	1
3	2	2	2	2	2	2	3	3	2
1	3	3	1	3	1	3	1	3	1
1	2	2	3	2	3	2	3	2	3
9	9	9	9	2	2	2	2	2	2
1	2	3	1	3	3	3	3	3	3
2	3	2	3	3	3	3	3	1	3
2	2	2	2	2	2	2	2	2	2
9	9	9	9	3	2	2	2	3	2
9	9	9	9	9	9	9	9	1	9
9	1	9	9	9	9	9	9	9	9
3	3	3	3	3	3	3	3	3	3
3	3	3	3	3	3	3	3	1	3
1	3	1	1	1	2	3	3	1	3
3	3	3	3	1	3	3	9	1	1
1	9	9	9	3	1	3	3	3	3
9	9	9	9	3	3	3	3	2	2
1	3	1	2	1	2	3	2	3	2
2	3	2	1	2	3	2	3	1	1
3	1	1	3	2	2	1	2	2	2
2	2	2	2	3	3	2	3	1	2
1	1	1	1	3	1	3	1	1	1
2	1	2	3	1	2	1	2	1	2
1	2	2	2	3	1	2	2	3	2
9	9	9	9	9	9	9	9	9	9
3	3	3	3	2	2	2	2	2	2
9	9	9	9	2	9	2	2	2	9
2	2	2	2	2	2	2	2	2	2
9	9	9	1	2	3	2	3	1	3
2	2	2	2	3	3	3	3	1	2
2	2	3	1	1	2	3	2	1	2
2	1	2	1	2	2	2	2	2	2
1	1	1	1	1	1	1	1	1	1
9	2	9	9	2	9	9	9	9	9
9	9	9	9	3	9	9	9	9	9
3	2	9	3	3	3	3	3	3	3
1	2	1	2	3	2	2	3	2	2

3	3	3	3	3	3	3	3	1	1
2	2	1	2	3	3	3	3	3	3
3	3	3	3	3	3	3	3	1	3
2	9	9	9	2	2	1	1	1	1
2	2	2	2	3	3	3	3	3	3
9	9	9	9	3	3	3	3	2	3
2	2	2	2	2	2	2	2	3	2
1	2	3	2	3	3	3	3	3	3
1	3	3	3	3	3	3	3	3	3
3	3	3	3	3	2	1	9	9	9
3	1	2	3	9	9	9	9	9	9
3	3	3	3	9	9	9	9	9	9
9	9	9	9	2	9	9	9	9	9
9	9	9	9	3	9	9	9	9	9
1	1	2	1	9	9	9	9	9	9
1	2	2	2	3	3	3	3	2	2
3	3	2	2	1	1	2	1	1	2
2	1	1	2	2	1	2	1	1	2
1	3	3	3	3	3	3	2	2	3
2	2	2	2	3	3	3	3	1	2
1	2	2	2	2	2	2	2	1	9
2	2	2	3	2	2	1	2	2	1
2	2	2	2	2	2	2	2	2	2
2	2	2	1	3	2	2	3	3	1
2	2	2	1	2	2	2	2	1	2
3	2	1	2	1	1	3	3	2	2
9	9	9	9	3	1	9	1	3	3
2	2	2	2	2	2	2	2	2	2
3	2	1	2	1	1	3	3	2	2
1	2	2	2	3	3	3	3	2	2
2	2	2	2	3	3	3	3	1	2
9	9	9	9	2	9	9	9	9	9
1	3	3	2	3	3	3	3	3	3
2	2	2	2	2	2	2	2	2	2
1	1	3	3	1	1	2	1	1	2
9	9	9	9	3	3	3	3	2	2
9	2	9	9	9	9	9	9	9	9
9	2	9	9	9	9	9	9	9	9
2	1	2	2	3	2	2	3	2	2
1	1	1	1	1	2	2	2	2	2
9	9	9	9	9	9	9	9	9	1
3	2	1	1	1	1	1	1	1	1
1	1	1	1	3	3	3	3	1	3
3	3	3	3	3	3	3	3	2	2
2	3	2	3	3	1	1	3	1	3
1	2	2	2	2	2	2	2	2	2
9	9	9	9	9	9	9	9	9	9

9	9	9	9	3	1	1	1	3	1
2	1	1	1	2	2	2	2	2	2
1	1	2	2	1	9	1	3	2	1
1	9	9	9	9	9	9	9	9	9
1	9	9	9	9	9	9	9	9	9
1	2	1	2	1	9	9	9	9	9
1	2	1	2	1	2	1	2	3	1
2	2	2	2	2	2	2	2	2	1
3	2	3	1	1	2	1	3	1	1
3	3	2	2	1	2	1	2	3	1
1	2	1	2	3	2	3	3	1	1
1	1	2	3	1	3	2	2	1	1
3	9	1	1	3	3	1	3	1	1
2	1	2	2	2	2	2	9	2	2
1	2	2	1	3	2	3	2	1	1
3	3	3	3	3	3	3	3	3	3
3	1	3	3	3	3	3	3	1	3
2	1	2	1	2	2	1	2	2	1
1	2	3	1	1	2	3	3	3	1
2	1	1	1	2	3	3	3	3	1
2	3	3	2	2	2	3	3	2	2
9	1	9	9	2	9	9	9	9	9
1	2	1	2	3	3	3	2	2	2
2	3	3	3	2	2	2	3	2	2
1	2	2	2	2	3	2	2	2	2
1	1	2	1	2	2	2	2	2	2
2	2	2	2	3	2	2	2	2	2
2	2	9	1	3	1	3	3	1	1
1	2	2	2	2	2	2	2	1	2
3	1	3	3	3	3	3	3	1	3
9	9	9	9	9	9	9	9	9	1
2	2	2	2	2	2	2	2	1	2
9	1	9	1	2	2	2	2	1	2
1	1	9	3	2	2	1	3	2	2
2	3	2	3	1	2	3	2	1	3
9	9	9	9	9	9	9	9	9	9
9	9	9	9	9	9	9	9	9	9
2	2	2	2	2	2	2	2	2	2
2	2	1	2	3	3	3	3	1	1
1	2	2	1	1	2	2	1	2	2
2	3	1	2	1	1	1	1	1	1
9	1	9	9	1	9	1	9	1	9
1	1	2	1	1	2	1	1	2	1
2	1	2	3	1	2	1	2	1	2
3	2	3	1	2	1	1	2	3	2
9	9	9	9	2	2	3	1	2	3
2	2	2	2	1	2	3	2	1	2

2	2	1	1	1	2	1	2	2	1
9	9	9	9	9	9	9	9	9	9
2	3	3	3	3	3	3	3	3	3
1	2	2	3	2	2	3	3	2	2
1	2	2	1	1	1	3	1	1	3
2	2	1	1	1	2	1	2	2	1
2	3	2	2	3	2	1	1	2	2
2	2	2	2	3	3	3	2	2	2
2	2	2	2	2	2	2	2	2	2
1	1	3	3	3	1	3	1	2	1
1	3	2	2	9	9	9	9	9	9
2	2	2	2	2	2	2	2	2	2
9	9	9	9	9	9	9	9	9	1
1	2	2	2	2	2	2	1	1	2
3	2	2	2	3	3	2	2	3	3
9	9	9	9	9	9	9	9	9	9
2	3	3	3	3	3	2	3	3	3
9	9	9	9	9	9	9	9	9	9
1	1	2	1	2	2	1	1	2	1
2	2	2	2	3	3	3	3	2	2
1	2	2	2	1	1	3	1	2	1
3	1	3	1	2	1	1	3	3	3
3	2	9	3	3	2	1	3	1	2
9	9	9	9	1	2	2	2	2	2
2	2	2	2	2	2	2	2	2	2
2	2	3	2	3	3	2	2	3	2
2	2	2	2	9	9	9	9	9	9
2	2	2	2	2	2	2	2	2	2
3	1	2	1	3	3	3	3	3	3
1	1	1	1	1	2	3	1	1	9
3	3	1	3	3	2	1	2	1	2
3	2	2	1	1	2	2	2	2	2
9	1	2	3	1	3	3	1	2	2
2	2	2	2	1	2	3	2	1	2
1	2	2	2	1	2	2	2	2	2
2	2	2	2	1	2	3	3	1	2
1	1	2	2	2	1	1	2	1	2
3	3	2	2	3	2	2	2	2	1
1	2	3	1	3	2	2	3	1	1
2	1	2	3	2	2	2	2	1	1
2	3	1	3	3	3	2	1	3	2
3	3	3	3	1	3	9	3	3	3
9	9	9	9	3	3	9	3	3	3
1	9	9	9	9	9	9	9	9	9
1	9	9	9	9	9	9	9	9	9
1	9	9	9	9	9	9	9	9	9
1	9	9	9	9	9	9	9	1	9

1	2	2	2	3	3	3	3	2	2
3	2	2	2	2	3	2	3	2	1
9	9	1	9	9	9	9	9	9	9
2	3	3	3	3	2	3	3	3	2
1	1	1	1	3	1	3	1	1	1
1	9	9	9	1	9	9	9	9	9
1	9	9	9	9	9	9	9	9	1
9	9	9	9	9	9	9	9	9	1
9	9	9	9	1	3	3	9	9	9
1	9	9	9	9	9	9	9	9	9
1	9	9	9	9	9	9	9	9	9
9	9	9	9	1	1	2	3	3	9
9	9	9	9	9	9	9	9	9	9
3	3	1	3	3	3	3	3	3	3
1	1	9	3	3	3	1	3	9	1
1	1	1	1	3	2	2	2	1	2
2	2	2	2	2	2	2	2	1	2
9	9	1	9	9	9	9	9	9	9
1	9	9	9	1	9	9	9	9	9
1	9	9	9	1	9	9	9	9	9
9	9	9	9	1	2	3	1	2	3
9	9	9	9	9	9	9	9	9	9
1	1	1	1	1	2	1	1	1	1
9	9	9	9	9	9	9	9	9	9
2	2	1	1	1	1	1	2	2	2
1	2	2	2	1	1	1	2	2	1
1	2	3	1	3	1	2	2	2	2
1	2	2	2	1	1	1	1	1	2
1	2	2	2	1	1	1	2	2	2
2	2	2	2	2	2	1	1	1	1
1	1	2	2	1	1	2	2	1	1
1	3	3	3	2	2	2	2	1	2
1	2	2	2	3	3	3	3	3	3
1	9	9	9	9	9	9	9	9	1
1	2	2	2	3	1	1	1	1	1
1	2	2	2	2	1	1	1	1	1
1	2	2	2	1	1	1	2	1	1
1	9	9	9	9	9	9	9	9	1
2	3	2	3	3	9	3	2	2	1
1	1	3	1	1	3	3	3	1	3
3	3	3	3	3	3	3	3	3	3
1	2	3	1	1	2	3	2	1	1
3	9	1	3	3	3	3	3	3	3
2	2	2	2	2	2	2	2	2	2
1	3	3	3	1	3	1	1	2	2
1	3	2	3	2	2	2	2	2	2
2	2	2	3	3	2	2	2	1	1

3	2	1	3	3	2	3	3	1	3
2	3	1	3	2	1	3	2	2	3
9	9	9	9	3	3	3	3	3	2
1	2	2	3	2	2	3	3	1	2
3	3	3	3	3	3	3	3	1	2
1	9	9	9	9	2	9	9	9	9
3	2	3	2	3	3	3	3	1	2
2	2	2	1	2	1	2	2	1	1
1	9	9	9	9	2	9	9	9	9
1	2	2	3	2	3	2	1	2	3
1	9	9	9	9	2	9	9	9	9
2	1	1	3	3	3	3	3	1	3
2	2	2	2	1	1	1	1	1	1
2	2	2	1	3	3	3	3	3	2
1	3	1	1	3	3	3	3	3	1
1	2	3	3	1	2	1	3	1	2
2	1	2	2	2	2	2	2	1	1
1	2	2	2	2	2	2	2	2	2
1	1	3	1	3	1	1	3	3	3
9	1	9	9	9	9	9	9	9	9
3	3	1	2	3	1	1	3	2	1
2	2	2	2	2	2	1	3	2	2
2	2	2	2	2	2	2	2	2	2
2	2	2	2	3	2	1	1	1	1
2	1	2	2	3	2	2	2	2	2
3	3	3	3	1	1	1	2	2	1
1	3	3	3	1	1	1	1	1	1
1	1	1	1	2	3	3	2	3	2
2	2	2	2	2	2	2	2	2	1
3	1	1	3	3	3	3	3	9	9
1	2	2	1	1	2	2	3	3	1
1	2	2	2	1	2	1	1	1	1
1	1	1	2	2	2	1	1	1	2
1	1	2	1	2	2	2	2	2	2
2	2	2	2	2	2	1	1	2	2
2	2	2	2	3	3	3	3	3	3
2	2	2	2	2	2	2	2	2	2
1	1	1	1	2	2	2	2	1	1
2	2	2	2	2	2	2	2	1	1
2	2	2	2	2	2	2	2	3	3
3	1	3	3	2	3	2	2	2	2
2	2	2	2	3	3	3	3	2	3
2	1	1	2	3	3	1	2	1	1
1	1	1	1	1	2	2	1	2	2
2	3	3	3	9	2	1	2	2	2
3	3	3	3	3	3	3	3	3	3
2	2	2	2	2	2	2	2	2	2

1	2	2	2	3	2	2	2	3	2
1	1	2	1	3	3	3	3	3	9
9	9	1	9	1	9	9	9	9	9
9	9	9	9	9	9	9	9	9	9
9	1	9	9	9	9	9	9	9	9
1	1	9	9	1	1	2	2	2	2
9	9	9	9	9	9	9	9	9	9
2	2	2	2	3	2	2	2	1	2
2	2	2	2	2	2	2	2	2	1
1	1	3	1	2	2	2	2	1	2
9	9	9	9	2	2	2	2	2	2
1	3	1	2	2	2	2	2	1	1
1	1	1	2	1	1	1	1	2	1
2	2	2	2	2	2	2	2	2	2
1	3	1	1	1	1	1	2	2	2
9	9	9	9	2	2	2	2	2	2
2	2	2	2	2	2	2	2	2	2
1	3	9	3	3	3	3	3	3	3
9	9	9	9	9	2	9	2	1	3
2	2	2	2	2	2	2	2	2	2
1	1	1	1	2	2	2	2	2	2
2	1	2	3	2	2	2	2	1	1
2	2	2	2	2	2	2	2	2	2
2	2	2	2	3	3	3	9	2	2
1	2	2	2	2	1	1	1	1	1
2	2	2	2	2	2	2	2	2	2
9	9	9	9	9	9	9	9	9	9
2	1	2	2	3	3	3	3	3	3
1	3	2	2	2	3	2	2	3	3
2	2	2	2	1	1	2	2	2	1
9	9	9	9	9	9	9	9	9	9
1	1	2	3	2	1	2	1	3	1
1	3	1	2	1	3	1	2	1	3
2	2	2	2	3	2	3	3	2	3
2	2	3	3	2	2	2	2	2	3
2	3	1	1	3	2	2	1	1	2
3	3	3	3	1	1	3	3	3	3
1	2	1	3	3	2	3	2	1	1
2	1	2	1	3	2	2	3	2	1
1	1	2	2	1	3	1	3	2	1
2	1	2	2	2	2	2	2	2	2
2	2	9	2	2	2	2	2	3	1
2	2	3	1	2	2	2	2	2	2
9	9	9	1	9	9	9	9	9	9
9	9	9	1	1	9	9	9	9	9
2	2	3	1	2	9	9	9	9	9
2	2	2	2	9	9	9	9	3	3

3	2	3	2	3	2	3	3	1	1
1	2	2	1	2	2	2	2	2	2
1	9	9	9	9	9	9	9	9	9
9	9	9	9	1	3	1	2	3	1
9	9	9	9	3	3	1	2	3	3
2	3	2	1	3	2	2	2	2	2
1	2	2	2	2	2	2	2	2	2
2	2	2	2	2	2	2	2	2	2
3	1	2	1	3	2	3	3	3	2
9	2	3	1	3	3	3	1	3	1
9	1	9	9	9	9	9	9	1	9
9	9	9	9	9	9	9	9	9	9
2	2	2	2	2	2	2	2	2	2
9	9	9	9	9	9	9	9	1	9
1	9	9	9	9	9	9	9	9	9
3	3	1	1	3	2	1	3	3	3
2	1	2	3	2	3	2	1	1	2
2	1	1	3	3	3	1	3	3	3
2	3	1	3	1	1	1	1	1	1
2	1	2	2	2	3	2	2	2	3
2	2	3	1	1	1	3	3	2	2
3	1	1	3	3	3	1	3	1	1
1	2	3	1	1	3	3	1	3	3
2	1	2	2	2	3	3	3	1	2
2	2	2	2	2	2	2	2	2	2
1	2	9	2	1	1	9	9	9	1
9	9	1	9	2	3	3	3	1	2
1	3	1	1	2	1	3	3	2	1
9	9	9	9	1	2	3	3	3	2
1	2	2	3	1	2	2	2	3	2
1	2	3	2	1	3	3	3	1	1
1	1	1	3	2	2	3	2	2	2
1	3	1	1	1	2	2	3	1	1
2	2	1	1	3	2	3	2	3	3
1	2	2	2	2	2	2	2	2	2
1	2	2	2	2	2	2	2	2	2
1	2	1	2	2	1	1	3	3	2
2	2	3	3	2	2	2	2	2	2
9	9	9	9	9	1	9	9	9	9
3	3	2	3	3	2	3	2	3	2
9	2	9	9	9	9	9	1	9	9
2	1	2	2	1	2	2	2	1	2
2	9	9	9	1	9	9	9	9	9
2	9	9	9	1	9	9	9	9	9
2	2	2	2	3	2	3	1	2	3
1	3	1	3	1	3	3	1	1	3
3	3	3	3	3	3	3	3	3	3

3	3	3	3	3	3	3	3	3	3
9	9	9	9	9	9	9	9	9	9
9	9	1	9	3	3	3	3	3	3
3	1	3	3	1	1	1	3	1	1
1	3	1	3	1	3	3	1	3	3
3	1	1	3	1	3	1	3	1	3
3	2	9	2	2	2	2	3	2	3
1	2	1	1	2	1	1	2	2	2
3	1	3	3	1	3	1	3	3	3
1	9	1	3	1	1	3	3	3	3
1	3	1	3	1	3	3	3	1	1
2	3	2	1	1	3	1	2	3	2
2	3	2	2	2	3	2	3	1	2
3	1	3	3	1	3	1	2	3	1
2	1	1	2	1	2	2	2	2	1
2	2	2	2	2	3	1	3	2	3
3	3	2	2	3	2	3	2	3	2
1	2	2	2	2	3	3	2	1	1
1	3	3	3	1	3	3	3	3	3
2	2	2	2	2	2	2	2	2	2
3	3	2	1	2	2	2	2	2	2
1	2	3	2	3	2	2	2	2	1
1	2	2	2	3	3	3	3	3	3
2	2	2	2	3	2	2	2	2	3
2	2	1	2	1	1	1	2	2	1
2	1	2	2	2	1	2	1	2	2
2	2	2	2	3	2	3	9	1	2
2	1	1	3	3	3	2	2	3	2
1	1	1	3	2	3	1	3	1	3
9	9	9	9	1	9	9	9	9	9
3	2	3	3	3	2	2	3	2	1
3	3	3	3	1	3	3	3	2	1
2	3	3	3	1	1	2	3	1	2
3	3	3	3	2	1	2	2	1	2
1	1	1	1	1	1	3	3	1	1
2	2	2	2	2	2	2	2	2	2
3	3	3	3	3	2	2	2	3	3
2	2	3	2	1	3	2	3	2	3
2	2	1	3	2	1	2	3	1	1
2	2	2	2	2	2	2	2	2	2
1	9	9	9	9	9	9	9	9	9
3	3	3	3	9	9	9	9	9	9
3	1	2	3	1	2	3	1	2	1
3	3	3	3	1	3	2	2	1	2
2	2	2	2	1	2	2	2	2	2
3	2	2	2	3	3	2	9	1	2
1	2	2	3	9	9	9	9	9	9

2	3	2	3	1	2	3	3	1	2
1	2	3	2	3	3	3	3	3	3
3	2	2	3	2	2	1	2	3	2
1	2	3	1	2	1	2	1	1	2
2	1	1	2	9	9	9	9	9	9
1	3	3	3	1	1	1	1	1	1
2	2	2	2	2	2	3	2	2	2
2	2	2	2	2	2	2	2	2	2
2	2	2	2	1	2	3	2	2	1
2	1	2	2	2	1	2	2	2	2
9	9	9	9	3	9	9	9	9	9
2	2	3	2	1	2	3	3	3	2
9	9	9	9	1	2	3	3	2	2
9	1	3	1	9	9	9	9	9	9
2	2	2	2	2	2	2	2	3	3
9	9	9	9	9	9	9	9	9	9
2	2	3	3	3	2	2	2	2	3
2	2	1	1	1	1	3	3	3	3
3	1	3	1	3	3	3	2	1	1
3	3	3	3	2	2	2	2	2	2
2	1	1	3	3	3	3	3	1	3
2	2	2	2	2	2	2	2	2	2
9	9	9	9	9	9	9	9	9	9
2	2	2	2	1	3	2	2	2	2
3	2	1	3	2	2	2	2	1	2
2	3	2	2	2	2	2	2	2	2
9	9	9	9	1	9	9	9	9	9
2	3	2	3	2	2	3	2	2	2
2	2	1	2	2	2	3	3	3	3
9	9	9	9	9	9	9	9	1	9
1	1	2	3	2	2	3	3	3	2
2	2	2	1	2	2	2	2	2	3
9	9	9	9	1	3	3	2	3	3
2	2	2	2	2	2	3	3	1	2
3	3	2	2	2	2	3	3	2	2
2	2	2	2	1	2	3	1	1	2
2	2	1	2	2	2	2	2	2	2
3	3	3	2	3	3	3	3	3	3
9	1	9	9	9	9	9	9	9	9
3	2	3	3	3	3	3	3	3	3
1	2	3	2	2	1	2	3	2	3
3	2	1	2	3	2	3	3	2	2
2	2	2	2	2	2	3	3	2	2
3	2	2	1	3	2	3	2	1	1
2	2	2	3	2	2	1	3	1	1
9	9	1	9	2	2	1	1	2	2
2	2	2	2	2	2	3	3	2	2

3	3	2	3	3	3	3	3	3	3
2	2	2	2	2	2	2	2	1	2
9	9	9	9	2	2	2	2	1	1
9	1	9	9	9	9	9	9	9	9
2	2	2	2	2	2	2	2	2	2
3	3	3	3	9	9	9	9	9	9
2	2	2	2	2	2	2	2	2	2
3	1	1	3	3	3	3	3	2	2
3	2	3	2	2	2	3	3	3	2
9	9	9	9	2	2	3	2	3	2
9	1	1	2	3	3	2	2	2	3
1	2	3	1	2	2	1	2	3	2
2	2	2	2	2	2	2	2	2	2
3	3	3	3	3	3	3	3	3	3
1	2	1	3	2	1	2	1	3	2
2	2	1	3	2	2	3	3	2	2
3	2	3	3	3	3	1	3	3	2
3	1	1	3	2	2	2	2	2	2
2	2	2	2	2	2	2	2	2	2
3	2	3	2	2	2	2	2	2	2
2	2	2	2	3	2	2	2	2	2
2	2	1	2	2	2	3	3	2	3
2	2	1	2	2	2	3	3	2	2
3	3	1	3	2	2	2	2	2	2
2	2	2	2	3	3	3	3	1	1
3	3	1	2	1	2	3	2	3	3
1	1	2	1	1	2	1	3	2	3
1	1	2	3	1	2	1	3	2	3
1	2	3	2	3	3	2	2	2	2
2	2	2	2	2	1	2	2	1	2
3	3	3	3	3	2	3	2	2	3
3	3	2	2	2	2	3	3	2	3
3	2	2	2	3	2	3	3	2	3
2	2	2	2	2	2	2	2	2	2
2	2	2	2	3	3	3	3	3	3
3	3	3	3	2	2	2	2	2	2
2	2	2	2	2	2	2	2	2	2
1	3	2	1	1	2	2	3	2	2
3	3	3	3	2	2	3	2	2	1
2	2	3	2	2	2	2	3	2	2
3	3	3	3	2	2	3	3	1	1
3	3	2	3	2	3	3	2	3	3
1	1	2	3	1	1	1	1	1	1
2	2	2	2	1	1	2	2	1	2
2	2	2	3	2	2	3	3	2	3
2	3	3	3	3	3	3	3	9	2
9	9	9	9	9	9	9	9	9	9

1	3	2	3	3	2	1	2	3	3
2	3	2	2	2	2	1	1	2	3
2	2	2	2	9	9	9	9	9	9
2	2	2	2	2	2	1	1	1	1
2	2	2	2	2	2	2	2	2	2
2	2	2	2	2	2	2	2	2	2
1	1	1	2	2	2	3	3	2	1
9	9	9	9	1	2	2	1	1	3
9	9	9	9	2	2	2	2	2	3
2	2	2	2	2	2	2	2	2	2
2	2	2	2	2	2	2	2	2	2
2	2	2	2	2	2	2	2	2	2
2	2	2	2	2	2	2	2	2	2
2	2	2	2	2	2	2	2	2	2
3	3	3	3	2	2	2	2	2	2
9	9	9	9	2	2	2	2	2	2
2	9	9	9	2	2	2	2	2	3
9	9	9	9	2	2	2	2	2	2
9	9	9	9	2	2	3	3	3	3
1	1	2	1	2	2	3	2	3	1
2	2	2	2	2	2	2	2	2	2
2	2	2	2	2	2	2	2	2	2
2	2	2	2	3	3	3	3	2	2
2	2	2	2	2	2	2	2	2	2
9	9	9	9	2	2	2	2	2	2
9	9	9	9	9	9	9	9	9	9
2	2	2	2	2	2	2	2	2	2
2	2	2	2	2	2	2	2	2	2
2	2	2	2	2	2	2	2	2	2
1	1	2	1	3	2	3	3	3	2
2	3	2	2	3	2	2	2	2	2
2	2	2	2	2	2	2	2	2	2
2	2	2	2	2	2	2	2	2	2
2	2	2	2	2	2	2	2	2	2
2	2	2	2	9	9	9	9	9	9
2	2	2	2	1	2	2	2	2	2
2	2	2	2	2	2	2	2	2	2
2	2	2	2	2	2	2	2	2	2
2	2	2	2	2	2	2	2	2	2
9	9	9	9	2	2	2	2	2	2
9	9	9	9	3	3	2	2	2	2
2	2	2	2	2	2	2	2	2	2
9	9	9	9	2	2	2	2	2	2
2	2	2	2	2	2	2	2	2	2
2	2	2	2	2	2	2	2	2	2
2	3	2	2	2	2	2	2	2	3
2	2	2	2	2	2	2	2	2	2

2	2	2	2	2	2	2	2	2	2
9	9	9	9	2	2	2	2	2	2
2	2	2	2	2	3	3	2	1	1
2	2	2	2	2	2	2	2	2	2
2	2	2	2	2	2	2	2	2	2
9	9	9	9	2	2	3	3	3	3
1	2	2	3	9	9	9	9	9	9
2	9	9	9	9	9	9	9	9	9
2	2	2	3	3	1	3	3	1	3
2	2	1	2	3	2	2	2	3	2
2	3	2	3	2	2	2	2	2	2
2	2	2	2	2	2	2	2	2	2
9	9	9	9	2	2	2	2	2	2
1	9	9	9	9	9	1	9	9	9
3	3	3	3	3	3	3	3	3	3
2	2	2	2	2	2	2	2	2	2
2	2	2	2	2	2	2	2	2	2
2	2	2	2	2	2	3	9	9	9
2	2	2	2	9	9	9	9	9	9
2	2	2	2	2	2	2	1	2	2
9	9	9	9	2	9	9	9	9	9
2	2	2	2	2	2	2	2	2	2
9	9	9	9	2	2	2	2	2	2
9	2	2	2	2	2	2	2	2	2
2	2	2	2	2	2	2	2	2	2
2	2	2	2	2	2	2	2	2	2
9	9	9	9	9	9	9	9	9	9
2	2	2	2	2	2	2	2	2	2
2	2	2	2	2	2	2	2	2	2
9	9	9	9	2	1	2	9	9	2
2	2	2	2	2	2	2	2	2	2
2	1	3	3	2	3	1	2	2	3
2	2	2	1	3	3	1	2	3	1
1	1	1	1	2	2	2	2	2	2
2	3	3	2	3	3	3	3	1	1
3	1	3	1	3	3	1	1	2	1
2	2	3	3	3	3	2	3	1	3
2	2	2	2	1	2	1	2	1	2
2	2	1	1	1	1	3	3	1	2
2	2	1	2	2	2	2	2	2	2
2	2	2	2	3	3	3	3	1	3
9	9	1	9	2	2	2	1	2	2
2	1	2	1	1	1	3	3	2	2
2	2	2	2	2	2	2	2	2	2
1	2	1	3	1	1	1	9	1	1
2	2	2	2	2	2	2	2	2	2
2	2	2	2	1	2	2	1	1	2

3	1	2	3	2	3	1	2	1	3
2	1	2	1	1	9	3	1	2	1
1	1	1	1	3	1	2	3	1	2
1	2	1	2	1	3	3	3	2	1
3	2	1	2	9	9	9	9	9	9
1	2	1	3	1	2	3	3	1	2
2	3	1	2	2	1	3	2	3	1
9	9	9	9	2	9	9	9	9	9
2	2	2	2	2	2	2	2	2	2
2	2	2	2	2	2	2	2	2	2
2	2	2	2	3	3	3	3	3	3
2	2	2	2	3	3	3	3	3	3
3	3	3	3	2	2	2	2	2	2
2	2	2	2	2	2	2	2	2	2
1	2	1	1	3	3	3	3	2	2
2	2	2	2	2	2	2	2	2	2
2	2	2	2	2	2	2	2	2	2
3	3	3	3	3	3	3	3	3	3
9	9	9	9	3	3	3	3	2	2
2	1	2	1	1	1	3	2	2	3
9	9	9	9	3	3	2	3	2	1
9	9	9	9	9	9	9	9	9	9
1	1	1	2	2	2	1	3	3	1
9	9	9	9	9	9	9	9	9	9
2	3	1	2	1	2	2	1	3	1
1	2	2	2	2	2	1	1	1	2
2	2	2	2	1	3	3	3	2	2
9	9	9	9	2	9	9	9	9	9
9	9	9	9	2	2	2	2	2	2
9	9	9	9	9	9	9	9	9	9
9	9	9	9	3	3	3	2	3	3
1	2	2	2	9	9	9	9	9	9
1	1	2	1	9	9	9	9	9	9
1	1	2	1	1	2	2	2	1	2
1	1	2	1	1	2	2	2	1	2
2	2	2	2	2	9	9	9	9	9
2	3	1	2	9	9	9	9	9	9
1	1	2	1	1	2	2	2	1	2
1	2	2	1	2	2	2	2	2	2
9	9	9	9	2	2	2	2	2	2
2	2	2	2	2	9	9	9	9	9
2	2	2	2	2	2	2	2	2	2
2	2	2	2	2	2	2	2	2	2
9	9	9	9	9	9	9	9	9	9
2	9	9	9	3	9	9	9	9	9
1	2	2	1	1	1	1	1	1	2
2	2	2	2	2	2	2	2	2	2

2	2	1	1	9	9	9	9	9	9
9	9	9	9	1	2	2	1	1	3
1	3	3	3	3	3	3	3	3	3
2	9	9	9	2	3	1	3	2	1
2	2	2	2	2	2	2	2	2	2
9	9	9	9	3	3	3	3	3	3
2	2	2	2	2	2	2	2	2	2
2	2	2	1	9	9	9	9	9	9
1	9	9	9	9	9	9	9	9	9
2	2	2	2	9	9	9	9	9	9
2	2	1	1	1	1	1	1	1	2
2	2	2	1	2	3	3	2	2	2
2	3	3	3	3	2	2	2	2	2
3	3	3	3	3	3	3	3	3	3
1	3	1	3	3	3	2	2	1	1
2	1	2	2	3	2	3	3	1	2
1	1	2	1	1	2	3	3	3	1
3	1	2	1	3	3	2	3	1	2
2	2	2	1	2	2	2	1	2	2
2	1	3	1	1	1	3	1	3	1
3	1	2	1	1	3	2	1	2	1
1	1	3	3	1	3	3	3	3	3
9	9	9	9	3	2	3	2	1	2
3	3	3	3	3	3	3	3	2	1
1	2	2	1	3	1	2	1	3	3
2	2	2	2	3	2	2	2	2	2
2	2	2	2	2	2	3	3	2	2
9	1	1	2	2	2	3	3	1	2
3	2	3	3	2	1	3	2	3	2
1	3	3	3	3	1	3	3	3	3
1	2	2	2	1	2	3	2	2	3
1	2	1	1	3	3	3	3	3	3
2	2	2	1	1	3	1	3	1	1
3	3	3	3	3	3	3	3	3	3
2	2	2	1	2	3	3	3	3	3
2	3	2	1	2	2	3	1	2	1
3	3	1	3	3	2	1	3	3	3
2	2	2	2	2	2	2	2	2	2
2	1	1	2	1	2	2	3	1	2
9	1	9	9	9	9	9	9	9	9
1	3	3	2	3	3	3	3	3	3
2	3	3	1	2	1	2	3	1	2
3	1	2	2	2	2	3	3	2	3
1	1	2	3	3	3	2	2	1	1
2	1	1	2	2	1	3	3	2	1
3	3	3	3	3	2	3	2	3	2
9	9	9	9	3	2	3	3	3	3

9	9	9	9	1	2	1	2	2	2
9	9	9	9	3	3	2	3	2	1
3	3	3	3	3	3	3	3	3	3
3	3	3	3	3	3	3	3	3	1
1	2	1	2	3	3	3	3	1	1
1	2	2	2	3	3	3	3	1	2
9	9	9	9	1	1	1	1	1	1
2	2	2	2	3	2	2	2	2	2
3	3	1	3	1	3	1	1	3	3
1	2	1	2	2	2	3	3	1	2
3	3	2	3	3	1	3	9	1	1
2	2	2	2	2	2	2	2	2	2
2	2	2	2	2	2	3	2	2	2
1	1	3	1	1	1	1	3	3	3
1	2	2	1	2	2	2	3	2	1
2	2	2	2	2	2	2	2	2	2
2	3	2	2	3	2	2	2	2	1
2	2	2	2	2	2	2	2	1	2
1	2	3	3	3	2	1	3	2	1
2	1	1	2	1	2	3	2	9	9
2	2	3	3	3	3	3	3	2	2
1	9	9	9	9	9	9	9	9	9
3	2	2	2	2	3	2	2	2	3
2	2	2	2	3	3	2	3	3	3
3	3	2	3	3	3	3	3	2	2
2	2	2	1	1	2	2	2	2	2
3	2	2	1	2	2	2	2	2	2
2	2	2	2	2	2	2	2	2	2
3	3	3	3	3	3	3	3	3	3
1	1	1	1	2	2	3	3	1	2
1	1	2	1	1	1	3	3	3	3
1	2	3	1	3	3	2	9	1	3
2	3	2	3	3	2	3	1	3	2
2	3	1	3	2	2	2	2	2	2
1	2	3	1	1	2	3	2	1	1
2	2	2	2	2	2	2	2	2	2
2	2	1	2	3	2	1	3	1	3
1	2	1	1	3	2	3	2	1	2
2	2	1	2	1	2	2	3	9	2
9	9	9	9	2	9	9	9	9	9
2	1	2	2	2	2	2	1	3	3
1	2	2	1	2	3	3	1	1	2
2	2	2	2	2	1	2	2	2	2
1	2	1	2	3	2	2	2	9	1
1	2	3	2	2	3	2	3	3	3
1	3	2	3	9	9	9	1	9	9
1	2	2	1	2	1	3	3	3	3

1	2	2	1	2	1	3	3	2	3
2	2	2	2	2	2	2	2	2	2
1	3	3	3	1	3	3	2	3	3
2	1	3	2	2	1	3	2	1	3
2	2	2	2	3	3	3	3	2	2
3	3	1	1	3	1	3	3	9	1
2	2	1	2	2	2	2	2	1	2
9	1	9	9	1	9	9	9	9	1
2	2	2	2	1	3	2	3	2	2
2	3	1	2	2	1	3	2	3	2
2	2	2	2	2	2	2	2	2	2
3	2	2	1	2	2	2	3	3	2
9	9	9	1	9	9	9	9	9	9
1	1	9	2	2	3	3	2	1	1
9	1	9	9	2	9	2	2	9	9
1	2	2	3	2	1	3	2	2	2
1	1	1	1	9	1	1	1	1	1
2	1	2	2	2	2	1	1	1	2
1	3	2	1	1	3	3	2	1	3
2	3	1	2	2	3	2	3	3	2
1	2	1	1	3	1	3	3	3	1
2	2	2	2	3	2	2	2	2	2
2	2	2	2	2	2	1	2	2	2
2	2	3	2	3	3	3	2	2	2
3	3	1	3	9	9	9	9	1	9
2	1	3	3	1	2	2	2	1	1
9	3	2	1	1	2	2	1	3	3
2	1	2	1	2	2	1	2	1	2
3	3	3	3	2	2	3	2	2	2
2	2	2	2	3	2	2	2	1	1
2	2	1	1	1	2	9	1	2	1
1	1	2	2	2	2	2	2	2	2
1	1	2	1	1	1	1	1	1	2
2	1	2	2	1	1	1	1	1	2
1	3	1	2	1	1	2	3	1	2
1	2	3	2	9	9	9	9	1	9
2	2	2	2	1	1	1	1	2	2
3	3	1	3	3	3	3	3	3	1
1	1	2	2	1	2	1	1	1	3
2	2	1	1	1	1	2	2	2	1
2	3	3	3	3	3	3	3	3	3
3	3	3	3	1	1	2	2	1	1
1	3	2	1	1	3	1	1	1	1
2	2	2	2	2	3	3	3	2	3
2	2	2	3	2	3	2	3	3	3
3	3	3	3	3	3	3	3	3	3
2	2	2	2	2	2	2	2	2	2

2	1	3	3	2	3	1	2	2	1
1	2	3	3	3	1	3	3	3	3
2	2	2	2	2	2	2	2	2	2
2	1	2	2	1	2	3	3	2	1
3	3	3	3	1	3	3	3	3	3
2	3	1	2	1	2	1	2	3	1
3	3	3	3	9	1	9	9	9	9
3	3	2	2	2	2	1	1	1	2
2	2	2	2	2	2	2	2	2	2
2	2	2	2	2	2	2	2	2	2
2	2	2	2	2	2	2	2	2	2
2	2	2	2	2	2	2	2	2	2
1	1	1	1	1	1	1	1	1	1
3	3	3	3	2	2	2	2	2	2
2	2	2	2	2	2	3	3	3	3
2	1	1	2	3	3	2	2	2	2
2	2	2	9	2	2	2	2	2	2
1	1	2	2	3	1	2	2	1	2
2	2	2	2	2	2	3	3	2	3
2	2	2	3	2	2	2	1	1	1
1	2	2	2	2	2	2	2	2	2
2	2	2	2	2	2	2	2	2	2
3	3	3	3	2	2	2	9	2	9
3	2	2	3	3	3	3	3	3	3
1	2	1	2	1	2	3	2	1	2
2	2	2	2	2	2	2	2	2	2
1	2	1	2	2	1	3	1	2	1
2	2	2	3	2	2	3	3	2	2
2	2	2	2	2	2	2	2	2	2
2	1	2	2	2	2	2	3	2	2
2	2	2	3	3	2	2	1	2	2
2	2	2	2	1	2	3	3	2	1
1	2	2	2	1	2	2	2	2	2
2	2	2	3	1	1	1	3	3	2
2	2	1	2	2	3	2	3	2	2
1	2	2	1	2	3	3	3	3	3
2	1	9	2	1	1	1	1	3	3
3	2	2	1	2	2	1	3	2	2
3	3	3	3	3	3	3	3	3	1
3	3	3	3	3	3	3	3	3	3
2	2	2	2	2	2	2	2	2	2
2	2	2	2	2	2	2	2	2	2
2	2	2	2	2	2	2	2	2	2
3	3	2	2	3	3	3	3	3	3
3	3	2	2	3	3	3	3	3	3
9	2	9	2	2	9	2	9	9	2
2	2	1	1	1	2	1	1	2	2

2	1	1	1	2	2	1	1	1	2
2	2	2	1	1	2	2	1	1	2
2	2	2	2	1	2	2	2	1	1
2	1	1	1	1	2	2	1	1	1
2	3	3	2	2	2	2	1	1	1
1	2	2	2	2	2	2	1	1	2
2	1	1	1	2	3	1	2	2	1
2	2	1	1	2	1	2	2	2	1
1	2	3	3	1	2	1	1	2	2
2	3	3	3	2	3	1	1	2	2
1	2	3	2	1	2	2	1	1	1
2	2	2	3	1	2	2	2	3	1
2	2	2	2	2	2	2	2	2	2
2	2	2	2	2	2	2	2	2	2
2	2	2	2	2	2	2	2	2	2
2	2	2	2	2	2	2	2	2	2
2	2	2	2	2	2	2	2	2	2
2	2	2	2	2	2	2	2	2	2
2	2	2	2	2	2	2	9	2	2
2	2	2	2	2	2	3	3	2	2
2	2	2	2	2	2	2	2	2	2
2	2	2	2	2	2	2	2	2	2
2	2	2	2	1	2	2	2	1	2
2	2	2	2	1	1	2	2	1	1
2	2	2	2	2	2	2	2	2	2
2	2	2	2	2	2	2	2	2	2
2	2	2	2	2	2	2	2	2	2
2	2	2	2	2	2	2	2	2	2
2	2	2	2	2	1	1	2	2	1
3	3	3	3	2	2	2	2	2	2
2	2	2	2	2	2	2	2	2	2
2	2	2	2	2	2	2	2	2	2
2	2	2	2	2	2	2	2	2	2
2	2	2	2	2	2	2	2	2	2
1	9	1	9	1	9	1	9	1	9
1	3	1	2	1	3	1	9	3	1
1	9	9	9	1	9	1	9	1	9
9	2	9	2	2	9	9	2	9	9
9	9	9	9	9	9	9	9	9	9
1	1	1	1	1	1	1	1	9	1
9	2	1	9	1	9	2	1	9	3
1	1	1	1	2	1	2	2	1	1
2	2	2	1	1	2	2	2	2	2
2	2	2	2	2	2	2	2	2	2
9	1	9	1	1	9	2	9	9	1
2	9	2	9	2	9	9	9	2	9
1	2	1	2	2	2	2	2	2	2

2	2	2	2	2	1	1	2	1	2
1	1	2	2	2	2	2	9	2	1
2	2	2	2	2	2	2	2	2	2
1	1	1	3	1	1	2	2	1	1
1	1	2	2	1	1	2	2	2	3
2	2	3	3	2	3	2	3	2	2
2	1	1	1	1	1	1	2	2	1
2	2	2	2	2	2	2	2	2	2
2	2	3	2	2	1	1	2	2	1
1	2	2	1	2	1	2	2	3	2
2	2	2	2	1	2	2	2	1	1
2	2	2	2	2	3	3	2	2	1
2	2	3	3	1	1	2	2	1	1
2	2	1	1	1	2	2	2	1	1
2	2	2	2	2	2	2	2	2	2
2	2	3	3	1	2	2	2	2	2
2	1	3	3	2	2	2	2	2	2
2	3	2	2	2	2	2	1	1	2
2	2	1	1	1	2	1	1	2	2
1	2	3	2	2	3	2	1	2	2
1	1	2	2	2	3	2	1	2	2
2	2	1	1	2	2	1	2	2	2
2	2	2	2	1	1	2	1	1	2
2	3	3	2	3	1	1	2	2	2
2	3	2	1	2	2	3	2	2	3
2	1	1	2	3	2	2	3	2	2
2	2	2	2	3	3	2	2	2	2
2	2	3	3	2	3	3	3	2	2
2	1	1	3	2	2	2	2	3	2
2	2	2	2	2	2	2	2	2	2
9	2	2	2	2	2	2	2	2	2
2	2	2	2	3	3	3	3	3	3
2	1	2	1	2	2	2	2	2	2
2	2	1	2	2	2	2	2	3	2
2	2	1	3	2	3	2	1	3	2
3	3	3	3	3	3	3	3	3	9
2	2	3	2	2	2	2	2	2	2
2	2	2	2	2	2	2	2	2	2
2	2	2	3	2	2	2	2	2	2
2	3	2	1	1	2	1	2	1	2
1	1	3	2	2	3	2	1	2	2
3	3	3	3	3	3	3	3	3	3
2	3	2	1	1	2	2	1	1	2
2	3	2	1	2	2	2	2	2	2
2	2	3	3	2	1	2	1	2	2
2	2	2	1	1	2	1	1	1	1
2	3	2	1	2	1	2	1	2	1

2	2	2	2	9	1	9	1	9	9
2	2	2	2	1	1	2	2	2	2
2	2	2	1	1	1	2	2	2	2
2	2	3	3	1	1	2	2	1	1
2	2	3	1	3	2	1	2	2	3
2	2	2	1	2	1	2	2	2	2
1	1	2	2	2	3	3	3	2	1
1	1	2	2	2	1	3	1	3	2
2	1	2	1	3	2	2	3	1	1
2	3	2	2	2	2	2	2	2	2
2	1	2	2	3	3	2	3	1	3
1	2	2	2	2	2	2	1	2	2
2	1	2	2	1	2	3	1	2	3
1	2	2	2	2	3	2	2	3	2
2	3	2	1	3	2	1	2	2	3
2	2	3	2	3	2	2	2	2	2
2	2	2	2	3	2	2	2	2	2
2	2	1	2	2	2	2	2	1	2
1	3	3	3	1	3	3	3	1	3
3	3	2	3	3	3	3	3	2	2
2	2	3	3	2	2	3	3	3	1
3	2	2	3	3	3	3	3	2	2
2	1	1	3	2	2	2	2	1	2
3	9	2	2	2	3	3	3	2	2
2	2	2	2	2	2	2	2	1	2
2	3	2	3	3	3	2	3	3	3
2	1	2	3	2	3	3	2	1	2
2	2	2	2	2	1	2	3	2	2
2	1	1	1	1	1	1	1	1	1
2	2	3	2	2	2	2	3	3	2
3	1	2	1	2	2	3	1	2	3
2	2	1	1	2	2	2	3	2	3
2	2	2	2	2	2	2	2	2	2
2	2	2	2	2	2	2	2	2	2
1	3	9	2	1	2	2	3	1	2
3	2	2	2	2	2	2	2	2	2
2	3	2	3	2	2	3	3	3	2
3	1	2	1	9	2	2	2	1	1
3	2	3	2	3	3	2	2	2	1
2	1	2	2	2	2	3	3	2	2
1	2	2	1	3	3	2	9	1	1
2	2	3	1	2	1	2	2	2	2
2	2	2	2	3	3	2	2	2	2
2	3	2	3	2	2	3	3	2	3
2	2	2	2	2	2	2	2	2	2
2	3	2	2	2	2	2	2	1	2
2	3	2	3	3	2	3	2	2	2

3	2	1	2	3	3	2	2	2	2
3	1	2	1	3	2	2	3	1	9
2	2	2	1	2	2	3	2	2	2
2	2	2	2	2	2	2	2	2	2
2	2	2	2	3	3	2	3	2	2
1	1	1	1	1	2	2	1	1	2
2	2	2	1	2	2	2	2	2	2
1	2	2	1	2	1	2	3	2	9
1	1	2	1	1	1	3	3	1	3
9	9	9	9	9	9	9	9	9	9
1	1	3	1	2	1	1	3	2	1
1	3	2	3	1	2	2	3	2	2
2	3	2	1	1	9	2	9	3	2
2	2	2	2	1	3	2	3	2	2
2	2	2	2	2	2	2	2	2	2
1	1	2	2	1	2	1	1	2	2
2	2	2	2	2	2	2	2	9	9
2	2	2	3	2	2	2	2	2	9
3	3	3	3	3	3	3	3	3	3
9	3	1	1	1	1	1	3	3	3
3	3	3	3	3	3	3	3	3	3
3	3	3	3	3	3	3	3	3	3
2	2	2	2	2	2	2	2	2	2
1	2	9	3	1	1	3	3	2	1
2	2	2	2	2	2	2	2	2	2
3	1	3	2	9	9	3	9	1	9
2	2	2	3	3	2	2	2	2	2
2	2	2	2	2	2	2	2	2	2
2	2	3	2	2	3	1	2	1	3
9	3	2	1	1	3	2	1	3	1
3	1	2	3	3	3	3	3	2	9
2	3	2	2	1	2	2	2	2	2
1	2	2	2	2	2	3	2	3	2
3	3	3	3	2	3	3	3	3	3
1	2	2	2	2	2	3	2	1	2
2	1	2	3	2	1	1	3	2	2
1	3	3	2	2	1	1	3	2	2
2	1	2	3	2	1	1	9	2	2
2	2	2	2	2	2	2	2	2	2
3	3	2	2	2	2	3	2	2	3
1	2	2	1	2	2	2	2	2	3
2	3	3	3	3	3	1	3	3	3
2	2	2	2	3	3	3	3	2	3
1	1	1	1	3	3	3	3	9	1
3	3	3	3	3	3	3	3	3	3
1	1	3	1	3	3	3	2	1	2
1	2	2	2	2	1	2	1	1	2

2	1	2	3	2	2	2	1	1	1
3	3	2	1	3	1	3	1	1	9
2	2	1	1	2	2	3	2	9	2
2	2	2	2	2	2	2	2	2	2
2	1	3	3	3	2	3	9	3	2
9	9	2	9	9	9	9	9	9	9
2	2	2	2	2	2	2	2	2	2
2	2	2	2	2	2	2	2	2	2
1	1	2	1	2	3	2	2	2	2
2	2	2	2	2	2	2	3	2	2
2	1	1	1	2	1	2	2	1	1
2	2	2	2	2	2	1	3	3	2
9	9	9	9	9	9	9	9	9	9
9	9	9	9	9	9	9	9	1	9
9	9	9	1	9	9	9	9	9	9
2	2	2	1	2	2	2	2	2	3
1	2	2	2	1	1	1	1	1	2
2	2	2	3	1	2	2	3	3	1
2	2	2	1	3	3	3	3	2	2
2	2	2	2	2	2	2	2	3	3
3	3	2	2	3	2	3	3	3	3
9	1	2	3	3	3	2	2	2	2
2	1	2	2	3	3	2	2	2	2
2	2	2	2	3	2	2	2	2	2
3	3	2	1	1	3	1	3	3	1
9	1	2	9	2	2	1	9	2	1
3	3	2	1	1	3	1	3	3	1
9	1	9	9	9	9	9	9	9	9
2	2	2	2	9	9	9	9	9	9
3	1	9	2	1	3	3	3	3	3
2	2	3	2	2	2	3	3	2	3
2	2	3	1	3	3	2	2	1	2
3	3	3	3	3	3	3	3	3	2
1	1	3	1	3	2	2	2	2	1
2	2	2	2	3	3	3	3	3	3
3	2	1	2	2	3	3	2	2	3
2	1	3	3	3	3	3	3	2	2
2	3	2	2	3	3	3	3	3	3
3	3	1	1	3	3	3	3	9	3
3	3	3	3	3	3	3	2	1	2
2	1	2	3	1	1	2	2	2	3
2	2	2	3	2	2	3	2	2	1
2	2	3	3	2	3	2	2	2	2
2	1	2	1	2	2	1	2	2	2
2	2	1	2	2	2	2	2	2	2
2	2	2	2	2	2	2	2	1	2
2	2	3	3	2	1	1	1	2	2

2	2	2	2	1	1	1	1	1	2
2	2	2	2	1	3	3	3	1	2
3	1	9	2	9	9	9	9	9	9
9	3	2	2	3	1	2	3	2	3
2	3	2	1	2	2	3	9	2	2
2	1	3	2	3	1	2	2	1	3
3	2	3	3	3	3	3	2	2	2
2	3	1	2	3	2	1	2	3	3
1	3	3	3	3	3	3	3	3	3
2	2	2	2	2	2	2	2	1	2
2	2	2	2	2	2	2	2	3	2
9	9	9	9	9	9	9	9	9	9
1	2	3	1	1	2	1	3	3	2
1	2	2	2	2	2	2	2	2	1
9	9	1	9	3	2	1	2	2	2
2	1	9	1	3	3	3	9	2	9
3	3	3	3	3	3	3	3	3	3
1	3	2	1	2	2	1	2	3	1
2	3	1	2	3	1	2	2	1	1
3	1	3	3	3	3	3	3	9	3
2	2	2	2	2	2	2	2	2	2
3	3	3	3	3	3	3	3	3	3
1	1	2	2	3	1	1	3	3	3
2	1	2	3	1	3	2	2	2	2
2	2	2	2	2	2	2	2	2	2
2	1	9	2	3	2	2	2	3	2
9	2	9	9	2	9	9	9	9	9
2	3	1	1	2	3	1	3	1	2
3	2	2	2	2	2	9	9	9	9
9	1	9	9	9	9	9	9	9	9
2	9	9	9	9	9	2	9	9	9
1	1	2	1	2	1	2	2	2	2
1	1	1	1	1	1	3	3	3	3
3	3	2	2	3	2	3	3	2	2
9	9	9	9	3	1	2	3	1	3
2	3	1	2	2	2	1	1	1	1
3	2	2	2	1	2	1	1	1	1
2	1	2	2	1	2	2	3	3	3
2	2	2	2	2	1	2	1	1	2
2	2	2	2	2	2	2	3	1	2
2	9	2	9	9	9	9	9	9	9
3	3	1	3	3	3	3	3	3	3
2	2	2	1	9	2	2	2	3	2
1	3	2	3	3	3	3	2	1	2
1	3	2	3	3	3	3	3	1	2
3	2	2	3	3	3	3	3	1	2
1	2	9	2	2	3	3	3	2	2

3	2	2	2	2	2	3	2	2	2
1	3	2	3	1	3	1	1	2	1
1	9	9	9	9	9	9	9	9	9
9	1	9	9	9	9	9	9	9	9
9	1	9	9	9	9	9	9	9	9
9	9	9	9	3	3	3	9	1	1
2	1	1	3	2	2	3	2	1	1
2	2	2	2	2	2	2	2	2	2
2	2	2	2	2	2	2	2	1	2
2	1	2	3	3	2	3	3	1	1
1	1	1	1	2	2	2	9	1	2
2	2	2	2	3	3	2	2	2	2
2	2	2	2	2	2	1	2	2	1
2	3	2	2	2	2	2	2	2	2
2	2	3	3	3	3	3	3	3	3
9	9	9	9	9	9	9	9	9	9
2	9	9	3	2	9	2	2	2	3
2	1	1	3	3	2	3	3	1	1
2	3	2	2	3	2	3	3	1	3
9	9	9	9	9	9	9	9	9	9
9	9	9	1	1	9	9	9	9	9
9	9	9	9	1	3	1	3	3	3
1	1	2	1	1	2	1	2	3	3
1	2	2	1	9	9	9	9	9	9
9	2	9	9	1	1	3	9	3	3
1	9	9	9	9	9	9	9	9	9
1	2	1	3	3	3	3	3	2	1
1	2	3	3	3	3	3	3	2	1
2	2	1	1	2	3	2	3	3	3
2	2	2	2	2	2	2	3	1	2
2	2	3	3	2	2	2	2	2	2
2	1	2	2	3	2	2	2	1	2
2	2	2	2	2	2	2	2	2	2
1	3	3	3	3	3	3	3	2	2
3	3	2	3	2	2	2	2	2	2
1	3	3	3	1	3	3	3	1	1
2	2	2	2	2	2	2	2	1	2
2	1	1	1	1	1	3	3	3	3
2	2	2	2	1	3	3	3	3	2
3	3	2	2	3	2	2	2	2	2
1	2	2	2	2	1	1	2	2	3
2	2	2	2	3	2	2	2	2	1
2	2	2	2	1	1	2	2	1	1
2	9	9	9	9	9	9	9	9	9
9	9	9	9	9	9	9	2	9	9
3	3	3	1	3	3	3	3	3	3
2	2	2	2	1	1	1	1	1	2

1	1	1	1	2	2	2	2	2	2
3	2	2	2	2	1	3	3	1	2
2	1	2	3	2	1	2	2	2	2
2	2	2	2	2	2	2	2	2	2
1	2	2	3	1	1	1	2	2	2
3	1	2	1	2	3	3	2	3	2
2	2	2	2	3	2	3	9	1	2
2	3	1	2	1	2	1	2	3	1
9	9	9	9	9	9	9	9	9	9
2	1	2	1	3	2	1	1	9	1
2	2	2	2	2	2	2	2	2	2
1	2	1	1	2	2	2	3	3	1
2	3	1	2	1	2	3	1	2	3
2	2	2	2	2	2	2	2	2	2
2	2	2	2	2	2	2	2	2	2
2	3	2	3	2	1	3	1	2	3
1	2	3	1	1	2	3	1	2	3
2	1	2	3	3	2	3	1	2	3
2	1	1	1	2	1	2	2	2	2
2	2	2	2	2	2	2	2	2	2
2	2	2	2	1	1	2	1	2	2
3	3	3	3	3	3	3	3	3	3
2	2	3	3	2	2	2	3	3	2
3	3	2	3	1	2	2	3	2	2
2	2	2	2	2	2	2	2	2	2
3	3	3	3	3	3	3	3	2	2
2	2	2	2	2	2	2	2	2	2
2	1	3	1	1	1	2	3	9	2
2	2	2	2	2	2	2	1	3	9
1	1	2	2	9	9	9	9	9	9
3	1	2	2	1	1	2	1	2	2
9	9	9	9	9	9	9	9	9	9
3	3	3	3	3	3	3	3	3	3
2	1	2	1	1	1	1	1	1	1
2	1	2	3	1	2	2	2	3	2
1	2	3	2	2	1	3	1	2	2
2	1	1	3	3	2	1	2	3	9
2	2	2	2	2	3	3	2	1	2
2	2	2	2	2	2	2	2	2	2
1	2	3	1	1	2	3	3	3	2
1	3	9	2	2	3	2	3	1	2
9	1	9	9	9	9	9	9	2	9
1	2	2	2	2	2	2	2	2	2
3	2	2	2	3	2	2	2	2	2
2	2	2	2	2	2	2	2	2	2
2	2	2	2	2	2	3	3	2	2
1	2	2	2	1	2	2	1	2	1

9	9	9	9	1	1	1	3	3	9
2	2	3	3	2	1	3	3	2	2
2	2	1	2	2	1	3	3	1	3
9	1	3	3	2	3	3	3	3	3
1	2	3	3	3	2	3	3	1	2
2	2	2	2	2	2	2	2	2	2
3	3	3	1	1	3	3	3	3	3
2	3	2	3	2	2	2	2	3	1
9	2	9	3	9	2	9	3	9	9
3	3	1	3	1	2	2	2	2	3
3	3	2	3	2	3	3	3	2	2
2	1	9	3	2	3	3	2	3	2
1	3	3	2	9	9	9	9	9	9
3	3	3	2	2	3	1	3	2	2
2	2	1	3	2	3	2	3	2	2
1	3	3	3	3	1	3	3	2	1
9	9	9	9	9	9	9	9	9	9
3	2	3	2	1	3	2	1	3	1
1	2	2	3	3	1	2	2	2	2
2	2	2	2	2	2	2	2	1	2
2	1	2	1	1	2	1	2	1	2
3	3	3	3	3	3	3	3	3	3
2	2	2	3	2	2	2	2	2	2
2	2	3	3	3	3	3	3	2	2
2	2	3	1	2	2	3	2	2	3
2	2	2	2	2	2	2	2	2	2
2	2	2	2	2	2	2	2	2	2
3	3	2	2	3	2	3	3	3	2
2	3	2	2	3	1	1	2	1	2
3	2	2	1	2	2	2	2	3	1
1	1	2	2	1	2	1	1	1	1
2	2	2	2	3	3	3	3	2	2
2	2	2	2	1	2	1	2	1	2
2	2	2	2	2	2	2	2	2	1
1	2	1	2	2	2	3	2	2	3
9	9	2	9	9	9	9	9	9	2
3	3	3	3	2	3	3	3	3	3
3	2	1	2	3	3	2	3	2	2
9	9	1	9	1	3	9	2	1	2
2	2	2	2	2	2	2	2	2	2
3	3	3	3	2	3	3	3	3	3
2	2	1	3	2	2	3	3	1	3
2	2	2	2	2	2	2	2	2	2
2	2	2	2	2	2	2	2	2	2
9	9	9	9	1	1	1	2	1	2
2	1	2	3	3	2	2	3	2	3
3	3	3	3	3	3	3	9	9	9

2	2	2	2	9	9	9	9	9	9
2	2	2	2	3	3	3	3	2	3
3	3	3	3	3	3	3	3	3	3
9	9	9	9	1	3	3	3	3	3
2	2	3	3	3	2	2	2	2	2
2	1	2	2	2	1	2	3	2	2
2	2	2	2	2	2	2	2	2	2
3	1	3	1	2	2	2	2	2	9
3	3	3	3	3	3	3	3	3	3
3	3	3	3	1	2	3	3	3	3
1	3	2	2	3	3	3	3	3	3
2	3	9	3	3	1	2	3	1	2
1	2	3	3	3	1	2	2	2	2
2	1	3	2	2	3	3	9	2	2
2	2	2	2	3	3	3	3	2	3
1	1	1	1	1	2	2	2	1	3
9	9	2	9	9	9	9	9	1	9
9	9	9	9	2	9	9	9	9	9
9	9	9	9	3	3	3	3	3	3
9	9	9	9	2	9	9	9	9	9
2	2	2	2	3	3	3	9	2	3
1	1	1	1	1	2	2	1	1	2
2	3	3	2	3	2	3	2	3	2
3	3	3	3	3	3	3	3	3	3
2	3	2	2	1	2	2	1	2	1
9	9	9	1	9	9	9	9	9	9
9	9	9	9	9	9	9	9	9	9
2	2	3	3	2	2	2	2	2	3
9	9	9	9	9	9	9	9	9	9
9	9	9	9	9	9	9	9	9	9
9	9	9	9	9	9	9	9	9	9
9	9	9	9	9	9	9	9	9	9
9	9	9	9	9	9	9	9	9	9
1	2	2	2	1	2	2	2	3	1
3	2	3	1	2	1	3	2	3	1
1	2	2	2	2	9	9	9	9	9
9	9	9	9	9	9	9	9	9	9
9	9	9	9	3	3	3	3	3	2
1	1	9	1	1	9	1	9	1	9
1	1	1	1	2	2	2	2	2	2
2	2	2	2	2	2	2	2	2	2
1	2	1	2	3	2	2	2	3	1
1	2	2	2	2	2	2	2	1	2
2	2	2	2	2	2	2	2	2	2
2	1	2	1	2	1	1	2	2	1
2	3	2	2	1	2	1	3	2	2
1	2	3	2	1	2	2	3	1	2

2	2	2	2	2	2	2	2	2	2
2	2	2	2	2	2	2	2	2	2
2	2	2	2	2	2	3	3	2	2
2	2	3	1	1	2	3	1	2	3
2	3	1	1	1	1	1	2	2	2
1	3	1	1	1	1	2	3	9	3
2	9	1	3	3	1	2	9	2	2
2	2	2	2	2	2	2	2	2	2
2	2	2	2	2	2	2	2	2	2
2	2	2	2	9	9	9	9	1	3
3	1	2	1	3	2	2	2	2	2
3	3	2	2	1	2	1	2	2	2
1	2	3	1	1	2	3	3	9	1
1	2	2	2	2	1	3	9	2	1
3	3	3	3	1	2	2	2	1	2
1	1	1	1	3	3	3	3	3	1
1	2	2	3	2	2	3	3	1	1
3	2	2	2	9	9	9	9	9	9
2	2	2	2	2	3	2	9	2	1
2	3	2	2	2	2	2	3	3	1
9	9	9	2	9	9	9	9	9	1
3	3	3	3	3	2	3	3	9	2
1	1	1	1	1	3	3	3	3	3
9	9	9	9	3	3	1	1	1	1
2	9	9	9	1	9	9	9	9	9
2	9	9	9	9	2	9	9	9	9
1	2	2	3	1	2	2	1	3	2
3	2	2	1	3	2	2	3	3	3
9	9	9	9	9	9	9	9	9	9
2	2	3	2	1	2	2	2	3	3
3	1	3	1	1	3	1	3	9	1
1	3	3	3	3	3	3	3	3	3
3	1	2	1	1	2	2	2	1	1
1	2	2	2	2	3	3	3	2	1
1	2	2	2	1	3	3	2	3	2
2	2	2	2	2	2	2	2	2	2
1	1	1	3	3	3	1	3	2	1
2	2	2	1	2	2	3	3	2	3
2	3	1	2	3	2	2	2	3	2
1	2	1	3	3	2	3	9	9	9
9	9	9	9	2	9	1	3	9	2
2	2	3	2	2	2	1	2	3	2
3	3	3	3	2	2	2	2	2	2
2	2	2	1	2	3	3	3	2	2
9	1	9	9	3	2	1	3	1	2
1	1	1	1	1	1	1	1	1	1
2	2	2	2	3	3	2	2	3	3

2	3	9	1	3	1	3	3	3	1
2	3	2	3	3	2	3	2	2	2
1	2	2	2	2	2	3	3	2	2
1	1	2	2	3	2	2	2	2	2
2	2	2	2	2	2	2	3	3	3
9	9	9	9	2	2	2	3	3	3
1	2	3	2	2	3	2	1	9	1
2	2	2	2	2	2	3	9	1	2
2	2	2	2	3	2	2	3	1	2
2	2	1	1	2	2	3	2	2	2
1	1	2	3	3	3	3	3	1	2
1	3	1	1	1	3	2	1	2	2
9	9	9	9	9	9	9	9	1	9
2	2	3	1	1	3	2	1	2	1
1	1	2	1	3	2	2	3	3	2
1	3	3	3	1	3	3	3	3	3
3	1	3	3	3	3	3	1	3	3
2	2	2	1	2	2	2	2	2	2
2	3	2	3	3	2	3	9	2	2
1	1	1	1	1	2	2	2	2	9
2	2	1	2	2	2	2	2	1	2
3	2	2	2	2	2	2	2	2	2
1	1	1	1	1	3	1	1	1	1
1	1	2	1	2	2	2	1	2	1
1	1	1	1	1	1	1	1	1	1
9	9	9	9	1	1	1	1	1	1
3	3	3	2	2	2	3	3	2	2
2	3	3	3	3	3	3	3	3	3
3	3	3	3	3	3	3	3	3	3
2	2	2	2	2	2	3	2	3	2
1	2	1	2	1	2	3	3	3	3
9	9	9	9	9	9	9	9	9	9
2	2	2	2	2	2	2	2	2	2
2	1	3	2	1	3	2	9	1	3
1	2	1	2	2	1	2	1	1	2
3	3	1	3	1	3	3	1	3	1
1	2	2	2	1	2	2	2	2	2
3	3	3	3	2	3	2	2	3	1
1	1	2	3	1	3	3	1	1	2
2	2	2	2	2	2	2	2	2	2
9	9	1	2	3	2	2	2	3	2
3	2	2	2	2	2	2	2	3	3
3	1	2	2	2	2	3	2	3	2
9	9	9	9	1	3	3	1	2	2
3	3	1	3	3	3	3	3	3	2
2	2	2	2	1	2	2	2	1	3
2	2	2	2	1	2	2	2	2	2

2	1	3	3	3	2	2	1	2	2
9	9	1	9	9	9	9	9	9	9
9	9	9	9	9	9	9	9	9	9
2	2	3	3	3	2	2	2	3	2
3	3	1	3	9	9	9	1	9	9
9	9	9	1	9	9	9	9	1	9
9	9	1	9	9	9	9	9	1	9
1	1	3	1	1	9	2	2	2	2
1	2	2	3	1	3	3	1	1	2
2	2	2	2	3	3	2	3	2	2
2	2	1	2	2	2	2	2	2	2
1	2	3	1	2	2	2	2	2	2
2	2	2	2	1	2	2	2	2	2
2	2	2	2	1	2	2	2	2	2
2	2	2	2	1	2	2	2	2	2
2	2	2	2	2	2	2	2	2	2
2	3	3	3	2	2	2	2	2	3
2	2	2	2	2	2	2	2	2	1
2	2	2	2	2	2	2	2	2	2
2	3	1	2	2	2	2	3	3	2
2	1	2	2	2	2	2	2	2	2
1	1	1	1	1	2	1	1	1	1
1	9	9	9	9	9	9	1	9	9
2	2	2	2	2	2	2	2	2	2
2	1	2	2	3	9	2	1	2	2
2	2	1	3	2	1	1	1	1	1
3	2	2	2	1	2	2	2	3	3
1	2	3	9	2	2	1	3	3	2
2	3	3	2	2	2	3	3	2	1
2	2	2	2	2	2	3	1	1	1
2	2	2	2	2	2	2	2	2	2
1	2	9	2	2	2	2	3	2	2
2	2	2	2	1	2	2	3	1	2
1	1	2	3	1	3	2	3	1	1
2	3	3	3	3	3	3	3	3	3
2	9	9	9	2	2	2	3	3	3
2	1	3	2	2	2	2	2	2	2
2	2	2	2	2	2	2	2	2	2
2	2	2	2	2	2	2	2	2	2
9	9	1	9	2	3	3	2	9	2
9	1	9	9	2	2	2	2	2	2
2	3	2	1	1	2	3	2	2	1
1	2	2	2	2	1	3	3	3	3
1	2	3	2	1	1	2	2	1	2
1	2	1	1	3	2	2	3	2	2
2	2	2	2	2	2	2	2	2	2
2	2	2	2	2	2	2	2	2	2

2	2	2	2	1	1	2	2	1	2
9	9	9	1	1	9	3	9	2	9
1	9	9	9	2	2	2	2	2	2
1	3	2	2	2	2	2	2	3	3
2	1	1	1	3	1	3	3	3	3
2	2	2	2	3	2	3	2	1	9
3	3	3	3	3	3	3	3	3	3
2	2	1	1	2	2	3	3	2	2
3	1	3	2	2	9	2	9	2	9
2	2	2	2	2	2	2	2	2	2
2	1	3	3	2	2	2	2	2	2
1	2	2	3	2	2	2	2	2	2
2	2	2	2	2	2	2	2	2	2
2	2	1	2	1	3	9	1	1	1
3	3	3	3	3	3	2	3	2	2
1	2	2	2	2	2	2	2	2	2
2	1	3	3	2	2	2	2	2	2
9	9	9	9	9	9	9	9	9	9
1	2	2	2	2	2	2	2	2	2
1	1	1	1	1	1	1	2	1	1
2	2	1	1	1	3	3	3	1	1
2	2	2	2	2	2	3	3	2	3
1	3	2	3	2	3	3	1	2	2
2	2	2	2	2	2	2	1	2	2
2	1	2	1	3	3	1	1	1	2
2	2	3	3	1	2	2	3	2	1
2	2	2	2	2	2	2	2	2	2
3	1	2	2	3	2	3	3	3	2
2	1	2	3	3	2	2	2	1	2
2	3	1	1	3	2	3	3	3	2
1	3	2	1	2	3	2	2	2	2
2	2	2	2	1	3	3	3	1	2
1	2	3	2	3	3	2	1	2	3
1	3	2	1	9	9	9	9	9	9
3	1	3	3	1	3	3	3	1	1
3	1	2	1	3	2	1	2	3	3
1	3	2	3	1	2	2	3	3	2
2	2	2	2	2	2	2	3	1	1
1	1	2	9	2	2	2	2	2	2
1	2	2	2	1	2	3	3	3	2
3	2	1	2	9	9	9	9	9	9
1	3	3	3	3	2	1	3	2	1
9	9	9	9	9	9	9	9	9	9
3	1	2	3	1	3	3	3	3	2
2	2	2	2	2	1	1	2	2	1
1	2	3	2	3	3	3	3	2	2
9	2	2	2	3	2	2	2	3	2

2	3	3	3	3	3	3	3	1	2
3	1	3	3	9	9	9	9	9	9
2	2	2	2	3	3	3	2	2	2
2	2	2	2	2	2	2	3	1	2
3	3	3	3	2	2	2	2	2	2
1	2	1	2	2	1	2	1	2	1
2	3	1	2	2	1	1	3	2	1
1	1	1	1	1	1	1	1	1	1
1	1	1	1	2	2	2	2	2	2
2	1	1	2	2	9	9	9	9	9
2	2	2	2	2	2	2	2	2	2
2	2	2	2	2	2	2	2	2	2
9	9	9	9	3	3	3	1	3	3
9	9	9	9	3	3	3	1	2	2
2	3	1	3	2	1	3	3	3	2
2	3	1	2	3	2	2	2	1	2
2	2	2	2	3	3	3	3	3	9
3	2	3	3	3	3	3	3	2	2
1	1	1	2	3	3	3	3	9	2
1	1	1	1	1	1	1	1	1	1
1	2	2	2	2	2	2	2	2	2
9	3	9	9	9	9	3	9	9	9
9	9	9	9	3	2	3	3	3	2
2	3	2	1	3	1	2	3	1	2
9	9	9	9	1	2	3	1	2	3
9	1	9	9	9	9	9	9	1	9
2	2	2	2	2	2	2	2	2	2
9	1	9	9	1	1	9	9	9	9
2	2	2	3	3	1	3	3	3	1
3	1	3	3	1	2	1	3	1	2
3	1	2	1	2	2	2	3	3	3
1	9	9	9	9	9	9	9	9	2
9	9	9	9	2	3	3	3	1	2
3	2	2	1	3	2	3	2	2	1
3	2	2	3	3	3	1	3	3	2
3	2	2	3	3	3	1	3	2	3
2	2	2	1	2	2	2	2	1	1
9	9	9	1	9	9	9	9	9	9
3	2	2	2	2	3	3	3	3	2
2	3	1	2	2	2	2	9	1	9
2	2	2	2	2	3	2	2	1	2
2	1	2	2	2	2	1	9	2	9
9	1	9	9	3	3	1	9	2	9
9	9	9	9	9	9	9	9	9	9
9	9	9	9	1	2	2	2	2	2
9	9	9	9	1	1	2	2	1	2
9	9	9	9	9	9	9	9	9	9

1	3	2	2	2	3	3	3	3	3
9	9	9	9	9	1	9	9	9	9
2	1	2	3	2	2	1	3	1	2
2	3	2	1	2	3	2	3	2	1
2	1	1	2	3	3	3	3	1	2
2	2	2	2	2	2	2	2	2	2
1	2	3	1	1	2	1	3	2	1
1	1	1	1	2	2	2	2	2	2
3	3	3	3	2	2	2	2	2	2
9	9	9	9	9	9	9	9	9	9
1	1	1	1	3	3	2	3	1	2
1	2	2	1	1	2	2	1	2	3
3	1	3	1	1	3	1	3	1	3
1	2	2	1	1	2	1	2	1	3
2	2	2	2	2	2	2	2	2	2
1	2	1	1	1	2	1	1	1	1
9	1	1	1	1	1	1	1	1	1
3	1	2	2	2	1	2	2	3	3
2	2	2	3	1	2	2	2	1	2
1	1	2	2	1	2	2	2	1	2
9	9	9	9	9	9	9	9	9	9
2	2	3	3	2	2	1	1	2	2
2	2	2	2	1	1	2	2	2	2
2	2	3	2	1	2	2	2	2	1
1	2	2	2	1	1	2	2	2	2
2	2	2	2	1	1	2	2	2	2
2	2	2	2	2	2	2	2	2	2
3	3	3	3	2	2	2	3	3	3
2	2	2	2	1	2	2	3	2	2
2	2	2	2	1	2	2	2	2	2
9	9	9	9	9	9	9	9	9	9
9	9	9	9	9	9	9	9	9	9
9	9	9	9	9	9	9	9	9	9
2	1	1	1	3	3	3	3	2	9
3	1	3	3	3	3	3	3	3	9
1	2	2	2	2	2	2	2	2	2
2	2	1	1	1	2	2	2	1	2
3	1	1	1	3	2	3	3	2	2
2	1	1	1	3	3	2	2	1	1
2	2	2	2	2	3	3	3	2	2
3	2	3	3	3	3	3	3	3	3
3	1	2	2	2	3	3	2	2	2
2	2	2	2	2	2	2	2	2	2
1	3	3	3	3	3	1	3	2	2
2	3	1	1	2	2	2	2	2	2
3	9	1	1	3	3	3	3	3	3
9	9	9	9	3	3	3	3	2	1

2	2	2	2	3	3	3	3	2	2
2	2	2	2	2	2	2	9	2	2
2	3	2	3	3	3	3	3	2	2
1	2	1	3	1	3	3	1	1	1
3	3	3	3	3	3	3	3	3	3
9	9	9	9	3	2	2	2	1	2
2	2	3	1	2	2	2	2	1	2
9	9	9	1	1	9	9	9	9	9
9	9	9	9	9	9	9	9	1	9
9	9	9	9	9	9	9	9	9	9
9	9	1	9	1	9	9	9	9	9
1	9	9	9	9	1	9	9	9	9
1	2	2	2	1	2	2	2	2	2
3	1	9	2	9	9	9	9	9	9
2	2	3	2	2	2	3	3	2	3
2	3	1	1	2	2	2	2	2	2
1	2	2	1	1	1	1	2	2	2
1	2	2	1	1	1	1	2	2	2
2	2	2	2	3	2	3	3	1	2
1	3	2	1	2	3	3	3	3	1
2	2	2	2	2	2	2	2	1	2
2	3	2	2	2	3	2	2	3	2
3	1	2	1	9	9	9	9	1	9
9	9	9	9	3	3	3	3	3	3
1	1	1	1	1	3	2	3	2	1
1	1	1	1	1	1	3	2	1	2
2	2	2	2	3	3	2	3	2	2
2	2	2	2	3	3	2	3	2	2
2	2	2	2	2	2	2	2	2	2
2	1	2	2	1	2	2	2	2	2
2	2	2	2	1	2	2	2	2	2
1	3	2	1	2	3	3	3	3	1
2	2	1	2	3	3	3	3	2	2
1	1	1	2	1	1	2	1	1	2
9	9	9	9	2	9	2	9	2	9
2	3	3	1	3	3	3	3	2	2
2	3	3	1	2	3	3	3	3	2
1	2	3	1	3	3	3	3	3	2
1	2	2	2	2	2	2	3	2	2
2	2	2	1	2	2	2	2	3	2
9	1	9	9	1	9	9	9	9	9
9	9	9	2	9	9	9	9	9	9
9	9	9	1	9	9	9	9	9	9
2	2	2	2	3	3	2	2	2	2
9	9	9	9	9	9	9	9	9	1
1	9	9	9	2	2	2	2	2	2
2	1	2	2	3	2	1	3	2	1

2	2	2	3	2	2	1	1	2	2
1	2	2	2	2	2	2	2	3	2
2	1	9	3	3	1	2	3	1	2
2	3	2	1	3	2	3	2	3	1
3	2	3	3	2	3	2	2	3	3
3	1	3	3	2	2	2	2	2	2
3	3	1	3	1	3	1	3	2	3
3	3	2	1	3	2	2	2	3	3
3	3	1	3	3	3	3	3	3	1
1	2	1	1	1	2	3	1	2	2
2	2	2	2	2	3	3	3	3	2
2	1	2	1	2	2	1	2	2	3
3	2	1	3	3	3	1	1	2	3
2	2	9	2	3	3	3	3	2	2
2	1	2	1	1	2	3	1	1	2
1	2	2	1	2	2	2	2	2	2
2	3	2	2	3	1	3	1	1	2
2	2	2	2	2	2	2	2	2	2
2	2	2	2	1	2	2	2	1	2
1	2	3	2	3	3	2	3	2	2
2	2	2	3	1	1	2	1	1	2
1	2	2	2	2	3	3	3	2	2
2	2	2	2	1	2	2	2	1	2
2	1	2	3	2	2	3	3	2	2
1	1	2	2	3	3	3	3	3	9
2	2	2	2	3	3	3	3	2	2
2	2	2	2	2	2	2	2	2	2
1	1	1	1	1	3	3	3	1	3
1	2	2	1	2	2	2	2	2	2
1	2	2	2	2	2	1	1	2	2
1	2	2	2	2	1	1	2	2	2
1	2	3	3	1	2	3	3	3	3
9	9	9	9	1	1	2	2	2	2
1	2	2	2	3	3	2	2	3	3
1	2	3	1	1	1	2	3	2	1
2	2	2	2	2	2	2	2	2	2
1	2	2	2	1	1	2	2	2	3
2	2	2	2	2	2	2	2	2	2
3	1	2	9	1	3	9	3	1	3
1	1	1	1	2	2	2	2	2	2
2	1	1	1	2	1	2	1	2	1
2	1	1	1	3	3	9	2	1	2
2	2	2	2	3	3	3	3	1	2
2	1	2	3	3	3	3	3	1	3
1	9	9	9	2	1	2	1	3	3
3	1	2	1	2	2	2	2	2	2
3	2	2	3	3	3	1	3	3	2

3	2	2	3	3	3	1	3	3	2
2	2	2	2	1	1	2	2	2	2
2	2	2	2	2	1	2	2	2	2
2	2	2	2	1	2	2	1	2	2
1	2	2	2	1	1	2	2	2	2
2	2	2	3	1	2	2	1	3	2
2	2	3	3	1	2	2	3	2	2
2	2	2	2	2	2	2	2	2	2
1	3	3	3	3	3	3	2	1	3
1	2	1	2	1	1	2	1	3	3
3	1	3	3	1	3	3	3	1	1
2	2	2	3	3	2	2	2	3	3
1	1	1	1	2	2	2	2	2	2
1	2	2	2	1	2	2	2	2	2
9	9	9	9	3	3	3	3	3	3
2	1	2	2	9	9	9	2	1	1
1	2	2	2	3	1	3	3	3	3
2	2	2	2	2	2	2	3	2	2
1	1	2	2	2	2	2	2	9	2
2	2	2	2	3	3	3	3	3	2
2	2	1	2	2	2	1	3	1	1
3	3	3	3	1	1	3	1	3	2
3	2	2	3	3	2	2	2	2	3
2	2	3	3	3	2	2	3	2	2
2	9	2	3	1	2	1	1	2	1
1	1	2	1	1	3	3	3	3	2
1	2	1	1	2	1	2	1	1	1
2	2	2	1	2	3	2	3	2	2
2	3	3	3	2	1	1	2	2	2
2	2	2	2	3	2	3	3	3	2
3	3	3	3	1	2	2	2	3	3
2	2	2	2	1	3	3	3	3	1
2	3	2	3	2	2	3	1	2	3
1	2	2	1	1	2	1	3	3	2
2	1	2	2	2	2	2	2	1	2
2	2	2	2	3	1	3	3	2	2
1	2	1	1	1	3	2	2	1	2
1	2	3	2	1	1	2	1	3	3
1	1	1	2	1	2	1	2	3	2
2	2	1	2	3	3	3	2	1	2
1	2	2	2	3	3	3	3	3	2
1	2	2	3	3	3	3	3	2	1
2	3	2	2	2	2	2	2	1	2
1	2	2	1	1	2	3	1	2	2
2	2	2	2	3	3	2	3	3	3
3	2	2	3	2	1	2	1	2	9
2	2	2	2	3	2	2	2	2	2

2	2	3	2	3	2	2	2	3	3
1	2	2	2	2	2	2	3	3	2
1	3	3	1	1	2	2	2	2	3
1	3	3	3	3	3	3	3	3	2
1	1	2	3	3	3	2	3	2	1
2	2	2	2	2	2	2	2	2	2
3	2	3	2	2	3	2	2	2	2
2	1	1	1	2	1	1	1	3	3
1	1	9	2	2	3	2	9	1	3
2	2	2	2	3	1	3	3	3	3
3	1	2	3	3	2	3	3	2	3
1	2	2	2	3	3	3	3	3	2
1	1	2	2	3	2	3	1	3	2
3	3	3	3	3	2	2	2	3	2
2	2	2	2	3	3	3	3	3	3
2	2	2	2	3	2	3	3	1	2
2	2	2	2	2	2	2	2	2	2
3	3	1	3	1	3	3	3	3	1
2	3	2	1	1	2	3	2	1	2
3	2	2	2	2	2	2	2	2	2
2	2	2	2	1	2	2	2	1	1
2	2	2	3	1	2	2	3	1	2
2	2	3	3	1	2	2	2	1	2
2	2	2	2	2	2	2	2	3	2
2	2	2	2	2	2	2	2	3	2
2	2	2	2	3	2	3	3	1	2
1	2	2	2	3	3	3	3	3	2
2	2	2	2	3	1	3	3	3	3
1	1	1	2	1	2	1	2	3	2
3	2	2	2	2	2	2	2	2	2
1	2	1	1	1	3	2	2	1	2
9	9	9	9	9	9	9	9	9	9
2	2	2	2	1	1	2	2	1	2
2	2	1	2	2	2	2	2	2	2
1	1	2	2	1	1	3	3	3	3
2	1	2	3	3	1	3	3	2	3
1	1	1	1	1	1	3	3	3	3
2	3	1	2	1	2	3	1	2	3
2	2	2	2	2	2	2	2	2	2
2	2	2	2	2	1	3	2	1	2
1	1	1	1	2	2	2	3	3	1
1	1	1	1	2	2	2	3	3	1
3	2	1	1	3	2	3	3	9	1
3	9	9	3	3	3	9	1	2	9
1	2	3	2	2	1	1	2	3	2
2	2	2	2	3	3	1	9	3	2
3	1	2	2	3	2	2	2	3	2

3	1	2	2	2	1	9	3	1	2
3	3	1	1	3	3	3	3	1	3
1	2	1	1	1	1	1	1	9	1
2	2	2	2	3	3	3	3	2	1
3	2	2	2	3	3	3	2	2	2
1	2	2	2	2	2	2	2	2	2
1	2	2	3	2	2	9	1	1	1
9	9	9	9	3	9	9	9	9	9
9	9	9	9	2	9	9	9	9	9
9	9	9	9	3	9	9	9	9	9
1	1	2	2	2	2	3	1	2	2
2	2	1	1	2	1	3	1	2	1
2	3	2	1	1	2	2	3	3	3
2	3	2	1	1	2	2	3	3	3
2	3	2	1	1	2	2	3	3	3
3	1	3	3	2	2	2	2	2	2
2	2	2	3	1	3	1	2	2	2
1	2	1	2	3	3	2	1	1	2
2	3	2	3	2	1	2	3	1	1
2	1	1	3	3	2	3	3	2	3
1	2	1	3	3	2	1	1	2	1
3	3	2	3	2	1	2	1	3	2
2	2	2	2	2	2	2	2	2	2
2	2	2	2	1	3	1	2	2	2
2	2	2	1	3	1	3	2	2	2
2	3	1	3	2	2	2	2	3	3
2	2	3	3	1	3	1	3	2	2
2	2	2	2	2	2	1	1	3	3
2	2	1	2	3	2	2	2	2	2
2	2	1	2	3	2	2	2	2	2
2	3	2	1	1	2	2	3	3	3
2	3	2	1	1	2	2	3	3	3
3	1	3	3	2	2	2	2	2	2
2	2	2	2	1	3	1	3	3	3
3	1	3	3	1	3	1	3	3	3
2	2	2	2	2	2	2	2	2	2
3	1	3	3	2	2	2	2	2	2
2	2	2	2	1	3	1	3	3	3
2	2	3	3	2	2	2	2	3	3
9	9	9	9	1	9	9	9	9	9
1	3	2	2	2	2	2	1	1	1
2	2	1	1	2	2	2	3	3	3
2	2	1	1	2	2	2	3	3	3
2	2	1	1	2	2	2	3	3	3
2	2	1	1	2	2	2	3	3	3
9	9	1	9	3	2	3	3	9	2
2	1	1	3	3	3	3	9	1	2

3	1	2	3	2	3	3	2	3	2
1	2	2	2	3	1	3	2	2	2
1	3	1	2	2	1	1	3	2	2
2	2	1	1	3	2	2	2	1	2
2	3	3	2	3	2	3	3	2	1
1	2	3	1	3	2	2	9	1	2
9	3	9	9	3	2	2	9	1	2
1	3	1	1	1	3	1	3	3	3
9	3	9	9	9	9	9	9	1	9
9	9	9	9	9	9	9	9	9	9
3	1	3	3	1	3	1	3	3	3
9	2	2	2	3	9	9	1	9	1
9	2	2	2	3	2	9	1	9	1
1	3	1	1	3	1	3	1	1	1
1	2	1	1	2	2	2	2	2	2
2	2	2	2	2	9	2	9	2	2
3	9	2	2	3	2	2	2	2	2
9	9	9	9	2	9	9	9	9	9
9	9	9	9	9	9	3	9	9	9
9	3	9	9	9	9	9	9	9	9
1	2	1	2	1	3	2	2	2	2
1	2	2	2	2	2	2	2	2	2
1	3	1	1	3	1	3	1	1	1
2	2	2	2	2	2	2	1	2	3
2	9	9	9	9	9	9	1	9	9
9	9	9	9	2	9	9	9	9	9
2	2	2	2	1	3	1	2	2	2
9	9	9	9	2	9	9	9	9	9
2	2	2	2	2	2	3	2	2	2
2	9	9	9	9	9	9	9	9	9
3	3	2	1	3	3	3	1	3	1
1	9	9	9	3	9	9	9	9	9
1	2	1	2	3	1	3	1	1	1
2	2	2	2	2	1	3	1	1	1
1	3	1	1	3	1	3	1	1	1
2	2	2	2	3	1	3	1	1	1
2	2	2	2	2	2	2	2	2	2
2	2	2	2	2	2	2	2	1	1
1	2	2	2	3	1	3	1	1	1
1	9	9	9	3	9	9	9	9	9
1	9	9	9	3	9	9	9	9	9
1	9	9	9	3	9	9	9	9	9
9	9	9	9	3	9	9	9	9	9
1	9	9	9	3	9	9	9	9	9
9	9	9	9	2	9	9	9	9	9
1	2	2	1	3	2	1	1	1	2
3	3	2	3	3	2	3	2	3	1

9	9	9	9	9	9	9	9	9	9
2	2	9	9	2	2	2	2	2	2
9	2	9	9	9	9	9	9	2	9
2	2	2	2	2	2	2	2	1	1
9	3	9	9	9	9	3	9	1	1
1	1	2	2	3	2	2	1	2	3
9	9	9	9	2	9	9	9	9	9
3	1	3	3	1	3	1	3	3	3
2	2	2	2	2	2	2	2	2	2
9	3	9	9	3	9	9	9	9	9
1	2	9	2	1	1	3	2	1	9
1	9	9	9	3	9	9	9	9	9
1	9	9	9	3	9	9	9	9	9
1	9	9	9	9	9	9	9	9	9
1	3	1	1	2	2	2	1	1	1
1	9	9	9	3	9	9	9	9	9
2	2	1	2	1	2	1	2	2	2
1	9	9	9	3	9	9	9	9	9
2	2	2	2	2	2	2	2	2	2
1	3	1	1	3	1	3	2	2	2
1	3	1	1	3	2	3	3	1	3
2	3	2	1	3	2	1	1	2	1
1	3	1	1	3	1	3	1	1	1
3	1	3	3	1	3	1	1	1	1
1	3	2	2	3	2	2	1	3	2
1	2	2	3	3	2	1	1	1	2
2	2	2	2	3	1	3	2	2	2
2	2	2	3	3	1	2	2	2	2
2	2	2	2	2	2	1	3	2	1
2	2	2	3	3	3	2	2	1	1
2	2	2	3	3	2	1	2	1	2
2	1	2	1	3	2	1	2	1	2
9	2	9	9	9	9	9	9	9	1
9	9	9	9	9	1	9	9	9	9
1	9	9	9	9	9	9	9	9	9
9	3	9	9	9	9	3	9	9	9
9	2	9	9	9	9	9	9	9	1
9	9	9	1	9	9	9	9	9	2
9	3	9	9	9	9	9	2	9	9
9	9	9	9	2	9	9	9	9	9
9	9	9	9	2	9	9	9	9	9
9	9	9	9	2	9	9	9	9	9
9	9	9	9	2	9	9	9	9	9
9	9	9	9	2	9	9	9	9	9
9	9	9	9	2	9	9	9	9	9
9	3	9	9	9	9	9	9	9	9
9	3	9	9	9	9	9	9	9	1

1	2	1	1	3	1	2	2	2	1
3	1	3	3	1	3	2	3	9	9
9	2	9	9	9	9	9	1	9	9
3	2	2	1	2	1	1	2	1	2
9	9	9	9	3	9	9	9	9	9
9	9	9	9	3	9	9	9	9	9
9	9	9	9	3	9	9	9	9	9
9	9	9	9	3	9	9	9	9	9
9	9	9	9	3	9	9	9	9	9
2	2	2	2	2	2	2	2	2	2
2	3	2	1	3	2	3	2	1	2
2	3	2	1	2	1	2	1	2	1
2	2	2	2	2	2	2	2	2	2
1	2	2	3	3	1	2	2	3	3
1	2	2	3	3	1	2	2	3	2
1	2	2	2	1	2	2	2	3	1
9	9	9	9	3	9	9	9	9	9
9	9	9	9	2	9	9	9	9	9
9	9	9	9	3	9	9	9	9	9
9	9	9	9	2	9	9	9	9	9
9	2	9	9	9	1	9	9	9	9
9	9	9	9	2	9	9	9	9	9
1	1	9	1	9	1	1	9	2	2
9	1	3	9	1	3	9	9	9	3
9	9	9	9	9	2	9	9	2	9
9	9	2	9	9	9	9	2	9	9
9	9	9	9	9	9	9	9	9	9
2	3	2	3	2	2	2	2	1	3
2	3	3	3	1	2	3	2	2	3
9	9	9	1	9	9	9	9	9	9
2	2	2	2	2	2	2	2	2	2
3	3	1	1	3	1	2	1	2	1
2	2	2	2	2	2	2	2	2	2
9	9	9	9	9	9	9	9	9	1
2	2	2	2	2	2	2	2	1	2
2	2	2	2	2	2	2	2	2	2
9	9	9	9	9	9	9	2	9	9
9	3	9	1	2	1	2	2	9	1
9	9	9	9	9	9	9	9	9	9
3	1	2	3	1	2	3	2	3	2
3	1	3	3	2	2	2	2	3	2
2	2	2	3	2	2	2	1	1	2
1	1	2	1	3	3	1	2	1	2
2	9	9	9	9	2	9	9	9	9
9	9	9	9	9	9	9	9	9	9
2	2	2	2	2	2	2	2	2	2
9	9	9	9	9	9	9	9	9	9

1	3	2	1	3	2	1	2	1	2
2	2	2	2	2	2	2	1	2	2
2	2	2	2	2	2	2	2	1	2
2	2	2	2	2	2	2	1	1	2
2	2	2	2	2	2	3	2	3	2
1	2	2	3	2	2	3	3	2	3
1	3	1	3	1	3	3	3	1	3
1	3	1	1	3	2	3	2	1	2
1	9	9	9	9	9	9	9	9	9
2	2	2	2	2	2	2	2	9	9
2	2	2	2	2	2	2	2	2	2
3	1	3	3	1	3	1	1	1	1
1	1	3	1	3	1	3	1	3	1
2	2	2	2	1	3	2	2	2	2
1	2	2	1	3	1	2	2	2	1
2	2	3	1	2	2	2	2	1	2
2	2	2	2	2	2	2	2	2	2
2	2	2	2	2	2	2	2	2	2
1	2	3	3	3	2	2	2	2	2
2	2	2	2	3	2	3	2	1	2
2	2	2	2	2	2	2	2	2	2
2	2	2	2	1	3	2	2	2	2
9	9	9	9	2	2	1	3	3	2
2	2	2	2	2	2	1	2	2	2
2	2	2	2	3	3	2	2	1	1
9	9	9	9	2	2	2	2	1	2
1	2	2	2	3	3	1	2	2	1
2	2	2	2	2	2	2	2	1	1
2	2	2	2	2	2	2	3	2	2
2	2	2	2	2	2	2	2	2	2
2	2	2	2	2	2	2	2	2	2
2	2	2	2	2	2	2	2	2	2
2	2	2	2	2	2	2	2	2	2
2	2	2	2	2	2	2	2	2	2
2	2	2	2	2	2	2	2	2	2
2	2	2	2	2	2	2	2	2	2
3	1	3	3	2	2	2	2	2	2
9	9	2	9	9	9	9	9	9	9
1	3	1	1	3	2	3	1	1	2
1	9	2	9	1	2	1	2	3	3
1	2	2	1	2	1	2	2	1	3
1	9	9	9	9	9	9	9	9	9
3	1	3	3	3	1	3	1	1	1
2	2	2	2	2	3	1	3	2	3
2	1	1	1	3	2	2	1	2	3
2	2	1	1	1	2	2	1	1	1
2	2	2	2	1	3	1	3	2	2

9	9	9	9	9	9	9	9	9	9
2	2	2	2	2	2	2	2	2	2
2	3	2	3	3	2	1	2	1	2
2	2	2	2	2	2	3	1	2	2
2	2	2	2	2	2	2	2	2	2
1	9	9	9	9	9	9	9	9	9
2	2	2	2	2	2	2	2	2	2
2	2	2	2	3	2	2	2	1	1
2	2	2	2	1	3	2	2	2	2
2	1	2	2	2	2	1	3	2	3
3	2	2	2	2	3	1	3	2	2
2	2	2	2	2	2	2	2	2	2
1	3	3	1	3	1	2	1	2	1
3	1	3	3	1	3	3	3	3	2
9	9	9	9	3	2	1	2	2	2
2	2	2	2	2	2	2	2	2	2
2	2	2	2	2	2	2	2	2	2
9	9	3	9	9	3	9	9	9	9
2	2	2	2	2	2	2	2	2	2
2	2	2	2	2	2	2	2	3	2
2	2	2	2	2	2	2	2	2	2
3	3	2	3	2	2	1	2	2	1
2	2	2	2	2	2	2	2	2	2
3	3	2	2	1	3	1	3	3	3
9	9	2	9	9	9	9	9	9	9
2	2	2	2	2	2	2	2	2	2
2	2	2	2	2	2	2	2	2	2
2	2	2	2	2	2	2	2	2	2
9	9	9	9	1	2	3	2	1	1
2	2	2	2	1	3	2	2	3	3
2	3	2	1	3	3	3	2	1	1
9	9	9	9	9	9	9	9	9	9
2	2	2	2	2	2	2	2	2	2
2	2	2	2	2	2	2	2	2	2
2	2	2	2	2	2	1	2	3	2
2	2	2	2	2	2	2	2	1	2
3	2	2	1	3	3	2	2	2	2
1	2	2	1	1	3	1	2	2	2
2	2	2	2	1	2	1	2	2	2
1	2	2	2	3	2	2	2	2	1
1	3	1	1	2	2	2	2	2	2
2	2	1	2	2	1	1	1	1	1
1	2	2	2	2	2	2	2	1	1
9	3	9	9	9	9	9	9	9	1
2	2	2	2	3	2	2	2	1	2
2	9	1	3	3	3	1	2	2	1
2	2	2	2	2	9	9	9	9	9

2	2	2	2	2	2	2	2	2	2
1	3	2	1	2	1	1	3	2	3
2	2	2	2	2	2	2	2	2	2
2	2	2	2	2	2	2	2	2	2
2	2	2	2	2	2	2	3	2	1
2	2	2	2	1	3	2	2	2	2
2	2	2	2	2	2	2	2	2	2
2	2	2	2	2	2	2	2	2	2
2	2	2	2	2	2	2	2	2	2
2	2	2	2	2	2	2	2	2	2
2	2	2	2	2	2	2	2	2	2
3	2	3	3	1	1	1	3	3	3
9	9	9	9	9	9	9	9	9	9
3	1	1	1	1	1	1	3	1	1
9	9	9	9	3	9	9	1	9	9
9	3	9	9	9	1	9	9	9	9
2	9	2	1	1	2	1	2	1	2
2	2	2	2	2	1	2	3	1	2
9	9	9	9	9	9	9	1	9	9
2	2	2	1	3	1	3	2	2	1
9	3	9	9	9	9	9	9	9	2
9	9	9	9	3	9	9	9	9	9
9	9	9	9	2	2	2	9	3	1
3	3	2	1	2	2	3	1	1	2
3	1	3	3	1	3	1	3	3	3
9	1	9	9	9	1	9	9	9	9
3	1	3	1	1	3	3	3	3	3
2	2	2	2	2	3	2	2	3	3
3	3	1	1	1	3	3	1	1	1
9	9	9	9	9	1	9	9	9	9
2	2	2	2	2	2	3	1	2	2
2	2	2	1	9	9	9	9	9	9
2	2	2	2	1	3	2	2	1	1
9	2	9	9	9	9	2	2	2	9
9	2	9	9	2	3	3	1	9	9
9	2	2	1	2	2	3	1	3	1
1	3	2	2	3	1	3	1	1	3
2	3	2	2	2	2	3	1	2	2
3	1	3	3	2	2	3	2	2	2
9	9	9	9	9	9	3	9	9	9
2	2	2	2	2	2	3	3	2	2
1	3	2	3	2	1	3	1	1	1
3	2	3	2	1	3	1	3	3	2
2	2	2	2	2	2	2	2	2	2
2	9	3	2	2	2	2	3	1	2
9	3	9	9	9	9	9	1	9	9
2	2	2	2	2	2	1	2	2	3

3	1	2	3	2	1	1	1	3	3
9	9	9	9	9	9	9	2	9	9
9	9	1	9	9	9	3	9	9	9
9	3	9	9	3	9	9	9	1	9
3	3	3	3	2	2	2	1	2	2
9	9	9	1	3	9	9	9	9	9
3	3	1	3	1	1	3	1	1	3
2	2	2	2	2	3	2	2	2	3
2	3	3	1	1	2	1	2	2	2
9	3	9	9	9	9	9	3	9	9
9	9	9	1	2	9	9	9	9	9
9	9	9	9	9	9	9	1	9	9
9	3	9	9	2	2	2	2	2	2
2	3	2	3	1	2	3	1	1	3
9	3	9	9	9	9	9	9	9	9
2	1	2	3	2	1	3	2	2	9
2	2	2	2	2	2	2	2	2	2
9	9	9	9	3	9	9	9	9	9
1	9	9	9	9	9	9	9	9	9
2	2	1	3	2	1	3	2	2	3
1	2	2	2	3	2	2	2	2	2
1	3	3	1	1	3	1	3	3	1
1	2	2	2	2	2	2	2	2	2
1	3	1	1	2	2	2	1	1	1
1	3	2	1	3	2	3	1	1	2
9	9	1	9	9	9	3	1	9	9
1	3	1	2	3	2	3	1	1	2
2	3	2	2	1	2	1	2	1	2
1	9	2	1	1	3	3	1	1	1
2	2	2	2	1	3	1	2	2	1
9	9	9	2	9	9	9	1	9	9
1	2	2	2	3	2	3	1	2	2
2	2	1	3	1	3	2	1	1	1
9	9	9	9	2	9	9	9	9	9
3	9	1	3	1	3	1	1	3	1
9	3	9	9	9	9	9	9	9	9
1	3	2	2	2	2	3	2	2	2
9	9	9	9	9	9	9	9	1	9
1	9	9	9	1	3	1	1	3	3
3	1	3	3	1	3	1	3	3	3
1	9	9	9	9	9	9	1	9	9
9	9	9	9	9	9	9	9	9	9
9	9	9	9	2	2	2	2	2	9
1	9	9	9	9	1	9	9	9	9
3	9	9	2	2	3	9	1	3	2
2	2	2	2	2	2	3	2	1	2
2	1	3	3	1	3	1	3	3	2

1	9	9	9	9	9	9	9	9	9
9	9	9	9	9	9	9	9	9	9
9	9	9	9	9	9	3	1	9	9
9	9	9	9	1	9	9	9	9	9
9	9	9	9	9	9	9	9	9	9
9	9	9	9	9	9	9	1	1	9
1	9	9	9	3	9	9	9	9	9
3	3	3	1	1	3	1	9	9	9
2	2	2	2	2	2	3	2	2	2
2	2	2	2	1	2	2	3	2	2
2	2	2	2	1	3	3	3	3	2
3	3	2	1	3	2	3	1	1	2
9	3	9	9	9	9	9	1	9	9
9	3	3	1	1	3	3	1	2	1
1	1	1	1	9	2	3	1	3	1
2	2	2	2	9	9	3	3	3	3
2	2	2	2	2	3	3	1	2	2
9	9	9	9	9	9	9	9	9	9
2	3	2	2	2	2	3	2	2	3
9	9	9	9	2	9	9	9	9	9
9	9	9	9	2	9	9	1	9	9
9	3	9	9	9	9	9	1	9	9
2	2	2	2	2	2	2	2	2	2
2	2	2	2	3	2	3	2	3	2
2	2	2	2	2	2	2	2	2	2
2	2	2	2	1	3	2	3	2	1
9	9	9	1	3	9	9	9	9	1
2	3	2	3	3	2	1	3	1	2
2	3	1	2	2	2	3	1	1	2
2	2	2	2	2	2	2	2	2	2
3	3	2	2	1	3	1	1	2	3
2	3	1	3	3	1	3	1	1	2
1	9	9	9	9	9	9	9	9	1
2	2	1	3	1	3	1	1	1	2
1	1	3	3	1	3	3	3	3	3
9	3	9	9	9	9	9	9	9	9
3	3	2	1	1	3	1	1	2	1
2	2	2	2	2	2	1	2	3	1
2	2	2	1	1	3	3	3	1	2
2	1	1	1	1	3	1	3	1	1
1	2	1	2	1	1	3	2	1	1
2	3	2	2	2	2	2	2	1	2
3	3	9	2	9	9	1	1	2	9
9	9	9	1	9	9	9	1	9	9
2	2	2	2	2	2	2	2	3	2
2	3	2	2	1	3	1	1	2	1
1	2	2	3	3	1	2	2	1	2

9	9	9	9	9	9	3	1	9	9
1	1	1	1	3	1	3	1	1	1
1	1	1	1	3	1	3	1	1	1
2	2	2	2	3	2	3	1	2	1
9	1	3	3	2	9	1	3	9	2
3	3	3	3	1	2	2	3	2	2
9	9	1	9	1	3	3	1	3	1
2	2	1	2	1	3	3	3	3	2
2	2	2	2	3	2	3	1	2	2
9	2	9	9	2	2	1	3	2	3
2	2	2	2	2	2	3	1	2	2
9	3	9	9	9	9	9	1	9	9
9	3	9	9	3	9	9	1	9	1
3	1	3	3	2	3	1	1	2	3
1	9	9	9	9	9	9	9	9	9
1	3	3	2	2	3	3	2	1	1
9	3	9	9	9	9	9	1	9	9
3	2	3	2	3	3	2	3	1	2
3	2	2	2	2	2	1	3	3	1
9	9	9	9	9	9	9	1	9	9
2	2	2	2	2	2	2	2	2	2
2	2	2	2	3	3	1	1	2	3
9	9	9	9	9	9	9	1	9	9
3	3	3	1	3	1	1	1	1	1
2	9	1	1	2	9	2	9	2	2
3	1	2	2	3	2	2	1	2	2
2	2	3	2	1	2	3	1	2	3
2	2	2	2	2	2	3	1	1	1
3	9	1	9	3	3	3	1	2	3
2	2	1	1	3	1	2	1	2	3
9	9	1	9	9	9	9	1	9	9
2	1	1	2	3	1	2	3	1	2
2	2	2	2	3	2	3	2	2	2
2	2	2	2	3	2	3	2	2	2
1	3	9	2	3	1	3	3	1	1
1	9	9	9	9	9	9	9	9	9
2	2	2	1	1	3	1	3	3	3
3	2	1	1	1	3	3	3	2	2
2	2	2	1	1	3	1	3	1	2
1	1	3	2	2	2	2	2	2	2
2	2	2	2	1	2	3	2	2	2
2	2	2	1	1	3	3	2	2	3
9	9	9	1	1	1	3	3	1	9
9	9	9	9	9	9	3	9	9	9
2	9	1	1	3	2	3	2	2	2
1	9	9	9	9	9	9	9	9	1
1	3	1	1	3	1	3	1	1	1

9	9	9	9	3	9	9	9	9	9
9	3	1	1	3	1	3	1	1	1
3	2	2	2	2	2	2	1	2	1
9	9	9	9	3	1	2	9	9	9
1	3	3	2	2	2	3	1	1	3
2	2	2	2	2	2	2	2	2	2
2	2	2	2	2	2	2	2	2	2
2	1	3	1	3	1	2	1	1	1
1	3	1	1	3	1	3	1	1	2
9	9	9	1	9	9	9	9	9	9
9	9	9	9	2	9	2	9	9	9
2	3	2	1	3	1	3	1	1	1
9	3	9	9	9	9	9	9	9	1
1	2	2	1	2	2	2	1	1	1
3	1	3	3	2	3	3	1	1	1
2	2	2	2	2	2	2	1	2	1
2	1	2	3	1	3	2	3	3	3
3	1	3	3	1	3	1	3	3	3
9	9	9	9	9	9	9	1	9	9
9	9	9	1	9	9	9	1	9	9
2	3	2	2	1	3	2	1	1	1
3	3	2	1	2	2	3	1	3	1
3	1	3	3	1	3	1	3	3	3
1	2	9	3	2	1	2	1	1	1
2	3	2	2	1	3	1	1	1	2
9	3	9	9	1	3	9	1	3	2
1	9	9	9	1	3	1	1	3	3
3	1	3	3	1	3	1	1	1	1
1	2	2	2	3	2	3	1	1	1
9	9	9	9	9	9	9	9	9	9
9	3	9	9	9	9	9	9	9	1
2	3	2	2	2	2	1	1	1	1
3	3	2	2	1	3	1	2	3	1
1	9	9	2	1	3	2	9	1	1
2	3	1	2	2	2	2	3	1	2
9	3	9	9	9	9	9	9	9	1
9	9	9	9	2	9	9	9	9	9

FamilyBrea	EarlyInterc	UTI	BSEnoneec	BSEnecessa	BSEshy	BSEpainful	BSEfearofC	BSEtimewa	BSEdirty
1	1	1	1	1	1	2	3	2	1
3	1	1	1	1	1	1	3	2	1
2	2	2	1	1	3	3	3	3	3
9	1	1	9	1	1	1	3	2	1
9	9	9	1	1	1	9	9	9	9
3	1	1	1	1	1	1	3	2	1
2	1	2	3	1	3	3	3	3	3
1	3	9	1	2	3	1	3	3	3
9	9	9	1	9	3	2	2	3	3
9	9	9	3	9	9	9	9	9	9
9	9	9	2	2	2	2	2	9	9
9	1	9	2	2	1	1	1	1	1
2	2	2	1	1	3	3	3	3	3
3	3	2	2	1	3	2	2	2	3
3	3	2	1	9	3	3	3	3	3
1	3	2	2	1	3	3	3	3	3
2	3	2	3	2	9	1	1	3	2
1	3	2	2	1	3	3	3	3	3
1	3	2	2	1	3	1	3	3	3
3	2	2	3	1	3	3	2	1	1
2	3	3	1	1	3	3	3	3	3
3	3	3	1	3	3	3	3	3	3
3	9	9	2	1	3	3	2	3	3
2	3	3	1	1	3	3	3	3	3
2	2	2	1	1	3	3	2	3	3
2	2	2	1	1	2	1	2	3	2
2	2	2	1	1	3	1	3	3	3
2	1	2	3	1	3	1	3	3	3
3	2	3	3	1	3	3	1	3	3
2	2	2	1	2	1	2	2	2	2
2	2	2	1	2	1	2	2	2	2
3	2	2	3	1	3	3	3	3	3
1	2	1	3	1	3	3	3	3	3
3	2	2	3	1	3	3	3	3	3
3	2	2	3	1	3	3	3	3	3
3	2	2	3	9	3	3	3	3	3
3	2	2	3	1	3	3	3	3	3
1	1	3	3	1	3	3	3	3	3
9	9	9	9	9	9	9	9	9	9
9	9	9	3	1	3	3	1	3	3
1	2	2	2	1	1	3	3	3	3
9	9	9	1	2	3	3	2	3	2
3	3	3	3	1	3	3	9	3	3
3	3	2	9	1	3	9	9	3	3
3	1	3	3	1	3	3	2	3	3
3	1	3	3	1	3	3	3	3	3

3	1	1	3	1	3	2	3	3	3
9	9	9	3	1	1	3	3	3	3
3	3	2	9	1	3	3	3	3	3
3	1	1	2	1	1	2	1	3	3
9	9	9	3	1	3	1	3	3	3
3	3	1	3	1	3	3	3	3	3
3	3	3	3	1	3	3	3	3	3
9	9	9	1	1	3	3	9	3	3
9	9	9	9	1	9	9	9	9	9
3	1	2	2	1	1	2	3	3	2
9	9	9	1	9	9	9	9	9	9
9	9	9	2	9	3	3	3	3	3
3	2	1	2	1	3	3	1	3	3
9	9	9	2	1	3	3	2	3	3
2	1	2	1	1	3	3	3	3	3
3	3	3	2	1	3	3	3	3	3
3	2	2	3	1	3	3	9	3	3
9	9	9	3	1	3	3	3	3	3
3	3	3	3	1	3	3	2	3	3
1	3	3	1	1	1	3	1	1	2
3	3	3	3	3	3	1	3	3	1
3	3	3	3	3	3	1	3	3	1
3	3	3	3	3	3	1	3	3	1
9	9	3	3	1	3	3	3	3	3
3	1	3	9	1	9	1	9	9	9
9	1	2	9	1	1	9	1	9	9
9	9	9	3	1	3	3	3	3	3
3	1	1	1	1	3	1	2	3	3
1	1	3	3	1	3	3	3	3	3
2	1	1	3	1	3	3	1	2	3
1	1	2	9	1	1	1	1	1	9
3	3	1	1	1	3	3	3	3	3
2	1	9	9	1	1	2	2	2	3
1	1	1	3	2	3	3	3	3	3
1	2	1	3	1	1	2	3	3	3
9	9	9	2	1	3	3	3	3	3
1	3	3	3	3	3	1	1	1	1
2	1	2	3	1	3	3	3	3	3
9	9	9	3	1	3	3	3	3	3
3	1	1	3	1	3	3	3	3	3
1	1	2	9	1	3	2	3	3	3
3	2	1	1	1	3	3	3	3	3
3	3	1	1	1	3	3	3	3	3
1	2	1	3	1	3	1	3	3	3
3	2	1	3	1	3	3	2	3	3
9	9	9	1	1	3	3	3	3	3
9	9	9	1	1	3	3	1	1	1

3	3	2	3	1	3	3	3	3	3
2	2	2	3	1	3	1	3	3	3
9	9	9	3	1	3	1	1	3	1
3	2	2	3	1	3	1	3	3	3
2	2	2	3	1	3	3	3	3	3
2	2	2	2	1	9	9	9	9	9
3	2	2	3	1	3	3	3	3	3
3	1	2	3	1	3	3	3	1	3
3	2	2	9	1	9	9	9	9	9
3	1	9	2	1	3	3	3	3	3
2	1	1	1	1	1	1	3	2	3
2	3	2	3	1	3	3	2	3	3
2	3	3	9	9	9	9	9	9	9
9	9	9	9	9	9	9	9	9	9
3	1	2	3	1	3	1	2	3	1
9	9	9	1	1	3	3	2	3	3
3	2	2	3	1	3	2	3	3	3
2	2	2	3	1	3	2	3	3	2
2	2	2	3	3	3	3	3	3	3
2	2	2	3	1	3	2	2	2	1
3	1	2	3	1	3	3	3	3	9
9	9	9	1	1	3	3	3	3	3
2	2	2	3	2	2	2	2	2	2
1	9	1	1	1	3	3	3	3	1
2	2	3	1	3	1	9	9	1	2
2	2	2	3	1	2	2	2	2	2
9	9	9	3	1	3	3	3	3	3
9	9	9	3	9	9	9	9	9	9
2	3	2	3	1	3	3	3	3	3
9	9	9	1	1	3	2	2	2	2
2	2	2	2	1	1	2	2	3	2
2	2	2	1	1	2	1	2	2	1
3	2	2	2	2	3	1	3	1	2
2	3	3	3	1	3	3	1	3	3
9	9	9	1	1	3	2	2	2	2
3	3	3	3	1	1	3	3	1	3
1	2	3	3	1	3	3	1	3	2
2	2	2	3	2	3	2	3	3	1
9	9	9	1	9	9	9	9	9	9
1	1	1	1	1	3	3	3	3	3
2	3	2	2	1	3	3	3	3	3
2	3	2	2	1	3	3	3	3	3
2	2	2	1	1	2	1	2	2	1
2	2	2	3	3	3	2	3	1	1
2	2	2	1	1	3	3	1	3	2
3	2	2	3	1	3	1	1	3	3
1	2	2	1	1	3	3	1	3	1

9	9	1	2	2	1	9	9	9	9
3	2	2	3	1	3	3	3	3	3
3	3	2	3	1	3	1	3	3	3
2	3	2	2	1	3	3	3	3	3
2	3	2	2	2	2	1	3	3	3
2	1	2	2	1	9	1	3	3	3
2	3	1	3	1	2	1	1	3	1
2	2	2	3	1	3	3	3	3	3
2	2	2	1	1	9	2	2	3	2
2	2	2	2	1	2	2	2	2	2
3	2	2	3	1	3	3	2	2	3
3	2	9	3	1	3	3	2	2	3
9	9	9	9	1	9	1	9	9	9
2	2	2	3	1	3	3	3	3	3
3	3	2	3	1	3	1	3	3	3
3	3	2	1	2	3	1	1	1	1
2	2	2	1	2	3	1	3	1	2
2	3	2	1	1	3	3	1	1	3
1	9	9	1	9	9	9	9	9	9
2	1	1	2	1	3	3	1	3	3
9	1	9	3	1	3	3	1	3	3
3	1	1	3	1	3	3	1	3	3
2	1	1	1	1	3	3	3	3	3
3	1	3	2	1	3	3	1	3	3
3	1	3	2	1	3	3	3	3	3
9	1	9	3	1	1	3	1	3	3
1	1	1	2	1	3	3	1	3	3
9	1	9	2	1	3	9	3	3	3
3	1	3	3	1	2	3	1	3	3
3	1	1	3	1	3	3	1	3	3
2	3	1	3	1	3	1	1	3	3
3	1	1	1	2	3	2	2	3	3
1	1	9	2	1	3	3	1	3	3
1	1	9	3	1	9	3	3	1	9
1	1	1	3	1	9	3	3	1	9
1	1	2	3	1	3	3	1	3	3
2	1	2	2	3	3	3	1	3	3
2	1	2	3	1	3	9	1	3	3
1	1	2	3	1	3	3	3	3	3
3	2	3	3	1	3	3	1	3	3
3	1	3	3	1	3	3	1	3	3
9	1	3	2	1	3	3	1	3	3
9	1	9	3	1	1	3	1	3	3
3	1	3	2	1	3	3	1	3	3
3	1	3	3	1	3	3	1	3	3
3	1	3	2	1	3	3	1	3	3
3	1	3	2	1	3	3	1	3	3

3	1	3	9	1	3	3	1	3	3
1	9	9	3	1	3	3	1	3	3
9	1	9	3	1	3	3	1	3	3
9	1	9	3	1	3	3	1	3	3
3	1	3	3	1	3	3	1	3	3
1	1	1	3	1	3	3	1	3	3
3	1	9	3	1	3	3	1	3	3
2	3	3	3	1	3	3	3	3	3
3	1	1	3	1	3	3	3	3	3
9	9	9	3	1	3	3	3	3	3
3	3	3	3	1	1	3	3	3	3
9	9	9	3	1	3	3	2	3	3
3	1	1	3	1	3	3	3	3	3
3	1	1	3	1	1	3	1	3	3
1	1	1	1	1	3	3	3	3	3
1	2	2	1	1	3	3	3	3	3
2	2	9	2	1	1	1	1	2	1
1	1	1	2	2	1	1	1	1	1
3	2	2	2	1	3	3	3	3	3
2	2	2	1	1	3	3	3	3	3
1	2	1	2	1	3	2	2	1	1
3	9	2	2	1	3	3	3	3	3
3	1	2	2	1	1	2	2	2	1
9	1	9	2	1	3	2	2	1	2
2	2	2	2	1	3	3	3	3	3
2	3	9	2	1	2	2	2	2	2
2	2	2	1	1	3	3	3	3	3
2	3	9	2	1	2	2	2	2	2
3	3	3	3	9	1	1	2	2	2
2	3	3	2	1	3	3	3	3	3
2	2	2	3	2	2	2	2	2	2
2	2	2	3	2	2	2	2	2	2
2	3	3	2	1	3	3	3	3	3
1	1	2	1	1	2	3	3	3	1
2	2	2	1	1	2	1	2	2	2
1	2	2	1	1	2	2	2	2	1
2	2	1	2	1	1	1	1	2	1
2	3	2	1	1	2	3	2	3	3
2	3	3	1	1	3	3	3	3	3
3	1	2	2	1	3	3	3	3	2
2	2	2	1	2	2	2	3	2	1
2	2	2	1	1	3	3	3	3	3
9	9	9	3	1	3	3	9	9	9
9	9	9	3	1	3	3	9	9	9
1	1	1	1	3	3	3	1	1	1
2	1	2	1	2	1	1	2	3	1
3	2	2	1	1	1	1	1	2	1

2	3	2	2	1	1	1	2	3	2
3	3	2	2	1	1	3	3	3	2
2	2	2	1	1	3	3	3	3	3
2	2	2	2	2	2	1	2	2	1
2	2	1	1	1	3	3	1	3	3
3	2	2	3	1	3	3	3	3	3
3	3	3	3	1	3	3	3	3	2
3	2	2	3	3	2	1	1	2	2
3	3	1	3	1	3	3	3	3	3
3	3	3	9	1	1	1	3	3	3
2	2	2	1	3	1	2	2	2	2
3	3	2	1	1	3	3	3	3	3
3	3	1	1	1	3	3	3	3	3
2	2	2	2	3	2	2	2	3	2
3	1	2	2	1	3	1	2	3	3
3	3	3	3	1	3	3	3	3	3
3	3	2	9	2	3	3	2	1	2
2	3	2	1	2	2	9	2	2	2
3	2	2	3	3	3	3	3	2	3
3	3	2	1	2	1	1	3	9	1
2	2	1	2	2	1	1	1	1	1
2	2	2	2	1	2	1	2	2	2
3	3	2	9	1	3	3	2	3	2
2	2	2	2	1	3	2	1	3	3
9	9	9	9	1	9	9	9	9	9
2	2	2	2	2	2	2	2	2	2
2	2	2	2	2	2	2	2	2	2
2	3	1	1	3	3	3	3	3	3
2	1	2	1	1	3	2	2	1	2
1	2	2	2	1	3	1	3	2	2
9	9	9	9	1	9	9	9	9	1
2	3	3	3	3	3	3	3	1	3
9	9	2	3	3	3	3	1	1	1
3	2	2	2	1	1	1	9	3	3
9	9	9	1	9	9	9	9	9	1
2	2	2	1	1	2	1	2	2	1
2	3	2	2	1	3	2	3	3	3
1	1	2	1	1	3	3	3	3	2
3	3	1	3	1	1	1	1	1	3
2	2	3	3	2	3	1	3	3	3
2	2	2	2	1	3	3	3	3	3
2	2	2	1	1	3	3	3	2	2
3	3	2	2	2	1	3	3	3	3
2	2	2	1	1	3	2	3	2	2
2	2	1	2	1	2	3	3	2	3
2	3	1	1	3	2	3	3	3	3
3	2	1	1	1	3	2	3	3	2

2	1	1	2	1	1	2	2	2	9
2	1	1	2	1	3	3	3	3	3
2	2	1	3	1	3	3	3	3	3
2	1	2	3	3	3	2	2	3	3
1	2	2	3	2	3	1	3	1	3
2	1	1	1	1	3	2	3	1	3
2	2	1	2	1	3	3	3	3	3
9	9	9	3	1	3	3	3	3	3
2	2	1	3	1	3	1	2	3	3
3	3	2	3	1	3	2	2	2	2
2	2	2	2	2	2	2	2	2	2
3	2	2	3	1	3	3	3	3	3
1	1	1	1	1	1	1	1	1	1
3	3	2	1	1	3	3	3	3	3
1	3	1	1	9	3	1	3	1	1
3	3	2	3	3	3	3	3	3	3
2	3	1	2	1	3	1	3	3	3
3	1	1	2	1	1	1	1	2	1
1	1	1	1	2	1	2	3	1	2
1	1	2	1	1	3	9	9	9	9
3	2	3	3	1	3	3	3	2	3
2	2	2	3	1	3	3	3	3	2
3	1	2	3	1	3	2	3	3	3
2	2	2	1	1	3	1	1	2	3
2	2	2	1	1	3	2	3	2	2
9	9	9	3	1	3	2	3	3	1
2	2	1	1	1	3	3	3	9	2
3	3	1	3	1	3	2	2	3	3
3	2	2	1	1	3	3	3	3	2
3	3	2	9	1	3	3	2	3	2
2	1	2	3	1	3	1	1	3	3
9	9	9	1	3	3	3	3	3	1
9	9	1	3	1	3	3	2	3	3
3	3	2	3	3	3	1	3	3	3
3	3	1	3	1	3	3	3	3	3
3	3	3	3	1	3	3	1	3	1
2	2	2	1	1	1	3	3	3	3
3	3	1	1	1	3	3	3	3	3
3	2	2	3	1	3	3	3	1	3
9	9	9	1	3	9	9	3	3	9
1	2	3	1	1	1	3	1	3	3
2	2	2	3	1	3	3	1	3	1
2	2	1	3	1	3	3	2	2	9
3	3	2	1	3	2	3	3	3	3
3	1	2	1	1	3	3	3	3	3
1	2	2	1	1	3	3	3	3	3
1	2	3	3	1	3	3	3	3	3

9	9	9	3	1	3	3	2	3	1
9	9	9	3	1	3	3	2	3	1
9	9	9	9	1	9	9	9	9	9
3	3	2	3	1	3	3	3	3	3
9	9	9	9	9	9	9	9	9	9
2	2	2	1	1	3	3	3	2	3
1	2	1	3	1	3	2	1	3	3
3	3	3	3	9	3	3	3	3	3
3	1	2	2	1	3	3	1	3	3
1	2	2	1	1	1	2	3	3	3
3	2	2	3	2	2	1	1	3	2
3	1	1	3	1	2	3	3	3	3
3	2	1	3	1	3	2	3	3	3
3	2	2	3	2	2	1	1	3	2
3	2	2	3	2	2	1	1	3	2
3	3	3	2	1	3	1	1	3	1
1	3	2	3	2	2	1	1	3	2
3	1	1	3	1	2	3	3	3	1
3	1	1	3	1	3	3	3	3	1
2	1	1	1	3	3	3	2	3	3
3	2	2	1	3	3	1	3	3	3
2	1	2	3	1	3	3	3	3	3
2	1	2	3	1	3	3	3	3	3
1	1	2	2	1	3	3	3	3	3
1	2	2	1	2	3	3	3	1	2
3	3	2	1	1	2	1	2	2	1
3	3	2	3	1	3	2	2	3	3
3	2	2	1	3	1	2	2	2	2
3	2	2	2	2	3	2	2	3	1
3	3	2	2	1	3	2	1	1	2
3	3	2	3	2	3	1	3	2	2
3	2	2	3	2	3	1	3	2	1
1	2	2	1	1	1	3	3	3	3
2	2	2	2	1	2	2	1	2	1
2	2	2	2	1	2	2	1	2	1
2	3	1	1	3	3	2	3	2	3
3	3	2	3	1	3	3	3	3	3
3	1	1	3	1	3	2	2	3	2
3	1	1	3	1	2	2	2	2	2
3	1	2	3	1	2	2	1	3	3
2	2	2	3	2	2	3	3	3	3
1	1	2	1	2	3	2	2	3	3
2	1	1	3	1	3	3	3	3	3
2	1	2	1	2	3	2	2	3	2
2	1	3	1	3	1	2	2	3	3
2	1	1	2	1	3	3	2	3	3
2	2	1	2	1	3	2	3	2	3

1	2	1	3	3	3	2	2	3	3
2	1	3	1	1	3	1	3	1	3
9	9	9	2	1	1	2	3	3	2
2	2	2	1	1	3	9	1	1	1
1	3	2	3	1	3	3	3	3	3
3	2	2	3	1	3	3	3	3	1
2	2	2	1	1	3	1	2	2	2
3	2	2	3	1	3	3	3	3	3
3	2	2	3	1	3	3	3	3	3
1	2	1	3	1	1	1	1	1	1
1	2	2	3	1	2	2	1	3	1
1	3	1	3	1	1	3	1	3	3
1	2	2	2	1	3	3	3	3	3
3	1	2	1	2	3	3	3	3	2
3	1	2	1	2	3	3	3	3	2
1	1	2	1	1	3	3	3	3	3
1	3	3	1	3	1	1	1	3	1
3	3	2	2	1	1	2	3	3	3
3	3	2	2	1	3	2	3	3	3
1	9	9	2	3	2	2	3	3	2
3	3	3	3	1	3	1	3	3	3
2	2	2	1	1	2	2	1	1	3
2	3	2	2	1	2	3	1	3	3
2	2	1	3	1	3	2	1	2	1
1	1	1	3	1	3	1	2	3	3
2	1	1	2	1	3	2	2	3	2
3	3	2	1	1	3	3	3	3	3
1	2	2	3	1	3	3	3	3	3
3	2	3	1	1	1	2	3	2	1
9	9	9	1	1	3	1	1	3	9
2	2	2	1	2	3	3	3	2	2
3	3	3	1	1	3	3	1	3	3
2	2	2	1	2	3	3	3	2	2
3	3	2	3	1	3	3	3	3	3
3	1	1	3	1	3	3	3	3	3
2	3	1	1	1	1	1	2	1	1
3	3	3	2	2	2	3	3	3	2
1	1	3	1	2	1	3	3	3	3
2	2	2	2	2	2	3	3	3	3
1	1	1	3	1	1	1	1	3	1
2	3	2	1	2	1	1	2	1	2
3	1	1	1	1	1	1	3	2	1
2	3	1	1	1	3	3	2	3	1
2	2	2	3	3	3	1	3	1	1
3	3	3	9	9	9	9	9	9	9
2	1	1	3	1	3	3	1	3	2
3	3	3	3	1	3	3	1	3	3

2	1	1	3	1	3	3	1	3	2
1	1	3	2	1	3	1	1	3	3
3	1	3	3	1	3	3	1	3	3
2	1	1	2	1	3	3	1	3	3
9	9	9	3	1	3	3	1	3	3
3	1	2	1	1	3	3	1	3	3
2	2	2	9	1	3	3	1	3	3
1	1	1	3	1	3	3	3	3	3
2	2	2	3	1	3	3	1	3	3
3	1	3	2	1	3	3	1	3	3
3	1	1	2	1	3	3	3	3	3
2	1	1	3	1	9	9	1	3	3
3	1	3	3	1	3	3	1	3	3
3	1	3	2	1	3	3	1	3	3
3	1	3	3	1	3	3	1	3	3
3	1	3	3	1	1	3	1	3	3
2	2	2	3	2	1	3	1	3	3
2	2	2	3	1	3	3	1	3	3
3	1	3	3	1	3	3	1	3	3
3	1	3	3	1	3	3	1	3	3
3	1	1	2	1	3	1	1	3	1
1	1	1	2	1	1	1	1	3	3
3	1	1	3	1	3	3	1	3	3
3	1	1	2	1	1	1	2	3	3
3	1	3	3	1	3	3	1	3	3
3	1	9	3	1	3	3	1	3	3
3	1	3	2	1	1	3	1	3	3
3	1	1	3	1	3	3	1	3	1
3	3	3	3	1	3	3	9	3	3
3	1	2	2	1	3	3	1	3	3
9	3	3	2	1	3	3	1	3	3
2	1	3	2	1	3	3	1	3	3
1	1	3	2	1	3	3	1	3	3
1	1	3	3	1	3	3	1	3	3
9	1	9	3	1	3	3	1	3	3
9	9	9	3	1	3	3	1	3	3
2	1	2	1	1	3	3	3	3	2
3	1	1	3	1	3	3	1	3	3
3	1	3	3	1	3	3	1	3	3
1	1	1	3	1	3	3	1	3	3
9	1	9	2	1	3	3	1	3	3
2	2	1	3	1	3	3	3	3	3
9	9	9	2	1	3	3	2	2	9
2	2	2	3	1	3	2	2	3	3
9	9	9	3	1	3	3	3	3	3
2	2	2	2	1	3	3	2	3	2
3	9	9	3	1	3	9	2	3	3

2	2	2	3	3	3	2	2	2	2
3	1	1	3	1	3	3	3	2	1
3	1	1	3	1	3	3	3	2	1
9	3	1	3	1	3	3	3	2	3
2	3	1	3	1	3	3	3	3	3
1	9	9	1	2	2	3	3	3	3
3	2	2	1	1	3	1	2	3	3
1	1	2	2	3	2	1	2	2	2
1	1	2	3	1	3	3	3	3	3
2	2	2	2	1	1	1	1	1	1
2	2	1	1	1	1	2	3	2	1
1	9	9	9	9	9	9	9	9	1
9	9	9	9	1	9	9	9	9	9
3	2	2	2	1	3	1	2	3	3
9	9	9	9	9	9	9	9	9	9
9	9	9	9	1	9	9	9	9	9
9	9	9	1	9	9	9	9	9	9
9	9	9	9	9	9	9	9	9	9
9	9	9	9	9	9	9	9	9	9
9	9	9	3	1	3	2	3	3	3
9	9	1	1	9	1	9	1	3	3
3	3	3	1	9	1	3	3	3	3
3	3	2	3	1	2	3	3	3	2
9	1	9	9	1	9	9	9	9	9
9	9	9	9	9	9	9	9	9	9
9	1	1	3	1	3	3	3	3	1
9	9	9	9	9	9	9	9	9	9
2	1	1	3	2	3	1	2	2	3
2	1	1	3	1	3	1	9	9	1
1	9	9	9	9	9	9	9	9	1
9	9	9	9	9	9	9	9	9	9
2	1	2	2	1	3	3	1	3	3
2	2	2	2	1	1	2	2	2	2
1	3	3	1	1	3	3	3	3	3
1	2	2	3	2	3	2	2	1	1
2	3	2	3	1	3	3	3	3	3
3	2	1	3	1	3	2	2	2	2
3	1	3	3	1	3	3	3	3	3
3	3	3	2	2	2	2	2	2	2
3	3	2	2	1	3	2	1	3	3
3	1	2	3	1	3	2	3	3	3
1	1	1	3	1	3	3	3	3	3
1	2	1	3	1	3	3	1	3	2
1	1	1	3	1	2	3	3	3	3
2	1	1	3	1	3	3	2	3	3
3	1	2	2	1	3	3	3	3	3
3	2	2	3	1	3	3	2	3	3

3	1	2	1	1	3	2	2	2	3
9	9	9	3	1	3	3	3	3	3
1	3	2	3	1	3	3	3	3	3
3	2	2	3	1	1	1	2	3	2
3	1	1	3	3	3	3	2	3	2
2	2	2	1	2	3	2	2	2	3
3	3	3	2	1	3	3	1	3	3
2	1	1	2	1	3	3	3	2	1
1	1	1	3	1	3	1	1	2	1
3	2	2	2	1	1	2	2	2	2
3	2	2	2	1	1	2	2	2	2
2	2	2	1	1	3	3	2	3	3
3	3	2	2	2	3	2	1	2	2
3	3	2	2	2	3	2	1	2	2
3	3	2	3	2	1	1	3	3	1
2	2	2	1	1	2	2	2	2	2
2	2	1	1	2	3	2	2	2	3
2	2	2	1	1	3	1	2	1	1
2	2	2	2	1	3	1	1	3	3
3	2	2	3	1	3	2	3	3	2
3	3	2	3	1	3	1	3	3	1
2	1	2	3	1	3	3	3	3	3
3	1	1	3	1	1	3	3	3	3
3	1	2	3	1	1	3	3	3	3
3	2	2	3	2	2	1	1	3	2
3	1	2	2	1	3	2	2	2	3
3	3	3	1	1	3	3	3	3	3
1	3	1	1	1	3	3	3	9	1
1	1	2	3	1	1	2	1	1	1
3	1	3	2	1	2	3	2	3	2
1	3	2	1	1	3	1	1	1	1
2	1	2	9	9	9	9	9	9	9
2	3	2	2	2	1	2	1	2	2
3	2	2	2	3	3	3	3	3	3
3	3	1	3	1	1	1	1	3	2
9	9	2	9	2	9	9	9	9	9
2	2	2	2	2	1	2	1	3	3
3	1	1	3	1	3	1	3	3	3
2	2	2	2	1	2	2	3	3	3
2	1	3	3	1	3	3	3	3	3
3	2	3	3	1	3	3	3	3	2
2	1	2	1	2	3	2	3	1	1
9	9	9	1	1	3	3	3	3	3
2	3	2	3	1	3	3	2	3	3
3	1	2	3	1	3	3	3	3	3
3	2	3	2	2	9	9	9	9	9
9	9	9	9	1	9	9	9	9	9

9	2	9	9	1	9	9	9	9	9
9	1	9	9	9	9	9	9	9	9
9	9	9	9	1	9	9	9	9	9
9	9	9	1	1	3	1	3	3	3
9	9	1	3	1	3	3	3	3	1
1	9	1	1	1	1	9	9	9	9
9	9	1	1	1	1	1	1	9	9
9	1	1	9	1	9	9	9	9	9
9	1	1	9	1	1	9	9	9	9
9	9	9	1	9	9	9	9	9	9
9	9	9	1	9	9	9	3	9	3
9	9	9	1	9	9	9	9	9	9
9	9	1	9	1	9	9	9	9	9
9	9	9	9	1	9	9	9	9	9
9	9	9	1	1	1	1	3	3	3
9	9	9	1	9	9	9	9	9	9
9	1	1	1	9	9	9	9	9	9
9	9	1	1	1	3	1	3	3	3
2	2	2	2	2	2	2	2	2	2
3	1	1	3	2	1	1	2	1	1
9	9	9	1	1	1	1	1	1	1
1	2	3	1	1	3	1	2	3	1
1	1	9	3	1	1	2	2	3	1
3	1	1	1	1	3	3	3	3	3
3	1	2	2	2	3	3	3	3	3
1	3	2	1	3	1	2	2	2	3
1	2	2	1	2	3	2	3	3	2
2	2	3	1	3	3	9	2	3	3
2	2	2	1	3	3	2	2	2	1
2	2	2	1	1	2	2	9	3	3
1	1	1	1	1	3	3	3	3	3
3	2	3	2	1	3	3	3	3	3
2	2	2	1	1	3	1	1	3	3
1	3	3	9	9	9	9	9	9	9
2	1	2	1	1	1	2	1	2	3
2	2	2	1	1	1	1	2	1	2
3	2	1	2	2	2	1	2	3	2
2	3	3	3	3	1	2	1	2	1
9	9	9	1	3	1	1	1	1	1
2	1	2	1	1	3	1	2	1	3
1	1	9	1	3	1	2	2	1	2
1	1	1	9	1	3	3	3	1	3
2	2	2	3	3	3	1	2	1	1
3	1	3	1	3	1	1	1	1	1
1	3	3	3	3	3	3	3	3	3
3	2	2	3	3	3	1	2	1	1
1	1	1	9	2	2	2	3	3	1

3	1	3	1	3	1	3	9	9	1
3	3	3	1	1	2	1	2	2	1
3	3	1	1	9	3	3	1	1	3
1	1	2	2	1	1	2	1	2	1
2	2	2	1	2	3	3	2	1	3
2	2	2	1	2	3	3	2	1	3
1	1	1	1	1	3	2	2	1	2
3	1	3	1	3	1	3	9	9	1
1	1	1	1	1	1	2	1	2	1
9	9	9	1	1	3	3	3	3	3
1	2	3	1	2	3	1	2	1	3
3	1	1	1	1	3	3	3	3	2
2	3	2	2	1	3	2	1	2	2
3	1	2	2	1	1	2	1	2	1
2	2	2	1	2	2	1	3	1	2
1	2	3	3	1	3	2	3	3	2
1	1	1	1	1	1	1	1	1	1
3	3	3	3	3	9	9	9	9	9
1	2	2	1	1	3	3	3	3	3
3	1	3	3	1	3	3	1	3	3
1	1	1	1	3	3	3	3	3	3
2	3	2	2	1	2	3	3	3	3
2	1	3	1	1	1	2	3	2	2
2	3	3	2	1	3	1	3	2	3
3	2	2	3	3	2	2	2	2	2
1	3	3	1	3	1	1	3	1	3
3	1	3	3	1	3	3	1	3	3
3	1	1	3	1	3	3	3	3	3
1	1	1	9	2	2	2	9	3	1
9	1	9	1	3	3	3	3	3	3
2	1	2	3	3	3	3	3	3	3
9	9	9	3	3	3	3	3	9	1
3	1	1	3	1	3	3	3	3	3
3	2	9	3	1	3	3	3	3	3
9	2	9	3	1	3	3	3	3	9
9	1	9	1	3	3	2	3	3	3
9	1	9	1	3	3	3	3	3	3
2	2	2	1	1	3	2	3	3	1
2	2	2	1	1	3	2	2	2	2
2	2	2	1	1	3	2	2	2	2
2	1	2	1	1	3	2	3	2	1
2	2	2	3	3	3	1	2	1	1
2	3	1	3	1	2	1	2	1	2
2	1	2	1	1	3	2	3	2	1
2	3	1	3	1	2	1	2	1	2
1	1	2	1	3	3	1	1	1	2
3	3	3	3	1	1	1	1	3	1

3	3	3	3	1	1	1	3	3	3
3	2	2	3	1	3	3	2	3	2
1	1	1	3	1	3	3	3	3	3
2	1	2	1	2	1	2	2	1	1
2	2	2	3	1	3	3	3	3	3
9	3	3	1	2	1	2	1	3	1
2	3	2	1	3	1	2	2	2	1
1	9	9	1	1	3	3	3	3	3
3	3	3	1	1	3	3	3	3	3
2	2	2	2	1	3	2	3	3	3
3	3	3	3	3	3	3	3	3	3
9	1	9	1	3	1	3	3	3	3
1	2	1	1	3	3	1	1	1	2
1	1	2	1	3	3	1	1	1	2
1	1	2	1	3	3	1	1	1	2
1	1	2	1	3	1	3	2	3	2
1	1	2	1	3	3	1	1	1	2
1	1	2	1	3	3	1	1	1	2
1	1	2	1	3	3	1	1	1	2
3	1	1	1	3	1	1	1	3	1
3	2	1	3	3	1	2	3	1	1
2	3	3	2	2	2	2	2	2	3
1	3	2	1	3	3	9	1	3	1
2	2	3	3	1	3	2	1	2	2
1	1	1	3	9	3	3	1	2	3
3	3	1	1	3	3	3	1	3	3
2	2	2	1	2	1	2	1	2	1
1	3	1	2	1	3	3	1	3	3
9	9	9	1	9	9	9	9	9	9
3	3	3	3	3	3	3	3	3	3
1	1	1	1	1	3	3	3	1	1
3	2	3	2	9	1	3	2	1	3
1	2	3	3	1	2	1	2	1	2
2	2	3	1	2	1	1	1	2	2
2	3	3	1	3	3	2	3	3	3
1	3	2	1	1	1	3	3	3	3
2	9	9	3	2	1	1	1	2	3
2	2	2	1	1	2	1	1	1	2
2	3	2	1	3	2	1	1	1	2
1	1	1	1	1	1	3	3	1	1
1	1	2	1	3	3	1	1	2	1
3	1	1	1	1	1	9	1	3	3
3	2	3	1	2	1	3	2	1	2
3	1	1	3	1	3	3	3	3	3
9	9	9	1	1	3	3	2	3	3
1	1	1	1	9	1	2	1	2	1
2	2	2	1	1	1	1	1	1	2

9	9	9	3	1	3	2	3	3	3
9	9	9	1	9	1	9	9	9	9
9	9	9	1	9	9	9	9	9	9
1	3	3	1	3	1	1	3	1	3
2	2	2	1	1	3	3	2	1	3
2	2	2	1	2	3	3	2	1	3
1	3	3	1	1	3	1	2	1	1
2	2	2	1	2	3	3	2	1	3
2	2	2	1	2	3	3	9	9	3
2	2	2	1	2	3	3	2	1	3
2	3	2	2	1	3	1	1	1	2
2	1	2	3	2	3	1	1	3	3
1	1	2	1	1	1	1	2	1	2
2	1	3	1	1	1	1	3	1	3
2	3	1	3	3	1	1	3	3	1
1	3	1	1	1	1	3	3	2	3
3	3	3	3	1	1	2	3	3	3
9	9	9	3	1	9	9	9	9	9
9	9	9	3	1	9	9	9	9	9
1	1	1	2	1	3	3	1	3	3
2	1	3	1	1	3	1	1	1	1
3	2	2	1	3	3	2	2	2	2
3	3	3	1	1	1	2	1	1	1
2	2	2	2	2	2	2	2	2	2
3	3	3	3	3	3	3	3	3	3
9	9	9	3	9	2	2	2	2	2
9	9	9	3	9	2	2	2	2	2
3	1	2	3	1	2	3	1	3	3
3	3	3	1	1	1	2	1	1	1
2	2	2	2	2	2	2	2	2	2
2	3	3	3	1	9	9	9	9	9
2	1	1	1	3	2	2	1	2	1
1	1	1	1	1	3	9	1	1	2
9	9	9	3	9	2	2	2	2	2
3	2	1	1	3	3	1	1	2	1
3	3	3	1	3	3	9	1	2	2
2	1	1	1	3	1	2	2	2	1
9	9	9	1	3	3	1	1	1	1
1	3	9	1	9	1	9	2	9	9
2	2	2	3	1	1	1	1	1	1
9	9	9	1	3	1	1	1	1	1
2	2	2	3	1	1	2	2	2	2
2	2	2	2	1	3	3	2	2	2
2	2	2	2	2	2	2	2	3	2
2	2	2	2	2	2	1	3	1	1
2	2	2	2	2	2	2	2	2	2
3	2	2	1	3	3	3	3	3	1

2	2	2	1	2	3	3	2	2	2
1	2	2	2	2	2	2	2	2	2
1	2	1	1	3	3	3	3	9	9
1	2	2	9	9	9	9	9	9	9
9	9	9	1	9	9	9	9	9	9
2	1	2	2	1	2	2	1	2	1
2	2	2	1	1	3	2	2	2	2
9	9	9	3	3	3	3	3	3	9
2	1	2	1	1	1	2	1	2	3
9	9	9	9	3	9	9	9	9	9
2	2	2	2	2	2	2	2	2	2
1	1	1	1	1	3	3	1	1	1
9	1	9	1	1	3	3	3	1	1
2	2	2	2	2	2	2	2	2	2
1	1	2	1	2	3	1	1	2	1
2	2	2	3	3	2	2	2	2	2
2	2	2	2	2	2	2	2	2	2
2	1	1	1	1	1	3	1	2	3
9	1	1	1	1	9	9	9	9	9
1	1	2	1	3	3	3	3	3	3
9	9	9	2	9	9	9	9	9	9
1	2	2	1	3	1	2	1	1	1
2	2	2	2	2	2	2	2	2	2
2	2	2	1	2	1	2	2	1	1
2	2	2	1	1	3	3	3	2	2
2	3	3	1	2	1	2	3	1	1
3	3	3	2	9	9	9	9	9	9
2	2	2	9	1	9	9	9	9	9
2	2	2	2	2	2	2	2	2	2
3	3	2	3	1	2	2	3	3	3
9	9	9	9	1	9	9	9	9	9
2	3	2	1	1	1	2	3	3	3
3	1	3	3	3	3	3	3	3	3
2	2	2	1	1	2	2	2	2	3
2	1	2	1	2	1	1	1	1	2
1	1	1	1	1	1	1	1	1	3
2	2	2	2	2	2	2	2	2	2
3	1	2	1	2	2	3	3	1	2
1	1	1	2	2	1	1	2	2	2
1	2	3	1	2	3	3	3	3	3
2	3	2	1	1	3	3	2	3	3
2	2	2	2	2	2	2	2	2	2
2	3	1	3	1	1	3	3	3	3
2	1	1	1	1	3	3	3	3	3
2	2	2	1	2	2	2	2	2	2
3	3	3	3	3	3	3	3	3	3
3	2	2	1	2	3	9	1	2	2

3	1	2	1	1	2	2	2	2	2
2	2	2	2	2	2	2	2	2	2
1	3	3	1	2	2	2	2	2	2
2	1	2	2	2	2	2	2	2	2
2	1	1	1	2	3	3	1	3	3
1	1	2	1	1	3	1	2	1	3
2	2	2	3	2	2	2	2	2	2
1	3	3	2	2	2	2	1	2	3
1	2	3	1	1	3	3	3	3	2
9	9	9	1	1	3	1	2	1	3
3	3	1	1	3	3	1	1	3	2
9	9	9	9	2	9	9	9	9	9
9	1	9	9	1	9	9	9	9	9
2	1	2	1	1	1	2	1	2	2
2	2	2	1	2	1	2	2	3	3
3	1	3	1	3	1	1	2	1	1
2	2	2	9	1	2	2	2	2	2
1	3	1	3	1	3	3	1	1	3
2	1	3	1	2	2	1	1	2	1
2	1	2	1	1	1	2	1	2	2
1	9	9	1	1	1	3	3	3	3
1	1	1	1	2	2	1	2	1	3
2	2	2	1	2	3	3	2	3	2
2	1	2	2	1	1	3	3	3	9
9	3	3	1	3	1	3	3	3	3
2	1	2	2	1	1	3	3	3	9
2	2	2	2	2	2	2	2	2	2
9	9	9	9	1	9	9	9	9	9
2	2	2	1	1	2	2	2	2	2
2	1	2	1	2	2	2	2	3	1
1	1	1	1	1	1	1	1	1	1
1	1	1	1	1	1	1	1	1	1
1	2	1	1	1	1	2	1	1	1
1	2	1	1	2	2	1	1	2	1
9	9	9	1	1	1	3	1	1	2
3	1	1	1	3	1	9	1	1	1
9	1	9	3	1	3	3	3	3	3
1	1	1	1	1	1	1	1	1	1
2	2	2	1	1	2	2	2	2	2
2	2	2	1	1	2	2	2	2	2
2	1	2	3	1	2	3	3	1	2
1	1	1	2	3	3	2	2	1	1
2	1	3	1	9	9	9	9	9	9
2	2	2	1	1	2	1	2	1	1
2	2	2	1	1	2	2	2	2	2
1	1	1	1	1	2	3	1	1	2
3	1	3	2	2	2	2	2	3	2

1	1	1	1	1	3	1	1	1	9
3	3	9	3	1	3	3	3	3	3
2	2	2	3	1	3	3	2	3	3
9	1	9	9	9	1	9	9	9	9
1	9	9	3	9	2	9	9	9	9
2	1	2	1	1	2	3	1	2	3
9	1	9	1	1	2	2	2	2	2
3	1	1	3	1	3	3	3	3	3
3	3	1	1	9	9	9	9	2	2
2	2	2	2	2	2	2	2	2	2
9	1	9	1	9	9	9	9	9	9
2	3	3	1	2	2	2	2	2	3
2	1	2	2	2	2	2	2	2	2
2	1	1	2	1	2	2	2	2	2
2	2	2	1	1	2	2	2	2	2
2	1	3	2	3	2	1	2	3	2
2	3	2	1	2	2	1	3	2	1
9	1	1	1	9	9	9	9	9	9
9	9	9	1	9	9	9	9	9	9
2	1	2	2	2	2	2	2	2	2
2	2	2	1	2	2	2	2	2	2
3	1	2	1	3	9	3	3	3	1
9	9	9	1	3	3	9	9	9	9
1	1	1	1	9	2	2	2	2	2
3	1	2	2	1	2	2	3	1	2
1	3	1	3	3	3	3	3	3	3
2	9	2	1	1	3	3	2	3	3
2	1	3	3	3	3	1	1	3	1
3	3	3	3	1	2	9	2	2	2
3	2	1	1	3	3	2	3	3	3
1	1	1	1	1	3	3	3	2	2
2	1	2	1	1	3	3	2	3	3
3	2	2	3	3	2	3	3	3	3
3	3	3	1	3	3	3	3	3	3
9	9	9	1	1	1	2	2	3	2
2	1	3	1	1	3	1	1	1	2
1	2	1	1	1	1	3	1	1	2
3	3	3	1	3	3	1	1	1	3
1	9	3	2	1	1	9	3	2	9
3	3	3	1	3	3	3	3	3	3
1	1	1	1	3	1	1	3	1	1
9	1	9	1	1	1	2	1	3	1
1	1	1	1	1	1	3	1	1	3
3	1	3	1	1	3	3	3	3	3
9	9	1	3	1	3	1	3	3	1
2	2	2	1	1	2	3	3	2	1
1	1	2	1	1	2	2	1	2	2

1	1	3	1	3	3	3	1	1	1
3	3	3	3	3	3	3	3	3	3
9	1	9	1	1	1	2	1	3	1
1	1	1	2	3	3	1	2	1	1
2	2	2	3	3	2	2	1	1	1
3	1	1	2	2	2	2	2	2	2
3	3	3	2	3	3	1	3	2	1
2	2	2	1	1	1	1	1	1	1
2	2	2	1	1	1	1	1	1	1
1	3	2	2	1	3	1	2	1	1
2	1	2	2	3	1	2	2	3	1
3	2	3	1	1	1	1	2	3	3
3	3	3	3	3	1	1	1	1	1
2	2	2	3	1	3	2	3	2	3
2	2	2	2	1	1	2	2	2	2
3	3	3	2	3	3	1	3	2	1
3	3	1	3	1	1	3	3	3	3
1	1	2	1	3	2	2	2	2	2
1	3	2	2	1	1	2	2	3	2
9	9	9	9	1	9	9	9	9	9
9	1	9	1	9	9	9	9	9	9
3	3	3	1	1	3	1	1	1	1
3	3	3	1	3	3	1	3	3	1
3	3	1	3	3	3	3	3	3	3
3	3	3	1	3	3	3	1	3	3
1	1	3	1	3	3	3	1	1	1
3	1	2	1	1	3	1	1	3	3
1	1	2	3	2	1	2	1	1	2
2	1	1	3	1	1	3	3	1	1
3	1	3	1	3	1	3	3	3	3
3	2	1	3	1	3	2	2	2	2
3	1	3	1	1	1	1	1	1	1
3	1	2	2	1	2	2	1	2	2
1	2	2	1	1	1	1	3	3	3
1	1	9	2	1	3	2	2	3	1
3	2	2	1	2	2	1	2	1	2
1	2	2	1	2	9	1	1	1	2
2	2	2	1	1	3	2	2	2	2
2	2	2	9	1	2	2	1	3	2
2	2	1	1	1	3	3	1	3	3
2	1	3	1	2	1	3	1	2	1
2	2	2	2	2	2	2	2	2	2
1	3	3	1	3	3	3	2	1	1
9	9	9	2	1	9	9	9	9	9
9	9	9	3	9	9	9	9	9	9
3	1	2	3	1	3	3	3	3	3
3	1	1	1	1	3	2	3	3	3

1	1	3	1	9	3	3	3	3	3
3	3	1	3	1	3	2	3	2	3
3	3	2	1	1	3	3	3	3	3
3	2	1	1	2	1	1	2	3	3
3	3	3	2	2	3	3	3	3	3
3	3	3	3	3	3	2	3	3	3
2	2	2	3	9	2	2	2	2	3
3	3	3	2	1	3	3	3	3	3
3	3	2	3	1	3	3	1	3	3
9	9	9	1	3	3	1	3	3	3
9	9	9	1	3	3	2	3	3	3
9	9	9	1	3	3	2	3	3	3
9	9	9	1	3	2	9	9	9	9
9	9	9	1	9	9	9	9	9	9
9	9	9	1	3	3	2	3	3	3
2	3	2	3	1	3	3	3	3	3
9	2	1	1	3	3	3	1	1	2
2	2	2	1	2	2	2	2	2	2
3	1	2	1	1	3	9	1	3	3
1	2	1	1	1	3	3	3	3	3
2	3	1	1	3	3	3	3	3	3
3	2	3	2	1	3	2	9	2	1
2	2	2	1	1	2	2	2	1	2
1	1	2	3	1	3	2	3	3	3
2	2	2	1	1	2	9	1	2	2
2	3	1	1	1	1	3	1	3	3
3	3	2	3	1	3	3	3	3	3
1	1	1	1	3	1	3	3	1	1
2	3	1	1	1	1	3	1	3	3
2	3	2	3	1	3	3	3	3	3
1	2	1	1	1	3	3	3	3	3
9	9	9	1	3	2	9	9	9	9
3	3	1	1	1	1	3	3	3	2
2	2	2	1	1	3	2	3	3	3
1	2	2	1	3	1	1	1	1	1
2	2	2	1	1	3	3	3	3	3
9	9	2	1	2	3	2	3	3	2
9	9	2	1	2	3	3	2	3	3
3	2	2	1	2	3	1	2	2	1
2	2	2	2	2	2	2	2	2	2
9	9	9	3	1	3	3	3	3	1
1	1	2	1	1	3	3	3	3	3
3	3	2	3	1	3	3	3	3	3
2	2	2	2	2	1	2	1	2	2
3	1	3	1	3	1	3	1	3	1
1	1	2	3	2	3	2	2	3	2
9	9	1	9	1	9	9	9	9	9

3	3	1	1	1	2	2	2	2	2
2	2	2	1	1	3	2	2	3	3
9	1	1	1	1	1	1	1	1	2
9	1	9	1	2	1	3	2	3	3
1	9	9	3	1	3	3	1	3	3
9	9	9	9	1	9	9	9	9	9
2	1	3	1	1	1	1	2	1	3
2	3	2	1	1	3	3	2	3	3
2	2	3	1	2	3	2	2	3	2
2	1	3	1	1	1	1	2	3	3
3	2	1	1	1	3	2	3	3	3
3	2	1	1	2	1	2	9	1	2
1	2	2	3	1	3	1	2	2	2
2	2	1	1	1	3	3	3	3	3
2	2	2	2	2	1	3	2	2	3
3	3	3	1	1	3	3	3	3	3
3	3	1	3	1	3	2	3	3	2
3	2	2	1	1	3	3	3	3	3
2	1	2	3	1	3	3	3	3	3
3	1	1	1	2	1	2	1	3	3
2	2	2	1	2	3	3	3	3	3
9	9	9	1	2	2	1	1	2	1
3	1	2	1	2	1	1	1	2	1
3	2	2	2	2	2	2	2	2	2
2	2	2	1	2	3	2	2	3	2
2	2	2	9	1	1	9	9	9	9
2	2	2	1	1	2	3	3	2	2
3	2	1	3	1	1	1	1	2	3
1	2	1	1	3	3	3	3	3	3
3	3	3	3	1	3	3	3	3	1
9	9	9	1	9	9	3	9	9	9
2	2	2	2	2	2	2	2	2	1
2	2	1	1	1	1	9	9	9	9
3	3	3	1	1	1	2	1	2	1
2	1	2	1	3	1	2	3	2	2
9	1	9	1	9	1	9	9	9	9
9	9	9	9	9	9	9	9	9	9
2	2	2	2	2	2	2	2	2	2
3	2	2	2	2	1	2	3	3	1
2	1	3	1	2	1	2	3	2	2
1	1	1	1	2	2	3	1	3	3
1	1	1	1	1	1	1	1	1	1
1	2	1	3	1	2	1	1	1	2
1	2	3	1	2	1	2	3	1	2
1	3	2	1	9	3	2	1	3	2
1	2	3	9	9	9	9	9	9	9
3	2	1	3	3	2	1	3	2	1

1	2	1	2	1	1	1	2	1	2
9	9	9	9	9	9	9	9	9	9
3	3	3	3	3	1	3	3	3	3
3	1	2	1	1	1	1	1	2	1
3	2	3	3	1	3	1	1	3	1
1	2	1	2	1	1	1	2	1	2
1	3	2	1	9	9	9	9	9	9
3	9	2	1	1	3	2	3	3	3
2	2	9	1	1	1	1	1	1	1
3	1	2	1	3	3	1	3	1	2
9	9	9	9	9	9	9	9	9	9
2	2	2	1	9	9	9	9	9	9
9	9	9	9	1	9	9	9	9	9
1	1	1	1	3	3	3	1	1	1
3	2	2	2	1	3	1	2	1	3
9	9	9	1	1	3	2	2	3	3
3	3	3	9	1	3	3	3	3	3
9	9	1	9	1	9	9	9	9	9
3	1	2	2	1	3	3	2	3	3
3	3	3	2	3	2	1	2	3	2
3	2	1	2	1	3	3	1	3	3
3	3	3	1	1	2	2	1	1	1
3	3	1	2	3	1	2	3	1	2
2	2	2	2	1	3	3	2	3	3
2	2	2	3	1	3	2	2	2	2
2	1	1	1	1	2	1	2	2	3
9	9	9	9	9	9	9	9	9	9
2	2	2	1	1	3	3	3	3	3
2	3	1	2	1	3	2	3	2	3
3	3	3	2	2	1	3	2	2	2
3	3	2	3	2	3	3	3	3	3
2	2	2	2	1	1	2	1	3	3
1	3	1	1	3	1	2	9	2	3
3	2	2	1	3	3	3	2	2	2
2	2	2	3	1	2	1	1	1	1
3	1	1	3	1	3	1	2	3	2
1	1	2	1	2	1	2	2	1	1
3	3	3	1	3	1	1	1	1	1
3	1	2	1	3	3	2	2	3	3
2	1	1	1	1	3	1	1	2	1
1	3	3	3	9	2	3	2	3	1
3	3	3	3	3	3	3	3	3	3
3	1	3	1	9	1	9	3	3	3
9	1	9	1	1	1	3	3	3	3
9	9	9	1	1	3	3	3	3	3
9	1	9	1	1	3	3	3	3	3
9	9	9	1	1	1	3	3	3	3

2	1	2	1	1	3	3	2	2	2
3	2	2	2	3	1	2	2	2	3
1	1	9	2	1	3	3	3	3	3
1	2	2	3	1	3	3	3	3	3
3	1	3	1	1	1	1	1	1	1
9	9	9	3	1	3	3	3	3	3
9	9	9	3	3	3	3	3	3	1
9	9	9	1	1	3	3	3	3	3
9	9	1	1	1	1	3	3	3	3
9	1	9	1	1	9	3	3	3	3
9	1	9	9	1	9	3	3	9	9
2	1	2	3	3	3	3	3	3	3
9	9	1	1	1	1	3	3	3	3
1	1	3	1	3	3	1	1	1	1
3	1	3	1	3	3	2	3	3	3
2	1	2	3	1	3	2	3	3	3
2	2	2	3	1	3	9	3	3	3
9	1	9	3	1	3	3	3	9	1
9	9	9	3	1	3	3	3	3	3
9	9	9	1	1	1	1	3	3	3
1	3	2	1	3	2	3	1	1	2
9	9	9	1	1	3	3	3	3	3
1	1	2	1	1	3	3	3	3	3
9	9	1	1	1	3	3	3	3	3
2	2	1	1	1	3	3	3	3	3
1	2	2	1	1	3	3	3	3	3
2	2	2	2	3	1	3	1	3	3
1	1	1	1	1	1	3	3	3	3
1	1	1	1	1	3	3	3	3	3
1	1	1	1	1	3	3	3	3	3
1	1	1	1	1	3	3	3	3	3
2	2	2	3	1	3	3	3	3	3
3	3	3	1	1	9	2	2	2	2
9	9	9	1	1	1	3	3	3	3
1	1	1	1	1	1	3	3	3	3
1	1	1	9	1	3	3	3	3	3
3	3	3	1	1	3	3	3	3	3
9	9	9	1	1	9	3	3	3	3
3	3	3	3	1	3	3	3	3	3
3	3	3	1	1	1	3	9	1	3
3	3	3	2	3	2	3	1	2	2
3	1	3	3	1	3	3	1	3	1
1	3	1	1	9	3	2	3	3	3
2	2	2	1	2	1	1	2	2	2
1	1	1	1	1	1	1	1	3	1
3	2	2	1	1	1	1	1	2	3
3	2	3	1	3	3	2	2	3	3

3	2	3	3	3	3	2	3	3	3
3	3	2	3	3	3	2	3	3	2
2	2	3	1	2	3	1	2	9	2
1	2	2	2	1	3	2	2	3	3
1	3	2	9	1	3	2	3	3	3
9	9	9	2	9	9	9	9	9	9
1	2	3	3	1	3	2	2	3	3
2	2	2	1	3	3	2	2	1	3
9	9	9	1	9	9	9	9	9	9
3	2	2	1	1	9	9	9	9	9
9	9	9	1	9	9	9	9	9	9
2	9	9	9	9	9	9	9	9	9
2	2	2	2	1	9	1	1	2	2
1	2	1	3	2	2	9	1	9	2
1	1	1	1	1	3	3	3	9	3
1	3	3	3	1	3	2	1	1	1
1	2	1	3	2	3	1	2	1	1
2	1	2	3	1	3	3	3	3	3
1	3	1	1	3	1	1	1	1	1
9	1	9	1	9	9	9	9	9	9
2	3	1	1	3	1	1	1	1	1
1	2	3	1	1	3	2	3	3	3
2	2	2	9	1	3	2	2	3	3
1	1	1	1	1	1	1	1	1	1
2	1	2	3	1	3	2	2	3	3
1	1	1	3	1	3	3	3	3	3
2	2	2	1	3	1	1	9	9	3
3	2	3	2	3	2	2	2	2	3
2	2	2	2	1	2	2	1	2	2
1	1	3	2	9	9	9	9	1	9
1	3	1	1	1	1	1	3	1	3
1	1	1	1	1	1	1	1	1	1
2	2	1	1	1	3	1	1	1	1
1	2	2	2	1	1	1	1	1	1
1	1	1	1	1	3	3	3	3	3
3	2	1	3	2	2	2	2	3	3
2	2	2	2	2	1	2	2	2	2
1	1	1	1	2	2	2	1	1	1
1	1	1	1	2	2	2	1	1	1
3	3	3	1	2	2	2	2	2	2
1	2	1	1	3	3	1	3	3	1
2	3	2	1	1	3	2	2	2	2
3	2	2	2	1	3	3	1	3	3
2	2	2	1	1	3	3	2	2	1
3	1	2	3	3	3	3	3	3	2
3	3	3	3	3	3	3	3	3	3
2	2	2	2	1	3	1	2	3	3

2	2	2	9	9	9	9	9	9	9
2	3	1	2	1	3	3	3	3	3
9	9	9	9	9	9	3	9	9	9
9	9	1	1	9	9	9	9	9	9
9	1	9	1	9	9	9	9	9	9
2	2	2	1	1	3	2	9	1	1
9	1	9	9	2	9	9	9	9	9
3	1	2	1	1	3	2	1	3	3
2	2	2	2	2	3	3	2	3	1
2	2	2	1	3	2	2	2	2	2
2	2	2	1	1	3	3	3	3	3
1	3	1	1	3	2	1	1	1	1
1	1	1	1	2	1	1	1	1	1
2	2	2	1	3	1	3	3	3	3
2	1	1	1	3	3	1	1	1	1
2	1	2	1	1	1	2	1	1	2
2	2	2	1	1	1	2	2	2	2
3	3	3	3	3	3	3	3	3	3
2	3	2	3	1	1	3	1	2	2
2	2	2	1	1	1	1	1	1	1
2	2	2	2	3	3	2	3	2	1
2	1	1	1	2	1	1	1	1	2
2	2	2	2	2	1	2	2	2	2
2	2	2	3	3	1	1	1	2	2
1	1	1	2	1	3	3	2	2	2
2	2	2	1	2	2	2	2	2	2
9	9	1	1	2	3	1	1	1	1
3	1	3	1	3	3	3	1	3	3
3	2	3	1	2	3	2	3	2	2
1	1	1	1	2	1	1	2	2	2
9	9	9	2	9	9	9	9	9	9
2	1	3	2	3	2	3	2	3	2
1	2	1	1	1	1	2	1	3	1
3	3	2	2	1	3	3	2	3	2
3	3	3	1	3	3	3	2	2	1
1	3	2	1	2	3	3	3	2	3
3	1	1	1	1	1	1	1	3	1
3	1	1	1	9	1	3	3	1	1
2	3	3	1	2	3	1	2	1	1
2	2	2	2	1	3	1	1	2	3
2	2	2	1	1	3	2	9	2	2
2	2	2	1	1	2	2	2	2	2
2	2	2	3	1	1	3	1	9	9
9	1	9	1	9	9	9	9	9	9
9	9	9	9	1	1	9	9	9	9
9	9	9	9	9	9	9	9	9	9
9	9	3	2	1	3	3	2	3	3

3	2	2	2	1	3	3	2	3	3
2	2	2	2	2	1	1	2	1	1
9	1	9	1	3	1	3	3	1	1
2	3	1	1	3	3	3	3	3	3
1	9	3	1	1	3	9	3	9	9
2	3	2	1	2	2	2	9	3	2
2	2	2	2	1	3	3	3	3	3
2	2	2	2	1	3	2	3	3	3
1	2	2	3	2	2	2	2	2	2
3	1	1	3	3	1	1	3	3	3
9	9	1	9	1	9	9	9	9	9
9	9	1	2	9	1	1	2	3	3
2	2	2	1	1	2	1	2	9	1
9	9	9	2	1	2	1	3	2	3
1	9	9	1	9	1	9	9	9	9
3	1	3	3	1	1	1	3	9	3
2	1	2	2	1	3	1	2	1	3
1	3	2	3	1	3	3	3	3	3
9	1	1	2	9	1	1	1	1	1
3	2	1	1	3	2	1	2	2	2
2	2	1	3	2	2	3	2	1	2
3	1	3	1	3	1	3	1	3	1
3	1	1	1	3	3	1	3	1	1
1	3	3	3	2	3	3	3	2	2
3	1	3	2	2	2	2	2	2	2
9	9	9	3	3	3	3	3	3	3
1	3	3	3	2	3	3	3	2	2
3	1	2	1	1	3	3	1	1	2
1	3	3	3	2	3	2	3	2	3
3	1	2	3	3	2	2	2	2	1
3	3	1	3	1	3	3	3	1	1
3	2	2	3	2	2	2	3	1	1
2	1	3	2	1	1	1	2	1	2
9	2	2	1	1	3	2	2	3	3
3	1	1	2	3	1	3	3	2	1
1	1	9	3	2	2	1	2	2	1
2	2	2	2	2	1	2	2	2	2
2	2	3	1	3	3	1	1	3	3
9	9	9	1	9	9	9	9	9	9
3	3	3	1	1	2	2	3	2	2
9	9	9	3	9	9	9	9	9	9
2	1	2	1	3	9	9	9	9	9
9	9	9	1	3	2	3	2	2	1
9	9	9	1	3	2	3	1	2	2
3	3	3	2	3	1	1	3	3	3
3	1	1	1	3	1	3	1	1	1
3	3	3	2	2	3	3	3	3	3

3	3	3	1	3	3	3	3	3	3
3	9	9	2	1	9	3	9	9	9
3	1	3	3	3	3	3	3	3	3
1	1	1	3	1	3	1	3	3	1
3	1	3	3	1	3	3	3	3	3
3	1	3	1	3	1	1	1	1	1
3	2	1	1	9	2	2	1	1	1
1	1	2	1	1	2	2	2	2	2
1	1	3	1	3	3	3	3	3	3
3	3	1	3	2	3	2	9	1	3
3	3	1	1	1	3	3	1	1	1
3	1	2	3	1	3	3	2	2	1
3	2	3	1	3	1	1	2	1	2
1	2	3	9	1	3	3	3	3	3
1	2	1	1	1	1	1	2	1	2
1	2	2	2	3	3	1	1	2	3
1	1	2	1	3	3	1	2	2	2
3	3	2	1	3	1	3	3	2	3
1	3	1	1	1	3	3	3	3	3
2	2	2	2	2	2	2	2	2	2
2	2	2	3	1	3	1	3	3	3
3	3	1	1	1	2	2	3	1	2
3	3	3	3	3	3	3	3	3	3
1	1	1	1	1	1	3	1	3	3
3	2	2	3	1	3	3	1	2	3
1	2	3	1	1	2	3	3	1	1
3	1	2	1	2	2	3	1	2	3
3	3	3	3	1	2	2	2	3	3
1	1	2	2	1	3	1	2	1	3
9	9	9	9	1	3	9	9	9	9
2	1	3	1	2	3	2	2	3	1
3	1	2	1	1	1	1	1	1	1
1	3	2	3	1	3	3	1	2	3
1	2	2	2	2	3	3	3	1	3
1	1	1	3	3	3	1	2	1	1
2	2	2	2	9	3	3	3	3	3
3	2	2	1	1	3	1	3	3	3
3	1	2	1	3	2	2	2	2	2
1	3	2	1	3	1	2	2	2	1
2	2	1	1	1	3	3	3	3	2
1	9	9	9	9	9	9	9	9	9
9	9	9	3	9	3	2	2	3	9
2	3	2	1	2	1	2	3	1	2
3	1	1	3	1	3	2	1	3	3
3	3	2	1	1	2	2	2	2	2
3	3	1	1	3	1	3	3	3	3
9	9	9	9	9	9	9	9	9	9

1	1	2	1	3	1	1	2	1	1
1	3	3	1	1	1	3	3	3	3
1	2	1	3	3	3	3	3	3	3
3	2	1	1	2	1	2	1	2	3
9	9	9	9	9	9	9	9	9	9
1	1	1	1	9	1	1	1	1	1
2	2	2	1	1	3	2	3	3	3
2	2	2	2	2	2	2	2	2	2
2	1	2	2	3	3	1	1	1	2
1	1	1	1	3	3	2	1	3	2
9	9	9	1	3	3	3	3	3	3
3	2	3	1	2	1	2	2	2	1
3	2	3	1	2	3	2	2	3	1
9	9	9	9	9	9	9	9	9	9
2	3	3	2	1	2	2	2	2	2
9	9	9	9	9	9	9	9	9	9
3	2	3	1	2	2	3	3	2	2
1	1	2	1	3	3	3	3	3	3
2	3	3	1	1	3	2	3	1	1
1	2	2	1	1	2	2	2	2	2
3	3	3	1	1	1	3	1	1	3
2	2	2	3	1	2	2	3	3	3
9	1	9	9	1	9	9	9	9	9
2	2	2	1	3	2	1	3	2	1
2	1	2	1	1	3	2	2	2	2
2	2	2	1	1	2	2	2	2	2
9	9	9	1	9	3	9	3	9	3
2	2	2	2	1	2	2	2	1	2
2	2	2	3	3	1	2	2	2	1
9	9	9	9	1	9	9	9	9	9
1	1	3	3	1	3	2	3	3	1
1	1	1	3	3	2	2	3	1	2
1	3	1	3	3	3	1	1	3	1
1	2	2	1	1	3	2	3	3	2
1	1	1	1	3	3	2	3	3	3
3	2	2	3	3	3	3	3	3	3
3	1	1	1	1	1	2	3	3	1
1	1	3	1	1	3	3	3	3	1
1	9	9	1	2	9	9	9	9	9
3	3	3	2	2	3	3	3	3	3
2	1	2	2	1	3	3	2	1	3
3	1	3	1	1	3	3	3	3	2
3	2	2	1	1	3	3	3	3	3
1	2	1	1	1	3	9	2	2	3
2	1	2	3	3	3	3	3	3	3
3	2	2	2	1	2	1	1	3	2
3	2	2	1	3	3	2	3	2	1

1	2	1	2	2	2	2	2	2	2
2	2	2	3	1	3	2	2	2	3
2	2	1	2	2	2	1	1	1	1
9	1	9	1	1	2	2	2	2	3
2	1	2	2	3	2	1	1	2	1
9	1	9	9	1	9	9	9	9	9
2	2	2	2	2	2	2	2	2	2
1	9	1	3	1	1	3	2	3	3
2	2	3	1	1	2	3	3	3	3
2	1	2	3	1	3	2	3	3	2
3	1	2	9	3	3	2	2	2	1
3	1	1	1	2	1	1	2	1	3
2	2	2	2	2	2	2	2	2	2
3	3	3	3	3	3	3	3	3	3
3	3	3	3	1	3	1	1	2	1
2	1	3	3	3	2	2	3	1	1
3	3	3	2	3	2	1	1	1	1
2	2	2	3	1	1	2	9	3	3
2	3	3	1	1	3	3	3	3	3
2	2	2	1	1	2	2	2	1	2
3	2	2	1	1	1	3	3	1	1
1	3	2	3	1	3	2	3	1	2
3	1	2	3	1	3	2	3	1	2
2	2	2	3	3	3	1	3	1	3
1	1	2	1	1	3	3	3	3	3
1	2	2	2	2	1	1	1	2	3
3	1	2	3	1	3	2	3	3	3
3	1	2	3	1	3	2	3	3	3
2	2	2	2	3	3	2	2	2	2
2	2	2	1	1	3	1	1	3	3
2	3	3	1	3	3	2	2	2	2
2	3	2	3	1	3	2	3	1	2
3	3	1	3	1	3	3	3	3	3
2	2	2	1	1	3	1	3	3	3
3	3	3	1	3	1	1	3	1	1
2	2	2	1	9	3	2	2	1	1
2	2	2	1	3	3	2	2	1	1
2	1	3	1	2	2	1	1	2	3
3	1	2	1	3	1	2	1	1	1
2	1	2	2	2	2	2	2	2	2
2	1	3	1	2	3	2	3	1	3
1	2	2	3	1	3	1	3	3	3
1	1	1	1	1	2	1	2	2	3
1	1	2	1	1	1	2	2	2	1
1	3	3	3	1	3	2	1	3	1
1	3	1	1	1	3	3	3	3	3
9	1	9	3	1	3	3	3	2	2

2	2	1	2	1	3	2	2	1	2
3	3	1	3	2	2	2	2	2	2
9	9	9	1	1	2	2	3	3	3
2	2	2	3	3	2	2	2	2	2
2	2	2	1	1	9	3	3	3	9
2	2	2	2	1	3	2	2	2	2
3	2	2	2	1	3	3	3	3	3
2	1	2	3	1	3	3	3	3	3
2	1	2	1	1	3	2	3	2	1
2	3	2	1	2	3	2	2	3	3
2	2	2	1	2	3	2	2	3	3
2	2	2	1	1	3	1	1	1	2
2	2	2	1	3	3	2	2	2	2
2	2	2	3	1	3	3	1	3	3
2	2	2	1	3	1	1	1	1	1
2	2	2	1	1	2	2	3	3	2
2	1	2	2	2	2	3	3	3	3
2	2	2	1	1	3	1	3	2	2
1	1	2	1	1	1	2	3	3	2
1	2	2	2	1	3	2	1	3	3
2	2	2	1	2	2	2	2	2	2
2	2	2	3	2	2	2	2	2	2
3	3	2	1	2	3	2	2	2	1
2	2	2	2	1	3	2	3	2	2
2	2	2	1	1	2	2	1	3	2
9	1	9	2	1	3	2	1	3	1
2	2	2	1	1	2	2	2	2	2
2	2	2	2	2	2	2	2	2	2
2	2	2	2	1	2	2	2	2	2
3	2	1	3	1	3	3	3	3	3
2	2	2	2	2	2	2	2	2	2
2	2	2	2	2	2	2	2	2	2
2	2	2	2	2	2	2	2	2	2
2	2	2	2	1	2	2	2	2	2
9	9	9	1	9	3	3	9	9	9
2	2	2	1	1	2	2	2	2	2
2	2	2	1	1	2	2	2	2	2
2	2	2	1	2	2	2	2	2	2
2	2	2	2	2	2	2	2	2	2
2	2	2	1	1	3	2	3	3	3
2	2	2	2	2	2	2	2	2	2
2	2	2	1	1	3	3	3	3	1
2	2	2	1	1	3	3	3	3	3
2	2	2	2	2	2	9	2	2	2
2	2	2	1	2	2	2	2	2	2
2	3	1	1	1	3	2	3	2	1
2	2	2	1	1	9	3	3	3	9

2	2	2	2	2	2	2	2	2	2
2	1	2	2	2	2	2	2	2	2
1	1	1	1	1	3	3	3	3	1
2	2	2	2	2	1	2	2	2	2
2	2	2	2	2	2	2	2	2	2
3	2	1	2	3	3	1	3	2	2
9	1	9	1	1	3	3	3	3	3
9	2	9	9	2	3	3	3	3	3
1	1	3	1	1	3	3	1	1	3
2	1	2	2	1	3	3	3	3	3
2	1	1	1	1	3	3	3	1	3
2	1	2	1	3	2	2	2	2	2
2	1	2	1	1	3	3	3	3	2
9	1	1	1	3	1	1	3	9	9
1	1	3	1	1	1	1	1	3	1
2	2	2	2	1	3	2	1	3	3
2	2	2	3	2	2	2	2	2	2
9	9	9	1	1	2	3	2	3	3
9	9	9	1	1	1	1	1	1	1
2	2	2	1	1	3	2	2	3	2
9	9	9	1	1	3	3	3	9	2
2	2	2	2	2	2	2	2	2	2
2	2	2	1	2	3	2	3	3	3
2	2	2	2	2	2	2	2	2	2
2	2	2	1	2	2	2	2	2	2
2	2	2	1	2	2	2	2	2	2
9	1	9	9	1	9	9	9	9	9
2	2	2	1	3	2	2	2	2	2
2	2	2	1	2	2	2	2	2	2
9	1	9	2	1	3	2	1	3	9
2	2	2	2	1	3	2	1	3	3
1	2	2	1	3	1	2	1	3	2
1	3	3	3	3	1	2	3	2	1
2	2	2	1	1	1	2	1	3	2
2	1	1	2	1	3	3	3	3	2
3	2	1	2	2	3	1	1	1	3
3	3	2	3	1	3	2	3	3	3
1	2	2	3	1	3	2	3	3	3
1	1	1	3	1	3	3	3	3	3
3	1	2	1	2	2	2	3	2	1
3	3	3	3	1	3	3	3	3	3
2	2	2	3	2	3	2	2	3	3
3	1	1	1	1	3	2	2	3	3
2	2	2	2	2	2	2	2	2	2
1	1	1	2	3	3	1	2	3	3
1	1	1	1	2	1	1	3	2	1
2	1	1	3	1	3	2	1	1	2

2	1	2	2	1	1	2	9	3	1
1	3	2	1	2	1	2	1	3	3
3	2	1	1	1	3	2	2	3	3
3	2	1	1	1	3	1	3	3	3
9	9	9	3	2	1	2	9	1	3
2	1	1	3	2	1	9	1	2	1
1	1	1	1	1	2	1	1	3	1
9	9	9	3	1	2	9	9	9	9
2	2	2	2	1	2	2	2	2	1
2	2	2	2	2	2	2	2	2	2
3	3	1	1	3	3	1	1	1	1
3	3	1	1	3	3	1	1	1	1
2	2	2	1	2	3	3	3	3	3
1	2	2	3	1	1	2	1	2	2
2	3	3	3	2	3	2	3	2	2
2	2	2	1	2	3	2	2	3	3
2	2	2	2	1	1	2	1	2	1
9	1	9	1	1	3	3	2	2	3
2	1	1	2	2	3	2	2	3	3
1	1	2	1	3	3	3	3	9	3
2	2	2	1	3	3	3	3	3	2
9	9	9	2	2	2	2	2	2	1
1	1	2	1	3	3	2	2	2	3
9	2	9	9	1	9	9	9	9	9
3	2	1	1	3	1	2	3	1	1
1	1	2	1	1	1	2	1	1	3
1	1	1	1	2	2	1	3	2	1
9	9	9	1	3	2	2	2	2	2
2	2	2	2	1	2	2	2	2	2
9	9	9	9	1	9	9	9	9	9
3	1	3	1	3	3	3	3	2	3
9	9	9	9	9	9	9	9	9	9
9	9	9	9	9	9	9	9	9	9
2	1	1	3	1	3	2	2	2	2
2	1	1	3	1	3	2	2	2	2
9	9	9	1	1	3	1	1	1	1
9	9	9	3	3	1	2	3	1	1
2	1	1	3	1	3	2	2	2	2
2	2	2	1	1	1	1	3	1	3
2	2	2	1	1	1	1	3	1	3
9	9	9	9	3	9	9	9	9	9
2	2	2	1	1	3	3	3	3	3
2	2	2	1	2	2	2	2	1	1
9	9	9	9	9	9	9	9	9	9
9	9	9	3	9	9	9	9	9	9
2	2	1	1	2	1	2	2	1	1
2	2	2	2	9	2	2	2	2	2

9	9	9	1	1	3	3	3	1	1
2	1	2	3	1	3	3	3	3	3
3	3	3	2	1	3	3	1	3	3
2	1	1	9	9	9	9	9	9	9
2	2	2	2	2	2	2	2	2	2
3	3	3	1	9	3	3	3	3	3
2	2	2	2	2	2	2	2	2	2
9	9	9	1	1	3	3	3	3	3
9	9	9	1	9	9	9	9	9	9
9	9	9	2	2	2	2	3	2	2
2	2	1	2	2	3	1	3	2	3
2	2	2	3	1	1	3	1	2	2
3	3	1	2	3	3	1	2	1	2
3	3	3	3	2	3	3	3	3	3
3	2	3	1	3	3	2	3	2	2
1	1	2	3	1	3	3	1	3	3
3	1	1	3	3	3	3	3	3	3
1	2	1	2	1	1	1	2	3	2
2	2	2	1	2	2	2	2	2	2
1	3	1	3	1	2	3	1	2	2
3	1	2	3	2	2	3	3	2	2
1	1	1	1	3	1	1	1	1	1
2	1	2	1	1	1	1	1	1	3
2	3	3	1	3	3	2	2	2	2
3	3	3	3	1	1	1	3	3	3
2	2	2	2	2	2	1	2	2	2
2	2	3	1	3	3	2	3	3	3
2	1	2	2	1	3	3	3	3	1
2	1	2	1	3	1	2	2	3	1
3	3	3	2	3	1	2	3	1	1
3	2	2	1	1	1	1	1	1	1
3	3	2	1	2	3	3	3	3	3
3	1	1	2	1	3	3	2	3	3
3	3	3	1	9	3	3	3	3	3
3	1	2	1	2	2	1	2	2	2
2	1	2	3	2	3	1	2	3	2
3	3	3	3	1	1	1	3	3	3
2	2	2	1	1	9	1	1	1	9
1	1	1	1	3	1	1	1	1	1
1	9	9	1	3	3	2	1	1	1
3	3	1	1	1	2	9	9	2	2
3	2	1	2	1	1	2	3	1	2
3	3	2	3	3	3	2	3	2	3
2	3	2	1	1	3	3	1	3	1
1	1	1	1	3	3	2	3	3	1
3	2	2	1	3	2	2	2	2	2
3	3	3	2	2	2	2	2	2	9

1	2	2	1	2	1	3	2	1	2
2	2	3	1	1	1	2	3	3	3
3	3	3	2	1	1	1	1	2	3
3	3	3	1	2	3	3	3	3	2
1	1	1	1	1	1	1	3	3	3
1	3	2	3	3	1	1	2	1	1
1	1	1	9	9	9	9	9	9	9
3	2	2	2	1	3	2	1	2	3
3	3	3	3	1	3	1	3	3	3
3	2	1	1	3	1	1	2	3	1
3	3	3	3	2	3	1	3	3	2
2	2	2	1	3	2	3	2	1	2
2	2	2	2	2	2	2	2	2	2
1	1	1	1	1	3	3	1	3	1
1	1	1	3	1	3	3	3	3	3
2	2	2	2	2	3	2	2	1	2
2	1	2	2	2	2	2	3	2	2
3	2	2	1	1	1	2	1	2	3
1	3	2	3	3	3	1	1	3	1
1	2	2	1	1	3	3	3	2	3
2	1	1	3	1	3	1	1	1	1
9	2	9	3	2	3	2	2	2	3
3	3	3	3	2	1	1	2	3	3
2	2	2	2	3	3	2	2	2	3
2	2	2	3	1	3	2	2	3	2
2	2	2	1	1	3	3	3	3	3
2	2	2	3	9	2	2	2	2	2
1	1	2	1	3	2	1	2	1	2
3	3	3	2	3	3	3	2	1	3
3	2	1	3	1	3	2	2	3	3
3	3	3	1	3	3	3	3	1	1
3	1	1	3	1	3	3	2	3	3
2	2	3	2	1	1	2	3	2	2
2	1	1	1	3	3	3	3	3	3
2	1	3	1	1	1	2	3	1	2
2	2	2	3	1	1	3	1	3	3
1	1	1	3	1	3	1	1	3	3
2	1	1	1	3	3	2	2	3	1
1	3	2	1	3	3	3	2	1	1
9	9	9	3	9	1	9	9	9	9
2	3	2	1	1	3	2	3	2	3
3	2	1	1	1	2	1	3	3	1
2	2	2	1	2	2	2	2	2	3
1	1	1	1	1	3	1	1	3	3
3	3	1	3	1	3	1	3	3	3
9	9	9	3	1	3	2	3	3	3
1	2	2	3	2	2	1	1	2	3

2	2	2	3	2	2	1	2	1	2
2	2	2	2	2	2	2	2	2	2
3	3	1	3	3	3	3	3	3	3
3	2	1	2	1	1	2	1	3	2
2	2	2	9	1	9	9	9	9	9
1	3	3	9	9	9	9	9	9	9
1	3	3	1	9	9	9	9	9	9
1	1	1	1	1	3	2	2	2	2
2	2	2	9	9	9	9	9	9	9
3	1	3	1	3	1	1	1	1	2
1	1	2	1	3	2	1	1	1	2
3	3	2	1	3	3	1	1	3	1
9	1	9	9	1	2	3	1	3	1
2	1	1	9	1	3	3	3	3	3
9	9	9	1	9	9	2	2	2	2
2	2	2	3	1	3	3	2	3	2
1	1	9	9	9	3	2	2	1	1
2	2	1	3	3	1	1	1	1	1
3	1	3	3	3	3	3	3	3	3
2	2	1	3	2	3	1	3	2	3
3	2	1	1	1	1	1	1	1	1
1	2	2	2	1	2	3	3	3	3
2	2	2	1	3	3	1	3	1	2
3	3	2	1	3	3	3	2	3	3
9	9	9	3	1	3	2	3	3	2
1	1	2	2	2	3	3	2	1	1
2	3	2	2	1	2	3	2	3	1
3	2	1	2	3	3	2	2	3	1
3	1	3	3	3	3	3	3	3	3
2	2	2	1	1	9	3	1	2	3
2	1	2	9	2	1	2	2	1	2
2	2	2	1	2	2	2	2	2	2
1	1	1	1	3	1	1	1	1	1
2	2	1	1	1	3	3	3	3	3
3	1	2	3	1	1	2	3	2	1
9	9	9	2	3	1	2	3	3	3
2	2	1	2	1	1	1	1	1	1
1	3	1	1	3	3	3	3	3	3
2	2	1	1	1	3	3	3	3	3
1	1	2	1	1	2	2	2	1	1
3	3	3	2	2	3	3	3	3	3
1	2	2	2	1	1	1	2	2	2
3	3	1	1	3	3	2	1	2	3
3	1	3	1	3	2	1	1	2	2
2	1	1	1	3	1	3	3	3	2
3	3	3	2	3	1	3	3	3	3
2	2	2	1	3	3	2	1	1	2

3	1	1	1	3	3	2	1	1	1
3	1	3	1	2	3	2	2	2	2
2	2	2	2	2	2	2	2	3	1
2	2	1	1	2	2	1	1	2	3
3	3	3	1	1	3	3	1	1	1
3	2	1	1	2	3	1	2	1	2
9	9	9	1	9	2	9	9	9	9
3	2	1	1	3	2	2	2	1	2
2	2	2	2	1	3	2	1	3	3
2	2	2	2	1	3	2	1	3	3
2	2	2	3	3	3	3	3	3	3
2	2	2	2	2	2	2	2	2	2
1	1	1	1	2	2	2	2	1	1
2	2	2	1	9	2	2	2	2	3
3	3	3	2	1	3	3	2	3	3
2	3	1	1	1	2	2	2	3	3
2	2	2	3	1	3	2	2	1	3
3	3	2	2	2	2	2	2	3	2
1	2	2	1	3	1	1	2	2	2
2	1	2	1	3	3	3	1	2	1
2	2	2	2	2	1	1	2	2	2
2	2	2	2	1	3	2	1	3	3
2	2	2	2	2	2	2	2	2	2
3	3	3	1	2	1	1	1	1	3
1	2	1	2	1	2	1	2	3	1
2	2	2	2	2	2	2	2	2	2
2	2	1	2	3	2	1	3	2	1
3	3	2	2	2	2	2	3	3	2
2	2	2	1	1	3	1	1	3	3
3	2	2	1	2	3	3	3	2	1
3	1	3	1	1	3	2	2	3	1
2	3	2	1	1	1	2	9	1	3
3	2	2	1	2	1	1	1	2	2
2	1	3	2	2	3	9	2	2	2
1	1	3	3	1	3	3	3	3	3
2	2	2	2	1	1	3	1	9	1
2	1	2	1	1	1	2	2	1	1
2	2	2	3	3	3	2	2	3	2
3	3	3	3	3	3	1	1	1	3
3	3	3	3	3	3	3	3	3	1
2	2	2	2	2	2	2	2	2	2
2	2	2	2	2	2	2	2	2	2
2	2	2	2	2	2	2	2	2	2
3	3	3	3	3	3	3	3	3	3
3	3	3	3	3	3	3	3	3	3
9	9	2	2	9	9	9	2	9	9
1	1	1	1	2	1	1	1	2	2

2	1	1	2	2	1	1	1	9	2
2	2	1	2	2	1	1	1	1	1
1	2	2	2	1	1	2	1	1	2
2	2	2	2	1	1	1	1	1	2
1	1	1	1	2	2	1	1	1	2
3	2	1	1	3	2	2	1	1	1
1	2	1	1	3	2	2	1	1	2
1	2	2	1	3	1	2	1	1	1
2	1	1	1	2	1	1	1	1	1
2	2	1	2	1	2	2	1	1	1
2	2	1	2	3	2	2	2	1	2
2	3	3	2	1	9	2	9	9	2
2	2	2	9	3	2	2	2	2	2
2	2	2	9	3	2	2	2	2	2
2	2	2	9	3	2	2	2	2	2
2	2	2	9	3	2	2	2	2	2
2	2	2	9	3	2	2	2	2	2
2	2	2	9	3	2	2	2	2	2
2	2	2	2	9	9	2	9	9	2
2	2	2	2	2	2	2	1	1	2
2	2	2	2	2	9	2	2	2	2
2	2	2	2	2	2	2	2	2	2
2	2	2	2	2	2	2	2	2	2
1	1	2	2	2	2	2	2	9	1
2	2	2	2	3	2	2	2	2	2
2	2	2	2	3	2	2	2	2	2
2	2	2	2	2	2	2	2	2	2
2	2	2	1	9	2	2	2	2	2
1	1	1	1	3	1	2	3	2	2
2	9	9	2	2	1	2	2	1	2
2	2	2	2	2	2	2	2	2	2
2	2	2	9	3	2	2	2	2	2
2	2	2	2	3	2	2	2	2	2
2	2	2	9	3	2	2	2	2	2
1	9	9	1	9	3	1	3	2	1
9	3	1	9	1	9	1	2	9	1
1	9	1	3	1	9	1	2	9	3
2	9	2	2	9	2	9	2	9	2
9	9	9	9	9	9	9	9	9	9
2	9	1	1	9	1	9	1	9	1
1	9	1	1	9	2	9	3	1	3
2	2	1	2	1	1	3	3	2	2
2	2	1	1	3	1	2	3	1	1
2	2	2	2	2	2	2	2	2	2
9	3	1	1	9	9	2	9	9	1
9	9	2	9	2	9	9	2	9	9
2	1	2	2	1	1	1	2	1	1

2	1	2	2	1	1	2	3	3	2
1	1	2	2	2	1	1	1	2	1
2	2	2	1	2	2	2	2	1	1
1	2	1	1	3	1	2	2	2	1
1	2	2	1	3	1	1	2	1	1
2	3	3	1	3	2	2	2	1	1
2	1	1	3	1	2	3	2	1	1
1	1	1	2	1	1	2	2	2	3
1	1	2	2	3	2	2	2	2	2
1	1	2	1	3	1	2	2	1	2
1	3	3	2	1	1	2	2	2	2
1	2	2	3	1	1	1	2	3	2
2	2	2	3	1	1	2	1	1	1
1	2	2	2	1	1	2	2	2	1
1	2	2	3	1	2	1	1	1	2
2	2	2	3	1	1	2	2	2	2
2	2	2	9	1	3	3	3	3	3
2	1	1	2	3	3	1	2	2	1
1	1	1	1	3	3	2	1	2	2
2	1	2	2	9	2	2	1	2	2
3	2	2	2	3	1	2	2	1	2
1	1	2	2	1	1	2	2	3	2
1	1	2	1	3	2	1	2	1	2
1	1	2	1	3	2	2	1	2	3
2	2	2	3	1	1	3	2	2	2
2	2	3	3	2	2	2	1	1	2
2	2	1	3	1	3	2	1	2	2
1	2	2	1	3	3	1	3	3	1
2	2	2	2	1	9	2	3	2	2
2	2	2	2	2	2	2	2	2	2
2	2	2	2	9	2	2	2	2	2
3	3	3	3	3	9	3	3	3	3
2	2	2	1	3	1	3	1	2	1
2	2	2	3	1	3	3	3	3	3
2	1	2	1	3	2	2	2	2	2
3	3	3	3	3	3	9	3	3	3
2	2	2	2	3	2	2	2	2	2
2	2	2	2	2	2	2	2	2	2
2	2	2	1	3	2	2	2	2	2
1	2	2	1	2	1	1	1	2	2
1	1	2	2	3	2	2	1	1	2
3	3	3	2	2	2	2	2	2	2
2	3	3	2	1	2	1	1	3	2
2	2	3	3	1	2	1	1	2	3
1	2	3	2	2	1	2	2	2	2
1	2	2	1	2	1	2	1	2	1
1	2	1	1	2	1	2	2	1	1

1	9	9	2	9	9	9	9	9	9
2	1	2	1	2	1	1	1	2	2
2	2	2	1	2	1	2	2	3	2
2	2	2	1	2	2	1	1	1	2
3	1	3	1	2	3	2	3	3	2
2	1	1	3	1	3	3	3	3	3
2	1	3	3	1	3	3	3	3	3
3	2	1	2	1	1	3	2	3	3
1	1	1	1	1	3	3	3	3	3
1	3	2	1	1	2	2	2	2	2
2	2	3	1	1	1	2	3	2	3
1	2	2	3	1	1	2	2	3	3
1	3	2	3	2	1	2	3	1	2
1	1	2	2	2	3	2	1	2	2
3	1	3	1	2	3	2	3	2	1
2	2	2	1	1	3	3	1	3	3
2	2	2	1	1	3	3	1	3	3
2	2	2	1	1	2	2	1	2	1
3	1	2	1	3	3	3	1	3	1
3	3	2	1	3	1	2	2	1	2
2	2	3	9	1	9	9	9	9	9
3	3	2	1	3	2	2	2	2	2
1	3	1	1	2	3	1	2	3	2
2	2	3	1	2	3	3	3	3	3
3	2	2	3	3	3	2	3	3	3
2	2	2	3	2	1	2	1	2	1
3	1	2	2	1	3	3	1	2	3
1	2	1	1	3	3	3	1	3	2
1	1	1	1	1	1	1	1	1	1
2	1	1	1	1	2	1	1	3	2
2	1	3	2	1	1	2	2	3	3
1	1	1	1	2	3	3	3	3	3
2	2	2	2	2	2	2	2	2	2
2	1	2	2	1	2	2	2	2	2
3	1	2	1	9	3	3	3	3	3
1	2	1	1	1	2	3	3	3	3
1	1	2	1	2	1	3	3	2	2
2	1	2	3	1	1	2	2	2	2
2	1	2	1	1	1	3	3	9	3
2	2	3	1	2	3	1	3	2	3
2	2	2	1	2	3	2	3	3	3
2	3	3	2	2	3	9	2	2	2
2	2	2	3	1	3	3	2	3	2
1	2	2	3	3	1	2	1	2	1
2	2	2	2	2	2	2	2	2	1
1	1	2	1	1	1	3	1	3	1
3	1	1	1	1	2	1	1	3	1

1	3	3	1	1	3	3	3	3	3
1	1	2	1	1	3	3	3	3	3
1	2	2	3	2	2	2	2	2	2
1	1	2	1	3	3	2	2	3	2
3	1	1	1	3	3	3	1	3	3
3	2	1	1	1	1	1	1	1	1
2	2	2	3	1	3	3	3	3	3
1	3	3	1	9	2	1	2	1	2
1	3	3	1	1	3	3	3	3	3
9	9	9	9	9	9	9	9	9	9
3	1	2	1	9	1	2	1	1	2
2	1	1	1	3	1	2	3	2	1
1	2	3	9	9	1	3	2	2	1
2	2	2	1	1	3	1	1	2	2
2	2	2	1	3	3	1	3	3	1
2	2	2	2	2	2	2	2	1	1
3	1	1	2	2	2	2	3	2	2
3	1	1	1	1	2	2	2	2	2
3	3	3	3	3	3	3	3	3	1
3	3	3	1	1	1	3	3	3	3
3	3	3	3	3	3	3	3	3	3
3	3	3	3	3	3	3	3	3	3
2	2	2	1	2	3	1	3	2	3
3	3	2	2	3	3	2	2	9	3
2	2	2	2	2	2	2	2	2	2
3	9	9	3	9	1	9	9	2	9
2	2	2	1	2	2	1	2	1	1
2	2	2	1	1	2	2	2	2	2
2	2	1	2	3	2	2	2	2	2
2	3	1	1	1	1	2	1	3	2
2	3	1	3	1	3	3	3	1	3
1	1	1	2	2	3	1	2	2	2
1	2	3	2	2	2	3	2	3	1
3	3	3	3	3	3	3	3	3	3
3	1	2	1	1	3	3	3	3	3
3	1	1	1	1	2	2	2	2	2
3	1	1	1	1	2	2	2	2	2
3	1	1	1	1	2	2	2	2	2
2	2	2	1	3	2	2	2	2	2
2	2	2	2	3	2	2	2	2	2
1	1	1	1	2	3	1	1	1	2
3	3	3	3	1	3	1	3	3	3
1	3	2	1	3	2	2	2	2	2
1	3	3	3	1	3	3	3	3	3
3	3	3	1	2	3	3	3	3	3
1	1	1	3	1	3	2	2	3	3
2	1	1	1	1	1	1	2	3	2

2	2	2	1	3	3	3	2	2	2
1	3	2	3	1	1	3	9	1	1
3	3	3	1	1	3	3	3	3	3
2	2	2	2	2	2	2	2	2	2
2	2	2	3	3	3	3	2	2	3
9	9	9	9	2	9	2	9	1	3
2	2	2	2	2	2	2	2	2	2
2	2	2	2	2	2	2	2	2	2
2	1	2	3	1	3	2	1	2	3
2	3	3	1	2	2	3	3	3	2
2	2	1	1	2	1	1	2	3	2
1	1	3	1	2	1	3	2	3	3
9	1	9	1	9	9	9	9	9	1
9	9	9	9	9	9	9	1	9	9
9	2	9	9	9	9	9	9	9	9
3	1	3	3	3	1	3	2	2	3
2	1	2	1	3	2	2	2	3	2
9	2	1	1	1	3	1	1	9	2
3	1	2	1	1	3	3	1	3	3
2	1	2	3	1	3	3	3	3	3
3	3	3	2	3	3	3	3	3	1
1	1	1	1	3	3	3	3	3	3
1	1	1	1	1	3	3	3	3	3
2	1	2	2	2	2	2	2	2	2
3	1	1	3	1	3	3	1	3	3
2	2	2	1	1	3	2	2	2	2
3	1	1	3	1	3	3	1	3	3
9	9	2	3	3	3	2	2	3	9
9	9	9	3	3	3	3	3	3	3
1	1	3	3	1	3	3	3	3	3
2	3	2	1	3	3	2	3	2	3
3	2	1	9	1	3	2	3	3	2
3	3	1	2	1	3	9	2	2	2
1	3	1	2	1	1	2	3	3	3
3	3	3	3	1	3	3	3	3	3
3	3	3	1	9	3	3	3	3	3
1	2	3	3	1	2	2	2	2	2
2	3	2	1	2	1	3	3	3	3
3	1	3	1	1	3	3	2	2	2
3	3	2	1	1	3	3	3	3	3
3	2	2	3	1	1	1	2	2	1
2	2	3	3	1	3	3	3	3	3
2	2	1	1	3	3	1	3	3	3
2	1	2	3	1	3	2	3	2	2
2	2	2	1	1	3	2	3	3	2
2	3	3	1	1	3	3	3	3	3
1	2	2	3	1	3	2	3	2	2

2	2	1	2	2	2	2	2	2	1
1	2	1	3	3	3	3	3	1	2
9	9	1	9	1	9	9	9	9	9
3	2	1	1	2	2	3	2	3	3
2	1	2	3	1	2	2	3	3	3
3	1	2	2	3	3	1	1	3	1
2	2	2	2	1	3	2	3	3	3
3	3	3	1	2	3	2	2	1	1
3	1	3	3	3	1	2	3	2	2
2	2	3	3	3	3	3	3	3	3
2	9	1	3	2	2	2	3	2	3
1	9	9	1	3	1	3	9	3	1
1	2	1	1	1	1	2	3	3	3
1	1	2	2	1	9	3	2	9	3
1	1	2	2	1	3	2	3	3	3
9	9	9	3	1	3	9	2	3	1
3	3	3	3	1	3	3	3	3	3
2	3	3	2	1	3	3	3	1	2
3	2	1	3	1	3	3	3	3	3
1	3	3	3	1	3	1	3	3	3
2	2	2	2	1	3	2	2	3	1
3	3	3	9	9	9	9	9	9	9
3	3	3	3	3	3	3	3	3	3
3	2	1	1	2	1	3	2	2	3
2	2	2	1	1	3	3	3	3	3
1	3	2	3	9	3	9	9	9	9
9	9	9	2	1	3	2	9	1	2
1	2	1	1	1	3	1	2	1	3
9	9	9	9	1	9	9	9	9	9
9	1	9	1	9	2	9	9	9	9
9	9	9	9	1	9	9	9	9	9
3	1	2	2	1	3	2	2	3	3
3	3	3	3	1	1	3	1	3	1
1	3	2	3	1	3	2	3	3	1
2	1	3	3	1	3	1	3	2	3
2	2	1	2	1	3	3	9	3	3
2	2	1	2	1	3	3	9	3	3
3	3	3	1	2	1	2	2	2	2
2	1	3	2	1	3	2	1	2	1
2	2	2	3	1	2	1	2	2	2
9	2	9	1	1	9	3	3	3	3
1	3	1	3	3	3	1	1	3	3
2	2	3	3	1	3	1	3	3	3
3	1	1	1	2	1	2	3	3	2
3	1	1	1	2	1	2	3	3	2
3	2	2	1	2	1	1	2	2	3
2	1	2	1	1	1	2	3	3	2

2	2	1	3	1	3	3	1	3	3
1	2	3	1	1	2	2	1	1	3
9	1	9	3	1	3	3	3	3	3
1	9	9	3	1	3	1	3	3	3
1	9	9	3	1	3	3	3	3	3
3	3	1	3	1	3	3	3	3	3
3	1	1	2	3	1	1	3	3	1
2	2	2	3	2	2	2	2	9	2
1	1	1	9	9	9	9	9	9	9
1	2	2	3	1	3	3	1	2	3
2	2	2	2	2	3	3	2	2	2
3	2	1	3	1	3	1	2	2	3
1	2	1	3	3	9	2	9	3	9
2	1	1	1	1	3	3	2	3	3
3	3	2	2	3	3	2	9	9	9
9	9	9	9	9	9	9	9	9	9
3	1	1	1	3	2	2	1	2	3
1	2	2	2	1	3	3	1	1	1
2	2	1	9	9	9	9	9	9	9
9	9	9	1	2	9	1	9	9	9
9	9	9	9	1	9	1	9	9	9
3	3	3	2	1	3	3	3	3	3
9	9	9	9	9	3	3	3	3	3
9	9	9	9	9	9	9	9	9	9
3	3	1	3	1	3	3	3	3	3
9	9	2	2	1	2	2	1	2	1
3	3	3	2	1	3	3	3	3	3
3	3	3	3	1	3	3	3	3	3
3	3	3	3	1	3	3	3	3	3
3	2	3	3	3	3	3	3	3	3
2	2	2	1	1	2	2	2	2	2
2	1	2	1	3	3	1	3	3	3
2	2	2	2	2	2	3	2	3	3
3	3	3	3	1	3	3	2	3	3
2	2	2	1	1	2	1	2	2	2
3	3	3	1	1	3	3	3	3	3
3	2	2	3	3	3	1	3	3	3
3	3	1	3	1	3	3	3	3	3
2	2	2	3	1	3	2	2	3	3
2	1	2	3	1	3	3	1	9	3
2	2	1	1	1	1	2	2	2	3
1	1	1	1	3	2	2	1	2	2
2	2	2	1	2	2	2	1	2	2
9	9	1	1	9	3	9	9	9	9
9	9	9	2	9	9	9	9	9	9
1	3	3	3	1	3	1	3	3	1
3	3	3	1	1	1	1	1	1	3

2	1	2	2	1	2	2	1	2	2
3	2	2	3	1	3	2	2	2	2
2	2	2	2	1	2	2	2	3	2
2	2	2	2	3	1	1	1	3	1
2	2	2	3	1	2	2	1	1	2
3	3	2	1	1	1	3	1	2	3
1	2	3	2	1	2	3	1	3	3
2	3	2	1	2	3	1	2	3	1
9	9	9	9	9	9	9	9	9	9
1	2	2	1	2	2	1	2	1	2
2	2	2	2	9	2	2	2	2	2
2	1	1	2	1	3	2	3	3	3
1	2	1	1	2	1	2	3	1	2
2	2	2	2	2	2	2	2	2	2
2	2	2	2	9	3	2	2	2	2
1	2	2	2	2	3	2	1	2	2
1	2	3	1	2	3	1	2	3	1
1	2	3	3	2	2	2	2	2	2
2	2	3	2	1	1	1	1	1	3
2	2	2	3	3	3	3	3	3	3
3	1	1	1	3	1	1	2	3	3
3	3	3	3	3	3	3	3	3	3
2	2	2	2	2	2	3	3	2	2
2	1	3	2	2	2	1	2	3	2
3	3	3	2	3	2	2	2	2	2
3	3	2	2	3	3	9	9	9	9
2	2	2	3	1	2	3	3	1	2
9	1	3	1	2	1	1	1	1	2
1	2	1	1	2	2	1	3	2	1
9	9	9	9	9	9	9	9	9	9
2	2	3	1	1	2	2	2	3	3
9	9	9	9	9	9	9	9	9	9
3	3	3	3	3	3	3	3	3	3
1	1	1	2	1	1	9	1	1	2
3	2	3	1	2	3	1	3	1	2
1	2	1	1	2	1	2	1	2	1
2	1	2	1	2	3	2	1	1	2
2	2	2	1	2	1	2	9	1	2
2	2	2	2	2	2	2	2	2	2
1	2	3	1	2	2	2	2	2	2
3	1	2	2	3	2	3	1	3	2
9	9	9	9	1	9	9	9	9	9
2	2	2	2	2	2	1	2	2	2
3	2	2	1	1	3	2	3	2	3
2	2	2	2	1	2	2	9	2	3
3	1	2	3	1	3	3	3	2	3
1	2	3	3	1	3	3	2	1	3

1	9	1	1	3	3	3	3	3	9
2	2	1	3	3	3	2	2	3	3
1	3	2	1	3	1	3	1	3	1
3	1	2	1	2	3	1	1	2	2
3	2	2	1	2	2	1	2	3	3
2	2	2	2	2	2	2	2	2	2
3	1	1	3	3	3	3	1	1	1
2	1	2	3	1	3	3	1	1	1
9	1	9	9	1	9	9	9	9	9
3	2	2	1	1	3	2	2	1	1
3	2	2	3	1	3	1	3	3	3
1	3	2	3	3	3	1	3	3	2
9	9	9	9	9	9	9	9	9	9
2	2	2	1	1	3	2	2	3	1
3	1	1	3	3	1	1	1	1	1
1	3	3	2	1	1	1	1	2	3
9	1	9	3	3	3	1	3	1	1
3	2	1	1	3	2	3	1	2	3
2	3	3	3	1	3	1	1	1	1
2	2	2	2	1	1	1	2	2	1
1	2	1	1	2	1	2	1	2	1
3	3	3	3	3	3	3	3	3	3
2	2	2	1	1	1	2	2	2	3
2	3	2	3	1	3	3	2	2	3
3	2	1	1	1	3	3	2	3	3
3	1	2	3	1	3	3	3	2	3
3	3	2	3	1	3	3	3	2	3
3	3	3	3	2	2	1	2	3	1
1	3	3	2	1	3	1	1	2	1
2	1	2	3	1	3	3	3	3	3
1	2	2	2	2	2	2	2	2	2
3	1	2	2	2	2	2	2	3	1
2	2	2	1	1	2	1	2	2	2
1	1	1	2	1	3	3	3	3	3
3	2	2	2	1	3	2	3	2	2
1	2	9	1	9	9	9	9	9	9
3	3	3	1	2	3	3	3	1	3
3	2	2	1	3	3	2	3	1	2
2	3	1	3	1	3	3	3	9	9
2	2	2	2	2	2	2	2	2	2
3	3	3	1	2	3	3	3	1	3
3	1	2	1	2	3	1	1	3	3
2	2	1	1	1	3	3	3	3	3
2	2	2	1	1	3	9	1	2	2
1	1	2	9	9	9	9	9	9	9
3	1	3	3	1	3	2	3	1	2
9	9	9	1	1	3	1	9	9	9

1	9	9	1	1	3	3	3	3	3
1	9	3	2	3	3	1	2	2	2
1	3	1	1	3	3	1	1	3	1
3	1	1	3	3	3	3	3	3	1
2	2	2	3	2	2	1	1	2	2
1	1	3	2	9	2	1	1	1	2
2	2	2	2	2	2	2	2	2	2
1	1	9	9	9	1	1	1	1	1
3	3	3	2	3	1	3	2	3	1
3	3	3	2	2	2	1	2	1	1
3	3	3	3	3	3	3	3	3	3
3	2	2	1	2	3	2	3	1	3
2	2	2	1	2	3	3	3	3	2
2	2	3	1	2	2	3	1	3	3
1	9	3	2	3	3	1	2	2	2
2	2	2	3	3	2	1	2	3	9
9	9	9	3	1	3	2	3	3	3
9	9	9	9	9	9	9	9	9	9
3	3	3	9	9	9	9	9	9	9
9	9	9	3	9	2	1	3	2	1
1	2	3	1	2	2	2	2	2	2
1	9	3	1	2	2	1	2	1	1
2	2	3	1	1	1	3	2	3	3
3	3	3	2	2	2	2	2	2	2
2	2	3	1	2	1	3	2	1	2
2	9	9	2	9	2	2	2	3	2
9	9	9	2	9	9	9	9	2	9
2	2	2	2	2	2	2	1	2	2
9	9	9	9	9	9	9	9	9	9
9	9	9	9	9	9	9	9	9	9
9	9	9	9	9	9	9	9	9	9
9	9	9	9	9	9	9	9	9	9
9	9	9	9	9	9	9	9	9	9
1	1	2	1	2	2	2	2	1	2
3	1	2	2	3	3	1	2	3	1
9	9	9	2	1	2	3	2	3	2
9	9	9	9	9	9	9	9	9	9
2	2	2	3	1	3	2	1	3	3
1	9	1	9	9	9	9	1	1	1
2	2	2	2	2	2	2	2	2	2
2	2	2	2	2	2	2	2	2	2
2	2	1	2	1	3	2	3	3	3
1	1	2	1	1	2	2	2	2	2
2	2	2	1	2	2	2	2	2	2
1	1	2	1	1	1	1	1	1	2
1	1	2	1	2	1	2	2	2	1
3	2	3	2	3	1	2	3	2	1

2	2	2	2	2	2	2	2	2	2
2	2	2	2	2	2	2	2	2	2
3	2	2	9	1	9	9	9	9	9
1	2	3	1	2	1	1	1	2	2
3	3	3	1	2	1	1	2	2	3
3	3	1	1	1	1	1	1	3	3
2	9	3	1	9	1	9	9	2	9
2	2	2	9	1	2	2	2	2	2
2	2	2	2	2	2	2	2	2	2
2	1	2	2	2	2	2	2	2	2
2	2	2	3	1	3	3	3	3	3
2	2	3	2	9	2	2	2	1	1
2	9	9	9	9	9	9	9	9	9
2	3	2	1	1	1	1	1	1	1
9	3	2	2	3	1	2	1	9	3
1	1	1	1	1	1	1	3	3	1
2	2	3	1	2	2	3	2	2	1
9	9	9	9	9	9	9	9	9	9
2	3	2	3	1	2	3	2	2	2
3	2	2	1	2	2	2	2	2	2
9	9	9	1	9	9	9	9	9	9
1	3	2	3	3	1	2	1	3	9
3	1	2	1	2	3	2	2	2	2
1	1	1	9	1	9	9	9	9	9
9	9	9	3	3	1	9	9	9	9
9	9	9	9	2	9	9	9	9	9
1	1	2	2	2	2	2	2	3	3
2	1	1	2	2	3	3	3	3	2
9	9	9	9	1	9	9	9	9	9
2	2	2	2	2	2	2	2	2	2
3	1	3	1	3	1	3	1	3	1
3	1	1	3	1	3	3	3	3	3
3	1	1	1	1	3	2	3	3	3
3	1	1	1	1	3	1	1	1	1
1	1	2	9	9	9	9	9	9	9
2	2	2	3	3	3	3	3	3	3
3	1	1	1	1	1	1	3	1	3
3	2	2	1	1	3	2	2	2	3
1	1	1	1	2	1	2	3	1	2
9	9	9	1	9	9	1	1	1	1
9	1	3	2	1	2	3	2	1	2
3	2	2	3	1	3	3	3	3	3
2	2	2	2	3	3	2	2	3	2
2	3	2	2	1	3	2	3	3	3
3	1	2	9	1	3	3	3	3	3
1	1	1	1	1	1	1	1	1	1
3	3	3	1	1	1	3	3	3	1

2	3	1	1	2	1	2	3	1	2
2	9	1	1	1	3	3	3	2	2
1	1	3	3	1	1	1	2	3	3
2	1	3	3	1	2	9	3	3	3
3	2	2	1	2	2	1	1	1	9
3	2	2	1	2	2	1	1	1	9
2	3	3	3	1	3	3	3	2	3
2	3	1	1	3	3	3	3	3	3
3	2	3	3	1	3	2	2	3	3
1	3	2	3	3	3	3	1	1	1
3	2	2	3	1	3	3	3	2	3
3	2	2	3	1	1	2	1	1	1
2	9	3	3	3	3	3	3	3	3
3	1	3	3	2	3	3	2	2	2
1	1	1	3	1	3	1	3	3	1
3	3	3	3	1	3	3	1	1	3
3	3	3	1	1	3	3	3	3	3
2	2	2	1	3	3	3	3	3	3
3	2	2	3	2	3	2	2	2	3
1	1	1	3	1	2	2	2	2	2
2	1	2	3	2	1	2	2	1	1
2	9	2	2	1	3	2	2	3	2
1	1	1	1	1	1	1	3	9	1
3	9	3	1	1	1	1	1	1	1
1	1	1	1	1	1	1	1	1	1
1	1	1	1	1	1	1	1	1	1
3	9	2	1	1	2	2	2	2	3
1	1	3	3	3	2	3	1	3	2
1	1	3	2	2	2	2	2	2	2
3	2	2	3	1	3	3	3	3	2
3	3	3	1	3	3	3	2	3	1
9	9	9	1	1	2	9	3	1	2
2	2	2	2	2	2	2	2	2	2
2	1	2	3	3	1	3	1	3	2
2	3	3	2	2	1	2	1	3	1
3	3	3	3	1	1	1	2	3	1
2	2	2	1	3	2	2	2	2	2
3	1	1	3	3	3	3	3	3	3
2	1	2	1	1	1	1	3	3	2
2	2	2	1	3	3	2	2	2	2
2	2	2	3	3	2	2	2	2	3
2	2	3	2	3	2	3	3	2	2
2	3	2	1	1	1	3	3	3	3
2	3	3	3	3	2	1	2	1	9
1	1	1	2	3	3	1	2	2	2
2	2	2	3	3	2	1	2	3	2
1	1	1	3	3	1	1	2	3	1

1	1	1	1	2	3	1	1	1	2
9	9	1	1	1	9	9	9	1	9
9	9	1	1	1	9	9	1	1	9
2	2	2	2	3	3	3	3	3	2
9	9	9	1	9	9	9	9	9	9
9	9	1	1	1	9	9	9	9	9
9	9	9	9	9	9	9	9	9	9
3	3	3	1	2	1	1	1	2	1
1	3	1	2	1	3	3	2	3	2
3	3	2	2	1	2	3	3	3	2
2	2	2	2	3	3	2	2	2	2
2	2	2	2	2	3	3	2	2	1
2	2	3	1	9	3	3	3	3	3
2	2	3	1	9	3	3	3	3	3
2	2	2	2	1	2	2	2	2	2
2	2	2	2	2	2	2	2	2	2
3	3	2	2	3	3	9	3	3	3
1	2	2	1	1	3	3	2	3	1
3	3	2	2	1	3	3	3	3	3
2	1	2	1	1	2	1	2	1	2
2	2	2	3	1	3	3	2	3	3
2	1	1	1	1	1	1	1	1	1
9	9	9	1	2	2	2	2	2	2
2	2	2	2	2	2	2	2	2	2
2	2	2	2	2	2	2	2	2	2
1	2	1	1	3	3	2	9	1	1
3	3	2	1	2	1	1	1	2	2
1	1	2	9	9	9	9	9	9	9
3	2	2	2	3	3	2	1	2	3
2	3	2	1	3	1	1	1	1	1
2	1	2	1	1	3	3	3	2	2
1	1	2	3	1	3	1	1	1	1
3	2	3	2	1	3	3	1	2	3
1	2	2	1	1	3	1	2	3	2
3	3	3	3	1	3	3	2	3	3
3	2	2	2	9	2	9	2	2	2
2	2	2	2	2	2	2	2	2	2
2	2	2	2	1	2	2	2	2	2
2	2	2	1	1	2	2	2	2	3
3	9	2	9	9	9	9	9	9	9
2	2	2	2	2	3	2	1	3	3
1	2	3	1	2	3	2	1	1	2
1	2	1	3	3	1	1	2	1	1
1	1	2	2	1	2	1	1	2	1
1	1	1	1	2	3	1	1	1	1
2	2	2	2	2	2	2	2	2	2
2	2	2	3	1	3	2	2	2	2

1	1	2	2	1	2	1	1	1	2
9	9	9	3	9	9	9	9	9	9
1	2	2	3	1	1	1	2	9	2
3	3	3	3	1	1	1	2	3	9
3	3	3	3	1	2	2	2	1	3
1	2	2	2	1	3	2	2	3	3
3	3	3	3	3	2	3	3	2	2
2	1	1	2	1	3	3	3	3	3
2	3	1	1	1	3	2	9	2	3
2	2	2	1	2	3	3	2	2	1
3	1	2	3	1	3	1	3	2	3
2	2	2	2	3	3	3	3	3	3
2	2	2	1	2	3	3	2	2	1
1	1	2	1	1	1	1	1	1	1
2	2	2	1	2	3	2	2	3	2
2	2	2	1	1	3	3	3	3	3
2	2	1	3	1	3	2	1	1	3
9	1	9	3	1	3	3	3	3	3
2	2	2	3	2	2	2	9	9	9
1	1	1	1	1	1	1	1	2	1
1	2	1	1	1	3	2	3	2	1
3	2	2	3	1	3	3	3	3	3
2	1	2	2	1	3	2	3	2	2
2	2	2	2	2	2	1	2	1	1
1	1	2	2	1	1	1	1	2	2
2	3	2	1	1	2	9	2	3	2
1	1	3	1	1	3	3	3	3	3
3	2	2	3	1	3	9	2	1	3
3	2	3	1	2	1	3	2	1	3
3	3	2	3	1	2	1	3	3	2
3	2	2	2	1	2	2	2	2	2
2	3	3	2	1	1	2	2	3	2
3	2	2	1	3	3	1	1	1	3
9	9	9	1	2	3	1	2	3	1
1	3	1	3	1	3	3	3	3	3
1	2	2	2	3	2	3	1	2	3
3	2	3	1	3	3	3	3	3	3
2	1	1	3	2	2	2	3	1	1
2	2	2	9	1	3	2	2	3	3
2	2	3	3	1	3	2	2	3	3
9	9	9	9	9	9	9	9	9	9
2	3	1	1	2	2	3	3	2	1
9	9	9	1	1	3	3	3	3	3
3	3	3	3	1	3	3	3	3	3
2	2	2	1	1	3	3	3	3	3
1	1	1	1	1	3	1	3	3	1
3	2	2	2	3	3	2	3	3	3

3	1	2	1	3	2	1	3	3	3
9	9	2	9	9	9	9	9	9	9
1	1	1	1	3	1	2	2	2	1
3	2	2	1	1	3	3	3	3	3
2	2	2	1	2	2	2	2	2	2
2	1	2	2	2	2	2	2	2	2
2	3	2	1	2	3	2	3	2	3
1	1	1	1	1	1	1	1	1	1
2	2	2	2	2	2	2	2	2	2
9	9	9	3	3	3	1	1	2	1
2	2	2	1	9	9	9	9	9	9
3	3	3	2	1	3	2	3	3	3
3	1	3	1	1	1	1	1	1	1
2	2	2	2	3	1	1	1	1	1
3	3	3	1	3	3	3	3	3	1
3	1	1	2	1	3	2	3	3	3
1	9	3	2	3	3	1	3	2	1
2	3	2	1	1	1	2	3	9	1
2	1	1	1	1	3	1	3	2	3
1	1	2	1	3	1	2	2	9	1
2	2	2	1	2	2	1	1	1	1
9	9	9	1	3	2	2	3	9	1
3	1	2	3	2	1	1	1	2	3
3	1	3	1	3	2	1	2	1	1
1	2	3	1	2	2	2	2	1	1
9	9	9	3	2	3	3	2	3	3
2	2	2	1	1	3	3	3	3	3
9	9	9	1	1	1	2	1	1	1
1	9	1	2	1	1	2	3	3	1
2	2	2	1	3	3	3	1	3	3
2	1	1	2	1	2	1	1	3	2
3	2	3	1	2	1	1	3	2	2
3	2	3	1	9	2	3	3	2	1
3	2	2	3	1	3	2	3	3	2
3	3	2	1	1	1	3	1	3	2
3	3	3	1	1	1	3	1	3	2
2	2	1	2	1	2	1	1	3	2
9	9	2	9	9	9	1	9	9	9
3	2	1	3	3	2	3	3	3	3
2	2	2	3	1	3	2	3	3	3
3	2	1	1	3	3	2	1	2	3
1	2	2	2	1	1	3	3	2	2
1	3	2	1	1	3	2	2	2	2
9	9	9	9	9	9	9	9	9	9
2	2	2	1	1	1	1	1	1	1
2	2	2	1	2	1	1	1	1	2
1	9	9	1	3	3	3	2	9	1

3	1	3	3	1	3	3	1	3	3
9	9	9	2	9	9	9	9	9	9
1	3	2	2	3	1	2	1	3	2
1	2	2	2	1	3	2	2	3	3
2	2	1	1	1	2	1	2	2	2
2	3	3	2	1	3	3	3	3	3
3	2	1	3	3	2	2	1	1	1
2	2	2	1	3	3	3	3	3	3
2	2	2	1	3	1	3	3	3	3
9	9	9	9	9	9	9	9	9	9
3	1	1	3	1	2	3	3	3	2
2	1	3	1	3	2	1	1	1	1
1	3	1	1	3	1	3	1	3	1
1	2	1	3	2	1	2	1	3	1
2	2	2	2	1	3	3	3	3	2
1	1	1	3	1	2	1	1	1	1
1	1	1	1	1	1	3	1	1	2
3	1	2	1	2	2	1	3	2	2
2	2	2	1	2	1	1	2	2	2
2	2	2	1	2	2	2	1	2	2
9	9	9	9	9	9	9	9	9	9
2	2	2	1	2	1	1	1	2	2
2	2	3	2	2	2	2	2	1	2
1	1	1	1	2	2	1	2	2	2
2	1	1	1	2	2	2	2	2	2
2	2	2	2	2	2	2	2	2	2
2	2	2	2	2	1	1	1	1	2
3	2	2	2	3	3	2	2	3	3
1	2	2	2	2	2	2	2	3	2
2	2	2	3	2	1	1	2	2	2
9	9	9	9	9	9	9	9	9	9
9	9	9	9	9	9	9	9	9	9
9	9	9	9	9	9	9	9	9	9
1	2	2	2	1	3	3	3	3	3
1	3	3	2	1	3	3	2	3	3
2	2	2	9	2	3	2	2	3	3
2	1	1	1	3	1	1	1	1	1
1	1	1	1	2	3	1	1	2	3
1	1	1	1	3	1	1	1	1	2
3	2	2	1	1	3	2	2	3	3
3	3	3	1	3	3	3	1	1	1
1	2	1	3	1	3	2	3	3	3
1	1	2	2	1	3	2	2	2	2
1	3	2	1	1	3	3	3	3	3
1	2	2	2	3	1	2	2	3	3
3	1	1	1	1	2	3	3	3	3
3	1	3	1	1	3	3	3	3	1

3	3	2	1	2	1	1	1	2	1
2	2	2	1	1	3	1	1	2	1
3	3	2	1	2	1	1	2	2	3
1	1	3	3	3	1	1	3	1	3
3	3	3	3	1	3	3	3	9	9
2	3	1	1	1	1	3	3	3	2
2	2	2	3	3	3	1	1	1	1
9	9	9	1	9	9	9	9	1	9
9	9	9	1	3	9	9	9	9	9
1	9	9	1	1	3	1	1	1	9
9	9	9	1	2	2	2	9	9	1
9	9	9	1	9	9	9	1	9	9
2	2	2	1	1	3	1	2	3	2
9	9	9	2	3	1	1	1	2	2
1	3	3	1	1	3	3	3	2	3
2	2	2	3	1	3	3	3	3	3
2	1	2	1	1	1	2	1	2	2
2	1	2	1	1	1	2	1	2	2
2	2	3	1	1	3	3	3	3	3
9	2	1	3	3	1	2	1	2	3
2	2	2	3	2	3	2	2	2	3
2	2	2	1	1	3	2	2	3	2
9	9	9	3	1	3	1	1	1	3
3	3	3	1	3	2	2	2	2	2
2	3	2	3	1	3	2	3	2	3
3	1	1	3	1	3	1	1	2	3
2	2	2	1	1	3	3	2	2	2
2	2	2	1	1	3	2	1	3	3
2	2	2	1	2	2	2	2	2	2
3	3	3	1	2	2	1	2	3	3
2	2	3	1	9	3	3	3	3	3
3	2	1	3	3	1	1	1	1	2
3	3	2	3	1	3	1	2	3	2
2	2	3	1	1	3	3	2	1	2
2	9	9	2	9	2	3	9	9	9
3	3	3	3	2	3	2	3	3	3
2	1	1	1	1	3	2	2	3	3
2	2	2	1	1	3	3	3	2	2
3	2	2	3	1	3	2	1	3	3
2	2	2	2	1	3	2	2	2	2
9	9	9	3	3	3	1	1	1	1
9	9	9	9	1	9	9	9	9	9
9	9	9	9	1	9	9	9	9	9
2	2	2	3	1	3	2	2	3	3
9	9	9	3	1	2	2	3	2	1
2	2	2	3	1	3	1	2	3	3
2	2	2	3	2	2	3	3	3	3

2	1	2	1	1	3	2	2	2	2
1	1	1	3	3	2	2	2	1	2
2	2	3	3	3	3	1	1	1	2
3	3	1	1	3	3	2	3	2	1
3	3	3	2	2	2	2	2	2	2
2	2	2	3	1	3	3	3	3	3
1	2	1	1	1	3	9	2	3	2
3	3	3	1	3	3	2	1	1	1
3	2	2	3	1	3	1	1	3	3
1	1	2	2	1	1	2	3	1	1
2	2	2	3	1	3	3	2	3	3
3	1	2	1	3	3	3	2	1	3
3	1	3	3	1	3	3	3	3	3
3	2	3	1	1	3	3	3	1	3
1	2	2	3	1	3	2	2	3	2
2	2	2	3	1	3	2	2	2	2
2	1	1	3	1	2	1	2	2	2
2	2	2	2	2	2	2	2	2	2
2	2	2	1	2	2	1	2	2	1
2	2	2	2	1	2	2	3	3	2
1	2	2	1	3	3	2	1	1	2
2	1	2	2	1	3	3	3	3	2
3	3	2	1	2	1	1	3	3	1
3	2	2	1	2	2	3	3	3	3
1	1	3	1	3	3	3	3	3	3
3	3	2	1	1	3	3	3	3	3
1	2	2	2	1	2	3	1	2	3
1	3	1	3	1	3	3	3	3	3
2	2	1	2	2	2	2	1	2	3
2	2	2	1	3	1	1	2	2	3
3	2	3	9	9	9	9	9	9	9
3	1	3	1	2	3	3	3	3	1
2	2	2	1	2	1	1	1	1	2
3	3	3	2	1	2	2	1	1	2
2	1	2	1	9	2	2	3	1	2
2	2	2	2	2	1	1	1	1	2
3	2	2	1	2	2	2	2	2	2
2	2	2	1	1	3	2	2	2	2
3	1	1	1	3	1	1	9	1	1
1	2	2	9	1	9	1	3	3	3
1	1	1	1	1	3	3	3	9	3
3	2	2	3	1	3	3	2	3	3
2	2	2	2	2	2	2	2	2	1
1	1	1	3	1	3	2	1	2	3
2	1	2	3	1	3	2	3	1	2
2	2	2	2	1	2	2	2	2	2
3	3	2	1	1	1	3	1	3	2

3	3	2	1	1	1	3	1	3	2
2	2	2	1	2	1	2	2	2	2
2	3	3	1	1	3	3	3	3	3
2	2	1	3	2	1	1	1	2	2
2	3	3	1	2	1	2	2	2	2
2	2	2	2	2	1	1	2	2	2
1	1	1	1	2	1	1	1	2	2
3	2	2	1	1	3	2	3	1	2
2	3	3	1	2	2	3	2	1	1
3	3	1	1	1	3	1	3	2	1
1	3	1	3	1	3	3	3	3	3
2	2	1	2	3	3	1	3	3	2
2	2	2	2	2	1	1	1	1	1
1	1	2	2	1	3	2	2	2	2
3	3	3	1	1	3	3	3	1	3
9	9	9	9	2	3	2	3	3	3
3	3	3	1	2	2	1	1	1	3
2	2	2	1	2	1	1	1	2	2
2	2	2	1	1	1	1	1	1	1
2	2	3	1	1	3	3	3	3	3
1	1	2	2	1	3	3	2	3	3
3	3	3	3	1	1	3	1	3	1
3	3	2	1	3	2	1	1	1	2
2	2	2	1	3	3	1	1	1	1
2	2	1	1	1	2	2	1	1	2
3	2	2	1	3	1	3	2	1	1
2	1	1	3	2	1	2	1	3	3
2	2	2	1	1	3	2	2	3	9
3	3	3	3	3	1	3	3	3	3
3	2	2	3	2	3	3	3	2	3
3	3	3	3	3	3	3	3	3	3
1	1	1	3	1	3	3	3	3	3
2	3	2	1	2	2	2	2	2	2
1	1	3	1	3	3	3	3	3	3
1	3	2	2	9	2	9	1	3	2
3	2	2	3	3	1	1	1	1	2
3	2	2	1	3	3	1	2	1	2
1	2	2	1	3	3	2	1	2	3
1	2	1	1	1	1	3	3	3	3
3	2	2	9	1	3	1	1	3	3
2	1	3	2	1	3	1	1	3	3
3	1	1	3	1	3	1	1	3	3
2	2	2	3	1	1	1	1	3	3
1	1	2	3	1	3	3	3	3	3
3	3	3	3	1	3	2	3	3	3
1	2	2	1	2	1	2	3	2	3
2	2	2	1	2	2	2	3	1	3

3	3	3	1	3	2	1	3	2	2
1	1	2	1	3	2	1	3	9	1
2	2	2	1	2	2	2	1	1	2
2	1	3	2	1	9	1	1	3	3
3	1	3	3	3	3	1	3	1	3
2	2	2	2	2	2	2	2	2	2
3	2	2	1	1	2	2	2	2	1
3	3	1	1	1	1	3	2	3	1
1	1	9	3	3	1	9	1	1	3
3	3	3	3	1	2	2	2	2	2
1	1	1	1	2	2	1	1	1	1
3	3	2	1	1	3	3	3	3	1
1	1	1	3	1	1	2	2	1	3
3	9	2	1	1	3	2	2	2	2
3	2	1	1	1	1	2	2	2	2
2	2	1	1	1	3	3	3	3	3
2	2	2	3	1	3	2	2	2	2
1	1	1	3	1	2	2	2	2	2
3	2	1	1	3	1	2	3	2	1
2	2	2	1	2	3	1	3	1	3
2	2	2	1	1	1	2	2	2	3
2	2	2	1	3	2	2	2	1	2
2	2	2	2	2	2	2	2	2	2
2	2	2	1	2	2	2	2	1	1
2	2	2	1	2	2	2	2	1	1
2	2	1	1	1	3	3	3	3	3
3	3	2	1	1	3	3	3	3	1
3	3	3	3	1	2	2	2	2	2
1	2	1	1	1	1	3	3	3	3
2	2	2	1	2	3	1	3	1	3
3	2	2	1	3	3	1	2	1	2
9	9	9	9	9	9	9	9	9	9
3	3	3	2	1	2	2	2	3	2
2	2	1	1	1	3	3	3	1	3
1	1	1	1	3	1	3	3	3	3
2	1	1	2	1	1	2	3	3	1
1	1	1	3	3	1	3	3	3	3
1	2	3	1	3	1	2	3	1	2
2	2	2	3	1	3	3	3	3	3
3	2	1	1	2	1	2	3	2	1
3	1	2	3	3	3	2	3	1	1
3	1	2	3	3	3	2	3	1	1
3	2	3	1	3	2	1	1	1	1
3	3	2	2	1	2	9	2	2	2
2	1	2	1	2	2	3	1	2	2
1	3	2	9	2	1	1	3	3	3
2	3	1	1	1	3	3	3	3	1

2	2	1	1	1	2	2	1	1	1
3	1	1	1	1	3	3	3	3	3
1	1	1	3	9	9	1	1	1	1
3	2	2	1	2	3	2	3	3	1
2	2	2	2	1	3	2	3	2	3
2	2	2	3	1	3	3	3	3	3
1	1	1	1	1	3	3	1	3	3
9	9	9	1	9	9	9	9	1	9
9	9	9	1	9	1	9	9	9	9
9	9	9	1	9	2	9	9	9	9
3	2	1	1	2	1	1	1	1	1
3	1	2	2	2	2	2	2	1	1
1	3	3	3	3	3	3	1	3	3
1	3	3	3	3	3	3	1	3	3
1	3	3	3	3	3	3	1	3	3
2	2	2	2	2	1	1	1	1	2
2	2	2	1	3	2	2	2	2	2
9	2	2	1	2	2	1	3	1	2
2	3	1	2	3	2	2	1	2	2
3	2	3	1	2	1	2	1	2	2
2	1	2	1	2	1	3	1	2	1
2	2	2	1	3	1	2	1	2	2
2	2	2	1	3	1	1	1	1	1
2	2	2	1	3	2	2	3	3	3
2	2	2	2	3	1	1	1	3	3
1	3	3	3	3	2	1	3	3	3
2	2	2	1	2	2	2	2	1	1
3	2	2	1	3	1	2	3	3	3
2	2	3	1	3	1	2	1	1	1
2	2	3	1	3	1	2	1	1	1
1	3	3	3	3	3	3	1	3	3
1	3	3	3	3	3	3	1	3	3
2	2	2	2	2	2	2	2	2	2
1	3	3	2	2	2	2	2	2	2
1	3	3	2	2	2	2	2	2	2
2	2	2	2	2	2	2	2	2	2
2	2	2	2	2	2	2	2	2	2
1	3	3	3	1	2	2	2	2	2
1	3	3	3	1	2	2	2	2	2
9	9	9	3	1	3	3	3	3	3
2	2	2	1	3	1	1	1	2	2
3	2	1	1	3	3	3	3	3	3
3	2	1	1	3	3	3	3	3	3
3	2	1	1	3	3	3	3	3	3
3	2	1	1	3	3	3	3	3	3
9	3	1	2	3	9	1	1	1	3
9	1	3	1	1	1	3	2	3	1

1	1	3	1	2	1	3	1	3	2
2	2	2	1	3	2	2	3	3	2
1	3	3	1	3	1	2	1	2	3
2	1	1	1	2	1	2	1	1	2
3	2	1	1	3	2	1	3	1	2
3	1	1	1	9	2	2	3	1	3
3	2	3	3	2	1	2	1	2	1
1	3	3	3	1	2	9	2	1	1
9	9	9	3	9	9	9	9	9	9
9	9	9	9	9	9	9	9	9	9
1	3	3	3	1	3	3	3	3	3
9	1	2	1	3	3	2	1	9	2
9	1	2	1	3	3	2	1	9	2
3	1	1	3	1	1	1	1	1	1
2	2	2	1	9	3	9	9	9	9
2	2	2	2	2	2	2	2	2	2
2	2	2	1	2	1	3	3	2	2
9	9	9	1	9	9	9	9	9	9
9	9	9	2	2	2	9	9	9	9
9	1	9	9	3	9	9	9	9	9
2	1	1	1	3	1	1	2	2	2
3	1	2	1	3	3	3	2	3	2
2	2	2	3	3	1	2	1	1	1
2	2	2	9	3	3	3	3	3	3
9	9	9	1	9	2	9	9	9	9
9	9	9	1	3	9	9	9	9	9
2	2	2	1	3	3	3	2	2	2
9	9	9	3	2	1	1	1	3	2
2	2	1	2	3	3	3	3	3	3
9	9	9	9	9	9	9	9	9	9
3	1	1	3	3	1	1	3	1	2
9	9	9	1	9	9	9	9	9	9
3	1	1	2	2	2	9	1	1	1
3	1	1	2	2	2	2	2	2	2
3	1	1	1	3	1	1	1	1	1
3	1	1	1	3	1	1	1	1	1
3	1	1	2	2	2	2	2	2	2
3	1	1	3	1	2	2	2	2	2
2	2	2	1	1	2	2	2	2	1
9	9	9	1	9	9	9	9	9	9
9	9	9	1	9	9	9	9	9	9
9	9	9	1	9	9	9	9	9	9
9	9	9	1	9	9	9	9	9	9
9	9	9	1	9	9	9	9	9	9
9	9	9	3	9	2	9	9	9	9
1	1	2	1	1	2	1	1	1	1
2	3	1	1	1	1	2	3	1	2

3	1	9	9	9	9	9	9	9	9
3	2	2	2	2	3	3	1	2	2
9	9	9	9	3	9	9	9	1	9
3	1	1	1	3	3	3	3	3	3
3	9	9	2	3	3	3	1	3	3
2	2	2	1	3	3	3	3	3	3
9	9	9	9	3	9	9	9	9	9
1	3	3	3	1	3	3	3	3	3
1	1	2	3	3	3	1	1	3	3
9	9	9	1	9	9	9	9	9	9
2	3	2	1	9	2	2	9	2	1
3	9	9	9	3	3	3	3	3	3
9	9	9	1	9	9	9	9	9	9
9	9	9	1	9	9	9	9	9	9
3	1	1	1	2	2	2	1	2	1
9	9	9	1	9	9	9	9	9	9
3	2	2	1	3	3	3	3	3	3
9	9	9	1	9	9	9	9	9	9
2	2	2	1	3	3	2	1	2	3
1	3	3	1	2	1	2	1	3	1
3	2	1	1	1	3	3	3	3	3
1	2	3	1	2	1	2	3	3	2
3	1	1	2	2	2	2	2	1	3
2	2	2	2	2	3	3	3	3	3
1	1	2	3	3	3	2	2	3	3
2	1	2	2	3	1	2	1	1	2
3	1	1	1	3	1	2	2	2	2
2	2	3	2	1	1	1	2	2	2
2	3	2	1	3	1	1	2	2	1
3	2	2	1	3	3	3	3	3	3
1	2	1	1	2	1	2	3	2	1
1	2	1	1	2	1	2	3	2	3
9	9	9	9	9	9	9	9	9	9
9	9	9	1	9	9	9	9	9	9
3	9	9	1	9	9	9	9	9	9
9	9	9	1	9	9	9	9	9	9
9	9	9	9	9	9	9	9	9	9
9	9	9	9	9	9	9	9	9	9
9	9	9	2	9	9	9	9	9	9
9	9	9	2	9	9	9	9	9	9
9	9	9	2	9	9	9	9	9	9
9	9	9	2	9	9	9	9	9	9
9	9	9	2	9	9	9	9	9	9
9	9	9	2	9	9	9	9	9	9
9	9	9	2	9	9	9	9	9	9
3	9	1	3	3	1	1	2	1	2
9	9	9	1	3	1	1	2	2	1

3	1	1	1	3	2	1	1	1	2
2	2	2	3	9	9	9	3	3	3
9	9	9	1	9	9	1	9	9	9
3	2	3	3	3	1	1	1	2	2
9	9	9	1	9	1	9	9	9	9
9	9	9	1	9	1	9	9	9	9
9	9	9	1	9	1	9	9	9	9
9	9	9	1	9	1	9	9	9	9
9	9	9	1	9	1	9	9	9	9
2	2	2	2	2	1	1	2	1	2
3	2	1	2	3	2	2	1	2	1
2	1	2	2	2	2	2	2	2	2
2	2	2	2	2	2	2	2	2	2
1	2	2	3	3	2	2	2	3	3
2	1	1	1	2	3	3	2	2	1
3	2	2	2	1	1	1	1	1	1
9	9	9	1	9	1	9	9	9	9
9	9	9	2	9	9	2	9	9	9
9	9	9	1	9	9	2	9	9	9
9	9	9	9	9	1	3	9	9	9
9	9	9	1	9	9	9	9	2	9
9	2	9	1	9	9	9	2	9	9
9	2	9	1	9	2	9	2	9	3
1	9	9	3	9	2	9	9	9	9
2	9	9	2	2	2	2	2	2	9
9	9	9	9	2	9	9	9	9	9
9	9	9	1	1	9	1	9	9	9
2	2	2	2	2	2	2	2	1	1
1	3	2	3	2	2	3	2	3	1
9	1	9	1	9	9	9	9	9	9
2	2	2	3	9	9	9	9	9	9
1	1	1	2	1	1	3	3	3	2
2	2	2	1	3	3	3	3	3	3
9	9	9	1	2	2	9	1	2	1
3	2	3	3	3	3	3	3	3	3
2	2	2	1	2	2	2	2	2	2
9	9	9	3	2	1	2	1	1	1
2	9	1	9	3	3	2	9	9	2
2	9	9	1	9	9	9	9	9	9
1	2	2	1	1	2	3	1	3	1
2	2	2	2	2	3	2	2	2	2
2	2	1	1	3	2	3	3	3	3
2	2	2	1	3	2	1	3	2	1
9	9	9	3	2	2	2	2	2	1
3	9	9	9	3	3	3	2	3	3
2	2	2	2	2	2	2	2	2	2
9	1	9	9	3	9	9	9	9	9

1	1	2	3	2	3	1	9	2	3
3	1	2	3	1	3	3	3	3	1
2	2	2	3	3	3	3	3	3	3
1	1	1	3	3	3	3	2	3	3
2	2	1	1	3	2	2	3	3	3
2	1	2	1	2	2	1	1	2	3
1	1	1	1	3	3	3	3	3	3
1	3	3	1	3	3	1	2	2	2
3	9	9	1	3	1	1	3	1	3
9	9	9	9	9	9	9	9	9	9
1	3	2	1	2	3	2	2	1	1
3	3	3	3	3	3	3	1	3	3
3	1	1	3	3	3	3	3	3	3
3	1	2	1	2	2	2	2	2	2
3	1	1	1	3	1	1	2	2	2
3	3	2	1	2	3	2	1	1	1
2	2	2	1	2	3	3	2	3	3
2	1	1	1	3	2	2	2	1	2
2	2	2	1	3	1	3	2	3	2
1	1	2	1	2	1	2	3	1	2
2	2	2	3	3	9	9	9	9	9
2	2	1	1	3	3	3	3	3	3
2	2	2	1	3	3	3	2	2	2
1	1	2	3	3	1	3	3	3	3
1	2	1	2	2	3	3	3	3	3
2	1	2	3	3	3	3	1	3	3
3	1	2	2	3	3	2	3	3	3
2	1	1	1	3	3	2	3	3	1
1	3	3	3	3	3	2	3	3	3
2	2	2	2	2	2	2	2	2	2
2	2	2	2	2	2	2	2	2	2
2	2	2	2	2	2	2	2	2	2
2	2	2	2	2	2	2	2	2	2
2	2	2	2	2	2	2	2	2	2
2	2	2	2	2	2	2	2	2	2
2	2	2	2	2	2	2	2	2	2
2	2	2	3	3	2	2	2	2	2
9	9	9	2	9	2	9	9	9	9
3	1	1	1	3	1	2	1	2	1
3	3	1	2	3	3	3	3	3	3
1	2	3	2	1	1	2	1	2	1
9	1	9	9	9	9	9	9	9	9
3	1	1	1	1	1	1	1	1	1
3	2	2	1	3	3	2	3	3	3
2	3	1	2	3	2	1	2	3	3
1	2	3	1	3	3	3	1	1	2
2	1	2	2	3	3	3	3	3	3

9	9	9	2	1	2	2	2	1	1
3	2	2	1	3	3	3	3	3	3
1	2	1	2	2	1	1	2	2	3
2	2	2	2	3	2	1	2	1	1
2	2	2	1	3	2	2	2	2	2
9	9	9	9	9	9	9	9	9	9
1	1	3	1	1	3	1	2	2	1
3	1	1	2	3	3	3	2	3	3
3	3	2	3	3	3	2	3	2	2
3	1	1	1	1	3	2	1	3	1
2	2	2	1	1	1	3	2	1	2
2	2	2	1	2	3	3	3	3	3
3	1	1	3	1	2	1	1	3	2
3	3	3	3	3	3	3	3	3	3
1	3	2	1	2	1	1	1	3	3
2	2	2	2	2	2	2	2	2	2
2	2	2	2	3	3	2	3	3	3
9	9	9	1	9	9	9	9	9	9
2	2	2	3	3	2	2	3	3	3
3	1	3	1	2	1	2	3	3	3
2	2	2	2	2	2	1	2	1	2
2	2	2	1	2	1	2	2	1	2
2	2	2	1	3	2	2	2	2	2
1	1	1	1	1	3	3	3	3	3
9	9	2	1	3	3	2	2	3	3
2	2	2	1	3	2	2	2	2	2
2	2	2	1	1	3	2	2	2	1
2	2	2	2	2	3	2	2	2	3
2	3	2	1	9	9	9	9	9	9
1	2	2	2	3	3	2	2	1	2
3	3	1	2	3	1	1	3	3	3
9	1	9	1	9	9	9	9	9	9
2	2	2	1	1	3	2	3	2	3
2	2	2	1	2	2	2	2	2	1
2	3	3	3	1	3	3	3	3	2
2	2	2	3	3	3	3	3	3	3
2	2	2	1	3	2	1	2	1	2
1	1	1	2	1	3	3	3	3	3
2	1	2	1	2	3	2	2	2	2
3	1	1	3	1	3	3	1	3	3
2	2	2	3	3	3	2	2	3	3
1	3	2	1	3	1	3	1	3	2
2	2	1	1	3	3	3	3	3	3
9	9	9	1	9	9	9	9	9	9
2	2	1	3	3	3	3	1	3	3
3	3	2	3	1	3	3	3	1	1
9	9	9	1	9	9	9	9	9	9

2	2	2	1	2	2	2	2	2	2
3	2	2	1	3	3	3	3	3	2
2	2	2	2	3	3	2	1	3	3
2	2	2	2	3	2	2	2	2	2
3	1	1	2	2	2	2	2	2	2
3	2	2	1	1	2	2	2	2	2
2	2	2	2	2	2	2	2	2	2
2	2	2	3	3	3	3	3	3	3
2	2	2	1	2	2	2	2	2	2
2	2	2	3	2	2	2	2	2	2
2	2	2	1	3	1	2	2	2	2
2	1	1	3	2	2	3	2	3	1
9	9	9	9	9	9	9	9	9	9
3	1	3	1	3	1	3	9	3	3
9	9	1	2	3	2	2	3	3	3
9	9	9	3	9	3	3	3	3	9
2	2	2	2	1	2	3	1	2	3
1	3	3	3	2	2	3	3	3	3
9	9	9	9	3	9	9	9	9	9
1	1	3	3	3	3	3	1	2	3
3	1	3	1	3	3	3	3	3	3
9	9	9	1	1	3	2	2	3	2
2	9	3	2	3	2	2	2	2	2
3	1	2	2	3	3	3	3	3	3
3	3	3	3	1	3	3	3	3	3
9	9	9	9	3	9	1	9	9	9
1	1	3	3	3	3	3	3	3	3
2	1	2	1	3	2	1	2	2	2
3	1	3	1	3	3	3	3	1	1
9	9	9	1	3	3	2	3	3	3
2	2	1	3	3	3	3	3	3	3
9	9	9	1	2	2	2	2	2	2
2	3	1	2	3	3	3	3	3	3
9	9	9	1	3	2	3	3	3	3
9	9	9	3	3	3	9	3	3	3
1	1	2	3	3	3	3	1	3	3
3	1	1	1	3	1	3	3	3	3
3	2	3	1	3	1	3	3	3	3
2	2	2	3	3	3	1	2	3	3
9	9	9	1	9	9	9	9	9	9
1	3	2	2	3	3	3	3	3	3
2	1	1	3	3	3	9	3	3	3
1	3	3	2	1	1	2	3	2	2
2	2	2	1	3	3	3	3	3	3
3	2	1	1	2	2	1	3	2	2
9	1	9	3	3	3	3	3	9	2
1	3	2	3	3	3	3	3	3	3

2	1	2	3	3	3	3	2	2	2
9	9	9	1	9	9	9	9	9	9
9	9	9	3	3	3	3	2	3	3
9	9	9	9	9	9	9	1	9	9
3	2	2	3	1	3	3	3	1	1
9	9	9	3	3	2	9	9	9	9
3	1	1	1	3	1	3	3	1	3
1	2	3	1	3	3	3	3	3	3
1	2	1	3	3	3	3	9	3	3
9	9	9	3	3	3	3	3	3	3
9	9	9	2	9	1	9	9	9	9
9	1	1	9	3	9	9	9	9	9
2	2	2	9	3	9	3	9	1	3
1	1	2	3	3	2	1	2	1	1
9	1	9	9	3	9	9	9	9	9
3	2	2	9	9	9	9	9	9	9
3	2	2	3	3	3	3	3	3	3
9	9	9	1	2	3	1	1	9	3
9	9	1	9	9	9	9	9	9	9
1	2	3	9	3	3	1	3	3	3
2	2	2	3	3	3	3	3	2	2
1	1	1	2	3	3	3	1	3	3
3	2	2	2	3	3	3	3	3	3
2	1	1	9	3	3	1	3	3	3
1	2	2	1	3	3	3	3	3	3
3	1	9	2	3	1	2	1	3	3
3	1	2	3	3	3	3	2	3	2
3	1	2	3	3	3	3	2	3	3
3	1	2	1	3	3	3	3	3	3
1	2	3	3	3	3	1	1	3	3
9	9	9	3	3	3	9	9	9	9
3	1	2	9	1	1	2	2	1	1
1	2	1	1	3	3	3	3	3	3
9	9	9	2	9	9	9	9	9	9
1	1	3	1	3	3	3	3	3	3
3	9	9	1	9	3	2	3	3	3
3	2	1	3	1	3	3	3	3	3
9	9	9	3	2	1	1	1	1	1
1	1	3	3	3	3	3	3	3	3
1	3	3	3	3	3	3	3	3	3
9	9	9	3	3	3	3	3	3	3
9	1	9	9	3	9	9	9	9	9
9	1	9	1	3	3	3	3	3	3
9	9	9	1	3	1	3	1	3	3
2	2	2	1	3	2	2	2	2	2
2	2	2	2	3	3	1	2	2	3
2	2	2	3	3	2	3	3	3	2

9	9	9	3	3	3	9	3	3	3
9	1	9	9	9	1	3	3	1	3
3	9	9	1	3	9	9	9	9	9
9	9	9	9	2	2	9	2	9	9
9	1	9	9	3	9	9	9	9	9
9	9	9	9	3	9	9	9	9	9
9	9	9	2	3	2	3	3	3	3
9	9	9	1	3	3	3	3	3	3
3	2	2	3	2	3	3	3	3	3
2	3	2	3	3	3	3	3	3	3
2	3	2	2	3	3	3	3	3	3
3	2	1	3	3	3	3	2	3	3
9	9	9	1	3	1	1	1	1	1
3	3	3	3	3	3	3	3	3	3
3	1	3	1	1	3	3	3	3	3
1	2	2	3	3	3	1	1	3	3
1	1	1	3	3	3	1	3	3	3
3	9	9	9	3	9	9	9	9	9
2	1	2	1	3	1	3	3	3	3
9	9	9	2	9	9	9	9	9	9
9	9	9	9	3	9	9	9	9	9
9	9	9	9	3	9	9	9	9	9
2	2	2	2	3	3	2	2	2	2
3	1	2	3	3	3	3	3	3	3
2	2	2	2	2	2	2	2	2	2
2	3	2	2	3	3	3	3	3	3
3	9	9	1	9	9	9	9	9	9
3	2	3	2	3	3	1	2	3	2
3	1	2	2	3	3	2	2	2	3
2	2	2	9	9	9	9	9	9	9
1	1	2	2	3	2	2	3	3	2
3	1	3	3	3	3	1	1	3	3
9	9	9	1	3	3	3	1	3	1
3	1	2	3	3	3	2	3	1	3
1	3	3	3	3	3	3	3	3	3
9	9	9	9	3	9	9	9	9	9
1	1	1	3	3	3	3	3	3	3
3	1	2	3	3	3	3	3	3	3
2	1	3	1	3	3	2	3	2	3
1	2	2	1	3	3	3	3	3	3
2	1	2	1	3	3	3	1	3	2
2	1	2	3	3	3	3	3	3	3
9	1	2	1	3	3	1	3	3	3
9	9	9	3	3	3	3	3	3	3
2	2	2	3	3	3	3	3	1	1
2	1	2	1	3	3	3	3	3	3
1	1	1	3	3	3	3	2	3	3

9	9	9	9	3	9	9	9	9	9
3	1	1	3	3	3	3	3	3	3
3	1	1	3	3	3	3	3	3	3
1	1	2	3	3	1	3	1	3	3
9	9	2	3	1	2	9	2	9	2
1	1	3	1	3	1	1	2	1	2
3	3	3	3	3	3	3	3	3	3
2	3	3	3	3	3	2	3	3	3
2	1	2	3	3	3	2	2	2	3
3	1	2	3	3	3	3	3	3	3
1	2	2	1	3	1	1	2	3	3
9	9	9	1	3	3	3	3	3	3
3	9	9	9	3	9	9	1	9	9
1	1	1	1	3	3	3	3	2	3
9	9	1	1	3	3	2	2	3	3
3	1	3	2	3	3	3	2	3	3
9	1	1	9	3	9	9	9	9	9
1	2	3	1	1	2	1	3	2	3
1	3	3	3	3	1	1	2	2	2
9	9	9	3	3	3	3	3	1	3
2	2	2	2	2	2	2	2	2	2
1	1	2	3	3	3	3	3	3	3
9	9	9	9	3	9	9	9	9	9
3	2	1	3	3	3	3	3	3	1
2	2	2	2	2	2	9	3	2	2
2	1	2	1	3	3	3	3	3	3
2	1	2	1	3	3	3	3	3	9
2	1	2	3	3	3	2	3	3	3
3	1	3	3	3	3	3	3	3	3
1	3	3	1	2	1	1	2	2	1
9	9	9	1	3	9	9	9	9	9
1	3	3	1	3	2	3	1	2	2
2	2	2	3	3	3	1	2	3	3
2	2	2	3	3	3	1	2	3	3
3	1	2	3	3	1	3	3	3	3
9	1	9	9	3	9	9	9	9	9
1	3	3	3	3	3	3	3	3	3
2	9	2	3	3	3	3	3	3	3
3	2	1	3	3	3	3	3	3	3
2	2	2	1	1	3	1	2	2	2
3	2	2	3	3	3	3	2	3	3
2	3	2	3	3	3	3	3	3	3
3	9	9	9	3	9	9	9	9	9
9	9	9	3	3	3	3	3	3	3
3	1	1	1	2	3	3	2	3	3
9	9	9	1	3	1	1	3	1	3
3	1	1	3	1	2	1	2	1	3

9	9	9	3	2	2	1	2	2	2
3	1	1	1	9	1	1	1	1	1
1	3	3	2	2	2	2	9	1	9
9	9	9	1	3	3	3	3	1	1
3	1	2	3	3	3	3	3	3	3
2	2	2	3	1	2	2	2	2	2
2	2	2	3	1	3	3	3	3	3
2	1	2	9	9	9	9	9	9	9
3	1	1	3	3	3	1	3	3	3
3	9	9	3	3	3	3	3	3	3
9	9	3	1	3	3	3	3	3	3
1	1	1	2	3	3	3	1	3	3
9	1	9	1	3	3	3	3	3	3
3	2	1	3	3	3	3	1	2	3
1	1	1	2	3	3	3	3	3	3
2	1	2	2	3	3	3	3	3	3
1	2	1	2	3	2	1	2	3	3
1	1	3	1	3	3	3	2	3	3
9	9	9	3	3	3	3	3	3	3
9	9	9	3	3	3	3	3	3	3
2	1	1	3	3	3	3	2	3	3
3	1	2	2	3	3	3	3	3	3
1	3	3	2	3	3	3	3	3	3
1	1	1	2	3	3	3	3	1	1
1	1	2	3	3	3	3	3	3	3
1	3	3	2	3	3	3	3	3	3
1	1	3	3	3	3	3	3	3	3
1	1	1	3	3	3	2	3	3	3
3	1	3	3	3	2	3	1	2	2
9	9	9	3	3	3	3	3	3	3
9	9	9	1	9	1	9	1	3	2
2	1	1	2	2	1	3	1	3	3
2	3	3	1	2	2	1	3	3	2
1	1	2	9	3	3	3	2	1	2
2	3	1	3	3	3	2	2	3	3
9	9	9	9	3	9	9	9	9	9
9	9	9	2	9	1	2	3	2	1

BSEdisliket	BSElumpse	CaBreastCo	CaBreastfr	CaBreastTr	BSEdetects	EverDoneB	StandMirro	Liedown	RhandtoLb
1	1	1	1	1	1	1	1	2	3
1	3	1	1	3	1	1	3	3	1
3	1	1	1	2	1	2	3	1	1
1	3	1	1	3	1	1	3	3	1
9	9	9	9	9	1	9	1	9	9
1	3	1	1	3	1	1	1	3	1
3	1	2	1	2	1	1	9	1	1
3	1	1	2	2	1	1	1	2	1
3	2	1	2	3	1	3	1	9	9
9	9	1	1	3	9	9	2	2	9
9	9	2	2	2	2	1	1	9	1
3	3	1	1	2	2	1	2	1	1
2	1	2	2	2	2	1	3	1	1
2	2	1	2	2	1	1	2	1	1
1	1	1	2	2	1	1	1	1	1
3	1	1	1	3	1	2	1	1	2
1	1	1	1	2	2	2	1	2	1
3	1	1	1	3	1	2	1	1	2
3	1	3	1	3	1	2	1	1	2
3	2	1	2	2	2	1	1	2	1
3	1	1	2	3	1	2	1	1	1
3	1	1	2	3	1	2	1	1	1
3	9	2	2	2	1	4	1	1	1
9	1	1	3	3	1	4	9	1	1
3	1	1	1	2	2	3	1	1	2
2	1	2	2	3	1	2	2	1	1
2	1	1	3	3	1	1	3	1	2
3	1	1	1	3	1	2	1	1	1
3	1	1	1	3	1	4	3	1	1
1	1	1	1	3	1	1	3	1	1
1	1	1	1	3	1	1	3	1	1
3	1	1	1	2	1	1	1	2	1
3	1	1	1	1	1	3	2	1	1
3	1	1	1	2	1	3	1	2	1
3	1	1	1	3	1	1	1	3	1
3	1	1	1	2	1	3	1	2	1
3	1	1	1	3	1	1	1	3	1
3	1	1	1	3	1	4	1	1	1
9	9	9	9	9	9	9	3	1	1
3	1	1	1	3	1	1	3	1	1
3	1	1	1	2	1	5	3	1	1
1	1	1	1	2	1	1	1	2	1
3	9	1	1	3	2	2	2	3	2
3	1	1	1	3	1	4	1	3	1
3	1	1	1	3	1	2	3	3	1
3	1	2	1	2	2	2	1	9	9

3	1	1	1	9	1	1	1	1	2
3	1	1	1	3	1	4	3	3	1
3	1	1	1	3	1	4	9	1	1
3	1	1	3	1	1	2	2	1	1
3	1	1	1	3	1	2	1	1	1
3	1	1	1	3	1	5	1	1	1
3	1	1	3	3	1	5	3	1	1
3	1	1	1	3	1	5	1	3	1
9	1	1	1	9	1	4	1	9	1
3	1	2	3	3	1	4	1	3	1
9	9	1	9	9	9	1	9	9	9
3	1	1	1	1	1	3	1	1	1
1	1	2	1	1	1	5	1	3	1
2	1	1	2	1	1	6	1	9	1
1	1	1	1	3	1	5	1	3	2
3	1	1	1	9	1	4	1	2	1
3	1	1	1	3	1	5	1	2	1
3	1	1	1	3	1	4	1	1	1
3	1	1	1	3	2	2	2	3	2
3	3	1	1	3	1	1	1	1	1
3	1	1	1	3	1	5	1	9	9
1	1	1	1	3	1	5	3	1	9
3	1	1	1	3	1	4	9	9	9
3	1	1	1	3	1	2	1	3	1
9	1	9	1	1	1	4	9	1	9
1	1	1	1	9	1	3	1	1	1
3	1	1	1	9	1	6	1	3	1
3	1	1	1	3	1	4	3	3	1
3	1	1	2	3	1	3	1	1	1
3	1	1	1	3	1	3	1	3	1
1	1	3	3	3	3	1	1	1	1
3	1	3	3	3	3	3	1	1	1
1	1	1	2	2	2	9	1	1	1
3	1	1	3	3	1	4	2	3	1
3	1	1	1	1	1	2	1	1	1
3	1	1	1	1	1	3	1	1	1
1	1	1	1	1	1	1	1	1	1
3	1	1	1	3	1	1	2	3	1
3	1	1	1	3	1	3	9	9	1
3	1	1	1	3	1	2	1	3	1
3	1	1	1	2	1	1	1	9	1
3	1	1	1	3	1	5	1	2	1
3	1	1	1	3	1	1	3	3	1
3	1	1	2	3	1	4	1	1	1
3	1	9	9	9	1	4	1	3	1
9	1	9	9	9	9	3	1	1	1
1	1	9	1	9	1	3	1	9	1

3	1	1	2	2	1	1	2	1	1
3	1	1	2	2	1	1	2	1	1
1	1	1	1	3	1	5	1	1	1
3	1	1	2	2	1	1	2	1	1
3	1	1	3	3	1	2	9	9	1
1	1	9	9	9	9	2	1	1	1
3	1	1	1	3	1	3	1	1	1
3	1	1	3	3	1	2	1	3	1
9	1	1	1	3	1	3	1	1	1
3	1	1	1	3	1	4	1	3	1
3	1	1	1	2	1	2	1	2	1
3	1	2	2	2	1	9	2	1	1
9	1	9	9	9	1	3	1	3	1
2	1	1	1	1	1	1	1	3	1
3	1	2	1	1	1	1	1	2	1
1	1	1	2	3	1	4	2	1	1
3	1	1	1	2	2	2	9	1	1
2	1	2	2	3	2	2	1	1	1
3	1	1	1	3	1	2	1	1	1
1	1	1	1	3	1	2	9	9	9
3	1	1	1	3	1	2	3	1	1
3	1	1	1	3	1	5	1	9	1
2	2	2	2	2	2	4	2	2	2
1	1	1	1	3	1	1	3	3	1
1	1	1	2	1	1	9	1	1	1
2	1	1	2	2	1	1	3	1	1
3	1	1	1	3	1	2	1	3	1
9	1	1	9	9	9	2	1	3	1
3	1	2	2	3	2	3	3	2	2
1	1	1	1	1	1	1	2	2	2
2	1	2	2	2	1	1	1	2	2
1	2	2	2	3	2	2	3	2	2
2	1	1	2	9	1	1	2	2	2
2	1	2	3	2	2	3	2	3	2
1	1	1	1	1	1	1	2	2	2
3	1	1	3	3	1	2	3	3	3
1	1	1	1	2	1	1	1	3	2
2	1	2	2	3	1	1	3	1	1
9	1	1	1	9	9	2	9	1	9
3	1	1	1	3	1	6	1	1	1
3	1	1	1	3	1	5	1	1	1
3	1	1	1	3	1	5	1	1	1
1	2	2	2	3	2	2	3	2	2
1	1	1	3	3	1	1	1	2	1
2	2	2	2	2	1	3	2	2	1
3	1	1	1	2	1	4	3	1	1
2	1	1	2	2	1	3	2	2	2

9	1	1	9	9	9	1	2	3	3
3	1	1	1	3	1	1	1	3	1
3	1	1	1	2	1	1	1	2	1
3	9	1	1	3	1	5	1	1	1
3	3	3	2	2	2	5	1	1	1
1	1	1	1	3	1	2	9	1	1
2	3	1	3	2	1	2	3	3	2
3	1	1	2	2	1	4	2	3	1
2	2	2	1	2	2	1	2	2	2
2	1	2	2	2	2	1	2	2	2
2	1	2	1	3	1	1	3	2	2
2	1	2	1	3	1	1	3	2	2
9	9	9	1	3	2	4	1	9	9
3	1	1	1	1	1	2	1	3	1
3	1	1	1	2	1	1	1	2	1
1	1	1	1	2	2	1	1	2	1
3	1	1	1	3	1	1	9	9	1
1	1	1	1	3	1	4	1	3	1
9	9	9	9	1	9	1	9	1	9
3	1	1	1	3	1	1	1	1	1
3	1	1	1	3	1	1	1	9	9
3	1	1	1	3	1	2	1	1	1
3	1	1	1	3	1	1	1	2	1
3	1	1	1	3	1	1	1	1	1
3	1	1	1	3	1	1	1	1	1
3	1	1	1	3	1	1	1	1	1
3	1	1	1	3	1	1	1	1	1
3	1	1	1	3	1	1	1	1	1
3	1	1	1	3	1	1	1	1	1
3	1	1	1	3	1	1	1	1	1
1	1	1	1	3	1	5	1	1	9
1	1	1	1	2	1	1	1	2	3
3	1	1	1	3	1	1	9	9	9
9	1	1	1	3	1	1	1	1	1
9	1	1	1	3	1	1	1	1	1
3	1	1	1	3	1	2	1	1	1
3	3	1	1	3	1	1	1	1	1
3	1	1	1	3	1	1	1	1	1
3	1	1	1	3	1	1	1	9	1
3	1	1	1	3	1	1	1	1	1
3	1	1	1	3	1	1	1	1	1
3	1	1	1	3	1	5	1	1	1
3	1	1	1	3	1	1	9	9	9
3	1	1	1	3	1	1	1	1	1
3	1	1	1	3	1	1	1	1	1
3	1	1	1	3	1	1	1	1	1
3	1	1	1	3	1	1	1	1	1

3	1	1	1	3	1	1	1	1	1
3	1	1	1	3	1	1	1	1	1
3	1	1	1	3	1	1	1	1	1
3	1	1	1	3	1	1	1	1	1
3	1	1	1	3	1	1	1	1	1
3	1	1	1	3	1	2	1	1	1
3	1	1	1	3	1	1	1	1	1
3	1	1	1	9	1	5	1	3	1
3	1	1	1	3	1	2	1	3	1
3	9	1	1	3	1	9	3	1	1
1	1	3	3	3	1	2	1	3	1
3	1	1	1	1	1	3	1	1	1
3	1	1	1	3	1	2	1	3	1
3	1	1	1	3	1	1	9	9	9
2	1	1	1	2	1	5	3	3	1
2	1	1	2	3	1	4	1	2	1
1	1	2	2	2	1	1	1	1	2
1	1	1	1	1	1	1	1	1	1
3	1	1	1	3	1	3	2	1	1
3	1	1	2	2	1	4	2	2	2
1	1	1	1	3	2	1	1	2	1
3	3	1	1	2	1	1	2	2	2
3	1	1	1	2	1	1	1	2	1
2	1	1	1	2	2	3	1	2	1
3	2	1	1	2	1	1	2	2	2
2	2	1	1	2	1	1	2	2	2
3	1	1	2	2	1	4	2	2	2
2	2	1	1	2	1	1	2	2	2
2	2	2	2	2	2	1	2	2	2
3	1	1	1	3	1	2	1	1	1
2	1	1	1	3	2	1	2	2	2
2	1	1	1	3	2	1	2	2	2
3	1	1	1	3	1	2	1	1	1
1	1	1	1	3	1	1	1	3	1
2	1	1	2	2	1	2	1	2	2
1	1	1	1	1	1	1	2	2	2
1	1	2	2	2	1	1	1	1	2
1	1	1	1	2	1	5	1	2	1
3	1	1	1	3	1	2	3	2	2
1	1	3	1	2	1	3	2	2	3
2	3	1	2	2	2	1	1	2	3
3	1	1	2	2	1	1	2	2	2
9	9	1	9	9	9	3	9	9	9
9	9	1	9	9	9	3	9	9	9
1	1	1	1	1	1	1	1	2	2
2	1	1	1	2	1	1	3	2	1
1	1	1	1	3	1	1	2	9	9

3	1	1	1	2	1	1	1	1	1
3	1	1	1	2	1	1	1	1	1
3	1	1	1	2	1	1	2	2	2
1	1	1	3	2	1	1	2	2	2
3	1	3	1	3	1	1	2	1	2
2	1	1	2	3	1	4	1	1	1
2	1	1	1	3	3	3	1	3	1
2	1	1	3	3	2	7	2	3	2
3	1	1	1	3	1	5	1	3	2
3	1	1	3	3	3	3	3	3	3
2	1	1	1	2	2	1	1	2	1
3	1	3	3	2	1	3	3	3	1
3	1	3	3	2	1	3	3	3	1
3	2	2	2	2	2	2	2	3	1
3	1	1	2	2	1	3	1	3	1
3	1	1	1	3	1	2	2	3	1
3	1	1	2	3	2	3	3	3	1
2	2	1	2	3	2	1	2	2	2
2	1	1	1	3	3	1	3	1	1
1	1	1	1	3	2	1	1	2	1
1	1	1	2	1	1	1	1	1	2
2	1	1	1	2	1	2	1	2	1
3	1	1	2	3	1	4	2	3	1
2	1	2	1	3	1	3	1	1	2
9	1	2	1	9	9	1	1	9	1
2	2	2	2	2	2	1	2	2	2
2	2	2	2	2	2	1	2	2	2
3	3	1	9	2	1	1	1	1	1
2	2	2	2	2	2	2	9	9	9
3	1	2	2	2	2	1	9	1	1
9	9	1	9	9	9	2	1	9	9
3	1	2	2	2	1	2	1	1	1
1	1	3	1	1	1	1	2	1	1
3	1	3	2	2	1	2	1	1	1
9	9	9	9	1	9	1	1	9	9
1	2	2	2	3	2	2	3	2	2
3	1	1	1	3	1	1	2	1	1
1	2	1	1	3	1	4	1	3	1
3	1	1	2	2	1	1	2	1	1
3	1	1	3	3	1	2	2	1	1
3	3	1	3	2	1	3	3	2	1
2	1	1	1	2	1	4	1	3	2
3	1	1	2	2	1	5	1	3	1
2	1	1	1	3	1	3	2	2	2
2	1	1	1	2	1	1	1	9	9
9	1	1	1	2	1	3	2	2	2
3	1	2	1	1	2	5	3	2	1

9	9	9	9	9	9	3	9	9	9
3	1	1	1	2	1	2	1	3	2
3	1	1	1	3	1	5	2	2	1
3	1	1	1	2	1	3	1	2	1
3	1	1	3	2	1	1	2	3	2
2	1	1	1	3	1	1	1	2	1
3	1	1	1	1	1	1	1	2	2
3	1	1	1	3	3	3	1	1	1
3	1	1	3	3	1	4	1	9	1
3	1	2	2	2	2	2	2	1	2
2	2	2	2	2	2	1	2	2	2
3	1	1	2	3	1	3	1	2	2
1	1	1	1	1	1	2	9	9	9
1	3	1	1	3	1	2	3	1	1
1	1	3	1	1	1	2	1	1	3
3	1	1	1	3	1	1	1	3	2
3	1	1	1	1	1	1	2	2	1
2	1	1	1	1	2	6	1	1	1
1	2	1	2	1	1	1	1	3	1
9	9	9	9	9	9	9	9	9	9
1	1	2	2	2	3	1	2	3	2
3	1	1	2	2	1	4	1	2	9
3	1	1	1	3	1	4	1	3	1
1	1	2	2	2	2	1	2	2	2
2	1	1	1	3	1	3	2	2	2
2	1	1	3	3	1	4	3	3	1
3	1	1	1	2	1	3	3	2	1
3	1	1	1	3	1	1	2	2	3
3	1	1	1	3	1	3	3	1	1
1	2	1	1	3	1	1	3	2	1
3	1	1	1	3	1	2	1	2	1
3	1	2	3	3	1	1	1	3	1
3	1	1	1	3	1	4	9	9	9
3	1	1	1	3	1	1	3	3	1
3	1	2	1	3	1	1	1	2	1
1	1	1	3	3	1	1	3	1	1
3	1	1	1	3	1	5	3	3	1
3	1	1	1	3	1	4	3	1	1
3	1	1	3	3	1	4	3	1	1
3	1	2	2	2	1	1	2	2	1
1	1	1	3	3	1	2	2	3	2
9	1	1	1	2	1	4	3	1	1
9	9	1	2	2	2	5	9	9	1
3	1	1	2	3	1	1	2	2	2
3	1	3	3	3	1	2	1	3	1
3	1	1	1	2	1	2	2	1	1
3	1	1	2	3	1	5	1	1	1

3	1	1	1	2	1	2	3	2	1
3	1	1	1	2	1	2	3	2	1
9	1	9	9	9	1	2	9	9	9
3	1	1	3	3	1	3	1	1	1
9	1	1	9	9	9	1	9	9	9
3	1	1	2	2	1	4	2	2	1
3	1	1	1	2	1	4	1	2	1
3	9	3	9	3	9	1	9	9	1
3	1	1	1	3	1	1	1	1	1
3	1	1	2	1	1	2	1	2	1
2	1	1	1	3	1	1	1	3	2
1	1	2	2	3	2	1	3	3	2
3	1	2	1	3	1	4	1	3	1
2	1	1	1	3	1	2	1	2	2
2	1	1	1	3	1	1	1	3	1
1	1	1	3	3	1	2	3	2	2
2	1	1	1	3	1	1	1	3	1
2	1	1	2	3	1	2	3	3	2
1	1	1	1	3	1	6	3	3	1
3	1	1	2	2	1	1	2	2	1
1	1	1	3	3	1	1	3	2	1
3	1	1	1	2	1	2	3	3	2
3	1	1	1	3	1	5	3	3	2
3	1	1	2	3	1	4	1	2	3
1	1	1	1	1	2	1	2	2	2
2	1	1	2	3	2	1	2	1	1
3	1	2	1	2	1	1	1	1	1
1	2	2	2	2	2	1	1	1	2
3	1	1	2	2	1	1	2	2	2
2	1	1	3	3	1	2	2	1	1
3	1	1	1	2	1	3	2	3	1
3	1	1	1	2	1	3	2	3	1
3	2	2	3	2	3	1	3	3	2
2	1	2	2	2	2	1	2	2	2
2	1	2	2	2	2	1	2	2	2
3	1	1	1	1	1	1	1	3	2
1	1	2	1	2	1	3	1	3	1
2	1	1	1	1	1	2	9	1	9
2	1	2	2	2	1	1	2	2	2
2	1	1	3	3	3	4	2	3	1
3	1	1	3	1	2	2	2	1	2
3	1	2	2	2	1	2	1	1	2
3	1	1	1	1	1	1	1	2	1
3	1	2	2	2	1	1	1	2	2
3	1	1	1	2	1	1	1	1	2
3	1	1	1	3	1	5	2	1	1
2	1	1	1	3	1	1	2	2	2

3	1	1	1	2	1	1	3	3	2
3	1	1	1	2	1	3	1	2	1
2	1	1	2	3	2	2	1	2	2
1	2	1	1	2	1	1	1	3	1
3	1	1	1	2	1	1	3	1	3
2	1	1	2	2	1	5	3	3	2
2	1	1	2	2	2	1	1	3	2
3	1	1	2	3	1	3	1	2	2
3	1	1	2	3	1	3	3	3	2
1	1	1	1	2	2	1	2	2	1
2	1	1	2	2	1	4	1	1	1
1	1	1	2	2	1	2	1	3	1
3	1	1	1	1	1	2	3	3	1
2	2	2	1	3	2	1	2	2	2
2	2	2	1	3	2	1	2	2	2
1	1	1	1	2	1	1	2	1	1
3	1	3	1	2	1	1	1	2	1
3	1	1	1	2	1	1	1	2	2
3	1	1	1	2	1	1	1	2	2
3	1	1	1	3	1	1	9	1	9
3	1	1	1	1	1	2	1	1	1
3	1	1	1	3	1	1	2	1	2
3	1	1	1	3	1	3	9	9	9
1	1	1	2	1	1	1	2	2	2
3	1	1	1	1	1	2	1	2	1
3	1	2	1	3	1	4	3	3	1
1	2	1	2	3	1	2	3	3	1
1	9	1	3	2	3	9	9	9	9
1	1	2	2	1	1	1	3	3	2
1	1	1	1	2	2	1	2	1	3
2	1	2	2	2	2	1	1	2	1
3	1	2	2	2	1	3	1	2	2
2	1	2	2	2	2	6	2	2	2
3	1	1	1	3	3	9	1	1	1
3	1	1	1	1	1	1	1	1	1
1	1	2	2	1	2	2	1	9	1
3	2	3	3	3	3	1	2	3	3
1	1	1	1	3	1	1	3	2	2
2	2	2	2	2	2	1	3	2	2
1	1	1	1	2	1	4	1	3	1
1	1	2	3	2	3	1	1	2	2
1	3	1	1	3	1	9	1	3	1
1	1	1	1	2	1	3	1	1	2
3	1	1	1	2	3	1	3	1	3
9	9	9	9	9	9	9	9	9	9
2	1	1	1	3	1	5	1	1	1
3	3	1	1	3	1	1	9	9	9

1	9	1	1	3	1	5	1	1	1
3	1	1	1	3	1	2	1	1	1
3	1	1	1	3	1	1	1	1	1
3	1	1	1	3	1	1	1	1	1
3	1	1	1	3	1	1	1	1	1
3	1	1	1	3	1	2	1	1	1
3	1	1	1	3	1	1	1	3	1
3	1	1	1	1	1	1	1	3	1
3	1	1	1	3	1	1	1	3	1
3	1	1	1	3	1	1	1	1	1
9	3	1	3	2	2	1	1	3	1
3	1	9	1	3	1	1	1	1	1
3	1	1	1	3	1	1	1	3	1
3	1	1	1	3	1	1	1	3	1
3	1	1	1	3	1	1	1	1	1
3	1	1	1	3	1	1	1	3	1
3	1	1	1	3	1	1	1	3	1
3	1	1	1	3	1	1	1	3	1
3	1	1	1	3	1	1	1	3	1
3	1	1	1	3	1	1	1	9	1
3	1	1	1	3	1	1	1	3	1
3	1	1	1	3	1	1	1	1	1
3	1	1	1	3	1	1	1	1	1
3	1	1	1	3	1	1	1	1	1
3	1	1	1	3	1	1	1	1	1
3	1	1	1	3	1	1	1	1	1
3	1	1	1	3	1	1	1	1	1
3	1	1	1	3	1	1	1	1	1
3	1	1	1	3	1	1	1	1	1
3	1	1	1	3	1	1	1	1	1
3	1	1	1	3	1	2	1	3	1
3	1	1	1	3	1	1	1	3	1
3	1	1	1	3	1	3	1	1	1
3	1	1	1	3	1	3	1	1	1
3	1	1	1	3	1	1	1	1	1
3	1	1	1	3	1	2	1	1	1
2	1	1	1	3	1	5	1	2	1
3	1	1	1	3	1	1	1	1	1
3	1	1	1	3	1	1	1	1	1
3	1	1	1	3	1	1	1	1	1
3	1	1	1	3	1	2	1	1	1
3	1	2	1	2	1	5	2	2	1
1	1	1	1	2	1	3	1	1	2
3	1	1	1	2	1	1	2	2	1
2	1	1	2	2	2	1	2	9	9
2	1	2	2	2	2	2	1	3	1
3	1	1	1	3	1	4	3	3	1

2	2	1	2	2	2	1	2	2	2
1	1	1	1	2	1	4	1	3	1
1	1	1	1	2	1	4	1	3	1
3	1	1	1	2	1	2	2	1	1
2	1	1	1	2	1	3	1	2	2
1	1	1	2	2	3	1	2	1	2
2	1	1	1	2	3	3	1	3	1
2	2	2	2	1	2	1	2	2	1
3	1	1	3	2	1	3	2	2	1
1	1	1	2	2	2	1	2	2	2
1	1	1	1	2	1	9	1	3	1
9	9	9	9	9	1	1	1	9	9
9	1	1	1	9	9	4	9	9	1
2	1	2	2	2	2	1	2	2	2
1	9	9	9	9	1	1	9	9	9
1	9	9	9	9	1	3	1	9	9
9	9	9	9	9	1	1	1	9	9
9	1	1	9	9	9	1	1	9	9
9	1	1	9	9	9	1	9	9	1
3	1	9	1	3	1	2	1	3	1
3	3	3	3	3	1	3	1	3	3
9	3	9	3	3	1	3	1	3	3
2	1	1	1	3	1	5	1	3	1
9	9	1	9	9	9	3	1	9	9
9	1	9	9	9	1	3	9	9	9
2	1	1	1	3	1	2	1	1	1
9	1	9	9	9	1	3	9	9	1
2	1	1	2	3	1	2	1	3	1
2	1	1	1	3	1	4	1	3	1
9	9	9	9	9	1	1	9	9	1
9	1	1	9	9	1	3	1	9	9
3	2	1	1	3	1	5	1	2	1
2	1	2	3	2	2	1	2	2	2
3	1	1	1	2	1	3	1	3	2
2	1	1	1	1	2	3	1	2	1
2	1	2	1	3	1	2	1	3	1
3	1	1	1	3	1	1	1	2	2
3	1	3	2	3	1	1	3	3	3
2	2	2	2	2	2	1	2	2	2
3	1	1	1	2	1	4	2	3	1
2	3	1	2	3	1	3	9	9	9
3	1	1	1	1	1	3	3	2	1
1	1	1	1	1	1	1	2	1	1
1	1	1	1	3	1	4	2	2	1
2	1	1	1	2	1	3	1	3	1
9	1	1	2	2	2	3	1	3	2
3	1	1	1	3	1	3	1	3	1

1	1	1	2	2	1	2	9	9	9
3	1	1	1	2	1	1	3	3	1
2	1	1	1	1	1	1	3	3	3
3	1	1	1	2	2	1	1	1	2
3	1	1	2	3	2	1	1	3	2
2	1	1	2	2	2	1	2	2	2
3	1	1	3	3	1	1	1	3	2
2	1	1	1	3	1	1	1	1	1
2	1	1	1	1	1	4	1	2	2
2	1	1	2	3	2	1	2	2	2
2	1	1	2	3	2	2	2	3	1
3	1	2	1	2	1	4	1	3	1
2	2	2	3	2	2	1	2	2	2
2	2	2	3	2	2	1	2	2	2
1	2	1	2	3	1	1	1	1	2
2	2	1	2	3	1	1	1	2	2
3	1	2	1	3	1	5	1	2	2
1	1	1	1	1	1	1	2	2	2
3	1	2	1	2	2	3	2	3	1
1	1	1	1	3	1	1	1	3	1
3	1	1	1	3	1	4	3	3	1
3	1	1	1	3	1	2	3	3	2
3	1	1	1	3	1	2	1	3	1
3	1	1	1	3	1	2	1	3	1
2	1	1	1	3	1	2	1	2	1
3	1	1	1	2	1	5	2	2	1
1	1	1	3	3	1	2	1	1	1
2	1	1	3	3	1	2	1	1	1
3	1	1	1	2	1	1	1	2	2
3	1	1	3	3	2	1	2	2	1
1	1	1	1	2	1	9	1	3	1
9	1	1	1	2	1	9	2	9	2
1	2	1	2	3	2	1	3	3	3
1	1	2	3	3	1	4	3	9	1
2	1	1	1	3	3	2	1	2	1
9	9	2	9	9	9	1	2	9	9
2	1	1	3	3	1	1	2	1	1
3	1	1	1	3	1	3	1	1	1
2	2	3	2	2	2	1	2	2	2
3	1	1	1	3	1	3	1	2	1
2	1	1	2	3	1	3	1	2	9
2	1	1	1	2	1	1	1	2	2
3	1	3	1	3	1	4	3	3	3
3	1	2	2	2	1	9	2	1	1
3	1	1	1	1	1	3	1	3	1
9	9	1	2	3	1	3	1	3	3
9	1	1	9	9	9	3	9	9	1

9	1	9	9	9	1	1	1	9	9
9	1	9	9	9	1	1	1	9	9
9	9	1	9	9	9	1	9	9	1
3	1	1	1	3	1	7	1	9	9
3	1	1	1	3	1	3	1	9	9
9	9	9	9	1	9	2	1	9	9
9	9	2	2	2	2	1	1	9	9
9	1	9	9	9	1	1	1	9	9
9	9	9	9	9	1	2	1	9	9
9	9	9	9	9	1	1	1	9	9
9	1	1	3	3	1	1	1	3	3
9	9	9	9	9	1	1	1	9	9
9	9	9	9	9	1	1	1	9	9
9	9	1	9	9	9	3	1	9	9
2	2	1	9	1	1	1	1	9	9
9	9	1	9	9	9	1	1	9	9
9	9	9	9	9	9	1	1	9	9
3	1	1	1	3	1	1	1	9	9
2	2	2	2	2	2	1	2	2	2
2	1	1	1	1	1	1	1	1	1
1	1	1	1	1	1	1	1	1	1
1	1	1	2	2	1	1	3	3	3
1	1	2	1	1	1	1	1	2	2
1	1	1	1	1	1	2	1	3	1
3	1	2	2	3	1	1	2	2	2
2	2	2	1	2	3	5	1	3	2
2	1	1	1	2	1	1	2	2	2
2	2	1	2	2	1	1	1	3	2
9	1	2	2	2	2	1	2	2	2
2	2	3	9	2	2	1	2	2	2
3	3	1	1	1	1	1	3	3	3
3	1	2	2	3	1	4	1	3	2
3	1	1	2	3	1	1	2	2	2
1	9	1	3	3	1	1	1	3	3
3	1	1	1	2	1	1	1	2	1
2	2	1	2	2	2	1	1	2	2
2	2	1	2	3	2	1	2	3	9
1	1	2	3	1	3	2	2	3	1
1	1	1	1	2	1	1	2	1	2
3	1	1	1	2	1	1	1	3	1
1	2	1	2	3	2	9	2	2	9
3	1	1	1	1	1	3	1	3	1
2	2	1	1	3	1	3	3	1	1
3	1	1	1	3	1	2	1	2	1
3	1	1	1	1	1	1	1	3	1
2	2	1	1	3	3	1	9	9	9
2	3	1	2	2	2	1	1	2	2

1	2	1	1	3	2	1	1	3	1
1	1	1	1	2	2	2	1	3	1
1	1	3	1	3	1	1	1	3	1
1	1	1	1	1	1	1	1	2	1
3	1	2	1	3	1	1	3	1	1
3	1	2	1	3	1	1	3	1	1
2	1	1	1	1	2	4	2	1	2
1	2	1	1	3	2	1	1	3	1
1	1	1	2	1	3	1	1	9	1
3	1	1	1	1	1	1	1	3	1
2	1	1	2	1	3	1	2	3	1
2	1	1	1	1	1	3	1	3	1
2	2	2	2	2	2	9	2	2	3
1	1	2	3	1	3	2	1	2	3
2	1	1	3	1	3	1	2	2	2
1	1	1	2	3	1	2	1	2	1
1	1	1	1	1	1	4	1	1	1
9	9	1	9	9	9	1	9	9	9
1	1	1	2	1	1	3	1	3	1
3	1	1	1	3	1	1	3	3	3
3	3	1	2	9	1	1	1	1	3
2	2	2	3	3	3	9	2	2	3
2	3	1	2	3	3	2	1	1	2
3	2	1	3	2	1	2	1	3	1
2	1	1	1	3	1	1	2	2	2
3	1	1	1	3	3	1	1	3	3
3	1	1	1	3	1	1	1	3	3
3	3	1	1	3	1	3	3	1	1
2	3	1	2	2	2	1	1	1	2
1	1	1	1	3	3	3	1	3	1
3	1	1	1	3	1	4	3	1	1
3	1	1	1	3	3	1	3	3	3
2	1	1	1	2	1	1	1	1	2
1	1	1	1	3	3	1	1	3	1
1	1	1	1	3	3	1	1	3	1
1	1	1	1	2	1	1	1	3	1
1	1	1	1	3	2	4	1	3	1
9	9	9	9	9	9	9	9	9	9
1	1	1	1	1	1	1	2	2	2
1	1	1	1	1	1	1	9	9	9
1	1	9	9	9	9	9	9	9	9
2	2	2	9	2	3	9	3	9	3
3	1	1	1	2	3	1	1	3	3
1	1	9	9	9	9	9	9	9	9
3	1	1	1	2	3	1	1	3	3
2	2	1	2	2	2	9	2	3	1
1	1	1	1	1	1	1	1	3	1

3	1	1	1	3	1	1	1	3	3
2	1	1	1	2	1	3	1	1	1
1	1	1	1	1	1	1	1	3	2
1	1	1	1	2	2	1	1	2	2
3	1	3	2	2	1	4	2	2	2
3	2	1	3	2	3	2	3	9	1
2	2	1	2	3	2	1	9	9	9
3	1	1	3	3	1	2	1	3	1
3	3	3	3	2	1	1	3	3	3
3	1	1	1	1	1	5	3	3	1
3	3	3	3	3	3	1	3	3	3
3	1	1	1	3	1	1	1	3	1
2	2	1	2	2	1	1	2	3	1
2	2	1	2	2	2	1	2	3	1
2	2	1	2	2	2	1	2	3	3
3	1	1	2	2	2	1	2	3	2
2	2	1	2	2	2	1	2	3	1
2	2	1	2	2	2	1	2	3	1
2	2	1	2	2	2	1	2	3	1
3	1	1	1	1	1	1	1	3	1
2	3	2	1	3	2	4	1	3	2
3	3	1	2	3	3	9	1	3	2
1	1	1	2	2	2	3	2	2	1
1	1	1	1	2	1	1	1	2	2
2	3	3	3	3	3	1	1	3	3
3	2	1	2	2	2	1	2	1	3
2	1	1	2	2	2	1	1	9	9
3	1	1	1	1	1	1	1	1	1
9	9	9	9	9	1	3	9	9	1
3	3	3	3	3	3	7	1	3	3
1	1	1	2	1	3	1	3	3	1
1	2	2	3	2	3	7	2	9	3
1	3	2	1	3	2	3	2	1	3
3	3	1	2	1	2	1	2	3	3
3	3	3	3	3	3	1	3	3	3
3	1	2	1	1	1	1	3	3	3
1	1	2	1	1	1	1	1	9	9
2	1	1	1	2	1	1	1	3	1
2	1	1	1	2	1	1	2	1	2
1	1	1	1	1	1	1	1	1	1
1	2	1	1	1	2	1	1	2	2
1	1	1	1	2	1	1	1	2	1
1	3	3	1	2	1	1	1	2	3
3	1	1	1	1	1	3	3	1	1
2	1	2	3	3	1	2	1	1	1
2	1	1	2	1	2	1	1	1	1
9	3	1	1	1	1	1	1	1	1

3	1	1	2	3	1	3	1	3	2
9	9	9	9	1	9	1	1	9	9
9	9	1	9	9	9	1	1	9	9
3	1	1	1	3	3	1	3	1	3
3	1	2	1	3	1	1	1	3	2
3	1	2	1	3	1	1	3	1	1
3	1	1	3	1	1	2	2	3	2
3	1	2	1	3	1	1	3	1	1
3	1	2	1	3	1	1	9	1	1
3	1	2	1	3	2	1	1	3	2
2	1	1	1	1	2	1	1	2	3
3	1	2	3	3	1	3	1	1	1
1	1	1	1	2	1	3	1	2	2
3	2	1	1	3	1	2	1	3	1
3	1	1	1	3	1	1	3	3	1
1	1	1	1	3	1	1	2	1	2
3	3	1	2	3	1	1	1	2	3
9	9	9	1	9	9	9	1	9	9
9	9	9	1	9	9	1	1	9	9
3	1	1	1	1	9	1	1	1	2
2	2	3	1	1	1	1	1	1	1
2	2	1	1	1	2	1	2	1	2
1	1	1	1	1	1	9	1	1	1
2	2	2	2	2	2	9	2	2	2
3	3	3	3	3	3	9	3	3	3
2	1	2	1	3	1	1	2	2	3
2	1	2	1	3	1	1	2	2	3
2	1	1	1	3	1	1	3	1	1
1	1	1	1	1	1	1	1	1	1
2	2	2	2	2	2	1	2	2	2
9	9	2	1	2	2	1	2	3	2
1	1	1	1	2	1	1	1	2	1
1	3	1	1	1	1	1	9	1	9
2	1	2	1	3	1	1	2	2	3
1	1	1	1	3	1	4	1	3	1
2	2	2	2	2	2	9	2	2	2
1	2	1	1	2	1	1	1	2	2
3	1	1	1	1	1	1	9	9	9
3	9	9	2	9	1	1	1	9	9
1	1	1	1	3	1	1	1	3	1
1	1	1	1	1	3	1	3	3	3
2	2	2	2	2	2	9	2	2	2
2	2	2	2	2	2	1	1	2	2
2	3	2	3	2	2	1	2	2	2
1	2	1	2	2	2	1	2	2	2
2	2	2	2	2	2	9	2	2	2
2	1	3	1	2	3	9	9	9	9

2	2	2	2	2	2	1	9	9	9
2	2	2	2	3	2	1	2	2	2
9	9	1	1	1	1	9	9	9	9
9	9	1	1	3	1	9	9	9	9
9	9	9	9	9	9	1	1	9	9
2	3	1	2	1	3	1	3	2	1
2	1	1	2	2	2	1	2	2	2
3	1	1	1	3	1	1	3	3	3
1	2	1	2	1	2	1	1	1	1
9	9	9	2	9	9	1	3	9	9
2	2	2	2	2	2	9	2	2	2
2	1	1	1	2	1	4	1	2	1
1	1	1	1	2	1	9	1	2	1
2	2	1	1	2	1	1	2	2	2
1	1	1	1	3	1	1	1	3	3
2	2	2	2	2	2	1	2	2	2
2	2	1	1	2	1	1	2	2	2
2	3	3	3	1	1	1	2	2	2
9	9	1	1	3	1	1	2	2	2
3	2	1	1	1	1	1	3	3	1
9	9	1	9	9	9	1	9	1	9
1	1	1	1	2	3	1	1	3	1
2	2	2	2	2	2	1	2	2	2
1	3	1	2	2	1	1	3	3	1
2	2	1	1	3	1	1	2	2	2
2	2	1	2	3	2	2	2	3	2
9	9	9	9	9	1	1	9	9	9
9	9	9	9	9	9	1	9	1	9
2	2	1	2	2	2	1	3	3	3
3	1	1	2	1	1	1	2	2	2
9	9	1	9	9	1	1	9	1	9
1	1	1	1	2	1	1	1	3	1
3	1	3	3	3	1	2	1	3	1
3	1	2	2	2	2	1	1	2	2
3	1	2	2	2	2	1	1	2	1
1	3	1	1	1	1	2	1	3	1
2	2	2	2	2	2	1	2	2	2
1	1	1	2	2	1	4	1	1	2
2	2	1	1	2	1	1	2	2	2
2	1	1	1	2	1	4	1	2	9
2	1	1	2	2	2	1	2	2	2
2	2	1	2	2	2	1	2	2	2
3	1	1	1	3	2	1	2	3	2
1	1	1	1	3	1	1	1	2	1
2	1	2	2	2	1	1	2	2	2
3	3	3	3	3	3	9	3	3	3
1	2	2	1	2	2	1	2	2	2

2	2	1	2	2	2	1	2	2	2
2	2	2	2	2	2	5	2	2	2
2	2	2	2	1	2	1	2	2	2
2	2	2	2	2	2	1	2	2	2
3	1	1	2	2	2	2	2	2	1
3	1	1	1	1	2	4	3	3	1
2	2	2	2	1	2	1	2	2	2
1	1	1	1	3	1	1	1	2	1
3	1	1	1	1	1	1	2	2	2
3	1	2	2	2	2	1	3	3	1
1	1	1	1	3	1	6	1	3	2
9	9	2	9	9	9	9	9	2	9
9	9	9	1	9	9	1	9	9	9
2	2	2	1	2	1	1	2	2	2
3	3	1	2	2	2	1	1	2	2
1	1	1	3	1	1	1	2	1	3
2	1	2	2	2	1	1	2	1	2
3	1	1	1	1	1	1	1	3	1
2	1	1	2	2	3	1	1	2	2
2	1	2	2	2	3	3	2	3	2
3	2	3	3	1	1	1	3	3	3
3	3	1	1	1	1	1	1	2	2
2	1	1	2	2	1	1	2	2	2
3	1	1	2	2	1	1	1	1	1
3	1	3	3	3	1	1	3	3	3
3	1	1	2	2	1	1	1	9	1
2	2	1	1	2	1	1	2	2	2
9	1	2	1	2	1	1	2	1	1
2	2	1	3	2	1	1	2	2	2
2	1	1	2	3	1	1	2	3	2
1	1	1	1	1	1	2	1	2	1
1	1	1	3	1	1	1	1	1	1
1	1	1	1	1	1	2	3	3	3
1	2	1	1	1	1	1	2	2	1
1	2	1	1	3	2	1	3	3	3
1	1	1	1	3	1	1	3	3	1
3	1	1	1	3	1	2	1	9	9
1	1	1	1	1	1	1	1	1	1
2	2	2	2	2	2	1	2	2	2
2	2	2	2	2	2	1	2	2	2
1	1	2	1	3	1	1	1	3	2
1	1	1	1	1	1	1	1	1	1
9	2	1	1	1	1	1	1	2	3
1	1	1	2	2	1	1	1	2	2
2	2	2	2	2	2	1	2	2	2
3	1	1	2	1	3	1	1	2	3
2	2	2	1	3	2	1	3	3	2

1	9	1	3	1	1	1	3	3	1
3	1	3	3	2	2	1	3	3	3
3	1	1	1	2	1	1	1	3	2
9	9	9	1	9	9	5	9	9	9
9	9	2	9	9	1	2	1	2	9
2	1	3	1	2	3	1	2	2	2
2	1	2	1	2	1	1	2	2	2
3	1	1	1	3	1	1	3	3	3
9	9	2	1	1	1	9	2	3	2
2	2	2	2	2	2	1	2	2	2
9	9	9	9	1	9	1	9	9	1
3	2	1	1	2	3	1	1	2	3
2	2	2	2	2	2	9	2	2	2
2	2	2	2	2	1	1	2	2	2
2	2	2	2	2	2	1	2	2	2
2	3	2	3	1	3	1	2	3	3
2	3	1	2	2	3	4	2	1	2
9	9	9	9	9	1	1	1	9	9
9	9	9	2	9	9	4	9	9	9
2	2	2	2	2	2	9	2	2	2
2	2	2	2	2	2	1	3	2	2
1	2	2	2	2	2	9	2	2	2
9	9	9	9	9	1	1	9	9	9
2	1	1	2	1	1	1	2	9	9
2	3	1	2	3	1	2	1	2	2
3	1	3	1	3	1	1	1	3	1
3	1	3	2	3	1	3	1	3	2
3	1	1	1	3	3	1	1	1	1
2	2	1	3	3	3	1	1	3	1
9	2	1	1	3	2	1	1	2	1
3	1	1	1	3	1	4	1	2	1
3	1	1	1	3	1	3	1	3	1
3	1	1	1	3	1	3	1	3	1
3	3	3	3	9	3	1	3	3	3
3	1	1	1	3	1	1	1	9	1
1	1	1	1	2	1	1	3	2	1
1	1	1	1	1	1	2	1	1	1
3	3	3	3	3	3	1	3	3	3
1	9	2	9	1	9	1	9	1	9
3	3	3	1	3	3	1	3	3	3
3	1	1	3	3	3	6	1	3	3
3	1	1	1	3	1	2	1	9	3
1	1	1	1	1	1	1	1	3	3
1	1	3	1	3	1	1	2	2	2
1	1	1	1	2	1	1	1	2	1
1	1	2	3	2	1	1	3	3	1
2	1	2	1	1	1	1	1	2	1

1	3	2	2	2	2	1	2	2	2
2	2	9	1	3	2	1	1	2	1
3	1	1	1	9	1	2	1	9	9
3	1	1	3	3	1	7	1	1	1
2	1	1	1	2	1	1	3	2	2
2	2	1	3	3	1	1	2	2	2
1	1	2	2	2	2	1	2	2	2
1	1	2	2	2	2	1	9	9	1
1	1	2	2	2	2	1	9	9	1
2	1	1	2	3	2	1	2	2	1
1	1	1	1	2	2	1	1	9	9
1	2	1	1	3	1	1	1	2	1
1	1	1	1	3	3	1	1	3	1
2	3	1	2	3	3	9	2	1	3
2	1	1	2	3	2	2	1	3	1
1	1	2	2	2	2	1	2	2	2
1	1	1	1	3	1	1	1	1	1
2	1	1	2	1	1	1	2	2	2
3	1	1	3	2	1	1	1	2	1
9	1	9	1	9	9	1	9	9	9
9	9	1	9	9	9	1	1	9	9
1	1	1	1	1	1	2	1	3	1
3	1	1	3	3	1	1	3	3	1
3	3	3	3	3	3	1	1	1	3
3	3	3	3	3	3	1	3	3	3
1	3	1	3	3	1	1	2	2	2
1	1	1	1	3	1	1	1	3	1
3	1	1	1	2	1	1	1	2	3
3	1	1	1	1	1	1	1	1	3
2	3	1	1	1	1	1	1	2	2
3	1	1	2	2	2	4	1	2	1
1	1	1	1	3	1	3	3	1	1
1	1	1	1	2	1	3	1	2	2
9	9	1	1	2	2	3	2	1	1
2	1	1	1	2	2	2	1	3	2
1	1	1	1	2	9	2	2	2	2
1	1	1	1	2	1	1	2	2	2
2	2	1	2	3	2	1	2	2	2
2	3	1	3	3	1	1	2	2	3
3	1	1	3	2	1	1	3	3	3
2	1	1	2	1	2	1	1	2	1
2	2	2	2	2	2	1	2	2	2
1	1	1	1	1	1	1	1	1	1
9	9	2	9	9	9	1	2	9	9
9	9	3	9	9	9	1	3	2	2
3	1	3	1	3	1	3	1	3	3
1	1	1	1	2	1	1	1	2	1

3	1	1	1	2	1	4	1	3	2
3	1	2	1	2	2	2	1	3	3
1	1	2	1	3	1	7	1	3	2
3	1	1	1	3	1	5	2	1	1
3	3	1	3	2	2	1	2	2	2
3	2	2	3	2	3	1	3	2	3
2	2	2	9	9	9	1	2	2	2
3	1	1	2	2	1	1	3	3	3
3	3	1	3	3	3	3	1	3	1
3	1	1	1	1	1	1	1	3	2
3	1	1	1	1	1	1	1	3	2
3	1	1	1	1	1	1	1	3	2
9	9	2	9	9	9	1	9	9	1
9	9	1	9	9	9	2	9	9	9
3	1	1	1	1	1	1	1	3	2
1	1	2	3	2	1	1	2	2	2
1	1	1	1	1	1	2	1	1	9
2	2	2	2	2	2	1	2	2	2
3	1	1	1	1	1	5	2	3	1
1	1	1	3	3	1	1	2	2	2
3	9	1	1	2	2	1	2	2	1
2	3	2	2	2	3	2	1	3	2
2	1	9	9	9	9	1	2	2	2
3	1	1	2	3	2	2	2	3	2
2	1	2	1	2	2	1	2	2	2
1	1	2	2	2	1	2	3	3	1
3	1	1	2	3	1	2	1	2	3
1	2	3	3	1	1	4	1	1	1
1	1	2	2	2	1	2	3	3	1
1	1	2	3	2	1	1	2	2	2
1	1	1	3	3	1	1	2	2	2
9	9	2	9	9	9	1	9	9	1
1	1	1	2	3	2	3	1	3	1
3	1	1	1	2	2	1	2	2	2
2	2	1	1	2	1	1	1	3	1
3	1	1	1	2	1	1	1	1	1
2	1	3	2	2	2	2	1	1	1
2	2	1	9	9	9	3	1	9	9
1	1	1	1	3	2	1	1	2	2
2	2	1	2	2	2	2	2	2	2
1	1	1	1	3	1	2	1	3	1
1	1	2	3	1	1	1	1	3	2
3	1	1	1	3	1	3	1	3	2
2	2	1	1	2	2	1	2	2	2
1	1	1	1	1	1	3	1	1	3
2	2	2	2	2	2	1	2	2	2
9	9	9	1	9	9	1	9	9	9

2	2	2	2	2	2	2	3	1	2
1	1	1	1	2	1	1	2	2	2
1	1	1	1	1	1	1	9	9	9
3	1	1	1	2	1	2	1	9	9
3	1	1	1	2	1	2	1	2	1
9	9	1	9	9	9	1	9	9	9
1	2	1	2	1	1	1	1	1	1
3	1	2	1	3	1	3	1	3	1
3	2	1	1	2	3	9	1	2	3
1	2	1	2	3	2	1	1	1	2
2	1	1	1	3	1	4	3	3	2
2	1	1	2	1	2	1	1	3	2
2	1	1	1	3	1	2	2	2	1
3	1	1	1	2	2	1	1	3	1
2	1	1	1	2	1	1	2	2	2
1	9	3	3	3	2	3	2	2	3
3	1	1	1	3	1	1	1	3	1
3	1	1	1	3	1	5	2	2	1
1	1	1	1	2	3	4	1	2	3
3	1	1	1	3	1	2	1	1	1
3	1	1	1	2	1	3	1	3	2
2	1	2	2	2	2	1	2	2	2
1	1	1	1	3	2	3	1	2	3
2	2	3	3	3	3	1	2	2	2
1	1	1	1	1	1	2	2	2	2
9	9	1	1	2	1	1	1	2	2
3	1	1	2	2	1	1	2	2	2
3	1	1	3	1	2	1	2	2	2
3	1	1	2	2	1	3	1	3	2
3	3	1	3	3	3	1	9	9	9
9	9	1	1	1	3	3	9	9	1
3	2	1	1	2	2	1	2	2	2
9	9	1	1	2	1	1	9	1	1
1	1	2	1	2	1	1	1	2	3
3	2	1	2	3	2	1	1	2	3
9	9	9	9	9	9	2	9	9	9
9	9	9	9	9	9	9	9	9	9
2	2	2	2	2	2	1	2	2	2
1	1	1	1	3	1	1	2	2	2
3	1	1	2	1	1	1	1	2	1
3	3	1	1	1	1	1	1	1	1
1	1	2	2	2	2	9	1	1	2
1	1	1	1	1	2	1	3	1	2
1	2	1	2	1	3	2	1	2	1
1	2	1	2	3	1	1	1	3	2
9	9	9	9	9	9	9	9	9	9
1	3	3	3	2	1	1	3	2	2

1	2	1	2	1	2	9	1	2	1
9	9	9	9	9	9	9	9	9	9
3	9	1	1	1	1	2	3	3	9
9	1	2	2	2	2	1	2	2	2
1	1	1	1	3	1	2	2	2	1
1	2	1	2	1	2	9	1	2	1
1	1	1	1	2	2	1	3	1	3
3	1	1	1	2	1	1	1	3	1
1	1	1	2	2	2	2	2	2	2
3	1	1	1	3	1	4	1	3	1
9	9	9	9	9	9	9	9	9	9
9	9	9	9	9	9	9	9	9	9
9	1	9	9	9	1	1	9	9	9
1	1	1	1	2	3	1	1	2	2
1	1	1	1	3	1	2	1	2	2
3	1	1	1	3	2	5	2	3	1
3	3	9	9	9	9	9	9	9	9
9	9	1	1	3	1	2	1	9	9
2	1	1	1	2	1	5	1	3	2
2	1	1	1	3	3	1	1	3	3
1	1	2	2	1	1	4	2	2	1
2	3	1	3	1	1	1	1	1	2
2	2	2	2	2	3	4	3	3	3
1	2	2	2	2	2	1	2	2	2
2	2	1	1	2	1	1	2	2	2
1	1	1	1	3	1	1	3	2	2
9	9	1	2	2	2	1	9	9	9
3	1	2	2	2	1	3	1	3	1
3	1	1	1	2	2	1	1	2	3
2	2	3	3	2	2	2	2	2	2
3	3	3	1	1	1	1	1	3	3
3	1	1	2	3	2	5	1	3	1
9	9	9	9	9	9	9	9	9	9
2	1	3	2	1	3	1	2	2	3
1	1	1	1	2	2	1	1	1	2
3	2	1	1	1	1	1	2	1	2
1	1	1	1	1	1	1	1	1	1
1	1	1	1	3	2	1	1	2	2
3	1	3	1	3	1	1	3	3	2
2	3	1	2	1	3	1	1	2	2
1	3	3	1	2	2	1	1	3	2
3	3	3	3	3	3	9	3	3	3
3	1	1	1	3	1	1	9	9	9
3	1	1	1	3	1	1	9	9	9
3	1	1	1	3	1	2	1	3	2
3	1	1	1	3	1	1	3	3	1
3	1	1	1	3	1	1	9	9	9

2	2	2	2	2	2	1	2	2	2
3	2	2	2	2	2	1	2	2	3
9	1	1	1	1	3	5	1	1	1
3	1	1	1	1	1	1	1	3	1
1	1	1	1	3	1	3	3	1	1
3	1	1	1	3	1	2	1	3	2
3	1	1	1	3	1	2	9	9	1
3	1	1	1	3	1	1	1	9	1
3	1	1	1	3	1	2	9	9	9
3	1	1	1	3	1	3	9	9	9
9	1	1	1	3	1	3	9	9	9
3	1	1	1	3	1	2	9	9	9
3	1	1	1	3	1	1	1	9	9
1	1	1	3	3	1	1	3	3	1
3	1	1	1	3	2	1	1	2	1
3	1	1	1	3	1	7	1	3	3
3	1	1	1	2	1	1	1	1	1
1	1	1	1	1	2	5	9	1	1
3	1	1	1	3	1	5	9	9	9
1	1	1	1	3	1	1	9	9	9
1	2	1	2	1	3	9	1	2	1
3	1	1	1	3	1	3	9	9	9
3	1	1	1	3	1	4	1	1	1
3	1	1	1	3	1	2	9	9	9
3	1	1	1	3	1	2	1	1	1
3	1	1	1	3	1	1	1	1	1
1	1	2	3	2	1	1	3	2	1
3	1	1	1	3	1	4	1	1	1
3	1	1	1	3	1	2	3	2	1
3	1	1	1	3	1	2	1	1	1
3	1	1	1	3	1	1	1	1	1
3	1	1	1	3	1	1	2	1	1
2	1	1	1	3	1	1	3	1	1
3	1	1	1	3	1	1	1	9	9
3	1	1	1	3	1	1	1	1	1
3	1	1	1	3	3	4	1	1	1
3	1	1	1	3	1	4	1	3	1
3	1	1	1	3	1	1	1	9	9
3	1	2	3	3	3	1	1	3	1
1	3	1	3	3	1	2	3	3	1
2	2	2	2	2	2	1	2	3	2
1	1	3	3	2	1	4	1	2	1
2	3	2	1	1	1	1	1	2	3
1	2	2	2	2	2	1	9	9	9
1	1	1	1	3	2	3	1	1	1
1	3	1	3	3	1	1	1	1	3
3	1	1	1	2	1	1	1	3	1

2	1	1	1	3	1	9	9	9	9
3	3	3	1	2	3	2	9	9	9
2	1	1	9	9	9	9	9	9	9
3	1	1	1	2	1	1	9	9	9
3	1	3	1	2	1	1	2	2	1
9	1	1	9	9	9	9	9	9	9
3	1	1	1	2	3	1	2	2	2
1	1	2	2	2	2	2	1	2	1
9	1	1	9	9	9	1	2	9	9
9	9	1	2	9	9	9	9	9	9
9	1	1	9	9	9	1	2	9	9
9	9	9	9	9	9	9	9	9	9
2	1	1	1	1	1	1	2	2	2
9	2	1	2	1	3	4	1	3	2
2	2	1	2	3	1	3	1	3	3
1	2	1	3	3	1	3	1	1	1
1	1	1	1	2	1	3	1	2	2
2	1	1	1	3	1	1	1	2	1
1	1	1	1	1	1	1	1	1	1
9	9	1	9	9	9	1	1	9	9
1	1	1	1	1	1	1	1	1	1
3	1	1	1	2	1	1	2	2	2
3	1	1	1	2	2	1	2	2	1
1	1	1	2	2	1	1	1	1	1
3	1	1	1	3	1	1	1	1	2
3	1	1	1	3	1	3	1	3	1
1	1	1	3	2	2	4	1	9	3
3	3	1	2	1	2	1	2	2	2
2	2	2	2	2	1	1	1	9	9
9	9	2	3	1	9	1	3	9	9
1	1	1	1	1	1	1	1	3	3
1	1	1	1	1	1	9	3	3	3
1	1	1	2	2	2	1	2	3	3
1	1	1	2	2	2	1	2	2	1
3	1	1	1	3	1	1	1	2	2
3	1	1	2	2	1	1	1	3	1
2	1	1	1	2	2	1	1	2	1
1	1	2	2	2	1	1	1	1	1
2	2	2	2	2	1	1	1	1	1
2	2	2	1	1	1	1	1	2	2
3	1	1	2	2	2	1	1	3	1
2	2	1	2	1	2	1	2	2	2
3	1	1	3	3	1	4	9	3	3
2	1	1	1	2	1	1	1	2	2
3	3	1	1	3	3	1	3	3	1
3	3	1	1	1	1	1	1	3	3
3	1	1	1	2	1	1	2	1	2

9	9	9	9	9	9	9	9	9	9
3	1	1	1	2	1	4	1	1	1
9	9	9	2	9	9	1	9	9	9
9	9	9	1	9	9	1	9	9	9
9	9	9	1	9	9	1	9	9	1
1	1	1	1	2	1	1	2	1	1
9	9	9	9	9	1	5	9	9	9
3	1	1	1	2	1	1	1	2	1
2	1	2	2	2	2	1	2	9	9
1	2	2	1	2	2	1	2	3	2
3	3	1	1	2	2	1	9	9	9
3	1	1	2	1	1	1	2	1	2
2	1	1	2	1	1	1	1	1	2
3	3	1	3	1	3	1	2	2	2
1	3	1	1	2	2	1	1	1	1
2	3	2	2	2	1	1	1	9	9
2	2	2	2	2	2	1	2	2	2
3	3	3	3	3	3	2	3	3	3
2	3	2	3	2	1	9	3	1	2
1	1	2	2	2	2	6	3	3	3
2	1	1	1	2	2	1	2	2	2
2	2	1	2	2	1	1	1	1	3
2	2	2	2	2	2	1	2	2	2
2	2	1	2	3	2	1	2	2	2
2	1	1	2	2	2	1	2	2	2
2	2	1	1	1	1	1	2	2	2
1	1	1	1	3	1	1	1	1	3
3	3	1	1	2	1	1	1	3	3
3	2	2	1	2	2	1	1	1	1
2	1	1	1	1	1	1	2	2	2
9	9	9	9	9	9	9	9	9	9
2	1	3	2	3	3	1	2	3	1
2	3	1	2	2	3	9	2	1	3
2	1	1	2	2	1	1	2	2	2
1	1	1	1	1	2	1	2	2	2
3	3	1	1	2	1	1	1	1	1
1	1	1	1	3	1	2	3	1	1
1	1	1	1	3	2	1	1	1	1
1	1	1	1	2	1	1	1	1	1
2	1	1	1	2	1	1	1	3	1
2	2	2	2	2	2	1	2	2	2
2	2	2	2	2	2	1	1	1	1
9	9	9	9	9	9	9	9	9	9
9	9	1	9	9	9	1	9	9	9
9	9	1	9	9	9	1	1	9	9
9	9	9	9	9	9	9	2	2	2
3	1	1	1	3	1	4	2	2	2

3	1	1	3	3	1	3	3	1	1
2	1	1	2	1	1	1	1	2	2
1	1	1	3	2	1	1	1	9	9
3	3	1	2	2	3	3	1	3	3
1	1	1	1	9	1	1	9	9	9
2	1	1	1	3	1	1	2	2	2
3	1	1	2	3	1	5	9	9	9
3	1	1	1	1	1	1	1	9	9
2	2	3	2	3	2	1	2	2	2
1	3	1	1	3	1	1	1	3	3
9	1	9	9	9	1	2	9	9	9
2	2	9	9	9	9	1	2	2	9
2	1	1	2	1	2	1	1	3	2
3	1	1	1	2	1	3	2	2	1
9	9	1	9	9	9	1	1	9	9
1	1	1	1	1	1	5	3	3	1
2	2	2	3	2	2	1	2	3	2
3	1	2	1	2	1	4	2	3	1
1	1	9	1	9	9	2	1	2	2
2	1	1	1	2	1	1	2	2	3
1	2	1	2	2	1	9	1	1	2
1	1	1	1	3	3	1	3	3	1
3	1	2	1	3	2	1	2	2	1
3	1	3	1	3	1	1	1	2	1
2	1	1	1	3	1	1	3	3	2
3	3	1	3	1	1	1	1	2	2
3	1	3	1	3	1	1	1	2	1
1	3	1	2	1	2	1	1	2	1
2	3	9	9	9	9	9	2	3	1
1	2	1	2	3	1	1	1	1	1
1	1	3	2	3	1	3	1	3	1
3	1	1	1	3	1	1	1	3	2
3	1	1	1	1	1	3	2	3	1
2	3	1	1	2	2	4	1	3	1
1	2	1	1	3	1	1	1	3	1
1	1	1	1	2	1	1	2	2	2
2	2	1	1	2	1	1	1	3	1
1	3	1	1	2	1	1	3	3	3
9	9	9	9	1	9	1	9	1	9
3	2	2	2	2	3	1	2	2	2
9	9	9	9	1	9	1	9	9	9
9	9	2	9	9	9	1	9	1	9
3	1	1	2	3	1	1	1	2	1
3	1	1	2	3	1	1	1	2	1
1	1	1	1	3	1	1	1	3	1
1	1	1	3	1	1	9	1	3	1
3	3	1	3	3	3	1	1	1	3

3	2	3	3	3	3	1	3	3	3
9	9	1	9	9	9	1	9	9	9
3	1	1	3	3	1	1	1	3	1
1	3	1	1	3	1	1	1	3	3
3	1	1	1	3	1	1	1	3	3
1	1	1	1	3	1	1	1	3	1
3	1	2	1	2	1	2	2	1	3
2	1	3	2	2	3	1	2	1	2
3	1	1	3	3	1	1	1	3	3
2	1	1	2	1	3	1	3	9	2
3	1	1	3	1	1	1	1	3	1
1	1	1	2	2	3	4	1	2	1
2	1	2	1	1	1	1	2	2	3
3	2	1	3	3	1	2	1	3	3
2	1	2	1	1	1	1	2	1	1
2	1	1	1	2	1	1	1	3	1
3	1	1	1	3	1	1	1	3	1
2	1	1	1	3	2	1	2	3	2
1	1	1	3	3	1	1	1	3	3
2	2	2	2	2	2	1	2	2	2
3	1	3	2	2	2	1	1	2	1
3	2	1	2	1	2	9	2	2	2
3	3	3	3	3	3	1	3	3	3
1	3	1	3	2	1	1	9	9	9
3	1	1	1	1	1	1	2	2	2
2	2	1	2	2	1	9	1	2	1
1	2	1	1	2	1	1	2	1	3
3	1	1	2	1	3	9	2	2	3
3	2	1	1	3	1	1	1	2	1
9	9	9	9	9	1	1	9	1	9
2	1	2	1	9	2	1	2	3	1
1	1	3	1	2	2	2	1	3	2
2	1	2	3	1	2	1	2	2	2
2	1	1	2	1	1	1	2	3	2
3	1	3	1	2	1	1	1	1	1
3	3	3	3	3	3	9	2	2	2
3	1	1	1	2	1	1	2	2	2
2	2	2	3	1	2	1	9	9	9
2	1	1	2	1	3	1	1	2	2
3	3	1	1	3	1	4	1	3	3
9	1	9	9	9	1	1	9	9	9
9	9	9	9	9	9	9	9	9	9
1	3	3	1	3	1	1	2	1	2
3	1	1	1	3	1	1	3	3	3
2	2	1	2	2	2	1	3	3	2
3	1	1	1	3	1	1	1	3	3
9	9	9	9	9	9	9	9	9	9

2	1	1	2	2	2	1	1	3	2
3	1	1	1	1	1	9	9	9	9
1	1	2	1	2	2	1	2	2	2
1	1	1	2	1	9	9	9	9	9
9	9	9	9	9	9	9	9	9	9
1	1	1	1	1	1	1	3	3	3
3	1	2	2	2	2	1	2	2	2
2	2	2	2	2	2	9	2	2	2
1	1	1	2	1	3	1	3	2	3
1	1	2	1	1	2	1	1	2	1
3	1	9	9	9	9	9	9	9	9
9	1	1	2	3	1	1	9	9	9
3	2	2	3	3	3	9	9	9	9
9	9	9	9	9	9	9	1	1	1
3	1	2	2	2	2	1	2	3	2
9	9	9	9	9	9	9	1	1	1
2	3	1	2	2	3	1	1	9	9
3	1	3	3	3	1	2	9	9	9
2	3	3	2	2	1	1	3	3	3
2	2	2	2	2	1	1	2	2	2
1	1	2	3	3	1	2	3	3	3
3	1	3	3	2	1	1	2	2	2
9	1	1	9	9	9	1	1	9	9
3	2	1	3	1	2	1	1	3	2
1	1	1	1	2	1	1	2	3	2
2	2	2	2	2	2	1	2	2	1
9	1	1	9	3	9	1	1	9	9
2	1	1	3	1	3	1	1	2	2
1	2	2	2	2	1	1	2	2	2
9	9	9	9	9	1	3	9	9	9
3	1	1	1	1	1	1	1	2	1
1	1	2	1	2	2	1	2	2	2
1	3	1	1	3	3	1	1	3	1
2	1	1	1	1	1	1	1	3	1
3	1	1	1	1	2	1	1	3	1
3	1	3	1	2	1	2	3	3	2
1	1	1	1	3	1	1	1	3	3
1	1	3	3	3	1	5	3	3	1
9	1	9	9	1	9	1	1	9	9
3	3	3	3	3	3	1	2	2	2
3	1	1	1	3	1	1	2	3	1
3	1	1	1	2	1	1	3	3	2
3	2	2	2	2	2	4	1	3	3
2	1	2	2	2	1	1	3	3	3
3	1	3	1	3	1	3	1	2	2
3	1	1	2	3	2	2	1	3	2
2	1	1	1	3	1	1	9	9	9

2	2	2	2	2	2	1	2	2	2
2	1	2	2	3	2	1	1	3	9
1	1	9	2	2	2	1	2	1	1
2	1	2	1	3	1	1	2	2	1
1	2	2	2	3	2	1	3	3	3
9	9	1	9	9	9	1	9	9	1
2	2	2	2	2	2	2	1	2	2
3	1	9	1	2	1	1	1	3	1
3	1	1	3	3	1	1	1	2	1
2	1	1	2	2	2	1	2	2	2
2	1	1	1	3	1	1	2	2	3
3	1	1	2	2	1	1	1	2	1
2	2	2	2	2	2	1	2	2	2
3	3	3	3	3	3	1	3	3	3
3	2	1	2	1	2	1	2	2	1
2	1	2	2	3	1	1	1	2	2
3	1	1	1	3	2	1	3	3	3
3	1	1	1	2	1	1	2	2	3
3	3	1	1	3	1	1	3	2	2
2	2	1	1	2	2	1	2	3	1
1	1	1	1	3	1	1	1	3	3
2	1	1	1	3	2	1	1	3	1
2	1	1	1	3	2	1	1	3	1
1	1	1	2	2	2	1	1	1	1
3	1	1	1	3	1	2	1	2	2
2	3	2	1	2	1	1	3	3	2
3	1	3	1	3	1	1	1	2	2
3	1	3	1	3	1	1	1	2	2
2	2	1	2	2	2	1	2	2	2
1	1	1	1	2	1	1	1	1	1
2	2	2	2	2	2	1	3	3	3
2	1	1	1	3	2	1	1	3	2
3	1	1	1	2	1	1	1	3	3
3	1	2	2	3	1	4	1	3	1
3	1	1	1	3	1	1	1	1	1
1	1	1	2	2	1	1	2	2	2
1	1	1	2	2	1	1	2	2	2
1	2	1	2	2	2	1	2	2	2
1	1	1	1	3	2	1	2	2	2
2	2	2	2	2	2	1	2	2	2
3	1	2	1	1	1	1	2	3	1
3	1	1	1	2	1	2	2	2	1
2	1	1	1	1	1	1	2	2	2
2	2	1	2	1	1	2	1	2	2
3	2	3	1	1	1	5	1	3	1
3	1	1	3	3	9	1	1	3	1
2	1	1	1	1	1	1	1	3	1

3	1	2	2	2	2	1	2	2	1
2	2	1	1	3	1	1	2	2	2
2	1	1	2	1	2	1	1	3	1
2	2	1	2	1	9	1	2	1	2
1	9	2	2	2	2	9	2	2	2
2	2	2	2	2	2	1	2	2	2
2	1	1	1	2	1	1	1	9	1
1	1	1	1	3	1	1	1	3	1
1	1	2	2	3	1	1	2	2	2
3	1	3	2	3	2	1	2	2	2
3	1	3	2	3	2	1	2	2	2
1	1	2	2	3	2	2	2	2	2
1	2	9	9	9	9	2	2	2	2
3	1	1	1	1	1	1	9	9	9
1	3	1	1	1	2	1	2	2	2
2	1	1	1	1	1	1	1	2	2
1	1	1	1	3	1	1	1	3	1
2	1	1	1	1	1	1	1	2	2
3	1	1	1	1	1	1	1	3	1
2	1	1	1	1	1	1	1	2	2
2	2	2	2	2	2	1	2	2	2
2	2	1	2	2	1	1	2	2	2
2	2	2	3	2	2	1	2	2	2
1	2	2	2	2	2	1	2	2	2
2	1	1	1	1	1	1	1	2	2
1	2	1	1	2	1	1	1	3	1
2	2	1	1	3	1	1	3	3	3
2	2	2	2	2	2	1	2	2	2
2	1	2	2	2	2	1	2	2	2
3	1	1	2	1	1	1	1	2	2
2	2	3	2	2	2	1	3	3	3
2	2	2	2	2	2	1	2	2	2
2	2	2	2	2	2	9	2	2	2
2	2	2	2	2	2	1	2	2	2
9	9	9	9	9	1	1	2	2	2
2	2	2	2	2	2	1	2	2	2
2	2	2	2	2	2	1	2	3	2
2	2	2	2	2	2	1	2	2	2
2	2	2	2	2	2	1	9	9	9
2	1	1	1	2	1	1	1	2	2
2	2	2	2	2	2	9	1	2	1
1	1	2	2	2	2	1	1	2	2
3	1	1	1	2	1	1	2	2	2
2	2	2	2	2	2	1	2	2	2
2	2	2	2	2	2	1	1	2	2
1	1	2	2	3	3	1	2	2	2
1	1	2	2	2	2	1	1	2	2

2	2	2	2	2	2	9	2	2	2
2	2	1	2	2	2	1	1	2	2
2	2	2	3	1	1	5	1	3	3
2	1	1	1	1	2	1	1	2	2
2	2	2	2	2	2	1	2	2	2
2	2	2	2	1	3	1	1	2	3
3	1	3	1	1	1	1	1	9	9
3	1	1	1	2	1	1	1	1	1
1	1	1	1	1	1	2	1	3	1
3	1	1	1	2	1	7	1	3	1
3	1	1	1	2	1	2	1	3	1
2	2	2	1	2	2	1	2	2	2
2	1	2	3	3	1	1	1	3	1
9	9	1	1	3	1	1	2	2	2
1	1	1	1	1	1	1	1	9	1
2	1	3	1	1	1	5	1	2	2
2	1	1	1	1	1	1	1	2	2
2	1	1	1	1	1	1	1	2	1
1	9	1	1	1	1	1	1	1	1
2	1	1	1	2	1	1	1	3	2
2	1	3	1	3	1	1	1	2	1
2	2	2	2	2	2	9	1	2	3
2	1	2	2	2	2	1	1	3	3
2	2	2	2	2	2	1	2	2	2
2	2	2	2	2	2	1	2	2	2
2	2	2	2	2	2	1	2	2	2
9	9	9	9	9	1	2	1	9	9
2	1	1	1	2	2	1	2	2	2
2	2	1	1	1	2	1	1	2	2
9	9	9	9	9	9	9	2	1	1
2	1	2	2	2	1	1	1	2	2
3	2	1	2	3	1	1	1	2	3
2	9	3	2	3	1	1	9	9	9
2	2	1	1	1	1	2	1	2	1
2	1	1	3	1	1	1	9	9	9
3	1	1	3	2	1	1	1	3	1
3	3	1	1	3	1	3	1	3	3
3	1	1	1	3	1	1	2	2	1
3	2	1	2	3	1	1	1	9	3
2	1	3	1	1	2	1	2	2	3
3	1	1	1	3	1	1	1	3	1
1	1	2	1	1	2	1	1	1	1
3	1	1	2	3	1	1	1	3	1
2	2	2	2	2	2	1	2	2	2
3	1	1	2	3	1	4	1	3	1
1	1	1	1	2	1	1	2	2	9
3	1	1	1	1	1	1	1	2	1

1	1	2	1	2	2	1	2	3	3
2	1	1	2	1	3	9	1	2	3
3	1	3	2	3	9	1	1	3	1
3	1	1	1	3	1	1	3	1	1
2	3	3	2	3	1	2	3	2	1
1	1	2	2	3	2	1	1	2	3
1	1	1	3	1	1	1	1	3	3
9	9	2	9	9	9	1	2	9	9
2	1	1	2	2	1	2	2	2	2
2	2	2	2	2	2	2	2	2	2
1	1	1	1	3	2	9	3	2	2
1	1	1	1	3	2	1	3	2	2
3	3	1	1	1	2	1	2	2	2
2	1	2	2	2	2	1	2	2	2
2	2	1	1	3	1	1	2	2	2
3	1	2	1	2	1	1	2	2	2
2	1	2	2	2	2	1	2	2	2
1	1	1	1	2	1	1	3	2	1
3	1	1	1	2	1	1	2	2	2
2	1	1	1	2	1	1	1	3	1
3	3	2	2	1	1	1	9	9	9
2	1	1	1	2	1	1	9	9	9
2	1	1	1	2	2	1	1	3	2
9	1	1	9	9	9	1	1	9	9
2	3	1	2	1	3	3	1	3	1
3	1	1	1	1	1	1	1	2	1
2	1	2	3	1	1	1	1	1	1
2	2	1	1	2	3	1	1	2	2
2	2	1	1	2	1	3	2	2	2
9	9	9	9	9	1	1	1	9	9
2	2	3	3	3	1	1	1	3	3
9	9	9	9	9	9	9	9	9	9
9	9	9	9	9	9	9	9	9	9
2	1	1	1	2	1	1	2	2	2
2	1	1	1	2	1	1	2	2	2
1	1	1	1	2	1	1	1	2	2
1	1	1	3	3	1	1	3	1	2
2	1	1	1	2	1	1	2	2	2
2	1	1	1	1	1	1	1	3	2
2	1	1	1	1	1	1	1	3	2
9	9	9	9	9	9	9	9	9	9
3	3	2	1	3	1	1	2	2	2
2	1	1	2	2	3	2	2	2	2
9	9	9	9	9	9	9	9	9	9
3	9	3	9	9	9	9	3	9	9
1	1	1	1	1	2	1	1	1	2
2	2	1	1	1	1	1	2	1	1

3	1	9	9	9	9	9	9	9	9
1	1	1	1	3	1	1	1	3	1
3	1	1	1	3	1	9	1	3	3
9	9	1	1	2	2	1	1	2	2
2	1	1	1	2	2	1	9	9	9
3	3	3	3	3	3	9	3	3	3
2	2	2	2	2	2	1	2	2	2
3	3	2	1	3	1	1	3	2	3
9	9	9	9	9	1	1	9	9	9
9	1	1	2	2	2	1	2	2	2
3	1	1	2	3	1	3	3	3	1
2	2	2	2	2	2	1	2	2	2
2	1	1	1	3	3	1	2	2	1
3	3	3	3	3	3	1	3	3	3
2	1	2	2	3	2	1	3	3	1
2	1	3	1	1	2	1	3	1	1
1	1	1	1	1	2	1	1	2	2
3	1	3	1	1	2	6	1	1	3
2	2	2	2	2	2	1	9	9	9
1	1	3	1	2	1	1	1	2	1
3	1	1	1	2	1	3	1	3	2
1	1	1	3	1	2	1	2	2	2
2	1	1	1	2	1	1	9	9	9
2	2	1	1	3	3	1	2	2	2
2	3	1	3	1	1	5	1	3	3
2	1	1	2	2	2	1	2	2	2
3	1	1	1	2	2	1	1	2	1
9	3	1	1	2	1	1	1	1	1
3	2	1	3	1	2	2	1	2	3
1	1	1	3	3	3	1	9	9	9
1	1	2	2	2	2	1	2	2	2
3	3	1	2	1	1	1	3	1	3
3	1	1	1	3	1	1	1	3	1
3	1	1	2	2	3	1	3	3	3
3	1	1	1	3	1	1	1	1	1
1	1	1	1	3	2	2	1	2	2
3	1	1	1	1	1	4	1	3	3
3	9	2	3	2	2	1	2	2	2
1	1	1	1	1	1	1	2	2	2
3	1	1	1	1	3	1	9	9	9
2	3	2	3	3	3	1	9	9	9
3	1	1	2	3	1	1	2	1	3
2	1	2	1	3	3	1	1	3	1
1	1	1	3	2	1	1	2	2	1
1	1	1	1	2	3	1	1	3	1
2	2	1	1	2	2	1	2	3	2
9	9	3	3	3	1	1	3	2	2

1	2	1	2	1	2	9	1	3	1
2	1	1	1	2	1	1	1	3	1
3	3	1	1	3	1	1	3	3	1
3	2	1	1	3	3	1	2	3	2
3	1	1	1	3	1	1	1	3	1
2	1	3	1	3	2	1	2	2	2
9	9	9	9	9	9	9	2	2	2
3	1	1	2	3	1	1	2	2	3
3	1	1	1	3	1	1	1	3	1
1	1	1	1	3	1	1	2	1	2
2	1	9	9	9	9	1	1	1	2
2	2	2	2	2	1	1	1	2	2
2	2	2	2	2	2	1	2	2	2
1	1	1	1	1	1	2	1	3	1
3	1	1	2	3	1	1	9	9	9
2	1	1	2	9	1	1	2	2	2
3	1	3	3	3	2	1	2	2	3
3	1	2	1	3	1	1	1	3	1
1	1	1	1	1	1	1	3	1	1
2	1	1	1	2	2	1	1	3	2
3	1	1	1	3	1	2	1	2	1
3	1	1	2	2	1	5	9	9	9
1	1	1	3	3	2	1	2	3	2
1	1	1	1	2	2	1	9	1	9
2	1	3	3	3	3	1	2	2	2
1	1	1	1	2	1	1	2	2	2
2	2	2	2	2	2	1	2	2	3
2	2	1	1	2	2	1	2	2	2
3	3	3	3	3	3	1	3	9	3
3	1	1	1	1	1	1	1	2	1
1	1	3	1	3	1	1	3	3	3
3	1	1	2	3	3	3	3	1	2
2	2	2	1	2	2	1	2	2	2
3	1	1	2	3	1	1	3	3	9
1	1	1	2	2	1	1	1	2	1
3	1	2	1	3	1	1	2	2	2
3	1	1	1	3	1	4	3	1	1
1	1	1	1	3	3	1	2	1	1
1	1	1	1	3	1	1	1	1	2
9	9	9	9	9	1	1	2	9	9
3	1	1	1	3	1	1	2	2	2
2	3	1	1	3	3	1	2	1	1
2	1	2	2	2	2	1	1	3	2
2	1	1	1	3	1	3	1	3	1
3	1	1	3	3	3	5	1	3	1
3	1	1	3	3	1	1	9	9	2
1	1	1	1	2	2	1	2	2	2

2	1	1	2	2	1	1	1	2	1
2	2	2	2	2	2	1	2	3	2
9	9	3	3	3	3	1	3	3	3
3	1	1	1	2	3	1	2	3	2
9	1	1	2	3	1	1	1	3	2
9	9	9	9	9	9	9	2	1	2
9	9	1	1	2	1	1	3	1	1
3	1	1	1	1	1	1	9	9	9
9	9	9	9	9	9	9	1	2	3
1	1	3	1	1	1	2	1	2	1
2	1	1	1	2	2	1	2	3	2
1	1	1	1	3	1	1	1	2	2
1	1	1	1	3	1	4	2	2	3
3	1	1	3	2	1	2	3	1	1
9	9	2	2	9	2	1	9	9	9
2	1	1	2	3	2	1	1	1	1
3	9	9	9	9	9	9	1	1	1
1	1	1	1	2	3	1	3	1	1
3	1	1	1	3	1	1	1	1	3
3	1	1	1	3	1	2	2	3	2
1	1	1	3	3	1	1	1	2	1
3	3	1	1	3	1	2	3	3	1
1	1	1	2	1	3	1	2	2	2
3	1	2	3	2	1	1	1	3	3
2	1	1	1	3	1	3	1	1	1
1	1	1	1	2	1	1	1	2	2
2	3	1	2	3	2	3	3	2	1
1	1	1	3	3	1	1	1	2	2
3	2	2	2	2	2	1	2	2	2
3	1	1	1	2	1	1	1	2	1
1	1	1	2	2	1	1	2	2	1
1	2	2	2	2	2	1	2	2	2
2	1	1	3	3	2	1	2	2	2
3	1	1	1	3	1	1	1	1	1
1	3	1	2	3	2	2	1	2	3
3	1	1	1	3	1	1	1	2	1
1	1	1	1	2	2	4	2	2	2
3	3	1	3	1	3	1	3	3	3
1	3	1	1	2	1	1	1	2	2
1	1	2	2	2	1	1	1	1	2
3	3	2	2	2	2	1	1	1	2
1	1	1	2	2	1	1	1	1	2
3	1	1	1	2	2	1	3	1	2
3	3	1	2	3	3	2	2	2	1
2	2	1	1	2	2	1	2	3	3
3	3	1	1	3	3	1	2	2	2
2	2	1	3	2	2	1	1	1	1

3	1	1	1	3	2	1	1	2	1
3	2	1	1	1	1	1	1	2	2
2	2	1	1	3	2	1	1	1	1
2	1	1	2	3	3	1	1	1	2
1	1	1	1	1	1	4	2	1	2
2	3	1	1	1	2	1	1	2	2
9	9	1	2	1	2	1	1	2	2
3	1	1	2	3	2	2	2	2	2
2	1	1	2	2	1	1	2	1	1
2	1	1	2	2	1	1	2	2	1
3	1	1	2	2	2	1	2	2	2
2	2	2	2	2	2	2	2	2	2
1	1	2	2	2	2	1	1	1	1
2	2	1	1	3	1	1	3	3	3
1	1	1	1	2	1	7	1	2	2
3	1	1	1	2	1	5	1	1	1
2	1	1	1	3	1	1	2	2	2
2	1	9	2	2	2	3	1	2	2
2	1	1	2	2	2	1	2	2	2
2	2	1	1	1	2	1	3	3	2
2	1	1	1	2	2	1	2	2	2
2	1	1	2	2	1	1	2	1	1
2	9	2	2	2	2	1	3	3	3
3	2	3	3	3	3	9	3	1	3
2	1	2	1	1	2	2	2	9	1
2	2	2	2	2	2	9	2	2	2
2	2	2	1	3	1	1	2	1	3
2	3	3	2	2	3	1	2	2	2
2	1	1	1	2	1	1	2	2	2
3	1	1	2	3	2	1	2	2	2
2	1	3	1	2	1	1	2	2	2
3	1	1	2	3	3	3	2	1	2
2	2	1	2	2	1	1	1	1	2
2	2	2	1	3	2	1	1	2	2
2	1	1	1	2	2	1	1	3	3
1	1	1	1	3	2	1	1	1	3
2	1	1	2	2	3	2	3	2	1
3	3	1	3	2	3	4	2	3	1
3	3	1	2	2	1	1	2	1	1
2	2	1	3	2	2	1	2	1	1
2	2	2	2	2	2	1	2	2	2
2	2	2	2	2	2	9	2	2	2
2	2	2	2	2	2	1	2	2	2
3	3	3	3	3	3	1	3	3	3
3	3	3	3	3	3	1	3	3	3
9	2	2	9	9	2	1	2	2	2
1	1	3	2	1	1	1	2	1	1

1	1	1	1	2	2	1	1	2	2
1	2	2	1	1	1	1	2	2	2
1	1	2	2	1	1	1	1	1	2
1	1	1	2	2	1	1	1	2	2
1	1	2	2	2	1	1	1	2	2
2	2	1	1	2	2	1	2	2	2
1	1	1	2	2	2	1	2	2	2
2	1	2	1	1	2	1	3	3	3
2	1	1	2	1	1	1	1	1	2
1	1	2	2	1	1	1	2	2	1
1	2	2	2	1	1	1	1	1	2
2	2	1	1	1	1	1	1	1	2
2	2	2	2	2	2	1	1	1	2
2	2	2	2	2	2	1	1	1	1
2	2	2	2	2	2	1	1	1	1
2	2	2	2	2	2	1	2	2	1
2	2	2	2	2	2	1	2	2	2
2	2	2	2	2	2	1	2	2	2
2	2	2	2	2	2	1	2	2	2
1	1	2	2	2	2	1	2	1	2
2	2	2	2	2	2	1	2	9	9
2	2	2	2	2	2	1	3	3	3
2	2	2	2	2	2	1	1	1	2
1	1	2	2	2	1	1	1	2	1
2	2	2	2	2	2	1	2	2	2
2	2	2	2	2	2	1	2	2	2
2	2	2	2	2	2	1	1	1	2
2	2	2	2	1	1	2	1	2	1
1	1	1	2	3	2	1	1	2	2
2	2	3	3	2	2	1	3	3	3
2	2	2	2	2	2	1	2	2	2
2	2	2	2	2	2	1	2	2	2
2	2	1	2	2	3	1	2	2	2
2	2	2	2	2	2	7	2	2	1
1	1	1	3	1	1	1	9	1	9
9	9	9	1	9	1	1	1	3	2
1	9	3	1	9	1	1	1	2	9
9	2	2	9	2	2	1	2	9	2
9	9	9	9	9	9	9	9	9	9
9	1	1	9	1	1	1	1	9	1
1	1	1	2	1	1	1	1	9	9
1	1	2	1	1	1	1	2	1	1
1	1	1	2	3	1	1	1	9	9
2	2	2	2	2	2	1	2	2	2
9	1	1	1	1	2	1	1	9	1
2	9	2	9	2	2	1	2	9	9
2	1	2	1	2	2	1	2	9	9

1	2	2	1	2	2	1	2	9	9
2	2	1	2	2	2	1	2	9	9
1	2	2	2	2	2	1	2	9	9
1	2	1	2	3	2	1	9	9	9
2	1	1	1	2	1	1	9	9	9
1	1	1	2	3	3	1	9	2	9
2	2	1	2	2	1	1	9	9	9
2	2	2	2	2	1	1	2	9	9
1	2	2	1	2	2	1	2	1	2
2	2	2	2	2	2	1	2	9	9
1	1	1	2	2	2	1	2	1	1
2	2	1	2	2	3	1	3	2	2
1	1	1	3	2	1	1	2	2	2
1	1	1	2	2	2	2	3	2	2
2	1	2	1	2	3	1	3	2	2
3	2	3	2	2	1	1	1	2	2
3	1	1	2	2	2	1	2	2	2
2	1	1	2	1	2	1	2	3	1
1	1	1	9	9	9	1	2	2	2
3	2	2	2	1	1	1	2	2	1
1	2	1	2	2	2	1	2	2	2
2	1	2	2	1	1	1	1	2	2
2	3	1	2	2	1	1	1	1	2
2	1	1	2	3	2	1	1	2	3
1	2	1	2	3	2	1	3	2	2
2	1	3	2	1	2	1	3	3	3
2	3	2	2	2	2	1	9	2	9
3	1	2	2	2	2	1	3	2	1
2	2	2	2	3	2	1	1	1	2
2	2	2	2	2	2	1	2	2	2
1	1	2	2	1	1	1	2	9	9
3	3	2	2	2	2	1	3	3	3
1	1	1	1	1	1	1	2	1	2
3	3	2	2	2	2	1	2	2	2
2	2	2	2	2	2	1	3	2	1
3	3	3	3	3	3	1	3	3	3
2	2	2	2	2	2	1	2	2	2
2	2	2	2	2	2	1	9	9	9
2	2	1	2	2	1	1	1	1	1
2	2	1	2	2	2	1	2	2	2
3	2	2	1	3	2	1	2	1	2
3	3	2	2	2	2	9	2	2	2
1	2	1	2	2	3	1	3	2	2
2	2	1	2	2	2	1	2	2	2
2	2	1	2	2	2	1	1	1	1
2	3	2	1	2	3	2	1	2	1
2	2	3	2	2	1	2	1	2	1

2	9	2	9	9	9	1	1	1	2
1	1	1	1	1	2	1	2	2	2
2	1	1	2	3	2	2	1	2	2
2	2	2	2	2	1	1	2	2	2
3	2	2	1	2	3	7	3	2	2
3	1	1	1	2	1	1	2	2	2
3	1	2	1	3	3	3	3	2	1
2	1	1	2	2	1	5	1	1	1
1	1	1	1	2	1	1	1	1	3
2	1	3	3	3	2	1	2	2	2
3	2	2	1	1	1	1	2	2	2
1	1	1	1	2	2	1	1	2	2
3	1	1	2	1	2	1	2	1	2
3	2	2	3	1	2	3	2	3	2
3	2	1	1	3	2	1	3	2	2
1	1	1	1	3	1	1	1	1	3
1	1	1	1	3	1	1	1	1	3
1	1	1	1	2	1	1	2	2	2
3	3	3	2	2	3	2	2	3	1
2	2	2	2	2	2	9	2	2	2
9	9	1	1	2	1	9	1	1	1
3	2	2	2	2	2	1	2	2	2
3	1	1	2	1	1	5	1	2	1
2	1	1	1	3	1	5	1	3	3
3	1	1	1	3	1	3	1	2	2
2	1	1	1	2	1	1	2	3	2
3	1	3	1	1	2	1	2	1	3
1	1	1	1	1	1	2	2	1	2
1	1	1	1	1	1	1	1	1	1
2	1	2	1	2	2	1	2	2	2
3	1	1	1	3	1	1	1	3	2
2	1	1	1	9	2	1	1	3	1
2	2	2	2	3	2	9	2	2	2
2	2	1	1	2	2	1	2	2	2
3	1	1	1	2	1	1	2	2	2
3	2	1	1	1	1	1	2	2	2
3	1	2	1	1	1	1	2	1	1
2	1	1	3	2	1	1	2	2	2
1	1	1	2	1	1	2	3	1	1
3	1	1	1	2	1	1	3	2	1
3	1	1	1	1	1	1	2	2	1
2	2	2	9	2	2	1	1	2	2
2	1	1	1	2	2	2	3	3	1
2	1	2	3	3	2	1	2	2	3
1	1	2	2	2	2	1	2	2	2
3	1	1	1	1	1	1	2	2	2
3	1	1	1	1	1	1	2	2	2

3	1	1	1	2	1	1	1	3	1
3	1	1	1	1	1	1	1	3	1
2	2	2	2	2	2	1	2	2	2
2	3	1	1	2	2	1	2	2	2
2	2	2	2	2	2	1	2	2	2
1	1	1	1	1	1	5	2	2	2
3	1	1	1	2	1	2	1	1	2
1	3	1	3	3	1	1	2	3	1
3	3	1	1	1	1	1	2	2	2
9	9	9	9	9	9	9	9	9	9
3	3	1	3	1	2	1	1	3	1
1	1	1	1	2	2	1	1	1	2
3	1	1	2	2	2	2	2	2	1
2	1	2	2	2	2	2	2	2	2
1	1	1	2	2	3	1	2	2	2
2	2	2	1	2	2	9	2	2	2
3	2	2	1	2	2	1	2	2	2
2	2	2	1	3	2	1	2	2	2
3	3	9	9	9	9	1	3	3	3
3	3	1	1	3	1	1	3	3	3
3	3	3	3	3	3	9	3	3	3
3	3	3	3	3	3	9	3	3	3
3	1	2	3	3	2	2	1	9	1
2	3	1	3	2	3	1	3	2	2
2	2	2	2	2	2	1	2	2	2
2	9	3	1	2	1	9	1	1	2
2	1	1	2	2	1	1	3	3	2
2	2	2	2	2	2	1	1	2	2
2	2	2	2	2	2	9	2	2	2
1	3	2	1	3	1	5	3	2	1
3	1	3	3	2	9	4	1	1	2
2	2	2	1	1	2	1	2	2	2
2	2	1	2	1	2	1	1	3	2
3	9	3	3	3	3	1	3	3	3
2	1	2	1	3	1	2	1	2	1
2	2	1	2	3	1	1	3	2	2
2	2	1	2	3	1	1	3	2	2
2	2	1	2	3	1	1	3	2	3
2	2	2	2	2	2	1	2	2	2
2	2	3	3	2	3	1	2	2	2
2	1	1	1	1	1	1	1	2	2
2	2	1	2	3	1	5	1	1	1
2	2	2	2	2	2	1	1	2	2
3	1	1	1	1	1	3	3	1	1
3	1	1	1	3	1	1	1	3	3
3	1	1	1	2	1	3	1	1	1
2	1	1	2	1	1	1	1	1	1

2	2	2	2	2	2	2	2	2	2
2	1	1	1	1	3	1	1	3	1
2	1	3	2	3	1	3	1	3	1
2	2	2	2	2	2	9	2	2	2
2	2	2	2	2	3	1	3	3	2
3	1	1	1	1	9	1	2	2	9
2	2	2	2	2	2	1	2	2	2
2	2	2	2	3	1	6	2	2	2
1	1	1	1	2	3	4	1	2	1
2	2	2	3	2	2	1	1	2	3
2	1	1	2	2	1	1	1	2	3
3	1	3	2	1	1	1	2	1	1
9	9	9	9	9	1	1	9	9	1
9	9	1	9	9	9	1	9	1	9
9	1	1	9	9	9	1	9	9	9
2	2	2	2	2	1	1	3	1	3
2	1	2	2	2	1	4	2	2	2
1	1	1	3	2	1	3	1	9	2
3	1	1	1	1	1	1	1	1	1
3	3	1	3	1	1	1	1	2	2
1	1	1	3	3	1	1	1	1	1
3	1	2	1	1	1	1	1	1	1
3	1	2	1	1	1	1	1	1	1
2	2	2	2	2	2	1	2	2	2
2	1	3	1	1	1	1	1	1	1
2	1	1	1	1	1	1	3	1	1
2	1	3	1	1	1	1	1	1	1
9	1	1	1	2	1	4	1	9	9
3	1	1	1	3	1	1	9	9	9
3	1	1	1	3	1	1	1	3	1
3	1	1	3	3	1	5	2	3	1
3	1	3	1	2	1	1	1	3	2
1	1	3	3	3	1	4	1	1	2
3	1	2	1	1	1	2	1	1	1
3	1	1	3	3	1	2	1	1	1
3	1	1	2	9	1	4	1	9	1
2	1	2	2	2	1	2	2	3	2
3	1	1	1	2	2	1	2	2	1
2	1	1	1	1	2	5	3	3	1
3	1	1	1	3	1	7	3	1	1
2	2	2	3	2	1	1	1	2	2
3	1	1	2	2	1	1	2	2	2
3	1	1	1	1	1	1	2	1	2
1	1	2	2	2	1	2	2	1	2
3	1	2	2	1	1	2	1	1	1
3	1	1	1	3	1	2	1	3	1
2	1	1	1	2	1	1	1	2	1

2	1	1	2	2	1	2	1	1	2
2	1	1	2	2	1	1	3	3	3
9	9	9	9	9	1	1	9	9	1
2	1	1	1	1	1	1	1	1	1
3	1	1	1	2	1	4	3	3	1
1	1	1	1	3	1	2	1	1	1
3	1	2	1	2	2	1	1	2	1
3	1	1	2	3	2	1	1	2	1
2	1	1	2	3	3	1	3	3	3
3	1	1	1	1	3	1	1	2	2
2	1	1	2	3	1	2	2	3	2
1	1	1	2	2	1	1	3	3	2
2	1	3	2	3	1	1	2	2	3
3	1	1	1	3	1	2	3	1	1
9	1	1	1	2	1	2	3	1	1
3	1	1	1	3	1	1	3	2	1
3	1	1	1	3	1	2	1	3	1
3	1	1	1	2	1	2	1	2	2
3	1	1	1	3	1	2	1	1	9
1	1	1	3	3	1	1	1	3	1
2	1	2	1	3	1	1	3	3	3
9	9	9	9	9	9	9	9	9	9
3	3	3	3	3	3	2	3	3	3
1	2	1	1	1	1	1	2	2	2
3	1	1	3	2	2	3	2	2	1
9	9	2	9	9	9	6	3	9	9
3	1	2	1	2	1	2	9	1	9
1	2	2	1	3	2	1	1	2	1
9	1	1	9	9	9	3	9	9	1
9	9	9	9	9	1	7	9	9	1
9	9	9	9	2	9	1	9	9	9
3	1	1	2	2	2	2	1	2	1
1	1	1	1	1	1	2	9	1	9
3	1	1	1	2	1	1	1	1	3
1	2	3	2	1	2	4	3	2	3
3	3	1	1	1	1	3	1	3	3
3	3	1	1	1	1	3	1	3	3
2	2	3	2	2	2	3	3	3	3
3	1	1	2	1	2	2	2	1	2
2	2	2	3	2	1	2	1	2	2
3	1	1	1	3	1	1	9	1	1
3	3	1	1	3	1	1	1	3	1
1	2	2	2	3	1	1	2	3	1
1	1	1	1	3	1	1	3	1	1
1	1	1	1	3	1	1	3	1	1
2	1	1	1	3	1	1	3	1	1
1	1	1	1	3	1	1	3	1	1

3	1	1	2	3	1	4	9	1	1
1	1	1	1	1	1	4	1	2	2
3	1	1	1	1	1	4	1	1	1
3	1	3	3	3	1	2	9	9	9
3	1	1	1	3	1	3	9	9	9
3	1	1	2	2	1	5	1	2	2
2	1	1	1	3	1	1	1	3	1
2	2	1	1	2	1	1	1	3	1
9	9	9	9	9	9	9	9	9	9
3	1	1	3	3	1	1	1	3	1
2	1	3	2	9	3	4	2	2	2
1	1	2	1	2	1	1	2	3	1
2	9	1	1	2	2	1	1	3	3
3	1	1	1	2	2	2	1	3	2
9	9	9	9	9	9	9	9	9	9
9	9	9	9	9	9	9	9	9	9
3	1	1	2	3	2	1	2	2	2
3	1	1	1	3	1	1	3	1	1
9	9	9	9	9	9	9	1	1	1
9	9	9	9	9	9	1	9	9	9
9	9	1	9	9	9	1	9	9	1
3	1	1	1	1	2	1	1	2	3
3	2	9	9	9	9	9	9	9	9
9	9	9	9	9	9	9	9	9	9
3	1	1	1	3	1	1	1	1	1
2	1	1	3	2	1	1	2	1	3
3	1	1	1	3	1	2	3	1	1
3	1	1	2	3	1	4	3	2	1
3	1	1	1	3	1	2	1	2	2
3	2	1	1	3	1	2	1	3	1
3	2	2	1	2	3	1	3	3	3
3	1	1	1	2	1	1	1	3	1
2	1	2	3	2	2	1	1	2	2
3	1	1	1	2	1	2	1	2	1
1	1	1	2	2	2	1	2	2	2
3	1	1	1	3	1	1	1	1	1
2	1	1	3	2	1	4	2	3	1
3	1	1	2	3	1	1	1	1	1
3	1	1	1	3	1	1	1	1	1
3	1	1	1	1	1	1	1	3	1
2	1	1	2	2	1	2	2	1	1
1	1	3	2	2	1	1	3	1	3
2	2	1	2	2	3	1	1	3	2
9	9	1	9	9	9	3	2	9	9
9	9	9	2	9	9	1	9	9	9
1	1	3	2	2	1	1	1	1	1
3	2	1	1	1	2	1	2	1	2

2	2	1	2	2	2	1	1	2	2
2	1	1	1	2	1	1	1	2	2
2	1	2	2	2	1	1	3	1	2
1	1	2	2	2	2	1	2	2	2
2	3	1	2	2	3	1	1	2	2
1	2	1	1	3	1	3	1	2	1
2	1	1	2	2	1	2	3	1	1
2	3	1	2	1	3	2	2	2	2
9	9	9	9	9	9	9	9	9	9
1	2	2	2	1	1	4	1	2	2
2	2	2	2	2	2	1	2	2	2
3	3	1	1	3	1	1	2	2	2
3	2	1	2	3	1	1	1	2	2
2	2	2	2	2	2	2	2	2	2
2	2	2	2	3	2	1	2	3	9
2	2	2	1	3	2	1	2	1	2
2	3	1	2	3	1	2	2	2	1
2	2	2	2	2	2	1	2	3	1
2	2	1	1	1	2	4	1	1	1
3	3	3	3	3	2	1	3	1	2
2	1	1	2	2	1	1	1	1	3
3	3	3	3	3	3	1	2	2	2
1	1	2	2	1	1	2	3	3	2
2	3	2	3	2	1	2	2	2	1
2	2	2	2	2	1	1	2	2	2
9	9	9	9	9	9	9	3	2	3
1	1	1	1	1	3	1	2	2	2
2	9	2	2	2	1	1	1	1	2
3	2	1	3	2	1	1	1	2	3
9	9	9	9	9	9	9	9	9	9
2	2	1	2	3	1	1	2	2	2
9	9	9	9	9	9	9	9	9	9
3	3	9	9	9	9	9	3	3	3
1	1	1	3	3	1	1	1	1	1
1	2	1	2	1	1	2	3	2	1
2	1	2	3	2	2	2	1	2	1
3	2	1	2	1	1	1	1	2	3
1	2	2	2	3	3	2	2	3	1
2	2	2	2	2	2	1	2	2	2
1	2	2	2	2	1	1	1	2	2
3	1	2	2	2	2	9	2	2	2
9	1	1	9	9	9	1	3	1	1
2	2	1	2	2	1	1	1	2	2
3	3	2	1	1	1	1	3	2	2
2	1	3	2	2	2	9	2	2	2
3	1	3	1	1	1	3	3	2	2
3	2	1	1	1	1	1	1	2	2

3	1	1	1	1	1	1	3	1	9
3	3	2	1	3	1	1	3	3	1
2	2	1	2	2	2	4	2	2	1
2	2	2	1	3	1	1	3	1	9
3	3	2	1	1	9	1	2	1	2
2	2	2	2	2	2	9	2	2	2
1	1	3	1	3	3	1	1	3	1
1	1	3	1	3	3	1	2	3	2
9	1	9	1	9	9	2	9	9	9
1	1	1	3	3	1	1	3	1	3
3	1	1	1	2	1	1	3	3	1
3	1	2	1	3	1	1	2	3	2
9	9	9	9	9	9	9	9	9	9
1	1	3	1	3	3	1	2	2	2
1	1	3	3	3	3	2	1	3	3
1	1	1	1	1	2	1	1	1	1
1	1	1	1	3	3	1	9	9	1
2	1	1	3	2	1	9	1	3	9
1	1	1	1	2	1	1	1	3	2
1	1	3	1	2	1	1	1	3	3
2	1	1	2	1	2	9	1	9	2
3	3	3	3	3	3	1	3	3	3
1	3	2	2	2	2	1	2	2	2
1	3	1	2	2	2	3	3	2	1
2	1	1	1	3	1	2	2	1	1
3	1	3	1	1	2	4	2	3	2
1	1	3	1	1	2	5	1	1	9
3	1	1	2	3	2	1	2	1	1
1	2	1	1	2	1	1	1	3	1
3	1	2	1	2	1	4	2	1	1
2	2	2	2	2	2	1	2	2	2
1	1	2	2	2	2	9	2	2	2
1	2	2	2	1	2	3	1	2	2
3	1	1	2	2	1	4	2	2	1
2	1	2	2	3	1	2	3	3	2
9	9	9	9	9	9	9	9	9	9
2	2	3	2	3	9	1	2	2	2
1	1	1	3	3	2	1	3	3	2
9	9	1	2	3	1	2	1	2	3
2	2	2	2	2	2	1	2	2	2
2	2	3	2	3	9	1	2	2	2
3	1	1	1	2	1	1	1	1	1
3	1	1	2	2	1	1	2	2	2
1	2	2	2	2	2	9	2	2	2
9	9	9	9	9	9	9	9	9	9
3	1	1	1	3	1	1	1	3	2
9	9	9	9	9	9	9	9	9	9

3	3	1	2	1	1	3	1	1	1
2	1	9	1	9	1	1	1	3	1
1	1	9	9	9	9	1	9	9	9
1	1	3	1	3	1	5	1	3	1
3	2	2	1	2	1	1	1	2	2
1	2	2	2	1	3	9	1	2	2
2	2	2	2	2	2	9	2	2	2
1	1	1	1	1	1	9	9	3	2
3	2	2	3	2	1	1	2	3	2
2	2	1	2	2	2	1	1	3	1
3	3	3	3	3	3	9	9	9	9
2	3	3	1	3	1	9	9	1	3
2	1	3	3	3	2	1	3	3	3
2	1	1	2	3	1	1	3	3	2
2	9	2	2	2	2	9	3	3	2
9	9	9	9	9	9	9	3	3	3
2	1	1	1	3	1	1	1	3	1
9	9	9	9	9	9	9	9	9	9
9	9	9	9	9	9	9	9	9	9
1	3	1	1	1	2	1	1	2	3
2	2	2	2	2	2	1	3	2	2
3	3	3	2	2	2	9	2	2	1
3	2	2	2	2	2	9	3	2	3
2	2	3	3	3	3	1	3	3	3
3	2	1	2	3	2	2	1	2	3
9	1	1	1	2	1	1	3	2	2
9	9	1	1	2	9	4	3	9	9
3	3	2	2	2	3	1	1	2	2
9	9	9	9	9	9	9	9	9	9
9	9	9	9	9	9	9	9	9	9
9	9	9	9	9	9	9	9	9	9
9	9	9	9	9	9	9	9	9	9
9	9	9	9	9	9	9	9	9	9
1	2	2	2	2	2	1	1	1	1
2	1	1	3	1	1	1	2	1	1
3	1	1	1	2	2	1	1	3	2
9	9	9	9	9	9	9	9	9	9
3	1	1	1	2	1	1	1	2	3
1	1	9	1	9	1	1	1	1	1
2	2	2	2	2	2	1	2	9	9
2	2	2	2	2	2	1	2	2	2
2	1	1	2	3	1	1	1	2	2
2	2	2	2	2	2	2	2	2	2
2	2	1	2	2	2	9	2	2	2
2	2	2	2	1	1	2	2	1	3
1	2	1	2	2	2	4	1	1	2
2	3	1	2	2	3	3	1	2	3

2	2	2	2	2	2	1	2	2	2
2	2	2	2	2	2	9	2	2	2
9	9	9	9	9	9	9	9	9	9
3	3	1	2	3	2	7	1	2	3
3	2	1	2	3	2	1	1	3	2
3	1	1	1	1	3	1	1	1	1
1	3	2	9	3	1	1	1	9	9
2	2	2	2	2	2	1	2	2	2
2	2	2	2	2	2	2	2	2	2
2	2	2	2	3	3	1	2	2	2
3	3	1	1	1	1	1	3	2	2
2	2	1	2	3	2	9	2	3	1
9	9	9	9	9	9	9	9	9	9
1	1	1	1	1	1	1	1	1	1
1	2	2	2	2	1	2	1	2	2
1	1	1	1	1	1	1	1	1	1
1	2	1	2	3	2	1	2	2	2
9	9	9	9	9	9	9	9	9	9
1	2	1	2	2	2	4	1	3	2
2	2	3	2	2	2	1	2	2	2
9	9	1	9	9	9	1	9	9	9
9	9	3	1	3	3	1	3	2	3
1	2	2	2	2	1	9	9	9	9
9	9	9	9	9	9	9	9	9	9
9	9	9	1	9	9	1	1	9	9
9	9	9	2	9	9	1	9	9	9
3	3	1	1	3	2	1	1	1	1
2	3	3	3	1	1	9	3	2	3
9	9	9	9	9	9	1	9	9	9
1	1	2	2	2	1	1	1	1	1
3	1	1	3	1	3	1	1	3	1
3	1	1	1	1	1	1	1	1	1
3	1	1	1	1	1	1	1	2	1
1	1	1	3	3	1	1	3	3	2
9	9	2	2	2	2	9	2	2	2
3	3	1	2	2	2	1	2	2	2
2	1	1	1	1	1	1	1	3	1
3	1	2	1	2	2	1	2	2	2
2	2	1	1	1	1	1	2	2	3
1	1	1	1	3	2	4	1	3	3
1	2	3	1	2	1	9	3	2	1
3	1	2	1	2	1	3	1	3	3
3	2	1	1	1	2	2	2	3	2
3	1	2	3	2	1	1	2	2	1
1	1	1	1	3	1	3	1	3	1
1	1	1	1	1	1	1	1	1	1
1	1	3	3	1	1	1	3	3	3

1	2	1	2	3	1	2	3	3	3
1	1	1	1	2	1	3	1	3	3
3	1	1	1	3	1	3	1	3	1
3	1	1	1	2	1	1	2	1	1
1	1	1	1	2	1	1	2	2	2
1	1	1	1	2	1	9	2	2	2
2	1	1	1	1	1	1	3	3	2
3	1	1	2	2	1	1	3	3	2
2	1	2	2	2	1	1	2	2	2
1	1	1	1	3	1	2	1	1	1
3	1	1	1	1	1	1	1	2	9
1	3	1	2	1	3	1	1	2	1
3	3	3	3	3	3	1	3	3	3
1	1	1	1	2	2	1	1	2	2
2	1	3	1	3	1	1	2	1	2
1	9	1	1	3	1	2	9	9	9
3	1	1	1	1	1	1	1	1	3
3	1	1	1	2	1	1	2	1	2
2	2	3	2	3	2	1	1	2	2
2	2	1	1	9	1	1	1	1	3
2	2	1	1	1	1	1	2	2	2
2	2	2	2	2	2	1	2	2	2
1	1	1	1	1	1	9	1	1	1
1	1	1	2	2	3	1	3	2	2
1	1	1	1	1	1	1	1	1	1
1	1	1	1	1	1	1	3	3	3
3	3	2	1	1	1	1	3	3	2
1	2	3	3	2	1	1	2	3	3
2	2	3	3	3	3	1	1	2	3
2	2	2	2	2	2	1	2	2	2
3	1	1	3	3	3	1	3	3	3
9	9	1	9	9	9	1	9	9	9
2	2	2	2	2	2	1	2	2	2
3	1	3	2	3	1	1	3	3	1
3	1	2	1	1	3	4	3	2	1
2	3	1	3	1	3	1	1	3	1
2	2	2	2	2	2	2	2	2	2
3	3	3	3	3	3	1	3	3	3
2	3	1	1	2	1	1	2	2	1
2	2	1	2	2	2	9	2	2	2
2	3	1	3	3	2	1	2	2	2
3	3	3	3	3	3	2	2	3	2
1	1	1	1	3	1	1	3	3	1
1	1	2	1	2	1	1	2	2	2
2	1	1	1	1	3	1	9	9	9
1	1	1	1	3	2	1	1	2	3
3	1	1	1	2	1	3	1	2	2

2	1	1	1	2	3	1	2	2	1
9	1	1	1	3	1	1	9	9	9
9	1	1	1	9	1	9	9	9	1
2	2	2	1	2	2	1	2	2	1
9	9	9	9	9	1	1	1	9	9
9	9	1	1	1	9	3	1	9	1
9	1	9	9	9	1	7	9	1	9
2	1	1	3	2	1	1	1	1	2
3	1	1	1	1	1	1	1	2	2
2	1	2	1	2	2	1	1	2	3
2	1	1	2	2	1	1	2	2	2
1	2	1	3	2	2	1	2	2	2
3	3	1	2	2	2	1	2	2	2
3	3	1	2	2	2	1	2	2	2
2	2	1	2	1	1	1	2	2	2
2	2	2	2	2	2	1	1	2	2
2	2	1	1	2	2	1	3	3	1
1	1	1	2	2	1	1	2	2	2
3	1	2	2	2	2	2	1	1	1
2	2	1	1	3	2	1	3	1	2
3	1	1	2	2	1	2	2	1	1
1	1	1	1	2	1	1	1	1	1
2	2	2	2	2	2	1	9	9	1
2	2	2	2	2	2	1	2	2	2
2	2	1	1	1	1	1	1	3	1
9	1	1	1	1	1	1	2	2	2
2	2	2	2	2	3	1	3	3	3
9	9	9	9	9	9	9	9	9	9
2	1	1	1	2	1	3	1	2	2
1	2	1	2	2	2	1	1	3	2
2	2	2	2	2	1	2	1	1	2
1	1	1	1	2	1	3	1	1	1
3	1	1	1	3	1	5	1	2	1
2	1	1	3	1	1	1	1	3	1
3	1	1	1	2	1	4	1	3	1
2	1	2	1	3	1	9	9	2	2
2	2	2	2	2	2	1	2	2	2
2	2	1	2	2	2	1	2	2	2
2	1	1	1	2	1	1	2	2	2
9	9	9	9	9	9	9	9	9	9
1	1	2	2	2	1	1	1	3	1
2	3	2	1	2	3	5	1	2	3
2	2	1	2	1	3	1	3	1	2
2	2	1	1	1	1	1	2	2	1
2	1	1	1	2	1	1	1	1	2
2	2	2	2	2	2	1	2	2	2
2	2	1	2	2	2	1	3	1	2

2	1	1	1	1	1	1	2	2	1
9	9	2	9	1	9	1	3	9	9
3	1	1	2	1	1	9	1	9	2
2	1	1	2	3	2	1	1	2	2
2	1	2	2	2	2	1	2	2	2
3	1	2	1	1	1	1	1	3	1
3	2	3	3	3	3	1	3	2	2
1	3	1	1	2	1	1	1	3	1
9	2	9	9	1	9	1	9	3	1
2	1	1	2	2	2	1	1	2	2
2	1	1	2	3	9	9	9	9	9
3	1	1	1	1	1	1	1	3	3
2	9	1	2	2	2	1	1	2	2
1	1	1	1	1	1	1	1	1	1
2	1	1	1	3	2	1	2	2	2
3	1	2	1	2	1	4	1	3	1
1	1	1	1	1	1	1	2	2	2
9	1	1	1	3	1	3	1	1	1
9	9	9	9	9	9	9	9	9	9
2	1	1	1	1	2	1	1	1	1
1	1	1	1	3	1	1	1	3	3
3	1	1	1	3	1	2	1	2	2
2	2	2	3	3	2	1	3	1	2
2	1	2	1	3	1	3	2	1	3
1	1	1	1	1	1	1	3	3	2
1	1	2	1	2	1	1	2	2	2
3	1	1	2	2	1	2	2	2	2
2	1	1	1	3	1	1	1	2	1
1	2	1	2	3	2	1	1	2	3
3	2	1	3	3	2	1	3	1	1
2	2	2	2	2	2	1	2	2	2
1	1	1	2	3	1	4	2	2	2
3	1	1	1	3	1	1	1	3	1
2	3	1	2	3	1	5	1	1	2
3	1	1	1	3	1	4	1	3	1
3	3	1	2	3	1	3	1	1	2
3	3	1	1	3	1	4	2	1	1
1	1	1	1	2	1	1	1	2	1
3	1	1	2	2	1	1	2	2	2
3	1	1	1	2	1	1	2	2	1
9	9	9	9	9	9	9	9	9	9
1	1	1	2	1	2	3	3	2	1
3	1	1	1	3	1	4	1	9	9
3	1	1	1	3	1	1	1	3	3
3	1	1	1	3	1	1	1	2	1
3	1	3	3	1	1	1	3	1	1
3	2	1	1	2	1	3	1	3	2

3	2	1	1	3	1	1	3	3	1
9	1	9	9	9	1	2	1	9	9
2	2	1	1	1	2	1	2	2	2
3	1	1	2	3	3	4	1	3	1
2	2	2	2	2	2	1	2	2	2
2	2	1	1	3	3	1	2	2	2
2	1	2	1	1	1	3	1	2	1
1	1	1	1	1	1	9	1	1	1
2	2	2	2	2	2	9	9	9	9
1	3	1	1	2	2	9	1	2	1
9	9	1	2	2	1	9	9	9	9
1	1	2	2	2	2	1	1	3	2
1	1	1	1	3	1	1	1	3	1
1	1	1	1	3	1	1	2	3	1
2	1	1	1	3	1	1	2	2	3
3	1	1	1	3	1	3	2	3	2
1	1	1	2	9	1	1	3	1	2
1	1	1	1	2	1	1	3	3	1
3	1	1	1	3	1	1	1	1	1
1	1	1	2	2	9	1	1	1	1
1	1	1	1	1	1	2	2	2	2
1	2	3	3	3	3	1	1	3	3
2	1	1	9	3	1	1	9	9	9
2	2	1	1	2	3	1	1	1	2
3	3	2	2	3	3	1	1	2	3
3	1	1	1	2	1	2	9	1	1
3	1	1	1	3	1	1	1	3	2
9	3	3	2	1	3	1	2	1	1
9	1	1	1	2	3	1	1	3	1
3	1	1	1	3	1	1	3	3	1
2	1	2	1	2	1	9	3	3	3
3	2	2	3	2	3	3	1	2	3
3	2	3	2	1	2	3	3	2	3
3	1	1	1	2	1	2	1	3	1
1	1	1	1	3	1	1	1	3	1
1	1	1	1	3	1	1	1	3	1
2	1	1	2	2	1	1	1	2	1
9	1	9	1	1	9	1	1	1	9
3	1	1	1	1	1	1	2	3	1
3	1	1	1	1	1	1	3	2	2
1	1	3	1	2	1	1	1	1	2
2	2	2	3	2	3	4	1	2	1
2	1	1	3	2	1	1	1	2	1
9	9	9	9	9	9	9	1	2	1
1	1	1	2	3	1	3	2	9	1
2	1	2	1	2	2	1	1	1	1
2	1	1	1	3	3	1	2	2	1

3	1	1	1	3	1	1	1	9	3
9	9	9	9	9	9	9	3	3	1
3	1	1	2	1	1	2	1	3	1
3	1	2	1	2	1	1	3	3	1
2	2	2	1	2	2	1	2	2	2
3	1	1	1	3	1	1	1	3	1
1	2	1	2	2	1	9	3	3	1
3	3	3	3	3	3	9	2	2	2
3	3	1	3	3	3	1	2	2	2
9	9	9	9	9	9	9	9	9	9
1	1	3	2	2	3	3	1	3	2
1	1	2	1	3	2	1	1	2	2
3	1	1	3	1	3	4	1	3	1
3	1	1	2	1	2	1	1	2	1
2	1	1	1	3	1	1	2	2	2
1	1	2	1	1	1	1	9	9	9
1	1	1	1	1	1	1	1	9	9
2	2	3	1	2	2	1	9	1	2
1	1	3	2	2	1	1	1	2	2
2	2	2	2	2	1	1	1	2	2
9	9	9	9	9	9	9	9	9	9
2	1	1	2	2	1	1	1	2	2
1	1	2	2	2	1	1	1	1	2
1	1	2	2	1	1	2	2	2	2
1	1	2	2	2	1	1	1	1	2
1	1	2	2	1	1	1	1	1	1
1	1	2	2	2	1	1	1	1	2
3	3	3	2	2	3	1	2	2	3
2	2	3	2	2	1	2	2	2	2
1	1	2	2	2	1	1	1	1	2
9	9	9	9	9	9	9	9	9	9
9	9	9	9	9	9	9	9	9	9
9	9	9	9	9	9	9	9	9	9
3	1	1	1	3	1	1	1	2	2
3	1	1	2	3	1	1	1	2	9
2	1	1	2	2	1	1	2	2	2
1	1	1	1	1	2	1	2	2	2
3	3	1	1	2	1	1	1	2	2
2	1	1	2	2	1	4	2	2	1
1	1	1	2	1	1	1	1	9	2
1	1	1	3	3	1	1	1	3	3
3	1	1	1	1	1	1	1	3	2
2	2	2	2	2	2	1	1	2	2
3	1	1	1	3	1	5	3	1	1
2	1	2	3	1	3	1	2	2	2
3	9	3	3	3	1	1	1	1	1
1	1	1	3	3	3	1	1	3	1

1	1	1	3	2	2	1	2	3	3
1	2	1	1	1	1	1	2	2	1
2	1	2	1	2	2	1	1	3	2
1	3	1	1	1	3	1	3	1	1
9	9	9	9	9	9	1	1	2	3
1	2	2	3	2	2	1	1	2	1
1	1	1	1	2	2	1	1	3	2
9	9	1	9	9	9	1	1	9	9
9	9	1	1	2	2	1	9	9	9
3	1	9	9	9	9	9	1	9	1
9	2	1	2	9	1	1	1	9	9
9	9	1	9	9	9	1	9	1	9
1	1	1	1	2	1	2	1	1	1
2	2	2	2	2	2	2	1	2	3
3	1	1	3	2	1	1	1	3	3
2	1	1	1	2	1	1	1	2	1
1	1	1	1	2	2	1	1	2	1
1	1	1	1	2	2	1	1	2	1
3	3	2	1	2	1	3	1	1	1
2	1	2	3	2	1	1	3	1	2
3	1	2	1	2	1	2	1	3	2
2	1	1	1	2	1	1	2	2	2
3	1	1	1	2	1	5	2	1	1
2	2	1	2	2	1	1	9	9	9
2	3	1	2	1	2	1	1	2	1
1	1	1	1	1	3	5	3	1	3
2	2	2	2	2	2	1	2	2	2
2	1	1	1	2	1	1	2	1	2
2	2	2	1	2	2	1	2	2	2
3	2	1	2	2	2	9	2	3	1
3	3	1	2	2	2	1	2	2	2
1	1	1	1	2	1	1	9	2	9
3	1	1	2	3	1	1	3	2	1
3	1	1	1	3	2	2	1	3	1
3	9	9	9	2	3	1	9	2	9
3	2	1	3	2	2	1	2	2	2
3	1	1	1	2	1	1	2	2	2
3	2	1	1	2	1	1	2	2	2
3	1	2	1	3	1	2	2	1	1
2	2	1	3	1	1	1	1	3	3
3	3	3	3	1	1	1	1	1	1
9	1	9	9	9	1	2	9	9	9
9	1	9	9	9	1	2	9	9	1
3	1	1	2	2	2	1	2	2	2
3	2	1	1	3	1	1	3	1	9
1	1	2	1	2	1	2	3	9	1
2	1	3	3	2	1	1	2	2	1

3	1	2	2	3	1	1	1	3	1
1	1	1	3	2	1	2	3	3	3
1	1	2	1	1	1	1	1	3	1
1	1	1	2	2	2	1	1	2	2
2	2	2	2	2	2	1	1	2	2
3	1	1	1	2	1	2	2	2	1
3	1	1	3	3	2	3	9	1	3
1	1	1	1	2	1	1	1	3	2
3	1	1	1	3	1	5	1	1	1
2	3	2	2	1	2	1	1	3	1
3	1	1	2	3	1	1	1	1	1
1	2	2	1	2	2	1	1	2	1
3	1	1	1	2	1	5	3	1	9
9	1	2	1	3	2	1	9	1	2
2	1	1	1	1	1	1	2	2	2
2	1	3	2	2	2	1	2	2	2
2	2	3	3	2	2	3	1	2	2
2	2	2	2	2	2	1	2	2	2
2	2	2	2	1	1	2	1	1	2
1	1	2	1	1	1	1	2	2	2
1	1	2	1	2	3	1	1	1	2
2	1	2	1	1	1	5	1	2	1
1	1	1	2	3	2	4	3	3	1
3	1	1	1	2	1	1	2	1	2
1	1	1	1	2	2	1	1	1	1
3	1	1	2	3	1	3	2	2	2
3	1	1	1	3	1	2	2	2	2
3	1	1	1	1	1	2	1	1	1
2	1	2	2	2	1	1	2	2	2
3	3	2	2	2	1	3	1	1	1
9	9	9	9	9	9	9	9	9	9
9	9	9	9	9	9	9	1	1	2
1	1	2	2	2	1	2	1	1	2
1	1	2	1	2	1	2	1	2	2
3	2	1	2	2	1	1	1	2	2
1	1	2	2	2	1	2	1	1	2
1	1	2	2	2	1	2	1	1	2
2	2	1	2	1	2	4	2	1	2
3	1	1	1	3	1	1	2	1	2
3	3	1	1	1	1	7	1	1	1
3	1	1	1	1	1	2	1	3	1
3	1	1	3	3	1	1	2	1	2
2	1	1	1	2	1	1	1	2	2
3	1	3	1	1	1	1	1	3	1
3	1	1	1	1	1	5	1	2	1
2	2	2	2	2	1	1	2	2	2
1	1	1	1	3	1	1	1	3	1

1	1	1	1	3	1	1	1	3	1
1	2	1	2	2	2	2	1	1	2
3	1	2	1	2	2	7	1	1	2
1	1	2	2	1	1	2	1	1	1
1	1	2	2	2	1	2	1	2	2
1	1	1	2	2	2	1	1	1	2
1	1	2	2	2	1	2	1	1	1
2	2	2	2	2	2	1	1	2	1
1	1	1	2	1	2	3	3	2	1
1	1	1	1	3	1	2	1	3	1
3	1	1	1	3	1	2	1	3	1
1	1	1	1	2	3	1	1	1	1
1	1	1	1	1	1	7	3	3	3
2	2	1	1	2	1	1	1	2	2
1	1	1	3	3	1	1	1	3	3
3	1	2	2	2	1	1	3	2	2
3	2	2	2	1	1	1	3	3	3
1	1	2	2	2	1	1	1	1	2
1	1	1	1	1	1	1	3	2	2
3	1	1	1	2	1	3	1	3	1
1	1	1	1	2	1	3	1	1	2
1	1	3	1	3	1	2	1	3	1
3	1	1	1	3	2	1	2	3	1
1	1	1	1	2	2	1	1	3	1
2	1	1	1	1	2	2	1	2	1
3	1	3	1	3	1	2	1	3	3
3	2	2	1	2	1	1	1	3	3
2	1	1	1	1	1	1	3	3	2
3	1	3	3	3	1	1	3	3	2
3	1	1	1	3	1	2	3	1	2
3	3	3	3	3	3	1	3	3	3
3	1	1	1	3	1	4	1	3	1
2	2	2	2	2	2	1	1	2	2
3	3	1	2	1	1	1	3	3	2
1	2	1	2	3	1	1	1	2	1
2	2	2	1	2	2	1	2	2	2
2	3	1	1	3	1	1	1	3	1
3	2	1	3	3	1	2	1	2	2
3	2	1	3	2	1	4	3	2	3
3	1	1	1	2	1	4	1	1	1
2	1	1	1	3	1	2	1	3	1
3	1	1	1	2	1	2	2	1	1
3	1	2	1	3	1	2	2	2	1
3	1	1	3	2	1	4	2	2	2
3	1	1	2	2	1	1	2	2	2
2	1	1	2	1	3	1	1	2	2
2	2	2	2	3	1	1	2	2	2

3	1	1	1	3	1	1	3	2	1
1	1	1	1	2	1	1	1	2	1
1	2	3	2	1	1	2	2	3	2
2	1	1	2	9	1	2	1	3	1
3	1	1	1	3	3	1	3	3	1
2	2	2	2	2	2	1	1	1	2
2	1	1	1	2	1	1	1	3	2
2	1	1	2	3	2	1	1	1	2
3	1	1	2	3	3	2	9	9	9
2	2	2	2	2	2	1	2	2	2
1	1	1	1	3	1	1	2	2	2
1	1	1	2	3	1	2	1	3	1
1	1	1	1	1	1	1	1	1	1
2	1	2	2	2	2	1	2	2	2
2	3	1	1	1	9	2	1	1	1
3	1	1	1	2	1	3	1	1	1
2	2	1	2	2	2	1	3	1	2
2	2	1	3	1	1	1	2	2	1
2	3	1	2	3	2	2	1	2	3
3	1	2	2	2	2	1	2	2	2
2	1	1	2	2	2	2	1	2	2
2	1	1	2	2	1	2	2	2	1
1	1	2	2	1	1	2	1	2	2
2	1	2	2	1	2	1	1	2	2
2	1	2	2	1	2	1	1	2	2
3	1	1	1	2	1	3	1	1	1
1	1	1	2	3	1	2	1	3	1
2	2	2	2	2	2	1	2	2	2
3	2	1	3	2	1	4	3	2	3
3	1	2	2	2	2	1	2	2	2
2	3	1	1	3	1	1	1	3	1
9	9	9	9	9	9	9	9	9	9
2	2	1	2	2	1	1	1	2	2
3	1	1	1	3	1	1	3	3	1
1	1	1	1	1	1	1	1	1	1
1	1	1	3	3	2	1	3	3	2
3	1	1	1	1	1	1	3	1	1
3	1	1	2	3	1	9	1	2	3
3	1	2	1	2	1	3	2	3	1
2	3	1	2	3	1	1	1	2	3
1	1	1	2	1	1	1	9	9	9
1	1	9	2	1	1	1	1	3	1
1	1	1	1	3	1	1	3	1	2
2	1	2	1	3	1	1	2	2	2
1	2	2	3	2	2	1	2	3	2
3	1	1	1	2	1	1	1	9	9
3	1	2	3	3	1	1	3	3	3

1	2	1	2	2	1	1	2	1	2
3	1	2	1	3	1	1	1	3	1
1	1	1	1	1	1	1	1	1	1
3	1	3	1	2	1	1	2	2	2
3	1	2	2	2	1	1	2	2	2
3	1	1	2	2	1	5	1	3	2
1	1	1	1	1	1	1	1	2	2
9	9	3	9	9	9	3	9	9	9
9	9	3	9	9	9	3	9	9	9
9	9	3	9	9	9	3	9	9	9
1	3	3	3	2	2	2	2	3	3
2	2	2	2	2	2	2	2	2	2
1	3	3	3	3	3	1	3	3	1
1	3	3	3	3	3	1	3	3	1
1	3	3	3	3	3	1	3	3	1
2	2	1	1	3	1	2	2	2	2
3	1	1	1	3	2	2	2	2	1
1	2	2	1	2	1	3	2	1	2
1	2	2	2	3	3	3	2	3	2
1	1	3	2	3	2	2	1	3	3
3	3	3	3	3	2	3	2	2	3
1	2	3	2	2	2	2	3	2	3
2	2	2	2	2	1	2	2	2	2
3	1	2	2	2	2	2	2	2	2
3	2	2	2	1	3	2	2	2	2
1	2	2	3	2	1	2	2	3	3
1	1	2	2	2	2	2	2	2	2
3	3	2	3	1	3	2	2	3	3
1	3	2	3	2	3	1	2	3	3
1	3	2	3	2	3	1	2	3	3
1	3	3	3	3	3	1	3	3	1
1	3	3	3	3	3	1	3	3	1
2	2	1	1	3	1	2	2	2	2
2	2	1	1	3	1	2	2	2	2
2	2	2	2	2	2	2	2	2	2
2	2	1	1	3	1	2	2	2	2
2	2	2	2	3	1	1	1	1	1
2	2	1	1	3	1	2	2	2	2
2	2	2	2	2	2	2	2	2	2
3	1	1	1	3	1	1	1	1	1
2	3	2	2	3	1	3	3	3	3
3	3	3	3	1	3	3	3	3	3
3	3	3	3	1	3	3	3	3	3
3	3	3	3	1	3	3	3	3	3
3	3	3	3	1	3	3	3	3	3
1	1	2	2	3	3	3	2	1	2
2	1	3	2	2	1	3	2	1	1

3	3	2	3	3	2	3	1	2	2
2	3	3	3	2	3	3	3	3	3
3	1	2	2	3	3	2	3	3	2
2	3	3	2	1	2	3	3	3	2
2	2	3	2	2	3	3	2	2	1
1	3	3	3	3	3	3	2	1	3
2	2	3	3	2	1	3	2	3	3
2	3	2	3	2	1	2	9	3	2
9	3	9	9	9	3	9	9	3	9
9	9	9	9	9	9	9	9	9	9
3	1	1	1	3	1	1	1	9	9
2	3	3	3	1	3	3	3	3	3
2	3	3	3	1	3	3	3	3	3
1	3	3	3	1	3	3	3	3	3
9	9	9	9	9	9	2	3	2	2
2	2	2	2	2	2	2	2	2	2
2	2	2	2	2	2	2	2	2	2
9	9	2	9	9	9	2	9	9	9
9	9	3	9	9	9	3	2	9	3
9	9	3	9	9	9	3	9	9	9
2	3	3	2	1	3	3	3	3	3
2	3	3	3	2	2	3	3	3	2
1	3	2	2	2	2	2	2	2	2
3	3	2	3	3	3	2	2	2	2
9	9	3	9	9	9	9	9	9	9
9	3	9	9	9	3	3	9	9	9
2	2	2	1	2	1	3	2	1	3
2	3	3	1	3	2	9	2	9	9
3	3	3	3	2	3	3	2	3	3
9	9	9	9	9	9	9	9	9	9
1	3	3	3	3	1	3	3	1	3
9	9	3	9	9	9	9	3	9	9
1	3	3	3	1	3	2	3	3	3
2	2	3	3	1	3	9	9	9	9
1	3	3	3	1	3	3	3	3	3
1	3	3	3	1	3	3	3	3	2
2	2	2	2	2	2	3	3	3	3
2	2	2	2	2	2	2	2	2	2
1	3	3	2	2	2	2	2	2	2
9	9	3	9	9	9	9	3	9	9
9	9	3	9	9	9	9	3	9	9
9	9	3	9	9	9	9	3	9	9
9	9	3	9	9	9	3	9	9	9
9	9	3	9	9	9	9	3	9	9
9	9	2	9	9	9	2	9	9	9
1	1	3	2	3	2	2	1	1	1
3	3	3	2	3	3	3	2	1	3

9	9	9	9	9	9	9	9	9	9
2	3	3	2	2	3	3	3	3	3
9	9	9	2	9	9	3	2	9	9
3	3	3	3	2	3	3	2	3	3
3	3	3	3	3	2	3	2	3	3
3	3	3	3	3	2	3	3	2	3
9	9	3	9	9	9	2	3	9	9
3	1	1	1	3	1	1	1	1	1
3	3	3	2	3	3	3	1	3	3
9	9	3	9	9	9	2	9	9	9
2	3	3	1	3	3	2	3	1	3
3	3	3	3	1	3	3	3	3	2
9	9	3	9	9	9	3	9	9	9
9	9	3	9	9	9	9	3	9	9
1	3	3	2	2	2	3	3	3	3
9	9	3	9	9	9	9	3	9	9
3	3	2	2	2	2	3	2	3	3
9	9	3	9	9	9	3	9	9	9
1	2	2	3	3	3	3	3	3	2
3	3	3	3	1	2	3	3	3	3
3	1	1	3	3	2	3	2	3	2
1	2	2	1	2	3	3	2	3	2
3	1	2	1	3	1	3	3	3	3
3	1	3	1	2	2	1	1	1	1
3	3	3	3	1	2	3	1	1	1
2	1	3	3	2	2	3	2	3	3
2	2	3	3	1	3	3	3	3	3
2	2	3	3	1	2	3	3	3	3
1	3	3	2	2	2	3	3	3	3
1	3	3	3	1	2	3	3	3	3
2	1	3	2	3	2	3	2	1	2
1	1	2	2	2	2	3	3	3	3
9	1	9	9	1	9	9	9	9	3
9	9	9	3	9	9	9	9	9	9
9	9	3	9	9	9	3	9	9	9
9	9	9	3	9	9	9	9	9	9
1	9	9	2	9	9	9	9	9	9
3	9	9	9	2	9	9	9	9	1
1	9	9	3	9	9	2	9	9	9
9	9	2	9	9	9	2	9	9	9
9	9	2	9	9	9	2	9	9	9
9	9	2	9	9	9	2	9	9	9
9	9	2	9	9	9	2	9	9	9
9	9	2	9	9	9	2	9	9	9
9	9	2	9	9	9	2	9	9	9
1	3	3	3	1	2	3	3	2	2
3	3	3	2	1	2	3	2	3	3

1	2	3	2	1	2	3	3	3	3
3	2	1	1	9	1	9	9	9	9
1	9	3	9	3	1	1	9	9	9
1	2	2	3	1	3	3	2	2	2
9	9	2	9	9	9	3	9	9	9
9	9	3	9	9	9	3	9	9	9
9	9	3	9	9	9	3	9	9	9
9	9	3	9	9	9	3	9	9	9
9	9	3	9	9	9	2	9	9	9
1	2	3	2	1	2	3	2	3	2
2	3	2	3	2	3	3	2	3	2
2	2	2	2	2	2	2	2	2	2
2	2	2	2	2	2	2	2	2	2
1	3	3	3	1	2	3	2	2	2
1	3	3	2	2	1	3	2	2	2
1	2	3	2	1	3	3	3	3	2
9	9	3	9	9	9	3	9	9	9
9	9	3	9	9	9	3	9	9	9
9	9	3	9	9	9	3	9	9	9
9	9	1	9	1	9	3	9	9	9
9	9	3	9	9	9	3	9	9	9
9	9	9	9	9	9	9	9	9	9
3	1	2	2	2	2	9	9	1	9
9	9	9	3	9	9	9	9	1	1
1	9	3	1	3	3	3	9	9	9
9	9	9	2	9	9	3	2	2	2
9	9	2	9	9	9	3	9	9	9
2	3	2	2	2	3	2	2	2	2
2	2	3	2	2	1	2	3	2	3
9	9	9	3	9	9	2	9	9	9
9	9	3	9	9	9	2	2	2	2
3	3	2	3	3	2	3	1	1	2
3	3	2	2	1	3	3	1	3	2
1	3	3	3	2	2	2	2	1	3
3	3	3	3	3	3	3	3	3	3
2	2	2	3	2	2	3	3	3	2
2	2	2	2	3	2	2	1	3	2
2	3	3	2	3	2	2	2	2	2
9	9	9	9	9	3	9	3	9	9
1	3	3	1	3	2	1	3	2	2
2	2	2	2	2	2	2	2	2	2
2	3	3	2	2	3	3	1	3	2
2	3	2	2	1	2	2	2	3	1
1	3	2	3	2	2	2	2	2	2
3	3	3	2	1	3	3	9	9	9
2	2	2	2	2	2	2	2	2	2
9	9	3	2	1	3	3	3	3	3

2	3	3	3	2	3	3	2	1	2
3	3	3	3	2	3	3	3	3	9
3	3	2	3	3	3	3	1	1	2
3	3	3	2	3	3	3	2	3	2
2	1	2	3	1	2	1	2	2	3
3	3	3	3	2	3	2	3	3	2
3	3	3	3	1	3	3	1	2	1
2	3	3	3	3	3	3	2	2	2
1	3	3	1	3	3	1	1	2	2
9	9	9	9	9	9	9	9	9	9
1	3	3	2	2	2	2	2	2	2
1	3	3	3	3	3	3	3	3	1
3	3	3	3	3	3	3	3	3	3
2	2	2	3	1	3	3	1	3	3
1	3	3	2	3	3	2	2	3	2
2	3	3	3	1	3	2	2	3	3
2	3	2	2	2	2	3	2	2	2
2	3	3	3	2	3	3	2	2	2
2	1	2	2	2	3	2	2	3	2
3	3	3	2	3	3	2	2	2	2
9	9	9	9	9	9	3	2	2	2
3	1	3	2	3	3	3	2	2	2
2	3	2	2	2	3	3	2	3	3
3	3	3	3	3	3	3	1	3	3
3	1	3	3	1	3	2	3	3	3
1	3	3	3	3	3	3	1	3	3
3	3	3	3	2	3	3	2	3	3
3	3	3	2	2	1	3	2	2	3
3	3	2	3	3	3	2	2	2	2
2	2	2	2	2	2	2	2	2	2
2	2	2	2	2	2	2	2	2	2
2	2	2	2	2	2	2	2	2	2
2	2	3	2	2	2	2	2	2	2
2	2	2	2	2	2	2	2	2	2
2	2	2	2	2	2	2	2	2	2
2	2	2	2	2	2	2	2	2	2
2	2	2	2	2	2	3	2	3	2
9	9	9	2	9	9	9	9	9	9
1	2	3	3	1	3	9	9	9	9
3	3	3	3	3	3	3	1	3	3
3	1	3	2	3	2	3	2	9	1
1	9	3	9	9	3	9	9	9	9
1	3	3	3	1	3	3	1	3	3
3	3	2	3	3	3	1	3	3	3
2	3	3	1	2	3	3	2	3	3
1	2	3	2	2	1	3	2	2	9
3	1	3	2	3	3	3	2	3	3

1	2	3	2	1	2	3	2	2	2
3	3	3	3	1	3	3	2	3	3
3	3	3	2	1	2	1	1	3	3
1	3	3	3	3	3	3	2	3	3
2	2	3	2	2	3	2	2	2	2
9	9	9	9	9	9	3	3	3	3
3	3	3	3	2	2	2	2	2	2
3	3	3	3	1	3	3	1	3	3
3	3	3	3	2	3	3	3	3	1
2	3	3	2	2	2	3	1	2	3
2	3	1	3	2	3	2	1	3	3
3	1	3	3	2	3	1	1	1	1
1	3	3	2	3	3	2	3	3	3
3	1	3	1	2	3	3	1	3	2
2	3	3	3	2	3	3	1	3	2
2	2	3	2	2	2	2	2	2	2
3	3	3	3	3	1	2	2	2	2
9	9	9	1	9	9	9	9	9	9
2	3	3	3	2	3	3	2	2	2
1	3	3	3	2	3	2	2	2	2
2	2	3	2	2	2	2	2	2	2
3	2	2	2	1	1	3	2	3	2
2	2	2	2	2	2	2	2	2	2
3	3	3	1	3	3	3	1	3	1
3	3	3	3	3	3	2	2	2	2
2	3	2	3	2	3	3	2	2	2
2	3	2	3	2	2	2	1	2	2
2	3	2	2	2	3	3	2	2	2
9	9	2	2	3	3	3	1	3	3
2	1	3	3	2	2	3	2	3	2
1	3	3	1	1	3	3	1	3	3
9	3	9	3	9	9	3	9	9	9
2	2	3	3	2	3	3	2	3	2
1	3	3	2	3	3	2	2	2	2
2	2	3	3	3	3	2	2	2	2
3	3	3	3	2	3	2	2	2	2
2	3	3	2	2	2	2	9	2	3
1	1	1	2	3	2	3	1	3	2
2	3	3	2	2	2	3	2	2	2
1	3	3	1	3	3	2	2	2	2
3	3	3	3	2	3	3	2	3	2
3	3	1	1	1	3	3	3	3	3
3	3	3	3	2	3	1	9	3	9
9	9	9	9	9	3	3	9	9	9
3	3	3	3	1	3	3	2	2	2
3	3	3	3	2	3	3	1	3	3
9	9	9	1	9	9	2	2	2	2

2	2	3	2	2	2	2	2	2	2
2	2	3	2	1	3	2	2	3	3
3	3	2	3	2	2	3	2	3	3
2	2	2	2	2	2	2	2	2	2
2	2	3	3	1	3	3	3	2	2
2	2	3	2	1	1	9	9	9	9
2	2	3	2	2	2	2	2	2	2
3	1	1	3	2	2	3	1	3	2
2	2	2	2	2	2	2	2	2	2
2	2	2	2	2	2	2	2	2	2
2	2	2	2	2	2	2	1	2	2
1	3	2	2	1	2	3	2	2	3
9	9	9	9	9	9	9	9	9	9
1	3	3	3	3	3	1	1	1	1
2	3	3	2	3	3	9	9	3	9
9	9	9	3	9	3	9	9	3	9
1	1	3	2	3	2	2	2	2	2
3	1	2	2	2	2	1	1	1	1
9	9	9	9	9	9	9	3	9	9
2	3	3	3	3	3	3	3	3	3
3	3	3	3	2	3	9	9	9	9
1	3	3	2	2	3	3	2	2	2
2	2	2	2	2	3	9	9	3	9
3	3	3	3	1	3	9	9	9	9
3	3	3	3	3	3	3	1	3	3
9	9	9	9	9	3	3	3	2	2
3	1	3	1	3	1	9	9	9	9
2	1	1	3	3	1	1	3	1	2
3	1	3	3	1	2	3	3	2	3
2	3	3	1	2	3	3	3	3	3
9	3	3	2	2	3	3	2	3	3
2	2	3	3	3	3	9	9	9	9
3	2	3	2	3	3	2	3	3	3
3	3	3	3	3	3	3	9	3	1
3	3	2	2	2	2	2	3	2	2
3	3	3	3	3	3	3	1	3	2
3	3	3	3	2	3	3	1	1	3
3	3	3	2	1	3	3	3	3	3
1	1	3	2	2	3	3	3	3	1
1	9	9	9	9	3	9	9	9	9
3	3	3	3	3	3	3	2	3	3
3	3	3	3	2	3	3	1	3	3
2	1	2	2	3	1	2	2	2	3
3	1	3	1	3	1	3	3	3	3
3	3	3	2	3	3	3	2	1	2
1	3	3	3	2	3	3	3	3	3
1	3	3	2	3	2	2	2	3	3

2	3	2	2	3	3	9	9	9	9
9	9	3	9	9	9	9	9	9	9
3	1	3	3	3	3	3	3	3	3
9	9	3	3	3	9	9	9	3	9
2	1	3	9	9	9	9	9	9	9
9	9	9	3	9	9	9	9	9	9
1	1	3	1	1	3	3	1	3	3
3	1	3	3	3	3	3	2	2	1
3	3	3	3	3	3	3	1	3	3
3	3	3	3	3	3	1	9	3	3
9	9	3	9	9	9	9	9	3	9
9	3	3	3	3	3	3	9	3	3
1	3	3	3	2	3	2	3	3	2
2	3	3	2	1	1	3	1	3	3
9	3	9	9	9	3	3	9	9	9
9	9	9	9	9	9	2	2	3	2
3	3	3	3	3	3	3	3	3	3
3	9	9	3	9	9	3	3	3	2
9	3	3	9	9	9	3	3	3	9
3	1	3	3	2	1	3	3	3	3
3	3	3	2	1	3	3	3	3	3
3	1	3	3	3	2	3	2	3	3
3	3	3	3	1	3	3	1	3	3
3	3	3	3	2	3	3	2	3	3
3	3	3	3	2	3	3	3	3	3
2	3	3	2	1	3	3	2	3	3
2	3	3	3	2	3	3	3	3	2
3	3	3	3	3	3	3	3	3	3
3	3	3	3	2	3	3	3	3	3
1	3	3	3	2	3	2	3	3	3
9	9	3	3	9	2	3	1	3	3
3	1	3	2	2	2	3	2	2	3
1	3	3	2	3	3	3	3	3	3
9	9	2	9	9	9	9	9	9	9
3	3	3	3	3	3	3	1	3	1
3	3	3	3	3	3	1	3	3	3
3	3	3	3	2	3	3	2	3	3
1	3	3	3	1	2	3	1	2	2
3	3	3	3	3	3	9	9	9	9
3	3	1	3	3	3	3	1	3	3
3	3	3	3	3	1	9	9	9	9
9	3	9	9	9	3	9	9	9	9
3	3	3	3	1	3	3	3	3	9
3	3	3	1	3	2	1	1	2	1
2	2	1	2	2	1	3	3	2	2
2	3	3	3	2	3	2	3	3	2
9	3	3	2	2	3	2	3	2	2

9	3	9	9	9	9	9	9	9	9
3	3	9	3	9	9	9	3	9	9
9	9	3	9	9	9	9	3	3	3
9	9	2	2	9	9	3	9	9	9
9	3	9	9	9	3	9	9	9	9
9	9	9	9	9	3	9	3	3	3
3	3	3	3	1	3	1	1	3	2
3	9	9	9	3	3	3	1	3	9
2	2	3	2	1	3	2	2	2	2
3	3	3	3	3	3	1	3	3	3
3	2	3	2	3	3	3	3	3	2
3	3	3	3	1	3	3	3	3	3
1	3	3	3	3	3	1	3	3	3
3	3	3	2	3	3	3	2	3	2
3	3	3	3	3	2	3	1	3	2
3	3	3	3	3	3	1	2	3	2
3	3	3	2	2	3	2	3	2	3
9	3	3	9	9	9	9	9	3	9
3	2	2	2	1	2	3	2	2	3
9	9	3	9	9	9	2	9	9	9
9	9	9	2	9	9	3	3	9	9
9	3	3	9	9	9	3	9	9	9
2	3	3	2	2	2	2	2	2	3
9	3	3	3	1	3	3	3	3	3
2	2	2	2	2	2	2	2	2	2
3	3	3	3	2	3	3	3	3	2
9	3	3	9	9	3	9	3	3	9
1	3	2	2	1	1	2	1	2	3
2	3	3	3	1	3	3	2	3	3
9	9	3	3	3	3	3	3	3	3
2	3	3	3	2	3	3	2	3	3
3	3	3	3	2	3	3	3	3	3
1	3	3	3	3	3	3	3	3	3
2	3	3	3	2	3	3	1	3	2
3	3	3	3	3	3	3	1	3	3
9	9	9	9	9	9	3	9	9	9
3	3	3	3	3	3	3	2	3	3
3	3	3	3	1	3	3	3	3	3
3	3	3	2	2	3	3	3	3	3
3	3	1	2	1	3	3	2	3	3
3	3	3	3	3	3	3	3	3	3
3	3	2	3	3	3	3	2	3	3
3	3	3	3	2	2	3	9	2	9
3	3	3	3	3	3	3	9	3	3
1	3	3	3	3	2	2	2	2	2
3	3	3	3	2	3	2	2	3	2
3	3	3	3	9	1	3	2	2	2

9	3	3	9	9	3	9	9	3	9
3	3	3	3	1	3	3	3	3	3
3	3	3	3	1	3	3	3	3	3
3	3	3	3	3	3	3	2	3	2
3	2	1	2	2	1	1	9	2	9
1	3	3	3	2	3	3	1	3	3
3	3	3	3	1	3	3	3	3	3
2	3	2	3	2	2	2	1	2	2
2	3	2	3	3	3	3	2	3	2
3	3	3	3	1	3	3	1	3	3
3	3	3	3	2	3	3	1	3	3
3	3	3	3	1	3	3	3	3	3
9	9	3	9	9	9	3	9	9	9
3	3	2	1	3	3	1	1	3	3
9	3	3	3	2	3	1	9	9	9
3	3	3	3	2	3	3	1	3	2
9	3	3	3	9	3	9	3	3	9
1	2	1	2	1	2	3	2	2	1
1	3	3	3	3	2	2	3	3	2
3	1	3	1	3	3	3	3	3	3
2	2	2	3	1	2	2	2	2	2
3	3	3	3	3	3	3	2	3	3
9	3	3	2	3	3	3	9	9	9
3	3	3	3	1	3	3	3	3	3
2	2	1	1	2	2	2	2	2	2
3	3	3	1	3	3	3	2	1	3
3	3	3	3	3	3	3	3	3	3
3	3	3	3	2	3	1	2	3	3
3	3	3	3	1	3	9	3	3	3
2	3	3	3	2	2	3	2	2	3
9	3	3	3	1	3	3	3	3	3
1	3	3	3	3	2	3	3	3	2
2	3	3	3	2	3	3	2	2	2
2	3	3	3	2	3	3	2	2	2
1	3	3	2	2	3	3	3	3	3
9	9	3	9	9	9	3	3	3	3
3	3	2	2	1	2	3	3	3	3
3	2	3	2	2	3	3	9	9	9
2	3	3	3	2	3	3	1	3	3
2	2	2	3	3	1	1	2	3	2
2	3	3	3	2	3	3	2	3	3
3	3	3	2	1	3	3	2	3	3
9	3	3	3	2	3	3	9	3	9
3	3	3	3	1	3	3	1	3	9
3	3	3	3	1	3	3	2	3	2
1	3	3	3	1	3	3	3	3	1
3	1	3	3	1	3	2	2	2	2

1	3	2	3	1	3	2	2	9	2
1	3	3	9	9	9	2	3	3	3
3	9	1	1	2	2	2	9	2	2
1	3	3	2	1	1	9	9	9	9
3	3	3	3	2	3	2	2	3	3
2	2	3	2	1	2	2	2	2	2
3	3	3	2	2	3	3	2	2	2
9	9	3	2	1	3	3	3	3	3
3	3	3	3	1	3	9	9	9	9
3	3	3	3	3	3	1	3	3	3
3	3	3	3	1	3	9	3	3	3
3	3	3	3	1	3	3	2	3	3
3	3	3	3	3	3	3	2	3	2
3	3	3	3	2	3	3	3	3	3
3	3	3	3	3	3	3	1	3	1
3	3	3	3	3	1	3	3	3	3
3	3	3	3	2	3	3	2	3	3
3	3	3	3	3	3	3	3	1	1
3	3	3	3	3	3	3	3	3	3
3	3	3	3	3	3	3	3	3	1
3	3	3	2	2	1	3	2	3	2
3	3	3	3	1	3	2	2	3	3
3	3	3	3	2	3	3	1	3	3
3	3	3	3	3	3	3	3	3	3
3	3	3	3	3	3	3	1	3	2
3	3	1	3	3	3	3	1	3	3
3	3	3	3	3	3	3	1	3	3
3	3	3	3	3	3	2	2	2	2
2	3	3	3	1	3	3	2	3	3
3	3	3	3	3	3	3	1	3	3
2	3	3	3	3	2	2	2	3	3
3	1	3	3	3	3	3	3	3	2
3	1	2	3	3	3	3	2	1	1
3	3	3	3	3	3	3	2	3	3
3	3	3	2	2	3	3	2	3	1
1	3	3	9	9	9	9	9	3	9
1	3	3	2	2	2	2	2	3	3

Fingerpads	Fingernails	Palms	Onefinger	Threemidd	FingersinCi	FingersVer	FingersWe	PressNippl	ExamineAr
2	3	2	2	2	2	3	2	2	2
3	2	1	2	1	1	1	3	2	1
2	1	2	2	2	3	2	2	2	1
3	2	1	2	1	1	1	3	2	9
9	9	9	9	9	9	9	9	9	9
3	2	1	2	1	1	1	3	2	1
2	2	2	2	2	2	2	2	1	2
1	2	3	2	2	1	2	2	1	1
2	9	2	9	9	2	9	9	9	1
1	9	9	9	9	9	9	9	9	9
9	2	9	2	2	1	2	2	2	2
3	3	3	1	2	2	2	2	1	2
1	2	2	1	2	2	2	2	2	2
2	3	2	3	2	1	3	2	1	1
2	2	2	2	2	2	2	2	2	2
2	3	1	3	3	2	2	2	1	1
2	3	2	2	1	1	2	1	1	1
2	3	1	3	3	2	2	2	1	1
2	3	1	3	3	2	2	2	1	2
1	3	2	3	3	2	1	2	3	9
1	2	1	1	3	1	1	1	1	1
1	2	1	1	3	1	1	1	1	1
2	1	9	2	2	9	9	9	1	9
1	3	1	1	2	1	1	2	1	1
2	2	2	2	2	2	2	2	2	2
2	3	2	2	2	2	2	2	2	2
2	2	2	2	2	2	2	2	2	2
2	3	2	3	2	2	1	2	1	1
3	3	1	3	9	1	1	1	1	1
3	3	1	3	2	2	2	2	2	2
3	3	1	3	2	2	2	2	2	2
1	3	1	3	2	9	1	2	1	1
1	3	3	2	1	1	2	2	1	2
1	3	1	3	2	1	2	1	1	1
1	3	3	3	2	9	1	2	1	1
1	3	1	3	2	1	2	1	1	1
1	3	3	3	2	9	1	2	1	1
1	3	3	3	9	1	1	1	1	1
3	3	1	3	1	1	1	1	1	1
3	3	1	3	9	1	1	1	1	1
3	2	1	2	1	1	1	1	1	1
2	2	2	3	9	2	2	2	2	2
1	3	1	3	2	2	1	2	1	3
1	3	1	3	3	1	1	3	1	9
3	3	1	3	1	3	2	1	1	1
9	9	9	9	9	9	9	9	9	9

2	3	3	3	2	3	2	1	1	3
2	1	2	2	1	1	1	1	2	2
9	9	9	9	1	1	1	1	1	1
1	3	2	2	2	1	1	2	1	1
2	3	2	3	2	1	9	1	1	1
1	3	1	3	3	1	1	1	1	1
3	3	3	3	1	1	3	3	1	3
1	3	3	9	1	1	1	1	1	1
9	9	9	9	9	1	9	9	9	1
3	2	3	1	2	1	1	3	2	3
9	9	9	9	9	9	9	9	9	9
3	3	3	1	1	1	1	1	1	1
1	3	2	3	1	1	1	1	2	2
2	9	1	9	3	2	1	9	2	9
3	2	1	2	3	1	1	1	2	1
1	3	3	3	2	1	1	1	3	2
2	2	2	3	1	1	2	2	1	1
2	9	1	1	9	1	1	1	1	1
1	3	1	3	2	2	1	2	1	3
3	2	2	3	1	2	1	2	1	2
9	9	9	9	9	1	9	9	9	9
9	9	9	9	3	1	3	3	1	3
9	9	9	9	9	1	9	9	9	9
2	1	2	3	3	1	2	9	1	1
9	1	9	9	9	9	1	9	9	1
1	3	1	2	3	1	1	1	1	1
1	3	1	3	3	1	1	1	1	1
1	2	1	3	2	1	1	1	2	2
3	3	3	3	1	1	1	1	1	2
1	2	1	2	2	1	1	9	1	1
1	2	1	2	3	1	1	1	1	1
2	1	1	3	3	1	3	1	3	1
1	3	1	2	3	1	1	1	1	1
3	3	1	3	2	1	1	2	3	1
2	2	3	3	2	1	2	2	1	1
3	3	3	2	1	1	1	1	9	1
1	1	1	1	1	1	1	1	1	1
2	2	3	3	1	1	1	1	3	1
1	3	1	3	3	1	1	1	1	1
1	3	1	3	3	1	1	1	1	1
1	2	3	3	1	2	1	9	1	1
3	3	3	3	3	9	9	9	9	1
1	3	1	1	1	1	1	3	3	3
1	1	1	1	2	1	1	1	1	1
3	3	1	3	3	1	1	2	1	3
1	3	3	9	9	1	1	2	2	3
1	9	9	9	9	9	9	9	9	9

2	3	2	2	1	1	1	1	1	1
2	2	2	2	2	1	1	1	2	1
2	3	3	3	1	1	1	1	1	1
2	2	2	2	1	1	1	1	2	1
1	3	3	1	1	1	1	3	2	1
1	2	2	2	2	1	1	2	1	1
1	1	2	3	1	1	1	1	1	1
3	1	3	3	1	1	2	2	1	1
2	3	2	1	1	1	2	2	1	1
1	2	3	3	1	1	1	1	1	1
1	1	1	3	3	1	2	2	2	1
2	3	2	2	2	1	1	1	1	2
1	1	3	3	1	1	1	1	1	2
1	3	3	3	1	1	1	1	1	1
2	3	3	1	2	1	1	2	1	2
2	3	1	2	3	1	2	1	2	1
2	2	1	2	2	2	2	1	2	2
1	3	2	1	1	1	3	3	1	3
2	1	2	1	1	1	1	1	1	1
9	9	9	9	9	9	9	9	9	9
2	2	2	3	2	1	1	1	1	2
1	3	1	3	3	1	1	2	1	3
2	2	3	1	2	2	2	2	2	2
3	3	3	3	1	1	9	9	9	9
1	1	3	3	1	1	1	1	3	3
1	3	2	2	2	1	2	2	2	2
1	9	3	3	2	2	1	2	1	2
3	2	1	3	2	1	2	1	1	2
2	2	2	2	2	2	2	2	2	2
2	2	3	2	2	2	2	2	2	3
2	2	2	2	2	2	2	2	2	2
2	3	2	1	2	2	2	2	2	2
1	3	1	3	2	1	1	3	1	3
2	2	2	2	2	3	2	3	2	2
2	2	2	2	2	2	2	2	2	2
3	2	1	3	9	2	2	1	2	2
1	2	2	2	1	9	1	2	2	2
1	3	1	3	3	3	2	2	2	1
9	1	2	9	9	9	9	9	9	9
3	3	1	3	2	3	2	2	1	2
2	2	3	3	1	1	2	1	3	3
2	2	3	3	1	1	1	2	1	3
2	3	2	2	2	2	2	2	2	2
3	3	2	2	2	1	1	1	1	2
2	2	3	2	2	1	2	2	2	2
1	2	3	2	2	1	1	2	1	1
2	2	1	2	2	1	2	2	1	2

3	3	3	3	3	3	3	3	3	3
1	3	1	2	3	2	1	2	1	2
1	3	2	2	2	2	1	1	1	1
2	2	3	3	1	1	1	2	1	3
1	1	1	1	1	1	1	1	1	1
1	3	3	2	1	1	1	2	1	2
2	3	3	3	3	3	2	2	3	3
2	3	1	3	3	2	2	2	1	2
2	2	2	2	2	2	2	2	2	2
2	2	2	2	2	2	2	2	2	2
3	2	2	9	3	2	2	2	3	2
3	3	2	2	3	2	2	2	3	2
9	9	9	9	9	9	1	9	9	9
1	1	2	2	9	1	1	2	2	2
1	3	2	2	2	2	1	1	1	1
1	3	2	2	2	2	1	1	1	1
9	9	9	9	9	9	9	9	9	9
1	3	3	1	1	1	1	1	3	1
9	9	9	9	9	9	9	9	9	9
1	1	1	3	3	1	1	1	1	1
9	9	9	9	9	9	1	1	1	1
1	1	1	3	3	1	1	1	1	1
1	1	1	3	3	1	1	1	1	1
1	1	1	3	3	1	1	1	1	1
1	1	1	3	3	1	1	1	1	1
1	1	1	3	3	1	1	1	1	1
1	1	1	3	3	1	1	1	1	1
1	1	3	3	9	1	1	1	1	1
1	1	1	3	3	1	1	1	1	1
1	1	1	3	3	1	1	1	1	1
2	3	3	2	2	3	1	3	2	9
2	2	2	2	2	1	1	2	2	3
9	9	9	9	9	9	9	9	9	9
1	1	1	3	3	1	1	1	1	1
1	1	1	3	3	1	1	1	1	1
1	1	1	3	3	1	1	1	1	1
1	1	1	3	3	1	1	1	1	1
1	1	1	3	3	1	1	1	1	1
1	1	3	3	1	1	1	1	1	1
9	1	1	1	3	1	1	1	1	1
1	1	1	3	3	1	1	1	1	1
1	1	1	3	3	1	1	1	1	1
9	9	9	9	9	9	9	9	9	9
1	1	1	1	3	3	1	1	1	1
1	1	1	3	3	1	1	1	1	1
1	1	3	1	1	1	1	1	1	1
1	1	1	3	3	1	1	1	1	1

1	1	1	3	3	1	1	1	1	1
1	1	1	1	3	1	1	1	1	1
1	1	1	1	3	1	1	1	1	1
1	1	1	3	3	1	1	1	1	1
1	1	1	1	1	1	1	1	1	1
1	1	1	2	2	1	1	1	1	1
1	1	1	3	3	1	1	1	1	1
1	3	3	3	1	1	1	1	1	1
1	3	1	3	1	1	1	1	1	1
1	3	2	3	1	1	1	1	1	2
1	3	3	3	1	1	1	1	1	1
1	3	3	3	1	1	3	1	3	1
1	3	1	3	3	1	1	1	1	1
9	9	9	3	3	1	1	1	1	1
2	2	1	1	3	1	2	2	2	2
2	2	1	3	3	2	1	1	1	1
2	2	2	1	1	1	1	1	1	1
1	1	1	1	1	1	1	1	1	1
2	2	1	2	2	1	2	2	1	2
2	2	2	2	2	2	2	2	2	2
1	2	2	3	1	1	1	1	2	1
2	2	2	2	2	2	2	2	2	2
3	2	1	2	2	1	2	1	3	3
1	3	1	2	2	2	1	2	1	2
2	2	2	2	2	2	2	2	2	2
2	2	2	2	2	2	2	2	2	2
2	2	2	2	2	2	2	2	2	2
2	2	2	2	2	2	2	2	2	2
2	2	2	2	2	2	2	2	2	2
2	2	1	2	2	2	2	2	1	2
2	2	2	2	2	2	1	1	2	1
2	2	2	2	2	2	1	1	2	1
2	2	2	2	2	1	2	2	1	2
2	3	3	2	2	2	1	1	3	1
2	2	1	2	2	2	2	1	1	1
2	2	2	2	2	2	2	2	2	2
2	2	2	1	1	1	1	2	2	1
1	2	3	3	3	1	1	1	2	1
2	2	2	2	2	2	1	2	2	2
3	3	1	3	3	2	3	1	1	1
2	1	2	2	2	2	2	2	2	2
2	2	2	2	2	2	2	2	2	2
9	9	9	9	9	9	9	9	9	9
9	9	9	9	9	9	9	9	9	9
3	3	3	2	2	2	3	2	2	1
2	1	3	2	1	3	2	2	1	1
9	9	9	9	9	9	9	9	9	9

2	2	2	2	2	1	2	2	1	2
2	2	2	2	2	1	2	2	1	2
2	2	2	2	2	2	1	2	2	2
2	3	2	2	2	2	2	1	1	2
3	2	1	2	1	2	2	1	2	2
1	2	1	2	3	1	1	2	1	1
1	3	1	3	3	2	1	2	1	1
2	3	2	2	2	2	2	2	1	1
2	2	3	3	3	1	1	2	1	1
3	3	3	3	3	1	1	3	3	3
1	2	2	2	2	2	3	2	2	2
2	3	3	3	1	1	2	1	1	1
3	2	3	3	3	1	1	2	1	1
3	3	3	2	2	2	2	2	1	2
3	3	1	3	2	2	2	2	1	1
3	2	3	3	3	2	3	2	2	2
2	3	3	2	2	2	2	1	2	1
2	2	2	2	2	2	2	2	2	2
2	3	3	3	2	2	3	2	2	2
1	2	2	2	2	2	2	1	1	3
2	2	1	3	3	2	2	2	1	1
2	2	2	2	2	1	1	2	1	1
3	3	3	2	2	2	2	2	1	2
2	2	2	2	2	2	3	3	3	2
9	9	1	9	9	9	9	9	1	9
2	2	2	2	2	2	2	2	2	2
2	2	2	2	2	2	2	2	2	2
2	2	3	9	2	1	9	1	2	9
9	9	9	9	9	9	9	9	9	9
1	2	2	2	2	2	1	2	2	2
9	9	9	9	9	9	1	9	9	9
1	2	2	2	9	9	9	1	1	9
1	3	3	1	3	1	3	2	3	3
2	1	2	2	2	1	2	1	2	1
9	9	9	9	9	9	9	9	9	9
2	3	2	1	2	2	2	2	2	2
2	1	2	2	2	1	2	2	1	1
1	3	2	3	2	3	2	1	2	2
1	2	2	3	1	1	1	1	1	1
1	3	1	3	2	1	1	2	3	1
3	3	1	3	3	2	2	2	1	1
2	3	2	2	2	1	1	1	1	2
2	3	1	2	1	1	1	1	1	1
2	2	2	2	2	2	2	2	1	2
9	9	9	9	9	9	9	9	9	9
2	2	9	1	2	2	2	2	1	1
2	3	2	3	1	1	2	2	1	1

9	9	9	9	9	9	9	9	9	9
1	1	2	3	1	9	9	9	9	9
1	2	1	2	1	1	1	1	1	2
1	2	1	3	9	1	1	1	1	1
2	2	2	2	2	1	3	3	3	2
3	1	2	3	2	1	1	3	1	3
1	1	2	2	2	2	2	1	1	1
1	3	1	2	3	1	1	1	1	1
1	2	2	2	2	2	3	1	1	1
2	1	2	2	2	2	2	2	2	2
2	2	2	2	2	2	2	2	2	2
2	3	1	3	2	1	1	1	2	1
9	9	9	9	9	9	9	9	9	9
1	3	3	1	1	1	1	1	3	3
1	1	3	1	3	3	1	3	3	3
1	2	2	2	9	1	1	2	2	1
1	3	3	1	1	1	1	1	2	2
2	2	1	2	1	1	1	2	1	2
2	2	2	1	1	2	3	1	2	1
9	9	9	9	9	9	9	9	9	9
3	1	2	3	2	1	2	1	3	1
2	3	2	3	1	1	1	1	1	1
3	2	3	3	1	1	2	2	1	1
2	2	2	2	2	2	2	2	2	2
2	2	2	2	2	2	2	2	1	2
9	9	9	9	1	1	1	2	1	1
3	3	3	2	1	2	1	1	1	2
1	3	9	3	9	1	1	2	2	1
3	3	1	3	3	2	1	2	2	1
1	3	3	1	1	1	1	1	2	1
1	1	3	3	1	1	1	1	2	2
2	3	3	3	3	2	3	3	3	2
9	9	9	9	9	1	9	9	9	9
3	2	3	1	1	1	3	3	3	1
3	3	1	2	1	1	1	2	2	2
1	3	3	3	1	3	2	2	1	1
1	3	1	3	3	1	1	1	1	1
1	3	3	3	3	1	1	1	1	1
1	3	3	3	1	1	1	1	1	1
1	3	3	3	1	3	1	2	1	1
3	3	3	2	2	1	3	3	3	3
1	3	3	3	1	1	1	1	1	1
2	2	2	3	2	3	2	2	3	3
2	2	2	2	2	2	2	2	2	2
1	3	3	3	3	3	3	2	1	3
2	2	2	2	2	2	1	2	1	1
3	3	3	3	1	1	1	3	1	1

1	3	3	2	2	1	1	2	2	1
1	3	3	2	2	1	1	2	2	1
9	9	9	9	9	9	1	9	9	9
3	3	3	3	3	1	1	2	1	1
9	9	9	9	9	9	9	9	9	9
2	3	3	3	2	1	2	2	1	1
1	1	2	1	1	1	1	1	1	1
1	3	9	9	9	9	1	9	2	2
9	1	1	3	3	1	1	1	1	1
2	3	1	3	2	3	2	2	1	3
2	2	2	3	3	1	2	2	3	2
3	3	3	2	2	2	2	2	2	2
1	3	2	3	3	2	2	2	1	1
3	2	2	2	2	1	1	2	2	3
3	2	2	3	3	1	2	2	3	2
1	2	2	3	1	2	2	1	1	1
3	2	2	3	3	1	2	2	2	2
1	1	1	1	2	2	2	2	1	3
1	1	1	1	3	1	1	1	1	1
1	2	2	2	2	2	2	2	2	2
2	2	3	9	2	1	1	3	2	1
2	3	2	3	1	1	1	2	1	3
2	3	1	3	3	1	1	2	2	2
3	3	3	3	3	1	1	1	1	2
2	2	2	2	2	2	2	2	2	2
2	2	2	2	2	2	2	2	2	2
2	3	1	2	2	2	2	2	1	1
2	3	2	3	2	2	2	2	2	2
2	3	2	2	3	2	2	1	1	2
1	1	1	1	2	1	1	2	1	1
2	3	2	2	2	2	2	2	2	1
2	3	2	2	2	2	2	2	2	1
3	3	2	2	1	2	3	2	3	1
2	2	2	2	2	2	2	2	2	2
2	2	2	2	2	2	2	2	2	2
3	3	2	2	2	1	1	2	3	2
3	3	2	3	3	2	2	3	1	1
9	9	9	9	9	1	1	9	1	9
2	2	2	2	2	2	2	2	2	2
2	2	3	2	2	2	2	3	2	1
2	2	1	2	2	1	2	2	2	2
2	2	1	2	2	2	1	2	1	2
2	2	2	2	3	1	1	1	1	2
2	2	2	2	2	2	2	2	1	2
2	2	1	2	2	1	2	2	1	2
1	3	1	2	2	1	1	2	1	1
2	2	2	2	2	2	2	2	2	2

1	3	3	3	3	2	2	1	2	3
1	2	3	2	1	1	1	1	2	2
1	2	2	2	2	3	3	2	3	3
2	2	1	2	2	1	2	2	2	2
2	3	2	2	2	1	2	2	1	2
2	3	2	3	3	1	1	2	2	2
2	2	1	2	2	2	1	9	2	9
2	3	1	3	3	1	1	1	1	1
2	3	2	2	1	1	1	1	2	1
2	3	1	2	2	1	1	2	1	3
2	1	2	1	2	1	2	2	2	2
1	1	1	2	2	1	1	1	1	2
3	3	1	3	3	1	1	2	2	1
2	2	2	2	2	2	2	2	2	2
2	2	2	2	2	2	2	2	2	2
1	1	1	1	2	1	1	1	2	1
3	3	1	3	3	1	2	2	1	2
2	2	2	2	2	1	1	1	1	1
2	2	2	2	2	1	1	1	1	1
9	9	9	9	9	9	9	9	9	9
2	2	1	2	2	1	1	2	1	2
2	2	2	2	2	2	2	2	2	2
9	9	9	9	9	9	9	9	9	9
2	2	2	2	2	2	2	2	2	2
1	3	3	2	2	1	1	1	1	1
3	3	9	1	2	9	2	3	1	2
2	2	2	2	2	2	2	2	3	1
9	9	9	9	9	9	9	9	9	9
2	2	2	2	1	2	2	2	2	2
1	2	1	3	2	1	3	1	2	3
2	1	2	2	2	2	2	2	2	2
2	1	3	3	2	3	1	1	2	3
2	1	2	2	2	2	2	2	2	2
1	3	1	1	2	1	1	1	1	1
1	1	2	1	1	1	1	1	1	1
3	2	1	2	1	2	1	1	1	1
3	3	3	3	3	3	3	3	3	2
1	2	1	2	3	2	1	3	2	1
1	2	2	2	2	2	2	1	3	2
1	3	1	2	2	1	1	2	2	1
1	1	2	3	2	2	3	2	1	2
3	2	1	2	1	1	1	3	2	1
1	3	2	3	3	1	1	1	1	1
2	3	3	3	2	3	3	3	3	3
9	9	9	9	9	9	9	9	9	9
1	1	3	3	1	1	1	1	1	1
9	9	9	9	9	1	1	1	9	1

1	1	1	1	1	1	1	1	1	1
1	1	1	3	3	1	1	1	1	1
1	1	1	3	3	1	1	1	1	1
1	1	1	3	3	1	1	1	1	1
1	1	1	1	1	1	1	1	1	1
1	1	1	3	3	1	1	1	1	1
1	1	1	3	3	1	1	1	1	1
1	1	1	3	3	1	1	1	1	1
1	1	1	1	3	3	1	1	1	1
1	1	1	1	3	1	1	1	1	1
2	1	1	2	2	9	1	2	1	1
1	1	1	3	3	1	1	1	1	1
1	1	1	3	3	1	1	1	1	1
1	1	1	3	3	1	1	1	1	1
1	1	1	3	3	1	1	1	1	1
1	1	1	3	3	1	1	1	1	1
1	2	2	2	2	1	2	2	1	2
1	1	3	3	1	1	1	1	1	1
1	1	1	3	3	1	1	1	1	1
1	1	1	3	3	1	1	1	1	1
1	1	1	3	3	1	1	1	1	1
1	1	1	3	3	1	1	1	1	1
1	1	1	3	3	1	1	1	1	1
1	1	1	3	3	1	1	1	1	1
1	1	1	3	3	1	1	1	1	1
1	1	1	3	3	1	1	1	1	1
1	1	1	3	3	1	1	1	1	1
1	1	1	3	3	1	1	1	1	1
1	1	3	1	3	3	1	1	1	1
1	1	1	3	3	1	1	1	1	1
1	1	1	3	3	1	1	1	1	1
1	1	1	3	3	1	1	1	1	1
1	1	1	3	3	1	1	1	1	1
1	1	1	3	3	1	1	1	1	1
1	1	1	3	3	1	1	1	1	1
1	1	1	3	3	1	1	1	1	1
1	3	1	3	2	1	1	1	1	1
1	1	1	1	3	1	1	1	1	1
1	1	1	3	3	1	1	1	1	1
1	1	1	3	3	1	1	1	1	1
9	1	1	3	3	1	1	1	1	1
2	2	1	2	2	2	2	2	1	1
1	2	2	3	2	2	3	1	1	1
2	2	2	2	2	2	2	2	2	2
9	9	9	9	9	9	9	9	9	9
2	2	2	2	2	1	1	2	2	2
1	3	1	3	3	1	1	1	2	1

2	2	2	2	2	2	2	2	2	2
1	2	1	2	2	1	2	1	1	2
1	2	1	2	2	1	2	1	1	2
2	2	3	3	1	1	1	1	1	1
1	1	1	3	1	1	1	1	1	1
2	3	2	2	2	1	2	1	1	1
2	2	2	2	2	2	2	2	2	2
2	2	2	2	2	2	2	2	3	2
2	3	3	2	2	1	1	1	2	1
2	2	2	2	2	1	2	2	2	2
1	2	1	2	2	1	2	1	1	2
9	9	9	9	9	9	9	9	9	9
9	1	2	9	2	1	9	9	9	1
2	2	2	2	2	2	1	2	2	2
9	1	9	9	9	9	9	9	9	9
9	9	9	9	9	9	1	9	9	9
9	9	9	9	9	9	9	9	9	9
9	9	9	9	9	9	9	9	9	9
9	9	9	9	9	9	9	9	9	9
1	3	3	3	2	2	2	1	2	3
3	3	3	3	3	3	3	3	3	9
3	3	3	3	3	3	3	3	3	1
2	3	2	1	3	1	2	1	2	2
9	9	9	9	9	9	9	9	9	9
9	9	9	1	9	9	9	9	9	9
9	9	9	9	1	9	9	9	1	9
9	9	9	9	9	9	9	9	9	9
1	2	3	1	3	3	2	2	1	2
1	3	2	2	1	2	2	2	1	2
9	9	9	9	9	9	9	9	9	9
9	9	9	9	9	9	9	9	9	9
2	2	1	2	2	2	2	2	2	2
2	2	2	2	2	2	2	2	2	2
3	1	1	3	3	1	1	1	1	3
1	2	1	2	2	2	2	1	1	2
2	2	3	3	3	2	2	2	1	1
1	2	1	3	3	2	2	2	9	1
3	3	2	3	3	3	3	2	2	9
2	2	2	2	2	2	2	2	2	2
1	3	1	3	2	1	2	1	1	2
9	9	9	9	9	9	9	9	1	9
1	3	3	3	3	1	1	3	2	3
1	2	1	1	1	1	1	2	2	2
2	1	1	3	2	1	2	1	2	1
3	3	1	3	3	3	2	2	3	3
2	2	2	2	2	1	1	1	2	2
1	3	2	3	3	1	1	1	1	2

9	9	9	9	9	9	9	9	9	9
2	2	2	2	2	2	2	2	2	1
3	3	3	3	3	2	2	2	2	2
3	3	2	2	2	1	1	2	1	1
2	3	1	2	2	1	1	2	1	1
2	2	2	2	2	2	2	2	2	2
2	2	2	2	2	2	2	2	2	2
1	1	1	2	1	1	1	2	1	1
3	3	1	3	3	2	1	1	1	1
2	2	2	2	2	2	2	2	2	2
1	3	2	2	2	2	2	2	2	2
2	3	1	3	2	2	2	1	1	2
2	2	2	2	2	2	2	2	2	2
2	2	2	2	2	2	2	2	2	2
2	2	2	2	2	2	2	2	2	1
2	2	2	2	2	2	2	2	2	2
2	2	2	2	2	1	1	2	2	2
2	2	2	2	2	2	2	2	2	2
2	3	3	3	3	2	2	2	1	1
1	3	2	3	2	1	1	1	1	2
2	3	3	1	1	1	1	1	2	2
2	3	1	3	3	2	2	2	2	2
1	3	1	3	3	1	1	1	1	1
1	3	1	3	3	1	1	1	1	1
2	2	2	2	2	2	1	1	2	2
2	2	2	2	2	1	1	2	2	2
3	3	3	1	1	3	3	1	1	1
3	3	3	2	1	2	3	1	2	1
2	3	1	1	2	1	1	1	1	1
3	2	3	3	3	2	2	2	2	3
1	1	3	1	1	1	2	2	2	2
2	9	9	9	9	9	9	9	9	2
2	3	3	3	2	2	2	2	2	2
3	3	1	3	3	3	2	2	2	2
1	3	1	3	3	2	1	2	1	1
9	9	9	9	9	9	9	9	9	9
2	2	2	2	2	2	2	2	2	2
1	3	3	3	1	3	3	1	1	1
2	2	2	2	2	2	2	2	2	2
2	1	3	3	1	1	1	2	2	1
9	9	9	9	9	1	1	2	9	1
1	2	3	3	2	1	1	1	1	2
1	3	3	3	3	1	1	1	1	1
2	3	2	2	2	1	1	1	1	2
1	3	1	3	3	1	1	1	1	2
2	1	2	1	2	2	2	2	2	2
9	9	9	9	9	9	9	9	9	9

9	9	9	9	9	9	9	9	9	9
9	9	9	9	9	9	9	9	9	9
9	9	9	9	9	9	9	9	9	9
9	9	9	9	9	9	9	9	9	9
9	9	9	9	9	9	9	9	9	9
1	9	9	9	9	9	9	9	9	9
9	9	9	9	9	9	9	9	9	9
9	9	9	9	9	9	9	9	9	9
9	9	9	9	9	9	9	9	9	9
9	9	9	9	9	9	9	9	9	9
1	3	3	1	3	9	9	1	9	9
9	9	9	9	9	9	9	9	9	9
9	9	9	9	9	9	9	9	9	9
9	9	9	9	9	9	9	9	9	9
9	9	9	9	9	9	9	9	9	9
9	9	9	9	9	9	9	9	9	9
9	9	9	9	9	9	9	9	9	9
9	9	9	9	9	9	9	9	9	9
2	2	2	2	2	2	2	2	2	2
1	2	1	1	1	1	2	1	1	1
1	1	1	1	1	1	1	1	1	1
1	2	2	2	2	3	2	1	2	2
9	2	1	1	2	1	2	2	2	3
1	1	1	3	1	3	1	1	1	1
2	2	2	3	3	3	2	3	2	2
2	1	1	2	3	3	2	1	3	1
2	2	2	2	2	2	2	2	2	2
2	2	2	2	3	3	2	2	2	3
2	2	2	2	2	2	2	2	2	2
1	9	2	2	3	2	1	2	2	3
1	2	1	1	1	1	1	1	2	3
2	1	1	3	3	2	2	2	1	3
2	3	3	3	2	2	2	2	2	9
2	3	3	3	3	1	1	3	1	3
2	2	2	2	2	2	2	2	1	1
2	2	1	3	3	3	3	3	2	2
1	2	2	2	2	2	2	2	2	2
2	1	2	2	3	1	3	2	1	2
1	1	1	2	2	2	2	1	1	1
2	1	2	3	3	1	1	1	1	1
2	1	2	2	1	2	1	2	1	2
1	1	3	1	3	1	1	3	1	3
1	1	1	3	1	1	1	1	9	1
1	1	2	3	1	1	1	1	3	1
1	3	2	2	2	2	2	2	2	2
9	9	9	9	9	9	9	9	9	9
2	3	1	1	2	2	3	1	2	3

2	3	3	3	3	1	2	2	1	2
1	1	9	2	9	9	9	9	9	9
3	1	3	1	1	3	1	1	3	1
2	2	9	9	9	9	9	9	9	9
2	1	3	2	3	2	2	2	2	2
2	3	2	2	2	2	2	2	2	2
2	2	9	9	9	9	9	9	9	9
2	3	3	3	1	2	2	1	2	2
3	1	2	1	1	1	3	1	1	1
1	3	3	3	3	1	1	2	1	1
2	9	1	1	2	1	1	1	1	1
1	2	2	1	2	1	1	1	2	2
3	2	2	2	2	2	2	2	2	3
1	2	3	1	2	3	1	2	3	1
2	2	2	2	2	2	2	2	2	2
3	1	1	1	3	2	2	2	1	1
1	1	2	2	1	1	1	1	2	1
9	9	9	9	9	9	9	9	9	9
1	3	9	2	3	1	2	1	2	2
3	3	3	3	3	1	3	3	3	3
2	2	2	3	3	3	3	3	3	3
3	2	2	2	2	2	2	2	2	3
3	3	3	3	3	2	2	2	2	2
3	2	2	9	3	1	3	3	3	3
2	2	2	2	2	2	2	2	2	2
1	1	3	1	3	3	1	1	3	1
3	3	3	3	3	2	3	3	3	3
3	1	3	3	3	3	3	1	1	3
2	1	3	2	1	9	1	1	1	1
2	3	1	3	3	1	1	2	1	1
1	3	1	3	3	1	1	1	1	1
3	3	3	3	3	3	9	9	9	9
1	1	2	2	2	1	3	2	1	1
3	3	2	3	3	1	2	1	2	9
2	3	3	9	3	1	9	9	1	9
2	3	1	3	3	1	2	2	1	1
2	3	1	3	3	1	2	2	1	1
9	9	9	9	9	9	9	9	9	9
2	2	2	2	2	9	9	9	9	9
9	9	9	9	9	9	9	9	9	9
9	9	9	9	9	9	9	9	9	9
2	3	2	1	2	2	3	2	2	3
2	3	1	2	3	1	2	3	1	2
9	9	9	9	9	9	9	9	9	9
2	3	1	2	3	1	2	3	1	2
2	3	1	3	2	1	1	1	2	2
1	3	3	3	1	1	3	3	1	1

3	3	3	3	3	3	3	3	3	3
1	2	1	2	2	1	1	2	1	1
2	2	3	3	3	3	3	2	2	3
2	3	2	2	3	1	2	2	1	1
2	2	2	2	2	2	2	2	2	2
9	1	9	9	1	9	1	9	1	9
9	9	9	9	9	9	9	9	9	9
3	3	1	3	2	2	2	1	1	2
3	3	3	3	3	3	3	3	3	3
1	3	3	3	1	1	1	2	2	2
3	3	3	3	3	3	3	3	3	3
1	3	3	1	3	1	3	1	1	1
2	2	1	2	9	2	2	3	2	2
2	3	1	2	2	1	1	1	1	2
2	3	1	2	2	1	1	1	1	2
2	3	2	2	9	2	2	3	2	2
2	3	1	2	2	1	1	1	1	2
2	3	1	2	2	1	1	1	1	2
2	3	1	2	2	1	1	1	1	2
3	3	3	3	3	3	3	3	1	3
1	1	2	9	1	3	2	1	3	1
3	1	2	3	3	3	3	3	3	3
3	3	1	3	3	1	1	1	1	3
2	2	2	3	2	2	2	3	3	3
3	3	3	3	3	3	3	3	3	3
2	3	1	2	2	1	1	1	1	2
9	9	1	9	9	9	9	9	9	9
2	2	3	3	3	1	1	1	1	3
9	9	9	9	9	9	9	9	9	9
3	3	3	3	3	3	3	3	3	3
2	1	1	2	2	1	1	2	1	1
2	2	3	2	3	2	3	2	2	3
1	2	3	2	1	3	1	3	2	3
3	3	3	3	3	3	3	3	3	3
3	3	3	3	3	3	3	3	3	3
3	3	3	3	9	9	9	9	9	9
9	9	9	9	9	9	9	9	9	9
2	1	1	1	1	1	2	1	1	1
1	2	2	1	1	1	1	2	2	2
1	3	3	3	1	2	2	2	1	2
2	2	3	2	2	9	3	9	1	2
3	3	1	2	2	1	2	1	3	1
9	9	1	9	2	1	1	1	9	2
1	3	1	3	3	3	3	1	1	1
2	1	1	3	3	2	2	1	1	1
1	1	1	1	1	1	1	1	1	1
2	1	2	2	2	2	2	2	2	2

1	3	3	3	3	2	2	1	1	1
9	9	9	9	9	9	9	9	9	9
9	9	9	9	9	9	9	9	9	9
1	3	3	1	3	1	3	1	3	1
2	2	2	2	2	2	2	2	2	2
2	1	3	2	3	2	2	2	2	2
9	3	2	9	2	2	2	2	2	2
2	3	1	3	2	2	2	2	2	2
2	3	1	3	2	1	2	2	2	2
1	3	2	1	2	1	3	2	1	3
2	2	1	3	9	2	3	2	2	2
2	3	3	2	1	1	2	1	1	1
2	3	1	3	2	2	2	1	1	2
1	1	1	3	1	2	3	3	2	1
3	3	3	3	3	1	1	1	1	2
3	2	2	1	2	3	2	1	2	1
1	2	3	1	2	3	1	2	3	1
9	9	9	9	9	9	9	9	9	9
9	9	9	9	9	9	9	9	9	9
2	3	2	3	2	3	1	2	3	1
1	1	1	9	2	2	2	1	1	3
2	2	2	2	2	2	2	2	1	2
1	1	1	1	1	1	1	1	1	1
2	2	2	2	2	2	2	2	2	2
3	3	3	3	3	3	3	3	3	3
2	9	2	2	2	3	2	3	3	9
2	3	2	2	3	2	3	3	9	2
1	3	3	3	1	1	1	1	1	1
1	1	1	1	1	1	1	1	1	1
2	2	2	2	2	2	2	2	2	2
3	1	2	3	2	2	3	3	2	1
1	2	3	3	2	1	2	1	1	1
9	9	9	9	9	9	9	9	9	9
2	9	2	2	3	2	3	3	9	2
2	2	2	2	2	1	2	1	2	1
2	2	2	2	2	2	2	2	2	2
2	2	2	2	2	2	2	1	1	2
9	9	9	9	9	9	9	9	9	9
9	9	9	9	1	3	9	9	2	9
1	1	1	2	2	1	2	2	1	1
3	3	3	3	3	3	3	3	3	3
2	2	2	2	2	2	2	2	2	2
2	2	2	2	2	2	2	2	2	2
2	2	2	2	2	2	2	2	2	2
2	2	2	2	2	2	2	2	2	2
2	2	2	2	2	2	2	2	2	2
9	9	9	9	9	9	9	9	9	9

9	9	9	9	9	9	9	9	9	9
2	2	2	2	2	2	2	2	2	2
9	9	9	9	9	9	9	9	9	9
9	9	9	9	9	9	9	9	9	9
9	9	9	9	9	9	9	9	9	9
1	1	3	3	1	2	1	2	3	1
2	2	2	2	2	2	2	2	2	2
3	3	3	3	3	3	3	3	3	3
1	1	1	1	1	2	3	2	3	2
9	9	9	9	9	9	9	9	9	9
2	2	2	2	2	2	2	2	2	2
2	2	1	2	2	2	2	2	1	2
1	2	2	2	2	2	2	2	1	1
2	2	2	2	2	2	2	2	2	2
1	1	1	3	3	3	1	1	1	2
2	2	2	2	2	2	2	2	2	2
2	2	2	2	2	2	2	2	2	2
2	2	2	2	2	2	2	2	2	2
1	2	1	2	2	1	2	2	1	1
1	1	2	2	3	3	3	3	3	3
9	9	9	9	9	9	9	9	9	9
2	2	2	3	3	3	3	2	2	2
2	2	2	2	2	2	2	2	2	2
1	3	1	2	2	1	1	1	1	2
2	2	2	2	2	2	2	2	2	2
3	3	2	3	3	2	3	2	3	2
9	9	9	9	9	9	9	9	1	9
9	9	9	9	9	9	9	9	9	9
3	3	3	3	3	3	3	3	3	3
2	2	2	2	2	2	2	2	2	2
9	9	9	9	9	9	9	9	9	9
3	3	1	2	2	2	2	2	1	1
1	3	3	3	3	1	1	3	1	3
2	1	2	2	2	2	2	2	2	2
1	1	1	1	1	1	1	1	1	1
1	3	3	3	3	3	3	3	3	3
2	2	2	2	2	2	2	2	2	2
2	1	2	1	2	1	3	2	3	1
2	2	2	2	2	2	2	2	2	2
9	9	1	9	9	9	9	9	9	9
2	2	2	2	2	2	2	2	2	2
2	2	2	2	2	2	2	2	2	2
3	3	3	3	3	3	3	1	1	1
1	3	1	3	2	2	2	2	2	2
2	2	2	2	2	2	2	2	2	2
3	3	3	3	3	3	3	3	3	3
2	2	2	2	2	2	2	2	2	2

2	2	2	2	2	2	2	2	2	2
2	2	2	2	2	2	2	2	2	2
2	2	2	2	2	2	2	2	2	2
2	2	2	2	2	2	2	2	2	2
3	3	3	2	2	2	2	2	2	2
1	3	2	2	2	1	2	3	1	3
2	2	2	2	2	2	2	2	2	2
3	2	2	1	2	1	3	2	1	2
2	2	2	2	2	2	2	2	3	2
1	2	2	2	2	1	2	2	2	2
3	3	3	1	3	3	1	1	1	2
9	9	9	9	9	9	9	9	9	9
9	9	9	9	1	9	9	9	9	9
2	2	2	2	2	2	2	2	2	2
2	2	2	2	2	2	2	2	2	2
2	2	2	3	3	3	3	3	2	2
2	3	3	2	3	2	3	3	2	3
1	1	9	3	3	1	2	2	1	1
1	2	3	2	1	2	3	2	2	1
3	1	2	2	3	2	2	2	3	3
3	3	3	3	3	3	1	1	1	3
2	2	2	2	2	2	2	2	2	2
2	2	2	2	2	2	2	2	2	2
2	2	3	3	3	3	3	3	1	1
3	3	3	1	1	1	1	1	3	3
9	9	9	9	9	9	9	9	1	1
2	2	2	2	2	2	2	2	2	2
1	2	1	2	2	1	1	2	1	1
2	2	2	2	2	2	9	9	9	9
2	2	3	3	3	2	3	3	1	2
9	1	2	2	1	1	1	1	1	1
3	2	1	1	1	2	2	3	3	1
3	3	3	3	3	3	3	1	1	2
1	2	2	2	1	1	1	1	1	1
3	3	3	3	3	3	3	3	3	3
1	1	1	1	1	3	3	1	1	1
9	9	9	9	9	9	9	9	9	9
1	1	1	1	1	1	1	1	1	1
2	2	2	2	2	2	2	2	2	2
2	2	2	2	2	2	2	2	2	2
2	1	3	3	3	1	2	3	1	2
1	2	2	2	2	1	1	2	1	1
2	3	2	2	3	3	2	2	1	2
2	1	2	2	2	2	2	2	2	2
2	2	2	2	2	2	2	2	2	2
2	1	3	2	1	3	2	3	2	3
3	2	2	3	2	3	2	2	2	2

1	2	9	1	3	2	3	2	3	2
3	3	3	3	3	3	3	3	3	3
2	3	1	2	2	3	2	2	3	3
1	9	9	9	9	9	9	9	9	9
2	9	9	9	9	9	9	9	9	9
2	2	3	3	3	2	2	3	2	3
2	2	2	2	2	2	2	2	2	2
3	3	3	3	3	3	3	3	3	3
3	1	2	2	2	2	2	2	2	2
2	2	2	2	2	2	2	2	2	2
9	9	9	9	9	9	9	9	9	9
2	1	2	2	3	1	3	2	3	2
2	2	2	2	2	2	2	2	2	2
2	2	2	2	2	2	1	9	1	2
2	2	2	2	2	2	2	2	2	2
3	3	3	3	9	2	2	2	2	2
3	2	2	1	2	3	2	2	1	2
9	9	9	9	9	9	9	9	9	9
9	9	2	9	9	9	9	9	9	9
2	2	2	2	2	2	1	2	2	1
2	2	2	2	2	2	2	2	2	2
2	2	2	2	2	2	2	2	2	2
9	9	9	9	9	9	9	9	9	9
9	9	9	9	9	9	9	9	9	9
2	3	1	2	3	3	1	2	3	2
3	3	3	1	3	3	3	3	3	3
2	3	3	3	3	2	3	2	3	3
1	3	3	3	2	1	2	1	1	1
3	3	3	3	3	3	3	3	3	3
2	2	3	3	3	1	2	2	1	2
1	1	1	2	3	1	1	1	1	1
2	3	3	3	3	3	3	1	2	2
1	3	2	3	2	1	2	9	2	3
3	3	3	3	3	3	3	3	3	3
1	1	1	1	1	1	1	1	1	1
1	2	3	3	2	1	2	3	1	1
1	1	1	1	1	1	1	1	3	3
3	3	3	3	3	3	3	3	3	3
9	1	9	9	1	9	9	1	9	9
3	3	3	3	3	3	3	3	3	3
3	3	3	3	3	1	1	1	3	1
1	1	3	3	1	1	3	1	1	1
3	3	3	3	3	1	1	1	3	3
2	2	2	2	2	2	2	2	2	2
1	1	2	2	2	1	1	1	1	2
1	1	1	2	1	1	1	1	3	3
1	2	2	2	2	1	1	2	1	2

2	2	2	2	2	2	2	2	2	2
2	1	2	2	2	2	2	2	2	2
1	1	3	1	1	9	9	1	1	1
3	3	1	3	2	1	1	1	2	1
3	3	3	3	3	3	3	3	3	3
2	2	2	2	2	2	2	2	2	2
2	2	2	2	2	2	2	2	2	2
9	9	9	9	9	9	9	9	9	9
9	9	9	9	9	9	9	9	9	9
3	1	2	2	2	2	2	2	2	2
9	9	9	9	9	9	9	9	9	9
1	3	3	3	3	1	2	2	2	3
3	3	1	3	3	9	1	1	1	1
1	3	3	1	3	2	2	3	2	2
2	2	1	2	2	3	2	2	2	1
2	2	2	2	2	2	2	2	2	2
1	3	1	3	3	1	1	1	1	1
2	2	2	2	2	2	2	2	2	2
3	3	2	3	2	2	2	2	3	2
9	1	9	9	9	9	9	9	9	9
9	9	9	9	9	9	9	9	9	9
1	1	1	1	1	3	3	3	3	3
1	1	3	3	3	3	1	1	1	3
2	2	2	2	2	1	1	1	1	1
3	3	3	3	3	3	3	3	3	3
2	2	2	2	2	2	2	2	2	2
1	2	1	3	9	1	2	1	1	1
2	3	3	3	2	2	1	1	1	2
3	2	1	1	1	1	1	3	2	2
1	2	1	3	3	1	2	2	1	3
2	2	2	2	2	2	2	2	2	2
1	1	1	3	1	1	1	1	3	1
2	1	2	2	3	1	2	2	2	1
3	2	1	2	2	2	1	3	2	1
2	1	2	2	2	2	2	1	2	2
2	2	2	2	2	2	2	2	2	2
2	2	2	2	2	2	2	2	2	2
2	2	2	2	2	2	2	2	2	2
2	2	2	3	3	3	9	2	1	1
2	3	1	2	3	1	2	3	1	2
2	3	2	1	3	2	1	2	1	2
2	2	2	2	2	2	2	2	2	2
1	1	1	1	1	1	1	1	1	1
9	9	9	9	9	9	9	9	9	9
2	2	2	2	2	2	2	2	2	2
3	3	1	3	2	2	2	2	2	2
1	3	1	2	3	2	1	1	1	1

3	3	2	2	3	1	1	2	1	2
3	3	3	2	2	2	2	3	1	2
3	3	1	2	3	1	1	3	3	2
2	3	1	3	3	1	1	1	1	2
2	2	2	2	2	2	2	2	2	2
2	2	2	2	2	2	2	3	2	2
3	3	3	2	2	2	2	2	2	2
2	2	2	2	2	2	2	9	1	2
1	1	1	1	1	1	1	1	1	1
2	2	2	3	2	2	2	1	1	2
2	2	2	2	2	3	2	2	2	1
3	3	2	2	2	3	2	2	2	1
9	9	9	9	9	9	9	9	9	9
1	9	9	9	9	9	9	9	9	9
2	3	2	3	3	3	2	2	2	1
3	3	3	3	3	2	2	2	2	3
9	9	9	9	9	9	9	9	9	9
2	2	2	2	2	2	2	1	1	3
2	1	3	1	1	3	2	2	3	1
2	2	2	2	2	2	2	2	2	2
2	1	3	3	3	2	1	1	2	1
1	9	2	1	2	3	2	3	3	2
2	2	2	2	2	2	2	2	2	1
1	3	2	3	1	1	1	1	2	2
2	2	2	2	2	2	2	2	2	2
3	3	2	2	3	2	1	2	2	1
3	3	3	3	3	3	3	3	3	3
1	2	2	2	2	9	2	2	2	9
3	3	2	2	3	2	1	2	2	1
3	3	3	3	2	2	2	2	3	2
2	2	2	2	2	2	2	2	2	2
9	9	9	9	9	9	9	9	9	9
3	1	1	3	3	1	1	3	2	3
2	2	2	2	2	2	2	2	2	2
1	2	3	1	3	3	2	2	2	1
1	2	2	2	2	1	1	1	2	1
1	2	2	1	2	1	1	1	2	1
9	9	9	9	9	9	9	9	9	9
2	2	2	2	2	2	2	2	2	2
2	2	2	2	2	2	2	2	2	2
1	3	3	3	3	1	3	2	1	1
3	2	2	2	3	1	2	3	2	3
2	2	1	2	2	1	1	1	1	1
2	2	2	2	2	2	2	2	2	2
1	1	9	1	1	3	9	2	2	1
2	2	2	2	2	2	2	2	2	2
9	9	9	9	9	9	1	9	9	9

2	2	2	2	2	2	2	2	2	2
2	2	2	2	2	2	1	1	1	2
9	9	9	9	9	1	9	9	9	9
9	9	9	9	9	9	9	9	9	9
2	2	2	2	1	1	1	1	1	1
9	9	9	9	9	9	9	9	9	1
2	2	2	3	3	3	1	1	1	2
1	1	1	2	3	2	1	1	3	2
2	2	2	3	2	2	1	3	1	2
2	3	3	2	2	1	1	2	2	3
2	2	9	1	2	2	1	1	2	2
1	2	1	2	1	2	2	1	2	1
2	2	1	2	2	1	1	1	1	1
1	2	2	2	2	1	1	2	2	2
1	2	1	3	2	1	2	1	1	1
3	3	3	3	3	3	3	3	3	3
1	3	1	3	3	3	3	2	2	2
1	3	2	3	1	1	1	1	1	2
3	3	1	2	2	3	1	1	1	1
1	3	3	3	1	1	1	3	2	3
1	2	2	3	3	2	3	2	2	2
2	2	2	1	2	1	1	1	2	2
3	3	3	3	3	3	2	1	1	1
2	2	2	2	2	2	2	2	2	2
2	2	2	2	2	2	1	2	1	2
1	2	2	2	2	2	2	2	1	1
2	2	2	2	2	2	2	2	2	2
2	2	2	2	2	2	2	2	2	2
1	1	1	3	1	2	3	2	2	2
9	9	9	9	9	9	9	9	9	9
9	9	9	9	9	9	9	9	9	9
1	2	1	2	2	2	2	2	2	2
9	9	9	9	9	9	9	9	9	9
1	2	3	2	1	3	1	3	1	2
2	2	1	2	1	2	3	2	1	2
9	9	9	9	9	9	9	9	9	9
9	9	9	9	9	9	9	9	9	9
2	2	2	2	2	2	2	2	2	2
2	2	2	9	9	9	9	9	9	3
1	2	2	2	1	1	1	3	2	2
1	1	1	1	1	1	1	1	1	1
1	3	1	3	1	2	1	2	1	1
1	1	1	2	1	2	1	1	1	1
2	1	2	2	3	2	1	2	2	3
1	2	3	1	2	2	3	1	2	1
9	9	9	9	9	9	9	9	9	9
1	3	2	1	3	2	2	1	3	2

2	1	2	2	1	2	1	1	2	2
9	9	9	9	9	9	9	9	9	9
9	9	9	9	9	9	9	9	9	9
2	2	2	2	2	2	2	2	2	2
2	3	1	3	2	1	1	1	1	1
2	1	2	2	1	2	1	1	2	2
1	2	2	2	2	1	1	1	1	3
1	2	2	2	2	2	2	2	2	1
2	2	2	2	2	2	2	2	2	2
1	3	9	3	3	1	1	3	1	1
9	9	9	9	9	9	9	9	9	9
9	9	9	9	9	9	9	9	9	9
1	9	9	9	9	9	9	9	9	9
2	2	2	2	2	1	1	3	2	2
1	2	1	2	2	2	1	2	3	3
2	2	2	2	2	9	9	9	9	9
9	9	9	9	9	9	9	9	9	9
9	9	9	9	9	1	9	9	9	9
2	2	9	3	2	1	1	1	1	1
1	1	9	3	3	3	3	2	1	1
1	1	2	1	1	1	1	2	1	2
2	2	3	3	2	2	1	1	1	2
3	3	3	3	3	3	3	3	3	3
2	2	2	2	2	2	2	2	2	2
2	2	2	2	2	2	2	2	2	2
2	2	3	3	2	1	1	2	2	2
9	9	9	9	9	9	9	9	9	9
3	3	2	3	2	2	1	2	1	1
1	2	3	1	2	3	2	1	2	1
2	2	2	2	2	2	2	2	2	2
1	3	3	3	1	1	1	3	1	1
2	2	1	2	2	1	1	1	1	2
9	9	9	9	9	9	9	9	9	9
3	1	1	1	3	2	1	2	1	3
2	2	2	2	2	2	2	2	2	2
2	2	2	2	2	2	2	2	2	2
1	2	2	2	2	1	1	2	2	1
2	2	2	2	2	2	2	2	2	2
2	1	2	1	1	1	1	1	1	2
1	2	3	1	2	1	2	2	1	2
3	2	2	3	1	1	3	2	1	3
3	3	3	3	3	3	3	3	3	3
9	9	9	9	9	9	9	9	9	9
9	9	9	9	9	1	9	9	9	9
2	9	2	2	2	1	2	2	2	2
1	3	1	3	9	9	9	9	9	9
9	9	9	9	9	9	9	9	9	9

2	2	2	2	2	2	2	2	2	2
2	3	3	2	2	2	2	1	2	1
9	3	1	3	3	2	1	2	1	1
1	3	1	2	2	2	2	2	2	1
1	1	1	1	3	1	1	1	3	1
3	3	3	3	3	3	3	9	9	9
9	9	9	9	9	9	9	9	9	9
3	3	3	3	3	1	3	1	1	3
9	9	9	9	9	9	9	9	9	9
9	9	9	9	9	9	9	9	9	9
9	9	9	9	9	9	9	9	9	9
9	9	9	9	9	9	9	9	9	9
9	9	9	9	9	9	9	9	9	9
3	1	3	3	1	3	3	3	3	3
1	2	3	3	1	1	1	1	1	1
2	2	2	2	2	2	1	2	1	1
1	1	1	1	1	1	1	2	2	2
1	3	9	9	9	1	9	9	9	9
9	9	9	9	9	9	9	9	9	9
9	9	9	1	9	9	9	9	9	9
1	1	9	1	9	3	2	9	1	3
9	9	9	9	9	9	9	9	9	9
1	1	1	1	1	1	1	1	1	1
9	9	9	9	9	9	9	9	9	9
1	1	1	1	2	1	1	2	2	2
2	2	1	1	1	1	1	2	2	2
3	2	1	2	2	3	3	3	2	3
1	1	1	1	1	1	2	2	2	2
1	1	1	1	1	1	1	1	1	1
2	2	1	1	1	1	2	2	2	2
1	1	1	1	1	1	1	1	1	2
2	1	1	1	1	2	2	1	1	1
1	1	2	2	2	2	1	1	1	1
9	9	9	9	9	9	9	9	9	9
2	2	2	2	1	1	1	1	1	1
1	1	1	2	2	2	1	1	1	1
3	3	1	2	2	2	3	3	3	3
9	9	9	9	9	9	9	9	9	9
1	1	1	2	2	1	1	2	1	3
3	3	3	3	3	3	1	1	3	3
3	2	2	2	2	2	2	2	2	2
2	3	1	3	2	1	2	1	1	9
1	1	3	9	9	9	9	9	9	9
9	9	9	9	9	9	9	9	9	9
1	1	1	1	3	1	3	3	1	1
1	2	9	1	3	2	9	3	3	3
2	3	2	3	2	3	1	1	1	2

9	9	9	9	9	9	9	9	9	9
9	9	9	9	9	9	9	9	9	9
9	9	9	9	9	9	9	9	9	9
9	9	9	9	9	9	9	9	9	9
2	3	2	3	2	1	1	3	1	2
9	9	9	9	9	9	9	9	9	9
2	2	2	2	2	2	2	2	2	2
1	1	1	2	1	9	9	9	9	9
9	9	9	9	9	9	9	9	9	9
9	9	9	9	9	9	9	9	9	9
9	9	9	9	9	9	9	9	9	9
9	9	9	9	9	9	9	9	9	9
2	2	2	2	2	1	1	1	1	1
2	2	1	3	1	3	2	2	3	1
3	3	3	3	3	1	1	9	2	2
1	1	1	3	2	1	1	2	1	3
1	1	1	1	1	2	2	2	1	1
2	2	1	2	2	1	1	1	1	2
1	1	2	2	2	2	1	1	1	1
9	9	9	9	9	9	9	9	9	9
1	1	1	1	2	2	2	2	2	2
1	2	1	3	3	1	1	3	3	3
2	2	2	2	2	2	2	2	2	2
3	9	1	9	2	1	3	1	1	2
1	2	2	2	1	1	2	1	1	1
1	1	3	3	3	3	3	3	3	3
2	9	9	1	9	9	9	2	9	9
1	1	1	3	3	3	2	2	2	2
9	9	9	9	9	9	9	9	9	9
9	9	9	9	9	9	9	9	9	9
3	3	3	3	1	1	3	1	1	9
3	3	3	3	3	3	3	3	3	3
3	3	3	3	3	3	3	3	3	3
1	1	1	1	1	2	2	1	2	2
2	2	1	1	1	1	2	2	1	1
2	2	2	2	2	1	1	2	2	2
2	2	2	2	2	2	2	2	2	2
1	2	2	2	2	1	1	1	1	1
1	2	2	2	1	1	1	1	1	2
2	2	2	2	2	2	2	2	2	2
1	3	3	3	3	1	1	1	1	1
2	2	2	2	2	2	2	2	2	2
3	3	3	3	3	3	3	1	1	3
2	2	1	2	2	1	2	2	1	1
2	3	1	2	2	3	2	1	1	2
3	3	3	3	3	3	3	3	3	3
2	2	2	2	2	2	2	2	1	2

9	9	9	9	9	9	9	9	9	9
1	1	1	2	2	3	3	2	2	2
9	2	9	9	9	9	9	9	9	9
9	9	9	9	9	9	9	9	9	9
9	9	9	9	9	9	9	9	9	9
1	2	2	1	1	1	1	2	1	1
9	9	9	9	9	9	9	9	9	9
2	3	1	2	3	2	2	2	1	3
9	9	9	9	9	9	9	9	9	9
2	2	3	2	2	2	2	2	2	2
9	9	9	9	9	9	9	9	9	9
2	2	2	2	2	2	2	2	2	2
1	1	1	2	2	2	1	1	1	1
2	2	2	2	2	2	2	2	2	2
1	1	2	2	1	1	2	2	1	1
9	9	9	9	9	9	9	9	9	9
2	2	2	2	2	2	2	2	2	2
3	3	3	3	3	3	3	3	3	3
2	3	1	9	9	9	9	9	9	9
3	3	3	3	3	3	3	3	3	3
2	2	2	2	2	2	2	2	2	2
3	3	1	1	2	2	2	2	2	1
2	2	2	2	2	2	2	2	2	2
2	2	2	2	2	2	2	2	2	2
1	1	1	1	1	1	1	1	1	1
2	2	2	2	2	2	2	2	2	2
1	3	3	1	3	1	3	2	2	3
3	3	1	2	3	1	3	3	3	3
1	2	2	2	2	2	1	1	1	1
2	1	1	1	1	1	2	2	2	2
9	9	9	9	9	9	9	9	9	9
2	3	1	2	2	3	1	2	1	3
1	2	3	2	1	2	2	3	1	2
2	2	2	2	2	2	2	2	2	2
3	3	2	2	2	2	2	2	2	2
2	2	2	1	1	1	2	1	1	1
1	3	3	3	3	3	1	1	1	1
1	2	2	2	2	2	1	1	1	2
2	1	1	1	1	1	1	2	2	2
2	2	2	2	2	1	1	2	2	1
2	2	2	2	2	2	2	2	2	2
1	1	1	1	1	1	1	1	1	1
9	9	9	9	9	9	9	9	9	9
9	9	9	9	9	9	9	9	9	9
9	9	9	9	9	9	9	9	9	9
2	9	1	2	9	2	2	2	1	9
2	2	2	2	2	2	2	2	1	1

1	3	3	3	1	1	1	1	1	1
2	2	2	2	2	2	2	1	2	2
9	9	9	9	9	9	9	9	9	9
2	3	1	2	3	1	2	1	3	2
9	9	9	9	9	9	9	9	9	9
2	2	2	2	2	3	2	3	2	3
1	9	9	9	9	9	9	9	9	9
9	9	9	9	9	9	9	9	9	9
2	2	2	2	2	1	2	2	2	2
3	3	1	1	1	1	1	1	1	2
9	9	9	9	9	9	9	9	9	9
9	9	9	9	9	9	9	9	9	9
1	2	1	1	2	2	2	2	1	2
2	3	1	3	3	2	2	2	1	9
9	9	9	9	9	9	9	9	9	9
1	1	1	1	1	1	3	1	2	9
1	2	3	2	2	1	3	1	2	3
1	3	1	3	2	2	2	3	1	1
2	2	2	2	2	2	2	2	2	3
1	2	3	3	3	2	2	1	1	1
3	3	2	2	1	1	2	2	1	3
1	3	1	3	1	1	1	3	1	1
2	3	3	3	3	1	3	1	1	2
2	2	2	3	3	2	1	1	1	2
2	2	2	2	2	2	2	2	2	2
2	2	9	1	1	1	3	2	2	2
2	2	2	3	3	2	1	1	1	3
1	3	2	2	3	2	1	1	2	2
3	3	9	9	3	2	3	2	2	3
2	2	2	1	2	2	2	1	2	1
3	3	9	1	3	3	1	2	3	1
3	3	3	2	3	2	2	2	3	1
2	9	9	9	9	9	9	9	9	9
3	3	1	3	9	3	3	2	2	3
1	3	2	3	2	3	1	2	1	1
2	2	2	2	2	2	2	2	2	2
3	3	1	3	3	3	2	2	2	2
2	3	3	3	3	1	2	2	1	3
9	9	9	9	9	9	9	9	9	9
2	2	3	2	3	2	3	2	9	2
9	9	9	9	2	9	9	9	9	9
9	9	9	9	9	9	9	9	9	9
2	2	2	2	2	2	3	2	2	2
2	2	1	2	2	1	9	9	9	9
3	3	1	3	3	2	2	3	3	1
1	3	1	1	1	3	1	3	1	1
3	3	3	3	1	1	3	3	9	1

3	3	3	3	3	3	3	3	3	3
9	9	9	9	9	9	9	9	9	9
1	3	3	3	3	1	3	3	3	1
1	3	1	3	3	1	3	1	1	1
3	3	1	3	3	3	3	3	1	3
1	1	3	1	3	3	1	2	2	2
1	3	9	1	2	2	1	3	1	3
2	2	2	2	2	2	2	2	2	2
3	1	1	3	3	3	1	1	3	2
1	3	1	3	2	3	1	2	3	2
3	1	3	1	1	3	1	1	1	2
3	2	3	1	2	3	2	1	3	2
2	2	3	3	3	2	2	3	2	3
3	3	3	2	1	2	1	2	3	1
2	2	2	2	1	1	1	3	1	1
1	3	3	3	3	2	1	1	1	2
1	3	3	2	3	1	1	2	2	3
3	3	2	1	3	2	1	1	1	1
3	3	1	3	3	3	3	1	1	1
2	2	2	2	2	2	2	2	2	2
1	2	1	1	2	2	2	2	2	2
2	2	2	2	2	2	3	3	3	3
3	3	3	3	3	3	3	3	3	3
9	9	9	9	9	9	9	9	9	9
3	3	2	2	2	1	1	1	1	1
2	1	2	2	1	2	1	1	1	2
2	1	3	2	1	3	2	1	3	2
3	3	2	3	3	2	2	3	3	3
1	1	1	3	3	1	1	1	1	1
9	9	9	9	9	9	9	9	9	9
2	3	2	2	2	2	2	2	2	1
1	2	3	1	2	3	2	3	1	2
3	3	3	9	9	1	2	3	1	2
2	3	3	2	3	2	2	2	1	1
3	1	1	1	9	2	9	1	3	3
2	2	2	2	2	2	2	2	2	2
2	2	2	2	9	3	3	3	3	1
9	9	9	9	9	9	9	9	9	9
3	2	2	2	1	2	3	2	2	2
2	3	3	1	2	3	1	2	3	2
9	9	9	9	9	9	9	9	9	9
9	9	9	9	9	9	9	9	9	9
2	3	1	2	1	2	2	3	2	1
3	3	1	3	3	3	1	3	3	3
2	2	2	2	2	2	2	2	2	2
1	9	3	3	3	2	2	2	2	3
9	9	9	9	9	9	9	9	9	9

2	3	2	1	2	2	3	3	3	2
9	9	9	9	9	9	9	9	9	9
3	3	2	3	3	2	2	2	3	1
9	9	9	9	9	9	9	9	9	9
9	9	9	9	9	9	9	9	9	9
3	3	3	3	3	3	3	3	3	3
2	2	2	2	2	2	2	2	2	2
2	2	2	2	2	2	2	2	2	2
3	3	3	3	1	1	1	1	1	1
3	3	3	3	3	2	3	2	3	3
9	9	9	9	9	9	9	9	9	9
9	9	9	9	9	9	9	9	9	9
9	9	9	9	9	9	9	9	9	9
1	2	2	2	2	1	1	2	2	2
2	2	2	2	2	2	2	2	2	2
2	2	2	2	2	2	2	2	2	2
9	9	9	9	9	9	9	9	9	9
9	9	9	9	9	9	9	9	9	9
3	3	3	3	3	3	3	3	3	3
2	2	2	2	2	2	2	2	2	2
3	3	3	3	3	3	3	3	3	3
2	2	2	2	2	2	2	2	2	2
9	9	9	9	9	9	9	9	9	9
1	2	2	1	3	2	1	2	3	1
3	1	2	3	2	2	1	1	2	2
2	1	2	3	3	3	2	2	3	2
9	9	9	9	9	9	9	9	9	9
2	2	2	2	2	2	2	2	2	2
2	2	2	2	2	2	2	2	2	2
9	9	9	9	9	9	9	1	9	9
1	3	3	3	2	2	3	1	1	9
2	2	2	2	2	2	2	2	2	2
3	1	3	1	3	1	3	1	3	1
1	3	2	2	3	1	2	2	2	1
1	3	3	3	3	3	1	1	1	1
3	2	2	1	2	2	2	1	3	2
3	3	3	3	3	3	3	1	1	1
1	3	3	3	3	3	3	3	1	3
9	9	9	9	9	9	9	9	9	9
2	2	2	2	2	2	2	2	2	2
2	3	1	2	2	3	2	3	1	3
2	2	1	2	2	3	3	1	2	2
3	3	3	3	3	3	3	3	3	2
3	3	3	3	3	2	2	2	1	1
2	2	1	2	2	2	2	1	3	1
2	2	2	3	2	3	2	1	2	2
9	9	9	9	9	9	9	9	9	9

2	2	2	2	2	2	2	2	3	3
2	3	2	2	2	2	2	2	2	2
1	2	1	2	2	1	1	1	1	2
3	3	9	2	2	2	2	2	2	2
3	3	3	3	2	3	9	2	1	2
9	9	9	9	9	9	9	9	9	9
2	2	2	2	2	2	2	2	2	2
2	2	1	2	9	2	2	2	2	2
2	2	2	3	1	2	1	2	1	2
2	2	2	2	2	2	2	2	2	2
2	2	3	2	2	2	2	2	2	2
3	1	3	9	3	3	2	2	1	2
2	2	2	2	2	2	2	2	2	2
3	3	3	3	3	3	3	3	3	3
3	3	2	2	3	3	3	3	2	2
3	2	1	2	3	2	2	1	3	2
3	3	2	2	3	3	3	3	3	3
1	2	3	2	3	2	3	3	2	1
2	2	2	2	2	2	2	2	2	2
3	3	3	2	2	2	2	2	2	2
3	3	3	3	3	1	1	2	1	1
2	3	3	3	2	2	3	2	2	1
2	3	3	3	2	2	3	2	2	1
1	2	2	2	1	1	1	1	1	3
1	2	2	2	2	2	2	2	1	1
3	2	3	3	2	2	2	2	2	3
2	2	2	2	1	9	1	2	2	2
2	2	2	2	1	9	1	2	2	2
2	2	2	2	2	2	2	2	2	2
1	2	2	2	2	1	1	1	1	1
2	2	2	2	3	2	3	2	3	2
3	3	2	9	3	2	2	3	2	3
3	3	3	3	3	3	1	1	1	1
2	3	1	2	2	2	2	3	3	3
1	3	3	2	2	2	3	3	2	2
2	2	2	2	2	2	2	2	2	2
2	2	2	2	2	2	2	2	2	2
2	2	2	2	3	1	1	2	3	2
2	2	2	2	2	2	2	2	2	2
2	2	2	2	2	2	2	2	2	2
2	2	3	3	2	1	1	2	1	1
1	3	3	3	3	2	2	3	1	1
2	2	2	2	2	2	2	2	2	2
2	2	2	1	2	2	2	2	2	2
2	3	2	2	2	2	3	2	1	2
1	3	1	1	3	2	1	1	1	1
2	3	1	2	2	2	1	1	1	3

3	2	3	2	2	1	2	3	2	1
2	2	2	2	2	2	2	2	2	2
2	3	2	2	2	2	2	2	1	2
1	2	2	2	2	2	2	2	2	2
2	2	2	2	2	2	2	2	2	2
2	2	2	2	2	2	2	2	2	2
2	3	1	2	2	1	1	1	1	2
1	2	2	3	2	2	1	2	1	1
2	2	2	2	2	2	2	2	2	2
2	3	3	3	2	1	2	2	2	2
2	3	3	3	2	1	2	2	2	2
2	2	2	2	2	2	2	2	2	2
2	2	2	2	2	2	2	2	2	2
9	9	9	9	9	9	9	9	9	9
2	2	2	2	2	2	2	2	2	2
2	1	2	2	2	1	2	2	1	2
1	2	2	3	2	2	1	2	2	2
2	2	2	2	2	1	1	2	2	2
1	1	1	3	3	1	1	1	1	1
1	2	1	3	2	1	2	1	2	1
2	2	2	2	2	2	2	2	2	2
2	2	2	2	2	2	2	2	2	2
2	2	2	3	2	3	2	3	2	2
2	2	2	2	2	2	2	2	2	2
2	2	1	2	2	1	2	2	2	1
1	3	2	3	3	9	2	2	2	2
3	3	3	3	2	2	2	2	2	2
2	2	2	2	2	2	2	2	2	2
2	2	2	2	2	2	2	2	2	2
1	2	2	2	2	2	2	1	1	1
3	3	3	3	3	3	3	3	3	3
2	2	2	2	2	2	2	2	2	2
2	2	2	2	2	2	2	2	2	2
2	2	2	2	2	2	2	2	2	2
2	2	2	2	2	2	2	2	2	2
2	2	2	2	2	2	2	2	2	2
2	2	2	2	2	2	2	2	2	2
2	2	2	2	2	2	2	2	2	2
9	9	9	9	9	9	9	9	9	9
2	1	2	2	2	1	2	2	2	2
2	2	1	2	2	1	1	1	1	2
2	2	1	2	2	2	2	2	2	2
2	2	2	2	2	2	2	2	2	2
2	2	2	2	2	2	2	2	2	2
2	2	2	2	2	2	2	2	2	2
2	2	2	2	2	2	2	2	2	2
2	2	2	2	2	2	2	2	2	2

2	2	2	2	2	2	2	2	2	2
2	2	2	2	2	2	2	2	2	2
1	1	2	3	3	1	1	3	1	1
2	2	2	2	2	2	2	2	2	2
2	2	2	2	2	2	2	2	2	2
2	2	1	2	2	1	2	2	2	2
9	9	9	9	9	9	9	9	9	9
1	3	3	3	3	1	2	1	1	3
1	3	3	3	3	3	1	1	1	1
2	3	3	3	1	2	2	1	1	1
1	2	1	2	2	1	1	1	1	1
3	3	2	3	3	3	2	2	2	2
2	2	2	3	2	1	1	2	1	2
2	2	2	2	2	2	2	2	2	2
1	3	1	3	1	2	2	2	1	1
2	2	2	2	2	2	2	2	2	2
2	2	2	2	2	2	2	2	2	2
1	2	2	2	1	1	1	1	1	2
1	1	1	2	2	1	1	1	1	1
2	2	2	3	1	2	2	2	2	2
1	3	1	3	1	1	1	1	1	1
1	2	2	2	2	3	1	2	2	2
2	2	2	2	2	2	2	2	2	2
2	2	2	2	2	2	2	2	2	2
2	2	2	2	2	2	2	2	2	2
2	2	2	2	2	2	2	2	2	2
9	9	9	9	9	9	9	9	9	9
2	2	2	2	2	2	2	2	2	2
2	2	2	2	2	2	2	2	2	2
2	2	2	2	2	2	2	2	2	2
2	2	2	2	2	2	2	2	2	2
2	2	2	2	2	2	2	2	2	3
9	9	9	9	9	9	9	9	9	9
1	2	1	1	2	3	3	3	3	3
9	9	9	9	9	9	9	9	9	9
1	1	3	3	2	1	1	1	1	1
2	3	3	3	3	3	3	2	2	2
1	2	1	1	2	1	2	1	1	1
2	2	2	2	2	2	2	2	2	2
3	3	3	3	3	3	3	3	3	3
2	2	2	2	1	1	1	1	1	2
2	2	2	1	2	2	2	2	2	2
1	2	1	2	3	1	1	2	1	2
2	2	2	2	2	2	2	2	2	2
2	1	1	1	2	1	2	1	1	1
2	2	2	2	2	2	2	2	2	2
1	1	1	1	2	1	2	1	2	1

2	2	1	2	3	2	2	3	2	1
1	2	1	1	2	3	1	1	2	1
2	2	3	1	9	2	3	3	3	2
3	2	3	3	2	2	1	2	1	2
2	2	3	2	3	2	2	2	2	2
1	2	3	1	2	3	2	2	2	2
2	2	2	1	1	1	1	1	3	2
9	9	9	9	9	9	9	9	9	9
2	2	2	2	2	2	2	2	2	2
2	2	2	2	2	2	2	2	2	2
2	2	2	2	2	2	9	1	2	2
2	2	2	2	2	2	2	1	2	2
2	2	2	9	9	9	9	9	9	9
2	2	2	2	2	2	2	2	2	2
2	2	2	2	2	2	2	2	2	2
2	2	2	2	2	2	2	2	2	2
2	2	1	2	2	1	1	2	1	1
2	3	2	1	2	1	3	3	3	2
2	2	2	2	2	2	2	2	2	2
2	3	1	3	9	2	2	2	2	1
9	9	9	9	9	9	9	9	9	9
9	9	9	9	9	9	9	9	9	9
2	3	3	3	3	3	2	3	1	2
9	9	9	9	9	9	9	9	9	9
2	2	1	3	1	3	1	2	1	2
1	3	1	2	2	1	1	3	1	1
1	1	1	1	1	1	1	1	1	1
2	2	2	2	2	2	2	1	1	1
2	2	2	2	2	2	2	2	2	2
9	9	9	9	9	9	9	9	9	9
2	3	3	3	3	2	2	3	3	3
9	9	9	9	9	9	9	9	9	9
9	9	9	9	9	9	9	9	9	9
2	2	2	2	2	2	2	2	2	2
2	2	2	2	2	2	2	2	2	2
2	2	2	2	2	2	2	2	2	2
2	2	9	1	9	2	3	2	2	3
2	2	2	2	2	2	2	2	2	2
2	2	1	2	2	2	2	2	2	2
2	2	1	2	2	2	2	2	2	2
9	9	9	9	9	9	9	9	9	9
2	2	2	2	2	2	2	2	2	2
2	2	2	2	2	2	2	2	2	2
9	9	9	9	9	9	9	9	9	9
9	9	9	9	9	3	9	3	9	2
2	1	1	1	1	2	1	1	1	2
1	2	2	1	1	1	1	1	2	2

9	9	9	9	9	9	9	9	9	9
1	2	2	3	2	2	1	2	1	1
3	3	3	3	3	3	3	3	1	3
2	2	2	2	2	2	2	2	2	2
9	9	9	9	9	9	9	9	9	9
3	3	3	3	3	3	3	3	3	3
2	2	2	2	2	2	2	2	2	2
3	9	2	3	2	2	2	2	3	3
9	9	9	9	9	9	9	9	9	9
2	2	2	2	2	2	2	2	2	2
3	3	3	3	3	1	1	1	1	1
2	2	2	2	2	2	2	2	2	2
2	2	2	2	2	2	1	2	3	3
3	3	3	3	3	3	3	3	3	3
9	9	9	9	9	9	9	9	9	9
2	3	3	3	3	1	2	1	1	3
2	2	2	2	2	1	1	1	1	1
2	3	2	1	3	1	2	1	3	2
9	1	9	9	9	9	9	9	9	9
3	2	1	3	3	1	1	3	2	2
1	3	1	2	1	3	1	2	1	3
2	2	2	2	2	2	2	2	2	2
9	9	9	9	9	9	9	9	9	9
2	2	2	2	2	2	2	2	2	2
1	1	1	1	1	1	1	3	3	1
2	2	2	2	2	2	2	2	2	2
2	2	2	3	2	2	3	1	1	2
1	3	3	3	3	1	1	1	1	1
2	3	2	1	3	1	2	2	2	3
9	9	9	9	9	9	9	9	9	9
2	2	2	2	2	2	2	2	2	2
1	2	2	1	2	2	2	2	1	2
3	3	3	3	3	3	1	3	1	1
3	3	3	3	3	3	3	3	3	3
1	1	1	1	1	2	1	1	1	1
2	3	2	2	1	1	1	1	2	2
3	3	1	9	3	3	3	3	3	3
2	2	2	2	2	2	2	2	2	9
2	1	2	3	1	1	2	2	1	1
9	9	9	9	9	9	9	9	9	9
9	9	9	9	9	9	9	9	9	9
1	3	1	2	3	1	3	1	2	3
2	3	3	3	2	2	1	1	1	2
2	2	3	2	1	2	1	2	1	2
2	3	2	1	3	1	2	2	2	1
2	2	2	1	1	2	2	2	1	3
2	2	2	3	2	2	3	3	2	2

1	3	3	3	1	1	3	1	1	1
2	1	9	3	2	2	1	3	1	2
2	3	2	2	2	2	2	2	1	1
2	2	2	2	3	3	3	3	3	3
1	3	1	3	3	1	1	3	1	1
2	2	2	2	2	2	2	2	2	2
2	2	2	2	2	2	2	2	2	2
3	2	3	3	3	3	2	2	2	2
1	3	1	3	3	1	1	1	1	1
2	2	2	2	2	2	2	2	2	2
2	1	2	9	1	9	1	2	1	2
2	2	2	1	2	2	1	1	2	1
2	2	2	2	3	2	2	3	2	2
1	3	1	3	3	1	1	1	1	1
9	9	9	9	9	9	9	9	9	9
2	2	2	2	2	2	2	2	2	2
2	3	3	2	3	2	3	3	2	2
3	2	2	2	2	2	2	2	2	2
1	3	3	3	2	2	1	1	3	1
3	2	1	3	1	1	1	2	1	2
2	3	1	3	3	1	2	1	1	2
9	9	9	9	9	9	9	9	9	1
2	2	9	2	2	1	2	2	3	3
9	9	9	9	9	9	9	9	9	9
2	2	2	2	2	2	2	2	2	2
2	2	2	2	9	3	3	3	9	2
2	1	2	3	2	1	2	3	2	2
2	2	2	2	2	2	2	2	2	2
3	3	3	3	3	3	3	3	3	3
2	2	1	2	2	2	2	2	1	1
3	3	3	3	3	3	3	3	1	3
2	3	1	2	3	3	3	2	1	1
3	3	2	3	2	3	3	2	3	2
3	3	3	3	3	2	1	1	2	1
1	2	2	3	3	1	2	1	2	1
2	2	2	2	2	2	2	2	2	2
3	3	3	3	3	1	1	1	1	1
3	3	3	3	3	2	2	2	2	1
2	2	2	2	2	2	2	2	2	2
9	9	9	9	9	9	9	9	9	9
2	2	3	2	2	2	3	3	3	2
1	1	2	3	1	2	1	3	1	2
3	3	3	3	2	3	2	3	2	3
1	3	2	3	2	1	1	1	1	2
3	3	3	3	2	3	3	1	1	3
9	9	9	9	9	9	9	9	9	9
2	2	2	3	3	2	2	3	2	3

2	1	2	1	1	2	1	2	1	2
3	2	2	2	3	2	2	2	2	2
3	3	3	3	3	3	1	1	1	3
1	3	2	2	3	1	2	3	1	2
1	2	2	2	2	2	2	2	1	2
2	1	9	2	1	1	2	2	2	1
2	3	3	3	9	2	2	2	1	3
9	9	9	9	9	9	9	9	9	9
9	9	9	9	9	9	9	9	9	9
3	2	9	1	2	1	2	1	2	1
2	2	2	3	3	2	2	2	2	2
2	3	2	2	2	2	2	2	3	1
2	3	3	3	3	2	1	1	3	2
3	3	1	3	3	3	1	3	1	1
2	9	9	9	9	9	9	9	9	9
3	3	1	2	2	3	3	2	2	1
1	2	1	1	1	2	2	2	2	2
3	2	3	3	3	3	3	2	2	3
3	3	1	3	3	1	1	3	1	3
3	1	3	3	1	2	2	2	3	2
2	3	2	3	2	1	1	1	1	2
1	3	3	3	3	1	1	1	2	1
2	1	2	1	1	1	2	2	1	1
1	3	3	3	3	1	2	3	3	3
2	3	1	2	3	1	1	1	2	1
2	2	2	2	2	1	2	1	1	1
2	3	1	2	3	2	2	3	2	3
1	1	1	2	2	1	1	1	2	1
2	2	2	2	2	2	2	2	2	2
2	2	2	2	2	2	2	2	1	1
2	1	1	1	2	2	2	1	2	2
2	2	2	2	2	2	2	2	2	2
2	2	2	2	2	2	2	2	2	2
1	1	2	2	2	2	1	2	2	2
1	1	1	2	2	2	3	3	3	1
1	3	1	3	2	1	1	2	1	2
2	1	2	1	1	1	2	2	2	1
3	3	3	3	3	3	3	3	3	9
1	1	1	1	2	1	1	2	1	2
2	2	1	1	1	1	1	1	2	2
2	3	3	3	3	2	2	2	2	2
1	1	1	1	2	2	3	1	1	1
1	2	3	3	3	1	3	3	3	2
2	2	3	3	2	2	2	2	2	2
3	3	3	2	2	1	1	2	2	2
2	2	2	2	2	2	2	1	1	2
1	2	2	1	1	1	3	2	2	1

3	3	2	3	3	1	1	2	1	3
2	1	2	2	2	1	1	2	2	2
2	2	2	2	2	3	2	1	2	3
1	1	1	1	2	2	2	1	1	1
3	3	3	3	1	2	2	1	1	3
1	1	2	3	3	2	2	1	1	1
2	3	2	2	1	1	1	1	2	1
1	1	2	2	1	2	2	1	2	2
1	2	2	2	2	2	2	1	2	1
1	1	2	2	2	2	2	2	1	2
2	2	2	2	2	2	2	2	2	2
2	2	2	2	2	2	2	2	2	2
1	1	1	1	1	1	1	1	1	1
3	3	3	3	3	3	3	3	3	3
2	2	2	2	2	2	2	2	1	1
2	1	2	2	2	2	2	2	2	1
2	2	2	9	9	9	9	9	1	1
2	2	2	9	1	1	2	2	2	2
2	2	2	2	2	2	2	2	2	2
2	2	1	1	1	2	3	2	3	1
2	2	2	2	2	2	2	2	2	2
1	2	2	2	2	2	2	1	2	1
3	3	3	3	3	3	3	3	3	3
3	3	3	2	9	2	3	2	3	3
2	9	1	2	1	2	1	1	2	1
2	2	2	2	2	2	2	2	2	2
2	2	3	1	1	1	1	2	3	2
2	3	3	3	3	2	2	2	2	3
2	2	2	2	2	2	2	2	2	1
2	2	2	2	2	2	2	1	1	2
2	2	3	2	2	2	2	1	1	3
1	2	3	3	2	1	1	3	2	2
2	2	1	1	1	1	1	2	2	2
2	2	2	2	3	3	3	3	2	2
3	3	3	3	3	2	2	3	1	2
2	3	3	1	3	2	2	2	2	2
1	2	2	3	3	1	1	1	1	2
2	2	2	3	3	2	2	2	2	3
1	1	1	1	1	1	1	3	3	1
1	2	1	2	1	1	1	1	1	1
2	2	2	2	2	2	2	2	2	2
2	2	2	2	2	2	2	2	2	2
2	2	2	2	2	2	2	2	2	2
3	3	3	3	3	3	3	3	3	3
3	3	3	3	3	3	3	3	3	3
2	2	1	1	2	2	2	2	3	3
1	1	1	1	1	1	1	1	1	1

1	1	2	2	2	2	3	3	2	1
1	1	2	2	2	1	1	2	2	1
2	2	2	1	1	1	2	2	1	1
3	3	2	2	1	1	2	3	2	2
2	1	1	1	2	1	1	2	2	1
1	1	1	2	2	2	1	1	1	1
1	1	1	1	1	1	1	2	2	2
3	3	2	2	1	1	1	2	2	2
2	2	2	1	1	1	2	2	2	1
1	2	2	1	2	2	1	1	2	2
2	2	1	1	1	2	2	2	2	2
2	2	1	1	2	2	1	1	1	1
2	2	2	2	1	1	1	1	1	1
1	2	1	1	2	1	1	2	1	1
1	1	1	2	2	2	1	1	1	1
1	2	2	1	1	1	2	2	1	1
2	2	2	2	2	2	2	2	2	2
2	2	2	2	2	2	2	2	2	2
2	2	2	2	2	2	2	2	9	2
2	3	3	3	2	1	1	1	2	2
9	2	9	9	9	9	9	2	9	9
3	3	3	3	3	3	3	3	3	3
2	2	1	1	1	1	1	1	2	2
1	2	1	1	2	1	1	1	1	2
2	1	1	1	2	2	1	1	1	1
2	2	2	2	2	2	2	2	2	2
2	2	2	1	1	1	1	2	1	1
1	1	2	1	1	2	2	1	1	2
2	1	1	1	2	1	1	2	2	3
2	1	2	2	1	2	2	2	2	2
1	1	1	1	1	1	2	2	1	1
2	2	2	2	2	2	2	2	2	2
2	2	2	2	2	2	2	2	2	2
1	1	2	2	2	1	1	2	2	1
1	9	3	1	9	1	9	1	9	3
3	3	3	9	1	2	3	9	1	3
1	9	2	1	9	1	9	1	9	3
9	2	9	2	9	2	9	2	9	9
9	9	9	9	9	9	9	9	9	9
9	1	9	1	9	1	1	1	9	1
9	2	9	9	9	9	9	9	9	9
1	2	2	1	1	1	1	1	2	2
9	1	9	9	9	1	9	9	9	1
2	2	2	2	2	2	2	2	2	2
9	1	9	1	9	1	1	9	1	9
2	9	9	2	9	9	2	9	9	2
9	9	2	9	9	9	9	2	9	9

9	2	9	9	9	2	9	9	9	2
9	9	9	2	9	9	9	2	9	9
2	9	9	2	9	9	2	9	9	2
9	9	9	9	9	9	9	9	9	9
1	2	9	9	9	9	9	1	3	9
9	9	9	9	9	2	9	9	9	9
9	9	9	9	9	9	9	9	9	9
9	9	9	9	9	9	9	9	9	9
1	2	1	2	1	2	1	2	1	2
9	9	9	9	2	9	9	9	9	2
2	1	1	1	2	1	1	2	1	1
1	1	2	2	2	1	1	3	2	2
2	1	1	2	2	1	1	1	1	2
1	1	1	1	2	2	1	1	2	1
2	1	1	2	2	1	1	1	2	2
2	1	2	2	1	1	2	2	1	1
2	2	2	2	2	2	2	2	2	2
1	1	1	2	1	1	2	1	1	2
1	1	2	3	2	1	2	2	3	2
1	2	1	1	1	2	1	2	1	2
1	2	2	1	1	2	2	1	2	2
1	2	2	1	2	2	1	2	2	1
2	1	2	2	1	2	1	2	2	1
3	2	2	1	2	2	3	2	2	1
2	1	2	2	3	2	2	1	2	2
3	3	2	2	1	2	3	2	2	1
9	9	9	9	9	9	9	9	2	9
2	2	2	3	1	2	2	2	2	2
3	2	1	2	2	3	2	1	2	3
2	2	2	2	2	2	2	3	3	3
9	9	9	9	9	9	9	9	9	9
3	3	3	3	3	3	1	3	2	2
2	3	2	2	1	2	2	1	2	2
2	2	2	2	2	2	2	2	2	2
2	2	2	2	3	2	2	1	2	2
3	9	3	3	3	3	3	3	3	2
2	2	2	2	2	2	2	2	2	2
9	9	9	9	9	9	9	9	9	9
2	2	1	1	2	1	2	2	2	2
2	2	2	2	2	2	2	3	2	2
1	1	2	2	1	2	1	1	2	3
2	2	2	2	2	2	2	2	2	2
2	1	2	2	2	2	1	1	1	3
2	2	2	3	3	2	2	2	2	2
1	2	1	2	1	2	2	1	2	2
1	2	1	2	1	2	1	2	2	1
1	2	1	2	1	1	1	2	1	2

2	2	3	2	2	1	1	2	3	2
2	2	2	1	1	1	2	1	2	2
2	1	1	2	2	2	1	2	2	2
2	2	2	2	2	2	2	2	2	1
2	3	2	3	2	3	2	1	3	2
2	1	2	2	2	1	1	1	1	1
2	1	3	3	2	2	1	2	2	2
2	3	1	3	1	2	1	1	1	1
2	2	2	2	2	1	9	1	1	1
2	3	2	2	2	2	2	2	2	2
3	3	3	2	2	2	2	3	2	2
2	2	2	2	2	2	2	2	2	2
3	1	3	2	1	1	2	3	1	2
2	3	2	1	2	3	2	2	3	1
2	3	2	3	2	3	2	2	1	2
3	3	9	2	3	2	1	1	1	3
3	3	2	2	3	2	3	1	1	9
2	2	2	2	2	2	2	2	2	2
3	3	3	2	3	3	2	3	2	3
2	2	2	2	2	2	2	2	2	2
2	2	2	2	2	1	1	2	2	2
2	2	2	2	2	2	2	2	2	2
1	3	2	3	2	1	1	1	1	2
3	2	2	2	9	2	2	2	2	9
2	2	2	2	2	2	2	1	1	1
2	3	3	3	3	3	3	3	3	3
2	2	3	3	1	2	2	1	2	3
2	2	2	2	2	1	1	2	2	1
1	1	1	1	1	1	1	1	1	1
2	2	2	2	2	2	2	2	2	2
2	2	2	2	2	3	2	1	1	1
2	2	2	2	2	3	2	3	2	2
2	2	3	2	2	2	9	1	2	2
2	2	2	2	2	2	2	2	2	2
2	2	2	2	9	1	2	1	2	9
2	2	2	2	2	1	1	2	2	2
2	2	2	3	3	3	2	2	2	2
2	2	2	2	2	2	2	2	1	1
2	2	3	3	2	2	3	2	1	3
2	1	3	3	3	1	2	1	3	3
2	1	2	1	2	1	2	1	2	1
2	2	2	2	3	3	3	2	3	2
2	3	2	2	3	2	1	1	1	1
2	3	3	3	2	2	3	2	3	3
2	2	2	2	2	2	2	2	2	2
2	2	2	2	2	2	2	2	2	2
2	2	2	2	2	1	2	3	2	2

1	3	2	2	3	1	1	3	1	3
3	3	1	3	2	1	2	1	1	2
2	2	2	2	2	2	2	2	2	2
2	2	2	2	2	2	2	2	2	2
2	2	2	2	2	2	2	2	2	2
2	2	3	2	2	2	2	2	2	2
2	2	2	2	2	1	1	2	2	1
2	2	1	3	1	2	3	2	1	2
2	2	2	2	2	2	2	2	2	2
9	9	9	9	9	9	9	9	9	9
2	2	9	1	2	2	3	1	2	3
2	2	1	1	1	1	1	2	2	2
1	3	2	2	2	1	2	1	3	2
2	2	2	2	2	1	1	2	2	2
2	2	2	2	2	2	2	2	2	2
2	2	1	2	2	1	2	2	2	2
2	2	2	2	2	2	2	2	2	2
2	2	2	2	2	2	2	2	2	2
3	3	3	3	3	3	3	3	3	3
3	1	1	1	1	1	1	3	3	1
3	3	3	3	3	3	3	3	3	3
3	3	3	3	3	3	3	3	3	3
2	2	2	2	2	2	3	3	3	3
3	2	1	3	2	3	2	1	1	1
2	2	2	2	2	2	2	2	2	2
2	1	1	2	2	2	1	1	2	2
3	2	1	1	1	1	2	2	2	3
2	1	1	1	1	2	2	2	2	2
2	2	2	2	2	2	2	2	2	2
3	2	1	2	2	1	2	1	2	2
2	2	3	1	1	1	1	2	1	1
2	3	2	9	2	2	2	2	2	3
3	2	3	1	2	2	2	1	2	3
3	3	3	3	3	3	3	3	3	3
2	3	2	2	2	2	2	1	1	1
2	2	2	2	3	3	3	1	2	2
2	2	3	3	2	1	1	1	2	9
2	3	3	3	2	1	2	2	9	1
2	2	2	2	2	2	2	2	2	2
2	2	2	2	2	2	2	2	3	2
2	2	2	2	2	2	2	2	1	2
2	1	1	3	1	1	1	3	1	1
2	2	2	2	2	2	2	2	2	2
1	3	3	3	1	1	1	1	1	1
3	3	3	1	3	3	3	3	3	3
2	2	1	3	3	1	1	2	1	3
1	1	1	2	3	3	2	2	1	1

3	2	2	2	2	2	2	3	3	3
3	3	3	3	1	3	3	1	1	1
2	3	3	3	3	3	2	2	2	2
2	2	3	2	2	2	2	2	2	2
3	2	2	1	3	3	3	3	2	3
9	9	9	9	9	9	9	9	9	9
2	2	2	2	2	2	2	2	2	2
2	2	2	2	2	2	2	2	2	2
3	3	1	2	2	2	2	2	1	1
2	3	3	3	3	3	3	3	3	3
2	1	2	2	1	2	3	1	1	1
2	2	2	3	1	2	2	2	2	3
9	9	9	9	9	9	9	9	9	9
9	9	9	9	9	9	9	9	9	9
9	9	9	9	2	9	9	9	9	9
3	2	2	2	2	3	2	2	3	2
2	2	2	3	3	1	2	2	2	2
2	2	9	3	9	3	9	2	9	3
3	2	2	9	2	1	1	1	1	2
2	3	2	3	2	2	1	2	1	2
2	2	2	3	3	3	3	3	3	3
1	2	1	2	1	1	1	1	1	1
2	2	2	2	3	1	1	1	1	1
2	2	2	2	2	2	2	2	2	2
2	3	3	3	3	1	2	3	2	1
2	1	2	2	2	1	1	2	2	2
2	3	3	3	3	1	2	3	2	1
9	9	9	9	9	9	9	9	9	9
9	9	9	9	9	9	1	9	9	9
1	1	3	1	3	3	3	3	1	1
2	2	2	3	2	1	2	1	1	1
3	2	1	2	3	2	2	3	1	1
3	3	1	3	3	1	2	1	1	1
1	2	9	3	3	1	3	2	1	1
1	3	2	3	3	2	2	2	1	1
2	3	3	2	2	2	2	2	2	2
2	2	2	3	3	1	3	3	3	2
2	2	2	2	2	2	2	2	2	2
1	1	3	3	2	2	1	2	2	2
3	3	3	3	3	1	1	3	1	1
2	1	1	2	2	2	3	2	2	1
2	2	2	2	3	2	2	1	2	2
2	2	2	2	3	2	2	2	1	1
2	3	2	2	2	2	2	2	2	2
1	3	3	3	2	2	2	2	2	1
1	1	2	2	2	2	2	2	2	1
2	2	1	2	2	1	2	2	3	2

1	1	1	1	1	1	1	1	1	1
3	3	3	2	2	3	2	2	2	2
9	9	9	9	9	9	9	9	9	9
2	3	1	3	3	1	2	1	1	1
3	2	1	3	2	1	1	2	1	1
3	3	1	3	2	2	2	2	2	2
2	3	2	2	1	1	2	2	1	2
3	3	1	2	1	2	3	2	1	2
3	3	3	3	3	3	3	3	3	3
2	2	2	2	3	2	2	1	2	2
3	3	2	3	2	2	2	2	2	2
2	2	2	2	2	2	2	1	2	9
3	3	1	2	2	3	2	3	3	3
2	9	1	3	3	2	1	1	1	1
2	2	2	2	2	2	2	2	1	1
2	1	3	3	3	3	2	3	2	1
3	3	9	9	3	1	3	3	3	3
2	2	3	2	3	1	2	2	1	3
1	2	2	2	9	1	2	2	2	3
3	3	3	3	3	1	1	1	1	3
3	3	3	3	3	3	3	3	1	2
9	9	9	9	9	9	9	9	9	9
3	3	3	3	3	3	3	3	2	1
1	3	2	1	3	2	1	3	2	1
2	2	2	2	1	2	2	2	2	2
9	9	9	9	9	9	9	9	9	9
9	9	9	9	9	1	9	9	9	9
3	2	2	3	2	1	3	1	2	3
9	9	9	9	9	9	9	9	9	9
9	9	9	9	9	9	9	9	9	9
9	9	9	9	9	2	9	9	9	9
1	2	2	2	2	2	1	2	2	1
9	9	9	9	9	9	9	9	9	9
2	1	2	3	1	2	3	1	2	3
1	3	1	3	1	2	3	2	1	3
3	3	3	3	3	3	3	3	3	3
3	3	3	3	3	3	3	3	3	3
2	2	1	1	1	1	1	1	2	2
2	2	2	2	2	2	2	2	2	1
3	2	2	1	1	2	2	1	1	2
9	9	9	9	9	1	1	9	1	9
3	2	2	2	2	2	2	2	2	1
2	3	2	2	2	2	2	2	2	2
1	3	2	9	1	1	1	1	2	1
1	3	2	9	1	1	1	1	2	1
1	2	3	2	1	1	1	1	2	1
1	3	2	1	2	1	1	1	1	2

1	3	1	3	1	1	3	2	2	2
3	2	1	2	9	2	1	2	3	3
1	3	1	3	1	1	3	3	3	1
9	9	9	9	9	9	9	9	9	9
9	9	9	9	9	1	9	9	9	9
1	3	1	2	9	1	2	1	1	9
1	3	1	3	1	2	3	1	1	1
1	2	9	9	9	9	9	9	9	9
9	9	9	9	9	9	9	9	9	9
2	1	3	3	1	1	1	3	1	1
2	2	2	2	2	2	2	2	2	2
2	2	2	2	2	2	2	2	2	2
1	3	3	3	3	3	3	2	2	2
2	2	2	2	9	9	9	9	9	9
9	9	9	9	9	9	9	9	9	9
9	9	9	9	9	9	9	9	9	9
2	2	2	2	2	3	2	2	2	3
2	3	3	3	1	1	1	3	1	2
2	2	2	2	2	1	1	2	1	1
9	9	9	9	9	9	9	9	9	9
9	9	9	9	9	9	9	9	9	9
1	2	3	1	2	2	2	1	1	1
9	9	9	9	9	9	9	9	9	9
9	9	9	9	9	9	9	9	9	9
1	1	3	3	1	1	2	1	1	2
3	3	3	3	3	1	2	2	2	3
1	1	2	1	1	1	1	2	2	1
1	1	3	3	2	1	1	1	3	3
2	2	2	2	2	1	1	1	1	2
2	2	2	2	9	1	1	2	2	2
3	3	3	3	3	3	3	3	3	3
1	2	2	2	2	2	2	2	1	1
1	2	2	2	2	1	2	2	1	2
2	2	3	3	3	9	1	1	1	2
2	2	2	2	2	2	2	2	2	2
3	3	3	1	3	1	1	1	1	3
2	2	2	2	1	2	2	1	1	1
2	2	3	2	2	1	1	1	2	2
2	3	3	2	2	1	2	2	3	2
1	1	1	3	3	2	2	1	1	2
1	2	2	2	2	2	2	2	1	2
1	1	1	1	1	2	2	2	1	1
2	1	1	1	1	2	2	2	1	1
9	9	9	9	9	9	9	9	9	9
9	9	9	2	9	9	9	9	9	9
1	1	1	2	3	1	2	2	2	2
2	2	2	2	3	2	2	2	1	1

2	1	2	2	2	2	2	2	3	3
3	3	1	2	2	2	2	2	2	2
2	2	2	2	2	2	2	3	3	2
2	2	2	2	2	2	2	2	2	2
2	2	2	2	2	2	2	1	1	1
2	3	1	1	3	2	2	1	1	3
1	3	2	2	3	3	2	3	1	2
1	2	1	2	3	1	2	1	2	1
9	9	9	9	9	9	9	9	9	9
1	1	1	1	1	1	2	2	1	1
2	2	2	2	2	2	2	2	2	2
2	2	2	2	2	2	2	2	2	2
2	2	2	2	2	2	1	2	3	3
2	2	2	2	2	2	2	2	2	2
2	3	3	3	3	3	3	3	2	2
1	2	3	2	2	3	2	2	3	2
2	3	1	2	3	2	3	1	2	3
2	3	1	2	3	1	2	3	1	2
2	2	2	2	2	2	2	3	3	3
2	1	2	2	3	3	1	2	3	2
3	2	2	2	1	2	1	3	2	3
2	2	2	2	2	2	2	2	2	2
3	2	3	2	2	3	2	2	2	1
1	1	3	2	1	1	3	2	2	1
2	2	2	2	2	2	2	2	2	2
1	9	9	3	3	3	2	2	2	2
2	2	2	2	2	2	2	2	2	2
2	3	2	9	2	1	1	2	9	2
1	2	3	1	2	3	1	2	3	1
9	9	9	9	9	9	9	9	9	9
1	3	2	2	1	1	1	2	1	1
9	9	9	9	9	9	9	9	9	9
3	3	3	9	9	9	9	9	9	9
1	3	3	3	3	1	1	1	1	1
2	1	2	1	3	1	3	2	1	3
3	2	1	3	2	1	2	3	2	1
3	2	1	1	2	3	3	2	1	1
2	2	2	2	3	3	3	3	2	2
2	2	2	2	2	2	2	2	2	2
2	2	2	2	1	1	2	2	1	1
2	1	1	1	3	3	3	2	2	2
1	3	3	2	2	1	1	1	2	1
2	2	2	2	2	2	2	2	2	2
2	2	2	2	2	2	2	2	2	3
1	3	2	9	9	1	2	2	9	1
2	2	2	2	2	2	2	2	1	2
2	2	2	3	2	3	2	3	2	2

3	3	3	3	3	2	2	2	1	1
2	2	2	2	2	2	2	2	2	2
2	2	3	1	2	2	2	1	2	2
9	9	9	2	9	9	9	9	9	9
1	3	2	2	3	3	1	2	1	2
2	2	2	2	2	2	2	2	2	2
3	1	3	3	1	3	3	1	1	1
2	3	3	2	2	1	2	2	1	3
9	9	9	9	9	1	9	9	9	9
2	2	2	2	2	2	2	2	2	2
3	3	3	3	3	1	1	1	2	2
1	2	1	3	1	1	1	2	1	2
9	9	9	9	9	9	9	9	9	9
2	2	2	2	2	2	2	2	2	2
3	3	9	1	3	2	1	2	3	2
3	3	3	1	3	2	1	1	2	3
9	9	9	9	9	9	9	9	9	9
9	9	9	9	9	9	9	9	9	9
1	2	2	1	3	3	1	3	1	1
3	3	3	3	3	2	2	2	1	3
1	2	1	2	1	2	1	1	2	1
3	3	3	3	3	3	3	3	3	3
2	2	2	2	2	2	2	2	2	2
3	2	3	2	1	2	2	2	2	1
2	1	3	1	9	1	2	3	1	1
2	3	2	3	3	1	1	2	2	2
2	3	2	3	3	2	2	2	2	2
2	3	2	3	2	3	1	1	1	3
2	2	3	3	1	1	1	2	1	1
3	3	1	3	3	2	2	2	1	2
2	2	2	2	2	2	2	2	2	2
2	2	2	2	2	2	2	3	3	3
9	1	2	2	1	2	2	1	2	3
2	2	2	2	2	2	1	2	2	2
2	2	1	2	2	2	2	2	1	1
9	9	9	9	9	9	9	9	9	9
1	2	2	2	2	2	3	3	2	2
3	3	3	3	3	2	2	2	2	2
3	3	1	1	1	2	2	1	2	3
1	2	2	2	2	2	2	2	2	2
1	2	2	2	2	2	3	3	2	2
2	2	2	2	2	1	2	2	1	1
2	2	2	2	2	2	2	2	2	2
2	2	2	2	2	2	2	2	2	2
9	9	9	9	9	9	9	9	9	9
1	3	1	2	3	2	2	1	2	1
9	9	9	9	9	9	9	9	9	9

2	2	2	2	2	1	1	2	2	2
1	9	3	2	1	2	1	1	1	1
9	9	9	9	9	9	9	9	9	9
1	3	3	3	3	1	1	1	1	3
1	1	1	2	2	1	1	2	2	1
1	2	3	2	2	1	1	2	2	1
2	2	2	2	2	2	2	2	2	2
9	9	9	1	9	9	9	2	3	9
1	2	3	2	3	1	2	1	2	3
2	2	1	2	2	1	1	1	1	2
9	9	9	9	9	9	9	9	9	9
1	3	1	3	3	1	3	1	3	1
3	3	2	1	2	9	2	9	2	3
9	3	2	2	2	3	2	3	3	2
2	2	3	9	3	2	3	2	2	2
9	3	3	9	3	9	2	2	2	2
2	2	2	1	2	2	1	3	3	3
9	9	9	9	9	9	9	9	9	9
9	9	9	9	9	9	9	9	9	9
2	1	3	2	2	2	1	3	2	1
3	9	1	2	1	2	3	2	1	2
3	3	1	1	1	2	2	3	3	3
2	9	2	2	3	2	3	2	3	2
3	3	3	3	3	3	3	3	3	3
2	1	2	3	2	1	2	3	2	1
2	2	2	2	2	2	2	2	1	3
9	9	9	9	9	9	9	9	9	9
1	2	2	3	3	2	2	3	3	3
9	9	9	9	9	9	9	9	9	9
9	9	9	9	9	9	9	9	9	9
9	9	9	9	9	9	9	9	9	9
9	9	9	9	9	9	9	9	9	9
9	9	9	9	9	9	9	9	9	9
1	2	1	1	1	1	1	1	2	2
2	1	2	1	2	3	2	2	3	1
2	3	2	2	2	2	3	2	2	2
9	9	9	9	9	9	9	9	9	9
1	3	2	3	3	2	2	2	2	2
1	1	9	1	1	1	9	1	1	1
9	9	9	9	9	9	9	9	9	9
2	2	2	2	2	2	2	2	2	2
2	2	1	2	2	2	2	2	2	1
2	2	2	1	2	1	1	2	2	2
2	2	2	2	2	2	2	2	2	2
1	2	3	1	2	3	1	2	3	2
1	3	2	2	1	2	2	3	2	1
2	3	2	3	2	1	1	2	2	1

2	2	2	2	2	2	2	2	2	2
2	2	2	2	2	2	2	2	2	2
9	9	9	9	9	9	9	9	9	9
1	2	3	1	2	3	1	2	3	1
3	1	1	1	1	2	2	2	2	3
1	1	2	2	1	1	3	3	3	3
3	1	9	2	9	2	9	9	2	9
2	2	2	2	2	2	2	2	2	2
2	2	2	2	2	2	2	2	2	2
2	2	2	2	2	2	2	2	2	2
2	2	2	2	2	2	2	2	2	2
1	2	2	3	2	2	1	2	2	1
9	9	9	9	9	9	9	9	9	9
1	1	1	1	1	1	1	1	1	1
2	3	2	3	9	3	2	1	2	1
1	3	3	3	3	1	1	3	1	3
3	3	3	1	1	1	2	2	2	3
9	9	9	9	9	9	9	9	9	9
2	1	3	1	2	2	3	2	3	2
2	3	3	2	3	2	9	9	9	9
9	9	9	9	9	9	9	9	9	9
1	2	2	3	3	1	3	1	1	2
9	9	9	9	9	9	9	9	9	9
9	9	9	9	9	9	9	9	9	9
9	9	9	9	9	9	9	9	9	9
9	9	9	9	9	9	9	9	9	9
2	2	1	2	2	1	2	2	1	1
3	3	3	2	2	3	2	2	1	2
9	9	9	9	9	9	9	9	9	9
1	1	1	2	2	1	1	2	2	1
3	1	3	1	3	1	3	1	3	1
3	3	3	1	3	3	3	1	1	1
1	2	2	3	2	2	2	2	1	1
2	1	2	2	2	2	2	2	2	2
2	2	2	2	2	2	2	2	2	2
2	2	2	2	2	2	2	2	2	2
3	1	1	1	3	1	3	1	3	1
2	2	2	2	2	2	2	2	2	2
2	2	2	2	3	2	3	2	9	2
1	1	3	3	1	1	2	2	1	1
9	2	9	1	2	1	2	9	2	1
2	3	2	3	3	2	2	2	1	1
2	2	2	2	2	2	2	2	2	2
1	3	1	3	2	1	1	2	9	9
1	1	2	1	3	2	1	1	1	1
1	1	1	1	1	1	1	1	1	1
3	3	3	3	3	3	3	2	2	2

3	3	1	3	3	1	1	2	2	2
2	2	3	2	3	3	3	2	2	2
3	3	1	3	3	1	3	2	2	2
1	3	2	2	1	1	1	2	1	1
2	2	2	2	2	2	2	2	2	2
2	2	2	2	2	2	2	2	2	2
2	2	2	2	2	2	2	2	2	2
2	3	3	3	3	3	3	3	3	3
2	2	2	2	2	2	2	2	2	2
1	3	3	3	3	3	1	2	1	1
9	3	1	1	2	2	2	2	1	2
1	1	3	2	1	3	1	2	1	3
3	3	3	3	3	3	3	3	3	3
2	3	2	2	3	1	2	3	1	2
1	3	2	1	1	2	2	2	1	1
9	9	9	9	9	9	9	9	9	9
3	1	3	1	3	1	1	1	3	1
2	2	2	2	2	2	2	2	2	2
3	2	2	3	9	2	3	2	2	2
3	3	3	3	3	3	3	3	3	3
2	2	2	2	2	2	2	2	2	2
2	2	2	2	2	2	2	2	2	2
1	1	1	1	1	1	1	1	1	1
3	3	3	2	3	2	2	3	2	3
1	1	1	1	1	1	1	1	1	1
3	3	3	3	3	3	3	3	3	3
2	3	2	2	2	3	2	2	3	2
2	3	3	2	3	2	3	2	3	2
1	3	2	2	2	1	2	2	3	3
2	2	2	2	2	2	3	3	3	2
3	3	3	3	3	3	3	3	3	3
9	9	9	9	9	9	9	9	9	9
2	2	2	2	2	2	2	2	2	2
3	2	9	1	9	1	2	3	1	2
2	1	3	2	1	3	1	2	3	3
2	3	2	1	3	1	3	1	3	1
2	2	2	2	2	2	2	2	2	2
3	3	3	3	3	3	3	3	3	3
1	1	3	3	3	2	2	1	2	1
2	2	2	1	2	2	2	2	2	2
2	2	2	2	2	2	2	2	2	3
2	3	2	3	2	2	3	1	1	1
2	2	3	2	2	1	1	1	2	2
3	2	2	1	1	2	2	1	1	1
9	9	9	9	9	9	9	9	9	9
1	2	3	1	2	3	1	2	1	1
2	2	2	2	2	2	2	2	2	2

1	2	2	2	2	2	1	1	1	1
9	9	9	1	9	1	1	1	1	3
9	9	9	1	9	1	1	1	1	3
1	2	2	2	2	1	2	2	2	2
9	9	9	9	9	9	9	9	9	9
9	1	9	9	9	9	9	9	1	1
9	9	9	9	9	9	9	9	9	9
2	2	2	2	2	2	2	2	2	2
1	2	1	2	2	2	1	3	1	2
2	2	2	3	2	3	3	2	1	2
2	2	2	2	2	2	2	2	2	2
2	2	2	2	2	2	2	2	2	2
2	2	2	2	2	2	2	2	2	2
2	2	2	2	2	2	2	2	2	2
2	2	2	2	2	2	2	2	2	2
2	2	2	2	2	2	2	2	2	2
1	1	2	3	3	1	1	3	3	3
2	2	2	2	2	2	2	2	2	2
2	3	2	2	2	2	2	2	2	2
2	2	2	3	9	2	3	1	2	1
2	2	2	1	2	1	2	2	2	1
1	1	1	1	1	1	1	1	1	2
9	2	9	1	9	9	9	9	9	9
2	2	2	2	2	2	2	2	2	2
1	3	2	1	2	1	1	1	1	1
2	2	2	2	1	1	1	1	1	1
2	2	2	1	2	2	3	2	2	1
9	9	9	9	9	9	9	9	9	9
2	2	1	2	3	2	1	1	1	1
2	2	2	2	2	2	2	2	2	2
2	2	3	2	2	2	2	2	2	2
1	2	1	1	1	1	1	1	1	2
2	3	2	1	3	2	1	1	1	2
1	2	2	3	1	1	2	2	2	2
2	2	3	3	1	1	1	1	1	1
2	2	2	2	3	3	3	3	3	2
2	2	2	2	2	2	2	2	2	2
2	2	2	2	2	2	2	2	2	2
2	2	2	2	2	2	2	2	1	1
9	9	9	9	9	9	9	9	9	9
1	3	2	9	1	2	1	2	2	2
3	2	2	1	2	2	2	1	2	3
1	1	1	2	2	3	2	1	1	2
1	2	2	2	2	1	1	1	1	1
2	2	1	3	3	3	3	3	2	2
2	2	2	2	2	2	2	2	2	2
2	2	2	2	2	2	2	2	2	2

1	2	2	2	2	1	1	1	1	1
9	2	9	9	9	1	9	9	9	9
2	2	1	2	2	2	2	2	2	2
3	2	3	3	1	2	3	1	2	3
2	2	2	2	2	2	2	2	2	2
3	3	1	2	3	3	2	2	1	2
3	2	3	2	3	2	3	2	3	3
1	2	2	3	3	1	1	1	1	1
3	9	9	2	3	9	9	9	1	9
2	2	3	3	3	1	2	2	2	2
9	9	9	9	9	9	9	1	1	2
3	2	1	3	3	3	3	1	1	1
2	2	2	2	2	2	2	2	2	2
1	1	1	1	1	2	1	3	1	2
2	2	2	2	2	2	2	2	1	2
2	2	2	3	1	1	1	1	1	2
2	2	2	2	2	2	2	1	1	2
1	3	1	3	9	1	1	1	1	1
9	9	9	9	9	9	9	9	9	9
1	2	1	2	1	1	1	1	1	1
2	2	1	1	2	2	1	2	1	1
2	2	3	3	1	1	2	2	2	1
2	2	2	2	2	2	3	2	2	2
1	2	1	1	2	3	1	2	1	3
2	2	2	2	2	2	2	2	2	2
1	2	1	1	2	2	3	2	2	3
2	2	2	2	2	1	1	1	1	2
2	2	2	2	2	2	2	1	1	2
2	1	2	3	2	2	1	2	3	2
1	1	3	1	3	3	3	3	3	3
2	2	2	2	2	2	2	2	2	2
3	3	2	2	2	2	1	2	3	1
2	3	2	1	2	1	3	3	1	2
3	1	2	3	1	2	3	1	3	2
1	3	3	3	3	1	3	1	1	1
3	3	2	1	1	1	3	1	1	1
3	2	2	1	1	1	1	2	2	2
1	2	2	2	2	2	1	1	2	2
2	2	2	2	2	2	2	2	2	2
3	3	3	3	2	1	1	1	1	1
9	9	9	9	9	9	9	9	9	9
2	3	1	3	2	2	1	2	3	2
9	9	3	3	3	1	3	1	1	9
1	1	2	3	1	1	3	1	1	2
3	3	1	3	3	1	1	1	1	1
1	1	3	3	1	1	1	3	3	3
2	2	3	2	3	1	2	2	3	2

3	2	2	2	3	2	3	2	3	1
9	9	9	9	9	9	9	9	9	9
2	2	2	2	2	2	2	2	2	2
1	9	3	2	3	9	9	9	9	9
2	2	2	2	2	2	2	2	2	2
2	2	2	2	2	2	2	2	2	2
1	2	1	3	1	1	1	2	1	1
1	1	1	1	1	1	1	1	1	1
9	9	9	9	9	9	9	9	9	9
2	3	1	2	1	2	1	2	1	3
9	9	9	9	9	9	9	9	9	9
2	3	2	3	3	2	2	2	2	2
1	2	1	2	2	2	2	1	1	1
3	3	3	2	2	2	2	2	1	1
3	3	3	3	3	2	2	2	2	2
3	2	1	2	3	2	2	3	1	2
2	3	3	3	3	3	3	2	1	1
1	3	3	3	3	1	1	1	1	1
1	3	3	3	2	3	3	1	2	2
1	1	1	1	1	1	1	1	1	1
2	2	2	2	1	1	1	1	1	1
3	9	1	2	2	3	2	3	1	3
9	9	9	9	9	9	9	9	9	9
3	1	3	1	3	1	3	1	1	1
1	2	3	1	2	3	1	2	3	1
2	3	3	3	1	1	1	1	1	1
1	3	3	3	2	1	1	1	1	1
3	3	2	3	2	2	1	1	1	2
2	2	2	2	2	2	2	2	1	2
2	3	2	3	2	1	1	1	1	2
2	1	1	1	2	1	1	2	2	2
1	2	3	2	9	2	9	2	2	3
2	1	3	2	3	2	2	2	3	2
2	2	1	3	2	1	2	1	1	1
1	3	3	3	2	3	1	2	3	3
3	3	3	3	2	3	2	2	3	3
1	1	2	2	2	1	2	1	1	1
9	9	9	9	9	9	9	9	9	9
2	2	2	2	2	2	2	2	2	2
2	2	2	3	2	2	3	2	9	1
2	2	2	2	1	1	1	1	2	2
1	1	2	2	2	2	1	2	1	1
2	2	2	2	3	2	3	2	1	2
2	1	2	2	3	1	2	2	3	9
1	1	1	1	2	2	1	2	1	2
3	3	1	3	1	1	1	1	1	2
1	2	1	2	3	1	2	1	1	1

1	3	1	3	1	1	2	1	1	3
9	9	9	9	9	9	9	9	9	9
2	3	2	1	3	2	1	3	2	3
2	3	2	1	1	3	2	2	1	3
2	2	2	2	2	2	2	2	2	2
2	3	3	3	2	2	1	2	1	3
2	3	2	3	1	2	9	2	9	2
2	2	2	2	2	2	2	2	2	2
2	2	2	2	2	2	2	2	2	2
9	9	9	9	9	9	9	9	9	9
3	3	3	2	3	3	1	1	1	3
1	1	2	2	3	3	2	3	1	2
3	1	3	1	3	1	3	1	3	1
3	2	1	2	1	2	1	3	1	3
2	2	2	2	2	2	2	2	2	2
9	9	9	9	9	9	9	9	9	9
9	9	9	9	9	9	9	9	9	9
2	1	1	1	1	2	2	2	2	1
2	1	2	2	2	2	2	2	2	2
1	2	1	1	2	2	2	2	2	2
9	9	9	9	9	9	9	9	9	9
2	1	2	2	2	1	1	1	1	1
2	2	2	1	1	1	1	2	2	2
1	1	2	2	2	1	1	1	2	3
2	2	2	2	1	1	2	2	2	1
2	2	2	2	3	3	3	3	2	2
2	1	1	1	1	2	2	2	1	1
3	3	2	3	3	3	3	3	3	3
1	1	2	2	2	2	3	2	2	2
2	2	3	3	3	3	3	3	3	3
9	9	9	9	9	9	9	9	9	9
9	9	9	9	9	9	9	9	9	9
9	9	9	9	9	9	9	9	9	9
1	3	1	9	3	1	2	1	1	1
9	1	2	2	2	1	2	1	1	2
2	2	2	2	2	2	2	2	1	2
2	2	2	2	2	2	2	2	2	2
2	9	2	2	9	1	1	1	1	2
1	2	2	1	2	2	2	2	1	2
2	2	2	2	2	2	2	2	2	2
3	3	3	3	3	2	1	3	1	1
2	1	3	3	1	1	1	1	1	1
2	2	2	2	2	2	2	2	1	1
1	3	3	9	1	1	1	3	3	3
2	2	2	2	2	2	2	2	1	1
1	1	1	1	1	1	1	1	1	1
3	3	1	3	3	3	1	3	1	3

3	3	3	3	3	3	3	3	3	3
2	2	2	2	2	2	2	2	1	1
2	2	2	3	2	1	3	2	3	3
1	1	3	3	1	1	1	1	1	1
3	3	3	3	3	2	9	9	9	2
1	2	2	2	3	1	3	2	1	2
3	3	3	3	3	1	2	2	1	2
9	9	9	9	9	9	9	9	9	9
9	9	9	9	9	9	2	9	9	9
9	9	9	9	9	9	9	9	9	9
9	9	9	1	9	9	9	9	9	9
9	9	9	9	9	9	9	9	9	9
1	1	1	1	1	1	1	1	1	1
2	2	2	2	1	1	2	2	1	2
3	3	3	3	3	2	3	2	1	3
2	3	2	3	1	1	1	1	2	2
1	2	1	2	2	1	1	1	1	1
1	2	1	2	2	1	1	1	1	2
2	2	1	3	2	2	2	2	2	2
2	3	2	3	1	3	2	3	2	1
2	2	2	2	2	2	2	2	1	2
2	3	2	2	3	2	2	2	2	2
2	3	1	3	3	1	1	2	1	1
9	9	9	9	9	9	9	9	9	9
1	2	3	2	1	2	3	2	1	2
3	3	3	3	3	3	1	1	2	1
2	2	2	2	2	2	2	2	2	2
2	2	1	2	3	2	2	2	2	3
2	2	2	2	2	2	2	2	2	2
2	3	2	2	3	3	2	2	2	2
2	2	2	2	2	2	2	2	2	2
9	9	9	9	9	9	9	9	9	9
2	3	1	3	3	2	2	2	1	2
1	2	1	3	2	1	2	2	3	1
9	9	9	9	9	9	9	9	2	9
2	2	2	2	2	2	2	2	2	2
2	2	2	2	2	2	2	2	2	2
2	2	2	2	2	2	2	2	2	2
2	2	2	2	2	2	2	2	1	1
3	3	1	3	3	3	3	2	2	2
1	1	1	3	3	3	3	3	3	3
9	9	9	9	9	9	9	9	9	9
9	9	9	9	9	9	9	9	9	9
2	2	2	2	2	2	2	2	2	2
1	9	9	1	9	2	9	1	9	2
3	3	1	3	3	3	3	2	1	1
1	3	3	2	2	2	2	2	2	2

1	3	1	3	2	1	2	1	2	1
2	2	2	3	3	2	2	2	2	2
2	3	3	3	3	1	1	1	2	2
3	2	3	2	2	3	2	2	2	2
2	2	2	2	2	2	2	2	2	2
1	3	3	3	9	1	1	1	1	2
2	3	1	3	3	2	3	2	2	2
2	2	2	1	2	3	2	1	2	3
2	3	1	3	3	3	3	3	1	1
2	1	2	3	1	2	2	1	3	2
1	2	2	2	2	1	2	3	2	1
2	2	1	2	2	2	2	2	1	3
1	1	3	3	3	3	3	1	1	9
2	2	2	2	2	2	1	2	2	1
2	2	2	2	2	2	2	2	2	2
2	2	2	2	2	2	2	2	2	2
1	1	3	3	3	2	2	2	2	3
2	2	2	2	2	2	2	2	2	2
2	2	1	1	1	2	2	2	2	2
2	2	1	1	1	1	3	3	2	2
2	1	2	2	3	2	2	1	2	2
1	3	1	3	3	3	2	2	1	2
2	2	2	2	2	2	2	2	1	2
2	3	2	3	2	2	2	2	2	1
2	2	2	1	3	3	2	2	2	1
2	2	2	2	2	2	2	2	2	2
2	2	2	2	2	2	2	2	2	2
1	1	1	1	1	1	1	1	1	1
2	2	2	2	2	2	2	2	2	2
2	2	3	2	2	3	2	2	2	3
9	9	9	9	9	9	9	9	9	9
2	3	2	2	1	1	2	1	1	2
2	2	2	2	1	2	2	2	2	1
2	1	2	2	2	2	3	3	2	2
1	3	9	2	1	2	2	1	2	2
2	2	2	2	2	1	1	2	2	2
2	2	1	2	3	2	2	1	2	2
2	2	2	2	2	2	3	2	1	3
2	3	2	1	2	2	1	9	1	2
1	1	1	1	1	1	1	1	2	2
1	3	3	3	3	1	1	1	1	1
2	2	2	2	2	1	2	2	1	2
2	2	2	2	2	2	2	1	1	2
3	3	3	2	1	3	2	1	2	2
1	1	2	9	2	1	2	1	2	2
2	2	2	2	2	2	2	2	2	2
1	3	3	3	2	3	1	2	3	3

1	3	3	3	2	3	1	2	3	3
2	2	1	2	2	2	2	2	2	2
2	1	1	2	2	1	2	2	1	1
2	2	2	1	1	2	3	2	1	2
3	2	2	1	2	2	2	1	2	2
2	2	1	1	2	2	2	2	2	2
1	1	1	2	2	1	2	2	1	1
2	2	2	2	2	2	2	2	2	1
3	2	1	3	2	1	3	2	1	3
1	3	1	2	2	2	1	2	2	3
1	3	3	3	9	1	9	1	1	1
2	2	2	2	2	2	2	2	2	2
3	3	3	3	3	3	3	3	3	3
2	2	2	2	2	2	2	2	1	2
3	3	3	3	3	3	1	3	1	1
2	2	2	2	2	3	3	2	1	1
3	3	3	3	2	2	3	1	1	2
2	2	3	3	2	2	1	2	2	3
2	2	3	3	9	3	2	9	2	2
2	3	1	3	1	1	1	2	1	2
2	2	3	3	3	2	2	3	2	3
3	3	3	3	3	3	3	3	3	3
2	2	2	3	2	2	2	2	1	3
2	1	2	2	2	1	2	2	2	2
2	1	2	1	3	2	1	2	2	3
2	2	3	1	3	1	2	3	2	2
1	2	2	2	1	1	1	2	2	1
2	2	2	2	2	1	2	2	2	2
2	2	2	2	1	1	3	2	1	2
2	2	2	2	2	1	2	2	2	1
3	3	3	3	3	3	3	3	3	3
1	3	3	3	3	1	9	1	1	1
2	2	2	3	2	3	2	3	2	3
1	2	3	2	2	1	2	2	1	1
3	2	1	2	3	1	2	1	2	3
2	3	2	3	3	2	2	2	2	2
2	3	2	1	2	1	1	1	2	2
1	2	1	2	3	2	3	3	2	2
1	1	1	2	3	1	1	2	1	2
2	3	1	3	2	2	2	1	2	2
1	3	1	3	3	3	3	1	2	3
2	3	1	3	3	2	2	1	3	2
1	1	2	2	2	2	2	2	2	3
2	3	1	2	1	2	2	1	1	1
2	2	2	2	2	2	2	2	2	2
1	2	1	1	2	2	2	1	3	2
2	2	2	2	2	2	2	2	2	2

2	1	1	3	3	3	2	1	2	1
1	2	3	9	3	3	2	1	2	1
1	2	2	2	2	2	2	2	2	1
1	2	1	3	3	3	3	1	2	3
2	3	3	3	3	2	2	3	1	2
2	2	1	2	2	2	3	3	2	2
2	2	2	2	2	2	2	2	2	2
2	2	1	1	1	1	1	1	1	1
9	9	9	9	9	9	9	9	9	9
2	2	2	2	2	2	2	2	2	2
2	2	2	2	2	2	2	1	1	1
2	2	2	3	3	1	1	1	1	3
1	1	1	2	2	3	2	2	2	3
2	2	2	2	2	2	2	2	2	3
1	1	1	1	1	1	1	1	1	1
2	2	1	3	2	1	2	1	1	2
2	2	2	2	2	2	2	2	2	2
2	2	1	1	2	2	1	2	1	1
2	1	2	3	2	1	2	3	2	1
2	2	2	2	2	2	2	2	2	2
1	2	2	2	2	1	2	2	2	3
1	2	2	2	2	2	2	2	2	1
2	3	2	2	2	2	2	3	2	2
3	2	2	2	3	2	2	2	2	2
3	2	2	2	3	1	1	1	1	1
2	2	1	3	2	1	2	1	1	2
2	2	2	3	3	1	1	1	1	3
2	2	2	2	2	2	2	2	2	2
1	1	1	2	3	1	1	2	1	2
2	2	2	2	2	2	2	2	2	2
2	3	2	1	2	1	1	1	2	2
9	9	9	9	9	9	9	9	9	9
2	2	2	3	2	2	2	1	2	2
3	3	3	1	3	1	1	1	1	3
1	3	3	3	3	9	2	2	2	2
2	2	2	3	3	2	3	3	1	2
1	3	3	3	1	1	1	1	1	1
3	2	1	2	3	3	3	1	1	2
2	1	2	1	2	2	2	2	1	1
2	1	2	1	2	3	1	2	3	1
1	2	1	2	2	1	1	1	2	1
2	9	1	2	1	1	1	1	2	1
1	1	1	3	2	1	2	1	1	3
2	2	2	2	2	1	1	1	1	1
1	2	1	2	2	3	2	2	3	2
9	9	9	9	9	9	9	9	9	9
3	3	3	2	2	2	2	2	2	2

3	2	2	2	3	2	2	2	1	2
3	3	1	3	3	3	3	3	3	3
1	1	1	1	1	1	1	1	1	1
2	2	2	2	2	2	2	2	2	2
2	2	2	2	2	2	2	2	2	2
2	2	1	2	2	2	2	2	2	3
1	2	1	2	2	1	1	2	2	1
9	9	9	9	9	9	9	9	9	9
9	9	9	9	9	9	3	9	9	9
9	9	9	9	9	9	9	9	9	9
9	1	2	3	3	2	3	2	3	3
1	1	1	3	3	2	3	3	3	2
3	1	3	3	3	3	3	3	3	3
3	1	3	1	3	3	3	3	3	3
3	1	3	1	3	3	3	3	3	3
3	3	3	1	2	2	2	2	2	1
3	3	3	1	2	2	2	2	2	2
2	3	2	3	2	3	3	2	3	2
3	2	1	2	3	1	2	3	1	2
2	1	2	3	2	2	3	2	1	2
3	1	2	3	1	2	1	3	1	3
2	1	2	3	3	2	3	2	1	2
2	2	2	1	1	1	1	1	1	1
2	3	3	1	1	3	3	3	3	3
2	2	1	3	3	3	3	1	1	1
3	3	3	2	2	2	2	2	3	3
2	2	2	2	2	2	2	1	1	1
2	2	2	3	3	3	2	2	3	3
1	1	1	3	3	3	3	3	3	3
1	1	1	3	3	3	3	3	3	3
3	1	3	1	3	3	3	3	3	3
3	1	3	1	3	3	3	3	3	3
2	2	2	2	2	2	2	2	2	2
2	2	2	2	2	2	2	2	2	2
2	2	2	2	2	2	2	2	2	2
2	2	2	2	2	2	2	2	2	2
3	3	3	1	1	1	1	1	1	1
2	2	2	2	2	2	2	2	2	2
2	2	2	2	1	1	1	1	1	1
3	3	3	1	1	1	1	1	1	1
2	2	2	2	2	3	3	3	3	3
2	1	3	3	3	3	3	3	3	3
2	1	3	3	3	3	3	3	3	3
2	1	3	3	3	3	3	3	3	3
2	1	3	3	3	3	3	3	3	3
3	9	2	9	1	2	3	1	3	9
2	9	3	2	1	9	1	9	2	9

9	3	1	1	3	1	2	1	3	2
1	2	2	2	2	2	1	1	1	1
1	2	2	1	1	2	1	2	1	2
1	2	1	2	3	3	2	3	2	3
3	2	2	3	2	1	2	3	2	2
1	1	1	2	1	3	2	1	3	3
2	3	9	2	3	3	2	3	3	1
9	1	2	3	2	3	2	3	2	3
9	9	9	9	9	9	9	9	9	9
9	9	9	9	9	9	9	9	9	9
9	9	9	9	9	9	9	9	9	9
9	1	1	3	9	9	3	3	3	9
9	1	1	3	3	3	2	3	3	9
1	9	1	2	3	3	3	3	3	3
2	2	2	2	2	2	2	2	2	2
2	2	2	2	2	2	2	2	2	2
2	2	2	2	2	2	2	2	2	2
9	9	9	9	9	9	9	9	9	9
2	9	3	1	1	1	2	2	2	2
9	9	9	9	9	9	9	9	9	9
2	2	2	2	3	3	3	2	3	3
3	2	1	1	3	2	3	3	2	3
2	2	2	2	2	2	2	2	2	2
2	2	2	2	2	2	2	2	2	2
9	9	9	9	9	2	9	9	9	9
9	9	9	9	9	9	9	9	9	9
2	3	1	2	1	3	2	1	3	2
9	9	9	9	9	9	9	9	9	9
2	2	2	2	3	3	3	3	3	3
9	9	9	9	9	9	9	9	9	9
1	3	1	2	3	3	1	3	3	1
9	9	9	9	9	9	9	9	9	9
1	1	1	3	3	3	3	3	3	3
9	9	9	9	9	9	9	9	9	9
1	1	2	2	2	2	2	2	2	2
2	2	2	2	2	2	2	2	2	2
1	1	1	3	3	3	2	2	2	2
2	2	2	2	2	2	2	2	3	3
2	2	2	3	3	3	3	1	1	1
9	9	9	9	9	9	9	9	9	9
9	9	9	9	9	9	9	9	9	9
9	9	9	9	9	9	9	9	9	9
9	9	9	9	9	9	9	9	9	9
9	9	9	9	9	9	9	9	9	9
9	9	9	9	9	9	9	9	9	9
3	2	2	2	3	2	2	2	3	2
2	3	1	2	1	3	2	1	3	2

9	9	9	9	9	9	9	9	9	9
1	1	1	3	3	3	3	3	3	3
9	2	9	9	9	9	2	9	9	9
1	2	2	3	3	3	3	3	2	3
2	2	1	3	3	3	3	3	3	3
2	3	2	3	2	2	1	2	2	1
9	9	9	9	9	9	9	9	9	9
3	3	3	1	9	3	2	3	1	1
1	1	3	1	3	3	3	3	3	3
9	9	9	9	9	9	9	9	9	9
2	3	2	9	3	2	2	3	3	2
1	3	1	1	3	3	1	3	3	3
9	9	9	9	9	9	9	9	9	9
9	9	9	9	9	9	9	9	9	9
1	1	1	3	3	3	3	3	3	3
9	9	9	9	9	9	9	9	9	9
3	1	2	3	3	3	3	3	3	3
9	9	9	9	9	9	9	9	9	9
2	2	2	2	2	2	2	2	2	2
1	2	2	2	2	1	1	1	1	1
9	1	2	9	3	9	3	2	3	3
1	2	3	2	3	3	2	2	3	2
3	3	3	1	1	1	1	2	2	1
3	3	3	1	1	2	2	3	3	3
3	3	3	1	1	2	2	3	3	2
2	2	3	2	3	2	1	2	3	2
1	1	1	3	3	3	3	3	3	3
1	1	1	3	3	3	2	2	2	3
1	1	2	3	3	2	2	2	3	3
2	2	2	2	3	2	3	2	9	3
1	2	3	2	3	2	1	2	3	2
1	1	1	3	3	3	3	3	3	3
9	9	9	9	9	9	9	9	9	9
9	1	9	9	9	9	9	9	9	9
9	9	9	9	9	9	9	9	9	9
9	9	9	3	9	9	9	9	9	9
9	9	9	9	9	9	9	9	9	9
9	9	9	9	9	9	9	9	9	9
9	9	9	9	9	9	9	9	9	9
9	9	9	9	9	9	9	9	9	9
9	9	9	9	9	9	9	9	9	9
9	9	9	9	9	9	9	9	9	9
9	9	9	9	9	9	9	9	9	9
9	9	9	9	9	9	9	9	9	9
9	9	9	9	9	9	9	9	9	9
1	1	2	3	2	1	3	2	3	1
1	2	1	3	2	1	3	2	3	3

1	2	2	2	3	3	3	3	3	2
9	9	9	9	1	9	9	1	9	9
3	9	9	9	1	9	9	9	1	9
2	2	1	2	3	2	2	2	2	3
9	9	9	9	9	9	9	9	9	9
9	9	9	9	9	9	9	9	9	9
9	9	9	9	9	9	9	9	9	9
9	9	9	9	9	9	9	9	9	9
9	9	9	9	9	9	9	9	9	9
1	2	1	2	3	2	3	2	3	2
1	2	1	2	3	2	3	2	3	2
2	2	2	2	2	2	2	2	2	2
2	2	2	2	2	2	2	2	2	2
2	1	2	3	1	1	3	2	2	2
1	1	1	1	1	1	1	2	2	2
2	3	3	2	2	2	3	3	3	2
9	9	9	9	9	9	9	9	9	9
9	9	9	9	9	9	9	9	9	9
9	9	9	9	9	9	3	9	9	9
1	9	9	9	9	2	9	9	9	1
9	9	9	9	9	3	9	9	9	9
9	9	9	9	9	9	9	9	9	9
9	9	9	1	9	2	9	1	9	2
9	9	1	9	1	1	9	1	9	9
9	9	9	9	9	9	9	9	9	9
2	2	2	2	2	9	3	3	3	1
9	9	9	9	9	9	9	9	9	9
2	2	2	2	2	2	2	2	2	2
3	2	3	1	2	2	1	1	2	3
9	9	9	9	9	9	9	9	9	9
2	2	2	2	2	2	2	2	2	2
1	2	1	3	2	1	3	3	1	2
2	2	2	2	2	2	2	2	2	2
2	2	2	2	2	2	2	2	2	2
1	1	1	3	3	3	3	3	2	3
2	2	2	2	2	2	2	3	2	2
2	1	2	2	3	1	2	1	2	1
9	1	1	9	3	3	9	3	2	2
9	9	9	9	9	9	9	9	9	9
2	2	2	2	2	2	2	2	2	2
2	2	2	2	2	2	2	2	2	2
3	3	3	2	2	3	2	3	2	2
2	1	3	2	3	1	3	1	3	2
2	2	2	2	2	2	2	2	2	2
9	9	9	9	9	9	9	9	9	9
2	2	2	2	2	2	2	2	2	2
3	3	1	9	9	9	9	9	9	9

1	2	1	1	1	1	3	3	2	2
2	1	2	2	3	3	3	3	2	3
2	2	2	2	2	2	2	2	2	2
3	2	3	2	3	2	1	2	3	2
2	2	2	2	2	2	2	2	2	2
2	1	2	3	2	3	3	2	2	3
1	3	3	1	3	3	1	3	3	3
2	2	2	2	2	2	2	2	2	1
2	2	2	2	3	3	3	3	3	3
9	9	9	9	9	9	9	9	9	9
2	2	2	2	2	2	2	2	2	2
3	3	3	1	3	3	3	3	1	1
3	3	3	3	3	3	3	3	3	3
2	1	2	2	2	3	2	3	3	3
2	2	2	2	3	3	2	3	3	3
3	2	2	3	2	2	1	1	1	2
2	2	2	2	2	2	2	2	2	2
2	2	2	2	2	2	2	2	2	2
3	3	2	2	1	3	3	2	2	2
2	2	2	2	2	2	2	3	3	2
2	2	2	2	2	2	2	2	2	2
2	2	2	2	2	2	2	2	2	2
2	2	2	2	3	2	3	3	3	2
3	3	3	1	3	3	3	3	2	2
1	1	1	3	3	3	2	2	2	3
3	1	2	2	3	3	3	3	3	3
3	3	2	9	3	1	1	3	3	3
2	1	2	2	3	2	3	3	2	3
2	2	3	1	2	2	2	1	2	2
2	2	2	2	2	2	2	2	2	2
2	2	2	2	2	2	2	2	2	2
2	2	2	2	2	2	2	2	2	2
2	2	2	2	2	2	2	2	2	2
2	2	2	2	2	2	2	2	2	2
2	2	2	2	2	2	2	2	2	2
2	2	2	2	2	2	2	2	2	2
1	2	1	2	2	2	1	2	1	2
9	9	9	9	9	9	9	9	9	9
9	9	9	9	9	9	9	9	9	9
1	3	3	1	3	3	1	3	3	1
9	1	2	1	3	2	3	1	2	3
9	1	9	9	9	9	9	9	9	9
1	1	1	3	3	3	3	3	3	3
2	3	3	3	3	2	3	3	3	3
2	1	3	3	2	3	2	1	3	2
9	9	9	9	9	9	9	9	9	9
1	1	2	1	3	3	3	3	3	3

1	1	2	2	3	2	3	2	3	1
2	2	2	2	3	3	3	3	3	3
3	3	2	3	2	1	3	2	1	3
2	2	1	3	3	3	2	3	3	3
2	2	2	2	2	2	2	2	2	2
1	1	1	3	3	9	9	9	9	9
2	2	2	2	2	2	2	2	2	2
1	3	3	1	3	3	3	3	3	3
3	2	3	1	2	2	2	3	2	3
3	1	3	1	3	3	2	2	3	3
2	1	3	1	1	3	3	3	2	3
2	2	3	3	2	2	2	3	2	3
1	2	2	3	3	3	3	2	3	3
2	2	2	2	2	2	2	3	2	2
3	1	3	1	2	2	2	3	3	3
2	2	2	2	2	2	2	2	2	2
2	2	2	2	2	2	2	2	2	2
9	9	2	9	9	9	9	9	9	9
2	2	2	2	3	3	3	3	3	3
2	2	2	1	1	1	2	2	2	3
2	2	2	2	2	2	2	2	2	2
1	2	1	2	3	1	3	2	2	3
2	2	2	2	2	2	2	2	2	2
3	3	3	1	1	1	1	1	3	1
2	2	2	2	2	2	2	3	2	3
2	2	2	2	2	2	2	2	2	2
2	2	2	2	2	2	2	2	2	2
2	2	2	2	2	2	2	2	2	2
2	2	2	2	3	3	3	3	3	2
2	3	2	9	2	2	2	3	2	3
1	1	1	3	1	1	3	3	3	3
9	9	9	9	9	9	9	9	9	9
2	1	3	1	3	2	3	3	2	3
2	2	2	2	2	2	2	2	2	2
2	2	2	2	2	2	2	2	2	3
2	2	2	2	2	2	2	2	2	2
3	2	9	2	9	2	1	2	2	2
3	3	3	1	3	2	1	3	3	2
2	2	2	3	2	2	2	1	1	2
2	2	2	2	2	2	2	2	2	2
2	1	2	2	2	2	2	2	2	2
1	1	3	1	3	3	3	3	3	3
9	1	3	1	3	3	3	3	3	3
9	9	9	9	9	9	9	9	9	9
2	2	2	2	3	2	3	3	2	2
2	3	3	2	3	2	3	3	3	3
2	2	2	2	2	2	2	2	2	2

2	2	2	2	2	2	2	2	2	2
1	2	2	2	2	2	2	2	2	2
2	2	2	2	2	3	2	3	2	2
2	2	2	2	2	2	2	2	2	2
2	2	2	2	2	2	2	2	2	2
9	9	9	9	9	9	9	9	9	9
2	2	2	2	2	2	2	2	2	2
2	2	2	2	2	2	2	2	2	1
2	2	2	2	2	2	2	2	2	2
2	2	2	2	2	2	2	2	2	3
2	2	2	2	2	2	2	2	2	2
1	1	1	3	3	3	2	2	2	2
9	9	9	9	9	9	9	9	9	9
1	1	1	1	1	1	3	3	3	3
9	9	9	9	9	3	3	3	3	2
9	9	9	9	9	9	9	9	9	9
2	9	1	3	3	3	3	2	2	2
3	3	3	1	1	1	1	1	1	1
9	9	9	9	9	9	9	9	9	9
2	1	2	2	3	2	1	3	3	2
9	9	9	9	9	9	9	9	9	9
2	3	3	2	2	2	2	2	2	3
2	9	2	9	1	2	9	1	9	2
9	9	9	9	9	9	9	9	9	9
3	9	3	3	3	3	3	1	1	1
9	9	9	2	1	1	2	9	3	9
9	9	9	9	9	9	9	9	9	9
3	3	2	3	1	1	2	3	2	1
3	3	3	3	3	3	3	3	3	3
9	1	2	9	9	9	9	9	9	9
2	2	2	2	3	3	3	3	2	3
9	9	9	9	9	9	9	9	9	9
1	1	1	3	2	2	2	3	3	1
3	1	3	1	2	3	2	2	2	2
2	2	2	1	2	2	2	2	2	2
2	1	2	1	2	2	1	3	3	3
1	3	3	1	3	3	3	3	3	3
3	1	2	2	3	3	3	3	3	2
3	1	3	1	1	1	2	3	3	3
9	9	9	9	9	9	9	3	9	9
3	1	3	2	3	2	2	3	3	3
2	3	2	2	2	3	3	3	3	3
1	1	1	3	2	2	2	2	3	3
1	2	2	2	2	2	2	2	2	2
1	1	2	2	2	2	1	3	1	2
3	2	1	3	3	3	3	3	3	3
2	3	2	2	3	3	2	1	3	2

9	9	9	9	9	9	9	9	9	9
9	9	9	9	9	9	9	9	3	9
1	1	1	3	3	3	3	3	3	3
9	9	9	9	9	9	9	9	9	9
9	9	9	9	9	9	9	9	9	9
9	9	9	9	9	9	9	9	9	9
3	1	1	3	1	3	3	3	3	1
3	2	2	2	2	2	2	2	2	1
3	3	3	2	3	3	3	2	3	3
3	3	3	3	1	3	1	3	3	3
9	9	9	3	3	3	9	3	3	9
3	3	1	9	3	3	3	3	3	3
2	2	2	3	3	3	3	3	3	2
1	1	1	3	2	3	3	2	2	3
9	9	9	9	9	9	9	9	9	9
2	2	2	3	2	2	2	3	2	2
3	3	3	3	3	3	3	3	3	3
2	1	1	3	3	3	3	3	3	3
9	9	9	9	9	9	9	9	9	9
1	2	1	1	2	3	2	9	3	9
1	2	2	2	3	3	2	3	3	3
2	3	1	3	3	3	3	3	3	2
3	3	3	3	3	3	3	3	3	3
2	1	3	3	3	3	3	3	3	3
2	1	2	3	3	3	3	3	3	3
3	2	2	2	2	3	3	3	3	3
2	1	2	3	3	3	3	2	3	3
3	1	2	3	3	3	3	3	3	3
1	1	1	3	3	3	3	3	3	3
3	3	2	3	3	2	3	3	3	3
1	3	9	9	9	9	9	9	9	9
1	1	1	3	2	2	3	2	2	2
3	1	3	9	3	3	2	2	2	3
9	9	9	9	3	9	9	9	9	9
3	1	3	1	3	3	1	3	3	3
1	1	3	2	3	3	3	2	3	3
2	2	2	2	2	3	3	3	3	3
2	2	3	2	2	3	2	2	2	3
9	9	9	9	9	9	9	9	9	3
1	3	9	3	3	1	9	9	9	9
9	9	9	9	9	9	9	9	9	3
9	9	9	9	9	9	9	9	9	3
3	3	1	3	3	3	3	3	3	1
2	2	3	2	2	2	2	2	2	1
1	2	2	2	2	2	2	2	2	2
2	2	2	2	3	2	2	3	3	3
2	2	2	2	2	2	2	2	2	2

9	9	9	9	9	9	9	9	9	9
9	9	9	9	9	9	9	9	9	9
9	9	9	9	9	9	9	3	9	9
9	3	9	9	9	2	9	9	9	9
9	9	9	9	9	9	9	9	9	3
9	9	9	9	9	9	9	9	9	9
2	2	2	2	2	2	2	2	2	1
9	9	9	9	9	9	1	3	3	3
2	2	2	2	2	2	2	2	2	2
3	3	3	2	3	3	2	1	3	3
2	3	3	1	2	2	3	2	2	2
3	1	3	2	2	2	3	3	3	3
1	1	3	1	3	3	3	1	1	3
3	1	2	1	3	3	3	3	3	1
2	1	2	9	3	1	2	2	3	2
3	3	2	3	3	2	2	2	2	2
3	3	3	1	1	9	1	2	1	2
9	9	9	9	9	9	9	9	9	9
2	2	1	2	3	2	3	2	1	2
9	9	9	9	9	9	9	9	9	9
9	9	9	9	9	9	9	9	9	9
9	9	9	9	9	9	9	9	9	9
2	2	2	2	2	2	2	3	2	2
2	2	2	1	3	3	3	2	2	3
2	2	2	2	2	2	2	2	2	2
2	3	3	3	1	3	2	2	3	2
9	9	9	9	9	9	3	9	9	9
1	2	1	1	2	2	3	1	2	1
2	3	2	2	2	2	3	3	3	3
2	2	2	2	3	3	3	3	2	2
3	2	3	3	3	3	2	3	2	3
3	2	2	2	3	3	3	2	3	3
3	1	3	3	3	3	3	3	3	3
3	2	3	3	3	2	2	3	3	2
3	3	3	1	3	3	3	3	3	3
9	9	9	9	9	9	9	9	9	9
1	3	1	9	3	3	3	3	3	2
1	1	2	2	3	3	2	3	3	3
1	3	3	1	3	3	2	3	3	2
3	3	3	3	3	3	2	2	2	3
3	2	9	3	2	2	2	3	3	3
3	3	3	1	3	2	1	3	3	2
1	9	2	9	1	2	1	9	3	9
3	1	3	3	3	3	3	1	2	2
2	2	2	2	2	2	2	2	2	2
3	3	3	3	3	3	2	2	3	3
2	2	3	9	1	9	3	3	3	3

9	9	9	9	3	3	9	9	9	3
1	1	1	3	3	3	3	3	3	3
1	1	1	3	3	3	3	3	3	3
2	2	2	2	3	3	3	3	3	2
9	2	9	2	9	2	9	9	1	1
2	2	1	3	2	2	3	3	3	3
3	1	3	1	3	3	3	3	3	3
3	2	3	1	1	2	2	2	2	1
3	2	2	2	2	2	2	2	1	1
1	2	3	2	3	3	2	3	2	3
3	2	2	3	3	2	2	3	3	3
2	2	2	3	3	3	3	3	2	3
1	1	1	3	3	3	3	3	3	3
3	1	3	1	3	3	3	3	3	2
9	9	9	9	9	9	9	9	9	9
3	1	2	2	3	2	2	3	3	3
9	9	9	9	3	3	3	3	3	3
9	2	9	3	1	3	2	1	9	3
2	3	2	2	1	2	2	2	2	2
2	3	1	2	3	3	2	2	3	2
2	2	2	2	2	2	2	2	2	2
2	2	2	2	3	3	3	3	3	3
9	9	9	9	9	9	9	9	9	9
1	3	3	3	3	3	1	3	3	3
2	9	1	3	2	3	2	2	2	2
1	2	2	2	2	2	3	3	3	2
1	9	3	3	2	3	2	2	9	2
3	2	2	3	3	3	2	3	3	3
1	9	9	9	3	3	3	3	3	3
1	2	1	2	1	1	3	2	9	1
1	1	9	9	3	3	9	3	9	3
2	3	3	2	3	3	2	1	9	2
2	2	2	2	2	2	2	2	2	2
2	2	2	2	2	2	2	2	2	2
2	1	1	3	3	3	3	2	2	3
9	1	3	1	9	9	9	9	9	9
1	1	9	2	2	2	1	3	2	3
9	9	9	9	9	9	9	9	9	9
2	3	3	1	3	3	2	3	3	3
2	2	2	1	1	1	1	1	1	1
3	2	2	3	3	2	3	3	3	3
3	2	1	2	3	1	2	3	2	3
9	1	9	9	9	9	9	3	9	9
3	3	3	1	9	9	9	3	3	3
2	1	2	2	3	2	2	3	9	3
9	3	1	1	3	3	1	1	3	3
2	2	2	2	2	2	2	2	2	2

2	2	2	2	2	2	2	3	3	3
1	9	2	3	3	3	3	3	3	3
9	2	9	2	9	3	3	3	3	3
9	9	9	9	9	9	9	9	9	9
3	3	3	2	3	3	2	2	3	2
2	2	2	2	2	2	2	2	2	2
2	2	2	2	2	2	2	2	2	2
1	1	1	3	3	3	3	3	3	3
9	9	9	9	2	3	3	3	3	3
3	3	3	3	3	3	3	1	3	3
3	3	1	3	9	3	1	1	1	1
3	2	3	1	3	3	3	3	3	3
2	1	2	2	3	3	3	3	3	3
3	2	2	2	3	3	2	3	3	2
3	1	1	3	3	3	3	3	3	1
3	1	2	2	3	3	3	3	3	3
1	2	2	1	1	2	3	3	3	3
3	1	3	1	1	1	1	1	1	1
3	3	3	2	3	3	1	1	3	3
3	3	3	1	1	1	3	3	3	3
1	2	2	3	3	3	3	2	3	3
3	3	3	3	3	3	3	3	3	3
3	1	3	3	3	1	3	3	2	3
3	3	3	2	3	3	3	2	3	3
2	1	3	2	2	2	2	2	3	2
3	2	3	1	3	3	3	3	3	3
3	1	3	3	3	3	3	3	3	3
2	2	2	2	2	2	2	2	2	2
1	2	2	3	3	3	3	3	3	3
3	3	3	1	3	3	1	3	1	1
1	3	2	1	3	2	3	9	9	3
2	1	3	1	3	3	3	3	3	3
3	2	2	1	1	2	2	1	1	2
1	2	1	2	2	3	2	3	3	3
2	3	3	2	2	2	3	3	3	3
9	9	9	9	9	9	9	9	9	9
2	2	2	3	1	3	2	3	1	2

BreastSizea	Nippledevi	Raisopposi	Undressto	HeardCerv	CervicalCA	PapSmear	HPVandVa	Papdetect	PapforMar
2	2	2	3	2	2	2	2	2	2
2	2	1	1	2	2	2	2	2	2
1	2	1	2	2	2	1	2	2	2
1	1	1	1	1	1	2	1	2	2
9	9	9	9	2	2	2	2	2	2
2	2	1	1	1	1	2	2	2	2
2	2	2	2	2	2	2	2	9	9
1	1	1	1	1	2	2	2	1	2
9	9	1	9	2	2	2	2	2	9
9	9	9	9	2	2	2	2	2	9
1	2	2	2	2	2	2	9	2	2
1	2	2	3	2	2	2	3	3	3
2	2	2	2	1	2	2	1	1	2
2	2	2	1	1	3	1	3	1	2
2	2	1	1	2	2	2	2	2	2
1	2	1	2	1	2	2	2	1	3
1	2	1	3	1	1	1	3	2	2
1	2	1	2	1	2	2	2	1	3
1	2	1	2	1	2	2	2	1	3
2	1	1	1	2	2	1	1	2	2
1	1	1	1	1	1	2	2	1	1
1	1	1	1	1	1	2	2	1	1
9	9	1	2	2	2	2	2	3	2
1	2	1	2	1	1	3	3	1	3
2	2	2	2	2	2	2	2	2	2
1	1	1	2	2	2	2	2	2	2
2	2	2	2	3	3	3	3	2	3
2	2	2	3	3	3	1	2	1	1
2	2	1	1	2	2	2	2	2	2
2	2	1	1	2	2	9	2	2	2
2	2	1	1	2	2	2	2	2	2
1	1	1	1	1	1	2	2	1	2
1	1	1	1	2	2	2	2	1	2
1	1	1	1	1	1	1	1	1	2
1	1	1	1	1	1	2	2	1	2
1	1	1	1	1	1	2	1	1	2
1	1	1	1	1	1	2	2	1	2
2	2	1	1	1	1	1	1	1	3
2	2	1	1	9	9	9	9	2	2
1	9	1	1	2	2	2	2	2	2
2	2	1	1	2	2	2	2	2	2
2	1	1	2	1	2	2	3	1	2
1	2	2	3	9	9	9	9	1	1
1	1	1	3	1	1	1	3	1	3
1	1	3	3	2	2	2	2	9	9
9	9	9	9	1	2	2	1	1	1

1	2	1	1	1	1	2	2	1	3
1	2	1	1	1	3	2	1	2	3
9	9	1	9	1	1	1	1	1	2
1	1	1	1	1	1	3	1	1	2
9	1	1	1	1	1	1	1	1	3
1	1	1	3	1	1	1	1	1	3
1	3	1	1	1	1	1	1	1	3
1	1	1	1	1	1	1	2	1	3
1	9	1	9	1	1	2	2	1	9
1	2	1	1	1	1	1	2	1	2
9	9	9	9	2	2	2	2	2	2
1	1	1	1	1	1	2	1	2	2
1	2	1	2	1	1	3	2	1	3
1	9	9	1	2	2	2	2	2	2
2	1	2	2	2	2	2	2	2	2
1	2	1	3	1	1	1	2	1	3
1	2	1	1	1	1	2	1	1	2
1	1	1	1	1	1	1	1	1	2
1	1	2	3	1	2	2	2	1	1
2	2	2	2	1	1	1	2	1	9
9	9	9	9	1	1	2	1	1	3
1	3	3	3	1	1	2	2	1	3
9	9	9	9	1	1	2	1	1	3
1	1	1	1	1	2	2	1	1	3
1	9	1	9	1	1	2	3	9	1
1	1	1	1	1	1	2	1	1	2
2	3	1	1	9	9	9	9	9	9
2	1	2	3	1	2	2	2	1	3
1	2	1	1	2	3	3	3	2	2
1	1	1	3	1	1	1	1	1	3
1	1	1	1	1	1	1	1	1	2
3	1	3	3	1	1	1	2	1	3
1	1	1	1	1	1	9	9	1	2
1	1	1	2	1	1	2	1	9	3
1	2	1	1	1	1	1	1	1	3
2	1	1	1	2	2	2	1	9	9
2	2	2	2	9	9	9	9	9	9
2	2	1	1	9	9	9	9	1	3
1	1	1	3	3	1	1	1	1	3
1	1	1	1	1	1	1	1	1	3
1	1	1	1	1	1	1	1	1	9
1	1	1	3	2	2	3	2	1	3
1	3	1	1	1	1	2	2	1	1
1	1	1	1	1	1	1	1	1	2
2	2	1	1	2	2	2	2	1	2
2	2	3	3	1	1	2	1	9	9
9	9	9	9	2	2	2	9	9	9

2	2	1	1	1	1	2	1	1	2
2	2	1	1	1	1	1	1	1	2
1	1	1	1	1	1	1	1	2	1
2	2	1	1	1	9	1	1	1	3
2	2	1	1	2	2	2	2	9	9
2	2	1	2	2	1	2	2	1	2
1	1	1	1	1	1	1	1	1	3
1	1	1	2	1	1	1	2	1	2
2	2	1	1	1	1	1	1	1	3
1	1	1	1	1	1	1	1	1	3
1	1	1	2	2	2	3	1	1	3
1	2	1	2	1	9	1	3	1	2
2	2	1	1	1	1	3	3	1	2
1	1	1	1	3	3	3	3	1	2
3	2	3	2	2	2	1	3	2	3
2	2	3	3	1	2	2	2	3	2
2	2	1	1	2	2	2	1	2	2
1	2	1	1	2	2	2	2	2	2
2	2	1	2	3	2	2	2	1	2
9	9	9	9	2	2	2	2	2	2
2	1	1	2	2	2	2	2	1	3
1	3	1	3	2	2	2	2	1	3
2	2	2	2	2	2	2	2	2	2
9	9	9	9	2	2	2	2	3	3
2	2	1	1	2	3	3	1	3	1
2	2	1	2	2	2	2	2	2	2
2	1	1	3	1	1	1	2	1	2
2	2	2	2	1	1	1	1	1	2
2	2	2	2	2	2	2	2	2	2
2	2	3	3	2	2	2	2	2	2
2	2	2	2	2	2	2	2	2	2
2	3	2	2	3	3	2	3	2	2
2	2	2	2	2	2	2	2	1	2
2	2	2	2	3	2	2	2	2	2
2	2	2	2	2	2	2	2	2	2
1	1	1	1	2	2	2	2	2	2
1	2	2	2	1	1	2	2	2	2
2	2	1	3	2	2	2	2	2	2
1	9	1	9	1	1	2	2	9	1
1	2	1	2	1	1	3	2	1	3
1	2	1	2	2	2	2	2	2	2
3	2	1	2	3	2	2	2	2	2
2	2	2	2	3	3	2	3	2	2
1	1	2	1	1	1	2	2	1	2
2	2	2	2	3	3	3	3	2	2
1	2	1	1	1	2	2	2	1	2
1	2	3	3	2	2	2	2	2	2

3	3	3	3	2	2	2	2	2	2
2	1	2	1	2	2	2	3	1	2
2	2	1	2	1	1	2	2	9	2
3	2	1	2	3	2	2	2	2	2
1	1	1	1	3	2	2	2	9	9
2	2	1	1	2	2	2	2	2	2
2	2	3	3	2	2	2	2	2	3
1	2	1	1	2	3	2	2	2	2
2	2	2	2	2	2	2	2	2	2
2	2	2	2	2	2	2	2	2	2
3	1	2	3	2	2	2	3	2	1
3	1	2	3	2	2	2	9	2	3
9	9	9	9	2	2	2	2	9	2
2	1	3	2	2	2	2	2	1	2
2	2	1	2	1	1	2	2	2	2
2	2	1	2	1	1	2	2	2	2
9	9	9	9	1	1	3	1	9	9
1	3	1	3	2	2	2	2	2	2
9	9	9	9	1	2	2	2	9	9
1	1	1	1	1	2	2	2	1	3
1	1	1	1	9	1	2	2	1	3
1	1	1	1	2	2	2	2	1	3
1	1	1	1	2	2	2	2	1	3
1	1	1	1	2	2	2	2	1	3
1	1	1	1	2	2	2	2	1	3
1	1	1	1	2	9	2	2	1	3
1	1	1	1	1	2	2	2	1	3
1	1	1	1	1	1	2	2	1	3
1	1	1	1	2	3	2	2	1	3
1	1	1	3	1	2	2	2	1	3
2	3	2	2	1	1	2	2	3	2
1	1	2	2	2	2	2	2	2	2
9	9	9	9	9	9	9	9	1	3
1	1	1	3	2	2	2	2	1	3
1	1	1	3	1	2	2	2	1	3
1	1	1	2	2	2	2	2	1	3
1	1	1	1	1	1	2	2	1	3
1	1	1	1	1	2	2	2	1	3
1	1	1	2	2	2	2	2	1	3
1	1	1	1	2	1	2	2	1	3
1	1	1	1	2	2	2	2	1	3
1	1	1	1	2	2	2	2	1	3
9	9	9	9	9	9	9	9	1	3
1	1	1	3	1	2	2	2	1	3
1	1	1	3	1	2	2	2	1	3
1	1	1	3	1	2	2	2	1	3
1	1	1	3	1	2	2	2	1	3

1	1	1	3	1	2	2	2	1	3
1	1	1	1	2	2	2	2	1	3
1	1	1	1	2	2	2	2	1	3
1	1	1	3	1	1	2	2	1	3
1	1	1	1	2	2	2	2	1	3
1	1	1	1	1	1	1	2	1	3
1	1	1	1	2	1	2	2	1	3
1	1	1	1	1	1	1	1	1	3
1	1	1	1	1	1	1	1	1	3
1	1	1	3	1	1	2	1	1	3
1	1	1	1	9	9	9	9	9	9
1	1	1	1	1	1	1	2	1	3
1	1	1	1	1	1	1	1	1	3
1	1	1	1	1	2	2	2	1	3
1	1	2	2	3	3	3	3	1	2
1	2	1	1	2	2	2	2	1	1
1	1	1	1	1	2	2	2	2	2
1	1	1	1	1	2	9	1	1	1
1	1	1	2	1	2	2	2	2	2
1	2	2	1	1	2	2	2	1	2
1	2	3	3	1	1	3	3	2	2
2	2	2	2	1	2	2	2	2	2
2	1	2	1	2	2	2	2	1	2
1	2	2	2	1	3	3	2	1	2
2	2	2	2	2	2	2	2	2	2
2	2	2	2	2	2	2	2	2	2
2	2	2	1	1	2	2	2	1	2
2	2	2	2	2	2	2	2	2	2
2	2	2	2	2	2	2	2	2	2
2	2	2	2	1	2	2	2	2	2
2	1	2	2	1	1	2	3	1	3
2	1	2	2	1	1	2	3	1	3
1	1	1	2	1	2	2	2	2	2
1	1	1	1	1	1	1	1	1	1
1	2	2	2	1	1	3	1	1	2
2	2	2	2	1	1	1	2	2	2
1	3	2	1	2	2	2	2	2	2
3	1	1	2	1	1	2	2	3	3
2	2	2	2	2	2	2	2	1	2
2	1	2	2	1	2	2	2	1	2
2	2	2	2	1	1	1	1	1	2
2	2	2	1	1	2	2	2	1	2
1	9	9	9	2	2	3	2	1	9
1	9	9	9	2	2	3	2	1	9
1	1	1	1	1	1	2	1	1	3
1	2	3	3	1	1	2	2	1	2
9	9	9	9	3	1	2	2	2	9

1	1	2	3	2	2	2	2	2	2
1	1	2	3	2	2	2	2	2	2
2	2	2	2	2	2	2	2	1	2
2	2	2	2	2	2	1	2	1	2
2	2	2	2	2	2	2	2	2	2
1	1	1	2	1	1	1	1	2	1
1	2	1	1	2	2	2	2	1	2
2	1	2	2	2	2	2	2	2	2
1	1	1	2	1	1	2	2	1	3
3	3	3	3	2	2	2	2	3	3
2	2	2	2	2	2	2	2	2	2
1	1	1	2	1	2	3	1	1	3
1	1	1	2	1	2	3	1	1	3
1	1	1	2	1	3	3	3	2	2
2	2	2	3	2	2	2	2	2	2
1	2	2	3	2	3	3	3	1	2
2	1	3	2	1	3	3	2	2	2
2	2	2	2	2	2	1	1	2	3
3	2	1	3	2	2	2	2	2	9
1	1	3	2	2	1	1	1	1	2
1	1	2	3	2	2	2	1	1	2
1	2	1	2	2	3	2	3	1	2
1	1	1	2	1	3	3	2	2	2
2	2	2	2	2	2	2	2	1	2
1	9	9	9	3	3	3	2	9	2
2	2	2	2	2	2	2	2	2	2
2	2	2	2	2	2	2	9	2	2
1	3	2	3	1	2	3	3	1	9
9	9	9	9	2	2	3	3	2	2
2	2	2	1	2	3	3	3	2	2
9	9	9	9	3	1	1	3	9	9
9	9	1	9	2	2	2	2	2	2
1	1	3	3	2	2	2	2	3	3
1	2	1	2	1	1	2	2	2	2
9	9	9	9	1	3	3	3	1	9
2	3	2	2	3	3	3	3	2	2
1	2	1	2	2	2	2	2	1	2
1	1	3	2	1	2	2	2	1	3
1	1	1	1	1	3	3	3	1	2
1	3	2	3	2	1	2	2	2	2
1	1	3	3	1	1	2	2	1	2
2	1	1	3	1	1	2	2	1	2
1	1	1	1	1	1	1	1	1	1
2	2	2	2	1	2	2	2	2	2
9	9	9	9	1	2	2	1	2	3
2	3	2	3	1	2	2	2	1	2
2	3	1	2	2	2	2	2	2	2

9	9	9	9	9	9	9	9	9	9
9	9	9	9	9	9	9	9	9	9
1	1	1	1	1	1	1	2	1	2
1	1	2	1	1	1	2	1	2	2
1	2	2	2	2	2	2	2	1	3
1	2	1	3	2	2	2	1	3	1
1	1	1	1	1	1	1	1	1	3
3	1	1	3	1	1	1	1	1	1
1	1	1	1	1	2	2	2	2	3
2	2	2	2	1	2	1	2	2	2
2	2	2	2	3	3	3	3	2	2
1	2	1	2	1	1	1	2	1	2
9	9	9	9	9	9	9	9	9	9
1	1	1	3	2	2	1	1	1	1
1	1	1	1	1	1	2	1	1	1
2	2	1	3	9	9	9	9	9	9
2	1	1	1	1	1	1	2	2	1
3	2	2	2	2	2	1	2	1	2
1	1	1	3	1	2	3	1	2	1
9	9	9	9	9	9	9	9	9	9
3	1	2	2	2	2	2	2	9	9
1	2	1	2	1	2	2	2	2	2
2	1	2	2	1	1	2	3	2	2
2	2	2	2	1	2	2	2	2	2
2	2	2	2	1	2	2	2	2	2
1	3	1	1	9	2	3	3	1	2
1	2	1	1	1	1	1	1	1	1
1	1	1	3	3	2	3	2	1	3
1	1	1	1	1	2	1	1	1	3
1	2	1	9	1	2	2	2	1	2
1	2	1	2	1	3	3	1	2	2
1	1	3	3	2	2	2	2	3	3
9	9	9	9	1	1	1	1	9	9
3	3	1	3	2	2	2	2	9	9
1	1	1	1	1	2	2	2	1	2
1	1	1	3	1	1	3	3	2	2
1	2	1	3	1	1	1	2	1	3
1	1	3	3	3	1	1	1	1	2
1	1	1	1	1	1	1	2	1	3
2	2	1	1	1	2	2	2	1	2
3	3	3	3	1	1	1	2	1	2
1	1	1	1	1	1	1	2	1	3
3	3	2	2	1	1	1	1	1	2
2	2	2	2	2	2	2	2	2	2
1	2	2	1	1	1	1	1	2	3
1	1	1	1	1	1	2	2	1	2
1	2	1	3	1	2	2	2	2	2

1	1	1	2	3	2	2	2	2	3
1	1	1	2	3	2	2	2	2	3
9	9	9	9	1	1	1	1	1	9
1	1	1	2	1	2	2	2	2	2
9	9	1	9	1	1	1	3	9	9
1	1	2	2	1	2	2	2	9	9
1	1	1	2	1	1	2	2	2	2
1	9	9	9	1	2	2	1	1	2
1	1	1	1	1	2	2	2	1	3
1	2	1	1	1	2	2	2	1	1
2	2	2	3	2	2	2	2	1	2
2	1	2	2	1	1	1	2	2	2
1	1	1	2	1	1	2	1	1	3
3	2	2	3	2	2	2	2	2	1
2	2	2	1	2	2	2	2	1	2
1	2	1	2	2	9	2	2	2	2
2	2	2	1	2	2	2	2	2	2
2	1	2	3	1	2	2	2	2	2
1	1	1	1	1	1	1	2	2	2
2	1	2	2	2	2	2	2	2	2
1	2	1	2	1	2	1	1	1	1
3	2	1	1	2	2	2	2	1	2
1	1	2	1	1	1	2	2	1	2
1	1	2	2	1	1	2	1	1	3
2	2	2	2	2	2	2	2	2	2
2	2	2	2	2	2	2	2	2	2
1	1	1	2	1	2	2	2	1	2
3	2	2	3	2	3	3	3	2	2
1	1	2	2	1	1	3	3	2	2
1	2	1	2	2	2	2	2	1	2
2	1	2	1	3	3	1	1	1	3
2	1	2	1	2	2	2	2	1	3
1	3	3	3	2	2	2	3	1	1
2	2	2	2	2	2	2	2	2	2
2	2	2	2	2	2	2	2	2	2
1	2	9	1	1	1	2	2	1	3
1	1	1	2	3	2	2	2	2	2
1	9	1	9	3	1	2	3	2	2
2	2	2	2	2	2	2	2	2	2
1	1	3	3	2	2	2	2	2	2
2	2	2	2	3	3	3	2	2	2
1	1	2	1	2	2	2	2	2	2
1	1	1	2	1	2	2	1	1	3
2	2	2	2	2	2	2	2	2	2
1	1	2	1	2	2	2	1	1	2
1	2	1	2	1	1	2	3	1	3
2	2	2	2	1	3	2	2	2	2

1	1	2	3	1	2	2	2	1	3
1	1	2	3	1	1	2	1	1	3
3	3	3	3	1	1	2	1	2	2
1	2	2	2	3	2	3	3	2	2
1	2	2	2	3	2	2	1	2	2
1	2	2	3	1	3	2	1	1	2
2	2	9	9	9	9	9	9	9	9
1	2	1	2	1	1	1	1	1	2
1	2	1	2	1	1	1	3	1	3
1	2	2	1	1	2	2	2	2	3
1	2	2	1	2	2	2	2	3	3
1	1	1	1	2	2	2	2	1	3
1	2	1	2	2	2	2	2	2	2
2	2	2	2	2	2	2	2	1	2
2	2	2	2	2	2	2	2	2	2
1	9	2	2	9	9	9	9	1	2
1	1	2	2	2	2	2	2	1	3
1	2	2	1	3	2	2	2	2	2
1	2	2	1	3	2	2	2	2	2
9	9	9	9	1	2	3	2	2	2
1	1	2	2	1	1	1	2	1	2
2	2	2	2	1	3	2	2	2	2
9	9	9	9	9	9	9	9	9	9
2	2	2	2	2	1	1	1	1	2
1	1	1	1	1	1	2	2	1	3
9	2	9	2	9	9	9	9	9	9
2	1	1	3	3	3	1	1	1	2
9	9	9	9	9	9	9	9	9	9
2	2	2	2	1	1	1	1	2	2
1	1	2	2	1	1	2	2	2	2
2	2	2	2	2	2	2	2	9	9
2	2	3	2	9	9	9	9	9	9
2	2	2	2	2	1	1	2	2	2
1	2	1	3	2	2	2	2	2	1
1	1	1	2	1	2	2	2	2	3
1	1	1	1	1	1	1	1	2	1
3	2	3	2	1	2	3	3	3	3
2	1	2	1	1	2	3	2	2	1
2	2	2	2	9	9	9	9	9	9
1	1	1	2	1	2	2	2	1	3
2	2	1	2	2	3	3	2	2	2
2	2	1	1	2	2	2	2	2	2
1	2	3	1	1	3	3	3	1	2
3	3	3	3	1	2	2	2	3	3
9	9	9	9	9	9	9	9	9	9
1	1	1	9	3	2	2	2	1	3
1	1	1	1	2	2	2	2	1	3

1	1	1	1	3	2	2	2	9	9
1	1	1	1	2	2	2	2	1	3
1	1	1	1	2	2	2	2	1	3
1	1	1	1	1	2	2	2	1	3
1	1	1	1	2	2	2	2	1	3
1	1	1	3	1	1	2	2	1	3
1	1	1	1	2	2	2	2	1	3
1	1	1	1	2	2	2	2	1	3
1	1	1	1	2	2	2	2	1	3
1	1	1	3	1	2	2	2	1	3
1	1	1	1	3	2	2	2	9	1
1	1	1	3	2	2	2	2	1	3
1	1	1	1	1	1	2	2	1	3
1	1	1	1	2	2	2	2	1	3
1	1	1	1	9	2	2	2	1	3
1	1	1	1	1	1	2	2	1	3
1	1	1	2	2	2	2	2	1	3
1	1	1	1	1	2	2	2	1	3
1	1	1	1	1	2	2	2	1	3
1	1	1	1	1	2	2	2	1	3
1	1	1	1	1	2	2	2	1	3
1	1	1	1	1	2	2	2	1	3
1	1	1	1	2	2	2	2	1	3
1	1	1	1	1	1	2	1	1	3
1	1	1	3	2	2	2	2	1	3
1	1	1	3	2	2	2	2	1	3
1	1	1	1	2	2	2	2	1	3
1	1	1	1	9	2	2	2	1	3
1	1	3	1	2	1	2	2	1	3
1	1	1	3	2	2	2	2	1	3
1	1	1	3	2	1	2	2	1	3
1	1	1	1	2	2	2	2	1	3
1	1	1	3	2	1	2	2	1	3
1	1	1	1	2	2	2	2	1	3
1	1	1	3	1	1	2	2	1	3
1	1	1	3	1	1	2	2	1	3
1	1	3	3	1	1	2	2	1	3
1	1	1	1	2	1	2	2	1	3
1	1	1	1	2	2	2	2	1	3
1	1	1	9	1	1	1	2	1	3
1	1	1	1	1	2	2	2	1	3
2	2	2	2	3	3	3	3	2	2
1	2	1	1	1	2	2	1	1	2
2	2	2	2	3	3	3	3	2	2
9	9	9	9	2	2	2	2	2	9
2	2	1	2	2	2	2	2	2	2
1	2	2	2	1	2	1	2	9	2

2	2	2	2	3	2	2	2	2	2
1	1	1	2	1	1	2	1	1	2
1	1	1	2	1	1	2	1	1	2
1	1	1	3	1	1	2	2	1	1
2	2	1	3	3	2	3	3	2	2
1	2	1	2	2	2	2	2	2	2
2	2	2	2	3	1	1	3	2	2
2	2	2	2	3	2	9	3	2	2
2	2	1	1	1	1	2	2	1	2
2	2	2	2	1	2	2	2	2	2
1	1	1	2	1	1	1	1	1	2
9	9	9	9	1	2	1	3	9	9
9	9	1	1	1	1	1	1	1	3
1	2	2	2	2	2	2	2	2	2
9	9	9	9	1	2	2	2	9	9
9	9	9	9	1	1	1	2	9	9
9	9	9	9	2	2	2	2	9	9
9	9	9	9	2	2	2	2	1	9
9	9	9	9	2	2	2	2	9	9
2	2	1	2	1	3	2	2	1	2
3	3	3	3	1	3	1	1	2	9
3	3	1	3	1	2	1	2	9	9
1	2	1	1	1	1	2	2	1	3
9	9	9	9	2	3	2	2	1	9
9	9	9	9	2	2	2	2	9	9
9	9	9	9	9	9	9	9	9	9
9	9	9	9	1	1	1	1	1	9
2	1	1	1	2	2	3	2	9	9
3	1	1	1	2	2	3	1	1	2
9	9	9	9	1	2	1	3	9	9
9	9	9	9	1	1	2	2	1	9
2	2	2	2	2	2	2	2	2	2
2	2	2	2	2	2	2	2	2	2
3	1	1	1	1	1	1	1	1	3
2	1	3	1	3	2	2	2	2	1
1	2	2	3	1	1	2	3	2	2
1	2	2	2	1	1	2	2	1	3
1	1	3	3	1	3	2	2	1	3
2	2	2	2	2	2	3	2	2	2
1	1	3	1	1	3	2	3	1	2
9	9	9	9	3	2	2	2	1	9
1	1	1	1	1	1	1	1	1	3
1	2	1	2	2	2	2	2	2	2
1	1	1	2	3	3	3	3	1	2
1	2	3	3	2	2	2	2	2	2
1	1	3	2	1	1	2	2	2	2
1	1	2	1	2	2	2	2	1	3

1	9	9	9	1	1	1	1	1	3
2	3	3	3	1	1	3	1	1	3
1	1	2	3	2	2	2	2	1	3
1	2	1	1	2	2	2	2	1	2
2	2	3	1	1	3	1	2	2	3
2	2	2	2	3	3	2	2	2	2
2	2	2	2	2	2	2	2	2	2
2	2	2	2	2	1	2	2	2	2
2	1	2	2	2	2	2	2	2	2
2	2	2	2	3	2	2	2	2	2
2	2	2	2	3	3	3	2	2	2
2	2	2	2	1	1	1	2	1	1
2	2	2	2	2	2	2	2	2	2
2	2	2	2	2	2	2	2	2	2
1	2	2	2	1	1	2	2	1	3
2	2	2	2	2	2	2	2	2	2
1	2	1	3	3	3	3	3	2	2
2	2	2	2	2	2	2	2	2	2
2	3	3	1	1	2	2	2	2	2
1	2	2	1	1	1	1	2	1	2
1	2	1	2	2	2	2	1	2	2
1	1	2	1	1	1	2	2	1	2
1	1	1	2	1	1	2	2	1	3
1	1	1	2	2	2	2	2	2	2
2	1	2	3	2	2	2	2	2	2
1	2	2	2	2	2	2	2	2	2
1	1	1	3	1	1	2	1	1	3
1	3	1	3	1	2	1	2	1	3
2	2	2	2	1	1	2	2	1	2
3	3	2	2	1	2	2	2	1	3
1	1	2	2	9	9	9	9	2	2
9	9	2	3	2	2	2	2	2	2
2	2	2	3	2	2	2	2	2	2
2	2	3	2	2	2	2	2	1	2
1	1	1	1	2	2	2	2	2	2
9	9	9	9	3	2	1	2	2	9
2	2	1	2	2	3	2	2	2	2
1	1	1	1	1	1	1	1	1	2
2	2	2	2	2	2	2	2	2	2
1	1	1	2	1	1	2	2	1	3
1	2	1	1	9	9	2	2	1	9
1	1	1	1	2	2	3	1	1	2
3	1	1	1	2	2	2	2	1	2
1	2	1	2	1	9	1	3	1	2
1	1	1	1	1	1	1	2	1	3
2	2	2	2	2	2	2	2	2	2
9	9	9	9	2	2	2	1	1	9

9	9	9	9	1	1	2	2	2	9
9	9	9	9	2	2	2	2	9	9
9	9	9	9	2	2	2	2	1	9
9	9	9	9	9	9	9	9	9	9
9	9	9	9	2	1	2	2	1	9
9	9	9	9	1	2	2	1	1	9
9	9	9	9	2	2	2	2	9	9
9	9	9	9	2	2	2	2	1	9
9	9	9	9	2	2	2	2	1	9
9	9	9	9	1	1	1	1	1	9
9	9	9	9	2	2	2	2	9	9
9	9	9	9	1	1	1	1	1	9
9	9	9	9	2	2	2	2	1	9
9	9	9	9	1	1	1	1	9	9
9	9	9	9	2	1	2	3	9	9
9	9	9	9	2	2	2	2	9	9
9	9	9	9	1	2	2	1	9	2
9	9	9	9	1	2	2	2	1	3
2	2	2	2	3	3	3	3	2	2
1	9	1	2	1	1	1	2	1	9
1	1	1	1	1	1	1	1	1	1
1	3	2	2	2	2	2	2	2	3
3	2	2	2	2	2	9	2	2	2
1	1	1	1	3	1	1	1	1	1
3	2	3	2	2	2	9	2	3	3
1	2	3	3	2	1	2	1	1	3
2	2	2	2	1	1	1	2	2	2
2	2	1	1	3	2	2	2	2	1
2	2	2	2	2	2	2	2	2	2
1	2	1	2	1	3	3	3	2	2
2	2	3	1	9	9	9	9	1	1
1	1	3	1	2	2	2	2	2	2
9	9	9	9	2	2	2	2	1	2
3	1	3	3	1	1	2	2	2	2
1	2	1	1	2	2	2	1	1	1
2	2	2	3	2	2	2	2	1	2
2	2	2	2	2	2	2	2	2	2
3	2	1	2	9	9	9	9	2	3
1	2	1	2	1	1	1	1	1	1
1	1	1	2	1	2	1	9	9	9
2	2	2	2	1	2	1	2	2	2
1	1	3	1	1	1	1	1	1	1
1	1	1	1	9	9	9	9	9	9
2	3	1	1	1	3	1	1	1	1
2	2	2	2	2	2	2	2	2	2
9	9	9	9	1	2	2	2	2	2
1	2	2	2	2	3	3	2	2	2

1	1	1	3	2	2	2	2	2	2
9	9	9	9	9	9	9	9	9	9
3	1	1	1	2	2	2	2	1	1
9	9	9	9	9	9	9	9	9	9
2	2	2	2	1	2	1	1	1	3
2	2	2	2	1	2	1	2	1	3
9	9	9	9	9	9	9	9	9	9
1	1	1	3	2	2	2	2	2	2
1	2	2	3	1	1	1	1	2	1
1	1	1	1	2	2	2	2	1	3
1	1	1	1	1	1	1	1	1	1
1	1	2	1	1	1	2	1	2	3
3	3	2	2	2	3	3	3	3	3
2	3	1	2	9	9	9	9	9	9
2	2	2	2	9	9	9	9	9	9
3	2	3	1	1	3	1	2	9	9
1	1	1	1	1	9	2	2	1	3
9	9	9	9	9	9	9	9	9	9
1	1	2	3	1	2	2	1	1	3
3	3	3	3	1	2	2	2	1	3
3	3	3	3	1	2	2	2	3	3
3	3	2	2	2	3	3	3	3	3
1	2	2	3	1	1	1	1	1	1
1	2	2	3	1	2	2	1	3	1
2	2	2	2	1	2	2	2	2	2
3	1	1	1	2	2	2	2	1	1
3	3	3	3	1	1	1	1	1	2
1	1	1	1	2	2	1	1	3	1
2	2	3	1	2	3	3	3	2	2
1	1	1	1	1	2	2	2	1	2
2	1	3	1	2	2	2	1	9	9
9	9	9	9	1	1	1	2	9	9
2	2	1	1	1	3	3	3	1	2
1	1	2	9	3	2	2	2	2	3
9	1	1	9	3	2	2	9	2	3
1	1	1	1	1	2	2	1	1	2
1	1	1	1	2	2	2	2	2	2
9	9	9	9	9	9	9	9	9	9
9	9	9	9	9	9	9	9	9	9
9	9	9	9	9	9	9	9	9	9
9	9	9	9	9	9	9	9	9	9
2	2	3	2	1	1	1	1	3	2
3	2	1	3	2	2	2	1	1	2
9	9	9	9	9	9	9	9	9	9
1	3	2	3	2	2	2	1	1	2
2	2	2	2	2	3	2	2	2	2
9	1	3	3	2	2	2	2	1	3

3	3	3	3	2	2	2	2	3	3
1	1	1	1	1	3	3	3	2	2
1	3	2	3	1	2	1	1	3	3
1	1	2	2	1	3	3	3	1	2
2	2	2	2	2	2	2	2	2	2
9	1	9	1	1	2	1	1	1	3
9	9	9	9	9	9	9	9	9	9
1	2	1	1	1	1	2	3	1	2
3	3	3	3	2	2	2	2	3	3
1	2	1	2	1	3	2	2	2	2
3	3	3	3	2	2	2	2	3	3
1	1	1	1	1	3	2	2	1	1
2	2	3	3	2	3	2	2	2	2
2	1	2	3	2	3	2	2	2	2
2	2	2	3	2	3	2	2	2	2
2	2	2	3	2	3	2	2	2	2
2	2	2	3	2	3	2	2	2	2
2	1	2	3	2	3	2	2	2	2
2	2	2	3	2	3	2	2	2	2
1	1	3	3	2	2	2	2	1	3
2	3	2	3	2	2	2	2	1	2
3	3	3	3	9	9	9	9	2	3
1	1	1	2	2	2	2	2	2	2
2	2	2	2	2	2	2	2	2	2
3	3	3	3	2	2	2	2	3	3
2	2	2	3	2	3	2	2	2	2
9	9	9	9	2	2	2	2	9	9
3	3	1	1	2	2	2	2	1	3
9	9	9	9	1	1	1	1	1	9
3	3	3	3	2	2	2	2	2	2
2	1	3	1	2	2	2	2	1	3
3	2	3	2	3	3	2	2	3	2
1	2	2	1	1	2	3	2	1	2
3	3	3	3	2	3	2	1	9	9
3	3	3	3	2	2	2	2	3	3
1	1	9	2	2	2	2	2	3	2
9	9	9	9	1	2	2	3	1	9
1	1	2	2	3	3	3	1	2	2
2	2	2	3	2	3	2	2	2	2
1	2	1	2	2	2	2	2	1	1
2	1	2	3	2	2	3	3	1	1
3	1	3	3	2	2	2	2	1	3
1	9	2	2	1	2	1	1	1	1
1	3	1	3	1	1	2	1	3	1
2	3	1	3	2	2	1	2	1	1
1	1	1	1	1	1	1	1	1	1
1	2	1	2	2	2	2	2	2	2

1	1	2	3	2	2	2	2	2	2
9	9	9	9	1	1	1	1	3	9
9	9	9	9	2	2	2	1	1	9
3	1	3	1	2	2	2	2	1	3
2	2	2	2	3	2	2	2	2	2
2	2	2	2	1	2	1	1	1	3
2	2	2	3	3	2	2	2	2	2
2	2	2	2	1	2	1	2	1	3
2	2	2	2	1	2	1	1	1	3
2	3	1	2	1	2	1	2	2	3
3	2	2	3	2	2	2	2	2	2
1	1	2	1	2	2	2	2	1	2
1	2	3	1	2	2	2	2	9	9
1	3	2	3	2	3	3	2	3	2
2	1	2	2	1	2	2	2	1	2
2	2	1	1	1	1	3	1	9	9
2	3	1	2	1	2	3	1	3	3
9	9	9	9	1	1	1	2	9	9
9	9	9	9	1	1	1	2	9	9
3	2	3	2	2	2	2	2	1	2
3	3	1	1	1	2	1	2	1	2
2	2	2	2	1	2	2	2	2	2
1	1	1	1	9	9	9	9	9	9
2	2	2	2	2	2	2	2	2	2
3	3	3	3	9	9	9	9	3	3
2	1	3	2	1	1	1	2	2	2
2	1	3	2	1	1	1	2	2	2
1	1	1	1	1	2	2	2	1	3
1	1	1	1	2	2	9	1	1	1
2	2	2	2	2	2	2	2	2	2
1	1	2	3	1	1	2	1	2	2
1	1	1	1	2	2	2	2	1	2
9	9	9	9	1	2	2	2	1	3
2	1	3	2	1	1	1	2	2	2
2	3	3	2	9	9	9	9	2	1
2	2	2	2	3	3	3	3	2	2
1	2	2	2	3	3	3	1	1	2
9	9	9	9	1	1	1	1	9	9
9	9	9	9	2	2	2	2	9	9
1	1	1	1	1	1	2	2	1	3
3	3	3	3	2	2	2	2	2	2
2	2	2	2	3	3	3	3	2	2
2	2	2	2	2	2	2	2	2	2
2	2	2	2	1	1	1	1	2	2
2	2	2	2	3	3	3	3	2	2
2	2	2	2	2	2	2	2	2	2
9	9	9	9	9	9	9	9	9	9

9	9	9	9	9	9	9	9	9	9
2	2	2	2	3	3	2	3	2	2
9	9	9	9	1	1	2	1	1	3
9	9	9	9	1	1	1	1	1	3
9	9	9	9	1	1	1	1	2	9
2	1	2	2	2	2	2	2	3	3
2	2	2	2	9	9	9	9	9	9
3	3	3	3	2	2	2	2	3	3
3	2	2	3	1	1	1	1	1	2
9	9	9	9	2	2	2	2	2	9
2	2	2	2	2	2	2	2	2	2
2	3	2	1	1	2	2	1	2	2
1	1	1	3	1	2	2	1	2	2
2	2	2	2	2	2	2	2	2	2
1	1	1	2	1	2	2	2	1	1
2	2	2	2	2	2	2	2	2	2
2	2	2	2	2	2	2	2	2	2
2	2	2	2	1	1	1	1	2	2
9	1	2	1	1	2	2	2	1	2
3	3	2	2	2	2	2	2	2	2
9	9	9	9	2	2	2	2	1	9
2	2	3	3	2	1	2	2	3	3
2	2	2	2	3	3	3	3	2	2
2	2	1	3	1	3	2	2	2	2
2	2	2	2	1	1	1	1	2	2
3	2	3	3	2	2	2	2	3	3
9	9	9	9	2	2	2	2	9	9
9	9	9	9	2	2	2	2	2	2
3	3	3	3	2	2	2	2	2	2
2	2	2	2	1	1	1	1	2	3
9	9	9	9	2	9	2	1	2	2
1	2	2	3	2	2	2	2	2	2
1	1	3	1	1	2	1	1	1	3
2	2	2	2	1	1	1	1	2	2
1	1	1	1	1	2	2	2	2	2
2	2	2	2	9	9	9	9	2	2
2	2	2	2	2	2	2	2	2	2
2	3	1	3	1	3	3	2	1	2
2	2	2	2	2	2	2	2	2	2
9	1	9	9	2	2	2	2	2	2
2	2	2	2	3	3	3	3	2	2
2	2	2	2	3	2	2	2	2	2
1	1	3	1	2	2	2	2	1	3
2	2	2	2	9	9	9	9	2	2
2	2	2	2	2	2	2	2	2	2
3	3	3	3	2	2	2	2	3	3
2	2	2	2	2	2	2	2	2	2

2	2	2	2	2	2	2	2	2	2
2	2	2	2	2	2	2	2	2	2
2	2	2	2	2	1	2	2	2	2
2	2	2	2	2	2	2	2	2	2
2	2	2	3	3	3	3	3	3	2
1	1	2	1	2	2	2	2	1	3
2	2	2	2	2	2	2	2	2	2
9	1	3	1	2	2	2	2	2	3
9	1	3	1	2	1	2	2	2	1
2	2	2	2	2	2	2	2	2	2
1	1	2	1	1	1	1	1	1	1
9	9	9	9	1	9	2	2	2	9
9	9	9	9	1	1	3	2	9	9
2	2	2	2	9	9	9	9	2	1
2	2	2	2	1	1	1	1	1	1
2	3	3	3	2	2	2	1	3	2
2	2	2	3	1	2	2	2	2	3
1	1	1	1	2	2	2	2	1	3
2	3	2	1	1	9	9	9	9	9
2	2	2	2	2	2	1	2	2	2
1	1	1	3	1	1	2	2	9	9
2	1	1	1	1	1	1	1	1	3
2	2	2	2	3	1	2	3	2	2
1	3	2	3	1	1	1	1	1	2
3	3	3	3	2	2	2	2	3	3
1	9	9	9	1	1	1	1	1	2
2	2	2	2	2	2	2	2	2	2
1	1	1	1	1	1	1	1	1	3
9	9	9	9	2	2	2	2	2	2
2	1	2	3	2	2	2	2	3	3
1	1	1	1	1	1	1	1	1	1
1	1	1	1	1	3	1	1	1	1
2	1	2	1	9	9	9	9	3	3
1	1	1	1	2	2	1	1	1	1
3	3	3	3	2	2	1	1	3	3
1	3	3	2	2	1	3	1	1	1
9	9	9	9	2	2	2	2	1	9
1	1	1	1	2	2	1	1	1	1
2	2	2	2	3	3	3	3	2	2
2	2	2	2	3	3	3	3	2	2
3	1	3	2	1	1	2	2	1	2
1	1	1	1	2	2	2	2	9	9
3	2	1	3	2	2	2	2	1	2
2	2	2	2	2	2	2	2	2	2
2	2	2	2	3	3	3	3	2	2
1	2	3	1	1	1	1	1	1	2
2	2	2	2	2	2	2	1	2	2

1	9	1	2	1	2	2	2	1	3
3	3	3	3	2	2	2	2	3	3
2	2	3	2	2	1	2	2	1	2
9	9	9	9	3	3	2	3	9	2
9	9	9	9	9	9	9	9	1	9
1	2	2	1	2	2	2	2	1	9
1	2	2	3	3	2	2	2	2	2
3	3	3	3	3	2	3	2	3	3
2	2	2	2	3	1	1	3	3	3
2	2	2	2	2	2	2	2	2	2
9	9	9	9	2	2	2	2	9	9
9	2	9	1	1	1	1	3	1	2
2	2	2	2	9	3	3	3	2	2
2	2	1	2	1	3	3	3	2	2
2	2	2	2	2	3	3	3	2	2
2	1	1	2	3	3	3	3	3	3
3	2	2	3	2	1	3	2	3	2
9	9	9	9	1	1	2	2	9	1
9	9	9	9	1	2	3	2	9	9
2	2	2	2	2	2	2	2	2	2
2	2	2	2	1	1	2	2	2	2
2	2	2	2	2	2	2	2	2	2
9	9	9	9	9	9	9	9	9	9
9	9	9	9	9	9	9	9	9	9
3	2	1	2	2	1	3	1	1	2
1	3	1	3	1	1	2	2	1	1
3	3	3	3	2	1	1	2	1	2
1	1	1	1	1	1	1	2	1	1
3	3	3	3	1	2	2	2	3	1
3	1	2	3	2	2	2	2	2	2
1	1	1	3	1	1	1	2	1	2
2	3	3	2	2	2	2	2	1	2
1	2	2	1	1	1	1	1	1	3
3	3	3	3	2	2	2	2	3	3
1	1	1	9	3	3	3	3	3	3
1	1	1	1	2	1	1	2	2	2
2	2	1	1	2	1	1	1	1	2
3	3	3	3	2	2	2	2	3	3
9	1	2	2	1	1	2	1	1	9
3	3	3	3	2	2	2	2	3	3
3	1	3	1	2	2	2	1	1	3
3	1	2	1	2	1	1	2	1	3
3	3	3	3	2	2	2	2	1	1
2	2	2	2	3	3	3	3	2	2
2	2	1	2	1	2	1	1	1	2
3	3	1	3	9	9	9	9	1	1
1	1	2	3	1	2	2	2	1	2

2	2	2	2	2	2	2	2	2	2
2	2	2	2	2	2	2	2	9	9
3	9	1	1	1	1	2	1	1	2
1	1	1	1	1	1	2	1	1	3
3	3	2	2	2	2	2	2	2	2
2	2	2	2	2	1	2	2	1	2
2	2	3	3	2	3	9	2	3	2
9	9	9	9	2	1	1	2	2	2
9	9	9	9	2	1	1	2	2	2
2	3	2	2	2	2	2	2	3	2
9	9	9	9	3	1	9	2	9	9
1	1	2	1	2	2	2	2	2	2
1	1	3	3	2	2	2	2	1	1
3	1	2	1	1	1	1	2	2	3
1	2	2	2	2	1	1	1	1	1
2	2	3	3	2	3	1	2	3	2
1	1	1	1	2	2	2	2	1	2
1	1	2	2	1	1	2	2	1	2
1	2	2	1	2	3	2	3	2	2
9	9	9	9	1	1	2	2	1	9
9	9	9	9	1	2	1	2	1	3
3	3	3	3	1	1	1	1	1	1
1	1	3	3	1	1	1	1	1	3
1	1	1	1	1	2	1	3	1	3
3	3	3	3	2	2	2	2	3	3
2	2	2	2	3	3	3	3	2	2
1	1	1	1	2	2	2	2	1	2
1	2	1	3	2	2	2	2	2	1
1	1	2	1	2	2	2	1	3	3
1	2	1	1	1	1	1	2	1	1
2	2	2	2	2	2	2	2	2	2
1	1	1	1	1	1	1	1	9	9
2	2	2	1	2	3	1	2	1	1
2	2	2	2	3	3	3	3	9	9
2	1	3	2	1	1	2	2	1	2
2	2	2	2	1	2	2	2	2	2
2	2	2	2	2	2	2	2	2	2
2	2	2	2	3	2	3	2	2	2
1	1	1	1	2	2	2	2	1	2
3	3	3	2	2	2	2	2	2	2
3	2	1	2	1	2	2	2	1	9
2	2	2	2	1	1	1	1	2	2
1	1	1	1	1	2	2	2	2	3
9	9	9	9	2	2	2	2	2	9
2	2	2	2	2	2	2	2	3	3
2	2	2	2	1	1	2	2	2	2
1	2	1	1	2	2	2	2	1	3

1	9	2	2	1	2	2	2	9	9
1	2	3	3	9	9	9	9	2	3
1	1	2	3	2	2	2	2	1	2
1	1	2	2	3	3	3	2	1	3
2	2	2	2	3	1	2	2	3	3
3	3	3	3	1	2	2	2	3	3
2	2	2	2	2	2	2	2	2	2
3	9	1	3	2	1	2	3	2	9
1	1	1	1	1	3	3	2	3	2
2	2	1	1	9	9	9	9	9	9
1	2	2	1	9	9	9	9	9	9
1	2	2	1	9	9	9	9	9	9
9	9	9	9	2	3	2	1	1	9
9	9	9	9	3	2	2	1	3	9
1	2	2	1	9	9	9	9	9	9
2	2	2	3	2	2	2	2	1	2
9	9	9	9	9	9	9	9	1	1
3	1	1	2	1	1	1	1	2	2
1	2	1	3	1	2	2	2	2	1
2	2	2	2	1	1	2	2	1	2
2	1	2	1	2	2	2	2	2	2
1	2	2	2	1	2	2	2	2	2
9	1	1	2	1	2	2	2	2	2
2	3	1	2	2	2	2	2	2	2
2	2	2	2	2	2	2	1	2	2
2	2	1	3	1	2	2	2	2	2
3	2	1	2	1	3	1	3	1	9
2	2	2	2	1	1	1	1	1	1
2	2	1	3	1	2	2	2	2	2
2	2	3	2	2	2	2	2	1	2
2	2	2	2	1	1	2	2	1	2
9	9	9	9	2	3	2	1	1	9
1	1	3	2	1	1	1	1	1	3
2	2	2	2	1	1	2	2	2	2
2	1	1	2	2	2	2	2	2	2
1	1	1	1	2	2	2	2	2	2
1	1	1	1	2	2	2	2	2	2
9	9	9	9	1	3	2	1	1	9
2	2	2	2	2	2	2	2	2	2
2	2	2	2	1	3	3	3	2	2
2	1	1	3	2	2	2	2	2	2
1	2	2	2	1	1	2	1	2	2
1	1	2	1	2	2	2	2	2	2
2	2	2	2	2	2	2	1	2	2
2	1	1	1	1	2	1	1	2	2
2	2	2	2	1	1	3	1	2	2
9	9	9	9	2	2	2	2	1	9

2	2	2	2	9	9	9	9	2	2
1	1	2	2	2	2	2	2	1	3
9	9	9	9	1	2	2	3	9	9
9	9	9	9	2	2	2	2	1	9
1	1	1	3	2	2	2	2	1	2
9	9	9	9	2	2	2	2	1	9
2	2	3	3	2	2	1	2	1	1
1	2	1	3	1	1	1	3	1	1
3	3	2	1	1	2	2	3	1	2
3	2	2	1	2	2	1	2	1	1
1	2	1	3	3	3	2	3	9	9
2	3	3	2	1	1	2	2	1	2
1	1	9	1	2	2	2	2	2	3
2	1	2	2	2	2	2	2	2	2
1	1	3	2	2	2	2	3	1	2
3	3	3	1	2	2	2	2	3	3
2	2	2	2	3	3	3	3	2	2
2	1	2	1	1	1	2	2	1	2
1	1	1	1	2	2	2	2	1	2
1	2	1	1	2	2	2	2	1	3
2	2	3	1	3	2	3	2	2	2
1	1	1	1	3	2	2	2	2	3
1	1	1	1	2	2	2	2	2	2
2	2	2	2	3	3	3	3	2	2
1	2	1	9	3	2	2	2	2	2
1	1	2	1	1	2	2	2	2	2
2	2	2	2	2	2	2	2	2	2
2	2	2	2	2	2	2	2	2	2
9	2	2	2	2	2	2	2	2	2
9	9	9	9	9	9	9	9	9	9
9	9	9	9	1	1	2	2	1	1
2	2	2	2	3	3	3	1	2	2
9	9	9	9	2	2	2	2	1	1
3	2	2	3	9	9	9	9	9	9
3	1	2	1	1	1	1	2	1	2
9	9	9	9	2	2	2	2	9	9
9	9	9	9	9	9	9	9	9	9
2	2	2	2	2	2	2	2	2	2
9	9	9	9	1	1	3	3	1	3
1	2	1	2	2	1	2	1	1	1
1	1	1	1	2	1	1	1	1	1
1	2	3	2	1	2	3	2	2	3
1	1	2	2	2	1	2	3	2	9
1	2	3	2	2	1	2	2	1	2
2	2	2	3	1	1	2	1	3	2
9	9	9	9	9	9	9	9	9	9
2	3	3	3	2	2	2	2	3	2

1	2	2	1	2	2	2	2	1	2
9	9	9	9	9	9	9	9	9	9
9	9	9	9	9	9	9	9	9	9
2	2	2	2	3	3	3	3	2	2
1	1	2	2	1	3	2	2	1	3
1	2	2	1	2	2	2	2	1	2
1	1	1	2	1	1	1	1	2	1
1	2	1	1	2	2	2	2	2	2
2	2	2	2	1	1	1	1	2	2
1	1	3	2	1	1	1	1	1	3
9	9	9	9	9	9	9	9	9	9
9	9	9	9	2	2	2	2	9	9
9	9	9	9	1	2	2	2	1	9
2	2	2	2	1	2	1	1	2	2
1	2	2	2	2	2	2	2	2	2
9	9	9	9	9	9	9	9	9	9
9	9	9	9	9	9	9	9	9	9
9	9	9	9	9	9	9	9	9	9
1	2	2	1	1	2	2	2	1	2
1	1	2	1	1	2	2	2	1	2
3	3	1	2	1	1	9	9	9	9
3	3	2	1	2	1	1	2	1	3
3	3	3	3	2	1	1	3	3	3
2	2	2	2	9	9	9	9	9	9
2	2	2	2	1	1	1	1	2	2
1	2	2	2	2	2	2	2	2	2
9	9	9	9	3	3	3	3	9	9
1	1	2	3	3	2	2	3	9	9
2	1	1	1	2	2	2	2	1	2
2	2	2	2	9	9	9	9	2	2
1	1	1	3	1	2	2	2	2	1
2	2	2	2	3	3	3	3	9	9
9	9	9	9	9	9	9	9	9	9
1	2	3	2	1	2	2	2	1	2
2	2	2	1	2	2	1	1	2	9
2	2	2	2	2	2	2	2	2	2
1	2	1	1	3	3	2	2	1	1
2	2	2	2	2	2	2	2	2	2
1	1	3	2	1	2	2	2	1	9
2	1	2	1	2	2	2	2	1	2
1	1	3	2	2	1	2	1	1	2
3	3	3	3	2	9	2	2	9	9
9	9	9	9	9	9	9	9	9	9
9	9	9	9	2	2	2	2	9	9
2	2	2	2	9	9	9	9	9	9
9	9	9	9	9	9	9	9	9	9
9	9	9	9	9	9	9	9	9	9

2	2	2	2	1	1	2	2	2	2
2	2	2	2	3	2	1	2	2	3
1	1	1	1	1	1	2	2	2	3
1	2	2	2	2	2	2	2	2	2
1	1	1	1	1	1	1	1	9	9
9	9	9	9	9	9	9	9	9	9
9	9	9	9	9	9	9	9	9	9
1	1	3	3	9	9	9	9	9	9
9	9	9	9	9	9	9	9	9	9
9	9	9	9	9	9	9	9	9	9
9	9	9	9	9	9	9	9	9	9
9	9	9	9	9	9	9	9	9	9
9	9	9	9	9	9	9	9	9	9
1	3	3	1	2	2	2	2	1	1
1	1	1	3	2	2	2	2	2	2
1	2	2	1	3	1	2	2	2	2
1	2	2	2	2	2	2	2	2	2
9	9	9	9	9	9	9	9	9	9
9	9	9	9	9	9	9	9	9	9
9	9	9	9	9	9	9	9	9	9
9	2	9	1	1	2	2	2	1	9
9	9	9	9	9	9	9	9	9	9
1	1	1	1	1	1	2	2	1	1
9	9	9	9	9	9	9	9	9	9
2	1	1	1	3	1	1	3	1	1
1	1	1	1	1	1	1	1	1	1
1	2	2	1	1	1	1	1	1	1
1	1	1	1	1	1	1	1	2	2
1	1	1	1	1	1	1	1	1	1
2	1	1	1	1	1	1	1	1	1
2	2	2	2	1	1	1	1	1	1
1	2	2	2	3	3	3	3	1	1
1	1	1	1	1	1	1	1	1	1
9	9	9	9	9	9	9	9	9	9
1	1	1	1	1	1	1	1	1	1
1	1	1	1	1	1	1	1	1	1
3	2	2	2	3	3	3	3	2	2
9	9	9	9	9	9	9	9	9	9
1	2	1	3	2	2	1	1	1	2
1	3	3	3	1	2	2	2	3	3
2	2	2	2	2	1	2	2	2	3
9	9	9	9	9	9	9	9	9	9
9	9	9	9	9	9	9	9	9	9
9	9	9	9	9	9	9	9	9	9
1	1	2	3	3	1	2	1	1	2
2	2	3	2	3	1	1	1	2	2
1	1	2	2	1	2	2	2	9	9

9	9	9	9	9	9	9	9	9	9
9	9	9	9	9	9	9	9	9	9
9	9	9	9	9	9	9	9	9	9
9	9	9	9	9	9	9	9	9	9
1	1	9	1	2	2	2	2	9	9
9	9	9	9	9	9	9	9	9	9
2	2	2	2	9	9	9	9	9	9
9	9	9	9	9	9	9	9	9	9
9	9	9	9	2	2	2	2	9	9
9	9	9	9	9	9	9	9	9	9
9	9	9	9	2	2	2	2	9	9
9	9	9	9	9	9	9	9	9	9
1	3	3	3	1	1	2	1	1	2
2	3	1	2	1	1	1	1	1	3
1	1	1	3	2	2	2	2	2	2
1	1	1	1	1	1	1	2	2	1
1	1	1	2	1	1	3	3	2	2
1	1	1	1	1	1	2	2	1	1
1	1	1	1	1	1	2	2	1	1
9	9	9	9	1	1	1	1	1	9
2	2	2	2	1	1	1	1	1	1
1	2	3	2	1	2	2	2	1	3
2	2	2	2	1	1	3	3	2	2
3	1	1	2	2	2	3	2	2	3
1	1	1	2	1	1	1	1	1	3
3	1	1	3	2	2	2	2	1	3
2	9	9	9	2	2	2	2	1	2
2	2	2	2	2	2	1	1	1	3
9	9	9	9	2	2	2	2	1	9
9	9	9	9	9	2	2	1	9	9
1	1	1	3	9	9	9	9	9	9
3	3	3	3	1	1	9	9	1	1
3	3	3	3	1	2	2	2	2	2
2	2	2	2	2	2	2	2	2	2
1	1	2	2	1	2	2	2	1	2
2	2	2	2	2	3	2	3	2	2
2	2	2	2	1	2	2	2	2	2
1	2	2	2	2	2	2	2	2	2
2	1	1	2	2	2	2	2	2	2
2	2	2	2	2	2	2	2	1	1
1	1	3	1	2	2	2	2	2	2
2	2	2	2	2	2	2	2	1	1
1	1	3	1	1	1	1	2	1	3
2	2	1	1	2	3	2	2	1	1
1	2	2	1	1	1	1	1	1	1
3	3	3	3	1	1	1	1	1	1
1	2	1	2	1	2	2	2	2	2

9	9	9	9	9	9	9	9	9	9
1	1	1	1	1	1	1	1	1	1
9	9	9	9	2	2	2	2	9	9
3	9	9	9	1	2	2	2	9	9
9	9	9	9	1	1	2	3	1	9
1	1	1	2	1	2	2	2	9	9
9	9	9	9	3	3	2	2	9	2
3	3	3	3	2	2	2	2	1	2
9	9	9	9	3	3	3	3	2	9
2	3	1	2	2	2	2	3	2	2
9	9	9	9	9	9	9	9	9	9
1	1	2	2	2	2	2	2	2	2
1	2	2	1	2	2	2	2	1	1
2	2	2	2	1	1	1	1	2	2
1	1	1	1	1	1	1	1	1	1
9	9	9	9	9	9	9	9	9	9
2	2	2	2	2	2	2	2	2	2
3	3	3	3	2	1	2	1	3	3
9	9	9	9	2	2	2	2	2	1
3	3	3	3	1	3	1	2	2	2
2	2	2	2	3	3	3	3	2	2
1	1	1	3	1	1	2	3	3	2
2	2	2	2	9	9	9	9	2	2
2	2	2	2	2	2	2	2	2	2
1	1	1	1	2	2	9	3	2	2
2	2	2	2	2	2	2	2	2	2
1	3	1	2	2	2	2	2	1	2
1	1	1	1	2	2	2	1	1	3
1	1	1	1	1	1	1	1	1	1
2	2	1	1	2	2	2	2	2	2
9	9	9	9	9	9	9	9	9	9
2	3	2	1	3	3	3	3	2	2
3	3	2	3	9	9	9	9	9	9
2	2	2	2	2	2	2	2	2	2
2	2	2	2	2	2	2	2	2	2
1	1	1	1	1	1	1	1	1	1
1	1	2	2	1	2	2	1	3	3
2	2	1	1	1	1	1	2	1	1
2	1	1	1	1	1	1	1	1	1
3	1	3	1	1	1	1	1	1	2
2	2	2	2	1	1	2	2	2	2
1	1	1	1	1	1	1	1	1	2
9	9	9	9	9	9	9	9	9	9
9	1	9	9	2	1	1	3	3	9
9	9	9	9	1	1	1	1	1	1
1	2	2	1	9	9	9	9	9	9
1	1	2	2	1	2	9	2	9	9

1	1	1	1	1	1	1	1	9	9
1	2	2	2	3	1	3	3	2	2
9	9	9	9	2	2	2	2	2	9
1	2	3	2	3	1	2	2	2	3
9	9	9	9	1	1	2	2	9	9
3	2	1	3	2	2	2	2	3	2
9	9	9	9	3	3	3	3	2	3
9	9	9	9	9	9	9	9	9	9
1	2	2	2	9	9	9	9	2	2
2	2	2	1	2	9	9	9	9	9
9	1	9	9	2	2	2	2	1	9
9	9	9	9	3	3	3	3	2	9
1	2	2	2	1	3	1	2	2	2
9	9	9	9	9	9	9	9	9	9
9	9	9	9	2	2	2	2	2	9
2	1	2	3	1	3	3	2	1	2
2	1	2	2	3	2	3	2	2	1
1	2	2	1	2	2	2	2	1	3
3	3	3	3	1	1	2	2	1	1
1	2	2	2	2	1	2	3	1	2
1	2	1	2	2	2	2	2	1	3
3	1	3	1	3	3	3	3	1	3
2	1	3	3	1	2	2	2	2	3
2	2	9	3	2	2	2	2	2	3
2	2	2	2	2	2	2	2	2	2
2	1	1	1	1	1	1	1	1	2
2	2	2	3	2	2	2	2	2	3
1	3	1	1	1	1	1	1	1	2
9	2	2	3	1	9	9	9	2	1
2	1	2	2	1	2	1	3	1	2
3	1	1	3	2	2	1	1	1	1
2	2	2	3	1	2	2	2	2	2
9	9	9	9	9	9	9	9	9	9
3	3	3	2	2	1	1	2	1	2
2	2	2	3	2	2	2	2	1	2
2	2	2	2	2	2	2	2	2	2
2	3	2	3	2	2	2	2	1	1
1	1	2	2	1	2	9	2	2	2
9	9	9	9	2	2	2	2	9	9
2	2	2	2	2	2	2	2	2	2
9	9	9	9	2	2	2	2	9	9
9	9	9	9	2	2	2	2	2	9
2	2	3	2	2	2	2	2	1	2
9	9	9	9	2	2	2	2	1	9
2	2	3	1	1	1	1	1	1	1
3	1	3	1	1	1	2	1	1	1
3	1	1	3	1	1	3	3	1	1

3	3	3	3	2	2	2	2	3	3
3	9	9	9	2	2	2	2	9	9
3	2	2	1	2	2	2	2	3	3
1	1	1	1	1	1	1	2	1	3
1	1	3	1	2	2	2	2	1	3
1	3	1	1	2	2	2	2	1	1
1	2	1	2	1	1	1	1	1	2
2	2	2	2	2	1	1	1	2	2
1	3	3	3	2	2	2	2	2	2
3	1	2	3	1	3	2	3	1	3
2	1	3	3	2	2	2	2	1	3
2	1	2	3	2	2	2	1	1	2
2	3	2	3	3	2	2	1	2	3
1	1	1	2	2	2	2	2	2	2
9	1	2	3	1	1	3	3	1	2
1	1	3	1	2	2	2	3	1	2
2	2	2	1	2	2	2	2	2	2
3	1	2	1	2	2	2	2	1	2
3	3	3	1	2	2	2	2	2	2
2	2	2	2	2	2	2	2	2	2
2	2	2	2	2	2	1	1	2	2
1	2	2	2	2	1	3	1	2	2
3	3	3	3	2	9	2	2	2	2
9	9	9	9	9	9	9	9	9	9
1	1	2	2	3	2	3	2	2	2
3	2	3	1	9	9	9	9	1	2
1	3	2	1	2	2	2	2	1	2
2	3	1	1	9	9	9	9	1	2
1	1	1	2	1	2	2	1	1	2
9	9	9	9	2	2	2	2	1	9
3	2	3	2	1	2	1	2	1	3
1	2	1	3	1	1	2	3	3	1
3	3	2	2	3	2	2	2	2	3
1	1	2	2	3	3	3	3	2	2
9	1	1	1	1	1	1	1	1	1
2	2	2	2	2	2	2	2	2	2
2	2	2	2	2	2	2	2	2	2
9	9	9	9	9	9	9	9	9	9
3	2	1	1	2	2	2	2	2	2
1	3	1	3	1	1	2	2	2	2
9	9	9	9	9	9	9	9	9	9
9	9	9	9	9	9	9	9	9	9
2	3	1	2	3	1	2	3	2	2
3	3	3	1	1	2	2	1	9	9
2	2	2	2	2	2	2	2	2	2
1	1	3	3	2	2	2	2	2	2
9	9	9	9	9	9	9	9	9	9

2	2	2	2	2	2	2	2	2	3
9	9	9	9	9	9	9	9	9	9
1	2	1	1	2	2	2	2	1	2
9	9	9	9	9	9	9	9	9	9
9	9	9	9	9	9	9	9	9	9
3	3	3	3	1	1	1	1	3	3
2	2	2	2	2	2	2	2	2	2
2	2	2	2	3	3	3	3	2	2
2	1	1	2	1	3	1	1	3	3
1	2	2	3	2	2	2	2	2	2
9	9	9	9	9	9	9	9	9	9
9	9	9	9	2	2	2	2	9	9
9	9	9	9	9	9	9	9	9	9
1	1	1	1	1	1	1	1	1	1
2	2	2	2	2	2	2	2	2	2
2	2	2	2	2	2	2	2	1	1
9	9	9	9	2	2	2	2	1	9
9	9	9	9	9	9	9	9	9	9
3	3	3	3	3	3	3	3	3	3
2	2	2	2	2	2	2	2	2	2
3	3	3	3	3	3	3	3	1	1
2	2	2	2	2	2	2	2	2	3
9	9	9	9	2	2	3	2	1	9
2	1	2	3	2	2	3	3	2	3
3	3	3	3	1	2	2	2	2	2
3	2	2	2	3	3	3	3	2	2
9	9	9	9	2	1	2	2	1	3
2	2	2	2	1	1	1	1	1	1
2	2	2	2	2	2	2	2	2	2
9	9	9	9	1	3	3	2	1	9
1	1	2	3	2	2	2	2	2	2
2	2	2	2	3	3	3	2	2	2
3	2	2	2	1	2	2	2	1	3
2	1	2	3	2	2	2	2	2	2
1	1	1	1	2	2	2	2	2	2
2	1	2	3	2	1	1	1	2	1
1	1	1	1	1	1	1	1	1	3
1	1	1	3	2	2	2	2	3	3
9	9	9	9	2	2	2	2	2	2
2	2	2	2	2	2	2	2	3	3
1	1	2	2	2	2	2	2	1	2
3	3	3	3	3	2	2	2	1	3
3	3	3	3	2	1	3	3	2	2
1	2	1	3	2	2	2	2	1	2
3	3	3	1	2	2	2	2	1	2
1	1	2	2	2	2	2	2	2	3
9	9	9	9	9	9	9	9	1	2

1	2	2	2	2	2	2	2	2	2
1	2	2	2	2	2	2	3	1	2
2	2	2	1	1	1	1	1	2	1
2	2	2	2	2	2	2	2	1	3
1	2	3	2	2	2	2	2	2	2
9	9	9	9	2	2	1	1	1	9
2	2	2	2	3	3	3	3	2	2
2	2	2	2	1	1	1	1	1	1
2	2	2	2	2	2	2	2	2	2
2	2	2	2	2	2	2	2	2	2
1	1	2	2	2	2	2	2	2	2
3	2	1	3	2	2	2	2	2	2
2	2	2	2	2	2	2	2	2	2
3	3	3	3	2	2	2	2	3	3
3	2	3	2	1	1	2	2	1	2
1	2	3	1	2	2	2	2	2	1
2	2	2	2	2	2	2	2	2	3
1	1	2	3	2	2	2	2	2	2
2	2	2	2	3	3	3	3	2	2
2	2	2	2	2	1	2	2	1	1
1	1	2	1	1	1	1	2	2	2
1	2	3	1	3	2	3	2	1	1
1	2	3	1	3	2	3	2	1	1
3	3	3	2	9	1	2	2	9	9
1	1	2	2	2	2	2	2	2	2
2	2	2	2	2	2	2	2	2	2
1	2	2	1	2	2	2	2	2	2
2	1	2	1	2	2	2	2	2	2
2	2	2	2	2	2	2	2	2	2
1	1	1	1	1	1	1	1	1	1
2	2	2	2	2	2	2	2	3	3
9	2	2	3	3	2	2	3	1	2
1	1	1	2	2	2	2	2	1	1
3	3	3	3	2	9	2	2	1	2
2	2	2	2	1	2	1	1	1	1
2	2	2	2	2	2	2	2	2	2
2	2	2	2	2	2	2	2	2	2
2	2	2	2	3	3	3	3	2	2
2	2	2	2	2	2	2	2	2	2
2	2	2	2	3	3	3	3	2	2
1	1	2	3	2	2	2	2	1	2
1	3	1	2	1	1	2	2	1	2
1	2	2	2	1	1	1	1	1	2
1	2	2	2	1	2	3	3	2	2
2	3	3	3	2	2	2	2	1	2
1	2	1	1	1	1	3	2	1	3
1	1	3	1	1	2	2	2	1	3

2	2	1	3	2	2	3	3	2	2
2	2	2	2	3	3	1	1	2	2
1	1	1	2	1	2	2	2	1	3
2	2	2	2	2	2	2	2	2	2
2	2	2	2	2	2	2	2	2	2
2	2	2	2	3	2	3	1	2	2
1	1	1	2	2	2	2	2	2	2
1	1	1	2	1	2	2	2	1	2
2	2	2	2	2	3	2	2	2	2
2	2	2	2	2	2	2	2	2	2
2	2	2	2	2	2	2	2	2	2
2	2	2	2	3	2	2	2	2	9
2	2	2	2	9	9	9	9	9	9
1	9	9	9	1	2	2	2	9	9
2	2	2	2	1	2	2	2	2	2
1	1	1	2	1	1	2	2	2	2
2	2	2	2	2	2	2	1	2	3
2	1	1	1	1	1	2	2	2	2
1	1	1	1	1	3	2	2	1	2
1	1	2	2	1	2	1	1	1	2
2	2	2	2	1	1	2	2	2	2
2	2	2	2	3	3	3	3	2	2
1	1	2	1	3	1	2	1	2	2
2	2	2	2	2	2	2	2	2	2
1	1	1	2	1	1	2	2	2	2
2	2	1	1	2	2	2	1	2	2
2	2	2	2	2	2	2	2	2	2
2	2	2	2	2	2	2	2	2	2
2	2	2	2	1	2	2	1	2	2
1	1	1	1	9	9	9	9	9	9
3	3	3	3	3	3	3	3	2	2
2	2	2	2	1	3	3	3	2	2
2	2	2	2	3	1	1	3	2	2
2	2	2	2	3	3	2	2	2	2
2	2	2	2	2	2	2	2	2	2
2	2	2	2	3	3	3	3	2	2
2	2	2	2	2	2	2	2	2	2
2	2	2	2	2	2	2	2	2	2
9	9	9	9	1	2	3	3	2	2
2	2	1	1	1	2	2	1	2	2
2	2	2	2	2	2	2	2	2	2
2	2	2	2	3	3	3	3	2	2
2	2	2	2	1	1	2	2	1	2
2	2	2	2	2	2	2	2	2	2
2	2	2	2	2	2	2	2	2	2
2	2	2	2	3	3	3	2	2	2
2	2	2	2	2	2	2	2	2	2

2	2	2	2	1	1	1	2	2	2
2	2	2	2	1	2	2	2	2	2
1	3	1	1	2	2	2	2	2	2
2	2	2	2	1	2	2	1	2	2
2	2	2	2	2	2	2	2	2	2
2	2	2	2	2	2	1	2	2	2
9	9	9	9	2	2	2	2	9	9
1	2	1	1	1	1	3	2	1	2
1	1	2	3	1	1	1	1	1	3
1	2	2	2	1	2	2	2	2	2
1	1	1	1	1	2	2	2	2	2
2	2	2	3	2	2	2	1	2	2
2	1	3	3	2	2	2	2	2	2
2	2	2	2	1	1	2	2	1	2
1	1	1	1	1	1	2	1	2	2
2	2	2	2	1	2	2	1	1	2
2	2	2	2	1	2	2	1	1	2
2	1	1	1	1	3	2	2	1	2
1	1	1	1	2	2	2	2	1	1
2	1	2	2	1	2	1	1	1	1
1	1	1	1	1	1	1	1	1	1
2	1	3	3	1	1	1	1	1	2
2	2	2	2	3	3	2	2	2	2
2	2	2	2	2	2	2	2	2	2
2	2	2	2	9	9	9	9	2	2
2	2	2	2	2	2	2	2	2	2
9	9	9	9	9	9	9	9	9	9
2	2	2	2	2	2	2	2	2	2
2	2	2	2	3	3	3	2	2	2
2	2	2	2	3	3	3	3	2	2
2	2	2	2	2	2	2	2	2	2
2	1	1	1	1	1	1	1	1	2
9	9	9	9	9	9	9	9	9	9
3	3	3	3	1	1	1	1	9	9
9	9	9	9	9	9	9	9	9	9
1	1	1	1	9	9	9	9	9	9
3	3	2	2	3	1	2	2	2	2
1	1	1	1	1	1	2	1	1	3
1	1	1	9	2	2	2	2	1	2
3	2	2	2	2	2	1	3	2	2
1	1	1	2	1	2	2	2	1	2
2	2	2	2	1	2	2	2	2	2
2	2	2	2	2	2	2	2	1	1
2	2	2	2	3	3	3	3	2	2
1	1	1	2	1	1	2	1	2	2
2	2	2	3	1	1	2	2	2	2
2	1	1	1	2	2	2	2	1	1

2	1	1	2	1	2	2	1	2	1
1	1	2	3	9	9	9	9	3	2
9	1	1	2	3	3	3	2	3	2
1	2	1	2	1	1	2	2	1	2
9	2	1	3	1	1	1	2	1	3
2	2	2	2	2	2	2	3	2	2
9	1	2	3	2	2	2	2	2	1
9	9	9	9	2	2	2	2	2	9
2	2	2	2	2	2	2	2	2	2
2	2	2	2	1	2	2	2	2	2
2	2	2	2	1	2	2	2	2	2
2	2	2	2	1	2	2	2	2	2
9	9	9	9	9	9	9	9	2	2
2	2	2	2	1	2	2	9	1	2
2	2	2	2	1	1	3	1	2	2
2	2	2	2	2	2	2	2	2	2
1	2	2	2	1	1	2	2	2	2
3	2	2	3	2	2	2	1	1	3
2	2	2	2	1	1	1	1	1	2
1	1	3	2	1	1	1	2	1	3
9	9	9	9	1	1	2	2	2	2
9	9	9	9	1	1	2	2	2	2
2	2	2	2	2	2	2	2	2	3
9	9	9	9	2	2	2	2	9	9
1	3	1	3	1	2	1	1	2	1
1	1	1	1	1	1	2	2	1	3
1	1	1	1	2	2	2	2	2	2
1	2	2	1	1	2	2	1	1	2
2	2	2	2	1	1	2	2	2	2
9	9	9	9	1	1	1	1	1	9
3	2	3	3	1	1	1	1	1	2
9	9	9	9	2	2	2	2	9	9
9	9	9	9	9	9	9	9	9	9
2	2	2	2	9	9	9	9	9	9
2	2	2	2	9	9	9	9	9	9
1	2	2	1	2	2	2	2	2	2
2	2	2	2	1	1	1	1	1	3
2	2	2	2	9	9	9	9	9	9
2	2	2	2	2	2	2	2	2	2
2	2	2	2	2	2	2	2	2	2
9	9	9	9	1	1	1	1	1	3
2	2	2	2	2	2	2	2	2	2
2	2	2	2	2	2	2	1	2	2
9	9	9	9	3	3	2	2	3	2
9	9	2	2	9	9	9	9	3	3
1	1	1	1	1	1	1	1	1	1
1	1	1	1	2	2	2	2	3	3

9	9	9	9	2	2	2	2	1	1
1	1	1	2	2	2	2	2	1	2
2	3	3	3	1	2	3	2	1	1
2	2	2	2	2	2	2	1	2	2
9	9	9	9	9	9	9	9	9	9
3	3	3	3	9	9	9	9	9	9
2	2	2	2	2	2	2	2	2	2
3	3	3	3	2	2	2	2	2	2
9	9	9	9	1	2	2	1	1	9
2	2	2	2	2	2	2	2	9	9
1	1	3	3	1	1	1	1	1	1
2	2	2	2	1	2	2	2	2	2
3	2	2	1	3	2	2	2	2	2
3	3	3	3	2	2	2	2	2	2
9	9	9	9	9	9	9	9	9	9
2	3	2	2	2	2	2	2	2	3
3	2	2	2	2	2	2	2	1	3
3	3	1	2	2	3	2	1	1	3
9	9	9	9	3	3	3	2	9	2
1	2	1	3	3	2	1	2	1	2
1	2	1	1	3	1	2	3	1	3
2	2	2	2	1	2	2	1	1	3
9	9	9	9	9	9	9	9	1	3
2	2	2	2	1	2	2	2	2	2
1	3	3	3	2	2	2	2	3	1
2	2	2	2	3	2	2	2	2	2
1	1	2	3	2	2	2	1	2	2
1	1	1	3	2	2	2	2	3	3
2	1	9	3	2	1	3	2	1	2
9	9	9	9	2	2	2	2	1	3
2	2	2	2	2	2	2	2	2	2
2	2	2	2	3	3	2	2	1	1
1	1	3	1	3	3	2	2	1	2
3	3	3	3	1	1	1	1	1	3
1	1	1	1	2	2	2	2	9	9
1	1	1	2	3	2	2	2	1	2
3	3	3	3	2	2	2	2	9	9
1	2	1	1	2	2	2	2	2	2
1	2	1	2	3	2	1	2	2	2
9	9	9	9	9	9	9	9	2	3
9	9	9	9	9	9	9	9	9	9
1	3	1	2	2	1	1	3	2	1
1	1	1	2	2	2	2	2	2	3
1	1	2	1	1	1	2	2	1	3
2	2	2	2	1	1	2	2	1	3
2	3	3	3	9	9	9	9	2	3
3	2	3	2	2	2	3	1	3	2

2	1	2	2	1	2	2	2	1	3
1	3	1	2	2	2	2	2	3	2
1	1	2	1	2	2	2	2	2	2
3	3	3	9	1	2	2	2	3	3
3	1	1	3	2	2	2	2	1	1
2	2	2	2	9	9	9	9	9	9
2	2	2	2	1	9	9	9	9	9
2	2	2	2	2	2	2	2	1	2
1	1	1	1	1	1	1	1	1	3
2	2	2	2	1	2	3	2	1	3
1	2	2	1	1	1	2	3	2	3
2	1	2	1	2	2	2	2	2	2
3	2	3	2	2	2	2	2	2	2
1	1	1	1	3	3	3	3	1	3
9	9	9	9	9	9	9	9	9	9
2	2	2	2	3	1	2	2	2	3
1	2	2	2	2	2	1	1	2	2
2	1	2	2	1	2	2	2	2	2
2	2	3	2	2	2	1	1	1	3
1	1	1	2	1	2	1	2	1	2
9	1	1	2	9	9	9	9	9	9
9	9	9	9	9	9	9	9	9	9
2	2	2	2	2	2	2	2	2	2
9	9	9	9	2	2	2	2	2	9
2	2	2	2	9	9	9	9	9	9
9	1	2	2	2	2	2	2	3	9
1	2	2	2	2	2	2	2	2	2
2	2	2	2	2	2	2	2	1	2
3	3	3	3	2	2	2	2	3	3
1	1	1	2	2	2	2	2	1	2
3	3	3	1	2	2	2	2	3	3
3	3	3	3	2	2	2	2	2	2
2	9	9	9	9	9	9	9	9	9
1	1	1	2	2	2	2	2	9	9
3	1	3	2	9	9	9	9	9	9
2	2	2	2	1	2	2	2	2	2
1	1	1	3	1	1	1	1	1	3
2	3	1	2	2	2	2	2	3	2
1	1	2	2	2	2	2	2	1	2
9	9	9	9	2	2	1	2	2	9
2	2	2	2	3	3	3	3	2	2
2	1	2	3	1	2	2	2	2	2
2	2	2	3	1	2	2	2	3	3
1	1	1	3	2	2	2	2	9	9
3	3	2	2	1	1	1	1	3	1
9	9	9	9	2	2	2	2	9	9
1	2	1	2	2	2	2	2	3	2

3	2	1	2	2	2	1	2	1	2
2	3	2	2	2	2	2	2	2	2
9	3	3	3	2	2	2	2	3	3
3	1	2	3	3	2	1	3	2	3
2	1	2	2	2	2	2	2	2	2
2	2	2	2	1	3	2	1	1	2
1	1	3	1	2	2	2	1	1	3
1	9	9	9	2	2	2	2	1	3
9	9	9	9	9	9	9	9	9	9
2	2	1	2	1	1	1	1	1	2
2	2	3	3	2	3	2	2	2	2
2	2	2	2	2	1	2	2	2	2
1	1	3	3	1	3	2	1	2	2
1	1	1	1	1	1	2	1	1	3
9	9	9	9	2	2	1	2	9	2
1	2	1	2	2	1	2	2	2	2
2	2	2	2	1	1	2	2	2	2
3	3	2	3	2	2	2	2	1	2
3	1	3	3	2	2	2	3	2	3
3	2	2	3	2	2	2	2	2	2
1	1	1	2	2	2	1	2	1	3
3	3	1	3	1	1	2	2	1	2
1	1	2	1	3	3	3	3	2	2
1	3	2	2	2	2	2	2	1	3
1	1	9	9	2	1	2	3	1	3
1	2	2	1	1	1	2	2	2	2
1	2	1	2	1	1	2	2	1	2
2	2	2	2	1	2	2	2	1	2
2	2	2	2	1	2	2	2	2	2
1	1	2	2	3	2	2	2	2	2
2	1	2	2	3	1	3	2	2	2
2	9	1	2	3	1	2	1	2	9
2	2	2	2	2	2	2	1	2	2
1	1	1	1	2	2	2	2	1	1
1	2	2	3	1	2	3	1	1	2
1	1	1	3	2	2	2	2	2	2
1	1	1	2	3	1	2	2	1	2
1	3	2	2	2	9	9	9	1	1
2	2	2	2	1	1	2	2	1	2
1	1	1	1	1	1	1	1	1	1
2	3	3	3	1	2	2	2	2	2
1	1	1	1	1	1	1	2	1	1
2	1	1	1	2	2	2	2	1	1
2	2	2	2	2	2	2	2	1	2
2	1	3	3	2	1	1	2	1	2
2	2	2	2	2	1	1	1	2	2
1	2	1	1	1	1	2	1	1	2

1	2	2	1	1	1	2	2	1	1
1	1	1	2	2	1	2	1	1	2
3	2	1	2	1	2	1	2	1	2
1	2	3	1	2	1	1	2	1	2
2	2	1	1	1	1	1	2	1	1
2	2	2	3	1	1	2	1	3	1
1	1	2	3	2	1	1	2	1	2
1	2	2	3	1	2	1	1	1	2
1	2	1	1	2	2	2	2	2	2
1	1	2	1	2	2	2	2	2	2
2	2	2	2	1	3	3	3	2	2
2	2	2	2	2	1	1	2	9	1
1	1	1	1	2	2	1	2	1	2
3	3	3	3	3	1	2	2	2	2
1	2	2	2	2	2	2	2	2	2
1	1	1	2	1	2	2	2	1	1
1	1	9	1	2	3	2	2	1	3
2	1	2	2	2	2	2	2	2	2
2	2	2	2	2	2	2	2	2	2
1	3	2	1	2	2	2	3	1	2
2	2	2	2	1	1	2	2	1	2
1	2	1	1	2	2	2	2	2	2
3	3	3	3	2	2	2	2	3	9
3	3	3	3	9	9	9	9	9	9
2	1	3	3	3	2	2	3	2	1
2	2	2	2	3	3	3	3	3	3
2	3	2	2	2	1	1	1	2	3
3	1	1	1	2	3	9	3	3	2
1	2	1	2	2	2	2	1	2	1
2	1	1	3	2	2	2	2	3	2
1	2	1	2	1	1	2	1	2	2
3	1	1	3	1	1	1	1	1	1
2	1	1	2	2	1	1	2	2	1
2	2	2	2	1	2	2	3	2	2
3	2	3	1	1	1	1	2	1	3
2	2	2	2	3	2	3	3	2	3
2	3	2	2	2	2	1	2	2	2
3	3	2	3	3	3	3	3	3	3
1	1	1	1	2	1	1	1	1	1
2	3	3	2	2	1	2	1	1	1
2	2	2	2	2	2	2	2	2	2
2	2	2	2	2	2	2	2	1	1
2	2	2	2	2	2	2	2	2	2
3	3	3	3	2	2	2	2	3	3
3	3	3	3	2	2	2	2	3	3
3	2	2	2	2	2	2	2	2	2
1	1	1	1	2	1	1	2	1	2

1	2	3	2	1	2	1	1	1	2
1	1	1	1	1	2	2	1	1	2
1	1	2	2	1	2	1	1	1	2
1	1	2	2	1	1	2	2	1	2
1	2	2	1	2	1	1	2	1	2
2	2	2	1	2	2	2	2	1	1
1	1	1	1	2	2	2	2	3	3
1	1	1	1	2	2	2	2	1	1
1	1	2	2	2	2	1	1	1	1
2	2	1	1	2	1	1	2	1	2
2	2	2	2	2	1	1	2	1	2
1	1	1	1	1	2	2	2	1	2
1	1	2	2	2	2	2	2	1	1
1	2	1	1	2	2	2	2	1	2
2	2	1	1	2	2	2	2	1	1
2	2	1	1	2	2	2	2	1	1
2	2	2	2	2	2	2	2	2	2
2	2	2	2	2	9	2	2	1	2
9	2	2	2	2	2	2	2	2	2
1	1	1	2	2	2	2	2	2	2
2	9	9	2	2	2	2	2	2	9
3	3	3	3	2	2	2	2	3	3
2	1	1	1	2	2	2	2	1	1
2	1	1	1	2	2	2	2	1	1
2	2	1	1	2	2	2	2	1	1
2	2	2	2	2	2	2	2	2	2
1	1	1	1	1	2	2	1	1	1
2	1	1	2	2	2	2	2	1	2
2	2	3	1	2	2	1	1	1	1
2	1	1	1	1	1	1	1	1	1
2	1	1	1	2	2	2	2	2	2
2	2	2	2	2	2	2	2	2	2
2	2	2	2	2	2	2	2	2	2
1	2	2	2	2	2	2	2	2	2
1	3	2	1	2	2	2	2	1	2
3	9	1	3	2	2	2	2	1	3
1	9	1	1	2	2	2	2	2	9
2	9	2	2	2	2	2	2	2	9
9	9	9	9	9	9	9	9	9	9
9	1	1	1	2	2	2	2	1	9
9	9	9	9	2	2	2	2	9	1
1	1	1	1	1	1	1	2	1	1
9	9	1	1	2	2	2	2	1	9
2	2	2	2	2	2	2	2	2	2
1	9	1	1	2	2	2	2	1	9
9	9	9	2	2	2	2	2	2	9
9	2	9	9	2	2	2	2	2	9

9	9	2	9	2	2	2	2	2	9
9	2	9	9	2	2	2	2	9	2
9	9	9	2	2	2	2	2	2	9
9	9	9	9	9	9	9	9	9	9
2	9	3	1	2	2	2	2	1	9
9	2	9	9	2	2	2	2	9	9
9	9	9	9	9	9	9	9	9	9
9	9	9	9	2	9	2	2	2	9
1	2	1	2	2	2	2	2	2	3
9	9	2	9	2	2	2	2	2	9
1	2	1	1	2	2	2	2	1	2
1	1	2	2	2	2	2	2	2	2
2	3	2	3	2	2	2	2	1	2
1	1	1	2	1	2	2	2	2	2
1	1	1	2	1	2	2	1	1	2
1	1	1	1	1	2	2	2	1	3
2	2	2	2	2	2	2	2	2	2
1	3	1	3	2	2	2	2	3	2
2	1	1	2	2	2	2	2	1	2
2	1	2	2	1	2	1	2	1	2
1	2	2	3	2	2	2	9	1	2
2	2	2	3	2	1	1	2	1	2
1	2	2	1	1	2	1	2	2	2
2	2	2	3	1	1	1	2	1	1
3	2	2	2	1	2	2	1	1	2
2	2	3	3	2	2	2	2	3	2
9	9	9	9	2	2	2	2	2	9
2	2	2	2	2	2	2	2	3	3
2	2	1	2	1	2	2	1	1	2
3	3	3	3	3	2	2	2	2	2
9	9	9	9	2	2	2	2	2	9
2	2	2	2	3	3	3	3	3	3
2	2	3	1	2	3	3	1	1	2
2	2	2	2	2	2	2	2	2	2
1	2	2	1	2	1	1	2	1	1
2	2	2	3	2	2	2	2	3	3
2	2	2	2	2	2	2	2	2	3
9	9	9	9	2	2	2	2	2	2
2	9	1	2	2	2	2	2	3	3
2	2	2	2	2	2	2	2	2	2
2	2	1	2	2	2	2	2	2	2
2	2	2	2	2	2	2	2	3	3
2	1	2	3	1	1	1	2	3	2
3	3	2	2	2	2	2	2	3	2
1	2	2	3	2	1	1	2	1	1
1	1	2	1	2	2	1	2	1	1
1	2	1	1	2	1	1	1	2	2

2	2	2	1	1	2	2	1	2	2
3	2	2	2	2	1	1	2	1	2
1	2	2	2	2	1	1	2	1	2
2	2	2	2	2	2	2	2	1	2
1	2	3	2	1	2	3	2	2	1
1	1	3	2	2	2	2	2	2	2
1	2	3	2	1	2	1	2	2	3
1	1	1	1	3	1	1	1	1	3
1	1	1	2	1	1	2	2	1	3
2	2	2	2	2	2	2	2	2	2
3	3	3	3	2	2	1	3	3	3
2	2	2	2	2	2	2	2	1	2
2	3	1	3	2	1	3	1	3	1
2	3	1	2	2	3	2	3	3	2
1	1	2	3	1	2	3	2	2	1
1	3	2	3	2	2	2	2	2	2
1	3	2	3	2	2	2	2	2	2
2	2	2	2	2	2	2	2	2	2
2	3	1	3	3	1	2	2	3	3
2	2	2	2	3	2	2	2	2	2
2	2	2	9	2	1	1	1	1	1
2	2	2	2	2	2	2	2	2	2
2	1	3	2	2	2	2	2	2	2
3	2	3	2	2	2	2	2	2	2
1	2	2	2	1	1	1	1	2	2
3	3	3	3	2	2	2	2	2	2
2	2	2	1	2	2	2	3	2	3
1	2	3	3	1	1	1	1	1	3
9	9	1	1	1	1	1	1	1	1
2	2	2	2	2	2	2	2	2	2
1	1	2	3	3	2	2	2	2	2
1	1	2	1	1	1	1	2	1	3
2	2	2	2	1	2	1	2	2	2
2	2	2	2	1	1	2	3	2	2
1	1	3	2	1	1	3	3	2	3
1	2	1	2	2	2	2	2	2	2
1	3	3	3	1	1	3	3	1	3
1	1	1	1	3	2	2	2	2	2
2	2	1	2	2	2	2	2	2	3
2	2	2	3	1	1	2	2	3	2
2	3	2	2	9	1	1	2	1	3
2	2	2	2	2	2	2	2	2	3
1	1	2	3	2	2	2	2	2	2
2	3	3	2	3	3	3	3	2	2
2	2	2	2	2	3	2	2	1	2
2	2	2	2	2	2	2	2	2	2
2	2	2	2	2	2	2	2	2	2

3	1	2	2	2	2	2	2	2	2
1	1	2	1	1	3	3	1	2	1
2	2	2	2	2	2	2	2	2	2
2	2	2	2	1	1	2	2	2	2
2	2	2	2	3	3	3	3	2	2
2	2	2	2	1	2	2	1	2	2
1	2	1	3	2	2	2	2	1	2
3	1	2	3	9	9	9	9	2	3
2	2	2	2	1	1	1	1	2	2
9	9	9	9	9	9	9	9	9	9
1	2	3	1	2	2	2	2	1	2
1	1	1	2	2	1	2	1	1	1
1	3	2	3	3	1	2	3	2	3
1	2	2	2	2	2	2	2	2	2
2	2	2	2	3	3	2	2	1	2
2	2	2	2	3	2	3	2	2	2
2	2	2	2	1	2	2	1	2	2
2	2	2	2	1	2	2	1	2	2
3	3	3	3	2	2	2	2	3	3
1	1	1	1	1	1	2	1	3	3
3	3	3	3	9	9	9	9	3	3
3	3	3	3	2	2	2	2	3	3
3	3	3	3	2	2	2	2	2	2
3	2	3	1	1	1	1	2	2	1
2	2	2	2	3	3	1	3	2	2
2	2	2	2	2	1	2	1	1	1
2	1	1	1	2	1	2	1	3	3
2	2	2	2	1	2	1	2	2	1
2	2	2	2	1	1	1	2	1	1
2	2	1	1	1	2	2	3	2	2
2	2	2	2	2	1	2	1	2	2
2	3	2	3	2	3	2	3	2	2
2	3	3	3	2	2	2	2	2	2
3	3	3	3	2	2	2	2	3	3
1	1	2	2	3	2	2	2	1	2
1	1	9	2	1	2	3	3	2	2
1	1	2	2	1	2	3	3	2	2
1	2	2	2	2	2	2	2	3	2
2	2	2	2	2	2	2	2	2	2
2	2	3	3	2	2	3	1	2	2
2	2	2	2	2	3	2	2	2	1
1	1	2	1	3	2	1	3	3	3
2	2	2	2	3	2	2	2	2	2
1	1	1	1	1	2	2	2	3	3
1	3	3	3	2	2	2	2	3	3
1	1	1	2	1	1	2	1	1	3
2	1	1	1	1	1	2	1	1	1

3	2	2	2	1	3	3	3	1	1
1	1	1	3	2	2	2	2	2	2
2	2	2	2	1	2	2	3	2	2
2	2	2	2	3	3	3	3	2	2
3	2	3	1	3	3	3	3	3	3
9	9	9	9	2	2	1	1	9	9
2	2	2	2	2	2	2	2	2	2
2	2	2	2	3	3	3	3	2	2
1	1	1	1	1	2	2	2	1	2
3	3	3	3	2	2	2	2	2	3
1	1	2	1	1	1	2	1	1	2
1	2	3	2	1	1	3	3	2	2
9	9	9	9	2	2	2	2	1	9
9	9	9	9	1	2	2	2	9	9
9	9	9	9	3	2	2	2	9	9
2	2	2	3	2	2	2	2	2	2
1	1	1	1	1	1	1	1	1	3
9	2	3	3	3	2	2	2	2	9
1	1	1	1	1	1	1	1	2	2
3	2	1	2	1	3	2	2	1	2
3	2	2	2	3	3	3	3	3	1
1	2	1	1	1	1	1	1	2	2
1	1	1	1	1	1	1	1	2	2
2	2	2	2	2	2	2	2	2	2
3	2	1	1	2	2	2	2	2	2
1	2	2	2	1	1	2	2	2	9
3	2	1	1	2	2	2	2	2	2
9	9	9	9	2	2	2	2	2	2
9	9	9	9	2	2	2	2	9	9
1	1	1	3	2	2	1	1	1	3
1	1	2	3	2	2	2	2	1	3
2	1	3	2	2	2	1	2	2	1
1	1	1	1	2	1	1	2	1	1
1	9	1	1	3	3	3	2	3	3
1	1	1	1	2	2	2	2	1	2
1	1	2	2	1	1	1	1	1	2
2	2	3	3	2	2	2	2	2	2
2	2	2	2	2	2	2	2	2	2
2	2	2	2	3	3	3	3	2	2
2	3	1	1	2	2	2	2	2	2
1	1	1	1	2	2	2	2	1	1
2	3	3	3	2	2	2	2	1	2
1	1	2	2	2	2	2	2	1	2
2	2	2	2	3	2	2	2	2	2
1	1	1	1	2	2	2	2	2	2
1	1	1	1	2	2	2	2	1	2
2	2	2	1	2	2	2	3	1	2

1	1	2	2	1	2	1	1	2	2
2	2	2	2	2	2	1	2	3	3
9	9	9	9	1	1	3	3	9	9
1	1	1	2	2	2	1	3	2	1
1	2	1	2	2	3	3	1	2	2
1	2	2	2	2	2	2	2	2	2
1	2	2	2	2	2	2	2	2	2
1	1	1	1	3	2	2	2	1	2
1	3	1	3	2	2	2	1	1	3
2	2	2	2	2	2	2	2	3	2
3	2	2	3	2	2	2	2	2	2
1	1	1	2	3	3	3	3	3	2
2	2	2	2	2	2	2	2	2	2
1	1	2	2	1	1	2	3	1	3
3	2	1	1	1	2	2	2	1	3
1	9	1	1	1	3	1	2	1	3
1	1	1	1	9	2	9	9	9	9
2	1	1	3	2	2	2	2	9	9
1	1	1	3	1	1	2	2	1	3
1	1	1	1	1	2	1	2	1	3
2	2	3	3	1	2	2	2	2	2
9	9	9	9	9	9	9	9	9	9
2	1	1	1	2	2	1	1	3	3
2	3	1	3	2	2	2	1	3	2
2	2	2	2	2	2	2	2	2	2
9	9	9	9	2	2	2	2	2	9
9	9	9	9	2	2	2	2	1	9
1	2	1	3	2	2	2	2	3	2
9	9	9	9	3	3	9	9	9	1
9	9	9	9	1	2	1	1	9	9
9	9	9	9	9	9	9	9	9	9
2	2	2	2	9	9	9	9	9	9
9	9	9	9	2	2	2	2	1	9
1	2	3	1	1	1	1	1	2	1
1	2	3	1	1	2	1	1	3	2
3	3	3	3	1	1	1	1	2	3
3	3	3	3	1	1	1	1	2	3
2	2	2	2	1	1	1	1	2	3
1	1	1	1	1	1	2	9	1	1
2	2	2	2	2	2	2	2	2	2
1	1	1	1	1	2	1	2	2	2
2	2	2	2	3	2	1	1	1	3
2	2	2	2	2	2	2	2	2	2
2	1	1	2	2	2	2	2	2	2
2	1	1	2	2	2	2	2	2	2
2	1	2	1	2	2	2	2	2	2
2	1	1	2	1	2	2	2	2	2

2	2	1	1	1	1	1	3	1	3
2	3	2	3	3	1	3	1	2	9
1	1	1	3	2	2	2	2	1	3
9	9	9	9	9	9	9	9	9	9
9	9	9	9	2	2	2	2	9	9
9	9	9	9	1	1	3	2	2	3
1	1	1	1	1	2	3	3	2	2
9	9	9	9	9	9	9	9	9	9
9	9	9	9	9	9	9	9	9	9
1	1	1	1	1	2	2	1	1	2
2	2	2	2	2	2	2	2	2	2
2	2	2	2	2	2	2	2	3	2
2	3	3	3	1	2	2	1	2	2
9	9	9	9	9	9	9	9	9	9
9	9	9	9	9	9	9	9	9	9
9	9	9	9	2	2	1	2	9	9
9	2	3	3	2	2	2	2	1	3
3	1	3	1	1	1	2	2	1	1
1	1	1	2	1	1	2	1	9	9
9	9	9	9	9	9	9	9	9	9
9	9	9	9	2	2	2	2	9	1
1	1	1	1	9	9	9	9	9	9
9	9	9	9	9	9	9	9	9	9
9	9	9	9	9	9	9	9	9	9
1	1	1	2	1	2	2	2	2	2
3	9	3	2	2	2	2	2	2	2
3	2	1	1	2	2	2	2	1	2
1	2	1	1	1	1	2	2	2	2
3	1	2	2	2	3	3	1	1	2
1	2	1	3	2	2	2	2	2	2
3	3	3	3	3	3	3	2	3	3
1	2	2	2	1	1	2	2	3	3
2	2	2	2	1	1	2	1	2	2
1	2	2	3	2	2	2	2	1	2
2	2	2	2	1	1	3	3	2	2
1	1	1	1	1	1	2	2	1	3
1	1	3	2	1	1	1	2	1	3
1	2	1	1	2	2	2	2	2	2
1	2	1	1	1	2	2	2	1	3
1	2	2	2	2	2	1	1	1	2
2	1	1	2	1	1	1	1	1	2
1	1	1	1	3	2	2	2	1	2
1	1	1	1	2	2	2	2	2	2
9	9	9	9	1	1	3	2	2	9
9	9	9	9	1	1	1	2	9	9
1	1	1	2	2	2	2	2	2	1
2	2	2	2	1	2	2	2	2	2

2	2	2	2	1	2	2	2	2	2
2	2	2	2	2	2	2	2	1	1
2	3	3	3	2	2	2	2	2	2
2	2	2	2	2	2	2	2	2	2
1	1	1	1	2	2	2	2	1	2
1	1	2	1	2	2	2	2	2	2
1	2	1	2	2	1	2	3	1	2
2	3	3	1	1	2	1	2	1	2
9	9	9	9	9	9	9	9	9	9
1	1	2	2	2	2	3	3	3	2
2	2	2	2	2	2	2	2	2	3
2	2	2	2	2	2	2	2	2	2
3	1	1	1	1	2	3	1	2	2
2	2	2	2	3	3	3	3	2	2
2	2	2	2	2	2	2	2	2	3
3	1	1	1	2	2	2	3	2	1
2	3	1	2	1	1	1	1	1	2
3	1	2	3	2	3	1	2	1	2
1	1	1	1	2	3	3	3	2	2
2	3	2	1	2	3	1	2	2	2
1	3	1	2	2	2	2	2	1	1
2	2	2	2	2	1	2	1	2	2
1	2	2	1	2	1	2	1	1	1
2	3	2	1	1	2	1	1	1	1
2	2	2	2	2	2	1	1	2	2
2	3	3	3	2	1	3	3	2	3
2	2	2	2	3	2	2	2	2	2
9	1	2	3	2	1	2	1	1	2
3	2	1	2	2	1	1	2	1	3
9	9	9	9	9	9	9	9	9	9
1	1	1	2	1	2	2	2	1	1
9	9	9	9	9	9	9	9	9	9
9	9	9	9	9	9	9	9	9	9
1	1	1	1	1	1	1	3	1	1
2	1	2	3	1	1	1	1	1	2
2	3	2	1	1	2	2	2	3	2
2	3	2	1	1	2	3	1	3	2
1	1	1	1	2	1	1	2	3	2
2	2	2	2	2	2	2	2	2	2
2	2	3	3	1	1	1	1	1	2
2	1	1	1	9	9	9	9	2	2
2	1	1	2	2	2	2	2	1	1
2	2	1	2	2	2	2	2	2	2
1	2	1	2	2	2	2	3	2	2
2	3	3	3	1	3	3	3	2	1
2	3	2	2	2	2	2	2	2	2
3	2	9	1	1	2	2	2	2	2

1	1	1	1	3	1	2	2	1	2
2	2	2	2	2	3	2	3	2	2
2	1	2	2	2	1	1	3	1	2
9	9	9	9	3	1	1	9	3	1
3	1	2	2	1	2	2	2	2	1
2	2	2	2	9	9	9	9	2	2
1	1	2	1	1	1	1	2	1	1
9	2	2	3	1	2	2	2	1	2
9	9	9	9	2	2	2	2	9	9
2	2	2	2	3	3	3	1	2	2
2	2	3	1	2	2	2	2	2	2
1	1	2	3	2	1	2	2	2	3
9	9	9	9	9	9	9	9	9	9
2	2	2	2	2	2	2	2	2	2
1	1	2	3	1	2	3	2	1	2
3	1	1	2	1	1	1	1	1	3
9	9	9	9	2	2	2	2	1	1
9	9	9	9	9	9	9	9	1	9
1	2	1	1	1	1	2	2	1	3
2	1	1	3	2	2	2	2	2	3
2	1	2	1	1	1	2	2	1	1
3	3	3	3	3	3	3	2	3	3
2	2	2	2	3	3	3	3	2	2
3	3	3	2	2	2	2	2	2	2
1	1	2	1	3	3	2	2	2	3
1	2	2	3	2	2	2	2	2	2
1	1	2	2	3	1	3	1	2	2
1	1	1	2	1	1	3	2	2	3
1	1	2	1	2	2	2	2	2	1
2	2	2	2	1	1	1	2	1	2
2	2	2	2	2	2	2	2	2	2
2	2	2	2	1	3	3	3	2	2
2	3	2	3	1	1	1	1	1	2
1	1	2	2	1	3	2	2	2	2
1	2	2	2	1	3	3	3	1	3
9	9	9	9	9	9	9	9	9	9
2	2	2	2	2	2	2	2	2	2
2	3	2	3	2	9	9	9	9	9
3	2	1	1	2	1	1	1	1	1
2	2	1	2	2	2	2	2	2	2
2	2	2	2	2	2	2	2	2	2
1	1	1	1	1	2	2	2	2	2
2	2	2	2	1	2	2	2	2	2
2	2	2	2	9	9	9	9	2	2
9	9	9	9	9	9	9	9	9	9
3	3	2	2	2	2	2	3	9	9
9	9	9	9	9	9	9	9	9	9

1	2	1	2	2	2	1	3	3	3
1	1	1	1	9	9	9	9	1	1
9	9	9	9	9	9	9	9	9	9
1	1	1	1	1	1	2	3	1	3
1	1	1	1	2	2	2	2	1	2
3	1	2	3	9	9	9	9	3	2
2	2	2	2	2	2	2	2	2	2
1	9	9	9	9	9	9	9	2	9
2	2	1	3	2	3	3	2	3	3
2	2	2	1	9	9	9	9	9	9
9	9	9	9	9	9	9	9	3	3
3	1	3	1	1	3	2	1	3	1
9	2	3	2	2	3	2	2	2	9
2	2	2	1	1	2	1	2	2	2
9	1	2	2	2	2	2	2	2	2
2	2	2	2	1	3	3	3	9	3
3	3	2	1	2	2	2	2	1	3
9	9	9	9	9	9	9	9	9	9
9	9	9	9	2	2	9	9	9	9
3	1	2	3	2	2	2	1	2	2
3	2	1	2	3	3	3	3	2	2
2	1	1	1	1	1	3	3	2	2
3	2	3	2	9	9	9	9	2	2
3	3	3	3	1	1	1	1	3	3
2	3	2	1	1	1	2	3	1	2
1	1	2	2	2	2	2	2	2	2
9	9	9	9	2	2	2	2	2	2
3	3	3	3	2	1	2	1	2	1
9	9	9	9	9	9	9	9	9	9
9	9	9	9	9	9	9	9	9	9
9	9	9	9	9	9	9	9	9	9
9	9	9	9	9	9	9	9	9	9
9	9	9	9	9	9	9	9	9	9
1	1	1	1	2	2	2	2	2	2
3	1	2	3	3	2	2	1	1	3
2	2	2	1	2	2	2	2	2	2
9	9	9	9	9	9	9	9	9	9
1	2	1	1	2	2	2	2	1	3
1	1	1	1	1	1	2	2	1	1
9	9	9	9	2	2	2	2	9	9
2	2	2	2	1	1	1	1	2	2
2	1	1	1	1	1	3	2	2	2
2	2	2	2	1	1	1	1	2	2
2	2	2	2	2	2	2	2	2	2
2	1	2	2	1	1	2	1	1	2
2	2	2	3	1	1	3	3	1	2
1	2	2	1	1	1	2	3	1	2

2	2	2	2	2	2	2	2	2	2
2	2	2	2	1	1	1	1	2	2
9	9	9	9	9	9	9	9	9	9
2	3	1	2	2	1	3	1	1	1
3	3	3	2	2	2	2	2	1	1
3	1	1	1	1	1	1	1	1	1
1	2	9	3	2	2	2	2	3	9
2	2	2	2	2	2	2	2	2	2
2	2	2	2	1	1	1	1	2	2
2	2	2	2	2	2	2	2	3	3
2	2	9	9	1	1	2	3	2	9
2	1	2	2	3	2	2	2	1	1
9	9	9	9	9	9	9	9	9	9
1	1	1	1	1	1	1	1	1	1
1	2	1	2	1	2	1	2	1	2
1	1	1	3	2	2	1	1	1	2
3	3	1	1	2	2	1	2	1	2
9	9	9	9	2	2	2	2	9	9
9	1	2	2	3	2	1	2	1	2
9	9	9	9	9	9	9	9	9	9
1	9	9	9	1	2	2	2	2	9
3	3	3	3	2	1	1	3	3	3
9	9	9	9	9	9	9	9	9	9
9	9	9	9	9	9	9	9	9	9
9	9	9	9	1	2	1	1	1	9
9	9	9	9	9	9	9	9	9	9
1	1	1	2	2	2	2	2	2	3
3	2	3	2	1	1	1	3	2	3
9	9	9	9	2	2	2	2	9	9
1	1	2	2	2	1	2	1	2	2
3	1	3	1	2	2	2	2	1	3
1	1	3	3	2	2	9	9	9	9
1	1	1	1	2	2	2	2	2	2
2	2	2	2	2	1	2	2	2	2
2	2	2	2	2	2	2	2	2	2
2	2	2	2	2	2	2	2	2	2
2	3	1	2	1	1	1	1	2	2
2	2	2	2	1	2	2	2	2	2
2	2	2	2	1	2	2	2	2	3
1	1	1	1	2	1	2	2	9	9
1	2	1	2	9	9	9	9	3	9
1	2	1	3	2	2	2	2	3	3
2	2	2	2	3	2	2	2	1	2
2	9	3	3	1	3	2	3	1	3
1	2	1	1	1	1	1	1	1	3
1	1	1	1	1	1	1	1	1	1
3	2	3	2	2	2	2	2	2	2

1	3	2	3	3	2	2	2	3	1
2	1	2	2	1	2	2	3	2	2
3	2	3	2	1	1	1	1	2	9
1	1	2	1	2	2	2	2	2	2
2	2	2	2	3	3	3	2	2	2
2	2	2	2	3	3	3	2	2	2
2	2	2	1	2	2	2	2	2	2
2	3	3	9	9	9	9	9	3	3
2	2	2	2	2	2	2	2	2	2
1	1	1	3	9	9	9	9	9	9
1	1	1	1	2	1	2	2	1	3
1	2	1	2	2	2	2	2	1	2
3	3	3	3	2	2	2	2	9	9
3	2	2	3	2	2	2	2	3	3
1	1	1	1	9	9	9	9	9	9
9	9	9	9	2	2	2	1	9	9
3	1	3	1	1	2	3	1	3	3
2	2	2	2	2	2	1	2	2	2
3	3	1	2	2	2	2	2	1	2
3	3	3	3	1	1	1	1	3	3
2	2	2	2	1	2	2	2	2	3
2	2	2	2	3	2	3	2	2	2
1	1	1	1	9	9	9	9	9	9
2	9	1	1	1	1	1	1	2	1
1	1	1	1	9	9	9	9	1	1
3	3	3	3	2	2	2	2	3	3
3	2	2	2	2	2	2	2	1	1
3	3	2	2	2	2	2	2	3	2
3	3	2	3	1	1	1	2	3	2
2	2	2	2	2	2	2	2	2	2
3	3	3	3	1	3	3	2	2	2
1	9	9	9	2	1	2	2	9	9
2	2	2	2	2	2	2	2	2	2
1	3	1	2	2	2	2	2	3	1
3	2	1	1	1	2	3	2	1	2
2	3	1	3	1	1	2	2	1	3
2	2	2	2	1	2	2	2	2	2
3	3	3	3	2	2	2	2	2	2
1	2	3	2	1	2	2	2	2	2
2	2	2	2	3	3	3	3	2	2
3	3	2	3	2	2	2	2	2	2
1	2	1	2	1	1	1	1	2	3
1	2	1	2	2	2	2	2	2	2
1	2	2	9	9	9	9	9	3	2
9	9	9	9	9	9	9	9	9	9
9	1	2	1	1	1	3	2	1	2
2	2	2	2	1	3	2	1	2	2

2	2	2	2	2	2	1	1	2	1
1	1	1	3	1	2	2	2	1	9
1	1	1	3	9	9	9	9	1	9
1	2	2	2	2	1	2	1	1	2
9	9	9	9	2	2	2	2	1	9
1	9	9	1	1	1	2	2	9	9
9	9	9	9	1	1	1	1	1	9
2	2	2	2	2	2	3	3	2	2
1	1	1	3	1	3	1	2	2	2
2	2	3	1	3	1	2	2	1	3
2	2	2	2	2	3	2	3	2	2
2	2	2	2	2	3	2	2	1	3
2	2	2	2	1	1	3	3	2	2
2	2	2	2	1	1	1	3	2	2
2	2	2	2	2	3	3	3	2	2
2	2	2	2	1	1	1	1	2	2
3	3	3	3	3	3	3	3	3	3
2	2	2	2	2	2	2	2	2	2
2	2	2	2	2	2	2	2	2	2
2	2	2	2	1	2	3	2	2	2
1	2	1	1	1	2	2	2	1	2
1	1	1	1	1	1	1	1	1	1
9	9	9	1	2	1	2	1	1	9
2	2	2	2	3	3	3	3	2	2
2	2	2	2	2	2	2	2	1	1
2	2	2	2	2	1	2	1	3	2
2	2	1	1	1	2	2	1	2	2
9	9	9	9	9	9	9	9	9	9
1	1	2	2	2	2	2	2	1	2
1	1	2	2	3	3	1	1	2	2
2	2	2	1	2	3	3	3	1	3
1	1	1	3	1	1	2	3	1	2
3	2	1	1	1	1	2	2	2	1
1	2	1	1	3	1	1	2	1	2
1	1	1	3	1	1	1	1	2	1
3	3	3	3	1	2	1	9	1	3
2	2	2	2	3	3	3	3	2	2
2	2	2	2	2	2	2	2	2	2
2	2	2	2	1	2	2	2	2	2
9	9	9	9	9	9	9	9	9	9
1	2	1	1	2	2	2	2	2	2
3	1	1	2	1	1	1	1	2	1
1	2	1	2	2	1	3	1	2	2
1	1	1	1	2	2	2	2	2	1
2	1	1	1	2	1	3	2	1	1
2	2	2	2	3	3	3	3	2	2
2	2	2	2	3	2	3	3	2	2

1	2	2	2	2	2	2	2	2	2
9	9	9	9	3	2	3	2	2	9
2	2	2	2	1	2	2	2	2	2
1	2	3	1	1	1	1	1	1	2
2	2	2	2	2	2	2	2	3	3
2	2	1	1	2	2	2	2	2	2
2	3	2	3	3	3	3	3	3	3
1	1	1	1	2	2	2	2	1	3
2	9	9	9	1	2	2	1	9	9
2	2	1	3	2	2	2	2	3	3
1	1	9	9	3	2	2	2	9	9
1	1	1	2	2	2	2	2	1	3
2	2	2	2	2	1	1	2	2	2
3	1	1	2	2	2	2	2	1	1
2	2	2	2	2	2	2	2	1	3
1	1	1	1	1	1	1	3	1	2
2	2	1	1	1	2	1	2	1	2
9	1	3	3	1	1	1	1	1	3
9	9	9	9	9	9	9	9	9	9
1	1	2	1	1	1	1	3	1	2
1	2	1	2	2	2	2	2	1	2
1	2	1	1	1	1	3	3	1	3
2	2	2	2	1	1	1	1	1	2
1	2	3	1	2	2	1	1	2	1
2	2	2	2	9	9	9	9	9	9
1	1	2	1	3	1	3	2	1	3
2	2	3	3	1	1	2	2	1	2
1	1	1	1	1	2	2	2	2	2
1	2	3	2	1	2	1	2	1	2
1	3	3	2	1	2	2	2	3	2
2	2	2	2	3	3	3	3	2	2
2	2	3	2	1	2	2	1	2	3
1	1	3	1	2	1	3	1	1	3
1	3	2	1	1	1	2	1	1	2
1	1	3	3	1	2	1	2	1	1
2	3	3	3	1	3	2	1	1	1
2	2	2	3	2	2	2	2	1	2
3	2	2	3	2	2	2	2	2	2
2	2	2	2	2	2	2	2	1	2
1	1	2	2	2	2	2	2	1	2
9	9	9	9	9	9	9	9	9	9
2	1	1	1	3	2	2	2	3	2
1	9	3	3	9	9	9	9	1	1
1	1	1	3	2	2	2	1	1	3
1	1	1	3	2	2	2	2	1	3
1	1	1	3	3	2	2	2	1	2
1	1	2	3	1	2	3	2	1	3

3	2	2	3	2	2	2	2	3	3
9	9	9	9	1	2	2	9	9	9
2	2	2	2	1	9	9	9	9	9
9	9	9	9	9	9	9	9	9	9
2	2	2	2	1	2	2	1	2	2
2	2	2	2	2	2	2	2	2	2
2	1	1	2	2	2	2	2	1	2
1	1	1	1	1	1	1	1	1	1
9	9	9	9	9	9	9	9	9	9
1	2	2	3	9	9	9	9	9	9
9	9	9	9	2	2	2	2	2	2
1	2	1	2	2	2	2	3	1	3
1	1	1	1	2	2	2	2	2	2
1	1	1	1	2	2	2	2	2	2
2	2	2	3	1	1	2	2	2	2
1	1	2	2	1	3	2	2	1	3
1	1	1	2	2	2	2	2	1	2
1	1	1	1	2	2	2	2	1	3
1	2	3	3	2	2	1	3	1	2
9	1	2	1	1	1	1	1	9	9
1	1	1	1	1	1	1	1	2	2
3	2	3	3	1	1	3	3	1	3
9	9	9	9	2	2	2	2	1	2
2	2	3	3	1	2	2	2	3	1
2	3	1	2	2	2	2	2	1	2
1	1	1	2	1	1	1	1	1	2
1	2	2	2	1	2	2	2	1	2
1	2	2	3	1	1	2	2	2	1
2	9	3	1	2	2	2	2	1	2
2	2	2	3	2	2	2	1	3	2
3	3	2	1	2	2	2	2	2	1
3	2	2	3	3	1	1	1	2	3
3	1	2	2	2	2	2	2	3	2
1	2	1	1	2	2	2	2	1	3
1	1	3	1	1	3	2	2	2	3
1	1	3	1	1	3	2	2	2	3
1	1	1	2	1	2	1	1	1	1
9	9	9	9	3	2	3	2	2	2
2	2	2	2	3	2	1	2	2	3
2	1	2	1	9	9	9	9	2	2
1	2	3	2	2	2	2	3	1	2
1	1	2	2	2	1	2	1	1	2
1	1	2	1	9	9	9	9	9	9
2	1	3	3	2	2	2	2	1	3
1	2	1	1	2	2	2	1	1	2
1	2	2	2	1	1	1	1	1	3
1	1	1	1	2	2	1	1	1	2

1	1	1	1	1	1	1	1	1	2
9	9	9	9	9	9	9	9	2	9
1	2	3	1	1	1	1	3	1	3
1	2	1	2	2	2	2	2	1	2
2	2	2	2	2	2	2	2	2	3
3	3	3	3	1	1	2	1	1	3
9	2	2	1	1	1	3	1	1	2
2	2	2	2	2	2	2	2	1	2
2	2	2	2	2	2	2	2	1	2
9	9	9	9	9	9	9	9	9	9
2	3	1	1	1	1	1	2	1	3
1	1	1	2	1	1	1	1	1	2
3	1	3	1	1	2	1	1	1	3
2	1	3	1	1	2	2	1	1	3
2	2	2	2	2	2	2	2	2	2
9	9	9	9	9	9	9	9	9	9
9	9	9	9	1	1	1	1	1	9
1	1	1	1	3	2	2	1	1	2
2	2	2	2	1	2	2	2	1	1
2	2	2	2	2	1	1	2	1	2
9	9	9	9	9	9	9	9	9	9
1	1	1	1	1	2	1	2	1	2
2	2	2	2	1	2	2	1	1	1
3	2	2	1	1	2	2	2	1	2
1	1	2	2	2	1	1	2	1	1
2	2	2	2	1	2	2	1	1	1
1	1	1	1	1	2	2	1	1	1
3	3	3	3	2	1	2	1	1	2
1	2	2	1	3	2	2	2	1	1
3	3	3	3	2	1	1	1	3	2
9	9	9	9	9	9	9	9	9	9
9	9	9	9	9	9	9	9	9	9
9	9	9	9	9	9	9	9	9	9
2	2	2	1	2	2	2	3	1	2
1	1	2	3	2	2	2	1	1	3
1	9	2	3	1	3	2	1	1	2
2	2	2	2	1	1	1	2	1	2
1	2	1	1	2	2	2	2	2	9
1	2	2	2	1	3	9	1	1	2
2	2	2	2	2	2	2	2	2	2
1	1	1	3	2	2	2	2	3	3
2	2	1	1	2	1	1	2	1	3
1	2	2	1	2	2	2	2	2	1
1	1	1	1	1	1	2	2	3	3
3	2	1	3	2	2	2	2	2	2
1	1	1	1	1	1	1	1	9	1
1	1	1	1	2	2	2	2	3	1

1	1	3	3	2	2	2	1	2	2
1	2	2	2	2	2	2	2	2	2
3	3	2	2	2	2	2	2	2	3
1	1	3	1	2	2	2	1	1	3
1	1	1	1	2	2	2	2	3	3
3	1	3	3	1	2	2	2	3	1
1	1	2	3	2	2	2	2	2	2
9	9	9	9	9	9	9	9	1	9
9	9	9	9	2	2	3	1	2	2
9	9	9	9	1	1	1	1	1	9
9	9	9	9	2	1	3	3	9	9
9	9	9	9	2	2	2	2	9	1
1	1	1	1	1	3	2	2	2	2
3	2	2	2	2	2	2	9	9	9
1	1	3	3	2	2	2	3	1	3
1	1	2	2	1	1	1	1	1	2
1	1	1	2	1	1	2	2	1	2
1	1	1	2	1	1	2	1	1	2
2	1	3	3	2	2	2	2	2	2
2	3	2	3	2	1	1	3	1	2
2	1	2	1	2	2	2	2	2	2
2	2	2	1	1	3	2	2	2	2
2	2	2	2	1	1	2	2	2	2
9	9	9	9	2	2	2	2	2	3
1	2	3	2	1	1	1	1	1	2
1	1	3	3	2	1	1	1	2	2
2	2	2	2	2	2	2	2	2	2
2	2	2	1	1	2	2	2	1	2
2	2	2	2	3	2	2	2	1	2
2	2	2	2	1	1	2	3	1	2
2	2	2	2	1	1	3	9	2	2
9	9	9	9	1	1	2	2	2	2
1	2	3	2	2	2	2	2	1	3
2	1	1	2	1	1	2	3	2	2
9	9	9	2	3	3	3	9	9	9
2	2	2	2	3	1	2	2	2	2
2	2	2	2	2	2	2	2	2	2
2	2	2	2	2	2	2	2	2	2
1	2	1	1	3	2	2	2	1	2
1	2	2	2	2	2	2	2	2	2
1	1	1	1	3	2	2	1	1	1
9	9	1	9	3	2	2	2	2	9
9	9	9	9	3	2	2	2	2	9
2	2	2	2	2	2	2	2	2	2
9	1	1	3	1	1	3	1	9	9
1	2	3	1	2	2	2	2	2	9
1	2	2	1	2	2	3	3	1	2

1	1	1	2	1	2	2	1	2	2
2	2	2	2	1	2	2	1	2	1
1	2	2	2	2	3	3	1	2	2
2	2	3	3	1	3	2	2	1	2
2	2	2	2	2	3	2	2	2	2
1	1	1	2	3	2	2	2	2	2
3	3	2	2	1	2	2	3	2	2
2	1	2	3	2	2	2	2	2	2
1	1	1	1	1	1	1	1	1	2
1	2	3	1	1	2	1	1	2	3
1	1	1	1	3	2	2	2	1	3
1	2	2	1	2	2	3	2	2	9
1	2	1	1	1	1	1	2	1	9
1	1	1	1	1	2	2	2	2	2
2	2	2	2	2	2	2	2	1	9
2	2	2	2	2	2	2	2	2	2
3	3	3	3	1	1	2	1	1	2
2	2	2	2	2	2	3	3	2	2
2	2	3	2	2	1	2	1	2	2
2	2	1	2	1	3	2	2	1	1
2	1	2	2	1	2	2	1	2	3
1	2	1	3	3	2	2	1	2	2
1	2	2	1	2	2	2	3	1	2
1	2	2	2	2	2	2	2	2	2
1	1	2	1	1	1	1	1	1	1
2	2	2	2	3	3	3	3	2	2
2	2	2	2	1	1	2	1	2	2
1	1	1	1	1	1	1	1	1	3
2	2	2	2	1	1	3	1	2	3
3	2	2	2	2	1	2	1	1	2
9	9	9	9	9	9	9	9	9	9
2	2	2	2	1	2	2	1	1	1
2	2	2	2	2	2	2	1	1	2
2	2	2	2	2	1	2	1	1	2
3	2	2	2	1	2	1	2	2	2
2	2	2	2	2	1	1	1	1	1
2	2	1	2	1	2	2	1	1	2
2	2	2	2	2	2	2	2	2	2
2	1	2	3	1	1	1	1	1	2
2	2	2	2	1	2	1	1	2	2
1	1	2	1	2	2	2	2	2	1
2	1	2	2	2	2	3	1	3	3
2	2	2	2	1	2	2	2	1	3
1	1	2	3	2	2	2	2	1	3
1	1	2	3	1	1	1	2	2	1
2	2	2	2	3	2	2	2	3	3
1	1	3	1	1	3	2	2	2	3

1	1	3	1	1	3	2	2	2	3
2	2	2	2	1	2	1	1	1	2
2	2	2	2	1	2	1	1	1	2
2	2	2	2	2	1	1	1	1	2
2	2	2	3	2	1	1	2	2	2
2	2	1	1	2	1	1	2	1	1
2	2	1	1	1	2	1	1	2	1
2	2	1	2	2	2	2	2	2	2
2	1	3	2	2	2	2	2	3	2
1	1	3	2	2	1	1	1	1	3
1	1	3	3	1	2	1	2	1	1
1	2	1	1	2	2	2	2	1	2
3	3	3	3	3	3	1	1	2	2
1	1	2	2	2	2	2	2	2	2
1	1	1	3	2	2	2	2	3	3
1	2	2	3	3	2	2	2	2	2
2	1	2	1	1	2	2	1	3	1
3	3	2	2	2	1	1	1	1	2
2	2	3	2	3	3	3	3	2	2
1	1	2	1	1	1	2	2	2	2
2	3	3	2	2	1	1	1	1	1
3	3	3	3	2	2	2	2	1	3
1	1	2	2	1	2	2	2	1	2
2	2	2	3	2	2	2	2	2	3
1	2	2	3	1	1	2	1	1	3
1	1	2	1	2	2	1	2	2	3
2	2	1	2	1	1	3	1	1	2
2	2	2	2	2	2	2	2	2	2
1	2	2	2	3	3	3	3	2	2
1	1	2	2	1	2	3	1	2	2
3	3	3	3	2	1	2	2	1	1
1	1	3	3	1	2	1	2	1	1
2	3	1	1	1	2	2	2	1	1
1	1	2	1	2	2	2	2	1	3
1	3	1	2	2	2	2	2	2	3
2	2	2	2	2	2	2	1	1	2
2	1	1	2	1	1	2	2	1	2
1	1	2	3	1	2	3	1	1	2
3	2	1	2	1	2	3	2	1	1
1	9	1	1	1	1	1	1	1	1
1	2	3	1	1	1	1	1	1	2
9	2	1	1	1	1	1	3	1	2
1	1	1	2	1	1	2	2	1	2
9	1	1	1	1	1	1	2	1	3
2	2	2	2	1	2	2	2	1	3
2	3	2	3	2	2	2	2	1	2
2	2	2	2	1	2	3	2	2	2

1	2	2	3	2	2	2	1	2	2
1	2	2	3	2	2	2	1	2	2
1	2	1	1	2	1	1	2	1	2
2	1	2	1	1	1	1	1	1	2
1	1	3	3	1	2	2	2	3	2
2	2	2	2	2	1	1	2	1	1
2	2	2	2	3	3	2	2	2	2
2	2	2	2	2	1	2	1	1	1
9	9	9	9	9	9	9	9	9	9
2	2	2	2	2	2	2	2	2	2
1	1	2	2	1	1	1	1	1	2
3	2	2	1	2	2	2	2	1	2
2	2	2	2	1	1	1	1	2	1
2	3	2	3	3	3	3	3	1	2
1	1	1	1	2	2	2	2	1	1
3	1	3	3	2	2	2	2	2	2
2	2	2	2	3	3	3	1	2	2
2	1	1	1	1	1	1	1	3	3
2	3	2	1	2	2	2	2	1	2
2	2	2	2	2	2	2	3	2	2
2	2	2	2	2	1	1	2	1	2
2	1	2	1	2	1	2	1	1	1
2	2	2	2	2	1	2	2	1	1
2	2	2	2	1	2	1	2	1	2
1	1	1	1	2	2	2	2	1	1
3	1	3	3	2	2	2	2	2	2
3	2	2	1	2	2	2	2	1	2
2	2	2	2	2	2	2	2	2	2
3	2	1	2	1	2	3	2	1	1
2	2	2	2	2	2	2	3	2	2
2	1	1	2	1	1	2	2	1	2
9	9	9	9	9	9	9	9	9	9
2	2	1	2	3	2	2	2	2	2
1	1	1	3	1	1	2	2	1	9
2	2	2	2	2	2	2	2	1	1
2	3	2	3	1	1	1	1	1	3
1	1	3	3	1	1	1	1	1	1
3	2	1	2	1	2	2	2	1	2
1	1	1	1	3	2	2	2	2	2
2	3	1	2	1	1	2	3	1	3
2	1	1	2	2	1	2	1	1	2
2	1	1	2	2	1	2	2	1	2
3	1	1	3	1	1	2	2	1	3
1	2	1	1	1	1	1	1	1	2
2	1	2	3	1	2	3	2	3	9
9	9	9	9	2	2	2	2	2	2
2	2	2	2	1	1	3	1	2	2

1	2	1	2	3	2	2	2	2	2
3	3	3	3	3	2	2	2	2	2
1	1	1	1	1	2	3	2	1	1
2	2	2	2	2	2	2	2	1	2
2	2	2	2	3	2	3	2	2	2
1	1	1	3	1	1	2	2	1	2
1	1	1	2	1	1	2	2	2	2
9	9	9	1	1	1	1	1	3	9
9	9	9	3	1	1	1	1	3	9
9	9	9	4	1	1	1	1	2	9
3	3	2	1	1	1	1	1	2	2
2	3	2	1	1	1	1	1	2	2
3	3	2	3	1	1	1	1	2	2
3	2	2	3	1	1	1	1	2	2
3	2	2	3	1	1	1	1	1	1
1	1	1	1	1	1	1	3	2	2
2	2	2	4	1	1	3	3	2	2
3	2	2	3	1	1	1	1	2	1
1	2	3	1	1	1	1	1	1	2
1	2	1	3	1	1	1	1	2	1
2	2	3	9	1	1	1	1	3	2
1	1	1	1	1	1	3	3	2	2
2	2	2	9	1	3	1	3	2	2
3	3	3	9	1	1	1	3	2	2
1	1	1	9	1	1	1	1	2	2
3	1	1	5	1	1	1	1	2	2
1	1	3	9	1	1	3	3	2	2
2	1	1	1	1	1	1	1	2	2
2	2	2	1	1	1	1	1	1	2
2	2	1	1	1	1	1	1	1	2
3	2	2	3	1	1	1	1	2	2
3	3	2	3	1	1	1	1	2	2
2	2	2	1	3	3	3	3	2	2
2	2	2	1	3	3	3	3	2	2
2	2	2	1	3	3	3	3	1	3
2	2	2	9	3	3	3	3	1	3
1	1	1	9	3	3	3	3	1	3
2	2	2	1	3	3	3	3	1	3
1	1	1	1	3	3	3	3	1	3
1	1	1	1	3	3	1	1	1	3
2	2	2	1	2	3	2	3	3	1
3	3	3	4	1	1	1	1	3	2
3	3	3	4	1	1	1	1	3	2
3	3	3	4	1	1	1	1	3	2
3	3	3	4	1	1	1	1	3	2
3	2	2	1	1	3	3	2	3	9
3	1	3	9	1	3	3	3	3	9

3	1	3	6	2	2	2	9	3	3
1	3	2	9	1	1	1	1	3	1
2	1	2	9	1	1	1	1	3	2
2	3	2	9	1	1	1	1	3	2
3	3	2	9	1	1	1	1	3	2
2	1	3	5	2	2	2	2	3	3
3	3	3	4	2	2	2	2	3	3
2	3	2	3	2	3	2	3	3	2
9	9	9	6	1	1	1	1	3	9
9	9	9	9	9	9	9	9	9	9
9	9	9	9	9	9	9	9	2	2
3	2	2	4	1	1	1	1	3	9
3	2	2	5	1	1	1	1	3	9
3	3	3	1	1	2	3	1	3	1
2	3	2	1	3	3	3	3	9	9
2	2	2	9	1	2	2	2	2	2
2	2	2	9	1	2	2	2	2	2
9	9	9	1	2	2	2	1	2	9
3	3	3	3	1	2	2	2	3	2
9	9	9	4	2	1	2	2	2	9
2	2	2	1	1	3	3	1	3	2
2	3	1	1	1	2	1	1	3	2
2	2	2	9	3	3	3	3	3	1
2	2	2	9	1	1	2	1	2	3
9	9	9	1	1	2	1	1	2	9
9	9	9	1	1	1	1	1	3	3
1	3	2	1	1	1	1	1	3	2
9	9	9	1	1	9	2	1	9	9
3	3	3	1	1	1	1	1	3	2
9	9	9	9	9	9	9	9	9	9
3	3	1	5	2	2	2	2	3	3
9	9	9	1	2	2	2	2	2	9
3	3	3	3	1	1	1	1	3	1
9	9	9	9	9	9	9	9	2	2
2	2	2	5	3	3	3	3	2	2
2	2	2	3	3	2	2	2	2	2
2	2	2	5	3	3	2	2	3	1
3	3	3	4	3	3	2	2	2	2
1	1	1	9	1	2	1	1	3	1
9	9	9	1	2	2	2	2	3	9
9	9	9	1	2	2	2	2	2	9
9	9	9	9	2	2	2	2	2	9
9	9	9	9	1	9	9	1	3	9
9	9	9	1	2	2	2	2	2	9
9	9	3	1	2	3	2	3	2	9
1	1	3	1	1	1	2	2	2	1
1	3	2	1	1	1	1	1	3	2

9	9	9	9	9	9	9	9	9	9
3	3	3	1	1	1	3	1	2	2
2	9	9	3	2	2	2	2	2	1
3	3	3	5	1	1	1	1	3	2
3	3	2	6	1	1	1	1	2	2
2	2	2	1	1	1	2	1	2	2
9	9	9	5	1	1	1	1	3	9
1	1	1	1	2	2	2	1	1	3
3	3	3	4	2	2	2	2	2	2
9	9	9	1	2	2	2	2	2	9
2	2	2	9	3	9	3	3	3	2
3	3	3	6	1	1	1	1	9	9
9	9	9	1	2	2	2	2	2	9
9	9	9	1	2	2	2	2	2	9
3	3	3	1	1	1	2	1	3	2
9	9	9	9	2	2	2	2	2	9
3	3	3	4	1	3	3	1	2	2
9	9	9	1	2	2	2	0	2	9
2	2	2	9	3	2	2	1	2	9
1	2	2	2	2	1	1	1	3	1
3	3	3	1	2	3	1	1	3	3
1	2	3	1	2	1	2	1	3	2
1	3	3	3	1	1	1	1	3	2
3	3	3	3	1	1	1	1	3	1
3	1	2	3	1	1	1	1	3	1
3	2	3	1	2	3	1	1	3	1
3	3	3	4	1	2	2	2	3	1
3	3	3	5	1	2	1	2	3	1
3	3	3	4	1	3	1	2	3	2
1	2	3	5	1	2	2	2	3	3
9	1	2	9	1	1	2	3	3	3
3	3	3	9	1	1	1	1	3	1
9	9	9	3	1	1	1	3	9	9
9	9	9	4	1	9	1	9	9	9
9	9	9	5	1	2	1	1	3	9
9	9	9	3	2	9	2	2	9	9
9	9	3	1	2	1	3	3	9	9
9	9	9	4	3	2	3	3	9	9
9	9	9	3	2	3	2	1	9	9
9	9	9	9	1	2	1	1	2	9
9	9	9	1	1	2	2	2	2	9
9	9	9	1	1	2	1	1	2	9
9	9	9	9	1	2	2	2	2	9
9	9	9	4	2	1	2	1	2	9
9	9	9	1	1	1	2	2	2	9
2	3	2	1	1	1	1	1	3	1
3	2	3	1	1	1	1	1	3	2

1	1	1	3	1	1	1	1	3	1
1	9	9	1	2	3	1	1	1	9
9	9	9	1	2	2	3	2	9	9
3	2	2	3	1	1	1	1	3	2
9	9	9	1	1	2	1	1	3	9
9	9	9	1	1	1	2	1	3	9
9	9	9	1	1	1	2	1	3	9
9	9	9	1	1	2	1	1	3	9
9	9	9	1	9	9	9	9	3	9
3	2	2	1	1	1	1	1	2	2
3	2	3	1	1	1	1	1	3	2
2	2	2	1	1	1	1	1	2	1
2	2	2	1	1	1	1	1	2	2
3	1	2	9	1	1	2	2	3	2
2	3	1	1	1	2	2	2	3	1
2	2	3	9	1	2	3	1	3	1
9	9	9	1	1	9	1	1	3	9
9	9	9	5	1	3	3	3	3	9
9	9	9	1	1	3	3	3	3	9
9	9	1	3	1	1	1	1	9	9
9	9	9	1	2	1	1	1	2	9
9	9	9	9	9	9	9	9	9	9
9	9	9	1	2	1	1	1	9	9
1	9	9	1	1	2	3	3	1	9
9	9	9	3	2	2	1	1	9	9
9	9	2	3	2	3	3	3	9	9
9	9	9	1	1	1	1	2	2	9
2	2	2	9	2	2	3	2	2	2
3	2	2	4	2	2	2	1	2	1
9	9	9	6	2	2	2	3	2	9
2	2	2	1	1	1	2	2	3	2
2	3	1	6	1	2	2	2	2	1
2	2	2	1	1	1	2	1	2	2
2	2	2	1	1	2	2	2	3	2
3	3	3	1	1	1	1	1	3	3
2	2	2	1	1	2	3	2	2	2
3	2	1	9	3	3	3	2	2	1
3	2	3	3	1	1	2	2	3	2
9	9	9	1	2	2	2	1	2	9
2	2	2	1	2	2	2	2	2	2
2	2	2	1	2	2	2	2	2	2
1	1	1	1	2	2	2	2	2	1
1	2	2	1	2	2	2	2	2	1
2	2	2	1	1	2	2	2	3	2
9	9	9	1	1	2	3	3	3	9
2	2	2	1	2	2	2	2	2	2
9	9	9	5	1	1	3	1	3	3

3	2	1	3	1	1	2	1	3	2
3	2	3	1	1	2	2	1	3	2
2	2	2	1	1	2	2	2	2	2
2	1	2	6	3	2	3	2	3	2
2	2	2	1	1	1	1	2	3	2
2	3	3	1	2	2	2	2	3	2
3	1	3	1	1	1	2	1	3	3
1	1	1	4	1	1	2	2	2	1
3	3	3	1	1	1	1	1	3	1
9	9	9	9	9	9	9	9	9	9
2	2	2	1	3	3	3	3	2	2
3	2	3	1	1	2	3	1	3	2
3	3	3	4	3	1	3	1	3	1
3	2	2	1	1	3	2	2	3	2
3	3	1	5	2	2	2	2	3	1
2	3	2	1	2	2	2	2	3	1
2	2	2	1	1	1	2	2	2	2
2	2	2	1	1	1	2	3	2	2
2	2	1	6	3	2	2	2	3	2
2	2	2	1	1	1	2	2	3	2
2	2	2	9	1	2	1	1	3	2
2	2	2	5	1	1	2	2	2	2
3	3	2	1	1	1	3	1	3	2
2	2	2	1	1	1	1	1	3	3
3	3	3	1	1	1	3	3	2	2
3	3	3	1	3	3	1	1	2	3
3	3	3	4	1	3	2	2	3	2
3	2	2	1	1	1	2	1	2	2
2	2	1	1	1	2	1	1	2	2
2	2	2	1	3	3	3	3	2	2
2	2	2	5	1	1	3	1	2	2
2	2	2	1	1	2	2	2	2	2
2	2	2	1	1	2	2	2	2	2
2	2	2	1	1	1	1	2	2	2
2	2	2	1	1	2	3	1	2	2
2	2	2	1	3	3	3	3	2	2
2	1	3	1	1	1	2	1	2	3
9	9	9	9	9	9	9	9	9	9
9	9	9	9	9	9	9	9	9	9
3	1	1	1	3	2	3	3	9	9
2	3	1	1	2	3	3	2	1	2
9	9	9	1	1	1	2	1	3	9
3	3	3	9	9	9	9	9	9	9
3	2	1	1	1	1	1	2	3	3
3	1	2	1	1	2	2	3	9	9
9	9	9	9	9	9	9	9	9	9
3	3	3	1	1	2	2	2	2	2

3	1	2	1	2	2	2	2	3	2
2	2	2	3	1	1	1	1	3	1
1	2	3	9	1	1	1	1	3	3
3	3	2	1	1	1	2	2	2	2
2	2	2	1	2	2	2	2	9	9
9	9	9	9	1	2	2	2	9	9
2	2	2	1	1	2	2	1	2	2
3	3	2	1	1	1	1	1	3	3
3	3	2	1	1	1	1	1	3	2
2	1	2	3	2	3	2	1	3	3
3	1	1	1	1	1	2	2	2	2
2	3	2	1	1	1	2	2	2	2
3	2	3	4	1	1	1	2	3	3
2	2	2	1	1	1	2	1	2	2
2	3	1	1	2	2	2	2	3	2
2	2	2	1	1	2	2	1	2	2
2	2	2	9	1	3	3	3	2	2
9	9	9	1	2	2	2	2	9	9
2	2	3	4	1	1	2	2	3	2
3	2	2	1	1	1	2	2	3	3
2	2	2	9	3	1	1	1	2	2
2	1	2	1	1	2	2	2	3	2
2	2	2	1	2	2	2	2	2	2
1	1	3	1	1	1	1	1	3	3
3	2	2	1	1	2	2	2	3	3
2	2	2	1	1	2	2	2	2	2
2	2	2	1	9	9	9	9	9	9
2	2	2	1	2	2	2	2	2	2
2	2	3	9	2	2	2	1	3	2
2	1	3	1	1	1	2	2	1	2
3	3	3	1	3	3	3	3	3	3
9	9	9	1	3	2	2	2	9	9
3	2	2	1	1	1	2	2	2	2
2	2	2	1	2	2	2	2	2	2
2	2	2	4	3	1	1	3	3	1
2	2	2	1	1	2	2	2	2	2
2	3	2	1	2	2	2	1	2	1
2	1	3	4	2	2	2	2	2	2
3	3	2	1	1	1	3	3	2	2
2	2	2	4	2	2	2	2	3	3
2	2	2	1	9	9	9	9	2	2
3	3	1	1	1	1	1	1	3	2
3	3	1	4	1	1	2	2	3	3
9	9	9	1	1	1	3	2	2	2
2	2	2	1	1	1	1	1	3	2
3	3	2	1	1	2	2	2	2	3
2	2	2	4	1	1	1	1	3	3

2	2	2	1	3	3	3	3	2	2
2	2	2	1	2	2	2	3	2	2
2	2	2	1	2	1	2	3	2	2
2	2	2	1	1	2	2	2	2	2
2	2	2	1	1	2	2	2	2	2
9	9	9	9	9	9	9	9	1	2
2	2	2	9	3	3	2	2	2	2
3	2	1	9	1	1	1	2	3	3
2	2	2	1	3	3	3	3	2	2
2	2	2	2	2	3	3	3	2	2
2	2	2	9	9	9	9	9	2	2
3	3	3	1	1	3	1	1	3	2
9	9	9	9	9	9	9	9	9	9
1	3	1	4	1	1	1	2	3	3
3	9	1	1	1	1	1	1	3	9
9	9	9	9	9	9	9	9	9	9
2	3	3	9	1	1	1	1	3	2
1	1	1	9	1	1	1	1	2	3
9	9	9	5	1	1	1	1	9	9
2	3	2	6	1	1	1	1	3	1
9	9	9	9	1	1	2	1	2	2
3	2	2	9	1	2	2	2	2	2
1	2	1	1	2	2	2	2	2	2
9	9	9	9	9	9	9	9	3	1
1	3	1	5	1	1	1	1	3	3
2	1	2	9	1	2	2	2	3	9
9	9	9	9	9	9	9	9	3	3
3	1	1	6	1	1	1	1	3	1
3	3	3	1	1	2	2	1	3	1
9	9	9	4	1	1	2	2	9	9
2	3	3	1	1	1	1	1	3	3
9	9	9	1	1	1	3	1	3	2
1	3	3	1	1	1	2	2	2	2
2	2	3	1	1	1	1	1	3	3
1	1	2	9	1	1	1	1	2	9
3	2	3	4	1	1	1	1	3	3
3	3	2	1	1	1	1	1	3	2
2	3	3	4	9	9	9	9	9	9
1	1	1	5	1	1	1	1	2	2
9	9	9	4	1	1	3	3	2	9
3	3	3	4	1	1	1	1	3	3
3	3	3	5	1	1	1	1	3	3
2	2	2	9	1	1	2	3	2	2
2	2	2	1	1	1	1	1	3	1
3	2	1	3	2	2	2	1	2	1
3	3	3	6	1	1	1	1	3	3
2	2	2	4	9	9	9	9	2	2

9	9	9	9	9	9	9	9	1	3
9	9	9	1	1	1	1	1	3	9
3	3	3	4	1	1	1	1	3	3
9	9	9	1	1	1	1	1	9	9
9	9	9	9	9	9	9	9	3	2
9	9	9	9	9	9	9	9	3	2
2	3	2	1	1	1	1	1	3	1
1	1	1	1	1	1	1	1	3	3
2	1	1	3	1	1	1	1	3	3
3	3	3	6	1	1	1	1	3	3
3	3	3	4	1	1	3	2	9	9
3	3	3	5	1	1	1	1	3	9
2	3	3	1	1	1	1	1	3	3
2	2	3	4	1	1	1	1	3	1
9	9	9	6	9	9	9	9	9	9
2	2	2	1	1	1	1	1	3	2
3	3	3	3	1	1	1	1	3	3
3	3	3	4	1	1	1	1	3	2
9	9	9	4	1	1	1	1	3	9
2	2	3	3	1	2	1	1	3	2
2	3	3	4	1	1	1	1	3	1
2	3	2	4	1	1	1	1	2	2
3	3	3	4	1	1	1	1	3	2
3	3	3	3	1	1	1	1	3	2
2	3	3	5	1	1	1	1	3	2
2	3	3	1	1	1	1	1	3	2
2	3	3	4	1	1	1	1	3	3
3	3	3	4	1	1	1	1	3	3
3	3	2	4	1	1	1	1	3	2
3	3	3	1	1	1	1	1	3	3
9	9	9	1	1	2	2	3	9	9
2	2	1	3	9	9	9	9	9	9
3	3	2	9	1	1	2	2	2	9
9	9	9	1	1	1	2	2	9	9
1	3	1	4	9	9	9	9	9	9
3	3	2	5	1	2	1	1	3	2
2	3	2	1	1	1	1	1	3	2
3	2	2	1	1	1	1	1	3	3
9	9	9	1	1	1	2	1	9	9
9	9	9	1	1	1	9	1	9	9
9	9	9	1	1	1	2	1	9	9
9	9	9	1	1	1	2	1	9	9
2	1	1	4	1	1	1	1	9	9
2	2	2	6	2	2	2	2	2	2
2	2	2	1	1	2	1	1	2	2
2	3	3	5	1	1	1	1	3	2
2	3	2	1	1	1	1	1	3	2

9	9	9	9	9	9	9	9	9	9
9	9	9	3	1	1	1	1	9	9
9	9	9	4	1	1	1	1	3	9
9	9	9	1	1	1	2	1	3	9
9	9	9	1	1	1	2	1	9	9
9	9	9	1	1	1	1	1	3	9
2	2	2	4	3	1	1	1	2	9
9	3	1	9	1	1	1	1	9	9
2	2	2	1	1	1	2	1	2	2
1	3	1	4	1	1	1	1	3	3
2	2	1	4	1	1	1	1	2	2
3	3	3	4	1	1	1	1	3	3
1	3	1	5	1	1	1	1	3	3
2	3	1	4	1	1	1	1	3	3
1	2	2	9	9	9	9	9	2	2
2	2	2	4	2	2	2	2	2	2
9	2	2	5	1	1	1	1	3	3
9	9	9	4	1	1	1	1	3	9
3	2	1	9	1	2	2	1	2	3
9	9	9	1	1	2	2	3	2	9
2	9	9	9	9	2	2	3	9	9
9	9	9	4	1	1	1	1	9	9
2	2	2	1	1	1	1	1	2	2
2	3	3	6	1	1	1	1	3	3
2	2	2	1	2	2	2	2	2	2
2	3	3	4	3	3	2	2	2	2
9	2	9	3	1	1	1	1	2	2
3	2	2	1	2	1	2	2	2	1
3	3	3	1	1	1	1	1	3	2
3	3	2	1	3	1	2	2	2	2
2	3	3	3	1	1	1	1	3	3
2	3	3	5	1	1	1	1	3	3
3	3	3	4	1	1	1	1	3	3
1	3	1	4	1	1	3	1	2	2
3	3	3	4	1	1	1	1	3	3
9	9	9	4	1	1	1	1	9	9
3	3	1	4	1	1	1	1	2	2
2	2	2	3	1	1	1	1	3	2
1	3	3	6	1	1	1	1	3	1
3	3	3	4	1	1	1	1	3	3
2	3	3	4	1	1	1	1	3	2
2	3	3	4	1	1	1	1	3	2
2	1	3	1	1	1	1	1	2	9
2	2	2	9	1	1	1	1	3	3
2	2	2	1	1	1	1	1	3	2
2	3	3	5	1	1	1	1	3	2
3	2	2	9	1	1	1	1	3	2

9	3	3	4	1	3	2	1	3	9
3	3	3	1	1	1	1	1	3	9
3	3	3	1	1	1	1	1	3	3
3	2	2	3	1	1	1	1	3	2
2	2	2	3	1	2	2	2	1	9
3	3	3	4	1	1	1	1	3	1
9	3	3	4	1	1	1	1	3	3
2	2	2	1	1	1	1	1	3	1
1	2	1	1	1	1	1	1	3	3
2	3	3	4	1	1	1	1	3	2
3	3	3	1	1	1	3	1	3	1
3	3	3	1	1	1	1	1	3	3
3	3	3	4	1	9	1	9	3	9
1	1	1	6	2	2	1	1	2	1
9	9	9	9	9	9	9	9	2	2
3	3	2	5	1	1	1	1	3	3
3	3	3	5	9	9	9	9	9	9
2	1	2	9	1	1	1	2	3	2
2	2	2	5	1	1	3	3	2	2
3	3	2	4	1	1	2	2	9	9
2	2	2	1	2	3	2	3	2	2
3	3	3	4	1	1	1	1	3	2
9	9	9	4	1	1	1	1	3	9
3	3	3	5	1	1	1	1	3	3
1	1	2	1	1	3	3	3	2	2
3	2	2	1	2	2	2	1	2	2
2	2	2	4	3	3	1	1	2	2
2	3	2	5	1	1	1	1	3	2
3	3	3	4	1	1	1	1	3	3
3	2	3	6	1	3	1	1	3	1
3	3	3	4	9	9	9	9	2	2
1	1	1	3	3	3	3	3	2	3
2	2	2	9	1	2	1	1	3	2
2	2	2	9	1	2	1	1	3	2
3	3	2	5	1	2	2	1	3	1
9	9	9	3	1	1	2	1	2	1
3	1	3	1	1	2	3	3	2	2
9	9	9	9	9	9	9	9	3	3
2	2	2	6	1	1	1	1	3	3
1	1	1	1	1	1	2	1	2	2
2	3	3	4	1	1	1	1	3	3
2	3	3	1	1	1	1	1	3	2
9	3	9	5	1	1	1	1	3	9
9	3	1	5	1	1	1	1	3	3
2	3	3	1	1	1	1	1	3	2
3	3	1	9	1	1	1	1	3	9
2	2	2	9	3	3	3	2	2	2

3	2	2	1	1	1	1	1	3	2
3	3	3	1	1	2	2	1	3	9
3	3	3	1	2	3	3	3	2	2
9	9	9	9	1	1	1	1	9	9
1	3	3	3	1	1	1	1	3	3
2	2	2	1	1	1	2	1	2	2
2	2	2	1	1	1	1	1	3	3
3	3	3	9	1	1	2	2	9	9
3	3	3	3	1	1	1	1	3	3
3	3	1	5	1	2	1	1	3	3
1	1	1	4	1	1	1	1	3	3
3	3	3	5	1	1	1	1	3	2
3	3	3	5	1	1	2	2	3	3
3	3	3	3	1	1	1	1	3	2
1	3	3	4	1	1	2	1	9	9
3	3	3	4	1	3	1	1	9	9
3	3	3	4	1	2	2	1	9	9
1	1	1	1	1	1	1	1	1	3
1	3	3	4	1	1	1	1	3	3
2	3	2	3	1	1	1	1	1	3
2	3	3	4	1	1	1	1	3	2
3	3	3	4	1	1	1	1	3	3
3	3	3	4	1	1	1	1	3	3
2	3	3	4	1	1	1	1	3	2
2	2	2	4	1	1	1	1	3	2
1	3	3	4	1	1	1	1	3	1
3	3	1	4	1	1	1	1	3	3
2	2	2	5	1	1	1	1	3	2
3	3	3	6	1	1	1	1	3	2
1	3	3	4	1	1	1	1	3	3
1	1	3	3	1	1	1	1	3	3
3	3	3	4	1	1	1	1	3	3
3	1	2	5	1	1	2	1	9	9
3	3	2	5	1	3	2	3	2	3
2	3	3	4	1	1	2	3	3	2
9	9	9	5	1	1	1	1	9	9
3	2	2	1	3	1	2	3	2	2

PapOverEi	PapTwoYe	PapfromM	PapHistory	PapTwoYe	PapOnceor	HPVVaccin	HPVVaccin	EarlyOnset	MultiplePa
2	2	2	2	2	2	2	2	2	2
1	3	3	3	2	1	1	1	2	1
2	2	2	2	2	2	2	2	2	2
1	3	3	3	2	1	1	1	2	1
1	3	3	3	2	1	1	1	2	1
9	3	3	3	2	1	1	1	1	1
2	9	9	9	9	9	9	9	9	9
2	2	2	3	1	2	1	1	1	1
2	9	2	9	2	9	2	2	9	2
9	9	9	9	9	9	9	9	9	2
2	2	3	2	2	2	2	2	3	2
3	3	3	3	3	3	3	2	2	2
2	3	2	1	2	2	1	2	2	2
2	2	2	2	2	2	2	2	2	2
2	2	2	1	2	1	1	1	2	1
2	2	3	3	2	3	1	2	2	2
9	1	2	1	2	2	3	2	1	2
2	2	3	3	2	3	1	2	2	2
2	2	3	3	2	3	1	2	2	2
2	2	2	2	2	2	2	2	3	2
1	1	2	2	1	1	1	1	3	3
1	1	2	2	1	1	1	1	3	3
9	2	2	2	2	2	2	2	2	2
3	3	3	3	1	3	1	1	3	3
2	2	2	2	2	2	2	2	2	2
2	2	2	2	2	2	2	2	2	2
2	1	1	1	1	3	1	1	2	2
1	2	2	2	1	3	1	1	1	1
2	2	2	2	2	2	2	2	2	2
2	2	2	2	2	2	2	2	2	2
2	2	2	2	2	2	2	2	2	2
2	1	2	2	2	2	2	1	2	1
1	1	3	3	2	3	1	1	1	1
2	1	1	2	1	2	1	1	2	1
2	1	2	2	2	2	2	2	2	2
2	1	1	2	1	2	1	1	2	1
2	1	2	2	2	2	2	2	2	2
1	3	3	3	1	3	1	1	1	1
2	2	2	2	2	2	2	2	2	2
2	2	2	2	2	2	2	2	2	2
2	2	2	2	2	2	2	2	2	2
2	1	2	2	2	2	2	2	1	1
2	3	3	9	2	1	1	1	1	1
1	3	3	3	3	3	1	1	3	3
1	2	2	3	3	3	2	3	1	1
2	3	3	9	2	1	1	2	1	1

1	1	2	3	2	3	2	1	1	1
1	2	2	3	1	2	1	1	1	2
2	2	2	2	2	2	1	1	1	1
1	2	2	3	1	2	1	2	1	1
3	2	3	3	2	2	1	1	1	1
3	3	3	3	1	3	1	1	1	1
3	3	3	3	1	3	1	1	1	1
1	9	1	1	9	9	1	1	1	1
1	9	1	1	9	9	1	1	1	1
3	2	1	2	3	1	1	3	1	1
2	2	2	2	2	2	2	2	2	2
1	2	2	2	2	2	1	2	1	1
1	2	3	3	1	3	2	2	1	1
2	2	2	2	2	2	2	2	2	1
2	2	2	2	2	2	2	2	1	1
1	2	3	3	1	3	2	2	1	1
1	2	3	2	1	3	1	1	1	1
1	1	2	1	1	3	1	1	1	1
2	3	3	2	1	1	1	2	1	1
9	9	9	9	9	9	9	9	1	2
3	3	3	3	1	3	1	1	1	1
3	3	3	3	1	3	1	1	1	1
3	3	3	3	1	3	1	1	1	1
1	1	2	2	1	2	1	1	1	1
9	9	1	9	9	1	9	1	1	1
1	1	1	1	1	1	9	1	1	2
9	9	9	9	9	9	9	9	2	2
1	2	2	3	1	2	1	1	1	3
2	2	2	2	2	2	2	2	2	2
2	2	2	1	1	3	1	1	1	1
9	9	9	9	9	9	9	9	1	9
3	3	3	1	3	3	3	3	3	3
9	9	9	9	9	9	9	9	1	9
3	3	3	2	3	3	1	1	1	1
2	2	3	3	1	3	1	1	1	3
9	9	9	9	9	9	9	9	9	9
9	9	9	9	9	9	9	9	9	9
3	2	2	2	2	2	1	1	1	1
1	3	1	3	3	1	1	1	3	3
1	1	3	3	1	3	1	1	1	1
1	1	2	2	1	3	1	1	1	1
9	9	9	9	9	9	9	9	1	9
1	3	3	3	1	3	1	3	1	1
1	2	2	2	1	3	1	1	1	1
1	2	2	2	1	3	1	1	1	1
9	9	9	9	9	9	9	9	9	9
9	9	9	9	9	9	9	9	9	9

2	2	3	1	1	2	1	1	3	2
3	2	3	2	2	3	1	1	1	1
2	2	1	1	1	1	1	1	1	1
2	1	2	2	2	2	2	1	2	2
9	9	9	9	9	9	9	9	9	9
2	2	2	2	2	2	1	1	1	9
3	2	3	1	1	3	1	1	1	1
3	1	2	2	2	3	2	2	1	1
1	1	3	3	1	3	1	1	1	1
1	2	3	3	1	3	1	1	9	9
1	1	1	2	9	2	1	1	1	1
2	1	3	3	3	2	3	1	2	3
1	1	1	1	2	3	9	9	3	1
2	2	2	1	2	9	9	9	1	9
1	2	2	2	1	2	1	2	2	1
2	1	3	2	1	2	2	3	2	2
2	2	2	2	2	2	2	1	2	2
2	2	2	2	2	2	2	1	1	2
1	2	2	1	2	2	9	9	2	2
2	2	2	2	2	2	2	2	2	2
3	3	3	3	1	3	1	1	2	1
1	1	2	2	1	3	2	2	1	1
2	2	2	2	2	2	2	2	1	2
3	3	3	3	3	3	3	3	3	3
2	3	2	2	1	2	2	1	1	1
2	2	2	2	2	2	2	2	2	2
1	2	3	1	2	3	1	2	2	2
1	2	3	2	2	3	1	1	1	2
2	2	2	2	2	2	2	2	2	2
2	2	2	2	2	2	2	2	1	1
2	2	2	2	2	2	2	2	2	2
2	2	2	2	2	2	2	2	2	2
1	2	2	2	2	2	1	1	1	1
3	3	2	2	2	2	2	2	2	2
2	2	2	2	2	2	2	2	1	1
2	2	2	2	2	2	1	2	1	1
2	2	2	2	2	2	2	2	2	2
2	2	2	2	2	2	2	2	2	2
9	9	9	2	9	9	1	9	9	9
1	1	3	3	3	3	1	1	1	1
2	2	2	2	2	2	2	2	2	2
2	2	2	2	2	2	2	2	2	2
2	2	2	2	2	2	2	2	2	2
1	2	1	1	3	2	2	1	2	3
1	2	2	2	2	1	2	2	1	1
2	2	2	2	2	2	1	2	2	2
2	2	2	2	2	2	2	2	2	2

2	2	2	1	1	1	1	2	2	2
1	2	1	2	2	2	2	2	2	2
9	2	1	2	2	2	2	2	2	2
2	2	2	2	2	2	2	2	2	2
9	9	9	9	9	9	9	9	1	1
2	2	2	2	2	2	2	2	1	1
3	3	2	2	3	3	3	2	2	1
2	2	2	2	2	2	2	2	2	2
2	2	2	2	2	2	2	2	2	2
2	2	2	2	2	2	2	2	2	2
2	2	3	2	2	2	2	3	3	2
2	3	2	2	2	2	1	1	2	2
9	9	9	9	9	9	9	9	1	1
2	2	2	2	2	2	2	2	2	1
2	2	2	2	2	2	2	2	1	1
2	2	2	2	2	2	2	2	1	1
9	9	9	9	9	9	9	9	9	9
2	2	2	2	2	2	2	2	2	2
9	9	1	9	9	9	9	9	9	9
3	1	3	3	2	3	1	1	1	1
1	1	3	3	1	3	1	1	1	1
1	1	3	3	2	3	2	1	1	1
1	1	3	3	1	3	1	1	1	1
1	3	3	2	1	3	3	1	1	1
1	1	3	3	2	3	3	1	1	1
1	1	3	9	9	9	9	9	1	1
1	1	3	3	2	3	1	1	1	1
1	1	3	3	2	3	1	1	1	1
1	1	3	3	2	3	1	1	9	1
1	1	3	3	2	3	1	1	1	1
1	2	2	1	2	2	2	2	2	3
2	2	2	2	2	2	2	2	2	2
1	1	3	3	2	3	1	1	1	1
1	1	3	3	2	3	1	1	1	1
1	1	3	2	1	3	1	1	1	1
1	1	3	2	1	3	1	1	1	1
1	1	3	3	2	3	1	1	1	1
1	1	3	3	2	3	1	1	1	1
1	1	3	1	2	3	1	1	1	1
1	1	3	3	2	3	1	1	1	1
1	1	3	3	1	3	1	1	1	1
1	1	3	3	2	3	3	1	1	1
1	1	3	3	2	3	1	1	1	1
1	1	3	3	2	3	1	1	1	1
1	1	3	3	2	3	2	1	1	1
1	1	3	3	2	3	1	1	1	1
1	1	3	3	2	3	1	1	1	1

1	1	3	3	2	3	3	1	1	1
1	1	3	3	2	3	3	1	1	1
1	1	3	3	2	3	3	2	1	1
1	1	3	3	3	3	1	1	1	1
3	1	3	1	2	3	3	1	1	1
1	1	3	3	2	3	1	1	1	1
1	1	3	3	2	3	1	1	1	1
1	3	3	3	1	3	1	1	3	1
1	1	3	3	1	3	1	1	1	1
3	3	3	3	3	3	1	1	1	1
9	9	9	9	9	9	9	9	9	9
1	2	3	3	1	3	1	2	1	1
1	1	3	3	1	3	1	1	1	1
1	1	3	3	2	3	1	1	1	1
2	2	2	1	1	1	1	2	2	1
2	2	2	2	1	2	2	1	1	1
1	2	2	2	2	2	1	1	2	2
1	1	1	1	1	1	1	1	1	1
2	2	2	2	2	2	2	2	1	1
2	2	2	2	2	2	1	1	1	1
2	1	2	1	1	2	1	1	1	1
2	2	2	2	2	2	2	2	2	2
1	2	1	2	3	2	1	2	1	1
1	2	2	2	2	2	1	1	1	1
2	2	2	2	2	2	2	2	2	2
2	2	2	2	2	2	2	2	2	2
2	2	2	2	2	2	1	2	1	1
2	2	2	2	2	2	2	2	2	2
2	2	2	2	2	2	2	2	2	2
2	2	2	2	2	2	2	2	1	1
1	1	2	2	3	3	1	1	1	1
1	1	2	2	3	3	1	1	1	1
2	2	2	2	2	2	2	2	1	1
1	2	2	2	2	2	2	2	1	1
1	2	1	1	1	2	2	2	1	1
2	2	2	2	2	2	2	2	2	2
2	2	2	2	2	2	2	2	2	2
3	3	3	1	1	2	1	1	1	1
1	1	2	2	2	2	2	2	1	1
2	2	2	3	3	2	1	2	1	1
1	2	1	3	3	3	3	3	3	2
2	2	2	2	2	9	1	1	1	1
9	9	9	9	9	9	9	9	2	9
9	9	9	9	9	9	9	9	2	9
3	2	2	3	2	2	1	2	2	1
1	1	3	1	1	2	1	1	1	1
9	9	9	9	9	9	9	9	9	9

2	2	2	2	2	2	2	2	2	2
2	2	2	2	2	2	2	2	2	2
1	2	2	2	1	2	1	2	1	1
2	2	2	2	1	2	1	1	2	2
1	2	1	1	3	3	3	2	1	2
1	2	3	2	3	2	1	2	2	1
2	2	2	2	2	2	1	1	2	2
2	2	2	2	2	2	2	1	2	2
3	3	3	1	1	3	1	1	1	3
3	3	3	3	3	3	3	3	2	2
2	2	2	2	2	2	2	2	2	1
3	3	2	2	2	2	2	2	1	3
3	3	2	2	2	2	2	2	1	3
2	2	2	2	2	3	1	2	2	2
2	2	2	2	2	2	2	2	2	2
2	2	2	1	2	3	1	2	1	2
2	2	2	2	3	3	1	1	1	2
1	3	3	2	1	3	1	2	1	1
9	9	9	9	9	9	9	9	9	9
2	2	2	2	1	3	1	3	9	1
2	2	2	2	1	3	1	1	1	1
2	2	2	2	2	2	2	1	2	1
2	2	2	2	2	3	1	1	2	2
2	2	2	3	1	3	1	2	2	2
9	9	9	9	9	9	9	9	9	9
2	2	2	2	2	2	2	2	2	2
2	2	2	2	2	2	2	2	2	2
1	2	2	2	1	3	2	1	1	9
2	2	2	2	2	2	2	2	2	1
2	2	2	2	2	2	2	2	2	2
1	9	9	9	9	9	9	9	9	1
3	3	3	3	3	3	3	3	3	3
3	3	3	3	3	3	3	3	3	3
2	2	2	2	2	2	2	2	2	2
9	9	9	9	9	9	9	9	1	1
2	2	2	2	2	2	2	2	2	2
2	2	3	3	1	3	1	2	1	2
1	1	3	3	2	2	3	1	1	1
2	2	2	2	2	2	2	2	2	2
2	2	2	3	1	3	2	2	2	2
1	2	2	3	2	2	1	1	1	2
2	2	2	3	1	3	1	1	3	3
1	1	2	2	2	2	2	2	3	3
2	2	2	2	2	2	2	2	2	2
1	2	3	3	2	2	1	2	2	1
2	2	1	1	2	2	2	2	2	2
2	2	2	2	2	2	2	2	2	2

9	9	9	9	9	9	9	9	9	9
9	9	9	9	9	9	9	9	9	9
1	9	9	9	9	9	9	9	9	9
1	1	3	1	1	3	1	1	1	1
3	2	3	2	2	3	2	2	2	2
2	1	2	3	1	2	3	1	2	1
2	1	2	2	1	3	1	1	1	1
1	1	1	1	1	1	1	1	1	1
2	9	3	2	1	1	1	1	1	2
2	2	2	2	2	2	2	2	2	2
2	2	2	2	2	2	2	2	2	2
3	2	2	2	2	3	2	2	9	9
9	9	9	9	9	9	9	9	9	9
1	1	1	1	1	1	1	1	1	3
1	3	3	3	3	1	1	1	3	3
9	9	9	9	9	9	9	9	9	9
1	2	2	2	1	1	2	2	2	2
2	2	1	3	2	1	2	2	2	3
1	1	1	1	1	1	1	3	3	1
9	9	9	9	9	9	9	9	9	9
9	9	9	9	9	9	9	9	9	9
2	2	3	2	2	2	2	2	2	2
3	3	2	2	3	2	1	2	2	1
2	2	2	2	2	2	2	2	1	1
2	2	2	2	2	2	2	2	2	2
2	2	2	3	1	3	2	2	2	1
1	2	2	1	1	2	1	1	2	3
2	2	3	3	2	3	2	2	1	1
2	2	2	3	1	3	1	2	2	3
1	2	2	3	1	3	1	1	2	1
2	2	2	2	2	2	2	2	2	2
2	2	3	3	3	2	3	2	3	1
9	9	9	9	9	9	9	9	9	9
9	9	9	9	9	9	9	9	9	9
2	2	3	3	2	3	1	3	1	1
3	2	3	1	1	3	3	2	1	3
1	1	3	3	1	3	2	2	1	2
3	1	2	1	1	2	1	1	1	3
1	1	2	1	1	3	1	1	1	3
2	2	2	1	2	9	1	2	2	2
3	2	3	2	2	1	1	3	3	2
1	1	3	1	1	3	1	1	3	3
2	2	2	2	2	2	2	2	1	2
2	2	2	2	2	2	2	2	2	2
2	3	3	3	3	3	1	1	3	3
1	9	3	3	2	3	2	2	2	1
2	2	2	2	2	2	2	2	2	2

1	2	3	2	2	2	2	1	9	9
1	2	3	2	2	2	2	1	9	9
9	9	9	9	9	9	9	9	1	9
2	2	2	2	2	2	2	2	2	2
9	9	9	9	9	9	9	1	9	9
9	9	9	9	9	9	9	9	9	9
1	1	2	2	1	3	1	1	1	1
2	2	9	3	9	3	1	9	9	9
1	1	3	3	1	3	1	1	1	1
2	2	3	1	1	2	1	2	1	1
1	2	2	2	3	1	2	1	1	2
2	2	2	2	2	2	2	2	2	2
2	2	3	1	1	3	1	1	1	2
2	2	2	2	3	2	2	2	1	2
1	2	3	3	2	3	1	1	1	1
2	2	2	3	2	3	2	1	2	2
2	2	2	2	2	2	2	2	2	2
2	2	2	2	2	2	2	2	2	1
2	2	2	2	2	2	2	2	2	2
2	2	2	2	2	2	2	2	2	2
2	2	3	1	1	3	1	1	2	3
1	2	3	2	2	3	1	1	1	2
1	2	2	3	2	2	1	2	1	1
2	1	2	3	2	2	1	1	1	1
2	2	2	2	2	2	2	2	2	2
3	2	3	2	3	2	3	2	2	2
2	2	2	2	2	2	2	2	2	2
2	2	2	2	2	2	2	2	2	2
2	2	2	2	2	1	1	1	2	2
2	1	1	2	1	2	1	1	1	1
3	3	2	2	2	3	2	2	2	3
3	3	2	2	2	3	2	2	2	2
2	1	1	3	2	2	1	2	2	2
2	2	2	2	2	2	2	2	2	2
2	2	2	2	2	2	2	2	2	2
1	2	2	1	1	3	1	1	2	1
2	2	2	2	2	2	1	1	1	2
2	2	2	2	2	2	2	2	2	2
2	2	2	2	2	2	2	2	2	2
2	2	2	2	2	2	2	2	2	2
2	2	2	2	2	2	2	2	2	2
2	2	2	2	2	2	2	2	1	1
1	2	1	1	1	2	1	1	1	1
2	2	2	2	2	2	2	2	1	1
1	2	2	2	2	2	1	1	2	2
1	2	2	2	1	3	1	2	1	1
2	2	2	2	2	2	2	2	2	2

3	3	3	2	2	2	1	2	1	1
2	3	3	1	2	2	3	1	1	2
2	2	2	2	2	2	2	2	2	2
2	2	2	2	2	2	2	2	2	2
1	2	2	2	2	2	2	2	2	2
3	3	2	2	1	2	1	2	2	2
9	9	9	9	9	9	9	9	9	9
3	2	2	1	2	2	2	2	2	2
2	2	2	2	2	2	2	2	2	2
1	2	1	2	3	2	2	3	1	2
1	2	1	2	1	2	1	2	1	2
3	3	3	3	1	3	3	1	1	1
2	2	2	2	2	2	2	2	2	1
2	2	2	2	2	2	2	2	2	2
2	2	2	2	2	2	2	2	2	2
1	2	1	1	1	1	1	1	1	1
3	3	3	3	1	3	1	1	3	3
2	2	2	2	2	2	2	2	2	2
2	2	2	2	2	2	2	2	2	2
2	2	2	2	2	2	2	2	2	2
1	2	2	1	1	2	1	2	2	2
2	2	2	2	2	2	2	2	1	1
9	9	9	9	9	9	9	9	9	9
2	2	2	2	2	2	2	2	2	2
1	2	3	3	1	3	1	3	2	1
9	9	9	9	9	9	9	9	9	9
2	2	1	2	1	2	1	1	2	2
9	9	9	9	9	9	9	9	9	9
2	2	2	2	2	2	2	2	1	2
1	1	2	2	1	2	1	1	1	2
9	9	9	9	9	9	9	9	9	9
9	9	9	9	9	9	9	9	9	9
2	2	2	2	2	2	2	2	2	2
2	1	1	1	2	2	1	1	1	1
1	9	1	1	2	9	9	9	9	9
2	1	2	1	1	2	1	2	1	1
3	3	3	3	3	3	3	3	3	3
2	2	2	2	1	2	2	2	2	1
9	9	9	9	9	9	9	9	9	9
1	2	2	3	1	3	1	1	1	1
2	3	2	1	2	2	2	2	2	1
1	3	3	3	2	1	1	1	2	1
2	1	3	2	1	2	3	2	1	2
3	3	3	3	3	3	3	3	2	2
9	9	9	9	9	9	9	9	9	9
1	1	3	3	2	3	1	1	1	1
1	1	3	3	2	3	1	1	1	1

9	9	9	9	9	9	9	9	9	9
1	1	3	3	2	3	1	1	1	1
1	1	3	3	2	3	1	1	1	1
1	1	3	3	2	3	1	1	1	1
3	1	3	3	2	3	1	1	1	1
1	1	3	3	2	3	1	1	1	1
1	1	3	3	2	3	1	1	1	1
1	1	3	3	2	3	1	1	1	1
1	1	3	3	2	3	1	1	1	1
1	1	3	3	2	3	1	1	1	1
3	3	2	2	2	2	2	2	1	1
1	2	3	2	1	1	1	1	1	1
1	1	3	3	1	3	1	1	1	1
1	1	3	3	2	3	3	1	1	1
1	1	3	3	2	3	1	1	1	1
3	1	3	3	2	3	1	1	1	1
1	1	2	3	2	3	2	1	1	1
1	3	3	3	3	3	1	1	1	1
1	1	3	3	2	3	1	1	1	1
1	1	3	3	2	3	1	1	1	1
1	1	3	3	1	3	1	1	1	1
1	1	3	3	2	3	1	1	1	1
1	1	3	3	2	3	1	1	1	1
1	2	3	3	1	3	1	1	1	1
1	1	3	3	2	3	1	1	1	1
1	1	3	3	2	3	1	1	1	1
1	1	3	3	2	3	1	1	1	1
1	1	3	3	2	3	2	1	1	1
1	1	3	3	2	1	1	1	1	1
1	1	3	3	1	3	1	1	1	1
1	1	3	3	2	3	2	1	1	1
1	1	3	3	1	3	1	1	1	1
1	1	3	3	2	3	1	1	1	1
3	1	3	3	2	3	1	1	1	1
1	1	3	3	3	3	1	1	1	1
1	1	3	3	2	3	1	1	1	1
1	1	3	3	2	3	1	1	1	1
1	1	3	3	2	3	1	1	1	1
1	1	3	3	2	3	1	1	1	1
1	1	3	3	2	3	1	1	1	1
1	1	3	3	2	3	1	1	1	1
2	2	2	2	2	2	2	2	2	2
1	2	3	2	1	3	1	1	1	1
2	2	2	2	2	2	2	2	2	2
9	9	9	9	9	9	9	9	2	9
2	2	2	2	2	2	2	2	2	2
9	9	9	9	9	9	9	9	2	9

2	2	2	2	2	2	2	2	2	2
1	2	3	2	3	3	1	1	1	1
1	2	3	2	3	3	1	1	1	1
1	1	3	3	1	2	1	1	1	1
2	2	2	2	2	2	2	2	2	2
1	3	2	1	1	3	2	2	2	2
2	2	2	2	2	2	2	2	2	2
3	2	2	2	2	1	2	2	2	3
2	2	2	3	2	3	1	1	1	1
2	2	2	2	2	2	2	2	2	2
1	2	3	2	3	3	1	1	1	1
9	1	9	9	9	9	9	9	9	9
1	1	3	3	9	9	1	9	1	1
2	2	2	2	2	2	2	2	2	2
9	9	1	9	9	9	9	9	1	9
1	9	9	9	9	9	9	9	1	9
9	9	9	9	9	1	9	9	9	9
9	9	9	9	9	9	9	9	9	9
9	9	9	9	9	9	1	9	9	9
3	2	1	2	1	2	1	2	1	1
2	9	1	1	9	1	9	9	1	1
9	1	9	9	9	9	9	9	1	1
2	3	3	3	1	3	2	2	1	1
9	9	9	9	9	9	9	9	9	9
1	9	9	9	9	9	9	9	1	9
9	9	9	9	9	9	9	9	9	9
9	9	9	9	9	9	9	9	9	9
9	9	9	9	9	9	9	9	9	9
1	2	3	1	2	3	1	1	2	1
9	1	9	9	9	9	9	9	9	9
9	9	9	9	9	9	9	9	1	9
2	2	2	2	2	2	2	1	2	2
2	2	2	2	2	2	2	2	2	2
1	1	3	3	1	3	1	1	1	1
1	1	2	1	1	2	2	1	1	1
1	2	2	2	1	2	2	1	2	2
2	2	2	3	1	3	2	1	1	2
3	2	9	3	3	3	1	1	1	1
2	2	2	2	2	2	2	2	2	2
1	2	2	3	1	3	1	1	1	2
9	9	9	9	9	9	9	9	9	9
2	2	3	2	1	3	1	1	1	1
2	2	2	2	2	2	2	2	2	2
1	1	3	3	1	3	1	1	1	1
2	2	2	2	2	2	2	2	2	2
2	2	2	2	2	2	2	2	2	2
1	2	3	2	2	3	1	1	1	1

2	2	3	2	1	3	1	2	1	3
1	1	3	2	2	3	1	1	1	1
3	3	3	3	1	3	1	2	2	2
2	2	1	1	2	3	1	1	1	1
3	1	3	1	2	2	1	2	1	1
2	2	2	2	2	2	2	2	2	2
3	2	2	3	2	2	1	1	1	1
2	2	2	2	2	2	2	2	2	2
2	2	2	2	2	2	2	2	2	2
2	2	2	2	2	2	2	2	2	2
2	2	2	2	2	2	2	3	2	2
1	2	3	2	2	2	1	2	1	1
2	2	2	2	2	2	2	2	2	2
2	2	2	2	2	2	2	2	2	2
1	2	3	3	1	2	1	2	2	2
2	2	2	2	2	2	2	2	2	2
2	2	2	2	2	2	2	2	2	2
2	2	2	2	2	2	2	2	2	2
2	2	2	2	2	2	2	2	1	1
1	2	3	1	1	3	1	2	1	2
1	2	2	2	2	2	2	2	2	2
1	2	2	3	2	2	2	2	1	1
1	3	2	3	1	3	2	2	1	1
2	2	2	3	1	2	1	2	1	1
2	2	2	2	2	2	2	2	2	2
1	2	2	2	2	2	2	1	2	2
1	1	3	1	1	3	9	1	1	3
1	1	3	1	3	1	3	1	1	3
1	2	3	3	1	3	1	1	1	3
3	3	3	3	3	3	3	3	2	3
2	2	2	2	2	2	2	2	2	2
2	2	2	2	2	2	2	2	9	2
2	2	2	2	2	2	2	2	2	2
2	3	1	3	2	2	3	2	2	2
2	2	2	2	2	2	2	2	2	2
9	9	9	9	9	9	9	9	2	9
2	2	2	2	2	2	2	2	2	2
3	1	2	2	1	3	1	2	1	1
2	2	2	2	2	2	2	2	2	1
1	2	3	3	1	3	2	1	1	1
9	9	9	9	9	9	1	9	2	2
2	2	2	3	1	2	1	1	3	1
2	2	2	3	1	2	1	1	3	1
2	1	3	3	3	2	3	1	1	1
1	2	3	3	1	3	1	1	1	1
2	2	2	2	2	2	2	2	2	2
9	9	9	9	9	9	9	9	2	9

9	9	9	9	9	9	9	9	2	9
9	9	9	1	9	9	9	9	9	9
9	9	9	9	9	9	9	9	9	9
9	9	9	9	9	9	9	9	9	9
9	9	9	9	9	9	9	9	9	9
9	9	9	9	9	9	9	9	1	1
9	2	9	9	9	9	9	9	9	9
9	9	9	9	9	9	9	9	9	1
9	9	9	9	9	9	9	9	9	1
9	9	9	9	9	9	9	9	2	9
9	9	9	9	9	9	9	9	1	9
9	9	9	9	9	9	9	9	2	9
9	9	9	9	9	9	9	9	9	9
1	9	9	9	9	9	9	9	9	1
9	9	9	9	9	9	9	1	9	9
9	9	9	9	9	9	9	1	9	9
9	9	9	9	9	9	9	9	1	9
1	3	3	3	3	3	1	1	1	1
2	2	2	2	2	2	2	2	2	2
9	9	9	9	9	9	9	9	1	9
1	1	1	1	1	1	1	1	1	1
1	2	2	1	3	2	2	3	1	2
1	1	2	2	2	1	1	2	2	3
1	3	2	1	1	1	1	1	1	1
3	2	3	2	3	2	3	2	2	3
2	2	2	1	3	3	3	2	3	1
2	2	2	2	2	2	2	2	2	2
2	2	3	1	2	1	2	3	2	3
2	2	2	2	2	2	2	2	2	2
2	2	2	2	2	2	2	2	2	2
1	1	2	2	2	3	3	3	1	1
2	2	2	2	2	2	2	2	1	1
2	2	2	2	2	2	2	2	2	2
1	3	3	3	2	2	2	2	1	1
2	3	1	2	3	1	2	3	1	2
2	2	3	2	3	2	3	2	1	2
2	2	2	2	2	2	2	2	2	2
2	3	2	3	2	1	1	1	9	9
1	1	1	1	1	1	1	1	1	3
9	9	9	9	9	9	9	9	9	9
2	2	2	2	2	2	2	2	2	2
1	1	1	1	1	1	1	1	3	3
9	9	9	9	9	9	9	9	9	9
1	1	1	3	2	1	1	1	1	1
2	2	2	2	2	2	2	2	2	2
2	2	2	2	2	2	2	2	2	2
2	2	2	3	3	2	2	2	2	2

2	2	2	2	2	2	2	2	1	1
9	9	9	9	9	9	9	9	9	9
3	1	1	3	1	3	1	1	1	3
9	9	9	9	9	9	9	9	9	9
1	2	2	2	2	2	2	2	2	2
1	2	2	2	2	2	2	2	2	2
9	9	9	9	9	9	9	9	9	9
2	2	2	2	2	2	2	2	1	1
3	1	2	1	1	1	1	3	1	1
1	1	2	1	1	3	3	1	2	3
1	1	1	1	1	1	1	1	1	1
1	2	2	2	1	1	1	1	2	2
3	2	2	3	3	2	2	2	2	3
9	9	9	9	9	9	9	9	9	9
9	9	9	9	9	9	9	9	9	9
9	9	9	9	9	9	9	9	9	9
1	1	3	3	2	3	1	1	1	1
9	9	9	9	9	9	9	9	9	9
2	9	3	3	1	3	2	2	2	3
2	1	2	3	1	3	1	2	1	1
3	3	3	2	2	2	1	2	1	1
3	2	2	3	3	3	2	2	2	3
1	1	1	1	1	1	1	1	2	2
3	2	1	3	2	3	1	3	1	2
2	2	2	2	2	2	2	2	2	1
3	1	2	1	2	1	2	1	1	3
3	3	1	2	3	3	3	3	3	9
3	3	1	1	1	3	1	1	1	3
2	2	2	2	2	1	1	2	2	1
2	2	2	3	3	3	2	2	1	1
9	9	9	9	9	9	9	9	9	9
9	1	9	9	9	9	9	9	9	9
1	1	2	2	2	2	2	2	2	1
1	1	1	2	2	9	2	2	1	2
1	1	9	9	2	9	2	9	1	3
2	2	2	1	2	2	1	2	1	1
2	3	2	9	1	3	9	1	1	1
9	9	9	9	9	9	9	9	9	9
9	9	9	9	9	9	9	9	9	9
9	9	9	9	9	9	9	9	9	9
9	9	9	9	9	9	9	9	9	9
3	2	2	1	2	1	2	2	2	1
3	1	2	3	1	2	3	3	2	1
9	9	9	9	9	9	9	9	9	9
3	1	2	1	3	1	2	3	2	2
3	2	2	3	1	2	1	2	1	2
3	3	1	1	3	1	3	3	1	1

3	3	3	3	3	3	3	3	3	3
1	2	2	2	2	2	2	2	2	1
3	3	2	2	3	3	2	2	3	3
2	1	3	2	1	2	2	2	1	2
2	2	2	2	2	2	2	2	2	2
2	9	1	2	3	9	3	2	1	9
9	9	9	9	9	9	9	9	9	9
3	3	9	2	2	3	1	2	3	3
3	3	3	3	3	3	3	3	3	3
1	2	3	3	1	3	1	2	1	1
3	3	3	3	3	3	3	3	3	3
1	1	3	1	1	3	1	1	1	1
3	2	2	3	1	2	1	2	1	2
3	2	2	3	1	2	1	2	1	2
3	2	2	3	1	2	1	2	1	2
3	2	2	3	1	2	1	2	2	1
3	2	2	3	1	2	1	2	1	2
3	2	2	3	1	2	1	2	1	2
3	2	2	3	1	2	1	2	1	2
2	2	1	3	1	2	1	1	1	1
3	2	1	2	1	2	3	3	1	2
2	2	3	2	3	2	9	2	2	3
2	2	2	3	2	3	2	3	2	3
2	2	2	2	2	3	3	3	2	2
3	3	3	3	3	3	3	3	3	3
3	2	2	3	3	2	1	2	1	2
9	9	1	9	9	9	9	9	9	9
1	1	3	3	1	3	3	1	9	9
9	9	9	9	9	9	9	9	9	9
2	2	2	2	2	2	2	2	2	2
2	3	3	3	3	1	1	1	2	1
3	2	3	2	3	2	2	3	2	3
1	3	2	1	3	1	2	1	2	1
9	9	1	9	9	9	9	9	9	9
3	3	3	3	3	3	3	3	3	3
2	2	2	3	3	3	1	2	1	1
9	9	2	9	1	3	9	2	2	9
2	1	2	2	2	1	9	1	2	1
3	2	2	3	1	2	1	2	2	3
2	1	2	3	1	1	1	1	1	1
1	1	2	2	3	1	1	2	2	3
2	1	2	2	9	2	3	2	2	2
2	1	1	3	2	3	1	2	2	1
3	3	1	1	1	3	1	1	1	3
1	2	2	1	1	1	1	2	2	1
1	1	1	1	1	1	1	1	1	1
2	2	2	2	2	2	2	3	2	3

2	2	2	2	2	2	2	2	2	2
9	9	9	9	9	9	9	9	2	9
9	9	9	9	9	9	9	9	9	9
1	1	3	1	1	3	1	3	1	3
9	1	2	1	2	3	1	2	2	2
1	2	2	2	2	2	2	2	2	2
9	1	1	1	1	1	1	1	1	1
1	2	2	2	2	2	2	2	2	2
1	2	2	2	2	2	2	2	2	2
1	2	3	2	1	2	3	3	2	2
3	2	2	3	2	9	9	2	9	9
2	2	1	2	1	3	1	1	1	1
9	9	9	9	9	9	9	9	9	9
3	1	1	3	1	3	1	1	1	1
1	1	1	2	1	3	1	1	1	1
9	9	9	9	9	9	9	9	9	9
3	3	3	3	3	3	3	3	3	3
9	9	9	9	9	9	9	9	9	9
9	9	9	9	9	9	9	9	9	9
3	1	2	3	1	2	3	1	1	2
1	1	1	1	1	1	1	3	1	1
2	2	2	2	2	2	2	2	2	2
9	9	9	9	9	9	9	9	9	9
2	2	2	2	2	2	2	2	2	2
3	3	3	3	3	3	3	3	3	3
3	2	1	2	1	3	1	1	1	1
3	2	1	2	1	3	1	1	1	1
1	2	3	3	1	3	1	3	1	1
1	1	1	1	1	1	1	1	1	1
2	2	2	2	2	2	2	2	2	2
3	2	1	1	1	3	2	2	3	2
2	3	2	2	2	2	3	1	1	1
1	3	2	2	1	2	3	3	1	3
3	2	1	2	1	3	1	1	1	1
1	2	3	2	2	1	1	2	1	1
2	2	2	2	2	2	2	2	2	2
2	2	2	1	1	1	2	2	2	2
9	9	9	9	9	9	9	9	9	9
9	9	2	9	3	9	9	1	9	9
9	1	1	1	2	1	2	1	1	1
2	2	2	2	2	2	2	2	2	2
2	2	2	2	2	2	2	2	2	2
2	2	2	2	2	2	2	2	2	9
2	2	2	2	2	2	2	2	2	2
2	2	2	2	2	2	2	2	2	2
2	2	2	2	2	2	2	2	2	2
9	9	9	9	9	9	9	9	9	9

9	9	9	9	9	9	9	9	9	9
2	2	2	2	2	2	2	2	2	2
1	2	2	3	1	3	1	1	1	1
1	1	2	2	2	3	1	1	1	1
9	9	9	9	9	9	9	9	9	1
2	2	3	2	1	2	3	2	1	2
9	9	9	9	9	9	9	9	9	9
3	3	3	3	3	3	3	3	1	1
1	2	1	1	2	2	1	2	1	2
9	9	9	9	9	9	9	9	9	9
2	2	2	2	2	2	2	2	2	2
2	1	2	2	2	3	2	1	1	1
2	1	2	2	2	3	2	1	1	1
2	2	2	2	2	2	2	2	2	2
3	3	3	2	2	2	1	1	1	1
2	2	2	2	2	2	2	2	2	2
2	2	2	2	2	2	2	2	2	2
2	2	2	2	2	2	2	2	3	3
1	3	2	2	1	3	2	1	1	1
1	1	3	3	1	1	2	2	1	1
9	9	9	9	9	9	9	9	9	9
3	3	3	3	3	3	3	3	2	2
2	2	2	2	2	2	2	9	2	2
2	2	2	2	2	2	2	2	1	1
2	2	2	2	2	2	2	2	2	2
3	3	3	3	3	3	3	3	2	2
9	9	9	9	9	9	2	9	9	1
2	2	2	2	2	2	2	2	2	2
2	2	2	2	2	2	2	2	2	2
2	3	2	1	2	3	2	2	3	2
2	2	2	2	2	2	2	2	2	2
2	2	2	2	2	2	2	2	2	2
1	3	1	1	1	3	3	1	1	1
2	2	2	2	2	2	2	2	2	2
2	2	2	2	2	2	2	2	2	2
2	2	3	3	1	3	1	1	1	1
2	2	2	2	2	2	2	2	2	2
3	1	2	2	2	1	2	1	1	2
2	2	2	2	2	2	2	2	2	2
2	2	2	2	2	2	2	2	2	2
2	2	2	2	2	2	2	2	2	2
2	2	2	2	2	2	2	2	2	2
1	3	3	3	3	3	1	1	1	1
2	2	2	2	2	2	2	2	2	2
2	2	2	2	2	2	2	2	2	2
3	3	3	3	3	3	3	3	3	3
2	2	2	2	2	2	2	2	2	2

2	2	2	2	2	2	2	2	2	2
2	2	2	2	2	2	2	2	2	2
2	2	2	2	2	2	2	2	2	2
2	2	2	2	2	2	2	2	2	2
2	2	2	2	2	2	2	2	2	1
3	1	3	1	2	3	1	1	1	1
2	2	2	2	2	2	2	2	2	2
1	3	2	1	3	2	2	2	2	2
1	2	3	3	1	3	1	1	3	1
2	2	2	2	2	2	2	2	2	2
1	1	3	3	1	3	1	1	1	3
9	9	9	9	9	9	9	9	9	2
9	2	9	9	9	9	9	9	2	9
1	2	2	2	1	3	1	2	2	1
1	1	3	3	3	3	3	3	2	2
2	2	2	2	3	3	3	3	2	2
3	2	2	2	2	3	3	2	2	2
1	9	2	2	2	2	2	2	1	1
9	9	9	9	9	9	9	9	9	9
3	1	2	2	2	3	2	3	3	2
9	9	9	9	9	9	9	9	9	9
2	3	2	1	2	3	2	1	1	2
2	2	2	2	2	2	2	2	1	1
2	2	2	2	2	2	2	2	1	1
3	3	3	3	3	3	3	1	1	1
2	2	3	3	1	3	1	1	1	1
2	2	2	2	2	2	2	2	2	2
1	2	2	3	1	3	1	2	2	1
2	2	2	2	2	2	1	1	2	2
3	2	2	3	2	3	2	3	1	2
1	1	1	1	1	1	1	1	1	1
1	1	1	1	1	1	1	1	3	3
3	3	3	3	3	3	3	3	1	1
1	1	1	1	1	1	1	1	1	1
3	3	3	3	3	3	3	3	3	3
3	3	3	1	1	1	1	3	1	3
9	9	9	9	9	9	9	9	9	9
1	1	1	1	1	1	1	1	1	1
2	2	2	2	2	2	2	2	2	2
2	2	2	2	2	2	2	2	2	2
1	2	2	1	1	3	2	1	2	2
9	9	1	9	9	9	9	9	1	1
3	2	1	3	2	2	2	1	1	2
2	2	2	2	2	2	2	2	2	2
2	2	2	2	2	2	2	2	2	2
2	3	1	2	2	1	3	2	3	3
3	2	2	2	2	1	2	2	1	1

3	1	1	2	1	2	3	1	1	3
3	3	3	3	3	3	3	3	3	3
3	2	1	3	1	2	3	1	3	2
9	9	9	9	9	9	9	9	9	2
9	9	9	9	9	9	9	9	9	9
2	1	2	3	1	2	1	3	3	3
2	2	2	2	2	2	2	2	2	2
3	3	3	3	3	3	3	3	3	1
3	3	3	3	3	3	3	3	2	2
2	2	2	2	2	2	2	2	2	2
9	9	9	9	9	9	9	1	1	9
2	2	3	9	2	3	9	2	2	2
2	2	2	2	2	2	2	2	2	2
2	2	2	2	1	3	3	3	2	2
2	2	2	2	2	2	2	2	2	2
3	3	3	2	2	1	1	1	3	2
1	2	3	2	2	3	3	2	2	3
9	9	9	9	9	9	9	9	1	9
9	9	9	9	9	9	9	2	9	9
2	2	2	2	2	2	2	2	2	2
2	2	2	2	2	2	2	2	2	2
2	2	2	2	2	2	2	2	2	2
9	9	9	9	9	9	9	9	9	9
9	9	9	9	9	9	9	9	9	9
2	3	1	2	3	1	2	3	1	2
1	1	1	1	1	3	1	1	1	1
2	2	2	1	1	1	1	1	2	3
1	1	1	1	1	3	1	1	1	1
3	3	3	1	1	3	3	1	3	1
9	1	2	3	1	2	3	1	2	1
1	2	2	1	1	3	2	1	1	2
3	1	2	2	1	2	3	1	2	3
1	2	2	3	1	3	1	2	1	1
3	3	3	3	3	3	3	3	3	3
3	3	3	3	3	3	3	3	3	3
3	3	3	2	2	3	2	3	1	1
1	3	2	2	2	2	1	2	1	2
3	3	3	3	3	3	3	3	3	3
9	3	1	3	1	9	1	9	1	2
3	3	3	3	3	3	3	3	3	3
3	1	3	1	1	3	1	3	9	1
1	3	1	1	1	2	1	3	1	1
1	1	1	1	1	1	1	1	1	3
2	2	2	2	2	2	2	2	2	2
2	2	1	2	2	2	2	2	2	1
1	1	1	1	1	1	1	1	2	2
1	1	2	2	2	2	1	1	2	1

2	2	2	2	2	2	2	2	2	2
3	2	9	2	9	3	2	3	3	2
1	2	3	1	9	1	9	1	1	2
1	1	3	1	1	1	1	1	3	3
2	2	2	2	2	2	2	2	2	2
1	2	2	2	2	3	2	2	1	3
3	3	2	2	2	2	2	2	2	2
2	2	2	2	2	2	2	2	2	2
2	2	2	2	2	2	2	2	2	2
2	2	2	2	2	2	2	2	2	2
9	9	9	3	9	9	9	9	9	1
2	2	2	2	2	2	2	2	2	2
3	1	1	3	1	3	1	3	1	1
1	2	3	1	2	2	3	3	2	1
1	1	1	1	1	1	1	1	2	2
3	3	2	2	2	2	2	2	1	1
1	2	3	3	1	3	1	1	1	1
2	2	2	2	1	2	1	1	1	1
3	2	2	1	1	3	1	1	1	1
9	9	9	9	9	9	9	9	9	9
1	1	1	3	1	1	1	1	1	1
1	1	1	1	3	2	2	3	1	1
3	3	3	3	3	3	1	1	3	3
3	3	3	3	3	3	3	3	1	1
3	3	3	3	3	3	3	3	3	3
2	2	2	2	2	2	2	2	1	1
1	1	2	1	1	2	1	1	1	1
2	3	1	2	1	2	3	1	1	2
2	1	1	2	3	1	2	2	3	1
2	1	2	1	1	1	1	1	1	1
2	2	2	2	2	2	2	2	2	2
9	9	9	9	9	9	9	9	9	9
2	1	2	2	2	2	1	3	2	3
9	9	9	9	9	9	9	9	9	9
2	2	3	2	1	2	2	2	1	2
2	2	2	2	2	2	2	2	2	2
2	2	2	2	2	2	2	2	2	2
2	2	2	2	2	2	2	2	2	2
2	2	2	2	2	3	3	3	2	2
1	1	2	2	3	1	2	3	1	2
2	2	1	2	1	2	3	1	1	2
2	2	2	2	2	2	2	2	2	2
3	3	2	2	1	2	2	3	1	2
9	9	9	9	9	9	9	9	2	9
3	3	3	3	3	3	3	3	3	3
2	2	2	2	2	2	2	2	2	2
2	2	2	2	1	3	1	1	1	1

9	9	9	9	9	9	9	9	9	9
2	2	1	2	2	9	2	3	2	2
2	3	3	3	3	3	3	3	1	2
1	2	3	2	1	3	1	1	1	1
3	3	3	3	3	3	3	3	2	2
2	2	2	2	9	2	3	2	2	2
2	2	2	9	9	9	9	9	9	9
1	1	3	3	2	3	2	2	2	3
2	2	2	2	2	2	2	2	2	2
9	9	9	9	9	9	9	9	9	9
9	9	9	9	9	9	9	9	9	9
9	9	9	9	9	9	9	9	9	9
9	9	9	9	9	9	9	9	9	2
9	9	9	9	9	9	9	9	3	9
9	9	9	9	9	9	9	9	9	9
2	2	2	2	1	3	1	1	1	2
2	1	1	2	2	1	2	2	1	9
2	2	2	2	2	2	2	2	2	2
3	2	2	2	2	1	3	1	1	2
2	2	2	1	1	2	1	1	1	1
2	2	2	2	2	2	2	2	1	3
1	2	2	2	3	2	2	2	2	3
3	2	1	1	1	3	1	2	1	1
3	3	2	3	2	3	2	1	1	1
2	2	2	2	2	2	2	2	2	2
2	1	2	1	2	3	1	3	2	3
3	9	1	3	9	1	3	3	2	2
1	1	1	1	1	1	1	1	1	1
2	1	2	1	2	3	1	3	2	3
2	2	2	2	1	3	1	1	1	2
2	2	2	1	1	2	1	1	1	1
9	9	9	9	9	9	9	9	9	2
1	1	3	1	2	2	1	3	1	3
2	2	2	2	2	2	2	2	2	2
2	2	2	2	2	2	2	2	2	2
2	2	2	2	2	2	2	2	2	2
2	2	2	2	2	2	2	2	2	2
9	9	9	9	9	9	9	9	2	9
2	2	2	2	2	2	2	2	2	2
2	2	2	2	2	2	2	2	2	2
2	2	2	2	2	2	2	2	2	2
2	2	2	2	2	2	2	2	2	2
2	2	2	2	2	2	2	2	2	2
2	2	2	1	3	3	3	3	1	1
2	2	2	2	2	2	2	2	1	2
2	2	2	2	2	2	2	2	2	2
9	9	9	9	9	9	9	9	9	9

2	2	2	2	2	2	2	2	2	2
2	2	2	2	2	2	2	2	2	2
9	9	1	9	9	9	9	9	9	9
9	9	9	9	9	9	9	9	9	9
1	1	2	1	1	1	2	2	1	2
9	9	9	9	9	9	9	9	1	9
2	2	3	3	1	2	3	1	1	1
2	3	2	3	3	2	1	1	3	2
3	3	2	1	1	2	3	2	9	9
2	2	3	3	2	2	1	1	1	1
9	9	9	9	9	9	9	9	9	9
1	2	1	2	2	1	1	3	2	1
3	3	2	2	2	2	2	2	2	2
2	2	2	2	2	2	2	2	2	2
2	2	2	3	2	2	1	1	2	1
3	3	3	2	3	1	2	3	3	3
2	2	2	2	2	2	2	2	2	2
2	2	2	2	2	2	2	2	2	2
1	2	2	1	3	3	1	2	1	2
1	2	3	3	3	3	3	3	1	1
2	2	2	1	3	3	2	2	3	3
2	2	2	2	2	2	2	2	2	2
1	1	1	2	2	2	1	1	1	3
2	2	2	2	2	2	2	2	3	3
2	2	2	2	2	2	2	2	2	2
2	2	2	2	2	2	2	2	1	1
2	2	2	2	2	2	2	2	2	2
2	2	2	2	2	2	2	2	2	2
2	2	2	2	2	2	2	2	2	2
9	9	9	9	9	9	9	9	9	9
1	1	9	1	9	1	1	1	1	3
2	2	2	2	2	2	2	2	2	2
1	1	1	1	1	1	1	1	9	9
9	9	9	9	9	9	9	9	9	9
3	3	2	1	3	2	1	1	1	2
9	9	9	9	9	9	9	9	9	9
9	9	9	9	9	9	9	9	9	9
2	2	2	2	2	2	2	2	2	2
3	3	3	3	3	3	1	3	9	1
2	1	2	1	1	3	3	2	1	1
1	1	1	1	1	1	1	1	1	1
1	2	3	2	1	1	2	3	1	2
9	9	9	9	9	9	9	9	1	9
2	1	2	2	3	1	2	3	1	2
1	2	2	2	1	3	2	1	2	2
9	9	9	9	9	9	9	9	9	9
2	2	3	1	2	2	3	3	3	3

2	2	1	2	2	2	1	2	1	2
9	9	9	9	9	9	9	9	9	9
9	9	9	9	9	9	9	9	9	9
2	2	2	2	2	2	2	2	2	2
1	2	1	1	1	3	2	1	1	1
2	2	1	2	2	2	1	2	1	2
3	1	2	1	2	3	1	2	1	3
2	2	2	2	2	2	2	2	1	3
2	2	2	2	2	2	2	2	2	2
1	1	3	1	2	1	1	1	1	1
9	9	9	9	9	9	9	9	9	9
9	9	9	9	9	9	9	9	9	9
9	9	9	9	9	9	9	9	1	9
2	2	2	2	2	2	2	2	1	1
1	2	3	2	3	2	3	3	2	1
9	9	9	9	9	9	9	9	9	9
9	9	9	9	9	9	9	9	9	9
9	9	9	9	9	9	9	9	9	9
1	1	2	2	1	2	1	1	1	1
2	2	2	2	2	2	2	2	1	1
9	9	9	9	9	9	9	9	9	9
1	1	1	3	3	2	2	1	1	3
3	3	3	3	3	3	3	3	3	3
9	9	9	9	9	9	9	9	9	9
2	2	2	2	2	2	2	2	2	2
2	2	2	2	2	2	2	2	2	2
9	9	9	9	9	9	9	9	9	9
9	9	9	9	9	9	9	9	9	9
2	2	2	2	2	2	2	2	2	2
2	2	2	2	2	2	2	2	2	2
1	1	2	1	1	2	3	1	1	1
9	9	9	9	9	9	9	9	9	9
9	9	9	9	9	9	9	9	9	9
2	2	2	2	3	3	3	3	1	1
1	2	9	2	1	2	1	2	2	2
2	2	2	2	2	2	2	2	2	2
2	2	2	1	1	1	2	2	2	2
2	2	2	2	2	2	2	2	2	2
2	1	2	1	2	1	2	1	3	2
1	3	1	2	3	1	2	3	1	2
2	3	3	1	2	2	2	3	2	3
9	9	9	9	9	9	9	9	9	9
9	9	9	9	9	9	9	9	9	9
9	9	1	9	9	9	9	9	9	9
9	9	9	9	9	9	9	9	9	9
9	9	9	9	9	9	9	9	9	9
9	9	9	9	9	9	9	9	9	9

2	2	2	2	2	2	2	2	2	2
2	3	2	3	2	3	2	3	2	3
2	2	2	2	2	1	9	1	1	1
2	2	2	2	2	2	2	2	2	2
9	9	9	9	9	9	9	9	9	9
9	9	9	9	9	9	9	9	9	9
9	9	9	9	9	9	9	9	9	9
9	9	9	9	9	9	9	9	9	9
9	9	9	9	9	9	9	9	9	9
9	9	9	9	9	9	9	9	9	9
9	9	9	9	9	9	9	9	9	9
9	9	9	9	9	9	9	9	9	9
9	9	9	9	9	9	9	9	9	9
3	3	1	1	1	1	1	1	1	1
1	1	2	2	2	2	1	1	1	1
2	2	2	2	2	2	2	2	2	2
2	2	2	2	2	2	2	2	2	2
9	9	9	9	9	9	9	9	9	9
9	9	9	9	9	9	9	9	9	9
9	9	9	9	9	9	9	9	9	9
9	9	9	9	2	9	9	9	1	9
9	9	9	9	9	9	9	9	9	9
1	2	2	2	2	2	2	2	2	2
9	9	9	9	9	9	9	9	9	9
1	1	1	1	1	1	1	2	2	2
1	1	1	1	2	2	2	2	1	1
1	1	1	1	1	1	1	1	2	2
2	1	1	1	1	1	1	1	1	1
1	2	2	2	1	1	1	1	2	2
2	2	1	1	1	2	2	1	1	2
1	2	2	2	1	1	1	1	1	1
1	2	2	2	1	1	1	1	2	2
1	1	1	1	1	1	1	1	1	1
9	9	9	9	9	9	9	9	9	9
1	2	1	1	1	1	1	1	2	2
1	1	2	2	2	1	1	1	1	1
2	2	2	2	2	2	2	2	1	1
9	9	9	9	9	9	9	9	9	9
2	2	2	2	2	2	2	2	3	3
1	1	3	1	3	1	1	1	3	3
3	3	3	3	3	3	3	3	3	3
9	9	9	9	9	9	9	9	9	9
9	9	9	9	9	9	9	9	9	9
9	9	9	9	9	9	9	9	9	9
1	9	9	9	9	9	9	9	9	9
2	2	2	2	3	2	1	3	2	3
9	9	9	9	9	9	9	9	9	9

9	9	9	9	9	9	9	9	9	9
9	9	9	9	9	9	9	9	9	9
9	9	9	9	9	9	9	9	9	9
9	9	9	9	9	9	9	9	9	9
9	9	9	9	9	9	9	9	9	9
9	9	9	9	9	9	9	9	9	9
9	9	9	9	9	9	9	9	9	9
9	9	9	9	9	9	9	9	9	9
9	9	9	9	9	9	9	9	9	9
9	9	9	9	9	9	9	9	9	9
2	9	9	9	9	9	9	9	9	9
9	9	9	9	9	9	9	9	9	9
2	2	1	2	2	2	1	1	1	2
1	2	3	2	9	1	1	2	1	1
2	1	1	1	2	2	2	2	1	1
3	2	1	2	3	2	2	1	1	1
2	2	2	2	2	2	2	2	2	2
1	2	2	1	1	1	3	3	3	3
1	2	2	1	1	1	1	2	1	1
9	9	9	9	9	9	9	9	1	9
1	1	1	1	2	2	2	1	1	1
2	1	3	1	1	2	2	1	1	1
2	2	2	2	2	2	2	2	2	2
1	1	1	2	1	1	2	3	1	2
1	2	2	2	1	1	1	1	2	1
3	3	3	3	3	3	1	1	3	3
9	9	2	9	3	2	3	2	1	2
2	2	2	2	2	2	2	2	1	3
9	9	9	9	9	9	9	9	9	1
9	9	9	9	9	9	9	9	3	3
9	9	9	9	9	9	9	9	9	9
1	1	1	1	1	1	1	1	2	2
2	2	2	2	2	2	2	2	2	2
2	1	1	1	1	1	1	2	2	1
1	1	1	1	2	2	1	1	1	2
2	2	2	2	2	2	2	2	2	2
2	2	2	2	2	2	2	2	2	2
2	1	1	1	1	1	1	2	2	1
1	1	2	2	1	2	2	2	2	1
1	2	2	2	1	1	1	2	1	1
2	2	2	2	2	2	2	2	2	2
2	2	1	1	1	2	1	1	1	1
3	3	3	3	1	3	1	1	1	3
1	2	2	2	1	1	1	1	2	1
1	1	2	2	2	1	1	1	1	1
2	2	2	1	1	1	1	1	1	1
2	2	2	2	2	2	2	2	2	2

9	9	9	9	9	9	9	9	9	9
1	3	2	2	2	3	1	1	1	1
9	9	2	9	9	9	9	9	9	9
3	9	9	9	9	9	9	9	9	9
9	9	9	9	9	9	9	9	1	9
9	9	9	9	9	9	9	9	9	9
9	9	9	9	9	9	9	9	9	9
2	2	2	2	2	2	2	2	3	3
9	9	9	9	9	9	9	9	2	9
2	2	2	2	2	2	2	2	2	2
9	9	9	9	9	9	9	9	9	9
2	1	2	1	2	1	2	2	9	9
1	2	2	1	1	1	1	1	1	1
2	2	2	2	2	2	2	2	2	2
1	1	2	2	2	2	2	1	1	1
9	9	9	9	9	9	9	9	9	9
2	2	2	2	2	2	2	2	2	2
3	3	3	3	3	3	3	3	3	3
3	1	2	3	1	2	1	9	9	9
2	2	2	2	2	2	2	2	1	1
2	2	2	2	2	2	2	2	2	2
2	2	3	2	3	2	2	3	3	2
2	2	2	2	2	2	2	2	2	2
2	2	2	2	2	2	2	2	2	2
2	2	2	1	2	2	2	2	2	2
2	2	2	2	2	2	2	2	2	2
3	1	2	2	3	3	1	1	3	3
3	1	1	1	3	3	3	3	1	3
1	1	1	1	1	1	1	1	1	1
2	2	2	1	1	1	1	1	2	2
9	9	9	9	9	9	9	9	9	9
2	2	2	2	2	2	2	2	3	3
9	9	9	9	9	9	9	9	9	9
2	2	2	2	2	2	2	2	2	2
2	2	2	2	2	2	2	2	2	2
2	2	2	1	1	2	1	1	2	2
3	3	3	3	3	3	3	3	3	1
1	2	2	1	1	2	1	1	1	1
1	1	1	1	1	1	1	1	1	1
1	1	1	3	2	2	1	1	2	2
2	2	2	2	2	2	2	2	2	2
2	1	1	2	2	2	2	2	1	1
9	9	9	9	9	9	9	9	9	9
9	9	9	9	9	9	9	9	9	9
9	9	9	9	9	9	9	9	1	9
9	9	9	9	9	9	9	9	9	9
9	9	9	9	9	9	9	9	9	9

9	9	9	9	9	9	9	9	9	9
2	2	2	2	2	2	2	2	2	2
9	9	9	9	9	9	9	9	2	9
1	2	1	1	2	1	2	2	2	1
9	9	9	9	9	9	9	9	9	9
3	3	2	2	1	2	1	3	1	2
3	3	3	3	3	9	2	1	2	2
9	9	9	9	9	9	9	9	9	9
9	9	9	9	9	9	9	9	9	9
9	9	9	9	9	9	9	9	9	9
9	9	9	9	9	9	9	9	9	9
9	9	9	9	9	9	9	9	9	9
1	1	1	2	2	1	2	3	1	2
9	9	9	9	9	9	9	9	9	9
9	9	9	9	9	9	9	9	9	9
1	1	2	3	1	2	3	1	2	1
2	3	2	1	2	3	2	2	1	3
2	2	2	2	1	3	1	2	2	1
1	1	2	2	3	3	3	3	2	3
2	2	1	1	1	2	3	2	1	1
2	1	1	2	2	3	1	2	2	3
1	1	3	3	1	3	1	1	1	3
2	2	1	1	3	1	3	2	1	1
3	1	1	2	1	1	2	2	1	2
2	2	2	2	2	2	2	2	2	2
2	2	2	2	2	2	1	1	1	1
3	3	2	2	2	2	2	2	2	2
1	2	2	2	1	3	1	2	1	2
2	1	1	2	1	2	2	2	2	3
2	1	2	1	2	3	3	3	1	2
3	3	3	1	1	3	1	1	3	3
2	2	2	3	2	2	2	2	1	2
9	9	9	9	9	9	9	9	9	9
3	2	1	3	2	1	1	2	1	2
1	2	2	2	1	3	1	2	1	1
2	2	2	2	2	2	2	2	1	1
1	2	1	2	2	2	2	2	2	2
3	1	2	2	3	1	2	2	1	1
9	9	9	9	9	9	9	1	9	9
2	2	2	2	2	2	2	2	1	2
9	9	9	2	9	9	9	9	9	9
9	9	9	9	9	9	9	9	1	9
2	2	2	3	2	1	2	3	2	3
9	9	9	9	9	9	9	9	1	9
2	1	3	2	1	1	2	1	1	1
1	3	3	1	1	3	3	1	1	1
1	1	1	1	1	1	1	1	2	2

3	3	3	3	3	3	3	3	3	3
9	9	9	9	9	1	9	9	9	9
9	3	3	3	3	3	3	3	2	2
1	3	1	3	1	3	1	1	1	1
1	3	3	3	1	3	1	1	1	1
3	1	3	1	1	3	1	1	1	1
3	1	3	2	3	1	2	1	1	2
2	2	2	2	1	3	1	3	1	2
2	2	2	2	2	2	2	2	2	2
2	3	1	2	3	1	2	3	2	2
1	2	3	1	3	2	1	1	1	2
3	3	2	1	1	3	2	2	2	3
1	2	3	1	3	2	3	1	3	1
2	2	2	2	2	2	2	2	2	2
2	1	3	2	1	2	1	2	1	1
1	2	3	2	3	1	2	1	1	2
2	2	2	2	2	2	2	2	2	2
2	2	2	1	1	3	1	3	1	3
2	2	2	2	2	2	2	2	2	2
2	2	2	2	2	2	2	2	2	2
2	2	2	2	2	2	2	2	2	2
2	1	1	1	1	3	3	3	1	1
2	2	2	2	2	2	2	2	1	2
9	9	9	9	9	9	9	9	9	9
2	2	2	2	2	2	2	2	2	2
2	1	1	1	1	1	2	1	1	2
3	3	2	1	2	3	2	1	2	2
2	1	3	2	2	1	1	2	3	3
1	3	2	1	2	1	1	3	1	1
9	9	9	9	9	9	9	9	9	9
3	2	1	3	2	3	1	2	2	3
1	3	1	2	3	2	1	2	2	3
2	2	3	3	2	1	3	2	3	2
2	2	2	2	2	3	2	2	2	2
2	3	1	2	1	3	1	1	3	3
2	2	2	2	2	2	2	2	2	2
2	2	2	2	2	2	2	2	3	3
9	9	9	9	9	9	9	9	9	9
2	3	3	1	2	2	1	3	1	2
2	2	3	2	2	2	2	2	2	3
9	9	9	9	9	9	9	9	9	9
9	9	9	9	9	9	9	9	9	9
2	2	2	2	2	2	2	2	2	2
9	9	9	9	9	9	9	9	9	9
2	2	2	2	2	2	2	2	2	2
2	2	2	2	2	2	2	2	1	1
9	9	9	9	9	9	9	9	9	9

2	1	2	2	1	2	2	3	2	2
9	9	9	9	9	9	9	9	9	9
2	2	2	2	3	2	1	2	1	3
9	9	9	9	9	9	9	9	9	9
9	9	9	9	9	9	9	9	9	9
3	3	3	3	3	3	3	3	3	3
2	2	2	2	2	2	2	2	2	2
2	2	2	2	2	2	2	2	2	2
3	3	2	2	2	2	2	2	1	1
2	2	2	2	2	2	2	2	2	2
9	9	9	9	9	9	9	9	9	9
9	9	9	9	9	9	9	9	9	9
9	9	9	9	9	9	9	9	9	9
1	1	2	2	2	1	1	1	2	1
2	2	2	2	2	2	2	2	2	2
1	2	2	2	1	1	1	1	1	1
9	9	9	9	9	9	9	9	2	9
9	9	9	9	9	9	9	9	9	9
3	3	3	3	3	3	3	3	1	3
2	2	2	2	2	2	2	2	2	2
1	2	2	1	1	1	1	1	1	1
3	3	3	3	3	3	3	3	3	3
9	9	9	9	9	9	9	9	1	9
2	3	3	2	3	2	2	3	2	3
2	2	3	3	3	3	3	3	1	1
2	2	3	3	2	2	3	3	2	2
9	9	1	9	9	9	1	2	9	9
1	1	1	1	1	1	1	1	1	2
2	2	2	2	2	2	2	2	2	2
9	9	9	9	9	9	9	9	9	9
2	2	1	1	2	3	3	3	3	1
2	2	2	2	2	2	2	2	2	2
1	3	1	2	1	2	2	2	1	1
2	2	2	3	3	2	3	1	2	2
2	2	2	2	2	2	2	2	2	2
3	2	2	2	1	2	9	1	2	1
2	2	2	2	1	3	1	1	1	1
3	3	3	3	3	3	1	3	1	1
2	2	2	2	2	2	2	2	2	2
3	3	3	3	3	3	3	3	3	3
1	1	3	2	1	2	1	2	2	1
1	9	2	2	1	3	1	1	1	1
2	2	2	2	2	2	2	2	2	2
2	2	2	2	2	2	2	3	2	2
1	3	1	3	1	3	1	3	1	3
2	1	1	3	2	1	3	2	1	2
2	2	2	2	2	3	1	1	2	2

2	2	2	2	2	2	2	2	2	3
2	2	2	2	2	2	2	2	2	2
2	1	2	2	1	2	1	1	1	1
2	2	2	1	2	2	1	2	1	2
2	2	2	2	2	2	2	2	1	1
9	9	9	9	9	9	9	9	9	9
2	2	2	2	2	2	2	2	2	2
1	1	2	2	2	2	1	1	1	1
2	2	2	2	2	2	2	2	2	2
2	2	2	2	2	2	2	2	1	1
3	2	2	2	2	2	1	2	1	1
2	2	2	2	2	2	2	2	2	2
2	2	2	2	2	2	2	2	2	2
3	3	3	3	3	3	3	3	3	3
2	3	3	3	2	2	3	2	2	9
2	3	2	1	3	2	1	2	2	1
2	3	2	2	3	2	3	2	3	2
2	1	3	2	3	2	1	3	2	2
2	2	2	2	2	2	2	2	2	2
2	2	1	3	1	2	2	1	2	1
2	2	2	2	2	2	2	2	2	2
2	2	3	2	3	3	1	2	2	2
2	2	3	2	3	3	1	2	2	2
9	9	9	9	9	9	9	9	1	1
2	2	2	2	2	9	1	1	1	1
2	2	2	2	2	2	2	2	2	2
2	2	2	2	2	3	1	1	1	1
2	2	3	3	2	3	2	3	1	1
2	2	2	2	2	2	2	2	2	2
1	1	1	1	1	1	1	1	1	1
3	2	2	2	3	3	3	2	2	2
2	3	3	2	3	2	2	3	2	3
1	1	3	3	3	1	1	1	2	3
2	2	3	2	3	3	3	2	3	3
1	1	3	3	3	1	1	1	2	2
2	2	2	2	2	2	2	2	2	2
2	2	2	2	2	2	2	2	2	2
2	2	2	2	1	2	2	2	2	2
2	2	2	2	2	2	2	2	2	1
2	2	2	2	2	2	2	2	2	9
3	2	3	3	1	3	1	3	1	1
1	2	2	3	1	3	1	2	1	1
2	2	2	2	2	2	2	2	2	1
2	2	2	2	2	2	2	2	2	2
2	3	2	2	2	1	2	3	1	2
1	3	3	3	2	3	1	1	1	1
1	2	2	2	1	3	2	2	1	1

2	2	2	2	2	2	2	2	1	2
2	2	2	2	2	2	2	2	9	2
2	2	2	9	1	3	1	2	1	1
2	2	2	2	2	2	2	2	2	2
2	2	2	2	2	2	2	2	1	3
2	2	2	2	2	2	2	2	2	2
2	2	2	2	2	2	1	1	1	1
1	1	2	2	1	2	1	1	1	1
2	2	2	2	2	2	2	2	2	2
2	2	2	2	2	2	2	2	2	2
2	2	2	2	2	2	2	2	2	2
9	9	9	9	9	9	9	9	9	9
9	9	9	9	9	9	9	9	9	9
2	9	9	9	9	9	9	9	9	1
2	2	2	2	2	2	2	2	1	1
2	2	2	2	2	2	2	2	1	1
1	1	2	2	1	3	1	1	1	1
2	2	2	2	2	2	2	2	1	1
1	1	3	2	1	3	1	1	1	1
1	1	2	1	2	1	1	2	1	1
2	2	2	2	2	2	2	3	1	1
2	2	2	2	2	2	2	2	9	2
2	2	2	2	2	2	2	2	2	2
2	2	2	2	2	2	2	2	2	2
2	2	2	2	2	2	2	2	1	1
2	2	2	1	2	1	1	1	9	9
2	2	2	2	2	2	2	2	1	1
2	2	2	2	2	2	2	2	2	2
2	2	2	2	2	2	2	2	1	1
9	9	9	9	9	9	9	9	9	9
2	2	2	2	2	2	2	2	2	2
2	2	2	2	2	2	2	2	2	2
2	2	2	2	2	2	2	2	1	9
2	2	2	2	2	2	2	2	2	2
2	2	2	2	2	2	2	2	1	1
2	2	2	2	2	2	2	2	2	2
2	2	2	2	2	2	2	2	2	2
2	2	2	2	2	2	2	2	2	2
2	2	2	2	2	2	2	2	2	2
2	2	2	2	2	2	2	2	1	1
2	2	2	2	2	2	2	2	2	2
2	2	2	2	2	2	2	2	2	2
2	2	2	2	2	2	2	2	1	1
2	2	2	2	2	2	2	2	2	1
2	2	2	2	2	2	2	2	2	2
2	2	2	3	2	2	3	2	1	1
2	2	2	2	2	2	2	2	2	2

2	2	2	2	2	2	2	2	1	1
2	2	2	2	2	2	2	2	1	1
2	2	2	2	2	2	2	2	1	1
2	2	2	2	2	2	2	2	2	2
2	2	2	2	2	2	2	2	2	2
1	2	2	2	2	2	2	2	2	2
9	9	9	9	9	9	9	9	9	9
1	2	2	1	1	3	1	1	1	1
1	1	2	1	2	2	2	2	1	2
2	2	2	2	2	2	2	2	1	1
2	2	2	2	2	2	2	2	1	1
2	2	2	2	2	2	2	2	2	2
2	2	2	2	2	2	2	2	1	1
1	9	9	9	9	9	9	1	1	9
2	2	2	2	2	2	1	1	1	1
2	2	2	2	2	2	2	2	1	1
1	1	1	2	2	2	1	1	2	1
1	1	2	2	2	3	2	2	1	1
1	1	1	1	1	1	1	1	1	1
1	2	2	2	2	2	2	2	1	1
1	2	2	3	3	2	2	3	2	1
2	2	9	1	2	2	2	2	1	2
2	2	2	2	2	2	2	2	1	1
2	2	2	2	2	2	2	2	2	2
2	2	2	2	2	2	2	2	2	2
2	2	2	2	2	2	2	2	2	2
9	9	9	9	9	9	9	9	9	9
2	2	2	2	2	2	2	2	2	2
2	2	2	2	2	2	2	2	1	1
2	2	2	2	2	2	2	2	2	2
2	2	2	2	2	2	2	2	2	2
2	2	1	1	2	2	2	2	1	2
9	9	9	9	9	9	9	9	9	9
9	9	9	9	9	9	9	9	9	9
9	9	9	9	9	9	9	9	9	9
9	9	9	9	9	9	9	9	9	9
2	2	2	3	3	3	3	3	1	1
2	3	3	3	3	3	1	1	1	2
1	1	2	2	1	3	3	2	2	1
2	2	2	2	2	2	2	2	2	3
2	3	3	3	2	3	1	2	1	1
2	2	2	2	2	2	2	2	1	1
1	2	2	1	2	1	2	1	1	1
2	2	2	2	2	2	2	2	2	2
2	2	2	2	2	2	2	1	1	1
2	2	2	2	2	2	2	2	1	1
1	1	2	1	2	1	2	1	1	2

3	1	2	3	1	2	9	2	1	1
1	1	2	3	1	9	1	2	1	2
2	9	1	3	1	2	1	1	1	1
1	2	1	2	1	2	1	2	1	2
2	3	2	3	2	1	1	1	2	1
2	2	2	2	3	1	2	2	1	2
1	2	3	2	1	3	1	1	1	1
9	9	9	9	9	9	9	9	2	9
2	2	2	2	2	2	2	2	2	2
2	2	2	2	2	2	2	2	2	2
2	2	2	2	2	2	2	2	2	2
2	2	2	2	2	2	2	2	2	2
2	2	2	2	2	2	2	2	2	2
2	1	2	1	2	3	1	1	1	1
2	2	2	2	2	2	2	2	1	1
2	2	2	2	2	2	2	2	2	2
2	2	2	2	2	2	2	2	2	2
3	2	2	1	1	2	1	1	1	9
1	2	3	3	2	3	1	1	1	1
1	2	3	3	2	3	1	1	1	1
3	3	3	2	3	3	2	2	2	3
2	2	2	2	2	2	2	2	1	2
1	2	3	3	1	3	2	1	1	1
9	9	9	9	9	9	9	9	9	9
3	1	3	1	2	1	3	3	3	1
1	2	2	2	1	3	1	1	1	1
2	2	2	2	2	2	2	2	2	1
1	2	2	2	2	3	2	1	2	2
2	2	2	2	2	2	2	2	2	2
9	9	9	9	9	9	9	9	9	1
2	2	3	3	3	3	3	3	3	1
9	9	9	9	9	9	9	9	9	9
9	9	9	9	9	9	9	9	9	9
9	9	9	9	9	9	9	9	9	9
9	9	9	9	9	9	9	9	9	9
2	2	2	2	2	2	2	2	2	2
2	3	3	3	3	3	3	3	1	3
9	9	9	9	9	9	9	9	9	9
2	2	2	2	2	2	2	2	2	2
2	2	2	2	2	2	2	2	2	2
1	2	3	3	2	3	2	2	3	3
2	2	2	2	2	2	2	2	1	1
2	2	2	2	2	2	1	1	1	1
3	1	2	3	1	2	3	1	3	2
9	9	9	9	9	9	9	9	3	9
2	1	1	1	2	1	1	1	1	2
3	3	3	3	3	3	3	3	3	3

1	1	1	1	1	1	2	1	1	2
1	1	2	2	1	2	1	1	1	1
2	1	1	1	1	1	1	1	1	1
2	2	2	2	2	2	2	2	1	1
9	9	9	9	9	9	9	9	9	9
9	9	9	9	9	9	9	9	9	9
2	2	2	2	2	2	2	2	2	2
2	2	2	2	2	2	2	2	3	3
9	9	9	9	9	9	9	9	9	9
9	9	9	9	9	9	9	9	9	9
2	2	1	2	2	2	1	2	9	1
2	1	1	1	1	1	1	1	2	2
2	2	1	1	3	3	2	2	3	3
2	3	2	2	3	2	3	2	3	3
9	9	2	9	9	9	9	9	3	3
2	3	1	2	1	2	3	1	1	1
1	2	2	1	2	3	1	1	1	1
3	1	1	2	1	2	2	3	2	1
9	9	9	9	9	9	9	9	9	9
3	2	2	1	1	2	3	1	2	9
2	1	2	1	3	1	2	1	1	1
1	1	1	1	2	1	2	1	1	2
1	2	2	1	1	2	1	1	1	2
2	2	2	2	2	2	2	2	2	2
1	1	1	3	3	3	3	3	1	1
2	2	2	2	2	2	2	2	2	2
1	2	3	1	2	3	2	2	2	2
2	2	2	2	2	2	2	2	2	2
3	2	2	2	3	1	2	2	1	2
3	3	1	3	3	9	1	3	3	1
2	2	2	2	2	2	2	2	2	2
2	1	2	2	2	2	1	2	2	2
2	3	3	3	1	3	3	3	3	1
2	2	2	1	1	3	3	2	1	1
1	9	9	9	9	9	9	9	9	9
1	1	1	2	1	2	2	1	2	1
9	9	9	9	9	9	9	9	9	9
2	2	2	2	2	2	2	2	2	2
1	2	2	2	1	2	2	2	9	9
2	1	1	2	1	3	1	1	1	1
9	9	9	9	9	9	9	9	9	9
3	1	3	2	1	3	2	1	1	2
1	2	3	2	3	2	2	2	2	3
1	2	3	2	1	3	1	1	2	2
1	2	3	2	1	3	1	1	2	2
3	2	3	3	3	3	3	3	2	2
2	3	2	3	3	2	2	3	2	3

2	1	2	3	3	2	1	2	2	2
3	3	2	3	3	3	2	3	9	9
2	2	2	2	2	2	2	2	2	2
3	3	3	3	3	3	3	3	3	3
1	1	3	1	1	3	3	1	1	1
9	9	9	9	9	9	9	9	9	9
9	9	9	9	9	9	9	9	9	9
2	2	3	2	2	2	1	2	2	2
1	1	3	3	3	3	1	1	1	1
2	2	2	2	2	2	2	2	3	3
1	2	1	3	2	3	2	2	2	1
3	2	1	2	2	1	2	1	2	1
2	3	2	2	2	2	3	2	2	2
1	1	3	3	1	1	1	1	1	1
9	9	9	9	9	9	9	9	9	9
3	3	3	3	3	3	3	3	3	3
2	2	2	2	2	2	2	2	2	3
2	2	2	2	2	2	2	2	2	2
2	1	2	3	1	1	2	3	1	2
1	2	3	2	1	2	2	2	1	2
9	9	9	9	9	9	9	9	9	9
9	9	9	9	9	2	9	9	9	9
2	2	3	2	2	3	3	2	1	1
9	9	9	9	9	9	9	9	9	9
9	9	9	9	9	9	9	9	9	9
2	2	3	2	3	2	3	2	3	3
3	1	2	3	2	3	3	3	2	3
2	2	2	2	2	2	2	2	2	2
3	3	3	3	3	3	3	3	3	3
1	1	2	2	1	2	1	1	1	1
3	3	3	3	3	3	3	3	3	3
2	2	2	2	2	2	2	2	2	2
9	9	9	9	9	9	9	9	9	9
9	9	9	9	9	9	9	9	9	9
9	9	9	9	9	9	9	9	9	9
2	2	2	2	2	2	2	2	2	2
3	3	3	1	1	3	1	1	1	3
2	2	3	1	1	1	3	1	2	2
1	1	3	3	2	3	1	1	2	1
9	9	9	9	9	9	9	9	9	2
2	2	3	3	2	2	2	3	2	2
2	1	2	1	2	3	3	1	2	1
3	2	2	2	2	3	3	3	2	2
9	9	9	9	9	9	9	9	1	1
2	3	2	3	2	1	2	3	3	2
9	9	9	9	9	9	9	1	1	1
2	2	3	2	3	2	3	2	1	1

1	2	1	2	1	2	1	2	2	1
3	3	3	2	2	2	3	3	2	2
3	3	1	1	3	3	1	1	3	3
1	2	3	1	2	3	1	2	1	2
2	2	2	2	2	2	2	2	3	3
2	2	2	2	2	2	2	2	2	2
3	1	2	2	2	2	1	1	1	2
1	1	3	2	3	3	2	2	1	1
9	9	9	9	9	9	9	9	9	9
1	2	3	2	1	2	2	1	1	3
2	3	3	2	3	2	2	3	1	2
2	2	2	2	2	2	2	2	2	2
2	2	3	3	1	3	1	1	1	1
1	1	3	1	1	3	1	1	1	2
9	9	9	9	9	9	9	9	9	2
3	3	2	2	1	3	2	1	2	2
2	1	1	1	3	2	2	1	1	1
3	1	2	3	1	2	1	2	1	2
1	2	1	3	2	1	3	1	1	3
2	2	2	2	2	2	2	2	2	2
1	1	1	1	1	2	2	1	2	1
2	2	2	2	2	2	2	2	2	2
2	1	2	1	1	1	1	1	1	1
1	2	2	2	2	3	1	1	1	1
1	2	2	2	1	3	2	1	1	1
2	3	2	2	3	3	3	1	1	1
3	2	2	3	2	2	3	2	2	1
2	2	2	2	2	2	2	2	2	1
2	2	2	2	2	2	2	2	2	2
2	2	2	2	2	2	2	2	2	2
2	1	9	1	2	2	1	1	2	2
3	2	9	1	2	3	9	9	2	2
2	2	2	2	2	2	2	2	2	2
1	2	2	1	1	2	2	2	1	2
3	1	3	2	1	2	3	1	1	2
2	2	2	2	2	2	2	2	2	1
1	1	2	2	1	1	2	2	1	1
1	2	2	1	1	1	1	1	1	2
1	2	3	3	2	3	2	1	1	2
2	2	1	1	1	1	1	1	1	1
3	3	3	3	3	3	3	2	3	3
2	1	1	1	1	1	2	2	1	2
2	2	2	1	2	2	2	2	1	1
2	2	1	1	2	1	1	1	2	2
2	2	2	1	1	1	1	2	1	1
2	2	1	1	1	2	2	1	2	2
3	3	2	1	2	1	1	1	1	2

2	2	2	1	1	1	2	2	2	2
2	1	2	2	2	1	2	2	2	3
1	1	2	1	1	2	2	1	1	1
1	3	3	1	1	2	2	1	1	2
1	1	1	2	2	1	2	1	2	1
1	2	3	3	1	1	2	1	1	2
2	3	2	1	2	2	2	1	1	2
2	1	1	2	2	1	1	1	1	1
2	2	2	2	2	2	2	2	2	2
2	2	2	2	2	2	2	2	2	2
2	2	2	2	2	2	2	2	2	2
3	1	2	2	2	2	2	3	3	3
3	2	1	2	2	2	1	2	2	1
1	3	3	1	2	2	2	2	2	2
2	2	2	2	2	2	2	2	2	2
1	1	1	1	1	1	1	1	2	2
2	1	3	2	1	3	2	2	2	1
2	2	2	3	2	3	2	2	2	2
2	2	2	2	2	2	2	2	2	1
3	2	1	2	2	2	2	2	3	2
2	2	2	2	2	2	2	2	2	2
2	2	2	2	2	2	2	2	2	2
2	9	1	2	2	2	1	3	3	3
9	9	9	9	9	9	9	9	9	9
2	1	2	1	2	9	2	1	1	1
3	3	3	3	3	3	3	3	2	2
2	3	2	3	2	3	1	1	1	1
2	3	2	2	1	1	2	3	3	3
2	2	2	2	2	1	1	1	1	1
2	2	3	3	2	3	2	1	2	2
1	3	2	1	2	1	2	2	2	3
2	2	3	3	2	2	3	1	1	2
1	1	2	2	1	1	1	2	1	2
2	2	2	2	2	2	2	2	2	2
2	3	3	3	3	3	3	1	1	1
3	3	2	3	2	3	3	1	2	3
1	2	1	2	2	2	2	2	2	2
3	3	3	3	3	2	2	2	2	2
1	1	1	1	2	3	3	3	3	3
2	2	1	1	2	1	1	1	1	2
2	2	2	2	2	2	2	2	2	2
1	1	1	1	3	3	2	2	2	2
2	2	2	2	2	2	2	2	2	2
3	3	3	3	3	3	3	3	3	3
3	3	3	3	3	3	3	3	3	3
2	2	2	2	2	2	2	2	2	1
2	1	1	2	1	1	2	1	1	2

2	1	1	2	3	2	1	1	1	2
2	1	1	1	2	2	1	1	1	2
1	1	2	2	2	1	2	2	1	2
2	1	1	2	2	3	3	2	1	2
2	3	3	2	2	1	1	1	1	2
2	2	1	1	1	2	2	1	1	1
3	3	2	2	2	1	3	3	3	3
2	2	2	1	1	2	2	2	1	1
2	2	2	1	1	1	1	1	1	1
2	1	1	1	2	2	2	1	1	2
2	2	1	1	2	3	2	2	1	2
2	1	1	2	2	1	1	1	1	2
2	2	1	1	1	2	2	2	1	1
1	1	2	1	1	2	2	2	1	1
2	2	1	1	1	1	1	2	1	1
2	1	1	2	1	1	2	2	1	1
2	2	2	2	2	2	3	3	2	2
2	1	1	1	2	2	2	2	1	1
2	2	2	9	2	9	9	9	2	9
2	2	2	2	2	2	2	2	2	2
9	2	9	9	9	2	9	2	2	9
3	3	3	3	3	3	3	3	3	3
2	2	2	1	2	1	1	1	1	1
1	2	2	1	1	1	1	2	1	1
2	3	2	1	2	2	2	3	1	2
1	1	1	2	2	1	1	1	1	1
1	1	2	2	1	1	1	1	1	1
2	1	1	2	1	1	1	2	1	1
2	2	1	1	1	2	1	1	1	1
2	1	2	1	2	1	2	1	1	2
1	1	2	2	1	1	1	2	2	1
2	2	2	2	2	2	2	2	2	2
3	2	2	2	3	2	2	2	2	2
2	1	2	3	2	2	1	2	2	2
2	1	1	3	2	1	2	2	2	1
1	3	3	2	1	3	2	1	2	2
1	9	2	1	3	9	2	9	9	9
2	9	2	9	2	9	2	9	2	9
9	9	9	9	9	9	9	9	9	9
9	9	1	9	3	1	9	1	1	2
9	9	9	2	9	9	9	9	9	9
1	2	2	1	1	2	1	1	1	1
9	2	9	9	1	9	1	1	1	9
2	2	2	2	2	2	2	2	2	2
2	9	9	1	9	9	1	9	1	9
9	2	9	9	2	9	9	2	2	9
9	9	2	9	9	9	9	2	2	9

9	9	2	9	9	2	9	9	9	2
9	9	9	2	9	9	9	2	9	2
9	2	9	9	2	9	9	2	2	9
9	9	9	9	9	9	9	9	9	9
2	9	9	3	1	9	3	1	9	9
2	9	9	9	9	2	9	9	9	9
9	9	9	9	9	9	9	9	9	9
9	9	9	9	9	9	9	9	2	9
3	2	1	1	1	1	2	3	2	3
2	9	2	9	2	9	2	2	2	9
2	1	1	2	2	2	1	1	1	2
1	1	2	2	1	1	2	2	2	2
2	1	1	2	2	1	1	2	1	2
1	1	1	2	1	1	2	2	1	2
1	1	2	1	2	3	2	2	1	2
2	2	2	1	2	3	2	2	1	3
9	2	2	2	2	2	2	2	2	2
9	1	2	1	2	2	1	2	1	2
2	3	2	1	2	2	1	2	1	2
2	1	2	1	2	3	2	3	2	2
1	2	2	1	2	2	1	2	1	2
2	3	2	2	1	2	2	2	2	2
1	1	2	2	3	2	2	1	1	2
2	2	1	2	2	2	1	2	2	1
2	3	2	2	1	2	2	2	1	2
2	3	2	2	2	3	3	2	3	3
2	9	2	2	2	9	2	2	2	9
3	3	3	3	2	2	2	3	2	2
3	2	1	2	3	2	2	2	2	2
2	2	2	2	2	2	2	2	2	3
9	9	9	9	9	9	9	9	2	9
3	3	3	3	3	3	3	3	3	3
2	1	2	1	2	3	2	1	1	1
2	2	2	2	2	2	2	2	2	2
2	2	1	1	2	2	1	1	1	1
3	3	3	3	3	3	3	3	3	3
3	2	2	2	2	2	2	2	2	3
2	2	2	2	2	2	2	2	2	9
3	3	3	3	3	2	2	3	3	2
1	1	2	2	1	2	2	1	2	1
1	1	1	2	1	1	1	2	2	1
3	3	3	3	3	3	3	3	3	3
1	2	2	1	2	3	1	2	2	2
1	2	2	3	2	1	2	2	2	2
2	2	1	1	2	2	2	3	2	2
1	2	2	2	1	1	1	1	1	2
2	2	1	3	2	1	1	1	1	2

2	1	2	2	1	2	2	1	1	1
3	2	2	2	1	2	2	2	1	2
2	2	2	1	2	2	2	2	1	1
2	3	2	2	1	2	2	1	2	2
3	2	1	2	1	3	2	3	2	3
2	2	2	1	2	2	1	1	1	1
2	2	2	2	2	3	2	1	1	3
2	2	3	2	2	2	1	2	1	1
1	1	3	3	2	3	1	1	1	1
2	2	3	2	1	3	2	2	2	2
2	3	2	3	2	2	2	3	3	3
2	2	2	2	2	2	2	2	2	2
2	1	2	1	3	1	3	1	1	2
2	3	3	1	2	1	2	2	3	3
1	2	3	2	1	3	1	2	1	1
2	2	2	2	2	2	2	2	2	2
2	2	2	2	2	2	2	2	2	2
2	2	2	2	2	2	2	2	2	1
2	3	3	1	3	3	2	1	3	3
2	2	2	2	2	2	2	2	2	2
2	2	3	2	2	2	2	2	1	2
2	2	2	2	2	2	2	2	2	2
1	1	3	3	2	1	3	2	1	1
3	2	3	2	3	2	3	2	2	1
1	2	2	2	2	2	2	2	1	1
2	2	2	2	2	3	3	3	3	3
2	2	1	2	3	2	2	2	2	2
2	2	2	1	2	2	3	2	1	2
1	2	2	2	2	3	3	3	2	2
2	2	2	2	2	2	2	2	2	2
1	1	3	2	2	3	1	1	1	1
1	1	3	3	1	3	1	1	1	1
2	2	2	2	2	2	2	2	2	3
2	2	2	2	2	2	2	2	2	2
2	2	2	2	2	2	2	1	1	2
2	2	2	2	2	2	2	2	2	2
1	2	3	1	2	3	2	1	2	1
2	2	2	2	2	2	2	2	2	2
2	3	2	2	3	3	2	2	2	1
1	3	3	3	2	3	2	1	2	3
2	1	3	1	1	3	1	1	1	1
2	2	2	2	2	2	2	2	2	1
2	2	2	2	2	2	2	2	2	2
2	2	3	3	2	2	3	3	2	2
2	2	2	2	2	2	2	2	2	2
2	2	2	2	2	2	2	2	2	1
2	2	2	2	2	2	2	2	2	3

2	2	2	2	2	2	2	2	2	2
9	2	2	2	1	2	2	1	1	1
2	2	2	2	2	2	2	2	2	2
2	2	2	2	2	2	2	2	2	2
2	2	2	2	2	2	2	2	2	2
2	2	2	2	2	2	2	2	2	1
2	2	2	2	2	2	2	2	2	2
1	2	3	1	3	2	2	2	2	3
2	2	2	2	2	2	2	2	2	2
9	9	9	9	9	9	9	9	9	9
2	1	2	2	3	1	2	2	1	3
2	1	2	1	2	1	1	2	1	1
1	2	3	1	2	3	1	2	2	1
2	2	2	2	2	2	2	2	2	2
2	2	2	2	2	2	2	2	2	2
2	2	1	2	2	2	2	2	2	1
2	2	2	2	2	2	2	2	2	2
2	2	2	2	2	2	2	2	2	2
3	3	3	3	3	3	3	3	3	3
9	1	1	1	1	1	1	1	3	3
3	3	3	3	3	3	3	3	3	3
3	3	3	3	3	3	3	3	3	3
2	2	2	2	2	2	2	2	2	2
1	1	2	2	2	3	3	2	2	2
2	2	2	2	1	2	2	2	2	2
1	2	2	2	1	1	1	1	1	1
3	3	1	2	2	2	1	2	2	2
2	2	2	2	2	2	2	3	2	1
2	1	1	1	1	1	1	1	1	1
1	9	9	9	9	9	9	9	9	9
1	2	1	2	1	1	2	1	1	1
2	3	2	3	3	2	3	2	2	3
2	1	3	2	2	3	3	3	3	2
3	3	3	3	3	3	3	3	3	3
1	2	2	2	2	2	2	2	1	1
3	2	2	3	3	2	2	2	2	1
3	2	2	3	3	2	2	2	2	1
1	2	2	3	1	2	2	1	2	1
2	2	2	2	2	2	2	2	2	2
2	2	2	2	3	3	3	2	2	2
2	2	2	2	2	2	2	2	1	2
3	3	3	3	3	3	3	3	1	1
2	1	2	2	2	2	2	2	2	2
3	3	3	3	3	3	3	3	1	1
3	3	3	3	3	3	3	3	3	3
2	3	3	1	1	3	3	3	1	1
1	1	2	1	1	1	1	1	1	1

1	2	2	2	2	2	2	2	2	2
2	2	3	3	3	3	3	2	2	2
1	2	3	2	2	2	2	2	2	3
2	2	2	2	2	2	2	2	2	2
3	3	3	3	3	3	3	3	2	2
9	9	2	9	9	9	9	9	1	9
2	2	2	2	2	2	2	2	2	2
2	2	2	2	2	2	2	2	2	2
1	1	3	3	1	3	1	1	1	1
3	3	3	3	3	3	3	3	2	2
2	3	2	1	1	1	1	1	1	1
2	2	2	2	2	2	2	2	1	1
9	9	9	9	9	9	9	9	1	9
1	9	9	9	9	9	9	9	9	1
1	9	9	9	9	9	9	9	9	9
2	2	3	2	2	2	2	3	1	2
2	2	3	2	2	2	2	2	1	2
9	9	9	9	9	9	9	9	3	9
2	2	2	2	1	3	1	1	1	1
2	2	3	3	2	3	1	1	1	1
1	1	1	2	2	2	2	1	1	1
2	2	2	2	1	2	1	1	1	1
2	2	2	2	2	2	1	2	1	1
2	2	2	2	2	2	2	2	2	2
2	2	2	2	2	2	2	2	2	2
2	2	2	2	2	2	2	2	2	2
2	2	2	2	2	2	2	2	2	2
2	2	2	2	2	2	2	2	1	1
9	9	9	9	9	9	9	9	9	9
3	3	3	3	3	3	1	3	3	3
2	2	2	2	1	2	1	1	1	3
3	2	1	3	2	1	2	1	2	2
2	2	1	2	2	2	1	1	1	1
3	3	3	2	2	2	2	2	2	2
2	2	2	2	2	2	1	2	2	2
1	1	2	2	1	1	2	2	1	1
2	2	1	2	3	2	2	2	2	2
2	2	2	2	2	2	2	2	2	2
2	2	2	2	2	2	2	2	2	3
2	2	2	2	2	2	2	2	2	2
2	1	1	1	1	1	2	1	2	1
2	2	1	2	2	2	1	2	2	2
2	3	2	2	2	2	2	2	2	1
2	2	3	2	2	2	2	2	2	2
2	2	2	2	2	2	2	2	2	2
2	2	2	2	2	2	2	2	2	2
2	2	3	2	2	2	2	2	2	2

1	1	2	2	2	2	1	1	1	1
2	1	1	1	3	2	2	2	2	2
9	9	9	9	9	9	9	9	9	9
3	1	3	2	1	3	1	2	2	1
3	2	1	1	1	3	1	1	1	1
2	2	2	2	2	2	2	2	2	2
2	2	2	2	2	2	2	2	2	2
1	2	3	2	1	3	1	1	1	1
1	3	3	3	1	3	1	1	2	2
1	2	2	3	2	2	2	2	2	2
2	2	2	2	2	2	2	2	1	1
2	2	2	2	2	2	2	2	2	2
1	2	3	1	2	3	1	3	1	2
1	1	2	3	1	3	1	1	1	1
2	9	3	3	1	2	2	1	1	1
1	3	2	3	1	2	1	2	1	1
9	9	9	9	9	9	9	9	9	9
9	9	9	9	9	9	9	9	2	3
2	2	3	1	1	3	1	1	1	3
1	1	1	1	1	1	1	1	1	1
2	2	2	2	2	2	2	2	2	2
9	9	9	9	9	9	9	9	9	9
3	1	1	1	1	1	1	3	3	3
1	3	1	3	2	1	3	2	3	2
2	2	2	2	2	2	2	2	2	2
9	9	9	9	9	9	9	9	2	9
9	9	9	9	9	9	9	9	2	9
3	1	2	1	2	3	2	2	1	2
9	9	9	9	9	9	9	9	2	9
9	9	1	9	9	9	9	9	9	9
9	9	2	9	9	9	9	9	9	9
9	9	9	9	9	9	9	9	9	9
9	9	9	9	9	9	9	9	1	9
1	2	3	2	3	1	1	1	1	1
3	3	2	1	3	2	3	1	1	2
1	2	9	2	2	1	2	3	1	2
1	2	3	2	2	1	2	3	1	2
3	3	3	2	2	2	2	2	2	2
2	2	2	3	2	2	2	2	2	2
2	2	2	1	1	1	1	1	3	3
2	2	2	2	2	2	2	2	2	2
3	3	3	3	1	3	1	3	1	3
2	2	2	2	2	3	3	2	2	2
2	2	2	2	2	2	2	2	1	2
2	2	2	2	2	2	2	2	1	2
2	2	2	2	2	2	2	2	1	2
1	1	1	2	1	2	2	2	2	2

2	2	1	9	1	3	1	1	1	2
1	2	1	3	1	2	3	2	2	1
3	1	3	3	3	3	3	3	1	3
9	9	9	9	9	9	9	9	9	9
9	9	9	9	9	9	9	9	9	9
1	9	2	2	3	2	1	1	2	2
1	2	2	2	2	2	2	1	1	1
9	9	9	9	9	9	9	9	9	9
9	9	9	9	9	9	9	9	9	9
1	1	3	2	1	3	1	2	1	2
2	2	2	2	2	2	2	2	2	2
9	1	3	2	2	2	2	2	2	2
2	1	1	1	1	2	2	2	2	2
9	9	9	9	9	9	9	9	9	9
9	9	9	9	9	9	9	9	9	9
9	9	9	9	9	9	9	9	9	9
2	2	2	3	1	2	9	1	1	1
9	9	9	9	9	9	9	9	9	9
9	9	9	9	9	9	9	9	9	9
9	9	9	9	9	9	9	9	9	9
9	9	9	9	9	9	9	9	9	1
9	9	9	9	9	9	9	9	9	9
9	9	9	9	9	9	9	9	9	9
9	9	9	9	9	9	9	9	9	9
2	2	2	2	2	2	2	2	2	2
2	2	2	2	2	2	2	2	1	2
3	2	3	2	1	3	2	2	2	2
2	2	2	2	2	2	2	2	2	2
2	3	3	3	2	3	1	1	2	3
2	2	2	2	2	2	2	2	1	1
3	3	3	3	3	3	3	3	2	2
3	3	3	3	3	3	3	3	2	2
2	2	2	2	2	2	2	2	2	2
3	2	2	3	2	2	1	1	2	2
2	2	2	2	2	2	2	2	2	2
1	3	3	3	3	3	3	1	1	1
2	2	2	2	2	2	2	2	1	1
2	2	2	2	2	2	2	2	2	2
1	3	3	3	1	3	2	1	2	2
2	2	2	2	2	2	2	2	2	2
2	2	2	2	2	2	2	2	1	1
2	2	2	2	2	2	2	3	1	2
1	2	2	1	1	1	1	1	2	2
9	9	9	9	9	9	9	9	2	9
9	9	2	9	9	9	9	9	2	9
2	2	2	2	1	1	1	2	2	1
1	2	2	3	2	2	2	2	2	1

1	1	1	1	1	2	2	2	2	1
2	2	1	1	1	2	2	2	1	1
1	1	2	2	3	2	2	2	1	2
2	2	2	2	2	2	2	2	2	2
2	2	2	2	2	2	2	2	2	1
3	2	3	2	3	2	1	1	1	1
2	2	2	1	2	3	1	2	2	2
3	1	2	3	1	2	3	1	1	2
9	9	9	9	9	9	9	9	9	9
2	2	2	3	2	3	2	3	2	1
1	2	2	2	2	2	2	2	2	2
2	2	2	2	2	2	2	2	2	2
2	2	2	2	2	2	2	2	1	3
2	2	2	2	2	2	2	2	2	2
2	2	3	2	3	2	3	2	2	3
2	1	2	1	3	2	3	3	2	3
3	2	3	2	3	1	2	3	2	3
3	1	2	3	1	2	3	1	1	2
2	2	2	2	2	3	3	3	2	2
2	2	2	2	2	2	2	2	1	1
1	1	1	2	3	2	3	2	1	1
2	2	2	2	2	2	2	2	2	2
2	2	2	2	2	2	2	2	2	2
2	2	3	3	1	1	2	3	1	1
2	2	2	2	2	2	2	2	2	2
2	2	3	3	2	2	1	1	2	1
2	2	2	2	2	2	2	2	2	2
9	1	2	2	9	2	2	2	2	2
2	1	2	3	1	2	3	2	1	2
9	9	9	9	9	9	9	9	9	9
1	2	1	2	1	1	2	2	2	2
9	9	9	9	9	9	9	9	9	9
9	9	9	9	9	9	9	9	9	9
1	1	1	1	1	1	1	1	1	1
1	2	2	3	1	2	1	3	1	3
3	2	3	1	2	1	2	2	3	2
1	1	1	2	3	2	3	1	1	2
3	2	3	2	3	2	2	2	2	3
2	2	2	2	2	2	2	2	2	2
1	2	1	1	1	1	2	2	1	2
1	1	3	2	1	1	2	3	3	2
2	1	2	2	1	2	1	1	1	2
2	2	2	2	2	2	2	2	2	2
1	2	2	2	2	2	2	2	2	2
1	2	2	1	2	3	3	3	3	2
2	2	2	2	2	2	2	2	2	2
2	2	2	2	2	2	2	2	2	2

2	1	1	1	2	1	1	2	1	1
2	1	3	2	2	1	2	2	2	2
2	2	2	1	2	2	2	2	2	2
2	1	2	1	9	9	9	9	1	2
2	3	3	1	2	3	2	1	1	2
2	2	2	2	2	2	2	2	2	2
2	2	2	2	2	2	2	2	2	2
2	1	2	2	3	2	3	1	2	2
1	9	9	9	9	9	9	9	9	9
2	2	2	2	2	2	2	2	2	2
2	2	2	2	2	2	2	2	2	2
1	1	2	2	1	3	1	2	2	1
9	9	9	9	9	9	9	9	9	9
2	1	2	2	2	2	2	1	1	2
2	3	2	2	2	2	9	9	9	9
3	3	1	3	3	3	1	1	1	1
1	1	9	9	9	9	9	9	1	1
2	3	9	1	2	9	3	1	1	3
1	3	3	3	2	3	2	1	2	1
2	2	2	2	1	3	1	9	1	1
2	1	2	1	2	1	2	1	1	1
3	3	3	3	3	3	3	3	3	3
2	2	2	2	2	2	2	2	2	2
2	2	2	2	2	2	2	2	2	2
2	1	2	3	1	2	1	2	2	1
2	2	2	2	2	2	2	2	2	2
2	2	2	2	2	2	2	2	2	2
3	3	3	2	2	2	2	2	2	3
3	1	2	1	1	3	1	1	1	1
2	2	2	2	1	3	2	2	1	1
2	2	2	2	2	2	2	2	1	1
2	2	2	2	2	2	2	2	2	2
2	1	2	1	2	2	1	3	2	2
2	2	2	2	2	2	2	2	2	2
2	2	2	3	2	3	2	2	2	2
9	9	9	9	9	9	9	9	9	9
2	2	2	2	2	2	2	2	2	2
9	9	9	9	9	9	9	9	9	9
2	3	2	1	2	3	2	1	2	1
2	2	1	3	2	1	2	3	1	2
2	2	2	2	2	2	2	2	2	2
1	1	2	2	1	2	1	2	9	9
2	2	2	2	2	2	2	2	2	2
2	2	2	2	2	2	2	2	2	2
9	9	9	9	9	9	9	9	9	9
9	9	9	9	9	9	9	9	9	9
9	9	9	9	9	9	9	9	9	9

3	3	3	3	2	2	2	2	3	3
2	1	1	1	2	9	1	3	2	3
9	9	9	9	9	9	9	9	9	9
1	1	1	1	1	2	2	1	1	1
2	2	1	1	1	1	2	2	2	2
2	3	1	2	2	3	3	2	1	1
2	2	2	2	2	2	2	2	2	2
3	2	9	1	9	1	9	9	9	9
3	3	3	3	3	3	3	3	3	3
9	9	9	9	9	9	9	9	9	9
3	3	3	3	3	3	3	3	3	3
3	1	3	1	3	1	3	1	1	3
1	2	2	2	2	3	1	2	9	1
3	9	2	3	2	2	2	2	1	1
2	2	2	2	2	2	2	2	2	2
3	3	3	3	3	3	3	3	9	3
3	3	3	2	3	3	1	1	3	3
9	9	9	9	9	9	9	9	9	9
9	9	9	9	9	9	9	9	9	9
2	2	3	1	1	2	1	2	3	1
2	2	2	2	2	2	2	2	1	2
2	2	2	2	2	2	2	2	1	1
2	2	2	2	2	2	2	2	2	2
3	3	3	3	3	3	3	3	3	3
3	2	1	2	3	2	1	2	1	2
2	2	2	2	3	3	2	1	1	1
2	2	9	2	2	2	2	1	2	1
2	2	1	2	2	3	3	3	2	2
9	9	9	9	9	9	9	9	9	9
9	9	9	9	9	9	9	9	9	9
9	9	9	9	9	9	9	9	9	9
9	9	9	9	9	9	9	9	9	9
9	9	9	9	9	9	9	9	9	9
2	2	2	2	2	2	2	2	3	2
2	3	2	1	2	1	3	1	1	2
2	2	2	2	2	2	2	2	9	9
9	9	9	9	9	9	9	9	9	9
1	2	1	3	1	3	3	2	9	9
1	1	9	1	1	9	1	1	1	9
9	9	9	9	9	9	9	9	9	9
2	2	2	2	2	2	2	2	2	2
2	2	9	9	1	2	9	1	2	1
2	2	1	2	1	2	1	2	2	1
2	2	2	2	2	2	2	2	2	2
1	2	3	2	2	1	2	3	1	2
2	1	3	2	2	3	1	2	1	2
3	2	2	1	2	3	2	3	1	2

2	2	2	2	2	2	2	2	3	3
2	2	2	2	2	2	2	2	2	2
9	9	9	9	9	9	9	9	9	9
1	1	1	2	2	3	3	3	1	1
1	2	2	2	3	3	3	1	1	1
1	1	1	3	1	1	2	1	1	1
1	9	2	9	2	3	1	2	1	9
2	2	2	2	2	2	2	2	2	2
2	2	2	2	2	2	2	2	2	2
2	3	2	1	1	2	2	3	1	1
3	2	2	2	2	2	2	2	2	2
2	3	2	2	2	1	2	2	3	2
9	9	9	9	9	9	9	9	9	9
1	1	1	1	1	1	1	1	1	1
2	2	2	1	2	3	2	2	1	2
3	1	1	1	1	1	2	2	1	2
2	3	3	2	2	1	1	1	1	1
9	9	9	9	9	9	9	9	9	9
2	2	2	1	3	2	3	2	3	2
9	9	9	9	9	9	9	9	9	9
9	9	9	9	9	9	9	9	9	9
3	2	1	1	2	3	3	3	1	2
9	9	9	9	9	9	9	9	9	9
9	9	9	9	9	9	9	9	9	9
9	9	9	9	9	9	9	9	9	1
9	9	9	9	9	9	9	9	9	9
1	2	1	2	2	2	2	2	1	1
1	2	3	2	2	3	2	2	2	2
9	9	9	9	9	9	9	9	9	9
2	2	2	3	3	2	2	1	2	2
1	3	1	3	1	3	1	3	1	3
9	9	9	9	9	9	9	9	9	9
2	2	2	2	2	2	2	2	2	2
2	2	2	2	2	2	2	2	2	2
2	2	2	2	2	2	2	2	2	2
2	2	3	2	2	2	3	2	2	3
2	3	2	2	2	2	1	2	1	3
2	2	2	2	2	2	2	2	2	2
2	2	2	2	2	2	2	2	1	1
9	9	9	9	9	9	9	9	9	9
1	2	9	1	3	2	3	3	3	2
1	1	3	3	2	2	1	1	1	1
2	2	2	2	2	2	2	2	2	2
1	3	3	1	2	3	1	1	1	1
1	1	3	1	2	2	1	2	3	1
1	1	1	1	1	1	1	1	1	1
2	1	2	2	2	3	1	2	2	2

1	1	1	1	1	1	3	2	2	2
1	1	2	2	2	2	2	2	2	2
1	3	1	2	3	2	3	2	1	2
2	2	2	3	2	2	2	2	2	2
2	2	2	2	2	2	2	2	1	1
2	2	2	2	2	2	2	2	1	1
2	2	2	2	2	2	2	2	2	2
2	2	2	2	2	2	2	2	3	3
2	2	2	2	2	2	2	2	2	2
9	9	9	9	9	9	9	9	9	9
3	3	3	3	1	3	1	1	1	1
1	3	1	3	2	1	2	3	1	2
9	9	9	9	9	9	9	9	9	9
3	2	1	2	1	2	1	2	2	1
9	9	9	9	9	9	9	9	9	9
9	9	9	9	9	9	9	9	9	9
3	3	3	1	1	1	1	1	3	3
2	2	2	2	2	2	2	2	2	2
2	2	2	2	2	9	2	3	2	3
2	2	2	2	2	2	2	2	1	1
1	1	2	2	2	2	2	2	1	1
2	2	2	2	2	2	2	2	2	2
9	9	9	9	9	9	9	9	9	9
2	2	1	2	1	2	1	2	1	2
1	1	1	1	1	1	1	1	1	1
3	3	3	3	3	3	3	3	3	3
2	3	3	2	1	1	2	3	1	3
2	2	3	2	2	3	2	2	2	3
3	2	2	2	3	3	3	3	2	1
2	2	2	2	2	2	2	2	2	2
2	2	2	2	2	2	2	2	1	1
1	9	9	9	9	9	9	9	9	9
2	2	2	2	2	2	2	2	2	2
2	3	2	2	3	1	2	3	3	1
1	3	1	2	1	2	3	1	3	3
3	1	1	3	1	3	1	2	1	3
2	2	2	2	2	2	2	2	2	2
2	2	2	2	2	2	2	2	2	2
1	2	3	1	3	2	3	2	3	3
2	2	2	3	3	3	3	3	2	2
3	2	3	3	2	3	2	2	3	3
2	2	2	2	2	2	3	3	2	3
2	1	1	1	1	3	1	3	2	2
3	2	3	2	2	3	2	3	3	2
9	9	9	9	9	9	9	9	9	9
3	1	2	3	1	2	3	1	1	2
1	2	2	2	3	3	3	3	3	3

2	1	2	1	1	2	2	2	1	1
9	3	3	1	3	9	1	1	1	3
3	9	1	9	3	9	3	9	1	3
2	1	2	2	2	2	2	2	2	2
9	9	9	9	9	9	9	9	1	9
9	9	9	9	9	9	9	9	9	9
9	9	9	9	9	9	9	9	1	9
1	2	2	2	2	2	2	2	1	2
1	1	2	3	1	3	1	1	1	3
2	2	3	3	1	3	2	2	2	2
2	2	2	2	2	2	2	2	1	1
3	3	3	2	3	3	1	2	2	2
2	2	2	2	2	2	2	2	2	2
2	2	2	2	2	2	2	2	2	2
2	2	2	2	2	2	2	2	2	2
2	2	2	2	2	2	1	3	2	2
2	2	3	2	3	3	3	3	2	2
2	2	2	2	2	2	2	2	2	2
2	2	2	2	2	2	2	2	2	2
3	2	2	2	1	2	1	3	2	2
2	2	2	2	2	2	1	2	2	2
1	1	1	2	2	2	1	1	9	9
1	9	2	9	1	9	2	9	1	2
2	2	2	2	2	2	2	2	2	2
1	1	1	1	1	1	1	1	1	1
2	2	2	1	1	3	3	3	3	2
2	2	2	2	2	2	2	2	2	2
9	9	9	9	9	9	9	9	9	9
1	2	2	2	2	2	1	1	1	2
2	1	2	2	1	2	2	2	2	2
1	2	3	2	2	3	1	2	1	1
2	2	2	1	1	2	2	2	2	1
2	1	1	2	3	2	1	2	2	1
1	2	3	2	1	3	2	1	2	1
2	2	3	2	2	3	1	1	1	1
1	3	3	3	1	3	2	2	2	9
2	2	2	2	2	2	2	2	2	2
2	2	2	2	2	2	2	2	1	2
2	2	2	2	2	2	2	2	2	2
9	9	9	9	9	9	9	9	9	9
2	2	2	2	2	2	2	2	1	1
1	2	2	3	3	1	2	2	1	2
2	1	1	2	3	3	2	1	2	1
3	2	2	2	2	2	2	2	2	2
1	1	2	2	2	3	3	3	1	1
2	2	2	2	2	2	2	2	2	2
2	2	2	2	2	2	2	2	2	2

2	2	2	2	2	2	2	2	2	2
1	3	9	9	9	9	1	3	3	9
2	2	2	2	2	2	2	2	2	2
3	1	2	3	1	2	3	1	1	2
1	3	2	2	2	1	1	2	2	2
2	2	2	2	2	2	2	2	2	2
3	3	3	3	3	3	3	3	2	2
1	2	3	2	1	3	1	1	1	1
9	9	9	9	9	9	9	1	9	9
3	3	3	3	2	2	2	2	2	2
1	9	9	9	9	9	9	1	1	1
2	2	2	2	2	2	2	2	2	2
1	2	2	2	2	2	2	2	1	1
2	1	2	1	2	3	1	2	9	9
2	3	3	2	2	3	2	2	1	1
2	2	2	2	2	2	2	2	2	2
2	1	2	2	3	2	2	2	2	2
1	3	1	1	1	3	1	9	1	9
9	9	9	9	9	9	9	9	9	9
1	1	1	1	1	2	1	2	1	1
1	1	2	1	1	3	1	1	1	2
2	2	2	3	1	3	1	1	2	2
2	2	2	2	2	2	2	2	1	1
3	1	2	1	3	1	2	2	1	2
9	9	9	9	9	9	9	9	9	9
2	2	2	3	2	2	1	1	2	2
1	2	2	2	2	2	2	2	1	2
2	2	2	2	2	2	2	2	1	1
2	2	3	2	1	2	2	3	2	1
2	2	2	2	2	2	2	2	2	2
2	2	2	2	2	2	2	2	2	2
1	2	2	2	1	1	3	1	2	3
1	1	2	3	1	3	2	1	2	2
3	1	2	3	2	1	2	2	3	1
1	1	2	2	1	2	9	1	1	1
1	2	2	3	1	3	3	2	1	3
2	3	2	1	2	2	2	2	2	1
2	2	2	2	2	2	2	2	2	2
2	2	2	2	2	2	2	2	2	1
2	2	2	2	2	2	2	2	2	2
9	9	9	9	9	9	9	9	9	9
3	1	2	1	2	3	1	2	2	2
1	1	2	2	1	2	2	1	1	1
3	1	2	3	1	3	1	2	1	3
1	1	3	2	2	2	1	1	1	1
1	2	2	2	9	1	1	1	1	1
2	2	2	2	2	3	1	1	2	2

2	2	3	2	3	2	3	2	3	2
9	9	1	9	9	9	9	9	9	9
9	9	9	9	9	9	9	9	9	9
9	9	9	9	9	9	9	9	9	9
2	2	2	2	2	2	2	2	2	2
2	2	2	2	2	2	2	2	2	2
1	2	1	1	1	2	1	1	2	1
1	1	1	1	1	1	1	1	1	1
9	9	9	9	9	9	9	9	9	9
9	9	9	9	9	9	9	9	9	9
2	2	2	2	2	2	2	2	1	1
2	2	2	2	2	2	1	1	2	3
2	2	2	2	2	2	2	2	2	2
2	2	2	2	2	2	2	2	2	2
2	2	2	2	2	2	1	1	2	3
1	3	2	3	2	3	1	1	1	1
1	3	3	1	1	3	1	3	1	1
2	2	3	3	2	3	1	1	1	2
2	2	2	2	2	2	1	1	1	1
9	9	9	9	9	9	9	9	9	9
2	2	2	2	2	2	2	2	1	3
2	1	3	3	2	3	1	1	1	1
2	2	2	2	2	2	2	2	1	1
3	1	3	1	3	1	3	2	1	3
3	1	2	3	1	2	3	2	1	2
2	2	2	2	2	2	1	1	1	1
1	2	2	3	2	2	1	2	1	2
1	2	3	2	1	3	2	1	1	2
2	2	3	1	2	3	1	2	2	1
2	2	2	2	2	2	2	2	1	1
1	1	1	2	1	3	1	2	2	2
2	1	3	1	2	3	2	1	1	2
2	2	2	1	3	1	2	3	2	3
2	2	3	2	1	1	1	2	1	1
2	3	3	2	1	2	2	1	1	1
2	3	3	2	3	2	2	1	1	1
2	1	2	2	1	2	9	1	1	1
9	9	2	3	1	9	1	1	1	1
2	2	2	2	2	2	2	2	1	2
2	1	2	1	1	2	3	3	1	1
1	2	2	2	1	1	2	1	2	2
2	2	1	2	2	2	2	2	1	1
9	9	9	9	9	9	9	9	9	9
1	1	1	3	1	3	1	1	1	1
1	2	2	1	2	1	2	1	1	2
1	2	2	2	1	3	1	1	1	1
1	2	2	1	2	1	2	1	1	2

1	2	3	3	1	3	1	1	1	1
9	9	9	9	9	9	9	9	2	9
2	3	2	1	2	3	1	2	1	3
1	2	2	2	2	2	1	1	2	1
2	2	2	2	2	2	2	2	2	3
1	1	3	3	1	3	1	1	1	1
3	2	2	1	2	3	1	2	2	1
2	9	2	1	9	3	9	2	2	2
2	3	2	9	1	3	9	2	2	2
9	9	9	9	9	9	9	9	9	9
2	2	2	2	1	2	1	3	1	2
1	1	2	1	2	2	2	1	1	1
1	3	1	3	1	3	1	3	1	3
2	1	2	3	1	2	3	1	1	2
2	2	2	2	2	2	2	2	2	2
9	9	9	9	9	9	9	9	9	9
9	9	9	9	9	9	9	9	9	9
2	2	2	3	1	2	3	3	2	2
2	2	2	3	3	3	3	3	2	2
2	2	1	1	1	2	2	2	1	2
9	9	9	9	9	9	9	9	9	9
2	2	1	1	1	2	2	2	1	1
2	2	2	2	2	2	2	2	1	1
2	1	2	2	1	1	1	2	1	1
2	2	2	2	1	1	2	2	1	2
2	2	2	1	1	1	1	1	1	1
2	2	1	2	2	2	1	1	1	2
2	1	2	2	2	3	3	3	2	2
1	1	1	2	2	2	1	1	1	1
1	1	2	1	2	2	1	1	1	2
9	9	9	9	9	9	9	9	9	9
9	9	9	9	9	9	9	9	9	9
9	9	9	9	9	9	9	9	9	9
2	2	2	3	2	2	2	1	2	2
1	2	2	3	1	3	1	1	1	1
2	2	2	2	1	2	2	2	2	2
1	1	2	2	1	2	2	1	1	1
2	2	2	2	2	2	2	2	1	1
1	2	1	1	1	1	2	1	1	2
2	2	2	2	2	2	2	2	3	2
3	3	3	3	3	3	3	3	1	1
1	1	3	2	1	3	1	1	1	1
2	2	2	2	1	1	2	2	1	2
2	2	2	2	2	2	2	2	1	1
2	2	2	2	2	2	2	2	2	2
1	1	1	1	1	1	1	1	1	1
3	3	1	3	3	3	3	3	1	1

2	2	2	1	2	2	2	2	9	9
2	2	2	2	2	2	2	2	2	2
2	2	2	3	2	2	2	3	9	9
1	3	3	1	3	1	1	1	1	1
3	3	3	3	1	3	3	3	3	3
2	3	2	2	3	2	1	2	2	3
1	1	2	2	2	2	2	2	1	1
9	9	9	9	9	9	9	9	1	9
2	2	2	2	2	2	2	2	2	2
9	9	9	9	9	9	9	9	1	9
9	1	1	1	9	9	9	9	1	9
9	9	9	9	9	9	9	9	9	9
2	2	2	1	2	2	2	2	1	1
9	9	9	9	9	9	9	9	9	9
2	2	2	2	3	2	1	2	1	2
2	2	2	3	2	3	1	2	1	1
2	1	1	1	2	1	1	1	1	1
1	1	1	1	1	1	1	1	1	1
2	2	2	2	2	2	2	2	2	2
3	2	3	3	2	3	2	3	1	1
2	2	2	2	2	2	2	2	2	1
2	2	2	2	2	2	2	2	2	2
2	2	2	3	1	3	1	1	1	1
9	3	9	2	3	9	3	2	2	2
1	2	1	2	1	2	3	2	2	1
2	1	3	1	1	1	1	1	1	2
2	2	2	2	2	2	2	2	2	2
1	2	3	3	2	3	1	1	2	2
2	2	2	1	2	2	2	2	2	2
2	2	2	3	2	2	3	2	2	2
2	2	2	2	2	2	2	2	2	2
3	2	3	3	2	2	2	2	2	2
1	2	3	3	1	3	1	1	1	3
3	3	1	2	1	2	1	2	2	2
9	9	9	9	9	9	9	9	3	9
2	2	2	2	2	2	2	2	2	2
2	2	2	2	2	2	2	2	2	2
2	2	2	2	2	2	2	2	2	1
2	2	2	2	2	2	2	2	2	2
2	2	2	2	2	2	2	2	2	2
1	1	1	3	3	1	1	3	1	3
9	9	9	9	9	9	9	9	9	2
9	9	9	9	9	9	9	9	9	2
2	2	2	2	2	2	2	2	2	2
9	9	1	9	9	9	9	9	9	9
2	2	2	9	2	2	2	2	2	2
2	1	2	2	2	2	2	2	1	1

2	2	2	2	2	2	2	2	2	2
1	1	1	1	2	2	1	1	1	2
1	1	3	2	2	1	2	3	2	3
2	2	2	2	3	2	2	2	2	2
2	2	2	2	2	2	2	2	2	2
2	1	1	2	2	3	2	2	2	2
2	3	3	2	2	2	2	2	2	2
2	2	2	2	2	2	2	2	1	1
2	2	2	1	1	2	1	1	1	1
1	2	1	3	1	2	3	2	1	3
1	2	2	2	1	3	2	2	1	1
3	1	3	2	1	2	2	2	2	3
3	3	1	3	1	3	1	3	3	1
2	2	2	2	2	2	2	2	2	2
1	2	2	1	3	2	1	2	2	1
2	2	2	2	2	2	2	2	2	2
2	2	1	1	2	2	1	2	1	2
2	2	2	2	2	2	2	2	2	2
1	2	2	1	2	2	2	2	2	2
1	1	1	1	1	1	2	1	2	2
2	2	3	2	2	2	2	1	2	2
2	2	2	2	2	2	2	2	2	2
2	2	2	3	2	2	1	2	2	2
2	2	2	2	2	2	2	2	2	2
1	1	1	2	2	2	2	1	1	1
2	2	2	2	2	2	2	2	2	2
2	2	2	2	2	2	2	2	2	2
2	1	2	2	1	1	1	1	1	1
2	3	3	3	1	3	3	1	1	1
2	3	2	2	2	1	2	2	1	2
9	9	9	9	9	9	9	9	9	9
2	2	3	2	2	1	1	2	1	2
2	2	1	2	2	1	2	2	1	2
2	2	3	3	2	2	2	2	2	2
2	2	1	2	2	2	2	2	2	2
2	2	2	2	3	2	2	2	2	1
2	1	2	2	3	2	2	2	1	2
2	2	3	2	2	2	1	2	2	2
3	1	2	3	2	1	2	3	1	2
2	2	2	2	1	2	2	2	3	3
1	1	1	2	1	1	1	1	1	1
2	3	2	2	2	2	2	2	2	2
1	3	3	3	1	3	1	1	1	1
1	1	1	2	2	3	3	2	1	1
1	1	1	2	1	1	1	1	1	1
3	3	1	1	1	1	1	1	1	2
2	3	3	2	2	2	2	1	1	1

2	3	3	2	1	2	2	1	1	1
2	3	3	3	2	2	1	1	1	1
2	3	2	2	1	2	2	3	1	2
2	3	2	2	1	2	2	2	1	2
2	1	2	2	2	2	2	1	1	1
2	2	3	3	2	2	2	2	1	2
1	1	1	2	2	2	1	1	1	2
2	2	2	2	2	2	2	2	2	2
1	3	2	1	2	3	2	2	3	2
1	1	2	1	1	3	1	1	1	2
1	1	2	2	1	2	9	1	1	1
2	2	2	3	3	2	2	2	1	9
2	2	2	2	2	2	2	2	3	3
2	2	2	2	2	2	2	2	2	2
3	3	3	3	3	2	3	3	1	1
2	2	2	2	2	2	2	2	1	1
1	1	1	1	1	2	3	2	2	2
2	2	3	1	2	2	2	3	1	1
2	2	2	2	2	2	2	2	2	3
3	3	3	3	9	3	2	2	3	3
2	2	2	1	1	2	2	2	1	1
3	3	3	3	3	3	3	3	1	3
2	2	2	2	2	2	2	2	2	2
1	2	3	3	2	2	2	2	1	1
2	1	2	2	3	1	2	1	1	3
2	3	2	3	1	2	1	2	1	1
2	3	2	2	2	2	2	2	2	2
2	1	2	2	2	2	2	2	2	2
1	2	2	2	1	2	1	1	2	2
2	2	2	2	2	2	2	2	2	2
2	2	1	1	2	2	1	1	2	2
1	2	2	1	9	2	9	1	1	1
1	1	2	1	3	3	3	3	1	1
2	1	2	1	1	3	1	1	1	1
1	2	1	3	2	1	3	2	2	1
1	3	2	2	2	2	2	3	1	1
3	1	2	1	2	1	2	3	2	2
2	2	3	3	3	1	1	1	1	1
3	2	1	2	2	1	2	1	2	3
2	2	2	3	1	9	1	1	2	2
1	2	3	3	1	2	2	1	2	1
3	3	3	2	2	3	2	3	2	1
2	2	2	2	2	2	2	2	1	2
1	2	2	3	1	3	1	1	1	1
1	1	3	3	2	2	1	2	1	2
3	2	2	2	2	2	2	2	1	2
2	2	2	2	2	2	2	2	1	2

2	2	2	2	2	2	2	2	2	2
2	2	2	2	2	2	2	1	1	2
2	2	1	2	2	1	1	2	2	2
2	2	2	1	1	3	1	1	2	1
2	2	1	3	3	2	2	2	1	2
2	2	2	1	2	2	2	3	1	2
2	2	2	2	2	2	2	2	2	2
1	2	2	2	2	2	2	1	1	2
9	9	9	9	9	9	9	9	9	9
2	2	2	2	2	2	2	2	2	2
2	1	3	1	1	3	1	1	1	1
2	2	2	2	2	2	2	2	2	2
1	1	1	1	1	1	1	1	2	2
3	1	2	3	1	2	3	1	2	3
1	1	1	1	1	1	1	1	1	1
2	2	2	2	2	2	2	2	2	2
2	2	2	2	2	2	2	2	2	2
3	3	3	3	3	3	3	3	1	1
3	2	1	2	3	2	1	2	1	2
2	2	2	2	2	9	9	9	2	2
2	2	1	2	2	2	3	3	2	2
2	3	3	3	2	2	2	1	1	2
2	2	2	2	2	2	2	2	2	2
2	1	2	2	3	2	2	2	1	2
1	1	1	1	1	1	1	1	1	1
2	2	2	2	2	2	2	2	2	2
2	2	2	2	2	2	2	2	2	2
2	2	2	2	2	2	2	2	2	2
3	2	1	2	2	1	2	1	2	3
2	2	2	2	2	9	9	9	2	2
3	1	2	1	2	1	2	3	2	2
9	9	9	9	9	9	9	9	9	9
2	2	2	2	2	2	2	2	2	2
3	9	1	9	3	9	1	9	1	3
1	2	2	2	2	1	1	1	1	1
2	2	2	1	1	9	1	1	2	1
1	3	1	3	1	1	1	1	1	1
3	1	2	3	1	2	3	1	1	2
2	2	2	2	2	2	2	2	2	2
2	1	3	2	1	3	2	1	2	1
1	1	2	2	2	2	1	1	1	2
1	1	2	2	2	2	1	1	1	2
2	1	2	1	2	3	1	1	1	1
1	1	3	2	1	3	3	1	1	2
2	2	3	2	2	2	2	2	2	2
2	2	2	2	2	2	2	2	2	2
2	2	2	2	2	2	2	2	2	2

2	1	2	3	2	1	3	2	1	2
2	2	2	2	2	2	2	2	1	1
1	1	1	1	1	1	1	1	1	1
2	3	2	2	2	3	1	1	2	2
2	2	2	2	2	2	2	2	2	2
2	2	2	2	2	3	1	1	1	1
1	1	2	2	2	3	1	1	1	1
9	9	9	9	9	9	9	9	3	9
9	9	9	9	9	9	9	9	3	9
9	9	9	2	9	9	9	9	3	9
3	2	2	1	3	1	3	2	2	2
3	3	2	1	3	2	2	3	2	2
3	3	1	2	1	3	1	2	3	2
1	3	1	1	2	3	1	1	3	2
3	3	3	1	3	2	1	3	3	3
2	2	3	3	1	3	1	1	2	2
2	2	2	3	1	3	1	1	2	2
2	2	1	3	2	1	1	3	3	2
3	1	1	2	1	2	1	2	3	2
2	1	2	1	1	1	2	3	2	1
2	3	2	1	1	1	1	2	3	2
2	3	3	1	1	2	2	1	2	1
2	2	2	2	2	1	3	3	3	3
2	2	2	1	3	1	3	3	2	2
2	3	1	1	3	3	1	1	2	2
2	2	2	3	1	3	1	1	2	2
2	2	1	1	3	1	3	3	2	2
2	2	2	2	2	2	2	2	9	3
2	3	1	1	2	2	2	2	2	3
2	3	1	1	3	1	3	3	2	3
1	3	1	1	2	3	1	1	3	2
1	3	1	1	2	3	1	1	3	2
2	2	2	2	2	2	2	2	1	1
2	2	2	2	2	2	2	2	1	1
1	1	3	3	1	3	1	1	1	1
1	1	3	3	1	3	1	1	1	1
1	1	3	3	1	3	1	1	2	2
1	1	3	3	1	3	1	1	2	2
1	1	3	3	1	3	1	1	2	2
1	1	3	3	1	3	1	1	1	1
3	3	1	1	2	2	2	2	3	3
2	2	2	1	2	1	2	2	2	3
2	2	2	1	2	1	2	2	2	3
2	2	2	1	2	1	2	2	2	3
2	2	2	1	2	1	2	2	2	3
2	2	1	2	9	2	9	2	3	9
2	2	3	2	9	3	2	2	3	9

9	3	2	3	1	1	3	2	3	2
2	2	3	3	2	2	3	3	3	3
3	2	1	2	3	2	3	2	2	1
3	3	2	1	2	1	2	3	2	2
2	3	2	1	2	3	1	1	1	2
3	1	1	1	1	1	3	3	9	9
3	3	2	1	3	3	3	3	3	3
1	2	1	2	1	2	2	1	2	9
9	9	9	9	9	9	9	9	3	9
9	9	9	9	9	9	9	9	9	9
2	2	2	2	2	2	2	2	2	2
9	3	1	9	3	9	2	2	3	9
3	2	2	2	2	9	3	3	9	3
3	3	1	1	3	1	3	3	3	3
9	9	9	9	9	9	9	9	9	9
2	2	2	2	2	2	2	2	2	2
2	2	2	2	2	2	2	2	2	2
9	9	9	9	9	9	9	9	2	9
3	1	2	1	1	2	3	1	2	2
2	9	9	9	9	9	9	9	3	9
3	3	2	2	2	2	2	2	3	3
1	3	1	2	2	3	1	3	3	2
3	3	1	1	2	2	2	1	2	2
2	2	2	2	2	2	2	2	9	3
9	9	9	9	9	9	9	9	3	9
9	9	9	9	9	9	9	9	9	9
1	3	2	3	3	2	1	3	3	2
9	9	9	9	9	1	9	9	9	9
3	2	2	3	3	2	3	3	3	3
9	9	9	9	9	9	9	9	9	9
3	1	1	1	3	3	3	1	3	3
9	9	9	9	9	9	9	9	2	9
3	3	1	2	2	1	3	2	3	3
2	2	2	2	3	1	3	3	3	3
2	2	2	2	3	1	3	3	3	3
2	2	2	2	2	2	2	2	3	3
3	2	2	2	2	2	2	2	3	3
2	2	2	2	2	2	2	2	2	2
2	2	2	2	2	2	2	2	3	3
9	9	9	9	9	9	9	9	2	9
9	9	9	9	9	9	9	9	2	9
9	9	9	9	9	9	9	9	2	9
9	9	9	9	9	9	9	9	3	9
9	9	9	9	9	9	9	9	2	9
9	9	9	9	9	9	9	9	1	9
2	3	3	2	3	2	3	2	2	1
1	3	2	3	3	2	1	3	3	2

9	9	9	9	9	9	9	9	9	9
2	2	2	2	2	2	2	2	3	3
1	3	2	3	2	1	2	1	2	2
2	2	2	1	2	3	2	2	3	1
3	2	2	9	2	2	1	3	2	2
1	2	3	2	1	2	1	2	3	3
9	9	9	9	9	9	9	9	3	9
1	1	3	3	1	3	1	1	1	1
2	2	2	2	2	2	2	2	2	2
9	9	9	9	9	9	9	9	2	9
3	1	2	3	3	2	9	3	1	3
9	9	9	9	9	9	9	9	9	9
9	9	9	9	9	9	9	9	2	9
9	9	9	9	9	9	9	9	2	9
3	2	1	1	3	1	3	3	3	3
9	9	9	9	9	9	9	9	2	9
2	2	2	1	2	3	3	3	3	3
9	9	9	9	9	9	9	9	2	9
3	1	2	1	2	2	1	3	2	3
3	2	2	2	2	1	3	3	3	3
3	2	1	2	3	3	3	2	3	3
9	3	2	3	3	2	1	2	1	2
2	2	2	3	1	3	1	1	3	3
3	3	2	3	2	2	1	1	3	2
3	1	3	3	2	2	2	2	1	2
2	3	2	3	2	1	2	3	2	2
3	3	1	1	3	1	3	3	3	3
3	2	2	3	2	2	3	3	2	2
2	3	3	3	1	2	3	3	2	2
2	3	1	2	3	2	3	3	2	3
3	1	1	2	3	2	3	2	3	2
3	3	1	1	3	1	3	3	3	3
9	9	9	1	9	9	9	9	9	9
9	9	9	9	3	9	9	9	9	9
9	9	9	9	9	9	9	9	9	9
9	9	9	1	9	9	9	9	9	9
9	9	9	9	9	9	9	3	9	9
9	9	9	9	9	9	9	1	9	9
9	9	1	9	9	9	9	9	9	9
9	9	9	9	9	9	9	9	2	9
9	9	9	9	9	9	9	9	2	9
9	9	9	9	9	9	9	9	2	9
9	9	9	9	9	9	9	9	2	9
9	9	9	9	9	9	9	9	2	9
9	9	9	9	9	9	9	9	2	9
3	2	1	2	1	3	1	3	3	3
3	3	1	2	3	2	3	3	3	2

3	3	1	2	2	1	3	2	3	3
9	9	9	2	9	9	9	9	9	9
9	3	9	9	9	1	9	9	3	9
3	2	2	1	2	1	2	2	2	3
9	9	9	9	9	9	9	9	2	9
9	9	9	9	9	9	9	9	3	9
9	9	9	9	9	9	9	9	3	9
9	9	9	9	9	9	9	9	3	9
9	9	9	9	9	9	9	9	3	9
2	2	2	2	2	2	2	2	2	2
3	2	1	2	3	2	3	2	3	2
2	3	2	1	2	1	2	3	3	2
2	2	2	2	2	2	2	2	2	2
2	2	3	3	1	2	2	3	3	3
2	2	2	1	3	2	2	1	3	2
3	2	2	2	1	3	2	1	3	3
9	9	9	9	9	9	9	9	3	9
9	9	9	9	9	9	9	9	3	9
9	9	9	9	9	9	9	9	3	9
9	9	9	1	9	3	9	1	2	9
9	9	9	9	9	9	9	9	3	9
9	3	9	9	9	9	9	9	2	9
9	2	9	3	9	3	9	9	1	9
9	9	3	9	9	3	9	1	9	9
1	9	1	3	9	2	9	9	3	9
9	3	9	9	9	9	9	9	9	9
9	9	9	9	9	9	9	9	2	9
2	2	2	2	2	2	2	2	2	2
1	2	2	2	2	2	1	1	2	2
9	9	9	9	9	9	9	9	9	9
2	2	2	2	2	2	2	2	2	3
1	2	2	1	1	1	2	2	2	3
2	2	2	2	2	2	2	2	2	2
2	3	2	2	2	2	2	2	2	3
3	2	3	3	3	3	3	3	3	3
2	2	2	2	2	2	2	2	2	3
2	2	2	3	2	9	3	1	3	2
2	2	2	2	2	2	2	2	2	2
9	9	9	9	9	9	9	9	9	9
2	2	2	2	2	2	2	2	2	2
2	2	2	2	2	2	2	2	2	2
1	3	2	1	1	2	3	2	2	3
1	3	3	2	3	2	1	2	2	2
2	2	2	2	2	2	3	3	3	3
9	9	9	9	9	9	9	9	3	9
2	2	2	2	2	2	2	2	2	2
3	2	2	2	3	3	3	3	3	3

1	3	2	3	3	2	3	2	3	2
2	2	2	3	3	3	3	3	3	3
2	2	2	2	2	2	2	2	3	3
1	1	2	1	2	3	2	2	3	2
1	2	1	2	1	2	3	2	3	2
3	3	2	2	3	3	3	3	3	3
1	1	3	3	1	3	3	1	3	3
3	3	1	1	2	2	2	2	2	2
3	3	1	1	3	1	3	3	3	3
9	9	9	9	9	9	9	9	9	9
2	2	2	2	2	2	2	2	2	2
1	1	2	3	2	2	3	3	3	3
3	3	1	1	3	1	3	3	3	3
3	3	2	1	3	2	3	3	3	3
2	3	2	1	3	2	2	3	2	2
3	2	1	3	1	9	3	2	3	3
2	2	2	2	2	2	2	2	2	2
2	2	2	2	2	2	2	2	2	2
2	2	3	2	2	1	2	2	3	2
2	2	2	3	2	1	2	2	2	2
3	2	2	2	3	3	3	3	3	3
2	2	2	2	2	2	2	2	2	2
2	2	2	2	2	2	2	2	2	3
3	3	3	3	1	3	3	3	3	3
2	2	2	2	2	2	2	2	2	1
3	3	3	3	2	3	2	3	3	3
3	2	2	3	2	2	2	3	3	3
2	2	2	2	2	2	2	2	3	3
2	2	2	3	2	2	2	2	2	2
2	2	2	2	2	2	2	2	2	2
2	2	2	2	2	2	2	2	2	2
2	2	2	2	2	2	2	2	2	2
2	2	2	2	2	2	2	2	3	3
2	2	2	2	2	2	2	2	2	2
2	2	2	2	2	2	2	2	2	2
2	2	2	2	2	2	2	2	2	2
3	2	1	1	1	3	3	2	2	2
9	9	9	9	9	9	9	9	9	9
9	9	9	9	9	9	9	9	9	9
9	9	9	9	9	9	9	9	9	9
2	2	3	1	2	1	3	3	3	1
9	9	9	9	9	9	9	9	9	3
9	9	9	9	9	9	9	9	9	9
2	3	3	3	3	3	3	3	9	9
9	9	9	9	9	9	9	9	9	9
9	9	9	9	9	9	9	9	9	9
2	2	2	2	2	2	2	2	3	3

2	3	2	1	3	2	1	3	3	2
3	3	2	1	3	2	3	3	3	3
9	9	9	9	9	9	9	9	9	9
2	2	2	2	2	2	2	2	2	2
9	9	9	9	9	9	9	9	9	9
9	9	9	9	9	9	9	9	9	9
2	2	2	2	2	2	2	2	2	2
1	1	3	3	1	3	3	3	3	3
2	2	1	1	3	2	3	2	3	3
2	2	3	1	2	1	2	1	2	3
2	2	1	3	2	3	2	2	1	1
2	2	2	2	2	2	2	2	3	2
3	3	2	2	3	2	2	2	3	2
2	2	2	2	2	2	2	2	1	1
3	3	3	2	2	3	3	2	3	3
2	2	2	2	2	2	2	2	2	3
2	2	2	2	2	2	2	2	2	3
9	9	9	1	9	9	9	9	9	9
3	2	2	3	3	3	3	3	3	3
1	1	3	3	2	2	1	2	2	2
2	2	2	2	2	2	2	2	2	3
2	1	2	1	2	3	2	2	3	1
2	2	2	2	2	2	2	2	2	2
1	1	3	3	3	3	3	3	3	3
2	2	2	3	3	2	3	2	2	2
2	2	2	2	2	2	2	2	2	2
9	9	9	9	9	9	9	9	9	9
2	2	2	2	2	2	2	3	3	3
3	2	1	1	3	3	3	3	1	3
2	2	2	2	1	2	1	1	1	2
2	3	1	1	2	2	3	3	3	3
3	9	9	9	9	9	9	9	9	9
2	2	2	2	2	2	2	2	2	2
2	2	2	2	2	2	2	2	2	2
3	3	2	2	1	3	3	3	3	3
2	2	2	2	2	2	2	2	2	2
2	2	2	2	3	1	1	1	3	1
2	2	2	2	2	2	2	2	2	2
2	2	3	3	2	3	2	2	3	2
3	1	1	1	3	3	1	3	3	3
2	2	2	2	2	2	2	2	2	2
3	3	1	1	3	1	3	3	3	3
2	2	3	3	3	3	3	3	3	3
2	9	9	9	9	9	9	9	9	9
3	3	2	2	2	2	2	2	3	2
3	2	2	2	1	3	1	2	3	3
1	9	2	2	3	2	3	2	2	2

2	2	2	2	2	2	2	2	2	2
2	2	2	2	2	2	2	2	2	2
2	2	2	2	2	2	2	2	2	2
2	2	2	2	2	2	2	2	2	2
2	2	2	2	2	2	2	2	3	3
2	2	2	2	2	3	2	2	2	2
2	2	2	2	2	2	2	2	2	2
2	2	3	2	2	2	2	2	3	3
2	2	2	2	2	2	2	2	2	2
2	2	2	2	2	2	2	2	2	2
2	2	2	2	2	2	2	2	2	2
3	2	2	3	3	3	3	3	3	3
9	9	9	9	9	9	9	9	9	9
3	3	3	1	3	3	1	1	1	3
9	9	9	9	9	9	3	9	9	9
9	9	9	9	9	9	9	9	9	9
2	3	2	1	2	2	3	2	2	2
2	1	2	3	2	3	2	1	2	2
3	9	9	9	9	9	9	9	9	3
3	2	3	3	3	2	1	3	3	2
2	2	2	2	2	2	2	2	2	2
2	2	2	2	2	2	2	2	3	3
2	2	2	2	2	2	2	2	2	2
3	3	3	3	3	3	3	1	3	3
1	1	3	3	3	3	3	3	1	1
9	9	9	9	9	9	9	9	2	9
1	1	3	1	1	3	3	1	1	3
3	2	1	1	2	2	3	3	1	3
3	3	2	2	1	9	3	1	3	3
9	9	9	9	9	9	9	9	9	9
3	2	3	3	3	3	3	3	3	3
2	2	2	2	2	2	2	2	3	3
2	2	2	2	2	2	2	2	2	2
3	2	2	2	2	1	3	1	3	3
9	2	9	9	9	9	9	9	2	9
1	2	3	3	3	3	3	1	1	2
2	3	1	1	3	1	3	3	3	3
9	9	9	9	9	9	9	9	9	9
2	2	2	2	2	2	2	2	2	2
9	9	9	9	9	9	9	9	9	3
2	1	3	3	2	3	3	2	2	3
3	2	2	2	3	3	3	2	3	3
2	2	2	2	3	1	3	2	2	2
2	2	2	2	2	1	3	3	3	2
2	3	2	2	2	3	2	1	3	2
2	3	2	3	3	3	2	2	2	3
2	2	2	2	2	2	2	2	2	3

1	1	3	3	1	3	3	1	9	3
9	9	9	9	9	9	9	9	2	3
3	2	2	2	3	3	3	3	3	3
9	9	9	9	9	9	3	9	3	3
3	3	2	3	2	1	3	3	2	3
2	2	1	1	2	1	1	1	3	3
2	3	3	1	3	1	2	3	3	1
1	1	3	3	1	3	1	1	3	1
1	1	3	3	1	3	3	1	3	3
3	3	3	3	3	3	3	3	3	3
9	9	9	9	9	9	9	9	9	9
3	3	9	9	3	9	3	2	3	3
3	2	2	3	3	3	3	3	3	3
2	3	1	2	3	3	3	1	3	3
9	9	9	9	9	9	9	9	9	9
2	2	2	2	3	1	2	3	3	3
2	2	2	3	2	3	3	1	3	3
1	9	9	9	9	9	9	9	2	2
9	9	9	9	9	9	9	9	9	2
2	2	1	2	3	2	3	1	3	2
2	2	2	1	3	1	2	2	3	3
2	2	2	2	2	2	2	2	2	2
3	3	2	2	3	1	3	3	3	3
3	2	2	2	3	3	3	3	3	3
3	3	2	2	3	2	3	3	3	3
2	2	3	3	2	3	3	3	3	3
3	2	2	2	3	2	3	3	3	3
3	1	2	2	3	3	3	3	3	3
3	3	3	3	3	3	3	3	3	3
3	3	2	2	3	3	3	3	3	3
9	9	9	2	9	9	9	9	9	3
9	9	9	9	9	9	9	9	9	9
9	9	9	9	9	9	9	9	3	2
9	9	9	9	9	9	9	9	9	9
9	9	9	9	9	9	9	9	9	9
2	3	2	2	3	2	3	3	3	3
3	2	2	2	2	2	2	2	2	2
2	2	2	2	1	1	3	2	3	3
9	9	9	9	9	9	3	9	9	3
9	9	9	9	9	9	9	9	9	9
9	9	9	9	9	9	3	9	9	3
9	9	9	9	9	9	3	9	9	3
9	9	9	9	9	9	9	9	1	1
2	2	2	2	2	2	2	2	2	2
3	2	9	2	3	3	2	3	3	9
2	3	2	2	3	2	3	3	3	3
1	2	2	2	3	2	3	3	9	3

9	9	9	9	9	9	9	9	9	9
9	9	1	9	9	9	9	9	9	3
9	9	9	9	9	9	9	9	3	3
9	9	9	9	9	9	9	9	3	9
9	9	9	9	9	9	3	9	9	3
9	9	9	9	9	9	9	9	9	9
2	1	2	2	2	2	2	2	2	3
9	9	9	9	9	9	9	9	9	9
2	2	2	2	2	2	2	2	2	2
3	2	2	2	2	3	3	2	1	2
2	2	2	2	2	2	2	2	2	3
3	3	2	2	3	2	3	3	3	3
1	1	3	3	3	3	3	1	3	3
2	3	2	2	2	2	3	3	2	3
2	2	2	2	9	1	1	3	3	3
3	2	3	3	2	3	2	1	9	2
2	2	2	1	2	2	3	2	3	3
9	9	9	9	9	9	3	9	3	9
2	3	2	1	2	1	2	2	3	9
9	2	9	9	9	9	9	9	2	9
2	9	9	9	9	9	9	9	9	9
9	9	9	9	3	9	9	9	9	9
2	2	2	2	2	2	2	2	2	3
3	2	1	2	2	2	3	3	3	3
2	2	2	2	2	2	2	2	9	2
2	2	2	2	2	2	2	2	2	2
2	2	2	9	9	9	9	9	3	3
2	1	2	1	1	1	2	1	3	2
2	2	3	3	2	3	3	2	3	3
1	2	2	2	2	2	2	2	3	3
2	2	3	2	2	1	3	3	3	3
3	3	2	2	3	3	3	3	3	3
3	3	1	3	3	1	3	3	3	3
2	2	2	2	2	2	3	3	3	3
3	2	3	3	2	3	3	2	3	3
9	9	9	9	9	9	9	9	3	9
2	3	2	2	3	3	3	3	3	3
2	2	2	2	2	3	3	2	3	3
2	3	2	1	3	2	1	1	3	3
2	2	2	3	3	3	3	2	3	3
2	3	2	1	3	3	3	3	3	3
3	1	2	2	2	9	9	9	3	3
2	9	1	9	2	3	2	2	2	3
3	1	2	2	2	3	1	2	9	9
1	2	3	2	2	2	2	1	1	2
2	2	2	2	2	2	2	2	2	2
2	9	2	1	2	2	2	2	2	2

9	9	9	9	9	9	3	9	9	3
3	3	9	9	3	9	3	3	3	3
3	3	3	3	3	3	3	3	3	3
2	2	2	3	3	2	3	3	3	3
2	9	9	2	9	2	9	2	2	9
3	2	1	3	3	2	2	2	3	3
3	3	3	3	3	3	3	3	3	3
3	3	2	2	3	2	3	3	3	3
2	1	3	3	1	3	2	1	3	3
3	3	2	2	3	2	3	3	3	3
3	3	2	2	3	2	3	3	3	3
3	3	3	3	3	3	3	3	3	3
3	9	9	9	9	1	9	3	3	9
3	1	2	1	1	1	3	3	3	3
2	2	2	2	2	2	2	2	2	2
3	2	3	1	3	2	3	3	3	3
9	9	9	9	9	9	9	9	9	9
1	2	2	2	1	2	3	2	3	2
2	2	2	2	2	2	2	2	2	2
9	9	9	9	9	9	9	9	9	9
2	2	2	2	2	2	2	2	2	2
2	2	2	3	3	2	3	2	3	3
9	9	9	9	9	9	9	9	3	9
1	3	3	3	1	3	1	1	3	1
2	2	2	2	2	2	2	2	2	3
2	2	3	3	2	2	1	2	2	2
2	2	1	2	2	2	1	1	2	1
3	2	2	2	3	2	3	2	3	3
3	2	3	3	1	3	3	1	3	3
2	3	1	2	2	1	1	2	3	2
2	2	2	2	2	2	2	2	3	3
1	2	2	1	2	1	1	2	2	2
2	2	2	2	2	2	2	2	2	2
2	2	2	2	2	2	2	2	2	2
3	2	1	2	2	2	2	2	3	3
3	3	1	2	3	1	3	3	3	3
2	2	1	1	3	3	1	1	2	2
2	2	2	2	2	2	3	2	2	2
2	2	3	2	3	3	3	2	2	3
2	2	2	2	2	2	2	2	2	2
3	2	2	2	3	3	3	2	3	3
1	2	3	2	1	2	3	2	3	3
9	9	9	9	9	9	9	3	9	9
3	3	1	1	3	3	3	3	3	3
3	3	2	2	3	2	3	3	3	3
3	3	9	9	3	9	3	3	3	3
3	3	1	1	3	2	3	2	2	2

2	2	9	1	2	2	2	3	2	2
9	9	9	9	9	9	9	9	3	9
2	2	2	2	2	2	2	2	2	2
9	9	9	9	9	9	9	9	9	9
3	2	2	2	2	2	3	3	2	3
2	2	2	2	2	2	2	2	2	2
3	1	3	3	1	3	2	3	3	3
9	9	9	9	9	9	9	9	3	3
3	3	2	3	2	3	3	3	3	3
3	3	3	1	3	3	3	3	3	3
3	1	3	3	1	3	1	1	9	9
3	3	2	3	3	3	1	3	3	3
3	1	3	3	3	3	3	3	3	2
2	3	3	1	3	2	3	3	2	3
9	9	9	9	9	9	9	9	9	9
9	9	9	9	9	9	9	9	9	9
9	9	9	9	9	9	9	9	9	9
1	1	3	3	1	3	1	1	1	1
3	1	3	2	2	2	2	2	3	3
3	1	3	1	1	2	3	3	2	2
2	2	1	2	1	1	2	2	3	2
2	3	2	3	3	2	3	2	3	3
2	1	3	3	3	3	3	2	3	3
3	2	2	3	3	3	3	3	3	3
2	2	2	2	2	2	3	1	3	3
3	1	2	3	3	3	3	3	3	3
3	1	3	3	1	3	3	3	3	3
2	3	2	1	2	1	3	3	3	3
3	3	2	3	1	2	3	3	3	3
1	1	3	3	1	3	1	3	3	3
9	9	9	9	9	9	9	9	9	9
3	2	2	3	3	3	3	3	3	3
9	9	9	9	9	9	9	9	9	9
3	2	1	3	2	3	3	2	2	3
2	3	3	2	3	3	3	3	3	3
9	9	9	9	3	9	9	9	9	9
3	2	3	2	3	2	2	2	2	2

Smoking	Alcohol	OverFourB	Hightfattyc	BirthPill	Spiritualatt	FamilyHxC	VaginalWa	UrineInfect	STD
2	2	2	2	2	2	2	2	2	2
3	3	3	2	1	2	3	1	1	1
2	2	2	2	2	2	2	2	2	2
3	3	3	2	1	2	3	1	1	1
3	3	3	2	1	2	3	1	1	1
3	3	3	2	1	2	3	1	1	1
2	9	9	9	9	9	9	9	9	9
1	1	2	1	3	2	1	2	1	2
2	9	2	2	9	2	9	2	9	2
9	9	9	9	9	9	9	9	9	9
2	2	2	2	2	2	2	2	3	3
1	1	2	2	2	3	3	3	3	2
2	2	2	2	2	2	2	2	2	2
2	2	2	1	2	2	1	1	2	3
2	1	2	2	2	1	1	1	2	1
2	2	3	2	2	3	1	2	2	2
3	1	2	1	2	3	1	2	1	2
2	2	3	2	2	3	1	2	2	2
2	2	3	2	2	3	1	2	2	2
1	1	1	2	3	3	3	3	3	3
1	1	3	1	2	2	2	2	2	2
1	1	3	2	2	2	2	2	2	2
2	2	2	2	2	2	2	2	2	2
3	3	3	3	3	3	3	3	3	2
2	2	2	2	2	2	2	2	2	2
2	2	2	2	2	2	2	2	2	2
2	2	2	2	2	2	2	2	2	2
1	1	1	1	2	1	2	1	2	2
2	2	2	2	2	2	2	2	2	2
2	2	2	2	2	2	2	2	2	2
2	2	2	2	2	2	2	2	2	2
1	1	2	2	2	1	1	1	1	1
2	2	2	2	2	2	1	1	1	1
1	1	2	2	2	1	1	1	1	1
1	1	2	2	2	3	2	1	1	1
1	1	2	2	2	1	1	1	1	1
1	1	2	2	2	3	2	1	1	1
1	1	1	2	1	2	1	1	2	1
2	2	2	2	2	2	2	2	2	2
2	2	2	2	2	2	2	2	2	2
2	2	2	2	2	2	2	2	2	2
2	2	2	1	2	2	2	2	2	2
3	2	9	2	3	3	3	1	1	1
1	1	3	3	3	3	1	1	3	3
1	1	3	3	2	1	3	1	2	2
3	2	3	3	3	3	3	1	1	2

3	3	3	1	2	3	3	2	1	1
2	2	2	1	2	1	1	9	1	1
2	2	2	3	1	3	3	1	1	1
1	1	3	1	1	3	3	1	1	1
2	1	3	2	2	1	3	1	1	1
3	3	3	3	1	3	3	1	1	1
3	3	3	3	1	3	3	1	1	1
1	1	3	9	1	9	9	1	1	1
9	9	9	9	9	9	9	9	1	1
2	1	3	1	2	3	2	1	1	1
2	2	2	9	9	9	2	2	2	2
2	2	2	2	1	3	2	2	2	2
2	2	3	2	1	2	3	2	1	1
2	1	2	2	1	2	1	2	2	2
1	1	2	2	1	2	2	2	2	1
1	1	3	2	2	3	2	2	2	2
2	2	2	2	1	2	3	1	2	2
2	2	2	2	1	3	2	1	1	1
3	3	2	3	2	3	3	1	1	2
1	1	2	2	3	3	1	2	3	2
3	3	3	3	3	3	3	1	1	1
3	3	3	3	3	3	3	1	1	1
3	3	3	3	3	3	3	1	1	1
3	3	2	2	2	3	3	1	2	2
9	9	1	9	9	9	1	9	9	1
1	1	3	2	2	3	2	1	1	1
1	1	3	1	3	3	1	1	1	1
3	3	2	2	1	3	3	1	1	3
2	2	2	2	2	2	2	2	2	2
1	1	2	2	1	2	2	1	1	1
1	1	3	3	3	3	3	2	9	1
1	1	1	3	3	1	1	1	1	1
1	1	3	2	2	9	2	1	1	1
1	1	3	2	2	3	2	1	1	1
1	1	3	1	2	3	1	1	1	3
9	9	9	9	9	9	9	9	9	9
9	9	9	9	9	9	9	9	9	9
1	1	2	2	2	2	2	2	1	1
3	3	3	3	1	3	3	1	1	1
1	1	3	1	1	1	3	1	1	1
9	1	2	2	1	3	9	1	9	1
1	1	9	1	2	2	3	2	1	1
3	3	3	3	3	3	3	3	1	1
1	1	3	1	1	2	2	1	1	1
1	1	3	3	1	1	2	2	1	1
9	9	9	9	9	9	9	9	9	9
9	9	9	9	9	9	9	9	9	9

2	3	3	2	2	1	2	1	2	2
2	2	2	2	1	2	2	2	1	2
1	2	3	1	2	2	2	1	1	1
2	1	3	3	2	3	2	2	1	2
9	9	9	9	9	9	9	9	9	9
9	1	9	9	9	2	9	9	9	9
1	1	3	2	1	1	3	1	1	1
3	1	3	3	1	3	2	1	1	1
3	3	3	3	3	3	3	1	1	2
1	1	9	1	9	9	9	1	2	9
2	2	3	2	3	3	2	1	1	2
1	3	2	1	2	3	3	2	2	2
3	3	3	3	2	2	2	2	1	1
9	9	9	9	9	9	1	1	9	9
2	3	1	3	2	1	2	2	2	3
1	3	2	3	2	1	2	1	3	2
2	2	2	2	2	2	2	2	2	1
2	2	2	3	2	2	1	2	2	1
2	2	2	2	2	2	2	2	2	2
2	2	3	3	2	1	2	2	1	2
3	1	1	1	2	3	3	2	2	2
2	2	2	3	2	2	2	1	1	1
3	2	2	2	3	3	3	3	3	3
3	3	3	3	3	3	3	3	3	3
3	3	2	1	1	2	2	1	2	1
1	1	2	2	2	2	2	2	2	2
2	2	1	2	1	2	3	1	2	2
2	2	1	2	1	2	2	2	2	2
2	2	2	2	2	2	2	2	2	2
3	2	3	1	2	2	1	1	2	2
2	2	2	2	2	2	2	2	2	2
2	2	2	2	2	2	2	2	2	2
2	2	3	2	2	1	2	2	2	1
2	2	2	2	2	2	2	2	2	2
3	2	3	1	2	2	1	1	2	2
1	1	3	3	2	2	2	2	2	2
2	2	2	2	2	2	2	2	2	2
2	2	2	2	2	2	2	2	2	2
1	9	9	9	9	1	9	9	9	1
3	3	3	3	2	1	3	1	1	3
2	2	2	2	2	2	2	2	2	2
2	2	2	2	2	2	2	2	2	2
2	2	2	2	2	2	2	2	2	2
1	1	2	3	2	3	3	2	2	2
1	1	2	2	2	3	2	2	2	2
1	1	2	2	2	3	3	2	2	2
2	2	2	2	2	2	2	2	2	2

2	2	2	2	2	2	2	2	2	2
2	2	3	1	2	2	2	2	2	2
2	2	3	1	2	2	2	2	2	2
2	2	2	2	2	2	2	2	2	2
1	1	1	1	1	1	1	1	1	1
3	2	3	3	1	2	2	1	1	1
3	9	2	3	3	2	3	2	3	3
2	2	2	2	2	2	2	2	2	2
2	2	2	2	2	2	2	2	2	2
2	2	2	2	2	2	2	2	2	2
2	2	2	9	2	2	3	2	2	2
2	3	2	2	2	2	2	3	3	2
3	3	9	9	9	9	3	3	9	3
2	2	2	2	2	2	2	2	2	2
2	2	2	3	2	1	2	2	1	1
2	2	2	3	2	1	2	2	1	1
9	9	9	9	9	9	9	9	9	9
1	1	3	2	2	3	3	2	2	2
1	9	9	9	9	9	9	9	9	9
1	1	3	2	1	2	1	1	1	1
1	1	3	2	1	2	1	1	1	1
1	1	3	2	1	3	3	1	1	1
1	1	1	3	1	1	1	1	1	1
1	1	3	2	3	2	1	1	1	1
1	1	1	2	1	2	1	1	1	1
1	1	3	2	1	2	1	1	1	1
1	1	3	2	1	2	1	1	1	1
1	1	3	2	1	2	3	1	1	1
1	1	1	2	1	3	1	1	1	1
1	1	1	2	1	2	1	1	1	1
2	2	1	2	2	3	2	2	1	2
2	2	2	2	2	2	2	2	2	2
1	1	3	2	1	2	1	1	1	1
1	1	1	2	1	1	1	1	1	1
1	1	3	2	1	3	1	1	1	1
1	1	3	2	1	2	3	1	1	1
1	1	3	2	1	2	1	1	1	1
1	1	3	2	1	2	1	1	1	1
1	1	3	2	1	2	1	1	1	1
1	1	3	2	1	1	1	1	1	1
1	1	3	2	1	3	1	1	1	1
1	1	1	2	1	2	1	1	1	1
1	1	3	2	1	2	1	1	1	1
1	1	3	2	1	2	1	1	1	1
1	1	3	2	1	1	2	1	1	1
1	1	9	2	1	2	1	1	1	2
1	1	3	2	1	2	1	1	1	1

1	1	3	2	1	2	1	1	1	1
1	1	3	2	1	1	1	1	1	1
1	1	3	2	1	1	1	9	1	1
1	1	3	2	1	2	3	1	1	1
1	1	3	2	1	1	1	1	1	1
1	1	3	1	1	1	1	1	1	1
1	1	3	2	1	3	3	1	1	2
3	3	3	3	2	2	2	2	1	1
1	1	3	1	1	1	3	1	1	1
2	2	2	3	1	3	3	1	3	3
9	9	9	9	9	9	9	9	9	9
1	1	2	2	1	2	2	1	1	1
1	1	3	1	1	1	3	1	1	1
1	1	3	2	1	3	3	1	1	1
2	1	3	2	2	3	2	1	1	1
1	1	3	3	2	1	2	1	1	1
2	2	2	2	2	2	2	2	2	2
1	1	1	1	1	1	1	1	1	1
3	3	2	2	2	1	1	2	1	1
2	2	2	2	2	2	2	2	2	2
2	2	2	1	1	2	1	2	1	1
2	2	2	2	2	2	2	2	2	2
2	2	2	1	2	2	2	2	1	1
2	2	2	2	2	1	2	1	2	2
2	2	2	2	2	2	2	2	2	2
2	2	2	2	2	2	2	2	2	2
2	2	2	2	2	2	2	2	2	2
2	2	2	2	2	2	2	2	2	2
2	2	2	2	2	3	2	1	2	2
3	3	2	2	2	1	1	2	1	1
1	1	2	1	2	2	2	2	2	2
1	1	2	1	2	2	2	2	2	2
3	3	2	2	2	1	1	2	2	1
1	1	2	2	2	1	3	1	2	2
2	2	2	1	1	2	1	2	1	2
2	2	2	2	2	2	2	2	2	2
2	2	2	2	2	2	2	2	2	2
2	2	3	2	1	1	1	1	2	1
1	1	2	2	1	2	2	2	2	2
1	1	2	1	2	3	2	3	2	2
2	2	2	2	2	2	2	2	2	2
2	2	2	2	2	2	2	2	2	2
9	9	9	9	9	9	9	9	9	9
9	9	9	9	9	9	9	9	9	9
2	1	2	3	3	1	1	1	1	1
2	2	2	2	1	2	2	3	1	1
9	9	9	9	1	9	9	9	9	9

2	2	2	2	2	2	2	2	2	2
2	2	2	2	2	2	2	2	2	2
1	1	2	2	1	3	2	1	2	2
2	2	2	2	2	2	3	2	2	2
2	2	2	2	3	3	2	3	3	2
3	3	2	3	2	3	2	2	1	2
1	1	2	2	2	2	2	1	1	2
2	2	2	2	2	2	2	2	2	2
1	1	3	3	2	3	2	2	2	2
2	1	2	2	2	2	2	2	2	2
2	2	2	1	2	2	2	2	2	1
3	3	2	2	2	2	2	2	2	2
3	3	2	2	2	2	2	2	2	2
1	2	3	2	1	2	2	3	2	2
2	2	2	2	2	2	2	2	2	2
1	2	2	2	2	3	3	2	2	2
2	3	1	2	2	2	1	1	3	2
1	1	3	2	2	2	3	2	2	1
9	9	9	9	9	1	9	9	9	1
1	1	1	2	3	3	9	1	2	1
1	1	1	1	3	9	2	1	1	1
1	1	2	2	2	2	2	2	2	2
1	2	3	2	2	2	2	1	1	2
3	2	3	2	3	3	2	3	2	2
2	2	9	9	9	9	2	9	9	9
2	2	2	2	2	2	2	2	2	2
2	2	2	2	2	2	2	2	2	2
2	9	2	9	1	9	2	9	2	9
2	1	3	3	2	2	2	2	2	2
2	2	2	2	2	2	2	2	2	2
9	1	9	9	9	9	9	9	9	9
3	3	3	2	2	2	2	2	2	2
3	3	3	3	3	3	3	3	3	3
1	1	2	2	2	3	3	2	2	2
9	9	9	9	9	9	9	9	9	9
2	2	2	2	2	2	2	2	2	2
2	3	3	3	2	3	3	1	1	2
3	1	2	2	2	1	2	1	1	1
2	2	2	2	2	2	2	2	2	2
2	2	3	3	2	2	3	2	2	2
3	3	3	3	3	2	2	2	2	2
3	2	2	2	1	3	3	1	2	2
1	1	1	1	1	1	1	1	9	9
2	2	2	2	2	2	2	2	2	2
1	2	3	2	2	2	3	1	2	2
2	2	2	2	2	2	3	2	2	2
2	1	2	2	2	2	2	2	2	2

9	9	9	9	9	9	9	9	9	9
9	9	9	9	9	9	9	9	9	9
9	9	9	9	9	9	9	9	9	9
1	1	2	2	2	3	2	1	2	1
1	1	2	2	2	2	9	2	1	2
2	2	2	3	2	2	2	1	2	3
1	1	3	1	1	3	2	2	2	1
1	1	1	1	1	1	1	1	1	1
2	2	3	2	2	2	3	1	1	1
2	2	2	2	2	2	2	2	2	2
2	2	2	2	2	2	2	2	2	2
9	9	9	9	9	9	9	9	9	9
9	9	9	9	9	9	9	9	9	9
1	1	3	3	1	3	3	1	1	1
3	3	9	1	1	1	1	3	3	9
9	9	9	9	9	9	9	9	9	9
3	3	3	3	2	2	2	9	9	9
1	1	2	1	2	1	3	2	3	2
2	3	2	1	3	1	1	1	1	2
9	9	9	9	9	9	9	9	9	9
9	9	9	9	9	9	9	9	9	9
2	2	2	2	2	2	2	2	2	2
1	1	3	2	3	3	3	2	2	2
1	1	3	3	1	2	3	2	2	2
2	2	2	2	2	2	2	2	2	2
1	1	2	1	2	1	1	1	1	3
3	3	1	2	1	1	1	1	2	1
2	3	3	3	3	3	3	1	1	1
3	3	3	1	2	1	3	1	2	1
2	1	3	1	2	3	3	1	2	2
2	2	2	2	2	2	2	2	2	2
1	3	2	3	2	3	2	1	2	2
9	9	9	9	9	9	9	9	9	9
9	9	9	9	9	9	9	9	9	9
2	2	2	2	2	2	2	1	1	1
1	3	3	3	1	3	2	2	3	2
2	1	2	2	2	3	2	2	2	1
1	1	3	3	1	1	3	1	1	1
3	3	3	1	3	3	3	1	1	3
2	2	2	2	2	2	2	2	1	1
1	1	2	1	2	2	2	2	1	1
3	3	9	1	3	3	3	1	1	1
3	3	2	2	2	2	2	2	2	2
2	2	2	2	2	2	2	2	2	2
3	3	3	2	2	3	3	3	2	2
1	2	2	2	2	2	2	2	2	2
2	2	2	2	2	2	1	2	2	2

9	9	9	9	9	9	9	9	9	9
9	9	9	9	9	9	9	9	9	9
9	9	9	9	9	9	9	9	9	9
2	2	2	2	2	2	2	1	1	2
9	9	9	9	1	9	9	9	9	9
9	9	9	9	9	9	9	9	9	9
1	1	2	2	1	2	2	1	1	1
9	9	9	9	9	9	9	9	9	9
1	1	3	2	1	2	3	1	1	1
1	1	2	3	2	1	1	2	2	1
2	2	3	2	2	2	3	2	2	2
2	2	3	2	2	2	2	2	2	2
3	3	3	2	3	2	3	1	1	1
2	2	2	2	2	2	2	3	1	2
2	2	3	2	3	2	2	2	2	1
2	1	2	3	3	3	3	2	2	2
2	2	2	2	2	2	2	2	2	2
1	2	2	2	2	2	2	2	2	2
2	2	2	2	2	2	2	2	2	2
2	2	2	2	2	2	2	2	2	2
1	1	1	2	2	1	1	1	2	2
1	1	3	2	1	2	2	1	2	1
1	1	3	2	2	1	3	2	2	1
2	2	3	2	2	3	1	1	1	1
2	2	2	2	2	2	2	2	2	2
3	2	1	2	3	2	2	2	2	3
2	2	2	2	2	2	2	2	2	2
2	2	2	2	2	2	3	2	2	2
1	2	3	3	2	2	2	1	2	2
1	1	2	2	2	1	2	1	1	1
3	3	2	3	3	1	3	3	3	3
1	2	3	2	1	2	3	2	2	2
1	9	2	3	1	1	1	3	2	2
2	2	2	2	2	2	2	2	2	2
2	2	2	2	2	2	2	2	2	2
3	2	3	2	1	3	2	1	2	1
2	2	2	2	2	2	3	2	2	2
1	1	2	1	2	1	1	2	2	2
2	2	2	2	2	2	2	2	2	2
2	2	2	2	2	2	2	2	2	2
2	1	2	2	2	2	2	2	2	2
1	1	2	2	2	1	2	2	2	2
1	1	1	1	2	2	1	1	1	1
1	1	2	2	2	2	2	2	2	2
3	2	3	3	3	1	2	2	2	1
2	1	2	3	2	3	2	1	1	1
2	2	2	2	2	2	2	2	2	2

3	3	3	3	3	2	1	2	1	3
2	2	1	2	2	1	2	1	2	2
2	2	2	2	2	2	2	2	2	1
2	2	2	2	2	2	2	2	2	2
1	1	2	1	2	2	1	1	2	2
2	2	2	3	2	2	2	2	2	1
9	9	9	9	9	9	9	9	9	9
2	2	2	2	2	2	2	2	2	2
3	3	3	2	2	2	2	2	2	2
3	2	2	2	2	3	3	1	1	1
2	2	1	2	1	2	1	2	1	2
2	2	3	1	1	2	2	1	2	1
1	1	2	2	2	2	2	2	2	2
2	2	2	2	2	2	2	2	2	2
2	2	2	2	2	2	2	2	2	2
1	1	2	1	1	1	1	1	1	1
1	1	3	3	3	1	1	3	3	1
2	2	2	2	2	2	2	2	2	2
2	2	2	2	2	2	2	2	2	2
3	3	3	3	2	2	2	1	2	3
1	1	1	2	2	1	1	1	2	1
3	3	2	2	2	2	2	2	2	2
9	9	9	9	9	9	9	9	9	9
2	2	2	2	2	2	2	2	2	2
1	1	3	2	1	3	1	1	1	1
9	9	9	9	9	9	9	9	9	9
1	3	3	2	2	3	2	3	3	1
9	9	9	9	9	9	9	9	9	9
2	2	2	2	2	2	2	9	2	2
3	2	1	2	3	2	2	1	1	1
9	9	9	9	9	9	9	9	9	9
9	9	9	9	9	9	9	9	9	9
2	2	2	2	2	2	2	2	2	2
2	2	1	1	1	1	2	1	2	1
9	9	9	9	9	9	9	9	9	9
3	2	1	3	1	2	2	1	1	1
3	2	3	3	3	3	3	3	3	3
2	1	2	1	1	2	1	2	2	1
9	9	9	9	9	9	9	9	9	9
1	2	2	1	2	3	2	1	1	2
2	2	2	2	2	2	2	3	2	2
3	3	3	2	1	2	3	1	1	1
2	2	3	3	2	2	3	1	2	3
2	2	2	2	2	2	2	2	2	2
9	9	9	9	9	9	9	9	9	9
1	1	1	2	1	2	2	1	1	1
1	1	1	2	1	1	1	1	1	1

9	9	9	9	9	9	9	9	9	9
1	1	3	1	1	1	1	1	1	1
1	1	1	2	1	9	1	1	1	1
1	1	3	2	1	2	1	1	1	1
1	1	3	1	1	3	1	1	1	1
1	1	3	2	1	2	3	1	1	1
1	1	3	2	2	2	1	1	3	2
1	1	3	1	1	1	3	1	1	1
1	1	2	2	1	2	1	1	1	1
1	1	3	2	1	2	1	1	1	2
1	1	2	2	2	3	2	2	1	2
1	1	1	2	2	2	1	1	1	1
1	1	3	2	1	3	2	1	1	1
1	1	3	2	1	2	1	1	1	1
1	1	1	2	1	2	1	1	1	1
1	1	1	2	1	3	3	1	1	1
1	1	3	2	1	2	1	1	2	1
1	1	3	2	1	2	1	1	1	1
1	1	1	2	1	2	1	1	1	1
1	1	1	2	1	2	1	1	1	1
1	1	3	2	1	1	3	1	1	1
1	1	1	2	1	1	1	1	1	2
1	1	3	2	1	1	3	1	1	1
1	1	3	2	2	2	1	1	1	1
1	1	3	2	1	2	3	1	1	1
1	1	3	2	1	2	3	1	1	1
1	1	3	2	1	1	3	1	1	1
1	1	9	2	1	2	3	1	1	1
1	1	3	2	1	1	2	1	1	1
1	1	3	2	1	2	1	1	1	1
1	1	3	3	1	1	1	1	1	1
1	1	1	2	1	2	2	1	1	1
1	1	3	2	1	1	3	1	1	1
1	1	1	2	1	2	1	1	1	1
1	1	3	2	1	2	1	1	1	1
1	1	3	2	2	2	1	1	1	1
1	1	1	3	9	2	2	1	1	1
1	1	3	2	1	1	1	1	1	1
1	1	1	2	1	1	1	1	1	1
1	1	3	2	1	1	1	1	1	1
1	1	3	2	1	2	3	1	1	1
2	2	2	2	2	2	2	2	2	2
2	2	2	2	1	3	2	1	1	1
2	2	2	2	2	2	2	2	2	2
9	9	9	9	9	9	9	9	9	9
2	2	2	2	2	2	2	2	2	2
9	9	9	9	9	9	9	9	9	9

2	2	2	2	2	2	2	2	2	2
1	1	2	2	1	3	2	1	1	1
1	1	2	2	1	3	2	1	1	1
1	1	3	2	2	2	3	1	1	1
2	2	2	2	2	2	2	2	2	2
1	1	2	3	2	3	1	1	2	1
2	2	2	2	2	2	2	2	2	2
2	1	2	2	2	2	1	2	2	1
2	2	2	2	2	3	1	2	2	1
2	2	2	2	2	2	2	2	2	2
1	1	2	2	1	3	2	1	1	1
9	9	9	9	9	9	9	1	9	9
1	1	3	3	1	2	2	1	1	1
2	2	2	2	2	2	2	2	2	2
9	9	9	9	9	9	9	9	9	9
9	9	9	9	9	9	9	9	9	9
9	9	9	9	9	9	9	1	9	9
9	9	9	9	9	9	9	9	9	9
9	9	9	9	9	9	9	9	9	1
2	1	2	2	1	3	2	1	1	1
3	3	1	1	1	3	1	1	3	3
1	3	9	3	1	1	3	1	9	9
2	2	3	2	2	1	2	2	2	1
9	9	9	9	9	9	9	1	9	9
9	9	9	9	9	9	9	9	9	9
9	9	9	9	9	9	9	9	9	9
9	9	9	9	9	9	9	9	9	9
9	9	9	9	9	9	9	9	9	9
3	3	2	3	1	2	2	1	1	1
9	9	9	9	9	9	9	1	9	9
9	9	9	9	9	9	9	9	9	9
2	2	2	2	2	2	2	2	2	2
2	2	2	2	2	2	2	2	2	2
1	1	3	3	3	3	3	1	3	1
2	2	2	2	2	2	1	1	1	1
2	2	3	1	2	3	2	2	2	1
1	1	3	2	2	2	3	1	2	2
2	2	3	1	1	2	3	1	2	1
2	2	2	2	2	2	2	2	2	2
2	2	3	3	2	1	2	2	2	2
9	9	9	1	9	9	9	9	9	9
2	2	3	2	2	2	1	1	1	1
2	2	2	2	2	2	2	2	2	2
2	2	2	2	1	2	2	2	1	1
1	1	2	2	2	2	2	2	2	1
2	2	2	2	2	2	2	2	2	2
2	1	2	1	1	2	3	2	2	2

2	3	2	2	2	2	9	1	1	1
1	1	2	2	2	2	2	2	2	1
2	2	2	2	2	2	2	2	2	2
1	1	2	2	2	2	2	2	2	1
2	2	2	2	1	3	3	1	1	1
2	2	2	2	2	2	2	2	2	2
2	2	2	2	3	3	3	2	1	1
2	2	2	2	2	2	2	2	2	2
2	2	2	2	2	2	1	2	1	1
2	2	2	2	2	2	2	2	2	2
2	2	2	2	2	2	2	2	2	2
2	1	3	2	2	2	3	2	2	1
2	2	2	2	2	2	2	2	2	2
2	2	2	2	2	2	2	2	2	2
2	2	3	2	2	3	3	1	2	2
2	2	2	2	2	2	2	2	2	2
2	2	2	2	2	2	2	2	2	2
2	2	2	2	2	2	2	2	2	2
2	2	2	2	2	2	2	2	2	1
3	3	3	2	2	3	2	1	2	2
1	1	2	1	1	2	2	2	2	2
1	1	3	2	2	1	2	2	2	1
1	1	3	3	2	3	3	1	1	2
1	1	3	3	2	2	3	1	1	2
3	3	3	3	3	3	2	2	2	2
2	2	2	2	2	2	2	2	2	2
1	1	3	3	1	3	3	9	1	1
1	3	3	1	2	1	1	2	1	3
2	2	3	2	1	1	1	1	1	1
2	2	2	2	3	2	3	2	3	2
2	2	2	2	2	2	2	2	2	2
1	2	2	2	2	2	2	2	2	2
2	2	2	2	2	2	2	2	2	2
2	2	3	2	2	2	3	2	2	2
2	2	2	2	2	2	2	2	2	2
9	9	9	9	9	9	9	9	9	9
2	2	2	2	2	2	2	2	2	2
1	1	2	2	3	2	2	2	1	1
2	1	3	2	2	1	2	2	2	2
3	3	3	1	1	2	2	1	1	2
2	9	3	2	9	2	3	1	1	1
1	1	2	1	2	1	2	3	2	2
1	1	2	1	2	1	2	3	2	2
2	1	2	1	2	3	3	2	2	2
2	1	1	2	1	3	3	1	1	1
2	2	2	2	2	2	2	2	2	2
9	9	9	9	9	9	9	9	9	9

9	9	9	9	9	9	9	9	9	9
9	9	9	9	1	9	9	9	9	9
9	9	9	9	9	9	9	1	9	9
9	9	9	9	9	9	3	3	1	1
9	9	9	9	1	9	9	9	9	9
9	9	9	9	9	9	9	9	9	9
9	9	9	9	2	9	9	9	9	9
9	9	9	9	9	9	9	9	9	9
9	9	9	9	9	9	9	9	9	9
9	9	9	9	9	9	9	9	9	9
9	9	9	9	9	9	9	9	9	9
9	9	9	9	9	9	9	9	9	9
9	9	9	9	9	9	9	2	9	9
9	9	9	9	9	9	9	9	9	9
9	9	9	9	9	9	9	9	9	9
9	9	9	9	9	9	9	9	9	9
9	9	9	9	9	9	9	9	9	9
1	1	3	3	1	1	3	3	1	1
2	2	2	2	2	2	2	2	2	2
9	9	9	9	9	9	9	9	9	9
1	1	1	1	1	1	1	1	1	1
3	1	2	2	1	2	3	3	2	1
3	2	2	2	1	2	1	1	2	2
1	1	1	1	1	1	1	1	1	1
1	2	3	1	2	1	3	2	2	3
2	1	2	3	2	2	2	2	3	2
2	9	1	2	2	2	2	2	2	2
3	2	3	2	1	3	2	3	2	2
2	2	2	2	2	2	2	2	2	2
2	2	2	2	2	2	2	2	2	2
1	1	1	1	1	2	2	2	2	3
1	1	3	3	2	3	3	1	3	1
2	2	2	2	2	2	2	2	2	2
3	3	3	3	3	3	1	1	1	1
3	1	2	3	1	2	3	1	2	3
2	2	2	2	2	2	2	3	3	3
2	2	2	2	2	2	2	2	2	2
9	9	2	3	2	9	9	9	9	9
3	3	1	1	1	3	9	1	9	1
9	9	9	9	9	9	9	9	9	9
2	2	2	2	2	2	2	2	2	2
3	1	2	2	3	3	2	2	2	3
9	9	9	9	9	9	9	9	9	9
1	1	1	1	1	1	3	2	2	1
2	2	2	2	2	2	2	2	2	2
2	2	2	2	2	2	2	2	2	2
1	1	1	2	2	2	2	2	3	3

2	2	3	3	3	3	3	2	2	1
9	9	9	9	9	9	9	9	9	9
1	3	3	1	3	1	3	3	1	3
9	9	9	9	9	9	9	9	9	9
2	2	2	2	2	2	2	2	2	2
2	2	2	2	2	2	2	2	2	2
9	9	9	9	9	9	9	9	9	9
2	2	3	3	3	3	3	2	2	1
1	1	3	1	2	3	1	2	3	2
3	3	3	3	1	3	3	3	3	3
1	1	1	1	1	2	3	1	2	3
1	1	3	2	3	1	1	1	1	1
3	2	2	3	9	2	3	2	3	2
9	9	9	9	9	9	9	9	9	9
9	9	9	9	9	9	9	9	9	9
9	9	9	9	9	9	9	9	9	9
1	3	3	3	1	3	3	1	3	3
9	9	9	9	9	9	9	9	9	9
1	1	3	2	2	3	3	1	1	1
1	1	1	3	3	3	3	1	3	3
2	1	3	2	2	1	1	1	1	1
3	2	2	3	9	2	3	2	3	2
2	2	2	2	2	2	2	2	2	2
3	9	9	9	9	9	9	9	9	9
1	2	2	2	2	2	2	2	2	2
3	1	3	1	3	1	3	1	3	1
1	3	3	3	3	3	3	3	3	3
3	3	3	9	3	3	3	1	1	1
1	1	1	2	1	2	2	2	1	1
2	2	2	2	2	2	2	1	1	1
9	9	9	9	9	9	9	9	9	9
9	9	9	9	1	9	9	9	9	9
1	1	2	2	2	2	2	2	2	1
1	3	3	2	2	3	3	2	1	2
1	9	2	9	9	9	9	9	9	3
2	2	2	2	2	2	2	1	1	1
3	3	3	3	3	3	2	2	2	2
9	9	9	9	9	9	9	9	9	9
9	9	9	9	9	9	9	9	9	9
9	9	9	9	9	9	9	9	9	9
9	9	9	9	9	9	9	9	9	9
2	3	3	1	2	3	2	3	2	9
3	1	2	1	3	2	1	3	1	2
9	9	9	9	9	9	9	9	9	9
3	1	2	1	3	3	2	1	2	3
1	3	3	1	1	1	1	2	2	2
1	1	3	3	3	1	1	3	1	3

3	3	3	3	3	3	3	3	3	3
3	3	3	3	2	1	3	3	3	3
3	3	3	9	3	3	3	3	3	3
1	2	2	2	2	2	2	2	2	2
2	2	2	2	2	2	2	2	2	2
9	9	9	9	1	3	9	2	9	9
9	9	9	9	9	9	9	9	9	9
1	1	3	1	2	3	2	2	1	2
3	3	3	3	3	3	3	3	3	3
1	1	3	3	1	3	3	2	2	2
3	3	3	3	3	3	3	3	3	3
1	1	1	1	9	9	1	1	1	1
1	1	3	1	1	1	1	2	2	2
1	1	3	2	1	1	2	2	2	2
1	1	3	1	1	1	1	2	2	2
1	1	2	2	1	1	1	2	2	2
1	1	3	1	1	1	1	2	2	2
1	1	3	1	1	1	1	2	2	2
1	1	3	1	1	1	1	2	2	2
1	2	3	2	2	3	3	2	2	3
3	2	3	2	3	2	3	1	1	1
3	3	3	9	2	2	2	2	2	9
3	3	3	3	3	2	3	3	3	3
2	3	3	2	2	2	3	3	3	2
3	3	3	3	3	3	3	3	3	3
1	1	3	1	1	1	1	2	2	2
9	2	9	9	9	9	9	9	9	9
9	9	9	9	1	9	9	9	9	9
9	9	9	9	9	9	9	1	9	9
2	2	2	2	2	2	2	2	2	2
3	1	2	1	1	2	1	2	3	2
3	2	3	2	3	2	3	3	2	3
3	2	1	2	1	2	1	3	2	1
9	9	9	9	1	9	9	9	9	9
3	3	3	3	3	3	3	3	3	3
1	2	2	1	2	3	3	2	2	2
9	3	9	9	1	9	9	9	9	2
1	1	2	1	2	1	2	1	2	1
1	1	2	1	1	1	1	2	2	2
1	1	3	2	1	1	1	1	1	1
1	1	2	1	2	3	1	1	1	2
2	2	3	2	2	2	2	2	2	9
1	2	1	2	3	1	9	2	1	2
3	3	3	1	1	3	1	1	1	3
1	1	3	3	1	2	3	2	2	3
1	1	1	1	1	1	1	1	1	1
9	2	3	2	2	9	2	2	2	3

2	2	2	2	2	2	2	2	2	2
9	9	9	9	9	9	9	9	9	9
1	9	9	9	9	9	9	9	9	9
1	1	3	3	1	1	3	1	3	3
2	2	2	2	2	2	2	2	2	2
2	2	2	2	2	2	2	2	2	2
1	1	1	1	1	1	1	1	1	1
2	2	2	2	2	2	2	2	2	2
2	2	2	2	2	2	2	2	2	2
3	1	3	1	1	2	2	2	3	3
9	9	9	9	9	9	9	9	9	9
3	2	2	2	3	3	3	1	2	2
9	9	9	9	9	9	9	9	9	9
3	1	3	3	3	3	1	1	1	1
1	1	3	2	2	2	1	1	1	1
9	9	9	9	9	9	9	9	9	9
3	3	3	3	3	3	2	2	2	2
9	1	9	9	9	9	9	9	9	9
9	1	9	9	9	9	9	9	9	9
3	1	2	3	1	2	3	1	2	3
1	2	1	1	1	1	1	1	1	1
2	2	2	2	2	2	2	2	2	2
9	9	9	9	9	9	9	9	9	9
2	2	2	2	2	2	2	2	2	2
3	3	3	3	3	3	3	3	3	3
1	1	3	3	2	3	1	3	9	1
1	1	3	3	2	3	1	3	9	1
1	1	3	3	1	1	3	2	1	1
1	1	1	1	1	1	1	1	1	1
2	2	2	2	2	2	2	2	2	2
3	3	3	1	3	2	3	3	2	3
1	1	1	1	2	2	1	1	1	1
2	1	1	1	1	2	2	1	1	1
1	1	3	3	2	3	1	3	1	2
2	9	1	1	3	9	2	1	1	1
2	2	2	2	2	2	2	2	2	2
2	2	2	2	2	2	2	2	2	2
9	9	9	9	9	9	9	9	9	9
1	9	2	9	9	3	9	9	9	9
1	1	1	1	3	3	3	1	3	3
2	2	2	2	2	2	2	2	2	2
2	2	2	2	2	2	2	2	2	2
9	9	9	9	9	9	9	9	9	9
2	2	2	2	2	2	2	2	2	2
2	2	2	2	2	2	2	2	2	2
2	2	2	2	2	2	2	2	2	2
9	9	9	9	9	9	9	9	9	9

9	9	9	9	9	9	9	9	9	9
2	2	1	2	2	3	2	2	2	2
2	2	2	2	2	1	2	1	1	1
1	1	2	1	2	2	9	9	1	2
9	9	9	9	9	9	9	9	9	9
3	1	2	3	1	2	3	2	2	2
9	9	9	9	9	9	9	9	9	9
1	1	3	1	1	3	1	1	1	1
2	1	1	2	3	1	2	2	1	2
2	9	9	9	9	9	9	9	9	9
2	2	2	2	2	2	2	2	2	2
1	2	2	2	1	1	2	2	1	1
1	2	2	2	1	1	2	2	1	1
2	2	2	2	2	2	2	2	2	2
1	1	1	2	1	1	1	3	3	3
2	2	2	2	2	2	2	2	2	2
2	2	2	2	2	2	2	2	2	2
3	3	3	3	3	3	3	3	3	3
2	2	2	3	2	2	2	1	1	2
3	3	3	1	2	2	2	2	3	3
1	9	9	9	9	9	9	9	9	9
2	2	2	2	2	2	2	2	3	3
2	2	2	2	2	2	2	2	2	2
2	2	2	2	2	2	2	2	2	2
2	3	3	3	3	3	3	3	3	3
2	2	2	2	2	2	2	2	2	2
9	9	9	9	9	9	9	9	9	9
9	9	9	9	9	9	9	9	9	9
2	2	2	2	2	2	2	2	2	2
3	2	1	3	3	9	2	2	3	3
2	2	2	2	2	2	2	2	2	2
2	2	2	2	2	2	2	2	2	2
3	3	3	3	3	3	3	1	1	1
2	2	2	2	2	2	2	2	2	2
2	2	2	2	2	2	2	2	2	2
2	1	2	2	2	2	2	2	2	2
2	2	2	2	2	2	2	2	2	2
2	2	2	2	3	2	3	2	1	2
2	2	2	2	2	2	2	2	2	2
2	2	2	2	2	2	2	2	2	2
2	2	2	2	2	2	2	2	2	2
2	2	2	2	2	2	2	2	2	2
1	1	3	3	1	3	3	1	1	1
2	2	2	2	2	2	2	2	2	2
2	2	2	2	2	2	2	2	2	2
3	3	3	3	3	3	3	3	3	3
2	2	2	2	2	2	2	2	1	2

2	2	2	2	2	2	2	2	2	2
2	2	2	2	2	2	2	2	2	2
2	2	2	2	2	2	2	2	2	2
2	2	2	2	2	2	2	2	2	2
1	1	2	2	2	1	2	2	2	2
1	1	2	2	1	1	1	9	2	1
2	2	2	2	2	2	2	2	2	2
1	3	1	9	2	1	2	2	1	2
1	1	3	3	1	3	1	1	1	1
2	2	2	2	2	2	2	2	2	2
3	3	3	1	1	3	3	1	1	1
9	9	9	9	9	9	9	9	9	9
9	9	9	9	9	9	9	9	9	9
1	2	2	2	3	9	1	1	2	9
2	2	2	2	2	2	2	2	2	2
2	2	2	2	2	3	3	3	3	3
2	2	2	2	2	3	3	3	3	3
1	1	3	1	3	2	1	1	1	1
9	9	9	9	9	9	9	9	9	9
3	2	2	2	1	2	2	2	2	3
9	9	9	9	9	9	9	9	9	9
2	2	1	1	1	1	1	1	2	9
2	2	2	2	1	1	1	1	1	1
1	1	1	1	1	1	1	1	1	1
1	1	3	3	2	2	2	2	3	3
1	1	1	1	1	1	3	1	1	1
2	2	2	2	2	2	2	2	2	2
2	2	2	2	1	2	3	2	2	1
2	2	2	2	2	2	2	2	2	2
3	1	2	2	3	3	3	3	3	3
1	1	1	1	1	2	1	1	1	1
3	3	3	3	3	3	3	3	3	3
1	1	1	1	1	1	1	1	1	1
1	1	2	2	1	1	2	2	2	1
3	3	3	3	3	3	3	3	3	3
3	3	1	3	1	3	3	3	3	3
9	9	9	9	9	9	9	9	9	1
1	1	1	1	1	1	1	1	1	1
2	2	2	2	2	2	2	2	2	2
2	2	2	2	2	2	2	2	2	2
2	2	3	3	3	3	2	2	2	3
9	9	9	9	9	9	9	9	9	9
3	1	2	1	2	2	1	2	2	3
2	2	2	2	2	2	2	2	2	2
2	2	2	2	2	2	2	2	2	2
3	3	3	3	1	2	2	3	3	2
1	1	2	3	2	2	2	3	2	1

2	1	1	2	3	2	1	1	2	1
3	3	3	3	3	3	3	3	3	3
1	2	2	1	2	3	2	1	2	3
9	9	9	9	9	9	9	9	9	9
9	9	9	9	9	9	9	9	9	9
3	3	3	3	2	3	3	3	2	1
2	2	2	2	2	2	2	2	2	2
1	1	3	1	1	3	1	1	1	1
2	2	2	2	2	2	2	2	2	2
2	2	2	2	2	2	2	2	2	2
9	9	9	9	9	9	9	9	9	9
2	2	2	2	2	2	3	3	3	3
2	2	2	2	2	2	2	2	2	2
9	3	3	1	3	1	2	2	3	2
2	2	2	2	2	2	2	2	2	2
9	1	1	9	1	9	1	2	9	2
2	2	9	3	2	2	2	2	2	2
9	9	9	9	9	9	9	9	9	9
9	9	9	1	9	9	9	9	9	9
2	2	2	2	2	2	2	2	2	2
2	2	2	2	2	2	2	2	2	2
2	2	2	2	2	2	2	2	2	2
9	9	9	9	9	9	9	9	9	9
9	9	9	9	9	9	9	9	9	9
3	1	2	3	1	2	3	1	3	2
1	1	3	3	3	1	3	3	1	1
2	2	2	2	2	2	3	1	1	1
1	1	3	3	3	1	3	1	2	1
1	1	3	3	3	1	3	3	3	1
1	1	2	3	2	3	3	2	2	3
2	2	2	2	2	2	2	1	1	1
1	2	3	3	3	3	2	3	2	1
3	2	1	2	9	3	3	1	2	2
1	1	3	3	3	3	3	3	3	3
3	3	3	3	3	3	3	3	3	3
3	2	2	3	3	2	2	3	3	2
1	1	2	2	2	1	1	1	3	1
1	1	3	3	3	3	3	3	3	3
1	2	3	3	1	3	2	1	9	1
3	3	3	3	3	3	3	3	3	3
1	1	3	9	1	3	1	1	1	1
1	1	3	1	3	3	3	3	1	1
1	1	1	3	1	3	1	3	3	1
2	2	2	2	2	2	2	2	2	2
1	2	2	2	2	2	2	2	2	2
1	1	2	2	2	1	1	2	1	1
1	1	2	2	1	2	1	1	1	1

2	2	2	2	2	2	2	2	2	2
3	3	3	3	3	3	3	3	3	3
1	2	1	2	1	1	2	1	2	9
3	3	3	3	3	3	3	3	3	3
2	2	2	2	2	2	2	2	2	2
2	2	1	2	1	3	2	1	2	2
2	2	2	2	2	2	2	2	2	2
2	2	2	2	2	2	2	2	2	2
2	2	2	2	2	2	2	2	2	2
2	2	2	2	2	2	2	2	2	2
9	9	9	9	9	9	9	9	9	9
2	2	2	2	2	2	2	2	2	2
3	3	3	3	3	3	1	1	1	3
1	1	2	2	2	2	2	1	1	2
1	1	2	2	2	1	2	1	1	2
1	2	1	2	1	2	2	2	2	9
1	1	3	1	3	3	3	3	3	3
2	2	2	2	2	1	1	2	2	2
1	2	3	2	3	2	1	2	2	1
9	9	9	1	9	9	9	9	9	9
1	1	3	1	1	1	1	1	1	1
1	2	3	3	3	2	1	2	1	1
3	3	3	1	1	3	3	3	1	1
1	1	3	3	3	3	3	1	1	1
3	3	3	3	3	3	3	3	3	3
2	2	2	2	2	2	2	2	2	2
1	9	3	2	2	1	2	1	1	1
2	1	2	1	1	2	1	3	1	3
2	2	1	2	1	3	3	2	1	3
2	1	1	1	1	1	1	2	2	1
2	2	2	2	2	2	2	2	2	2
9	9	9	9	9	9	9	9	9	9
1	2	2	2	1	2	1	2	1	2
9	9	9	9	9	9	9	9	9	9
2	2	2	1	2	2	2	2	2	2
2	2	2	2	2	2	2	2	2	2
2	2	2	2	2	2	2	2	2	2
2	2	2	2	2	2	2	2	2	2
2	2	2	2	2	2	2	3	2	2
1	2	3	1	2	3	1	3	1	2
1	3	2	1	2	1	3	1	2	3
2	2	2	2	2	2	2	2	2	2
3	1	2	3	1	2	3	1	2	3
9	9	9	9	9	9	9	9	9	9
3	3	3	3	3	3	3	3	3	3
2	2	2	2	2	2	2	2	2	2
1	1	3	2	2	2	2	3	1	1

9	9	9	9	9	9	9	9	9	9
2	2	2	2	2	2	2	2	2	2
2	3	2	3	3	3	3	3	3	3
2	2	2	2	3	1	3	2	2	1
2	2	2	2	2	3	3	2	2	2
3	3	2	2	2	3	3	3	3	2
9	9	9	9	9	9	9	9	9	9
3	2	9	2	2	2	2	2	2	9
2	2	2	2	2	2	2	2	2	2
9	9	9	9	9	9	9	9	9	9
9	9	9	9	9	9	9	9	9	9
9	9	9	9	9	9	9	9	9	9
9	9	9	9	9	9	9	9	9	9
9	9	9	9	9	9	9	9	9	9
9	9	9	9	9	9	9	9	9	9
2	2	3	2	2	3	2	2	1	1
1	1	2	9	1	9	2	2	9	2
1	1	2	2	2	2	2	2	2	2
1	1	3	2	1	2	3	1	3	2
2	3	2	1	2	2	1	1	1	1
2	3	2	1	2	1	3	1	2	3
2	1	2	2	2	3	2	2	1	2
1	1	3	3	3	1	1	1	1	1
2	2	3	3	1	3	2	1	1	1
2	2	2	2	2	2	2	2	2	2
1	2	2	2	2	2	2	2	1	2
2	2	2	2	2	2	2	2	2	2
1	1	1	1	1	1	1	1	1	1
1	2	2	2	2	2	2	2	1	2
2	2	3	2	2	3	2	2	1	1
2	3	2	1	2	2	1	1	1	1
9	9	9	9	9	9	9	9	9	9
1	1	3	1	3	3	3	3	1	3
2	2	2	2	2	2	2	2	2	2
2	2	2	2	2	2	2	2	2	2
2	2	2	2	2	2	2	2	2	2
2	2	2	2	2	2	2	2	2	2
9	9	9	9	9	9	9	9	9	9
2	2	2	2	2	2	2	2	2	2
2	2	2	2	2	2	2	2	2	2
2	2	2	2	2	2	2	2	2	2
2	2	2	2	2	2	2	2	2	2
2	2	2	2	2	2	2	2	2	2
1	1	1	2	2	2	2	2	3	3
2	2	2	2	2	2	2	2	2	2
2	2	2	2	2	2	2	2	2	2
9	9	9	9	9	9	9	1	9	9

2	2	2	2	2	2	2	2	2	2
2	2	2	2	2	2	2	2	2	2
9	9	9	9	9	9	9	9	9	1
9	9	9	9	9	9	9	9	9	9
2	2	2	2	2	2	2	2	2	2
9	9	9	9	9	9	9	9	9	9
1	1	1	2	3	1	2	3	2	1
1	2	2	2	3	3	2	3	2	2
9	9	9	9	9	9	9	9	9	9
1	1	2	2	2	2	3	3	3	2
9	9	9	9	9	9	9	9	9	9
2	1	1	2	1	2	3	3	2	2
2	2	2	2	2	2	2	2	2	2
2	2	2	2	2	2	2	2	2	2
3	1	2	2	1	2	3	3	1	2
3	3	3	3	3	3	3	3	3	3
2	2	2	2	2	2	2	2	2	2
2	2	2	2	2	2	2	2	2	2
1	1	2	2	1	1	2	3	2	3
1	1	1	2	1	3	3	2	2	1
3	3	3	3	3	3	3	1	1	1
2	2	2	2	2	2	2	2	2	2
3	3	2	2	3	2	1	1	1	1
3	3	3	3	3	3	3	3	3	3
2	2	2	2	2	2	2	2	2	2
1	1	2	2	2	2	3	1	1	1
2	2	2	2	2	2	2	2	2	2
2	2	2	2	2	2	2	2	2	2
2	3	2	2	2	3	2	2	2	2
9	9	9	9	9	9	9	9	9	9
3	3	3	1	1	1	9	1	9	9
2	2	2	2	2	2	2	2	2	2
9	9	9	9	9	9	9	9	9	9
9	9	9	9	9	9	9	9	9	9
3	1	1	3	2	1	2	3	3	2
9	1	9	9	9	9	9	9	9	9
9	9	9	9	9	9	9	9	9	9
2	2	2	2	2	2	2	2	2	2
1	9	9	1	9	9	9	9	9	1
2	1	1	2	2	1	3	3	2	2
1	1	1	1	1	1	1	1	1	1
9	3	2	2	3	1	2	3	1	2
9	9	9	9	9	9	9	9	9	9
2	3	1	2	1	3	2	1	2	3
1	2	2	3	2	2	1	3	2	3
9	9	9	9	9	9	9	9	9	9
2	1	2	3	2	1	1	2	2	3

2	1	1	2	1	2	1	1	1	2
9	9	9	9	9	9	9	9	9	9
9	9	9	9	9	9	9	9	9	9
2	2	2	2	2	2	2	2	2	2
2	2	1	3	1	3	3	3	3	1
2	1	1	2	1	2	1	1	1	2
2	9	1	9	1	9	1	1	1	2
3	3	3	3	3	3	1	1	1	1
2	2	2	2	2	2	2	2	2	2
3	3	1	2	2	2	3	1	1	3
9	9	9	9	9	9	9	9	9	9
9	9	9	9	9	9	9	9	9	9
9	9	9	9	9	9	9	9	9	9
2	1	2	2	2	2	2	2	1	2
2	2	2	2	2	2	2	2	2	2
9	9	9	9	9	9	9	9	9	9
9	9	9	9	9	9	9	9	9	9
9	9	9	9	9	9	9	9	9	9
1	1	1	2	1	2	2	2	2	1
1	1	2	2	2	2	2	2	2	2
9	9	9	9	9	9	9	9	9	9
3	3	3	2	2	1	1	1	3	3
3	3	3	3	3	3	3	3	3	3
9	9	9	9	9	9	9	9	9	9
2	2	2	2	2	2	2	2	2	2
1	1	2	3	2	3	3	2	2	2
9	9	9	9	9	9	9	9	9	9
9	9	9	9	9	9	9	9	9	9
2	2	2	1	2	2	1	2	9	1
2	1	1	2	2	1	1	2	2	2
1	1	1	1	2	3	3	2	2	3
9	9	9	9	9	9	9	9	9	9
9	9	9	9	9	9	9	9	9	9
1	1	1	1	1	1	1	1	1	1
2	2	9	2	9	1	1	1	1	1
2	2	2	2	2	2	2	2	2	2
2	2	2	2	2	1	2	1	1	1
2	2	2	2	2	2	2	2	2	2
9	1	3	3	2	3	1	9	2	1
2	2	1	3	1	2	3	2	3	2
1	1	2	3	3	2	2	2	2	1
9	9	9	9	9	9	9	9	9	9
9	9	9	9	9	9	9	9	9	9
9	9	9	9	9	1	9	9	9	9
9	9	9	9	9	9	9	9	9	9
9	9	9	9	9	9	9	9	9	9
9	9	9	9	9	9	9	9	9	9

2	2	2	2	2	2	2	2	2	2
2	3	2	3	2	3	2	3	2	3
1	1	2	2	2	3	1	1	1	3
2	2	2	2	2	2	2	2	2	2
9	9	9	9	9	9	9	9	9	9
9	9	9	9	9	9	9	9	9	9
9	9	9	9	9	9	9	9	9	9
9	9	9	9	9	9	9	9	9	9
9	9	9	9	9	9	9	9	9	9
9	9	9	9	9	9	9	9	9	9
9	9	9	9	9	9	9	9	9	9
9	9	9	9	9	9	9	9	9	9
9	9	9	9	9	9	9	9	9	9
1	3	3	1	3	3	3	1	3	1
1	1	9	9	9	9	9	9	9	9
1	2	2	1	2	2	2	2	2	2
2	2	2	2	2	2	2	2	2	2
9	9	9	9	9	9	9	9	9	9
9	9	9	9	9	9	9	9	9	9
9	9	9	9	9	9	9	9	9	9
9	9	9	9	2	3	9	9	9	9
9	9	9	9	9	9	9	9	9	9
2	2	1	1	1	1	1	1	1	1
9	9	9	9	9	9	9	9	9	9
2	2	2	1	1	1	1	1	2	1
1	1	2	2	2	2	2	2	2	2
2	2	2	1	1	2	2	2	2	2
1	1	1	1	2	2	2	1	1	1
2	1	1	1	1	2	2	2	1	1
1	1	1	2	2	2	2	1	1	1
1	1	2	2	2	2	2	2	2	2
2	2	1	1	2	2	2	2	2	2
1	1	1	1	1	1	1	1	1	1
9	9	9	9	9	9	9	9	9	9
1	1	1	2	2	2	3	3	2	2
1	1	2	2	2	1	1	1	1	1
1	1	1	1	1	1	1	1	1	1
9	9	9	9	9	9	9	9	9	9
3	3	3	3	1	3	3	3	3	3
3	3	3	3	3	3	3	3	3	3
3	3	3	3	3	3	3	3	3	3
9	9	9	9	9	9	9	9	9	9
9	9	9	9	9	9	9	9	9	9
9	9	9	9	9	9	9	9	9	9
9	9	9	9	9	9	9	9	9	9
9	2	2	2	2	2	2	2	2	2
9	9	9	9	9	9	9	9	9	9

9	9	9	9	9	9	9	9	9	9
9	9	9	9	9	9	9	9	9	9
9	9	9	9	9	9	9	9	9	9
9	9	9	9	9	9	9	9	9	9
9	9	9	9	9	9	9	9	9	9
9	9	9	9	9	9	9	9	9	9
9	9	9	9	9	9	9	9	9	9
9	9	9	9	9	9	9	9	9	9
9	9	9	9	9	9	9	9	9	9
9	9	9	9	9	9	9	9	9	9
3	9	9	9	9	9	9	9	9	9
9	9	9	9	9	9	9	9	9	9
1	1	1	1	1	1	1	1	1	2
1	2	2	1	1	1	1	2	1	1
1	1	2	2	2	2	2	1	1	2
2	2	2	1	2	1	2	1	2	2
2	1	2	1	2	1	1	2	1	1
3	2	1	2	2	2	3	3	3	2
1	1	1	2	2	2	2	2	2	2
9	9	9	9	9	9	9	9	9	9
1	2	2	2	1	1	1	1	1	1
2	2	2	1	2	2	1	2	1	2
2	2	2	2	2	2	2	2	2	2
1	1	1	1	2	1	1	1	1	1
2	2	2	1	1	1	2	1	1	2
1	1	3	3	1	3	3	1	1	1
2	2	1	9	2	2	2	2	2	2
3	3	3	3	3	3	3	3	3	3
9	9	9	9	9	9	9	9	9	9
9	1	3	3	9	9	9	9	9	9
9	9	9	9	9	9	9	9	9	9
1	1	1	2	2	1	1	2	2	1
2	1	1	1	1	2	2	2	2	2
2	2	2	1	1	2	2	1	1	1
2	1	1	1	2	2	2	1	1	2
2	2	2	2	2	2	2	2	2	2
2	2	2	1	1	1	1	1	1	1
1	1	1	1	1	2	2	2	2	2
2	2	1	2	1	1	1	1	2	2
1	1	1	2	2	2	1	1	1	2
2	2	2	2	2	2	2	2	2	2
1	1	2	2	2	1	1	1	1	1
1	1	3	1	1	3	3	1	2	2
1	1	2	1	1	1	2	1	1	1
1	1	1	2	2	2	2	1	1	1
1	1	1	1	1	1	1	1	1	1
2	2	2	2	2	2	2	2	2	2

9	9	9	9	9	9	9	9	9	9
2	2	2	2	2	1	2	2	2	2
9	9	9	2	9	9	9	9	9	9
9	2	9	9	9	9	9	9	9	9
9	9	9	9	9	9	9	9	9	9
9	9	9	9	9	9	9	9	9	9
9	9	9	9	9	9	9	9	1	9
3	3	3	2	2	2	2	9	3	3
9	9	9	9	9	9	9	9	9	9
2	2	2	2	2	2	2	2	2	2
9	9	9	9	9	9	9	9	9	9
9	9	9	9	9	9	9	9	9	9
1	2	2	2	1	1	1	1	2	1
2	2	2	2	2	2	2	2	2	2
1	1	1	1	1	1	1	1	2	2
9	9	9	9	9	9	9	9	9	9
2	2	2	2	2	2	2	2	2	2
3	3	3	3	3	3	3	3	3	3
9	9	9	9	9	9	9	9	9	9
1	1	1	1	1	1	1	1	1	1
2	2	2	2	2	2	2	2	2	2
3	2	3	3	2	2	2	2	3	3
2	2	2	2	2	2	2	2	2	2
2	2	2	2	2	2	2	2	2	2
2	1	1	2	2	2	2	2	2	2
1	1	2	2	2	2	2	2	2	2
3	3	3	3	2	9	2	3	1	2
3	3	3	2	2	2	2	2	1	2
1	1	1	1	1	1	1	1	1	1
2	1	1	1	1	1	1	1	1	1
9	9	9	9	9	9	9	9	9	9
3	3	3	3	2	2	2	2	2	2
9	9	9	9	9	9	9	9	9	9
2	2	2	2	2	2	2	2	2	2
2	2	2	2	2	2	2	2	2	2
2	1	1	1	1	1	2	2	2	2
3	3	3	3	3	3	3	3	3	3
1	1	1	2	2	2	1	1	2	2
1	1	1	1	1	1	1	1	1	1
2	3	3	1	1	1	2	1	2	2
2	2	2	2	2	2	2	2	2	2
1	1	2	2	2	1	1	1	1	1
9	9	9	9	9	9	9	9	9	9
9	9	9	9	9	9	9	9	9	9
9	9	9	9	9	9	9	9	9	9
9	9	9	9	9	9	9	9	9	9
9	9	9	9	9	9	9	9	9	9

9	9	9	9	9	9	9	9	9	9
2	2	2	2	2	2	2	2	2	2
9	9	9	9	9	9	9	9	9	9
2	1	3	1	2	1	2	3	1	2
9	9	9	9	9	9	9	9	9	9
2	1	2	1	1	2	2	3	2	3
2	2	2	2	3	3	2	2	2	3
9	9	9	9	9	9	9	9	9	9
9	9	9	9	9	9	9	9	9	9
9	9	9	9	9	9	9	9	9	9
9	9	9	9	9	9	9	1	9	9
9	9	9	9	9	9	9	9	9	9
2	2	1	2	1	1	1	2	3	2
9	9	9	9	9	9	9	9	9	9
2	9	9	9	9	9	9	9	9	9
3	2	1	2	1	3	2	1	1	3
2	3	3	2	2	3	2	2	1	3
2	2	3	3	2	2	1	2	2	1
3	3	3	2	2	2	2	2	2	2
3	2	2	2	1	1	3	2	2	3
2	1	3	3	2	1	1	2	3	1
3	1	3	3	1	3	3	1	1	1
2	3	1	1	1	2	3	1	1	2
2	3	1	2	1	2	1	2	3	2
2	2	2	2	2	2	2	2	2	2
1	1	1	1	1	2	2	2	1	2
2	2	2	2	2	2	2	2	2	2
2	2	9	2	1	1	1	1	1	1
2	2	2	1	9	1	2	1	2	3
2	2	2	2	1	1	1	2	2	2
3	1	1	2	2	2	2	2	1	9
2	3	2	2	2	2	3	2	2	2
9	9	9	9	9	9	9	9	9	9
3	1	2	9	2	9	1	3	2	1
2	1	2	2	2	2	2	1	1	1
2	2	2	2	2	2	2	1	2	1
2	2	2	2	3	3	3	2	2	2
2	2	2	3	2	3	3	1	2	2
9	9	9	9	9	9	2	9	9	9
3	1	2	3	2	1	2	3	2	1
9	9	9	9	9	9	9	1	9	9
9	9	9	9	9	9	9	9	9	9
2	3	2	1	3	1	2	2	2	2
9	9	9	9	9	9	9	9	9	9
1	1	1	1	1	1	1	1	1	1
3	1	3	1	1	1	3	1	1	3
9	3	3	3	3	3	3	3	3	3

2	2	3	3	3	2	3	3	3	3
1	9	9	9	9	9	9	9	9	9
2	2	2	2	2	2	2	2	2	2
3	1	1	3	1	1	3	3	1	1
3	3	3	3	3	3	3	1	1	1
1	1	3	3	3	1	1	1	3	1
9	1	3	9	1	3	1	2	3	2
9	3	1	2	1	2	3	1	2	1
2	2	2	2	2	2	2	2	2	2
2	2	2	2	2	2	2	2	2	2
1	1	1	1	3	3	2	2	1	1
3	2	1	2	3	2	3	2	2	1
3	2	9	1	2	3	2	1	2	3
2	2	2	2	2	2	2	2	2	2
1	1	3	1	1	2	2	2	2	1
3	1	2	2	1	2	1	2	2	1
2	2	2	2	2	2	2	2	2	2
1	1	3	3	2	2	2	2	1	1
2	2	2	2	2	2	2	2	2	2
2	2	2	2	2	2	2	2	2	2
2	2	2	2	2	2	2	2	2	2
1	1	1	2	3	3	2	3	2	3
9	9	9	9	9	9	9	9	9	9
9	9	9	9	9	9	9	9	9	9
2	2	2	2	2	2	2	2	2	2
3	2	1	2	2	1	2	3	2	1
3	1	3	1	2	2	3	3	2	1
2	2	1	1	1	2	3	3	3	1
1	1	2	1	3	1	1	1	1	1
9	9	9	9	9	1	9	9	9	9
1	2	1	2	3	1	2	2	2	2
2	1	2	2	3	2	3	3	2	1
3	9	2	3	3	2	2	3	2	3
1	1	2	2	2	2	2	3	2	2
3	3	2	1	1	3	3	1	1	3
2	2	2	2	2	2	2	2	2	2
3	3	9	3	3	3	2	2	2	2
9	9	9	9	9	9	9	9	9	9
1	1	1	2	3	3	2	2	1	2
1	1	2	3	1	3	3	3	2	2
9	9	9	9	9	9	9	9	9	9
9	9	9	9	9	9	9	9	9	9
2	9	1	2	2	3	2	1	2	2
9	9	9	9	9	9	9	9	9	9
2	2	2	2	2	2	2	2	2	2
1	1	2	3	2	2	3	1	1	1
9	9	9	9	9	9	9	9	9	9

2	3	3	2	2	2	2	3	2	3
9	9	9	9	9	9	9	9	9	9
1	1	2	3	3	3	2	2	2	1
9	9	9	9	9	9	9	9	9	9
9	9	9	9	9	9	9	9	9	9
3	3	3	3	3	3	3	3	3	3
2	2	2	2	2	2	2	2	2	2
2	2	2	2	2	2	2	2	2	2
1	1	1	1	1	1	1	1	1	1
2	2	2	2	2	2	2	2	2	2
9	9	9	9	9	9	9	9	9	9
9	9	9	9	9	9	9	9	9	9
9	9	9	9	9	9	9	9	9	9
1	1	1	1	1	1	1	1	1	1
2	2	2	2	2	2	2	2	2	2
1	1	1	1	1	1	1	1	1	1
9	9	9	9	9	9	9	9	9	9
9	9	9	9	9	9	9	9	9	9
3	3	3	3	3	3	1	1	1	1
2	2	2	2	2	2	2	2	2	2
1	2	2	2	1	1	1	1	1	1
3	3	3	3	3	3	3	3	2	2
9	9	9	9	9	9	9	9	9	9
2	2	3	3	3	3	2	2	2	2
1	1	1	1	2	2	1	2	2	2
2	2	3	3	3	3	2	2	2	2
9	9	9	1	9	9	1	9	9	9
2	2	1	1	1	1	2	2	2	2
2	2	2	2	2	2	2	2	2	2
1	9	9	9	9	9	9	9	9	9
1	1	3	2	2	3	1	2	2	1
2	2	2	2	2	2	2	2	2	2
3	3	3	3	1	1	1	2	2	2
2	2	2	1	2	3	3	3	3	1
2	2	2	2	2	2	2	2	2	2
2	2	2	1	2	3	3	1	2	2
1	2	3	3	2	2	2	1	1	1
3	3	3	3	3	3	1	1	1	1
2	2	2	2	2	2	2	2	2	2
3	3	3	3	3	3	3	3	3	3
1	1	3	3	2	2	2	1	1	1
2	2	2	2	2	2	2	2	2	3
3	3	3	3	3	3	3	3	3	9
2	2	2	3	3	3	2	2	2	2
2	1	3	1	3	2	3	1	3	1
3	2	1	2	3	2	2	1	1	2
1	1	2	2	2	1	3	2	2	2

9	2	2	3	2	3	2	2	3	3
2	2	2	2	2	2	2	2	2	2
2	1	2	1	1	1	2	2	1	1
3	3	3	3	3	2	2	2	2	2
1	1	2	2	2	1	2	2	2	2
1	9	9	9	9	9	9	9	9	9
2	2	2	2	2	2	2	2	2	2
2	2	1	1	1	1	2	2	2	1
2	2	2	2	2	1	2	2	2	2
2	2	2	3	2	2	2	2	2	2
2	2	2	2	2	2	2	2	2	1
2	2	2	3	2	1	3	1	1	1
2	2	2	2	2	2	2	2	2	2
3	3	3	3	3	3	3	3	3	3
3	2	2	2	3	1	2	2	3	3
3	2	1	3	2	2	1	2	3	1
3	2	3	2	3	2	3	2	3	2
1	1	2	1	3	3	2	2	2	2
2	2	2	2	2	2	2	2	2	2
2	9	2	3	2	2	2	2	1	2
2	2	2	2	2	2	2	2	2	2
3	1	3	2	2	2	2	2	2	2
3	3	3	3	3	3	3	3	3	3
1	1	1	1	9	1	3	3	3	3
1	1	3	3	1	2	1	2	1	1
2	2	2	2	2	2	2	2	2	2
1	2	2	2	2	3	3	2	1	1
1	2	2	2	2	3	3	2	1	1
2	2	2	2	2	2	2	2	2	2
1	1	1	1	1	2	2	1	1	1
2	2	3	3	3	3	2	2	2	2
3	2	2	3	2	2	3	2	3	2
3	3	3	3	2	1	1	1	1	2
3	3	3	3	3	3	3	3	3	2
3	3	1	1	1	2	1	1	1	1
2	2	2	2	2	2	2	2	2	2
2	2	2	2	2	2	2	2	2	2
2	2	2	2	2	3	1	2	1	1
1	1	2	2	2	2	2	2	2	3
1	1	3	2	2	2	2	2	2	2
2	2	2	2	1	3	3	2	2	1
1	1	3	3	3	2	2	1	1	1
2	2	2	2	2	1	1	1	1	1
2	2	2	2	2	2	1	2	2	2
2	2	3	3	3	3	2	2	1	2
1	1	3	2	1	3	1	2	1	1
1	1	2	3	2	3	2	1	2	2

2	2	3	2	2	1	1	2	2	1
2	2	3	3	3	3	3	3	3	3
1	1	2	2	2	3	2	1	2	1
2	2	2	2	2	2	2	2	2	2
3	2	2	2	2	2	2	2	2	2
2	2	2	2	2	2	2	2	2	2
1	1	3	2	1	2	2	2	1	1
2	2	1	1	1	2	2	1	1	1
2	2	2	2	2	2	2	2	2	2
2	2	2	2	2	2	2	2	2	2
2	2	2	2	2	2	2	2	2	2
9	9	9	9	9	9	9	9	9	9
9	9	9	9	9	9	9	9	9	9
9	9	9	9	9	9	9	9	9	9
1	1	2	1	2	2	1	2	1	1
1	2	2	2	2	2	2	2	2	1
2	2	1	1	1	2	2	1	1	1
1	1	2	2	2	2	2	2	2	2
1	1	3	1	1	1	1	1	1	1
1	1	3	2	2	2	1	1	2	1
1	2	3	2	2	1	2	3	2	2
2	2	3	3	3	3	3	3	3	3
2	2	2	2	2	2	2	2	2	2
2	2	2	2	2	2	2	2	2	2
1	1	2	2	2	2	2	2	2	2
1	1	2	3	1	2	1	2	9	2
1	1	2	2	2	2	2	2	1	1
2	2	2	2	2	2	2	2	2	2
2	2	2	2	2	2	2	2	2	2
9	9	9	9	9	9	9	9	9	9
2	2	2	2	2	2	2	2	2	2
2	2	2	2	2	2	2	2	2	2
2	1	1	2	1	3	1	1	2	2
2	2	2	2	2	2	2	2	2	2
1	9	9	9	9	9	9	9	9	9
2	2	2	2	2	2	2	2	2	2
2	2	2	2	2	2	2	2	2	2
2	2	2	2	2	2	2	2	2	2
2	2	2	2	2	2	2	2	2	2
2	2	2	2	2	2	1	1	2	1
2	2	2	2	2	2	2	2	2	2
2	2	2	2	2	2	2	2	2	2
1	1	2	1	1	1	1	1	1	1
1	1	2	2	2	2	1	2	2	1
2	2	2	2	2	2	2	2	2	2
1	1	2	3	2	2	2	2	2	2
2	2	2	2	2	2	2	2	2	2

2	2	2	2	1	1	2	3	3	2
1	1	2	2	2	2	2	2	2	2
1	1	3	3	3	2	1	1	1	1
2	2	2	2	2	2	2	2	2	2
2	2	2	2	2	2	2	2	2	2
2	2	2	3	3	3	2	2	2	2
9	9	9	9	9	9	9	9	9	9
1	1	2	1	2	2	1	1	1	1
3	2	1	1	1	1	1	1	2	2
2	2	2	2	2	3	2	2	1	1
1	1	2	2	2	2	2	1	1	1
2	2	2	2	2	2	2	2	2	2
1	2	2	2	2	2	2	2	1	1
1	1	9	9	9	9	1	9	9	1
1	1	2	2	2	3	1	1	3	1
1	2	2	1	2	2	1	1	2	1
1	2	1	2	2	2	1	1	1	2
1	1	3	2	2	2	2	2	2	2
1	1	1	1	1	1	1	1	1	1
1	2	2	2	2	2	2	3	3	2
1	1	1	1	1	1	2	2	2	2
2	2	1	1	2	2	3	3	2	3
2	2	2	2	2	2	2	2	2	2
2	2	2	2	2	2	2	2	2	2
2	2	2	2	2	2	2	2	2	2
2	2	2	2	2	2	2	2	2	2
9	9	9	9	9	9	9	9	9	9
2	2	2	2	2	2	2	2	2	2
1	1	2	2	2	2	2	2	2	1
2	2	2	2	2	2	2	2	2	2
2	2	2	2	2	2	2	2	2	2
2	1	2	2	2	1	1	2	1	2
9	9	9	9	9	9	9	9	9	9
9	9	9	9	9	9	9	9	9	9
9	9	9	9	9	9	9	9	9	9
9	9	9	9	9	9	9	9	9	9
3	1	2	1	2	3	2	3	2	2
1	1	3	1	2	2	2	1	1	1
1	1	2	1	2	1	1	1	1	1
2	2	2	2	9	2	3	2	2	3
1	3	1	3	1	3	1	2	1	3
1	2	2	2	2	2	2	2	2	2
2	1	2	2	2	1	2	2	2	2
2	2	2	2	2	2	2	2	2	2
1	1	2	2	1	1	3	2	1	1
1	1	2	2	2	2	2	1	2	1
1	1	2	1	2	2	1	1	2	1

2	3	1	2	2	3	1	2	2	1
1	3	2	2	1	1	2	3	2	3
1	2	3	2	1	2	2	2	1	2
1	2	1	1	1	1	1	1	1	1
3	3	2	1	1	2	3	2	3	2
3	1	2	3	3	2	1	2	3	2
1	1	3	3	1	1	1	1	1	1
9	9	9	9	9	9	9	9	9	9
2	2	2	2	2	2	2	2	2	2
2	2	2	2	2	2	2	2	2	2
2	2	2	2	2	2	2	2	2	2
2	2	2	2	2	2	2	2	2	2
2	2	2	2	2	2	2	2	2	2
1	1	2	2	2	1	2	2	2	2
2	2	2	2	1	2	2	1	1	1
2	2	2	2	2	2	2	2	2	2
2	2	2	2	2	2	2	2	2	2
3	2	2	3	3	3	1	1	1	1
2	2	3	2	2	1	2	2	2	1
2	2	9	3	3	3	1	1	2	2
2	2	2	2	3	2	2	2	2	3
2	2	2	2	2	2	2	1	2	1
1	1	3	2	3	2	2	1	1	1
2	2	9	9	9	9	9	9	9	9
2	3	1	1	2	3	1	3	2	9
1	1	2	1	2	2	2	1	1	1
1	1	1	2	2	1	1	2	1	1
1	1	2	2	2	1	2	1	2	2
2	2	2	2	2	2	2	2	2	2
9	9	9	9	9	9	9	9	9	9
3	2	2	3	3	3	3	3	3	3
9	9	9	9	9	9	9	9	9	9
9	9	9	9	9	9	9	9	9	9
9	9	9	9	9	9	9	9	9	9
9	9	9	9	9	9	9	9	9	9
2	2	2	2	2	2	2	2	2	2
3	1	2	2	2	2	2	2	2	2
9	9	9	9	9	9	9	9	9	9
2	2	2	2	2	2	2	2	2	2
2	2	2	2	2	2	2	2	2	2
2	2	2	2	2	3	2	1	2	3
1	2	2	2	2	2	2	2	2	2
2	2	2	2	2	2	2	2	2	2
1	3	2	1	3	2	1	3	2	1
9	9	9	9	9	3	9	3	9	9
1	1	1	1	2	1	1	1	1	1
3	3	3	3	3	3	3	3	3	3

2	1	1	1	2	2	1	1	1	1
2	2	1	1	1	2	2	1	1	1
1	1	2	1	1	1	1	1	1	1
1	2	2	2	2	1	2	2	2	2
9	9	9	9	9	9	9	9	9	9
9	9	9	9	9	9	9	9	9	9
2	2	2	2	2	2	2	2	2	2
3	3	3	3	3	3	3	3	3	3
1	9	9	9	9	9	9	1	9	9
9	9	9	9	9	9	9	9	9	9
1	1	2	2	2	2	3	2	9	1
2	1	1	1	1	2	2	2	1	1
3	2	2	1	1	1	1	1	2	2
3	3	3	3	3	3	3	3	3	3
3	3	3	1	3	3	3	3	3	3
2	2	3	3	3	3	1	2	3	2
1	1	3	2	2	2	3	1	1	2
2	2	1	3	2	3	1	3	2	3
3	9	9	9	9	9	9	9	9	9
1	1	3	2	1	3	3	3	2	1
2	3	1	1	3	1	2	3	1	2
1	2	2	1	2	2	1	2	2	1
3	3	3	2	9	1	1	2	1	9
1	1	2	2	2	2	2	2	2	2
1	1	1	1	3	3	3	3	3	3
3	3	2	2	2	2	2	2	2	2
2	3	3	2	2	2	2	2	1	2
2	2	2	2	2	2	2	2	2	2
9	3	1	9	2	3	1	3	3	1
1	1	3	3	3	3	3	1	3	9
2	2	2	2	2	2	2	2	2	2
2	2	2	2	2	1	1	1	2	1
1	1	3	1	1	1	3	1	1	1
1	1	3	3	1	3	2	3	1	1
1	9	9	9	9	9	9	9	9	9
1	2	2	2	2	2	1	1	1	1
9	9	9	9	9	9	9	9	9	9
2	2	2	2	2	2	2	2	2	2
9	9	9	9	9	9	9	9	9	9
1	1	1	1	1	1	1	1	2	1
9	9	9	9	9	9	9	9	9	9
3	1	2	3	3	3	2	1	2	1
1	3	2	1	2	2	2	3	2	3
1	1	2	1	1	2	1	2	2	2
1	1	2	1	1	2	1	2	2	2
1	3	1	2	3	3	3	2	1	2
2	3	3	2	3	2	3	2	2	2

1	2	2	1	3	1	2	3	1	2
9	9	9	9	9	9	9	9	9	9
1	1	3	2	2	1	2	2	2	2
1	1	3	2	9	3	3	2	3	3
1	1	3	1	1	1	1	1	1	1
9	9	9	9	9	9	9	9	9	9
9	9	9	9	9	9	9	9	9	9
2	2	2	1	2	2	2	2	2	2
3	1	1	1	1	3	1	1	1	3
1	1	3	3	3	3	3	3	9	3
1	1	1	3	1	1	1	2	2	2
3	2	1	2	3	1	2	3	2	1
2	2	2	3	2	3	2	2	3	2
1	1	3	1	1	1	1	1	1	1
9	9	9	9	9	9	9	9	9	9
3	3	3	3	3	3	3	3	3	3
3	3	3	2	2	1	2	3	2	3
2	2	2	2	2	2	2	2	2	2
3	1	2	3	1	1	1	2	3	3
3	2	1	3	2	1	2	3	2	2
9	9	9	9	9	9	9	9	9	9
9	9	9	9	9	9	9	9	9	2
3	3	1	2	3	1	1	2	2	2
9	9	9	9	9	9	2	9	9	9
9	9	9	9	9	9	9	9	9	9
3	3	2	3	9	2	3	3	2	9
3	3	2	2	3	2	3	2	2	2
2	2	2	2	2	2	2	2	2	2
3	3	3	3	3	3	3	3	3	3
1	1	2	1	2	2	1	2	1	1
3	3	3	3	3	3	3	3	3	3
2	2	2	2	2	2	2	1	3	2
9	9	9	9	9	9	9	9	9	9
9	9	9	9	9	9	9	9	9	9
9	9	9	9	9	9	9	9	9	9
1	2	2	2	2	1	2	2	2	2
1	1	3	1	3	3	1	1	1	3
3	1	2	1	3	1	2	1	3	1
2	2	3	2	2	3	2	2	2	2
9	9	9	9	9	9	9	9	9	9
2	2	2	2	2	2	2	2	2	2
2	1	3	1	2	3	1	2	3	1
3	3	2	3	2	3	2	3	2	3
1	1	2	1	1	9	9	9	9	9
1	1	2	2	3	2	1	2	3	2
3	3	1	3	3	3	3	1	3	1
1	1	3	3	2	3	3	3	2	1

3	1	2	3	1	2	3	2	1	2
2	3	3	3	3	2	2	2	2	3
3	3	3	3	9	1	1	1	1	3
3	1	2	3	1	2	3	1	3	1
1	1	3	2	2	3	3	2	2	1
2	2	2	2	2	2	1	1	2	2
2	1	1	9	2	1	2	2	1	2
2	2	2	2	2	2	1	1	1	1
9	9	9	9	9	9	9	9	9	9
2	1	2	1	2	2	1	1	1	1
2	2	2	2	2	2	2	2	2	2
2	2	2	3	3	3	3	3	2	2
1	2	3	2	1	3	3	1	2	1
1	1	3	1	1	1	1	2	3	2
9	9	9	9	9	9	9	9	9	9
2	3	3	3	3	2	2	1	3	2
1	1	2	1	1	1	2	2	3	1
3	1	1	2	2	2	3	3	3	2
2	1	3	2	1	1	3	1	3	2
2	2	2	2	2	2	2	2	2	2
1	1	1	1	1	2	2	1	2	1
2	2	2	2	2	2	1	2	2	2
1	2	1	2	1	1	1	1	2	1
1	1	2	2	2	3	2	2	2	2
3	9	2	1	2	2	2	2	2	2
1	1	2	3	1	3	3	2	2	1
2	2	3	2	2	3	2	1	1	2
1	1	3	3	2	3	2	2	2	1
2	2	2	2	2	2	2	2	2	2
2	2	2	2	2	2	2	2	1	2
2	2	2	2	2	2	9	1	1	2
2	2	2	2	2	2	2	2	2	2
2	2	2	2	2	2	2	2	2	2
1	1	2	3	3	3	3	3	3	3
3	1	3	1	2	3	3	1	1	2
1	1	3	1	1	1	3	2	2	2
2	2	1	1	1	2	2	1	1	1
2	2	2	2	1	1	2	2	1	1
3	3	3	3	1	3	2	1	1	1
1	2	2	2	1	1	1	1	3	3
3	3	3	3	3	9	2	2	2	2
2	2	1	1	2	2	1	1	1	2
1	2	2	2	2	1	2	2	2	2
1	1	1	1	1	1	1	1	1	1
1	2	2	2	2	1	1	1	1	2
1	2	1	1	2	2	2	2	1	1
2	3	3	2	1	1	1	1	1	2

1	1	1	2	2	1	2	2	2	2
3	2	1	1	1	2	2	2	1	1
1	2	2	2	1	1	1	2	1	1
3	3	3	3	2	2	2	3	3	2
1	2	1	2	2	2	1	1	2	1
3	1	2	2	3	3	1	1	2	1
2	1	1	2	2	1	2	3	3	2
1	2	2	2	1	1	1	1	2	2
1	1	2	2	1	3	2	2	2	2
1	1	2	2	1	3	2	2	2	2
2	2	2	2	2	2	2	2	2	2
3	3	3	3	1	1	1	1	1	1
1	1	1	1	1	1	1	1	1	1
2	2	2	2	2	2	2	2	2	2
2	2	2	2	2	2	2	2	2	2
2	2	2	2	2	2	2	2	2	2
1	1	2	2	2	2	2	1	1	1
3	3	3	3	9	2	2	2	2	2
1	1	3	3	2	2	3	2	2	2
9	1	2	3	2	1	2	2	2	1
1	1	2	2	2	2	2	1	1	1
1	1	2	2	1	3	2	2	2	2
3	3	3	3	3	3	1	2	9	2
9	9	9	9	9	9	9	9	9	9
2	2	2	2	2	2	2	9	1	2
2	2	2	2	2	2	2	2	2	2
1	1	1	2	2	3	3	2	1	2
3	3	3	3	3	3	3	3	3	3
3	3	3	3	1	2	1	1	1	1
1	1	3	3	2	3	3	1	1	1
2	2	2	2	1	2	1	2	3	1
1	3	2	2	3	1	3	1	3	2
2	1	2	2	1	1	1	1	1	2
3	2	2	2	2	2	2	2	2	2
3	3	3	3	3	3	3	3	3	3
2	1	3	2	3	2	1	2	2	2
2	2	2	2	2	2	2	2	2	2
2	2	2	2	2	2	2	2	1	1
3	3	3	3	3	3	3	2	1	1
1	1	1	1	2	2	1	1	2	1
2	2	2	2	2	2	2	2	2	2
2	2	2	2	2	2	2	2	2	3
2	2	2	2	2	2	2	2	2	2
3	3	3	3	3	3	3	3	3	3
3	3	3	3	3	3	3	3	3	3
1	1	1	3	3	2	3	3	2	2
2	1	1	2	1	1	1	2	2	1

2	3	3	2	1	1	2	3	2	1
3	3	2	1	2	2	2	1	1	1
2	2	3	2	1	1	2	1	1	1
2	1	2	2	2	1	1	1	2	2
2	3	3	2	2	1	1	1	1	1
2	2	1	1	2	1	2	2	1	1
3	3	2	2	2	3	3	3	3	3
2	2	2	1	1	1	1	1	2	2
2	2	1	1	1	2	2	1	1	2
2	2	1	1	1	2	2	2	1	1
3	3	2	1	1	2	2	3	2	2
2	2	2	2	2	2	1	1	1	1
2	2	2	1	1	2	2	2	1	1
1	2	2	1	1	1	1	2	2	1
1	2	2	1	1	1	2	2	1	1
1	2	2	1	1	1	2	2	1	1
2	3	3	3	2	2	2	3	2	2
1	1	1	1	1	1	1	2	2	3
9	9	2	9	9	9	9	2	9	9
1	1	2	2	1	1	2	2	1	1
9	9	2	9	9	2	9	9	9	2
3	3	3	3	3	3	3	3	3	3
1	1	2	1	1	1	1	2	2	1
1	1	2	2	2	2	2	1	1	1
2	1	1	2	2	2	1	1	2	2
2	2	2	1	1	1	2	2	1	1
2	2	1	1	1	1	2	2	1	1
2	2	1	1	2	2	1	1	1	1
2	1	2	2	1	1	2	1	1	1
1	1	2	2	1	1	2	1	2	2
1	2	2	3	2	1	2	3	2	1
2	2	2	2	2	2	2	2	2	2
3	3	2	2	3	2	2	2	3	3
1	2	2	3	2	2	1	2	2	1
2	2	3	3	2	2	2	1	2	2
2	1	3	2	1	9	3	3	1	3
2	3	1	9	9	2	9	9	1	9
2	9	2	9	2	9	9	2	9	2
9	9	9	9	9	9	9	9	9	9
3	1	9	2	1	9	1	9	1	9
2	9	9	1	9	9	9	9	9	9
1	2	1	1	1	2	1	1	2	2
9	9	1	9	9	2	1	9	9	1
2	2	2	2	2	2	2	2	2	2
2	9	9	1	3	9	9	1	9	9
9	9	2	9	9	9	2	9	9	9
9	9	9	2	9	9	9	9	9	2

9	9	9	9	2	9	9	9	2	9
9	9	9	9	2	9	9	9	2	9
9	2	9	9	2	9	9	9	2	9
9	9	9	9	9	9	9	9	9	9
1	2	9	9	9	1	2	9	3	2
2	9	9	9	9	9	2	9	9	9
9	9	9	9	9	9	9	9	9	9
9	9	9	9	9	9	9	9	9	9
1	2	1	2	2	1	1	2	1	1
9	2	9	9	2	9	2	9	2	9
2	1	1	2	2	1	1	2	2	1
1	1	1	2	2	1	1	2	2	2
2	1	2	3	2	2	1	1	2	2
2	1	1	2	1	2	1	2	2	1
2	1	1	2	2	1	1	2	1	1
2	2	2	3	2	2	1	1	2	3
2	2	2	2	2	2	2	2	9	2
2	2	1	2	2	1	1	2	2	3
1	1	2	2	1	2	2	1	2	1
3	3	2	2	3	2	2	3	2	3
1	2	2	1	2	2	1	1	2	2
1	2	2	1	2	2	2	1	2	2
2	1	2	1	2	2	9	1	2	2
2	2	1	2	2	2	3	2	2	3
2	3	2	2	2	1	1	2	1	1
2	2	3	3	2	2	3	2	3	2
9	9	2	9	9	2	9	9	2	9
2	2	2	3	2	2	2	2	3	3
2	2	3	3	2	2	1	2	2	1
3	3	3	3	3	3	3	3	3	3
9	9	9	9	9	9	9	9	9	9
3	3	2	2	2	2	2	2	2	2
2	2	3	2	2	1	2	2	1	2
2	2	2	2	2	2	2	2	2	2
2	2	2	2	2	3	2	2	2	2
3	3	3	2	3	2	3	2	3	3
3	2	2	3	2	3	3	2	2	3
9	9	9	9	2	9	2	9	9	9
2	2	1	2	2	2	1	2	1	2
2	2	3	2	1	2	2	3	2	2
2	1	1	2	2	1	1	2	2	2
3	3	3	3	3	3	3	3	3	3
1	2	2	2	1	1	2	2	3	2
1	1	2	2	1	2	2	2	3	2
2	3	2	2	2	2	2	1	2	2
2	1	1	2	1	1	1	1	2	2
2	1	2	2	1	1	2	2	1	1

2	2	2	1	1	2	2	1	1	2
2	2	1	2	2	3	2	2	2	1
2	2	2	1	2	2	1	2	2	2
2	1	1	2	2	1	1	2	2	2
2	3	2	3	2	1	2	2	1	3
1	1	2	2	2	2	2	1	1	1
2	2	2	3	2	3	2	2	3	2
1	1	1	2	2	2	2	1	2	1
2	2	3	1	1	1	2	1	1	1
2	3	2	2	3	2	2	2	2	3
3	3	3	2	3	3	2	2	3	3
1	1	1	2	2	1	1	1	2	1
3	1	2	3	1	3	1	2	1	2
2	2	2	1	2	3	2	2	2	1
2	2	3	2	2	1	2	3	1	1
2	2	2	2	2	2	2	2	2	2
2	2	2	2	2	2	2	2	2	2
1	1	2	1	2	2	2	2	2	2
2	3	3	2	1	3	2	1	1	3
2	2	2	2	2	2	2	2	2	2
1	1	2	2	2	1	1	2	2	2
2	2	2	2	2	2	2	2	2	2
2	2	2	2	3	3	1	1	1	1
1	2	3	1	2	3	2	3	2	2
1	1	3	1	3	3	3	3	1	1
3	3	3	2	2	2	2	2	3	3
1	1	2	2	2	1	3	2	2	2
1	2	3	2	2	1	1	1	1	1
2	2	2	2	2	2	2	1	2	2
2	2	2	2	2	2	2	2	2	2
3	3	2	2	1	1	1	2	2	1
1	1	3	3	3	3	3	1	3	1
2	2	2	3	2	2	2	1	2	2
2	2	2	2	2	2	2	2	2	2
3	3	3	1	1	2	9	2	2	1
2	2	2	2	3	1	1	2	2	1
1	1	3	3	2	2	1	1	2	1
2	2	2	2	2	2	2	2	2	1
1	2	3	2	1	3	2	1	2	1
3	3	3	2	2	3	2	2	2	2
2	2	2	2	2	3	2	1	2	1
2	2	2	2	2	2	2	2	2	2
2	2	2	2	2	2	2	2	2	2
3	2	3	3	3	3	3	2	2	3
2	2	2	2	2	2	2	2	2	2
1	1	2	2	2	2	2	2	2	2
1	1	2	1	1	2	2	2	2	2

2	2	2	2	2	2	2	2	2	2
2	2	3	1	1	2	1	1	2	1
2	2	2	2	2	2	2	2	2	2
2	2	2	2	2	2	2	2	2	2
2	2	2	2	2	2	2	2	2	2
1	2	2	2	2	2	2	2	2	2
2	2	2	2	2	2	2	2	2	2
2	3	1	2	1	2	3	1	3	1
2	2	2	2	2	2	2	2	2	2
9	9	9	9	9	9	9	9	9	9
2	2	2	2	3	3	1	1	1	1
2	2	1	1	2	1	1	2	2	2
3	2	1	3	2	1	3	2	1	3
2	2	2	2	2	2	2	2	2	2
2	2	2	2	2	2	2	2	2	2
1	2	2	2	2	2	2	2	2	2
2	2	2	2	2	2	2	2	2	2
2	2	2	3	2	3	2	3	2	2
3	3	3	3	3	3	3	3	3	3
9	1	1	1	1	1	1	1	1	1
3	3	3	3	3	3	3	3	3	3
3	3	3	3	3	3	3	3	3	3
2	2	2	2	2	2	2	2	2	2
1	3	1	1	1	2	3	2	2	3
2	2	2	2	9	1	2	1	3	2
1	1	2	2	2	3	1	1	1	1
2	2	1	2	2	2	2	2	2	2
2	2	2	1	1	2	2	2	2	2
1	2	2	2	2	1	1	1	1	2
9	9	9	9	9	9	9	9	9	9
2	2	2	2	3	3	3	2	2	2
2	3	2	3	2	3	2	3	2	3
2	3	2	3	2	2	3	2	3	2
3	3	3	3	3	3	3	3	3	3
1	2	2	2	2	2	2	2	2	2
3	1	2	3	9	1	2	1	2	2
1	1	2	2	2	3	3	3	2	2
9	1	1	2	2	2	3	3	2	2
2	2	2	2	2	2	2	2	2	2
2	3	3	3	2	2	2	2	2	2
3	2	1	1	3	2	1	2	3	1
1	1	2	2	2	2	2	2	2	2
2	2	2	2	2	2	2	2	2	2
1	1	3	3	1	3	3	1	2	2
3	3	3	3	3	3	3	3	3	3
1	1	1	3	3	3	1	1	1	1
2	2	1	1	1	2	2	1	1	1

2	2	3	3	3	2	2	2	2	2
2	2	3	3	3	3	3	3	3	3
3	3	3	3	3	3	3	3	3	3
2	2	2	2	2	2	2	2	2	2
2	2	2	2	3	1	1	1	1	1
9	9	9	9	9	9	9	9	9	9
2	2	2	2	2	2	2	2	2	2
2	2	2	1	2	2	2	2	2	2
1	2	2	2	2	2	2	1	1	1
2	3	3	3	3	3	3	3	3	3
2	2	3	2	1	2	1	1	1	2
1	1	2	3	2	3	1	2	3	2
9	9	9	9	9	9	9	9	9	9
9	9	9	9	9	9	9	9	9	9
9	9	9	9	9	9	9	9	9	2
2	3	2	2	3	2	2	2	3	3
2	2	2	2	2	2	2	2	2	2
9	9	9	9	9	9	9	9	9	9
1	1	3	2	2	1	1	1	1	1
1	1	2	2	2	3	3	2	2	1
1	2	2	2	1	2	2	3	1	2
1	1	3	2	2	1	1	1	1	1
1	1	3	2	2	1	1	1	1	1
2	2	2	2	2	2	2	2	2	2
2	2	2	2	2	2	2	2	2	2
3	3	2	2	3	9	2	9	2	2
2	2	2	2	2	2	2	2	2	2
1	1	3	2	2	1	1	1	2	1
9	9	9	9	9	9	9	9	9	9
1	1	3	1	3	1	3	3	3	3
3	3	2	1	2	3	3	3	3	3
1	2	2	2	3	1	1	1	1	1
2	2	1	2	2	2	2	1	1	1
2	2	2	2	2	2	2	2	2	2
2	2	2	3	2	2	2	1	1	1
2	2	3	3	2	3	2	2	2	2
2	2	1	2	2	3	2	2	2	3
2	2	2	2	2	2	2	2	2	2
2	9	2	2	2	2	2	2	2	2
2	2	2	2	2	2	2	2	2	2
1	2	1	2	2	1	2	2	2	1
2	2	1	2	3	2	2	1	2	2
2	2	2	2	2	3	2	1	2	2
2	2	2	2	2	2	2	2	3	2
2	2	2	2	2	2	2	2	2	2
2	2	2	2	2	2	2	2	2	2
1	2	2	2	1	3	2	2	3	3

2	2	2	2	1	1	2	2	1	1
1	1	2	2	2	2	1	1	3	2
9	9	9	9	9	9	9	9	9	9
2	2	3	2	1	3	1	2	2	2
1	1	3	2	2	1	1	1	1	1
2	2	2	2	2	2	2	2	2	2
2	2	2	2	2	2	2	2	2	2
2	3	3	2	1	2	2	3	1	2
1	1	2	2	2	2	2	2	1	2
2	1	1	1	1	1	1	1	2	2
1	2	2	2	2	2	2	2	2	2
2	2	2	2	2	2	2	2	2	2
3	2	3	1	2	2	3	2	3	2
1	1	3	1	2	2	2	1	1	1
1	1	3	2	2	2	9	2	1	1
3	3	2	1	2	3	1	1	3	1
9	9	9	9	9	9	9	9	9	9
3	3	3	3	3	2	2	2	2	2
3	3	3	1	1	1	3	3	9	1
1	1	1	1	3	3	1	1	1	1
2	2	2	2	2	2	2	2	2	2
9	9	9	9	9	9	9	9	9	9
3	3	3	3	9	1	2	1	2	2
1	3	1	3	2	1	2	1	2	1
2	2	2	2	2	2	2	2	2	2
9	9	9	9	9	9	9	9	9	9
9	9	9	9	9	9	9	9	9	9
2	2	3	9	1	2	2	3	1	1
9	9	9	9	9	9	9	9	9	9
9	1	9	9	9	9	9	9	9	9
9	9	9	9	9	2	9	9	9	9
9	9	9	9	9	9	9	9	9	9
9	9	9	9	9	9	9	9	9	9
1	2	2	2	3	3	3	1	1	1
2	1	2	3	1	2	3	2	1	3
1	2	2	2	2	3	9	2	9	2
1	2	2	2	2	3	1	2	1	2
2	9	2	1	1	1	1	1	1	1
1	1	1	2	2	2	2	2	2	2
2	2	2	1	1	1	2	2	2	2
2	2	2	2	2	2	2	2	2	2
1	3	3	3	3	3	1	2	1	2
2	2	3	3	2	2	2	2	2	3
2	1	3	1	2	3	2	1	1	3
2	1	3	2	3	2	1	2	3	2
2	2	2	2	2	2	3	2	2	2
2	9	3	3	2	3	2	2	2	2

1	1	3	2	1	2	3	1	1	1
1	1	2	1	2	3	1	2	1	3
1	3	3	3	3	3	9	9	9	9
9	9	9	9	9	9	9	9	9	9
9	9	9	9	9	9	9	9	9	9
9	1	1	1	1	1	1	2	1	3
1	3	3	2	2	1	3	1	1	1
9	9	9	9	9	9	9	9	9	9
9	9	9	9	9	9	9	9	9	9
2	2	2	2	2	1	1	1	1	1
1	1	2	1	2	2	2	1	2	2
2	2	2	2	2	2	2	2	2	2
2	1	1	1	1	2	2	2	2	2
9	9	9	9	9	9	9	9	9	9
9	9	9	9	9	9	9	9	9	9
9	9	9	9	9	9	9	9	9	9
2	3	3	3	2	3	1	1	1	3
9	9	9	9	9	9	9	9	9	9
9	9	9	9	9	9	9	9	9	9
9	9	9	9	9	9	9	9	9	9
9	9	9	9	9	9	9	9	9	9
9	9	9	9	9	9	9	9	9	9
9	9	9	9	9	9	9	9	9	9
9	9	9	9	9	9	9	9	9	9
2	2	2	2	2	2	2	2	2	2
2	2	3	2	2	3	3	2	2	1
2	2	2	3	3	3	3	2	2	2
2	2	2	2	2	2	2	2	2	2
1	1	3	3	3	3	3	2	3	1
2	2	2	2	2	3	2	2	9	1
2	2	2	2	2	2	2	2	2	2
2	2	2	2	2	2	2	2	2	2
2	2	2	2	2	2	2	2	2	2
2	2	2	2	2	2	2	2	2	2
3	3	2	3	2	3	2	2	2	3
1	1	3	3	2	3	3	1	1	1
2	3	3	2	2	1	3	1	1	1
2	2	2	2	2	2	2	2	2	2
2	2	2	2	2	2	2	2	2	2
2	2	2	2	2	2	2	2	2	2
1	1	2	2	2	1	2	1	1	1
2	2	2	2	3	2	2	2	3	2
1	2	2	2	2	2	2	2	2	2
9	9	9	9	9	9	9	9	9	9
9	9	9	9	9	9	9	9	9	9
2	2	2	2	2	1	2	2	2	2
2	2	2	2	2	2	2	2	2	2

1	1	1	1	1	1	1	1	1	1
1	2	2	1	1	1	1	1	1	1
2	3	2	2	2	3	3	3	2	2
2	2	2	2	2	2	2	2	2	2
2	2	2	1	2	2	2	2	3	2
3	2	3	3	2	3	2	1	1	1
1	1	3	1	2	3	1	2	2	3
3	1	2	3	1	2	3	1	3	1
9	9	9	9	9	9	9	9	9	9
1	1	2	1	2	1	2	1	2	1
2	2	2	2	2	2	2	2	2	2
2	3	3	3	2	1	3	2	2	3
1	3	1	2	3	2	2	2	2	2
2	2	2	2	2	2	2	2	2	2
2	3	2	3	2	3	2	3	2	3
2	2	3	2	3	1	3	2	3	1
1	2	3	2	3	2	2	3	3	1
3	1	2	3	1	2	3	1	2	3
2	2	2	2	2	2	2	2	2	2
1	1	1	1	1	1	1	1	1	1
1	2	2	1	2	3	3	3	2	1
2	2	2	2	2	2	2	2	2	2
1	1	1	2	2	2	3	2	2	3
2	3	1	2	1	1	2	1	2	2
2	2	2	2	2	2	2	2	2	2
3	1	3	2	3	1	2	1	3	3
2	2	2	2	2	2	2	2	2	2
2	1	2	2	2	1	2	2	2	3
3	2	3	1	2	3	1	2	3	2
9	9	9	9	9	9	9	9	9	9
1	1	1	1	2	2	1	2	2	1
9	9	9	9	9	9	9	9	9	9
9	9	9	9	9	9	9	9	9	9
1	1	1	1	1	1	1	1	1	1
2	1	2	1	3	1	3	2	1	3
2	2	1	2	1	3	3	2	2	2
3	2	3	1	2	3	1	2	3	1
2	1	1	2	2	2	2	2	2	2
2	2	2	2	2	2	2	2	2	2
2	2	3	3	3	3	3	3	3	2
1	1	2	3	2	1	2	1	9	2
1	1	1	2	2	1	1	1	1	2
2	2	2	2	2	2	2	2	2	2
2	2	2	1	2	2	2	2	2	2
1	3	3	1	9	9	2	2	2	2
2	2	2	2	2	2	2	2	2	2
2	2	2	2	2	2	2	2	2	2

1	1	2	2	3	1	1	1	1	1
2	2	2	2	2	2	2	2	2	2
2	2	2	2	2	2	2	2	2	2
1	9	2	1	2	2	3	2	1	1
1	1	2	3	1	1	2	2	3	2
2	2	2	2	2	2	2	2	2	2
2	2	2	2	2	2	2	2	2	2
1	2	3	2	2	1	2	3	2	2
9	9	9	9	9	9	9	1	9	9
2	2	2	2	2	2	2	2	2	2
2	2	2	2	2	2	2	2	2	2
3	2	3	2	2	3	1	2	3	2
9	9	9	9	9	9	9	9	9	9
2	2	3	2	2	2	3	2	2	2
9	9	9	9	9	9	9	9	9	9
3	3	3	3	1	3	1	2	2	3
1	2	2	2	2	2	1	1	1	1
2	3	9	1	3	2	3	1	9	1
1	3	3	3	3	3	2	2	2	3
1	1	3	1	2	3	2	2	2	2
2	1	2	1	9	1	9	1	1	2
3	3	3	3	3	3	3	3	3	3
2	2	2	2	2	2	2	2	2	2
3	2	2	2	3	3	2	2	2	2
1	3	1	1	2	3	2	1	2	1
2	2	2	2	2	2	2	2	2	2
2	2	2	2	2	2	2	2	2	2
1	2	2	2	2	2	2	2	2	2
1	2	2	1	2	3	1	1	2	1
2	2	2	2	2	3	2	2	2	2
1	1	1	1	1	1	1	1	1	1
2	2	2	2	2	2	2	2	2	2
1	2	2	3	2	2	3	2	2	2
1	2	2	2	2	1	2	2	2	2
1	1	2	2	2	2	3	2	2	2
9	9	9	9	9	9	9	9	9	9
2	2	2	3	2	2	2	2	2	2
9	9	9	9	9	9	9	9	9	9
2	3	2	2	1	2	3	2	1	2
2	2	2	2	2	2	2	2	2	2
2	2	2	3	2	2	2	2	2	2
9	9	9	9	9	9	9	9	9	9
2	2	2	2	2	2	2	2	2	2
2	2	2	2	2	2	2	2	2	2
9	9	9	9	9	9	9	9	9	9
9	9	9	9	9	9	9	9	9	9
9	9	9	9	9	9	9	9	9	9

3	3	3	9	2	2	2	2	2	3
1	1	2	1	9	1	2	3	2	2
9	9	9	9	9	9	9	9	9	9
1	1	3	3	3	3	3	1	2	1
1	2	2	2	2	1	1	1	1	2
2	3	2	2	2	2	1	1	1	2
2	2	2	2	2	2	2	2	2	2
2	9	9	9	2	9	9	9	2	9
3	3	3	3	3	3	3	3	3	3
9	9	9	9	9	9	9	9	9	9
3	3	3	3	3	3	3	3	3	3
1	3	1	3	1	3	1	3	1	3
1	1	3	9	3	3	3	3	2	1
1	1	2	2	2	2	3	2	2	3
2	2	2	2	2	2	2	2	2	2
9	3	3	3	3	9	3	3	3	9
3	3	3	2	1	3	2	2	2	3
9	9	9	9	9	9	9	9	9	9
9	9	9	9	9	9	9	9	9	9
3	1	3	2	1	3	1	2	3	2
3	2	1	2	3	2	1	2	3	2
1	1	1	1	1	1	3	3	3	3
2	2	2	2	2	2	2	2	2	2
3	3	3	3	3	3	3	3	3	3
3	2	1	2	3	2	1	2	3	2
1	1	3	2	2	3	2	1	1	1
1	1	9	9	9	9	2	2	1	1
2	2	2	1	1	2	2	2	3	3
9	9	9	9	9	9	9	9	9	9
9	9	9	9	9	9	9	9	9	9
9	9	9	9	9	9	9	9	9	9
9	9	9	9	9	9	9	9	9	9
9	9	9	9	9	9	9	9	9	9
2	3	2	2	2	2	2	3	3	2
1	1	9	1	9	2	1	2	3	2
9	9	9	9	9	9	9	9	9	9
9	9	9	9	9	9	9	9	9	9
3	9	2	3	9	2	3	1	9	1
1	1	1	9	2	1	1	1	1	1
9	9	9	9	9	9	9	9	9	9
2	2	2	2	2	2	2	2	2	2
1	1	2	2	2	3	2	2	1	1
1	2	2	2	2	1	1	2	2	2
2	2	2	2	2	2	2	2	2	2
3	1	2	3	1	2	2	2	3	3
3	2	2	2	2	3	1	1	2	2
3	1	2	1	2	1	2	1	2	2

3	3	3	3	3	3	3	3	3	3
2	2	2	2	2	2	2	2	2	2
9	9	9	9	9	9	9	9	9	9
2	2	3	3	1	1	2	2	3	3
1	1	2	2	2	2	3	3	3	3
1	1	1	1	1	1	1	3	3	3
2	3	9	9	1	9	2	2	9	2
2	2	2	2	2	2	2	2	2	2
2	2	2	2	2	2	2	2	2	2
1	2	2	2	9	9	9	9	9	9
1	1	2	2	3	2	2	2	2	3
1	2	1	2	2	1	9	1	2	1
9	9	9	9	9	9	9	9	9	9
1	1	1	1	1	1	1	1	1	1
2	1	2	2	1	2	3	3	3	3
2	2	3	1	1	1	1	1	1	2
1	2	2	2	3	3	3	1	1	2
9	9	9	9	9	9	9	9	9	9
2	1	2	2	3	1	2	2	2	3
9	9	9	9	9	9	9	9	9	9
2	9	9	9	9	9	9	9	9	9
2	1	3	3	3	3	9	2	2	2
9	9	9	9	9	9	9	9	9	9
9	9	9	9	9	9	9	9	9	9
9	9	9	9	9	9	9	9	9	9
9	9	9	9	9	9	9	9	9	9
1	2	2	2	1	2	2	1	2	1
3	2	2	1	3	3	2	3	3	3
9	9	9	9	9	9	9	9	9	9
2	2	2	2	3	3	3	3	3	3
1	3	1	3	1	3	1	3	1	3
9	9	9	9	9	9	9	9	9	9
2	2	2	2	2	2	2	2	2	2
2	2	2	2	2	2	2	2	2	2
2	2	2	2	2	2	2	2	2	2
2	2	2	2	2	2	2	2	2	2
3	2	2	1	3	2	1	3	2	3
2	2	2	2	2	2	2	2	2	2
1	1	2	2	2	1	1	3	2	1
9	9	9	9	9	9	9	9	9	9
9	1	2	9	1	9	1	9	1	9
2	2	2	2	2	3	3	1	1	1
1	1	3	9	2	2	2	2	2	2
1	1	3	2	2	1	2	2	2	2
2	1	3	2	2	3	2	2	3	1
1	1	1	1	1	1	1	1	1	1
2	2	2	2	2	2	3	2	2	2

2	3	3	2	3	2	1	1	1	1
1	1	2	1	2	2	2	1	1	2
3	2	3	1	2	1	3	1	2	2
2	2	2	2	2	2	2	2	2	2
1	2	2	2	2	2	2	2	2	2
1	2	2	2	2	2	2	2	2	2
2	2	2	2	2	2	2	2	2	2
3	3	2	2	3	3	3	3	2	3
2	2	2	2	2	2	2	2	2	2
9	9	9	9	9	9	9	9	9	9
2	2	3	3	2	3	2	1	1	1
3	1	1	2	1	2	1	2	1	3
9	9	9	9	9	9	9	9	9	9
3	1	2	3	2	1	2	2	2	2
9	9	9	9	9	9	9	9	9	9
9	9	9	9	9	9	9	9	9	9
1	1	3	1	3	3	3	1	1	1
2	2	2	2	2	2	2	2	2	2
2	3	2	3	2	3	2	3	2	3
1	1	2	2	2	2	2	2	2	2
1	1	2	9	1	1	1	1	1	1
2	2	2	2	2	2	2	2	2	2
9	9	9	9	9	9	9	9	9	9
3	1	2	3	1	1	1	1	1	1
1	1	1	1	1	1	1	1	1	1
3	3	3	3	3	3	3	3	3	3
2	2	1	2	9	2	3	1	2	3
2	2	2	2	1	1	1	2	2	2
1	1	1	2	1	2	2	1	1	1
2	2	2	2	2	2	2	2	2	2
1	1	3	3	3	1	3	3	3	1
1	9	9	9	9	9	9	9	9	9
2	2	2	2	2	2	2	2	2	2
2	3	2	1	3	2	1	2	3	1
3	3	2	1	2	1	2	3	1	2
2	1	1	3	2	1	3	1	3	2
2	2	2	2	2	2	2	2	2	2
2	2	2	2	2	2	2	2	2	2
2	2	1	1	2	2	3	2	2	2
2	2	3	3	2	2	2	2	2	2
3	2	3	3	3	2	2	2	2	2
1	1	1	1	1	2	1	2	2	1
2	2	2	2	2	2	2	2	2	2
2	3	3	3	2	1	1	2	2	9
9	9	9	9	9	9	9	9	9	9
3	1	2	3	1	2	3	1	2	1
3	2	2	3	3	3	3	3	3	3

1	1	2	2	2	2	2	2	2	2
1	1	3	3	3	1	3	1	1	3
1	1	3	3	3	1	3	1	1	3
2	2	1	2	2	1	1	2	2	2
9	9	9	9	9	9	9	9	9	9
9	9	9	9	9	9	9	9	9	9
9	9	9	9	9	9	9	9	9	9
2	2	1	2	2	2	2	2	2	2
1	1	2	1	2	1	2	1	1	2
1	1	3	2	2	3	3	2	2	2
2	2	1	2	2	2	3	1	1	1
1	1	3	2	1	3	3	2	2	3
2	2	2	2	2	2	2	2	2	2
2	2	2	2	2	2	2	2	2	2
2	2	2	2	2	2	2	2	2	2
2	2	2	2	1	1	2	2	2	2
2	2	2	2	2	3	3	3	3	3
2	2	2	2	2	2	2	2	2	2
2	2	2	2	2	2	2	2	2	2
1	2	3	1	3	2	1	3	2	1
2	2	2	2	2	2	2	2	2	2
9	9	9	1	9	9	9	9	9	9
1	2	9	1	9	1	2	1	2	9
2	2	2	2	2	2	2	2	2	2
1	2	2	2	2	1	3	2	2	2
1	2	2	2	2	2	2	2	2	2
2	2	2	2	2	2	2	2	2	2
9	9	9	9	9	9	9	9	9	9
2	1	3	2	1	1	3	2	1	3
2	2	2	1	2	2	2	1	2	1
2	2	2	2	2	1	2	2	2	1
1	2	3	2	3	1	1	1	1	1
2	2	3	1	2	9	1	2	1	3
1	1	3	1	1	1	1	3	1	2
2	2	3	3	2	3	3	2	2	3
3	2	3	2	2	3	3	2	2	2
2	2	2	2	2	2	2	2	2	2
2	2	2	2	2	2	2	2	2	2
2	2	2	2	2	2	2	2	2	2
9	9	9	9	9	9	9	9	9	9
1	2	2	2	1	2	2	1	2	1
3	3	2	2	2	2	3	3	3	3
1	1	1	2	2	3	3	2	1	1
2	2	2	2	2	2	2	2	2	2
1	2	2	2	3	3	3	1	1	2
2	2	2	2	2	2	2	2	2	2
2	2	2	2	2	2	2	2	2	2

2	2	2	2	2	2	2	2	2	2
9	9	9	9	2	9	9	9	9	1
2	2	2	2	2	2	2	2	2	2
3	1	2	3	1	2	1	1	1	2
2	2	3	1	2	2	2	2	2	2
2	2	2	2	2	2	2	2	2	2
3	2	3	2	3	2	3	2	3	2
2	2	2	2	2	3	2	1	1	1
9	9	9	9	9	9	9	1	9	9
2	2	2	2	2	2	2	2	2	1
2	2	2	1	2	2	3	2	2	2
2	2	2	2	2	2	2	2	2	2
2	2	2	2	2	2	2	2	2	2
1	1	3	3	3	3	3	3	3	3
2	2	2	2	2	2	2	2	2	2
2	2	2	2	2	2	2	2	2	2
2	2	3	3	3	2	2	1	1	2
9	9	9	9	9	9	9	9	9	9
9	9	9	9	9	9	9	9	9	9
1	1	1	1	1	1	1	2	1	2
2	1	1	1	1	1	2	2	2	3
2	3	3	2	3	3	3	2	2	2
2	2	2	2	2	2	2	2	2	2
3	1	2	2	2	2	1	3	1	3
9	9	9	9	9	9	9	9	9	9
3	3	2	2	2	3	2	1	2	2
2	2	2	2	2	2	2	2	2	2
2	2	2	2	2	2	3	2	2	2
2	2	3	2	2	1	1	1	1	2
2	2	2	2	2	2	2	2	2	2
2	2	2	2	2	2	2	2	2	2
2	1	2	2	2	3	1	2	3	2
1	1	2	3	2	1	3	2	2	1
2	9	2	3	1	2	3	1	2	1
2	2	2	2	2	2	1	1	1	1
3	3	3	2	1	2	1	2	3	3
2	2	3	2	2	2	2	3	3	2
2	2	2	2	2	2	2	2	2	2
2	2	2	2	2	2	2	2	2	2
2	2	2	2	2	2	2	2	2	2
9	9	9	9	9	9	9	9	9	9
1	1	2	3	3	3	1	1	2	1
2	2	2	2	2	9	1	1	1	1
3	3	3	3	1	3	1	1	1	1
1	1	1	1	2	2	1	1	1	1
1	2	2	2	2	1	3	2	2	1
1	2	3	2	2	3	2	1	1	1

3	2	3	1	2	1	2	2	3	2
9	9	9	9	9	9	9	2	9	9
9	9	9	9	9	9	9	9	9	9
9	9	9	9	9	9	9	9	9	9
2	2	2	2	2	2	2	2	2	2
2	2	2	2	2	2	2	2	2	2
2	2	2	2	2	1	1	2	1	1
1	1	1	1	1	1	1	1	1	1
9	9	9	9	9	9	9	9	9	9
9	9	9	9	9	9	9	9	9	9
2	2	2	2	1	2	2	1	2	2
2	2	3	2	2	2	3	2	3	3
2	2	2	2	2	2	2	2	2	2
2	2	2	2	2	2	2	2	2	2
3	3	2	2	3	1	9	2	2	3
2	2	2	2	3	2	3	1	1	1
2	2	3	3	3	3	2	1	1	1
2	2	2	2	2	3	2	1	1	1
1	1	9	2	2	2	2	2	1	9
9	9	9	9	9	9	9	9	9	9
1	1	1	1	3	3	3	1	1	2
2	2	3	3	1	2	3	2	1	1
1	1	2	3	2	3	3	3	3	1
1	2	3	1	2	3	2	1	2	3
2	2	2	2	9	1	1	1	1	1
1	1	2	1	2	2	2	2	2	1
9	1	1	2	2	2	3	1	1	1
2	3	1	2	2	1	2	2	2	1
1	1	3	3	1	9	1	2	1	2
1	2	2	2	2	1	2	2	1	1
1	1	2	2	1	2	1	1	1	1
3	9	2	3	1	3	2	3	2	3
2	1	2	3	1	3	1	3	1	2
2	2	3	3	1	3	3	2	3	1
1	2	3	3	2	3	3	1	1	2
1	2	3	2	2	3	3	1	1	2
1	1	2	2	1	2	2	1	1	1
1	1	2	2	1	2	2	2	2	1
2	2	3	2	1	2	2	1	1	1
1	1	1	2	2	1	1	1	1	2
1	1	3	1	2	2	3	1	1	2
1	1	3	2	2	2	1	2	2	2
9	9	9	9	9	9	9	9	9	9
1	3	3	2	2	2	2	2	1	1
2	1	2	1	2	2	1	2	1	9
3	3	3	1	1	1	3	1	1	1
2	2	1	2	9	1	1	2	1	1

2	2	3	3	3	3	3	1	1	1
9	9	9	9	9	9	9	9	9	9
2	1	2	1	2	3	1	2	1	3
1	1	3	3	2	2	2	2	2	2
1	2	2	2	2	2	2	2	2	2
3	3	3	3	2	3	3	2	1	1
3	1	2	1	3	2	3	2	3	1
2	2	2	2	2	2	2	2	2	2
2	2	2	2	2	2	2	2	2	2
9	9	9	9	9	9	9	9	9	9
2	2	2	9	2	1	2	2	2	2
2	2	2	2	1	2	1	1	1	2
1	3	1	3	1	3	1	3	1	3
1	3	2	1	3	2	1	3	2	1
2	2	2	2	2	2	2	2	2	2
9	9	9	9	9	9	9	9	9	9
1	9	9	9	9	9	9	9	9	9
2	2	9	1	1	2	9	3	3	3
2	2	2	2	3	3	3	2	2	2
2	2	1	2	2	2	2	2	2	2
9	9	9	9	9	9	9	9	9	9
2	2	2	2	1	1	1	2	2	2
2	2	2	2	2	2	2	2	2	2
1	2	2	2	1	1	2	2	2	2
2	2	2	2	2	2	2	2	2	2
2	2	2	1	1	1	1	2	1	1
2	1	1	2	2	2	1	2	2	2
2	2	2	2	3	3	2	2	2	3
2	2	2	1	2	2	1	1	1	2
2	3	2	2	1	1	2	2	3	2
9	9	9	9	9	9	9	9	9	9
9	9	9	9	9	9	9	9	9	9
9	9	9	9	9	9	9	9	9	9
2	2	1	3	2	2	3	2	2	2
1	2	2	3	3	2	2	1	1	1
2	2	3	2	2	2	3	2	1	1
1	1	3	3	3	3	2	1	2	1
2	2	3	3	3	3	3	2	2	3
2	1	2	2	1	2	1	1	2	1
2	2	2	2	2	2	2	2	2	2
1	1	3	3	2	3	3	3	3	1
1	2	2	1	2	1	2	2	2	2
2	2	2	2	2	2	2	2	2	1
3	3	1	3	3	3	3	1	1	1
2	2	2	2	2	2	2	2	2	2
1	1	1	1	1	1	1	1	1	1
1	1	3	3	1	1	3	3	3	3

9	9	9	9	9	9	9	9	9	9
2	2	2	2	2	2	2	2	2	2
9	9	9	9	9	9	9	9	9	9
1	1	3	3	3	3	1	9	9	9
3	3	3	3	3	3	3	3	3	3
2	3	1	2	3	2	1	2	3	2
1	1	1	1	2	2	3	1	1	2
9	9	9	9	9	9	9	9	9	9
2	2	2	2	2	2	2	2	2	2
9	9	9	9	9	9	9	9	9	9
9	2	9	1	9	9	9	9	2	9
9	9	9	1	9	9	9	9	9	9
2	1	2	2	1	9	1	1	1	2
9	9	9	9	9	9	9	9	9	9
3	3	3	2	3	3	2	1	1	2
2	2	2	2	2	2	2	2	2	2
2	2	1	2	1	2	2	1	1	1
2	2	1	2	1	2	2	1	1	1
2	2	2	2	2	2	2	2	2	2
2	2	1	2	2	2	3	3	2	9
1	2	1	2	2	2	2	1	2	2
2	2	2	2	2	2	2	2	2	1
2	2	1	2	2	2	2	1	1	1
2	3	3	3	2	2	2	3	2	3
3	2	3	2	1	2	3	2	3	2
3	2	1	2	2	2	3	3	1	1
2	2	2	2	2	2	2	2	2	2
3	3	3	3	3	2	3	1	2	2
2	2	2	2	2	2	2	2	2	2
2	2	2	3	3	2	2	2	1	2
2	2	2	2	2	2	2	2	2	2
1	1	3	2	2	3	2	2	2	2
2	3	3	2	3	1	3	2	1	1
1	2	3	3	1	2	1	1	1	1
2	2	9	9	9	2	9	9	9	2
2	2	2	3	2	3	3	1	1	3
2	2	2	3	2	3	3	1	1	3
1	1	3	3	2	3	3	3	3	3
2	2	2	2	2	2	2	2	2	2
2	2	2	3	2	2	2	2	2	2
1	3	1	1	1	3	3	1	1	1
9	9	9	9	9	9	9	9	9	9
9	9	9	9	9	9	9	9	9	9
2	2	2	2	2	2	2	2	2	2
9	9	1	9	9	9	9	9	9	9
2	2	2	2	2	2	2	2	2	2
2	2	2	2	2	3	1	2	1	2

2	2	2	2	2	2	2	2	2	2
2	1	1	2	2	1	1	2	2	2
1	2	1	3	1	2	3	1	2	3
2	2	2	2	2	3	2	2	1	2
2	2	2	2	2	2	2	2	3	3
2	3	3	2	2	2	3	3	3	3
3	1	1	2	2	3	2	2	2	2
1	1	2	2	2	2	1	2	2	2
3	3	2	3	2	3	3	1	1	1
2	2	3	2	1	2	3	1	2	3
3	3	3	2	3	2	2	2	2	1
1	2	3	2	2	2	3	2	1	2
1	3	3	3	3	3	3	1	3	3
2	3	3	9	2	3	9	2	2	9
1	2	3	3	2	2	1	1	2	2
2	2	2	2	2	2	2	2	2	2
2	2	2	2	2	2	2	1	1	1
2	2	2	2	2	2	2	2	2	2
2	2	2	2	2	2	2	2	3	3
1	1	2	2	2	2	2	1	1	1
2	2	2	2	3	3	2	2	1	1
2	2	2	2	3	2	2	2	2	2
2	2	2	2	2	1	3	1	1	1
2	2	2	2	2	2	2	2	2	2
1	1	3	3	1	1	1	1	1	1
2	2	2	2	2	2	2	2	2	2
2	2	2	2	2	2	2	2	2	2
1	1	3	2	2	2	2	2	9	1
1	1	2	2	2	2	2	1	1	1
2	3	2	2	2	3	2	2	2	2
9	9	9	9	9	9	9	9	9	9
2	2	1	2	2	2	2	2	2	3
2	2	1	2	2	2	2	2	2	2
2	2	2	2	2	2	2	2	2	2
1	2	2	3	2	2	2	3	2	2
2	2	3	2	2	1	2	2	3	2
2	2	3	2	2	1	2	2	3	2
2	2	3	2	2	2	2	2	2	9
3	2	1	2	3	2	1	2	3	1
1	3	3	3	3	3	3	3	3	3
1	1	2	2	2	3	1	2	1	1
1	1	2	1	2	2	2	2	2	1
3	3	3	1	1	1	3	1	1	1
2	1	2	2	3	3	1	1	1	1
1	1	2	2	2	1	2	3	3	2
2	2	2	2	2	2	2	3	3	3
1	2	3	3	2	3	3	1	1	2

1	2	3	3	2	3	3	1	1	2
2	2	2	2	3	3	3	2	2	2
2	2	3	2	1	2	2	3	2	2
3	2	2	2	2	2	3	2	2	2
2	2	2	3	2	2	2	2	1	1
2	2	3	3	3	2	2	2	3	2
2	2	2	3	3	3	3	3	3	3
2	2	2	2	2	2	2	2	2	2
1	3	2	1	3	2	3	1	3	2
3	2	2	1	2	3	1	1	1	1
2	2	2	2	2	1	1	1	1	9
1	1	2	2	2	3	2	2	2	1
3	3	3	3	3	3	3	3	3	3
2	1	2	2	2	1	1	1	2	2
1	1	1	1	1	1	1	3	3	3
2	2	2	2	1	2	2	2	2	2
2	3	3	2	2	9	1	1	1	3
2	2	3	2	2	2	2	3	3	2
2	3	3	3	2	2	2	2	2	2
3	2	2	3	3	2	2	2	2	3
2	2	2	2	2	2	2	3	3	3
3	3	3	3	3	3	3	3	3	3
2	2	2	2	2	2	2	2	2	2
1	1	3	3	3	3	3	2	2	2
2	1	2	1	3	1	2	1	2	3
1	2	3	3	2	2	2	2	2	1
2	2	1	1	2	3	3	2	1	2
2	2	2	2	2	2	2	2	2	1
1	2	2	1	1	2	2	1	2	1
2	2	2	2	2	2	9	2	2	2
1	1	2	2	1	1	1	1	1	2
2	2	2	2	2	9	1	1	1	1
2	2	2	1	1	1	1	1	1	2
1	3	3	3	2	2	2	1	1	1
2	3	9	1	2	1	3	2	9	1
1	1	3	3	3	3	3	3	2	2
2	2	2	1	1	1	1	3	2	3
1	2	2	2	2	1	3	2	2	1
1	3	2	1	2	1	3	1	2	3
2	2	2	2	1	2	3	2	1	2
1	3	3	3	3	3	2	2	3	3
2	3	3	3	2	3	3	1	1	1
2	2	2	2	2	2	2	2	2	2
2	2	1	3	2	3	1	1	1	1
2	2	3	3	2	3	2	2	2	2
1	1	2	3	2	2	1	2	2	1
2	2	2	2	2	2	2	2	2	2

2	2	2	2	2	2	2	2	2	2
2	2	2	2	2	2	2	2	2	2
2	2	1	2	2	2	2	2	2	3
1	3	3	3	2	3	9	2	3	2
2	2	2	3	3	2	2	2	3	2
3	2	2	3	2	2	2	2	2	2
2	2	2	2	2	2	2	2	2	2
2	2	2	3	2	2	2	3	3	2
9	9	9	9	9	9	9	9	9	9
2	2	2	1	2	2	2	2	2	2
1	3	2	3	3	3	2	1	1	1
2	2	2	2	2	2	2	2	2	2
3	3	3	3	3	3	3	3	3	3
2	3	2	3	2	3	3	2	3	2
1	1	1	1	1	1	1	1	1	1
2	2	2	2	2	2	2	2	2	2
2	2	2	2	2	2	2	2	2	2
1	1	1	1	1	1	1	1	1	2
3	2	1	2	3	2	1	2	3	2
2	2	2	2	2	2	2	2	2	2
2	2	1	2	2	2	3	2	2	2
1	2	2	2	2	1	1	2	1	2
2	2	2	2	2	2	2	2	2	2
2	2	2	2	3	2	2	3	2	2
1	1	1	1	1	1	1	1	1	1
2	2	2	2	2	2	2	2	2	2
2	2	2	2	2	2	2	2	2	2
2	2	2	1	2	2	2	2	2	2
1	3	2	1	2	1	3	1	2	3
2	2	2	2	2	2	2	2	2	2
2	2	2	1	1	1	1	3	2	3
9	9	9	9	9	9	9	9	9	9
2	2	2	2	2	2	2	2	2	2
1	1	3	3	3	1	3	1	1	3
2	2	2	2	2	2	2	2	2	2
1	1	3	1	2	1	3	1	1	1
1	1	3	3	3	1	1	1	1	1
3	1	2	3	1	2	3	1	2	3
2	2	2	2	2	2	2	2	2	2
3	2	1	2	3	2	1	2	3	1
1	1	2	2	2	1	2	1	1	1
1	1	2	2	2	2	1	2	1	1
3	3	3	2	2	9	3	1	1	1
2	1	2	2	1	3	2	1	1	1
3	2	3	2	2	3	3	1	2	2
2	2	2	2	2	2	1	2	3	2
1	2	2	2	2	2	3	2	2	2

1	3	2	1	2	1	2	2	2	1
1	1	3	2	3	1	3	2	1	1
1	1	1	1	1	1	1	1	1	1
2	2	2	2	1	3	1	1	2	1
2	2	2	2	2	2	2	2	2	2
1	1	2	2	2	2	2	2	2	1
2	2	1	2	1	3	1	1	1	1
9	9	9	9	9	1	9	9	9	9
9	9	9	9	9	9	9	9	9	9
9	9	9	9	9	9	9	9	9	9
3	1	2	1	3	2	1	3	2	1
3	1	2	2	3	1	1	2	1	2
3	3	3	1	2	3	3	1	1	1
3	3	3	1	1	2	3	3	1	1
2	1	1	1	2	1	1	1	2	3
2	2	2	2	2	3	3	3	3	3
2	2	1	3	1	3	3	3	1	1
3	2	1	2	2	3	2	1	2	9
3	2	3	2	3	1	9	2	3	3
2	1	3	2	3	3	2	1	3	2
1	1	2	3	3	2	3	1	2	1
2	2	1	1	2	1	2	3	2	1
3	1	3	1	3	1	1	1	2	2
2	3	1	3	1	3	1	1	1	1
2	2	2	3	1	3	3	3	2	2
2	2	3	1	3	1	1	1	1	1
2	2	2	2	2	2	3	3	3	3
3	1	2	1	2	3	3	3	3	3
3	1	3	1	3	9	1	1	1	1
3	1	3	1	3	9	1	1	1	1
3	3	3	1	2	3	3	1	1	1
3	1	2	1	9	2	3	2	1	1
1	3	1	3	1	3	3	3	3	3
1	3	1	3	1	3	3	3	3	3
1	3	1	3	1	3	3	3	3	3
1	3	1	3	1	3	3	3	3	3
2	2	2	2	2	2	2	2	2	2
2	2	2	2	2	2	2	2	2	2
2	2	2	2	2	2	2	2	2	2
1	3	1	3	1	3	3	3	3	3
3	1	2	2	2	2	3	3	3	3
2	3	1	3	1	3	2	2	1	1
2	3	2	3	1	3	2	2	1	1
2	3	2	3	1	3	2	2	1	1
2	3	2	3	1	3	2	2	1	1
1	1	3	2	1	9	1	2	2	3
3	2	9	2	2	9	2	9	2	9

1	2	1	2	2	3	2	2	2	3
2	2	1	3	2	2	1	1	2	2
2	1	2	2	1	2	2	1	2	2
3	1	2	1	2	3	3	3	2	2
3	2	1	2	3	2	3	2	2	1
9	9	9	9	9	9	9	9	9	9
1	2	3	2	3	1	2	1	1	1
3	2	3	2	1	2	1	2	1	2
9	9	9	9	9	9	9	9	9	9
9	9	9	9	9	9	9	9	9	9
2	2	2	2	2	2	2	2	2	2
3	2	3	2	2	9	1	9	1	3
3	1	3	2	2	2	9	1	2	2
3	1	3	1	3	1	1	1	1	1
9	9	9	9	9	9	9	9	9	9
2	2	2	2	2	2	2	2	2	2
2	2	2	2	2	2	2	2	2	2
9	9	9	9	9	9	9	9	9	9
9	1	2	2	2	1	1	2	1	2
9	9	9	9	9	1	9	9	9	9
3	1	2	2	2	2	2	1	1	1
1	2	2	3	1	3	1	1	1	1
2	2	2	2	2	2	2	2	2	2
2	3	3	3	1	3	2	2	2	2
9	9	9	9	9	9	9	9	9	9
9	9	9	9	9	9	9	9	9	9
1	1	2	3	3	2	3	1	2	3
3	9	9	9	9	9	9	9	9	9
1	3	2	2	2	3	2	1	1	1
9	9	9	9	9	9	9	9	9	9
3	3	3	3	3	3	1	3	1	1
9	9	9	9	9	9	9	9	9	9
3	1	3	1	3	2	9	1	2	1
3	1	3	1	3	1	1	1	1	1
3	1	3	1	3	1	1	1	1	1
3	2	2	2	2	2	2	2	2	2
3	1	3	1	3	1	2	2	2	2
2	1	3	1	3	1	1	1	1	1
3	1	3	1	3	2	2	2	3	3
9	9	9	9	9	9	9	9	9	9
9	9	9	9	9	9	9	9	9	9
9	9	9	9	9	9	9	9	9	9
9	9	9	9	9	9	9	9	9	9
9	9	9	9	9	9	9	9	9	9
9	9	9	9	9	9	9	9	9	9
3	1	3	1	3	3	3	2	3	1
1	1	2	3	3	2	3	1	2	3

9	9	9	9	9	9	9	9	9	9
2	2	3	2	2	2	2	1	2	2
2	2	2	2	1	3	3	3	3	1
2	1	2	3	3	2	3	1	2	3
2	2	2	2	2	3	3	3	2	1
2	2	3	3	2	3	3	3	3	2
9	9	9	9	9	9	9	9	9	9
1	3	1	3	1	3	3	3	3	3
2	2	2	2	2	2	2	2	2	2
9	9	9	9	9	9	9	9	9	9
2	2	2	2	1	9	2	1	2	1
9	9	9	9	9	9	9	9	9	9
9	9	9	9	9	9	9	9	9	9
9	9	9	9	9	9	9	9	9	9
3	1	3	1	3	1	1	1	1	1
9	9	9	9	9	9	9	9	9	9
2	2	1	3	1	2	2	2	1	1
9	9	9	9	9	9	9	9	9	9
2	2	1	1	2	1	2	3	1	3
3	1	3	1	3	9	2	2	2	2
3	1	3	1	2	2	2	2	3	3
3	2	2	1	2	3	2	1	2	3
3	1	3	1	3	1	1	1	1	1
2	2	2	2	2	2	1	1	1	2
2	1	3	1	3	1	1	1	1	1
3	1	2	3	2	2	1	2	1	2
3	1	3	2	2	2	2	1	2	2
2	2	2	1	3	3	3	3	2	2
2	2	1	2	2	2	3	3	3	3
3	3	2	2	3	1	1	2	2	1
1	2	3	2	1	2	1	2	3	2
3	1	3	1	3	1	1	1	1	1
9	9	9	9	2	9	9	9	9	9
9	9	9	9	9	9	1	9	9	9
3	9	9	9	9	9	9	9	9	9
9	9	9	1	9	9	9	9	9	9
9	9	2	9	9	9	9	9	9	9
9	9	9	2	9	9	9	9	9	9
1	9	9	9	9	9	9	9	9	9
9	9	9	9	9	9	9	9	9	9
9	9	9	9	9	9	9	9	9	9
9	9	9	9	9	9	9	9	9	9
9	9	9	9	9	9	9	9	9	9
9	9	9	9	9	9	9	9	9	9
9	9	9	9	9	9	9	9	9	9
2	3	1	2	3	1	2	2	1	1
3	1	2	2	2	3	1	1	1	1

3	2	3	2	1	3	3	2	1	1
9	3	9	9	2	9	9	9	1	9
9	9	9	9	3	9	9	9	9	9
3	1	3	2	2	1	1	2	2	2
9	9	9	9	9	9	9	9	9	9
9	9	9	9	9	9	9	9	9	9
9	9	9	9	9	9	9	9	9	9
9	9	9	9	9	9	9	9	9	9
9	9	9	9	9	9	9	9	9	9
2	2	2	2	2	2	2	2	2	2
3	2	3	2	3	2	1	2	1	2
3	2	3	2	3	2	1	2	1	2
2	2	2	2	2	2	2	2	2	2
2	2	1	3	2	1	1	1	1	2
2	2	3	3	1	3	2	1	1	1
2	3	1	2	1	3	2	2	2	3
9	9	9	9	9	9	9	9	9	9
9	9	9	9	9	9	9	9	9	9
9	9	9	9	9	9	9	9	9	9
9	2	9	9	9	2	9	9	2	9
9	9	9	9	9	9	9	9	9	9
9	3	9	9	9	3	9	9	3	9
9	3	9	9	1	9	3	9	9	9
9	9	9	9	9	9	9	9	9	9
9	9	9	2	9	9	2	9	3	9
9	9	9	2	9	9	9	9	9	9
9	9	9	9	9	9	9	1	9	9
2	2	2	2	2	2	2	2	2	2
1	3	1	2	2	3	3	1	1	2
3	9	9	9	9	9	9	9	9	9
2	2	2	2	2	1	1	2	2	2
3	1	3	2	3	2	3	2	2	3
2	2	2	2	2	2	2	2	2	2
2	2	2	2	2	2	2	2	2	2
3	1	2	1	2	2	1	1	2	1
3	1	3	2	2	2	2	2	1	1
2	3	2	2	1	2	2	1	2	1
3	2	2	2	2	1	2	2	2	2
2	9	9	9	9	9	9	9	9	9
2	2	2	2	2	2	2	2	2	2
2	2	2	2	2	2	2	2	2	2
2	2	1	1	2	3	2	2	1	2
2	2	2	2	2	3	1	2	3	2
3	1	2	2	2	2	2	2	2	2
9	9	9	9	9	9	9	9	9	9
2	2	2	2	2	2	2	2	2	2
3	1	2	2	2	2	2	2	1	1

3	2	1	2	2	3	2	2	1	2
2	2	2	2	3	3	1	1	2	1
3	1	3	2	2	2	2	2	2	2
3	2	3	2	1	2	2	3	2	1
1	2	3	2	1	2	1	1	2	3
3	2	2	1	2	2	1	2	1	1
3	3	1	1	1	1	1	1	1	3
3	1	2	2	2	2	2	2	2	2
3	1	3	1	3	3	1	1	1	1
9	9	9	9	9	9	9	9	9	9
2	2	2	2	2	2	2	2	2	2
3	1	2	3	2	3	3	1	1	1
3	1	3	1	3	1	1	1	1	1
3	1	1	2	2	3	1	2	1	1
3	1	3	1	3	2	2	1	1	1
3	1	2	2	2	2	2	2	3	1
2	2	2	2	2	2	2	2	2	2
2	2	2	2	2	2	2	2	2	2
2	2	2	2	2	2	2	2	2	2
2	2	2	1	3	2	2	2	2	2
2	2	2	1	3	3	1	1	1	1
2	2	2	1	2	3	2	2	2	2
3	2	2	3	2	2	3	1	2	2
3	1	2	2	2	3	3	1	2	1
3	1	3	1	3	1	1	1	1	1
2	2	2	2	2	3	2	1	1	1
3	1	3	1	2	1	1	1	1	1
3	2	2	2	2	2	2	1	1	1
3	1	2	2	2	2	3	2	2	2
2	2	2	2	2	2	2	2	2	2
2	2	2	2	2	2	2	2	2	2
2	2	2	2	2	2	2	2	2	2
2	2	2	2	2	2	2	2	2	2
2	2	2	2	2	2	2	2	2	2
2	2	2	2	2	2	2	2	2	2
2	2	2	2	2	2	2	2	2	2
2	2	1	3	1	3	1	1	1	1
9	9	9	9	9	9	9	9	9	9
9	9	9	9	9	9	9	9	9	9
9	9	9	9	9	9	9	9	9	9
2	1	3	1	1	2	1	2	2	3
9	9	9	9	9	9	9	9	9	9
9	9	9	9	9	9	9	9	9	9
9	9	9	9	9	9	9	9	9	9
9	9	9	9	9	9	9	9	9	9
9	9	9	9	9	9	9	9	9	9
3	2	2	2	2	2	2	1	1	1

9	1	1	1	3	2	1	2	1	2
2	2	2	2	2	2	1	1	1	1
9	9	9	9	9	9	9	9	9	9
2	3	1	1	2	2	3	3	3	3
9	9	9	9	9	9	9	9	9	9
9	9	9	9	9	9	9	9	9	9
2	2	2	2	2	2	2	2	2	2
3	1	2	1	2	2	1	1	1	1
3	2	2	2	2	2	2	1	2	1
2	3	3	3	2	2	2	1	3	1
2	2	1	2	2	3	3	3	2	2
2	2	2	3	1	3	2	2	2	2
3	1	1	1	1	3	2	1	1	1
1	3	1	3	1	3	3	3	3	3
3	1	1	3	2	2	3	1	2	1
3	2	2	2	2	1	2	2	2	1
3	1	3	2	9	1	2	2	2	1
9	9	9	9	9	1	9	9	9	9
2	2	2	2	2	2	2	1	1	1
2	2	2	2	2	2	1	2	2	2
3	2	2	2	2	1	1	2	2	2
2	1	2	3	2	1	2	3	2	1
2	2	2	2	2	2	2	2	2	2
3	1	1	3	1	3	3	1	3	1
2	1	2	2	2	2	3	1	1	1
2	2	2	2	2	2	2	2	2	2
9	9	9	9	9	9	9	9	9	9
2	2	2	2	2	2	2	2	1	1
3	1	1	1	3	1	3	1	2	1
3	2	2	3	2	2	3	2	2	3
3	2	1	2	3	3	1	1	2	1
3	9	9	9	9	9	9	9	9	9
2	2	2	2	2	2	2	2	2	2
2	2	2	2	2	2	2	2	2	2
3	1	2	2	2	2	2	2	2	2
2	2	2	2	2	2	2	2	2	2
2	2	3	2	1	1	2	2	1	1
2	2	2	2	2	2	2	2	2	2
3	1	1	3	2	3	3	3	2	2
3	1	1	3	1	3	1	1	1	1
2	2	2	2	2	1	2	2	2	2
2	2	2	2	2	2	2	2	2	9
3	1	3	1	3	3	2	1	1	1
9	1	9	9	9	9	9	9	9	9
3	1	2	1	3	2	1	1	2	2
3	1	1	3	3	3	3	2	1	2
2	1	2	1	3	1	2	1	2	1

2	2	2	2	2	2	2	2	2	2
2	2	2	2	2	2	2	2	2	2
2	2	2	2	2	2	2	2	2	2
2	2	2	2	2	2	2	2	2	2
3	1	2	2	2	2	2	1	1	1
1	3	1	2	2	3	1	2	2	2
2	2	2	2	2	2	2	2	2	2
3	2	2	2	1	2	2	2	2	2
2	2	2	2	2	2	2	2	2	2
2	2	2	2	2	2	2	2	2	2
3	2	2	2	2	2	2	2	2	2
3	1	3	2	2	1	2	2	1	2
9	9	9	9	9	9	9	9	9	9
3	3	3	1	1	2	1	3	1	1
3	1	9	9	9	9	9	1	9	9
9	9	9	9	9	9	9	9	9	9
2	2	2	2	2	2	1	1	1	1
2	2	2	2	2	2	2	2	2	2
9	9	9	9	9	9	9	9	9	9
3	2	3	1	3	3	1	1	2	2
2	2	2	2	2	2	2	2	2	2
3	1	2	2	2	2	2	2	2	2
2	2	2	2	2	2	2	2	2	2
2	2	2	1	2	2	1	1	1	1
1	1	1	3	1	3	1	1	1	3
9	9	9	9	9	9	9	9	9	9
3	1	3	3	1	3	3	3	3	3
2	2	2	3	1	3	1	2	2	3
1	2	3	1	1	3	2	1	2	1
9	9	9	9	9	9	9	9	9	9
3	1	3	2	2	2	2	1	2	1
3	2	2	2	2	9	3	1	1	1
2	2	2	2	2	2	2	2	2	2
3	1	3	1	3	3	2	1	2	1
2	9	2	2	9	9	9	9	2	2
2	2	3	2	2	3	3	2	3	2
3	1	3	3	9	1	1	1	1	1
9	9	9	9	9	9	9	9	9	9
2	2	2	2	2	2	2	2	2	2
9	9	9	9	9	9	9	9	9	9
3	1	3	2	2	3	3	1	1	1
3	1	3	2	2	2	2	1	1	1
2	2	2	2	2	1	2	2	2	1
2	2	2	2	3	1	1	1	1	1
1	2	2	3	2	2	1	1	2	2
2	2	3	1	1	3	2	1	2	2
3	1	3	2	2	3	3	1	1	2

1	3	1	3	1	3	3	3	3	3
1	2	3	2	1	1	3	1	3	1
3	1	2	2	3	1	2	1	1	1
9	9	9	9	9	9	9	9	9	1
3	1	3	2	2	2	2	1	1	1
3	1	1	3	1	2	1	1	3	2
3	1	2	2	3	1	2	1	1	1
1	1	2	2	2	2	2	3	3	3
2	1	2	2	2	3	2	1	1	1
1	2	3	2	3	3	3	1	1	1
9	9	9	9	9	9	9	9	9	9
9	9	3	9	3	9	9	1	1	1
2	2	3	2	3	2	2	1	1	1
3	1	3	2	3	1	1	2	2	1
9	9	9	9	9	9	9	9	9	9
3	1	3	1	3	2	1	2	1	2
2	2	3	2	2	2	1	1	1	1
2	2	2	2	2	2	2	2	2	2
9	9	9	9	9	9	9	9	9	9
2	2	2	1	3	2	2	1	1	1
3	1	2	2	1	3	2	1	2	1
2	2	2	2	2	2	2	2	2	2
2	2	3	2	3	2	1	1	1	1
3	1	3	1	3	2	2	1	1	1
3	1	3	1	3	3	2	1	1	1
2	2	2	2	3	3	2	2	2	2
3	1	3	1	2	2	2	2	1	1
3	1	3	1	3	2	3	1	1	1
3	1	3	1	3	3	2	1	1	1
2	2	3	2	3	2	3	1	1	1
2	9	9	2	9	3	9	3	9	3
9	9	9	9	9	9	9	9	9	9
3	1	3	1	3	1	3	1	1	2
9	9	9	9	9	9	9	9	9	9
9	9	9	9	9	9	9	9	9	9
3	1	3	1	2	2	1	1	1	1
2	2	2	2	2	2	2	2	2	2
3	1	2	1	3	1	1	1	1	1
9	9	9	9	9	9	9	9	9	9
9	9	9	9	9	9	9	9	9	9
9	9	9	9	9	9	9	9	9	9
9	9	9	9	9	9	9	9	9	9
9	9	9	9	9	9	9	9	9	9
2	2	2	2	2	2	2	2	2	2
2	9	3	2	3	2	1	2	2	1
3	2	3	2	2	1	2	1	1	2
3	1	3	9	9	9	9	1	9	9

9	9	9	9	9	9	9	9	9	9
9	9	9	9	9	9	9	9	9	9
9	9	9	9	9	9	9	9	9	9
9	9	9	9	9	9	9	9	9	9
9	9	9	9	9	9	9	9	9	9
9	9	3	9	9	9	9	9	9	9
2	2	3	1	1	3	2	1	3	2
9	9	9	9	9	9	9	9	9	9
2	2	2	2	2	2	2	2	2	2
2	2	9	9	9	9	9	9	9	9
2	2	2	3	2	2	2	1	1	1
3	1	3	1	1	1	1	1	1	1
3	1	3	3	1	1	1	1	1	1
3	1	3	2	2	2	3	2	2	1
3	2	1	2	1	2	2	2	2	1
2	9	3	1	3	1	1	1	2	2
2	1	3	2	2	3	3	1	2	2
3	9	9	9	9	9	9	9	9	1
2	1	2	2	3	2	3	2	1	2
2	9	2	2	9	9	9	9	2	2
9	2	9	9	9	9	9	9	9	9
9	9	3	9	9	9	9	9	9	9
3	1	2	2	2	2	2	2	2	2
3	1	3	2	2	3	1	2	2	2
9	9	9	9	9	9	9	9	9	9
2	2	2	2	2	2	2	2	2	2
3	9	9	9	9	9	1	9	9	9
1	1	2	3	2	3	1	1	2	3
3	1	3	1	2	2	1	1	2	2
3	1	3	2	2	3	1	2	2	2
2	3	3	2	2	2	2	1	2	2
3	1	3	2	2	2	2	1	1	1
3	1	3	1	9	3	3	3	1	1
3	1	3	2	3	2	2	3	1	1
2	2	3	2	2	3	2	3	1	1
9	9	9	9	9	9	9	9	9	9
3	1	3	2	3	3	3	1	1	1
2	1	2	2	3	2	2	1	1	1
3	2	2	2	1	2	1	1	2	3
2	1	1	2	2	2	3	2	1	1
2	1	3	2	3	3	3	1	1	1
1	3	3	2	2	2	2	2	9	1
2	2	1	3	2	2	2	3	3	9
9	9	9	9	9	9	9	9	9	9
2	3	1	2	2	2	3	2	3	3
2	2	2	2	2	2	3	2	2	1
9	1	2	2	9	1	3	3	2	1

3	1	9	9	9	9	9	9	9	9
3	1	3	1	3	9	1	1	1	1
3	1	3	1	3	3	1	1	1	1
3	1	3	2	3	2	3	1	1	1
2	9	2	9	1	9	2	2	3	2
3	2	3	2	3	1	3	1	2	2
3	1	3	3	1	3	1	1	1	1
3	2	9	1	2	9	9	1	1	1
3	1	3	1	2	3	1	2	2	2
2	1	2	3	1	2	1	1	1	1
3	2	3	2	3	1	3	1	1	1
2	2	3	2	1	2	3	1	1	1
9	9	9	9	9	9	9	9	9	9
2	2	3	2	1	3	2	1	2	1
2	2	2	2	2	2	2	2	1	1
2	2	1	2	3	2	1	1	1	1
9	9	9	9	9	9	9	9	9	9
1	2	2	9	1	2	9	2	1	9
1	3	2	2	2	3	2	2	2	2
9	9	9	9	9	9	9	9	9	9
2	2	2	2	2	2	2	2	2	2
2	1	3	1	1	2	3	1	1	1
9	9	9	9	9	9	9	9	9	9
1	3	1	3	1	1	1	1	1	3
3	1	2	3	2	2	2	2	2	2
1	3	1	2	2	2	3	3	2	2
1	1	3	1	1	3	3	2	1	3
3	1	3	1	2	2	2	2	2	2
1	1	3	3	1	3	3	1	1	1
3	2	1	2	3	2	3	3	1	2
3	1	3	1	3	3	1	2	2	2
2	3	1	3	3	1	2	1	2	2
2	2	2	2	2	2	2	2	2	2
2	2	2	2	2	2	2	2	2	2
3	1	3	1	2	3	3	1	1	1
3	1	1	2	2	2	1	1	1	1
1	2	2	1	3	1	1	2	2	2
3	2	2	2	2	2	2	2	2	2
2	3	2	3	2	9	2	9	1	2
2	2	2	2	2	2	2	2	2	2
2	2	3	2	2	2	2	1	2	1
3	1	3	1	2	2	2	1	2	2
3	9	9	9	9	9	9	1	9	9
1	1	3	1	3	3	1	1	1	1
3	2	2	2	3	2	1	1	2	1
3	1	3	1	3	3	1	1	1	1
2	1	2	2	2	2	1	2	2	2

2	1	2	2	2	2	2	2	2	2
9	9	9	9	9	9	9	9	9	9
2	2	2	2	2	2	2	2	2	2
9	9	9	9	9	9	9	9	9	9
3	1	3	1	2	2	2	2	2	2
2	2	2	2	2	2	2	2	2	2
3	1	3	2	2	2	2	2	2	1
3	1	3	1	3	1	2	1	1	1
3	1	3	1	2	3	1	1	1	1
3	1	3	3	1	3	1	1	1	1
3	1	9	9	9	9	9	9	9	9
3	1	3	1	2	3	3	1	1	1
2	2	2	2	3	3	3	2	2	2
3	1	3	1	3	3	2	1	2	2
9	9	9	9	9	9	9	9	9	9
3	9	3	9	3	9	2	9	9	9
9	9	9	9	9	9	9	9	9	9
1	3	1	3	1	3	3	3	3	3
1	3	3	3	3	3	3	1	1	1
2	3	1	2	2	2	3	2	2	1
3	3	3	1	3	2	3	1	1	3
3	1	3	2	3	3	2	3	2	1
3	1	1	2	1	1	3	1	1	1
2	2	3	1	2	2	3	1	1	1
3	1	3	1	3	1	1	1	2	2
1	3	3	3	1	3	3	1	1	1
1	3	3	3	1	3	3	1	3	3
3	1	3	1	3	2	3	2	1	1
3	1	3	1	3	3	1	1	1	1
1	9	3	3	1	3	3	3	3	3
9	9	9	9	9	9	9	9	9	9
2	2	3	1	3	3	2	1	1	1
9	9	9	9	9	9	9	9	9	9
3	1	3	2	2	3	3	2	2	2
2	2	3	3	1	3	2	1	1	1
9	9	9	9	3	9	9	9	9	9
2	3	9	3	3	1	2	2	2	2

Poison	InheritedC.	OffensiveP	PostCoitalE	Dysmenorr	Menorrhag	Asymptom	VaginalRas	Vaginalswe	WillLikePa
2	2	2	2	2	2	2	2	2	2
2	2	2	1	1	1	2	2	2	2
2	2	1	1	2	1	2	1	1	2
2	2	2	1	1	1	2	1	1	2
2	2	2	1	1	1	2	2	2	2
2	2	2	1	1	1	2	1	1	2
9	9	9	9	9	9	9	9	9	9
3	1	3	3	3	3	1	3	2	2
2	9	2	2	2	2	2	2	2	1
9	9	2	9	9	9	9	9	2	2
3	3	2	9	3	2	2	2	2	2
3	2	2	3	1	1	2	2	3	3
2	1	2	2	2	2	2	2	2	1
2	1	2	3	3	3	2	2	1	2
1	1	2	2	3	2	2	2	2	1
3	1	2	2	3	2	3	2	2	3
1	3	2	3	9	2	1	2	1	1
3	1	2	2	3	2	3	3	1	3
3	1	2	2	3	2	3	2	2	3
3	3	1	2	3	3	3	2	1	1
2	1	2	2	2	2	2	2	2	1
2	1	2	2	2	2	2	2	2	1
2	2	2	2	2	2	2	2	2	2
2	1	2	2	1	2	2	2	2	1
2	2	2	2	2	2	2	2	2	2
3	2	2	2	2	2	2	2	2	1
2	1	2	2	2	2	2	2	2	1
1	1	2	2	2	2	2	1	1	1
2	2	2	2	2	2	2	2	2	2
2	2	2	2	2	2	2	2	2	2
2	2	2	2	2	2	2	2	2	2
1	1	1	2	2	2	3	1	1	1
2	2	1	2	2	2	2	2	1	1
1	1	1	2	2	2	3	1	1	1
3	2	2	2	2	2	2	1	1	2
1	1	1	2	2	2	1	1	1	1
3	2	2	2	2	2	2	1	1	2
3	1	1	1	2	2	2	1	1	1
2	2	2	2	2	2	2	2	2	2
2	2	2	2	2	2	2	2	2	2
2	2	2	2	2	2	2	2	2	2
2	2	2	2	2	2	2	2	2	1
2	1	1	1	2	2	3	2	1	1
1	3	1	3	3	3	3	3	1	1
2	3	1	3	3	3	2	2	2	1
2	1	1	1	2	2	3	2	1	2

3	3	1	1	3	2	3	1	1	1
9	2	1	2	1	2	2	3	1	1
3	2	1	2	3	2	3	1	1	2
2	2	1	2	2	2	2	1	1	1
1	3	1	2	9	3	1	1	1	1
3	3	1	3	3	2	3	1	1	1
3	1	1	1	1	3	3	1	1	1
9	9	1	3	3	3	3	1	1	1
9	9	1	9	9	9	9	1	1	1
3	3	1	1	2	3	2	1	1	1
9	9	2	9	2	9	2	9	2	2
3	2	2	2	2	2	2	2	2	2
3	3	1	1	2	2	3	2	1	1
2	2	1	2	2	2	2	2	1	2
3	3	1	1	1	2	2	1	2	1
2	2	3	3	3	3	3	2	2	3
1	3	1	2	3	3	2	1	1	1
3	2	1	1	2	2	1	1	1	1
2	1	1	1	2	2	3	2	1	1
2	1	9	9	9	9	9	9	1	1
3	3	1	3	3	3	3	1	1	1
3	3	1	1	3	3	3	1	1	1
3	3	1	3	3	3	3	1	1	1
3	3	1	1	1	1	2	2	1	1
9	9	1	9	1	1	9	9	1	1
2	2	1	1	2	2	1	1	1	1
3	2	9	9	9	9	9	9	9	9
1	1	1	1	3	3	3	1	1	1
2	2	9	9	9	9	9	9	9	9
2	3	1	1	2	9	3	1	1	1
1	1	1	1	2	2	2	1	1	2
9	1	3	1	1	1	2	1	1	1
2	2	1	1	2	2	1	1	1	2
3	2	2	1	1	1	2	1	1	1
3	1	2	3	1	1	3	1	1	1
9	9	9	9	9	9	9	9	9	9
9	9	9	9	9	9	9	9	9	9
2	2	2	1	1	1	2	1	1	1
3	3	1	3	3	3	3	3	1	1
1	3	1	1	1	3	3	1	1	1
9	9	1	1	1	9	9	1	1	1
9	9	1	2	2	2	2	2	1	1
3	1	1	1	3	3	1	1	1	1
2	2	1	1	2	2	1	1	1	1
1	2	1	1	2	2	1	1	1	1
9	9	9	9	9	9	9	9	9	9
9	9	9	9	9	9	9	9	9	9

1	3	2	1	3	3	2	1	1	1
2	2	1	2	2	2	2	2	1	1
2	2	1	2	3	2	3	1	1	1
2	1	1	2	3	3	3	2	1	2
9	9	9	9	9	9	9	9	9	9
9	9	1	2	2	1	2	2	1	2
1	1	1	1	2	2	3	2	2	1
3	1	1	2	2	3	2	1	2	1
3	3	1	1	2	2	2	2	2	1
9	9	9	9	9	9	9	9	9	1
2	1	1	3	1	1	3	2	1	1
3	3	1	3	1	1	1	3	3	1
2	3	1	2	3	2	3	1	1	1
9	1	1	2	2	2	2	1	1	1
1	2	2	1	2	3	1	2	2	2
2	3	9	9	9	2	9	9	9	2
2	2	1	2	2	2	2	2	1	1
2	1	1	2	1	1	2	1	1	1
2	1	2	2	2	2	2	2	2	2
1	1	2	1	1	2	2	1	2	1
3	2	1	2	1	2	2	2	2	1
3	2	1	1	1	2	2	1	1	1
3	3	2	2	2	2	2	2	2	2
3	3	9	9	9	9	9	9	9	9
1	1	1	2	1	1	1	2	2	2
2	2	1	2	2	2	2	2	2	1
3	3	2	2	2	2	3	2	1	1
2	2	2	2	2	2	3	2	1	1
2	2	3	2	2	2	2	2	2	2
1	1	1	2	2	3	2	1	1	1
2	2	2	2	2	2	2	2	2	1
2	2	2	2	2	2	2	2	2	2
2	2	1	3	3	2	1	1	1	1
2	2	3	2	2	3	3	2	2	2
1	1	1	2	2	3	2	1	1	1
2	1	2	2	1	2	2	1	1	1
2	2	1	2	1	1	2	1	1	3
2	2	2	2	2	2	2	2	2	2
9	9	1	9	9	9	2	9	1	9
2	1	1	2	2	3	3	1	2	1
2	2	2	2	2	2	2	2	2	2
2	2	2	2	2	2	2	2	2	2
2	2	2	2	2	2	2	2	2	2
3	3	2	2	2	2	3	3	3	1
2	3	2	1	3	3	2	2	2	2
2	2	1	2	2	2	2	2	2	1
3	2	1	1	3	3	2	3	2	2

2	2	9	9	9	9	9	9	9	9
3	2	2	2	2	2	2	2	2	2
3	2	2	2	2	2	2	2	2	2
2	2	2	2	2	2	2	2	2	2
1	1	2	2	2	2	2	2	2	2
2	3	1	1	2	3	2	1	1	1
3	3	2	3	3	2	3	3	3	2
2	2	2	2	2	2	2	2	2	1
2	2	2	2	2	2	2	2	2	2
2	2	2	2	2	2	2	2	2	2
2	2	9	9	9	9	9	9	9	3
2	3	3	2	2	3	3	3	3	3
9	2	9	9	2	9	2	2	2	2
2	2	2	2	3	3	2	2	1	2
2	2	2	1	2	2	2	2	1	3
2	2	2	1	1	2	2	2	1	3
9	9	9	9	9	9	1	9	9	9
2	2	2	1	1	1	1	2	2	1
9	9	1	9	9	9	9	9	9	9
2	2	1	1	1	2	1	1	1	1
1	3	1	1	3	2	1	1	1	1
2	3	1	1	2	1	1	1	1	1
1	2	1	1	1	2	1	1	1	1
2	1	1	1	1	2	1	1	1	1
2	1	1	1	2	2	1	1	1	1
2	2	1	1	1	2	1	1	1	1
2	2	1	1	1	2	1	1	1	1
1	2	1	1	3	2	1	1	1	1
2	3	1	1	2	1	1	1	1	1
2	3	1	1	3	2	1	1	1	1
2	2	2	2	3	2	2	2	1	2
2	2	2	2	2	2	2	2	2	2
2	2	1	1	1	2	1	1	1	1
2	3	1	1	1	2	1	1	1	1
2	3	1	1	2	1	1	1	1	1
2	3	1	1	2	1	1	1	1	1
3	1	1	1	2	2	1	1	1	1
2	1	1	1	2	2	1	1	1	1
2	2	1	1	1	2	1	1	1	1
2	3	1	1	2	3	1	1	1	1
2	3	1	1	1	1	1	1	1	1
2	1	1	1	2	2	1	1	1	1
2	3	1	1	2	2	1	1	1	1
2	3	1	1	2	2	1	1	1	1
2	3	1	1	1	1	1	1	1	1
2	3	1	1	2	2	1	1	1	1
2	3	1	1	2	2	1	1	1	1

2	3	1	1	2	2	1	1	1	1
2	3	1	1	1	1	1	1	1	1
2	3	1	1	1	3	1	1	1	1
3	3	1	1	3	2	1	1	1	1
2	3	1	1	1	1	1	1	1	1
1	3	1	1	1	1	1	1	1	1
3	3	1	1	2	1	1	1	1	1
2	3	1	1	2	2	1	1	1	1
1	3	1	1	1	3	3	1	1	1
2	1	9	9	9	9	9	9	9	1
9	9	9	9	9	9	9	9	9	9
3	2	9	9	9	9	9	9	9	9
1	3	1	1	1	3	1	1	1	1
2	3	1	1	1	1	1	1	1	1
2	2	1	1	2	1	2	1	1	1
2	2	1	1	2	2	1	2	1	1
2	2	2	2	2	2	2	2	2	2
1	1	2	2	2	2	2	2	2	1
2	1	1	1	2	2	2	1	1	1
2	2	1	1	2	2	1	2	1	1
3	2	1	1	2	2	3	1	1	1
2	2	2	2	2	2	2	2	2	2
1	2	1	1	2	2	1	1	2	2
1	2	1	2	3	2	2	2	1	1
2	2	2	2	2	2	2	2	2	2
2	2	2	2	2	2	2	2	2	2
2	1	1	1	2	2	2	2	1	2
2	2	2	2	2	2	2	2	2	2
2	2	2	2	2	2	2	2	2	2
2	2	1	1	2	2	2	1	1	1
3	2	1	1	2	2	2	2	2	1
3	2	1	1	2	2	2	2	2	1
2	1	1	1	2	2	2	1	1	1
1	2	2	1	1	2	2	2	2	1
1	2	1	1	1	1	2	2	1	1
2	2	2	2	2	1	1	1	3	2
2	2	2	2	2	2	2	2	2	1
1	1	2	1	1	1	2	2	3	1
2	2	2	2	2	2	2	2	1	2
2	2	1	1	2	3	3	2	2	2
2	2	1	2	2	2	2	2	2	2
2	1	1	1	2	2	2	2	1	2
9	9	9	2	9	9	9	9	9	9
9	9	9	2	9	9	9	9	9	9
1	1	1	2	2	2	1	2	2	2
1	1	1	1	1	2	1	1	1	1
9	9	9	9	9	9	2	9	9	9

2	2	2	2	2	2	2	2	2	2
2	2	2	2	2	2	2	2	2	2
2	2	2	2	2	2	2	2	1	2
2	1	2	2	2	2	2	2	1	1
2	2	1	2	2	2	2	2	2	2
3	2	2	2	2	2	2	2	2	2
2	2	2	2	2	2	2	1	1	2
2	2	2	2	2	2	2	2	2	2
2	2	1	3	2	2	2	2	2	2
2	2	1	2	2	2	1	2	2	1
2	2	2	2	2	2	2	2	2	2
2	2	2	2	2	2	2	2	2	2
2	2	2	2	2	2	2	2	2	2
2	1	2	2	2	3	3	2	2	2
2	2	2	2	2	2	2	2	2	1
2	3	2	1	2	2	1	3	2	2
2	2	3	3	2	3	3	2	1	3
2	2	1	2	3	3	2	2	1	1
9	9	9	9	9	9	1	9	9	1
3	1	1	2	2	2	1	2	2	1
2	2	1	1	2	2	2	2	2	1
2	1	2	1	2	2	2	2	2	1
2	2	2	2	2	3	3	2	2	3
1	2	2	2	3	3	1	2	2	1
9	9	2	2	9	9	9	2	9	9
2	2	2	2	2	2	2	2	2	2
2	2	2	2	2	2	2	2	2	2
1	2	1	9	2	3	2	2	1	1
2	2	2	2	2	2	2	2	2	2
2	2	2	2	2	2	2	2	2	2
9	9	9	9	1	9	9	9	9	9
2	2	2	2	1	1	2	2	1	1
3	3	3	3	1	1	3	1	3	3
3	2	1	2	2	2	2	2	2	1
9	9	2	9	9	9	9	9	9	2
2	2	2	2	2	2	2	2	2	2
3	3	2	1	3	3	2	2	2	1
2	2	2	1	1	2	3	2	2	2
2	2	2	2	1	1	1	1	9	1
1	3	2	2	2	2	1	1	1	1
2	1	1	2	2	2	3	2	1	1
2	3	1	2	2	2	3	2	2	1
9	9	9	9	9	9	9	9	9	9
2	2	2	2	2	2	2	2	2	2
1	2	2	2	1	2	2	1	1	2
2	2	2	2	2	2	2	2	1	2
2	2	2	2	2	2	2	2	2	2

9	9	9	9	9	9	9	9	9	9
9	9	9	9	9	9	9	9	9	9
9	9	9	9	9	9	9	9	9	9
2	1	1	1	2	2	2	1	1	1
2	3	2	1	3	2	2	1	2	2
2	3	1	1	3	1	2	3	1	1
1	1	1	1	2	2	2	1	1	1
1	3	1	2	2	2	2	1	1	1
3	1	1	9	9	9	9	9	9	9
2	2	2	2	2	2	2	2	2	2
2	2	2	2	2	2	2	2	2	2
9	9	9	9	9	9	9	9	9	1
9	9	9	9	9	9	9	9	9	9
3	1	3	1	1	1	1	1	3	3
1	1	1	1	1	3	3	1	1	1
9	9	9	9	9	9	9	9	9	9
9	9	9	9	9	9	9	9	9	9
2	1	1	1	3	2	2	1	1	1
2	2	1	1	3	2	2	1	1	1
9	9	9	9	9	9	9	9	9	9
9	9	9	9	9	9	9	9	9	9
2	2	1	1	1	2	3	2	2	2
3	2	3	2	2	3	3	1	1	2
2	2	1	1	3	3	2	2	2	2
2	2	2	2	2	2	2	2	2	2
3	1	3	3	3	3	3	1	1	1
2	1	2	1	2	2	3	1	1	1
3	3	1	2	2	3	2	1	1	1
2	1	2	1	1	2	3	1	1	1
2	3	2	2	2	3	3	2	2	1
2	2	2	2	2	2	2	2	2	2
2	2	3	3	2	2	2	2	2	9
9	9	9	9	9	9	9	9	9	9
9	9	9	9	9	9	9	9	9	9
2	2	1	3	3	3	2	1	1	1
1	3	1	3	3	3	1	3	2	3
3	2	2	2	3	2	3	2	2	1
1	3	1	3	3	3	1	3	2	1
3	1	1	3	3	2	3	2	2	1
2	2	1	1	2	1	2	2	2	1
3	2	1	3	2	2	3	2	3	1
3	1	1	3	1	2	3	1	1	1
2	2	9	1	2	2	2	2	2	2
2	2	2	2	2	2	2	2	2	2
3	1	3	3	3	3	3	3	2	2
2	2	2	2	2	2	2	2	2	2
2	2	1	1	2	2	2	2	1	1

9	9	9	9	9	9	9	9	9	9
9	9	9	9	9	9	9	9	9	9
9	9	9	9	9	9	9	9	1	9
2	2	1	2	1	2	2	1	1	1
9	9	9	9	9	9	9	9	9	2
9	9	9	9	9	9	9	9	9	9
2	2	1	1	2	2	2	1	1	1
9	9	3	1	2	3	9	9	2	2
1	3	1	1	2	2	1	1	1	1
1	1	1	2	2	2	3	1	1	3
2	2	2	2	2	2	2	2	2	2
2	2	2	2	2	2	2	2	2	2
2	1	2	2	2	2	2	1	1	1
2	2	2	1	2	2	2	2	2	1
2	2	2	2	2	2	2	2	2	1
3	2	3	2	2	3	2	2	2	2
2	2	2	2	2	2	2	2	2	2
2	2	2	2	2	2	2	2	2	2
2	2	1	2	1	1	2	1	1	2
2	2	2	2	2	2	2	2	2	2
2	2	1	3	2	2	2	2	2	1
1	2	1	1	3	3	2	2	2	1
1	2	1	2	3	3	1	2	2	1
3	3	1	1	1	1	1	1	1	1
2	2	2	2	2	2	2	2	2	2
2	2	2	2	2	3	2	2	2	2
2	2	2	2	2	2	2	2	2	1
2	2	2	2	2	2	2	2	2	2
3	2	2	2	3	2	2	1	1	1
1	2	1	2	2	1	2	1	1	1
3	2	2	2	2	2	1	2	1	2
1	1	2	2	2	2	1	2	1	2
3	2	3	2	2	1	3	2	2	1
2	2	2	2	2	2	2	2	2	2
2	2	2	2	2	2	2	2	2	2
3	2	2	2	2	2	1	1	9	1
2	2	2	2	2	2	2	2	2	2
1	1	2	2	2	2	2	2	2	2
2	2	2	2	2	2	2	2	2	2
2	2	2	2	2	2	2	2	2	2
2	1	2	2	2	2	2	2	2	1
2	1	2	2	2	2	2	2	2	2
3	1	1	2	2	2	1	1	1	1
2	1	2	2	2	2	2	2	2	2
1	2	2	2	2	2	2	2	2	1
3	2	1	2	3	2	1	1	1	1
2	2	2	2	2	2	2	2	2	2

3	2	1	2	1	1	3	1	1	1
2	2	2	2	2	2	2	2	2	1
2	2	2	2	2	2	2	2	2	2
2	2	2	2	2	2	2	2	2	2
1	1	2	2	2	2	2	2	2	2
2	3	1	2	2	2	3	2	1	2
9	9	9	9	9	9	9	9	9	9
2	2	2	2	2	2	2	2	2	2
2	2	2	2	2	2	2	2	2	2
1	1	2	2	2	2	3	1	1	1
2	1	1	2	1	1	2	2	2	1
3	1	1	1	1	2	3	1	1	1
2	2	1	1	1	1	1	1	1	1
2	2	1	1	1	1	2	1	1	1
2	2	2	2	2	2	2	2	2	2
1	1	1	1	1	1	2	1	1	1
1	3	3	3	3	3	3	3	3	1
2	2	2	2	2	2	2	2	2	1
3	2	2	2	2	2	2	2	2	1
2	2	2	2	2	2	2	2	2	3
2	1	1	1	2	2	3	1	1	1
2	2	1	2	2	2	2	1	1	2
9	9	9	9	9	9	9	9	9	9
2	2	2	2	2	2	2	2	2	2
3	1	1	1	2	2	3	1	2	1
9	9	9	9	9	9	9	9	9	9
3	2	2	2	1	2	2	1	1	2
9	9	9	9	9	9	9	9	9	9
2	2	1	2	2	2	2	2	2	1
2	2	1	1	2	2	2	1	1	1
9	9	9	9	9	9	9	9	9	9
9	9	9	9	9	9	9	9	9	9
2	2	2	2	3	2	2	3	2	2
1	1	1	1	1	1	1	1	1	1
9	9	9	9	9	9	9	9	9	9
1	1	1	1	1	2	2	1	2	1
3	2	2	3	3	3	3	3	3	3
1	9	1	2	2	2	2	2	2	9
9	9	9	9	9	9	9	9	9	9
2	1	1	1	2	2	2	1	2	1
2	2	1	2	2	2	1	3	2	9
2	2	2	1	1	1	2	2	2	2
2	1	1	2	2	1	3	2	3	2
2	2	2	2	2	2	2	2	2	1
9	9	3	3	3	3	2	3	3	1
2	2	1	1	2	2	2	1	1	1
3	1	1	1	1	1	3	1	2	1

9	9	9	9	9	9	9	9	9	9
2	3	1	1	2	1	1	1	1	1
3	1	1	1	2	1	1	1	1	1
1	2	1	1	1	2	1	1	1	1
1	3	1	1	2	1	2	1	2	1
2	3	1	1	2	1	1	1	1	1
2	2	1	1	1	2	1	1	1	1
1	3	1	1	2	1	1	1	1	1
2	2	1	1	2	2	1	1	1	1
9	3	1	1	1	2	1	1	1	1
2	2	1	1	2	3	1	1	1	1
2	3	1	1	2	1	1	1	1	1
3	3	1	1	1	2	1	1	1	1
2	3	1	1	1	1	1	1	1	1
3	1	1	1	2	1	1	1	1	1
3	3	1	1	2	1	1	1	1	1
1	2	1	1	1	2	1	1	1	1
2	2	1	1	1	2	1	1	1	1
1	2	1	1	1	1	1	1	1	1
1	2	1	1	1	1	1	1	1	1
1	3	1	1	3	3	3	1	1	1
1	3	1	1	3	3	1	1	1	1
2	3	1	1	3	1	3	2	1	1
1	2	1	1	2	2	1	1	1	1
2	3	1	1	2	1	1	1	1	1
2	3	1	1	2	1	1	1	1	1
1	2	1	1	3	2	1	1	1	1
2	3	1	1	3	3	1	1	1	1
3	1	1	1	2	2	3	1	1	1
2	3	1	1	2	2	1	1	1	1
1	3	1	1	2	2	2	1	1	1
2	3	1	1	2	1	2	1	1	1
1	3	1	1	1	2	2	1	1	1
1	2	1	1	2	2	2	1	1	1
1	3	1	1	1	2	1	1	1	1
2	2	1	1	2	2	1	1	1	1
1	2	1	1	2	1	1	1	1	1
1	3	1	1	2	1	1	1	1	1
1	3	1	1	2	1	1	1	1	1
1	3	1	1	1	1	1	1	1	1
2	3	1	1	2	1	1	1	1	1
2	2	2	2	2	2	2	2	2	2
3	2	1	2	1	2	3	1	1	1
2	2	2	2	2	2	2	2	2	2
9	9	2	9	9	9	9	9	9	2
2	2	2	2	2	2	2	2	2	2
9	9	2	9	9	9	9	9	9	2

2	2	2	2	2	2	2	2	2	2
3	2	1	2	3	2	1	1	1	1
3	2	1	2	3	2	1	1	1	1
2	1	1	1	3	3	1	2	1	1
2	2	2	2	2	2	2	2	2	2
1	1	1	2	2	2	2	1	1	1
2	2	2	2	2	2	2	2	2	2
3	2	2	1	2	2	2	2	3	2
1	1	1	2	2	2	2	1	1	1
2	2	2	2	2	2	2	2	2	2
3	2	1	2	3	2	1	1	1	1
9	9	9	9	3	9	9	9	9	9
1	3	1	3	3	3	3	1	1	1
2	2	2	2	2	2	2	2	2	2
9	9	3	9	9	9	9	9	9	1
9	9	3	9	9	9	9	9	9	1
9	9	3	9	9	9	9	9	9	1
9	1	9	9	3	9	9	9	9	1
9	9	9	9	3	9	9	9	1	9
3	1	1	1	3	1	1	1	1	1
9	9	1	1	9	1	1	3	1	1
9	9	1	1	3	9	1	3	1	9
2	3	3	3	3	2	2	2	3	2
9	9	9	9	9	9	9	1	9	1
9	9	2	9	9	9	9	9	9	9
9	9	9	9	9	9	9	9	9	9
9	2	3	9	9	9	9	9	9	1
9	9	9	9	9	9	9	9	9	9
2	2	1	2	3	2	3	1	1	2
9	9	9	9	3	9	9	9	9	1
9	9	9	9	3	9	9	9	9	9
2	2	2	2	2	2	2	2	2	2
2	2	2	2	2	2	2	2	2	2
3	1	1	1	2	3	2	1	1	1
2	1	1	2	2	2	1	1	1	1
2	1	2	2	2	3	2	2	2	1
2	2	2	2	3	3	2	2	1	9
2	3	2	3	3	2	2	2	1	1
2	2	2	2	2	2	2	2	2	2
3	2	2	2	2	2	3	2	2	1
9	9	9	9	1	9	9	9	9	1
1	2	1	2	1	2	2	1	1	1
2	2	2	2	2	2	2	2	2	2
2	2	1	1	2	1	2	1	1	1
2	2	2	2	2	2	2	2	2	1
2	2	2	2	2	2	2	2	2	2
1	2	2	1	2	2	2	2	1	1

3	1	2	2	2	2	2	2	2	1
3	2	1	1	1	2	3	2	2	1
2	2	2	2	2	2	2	2	2	1
1	2	1	2	2	2	2	1	1	1
2	3	2	2	3	2	3	1	1	1
2	2	2	2	2	2	2	2	2	1
3	3	2	3	2	2	3	2	2	1
2	2	2	2	2	2	2	2	2	2
2	2	2	2	2	2	2	2	2	2
2	2	2	2	2	2	2	2	2	2
2	2	2	2	2	2	2	2	2	2
3	3	2	2	3	2	2	2	1	1
2	2	2	2	2	2	2	2	2	2
2	2	2	2	2	2	2	2	2	2
2	3	2	2	3	2	3	2	2	2
2	2	2	2	2	2	2	2	2	2
2	2	2	2	2	2	9	1	1	2
2	2	2	2	2	2	2	2	2	1
2	2	2	2	2	2	2	2	2	2
3	3	1	2	3	3	3	1	1	1
2	2	2	2	2	2	1	2	2	1
1	2	1	2	3	3	2	2	2	1
3	3	1	3	3	3	2	2	1	1
3	3	1	2	1	1	3	1	1	1
2	3	2	3	2	3	3	2	2	2
2	2	2	2	2	2	2	2	2	2
1	1	1	1	1	1	3	1	3	9
1	2	1	2	1	3	1	2	1	1
2	1	1	2	1	2	2	2	2	1
3	2	2	1	2	2	2	2	3	2
2	2	2	2	2	2	2	2	2	2
2	2	3	2	3	2	2	2	1	2
2	2	2	2	2	2	2	2	2	2
3	2	2	2	3	3	2	2	3	2
2	2	2	2	1	2	2	2	2	2
9	9	2	9	9	9	9	9	9	2
2	2	2	2	2	2	2	2	2	2
2	2	1	1	1	1	2	1	1	1
1	2	2	2	2	2	2	2	2	3
2	2	1	1	2	3	3	1	1	1
2	3	9	9	9	9	9	9	9	1
2	1	1	1	2	2	1	2	2	1
2	1	1	1	3	3	3	1	1	1
3	3	2	1	3	3	2	1	1	1
3	3	1	1	3	1	3	1	1	1
2	2	2	3	3	3	2	2	2	2
9	9	9	9	9	9	9	2	9	1

9	9	9	9	9	9	9	2	9	1
9	9	9	9	3	9	9	9	9	9
9	9	2	9	9	9	9	9	9	1
1	1	9	9	9	9	9	9	9	1
9	9	1	9	9	9	9	9	9	9
9	9	3	1	3	9	9	9	9	9
9	9	9	9	9	9	1	9	9	9
9	9	9	9	3	9	9	9	9	1
9	9	9	9	3	9	9	9	9	9
9	9	3	9	9	9	9	9	9	9
9	9	9	9	3	9	9	9	9	9
9	9	3	9	9	9	9	9	9	9
9	9	9	9	3	9	9	9	9	1
9	9	1	9	3	9	9	9	9	1
9	1	9	9	3	9	9	9	9	9
9	1	9	9	3	9	9	9	9	9
9	9	9	9	9	9	1	9	9	9
1	1	1	1	3	1	1	1	1	1
2	2	2	2	2	2	2	2	2	2
9	9	1	9	9	9	9	9	9	1
1	1	1	1	1	1	1	1	1	1
2	2	2	2	2	2	1	2	3	2
3	2	1	1	2	1	2	1	3	2
1	1	1	1	1	3	1	1	1	1
2	3	1	1	3	2	3	2	3	2
3	2	2	2	2	2	1	2	1	3
2	2	2	2	2	2	2	2	2	2
1	3	2	2	2	3	2	3	1	3
2	2	2	2	2	2	2	2	2	2
2	2	2	2	2	2	2	2	2	2
3	3	3	3	3	3	2	2	2	1
3	2	2	2	2	2	2	2	2	1
2	2	2	2	2	2	2	2	2	2
3	3	1	1	3	3	3	3	1	1
1	2	1	2	3	1	2	3	1	1
3	3	1	2	1	3	3	2	2	1
2	1	3	2	3	2	2	2	2	1
9	9	9	9	9	9	9	9	9	9
1	9	1	1	1	1	1	1	1	1
9	9	9	9	9	9	9	9	9	9
2	2	2	2	2	2	2	2	2	2
2	2	1	2	3	1	1	3	2	2
9	9	9	9	9	9	9	9	9	9
1	1	1	1	1	1	1	1	1	1
2	2	2	2	2	2	2	2	2	2
2	2	2	2	2	2	2	2	2	2
3	3	2	2	2	2	3	2	2	2

3	3	2	2	2	2	2	2	2	2
9	9	9	9	9	9	9	9	9	9
3	1	1	3	1	1	3	3	1	1
9	9	9	9	9	9	9	9	9	9
2	2	1	1	2	2	2	2	2	1
2	2	1	1	2	2	2	2	2	1
9	9	9	9	9	9	9	9	9	9
3	3	2	2	2	2	2	2	2	2
1	2	2	1	2	3	1	1	1	1
3	1	1	1	1	1	1	1	1	2
1	2	1	1	1	1	1	1	1	2
2	1	1	2	1	1	2	1	1	2
2	3	1	1	1	2	2	2	2	2
9	9	9	9	9	9	9	9	9	9
9	9	9	9	9	9	9	9	9	9
9	9	9	9	9	9	9	9	9	9
1	2	2	2	1	3	3	2	1	1
9	9	9	9	9	9	9	9	9	9
2	3	3	3	2	3	9	1	1	1
3	1	1	1	1	1	2	2	2	1
1	1	1	1	1	1	1	1	1	3
2	3	1	1	1	2	2	2	2	2
2	2	3	3	3	3	3	3	3	1
9	9	9	9	9	9	9	9	9	9
2	2	2	2	2	2	2	2	2	2
3	1	1	3	3	1	3	1	3	1
3	3	1	3	3	3	3	3	3	3
3	1	1	3	3	1	1	3	3	1
2	2	1	1	2	2	2	1	1	2
2	2	1	2	1	2	2	1	1	1
9	9	9	9	9	9	9	9	9	9
9	9	9	9	9	2	9	9	9	9
1	1	2	2	1	2	2	2	2	1
3	3	1	3	3	3	2	1	1	3
2	1	1	3	3	3	2	1	9	3
2	2	1	2	1	2	2	1	1	1
3	3	1	2	1	2	2	1	1	1
9	9	9	9	9	9	9	9	9	9
9	9	9	9	9	9	9	9	9	9
9	9	9	9	9	9	9	9	9	9
9	9	9	9	9	9	9	9	9	9
1	1	1	3	2	1	2	1	2	1
3	2	3	3	2	1	1	2	3	1
9	9	9	9	9	9	9	9	9	9
2	3	3	3	2	1	1	2	3	1
2	1	1	2	2	2	1	1	1	2
3	1	1	1	1	3	3	3	3	1

3	3	3	3	3	3	3	3	3	3
3	3	1	1	2	2	3	1	1	2
3	3	1	1	3	3	2	3	3	1
1	2	1	1	1	2	2	2	2	2
2	2	2	2	2	2	2	2	2	2
2	3	2	2	3	9	1	3	1	1
9	9	9	9	9	9	9	9	9	9
3	2	2	2	2	2	2	2	1	2
3	3	3	3	3	3	3	3	3	3
3	2	2	2	2	2	2	2	2	1
3	3	3	3	3	3	3	3	3	3
1	1	3	3	3	3	3	3	3	1
2	1	1	2	2	2	3	1	1	2
1	1	1	2	2	2	3	1	1	2
2	1	1	2	2	2	3	1	1	2
1	1	1	2	2	2	3	1	1	2
2	1	1	2	2	2	3	1	1	2
2	1	1	2	2	2	3	1	1	2
2	1	1	2	2	2	3	1	1	2
2	3	1	2	1	2	3	2	2	1
3	2	2	1	3	2	1	2	3	3
3	3	2	2	2	2	2	2	2	3
2	2	2	3	3	3	2	3	2	2
2	2	2	2	2	2	2	2	2	2
3	3	3	3	3	3	3	3	3	3
2	1	1	2	2	2	3	1	1	2
9	9	9	9	9	2	9	9	9	2
9	9	1	3	1	3	2	1	1	1
9	9	1	9	9	9	9	9	9	1
2	2	3	3	3	3	3	3	3	2
2	1	2	2	1	1	9	2	1	1
2	2	2	2	2	2	2	2	2	3
2	1	1	3	2	2	1	1	1	2
9	9	9	1	9	9	9	9	9	9
3	3	2	3	3	3	3	3	3	2
3	2	1	2	3	3	2	3	3	1
9	9	2	1	3	9	2	9	3	1
1	2	2	2	2	1	1	1	2	1
2	1	2	2	2	1	1	1	2	1
1	1	1	1	1	9	2	1	2	1
3	1	1	1	1	1	2	1	1	1
1	2	2	2	2	2	2	2	2	2
9	2	3	9	1	9	3	9	1	3
3	1	1	3	3	3	3	1	1	1
2	2	2	1	1	3	2	3	2	2
1	1	1	1	1	1	1	1	1	1
2	3	1	2	2	2	2	2	1	2

2	2	1	1	1	1	3	1	1	2
9	9	2	9	9	9	9	9	9	1
9	9	1	9	9	9	9	9	9	1
1	1	1	1	3	1	1	3	1	1
2	2	2	2	2	2	2	2	2	2
2	2	1	1	2	2	2	2	2	1
1	1	1	1	1	1	1	1	1	1
2	2	1	1	2	2	2	2	2	1
2	2	1	1	2	2	2	2	2	1
3	3	1	2	3	1	2	3	1	1
9	9	9	9	9	9	9	9	9	9
3	1	1	1	2	1	3	1	1	1
9	9	9	9	9	9	9	9	9	9
3	1	1	1	1	1	3	1	1	1
2	2	1	1	1	1	3	1	1	1
9	9	9	9	9	9	9	9	9	9
2	2	2	2	2	2	2	2	2	2
9	9	9	9	9	9	9	9	9	9
9	9	1	9	9	9	9	9	9	9
1	2	1	2	3	1	2	3	1	1
1	1	2	1	1	1	1	1	1	1
2	2	2	2	2	2	2	2	2	2
9	9	9	9	9	9	9	9	9	9
2	2	2	2	2	2	2	2	2	2
3	3	3	3	3	3	3	3	3	3
2	1	1	2	1	1	2	3	2	3
2	1	1	2	1	1	2	3	2	3
3	3	1	1	1	1	3	1	1	3
1	1	1	1	1	1	1	1	1	1
2	2	2	2	2	2	2	2	2	2
1	1	1	2	1	1	1	3	2	1
2	2	2	2	1	1	2	2	2	1
2	1	1	9	1	1	1	1	1	2
3	1	1	2	1	1	2	3	2	3
2	3	2	2	2	1	1	1	2	3
2	2	2	2	2	2	2	2	2	2
2	2	2	2	2	2	2	2	2	2
9	9	9	9	9	9	9	9	9	9
9	9	9	1	9	9	9	9	9	1
2	3	1	9	1	1	2	3	1	1
2	2	2	2	2	2	2	2	2	2
2	2	2	2	2	2	2	2	2	2
9	9	2	2	2	2	2	9	2	2
2	2	2	2	2	2	2	2	2	2
2	2	2	2	2	2	2	2	2	2
2	2	2	2	2	2	2	2	2	2
9	9	9	9	9	9	9	9	9	9

9	9	9	9	9	9	9	9	9	9
3	1	2	2	2	2	3	2	2	2
1	2	2	2	2	2	3	1	2	9
3	2	1	2	3	2	3	1	2	9
9	9	9	1	9	9	9	9	9	9
2	2	2	2	2	2	2	2	2	2
9	9	9	9	9	9	9	9	9	9
1	1	1	1	3	1	1	1	1	3
3	1	1	2	2	1	1	2	3	1
9	9	9	2	9	9	9	9	9	9
2	2	2	2	2	2	2	2	2	2
2	2	1	1	2	2	1	2	1	2
2	2	1	1	2	2	1	2	1	2
2	2	2	2	2	2	2	2	2	2
2	2	1	1	1	1	1	1	1	1
2	2	2	2	2	2	2	2	2	2
2	2	2	2	2	2	2	2	2	2
3	3	2	2	2	2	2	2	2	2
1	1	1	2	1	2	3	3	3	1
1	1	1	1	1	1	3	3	1	2
9	9	9	9	1	9	9	9	9	9
3	3	2	2	2	2	2	2	2	2
2	2	2	2	2	2	2	2	2	2
2	2	2	2	2	2	2	2	2	2
3	3	1	1	1	1	1	1	1	3
2	2	2	2	2	3	2	3	2	2
9	9	9	9	9	9	1	9	9	2
9	9	1	9	9	9	9	9	9	9
2	2	2	2	2	2	2	2	2	2
2	2	3	3	3	3	3	3	3	3
2	2	1	9	9	9	9	9	9	2
2	2	2	2	2	2	2	2	2	2
3	1	1	1	1	1	3	1	1	1
2	2	1	2	2	2	2	2	2	2
2	2	2	2	2	2	2	2	2	2
2	1	1	1	1	1	2	1	1	1
2	2	2	2	2	2	2	2	2	2
1	3	1	2	3	1	2	3	2	2
2	2	2	2	2	2	2	2	2	2
2	2	2	2	2	2	2	2	2	2
2	2	2	2	2	2	2	2	2	2
2	2	2	2	2	2	2	2	2	2
3	3	1	1	1	1	2	1	1	3
2	2	2	2	2	2	2	2	2	2
2	2	2	2	2	2	2	2	2	2
3	3	3	3	3	3	3	3	3	3
2	2	2	2	2	2	2	2	2	2

2	2	2	2	2	2	2	2	2	2
2	2	2	2	2	2	2	2	2	2
2	2	2	2	2	2	2	2	2	2
2	2	2	2	2	2	2	2	2	2
3	2	2	3	1	3	2	1	2	1
1	1	1	1	1	1	2	1	1	1
2	2	2	2	2	2	2	2	2	2
3	2	1	1	2	3	2	1	2	2
3	3	1	1	1	1	3	1	1	2
2	2	2	2	2	2	2	2	2	2
3	1	1	1	1	3	1	3	1	1
9	9	2	9	9	9	9	9	9	2
9	9	9	2	9	9	9	9	9	9
9	9	9	9	9	9	9	9	9	9
1	1	1	3	3	3	3	3	3	2
2	2	1	1	2	2	3	3	2	2
2	2	1	1	2	2	3	3	2	2
2	2	1	1	3	2	2	1	1	9
9	9	9	9	9	9	9	9	9	9
2	3	2	1	2	3	2	2	3	2
9	9	9	9	9	9	9	9	9	9
1	2	1	2	2	2	2	2	2	1
2	1	2	2	2	2	2	2	2	2
1	1	1	1	1	1	1	1	1	2
3	3	1	1	1	3	3	3	3	2
3	3	1	2	1	1	3	1	1	1
2	2	2	2	2	2	2	2	2	2
2	3	1	1	1	1	2	1	1	1
3	2	1	2	2	2	2	2	2	2
2	3	3	3	2	2	2	2	2	2
1	1	1	1	1	1	1	1	1	1
3	3	1	1	1	1	1	1	1	3
1	1	1	2	1	2	3	1	2	1
1	1	3	3	3	3	3	3	3	3
3	3	1	1	1	1	1	1	1	1
3	3	1	3	1	1	3	3	3	1
9	9	9	9	9	9	9	9	1	1
1	1	1	1	1	1	1	1	1	1
2	2	2	2	2	2	2	2	2	2
2	2	2	2	2	2	2	2	2	2
2	1	1	1	1	1	1	1	2	1
9	9	1	1	1	2	9	9	1	9
2	3	1	3	2	3	2	2	1	1
2	2	2	2	2	2	2	2	2	2
2	2	2	2	2	2	2	2	2	2
1	1	1	3	2	2	3	1	1	1
2	2	3	2	2	2	2	2	2	2

3	1	1	1	1	1	3	3	1	3
3	3	3	3	3	3	3	3	3	3
1	2	2	1	3	2	1	2	3	1
9	9	9	2	9	9	9	9	9	3
9	1	9	9	9	9	1	9	9	9
1	2	1	1	2	2	2	2	2	2
2	2	2	2	2	2	2	2	2	2
3	3	3	3	3	3	3	3	3	1
2	2	1	2	1	1	2	1	2	2
2	2	2	2	2	2	2	2	2	2
9	9	9	9	9	9	9	9	1	1
3	3	2	2	2	2	2	2	3	2
2	2	2	2	2	2	2	2	2	2
2	2	2	2	2	2	2	2	2	2
2	2	2	2	2	2	2	2	2	2
1	3	2	2	2	2	3	3	2	3
2	2	2	2	2	2	2	2	2	2
9	9	9	9	9	9	9	9	1	1
9	9	9	9	9	1	9	9	9	9
2	2	1	1	1	1	1	1	1	2
2	2	2	2	2	2	2	2	2	2
2	2	2	2	2	2	2	2	2	2
9	9	9	9	9	9	9	9	9	9
9	9	9	9	9	9	9	9	9	9
3	2	1	2	2	3	2	3	3	1
1	2	3	3	1	1	3	1	1	3
2	1	2	3	1	1	1	1	1	1
1	3	1	1	3	3	2	3	1	1
3	3	3	1	3	1	3	3	3	1
3	3	1	2	1	3	3	2	1	1
3	2	1	1	1	2	1	1	1	1
3	3	2	1	2	3	2	1	3	2
3	1	1	2	3	2	1	2	1	1
3	3	3	3	3	3	3	3	3	3
3	3	2	2	2	2	2	2	2	2
3	2	1	2	2	3	3	2	3	2
9	1	1	9	2	9	2	9	9	9
3	3	3	3	3	3	3	3	3	3
9	9	9	2	1	9	2	9	9	1
3	3	3	3	3	3	3	3	3	3
3	1	1	1	1	3	3	1	1	1
1	1	1	1	1	1	2	1	1	1
3	1	3	3	3	3	3	3	3	1
2	2	2	2	2	2	2	2	2	2
2	2	1	1	2	1	2	2	2	2
1	1	2	1	2	2	1	1	2	1
2	1	1	1	1	2	2	1	1	3

2	2	2	2	2	2	2	2	2	2
3	3	3	3	3	3	2	3	3	3
1	3	1	2	1	2	1	2	1	1
3	3	3	1	1	1	1	1	1	1
2	2	2	2	2	2	2	2	2	2
1	2	1	2	2	2	2	2	2	3
2	2	1	2	2	2	2	2	2	2
2	2	2	2	2	2	2	2	2	2
2	2	2	2	2	2	2	2	2	1
2	2	2	2	2	2	2	2	2	2
9	9	1	9	9	9	9	9	9	9
2	2	2	2	2	2	2	2	2	2
3	1	1	1	1	1	3	1	1	3
2	1	1	1	2	1	2	2	1	2
1	1	2	1	2	2	1	1	2	2
2	2	1	2	2	2	2	2	2	1
3	2	1	1	1	1	2	1	1	1
1	2	2	2	2	2	2	1	1	1
2	2	2	2	3	3	3	2	2	1
9	9	9	9	9	9	9	9	1	9
1	1	3	1	1	1	1	1	1	1
3	3	1	1	1	1	1	2	3	1
3	3	3	3	3	3	1	3	3	1
3	1	1	1	1	1	3	1	1	1
3	3	3	3	3	3	3	3	3	3
2	2	2	2	2	2	2	2	2	3
1	1	1	1	1	1	2	2	2	1
1	2	3	1	2	1	2	1	3	1
1	2	1	1	1	2	3	2	1	2
3	3	1	1	1	2	1	3	1	1
2	2	2	2	2	2	2	2	2	2
9	9	9	9	9	9	9	9	9	9
2	2	2	3	2	2	1	2	2	2
9	9	9	9	9	9	9	9	9	9
2	2	1	2	2	2	2	2	3	1
2	2	1	2	1	2	1	2	1	2
2	2	2	2	2	2	2	2	2	2
2	2	2	2	2	2	2	2	2	2
3	2	2	3	2	3	2	3	3	2
3	1	2	1	3	1	2	1	2	1
1	3	1	2	3	2	1	2	3	1
2	2	1	2	2	2	2	2	2	2
1	2	1	2	3	1	2	3	1	1
9	9	9	9	9	9	9	9	9	9
3	3	3	3	3	3	3	3	3	3
2	2	2	2	2	2	2	2	2	2
1	2	1	1	2	2	2	1	1	1

9	9	1	9	9	9	9	9	9	9
2	2	1	2	2	2	2	2	2	2
3	9	1	2	2	2	2	2	2	1
1	1	1	2	3	2	3	3	2	1
2	2	3	3	3	3	3	3	3	2
2	3	2	2	3	3	3	3	3	2
9	9	9	9	9	9	9	9	9	9
2	2	2	2	2	2	2	2	2	2
2	2	2	2	2	2	2	2	2	2
9	9	9	9	9	9	9	9	9	9
9	9	9	9	9	9	9	9	9	9
9	9	9	9	9	9	9	9	9	9
9	9	9	2	9	9	9	9	9	9
9	9	9	9	1	9	9	9	9	3
9	9	9	9	9	9	9	9	9	9
2	2	2	2	3	3	2	2	2	1
9	1	2	2	2	2	2	2	2	2
2	2	1	1	1	1	1	1	1	2
1	2	1	3	1	3	1	2	3	1
2	1	1	2	2	2	2	2	2	1
1	2	1	2	2	2	2	2	2	1
2	1	3	2	2	3	2	2	3	3
1	1	1	1	9	2	9	2	2	2
3	3	1	1	3	3	2	2	2	1
2	2	2	2	2	2	2	2	2	2
2	2	2	2	2	2	1	3	2	2
2	2	2	9	3	2	9	2	9	9
1	1	1	1	1	1	1	1	1	1
2	2	2	2	2	2	1	3	2	2
2	2	2	2	3	3	2	2	2	1
2	1	1	2	2	2	2	2	2	1
9	9	9	2	9	9	9	9	9	9
3	1	1	3	1	1	2	3	1	2
2	2	2	2	2	2	2	2	2	2
2	2	2	2	2	2	2	2	2	2
2	2	2	2	2	2	2	2	2	2
2	2	2	2	2	2	2	2	2	2
9	9	1	9	9	9	9	9	9	1
2	2	2	2	2	2	2	2	2	2
2	2	2	2	2	2	2	2	2	2
2	2	2	2	2	2	2	2	2	2
2	2	2	2	2	2	2	2	2	2
2	2	2	2	2	2	2	2	2	2
3	3	1	2	2	2	2	1	1	2
2	2	1	2	2	2	2	2	2	1
2	2	2	2	2	2	2	2	2	2
9	9	1	9	9	9	9	9	9	9

2	2	2	2	2	2	2	2	2	2
2	2	2	2	2	2	2	2	2	2
9	9	9	1	9	9	9	9	9	9
9	2	9	9	9	9	2	9	9	1
2	2	1	1	2	1	2	2	2	2
9	9	1	9	9	9	9	9	9	1
1	2	1	2	3	2	1	1	2	1
1	2	1	2	3	2	1	2	3	1
9	9	1	2	3	2	3	1	3	1
2	2	1	2	3	2	1	2	3	1
9	9	9	9	9	9	9	9	9	9
3	2	2	1	3	1	1	2	2	1
2	2	2	2	2	2	2	2	2	1
2	2	2	2	2	2	2	2	2	2
1	1	1	2	2	1	2	1	1	1
2	1	3	3	3	3	3	3	3	3
2	2	2	2	2	2	2	2	2	2
2	2	2	2	2	2	2	2	2	2
2	2	1	1	2	1	2	2	2	1
3	3	1	2	3	3	3	1	1	1
3	3	1	1	1	1	3	1	1	1
2	2	2	2	2	2	2	2	2	2
3	3	1	2	1	1	3	1	1	1
3	3	2	2	2	2	2	2	2	2
2	2	2	2	2	2	2	2	2	2
3	2	1	2	2	2	1	1	1	1
2	2	2	2	2	2	2	2	2	2
2	2	2	2	2	2	2	2	2	2
3	2	2	2	2	2	2	2	2	1
9	9	9	9	9	9	9	9	9	9
9	1	9	9	9	9	2	9	9	1
2	2	2	2	2	2	2	2	2	2
9	9	9	9	9	9	9	9	9	9
9	9	9	9	9	9	9	9	9	9
2	3	1	2	3	3	3	3	3	3
9	9	9	9	9	1	9	9	9	9
9	9	9	9	9	9	9	9	9	9
2	2	2	2	2	2	2	2	2	2
1	9	1	2	2	2	2	1	1	1
3	1	1	1	2	1	3	2	2	1
1	1	1	1	1	1	1	1	1	1
2	3	1	3	2	3	1	2	1	1
9	9	2	9	9	9	9	9	9	1
1	2	1	2	1	2	1	2	3	1
1	3	1	2	9	1	3	2	1	2
9	9	9	9	9	9	9	9	9	9
2	1	3	3	2	3	3	1	3	3

1	2	1	2	1	2	1	2	1	1
9	9	9	9	9	9	9	9	9	9
9	9	9	9	9	9	9	9	9	9
2	2	2	2	2	2	2	2	2	2
1	1	1	1	1	1	1	1	1	1
1	2	1	2	1	2	1	2	1	1
1	2	1	1	2	9	1	2	1	1
3	3	1	1	1	1	3	1	1	1
2	2	2	2	2	2	2	2	2	2
3	3	3	1	1	1	2	2	2	1
9	9	9	9	9	9	9	9	9	9
9	9	9	9	9	9	9	9	9	9
9	1	1	9	9	9	9	9	9	9
2	1	1	1	1	2	2	2	2	1
2	2	2	3	1	2	2	2	2	2
9	9	9	9	9	9	9	9	9	9
9	9	9	9	9	9	9	9	9	9
9	9	9	9	9	9	9	9	9	9
2	2	1	2	2	2	2	1	2	2
2	2	1	2	2	2	2	2	2	2
9	9	9	9	9	9	9	9	9	9
2	1	1	3	3	3	3	1	1	1
3	3	3	3	3	3	3	3	3	3
9	9	9	9	9	9	9	9	9	9
2	2	2	2	2	2	2	2	2	2
2	2	2	2	2	2	2	2	2	2
9	9	9	9	9	9	9	9	9	9
9	9	9	9	9	9	9	9	9	9
2	2	2	2	2	2	2	2	2	3
2	2	2	2	9	1	1	1	1	2
2	1	1	1	1	1	1	1	1	1
9	9	9	9	9	9	9	9	9	9
9	9	9	9	9	9	9	9	9	9
1	1	1	3	3	1	3	2	3	1
1	1	3	3	3	3	2	2	2	3
2	2	2	2	2	2	2	2	2	2
2	1	2	2	2	2	2	2	2	2
2	2	2	2	2	2	2	2	2	2
2	1	1	9	1	9	1	3	1	1
3	2	2	9	1	2	3	2	3	1
1	1	1	1	2	2	2	1	3	1
9	9	9	9	9	9	9	9	9	9
9	9	9	9	9	9	9	9	9	9
9	9	9	9	9	9	1	9	9	9
9	9	9	9	9	9	9	9	9	9
9	9	9	9	9	9	9	9	9	9
9	9	9	9	9	9	9	9	9	9

2	2	2	2	2	2	2	2	2	2
2	3	2	2	2	2	2	3	3	3
2	1	1	1	2	2	3	1	1	1
2	2	2	2	2	2	2	2	2	2
9	9	9	9	9	9	9	9	9	9
9	9	9	9	9	9	9	9	9	9
9	9	9	9	9	9	9	9	9	9
9	9	9	9	9	9	9	9	9	9
9	9	9	9	9	9	9	9	9	9
9	9	9	9	9	9	9	9	9	9
9	9	9	9	9	9	9	9	9	9
9	9	9	9	9	9	9	9	9	9
9	9	9	9	9	9	9	9	9	9
1	1	1	9	1	9	1	9	1	1
9	9	9	9	9	9	9	9	9	9
2	2	1	2	2	1	2	2	1	1
2	2	2	2	2	2	2	2	2	2
9	9	9	9	9	9	9	9	9	9
9	9	9	9	9	9	9	9	9	9
9	9	9	9	9	9	9	9	9	9
9	2	2	9	9	9	1	3	9	2
9	9	9	9	9	9	9	9	9	9
1	1	2	2	2	2	1	1	1	2
9	9	9	9	9	9	9	9	9	9
1	1	1	1	1	1	1	1	1	1
2	2	2	2	2	2	2	2	2	1
2	2	3	3	1	1	2	2	2	1
1	1	1	1	1	1	2	2	2	1
1	1	1	1	1	2	1	1	1	2
1	1	1	1	1	1	1	1	1	2
2	2	1	1	1	3	3	2	2	2
2	2	1	1	1	1	1	1	1	1
1	1	1	1	1	1	1	1	1	1
9	9	9	9	9	9	9	9	9	9
2	2	2	1	1	1	2	2	2	1
1	1	1	1	1	1	1	1	1	2
1	1	1	1	1	1	1	1	1	1
9	9	9	9	9	9	9	9	9	9
3	3	2	2	2	2	2	3	3	1
3	3	3	3	1	1	3	1	3	1
3	3	3	3	3	3	3	3	3	3
9	9	9	9	9	9	9	9	9	9
9	9	9	9	9	9	9	9	9	9
9	9	9	9	9	9	9	9	9	9
9	9	9	9	9	9	9	9	9	9
2	2	2	2	2	2	2	2	2	2
9	9	9	9	9	9	9	9	9	9

9	9	9	9	9	9	9	9	9	9
9	9	9	9	9	9	9	9	9	9
9	9	9	9	9	9	9	9	9	9
9	9	9	9	9	9	9	9	9	9
9	9	9	9	9	9	9	9	9	9
9	9	9	9	9	9	9	9	9	9
9	9	9	9	9	9	9	9	9	9
9	9	9	9	9	9	9	9	9	9
9	9	9	9	9	9	9	9	9	9
9	9	9	9	9	9	9	9	9	9
9	9	9	2	9	9	9	9	9	9
9	9	9	9	9	9	9	9	9	9
2	2	2	2	2	2	2	2	2	2
1	1	1	1	1	1	2	2	2	2
2	2	1	1	1	1	2	2	2	2
1	2	1	2	1	1	3	2	1	3
1	1	1	1	2	2	2	2	2	2
2	3	1	1	1	1	2	1	1	1
2	2	2	2	2	2	1	1	1	1
9	9	1	9	9	9	9	9	9	1
2	2	1	1	1	1	1	1	1	1
1	1	1	2	2	2	2	2	2	1
2	2	2	2	2	2	2	2	2	2
1	1	2	2	2	1	2	2	2	1
2	1	1	2	1	1	2	1	1	1
3	3	3	3	3	3	3	1	1	3
2	2	9	9	9	9	9	9	9	1
3	3	2	2	1	1	1	1	1	2
9	9	1	9	9	9	9	9	9	9
9	9	2	9	9	3	1	9	3	9
9	9	9	9	9	9	9	9	9	9
1	1	1	1	1	1	2	2	1	1
2	2	2	2	1	1	1	1	1	2
1	1	1	1	1	1	1	1	1	1
1	1	1	2	2	2	2	2	2	1
2	2	1	2	2	2	2	2	2	2
1	1	1	1	2	2	2	2	2	1
2	2	2	2	2	2	2	2	1	2
2	1	2	2	1	1	1	2	2	2
1	1	1	1	1	1	1	1	1	2
2	2	2	2	2	2	2	2	2	2
1	1	1	1	1	1	1	1	1	1
3	2	1	1	1	3	3	1	1	1
1	1	1	1	1	1	2	2	2	1
1	1	1	1	1	1	1	1	1	2
1	1	1	1	2	2	1	1	1	1
2	2	2	2	2	2	2	2	2	1

9	9	9	9	9	9	9	9	9	9
2	2	1	1	1	1	1	1	1	2
9	9	9	9	9	2	9	9	9	9
9	9	9	9	9	2	9	9	9	9
9	9	9	9	1	9	9	9	9	9
9	9	9	9	9	9	9	9	9	9
9	9	9	9	9	9	9	2	9	9
3	3	1	2	1	1	1	1	1	2
9	9	2	9	9	9	9	9	9	2
2	2	2	2	2	2	2	2	2	2
9	9	9	9	9	9	9	9	9	9
9	9	9	9	9	9	9	9	9	9
1	1	1	1	1	1	2	1	1	1
2	2	2	2	2	2	2	2	2	9
1	1	1	1	1	1	1	1	1	1
9	9	9	9	9	9	9	9	9	9
2	2	2	2	2	2	2	2	2	2
3	3	3	3	3	3	3	3	3	3
9	9	9	9	9	9	9	9	9	9
1	1	2	2	2	2	2	2	2	2
2	2	2	2	2	2	2	2	2	2
3	2	2	2	3	3	3	2	2	3
2	2	2	2	2	2	2	2	2	2
2	2	2	2	2	2	2	2	2	2
2	2	2	1	1	1	1	1	1	2
2	2	2	2	2	2	2	2	2	2
3	1	2	2	2	2	2	2	2	3
2	2	1	1	1	2	2	2	2	1
1	1	1	2	2	2	2	2	2	2
1	2	2	2	2	2	2	1	1	1
9	9	9	9	9	9	9	9	9	9
2	2	3	2	3	2	3	2	3	2
9	9	9	9	9	9	9	9	9	9
2	2	2	2	2	2	2	2	2	2
2	2	2	2	2	2	2	9	2	3
2	2	1	1	1	1	1	1	1	1
3	3	1	1	1	2	2	1	1	1
1	1	1	1	1	1	1	1	1	1
1	1	1	1	1	1	1	1	1	1
2	2	1	1	3	1	3	3	2	1
2	2	2	2	2	2	2	2	2	2
1	1	1	1	1	1	2	2	2	2
9	9	9	9	9	9	9	9	9	9
9	9	9	9	9	9	9	9	9	9
9	9	1	1	9	9	9	9	9	1
9	9	9	9	9	9	9	9	9	9
9	9	9	9	9	9	9	9	9	9

9	9	9	9	9	9	9	9	9	9
2	2	2	2	2	2	2	2	2	2
9	9	9	1	9	9	9	9	9	9
3	1	2	3	1	3	9	1	3	9
9	9	9	9	9	9	9	9	9	9
1	3	2	2	1	2	3	1	2	1
3	3	3	3	3	3	2	2	2	1
9	9	9	9	9	9	9	9	9	9
9	9	9	9	9	9	9	9	9	9
9	9	9	9	9	9	9	9	9	9
9	9	1	9	9	9	9	9	9	9
9	9	9	9	9	9	9	9	9	9
1	2	2	1	2	2	2	2	2	2
9	9	9	9	9	9	9	9	9	9
9	9	9	9	9	2	9	9	9	9
2	1	2	1	2	2	3	2	2	2
1	2	3	2	3	2	1	2	3	3
2	2	2	3	3	2	3	2	2	1
2	2	1	2	3	1	1	2	2	1
2	3	1	2	2	1	3	2	3	2
2	1	1	2	2	1	2	3	1	1
9	3	1	1	3	1	3	3	1	1
1	1	1	2	3	2	1	1	1	1
1	3	1	2	1	3	1	3	1	1
2	2	2	2	2	2	2	2	2	2
1	2	1	1	1	2	2	2	1	2
2	3	3	9	9	9	9	9	9	9
1	9	1	2	9	3	1	2	3	2
2	2	2	3	3	3	9	9	9	9
3	3	1	1	2	2	2	3	3	1
1	2	2	2	2	2	2	2	2	3
2	2	2	2	2	2	2	2	2	2
9	9	9	9	9	9	9	9	9	9
9	1	1	2	2	2	3	3	3	2
2	2	2	2	2	2	3	1	1	1
1	1	2	1	2	2	2	1	1	2
2	2	2	2	2	2	2	2	2	2
2	2	1	1	2	2	3	1	2	2
9	9	9	9	1	9	9	9	9	9
2	3	1	2	2	1	3	1	3	1
9	9	9	9	9	9	9	9	3	9
9	9	1	9	9	9	9	9	9	2
2	2	1	9	9	9	9	9	9	2
9	9	1	9	9	9	9	9	9	2
1	1	3	3	3	3	3	3	3	1
1	3	1	3	1	1	3	1	1	1
3	3	3	3	3	2	2	1	3	3

3	3	3	3	3	3	3	3	3	3
9	9	9	9	9	9	9	9	1	1
2	2	2	2	2	2	2	2	2	2
3	1	1	1	3	1	1	3	1	1
3	3	1	3	1	1	3	1	1	1
1	1	1	1	3	1	1	1	3	1
1	2	2	3	2	3	1	2	3	2
2	3	3	1	1	2	3	1	3	1
2	2	2	2	2	2	2	2	2	2
2	3	2	9	2	3	2	3	2	2
2	3	1	2	2	2	2	2	2	2
2	1	2	1	2	3	3	3	2	2
1	2	1	2	3	1	2	3	2	2
2	2	2	1	3	1	2	1	2	1
2	3	1	2	3	2	2	2	2	1
2	3	2	2	3	2	1	2	3	1
2	2	2	2	2	2	2	2	2	2
2	3	3	3	2	2	1	2	2	2
2	2	2	2	2	2	2	2	2	2
2	2	2	2	2	2	2	2	2	2
2	2	2	2	2	2	2	2	2	3
3	3	1	2	2	2	3	3	3	1
9	9	2	9	9	9	9	9	9	2
9	9	9	9	9	9	9	9	9	9
2	2	2	2	2	2	2	2	2	3
2	3	1	1	1	1	1	1	1	1
2	3	1	2	1	3	2	1	2	1
2	2	1	2	2	3	3	2	1	2
1	1	1	1	1	1	1	1	1	3
9	9	9	9	1	9	9	9	9	9
2	2	2	1	3	1	2	2	2	2
1	1	1	1	1	1	1	1	1	2
9	2	2	3	3	2	3	2	3	2
3	2	2	2	1	2	1	1	1	1
1	1	1	2	9	1	3	1	1	1
2	2	2	2	2	2	2	2	2	2
3	3	3	3	3	3	3	3	2	2
9	9	9	9	9	9	9	9	9	9
1	2	1	2	1	1	3	2	2	2
1	1	2	2	2	2	3	2	2	2
9	9	9	9	9	9	9	9	9	9
9	9	9	9	9	9	9	9	9	9
3	3	2	2	2	2	2	2	2	3
9	9	9	9	9	9	9	9	9	9
2	2	2	2	2	2	2	2	2	2
1	1	2	2	1	2	3	1	1	1
9	9	9	9	9	9	9	9	9	9

2	2	2	2	3	3	2	2	2	2
9	9	9	9	9	9	9	9	9	9
2	2	3	2	2	2	2	2	2	3
9	9	9	9	9	9	9	9	9	9
9	9	9	9	9	9	9	9	9	9
3	3	2	2	2	2	2	2	2	2
2	2	2	2	2	2	2	2	2	1
2	2	2	2	2	2	2	2	2	3
1	1	1	1	1	1	1	1	1	2
2	2	2	2	1	1	2	2	2	2
9	9	9	9	9	9	9	9	9	9
9	9	1	1	1	2	2	3	3	3
9	9	9	9	9	9	9	9	9	9
1	1	1	1	1	1	1	1	1	1
2	2	2	2	2	2	2	2	2	2
1	1	2	2	1	1	2	2	2	3
9	9	2	9	9	9	9	9	9	2
9	9	9	9	9	9	9	9	9	9
1	3	1	1	1	1	1	3	3	1
2	2	2	2	2	2	3	3	2	3
1	1	1	1	1	1	1	1	1	1
3	2	2	2	2	2	2	3	3	2
9	9	1	9	9	9	9	9	9	1
2	3	2	2	2	2	3	3	2	2
2	1	1	1	2	2	2	2	2	3
2	2	2	2	2	2	2	3	3	2
9	9	9	9	9	9	1	9	9	1
2	2	1	1	1	1	1	1	1	1
2	2	2	2	2	2	2	2	2	2
9	9	9	9	1	9	9	9	9	1
3	1	1	2	2	2	3	2	2	1
2	2	2	2	2	2	2	2	2	2
2	2	3	3	3	3	3	3	3	1
2	3	1	1	1	2	3	1	2	2
2	2	2	2	2	2	2	2	2	2
1	3	2	2	2	3	2	1	2	1
1	1	2	2	2	2	2	2	2	2
3	1	9	1	2	2	3	3	3	3
2	2	2	2	2	2	2	2	2	2
3	3	3	3	3	3	3	3	3	3
2	1	1	1	1	2	3	2	1	1
1	1	1	2	2	2	2	1	1	1
3	3	2	2	2	2	2	2	2	2
3	2	2	2	2	2	3	2	2	2
3	1	2	2	2	2	2	2	2	2
3	2	2	3	2	2	2	2	2	2
3	3	2	2	2	2	3	2	2	1

3	2	3	3	2	1	2	2	2	3
2	2	2	2	2	2	2	2	2	1
2	1	1	2	1	1	2	1	1	1
2	3	2	2	2	2	2	2	2	1
2	2	2	1	1	1	2	2	2	2
9	9	9	1	9	9	9	9	9	9
2	2	2	2	2	2	2	2	2	2
1	1	1	1	1	2	2	2	1	1
1	2	2	2	2	2	2	2	2	2
2	2	2	2	2	2	2	2	2	1
1	1	2	2	2	2	1	1	1	2
2	3	1	2	3	1	2	1	2	3
2	2	2	2	2	2	2	2	2	2
3	3	3	3	3	3	3	3	3	3
2	3	2	2	1	3	2	2	1	3
2	3	1	2	3	1	2	3	1	1
3	2	3	2	3	2	3	2	3	2
2	2	2	3	2	3	2	3	2	2
2	2	2	2	2	2	2	2	2	2
1	2	3	2	2	2	2	2	2	2
2	2	2	2	2	2	2	2	2	2
2	2	2	2	2	2	2	2	2	2
3	3	3	2	2	2	2	2	2	2
3	3	3	2	2	2	2	2	2	2
2	2	2	1	1	1	2	1	1	2
2	2	2	2	2	2	2	2	2	2
3	2	2	2	2	2	3	3	3	1
3	2	1	1	1	1	2	3	3	1
2	2	2	2	2	2	2	2	2	2
1	1	1	1	1	1	2	2	2	1
3	3	2	2	2	3	3	3	2	2
3	3	3	2	2	3	3	3	3	3
2	2	1	1	1	3	3	3	3	1
2	2	2	2	2	2	2	2	2	2
1	1	1	1	1	1	2	2	1	1
2	2	2	2	2	2	2	2	2	2
2	2	2	2	2	2	2	2	2	2
1	2	2	1	2	3	2	2	1	2
3	2	2	2	2	2	2	2	2	2
2	2	2	2	2	2	2	2	2	2
2	1	1	1	1	2	2	1	1	1
3	3	1	1	1	1	3	2	2	1
1	1	2	1	2	2	2	2	2	1
2	2	2	2	2	2	1	2	2	2
2	3	2	2	1	2	3	2	3	2
3	1	1	1	2	2	1	3	1	1
2	1	2	2	9	9	9	9	9	9

1	2	2	1	2	2	2	2	1	1
3	3	2	2	2	2	3	9	3	2
3	2	1	2	2	2	2	2	2	1
2	2	2	2	2	2	2	2	2	2
2	2	2	2	2	2	2	2	2	2
2	2	2	2	2	1	2	2	2	2
2	2	1	2	2	2	2	2	2	1
3	2	1	9	1	2	1	1	1	1
2	2	2	2	2	2	2	2	2	2
2	2	2	2	2	2	2	2	2	2
2	2	2	2	2	2	2	2	2	2
9	9	9	9	9	9	9	9	9	9
9	9	9	9	9	9	9	9	9	9
9	9	2	2	2	2	2	2	2	2
1	1	1	1	1	2	2	1	1	2
2	2	2	2	2	2	2	2	2	2
3	2	1	2	1	2	9	1	1	1
2	2	2	2	2	2	2	2	2	1
2	2	1	2	2	2	2	1	1	1
3	2	1	1	2	1	2	2	1	1
2	2	2	2	2	2	3	2	2	2
3	3	1	1	1	1	1	1	1	2
2	2	2	2	2	2	2	2	2	2
2	2	2	2	2	2	2	2	2	2
2	2	2	2	2	2	2	2	2	1
2	2	1	1	1	2	1	2	1	1
1	2	2	2	2	2	2	2	2	2
2	2	2	2	2	2	2	2	2	2
2	2	1	1	1	1	1	1	1	2
9	9	9	9	9	9	9	9	9	9
2	2	3	3	3	3	3	3	3	2
2	2	2	2	2	2	2	2	2	2
2	3	2	2	2	2	2	2	2	2
2	2	2	2	2	2	2	2	2	2
9	9	2	2	2	2	2	2	2	2
2	2	2	2	2	2	2	2	2	2
2	2	2	2	2	2	2	2	2	2
2	2	2	2	2	2	2	2	2	2
2	2	2	2	2	2	2	2	1	2
2	2	2	2	2	2	2	2	2	2
2	2	2	2	2	2	2	2	2	2
2	2	2	2	2	2	2	2	2	2
1	1	1	1	1	1	1	1	1	2
2	2	2	2	2	2	2	2	2	2
2	2	2	2	2	2	2	2	2	2
3	2	2	2	3	2	2	3	3	2
2	2	2	2	2	2	2	2	2	2

1	1	2	2	2	2	2	2	2	1
2	2	1	2	2	2	2	2	2	2
2	3	1	2	1	1	3	1	1	1
2	2	2	2	2	2	2	2	2	2
2	2	2	2	2	2	2	2	2	2
2	2	2	1	1	2	2	2	2	2
9	9	9	9	9	9	9	9	9	9
2	2	1	1	1	1	1	1	1	1
2	2	1	2	1	2	2	2	1	1
3	2	1	1	2	2	3	1	1	2
2	2	1	1	1	2	3	1	1	2
2	2	2	2	2	2	2	2	2	2
2	3	2	2	2	2	2	2	2	2
9	9	1	9	1	9	1	1	1	2
9	3	1	3	3	3	1	1	1	9
2	1	2	2	2	2	2	2	2	1
2	1	1	2	2	2	2	2	2	2
2	2	2	2	2	2	2	2	2	1
1	1	1	1	1	1	1	1	1	1
1	1	1	1	1	1	2	2	3	2
1	1	1	1	1	2	2	1	1	1
3	3	1	1	2	3	3	2	2	1
2	2	2	2	2	2	2	2	2	2
2	2	2	2	2	2	2	2	2	2
2	2	2	2	2	2	2	2	2	2
2	2	2	2	2	2	2	2	2	2
9	9	9	9	9	9	9	9	9	9
2	2	2	2	2	2	2	2	2	2
2	2	2	2	2	2	2	2	2	2
2	2	2	2	2	2	2	2	2	2
2	2	2	2	2	2	2	2	2	2
2	2	1	2	2	2	2	2	2	2
9	9	9	9	9	9	9	9	9	9
9	9	9	9	9	9	9	9	9	9
9	9	9	9	9	9	9	9	9	9
9	9	9	9	9	9	9	9	9	9
3	2	2	3	2	2	3	2	2	1
2	3	1	1	1	1	3	1	1	1
1	1	1	2	1	1	3	1	1	2
2	3	2	2	2	2	2	2	2	2
3	3	1	1	1	2	1	1	1	1
2	2	2	2	2	2	2	2	2	1
1	2	1	1	1	1	2	1	1	2
2	2	2	2	2	2	2	2	2	2
2	3	1	1	2	2	2	2	2	2
2	2	1	1	2	2	2	2	2	2
2	1	1	2	1	3	1	2	2	1

2	2	1	2	3	1	2	3	2	2
1	2	1	2	1	1	2	3	3	3
3	3	1	2	1	3	2	3	2	2
1	1	2	1	1	1	2	1	1	1
2	1	2	1	2	1	3	1	2	3
3	1	2	1	1	1	1	3	2	1
3	1	2	1	1	1	3	1	1	1
9	9	2	9	9	9	9	9	9	2
2	2	2	2	2	2	2	2	2	2
2	2	2	2	2	2	2	2	2	2
2	2	2	2	2	2	2	2	2	2
2	2	2	2	2	2	2	2	2	2
2	2	2	2	2	2	2	2	2	2
1	2	1	1	9	2	2	2	2	2
2	2	1	1	2	1	2	1	1	2
2	2	2	2	2	2	2	2	2	2
2	2	2	2	2	2	2	2	2	2
1	1	1	1	1	1	3	1	1	1
1	2	2	2	2	2	2	2	2	1
2	3	1	1	2	2	1	2	1	1
2	2	2	3	2	2	1	2	2	1
3	2	2	2	2	2	2	2	1	2
1	1	1	1	1	1	2	1	1	1
9	9	9	9	9	9	9	9	9	1
3	1	1	3	2	3	2	3	1	3
1	1	1	1	1	1	2	1	1	1
1	1	2	1	1	1	1	1	1	2
1	3	1	2	1	2	3	2	1	2
2	2	2	2	2	2	2	2	2	2
9	9	9	1	9	9	9	9	9	9
3	3	2	1	3	3	3	3	3	2
9	9	9	9	9	9	9	9	9	9
9	9	9	9	9	9	9	9	9	9
9	9	9	9	9	9	9	9	9	9
9	9	9	9	9	9	9	9	9	9
2	2	2	2	2	2	2	2	2	2
2	2	1	1	1	1	1	1	1	1
9	9	9	9	9	9	9	9	9	9
2	2	2	2	2	2	2	2	2	2
2	2	2	2	2	2	2	2	2	2
2	3	1	1	2	2	2	1	1	9
2	2	2	2	2	2	2	2	2	3
2	2	2	2	2	2	2	2	2	2
3	2	3	3	3	3	3	3	3	9
9	9	9	9	9	9	9	9	9	9
1	1	1	1	2	2	1	1	1	1
3	3	3	3	3	3	3	3	3	3

1	1	1	1	1	1	1	1	1	1
9	2	1	2	1	2	1	1	1	1
2	1	1	1	1	1	1	1	1	1
2	2	2	2	2	2	2	2	2	2
9	9	9	9	9	9	9	9	9	9
9	9	9	9	9	9	9	9	9	9
2	2	2	2	2	2	2	2	2	2
3	3	9	9	9	9	9	9	9	2
9	9	9	9	9	9	9	9	1	1
9	9	9	9	9	9	9	9	9	9
2	3	1	2	2	2	2	2	2	2
1	1	2	1	1	1	2	2	2	2
2	2	3	2	2	2	2	2	2	2
3	3	3	3	3	3	3	3	3	3
3	3	9	9	9	9	9	9	9	9
3	3	2	3	3	2	2	3	2	1
2	2	2	1	1	1	2	1	1	1
2	2	2	2	3	2	1	3	2	2
9	9	1	9	9	9	9	9	9	9
3	2	2	3	1	2	2	1	2	2
1	2	1	2	1	3	1	2	1	1
2	3	1	1	1	2	2	2	1	2
1	1	1	3	2	1	2	2	1	1
2	2	2	2	2	2	2	2	2	1
1	3	9	9	9	9	9	9	9	3
3	3	2	2	2	2	2	2	2	2
2	1	2	1	2	2	2	2	1	2
2	2	1	1	3	3	2	2	2	1
2	3	3	2	3	3	3	3	3	1
3	3	3	2	3	3	3	3	3	3
2	2	2	2	2	2	2	2	2	2
1	1	1	2	2	2	1	1	1	2
1	1	1	1	1	1	1	1	1	1
1	2	3	2	2	2	2	2	2	3
9	9	9	9	9	9	9	9	2	9
2	1	1	1	2	2	1	2	1	1
9	9	9	9	9	9	9	9	9	1
2	2	2	2	2	2	2	2	2	2
9	9	9	9	9	9	9	9	9	9
3	1	1	1	2	2	2	3	1	9
9	9	9	9	9	9	9	9	9	9
3	2	1	2	3	2	2	3	1	1
2	3	2	2	2	2	2	2	2	3
2	1	1	2	2	2	2	1	2	1
2	2	1	2	2	2	2	1	2	1
2	2	2	2	2	2	2	2	2	2
3	3	2	2	2	2	3	3	3	2

2	2	2	2	2	2	2	2	2	2
9	9	9	9	9	9	9	9	9	9
2	2	2	2	2	2	2	2	2	1
3	3	3	3	3	3	3	3	3	2
3	1	1	1	3	3	3	1	1	1
9	9	9	9	9	9	9	9	9	9
9	9	9	9	9	9	9	9	9	9
2	2	2	2	2	2	2	2	2	2
1	1	1	1	1	9	9	1	1	1
1	3	2	2	2	2	2	2	2	2
2	2	1	2	2	2	1	2	2	2
3	1	2	2	2	2	2	2	2	2
2	3	2	2	2	3	9	2	2	2
1	1	1	1	1	1	1	1	1	1
9	9	9	9	9	9	9	9	9	9
3	3	3	3	3	3	3	3	3	3
2	3	2	2	2	2	2	2	2	2
2	2	2	2	2	2	2	2	2	2
3	3	3	1	3	1	2	2	2	1
2	1	1	2	2	1	3	2	1	1
9	9	9	9	9	9	9	9	9	9
9	9	1	9	9	9	9	9	9	9
2	2	2	2	2	1	2	2	2	2
9	9	9	9	9	9	9	9	9	9
9	9	9	9	9	9	9	9	9	9
2	3	2	3	9	2	2	3	3	2
3	3	2	2	2	2	2	2	2	2
2	2	2	2	2	2	2	2	2	2
3	3	3	3	3	3	3	3	3	3
1	1	1	1	1	1	2	1	1	1
3	3	3	3	3	3	3	3	3	3
1	3	1	1	1	1	2	2	2	3
9	9	9	9	9	9	9	9	9	9
9	9	9	9	9	9	9	9	9	9
9	9	9	9	9	9	9	9	9	9
1	1	2	2	2	2	2	2	2	9
1	1	1	1	3	1	3	1	1	1
2	3	2	2	2	3	1	2	3	2
3	1	1	2	1	2	3	1	1	1
9	9	2	9	9	9	9	9	9	2
2	2	2	2	2	2	2	3	3	3
2	2	1	1	2	2	1	2	1	1
2	3	3	3	3	3	3	3	3	3
9	9	9	9	9	9	9	9	9	9
1	2	2	1	3	2	1	2	1	1
3	3	1	1	3	1	3	1	3	9
2	1	1	1	2	2	3	2	2	1

1	2	2	1	3	2	1	2	3	2
3	3	2	2	2	2	2	2	2	3
3	3	1	1	1	1	9	3	3	3
2	3	1	2	3	1	2	3	2	1
3	2	1	2	2	2	2	1	1	1
1	2	1	2	1	1	2	2	1	2
1	2	2	2	2	2	2	2	2	2
2	1	1	1	1	2	2	1	1	1
9	9	9	9	9	9	9	9	9	9
1	1	2	2	2	1	1	1	1	2
1	2	2	2	2	2	2	2	2	2
3	3	2	2	2	2	2	2	2	2
2	2	1	2	1	2	1	1	1	1
2	1	1	2	3	2	3	1	1	1
9	9	9	2	9	9	9	9	9	9
2	2	2	2	1	1	1	9	1	2
1	1	1	1	1	1	2	2	1	1
1	1	1	2	1	2	1	2	3	3
2	2	1	1	3	3	2	2	2	3
2	2	2	2	2	2	2	2	2	2
1	1	2	1	1	2	2	1	1	1
2	2	2	2	2	2	2	2	2	1
1	1	1	3	3	2	1	2	1	1
3	2	2	2	2	2	2	2	2	1
2	1	9	9	9	9	9	9	1	1
3	3	1	1	2	2	2	3	1	1
2	3	2	2	1	1	2	3	3	2
2	2	1	2	2	2	2	2	2	1
2	2	2	2	2	2	2	2	2	2
2	2	2	2	2	2	2	2	1	1
1	1	1	2	1	2	2	2	2	1
2	2	2	2	2	2	2	2	2	2
2	2	2	2	2	2	2	2	2	2
2	2	1	2	1	1	1	2	1	1
2	3	1	2	3	3	3	3	3	1
2	2	1	1	1	2	3	2	2	1
1	1	1	1	2	2	1	1	1	1
1	1	1	1	2	2	2	2	2	1
3	2	1	2	1	1	3	1	1	1
3	3	2	2	3	3	3	3	1	1
2	2	3	3	3	3	2	2	2	2
2	2	1	1	1	1	2	2	2	1
1	1	1	1	2	2	2	1	1	1
1	1	1	1	2	2	2	2	1	1
2	2	2	1	1	1	2	2	2	2
1	1	2	2	2	2	1	1	1	1
1	1	3	3	2	1	1	1	1	3

2	1	2	3	2	1	2	2	1	1
2	2	2	1	2	2	2	1	2	1
1	2	2	1	1	2	2	1	1	1
2	1	2	2	1	2	1	2	1	1
1	1	1	1	2	2	1	1	1	1
1	1	1	2	2	2	1	1	1	2
1	1	1	2	2	1	1	2	2	1
2	2	1	2	2	1	1	1	1	2
2	2	2	2	2	2	2	2	2	2
2	2	2	2	2	2	2	2	2	2
2	2	2	2	2	2	2	2	2	2
1	1	1	1	1	1	2	2	2	3
1	1	3	3	2	2	2	2	2	2
2	2	2	2	2	2	2	2	2	2
2	2	2	2	2	2	2	2	2	2
2	2	3	3	3	3	3	3	3	1
2	2	1	1	2	2	2	2	2	1
2	2	2	2	2	2	2	2	3	3
2	2	2	2	2	2	2	2	2	2
2	2	1	1	1	2	3	3	2	1
2	2	1	1	2	2	2	1	1	1
2	2	2	2	2	2	2	2	2	2
1	2	2	2	2	2	2	2	2	3
9	9	2	2	2	2	2	2	2	3
2	1	2	2	2	2	3	1	2	2
2	2	1	1	1	1	1	1	1	1
3	2	2	2	3	9	1	2	3	2
3	3	2	3	1	2	3	2	1	3
1	2	2	2	2	3	2	2	2	1
3	3	2	2	2	2	3	3	2	2
3	3	2	2	3	2	2	2	2	2
2	1	2	3	1	2	3	1	1	2
2	2	1	1	2	1	1	1	2	1
2	2	3	2	2	1	2	2	2	2
3	1	1	1	3	3	1	2	2	2
1	2	2	2	2	2	2	2	2	2
2	2	2	2	2	2	2	2	2	2
1	1	3	3	3	3	3	3	3	1
1	1	1	1	1	1	1	1	1	1
1	1	1	1	2	2	1	1	1	1
2	2	2	2	2	2	2	2	2	2
3	3	9	9	9	9	9	9	9	9
2	2	2	2	2	2	2	2	2	2
3	3	3	3	3	3	3	3	3	3
3	3	3	3	3	3	3	3	3	3
2	2	2	1	1	2	9	2	2	2
1	1	1	1	2	2	1	1	1	2

2	2	2	2	1	1	2	2	2	1
2	2	2	1	1	1	2	2	1	1
1	1	1	1	1	1	2	2	2	1
2	2	1	1	1	2	2	1	1	1
1	2	1	1	2	2	3	3	3	1
2	2	1	2	2	2	1	1	1	1
3	3	3	3	2	2	2	2	2	1
2	2	1	2	2	2	2	1	1	1
2	2	1	1	1	1	2	2	1	1
1	1	1	1	1	1	2	2	2	1
1	1	1	2	1	1	1	2	1	1
1	2	1	2	3	3	2	2	2	1
1	1	1	1	2	2	1	1	1	1
1	1	1	1	1	1	1	2	2	2
1	1	1	1	1	2	2	1	1	1
1	1	1	1	2	2	1	1	1	1
3	3	1	1	2	2	2	2	3	1
2	2	1	1	2	2	2	2	1	1
2	9	2	9	9	2	9	9	2	2
2	2	3	2	3	3	2	2	1	3
9	2	2	9	9	2	9	9	2	2
3	3	2	2	2	2	2	2	2	2
1	1	1	1	1	2	3	3	2	1
1	1	1	2	1	1	2	2	1	2
3	2	1	2	2	2	1	1	2	1
1	1	1	1	2	2	2	1	1	1
1	1	1	1	2	2	2	1	1	1
1	2	1	1	2	2	3	3	2	1
1	1	1	2	2	1	1	1	1	2
1	1	1	1	2	2	1	1	1	1
1	1	1	1	2	1	1	1	2	1
2	2	2	2	2	2	2	2	2	2
2	2	2	1	1	1	2	2	1	2
1	2	1	2	2	3	2	1	2	1
3	2	1	2	2	3	2	2	1	1
3	1	9	1	3	9	1	3	3	3
9	9	1	9	9	1	9	9	1	9
9	2	2	9	2	9	2	9	2	2
9	9	9	9	9	9	9	9	9	9
2	1	1	9	2	9	1	3	1	1
9	9	1	9	2	9	9	9	9	9
1	1	1	1	2	2	1	1	1	1
9	1	9	9	9	9	9	9	9	9
2	2	2	2	2	2	2	2	2	2
1	9	1	2	9	9	1	9	1	1
2	2	2	9	9	2	9	9	2	2
9	9	2	9	9	2	9	9	2	9

9	9	9	2	9	9	9	2	9	9
9	2	9	2	9	9	2	9	9	9
9	2	2	9	9	2	9	9	2	2
9	9	9	9	9	9	9	9	9	9
1	9	9	9	1	9	9	2	3	9
2	9	9	2	9	9	2	9	2	9
9	9	9	9	9	9	9	9	9	9
9	9	2	9	9	9	9	9	9	2
2	1	2	1	1	1	2	2	3	2
2	2	2	9	9	2	9	9	2	2
1	2	1	2	2	1	1	2	2	1
1	2	1	1	2	2	1	1	1	1
3	3	1	1	2	2	1	1	2	1
2	2	1	1	1	2	2	2	1	1
3	3	2	1	2	1	1	2	2	1
3	3	1	1	2	2	2	1	2	1
2	9	2	2	2	2	2	2	2	2
2	2	2	2	2	2	2	2	2	2
2	2	1	1	2	1	2	1	2	1
2	3	2	2	1	1	1	2	2	2
1	2	1	2	2	1	1	2	1	1
1	1	1	1	2	1	1	2	2	1
1	1	1	1	2	2	1	1	1	1
2	2	1	2	3	2	2	2	2	1
1	2	1	1	2	2	2	3	2	1
3	3	3	2	1	2	2	2	3	3
2	9	2	9	9	2	9	9	2	9
3	2	3	3	3	2	2	2	2	2
2	2	1	2	2	3	2	2	1	1
3	3	3	3	3	3	3	3	3	3
9	9	2	9	9	9	9	9	9	2
2	2	2	2	2	2	2	2	2	2
2	3	1	1	2	2	2	3	2	1
2	2	2	2	2	9	2	2	2	2
1	1	2	1	1	2	2	1	1	1
2	2	3	3	3	3	3	3	3	3
2	2	2	2	2	2	2	2	2	2
9	2	2	9	9	2	9	9	2	2
3	2	2	2	2	2	2	2	2	2
2	2	2	2	2	9	2	2	1	2
3	2	2	1	2	2	2	2	2	2
3	3	2	2	2	2	2	2	2	2
2	1	2	2	1	1	2	3	2	2
2	2	1	1	2	2	3	2	2	1
2	2	1	1	2	1	1	2	1	1
2	2	1	2	2	2	1	1	1	1
2	2	2	2	2	3	2	2	1	1

2	2	1	2	1	1	2	2	1	1
2	2	2	2	2	1	1	2	2	1
1	2	1	2	2	2	2	2	2	1
2	2	2	2	1	2	2	2	2	2
2	3	2	3	2	1	1	2	3	2
2	2	1	2	2	2	2	1	1	1
2	1	2	3	2	3	2	9	2	2
2	2	1	2	2	3	3	2	1	2
1	2	1	1	1	1	2	1	1	1
3	3	2	3	3	2	1	2	2	2
3	3	3	2	3	2	3	2	2	2
1	1	2	1	1	1	1	2	1	2
3	1	1	2	3	1	2	3	1	1
2	2	2	3	1	2	2	2	2	2
1	1	1	2	2	3	2	1	1	2
2	2	2	2	2	2	2	2	2	2
2	2	3	3	3	2	2	2	1	3
2	2	2	2	2	2	2	2	2	2
1	3	3	3	2	3	2	3	1	3
2	2	2	2	2	2	2	2	2	2
1	1	1	1	1	2	2	2	2	1
2	2	2	2	2	2	2	2	2	2
2	2	2	1	2	2	3	1	1	1
3	3	3	3	3	3	3	3	3	2
3	3	2	2	2	2	2	2	2	2
3	3	2	2	3	3	3	2	2	2
2	2	2	2	2	2	2	2	2	1
1	1	1	1	1	2	2	1	1	1
3	2	2	2	2	2	2	3	3	3
2	2	2	2	2	2	2	2	2	2
2	1	2	1	2	3	3	2	2	1
3	3	1	1	3	3	2	1	1	1
2	2	2	2	2	3	2	2	2	2
2	2	2	2	2	2	2	2	2	2
3	1	1	1	1	1	3	1	2	2
2	2	2	2	2	2	2	2	2	2
2	1	2	1	3	3	2	1	1	1
1	2	2	1	1	1	2	1	1	2
3	2	2	1	1	1	2	3	1	1
2	2	3	3	2	3	1	2	3	2
3	2	2	1	2	2	3	9	2	1
2	2	2	2	3	3	2	3	2	2
2	2	2	2	2	2	2	2	2	2
3	2	2	2	2	2	2	2	2	2
2	2	2	2	2	2	2	2	2	2
2	2	2	2	2	2	2	2	2	2
2	2	2	2	2	2	2	2	2	2

2	2	2	2	3	2	2	2	2	2
1	1	1	2	2	2	3	1	1	2
2	2	2	1	1	1	2	2	1	1
2	2	2	2	2	2	2	2	2	2
2	2	2	2	2	2	2	2	2	2
2	2	3	2	2	2	2	2	2	2
2	2	2	2	2	2	2	2	2	2
2	3	1	2	1	2	3	2	1	3
2	2	2	2	2	2	2	2	2	2
9	9	9	9	9	9	9	9	9	9
3	2	1	1	1	1	1	1	3	3
2	2	1	1	1	1	2	1	1	1
1	3	1	2	1	3	2	1	3	1
2	2	2	2	2	2	2	2	2	2
2	2	2	2	2	2	2	2	2	2
2	2	2	2	2	2	2	2	2	2
2	2	2	2	2	2	2	2	2	2
2	2	1	2	1	2	2	2	2	2
3	3	3	3	3	3	3	3	3	3
1	1	3	3	3	3	3	3	3	3
3	3	3	3	3	3	3	3	3	3
3	3	3	3	3	3	3	3	3	3
2	2	2	2	2	2	2	2	2	2
2	1	2	3	2	1	1	2	2	3
1	2	2	2	2	2	2	2	2	2
1	1	1	1	1	1	1	1	2	1
2	3	2	3	3	2	2	2	3	1
2	2	1	2	2	3	2	2	1	1
2	2	1	1	1	2	2	2	2	1
9	9	9	9	9	9	9	9	9	9
2	2	2	2	2	2	1	1	1	2
2	3	2	3	2	3	2	3	2	2
3	2	2	2	2	2	2	2	3	1
3	3	3	3	3	3	3	3	3	3
2	2	2	2	2	2	2	2	2	2
2	2	2	2	3	2	3	1	3	3
2	2	2	2	3	2	2	3	3	3
2	2	2	2	3	2	2	1	3	3
2	2	2	2	2	2	2	2	2	2
2	2	2	2	2	2	3	2	2	2
2	3	1	2	2	2	1	3	2	1
2	2	1	1	1	1	1	1	1	2
2	2	2	2	2	2	2	2	2	2
2	2	1	1	1	1	1	2	1	2
3	3	3	3	3	3	3	3	3	3
2	1	3	1	2	1	1	2	1	1
1	1	2	3	3	3	3	2	1	3

2	2	2	2	3	3	1	1	1	2
3	3	3	3	3	3	3	3	2	2
3	3	1	2	2	3	3	3	3	1
2	2	2	2	2	2	2	2	2	2
1	1	2	2	2	2	2	2	2	3
9	9	9	9	9	9	9	9	3	9
2	2	2	2	2	2	2	2	2	2
3	3	2	2	2	2	2	2	2	2
2	9	1	2	3	2	3	1	1	1
3	3	1	2	2	3	3	3	3	2
2	2	1	1	2	1	1	1	1	1
3	1	2	2	2	3	1	2	2	3
9	9	9	1	9	9	9	9	9	9
9	9	9	1	9	9	9	9	9	9
9	9	2	9	9	9	9	9	9	9
2	2	2	1	2	2	2	2	2	2
2	2	1	1	2	2	2	3	1	2
9	9	1	9	9	9	9	9	9	2
1	1	1	1	2	2	2	1	1	1
2	1	1	1	1	1	1	1	1	1
2	2	1	2	1	2	1	2	1	2
1	1	1	1	2	2	2	1	1	1
1	1	1	1	2	2	2	1	1	1
2	2	2	2	2	2	2	2	2	2
2	2	2	2	2	2	2	2	2	1
3	2	2	2	2	3	1	1	1	2
2	2	2	2	2	2	2	2	2	1
2	2	3	3	2	2	1	2	2	1
9	1	9	9	1	9	9	9	9	9
3	3	3	1	3	3	3	3	3	1
3	1	1	2	3	3	2	1	1	1
1	1	2	1	1	2	2	2	1	2
1	1	2	2	2	2	2	2	1	1
2	2	2	2	2	2	2	2	2	1
2	2	2	2	2	2	3	2	2	1
2	2	2	2	2	2	2	2	2	2
3	2	3	2	1	2	2	3	3	1
2	2	2	2	2	2	2	2	2	2
2	2	3	2	2	2	2	2	2	2
2	2	2	2	2	2	2	2	2	2
1	1	1	1	1	1	2	2	1	2
2	3	1	2	2	2	3	2	1	1
2	1	1	2	2	3	2	2	1	2
2	2	2	2	2	2	2	3	2	2
2	2	2	2	2	2	2	2	2	2
2	2	2	2	2	2	2	2	2	2
3	1	2	2	2	2	2	2	2	1

1	1	2	2	2	1	1	1	1	1
2	3	2	9	2	2	2	2	2	2
9	9	9	9	9	9	9	9	9	9
1	3	2	1	2	1	2	3	3	1
1	1	1	1	1	2	1	1	1	1
2	2	2	2	2	2	2	2	2	1
2	2	2	2	2	2	2	2	2	1
3	2	2	1	1	2	3	3	2	2
2	2	2	1	1	2	2	2	2	2
2	1	2	2	2	2	2	1	2	2
2	2	2	2	3	2	2	2	2	1
2	2	2	2	2	2	2	2	2	2
2	2	1	1	2	2	1	1	2	1
2	1	2	1	1	2	1	1	1	1
2	1	1	2	2	2	2	2	2	1
1	1	1	2	9	3	3	1	1	2
9	9	9	9	9	9	9	9	9	9
2	3	2	2	3	3	2	3	2	9
1	1	1	1	1	1	1	1	1	1
3	3	1	3	3	3	3	1	1	1
2	2	2	2	2	2	2	2	2	2
9	9	9	9	9	9	9	9	9	9
2	2	2	2	2	2	2	2	2	2
3	2	1	2	3	1	2	3	1	2
2	2	2	2	2	2	2	2	2	2
9	9	2	9	9	9	9	9	9	1
9	9	2	9	9	9	9	9	9	1
2	1	2	2	3	2	1	3	2	3
9	9	2	9	9	9	9	9	9	2
9	9	9	9	9	1	9	9	9	9
9	9	9	9	9	2	9	9	9	9
9	9	9	9	9	9	9	9	9	9
9	9	9	9	9	9	9	9	1	1
2	2	1	3	2	3	1	2	3	1
1	2	3	2	3	2	3	1	3	1
3	2	2	2	3	2	3	3	3	2
3	2	2	2	3	2	3	3	3	2
1	1	1	1	1	1	1	1	1	1
2	3	2	2	2	1	1	1	1	1
2	3	2	2	2	2	2	2	2	2
2	2	2	2	2	2	2	2	2	2
2	2	1	2	1	3	1	1	1	1
2	2	2	2	2	3	2	3	2	2
1	2	1	3	2	1	2	1	3	3
3	3	1	3	2	1	2	1	3	3
2	2	2	2	3	2	2	2	2	3
2	2	2	2	2	2	2	2	2	2

2	3	1	2	2	2	1	1	1	9
1	2	1	2	1	3	1	2	2	1
3	3	1	1	1	1	1	1	1	3
9	9	9	9	9	9	9	9	9	9
9	9	9	9	9	9	9	9	9	9
1	3	9	9	9	9	9	9	9	1
2	2	1	1	2	2	2	1	9	1
9	9	9	9	9	9	9	9	9	9
9	9	9	9	9	9	9	9	9	9
3	1	1	1	2	2	1	1	1	1
2	2	2	2	2	2	2	2	2	2
2	2	2	2	2	2	2	2	2	2
2	2	2	2	2	2	2	2	2	2
9	9	9	9	9	9	9	9	9	9
9	9	9	9	9	9	9	9	9	9
9	9	9	9	9	9	9	9	9	9
2	2	2	1	1	2	2	3	2	1
9	9	9	9	9	9	9	9	9	9
9	9	1	1	3	2	3	1	1	1
9	9	9	9	9	9	9	9	9	9
9	9	1	9	9	9	9	9	9	9
9	9	9	9	9	9	9	9	9	9
9	9	9	9	9	9	9	9	9	9
9	9	9	9	9	9	9	9	9	9
2	2	2	2	2	2	2	2	2	2
3	9	2	1	2	2	3	2	2	2
3	1	2	2	2	2	3	2	1	1
2	2	2	2	2	2	2	2	2	1
3	2	2	2	1	2	1	1	2	1
3	2	2	2	2	2	2	2	2	2
2	2	1	1	1	1	1	1	1	2
2	2	1	1	1	1	1	1	1	1
2	2	1	1	2	2	2	1	1	2
2	2	2	2	2	2	2	2	2	1
3	2	2	3	3	3	2	2	2	2
3	3	1	1	2	2	3	1	1	2
2	2	1	1	2	2	3	1	1	1
2	2	1	1	1	1	2	1	1	1
2	2	2	2	2	2	2	2	2	1
2	2	2	2	2	2	2	2	2	2
2	3	1	1	2	2	2	2	1	1
2	2	1	2	2	2	2	3	3	2
2	2	2	1	2	2	2	2	2	2
9	9	2	9	9	9	9	9	9	2
9	9	9	9	2	9	9	9	9	9
2	2	3	3	3	1	2	2	2	2
2	2	1	1	1	1	1	1	1	2

1	1	2	1	2	1	1	1	1	2
1	1	2	2	2	2	2	2	2	3
2	2	2	2	2	2	2	2	2	2
2	2	2	2	2	2	2	2	2	1
2	2	2	2	1	2	2	2	2	2
3	2	2	1	3	2	1	1	3	2
2	1	2	2	3	2	3	2	2	1
2	3	1	2	3	1	2	3	1	1
9	9	9	9	9	9	9	9	9	9
2	1	2	2	2	2	2	2	2	1
2	2	2	2	2	2	2	2	2	2
2	3	1	2	2	2	2	2	2	1
3	1	1	2	3	1	2	3	1	1
2	2	2	1	1	1	2	2	2	2
2	3	3	3	3	3	2	2	2	2
2	3	2	3	1	2	1	2	2	2
3	2	1	2	3	2	1	3	2	1
1	2	1	2	3	1	2	3	1	1
2	2	2	2	2	2	2	2	2	2
1	1	2	3	1	1	2	2	2	2
1	1	1	2	2	2	2	2	2	3
2	2	2	2	2	2	2	2	2	2
2	2	2	3	3	3	2	2	1	3
3	2	1	2	2	3	2	3	2	1
2	2	2	2	2	2	2	2	2	2
3	3	3	2	3	2	2	2	2	3
3	2	2	2	2	2	2	2	2	2
3	3	1	2	3	2	1	2	2	1
1	1	1	2	3	1	2	3	2	1
9	9	9	9	9	9	9	9	9	9
1	1	1	1	2	2	2	1	1	1
9	9	9	9	9	9	9	9	9	9
9	9	9	9	9	9	9	9	9	9
1	1	1	1	1	1	1	1	1	2
2	1	3	2	1	3	1	2	3	1
1	3	2	2	1	2	2	1	2	2
1	2	3	3	2	2	1	1	2	3
2	2	1	2	2	2	2	2	2	1
2	2	2	2	2	2	2	2	2	2
2	2	1	1	1	1	2	2	2	2
1	3	1	1	2	2	3	3	3	2
2	3	1	1	1	2	2	1	1	1
2	2	2	2	2	2	2	2	2	2
2	2	2	2	2	2	2	2	2	2
2	2	2	2	2	2	2	2	2	2
2	2	2	2	2	2	2	2	2	2
2	2	2	2	2	2	2	2	2	2

1	3	1	1	1	1	3	1	1	3
2	2	2	2	2	2	2	2	2	2
2	2	2	2	2	2	2	2	2	2
1	1	1	2	3	2	1	1	2	1
1	2	2	2	2	2	2	1	2	2
2	2	9	9	9	9	9	9	9	9
2	2	2	2	2	2	2	2	2	1
2	1	1	1	1	1	2	3	1	2
9	9	1	9	9	9	9	9	9	9
2	2	2	2	2	2	2	2	2	2
2	2	2	2	2	2	2	2	2	2
3	1	2	3	2	2	2	1	2	1
9	9	9	9	9	9	9	9	9	9
2	3	1	1	1	1	1	2	2	2
9	9	1	1	1	2	3	1	2	1
1	2	3	3	3	3	2	1	1	3
2	2	1	1	1	1	2	1	1	1
3	1	1	2	3	1	2	3	1	1
3	1	2	1	3	3	1	2	2	1
3	2	2	1	2	2	2	2	1	1
1	2	1	2	1	9	1	2	1	1
3	3	3	3	3	3	3	3	3	3
2	2	2	2	2	2	2	2	2	2
2	2	2	2	2	2	2	2	2	2
1	2	1	1	2	2	3	2	2	1
2	2	2	2	2	2	2	2	2	2
2	2	2	2	2	2	2	2	2	2
2	2	3	3	2	3	2	2	2	1
3	1	1	3	2	1	3	1	1	1
3	3	2	1	1	2	2	1	1	2
1	1	1	1	1	1	1	1	1	2
2	2	2	2	2	2	2	2	2	2
1	1	1	2	2	1	2	1	2	1
1	2	2	2	2	2	2	2	2	1
2	2	2	2	2	2	2	2	2	1
9	9	9	9	9	9	9	9	9	9
2	2	2	2	2	2	2	2	2	2
9	9	9	9	9	9	9	9	9	9
3	2	1	2	3	2	1	2	3	1
2	2	2	2	2	2	2	2	2	2
2	2	2	2	2	2	2	2	2	2
9	9	9	9	9	9	9	9	9	9
2	2	2	2	1	1	2	1	1	2
2	2	1	2	2	2	2	2	2	2
9	9	9	9	9	9	9	9	9	9
9	9	9	9	9	9	9	9	9	9
9	9	9	9	9	9	9	9	9	9

3	2	2	2	2	2	3	3	3	3
1	1	1	2	9	1	2	1	1	2
9	9	9	9	9	9	9	9	9	9
3	2	1	1	1	2	2	2	2	1
2	2	2	2	3	2	2	2	2	2
3	2	1	1	2	3	2	2	1	1
2	2	2	2	2	2	2	2	2	2
9	2	9	2	2	9	9	9	9	2
3	3	1	2	2	2	2	2	2	3
9	9	9	9	9	9	9	9	9	9
3	9	3	3	3	3	3	3	3	3
1	3	3	9	3	9	3	9	3	1
2	1	3	1	2	3	2	9	2	1
2	2	2	2	2	2	2	2	2	2
2	2	2	2	2	2	2	2	2	2
3	9	2	9	2	2	9	2	2	1
3	2	3	3	3	2	2	2	2	1
9	9	9	9	9	9	9	9	9	9
9	9	9	9	9	9	9	9	9	9
1	3	2	1	2	1	2	3	1	1
1	3	2	1	2	3	2	1	2	1
3	2	2	2	2	2	2	2	2	3
2	2	2	3	2	3	2	3	2	2
3	3	3	3	3	3	3	3	3	3
1	2	1	2	3	2	1	2	3	1
1	1	1	1	2	2	2	2	2	1
1	1	1	1	9	1	2	3	2	2
3	3	1	2	2	3	3	2	2	1
9	9	9	9	9	9	9	9	9	9
9	9	9	9	9	9	9	9	9	9
9	9	9	9	9	9	9	9	9	9
9	9	9	9	9	9	9	9	9	9
9	9	9	9	9	9	9	9	9	9
2	2	2	2	2	2	2	2	2	3
1	2	1	2	1	3	3	2	3	1
9	9	9	9	9	9	9	9	9	9
9	9	9	9	9	9	9	9	9	9
1	3	1	2	1	1	1	2	3	1
9	9	1	9	1	9	9	9	9	9
9	9	9	9	9	9	9	9	9	9
2	2	2	2	2	2	2	2	2	2
2	2	2	1	2	3	2	1	1	1
2	2	2	1	1	2	2	2	1	2
2	2	2	2	2	2	2	2	2	2
1	1	2	1	1	2	1	2	1	1
2	2	1	2	1	3	2	2	3	1
2	3	1	2	2	2	1	1	2	1

3	3	3	3	3	3	3	3	3	2
2	2	2	2	2	2	2	2	2	2
9	9	9	9	9	9	9	9	9	9
3	2	1	1	1	1	2	2	3	1
3	2	1	1	1	2	2	2	3	1
1	1	1	1	1	1	1	1	1	1
3	1	9	3	9	1	9	9	2	2
2	2	2	2	2	2	2	2	2	2
2	2	2	2	2	2	2	2	2	2
9	9	1	3	3	3	2	2	2	9
2	2	3	3	3	3	3	3	3	2
2	2	1	2	3	1	3	2	2	1
9	9	9	9	9	9	9	9	9	9
1	1	1	1	1	1	1	1	1	1
3	3	3	3	3	3	3	3	3	1
3	1	1	1	1	1	2	2	3	1
2	3	1	2	3	1	3	1	2	1
9	9	9	9	9	9	9	9	9	9
3	3	9	9	9	9	9	9	9	2
9	9	9	9	9	9	9	9	9	9
9	9	9	2	9	9	9	9	9	9
3	3	1	1	1	3	3	3	3	3
9	9	9	9	9	9	9	9	9	9
9	9	9	9	9	9	9	9	9	9
9	9	2	9	9	9	9	9	9	9
9	9	9	9	9	9	9	9	9	9
1	2	3	3	3	1	2	3	3	2
2	3	2	3	2	3	1	2	3	2
9	9	9	9	9	9	9	9	9	9
3	2	2	2	3	2	2	1	1	1
1	3	1	3	1	3	1	3	1	1
9	9	9	9	9	9	9	9	9	9
2	2	2	2	2	2	1	1	2	2
2	2	2	2	2	2	2	2	2	2
2	2	2	2	2	2	2	2	2	1
2	2	3	2	2	2	2	2	2	2
1	2	1	2	1	1	2	1	1	1
2	2	2	2	2	2	2	2	2	1
2	2	2	2	2	2	2	2	2	2
9	9	9	9	9	9	9	9	9	9
1	3	3	2	9	3	9	2	1	3
3	2	2	3	9	2	3	1	1	1
2	2	1	1	3	9	2	2	2	2
1	2	2	1	3	2	9	2	2	1
2	1	1	2	1	2	1	2	2	1
1	1	1	1	1	1	1	1	1	1
2	2	2	3	1	2	2	3	3	1

1	1	1	1	3	3	3	3	1	1
3	2	1	2	1	2	1	1	1	2
1	3	1	2	3	2	1	3	2	2
2	2	2	2	2	2	2	2	2	2
2	2	2	2	2	2	2	2	2	2
2	2	2	2	2	2	2	2	2	2
2	2	2	2	2	2	2	2	2	2
3	1	3	3	2	2	2	2	3	3
2	2	2	2	2	2	2	2	2	2
9	9	9	9	9	9	9	9	9	9
3	2	2	2	3	3	3	1	1	1
1	2	1	2	3	2	1	3	2	1
9	9	9	9	9	9	9	9	9	9
1	2	1	1	2	2	1	2	1	2
9	9	9	9	9	9	9	9	9	9
9	9	9	9	9	9	9	9	9	9
1	3	1	3	1	3	1	1	3	1
2	2	2	2	2	2	2	2	2	2
2	3	3	2	2	2	3	3	2	3
2	2	1	1	2	2	2	2	2	1
1	1	1	1	1	1	2	1	1	1
2	2	2	2	2	2	2	2	2	2
9	9	9	9	9	9	9	9	9	9
1	1	1	3	2	9	1	1	1	1
1	1	1	1	1	1	1	1	1	1
3	3	3	3	3	3	3	3	3	3
2	1	2	1	3	2	1	2	2	2
2	2	2	3	3	3	2	2	2	2
1	9	2	1	1	2	2	2	2	3
2	9	2	2	2	2	2	2	2	2
1	3	1	1	2	2	2	2	2	2
9	9	9	9	9	9	1	9	9	2
2	2	2	2	2	2	2	2	2	2
2	3	1	3	2	3	1	2	3	1
1	3	1	2	3	2	1	3	1	3
1	3	1	3	1	3	1	2	3	3
2	2	2	2	2	2	2	2	2	2
2	2	2	2	2	2	2	2	2	2
2	2	1	2	2	2	2	2	2	2
2	2	2	2	2	2	2	2	2	2
2	2	2	2	2	2	2	2	2	2
2	3	3	2	3	1	1	1	1	2
2	2	2	3	2	1	3	2	2	1
1	1	1	2	1	2	2	1	1	2
9	9	9	9	9	9	9	9	9	9
3	1	1	3	3	2	1	2	1	3
3	3	2	2	1	2	2	2	2	1

1	1	9	1	3	2	3	2	2	3
1	1	1	3	3	3	3	1	1	1
1	1	3	3	3	3	9	1	1	1
2	2	1	2	2	2	1	2	2	1
9	9	9	9	9	9	9	9	1	1
9	9	9	9	9	9	9	9	9	1
9	9	1	9	9	9	9	9	9	9
2	2	3	2	9	2	2	3	3	1
1	2	1	2	1	2	3	1	2	1
2	2	2	2	2	2	2	2	2	1
2	2	1	1	2	2	2	1	1	1
3	3	2	2	3	2	2	2	2	1
2	2	2	2	2	2	2	2	2	2
2	2	2	2	2	2	2	2	2	2
2	2	1	2	2	2	2	2	2	2
2	2	2	2	2	2	2	2	2	1
3	3	2	2	2	2	2	2	2	2
2	2	2	2	2	2	2	2	2	2
2	2	2	2	2	2	3	1	1	1
3	2	2	3	1	2	1	3	1	2
2	2	2	2	2	2	2	2	2	2
9	9	9	9	9	1	9	9	9	9
9	9	1	2	9	1	9	1	2	1
2	2	2	2	2	2	2	2	2	2
2	2	2	2	2	2	2	2	2	2
2	3	2	2	3	2	2	1	1	2
2	2	1	1	1	1	1	1	1	1
9	9	9	9	9	9	9	9	9	9
2	2	1	2	2	3	3	2	1	1
2	3	2	2	2	2	2	2	2	2
1	2	1	1	2	2	2	2	2	2
1	2	2	2	2	2	3	2	2	3
2	3	1	2	2	1	1	3	1	2
1	2	2	1	1	1	2	1	1	1
3	3	1	1	1	1	1	1	1	2
2	2	2	3	2	3	3	3	3	9
2	2	2	2	2	2	2	2	2	2
2	2	2	2	2	2	2	2	2	2
2	2	2	2	2	2	2	2	2	1
9	9	9	9	9	9	9	9	9	9
2	2	2	2	2	2	2	2	2	1
2	1	1	2	3	2	1	2	1	2
2	2	1	1	1	1	2	2	3	2
2	2	1	2	1	2	3	1	2	1
3	3	1	1	1	2	2	3	3	3
2	2	1	1	1	1	1	1	1	1
2	2	2	2	2	2	2	2	2	2

2	2	2	2	2	2	2	2	2	1
9	9	3	9	9	1	9	9	2	9
2	2	1	2	2	2	2	2	2	2
2	2	2	1	1	1	1	1	1	1
2	2	2	1	2	2	3	2	3	2
2	2	2	2	2	2	2	2	2	2
3	2	3	2	3	2	3	2	3	2
3	2	2	2	2	2	1	1	1	1
9	9	9	9	9	1	9	9	9	1
2	2	3	2	2	2	2	2	2	2
3	3	1	1	1	1	2	1	1	1
2	2	1	2	2	2	2	2	2	1
2	2	2	2	2	2	2	2	2	2
3	3	1	2	1	3	1	3	1	1
2	2	2	2	2	2	2	2	2	2
2	2	2	2	2	2	2	2	2	2
2	3	3	2	2	2	2	3	3	3
9	9	1	9	9	9	9	9	9	9
9	9	9	9	9	9	9	9	9	9
1	2	1	1	1	1	1	1	1	1
3	3	1	2	1	2	1	2	1	1
3	3	2	2	3	2	3	2	2	1
2	2	2	3	2	3	2	1	2	2
1	2	2	2	2	2	2	2	2	2
9	9	9	9	9	9	9	9	9	9
3	2	2	2	2	2	3	2	2	1
2	2	1	2	2	2	2	1	1	1
2	3	2	2	3	2	2	2	2	1
2	2	1	2	2	2	2	1	3	1
2	2	2	2	2	2	2	2	2	2
2	2	2	2	2	2	2	2	2	2
3	1	2	2	2	2	2	2	2	2
2	2	2	3	1	1	2	2	1	1
2	3	1	1	1	2	1	3	3	1
2	1	1	3	3	3	3	1	1	1
2	1	1	1	2	3	3	3	3	1
3	2	2	1	2	1	2	2	1	1
2	2	1	3	2	1	2	3	2	2
2	2	1	1	1	1	2	2	1	1
3	1	2	2	1	2	2	2	2	1
9	9	9	9	9	9	9	9	9	9
2	2	1	2	3	2	1	1	1	2
9	1	1	3	3	3	3	1	1	1
3	1	2	2	9	3	2	3	1	1
2	2	1	1	1	1	2	1	1	3
1	2	3	1	2	2	3	2	1	1
3	2	2	3	2	2	3	1	3	1

3	2	3	3	1	3	3	3	3	3
9	9	9	9	9	9	9	9	1	9
9	9	9	9	9	9	9	9	9	9
9	9	9	9	9	9	9	9	9	9
2	2	2	2	2	2	2	2	2	2
2	2	2	2	2	2	2	2	2	2
1	1	2	1	3	3	3	1	1	1
1	1	1	1	1	1	1	1	1	1
9	9	9	9	9	9	9	9	9	9
9	9	9	9	9	9	9	9	9	9
2	2	2	2	2	2	2	2	2	9
3	2	2	2	2	2	3	2	2	2
2	2	2	2	2	2	2	2	2	2
2	2	2	2	2	2	2	2	2	2
1	2	1	2	1	2	3	1	1	2
2	2	1	1	2	2	3	1	1	1
2	1	1	1	1	3	2	9	1	1
3	1	3	1	3	2	3	2	1	1
2	3	2	2	2	2	3	1	1	2
9	9	9	9	9	9	9	9	9	9
2	2	1	1	1	1	1	1	1	2
2	2	2	1	3	2	2	3	2	2
3	2	2	2	2	2	2	2	2	1
1	2	1	3	1	3	2	1	2	1
1	1	1	1	1	1	2	2	2	3
2	3	2	2	2	2	3	1	1	2
1	2	1	2	3	2	1	3	2	2
3	1	1	2	3	1	1	1	2	3
2	1	2	3	3	2	2	3	1	1
1	2	3	1	2	2	2	1	3	1
2	2	3	2	1	1	1	2	3	1
1	3	1	2	3	1	2	3	1	1
1	3	3	2	3	2	2	2	3	1
3	2	1	2	2	2	3	3	3	1
3	3	1	2	3	3	3	1	1	1
3	3	1	2	3	3	3	1	1	1
2	2	1	1	1	2	2	1	1	1
1	1	9	1	9	2	9	9	9	9
3	2	2	2	2	3	2	2	2	1
3	3	1	1	1	2	2	2	3	2
2	2	1	1	2	2	2	2	2	3
2	2	3	2	1	2	2	1	1	1
9	9	9	9	9	9	9	9	9	9
3	3	2	2	2	2	3	2	2	1
1	2	1	2	2	1	2	1	2	1
2	3	2	2	2	2	2	2	2	1
2	2	1	1	1	1	2	1	3	1

3	2	1	1	3	2	1	1	1	1
9	9	2	9	9	9	9	9	9	2
3	1	1	9	9	9	9	9	9	1
2	2	2	2	2	2	2	2	2	2
2	2	3	2	2	3	2	1	1	1
3	3	1	3	3	3	1	1	2	1
2	3	2	3	3	2	1	1	2	1
2	2	1	1	1	1	1	1	1	3
2	2	1	1	1	1	1	1	1	3
9	9	9	9	9	9	9	9	9	9
2	2	1	2	3	2	3	2	1	1
1	2	1	2	2	3	1	1	1	1
1	3	1	3	1	3	1	3	1	1
2	1	1	2	1	3	1	2	3	1
2	2	2	2	2	2	2	2	2	1
9	9	9	9	9	9	9	9	9	9
9	9	9	1	9	9	9	9	9	1
3	3	1	2	2	2	2	1	3	3
2	2	3	3	3	2	2	1	1	2
2	2	2	2	2	3	2	2	1	1
9	9	9	9	9	9	9	9	9	9
2	2	2	2	2	2	1	1	1	1
2	2	2	2	2	2	2	1	1	1
2	2	3	2	2	2	1	1	1	1
2	2	2	2	2	2	1	1	1	1
2	2	1	1	1	2	2	1	1	2
2	2	2	3	2	2	2	1	1	1
3	3	3	2	2	2	2	1	1	1
2	2	2	2	2	1	1	2	2	1
1	1	2	2	3	2	2	1	1	1
9	9	9	9	9	9	9	9	9	9
9	9	9	9	9	9	9	9	9	9
9	9	9	9	9	9	9	9	9	9
2	2	1	3	2	2	2	2	3	2
2	1	1	1	2	3	2	1	2	1
2	2	1	2	2	2	2	1	1	1
1	3	1	2	2	3	1	1	1	1
9	2	2	2	2	2	2	2	2	2
2	2	2	2	2	1	1	1	1	2
2	2	2	2	2	2	2	2	2	3
3	3	1	3	3	3	3	2	3	1
2	2	1	1	2	1	2	2	2	1
2	2	2	1	2	2	2	2	2	2
3	3	1	2	1	1	3	1	1	2
2	2	2	2	2	2	2	2	2	2
1	1	1	1	1	1	1	1	1	1
1	3	3	3	3	3	2	3	3	3

9	9	9	9	9	9	9	9	9	9
2	2	1	2	2	2	2	2	2	2
9	9	9	9	9	9	9	9	9	9
9	9	9	9	9	9	9	9	9	9
3	3	2	2	3	3	3	3	3	1
2	2	2	2	3	2	3	3	2	2
1	2	2	1	2	2	2	2	2	3
9	9	9	9	9	9	1	9	9	9
2	2	2	2	2	2	2	2	2	2
9	9	1	9	9	9	9	9	9	1
9	2	1	1	1	9	2	2	2	1
9	9	1	9	9	9	9	9	9	9
2	2	1	1	1	1	1	1	1	2
9	9	9	9	9	9	9	9	9	9
3	3	1	2	3	2	3	1	2	3
2	2	2	3	2	2	2	2	2	1
2	2	1	1	1	1	2	1	1	1
2	2	1	1	1	1	2	1	1	1
2	2	2	2	2	2	2	2	2	2
2	1	1	1	1	1	1	2	1	1
2	2	2	2	2	2	2	2	2	1
2	2	2	1	3	2	3	1	2	2
2	2	1	1	2	2	2	2	1	2
2	3	3	3	3	3	3	3	3	3
3	2	2	3	1	3	2	3	2	2
1	1	1	1	1	2	3	1	2	1
2	2	2	2	2	2	2	2	2	2
2	2	2	1	2	2	2	1	1	1
2	2	2	2	2	2	2	2	2	2
2	2	1	2	2	2	3	2	2	1
2	2	2	2	2	2	2	2	2	2
3	2	2	2	2	2	2	2	2	1
1	2	1	2	3	3	2	1	1	1
1	9	1	1	1	1	2	2	2	1
9	9	9	9	9	9	9	9	9	9
3	3	1	3	3	3	3	1	3	3
3	3	1	2	3	2	3	2	2	1
3	3	3	2	2	2	2	2	2	2
2	2	2	2	2	2	1	2	2	1
2	2	2	2	1	2	1	2	3	2
1	1	1	1	1	1	1	3	3	1
9	9	9	9	9	9	2	9	9	9
9	9	9	9	9	9	2	9	9	9
2	2	2	2	2	2	2	2	2	2
9	9	9	9	1	9	9	9	9	9
2	2	2	2	1	3	2	1	2	2
2	2	1	1	2	1	3	2	1	1

2	2	2	2	2	2	2	2	2	2
2	2	1	1	1	2	2	1	1	1
2	3	1	2	3	9	2	1	3	2
1	1	1	2	2	2	2	2	2	2
3	3	3	3	3	3	3	3	3	2
3	3	2	2	2	3	3	3	3	2
2	2	3	2	1	2	2	1	1	2
2	2	2	2	2	2	2	2	2	2
3	3	1	1	1	2	3	2	2	1
1	2	1	2	3	1	2	1	3	1
2	1	1	2	1	3	3	2	2	1
2	2	1	2	1	3	2	2	1	2
3	2	2	1	3	3	2	3	3	1
2	9	2	3	2	2	9	2	2	9
3	3	1	2	1	2	3	2	2	1
2	2	2	2	2	2	2	2	2	2
1	1	2	2	2	2	1	1	1	1
2	2	2	2	2	2	2	2	2	2
3	3	2	3	3	2	2	2	2	2
1	1	1	1	1	1	2	2	3	3
2	2	2	2	2	3	2	2	1	1
2	3	2	2	2	2	2	2	2	2
1	2	1	2	2	2	2	2	2	1
2	2	1	2	2	2	2	2	2	1
1	2	1	1	1	3	3	1	2	1
2	2	2	2	2	2	2	2	2	2
2	2	2	2	2	2	2	2	2	2
2	1	1	1	2	2	1	1	2	1
2	2	1	1	2	2	3	2	1	1
3	2	1	1	2	2	2	3	3	1
9	9	9	9	9	9	9	9	9	9
3	3	3	3	3	2	2	2	2	2
2	2	2	2	2	2	2	1	1	1
2	2	2	2	2	2	2	2	2	2
3	3	2	1	1	2	1	1	1	1
2	2	1	2	2	2	1	2	2	1
2	2	1	2	2	3	2	1	2	1
2	3	2	2	9	1	3	2	1	2
2	3	1	2	3	3	2	1	2	1
3	3	1	1	1	1	1	1	1	2
3	1	2	1	1	1	3	1	1	1
1	3	2	1	2	2	2	2	2	2
1	3	2	2	2	2	1	2	2	1
2	1	3	1	1	1	2	1	1	3
1	3	1	1	1	2	2	2	2	1
3	3	1	1	1	2	2	2	2	2
2	3	1	2	3	3	3	1	1	1

3	3	1	2	3	3	3	1	1	1
2	3	1	2	2	2	3	3	3	1
2	2	3	2	1	1	2	2	1	1
2	2	1	2	2	3	2	2	2	1
1	1	1	2	2	3	2	1	2	1
2	2	1	1	2	2	2	2	1	1
3	3	1	2	2	3	2	2	2	1
2	2	2	2	2	2	2	2	2	2
3	2	3	2	3	2	1	3	2	2
2	3	1	1	1	1	2	1	1	1
2	1	1	3	3	3	3	1	1	1
2	2	2	1	2	1	2	1	2	2
3	3	1	1	1	1	1	1	1	3
2	2	2	2	2	2	2	2	2	2
3	3	9	9	9	9	9	9	9	9
2	2	2	2	2	2	2	2	2	2
2	3	3	2	2	2	3	3	3	3
2	2	1	2	2	3	3	2	2	1
2	3	2	1	2	2	9	2	3	1
2	2	1	2	1	1	2	1	1	1
3	3	2	2	2	1	1	1	1	2
3	3	1	3	3	3	3	3	3	3
2	2	2	2	2	2	2	2	2	2
2	1	1	1	2	2	2	2	2	1
1	3	1	2	3	1	2	3	1	2
1	2	1	2	2	1	2	2	2	2
1	1	1	2	1	1	1	1	2	1
2	1	2	1	1	1	3	1	1	2
1	1	1	2	2	2	2	2	1	1
2	2	2	2	2	3	3	2	2	2
1	1	1	1	1	1	1	1	1	1
2	1	1	3	3	3	3	1	1	1
1	1	2	2	2	3	3	1	1	1
2	1	2	1	1	2	2	1	1	1
2	3	1	3	2	1	2	3	2	2
2	2	2	3	3	3	2	2	2	1
3	3	2	2	3	3	3	3	3	2
1	2	3	1	2	2	3	2	1	1
1	2	1	3	2	3	2	1	1	2
2	2	1	2	1	2	2	1	2	1
3	2	3	2	3	3	2	1	1	1
3	3	1	2	3	3	2	1	1	1
2	2	1	2	2	2	2	2	2	1
2	2	1	2	3	2	3	1	1	1
3	2	2	2	3	3	3	2	2	1
2	3	1	3	2	2	2	1	1	1
2	2	2	2	2	2	2	2	2	2

2	2	1	2	2	2	1	1	1	1
2	2	2	2	2	2	2	2	2	2
3	3	1	1	2	2	3	2	2	1
3	2	3	2	3	2	2	1	1	1
2	2	3	1	3	3	2	3	1	1
2	2	2	2	2	2	2	1	1	2
2	2	2	2	3	2	3	2	3	2
2	2	2	1	1	2	2	1	1	1
9	9	9	9	9	9	9	9	9	9
2	2	2	2	1	2	2	2	2	2
3	1	1	3	3	3	2	1	1	1
2	2	2	2	2	2	2	2	2	1
2	2	9	9	9	9	9	9	9	1
3	2	2	2	2	2	2	2	2	2
1	1	2	1	1	1	1	1	1	1
2	2	2	2	2	2	2	2	2	1
2	2	2	2	2	2	2	2	2	2
9	2	1	2	1	3	1	2	3	1
1	2	1	2	3	2	1	2	3	1
2	2	2	2	2	2	2	2	2	2
3	3	2	2	1	1	2	2	1	1
2	2	1	1	2	2	2	2	1	1
2	2	1	1	2	3	2	2	2	2
2	2	1	2	2	3	2	2	2	1
1	1	2	1	1	1	1	1	1	1
2	2	2	2	2	2	2	2	2	1
2	2	2	2	2	2	2	2	2	1
2	2	2	2	1	2	2	2	2	2
1	2	9	1	3	2	3	2	9	1
2	2	2	2	2	2	2	2	2	2
3	3	2	2	3	3	3	3	3	2
9	9	9	9	9	9	9	9	9	9
2	2	2	2	2	2	2	2	2	2
1	1	3	3	3	3	9	1	1	1
2	2	3	3	3	3	2	2	2	1
3	1	1	2	2	3	2	3	1	3
1	1	1	1	1	1	1	1	1	1
1	2	1	2	3	1	2	3	1	1
2	2	2	2	2	2	2	2	2	2
2	1	1	2	3	2	1	2	3	2
1	2	2	2	2	2	9	2	1	1
1	2	2	2	2	2	2	2	1	1
3	1	1	2	9	3	3	2	3	1
3	3	1	1	2	1	1	1	1	1
3	2	2	2	2	3	3	2	3	2
1	1	2	2	2	2	2	2	2	2
3	1	1	2	3	3	3	2	2	2

2	3	2	2	2	1	1	2	2	1
1	3	2	1	3	3	3	2	3	2
1	1	1	1	1	1	1	1	1	1
3	3	1	2	1	1	2	3	1	1
2	2	2	2	2	2	2	2	2	1
2	2	1	1	1	2	2	2	2	1
1	1	1	1	1	1	2	2	1	2
9	9	1	9	9	9	9	9	9	3
9	9	9	2	9	9	9	9	9	9
9	9	1	9	9	9	9	9	9	3
2	3	2	2	2	2	9	2	2	2
1	2	2	2	2	2	3	2	2	2
3	2	1	1	3	3	3	2	1	3
1	2	1	1	3	3	3	2	1	3
3	1	1	1	1	1	3	1	2	3
3	3	3	1	3	3	1	3	3	2
1	1	1	3	1	1	2	2	2	2
1	2	2	1	2	1	2	3	2	2
3	1	9	9	9	9	9	9	9	2
3	3	1	2	3	2	3	3	2	2
3	2	1	9	1	3	2	1	2	3
3	1	2	3	2	3	2	1	3	3
2	2	2	2	2	2	2	2	2	2
1	1	2	2	2	2	2	2	2	3
2	2	2	2	2	2	3	1	1	2
1	1	2	2	2	2	2	2	2	3
3	3	2	2	2	2	1	3	3	1
3	1	2	2	2	3	1	3	3	1
3	1	1	3	2	2	2	1	2	2
9	1	1	3	2	2	2	1	2	2
2	2	1	1	3	3	3	2	1	3
2	2	1	1	3	3	3	2	1	3
3	3	2	2	2	2	2	2	2	1
3	3	2	2	2	2	2	2	2	1
3	3	2	2	2	2	2	2	2	2
3	3	3	1	3	3	1	3	3	2
2	2	2	2	2	2	2	2	2	1
2	2	3	1	3	3	1	3	3	2
2	2	3	1	3	3	1	3	3	2
3	3	3	1	3	3	1	3	3	1
3	3	1	3	1	2	2	2	2	3
3	2	2	1	3	3	1	1	1	3
3	2	2	1	3	3	1	1	1	3
3	1	2	1	3	3	1	1	1	3
3	2	2	1	3	3	1	1	1	3
1	3	1	2	9	1	1	3	1	3
2	1	1	9	2	3	2	1	3	3

9	2	1	2	3	9	1	1	2	2
2	3	1	2	2	2	3	1	1	3
1	2	1	3	2	2	3	1	1	2
3	3	2	2	2	1	3	1	3	3
1	1	1	3	1	1	2	2	2	3
9	9	1	3	3	1	2	1	2	3
1	1	1	3	2	1	2	1	1	3
1	2	1	2	1	2	2	1	2	2
9	9	1	9	9	9	9	9	9	9
9	9	9	9	9	9	9	9	9	9
2	2	2	2	2	2	2	2	2	2
2	3	1	9	2	3	3	2	1	3
2	2	1	3	1	2	9	2	2	2
1	1	1	3	2	2	3	1	1	2
9	9	9	9	9	9	9	9	9	9
2	2	2	2	2	2	2	2	2	2
2	2	2	2	2	2	2	2	2	2
9	9	2	9	9	9	9	9	9	2
2	2	1	2	2	2	2	3	1	3
9	9	9	3	9	9	9	9	9	2
1	1	2	3	3	2	1	2	2	3
1	1	1	3	1	2	3	3	3	3
2	2	1	3	1	1	3	1	1	3
2	3	2	2	2	2	2	2	2	2
9	9	1	9	9	9	9	9	9	2
9	9	9	9	9	9	9	9	9	9
1	2	1	2	3	1	2	3	1	3
9	9	9	9	9	2	9	9	9	9
3	2	1	3	2	2	1	2	1	3
9	9	9	9	9	9	9	9	9	9
3	1	1	1	1	1	1	1	3	3
9	9	2	9	9	9	9	9	9	2
2	1	1	3	1	1	3	1	1	2
1	1	1	3	1	1	3	1	1	3
1	1	2	2	2	2	2	2	2	2
2	2	1	3	1	1	3	1	1	3
2	2	2	2	2	2	2	2	2	3
1	1	3	1	2	2	2	2	2	2
3	3	1	1	2	3	9	2	1	1
9	9	2	9	9	9	9	9	9	2
9	9	2	9	9	9	9	9	9	2
9	9	2	9	9	9	9	9	9	2
9	9	2	9	9	9	9	9	9	2
9	9	2	9	9	9	9	9	9	2
9	9	2	9	9	9	9	9	9	2
3	3	2	2	1	3	1	2	2	2
1	2	1	2	3	1	2	3	1	3

9	9	9	9	9	9	9	9	9	9
2	2	1	2	3	3	1	1	2	2
1	2	1	2	2	2	2	3	3	3
1	3	1	3	1	1	2	2	1	3
1	1	1	3	1	1	3	1	1	2
2	2	2	2	2	2	2	2	2	2
9	9	1	9	9	9	9	9	9	2
3	3	3	1	3	3	1	3	3	1
2	2	1	3	1	1	3	1	1	2
9	9	2	9	9	9	9	9	9	2
2	2	2	9	3	9	2	3	1	3
9	9	9	9	9	9	9	9	9	9
9	9	2	9	9	9	9	9	9	2
9	9	2	9	9	9	9	9	9	2
1	1	1	3	1	1	3	1	1	3
9	9	2	9	9	9	9	9	9	2
3	1	2	1	2	2	1	1	1	3
9	9	2	9	9	9	9	9	9	2
2	3	1	2	1	3	3	2	2	3
3	3	1	3	1	1	3	1	2	3
3	3	1	3	1	2	2	3	3	3
2	1	1	2	1	2	2	2	2	2
1	1	1	2	3	3	1	3	3	3
3	2	1	3	1	1	3	1	1	3
1	1	1	3	2	3	2	2	2	2
3	2	2	3	2	2	3	2	1	2
2	2	1	3	1	1	3	1	1	2
1	1	1	3	1	1	2	1	1	2
3	2	1	2	1	1	3	1	1	2
3	1	2	2	2	2	2	2	2	3
1	3	1	2	3	2	2	3	3	3
1	1	1	3	1	1	3	1	1	3
9	9	9	9	9	9	9	9	3	9
9	9	9	9	9	1	9	9	9	9
9	9	9	3	9	9	9	9	9	3
9	9	9	9	9	9	3	9	9	9
9	9	9	9	2	9	9	9	9	9
9	9	9	9	9	9	9	9	3	9
9	9	9	9	9	9	9	2	9	9
9	9	2	9	9	9	9	9	9	2
9	9	2	9	9	9	9	9	9	2
9	9	2	9	9	9	9	9	9	2
9	9	2	9	9	9	9	9	9	2
9	9	2	9	9	9	9	9	9	2
9	9	2	9	9	9	9	9	9	2
1	2	1	3	1	1	2	2	3	3
3	1	1	3	2	1	3	1	1	2

1	1	1	3	1	2	3	2	1	3
9	1	2	9	3	9	1	9	2	2
9	9	1	9	9	9	9	3	3	2
1	1	1	3	1	2	1	2	1	3
9	9	1	9	9	9	9	9	9	3
9	9	1	9	9	9	9	9	9	3
9	9	1	9	9	9	9	9	9	3
9	9	1	9	9	9	9	9	9	3
9	9	1	9	9	9	9	9	9	2
2	2	2	2	2	2	2	2	2	2
1	2	1	2	1	2	3	2	1	3
1	2	1	2	1	2	3	2	1	3
2	2	2	2	2	2	2	2	2	2
2	2	1	2	2	2	2	1	1	3
2	3	1	3	2	2	1	3	2	3
3	2	1	2	2	2	1	3	1	3
9	9	1	9	9	9	9	9	9	9
9	9	1	9	9	9	9	9	9	3
9	9	1	9	9	9	9	9	9	3
9	9	1	9	9	1	9	1	9	3
9	9	1	9	9	9	9	9	9	3
9	9	2	9	9	9	9	9	9	2
9	9	3	9	9	9	1	9	9	1
9	9	9	1	9	9	1	9	9	1
9	9	2	2	9	3	1	3	2	2
9	9	9	9	1	9	9	9	9	2
9	9	1	9	9	9	9	9	9	2
2	2	2	2	2	2	2	2	2	2
2	3	2	1	3	3	2	2	2	2
9	9	9	9	9	9	3	9	9	3
2	2	1	2	2	2	2	2	1	3
2	3	2	3	1	3	2	1	1	3
2	2	2	2	2	2	2	2	2	2
2	2	2	2	2	2	2	2	2	2
9	2	1	3	1	1	3	1	1	3
2	2	1	2	1	1	3	1	2	2
2	1	2	3	2	3	3	2	2	2
2	2	1	9	1	2	9	1	2	3
9	9	9	9	9	2	9	9	9	3
2	2	2	2	2	2	2	2	2	2
2	2	2	2	2	2	2	2	2	2
3	3	2	2	2	2	2	2	2	2
1	2	2	1	1	2	1	2	1	9
2	2	2	2	2	2	2	2	2	3
9	9	1	9	9	9	9	9	9	3
2	2	2	2	2	2	2	2	2	2
2	3	1	3	3	3	1	1	1	2

1	2	1	2	1	2	1	1	2	3
3	1	1	2	1	2	2	2	2	3
2	2	1	2	3	2	2	2	2	2
2	3	2	2	3	2	3	2	2	2
2	1	1	2	3	2	3	2	3	3
2	2	1	3	2	2	3	2	2	3
3	1	1	3	1	1	1	3	3	3
2	2	1	3	1	2	2	1	2	3
1	1	1	3	1	1	1	1	1	3
9	9	9	9	9	9	9	9	9	9
2	2	2	2	2	2	2	2	2	2
3	1	1	2	3	2	3	1	1	3
1	1	1	3	1	1	3	1	1	3
2	2	1	2	2	2	1	2	1	3
1	2	2	3	1	2	3	1	1	3
2	1	1	3	1	2	3	2	1	3
2	2	2	2	2	2	2	2	2	2
2	2	2	2	2	2	2	2	2	2
2	2	1	3	1	2	1	2	3	2
1	2	1	3	1	1	2	2	2	3
3	3	1	3	1	2	2	2	2	3
2	1	2	2	2	2	2	2	2	2
2	3	1	2	2	2	2	2	1	3
3	3	1	3	2	2	1	1	1	3
2	2	1	3	1	1	3	1	1	3
3	3	1	1	2	2	2	2	2	3
1	2	1	3	1	1	2	1	1	3
1	2	1	2	1	1	2	2	1	3
3	3	2	2	2	2	1	3	3	3
2	2	2	2	2	2	2	2	2	2
2	2	2	2	2	2	2	2	2	2
2	2	2	2	2	2	2	2	2	2
2	2	1	2	2	2	2	2	2	2
2	2	2	2	2	2	2	2	2	2
2	2	1	2	3	1	2	3	1	3
2	2	2	2	2	2	2	2	2	2
1	2	1	2	1	3	2	1	3	3
9	9	9	9	9	9	9	9	9	9
9	9	9	9	9	9	9	9	9	9
9	9	9	9	9	9	9	9	9	9
3	3	3	2	1	3	3	2	1	3
9	9	9	9	1	9	9	9	9	3
9	9	9	9	9	9	9	9	9	9
9	9	9	9	9	9	9	9	9	9
9	9	9	9	9	9	9	9	9	9
9	9	9	9	9	9	9	9	9	9
1	1	1	3	1	2	1	1	1	2

1	2	1	2	3	1	2	2	3	3
2	1	1	3	1	1	1	2	1	3
9	9	9	9	9	9	9	9	9	9
3	2	2	2	2	2	2	2	2	3
9	9	9	9	9	9	9	9	9	9
9	9	9	9	9	9	9	9	9	9
2	2	1	3	1	2	2	2	2	2
1	1	1	2	1	1	3	1	1	3
2	2	2	3	1	2	2	1	1	3
3	2	1	9	2	1	2	3	2	3
3	2	3	1	3	9	1	2	2	1
2	2	2	2	2	2	2	2	2	2
3	3	1	2	1	3	3	2	1	3
3	3	2	2	2	2	2	2	2	3
3	3	2	2	2	1	1	1	1	3
1	2	2	2	2	2	2	2	2	2
2	2	2	2	2	2	2	2	2	3
1	9	9	9	9	9	9	1	9	9
3	3	1	2	2	2	3	2	1	3
2	3	1	2	2	2	2	2	2	3
2	2	2	2	2	2	2	2	2	2
2	3	2	3	3	2	1	3	3	3
2	2	2	2	2	2	2	2	2	2
3	3	1	3	3	3	1	1	1	3
2	1	2	2	2	2	2	2	1	3
2	2	1	2	2	2	2	2	2	2
9	9	9	9	9	9	9	9	9	9
3	2	2	2	2	2	2	1	1	3
1	3	1	3	1	1	1	1	1	3
2	2	3	2	9	2	2	2	2	2
1	1	1	3	1	1	1	1	1	3
9	9	9	9	9	9	9	9	1	9
2	2	2	2	2	2	2	2	2	2
2	2	2	2	2	2	2	2	2	2
2	2	2	2	2	2	2	1	2	2
2	2	2	2	2	2	2	2	2	3
2	3	2	2	2	2	2	2	2	3
2	2	2	2	2	2	2	2	2	2
3	2	2	2	1	2	1	2	3	2
3	1	1	3	1	3	1	1	1	3
1	2	2	2	2	2	2	2	1	2
1	1	1	3	1	1	3	1	1	2
1	2	1	3	1	2	1	2	2	3
9	9	9	9	1	9	9	9	9	2
2	2	1	2	2	2	2	2	1	3
3	1	1	3	2	2	1	3	2	3
1	2	1	3	1	1	2	1	1	3

2	2	2	2	2	2	2	2	2	2
2	2	2	2	2	2	2	2	2	2
2	2	2	2	2	2	2	2	2	2
2	2	2	2	2	2	2	2	2	2
1	1	1	3	1	1	3	1	1	2
2	2	2	2	2	2	2	2	2	2
2	2	2	2	2	2	2	2	2	2
2	2	1	3	1	1	2	2	2	2
2	2	2	2	2	2	2	2	2	2
2	2	2	2	2	2	2	2	2	2
2	2	2	1	3	2	2	2	2	2
2	1	1	3	1	2	3	2	1	3
9	9	9	9	9	9	9	9	9	9
3	3	3	3	3	3	3	1	3	3
9	9	9	9	9	9	9	1	1	3
9	9	9	9	9	9	9	9	9	9
1	1	2	3	1	1	3	2	1	2
2	2	1	3	1	1	3	1	1	3
9	9	9	9	9	9	9	9	1	9
2	1	1	3	2	2	3	1	2	3
2	2	1	3	1	3	3	1	2	3
2	2	2	2	2	2	2	2	2	2
2	2	9	9	1	2	2	2	2	2
2	1	1	3	1	2	3	1	1	3
3	1	1	1	3	3	1	1	1	3
9	9	3	9	9	9	9	9	9	3
3	1	1	1	3	3	1	1	1	3
2	2	1	1	3	3	1	1	1	3
1	1	1	3	1	1	3	3	2	2
9	9	9	9	9	9	9	9	9	9
2	2	1	2	1	2	1	1	1	3
3	2	1	2	2	1	1	1	1	3
2	2	2	2	2	2	2	2	2	2
3	1	1	3	1	2	9	2	2	1
2	2	2	9	2	9	9	9	9	2
3	3	2	2	3	2	2	2	2	3
9	1	1	3	1	1	3	1	1	3
9	9	9	9	9	9	9	9	9	9
2	2	1	2	9	9	9	9	9	9
9	9	9	3	9	9	9	9	9	2
3	3	1	2	1	3	1	1	2	3
3	2	1	3	1	1	3	1	1	3
1	1	9	9	9	9	9	9	9	1
2	2	1	3	1	1	2	2	2	3
3	1	1	2	3	1	2	3	2	3
3	2	1	3	3	2	1	2	2	2
3	1	1	2	1	2	2	1	1	2

3	3	1	1	3	3	1	3	3	3
2	3	9	9	9	9	9	9	1	3
1	2	1	3	2	2	3	2	1	3
9	3	1	9	9	9	9	9	1	9
3	3	1	2	3	3	1	1	1	3
1	2	1	3	1	1	2	2	2	2
1	1	1	2	1	3	3	1	1	3
3	3	1	1	3	3	1	3	3	1
3	1	2	2	3	3	2	2	1	3
3	3	1	3	1	2	1	1	1	3
9	9	9	9	9	9	9	9	9	9
9	2	1	3	1	9	2	1	1	3
2	2	1	3	2	2	1	1	1	3
1	3	1	2	1	1	2	1	2	3
9	9	9	9	9	9	9	9	9	9
2	2	2	2	2	2	2	2	2	2
2	1	1	3	1	2	3	1	2	3
2	2	1	3	1	1	3	1	1	3
9	9	1	9	9	9	9	9	9	9
2	2	2	2	2	2	3	2	2	9
2	1	1	3	2	2	2	2	1	3
2	2	2	2	2	2	2	2	2	2
2	2	1	3	2	1	3	1	1	3
2	2	1	3	2	2	3	1	1	3
3	2	1	3	1	1	2	1	1	3
3	2	1	2	2	3	2	1	1	3
2	3	1	3	1	1	3	1	1	3
2	3	1	3	1	1	2	1	1	3
3	1	1	3	1	1	3	1	1	3
3	2	1	3	2	2	2	1	1	3
9	2	3	9	2	9	2	9	2	9
9	9	9	9	9	9	9	9	9	9
2	2	1	3	1	1	2	9	2	9
9	9	9	9	9	9	9	9	9	9
9	9	9	9	9	9	9	9	9	9
2	1	1	3	2	2	2	2	1	3
2	2	2	2	2	2	2	2	2	2
1	1	1	3	1	1	3	1	1	3
9	9	1	9	9	9	9	9	9	9
9	9	9	9	9	9	9	9	9	9
9	9	1	9	9	9	9	9	9	9
9	9	1	9	9	9	9	9	9	9
9	9	1	3	1	1	3	1	1	3
2	2	2	2	2	2	2	2	2	2
2	2	2	2	2	2	2	2	2	3
2	1	1	3	1	2	2	1	1	3
9	9	1	3	1	9	9	9	1	9

9	9	9	9	9	9	9	9	9	9
9	9	1	9	9	9	9	9	9	9
9	9	1	9	9	9	9	9	9	3
9	9	3	9	9	9	9	9	9	9
9	9	1	9	9	9	9	9	9	9
9	9	1	9	9	9	9	9	9	9
2	2	2	2	3	2	2	2	2	1
9	9	9	9	9	9	9	9	9	9
2	2	2	2	2	2	2	2	2	2
9	9	9	9	9	9	9	9	9	9
3	2	1	2	2	2	1	1	1	3
3	1	1	3	1	1	2	1	1	3
3	1	1	3	3	3	1	3	1	3
2	2	1	3	3	2	2	1	1	3
2	9	1	2	2	2	1	3	2	3
2	2	1	2	2	1	2	1	2	1
3	3	1	2	3	3	1	3	3	2
9	9	9	9	1	9	3	9	1	9
1	2	1	2	2	1	2	1	2	3
9	2	2	9	2	9	9	9	9	2
9	9	9	9	9	9	2	9	9	9
9	9	1	9	9	9	9	9	9	3
2	2	2	2	2	2	2	2	2	3
3	1	1	2	2	3	1	2	1	3
9	9	2	2	2	2	2	2	2	2
2	2	2	2	2	2	2	2	2	3
9	9	1	3	1	1	9	9	9	3
1	2	2	3	3	1	3	2	3	3
2	1	1	3	2	2	2	2	1	3
3	3	2	2	2	2	2	2	2	2
2	2	1	2	2	2	2	1	1	3
2	2	1	3	3	2	1	1	1	3
9	1	1	3	1	1	1	1	1	3
2	1	1	2	3	2	3	1	1	3
3	2	1	3	1	2	1	1	1	1
9	9	1	9	9	9	9	9	9	9
3	3	1	3	1	1	1	1	1	3
2	2	1	3	1	1	1	1	2	3
2	3	1	2	3	3	2	1	1	2
3	1	1	2	1	1	2	1	1	3
3	3	1	2	1	2	1	1	1	3
2	2	1	3	2	2	2	2	2	9
3	2	1	2	3	9	2	9	3	2
9	9	9	9	9	9	9	9	9	9
2	2	2	2	2	2	2	2	2	2
3	2	1	3	1	1	2	1	1	2
1	2	1	2	2	3	3	1	1	3

9	9	1	9	9	9	9	1	9	3
9	1	1	3	1	1	1	1	1	3
3	1	1	3	3	1	1	1	1	3
3	3	1	2	2	2	1	2	2	3
3	2	3	2	3	2	9	2	3	2
2	2	1	3	2	2	2	2	2	3
3	3	1	3	1	3	9	1	1	3
2	2	1	3	2	1	2	2	1	3
2	1	1	3	3	3	1	3	3	2
2	2	1	1	2	2	1	1	1	3
2	2	1	3	2	1	2	2	1	3
3	1	1	3	1	1	2	1	1	3
9	9	1	9	9	9	9	9	9	3
1	3	1	3	3	1	1	2	2	3
2	2	1	2	2	2	2	2	2	3
2	3	1	3	3	2	2	1	1	3
9	9	9	9	9	9	9	9	9	9
2	3	1	2	1	3	2	3	2	2
2	2	9	9	9	9	9	9	9	9
9	9	9	9	9	9	9	9	9	9
2	2	2	2	2	2	2	2	2	3
1	1	1	3	2	2	1	1	1	3
9	9	9	9	9	9	9	9	1	3
3	3	1	1	1	3	3	1	1	3
2	2	2	2	1	1	3	2	2	2
2	1	3	2	3	2	2	2	3	2
3	2	2	1	2	2	3	2	1	2
2	2	2	2	2	2	3	2	2	3
3	1	1	3	1	3	9	1	1	3
2	2	1	2	1	2	1	3	3	3
2	1	1	3	1	1	3	1	1	3
3	3	1	3	1	2	2	3	3	2
2	2	2	1	3	2	2	2	2	3
2	2	2	1	2	2	2	2	2	3
3	3	1	3	1	2	1	1	1	3
2	3	1	3	1	1	3	1	1	2
2	2	2	2	2	2	2	2	2	3
2	2	1	2	3	2	2	2	2	3
3	2	1	2	2	2	2	2	2	3
2	2	1	2	2	2	2	2	2	2
2	2	1	2	2	2	1	1	1	3
2	2	1	3	2	2	1	1	1	3
9	9	1	9	9	9	9	9	9	3
3	1	1	3	1	1	1	1	1	3
1	2	1	3	2	2	2	2	2	3
9	1	1	3	3	1	3	1	1	3
2	2	1	3	1	1	3	1	1	3

1	1	1	3	2	2	2	2	2	2
9	9	2	9	9	9	9	9	9	3
2	2	2	2	2	2	3	9	1	2
9	9	9	9	9	9	9	9	9	9
2	2	1	2	1	2	2	1	1	3
2	2	2	2	2	2	2	2	2	2
2	2	1	3	2	2	2	1	1	3
1	1	1	3	1	1	3	1	1	9
3	1	1	3	1	1	2	1	1	3
3	1	1	3	3	3	3	3	1	3
9	9	1	2	1	1	3	1	1	3
2	2	1	3	2	2	2	1	1	3
3	2	9	9	9	9	9	9	9	3
3	3	1	2	1	1	1	2	2	3
9	9	9	9	9	9	9	9	9	9
9	9	2	2	2	2	2	2	2	9
9	9	9	9	9	9	9	9	9	9
3	3	3	1	3	3	1	3	3	1
3	3	1	3	3	3	1	1	1	3
2	3	9	9	1	9	9	9	9	2
3	3	2	3	2	1	2	3	2	2
2	3	1	3	3	3	2	3	2	2
3	3	1	3	3	3	1	1	1	3
3	3	1	2	2	2	2	1	1	3
1	2	1	2	2	2	3	1	1	3
3	3	1	3	3	3	1	1	1	3
3	3	1	3	3	3	3	1	1	3
1	3	1	3	1	1	3	1	1	3
3	2	1	2	2	2	1	2	1	3
3	3	1	1	3	3	1	3	1	3
9	9	9	9	9	9	9	9	9	9
3	1	1	3	2	2	9	1	1	3
9	9	9	9	9	9	9	9	9	9
3	3	2	2	2	2	9	3	3	3
3	2	1	3	2	2	1	1	1	3
9	9	1	3	9	9	9	9	9	9
2	1	2	2	2	2	2	2	2	1

AfraidPapS	LiketoKnov	WilldoPapi	WillLikePa	Embarrase	WorriedAb	NoTimefor	PapSmearN	PapAgainst	LikeHPVVa
2	2	2	2	2	2	2	2	2	2
1	3	1	1	1	1	2	2	2	1
1	1	2	1	3	3	3	3	3	9
1	3	1	1	1	1	2	2	2	1
1	1	1	1	1	1	2	2	2	1
1	1	1	1	1	1	2	2	2	1
9	9	9	2	9	9	9	9	9	9
3	1	2	3	3	1	2	2	3	1
9	1	9	1	1	2	2	2	2	1
2	2	2	9	9	9	9	9	9	2
2	3	2	2	2	2	2	2	2	2
3	3	3	3	3	3	3	3	3	2
2	1	1	1	3	3	3	3	3	1
2	1	3	2	2	2	3	2	3	2
3	1	1	1	1	2	3	3	3	1
2	1	3	2	3	2	3	3	3	1
1	1	1	1	1	2	1	1	1	1
2	1	3	2	3	2	3	3	3	1
2	1	3	2	3	2	3	3	3	1
1	1	1	1	1	1	1	1	1	2
1	1	1	3	3	3	3	3	3	1
1	1	1	3	3	3	3	3	3	1
2	1	2	2	2	2	2	2	2	2
1	1	2	1	3	1	3	3	3	1
2	2	2	2	2	2	2	2	2	2
1	1	2	1	3	1	3	1	3	1
1	1	1	1	3	1	3	3	2	1
3	1	3	1	1	3	3	3	3	1
2	2	2	2	2	2	2	2	2	1
2	2	2	2	2	2	2	2	2	1
2	2	2	2	2	2	2	2	9	1
2	1	2	2	1	1	2	1	1	1
3	1	3	1	3	3	3	3	3	1
2	1	2	1	3	1	3	2	3	1
2	2	2	2	2	2	2	2	2	1
2	1	2	1	1	1	2	1	1	1
2	2	2	2	2	2	2	2	2	1
3	1	1	1	3	1	3	1	3	9
2	2	2	2	2	2	2	2	2	2
2	2	2	2	2	2	2	2	2	1
2	2	2	2	2	2	2	2	2	1
2	1	2	2	2	2	3	3	2	2
3	1	3	1	3	1	3	2	3	9
3	1	1	1	3	3	3	2	3	1
3	1	1	1	3	3	3	2	3	1
1	2	3	3	2	2	1	1	2	1

3	1	3	1	3	3	3	3	3	3
1	1	2	1	1	1	1	3	3	1
2	1	3	1	2	2	3	3	3	1
1	1	2	2	1	1	3	2	3	1
1	9	2	1	3	2	3	3	3	1
3	1	3	1	3	3	3	3	3	1
3	1	3	1	3	3	3	3	3	3
3	1	1	1	3	3	3	3	3	1
9	1	9	1	9	9	9	9	9	1
1	1	3	1	1	2	3	3	3	3
9	9	2	9	2	9	2	9	2	2
2	1	3	3	3	2	3	3	3	1
1	1	2	2	3	1	3	3	3	1
2	2	2	2	2	2	2	2	2	9
1	1	1	2	3	2	3	3	3	1
1	1	3	3	3	1	3	3	3	1
3	1	3	1	3	3	3	3	3	1
3	1	3	1	3	3	3	3	3	9
1	1	1	1	2	1	2	3	3	1
9	9	9	9	9	9	9	9	9	1
3	1	3	3	3	3	3	3	3	3
3	1	3	3	3	3	3	3	3	3
3	1	3	3	3	3	3	3	3	3
3	1	3	1	1	1	2	3	3	9
9	1	9	9	9	9	9	9	9	1
1	1	1	1	1	1	2	2	3	9
9	9	9	9	9	9	9	9	9	9
2	1	1	2	3	1	1	3	1	1
9	9	9	9	9	9	9	9	9	1
1	1	2	1	3	3	3	3	3	1
1	1	2	1	1	3	2	1	2	3
3	1	2	1	3	3	1	2	2	1
9	1	9	1	9	1	2	2	3	2
3	1	3	1	3	3	3	3	3	1
1	1	3	1	3	1	3	3	3	1
9	9	9	9	9	9	9	9	9	9
9	9	9	9	9	9	9	9	9	9
3	1	3	1	3	3	3	3	3	1
3	1	3	1	3	3	3	3	3	1
3	1	3	1	3	3	1	3	1	1
9	9	9	9	9	9	9	9	9	1
3	1	9	1	3	3	1	2	3	1
3	1	1	1	3	3	3	3	3	1
2	1	1	1	3	2	3	3	3	1
2	1	1	1	3	2	3	3	3	1
9	9	9	9	9	9	9	9	9	9
9	9	9	9	9	9	9	9	9	9

1	1	1	2	1	3	3	3	3	9
3	1	3	2	3	3	3	3	3	2
3	1	1	2	1	1	1	3	3	1
1	1	2	1	3	3	3	3	3	1
9	9	9	9	9	9	9	9	9	9
1	1	2	2	2	2	2	2	2	2
3	1	3	1	3	3	3	3	3	1
3	1	3	1	3	1	3	3	3	1
3	1	1	2	1	1	2	3	3	1
2	1	2	1	3	3	3	3	3	1
1	1	3	1	2	1	3	2	2	1
1	1	1	2	2	3	3	2	2	1
3	1	3	1	3	3	3	1	3	1
1	1	2	2	2	1	2	2	2	1
1	2	1	2	1	1	2	3	2	2
2	1	1	1	3	2	1	2	2	2
2	1	3	3	3	2	3	3	3	1
2	1	2	1	2	2	2	2	2	1
2	2	2	2	2	2	2	2	2	9
3	1	3	1	3	3	3	3	3	1
3	1	2	1	3	3	3	3	3	1
9	1	3	1	3	3	3	3	3	9
2	2	2	2	2	2	2	2	2	1
9	9	9	9	9	9	9	9	9	1
2	2	1	1	1	1	1	2	2	2
2	1	3	2	1	2	3	3	3	1
3	1	1	2	3	3	3	3	3	1
3	1	1	2	3	3	3	3	3	1
2	2	2	2	2	2	2	2	2	2
2	1	3	1	3	1	2	2	3	1
9	1	2	1	3	2	2	2	3	1
2	2	2	2	2	2	2	2	2	2
2	1	3	1	3	2	3	2	3	1
1	1	2	2	2	2	2	2	2	2
2	1	2	1	3	1	2	3	3	1
2	1	1	2	2	1	2	1	2	1
1	1	2	1	3	3	2	2	2	1
2	2	2	2	2	2	2	2	2	1
2	9	3	9	1	1	9	2	1	9
3	1	3	1	3	3	3	3	3	1
2	2	2	2	2	2	2	2	1	1
2	2	2	2	2	2	2	2	1	1
2	2	2	2	2	2	2	2	2	2
3	1	3	3	3	3	3	1	3	1
1	1	2	2	1	1	2	3	2	2
2	1	1	1	3	3	3	3	3	1
2	2	2	2	2	2	2	2	2	1

9	9	9	9	9	9	9	9	9	3
2	2	1	1	1	2	3	3	2	2
2	2	1	1	1	2	3	3	2	2
2	2	2	2	2	2	2	2	1	1
2	2	2	2	2	2	2	2	1	1
3	1	3	1	3	3	3	3	3	9
1	3	3	3	3	3	3	3	3	3
2	1	3	2	3	3	3	3	3	1
2	2	2	2	2	2	2	2	2	2
2	2	2	2	2	2	2	2	2	2
2	3	2	2	2	3	2	3	2	9
2	2	2	2	2	3	2	3	2	9
2	1	3	2	2	2	2	9	1	2
2	1	2	2	3	3	3	2	3	1
3	3	1	3	1	3	3	1	3	3
3	3	1	3	1	3	3	1	3	3
1	9	9	9	9	9	9	9	9	9
1	1	3	3	3	1	3	3	1	3
9	1	9	9	9	9	9	9	9	9
2	1	3	1	3	1	3	3	3	1
2	1	3	1	3	1	3	3	3	1
2	1	3	1	3	1	3	3	3	1
2	1	3	1	3	3	3	3	3	1
2	1	3	1	3	1	3	3	3	1
2	1	3	1	3	1	3	3	3	1
2	1	3	1	1	1	3	3	3	1
2	1	3	1	1	1	3	3	3	1
3	1	3	1	3	1	3	3	3	1
2	1	3	1	3	1	3	3	3	1
1	1	3	1	3	1	3	3	3	1
2	3	1	3	2	2	2	2	2	1
2	2	2	2	2	2	2	2	2	2
2	1	3	1	1	1	3	3	3	1
9	1	3	1	3	1	3	3	3	1
1	1	3	1	3	1	3	3	3	1
2	1	3	1	3	1	3	3	3	1
2	1	3	1	3	1	3	3	3	1
2	1	3	1	3	1	3	3	3	1
2	1	3	1	3	1	3	3	3	1
3	1	3	1	2	1	3	3	3	1
2	1	1	3	3	1	3	3	3	1
2	1	3	1	3	1	3	3	3	1
2	1	3	1	3	1	3	3	3	1
2	1	3	2	3	1	3	3	3	1
2	1	3	1	1	1	3	3	3	1
2	1	3	2	3	1	3	3	3	1
2	1	3	2	3	1	3	3	3	1

2	1	2	3	1	1	3	3	3	1
9	1	3	1	3	1	3	3	3	1
3	1	1	1	1	1	3	3	3	1
3	1	3	1	1	1	1	3	3	1
2	1	3	1	3	1	3	3	3	1
3	1	3	1	1	1	3	3	9	1
3	3	3	1	1	1	3	3	3	1
3	1	3	1	3	3	3	3	3	1
3	1	3	1	3	3	1	3	1	1
3	1	3	1	3	3	3	3	3	1
9	9	9	9	9	9	9	9	9	9
9	9	9	9	9	9	9	9	9	9
1	1	3	1	3	1	1	1	3	1
1	1	3	1	3	1	3	3	3	1
2	1	1	1	2	1	2	2	2	9
2	1	1	2	2	2	1	3	3	1
2	2	2	2	2	2	2	2	2	2
1	1	1	1	1	1	1	1	1	1
2	1	2	1	3	3	3	2	3	3
3	1	3	1	3	3	3	2	3	1
2	1	1	1	1	2	2	1	1	2
2	2	2	2	2	2	2	2	2	2
1	1	2	2	3	1	3	2	3	1
2	1	1	3	1	1	1	2	3	1
2	2	2	2	2	2	2	2	2	2
2	2	2	2	2	2	2	2	2	2
3	1	3	1	3	3	2	3	3	1
2	2	2	2	2	2	2	2	2	2
2	2	2	2	2	2	2	2	2	2
2	1	2	1	2	3	3	3	3	3
3	1	3	1	3	3	3	3	3	1
3	1	3	1	3	3	3	3	3	1
2	1	1	1	3	3	3	3	3	3
2	2	2	2	1	1	1	2	2	1
2	1	1	1	2	1	2	1	2	1
1	1	1	1	2	2	2	2	1	2
2	2	1	1	2	2	2	1	2	2
1	1	1	1	1	1	1	2	3	1
2	1	2	2	2	2	2	2	2	2
2	3	1	2	3	2	2	2	3	1
2	2	2	2	2	2	2	2	2	1
3	1	3	1	3	3	2	3	3	1
9	9	9	3	9	9	9	9	9	1
9	9	9	3	9	9	9	9	9	1
1	3	1	2	1	2	1	2	1	2
2	1	3	1	2	3	1	1	3	1
1	9	9	9	9	9	9	9	9	1

2	2	2	2	2	2	2	2	2	1
2	2	2	2	2	2	2	2	2	1
2	1	2	2	2	2	2	2	3	2
2	1	1	2	1	2	2	2	2	1
1	1	1	1	1	2	3	1	2	3
2	2	2	2	2	2	2	2	2	2
2	2	2	2	2	2	2	2	2	2
2	1	2	2	1	2	2	2	2	2
2	9	1	2	1	2	2	2	2	1
2	2	1	1	2	2	2	2	2	2
2	1	2	2	2	2	2	2	2	1
2	2	2	2	2	2	2	2	2	2
2	2	2	2	2	2	2	2	2	2
2	3	3	2	2	2	2	2	2	2
2	1	1	1	3	3	3	3	3	1
2	1	3	2	1	2	2	2	2	2
3	3	2	1	2	2	3	2	2	2
2	1	2	2	2	3	3	2	3	1
9	1	9	9	9	9	9	9	9	1
1	1	3	1	3	1	3	3	3	1
1	3	9	1	1	1	1	1	3	1
2	1	1	2	2	2	2	2	2	1
2	3	3	2	2	2	2	2	2	2
9	9	9	9	9	9	9	9	9	1
2	1	9	1	9	1	9	1	9	9
1	1	2	2	2	2	2	2	2	2
1	1	2	2	2	2	2	2	2	2
3	2	2	1	3	2	1	2	2	1
2	2	2	2	2	2	2	2	2	9
2	2	2	2	2	2	2	2	2	1
9	1	9	9	9	9	9	9	2	2
1	1	2	1	3	1	3	3	3	1
3	3	1	3	1	1	1	1	3	1
1	1	3	1	1	1	3	2	3	1
9	9	9	9	9	9	9	9	9	2
2	2	2	2	2	2	2	2	2	2
3	1	3	1	2	1	3	3	3	1
2	1	3	1	3	3	3	3	3	2
1	1	2	1	2	1	2	2	1	1
3	1	2	3	3	2	3	3	3	1
2	1	1	1	1	1	1	1	1	1
3	1	2	1	3	1	3	1	3	1
9	9	9	9	9	9	9	9	9	9
2	2	2	2	2	2	2	2	2	2
3	1	2	2	2	1	1	1	2	2
2	2	2	2	3	3	3	1	1	2
2	2	2	2	2	2	2	2	2	1

9	9	9	9	9	9	9	9	9	9
9	9	9	9	9	9	9	9	9	9
9	9	9	9	9	9	9	9	9	9
1	1	3	1	2	2	3	3	3	1
3	2	2	2	3	2	2	3	3	2
3	1	1	3	3	3	3	2	1	1
3	1	3	1	3	3	2	3	3	1
3	1	1	1	1	1	1	1	3	1
9	9	9	9	9	9	9	9	9	9
2	2	2	2	2	2	2	2	2	2
2	2	2	2	2	2	2	2	2	2
2	1	1	1	2	2	3	2	2	1
9	9	9	9	9	9	9	9	9	9
3	1	3	3	3	3	3	3	3	3
3	3	1	3	1	9	1	9	1	1
9	9	9	9	9	9	9	9	9	9
9	9	9	9	9	9	9	9	9	9
1	1	1	1	3	1	3	2	2	1
1	1	1	1	2	1	1	1	1	1
9	9	9	9	9	9	9	9	9	9
9	9	9	9	9	9	9	9	9	9
3	1	2	1	3	1	3	2	3	1
2	1	2	2	3	2	1	1	2	1
1	1	1	1	1	3	1	1	3	3
2	2	2	2	2	2	2	2	9	2
3	1	3	1	3	3	3	3	3	1
1	3	1	2	1	3	1	2	1	1
3	1	2	1	3	3	3	3	3	1
3	1	3	1	3	1	3	3	3	1
3	1	3	1	3	3	3	3	3	1
2	2	2	2	2	2	2	2	2	2
9	9	9	9	9	9	9	9	9	9
9	9	9	9	9	9	9	9	9	9
9	9	9	9	9	9	9	9	9	2
3	1	3	2	3	3	3	3	3	1
1	1	1	1	1	3	1	2	1	9
1	1	3	1	3	2	3	3	3	1
1	1	1	1	1	1	3	1	3	1
3	1	3	1	3	3	3	3	3	1
2	1	2	1	2	2	3	3	3	2
2	1	1	2	1	2	2	1	2	1
1	1	3	1	1	1	3	3	3	1
2	1	2	2	2	2	2	2	9	1
2	1	2	2	2	2	2	2	2	9
2	1	1	1	3	3	3	3	3	1
2	2	2	2	2	2	2	2	2	2
1	1	3	1	3	1	3	3	3	1

9	9	9	9	9	9	9	9	9	9
9	9	9	9	9	9	9	9	9	1
9	1	9	9	9	9	9	9	9	1
2	1	3	1	3	3	3	3	3	1
9	9	9	9	9	9	9	9	9	1
9	9	9	9	9	9	9	9	9	9
2	1	1	2	2	3	3	3	3	1
9	1	1	2	2	1	9	9	9	2
2	1	3	1	1	1	3	3	3	1
2	1	2	2	3	2	3	3	3	1
2	2	2	2	2	2	2	2	2	2
2	2	2	2	2	2	2	2	2	3
2	1	3	1	3	3	2	2	3	1
1	1	2	3	1	2	3	3	2	3
2	1	3	1	2	2	3	3	2	1
2	1	2	2	2	3	2	3	1	2
2	2	2	2	2	2	9	2	2	2
2	3	3	2	2	2	2	3	2	2
2	2	2	2	2	2	2	2	2	2
2	2	2	2	2	2	2	2	3	2
3	2	2	1	3	3	2	3	3	1
3	1	3	1	3	3	3	3	3	1
2	1	3	1	3	1	3	3	3	1
2	1	2	1	2	2	2	3	3	1
2	2	2	2	2	2	2	2	2	2
2	1	3	2	2	1	1	2	2	2
2	2	2	2	2	2	2	2	3	1
2	2	2	2	2	2	2	2	2	2
1	1	3	2	2	1	2	3	2	1
2	1	1	2	1	1	1	2	2	1
1	1	1	2	3	2	2	3	3	2
1	1	1	2	3	2	2	3	3	2
1	2	2	3	3	3	2	3	1	2
2	2	2	2	2	2	2	2	2	9
2	2	2	2	2	2	2	2	2	9
2	3	2	1	3	3	2	3	3	1
2	1	2	2	2	2	2	2	3	1
2	2	2	2	2	2	2	2	2	9
2	2	2	2	2	2	2	2	2	2
2	2	2	2	2	2	2	2	3	2
2	2	2	1	2	2	3	3	3	2
2	1	2	2	2	2	2	2	2	1
3	1	1	1	3	3	3	3	3	2
2	1	2	2	2	2	2	2	2	1
2	2	1	2	2	1	2	1	2	1
1	1	2	2	1	2	3	3	3	2
2	2	2	2	2	2	2	2	2	1

2	1	3	1	1	3	3	3	2	1
2	9	1	3	3	3	3	3	3	1
2	2	2	2	2	2	2	2	2	2
2	2	2	2	2	2	2	2	2	2
2	2	2	2	2	2	2	2	2	1
2	2	2	2	2	2	2	2	2	2
9	9	9	9	9	9	9	9	9	9
2	2	2	2	2	2	2	2	2	1
2	2	2	2	2	2	2	2	2	1
1	2	2	3	3	1	1	2	2	1
1	1	2	2	1	2	1	2	2	1
1	1	3	1	1	1	3	3	3	1
1	1	1	1	1	1	1	1	1	1
1	1	1	1	3	1	1	2	3	1
2	2	2	2	2	2	2	2	2	2
1	1	1	2	2	1	1	1	1	1
2	1	3	1	3	1	3	3	3	1
2	1	1	1	1	1	2	3	3	1
2	1	1	1	1	1	2	3	3	1
2	9	2	2	2	3	2	3	1	2
3	1	1	1	3	1	2	1	1	2
2	1	2	2	2	2	2	2	2	2
9	9	9	9	9	9	9	9	9	9
2	2	2	2	2	2	2	2	2	2
1	1	2	1	1	1	2	3	3	1
9	9	9	9	9	9	9	9	9	9
1	1	2	3	2	1	2	2	2	1
9	9	9	9	9	9	9	9	9	9
1	2	2	2	2	2	2	2	2	2
2	1	2	1	2	1	2	1	2	1
9	9	9	9	9	9	9	9	9	9
9	9	9	9	9	9	9	9	9	9
2	2	2	2	2	2	2	2	2	2
1	1	1	2	2	1	1	1	1	2
9	9	9	9	9	9	9	9	9	9
1	2	2	1	1	1	1	1	2	1
3	2	3	3	2	3	3	3	3	3
1	1	1	2	1	2	1	9	9	2
9	9	9	9	9	9	9	9	9	9
1	1	3	1	2	1	1	3	3	1
9	9	9	9	9	9	9	9	9	9
1	3	1	1	1	1	1	2	2	1
2	2	2	2	2	2	2	2	2	2
3	1	1	1	1	1	1	1	1	2
1	1	1	9	9	9	9	9	9	1
1	1	3	1	3	1	3	3	3	1
3	1	3	1	3	1	3	3	3	1

9	9	9	9	9	9	9	9	9	9
2	1	3	1	1	1	1	3	3	1
2	1	3	1	3	1	3	3	3	1
1	9	3	1	3	1	3	3	3	1
2	1	3	1	3	1	3	3	3	1
1	1	3	1	1	1	3	3	3	1
2	1	3	1	2	1	3	3	3	1
3	1	3	1	3	1	3	3	3	1
3	1	3	1	3	1	3	3	3	1
2	2	3	1	1	1	3	3	3	1
2	1	2	1	3	1	2	2	3	1
1	1	3	1	1	1	3	3	3	1
3	1	3	1	1	1	3	3	3	1
2	1	3	1	1	1	3	3	3	1
2	1	3	1	3	1	3	3	3	1
2	1	3	1	3	1	3	3	3	1
2	1	3	1	1	1	3	3	3	1
2	1	3	1	1	1	3	3	3	1
2	1	3	1	1	1	3	3	3	1
2	1	3	1	1	1	3	3	3	1
1	1	3	1	3	1	3	3	3	1
2	1	3	1	3	1	3	3	3	1
2	1	3	1	3	1	3	3	3	1
2	1	3	1	1	1	3	3	3	1
2	1	3	1	3	1	3	3	3	1
2	1	3	1	3	1	3	3	3	1
2	1	3	1	3	1	3	3	3	1
1	1	3	1	3	1	3	3	3	1
2	1	3	1	1	1	3	1	3	1
1	1	3	1	2	1	3	3	3	1
2	1	3	1	1	1	3	3	3	1
3	1	3	1	3	1	3	3	3	1
2	1	3	1	3	1	3	3	3	1
2	1	3	1	3	1	3	3	3	1
2	3	1	1	3	1	3	3	3	1
2	1	2	1	3	1	3	3	3	1
3	1	3	1	3	1	3	9	3	1
3	1	3	1	3	1	3	3	3	1
3	1	3	1	1	1	3	3	3	1
3	1	3	1	1	1	3	3	3	1
2	1	3	1	1	1	3	3	3	1
2	2	2	2	2	2	2	2	2	2
1	1	1	1	2	1	3	3	3	1
2	2	2	2	2	2	2	2	2	2
9	9	9	9	9	9	9	9	9	2
2	2	2	2	2	2	2	2	2	2
1	1	2	3	3	1	1	2	3	1

2	2	2	2	2	2	2	2	2	2
2	1	3	1	1	3	2	3	3	1
2	1	3	1	1	3	2	3	3	1
2	1	1	1	2	2	3	2	2	1
2	2	2	2	2	2	2	2	2	1
2	9	9	9	9	9	9	9	9	9
2	2	2	2	2	2	2	2	2	2
1	2	2	2	2	2	2	2	2	1
2	1	1	1	3	1	3	3	3	1
2	1	2	2	2	2	2	2	2	2
2	1	3	1	1	3	2	3	3	1
1	9	9	9	9	9	9	9	9	1
9	9	9	9	9	9	9	9	9	1
2	1	2	1	2	2	2	2	2	2
9	9	9	9	9	9	9	9	9	1
9	9	9	9	9	9	9	9	9	1
9	9	9	9	9	9	9	9	9	1
9	9	9	9	9	9	9	9	9	1
9	9	1	9	9	9	9	9	9	1
3	2	1	1	3	1	3	3	1	1
3	3	9	1	1	9	9	3	3	9
9	9	9	9	1	1	9	9	9	9
2	1	1	1	1	1	3	1	3	1
9	9	9	9	9	9	9	9	9	1
2	9	9	9	9	9	9	9	9	9
9	9	9	9	9	9	9	9	9	9
9	9	9	9	9	9	9	9	9	1
9	9	9	9	9	9	9	9	9	9
3	1	2	1	3	1	3	3	3	1
9	9	9	9	9	9	9	9	9	1
9	9	9	9	9	9	9	3	9	1
2	1	2	2	2	2	2	2	3	1
2	2	2	2	2	2	2	2	2	2
3	1	3	1	3	3	3	3	3	1
2	1	1	2	2	1	1	2	2	1
2	1	2	1	2	2	2	2	3	1
3	1	3	1	3	1	3	2	3	1
3	1	3	1	3	3	3	3	3	1
2	2	2	2	2	2	2	2	2	2
2	1	1	2	3	3	3	2	3	1
2	1	3	1	2	3	2	3	2	1
1	1	3	1	1	1	3	3	3	1
2	2	2	2	2	2	2	2	2	2
3	1	2	1	1	2	2	3	3	9
3	1	1	2	3	1	3	2	3	2
2	2	2	2	2	2	2	2	2	9
1	1	3	1	3	2	3	3	3	1

1	1	1	2	1	2	3	2	2	1
3	1	3	1	3	3	3	3	3	1
2	1	3	1	3	3	3	3	3	1
1	1	1	2	2	1	3	3	3	1
3	1	2	1	3	2	3	1	2	1
2	1	2	2	2	2	2	2	2	1
3	1	2	1	1	1	3	2	3	1
2	2	2	2	2	2	2	2	2	2
2	2	2	2	2	2	2	2	2	2
2	2	2	2	2	2	2	2	2	2
2	2	2	2	2	2	2	2	2	2
2	1	3	2	1	3	3	3	3	2
2	2	2	2	2	2	2	2	2	2
2	2	2	2	2	2	2	2	2	2
2	1	3	2	1	2	3	2	2	2
2	2	2	2	2	2	2	2	2	2
2	2	2	2	2	2	2	2	2	2
2	1	2	2	2	2	2	2	2	2
2	1	1	2	2	2	2	2	3	1
3	1	1	1	3	3	2	3	3	1
2	1	2	2	2	2	3	2	2	2
2	1	3	1	3	1	3	3	3	1
1	1	3	1	3	3	3	3	3	1
2	1	1	1	3	3	2	2	3	1
2	1	2	3	1	1	2	2	3	2
2	1	2	2	2	2	2	2	2	1
1	1	3	1	1	3	1	3	3	9
2	1	2	1	3	1	1	2	1	1
1	1	3	1	3	1	3	3	2	1
2	2	3	3	2	3	2	3	2	2
2	2	2	2	2	2	2	2	2	2
2	1	1	1	1	2	1	2	2	1
1	1	2	2	2	2	2	2	2	2
2	2	2	2	3	3	2	2	2	3
2	2	2	2	2	2	2	2	2	2
9	9	9	9	9	9	9	9	9	2
2	1	3	1	2	1	3	2	3	1
3	1	3	1	3	3	3	3	3	1
2	1	2	2	2	2	2	2	2	3
3	1	2	1	3	3	3	3	3	9
2	1	9	9	9	9	9	9	9	9
1	1	2	1	2	2	2	2	3	1
3	1	2	1	3	3	3	2	3	1
1	1	1	2	2	3	3	2	2	1
1	1	2	1	3	3	3	3	3	1
2	2	2	2	2	2	2	2	2	1
9	9	9	9	9	9	9	9	9	1

9	9	9	9	9	9	9	9	9	1
9	9	9	9	9	9	9	1	9	9
9	9	9	9	9	9	9	9	9	1
1	1	1	1	3	3	3	1	3	1
9	9	1	9	9	9	9	9	9	1
9	9	1	9	9	9	9	9	9	1
9	9	9	9	9	9	9	1	9	1
9	9	9	9	9	9	9	9	9	1
9	9	1	9	9	9	9	9	9	1
9	9	1	9	9	9	9	9	9	1
9	9	9	9	3	9	9	9	9	1
9	1	9	9	9	9	9	9	9	1
9	9	9	9	9	9	9	9	9	1
9	9	9	9	9	9	9	9	9	1
9	9	9	9	9	9	9	9	3	1
9	9	9	9	9	9	9	9	9	1
9	9	9	9	9	9	9	1	9	1
1	1	1	1	3	3	3	1	3	1
2	2	2	2	2	2	2	2	2	2
9	9	9	9	9	9	9	9	9	1
1	1	1	1	1	1	1	1	1	1
1	3	2	2	1	3	1	2	3	2
1	1	2	1	1	2	2	2	2	1
1	1	1	1	3	2	1	1	1	1
2	2	3	3	2	2	3	2	3	1
2	1	2	1	2	1	2	2	2	3
2	2	2	2	2	2	2	2	2	2
3	9	2	1	2	1	1	2	3	3
2	2	2	2	2	2	2	2	2	2
2	2	2	2	2	2	2	2	2	1
1	1	2	2	3	3	3	3	2	1
2	1	2	2	3	2	2	2	3	1
2	2	2	2	2	2	2	2	2	2
2	3	1	3	3	3	3	3	3	1
2	3	1	2	3	1	2	3	1	1
1	2	2	2	2	2	2	2	2	1
2	2	2	2	2	2	2	2	2	2
9	9	9	9	9	9	9	9	9	9
1	1	1	1	1	1	3	1	1	1
9	9	9	9	9	9	9	9	9	9
2	2	3	3	3	3	3	3	3	2
3	2	3	2	2	2	3	2	2	2
9	9	9	9	9	9	9	9	9	9
3	1	1	3	1	1	1	1	1	1
2	2	2	2	2	2	2	2	2	2
2	2	2	2	2	2	2	2	2	9
2	2	2	3	2	3	2	2	2	3

2	2	2	2	2	2	2	2	2	2
9	9	9	9	9	9	9	9	9	9
1	3	1	3	1	3	1	3	1	1
9	9	9	9	9	9	9	9	9	9
1	1	1	1	1	1	1	1	1	1
1	1	1	1	1	1	1	1	1	1
9	9	9	9	9	9	9	9	9	9
2	2	2	2	2	2	2	2	2	2
2	1	1	2	1	2	1	2	3	3
2	2	2	2	2	2	2	2	2	9
2	2	9	3	2	1	1	2	2	9
1	1	2	1	2	1	1	1	3	1
2	3	3	3	3	3	2	2	2	2
9	9	9	9	9	9	9	9	9	9
9	9	9	9	9	9	9	9	9	9
9	9	9	9	9	9	9	9	9	9
2	1	1	2	1	2	3	3	3	1
9	9	9	9	9	9	9	9	9	9
2	1	3	1	3	1	3	2	3	3
3	1	3	2	3	3	3	3	3	1
3	3	3	3	3	3	3	3	3	2
2	3	3	3	3	3	2	2	2	2
1	1	1	1	1	1	1	1	1	3
9	9	9	9	9	9	9	9	9	9
2	2	2	2	2	2	2	2	2	2
3	1	1	3	1	3	1	3	1	1
1	3	3	3	3	3	3	3	3	3
1	1	3	3	1	1	3	1	3	1
2	2	2	1	1	1	1	1	1	2
3	1	3	1	3	3	3	3	3	1
9	9	9	9	9	9	9	9	9	9
9	9	9	1	9	9	9	9	9	9
2	1	2	1	3	3	3	2	3	1
1	1	1	2	1	1	9	3	3	2
9	1	9	2	1	9	1	9	3	2
2	1	3	1	1	3	3	3	3	1
3	1	3	1	3	3	3	3	3	1
9	9	9	9	9	9	9	9	9	9
9	9	9	9	9	9	9	9	9	9
9	9	9	9	9	9	9	9	9	9
9	9	9	9	9	9	9	9	9	9
2	3	2	1	1	1	1	1	1	2
1	2	2	3	3	2	3	1	2	3
9	9	9	9	9	9	9	9	9	9
1	2	2	3	3	2	3	1	2	2
2	1	2	3	1	1	2	1	3	1
1	1	1	1	1	3	3	3	3	1

3	3	3	3	3	3	3	3	3	1
2	1	3	2	2	2	2	2	2	2
3	2	3	3	2	3	3	3	3	3
2	2	2	2	2	2	2	2	2	2
2	2	2	2	2	2	2	2	2	3
1	1	1	1	1	1	9	1	1	1
9	9	9	9	9	9	9	9	9	9
2	1	2	2	2	2	3	3	3	1
3	2	9	2	3	1	1	2	9	2
2	1	3	2	2	2	3	2	3	9
3	3	3	3	3	3	3	3	3	3
3	1	1	1	1	3	3	1	3	1
2	1	3	3	3	1	1	2	3	1
2	1	2	3	1	1	1	2	3	1
2	1	3	2	3	1	1	2	3	1
2	1	2	2	3	2	1	2	1	2
2	1	3	2	3	1	1	2	3	1
2	1	3	2	3	1	1	2	3	1
2	1	3	2	3	1	1	2	3	1
1	3	3	2	1	1	2	1	1	1
1	2	3	1	3	1	3	2	1	3
3	3	3	3	3	3	3	3	3	1
3	2	2	2	2	3	2	2	1	1
2	2	2	2	2	2	2	2	2	2
3	3	3	3	3	3	3	3	3	3
2	1	3	2	3	1	1	2	3	1
1	1	3	9	2	2	9	1	9	9
3	1	3	1	1	1	3	3	3	1
9	9	9	9	9	9	9	9	9	9
2	2	2	2	2	2	2	2	3	2
2	1	2	2	1	3	9	2	1	1
3	2	3	2	3	2	2	3	2	3
9	1	2	3	2	1	2	1	2	1
9	9	9	9	2	9	9	9	9	9
3	3	3	3	3	3	3	3	3	2
3	2	3	3	3	3	3	3	3	1
3	9	2	9	3	1	2	3	3	1
2	1	1	1	2	1	2	2	2	1
2	1	1	1	2	1	2	2	2	1
1	1	2	1	1	1	1	3	1	1
2	1	2	2	1	1	1	1	1	3
2	2	2	2	2	2	2	9	2	2
1	2	2	1	2	1	2	1	2	1
3	1	3	3	1	3	3	3	3	1
3	2	1	2	1	1	1	1	1	9
1	1	1	1	1	1	1	1	9	1
3	3	2	2	3	2	9	2	2	3

2	2	2	2	2	2	2	2	2	1
9	9	9	9	9	9	9	9	9	1
9	9	9	9	9	9	9	9	9	9
3	1	3	1	3	1	3	1	1	1
2	2	2	2	2	2	2	2	2	2
1	1	1	1	1	1	1	1	1	1
1	2	2	2	2	2	2	2	2	1
1	1	1	1	1	1	1	1	1	1
1	1	1	1	1	1	1	1	1	1
2	2	2	2	2	3	3	3	1	9
9	9	9	9	9	9	9	9	9	9
1	1	3	1	2	1	3	3	3	1
9	9	9	9	9	9	9	9	9	9
3	1	3	1	1	1	1	3	2	1
2	1	2	1	3	1	3	3	3	1
9	9	9	9	9	9	9	9	9	9
2	2	2	2	1	1	1	1	2	1
1	9	9	9	9	9	9	9	9	2
1	9	9	9	9	9	9	9	9	2
2	3	1	2	1	2	3	1	2	1
1	1	1	1	1	1	1	3	1	1
2	2	2	2	2	3	3	3	3	2
9	9	9	9	9	9	9	9	9	9
2	2	2	2	2	2	2	2	2	2
3	3	3	3	3	3	3	3	9	3
2	1	3	1	1	2	1	1	3	1
2	1	3	1	1	2	1	1	3	1
2	1	3	3	3	3	3	3	3	1
1	1	1	1	1	1	1	1	1	1
2	2	2	2	2	2	2	2	2	2
2	3	1	3	2	2	1	2	2	2
2	2	1	2	3	3	2	1	2	1
2	1	1	2	2	1	1	2	1	2
2	1	3	1	1	3	1	1	3	1
1	3	2	1	1	3	2	2	1	1
2	2	2	2	2	2	2	2	2	2
2	2	2	2	2	2	2	2	2	1
9	9	9	9	9	9	9	9	9	9
9	9	9	9	9	9	9	9	9	9
2	1	1	1	3	3	1	3	3	1
2	2	2	2	2	2	2	2	2	2
2	2	2	2	2	2	2	2	2	2
2	2	2	2	2	2	2	2	2	2
2	2	2	2	2	2	2	2	2	2
2	2	2	2	2	2	2	2	2	2
2	2	2	2	2	2	2	2	2	2
9	9	9	9	9	9	9	9	9	9

9	9	9	9	9	9	9	9	9	9
2	2	2	2	2	2	2	2	2	2
9	9	9	9	9	9	9	9	9	9
9	9	9	9	9	9	9	9	9	9
1	9	9	9	9	9	9	9	9	9
2	2	2	2	2	2	2	2	2	2
9	9	9	9	9	9	9	9	9	9
3	3	3	3	3	3	3	3	3	3
2	2	3	2	2	1	2	1	1	1
9	2	9	9	9	9	9	9	9	9
2	2	2	2	2	2	2	2	2	2
2	3	2	2	1	2	1	2	1	1
2	3	2	2	1	2	1	2	1	1
2	2	2	2	2	2	2	2	2	2
1	1	1	2	2	2	1	1	1	3
2	2	2	2	2	2	2	2	2	2
2	2	2	2	2	2	2	2	2	2
2	2	2	3	3	3	3	2	1	9
2	2	1	2	2	1	3	3	2	1
3	1	2	3	2	2	2	3	1	3
9	1	9	9	9	9	9	9	9	9
2	2	2	2	2	3	3	3	3	3
2	2	2	2	2	2	2	2	2	2
2	1	3	2	2	2	2	2	2	1
3	3	3	3	3	3	3	3	1	9
3	2	3	2	3	2	3	2	3	2
9	9	9	9	9	9	9	9	9	9
9	1	9	9	9	9	9	9	9	1
2	2	2	2	2	2	2	2	2	2
3	3	3	3	3	3	3	3	2	2
2	2	2	2	2	2	2	2	2	1
2	2	2	2	2	2	2	2	2	1
3	1	1	1	1	3	3	1	3	1
2	2	2	2	2	2	2	2	2	2
2	2	2	2	2	2	2	2	2	2
1	1	1	1	1	1	3	1	1	1
2	2	2	2	2	2	2	2	2	2
2	3	1	3	1	3	2	3	2	1
2	2	2	2	2	2	2	2	2	2
2	2	2	2	2	2	2	2	2	2
2	2	2	2	2	2	2	2	2	9
2	2	2	2	2	2	2	2	2	2
3	3	3	1	2	1	2	3	3	1
2	2	2	2	2	2	2	2	2	2
2	2	2	2	2	2	2	2	2	2
3	3	3	3	3	3	3	3	3	3
2	2	2	2	2	2	2	2	2	2

2	2	2	2	2	2	2	2	2	2
2	2	2	2	2	2	2	2	2	2
2	2	2	2	2	2	2	2	2	2
2	2	2	2	2	2	2	2	2	2
2	2	1	3	2	1	2	1	2	1
1	1	1	2	3	3	2	1	9	2
2	2	2	2	2	2	2	2	2	2
1	2	3	2	1	2	1	1	2	1
2	2	2	2	2	2	2	2	2	2
2	2	2	2	2	2	2	2	2	2
1	1	3	1	3	1	1	1	3	1
9	9	9	9	9	9	9	9	9	2
2	9	9	9	9	9	9	9	9	9
9	9	9	9	9	9	9	9	9	9
2	3	3	3	3	3	3	3	1	1
2	2	3	3	3	1	1	1	2	3
2	2	3	3	3	1	1	1	2	3
1	1	3	1	3	2	3	3	3	1
9	9	9	9	9	9	9	9	9	9
2	1	2	2	3	3	2	3	3	3
9	9	9	9	9	9	9	9	9	9
2	2	2	2	2	2	2	2	2	1
2	2	2	2	2	2	2	2	2	1
2	2	1	1	1	1	1	1	1	9
2	2	2	2	2	2	2	1	1	3
1	9	2	2	2	2	2	2	2	1
2	2	2	2	2	2	2	2	1	2
2	2	2	2	2	2	2	2	3	1
2	2	2	2	2	2	2	2	2	2
3	2	1	2	1	2	2	1	1	1
1	1	1	1	1	1	2	1	9	1
3	3	3	1	1	1	1	1	1	1
2	1	2	1	2	1	1	2	2	9
3	3	3	3	3	3	3	3	3	3
3	1	2	1	1	2	1	3	2	1
3	3	3	1	1	1	1	1	1	1
3	1	3	1	3	3	3	3	3	1
1	1	1	1	1	1	1	1	1	1
2	2	2	2	2	2	2	2	2	2
2	2	2	2	2	2	2	2	2	2
2	3	3	2	1	2	3	1	1	1
1	2	1	9	2	9	2	2	2	1
2	1	2	2	1	2	2	1	1	1
2	2	2	2	2	2	2	2	2	2
2	2	2	2	2	2	2	2	2	2
2	1	2	3	2	3	2	1	2	1
2	2	2	2	2	2	2	2	2	2

1	9	1	1	3	1	2	3	1	1
3	3	3	3	3	3	3	3	3	3
2	3	1	2	3	1	2	3	2	1
9	9	9	9	9	9	9	9	9	9
9	9	9	9	9	1	9	9	9	1
3	1	3	3	2	3	2	1	2	3
2	2	2	2	2	2	2	2	2	2
1	1	1	3	3	3	1	1	2	3
2	2	2	2	2	2	2	9	3	2
2	2	2	2	2	2	2	2	9	2
9	9	9	9	9	9	9	9	9	9
2	2	2	2	3	3	3	3	3	2
2	2	2	2	2	2	2	2	2	2
2	2	2	2	2	2	2	2	2	3
2	2	2	2	2	2	2	2	2	2
2	9	2	2	2	3	3	2	3	2
2	2	2	2	2	2	2	2	2	2
9	9	9	9	9	9	9	9	9	1
9	9	9	2	9	9	9	9	9	9
2	2	2	2	2	2	2	2	2	2
2	2	2	2	2	2	2	2	2	2
2	2	2	2	2	2	2	2	2	2
9	9	9	9	9	9	9	9	9	9
9	9	9	9	9	9	9	9	9	9
2	3	2	2	2	2	2	2	2	1
1	1	1	3	1	3	1	1	3	3
2	1	1	1	1	1	1	1	1	1
3	1	1	1	3	3	2	3	3	1
2	1	2	2	2	2	2	2	2	1
2	9	2	9	1	2	3	1	3	1
2	2	1	1	1	2	1	2	3	1
3	1	2	3	1	2	1	3	1	2
3	3	2	1	3	3	1	3	3	1
3	3	3	3	3	3	3	3	3	3
2	2	2	2	2	2	2	2	9	9
2	2	3	3	3	2	1	3	1	2
1	1	2	1	1	2	1	1	1	1
3	3	3	3	3	3	3	3	3	3
9	3	2	1	9	2	1	9	1	2
3	3	3	3	3	3	3	3	3	3
1	1	1	1	3	1	9	3	3	1
1	2	2	3	3	1	1	3	2	1
1	1	1	1	1	1	1	1	1	3
2	2	2	2	2	2	2	2	2	2
2	1	1	1	2	2	3	3	3	1
3	1	1	2	1	1	3	1	2	1
3	3	2	2	2	2	2	2	2	1

2	2	2	2	2	2	2	2	2	2
3	1	2	1	3	3	3	3	1	2
2	1	3	1	1	1	3	1	1	9
1	1	1	1	1	1	1	1	1	1
2	2	2	2	2	2	2	2	2	2
2	3	2	2	2	2	2	2	2	2
2	2	2	2	2	2	2	2	2	2
2	2	2	2	2	2	2	2	2	2
1	1	1	1	1	1	1	1	1	2
3	2	3	2	2	2	3	2	2	1
1	9	9	9	9	9	9	9	9	1
2	2	2	2	2	2	2	2	2	1
1	1	3	3	3	3	3	3	3	3
1	1	1	2	2	3	2	1	2	1
1	2	1	1	1	3	1	1	1	1
1	1	1	3	2	2	2	2	2	2
3	1	3	1	1	1	3	3	3	1
2	1	2	2	2	2	2	2	2	1
2	1	3	3	3	2	2	3	3	1
9	9	9	1	9	9	9	9	9	1
1	1	3	1	1	1	1	1	1	1
1	3	3	3	3	3	3	3	2	1
3	1	3	1	3	1	1	3	3	1
1	1	1	1	1	1	3	1	1	1
3	3	3	3	3	3	3	3	3	3
3	3	3	3	3	3	3	3	3	3
2	1	2	1	2	1	2	1	1	1
2	3	1	2	1	2	1	2	2	2
1	3	1	2	1	2	3	2	2	1
1	9	1	2	2	1	1	1	1	1
2	2	2	2	2	2	2	2	2	9
9	9	9	9	9	9	9	9	9	9
2	2	1	1	1	2	1	2	2	1
9	9	9	9	9	9	9	9	9	9
2	1	2	2	1	1	2	2	2	2
2	2	2	2	2	2	2	2	2	2
2	2	2	2	2	2	2	2	2	2
2	2	2	2	2	2	2	2	2	1
3	3	2	2	2	3	3	2	3	2
2	2	3	2	1	2	3	2	3	9
2	1	2	2	1	2	3	3	3	1
2	2	2	2	2	2	2	2	2	2
2	3	3	2	1	1	1	2	2	1
9	9	9	9	9	9	9	9	9	9
3	3	3	3	3	3	3	3	3	3
2	2	2	2	2	2	2	2	2	2
2	9	1	1	2	2	2	2	2	9

9	9	9	9	9	9	9	9	9	9
1	1	2	2	2	2	2	2	2	2
1	2	3	2	1	2	2	1	1	1
2	1	1	2	3	3	2	1	3	1
2	2	2	2	2	2	2	2	2	2
2	2	2	2	3	2	2	2	2	9
9	9	9	9	9	9	9	9	9	9
2	2	2	2	3	3	2	3	3	1
2	2	2	2	2	2	2	2	2	2
9	9	9	9	9	9	9	9	9	9
9	9	9	9	9	9	9	9	9	9
9	9	9	9	9	9	9	9	9	9
9	9	1	9	9	9	9	9	9	9
9	9	9	9	9	9	9	9	9	2
9	9	9	9	9	9	9	9	9	9
1	1	2	2	3	1	2	3	3	2
2	2	2	2	2	2	2	2	2	2
2	2	2	2	2	2	2	2	2	2
2	9	1	3	2	1	3	1	2	1
2	1	2	1	1	2	3	1	1	2
2	1	2	3	2	2	2	2	2	1
2	2	1	2	2	2	2	3	2	2
2	2	2	2	1	2	2	2	2	1
3	1	1	3	3	1	3	3	3	1
2	2	2	2	2	2	2	2	2	2
2	2	2	2	2	1	2	3	2	2
9	9	9	9	9	9	9	9	9	9
1	1	1	1	1	1	1	1	1	1
2	2	2	2	2	1	2	3	2	2
1	1	2	2	3	1	2	3	3	2
2	1	2	1	1	2	3	1	1	2
9	9	1	9	9	9	9	9	9	9
1	1	1	1	1	3	3	1	1	1
2	2	2	2	2	2	2	2	2	2
2	2	2	2	2	2	2	2	2	2
2	2	2	2	2	2	2	2	2	2
2	2	2	2	2	2	2	2	2	9
9	9	9	9	9	9	9	9	3	1
2	2	2	2	2	2	2	2	2	2
2	2	2	2	2	2	2	2	2	2
2	2	2	2	2	2	2	2	2	2
2	2	2	2	2	2	2	2	2	9
2	2	2	2	2	2	2	2	2	2
3	3	2	2	2	2	2	2	2	1
1	2	2	2	2	2	2	2	2	9
2	2	2	2	2	2	2	2	2	2
9	9	9	1	9	9	9	9	9	1

2	2	2	2	2	2	2	2	2	3
2	2	2	2	2	2	2	2	2	1
9	9	9	1	9	9	9	9	9	9
9	9	9	9	9	9	9	9	9	1
2	2	2	2	2	2	2	2	2	1
9	9	9	9	9	9	9	9	9	1
1	1	2	2	2	2	2	2	3	1
2	1	2	3	2	3	1	2	1	1
2	3	2	2	1	3	2	1	9	1
2	3	2	1	2	3	2	1	2	1
9	2	2	2	2	2	2	2	2	3
2	1	1	2	2	1	2	1	3	1
1	1	1	2	1	2	2	2	1	2
2	2	2	2	2	2	2	2	2	9
9	9	9	9	9	9	9	9	9	9
3	3	3	3	3	3	3	3	3	3
2	2	2	2	2	2	2	2	2	2
2	2	2	2	2	2	2	2	2	2
2	1	1	2	2	3	1	2	3	1
1	1	3	1	2	2	3	3	3	1
3	1	1	1	3	1	3	1	3	1
2	2	2	2	2	2	2	2	2	2
3	1	1	1	3	1	3	3	3	1
2	2	2	2	2	2	2	2	2	2
2	2	2	2	2	2	2	2	2	2
2	1	2	1	2	1	2	2	3	1
2	2	2	2	2	2	2	2	2	2
2	2	2	2	2	2	2	2	2	2
1	1	1	2	1	1	2	2	2	1
9	9	9	9	9	9	9	9	9	9
9	9	9	9	9	9	9	9	9	1
2	2	2	2	2	2	2	2	2	2
9	9	9	9	9	9	9	9	9	9
9	9	9	9	9	9	9	9	9	9
2	1	2	3	2	2	2	3	3	1
9	9	9	9	9	9	9	9	9	9
9	9	9	9	9	9	9	9	9	9
2	2	2	2	2	2	2	2	2	2
1	1	3	1	2	2	3	3	3	1
1	1	2	2	2	2	2	2	1	1
1	1	1	1	1	1	1	1	1	1
2	3	1	3	1	2	2	1	3	2
9	9	9	9	9	9	9	9	9	2
2	1	2	2	3	2	2	3	1	1
3	1	3	2	1	2	3	1	2	9
9	9	9	9	9	9	9	9	9	9
2	1	2	1	2	1	1	3	3	2

2	1	2	2	1	1	9	1	1	1
9	9	9	9	9	9	9	9	9	9
9	9	9	9	9	9	9	9	9	9
2	2	2	2	2	2	2	2	2	2
1	1	3	3	1	1	1	1	1	1
2	1	2	2	1	1	9	1	1	1
2	9	1	3	9	1	3	1	2	1
3	1	1	1	3	3	3	3	3	1
2	2	2	2	2	2	2	2	2	2
3	1	1	1	3	3	3	3	3	2
9	9	9	9	9	9	9	9	9	9
9	9	9	9	9	9	9	9	9	9
1	9	9	9	9	9	9	9	9	1
9	2	2	2	2	9	1	1	9	9
3	2	2	2	2	2	2	2	2	1
9	9	9	9	9	9	9	9	9	9
9	9	9	9	9	9	9	9	9	9
9	9	9	9	9	9	9	9	9	9
1	2	2	2	2	2	2	2	2	1
2	2	2	2	2	2	2	2	2	1
9	9	9	9	9	9	9	9	9	9
1	1	1	1	3	3	3	2	1	1
3	3	3	3	3	3	3	2	9	2
9	9	9	9	9	9	9	9	9	9
2	2	2	2	2	2	2	2	2	2
2	2	2	2	2	2	2	2	9	2
9	9	9	9	9	9	9	9	9	9
9	9	9	9	9	9	9	9	9	9
2	3	2	1	2	3	3	3	3	3
2	2	2	2	2	2	2	2	2	2
1	1	1	1	1	1	1	1	1	1
9	9	9	9	9	9	9	9	9	9
9	9	9	9	9	9	9	9	9	9
2	1	3	3	1	2	2	2	2	2
2	2	3	3	3	3	2	2	9	2
2	2	2	2	2	2	2	2	2	2
2	3	3	3	3	3	3	2	2	2
2	2	2	2	2	2	2	2	2	2
2	1	9	2	1	2	1	2	2	1
2	1	2	2	1	2	1	2	1	1
2	2	3	3	1	1	1	1	2	2
9	9	9	9	9	9	9	9	9	9
9	9	9	9	9	9	9	9	9	9
9	9	9	9	1	9	9	9	9	9
9	9	9	9	9	9	9	9	9	9
9	9	9	9	9	9	9	9	9	9
9	9	9	9	9	9	9	9	9	9

2	2	2	2	2	2	2	2	2	2
3	3	2	3	2	3	2	3	2	3
1	1	3	1	3	3	3	3	3	1
2	2	2	2	2	2	2	2	2	1
9	9	9	9	9	9	9	9	9	9
9	9	9	9	9	9	9	9	9	9
9	9	9	9	9	9	9	9	9	9
9	9	9	9	9	9	9	9	9	9
9	9	9	9	9	9	9	9	9	9
9	9	9	9	9	9	9	9	9	9
9	9	9	9	9	9	9	9	9	9
9	9	9	9	9	9	9	9	9	9
9	9	9	9	9	9	9	9	9	9
3	3	1	1	1	3	3	1	1	1
9	9	9	9	9	9	9	9	9	9
2	1	1	1	3	1	2	3	2	2
2	2	2	2	2	2	2	2	2	2
9	9	9	9	9	9	9	9	9	9
9	9	9	9	9	9	9	9	9	9
9	9	9	9	9	9	9	9	9	9
9	9	1	9	9	9	9	9	9	9
9	9	9	9	9	9	9	9	9	9
2	2	2	1	1	1	1	1	1	1
9	9	9	9	9	9	9	9	9	9
1	1	1	1	1	1	1	1	1	2
3	3	3	1	1	3	3	3	3	1
1	1	2	2	2	3	3	3	3	2
1	1	1	2	2	2	1	1	1	1
2	1	1	1	1	1	1	1	2	1
1	1	1	1	2	2	1	1	1	1
2	2	2	2	2	2	2	2	2	2
1	1	1	1	1	1	1	1	1	1
1	1	1	1	1	1	1	1	1	1
9	9	9	9	9	9	9	9	9	9
1	1	2	2	2	1	1	1	1	1
1	1	1	2	1	1	2	2	2	2
1	1	1	1	1	1	1	1	1	1
9	9	9	9	9	9	9	9	9	9
2	2	2	2	2	3	3	3	3	1
2	2	2	1	2	3	3	1	1	1
3	3	3	3	3	3	3	3	3	1
9	9	9	9	9	9	9	9	9	9
9	9	9	9	9	9	9	9	9	9
9	9	9	9	9	9	9	9	9	9
9	9	9	9	9	9	9	9	9	9
2	2	2	2	2	2	2	2	2	1
9	9	9	9	9	9	9	9	9	9

9	9	9	9	9	9	9	9	9	9
9	9	9	9	9	9	9	9	9	9
9	9	9	9	9	9	9	9	9	9
9	9	9	9	9	9	9	9	9	9
9	9	9	9	9	9	9	9	9	9
9	9	9	9	9	9	9	9	9	9
9	9	9	9	9	9	9	9	9	9
9	9	9	9	9	9	9	9	9	9
9	9	9	9	9	9	9	9	9	9
9	9	9	9	9	9	9	9	9	9
9	9	1	9	9	9	9	9	9	9
9	9	9	9	9	9	9	9	9	9
2	2	1	2	1	1	1	1	1	1
2	2	2	2	2	2	1	1	1	1
2	2	1	1	1	1	2	2	2	1
3	2	2	1	1	1	2	2	2	1
1	1	2	2	2	2	2	2	2	2
1	1	1	1	1	1	2	1	1	2
2	2	1	1	1	1	1	1	1	1
9	9	9	9	9	9	9	9	9	1
1	1	1	1	1	2	2	2	1	1
3	1	2	2	3	3	3	3	3	1
2	2	2	2	2	2	2	2	2	2
2	2	1	2	2	1	1	1	1	1
2	1	1	2	1	2	1	2	1	1
3	3	3	3	3	3	3	3	3	1
9	1	3	1	2	2	2	9	2	1
2	2	2	2	2	1	1	1	1	2
9	1	9	9	9	9	9	9	9	1
9	9	9	9	9	9	9	9	9	9
9	9	9	9	9	9	9	9	9	9
1	1	1	1	2	2	2	1	1	1
2	1	2	1	1	1	2	2	2	2
1	1	1	1	1	1	1	1	1	2
2	2	1	1	1	1	1	1	1	1
2	2	2	2	2	2	2	2	2	2
2	1	1	2	2	2	2	2	2	1
1	1	1	1	1	1	1	1	1	2
1	2	2	2	1	1	1	1	1	2
1	1	1	1	1	1	1	1	1	1
2	2	2	2	2	2	2	2	2	2
1	2	2	2	1	1	1	1	1	1
1	1	3	1	1	1	3	3	3	1
1	2	2	2	2	1	2	2	3	1
2	2	2	2	1	1	1	1	1	1
2	1	1	1	1	1	1	1	1	1
2	2	2	1	3	2	2	2	3	1

9	9	9	9	9	9	9	9	9	9
2	2	2	2	2	2	2	2	2	1
9	9	9	9	2	9	9	9	9	9
9	9	9	1	9	9	9	9	9	9
1	9	9	9	9	9	9	9	9	9
9	9	9	9	9	9	9	9	9	9
9	9	9	9	9	9	9	9	9	9
2	3	3	3	3	3	3	2	2	1
9	9	9	9	9	9	9	9	2	2
2	2	2	2	2	2	2	2	2	2
9	9	9	9	9	9	9	9	9	9
9	9	9	9	9	9	9	9	9	9
1	1	1	1	1	2	2	2	1	1
9	9	9	9	9	9	9	9	9	9
1	1	1	1	1	1	1	1	1	1
9	9	9	9	9	9	9	9	9	9
2	2	2	2	2	2	2	2	2	2
3	3	3	3	1	3	3	3	3	3
9	9	9	9	9	9	9	9	9	9
2	2	2	2	2	2	2	2	2	2
2	2	2	2	2	2	2	2	2	2
2	2	2	2	2	2	2	3	2	2
2	2	2	2	2	2	2	2	2	2
2	2	2	2	2	2	2	2	2	2
1	1	1	1	1	1	1	1	2	2
2	2	2	2	2	2	2	2	2	2
2	2	9	2	3	1	2	1	2	1
2	1	2	1	2	1	2	2	1	1
2	2	2	2	2	2	2	2	2	2
1	1	1	1	1	1	1	2	2	1
9	9	9	9	9	9	9	9	9	9
3	2	3	2	3	2	2	3	9	2
9	9	9	9	9	9	9	9	9	9
2	2	2	2	2	2	2	2	2	2
3	2	2	2	2	2	2	2	2	2
1	1	1	1	1	1	1	1	1	2
1	2	2	1	1	2	2	2	2	1
1	1	1	1	1	1	1	1	1	1
1	1	1	1	1	1	1	1	1	1
1	1	1	1	1	1	1	1	1	9
2	2	2	2	2	2	2	2	2	1
2	2	1	1	1	2	2	2	2	1
9	9	9	9	9	9	9	9	9	9
9	9	9	9	9	9	9	9	9	9
1	9	9	9	9	9	9	9	9	1
9	9	9	9	9	9	9	9	9	9
9	9	9	9	9	9	9	9	9	9

9	9	9	9	9	9	9	9	9	9
2	2	2	2	2	2	2	2	2	2
9	1	9	9	9	9	9	9	9	1
9	9	9	9	9	9	9	9	9	9
9	9	9	9	9	9	9	9	9	9
2	9	1	2	1	2	3	1	2	2
3	1	2	1	3	3	3	3	3	1
9	9	9	9	9	9	9	9	9	9
9	9	9	9	9	9	9	9	9	9
9	9	9	9	9	9	9	9	9	9
9	1	9	9	9	9	9	9	3	1
9	9	9	9	9	9	9	9	9	9
2	2	2	2	2	2	2	2	2	2
9	9	9	9	9	9	9	9	9	9
9	9	9	9	9	9	9	1	9	9
1	2	2	3	3	3	2	2	3	1
2	1	2	2	3	1	2	2	2	2
3	1	3	1	3	3	3	3	3	1
1	1	1	1	2	2	2	2	2	1
2	1	1	1	3	3	2	2	3	1
2	3	3	2	1	2	3	3	1	1
3	1	1	3	1	1	3	1	1	1
1	9	1	2	1	2	2	1	2	1
2	2	2	3	2	3	3	2	9	9
2	2	2	2	2	2	2	2	2	2
2	2	2	2	1	1	1	1	2	1
2	9	9	9	9	9	9	9	9	9
2	2	2	2	1	1	1	3	3	1
9	9	9	9	9	9	9	9	9	2
1	2	2	2	2	2	2	2	2	1
3	3	3	3	3	3	3	3	1	2
2	2	2	2	2	2	3	2	2	2
9	9	9	9	9	9	9	9	9	9
3	2	3	2	2	3	9	2	3	2
3	1	3	1	3	3	3	1	3	1
2	2	2	2	2	2	2	2	2	1
2	2	2	2	2	2	2	2	2	2
1	3	2	1	1	3	3	2	2	1
9	9	1	9	9	9	9	9	9	9
2	3	2	1	2	3	2	1	2	9
9	9	9	9	9	2	9	9	9	9
9	9	9	9	9	9	9	9	9	1
2	2	2	2	2	2	2	2	3	1
9	9	9	9	9	9	9	9	9	9
1	1	1	1	1	1	1	1	1	1
1	3	3	1	1	3	1	1	3	1
3	3	3	3	3	3	3	3	3	3

3	3	3	3	3	3	3	3	3	3
9	9	9	9	9	9	9	9	9	9
2	2	2	2	2	2	2	1	2	2
1	1	1	1	1	3	3	3	3	1
3	1	3	1	3	3	3	3	3	1
1	3	1	1	3	1	3	1	1	1
3	1	3	1	3	1	2	3	1	1
3	9	2	1	2	1	3	2	3	9
2	2	2	2	2	3	2	3	2	2
3	9	2	3	2	3	2	3	2	2
2	2	2	2	3	3	1	3	1	1
1	2	3	2	1	2	3	2	1	1
1	2	3	1	2	2	3	2	2	2
2	3	1	2	1	2	2	2	2	1
2	2	3	2	1	2	2	2	2	1
2	1	2	3	3	2	1	2	3	1
2	2	2	2	2	2	2	2	2	2
1	2	1	2	1	2	2	1	2	1
2	2	2	2	2	2	2	2	2	2
2	2	2	2	2	2	2	2	2	2
3	9	1	1	3	3	3	3	3	1
2	2	2	1	1	1	3	3	3	1
9	9	9	9	9	9	9	9	9	3
9	9	9	9	9	9	9	9	9	9
3	2	2	2	1	1	1	1	1	2
1	1	1	2	1	2	1	2	2	2
2	3	3	2	1	2	1	2	1	9
3	3	2	2	2	2	1	1	2	1
2	9	1	1	1	1	3	3	1	1
9	1	9	9	9	9	9	9	9	9
3	3	1	2	3	2	1	3	3	1
2	2	2	2	2	2	2	2	2	3
2	3	3	1	2	1	2	3	3	2
2	1	1	1	3	1	3	2	2	1
2	3	1	1	1	1	1	1	3	9
2	2	2	2	2	2	2	2	2	2
2	2	2	2	2	2	2	2	2	2
9	9	9	9	9	9	9	9	9	9
2	2	2	1	1	2	3	3	3	2
2	2	2	2	2	2	2	2	2	1
9	9	9	9	9	9	9	9	9	9
9	9	9	9	9	9	9	9	9	9
3	2	2	2	2	2	1	1	2	1
9	9	9	9	9	9	9	9	9	9
2	2	2	2	2	2	2	2	2	2
3	1	1	1	1	3	1	3	3	2
9	9	9	9	9	9	9	9	9	9

3	2	3	2	2	2	3	2	2	2
9	9	9	9	9	9	9	9	9	9
3	3	3	3	3	3	3	3	3	1
9	9	9	9	9	9	9	9	9	9
9	9	9	9	9	9	9	9	9	9
2	2	2	2	2	2	2	2	2	2
3	1	1	2	3	3	3	3	3	1
2	2	2	2	2	2	2	2	2	2
2	2	2	2	2	2	2	2	2	2
2	2	2	2	2	2	2	2	2	2
9	9	9	9	9	9	9	9	9	9
3	1	3	1	3	3	2	1	1	1
9	9	9	9	9	9	9	9	9	9
1	1	1	2	2	1	1	1	1	1
2	2	2	2	2	2	2	2	2	2
3	3	3	2	2	2	1	1	1	2
9	9	9	9	9	9	9	9	9	2
9	9	9	9	9	9	9	9	9	9
1	1	1	1	1	1	3	3	2	3
3	3	2	2	2	2	3	3	3	3
1	1	1	2	2	1	1	1	1	2
1	1	2	3	3	3	3	3	3	1
9	9	9	9	9	9	9	9	9	9
2	2	2	3	3	3	3	2	2	3
3	2	3	3	3	3	3	3	1	3
2	2	2	2	2	2	3	3	3	2
9	9	9	9	9	9	9	9	9	1
1	1	1	1	1	1	1	1	1	1
2	2	2	2	2	2	2	2	2	2
9	9	9	9	9	9	9	9	9	1
2	3	3	2	1	1	2	3	2	1
2	2	2	2	2	2	2	2	3	1
1	1	1	3	3	3	2	2	1	1
2	2	2	2	2	2	2	2	2	2
2	2	2	2	2	2	2	2	2	2
2	1	2	2	3	1	1	2	3	1
2	1	1	2	2	2	2	3	1	2
1	1	3	2	2	2	2	1	3	1
2	2	2	2	2	2	2	2	2	1
3	3	3	3	3	3	3	3	3	3
1	1	3	1	1	2	3	2	3	1
3	3	1	3	3	3	3	3	3	1
2	1	2	2	3	1	3	2	3	9
2	2	2	2	2	2	2	2	2	2
2	2	2	2	2	2	2	2	2	2
2	2	2	2	2	2	2	2	2	2
2	1	1	3	2	3	1	2	1	1

1	1	2	2	2	2	2	2	2	2
2	2	2	2	2	2	3	2	3	1
1	1	1	2	1	2	1	1	1	1
2	2	2	2	2	2	2	2	2	2
2	2	2	2	3	2	3	2	1	2
1	9	9	9	9	9	9	9	9	1
2	2	2	2	2	2	2	2	2	2
2	2	2	1	1	2	1	1	1	1
2	2	2	2	2	2	2	2	2	2
2	1	2	3	3	2	2	2	2	1
2	2	2	2	2	2	3	2	2	9
1	2	3	1	2	3	1	2	3	1
2	2	2	2	2	2	2	2	2	2
3	3	3	3	3	3	3	3	3	3
3	2	2	2	2	2	2	3	2	2
2	2	1	2	2	1	2	1	2	1
3	2	3	1	2	1	2	3	2	3
1	3	2	3	1	2	3	2	2	1
2	2	2	2	2	2	2	2	2	2
2	1	1	1	1	2	2	2	1	2
2	2	2	2	2	2	2	2	2	1
2	2	2	2	2	2	2	2	2	3
2	2	2	2	1	1	1	3	3	1
2	2	2	2	2	2	2	2	2	1
2	1	2	2	2	2	2	2	2	1
2	1	2	2	2	3	2	2	2	2
2	1	1	1	3	2	3	1	1	2
2	1	1	2	2	3	3	1	1	2
2	2	2	2	2	2	2	2	2	2
1	2	2	2	1	1	1	1	1	1
3	3	3	2	3	2	3	2	2	2
3	3	3	2	2	3	2	3	2	3
1	1	1	1	1	2	3	3	3	1
2	2	2	2	2	2	2	2	2	2
1	1	1	2	2	1	1	1	1	1
2	2	2	2	2	2	2	2	2	2
2	3	3	3	3	3	3	3	1	2
2	2	1	1	1	1	1	2	3	2
2	2	2	2	2	2	2	2	2	2
2	2	2	2	2	2	2	2	2	2
2	3	1	2	2	1	3	2	2	1
2	1	1	2	1	2	1	3	3	2
2	2	2	2	2	2	2	2	9	2
2	2	2	2	2	2	2	2	2	2
2	1	2	2	3	3	1	2	3	2
1	1	1	1	1	1	3	3	3	1
9	9	9	9	9	9	9	9	9	9

1	2	1	2	3	2	2	3	1	1
2	2	2	2	2	3	9	3	1	1
2	1	3	2	3	3	3	3	3	1
2	2	2	2	2	2	2	2	2	2
2	2	2	2	2	2	2	2	2	2
2	2	2	2	2	2	2	2	2	2
3	1	2	2	1	2	2	3	2	1
2	2	2	3	3	1	2	1	2	1
2	2	2	2	2	2	2	2	1	2
2	2	2	2	2	2	2	2	3	2
2	2	2	2	2	2	2	2	2	2
9	9	9	9	9	9	9	9	9	9
9	9	9	9	9	9	9	9	9	9
2	2	2	2	2	2	2	2	2	3
2	1	1	2	2	1	2	1	1	2
2	1	2	2	2	2	2	2	2	1
1	1	1	1	3	3	3	3	3	1
2	1	1	1	1	2	2	2	2	1
1	1	2	2	2	2	3	3	3	1
2	1	3	1	3	3	2	2	3	1
2	2	2	2	2	2	2	2	2	9
2	2	2	2	2	2	2	2	2	2
2	2	2	2	2	2	2	2	3	2
2	2	2	2	2	2	2	2	2	2
2	1	1	1	2	2	2	2	2	1
2	2	2	2	2	2	2	2	2	1
2	2	2	2	2	2	2	2	1	2
2	2	2	2	2	2	2	2	2	2
2	2	2	2	2	2	2	2	2	1
9	9	9	9	9	9	9	9	9	9
2	2	2	2	3	3	3	3	3	3
2	2	2	2	2	2	2	2	2	9
2	2	2	2	2	2	3	2	3	2
2	2	2	2	2	2	2	2	2	3
2	2	1	2	2	2	2	2	2	2
2	2	2	2	2	2	2	2	2	2
2	2	2	2	2	2	2	2	2	9
2	2	2	2	2	2	2	2	2	2
2	2	2	2	2	2	2	2	2	2
2	2	2	2	2	2	2	2	2	1
2	2	2	2	2	2	2	2	2	2
2	2	2	2	2	2	2	2	2	2
2	2	2	2	2	2	2	2	2	2
2	1	2	2	3	1	2	2	3	2
2	2	2	2	2	2	2	2	2	2
2	2	2	2	2	2	2	2	2	2
2	2	2	2	2	2	2	2	2	2

2	3	3	3	1	2	2	1	2	2
2	2	2	2	2	2	2	2	2	1
2	1	2	1	3	1	3	3	3	1
2	2	2	2	2	2	2	2	2	2
2	2	2	2	2	2	2	2	2	2
2	2	2	2	2	2	2	2	2	3
9	9	9	9	9	9	9	9	9	9
3	1	3	1	3	3	3	3	3	1
1	1	1	2	1	1	1	1	2	1
2	1	2	2	3	3	2	3	3	1
2	2	2	2	2	2	2	2	2	1
2	2	2	2	2	2	2	2	2	9
2	2	2	2	2	2	2	2	2	2
2	2	2	2	2	2	2	2	2	1
2	9	9	9	9	9	9	9	9	1
2	2	2	2	2	2	2	2	2	9
2	2	2	2	2	2	2	2	2	9
2	1	1	3	3	1	3	2	3	1
1	1	1	1	1	1	1	1	1	1
2	2	2	2	2	2	2	2	3	9
1	1	1	1	1	2	2	1	1	1
2	2	2	3	3	3	1	1	2	2
2	2	2	2	2	2	2	2	2	2
2	2	2	2	2	2	2	2	2	2
2	2	2	2	2	2	2	2	9	2
2	2	2	2	2	2	2	2	2	2
9	9	9	9	9	9	9	9	9	9
2	2	2	2	2	2	2	2	2	1
2	2	2	2	2	2	2	2	2	9
2	2	2	2	2	2	2	2	2	9
2	2	2	2	2	2	2	2	2	2
2	2	2	2	2	2	2	1	1	1
9	9	9	9	9	9	9	9	9	9
9	9	9	9	9	9	9	9	9	9
9	9	9	9	9	9	9	9	9	9
9	9	9	9	9	9	9	9	9	9
1	1	1	1	2	1	1	1	1	1
2	1	2	2	3	3	3	3	3	1
2	1	2	1	3	1	3	1	3	9
3	3	2	3	2	2	3	2	2	2
1	1	2	3	2	3	3	3	3	2
2	1	2	2	2	2	2	2	2	2
2	2	2	2	2	2	2	2	2	1
2	2	2	2	2	2	2	2	2	2
2	2	2	2	2	2	2	2	2	1
2	2	2	2	2	2	2	2	2	1
1	2	1	2	1	2	1	3	1	1

1	3	2	3	2	1	2	1	1	1
3	2	1	1	2	2	1	2	1	1
3	1	1	2	1	2	2	1	1	2
1	1	1	1	1	1	1	1	1	2
2	1	3	1	2	1	1	2	1	3
2	3	3	2	1	2	3	2	1	1
3	1	1	1	3	1	2	2	3	1
9	9	9	9	9	9	9	9	2	2
2	2	2	2	2	2	2	2	2	2
2	2	2	2	2	2	2	2	2	2
2	2	2	2	2	2	2	2	2	1
2	2	2	2	2	2	2	2	2	1
2	2	2	2	2	2	2	2	2	2
2	2	2	2	2	2	2	2	1	9
2	2	2	2	2	2	2	2	2	2
2	2	2	2	2	2	2	2	2	2
2	2	2	2	2	2	2	2	2	2
3	3	2	1	1	3	2	3	2	1
2	1	1	1	1	1	3	3	2	1
2	1	1	2	3	2	3	3	3	1
3	1	3	2	3	3	3	2	3	1
3	1	3	1	2	2	3	3	2	1
1	1	1	1	3	3	3	3	3	1
9	9	9	9	9	9	9	9	3	9
3	3	3	3	3	3	3	3	3	3
1	1	3	1	1	1	1	1	3	1
2	2	2	2	2	2	2	2	2	1
2	1	1	1	1	1	1	2	2	1
2	2	2	2	2	2	2	2	2	1
9	1	9	9	9	9	9	9	9	9
2	1	3	3	2	2	2	3	3	3
9	9	9	9	9	9	9	9	9	9
9	9	9	9	9	9	9	9	9	9
9	9	9	9	9	9	9	9	9	9
9	9	9	9	9	9	9	9	9	9
2	2	2	2	2	2	2	2	2	2
1	2	2	1	2	9	1	1	1	1
9	9	9	9	9	9	9	9	9	9
2	2	2	2	2	2	2	2	2	1
2	2	2	2	2	2	2	2	2	1
9	9	9	9	9	9	9	9	9	9
3	3	3	3	3	3	3	3	2	3
2	2	2	2	2	2	2	2	2	2
9	9	9	9	9	9	9	9	9	3
9	9	9	9	9	9	9	9	9	9
1	1	1	1	1	1	2	2	2	2
3	3	3	3	3	3	3	3	3	2

1	1	1	1	1	1	1	1	1	1
1	1	1	1	3	3	3	3	3	1
2	2	1	1	1	2	1	1	1	3
2	2	2	2	2	2	2	2	2	2
9	9	9	9	9	9	9	9	9	9
9	9	9	9	9	9	9	9	9	9
2	2	2	2	2	2	2	2	2	2
2	2	2	2	3	3	3	3	2	2
9	9	9	9	9	9	9	9	9	1
9	9	9	9	9	9	9	9	9	9
1	1	3	3	2	1	1	1	1	1
1	1	2	2	1	1	2	1	1	2
3	1	1	1	1	2	2	2	1	9
3	3	3	3	3	3	3	3	3	3
9	9	9	9	9	9	9	9	9	1
2	1	3	3	3	2	1	3	3	2
3	1	1	2	3	3	2	3	3	1
2	2	2	2	2	2	2	2	2	1
9	9	1	9	9	9	9	9	9	9
3	1	1	1	2	1	3	3	2	2
2	3	1	3	1	2	2	1	3	1
3	2	3	2	2	2	2	2	2	1
1	3	1	1	2	1	3	1	2	1
2	1	1	1	2	1	3	2	2	1
3	3	3	3	3	3	3	3	3	1
2	1	2	2	2	2	2	2	2	2
3	2	2	1	2	2	1	3	3	9
3	1	2	1	9	1	3	3	3	1
3	2	1	2	1	3	2	2	2	2
3	1	3	1	3	3	3	3	3	3
2	2	2	2	2	2	2	2	2	2
2	1	1	1	1	1	1	1	3	1
2	3	2	1	2	3	2	1	2	1
3	3	3	1	3	3	1	1	3	3
9	9	9	9	1	9	9	9	9	9
1	1	2	2	1	1	2	2	3	1
3	3	3	3	3	9	9	9	9	1
2	2	2	2	2	2	2	2	2	2
9	9	9	9	9	9	9	9	9	9
9	9	9	9	9	9	9	9	9	9
9	9	9	9	9	9	9	9	9	9
9	1	2	2	3	2	1	2	2	2
1	2	1	2	1	2	2	3	1	3
1	1	2	2	1	1	2	3	3	1
1	9	1	2	2	1	2	3	3	1
2	3	3	2	2	3	2	1	2	3
3	3	3	2	2	3	3	2	3	3

2	2	2	2	2	2	2	2	2	2
9	9	9	9	9	9	9	9	9	9
1	1	3	1	3	1	3	2	3	1
3	3	3	3	3	3	3	3	2	3
3	1	3	1	3	1	1	3	3	9
9	9	9	9	9	9	9	9	9	9
9	9	9	9	9	9	9	9	9	1
2	1	2	2	2	3	3	2	2	9
1	9	9	1	2	9	9	1	2	1
2	2	2	2	2	2	2	2	2	2
2	2	1	1	1	1	1	2	2	2
2	2	2	2	2	3	2	1	9	2
2	2	2	2	2	3	2	3	2	2
1	1	1	1	3	3	3	3	3	1
9	9	9	9	9	9	9	9	9	9
3	1	1	3	3	3	1	3	3	1
2	2	2	2	2	2	2	2	9	2
2	2	2	2	2	2	2	2	2	2
2	3	2	3	3	3	1	2	1	1
2	1	3	2	1	2	1	3	3	1
9	9	9	9	9	9	9	9	9	9
9	9	9	9	2	9	9	9	9	1
2	2	2	1	2	2	2	2	2	2
9	9	9	9	9	9	9	9	9	9
9	9	9	9	9	9	9	9	9	9
2	2	3	3	3	3	2	3	2	3
2	2	2	2	2	2	2	2	2	3
2	1	2	2	3	3	3	3	3	1
3	3	3	3	3	3	3	3	3	3
1	1	1	1	2	1	3	2	3	1
3	3	3	3	3	3	3	3	3	3
1	1	3	3	3	3	3	3	3	1
9	9	9	9	9	9	9	9	9	9
9	9	9	9	9	9	9	9	9	9
9	9	9	9	9	9	9	9	9	9
1	1	1	2	1	2	3	1	3	1
2	1	1	1	2	1	2	1	2	1
2	3	1	2	2	3	1	2	2	1
1	1	3	1	3	1	3	3	3	1
9	9	9	9	9	9	9	9	9	2
3	2	2	2	2	2	2	2	2	2
2	3	3	1	2	3	2	3	2	3
3	3	3	3	3	3	3	3	3	3
9	9	9	9	9	9	9	9	9	9
2	3	2	3	1	2	2	1	1	9
9	9	9	1	9	9	9	9	3	1
2	1	1	2	1	2	3	2	2	2

1	2	1	2	1	2	1	2	1	1
2	2	2	3	3	3	2	2	2	2
1	1	9	2	1	3	1	3	3	3
2	3	1	2	3	1	2	3	1	1
3	1	3	1	3	3	3	3	3	1
2	1	2	1	1	2	2	2	2	1
2	1	2	1	2	1	2	1	1	2
1	1	3	1	3	2	3	3	3	1
9	9	9	9	9	9	9	9	9	9
2	2	2	2	2	2	2	2	2	1
2	2	2	2	2	2	2	2	2	3
2	2	2	2	2	2	2	2	2	2
2	1	3	1	2	3	2	3	3	1
2	1	1	1	3	2	1	2	3	1
2	9	9	9	9	9	9	9	9	2
1	2	2	1	2	1	1	2	1	1
1	1	1	1	2	3	1	2	3	9
2	3	2	2	3	2	3	2	3	2
3	1	2	1	1	3	2	1	3	2
2	1	2	1	3	3	3	3	2	2
2	1	1	1	2	1	2	1	1	1
1	1	1	3	1	2	3	3	3	1
1	1	1	2	1	1	1	1	1	2
3	1	1	1	3	3	3	3	3	1
9	9	9	9	9	9	9	9	9	1
1	1	2	2	1	2	1	2	1	1
1	2	3	1	2	3	1	3	3	2
2	1	2	1	2	2	2	1	3	1
2	2	2	2	2	2	2	2	2	2
1	1	1	2	2	1	3	3	3	1
2	1	2	1	2	2	2	2	2	1
2	2	2	2	2	2	2	2	2	2
2	2	2	2	2	2	2	2	2	1
1	1	1	1	2	2	2	1	1	2
2	3	3	1	1	2	3	3	3	1
2	1	1	2	3	3	3	3	3	1
2	2	1	1	2	1	1	1	2	1
1	2	2	2	3	2	1	1	1	1
1	9	1	2	1	1	1	2	3	1
1	2	2	3	3	2	2	2	2	1
2	3	3	3	3	3	2	2	2	3
1	1	1	1	2	2	2	2	2	1
2	2	2	1	1	2	2	2	2	1
2	2	2	1	1	2	2	1	1	2
2	2	1	1	1	1	1	1	2	2
1	1	1	1	2	2	2	2	1	1
3	3	1	2	2	1	2	2	2	1

1	1	2	2	2	1	1	1	1	1
1	2	2	1	1	1	2	2	1	1
2	2	2	1	1	1	2	1	1	1
2	2	2	1	1	2	2	2	1	2
1	1	2	2	1	1	2	2	1	1
2	1	1	2	1	2	1	2	1	1
3	2	2	1	2	1	2	2	1	1
1	2	2	2	1	2	2	2	1	1
2	1	2	2	2	2	2	2	3	2
2	1	2	2	2	2	2	2	3	2
2	2	2	2	2	2	2	2	2	2
3	3	3	3	1	1	1	1	1	1
1	3	2	1	2	2	2	3	2	1
2	2	2	2	2	2	2	2	3	3
2	2	2	2	2	2	2	2	3	2
1	1	1	1	1	1	1	1	1	2
2	1	3	1	3	1	3	3	3	1
2	3	9	2	2	2	3	3	2	2
2	2	2	2	2	2	2	2	2	1
2	2	2	2	1	2	3	2	2	1
2	2	2	2	2	2	2	2	2	2
2	1	2	2	2	2	2	2	3	2
3	9	2	3	2	3	2	3	2	2
2	3	2	2	3	3	3	1	3	3
2	9	1	2	1	2	2	1	2	2
1	1	1	1	1	1	1	1	1	2
1	2	3	2	1	2	1	2	2	2
1	3	1	3	2	1	2	1	2	2
1	1	1	3	1	1	1	1	1	1
2	1	1	3	1	1	1	2	3	1
1	1	2	2	2	2	2	2	2	2
1	1	2	2	3	3	1	3	3	2
1	1	2	2	3	3	2	1	1	1
2	2	2	2	2	2	2	2	2	1
2	1	3	3	2	2	3	2	3	1
2	2	2	2	2	2	2	2	9	2
2	2	2	2	2	2	2	2	2	3
1	1	1	1	1	1	2	2	2	3
1	1	1	1	3	3	2	2	2	1
1	1	1	2	2	1	1	1	1	1
2	2	2	2	2	2	2	2	2	2
9	9	9	9	9	9	9	9	9	9
2	2	2	2	2	2	2	2	2	2
3	3	3	3	3	3	3	3	3	3
3	3	3	3	3	3	3	3	3	3
1	2	2	2	2	1	1	1	2	2
1	1	1	1	1	2	2	1	1	1

2	2	3	2	1	1	1	2	2	1
2	2	1	1	2	2	1	1	1	1
2	2	2	2	1	1	2	2	1	2
1	1	1	1	1	1	2	2	1	1
1	2	2	2	1	1	2	2	1	1
2	2	1	1	1	2	2	1	1	1
2	1	1	1	2	2	2	2	2	2
1	1	1	2	2	2	1	1	2	1
1	1	1	1	1	2	2	2	1	1
2	2	2	1	1	2	2	2	2	2
2	1	1	2	1	2	1	1	2	3
2	2	2	2	1	1	2	2	1	1
1	1	2	3	3	2	2	1	1	1
2	1	1	1	1	1	1	2	2	1
1	2	2	1	1	1	1	1	2	1
1	2	1	1	1	2	1	1	1	1
2	2	2	3	3	3	2	2	3	2
1	2	2	2	2	2	1	1	1	3
9	9	9	2	9	2	9	9	2	2
2	1	2	1	2	1	2	1	1	3
9	9	9	2	9	2	9	9	2	2
2	2	2	2	2	2	2	2	2	2
1	2	2	2	3	3	2	2	1	1
2	2	3	2	1	2	1	2	3	1
2	2	3	3	2	1	1	2	2	1
2	2	2	1	1	2	1	1	2	2
1	1	2	2	1	1	2	1	1	1
1	2	2	3	3	2	1	1	2	1
1	1	2	1	1	1	1	1	1	1
2	3	2	1	1	2	1	1	1	1
1	1	2	1	1	2	1	1	1	1
2	2	2	2	2	2	2	2	2	2
2	2	2	1	1	2	2	2	1	2
2	3	2	1	2	3	2	1	2	3
2	3	2	1	2	2	3	2	2	1
1	9	3	1	3	1	9	1	2	1
1	9	2	9	9	1	9	9	1	3
9	2	9	2	9	2	9	2	2	2
9	9	9	9	9	9	9	9	9	9
2	3	1	9	2	1	9	1	1	1
9	1	9	9	9	9	9	9	9	9
1	1	2	2	1	1	1	1	2	1
9	9	9	9	9	9	9	9	9	9
2	2	2	2	2	2	2	2	2	2
2	9	3	1	9	1	9	1	1	1
9	9	2	9	2	9	2	9	2	2
2	9	9	9	2	9	9	2	9	9

9	2	9	9	9	2	9	9	2	2
2	9	9	2	9	9	9	2	2	2
9	9	2	9	2	9	9	2	2	2
9	9	9	9	9	9	9	9	9	9
9	1	9	2	9	9	1	9	9	9
2	9	9	2	9	9	2	9	2	2
9	9	9	9	9	9	9	9	9	9
9	9	9	9	9	9	9	9	9	2
1	1	2	2	2	3	2	2	2	2
9	9	9	2	9	9	2	9	2	2
2	2	1	2	1	1	2	2	1	1
2	2	1	1	2	2	1	1	2	2
2	2	3	2	1	2	2	2	1	1
2	2	2	1	2	2	2	3	2	1
2	2	1	1	2	3	2	1	1	1
2	2	1	2	2	1	2	2	3	1
2	2	2	2	2	2	2	2	2	2
2	2	9	2	2	2	2	2	2	1
2	2	1	2	1	2	2	2	3	1
2	1	2	3	2	2	2	3	2	3
2	2	3	2	2	1	2	2	3	2
1	2	2	3	2	2	1	2	2	1
1	2	2	2	2	2	2	1	1	3
2	3	2	1	2	2	3	2	2	1
2	3	2	2	2	1	2	2	3	2
2	1	2	2	2	1	1	2	2	3
2	9	9	2	9	9	9	9	2	2
2	2	2	2	2	2	3	3	3	3
2	2	3	3	2	1	2	2	3	1
3	3	3	3	3	3	3	3	3	3
9	9	9	9	9	9	9	9	9	2
1	2	1	1	2	1	2	2	1	2
2	2	1	2	2	3	2	2	1	1
2	2	2	2	2	2	2	2	2	2
2	2	1	2	2	1	1	1	1	1
3	3	3	3	3	3	3	3	3	3
2	2	2	2	2	2	2	2	2	2
9	9	9	2	9	9	2	9	2	2
2	2	2	2	2	2	2	2	2	2
2	2	2	2	2	2	2	2	2	2
1	2	2	2	1	2	2	2	2	2
2	2	2	2	2	2	2	2	2	3
2	2	3	2	1	1	2	3	2	1
2	3	2	1	2	2	1	2	2	1
2	2	1	1	2	2	2	2	1	1
2	3	2	1	2	3	2	2	2	1
2	3	2	2	2	2	1	2	2	1

2	2	2	3	2	2	1	2	2	1
2	2	3	2	2	2	2	1	1	1
1	2	2	1	1	2	2	2	2	1
2	2	2	1	2	2	2	2	2	2
1	1	2	3	2	3	2	1	3	2
2	1	1	2	1	1	2	2	3	1
3	2	2	2	2	1	3	2	2	2
3	1	1	1	3	2	3	3	3	1
3	1	1	1	3	1	2	3	3	1
2	2	2	3	2	3	2	2	2	2
1	1	1	2	3	2	1	2	2	2
1	1	1	1	1	2	3	2	9	1
2	2	3	1	2	3	2	3	2	2
2	2	3	2	2	1	2	1	2	2
1	1	1	1	2	2	3	3	2	9
2	2	2	2	2	2	2	2	2	2
2	2	2	2	2	2	2	2	3	2
2	2	2	2	2	2	2	2	2	1
2	9	3	3	3	3	1	3	3	2
2	2	2	2	2	2	2	2	2	2
2	1	1	2	1	2	1	1	1	1
2	2	2	2	2	2	2	2	2	2
2	1	2	1	2	3	3	2	2	1
2	2	2	2	2	2	2	2	2	2
2	2	2	1	3	3	3	3	3	2
2	2	2	3	3	2	2	2	2	2
2	1	2	3	2	1	2	3	2	2
1	1	2	2	1	1	3	2	3	2
3	3	2	2	2	2	3	3	2	2
2	2	2	2	2	2	2	2	2	1
2	1	2	2	3	2	2	3	3	2
3	1	3	1	3	1	3	1	3	1
1	2	1	2	1	2	2	1	2	1
2	2	2	2	2	2	2	2	2	2
3	3	3	3	1	2	3	2	3	2
2	2	2	2	2	2	2	2	2	3
3	1	2	2	2	2	2	3	3	1
1	1	2	2	2	2	2	2	2	1
3	1	2	3	3	3	2	3	3	1
2	1	3	3	1	2	3	3	3	2
2	1	3	1	3	1	3	3	3	1
2	1	2	2	2	2	2	2	2	2
2	2	2	2	2	2	2	2	2	2
3	3	2	2	2	3	3	2	2	2
2	2	2	2	2	2	2	2	2	2
2	2	2	2	2	2	2	2	2	2
2	2	2	2	2	2	2	2	2	2

2	2	2	2	2	2	2	2	3	2
2	1	1	1	3	1	1	1	2	1
2	1	2	2	2	1	2	2	2	2
2	2	2	2	2	2	2	2	2	2
2	2	2	2	2	2	2	2	2	2
2	2	2	2	2	2	2	2	2	1
2	2	2	2	2	2	2	2	2	2
2	2	3	3	1	1	2	3	2	9
2	2	2	2	2	2	2	2	2	2
9	9	9	9	9	9	9	9	9	9
3	3	3	2	2	2	1	3	2	1
1	1	1	1	1	2	1	1	1	3
2	9	1	2	2	3	3	1	1	2
2	2	2	2	2	2	2	2	2	9
2	2	2	2	2	2	2	2	2	2
2	2	2	2	2	2	2	2	2	2
2	2	2	2	2	2	2	2	2	2
2	2	2	2	2	2	2	2	2	2
3	3	3	3	3	3	3	3	3	9
9	1	1	1	1	1	1	1	1	3
3	3	3	3	3	3	3	3	3	3
3	3	3	3	3	3	3	3	3	9
2	2	2	2	2	2	2	2	2	2
1	2	3	1	1	1	3	2	2	3
2	2	2	2	2	2	2	2	2	2
2	2	1	1	2	1	2	2	2	1
2	1	1	1	1	3	2	2	3	1
2	2	1	1	1	1	1	1	1	1
1	2	2	2	2	2	1	1	1	1
9	9	9	9	9	9	9	9	9	9
2	3	2	3	2	2	1	1	1	2
3	2	3	2	3	2	2	3	2	2
1	2	2	2	3	3	3	3	3	2
3	3	3	3	3	3	3	3	3	3
2	1	2	2	2	2	2	2	3	2
2	2	2	2	3	2	2	2	2	2
2	2	3	2	2	3	2	2	2	2
2	2	3	2	1	3	2	2	2	2
2	2	2	2	2	2	2	2	3	1
2	2	2	2	3	3	2	2	2	2
2	1	1	1	2	2	3	2	1	1
2	2	2	2	2	2	2	2	2	1
2	2	2	2	2	2	2	2	2	2
2	2	2	2	2	2	2	2	2	1
3	3	3	3	3	3	3	3	3	9
2	1	3	1	1	2	3	3	2	1
3	3	3	3	2	2	1	1	1	1

2	2	2	2	3	3	3	1	1	2
2	2	2	2	2	3	3	3	3	2
3	2	2	3	1	3	2	3	3	2
2	2	2	2	2	2	2	2	2	2
3	3	3	3	2	3	3	3	2	3
9	9	9	9	9	9	9	9	9	9
2	2	2	2	2	2	2	2	2	2
2	2	2	2	2	2	2	2	2	2
1	1	1	2	1	1	3	3	3	1
2	2	3	3	3	3	3	3	2	2
1	1	1	2	1	1	2	1	1	1
2	1	2	2	2	2	2	2	2	2
9	1	9	9	9	9	9	9	9	9
1	9	9	9	9	9	9	9	9	9
2	9	9	9	9	9	9	9	9	9
2	2	1	2	2	2	2	3	1	2
2	1	2	2	2	2	1	1	1	1
9	9	9	9	9	9	9	9	9	1
3	1	3	1	3	3	3	3	3	1
2	1	3	1	3	3	3	2	3	1
2	2	3	1	3	2	9	2	2	2
3	1	3	1	3	3	3	3	3	1
3	1	3	1	3	3	3	3	3	1
2	2	2	2	2	2	2	2	2	2
1	1	3	1	2	2	2	2	2	1
2	2	2	2	2	2	2	2	2	2
3	1	3	1	2	2	2	2	2	1
2	1	1	2	1	1	1	3	3	1
9	9	1	9	9	9	9	9	9	9
3	1	3	1	3	3	3	3	3	3
3	3	3	1	3	3	3	3	3	1
1	1	2	1	1	2	1	1	2	1
1	1	1	1	1	1	1	1	1	2
3	1	1	2	2	2	2	1	2	2
3	1	1	2	3	2	3	2	3	1
1	1	3	1	1	1	3	3	3	1
2	2	3	2	2	2	2	3	3	1
2	2	2	2	3	3	3	3	3	1
2	2	2	2	2	2	2	2	2	2
2	1	1	2	1	2	2	3	3	1
1	1	2	2	1	1	1	1	1	1
2	2	2	2	2	2	2	2	3	1
2	1	2	2	3	2	2	2	2	2
2	1	2	1	2	3	3	3	3	2
2	2	2	2	2	3	9	2	2	1
2	2	2	2	2	3	3	9	3	1
1	1	2	1	1	1	3	2	1	1

1	1	1	1	1	2	1	1	1	1
2	2	2	2	1	2	2	2	9	3
9	9	9	9	9	9	9	9	9	1
2	3	1	2	3	1	2	3	1	1
1	9	2	2	2	1	2	2	3	1
2	1	2	2	2	2	2	2	2	1
2	1	1	1	2	2	2	2	2	1
2	9	1	3	2	1	3	2	3	1
2	2	1	2	2	1	3	3	2	1
2	2	1	2	2	2	2	2	2	2
2	2	2	2	2	2	2	2	3	2
2	2	2	2	2	2	2	2	1	2
1	2	2	3	3	2	2	2	1	2
1	1	1	1	1	3	3	3	3	1
2	1	1	1	2	2	3	3	3	1
2	9	3	2	3	3	2	2	2	1
9	9	9	9	9	9	9	9	9	9
9	9	9	9	9	9	9	9	9	9
3	1	3	1	3	3	3	3	3	1
1	1	1	1	3	1	3	3	3	1
2	2	2	2	2	2	2	2	2	1
9	9	9	9	9	9	9	9	9	9
2	2	2	2	2	2	2	2	2	2
3	1	1	2	2	3	1	2	3	2
2	2	2	2	2	2	2	2	2	2
9	9	9	9	9	9	9	9	9	1
9	9	9	9	9	9	9	9	9	1
2	1	2	2	3	2	1	3	2	2
9	1	9	1	9	9	9	9	3	2
9	9	9	1	9	1	9	9	9	9
9	2	9	9	9	9	9	9	9	9
9	9	9	9	9	9	9	9	9	9
9	9	9	9	9	9	9	9	9	1
2	1	3	2	3	1	2	3	1	3
2	1	3	1	3	1	2	1	2	3
3	2	3	9	2	1	2	2	1	2
3	2	3	2	3	1	2	2	1	2
1	1	1	1	1	1	1	1	1	3
1	2	2	2	3	2	2	2	2	1
2	2	2	1	1	1	1	1	1	3
2	2	2	2	2	2	2	2	2	2
2	1	1	2	1	1	2	2	3	1
2	2	2	1	2	1	2	3	2	2
1	2	3	1	3	2	1	3	1	1
1	2	3	1	3	2	1	3	1	1
1	2	3	1	3	2	1	3	1	1
2	2	2	2	2	2	2	2	2	2

9	9	9	9	9	9	9	9	9	9
2	2	2	1	1	1	3	2	9	2
3	3	3	3	3	3	3	3	3	1
9	9	9	9	9	9	9	9	9	9
9	9	9	9	9	9	9	9	9	9
3	9	2	2	2	9	1	2	2	2
1	1	1	2	3	1	3	3	3	1
9	9	9	9	9	9	9	9	9	9
9	9	9	9	9	9	9	9	9	9
2	1	2	3	3	1	3	1	3	1
2	1	2	2	2	2	2	2	2	1
2	2	2	2	2	2	2	2	9	2
2	2	2	2	2	2	2	2	2	1
9	9	9	9	9	9	9	9	9	9
9	9	9	9	9	9	9	9	9	9
9	9	9	9	9	9	9	9	9	9
1	1	2	2	1	1	3	2	3	9
9	9	9	9	9	9	9	9	9	9
3	1	3	1	3	3	3	3	3	1
9	9	9	9	9	9	9	9	9	9
1	9	9	9	9	9	9	9	9	9
9	9	9	9	9	9	9	9	9	9
9	9	9	9	9	9	9	9	9	9
9	9	9	9	9	9	9	9	9	9
2	2	2	2	2	2	2	2	3	9
2	1	1	1	2	2	3	2	3	9
1	1	1	1	2	2	3	3	3	1
3	1	1	2	2	2	2	2	2	2
2	1	1	1	3	3	3	3	3	1
2	2	2	2	2	2	2	2	2	2
3	3	3	3	3	3	3	3	3	2
1	1	1	1	1	2	2	2	3	2
1	1	2	1	3	3	3	3	3	1
1	1	2	2	1	1	2	3	3	1
2	1	1	2	3	2	3	2	3	1
1	1	3	2	1	3	1	3	3	1
1	1	2	2	1	1	3	2	3	1
2	2	2	2	2	2	2	2	3	1
3	9	1	1	3	1	3	3	3	1
2	2	2	2	2	2	2	2	2	1
2	1	2	2	2	1	2	2	2	1
2	1	2	2	2	1	2	2	2	1
2	1	2	2	2	2	2	2	2	9
9	9	9	9	9	9	9	9	9	1
9	9	9	2	9	9	9	9	9	9
2	2	3	2	2	2	2	2	2	2
1	2	2	2	1	1	1	1	1	2

1	2	2	2	2	2	1	1	1	2
3	1	3	3	2	2	2	1	1	2
2	2	2	3	2	2	1	1	1	2
2	1	1	2	3	1	3	2	3	2
2	1	2	2	2	2	2	2	2	1
1	1	3	3	1	1	1	1	3	1
2	1	1	2	2	1	3	2	3	2
2	1	3	1	2	3	3	3	3	9
9	9	9	9	9	9	9	9	9	9
1	1	2	1	2	2	1	1	1	1
2	2	2	2	2	2	2	2	2	2
1	1	1	1	1	1	1	1	1	1
2	3	1	2	3	1	2	3	1	1
2	9	2	1	3	3	1	2	2	1
2	2	2	2	2	2	3	3	2	2
1	2	1	1	1	2	1	2	2	2
2	3	2	3	2	2	1	1	2	1
2	3	1	2	3	1	2	3	1	1
2	2	2	2	2	2	2	2	2	2
1	2	1	2	2	2	9	1	2	2
3	3	3	2	2	2	2	1	1	1
2	2	2	2	2	2	2	2	9	2
2	2	2	3	2	1	2	2	1	2
2	9	3	2	2	1	2	2	1	1
2	2	2	2	2	2	2	2	2	2
2	2	2	1	2	2	2	2	2	2
2	2	2	2	2	2	2	2	2	1
2	9	1	2	1	3	2	2	2	2
2	3	1	2	3	1	2	3	2	1
9	9	9	9	9	9	9	9	9	9
1	2	2	2	1	1	1	2	1	1
9	9	9	9	9	9	9	9	9	9
9	9	9	9	9	9	9	9	9	9
1	1	9	1	1	1	3	3	1	1
2	1	2	1	3	1	3	1	3	3
2	3	9	3	3	2	1	2	2	3
2	1	1	2	3	3	1	1	2	3
2	1	2	2	2	2	2	2	2	3
2	2	2	2	2	2	2	2	2	2
2	2	2	1	1	1	1	1	1	1
2	2	3	3	3	1	1	1	3	1
2	3	1	2	1	1	1	2	1	1
2	2	2	2	2	2	2	2	2	2
2	2	2	2	2	2	2	2	2	9
2	2	2	2	2	2	2	2	2	2
1	1	1	1	2	2	1	2	2	2
2	2	2	2	2	2	2	2	2	1

2	1	1	2	3	1	3	2	3	1
2	2	2	2	1	2	2	1	3	2
2	2	2	2	2	2	2	2	2	1
3	2	2	2	2	2	3	3	3	3
2	2	2	2	3	1	2	2	1	2
9	9	9	9	9	9	9	9	9	9
3	1	2	1	1	1	1	1	2	1
3	1	2	2	1	1	1	2	1	1
9	1	9	9	9	9	9	9	9	9
2	2	2	2	2	2	2	2	2	2
2	2	2	2	2	2	2	2	2	2
2	9	1	2	3	2	3	2	3	1
9	9	9	9	9	9	9	9	9	9
2	3	2	2	2	3	2	1	1	1
3	1	3	1	3	3	3	3	3	1
2	1	1	1	1	1	1	1	3	1
1	1	1	1	2	1	2	1	2	1
2	3	1	2	3	1	2	3	2	1
1	1	1	1	1	1	3	3	3	2
2	2	2	2	2	3	3	3	3	3
2	1	1	3	1	2	1	2	1	1
3	3	3	3	3	3	3	3	3	2
2	2	2	2	2	2	2	2	2	2
1	1	1	1	1	1	1	3	2	2
1	1	2	3	1	1	3	1	1	1
2	2	2	2	2	2	2	2	2	9
2	2	2	2	2	2	2	2	2	2
2	1	1	2	1	1	3	3	3	1
2	1	1	3	2	3	2	3	2	1
1	2	2	1	2	1	2	1	2	2
2	2	2	2	2	2	2	2	2	3
2	2	2	2	2	2	2	2	2	2
2	2	2	1	2	1	3	2	2	1
2	1	1	2	2	2	1	2	2	1
2	1	1	2	2	2	3	2	2	1
9	9	9	9	9	9	9	9	9	9
2	2	2	2	2	2	2	2	2	2
9	9	9	9	9	9	9	9	9	9
2	2	3	3	1	1	2	2	2	1
2	1	2	2	1	2	2	1	2	2
2	2	2	2	2	2	2	2	2	2
9	9	9	9	9	9	9	9	9	9
2	1	2	2	3	3	2	2	2	1
2	2	2	2	2	2	2	2	9	2
9	9	9	9	9	9	9	9	9	9
9	9	9	9	9	9	9	9	9	9
9	9	9	9	9	9	9	9	9	9

3	3	2	2	2	3	3	3	2	3
1	2	9	1	2	1	1	2	1	2
9	9	9	9	9	9	9	9	9	9
1	1	1	1	3	3	2	1	3	9
2	2	2	2	2	2	2	2	2	2
2	3	2	2	2	1	1	3	2	1
2	2	2	2	2	2	2	2	2	2
2	2	2	2	2	2	2	2	9	2
2	3	2	3	3	3	3	3	3	1
9	9	9	9	9	9	9	9	9	9
3	3	3	3	3	3	3	3	3	3
9	1	3	9	1	9	9	1	9	2
3	1	2	2	3	2	2	2	2	2
3	1	2	2	2	3	3	2	2	1
2	2	2	2	2	2	2	2	2	2
1	9	1	1	9	1	1	1	9	2
2	1	2	2	2	2	3	2	3	1
9	9	9	9	9	9	9	9	9	9
9	9	9	9	9	9	9	9	9	9
2	3	2	2	1	2	1	2	1	1
2	3	2	1	2	3	1	3	9	1
3	3	3	3	3	3	3	3	1	1
3	2	3	2	3	2	3	2	3	2
3	3	3	3	3	3	3	3	3	3
2	3	2	1	2	3	2	1	2	1
1	1	1	1	3	3	3	2	2	1
2	2	2	2	2	2	2	1	9	1
2	2	2	3	2	2	3	3	3	1
9	9	9	9	9	9	9	9	9	9
9	9	9	9	9	9	9	9	9	9
9	9	9	9	9	9	9	9	9	9
9	9	9	9	9	9	9	9	9	9
9	9	9	9	9	9	9	9	9	9
2	3	3	3	3	3	3	3	3	2
9	2	2	1	3	3	1	3	1	2
9	9	9	9	9	9	9	9	9	9
9	9	9	9	9	9	9	9	9	9
2	1	1	1	3	1	3	2	3	1
9	9	9	9	9	9	9	9	9	9
9	9	9	9	9	9	9	9	9	9
2	2	2	2	2	2	2	2	2	2
2	1	2	2	2	3	3	2	3	2
2	3	1	2	2	2	2	3	1	1
2	2	2	2	2	2	2	2	2	2
2	2	1	2	2	2	1	1	2	2
2	1	1	2	2	1	2	2	1	1
2	1	1	1	2	1	2	2	2	1

2	2	2	2	2	2	2	2	2	3
2	2	2	2	2	2	2	2	2	2
9	9	9	9	9	9	9	9	9	9
2	2	2	3	3	3	1	1	1	1
1	1	1	2	2	2	2	3	3	1
2	1	2	1	3	3	1	2	1	1
9	9	9	9	2	9	9	2	3	3
2	2	2	2	2	2	2	2	2	9
2	2	2	2	2	2	2	2	2	1
9	9	9	9	9	9	9	9	9	9
2	2	2	2	2	2	2	2	2	2
2	3	2	1	2	2	2	2	2	1
9	9	9	9	9	9	9	9	9	9
1	1	1	1	1	1	1	1	1	1
1	1	1	1	1	2	2	1	2	1
2	3	1	1	1	1	2	3	2	1
2	3	1	2	3	1	2	3	1	1
9	9	9	9	9	9	9	9	9	9
1	3	2	2	2	2	2	1	3	1
9	9	9	9	9	9	9	9	9	9
9	2	9	9	9	9	9	9	9	9
2	3	2	3	2	3	1	1	1	1
9	9	9	9	9	9	9	9	9	1
9	9	9	9	9	9	9	9	9	9
9	2	9	9	9	9	9	9	9	1
9	9	9	9	9	9	9	9	9	9
1	1	1	3	2	2	3	1	3	3
3	2	1	2	3	2	1	2	3	9
9	9	9	9	9	9	9	9	9	9
1	2	2	2	2	2	2	2	2	2
3	1	3	1	3	1	3	1	3	1
9	9	9	9	9	9	9	9	9	9
2	2	2	2	2	2	2	2	2	2
1	9	3	2	1	3	2	2	2	2
2	1	1	1	1	1	1	1	1	2
2	2	2	2	2	2	2	2	2	2
2	9	2	1	2	2	1	2	1	2
2	1	3	1	1	1	3	3	3	1
3	2	3	2	2	2	2	2	2	2
9	9	9	9	9	9	9	9	9	9
2	9	1	2	3	2	1	2	1	2
3	1	3	2	1	2	3	3	3	1
2	2	2	2	2	2	2	2	2	3
2	1	1	1	3	3	1	3	3	2
1	2	9	1	3	3	3	3	3	2
1	1	1	1	1	1	1	1	1	1
2	1	1	3	3	3	2	2	3	2

1	3	3	9	3	3	3	3	3	1
2	1	2	1	1	2	2	2	2	1
9	3	2	3	2	1	3	2	3	2
2	2	2	2	2	2	2	2	3	2
2	3	3	2	2	2	2	2	2	2
2	2	3	3	2	2	3	2	3	2
2	2	2	2	2	2	2	2	2	2
3	3	3	3	3	3	3	3	3	3
1	1	2	2	2	2	2	2	3	1
9	9	9	9	9	9	9	9	9	9
3	9	1	3	3	3	3	3	3	1
2	3	2	1	2	2	1	2	3	1
9	9	9	9	9	9	9	9	9	9
2	2	2	3	1	2	1	2	2	1
9	9	9	9	9	9	9	9	9	9
9	9	9	9	9	9	9	9	9	9
1	1	1	1	1	3	3	3	3	1
2	2	2	2	2	2	2	2	2	2
2	3	9	2	3	2	2	3	2	1
1	2	2	2	2	2	2	2	2	1
2	1	2	1	1	1	1	1	2	1
2	2	2	2	2	2	2	2	2	2
9	9	9	9	9	9	9	9	9	9
1	1	1	1	2	2	3	1	2	2
1	1	1	1	1	1	1	1	1	1
3	3	3	3	3	3	3	3	3	3
2	2	3	2	2	3	2	1	9	1
2	2	3	2	2	2	2	2	3	1
3	3	3	2	2	2	3	1	1	1
2	2	2	2	2	2	2	2	2	2
2	2	2	2	2	2	2	2	2	3
9	9	9	9	9	9	9	9	9	9
2	2	2	2	2	2	2	2	2	2
2	3	3	2	3	2	3	3	2	3
2	9	1	2	1	3	2	1	2	1
1	1	3	1	3	1	3	1	9	1
2	2	2	2	2	2	2	2	2	2
2	2	2	2	2	2	2	2	2	2
1	2	2	2	2	2	2	2	2	2
2	2	2	2	2	2	2	2	2	2
2	2	2	2	2	2	2	2	2	2
1	1	1	2	2	3	3	3	3	2
2	3	1	2	3	1	2	3	1	1
2	3	1	1	3	3	2	2	3	2
9	9	9	9	9	9	9	9	9	9
2	1	2	1	3	2	1	2	3	9
1	2	2	2	2	1	1	1	1	1

3	2	1	1	2	1	2	1	1	1
3	1	3	1	3	3	3	3	3	3
3	1	3	1	3	3	3	3	3	1
2	2	1	2	2	1	2	2	2	1
9	9	9	9	9	9	9	9	9	1
9	9	9	9	9	9	9	9	9	1
9	9	9	9	1	9	9	9	9	9
2	2	2	2	1	2	2	2	2	2
2	1	3	1	3	1	2	2	1	1
1	1	1	3	2	2	3	1	2	2
1	1	2	2	1	1	2	3	3	1
2	1	1	3	1	1	3	2	2	1
2	2	2	2	2	2	2	2	2	2
2	2	2	2	2	2	2	2	2	2
2	2	2	2	2	2	2	2	2	2
1	1	1	1	1	1	1	1	2	1
2	2	2	2	2	2	2	2	9	2
2	2	2	2	2	2	2	2	2	2
3	1	1	2	3	2	3	3	3	1
1	2	3	2	3	2	1	3	2	2
2	2	2	2	2	2	2	2	2	2
9	1	9	9	9	9	9	9	9	9
9	9	1	9	1	2	9	1	2	2
2	2	2	2	2	2	2	2	2	2
2	2	2	2	2	2	2	2	2	2
2	2	2	3	3	2	2	1	1	2
1	2	2	1	1	2	2	1	1	2
9	9	9	9	9	9	9	9	9	9
1	1	2	1	3	1	3	2	3	1
2	2	2	2	1	1	2	1	1	2
1	1	3	1	3	3	3	3	3	1
1	1	1	1	1	1	3	3	3	1
1	3	2	1	1	2	1	2	1	2
1	1	2	1	3	2	2	2	3	1
1	1	3	1	3	1	3	3	3	1
2	2	2	3	3	3	3	3	2	2
2	2	2	2	2	2	2	2	2	2
2	2	2	2	2	2	2	2	2	2
2	2	1	1	1	2	2	2	3	2
9	9	9	9	9	9	9	9	9	9
1	1	3	2	3	1	2	3	3	1
3	2	1	1	2	1	3	2	1	1
2	2	2	2	1	3	3	1	1	1
2	3	1	2	1	2	3	2	1	1
3	2	2	1	1	2	3	1	2	1
1	1	1	1	1	1	1	1	1	1
2	2	2	2	2	2	2	2	2	2

1	2	1	2	1	1	3	3	3	1
1	9	3	9	1	3	9	9	2	1
1	1	2	2	2	2	2	2	2	2
1	1	1	1	1	1	2	3	3	1
2	2	2	2	2	2	2	2	2	2
2	2	2	2	2	2	2	2	2	1
3	2	3	2	3	2	3	2	3	2
2	1	3	1	1	3	3	3	3	1
9	9	9	9	9	9	9	9	9	1
2	2	2	2	2	2	2	2	2	2
3	1	2	2	3	3	3	3	3	1
2	1	3	1	3	3	3	3	3	3
2	2	2	2	2	2	2	2	2	2
3	1	2	1	1	1	1	3	3	2
1	1	2	1	3	3	3	2	3	3
3	1	3	1	3	3	3	2	3	1
3	3	3	3	2	2	3	2	1	2
1	9	9	9	9	9	9	9	9	9
9	9	9	9	9	9	9	9	9	9
1	1	1	1	1	2	1	2	2	1
3	1	1	1	3	1	3	2	3	1
2	1	2	2	3	1	3	3	3	1
2	2	2	2	2	2	2	2	3	2
2	2	2	2	1	1	2	3	1	2
9	9	9	9	9	9	9	9	9	9
1	1	1	2	1	1	3	3	3	1
1	2	1	2	3	1	2	3	3	1
2	1	2	2	3	3	3	3	3	1
1	1	1	2	2	2	3	3	3	1
2	2	2	2	2	2	2	2	2	1
2	2	2	2	2	2	2	2	2	2
1	2	2	2	2	1	2	3	2	1
1	2	1	2	3	3	3	1	2	3
2	3	1	2	3	1	2	1	2	1
3	1	3	3	3	3	3	1	3	1
1	2	3	2	1	1	3	1	9	1
1	2	1	1	2	3	2	2	2	2
2	2	2	2	2	2	2	2	2	1
1	1	3	1	1	3	3	3	3	1
2	1	2	2	3	2	3	2	3	2
9	9	9	9	9	9	9	9	9	9
3	1	1	1	1	2	3	2	1	3
3	9	1	3	3	3	3	1	3	9
3	1	3	1	3	3	3	3	3	1
3	3	3	3	3	3	3	3	3	3
1	1	1	2	1	1	3	2	3	1
3	1	1	2	1	1	1	2	3	1

2	1	3	2	3	2	3	2	3	3
9	9	9	9	1	9	9	9	9	1
9	9	9	9	9	9	9	9	9	9
9	9	9	9	9	9	9	9	9	9
2	2	2	2	2	2	2	2	2	2
2	2	2	2	2	2	2	2	2	2
1	1	2	2	2	1	2	2	2	1
1	1	1	1	1	1	1	1	1	1
9	9	9	9	9	9	9	9	9	1
9	9	9	9	9	9	9	9	9	1
9	9	9	9	9	9	9	9	9	2
2	1	1	1	3	2	3	3	3	1
2	2	2	2	2	2	2	2	2	1
2	2	2	2	2	2	2	2	2	1
2	1	2	2	2	2	2	2	3	1
1	1	1	1	2	2	3	3	2	1
3	1	3	1	2	1	9	2	3	1
2	1	1	2	2	1	3	3	3	1
1	1	1	1	3	9	3	3	3	1
9	9	9	9	9	9	9	9	9	9
2	2	2	2	2	2	2	2	2	3
3	1	2	2	1	3	2	3	1	2
1	1	1	2	1	1	3	3	3	1
2	3	2	1	2	2	1	2	2	3
3	3	3	3	2	2	2	3	3	9
1	1	1	1	3	1	3	3	3	1
2	2	3	3	3	3	2	2	2	9
2	1	2	3	1	1	2	3	1	1
1	1	2	1	1	9	2	9	3	1
1	1	1	2	3	3	3	2	3	1
1	1	1	1	1	1	1	1	2	3
3	2	1	2	2	3	3	2	3	1
2	2	1	2	3	3	2	2	2	1
2	1	3	1	3	3	3	3	3	1
1	1	3	2	1	1	3	2	3	1
1	1	3	2	1	1	3	2	3	1
1	1	2	1	2	1	2	1	2	1
2	1	3	1	2	1	2	2	3	2
2	1	1	1	3	3	2	3	3	1
2	9	1	2	3	1	3	1	2	1
2	1	2	1	3	3	3	3	3	1
1	1	1	1	1	2	2	2	2	2
9	9	9	9	9	9	9	9	9	9
3	1	2	1	3	3	3	3	3	1
1	1	1	1	1	1	2	1	1	1
3	3	3	1	1	3	3	3	3	9
1	1	1	2	1	1	1	1	1	1

1	1	1	1	3	1	1	3	3	1
9	9	9	9	9	9	9	9	9	2
2	1	3	3	3	2	1	2	3	1
1	1	2	1	2	2	2	2	3	9
1	1	2	2	2	2	2	2	2	1
3	1	3	2	3	3	3	1	3	1
2	1	1	1	1	3	3	1	2	1
3	3	3	3	3	3	3	3	3	1
3	1	3	3	3	3	3	3	3	1
9	9	9	9	9	9	9	9	9	9
2	1	1	2	2	2	2	2	3	2
1	1	1	2	2	1	2	1	2	1
3	1	3	1	3	1	3	1	3	1
2	3	1	3	1	3	1	3	1	1
2	1	1	2	3	3	1	1	3	1
9	9	9	9	9	9	9	9	9	9
9	9	9	9	9	9	9	9	9	1
1	2	1	2	2	9	2	1	1	1
2	2	3	3	3	3	2	2	1	1
2	2	2	2	2	2	2	2	2	2
9	9	9	9	9	9	9	9	9	9
1	1	1	1	1	2	2	2	1	1
1	2	2	2	2	2	1	1	1	2
2	2	2	1	1	1	1	1	1	1
1	1	2	2	2	2	2	2	2	2
2	1	2	2	2	1	1	2	2	1
1	1	2	2	2	1	1	2	2	1
1	2	2	1	1	2	2	2	2	2
1	1	1	2	1	2	2	1	1	1
1	2	2	1	1	1	2	2	3	2
9	9	9	9	9	9	9	9	9	9
9	9	9	9	9	9	9	9	9	9
9	9	9	9	9	9	9	9	9	9
1	1	2	2	2	2	2	2	2	2
3	1	1	2	3	2	3	3	3	1
2	9	1	2	2	2	3	2	2	1
1	1	1	2	1	1	2	2	2	3
2	2	2	2	2	2	2	2	9	1
3	1	2	2	1	1	3	2	2	2
3	3	3	3	3	3	3	3	3	1
1	1	1	1	1	1	3	3	3	3
9	1	1	2	3	1	3	3	3	1
2	2	2	2	2	2	2	2	2	2
2	2	2	2	2	3	3	3	2	2
2	2	2	2	2	2	2	2	2	2
1	1	1	1	1	1	1	1	1	1
3	3	3	3	3	3	3	1	1	9

9	9	9	9	9	9	9	9	9	9
2	2	2	2	1	1	2	1	2	3
9	9	9	9	9	9	9	9	9	9
9	9	9	9	9	9	9	9	9	9
3	1	1	3	3	3	3	3	1	3
3	2	2	2	1	2	1	2	2	9
3	3	3	3	2	2	2	2	2	1
9	9	9	1	9	9	9	9	1	9
2	2	2	2	2	2	2	2	2	1
9	9	9	9	9	9	9	9	9	1
1	1	9	1	2	9	2	9	1	1
9	2	9	9	9	9	9	9	9	9
1	2	1	2	2	2	2	2	2	2
9	9	9	9	9	9	9	9	9	9
2	1	1	3	3	3	3	1	3	1
2	1	2	1	3	3	3	3	3	1
1	1	1	1	1	1	2	1	2	1
1	1	1	1	1	1	2	1	2	1
2	2	2	2	2	2	2	2	2	1
2	1	2	3	2	1	2	3	1	1
2	1	2	1	2	2	2	3	3	1
2	1	2	1	2	1	2	2	3	2
1	1	2	2	1	1	3	2	3	1
3	3	3	3	3	3	3	3	3	3
1	9	2	1	1	2	1	2	1	3
1	1	1	3	3	1	2	2	2	1
2	2	2	2	2	2	2	2	2	2
1	1	2	1	1	1	3	3	3	1
2	2	2	2	2	2	2	2	2	2
2	2	2	3	2	2	2	2	9	1
2	2	2	2	2	2	2	2	2	2
1	1	1	2	1	1	2	2	2	1
1	1	1	2	3	2	3	3	3	1
1	1	2	2	2	2	1	2	2	2
9	9	9	9	9	9	9	9	9	9
2	1	3	2	3	3	3	2	3	1
2	1	2	2	3	2	2	3	2	2
3	1	2	2	3	2	2	2	3	2
2	1	1	1	3	3	3	3	3	1
3	2	1	2	1	2	1	2	2	2
1	1	1	1	1	1	1	1	1	1
9	1	9	9	9	9	9	9	9	9
9	1	9	9	9	9	9	9	9	9
2	1	2	2	3	2	3	2	2	1
9	9	9	1	9	9	9	9	9	1
2	2	2	2	2	2	2	2	2	2
2	1	3	2	1	2	3	1	3	1

2	2	2	2	2	2	2	2	2	2
2	2	2	2	2	2	2	2	2	2
2	2	3	1	3	2	1	3	2	1
2	2	2	2	2	2	2	2	2	1
3	2	3	3	3	3	3	3	3	2
2	3	3	2	2	3	3	2	3	2
1	2	2	3	2	1	2	2	2	2
2	2	2	1	1	1	2	2	2	2
3	1	3	1	3	3	3	3	3	1
2	3	2	1	2	1	1	2	1	1
3	1	1	1	3	3	2	2	3	1
2	2	2	2	1	1	1	1	2	1
3	1	3	1	3	3	3	3	3	1
1	2	2	2	1	1	2	2	2	2
2	3	2	1	2	3	2	1	2	1
2	2	2	2	2	2	2	2	2	2
1	1	1	1	2	2	2	1	2	3
2	2	2	2	2	2	2	2	9	2
2	2	2	2	3	3	3	3	3	2
2	2	1	2	2	2	3	3	2	2
2	2	3	2	2	3	2	2	3	2
2	2	2	2	2	2	2	2	2	9
3	1	3	1	2	3	3	3	3	1
2	1	2	2	2	2	3	2	3	1
1	1	1	1	1	1	1	1	1	1
2	2	2	2	2	2	2	2	2	2
2	2	2	2	2	2	2	2	2	2
1	1	1	2	2	2	2	1	1	1
2	1	3	1	3	3	2	3	3	1
2	2	1	2	2	3	2	2	2	1
9	9	9	9	9	9	9	9	9	9
2	2	2	1	1	2	2	3	2	3
1	2	2	2	1	1	2	2	2	1
3	2	2	2	3	2	2	3	2	2
2	1	1	1	2	1	2	1	1	1
2	2	2	1	2	2	3	2	2	1
2	2	2	3	2	2	2	2	3	1
2	2	2	2	2	2	2	2	2	2
2	3	3	2	1	2	3	2	1	1
2	2	2	2	2	2	1	2	3	1
1	1	1	1	3	1	1	3	3	1
2	1	2	2	3	2	3	1	2	2
3	1	3	1	1	1	3	3	3	1
1	2	1	1	2	1	1	2	2	1
2	2	1	3	3	1	2	3	3	1
2	2	2	2	2	2	2	2	2	2
1	1	3	2	1	1	3	2	3	1

1	1	3	2	1	1	3	2	3	1
2	2	2	1	2	2	2	2	2	1
2	2	2	1	2	2	1	1	1	1
2	3	2	2	2	2	2	2	2	1
1	2	2	2	1	2	2	2	2	1
2	2	2	2	1	1	2	2	2	1
2	2	2	3	2	2	2	2	2	1
2	2	2	2	2	2	2	2	2	2
3	2	2	3	2	3	2	1	2	2
2	1	1	1	3	3	1	3	3	1
3	1	3	9	3	3	3	1	1	1
2	9	2	1	1	3	2	2	2	2
3	3	3	3	3	3	3	3	3	3
2	2	2	2	2	2	2	2	2	1
9	9	9	9	9	9	9	9	9	3
2	2	2	2	2	2	2	2	2	2
2	2	9	2	3	3	3	3	1	1
2	2	3	2	3	2	3	2	2	1
2	9	1	2	1	2	1	2	2	2
2	1	2	1	3	3	3	2	3	1
2	2	1	2	2	1	2	2	2	1
3	3	3	3	3	3	3	3	3	3
2	2	2	2	2	2	2	2	2	2
1	1	1	1	1	1	3	2	3	1
3	1	2	3	1	2	3	1	2	1
1	1	2	2	1	2	1	2	2	1
2	2	2	1	1	2	2	2	1	1
2	1	2	2	1	1	2	3	2	2
1	1	2	2	3	1	2	3	2	1
2	2	2	2	2	2	2	2	2	2
1	1	1	2	2	3	3	3	1	1
3	1	3	3	3	3	9	1	3	1
1	1	1	2	2	2	1	1	2	1
3	1	2	2	2	2	3	2	2	1
1	3	1	2	1	2	3	1	2	2
3	2	2	1	1	1	2	2	3	2
2	2	2	1	1	1	1	2	2	1
1	2	2	1	3	1	3	2	3	1
2	3	1	2	3	1	9	1	2	3
2	1	3	1	3	3	3	3	3	1
1	1	1	9	3	1	3	3	3	1
1	1	3	1	3	3	3	3	3	1
3	1	1	1	1	1	1	1	3	1
3	1	3	1	3	3	3	3	3	1
2	1	3	1	3	2	3	3	3	1
2	3	1	2	2	1	2	3	1	1
2	2	2	2	2	2	2	2	2	2

1	1	2	2	1	1	2	1	2	1
2	2	2	2	2	2	2	2	2	2
1	2	2	3	2	3	2	2	3	1
1	1	1	9	3	1	3	3	3	1
2	2	1	2	2	2	2	2	2	1
2	2	2	2	2	2	2	2	2	2
2	2	2	2	2	2	2	2	2	2
2	2	2	2	1	2	2	2	2	1
9	9	9	9	9	9	9	9	9	9
1	2	2	2	2	2	2	2	3	2
2	1	2	1	2	1	3	3	3	1
2	2	2	2	2	3	3	3	3	1
2	1	1	1	1	2	2	2	2	2
2	2	2	2	2	2	2	2	2	2
1	1	2	1	1	1	1	1	1	1
1	1	1	2	3	1	3	3	3	1
2	2	2	2	2	2	2	2	2	2
2	3	2	1	2	3	2	1	2	1
1	1	1	1	1	1	1	1	1	3
2	2	2	2	2	2	2	2	2	2
2	2	2	3	2	3	2	2	3	3
2	2	2	1	2	2	2	1	2	3
2	2	2	2	2	2	2	2	2	1
1	2	2	1	2	2	2	2	2	1
1	1	2	1	1	1	1	1	1	1
1	1	1	2	3	1	3	3	3	1
2	2	2	2	2	3	3	3	3	1
2	1	2	2	2	2	2	2	2	2
1	2	2	3	1	2	3	1	9	3
2	2	2	2	2	2	2	2	2	2
2	2	2	1	1	1	1	2	2	1
9	9	9	9	9	9	9	9	9	9
2	2	2	2	2	2	2	2	2	1
3	1	3	1	3	3	3	3	3	1
1	1	2	2	2	2	2	3	3	1
1	1	2	3	1	2	1	3	1	1
1	1	3	3	3	3	3	3	3	1
2	3	1	2	3	1	2	3	1	1
2	2	2	2	2	2	2	2	2	2
1	3	1	2	3	1	2	1	2	2
2	1	2	1	3	2	3	2	2	1
2	1	2	1	3	2	3	2	2	1
3	1	2	2	3	2	3	3	1	1
2	1	1	1	1	1	2	3	3	1
2	3	3	2	2	3	2	3	2	2
2	2	3	3	3	3	3	3	3	3
3	3	3	3	2	3	3	2	3	1

2	2	1	2	2	1	2	1	2	1
2	2	1	1	3	1	3	2	2	1
1	1	1	1	1	1	1	1	1	1
2	2	2	1	3	3	3	3	3	1
2	1	1	1	3	1	2	2	3	1
2	1	2	1	2	2	2	2	2	1
2	1	1	2	1	1	1	2	2	1
9	9	9	9	9	9	9	9	9	3
9	3	9	9	9	9	9	9	9	3
9	9	9	9	9	9	9	9	9	3
2	2	1	3	2	3	3	2	2	2
2	3	1	2	1	1	1	2	2	2
2	3	1	2	3	3	1	3	1	3
2	3	1	3	2	3	3	1	1	3
1	3	2	1	3	1	1	1	1	3
2	2	2	2	2	2	2	2	1	1
2	2	2	1	3	3	3	3	3	2
1	2	3	2	2	1	3	2	2	3
1	1	9	2	2	3	9	2	1	1
1	2	1	2	3	2	1	3	1	2
2	2	3	3	2	2	3	1	1	3
2	3	2	3	3	2	3	1	1	3
2	1	3	1	3	1	1	1	1	3
1	3	1	2	2	2	2	2	2	2
3	1	3	1	3	1	1	1	1	2
1	3	2	1	3	2	2	2	2	2
3	1	2	2	1	1	1	2	2	3
3	1	3	3	1	1	1	2	2	3
2	3	2	2	2	1	2	2	2	1
2	3	2	2	2	1	2	2	2	1
2	3	1	3	2	3	3	1	1	3
2	3	1	3	2	3	3	1	9	3
3	1	3	1	3	3	3	3	3	9
3	1	3	1	3	3	3	3	3	1
2	2	2	2	2	2	2	2	2	1
2	2	2	2	2	2	2	2	2	9
3	1	3	1	3	3	3	3	3	9
2	2	2	2	2	2	2	2	2	2
2	2	2	2	2	2	2	2	2	2
3	1	3	1	3	3	3	3	3	1
1	3	2	2	2	2	1	1	1	3
3	3	1	3	1	3	3	3	3	3
3	3	1	3	1	3	3	3	3	3
3	3	1	3	1	3	3	3	3	3
3	3	1	3	1	3	3	3	3	3
1	3	2	2	9	1	9	3	3	3
2	9	3	9	2	9	2	3	2	3

1	2	3	9	1	9	3	2	2	3
2	2	1	3	2	1	3	3	2	3
2	1	3	1	1	1	1	1	1	9
1	3	2	2	2	3	3	3	1	9
2	3	2	1	2	1	2	2	1	3
3	2	1	3	1	3	1	2	1	3
1	2	2	1	1	1	1	1	1	3
1	2	3	2	1	2	1	2	1	9
9	3	9	9	9	9	9	9	9	3
9	9	9	9	9	9	9	9	9	9
2	2	2	2	2	2	2	2	2	2
9	2	1	9	1	2	2	1	2	3
2	2	2	2	2	2	2	2	2	3
2	3	2	3	3	1	1	1	1	3
9	9	9	9	9	9	9	9	9	9
2	2	2	2	2	2	2	2	2	2
2	2	2	2	2	2	2	2	2	2
9	9	9	9	9	9	9	9	9	2
2	2	2	2	2	2	2	2	2	2
9	9	9	9	2	9	9	9	9	3
1	2	3	3	2	2	2	3	2	3
2	1	3	1	3	2	2	2	1	3
2	2	2	2	2	2	2	2	2	3
2	9	3	2	2	2	3	9	9	2
9	9	9	9	9	9	9	9	9	2
9	9	9	9	9	9	9	9	9	9
2	1	1	2	3	1	2	3	1	3
9	3	9	9	9	9	9	9	9	9
3	1	3	3	3	3	3	3	3	3
9	9	9	9	9	9	9	9	9	9
1	1	1	3	1	3	1	3	1	3
9	9	9	9	9	9	9	9	9	2
2	1	1	3	1	1	1	1	1	3
1	3	1	3	1	1	1	1	1	1
2	2	2	2	2	2	2	2	1	3
1	3	1	3	1	1	1	1	1	3
1	3	1	3	2	2	3	3	3	3
1	3	1	3	1	1	2	2	2	3
3	1	3	1	3	2	2	2	1	3
9	9	9	9	9	9	9	9	9	2
9	9	9	9	9	9	9	9	9	2
9	9	9	9	9	9	9	9	9	2
9	9	9	9	9	9	9	9	9	2
9	9	9	9	9	9	9	9	9	2
9	9	9	9	9	9	9	9	2	9
3	3	1	1	3	2	1	2	1	1
2	1	1	2	3	1	2	3	1	3

9	9	9	9	9	9	9	9	9	9
2	2	2	2	2	2	2	2	2	2
1	3	2	1	1	2	3	1	2	3
1	2	2	3	2	1	1	2	3	3
2	2	2	2	2	2	2	2	2	2
2	2	2	2	2	2	2	2	2	2
9	9	9	9	9	9	9	9	9	2
3	1	3	1	3	3	3	3	3	1
2	2	2	2	2	2	2	2	2	3
9	9	9	9	9	9	9	9	9	2
2	9	1	2	9	1	9	2	1	3
9	9	9	9	9	9	9	9	9	9
9	9	9	9	9	9	9	9	9	2
9	9	9	9	9	9	9	9	9	2
1	3	1	3	1	1	1	1	1	3
9	9	9	9	9	9	9	9	9	2
3	3	1	3	3	3	3	3	3	3
9	9	9	9	9	9	9	9	9	2
2	1	2	3	2	1	3	2	1	2
1	3	1	2	2	2	3	3	3	3
1	3	3	1	2	2	2	1	2	3
3	2	1	2	3	2	1	2	2	2
3	3	2	2	1	1	3	3	2	2
1	3	1	3	1	1	1	1	1	3
3	2	2	2	2	2	2	2	3	3
1	2	2	3	2	1	2	1	2	3
2	2	2	2	2	2	9	1	2	2
2	2	2	2	2	1	1	1	2	3
1	3	1	3	2	2	2	3	3	2
2	3	1	3	1	1	1	2	2	3
2	3	2	2	1	2	2	9	1	3
1	3	1	3	1	1	1	1	1	3
9	9	9	9	9	9	9	9	1	9
9	9	9	9	1	9	9	9	9	9
9	9	9	9	9	9	9	9	9	9
9	9	9	9	1	9	9	9	9	9
1	9	9	9	9	9	9	9	9	9
9	9	9	9	9	9	2	9	9	9
9	9	9	9	3	9	9	9	9	9
9	9	9	9	9	9	9	9	9	2
9	9	9	9	9	9	9	9	9	2
9	9	9	9	9	9	9	9	9	2
9	9	9	9	9	9	9	9	9	2
9	9	9	9	9	9	9	9	9	2
9	9	9	9	9	9	9	9	9	2
1	2	2	3	3	1	2	3	2	3
1	3	2	3	2	1	1	1	2	3

1	3	1	2	3	3	3	2	1	3
9	2	9	9	9	2	9	9	9	2
9	9	9	2	9	2	9	9	9	9
1	3	1	3	2	2	2	2	1	3
9	9	9	9	9	9	9	9	9	2
9	9	9	9	9	9	9	9	9	3
9	9	9	9	9	9	9	9	9	3
9	9	9	9	9	9	9	9	9	9
9	9	9	9	9	9	9	9	9	3
2	2	2	2	2	2	2	2	2	3
2	3	2	3	2	1	2	1	2	3
2	3	2	3	2	1	2	1	2	3
2	2	2	2	2	2	2	2	2	2
1	2	2	2	2	1	3	3	3	9
1	2	2	1	3	3	2	2	2	3
1	2	1	3	2	3	3	2	1	3
1	9	9	9	9	9	9	9	1	3
9	9	9	9	9	9	9	9	3	3
9	9	9	9	9	9	9	9	9	3
9	3	1	3	1	1	9	9	1	3
9	9	9	9	9	9	9	9	9	9
9	9	9	1	9	9	3	9	9	9
9	9	9	9	3	9	9	3	9	1
2	9	9	9	9	2	3	9	9	1
3	1	3	2	9	9	2	9	9	3
9	9	9	9	9	9	9	9	9	9
9	9	9	9	9	9	9	9	1	9
2	2	2	2	2	2	2	2	2	1
3	2	2	3	2	3	2	1	2	1
9	9	9	9	9	9	9	9	9	3
2	2	2	2	2	2	2	2	2	9
1	2	1	1	2	1	1	1	1	1
2	2	2	2	2	2	2	2	2	2
2	2	2	2	2	2	2	2	1	3
3	3	2	2	3	2	3	3	3	9
2	2	2	2	2	2	2	2	2	2
2	1	1	1	2	2	3	2	1	2
2	2	9	2	2	2	2	9	2	2
9	9	9	9	9	9	9	9	9	2
2	2	2	2	2	2	2	2	2	2
2	2	2	2	2	2	2	2	2	2
2	2	2	2	2	2	2	2	2	2
9	9	9	9	9	9	9	9	9	2
2	3	2	2	2	2	2	2	2	3
9	9	9	9	9	9	9	9	9	9
2	2	2	2	2	2	2	2	2	2
2	2	2	2	1	2	2	3	2	2

2	2	2	3	2	1	2	3	1	3
3	3	3	3	3	3	3	3	3	3
2	2	2	2	2	2	2	2	2	3
1	3	1	2	3	1	2	1	2	3
2	1	2	3	2	3	2	1	2	3
1	3	2	2	1	1	1	2	3	3
1	3	1	1	1	1	1	3	3	3
1	3	1	3	2	2	2	1	2	2
1	9	1	3	3	1	3	3	3	3
9	9	9	9	9	9	9	9	9	9
2	2	2	2	2	2	2	2	2	2
2	3	1	3	1	1	3	3	3	3
1	3	1	3	1	1	1	1	1	3
2	2	2	2	2	2	2	2	2	2
2	3	2	2	1	1	1	2	1	3
2	3	2	2	1	2	1	2	3	3
2	2	2	2	2	2	2	2	2	2
2	2	2	2	2	2	2	2	2	2
3	2	2	3	2	3	2	2	2	2
2	3	2	3	2	3	1	2	2	3
1	3	1	2	2	2	2	3	3	3
2	2	2	2	3	3	3	2	3	3
2	3	1	2	2	2	2	2	2	3
3	3	3	2	2	2	3	3	3	3
2	2	2	2	2	2	2	2	2	2
2	3	3	2	1	1	3	3	3	1
3	3	2	3	3	3	3	3	3	3
2	3	2	3	2	2	3	2	3	3
3	2	2	2	2	2	3	2	3	2
2	2	2	2	2	2	2	2	2	2
2	2	2	2	2	2	2	2	2	2
2	2	2	2	2	2	2	2	2	2
2	2	2	2	2	2	2	2	2	2
2	2	2	2	2	2	2	2	2	2
2	1	1	2	3	2	3	1	2	2
2	2	2	2	2	2	2	2	2	2
2	3	3	3	2	3	2	1	2	2
9	9	9	9	9	9	9	9	9	9
9	9	9	9	9	9	9	9	9	9
9	9	9	9	9	9	9	9	9	9
3	2	3	3	2	1	2	1	2	3
9	9	9	9	9	9	9	9	9	9
9	9	9	9	9	9	9	9	9	9
9	9	9	9	9	9	9	9	9	9
9	9	9	9	9	9	9	9	9	9
9	9	9	9	9	9	9	9	9	9
2	2	2	2	2	2	2	3	3	3

1	2	9	3	1	2	3	1	1	3
2	3	1	2	2	2	2	2	2	3
9	9	9	9	9	9	9	9	9	9
2	2	2	2	2	2	2	2	2	1
9	9	9	9	9	9	9	9	9	9
9	9	9	9	9	9	9	9	9	9
2	2	2	2	2	2	2	2	2	3
2	3	2	3	1	3	2	3	3	3
3	3	1	9	2	2	2	3	2	3
3	2	1	2	1	3	1	3	1	3
3	2	2	2	2	2	3	2	2	2
2	2	2	2	2	2	2	2	2	2
2	3	1	2	1	1	1	1	3	3
2	1	1	2	3	1	2	2	2	3
2	3	3	3	3	1	3	3	2	3
2	2	2	2	2	2	2	2	2	2
3	3	1	1	1	3	3	3	3	3
9	9	9	9	9	9	9	1	9	9
3	3	3	3	3	2	3	3	3	3
2	3	3	3	3	2	3	3	3	3
3	2	2	2	1	2	3	2	2	2
2	1	3	2	2	2	2	1	2	2
2	2	2	2	2	2	2	2	2	2
3	3	3	3	3	3	3	3	3	3
1	3	3	1	3	3	3	2	3	3
2	3	2	2	2	2	2	2	2	2
9	9	9	9	9	9	9	9	9	9
2	3	2	2	1	3	2	3	3	3
1	3	2	3	3	3	3	3	3	3
2	1	3	2	2	3	2	2	2	2
3	3	1	1	3	3	1	3	3	3
9	3	9	9	9	9	9	9	9	3
2	2	2	2	2	2	2	2	2	3
2	2	2	2	2	2	2	2	2	2
2	3	2	2	3	3	3	3	3	3
2	3	2	2	2	2	2	9	2	3
1	2	2	1	2	3	2	1	9	9
2	2	2	2	2	2	2	2	2	2
1	3	3	2	2	2	1	2	3	2
3	3	1	3	3	3	3	1	3	3
2	2	2	2	2	2	2	2	3	3
1	3	1	3	1	1	2	1	2	1
3	3	1	1	3	3	3	3	3	3
9	9	9	9	9	9	9	9	9	3
1	3	1	3	1	1	3	1	9	3
1	3	1	3	3	1	3	3	3	3
3	3	1	3	3	2	2	2	2	9

2	2	2	2	2	2	2	2	2	2
2	2	2	2	2	2	2	2	2	2
2	2	2	2	2	2	2	2	2	2
2	2	2	2	2	2	2	2	2	2
2	2	2	2	2	2	2	2	2	2
3	3	2	2	2	2	2	3	3	3
2	2	2	2	2	2	2	2	2	9
2	2	2	2	2	2	2	2	9	2
2	2	2	2	2	2	2	2	2	2
2	2	2	2	2	2	2	2	2	2
1	2	2	2	2	2	2	2	2	9
1	3	2	2	3	3	2	2	3	3
9	9	9	9	9	9	9	9	9	9
3	3	1	1	1	1	3	1	1	3
9	9	9	2	3	9	9	9	9	2
9	9	9	9	9	9	9	9	9	9
2	2	1	3	1	1	2	2	2	2
1	3	1	3	1	1	1	1	1	2
9	3	9	9	9	9	9	9	9	9
1	3	2	2	1	1	1	1	1	3
3	3	1	1	1	3	3	3	3	9
2	3	2	2	2	2	2	2	2	1
2	2	2	2	2	2	2	2	2	1
2	3	3	3	3	2	3	1	3	3
3	3	3	3	3	3	3	3	3	3
9	9	9	9	9	9	9	9	9	9
3	1	3	3	3	3	3	3	3	3
3	1	3	3	3	3	3	3	3	3
1	3	3	3	2	1	3	3	1	3
9	9	9	9	9	9	9	9	9	9
3	3	1	2	3	1	2	2	3	9
1	3	9	9	2	2	2	3	3	9
2	2	2	2	2	2	2	2	2	2
2	9	1	2	2	1	1	1	3	2
9	2	9	9	2	9	2	9	2	2
3	3	3	3	3	3	3	3	3	3
3	3	1	3	3	1	1	1	1	9
9	9	9	9	9	9	9	9	9	9
9	9	9	9	9	9	9	9	9	9
9	9	9	9	9	9	9	9	9	2
3	3	3	3	3	3	3	3	3	3
3	9	2	1	3	3	3	3	3	3
2	1	1	3	1	2	2	2	1	2
1	3	1	2	2	2	1	1	1	9
2	1	1	3	1	1	1	2	3	3
3	3	3	2	1	3	1	1	3	3
2	3	2	3	3	1	2	1	3	2

3	1	3	1	3	3	3	3	3	3
3	2	3	3	2	3	1	2	3	3
2	3	3	3	2	2	3	3	3	3
1	9	9	9	9	9	9	9	9	3
3	3	1	3	3	1	1	2	3	3
2	2	2	3	1	1	2	2	2	9
2	3	3	3	1	2	1	2	1	3
2	2	2	2	2	2	3	3	3	2
3	3	1	2	3	3	3	3	3	3
3	3	3	3	3	3	3	3	3	3
9	9	9	9	9	9	9	9	9	9
9	3	9	9	9	9	9	9	9	3
1	3	1	3	1	1	2	3	3	3
2	3	1	2	1	1	2	2	1	3
9	9	9	9	9	9	9	9	9	3
2	2	2	2	2	2	2	2	2	2
3	3	3	3	3	3	3	3	3	3
1	3	1	3	1	1	1	1	1	2
9	3	9	9	9	9	9	9	9	3
2	3	1	3	2	1	3	1	1	3
2	2	2	2	2	2	2	2	2	3
2	2	2	2	2	2	2	2	2	2
2	3	3	3	3	3	3	3	3	3
2	3	2	2	3	2	3	3	3	3
3	3	1	2	3	3	3	3	3	9
2	3	3	2	2	2	2	2	3	3
2	3	1	2	1	2	1	2	3	3
3	3	3	3	3	3	3	3	3	3
3	3	1	3	1	1	2	2	3	9
3	3	1	1	3	1	3	3	3	3
2	1	9	1	9	3	3	9	9	3
9	9	9	9	9	9	9	9	9	9
9	9	9	9	9	9	9	9	9	9
9	9	9	9	9	9	9	9	9	9
9	9	9	9	9	9	9	9	9	9
2	9	2	3	2	2	2	2	2	3
2	2	2	2	2	2	2	2	2	2
1	3	1	3	1	9	9	9	9	9
9	3	9	9	9	9	9	9	9	9
9	9	9	9	9	9	9	9	9	9
9	3	9	9	9	9	9	9	9	3
9	3	9	9	9	9	9	9	9	3
3	3	3	3	3	3	3	3	3	9
1	3	1	2	2	2	2	2	1	9
1	3	2	2	2	2	2	2	1	1
2	3	1	2	2	2	2	2	2	3
9	3	9	9	9	9	9	9	9	9

9	9	9	9	9	9	9	9	9	9
9	3	9	9	9	9	9	9	9	3
9	9	9	9	9	9	9	9	9	3
9	3	9	9	9	9	9	9	9	3
9	3	9	9	9	9	9	9	9	3
9	9	9	3	9	9	9	9	9	9
1	2	2	2	2	2	1	1	1	9
9	9	9	9	9	9	9	9	9	9
2	2	2	2	2	2	2	2	2	2
9	9	9	9	9	9	9	9	9	3
3	3	2	3	3	3	3	3	3	2
3	3	3	3	2	1	3	3	3	9
1	3	3	1	3	1	1	1	3	3
3	3	2	2	2	2	2	2	2	3
2	3	3	2	2	2	1	2	1	2
3	2	3	1	2	2	2	1	1	3
9	3	1	2	1	2	3	2	2	9
9	9	9	3	9	9	9	9	9	3
2	9	2	3	1	2	3	2	1	9
9	2	9	9	9	9	2	9	2	2
9	2	9	9	9	9	9	9	9	9
9	9	9	9	9	9	9	9	9	3
2	3	1	3	2	2	2	2	2	2
3	3	3	3	2	3	3	3	3	9
2	2	2	2	2	2	2	2	2	2
3	3	1	2	3	3	3	3	3	3
9	3	1	3	1	1	1	9	9	9
1	2	3	3	2	1	1	2	3	9
2	3	2	2	3	2	3	2	3	3
2	2	2	2	2	2	2	2	2	2
2	3	2	3	2	2	2	2	3	3
3	3	3	3	3	1	3	3	3	3
1	3	1	3	3	1	1	1	3	3
2	3	3	3	2	2	3	2	3	3
1	1	1	1	3	3	3	3	3	3
9	9	9	9	9	9	9	9	9	3
3	3	3	3	3	3	3	3	3	3
3	3	3	3	1	3	3	3	3	3
3	2	1	3	2	3	2	3	3	2
3	3	2	2	3	3	3	3	3	9
1	3	3	2	2	1	3	3	2	3
2	2	9	3	9	9	9	9	3	3
1	3	1	3	1	3	3	3	2	3
9	9	9	9	9	9	9	9	9	9
3	2	3	2	2	2	3	3	2	2
2	2	2	2	2	2	2	2	2	3
2	2	1	1	2	2	2	2	2	3

9	3	9	9	9	9	9	9	9	9
3	3	1	3	1	1	1	1	1	9
3	3	1	3	1	1	1	1	1	3
3	3	3	2	3	2	3	3	3	9
3	2	3	2	3	2	3	2	2	1
2	2	1	2	2	2	2	2	2	2
3	3	2	3	1	2	3	3	3	3
2	3	1	2	2	1	2	1	3	3
2	3	2	2	2	2	2	2	3	3
3	3	1	3	3	3	3	3	3	3
2	3	1	2	2	1	2	1	3	3
3	3	1	1	1	3	1	1	1	3
3	3	9	9	9	1	9	1	9	3
2	2	1	1	3	3	3	3	3	3
2	3	2	2	2	2	2	2	3	2
1	3	1	3	2	1	3	2	1	3
9	9	9	9	9	9	9	9	9	9
2	3	9	2	3	2	1	3	2	3
9	9	9	9	9	9	9	9	9	9
9	9	9	9	9	9	9	9	9	9
9	3	1	3	2	1	2	2	2	3
1	3	3	3	3	3	3	3	3	3
9	9	9	9	9	9	9	9	9	3
3	3	3	3	3	3	3	1	3	3
1	3	1	3	1	1	1	1	1	2
2	2	2	2	2	3	3	2	3	2
2	2	1	2	2	1	2	2	1	1
1	3	3	3	1	2	3	2	3	9
3	3	1	3	3	3	3	3	3	3
2	1	1	2	2	2	2	2	9	9
3	3	3	3	3	3	3	3	3	3
2	2	1	1	2	3	2	3	2	3
1	3	3	3	3	3	3	2	3	3
1	3	3	3	3	3	3	2	3	3
2	3	1	1	3	3	3	3	3	9
1	3	3	3	1	1	1	1	1	1
1	3	2	2	2	2	2	2	9	2
2	3	3	3	3	3	3	3	3	3
2	1	2	2	2	3	3	1	2	3
2	2	2	2	2	2	2	2	2	2
2	3	2	2	3	2	2	3	3	9
2	3	2	2	3	2	2	1	3	3
9	9	9	9	9	9	9	9	9	3
3	3	9	1	3	3	3	3	3	9
2	3	1	3	2	2	3	2	2	3
9	3	1	3	1	1	1	1	1	3
1	3	1	3	1	1	2	2	2	3

1	2	2	3	1	2	2	3	2	2
9	9	9	9	9	9	9	9	9	9
9	9	3	9	2	3	3	3	3	2
9	9	9	9	9	9	9	9	9	9
3	3	3	3	3	3	3	3	3	3
2	2	2	2	2	2	2	2	2	2
2	2	2	2	2	2	2	2	2	9
9	9	9	9	9	9	9	9	9	2
3	3	3	3	3	3	3	3	3	3
3	3	3	3	3	3	3	3	3	3
9	9	9	9	9	1	9	9	9	3
2	3	2	2	2	1	3	3	3	3
2	3	1	3	1	1	1	1	1	3
1	3	1	3	2	1	2	3	3	3
9	9	9	9	9	9	9	9	9	9
9	9	9	9	9	9	9	9	9	2
9	9	9	9	9	9	9	9	9	9
3	1	3	1	3	3	3	3	3	1
3	3	3	3	3	3	3	3	3	3
3	3	1	3	3	3	1	2	3	3
2	3	1	2	1	1	1	1	3	9
2	3	3	2	3	3	3	3	3	3
3	3	3	3	3	3	3	3	3	3
2	3	3	3	3	3	3	2	3	3
2	2	2	2	2	2	3	3	2	2
3	1	3	2	3	3	3	3	3	3
3	3	3	3	3	3	3	3	3	3
2	2	2	2	2	2	2	2	3	3
1	3	2	2	2	2	3	3	3	3
1	3	3	3	3	3	3	1	3	3
9	9	9	9	9	9	9	9	9	9
1	3	1	1	1	3	1	3	3	3
9	9	9	9	9	9	9	9	9	9
2	3	3	2	3	2	3	2	3	3
2	3	1	1	3	2	3	3	3	3
9	9	9	9	9	9	9	9	9	9
1	2	2	2	2	2	2	2	1	2

AfraidofHP	KnowMore	HPVVaccin	HPVVaccin	Embarrass	TooBusyfo	HPVVaccin	HPVAgains	HeardCxCA	CxScreen2
2	2	2	2	2	2	2	2	2	2
1	1	1	1	1	2	2	2	2	2
9	9	9	9	9	9	9	9	2	2
1	1	1	1	1	2	2	2	1	1
1	1	1	1	1	2	2	2	2	2
1	1	1	1	1	2	2	2	1	1
9	9	9	2	9	9	9	9	2	2
2	1	1	1	3	2	3	3	1	2
2	2	1	2	2	2	2	2	2	2
9	9	9	9	9	9	9	9	2	2
2	3	2	3	2	2	2	2	2	2
2	2	2	3	1	1	3	1	2	2
3	1	1	1	3	3	3	3	1	2
1	1	3	2	2	3	3	3	1	2
2	1	2	1	3	3	3	3	2	2
2	1	3	2	3	3	3	3	1	2
1	3	2	1	2	2	3	2	1	1
2	1	3	2	3	3	3	3	1	2
2	1	3	2	3	3	3	3	1	2
3	3	1	1	1	1	1	3	2	2
2	1	1	1	1	2	1	3	1	1
2	1	1	1	1	2	1	3	1	1
2	2	2	2	2	2	2	2	2	2
1	1	3	1	1	3	1	3	1	1
2	2	2	2	2	2	2	2	2	2
1	1	2	1	2	3	2	3	2	2
1	1	1	1	3	3	3	2	2	2
1	1	3	1	3	3	3	3	2	2
2	2	2	2	2	2	9	2	2	2
2	2	2	2	2	2	2	2	2	2
2	2	2	2	2	2	2	2	2	2
2	1	2	2	2	2	2	2	1	1
3	1	3	1	3	3	3	3	2	2
2	1	2	1	3	3	3	3	1	1
2	2	2	2	2	2	2	2	1	1
2	1	2	1	3	2	1	1	1	1
3	2	2	2	2	2	2	2	1	1
9	9	9	9	9	9	9	9	1	1
2	2	2	2	2	2	1	2	#NULL!	#NULL!
2	1	1	2	2	2	2	2	2	2
2	2	2	2	2	2	1	2	2	2
2	1	2	2	2	3	2	2	1	2
9	9	9	9	9	9	9	9	#NULL!	#NULL!
3	1	3	1	3	3	1	3	1	1
3	1	1	1	3	3	1	3	2	2
1	2	2	3	1	2	2	1	1	2

3	9	3	3	3	3	3	3	1	1
3	1	1	1	1	1	3	3	1	2
2	2	3	1	2	3	3	3	1	1
1	1	1	2	1	3	2	3	1	1
1	1	3	1	3	3	3	3	1	1
3	1	2	1	3	3	3	3	1	1
3	1	3	1	3	3	3	3	1	1
3	1	1	1	3	3	3	3	1	1
9	1	9	9	9	9	9	9	1	1
2	1	2	1	3	3	2	3	1	1
9	2	2	9	2	2	2	9	2	2
2	1	3	3	3	3	3	3	1	1
2	1	2	2	3	3	3	3	1	1
9	9	9	9	9	9	9	9	2	2
1	1	1	1	3	3	3	3	2	2
1	1	2	2	3	2	3	3	1	1
3	1	3	1	3	3	3	3	1	1
9	9	9	9	9	9	9	9	1	1
3	1	1	2	1	2	3	3	1	2
9	1	2	2	1	1	2	9	1	1
3	1	3	3	1	3	3	3	1	1
3	1	3	3	3	1	1	3	1	1
3	1	3	3	1	3	3	3	1	1
9	9	9	9	9	9	9	9	1	2
9	1	9	9	9	9	9	9	1	1
9	1	9	1	3	1	3	3	1	1
9	9	9	9	9	9	9	9	#NULL!	#NULL!
2	1	1	1	2	3	3	3	1	2
1	1	3	1	3	3	3	3	2	2
2	1	2	1	3	3	3	3	1	1
1	2	1	2	1	2	1	1	1	1
2	1	3	1	3	1	2	3	1	1
9	1	1	1	1	2	2	1	1	1
3	1	3	1	3	3	3	3	1	1
2	1	3	1	3	3	3	3	1	1
9	9	9	9	9	9	9	9	2	2
9	9	9	9	9	9	9	9	#NULL!	#NULL!
3	1	3	1	3	3	3	3	#NULL!	#NULL!
3	1	3	1	3	3	3	3	2	1
1	1	1	1	3	3	1	3	1	1
3	1	2	2	3	3	3	3	1	1
1	1	2	9	3	9	9	3	2	2
3	1	3	1	3	3	1	3	1	1
2	1	1	1	3	2	3	3	1	1
2	1	1	1	3	2	3	3	2	2
9	9	9	9	9	9	9	9	1	1
9	9	9	9	9	9	9	9	2	2

9	9	9	9	9	9	9	9	1	1
3	1	3	2	3	3	9	3	1	1
1	1	1	2	1	3	3	3	1	1
2	1	1	3	3	3	3	1	1	#NULL!
9	9	9	9	9	9	9	9	2	2
1	2	2	2	2	2	2	2	2	1
3	1	3	1	3	3	3	3	1	1
1	1	3	1	3	3	3	3	1	1
1	1	1	2	1	3	3	3	1	1
3	1	3	1	3	3	3	3	1	1
3	1	3	1	2	3	2	2	2	2
3	1	2	1	3	3	3	3	1	#NULL!
1	1	2	2	2	2	3	3	1	1
1	1	2	2	2	2	3	3	2	2
2	1	2	2	3	2	2	1	2	2
2	1	1	2	3	2	2	2	1	2
2	1	3	3	3	3	3	3	2	2
2	1	2	2	2	2	3	3	2	2
9	9	9	9	9	9	9	9	2	2
3	1	3	1	3	3	3	3	2	2
9	1	3	1	3	3	3	3	2	2
9	9	9	9	9	9	9	9	2	2
1	2	2	3	2	2	1	2	2	2
3	1	1	3	1	3	1	1	2	2
1	1	1	2	2	1	2	1	2	2
2	1	3	2	2	3	3	3	2	2
3	1	1	1	3	3	3	3	1	1
3	1	1	2	3	3	3	3	1	1
2	2	2	2	2	2	2	2	2	2
3	1	3	1	3	3	3	3	2	2
2	1	1	3	1	9	3	3	2	2
2	2	2	2	2	2	2	1	2	2
3	1	3	1	3	3	3	3	2	2
2	2	2	2	2	2	2	2	2	2
3	1	3	1	3	2	3	3	2	2
2	1	1	2	2	2	2	2	2	2
1	1	2	1	3	2	2	2	1	1
2	2	3	1	3	2	3	3	2	2
1	1	2	3	2	1	2	9	1	1
3	1	3	3	3	3	3	3	1	1
1	1	2	1	3	3	3	3	2	2
1	1	2	1	3	3	3	3	2	2
2	2	2	2	2	2	2	2	2	2
3	1	3	2	3	3	3	3	1	1
1	1	3	3	2	2	3	2	2	2
1	1	1	1	3	3	3	3	1	2
2	1	2	3	3	1	3	2	2	2

1	2	2	2	3	3	2	3	2	2
2	1	1	2	1	3	3	2	2	2
2	1	1	2	1	3	3	2	1	1
1	1	2	1	3	3	3	3	2	2
1	1	2	1	3	3	3	3	2	2
9	9	9	9	9	9	9	9	2	2
3	2	2	3	3	3	3	2	2	2
3	1	3	1	3	3	3	3	2	2
2	2	2	2	2	2	2	2	2	2
2	2	2	2	2	2	2	2	2	2
9	9	9	9	9	9	9	9	2	2
9	9	9	9	9	9	9	9	2	2
2	2	9	9	2	9	2	9	2	2
1	1	2	2	3	3	3	3	2	2
2	2	2	3	3	3	3	3	1	1
2	2	2	3	3	3	3	3	1	1
9	9	9	9	9	9	9	9	1	1
1	1	3	3	3	3	3	1	2	2
9	9	1	9	9	9	9	9	1	2
2	1	3	1	1	3	3	1	1	2
2	1	3	1	3	3	3	3	#NULL!	1
2	1	3	1	3	3	3	3	2	2
2	1	3	1	3	3	3	3	2	2
2	1	3	1	1	3	3	3	2	2
2	1	3	1	3	3	3	3	2	2
2	1	3	1	3	3	3	3	2	#NULL!
2	1	3	1	3	3	3	3	1	2
2	1	3	1	3	3	3	3	1	1
2	1	3	1	3	3	3	3	2	2
2	1	3	1	3	3	3	3	1	2
2	3	2	2	1	2	9	2	1	1
2	2	2	2	2	2	2	2	2	2
2	1	3	1	3	3	3	3	#NULL!	#NULL!
2	1	3	1	3	3	3	3	2	2
2	1	3	1	3	3	3	3	1	2
2	1	3	1	3	3	3	3	2	2
2	1	3	1	3	3	3	3	1	1
2	1	3	1	3	3	3	3	1	2
2	1	3	1	3	3	3	3	2	2
2	1	3	1	3	3	3	3	2	1
2	1	3	1	3	3	3	3	2	2
2	1	3	1	3	3	3	3	2	2
2	1	3	1	3	3	3	3	#NULL!	#NULL!
2	1	3	1	3	3	3	3	1	2
2	1	3	1	3	3	3	3	1	2
2	1	3	1	3	3	3	3	1	2
2	1	3	1	3	3	3	3	1	2

2	1	3	1	3	3	3	3	1	2
2	1	3	1	1	3	3	3	2	2
2	1	3	1	3	1	3	3	2	2
2	1	3	1	1	3	1	1	1	1
2	1	3	1	1	3	3	3	2	2
2	1	3	1	3	3	3	3	1	1
2	1	3	1	3	3	3	3	2	1
3	1	3	1	3	3	3	3	1	1
1	1	1	1	3	3	1	3	1	1
3	1	3	3	3	3	3	3	1	1
9	9	9	9	9	9	9	9	#NULL!	#NULL!
9	9	9	9	9	9	9	9	1	1
1	9	1	1	3	1	1	3	1	1
2	1	3	1	3	3	3	3	1	2
9	9	9	9	9	9	9	9	2	2
1	1	1	2	1	1	3	3	2	2
2	2	2	2	2	2	2	1	1	2
1	1	1	1	1	1	1	1	1	2
2	1	2	2	1	3	2	3	1	2
2	1	3	1	3	3	3	3	1	2
1	1	1	2	2	2	1	1	1	1
2	2	2	2	2	2	2	2	1	2
2	1	1	1	2	1	2	3	2	2
2	1	1	3	2	1	2	3	1	2
2	2	2	2	2	2	2	2	2	2
2	2	2	2	2	2	2	2	2	2
2	1	2	2	1	3	3	3	1	2
2	2	2	2	2	2	2	2	2	2
2	2	2	2	2	2	2	2	2	2
2	1	2	2	1	3	3	3	1	2
3	1	3	1	3	3	3	3	1	1
3	1	3	1	3	3	3	3	1	1
3	1	2	2	1	3	3	3	1	2
1	1	1	1	2	2	2	3	1	1
1	1	2	1	2	2	2	2	1	1
2	2	2	2	2	2	2	2	1	1
2	2	2	2	2	2	2	1	2	2
1	1	3	2	3	2	2	3	1	1
2	1	2	2	2	2	2	2	2	2
1	1	2	2	1	2	1	1	1	2
1	2	2	2	2	2	2	2	1	1
2	1	2	1	3	3	3	3	1	2
9	9	9	9	9	9	9	9	2	2
9	9	9	9	9	9	9	9	2	2
2	1	1	2	1	2	2	2	1	1
3	1	3	1	3	3	3	3	1	1
9	9	9	9	9	9	9	9	2	1

1	1	1	1	1	1	1	2	2	2
1	1	1	1	1	1	1	2	2	2
2	1	2	2	2	2	2	3	2	2
2	1	1	1	1	2	2	2	2	2
1	3	3	3	1	2	1	1	2	2
2	2	2	2	2	2	2	2	1	1
9	2	9	2	9	9	9	3	2	2
2	1	2	2	1	2	2	2	2	2
2	2	2	2	1	2	3	2	1	1
2	2	2	2	2	2	2	2	2	2
2	1	2	2	2	2	2	2	2	2
2	2	2	2	2	2	2	2	1	2
2	2	2	2	2	2	2	2	1	2
3	1	2	1	1	3	3	2	1	2
3	3	1	1	3	3	3	3	2	2
1	3	2	2	3	1	2	2	2	2
3	1	2	3	2	3	3	2	1	2
3	1	1	2	3	2	1	3	2	2
9	1	9	9	9	9	1	3	2	2
3	1	3	1	3	3	3	3	2	1
1	1	1	1	1	1	1	3	2	2
2	1	1	2	2	2	2	2	2	2
3	1	1	1	3	3	2	3	1	2
9	9	2	2	9	9	9	3	2	2
9	1	9	9	1	9	9	9	2	2
2	2	2	2	2	2	2	2	2	2
2	2	2	2	2	2	2	2	2	2
9	2	9	2	1	3	2	2	1	2
9	9	9	9	9	9	9	9	2	2
2	1	2	1	3	2	3	3	2	2
9	9	9	9	9	9	9	9	2	1
3	3	9	1	3	3	2	3	2	2
1	1	1	1	1	1	2	1	2	2
1	1	3	1	1	3	2	3	1	1
9	9	9	9	9	9	9	9	1	2
2	2	2	2	2	2	2	1	2	2
2	1	3	1	2	3	3	3	2	2
3	1	1	1	2	3	2	3	1	2
1	1	1	1	2	2	2	2	1	2
3	1	1	1	3	3	3	3	2	1
1	1	1	1	1	1	1	1	1	1
2	1	3	1	1	1	3	3	1	1
9	9	9	9	9	9	9	9	1	1
2	2	2	2	2	2	2	2	1	2
2	1	2	1	2	1	2	1	1	2
1	2	3	1	2	2	2	2	1	2
1	1	1	2	3	2	2	3	2	2

9	9	9	9	9	9	9	9	#NULL!	#NULL!
9	9	9	9	9	9	9	9	#NULL!	#NULL!
9	9	9	9	9	9	9	9	1	1
2	1	3	1	3	3	3	3	1	1
1	2	3	2	3	2	3	9	2	2
2	2	3	1	2	3	2	2	2	2
2	1	1	1	2	2	2	3	1	1
3	1	3	1	3	3	1	3	1	1
9	9	9	9	9	9	9	9	1	2
2	2	2	2	2	2	2	2	1	2
2	2	2	2	2	2	2	2	2	2
2	1	3	2	2	2	3	3	1	1
9	9	9	9	9	9	9	9	#NULL!	#NULL!
3	3	3	3	3	3	3	3	2	2
3	9	1	3	1	1	1	3	1	1
9	9	9	9	9	9	9	9	#NULL!	#NULL!
9	9	9	9	9	9	9	9	1	1
1	1	1	1	1	3	2	2	2	2
1	1	1	1	2	2	1	1	1	2
9	9	9	9	9	9	9	9	#NULL!	#NULL!
9	9	9	9	9	9	9	9	2	2
3	1	2	1	2	2	2	2	1	2
1	1	1	1	1	1	3	3	1	1
1	1	2	2	2	2	2	2	1	2
2	2	2	2	2	2	2	2	1	2
3	1	1	1	3	3	3	3	#NULL!	2
2	1	1	1	1	2	1	2	1	1
2	1	2	1	3	3	3	3	2	2
2	1	3	1	1	3	3	1	1	2
3	1	3	1	3	3	3	3	1	2
2	2	2	2	2	2	2	2	1	2
9	9	9	9	9	9	9	9	2	2
9	9	9	9	9	9	9	9	1	1
2	2	2	2	2	2	2	3	2	2
3	1	3	2	3	3	3	3	1	2
1	1	1	2	1	3	1	3	1	1
3	1	3	1	3	3	3	3	1	1
1	1	3	1	3	3	3	3	2	1
3	1	3	1	3	3	1	3	1	1
2	1	3	1	2	3	3	3	1	2
2	1	2	3	2	2	1	9	1	1
1	1	3	1	1	3	3	3	1	1
9	3	2	2	3	9	2	2	1	1
9	9	9	9	9	9	9	9	2	2
3	1	3	1	3	3	3	3	1	1
2	2	2	2	2	2	2	2	1	1
3	1	3	1	1	3	3	3	1	2

9	9	9	9	9	9	9	9	2	2
2	1	3	1	3	2	2	3	2	2
9	9	9	9	9	9	9	9	1	1
3	1	3	1	3	3	3	3	1	2
9	9	9	9	9	9	9	9	1	1
9	9	9	9	9	9	9	9	1	2
3	1	1	2	2	3	3	3	1	1
2	9	3	9	2	1	2	9	1	2
2	1	3	1	3	3	3	3	1	2
2	1	1	1	3	3	3	3	1	2
2	2	2	2	2	2	2	2	2	2
2	1	3	2	3	3	2	2	1	1
2	1	2	1	3	2	3	3	1	1
2	2	1	1	2	3	2	2	2	2
2	1	3	1	3	3	3	2	2	2
3	2	2	2	3	3	2	2	2	#NULL!
2	2	2	2	2	2	2	2	2	2
2	2	2	2	2	2	2	2	1	2
2	2	2	2	2	2	2	2	1	1
2	2	2	2	2	2	2	3	2	2
3	1	3	2	3	3	3	3	1	2
3	1	3	1	3	3	3	3	2	2
3	1	3	1	3	3	3	3	1	1
3	1	2	1	1	3	1	3	1	1
2	2	2	2	2	2	2	2	2	2
1	1	2	2	2	2	2	2	2	2
2	1	2	2	2	3	2	3	1	2
2	2	2	2	2	2	2	2	2	2
2	1	2	3	2	2	2	3	1	1
2	1	1	2	1	1	1	2	2	2
1	1	2	2	3	2	2	2	2	2
1	1	2	2	3	2	3	3	2	2
2	1	1	1	1	1	1	1	2	2
9	9	9	9	9	9	9	9	2	2
9	9	9	9	9	9	9	9	2	2
3	1	2	1	3	2	2	3	1	1
2	1	2	2	2	2	3	9	2	2
9	9	9	9	9	9	9	9	2	1
2	2	2	2	2	2	2	2	2	2
2	2	2	2	2	2	2	2	2	2
2	2	2	2	2	2	2	2	2	2
2	1	2	2	2	2	2	2	2	2
2	1	2	1	3	3	2	2	1	2
2	1	2	2	2	2	2	2	2	2
2	1	2	2	2	1	3	2	2	2
1	1	3	2	1	2	3	3	1	1
2	1	1	1	2	3	2	2	1	2

2	3	3	1	3	3	2	3	1	2
2	1	1	3	3	3	2	3	1	1
2	2	2	2	2	2	2	9	1	1
2	2	2	2	2	2	2	2	2	2
3	1	2	1	3	3	3	3	2	2
2	2	9	2	2	2	2	2	1	2
9	9	9	9	9	9	9	9	#NULL!	#NULL!
3	1	1	1	1	2	3	3	1	1
3	1	1	2	3	3	1	2	1	1
1	1	1	1	3	3	3	3	1	2
2	2	2	2	1	1	2	3	2	2
1	1	1	1	1	3	1	3	2	2
2	2	2	2	2	2	2	2	2	2
1	1	1	1	3	1	2	3	2	2
2	2	2	2	2	2	2	2	2	2
1	2	9	1	1	1	1	1	#NULL!	#NULL!
3	1	1	3	1	1	1	3	2	2
2	1	1	2	1	2	2	3	2	2
2	1	1	1	1	3	2	3	2	2
9	2	2	2	2	2	2	2	1	2
1	1	1	2	1	1	2	2	1	1
2	2	2	2	2	2	2	2	1	2
9	9	9	9	9	9	9	9	#NULL!	#NULL!
2	2	2	2	2	2	2	9	2	1
1	1	1	1	1	1	3	3	1	1
9	9	9	9	9	9	9	9	#NULL!	#NULL!
2	1	1	2	2	3	3	2	2	2
9	9	9	9	9	9	9	9	#NULL!	#NULL!
2	2	2	2	2	2	2	2	1	1
2	1	2	1	2	2	2	2	1	1
9	9	9	9	9	9	9	9	2	2
9	9	9	9	9	9	9	9	#NULL!	#NULL!
2	2	2	2	2	2	2	2	2	1
1	1	1	1	1	2	1	2	2	2
9	9	9	9	9	9	9	9	1	2
1	1	2	2	1	2	2	1	1	1
3	2	2	3	3	3	3	2	1	2
1	2	1	2	1	2	3	1	1	2
9	9	9	9	9	9	9	9	#NULL!	#NULL!
1	1	3	1	1	1	3	3	1	2
9	9	9	9	9	9	9	9	2	2
1	1	1	1	1	2	2	2	2	2
2	2	2	2	2	2	2	3	1	2
2	2	2	2	2	2	2	2	1	2
1	1	1	1	3	3	3	3	#NULL!	#NULL!
2	9	3	1	3	3	3	3	2	2
2	1	3	1	3	9	9	9	2	2

9	9	9	9	9	9	9	9	2	2
2	1	3	1	3	3	1	3	2	2
2	1	3	1	3	3	1	3	2	2
2	1	3	1	3	3	3	1	1	2
2	1	3	1	3	3	3	3	2	2
2	1	3	1	3	3	3	3	1	1
2	1	3	1	3	3	3	3	2	2
2	1	3	1	3	3	1	3	2	2
2	1	3	1	3	3	1	3	2	2
2	1	1	3	3	3	2	3	1	2
2	2	1	1	2	2	1	1	2	2
2	1	3	1	1	3	1	1	2	2
2	1	3	1	3	3	3	3	1	1
2	1	3	1	3	3	1	3	2	2
2	1	3	1	3	3	1	3	#NULL!	2
2	1	3	1	3	3	3	3	1	1
2	1	3	1	3	3	2	2	2	2
2	1	3	1	3	3	1	3	1	2
2	1	3	1	1	3	1	3	1	2
2	1	3	1	1	3	1	3	1	2
1	1	3	1	3	3	3	3	1	2
2	1	3	1	3	3	3	3	1	2
2	1	3	1	1	3	1	3	2	2
2	1	3	1	3	3	1	3	1	1
2	1	3	1	3	3	3	3	2	2
2	1	3	1	3	3	3	3	2	2
2	1	3	1	3	3	3	3	2	2
2	1	3	1	3	3	3	3	#NULL!	2
2	1	3	1	3	3	3	1	2	1
2	1	3	1	2	3	3	3	2	2
2	1	3	1	3	3	3	3	2	1
2	1	3	1	3	3	2	3	2	2
2	1	3	1	3	3	3	3	2	1
2	1	3	1	3	3	3	3	2	2
2	1	3	1	3	3	3	3	1	1
2	1	3	1	3	3	3	3	1	1
3	1	2	1	3	3	2	3	1	1
2	1	3	1	3	3	3	3	2	1
2	1	3	1	3	3	3	3	2	2
2	1	3	1	3	3	3	3	1	1
2	1	3	1	3	3	3	3	1	2
2	2	2	2	2	2	2	2	2	2
2	1	1	1	1	3	3	3	1	2
2	2	2	2	2	2	2	2	2	2
9	9	9	9	9	9	9	9	2	2
2	2	2	2	2	2	2	2	2	2
1	1	1	2	3	3	3	3	1	2

2	2	2	2	2	2	2	2	2	2
3	3	3	1	2	3	3	1	1	1
3	3	3	1	2	3	3	1	1	1
2	2	3	2	2	2	2	2	1	1
2	1	3	1	3	1	2	2	2	2
9	9	9	9	9	9	9	9	2	2
2	2	2	2	2	2	2	2	2	1
2	2	1	2	3	2	2	2	2	2
3	1	2	1	3	3	3	3	1	1
2	1	2	2	2	2	2	2	1	2
3	3	3	1	2	3	3	1	1	1
9	9	9	9	9	9	9	9	1	2
3	1	1	2	1	2	3	3	1	1
1	1	2	2	2	2	2	2	2	2
9	9	9	9	9	9	9	9	1	2
9	9	9	9	9	9	9	9	1	1
9	9	9	9	9	9	9	9	2	2
9	9	9	9	9	9	9	9	2	2
9	9	9	9	9	9	9	9	2	2
3	1	2	1	1	3	1	3	1	2
9	9	9	9	9	9	9	9	1	2
9	9	9	9	9	9	9	9	1	2
3	1	2	1	2	1	1	1	1	1
9	9	9	9	9	9	9	9	2	2
9	1	9	9	9	9	9	9	2	2
9	9	9	9	9	9	9	9	#NULL!	#NULL!
9	9	9	9	9	9	9	9	1	1
9	9	9	9	9	9	9	9	2	2
2	1	2	1	3	3	3	3	2	2
9	9	9	9	9	9	9	9	1	2
3	9	9	9	9	9	9	9	1	1
1	1	2	1	2	2	2	3	2	2
2	2	2	2	2	2	2	2	2	2
3	1	3	1	3	3	3	3	1	1
2	1	1	2	1	2	2	2	2	2
2	1	2	1	2	2	2	2	1	1
3	1	3	1	3	2	2	3	1	1
3	1	3	1	3	3	3	3	1	2
2	2	2	2	2	2	2	2	2	2
2	1	2	1	3	3	3	3	1	2
2	2	2	2	2	3	2	2	2	2
2	1	3	1	3	3	3	3	1	1
2	2	2	2	2	2	2	2	2	2
9	9	9	9	9	9	9	9	2	2
2	1	1	2	2	3	2	3	2	2
9	9	9	9	9	9	9	9	1	1
2	2	3	1	3	3	3	3	2	2

2	1	1	2	2	2	1	2	1	1
3	1	3	1	3	3	3	3	1	1
2	1	3	1	3	3	3	3	2	2
1	1	1	2	2	2	2	3	2	2
3	1	2	1	2	2	1	2	1	2
2	2	2	1	2	2	2	2	2	2
3	1	2	3	2	3	2	3	2	2
2	2	2	2	2	2	2	2	2	1
2	2	2	2	2	2	2	2	2	2
2	2	2	2	2	2	2	2	2	2
2	2	2	2	2	2	2	2	2	2
2	1	3	3	2	3	3	3	1	1
2	2	2	2	2	2	2	2	2	2
2	2	2	2	2	2	2	2	2	2
3	1	3	2	3	3	3	2	1	1
2	2	2	2	2	2	2	2	2	2
2	2	2	2	2	2	2	2	2	2
2	2	2	1	2	2	2	2	2	2
2	1	1	2	2	3	2	3	1	2
3	1	1	1	3	2	3	3	1	1
2	2	2	1	2	2	2	2	2	2
3	1	3	1	3	3	3	3	1	1
2	1	1	1	3	3	3	3	1	1
2	1	1	1	3	3	3	3	2	2
3	2	2	2	1	1	2	3	2	2
2	1	2	1	2	2	2	2	2	2
9	9	9	9	9	9	9	9	1	1
2	1	1	2	1	1	2	1	1	2
1	1	3	1	1	3	1	2	1	1
2	3	3	3	3	3	3	2	1	2
2	2	2	2	2	2	2	9	#NULL!	#NULL!
2	1	1	2	2	1	2	2	2	2
2	1	2	2	2	2	2	2	2	2
2	1	2	2	3	2	2	2	2	2
2	2	2	2	2	2	2	3	2	2
9	9	9	9	9	9	9	9	2	2
2	1	3	1	2	3	3	3	2	2
3	1	3	1	3	3	3	3	1	1
2	1	3	2	2	2	2	2	2	2
9	9	9	9	9	9	9	9	1	1
9	1	9	9	9	9	9	9	#NULL!	#NULL!
1	1	2	1	2	2	2	3	2	2
1	1	2	1	2	2	2	3	2	2
2	1	2	3	2	1	2	3	1	#NULL!
3	1	3	1	3	3	3	3	1	1
2	2	2	2	2	2	2	2	2	2
9	9	9	9	9	9	9	9	2	2

9	9	9	9	9	9	9	9	1	1
9	9	9	1	9	9	9	9	2	2
9	9	9	9	9	9	9	9	2	2
9	9	9	9	9	9	9	9	#NULL!	#NULL!
9	9	9	9	9	9	9	9	2	1
9	9	9	9	9	9	9	9	1	2
9	9	9	9	9	9	9	9	2	2
9	9	9	9	9	9	9	9	2	2
9	9	9	9	9	9	9	9	2	2
9	9	9	9	9	9	9	9	1	1
9	9	9	9	9	9	9	9	2	2
9	9	9	9	9	9	9	9	1	1
9	9	9	9	9	9	9	9	2	2
9	9	9	9	9	9	9	9	1	1
9	9	9	1	9	9	9	9	2	1
9	9	9	9	9	9	9	9	2	2
9	9	9	9	9	9	9	9	1	2
9	9	1	3	3	1	1	3	1	2
2	2	2	2	2	2	2	2	2	2
9	9	9	9	9	9	9	9	1	1
1	1	1	1	1	1	1	1	1	1
2	2	3	3	2	1	2	1	2	2
2	1	1	2	1	2	2	2	2	2
1	1	1	1	1	1	1	1	2	1
2	1	2	2	2	3	2	2	2	2
3	3	3	2	2	2	2	2	2	1
2	2	2	2	2	2	2	2	1	1
2	3	2	1	2	2	1	2	2	2
2	2	2	2	2	2	2	2	2	2
1	1	1	1	1	1	1	1	1	2
1	1	1	2	2	2	3	3	#NULL!	#NULL!
3	1	2	1	3	2	3	3	2	2
2	2	2	2	2	2	2	2	2	2
3	1	1	1	3	3	3	3	1	1
2	1	1	1	2	2	3	3	2	2
1	1	2	2	1	2	1	3	2	2
2	2	2	2	2	2	2	2	2	2
9	9	9	9	9	9	9	9	#NULL!	#NULL!
1	1	1	1	1	1	1	1	1	1
9	9	9	9	9	9	9	9	1	2
3	3	3	2	2	3	2	3	1	2
2	2	3	3	2	9	2	2	1	1
9	9	9	9	9	9	9	9	#NULL!	#NULL!
1	1	1	1	1	1	1	3	1	2
2	2	3	3	2	2	2	2	2	2
9	9	9	9	9	9	9	9	1	2
3	3	3	3	3	3	3	3	2	2

2	2	2	2	2	2	2	2	2	2
9	9	9	9	9	9	9	9	#NULL!	#NULL!
3	1	1	1	3	1	3	1	2	2
9	9	9	9	9	9	9	9	#NULL!	#NULL!
3	1	1	2	3	3	3	3	1	2
3	1	1	2	3	3	3	3	1	2
9	9	9	9	9	9	9	9	#NULL!	#NULL!
2	2	2	2	2	2	2	2	2	2
1	2	1	2	1	2	1	2	1	1
9	9	9	9	9	9	9	9	2	2
9	9	9	9	9	9	9	9	1	1
1	1	1	2	2	1	1	2	1	1
2	3	3	2	9	3	3	2	2	2
9	9	9	9	9	9	9	9	#NULL!	#NULL!
9	9	9	9	9	9	9	9	#NULL!	#NULL!
9	9	9	9	9	9	9	9	1	2
2	1	1	2	3	2	3	3	1	#NULL!
9	9	9	9	9	9	9	9	#NULL!	#NULL!
2	1	3	1	3	3	3	1	1	2
3	1	9	3	3	3	3	3	1	2
3	3	3	3	3	3	3	3	1	2
2	3	3	2	2	3	3	2	2	2
3	3	3	3	3	3	3	2	1	1
9	9	9	9	9	9	9	9	1	2
2	2	2	2	2	2	2	2	1	2
1	3	1	3	1	1	3	1	2	2
1	3	3	3	3	3	3	3	1	1
3	1	1	1	3	3	3	3	2	2
1	2	1	2	1	2	2	1	2	2
3	1	3	1	3	3	3	3	1	2
9	9	9	9	9	9	9	9	2	2
9	9	9	1	9	9	9	9	1	1
2	1	1	1	1	3	3	3	1	2
1	2	1	2	1	2	1	1	2	2
9	1	2	9	1	1	2	2	2	2
3	1	3	1	3	3	3	3	1	2
3	1	3	1	3	3	3	3	2	2
9	9	9	9	9	9	9	9	#NULL!	#NULL!
9	9	9	9	9	9	9	9	#NULL!	#NULL!
9	9	9	9	9	9	9	9	#NULL!	#NULL!
9	9	9	9	9	9	9	9	#NULL!	#NULL!
3	2	2	3	1	1	2	2	1	1
1	1	3	3	1	1	2	1	2	2
9	9	9	9	9	9	9	9	#NULL!	#NULL!
1	1	3	3	2	1	2	3	2	2
2	1	1	1	3	1	1	3	2	2
1	1	3	3	3	3	3	3	2	2

3	1	1	3	3	3	3	3	2	2
2	2	3	2	1	3	1	3	1	2
2	2	3	3	3	3	3	3	1	2
2	2	2	2	2	2	2	2	1	2
3	3	3	2	3	3	3	3	2	2
1	1	1	1	1	1	1	1	1	2
9	9	9	9	9	9	9	9	#NULL!	#NULL!
2	1	1	2	2	3	3	9	1	1
3	3	3	3	2	2	2	3	2	2
9	9	9	9	9	9	9	9	1	2
3	3	3	3	3	3	3	3	2	2
3	3	9	1	2	9	2	9	1	2
2	1	1	1	3	1	1	3	2	2
2	1	1	1	3	1	1	3	2	2
2	1	1	1	3	1	1	3	2	2
2	2	2	3	3	3	1	3	2	2
2	1	1	1	3	1	1	3	2	2
2	1	1	1	3	1	1	3	2	2
2	1	1	1	3	1	1	3	2	2
1	2	3	3	1	1	3	1	2	2
1	2	1	2	1	3	2	3	2	2
1	1	1	1	1	2	2	2	#NULL!	#NULL!
1	1	3	3	3	3	2	1	2	2
2	2	2	2	2	2	2	9	2	2
3	3	3	3	3	3	3	3	2	2
2	2	3	1	1	3	2	1	2	2
9	9	9	9	9	9	9	9	2	2
3	1	3	1	3	3	3	3	2	2
9	9	9	9	9	9	2	9	1	1
2	2	2	2	2	2	2	3	2	2
1	2	1	3	2	9	1	2	2	2
2	3	3	2	3	2	3	2	2	2
2	3	2	1	2	1	2	2	1	2
9	9	9	2	9	9	9	9	2	2
2	2	3	3	3	2	2	2	2	2
3	1	9	2	2	3	2	3	2	2
3	2	9	1	3	9	9	3	1	2
2	1	1	1	2	2	2	3	2	2
2	1	1	1	2	2	2	3	2	2
1	1	1	1	1	3	1	1	2	2
1	1	2	3	1	2	1	1	2	2
2	2	2	2	2	2	2	2	2	2
2	3	1	2	3	1	2	2	1	2
3	1	1	1	3	3	3	3	1	1
9	9	9	9	9	9	9	9	2	2
1	1	1	1	1	1	1	1	1	1
2	3	2	3	2	3	2	2	2	2

2	1	1	3	1	1	3	3	2	2
9	9	9	9	9	9	9	9	1	1
9	9	1	9	9	9	9	9	2	2
3	1	3	1	3	1	3	1	2	2
2	2	2	2	2	2	2	2	2	2
3	1	1	2	3	3	3	3	1	2
1	1	1	2	2	2	2	2	2	2
3	1	1	2	3	3	3	3	1	2
3	1	1	2	3	3	3	3	1	2
9	9	9	9	9	9	9	9	1	2
9	9	9	9	9	9	9	9	2	2
2	1	3	1	3	3	3	3	2	2
9	9	9	9	9	9	9	9	2	2
1	1	1	3	1	3	1	1	2	2
2	1	2	1	2	3	3	3	1	2
9	9	9	9	9	9	9	9	1	1
2	2	2	2	2	2	2	2	1	2
9	9	9	9	9	9	9	9	1	1
9	9	9	9	9	9	9	9	1	1
2	3	1	2	3	1	2	3	2	2
1	1	1	1	1	3	1	1	1	2
2	2	3	3	3	3	2	3	1	2
9	9	9	9	9	9	9	9	#NULL!	#NULL!
2	2	2	2	2	2	2	2	2	2
3	3	3	3	3	3	3	3	#NULL!	#NULL!
2	2	1	1	1	2	3	3	1	1
2	2	3	1	1	1	2	3	1	1
3	1	3	1	3	3	3	3	1	2
1	1	1	1	1	1	1	1	2	2
2	2	2	2	2	2	2	2	2	2
3	3	1	1	1	1	1	2	1	1
2	1	1	2	3	2	1	2	2	2
1	1	1	1	2	2	1	1	1	2
2	2	3	1	1	1	2	2	1	1
1	1	2	2	3	2	1	2	#NULL!	#NULL!
2	2	2	2	2	2	2	2	2	2
2	1	1	1	2	1	3	3	2	2
9	1	9	9	9	9	9	9	1	1
1	9	9	9	9	9	9	9	2	2
1	1	1	3	3	1	2	1	1	1
2	2	2	2	2	2	2	2	2	2
2	2	2	2	2	2	2	2	2	2
2	2	2	2	2	2	2	3	2	2
2	2	2	2	2	2	2	2	1	1
2	2	2	2	2	2	2	2	2	2
2	2	2	2	2	2	2	2	2	2
9	9	9	9	9	9	9	9	#NULL!	#NULL!

9	9	9	9	9	9	9	9	#NULL!	#NULL!
2	2	2	2	2	2	2	2	2	2
9	9	9	9	9	9	9	9	1	1
9	9	9	9	9	9	9	9	1	1
9	9	9	9	9	1	9	9	1	1
2	2	2	2	2	2	2	2	2	2
9	9	9	9	9	9	9	9	#NULL!	#NULL!
3	3	3	3	3	3	3	3	2	2
2	1	2	2	2	2	2	2	1	1
9	2	9	9	9	9	9	9	2	2
2	2	2	2	2	2	2	2	2	2
1	2	2	1	2	1	1	2	1	2
1	2	2	1	2	1	1	2	1	2
2	2	2	2	2	2	2	2	2	2
1	1	1	3	1	1	1	1	1	2
2	2	2	2	2	2	2	2	2	2
2	2	2	2	2	2	2	1	2	2
9	9	9	9	9	9	9	9	1	1
1	1	2	1	2	2	2	3	1	2
3	3	3	3	3	3	3	3	2	2
9	1	9	9	9	9	9	9	2	2
3	3	3	3	3	3	3	3	2	1
2	2	2	2	2	2	2	2	2	2
2	1	3	3	3	3	3	2	1	2
9	9	9	9	9	9	9	9	1	1
3	2	3	2	3	2	3	1	2	2
9	9	1	9	9	9	9	9	2	2
9	9	9	9	9	9	9	9	2	2
2	2	2	2	2	2	2	2	2	2
2	1	1	1	1	3	3	2	1	1
9	9	9	9	9	9	9	9	2	#NULL!
3	1	2	1	3	3	3	3	2	2
3	1	1	1	3	3	1	3	1	2
2	2	2	2	2	2	2	2	1	1
2	2	2	2	2	2	2	2	1	2
1	1	1	1	1	3	3	1	#NULL!	#NULL!
2	2	2	2	2	2	2	2	2	2
2	9	1	3	2	2	2	1	1	2
2	2	2	2	2	2	2	2	2	2
2	2	2	2	2	2	2	2	2	2
9	9	9	9	9	9	9	9	2	2
2	2	2	2	2	2	2	2	2	2
1	3	3	3	1	3	3	3	2	2
2	2	2	2	2	2	2	2	#NULL!	#NULL!
2	2	2	2	2	2	2	2	2	2
3	3	3	3	3	3	3	3	2	2
2	2	2	2	2	2	2	3	2	2

2	2	2	2	2	2	2	2	2	2
2	2	2	2	2	2	2	2	2	2
2	2	2	2	2	2	2	2	2	1
2	2	2	2	2	2	2	2	2	2
2	3	1	2	1	2	3	3	2	2
1	1	1	2	3	1	1	2	2	2
2	2	2	2	2	2	2	2	2	2
2	3	1	3	2	9	2	2	2	2
2	2	2	2	2	2	2	2	2	1
2	2	2	2	2	2	2	2	2	2
1	1	1	1	3	1	1	1	1	1
9	9	9	9	9	9	9	9	1	#NULL!
9	2	9	9	9	9	9	9	1	1
9	9	9	9	9	9	9	9	#NULL!	#NULL!
1	1	1	1	1	1	1	1	1	1
3	3	3	3	3	3	3	3	2	2
3	3	3	3	3	3	3	3	1	2
3	1	3	1	3	3	3	3	2	2
9	9	9	9	9	9	9	9	1	#NULL!
2	2	3	2	2	3	3	1	2	2
9	9	9	9	9	9	9	9	1	1
2	1	2	2	2	2	2	2	1	1
2	1	2	1	2	2	3	2	2	1
9	9	9	9	9	9	9	9	1	1
3	3	2	2	2	2	2	1	2	2
2	1	9	1	2	1	3	3	1	1
2	2	2	2	2	2	2	1	2	2
2	1	2	1	2	3	3	3	1	1
2	2	2	2	2	2	2	2	2	2
1	2	1	2	1	2	2	1	2	2
1	1	2	2	2	1	1	1	1	1
1	1	1	3	3	3	3	3	1	2
9	9	9	9	9	9	9	9	#NULL!	#NULL!
3	3	3	3	3	3	3	3	2	2
1	1	1	1	1	1	3	1	2	2
1	3	3	3	3	3	3	1	2	1
3	1	1	2	3	3	3	3	2	2
1	1	1	1	1	1	1	1	2	2
2	2	2	2	2	2	2	2	2	2
2	2	2	2	2	2	2	2	2	2
3	3	2	1	3	1	2	1	1	1
1	1	1	1	1	1	1	1	2	2
1	1	1	1	3	1	2	2	2	2
2	2	2	2	2	2	2	2	2	2
2	2	2	2	2	2	2	2	2	2
2	1	3	2	1	2	1	1	1	1
2	2	3	2	2	2	2	2	2	2

2	1	2	2	2	1	1	1	1	2
3	3	3	3	3	3	3	3	2	2
2	3	2	1	2	3	1	2	2	1
9	9	9	9	1	9	9	9	2	2
9	9	9	9	9	9	9	9	#NULL!	#NULL!
1	2	1	1	1	2	1	3	2	2
2	2	2	2	2	2	2	2	2	2
9	2	2	9	2	9	1	3	2	2
3	1	2	3	1	3	1	1	2	1
2	2	2	2	2	2	2	2	2	2
9	9	9	9	9	9	1	9	2	2
2	2	2	2	3	3	9	1	1	1
2	2	2	2	2	2	2	2	#NULL!	2
3	2	3	2	3	3	2	2	1	2
2	2	2	2	2	2	2	2	2	2
2	3	9	3	2	3	2	3	2	2
2	2	2	2	2	2	2	2	2	1
9	9	9	9	9	9	9	9	1	1
9	9	9	9	2	9	9	9	1	2
2	2	2	2	2	2	2	2	2	2
2	2	2	2	2	2	2	2	1	1
2	2	2	2	2	2	2	2	2	2
9	9	9	9	9	9	9	9	#NULL!	#NULL!
9	9	9	9	9	9	9	9	#NULL!	#NULL!
1	1	1	1	1	1	1	1	2	1
3	3	3	1	3	3	3	3	1	1
2	2	3	3	3	3	2	2	2	1
3	1	3	1	3	1	3	3	1	1
2	3	1	9	1	2	2	2	1	2
2	3	1	2	3	1	2	3	2	2
2	1	1	2	1	2	9	1	1	1
1	1	3	2	1	2	3	2	2	2
3	3	2	1	3	1	3	3	1	1
3	3	3	3	3	3	3	3	2	2
9	9	9	9	9	9	9	9	2	2
3	2	1	1	2	3	3	2	2	1
3	1	1	1	1	1	1	1	2	1
3	3	3	3	3	3	3	3	2	2
9	1	9	2	1	9	9	3	1	1
3	3	3	3	3	3	3	3	2	2
9	9	9	9	9	9	9	9	2	2
1	1	2	2	3	3	1	1	2	1
3	3	3	3	3	3	3	3	2	2
2	2	2	2	2	2	2	2	2	2
1	1	3	1	3	3	3	3	1	2
2	1	1	2	2	1	1	2	#NULL!	#NULL!
2	1	2	1	1	1	2	3	1	2

2	2	2	2	2	2	2	2	2	2
3	3	3	3	2	2	3	1	2	2
9	9	9	9	9	9	9	9	1	1
1	1	1	1	1	1	1	1	1	1
2	2	2	2	2	2	2	2	2	2
2	2	2	2	2	2	2	2	2	1
2	2	2	2	2	2	2	2	2	2
2	2	2	2	2	2	2	2	2	1
2	2	2	2	2	2	2	2	2	1
2	1	2	1	2	1	3	2	2	2
9	9	9	9	9	9	9	9	2	1
3	1	1	3	1	3	3	2	2	2
1	1	3	3	1	3	3	3	2	2
2	1	1	2	3	1	2	3	1	1
1	9	2	1	2	1	2	1	2	1
2	2	2	2	2	2	2	2	2	2
2	1	3	1	1	3	3	3	2	2
2	1	2	2	2	2	2	2	1	1
2	1	2	1	3	3	3	3	2	2
9	9	9	9	9	9	9	9	1	1
3	3	3	3	3	3	1	3	1	2
1	1	1	1	1	1	1	1	1	1
3	1	3	3	3	1	3	3	1	1
1	1	1	1	1	1	1	1	1	2
3	3	3	3	3	3	3	3	2	2
3	3	3	3	3	3	3	3	2	2
1	1	1	1	1	1	1	3	2	2
2	1	3	1	3	1	2	3	2	2
1	1	2	1	1	1	3	1	2	2
1	2	2	2	1	2	1	1	1	1
9	9	9	9	9	9	9	9	2	2
9	9	9	9	9	9	9	9	1	1
1	3	2	2	1	2	2	1	2	2
9	9	9	9	9	9	9	9	2	2
2	2	2	2	2	2	2	2	1	1
2	2	2	2	2	2	2	2	1	2
2	2	2	2	2	2	2	3	2	2
2	2	2	2	2	2	2	3	2	2
3	2	2	3	3	3	3	3	2	2
9	9	9	9	9	9	9	9	2	2
2	3	2	1	2	2	1	2	1	2
2	2	2	2	2	2	2	2	1	1
2	2	2	2	2	2	1	1	1	2
9	9	9	9	9	9	9	9	2	2
3	3	3	3	3	3	3	3	2	2
2	2	2	2	2	2	2	2	1	1
9	9	9	9	9	9	9	9	2	2

9	9	9	9	9	9	9	9	1	2
1	2	2	2	3	2	2	2	#NULL!	#NULL!
2	2	2	9	1	2	2	2	2	2
2	1	1	2	2	2	1	3	2	2
2	2	2	2	2	2	2	2	2	1
9	9	9	9	9	9	9	9	1	2
9	9	9	9	9	9	9	9	2	2
1	2	2	3	3	3	3	3	2	1
2	2	2	2	2	2	2	2	1	2
9	9	9	9	9	9	9	9	#NULL!	#NULL!
9	9	9	9	9	9	9	9	#NULL!	#NULL!
9	9	9	9	9	9	9	9	#NULL!	#NULL!
1	9	9	9	9	9	9	9	2	2
9	9	9	9	9	9	9	9	2	2
9	9	9	9	9	9	9	9	#NULL!	#NULL!
2	2	9	2	2	2	2	3	2	2
2	2	2	2	2	2	2	2	#NULL!	#NULL!
2	2	2	2	2	2	2	2	1	1
2	3	1	2	1	1	2	2	1	2
2	2	2	2	2	2	2	2	1	1
2	2	2	9	1	2	3	2	2	2
2	1	2	3	3	2	3	1	1	2
1	1	1	1	1	3	2	2	1	2
3	1	1	3	2	3	3	3	2	2
2	2	2	2	2	2	2	2	2	2
2	2	2	2	2	2	2	2	1	2
9	9	9	9	9	9	9	9	1	2
1	1	1	1	1	1	1	1	1	1
2	2	2	2	2	2	2	2	1	2
2	2	9	2	2	2	2	3	2	2
2	2	2	2	2	2	2	2	1	1
1	9	9	9	9	9	9	9	2	2
3	1	1	1	1	3	2	2	1	1
2	2	2	2	2	2	2	2	1	1
2	2	2	2	2	2	2	2	2	2
2	2	2	2	2	2	2	2	2	2
9	9	9	9	9	9	9	9	2	2
9	9	9	9	9	9	9	3	1	2
2	2	2	2	2	2	2	2	2	2
2	2	2	2	2	2	2	2	1	2
2	2	2	2	2	2	2	2	2	2
9	9	9	9	9	9	9	9	1	1
2	2	2	2	2	2	2	2	2	2
1	2	2	2	2	2	2	2	2	2
9	9	9	9	9	9	9	9	1	2
2	2	2	2	2	2	2	2	1	1
9	1	9	9	9	9	9	9	2	2

2	2	2	1	2	2	2	3	#NULL!	#NULL!
2	1	1	1	2	2	1	1	2	2
9	9	9	9	9	9	2	9	1	2
9	9	9	9	9	9	9	9	2	2
2	2	2	2	2	2	2	2	2	2
9	9	9	9	9	9	9	9	2	2
2	3	3	3	2	2	2	2	2	2
1	1	2	2	1	2	2	1	1	1
2	3	2	2	2	3	2	1	1	2
2	3	2	1	2	3	2	1	2	2
2	1	3	2	3	2	3	3	2	2
2	1	2	3	3	3	2	2	1	1
2	2	2	2	2	2	2	2	2	2
9	9	9	9	9	9	9	9	2	2
9	9	9	9	9	9	9	9	2	2
3	3	3	3	3	3	3	3	2	2
2	2	2	2	2	2	2	2	2	2
2	2	2	2	2	2	2	2	1	1
2	1	1	2	1	1	2	3	2	2
1	1	1	1	1	3	3	3	2	2
9	9	9	9	9	9	9	9	2	2
2	2	2	2	2	2	2	2	2	2
3	1	1	1	3	3	3	3	2	2
2	2	2	2	2	2	2	2	2	2
2	2	2	2	2	2	2	2	2	2
3	1	3	1	3	3	1	3	1	2
2	2	2	2	2	2	2	2	2	2
2	2	2	2	2	2	2	2	2	2
1	1	1	2	1	3	3	2	2	2
9	9	9	9	9	9	9	9	#NULL!	#NULL!
9	9	9	9	9	9	9	9	1	1
2	2	2	2	2	2	2	2	2	2
9	9	9	9	9	9	9	9	2	2
9	9	9	9	9	9	9	9	#NULL!	#NULL!
2	3	3	3	2	1	1	2	1	1
9	9	9	9	9	9	9	9	2	2
9	9	9	9	9	9	9	9	#NULL!	#NULL!
2	2	2	2	2	2	2	2	2	2
3	1	3	1	2	3	3	3	1	1
1	1	1	1	2	2	3	9	2	1
1	1	1	1	1	1	1	1	2	1
3	1	2	2	3	1	1	1	1	2
9	9	9	9	9	9	9	9	2	1
2	1	2	1	2	2	2	3	2	1
9	9	9	9	9	9	9	9	1	1
9	9	9	9	9	9	9	9	#NULL!	#NULL!
1	1	2	2	2	2	2	3	2	2

2	2	1	2	2	1	2	1	2	2
9	9	9	9	9	9	9	9	#NULL!	#NULL!
9	9	9	9	9	9	9	9	#NULL!	#NULL!
2	2	2	2	2	2	2	2	2	2
1	1	1	1	1	3	3	3	1	2
2	2	1	2	2	1	2	1	2	2
2	9	2	1	1	2	3	1	1	1
3	1	1	1	3	3	3	3	2	2
2	2	2	2	2	2	2	2	1	1
3	3	3	1	3	3	3	3	1	1
9	9	9	9	9	9	9	9	#NULL!	#NULL!
9	9	9	9	9	9	9	9	2	2
9	9	9	9	9	9	9	9	1	2
9	9	9	9	9	9	9	9	1	2
3	2	2	2	2	3	1	2	2	2
9	9	9	9	9	9	9	9	#NULL!	#NULL!
9	9	9	9	9	9	9	9	#NULL!	#NULL!
9	9	9	9	9	9	9	9	#NULL!	#NULL!
2	1	1	1	2	2	2	1	1	2
1	1	1	1	1	1	2	3	1	2
9	9	9	9	9	9	9	9	1	1
3	3	3	3	3	2	2	1	2	1
2	2	2	2	2	2	2	9	2	1
9	9	9	9	9	9	9	9	#NULL!	#NULL!
2	2	2	2	2	2	2	2	1	1
2	1	2	2	2	2	2	2	2	2
9	9	9	9	9	9	9	9	2	2
9	9	9	9	9	9	9	9	2	2
2	2	2	2	2	2	2	2	2	2
2	2	2	2	2	2	2	2	#NULL!	#NULL!
1	1	1	1	1	1	1	1	1	2
9	9	9	9	9	9	9	9	2	2
9	9	9	9	9	9	9	9	#NULL!	#NULL!
2	3	1	2	3	1	2	2	1	2
1	2	2	2	1	2	1	2	2	2
2	2	1	3	2	1	3	9	2	2
2	2	2	2	2	2	2	2	2	2
2	2	2	2	2	2	2	2	2	2
9	1	2	2	1	2	1	2	1	2
2	3	1	2	3	1	2	2	2	2
1	3	3	3	1	1	1	2	2	1
9	9	9	9	9	9	9	9	2	#NULL!
9	9	9	9	9	9	9	9	#NULL!	#NULL!
9	9	9	1	9	9	9	9	2	2
9	9	9	9	9	9	9	9	#NULL!	#NULL!
9	9	9	9	9	9	9	9	#NULL!	#NULL!
9	9	9	9	9	9	9	9	#NULL!	#NULL!

2	2	2	2	2	2	2	2	1	1
3	2	2	2	2	3	3	3	2	2
3	3	1	3	3	3	3	3	1	1
3	1	3	1	3	3	3	3	2	2
9	9	9	9	9	9	9	9	1	1
9	9	9	9	9	9	9	9	#NULL!	#NULL!
9	9	9	9	9	9	9	9	#NULL!	#NULL!
9	9	9	9	9	9	9	9	#NULL!	#NULL!
9	9	9	9	9	9	9	9	#NULL!	#NULL!
9	9	9	9	9	9	9	9	#NULL!	#NULL!
9	9	9	9	9	9	9	9	#NULL!	#NULL!
9	9	9	9	9	9	9	9	#NULL!	#NULL!
9	9	9	9	9	9	9	9	#NULL!	#NULL!
3	1	3	1	3	1	3	1	2	2
9	9	9	9	9	9	9	9	2	2
2	2	2	1	1	1	1	1	2	1
2	1	2	2	2	2	2	2	2	2
9	9	9	9	9	9	9	9	#NULL!	#NULL!
9	9	9	9	9	9	9	9	#NULL!	#NULL!
9	9	9	9	9	9	9	9	#NULL!	#NULL!
9	9	9	9	9	9	9	9	1	2
9	9	9	9	9	9	9	9	#NULL!	#NULL!
1	1	1	1	1	1	1	1	1	1
9	9	9	9	9	9	9	9	#NULL!	#NULL!
2	2	2	2	1	1	1	1	2	1
1	1	1	1	3	3	1	1	1	1
1	1	1	1	2	2	2	3	1	1
1	1	2	2	1	1	1	1	1	1
1	1	2	2	1	1	1	1	1	1
1	1	1	1	1	2	2	1	1	1
2	2	2	2	2	2	2	2	1	1
1	1	1	1	1	1	1	1	2	2
1	1	1	1	1	1	1	1	1	1
9	9	9	9	9	9	9	9	#NULL!	#NULL!
1	1	2	2	2	1	1	1	1	1
1	1	1	1	1	1	1	1	1	1
2	2	2	2	1	1	1	1	2	2
9	9	9	9	9	9	9	9	#NULL!	#NULL!
3	3	3	3	3	3	3	3	2	2
1	1	1	1	3	3	3	3	1	2
2	3	2	2	2	2	2	2	2	1
9	9	9	9	9	9	9	9	#NULL!	#NULL!
9	9	9	9	9	9	9	9	#NULL!	#NULL!
9	9	9	9	9	9	9	9	#NULL!	#NULL!
9	9	9	9	9	9	9	9	2	1
3	2	2	2	2	2	2	2	2	1
9	9	9	9	9	9	9	9	1	2

9	9	9	9	9	9	9	9	#NULL!	#NULL!
9	9	9	9	9	9	9	9	#NULL!	#NULL!
9	9	9	9	9	9	9	9	#NULL!	#NULL!
9	9	9	9	9	9	9	9	#NULL!	#NULL!
9	9	9	9	9	9	9	9	2	2
9	9	9	9	9	9	9	9	#NULL!	#NULL!
9	9	9	9	9	9	9	9	#NULL!	#NULL!
9	9	9	9	9	9	9	9	#NULL!	#NULL!
9	9	9	9	9	9	9	9	2	2
9	9	9	9	9	9	9	9	#NULL!	#NULL!
2	9	9	9	9	9	9	9	2	2
9	9	9	9	9	9	9	9	#NULL!	#NULL!
2	2	2	2	2	1	1	1	1	1
1	2	2	2	1	1	1	1	1	1
1	1	1	2	2	2	2	1	2	2
3	1	1	1	1	2	3	2	1	1
2	1	1	1	1	1	1	2	1	1
2	1	1	1	2	1	1	1	1	1
1	1	1	1	1	2	2	2	1	1
9	9	9	9	9	9	9	9	1	1
1	1	1	1	1	1	1	1	1	1
3	1	2	2	3	3	1	3	1	2
2	1	1	1	1	1	1	2	1	1
1	1	1	2	1	1	1	1	2	2
1	2	2	1	1	1	1	1	1	1
3	1	1	1	1	1	1	3	2	2
3	1	9	3	2	1	3	9	2	2
1	1	1	2	1	1	1	1	2	2
9	9	9	9	9	9	9	9	2	2
9	9	9	9	1	9	3	3	#NULL!	2
9	9	9	9	9	9	9	9	#NULL!	#NULL!
2	1	1	1	2	2	2	1	1	1
2	1	2	2	2	2	2	2	1	2
2	2	2	1	1	1	1	1	2	2
2	1	1	2	2	1	1	2	1	2
2	2	2	2	2	2	2	2	2	2
1	1	1	2	2	2	2	2	1	2
1	1	1	1	1	1	1	1	2	2
1	1	1	1	1	1	1	1	2	2
1	1	1	1	1	1	1	1	2	2
2	2	2	2	2	2	2	2	2	2
1	1	1	1	1	1	1	9	2	2
1	1	3	1	1	3	3	3	1	1
1	1	1	1	2	2	3	3	2	2
1	1	1	1	2	2	2	1	1	1
1	1	2	2	2	1	1	1	1	1
2	1	2	2	3	3	1	3	1	2

9	9	9	9	9	9	9	9	#NULL!	#NULL!
1	1	1	1	1	1	1	1	1	1
9	9	1	9	9	9	9	9	2	2
9	9	9	2	9	9	9	9	1	2
9	9	9	1	9	9	9	9	1	1
9	9	9	9	9	9	9	9	1	2
9	9	9	9	9	9	9	9	2	2
2	9	3	2	1	2	3	1	2	2
9	9	9	9	9	9	9	9	2	2
2	2	2	2	2	2	2	2	2	2
9	9	9	9	9	9	9	9	#NULL!	#NULL!
9	9	9	9	9	9	9	9	2	2
1	1	1	1	1	1	1	1	2	2
9	9	9	9	9	9	9	9	1	1
1	1	1	1	2	2	1	1	1	1
9	9	9	9	9	9	9	9	#NULL!	#NULL!
2	2	1	1	1	3	3	9	2	2
3	3	3	3	3	3	3	3	2	1
9	9	9	9	9	9	9	9	2	2
2	2	2	2	2	2	2	2	1	2
2	2	2	2	2	2	2	2	2	2
2	2	2	2	2	2	3	2	1	1
2	2	9	2	2	2	2	2	#NULL!	#NULL!
2	2	2	2	2	2	2	2	2	2
1	1	1	1	1	1	1	2	2	2
2	2	2	2	2	2	2	2	2	2
2	3	1	2	3	3	2	2	2	2
2	2	3	1	1	1	2	2	2	2
2	2	2	2	2	2	2	2	1	1
1	1	1	1	2	2	2	2	2	2
9	9	9	9	9	9	9	9	#NULL!	#NULL!
3	3	2	2	3	2	2	2	2	2
9	9	9	9	9	9	9	9	#NULL!	#NULL!
2	2	2	2	2	2	2	2	2	2
2	2	2	2	2	2	2	2	2	2
1	1	1	1	1	1	1	1	1	1
1	1	1	2	2	2	2	2	1	2
1	1	1	1	1	1	1	1	1	1
1	1	1	1	1	1	1	1	1	1
9	9	9	9	9	9	9	9	1	1
9	1	1	1	1	3	3	3	1	1
1	2	2	2	1	1	1	1	1	1
9	9	9	9	9	9	9	9	#NULL!	#NULL!
9	9	9	9	9	9	9	9	2	1
1	9	9	9	9	9	9	9	1	1
9	9	9	9	9	9	9	9	#NULL!	#NULL!
9	9	9	9	9	9	9	9	1	2

9	9	9	9	9	9	9	9	1	1
2	2	2	2	2	2	2	2	2	1
9	9	9	9	9	9	9	9	2	2
9	9	9	9	9	9	9	9	2	1
9	9	9	9	9	9	9	9	1	1
2	1	1	2	3	1	2	2	2	2
3	3	1	1	3	3	3	3	2	2
9	9	9	9	9	9	9	9	#NULL!	#NULL!
9	9	9	9	9	9	9	9	#NULL!	#NULL!
9	9	9	9	9	9	9	9	2	#NULL!
9	9	9	9	9	9	9	3	2	2
9	9	9	9	9	9	9	9	2	2
2	1	2	2	2	2	2	2	1	2
9	9	9	9	9	9	9	9	#NULL!	#NULL!
9	9	9	9	9	9	1	9	2	2
2	1	3	2	1	2	3	2	1	2
1	2	2	3	1	2	3	1	2	2
3	1	3	1	3	3	3	3	2	2
2	2	2	3	3	2	3	3	1	1
2	3	1	1	3	2	3	1	2	1
2	3	3	2	2	1	2	2	2	2
3	1	1	1	1	3	3	1	2	2
1	2	2	3	3	3	2	1	1	2
9	9	9	9	9	9	9	9	2	2
2	2	2	2	2	2	2	2	2	2
1	1	2	1	1	1	3	1	1	1
9	1	9	9	9	9	9	9	2	2
2	2	2	1	1	1	1	2	1	1
1	1	2	9	1	2	2	1	1	#NULL!
1	1	2	2	1	1	2	3	1	2
1	1	1	3	3	1	1	2	2	2
2	3	2	2	3	2	2	2	1	2
9	9	9	9	9	9	9	9	#NULL!	#NULL!
3	1	3	2	1	3	1	2	2	1
3	3	3	1	3	3	3	3	2	2
1	1	1	2	2	2	2	9	2	2
2	3	3	3	2	2	2	2	2	2
1	3	3	3	3	2	2	2	1	2
1	9	9	9	9	9	9	9	2	2
9	9	9	9	9	9	9	9	2	2
9	9	9	9	2	9	9	9	2	2
9	9	9	9	9	9	9	9	2	2
2	2	2	2	2	2	3	3	2	2
9	9	9	2	9	9	9	9	2	2
1	1	1	1	1	1	1	1	1	1
1	1	3	3	1	3	1	3	1	1
1	3	3	3	1	1	1	3	1	1

3	3	3	3	3	3	3	3	2	2
1	9	1	9	9	9	1	9	2	2
2	2	2	2	2	2	1	2	2	2
3	1	3	1	1	1	3	3	1	1
1	3	1	3	1	3	1	3	2	2
1	1	1	3	1	3	1	1	2	2
3	9	2	1	3	2	1	3	1	1
9	9	9	9	9	9	9	9	2	1
3	3	3	3	3	2	2	2	2	2
3	2	3	2	3	2	2	2	1	2
1	2	1	1	1	2	3	3	2	2
2	3	2	1	2	3	2	2	2	2
1	3	2	3	2	1	2	3	2	2
2	3	1	2	3	1	2	3	2	2
2	3	1	2	1	2	3	1	1	1
2	2	2	2	2	2	2	2	2	2
2	2	2	2	2	2	2	2	2	2
2	1	2	1	2	1	3	2	2	2
2	2	2	2	2	2	2	2	2	2
2	2	2	2	2	2	2	2	2	2
3	1	1	3	3	3	2	3	2	2
1	1	2	2	2	2	3	3	2	1
3	3	9	9	9	9	9	9	2	#NULL!
9	9	9	9	9	9	9	9	#NULL!	#NULL!
2	2	2	2	3	3	3	1	2	2
1	2	2	1	2	2	1	1	#NULL!	#NULL!
9	9	9	9	9	9	9	9	2	2
1	1	3	1	2	1	1	3	#NULL!	#NULL!
3	1	2	1	1	3	3	9	1	2
9	1	9	9	9	9	9	9	2	2
2	9	1	3	2	3	2	2	1	2
3	3	3	3	3	3	3	3	1	1
1	3	1	2	2	3	2	2	2	2
2	1	3	2	3	2	2	3	2	2
9	9	9	9	9	9	9	9	1	1
2	2	2	2	2	2	2	2	2	2
2	2	2	2	2	2	2	2	2	2
9	9	9	9	9	9	9	9	#NULL!	#NULL!
2	2	1	1	2	2	2	3	2	2
2	1	2	2	2	2	2	2	1	1
9	9	9	9	9	9	9	9	#NULL!	#NULL!
9	9	9	9	9	9	9	9	#NULL!	#NULL!
2	2	3	1	2	1	2	1	2	1
9	9	9	9	9	9	9	9	1	2
2	2	2	2	2	2	2	2	2	2
3	1	1	1	3	1	3	3	2	2
9	9	9	9	9	9	9	9	#NULL!	#NULL!

2	3	2	2	2	3	2	2	2	2
9	9	9	9	9	9	9	9	#NULL!	#NULL!
1	1	1	1	1	1	1	1	2	2
9	9	9	9	9	9	9	9	#NULL!	#NULL!
9	9	9	9	9	9	9	9	#NULL!	#NULL!
2	2	2	2	2	2	2	2	1	1
3	9	1	2	3	3	3	3	2	2
2	2	2	2	2	2	2	2	2	2
2	2	2	2	2	2	2	1	1	2
2	2	2	2	2	2	2	2	2	2
9	9	9	9	9	9	9	9	#NULL!	#NULL!
2	2	1	2	2	3	3	1	2	2
9	9	9	9	9	9	9	9	#NULL!	#NULL!
1	1	1	1	2	2	2	1	1	1
2	2	2	2	2	2	2	2	2	2
2	2	1	1	1	1	1	1	2	2
9	9	9	9	9	9	9	1	2	2
9	9	9	9	9	9	9	9	#NULL!	#NULL!
3	2	2	2	2	2	3	3	2	2
3	3	2	2	2	3	3	2	2	2
2	2	1	1	1	1	2	2	2	2
1	1	2	2	3	3	3	3	2	2
1	9	9	9	9	9	9	9	2	2
2	3	2	3	2	2	3	3	2	2
3	3	3	3	3	3	3	3	1	2
2	2	2	2	2	2	2	2	2	2
9	9	9	9	9	9	9	9	2	1
1	1	1	1	1	1	1	1	1	1
2	2	2	2	2	2	2	2	2	2
9	9	9	9	9	9	9	9	1	2
3	1	2	3	1	3	1	3	2	2
3	1	1	1	3	2	3	3	2	2
3	1	1	1	2	2	3	3	1	2
2	2	2	2	2	2	2	2	2	2
2	2	2	2	2	2	2	2	2	2
2	3	1	2	3	1	2	3	2	1
1	1	2	2	2	1	2	3	1	1
2	1	2	2	2	2	2	3	2	2
2	2	2	2	2	2	2	3	2	2
3	3	3	3	3	3	3	3	2	2
1	1	3	1	1	3	2	3	2	2
3	1	3	1	3	3	3	3	2	2
9	9	9	9	9	9	9	9	2	1
2	2	2	2	2	2	2	2	2	2
2	2	2	2	2	2	2	3	2	2
2	2	2	2	2	2	3	3	2	2
2	1	1	3	3	1	3	1	#NULL!	#NULL!

3	3	3	3	3	3	2	3	2	2
2	2	2	1	2	2	2	3	2	2
1	1	2	2	1	1	1	1	1	1
2	2	2	2	2	2	2	2	2	2
2	2	1	2	2	2	2	1	2	2
9	9	9	9	9	9	9	9	2	2
2	2	2	2	2	2	2	2	2	2
1	1	1	1	1	1	1	1	1	1
2	2	2	2	2	2	2	3	2	2
2	2	2	2	2	2	2	2	2	2
3	1	2	2	2	2	2	3	2	2
2	3	1	2	3	1	2	3	2	2
2	2	2	2	2	2	2	2	2	2
3	3	3	3	3	3	3	3	2	2
2	3	2	2	2	3	3	1	1	1
2	2	2	3	2	2	3	2	2	2
2	1	2	3	2	2	1	2	2	2
2	1	3	1	1	1	2	2	2	2
2	2	2	2	2	2	2	2	2	2
2	9	1	3	2	1	2	1	2	1
2	2	2	2	2	2	2	2	1	1
3	2	2	2	2	3	2	2	2	2
1	1	3	3	3	2	2	2	2	2
2	2	2	2	2	2	2	2	#NULL!	1
3	1	3	1	3	3	3	3	2	2
2	2	2	2	2	2	2	2	2	2
3	3	3	3	3	1	1	3	2	2
3	3	3	3	3	1	1	3	2	2
2	2	2	2	2	2	2	2	2	2
1	2	2	2	1	1	1	1	1	1
2	2	3	3	3	2	2	2	2	2
3	2	2	3	2	3	2	3	2	2
1	1	1	2	3	3	3	3	2	2
2	2	2	2	2	2	2	2	2	#NULL!
1	1	1	1	1	2	1	1	1	2
2	2	2	2	2	2	2	2	2	2
2	2	2	2	2	2	2	2	2	2
2	2	3	3	3	3	2	3	2	2
2	2	2	2	2	2	2	2	2	2
2	2	2	2	2	2	2	9	2	2
2	2	2	2	2	2	1	3	2	2
3	3	3	3	3	3	2	3	1	1
3	1	2	3	2	3	2	2	1	1
2	2	2	2	2	2	2	2	1	2
1	2	2	3	1	2	3	2	2	2
3	1	1	1	1	2	2	3	1	1
9	9	9	9	9	9	9	9	1	2

2	2	2	3	2	1	2	3	2	2
1	1	2	2	9	3	3	2	2	2
2	1	2	2	3	3	3	3	1	2
2	2	2	2	2	2	2	2	2	2
2	2	2	2	2	2	2	2	2	2
2	2	2	2	2	2	2	2	2	2
2	1	3	9	9	9	9	9	2	2
2	2	2	1	3	3	2	2	1	2
2	2	2	2	2	2	2	1	2	2
2	2	3	1	1	3	2	3	2	2
2	2	2	2	2	2	2	2	2	2
9	9	9	9	9	9	9	9	2	2
9	9	9	9	9	9	9	9	#NULL!	#NULL!
2	2	2	2	2	2	2	2	1	2
2	1	1	1	1	3	3	1	1	2
2	1	1	2	2	2	2	2	1	1
3	1	2	1	3	3	3	3	2	2
2	1	1	2	2	2	2	2	1	1
1	1	2	2	2	3	3	3	1	2
2	1	2	1	3	2	3	2	1	2
9	9	9	9	9	9	9	9	1	1
2	2	2	2	2	3	3	1	2	2
2	2	2	2	2	2	2	3	2	1
2	2	2	2	2	2	2	2	2	2
2	1	1	2	2	2	2	2	1	1
9	9	9	9	9	9	9	9	2	2
2	2	2	2	2	2	2	2	2	2
2	2	2	2	2	2	2	2	2	2
1	2	2	2	2	2	2	2	1	2
9	9	9	9	9	9	9	9	#NULL!	#NULL!
3	3	3	3	3	3	3	3	2	2
9	9	9	9	9	9	9	9	1	2
2	2	3	1	2	2	2	2	2	1
2	2	2	2	2	2	2	2	2	2
2	2	1	2	2	2	2	3	2	2
2	2	2	2	2	2	2	2	2	2
9	9	9	9	9	9	9	9	2	2
2	2	2	2	2	2	2	2	2	2
2	2	2	2	2	2	2	2	1	2
2	2	2	2	2	2	2	2	1	2
2	2	2	2	2	2	2	2	2	2
2	2	2	2	2	2	2	2	2	2
2	2	2	2	2	2	2	2	1	1
2	2	2	2	3	2	2	3	2	2
2	2	2	2	2	2	2	2	2	2
2	3	2	3	3	2	2	2	2	2
2	2	2	2	2	2	2	2	2	2

2	2	2	2	2	2	2	2	1	1
2	1	2	2	2	2	2	2	1	2
3	1	3	1	3	3	3	3	2	2
2	2	2	2	2	2	2	2	1	2
2	2	2	2	2	2	2	2	2	2
2	1	3	2	2	2	2	2	2	2
9	9	9	9	9	9	9	9	2	2
3	1	2	1	3	3	3	3	1	1
1	1	1	2	1	1	2	3	1	1
3	1	3	1	3	3	3	3	1	2
2	1	3	1	3	3	3	3	1	2
9	9	9	9	9	9	9	9	2	2
2	2	2	2	2	2	2	2	2	2
1	1	1	2	2	2	2	9	1	1
9	9	9	9	9	9	9	9	1	1
9	9	9	9	9	9	9	9	1	2
9	9	9	9	9	9	9	9	1	2
1	1	1	3	3	3	2	3	1	2
1	1	1	1	1	1	1	1	2	2
9	9	9	9	9	9	9	9	1	2
1	1	1	1	2	1	1	1	1	1
2	2	1	2	3	3	1	1	1	1
2	2	2	2	2	2	2	2	2	2
2	2	2	2	2	2	2	2	2	2
2	2	2	2	2	2	2	9	#NULL!	#NULL!
2	2	2	2	2	2	2	2	2	2
9	9	9	9	9	9	9	9	#NULL!	#NULL!
3	1	1	2	3	3	2	2	2	2
9	9	9	9	9	9	9	9	2	2
9	9	9	9	9	9	9	9	2	2
2	2	2	2	2	2	2	2	2	2
2	1	1	2	1	1	1	1	1	1
9	9	9	9	9	9	9	9	#NULL!	#NULL!
9	9	9	9	9	9	9	9	1	1
9	9	9	9	9	9	9	9	#NULL!	#NULL!
9	9	9	9	9	9	9	9	#NULL!	#NULL!
2	2	2	2	2	2	3	3	2	1
3	1	2	2	1	3	3	3	1	1
9	9	9	9	9	9	9	9	2	2
2	2	3	3	2	2	2	2	2	2
2	1	2	3	2	2	3	3	1	2
3	1	2	2	2	1	2	3	1	2
2	1	2	1	2	2	1	2	2	2
2	2	2	2	2	2	2	2	2	2
1	2	2	2	2	2	2	1	1	1
2	1	2	1	2	2	2	2	1	1
2	1	2	1	3	1	2	1	2	2

2	1	3	2	1	3	2	2	1	2
2	1	1	2	3	3	3	2	#NULL!	#NULL!
1	9	2	3	1	3	2	1	2	2
2	2	1	2	1	2	1	3	1	1
1	3	2	1	3	2	2	3	1	1
2	2	3	2	3	2	2	2	2	2
3	1	1	1	2	3	3	3	2	2
9	9	9	9	9	9	9	2	2	2
2	2	2	2	2	2	2	2	2	2
2	2	2	2	2	2	2	2	1	2
2	9	1	2	2	2	3	3	1	2
2	1	2	2	2	2	3	3	1	2
2	2	2	1	2	3	2	3	#NULL!	#NULL!
9	9	9	9	9	9	9	9	1	2
2	2	2	2	2	2	2	2	1	1
2	2	2	2	2	2	2	2	2	2
2	2	2	2	2	2	2	2	1	1
3	1	2	1	1	3	3	3	2	2
2	1	1	1	3	3	1	2	1	1
2	1	1	2	2	3	3	3	1	1
2	1	1	1	3	3	2	3	1	1
3	1	3	1	2	3	3	3	1	1
1	1	3	2	3	3	3	3	2	2
9	1	2	3	3	3	3	3	2	2
1	3	3	2	3	9	3	2	1	2
1	1	3	1	1	1	1	3	1	1
1	1	1	1	1	1	1	1	2	2
2	1	1	1	1	1	2	2	1	2
3	1	1	1	3	3	3	2	1	1
9	1	9	9	9	9	9	9	1	1
3	1	3	3	3	3	3	3	1	1
9	9	9	9	9	9	9	9	2	2
9	9	9	9	9	9	9	9	#NULL!	#NULL!
9	9	9	9	9	9	9	9	#NULL!	#NULL!
9	9	9	9	9	9	9	9	#NULL!	#NULL!
2	2	2	2	2	2	2	2	2	2
1	1	1	1	2	2	2	3	1	1
9	9	9	9	9	9	9	9	#NULL!	#NULL!
1	2	2	1	1	2	2	1	2	2
1	2	2	1	1	2	2	1	2	2
9	9	9	9	9	9	9	9	1	1
3	3	3	3	3	3	3	3	2	2
2	2	2	2	2	2	2	2	2	2
3	3	3	2	2	2	3	2	2	2
9	9	9	9	9	9	9	9	#NULL!	#NULL!
1	1	1	1	1	1	1	1	1	1
2	2	2	2	2	2	2	2	2	2

1	1	1	1	1	1	1	1	2	2
3	1	2	1	3	3	3	3	2	2
3	3	2	1	1	1	1	2	1	2
2	2	2	2	2	2	2	2	2	2
9	9	9	9	9	9	9	9	#NULL!	#NULL!
9	9	9	9	9	9	9	9	#NULL!	#NULL!
2	2	2	2	2	2	2	2	2	2
2	2	2	2	2	2	2	2	2	2
9	9	9	9	9	9	9	9	1	2
9	9	9	9	9	9	9	9	2	2
1	1	1	1	2	2	2	1	1	1
1	1	1	1	1	1	1	1	1	2
9	9	9	9	9	9	9	9	2	2
2	3	2	3	2	3	3	2	2	2
1	1	9	9	9	9	9	9	#NULL!	#NULL!
3	1	2	2	3	1	1	2	2	2
3	1	2	2	3	2	3	3	2	2
1	3	2	1	2	2	2	3	2	2
9	9	9	9	9	3	9	9	2	2
3	1	3	3	1	1	2	3	2	2
2	1	3	2	1	1	3	1	2	1
1	1	1	1	1	1	1	1	1	2
2	1	1	1	3	3	3	2	#NULL!	#NULL!
2	1	1	1	2	2	2	2	1	2
1	1	1	3	3	3	3	3	2	2
2	2	9	2	2	2	2	2	2	2
9	9	9	9	9	9	9	9	2	2
3	1	1	1	1	3	3	3	2	2
1	2	2	3	1	1	2	2	2	1
3	3	1	3	1	3	1	3	2	2
2	2	2	2	2	2	2	2	2	2
3	1	3	2	2	2	3	3	2	2
2	3	2	1	2	3	2	1	2	2
3	1	3	2	3	3	3	3	1	1
9	9	9	1	9	9	9	3	2	2
1	2	1	2	1	2	2	3	2	2
3	1	1	3	3	1	3	3	2	2
2	2	2	2	2	2	2	2	2	2
9	9	9	9	9	9	9	9	2	2
9	9	9	9	9	9	9	9	#NULL!	#NULL!
9	9	9	9	9	9	9	9	#NULL!	#NULL!
2	2	3	1	2	3	2	9	2	1
2	2	1	2	2	1	1	2	2	2
1	1	2	1	1	3	3	3	1	1
1	1	2	1	1	3	3	3	1	1
3	3	3	3	2	1	1	2	#NULL!	#NULL!
2	3	2	3	2	3	3	3	2	2

2	2	2	2	2	2	2	2	1	2
9	9	9	9	9	9	9	9	2	2
2	2	2	2	2	2	2	2	2	2
3	3	2	3	2	2	3	3	1	2
9	9	9	9	9	9	9	9	2	2
9	9	9	9	9	9	9	9	#NULL!	#NULL!
1	1	1	1	1	1	3	3	1	#NULL!
9	9	9	9	9	9	9	9	2	2
1	9	3	3	9	1	1	9	1	1
2	2	2	2	2	2	2	2	1	2
2	2	2	1	2	2	2	2	1	1
1	3	1	2	3	1	2	3	2	2
2	2	2	3	2	2	3	2	2	2
3	1	3	3	1	3	3	3	2	2
9	9	9	9	9	9	9	9	#NULL!	#NULL!
3	1	1	3	1	1	3	3	2	1
2	2	2	2	2	2	2	2	2	2
2	2	2	2	2	2	2	2	1	2
3	2	2	2	1	1	1	3	2	2
2	9	1	3	2	2	1	3	1	2
9	9	9	9	9	9	9	9	#NULL!	#NULL!
9	9	9	9	9	9	9	9	#NULL!	#NULL!
3	3	2	3	3	2	2	3	2	2
9	9	9	9	9	9	9	9	2	2
9	9	9	9	9	9	9	9	#NULL!	#NULL!
3	2	3	2	3	2	3	2	2	2
3	2	2	2	3	2	1	2	2	2
2	1	2	1	1	2	2	3	2	2
3	3	3	3	3	3	3	3	2	2
2	1	1	1	2	3	3	3	2	2
3	3	3	3	3	3	3	3	2	2
2	1	1	1	3	3	3	3	2	2
9	9	9	9	9	9	9	9	#NULL!	#NULL!
9	9	9	9	9	9	9	9	2	2
9	9	9	9	9	9	9	9	#NULL!	#NULL!
1	1	1	1	1	3	1	3	1	2
3	1	1	1	1	3	3	1	1	1
2	2	9	3	3	2	2	2	2	2
3	1	3	1	3	3	3	3	2	2
9	9	9	9	9	9	9	9	2	2
2	2	2	3	3	3	2	2	2	2
2	3	2	2	1	2	2	1	1	2
3	3	3	3	3	3	3	9	1	2
9	9	9	9	9	9	9	9	2	2
9	9	9	9	9	9	9	9	1	1
3	1	3	1	3	3	3	3	2	2
1	2	3	2	1	3	2	3	2	2

2	1	3	1	2	1	1	2	2	2
2	3	3	3	3	2	2	2	2	2
3	3	3	3	1	9	3	1	2	2
2	3	1	2	3	1	2	3	2	2
3	1	3	1	3	3	3	3	2	2
1	1	2	2	1	2	2	2	1	2
2	2	2	1	2	2	1	9	2	2
1	1	3	1	3	3	3	3	2	2
9	9	9	9	9	9	9	9	#NULL!	#NULL!
1	2	3	2	2	2	2	9	1	1
2	1	2	2	2	2	2	2	2	2
2	2	2	2	2	2	2	2	2	1
3	1	3	1	3	3	3	3	1	2
2	1	1	1	2	2	3	3	1	1
9	9	9	9	9	9	9	9	2	2
2	3	3	3	3	3	3	3	2	1
1	1	2	1	1	1	1	2	1	1
2	3	3	3	2	2	2	2	2	2
3	1	2	1	3	2	2	2	2	2
2	2	2	2	2	2	2	2	2	2
1	2	1	1	2	1	2	2	2	2
1	1	1	3	1	3	3	3	1	1
3	2	2	1	2	2	1	9	2	2
3	3	3	3	3	3	3	3	2	2
9	9	9	9	9	9	9	9	2	1
2	3	1	1	1	1	1	1	1	1
1	3	2	2	1	3	1	2	1	1
2	1	2	2	2	2	2	2	1	2
2	2	2	2	2	2	2	2	1	2
1	1	3	1	3	3	3	3	2	2
2	1	2	2	2	2	2	2	2	1
2	2	2	2	2	2	2	2	2	1
1	1	3	2	3	3	3	3	2	2
2	1	2	2	2	1	1	1	2	2
2	3	2	3	2	2	3	3	1	2
3	1	1	2	3	2	3	3	2	2
1	1	1	1	2	2	2	1	2	1
1	1	1	1	1	1	2	1	2	#NULL!
3	1	1	9	3	2	1	3	1	1
1	2	2	2	2	3	3	2	1	1
2	3	3	3	3	3	2	2	1	2
1	1	1	2	2	3	3	3	1	1
1	2	2	2	1	1	1	1	2	2
2	2	2	2	1	2	2	2	2	2
2	2	2	1	1	1	1	1	2	1
2	1	1	1	1	2	1	1	2	1
1	1	2	1	1	2	1	1	1	1

1	2	2	1	2	2	2	1	1	1
2	2	1	2	1	1	2	1	2	1
2	1	3	1	1	2	2	1	1	2
1	1	2	1	2	1	3	1	2	1
2	2	2	2	1	1	1	1	1	1
2	1	1	1	2	2	1	1	1	1
2	1	2	1	2	2	3	3	2	1
1	1	1	1	2	2	2	1	1	2
2	2	2	2	2	2	2	3	2	2
2	2	2	2	2	2	2	3	2	2
2	2	2	2	2	2	2	2	1	2
1	1	1	2	2	2	3	3	2	1
1	2	2	3	3	3	3	2	2	2
3	3	3	3	3	3	3	3	2	1
2	2	2	2	2	2	2	3	2	2
2	2	2	2	2	2	2	2	1	2
2	1	3	2	3	3	3	3	2	2
3	9	2	2	3	2	3	2	2	2
2	1	1	1	2	2	3	2	2	2
2	3	2	2	2	2	3	3	2	2
2	2	2	2	2	2	2	2	1	1
2	2	2	2	2	2	2	3	2	2
3	2	3	2	3	2	3	2	2	2
3	3	9	2	2	3	3	1	#NULL!	#NULL!
9	1	2	1	2	2	2	1	2	2
2	2	9	1	1	1	3	3	2	2
1	9	1	2	1	2	1	9	2	1
3	1	3	2	1	9	2	1	2	2
2	1	1	3	2	1	2	2	2	2
2	1	1	2	1	1	3	1	2	2
2	2	2	2	2	2	2	3	1	1
3	1	3	1	2	3	1	3	1	1
1	2	1	1	1	2	1	1	2	1
2	1	2	3	2	2	1	2	1	2
2	2	2	1	3	3	3	2	1	1
2	2	2	2	2	2	2	2	2	2
3	3	3	3	3	3	3	3	2	2
3	2	2	2	2	3	3	1	2	2
1	1	1	3	1	3	1	1	2	1
1	2	2	3	3	2	2	1	2	1
2	2	2	2	2	2	2	2	2	2
9	9	9	9	9	9	9	9	2	2
2	2	2	2	2	2	2	2	2	2
3	3	3	3	3	3	3	3	2	2
3	3	3	3	3	3	3	3	2	2
3	3	3	2	2	2	2	2	2	2
1	1	1	1	2	2	1	1	2	1

1	1	1	2	3	2	2	2	1	2
2	2	1	1	2	1	2	1	1	2
1	1	2	1	2	2	1	2	1	2
1	1	1	1	2	2	1	1	1	1
2	2	1	1	1	2	1	1	2	1
1	2	2	2	1	1	1	2	2	2
2	2	2	2	2	2	2	2	2	2
2	2	1	1	2	2	2	1	2	2
2	1	1	1	2	2	2	1	2	2
2	2	1	1	1	1	2	3	2	1
3	2	1	2	2	3	3	2	2	1
2	9	3	2	2	1	1	2	1	2
2	2	2	1	1	1	1	2	2	2
1	2	2	1	1	2	2	1	2	2
1	1	2	1	1	1	2	1	2	2
1	1	1	2	2	1	1	1	2	2
2	2	2	2	2	3	2	1	2	2
3	3	2	2	2	1	1	1	2	#NULL!
9	9	2	9	9	2	9	2	2	2
2	1	2	1	2	1	3	3	2	2
9	9	2	9	2	9	2	2	2	2
2	2	2	2	2	2	2	2	2	2
1	2	2	3	3	2	2	1	2	2
2	1	1	2	1	2	2	1	2	2
2	2	2	3	2	1	2	3	2	2
1	1	2	2	1	1	1	2	2	2
2	1	1	1	2	1	1	1	1	2
2	2	1	1	2	1	1	1	2	2
1	1	2	1	1	2	2	1	2	2
1	2	1	1	1	2	1	1	1	1
1	1	1	2	1	2	1	1	2	2
2	2	2	2	2	2	2	2	2	2
1	2	2	1	2	1	2	2	2	2
2	2	1	2	2	1	1	2	2	2
2	3	2	1	2	2	3	2	2	2
1	2	2	3	1	9	1	1	2	2
1	9	3	1	1	2	9	1	2	2
9	2	9	2	9	2	9	2	2	2
9	9	9	9	9	9	9	9	#NULL!	#NULL!
9	9	1	9	1	9	1	1	2	2
2	1	9	9	9	9	9	9	2	2
1	2	1	1	1	2	2	1	1	1
9	9	9	9	9	9	9	9	2	2
2	2	2	2	2	2	2	2	2	2
3	2	9	1	9	1	9	1	2	2
9	2	9	2	9	2	9	2	2	2
9	9	9	9	9	9	9	9	2	2

9	9	2	9	9	2	9	2	2	2
9	9	9	2	9	9	9	2	2	2
9	9	2	9	9	2	9	2	2	2
9	9	9	9	9	9	9	9	#NULL!	#NULL!
9	1	2	9	9	1	2	9	2	2
9	9	2	9	9	9	2	2	2	2
9	9	9	9	9	9	9	9	#NULL!	#NULL!
9	9	9	9	9	9	9	9	2	#NULL!
1	1	1	2	1	1	1	2	2	2
9	2	9	2	9	2	9	2	2	2
3	2	1	2	2	1	1	2	2	2
2	1	1	2	3	2	2	1	2	2
1	2	2	1	1	1	2	2	2	2
2	2	2	1	2	2	2	1	1	2
2	2	2	1	1	2	2	1	1	2
3	2	2	1	2	2	2	3	1	2
2	2	2	2	2	2	2	3	2	2
2	1	1	2	1	1	2	2	2	2
2	2	1	1	2	2	1	1	2	2
2	2	2	3	2	2	2	1	1	2
2	1	2	2	2	1	2	2	2	2
2	2	1	1	2	2	1	2	2	1
2	2	3	2	2	2	1	1	1	2
2	2	3	2	2	2	2	1	1	1
2	1	1	2	2	3	2	1	1	2
2	2	2	2	1	1	2	3	2	2
9	9	9	2	9	9	2	2	2	2
3	3	3	3	9	3	3	3	2	2
2	2	1	2	2	3	2	2	1	2
3	3	3	3	3	3	3	3	2	2
9	9	9	9	9	9	9	9	2	2
2	1	2	3	2	2	1	3	2	2
2	2	3	2	2	1	2	2	2	2
2	2	2	2	2	2	2	2	2	2
2	1	2	1	2	1	2	1	2	1
3	3	3	3	3	3	3	3	2	2
2	2	2	2	2	2	2	2	2	2
2	9	9	2	9	9	2	2	2	2
2	2	2	2	2	2	2	2	2	2
2	1	2	2	2	2	2	1	2	2
1	2	2	1	2	2	3	2	2	2
3	3	3	3	3	3	3	3	2	2
1	1	2	2	3	2	2	3	1	1
1	2	2	3	2	1	2	2	2	2
1	1	1	1	2	1	2	3	2	1
2	2	1	1	1	2	2	3	2	2
1	1	1	2	1	2	2	1	2	1

1	2	1	2	1	2	2	3	1	2
2	2	1	1	2	2	2	2	2	1
1	1	1	1	2	2	1	1	2	1
2	2	2	3	2	2	2	2	2	2
3	1	2	3	2	1	2	3	1	2
2	1	1	1	2	2	3	3	2	2
1	1	2	2	2	1	2	2	1	2
3	2	2	3	3	3	1	3	2	1
3	1	2	1	3	2	3	3	1	1
2	3	2	2	2	3	2	3	2	2
1	2	2	3	2	2	2	3	2	2
3	1	1	2	2	3	1	3	2	2
3	2	1	3	1	3	1	2	2	1
2	2	1	2	3	2	1	2	2	2
9	9	9	9	9	9	9	9	1	2
2	2	2	2	2	2	2	2	2	2
2	2	2	2	2	2	2	3	2	2
1	1	1	2	1	2	2	1	2	2
3	2	3	2	2	2	3	3	2	1
2	2	2	2	2	2	2	2	2	2
1	2	2	2	1	2	3	3	2	1
2	2	2	2	2	2	2	2	2	2
2	1	1	2	2	3	2	2	2	2
2	2	2	3	2	3	3	3	2	2
2	2	2	2	2	2	2	2	1	1
3	3	2	2	2	3	3	2	2	2
1	2	1	2	3	2	2	2	2	2
1	1	1	2	1	2	1	3	1	1
2	2	9	3	3	3	3	2	1	1
2	3	2	2	2	3	2	3	2	2
2	1	2	2	1	3	3	3	2	2
3	1	1	2	1	3	1	2	1	1
1	2	9	2	3	2	2	2	1	2
2	2	2	2	2	2	2	2	1	1
3	3	9	1	3	3	3	3	1	1
1	1	2	2	2	2	2	3	2	2
3	1	2	2	2	2	3	3	1	1
1	1	1	2	2	2	2	2	2	2
3	1	2	1	3	2	3	3	2	2
2	1	3	1	2	1	2	3	1	1
2	1	3	1	2	3	3	3	#NULL!	1
2	1	2	2	2	2	2	9	2	2
2	2	2	2	2	2	2	2	2	2
2	3	2	2	2	2	3	2	2	2
2	1	2	1	2	2	2	2	2	2
2	2	2	2	2	2	2	2	2	2
2	2	2	2	2	2	2	2	2	2

2	2	2	2	2	2	2	2	2	2
2	1	1	2	3	1	3	3	1	2
2	1	2	1	1	2	2	2	2	2
2	2	2	2	2	2	2	2	1	1
2	2	2	2	2	2	2	2	2	2
1	1	1	1	1	1	1	1	1	2
2	2	2	2	2	2	2	2	2	2
9	9	9	9	9	9	9	9	#NULL!	#NULL!
2	2	2	2	2	2	2	2	1	1
9	9	9	9	9	9	9	9	#NULL!	#NULL!
1	2	3	1	2	3	1	2	2	2
2	2	2	2	2	2	1	1	2	1
3	9	1	3	9	2	3	1	2	1
9	9	9	9	9	9	9	9	2	2
3	1	3	1	3	3	2	2	2	2
2	2	2	2	2	2	2	2	2	2
2	2	2	2	2	2	2	2	1	2
2	3	3	3	3	2	2	2	1	2
9	9	9	9	9	9	9	9	2	2
3	3	9	1	1	1	1	1	1	1
3	3	3	3	3	3	3	3	#NULL!	#NULL!
9	9	9	9	9	9	9	9	2	2
2	2	2	2	2	2	2	2	2	2
1	1	3	3	2	2	3	3	1	1
2	2	2	2	2	2	2	2	2	2
1	2	2	3	2	2	2	1	2	1
2	1	1	2	1	3	2	3	2	1
1	2	2	1	2	2	2	2	1	2
1	2	2	2	1	1	1	1	1	1
9	9	9	9	9	9	9	9	1	2
2	2	2	1	2	3	2	1	2	1
2	3	2	3	2	3	2	2	2	2
2	2	2	2	2	2	2	2	2	2
3	3	3	3	3	3	3	3	2	2
2	2	2	2	2	2	2	2	2	2
2	2	2	2	2	3	1	2	1	2
2	2	2	2	2	2	2	2	1	2
3	1	3	2	3	1	2	2	2	2
1	1	1	1	3	3	3	3	2	2
2	2	2	2	3	3	3	2	2	2
1	3	2	1	2	3	2	2	2	2
2	1	1	1	1	1	3	1	2	2
2	2	2	2	2	2	2	2	2	2
2	1	1	1	1	1	3	1	1	2
9	9	9	9	9	9	9	9	2	2
1	1	1	1	3	3	2	2	1	1
1	1	2	1	1	2	1	1	1	1

2	2	2	2	2	2	2	2	1	2
2	2	2	2	2	3	3	3	2	2
2	2	2	2	2	2	2	2	1	2
2	1	2	1	1	1	1	2	2	2
3	2	9	1	2	2	3	1	2	2
9	9	9	9	9	9	9	9	2	2
2	2	2	2	2	2	2	2	2	2
2	2	3	3	2	2	2	2	2	2
1	1	1	2	2	3	3	3	1	2
3	3	2	2	2	2	2	2	2	2
2	1	1	2	1	1	2	1	1	1
2	2	2	2	2	2	2	2	1	1
9	1	9	9	9	9	9	9	2	2
1	9	9	9	9	9	9	9	1	2
9	2	9	9	9	9	9	9	2	2
2	1	2	2	2	2	2	2	2	2
3	2	2	1	2	2	2	1	1	1
9	9	9	9	9	9	9	9	2	2
3	1	3	1	3	3	3	3	1	1
3	1	3	1	3	2	3	3	1	2
3	1	3	3	1	1	1	3	2	2
9	2	1	3	1	3	3	3	1	1
3	3	3	1	3	3	3	3	1	1
2	2	2	2	2	2	2	2	2	2
1	1	2	2	2	2	2	2	2	2
3	1	1	1	3	2	3	2	1	1
1	1	2	2	2	2	2	2	2	2
3	1	1	1	3	3	3	2	2	2
9	9	1	9	9	9	9	9	2	2
3	2	3	3	2	3	2	2	2	2
3	1	2	1	2	2	3	3	2	2
2	3	2	2	1	2	2	1	2	2
2	2	1	1	1	1	1	2	2	1
2	2	1	1	1	1	3	2	2	2
2	1	1	2	3	3	3	3	2	2
1	1	3	1	3	3	1	3	1	1
2	2	2	3	2	2	2	3	2	2
3	1	1	2	3	3	3	3	2	2
2	2	2	2	2	2	2	2	2	2
1	1	1	2	3	3	3	3	2	2
2	2	2	2	1	2	2	2	2	2
2	2	2	1	2	2	2	3	2	2
2	2	1	3	2	2	1	2	2	2
3	1	2	2	2	2	2	2	2	2
2	1	2	2	2	3	3	3	2	2
2	1	2	2	2	3	2	3	2	2
1	1	2	2	1	3	2	2	2	2

1	1	1	2	2	2	1	1	1	2
3	3	3	3	3	3	3	3	2	2
9	1	9	3	1	9	2	3	1	1
2	3	2	1	3	1	2	3	2	2
2	2	2	1	3	2	1	3	2	2
2	1	2	2	3	2	2	2	2	2
2	1	2	1	2	2	3	2	2	2
3	1	3	1	2	3	3	3	2	2
2	1	3	2	1	3	2	2	2	2
2	1	2	2	2	2	2	2	2	2
2	2	2	2	2	2	2	2	2	2
1	1	1	1	1	1	1	1	2	2
3	2	3	1	2	3	1	2	2	2
1	1	1	1	3	2	1	3	1	1
2	1	9	1	3	9	3	3	1	2
3	1	3	1	1	3	1	1	1	2
9	9	9	9	9	9	9	9	#NULL!	2
9	9	9	9	9	9	9	9	2	2
3	1	3	1	3	3	3	3	1	1
1	1	1	1	3	3	3	3	1	2
2	1	2	1	1	1	3	3	1	2
9	9	9	9	9	9	9	9	#NULL!	#NULL!
2	2	2	3	3	3	3	2	2	2
3	1	1	2	2	3	2	3	2	2
2	2	2	2	2	2	2	9	2	2
9	9	9	9	9	9	9	9	2	2
9	9	9	9	9	9	9	9	2	2
3	2	3	1	2	2	3	2	2	2
2	9	9	9	9	9	9	3	2	2
9	9	9	9	1	9	9	9	1	2
9	9	9	2	9	9	9	9	#NULL!	#NULL!
9	9	9	9	9	9	9	9	#NULL!	#NULL!
9	9	9	9	9	9	9	9	2	2
2	1	1	1	2	2	3	3	1	1
2	3	1	2	3	1	2	3	1	2
9	3	2	1	2	1	2	2	1	1
9	3	2	1	2	1	2	2	1	1
3	3	3	3	9	2	2	2	1	1
2	2	1	1	2	2	1	1	1	1
3	3	2	2	2	2	2	2	2	2
2	2	2	2	2	2	2	2	1	2
2	1	1	2	1	2	3	3	2	2
2	1	2	2	2	2	2	2	2	2
2	3	1	2	2	1	3	2	2	2
2	3	1	2	2	1	3	2	2	2
2	3	1	2	2	1	3	2	2	2
2	3	2	1	2	2	3	2	1	2

9	9	9	9	9	9	9	9	1	1
2	1	2	2	1	2	1	9	2	1
3	1	3	2	1	1	2	2	2	2
9	9	9	9	9	9	9	9	#NULL!	#NULL!
9	9	9	9	9	9	9	9	2	2
2	1	1	2	9	2	2	3	1	1
1	1	1	2	2	3	3	3	1	2
9	9	9	9	9	9	9	9	#NULL!	#NULL!
9	9	9	9	9	9	9	9	#NULL!	#NULL!
2	1	2	3	3	3	3	3	1	2
2	1	3	2	2	2	2	3	2	2
2	2	2	2	2	2	2	2	2	2
1	1	1	2	2	2	2	2	1	2
9	9	9	9	9	9	9	9	#NULL!	#NULL!
9	9	9	9	9	9	9	9	#NULL!	#NULL!
9	9	9	9	9	9	9	9	2	2
9	9	9	9	9	9	9	9	2	2
9	9	9	9	9	9	9	9	1	1
3	1	3	1	3	3	3	3	1	1
9	9	9	9	9	9	9	9	#NULL!	#NULL!
1	9	9	9	9	9	9	9	2	2
9	9	9	9	9	9	9	9	#NULL!	#NULL!
9	9	9	9	9	9	9	9	#NULL!	#NULL!
9	9	9	9	9	9	9	9	#NULL!	#NULL!
9	9	9	9	9	9	9	9	1	2
9	9	9	9	9	9	9	9	2	2
2	1	3	3	1	3	3	3	2	2
2	2	2	2	2	2	2	2	1	1
3	1	3	1	3	1	3	3	2	2
2	2	2	2	2	2	2	2	2	2
2	2	2	2	2	2	2	2	2	2
2	1	1	3	2	2	1	2	1	1
2	1	2	1	2	2	2	3	1	1
1	1	1	2	1	2	3	3	2	2
1	1	1	2	3	3	2	3	1	1
1	1	3	1	1	1	3	1	1	1
1	1	1	2	1	2	2	2	1	1
2	1	3	1	3	3	3	3	2	2
3	1	1	1	3	3	3	3	1	2
1	1	2	1	3	3	3	1	2	2
1	1	1	1	2	2	1	2	1	1
2	2	2	3	2	2	2	1	2	2
9	1	9	9	9	9	9	9	2	2
9	9	9	9	9	9	9	9	1	1
9	2	9	9	9	9	9	9	1	1
1	1	2	2	2	2	2	2	2	2
1	2	2	2	2	1	1	1	1	2

1	2	2	2	3	2	2	2	1	2
2	2	2	2	2	2	2	2	2	2
2	2	2	2	2	2	1	1	2	2
2	1	2	2	1	2	2	3	2	2
1	2	2	1	2	2	2	2	2	2
1	1	3	1	3	1	3	3	2	2
1	1	1	2	2	3	2	3	2	1
9	9	9	9	9	9	9	9	1	2
9	9	9	9	9	9	9	9	#NULL!	#NULL!
1	1	1	1	2	2	2	2	2	2
2	2	2	2	2	2	2	2	2	2
3	1	2	1	3	3	3	3	2	2
2	3	1	2	3	1	2	3	1	2
1	1	2	3	1	1	1	3	2	2
2	2	2	2	2	2	2	2	2	2
1	2	3	2	1	2	1	2	2	2
3	2	2	1	2	3	2	3	1	1
2	3	1	2	3	1	2	3	2	2
2	2	2	2	2	2	2	2	2	2
2	2	2	2	2	2	2	2	2	2
2	1	2	1	2	3	3	3	2	2
2	2	2	2	2	2	2	2	2	1
2	2	2	2	3	2	2	1	2	1
3	2	1	2	1	1	2	1	1	2
2	2	2	2	2	2	2	2	2	2
3	3	2	2	2	2	2	3	2	1
3	1	1	2	3	2	3	3	2	2
3	9	2	3	2	2	2	1	2	1
2	3	1	2	3	1	2	3	2	1
9	9	9	9	9	9	9	9	#NULL!	#NULL!
2	1	2	1	1	1	2	2	1	2
9	9	9	9	9	9	9	9	#NULL!	#NULL!
9	9	9	9	9	9	9	9	#NULL!	#NULL!
1	1	1	1	3	2	1	2	1	1
1	2	1	3	1	3	1	2	1	1
2	3	2	3	2	2	3	2	1	2
2	1	2	2	3	2	1	2	1	2
3	3	3	3	2	2	2	1	2	1
2	2	2	2	2	2	2	2	2	2
1	1	1	1	2	2	2	2	1	1
1	1	2	2	2	1	1	2	#NULL!	#NULL!
1	1	3	1	3	3	3	3	2	2
2	1	1	2	2	2	2	2	2	2
9	9	9	9	9	9	9	9	2	2
3	2	2	2	2	2	2	2	1	2
2	2	2	2	2	2	2	2	2	2
3	1	1	2	1	2	3	3	1	2

3	1	1	2	3	3	2	3	2	1
2	3	2	3	2	1	2	1	2	2
2	2	2	2	2	2	2	2	2	1
1	2	2	2	1	1	1	1	2	1
2	3	2	2	1	1	2	3	1	2
9	9	9	9	9	9	9	9	#NULL!	#NULL!
1	1	1	1	1	1	1	1	1	1
2	2	1	3	3	2	2	3	1	2
9	9	9	1	9	9	9	9	2	2
2	2	2	2	2	2	2	2	2	2
2	2	2	2	2	2	2	2	2	2
2	1	2	3	9	9	9	9	2	1
9	9	9	9	9	9	9	9	#NULL!	#NULL!
1	2	2	3	3	2	1	2	2	2
2	1	2	1	3	3	3	3	1	2
1	1	3	2	2	3	3	3	1	1
2	1	1	2	2	2	2	2	2	2
2	3	1	2	3	1	2	3	#NULL!	#NULL!
1	1	1	1	1	3	3	1	1	1
1	1	2	2	2	3	3	3	2	2
2	1	2	1	2	1	2	1	1	1
2	2	2	2	2	2	2	2	2	2
2	2	2	2	2	2	2	2	2	2
2	2	2	2	2	2	2	2	2	2
2	1	2	3	1	2	2	2	2	2
2	2	2	2	2	2	2	2	2	2
2	2	2	2	2	2	2	2	2	1
2	1	2	1	1	3	3	3	1	1
2	1	1	3	3	2	2	2	2	2
1	2	1	2	2	2	1	3	1	1
3	3	3	3	3	3	3	3	2	2
2	2	2	2	2	2	2	3	1	2
2	1	2	1	3	2	2	2	1	1
2	2	2	2	2	2	2	2	1	2
2	1	2	1	2	3	3	3	1	2
9	9	9	9	9	9	9	9	#NULL!	#NULL!
3	3	2	2	2	2	2	2	2	2
9	9	9	9	9	9	9	9	2	#NULL!
1	1	3	1	3	1	2	1	2	1
2	2	2	2	2	2	2	2	2	2
3	3	2	2	2	2	2	2	2	2
9	9	9	9	9	9	9	9	1	2
3	1	9	1	9	3	2	3	1	2
2	2	2	2	2	2	2	2	#NULL!	#NULL!
9	9	9	9	9	9	9	9	#NULL!	#NULL!
9	9	9	9	9	9	9	9	2	2
9	9	9	9	9	9	9	9	#NULL!	#NULL!

3	3	3	3	3	3	3	3	2	2
2	2	2	2	2	2	2	2	#NULL!	#NULL!
9	9	9	9	9	9	9	9	#NULL!	#NULL!
9	9	9	9	9	9	9	9	1	1
3	2	2	2	2	2	2	2	2	2
2	3	2	1	2	1	2	3	#NULL!	#NULL!
2	2	2	2	2	2	2	2	2	2
2	2	2	2	2	2	2	9	#NULL!	#NULL!
1	2	2	2	2	2	2	3	2	2
9	9	9	9	9	9	9	9	#NULL!	#NULL!
3	3	3	3	3	3	3	3	#NULL!	#NULL!
9	1	9	9	1	9	2	1	1	2
2	2	1	3	3	1	2	2	2	2
2	2	2	3	2	2	2	2	1	2
2	2	2	2	2	2	2	2	2	2
2	9	2	2	2	3	3	3	1	2
2	1	3	2	3	3	2	3	2	2
9	9	9	9	9	9	9	9	#NULL!	#NULL!
9	9	9	9	9	9	9	9	2	2
3	2	1	2	3	2	1	3	2	2
3	2	1	2	3	2	1	3	2	2
2	9	3	2	1	1	2	9	1	1
3	2	3	2	3	2	3	2	#NULL!	#NULL!
3	3	3	3	3	3	3	3	1	1
2	3	2	1	2	3	2	1	1	1
1	1	1	1	2	2	2	2	2	2
1	9	9	9	9	9	3	2	2	2
1	2	2	2	3	3	2	2	2	1
9	9	9	9	9	9	9	9	#NULL!	#NULL!
9	9	9	9	9	9	9	9	#NULL!	#NULL!
9	9	9	9	9	9	9	9	#NULL!	#NULL!
9	9	9	9	9	9	9	9	#NULL!	#NULL!
9	9	9	9	9	9	9	9	#NULL!	#NULL!
2	2	2	2	2	2	2	2	2	2
9	1	1	2	9	3	2	1	2	2
9	9	9	9	9	9	9	9	2	2
9	9	9	9	9	9	9	9	#NULL!	#NULL!
2	1	1	1	3	3	2	3	2	2
9	9	9	9	9	9	9	9	1	1
9	9	9	9	9	9	9	9	2	2
2	2	2	2	2	2	2	2	1	1
3	1	1	2	3	3	3	3	1	1
1	1	1	2	2	3	2	2	1	1
2	2	2	9	2	2	2	2	2	2
2	1	1	3	3	3	3	2	1	1
2	3	2	1	2	2	2	1	1	1
2	3	2	2	1	2	2	2	1	1

3	3	3	3	3	3	3	3	2	2
2	2	2	2	2	2	2	2	1	1
9	9	9	9	9	9	9	9	#NULL!	#NULL!
1	2	2	2	3	3	2	1	2	1
1	2	2	3	3	1	2	3	2	2
1	1	1	1	1	2	2	1	1	1
9	1	2	3	9	1	9	2	2	2
9	9	9	9	9	9	9	9	2	2
1	1	1	1	1	1	1	1	1	1
9	9	9	9	9	9	9	9	2	2
1	2	3	2	3	2	2	2	1	1
2	3	2	2	3	3	2	2	2	2
9	9	9	9	9	9	9	9	#NULL!	#NULL!
1	1	1	1	1	1	1	1	1	1
2	1	2	2	3	3	3	3	1	2
1	1	1	2	2	2	3	2	2	2
2	3	1	2	3	1	2	3	2	2
9	9	9	9	9	9	9	9	2	2
2	2	2	1	2	2	2	3	2	2
9	9	9	9	9	9	9	9	#NULL!	#NULL!
9	2	9	9	9	9	9	9	1	2
3	2	1	1	1	3	2	1	2	1
1	1	1	9	9	9	9	9	#NULL!	#NULL!
9	9	9	9	9	9	9	9	#NULL!	#NULL!
9	9	9	9	9	9	9	9	1	2
9	9	9	9	9	9	9	9	#NULL!	#NULL!
3	3	3	1	1	1	1	3	2	2
9	9	9	9	9	9	9	9	1	1
9	9	9	9	9	9	9	9	2	2
2	2	2	2	2	2	2	1	2	1
3	1	3	1	3	1	3	1	2	2
9	9	9	9	9	9	9	9	2	2
2	2	2	1	3	2	2	3	2	2
2	2	2	2	2	2	1	1	2	1
2	2	2	2	2	2	2	3	2	2
2	2	2	2	2	2	2	2	2	2
2	3	2	2	2	2	2	2	1	1
2	1	9	1	2	3	3	3	1	2
2	2	2	2	2	2	2	3	1	2
9	9	9	9	9	9	9	9	2	1
3	1	2	1	3	2	1	2	#NULL!	#NULL!
3	1	2	3	2	3	3	3	2	2
1	1	2	3	3	3	2	2	2	2
2	1	1	1	2	1	2	3	1	2
1	3	1	2	3	2	3	3	1	1
1	1	1	1	1	1	1	1	1	1
3	1	1	2	2	2	3	3	2	2

2	1	2	3	1	2	1	1	2	2
3	1	2	2	1	2	2	1	1	2
2	3	2	3	1	3	2	3	1	1
2	1	2	3	2	2	2	3	2	2
2	2	2	2	2	2	2	2	2	2
2	2	2	2	2	2	2	2	2	2
2	2	2	2	2	2	2	2	2	2
3	1	3	2	3	3	3	3	#NULL!	#NULL!
1	1	2	2	2	2	2	3	2	2
9	9	9	9	9	9	9	9	#NULL!	#NULL!
3	1	2	2	3	3	3	3	2	1
2	9	2	2	2	2	2	2	2	2
9	9	9	9	9	9	9	9	2	2
2	3	1	2	2	1	2	3	2	2
9	9	9	9	9	9	9	9	#NULL!	#NULL!
9	9	9	9	9	9	9	9	2	2
1	1	1	1	1	1	1	3	1	2
2	2	2	2	2	2	2	2	2	2
2	2	3	2	3	2	3	2	2	2
2	2	2	2	2	2	2	9	1	1
3	1	1	1	1	2	1	1	1	2
2	2	2	2	2	2	2	2	2	2
9	9	9	9	9	9	9	9	#NULL!	#NULL!
1	2	3	2	1	2	1	1	1	1
1	1	1	1	1	1	1	1	#NULL!	#NULL!
3	3	3	3	3	3	3	3	2	2
2	2	1	2	2	2	2	9	2	2
9	1	1	9	1	1	2	1	2	2
2	1	1	2	1	1	2	2	1	1
2	2	2	2	2	2	2	2	2	2
3	3	3	3	3	3	3	3	1	2
9	1	9	9	9	9	9	9	2	1
2	2	2	2	2	2	2	2	2	2
3	3	3	3	3	3	3	3	2	2
1	3	3	2	2	1	2	2	1	2
1	1	3	3	3	2	2	2	1	1
2	2	2	2	2	2	2	2	1	2
2	2	2	2	2	2	2	2	2	2
2	2	2	2	2	2	2	2	1	2
2	2	2	2	2	2	2	2	2	2
2	2	2	2	2	2	2	2	2	2
2	2	3	3	3	3	3	3	1	1
2	3	1	2	3	1	2	3	2	2
1	3	2	1	1	1	2	3	#NULL!	#NULL!
9	9	9	9	9	9	9	9	#NULL!	#NULL!
9	9	9	9	9	9	9	9	1	1
1	1	1	2	2	2	3	3	1	2

2	1	3	2	1	2	2	2	2	2
3	1	3	1	3	3	3	3	1	2
3	1	3	1	3	3	3	3	#NULL!	#NULL!
2	2	2	2	2	2	2	2	2	1
9	9	9	9	9	9	9	9	2	2
9	9	9	9	9	9	9	9	1	1
9	9	9	9	1	9	9	9	1	1
2	2	2	2	2	2	2	2	2	2
2	1	3	1	3	3	3	3	1	2
2	2	2	2	2	2	2	2	2	1
1	1	1	1	1	2	3	3	2	2
9	1	1	3	1	2	2	2	2	2
2	2	2	2	2	2	2	2	1	1
2	2	2	2	2	2	2	2	1	1
2	2	2	2	2	2	2	2	2	2
1	3	2	2	2	1	2	1	1	1
2	2	2	2	3	3	3	2	2	2
2	2	2	2	2	2	2	2	2	2
3	1	1	2	3	3	3	3	2	2
1	3	1	2	3	1	2	3	1	2
2	2	2	2	2	2	2	2	1	2
9	1	9	9	9	9	9	9	1	1
2	2	2	2	2	2	2	1	2	1
2	2	2	2	2	2	2	2	2	2
3	2	3	2	2	2	2	2	2	2
2	2	2	2	3	2	2	1	2	1
2	1	2	2	3	2	2	1	1	2
9	9	9	9	9	9	9	9	#NULL!	#NULL!
1	1	2	1	3	2	2	3	2	2
1	2	2	2	2	2	1	2	2	2
2	1	3	1	2	2	2	3	2	2
1	1	1	1	1	3	1	3	1	1
1	1	2	1	2	3	2	2	1	1
1	1	1	1	3	2	2	3	2	1
3	1	3	1	3	3	3	3	1	1
2	2	3	3	3	2	2	2	1	2
2	2	2	2	2	2	2	2	2	2
2	2	2	2	2	2	2	2	2	2
2	2	2	2	2	2	2	3	1	2
9	9	9	9	9	9	9	9	#NULL!	#NULL!
3	1	3	1	3	3	3	3	2	2
2	3	2	1	2	1	2	3	1	1
2	2	1	1	2	1	2	2	2	1
2	3	1	2	3	1	2	3	2	2
1	2	2	3	3	1	2	3	2	1
1	1	1	1	1	1	1	1	2	2
2	2	2	2	2	2	2	2	2	2

1	1	1	9	1	3	3	3	2	2
9	9	1	9	9	9	1	3	2	2
2	2	2	2	2	2	2	2	1	2
1	2	3	1	1	1	1	1	1	1
2	2	2	2	2	2	2	2	2	2
2	2	9	3	2	2	2	2	2	2
3	2	3	2	3	2	3	2	2	2
2	1	3	1	1	3	3	3	2	2
9	9	9	9	9	9	9	9	1	2
2	2	2	2	2	2	2	2	2	2
3	1	3	1	3	3	3	3	2	2
1	1	3	1	3	3	3	3	2	2
2	2	2	2	2	2	2	2	2	1
2	3	3	1	1	3	2	1	2	2
2	1	2	2	3	3	3	3	2	2
3	1	3	2	3	3	3	3	1	1
2	2	2	2	3	2	3	3	1	2
9	9	9	1	9	9	9	9	1	1
9	9	9	9	9	9	9	9	#NULL!	#NULL!
3	1	1	2	2	2	1	3	1	1
3	1	1	1	3	2	3	3	2	2
2	1	2	2	2	3	3	3	1	1
2	2	2	2	2	2	2	3	1	1
1	2	3	1	2	1	2	3	2	2
9	9	9	9	9	9	9	9	#NULL!	#NULL!
1	1	1	2	1	3	3	3	2	1
1	1	1	2	3	2	3	3	1	1
2	1	2	2	3	3	3	3	1	2
2	2	2	3	3	3	2	3	1	2
2	1	1	2	2	2	2	2	1	2
2	2	2	2	2	2	2	2	2	2
2	2	2	2	3	2	3	2	1	2
1	1	2	3	3	1	2	3	2	1
1	1	2	3	2	3	2	1	1	1
3	1	3	1	3	3	1	3	1	2
3	2	1	1	2	3	1	3	1	2
3	1	2	2	2	3	2	3	2	2
2	1	2	2	2	2	2	3	2	2
2	1	2	1	2	2	1	3	2	2
2	1	9	3	3	3	2	3	2	2
9	9	9	9	9	9	9	9	#NULL!	#NULL!
9	2	2	1	1	2	3	1	2	2
9	9	9	9	9	9	9	9	#NULL!	#NULL!
3	1	3	1	3	3	3	3	2	2
3	3	3	3	3	3	3	3	2	2
1	1	1	2	1	3	3	3	2	2
2	1	1	1	1	2	3	3	1	2

3	3	1	3	1	1	2	3	2	2
9	9	9	9	9	9	9	9	1	2
9	9	9	9	9	9	9	9	1	#NULL!
9	9	9	9	9	9	9	9	#NULL!	#NULL!
2	2	2	2	2	2	2	2	1	2
2	2	2	2	2	2	2	2	2	2
2	1	1	1	1	2	2	3	2	2
1	1	1	1	1	1	1	1	1	1
2	2	2	2	2	2	2	2	#NULL!	#NULL!
2	1	3	1	3	1	2	3	#NULL!	#NULL!
2	2	2	2	2	2	2	2	2	2
2	1	3	1	3	3	3	3	2	2
2	2	2	2	2	2	2	2	2	2
2	2	2	2	2	2	2	2	2	2
2	1	2	3	2	1	2	3	1	1
2	1	2	1	2	3	3	2	1	2
2	1	3	2	3	3	3	3	2	2
2	1	1	2	3	2	3	3	2	2
1	1	9	2	1	3	3	3	2	2
9	9	9	9	9	9	9	9	1	1
3	3	3	3	3	3	3	1	1	1
1	1	1	3	1	2	1	1	1	1
1	1	1	2	1	3	3	3	2	2
2	1	2	3	2	1	2	3	1	2
9	9	9	9	9	9	9	9	2	2
1	1	2	1	3	3	3	3	1	1
9	9	9	9	9	9	9	9	1	2
2	1	2	1	2	1	2	1	1	1
1	1	3	2	1	2	1	2	2	2
2	1	1	2	3	3	3	3	2	2
2	1	2	1	2	2	3	2	2	2
1	1	2	2	2	2	2	3	2	1
2	2	2	2	3	3	3	2	2	2
3	1	3	2	3	3	3	3	2	2
1	1	1	2	3	3	3	3	1	2
1	1	3	2	1	3	3	3	1	2
2	1	1	1	1	1	2	2	1	2
1	1	2	1	2	2	2	3	2	2
3	1	1	1	3	3	2	3	2	2
3	1	3	2	3	3	2	2	#NULL!	#NULL!
3	1	1	1	3	3	3	3	2	2
1	3	2	2	1	3	2	2	2	1
9	9	9	9	9	9	9	9	#NULL!	#NULL!
3	1	3	2	3	3	3	3	2	2
1	1	1	1	1	1	1	1	2	2
9	9	9	9	9	9	9	9	1	1
1	1	3	2	1	1	1	1	2	2

1	1	3	1	3	3	3	3	1	1
9	9	9	9	9	9	9	9	#NULL!	#NULL!
3	2	1	3	2	1	2	3	1	1
9	9	9	9	9	9	9	9	2	2
2	1	2	2	2	2	2	2	2	2
3	1	3	1	3	3	1	3	1	1
2	9	2	9	2	3	2	1	1	1
1	1	1	1	1	1	1	2	2	2
1	1	1	1	1	1	1	2	2	2
9	9	9	9	9	9	9	9	#NULL!	#NULL!
1	2	1	2	1	2	2	2	1	1
1	1	1	1	1	1	1	1	1	1
3	1	3	1	3	1	3	1	1	2
2	1	3	1	2	1	3	1	1	2
3	1	1	3	3	1	1	3	2	2
9	9	9	9	9	9	9	9	#NULL!	#NULL!
9	9	9	9	9	9	9	9	1	1
2	3	2	1	3	2	2	1	2	2
2	2	2	3	2	2	2	1	1	2
2	2	2	2	2	2	2	1	2	1
9	9	9	9	9	9	9	9	#NULL!	#NULL!
1	1	1	1	2	2	1	1	1	2
2	2	2	2	2	2	2	1	1	2
1	2	2	2	1	1	1	1	1	2
2	2	2	2	2	2	1	1	2	1
1	2	2	1	1	1	2	3	1	2
2	2	2	2	2	2	2	1	1	2
2	2	2	2	2	2	2	2	2	1
2	2	2	2	3	3	3	3	2	2
3	2	2	1	2	2	1	1	2	1
9	9	9	9	9	9	9	9	#NULL!	#NULL!
9	9	9	9	9	9	9	9	#NULL!	#NULL!
9	9	9	9	9	9	9	9	#NULL!	#NULL!
2	3	1	2	2	3	2	2	2	2
2	1	1	2	3	3	3	3	2	2
2	1	2	2	3	2	3	2	1	2
1	1	1	3	1	1	2	2	1	1
2	1	1	1	3	3	3	3	2	2
3	2	2	2	3	3	2	2	1	2
2	2	2	2	2	2	2	2	2	2
1	1	3	3	1	3	2	3	2	2
1	1	1	2	3	3	3	3	2	1
2	2	2	2	2	2	2	2	2	2
2	2	2	2	2	2	2	2	1	1
2	2	2	2	2	2	2	2	2	2
1	1	1	1	1	1	1	1	1	1
9	9	9	9	9	9	9	9	2	2

9	9	9	9	9	9	9	9	2	2
3	1	2	2	2	2	2	2	2	2
9	9	9	9	9	9	9	9	2	2
9	9	9	9	9	9	9	9	2	2
3	1	3	3	3	3	3	3	2	2
9	9	9	9	9	9	9	9	1	2
1	1	1	2	1	2	1	2	2	2
9	9	9	1	9	9	9	1	#NULL!	#NULL!
3	1	2	2	3	2	3	2	2	2
9	9	9	9	9	9	9	9	1	1
2	9	9	1	2	9	1	1	2	1
9	1	9	9	9	9	9	9	2	2
2	2	2	2	2	2	2	2	1	2
9	9	9	9	9	9	9	9	2	2
3	1	1	1	3	3	3	3	2	2
3	1	1	1	3	3	3	3	1	1
1	1	2	1	1	1	1	2	1	1
1	1	2	1	1	1	1	2	1	1
1	1	2	3	2	2	2	3	2	2
2	2	1	2	1	2	1	2	2	1
2	1	2	1	2	3	3	3	2	2
2	2	1	2	2	2	2	3	1	2
2	1	2	2	1	2	3	3	1	1
3	3	3	3	3	3	3	3	2	2
2	1	3	1	3	2	3	1	1	1
2	1	1	2	2	1	2	2	2	1
2	2	2	2	2	2	2	2	2	2
1	1	3	1	1	3	3	3	1	2
2	2	2	2	2	2	2	2	2	2
2	2	2	2	3	3	3	2	1	1
2	2	2	2	2	2	2	2	1	1
1	1	1	2	1	2	2	2	1	1
2	1	1	2	3	3	3	3	2	2
1	1	1	2	3	3	2	3	1	1
9	9	9	9	9	9	9	9	2	2
3	1	3	3	3	3	3	3	2	1
2	2	2	2	2	2	2	2	2	2
1	2	2	2	2	3	2	3	2	2
3	1	1	1	3	3	3	3	2	2
2	1	1	2	2	2	2	9	2	2
1	1	1	1	1	1	1	1	2	2
9	1	9	9	9	9	9	9	2	2
9	1	9	9	9	9	9	9	2	2
2	1	3	2	3	3	2	2	2	2
3	1	9	3	1	9	1	1	1	1
2	2	2	2	2	2	2	2	2	2
2	1	3	2	1	2	3	3	2	2

2	2	2	2	2	2	2	2	1	2
1	1	2	1	1	2	2	1	1	2
3	2	9	1	3	2	2	1	2	2
2	1	2	2	2	2	2	2	1	2
3	3	2	2	3	3	3	3	2	2
2	3	3	2	2	3	2	2	2	2
1	1	2	3	3	3	3	3	1	2
2	2	2	2	2	2	2	2	2	2
3	1	3	1	3	3	3	3	1	1
3	2	1	2	1	2	1	1	1	2
3	1	1	1	3	3	2	3	2	2
1	2	2	2	2	2	2	2	2	2
3	1	3	1	3	3	3	3	1	1
2	2	2	2	2	2	2	3	1	2
2	3	2	1	2	3	2	1	2	2
2	2	2	2	2	2	2	2	2	2
3	3	3	1	3	3	3	3	1	1
2	2	2	2	2	2	2	2	2	2
2	2	2	2	2	2	3	2	2	1
2	1	2	2	2	2	2	2	1	2
2	2	2	2	1	2	2	2	1	2
9	9	9	9	9	9	9	9	2	2
3	1	3	1	2	3	3	3	2	2
2	2	2	2	2	9	1	2	2	2
1	1	2	2	2	3	3	3	1	1
2	2	2	2	2	2	2	2	2	2
2	2	2	2	2	2	2	2	1	1
1	1	1	2	2	2	1	1	1	1
2	1	1	1	3	2	3	3	1	1
2	2	3	2	2	2	2	2	2	1
9	9	9	9	9	9	9	9	#NULL!	#NULL!
2	2	1	2	2	2	2	2	1	2
2	2	2	2	1	2	2	2	2	2
2	1	2	2	2	1	2	2	2	1
2	2	2	2	2	3	2	2	1	2
2	2	1	2	2	2	3	2	2	1
2	2	2	3	2	2	2	3	1	2
2	2	2	2	2	2	2	3	2	2
2	2	3	1	2	3	2	1	1	1
1	1	1	1	1	1	2	2	1	2
1	1	1	1	1	3	3	1	2	2
3	2	3	2	3	2	2	2	2	2
3	1	3	1	1	3	3	3	1	2
1	1	3	2	3	3	3	3	2	2
2	1	3	1	1	3	3	3	1	1
2	2	2	2	2	2	2	2	2	2
2	1	1	2	3	3	3	3	1	2

1	1	3	2	1	3	3	3	1	2
2	2	2	1	2	2	2	3	1	2
1	1	2	2	1	2	2	1	1	2
2	2	2	1	1	2	2	1	2	1
2	2	2	3	3	2	2	2	2	1
1	1	1	2	2	2	2	1	2	1
2	2	2	3	2	2	2	3	1	2
2	2	2	2	2	2	2	2	2	2
3	2	3	2	3	2	1	1	2	2
2	1	1	1	3	3	3	3	2	1
3	1	3	1	9	3	1	3	1	2
1	3	1	2	3	1	2	1	2	2
3	3	3	2	2	2	2	2	2	2
2	1	2	2	2	2	2	2	2	2
1	3	3	1	3	2	3	3	2	2
2	1	2	2	2	2	2	3	2	2
2	2	2	1	2	2	1	2	1	2
2	2	2	2	3	2	2	3	2	1
2	3	2	1	2	2	1	3	2	2
2	1	3	2	3	3	3	3	1	1
1	2	2	2	1	2	2	2	2	1
3	3	3	3	3	3	3	3	2	2
2	2	2	2	2	2	2	2	1	2
1	1	1	3	1	3	3	3	2	2
9	1	1	1	2	2	3	3	1	1
2	1	1	2	1	3	3	3	2	2
2	2	1	1	1	2	2	2	1	1
1	1	2	1	2	2	3	3	2	2
2	1	2	1	3	1	3	2	2	2
2	2	1	2	1	2	2	2	1	2
1	1	1	1	1	1	1	1	2	1
3	1	3	1	3	3	1	2	1	2
2	2	2	1	2	2	1	3	1	2
1	1	3	2	2	3	1	3	2	2
1	2	3	1	2	3	2	2	2	2
1	1	1	1	2	2	2	3	2	2
2	2	2	1	2	3	3	1	1	1
1	1	1	1	2	3	3	3	1	2
3	1	1	2	1	2	1	2	1	2
3	1	3	1	3	3	3	3	1	1
1	1	3	1	3	3	3	9	1	1
2	1	3	3	2	3	3	3	1	1
1	1	1	1	1	1	1	3	1	1
3	1	3	1	1	3	3	3	1	1
2	1	3	1	3	3	3	3	1	2
1	2	3	2	1	2	2	1	2	2
2	2	2	2	2	2	2	2	1	2

2	2	2	2	2	2	2	2	2	2
2	2	2	2	2	2	2	2	2	2
2	2	1	2	2	2	3	2	2	1
1	1	3	3	1	3	3	3	1	1
3	1	3	2	3	3	3	3	1	2
1	2	2	2	2	2	3	2	2	1
2	2	2	2	2	2	2	2	2	2
2	2	1	2	2	2	3	2	2	1
9	9	9	9	9	9	9	9	#NULL!	#NULL!
2	1	3	2	2	3	1	9	2	2
1	1	1	2	1	1	1	2	1	1
3	1	1	1	3	1	3	3	2	2
2	2	2	2	2	2	2	2	1	1
2	2	2	2	2	2	2	2	2	2
1	1	1	1	1	1	1	1	2	2
1	1	1	2	3	3	3	3	2	2
2	2	2	2	2	2	2	2	2	2
2	3	2	1	2	3	2	1	1	1
3	3	3	3	3	3	3	3	2	2
2	2	2	2	2	2	2	2	2	2
2	2	3	2	2	3	3	3	2	1
3	3	2	2	2	1	2	1	2	1
2	3	2	2	3	3	3	3	2	1
2	2	2	3	2	2	2	2	1	2
1	1	1	1	1	1	1	1	2	2
1	1	1	2	3	3	3	3	2	2
3	1	1	1	3	1	3	3	2	2
2	1	3	2	2	3	1	9	2	2
3	1	1	2	1	2	1	2	1	2
2	2	2	2	2	2	2	2	2	2
2	2	2	1	2	3	3	1	1	1
9	9	9	9	9	9	9	9	#NULL!	#NULL!
2	2	2	2	2	2	2	2	2	2
3	1	3	1	3	3	3	3	1	1
2	2	2	2	1	2	3	2	2	2
2	1	2	2	3	1	2	1	1	1
3	3	3	3	3	3	3	3	1	1
2	3	1	2	3	1	2	3	1	2
2	2	2	2	2	2	2	2	2	2
2	2	3	3	3	1	1	1	1	1
2	1	2	1	3	3	3	3	2	1
2	1	1	2	1	3	3	3	2	1
2	1	1	1	2	2	3	2	1	1
1	1	9	2	2	2	2	3	1	1
3	2	3	2	2	2	3	2	1	2
3	3	3	3	3	3	3	3	2	2
3	1	3	2	3	9	3	3	1	1

2	2	1	3	2	3	1	1	2	2
3	1	1	2	3	3	3	2	2	2
1	1	1	1	1	1	1	1	1	2
2	1	2	1	3	3	3	3	2	2
2	2	2	1	2	3	2	3	2	2
3	1	2	1	2	3	3	3	1	1
1	1	1	2	1	1	1	2	1	1
9	9	9	9	9	9	9	1	1	1
9	9	9	9	9	9	9	1	1	1
9	9	9	9	9	9	9	1	1	1
1	3	1	2	2	2	1	2	1	1
1	3	1	2	1	2	1	2	1	1
1	3	1	2	1	2	1	1	1	1
1	3	1	2	1	2	1	1	1	1
1	3	1	3	2	1	2	1	1	1
3	1	3	1	3	3	2	1	1	1
2	2	2	1	3	3	3	3	1	1
3	3	9	2	9	1	2	2	1	1
1	3	2	3	2	3	2	2	1	1
3	2	1	2	2	1	2	2	1	1
2	1	2	3	2	1	3	2	1	1
2	1	1	2	1	3	1	2	1	1
1	2	2	2	2	2	2	2	1	2
2	2	1	3	1	1	1	9	1	1
2	2	1	3	1	3	3	3	1	1
1	3	1	3	3	3	3	2	1	1
2	2	2	1	3	3	3	9	1	1
1	2	2	2	2	3	3	3	1	1
1	3	1	3	1	2	2	2	1	1
1	3	1	2	3	2	2	2	1	1
1	3	1	2	1	2	1	1	1	1
1	3	1	2	1	2	1	1	1	1
9	9	9	9	9	9	9	9	2	2
3	1	3	1	3	3	3	2	2	2
3	1	3	1	3	3	3	3	2	2
9	9	9	9	9	9	9	9	2	2
9	9	9	9	9	9	9	9	2	2
2	2	2	2	2	2	2	2	2	2
2	2	2	2	2	2	3	3	2	2
3	1	3	1	3	3	3	3	2	2
1	3	1	2	2	2	2	2	2	2
3	3	1	3	3	3	3	3	1	1
3	3	1	3	3	3	3	3	1	1
3	3	1	3	3	3	3	3	1	1
3	3	1	3	3	3	3	3	1	1
2	1	2	9	2	9	2	1	1	2
9	2	9	3	2	2	3	1	1	2

9	9	2	1	1	1	2	3	2	2
1	2	2	1	3	2	2	1	1	1
9	9	9	9	9	9	9	9	1	1
9	9	9	9	9	9	9	9	1	1
2	2	1	3	2	1	1	2	1	1
1	1	1	3	3	1	1	2	2	2
3	3	2	3	1	2	1	1	2	2
1	2	3	3	1	2	1	2	2	2
9	9	9	9	9	9	9	9	1	1
9	9	9	9	9	9	9	9	#NULL!	#NULL!
2	2	2	2	2	2	2	2	#NULL!	#NULL!
3	9	9	3	9	1	9	2	1	1
2	2	2	2	2	2	2	1	1	1
1	3	1	3	1	1	1	1	1	2
9	9	9	9	9	9	9	9	2	2
2	2	2	2	2	2	2	2	1	2
2	2	2	2	2	2	2	2	1	2
9	9	9	9	9	9	9	9	2	2
2	2	2	2	2	2	2	2	1	2
9	9	9	9	9	9	9	9	2	1
2	3	3	3	3	3	3	2	1	2
2	2	1	2	3	3	1	1	1	2
1	3	1	3	1	1	1	1	2	2
2	2	9	3	3	3	3	3	1	1
9	9	9	9	9	9	9	3	1	2
9	9	9	9	9	9	9	9	1	1
2	1	1	2	3	1	2	3	1	1
9	3	9	9	9	9	9	9	1	#NULL!
3	1	3	3	3	3	3	3	1	1
9	9	9	9	9	9	9	9	#NULL!	#NULL!
3	2	3	1	1	2	2	1	2	2
9	9	9	9	9	9	9	9	2	2
2	3	2	2	1	2	1	1	1	1
3	1	1	3	1	1	2	2	#NULL!	#NULL!
1	3	1	3	1	1	1	1	2	2
1	3	1	3	1	1	1	1	2	2
1	3	1	3	1	1	1	1	2	2
1	3	1	3	1	1	2	1	2	2
1	3	1	3	1	1	1	1	1	2
9	9	9	9	9	9	9	9	2	2
9	9	9	9	9	9	9	9	2	2
9	9	9	9	9	9	9	9	2	2
9	9	9	9	9	9	9	9	1	#NULL!
9	9	9	9	9	9	9	9	2	2
9	9	9	9	9	9	9	9	2	2
1	3	2	1	2	1	2	1	1	1
2	1	1	2	3	1	2	3	1	1

9	9	9	9	9	9	9	9	#NULL!	#NULL!
2	3	2	2	2	1	3	2	1	1
2	3	1	1	3	3	3	3	2	2
1	3	1	2	2	2	2	2	1	1
2	2	2	2	2	2	2	2	1	1
2	2	2	2	2	2	2	2	1	1
9	9	9	9	9	9	9	9	1	1
3	1	3	1	3	3	3	3	2	2
1	3	1	3	1	3	3	3	2	2
9	9	9	9	9	9	9	9	2	2
9	9	9	9	9	9	9	9	2	#NULL!
9	9	9	9	9	9	9	9	1	1
9	9	9	9	9	9	9	9	2	2
9	9	9	9	9	9	9	9	2	2
1	3	1	3	1	1	1	1	1	1
9	9	9	9	9	9	9	9	2	2
1	3	1	3	3	3	3	3	1	2
9	9	9	9	9	9	9	9	2	2
3	3	3	3	2	1	2	1	2	2
1	3	2	2	3	3	3	2	2	1
1	3	2	2	2	3	3	2	2	2
3	9	2	1	3	1	2	2	2	1
2	2	3	1	3	3	3	1	1	1
1	3	1	3	1	1	1	1	1	1
1	3	1	3	1	1	1	1	1	1
2	1	1	2	2	3	2	1	2	2
2	9	3	1	1	2	2	2	1	2
1	2	2	2	1	1	1	2	1	2
2	2	2	2	2	2	2	3	1	2
9	1	1	2	1	1	1	3	1	2
2	1	2	3	2	3	2	1	1	1
1	3	1	3	1	1	1	2	1	1
9	9	9	9	1	9	9	9	1	1
9	9	9	3	9	9	9	9	1	#NULL!
1	9	9	9	9	9	9	9	1	2
9	3	9	9	9	9	9	9	2	#NULL!
9	9	9	9	9	9	2	9	2	1
9	9	9	9	9	9	2	9	2	2
9	9	9	2	9	9	9	9	2	2
9	9	9	9	9	9	9	9	1	2
9	9	9	9	9	9	9	9	1	2
9	9	9	9	9	9	9	9	1	2
9	9	9	9	9	9	9	9	1	2
9	9	9	9	9	9	9	9	2	1
9	9	9	9	9	9	9	9	1	1
1	3	2	3	1	2	1	2	1	1
2	3	1	3	2	1	2	1	1	1

1	2	1	2	3	1	1	1	1	1
9	1	9	9	3	9	3	9	2	2
9	9	9	9	9	9	9	9	2	2
2	3	9	2	1	1	1	2	1	1
9	9	9	9	9	9	9	9	1	2
9	9	9	9	9	9	9	9	1	1
9	9	9	9	9	9	9	9	1	1
9	9	2	9	9	9	9	9	1	2
9	9	9	9	9	9	9	9	#NULL!	#NULL!
2	3	2	3	2	1	2	1	1	1
2	3	2	3	2	1	2	2	1	1
2	3	2	3	2	1	2	1	1	1
2	2	2	2	2	2	2	2	1	1
9	9	9	9	9	9	9	9	1	1
1	2	2	2	1	1	1	2	1	2
2	2	2	3	3	3	3	1	1	2
9	9	9	9	9	9	9	2	1	#NULL!
9	9	9	9	9	9	9	2	1	2
9	9	9	9	9	9	9	9	1	2
9	9	1	3	1	9	9	1	1	1
9	9	9	9	9	9	9	9	2	1
9	9	9	9	9	9	9	9	#NULL!	#NULL!
9	9	3	9	9	9	9	9	2	1
9	9	3	9	3	9	9	9	1	2
9	2	2	2	3	3	9	9	2	2
9	9	9	3	9	9	9	9	2	2
9	9	9	9	9	9	9	9	1	1
2	2	2	2	2	2	2	2	2	2
3	2	3	2	3	2	1	2	2	2
9	9	9	9	9	9	9	9	2	2
9	9	9	9	9	9	9	9	1	1
2	3	1	2	3	1	2	2	1	2
2	2	2	2	2	2	2	3	1	1
1	3	2	1	3	2	2	2	1	2
3	3	3	3	2	3	3	3	1	1
1	2	2	2	2	2	2	2	1	2
1	1	2	2	3	2	1	2	2	2
9	3	2	2	2	2	1	1	1	1
9	9	9	9	9	9	9	9	2	2
2	2	2	2	2	2	2	2	2	2
2	2	2	2	2	2	2	2	2	2
2	2	2	2	2	2	2	2	2	2
3	3	3	3	2	3	1	2	2	2
1	3	1	2	2	1	2	2	1	2
9	3	9	9	9	9	9	9	1	2
2	2	2	2	2	2	2	2	2	2
2	3	2	2	2	2	3	3	1	1

2	3	2	3	2	3	3	3	1	1
3	3	3	3	3	3	3	3	1	2
3	3	1	2	2	2	2	2	1	2
2	2	3	2	1	2	2	1	2	2
2	1	2	3	2	3	2	1	1	1
2	2	1	3	2	1	1	1	2	2
3	3	3	1	1	1	3	3	1	1
2	3	1	3	2	2	2	2	1	1
1	3	1	1	3	3	1	3	1	1
9	9	9	9	9	9	9	9	#NULL!	#NULL!
2	2	2	2	2	2	2	2	2	2
2	3	1	3	1	2	3	3	1	2
1	3	1	3	3	1	1	9	2	1
2	2	2	2	2	2	2	2	1	2
2	3	1	2	1	1	1	1	2	2
1	3	3	2	3	3	3	3	2	2
2	2	2	2	2	2	2	2	1	1
2	2	2	2	2	2	2	9	1	1
1	2	1	2	1	2	2	2	2	2
1	3	1	3	1	1	1	3	1	1
2	3	2	2	2	2	2	3	1	2
3	1	3	2	3	2	2	3	1	1
2	3	1	2	2	2	3	2	1	1
3	3	3	3	3	3	3	3	1	1
2	2	1	1	2	2	3	3	1	1
2	1	2	1	3	1	3	3	2	2
3	3	2	3	3	3	3	3	1	2
3	3	2	3	2	2	1	3	1	1
2	3	3	3	2	3	3	3	1	2
2	2	2	2	2	2	2	2	2	2
2	2	2	2	2	2	2	2	1	1
2	2	2	2	2	2	2	3	1	2
2	2	2	2	2	2	2	2	1	2
2	2	2	2	2	2	2	2	1	1
2	2	2	3	2	3	1	2	1	2
2	2	2	2	2	2	2	2	2	2
2	2	2	3	2	1	3	2	1	1
9	9	9	9	9	9	9	9	#NULL!	#NULL!
9	9	9	9	9	9	9	9	#NULL!	#NULL!
9	9	9	9	9	9	9	9	2	2
2	2	1	1	1	3	1	2	2	2
9	9	9	9	9	9	9	9	1	1
9	9	9	9	9	9	9	9	#NULL!	#NULL!
9	9	9	9	9	9	9	9	1	1
9	9	9	9	9	9	9	9	1	2
9	9	9	9	9	9	9	9	#NULL!	#NULL!
3	3	1	1	3	3	1	3	1	2

2	2	2	2	1	1	2	3	2	2
2	3	1	2	2	2	2	2	1	1
9	9	9	9	9	9	9	9	1	1
3	1	3	2	2	2	2	1	1	1
9	9	9	9	9	9	9	9	2	2
9	9	9	9	9	9	9	9	1	2
1	3	2	2	2	2	2	9	1	2
2	3	3	3	3	2	3	3	1	1
2	9	1	3	2	9	2	2	1	1
2	9	1	1	2	2	1	1	2	2
2	2	2	9	1	1	1	1	1	1
2	2	2	2	2	2	2	2	1	1
1	2	1	3	1	2	3	3	1	1
2	1	9	3	2	2	9	2	1	1
1	3	2	3	2	3	3	3	2	2
1	3	2	2	2	2	2	2	1	2
3	3	1	3	3	3	1	3	1	2
9	9	9	9	9	1	9	9	2	2
3	3	3	3	3	3	3	3	1	1
2	2	2	3	3	3	3	3	1	1
2	2	2	2	2	2	2	1	2	1
2	2	2	2	2	2	2	2	1	2
2	2	2	2	2	2	2	2	2	2
3	3	3	3	3	3	3	3	1	1
3	2	2	2	2	3	3	3	1	2
2	2	2	2	2	2	2	2	1	2
9	9	9	9	9	9	9	9	#NULL!	#NULL!
2	3	2	2	3	2	3	3	2	2
3	3	1	3	2	2	2	2	2	2
2	2	3	2	3	2	2	3	1	1
3	3	1	1	3	3	1	3	2	2
9	9	9	9	9	9	9	9	2	2
2	3	2	2	2	2	2	2	1	1
2	2	2	2	2	2	2	2	2	2
1	3	2	2	2	3	3	3	2	1
3	3	3	3	3	3	3	3	1	2
9	9	9	9	9	9	9	9	2	2
2	2	2	2	2	2	2	3	2	2
2	3	2	2	3	2	2	3	1	1
3	3	1	3	3	3	1	3	2	2
1	3	2	2	3	2	2	3	#NULL!	#NULL!
3	3	1	2	3	3	2	1	1	1
3	3	1	1	3	3	3	3	1	1
9	9	9	9	9	9	9	9	1	1
2	3	1	3	3	3	1	3	1	1
1	3	1	3	3	3	3	3	1	2
9	9	9	9	9	9	9	9	1	1

2	2	2	2	2	2	2	2	2	2
2	2	2	2	2	2	2	2	2	2
2	2	2	2	2	2	2	2	2	1
2	2	2	2	2	2	2	2	1	2
2	2	2	2	2	2	2	2	1	2
3	2	2	2	3	3	2	2	#NULL!	#NULL!
9	9	9	9	9	9	9	9	2	2
2	2	2	2	2	2	2	2	1	1
2	2	2	2	2	2	2	2	2	2
2	2	2	2	2	2	2	2	2	2
9	9	9	9	9	9	9	9	#NULL!	#NULL!
2	1	2	2	3	3	3	3	1	2
9	9	9	9	9	9	9	9	#NULL!	#NULL!
1	3	1	1	1	1	1	1	1	1
3	3	9	3	2	3	2	3	1	1
9	9	9	9	9	9	9	9	#NULL!	#NULL!
2	2	2	2	2	2	2	2	1	1
3	2	3	2	2	3	2	3	1	1
1	9	9	9	9	9	9	9	1	1
1	3	2	2	1	1	1	2	1	1
9	9	9	9	9	9	9	9	1	1
9	2	2	2	2	2	2	3	1	2
2	2	2	2	2	2	2	2	2	2
3	3	2	3	3	3	3	3	#NULL!	#NULL!
3	3	1	1	1	3	3	3	1	1
9	9	9	9	9	9	9	9	1	2
3	1	3	3	3	3	3	3	#NULL!	#NULL!
3	1	9	3	3	3	3	3	1	1
1	3	1	3	1	1	3	2	1	2
9	9	9	9	9	9	9	9	1	1
9	9	9	9	9	9	9	9	1	1
2	3	9	2	1	2	3	3	1	1
2	2	2	2	2	2	2	2	1	1
3	3	2	3	2	2	2	1	1	1
9	3	9	9	9	9	2	2	1	1
3	3	3	3	3	3	3	3	1	1
9	9	9	9	9	9	9	9	1	1
9	9	9	9	9	9	9	9	#NULL!	#NULL!
9	9	9	9	9	9	9	9	1	1
9	9	9	9	9	9	9	9	1	1
3	3	3	3	3	3	3	3	1	1
3	3	3	1	3	3	3	3	1	1
2	3	1	2	2	1	2	1	1	1
9	9	9	9	9	9	9	9	1	1
2	1	3	2	2	2	1	1	2	2
3	1	9	3	3	3	3	3	1	1
3	3	2	3	2	2	3	3	#NULL!	#NULL!

3	1	3	1	3	3	3	3	#NULL!	#NULL!
2	3	1	3	3	3	3	1	1	1
2	3	2	3	2	3	3	3	1	1
9	9	9	9	9	9	9	9	1	1
3	3	1	3	3	1	2	3	#NULL!	#NULL!
9	9	9	9	9	9	9	9	#NULL!	#NULL!
1	1	1	3	1	2	1	1	1	1
2	1	2	1	2	3	2	3	1	1
1	3	1	2	3	3	3	3	1	1
3	3	3	3	3	3	3	3	1	1
9	9	9	9	9	9	9	9	1	1
9	3	1	9	9	9	9	9	1	1
1	3	1	3	1	3	3	3	1	1
1	2	1	2	1	2	1	9	1	1
9	9	9	9	9	9	9	9	#NULL!	#NULL!
2	2	2	2	2	2	2	2	1	1
3	3	3	3	3	3	3	3	1	1
2	2	2	2	2	2	2	2	1	1
9	9	9	9	9	9	9	9	1	1
1	3	9	2	2	2	2	9	1	2
2	3	3	3	3	3	3	3	1	1
2	2	2	2	2	2	2	2	1	1
3	3	3	3	3	3	3	3	1	1
2	3	2	2	1	3	3	3	1	1
9	9	9	9	9	9	9	9	1	1
2	3	2	3	2	2	2	3	1	1
3	3	1	2	2	2	1	3	1	1
3	3	3	3	3	3	3	3	1	1
9	9	9	9	9	9	9	9	1	1
2	3	1	1	3	3	3	3	1	1
2	1	9	2	2	3	2	2	1	2
9	9	9	9	9	9	9	9	#NULL!	#NULL!
9	9	9	9	9	9	9	9	1	1
9	9	9	9	9	9	9	9	1	1
9	9	9	9	9	9	9	9	#NULL!	#NULL!
2	3	2	2	2	2	2	2	1	2
2	2	2	2	2	2	2	2	1	1
9	9	9	9	9	9	9	9	1	1
9	9	9	9	9	9	9	9	1	1
9	9	9	9	9	9	9	9	1	1
9	9	9	9	9	9	9	9	1	1
9	9	9	9	9	9	9	9	1	1
9	9	9	9	9	9	9	9	1	1
9	3	2	3	1	2	9	3	2	2
2	3	2	2	2	2	2	2	1	2
2	3	2	3	2	2	2	2	1	1
9	3	9	9	9	9	9	9	1	1

9	9	9	9	9	9	9	9	#NULL!	#NULL!
9	9	9	9	9	9	9	9	1	1
9	9	9	3	9	9	9	9	1	1
9	9	9	9	9	9	9	9	1	1
9	9	9	9	9	9	9	9	1	1
9	3	9	9	9	9	9	9	1	1
9	9	9	9	9	9	9	9	2	1
9	9	9	9	9	9	9	9	1	1
2	2	2	2	2	2	2	2	1	1
3	3	3	3	3	3	3	3	1	1
2	3	1	2	2	2	3	3	1	1
9	9	9	9	9	9	9	9	1	1
1	3	3	1	1	3	1	1	1	1
3	1	2	2	2	2	2	2	1	1
2	3	2	3	2	2	2	2	#NULL!	#NULL!
2	1	2	3	1	2	2	1	2	2
9	9	9	9	9	9	9	9	1	1
1	3	9	3	9	9	2	3	1	1
9	9	9	9	9	9	9	9	1	2
9	3	9	9	9	9	2	2	1	2
9	9	9	9	9	9	9	9	#NULL!	2
9	9	9	9	9	9	9	9	1	1
2	3	2	2	2	2	2	2	1	1
9	9	9	9	9	9	9	9	1	1
2	2	2	2	2	2	2	2	2	2
3	9	1	2	3	3	3	3	2	2
9	9	9	9	9	9	9	9	1	1
9	9	9	9	9	9	9	9	2	1
2	3	2	2	2	3	2	3	1	1
2	2	2	2	2	2	2	2	2	1
2	3	2	3	2	2	2	2	1	1
3	3	3	3	3	3	3	3	1	1
1	3	1	3	3	1	1	3	1	1
2	3	3	3	3	2	3	3	1	1
3	3	3	3	3	3	3	3	1	1
9	3	1	9	9	9	9	9	1	1
1	3	3	3	3	3	3	3	1	1
3	3	3	3	3	3	3	3	1	1
2	3	1	2	2	3	2	3	1	1
9	9	9	9	9	9	9	9	1	1
1	9	3	2	3	3	3	2	1	1
3	9	9	9	9	9	9	9	1	1
2	9	3	9	2	9	2	3	1	1
9	9	9	9	9	9	9	9	1	1
2	1	2	1	2	2	2	2	1	1
3	3	3	3	3	3	3	3	1	1
2	1	1	2	2	2	2	2	1	1

9	9	9	9	9	9	9	9	1	2
9	9	9	9	9	9	9	9	1	1
3	3	1	3	3	3	3	3	1	1
9	9	9	9	9	9	9	9	1	1
9	9	2	1	2	3	3	2	1	2
2	2	2	2	2	2	2	2	1	1
3	3	3	3	3	3	3	3	1	1
2	3	1	2	2	2	2	3	1	1
2	3	2	2	2	2	2	3	1	1
3	3	1	3	3	3	3	3	1	1
2	3	1	2	2	2	2	3	1	1
3	3	1	3	1	1	1	3	1	1
9	9	9	9	3	9	1	2	1	#NULL!
3	3	3	1	3	3	1	1	2	2
2	2	2	2	2	2	2	3	#NULL!	#NULL!
1	3	1	3	1	1	1	3	1	1
9	9	9	9	9	9	9	9	#NULL!	#NULL!
2	2	3	3	2	3	1	3	1	1
9	9	9	9	9	9	9	9	1	1
9	9	9	9	9	9	9	9	1	1
2	3	1	2	2	1	1	2	2	2
1	3	3	3	3	3	3	3	1	1
9	9	9	9	9	9	9	9	1	1
3	3	1	3	3	1	3	3	1	1
2	2	1	2	2	2	2	2	1	2
2	9	1	2	2	2	2	3	2	2
1	3	1	1	2	1	1	3	2	2
9	9	9	9	9	9	9	9	1	1
3	3	3	3	3	3	3	3	1	1
9	9	9	9	9	9	9	9	1	2
3	3	3	3	3	3	3	3	#NULL!	#NULL!
2	2	3	1	2	3	3	2	2	2
3	2	3	3	3	3	3	3	1	2
3	3	3	3	3	3	3	3	1	2
9	9	9	9	9	9	9	9	1	2
1	3	2	3	3	1	2	1	1	1
2	2	2	3	2	1	2	2	1	2
2	2	9	3	2	1	2	1	#NULL!	#NULL!
2	9	1	2	2	2	2	3	1	1
2	2	2	2	2	2	2	2	1	1
9	9	9	9	9	9	9	9	1	1
3	3	3	2	1	3	1	3	1	1
9	3	1	9	9	9	9	9	1	1
9	9	9	9	9	9	9	9	1	1
2	3	1	3	2	2	1	3	1	1
9	3	1	3	9	9	9	9	1	1
1	3	1	3	1	1	1	1	2	2

2	2	2	2	2	2	2	2	1	1
9	9	9	9	9	9	9	9	1	2
2	3	1	3	1	1	2	2	2	2
9	9	9	9	9	9	9	9	1	1
3	3	3	3	3	3	3	3	1	1
2	2	2	2	2	2	2	2	1	1
9	9	9	9	9	9	9	9	1	1
1	3	1	3	1	1	1	1	1	1
2	3	1	3	2	9	3	3	1	1
3	3	3	3	3	3	3	3	1	2
3	9	9	9	9	9	9	9	1	1
2	3	2	2	2	3	3	3	1	1
1	3	3	3	1	1	1	1	1	1
3	3	1	3	2	2	3	3	1	1
9	9	9	9	9	9	9	9	1	1
2	3	2	2	3	3	2	3	1	2
9	9	9	9	9	9	9	9	1	2
3	1	3	1	3	3	3	3	1	1
3	3	3	3	3	3	3	3	1	1
2	3	3	3	3	3	2	3	1	1
9	9	9	9	9	9	9	9	1	1
2	3	3	2	3	3	1	3	1	1
3	3	3	3	3	3	3	3	1	1
3	3	3	3	3	3	3	3	1	1
3	2	3	2	3	2	2	2	1	1
2	3	3	1	3	3	3	3	1	1
3	3	3	1	3	3	3	3	1	1
3	3	1	3	2	3	3	3	1	1
2	3	2	2	2	2	2	2	1	1
1	3	3	3	3	1	3	3	1	1
9	9	9	9	9	9	9	9	1	1
3	3	1	3	2	3	3	3	1	1
9	9	9	9	9	9	9	9	1	1
2	3	2	2	3	3	3	3	1	2
2	3	1	3	3	3	3	3	1	1
9	9	9	9	9	9	9	9	1	1
1	3	1	2	2	1	1	2	2	1

HeardSme	HeardHPV	PadDetCaC	PapMarry2	PapOver18	Pap2yearsS	PapMeno2	PapFromH	Pap2Yearly	PapOnce2
2	2	2	2	2	2	2	2	2	2
2	2	2	2	2	1	2	2	2	1
1	2	2	2	2	2	2	2	2	2
2	1	2	2	2	1	2	2	2	1
2	2	2	2	2	1	2	2	2	1
2	2	2	2	#NULL!	1	2	2	2	1
2	2	#NULL!	#NULL!	2	#NULL!	#NULL!	#NULL!	#NULL!	#NULL!
2	2	2	2	2	2	2	2	2	2
2	2	2	#NULL!	2	#NULL!	2	#NULL!	2	#NULL!
2	2	2	#NULL!	#NULL!	#NULL!	#NULL!	#NULL!	#NULL!	#NULL!
2	#NULL!	2	2	2	2	2	2	2	2
2	2	1	2	1	1	2	2	1	2
2	1	2	2	2	1	2	1	2	2
1	2	2	2	2	2	2	2	2	2
2	2	2	2	2	2	2	1	2	1
2	2	2	2	2	2	2	2	2	2
1	2	2	2	#NULL!	2	2	1	2	2
2	2	2	2	2	2	2	2	2	2
2	2	2	2	2	2	2	2	2	2
1	1	2	2	2	2	2	2	2	2
2	2	2	1	2	2	2	2	2	1
2	2	2	1	2	2	2	2	2	1
2	2	1	2	#NULL!	2	2	2	2	2
2	2	2	2	1	1	2	2	2	2
2	2	2	2	2	2	2	2	2	2
2	2	2	2	2	2	2	2	2	2
2	2	2	2	2	2	1	1	2	2
1	2	2	1	2	2	2	2	2	2
2	2	2	2	2	2	2	2	2	2
#NULL!	2	2	2	2	2	2	2	2	2
2	2	2	2	2	2	2	2	2	2
2	2	2	2	2	2	2	2	2	2
2	2	2	2	2	2	2	2	2	2
1	1	2	2	2	2	1	2	2	2
2	2	2	2	2	2	2	2	2	2
2	1	2	2	2	2	1	2	2	2
2	2	2	2	2	2	2	2	2	2
1	1	2	2	2	1	2	2	2	2
#NULL!	#NULL!	2	2	2	2	2	2	2	2
2	2	2	2	2	2	2	2	2	2
2	2	2	2	2	2	2	2	2	2
2	2	2	2	2	2	2	2	2	2
#NULL!	#NULL!	2	1	2	1	2	#NULL!	2	1
1	2	2	2	2	1	2	2	1	2
2	2	#NULL!	#NULL!	2	2	2	2	1	2
2	1	2	1	2	1	2	#NULL!	2	1

2	2	2	2	2	2	2	2	2	2
2	1	2	2	2	2	2	2	2	2
1	1	2	2	2	2	2	2	2	2
2	1	2	2	2	2	2	2	2	2
1	1	2	2	1	2	2	2	2	2
1	1	2	2	1	1	2	2	2	2
1	1	2	2	1	1	2	2	2	2
1	2	2	2	2	#NULL!	1	1	#NULL!	#NULL!
2	2	2	#NULL!	2	#NULL!	1	1	#NULL!	#NULL!
1	2	2	2	1	2	1	2	1	1
2	2	2	2	2	2	2	2	2	2
2	1	2	2	2	2	2	2	2	2
2	2	2	2	2	2	2	2	2	2
2	2	2	2	2	2	2	2	2	2
2	2	2	2	2	2	2	2	2	2
1	2	2	2	2	2	2	2	2	2
2	1	2	2	2	2	2	2	2	2
1	1	2	2	2	2	2	1	2	2
2	2	2	1	2	1	2	2	2	1
1	2	2	#NULL!	#NULL!	#NULL!	#NULL!	#NULL!	#NULL!	#NULL!
2	1	2	2	1	1	2	2	2	2
2	2	2	2	1	1	2	2	2	2
2	1	2	2	1	1	2	2	2	2
2	1	2	2	2	2	2	2	2	2
2	2	#NULL!	1	#NULL!	#NULL!	1	#NULL!	#NULL!	1
2	1	2	2	2	2	1	1	2	1
#NULL!	#NULL!	#NULL!	#NULL!	#NULL!	#NULL!	#NULL!	#NULL!	#NULL!	#NULL!
2	2	2	2	2	2	2	2	2	2
2	2	2	2	2	2	2	2	2	2
1	1	2	2	2	2	2	1	2	2
1	1	2	2	#NULL!	#NULL!	#NULL!	#NULL!	#NULL!	#NULL!
1	2	2	2	1	1	2	1	1	2
#NULL!	#NULL!	2	2	#NULL!	#NULL!	#NULL!	#NULL!	#NULL!	#NULL!
2	1	#NULL!	2	1	1	2	2	1	2
1	1	2	2	2	2	2	2	2	2
2	1	#NULL!	#NULL!	#NULL!	#NULL!	#NULL!	#NULL!	#NULL!	#NULL!
#NULL!	#NULL!	#NULL!	#NULL!	#NULL!	#NULL!	#NULL!	#NULL!	#NULL!	#NULL!
#NULL!	#NULL!	2	2	1	2	2	2	2	2
1	1	2	2	2	1	1	2	1	1
1	1	2	2	2	2	2	2	2	2
1	1	2	#NULL!	2	2	2	2	2	2
2	2	2	2	#NULL!	#NULL!	#NULL!	#NULL!	#NULL!	#NULL!
2	2	2	1	2	1	2	2	2	2
1	1	2	2	2	2	2	2	2	2
2	2	2	2	2	2	2	2	2	2
2	1	#NULL!	#NULL!	#NULL!	#NULL!	#NULL!	#NULL!	#NULL!	#NULL!
2	#NULL!	#NULL!	#NULL!	#NULL!	#NULL!	#NULL!	#NULL!	#NULL!	#NULL!

2	1	2	2	2	2	2	1	2	2
1	1	2	2	1	2	2	2	2	2
1	1	2	1	2	2	1	1	2	1
1	1	2	2	2	2	2	2	2	2
2	2	#NULL!	#NULL!	#NULL!	#NULL!	#NULL!	#NULL!	#NULL!	#NULL!
2	2	2	2	2	2	2	2	2	2
1	1	2	2	1	2	2	1	2	2
1	2	2	2	1	2	2	2	2	2
1	1	2	2	2	2	2	2	2	2
1	1	2	2	2	2	2	2	2	2
2	1	2	2	2	2	1	2	#NULL!	2
1	2	2	2	2	2	2	2	1	2
2	2	2	2	2	2	1	1	2	2
2	2	2	2	2	2	2	1	2	#NULL!
1	2	2	2	2	2	2	2	2	2
2	2	1	2	2	2	2	2	2	2
2	1	2	2	2	2	2	2	2	2
2	2	2	2	2	2	2	2	2	2
2	2	2	2	2	2	2	1	2	2
2	2	2	2	2	2	2	2	2	2
2	2	2	2	1	1	2	2	2	2
2	2	2	2	2	2	2	2	2	2
2	2	2	2	2	2	2	2	2	2
2	2	1	2	1	1	2	2	1	2
2	1	1	1	2	1	2	2	2	2
2	2	2	2	2	2	2	2	2	2
1	2	2	2	2	2	2	1	2	2
1	1	2	2	2	2	2	2	2	2
2	2	2	2	2	2	2	2	2	2
2	2	2	2	2	2	2	2	2	2
2	2	2	2	2	2	2	2	2	2
2	2	2	2	2	2	2	2	2	2
2	2	2	2	2	2	2	2	2	2
2	2	2	2	1	1	2	2	2	2
2	2	2	2	2	2	2	2	2	2
2	2	2	2	2	2	2	2	2	2
2	2	2	2	2	2	2	2	2	2
2	2	2	2	2	2	2	2	2	2
2	2	#NULL!	1	#NULL!	#NULL!	#NULL!	2	#NULL!	#NULL!
2	2	2	2	2	2	2	2	1	2
2	2	2	2	2	2	2	2	2	2
2	2	2	2	2	2	2	2	2	2
2	2	2	2	2	2	2	2	2	2
2	2	2	2	2	2	1	1	1	2
2	2	2	2	2	2	2	2	2	1
2	2	2	2	2	2	2	2	2	2
2	2	2	2	2	2	2	2	2	2

2	2	2	2	2	2	2	1	2	1
2	2	2	2	2	2	1	2	2	2
2	2	#NULL!	2	#NULL!	2	1	2	2	2
2	2	2	2	2	2	2	2	2	2
2	2	#NULL!	#NULL!	#NULL!	#NULL!	#NULL!	#NULL!	#NULL!	#NULL!
2	2	2	2	2	2	2	2	2	2
2	2	2	2	1	1	2	2	1	2
2	2	2	2	2	2	2	2	2	2
2	2	2	2	2	2	2	2	2	2
2	2	2	2	2	2	2	2	2	2
2	2	2	1	2	2	2	2	2	2
2	#NULL!	2	2	2	1	2	2	2	2
2	2	#NULL!	2	#NULL!	#NULL!	#NULL!	#NULL!	#NULL!	#NULL!
2	2	2	2	2	2	2	2	2	2
2	2	2	2	2	2	2	2	2	2
2	2	2	2	2	2	2	2	2	2
2	1	#NULL!	#NULL!	#NULL!	#NULL!	#NULL!	#NULL!	#NULL!	#NULL!
2	2	2	2	2	2	2	2	2	2
2	2	#NULL!	#NULL!	#NULL!	#NULL!	1	#NULL!	#NULL!	#NULL!
2	2	2	2	1	2	2	2	2	2
2	2	2	2	2	2	2	2	2	2
2	2	2	2	2	2	2	2	2	2
2	2	2	2	2	2	2	2	2	2
2	2	2	2	2	1	2	2	2	2
2	2	2	2	2	2	2	2	2	2
2	2	2	2	2	2	2	#NULL!	#NULL!	#NULL!
2	2	2	2	2	2	2	2	2	2
2	2	2	2	2	2	2	2	2	2
2	2	2	2	2	2	2	2	2	2
2	2	2	2	2	2	2	2	2	2
2	2	1	2	2	2	2	1	2	2
2	2	2	2	2	2	2	2	2	2
#NULL!	#NULL!	2	2	2	2	2	2	2	2
2	2	2	2	2	2	2	2	2	2
2	2	2	2	2	2	2	2	2	2
2	2	2	2	2	2	2	2	2	2
2	2	2	2	2	2	2	2	2	2
2	2	2	2	2	2	2	2	2	2
2	2	2	2	2	2	2	1	2	2
2	2	2	2	2	2	2	2	2	2
2	2	2	2	2	2	2	2	2	2
2	2	2	2	2	2	2	2	2	2
#NULL!	#NULL!	2	2	2	2	2	2	2	2
2	2	2	2	2	2	2	2	2	2
2	2	2	2	2	2	2	2	2	2
2	2	2	2	2	2	2	2	2	2
2	2	2	2	2	2	2	2	2	2

2	2	2	2	2	2	2	2	2	2
2	2	2	2	2	2	2	2	2	2
2	2	2	2	2	2	2	2	2	2
2	2	2	2	2	2	2	2	1	2
2	2	2	2	1	2	2	1	2	2
1	2	2	2	2	2	2	2	2	2
2	2	2	2	2	2	2	2	2	2
1	1	2	2	2	1	2	2	2	2
1	1	2	2	2	2	2	2	2	2
2	1	2	2	1	1	2	2	1	2
#NULL!	#NULL!	#NULL!	#NULL!	#NULL!	#NULL!	#NULL!	#NULL!	#NULL!	#NULL!
1	2	2	2	2	2	2	2	2	2
1	1	2	2	2	2	2	2	2	2
2	2	2	2	2	2	2	2	2	2
2	2	2	2	2	2	2	1	2	1
2	2	2	1	2	2	2	2	2	2
2	2	2	2	2	2	2	2	2	2
#NULL!	1	2	1	2	2	1	1	2	1
2	2	2	2	2	2	2	2	2	2
2	2	2	2	2	2	2	2	2	2
2	2	2	2	2	2	2	1	2	2
2	2	2	2	2	2	2	2	2	2
2	2	2	2	2	2	1	2	1	2
2	2	2	2	2	2	2	2	2	2
2	2	2	2	2	2	2	2	2	2
2	2	2	2	2	2	2	2	2	2
2	2	2	2	2	2	2	2	2	2
2	2	2	2	2	2	2	2	2	2
2	2	2	2	2	2	2	2	2	2
2	2	2	2	2	2	2	2	2	2
2	2	2	2	2	2	2	2	1	2
2	2	2	2	2	2	2	2	1	2
2	2	2	2	2	2	2	2	2	2
1	1	2	1	2	2	2	2	2	2
2	1	2	2	2	2	1	1	2	2
1	2	2	2	2	2	2	2	2	2
2	2	2	2	2	2	2	2	2	2
2	2	1	2	1	1	2	1	2	2
2	2	2	2	2	2	2	2	2	2
2	2	2	2	2	2	2	2	1	2
1	1	2	2	2	2	1	2	1	2
2	2	2	2	2	2	2	2	2	#NULL!
2	2	2	#NULL!	#NULL!	#NULL!	#NULL!	#NULL!	#NULL!	#NULL!
2	2	2	#NULL!	#NULL!	#NULL!	#NULL!	#NULL!	#NULL!	#NULL!
2	1	2	2	1	2	2	2	2	2
2	2	2	2	2	2	2	1	2	2
2	2	2	#NULL!	#NULL!	#NULL!	#NULL!	#NULL!	#NULL!	#NULL!

2	2	2	2	2	2	2	2	2	2
2	2	2	2	2	2	2	2	2	2
2	2	2	2	2	2	2	2	2	2
1	2	2	2	2	2	2	2	2	2
2	2	2	2	2	2	1	1	1	2
1	1	2	1	2	2	2	2	1	2
2	2	2	2	2	2	2	2	2	2
2	2	2	2	2	2	2	2	2	2
2	2	2	2	1	1	2	1	2	2
2	2	1	2	1	1	2	2	1	2
2	2	2	2	2	2	2	2	2	2
2	1	2	2	1	1	2	2	2	2
2	1	2	2	1	1	2	2	2	2
2	2	2	2	2	2	2	2	2	2
2	2	2	2	2	2	2	2	2	2
2	2	2	2	2	2	2	1	2	2
2	2	2	2	2	2	2	2	1	2
1	1	2	2	2	1	2	2	2	2
2	2	2	#NULL!	#NULL!	#NULL!	#NULL!	#NULL!	#NULL!	#NULL!
1	1	2	2	2	2	2	2	2	2
2	1	2	2	2	2	2	2	2	2
2	2	2	2	2	2	2	2	2	2
2	2	2	2	2	2	2	2	2	2
2	2	2	2	2	2	2	2	2	2
2	2	#NULL!	2	#NULL!	#NULL!	#NULL!	#NULL!	#NULL!	#NULL!
2	2	2	2	2	2	2	2	2	2
2	#NULL!	2	2	2	2	2	2	2	2
2	2	2	#NULL!	2	2	2	2	2	2
2	2	2	2	2	2	2	2	2	2
2	2	2	2	2	2	2	2	2	2
1	2	#NULL!	#NULL!	2	#NULL!	#NULL!	#NULL!	#NULL!	#NULL!
2	2	2	2	1	1	2	2	1	2
2	2	1	2	1	1	2	2	1	2
2	2	2	2	2	2	2	2	2	2
2	2	2	#NULL!	#NULL!	#NULL!	#NULL!	#NULL!	#NULL!	#NULL!
2	2	2	2	2	2	2	2	2	2
2	2	2	2	2	2	2	2	2	2
2	2	2	2	2	2	2	2	2	2
2	2	2	2	2	2	2	2	2	2
2	2	2	2	2	2	2	2	2	2
2	2	2	2	2	2	2	2	2	2
2	2	2	2	2	2	2	2	2	2
1	1	2	1	2	2	2	2	2	2
2	2	2	2	2	2	2	2	2	2
2	1	2	2	2	2	2	2	2	2
2	2	2	2	2	2	1	1	2	2
2	2	2	2	2	2	2	2	2	2

#NULL!	#NULL!	#NULL!	#NULL!	#NULL!	#NULL!	#NULL!	#NULL!	#NULL!	#NULL!
#NULL!	#NULL!	#NULL!	#NULL!	#NULL!	#NULL!	#NULL!	#NULL!	#NULL!	#NULL!
1	2	2	2	2	#NULL!	#NULL!	#NULL!	#NULL!	#NULL!
2	1	2	2	2	2	2	1	2	2
2	2	2	2	1	2	2	2	2	2
2	1	1	1	2	2	2	2	2	2
1	1	2	2	2	2	2	2	2	2
1	1	2	1	2	2	1	1	2	1
2	2	2	2	2	#NULL!	2	2	2	1
1	2	2	2	2	2	2	2	2	2
2	2	2	2	2	2	2	2	2	2
1	2	2	2	1	2	2	2	2	2
#NULL!	#NULL!	#NULL!	#NULL!	#NULL!	#NULL!	#NULL!	#NULL!	#NULL!	#NULL!
1	1	2	1	2	2	1	1	2	1
2	1	2	1	2	1	2	2	1	1
#NULL!	#NULL!	#NULL!	#NULL!	#NULL!	#NULL!	#NULL!	#NULL!	#NULL!	#NULL!
1	2	2	1	2	2	2	2	2	1
1	2	2	2	2	2	1	2	2	1
2	1	2	1	2	2	1	1	2	1
#NULL!	#NULL!	#NULL!	#NULL!	#NULL!	#NULL!	#NULL!	#NULL!	#NULL!	#NULL!
2	2	#NULL!	#NULL!	#NULL!	#NULL!	#NULL!	#NULL!	#NULL!	#NULL!
2	2	2	2	2	2	2	2	2	2
2	2	2	2	1	1	2	2	1	2
2	2	2	2	2	2	2	2	2	2
2	2	2	2	2	2	2	2	2	2
2	2	2	2	2	2	2	2	2	2
1	1	2	1	2	2	2	1	2	2
2	2	2	2	2	2	2	2	2	2
1	1	2	2	2	2	2	2	2	2
2	2	2	2	2	2	2	2	2	2
2	1	2	2	2	2	2	2	2	2
2	2	1	2	2	2	2	2	1	2
1	1	#NULL!	#NULL!	#NULL!	#NULL!	#NULL!	#NULL!	#NULL!	#NULL!
2	2	#NULL!	#NULL!	#NULL!	#NULL!	#NULL!	#NULL!	#NULL!	#NULL!
2	2	2	2	2	2	2	2	2	2
2	2	2	2	1	2	2	1	2	2
1	2	2	2	2	2	2	2	2	2
1	1	2	2	1	2	2	1	2	2
1	2	2	2	2	2	2	1	2	2
2	2	2	2	2	2	2	1	2	#NULL!
1	2	2	2	1	2	2	2	2	1
1	2	2	2	2	2	2	1	2	2
1	1	2	2	2	2	2	2	2	2
2	2	2	2	2	2	2	2	2	2
1	1	2	2	2	1	2	2	1	2
2	2	2	2	2	#NULL!	2	2	2	2
2	2	2	2	2	2	2	2	2	2

2	2	2	2	2	2	2	2	2	2
2	2	2	2	2	2	2	2	2	2
1	1	2	#NULL!	#NULL!	#NULL!	#NULL!	#NULL!	#NULL!	#NULL!
2	2	2	2	2	2	2	2	2	2
1	2	#NULL!	#NULL!	#NULL!	#NULL!	#NULL!	#NULL!	#NULL!	#NULL!
2	2	#NULL!	#NULL!	#NULL!	#NULL!	#NULL!	#NULL!	#NULL!	#NULL!
2	2	2	2	2	2	2	2	2	2
2	1	2	2	2	2	#NULL!	2	#NULL!	2
2	2	2	2	2	2	2	2	2	2
2	2	2	1	2	2	2	1	2	2
2	2	2	2	2	2	2	2	1	1
1	2	2	2	2	2	2	2	2	2
2	1	2	2	2	2	2	1	2	2
2	2	2	1	2	2	2	2	1	2
2	2	2	2	2	2	2	2	2	2
2	2	2	2	2	2	2	2	2	2
2	2	2	2	2	2	2	2	2	2
2	2	2	2	2	2	2	2	2	2
1	2	2	2	2	2	2	2	2	2
2	2	2	2	2	2	2	2	2	2
1	1	2	1	2	2	2	1	2	2
2	2	2	2	2	2	2	2	2	2
2	2	2	2	2	2	2	2	2	2
2	1	2	2	2	2	2	2	2	2
2	2	2	2	2	2	2	2	2	2
2	2	2	2	1	2	2	2	1	2
2	2	2	2	2	2	2	2	2	2
2	2	2	2	2	2	2	2	2	2
2	2	2	2	2	2	2	2	2	1
2	2	2	2	2	2	1	2	2	2
1	1	2	2	1	1	2	2	2	2
2	2	2	2	1	1	2	2	2	2
2	2	2	1	2	2	1	2	2	2
2	2	2	2	2	2	2	2	2	2
2	2	2	2	2	2	2	2	2	2
2	2	2	2	2	2	2	1	2	2
2	2	2	2	2	2	2	2	2	2
2	2	2	2	2	2	2	2	2	2
2	2	2	2	2	2	2	2	2	2
2	2	2	2	2	2	2	2	2	2
2	2	2	2	2	2	2	2	2	2
2	2	2	2	2	2	2	2	2	2
2	1	2	2	2	2	1	1	2	2
2	2	2	2	2	2	2	2	2	2
2	1	2	2	2	2	2	2	2	2
2	2	2	2	2	2	2	2	2	2
2	2	2	2	2	2	2	2	2	2

2	2	2	2	1	1	2	2	2	2
2	1	2	2	2	1	2	1	2	2
2	1	2	2	2	2	2	2	2	2
2	2	2	2	2	2	2	2	2	2
2	1	2	2	2	2	2	2	2	2
2	1	2	2	1	1	2	2	2	2
#NULL!	#NULL!	#NULL!	#NULL!	#NULL!	#NULL!	#NULL!	#NULL!	#NULL!	#NULL!
1	1	2	2	1	2	2	1	2	2
1	2	2	2	2	2	2	2	2	2
2	2	2	2	2	2	1	2	1	2
2	2	1	2	2	2	1	2	2	2
2	2	2	2	1	1	2	2	2	2
2	2	2	2	2	2	2	2	2	2
2	2	2	2	2	2	2	2	2	2
2	2	2	2	2	2	2	2	2	2
#NULL!	#NULL!	2	2	2	2	1	1	2	1
2	2	2	2	1	1	2	2	2	2
2	2	2	2	2	2	2	2	2	2
2	2	2	2	2	2	2	2	2	2
2	2	2	2	2	2	2	2	2	2
1	2	2	2	2	2	2	1	2	2
2	2	2	2	2	2	2	2	2	2
#NULL!	#NULL!	#NULL!	#NULL!	#NULL!	#NULL!	#NULL!	#NULL!	#NULL!	#NULL!
1	1	2	2	2	2	2	2	2	2
2	2	2	2	2	2	2	2	2	2
#NULL!	#NULL!	#NULL!	#NULL!	#NULL!	#NULL!	#NULL!	#NULL!	#NULL!	#NULL!
1	1	2	2	2	2	1	2	2	2
#NULL!	#NULL!	#NULL!	#NULL!	#NULL!	#NULL!	#NULL!	#NULL!	#NULL!	#NULL!
1	1	2	2	2	2	2	2	2	2
2	2	2	2	2	2	2	2	2	2
2	2	#NULL!	#NULL!	#NULL!	#NULL!	#NULL!	#NULL!	#NULL!	#NULL!
#NULL!	#NULL!	#NULL!	#NULL!	#NULL!	#NULL!	#NULL!	#NULL!	#NULL!	#NULL!
1	2	2	2	2	2	2	2	2	2
2	2	2	1	2	2	1	1	2	2
2	2	2	2	2	#NULL!	1	1	2	#NULL!
1	1	2	1	2	2	2	1	2	2
2	2	1	2	1	1	2	2	1	2
2	2	2	1	2	2	2	2	2	2
#NULL!	#NULL!	#NULL!	#NULL!	#NULL!	#NULL!	#NULL!	#NULL!	#NULL!	#NULL!
2	2	2	2	2	2	2	2	2	2
2	2	2	2	2	1	2	1	2	2
2	2	2	2	2	1	2	2	2	1
2	2	2	2	2	2	2	2	2	2
2	2	1	2	1	1	2	2	1	2
#NULL!	#NULL!	#NULL!	#NULL!	#NULL!	#NULL!	#NULL!	#NULL!	#NULL!	#NULL!
2	2	2	2	2	2	2	2	2	2
2	2	2	2	2	2	2	2	2	2

2	2	#NULL!	#NULL!	#NULL!	#NULL!	#NULL!	#NULL!	#NULL!	#NULL!
2	2	2	2	2	2	2	2	2	2
2	2	2	2	2	2	2	2	2	2
2	2	2	2	2	2	2	2	2	2
2	2	2	2	1	2	2	2	2	2
2	2	2	2	2	2	2	2	2	2
2	2	2	2	2	2	2	2	2	2
2	2	2	2	2	2	2	2	2	2
2	2	2	2	2	2	2	2	2	2
2	2	2	2	2	2	2	2	2	2
2	2	#NULL!	1	1	1	2	2	2	2
2	2	2	2	2	2	2	2	2	1
2	2	2	2	2	2	2	2	2	2
2	2	2	2	2	2	2	2	2	2
2	2	2	2	2	2	2	2	2	2
2	2	2	2	1	2	2	2	2	2
2	2	2	2	2	2	2	2	2	2
2	2	2	2	2	1	2	2	1	2
2	2	2	2	2	2	2	2	2	2
2	2	2	2	2	2	2	2	2	2
2	2	2	2	2	2	2	2	2	2
2	2	2	2	2	2	2	2	2	2
2	2	2	2	2	2	2	2	2	2
2	1	2	2	2	2	2	2	2	2
2	2	2	2	2	2	2	2	2	2
2	2	2	2	2	2	2	2	2	2
2	2	2	2	2	2	2	2	2	2
2	2	2	2	2	2	2	2	2	2
2	2	2	2	2	2	2	2	2	1
2	2	2	2	2	2	2	2	2	2
2	2	2	2	2	2	2	2	2	2
2	2	2	2	2	2	2	2	2	2
2	2	2	2	2	2	2	2	2	2
2	2	2	2	1	2	2	2	2	2
2	2	2	2	2	2	2	2	1	2
2	2	2	2	2	2	2	2	2	2
2	2	2	2	2	2	2	2	2	2
2	2	2	2	2	2	2	2	2	2
2	2	2	2	2	2	2	2	2	2
1	2	2	2	2	2	2	2	2	2
2	2	2	2	2	2	2	2	2	2
2	2	2	2	2	2	2	2	2	2
2	1	2	2	2	2	2	2	2	2
2	2	2	2	2	2	2	2	2	2
2	2	2	#NULL!	#NULL!	#NULL!	#NULL!	#NULL!	#NULL!	#NULL!
2	2	2	2	2	2	2	2	2	2
1	2	#NULL!	2	#NULL!	#NULL!	#NULL!	#NULL!	#NULL!	#NULL!

2	2	2	2	2	2	2	2	2	2
2	1	2	2	2	2	2	2	1	2
2	1	2	2	2	2	2	2	1	2
2	2	2	1	2	2	2	2	2	2
2	2	2	2	2	2	2	2	2	2
2	2	2	2	2	1	2	1	2	2
1	2	2	2	2	2	2	2	2	2
#NULL!	2	2	2	1	2	2	2	2	1
2	2	2	2	2	2	2	2	2	2
2	2	2	2	2	2	2	2	2	2
1	1	2	2	2	2	2	2	1	2
1	2	#NULL!	#NULL!	#NULL!	2	#NULL!	#NULL!	#NULL!	#NULL!
1	1	2	2	2	2	2	2	#NULL!	#NULL!
2	2	2	2	2	2	2	2	2	2
2	2	#NULL!	#NULL!	#NULL!	#NULL!	1	#NULL!	#NULL!	#NULL!
1	2	#NULL!	#NULL!	2	#NULL!	#NULL!	#NULL!	#NULL!	#NULL!
2	2	#NULL!	#NULL!	#NULL!	#NULL!	#NULL!	#NULL!	#NULL!	1
2	2	2	#NULL!	#NULL!	#NULL!	#NULL!	#NULL!	#NULL!	#NULL!
2	2	#NULL!	#NULL!	#NULL!	#NULL!	#NULL!	#NULL!	#NULL!	#NULL!
2	2	2	2	1	2	1	2	2	2
1	1	2	#NULL!	2	#NULL!	1	1	#NULL!	1
1	2	#NULL!	#NULL!	#NULL!	2	#NULL!	#NULL!	#NULL!	#NULL!
2	2	2	2	2	1	2	2	2	2
2	2	2	#NULL!	#NULL!	#NULL!	#NULL!	#NULL!	#NULL!	#NULL!
2	2	#NULL!	#NULL!	2	#NULL!	#NULL!	#NULL!	#NULL!	#NULL!
#NULL!	#NULL!	#NULL!	#NULL!	#NULL!	#NULL!	#NULL!	#NULL!	#NULL!	#NULL!
1	1	2	#NULL!	#NULL!	#NULL!	#NULL!	#NULL!	#NULL!	#NULL!
2	2	#NULL!	#NULL!	#NULL!	#NULL!	#NULL!	#NULL!	#NULL!	#NULL!
2	1	2	2	2	2	2	1	2	2
1	2	#NULL!	#NULL!	#NULL!	2	#NULL!	#NULL!	#NULL!	#NULL!
2	2	2	#NULL!	#NULL!	#NULL!	#NULL!	#NULL!	#NULL!	#NULL!
2	2	2	2	2	2	2	2	2	2
2	2	2	2	2	2	2	2	2	2
1	1	2	2	2	2	2	2	2	2
2	2	2	1	2	2	2	1	2	2
2	2	2	2	2	2	2	2	2	2
2	2	2	2	2	2	2	2	2	2
2	2	2	2	1	2	#NULL!	2	1	2
2	2	2	2	2	2	2	2	2	2
2	2	2	2	2	2	2	2	2	2
2	2	2	#NULL!	#NULL!	#NULL!	#NULL!	#NULL!	#NULL!	#NULL!
1	1	2	2	2	2	2	2	2	2
2	2	2	2	2	2	2	2	2	2
2	2	2	2	2	2	2	2	2	2
2	2	2	2	2	2	2	2	2	2
2	2	2	2	2	2	2	2	2	2
2	2	2	2	2	2	2	2	2	2

1	1	2	2	2	2	2	2	2	2
2	1	2	2	2	2	2	2	2	2
2	2	2	2	1	1	2	2	2	2
2	2	2	2	2	2	1	1	2	2
1	2	2	2	1	2	2	1	2	2
2	2	2	2	2	2	2	2	2	2
2	2	2	2	1	2	2	2	2	2
2	2	2	2	2	2	2	2	2	2
2	2	2	2	2	2	2	2	2	2
2	2	2	2	2	2	2	2	2	2
2	2	2	2	2	2	2	2	2	2
1	2	2	1	2	2	2	2	2	2
2	2	2	2	2	2	2	2	2	2
2	2	2	2	2	2	2	2	2	2
2	2	2	2	2	2	2	2	2	2
2	2	2	2	2	2	2	2	2	2
2	2	2	2	2	2	2	2	2	2
2	2	2	2	2	2	2	2	2	2
2	2	2	2	2	2	2	2	2	2
1	2	2	2	2	2	2	1	2	2
2	1	2	2	2	2	2	2	2	2
2	2	2	2	2	2	2	2	2	2
2	2	2	2	2	1	2	2	2	2
2	2	2	2	2	2	2	2	2	2
2	2	2	2	2	2	2	2	2	2
2	2	2	2	2	2	2	2	2	2
2	1	2	2	2	2	2	1	2	2
1	2	2	2	2	2	2	1	1	1
2	2	2	2	2	2	2	2	2	2
2	2	2	2	1	1	2	2	1	2
#NULL!	#NULL!	2	2	2	2	2	2	2	2
2	2	2	2	2	2	2	2	2	2
2	2	2	2	2	2	2	2	2	2
2	2	2	2	2	1	1	2	2	2
2	2	2	2	2	2	2	2	2	2
1	2	2	#NULL!	#NULL!	#NULL!	#NULL!	#NULL!	#NULL!	#NULL!
2	2	2	2	2	2	2	2	2	2
1	1	2	2	1	2	2	2	2	2
2	2	2	2	2	2	2	2	2	2
2	2	2	2	2	2	2	2	2	2
2	2	2	#NULL!	#NULL!	#NULL!	#NULL!	#NULL!	#NULL!	#NULL!
2	1	2	2	2	2	2	2	2	2
2	2	2	2	2	2	2	2	2	2
1	2	2	2	2	2	2	2	1	2
1	2	2	2	2	2	2	2	2	2
2	2	2	2	2	2	2	2	2	2
2	1	2	#NULL!	#NULL!	#NULL!	#NULL!	#NULL!	#NULL!	#NULL!

2	2	2	#NULL!	#NULL!	#NULL!	#NULL!	#NULL!	#NULL!	#NULL!
2	2	#NULL!	#NULL!	#NULL!	#NULL!	#NULL!	1	#NULL!	#NULL!
2	2	2	#NULL!	#NULL!	#NULL!	#NULL!	#NULL!	#NULL!	#NULL!
#NULL!	#NULL!	#NULL!	#NULL!	#NULL!	#NULL!	#NULL!	#NULL!	#NULL!	#NULL!
2	2	2	#NULL!	#NULL!	#NULL!	#NULL!	#NULL!	#NULL!	#NULL!
2	1	2	#NULL!	#NULL!	#NULL!	#NULL!	#NULL!	#NULL!	#NULL!
2	2	#NULL!	#NULL!	#NULL!	2	#NULL!	#NULL!	#NULL!	#NULL!
2	2	2	#NULL!	#NULL!	#NULL!	#NULL!	#NULL!	#NULL!	#NULL!
2	2	2	#NULL!	#NULL!	#NULL!	#NULL!	#NULL!	#NULL!	#NULL!
1	1	2	#NULL!	#NULL!	#NULL!	#NULL!	#NULL!	#NULL!	#NULL!
2	2	#NULL!	#NULL!	#NULL!	#NULL!	#NULL!	#NULL!	#NULL!	#NULL!
1	1	2	#NULL!	#NULL!	#NULL!	#NULL!	#NULL!	#NULL!	#NULL!
2	2	2	#NULL!	#NULL!	#NULL!	#NULL!	#NULL!	#NULL!	#NULL!
1	1	#NULL!	#NULL!	2	#NULL!	#NULL!	#NULL!	#NULL!	#NULL!
2	2	#NULL!	#NULL!	#NULL!	#NULL!	#NULL!	#NULL!	#NULL!	#NULL!
2	2	#NULL!	#NULL!	#NULL!	#NULL!	#NULL!	#NULL!	#NULL!	#NULL!
2	1	#NULL!	2	#NULL!	#NULL!	#NULL!	#NULL!	#NULL!	#NULL!
2	2	2	2	2	1	2	2	1	2
2	2	2	2	2	2	2	2	2	2
1	2	2	#NULL!	#NULL!	#NULL!	#NULL!	#NULL!	#NULL!	#NULL!
1	1	2	1	2	2	1	1	2	1
2	2	2	2	2	2	2	1	1	2
#NULL!	2	2	2	2	2	2	2	2	1
1	1	2	1	2	1	2	1	2	1
#NULL!	2	1	2	1	2	2	2	1	2
2	1	2	2	2	2	2	1	1	2
1	2	2	2	2	2	2	2	2	2
2	2	2	1	2	2	2	1	2	1
2	2	2	2	2	2	2	2	2	2
2	2	2	2	2	2	2	2	2	2
#NULL!	#NULL!	2	1	2	2	2	2	2	2
2	2	2	2	2	2	2	2	2	2
2	2	2	2	2	2	2	2	2	2
2	2	2	2	2	1	2	2	2	2
2	1	2	1	2	1	1	2	1	1
2	2	2	2	2	2	2	2	1	2
2	2	2	2	2	2	2	2	2	2
#NULL!	#NULL!	2	2	2	1	2	2	2	1
1	1	2	1	2	2	1	1	2	1
1	#NULL!	#NULL!	#NULL!	#NULL!	#NULL!	#NULL!	#NULL!	#NULL!	#NULL!
1	2	2	2	2	2	2	2	2	2
1	1	2	1	2	2	1	1	2	1
#NULL!	#NULL!	#NULL!	#NULL!	#NULL!	#NULL!	#NULL!	#NULL!	#NULL!	#NULL!
1	1	2	1	2	2	1	2	2	1
2	2	2	2	2	2	2	2	2	2
2	2	2	2	2	2	2	2	2	2
2	2	2	2	2	2	2	2	1	2

2	2	2	2	2	2	2	2	2	2
#NULL!	#NULL!	#NULL!	#NULL!	#NULL!	#NULL!	#NULL!	#NULL!	#NULL!	#NULL!
2	2	2	1	1	2	1	2	2	2
#NULL!	#NULL!	#NULL!	#NULL!	#NULL!	#NULL!	#NULL!	#NULL!	#NULL!	#NULL!
1	1	2	2	2	2	2	2	2	2
1	2	2	2	2	2	2	2	2	2
#NULL!	#NULL!	#NULL!	#NULL!	#NULL!	#NULL!	#NULL!	#NULL!	#NULL!	#NULL!
2	2	2	2	2	2	2	2	2	2
1	1	2	1	1	2	2	1	2	1
2	2	2	2	2	2	2	1	2	2
1	1	2	1	2	2	1	1	2	1
2	1	2	2	2	2	2	2	2	1
2	2	1	2	1	2	2	2	1	2
#NULL!	#NULL!	#NULL!	#NULL!	#NULL!	#NULL!	#NULL!	#NULL!	#NULL!	#NULL!
#NULL!	#NULL!	#NULL!	#NULL!	#NULL!	#NULL!	#NULL!	#NULL!	#NULL!	#NULL!
1	2	#NULL!	#NULL!	#NULL!	#NULL!	#NULL!	#NULL!	#NULL!	#NULL!
2	2	2	2	2	2	2	2	2	2
#NULL!	#NULL!	#NULL!	#NULL!	#NULL!	#NULL!	#NULL!	#NULL!	#NULL!	#NULL!
2	1	2	2	2	#NULL!	2	2	2	2
2	2	2	2	2	2	2	2	2	2
2	2	1	2	1	1	2	2	2	2
2	2	1	2	1	2	2	2	1	2
1	1	2	1	2	2	1	1	2	1
2	1	1	1	1	2	1	2	2	2
2	2	2	2	2	2	2	2	2	2
2	2	2	1	1	2	2	1	2	1
1	1	2	2	1	1	1	2	1	2
1	1	1	1	1	1	1	1	2	2
2	2	2	2	2	2	2	2	2	1
2	2	2	2	2	2	2	2	1	2
2	1	#NULL!	#NULL!	#NULL!	#NULL!	#NULL!	#NULL!	#NULL!	#NULL!
1	2	#NULL!	#NULL!	#NULL!	2	#NULL!	#NULL!	#NULL!	#NULL!
2	2	2	2	2	2	2	2	2	2
2	2	2	2	2	2	1	2	2	#NULL!
2	#NULL!	2	2	2	2	#NULL!	#NULL!	2	#NULL!
2	1	2	2	2	2	2	1	2	2
2	2	2	2	2	1	2	#NULL!	2	2
#NULL!	#NULL!	#NULL!	#NULL!	#NULL!	#NULL!	#NULL!	#NULL!	#NULL!	#NULL!
#NULL!	#NULL!	#NULL!	#NULL!	#NULL!	#NULL!	#NULL!	#NULL!	#NULL!	#NULL!
#NULL!	#NULL!	#NULL!	#NULL!	#NULL!	#NULL!	#NULL!	#NULL!	#NULL!	#NULL!
#NULL!	#NULL!	#NULL!	#NULL!	#NULL!	#NULL!	#NULL!	#NULL!	#NULL!	#NULL!
1	1	1	2	1	2	2	1	2	1
2	1	2	2	1	2	2	2	2	2
#NULL!	#NULL!	#NULL!	#NULL!	#NULL!	#NULL!	#NULL!	#NULL!	#NULL!	#NULL!
2	1	2	2	1	2	2	1	1	1
2	2	2	2	1	2	2	2	2	2
2	2	2	2	1	1	1	1	1	1

2	2	1	2	1	1	2	2	1	2
2	2	2	2	2	2	2	2	2	2
1	1	1	2	1	1	2	2	1	2
2	2	2	2	2	2	2	2	2	2
2	2	2	2	2	2	2	2	2	2
1	1	2	2	2	#NULL!	1	2	1	#NULL!
#NULL!	#NULL!	#NULL!	#NULL!	#NULL!	#NULL!	#NULL!	#NULL!	#NULL!	#NULL!
2	2	2	2	1	1	#NULL!	2	2	2
2	2	1	2	1	1	2	2	1	2
2	2	2	2	2	2	2	2	2	2
2	2	1	2	1	1	2	2	1	2
2	2	2	1	2	2	2	1	2	2
2	2	2	2	1	2	2	2	2	2
2	2	2	2	1	2	2	2	2	2
2	2	2	2	1	2	2	2	2	2
2	2	2	2	1	2	2	2	2	2
2	2	2	2	1	2	2	2	2	2
2	2	2	2	1	2	2	2	2	2
2	2	2	2	1	2	2	2	2	2
2	2	2	2	2	2	1	2	2	2
2	2	2	2	1	2	1	2	2	2
#NULL!	#NULL!	2	2	2	2	2	2	1	2
2	2	2	2	2	2	2	2	2	2
2	2	2	2	2	2	2	2	2	2
2	2	1	2	1	1	2	2	1	2
2	2	2	2	1	2	2	2	1	2
2	2	#NULL!	#NULL!	#NULL!	#NULL!	1	#NULL!	#NULL!	#NULL!
2	2	2	2	2	2	2	2	2	2
1	1	2	#NULL!	#NULL!	#NULL!	#NULL!	#NULL!	#NULL!	#NULL!
2	2	2	2	2	2	2	2	2	2
2	2	2	2	2	1	2	2	1	1
2	2	1	2	1	2	2	2	1	2
2	2	2	2	2	1	2	1	1	1
2	1	#NULL!	#NULL!	#NULL!	#NULL!	1	#NULL!	#NULL!	#NULL!
2	2	1	2	1	1	2	2	1	2
2	2	1	2	2	2	2	2	1	2
2	2	2	#NULL!	#NULL!	#NULL!	2	#NULL!	2	2
2	1	2	2	2	2	2	2	2	1
2	2	2	2	1	2	2	2	2	2
2	2	2	1	2	2	2	2	2	1
2	2	2	1	2	2	2	2	1	1
2	2	2	2	2	2	2	2	#NULL!	2
1	1	2	1	2	2	1	2	2	2
2	1	1	1	1	1	1	1	2	2
1	2	2	1	2	2	2	1	2	1
1	1	2	1	2	2	1	1	2	1
2	2	2	2	2	2	2	2	2	2

2	2	2	2	2	2	2	2	2	2
1	1	1	#NULL!	#NULL!	#NULL!	#NULL!	#NULL!	#NULL!	#NULL!
2	1	2	#NULL!	#NULL!	#NULL!	#NULL!	#NULL!	#NULL!	#NULL!
2	2	2	2	2	2	2	1	2	2
2	2	2	2	#NULL!	2	2	1	2	2
1	1	2	2	2	2	2	2	2	2
2	2	2	2	#NULL!	2	1	1	2	1
1	2	2	2	2	2	2	2	2	2
1	1	2	2	2	2	2	2	2	2
1	2	2	2	2	2	2	2	2	2
2	2	2	2	1	2	2	2	2	#NULL!
2	2	2	2	2	2	1	2	2	2
2	2	#NULL!	#NULL!	#NULL!	#NULL!	#NULL!	#NULL!	#NULL!	#NULL!
2	2	1	2	1	2	1	2	2	2
2	2	2	2	2	2	1	2	2	2
2	1	#NULL!	#NULL!	#NULL!	#NULL!	#NULL!	#NULL!	#NULL!	#NULL!
2	1	1	2	1	1	2	2	1	2
1	2	#NULL!	#NULL!	#NULL!	#NULL!	#NULL!	#NULL!	#NULL!	#NULL!
1	2	#NULL!	#NULL!	#NULL!	#NULL!	#NULL!	#NULL!	#NULL!	#NULL!
2	2	2	2	1	2	2	2	2	2
1	2	2	2	2	2	1	1	2	1
2	2	2	2	2	2	2	2	2	2
#NULL!	#NULL!	#NULL!	#NULL!	#NULL!	#NULL!	#NULL!	#NULL!	#NULL!	#NULL!
2	2	2	2	2	2	2	2	2	2
#NULL!	#NULL!	1	2	1	1	2	2	1	2
1	2	2	2	1	2	1	2	2	2
1	2	2	2	1	2	1	2	2	2
2	2	2	2	2	2	2	2	2	2
#NULL!	1	2	1	2	2	1	1	2	1
2	2	2	2	2	2	2	2	2	2
2	1	2	2	1	2	1	1	2	2
2	2	2	2	2	1	2	2	2	2
2	2	2	2	2	1	2	2	2	2
1	2	2	2	1	2	1	2	2	2
#NULL!	#NULL!	2	1	2	2	2	2	2	1
2	2	2	2	2	2	2	2	2	2
2	1	2	2	2	2	2	1	2	1
1	1	#NULL!	#NULL!	#NULL!	#NULL!	#NULL!	#NULL!	#NULL!	#NULL!
2	2	#NULL!	#NULL!	#NULL!	#NULL!	2	#NULL!	1	#NULL!
2	2	2	2	#NULL!	2	1	1	2	1
2	2	2	2	2	2	2	2	2	2
2	2	2	2	2	2	2	2	2	2
2	2	2	2	2	2	2	2	2	2
1	1	2	2	2	2	2	2	2	2
2	2	2	2	2	2	2	2	2	2
2	2	2	2	2	2	2	2	2	2
#NULL!	#NULL!	#NULL!	#NULL!	#NULL!	#NULL!	#NULL!	#NULL!	#NULL!	#NULL!

#NULL!	#NULL!	#NULL!	#NULL!	#NULL!	#NULL!	#NULL!	#NULL!	#NULL!	#NULL!
2	2	2	2	2	2	2	2	2	2
2	1	2	2	2	2	2	2	2	2
1	1	2	2	2	2	2	2	2	2
1	1	2	#NULL!	#NULL!	#NULL!	#NULL!	#NULL!	#NULL!	#NULL!
2	2	1	2	2	2	2	2	2	2
#NULL!	#NULL!	#NULL!	#NULL!	#NULL!	#NULL!	#NULL!	#NULL!	#NULL!	#NULL!
2	2	1	2	1	1	2	2	1	2
1	1	2	2	2	2	1	1	2	2
2	2	2	#NULL!	#NULL!	#NULL!	#NULL!	#NULL!	#NULL!	#NULL!
2	2	2	2	2	2	2	2	2	2
2	1	2	2	2	2	2	2	2	2
2	1	2	2	2	2	2	2	2	2
2	2	2	2	2	2	2	2	2	2
2	2	2	1	1	1	2	2	2	2
2	2	2	2	2	2	2	2	2	2
2	2	2	2	2	2	2	2	2	2
1	1	2	2	2	2	2	2	2	2
2	2	2	2	2	1	2	2	2	2
2	2	2	2	2	2	2	2	2	1
2	2	2	#NULL!	#NULL!	#NULL!	#NULL!	#NULL!	#NULL!	#NULL!
2	2	1	2	1	1	2	2	1	2
2	2	2	2	2	2	2	2	2	2
2	2	2	2	2	2	2	2	2	2
1	1	2	2	2	2	2	2	2	2
2	2	1	2	1	1	2	2	1	2
2	2	#NULL!	#NULL!	#NULL!	#NULL!	#NULL!	#NULL!	#NULL!	#NULL!
2	2	2	2	2	2	2	2	2	2
2	2	2	2	2	2	2	2	2	2
1	1	2	2	2	1	2	1	2	2
2	1	2	2	2	2	2	2	2	2
2	2	2	2	2	2	2	2	2	2
1	1	2	2	2	1	1	1	2	2
1	1	2	2	2	2	2	2	2	2
2	2	2	2	2	2	2	2	2	2
#NULL!	#NULL!	2	2	2	2	2	2	2	2
2	2	2	2	2	2	2	2	2	2
2	2	2	2	1	2	2	2	2	1
2	2	2	2	2	2	2	2	2	2
2	2	2	2	2	2	2	2	2	2
2	2	2	2	2	2	2	2	2	2
2	2	2	2	2	2	2	2	2	2
2	2	2	2	2	1	2	2	1	2
#NULL!	#NULL!	2	2	2	2	2	2	2	2
2	2	2	2	2	2	2	2	2	2
2	2	1	2	1	1	2	2	1	2
2	2	2	2	2	2	2	2	2	2

2	2	2	2	2	2	2	2	2	2
2	2	2	2	2	2	2	2	2	2
2	2	2	2	2	2	2	2	2	2
2	2	2	2	2	2	2	2	2	2
2	2	1	2	2	2	2	2	2	2
2	2	2	2	1	2	2	1	2	2
2	2	2	2	2	2	2	2	2	2
2	2	2	2	2	1	2	1	1	2
2	2	2	1	2	2	2	2	2	2
2	2	2	2	2	2	2	2	2	2
1	1	2	1	2	2	2	2	2	2
2	2	2	#NULL!	#NULL!	#NULL!	#NULL!	#NULL!	#NULL!	#NULL!
2	2	#NULL!	#NULL!	#NULL!	2	#NULL!	#NULL!	#NULL!	#NULL!
#NULL!	#NULL!	2	1	2	2	2	2	2	2
1	1	2	1	2	2	2	2	1	2
2	1	1	2	2	2	2	2	1	2
2	2	2	2	1	2	2	2	2	2
2	2	2	2	2	#NULL!	2	2	2	2
#NULL!	#NULL!	#NULL!	#NULL!	#NULL!	#NULL!	#NULL!	#NULL!	#NULL!	#NULL!
1	2	2	2	1	2	2	2	2	2
2	2	#NULL!	#NULL!	#NULL!	#NULL!	#NULL!	#NULL!	#NULL!	#NULL!
1	1	2	2	2	1	2	1	2	2
2	2	2	2	2	2	2	2	2	2
1	1	2	2	2	2	2	2	2	2
2	2	1	2	1	1	2	2	1	2
1	1	2	2	2	2	2	2	2	2
2	2	2	2	2	2	2	2	2	2
1	1	2	2	2	2	2	2	2	2
2	2	2	2	2	2	2	2	2	2
2	2	1	2	1	2	2	2	2	2
1	1	2	1	2	2	1	1	2	1
1	1	2	1	2	2	1	1	2	1
#NULL!	#NULL!	1	2	1	1	2	2	1	2
1	1	2	1	2	2	1	1	2	1
1	1	1	2	1	1	2	2	1	2
2	1	2	1	1	1	2	1	2	1
2	2	2	#NULL!	#NULL!	#NULL!	#NULL!	#NULL!	#NULL!	#NULL!
1	1	2	1	2	2	1	1	2	1
2	2	2	2	2	2	2	2	2	2
2	2	2	2	2	2	2	2	2	2
2	2	2	2	2	2	2	1	2	2
2	2	#NULL!	#NULL!	#NULL!	#NULL!	1	#NULL!	#NULL!	#NULL!
2	2	2	2	1	2	1	2	2	2
2	2	2	2	2	2	2	2	2	2
2	2	2	2	2	2	2	2	2	2
1	1	2	2	2	1	1	2	2	1
2	1	2	2	1	2	2	2	2	1

2	2	2	2	1	2	1	2	2	2
2	2	1	2	1	1	2	2	1	2
2	2	2	2	1	2	1	2	2	2
2	2	#NULL!	2	#NULL!	#NULL!	#NULL!	#NULL!	#NULL!	#NULL!
#NULL!	#NULL!	2	#NULL!	#NULL!	#NULL!	#NULL!	#NULL!	#NULL!	#NULL!
2	2	2	#NULL!	2	2	2	2	2	2
2	2	2	2	2	2	2	2	2	2
2	2	1	2	1	1	2	2	1	2
1	2	1	2	1	1	2	2	1	2
2	2	2	2	2	2	2	2	2	2
2	2	#NULL!	#NULL!	#NULL!	#NULL!	#NULL!	#NULL!	#NULL!	#NULL!
1	2	2	2	2	2	2	#NULL!	2	2
2	2	2	2	2	2	2	2	2	2
2	2	2	2	2	2	2	2	2	2
2	2	2	2	2	2	2	2	2	2
2	2	1	2	1	1	2	2	2	1
2	2	1	2	2	2	2	2	2	2
2	2	#NULL!	1	#NULL!	#NULL!	#NULL!	#NULL!	#NULL!	#NULL!
2	2	#NULL!	#NULL!	#NULL!	#NULL!	#NULL!	#NULL!	#NULL!	#NULL!
2	2	2	2	2	2	2	2	2	2
2	2	2	2	2	2	2	2	2	2
2	2	2	2	2	2	2	2	2	2
#NULL!	#NULL!	#NULL!	#NULL!	#NULL!	#NULL!	#NULL!	#NULL!	#NULL!	#NULL!
#NULL!	#NULL!	#NULL!	#NULL!	#NULL!	#NULL!	#NULL!	#NULL!	#NULL!	#NULL!
2	1	2	2	2	1	1	2	1	1
2	2	2	1	2	2	1	1	2	2
1	2	2	2	2	2	2	1	2	1
1	2	2	1	2	2	1	1	2	2
2	2	1	1	1	1	2	1	2	2
2	2	2	2	#NULL!	2	2	2	2	2
1	2	2	2	2	2	2	1	2	2
2	2	2	2	1	2	2	2	2	2
1	1	2	2	2	2	2	2	2	2
2	2	1	2	1	1	2	2	1	2
2	2	1	2	1	1	2	2	1	2
1	2	2	2	1	1	2	2	2	2
1	1	2	2	2	1	2	2	2	2
2	2	1	2	1	1	2	2	1	2
2	1	2	#NULL!	#NULL!	1	1	2	2	#NULL!
2	2	1	2	1	1	2	2	1	2
2	1	2	2	1	2	2	1	2	2
1	2	2	2	2	1	1	1	2	2
2	2	2	1	2	2	1	1	2	1
2	2	2	2	2	2	2	2	2	2
1	1	2	2	2	2	1	2	2	2
#NULL!	#NULL!	2	1	2	2	1	1	2	1
2	2	2	2	2	2	2	2	2	2

2	2	2	2	2	2	2	2	2	2
2	2	#NULL!	#NULL!	1	2	#NULL!	2	#NULL!	2
2	1	2	2	2	2	2	1	#NULL!	1
2	1	2	2	2	2	2	1	2	1
2	2	2	2	2	2	2	2	2	2
2	2	2	2	2	2	2	2	2	2
#NULL!	2	1	2	1	1	2	2	2	2
1	2	2	2	2	2	2	2	2	2
1	2	2	2	2	2	2	2	2	2
2	2	1	2	2	2	2	2	2	2
#NULL!	2	#NULL!	#NULL!	#NULL!	#NULL!	#NULL!	2	#NULL!	#NULL!
2	2	2	2	2	2	2	2	2	2
2	2	2	1	1	2	1	2	2	2
1	2	2	2	2	2	2	1	2	2
1	1	2	1	2	2	1	1	2	1
1	2	1	2	1	1	2	2	2	2
2	2	2	2	2	2	2	2	2	2
2	2	2	2	2	2	2	2	2	2
2	2	2	2	1	2	2	1	2	2
2	2	2	#NULL!	#NULL!	#NULL!	#NULL!	#NULL!	#NULL!	#NULL!
1	2	2	2	2	2	1	2	2	1
1	1	2	1	2	2	1	1	1	2
1	1	2	2	1	1	2	2	1	2
1	2	2	2	1	1	2	2	1	2
2	2	1	2	1	1	2	2	1	2
2	2	2	2	2	2	2	2	2	2
2	2	2	2	2	2	2	1	2	2
2	2	2	1	2	1	1	2	2	2
2	1	1	2	2	2	1	2	1	1
1	2	2	1	2	2	2	1	2	1
2	2	2	2	2	2	2	2	2	2
1	1	#NULL!	#NULL!	#NULL!	#NULL!	#NULL!	#NULL!	#NULL!	#NULL!
1	2	2	1	2	2	2	2	2	2
2	2	#NULL!	#NULL!	#NULL!	#NULL!	#NULL!	#NULL!	#NULL!	#NULL!
2	2	2	2	2	2	2	2	2	2
2	2	2	2	2	2	2	2	2	2
2	2	2	2	2	2	2	2	2	2
2	2	2	2	2	2	2	2	2	2
2	2	2	2	2	2	2	2	2	2
2	2	2	2	2	2	2	2	1	1
2	2	2	#NULL!	2	2	1	2	2	2
1	1	2	2	2	2	2	2	2	2
2	2	2	2	1	1	2	2	2	2
2	2	2	#NULL!	#NULL!	#NULL!	#NULL!	#NULL!	#NULL!	#NULL!
2	2	1	2	1	1	2	2	1	2
2	2	2	2	2	2	2	2	2	2
2	2	2	2	2	2	2	2	2	2

2	2	#NULL!	#NULL!	#NULL!	#NULL!	#NULL!	#NULL!	#NULL!	#NULL!
#NULL!	#NULL!	2	2	2	2	1	2	2	#NULL!
2	2	2	2	2	1	2	2	1	2
2	2	2	2	2	2	2	2	2	2
2	2	1	2	1	1	2	2	1	2
2	2	1	2	2	2	2	2	#NULL!	2
2	2	2	2	2	2	2	#NULL!	#NULL!	#NULL!
2	2	2	#NULL!	2	2	2	2	2	2
2	2	1	2	2	2	2	2	2	2
#NULL!	#NULL!	#NULL!	#NULL!	#NULL!	#NULL!	#NULL!	#NULL!	#NULL!	#NULL!
#NULL!	#NULL!	#NULL!	#NULL!	#NULL!	#NULL!	#NULL!	#NULL!	#NULL!	#NULL!
#NULL!	#NULL!	#NULL!	#NULL!	#NULL!	#NULL!	#NULL!	#NULL!	#NULL!	#NULL!
2	1	2	#NULL!	#NULL!	#NULL!	#NULL!	#NULL!	#NULL!	#NULL!
2	1	1	#NULL!	#NULL!	#NULL!	#NULL!	#NULL!	#NULL!	#NULL!
#NULL!	#NULL!	#NULL!	#NULL!	#NULL!	#NULL!	#NULL!	#NULL!	#NULL!	#NULL!
2	2	2	2	2	2	2	2	2	2
#NULL!	#NULL!	2	1	2	2	1	2	2	1
1	1	2	2	2	2	2	2	2	2
2	2	2	1	1	2	2	2	2	1
2	2	2	2	2	2	2	1	2	2
2	2	2	2	2	2	2	2	2	2
2	2	2	2	2	2	2	2	1	2
2	2	2	2	1	2	1	1	2	2
2	2	2	2	1	1	2	2	2	2
2	1	2	2	2	2	2	2	2	2
2	2	2	2	2	2	2	1	2	2
1	2	2	#NULL!	1	#NULL!	1	2	#NULL!	1
1	1	2	1	2	2	1	1	2	1
2	2	2	2	2	2	2	1	2	2
2	2	2	2	2	2	2	2	2	2
2	2	2	2	2	2	2	1	2	2
2	1	2	#NULL!	#NULL!	#NULL!	#NULL!	#NULL!	#NULL!	#NULL!
1	1	2	2	2	2	2	1	2	2
2	2	2	2	2	2	2	2	2	2
2	2	2	2	2	2	2	2	2	2
2	2	2	2	2	2	2	2	2	2
2	2	2	2	2	2	2	2	2	2
2	1	2	#NULL!	#NULL!	#NULL!	#NULL!	#NULL!	#NULL!	#NULL!
2	2	2	2	2	2	2	2	2	2
2	2	2	2	2	2	2	2	2	2
2	2	2	2	2	2	2	2	2	2
2	1	2	2	2	2	2	2	2	2
2	2	2	2	2	2	2	2	2	2
2	1	2	2	2	2	2	1	1	2
1	1	2	2	2	2	2	2	2	2
2	1	2	2	2	2	2	2	2	2
2	2	2	#NULL!	#NULL!	#NULL!	#NULL!	#NULL!	#NULL!	#NULL!

#NULL!	#NULL!	2	2	2	2	2	2	2	2
2	2	2	2	2	2	2	2	2	2
2	2	#NULL!	#NULL!	#NULL!	#NULL!	1	#NULL!	#NULL!	#NULL!
2	2	2	#NULL!	#NULL!	#NULL!	#NULL!	#NULL!	#NULL!	#NULL!
2	2	2	2	2	2	2	1	2	1
2	2	2	#NULL!	#NULL!	#NULL!	#NULL!	#NULL!	#NULL!	#NULL!
1	2	2	1	2	2	2	2	2	2
1	2	2	1	2	1	2	2	1	2
2	2	2	2	1	1	2	1	2	2
1	2	2	1	2	2	2	2	2	2
2	2	#NULL!	#NULL!	#NULL!	#NULL!	#NULL!	#NULL!	#NULL!	#NULL!
2	2	2	2	2	2	1	2	2	1
2	2	2	2	1	1	2	2	2	2
2	2	2	2	2	2	2	2	2	2
2	2	2	2	2	2	2	2	2	2
2	2	1	2	1	1	2	2	1	1
2	2	2	2	2	2	2	2	2	2
2	2	2	2	2	2	2	2	2	2
2	2	2	2	2	2	2	1	1	2
2	2	2	2	2	2	2	2	1	2
2	2	2	2	2	2	2	1	1	2
2	2	2	2	2	2	2	2	2	2
2	2	2	2	2	2	1	2	2	2
2	2	2	2	2	2	2	2	2	2
2	2	2	2	2	2	2	2	2	2
2	2	2	2	2	2	2	2	2	2
2	2	2	2	2	2	2	2	2	2
2	2	2	2	2	2	2	2	2	2
2	2	2	2	2	2	2	2	2	2
#NULL!	#NULL!	#NULL!	#NULL!	#NULL!	#NULL!	#NULL!	#NULL!	#NULL!	#NULL!
2	2	2	1	2	2	#NULL!	1	#NULL!	1
2	1	2	2	2	2	2	2	2	2
2	2	2	1	2	2	1	1	2	1
#NULL!	#NULL!	#NULL!	#NULL!	#NULL!	#NULL!	#NULL!	#NULL!	#NULL!	#NULL!
1	2	2	2	1	1	2	1	1	2
2	2	#NULL!	#NULL!	#NULL!	#NULL!	#NULL!	#NULL!	#NULL!	#NULL!
#NULL!	#NULL!	#NULL!	#NULL!	#NULL!	#NULL!	#NULL!	#NULL!	#NULL!	#NULL!
2	2	2	2	2	2	2	2	2	2
2	2	2	2	1	1	2	2	1	2
2	1	2	1	2	2	2	1	2	2
1	1	2	1	2	2	1	1	2	1
2	2	2	2	2	2	2	2	2	1
2	2	2	#NULL!	#NULL!	#NULL!	#NULL!	#NULL!	#NULL!	#NULL!
2	2	2	2	2	2	2	2	1	1
2	1	1	2	2	2	2	2	2	2
#NULL!	#NULL!	#NULL!	#NULL!	#NULL!	#NULL!	#NULL!	#NULL!	#NULL!	#NULL!
2	2	1	2	2	2	2	1	2	2

2	2	2	2	2	2	1	2	2	2
#NULL!	#NULL!	#NULL!	#NULL!	#NULL!	#NULL!	#NULL!	#NULL!	#NULL!	#NULL!
#NULL!	#NULL!	#NULL!	#NULL!	#NULL!	#NULL!	#NULL!	#NULL!	#NULL!	#NULL!
2	2	2	2	2	2	2	2	2	2
2	2	2	2	2	2	1	1	2	2
2	2	2	2	2	2	1	2	2	2
1	1	2	1	1	2	2	1	2	2
2	2	2	2	2	2	2	2	2	2
1	1	2	2	2	2	2	2	2	2
1	1	2	2	2	2	2	1	2	1
#NULL!	#NULL!	#NULL!	#NULL!	#NULL!	#NULL!	#NULL!	#NULL!	#NULL!	#NULL!
2	2	#NULL!	#NULL!	#NULL!	#NULL!	#NULL!	#NULL!	#NULL!	#NULL!
2	2	2	#NULL!	#NULL!	#NULL!	#NULL!	#NULL!	#NULL!	#NULL!
1	1	2	2	2	2	2	2	2	2
2	2	2	2	2	2	2	2	1	2
#NULL!	#NULL!	#NULL!	#NULL!	#NULL!	#NULL!	#NULL!	#NULL!	#NULL!	#NULL!
#NULL!	#NULL!	#NULL!	#NULL!	#NULL!	#NULL!	#NULL!	#NULL!	#NULL!	#NULL!
#NULL!	#NULL!	#NULL!	#NULL!	#NULL!	#NULL!	#NULL!	#NULL!	#NULL!	#NULL!
2	2	2	2	2	2	2	2	2	2
2	2	2	2	2	2	2	2	2	2
#NULL!	#NULL!	#NULL!	#NULL!	#NULL!	#NULL!	#NULL!	#NULL!	#NULL!	#NULL!
1	2	2	2	2	2	1	2	1	2
1	2	1	2	1	1	2	2	1	2
#NULL!	#NULL!	#NULL!	#NULL!	#NULL!	#NULL!	#NULL!	#NULL!	#NULL!	#NULL!
1	1	2	2	2	2	2	2	2	2
2	2	2	2	2	2	2	2	2	2
2	2	#NULL!	#NULL!	#NULL!	#NULL!	#NULL!	#NULL!	#NULL!	#NULL!
2	2	#NULL!	#NULL!	#NULL!	#NULL!	#NULL!	#NULL!	#NULL!	#NULL!
2	2	2	2	2	2	2	2	2	2
#NULL!	#NULL!	2	2	2	2	2	2	2	2
2	2	2	1	2	2	2	1	2	2
2	2	#NULL!	#NULL!	#NULL!	#NULL!	#NULL!	#NULL!	#NULL!	#NULL!
#NULL!	#NULL!	#NULL!	#NULL!	#NULL!	#NULL!	#NULL!	#NULL!	#NULL!	#NULL!
2	2	2	2	2	2	2	2	1	2
1	1	2	#NULL!	2	2	#NULL!	2	2	2
2	2	2	2	2	2	2	2	2	2
2	2	2	1	2	2	2	1	2	1
2	2	2	2	2	2	2	2	2	2
2	2	2	#NULL!	2	2	2	1	2	1
2	2	2	2	2	1	1	2	1	1
2	1	2	2	2	1	2	1	2	2
2	2	#NULL!	#NULL!	#NULL!	#NULL!	#NULL!	#NULL!	#NULL!	#NULL!
#NULL!	#NULL!	#NULL!	#NULL!	#NULL!	#NULL!	#NULL!	#NULL!	#NULL!	#NULL!
2	2	#NULL!	#NULL!	#NULL!	#NULL!	1	#NULL!	#NULL!	#NULL!
#NULL!	#NULL!	#NULL!	#NULL!	#NULL!	#NULL!	#NULL!	#NULL!	#NULL!	#NULL!
#NULL!	#NULL!	#NULL!	#NULL!	#NULL!	#NULL!	#NULL!	#NULL!	#NULL!	#NULL!
#NULL!	#NULL!	#NULL!	#NULL!	#NULL!	#NULL!	#NULL!	#NULL!	#NULL!	#NULL!

2	2	2	2	2	2	2	2	2	2
1	2	2	2	2	1	2	2	2	2
2	2	2	2	2	2	2	2	2	1
2	2	2	2	2	2	2	2	2	2
1	1	#NULL!	#NULL!	#NULL!	#NULL!	#NULL!	#NULL!	#NULL!	#NULL!
#NULL!	#NULL!	#NULL!	#NULL!	#NULL!	#NULL!	#NULL!	#NULL!	#NULL!	#NULL!
#NULL!	#NULL!	#NULL!	#NULL!	#NULL!	#NULL!	#NULL!	#NULL!	#NULL!	#NULL!
#NULL!	#NULL!	#NULL!	#NULL!	#NULL!	#NULL!	#NULL!	#NULL!	#NULL!	#NULL!
#NULL!	#NULL!	#NULL!	#NULL!	#NULL!	#NULL!	#NULL!	#NULL!	#NULL!	#NULL!
#NULL!	#NULL!	#NULL!	#NULL!	#NULL!	#NULL!	#NULL!	#NULL!	#NULL!	#NULL!
#NULL!	#NULL!	#NULL!	#NULL!	#NULL!	#NULL!	#NULL!	#NULL!	#NULL!	#NULL!
#NULL!	#NULL!	#NULL!	#NULL!	#NULL!	#NULL!	#NULL!	#NULL!	#NULL!	#NULL!
#NULL!	#NULL!	#NULL!	#NULL!	#NULL!	#NULL!	#NULL!	#NULL!	#NULL!	#NULL!
2	2	2	1	1	1	1	1	2	1
2	2	2	2	2	2	2	2	2	2
2	2	2	2	2	2	2	2	2	2
2	2	2	2	2	2	2	2	2	2
#NULL!	#NULL!	#NULL!	#NULL!	#NULL!	#NULL!	#NULL!	#NULL!	#NULL!	#NULL!
#NULL!	#NULL!	#NULL!	#NULL!	#NULL!	#NULL!	#NULL!	#NULL!	#NULL!	#NULL!
#NULL!	#NULL!	#NULL!	#NULL!	#NULL!	#NULL!	#NULL!	#NULL!	#NULL!	#NULL!
2	2	2	#NULL!	#NULL!	#NULL!	#NULL!	#NULL!	2	#NULL!
#NULL!	#NULL!	#NULL!	#NULL!	#NULL!	#NULL!	#NULL!	#NULL!	#NULL!	#NULL!
2	2	2	1	2	2	2	2	2	2
#NULL!	#NULL!	#NULL!	#NULL!	#NULL!	#NULL!	#NULL!	#NULL!	#NULL!	#NULL!
1	2	2	1	2	2	1	1	2	1
1	1	2	1	2	2	1	1	2	2
1	1	2	1	2	2	1	1	2	1
1	1	2	2	2	2	1	1	2	1
1	1	2	1	2	2	2	2	2	1
1	1	2	1	2	2	1	1	2	2
1	1	2	1	2	2	2	2	2	1
2	2	2	1	2	2	2	2	2	1
1	1	2	1	2	2	1	1	2	1
#NULL!	#NULL!	#NULL!	#NULL!	#NULL!	#NULL!	#NULL!	#NULL!	#NULL!	#NULL!
1	1	2	1	2	2	1	1	2	1
1	1	2	1	2	2	2	2	2	1
2	2	2	2	2	2	2	2	2	2
#NULL!	#NULL!	#NULL!	#NULL!	#NULL!	#NULL!	#NULL!	#NULL!	#NULL!	#NULL!
1	1	2	2	2	2	2	2	2	2
2	2	1	2	2	2	2	1	1	1
2	2	2	2	1	1	2	2	1	2
#NULL!	#NULL!	#NULL!	#NULL!	#NULL!	#NULL!	#NULL!	#NULL!	#NULL!	#NULL!
#NULL!	#NULL!	#NULL!	#NULL!	#NULL!	#NULL!	#NULL!	#NULL!	#NULL!	#NULL!
#NULL!	#NULL!	#NULL!	#NULL!	#NULL!	#NULL!	#NULL!	#NULL!	#NULL!	#NULL!
2	1	2	2	2	#NULL!	#NULL!	#NULL!	#NULL!	#NULL!
1	1	2	2	2	2	2	2	1	2
2	2	#NULL!	#NULL!	#NULL!	#NULL!	#NULL!	#NULL!	#NULL!	#NULL!

#NULL!	#NULL!	#NULL!	#NULL!	#NULL!	#NULL!	#NULL!	#NULL!	#NULL!	#NULL!
#NULL!	#NULL!	#NULL!	#NULL!	#NULL!	#NULL!	#NULL!	#NULL!	#NULL!	#NULL!
#NULL!	#NULL!	#NULL!	#NULL!	#NULL!	#NULL!	#NULL!	#NULL!	#NULL!	#NULL!
#NULL!	#NULL!	#NULL!	#NULL!	#NULL!	#NULL!	#NULL!	#NULL!	#NULL!	#NULL!
2	2	#NULL!	#NULL!	#NULL!	#NULL!	#NULL!	#NULL!	#NULL!	#NULL!
#NULL!	#NULL!	#NULL!	#NULL!	#NULL!	#NULL!	#NULL!	#NULL!	#NULL!	#NULL!
#NULL!	#NULL!	#NULL!	#NULL!	#NULL!	#NULL!	#NULL!	#NULL!	#NULL!	#NULL!
#NULL!	#NULL!	#NULL!	#NULL!	#NULL!	#NULL!	#NULL!	#NULL!	#NULL!	#NULL!
2	2	#NULL!	#NULL!	#NULL!	#NULL!	#NULL!	#NULL!	#NULL!	#NULL!
#NULL!	#NULL!	#NULL!	#NULL!	#NULL!	#NULL!	#NULL!	#NULL!	#NULL!	#NULL!
2	2	#NULL!	#NULL!	2	#NULL!	#NULL!	#NULL!	#NULL!	#NULL!
#NULL!	#NULL!	#NULL!	#NULL!	#NULL!	#NULL!	#NULL!	#NULL!	#NULL!	#NULL!
2	1	2	2	2	2	1	2	2	2
1	1	2	2	2	2	2	2	#NULL!	1
2	2	2	2	2	2	1	1	2	2
1	2	2	1	1	2	1	2	1	2
2	2	2	2	2	2	2	2	2	2
2	2	2	1	2	2	2	1	2	1
2	2	2	1	2	2	2	1	2	1
1	1	2	#NULL!	#NULL!	#NULL!	#NULL!	#NULL!	#NULL!	#NULL!
1	1	2	1	2	2	1	1	2	2
2	2	2	2	2	2	2	1	2	2
2	2	2	2	2	2	2	2	2	2
2	2	2	2	2	2	1	2	2	1
1	1	2	2	2	2	2	2	2	1
2	2	2	2	1	1	2	2	1	2
2	2	2	2	#NULL!	#NULL!	2	#NULL!	1	2
1	1	2	2	2	2	2	2	2	2
2	2	2	#NULL!	#NULL!	#NULL!	#NULL!	#NULL!	#NULL!	#NULL!
2	1	#NULL!	#NULL!	#NULL!	#NULL!	#NULL!	#NULL!	#NULL!	#NULL!
#NULL!	#NULL!	#NULL!	#NULL!	#NULL!	#NULL!	#NULL!	#NULL!	#NULL!	#NULL!
#NULL!	#NULL!	2	1	2	2	1	1	2	1
2	2	2	2	2	2	2	2	2	2
2	2	2	2	2	2	1	1	2	1
2	2	2	2	2	2	1	1	2	2
2	2	2	2	2	2	2	2	2	2
2	2	2	2	2	2	2	2	2	2
2	2	2	2	2	2	1	1	2	1
2	2	2	2	2	2	2	2	2	2
2	2	2	1	2	2	2	2	2	1
2	2	2	2	2	2	2	2	2	2
2	2	2	1	2	2	1	1	2	2
1	2	2	2	1	1	2	2	2	2
2	2	2	1	2	2	2	2	2	1
1	1	2	1	2	2	2	2	2	1
1	1	2	1	2	2	2	1	2	1
2	2	2	2	2	2	2	2	2	2

#NULL!	#NULL!	#NULL!	#NULL!	#NULL!	#NULL!	#NULL!	#NULL!	#NULL!	#NULL!
1	1	2	1	2	1	2	2	2	2
2	2	#NULL!	#NULL!	#NULL!	#NULL!	2	#NULL!	#NULL!	#NULL!
2	2	#NULL!	#NULL!	1	#NULL!	#NULL!	#NULL!	#NULL!	#NULL!
2	2	2	#NULL!	#NULL!	#NULL!	#NULL!	#NULL!	#NULL!	#NULL!
2	2	#NULL!	#NULL!	#NULL!	#NULL!	#NULL!	#NULL!	#NULL!	#NULL!
2	2	#NULL!	2	#NULL!	#NULL!	#NULL!	#NULL!	#NULL!	#NULL!
2	2	2	2	2	2	2	2	2	2
2	2	2	#NULL!	#NULL!	#NULL!	#NULL!	#NULL!	#NULL!	#NULL!
2	2	2	2	2	2	2	2	2	2
#NULL!	#NULL!	#NULL!	#NULL!	#NULL!	#NULL!	#NULL!	#NULL!	#NULL!	#NULL!
2	2	2	2	2	2	2	1	2	1
2	2	2	1	2	2	2	1	2	1
1	1	2	2	2	2	2	2	2	2
1	1	2	1	2	2	2	2	2	2
#NULL!	#NULL!	#NULL!	#NULL!	#NULL!	#NULL!	#NULL!	#NULL!	#NULL!	#NULL!
2	2	2	2	2	2	2	2	2	2
2	1	1	2	1	1	2	2	1	2
2	2	2	1	1	2	2	2	2	2
1	2	2	2	2	2	2	2	2	2
2	2	2	2	2	2	2	2	2	2
2	2	1	2	2	2	2	2	1	2
#NULL!	#NULL!	2	2	2	2	2	2	2	2
2	2	2	2	2	2	2	2	2	2
#NULL!	2	2	2	2	2	2	1	2	2
2	2	2	2	2	2	2	2	2	2
2	2	2	2	1	2	2	2	1	2
2	1	2	2	1	2	1	1	1	2
1	1	2	1	2	2	1	1	2	1
2	2	2	2	2	2	2	1	2	1
#NULL!	#NULL!	#NULL!	#NULL!	#NULL!	#NULL!	#NULL!	#NULL!	#NULL!	#NULL!
2	2	2	2	2	2	2	2	2	2
#NULL!	#NULL!	#NULL!	#NULL!	#NULL!	#NULL!	#NULL!	#NULL!	#NULL!	#NULL!
2	2	2	2	2	2	2	2	2	2
2	2	2	2	2	2	2	2	2	2
1	1	2	1	2	2	2	1	2	2
2	1	1	2	1	1	2	2	1	2
1	2	2	1	2	2	2	1	2	2
1	1	2	1	2	2	1	1	2	1
1	1	2	2	2	2	1	2	2	2
2	2	2	2	2	2	2	2	2	2
1	1	2	2	2	2	1	2	2	2
#NULL!	#NULL!	#NULL!	#NULL!	#NULL!	#NULL!	#NULL!	#NULL!	#NULL!	#NULL!
1	2	1	#NULL!	#NULL!	#NULL!	#NULL!	#NULL!	#NULL!	#NULL!
1	1	2	1	#NULL!	#NULL!	#NULL!	#NULL!	#NULL!	#NULL!
#NULL!	#NULL!	#NULL!	#NULL!	#NULL!	#NULL!	#NULL!	#NULL!	#NULL!	#NULL!
#NULL!	2	#NULL!	#NULL!	#NULL!	#NULL!	#NULL!	#NULL!	#NULL!	#NULL!

1	1	#NULL!	#NULL!	#NULL!	#NULL!	#NULL!	#NULL!	#NULL!	#NULL!
2	2	2	2	2	2	2	2	2	2
2	2	2	#NULL!	#NULL!	#NULL!	#NULL!	#NULL!	#NULL!	#NULL!
2	2	2	2	2	2	1	1	2	1
2	2	#NULL!	#NULL!	#NULL!	#NULL!	#NULL!	#NULL!	#NULL!	#NULL!
2	2	1	2	1	1	2	2	2	2
2	2	2	2	1	1	2	2	1	#NULL!
#NULL!	#NULL!	#NULL!	#NULL!	#NULL!	#NULL!	#NULL!	#NULL!	#NULL!	#NULL!
#NULL!	#NULL!	2	2	#NULL!	#NULL!	#NULL!	#NULL!	#NULL!	#NULL!
#NULL!	#NULL!	#NULL!	#NULL!	#NULL!	#NULL!	#NULL!	#NULL!	#NULL!	#NULL!
2	2	2	#NULL!	#NULL!	#NULL!	#NULL!	#NULL!	#NULL!	#NULL!
2	2	2	#NULL!	#NULL!	#NULL!	#NULL!	#NULL!	#NULL!	#NULL!
1	2	2	2	2	2	1	2	2	1
#NULL!	#NULL!	#NULL!	#NULL!	#NULL!	#NULL!	#NULL!	#NULL!	#NULL!	#NULL!
2	2	2	#NULL!	#NULL!	#NULL!	#NULL!	#NULL!	#NULL!	#NULL!
2	2	2	2	2	2	2	2	2	2
2	2	2	1	2	1	2	1	2	2
2	2	2	2	2	2	2	2	2	2
2	2	2	1	2	2	2	2	1	2
2	2	2	2	2	2	1	1	2	2
2	2	2	2	2	2	1	2	2	2
2	2	2	2	2	2	2	2	2	2
2	2	2	2	2	2	1	1	1	1
2	2	2	2	1	2	1	2	2	1
2	2	2	2	2	2	2	2	2	2
1	1	2	2	2	2	2	2	2	2
2	2	2	2	1	1	2	2	2	2
1	1	2	2	2	2	2	2	2	2
#NULL!	#NULL!	2	1	2	2	1	2	2	2
1	2	2	2	2	2	2	1	2	2
1	1	2	1	1	1	2	1	2	2
2	2	2	2	2	2	2	2	2	2
#NULL!	#NULL!	#NULL!	#NULL!	#NULL!	#NULL!	#NULL!	#NULL!	#NULL!	#NULL!
1	2	2	2	1	2	1	2	2	1
2	2	2	2	2	2	2	2	2	2
2	2	2	2	2	2	2	2	2	2
2	2	2	1	2	2	1	2	2	2
#NULL!	2	2	2	1	2	2	2	1	1
2	2	#NULL!	#NULL!	#NULL!	#NULL!	#NULL!	#NULL!	#NULL!	#NULL!
2	2	2	2	2	2	2	2	2	2
2	2	#NULL!	#NULL!	#NULL!	#NULL!	#NULL!	2	#NULL!	#NULL!
2	2	2	#NULL!	#NULL!	#NULL!	#NULL!	#NULL!	#NULL!	#NULL!
2	2	2	2	2	2	2	2	2	1
2	2	2	#NULL!	#NULL!	#NULL!	#NULL!	#NULL!	#NULL!	#NULL!
1	1	2	1	2	2	2	2	2	1
2	1	2	1	2	1	2	1	2	2
2	2	2	1	2	2	1	1	2	1

2	2	1	2	1	1	2	2	1	2
2	2	#NULL!	#NULL!	#NULL!	#NULL!	#NULL!	#NULL!	#NULL!	1
2	2	1	2	#NULL!	1	2	2	1	2
1	2	2	2	2	1	1	2	2	2
2	2	2	2	2	1	2	2	2	2
2	2	2	1	1	2	2	1	2	2
1	1	2	2	1	2	2	2	1	1
1	1	2	2	2	2	2	2	2	2
2	2	2	2	2	2	2	2	2	2
2	2	2	2	2	1	1	2	1	1
2	2	2	2	2	2	2	1	1	2
2	1	2	2	1	1	2	1	2	2
2	1	2	2	2	2	2	1	1	2
2	2	2	2	2	2	2	2	2	2
2	2	2	2	2	2	2	2	2	2
2	2	2	2	2	2	2	2	1	1
2	2	2	2	2	2	2	2	2	2
2	2	2	2	2	2	2	1	2	2
2	2	2	2	2	2	2	2	2	2
2	2	2	2	2	2	2	2	2	2
1	1	2	2	2	2	2	2	2	2
2	1	2	2	2	2	1	1	2	2
2	2	2	2	2	2	2	2	2	2
#NULL!	#NULL!	#NULL!	#NULL!	#NULL!	#NULL!	#NULL!	#NULL!	#NULL!	#NULL!
2	2	2	2	2	2	2	2	2	2
#NULL!	#NULL!	2	2	2	2	1	1	2	1
2	2	2	2	1	1	2	1	2	2
#NULL!	#NULL!	2	2	2	2	2	2	2	1
2	1	2	2	2	1	2	1	2	1
2	2	2	#NULL!	#NULL!	#NULL!	#NULL!	#NULL!	#NULL!	#NULL!
1	2	2	2	1	2	1	2	2	2
2	2	1	1	2	1	1	2	1	2
2	2	2	2	2	2	2	2	2	1
2	2	2	2	2	2	2	2	2	2
1	1	2	1	2	1	1	2	2	2
2	2	2	2	2	2	2	2	2	2
2	2	2	2	2	2	2	2	2	2
#NULL!	#NULL!	#NULL!	#NULL!	#NULL!	#NULL!	#NULL!	#NULL!	#NULL!	#NULL!
2	2	2	2	2	1	2	1	2	2
2	2	2	2	2	2	2	2	2	2
#NULL!	#NULL!	#NULL!	#NULL!	#NULL!	#NULL!	#NULL!	#NULL!	#NULL!	#NULL!
#NULL!	#NULL!	#NULL!	#NULL!	#NULL!	#NULL!	#NULL!	#NULL!	#NULL!	#NULL!
2	2	2	2	2	2	2	2	2	2
2	1	#NULL!	#NULL!	#NULL!	#NULL!	#NULL!	#NULL!	#NULL!	#NULL!
2	2	2	2	2	2	2	2	2	2
2	2	2	2	2	2	2	2	2	2
#NULL!	#NULL!	#NULL!	#NULL!	#NULL!	#NULL!	#NULL!	#NULL!	#NULL!	#NULL!

2	2	2	2	2	2	2	2	2	2
#NULL!	#NULL!	#NULL!	#NULL!	#NULL!	#NULL!	#NULL!	#NULL!	#NULL!	#NULL!
2	2	2	2	2	2	2	2	1	2
#NULL!	#NULL!	#NULL!	#NULL!	#NULL!	#NULL!	#NULL!	#NULL!	#NULL!	#NULL!
#NULL!	#NULL!	#NULL!	#NULL!	#NULL!	#NULL!	#NULL!	#NULL!	#NULL!	#NULL!
1	1	1	2	1	1	2	2	1	2
2	2	2	2	2	2	2	2	2	2
2	2	2	2	2	2	2	2	2	2
1	1	1	2	1	1	2	2	2	2
2	2	2	2	2	2	2	2	2	2
#NULL!	#NULL!	#NULL!	#NULL!	#NULL!	#NULL!	#NULL!	#NULL!	#NULL!	#NULL!
2	2	#NULL!	#NULL!	#NULL!	#NULL!	#NULL!	#NULL!	#NULL!	#NULL!
#NULL!	#NULL!	#NULL!	#NULL!	#NULL!	#NULL!	#NULL!	#NULL!	#NULL!	#NULL!
1	1	2	1	2	2	2	2	2	1
2	2	2	2	2	2	2	2	2	2
2	2	2	1	2	2	2	2	2	1
2	2	2	#NULL!	#NULL!	#NULL!	#NULL!	#NULL!	#NULL!	#NULL!
#NULL!	#NULL!	#NULL!	#NULL!	#NULL!	#NULL!	#NULL!	#NULL!	#NULL!	#NULL!
2	2	1	2	1	1	2	2	1	2
2	2	2	2	2	2	2	2	2	2
2	2	2	1	2	2	2	1	2	1
2	2	2	2	1	1	2	2	1	2
2	2	2	#NULL!	#NULL!	#NULL!	#NULL!	#NULL!	#NULL!	#NULL!
2	2	2	2	2	1	2	2	1	2
2	2	2	2	2	2	2	2	1	2
2	2	2	2	2	2	2	2	2	2
2	2	2	2	#NULL!	#NULL!	1	#NULL!	#NULL!	#NULL!
1	1	2	1	2	2	1	1	2	1
2	2	2	2	2	2	2	2	2	2
2	2	2	#NULL!	#NULL!	#NULL!	#NULL!	#NULL!	#NULL!	#NULL!
2	2	2	2	2	2	1	1	2	2
2	2	2	2	2	2	2	2	2	2
2	2	2	2	2	1	1	2	2	2
2	2	2	2	2	2	2	2	1	2
2	2	2	2	2	2	2	2	2	2
1	1	2	1	1	2	2	2	2	2
1	1	2	2	2	2	2	2	2	2
2	2	1	2	1	1	2	2	1	2
2	2	2	2	2	2	2	2	2	2
2	2	1	2	1	1	2	2	1	2
2	2	2	2	2	2	2	2	2	2
2	2	2	2	2	#NULL!	2	2	2	2
2	2	2	2	2	2	2	2	2	2
2	2	2	2	2	2	2	2	2	2
2	2	2	2	2	1	1	2	2	2
2	2	2	2	2	2	1	2	2	1
#NULL!	#NULL!	2	2	2	2	2	2	2	2

2	2	2	2	2	2	2	2	2	2
2	2	2	2	2	2	2	2	2	2
1	1	2	1	2	2	2	2	2	2
2	2	2	2	2	2	2	1	2	2
2	2	2	2	2	2	2	2	2	2
1	1	2	#NULL!	#NULL!	#NULL!	#NULL!	#NULL!	#NULL!	#NULL!
2	2	2	2	2	2	2	2	2	2
1	1	2	1	2	2	2	2	2	2
2	2	2	2	2	2	2	2	2	2
2	2	2	2	2	2	2	2	2	2
2	2	2	2	1	2	2	2	2	2
2	2	2	2	2	2	2	2	2	2
2	2	2	2	2	2	2	2	2	2
2	2	1	2	1	1	2	2	1	2
2	2	2	2	2	1	2	2	2	2
2	2	2	1	2	1	2	1	1	2
2	2	2	2	2	1	2	2	1	2
2	2	2	2	2	2	2	2	1	2
2	2	2	2	2	2	2	2	2	2
2	2	2	1	2	2	1	2	2	2
1	2	2	2	2	2	2	2	2	2
2	2	2	1	2	2	2	2	1	2
2	2	2	1	2	2	2	2	1	2
2	2	#NULL!	#NULL!	#NULL!	#NULL!	#NULL!	#NULL!	#NULL!	#NULL!
2	2	2	2	2	2	2	2	2	#NULL!
2	2	2	2	2	2	2	2	2	2
2	2	2	2	2	2	2	2	2	2
2	2	2	2	2	2	2	2	2	2
2	2	2	2	2	2	2	2	2	2
1	1	2	1	2	2	1	1	2	1
2	2	1	2	1	2	2	2	1	2
2	2	2	2	2	1	2	2	1	2
2	2	2	1	2	2	2	2	1	1
2	2	2	2	2	2	2	2	1	2
1	1	2	1	2	2	2	2	1	1
2	2	2	2	2	2	2	2	2	2
2	2	2	2	2	2	2	2	2	2
2	2	2	2	2	2	2	2	2	2
2	2	2	2	2	2	2	2	2	2
2	2	2	2	2	2	2	2	2	2
2	2	2	2	1	2	2	2	2	2
2	2	2	2	2	2	2	2	2	2
1	1	2	2	2	2	2	2	2	2
2	2	2	2	2	2	2	2	2	2
2	2	2	2	2	1	2	2	2	1
2	2	2	2	2	1	2	2	2	2
2	2	2	2	2	2	2	2	2	2

2	2	2	2	2	2	2	2	2	2
1	1	2	2	2	2	2	2	2	2
2	2	2	2	2	2	2	#NULL!	2	2
2	2	2	2	2	2	2	2	2	2
2	2	2	2	2	2	2	2	2	2
2	1	2	2	2	2	2	2	2	2
2	2	2	2	2	2	2	2	2	2
2	2	2	2	2	2	2	2	2	2
2	2	2	2	2	2	2	2	2	2
2	2	2	2	2	2	2	2	2	2
2	2	2	2	2	2	2	2	2	2
2	2	2	#NULL!	#NULL!	#NULL!	#NULL!	#NULL!	#NULL!	#NULL!
#NULL!	#NULL!	#NULL!	#NULL!	#NULL!	#NULL!	#NULL!	#NULL!	#NULL!	#NULL!
2	2	#NULL!	#NULL!	2	#NULL!	#NULL!	#NULL!	#NULL!	#NULL!
2	2	2	2	2	2	2	2	2	2
2	2	2	2	2	2	2	2	2	2
2	1	2	2	2	2	2	2	2	2
2	2	2	2	2	2	2	2	2	2
2	2	2	2	2	2	2	2	2	2
1	1	2	2	2	2	2	1	2	1
2	2	2	2	2	2	2	2	2	2
2	2	2	2	2	2	2	2	2	2
2	1	2	2	2	2	2	2	2	2
2	2	2	2	2	2	2	2	2	2
2	2	2	2	2	2	2	2	2	2
2	1	2	2	2	2	2	1	2	1
2	2	2	2	2	2	2	2	2	2
2	2	2	2	2	2	2	2	2	2
2	1	2	2	2	2	2	2	2	2
#NULL!	#NULL!	#NULL!	#NULL!	#NULL!	#NULL!	#NULL!	#NULL!	#NULL!	#NULL!
2	2	2	2	2	2	2	2	2	2
2	2	2	2	2	2	2	2	2	2
1	2	2	2	2	2	2	2	2	2
2	2	2	2	2	2	2	2	2	2
2	2	2	2	2	2	2	2	2	2
2	2	2	2	2	2	2	2	2	2
2	2	2	2	2	2	2	2	2	2
2	2	2	2	2	2	2	2	2	2
2	2	2	2	2	2	2	2	2	2
2	1	2	2	2	2	2	2	2	2
2	2	2	2	2	2	2	2	2	2
2	2	2	2	2	2	2	2	2	2
2	2	2	2	2	2	2	2	2	2
2	2	2	2	2	2	2	2	2	2
2	2	2	2	2	2	2	2	2	2
2	2	2	2	2	2	2	2	2	2
2	2	2	2	2	2	2	2	2	2

1	2	2	2	2	2	2	2	2	2
2	2	2	2	2	2	2	2	2	2
2	2	2	2	2	2	2	2	2	2
2	1	2	2	2	2	2	2	2	2
2	2	2	2	2	2	2	2	2	2
1	2	2	2	2	2	2	2	2	2
2	2	#NULL!	#NULL!	#NULL!	#NULL!	#NULL!	#NULL!	#NULL!	#NULL!
2	2	2	2	2	2	2	1	2	2
1	1	2	2	2	2	2	1	2	2
2	2	2	2	2	2	2	2	2	2
2	2	2	2	2	2	2	2	2	2
2	1	2	2	2	2	2	2	2	2
2	2	2	2	2	2	2	2	2	2
2	2	2	2	2	#NULL!	#NULL!	#NULL!	#NULL!	#NULL!
2	1	2	2	2	2	2	2	2	2
2	1	2	2	2	2	2	2	2	2
2	1	2	2	2	2	1	2	2	2
2	2	2	2	2	2	2	2	2	2
2	2	2	1	2	2	1	1	2	1
1	1	2	1	2	2	2	2	2	2
1	1	2	1	2	2	2	2	1	2
1	1	2	2	2	2	#NULL!	1	2	2
2	2	2	2	2	2	2	2	2	2
2	2	2	2	2	2	2	2	2	2
#NULL!	#NULL!	2	2	2	2	2	2	2	2
2	2	2	2	2	2	2	2	2	2
#NULL!	#NULL!	#NULL!	#NULL!	#NULL!	#NULL!	#NULL!	#NULL!	#NULL!	#NULL!
2	2	2	2	2	2	2	2	2	2
2	2	2	2	2	2	2	2	2	2
2	2	2	2	2	2	2	2	2	2
2	2	2	2	2	2	2	2	2	2
1	1	2	2	2	2	1	1	2	2
#NULL!	#NULL!	#NULL!	#NULL!	#NULL!	#NULL!	#NULL!	#NULL!	#NULL!	#NULL!
1	1	#NULL!	#NULL!	#NULL!	#NULL!	#NULL!	#NULL!	#NULL!	#NULL!
#NULL!	#NULL!	#NULL!	#NULL!	#NULL!	#NULL!	#NULL!	#NULL!	#NULL!	#NULL!
#NULL!	#NULL!	#NULL!	#NULL!	#NULL!	#NULL!	#NULL!	#NULL!	#NULL!	#NULL!
2	2	2	2	2	2	2	2	1	2
2	1	2	2	2	1	2	2	1	2
2	2	2	2	2	2	2	2	2	2
1	2	2	2	2	2	2	2	2	2
2	2	2	2	2	1	2	2	2	2
2	2	2	2	2	2	2	2	2	2
2	2	2	1	2	2	2	1	2	1
2	2	2	2	2	2	2	2	2	2
2	1	2	2	2	2	2	2	2	2
2	2	2	2	2	2	2	2	2	2
2	2	2	1	2	2	2	1	2	1

2	1	2	1	1	2	2	2	2	2
#NULL!	#NULL!	1	2	2	2	2	2	2	#NULL!
2	2	1	2	2	#NULL!	1	2	2	2
2	2	2	2	2	2	1	2	2	2
1	2	2	2	2	1	2	2	2	1
2	2	2	2	2	2	2	2	1	1
2	2	2	1	2	2	2	2	2	2
2	2	2	#NULL!	#NULL!	#NULL!	#NULL!	#NULL!	#NULL!	#NULL!
2	2	2	2	2	2	2	2	2	2
2	2	2	2	2	2	2	2	2	2
2	2	2	2	2	2	2	2	2	2
2	2	2	2	2	2	2	2	2	2
#NULL!	#NULL!	2	2	2	2	2	2	2	2
2	#NULL!	2	2	2	2	2	1	2	2
2	1	2	2	2	2	2	2	2	2
2	2	2	2	2	2	2	2	2	2
2	2	2	2	2	2	2	2	2	2
2	1	2	2	1	2	2	1	2	2
1	1	2	2	2	2	2	2	2	2
1	2	2	2	2	2	2	2	2	2
2	2	2	2	1	1	2	2	1	2
2	2	2	2	2	2	2	2	2	2
2	2	2	2	2	2	2	2	2	2
2	2	#NULL!	#NULL!	#NULL!	#NULL!	#NULL!	#NULL!	#NULL!	#NULL!
1	1	2	1	1	2	2	1	2	1
2	2	2	2	2	2	2	2	2	2
2	2	2	2	2	2	2	2	2	2
2	1	2	2	2	2	2	2	2	2
2	2	2	2	2	2	2	2	2	2
1	1	2	#NULL!	#NULL!	#NULL!	#NULL!	#NULL!	#NULL!	#NULL!
1	1	2	2	2	2	2	2	1	2
2	2	#NULL!	#NULL!	#NULL!	#NULL!	#NULL!	#NULL!	#NULL!	#NULL!
#NULL!	#NULL!	#NULL!	#NULL!	#NULL!	#NULL!	#NULL!	#NULL!	#NULL!	#NULL!
#NULL!	#NULL!	#NULL!	#NULL!	#NULL!	#NULL!	#NULL!	#NULL!	#NULL!	#NULL!
#NULL!	#NULL!	#NULL!	#NULL!	#NULL!	#NULL!	#NULL!	#NULL!	#NULL!	#NULL!
2	2	2	2	2	2	2	2	2	2
1	1	2	2	2	1	2	2	1	2
#NULL!	#NULL!	#NULL!	#NULL!	#NULL!	#NULL!	#NULL!	#NULL!	#NULL!	#NULL!
2	2	2	2	2	2	2	2	2	2
2	2	2	2	2	2	2	2	2	2
1	1	2	2	2	2	2	2	2	2
2	2	2	2	2	2	2	2	2	2
2	1	2	2	2	2	2	2	2	2
2	2	1	2	1	2	2	2	2	2
#NULL!	#NULL!	1	2	#NULL!	#NULL!	#NULL!	#NULL!	#NULL!	#NULL!
1	1	2	1	2	2	1	1	2	1
2	2	1	2	1	1	2	2	1	2

2	2	2	1	2	2	1	1	2	1
2	2	2	2	2	2	2	2	2	2
2	2	2	1	2	2	1	1	2	1
2	1	2	2	2	2	2	2	2	2
#NULL!	#NULL!	#NULL!	#NULL!	#NULL!	#NULL!	#NULL!	#NULL!	#NULL!	#NULL!
#NULL!	#NULL!	#NULL!	#NULL!	#NULL!	#NULL!	#NULL!	#NULL!	#NULL!	#NULL!
2	2	2	2	2	2	2	2	2	2
2	2	2	2	2	2	2	2	2	2
2	1	2	#NULL!	#NULL!	#NULL!	#NULL!	#NULL!	#NULL!	#NULL!
2	2	#NULL!	#NULL!	#NULL!	#NULL!	#NULL!	#NULL!	#NULL!	#NULL!
1	1	2	1	2	2	1	2	2	2
2	2	2	2	2	2	1	1	2	1
2	2	2	2	2	2	1	1	1	2
2	2	2	2	2	1	2	2	1	2
#NULL!	#NULL!	#NULL!	#NULL!	#NULL!	#NULL!	2	#NULL!	#NULL!	#NULL!
2	2	2	2	2	1	1	2	2	2
2	2	2	2	2	2	2	1	2	2
2	1	2	2	1	2	1	2	2	2
2	2	#NULL!	2	#NULL!	#NULL!	#NULL!	#NULL!	#NULL!	#NULL!
1	2	2	2	1	2	2	1	2	2
2	2	2	2	2	2	2	1	1	1
2	1	2	2	2	2	1	1	2	1
#NULL!	#NULL!	2	2	2	2	2	1	2	2
2	2	2	2	2	2	2	2	2	2
2	2	1	1	2	2	1	2	1	2
2	2	2	2	2	2	2	2	2	2
2	1	2	2	2	2	2	1	2	2
2	2	1	2	2	2	2	2	2	2
2	2	2	2	1	2	2	2	1	1
2	2	2	2	1	1	1	2	1	#NULL!
2	2	2	2	2	2	2	2	2	2
2	2	2	1	2	2	2	2	2	2
2	2	2	2	2	1	2	2	2	2
1	1	2	2	2	2	2	1	2	2
2	2	#NULL!	#NULL!	2	#NULL!	#NULL!	#NULL!	#NULL!	#NULL!
2	2	2	2	2	2	1	2	2	2
2	2	#NULL!	#NULL!	#NULL!	#NULL!	#NULL!	#NULL!	#NULL!	#NULL!
2	2	2	2	2	2	2	2	2	2
1	2	2	2	2	2	2	2	2	2
#NULL!	#NULL!	2	2	2	2	1	2	2	2
#NULL!	#NULL!	#NULL!	#NULL!	#NULL!	#NULL!	#NULL!	#NULL!	#NULL!	#NULL!
1	2	2	1	1	2	2	2	2	2
2	2	2	2	2	2	2	2	1	2
2	2	2	2	2	2	2	2	2	2
2	2	2	2	2	2	2	2	2	2
#NULL!	#NULL!	2	2	1	2	2	2	1	2
2	1	1	2	2	1	2	2	1	2

2	2	2	2	2	2	2	2	1	2
2	2	1	2	1	1	2	2	1	2
2	2	2	2	2	2	2	2	2	2
2	2	1	2	1	1	2	2	1	2
2	2	2	1	2	2	2	1	2	2
#NULL!	#NULL!	#NULL!	#NULL!	#NULL!	#NULL!	#NULL!	#NULL!	#NULL!	#NULL!
#NULL!	#NULL!	#NULL!	#NULL!	#NULL!	#NULL!	#NULL!	#NULL!	#NULL!	#NULL!
2	2	2	2	2	2	2	2	2	2
1	1	2	2	2	2	2	2	1	2
2	2	2	2	2	2	2	2	2	2
2	2	2	2	2	2	1	2	2	2
2	2	2	2	1	2	1	2	2	1
2	2	2	2	2	1	2	2	2	2
2	2	2	2	2	2	2	2	2	1
#NULL!	#NULL!	#NULL!	#NULL!	#NULL!	#NULL!	#NULL!	#NULL!	#NULL!	#NULL!
2	2	2	2	1	1	2	2	1	2
1	1	2	2	2	2	2	2	2	2
2	2	2	2	2	2	2	2	2	2
1	1	2	2	2	2	2	2	2	1
1	2	2	2	2	2	2	2	2	2
#NULL!	#NULL!	#NULL!	#NULL!	#NULL!	#NULL!	#NULL!	#NULL!	#NULL!	#NULL!
#NULL!	#NULL!	#NULL!	#NULL!	#NULL!	#NULL!	#NULL!	#NULL!	#NULL!	2
2	2	2	2	2	2	2	2	2	2
2	2	2	#NULL!	#NULL!	#NULL!	#NULL!	#NULL!	#NULL!	#NULL!
#NULL!	#NULL!	#NULL!	#NULL!	#NULL!	#NULL!	#NULL!	#NULL!	#NULL!	#NULL!
2	2	1	#NULL!	2	2	2	2	1	2
2	2	2	2	1	2	2	2	2	2
2	2	2	2	2	2	2	2	2	2
2	2	1	2	1	1	2	2	1	2
2	2	2	2	2	2	2	2	2	2
2	2	1	2	1	1	2	2	1	2
2	2	2	2	2	2	2	2	2	2
#NULL!	#NULL!	#NULL!	#NULL!	#NULL!	#NULL!	#NULL!	#NULL!	#NULL!	#NULL!
2	2	#NULL!	#NULL!	#NULL!	#NULL!	#NULL!	#NULL!	#NULL!	#NULL!
#NULL!	#NULL!	#NULL!	#NULL!	#NULL!	#NULL!	#NULL!	#NULL!	#NULL!	#NULL!
2	2	2	2	2	2	2	2	2	2
1	1	2	2	1	1	2	1	2	2
2	2	1	2	2	2	2	1	2	1
2	2	2	2	2	2	2	2	2	2
1	2	2	#NULL!	#NULL!	#NULL!	#NULL!	#NULL!	#NULL!	#NULL!
2	2	2	2	2	2	2	2	2	2
2	2	2	2	2	2	2	1	2	2
2	2	1	2	1	2	2	2	2	2
2	2	#NULL!	#NULL!	#NULL!	#NULL!	#NULL!	#NULL!	#NULL!	#NULL!
1	1	1	1	2	1	2	2	2	1
2	2	#NULL!	#NULL!	#NULL!	#NULL!	#NULL!	#NULL!	#NULL!	#NULL!
2	2	1	2	2	2	2	2	1	2

1	2	2	2	2	2	1	2	2	2
2	2	2	2	1	1	2	2	2	2
2	2	1	2	1	1	1	1	1	2
1	2	2	2	2	2	2	1	2	2
2	2	2	2	2	2	2	2	2	2
2	1	2	2	2	2	2	2	2	2
2	1	2	2	1	2	2	2	2	2
2	2	2	2	2	2	2	2	1	2
#NULL!	#NULL!	#NULL!	#NULL!	#NULL!	#NULL!	#NULL!	#NULL!	#NULL!	#NULL!
1	1	2	2	2	2	2	2	2	2
2	2	2	2	2	1	2	2	1	2
2	2	2	2	2	2	2	2	2	2
2	1	2	2	2	2	2	2	2	2
2	1	2	2	2	2	2	1	2	2
1	2	#NULL!	2	#NULL!	#NULL!	#NULL!	#NULL!	#NULL!	#NULL!
2	2	2	2	1	1	2	2	2	2
2	2	2	2	2	2	1	1	1	2
2	2	2	2	1	2	2	2	2	2
2	2	2	2	2	2	1	2	2	1
2	2	2	2	2	2	2	2	2	2
1	2	2	2	2	2	1	1	2	2
2	2	2	2	2	2	2	2	2	2
2	2	2	2	2	2	2	1	2	1
2	2	2	2	2	2	2	2	2	2
2	2	2	2	2	2	2	2	2	2
2	2	2	2	2	1	2	2	1	2
2	2	2	2	1	2	2	2	2	2
2	2	2	2	2	2	2	2	2	2
2	2	2	2	2	2	2	2	2	2
2	2	2	2	2	2	2	2	2	2
2	2	2	2	2	2	#NULL!	1	2	2
2	1	2	#NULL!	1	2	#NULL!	1	2	2
2	1	2	2	2	2	2	2	2	2
2	2	2	1	2	2	2	1	2	2
2	1	2	2	1	2	2	2	2	2
2	2	2	2	2	2	2	2	2	2
2	2	2	2	2	2	2	2	2	1
#NULL!	#NULL!	2	1	2	2	2	1	2	1
2	2	2	2	2	2	2	2	2	2
1	1	2	1	2	2	1	1	2	1
2	2	2	2	1	1	2	2	1	2
1	2	2	1	2	2	1	1	2	1
2	2	2	1	2	2	2	1	2	2
2	2	2	2	2	2	1	1	2	1
1	2	2	2	2	2	2	1	2	1
1	1	2	2	2	2	1	1	2	2
2	1	2	2	1	1	2	1	2	1

2	2	2	1	2	2	2	1	2	1
2	1	2	2	2	2	2	2	2	1
1	2	2	2	2	2	2	1	2	2
1	2	2	2	2	1	2	1	2	2
1	2	2	1	2	2	1	2	2	1
2	1	1	1	2	2	2	2	2	1
1	2	2	2	2	1	2	1	2	2
1	1	2	2	2	2	1	2	2	1
2	2	2	2	2	2	2	2	2	2
2	2	2	2	2	2	2	2	2	2
2	2	2	2	2	2	2	2	2	2
1	2	#NULL!	1	1	2	2	2	2	2
1	2	2	2	1	2	1	2	2	2
2	2	2	2	2	1	2	1	2	2
2	2	2	2	2	2	2	2	2	2
2	2	2	1	2	2	1	1	2	1
2	2	2	2	2	2	2	2	2	2
2	2	2	2	2	2	2	2	2	2
2	2	2	2	2	2	2	2	2	2
2	2	2	2	1	2	1	2	2	2
2	2	2	2	2	2	2	2	2	2
2	2	2	2	2	2	2	2	2	2
2	2	1	#NULL!	2	#NULL!	1	2	2	2
#NULL!	#NULL!	#NULL!	#NULL!	#NULL!	#NULL!	#NULL!	#NULL!	#NULL!	#NULL!
2	2	2	1	2	2	2	1	2	#NULL!
2	2	1	2	1	1	2	2	1	2
1	1	2	2	2	1	2	2	2	2
#NULL!	2	1	2	2	1	2	2	2	1
2	1	2	1	2	2	2	2	2	1
2	2	1	2	2	2	2	2	2	2
2	1	2	2	2	1	2	1	2	1
1	1	2	1	2	2	2	2	2	2
1	2	2	1	2	2	2	2	2	1
2	2	2	2	2	2	2	2	2	2
1	2	2	2	2	1	2	2	1	2
2	2	2	2	1	1	2	2	2	2
1	2	2	2	2	2	1	2	2	2
2	2	1	2	1	1	2	2	1	2
1	1	2	1	2	2	1	1	2	2
2	1	2	1	2	2	1	1	2	1
2	2	2	2	2	2	2	2	2	2
2	2	2	1	2	2	1	1	1	2
2	2	2	2	2	2	2	2	2	2
2	2	1	2	1	1	2	2	1	2
2	2	1	2	1	1	2	2	1	2
2	2	2	2	2	2	2	2	2	2
1	2	2	2	2	2	1	2	2	1

1	1	2	2	2	2	1	2	1	2
2	1	2	2	2	2	1	1	2	2
1	1	2	2	2	2	2	2	2	1
2	2	2	2	2	2	1	2	2	2
1	2	2	2	2	1	2	2	2	1
2	2	2	1	2	2	1	1	2	2
2	2	1	2	1	1	2	2	2	1
2	2	2	1	2	2	2	1	2	2
1	1	2	1	2	2	2	1	2	1
1	2	2	2	2	2	1	1	2	2
1	2	2	2	2	2	1	1	2	2
2	2	2	2	2	2	1	2	2	1
2	2	2	1	2	2	1	1	2	2
2	2	2	2	2	2	2	1	2	2
2	2	2	1	2	2	1	1	2	1
2	2	2	1	2	2	1	2	2	1
2	2	2	2	2	2	2	2	2	2
2	2	2	2	2	2	1	1	2	2
2	2	2	2	2	2	2	#NULL!	2	#NULL!
2	2	2	2	2	2	2	2	2	2
2	2	2	#NULL!	#NULL!	2	#NULL!	#NULL!	#NULL!	2
2	2	1	2	1	1	2	2	1	2
2	2	2	1	2	2	2	1	2	1
2	2	2	1	2	2	2	1	2	1
2	2	2	1	2	1	2	1	2	2
2	2	2	2	2	2	1	2	2	1
2	1	2	1	2	2	2	2	2	1
2	2	2	2	2	2	1	2	2	1
1	1	2	1	2	2	1	1	2	2
1	1	2	1	2	2	2	1	2	1
2	2	2	2	2	2	2	2	2	1
2	2	2	2	2	2	2	2	2	2
2	2	2	2	1	2	2	2	1	2
2	2	2	2	2	2	2	2	2	2
2	2	2	2	2	2	1	2	2	1
2	2	2	2	2	1	2	2	2	2
2	2	2	#NULL!	2	#NULL!	2	1	1	#NULL!
2	2	2	#NULL!	2	#NULL!	2	#NULL!	2	#NULL!
#NULL!	#NULL!	#NULL!	#NULL!	#NULL!	#NULL!	#NULL!	#NULL!	#NULL!	#NULL!
2	2	2	#NULL!	#NULL!	#NULL!	1	#NULL!	1	1
2	2	#NULL!	1	#NULL!	#NULL!	#NULL!	2	#NULL!	#NULL!
1	2	2	1	2	2	2	1	2	2
2	2	2	#NULL!	#NULL!	2	#NULL!	#NULL!	2	#NULL!
2	2	2	2	2	2	2	2	2	2
2	2	2	#NULL!	2	#NULL!	#NULL!	1	#NULL!	#NULL!
2	2	2	#NULL!	#NULL!	2	#NULL!	#NULL!	2	#NULL!
2	2	2	#NULL!	#NULL!	#NULL!	2	#NULL!	#NULL!	#NULL!

2	2	2	#NULL!	#NULL!	#NULL!	2	#NULL!	#NULL!	2
2	2	#NULL!	2	#NULL!	#NULL!	#NULL!	2	#NULL!	#NULL!
2	2	2	#NULL!	#NULL!	2	#NULL!	#NULL!	2	#NULL!
#NULL!	#NULL!	#NULL!	#NULL!	#NULL!	#NULL!	#NULL!	#NULL!	#NULL!	#NULL!
2	2	2	#NULL!	2	#NULL!	#NULL!	2	2	#NULL!
2	2	#NULL!	#NULL!	2	#NULL!	#NULL!	#NULL!	#NULL!	2
#NULL!	#NULL!	#NULL!	#NULL!	#NULL!	#NULL!	#NULL!	#NULL!	#NULL!	#NULL!
2	2	2	#NULL!	#NULL!	#NULL!	#NULL!	#NULL!	#NULL!	#NULL!
2	2	2	2	1	2	1	1	2	1
2	2	2	#NULL!	2	#NULL!	2	#NULL!	2	#NULL!
2	2	2	2	2	2	1	2	2	2
2	2	2	2	2	2	2	2	2	1
2	2	2	2	2	2	1	2	2	1
2	2	2	2	2	2	1	2	2	1
2	1	2	2	2	2	2	1	2	2
2	2	2	2	2	2	2	1	2	2
2	2	2	2	#NULL!	2	2	2	2	2
2	2	1	2	#NULL!	2	2	1	2	2
2	2	2	2	2	1	2	1	2	2
1	2	2	2	2	2	2	1	2	2
2	#NULL!	2	2	2	2	2	1	2	2
1	2	2	2	2	1	2	2	2	2
1	2	2	2	2	2	2	2	1	2
1	2	2	1	2	2	1	2	2	2
2	1	2	2	2	1	2	2	2	2
2	2	1	2	2	1	2	2	2	2
2	2	2	#NULL!	2	#NULL!	2	2	2	#NULL!
2	2	1	2	1	1	2	2	2	2
2	1	2	2	1	2	1	2	1	2
2	2	2	2	2	2	2	2	2	2
2	2	2	#NULL!	#NULL!	#NULL!	#NULL!	#NULL!	#NULL!	#NULL!
2	2	1	2	1	1	2	2	1	2
2	1	2	2	2	2	2	1	2	2
2	2	2	2	2	2	2	2	2	2
1	2	2	1	2	2	1	1	2	2
2	2	1	2	1	1	2	2	1	2
2	2	2	2	1	2	2	2	2	2
2	2	2	2	2	2	2	2	2	2
2	2	1	2	1	1	2	2	1	2
2	2	2	2	2	2	2	2	2	2
2	2	2	2	2	2	1	2	2	1
2	2	1	2	1	1	2	2	1	2
1	2	1	2	2	2	2	1	2	2
2	2	1	2	2	2	2	2	2	1
1	2	2	1	2	2	1	1	2	2
1	2	2	1	2	2	2	2	2	1
1	1	2	2	2	2	1	2	2	1

2	1	2	2	2	2	2	2	2	2
1	2	2	2	1	2	2	2	2	2
1	2	2	2	2	2	2	1	2	2
2	2	2	2	2	1	2	2	2	2
2	2	2	1	1	2	1	2	2	2
2	2	2	2	2	2	2	1	2	2
1	2	2	2	2	2	2	2	2	2
1	1	2	2	2	2	2	2	2	2
2	2	2	2	2	2	2	2	2	2
2	2	2	2	2	2	2	2	2	2
1	2	1	2	2	1	2	2	2	2
2	2	2	2	2	2	2	2	2	2
2	1	1	1	2	2	2	1	1	1
2	2	1	2	2	1	2	1	2	1
2	2	2	1	2	2	2	2	2	2
2	2	2	2	2	2	2	2	2	2
2	2	2	2	2	2	2	2	2	2
2	2	2	2	2	2	2	2	2	2
2	2	1	2	2	1	2	1	1	2
2	2	2	2	2	2	2	2	2	2
1	1	2	1	2	2	2	2	2	2
2	2	2	2	2	2	2	2	2	2
2	2	2	2	2	2	2	2	2	1
2	2	2	2	1	2	2	2	1	2
1	1	2	2	2	2	2	2	2	2
2	2	2	2	2	2	2	2	2	2
2	2	2	2	2	2	1	2	1	2
1	1	2	2	2	2	2	1	2	2
1	1	2	1	2	2	2	2	2	2
2	2	2	2	2	2	2	2	2	2
2	2	2	2	2	2	2	2	2	2
1	2	2	2	2	2	2	2	2	2
1	2	2	2	2	2	2	2	2	2
2	2	2	2	2	2	2	2	2	2
2	2	2	2	2	2	2	2	2	2
2	2	2	2	2	2	2	2	2	2
2	2	2	2	2	2	2	1	2	2
2	2	2	2	2	2	2	2	2	2
2	2	2	2	2	1	2	2	1	2
2	2	1	2	2	1	2	2	2	2
1	2	2	2	2	2	2	1	2	2
2	2	2	2	2	2	2	2	2	2
2	2	2	2	2	2	2	2	2	2
2	2	2	2	2	2	2	2	2	2
2	2	2	2	2	2	2	2	2	2
2	2	2	2	2	2	2	2	2	2
2	2	2	2	2	2	2	2	2	2

2	2	2	2	2	2	2	2	2	2
2	1	2	1	#NULL!	2	2	2	2	2
2	2	2	2	2	2	2	2	2	2
2	2	2	2	2	2	2	2	2	2
2	2	2	2	2	2	2	2	2	2
2	1	2	2	2	2	2	2	2	2
2	2	2	2	2	2	2	2	2	2
#NULL!	#NULL!	2	2	2	2	2	1	1	2
1	1	2	2	2	2	2	2	2	2
#NULL!	#NULL!	#NULL!	#NULL!	#NULL!	#NULL!	#NULL!	#NULL!	#NULL!	#NULL!
2	2	2	2	2	2	2	2	1	1
2	1	2	1	2	2	2	1	2	1
2	2	2	2	2	2	2	1	2	2
2	2	2	2	2	2	2	2	2	2
2	2	2	2	2	2	2	2	2	2
2	2	2	2	2	2	1	2	2	2
2	1	2	2	2	2	2	2	2	2
2	1	2	2	2	2	2	2	2	2
2	2	1	2	1	1	2	2	1	2
2	1	1	2	#NULL!	2	1	1	2	1
#NULL!	#NULL!	1	2	1	1	2	2	1	2
2	2	1	2	1	1	2	2	1	2
2	2	2	2	2	2	2	2	2	2
1	2	2	1	2	2	2	2	2	2
1	2	2	2	2	2	2	2	2	2
2	1	2	1	2	2	2	2	2	1
2	1	1	2	1	1	1	2	2	2
1	2	2	1	2	2	2	2	2	2
1	2	2	1	2	2	1	1	2	1
2	2	2	2	2	#NULL!	#NULL!	#NULL!	#NULL!	#NULL!
2	1	2	2	2	2	1	2	2	1
2	2	2	2	2	1	2	2	1	2
2	2	2	2	2	2	2	2	2	2
2	2	1	2	1	1	2	2	1	2
2	2	2	2	2	2	2	2	2	2
2	2	2	2	1	2	2	2	1	2
2	2	2	2	1	2	2	2	1	2
2	2	1	2	2	2	2	2	2	2
2	2	2	2	2	2	2	2	2	2
2	1	2	2	2	2	2	2	1	2
2	2	2	1	2	2	2	2	2	2
1	2	1	2	1	1	2	2	1	2
2	2	2	2	2	2	2	2	2	2
2	2	1	2	1	1	2	2	1	2
2	2	1	2	1	1	2	2	1	2
2	1	2	2	2	1	2	1	2	2
2	1	2	1	2	2	2	1	2	1

2	2	2	1	2	2	2	2	2	2
2	2	2	2	2	2	2	2	1	2
2	2	2	2	2	2	2	2	2	2
2	2	2	2	2	2	2	2	2	2
2	2	1	2	1	1	2	2	1	2
1	1	#NULL!	#NULL!	#NULL!	#NULL!	2	#NULL!	#NULL!	#NULL!
2	2	2	2	2	2	2	2	2	2
2	2	2	2	2	2	2	2	2	2
2	2	2	2	2	2	2	2	2	2
2	2	2	2	1	1	2	2	1	2
2	1	2	2	2	1	2	1	2	1
2	2	2	2	2	2	2	2	2	2
2	2	2	#NULL!	#NULL!	#NULL!	#NULL!	#NULL!	#NULL!	#NULL!
2	2	#NULL!	#NULL!	2	#NULL!	#NULL!	#NULL!	#NULL!	#NULL!
2	2	#NULL!	#NULL!	2	#NULL!	#NULL!	#NULL!	#NULL!	#NULL!
2	2	2	2	2	2	2	2	2	2
1	1	2	2	2	2	2	2	2	2
2	2	2	#NULL!	#NULL!	#NULL!	#NULL!	#NULL!	#NULL!	#NULL!
1	1	2	2	2	2	2	2	2	2
2	2	2	2	2	2	2	2	2	2
2	2	1	1	2	2	1	2	2	2
1	1	2	2	2	2	2	2	2	2
1	1	2	2	2	2	2	2	2	2
2	2	2	2	2	2	2	2	2	2
2	2	2	2	2	2	2	2	2	2
2	2	2	#NULL!	2	2	2	2	2	2
2	2	2	2	2	2	2	2	2	2
2	2	2	2	2	2	2	2	2	2
2	2	#NULL!	#NULL!	#NULL!	#NULL!	#NULL!	#NULL!	#NULL!	#NULL!
1	1	2	2	1	1	2	2	1	2
2	2	2	2	2	2	2	2	2	2
1	2	2	1	1	2	1	2	2	1
1	2	2	1	2	2	1	2	2	2
2	2	1	2	1	1	2	2	2	2
2	2	2	2	2	2	2	2	2	2
1	1	2	2	2	2	2	2	2	1
2	2	2	2	2	2	1	2	1	2
2	2	2	2	2	2	2	2	2	2
2	2	2	2	2	2	2	2	2	2
2	2	2	2	2	2	2	2	2	2
2	2	2	1	2	2	1	1	2	1
2	2	2	2	2	2	1	2	2	2
2	2	2	2	2	1	2	2	2	2
2	2	2	2	2	2	2	2	2	2
2	2	2	2	2	2	2	2	2	2
2	2	2	2	2	2	2	2	2	2
2	2	2	2	2	2	2	2	2	2

1	1	2	2	2	2	2	2	2	2
1	2	1	2	2	2	1	1	1	2
2	2	#NULL!	#NULL!	#NULL!	#NULL!	#NULL!	#NULL!	#NULL!	#NULL!
1	2	2	1	1	2	2	2	2	2
2	1	2	2	1	2	1	1	2	2
2	2	2	2	2	2	2	2	2	2
2	2	2	2	2	2	2	2	2	2
2	2	2	2	2	2	2	2	2	2
2	1	2	2	2	1	2	2	2	2
2	2	1	2	2	2	2	2	2	2
2	2	2	2	2	2	2	2	2	2
2	2	1	2	2	2	2	2	2	2
2	2	2	2	2	2	2	1	2	2
2	2	2	2	2	2	2	2	2	2
2	2	2	2	2	#NULL!	2	2	2	2
1	2	2	2	2	1	2	2	2	2
#NULL!	#NULL!	#NULL!	#NULL!	#NULL!	#NULL!	#NULL!	#NULL!	#NULL!	#NULL!
2	2	#NULL!	#NULL!	#NULL!	#NULL!	#NULL!	#NULL!	#NULL!	#NULL!
2	2	2	2	2	2	2	1	2	2
1	2	2	2	2	2	1	1	2	1
2	2	2	2	2	2	2	2	2	2
#NULL!	#NULL!	#NULL!	#NULL!	#NULL!	#NULL!	#NULL!	#NULL!	#NULL!	#NULL!
1	1	1	2	1	2	1	1	2	1
2	1	1	2	2	1	1	2	2	1
2	2	2	2	2	2	2	2	2	2
2	2	2	#NULL!	#NULL!	#NULL!	#NULL!	#NULL!	#NULL!	#NULL!
2	2	2	#NULL!	#NULL!	#NULL!	#NULL!	#NULL!	#NULL!	#NULL!
2	2	1	2	1	2	2	1	2	2
#NULL!	#NULL!	#NULL!	1	#NULL!	#NULL!	#NULL!	#NULL!	#NULL!	#NULL!
1	1	#NULL!	#NULL!	#NULL!	#NULL!	1	#NULL!	#NULL!	#NULL!
#NULL!	#NULL!	#NULL!	#NULL!	#NULL!	#NULL!	2	#NULL!	#NULL!	#NULL!
#NULL!	#NULL!	#NULL!	#NULL!	#NULL!	#NULL!	#NULL!	#NULL!	#NULL!	#NULL!
2	2	2	#NULL!	#NULL!	#NULL!	#NULL!	#NULL!	#NULL!	#NULL!
1	1	2	1	2	2	2	2	1	1
1	1	1	2	1	1	2	1	1	2
1	1	2	2	2	2	#NULL!	2	2	1
1	1	2	2	2	2	2	2	2	1
1	1	2	2	1	1	2	2	2	2
2	#NULL!	2	1	2	2	2	2	2	2
2	2	2	2	2	2	2	1	2	1
1	2	2	2	2	2	2	2	2	2
1	1	2	2	1	1	2	2	2	2
2	2	2	2	2	2	2	2	2	2
2	2	2	2	2	2	2	2	2	2
2	2	2	2	2	2	2	2	2	2
2	2	2	2	2	2	2	2	2	2
2	2	2	2	2	2	1	2	2	2

1	2	2	2	2	2	1	#NULL!	2	2
2	1	2	#NULL!	2	2	1	2	2	2
2	2	2	2	1	2	2	2	1	2
#NULL!	#NULL!	#NULL!	#NULL!	#NULL!	#NULL!	#NULL!	#NULL!	#NULL!	#NULL!
2	2	#NULL!	#NULL!	#NULL!	#NULL!	#NULL!	#NULL!	#NULL!	#NULL!
2	2	2	2	2	#NULL!	2	2	1	2
2	2	2	2	2	2	2	2	2	2
#NULL!	#NULL!	#NULL!	#NULL!	#NULL!	#NULL!	#NULL!	#NULL!	#NULL!	#NULL!
#NULL!	#NULL!	#NULL!	#NULL!	#NULL!	#NULL!	#NULL!	#NULL!	#NULL!	#NULL!
2	1	2	2	2	2	2	2	2	2
2	2	2	2	2	2	2	2	2	2
2	2	1	2	#NULL!	2	2	2	2	2
2	1	2	2	2	2	1	1	2	2
#NULL!	#NULL!	#NULL!	#NULL!	#NULL!	#NULL!	#NULL!	#NULL!	#NULL!	#NULL!
#NULL!	#NULL!	#NULL!	#NULL!	#NULL!	#NULL!	#NULL!	#NULL!	#NULL!	#NULL!
1	2	#NULL!	#NULL!	#NULL!	#NULL!	#NULL!	#NULL!	#NULL!	#NULL!
2	2	2	2	2	2	2	2	2	2
2	2	2	1	#NULL!	#NULL!	#NULL!	#NULL!	#NULL!	#NULL!
2	1	#NULL!	#NULL!	#NULL!	#NULL!	#NULL!	#NULL!	#NULL!	#NULL!
#NULL!	#NULL!	#NULL!	#NULL!	#NULL!	#NULL!	#NULL!	#NULL!	#NULL!	#NULL!
2	2	#NULL!	1	#NULL!	#NULL!	#NULL!	#NULL!	#NULL!	#NULL!
#NULL!	#NULL!	#NULL!	#NULL!	#NULL!	#NULL!	#NULL!	#NULL!	#NULL!	#NULL!
#NULL!	#NULL!	#NULL!	#NULL!	#NULL!	#NULL!	#NULL!	#NULL!	#NULL!	#NULL!
#NULL!	#NULL!	#NULL!	#NULL!	#NULL!	#NULL!	#NULL!	#NULL!	#NULL!	#NULL!
2	2	2	2	2	2	2	2	2	2
2	2	2	2	2	2	2	2	2	2
2	2	2	2	1	2	2	2	2	2
2	2	2	2	2	2	2	2	2	2
2	1	2	2	2	1	2	2	2	2
2	2	2	2	2	2	2	2	2	2
2	2	1	2	1	1	2	2	1	2
2	2	1	2	1	1	2	2	1	2
2	1	2	2	2	2	2	2	2	2
2	2	2	2	1	2	2	2	2	2
2	2	2	2	2	2	2	2	2	2
2	2	2	2	2	1	2	2	1	2
1	2	2	2	2	2	2	2	2	2
2	2	2	2	2	2	2	2	2	2
2	2	2	2	2	1	2	2	2	2
1	1	2	2	2	2	2	2	2	2
1	1	2	2	2	2	2	2	2	2
2	2	2	2	2	2	2	2	2	2
2	2	2	2	2	2	2	1	2	1
2	2	2	#NULL!	#NULL!	#NULL!	#NULL!	#NULL!	#NULL!	#NULL!
1	2	#NULL!	#NULL!	#NULL!	#NULL!	2	#NULL!	#NULL!	#NULL!
2	2	2	1	2	2	2	2	2	1
2	2	2	2	2	2	2	2	2	2

2	2	2	2	2	2	1	1	2	2
2	2	2	1	2	2	1	1	2	2
2	2	2	2	2	2	2	2	1	2
2	2	2	2	2	2	2	2	2	2
2	2	2	2	2	2	2	2	2	2
2	2	2	2	1	2	2	2	1	2
2	2	2	2	2	2	2	1	2	2
1	2	2	2	1	2	2	2	2	2
#NULL!	#NULL!	#NULL!	#NULL!	#NULL!	#NULL!	#NULL!	#NULL!	#NULL!	#NULL!
2	2	1	2	2	2	2	2	2	2
2	2	2	2	2	2	2	2	2	2
2	2	2	2	2	2	2	2	2	2
2	1	2	2	2	2	2	2	2	2
2	2	2	2	2	2	2	2	2	2
2	2	2	2	2	2	2	2	1	2
2	2	2	1	2	2	2	1	1	2
1	1	2	2	1	2	2	2	1	1
1	2	2	2	1	2	2	2	2	2
2	2	2	2	2	2	2	2	2	2
1	2	2	2	2	2	2	2	2	2
2	2	2	1	2	2	1	2	1	2
2	1	2	2	2	2	2	2	2	2
2	1	2	1	2	2	2	2	2	2
1	1	2	1	2	2	2	2	2	1
1	1	2	2	2	2	2	2	2	2
2	2	2	2	2	2	2	2	2	2
2	2	2	2	2	2	2	2	2	2
2	1	2	2	#NULL!	2	2	2	#NULL!	2
1	2	2	2	2	2	2	2	2	2
#NULL!	#NULL!	#NULL!	#NULL!	#NULL!	#NULL!	#NULL!	#NULL!	#NULL!	#NULL!
2	2	2	1	2	2	1	2	2	1
#NULL!	#NULL!	#NULL!	#NULL!	#NULL!	#NULL!	#NULL!	#NULL!	#NULL!	#NULL!
#NULL!	#NULL!	#NULL!	#NULL!	#NULL!	#NULL!	#NULL!	#NULL!	#NULL!	#NULL!
1	2	2	1	2	2	1	1	2	1
1	1	2	2	2	2	2	2	2	2
2	2	1	2	1	2	2	1	2	1
2	1	1	2	2	2	1	2	1	2
1	2	1	2	1	2	2	2	1	2
2	2	2	2	2	2	2	2	2	2
1	1	2	2	2	2	1	1	2	1
#NULL!	#NULL!	2	2	2	2	2	2	2	1
2	2	2	1	2	2	2	2	2	2
2	2	2	2	2	2	2	2	2	2
2	2	2	2	2	2	2	2	2	2
2	2	2	1	2	2	2	1	2	2
2	2	2	2	2	2	2	2	2	2
2	2	2	2	2	2	2	2	2	2

2	2	2	2	2	2	1	1	2	1
2	2	2	2	2	2	2	2	2	1
1	2	2	2	2	2	2	1	2	2
1	#NULL!	1	1	2	2	2	1	#NULL!	#NULL!
2	2	2	1	2	1	2	1	2	2
#NULL!	#NULL!	2	2	2	2	2	2	2	2
1	2	2	1	2	2	2	2	2	2
2	2	2	2	2	2	2	2	1	2
2	2	#NULL!	#NULL!	2	#NULL!	#NULL!	#NULL!	#NULL!	#NULL!
2	1	2	2	2	2	2	2	2	2
2	2	2	2	2	2	2	2	2	2
2	2	2	2	2	2	2	2	2	2
#NULL!	#NULL!	#NULL!	#NULL!	#NULL!	#NULL!	#NULL!	#NULL!	#NULL!	#NULL!
2	2	2	2	2	2	2	2	2	2
2	2	2	2	2	1	2	2	2	2
1	1	2	2	1	1	1	2	1	2
2	2	2	1	2	2	#NULL!	#NULL!	#NULL!	#NULL!
#NULL!	#NULL!	2	#NULL!	2	1	#NULL!	1	2	#NULL!
2	2	2	2	2	1	2	2	2	2
2	2	2	2	2	2	2	2	2	2
2	2	2	1	2	2	2	1	2	1
2	2	1	2	1	1	2	2	1	2
2	2	2	2	2	2	2	2	2	2
2	2	2	2	2	2	2	2	2	2
2	2	2	2	2	2	2	2	2	2
2	2	2	2	2	2	2	2	2	2
2	1	2	2	2	2	2	2	2	2
2	2	2	2	1	1	2	2	2	2
2	2	2	1	1	2	2	1	2	2
1	2	2	2	2	2	2	2	2	2
2	2	2	2	2	2	2	2	2	2
2	2	2	2	2	2	2	2	2	2
1	1	2	2	2	2	2	1	2	2
2	2	2	2	2	2	2	2	2	2
2	2	2	2	2	2	2	2	2	2
#NULL!	#NULL!	#NULL!	#NULL!	#NULL!	#NULL!	#NULL!	#NULL!	#NULL!	#NULL!
2	2	2	2	2	2	2	2	2	2
#NULL!	#NULL!	#NULL!	#NULL!	#NULL!	#NULL!	#NULL!	#NULL!	#NULL!	#NULL!
1	1	2	1	2	1	2	1	2	2
2	2	2	2	2	2	1	2	2	1
2	2	2	2	2	2	2	2	2	2
2	2	2	2	2	2	2	2	2	2
2	2	2	2	2	2	2	2	2	2
#NULL!	#NULL!	2	2	2	2	2	2	2	2
#NULL!	#NULL!	#NULL!	#NULL!	#NULL!	#NULL!	#NULL!	#NULL!	#NULL!	#NULL!
2	2	#NULL!	#NULL!	#NULL!	#NULL!	#NULL!	#NULL!	#NULL!	#NULL!
#NULL!	#NULL!	#NULL!	#NULL!	#NULL!	#NULL!	#NULL!	#NULL!	#NULL!	#NULL!

1	2	1	2	1	1	2	2	2	2
#NULL!	#NULL!	2	1	2	2	1	1	2	#NULL!
#NULL!	#NULL!	#NULL!	#NULL!	#NULL!	#NULL!	#NULL!	#NULL!	#NULL!	#NULL!
2	2	2	2	2	2	1	1	2	2
2	2	2	2	2	2	1	1	2	1
#NULL!	#NULL!	1	2	2	1	1	2	2	2
2	2	2	2	2	2	2	2	2	2
#NULL!	#NULL!	2	#NULL!	1	2	#NULL!	1	#NULL!	1
2	2	1	2	1	1	2	2	1	2
#NULL!	#NULL!	#NULL!	#NULL!	#NULL!	#NULL!	#NULL!	#NULL!	#NULL!	#NULL!
#NULL!	#NULL!	1	2	1	1	2	2	1	2
2	1	1	1	1	2	2	1	1	1
2	2	2	#NULL!	2	2	2	2	2	2
1	2	2	2	1	#NULL!	2	2	2	2
2	2	2	2	2	2	2	2	2	2
2	2	#NULL!	2	1	1	2	2	1	2
2	2	2	2	1	1	2	2	1	2
#NULL!	#NULL!	#NULL!	#NULL!	#NULL!	#NULL!	#NULL!	#NULL!	#NULL!	#NULL!
#NULL!	#NULL!	#NULL!	#NULL!	#NULL!	#NULL!	#NULL!	#NULL!	#NULL!	#NULL!
2	1	2	2	2	2	2	1	2	2
2	2	2	2	2	2	2	2	2	2
2	2	2	2	2	2	2	2	2	2
#NULL!	#NULL!	2	2	2	2	2	2	2	2
1	1	1	2	1	1	2	2	1	2
2	2	2	2	1	2	1	2	1	2
2	2	2	2	2	2	2	2	1	2
2	2	2	2	2	2	#NULL!	2	2	2
2	1	2	1	2	2	1	2	2	2
#NULL!	#NULL!	#NULL!	#NULL!	#NULL!	#NULL!	#NULL!	#NULL!	#NULL!	#NULL!
#NULL!	#NULL!	#NULL!	#NULL!	#NULL!	#NULL!	#NULL!	#NULL!	#NULL!	#NULL!
#NULL!	#NULL!	#NULL!	#NULL!	#NULL!	#NULL!	#NULL!	#NULL!	#NULL!	#NULL!
#NULL!	#NULL!	#NULL!	#NULL!	#NULL!	#NULL!	#NULL!	#NULL!	#NULL!	#NULL!
#NULL!	#NULL!	#NULL!	#NULL!	#NULL!	#NULL!	#NULL!	#NULL!	#NULL!	#NULL!
2	2	2	2	2	2	2	2	2	2
2	1	2	2	2	1	2	1	2	1
2	2	2	2	2	2	2	2	2	2
#NULL!	#NULL!	#NULL!	#NULL!	#NULL!	#NULL!	#NULL!	#NULL!	#NULL!	#NULL!
2	2	2	2	2	2	1	2	2	2
2	2	2	1	2	2	#NULL!	1	2	#NULL!
2	2	#NULL!	#NULL!	#NULL!	#NULL!	#NULL!	#NULL!	#NULL!	#NULL!
1	1	2	2	2	2	2	2	2	2
2	2	2	2	2	2	#NULL!	#NULL!	2	2
1	1	2	2	2	2	1	2	2	2
2	2	2	2	2	2	2	2	2	2
2	1	2	2	2	2	2	2	2	1
2	2	2	2	2	2	2	2	2	2
2	2	2	2	1	2	2	1	2	2

2	2	2	2	2	2	2	2	2	2
1	1	2	2	2	2	2	2	2	2
#NULL!	#NULL!	#NULL!	#NULL!	#NULL!	#NULL!	#NULL!	#NULL!	#NULL!	#NULL!
2	1	2	1	2	2	1	2	2	2
2	2	2	1	2	2	2	2	1	2
1	1	2	1	2	2	1	2	2	1
2	2	1	#NULL!	2	#NULL!	2	#NULL!	2	2
2	2	2	2	2	2	2	2	2	2
1	1	2	2	2	2	2	2	2	2
2	2	1	2	2	1	2	1	2	2
2	2	2	#NULL!	1	2	2	2	2	2
2	2	2	1	2	1	2	2	2	1
#NULL!	#NULL!	#NULL!	#NULL!	#NULL!	#NULL!	#NULL!	#NULL!	#NULL!	#NULL!
1	1	2	1	2	2	1	1	2	1
1	2	2	2	2	2	2	1	2	2
1	1	2	2	1	2	1	1	2	1
1	2	2	2	2	1	2	2	2	1
2	2	#NULL!	#NULL!	#NULL!	#NULL!	#NULL!	#NULL!	#NULL!	#NULL!
1	2	2	2	2	2	2	1	1	2
#NULL!	#NULL!	#NULL!	#NULL!	#NULL!	#NULL!	#NULL!	#NULL!	#NULL!	#NULL!
2	2	2	#NULL!	#NULL!	#NULL!	#NULL!	#NULL!	#NULL!	#NULL!
1	2	1	2	1	2	1	1	2	2
#NULL!	#NULL!	#NULL!	#NULL!	#NULL!	#NULL!	#NULL!	#NULL!	#NULL!	#NULL!
#NULL!	#NULL!	#NULL!	#NULL!	#NULL!	#NULL!	#NULL!	#NULL!	#NULL!	#NULL!
1	1	2	#NULL!	#NULL!	#NULL!	#NULL!	#NULL!	#NULL!	#NULL!
#NULL!	#NULL!	#NULL!	#NULL!	#NULL!	#NULL!	#NULL!	#NULL!	#NULL!	#NULL!
2	2	2	2	2	2	1	2	2	2
1	2	2	2	2	2	2	2	2	2
2	2	#NULL!	#NULL!	#NULL!	#NULL!	#NULL!	#NULL!	#NULL!	#NULL!
2	1	2	2	2	2	2	2	1	2
2	2	2	2	2	1	1	2	2	2
#NULL!	#NULL!	#NULL!	#NULL!	#NULL!	#NULL!	#NULL!	#NULL!	#NULL!	#NULL!
2	2	2	2	2	2	2	2	2	2
2	2	2	2	2	2	2	2	2	2
2	2	2	2	2	2	2	2	2	2
2	2	2	2	2	2	2	2	2	2
1	1	2	2	2	1	2	2	2	2
2	2	2	2	2	2	2	2	2	2
2	2	2	2	2	2	2	2	2	2
2	2	#NULL!	#NULL!	#NULL!	#NULL!	#NULL!	#NULL!	#NULL!	#NULL!
#NULL!	#NULL!	1	#NULL!	2	2	#NULL!	1	1	2
2	2	1	2	2	2	2	2	2	2
2	2	2	2	2	2	2	2	2	2
2	2	2	2	2	1	2	1	2	2
1	1	2	2	2	2	2	1	2	2
1	1	2	1	2	2	1	1	2	1
2	2	2	2	2	2	2	2	2	2

2	2	1	1	2	2	1	1	2	1
2	2	2	2	2	2	2	2	2	2
1	1	2	#NULL!	2	1	1	2	1	2
2	2	2	2	2	2	2	2	2	2
2	2	2	2	2	2	2	2	2	2
2	2	2	2	2	2	2	2	2	2
2	2	2	2	2	2	2	2	2	2
#NULL!	#NULL!	1	2	2	2	2	2	2	2
2	2	2	2	2	2	2	2	2	2
#NULL!	#NULL!	#NULL!	#NULL!	#NULL!	#NULL!	#NULL!	#NULL!	#NULL!	#NULL!
2	2	2	2	1	1	2	2	2	2
2	2	2	2	2	1	1	2	2	1
2	2	#NULL!	#NULL!	#NULL!	#NULL!	#NULL!	#NULL!	#NULL!	#NULL!
2	2	1	2	1	2	1	2	2	2
#NULL!	#NULL!	#NULL!	#NULL!	#NULL!	#NULL!	#NULL!	#NULL!	#NULL!	#NULL!
2	1	#NULL!	#NULL!	#NULL!	#NULL!	#NULL!	#NULL!	#NULL!	#NULL!
2	1	1	2	1	1	2	1	2	1
1	2	2	2	2	2	2	2	2	2
2	2	2	2	2	2	2	2	2	#NULL!
1	1	1	2	2	2	2	2	2	2
2	2	2	2	2	2	2	2	2	2
2	2	2	2	2	2	2	2	2	2
#NULL!	#NULL!	#NULL!	#NULL!	#NULL!	#NULL!	#NULL!	#NULL!	#NULL!	#NULL!
1	1	2	1	2	2	1	2	2	2
#NULL!	#NULL!	2	1	2	2	1	1	2	1
2	2	1	2	1	1	2	2	1	2
2	2	2	1	2	1	2	2	2	1
2	2	1	2	2	2	2	2	2	2
1	2	1	2	1	2	2	2	1	2
2	2	2	2	2	2	2	2	2	2
2	2	2	2	2	2	2	2	2	2
2	2	#NULL!	#NULL!	2	#NULL!	#NULL!	#NULL!	#NULL!	#NULL!
2	2	2	2	2	2	2	2	2	2
2	2	1	1	2	1	2	2	1	1
2	2	2	2	2	1	1	2	2	2
2	2	2	2	1	2	1	2	2	2
2	2	2	2	2	2	2	2	2	2
2	2	2	2	2	2	2	2	2	2
2	2	2	2	2	2	2	1	1	2
2	2	2	2	2	2	2	2	1	2
2	2	2	2	1	2	2	2	2	2
1	1	2	2	2	2	2	2	2	2
2	2	2	2	2	2	1	1	2	2
#NULL!	#NULL!	1	2	1	2	2	2	2	2
#NULL!	#NULL!	#NULL!	#NULL!	#NULL!	#NULL!	#NULL!	#NULL!	#NULL!	#NULL!
2	2	2	2	1	2	2	2	2	2
2	1	2	2	2	2	2	2	1	2

1	1	2	1	2	2	2	1	2	2
2	2	2	#NULL!	#NULL!	1	2	1	1	#NULL!
#NULL!	#NULL!	2	#NULL!	1	#NULL!	1	#NULL!	1	#NULL!
2	1	2	2	2	2	2	2	2	2
2	2	2	#NULL!	#NULL!	#NULL!	#NULL!	#NULL!	#NULL!	#NULL!
2	2	#NULL!	#NULL!	#NULL!	#NULL!	#NULL!	#NULL!	#NULL!	#NULL!
1	1	2	#NULL!	#NULL!	#NULL!	#NULL!	#NULL!	#NULL!	#NULL!
2	2	2	2	2	2	2	2	2	2
1	2	2	2	2	2	2	2	2	2
2	2	2	2	2	2	2	2	2	2
2	2	2	2	2	2	2	2	2	2
2	2	2	2	1	1	2	2	1	2
2	2	2	2	2	2	2	2	2	2
1	2	2	2	2	2	2	2	2	2
2	2	2	2	2	2	2	2	2	2
1	1	2	2	2	2	2	2	2	2
2	2	1	2	2	2	2	2	1	2
2	2	2	2	2	2	2	2	2	2
2	2	2	2	2	2	2	2	2	2
2	2	2	2	1	2	2	2	2	2
2	2	2	2	2	2	2	2	2	2
1	1	2	1	2	2	1	2	2	2
2	1	2	#NULL!	2	#NULL!	2	#NULL!	2	#NULL!
2	2	2	2	2	2	2	2	2	2
2	2	2	1	2	2	1	1	2	1
2	1	1	2	2	2	2	1	2	2
2	1	2	2	2	2	2	2	2	2
#NULL!	#NULL!	#NULL!	#NULL!	#NULL!	#NULL!	#NULL!	#NULL!	#NULL!	#NULL!
2	2	2	2	2	2	2	2	2	2
1	1	2	2	2	2	2	2	2	2
2	2	2	2	2	2	2	2	2	2
2	2	2	2	2	2	2	1	2	2
2	2	2	1	2	2	1	2	1	2
1	2	2	2	2	2	2	2	2	2
1	1	2	1	2	2	2	2	2	2
1	#NULL!	2	2	2	1	2	2	2	2
2	2	2	2	2	2	2	2	2	2
2	2	2	2	2	2	2	2	2	2
2	2	2	2	2	2	2	2	2	2
#NULL!	#NULL!	#NULL!	#NULL!	#NULL!	#NULL!	#NULL!	#NULL!	#NULL!	#NULL!
2	2	2	2	2	2	2	2	2	2
1	1	2	1	2	2	2	2	1	1
2	1	2	2	2	2	1	2	1	2
2	2	2	1	1	2	2	2	2	2
2	2	2	1	2	2	2	2	2	2
2	2	2	2	2	2	2	2	2	2
2	2	2	2	2	2	2	2	2	2

2	2	2	2	2	2	2	2	2	2
2	2	2	#NULL!	2	1	#NULL!	#NULL!	#NULL!	#NULL!
2	2	2	2	2	2	2	2	2	2
1	1	2	2	1	2	2	2	2	2
2	2	1	2	2	1	2	2	2	1
2	2	2	2	2	2	2	2	2	2
2	2	1	2	1	1	2	2	1	2
2	2	2	2	2	2	2	2	2	2
2	1	#NULL!	#NULL!	#NULL!	#NULL!	#NULL!	#NULL!	#NULL!	#NULL!
2	2	1	2	1	1	2	2	2	2
2	2	#NULL!	#NULL!	2	#NULL!	#NULL!	#NULL!	#NULL!	#NULL!
2	2	2	2	2	2	2	2	2	2
1	2	2	2	2	2	2	2	2	2
2	2	2	1	2	2	2	1	2	2
2	2	2	2	2	1	2	2	2	2
1	2	2	2	2	2	2	2	2	2
1	2	2	2	2	2	2	2	1	2
1	1	2	2	2	1	1	1	2	2
#NULL!	#NULL!	#NULL!	#NULL!	#NULL!	#NULL!	#NULL!	#NULL!	#NULL!	#NULL!
1	2	2	2	2	2	1	1	2	2
2	2	2	2	2	2	2	1	2	2
2	2	2	2	2	2	2	2	2	2
1	1	2	2	2	2	2	2	2	2
1	1	2	1	1	2	2	1	1	1
#NULL!	#NULL!	#NULL!	#NULL!	#NULL!	#NULL!	#NULL!	#NULL!	#NULL!	#NULL!
2	2	2	2	2	2	2	2	2	2
2	2	2	2	2	2	2	2	2	2
2	2	2	2	2	2	2	2	2	2
1	2	2	2	2	2	2	2	2	2
2	2	1	2	2	2	2	2	2	2
2	2	2	2	2	2	2	2	2	2
2	1	2	2	2	2	2	2	2	1
2	1	2	2	2	2	2	2	2	2
2	1	2	2	1	2	2	2	2	1
1	2	2	1	2	2	2	2	2	2
2	1	2	1	2	2	2	2	2	2
2	2	2	2	2	1	2	1	2	2
2	2	2	2	2	2	2	2	2	2
2	2	2	2	2	2	2	2	2	2
2	2	2	2	2	2	2	2	2	2
#NULL!	#NULL!	#NULL!	#NULL!	#NULL!	#NULL!	#NULL!	#NULL!	#NULL!	#NULL!
2	2	1	2	1	2	2	1	2	2
#NULL!	#NULL!	2	1	2	2	2	2	2	2
2	1	2	2	1	2	2	2	2	2
2	2	2	2	2	2	2	2	2	2
2	2	2	2	2	2	2	2	#NULL!	1
2	2	2	2	2	2	2	2	2	2

2	2	1	2	2	2	2	2	1	2
2	#NULL!	#NULL!	#NULL!	#NULL!	#NULL!	1	#NULL!	#NULL!	#NULL!
#NULL!	#NULL!	#NULL!	#NULL!	#NULL!	#NULL!	#NULL!	#NULL!	#NULL!	#NULL!
#NULL!	#NULL!	#NULL!	#NULL!	#NULL!	#NULL!	#NULL!	#NULL!	#NULL!	#NULL!
2	1	2	2	2	2	2	2	2	2
2	2	2	2	2	2	2	2	2	2
2	2	2	2	2	2	1	1	2	2
1	1	2	1	2	2	1	1	2	1
#NULL!	#NULL!	#NULL!	#NULL!	#NULL!	#NULL!	#NULL!	#NULL!	#NULL!	#NULL!
#NULL!	#NULL!	#NULL!	#NULL!	#NULL!	#NULL!	#NULL!	#NULL!	#NULL!	#NULL!
2	2	2	2	2	2	2	2	2	2
2	2	2	2	2	2	2	2	2	2
2	2	2	2	2	2	2	2	2	2
2	2	2	2	2	2	2	2	2	2
2	2	2	2	2	2	2	2	2	2
2	2	2	2	2	1	2	2	2	2
2	2	2	2	2	1	2	1	2	2
2	2	2	2	2	2	2	2	2	2
1	2	2	2	2	2	2	2	2	2
1	1	#NULL!	#NULL!	#NULL!	#NULL!	#NULL!	#NULL!	#NULL!	#NULL!
1	1	2	2	2	2	2	2	2	2
2	2	2	2	2	2	2	2	2	2
2	2	2	2	2	2	2	2	2	2
2	2	1	1	1	2	2	1	1	1
2	2	2	2	1	2	2	2	2	2
1	1	2	2	2	2	2	2	2	2
2	2	2	2	2	2	2	2	2	2
2	2	2	1	2	2	2	2	2	2
2	2	2	2	2	2	2	1	2	2
2	1	1	2	2	2	2	2	2	2
2	2	2	1	2	2	1	2	2	2
1	1	2	2	2	2	2	1	2	2
2	2	1	2	2	2	2	1	1	1
2	2	2	2	2	2	2	2	2	1
2	2	2	2	2	1	2	2	2	2
2	2	2	2	2	1	2	2	1	2
1	1	2	1	2	2	2	2	2	2
2	2	2	2	#NULL!	#NULL!	2	2	2	#NULL!
1	2	2	2	2	2	2	2	2	2
#NULL!	#NULL!	2	2	2	2	2	1	2	2
2	2	2	2	2	2	2	2	2	1
2	1	2	2	2	2	1	2	2	2
#NULL!	#NULL!	#NULL!	#NULL!	#NULL!	#NULL!	#NULL!	#NULL!	#NULL!	#NULL!
2	2	2	2	2	2	1	2	2	2
2	1	2	2	2	2	2	1	2	1
1	1	2	2	2	2	2	2	2	2
1	1	2	2	2	2	2	1	2	1

1	1	2	2	2	2	2	2	2	2
#NULL!	#NULL!	2	#NULL!	#NULL!	#NULL!	#NULL!	#NULL!	#NULL!	#NULL!
1	2	2	2	2	1	2	1	2	2
2	2	2	2	2	2	2	2	2	2
2	2	2	2	2	2	2	2	2	2
2	1	2	2	2	2	2	2	2	2
2	1	2	2	1	2	2	1	2	2
2	2	2	2	2	#NULL!	2	1	#NULL!	2
2	2	2	2	2	1	2	#NULL!	2	2
#NULL!	#NULL!	#NULL!	#NULL!	#NULL!	#NULL!	#NULL!	#NULL!	#NULL!	#NULL!
1	2	2	2	2	2	2	2	2	2
1	1	2	2	2	2	2	1	2	2
1	1	2	2	2	1	1	2	2	2
2	1	2	2	2	2	2	2	2	2
2	2	2	2	2	2	2	2	2	2
#NULL!	#NULL!	#NULL!	#NULL!	#NULL!	#NULL!	#NULL!	#NULL!	#NULL!	#NULL!
1	1	2	#NULL!	#NULL!	#NULL!	#NULL!	#NULL!	#NULL!	#NULL!
2	1	2	2	2	2	2	2	2	2
2	2	2	1	2	2	2	2	1	2
1	2	2	2	2	2	1	1	2	2
#NULL!	#NULL!	#NULL!	#NULL!	#NULL!	#NULL!	#NULL!	#NULL!	#NULL!	#NULL!
1	2	2	2	2	2	1	1	2	2
2	1	2	1	2	2	2	2	2	2
2	2	2	2	2	2	2	2	2	1
1	2	2	1	2	2	2	2	2	1
2	1	2	1	2	2	2	1	2	1
2	1	2	1	2	2	1	2	2	2
2	1	2	2	2	2	2	2	2	2
2	2	2	1	2	2	1	2	2	2
1	1	1	2	2	2	2	1	2	2
#NULL!	#NULL!	#NULL!	#NULL!	#NULL!	#NULL!	#NULL!	#NULL!	#NULL!	#NULL!
#NULL!	#NULL!	#NULL!	#NULL!	#NULL!	#NULL!	#NULL!	#NULL!	#NULL!	#NULL!
#NULL!	#NULL!	#NULL!	#NULL!	#NULL!	#NULL!	#NULL!	#NULL!	#NULL!	#NULL!
2	2	2	2	2	2	2	2	2	2
2	1	2	2	2	2	2	2	2	2
2	1	2	2	2	2	2	2	2	2
1	2	2	2	2	2	2	2	2	2
2	2	2	#NULL!	2	2	2	2	2	2
#NULL!	1	2	2	2	2	1	1	2	1
2	2	2	2	2	2	2	2	2	2
2	2	1	2	1	1	2	2	1	2
1	2	2	2	2	2	2	2	2	2
2	2	2	1	2	2	2	2	2	1
2	2	1	2	2	2	2	2	2	2
2	2	2	2	2	2	2	2	2	2
1	1	#NULL!	1	2	2	1	1	2	1
2	2	1	1	1	1	1	2	1	2

2	1	2	2	2	2	2	1	2	2
2	2	2	2	2	2	2	2	2	2
2	2	2	2	2	2	2	2	2	2
2	1	2	2	2	1	2	1	1	1
2	2	1	2	1	1	2	2	2	2
2	2	1	1	2	1	2	2	1	2
2	2	2	2	2	2	2	2	2	2
#NULL!	#NULL!	2	#NULL!	#NULL!	#NULL!	#NULL!	#NULL!	#NULL!	#NULL!
2	1	2	2	2	2	2	2	2	2
1	1	2	#NULL!	#NULL!	#NULL!	#NULL!	#NULL!	#NULL!	#NULL!
2	2	#NULL!	#NULL!	#NULL!	2	1	1	#NULL!	#NULL!
2	2	#NULL!	1	#NULL!	#NULL!	#NULL!	#NULL!	#NULL!	#NULL!
2	2	2	2	2	2	2	1	2	2
2	#NULL!	#NULL!	#NULL!	#NULL!	#NULL!	#NULL!	#NULL!	#NULL!	#NULL!
2	2	2	2	2	2	2	2	1	2
1	1	2	2	2	2	2	2	2	2
2	2	2	2	2	2	1	1	2	1
2	1	2	2	2	2	1	1	2	1
2	2	2	2	2	2	2	2	2	2
1	2	2	2	1	2	2	2	2	2
2	2	2	2	2	2	2	2	2	2
2	2	2	2	2	2	2	2	2	2
2	2	2	2	2	2	2	2	2	2
2	2	2	2	#NULL!	1	#NULL!	2	1	#NULL!
1	1	2	2	2	2	1	2	2	2
1	1	2	2	2	2	2	1	2	1
2	2	2	2	2	2	2	2	2	2
2	2	2	2	2	2	2	2	2	2
2	2	2	2	2	2	2	1	2	2
2	2	2	2	2	2	2	2	2	2
2	#NULL!	2	2	2	2	2	2	2	2
2	2	2	2	1	2	2	2	2	2
2	2	2	2	2	2	2	2	2	2
2	2	2	2	1	1	1	2	2	2
2	#NULL!	#NULL!	#NULL!	#NULL!	#NULL!	#NULL!	#NULL!	#NULL!	#NULL!
2	2	2	2	2	2	2	2	2	2
2	2	2	2	2	2	2	2	2	2
2	2	2	2	2	2	2	2	2	2
2	2	2	2	2	2	2	2	2	2
2	2	2	2	2	2	2	2	2	2
2	1	2	1	2	2	1	2	1	1
2	2	2	#NULL!	#NULL!	#NULL!	#NULL!	#NULL!	#NULL!	#NULL!
2	2	2	#NULL!	#NULL!	#NULL!	#NULL!	#NULL!	#NULL!	#NULL!
2	2	2	2	2	2	2	2	2	2
2	1	#NULL!	#NULL!	#NULL!	#NULL!	1	#NULL!	#NULL!	#NULL!
2	2	2	#NULL!	2	2	2	#NULL!	2	2
2	2	2	2	2	2	2	2	2	2

2	1	2	2	2	2	2	2	2	2
2	1	2	1	2	2	1	1	2	2
2	1	2	2	2	2	2	2	2	1
2	2	2	2	2	2	2	2	1	2
2	2	2	2	2	2	2	2	2	2
2	2	2	2	2	2	1	2	2	2
2	2	2	2	2	1	2	2	2	2
2	2	2	2	2	2	2	2	2	2
1	1	2	2	2	2	2	1	2	2
1	1	2	2	2	2	1	2	2	2
2	2	2	2	2	2	2	2	2	2
2	2	2	#NULL!	1	2	2	2	2	2
1	2	2	#NULL!	1	1	1	2	2	2
2	2	2	2	2	2	2	2	2	2
2	2	2	#NULL!	2	2	2	1	1	2
2	2	2	2	2	2	2	2	2	2
2	1	2	2	2	2	1	1	2	2
2	2	2	2	2	2	2	2	2	2
2	1	2	2	2	2	2	1	2	2
2	2	2	1	2	2	1	1	2	1
2	1	2	2	2	2	2	2	2	2
2	1	2	2	2	2	2	2	2	2
2	2	2	2	2	2	2	2	2	2
2	2	2	2	2	2	2	2	2	2
1	1	2	1	2	2	1	2	2	2
2	2	2	2	2	2	2	2	2	2
2	1	2	2	2	2	2	2	2	2
1	1	2	2	2	2	2	2	2	1
2	1	2	2	2	1	2	2	2	2
2	1	2	2	2	1	2	2	2	1
#NULL!	#NULL!	#NULL!	#NULL!	#NULL!	#NULL!	#NULL!	#NULL!	#NULL!	#NULL!
2	1	2	1	2	2	2	2	2	1
2	1	2	2	2	2	1	2	2	1
2	1	2	2	2	2	2	2	2	2
1	2	2	2	2	2	1	2	2	2
1	1	2	1	2	2	2	2	1	2
2	1	2	2	2	2	2	2	1	2
2	2	2	2	2	2	2	2	2	2
1	1	2	2	1	2	2	2	2	1
1	1	2	2	2	2	2	2	2	2
2	2	2	1	2	2	1	2	2	1
2	1	1	2	2	1	2	2	2	2
2	2	2	2	2	1	2	2	2	2
2	2	2	2	2	2	1	2	2	2
1	2	2	1	2	2	1	2	2	1
2	2	1	2	1	1	1	1	2	1
2	2	2	2	2	1	2	2	2	2

2	2	2	2	2	1	2	2	2	2
1	1	2	2	2	1	2	2	2	2
1	1	2	2	2	1	2	2	2	2
1	1	2	2	2	1	2	2	2	2
1	2	2	2	2	2	2	2	2	2
1	2	2	1	2	2	2	2	2	2
1	1	2	1	2	2	1	2	2	2
2	2	2	2	2	2	2	2	2	2
2	2	1	2	2	1	2	1	2	2
1	1	2	2	2	2	2	1	2	2
1	2	2	1	2	2	2	2	2	2
2	2	2	2	2	2	2	2	1	2
1	1	2	2	2	2	2	2	2	2
2	2	2	2	2	2	2	2	2	2
2	2	1	2	1	1	2	2	1	2
2	2	2	2	2	2	2	2	2	2
2	1	1	1	2	2	1	1	2	2
1	1	2	2	2	2	2	1	2	2
2	2	2	2	2	2	2	2	2	2
2	2	2	2	1	1	2	2	#NULL!	2
1	1	2	1	2	2	2	1	2	2
2	2	2	2	1	1	2	2	1	2
2	2	2	2	2	2	2	2	2	2
2	2	2	2	2	2	2	2	2	2
2	1	2	2	2	2	2	2	1	1
1	2	2	2	2	1	2	2	2	2
2	1	2	2	2	1	2	2	2	2
2	2	2	2	2	2	2	2	2	2
2	2	2	2	2	2	2	2	2	2
2	1	2	2	2	2	2	2	2	2
2	2	2	1	2	2	1	1	2	2
1	2	2	1	2	2	2	1	#NULL!	2
2	2	2	1	2	2	2	1	1	2
2	2	2	2	2	2	2	1	2	2
2	2	2	2	2	2	1	2	2	1
2	1	2	2	2	1	2	2	2	2
2	2	2	2	1	2	2	1	2	1
2	1	2	2	2	2	2	2	1	1
2	2	2	1	1	2	1	2	2	1
1	1	2	1	2	2	2	2	2	#NULL!
1	1	2	2	2	2	2	2	2	2
1	2	2	2	1	1	2	2	2	2
2	2	2	2	2	2	2	2	2	2
1	2	2	2	2	2	2	2	2	2
2	2	2	2	2	2	2	2	2	2
2	2	2	2	1	2	2	2	2	2
2	2	2	2	2	2	2	2	2	2

2	1	2	2	2	2	2	2	2	2
2	1	2	2	2	2	2	2	2	2
1	2	2	2	2	2	1	2	2	1
1	1	2	2	2	2	2	1	2	2
2	2	1	2	2	2	1	2	1	2
1	2	2	1	2	2	2	1	2	2
2	2	2	2	2	2	2	2	2	2
2	1	2	1	2	2	2	2	2	2
#NULL!	#NULL!	#NULL!	#NULL!	#NULL!	#NULL!	#NULL!	#NULL!	#NULL!	#NULL!
2	2	2	2	2	2	2	2	2	2
1	1	2	2	2	2	2	1	2	2
2	2	2	2	2	2	2	2	2	2
1	1	2	1	2	2	1	1	2	1
2	2	2	2	1	2	2	2	2	2
2	2	2	1	2	2	1	1	2	1
2	2	2	2	2	2	2	2	2	2
2	1	2	2	2	2	2	2	2	2
1	1	1	2	1	1	2	2	1	2
2	2	2	2	1	2	1	2	1	2
2	2	2	2	2	2	2	2	2	#NULL!
1	2	2	2	2	2	1	2	2	2
2	1	2	1	2	1	2	2	2	2
2	2	2	1	2	2	2	2	2	2
1	2	2	2	2	2	2	2	1	2
2	2	2	1	2	2	1	1	2	1
2	2	2	2	2	2	2	2	2	2
2	2	2	2	2	2	2	2	2	2
2	2	2	2	2	2	2	2	2	2
2	2	2	1	1	2	1	2	2	1
2	2	2	2	2	2	2	2	2	#NULL!
2	2	2	2	1	2	2	1	2	1
#NULL!	#NULL!	#NULL!	#NULL!	#NULL!	#NULL!	#NULL!	#NULL!	#NULL!	#NULL!
2	2	2	2	2	2	2	2	2	2
2	2	2	#NULL!	1	#NULL!	1	#NULL!	1	#NULL!
2	2	2	1	2	2	2	2	2	1
1	1	2	2	2	2	2	1	2	#NULL!
1	1	2	1	2	1	1	2	2	1
2	2	2	2	1	2	2	2	2	2
2	2	2	2	2	2	2	2	2	2
2	2	2	2	2	2	2	2	2	2
2	1	2	2	2	2	2	2	2	2
2	2	2	2	2	2	2	2	2	2
2	2	2	2	2	2	2	1	2	2
1	1	2	2	2	2	2	2	2	2
2	2	1	#NULL!	2	2	2	2	2	2
2	2	2	2	2	2	2	2	2	2
2	1	2	2	2	2	2	2	2	2

2	2	2	2	2	2	2	2	2	1
2	2	2	2	2	2	2	2	2	2
2	2	2	1	2	2	1	1	2	1
2	2	2	2	2	1	2	2	2	2
2	2	2	2	2	2	2	2	2	2
2	2	2	2	2	2	2	2	2	2
2	2	2	2	2	2	2	2	2	2
1	1	1	#NULL!	#NULL!	#NULL!	#NULL!	#NULL!	#NULL!	#NULL!
1	1	1	#NULL!	#NULL!	#NULL!	#NULL!	#NULL!	#NULL!	#NULL!
1	1	2	#NULL!	#NULL!	#NULL!	#NULL!	2	#NULL!	#NULL!
1	1	2	2	1	2	2	1	1	1
1	1	2	2	1	1	2	1	1	2
1	1	2	2	1	1	1	2	2	2
1	1	2	2	2	1	1	1	2	2
1	1	2	1	1	1	2	1	1	2
1	2	2	2	2	2	2	2	2	2
2	2	2	2	2	2	2	2	2	2
1	1	2	1	2	2	1	2	2	1
1	1	2	2	1	2	1	2	2	2
1	1	2	1	2	2	2	1	2	1
1	1	1	2	2	1	2	1	2	1
2	2	2	2	2	1	2	1	2	2
1	2	2	2	2	2	2	2	2	1
1	2	2	2	2	2	2	1	1	1
1	1	2	2	2	1	1	1	1	2
1	1	2	2	2	2	2	2	2	2
2	2	2	2	2	2	1	1	1	1
1	1	2	2	2	2	2	2	2	2
1	1	2	2	2	1	1	1	2	2
1	1	2	2	2	1	1	1	1	1
1	1	2	2	2	1	1	1	2	2
1	1	2	2	2	1	1	1	2	2
2	2	2	2	2	2	2	2	2	2
2	2	2	2	2	2	2	2	2	2
2	2	2	2	2	2	2	2	2	2
2	2	2	2	2	2	2	2	2	2
2	2	2	2	2	2	2	2	2	2
2	2	2	2	2	2	2	2	2	2
2	2	2	2	2	2	2	2	2	2
1	1	2	2	2	2	2	2	2	2
2	2	1	1	1	1	1	1	2	2
1	1	1	2	2	2	2	1	2	1
1	1	1	2	2	2	2	1	2	1
1	1	1	2	2	2	2	1	2	1
1	1	1	2	2	2	2	1	2	1
2	2	1	#NULL!	2	2	1	2	#NULL!	2
2	2	1	#NULL!	2	2	2	2	#NULL!	2

2	#NULL!	1	2	#NULL!	1	2	2	2	1
1	1	1	1	2	2	2	2	2	2
1	1	1	2	1	2	1	2	1	2
1	1	1	2	1	1	2	1	2	1
1	1	1	2	2	1	2	1	2	2
2	2	1	2	1	2	1	1	2	1
2	2	1	2	1	1	2	1	1	2
2	2	1	2	2	2	1	2	2	2
1	1	1	#NULL!	#NULL!	#NULL!	#NULL!	#NULL!	#NULL!	#NULL!
#NULL!	#NULL!	#NULL!	#NULL!	#NULL!	#NULL!	#NULL!	#NULL!	#NULL!	#NULL!
#NULL!	#NULL!	2	2	2	2	2	2	2	2
1	1	1	#NULL!	#NULL!	1	1	#NULL!	1	#NULL!
1	1	1	#NULL!	1	2	2	2	2	#NULL!
2	1	1	1	1	1	1	1	1	1
2	2	#NULL!	#NULL!	#NULL!	#NULL!	#NULL!	#NULL!	#NULL!	#NULL!
2	2	2	2	2	2	2	2	2	2
2	2	2	2	2	2	2	2	2	2
2	1	2	#NULL!	#NULL!	#NULL!	#NULL!	#NULL!	#NULL!	#NULL!
2	2	1	2	1	2	2	1	2	2
2	2	2	#NULL!	2	#NULL!	#NULL!	#NULL!	#NULL!	#NULL!
2	1	1	2	1	1	2	2	2	2
1	1	1	2	2	1	1	2	2	2
2	2	1	1	1	1	1	1	2	2
2	1	2	2	2	2	2	2	2	2
1	1	2	#NULL!	#NULL!	#NULL!	#NULL!	#NULL!	#NULL!	#NULL!
1	1	1	2	#NULL!	#NULL!	#NULL!	#NULL!	#NULL!	#NULL!
1	1	1	2	2	1	2	2	1	2
2	1	#NULL!	#NULL!	#NULL!	#NULL!	#NULL!	#NULL!	#NULL!	1
1	1	1	2	1	2	2	2	1	2
#NULL!	#NULL!	#NULL!	#NULL!	#NULL!	#NULL!	#NULL!	#NULL!	#NULL!	#NULL!
2	2	1	2	1	2	1	1	1	2
2	2	2	#NULL!	#NULL!	#NULL!	#NULL!	#NULL!	#NULL!	#NULL!
1	1	1	1	1	1	1	2	2	1
#NULL!	#NULL!	2	2	2	2	2	2	1	1
2	2	2	2	2	2	2	2	1	1
2	2	2	2	2	2	2	2	2	2
2	2	1	1	1	2	2	2	2	2
2	2	2	2	2	2	2	2	2	2
1	1	1	1	2	2	2	2	2	2
2	2	1	#NULL!	#NULL!	#NULL!	#NULL!	#NULL!	#NULL!	#NULL!
2	2	2	#NULL!	#NULL!	#NULL!	#NULL!	#NULL!	#NULL!	#NULL!
2	2	2	#NULL!	#NULL!	#NULL!	#NULL!	#NULL!	#NULL!	#NULL!
#NULL!	1	1	#NULL!	#NULL!	#NULL!	#NULL!	#NULL!	#NULL!	#NULL!
2	2	2	#NULL!	#NULL!	#NULL!	#NULL!	#NULL!	#NULL!	#NULL!
2	2	2	#NULL!	#NULL!	#NULL!	#NULL!	#NULL!	#NULL!	#NULL!
2	2	2	1	2	1	2	2	1	2
1	1	1	2	2	1	2	2	1	2

#NULL!	#NULL!	#NULL!	#NULL!	#NULL!	#NULL!	#NULL!	#NULL!	#NULL!	#NULL!
2	1	2	2	2	2	2	2	2	2
2	2	2	1	2	1	2	2	2	1
1	1	1	2	2	2	2	1	2	2
1	1	2	2	1	2	2	#NULL!	2	2
2	1	2	2	2	2	2	2	2	2
1	1	1	#NULL!	#NULL!	#NULL!	#NULL!	#NULL!	#NULL!	#NULL!
2	1	2	2	2	2	2	2	2	2
2	2	2	2	2	2	2	2	2	2
2	2	2	#NULL!	#NULL!	#NULL!	#NULL!	#NULL!	#NULL!	#NULL!
2	2	1	2	1	2	2	2	1	2
1	1	#NULL!	#NULL!	#NULL!	#NULL!	#NULL!	#NULL!	#NULL!	#NULL!
2	2	2	#NULL!	#NULL!	#NULL!	#NULL!	#NULL!	#NULL!	#NULL!
2	2	2	#NULL!	#NULL!	#NULL!	#NULL!	#NULL!	#NULL!	#NULL!
2	1	1	2	1	2	1	1	1	1
2	2	2	#NULL!	#NULL!	#NULL!	#NULL!	#NULL!	#NULL!	#NULL!
2	1	2	2	2	2	2	1	2	2
2	#NULL!	2	#NULL!	#NULL!	#NULL!	#NULL!	#NULL!	#NULL!	#NULL!
2	1	2	#NULL!	1	2	2	1	2	2
1	1	1	1	1	2	2	2	2	1
1	1	1	2	1	2	1	2	1	2
2	1	1	2	#NULL!	1	2	2	1	2
1	1	1	2	2	2	2	2	2	2
1	1	1	1	1	1	2	2	2	2
1	1	1	1	1	2	2	2	2	2
1	1	1	1	2	1	2	2	2	1
2	2	1	1	1	1	1	1	1	1
1	2	1	1	1	2	2	2	2	2
1	2	1	2	2	1	2	2	2	2
2	2	1	2	2	1	1	2	1	2
2	2	1	2	1	2	1	2	1	2
1	1	1	1	1	1	1	1	1	1
1	2	#NULL!	#NULL!	#NULL!	#NULL!	#NULL!	1	#NULL!	#NULL!
1	#NULL!	#NULL!	#NULL!	#NULL!	#NULL!	#NULL!	#NULL!	1	#NULL!
1	1	1	#NULL!	#NULL!	#NULL!	#NULL!	#NULL!	#NULL!	#NULL!
2	2	#NULL!	#NULL!	#NULL!	#NULL!	#NULL!	1	#NULL!	#NULL!
2	2	#NULL!	#NULL!	#NULL!	#NULL!	#NULL!	#NULL!	#NULL!	#NULL!
2	2	#NULL!	#NULL!	#NULL!	#NULL!	#NULL!	#NULL!	#NULL!	#NULL!
2	1	#NULL!	#NULL!	#NULL!	#NULL!	1	#NULL!	#NULL!	#NULL!
1	1	2	#NULL!	#NULL!	#NULL!	#NULL!	#NULL!	#NULL!	#NULL!
2	2	2	#NULL!	#NULL!	#NULL!	#NULL!	#NULL!	#NULL!	#NULL!
1	1	2	#NULL!	#NULL!	#NULL!	#NULL!	#NULL!	#NULL!	#NULL!
2	2	2	#NULL!	#NULL!	#NULL!	#NULL!	#NULL!	#NULL!	#NULL!
2	1	2	#NULL!	#NULL!	#NULL!	#NULL!	#NULL!	#NULL!	#NULL!
2	2	2	#NULL!	#NULL!	#NULL!	#NULL!	#NULL!	#NULL!	#NULL!
1	1	1	1	1	2	1	2	2	2
1	1	1	2	1	1	1	2	1	2

1	1	1	1	1	1	1	2	2	1
1	1	2	#NULL!	#NULL!	#NULL!	#NULL!	2	#NULL!	#NULL!
2	2	#NULL!	#NULL!	#NULL!	1	#NULL!	#NULL!	#NULL!	1
1	1	1	2	1	2	2	1	2	1
1	1	1	#NULL!	#NULL!	#NULL!	#NULL!	#NULL!	#NULL!	#NULL!
2	1	1	#NULL!	#NULL!	#NULL!	#NULL!	#NULL!	#NULL!	#NULL!
2	1	1	#NULL!	#NULL!	#NULL!	#NULL!	#NULL!	#NULL!	#NULL!
1	1	1	#NULL!	#NULL!	#NULL!	#NULL!	#NULL!	#NULL!	#NULL!
#NULL!	#NULL!	1	#NULL!	#NULL!	#NULL!	#NULL!	#NULL!	#NULL!	#NULL!
1	1	2	2	2	2	2	2	2	2
1	1	1	2	1	2	1	2	1	2
1	1	2	1	2	1	2	1	2	1
1	1	2	2	2	2	2	2	2	2
2	2	1	2	2	2	2	2	2	2
2	2	1	1	2	2	2	1	1	2
2	1	1	1	1	2	2	2	2	2
1	1	1	#NULL!	#NULL!	#NULL!	#NULL!	#NULL!	#NULL!	#NULL!
2	2	1	#NULL!	#NULL!	#NULL!	#NULL!	#NULL!	#NULL!	#NULL!
2	2	1	#NULL!	#NULL!	#NULL!	#NULL!	#NULL!	#NULL!	#NULL!
1	1	#NULL!	#NULL!	#NULL!	#NULL!	#NULL!	1	#NULL!	2
1	1	2	#NULL!	#NULL!	#NULL!	#NULL!	#NULL!	#NULL!	#NULL!
#NULL!	#NULL!	#NULL!	#NULL!	#NULL!	1	#NULL!	#NULL!	#NULL!	#NULL!
1	1	#NULL!	#NULL!	#NULL!	2	#NULL!	2	#NULL!	2
2	2	2	#NULL!	#NULL!	#NULL!	2	#NULL!	#NULL!	2
1	1	#NULL!	#NULL!	2	#NULL!	1	2	#NULL!	2
2	2	#NULL!	#NULL!	#NULL!	1	#NULL!	#NULL!	#NULL!	#NULL!
1	2	2	#NULL!	#NULL!	#NULL!	#NULL!	#NULL!	#NULL!	#NULL!
2	2	2	2	2	2	2	2	2	2
2	1	2	1	2	2	2	2	2	2
2	2	2	#NULL!	#NULL!	#NULL!	#NULL!	#NULL!	#NULL!	#NULL!
2	2	1	2	2	2	2	2	2	2
2	2	2	1	2	2	2	1	2	1
2	1	2	2	2	2	2	2	2	2
2	2	1	2	2	1	2	2	2	2
1	1	1	2	1	2	2	2	1	2
2	2	2	2	2	2	2	2	2	2
2	2	2	1	2	2	2	2	2	#NULL!
2	2	1	2	2	2	2	2	2	2
2	1	2	#NULL!	#NULL!	#NULL!	#NULL!	#NULL!	#NULL!	#NULL!
2	2	2	2	2	2	2	2	2	2
2	2	2	2	2	2	2	2	2	2
2	2	2	1	2	1	2	1	2	2
2	2	2	1	2	1	2	2	1	2
2	2	1	2	2	2	2	2	2	2
2	2	1	#NULL!	#NULL!	#NULL!	#NULL!	#NULL!	#NULL!	#NULL!
2	2	2	2	2	2	2	2	2	2
2	1	1	2	1	2	2	2	1	2

2	1	1	2	2	1	2	2	1	2
2	1	1	2	2	2	2	2	1	2
2	2	2	2	2	2	2	2	2	2
2	2	1	2	2	2	2	1	2	2
1	2	1	2	2	2	1	2	2	2
2	2	1	2	1	1	2	2	1	2
2	1	1	2	2	2	2	2	2	2
2	2	2	1	1	1	1	1	2	2
1	1	1	1	1	1	1	1	1	1
#NULL!	#NULL!	#NULL!	#NULL!	#NULL!	#NULL!	#NULL!	#NULL!	#NULL!	#NULL!
2	2	2	2	2	2	2	2	2	2
2	1	1	2	2	2	2	2	2	2
2	1	1	1	1	1	1	1	1	1
2	2	1	2	1	1	2	1	1	2
2	2	1	1	2	1	2	1	1	2
2	2	1	1	1	2	1	2	2	#NULL!
2	2	2	2	2	2	2	2	2	2
2	2	2	2	2	2	2	2	2	2
2	2	1	2	2	2	2	2	2	1
2	2	1	2	2	2	2	2	2	1
1	1	1	2	1	2	2	2	1	2
2	2	2	2	2	2	2	2	2	2
2	1	1	2	2	2	2	2	2	2
1	1	1	2	1	1	2	2	2	2
2	2	2	2	2	2	2	2	2	2
1	1	2	2	1	1	2	2	2	2
2	2	1	2	1	2	2	2	2	2
2	1	2	2	2	2	2	2	2	2
1	1	2	2	2	2	2	2	2	2
2	2	2	2	2	2	2	2	2	2
2	1	2	2	2	2	2	2	2	2
2	2	2	2	2	2	2	2	2	2
2	2	2	2	2	2	2	2	2	2
1	2	2	2	2	2	2	2	2	2
2	1	2	2	2	2	2	2	2	2
2	2	2	2	2	2	2	2	2	2
2	1	2	2	1	2	1	1	2	2
#NULL!	#NULL!	#NULL!	#NULL!	#NULL!	#NULL!	#NULL!	#NULL!	#NULL!	#NULL!
#NULL!	#NULL!	#NULL!	#NULL!	#NULL!	#NULL!	#NULL!	#NULL!	#NULL!	#NULL!
2	2	#NULL!	#NULL!	#NULL!	#NULL!	#NULL!	#NULL!	#NULL!	#NULL!
2	2	2	2	2	2	2	1	2	1
2	1	1	#NULL!	#NULL!	#NULL!	#NULL!	#NULL!	#NULL!	#NULL!
#NULL!	#NULL!	#NULL!	#NULL!	#NULL!	#NULL!	#NULL!	#NULL!	#NULL!	#NULL!
1	2	1	2	2	1	2	2	1	2
2	2	#NULL!	#NULL!	#NULL!	#NULL!	#NULL!	#NULL!	#NULL!	#NULL!
#NULL!	#NULL!	#NULL!	#NULL!	#NULL!	#NULL!	#NULL!	#NULL!	#NULL!	#NULL!
2	2	2	2	2	2	2	2	2	2

2	2	1	2	2	1	2	1	1	2
1	1	1	1	1	1	2	1	1	2
1	1	1	2	#NULL!	#NULL!	#NULL!	#NULL!	#NULL!	#NULL!
2	2	2	2	2	2	2	2	2	2
2	2	#NULL!	#NULL!	#NULL!	#NULL!	#NULL!	#NULL!	#NULL!	#NULL!
2	2	#NULL!	#NULL!	#NULL!	#NULL!	#NULL!	#NULL!	#NULL!	#NULL!
2	1	2	2	2	2	2	2	2	2
1	1	1	2	2	2	2	2	2	2
1	1	1	2	2	2	1	1	1	2
2	1	1	2	2	2	2	1	2	1
2	2	2	2	2	2	1	2	2	2
2	2	2	2	2	2	2	2	2	2
1	2	1	2	1	1	2	2	1	2
2	1	2	2	2	2	2	2	2	2
2	2	1	2	1	1	2	2	2	2
2	1	2	2	2	2	2	2	2	2
2	2	2	2	2	2	2	2	2	2
2	2	#NULL!	#NULL!	#NULL!	#NULL!	#NULL!	1	#NULL!	#NULL!
2	2	1	2	1	2	2	2	1	2
2	2	1	2	2	2	2	2	2	2
1	1	2	2	2	2	2	2	2	2
2	2	1	2	2	2	2	1	2	2
2	2	2	2	2	2	2	2	2	2
1	1	1	2	2	2	2	2	1	2
2	2	1	2	2	2	2	2	1	2
2	2	2	2	2	2	2	2	2	2
#NULL!	#NULL!	#NULL!	#NULL!	#NULL!	#NULL!	#NULL!	#NULL!	#NULL!	#NULL!
2	2	2	2	2	2	2	2	2	2
2	1	1	2	1	2	1	1	1	2
2	2	2	2	2	2	2	2	2	2
2	2	1	2	2	1	1	1	2	2
2	2	#NULL!	#NULL!	1	#NULL!	#NULL!	#NULL!	#NULL!	#NULL!
2	2	2	2	2	2	2	2	2	2
2	2	2	2	2	2	2	2	2	2
1	2	1	1	1	1	2	2	2	2
2	2	2	2	2	2	2	2	2	2
2	1	2	1	2	2	2	2	1	1
2	2	2	2	2	2	2	2	2	2
2	2	2	2	2	2	2	2	2	2
2	2	1	2	1	2	1	1	1	2
#NULL!	#NULL!	2	2	2	2	2	2	2	2
1	1	1	2	1	1	1	1	1	1
2	2	1	2	2	2	2	2	1	2
2	2	2	2	2	#NULL!	#NULL!	#NULL!	#NULL!	#NULL!
1	1	1	2	1	1	2	2	2	2
2	2	2	2	1	2	2	2	2	2
1	1	1	2	2	#NULL!	2	2	1	2

2	2	2	2	2	2	2	2	2	2
2	2	2	2	2	2	2	2	2	2
2	2	2	2	2	2	2	2	2	2
2	2	2	2	2	2	2	2	2	2
2	2	2	2	2	2	2	2	2	2
#NULL!	#NULL!	2	2	2	2	2	2	2	2
2	2	2	2	2	2	2	2	2	2
1	2	1	2	2	2	2	2	2	2
2	2	2	2	2	2	2	2	2	2
2	2	2	2	2	2	2	2	2	2
#NULL!	#NULL!	2	2	2	2	2	2	2	2
1	1	1	2	1	2	2	2	1	2
#NULL!	#NULL!	#NULL!	#NULL!	#NULL!	#NULL!	#NULL!	#NULL!	#NULL!	#NULL!
1	2	1	2	1	1	2	1	1	2
1	1	1	#NULL!	#NULL!	#NULL!	#NULL!	#NULL!	#NULL!	#NULL!
#NULL!	#NULL!	#NULL!	#NULL!	#NULL!	#NULL!	#NULL!	#NULL!	#NULL!	#NULL!
1	1	1	2	2	1	2	1	2	2
1	1	2	2	2	2	2	2	2	2
1	1	#NULL!	#NULL!	1	#NULL!	#NULL!	#NULL!	#NULL!	#NULL!
1	1	1	1	1	2	2	2	1	2
2	1	2	2	2	2	2	2	2	2
2	2	2	2	2	2	2	2	2	2
2	2	2	2	2	2	2	2	2	2
#NULL!	#NULL!	1	1	1	1	2	2	1	2
1	1	1	2	2	2	2	2	1	2
2	2	1	#NULL!	#NULL!	#NULL!	#NULL!	#NULL!	#NULL!	#NULL!
#NULL!	#NULL!	1	2	2	2	2	1	2	2
1	1	1	1	1	2	1	1	2	2
2	1	1	1	1	1	2	2	2	#NULL!
2	2	#NULL!	#NULL!	#NULL!	#NULL!	#NULL!	#NULL!	#NULL!	#NULL!
1	1	1	2	1	2	2	2	1	2
2	1	1	2	2	2	2	2	2	2
2	2	2	2	2	2	2	2	2	2
1	1	1	2	1	2	2	2	2	1
1	1	2	#NULL!	#NULL!	2	#NULL!	#NULL!	#NULL!	#NULL!
1	1	1	2	2	2	2	2	1	2
1	1	1	2	2	1	1	1	1	1
#NULL!	#NULL!	#NULL!	#NULL!	#NULL!	#NULL!	#NULL!	#NULL!	#NULL!	#NULL!
1	1	2	2	2	2	2	2	2	2
2	2	2	#NULL!	#NULL!	#NULL!	#NULL!	#NULL!	#NULL!	#NULL!
1	1	1	2	2	2	2	2	2	2
1	1	1	2	1	2	2	2	1	2
2	2	2	2	2	2	2	2	1	1
1	1	1	1	2	2	2	2	2	1
2	1	2	1	2	1	2	2	2	2
1	1	1	2	2	1	2	2	1	2
#NULL!	#NULL!	2	2	2	2	2	2	2	2

#NULL!	#NULL!	2	2	2	2	2	2	2	2
1	1	1	#NULL!	#NULL!	#NULL!	#NULL!	#NULL!	#NULL!	#NULL!
1	1	1	2	1	2	2	2	1	2
1	1	#NULL!	#NULL!	#NULL!	#NULL!	#NULL!	#NULL!	#NULL!	#NULL!
#NULL!	#NULL!	1	2	1	1	2	2	2	1
#NULL!	#NULL!	1	2	2	2	1	1	2	1
1	1	1	1	2	1	2	1	1	1
1	1	1	2	2	2	2	2	2	2
1	1	1	2	2	2	2	2	2	2
1	1	1	2	1	1	2	2	1	2
2	2	#NULL!	#NULL!	#NULL!	#NULL!	#NULL!	#NULL!	#NULL!	#NULL!
1	1	1	#NULL!	1	1	#NULL!	#NULL!	1	#NULL!
1	1	1	2	1	2	2	2	1	2
1	1	1	1	2	1	1	2	1	2
#NULL!	#NULL!	#NULL!	#NULL!	#NULL!	#NULL!	#NULL!	#NULL!	#NULL!	#NULL!
1	1	1	2	2	2	2	2	1	1
1	1	1	2	2	2	2	2	2	2
1	1	1	2	2	#NULL!	#NULL!	#NULL!	#NULL!	#NULL!
1	1	1	#NULL!	#NULL!	#NULL!	#NULL!	#NULL!	#NULL!	#NULL!
1	1	1	2	2	2	1	2	1	2
1	1	1	1	2	2	2	1	1	1
1	1	2	2	2	2	2	2	2	2
1	1	1	2	1	1	2	2	1	1
1	1	1	2	1	2	2	2	1	2
1	1	1	2	1	1	2	2	1	2
1	1	1	2	2	2	2	2	2	2
1	1	1	2	1	2	2	2	1	2
1	1	1	2	1	2	2	2	1	2
1	1	1	2	1	1	2	2	1	2
1	1	1	2	1	1	2	2	1	2
2	2	#NULL!	#NULL!	#NULL!	#NULL!	#NULL!	2	#NULL!	#NULL!
#NULL!	#NULL!	#NULL!	#NULL!	#NULL!	#NULL!	#NULL!	#NULL!	#NULL!	#NULL!
2	2	2	#NULL!	#NULL!	#NULL!	#NULL!	#NULL!	#NULL!	#NULL!
2	2	#NULL!	#NULL!	#NULL!	#NULL!	#NULL!	#NULL!	#NULL!	#NULL!
#NULL!	#NULL!	#NULL!	#NULL!	#NULL!	#NULL!	#NULL!	#NULL!	#NULL!	#NULL!
1	1	1	2	2	1	2	2	1	2
1	1	1	2	1	2	2	2	2	2
1	1	1	2	2	2	2	2	2	1
2	1	#NULL!	#NULL!	#NULL!	#NULL!	#NULL!	#NULL!	#NULL!	#NULL!
#NULL!	1	#NULL!	#NULL!	#NULL!	#NULL!	#NULL!	#NULL!	#NULL!	#NULL!
2	1	#NULL!	#NULL!	#NULL!	#NULL!	#NULL!	#NULL!	#NULL!	#NULL!
2	1	#NULL!	#NULL!	#NULL!	#NULL!	#NULL!	#NULL!	#NULL!	#NULL!
1	1	#NULL!	#NULL!	#NULL!	#NULL!	#NULL!	#NULL!	#NULL!	#NULL!
2	2	2	2	2	2	2	2	2	2
1	1	2	2	1	2	#NULL!	2	1	2
1	1	1	2	2	1	2	2	1	2
1	1	1	2	2	2	2	2	1	2

#NULL!	#NULL!	#NULL!	#NULL!	#NULL!	#NULL!	#NULL!	#NULL!	#NULL!	#NULL!
1	1	#NULL!	#NULL!	#NULL!	#NULL!	1	#NULL!	#NULL!	#NULL!
1	1	1	#NULL!	#NULL!	#NULL!	#NULL!	#NULL!	#NULL!	#NULL!
2	1	1	#NULL!	#NULL!	#NULL!	#NULL!	#NULL!	#NULL!	#NULL!
2	1	#NULL!	#NULL!	#NULL!	#NULL!	#NULL!	#NULL!	#NULL!	#NULL!
1	1	1	#NULL!	#NULL!	#NULL!	#NULL!	#NULL!	#NULL!	#NULL!
1	1	2	#NULL!	2	2	2	2	2	2
1	1	#NULL!	#NULL!	#NULL!	#NULL!	#NULL!	#NULL!	#NULL!	#NULL!
2	1	2	2	2	2	2	2	2	2
1	1	1	2	1	2	2	2	2	2
1	1	2	2	2	2	2	2	2	2
1	1	1	2	1	1	2	2	1	2
1	1	1	2	2	2	2	2	1	2
1	1	1	2	2	1	2	2	2	2
#NULL!	#NULL!	2	2	2	2	2	2	#NULL!	1
2	2	2	2	1	2	2	2	2	2
1	1	1	2	2	2	2	1	2	2
1	1	1	#NULL!	#NULL!	#NULL!	#NULL!	#NULL!	#NULL!	#NULL!
2	1	2	2	2	1	2	1	2	1
2	2	2	#NULL!	#NULL!	2	#NULL!	#NULL!	#NULL!	#NULL!
2	2	#NULL!	#NULL!	2	#NULL!	#NULL!	#NULL!	#NULL!	#NULL!
1	1	#NULL!	#NULL!	#NULL!	#NULL!	#NULL!	#NULL!	1	#NULL!
1	1	2	2	2	2	2	2	2	2
1	1	1	2	1	2	1	2	2	2
2	2	2	2	2	2	2	2	2	2
2	2	2	2	2	2	2	2	2	2
1	1	2	2	2	2	2	#NULL!	#NULL!	#NULL!
2	2	2	1	2	2	2	1	2	1
1	1	1	2	2	2	2	2	2	2
2	2	2	2	2	2	2	2	2	2
1	1	1	2	2	2	2	2	2	1
1	1	1	2	1	1	2	2	1	2
1	1	1	2	1	1	1	2	1	1
2	1	2	2	2	2	2	2	2	2
1	1	1	2	1	2	2	2	2	2
1	1	#NULL!	#NULL!	#NULL!	#NULL!	#NULL!	#NULL!	#NULL!	#NULL!
1	1	2	2	2	1	2	2	1	2
1	1	1	2	2	2	2	2	2	2
1	1	1	1	2	1	2	1	1	2
1	1	1	2	2	2	2	2	1	2
1	1	1	2	2	1	2	1	1	2
1	1	1	2	1	2	2	2	2	#NULL!
1	1	2	#NULL!	2	#NULL!	1	#NULL!	2	2
1	1	1	2	1	2	2	2	2	2
1	1	1	2	2	2	2	2	2	2
1	1	1	2	2	2	2	2	2	2
1	1	1	2	2	#NULL!	2	1	2	2

2	1	1	#NULL!	#NULL!	#NULL!	#NULL!	#NULL!	#NULL!	#NULL!
1	1	1	#NULL!	1	1	#NULL!	#NULL!	1	#NULL!
1	1	1	2	1	1	2	2	1	2
1	1	1	2	2	2	2	2	1	2
2	2	2	#NULL!	2	#NULL!	#NULL!	2	#NULL!	2
1	1	1	1	1	2	1	2	1	2
1	1	1	2	1	1	2	2	1	2
1	1	1	1	1	1	2	2	1	2
1	1	1	2	2	2	2	2	2	2
1	1	1	2	1	1	2	2	1	2
2	1	1	1	1	1	2	2	1	2
1	1	1	2	1	1	2	2	1	2
1	#NULL!	1	#NULL!	1	#NULL!	#NULL!	#NULL!	#NULL!	1
1	1	2	1	1	2	2	1	2	1
#NULL!	#NULL!	2	2	2	2	2	2	2	2
1	1	1	2	1	2	2	1	1	2
#NULL!	#NULL!	#NULL!	#NULL!	#NULL!	#NULL!	#NULL!	#NULL!	#NULL!	#NULL!
1	2	1	2	2	2	2	2	2	2
2	2	2	2	2	2	2	2	2	2
2	2	#NULL!	#NULL!	#NULL!	#NULL!	#NULL!	#NULL!	#NULL!	#NULL!
2	2	2	2	2	2	2	2	2	2
1	1	1	2	2	2	2	2	1	2
1	1	1	#NULL!	#NULL!	#NULL!	#NULL!	#NULL!	#NULL!	#NULL!
1	1	1	2	2	1	2	2	2	2
2	2	2	2	2	2	2	2	2	2
2	1	2	2	2	2	2	2	2	2
1	1	2	2	2	2	1	2	2	2
1	1	1	2	1	2	2	2	1	2
1	1	1	2	1	2	2	2	2	2
1	1	1	1	2	1	1	2	2	1
#NULL!	#NULL!	2	2	2	2	2	2	2	2
2	2	2	2	2	2	2	1	2	1
1	1	1	2	2	2	2	2	2	2
1	1	1	2	2	2	2	2	2	2
2	1	1	1	1	2	1	2	2	2
2	1	2	1	1	1	1	2	1	1
2	2	2	2	2	2	1	1	1	2
#NULL!	#NULL!	1	2	2	2	2	2	2	2
1	1	1	2	2	2	2	2	1	2
2	1	2	2	2	2	2	2	2	2
1	1	1	2	1	2	2	2	1	2
1	1	1	2	2	2	2	2	2	2
1	1	1	#NULL!	#NULL!	#NULL!	#NULL!	#NULL!	#NULL!	#NULL!
1	1	1	2	1	1	1	1	1	2
1	1	1	2	1	1	2	2	1	2
1	1	1	#NULL!	1	1	#NULL!	#NULL!	1	#NULL!
2	2	2	2	1	1	1	1	1	2

1	1	1	2	2	2	#NULL!	1	2	2
2	1	1	#NULL!	#NULL!	#NULL!	#NULL!	#NULL!	#NULL!	#NULL!
2	2	2	2	2	2	2	2	2	2
1	1	#NULL!	#NULL!	#NULL!	#NULL!	#NULL!	#NULL!	#NULL!	#NULL!
1	1	1	2	1	2	2	2	2	2
2	1	2	2	2	2	2	2	2	2
1	1	1	2	1	2	2	2	2	2
2	2	#NULL!	#NULL!	#NULL!	#NULL!	#NULL!	#NULL!	#NULL!	#NULL!
1	1	1	2	1	1	2	2	2	2
1	1	1	2	1	1	2	1	1	2
1	1	1	2	1	2	2	2	2	2
1	1	1	2	1	1	2	2	1	2
2	2	1	2	1	2	2	2	1	2
1	1	1	2	2	1	2	1	1	2
2	1	#NULL!	#NULL!	#NULL!	#NULL!	#NULL!	#NULL!	#NULL!	#NULL!
1	1	#NULL!	#NULL!	#NULL!	#NULL!	#NULL!	#NULL!	#NULL!	#NULL!
2	1	#NULL!	#NULL!	#NULL!	#NULL!	#NULL!	#NULL!	#NULL!	#NULL!
1	1	2	2	2	2	2	2	2	2
1	1	1	2	1	2	2	2	2	2
1	1	2	2	1	2	2	1	2	2
1	1	1	2	2	2	1	2	2	1
1	1	1	2	2	1	2	2	1	2
1	1	1	2	2	2	2	2	1	2
1	1	1	2	1	2	2	2	1	2
1	1	1	2	2	2	2	2	2	2
1	1	1	1	1	2	2	2	1	2
1	1	1	2	1	2	2	2	2	2
1	1	1	2	2	1	2	1	2	1
1	1	1	2	1	1	2	2	2	2
1	1	1	2	2	2	2	2	2	2
1	1	1	2	#NULL!	#NULL!	#NULL!	#NULL!	#NULL!	#NULL!
1	1	1	2	1	2	2	2	1	2
2	1	#NULL!	#NULL!	#NULL!	#NULL!	#NULL!	#NULL!	#NULL!	#NULL!
2	2	2	2	1	2	1	2	2	2
2	2	1	2	2	1	2	2	1	2
1	1	#NULL!	#NULL!	#NULL!	#NULL!	#NULL!	#NULL!	1	#NULL!
2	2	2	2	1	2	2	2	1	2

HPVPrever	HPVb4Sex	EarlySex2	Multipartn	Smoke2	Alcohol2	Over4Baby	HighFat2	BirthPill2	Spirit2
2	2	2	2	2	2	2	2	2	2
2	2	2	2	1	2	1	2	2	2
2	2	2	2	2	2	2	2	2	2
2	2	2	2	1	2	1	2	2	2
2	2	2	2	1	2	1	2	2	2
2	2	2	2	1	2	1	2	2	2
#NULL!	#NULL!	#NULL!	#NULL!	2	#NULL!	#NULL!	#NULL!	#NULL!	#NULL!
2	2	2	2	2	1	2	1	1	2
2	2	#NULL!	2	2	#NULL!	2	2	#NULL!	2
#NULL!	#NULL!	#NULL!	2	#NULL!	#NULL!	#NULL!	#NULL!	#NULL!	#NULL!
2	2	1	2	2	2	2	2	2	2
1	2	2	2	2	1	2	2	2	2
2	2	2	2	2	2	2	2	2	2
2	2	2	2	2	2	2	1	2	2
2	2	2	2	2	1	2	2	2	1
2	2	2	2	2	2	1	2	2	2
1	2	2	2	1	1	2	1	2	2
2	2	2	2	2	2	1	2	2	2
2	2	2	2	2	2	1	2	2	2
2	2	1	2	2	1	2	2	1	2
2	2	1	1	2	1	1	1	2	2
2	2	1	1	2	1	1	2	2	2
2	2	2	2	2	2	2	2	2	2
2	2	1	1	1	2	1	2	1	2
2	2	2	2	2	2	2	2	2	2
2	2	2	2	2	2	2	2	2	2
2	2	2	2	2	2	2	2	2	2
2	2	2	2	2	1	2	1	2	1
2	2	2	2	2	2	2	2	2	2
2	2	2	2	2	2	2	2	2	2
2	2	2	2	2	2	2	2	2	2
2	2	2	2	2	1	2	2	2	1
2	2	2	2	2	2	2	2	2	2
2	2	2	2	2	1	2	2	2	1
2	2	2	2	2	1	2	2	2	2
2	2	2	2	2	1	2	2	2	1
2	2	2	2	2	1	2	2	2	2
2	2	2	2	2	1	2	2	2	2
2	2	2	2	2	2	2	2	2	2
2	2	2	2	2	2	2	2	2	2
2	2	2	2	2	2	2	2	2	2
2	2	2	2	2	2	2	1	2	2
2	2	2	2	1	2	#NULL!	2	1	2
2	2	1	1	2	1	1	2	1	2
2	1	2	2	2	1	1	2	2	1
2	2	2	2	1	2	1	2	1	2

2	2	2	2	1	2	1	1	2	2
2	2	2	2	2	2	2	1	2	1
2	2	2	2	2	2	2	2	2	2
2	2	2	2	2	1	1	1	2	2
2	2	2	2	2	1	1	2	2	1
2	2	2	2	1	2	1	2	2	2
2	2	2	2	1	2	1	2	2	2
2	2	2	2	2	1	1	#NULL!	2	#NULL!
2	2	2	2	#NULL!	#NULL!	#NULL!	#NULL!	#NULL!	#NULL!
2	1	2	2	2	1	1	1	2	2
2	2	2	2	2	2	2	#NULL!	#NULL!	#NULL!
2	2	2	2	2	2	2	2	2	2
2	2	2	2	2	2	1	2	2	2
2	2	2	2	2	1	2	2	2	2
2	2	2	2	2	1	2	2	2	2
2	2	2	2	2	1	1	2	2	2
2	2	2	2	2	2	2	2	2	2
2	2	2	2	2	2	2	2	2	2
2	2	2	2	1	2	2	2	2	2
#NULL!	#NULL!	2	2	2	1	2	2	1	2
2	2	2	2	1	2	1	2	1	2
2	2	2	2	1	2	1	2	1	2
2	2	2	2	1	2	1	2	1	2
2	2	2	2	1	2	2	2	2	2
#NULL!	2	2	2	#NULL!	#NULL!	2	#NULL!	#NULL!	#NULL!
#NULL!	2	2	2	2	1	1	2	2	2
#NULL!	#NULL!	2	2	2	1	1	1	1	2
2	2	2	1	1	2	2	2	2	2
2	2	2	2	2	2	2	2	2	2
2	2	2	2	2	1	2	2	2	2
#NULL!	#NULL!	2	#NULL!	2	1	1	2	1	2
1	1	1	1	2	1	2	2	1	1
#NULL!	#NULL!	2	#NULL!	2	1	1	2	2	#NULL!
2	2	2	2	2	1	1	2	2	2
2	2	2	1	2	1	1	1	2	2
#NULL!	#NULL!	#NULL!	#NULL!	#NULL!	#NULL!	#NULL!	#NULL!	#NULL!	#NULL!
#NULL!	#NULL!	#NULL!	#NULL!	#NULL!	#NULL!	#NULL!	#NULL!	#NULL!	#NULL!
2	2	2	2	2	1	2	2	2	2
2	2	1	1	1	2	1	2	2	2
2	2	2	2	2	1	1	1	2	1
2	2	2	2	#NULL!	1	2	2	2	2
#NULL!	#NULL!	2	#NULL!	2	1	#NULL!	1	2	2
2	1	2	2	1	2	1	2	1	2
2	2	2	2	2	1	1	1	2	2
2	2	2	2	2	1	1	2	2	1
#NULL!	#NULL!	#NULL!	#NULL!	#NULL!	#NULL!	#NULL!	#NULL!	#NULL!	#NULL!
#NULL!	#NULL!	#NULL!	#NULL!	#NULL!	#NULL!	#NULL!	#NULL!	#NULL!	#NULL!

2	2	1	2	2	2	1	2	2	1
2	2	2	2	2	2	2	2	2	2
2	2	2	2	2	2	1	1	2	2
2	2	2	2	2	1	1	2	2	2
#NULL!	#NULL!	#NULL!	#NULL!	#NULL!	#NULL!	#NULL!	#NULL!	#NULL!	#NULL!
2	2	2	#NULL!	#NULL!	1	#NULL!	#NULL!	#NULL!	2
2	2	2	2	2	1	1	2	2	1
2	2	2	2	1	1	1	2	2	2
2	2	2	2	1	2	1	2	1	2
2	2	#NULL!	#NULL!	2	1	#NULL!	1	#NULL!	#NULL!
2	2	2	2	2	2	1	2	1	2
1	2	2	1	2	2	2	1	2	2
#NULL!	#NULL!	1	2	1	2	1	2	2	2
#NULL!	#NULL!	2	#NULL!	#NULL!	#NULL!	#NULL!	#NULL!	#NULL!	#NULL!
2	2	2	2	2	2	2	2	2	1
2	1	2	2	2	2	2	2	2	1
2	2	2	2	2	2	2	2	2	2
2	2	2	2	2	2	2	2	2	2
#NULL!	#NULL!	2	2	2	2	2	2	2	2
2	2	2	2	2	2	1	2	2	1
2	2	2	2	1	1	2	1	2	2
2	2	2	2	2	2	2	2	2	2
2	2	2	2	1	2	2	2	1	2
1	1	1	1	1	2	1	2	1	2
2	2	2	2	1	2	2	1	2	2
2	2	2	2	2	1	2	2	2	2
2	2	2	2	2	2	2	2	2	2
2	2	2	2	2	2	2	2	2	2
2	2	2	2	2	2	2	2	2	2
2	2	2	2	1	2	1	1	2	2
2	2	2	2	2	2	2	2	2	2
2	2	2	2	2	2	2	2	2	2
2	2	2	2	2	2	1	2	2	1
2	2	2	2	2	2	2	2	2	2
2	2	2	2	1	2	1	1	2	2
2	2	2	2	2	1	1	2	2	2
2	2	2	2	2	2	2	2	2	2
2	2	2	2	2	2	2	2	2	2
2	#NULL!	#NULL!	#NULL!	2	#NULL!	#NULL!	#NULL!	#NULL!	1
2	2	2	2	1	2	1	2	2	1
2	2	2	2	2	2	2	2	2	2
2	2	2	2	2	2	2	2	2	2
2	2	2	2	2	2	2	2	2	2
2	2	2	1	2	1	2	2	2	2
2	2	2	2	2	1	2	2	2	2
2	2	2	2	2	1	2	2	2	2
2	2	2	2	2	2	2	2	2	2

2	2	2	2	2	2	2	2	2	2
2	2	2	2	2	2	1	1	2	2
2	2	2	2	2	2	1	1	2	2
2	2	2	2	2	2	2	2	2	2
#NULL!	#NULL!	2	2	2	1	2	1	2	1
2	2	2	2	1	2	1	2	2	2
1	2	2	2	1	#NULL!	2	2	1	2
2	2	2	2	2	2	2	2	2	2
2	2	2	2	2	2	2	2	2	2
2	2	2	2	2	2	2	2	2	2
2	1	1	2	2	2	2	#NULL!	2	2
2	2	2	2	2	2	2	2	2	2
#NULL!	#NULL!	2	2	1	2	#NULL!	#NULL!	#NULL!	#NULL!
2	2	2	2	2	2	2	2	2	2
2	2	2	2	2	2	2	2	2	1
2	2	2	2	2	2	2	2	2	1
#NULL!	#NULL!	#NULL!	#NULL!	#NULL!	#NULL!	#NULL!	#NULL!	#NULL!	#NULL!
2	2	2	2	2	1	1	2	2	2
#NULL!	#NULL!	#NULL!	#NULL!	2	#NULL!	#NULL!	#NULL!	#NULL!	#NULL!
2	2	2	2	2	1	1	2	2	2
2	2	2	2	2	1	1	2	2	2
2	2	2	2	2	1	1	2	2	2
2	2	2	2	2	1	2	2	2	1
1	2	2	2	2	1	1	2	1	2
1	2	2	2	2	1	2	2	2	2
#NULL!	#NULL!	2	2	2	1	1	2	2	2
2	2	2	2	2	1	1	2	2	2
2	2	2	2	2	1	1	2	2	2
2	2	#NULL!	2	2	1	2	2	2	2
2	2	2	2	2	1	2	2	2	2
2	2	2	1	2	2	2	2	2	2
2	2	2	2	2	2	2	2	2	2
2	2	2	2	2	1	1	2	2	2
2	2	2	2	2	1	2	2	2	1
2	2	2	2	2	1	1	2	2	2
2	2	2	2	2	1	1	2	2	2
2	2	2	2	2	1	1	2	2	2
2	2	2	2	2	1	1	2	2	2
2	2	2	2	2	1	1	2	2	2
2	2	2	2	2	1	1	2	2	1
2	2	2	2	2	1	1	2	2	2
1	2	2	2	2	1	2	2	2	2
2	2	2	2	2	1	1	2	2	2
2	2	2	2	2	1	1	2	2	2
2	2	2	2	2	1	1	2	2	1
2	2	2	2	2	1	#NULL!	2	2	2
2	2	2	2	2	1	1	2	2	2

1	2	2	2	2	1	1	2	2	2
1	2	2	2	2	1	1	2	2	1
1	2	2	2	2	1	1	2	2	1
2	2	2	2	2	1	1	2	2	2
1	2	2	2	2	1	1	2	2	1
2	2	2	2	2	1	1	1	2	1
2	2	2	2	2	1	1	2	2	2
2	2	1	2	1	2	1	2	2	2
2	2	2	2	2	1	1	1	2	1
2	2	2	2	2	2	2	2	2	2
#NULL!	#NULL!	#NULL!	#NULL!	#NULL!	#NULL!	#NULL!	#NULL!	#NULL!	#NULL!
2	2	2	2	2	1	2	2	2	2
2	2	2	2	2	1	1	1	2	1
2	2	2	2	2	1	1	2	2	2
2	2	2	2	2	1	1	2	2	2
2	2	2	2	2	1	1	2	2	1
2	2	2	2	2	2	2	2	2	2
2	2	2	2	2	1	2	1	2	1
2	2	2	2	1	2	2	2	2	1
2	2	2	2	2	2	2	2	2	2
2	2	2	2	2	2	2	1	2	2
2	2	2	2	2	2	2	2	2	2
2	2	2	2	2	2	2	1	2	2
2	2	2	2	2	2	2	2	2	1
2	2	2	2	2	2	2	2	2	2
2	2	2	2	2	2	2	2	2	2
2	2	2	2	2	2	2	2	2	2
2	2	2	2	2	2	2	2	2	2
2	2	2	2	2	2	2	2	2	2
2	2	2	2	1	2	2	2	2	1
2	2	2	2	2	1	2	1	2	2
2	2	2	2	2	1	2	1	2	2
2	2	2	2	1	2	2	2	2	1
2	2	2	2	2	1	2	2	2	1
2	2	2	2	2	2	2	1	2	2
2	2	2	2	2	2	2	2	2	2
2	2	2	2	2	2	2	2	2	2
2	2	2	2	2	2	1	2	2	1
2	2	2	2	2	1	2	2	2	2
2	2	2	2	2	1	2	1	2	2
1	1	1	2	2	2	2	2	2	2
2	2	2	2	2	2	2	2	2	2
#NULL!	#NULL!	2	#NULL!	#NULL!	#NULL!	#NULL!	#NULL!	#NULL!	#NULL!
#NULL!	#NULL!	2	#NULL!	#NULL!	#NULL!	#NULL!	#NULL!	#NULL!	#NULL!
2	2	2	2	2	1	2	2	1	1
2	2	2	2	2	2	2	2	2	2
#NULL!	#NULL!	#NULL!	#NULL!	#NULL!	#NULL!	#NULL!	#NULL!	2	#NULL!

2	2	2	2	2	2	2	2	2	2
2	2	2	2	2	2	2	2	2	2
2	2	2	2	2	1	2	2	2	2
2	2	2	2	2	2	2	2	2	2
1	2	2	2	2	2	2	2	1	2
2	2	2	2	1	2	2	2	2	2
2	2	2	2	2	1	2	2	2	2
2	2	2	2	2	2	2	2	2	2
2	2	2	1	2	1	1	2	2	2
1	1	2	2	2	1	2	2	2	2
2	2	2	2	2	2	2	1	2	2
2	2	2	1	1	2	2	2	2	2
2	2	2	1	1	2	2	2	2	2
2	2	2	2	2	2	1	2	2	2
2	2	2	2	2	2	2	2	2	2
2	2	2	2	2	2	2	2	2	2
2	2	2	2	2	2	2	2	2	2
2	2	2	2	2	1	1	2	2	2
#NULL!	#NULL!	#NULL!	#NULL!	#NULL!	#NULL!	#NULL!	#NULL!	#NULL!	1
2	1	#NULL!	2	2	1	2	2	1	2
2	2	2	2	2	1	2	1	1	#NULL!
2	2	2	2	2	1	2	2	2	2
2	2	2	2	2	2	1	2	2	2
2	2	2	2	1	2	1	2	1	2
#NULL!	#NULL!	#NULL!	#NULL!	2	2	#NULL!	#NULL!	#NULL!	#NULL!
2	2	2	2	2	2	2	2	2	2
2	2	2	2	2	2	2	2	2	2
2	2	2	#NULL!	2	#NULL!	2	#NULL!	2	#NULL!
2	2	2	2	2	1	1	2	2	2
2	2	2	2	2	2	2	2	2	2
#NULL!	#NULL!	#NULL!	2	#NULL!	1	#NULL!	#NULL!	#NULL!	#NULL!
1	1	1	1	1	2	1	2	2	2
1	1	1	1	1	2	1	2	1	2
2	2	2	2	2	1	2	2	2	2
#NULL!	#NULL!	2	2	#NULL!	#NULL!	#NULL!	#NULL!	#NULL!	#NULL!
2	2	2	2	2	2	2	2	2	2
2	2	2	2	2	2	1	2	2	2
1	2	2	2	1	1	2	2	2	1
2	2	2	2	2	2	2	2	2	2
2	2	2	2	2	2	1	2	2	2
2	2	2	2	1	2	1	2	1	2
2	2	1	1	1	2	2	2	2	2
2	2	1	1	2	1	2	1	2	1
2	2	2	2	2	2	2	2	2	2
2	2	2	2	2	2	1	2	2	2
2	2	2	2	2	2	2	2	2	2
2	2	2	2	2	1	2	2	2	2

#NULL!	#NULL!	#NULL!	#NULL!	#NULL!	#NULL!	#NULL!	#NULL!	#NULL!	#NULL!
#NULL!	#NULL!	#NULL!	#NULL!	#NULL!	#NULL!	#NULL!	#NULL!	#NULL!	#NULL!
#NULL!	#NULL!	#NULL!	#NULL!	#NULL!	#NULL!	#NULL!	#NULL!	#NULL!	#NULL!
2	2	2	2	2	1	2	2	2	2
2	2	2	2	2	1	2	2	2	2
1	2	2	2	2	2	2	2	2	2
2	2	2	2	2	1	1	1	2	2
2	2	2	2	2	1	2	1	2	1
2	2	2	2	2	2	1	2	2	2
2	2	2	2	2	2	2	2	2	2
2	2	2	2	2	2	2	2	2	2
2	2	#NULL!	#NULL!	#NULL!	#NULL!	#NULL!	#NULL!	#NULL!	#NULL!
#NULL!	#NULL!	#NULL!	#NULL!	#NULL!	#NULL!	#NULL!	#NULL!	#NULL!	#NULL!
2	2	2	1	2	1	1	2	2	2
2	2	1	1	1	2	#NULL!	1	2	1
#NULL!	#NULL!	#NULL!	#NULL!	#NULL!	#NULL!	#NULL!	#NULL!	#NULL!	#NULL!
2	2	2	2	1	2	1	2	2	2
2	2	2	1	2	1	2	1	2	1
2	1	1	2	2	2	2	1	1	1
#NULL!	#NULL!	#NULL!	#NULL!	#NULL!	#NULL!	#NULL!	#NULL!	#NULL!	#NULL!
#NULL!	#NULL!	#NULL!	#NULL!	#NULL!	#NULL!	#NULL!	#NULL!	#NULL!	#NULL!
2	2	2	2	2	2	2	2	2	2
2	2	2	2	2	1	1	2	1	2
2	2	2	2	2	1	1	2	2	2
2	2	2	2	2	2	2	2	2	2
2	2	2	2	2	1	2	1	2	1
2	2	2	1	1	2	2	2	2	1
2	2	2	2	2	2	1	2	1	2
2	2	2	1	1	2	1	1	2	1
2	2	2	2	2	1	1	1	2	2
2	2	2	2	2	2	2	2	2	2
1	2	1	2	2	2	2	2	2	2
#NULL!	#NULL!	#NULL!	#NULL!	#NULL!	#NULL!	#NULL!	#NULL!	#NULL!	#NULL!
#NULL!	#NULL!	#NULL!	#NULL!	#NULL!	#NULL!	#NULL!	#NULL!	#NULL!	#NULL!
2	1	2	2	2	2	2	2	2	2
1	2	2	1	2	2	1	2	2	2
2	2	2	2	2	1	2	2	2	2
2	2	2	1	2	1	1	2	2	1
2	2	2	1	1	2	1	1	1	2
2	2	2	2	2	2	2	2	2	2
2	1	1	2	2	1	2	1	2	2
2	2	1	1	1	2	#NULL!	1	1	2
2	2	2	2	1	2	2	2	2	2
2	2	2	2	2	2	2	2	2	2
2	2	1	1	1	2	1	2	2	2
2	2	2	2	2	2	2	2	2	2
2	2	2	2	2	2	2	2	2	2

2	2	#NULL!	#NULL!	#NULL!	#NULL!	#NULL!	#NULL!	#NULL!	#NULL!
2	2	#NULL!	#NULL!	#NULL!	#NULL!	#NULL!	#NULL!	#NULL!	#NULL!
#NULL!	#NULL!	2	#NULL!	#NULL!	#NULL!	#NULL!	#NULL!	#NULL!	#NULL!
2	2	2	2	2	2	2	2	2	2
#NULL!	2	#NULL!	#NULL!	#NULL!	#NULL!	#NULL!	#NULL!	2	#NULL!
#NULL!	#NULL!	#NULL!	#NULL!	#NULL!	#NULL!	#NULL!	#NULL!	#NULL!	#NULL!
2	2	2	2	2	1	2	2	2	2
2	#NULL!	#NULL!	#NULL!	#NULL!	#NULL!	#NULL!	#NULL!	#NULL!	#NULL!
2	2	2	2	2	1	1	2	2	2
2	2	2	2	2	1	2	2	2	1
2	2	2	2	2	2	1	2	2	2
2	2	2	2	2	2	1	2	2	2
2	2	2	2	1	2	1	2	1	2
2	2	2	2	2	2	2	2	2	2
2	2	2	2	2	2	1	2	1	2
2	2	2	2	2	1	2	2	1	2
2	2	2	2	2	2	2	2	2	2
2	2	2	2	2	2	2	2	2	2
2	2	2	2	2	2	2	2	2	2
2	2	2	2	2	2	2	2	2	2
2	2	2	1	2	1	2	2	2	1
2	2	2	2	2	1	1	2	2	2
2	2	2	2	2	1	1	2	2	1
2	2	2	2	2	2	1	2	2	2
2	2	2	2	2	2	2	2	2	2
1	2	2	2	1	2	2	2	1	2
2	2	2	2	2	2	2	2	2	2
2	2	2	2	2	2	2	2	2	2
2	2	2	2	2	2	1	2	2	2
2	2	2	2	2	1	2	2	2	1
2	2	2	1	1	2	2	2	1	1
2	2	2	2	2	2	1	2	2	2
2	2	2	2	2	#NULL!	2	2	2	1
2	2	2	2	2	2	2	2	2	2
2	2	2	2	2	2	2	2	2	2
2	2	2	2	1	2	1	2	2	2
2	2	2	2	2	2	2	2	2	2
2	2	2	2	2	1	2	1	2	1
2	2	2	2	2	2	2	2	2	2
2	2	2	2	2	2	2	2	2	2
2	2	2	2	2	1	2	2	2	2
2	2	2	2	2	1	2	2	2	1
2	2	2	2	2	1	2	1	2	2
2	2	2	2	2	1	2	2	2	2
2	2	2	2	1	2	1	2	1	1
2	2	2	2	2	1	2	2	2	2
2	2	2	2	2	2	2	2	2	2

2	2	2	2	1	2	1	2	1	2
1	2	2	2	2	2	2	2	2	1
2	2	2	2	2	2	2	2	2	2
2	2	2	2	2	2	2	2	2	2
2	2	2	2	2	1	2	1	2	2
2	2	2	2	2	2	2	2	2	2
#NULL!	#NULL!	#NULL!	#NULL!	#NULL!	#NULL!	#NULL!	#NULL!	#NULL!	#NULL!
2	2	2	2	2	2	2	2	2	2
2	2	2	2	1	2	1	2	2	2
2	1	2	2	1	2	2	2	2	2
2	2	2	2	2	2	2	2	2	2
1	2	2	2	2	2	1	1	2	2
2	2	2	2	2	1	2	2	2	2
2	2	2	2	2	2	2	2	2	2
2	2	2	2	2	2	2	2	2	2
2	2	2	2	2	1	2	1	2	1
2	2	1	1	2	1	1	2	1	1
2	2	2	2	2	2	2	2	2	2
2	2	2	2	2	2	2	2	2	2
2	2	2	2	1	2	1	2	2	2
2	2	2	2	2	1	2	2	2	1
2	2	2	2	1	2	2	2	2	2
#NULL!	#NULL!	#NULL!	#NULL!	#NULL!	#NULL!	#NULL!	#NULL!	#NULL!	#NULL!
2	2	2	2	2	2	2	2	2	2
2	1	2	2	2	1	1	2	2	2
#NULL!	#NULL!	#NULL!	#NULL!	#NULL!	#NULL!	#NULL!	#NULL!	#NULL!	#NULL!
2	2	2	2	2	2	1	2	2	2
#NULL!	#NULL!	#NULL!	#NULL!	#NULL!	#NULL!	#NULL!	#NULL!	#NULL!	#NULL!
2	2	2	2	2	2	2	2	2	2
2	2	2	2	1	2	2	2	1	2
#NULL!	#NULL!	#NULL!	#NULL!	#NULL!	#NULL!	#NULL!	#NULL!	#NULL!	#NULL!
#NULL!	#NULL!	#NULL!	#NULL!	#NULL!	#NULL!	#NULL!	#NULL!	#NULL!	#NULL!
2	2	2	2	2	2	2	2	2	2
2	2	2	2	2	2	2	1	2	1
#NULL!	#NULL!	#NULL!	#NULL!	#NULL!	#NULL!	#NULL!	#NULL!	#NULL!	#NULL!
2	2	2	2	1	2	2	2	2	2
1	1	1	1	1	2	1	2	1	2
2	2	2	2	2	1	2	1	2	2
#NULL!	#NULL!	#NULL!	#NULL!	#NULL!	#NULL!	#NULL!	#NULL!	#NULL!	#NULL!
2	2	2	2	2	2	2	1	2	2
2	2	2	2	2	2	2	2	2	2
2	2	2	2	1	2	1	2	2	2
1	2	2	2	2	2	1	2	2	2
1	1	2	2	2	2	2	2	2	2
#NULL!	#NULL!	#NULL!	#NULL!	#NULL!	#NULL!	#NULL!	#NULL!	#NULL!	#NULL!
2	2	2	2	2	1	2	2	2	2
2	2	2	2	2	1	2	2	2	1

#NULL!	#NULL!	#NULL!	#NULL!	#NULL!	#NULL!	#NULL!	#NULL!	#NULL!	#NULL!
2	2	2	2	2	1	1	1	2	1
2	2	2	2	2	1	2	2	2	#NULL!
2	2	2	2	2	1	1	2	2	2
2	2	2	2	2	1	1	1	2	2
2	2	2	2	2	1	1	2	2	2
2	2	2	2	2	1	1	2	2	2
2	2	2	2	2	1	1	1	2	1
2	2	2	2	2	1	2	2	2	2
2	2	2	2	2	1	1	2	2	2
2	2	2	2	2	1	2	2	2	2
2	2	2	2	2	1	2	2	2	2
2	2	2	2	2	1	1	2	2	2
1	2	2	2	2	1	1	2	2	2
2	2	2	2	2	1	2	2	2	2
2	2	2	2	2	1	2	2	2	2
2	2	2	2	2	1	1	2	2	2
2	2	2	2	2	1	1	2	2	2
2	2	2	2	2	1	2	2	2	2
2	2	2	2	2	1	2	2	2	2
2	2	2	2	2	1	1	2	2	1
2	2	2	2	2	1	2	2	2	1
2	2	2	2	2	1	1	2	2	1
2	2	2	2	2	1	1	2	2	2
2	2	2	2	2	1	1	2	2	2
2	2	2	2	2	1	1	2	2	2
2	2	2	2	2	1	1	2	2	1
2	2	2	2	2	1	#NULL!	2	2	2
2	2	2	2	2	1	1	2	2	1
2	2	2	2	2	1	1	2	2	2
2	2	2	2	2	1	1	2	2	1
2	2	2	2	2	1	2	2	2	2
2	2	2	2	2	1	1	2	2	1
2	2	2	2	2	1	2	2	2	2
2	2	2	2	2	1	1	2	2	2
2	2	2	2	2	1	1	2	2	2
2	2	2	2	2	1	2	2	#NULL!	2
2	2	2	2	2	1	1	2	2	1
2	2	2	2	2	1	2	2	2	1
2	2	2	2	2	1	1	2	2	1
2	2	2	2	2	1	1	2	2	2
2	2	2	2	2	2	2	2	2	2
2	2	2	2	2	2	2	2	2	2
2	2	2	2	2	2	2	2	2	2
#NULL!	#NULL!	2	#NULL!	#NULL!	#NULL!	#NULL!	#NULL!	#NULL!	#NULL!
2	2	2	2	2	2	2	2	2	2
#NULL!	#NULL!	2	#NULL!	#NULL!	#NULL!	#NULL!	#NULL!	#NULL!	#NULL!

2	2	2	2	2	2	2	2	2	2
2	2	2	2	2	1	2	2	2	2
2	2	2	2	2	1	2	2	2	2
2	2	2	2	2	1	1	2	2	2
2	2	2	2	2	2	2	2	2	2
2	2	2	2	2	1	2	2	2	2
2	2	2	2	2	2	2	2	2	2
2	2	2	1	2	1	2	2	2	2
2	2	2	2	2	2	2	2	2	2
2	2	2	2	2	2	2	2	2	2
2	2	2	2	2	1	2	2	2	2
#NULL!	#NULL!	#NULL!	#NULL!	#NULL!	#NULL!	#NULL!	#NULL!	#NULL!	#NULL!
2	#NULL!	2	2	2	1	1	2	2	2
2	2	2	2	2	2	2	2	2	2
[truncated: 367,452 more chars]
