# Supplementary material for: Outcomes from integrating anti-cervical cancer teachings into the curriculum of high schools in a South-Eastern Nigerian State
Source: BMC Public Health. 2022 Oct 14;22:1914. doi: 10.1186/s12889-022-14231-4 (PMC9562070; doi:10.1186/s12889-022-14231-4)
Supplement: Supplementary file 9 — Additional file 9. [file 12889_2022_14231_MOESM9_ESM.pdf]

Comparing Phases 2 and 4 interventions for anti-cervical cancer teachings for high schools in Southeast Nigeria

Phase	ID	Gender	Age	Age2	StateofSch	ClassofStu	SchoolGen	HeardofBr	HeardofBS
2	1	1	16	2	2	2	3	2	2
2	2	1	16	2	1	2	3	2	2
2	3	1	15	2	2	1	3	2	2
2	4	2	15	2	9	2	3	2	2
2	5	2	18	2	2	3	3	9	1
2	6	2	16	2	2	2	3	1	1
2	7	2	16	2	2	2	1	2	2
2	8	2	15	2	2	2	1	1	1
2	9	2	15	2	2	2	1	1	1
2	10	2	15	2	2	2	1	1	1
2	11	2	14	1	2	2	1	1	1
2	12	2	15	2	2	2	1	2	1
2	13	2	14	1	2	2	1	1	2
2	14	2	17	2	2	2	1	1	2
2	15	2	15	2	2	2	1	1	2
2	16	2	16	2	2	2	1	2	1
2	17	2	0	#NULL!	2	2	1	1	1
2	18	2	16	2	2	2	1	1	2
2	19	2	17	2	2	2	1	1	2
2	20	2	15	2	2	2	1	1	1
2	21	2	17	2	2	3	1	1	1
2	22	2	17	2	2	3	1	1	1
2	23	2	17	2	2	3	1	1	1
2	24	2	17	2	2	3	1	2	1
2	25	2	14	1	2	1	1	3	3
2	26	2	14	1	2	1	1	2	2
2	27	2	13	1	2	1	1	1	1
2	28	2	15	2	2	1	1	1	1
2	29	2	14	1	2	1	1	1	1
2	30	2	15	2	2	1	1	1	2
2	31	2	13	1	2	1	1	1	3
2	32	2	15	2	2	1	1	1	1
2	33	2	14	1	2	1	3	1	2
2	34	2	16	2	2	1	1	1	2
2	35	2	13	1	2	1	1	1	2
2	36	2	15	2	2	1	1	1	2
2	37	2	15	2	2	1	1	1	1
2	38	2	15	2	2	1	1	1	2
2	39	2	15	2	2	1	1	1	1
2	40	2	15	2	2	2	1	1	2
2	41	2	15	2	2	3	3	1	1
2	42	2	16	2	2	3	3	1	2
2	43	2	15	2	2	3	3	1	1
2	44	2	17	2	2	3	3	1	1
2	45	2	13	1	2	1	3	1	1
2	46	9	14	1	2	1	3	1	1

2	47	2	14	1	2	1	3	1	1
2	48	2	10	1	2	1	9	1	1
2	49	2	14	1	2	1	1	1	1
2	50	2	13	1	2	1	3	1	1
2	51	2	16	2	2	1	3	1	1
2	52	2	14	1	2	1	3	1	2
2	53	1	16	2	2	1	3	1	2
2	54	2	18	2	2	1	3	2	2
2	55	2	29	3	2	1	3	1	1
2	56	1	15	2	2	1	3	1	1
2	57	2	13	1	2	1	3	1	2
2	58	2	14	1	2	1	3	2	1
2	59	1	16	2	2	2	2	2	2
2	60	1	15	2	2	1	3	1	2
2	61	2	15	2	2	2	3	1	1
2	62	2	14	1	2	2	3	2	1
2	63	1	15	2	2	1	3	1	2
2	64	2	16	2	2	1	3	1	2
2	65	2	14	1	2	1	3	1	2
2	66	1	0	#NULL!	2	1	3	3	3
2	67	2	16	2	2	1	3	3	3
2	68	2	16	2	2	1	3	1	1
2	69	2	16	2	2	2	3	1	1
2	70	1	16	2	2	2	3	1	2
2	71	1	15	2	2	2	3	2	2
2	72	9	0	#NULL!	2	1	3	2	2
2	73	2	15	2	2	1	3	1	2
2	74	1	15	2	2	1	3	1	2
2	75	2	15	2	2	1	3	1	2
2	76	2	15	2	2	1	3	1	1
2	77	2	13	1	2	1	3	1	1
2	78	2	14	1	2	1	3	1	2
2	79	2	15	2	2	1	3	1	2
2	80	1	13	1	2	1	3	1	1
2	81	2	0	#NULL!	2	1	3	1	2
2	82	1	16	2	2	1	3	1	2
2	83	2	14	1	2	1	3	1	1
2	84	2	14	1	2	1	3	1	2
2	85	2	15	2	2	1	3	1	2
2	86	1	15	2	2	1	3	1	1
2	87	2	16	2	2	1	3	1	1
2	88	9	15	2	2	1	3	1	1
2	89	1	16	2	2	1	3	1	1
2	90	2	13	1	2	1	3	1	2
2	91	1	16	2	2	1	3	1	2
2	92	1	18	2	2	1	3	1	1
2	93	2	16	2	2	1	3	1	1

2	94	2	19	2	2	1	3	1	2
2	95	2	15	2	2	1	3	1	3
2	96	1	16	2	2	1	3	1	3
2	97	2	11	1	2	1	3	1	2
2	98	1	19	2	2	1	3	1	1
2	99	2	16	2	2	1	3	1	1
2	100	2	15	2	2	1	3	1	2
2	101	1	19	2	2	1	3	1	1
2	102	2	15	2	2	1	3	1	1
2	103	2	16	2	2	1	3	1	3
2	104	2	14	1	2	1	3	1	2
2	105	2	15	2	2	2	3	1	1
2	106	2	15	2	2	2	3	2	1
2	107	2	16	2	2	1	3	1	1
2	108	2	16	2	2	1	3	2	2
2	109	2	15	2	2	1	3	2	9
2	110	1	17	2	2	2	3	1	3
2	111	2	16	2	2	1	3	1	2
2	112	2	17	2	2	1	3	1	1
2	113	1	14	1	2	1	3	1	1
2	114	2	14	1	2	1	3	1	2
2	115	2	15	2	2	1	3	1	1
2	116	1	15	2	2	2	3	1	1
2	117	9	17	2	2	1	3	3	3
2	118	2	15	2	2	1	3	2	2
2	119	2	16	2	2	1	3	1	2
2	120	1	15	2	2	2	3	1	2
2	121	2	16	2	2	1	3	1	2
2	122	2	15	2	2	1	3	1	1
2	123	1	14	1	2	1	3	1	1
2	124	2	15	2	2	2	3	2	1
2	125	2	16	2	2	2	3	2	2
2	126	2	16	2	2	2	3	2	1
2	127	2	17	2	2	2	1	1	1
2	128	2	16	2	2	2	3	1	1
2	129	2	15	2	2	2	3	1	1
2	130	1	16	2	2	2	3	1	1
2	131	2	15	2	2	1	3	1	2
2	132	2	14	1	2	1	3	1	2
2	133	2	13	1	2	1	3	3	3
2	134	2	16	2	2	2	3	2	1
2	135	9	0	#NULL!	9	9	9	9	9
2	136	1	15	2	2	2	3	2	1
2	137	1	15	2	2	2	3	2	1
2	138	9	14	1	2	1	3	1	1
2	139	2	14	1	2	1	3	1	1
2	140	2	16	2	2	1	3	1	2

2	141	2	19	2	2	1	3	1	2
2	142	1	13	1	2	1	3	1	2
2	143	1	15	2	2	2	3	2	1
2	144	1	15	2	2	1	3	1	1
2	145	1	17	2	2	1	3	1	2
2	146	1	17	2	2	1	3	1	2
2	147	2	16	2	2	1	9	1	2
2	148	2	19	2	2	1	3	2	2
2	149	1	15	2	2	1	3	1	2
2	150	1	16	2	2	1	3	1	2
2	151	1	16	2	2	1	3	1	9
2	152	2	14	1	2	1	3	1	1
2	153	2	0	#NULL!	2	1	3	1	2
2	154	1	20	3	2	1	3	2	2
2	155	1	16	2	2	2	3	2	1
2	156	1	16	2	2	2	3	2	1
2	157	1	16	2	2	2	3	2	1
2	158	1	16	2	2	2	3	2	1
2	159	1	17	2	2	1	3	1	2
2	160	2	15	2	2	1	3	1	2
2	161	2	16	2	2	1	3	2	2
2	162	1	17	2	2	3	2	1	1
2	163	1	19	2	2	3	2	2	2
2	164	1	19	2	2	3	2	3	3
2	165	1	18	2	2	3	2	1	3
2	166	1	15	2	2	1	3	1	2
2	167	1	19	2	2	3	2	1	2
2	168	1	15	2	2	1	3	1	2
2	169	1	16	2	2	1	3	1	2
2	170	1	16	2	2	2	3	1	2
2	171	9	0	#NULL!	9	9	9	9	9
2	172	1	14	1	2	1	3	2	2
2	173	1	18	2	2	1	3	1	3
2	174	1	19	2	9	2	9	9	9
2	175	1	13	1	2	1	3	1	1
2	176	2	18	2	2	1	3	1	2
2	177	1	40	3	2	1	2	1	1
2	178	2	14	1	2	1	3	1	1
2	179	2	15	2	2	2	3	1	1
2	180	2	17	2	1	3	1	2	1
2	181	1	17	2	1	1	1	1	1
2	182	2	14	1	2	1	3	1	1
2	183	1	19	2	2	1	3	1	2
2	184	9	0	#NULL!	9	9	9	9	9
2	185	9	0	#NULL!	9	9	9	9	9
2	186	2	15	2	2	2	3	2	1
2	187	1	17	2	2	1	2	1	3

2	188	1	17	2	2	1	2	3	2
2	189	1	0	#NULL!	2	3	2	1	1
2	190	1	18	2	2	3	2	1	1
2	191	1	17	2	2	3	2	1	3
2	192	1	17	2	2	3	2	1	2
2	193	1	18	2	2	3	2	1	3
2	194	1	17	2	2	3	2	1	1
2	195	1	18	2	2	3	2	2	2
2	196	9	0	#NULL!	2	3	2	1	3
2	197	1	18	2	2	1	2	2	2
2	198	1	14	1	2	1	2	1	2
2	199	9	0	#NULL!	2	1	1	1	2
2	200	1	17	2	2	1	2	1	2
2	201	1	18	2	9	9	9	9	9
2	202	1	20	3	2	2	2	3	1
2	203	1	16	2	2	1	2	1	1
2	204	9	0	#NULL!	2	2	9	1	9
2	205	1	18	2	9	1	2	1	1
2	206	1	18	2	2	3	2	3	2
2	207	1	18	2	2	3	3	1	2
2	208	1	17	2	2	3	2	1	2
2	209	1	17	2	2	3	2	1	2
2	210	1	17	2	2	3	2	1	2
2	211	9	19	2	2	3	2	1	2
2	212	1	19	2	2	3	2	1	2
2	213	1	17	2	2	1	2	1	2
2	214	9	9	1	2	1	2	1	1
2	215	1	17	2	2	1	2	1	1
2	216	9	0	#NULL!	9	9	9	9	9
2	217	1	16	2	2	1	2	1	1
2	218	1	18	2	2	1	2	1	2
2	219	1	18	2	2	3	2	1	1
2	220	1	18	2	2	3	2	1	2
2	221	1	19	2	2	3	2	2	2
2	222	1	18	2	2	3	3	2	2
2	223	1	14	1	2	1	2	2	1
2	224	1	14	1	2	1	2	1	2
2	225	1	15	2	2	1	2	1	3
2	226	1	14	1	2	1	2	1	2
2	227	1	19	2	2	1	1	1	2
2	228	1	17	2	2	1	2	1	1
2	229	1	13	1	2	1	2	1	2
2	230	1	15	2	2	1	2	1	2
2	231	1	15	2	2	1	2	2	2
2	232	1	17	2	2	1	2	1	3
2	233	9	0	#NULL!	9	1	2	2	1
2	234	1	0	#NULL!	2	1	2	2	2

2	235	9	0	#NULL!	2	3	2	1	1
2	236	1	19	2	2	3	2	1	1
2	237	1	22	3	2	3	2	1	1
2	238	1	17	2	2	3	2	1	1
2	239	1	14	1	2	3	2	1	3
2	240	1	17	2	2	3	2	1	2
2	241	1	17	2	2	3	2	9	1
2	242	1	40	3	2	1	2	2	1
2	243	1	16	2	2	1	2	1	2
2	244	1	14	1	2	1	2	1	2
2	245	1	17	2	2	1	2	1	3
2	246	1	15	2	2	1	2	1	3
2	247	1	14	1	2	1	2	1	2
2	248	1	18	2	2	3	2	1	2
2	249	1	16	2	2	2	3	2	2
2	250	9	0	#NULL!	9	9	9	9	9
2	251	1	14	1	2	1	2	2	2
2	252	1	14	1	2	1	2	2	2
2	253	1	16	2	2	2	2	2	2
2	254	9	0	#NULL!	9	9	9	9	9
2	255	9	0	#NULL!	9	9	9	9	9
2	256	1	14	1	2	2	2	2	2
2	257	1	16	2	2	2	2	2	2
2	258	1	17	2	2	3	2	3	3
2	259	1	16	2	2	2	2	2	2
2	260	1	16	2	2	2	2	2	2
2	261	1	16	2	2	2	2	2	2
2	262	9	0	#NULL!	9	9	9	9	9
2	263	9	0	#NULL!	9	9	9	9	9
2	264	1	16	2	2	2	2	1	2
2	265	9	0	#NULL!	9	9	9	9	9
2	266	1	18	2	2	3	2	2	2
2	267	1	16	2	2	2	3	2	2
2	268	1	19	2	2	3	2	2	2
2	269	1	15	2	2	1	2	2	2
2	270	1	17	2	2	2	2	2	2
2	271	1	18	2	2	3	2	2	2
2	272	1	19	2	2	3	2	2	2
2	273	1	17	2	2	3	2	2	2
2	274	1	0	#NULL!	2	2	9	1	1
2	275	9	0	#NULL!	9	9	9	9	9
2	276	9	0	#NULL!	2	2	2	1	2
2	277	1	10	1	1	1	1	1	1
2	278	1	17	2	2	2	2	1	2
2	279	1	17	2	1	2	2	1	1
2	280	1	17	2	2	2	2	1	2
2	281	9	0	#NULL!	2	2	2	1	2

2	282	1	18	2	2	2	2	1	2
2	283	1	18	2	2	2	2	1	2
2	284	1	17	2	2	2	2	1	3
2	285	1	19	2	9	2	2	1	1
2	286	1	0	#NULL!	1	3	1	1	1
2	287	1	13	1	2	1	2	1	1
2	288	1	15	2	2	1	2	1	2
2	289	1	15	2	2	1	2	1	2
2	290	9	0	#NULL!	9	9	9	9	9
2	291	1	20	3	1	1	2	2	2
2	292	9	0	#NULL!	9	9	9	9	9
2	293	9	0	#NULL!	9	9	9	9	9
2	294	2	16	2	2	3	1	1	1
2	295	1	19	2	2	2	2	1	1
2	296	2	16	2	2	2	1	1	1
2	297	1	18	2	2	2	2	2	2
2	298	2	15	2	2	3	1	1	1
2	299	1	18	2	2	3	2	1	2
2	300	1	17	2	2	3	2	1	3
2	301	1	19	2	2	3	2	2	2
2	302	1	27	3	2	3	2	1	1
2	303	1	18	2	2	3	2	1	2
2	304	1	20	3	2	3	2	1	1
2	305	1	18	2	2	3	2	1	1
2	306	1	15	2	2	3	2	1	1
2	307	1	20	3	2	3	2	1	1
2	308	1	12	1	2	3	2	1	2
2	309	1	18	2	2	3	2	1	1
2	310	1	19	2	2	3	2	3	2
2	311	1	18	2	2	3	2	1	2
2	312	1	17	2	2	3	2	1	2
2	313	1	18	2	2	3	3	1	1
2	314	1	19	2	2	3	2	3	2
2	315	1	20	3	2	3	2	3	3
2	316	1	20	3	2	3	2	2	2
2	317	1	20	3	2	3	2	1	1
2	318	1	16	2	2	3	2	3	2
2	319	1	18	2	2	2	2	1	2
2	320	9	19	2	2	2	2	2	2
2	321	9	18	2	2	2	2	2	2
2	322	1	18	2	2	2	2	2	1
2	323	9	0	#NULL!	2	2	2	1	2
2	324	1	19	2	2	2	2	2	2
2	325	1	18	2	2	2	2	1	2
2	326	1	17	2	2	2	2	1	2
2	327	2	13	1	2	2	2	2	2
2	328	1	15	2	2	2	2	1	1

2	329	1	13	1	2	2	2	1	2
2	330	1	18	2	2	1	2	1	2
2	331	9	16	2	2	2	2	1	2
2	332	1	18	2	2	2	2	1	2
2	333	1	21	3	2	2	2	1	1
2	334	1	18	2	2	2	2	2	9
2	335	1	18	2	2	1	2	1	2
2	336	1	17	2	2	1	2	1	2
2	337	1	15	2	2	1	2	2	1
2	338	9	28	3	2	1	2	1	1
2	339	1	16	2	2	2	2	1	2
2	340	1	17	2	2	2	2	1	2
2	341	1	16	2	2	2	2	2	2
2	342	1	17	2	2	2	2	2	2
2	343	1	19	2	2	2	2	1	1
2	344	1	17	2	2	2	2	1	2
2	345	1	16	2	2	2	2	2	2
2	346	1	18	2	2	1	3	2	2
2	347	1	16	2	2	1	3	1	1
2	348	1	15	2	2	1	3	1	2
2	349	2	14	1	2	1	3	1	1
2	350	2	14	1	2	1	3	1	1
2	351	1	14	1	2	1	3	2	2
2	352	2	14	1	2	1	3	2	2
2	353	2	15	2	2	1	3	1	1
2	354	2	16	2	2	1	1	1	2
2	355	2	17	2	2	1	3	9	2
2	356	1	16	2	2	1	3	1	2
2	357	1	14	1	2	1	3	1	3
2	358	1	17	2	2	1	3	1	1
2	359	1	14	1	2	1	3	1	3
2	360	1	15	2	2	1	3	1	2
2	361	9	17	2	2	1	3	1	1
2	362	2	16	2	2	3	3	2	2
2	363	1	19	2	2	3	3	2	2
2	364	2	16	2	2	3	3	1	3
2	365	1	0	#NULL!	2	3	3	1	3
2	366	2	18	2	2	3	3	3	2
2	367	2	16	2	2	3	3	1	1
2	368	1	12	1	2	3	3	1	1
2	369	2	22	3	2	3	3	1	2
2	370	2	16	2	2	2	3	9	1
2	371	2	16	2	2	2	3	1	1
2	372	2	17	2	2	2	3	1	3
2	373	1	16	2	9	2	3	1	1
2	374	2	17	2	2	2	3	1	1
2	375	1	16	2	2	2	3	1	1

2	376	1	16	2	2	2	3	1	1
2	377	1	15	2	2	2	3	1	1
2	378	2	14	1	1	1	3	1	1
2	379	2	14	1	2	2	3	2	2
2	380	1	20	3	1	3	2	2	2
2	381	2	18	2	2	3	3	1	2
2	382	1	0	#NULL!	2	2	3	1	3
2	383	2	0	#NULL!	2	2	3	1	1
2	384	1	18	2	2	2	3	2	1
2	385	2	15	2	2	2	3	1	1
2	386	2	16	2	2	2	3	1	1
2	387	2	16	2	2	2	3	1	1
2	388	2	16	2	2	2	3	1	1
2	389	2	15	2	2	2	3	1	1
2	390	2	15	2	2	2	3	1	1
2	391	2	16	2	2	2	3	3	2
2	392	9	0	#NULL!	9	9	9	9	9
2	393	2	15	2	2	2	1	2	2
2	394	2	15	2	2	1	1	1	2
2	395	2	14	1	2	2	3	1	1
2	396	2	16	2	2	2	3	1	1
2	397	2	14	1	2	2	3	2	2
2	398	2	15	2	2	1	1	1	2
2	399	2	15	2	2	1	1	1	2
2	400	2	14	1	2	1	1	1	1
2	401	2	15	2	2	1	1	1	2
2	402	2	15	2	2	1	1	1	1
2	403	2	19	2	2	1	1	1	2
2	404	2	15	2	2	1	1	2	2
2	405	2	15	2	2	1	1	1	2
2	406	9	0	#NULL!	9	9	9	9	9
2	407	2	14	1	2	1	1	1	1
2	408	2	15	2	2	1	1	1	2
2	409	2	17	2	2	2	1	1	2
2	410	2	16	2	2	2	1	1	2
2	411	2	16	2	2	2	1	2	1
2	412	2	17	2	2	2	1	1	2
2	413	2	16	2	2	2	1	1	3
2	414	2	17	2	2	2	1	1	1
2	415	2	16	2	2	1	1	1	2
2	416	2	15	2	2	1	1	1	1
2	417	2	15	2	2	1	1	1	1
2	418	2	15	2	2	1	1	1	1
2	419	2	15	2	2	1	1	1	2
2	420	9	0	#NULL!	9	9	9	9	9
2	421	2	15	2	9	1	1	1	1
2	422	2	15	2	2	1	1	1	2

2	423	2	14	1	2	1	1	1	1
2	424	2	14	1	2	1	1	1	1
2	425	2	15	2	2	1	1	1	1
2	426	2	15	2	2	1	1	1	1
2	427	2	18	2	2	3	1	2	1
2	428	2	18	2	2	3	1	1	1
2	429	1	16	2	2	2	3	2	2
2	430	1	16	2	2	2	3	2	2
2	431	1	17	2	2	2	3	3	3
2	432	1	14	1	2	1	3	9	9
2	433	1	14	1	2	1	3	1	2
2	434	1	14	1	2	1	3	2	2
2	435	1	15	2	2	1	3	1	2
2	436	1	14	1	2	1	3	2	2
2	437	1	15	2	2	1	3	1	2
2	438	1	20	3	2	3	3	2	2
2	439	1	19	2	2	3	3	3	3
2	440	1	19	2	2	3	3	1	1
2	441	2	18	2	2	3	3	1	1
2	442	1	18	2	2	3	3	1	1
2	443	1	18	2	2	3	3	9	9
2	444	1	18	2	2	3	3	2	2
2	445	2	19	2	2	3	3	2	2
2	446	1	19	2	2	3	3	2	2
2	447	9	0	#NULL!	9	9	9	9	9
2	448	2	18	2	2	2	3	1	1
2	449	2	17	2	2	2	3	1	1
2	450	2	18	2	2	2	3	1	1
2	451	2	18	2	2	2	3	1	1
2	452	2	17	2	2	2	3	2	2
2	453	2	17	2	2	2	3	3	3
2	454	2	17	2	2	2	3	1	1
2	455	2	16	2	2	2	3	2	2
2	456	2	17	2	2	2	3	3	3
2	457	2	18	2	2	2	3	2	2
2	458	9	0	#NULL!	9	9	9	9	9
2	459	2	17	2	2	2	3	3	3
2	460	1	14	1	2	1	3	2	2
2	461	2	15	2	2	1	1	1	1
2	462	2	15	2	2	1	1	1	2
2	463	2	13	1	2	1	1	1	3
2	464	2	17	2	2	1	1	1	1
2	465	2	15	2	2	1	1	1	1
2	466	2	15	2	2	1	1	1	2
2	467	9	0	#NULL!	9	1	1	1	2
2	468	2	15	2	2	1	1	1	1
2	469	2	15	2	2	1	1	1	1

2	470	2	16	2	2	1	1	1	1
2	471	2	14	1	2	1	1	1	2
2	472	2	14	1	2	1	1	1	1
2	473	2	13	1	2	1	1	1	1
2	474	2	14	1	2	1	1	1	2
2	475	2	15	2	2	1	1	1	3
2	476	9	0	#NULL!	9	9	9	9	9
2	477	2	0	#NULL!	2	1	1	1	3
2	478	2	15	2	2	1	1	1	1
2	479	2	16	2	2	1	1	1	2
2	480	2	14	1	9	1	1	1	2
2	481	2	14	1	2	1	1	1	2
2	482	2	13	1	2	1	1	1	2
2	483	9	14	1	2	1	1	2	2
2	484	9	18	2	2	1	1	1	2
2	485	2	17	2	2	3	1	1	1
2	486	2	15	2	2	1	1	1	2
2	487	2	16	2	2	1	1	1	2
2	488	2	16	2	2	1	1	1	2
2	489	2	14	1	2	1	1	1	3
2	490	2	15	2	2	1	1	2	2
2	491	2	14	1	2	1	1	1	3
2	492	2	15	2	2	1	1	1	2
2	493	2	12	1	2	1	1	1	1
2	494	2	16	2	2	1	1	1	3
2	495	2	16	2	2	1	1	1	1
2	496	2	14	1	2	1	1	1	1
2	497	2	14	1	2	1	1	1	2
2	498	2	15	2	1	1	1	1	1
2	499	2	17	2	2	1	1	1	1
2	500	2	18	2	2	1	1	1	2
2	501	9	0	#NULL!	2	1	1	2	2
2	502	2	17	2	2	1	1	1	1
2	503	2	16	2	2	1	1	1	1
2	504	2	16	2	2	1	1	1	1
2	505	2	16	2	2	1	1	1	1
2	506	2	15	2	2	1	1	9	1
2	507	2	15	2	2	1	1	1	2
2	508	2	15	2	2	1	1	1	3
2	509	2	13	1	2	1	1	1	1
2	510	2	15	2	2	1	1	1	3
2	511	2	15	2	2	1	3	1	3
2	512	2	14	1	2	1	1	1	2
2	513	2	15	2	2	1	1	1	2
2	514	2	16	2	2	1	1	1	1
2	515	2	16	2	2	1	1	2	2
2	516	2	16	2	2	1	1	1	1

2	517	2	14	1	2	1	1	1	3
2	518	2	14	1	2	1	1	1	1
2	519	2	15	2	2	1	1	1	2
2	520	2	16	2	2	1	1	1	1
2	521	2	14	1	2	1	1	1	2
2	522	2	0	#NULL!	9	2	9	1	9
2	523	9	0	#NULL!	9	2	9	1	9
2	524	2	16	2	2	3	1	1	1
2	525	2	18	2	2	3	1	1	1
2	526	2	0	#NULL!	2	3	1	1	1
2	527	2	15	2	2	3	1	1	3
2	528	2	12	1	2	3	1	1	1
2	529	2	17	2	2	3	1	1	1
2	530	2	17	2	2	3	1	2	2
2	531	2	17	2	2	3	1	2	1
2	532	1	16	2	2	2	3	2	2
2	533	1	17	2	2	2	3	3	3
2	534	1	0	#NULL!	2	3	3	9	9
2	535	1	18	2	2	3	3	2	2
2	536	1	18	2	2	3	3	3	3
2	537	1	19	2	2	3	3	2	2
2	538	1	18	2	2	3	3	9	9
2	539	1	19	2	2	3	3	2	2
2	540	1	0	#NULL!	2	2	3	2	2
2	541	1	16	2	2	2	3	2	2
2	542	1	16	2	2	2	3	2	2
2	543	1	17	2	2	2	3	9	9
2	544	2	16	2	2	2	3	1	1
2	545	1	17	2	2	2	3	2	2
2	546	1	16	2	2	2	3	2	2
2	547	1	17	2	2	2	3	2	2
2	548	1	17	2	2	2	3	2	2
2	549	1	16	2	2	2	3	2	2
2	550	1	17	2	2	2	3	2	2
2	551	1	19	2	2	3	3	3	3
2	552	1	20	3	2	3	3	2	2
2	553	1	19	2	2	3	3	2	2
2	554	2	18	2	2	3	3	9	9
2	555	2	17	2	2	3	3	2	2
2	556	2	18	2	2	3	3	3	3
2	557	2	18	2	2	3	3	1	1
2	558	2	20	3	2	3	3	1	1
2	559	2	19	2	2	3	3	2	2
2	560	2	18	2	2	3	3	3	3
2	561	1	14	1	2	1	3	2	2
2	562	1	15	2	2	1	3	2	2
2	563	9	0	#NULL!	9	9	9	9	9

2	564	2	18	2	2	2	3	2	2
2	565	2	17	2	2	2	3	3	3
2	566	2	17	2	2	2	3	2	2
2	567	2	16	2	2	2	3	1	1
2	568	2	13	1	2	1	3	2	2
2	569	2	14	1	2	1	3	2	2
2	570	2	15	2	2	1	3	2	2
2	571	2	15	2	2	1	3	1	2
2	572	9	0	#NULL!	9	9	9	9	9
2	573	9	0	#NULL!	9	9	9	9	9
2	574	9	0	#NULL!	9	9	9	9	9
2	575	9	0	#NULL!	9	9	9	9	9
2	576	9	0	#NULL!	9	9	9	9	9
2	577	9	0	#NULL!	9	9	9	9	9
2	578	1	14	1	2	1	3	2	2
2	579	1	15	2	2	1	3	2	2
2	580	1	15	2	2	1	3	1	2
2	581	2	16	2	2	2	3	2	2
2	582	1	14	1	2	1	3	2	2
2	583	1	15	2	2	1	3	2	2
2	584	1	15	2	2	1	3	2	2
2	585	2	14	1	2	1	3	1	2
2	586	2	15	2	2	1	3	1	2
2	587	1	15	2	2	1	3	2	2
2	588	1	14	1	2	1	3	2	2
2	589	1	15	2	2	1	3	1	2
2	590	2	18	2	2	3	3	3	3
2	591	2	19	2	2	2	3	2	2
2	592	2	0	#NULL!	2	2	3	2	2
2	593	2	18	2	2	2	3	1	1
2	594	2	16	2	2	2	3	1	1
2	595	2	17	2	2	2	3	2	2
2	596	2	17	2	2	2	3	3	3
2	597	2	17	2	2	2	3	1	1
2	598	2	18	2	2	2	3	1	1
2	599	1	14	1	2	1	3	1	2
2	600	9	18	2	2	3	3	2	2
2	601	2	0	#NULL!	2	3	3	2	2
2	602	2	19	2	2	3	3	3	3
2	603	2	19	2	2	3	3	2	2
2	604	2	18	2	2	3	3	2	2
2	605	2	18	2	2	3	3	2	2
2	606	2	18	2	2	3	3	2	2
2	607	2	18	2	2	3	3	1	1
2	608	1	19	2	2	3	3	2	2
2	609	2	17	2	2	3	3	2	2
2	610	1	18	2	2	3	3	2	2

2	611	2	18	2	2	3	3	3	3
2	612	1	19	2	2	3	3	9	9
2	613	1	17	2	2	3	3	2	2
2	614	1	20	3	2	3	3	2	2
2	615	1	18	2	2	3	3	9	9
2	616	1	19	2	2	3	3	2	2
2	617	2	18	2	2	3	3	1	1
2	618	2	18	2	2	3	3	2	2
2	619	2	18	2	2	3	3	1	1
2	620	1	19	2	2	3	3	1	1
2	621	1	18	2	2	3	3	2	2
2	622	1	19	2	2	3	3	2	2
2	623	2	17	2	2	3	1	1	2
2	624	2	16	2	2	3	1	2	2
2	625	2	15	2	2	1	1	1	2
2	626	2	15	2	2	1	1	1	1
2	627	2	16	2	2	1	1	2	2
2	628	2	15	2	2	1	1	1	2
2	629	2	14	1	2	1	1	1	2
2	630	2	16	2	2	3	1	2	2
2	631	2	17	2	2	2	1	1	1
2	632	2	15	2	2	2	1	1	2
2	633	2	14	1	2	2	1	1	3
2	634	2	16	2	2	2	1	1	3
2	635	2	15	2	2	3	1	1	1
2	636	2	17	2	2	3	1	1	1
2	637	2	17	2	2	3	1	1	1
2	638	2	16	2	2	3	1	1	1
2	639	2	16	2	2	3	1	2	1
2	640	2	16	2	2	2	1	1	2
2	641	2	16	2	2	2	1	1	3
2	642	2	16	2	2	2	1	2	2
2	643	2	15	2	2	2	1	1	1
2	644	2	14	1	2	1	1	1	2
2	645	2	14	1	2	2	1	1	9
2	646	9	0	#NULL!	9	9	9	9	9
2	647	9	0	#NULL!	9	9	9	9	9
2	648	1	19	2	2	3	3	1	1
2	649	1	15	2	2	1	3	2	2
2	650	1	18	2	2	3	3	2	2
2	651	2	16	2	2	2	3	1	1
2	652	2	18	2	2	3	3	1	1
2	653	1	18	2	2	3	3	1	1
2	654	1	16	2	2	2	3	2	2
2	655	2	14	1	2	1	3	2	2
2	656	1	16	2	2	2	3	2	2
2	657	2	16	2	2	2	3	1	3

2	658	2	15	2	2	2	3	1	1
2	659	2	16	2	2	2	3	1	2
2	660	2	15	2	2	2	3	1	1
2	661	2	16	2	2	2	3	1	2
2	662	2	16	2	9	9	9	9	9
2	663	2	15	2	2	2	3	1	1
2	664	2	15	2	2	2	3	1	2
2	665	1	18	2	2	3	3	2	2
2	666	1	17	2	2	2	3	2	2
2	667	1	18	2	2	3	3	1	1
2	668	2	18	2	2	3	3	1	1
2	669	2	16	2	2	2	3	1	1
2	670	1	14	1	2	2	3	1	1
2	671	1	17	2	2	2	3	1	2
2	672	9	0	#NULL!	9	9	9	9	9
2	673	1	16	2	2	2	3	1	3
2	674	1	16	2	2	2	3	1	2
2	675	2	17	2	2	2	3	1	3
2	676	2	14	1	2	2	3	1	1
2	677	2	16	2	2	2	3	1	1
2	678	2	16	2	2	2	3	1	3
2	679	2	15	2	9	9	9	9	9
2	680	9	16	2	2	2	3	1	1
2	681	2	16	2	2	2	3	1	2
2	682	1	15	2	2	2	3	1	3
2	683	2	16	2	2	2	3	1	2
2	684	2	16	2	2	2	3	1	1
2	685	2	15	2	2	2	3	1	3
2	686	2	15	2	2	2	3	1	3
2	687	2	17	2	2	2	3	1	1
2	688	2	15	2	2	2	3	1	2
2	689	2	16	2	9	9	9	9	9
2	690	1	16	2	2	2	3	1	2
2	691	9	0	#NULL!	9	9	9	9	9
2	692	1	16	2	2	2	3	1	3
2	693	2	15	2	2	2	3	1	2
2	694	1	14	1	2	2	3	1	1
2	695	1	18	2	2	3	3	1	1
2	696	1	16	2	2	2	3	1	2
2	697	1	17	2	2	2	3	2	2
2	698	2	16	2	2	1	3	2	2
2	699	2	14	1	2	1	3	1	3
2	700	1	16	2	2	2	3	1	1
2	701	2	16	2	2	2	3	1	2
2	702	1	15	2	2	2	3	1	3
2	703	1	16	2	2	2	3	1	3
2	704	1	18	2	2	2	3	1	3

2	705	1	15	2	2	2	3	2	2
2	706	1	20	3	2	2	2	1	1
2	707	9	0	#NULL!	2	2	3	2	2
2	708	2	16	2	9	9	9	9	9
2	709	9	16	2	2	2	3	1	1
2	710	1	17	2	2	2	3	2	1
2	711	1	17	2	2	2	2	1	1
2	712	1	19	2	2	1	3	2	2
2	713	2	16	2	9	9	9	9	9
2	714	2	14	1	2	1	3	1	1
2	715	2	17	2	2	1	3	1	2
2	716	2	15	2	2	1	3	1	9
2	717	1	17	2	2	1	3	1	3
2	718	2	15	2	2	2	3	1	1
2	719	2	16	2	2	2	3	1	9
2	720	2	16	2	2	1	3	2	2
2	721	2	16	2	2	1	3	2	2
2	722	1	16	2	2	1	2	1	2
2	723	1	15	2	2	1	1	2	2
2	724	2	15	2	2	1	3	1	2
2	725	2	16	2	9	9	9	9	9
2	726	1	17	2	2	2	3	1	1
2	727	1	17	2	2	2	3	1	1
2	728	1	15	2	9	9	9	9	9
2	729	1	17	2	2	2	3	1	1
2	730	1	15	2	2	2	3	1	1
2	731	2	15	2	2	1	3	1	1
2	732	1	15	2	2	1	3	1	2
2	733	1	15	2	2	1	3	1	2
2	734	1	15	2	9	1	3	3	3
2	735	1	12	1	2	1	3	1	1
2	736	1	16	2	2	1	3	2	2
2	737	1	0	#NULL!	9	9	9	9	9
2	738	1	15	2	2	1	3	1	3
2	739	2	15	2	2	1	3	1	2
2	740	9	16	2	9	9	9	9	9
2	741	1	14	1	2	1	3	1	1
2	742	1	10	1	9	9	9	9	9
2	743	2	0	#NULL!	9	9	9	9	9
2	744	2	0	#NULL!	2	1	3	1	3
2	745	2	14	1	2	1	3	1	2
2	746	2	10	1	2	1	3	1	2
2	747	2	14	1	2	1	3	1	2
2	748	1	15	2	2	1	3	1	3
2	749	2	15	2	9	9	9	9	9
2	750	2	15	2	2	1	3	1	2
2	751	2	15	2	2	1	3	1	3

2	752	2	16	2	2	1	3	1	1
2	753	2	15	2	2	1	3	1	2
2	754	2	15	2	2	1	3	1	3
2	755	1	16	2	2	1	3	1	2
2	756	2	14	1	2	1	3	1	2
2	757	2	15	2	2	1	3	1	2
2	758	2	15	2	2	1	3	1	2
2	759	1	15	2	2	1	3	1	3
2	760	1	15	2	2	1	3	1	3
2	761	9	0	#NULL!	9	9	9	9	9
2	762	1	16	2	2	1	2	1	2
2	763	1	17	2	2	1	2	1	2
2	764	1	15	2	2	1	2	3	2
2	765	1	16	2	2	1	2	3	1
2	766	1	10	1	2	1	2	1	2
2	767	1	12	1	2	1	2	2	1
2	768	1	0	#NULL!	2	1	2	1	1
2	769	1	17	2	2	1	2	1	1
2	770	9	0	#NULL!	9	9	9	9	9
2	771	1	16	2	2	1	2	1	2
2	772	1	17	2	2	1	2	1	3
2	773	1	17	2	2	1	2	1	2
2	774	1	16	2	2	1	2	2	2
2	775	1	17	2	9	9	9	9	9
2	776	1	18	2	2	1	2	1	1
2	777	1	17	2	2	1	2	1	1
2	778	9	0	#NULL!	2	1	2	1	2
2	779	1	16	2	2	1	2	1	1
2	780	1	18	2	2	1	2	1	9
2	781	1	18	2	2	1	2	1	2
2	782	1	18	2	2	1	2	1	3
2	783	1	17	2	2	1	2	1	1
2	784	1	18	2	2	1	2	1	2
2	785	1	18	2	2	1	2	1	3
2	786	1	16	2	2	1	2	1	2
2	787	1	15	2	2	1	2	1	1
2	788	1	17	2	2	2	2	2	2
2	789	1	17	2	2	2	2	1	2
2	790	1	0	#NULL!	2	2	2	1	1
2	791	1	14	1	2	1	2	2	3
2	792	1	0	#NULL!	2	1	1	1	1
2	793	1	15	2	2	1	2	2	2
2	794	1	15	2	2	1	2	3	2
2	795	1	16	2	2	1	2	1	2
2	796	1	15	2	2	1	2	3	3
2	797	1	16	2	2	1	2	1	2
2	798	1	17	2	2	3	2	1	2

2	799	1	15	2	9	3	2	2	2
2	800	1	18	2	2	3	2	1	2
2	801	1	19	2	2	3	2	3	3
2	802	1	16	2	2	3	2	1	9
2	803	1	18	2	2	3	2	2	2
2	804	1	18	2	2	3	2	1	3
2	805	1	18	2	2	3	2	1	2
2	806	1	20	3	2	3	2	3	3
2	807	1	15	2	2	2	2	1	2
2	808	1	16	2	2	2	2	2	2
2	809	2	18	2	2	1	3	1	1
2	810	2	15	2	9	1	3	1	2
2	811	2	14	1	2	3	3	1	1
2	812	2	17	2	2	3	3	1	1
2	813	2	17	2	2	3	3	1	2
2	814	2	16	2	2	3	3	1	2
2	815	2	16	2	2	3	3	1	2
2	816	2	17	2	2	3	3	1	1
2	817	2	17	2	2	3	3	1	3
2	818	2	16	2	2	3	3	1	1
2	819	2	17	2	2	3	3	3	2
2	820	2	17	2	2	3	3	1	1
2	821	2	0	#NULL!	2	3	3	1	2
2	822	2	17	2	2	3	3	1	2
2	823	2	16	2	2	3	3	1	1
2	824	2	19	2	2	3	3	1	1
2	825	2	16	2	2	3	3	1	2
2	826	2	17	2	2	3	3	1	2
2	827	2	15	2	2	1	3	1	2
2	828	9	0	#NULL!	2	1	3	1	2
2	829	9	0	#NULL!	9	9	9	9	9
2	830	2	15	2	2	1	3	9	9
2	831	2	11	1	2	1	3	1	1
2	832	2	15	2	2	1	3	1	1
2	833	2	15	2	2	1	3	1	1
2	834	1	14	1	2	1	3	1	2
2	835	1	14	1	2	1	3	1	1
2	836	1	14	1	2	1	3	1	1
2	837	1	0	#NULL!	2	1	3	1	1
2	838	2	15	2	2	1	3	1	3
2	839	1	14	1	2	1	3	1	1
2	840	2	16	2	2	1	3	1	1
2	841	1	16	2	2	1	3	2	2
2	842	2	15	2	2	1	3	1	1
2	843	2	14	1	2	1	3	1	1
2	844	2	14	1	2	1	3	1	1
2	845	2	13	1	2	1	3	1	2

2	846	2	14	1	2	1	3	1	2
2	847	2	15	2	2	1	3	1	1
2	848	2	14	1	2	1	3	1	2
2	849	1	0	#NULL!	2	1	2	1	2
2	850	2	16	2	2	1	1	1	1
2	851	2	15	2	2	2	1	1	2
2	852	2	14	1	2	2	1	1	1
2	853	2	15	2	2	2	1	1	2
2	854	2	16	2	2	2	1	1	2
2	855	2	14	1	2	2	1	1	1
2	856	2	15	2	2	2	1	1	2
2	857	2	16	2	2	2	1	1	1
2	858	2	15	2	2	2	1	1	1
2	859	2	15	2	2	2	1	2	2
2	860	2	16	2	2	2	1	1	2
2	861	2	16	2	2	2	1	1	1
2	862	2	16	2	9	9	9	9	9
2	863	2	16	2	2	2	1	2	2
2	864	2	15	2	2	2	1	2	2
2	865	2	15	2	2	2	1	2	2
2	866	2	0	#NULL!	2	3	1	1	1
2	867	2	15	2	2	2	3	2	2
2	868	1	19	2	2	3	2	1	9
2	869	1	23	3	9	3	9	9	9
2	870	1	15	2	2	2	2	1	2
2	871	1	15	2	2	1	2	1	3
2	872	1	15	2	2	1	2	2	2
2	873	1	16	2	2	1	2	1	2
2	874	1	16	2	2	1	2	1	3
2	875	1	16	2	2	1	2	2	2
2	876	1	15	2	2	1	2	2	2
2	877	1	0	#NULL!	9	9	9	9	9
2	878	1	19	2	2	2	2	1	3
2	879	1	17	2	2	2	2	1	2
2	880	1	16	2	1	1	3	1	1
2	881	1	18	2	2	2	2	1	1
2	882	1	17	2	9	2	2	1	1
2	883	1	19	2	2	2	9	9	9
2	884	1	16	2	2	2	2	1	2
2	885	1	16	2	2	1	3	1	3
2	886	1	15	2	2	1	3	1	1
2	887	1	17	2	2	2	3	3	3
2	888	1	16	2	2	2	3	2	2
2	889	2	15	2	2	1	3	1	1
2	890	2	16	2	2	2	3	1	1
2	891	1	15	2	2	1	3	1	1
2	892	1	16	2	2	2	3	2	2

2	893	1	15	2	2	1	3	2	2
2	894	2	18	2	2	3	3	1	1
2	895	2	17	2	2	3	3	1	1
2	896	1	15	2	2	9	3	9	9
2	897	2	14	1	2	1	3	2	2
2	898	1	16	2	2	2	3	2	2
2	899	2	14	1	2	1	3	2	2
2	900	2	15	2	2	1	3	2	2
2	901	1	18	2	2	3	3	2	2
2	902	2	15	2	2	2	3	1	1
2	903	2	16	2	2	2	3	2	2
2	904	2	18	2	2	3	3	1	1
2	905	2	18	2	2	3	3	1	1
2	906	1	17	2	2	3	3	2	2
2	907	2	14	1	2	1	3	1	1
2	908	1	17	2	2	3	3	2	2
2	909	2	15	2	2	2	3	1	1
2	910	1	16	2	2	2	3	2	2
2	911	2	13	1	2	1	3	2	2
2	912	2	16	2	2	2	3	1	1
2	913	2	16	2	2	1	3	1	2
2	914	2	15	2	2	1	3	1	1
2	915	2	15	2	2	1	3	1	1
2	916	1	16	2	2	2	3	1	2
2	917	9	0	#NULL!	2	1	3	1	2
2	918	9	0	#NULL!	9	9	9	9	9
2	919	1	15	2	2	1	3	2	2
2	920	2	14	1	2	1	3	1	2
2	921	2	15	2	2	1	2	1	1
2	922	2	15	2	2	1	3	1	2
2	923	1	16	2	2	1	3	1	2
2	924	1	15	2	2	1	3	3	3
2	925	1	25	3	2	3	2	1	2
2	926	9	17	2	2	3	2	1	1
2	927	1	15	2	2	1	2	1	2
2	928	9	0	#NULL!	2	1	2	1	1
2	929	1	15	2	2	1	2	3	3
2	930	1	16	2	2	1	2	1	2
2	931	1	14	1	2	1	2	1	2
2	932	1	15	2	2	1	2	1	2
2	933	1	14	1	2	1	2	1	1
2	934	1	0	#NULL!	9	1	2	1	2
2	935	1	18	2	2	1	2	1	2
2	936	1	16	2	2	1	2	2	1
2	937	1	18	2	2	1	2	1	2
2	938	1	19	2	9	1	1	9	9
2	939	1	16	2	2	1	2	1	1

2	940	1	17	2	2	1	2	3	2
2	941	1	17	2	2	1	2	1	3
2	942	2	15	2	2	1	3	1	1
2	943	1	16	2	2	1	3	1	1
2	944	1	17	2	2	3	3	1	1
2	945	1	15	2	2	1	3	1	1
2	946	2	16	2	2	1	3	1	1
2	947	1	16	2	2	2	3	1	1
2	948	1	14	1	2	1	3	2	2
2	949	2	15	2	2	1	3	1	1
2	950	1	15	2	2	1	3	1	1
2	951	2	16	2	2	2	3	1	1
2	952	2	16	2	2	2	3	1	1
2	953	2	18	2	2	3	3	1	1
2	954	1	17	2	2	3	3	1	1
2	955	2	16	2	2	2	3	1	1
2	956	2	14	1	2	1	3	1	1
2	957	2	16	2	2	1	3	1	1
2	958	2	16	2	2	2	3	1	1
2	959	1	16	2	2	1	3	2	2
2	960	2	15	2	2	1	3	1	1
2	961	1	17	2	2	2	3	2	2
2	962	9	0	#NULL!	9	9	9	9	9
2	963	1	18	2	2	3	3	1	1
2	964	2	16	2	9	2	3	1	1
2	965	2	14	1	2	1	3	1	1
2	966	2	15	2	2	1	3	1	1
2	967	2	15	2	2	1	3	1	1
2	968	2	15	2	2	1	3	3	3
2	969	2	16	2	2	3	3	1	1
2	970	1	16	2	2	3	3	3	3
2	971	1	18	2	2	3	3	3	3
2	972	2	17	2	2	3	3	1	1
2	973	2	16	2	2	2	3	1	1
2	974	1	15	2	2	1	3	1	1
2	975	1	16	2	2	2	3	3	3
2	976	2	15	2	2	1	3	1	1
2	977	2	14	1	2	1	3	1	1
2	978	1	16	2	2	9	3	9	9
2	979	1	16	2	2	3	3	2	2
2	980	1	16	2	2	2	3	1	1
2	981	1	17	2	2	2	3	2	2
2	982	2	17	2	2	3	3	1	1
2	983	1	17	2	2	2	3	2	2
2	984	2	15	2	2	1	3	1	1
2	985	1	16	2	2	2	3	2	2
2	986	2	18	2	2	3	3	1	1

2	987	2	15	2	1	1	3	1	1
2	988	1	15	2	2	1	3	2	2
2	989	2	16	2	2	2	3	1	1
2	990	2	15	2	2	2	1	1	1
2	991	2	19	2	2	3	3	1	1
2	992	1	17	2	2	2	3	2	2
2	993	2	17	2	2	3	3	1	1
2	994	2	18	2	2	3	3	1	1
2	995	1	16	2	2	3	3	1	1
2	996	2	16	2	2	2	3	1	1
2	997	1	17	2	2	3	3	3	3
2	998	1	16	2	2	2	3	1	1
2	999	1	15	2	2	1	3	3	3
2	1000	2	15	2	2	1	1	3	1
2	1001	2	16	2	2	2	3	1	1
2	1002	1	16	2	2	2	3	3	3
2	1003	2	15	2	2	1	3	1	1
2	1004	9	0	#NULL!	9	9	9	9	9
2	1005	1	16	2	2	2	2	1	2
2	1006	2	15	2	1	1	1	1	1
2	1007	2	16	2	1	1	1	1	1
2	1008	2	16	2	1	1	1	1	1
2	1009	2	15	2	1	1	1	1	1
2	1010	2	15	2	1	1	1	1	1
2	1011	2	15	2	1	1	1	1	1
2	1012	2	19	2	1	1	1	1	1
2	1013	2	14	1	1	1	1	1	1
2	1014	2	15	2	1	1	1	1	1
2	1015	2	15	2	1	1	1	1	1
2	1016	2	14	1	1	1	1	1	1
2	1017	2	17	2	1	1	1	1	1
2	1018	2	15	2	1	1	1	1	1
2	1019	2	13	1	1	1	1	1	1
2	1020	2	15	2	1	1	1	1	1
2	1021	2	0	#NULL!	1	1	1	1	1
2	1022	2	17	2	1	1	1	1	1
2	1023	2	14	1	1	1	1	1	1
2	1024	2	14	1	1	1	1	1	1
2	1025	2	14	1	1	1	1	1	1
2	1026	2	13	1	1	1	1	1	1
2	1027	2	13	1	1	1	1	1	1
2	1028	2	15	2	1	1	1	1	1
2	1029	2	15	2	1	1	1	1	1
2	1030	2	15	2	1	1	1	1	1
2	1031	2	0	#NULL!	9	9	9	9	9
2	1032	2	17	2	1	1	1	1	3
2	1033	2	15	2	2	1	1	1	1

2	1034	2	15	2	1	1	1	3	3
2	1035	2	14	1	1	1	1	1	1
2	1036	9	0	#NULL!	9	9	9	9	9
2	1037	2	15	2	1	1	1	1	2
2	1038	2	16	2	1	1	1	2	2
2	1039	2	13	1	1	1	1	1	1
2	1040	2	14	1	1	1	1	1	2
2	1041	2	14	1	1	1	1	1	1
2	1042	9	0	#NULL!	9	9	9	9	9
2	1043	2	15	2	2	1	1	1	1
2	1044	2	15	2	1	1	1	1	2
2	1045	2	16	2	1	1	1	1	1
2	1046	2	14	1	1	1	1	1	1
2	1047	2	14	1	1	1	1	1	1
2	1048	2	12	1	1	1	1	1	1
2	1049	2	16	2	1	1	1	1	9
2	1050	2	15	2	1	1	1	2	2
2	1051	2	13	1	2	1	1	1	9
2	1052	2	16	2	2	1	1	9	9
2	1053	2	16	2	1	1	1	1	1
2	1054	2	17	2	1	1	1	1	1
2	1055	2	15	2	1	1	1	1	1
2	1056	2	14	1	1	1	1	1	1
2	1057	2	16	2	1	1	1	1	1
2	1058	2	15	2	1	1	1	1	1
2	1059	2	13	1	1	1	1	1	1
2	1060	2	14	1	1	1	1	1	1
2	1061	2	13	1	1	1	1	1	1
2	1062	2	14	1	1	1	1	1	1
2	1063	2	15	2	1	1	1	1	1
2	1064	2	16	2	1	1	1	1	1
2	1065	2	13	1	1	1	1	1	1
2	1066	2	15	2	1	1	1	1	1
2	1067	2	15	2	1	1	1	1	1
2	1068	2	15	2	1	1	1	1	1
2	1069	2	16	2	1	1	1	1	2
2	1070	2	15	2	1	1	1	1	1
2	1071	2	13	1	9	1	1	1	1
2	1072	2	13	1	1	1	1	1	1
2	1073	2	15	2	1	1	1	1	1
2	1074	2	15	2	1	1	1	1	1
2	1075	2	14	1	1	1	1	1	1
2	1076	2	15	2	1	1	1	1	1
2	1077	2	14	1	1	1	1	1	1
2	1078	2	15	2	2	1	1	1	2
2	1079	2	15	2	1	1	1	1	1
2	1080	2	15	2	1	1	1	1	1

2	1081	2	15	2	1	1	1	1	1
2	1082	2	15	2	1	1	1	1	1
2	1083	2	16	2	1	1	1	1	3
2	1084	2	14	1	1	1	1	1	1
2	1085	2	15	2	1	1	1	1	1
2	1086	2	14	1	1	1	1	1	1
2	1087	2	15	2	1	1	1	1	3
2	1088	9	0	#NULL!	9	9	9	9	9
2	1089	2	14	1	1	1	1	1	1
2	1090	2	15	2	1	1	1	1	1
2	1091	2	14	1	1	1	1	1	1
2	1092	2	14	1	1	1	1	1	1
2	1093	2	15	2	1	1	1	1	1
2	1094	2	15	2	1	1	1	1	1
2	1095	2	15	2	1	1	1	1	1
2	1096	2	13	1	1	1	1	1	1
2	1097	2	16	2	1	1	1	1	1
2	1098	2	15	2	1	1	1	1	1
2	1099	2	15	2	1	1	1	1	1
2	1100	2	14	1	1	1	1	1	1
2	1101	2	14	1	1	1	1	1	1
2	1102	9	0	#NULL!	9	9	9	9	9
2	1103	2	15	2	1	1	1	1	1
2	1104	2	14	1	1	1	1	1	1
2	1105	2	14	1	1	1	1	1	1
2	1106	2	15	2	1	1	1	1	1
2	1107	2	16	2	1	1	1	1	1
2	1108	2	15	2	1	1	1	1	1
2	1109	2	16	2	1	1	1	1	1
2	1110	2	15	2	1	1	1	1	1
2	1111	2	14	1	1	1	1	1	1
2	1112	2	15	2	1	1	1	1	1
2	1113	2	16	2	1	1	1	1	2
2	1114	2	16	2	1	1	1	1	1
2	1115	2	15	2	1	1	1	1	1
2	1116	2	0	#NULL!	1	1	1	1	1
2	1117	2	15	2	1	1	1	1	1
2	1118	2	15	2	1	1	1	1	1
2	1119	2	16	2	1	1	1	1	1
2	1120	2	15	2	1	1	1	1	1
2	1121	2	16	2	1	1	1	1	1
2	1122	2	16	2	1	1	1	1	1
2	1123	2	14	1	1	1	1	1	1
2	1124	2	14	1	1	1	1	1	1
2	1125	2	15	2	1	1	1	1	1
2	1126	2	0	#NULL!	1	1	1	1	1
2	1127	2	14	1	1	1	1	1	1

2	1128	2	15	2	1	1	1	1	1
2	1129	2	13	1	1	1	1	1	1
2	1130	2	14	1	1	1	1	1	1
2	1131	2	15	2	1	1	1	1	1
2	1132	2	15	2	1	1	1	1	1
2	1133	2	15	2	1	1	1	1	1
2	1134	2	13	1	1	1	1	1	1
2	1135	2	18	2	1	1	1	1	1
2	1136	2	14	1	1	1	1	1	1
2	1137	2	14	1	1	1	1	1	1
2	1138	2	13	1	1	1	1	1	1
2	1139	2	17	2	1	1	1	1	1
2	1140	2	16	2	1	1	1	1	1
2	1141	2	17	2	1	1	1	1	1
2	1142	2	15	2	1	1	1	1	1
2	1143	2	0	#NULL!	1	1	1	1	1
2	1144	2	14	1	1	1	1	1	1
2	1145	2	17	2	1	1	1	1	1
2	1146	2	17	2	1	1	1	1	1
2	1147	9	0	#NULL!	9	9	9	9	9
2	1148	2	14	1	1	1	1	1	1
2	1149	2	15	2	1	1	1	1	1
2	1150	2	15	2	1	1	1	1	1
2	1151	2	13	1	1	1	1	1	1
2	1152	2	14	1	1	1	1	1	2
2	1153	2	16	2	1	1	1	1	1
2	1154	2	14	1	1	1	1	1	1
2	1155	2	15	2	1	1	1	1	1
2	1156	2	13	1	1	1	1	1	1
2	1157	2	16	2	1	1	1	1	1
2	1158	2	15	2	1	1	1	1	1
2	1159	2	14	1	1	1	1	1	1
2	1160	2	15	2	1	1	1	1	1
2	1161	2	14	1	1	1	1	1	1
2	1162	2	14	1	1	1	1	1	1
2	1163	2	14	1	1	1	1	1	1
2	1164	2	13	1	1	1	1	1	1
2	1165	2	13	1	1	1	1	1	1
2	1166	2	14	1	1	1	1	1	1
2	1167	2	16	2	1	1	1	1	1
2	1168	2	16	2	1	1	1	1	1
2	1169	9	0	#NULL!	9	9	9	9	9
2	1170	2	15	2	1	1	1	1	1
2	1171	2	16	2	1	1	1	1	1
2	1172	2	15	2	1	1	1	1	1
2	1173	2	17	2	1	1	1	1	1
2	1174	2	17	2	1	1	1	1	1

2	1175	2	18	2	1	1	1	1	1
2	1176	2	15	2	1	1	1	1	1
2	1177	2	14	1	1	1	1	1	1
2	1178	2	15	2	1	1	1	1	1
2	1179	2	15	2	1	1	1	1	1
2	1180	2	15	2	1	1	1	1	1
2	1181	2	14	1	1	1	1	1	1
2	1182	2	19	2	1	1	1	1	1
2	1183	2	15	2	1	1	1	1	1
2	1184	2	13	1	1	1	1	1	1
2	1185	2	15	2	1	1	1	1	1
2	1186	2	13	1	1	1	1	1	1
2	1187	2	15	2	1	1	1	1	1
2	1188	2	17	2	1	1	1	1	1
2	1189	2	16	2	1	1	1	1	1
2	1190	2	14	1	1	1	1	1	1
2	1191	2	14	1	1	1	1	1	1
2	1192	2	16	2	1	1	1	1	1
2	1193	2	14	1	1	1	1	1	1
2	1194	2	14	1	1	1	1	1	1
2	1195	2	15	2	1	1	1	1	1
2	1196	2	14	1	1	1	1	1	1
2	1197	2	16	2	2	1	1	1	1
2	1198	2	13	1	1	1	1	1	1
2	1199	2	14	1	1	1	1	1	1
2	1200	2	14	1	1	1	1	1	1
2	1201	2	14	1	1	1	1	1	1
2	1202	2	15	2	1	1	1	1	2
2	1203	9	0	#NULL!	1	1	1	1	9
2	1204	2	14	1	1	1	1	1	3
2	1205	2	14	1	1	1	1	1	1
2	1206	2	15	2	9	1	1	1	1
2	1207	2	16	2	1	1	1	1	2
2	1208	2	17	2	9	1	1	1	1
2	1209	2	15	2	1	1	1	1	2
2	1210	2	16	2	1	1	1	1	1
2	1211	2	14	1	1	1	1	1	3
2	1212	2	15	2	1	1	1	1	3
2	1213	2	14	1	9	1	1	9	9
2	1214	2	16	2	1	1	1	1	2
2	1215	2	15	2	2	1	1	1	2
2	1216	2	14	1	1	1	1	1	2
2	1217	2	14	1	1	1	1	1	1
2	1218	2	17	2	1	1	1	1	1
2	1219	2	14	1	1	1	1	1	1
2	1220	2	15	2	1	1	1	1	1
2	1221	9	16	2	1	1	1	2	2

2	1222	2	15	2	1	1	1	1	1
2	1223	2	14	1	1	1	1	1	1
2	1224	2	15	2	1	1	1	1	2
2	1225	9	14	1	2	1	1	1	1
2	1226	2	15	2	1	1	1	1	1
2	1227	2	15	2	1	1	1	1	2
2	1228	2	13	1	1	1	1	1	1
2	1229	2	16	2	1	1	1	1	1
2	1230	2	14	1	1	1	1	1	1
2	1231	2	0	#NULL!	1	1	1	1	1
2	1232	9	9	1	9	9	9	9	9
2	1233	2	16	2	1	1	3	9	9
2	1234	2	15	2	1	1	1	1	1
2	1235	2	17	2	1	1	3	1	1
2	1236	1	15	2	1	1	3	1	2
2	1237	2	16	2	1	1	3	1	2
2	1238	2	22	3	1	1	3	1	2
2	1239	2	16	2	1	1	3	1	1
2	1240	2	15	2	1	1	3	2	3
2	1241	1	15	2	1	1	3	1	2
2	1242	2	15	2	1	1	3	1	1
2	1243	2	15	2	1	1	3	2	2
2	1244	2	14	1	1	1	3	1	2
2	1245	2	14	1	1	1	3	1	3
2	1246	2	15	2	1	1	3	1	2
2	1247	2	15	2	1	1	3	1	1
2	1248	2	17	2	1	1	3	1	2
2	1249	2	13	1	1	1	3	1	2
2	1250	2	14	1	2	1	3	1	1
2	1251	2	16	2	1	1	3	1	1
2	1252	1	16	2	1	1	3	2	2
2	1253	2	15	2	1	1	3	1	2
2	1254	2	15	2	1	1	3	1	2
2	1255	2	15	2	1	1	3	1	2
2	1256	1	14	1	1	1	3	1	1
2	1257	2	15	2	9	1	3	1	2
2	1258	2	14	1	2	1	3	1	3
2	1259	2	15	2	1	1	3	1	1
2	1260	2	14	1	1	1	3	1	1
2	1261	2	16	2	1	1	3	1	2
2	1262	9	0	#NULL!	1	1	3	2	3
2	1263	1	17	2	1	1	3	2	2
2	1264	2	15	2	2	1	3	2	1
2	1265	9	16	2	2	1	3	1	1
2	1266	2	15	2	1	1	1	1	2
2	1267	2	15	2	1	1	1	1	2
2	1268	2	14	1	1	1	3	2	3

2	1269	2	17	2	2	1	3	1	2
2	1270	1	15	2	1	1	3	2	2
2	1271	2	20	3	1	1	3	1	2
2	1272	2	15	2	1	1	3	1	1
2	1273	1	15	2	1	1	3	1	2
2	1274	2	13	1	1	1	3	2	2
2	1275	2	14	1	1	1	3	1	1
2	1276	2	14	1	1	1	3	2	2
2	1277	2	14	1	1	1	3	2	2
2	1278	2	15	2	1	1	3	1	2
2	1279	2	14	1	1	1	3	1	1
2	1280	1	15	2	1	1	3	2	2
2	1281	1	14	1	1	1	3	1	1
2	1282	2	14	1	1	1	3	2	2
2	1283	2	19	2	1	1	3	1	2
2	1284	2	16	2	1	1	3	1	1
2	1285	2	14	1	1	1	3	2	2
2	1286	1	15	2	2	1	3	2	2
2	1287	1	18	2	1	1	3	3	2
2	1288	1	14	1	1	1	3	1	3
2	1289	1	17	2	1	1	3	1	2
2	1290	2	14	1	1	1	3	2	2
2	1291	1	13	1	1	1	3	1	1
2	1292	2	14	1	1	1	3	1	2
2	1293	1	14	1	1	1	3	1	3
2	1294	2	14	1	1	1	3	1	2
2	1295	2	17	2	1	1	3	2	2
2	1296	2	15	2	2	1	3	2	1
2	1297	2	19	2	1	1	3	1	3
2	1298	2	15	2	1	1	3	1	1
2	1299	2	14	1	1	1	3	1	1
2	1300	9	0	#NULL!	9	9	9	9	9
2	1301	2	15	2	1	1	3	2	2
2	1302	2	14	1	1	1	3	1	1
2	1303	2	14	1	1	1	3	1	2
2	1304	9	0	#NULL!	9	9	9	9	9
2	1305	2	16	2	1	1	3	1	1
2	1306	2	15	2	1	1	1	1	1
2	1307	9	0	#NULL!	9	9	9	9	9
2	1308	2	15	2	2	1	3	1	1
2	1309	1	15	2	1	1	3	2	2
2	1310	2	16	2	1	1	3	1	1
2	1311	1	17	2	1	1	3	1	1
2	1312	2	15	2	1	1	3	1	1
2	1313	1	15	2	1	1	3	1	1
2	1314	2	14	1	1	1	1	1	1
2	1315	2	12	1	1	1	1	1	3

2	1316	2	13	1	1	1	3	1	1
2	1317	1	14	1	1	1	3	1	1
2	1318	2	16	2	1	1	3	1	3
2	1319	9	0	#NULL!	9	9	9	9	9
2	1320	2	15	2	1	1	3	1	1
2	1321	2	16	2	1	1	3	1	1
2	1322	2	14	1	1	1	3	1	1
2	1323	1	15	2	1	1	3	3	3
2	1324	1	14	1	1	1	3	1	3
2	1325	2	15	2	1	1	3	3	2
2	1326	9	0	#NULL!	9	9	9	9	9
2	1327	1	19	2	1	1	3	3	3
2	1328	1	14	1	1	1	3	2	2
2	1329	1	17	2	1	1	3	1	2
2	1330	9	0	#NULL!	9	9	9	9	9
2	1331	2	15	2	1	1	3	3	1
2	1332	1	17	2	1	1	3	1	1
2	1333	2	14	1	1	1	3	1	1
2	1334	2	14	1	1	1	3	2	2
2	1335	1	18	2	1	1	3	1	2
2	1336	2	14	1	1	1	3	1	1
2	1337	2	16	2	1	1	3	1	2
2	1338	9	0	#NULL!	9	9	9	9	9
2	1339	9	0	#NULL!	9	9	9	9	9
2	1340	2	16	2	1	1	3	1	1
2	1341	2	15	2	1	1	3	1	3
2	1342	1	14	1	1	1	3	1	2
2	1343	2	13	1	1	1	3	1	3
2	1344	2	15	2	1	1	3	1	1
2	1345	2	14	1	1	1	3	1	1
2	1346	2	12	1	1	1	1	1	1
2	1347	1	15	2	1	1	3	2	2
2	1348	1	14	1	1	1	3	2	2
2	1349	1	19	2	1	1	9	2	1
2	1350	1	15	2	1	1	3	2	1
2	1351	2	15	2	1	1	3	9	9
2	1352	1	15	2	1	1	3	1	1
2	1353	1	14	1	1	1	3	1	1
2	1354	2	15	2	1	1	3	1	1
2	1355	2	15	2	1	1	3	1	2
2	1356	2	17	2	1	1	3	1	1
2	1357	2	14	1	1	1	3	1	1
2	1358	2	16	2	1	1	3	1	3
2	1359	1	14	1	1	1	3	1	2
2	1360	1	15	2	1	1	3	2	2
2	1361	1	14	1	1	1	2	2	2
2	1362	1	14	1	1	1	3	2	1

2	1363	2	15	2	1	1	3	3	2
2	1364	2	16	2	1	1	3	1	2
2	1365	2	15	2	1	1	3	1	2
2	1366	1	17	2	1	1	3	1	2
2	1367	2	18	2	1	1	3	2	2
2	1368	2	14	1	1	1	3	1	1
2	1369	2	19	2	1	1	3	1	3
2	1370	1	16	2	1	1	3	1	1
2	1371	2	15	2	1	1	3	1	1
2	1372	2	16	2	1	1	3	2	3
2	1373	1	18	2	1	1	3	1	2
2	1374	1	17	2	1	1	3	1	1
2	1375	1	19	2	2	1	3	2	2
2	1376	1	16	2	1	1	3	3	3
2	1377	1	18	2	1	1	3	1	2
2	1378	2	14	1	1	1	3	1	1
2	1379	2	15	2	1	1	3	2	2
2	1380	1	15	2	1	1	3	1	2
2	1381	2	16	2	1	1	3	2	2
2	1382	2	15	2	1	1	3	1	2
2	1383	1	12	1	1	1	3	1	2
2	1384	2	14	1	1	1	3	2	3
2	1385	2	15	2	1	1	3	2	9
2	1386	1	15	2	1	1	3	1	1
2	1387	2	17	2	1	1	3	1	3
2	1388	9	0	#NULL!	9	9	9	9	9
2	1389	2	14	1	1	1	3	1	2
2	1390	1	16	2	1	1	3	2	2
2	1391	1	19	2	1	1	3	1	1
2	1392	2	16	2	1	1	3	1	3
2	1393	2	14	1	1	1	3	1	2
2	1394	2	14	1	1	1	3	1	2
2	1395	2	15	2	1	1	3	1	2
2	1396	1	16	2	1	1	3	1	1
2	1397	1	17	2	1	1	3	1	1
2	1398	2	50	3	9	9	9	9	9
2	1399	1	15	2	1	1	3	2	2
2	1400	2	14	1	1	1	3	1	9
2	1401	2	0	#NULL!	1	1	3	1	1
2	1402	2	14	1	1	1	3	1	2
2	1403	1	16	2	1	1	3	1	1
2	1404	1	15	2	1	1	3	3	3
2	1405	2	14	1	1	1	3	1	1
2	1406	1	12	1	1	1	3	1	3
2	1407	1	14	1	1	1	3	1	3
2	1408	2	14	1	1	1	1	1	1
2	1409	1	16	2	2	1	3	2	3

2	1410	2	14	1	1	1	3	1	1
2	1411	1	15	2	1	1	3	1	2
2	1412	2	14	1	1	1	3	1	1
2	1413	2	14	1	1	1	3	1	2
2	1414	1	17	2	1	1	3	1	3
2	1415	1	2	1	1	1	3	1	3
2	1416	1	14	1	1	1	3	1	1
2	1417	1	14	1	1	1	3	1	1
2	1418	2	13	1	1	1	3	1	1
2	1419	2	14	1	1	1	3	1	1
2	1420	2	15	2	1	1	3	1	2
2	1421	1	14	1	1	1	3	3	3
2	1422	2	15	2	1	1	3	1	2
2	1423	2	16	2	1	1	3	1	1
2	1424	1	17	2	1	1	3	1	2
2	1425	2	14	1	1	1	3	1	1
2	1426	2	15	2	1	1	3	3	2
2	1427	2	14	1	1	1	3	1	1
2	1428	2	15	2	1	1	3	1	3
2	1429	2	16	2	1	1	3	1	2
2	1430	2	15	2	1	1	3	1	1
2	1431	1	17	2	1	3	3	1	1
2	1432	2	17	2	1	3	3	1	1
2	1433	1	16	2	1	3	3	1	1
2	1434	1	18	2	1	3	3	3	2
2	1435	2	17	2	1	3	3	1	1
2	1436	2	16	2	1	3	3	1	1
2	1437	2	16	2	1	3	3	1	1
2	1438	2	16	2	1	3	3	1	1
2	1439	2	16	2	1	3	3	1	1
2	1440	2	15	2	1	3	3	1	1
2	1441	2	16	2	1	3	3	1	2
2	1442	2	17	2	1	3	3	1	2
2	1443	2	16	2	1	3	3	1	1
2	1444	1	14	1	2	3	3	1	1
2	1445	2	17	2	1	3	3	1	1
2	1446	2	17	2	1	3	3	1	3
2	1447	2	16	2	1	3	3	1	1
2	1448	1	35	3	1	1	3	1	1
2	1449	1	10	1	1	2	3	3	1
2	1450	1	20	3	1	1	3	1	1
2	1451	9	0	#NULL!	9	9	9	9	9
2	1452	9	0	#NULL!	9	9	9	9	9
2	1453	2	25	3	2	1	1	1	1
2	1454	1	30	3	2	1	2	1	1
2	1455	9	0	#NULL!	9	9	9	9	9
2	1456	1	28	3	1	1	3	1	1

2	1457	9	0	#NULL!	9	9	9	9	9
2	1458	1	0	#NULL!	1	3	3	1	1
2	1459	1	25	3	1	3	3	2	2
2	1460	1	30	3	1	3	3	2	2
2	1461	2	17	2	1	3	3	1	1
2	1462	2	17	2	1	3	3	1	1
2	1463	1	16	2	2	3	3	1	3
2	1464	1	29	3	1	3	3	1	1
2	1465	1	13	1	1	3	3	1	1
2	1466	9	0	#NULL!	9	9	9	9	9
2	1467	2	20	3	1	1	1	1	1
2	1468	2	15	2	1	3	3	2	1
2	1469	2	14	1	1	3	1	9	9
2	1470	1	20	3	1	3	3	1	1
2	1471	1	18	2	1	3	3	1	1
2	1472	9	0	#NULL!	9	9	9	9	9
2	1473	9	0	#NULL!	9	9	9	9	9
2	1474	2	13	1	1	3	3	1	1
2	1475	2	13	1	2	3	3	1	2
2	1476	2	17	2	1	3	3	1	1
2	1477	1	8	1	2	1	1	2	3
2	1478	2	19	2	1	3	3	1	1
2	1479	2	16	2	1	3	3	1	3
2	1480	2	18	2	1	3	3	3	1
2	1481	1	17	2	1	3	3	2	2
2	1482	2	18	2	1	3	1	1	1
2	1483	2	16	2	1	3	3	1	1
2	1484	2	17	2	1	3	3	9	1
2	1485	9	0	#NULL!	9	9	9	9	9
2	1486	2	17	2	1	3	3	2	2
2	1487	1	19	2	1	3	3	1	1
2	1488	2	16	2	1	3	3	1	2
2	1489	2	18	2	1	3	3	3	3
2	1490	1	16	2	2	3	3	1	1
2	1491	2	14	1	1	3	3	1	1
2	1492	2	13	1	1	3	3	1	2
2	1493	2	13	1	1	3	1	2	2
2	1494	2	17	2	9	3	3	1	2
2	1495	1	40	3	1	3	3	2	2
2	1496	2	15	2	1	3	3	1	1
2	1497	1	20	3	1	3	1	3	3
2	1498	2	14	1	1	3	3	1	1
2	1499	1	12	1	1	3	3	2	3
2	1500	2	14	1	2	3	3	1	1
2	1501	2	12	1	1	3	3	3	3
2	1502	9	0	#NULL!	1	2	2	1	1
2	1503	2	15	2	1	3	3	1	1

2	1504	2	16	2	1	3	3	2	3
2	1505	2	16	2	1	3	3	1	2
2	1506	1	17	2	1	3	3	3	3
2	1507	2	17	2	1	3	3	1	2
2	1508	9	0	#NULL!	2	1	3	1	2
2	1509	2	18	2	1	3	3	2	2
2	1510	2	17	2	1	3	3	2	1
2	1511	2	17	2	1	3	3	1	2
2	1512	2	19	2	9	3	3	1	2
2	1513	2	16	2	1	3	3	1	2
2	1514	2	16	2	1	3	3	1	2
2	1515	2	14	1	1	3	3	1	1
2	1516	9	0	#NULL!	1	3	3	1	1
2	1517	2	15	2	1	3	3	2	2
2	1518	2	18	2	1	3	3	1	2
2	1519	2	17	2	1	3	3	1	2
2	1520	2	16	2	1	3	3	1	2
2	1521	2	17	2	1	3	3	1	2
2	1522	2	16	2	2	1	3	1	1
2	1523	2	17	2	1	3	3	1	2
2	1524	2	19	2	1	3	3	3	1
2	1525	2	16	2	1	3	3	1	1
2	1526	1	15	2	1	3	3	1	2
2	1527	9	0	#NULL!	9	9	9	9	9
2	1528	1	17	2	1	3	3	9	9
2	1529	1	18	2	1	3	3	1	2
2	1530	1	18	2	1	3	3	1	2
2	1531	1	19	2	1	3	3	1	2
2	1532	1	20	3	1	3	3	1	1
2	1533	2	19	2	1	3	3	1	1
2	1534	2	17	2	1	3	3	2	2
2	1535	2	16	2	1	3	3	1	1
2	1536	1	20	3	1	3	3	2	2
2	1537	2	18	2	1	3	3	3	3
2	1538	2	17	2	1	3	3	1	1
2	1539	2	16	2	1	3	3	1	1
2	1540	1	15	2	1	3	3	1	2
2	1541	9	18	2	1	3	3	1	2
2	1542	1	13	1	1	3	3	1	1
2	1543	1	18	2	1	3	3	1	1
2	1544	1	16	2	2	3	3	1	3
2	1545	1	16	2	1	3	3	2	2
2	1546	2	19	2	1	3	3	1	1
2	1547	2	16	2	1	3	3	1	1
2	1548	2	17	2	1	3	3	2	2
2	1549	2	18	2	1	3	3	1	2
2	1550	1	0	#NULL!	1	3	3	1	2

2	1551	1	19	2	1	3	3	1	1
2	1552	1	17	2	1	3	3	3	9
2	1553	1	18	2	1	3	3	1	1
2	1554	9	0	#NULL!	9	9	9	9	9
2	1555	1	17	2	1	3	3	3	2
2	1556	2	0	#NULL!	1	3	3	1	2
2	1557	9	0	#NULL!	9	9	9	9	9
2	1558	9	0	#NULL!	9	9	9	9	9
2	1559	9	0	#NULL!	9	9	9	9	9
2	1560	2	14	1	1	3	3	1	1
2	1561	2	18	2	1	3	3	1	1
2	1562	2	17	2	1	3	3	1	1
2	1563	2	17	2	1	3	3	1	1
2	1564	1	17	2	1	3	3	3	3
2	1565	2	13	1	1	3	3	1	1
2	1566	1	17	2	1	3	3	1	1
2	1567	2	17	2	1	3	3	1	1
2	1568	2	16	2	2	3	3	1	2
2	1569	2	16	2	1	3	3	2	1
2	1570	2	16	2	1	3	3	1	2
2	1571	2	15	2	1	3	3	1	2
2	1572	2	17	2	1	3	3	1	2
2	1573	2	15	2	2	3	3	1	2
2	1574	2	16	2	1	3	3	1	1
2	1575	1	17	2	1	3	3	1	1
2	1576	2	18	2	2	3	3	2	2
2	1577	2	16	2	2	3	3	2	1
2	1578	2	15	2	1	3	3	1	1
2	1579	9	0	#NULL!	9	9	9	9	9
2	1580	1	17	2	1	3	3	2	1
2	1581	9	0	#NULL!	9	9	9	9	9
2	1582	1	17	2	1	3	3	1	1
2	1583	1	17	2	1	3	3	3	3
2	1584	1	19	2	1	2	3	1	1
2	1585	2	17	2	1	2	1	2	2
2	1586	1	16	2	1	2	3	2	2
2	1587	2	16	2	1	2	3	1	2
2	1588	2	16	2	2	2	3	1	1
2	1589	9	16	2	1	2	3	1	1
2	1590	9	0	#NULL!	9	9	9	9	9
2	1591	2	16	2	1	2	3	1	1
2	1592	1	18	2	1	2	3	1	2
2	1593	2	0	#NULL!	1	2	3	2	2
2	1594	2	17	2	1	2	3	1	1
2	1595	2	16	2	1	2	3	1	1
2	1596	2	16	2	1	2	3	2	2
2	1597	2	14	1	1	2	3	1	2

2	1598	9	0	#NULL!	9	9	9	9	9
2	1599	2	0	#NULL!	1	2	3	1	2
2	1600	2	17	2	1	2	3	1	1
2	1601	9	0	#NULL!	9	9	9	9	9
2	1602	9	0	#NULL!	9	9	9	9	9
2	1603	9	0	#NULL!	9	9	9	9	9
2	1604	9	0	#NULL!	9	9	9	9	9
2	1605	9	0	#NULL!	9	9	9	9	9
2	1606	9	0	#NULL!	9	9	9	9	9
2	1607	9	0	#NULL!	9	9	9	9	9
2	1608	9	0	#NULL!	9	9	9	9	9
2	1609	9	0	#NULL!	9	9	9	9	9
2	1610	9	0	#NULL!	9	9	9	9	9
2	1611	9	0	#NULL!	9	9	9	9	9
2	1612	9	0	#NULL!	9	9	9	9	9
2	1613	9	0	#NULL!	9	9	9	9	9
2	1614	9	0	#NULL!	9	9	9	9	9
2	1615	9	0	#NULL!	9	9	9	9	9
2	1616	9	0	#NULL!	9	9	9	9	9
2	1617	9	0	#NULL!	9	9	9	9	9
2	1618	9	0	#NULL!	9	9	9	9	9
2	1619	9	0	#NULL!	9	9	9	9	9
2	1620	9	0	#NULL!	9	9	9	9	9
2	1621	1	16	2	1	2	3	1	1
2	1622	2	15	2	1	2	3	1	2
2	1623	2	15	2	1	2	3	1	1
2	1624	2	15	2	1	2	3	1	1
2	1625	2	14	1	1	2	3	1	2
2	1626	2	15	2	1	2	3	1	1
2	1627	1	15	2	1	2	3	1	1
2	1628	2	17	2	1	2	3	1	1
2	1629	2	18	2	1	2	3	1	3
2	1630	1	16	2	1	2	3	2	2
2	1631	2	15	2	1	2	3	1	1
2	1632	2	16	2	1	2	3	1	1
2	1633	2	14	1	1	2	3	1	1
2	1634	2	15	2	1	2	3	1	1
2	1635	2	16	2	1	2	3	1	1
2	1636	2	16	2	1	2	3	1	1
2	1637	1	16	2	1	2	3	1	1
2	1638	2	19	2	1	2	3	1	1
2	1639	1	18	2	1	2	3	1	1
2	1640	2	14	1	1	2	3	1	1
2	1641	2	17	2	1	2	3	1	1
2	1642	2	15	2	1	2	3	1	2
2	1643	1	17	2	1	2	2	1	2
2	1644	1	16	2	1	2	3	1	1

2	1645	2	14	1	1	2	3	1	1
2	1646	2	17	2	1	2	3	1	1
2	1647	2	16	2	1	2	3	1	1
2	1648	2	14	1	1	2	3	1	1
2	1649	2	15	2	2	2	2	1	1
2	1650	2	15	2	1	2	3	1	2
2	1651	2	15	2	1	2	3	1	1
2	1652	2	17	2	1	2	3	1	1
2	1653	2	16	2	1	2	3	1	2
2	1654	2	18	2	1	2	3	1	2
2	1655	2	16	2	1	2	3	1	1
2	1656	2	16	2	1	2	3	1	1
2	1657	2	16	2	1	2	3	1	1
2	1658	1	16	2	1	2	3	1	1
2	1659	2	14	1	1	2	3	1	1
2	1660	2	15	2	1	2	3	1	2
2	1661	2	16	2	1	2	3	1	1
2	1662	1	16	2	1	2	3	1	2
2	1663	2	16	2	1	2	3	1	1
2	1664	1	14	1	1	2	3	1	1
2	1665	2	17	2	1	2	3	1	1
2	1666	1	17	2	1	2	3	1	3
2	1667	2	15	2	1	2	3	1	1
2	1668	2	17	2	1	2	3	1	1
2	1669	1	17	2	1	2	3	1	1
2	1670	2	19	2	2	2	1	1	1
2	1671	1	0	#NULL!	1	2	3	1	1
2	1672	2	15	2	1	2	3	1	2
2	1673	2	16	2	1	2	3	1	1
2	1674	1	16	2	1	2	3	1	1
2	1675	2	16	2	1	2	1	1	2
2	1676	2	16	2	2	2	3	1	1
2	1677	2	15	2	1	2	3	1	2
2	1678	2	17	2	1	2	3	1	2
2	1679	2	16	2	1	2	3	1	1
2	1680	1	17	2	1	2	3	1	2
2	1681	2	18	2	1	2	3	1	1
2	1682	2	17	2	1	2	3	3	2
2	1683	9	16	2	1	2	2	2	1
2	1684	1	16	2	1	2	3	1	1
2	1685	2	14	1	1	2	3	1	1
2	1686	2	15	2	1	2	3	1	1
2	1687	2	16	2	1	2	3	1	2
2	1688	2	17	2	1	2	3	1	2
2	1689	2	17	2	1	2	3	1	1
2	1690	2	15	2	1	2	3	1	1
2	1691	2	16	2	1	2	3	1	1

2	1692	2	15	2	1	2	3	1	1
2	1693	2	17	2	1	3	3	1	1
2	1694	2	15	2	1	2	3	1	2
2	1695	2	16	2	1	2	3	1	1
2	1696	1	15	2	1	2	3	1	1
2	1697	2	9	1	1	2	3	1	2
2	1698	2	18	2	1	2	3	1	1
2	1699	2	16	2	1	2	3	1	1
2	1700	2	15	2	1	2	3	3	3
2	1701	1	18	2	1	2	3	1	1
2	1702	1	16	2	1	2	3	1	1
2	1703	2	16	2	1	2	3	1	1
2	1704	9	15	2	1	2	3	1	1
2	1705	2	16	2	1	2	3	1	1
2	1706	2	19	2	1	2	3	1	1
2	1707	1	14	1	1	2	3	1	1
2	1708	2	17	2	1	2	3	1	1
2	1709	2	17	2	1	2	3	1	1
2	1710	1	18	2	1	2	3	1	1
2	1711	2	15	2	1	2	3	1	1
2	1712	2	14	1	1	2	3	1	1
2	1713	2	14	1	1	2	3	1	1
2	1714	1	16	2	1	2	3	2	3
2	1715	2	17	2	1	2	3	1	1
2	1716	2	15	2	1	2	3	1	1
2	1717	2	16	2	1	2	3	3	9
2	1718	2	0	#NULL!	2	2	3	1	2
2	1719	2	16	2	1	2	3	1	1
2	1720	2	17	2	1	2	3	1	2
2	1721	2	14	1	1	2	3	1	1
2	1722	9	0	#NULL!	9	9	9	9	9
2	1723	9	0	#NULL!	9	9	9	9	9
2	1724	9	0	#NULL!	9	9	9	9	9
2	1725	9	0	#NULL!	9	9	9	9	9
2	1726	9	0	#NULL!	9	9	9	9	9
2	1727	9	0	#NULL!	9	9	9	9	9
2	1728	9	0	#NULL!	9	9	9	9	9
2	1729	9	0	#NULL!	9	9	9	9	9
2	1730	9	0	#NULL!	9	9	9	9	9
2	1731	9	0	#NULL!	9	9	9	9	9
2	1732	9	0	#NULL!	9	9	9	9	9
2	1733	9	0	#NULL!	9	9	9	9	9
2	1734	9	0	#NULL!	9	9	9	9	9
2	1735	9	0	#NULL!	9	9	9	9	9
2	1736	9	0	#NULL!	9	9	9	9	9
2	1737	9	0	#NULL!	9	9	9	9	9
2	1738	9	0	#NULL!	9	9	9	9	9

2	1739	9	0	#NULL!	9	9	9	9	9
2	1740	9	0	#NULL!	9	9	9	9	9
2	1741	9	0	#NULL!	9	9	9	9	9
2	1742	9	0	#NULL!	9	9	9	9	9
2	1743	9	0	#NULL!	9	9	9	9	9
2	1744	9	0	#NULL!	9	9	9	9	9
2	1745	9	0	#NULL!	9	9	9	9	9
2	1746	9	0	#NULL!	9	9	9	9	9
2	1747	9	0	#NULL!	1	2	3	1	1
2	1748	9	0	#NULL!	9	9	9	9	9
2	1749	9	0	#NULL!	9	9	9	9	9
2	1750	9	0	#NULL!	9	9	9	9	9
2	1751	9	0	#NULL!	9	9	9	9	9
2	1752	9	0	#NULL!	9	9	9	9	9
2	1753	9	0	#NULL!	9	9	9	9	9
2	1754	9	0	#NULL!	9	9	9	9	9
2	1755	9	0	#NULL!	9	9	9	9	9
2	1756	9	0	#NULL!	9	9	9	9	9
2	1757	9	0	#NULL!	9	9	9	9	9
2	1758	2	0	#NULL!	1	2	3	1	1
2	1759	9	0	#NULL!	9	9	9	9	9
2	1760	9	0	#NULL!	9	9	9	9	9
2	1761	9	0	#NULL!	9	9	9	9	9
2	1762	9	0	#NULL!	9	9	9	9	9
2	1763	9	0	#NULL!	9	9	9	9	9
2	1764	9	0	#NULL!	9	9	9	9	9
2	1765	9	0	#NULL!	9	9	9	9	9
2	1766	9	0	#NULL!	9	9	9	9	9
2	1767	9	0	#NULL!	9	9	9	9	9
2	1768	9	0	#NULL!	9	9	9	9	9
2	1769	9	0	#NULL!	9	9	9	9	9
2	1770	9	0	#NULL!	9	9	9	9	9
2	1771	9	0	#NULL!	9	9	9	9	9
2	1772	9	0	#NULL!	9	9	9	9	9
2	1773	9	0	#NULL!	9	9	9	9	9
2	1774	1	16	2	1	2	3	1	1
2	1775	2	17	2	1	2	3	1	1
2	1776	2	16	2	1	2	3	1	1
2	1777	2	17	2	1	2	3	1	1
2	1778	1	17	2	1	2	3	3	3
2	1779	2	17	2	1	2	3	1	1
2	1780	1	15	2	1	2	3	1	1
2	1781	2	17	2	1	2	3	1	1
2	1782	1	15	2	1	2	3	2	2
2	1783	1	15	2	1	2	3	1	3
2	1784	1	16	2	1	2	2	1	2
2	1785	2	16	2	1	2	3	1	1

2	1786	2	15	2	1	2	3	1	1
2	1787	2	0	#NULL!	1	2	3	1	1
2	1788	2	16	2	1	2	3	1	1
2	1789	2	14	1	1	2	3	1	1
2	1790	2	15	2	1	2	3	1	1
2	1791	2	14	1	1	2	3	1	1
2	1792	1	14	1	1	2	3	1	1
2	1793	2	16	2	1	2	3	1	1
2	1794	1	17	2	1	2	3	2	2
2	1795	1	16	2	1	2	3	1	2
2	1796	2	14	1	1	2	3	1	1
2	1797	2	17	2	1	2	3	1	1
2	1798	1	19	2	1	2	3	1	1
2	1799	2	16	2	1	2	3	1	1
2	1800	2	16	2	1	2	3	1	1
2	1801	1	18	2	1	2	3	1	1
2	1802	1	0	#NULL!	1	2	3	1	1
2	1803	1	16	2	2	2	3	1	3
2	1804	2	14	1	1	2	3	1	3
2	1805	2	15	2	1	2	3	1	1
2	1806	2	17	2	1	2	3	1	2
2	1807	2	15	2	2	2	3	1	1
2	1808	2	16	2	1	2	3	1	1
2	1809	2	15	2	1	2	3	1	1
2	1810	1	17	2	1	2	3	2	2
2	1811	1	16	2	1	3	2	1	2
2	1812	2	16	2	1	3	2	2	2
2	1813	1	15	2	1	3	2	2	2
2	1814	1	15	2	1	3	2	1	1
2	1815	1	0	#NULL!	1	3	2	1	1
2	1816	1	17	2	1	3	2	1	1
2	1817	1	0	#NULL!	1	3	2	1	1
2	1818	1	0	#NULL!	1	3	2	1	1
2	1819	1	18	2	1	3	2	1	2
2	1820	1	18	2	1	3	2	1	1
2	1821	1	18	2	1	3	2	1	2
2	1822	1	18	2	1	3	2	1	1
2	1823	1	18	2	1	3	2	1	1
2	1824	1	18	2	1	3	2	1	1
2	1825	1	18	2	1	3	2	1	2
2	1826	1	18	2	1	3	2	1	1
2	1827	2	18	2	2	3	1	1	1
2	1828	1	16	2	1	3	2	1	1
2	1829	1	17	2	1	2	2	1	2
2	1830	1	14	1	2	3	2	2	2
2	1831	1	14	1	1	3	2	2	1
2	1832	1	12	1	1	3	2	2	2

2	1833	1	16	2	1	3	2	2	2
2	1834	1	16	2	1	3	2	2	2
2	1835	1	30	3	1	3	2	2	2
2	1836	1	15	2	1	3	2	2	2
2	1837	1	17	2	1	3	2	1	1
2	1838	1	16	2	1	3	2	2	2
2	1839	1	14	1	1	3	2	1	2
2	1840	1	18	2	1	3	2	1	3
2	1841	1	14	1	1	3	2	2	2
2	1842	1	16	2	1	3	2	1	2
2	1843	1	18	2	1	3	2	1	1
2	1844	1	17	2	1	3	2	2	2
2	1845	1	14	1	1	3	2	2	2
2	1846	1	14	1	1	3	2	1	2
2	1847	1	18	2	1	3	2	2	2
2	1848	1	18	2	1	3	2	2	2
2	1849	1	18	2	1	3	2	1	1
2	1850	1	19	2	1	3	2	1	2
2	1851	1	18	2	1	3	2	1	2
2	1852	1	18	2	1	3	2	3	2
2	1853	1	18	2	1	3	2	2	1
2	1854	1	18	2	1	3	2	2	2
2	1855	1	16	2	1	3	2	1	2
2	1856	1	19	2	1	3	2	1	2
2	1857	1	14	1	1	3	2	2	2
2	1858	1	18	2	1	3	2	2	2
2	1859	1	18	2	1	3	2	1	3
2	1860	1	19	2	1	3	2	1	2
2	1861	1	17	2	1	3	2	2	2
2	1862	1	18	2	1	3	2	1	2
2	1863	1	18	2	1	3	2	1	2
2	1864	1	16	2	1	3	2	2	2
2	1865	1	16	2	1	3	2	2	2
2	1866	1	18	2	1	3	2	2	2
2	1867	1	16	2	1	3	2	1	2
2	1868	1	13	1	1	3	2	2	2
2	1869	1	20	3	1	3	2	1	9
2	1870	1	14	1	1	3	2	1	2
2	1871	1	14	1	1	3	2	1	2
2	1872	1	14	1	1	3	2	1	2
2	1873	1	15	2	1	3	2	2	2
2	1874	1	14	1	1	3	2	2	2
2	1875	1	12	1	1	3	2	2	2
2	1876	1	14	1	1	3	2	2	2
2	1877	1	13	1	1	3	2	3	3
2	1878	1	18	2	1	3	2	2	2
2	1879	2	18	2	1	3	1	2	2

2	1880	2	18	2	1	3	1	1	1
2	1881	1	15	2	1	3	2	2	2
2	1882	1	17	2	1	3	2	1	1
2	1883	1	23	3	1	3	2	1	2
2	1884	1	20	3	1	3	2	1	2
2	1885	1	29	3	9	3	2	2	2
2	1886	1	19	2	1	3	2	1	2
2	1887	1	12	1	1	3	1	3	3
2	1888	9	0	#NULL!	1	3	2	9	9
2	1889	1	19	2	1	3	2	2	3
2	1890	1	17	2	1	3	2	1	1
2	1891	1	18	2	1	3	2	1	2
2	1892	1	15	2	1	3	2	2	2
2	1893	1	20	3	1	3	2	1	1
2	1894	2	19	2	1	3	1	2	2
2	1895	2	19	2	1	3	1	2	2
2	1896	1	19	2	1	3	9	9	9
2	1897	1	20	3	1	3	2	1	1
2	1898	1	19	2	1	3	2	1	1
2	1899	1	18	2	1	3	2	1	1
2	1900	1	14	1	1	3	2	2	2
2	1901	1	17	2	1	3	2	1	2
2	1902	1	19	2	1	3	2	1	2
2	1903	1	17	2	1	3	2	2	2
2	1904	1	18	2	1	3	2	1	2
2	1905	1	18	2	1	3	2	1	2
2	1906	1	17	2	1	3	2	1	1
2	1907	1	19	2	2	3	2	1	3
2	1908	2	23	3	2	3	2	2	2
2	1909	1	18	2	1	3	2	2	1
2	1910	1	18	2	9	9	9	9	9
2	1911	1	17	2	1	3	2	1	1
2	1912	1	19	2	9	9	9	9	9
2	1913	1	17	2	1	3	2	2	2
2	1914	1	17	2	1	3	2	1	1
2	1915	1	17	2	1	3	2	1	1
2	1916	1	19	2	1	3	2	1	1
2	1917	1	16	2	1	3	2	1	2
2	1918	1	17	2	9	9	9	9	9
2	1919	1	16	2	1	3	2	1	2
2	1920	1	17	2	1	3	2	1	2
2	1921	1	17	2	1	3	2	1	2
2	1922	1	20	3	9	2	2	1	1
2	1923	1	14	1	1	2	2	1	1
2	1924	1	20	3	1	2	2	1	1
2	1925	1	15	2	1	2	9	9	9
2	1926	1	18	2	1	3	2	1	2

2	1927	1	14	1	1	2	2	1	1
2	1928	1	19	2	1	2	2	1	1
2	1929	1	16	2	1	3	2	2	2
2	1930	1	18	2	1	2	2	1	2
2	1931	1	21	3	1	2	2	1	1
2	1932	1	16	2	9	9	9	9	9
2	1933	1	15	2	1	2	2	2	1
2	1934	1	18	2	1	2	2	1	1
2	1935	1	18	2	1	2	2	1	1
2	1936	9	0	#NULL!	1	2	2	2	2
2	1937	1	19	2	1	2	2	2	2
2	1938	1	19	2	2	2	1	2	1
2	1939	1	17	2	1	2	2	2	2
2	1940	1	18	2	1	2	2	1	1
2	1941	1	17	2	2	2	2	1	1
2	1942	1	17	2	1	2	2	1	2
2	1943	1	17	2	1	2	2	1	1
2	1944	1	18	2	1	2	2	1	1
2	1945	1	16	2	1	2	2	1	1
2	1946	1	15	2	1	2	2	1	1
2	1947	1	16	2	1	2	2	1	1
2	1948	1	16	2	1	2	3	1	1
2	1949	1	16	2	1	2	2	1	1
2	1950	1	17	2	1	2	2	1	2
2	1951	1	30	3	1	2	3	1	1
2	1952	1	17	2	1	2	2	2	2
2	1953	1	14	1	1	2	2	2	2
2	1954	1	15	2	1	2	2	1	1
2	1955	1	18	2	1	2	2	2	1
2	1956	1	18	2	1	3	3	1	1
2	1957	9	0	#NULL!	9	9	9	9	9
2	1958	1	25	3	1	3	3	2	2
2	1959	1	18	2	1	2	2	2	2
2	1960	1	16	2	1	2	2	1	2
2	1961	1	17	2	1	2	2	1	2
2	1962	1	16	2	1	2	3	1	1
2	1963	1	16	2	1	2	2	2	1
2	1964	1	19	2	1	2	2	1	1
2	1965	1	18	2	1	2	2	3	3
2	1966	1	29	3	1	2	2	3	3
2	1967	1	18	2	1	3	2	1	1
2	1968	1	22	3	1	2	2	1	1
2	1969	1	18	2	1	2	2	1	2
2	1970	1	16	2	1	2	2	2	2
2	1971	1	17	2	1	2	2	1	1
2	1972	1	16	2	1	2	2	1	1
2	1973	1	18	2	1	2	2	1	1

2	1974	1	15	2	1	2	3	1	1
2	1975	1	16	2	1	2	2	1	1
2	1976	1	15	2	1	2	2	1	1
2	1977	1	27	3	1	3	2	1	1
2	1978	1	16	2	1	2	2	1	1
2	1979	1	16	2	1	2	2	1	1
2	1980	1	18	2	1	2	2	1	1
2	1981	1	16	2	1	1	1	1	1
2	1982	1	16	2	1	2	2	1	2
2	1983	1	17	2	1	2	2	1	1
2	1984	1	12	1	1	2	2	1	2
2	1985	1	25	3	2	3	3	3	1
2	1986	1	15	2	1	2	2	1	2
2	1987	1	15	2	1	2	2	1	2
2	1988	1	50	3	2	3	1	2	2
2	1989	1	20	3	2	3	1	2	2
2	1990	1	18	2	1	2	2	2	2
2	1991	1	16	2	1	2	2	1	1
2	1992	1	5	1	2	3	1	9	9
2	1993	9	17	2	1	2	2	1	2
2	1994	1	15	2	1	2	2	1	1
2	1995	1	17	2	1	2	2	1	1
2	1996	1	13	1	1	2	2	2	1
2	1997	1	16	2	1	2	2	1	2
2	1998	1	13	1	1	2	2	2	2
2	1999	1	17	2	1	2	2	1	1
2	2000	1	16	2	1	2	2	1	3
2	2001	1	16	2	1	2	2	1	2
2	2002	1	18	2	1	2	2	1	1
2	2003	1	15	2	1	2	2	2	2
2	2004	9	0	#NULL!	9	9	9	9	9
2	2005	1	16	2	1	2	2	1	2
2	2006	1	39	3	1	2	2	3	3
2	2007	1	25	3	1	2	2	1	1
2	2008	1	15	2	1	2	2	1	1
2	2009	9	0	#NULL!	9	9	9	9	9
2	2010	1	16	2	1	2	2	3	2
2	2011	1	18	2	1	2	3	3	3
2	2012	1	16	2	1	2	2	1	3
2	2013	1	19	2	1	2	2	1	1
2	2014	1	16	2	1	2	2	1	1
2	2015	1	12	1	1	2	2	2	2
2	2016	9	0	#NULL!	9	9	9	9	9
2	2017	1	13	1	1	2	2	2	2
2	2018	1	14	1	1	2	2	1	3
2	2019	1	15	2	1	2	2	1	1
2	2020	1	15	2	1	2	2	2	1

2	2021	1	17	2	1	2	2	1	1
2	2022	1	16	2	1	2	2	1	2
2	2023	1	12	1	1	2	2	2	3
2	2024	1	16	2	1	2	2	1	1
2	2025	1	14	1	2	1	2	1	2
2	2026	1	13	1	9	1	2	2	2
2	2027	1	14	1	1	1	2	1	1
2	2028	1	13	1	1	1	2	1	2
2	2029	9	0	#NULL!	9	9	9	9	9
2	2030	1	13	1	1	1	2	1	2
2	2031	1	15	2	1	1	2	1	2
2	2032	1	14	1	1	1	2	2	2
2	2033	1	14	1	1	1	2	1	2
2	2034	1	13	1	1	1	2	1	2
2	2035	1	16	2	1	1	2	2	2
2	2036	1	12	1	1	1	2	1	2
2	2037	1	14	1	1	1	2	2	3
2	2038	1	13	1	1	1	2	1	2
2	2039	1	14	1	1	1	2	1	2
2	2040	1	14	1	1	1	2	1	1
2	2041	1	14	1	1	1	2	1	1
2	2042	1	15	2	1	1	2	2	2
2	2043	1	12	1	1	1	2	2	2
2	2044	1	15	2	2	1	2	1	1
2	2045	1	14	1	1	1	1	1	1
2	2046	9	0	#NULL!	9	9	9	9	9
2	2047	1	13	1	1	1	2	1	2
2	2048	1	14	1	1	1	2	1	1
2	2049	1	14	1	1	1	2	1	2
2	2050	1	14	1	1	1	2	1	2
2	2051	1	13	1	1	1	2	1	2
2	2052	9	0	#NULL!	9	9	9	9	9
2	2053	1	14	1	9	1	2	1	2
2	2054	1	14	1	1	1	2	1	2
2	2055	1	11	1	1	1	2	1	2
2	2056	1	13	1	1	1	2	1	2
2	2057	9	0	#NULL!	1	1	2	2	2
2	2058	1	15	2	1	1	2	2	2
2	2059	1	13	1	1	3	2	3	3
2	2060	1	15	2	1	1	2	1	2
2	2061	1	14	1	1	1	2	1	2
2	2062	1	15	2	1	1	2	2	2
2	2063	1	15	2	1	1	2	1	2
2	2064	1	14	1	1	9	2	2	2
2	2065	1	14	1	1	9	2	2	2
2	2066	1	14	1	1	1	3	1	2
2	2067	1	15	2	2	3	3	2	2

2	2068	1	14	1	9	9	9	9	9
2	2069	1	15	2	1	1	2	1	1
2	2070	1	14	1	1	1	2	1	2
2	2071	1	15	2	1	1	2	1	2
2	2072	1	16	2	1	1	2	1	2
2	2073	1	16	2	1	1	2	1	2
2	2074	1	16	2	1	1	2	1	2
2	2075	1	0	#NULL!	2	1	2	1	2
2	2076	1	16	2	1	1	2	1	2
2	2077	1	14	1	1	1	2	1	1
2	2078	1	16	2	1	1	2	1	1
2	2079	1	14	1	2	1	2	2	2
2	2080	1	14	1	1	1	2	1	2
2	2081	1	15	2	1	1	2	1	1
2	2082	9	0	#NULL!	1	1	1	1	1
2	2083	1	15	2	1	1	2	3	3
2	2084	1	15	2	2	1	2	1	1
2	2085	1	14	1	1	1	2	1	2
2	2086	1	16	2	1	1	2	1	2
2	2087	1	15	2	1	1	2	1	1
2	2088	9	0	#NULL!	9	9	9	9	9
2	2089	9	0	#NULL!	1	1	2	1	1
2	2090	1	14	1	1	1	2	1	3
2	2091	9	0	#NULL!	9	9	9	9	9
2	2092	1	16	2	1	1	2	2	2
2	2093	1	14	1	1	1	2	2	2
2	2094	1	15	2	1	1	2	2	1
2	2095	1	16	2	1	1	2	1	2
2	2096	1	15	2	1	1	2	1	1
2	2097	1	15	2	1	1	2	1	1
2	2098	1	15	2	1	1	2	1	2
2	2099	1	15	2	1	1	2	1	2
2	2100	9	18	2	1	1	2	1	1
2	2101	1	15	2	1	1	2	2	2
2	2102	1	13	1	1	1	2	3	3
2	2103	9	0	#NULL!	1	1	2	2	2
2	2104	9	0	#NULL!	1	1	2	1	2
2	2105	1	15	2	1	1	2	1	2
2	2106	1	16	2	1	1	2	1	1
2	2107	1	16	2	1	1	2	1	1
2	2108	1	16	2	1	1	2	1	1
2	2109	1	15	2	1	1	2	1	2
2	2110	1	15	2	1	1	2	1	1
2	2111	1	15	2	1	1	2	1	2
2	2112	1	16	2	1	1	2	1	2
2	2113	9	17	2	1	2	2	1	1
2	2114	1	18	2	1	1	2	1	2

2	2115	9	13	1	1	1	2	1	2
2	2116	1	14	1	1	1	2	1	1
2	2117	1	15	2	1	1	2	1	1
2	2118	9	0	#NULL!	1	1	2	2	2
2	2119	1	15	2	1	1	2	2	2
2	2120	1	15	2	1	1	2	2	2
2	2121	1	15	2	1	1	1	1	1
2	2122	1	15	2	1	1	2	2	2
2	2123	1	13	1	1	1	2	1	3
2	2124	1	13	1	1	1	2	1	1
2	2125	1	13	1	1	1	2	2	2
2	2126	1	13	1	1	1	2	1	2
2	2127	1	18	2	1	1	2	2	2
2	2128	1	18	2	2	1	2	1	2
2	2129	1	19	2	2	1	1	2	2
2	2130	1	15	2	1	1	2	2	3
2	2131	1	15	2	1	1	2	1	2
2	2132	1	13	1	1	1	2	1	2
2	2133	1	14	1	1	1	2	3	3
2	2134	1	15	2	1	1	2	1	2
2	2135	1	14	1	1	1	2	1	2
2	2136	1	12	1	1	1	2	2	2
2	2137	1	17	2	1	1	2	2	2
2	2138	1	14	1	1	1	2	1	3
2	2139	1	14	1	1	1	2	1	2
2	2140	1	14	1	1	1	2	2	2
2	2141	1	14	1	2	1	2	1	2
2	2142	1	15	2	1	1	2	1	2
2	2143	1	14	1	1	1	2	1	2
2	2144	1	29	3	2	1	2	1	2
2	2145	1	15	2	1	1	2	1	1
2	2146	1	0	#NULL!	1	1	2	3	3
2	2147	1	14	1	1	1	1	1	1
2	2148	1	19	2	2	1	2	1	1
2	2149	1	13	1	1	1	2	1	2
2	2150	1	15	2	1	1	2	1	2
2	2151	1	14	1	1	1	2	1	1
2	2152	1	14	1	1	1	2	1	2
2	2153	1	15	2	1	1	2	1	2
2	2154	1	15	2	1	1	2	1	2
2	2155	1	15	2	1	1	2	2	2
2	2156	1	14	1	1	1	2	1	2
2	2157	1	14	1	1	1	2	1	1
2	2158	1	15	2	1	1	2	1	2
2	2159	9	0	#NULL!	9	9	9	9	9
2	2160	1	16	2	1	9	9	1	1
2	2161	1	19	2	2	1	2	1	1

2	2162	1	15	2	1	1	2	1	2
2	2163	1	16	2	1	1	2	1	3
2	2164	1	15	2	1	1	2	1	3
2	2165	1	14	1	1	1	2	1	2
2	2166	1	14	1	1	1	2	1	2
2	2167	1	13	1	1	1	2	1	2
2	2168	1	13	1	1	1	2	2	3
2	2169	1	14	1	1	1	2	1	1
2	2170	1	13	1	1	1	2	1	2
2	2171	1	14	1	1	1	2	1	2
2	2172	1	14	1	1	1	2	1	3
2	2173	1	17	2	1	1	2	1	2
2	2174	1	14	1	1	1	2	1	1
2	2175	1	14	1	1	1	2	1	2
2	2176	1	14	1	9	1	2	1	2
2	2177	1	13	1	1	1	2	1	2
2	2178	1	12	1	1	1	2	3	3
2	2179	1	14	1	1	1	2	1	1
2	2180	1	14	1	1	1	2	1	2
2	2181	1	13	1	1	1	2	1	2
2	2182	1	14	1	1	1	2	1	2
2	2183	1	14	1	1	1	2	1	1
2	2184	1	0	#NULL!	1	1	2	1	2
2	2185	1	15	2	1	1	2	1	2
2	2186	1	15	2	1	1	2	2	2
2	2187	1	13	1	1	1	2	2	2
2	2188	1	15	2	1	1	2	2	1
2	2189	1	14	1	1	1	2	1	1
2	2190	1	14	1	1	1	2	1	1
2	2191	1	14	1	1	1	2	1	2
2	2192	1	15	2	1	1	2	1	2
2	2193	1	15	2	1	1	2	1	2
2	2194	1	30	3	1	3	2	1	1
2	2195	1	14	1	1	1	2	2	2
2	2196	9	0	#NULL!	1	1	2	1	2
2	2197	9	0	#NULL!	1	1	2	1	1
2	2198	1	14	1	1	1	2	2	3
2	2199	1	15	2	1	1	2	2	2
2	2200	1	14	1	1	1	2	1	2
2	2201	1	14	1	1	1	2	1	1
2	2202	1	17	2	1	1	2	1	1
2	2203	1	15	2	1	1	2	1	2
2	2204	1	14	1	1	1	2	1	2
2	2205	1	15	2	1	1	2	1	2
2	2206	1	13	1	1	1	2	1	1
2	2207	1	16	2	1	1	2	2	1
2	2208	1	17	2	1	1	2	2	1

2	2209	1	2	1	1	1	2	2	2
2	2210	1	13	1	1	1	2	1	2
2	2211	1	16	2	1	1	2	1	1
2	2212	1	16	2	1	1	2	1	2
2	2213	1	15	2	1	1	2	1	1
2	2214	1	17	2	1	1	2	1	2
2	2215	1	14	1	1	1	2	1	2
2	2216	1	14	1	1	1	2	1	2
2	2217	1	13	1	1	1	2	1	2
2	2218	1	14	1	1	1	2	1	1
2	2219	1	12	1	1	1	2	2	2
2	2220	1	13	1	2	1	2	1	1
2	2221	1	13	1	1	1	2	1	1
2	2222	1	15	2	1	1	2	2	2
2	2223	1	15	2	1	1	2	2	2
2	2224	1	0	#NULL!	1	1	2	1	2
2	2225	1	14	1	1	1	2	2	2
2	2226	1	13	1	1	1	2	2	2
2	2227	1	15	2	1	1	2	2	2
2	2228	1	14	1	1	1	2	1	2
2	2229	2	0	#NULL!	1	3	1	1	1
2	2230	2	19	2	1	3	1	1	1
2	2231	2	18	2	1	3	1	1	1
2	2232	2	16	2	1	3	1	1	1
2	2233	2	16	2	1	3	1	1	1
2	2234	2	0	#NULL!	1	3	1	1	1
2	2235	2	16	2	1	3	1	1	1
2	2236	2	16	2	1	3	1	1	1
2	2237	2	16	2	1	3	1	1	1
2	2238	9	0	#NULL!	9	9	9	9	9
2	2239	2	17	2	1	3	1	1	1
2	2240	9	0	#NULL!	1	3	1	1	1
2	2241	2	15	2	1	3	1	1	1
2	2242	2	16	2	1	3	1	1	1
2	2243	2	16	2	1	3	1	1	1
2	2244	2	16	2	1	3	1	1	1
2	2245	2	0	#NULL!	1	3	1	1	1
2	2246	2	16	2	1	3	1	1	1
2	2247	2	18	2	1	3	1	1	1
2	2248	2	18	2	1	3	1	1	1
2	2249	2	17	2	1	3	1	1	1
2	2250	2	16	2	1	3	1	1	1
2	2251	2	17	2	1	3	1	1	1
2	2252	2	20	3	1	3	1	1	1
2	2253	2	17	2	1	3	1	1	1
2	2254	2	17	2	1	3	1	1	1
2	2255	2	17	2	1	3	1	1	1

2	2256	2	17	2	2	3	1	1	1
2	2257	2	17	2	1	3	1	1	1
2	2258	2	19	2	1	3	1	1	1
2	2259	2	16	2	1	3	1	1	1
2	2260	2	17	2	1	3	1	1	1
2	2261	2	16	2	1	3	1	1	1
2	2262	2	18	2	1	3	1	1	1
2	2263	2	16	2	1	3	1	1	1
2	2264	2	16	2	1	3	1	1	1
2	2265	2	16	2	1	3	1	1	1
2	2266	2	17	2	1	3	9	1	1
2	2267	2	18	2	1	3	1	1	1
2	2268	2	17	2	1	3	1	1	1
2	2269	9	0	#NULL!	9	9	9	1	1
2	2270	9	0	#NULL!	1	9	9	1	1
2	2271	2	16	2	1	3	1	1	1
2	2272	2	18	2	1	3	1	1	1
2	2273	2	18	2	1	3	1	1	1
2	2274	2	15	2	1	9	9	1	1
2	2275	2	15	2	1	3	1	1	1
2	2276	2	16	2	1	3	1	1	1
2	2277	2	17	2	1	3	1	1	1
2	2278	2	17	2	1	3	1	1	1
2	2279	2	18	2	1	3	1	1	1
2	2280	9	0	#NULL!	1	3	1	1	1
2	2281	2	16	2	1	3	1	1	1
2	2282	2	16	2	1	3	1	1	1
2	2283	2	17	2	1	3	1	1	1
2	2284	2	17	2	1	3	1	1	1
2	2285	2	17	2	1	3	1	1	1
2	2286	9	0	#NULL!	1	3	9	1	2
2	2287	2	17	2	1	3	1	1	1
2	2288	2	17	2	1	3	1	1	1
2	2289	2	16	2	1	3	1	1	1
2	2290	2	17	2	1	3	1	1	1
2	2291	2	16	2	1	3	1	1	1
2	2292	2	18	2	1	3	1	1	1
2	2293	2	18	2	1	3	1	1	1
2	2294	2	17	2	1	3	1	1	1
2	2295	2	17	2	1	3	1	1	1
2	2296	2	16	2	1	3	1	1	1
2	2297	2	17	2	1	3	1	1	1
2	2298	2	16	2	1	3	1	1	1
2	2299	2	15	2	1	3	1	1	1
2	2300	9	0	#NULL!	9	9	9	9	9
2	2301	2	15	2	1	3	1	1	1
2	2302	2	16	2	1	3	1	1	1

2	2303	2	17	2	1	3	1	1	1
2	2304	2	16	2	1	3	1	1	1
2	2305	2	16	2	1	3	1	1	1
2	2306	2	17	2	1	3	1	1	1
2	2307	2	16	2	1	3	1	1	1
2	2308	2	17	2	1	3	1	1	2
2	2309	2	17	2	1	3	1	1	1
2	2310	2	19	2	1	3	1	1	1
2	2311	2	18	2	1	3	1	1	1
2	2312	2	19	2	1	3	1	1	1
2	2313	2	17	2	1	3	1	1	2
2	2314	2	20	3	1	3	1	1	1
2	2315	2	17	2	1	3	1	2	1
2	2316	2	17	2	1	3	1	1	2
2	2317	2	18	2	1	3	1	1	1
2	2318	2	17	2	1	3	1	1	1
2	2319	2	16	2	1	3	1	1	1
2	2320	2	16	2	1	3	1	1	1
2	2321	2	17	2	1	3	1	1	1
2	2322	2	16	2	1	3	1	1	1
2	2323	2	17	2	1	3	1	1	1
2	2324	2	17	2	1	3	1	1	1
2	2325	2	15	2	2	3	1	1	1
2	2326	2	18	2	1	3	1	1	1
2	2327	2	16	2	1	3	1	1	1
2	2328	2	16	2	1	3	1	1	1
2	2329	2	17	2	1	3	1	1	2
2	2330	2	17	2	1	3	1	9	1
2	2331	9	18	2	1	3	1	1	1
2	2332	2	0	#NULL!	1	3	1	1	1
2	2333	2	17	2	1	3	1	1	1
2	2334	2	18	2	1	3	1	1	2
2	2335	2	17	2	1	3	1	2	1
2	2336	2	17	2	1	3	1	1	1
2	2337	2	17	2	1	3	1	1	1
2	2338	2	17	2	1	3	1	1	1
2	2339	9	16	2	2	3	1	1	1
2	2340	9	17	2	1	3	1	1	1
2	2341	9	17	2	1	3	1	1	1
2	2342	2	17	2	1	3	1	2	1
2	2343	2	16	2	1	3	1	1	1
2	2344	2	16	2	1	3	1	1	1
2	2345	2	17	2	1	3	1	1	1
2	2346	2	16	2	9	3	1	1	1
2	2347	2	15	2	1	3	1	2	2
2	2348	2	16	2	1	3	1	1	1
2	2349	2	18	2	1	3	1	1	1

2	2350	2	16	2	1	3	1	1	1
2	2351	2	18	2	1	3	1	1	1
2	2352	2	17	2	1	3	1	1	1
2	2353	2	16	2	1	3	1	1	1
2	2354	2	15	2	1	3	1	1	1
2	2355	2	0	#NULL!	2	3	1	1	1
2	2356	2	16	2	1	3	1	9	1
2	2357	2	16	2	1	3	1	1	1
2	2358	2	17	2	1	3	1	1	1
2	2359	2	16	2	1	3	1	1	1
2	2360	2	15	2	1	3	1	1	1
2	2361	2	15	2	1	3	1	1	1
2	2362	2	17	2	1	3	1	1	1
2	2363	2	15	2	1	3	1	1	1
2	2364	2	16	2	1	3	1	1	1
2	2365	2	16	2	1	3	1	1	1
2	2366	2	16	2	1	3	1	1	1
2	2367	2	16	2	1	3	1	1	1
2	2368	2	17	2	1	3	1	1	1
2	2369	2	16	2	1	3	1	1	1
2	2370	2	16	2	1	3	1	1	1
2	2371	2	17	2	1	3	1	1	1
2	2372	9	0	#NULL!	1	3	1	1	1
2	2373	9	19	2	1	3	1	1	1
2	2374	2	16	2	1	3	1	1	1
2	2375	2	16	2	1	3	1	1	1
2	2376	2	15	2	1	3	1	1	1
2	2377	2	16	2	1	3	1	1	1
2	2378	2	15	2	1	3	1	1	1
2	2379	2	17	2	1	3	1	1	1
2	2380	2	15	2	2	3	1	1	1
2	2381	2	18	2	1	3	1	1	1
2	2382	2	15	2	1	3	1	1	1
2	2383	2	16	2	1	3	1	1	1
2	2384	2	16	2	1	3	1	1	1
2	2385	2	0	#NULL!	1	3	1	1	1
2	2386	2	17	2	1	3	1	1	1
2	2387	9	17	2	1	3	1	1	1
2	2388	2	17	2	1	3	1	1	1
2	2389	2	15	2	1	3	1	1	1
2	2390	2	17	2	2	3	1	1	1
2	2391	2	16	2	1	3	1	1	1
2	2392	2	16	2	1	3	1	1	1
2	2393	2	16	2	1	3	1	1	1
2	2394	2	16	2	1	3	1	1	1
2	2395	2	18	2	9	3	1	1	1
2	2396	2	16	2	1	3	1	1	1

2	2397	2	0	#NULL!	1	3	1	1	1
2	2398	2	17	2	1	3	1	1	1
2	2399	9	0	#NULL!	9	9	9	9	9
2	2400	2	18	2	1	3	1	1	1
2	2401	2	18	2	1	3	1	1	1
2	2402	2	16	2	1	3	1	1	1
2	2403	2	18	2	1	3	1	1	1
2	2404	2	16	2	1	3	1	1	1
2	2405	2	18	2	1	3	1	1	1
2	2406	2	17	2	1	3	1	1	1
2	2407	2	17	2	2	3	1	1	1
2	2408	2	17	2	1	3	1	1	1
2	2409	2	19	2	1	3	1	1	1
2	2410	2	17	2	1	3	1	1	1
2	2411	2	18	2	9	3	1	1	1
2	2412	2	17	2	1	3	1	1	1
2	2413	2	18	2	1	3	1	1	1
2	2414	2	16	2	1	3	1	1	1
2	2415	2	17	2	1	3	1	1	1
2	2416	2	17	2	1	3	1	1	1
2	2417	2	17	2	2	3	1	1	1
2	2418	2	15	2	1	3	1	1	1
2	2419	2	16	2	1	3	1	1	1
2	2420	2	19	2	1	3	1	1	1
2	2421	2	17	2	1	3	1	1	1
2	2422	2	17	2	1	3	1	1	1
2	2423	2	16	2	1	3	1	1	1
2	2424	2	15	2	1	3	1	1	1
2	2425	2	18	2	1	3	1	1	1
2	2426	2	16	2	1	3	1	1	1
2	2427	2	15	2	2	3	1	1	1
2	2428	2	17	2	1	3	1	1	1
2	2429	2	0	#NULL!	9	3	1	1	1
2	2430	2	17	2	1	3	1	1	1
2	2431	2	17	2	1	3	1	2	1
2	2432	2	16	2	1	3	1	1	1
2	2433	2	17	2	1	3	1	2	1
2	2434	2	17	2	1	3	1	1	1
2	2435	2	17	2	1	3	1	1	1
2	2436	2	17	2	1	3	1	1	1
2	2437	2	17	2	1	3	9	1	1
2	2438	2	16	2	1	3	1	1	1
2	2439	2	17	2	1	3	1	1	1
2	2440	2	17	2	1	3	1	1	1
2	2441	2	16	2	1	3	1	1	1
2	2442	2	18	2	1	3	1	1	1
2	2443	2	15	2	1	3	1	1	1

2	2444	2	17	2	1	3	1	1	1
2	2445	2	16	2	1	3	1	1	1
2	2446	2	16	2	1	3	1	1	1
2	2447	2	18	2	1	3	1	1	1
2	2448	2	17	2	1	3	1	1	1
2	2449	2	16	2	1	3	1	1	1
2	2450	2	15	2	1	3	1	1	1
2	2451	2	17	2	2	3	1	1	1
2	2452	2	16	2	1	3	1	2	1
2	2453	2	18	2	1	3	1	1	1
2	2454	2	15	2	1	3	1	1	1
2	2455	2	17	2	1	3	1	1	1
2	2456	2	17	2	1	3	1	1	1
2	2457	2	16	2	1	3	1	1	1
2	2458	2	17	2	1	3	1	1	1
2	2459	2	18	2	1	3	1	1	1
2	2460	2	16	2	1	3	1	1	1
2	2461	2	18	2	1	3	1	1	1
2	2462	2	18	2	1	3	1	1	1
2	2463	2	18	2	1	3	1	1	1
2	2464	2	15	2	1	3	1	1	1
2	2465	2	15	2	1	3	1	1	1
2	2466	2	16	2	1	3	1	1	1
2	2467	2	19	2	1	3	1	1	1
2	2468	2	17	2	1	3	1	1	1
2	2469	2	17	2	1	3	1	1	1
2	2470	2	15	2	1	3	1	1	1
2	2471	2	17	2	1	3	1	1	1
2	2472	2	18	2	1	3	1	1	1
2	2473	2	15	2	1	3	1	1	1
2	2474	2	17	2	1	3	1	1	1
2	2475	2	16	2	1	3	1	1	1
2	2476	2	15	2	1	3	1	1	1
2	2477	2	14	1	1	3	1	1	2
2	2478	2	17	2	1	3	1	1	1
2	2479	2	17	2	1	3	1	1	1
2	2480	2	15	2	1	3	1	1	1
2	2481	2	15	2	1	3	1	1	1
2	2482	9	0	#NULL!	1	3	1	1	1
2	2483	2	18	2	1	3	1	1	2
2	2484	2	17	2	1	3	1	1	1
2	2485	9	18	2	1	3	1	1	1
2	2486	2	16	2	1	3	1	2	1
2	2487	2	16	2	1	3	1	1	1
2	2488	2	19	2	1	3	1	1	1
2	2489	2	17	2	1	3	1	1	1
2	2490	2	14	1	1	2	1	1	1

2	2491	2	16	2	1	2	1	1	1
2	2492	2	15	2	1	2	1	1	1
2	2493	2	17	2	1	2	1	1	1
2	2494	2	14	1	1	2	1	1	3
2	2495	2	15	2	1	2	1	1	3
2	2496	2	12	1	1	2	1	1	1
2	2497	2	15	2	1	2	1	1	1
2	2498	2	14	1	1	2	1	1	1
2	2499	2	16	2	1	2	1	1	1
2	2500	2	15	2	1	2	1	1	1
2	2501	2	15	2	1	2	1	1	1
2	2502	2	17	2	1	2	1	1	1
2	2503	2	17	2	1	2	1	1	1
2	2504	2	15	2	1	2	1	2	1
2	2505	2	14	1	1	2	1	1	2
2	2506	2	17	2	1	2	1	1	1
2	2507	2	16	2	1	2	1	1	1
2	2508	2	15	2	1	2	1	1	1
2	2509	2	16	2	1	2	1	1	1
2	2510	2	15	2	1	2	1	1	1
2	2511	2	15	2	1	2	1	1	1
2	2512	2	17	2	1	2	1	1	1
2	2513	2	16	2	1	2	1	1	1
2	2514	2	16	2	1	2	1	1	1
2	2515	2	15	2	1	2	1	1	1
2	2516	2	16	2	1	2	1	1	1
2	2517	2	15	2	1	2	1	2	2
2	2518	2	15	2	1	2	1	1	1
2	2519	2	16	2	1	2	1	1	1
2	2520	2	18	2	2	2	1	2	9
2	2521	2	15	2	1	2	1	1	1
2	2522	2	14	1	1	2	1	1	1
2	2523	2	16	2	1	2	1	1	2
2	2524	2	16	2	1	2	1	1	1
2	2525	2	16	2	1	2	1	2	1
2	2526	2	17	2	1	2	1	1	1
2	2527	2	16	2	1	2	1	2	1
2	2528	2	17	2	1	2	1	2	1
2	2529	2	17	2	1	2	1	1	1
2	2530	2	16	2	1	2	1	1	1
2	2531	2	16	2	1	2	1	1	1
2	2532	2	15	2	1	2	1	1	1
2	2533	2	15	2	1	2	1	1	1
2	2534	2	16	2	1	2	1	1	1
2	2535	2	17	2	2	2	1	1	1
2	2536	2	17	2	1	2	1	2	1
2	2537	2	18	2	1	2	1	1	1

2	2538	2	16	2	1	2	1	1	1
2	2539	2	15	2	1	2	1	1	9
2	2540	2	19	2	1	2	1	1	1
2	2541	2	16	2	1	2	1	1	1
2	2542	2	16	2	1	2	1	1	1
2	2543	2	18	2	1	2	1	1	1
2	2544	2	18	2	9	9	9	9	9
2	2545	2	17	2	1	2	1	1	1
2	2546	2	15	2	1	2	1	1	1
2	2547	2	16	2	1	2	1	1	1
2	2548	2	17	2	1	2	1	1	1
2	2549	2	15	2	1	2	1	1	1
2	2550	2	16	2	1	2	1	1	1
2	2551	2	15	2	1	2	1	1	1
2	2552	2	16	2	1	2	1	1	1
2	2553	2	15	2	1	2	1	1	1
2	2554	2	15	2	1	2	1	1	1
2	2555	2	16	2	2	2	1	1	3
2	2556	2	15	2	1	2	1	1	1
2	2557	2	17	2	1	2	1	2	2
2	2558	2	15	2	1	2	1	1	1
2	2559	2	17	2	1	2	1	1	2
2	2560	2	16	2	1	2	1	1	1
2	2561	2	14	1	1	2	1	1	1
2	2562	2	15	2	1	2	1	1	1
2	2563	2	15	2	1	2	1	1	1
2	2564	2	14	1	2	2	3	2	1
2	2565	2	15	2	1	2	1	1	1
2	2566	2	15	2	1	2	1	1	1
2	2567	2	16	2	1	2	1	1	1
2	2568	2	15	2	1	2	1	1	1
2	2569	2	15	2	1	2	1	1	1
2	2570	2	17	2	1	2	1	1	3
2	2571	2	16	2	1	2	1	1	1
2	2572	2	16	2	1	2	1	1	1
2	2573	2	16	2	1	2	1	1	1
2	2574	2	14	1	1	2	1	1	1
2	2575	2	15	2	1	2	1	1	1
2	2576	2	15	2	1	2	1	1	1
2	2577	2	15	2	1	2	1	1	1
2	2578	2	16	2	1	2	1	1	1
2	2579	2	17	2	1	2	1	1	2
2	2580	2	20	3	1	2	1	1	2
2	2581	2	17	2	1	2	1	3	3
2	2582	2	15	2	1	2	1	3	1
2	2583	2	17	2	1	2	1	1	1
2	2584	2	16	2	1	2	1	1	1

2	2585	2	16	2	2	2	1	1	1
2	2586	2	15	2	1	2	1	1	1
2	2587	2	18	2	1	2	1	1	1
2	2588	2	14	1	1	2	1	3	1
2	2589	2	15	2	1	2	1	1	1
2	2590	2	17	2	1	2	1	3	3
2	2591	2	16	2	1	2	1	1	1
2	2592	2	15	2	1	2	1	1	1
2	2593	2	16	2	1	2	1	1	1
2	2594	2	16	2	1	2	1	1	1
2	2595	2	16	2	1	2	1	1	1
2	2596	2	21	3	1	2	1	1	1
2	2597	2	15	2	1	2	1	1	1
2	2598	2	16	2	1	2	1	1	1
2	2599	2	16	2	1	2	1	1	1
2	2600	2	16	2	1	2	1	1	1
2	2601	2	15	2	1	2	1	1	1
2	2602	2	13	1	1	2	1	1	1
2	2603	2	15	2	1	2	1	1	2
2	2604	2	15	2	1	2	1	1	1
2	2605	2	15	2	1	2	1	1	1
2	2606	2	16	2	1	2	1	1	1
2	2607	2	16	2	1	2	1	1	2
2	2608	2	15	2	1	2	1	1	2
2	2609	2	15	2	1	2	9	1	2
2	2610	2	14	1	1	2	1	1	1
2	2611	2	14	1	1	2	1	1	2
2	2612	2	15	2	1	2	1	1	2
2	2613	2	15	2	1	2	1	1	1
2	2614	2	16	2	1	2	1	1	1
2	2615	2	14	1	2	2	1	1	2
2	2616	2	15	2	1	2	1	1	1
2	2617	2	0	#NULL!	1	2	1	1	1
2	2618	2	16	2	1	2	1	1	1
2	2619	2	16	2	1	2	1	1	2
2	2620	2	17	2	1	2	1	1	1
2	2621	2	15	2	1	2	1	1	3
2	2622	2	14	1	2	2	1	1	1
2	2623	2	15	2	1	2	1	1	1
2	2624	2	17	2	1	2	1	1	1
2	2625	2	15	2	1	2	1	1	1
2	2626	2	14	1	2	2	1	1	1
2	2627	2	17	2	1	2	1	1	1
2	2628	2	15	2	2	2	1	1	1
2	2629	2	16	2	1	2	1	1	1
2	2630	2	15	2	1	2	1	1	1
2	2631	2	0	#NULL!	2	2	1	1	1

2	2632	2	15	2	1	2	1	1	1
2	2633	2	14	1	1	2	1	1	2
2	2634	2	15	2	1	2	1	1	1
2	2635	2	15	2	1	2	1	1	1
2	2636	2	15	2	1	2	1	1	1
2	2637	2	16	2	1	2	1	1	1
2	2638	2	14	1	1	2	1	1	1
2	2639	2	13	1	1	2	1	1	1
2	2640	2	16	2	1	2	1	1	1
2	2641	2	15	2	1	2	1	1	1
2	2642	2	14	1	1	2	1	1	1
2	2643	2	14	1	1	2	1	1	1
2	2644	2	15	2	1	2	1	1	1
2	2645	2	16	2	1	2	1	1	1
2	2646	2	14	1	1	2	1	1	1
2	2647	2	17	2	1	2	1	1	2
2	2648	2	15	2	1	2	1	1	1
2	2649	2	14	1	1	2	1	1	1
2	2650	2	16	2	1	2	1	1	1
2	2651	2	15	2	1	2	1	1	1
2	2652	2	16	2	2	2	1	1	1
2	2653	2	15	2	1	2	1	1	1
2	2654	2	16	2	1	2	1	1	1
2	2655	2	16	2	2	2	1	1	1
2	2656	2	14	1	1	2	1	1	1
2	2657	2	16	2	1	2	1	1	1
2	2658	2	15	2	1	2	1	1	1
2	2659	2	17	2	1	2	1	1	1
2	2660	2	16	2	2	2	1	1	1
2	2661	2	16	2	1	2	1	1	1
2	2662	2	15	2	1	2	1	1	1
2	2663	2	17	2	1	2	1	1	1
2	2664	2	15	2	1	2	1	1	2
2	2665	2	15	2	1	2	1	1	2
2	2666	2	16	2	1	2	1	1	1
2	2667	2	15	2	1	2	1	1	2
2	2668	2	16	2	1	2	1	1	2
2	2669	2	20	3	1	2	1	1	2
2	2670	2	12	1	1	2	1	1	1
2	2671	2	18	2	1	2	1	1	1
2	2672	2	16	2	1	2	1	1	1
2	2673	2	16	2	1	2	1	1	1
2	2674	2	15	2	1	2	1	1	1
2	2675	2	18	2	2	2	1	1	1
2	2676	2	17	2	1	1	1	2	2
2	2677	2	15	2	1	2	1	1	3
2	2678	2	16	2	1	2	1	1	1

2	2679	2	14	1	1	2	1	1	1
2	2680	2	14	1	1	2	1	1	1
2	2681	2	16	2	1	2	1	1	1
2	2682	2	14	1	1	2	1	1	1
2	2683	2	15	2	1	2	1	1	2
2	2684	2	15	2	1	2	1	1	1
2	2685	2	16	2	1	2	1	1	1
2	2686	2	15	2	1	2	1	1	1
2	2687	2	15	2	1	2	1	1	2
2	2688	2	16	2	1	2	1	1	2
2	2689	2	0	#NULL!	1	2	1	1	1
2	2690	2	17	2	1	2	1	1	1
2	2691	2	18	2	1	2	1	1	1
2	2692	9	15	2	1	2	1	1	1
2	2693	2	16	2	1	2	1	1	1
2	2694	2	15	2	1	2	1	1	1
2	2695	2	17	2	1	2	1	1	1
2	2696	2	19	2	1	2	1	1	1
2	2697	2	16	2	1	2	1	1	1
2	2698	2	16	2	1	2	1	1	1
2	2699	2	16	2	1	2	1	1	1
2	2700	2	15	2	1	2	1	1	1
2	2701	2	16	2	1	2	1	1	1
2	2702	2	16	2	1	2	1	1	1
2	2703	2	15	2	1	2	1	1	1
2	2704	9	18	2	1	2	1	1	1
2	2705	2	16	2	1	2	1	1	1
2	2706	2	19	2	1	2	1	1	1
2	2707	2	16	2	1	2	1	1	1
2	2708	2	15	2	1	2	1	1	1
2	2709	2	15	2	1	2	1	1	1
2	2710	2	17	2	1	2	1	1	1
2	2711	2	15	2	1	2	1	1	1
2	2712	2	15	2	1	2	1	1	1
2	2713	9	16	2	1	2	1	1	1
2	2714	2	17	2	1	2	1	1	1
2	2715	2	16	2	2	2	1	1	1
2	2716	2	17	2	1	2	1	1	1
2	2717	2	15	2	1	2	1	1	1
2	2718	2	15	2	1	2	1	1	1
2	2719	2	15	2	1	2	1	1	1
2	2720	2	15	2	1	2	1	1	1
2	2721	2	15	2	1	2	1	1	2
2	2722	2	17	2	1	2	1	2	2
2	2723	2	16	2	1	2	1	1	1
2	2724	2	15	2	1	2	1	1	1
2	2725	2	15	2	1	2	1	1	1

2	2726	2	16	2	1	2	1	1	1
2	2727	2	15	2	1	2	1	1	1
2	2728	2	16	2	1	2	1	1	1
2	2729	2	16	2	1	2	1	1	1
2	2730	2	16	2	1	2	1	1	1
2	2731	2	17	2	1	2	1	1	1
2	2732	2	16	2	1	2	1	1	1
2	2733	2	16	2	1	2	1	1	1
2	2734	2	16	2	1	2	1	1	1
2	2735	2	15	2	1	2	1	9	9
2	2736	2	15	2	1	2	1	1	1
2	2737	2	16	2	1	2	1	1	1
2	2738	2	15	2	1	2	1	1	1
2	2739	2	16	2	1	2	1	1	1
2	2740	2	16	2	1	2	1	1	1
2	2741	2	16	2	1	2	1	1	1
2	2742	2	14	1	1	2	1	1	2
2	2743	2	15	2	1	1	1	1	1
2	2744	2	16	2	1	2	1	1	1
2	2745	2	14	1	1	2	1	1	9
2	2746	2	18	2	1	2	3	3	1
2	2747	2	15	2	2	2	1	1	1
2	2748	2	15	2	1	2	1	1	1
2	2749	2	15	2	1	2	1	1	1
2	2750	2	15	2	2	2	1	1	1
2	2751	2	17	2	1	2	1	1	1
2	2752	2	16	2	1	2	1	1	2
2	2753	2	18	2	1	2	1	2	2
2	2754	2	17	2	1	2	1	1	1
2	2755	1	18	2	9	2	1	1	2
2	2756	2	16	2	1	2	1	1	2
2	2757	2	16	2	2	2	1	1	1
2	2758	2	16	2	1	2	1	1	3
2	2759	2	15	2	1	2	1	1	1
2	2760	2	16	2	1	2	1	1	1
2	2761	2	15	2	1	2	1	1	1
2	2762	2	15	2	1	2	1	1	1
2	2763	2	18	2	1	2	1	1	1
2	2764	2	16	2	2	2	1	1	1
2	2765	2	15	2	1	2	1	1	1
2	2766	2	17	2	1	2	1	1	1
2	2767	2	20	3	1	2	1	1	1
2	2768	2	16	2	1	2	1	1	2
2	2769	2	16	2	1	2	1	1	1
2	2770	2	18	2	1	2	1	1	2
2	2771	2	18	2	1	2	1	1	1
2	2772	2	16	2	1	2	1	1	1

2	2773	2	15	2	1	2	1	1	1
2	2774	2	16	2	1	2	1	1	1
2	2775	2	15	2	1	2	1	1	1
2	2776	2	16	2	1	2	1	1	2
2	2777	2	16	2	2	2	1	1	2
2	2778	2	16	2	1	2	1	1	1
2	2779	2	16	2	1	2	1	1	2
2	2780	2	15	2	1	2	1	1	1
2	2781	2	15	2	1	2	1	1	1
2	2782	2	19	2	1	2	1	2	2
2	2783	2	17	2	1	2	1	1	1
2	2784	2	13	1	1	2	1	1	2
2	2785	2	17	2	1	2	1	1	2
2	2786	2	14	1	1	2	1	1	2
2	2787	2	17	2	1	2	1	1	3
2	2788	2	15	2	1	2	1	1	2
2	2789	2	16	2	1	2	1	1	1
2	2790	2	15	2	1	2	1	1	1
2	2791	2	15	2	1	2	1	1	1
2	2792	2	15	2	1	2	1	1	1
2	2793	2	17	2	2	2	1	1	1
2	2794	2	16	2	1	2	1	1	1
2	2795	2	18	2	1	2	1	1	1
2	2796	2	16	2	1	2	1	1	2
2	2797	2	14	1	1	2	1	1	1
2	2798	2	15	2	1	2	1	1	1
2	2799	2	15	2	1	2	1	1	1
2	2800	2	16	2	1	2	1	1	1
2	2801	2	16	2	1	2	1	1	2
2	2802	2	16	2	1	2	1	1	1
2	2803	2	16	2	2	2	1	1	1
2	2804	2	17	2	1	2	1	1	2
2	2805	2	17	2	1	2	1	1	1
2	2806	2	15	2	1	2	1	1	2
2	2807	2	14	1	1	2	1	1	1
2	2808	2	18	2	1	2	1	1	2
2	2809	2	16	2	1	2	1	1	2
2	2810	2	16	2	1	2	1	1	1
2	2811	2	15	2	1	2	1	1	3
2	2812	9	0	#NULL!	9	9	9	9	9
2	2813	2	14	1	1	2	1	1	1
2	2814	2	15	2	1	2	1	1	1
4	1	2	16	2	1	3	3	1	2
4	2	2	18	2	1	3	3	1	1
4	3	2	18	2	1	3	3	1	1
4	4	2	18	2	1	3	3	1	1
4	5	2	18	2	1	3	3	1	1

4	6	2	18	2	1	3	3	1	1
4	7	2	18	2	1	3	3	1	1
4	8	2	17	2	1	3	3	1	1
4	9	9	9	1	9	9	9	1	1
4	10	2	15	2	1	3	3	1	1
4	11	2	17	2	1	3	3	1	1
4	12	1	17	2	1	3	3	1	1
4	13	2	19	2	1	3	3	1	1
4	14	1	19	2	1	3	3	1	1
4	15	2	18	2	1	3	3	1	1
4	16	2	18	2	1	3	3	1	1
4	17	2	18	2	1	3	3	1	3
4	18	2	18	2	1	3	3	1	1
4	19	2	18	2	1	3	3	1	1
4	20	2	18	2	1	3	3	1	1
4	21	2	18	2	1	3	3	1	1
4	22	2	18	2	1	3	3	1	1
4	23	2	18	2	1	3	3	1	1
4	24	2	17	2	1	3	3	1	1
4	25	2	17	2	1	3	3	1	1
4	26	9	9	1	9	9	9	9	9
4	27	9	9	1	9	9	9	3	3
4	28	9	9	1	9	9	9	3	3
4	29	9	9	1	9	9	9	3	3
4	30	9	9	1	9	9	9	3	3
4	31	9	9	1	9	9	9	1	1
4	32	9	9	1	9	9	9	1	1
4	33	9	9	1	9	9	9	1	1
4	34	1	15	2	1	3	3	3	1
4	35	2	16	2	1	3	3	1	1
4	36	2	16	2	1	3	3	1	1
4	37	2	16	2	1	3	3	1	1
4	38	2	16	2	1	3	3	1	1
4	39	1	18	2	1	3	3	1	3
4	40	1	13	1	1	3	3	1	3
4	41	1	15	2	1	3	3	1	3
4	42	9	9	1	1	3	3	1	1
4	43	9	9	1	1	3	3	1	1
4	44	9	9	1	1	3	3	1	1
4	45	9	9	1	1	3	3	1	1
4	46	2	18	2	1	3	3	2	2
4	47	2	17	2	1	3	3	1	2
4	48	1	15	2	1	3	3	1	2
4	49	2	18	2	1	3	3	1	1
4	50	1	17	2	1	3	3	1	1
4	51	2	20	3	1	3	3	2	2
4	52	2	19	2	1	3	3	1	1

4	53	2	18	2	1	3	3	1	1
4	54	1	20	3	9	9	9	1	1
4	55	1	18	2	1	3	3	1	2
4	56	2	15	2	1	3	3	1	3
4	57	9	9	1	1	3	3	1	1
4	58	1	21	3	1	3	3	1	2
4	59	2	18	2	1	3	3	1	1
4	60	2	17	2	1	3	3	1	1
4	61	2	19	2	1	3	3	1	2
4	62	1	18	2	1	3	3	1	1
4	63	1	19	2	1	3	3	3	3
4	64	1	17	2	1	3	3	1	1
4	65	1	18	2	1	3	3	1	1
4	66	2	18	2	1	3	3	1	1
4	67	2	18	2	1	3	3	1	1
4	68	2	16	2	1	3	3	1	2
4	69	2	17	2	1	3	3	1	1
4	70	1	18	2	1	3	3	1	3
4	71	2	20	3	1	3	3	1	2
4	72	2	18	2	1	3	3	1	1
4	73	2	18	2	1	3	3	1	1
4	74	9	9	1	1	1	1	1	1
4	75	9	9	1	1	1	1	1	1
4	76	9	9	1	9	9	9	9	9
4	77	9	9	1	1	1	1	1	1
4	78	2	19	2	1	3	3	3	3
4	79	9	9	1	1	1	3	1	1
4	80	1	16	2	1	3	3	1	1
4	81	2	18	2	1	3	3	1	1
4	82	2	18	2	1	3	3	1	1
4	83	2	17	2	1	3	3	1	1
4	84	2	18	2	1	3	3	1	1
4	85	9	9	1	9	9	9	9	9
4	86	1	18	2	1	3	3	1	1
4	87	2	18	2	1	3	3	1	1
4	88	2	18	2	1	3	3	1	2
4	89	2	15	2	1	3	3	1	1
4	90	2	17	2	1	3	3	1	1
4	91	2	17	2	1	3	3	1	1
4	92	2	18	2	1	3	3	1	1
4	93	2	18	2	1	3	3	1	1
4	94	2	17	2	1	3	3	1	1
4	95	2	16	2	1	3	3	1	1
4	96	2	18	2	1	3	3	1	1
4	97	1	18	2	1	3	3	1	1
4	98	2	19	2	1	3	3	1	1
4	99	2	18	2	1	3	3	1	1

4	100	2	18	2	1	3	3	1	1
4	101	2	18	2	1	3	3	1	1
4	102	1	18	2	1	3	3	1	2
4	103	2	18	2	1	3	3	1	1
4	104	1	16	2	1	3	3	1	1
4	105	2	18	2	1	3	3	1	1
4	106	9	9	1	1	3	3	1	1
4	107	1	14	1	1	3	3	2	1
4	108	1	15	2	1	3	3	1	1
4	109	1	13	1	1	2	1	2	2
4	110	1	16	2	1	3	3	1	1
4	111	1	16	2	1	3	3	1	1
4	112	1	16	2	1	3	3	1	1
4	113	2	37	3	1	2	2	1	3
4	114	2	16	2	1	3	3	1	1
4	115	2	18	2	1	3	3	1	1
4	116	2	17	2	1	3	3	1	1
4	117	2	16	2	1	3	3	1	1
4	118	1	20	3	1	3	3	1	1
4	119	1	20	3	1	3	3	1	1
4	120	1	18	2	1	2	3	2	2
4	121	2	9	1	1	3	3	1	1
4	122	2	9	1	1	3	3	1	2
4	123	2	9	1	1	3	3	1	1
4	124	2	14	1	1	1	3	3	3
4	125	1	19	2	1	3	3	1	1
4	126	2	15	2	1	3	3	2	2
4	127	2	20	3	1	3	3	1	1
4	128	2	20	3	1	3	3	1	2
4	129	2	20	3	1	3	3	1	2
4	130	2	20	3	1	3	3	1	2
4	131	2	20	3	1	3	3	1	2
4	132	2	20	3	1	3	3	1	1
4	133	2	20	3	1	3	3	1	1
4	134	2	18	2	1	3	3	1	1
4	135	2	17	2	1	3	3	1	1
4	136	1	16	2	1	3	3	1	1
4	137	2	21	3	1	3	3	1	3
4	138	9	9	1	9	9	9	1	1
4	139	2	19	2	1	3	3	1	1
4	140	1	18	2	1	3	3	1	1
4	141	1	17	2	9	3	3	1	1
4	142	1	18	2	1	3	3	1	1
4	143	1	19	2	1	3	3	1	1
4	144	2	16	2	1	3	3	1	2
4	145	2	16	2	1	3	3	1	1
4	146	2	16	2	1	3	3	1	1

4	147	2	16	2	1	3	3	1	2
4	148	2	18	2	1	3	3	1	1
4	149	2	18	2	1	3	3	1	1
4	150	2	18	2	1	3	3	1	1
4	151	2	19	2	1	1	3	2	2
4	152	2	15	2	1	1	1	1	1
4	153	2	18	2	1	3	1	1	1
4	154	1	20	3	1	3	3	1	1
4	155	9	9	1	9	9	9	9	9
4	156	9	9	1	9	9	9	9	9
4	157	2	19	2	1	3	3	3	2
4	158	2	18	2	9	3	3	1	1
4	159	2	15	2	1	3	3	1	1
4	160	1	20	3	1	3	3	2	2
4	161	2	18	2	1	3	3	1	1
4	162	1	18	2	1	3	2	1	3
4	163	1	9	1	1	3	2	2	2
4	164	1	18	2	1	3	2	1	1
4	165	1	18	2	1	3	2	1	1
4	166	1	17	2	1	3	2	1	2
4	167	1	18	2	1	3	2	2	2
4	168	1	18	2	1	3	2	1	1
4	169	1	17	2	1	3	2	1	1
4	170	1	20	3	1	3	2	1	3
4	171	1	18	2	1	3	2	1	2
4	172	1	18	2	1	3	2	1	1
4	173	1	18	2	1	3	2	2	2
4	174	1	18	2	1	3	2	1	1
4	175	1	18	2	1	3	2	1	2
4	176	1	17	2	1	3	2	1	2
4	177	1	9	1	1	3	2	1	2
4	178	1	18	2	1	3	2	1	1
4	179	1	18	2	1	3	2	1	1
4	180	1	20	3	1	3	2	1	2
4	181	1	18	2	1	3	2	1	1
4	182	1	20	3	1	3	2	1	3
4	183	1	17	2	1	3	2	1	2
4	184	1	17	2	1	3	2	1	1
4	185	1	16	2	1	3	2	1	1
4	186	1	18	2	1	3	2	1	2
4	187	1	18	2	1	3	2	1	1
4	188	1	9	1	1	3	2	1	1
4	189	1	18	2	1	3	2	1	2
4	190	1	19	2	1	3	2	1	1
4	191	1	20	3	1	3	2	1	2
4	192	1	18	2	1	3	2	1	3
4	193	1	18	2	1	3	2	1	1

4	194	1	18	2	1	3	2	1	1
4	195	1	17	2	1	3	2	1	1
4	196	1	18	2	1	3	2	1	1
4	197	1	16	2	1	3	2	1	3
4	198	1	18	2	1	3	2	1	2
4	199	1	16	2	1	3	2	1	3
4	200	1	17	2	1	3	2	1	3
4	201	1	18	2	1	3	2	1	2
4	202	1	17	2	1	3	2	1	1
4	203	1	18	2	1	3	2	1	1
4	204	1	17	2	1	3	2	1	1
4	205	1	19	2	1	3	2	1	1
4	206	1	18	2	1	3	2	1	1
4	207	1	17	2	1	3	2	1	1
4	208	1	18	2	1	3	2	1	2
4	209	1	16	2	1	3	2	1	1
4	210	1	18	2	1	3	2	1	1
4	211	1	23	3	1	3	2	1	2
4	212	1	18	2	1	3	2	1	1
4	213	1	18	2	1	3	2	1	2
4	214	1	17	2	1	3	2	1	1
4	215	1	18	2	1	3	2	1	1
4	216	1	17	2	1	3	2	1	2
4	217	1	20	3	1	3	2	1	3
4	218	1	17	2	1	3	2	1	1
4	219	1	17	2	1	3	2	1	2
4	220	1	14	1	1	3	2	1	1
4	221	1	18	2	1	3	2	1	1
4	222	1	18	2	1	3	2	2	2
4	223	1	17	2	1	3	2	1	1
4	224	1	19	2	1	3	2	1	1
4	225	1	18	2	1	3	2	1	1
4	226	1	18	2	1	3	2	1	2
4	227	1	20	3	1	3	2	1	1
4	228	1	17	2	1	3	2	1	3
4	229	1	18	2	1	3	2	1	2
4	230	1	9	1	1	3	2	1	1
4	231	1	18	2	1	3	2	1	1
4	232	1	18	2	1	3	2	1	1
4	233	1	18	2	1	3	2	2	2
4	234	1	18	2	1	3	2	1	1
4	235	1	18	2	1	3	2	1	2
4	236	1	18	2	1	3	2	1	1
4	237	1	18	2	1	3	2	1	3
4	238	1	18	2	1	3	2	1	1
4	239	1	18	2	1	3	2	1	1
4	240	1	17	2	1	3	2	1	1

4	241	1	18	2	1	3	2	1	1
4	242	1	18	2	1	3	2	1	1
4	243	1	16	2	1	3	2	2	2
4	244	1	20	3	1	3	2	1	1
4	245	1	19	2	1	3	2	1	1
4	246	1	18	2	1	3	2	1	2
4	247	1	19	2	1	3	2	1	2
4	248	1	18	2	1	3	2	1	1
4	249	1	20	3	1	3	2	1	1
4	250	1	17	2	1	3	2	1	1
4	251	1	19	2	1	3	2	2	2
4	252	1	18	2	1	3	2	1	1
4	253	1	18	2	1	3	2	1	2
4	254	1	9	1	1	3	2	1	1
4	255	1	19	2	1	3	2	1	2
4	256	1	18	2	1	3	2	1	2
4	257	1	17	2	1	3	2	1	1
4	258	1	18	2	1	3	2	1	2
4	259	1	16	2	1	3	2	1	2
4	260	1	19	2	1	3	2	1	2
4	261	1	17	2	1	3	2	1	2
4	262	1	18	2	1	3	2	1	2
4	263	1	18	2	1	3	2	3	3
4	264	1	18	2	1	3	2	1	1
4	265	1	18	2	1	3	2	1	2
4	266	1	18	2	1	3	2	1	1
4	267	1	18	2	1	3	2	1	1
4	268	1	18	2	1	3	2	2	2
4	269	1	18	2	1	3	2	1	1
4	270	1	19	2	1	3	2	1	1
4	271	1	18	2	1	3	2	1	1
4	272	1	19	2	1	3	2	1	1
4	273	1	20	3	1	3	2	1	1
4	274	1	19	2	1	3	2	2	2
4	275	1	18	2	1	3	2	1	2
4	276	1	18	2	1	3	2	1	3
4	277	1	17	2	1	3	2	1	1
4	278	1	19	2	1	3	2	1	1
4	279	1	19	2	1	3	2	1	1
4	280	1	17	2	1	3	2	1	2
4	281	1	18	2	1	3	2	1	2
4	282	1	17	2	1	3	2	1	2
4	283	1	16	2	1	3	2	1	1
4	284	1	16	2	1	3	2	2	2
4	285	1	17	2	1	3	2	1	1
4	286	1	18	2	1	3	2	2	2
4	287	2	19	2	1	3	1	1	1

4	288	2	9	1	1	3	1	1	1
4	289	2	17	2	1	3	1	1	1
4	290	2	18	2	1	3	1	1	1
4	291	2	18	2	1	3	1	1	1
4	292	2	21	3	1	3	1	1	1
4	293	2	17	2	1	3	1	1	1
4	294	2	17	2	1	3	1	1	1
4	295	2	19	2	1	3	3	1	1
4	296	2	16	2	1	3	1	1	1
4	297	2	19	2	1	3	1	1	1
4	298	2	18	2	1	3	1	2	1
4	299	2	16	2	1	3	1	1	1
4	300	2	16	2	1	3	1	1	1
4	301	2	18	2	1	3	1	2	2
4	302	2	16	2	1	3	1	1	1
4	303	2	17	2	1	3	1	1	2
4	304	2	16	2	1	3	1	1	1
4	305	2	17	2	1	3	1	1	1
4	306	2	18	2	1	3	1	1	1
4	307	2	17	2	1	3	1	1	1
4	308	2	18	2	1	3	1	1	1
4	309	2	19	2	1	3	1	1	1
4	310	2	17	2	1	3	1	1	1
4	311	2	17	2	1	3	3	1	1
4	312	2	17	2	1	3	1	1	1
4	313	2	17	2	1	3	1	1	1
4	314	2	18	2	1	3	1	1	1
4	315	2	19	2	1	3	1	1	2
4	316	2	18	2	1	3	1	1	1
4	317	2	19	2	1	3	1	1	1
4	318	2	19	2	1	3	3	1	1
4	319	2	18	2	1	3	1	1	1
4	320	2	17	2	1	3	1	1	1
4	321	2	17	2	1	3	1	1	1
4	322	2	16	2	1	3	1	1	1
4	323	2	18	2	1	3	1	1	1
4	324	2	16	2	1	3	1	1	1
4	325	2	18	2	1	3	1	1	1
4	326	2	17	2	1	3	1	1	1
4	327	2	16	2	1	3	1	1	1
4	328	2	16	2	1	3	1	1	1
4	329	2	19	2	1	3	1	1	1
4	330	2	18	2	1	3	1	1	1
4	331	2	18	2	1	3	1	1	1
4	332	2	16	2	1	3	1	1	1
4	333	2	19	2	1	3	1	1	1
4	334	2	17	2	1	3	1	1	1

4	335	2	17	2	1	3	1	1	1
4	336	2	16	2	1	3	1	1	1
4	337	2	16	2	1	3	1	1	1
4	338	9	9	1	9	9	9	3	3
4	339	2	18	2	1	3	1	1	1
4	340	2	18	2	1	3	1	1	1
4	341	2	17	2	1	3	1	1	1
4	342	9	9	1	9	9	9	1	1
4	343	2	17	2	1	3	1	1	1
4	344	2	18	2	1	3	1	1	1
4	345	2	18	2	1	3	1	1	1
4	346	2	17	2	1	3	1	1	1
4	347	2	18	2	1	3	1	1	1
4	348	2	16	2	1	3	1	1	1
4	349	2	17	2	1	3	1	1	1
4	350	2	18	2	1	3	1	1	1
4	351	2	17	2	1	3	1	1	1
4	352	2	18	2	1	3	1	1	1
4	353	2	9	1	1	3	1	1	2
4	354	2	17	2	1	3	1	2	2
4	355	2	9	1	1	3	1	1	1
4	356	2	18	2	1	3	1	1	1
4	357	2	19	2	1	3	1	1	1
4	358	2	17	2	1	3	1	1	1
4	359	2	17	2	1	3	1	1	1
4	360	2	9	1	1	3	1	1	1
4	361	2	18	2	1	3	1	1	1
4	362	2	9	1	1	3	1	1	1
4	363	2	18	2	1	3	1	1	1
4	364	2	17	2	1	3	1	1	1
4	365	2	16	2	1	3	1	1	1
4	366	2	18	2	1	3	1	1	2
4	367	2	17	2	9	3	1	2	9
4	368	2	17	2	1	3	1	1	1
4	369	2	18	2	1	3	1	1	1
4	370	2	17	2	1	3	2	1	1
4	371	2	18	2	1	3	1	1	1
4	372	2	18	2	1	3	1	1	1
4	373	2	17	2	1	3	1	1	1
4	374	2	16	2	1	3	1	1	1
4	375	2	17	2	1	3	1	1	1
4	376	2	18	2	1	3	1	1	1
4	377	2	21	3	1	3	1	1	1
4	378	2	17	2	1	3	1	1	2
4	379	2	18	2	1	3	1	1	1
4	380	2	17	2	1	3	1	1	1
4	381	2	21	3	1	3	1	1	1

4	382	2	18	2	1	3	1	1	1
4	383	2	18	2	1	3	1	1	1
4	384	2	17	2	1	3	1	1	1
4	385	2	18	2	1	3	1	2	1
4	386	2	17	2	1	3	1	1	1
4	387	2	17	2	1	3	1	1	1
4	388	2	9	1	1	3	1	1	1
4	389	2	18	2	1	3	1	1	1
4	390	2	16	2	1	3	1	1	1
4	391	2	18	2	1	3	1	1	1
4	392	2	17	2	1	3	1	1	1
4	393	2	17	2	1	3	1	1	1
4	394	2	16	2	1	3	1	2	2
4	395	2	19	2	1	3	1	1	1
4	396	2	18	2	1	3	1	1	1
4	397	2	18	2	1	3	1	1	1
4	398	2	16	2	1	3	1	1	1
4	399	2	18	2	1	3	1	1	2
4	400	2	17	2	1	3	1	1	1
4	401	2	17	2	1	3	1	1	1
4	402	2	18	2	1	3	1	1	1
4	403	2	17	2	1	3	1	1	1
4	404	2	17	2	1	3	1	1	1
4	405	2	18	2	1	3	1	1	1
4	406	2	18	2	1	3	1	1	1
4	407	2	16	2	1	3	1	1	1
4	408	2	16	2	1	3	1	1	1
4	409	2	18	2	1	3	1	1	1
4	410	2	16	2	1	3	1	1	1
4	411	2	18	2	1	3	1	1	1
4	412	2	18	2	1	3	1	1	1
4	413	2	9	1	1	3	1	1	1
4	414	2	17	2	1	3	1	1	1
4	415	2	17	2	1	3	1	1	1
4	416	2	17	2	1	3	1	1	1
4	417	2	16	2	1	3	1	1	1
4	418	2	19	2	1	3	1	1	1
4	419	2	18	2	1	3	1	1	1
4	420	2	9	1	1	3	1	1	1
4	421	2	21	3	1	3	1	1	1
4	422	2	19	2	1	3	1	1	1
4	423	2	18	2	1	3	1	1	1
4	424	2	16	2	1	3	1	1	1
4	425	2	18	2	1	3	1	1	1
4	426	2	20	3	1	3	1	1	1
4	427	2	17	2	1	3	1	1	1
4	428	2	18	2	1	3	1	1	1

4	429	2	20	3	1	1	3	1	1
4	430	2	15	2	1	3	1	1	1
4	431	2	19	2	1	3	1	1	1
4	432	2	17	2	1	3	1	1	1
4	433	2	17	2	1	3	1	1	1
4	434	2	9	1	1	3	1	1	1
4	435	2	20	3	1	3	1	1	1
4	436	2	17	2	1	3	1	1	1
4	437	2	18	2	1	3	1	1	2
4	438	2	18	2	1	3	1	1	1
4	439	2	18	2	1	3	1	1	1
4	440	2	18	2	1	3	1	1	1
4	441	2	19	2	1	3	1	3	3
4	442	2	18	2	1	3	1	1	2
4	443	2	18	2	1	3	1	2	1
4	444	2	17	2	1	3	1	1	1
4	445	2	18	2	1	3	1	1	1
4	446	2	19	2	9	3	1	1	1
4	447	2	9	1	1	3	1	1	1
4	448	2	17	2	1	3	1	1	3
4	449	2	19	2	1	3	1	1	2
4	450	2	17	2	1	3	1	1	2
4	451	2	18	2	1	3	1	1	1
4	452	2	18	2	1	3	1	1	1
4	453	2	19	2	1	3	1	1	1
4	454	2	18	2	1	3	1	1	1
4	455	2	17	2	1	3	1	1	1
4	456	2	18	2	1	3	1	1	1
4	457	2	17	2	1	3	1	1	1
4	458	2	16	2	1	3	1	1	1
4	459	2	18	2	1	3	1	1	1
4	460	2	17	2	1	3	1	1	1
4	461	2	17	2	1	3	1	1	1
4	462	2	19	2	1	3	1	1	1
4	463	2	17	2	1	3	1	1	1
4	464	2	17	2	1	3	1	1	1
4	465	2	17	2	1	3	1	1	1
4	466	2	19	2	1	3	1	1	1
4	467	2	17	2	1	3	1	1	1
4	468	2	16	2	1	3	1	1	1
4	469	2	17	2	1	3	1	1	1
4	470	2	17	2	1	3	1	1	1
4	471	2	9	1	1	3	1	1	1
4	472	2	21	3	1	3	1	1	1
4	473	2	17	2	1	3	1	1	1
4	474	2	18	2	1	3	1	1	1
4	475	2	16	2	1	3	1	1	1

4	476	2	16	2	1	3	1	1	1
4	477	2	19	2	1	3	1	1	1
4	478	2	18	2	1	3	1	1	1
4	479	2	18	2	1	3	1	2	1
4	480	2	17	2	1	3	1	1	1
4	481	2	18	2	1	3	1	1	1
4	482	2	18	2	1	3	1	1	1
4	483	2	18	2	1	3	1	1	1
4	484	2	17	2	1	3	1	1	1
4	485	2	18	2	1	3	1	1	1
4	486	2	17	2	1	3	1	1	1
4	487	2	19	2	1	3	1	1	1
4	488	2	19	2	1	3	1	1	1
4	489	2	17	2	1	3	1	1	1
4	490	2	18	2	1	3	1	1	1
4	491	2	19	2	1	3	1	1	1
4	492	2	17	2	1	3	1	1	1
4	493	2	15	2	1	3	1	1	1
4	494	2	18	2	1	3	1	1	1
4	495	2	18	2	1	3	1	1	1
4	496	2	17	2	1	3	1	1	1
4	497	2	17	2	1	3	1	1	1
4	498	2	18	2	1	3	1	1	1
4	499	2	18	2	1	3	1	1	1
4	500	2	18	2	1	3	1	1	3

PainBreast	ChestPain	Headache	BreastLum	ArmpitLum	DischargeC	DischargeP	DischargeS	Women20	WomenMa
2	2	2	2	2	2	2	2	2	2
2	2	2	2	2	2	2	2	2	2
2	2	2	2	2	2	2	2	2	2
3	3	3	3	3	3	3	3	2	2
1	1	2	2	2	3	3	3	1	2
2	2	2	2	2	2	2	2	2	2
1	2	2	3	2	3	1	2	3	2
2	1	1	1	2	2	2	2	1	3
2	1	1	2	9	1	2	2	1	2
1	1	1	1	2	1	1	2	2	1
2	2	2	2	2	2	2	2	3	3
1	1	2	2	2	3	2	1	1	1
1	2	1	1	2	1	2	2	2	2
3	2	1	1	2	2	1	1	3	3
2	2	2	2	2	2	2	2	2	2
2	2	1	2	2	2	2	2	2	2
1	2	1	3	2	2	2	2	2	2
1	3	3	3	3	3	3	3	3	3
1	2	2	1	3	2	2	2	2	2
1	2	2	1	2	3	2	1	2	3
1	2	2	1	1	1	3	1	1	3
1	1	1	1	1	1	2	2	1	3
1	1	2	1	1	1	1	1	1	3
1	1	1	1	2	2	2	1	1	2
2	2	2	2	2	2	2	2	2	2
1	3	3	1	3	1	3	3	3	3
1	9	9	9	9	9	9	9	9	9
1	1	2	1	1	1	1	3	1	3
1	2	3	1	2	2	2	2	1	3
1	1	3	3	3	1	1	1	1	2
3	3	3	1	2	2	2	3	3	3
1	3	3	1	3	2	2	2	3	3
1	1	2	1	2	1	9	2	3	3
1	1	1	1	2	1	3	3	1	2
1	2	1	1	1	9	9	9	1	2
3	1	1	2	2	9	9	2	3	3
1	1	3	2	3	2	1	2	1	1
2	3	3	1	3	2	2	1	2	3
1	2	2	1	2	2	2	2	1	3
9	1	3	2	2	1	3	2	2	2
1	2	3	1	2	2	2	2	3	3
1	9	9	9	9	9	9	9	3	9
9	9	9	9	9	9	9	9	1	9
1	2	2	1	1	2	2	2	1	3
1	2	2	1	2	2	3	2	3	3
3	2	1	3	3	3	3	3	9	9

1	1	2	2	2	1	1	2	1	1
1	2	2	2	2	1	1	2	1	2
1	2	2	2	2	2	2	2	1	2
2	2	2	1	2	2	1	2	2	1
1	3	3	1	3	1	1	9	3	3
1	3	3	1	3	1	1	3	1	3
2	2	2	3	2	2	2	3	2	2
1	2	3	1	2	3	3	2	2	1
1	1	1	1	3	3	3	1	1	3
3	2	1	3	3	3	2	1	3	1
9	9	9	1	9	9	9	9	9	2
1	1	2	2	2	2	3	2	1	1
2	2	2	1	1	1	1	1	1	1
1	1	1	1	1	1	1	1	1	1
1	1	1	2	2	1	1	2	1	1
1	1	2	2	1	1	1	2	1	1
2	2	2	2	9	1	2	2	3	2
3	1	3	1	9	3	3	1	1	2
1	3	2	1	3	2	2	2	2	2
2	3	3	3	3	3	3	3	2	2
3	1	3	3	3	3	3	9	3	3
1	2	1	1	2	2	1	2	2	3
1	1	2	2	2	2	1	1	1	2
1	1	2	2	3	3	3	3	2	1
1	2	2	3	1	2	2	2	1	1
2	2	2	1	1	1	2	1	2	2
1	1	2	9	2	2	1	1	3	1
1	1	1	1	2	2	1	2	2	3
1	1	2	1	2	2	1	1	1	1
1	3	3	1	2	9	1	9	1	3
2	2	2	2	2	2	2	2	9	9
3	3	3	2	1	3	3	3	1	3
1	3	3	1	3	1	1	1	3	3
1	2	2	2	1	1	1	1	1	2
1	2	2	1	1	9	9	9	2	3
1	1	3	1	2	1	1	1	1	3
2	3	3	1	3	3	2	1	3	2
1	2	2	1	1	2	2	2	1	2
1	3	3	1	3	1	3	3	1	3
2	2	2	1	1	2	2	2	1	2
2	3	3	1	3	2	2	3	1	2
1	9	1	1	1	1	1	1	3	3
9	9	9	9	9	9	9	9	1	3
2	2	2	1	2	2	2	2	1	2
1	1	2	1	2	2	3	3	1	2
1	1	1	1	1	1	1	1	1	3
1	2	2	1	2	2	2	2	1	3

2	1	3	2	1	2	1	3	1	2
3	1	1	3	3	3	1	3	3	3
1	1	2	1	1	2	2	2	1	2
2	1	1	1	2	3	3	2	1	2
2	2	2	3	2	1	1	1	1	2
1	2	2	1	2	2	3	2	1	2
2	2	1	2	2	2	2	2	1	2
3	3	3	3	3	3	3	3	1	3
1	2	3	2	2	2	2	2	2	3
1	2	2	1	9	2	3	1	1	2
1	2	2	1	3	1	2	3	3	1
1	3	3	1	2	2	1	1	3	3
1	2	2	1	2	2	2	2	1	1
1	2	2	1	2	2	2	2	2	2
1	2	2	1	2	1	1	1	2	1
1	2	2	1	2	1	1	1	2	1
3	3	3	1	3	2	2	1	1	3
2	3	3	3	3	2	2	2	2	2
1	1	3	1	3	1	9	2	2	2
2	2	2	1	2	2	1	1	1	2
1	1	3	3	3	3	3	3	3	1
1	1	2	1	1	1	2	2	1	3
1	1	1	1	1	2	1	1	1	1
1	2	1	2	1	2	9	2	1	2
3	3	1	3	3	3	3	3	3	3
2	1	3	2	2	1	3	2	3	3
3	2	1	1	2	2	1	1	1	1
1	2	1	2	1	2	2	2	1	1
1	2	1	2	2	2	2	2	1	1
1	2	3	1	2	2	2	3	1	3
1	1	1	2	2	1	1	1	1	1
3	3	3	3	3	3	3	3	1	2
2	1	2	3	1	2	2	9	3	3
1	2	1	1	2	2	2	2	1	2
1	2	1	3	3	3	3	1	1	3
2	2	2	2	2	2	2	2	1	1
1	1	2	2	1	2	9	2	1	9
1	1	9	1	9	1	9	1	1	3
1	1	1	1	3	2	1	3	1	3
3	3	3	3	3	3	3	3	1	3
1	1	2	2	2	2	1	2	1	1
9	9	9	9	9	9	9	9	9	9
1	2	2	1	1	2	2	2	1	1
2	2	2	1	1	2	1	1	2	1
1	3	3	1	3	2	3	1	1	3
1	2	2	1	2	2	2	1	2	3
1	1	9	1	2	2	2	1	1	3

1	3	3	1	1	3	1	3	1	1
3	3	3	3	3	3	3	3	3	3
3	3	3	3	3	3	3	3	3	3
1	2	2	2	2	2	2	2	1	3
1	1	2	1	2	1	2	2	1	2
3	3	3	3	3	3	3	3	3	3
1	3	3	1	3	1	9	2	1	9
1	1	3	2	2	3	2	1	3	3
9	9	9	9	9	9	9	9	2	2
1	2	3	2	2	2	2	2	2	2
1	1	1	1	1	1	1	1	2	3
1	3	3	1	3	1	3	2	2	2
2	2	2	9	2	2	2	2	2	2
9	1	3	2	2	2	2	2	3	3
1	1	2	3	2	2	2	2	1	1
2	1	1	1	1	1	1	1	1	1
1	1	1	1	2	2	1	1	1	1
1	1	2	2	2	2	1	1	1	1
1	1	2	1	2	2	2	2	2	2
1	1	3	2	2	1	2	2	3	3
1	9	9	9	9	9	9	9	2	9
1	2	3	9	1	2	2	2	2	3
3	3	2	2	2	2	3	2	2	3
1	1	2	1	1	2	9	2	2	1
1	2	2	1	2	3	3	3	1	2
3	3	1	3	3	3	3	3	3	3
1	2	3	1	2	1	2	2	1	3
1	1	3	1	1	2	2	2	3	3
1	1	2	1	1	2	2	2	2	3
1	2	2	2	1	1	1	1	1	1
9	9	9	9	9	9	9	9	9	9
2	2	2	2	2	2	2	2	2	2
1	2	2	2	1	1	1	2	1	2
1	1	1	1	1	1	1	1	1	1
1	1	2	1	2	2	2	2	1	2
1	1	1	1	1	1	1	1	1	1
1	1	1	1	1	1	1	1	1	1
1	2	3	1	1	2	2	1	2	3
1	3	3	3	3	1	3	1	1	3
1	1	1	1	2	2	2	2	2	3
2	1	2	1	2	1	2	1	3	9
2	2	2	2	2	2	2	2	2	2
1	2	2	3	1	1	1	1	1	1
9	9	9	9	9	9	9	9	9	9
9	9	9	9	9	9	9	9	9	9
1	1	2	2	1	1	2	2	1	1
2	2	1	1	1	2	2	2	1	2

9	9	1	9	9	9	9	9	9	9
9	9	9	1	9	9	9	9	1	9
1	1	2	1	2	1	1	1	1	3
9	9	9	1	9	9	9	9	1	9
2	3	3	1	1	2	3	2	3	3
1	1	1	2	2	2	2	2	1	3
2	2	9	1	2	2	2	2	1	3
1	2	1	3	3	1	3	1	3	1
1	1	3	1	2	1	1	1	2	1
9	9	9	9	9	1	9	9	9	9
1	9	9	9	9	9	9	9	9	9
1	9	9	9	9	9	9	9	9	2
1	1	1	2	2	1	1	1	1	1
2	3	1	1	2	2	3	3	2	2
9	2	9	9	9	9	9	9	9	9
1	1	2	1	1	2	2	2	1	3
3	3	3	3	3	3	3	3	3	3
2	2	2	1	1	1	1	1	2	1
1	1	2	1	2	1	1	1	1	2
2	3	2	1	2	2	2	3	1	2
2	2	2	1	2	2	2	1	1	3
1	2	3	1	3	1	2	2	3	2
3	1	1	3	9	3	3	3	2	2
1	1	3	1	2	2	1	1	1	3
2	3	3	2	1	2	2	2	2	3
2	3	1	2	2	2	2	2	2	1
1	1	1	2	2	2	2	2	2	2
1	2	2	2	2	3	3	3	2	2
9	9	9	9	9	9	9	9	9	9
1	1	2	1	1	1	2	2	1	2
1	2	2	2	2	2	2	2	2	2
1	2	1	2	1	1	2	1	1	1
3	3	3	2	3	3	9	2	1	2
1	1	1	2	2	2	2	1	1	2
1	2	9	1	3	1	3	1	1	3
1	1	1	2	1	1	1	1	2	1
1	3	1	2	3	2	1	1	3	1
1	2	2	1	1	1	1	1	2	1
1	3	1	2	3	2	1	1	3	1
1	1	1	1	1	1	2	2	2	2
1	1	1	2	2	1	1	2	2	1
1	2	2	2	1	1	3	3	1	3
3	3	3	3	3	3	3	3	1	2
1	1	1	2	2	1	1	1	1	1
2	2	2	2	2	2	2	2	1	2
1	1	3	1	3	2	1	2	1	1
3	3	3	3	3	3	3	3	9	9

1	1	1	1	2	1	3	1	1	3
1	1	1	1	2	1	1	2	3	3
1	1	1	1	2	2	3	3	1	1
1	3	2	1	3	1	2	1	1	3
1	1	2	2	2	2	2	2	3	3
1	2	3	2	2	2	2	2	2	3
1	1	3	2	2	1	1	1	1	2
1	1	2	2	2	2	2	2	3	2
1	2	2	1	1	1	1	1	2	2
1	2	1	1	1	1	1	1	1	1
1	1	1	1	1	1	1	1	1	1
1	1	1	1	1	1	1	1	2	3
2	9	9	9	9	9	9	9	2	9
2	2	2	9	2	2	2	2	2	2
9	9	3	1	9	9	2	1	2	9
9	9	9	9	9	9	9	9	9	9
9	1	9	9	1	9	9	9	2	9
9	2	1	9	9	9	9	9	9	2
2	9	2	9	9	2	9	2	9	1
9	9	9	9	9	9	9	9	9	9
9	9	9	9	9	9	9	9	9	9
2	2	2	2	2	2	2	2	2	2
2	2	2	2	2	2	2	2	2	2
3	3	3	3	3	3	3	3	2	3
2	2	1	1	2	2	2	2	2	2
9	2	9	9	2	9	2	2	9	9
2	2	2	2	2	2	2	2	2	2
9	9	9	9	9	9	9	9	9	9
9	9	9	9	9	9	9	9	9	9
1	1	2	2	2	2	2	2	2	2
9	9	2	9	9	9	9	9	9	9
9	9	9	9	9	9	9	9	9	9
9	9	9	9	9	9	9	9	9	9
9	2	9	9	2	9	9	2	2	9
9	9	2	1	9	3	1	9	9	2
9	9	1	9	9	3	1	9	9	3
9	9	9	9	9	9	9	9	9	9
9	2	9	9	9	2	9	9	9	2
9	9	3	1	3	9	2	1	9	2
3	9	9	9	9	9	9	9	3	9
9	9	9	9	2	9	9	9	9	9
1	9	9	9	9	9	9	9	1	9
3	3	3	3	3	3	3	3	3	3
3	1	3	1	1	1	1	1	2	2
1	2	3	1	2	3	1	1	2	1
1	1	1	1	1	1	1	1	1	1
9	1	9	9	9	9	9	9	9	9

9	9	9	1	9	9	9	9	1	9
1	9	9	9	9	9	9	9	1	9
1	1	2	1	3	2	3	2	3	2
1	3	9	3	2	9	2	3	3	2
2	2	2	2	2	2	2	2	1	1
1	2	2	1	2	2	2	2	2	2
1	1	2	1	2	1	3	2	1	3
1	3	1	1	1	1	1	1	3	3
1	2	1	1	1	1	1	1	9	9
1	1	2	1	2	2	2	2	1	2
9	9	9	9	9	9	9	9	9	9
9	9	9	9	9	9	9	9	9	9
1	9	9	9	9	9	9	9	9	1
1	1	2	2	1	2	2	1	1	1
1	1	1	1	1	1	1	1	1	1
1	1	1	2	2	1	2	1	2	1
9	9	9	9	9	9	9	9	9	9
1	1	2	1	2	2	2	2	2	2
1	1	2	3	1	1	1	1	1	1
1	1	1	1	1	1	1	1	1	3
1	3	3	1	3	1	1	3	3	3
2	2	3	2	2	2	2	2	1	3
3	3	3	2	2	1	1	2	1	3
1	1	1	1	1	1	1	1	2	2
2	3	1	1	1	1	1	1	2	2
2	2	2	2	2	2	2	2	2	2
2	2	2	2	2	2	2	2	2	2
1	2	3	1	2	3	1	2	1	2
2	2	2	2	2	2	2	2	3	3
1	1	1	1	1	1	1	1	2	2
2	2	2	2	2	2	2	2	3	2
2	2	2	2	2	2	2	2	2	2
2	3	2	1	2	3	2	3	2	2
2	2	2	2	2	2	2	2	2	2
3	3	9	3	3	3	3	3	3	3
1	1	2	2	2	2	2	2	1	3
3	2	2	2	2	1	1	1	9	9
2	1	2	3	3	2	3	2	3	1
3	3	3	3	3	3	3	3	3	3
3	3	3	3	3	3	3	3	3	3
2	2	2	2	2	2	2	9	2	2
2	1	2	1	3	1	2	3	1	1
1	1	1	1	1	1	1	2	1	3
2	1	1	3	1	2	3	3	3	1
2	2	2	2	2	2	2	2	1	1
2	2	2	2	2	2	2	2	2	2
2	2	2	2	2	2	2	2	1	1

1	1	2	1	3	2	1	2	2	2
1	1	3	1	1	2	2	2	9	9
9	9	9	1	9	9	9	9	9	1
1	3	2	1	2	3	2	3	1	2
1	9	9	9	9	9	9	9	9	9
2	2	1	2	3	2	1	2	2	2
1	9	9	9	9	9	9	9	9	9
2	9	9	9	9	9	9	9	3	9
2	2	2	2	2	2	2	2	2	2
2	9	9	9	9	9	9	9	1	9
1	9	9	9	9	9	9	9	9	1
1	9	9	9	9	9	9	9	9	2
2	9	9	9	9	9	9	9	9	9
9	9	2	9	9	9	9	9	9	1
1	9	9	9	9	9	9	9	1	9
9	9	2	9	9	9	9	9	9	9
2	2	1	2	1	3	2	1	2	3
1	9	2	1	3	2	1	3	2	1
1	2	1	1	2	3	1	2	1	3
1	2	2	2	1	1	2	2	2	2
1	2	3	1	2	1	2	3	3	3
1	2	2	1	2	1	2	1	1	3
1	1	3	1	3	1	1	2	1	2
2	1	2	1	2	3	2	2	2	3
2	9	2	1	2	3	3	3	1	3
3	3	3	3	3	3	3	3	3	3
2	2	2	2	2	2	2	2	9	3
1	2	2	2	3	2	3	2	1	3
1	1	2	1	2	2	2	2	2	2
1	2	2	1	1	1	1	1	1	2
1	1	2	1	1	2	1	1	1	3
2	2	2	2	2	2	2	2	2	2
1	2	2	1	1	1	1	2	1	1
1	1	1	1	1	1	1	1	1	9
9	9	9	9	9	9	9	9	2	3
2	2	3	1	2	1	2	2	1	3
3	3	3	3	3	3	3	3	9	9
1	1	1	2	2	2	2	2	1	2
1	1	9	2	2	2	2	2	2	9
2	2	2	2	2	2	2	2	2	2
1	9	9	9	9	9	9	9	1	9
3	3	3	2	3	2	3	3	3	3
9	9	9	1	9	9	9	9	1	9
1	3	2	1	2	1	3	1	2	3
1	1	2	2	2	2	2	2	2	2
1	1	2	9	2	2	1	3	1	2
1	1	1	1	1	1	1	1	2	2

1	1	1	2	2	3	2	1	3	2
2	9	9	1	9	9	9	9	9	9
1	9	9	2	2	9	9	9	2	9
1	2	9	3	2	3	2	3	1	2
2	2	2	2	2	2	2	2	3	3
1	9	2	2	2	2	2	2	2	2
1	3	3	1	3	2	2	2	1	2
1	1	2	1	2	2	2	1	2	2
9	9	9	9	9	9	9	9	9	9
1	1	3	1	2	2	3	2	2	3
1	1	3	1	2	1	2	1	1	3
1	9	9	9	9	9	9	9	1	9
1	1	1	1	1	2	2	1	3	2
1	9	9	9	9	9	9	9	1	9
1	2	2	1	2	1	2	2	1	3
1	1	2	1	2	1	2	2	2	3
9	9	9	9	9	9	9	9	9	9
2	9	9	9	9	9	9	9	2	9
9	9	9	1	9	9	9	9	1	9
1	3	3	1	3	2	3	3	3	3
2	2	2	2	2	2	2	2	2	2
1	3	1	3	3	3	2	2	2	2
1	2	1	2	2	2	2	2	2	1
9	9	9	9	1	9	9	9	1	9
1	2	1	2	2	1	2	2	1	3
2	2	2	2	1	2	2	2	1	1
2	2	2	1	2	2	1	1	1	1
1	1	1	2	1	2	1	2	2	1
2	2	1	2	3	3	3	3	3	2
1	9	9	9	9	9	9	9	1	9
9	9	9	9	9	9	9	9	9	9
1	2	1	1	1	1	1	1	1	1
1	1	1	1	1	1	2	2	1	3
1	1	1	1	1	1	9	9	9	3
1	2	1	1	1	1	2	2	1	3
1	2	2	1	1	1	1	1	2	2
1	1	2	1	1	1	9	9	1	3
1	2	2	1	1	1	1	1	2	1
3	2	9	3	1	2	2	9	2	3
2	2	9	1	1	2	2	1	2	1
2	2	2	1	2	2	2	2	1	3
1	3	3	1	1	2	3	2	2	2
3	3	3	3	3	3	2	2	2	2
2	2	1	1	2	1	2	1	2	3
1	1	1	1	1	1	1	1	2	2
1	1	1	1	1	1	1	1	2	2
3	3	3	3	3	3	3	3	2	2

1	2	2	2	2	2	2	2	2	2
2	9	9	1	9	9	9	9	1	9
3	1	9	9	9	9	9	9	9	9
9	9	9	1	9	9	9	9	1	9
1	1	2	2	3	1	2	9	1	2
1	1	1	2	1	1	1	1	3	3
2	2	2	9	9	2	9	2	2	2
2	9	2	2	9	2	9	2	2	9
9	9	9	9	9	9	9	9	9	9
2	9	9	9	2	9	9	9	9	9
1	2	2	1	2	2	2	2	1	2
2	2	2	2	2	2	2	2	2	2
1	2	2	2	2	2	2	2	2	2
2	2	2	2	2	2	2	2	2	2
1	9	9	1	9	9	9	9	2	2
2	9	9	9	9	9	9	9	2	9
3	3	3	3	3	3	3	3	3	3
1	9	9	9	9	9	9	9	1	9
2	9	9	9	9	9	9	9	2	9
2	9	9	9	9	9	9	9	2	9
9	9	9	9	9	9	9	9	9	9
2	9	9	9	9	9	9	9	2	9
2	2	2	2	2	2	2	2	2	2
2	2	2	2	2	2	2	2	2	2
9	9	9	9	9	9	9	9	9	9
1	9	9	9	9	9	9	9	1	9
2	2	1	1	1	1	1	1	1	9
1	1	9	1	1	9	9	9	1	9
1	3	3	1	1	3	3	3	1	3
2	2	2	2	2	2	2	2	2	9
2	2	2	2	9	9	9	9	1	9
1	2	3	1	1	3	3	3	1	3
1	9	1	9	1	9	1	9	1	9
2	9	2	9	9	9	1	9	1	9
2	2	2	2	2	2	2	2	2	2
3	3	3	3	2	2	2	2	1	9
2	2	2	2	2	2	2	2	2	2
2	2	2	2	2	2	2	2	2	2
1	1	1	1	1	2	2	2	1	2
2	1	1	2	2	2	9	2	2	2
9	9	9	9	9	1	9	9	1	9
1	3	3	1	1	2	2	1	2	3
1	3	3	1	2	1	2	3	2	1
1	1	1	2	2	3	9	3	3	3
3	3	3	1	1	1	1	1	3	3
1	2	3	1	2	2	1	2	2	1
1	2	3	1	2	2	3	2	1	3

1	9	9	9	9	9	9	9	1	9
1	3	3	9	1	1	2	1	3	1
1	2	2	2	2	2	2	2	2	2
1	9	9	1	9	1	9	1	1	9
1	2	2	2	2	2	2	2	2	2
1	1	3	3	2	1	3	2	1	3
1	2	1	2	2	1	1	1	3	3
1	1	3	3	2	1	3	2	1	3
1	3	3	1	3	1	1	1	3	3
1	2	2	1	1	1	9	1	2	2
1	2	2	2	2	2	2	2	2	2
9	9	9	9	9	9	9	9	1	2
2	9	9	1	3	9	9	9	9	9
1	2	3	1	2	2	1	1	1	2
9	9	9	2	9	2	9	9	9	2
1	1	2	2	2	2	1	1	2	2
2	3	3	1	1	2	2	3	1	2
2	2	2	1	2	2	2	2	2	3
1	1	3	1	1	3	9	2	2	3
1	1	2	2	2	1	1	2	1	3
2	9	9	9	9	9	9	9	2	9
2	3	1	3	3	2	3	1	1	3
3	2	3	1	3	3	3	3	3	3
1	9	9	9	9	9	9	9	1	9
1	9	9	1	2	1	9	9	9	9
1	1	1	1	1	1	1	1	1	1
9	9	9	9	1	9	9	9	1	9
1	2	2	1	1	1	1	1	2	2
9	9	9	9	9	9	9	9	9	9
1	9	9	9	9	9	9	9	9	1
1	1	2	2	2	2	2	2	2	2
3	2	1	2	1	3	2	3	2	3
1	2	1	1	1	1	2	3	2	2
1	1	2	1	2	1	1	1	1	1
1	1	2	2	2	1	3	1	1	3
2	2	3	2	3	2	9	9	2	3
2	3	3	2	2	2	2	3	3	1
1	1	1	1	1	1	1	1	1	1
1	2	2	1	2	2	2	2	3	3
2	2	2	2	2	2	2	2	2	2
1	9	3	1	2	2	2	2	1	9
1	9	9	1	2	2	2	2	1	3
1	2	1	3	1	1	2	3	3	3
1	2	3	2	1	1	2	2	3	3
1	9	1	3	3	3	3	3	3	2
1	1	1	3	2	1	2	2	2	1
1	1	3	3	3	3	1	1	3	3

1	9	9	9	9	9	9	9	9	9
1	2	3	1	2	2	2	2	2	2
1	3	3	1	2	2	3	2	1	2
1	2	3	1	1	1	2	1	3	3
1	2	2	2	2	2	2	2	1	2
1	1	3	9	9	9	9	9	9	9
1	9	1	9	9	9	9	9	9	9
1	2	2	2	1	2	2	2	2	2
1	1	2	1	1	2	2	2	2	2
1	2	2	1	2	2	2	2	2	2
2	2	3	2	2	2	2	2	2	2
1	2	3	2	2	2	2	2	3	2
1	2	2	1	2	1	2	2	2	3
2	2	2	2	2	2	2	2	2	2
1	3	3	3	3	2	3	2	3	2
2	9	9	2	9	2	9	2	2	9
9	9	9	9	9	2	9	9	2	9
9	9	9	9	9	9	9	9	9	9
1	9	9	9	9	9	9	9	1	9
2	2	2	2	2	2	2	2	2	9
2	9	9	9	9	9	9	9	2	9
9	9	9	9	9	9	9	9	9	9
2	2	2	2	2	2	2	2	2	2
2	9	9	9	9	9	9	9	2	9
9	9	9	9	9	9	9	9	9	9
3	9	9	9	9	9	9	9	3	9
9	9	9	9	9	9	9	9	9	9
1	1	1	1	1	1	1	1	1	1
3	9	9	9	9	9	9	2	2	9
3	9	9	9	9	9	9	3	3	9
3	9	9	9	9	9	9	9	3	9
2	2	2	2	2	2	2	2	2	2
1	1	1	1	1	1	1	1	1	1
3	9	9	9	9	9	9	9	3	9
3	3	3	3	3	3	3	3	2	2
9	9	9	9	9	9	9	9	9	9
2	9	9	9	9	9	9	9	2	9
9	9	9	9	9	9	9	9	9	9
2	9	1	9	9	2	9	9	1	9
1	9	9	9	9	9	9	9	1	9
2	2	2	2	2	2	2	2	2	2
2	9	9	9	9	9	9	9	2	9
2	9	9	9	9	9	9	9	2	9
2	9	9	9	9	9	9	9	9	9
2	2	2	2	2	2	2	2	2	2
2	2	2	2	2	2	2	2	2	2
9	9	9	9	9	9	9	9	9	9

1	2	1	1	9	1	1	1	1	1
2	1	9	1	2	1	2	2	2	2
2	9	9	9	2	9	9	9	1	9
1	3	3	1	2	2	2	2	1	2
2	9	9	9	9	9	9	9	2	9
2	9	9	9	9	9	9	9	2	9
2	9	9	9	9	9	9	9	2	9
3	9	9	9	9	9	9	9	2	9
9	9	9	9	9	9	9	9	9	9
9	9	9	9	9	9	9	9	9	9
9	9	9	9	9	9	9	9	9	9
9	9	9	9	9	9	9	9	9	9
9	9	9	9	9	9	9	9	9	9
9	9	9	9	9	9	9	9	9	9
2	2	2	2	2	2	2	2	2	2
2	2	2	2	2	2	2	2	2	2
1	2	2	1	2	2	2	2	1	2
2	2	2	2	2	2	2	2	2	2
2	2	2	2	2	2	2	2	2	2
2	2	2	2	2	2	2	2	2	2
2	2	2	2	2	2	2	2	2	2
2	9	9	9	9	9	9	9	2	9
1	9	9	9	9	9	9	9	1	9
2	2	2	2	2	2	2	2	2	2
2	2	2	2	2	2	2	2	2	2
1	9	9	1	2	2	2	2	1	2
9	9	9	9	9	9	9	9	9	9
2	2	2	2	2	2	2	2	3	3
9	9	9	9	9	9	9	9	9	9
1	1	1	1	1	1	1	1	2	2
1	1	1	1	1	1	1	1	1	1
2	2	3	2	1	2	3	2	1	2
2	1	1	2	2	2	2	2	1	1
2	3	3	1	9	2	2	2	1	1
2	2	2	2	2	2	2	2	2	1
1	9	9	1	9	9	9	9	1	9
3	9	9	9	9	9	9	3	3	9
2	9	9	9	9	9	9	9	2	9
1	1	1	1	1	1	1	1	1	1
3	9	9	9	9	9	9	3	3	9
2	9	9	9	9	9	9	9	2	9
9	9	9	9	9	9	9	9	9	9
2	2	2	2	2	2	2	2	2	2
1	9	9	1	1	9	9	9	1	9
1	9	1	9	1	9	1	2	9	1
2	9	9	9	9	9	9	2	2	9
2	9	9	9	2	9	9	2	2	9

3	9	9	9	9	9	9	9	2	9
9	9	9	9	9	9	9	9	9	9
3	9	9	9	9	9	9	3	3	9
3	9	9	9	9	9	9	9	3	9
9	9	9	9	9	9	9	9	9	9
2	9	9	9	9	9	9	9	2	9
1	9	9	1	1	9	9	9	1	9
2	9	9	9	9	9	9	9	2	9
2	2	2	9	2	2	2	2	2	9
1	9	9	9	9	9	9	9	1	1
2	9	9	9	9	9	9	9	2	9
9	9	9	9	9	9	9	9	9	9
1	3	3	1	1	1	3	3	3	3
9	9	9	9	9	9	9	9	9	9
1	1	2	1	2	3	1	1	3	3
3	2	3	1	2	1	2	2	2	3
9	9	9	1	9	9	9	9	9	9
9	9	9	1	9	9	9	9	9	9
1	3	2	2	2	2	9	1	1	3
2	2	2	1	2	2	9	9	2	2
1	1	3	2	2	2	3	2	1	3
9	9	1	9	9	9	9	9	2	9
1	2	2	3	2	3	1	1	3	3
1	2	2	3	2	3	1	2	2	2
1	2	9	1	2	2	2	2	2	3
1	2	2	1	2	2	2	2	2	3
1	2	2	1	1	2	2	2	3	3
2	2	1	2	2	1	2	2	3	3
2	3	3	9	9	9	9	9	1	1
1	2	1	1	1	2	9	1	2	2
2	9	9	9	9	9	9	9	2	9
2	9	9	9	9	9	9	9	1	9
1	2	1	1	3	1	1	1	1	3
9	9	9	9	9	9	2	9	9	9
1	3	3	1	2	9	9	9	1	9
9	9	9	9	9	9	9	9	9	9
9	9	9	9	9	9	9	9	9	9
9	2	9	2	9	9	2	9	9	2
9	9	9	9	9	9	9	9	9	9
2	2	9	2	9	2	9	2	2	9
9	2	9	9	2	9	9	2	2	9
2	2	2	2	2	2	2	2	2	2
2	2	2	2	2	2	2	2	2	2
2	2	2	2	2	2	2	2	2	2
2	2	2	2	2	2	2	2	2	2
2	2	2	2	2	2	2	2	2	2
3	1	1	2	3	2	1	2	2	2

1	2	1	1	3	1	2	3	2	2
2	3	2	1	2	2	2	2	2	2
1	2	1	1	1	1	2	2	2	3
1	2	1	3	2	1	2	1	3	3
3	3	1	3	3	3	3	3	3	3
1	1	2	3	2	2	2	1	1	3
1	2	2	1	2	2	2	3	1	2
3	1	1	3	3	3	3	3	3	3
2	2	2	2	2	2	2	2	2	2
2	2	2	2	2	2	2	2	2	2
2	2	2	2	2	2	2	2	2	2
2	2	2	2	2	2	2	2	2	2
1	2	1	2	3	3	2	1	1	3
2	1	3	1	3	2	1	2	1	3
1	2	2	2	2	2	3	3	3	2
2	2	2	2	2	2	2	2	2	2
3	1	3	3	3	3	3	3	2	3
1	2	3	1	3	2	3	2	1	2
1	2	2	9	1	2	2	3	1	3
1	3	2	1	2	1	3	2	3	2
2	1	1	2	1	2	1	2	2	1
1	3	2	1	1	1	2	2	2	3
2	1	1	2	1	3	2	1	2	3
1	2	2	1	2	1	2	2	3	1
2	2	1	1	2	2	1	1	1	3
1	2	3	2	2	2	3	3	2	3
2	2	3	2	3	3	3	3	2	3
3	2	3	2	3	2	2	3	2	3
1	3	3	1	3	2	2	3	1	1
1	1	2	1	3	1	3	3	1	3
1	1	2	2	2	1	3	3	3	3
2	1	1	2	2	3	2	2	1	2
3	2	3	3	3	3	3	3	3	3
1	1	3	1	1	1	2	3	3	3
9	9	9	1	9	9	9	9	9	9
2	1	3	3	3	2	3	3	2	3
1	2	2	1	3	3	2	2	1	3
1	1	1	1	1	1	1	1	1	1
1	2	2	1	3	3	2	2	1	3
3	2	1	3	2	9	9	9	9	9
3	2	9	2	2	2	2	2	2	2
3	3	1	3	3	3	3	3	1	9
1	1	2	2	3	2	1	3	1	3
1	2	2	2	3	2	1	3	1	3
3	3	3	2	2	3	2	3	2	3
3	3	1	3	3	3	3	3	2	3
3	2	3	2	2	3	2	3	2	1

3	3	3	3	3	3	3	3	2	1
3	3	3	3	3	3	3	3	3	3
1	1	2	2	2	2	3	3	1	1
1	2	1	3	3	2	3	2	3	3
1	9	2	9	1	2	2	3	2	1
1	1	3	1	2	1	1	2	1	3
2	3	2	1	1	1	2	2	1	2
2	3	2	3	9	2	3	1	3	1
1	3	3	1	3	1	1	1	1	3
1	2	2	2	2	1	3	3	1	3
2	2	2	1	2	1	2	2	2	3
3	2	3	2	2	2	2	3	3	2
1	2	9	2	1	9	1	2	2	2
2	2	1	1	3	2	2	2	1	1
1	3	2	2	1	1	2	3	2	1
2	2	2	2	2	2	2	2	2	3
2	2	2	2	9	1	2	2	2	3
2	2	2	2	2	2	2	2	3	3
3	2	2	3	3	3	3	3	2	9
3	3	1	3	3	3	3	3	2	3
1	2	1	1	2	1	2	2	1	1
1	1	2	2	1	1	2	2	2	1
3	3	2	3	3	3	3	2	2	3
1	2	1	3	2	3	2	1	1	1
3	2	3	3	3	3	3	3	3	3
3	3	1	2	1	2	3	2	1	2
2	3	1	2	3	2	2	1	2	2
3	2	2	2	1	2	2	2	2	2
2	3	9	2	3	2	2	2	3	3
2	2	2	2	2	2	2	2	2	2
3	3	3	3	3	3	3	3	2	3
3	9	9	9	9	9	9	9	3	9
1	2	2	2	1	2	1	2	2	3
1	2	1	1	2	2	2	1	3	3
1	3	2	1	3	1	2	3	1	3
3	1	3	3	2	3	2	2	3	1
1	2	1	1	1	1	2	1	3	3
3	1	2	3	1	2	2	1	2	3
1	3	9	1	1	1	3	3	3	3
1	2	2	1	2	2	1	2	2	3
1	2	1	1	1	2	2	2	1	3
2	2	2	2	2	2	2	2	2	2
2	2	2	2	2	2	2	2	2	2
2	2	2	2	2	2	2	2	2	2
1	2	2	2	1	2	1	2	2	3
1	1	1	2	2	2	2	1	1	2
2	2	2	2	3	2	2	2	3	3

2	3	2	2	2	2	3	2	2	2
1	1	2	2	2	1	2	2	1	2
2	2	2	2	2	2	2	2	3	3
1	1	1	2	1	1	3	1	3	3
1	1	1	9	2	1	2	2	9	9
1	1	2	1	2	1	2	2	1	2
1	1	2	2	2	1	2	2	1	2
1	2	1	1	2	1	2	9	1	3
3	3	2	1	2	2	3	2	3	3
2	9	9	9	9	9	9	9	9	9
1	1	3	1	2	2	1	2	3	2
1	1	3	1	2	2	1	2	3	2
9	9	9	1	9	9	9	9	9	9
9	9	9	1	9	9	9	9	2	9
1	1	2	2	2	3	1	2	3	2
9	9	9	9	9	9	9	9	9	9
9	3	1	3	3	3	3	3	1	9
1	9	9	9	9	9	9	9	1	9
1	9	9	9	9	9	9	9	9	9
3	3	3	1	3	2	3	2	3	3
2	9	3	2	2	2	2	2	1	3
2	2	9	9	9	1	9	9	1	9
1	2	1	1	2	3	1	2	1	1
1	1	1	2	2	2	2	3	2	2
2	2	2	2	2	2	2	2	1	2
1	1	1	2	2	2	2	3	3	2
1	1	2	1	1	2	2	3	2	2
3	9	9	9	9	9	9	9	1	3
2	2	2	2	2	2	2	2	2	2
2	2	3	1	2	1	2	2	2	3
3	3	3	1	1	9	9	9	9	9
1	1	2	3	3	2	2	2	1	1
1	2	3	1	2	1	3	2	1	3
2	2	2	2	2	2	2	2	3	2
2	2	2	2	2	2	2	2	2	2
1	1	1	2	9	2	9	3	3	2
9	1	9	9	9	9	9	9	1	9
3	3	1	2	2	2	2	2	1	3
1	1	2	2	1	2	2	2	2	3
3	3	3	3	3	3	3	3	1	1
1	2	2	1	1	1	2	2	2	2
1	2	9	1	2	1	2	3	2	3
2	1	1	2	2	2	2	2	2	2
2	1	1	1	2	2	2	2	2	2
3	2	3	1	3	3	3	2	1	3
9	9	9	9	9	9	9	9	9	9
2	2	1	2	2	2	3	3	3	3

2	3	1	2	2	1	1	2	3	1
1	1	1	3	3	3	3	3	2	2
2	3	3	1	1	2	2	3	3	9
2	2	3	1	2	1	2	1	1	2
2	3	9	3	3	3	3	3	3	2
9	9	9	9	1	1	1	1	1	1
9	9	9	1	9	9	9	9	1	9
3	3	3	3	3	3	3	3	3	3
2	3	3	3	3	3	3	3	3	3
1	2	2	2	2	2	2	2	3	3
9	1	1	2	2	2	2	2	2	2
1	3	3	1	1	3	3	3	1	2
2	3	3	2	2	2	1	1	1	2
9	9	9	9	9	1	9	9	3	3
9	9	9	2	9	9	9	9	1	9
9	9	9	1	9	9	9	9	9	9
2	2	2	1	1	1	1	1	3	3
1	1	1	1	3	9	1	1	9	3
1	2	3	1	2	2	2	2	2	3
2	2	2	2	2	2	2	2	2	3
1	1	1	2	2	3	3	2	2	2
1	1	1	1	1	1	1	9	1	3
1	2	2	1	1	1	2	1	2	2
1	2	2	1	2	2	2	2	2	2
1	1	3	1	1	1	3	1	1	2
3	2	2	2	2	2	2	2	2	2
2	2	2	2	2	2	2	2	2	3
2	9	9	9	9	9	9	9	2	9
2	9	2	3	2	3	2	1	1	3
3	3	3	3	3	3	3	3	2	2
1	1	1	1	1	1	1	1	1	1
1	1	2	1	2	2	1	2	3	3
1	1	2	1	2	1	1	9	1	2
1	2	2	1	1	1	2	2	2	2
1	1	2	2	1	1	1	1	2	2
2	2	2	2	2	2	2	2	1	2
2	2	2	2	2	2	2	2	1	1
1	3	2	1	3	3	3	1	1	3
1	2	3	1	3	2	3	2	3	3
1	1	1	1	2	2	3	3	3	3
1	9	9	9	9	9	9	9	9	9
2	1	2	1	2	1	2	1	2	2
1	2	2	1	2	2	2	2	1	3
1	2	2	1	2	2	2	2	3	3
2	1	1	2	2	2	2	2	2	2
1	2	1	1	2	2	2	2	1	1
3	9	9	9	9	9	9	9	3	9

1	2	1	2	3	2	1	2	3	3
1	2	1	1	2	9	1	2	3	9
3	3	1	3	3	3	3	3	1	2
1	2	2	1	2	2	2	2	1	3
2	2	9	1	3	1	2	1	3	2
1	3	3	1	2	1	3	2	1	3
1	2	3	1	1	1	2	3	1	3
1	9	9	9	9	9	9	9	1	9
1	9	9	9	9	9	9	9	1	9
9	9	9	1	9	9	9	9	9	1
1	2	9	1	2	2	2	1	2	2
1	1	1	1	1	1	1	1	3	3
1	1	1	1	1	1	2	2	1	2
1	9	9	9	9	9	9	9	2	9
1	1	1	3	2	2	2	2	2	2
2	2	2	1	1	1	9	9	1	9
1	2	9	2	2	2	9	1	3	2
9	9	9	1	9	9	9	9	9	9
9	9	9	1	9	9	9	9	9	9
1	9	9	9	9	9	9	9	9	9
1	1	1	1	1	1	1	1	1	1
1	1	2	2	2	1	1	1	3	3
3	2	2	3	2	3	2	2	1	2
3	3	3	3	3	3	3	3	2	2
1	1	9	9	9	9	1	1	2	1
1	1	3	3	3	1	1	2	1	3
1	1	1	3	3	3	3	3	1	3
2	9	9	9	9	9	9	9	2	9
2	2	2	2	2	2	2	3	1	2
1	3	9	1	3	1	3	1	1	3
3	2	1	3	3	3	3	3	3	1
2	9	9	9	9	9	9	9	1	9
1	1	1	1	1	2	2	3	3	3
1	9	9	9	9	9	9	9	3	9
1	9	9	9	9	9	9	9	1	9
1	1	2	2	1	2	2	2	2	2
1	9	9	9	9	9	9	9	1	9
1	1	1	1	1	1	1	9	1	2
1	2	2	1	2	3	2	2	3	3
2	2	2	2	2	2	2	2	2	9
2	2	2	2	2	2	2	2	2	2
2	2	2	2	2	2	2	2	2	2
3	3	3	3	3	3	3	3	3	3
2	2	2	2	2	2	2	2	2	2
1	1	1	1	1	1	1	1	2	2
1	1	1	1	1	1	1	9	1	1
2	2	2	2	2	2	2	2	1	3

9	3	1	3	9	1	3	1	9	1
2	9	2	2	9	2	2	2	9	2
2	9	2	8	2	2	2	9	9	2
9	9	9	9	9	9	9	9	9	9
9	1	3	1	9	1	9	1	1	9
1	1	3	2	1	1	9	1	1	2
9	3	1	9	9	3	1	9	9	2
9	2	9	2	9	9	2	9	2	3
9	2	9	9	9	2	9	2	2	9
1	1	1	1	1	1	1	1	1	1
2	2	2	2	2	2	2	9	2	2
2	9	9	2	9	9	2	9	2	9
2	2	2	2	2	2	2	2	2	2
3	3	3	3	3	3	3	3	3	3
2	2	2	2	2	2	2	2	2	2
9	9	9	9	9	9	9	9	9	9
2	9	2	9	9	2	9	2	2	9
2	2	2	2	2	2	2	2	2	2
9	2	1	9	2	1	2	9	9	1
2	2	2	2	2	2	2	2	2	2
2	2	2	2	2	2	2	2	2	2
2	2	2	2	2	2	2	2	2	2
1	2	1	3	1	1	2	3	1	2
1	1	9	2	1	1	1	1	1	2
3	3	3	3	3	3	3	3	2	2
3	3	3	3	3	3	3	3	3	3
2	3	1	3	1	2	1	2	3	3
1	2	2	1	2	2	3	2	2	3
1	2	1	3	2	1	2	3	3	3
1	2	2	1	2	2	2	2	2	3
3	3	3	3	3	3	3	3	2	2
1	2	2	2	2	2	2	2	3	2
2	3	3	2	2	2	2	1	2	2
1	1	1	2	2	1	2	2	3	3
1	2	2	1	2	1	1	1	1	3
9	1	9	9	9	9	9	9	1	1
3	2	3	1	3	3	3	2	2	3
1	3	3	1	3	1	2	2	1	3
2	2	2	2	2	2	2	3	2	1
2	2	3	2	2	2	2	2	2	3
1	1	1	3	3	3	3	3	1	3
1	1	3	2	2	3	2	3	3	3
1	1	3	1	2	3	1	2	3	2
9	9	9	9	9	9	9	9	9	9
1	3	3	2	1	2	1	1	1	9
1	1	1	2	2	9	9	3	3	2
1	3	1	3	3	3	3	3	1	3

3	1	2	1	2	2	2	2	2	1
3	9	3	3	2	2	2	2	2	2
1	1	1	1	1	1	1	1	1	1
2	2	2	2	2	2	2	2	2	2
1	1	1	1	2	2	2	2	1	1
2	2	2	2	2	2	2	2	2	2
1	1	1	1	1	1	2	1	1	1
1	1	9	1	1	3	3	1	1	3
3	3	3	3	3	3	3	3	3	3
1	2	1	1	1	1	1	2	1	2
1	1	1	1	1	1	9	9	1	1
1	1	1	1	1	1	1	1	1	1
1	1	1	1	1	1	1	1	1	1
1	1	1	1	1	1	1	1	1	1
1	1	1	1	2	2	2	2	1	1
1	1	1	1	1	1	1	1	2	2
1	1	1	2	2	2	1	1	1	2
1	1	2	2	1	2	2	2	1	1
1	1	1	1	1	1	1	1	1	1
2	2	2	2	2	2	2	2	2	2
1	9	9	9	9	9	9	9	1	2
1	1	1	2	2	2	2	2	2	2
9	9	9	9	9	9	9	9	9	9
1	1	1	1	1	1	1	1	1	1
1	1	9	1	1	1	1	1	1	9
1	2	1	1	1	1	1	1	2	1
1	2	1	1	1	1	2	1	1	1
1	1	1	2	1	1	1	1	1	1
2	2	9	2	2	2	2	2	2	9
1	1	1	1	9	1	1	1	1	9
2	2	2	2	2	2	2	2	2	2
2	2	2	2	2	2	2	2	2	2
1	1	1	1	1	1	9	1	1	1
1	1	1	1	1	1	1	1	1	1
1	1	1	1	1	2	2	2	1	1
2	2	2	2	2	2	2	2	2	2
1	2	1	2	1	2	1	2	1	2
1	1	1	1	2	2	1	1	1	1
9	9	9	9	9	9	9	9	9	9
2	2	2	2	2	2	2	2	2	2
2	2	2	2	2	2	2	2	2	2
2	2	2	2	2	2	2	2	2	2
1	1	1	1	1	1	1	1	1	1
2	2	2	2	2	2	2	2	2	2
1	1	1	1	2	1	2	1	1	1
2	2	2	2	2	2	2	2	2	2
1	1	1	1	1	1	1	1	1	1

1	2	1	1	2	1	1	1	1	2
2	2	2	2	2	2	2	2	2	9
1	1	1	1	1	1	1	1	1	1
1	1	1	9	1	1	1	9	1	9
1	1	1	1	1	1	1	1	1	1
2	2	2	2	2	2	2	2	2	2
1	1	1	1	1	1	1	1	1	1
1	1	1	1	1	1	1	1	1	1
2	9	2	2	9	2	2	2	2	9
1	1	1	1	1	1	1	1	1	1
2	2	9	2	2	2	2	2	9	2
1	9	1	1	1	1	9	1	1	9
2	2	2	2	2	2	2	2	2	9
1	1	1	1	1	1	1	1	1	1
1	1	1	1	1	1	1	1	1	1
2	2	2	2	2	2	2	2	2	2
1	1	1	1	1	1	9	9	1	1
9	9	9	9	9	9	9	9	9	9
2	2	2	2	2	2	2	2	2	2
1	3	1	1	1	1	3	3	1	3
1	3	2	1	1	1	1	3	1	3
1	3	2	1	1	1	1	3	1	3
1	3	2	1	1	1	3	1	1	3
1	3	2	1	1	1	3	3	1	3
1	3	2	1	1	1	3	3	1	3
1	3	2	1	1	1	3	3	1	9
1	3	2	1	1	1	9	3	1	3
1	3	1	1	1	1	3	3	1	9
1	3	2	1	1	1	3	3	1	3
1	3	1	1	1	1	3	3	1	3
1	3	2	1	1	1	3	1	1	3
1	3	2	1	1	1	3	3	1	3
1	3	2	1	1	1	3	3	1	3
1	3	2	1	1	1	3	3	1	3
1	3	1	9	9	1	9	3	1	3
3	3	1	2	1	1	2	3	1	3
1	3	2	1	1	1	3	3	1	3
1	1	2	1	1	1	3	3	1	3
1	3	2	1	1	1	3	3	1	3
1	3	2	1	1	1	3	3	1	3
3	3	2	1	1	1	3	3	1	3
1	3	2	1	1	1	3	3	1	3
1	3	2	1	1	1	3	3	1	3
9	9	1	1	1	3	3	3	1	3
9	9	9	9	9	9	9	9	9	9
9	9	9	9	9	9	9	9	9	9
9	9	9	9	9	9	9	9	9	9

9	9	9	9	9	9	9	9	9	9
9	9	9	9	9	9	9	9	3	3
9	9	9	9	9	9	9	9	9	9
9	9	9	9	9	9	9	9	9	9
9	9	9	9	9	9	9	9	9	9
9	9	9	9	9	9	9	9	9	9
9	9	9	9	9	9	9	9	9	9
9	9	9	9	9	9	9	9	9	9
9	9	9	9	9	9	9	9	9	9
9	9	9	9	9	9	9	9	1	9
9	9	9	9	9	9	9	9	1	9
9	9	9	9	9	9	9	9	9	9
9	9	9	9	9	9	9	9	1	1
9	9	9	9	9	9	9	9	1	9
9	9	9	9	9	9	9	9	9	9
9	9	9	9	9	9	9	9	9	9
9	9	9	9	9	9	9	9	1	1
9	9	9	9	9	9	9	9	1	9
9	9	9	9	9	9	9	9	9	9
1	3	2	1	1	1	3	3	1	3
1	3	2	1	1	1	3	3	1	3
1	3	2	1	1	1	3	1	1	3
1	3	2	1	9	1	3	3	1	3
1	3	1	1	1	1	3	3	1	3
1	3	2	1	1	1	3	3	1	3
1	3	2	1	1	1	3	3	1	3
1	3	1	1	1	1	3	3	1	3
1	3	2	1	1	1	3	3	1	3
3	2	1	1	9	9	3	3	1	3
3	3	9	9	1	1	1	1	1	3
1	3	1	1	1	1	3	3	1	3
1	3	3	9	9	1	3	3	1	3
1	3	2	1	1	1	9	3	1	3
1	3	3	1	1	1	3	3	1	3
1	3	1	1	1	1	3	3	1	3
2	9	1	1	9	2	9	3	3	9
3	2	2	1	1	1	3	9	1	3
1	3	3	3	9	1	3	3	1	3
1	3	3	9	9	1	3	3	1	3
9	9	9	9	9	1	9	9	1	3
3	3	1	1	1	1	3	3	1	3
1	3	2	1	1	1	3	3	1	3
3	3	2	1	9	1	3	1	1	3
1	3	2	1	1	1	3	3	1	3
3	3	2	1	1	1	3	3	1	3
1	3	1	1	1	1	3	3	1	3
1	3	1	1	1	1	3	3	1	3

1	3	2	1	1	1	1	1	1	3
1	3	1	1	1	1	3	3	1	3
1	3	1	1	1	1	3	3	1	3
1	3	2	1	1	1	3	3	1	3
1	3	1	1	1	1	3	3	1	3
1	3	2	1	1	1	3	3	1	3
1	3	1	1	1	1	3	3	1	3
1	3	3	1	1	1	1	3	1	3
1	3	3	1	1	1	1	3	1	3
3	2	1	9	9	1	3	9	1	3
1	3	2	1	1	1	3	1	1	3
3	2	1	3	9	9	9	9	1	3
1	3	2	1	1	1	3	3	1	3
9	3	2	1	1	3	3	3	1	3
3	3	2	1	9	9	9	9	1	3
1	3	2	3	2	3	3	3	1	3
1	3	2	1	1	1	3	3	1	3
1	3	2	1	1	1	3	3	1	3
1	2	1	1	1	2	3	3	1	3
1	3	2	1	1	1	3	3	1	3
1	3	2	1	1	1	3	3	3	3
1	3	2	1	1	1	9	3	1	3
1	2	1	1	1	1	3	3	1	3
3	3	2	1	1	1	3	3	1	3
1	3	2	1	1	1	3	3	1	3
1	3	2	1	1	1	3	3	1	3
3	1	1	1	3	3	3	1	1	3
1	3	2	1	1	1	3	3	3	3
1	3	3	1	1	1	1	3	1	3
1	3	3	9	1	1	1	3	1	3
3	2	1	1	9	9	3	3	1	3
3	3	2	1	1	1	9	3	1	3
3	3	2	1	1	1	9	3	1	3
1	3	1	1	1	1	3	3	1	3
9	9	9	9	9	9	9	9	1	3
3	2	3	1	1	1	3	3	1	3
1	3	2	1	1	1	3	3	1	3
3	3	1	1	1	3	3	3	1	3
9	9	1	9	9	9	9	9	1	3
9	9	9	9	9	9	9	9	1	3
3	2	1	1	1	3	3	3	1	3
1	3	2	1	1	1	1	1	1	3
3	3	2	1	1	1	1	1	1	3
1	3	1	1	1	1	3	3	1	3
1	3	1	1	1	1	3	3	1	3
1	3	2	1	1	1	3	3	1	3
1	3	1	1	1	1	3	3	1	3

1	3	1	1	2	3	9	3	1	3
1	3	2	1	1	1	3	3	1	3
1	3	1	1	1	1	3	3	1	3
1	3	2	1	1	1	3	3	1	3
1	3	2	1	1	1	3	3	1	3
1	3	2	1	1	1	3	3	1	3
1	3	2	1	1	1	3	3	1	3
1	3	2	1	1	1	3	3	1	3
1	2	1	1	1	1	3	3	1	3
1	3	2	1	1	1	3	3	1	3
1	3	1	1	1	1	3	3	1	3
1	3	1	1	1	1	3	3	1	3
1	3	2	1	1	1	3	3	1	3
1	3	2	1	1	1	3	3	1	3
1	9	3	1	1	1	1	1	3	3
1	3	2	1	1	1	1	1	3	3
1	3	2	1	1	1	3	3	1	3
1	3	1	1	1	1	3	3	3	3
1	3	2	1	1	1	3	3	1	3
9	9	9	9	9	9	9	9	9	9
1	3	2	1	1	3	3	3	1	3
1	3	2	9	1	1	1	1	1	3
1	3	2	1	1	1	3	1	1	3
1	3	1	1	1	1	3	3	1	3
9	9	9	9	9	9	9	9	3	3
1	3	2	1	1	1	3	3	3	3
1	3	2	1	1	1	3	3	1	3
1	3	3	1	1	1	3	3	1	3
1	3	2	1	1	1	3	3	1	3
1	3	2	1	1	1	3	3	1	3
1	3	2	1	1	1	3	3	1	3
1	3	1	1	1	1	3	1	1	3
1	3	2	1	1	3	3	3	1	3
1	3	2	1	1	3	3	3	1	3
1	3	1	1	1	1	3	3	1	3
1	3	1	1	1	3	3	3	1	3
1	3	1	1	1	3	3	3	1	3
1	3	2	1	1	1	3	3	1	3
1	3	1	1	1	1	3	3	1	3
1	3	1	1	1	1	3	3	1	3
1	3	2	1	1	1	3	3	1	3
9	9	9	9	9	9	9	9	9	9
1	3	1	1	1	1	3	3	1	3
1	3	3	1	1	1	3	3	1	3
1	3	2	1	1	1	3	3	1	3
1	3	1	1	1	1	3	3	1	3
1	3	1	1	1	1	3	3	1	3

1	3	2	1	1	1	3	3	1	3
1	3	2	1	1	1	3	3	1	3
1	3	1	1	1	1	3	3	1	3
1	3	2	1	1	1	3	3	1	3
1	3	1	1	1	1	3	3	1	3
1	3	3	1	1	1	3	3	1	3
1	3	2	1	1	1	3	3	1	3
1	3	2	1	9	1	9	3	1	3
1	3	2	1	1	1	3	3	1	3
1	3	1	1	1	3	3	3	1	3
1	9	9	9	9	1	3	3	1	3
1	3	2	1	1	1	3	3	1	3
1	3	1	1	1	1	3	3	1	3
1	3	2	1	1	2	3	3	1	3
3	2	1	1	1	1	3	3	1	3
1	3	1	1	1	1	3	3	1	3
1	3	2	1	1	1	3	3	1	3
1	3	2	1	1	1	3	3	1	3
1	3	2	1	1	1	3	3	1	3
1	3	2	1	1	1	3	3	1	3
1	3	2	1	1	1	3	3	1	3
1	3	2	1	1	1	1	3	1	3
3	1	9	1	1	1	3	3	1	3
3	1	2	1	1	1	3	3	1	3
1	3	2	1	1	1	1	3	1	3
1	3	2	1	1	1	1	1	1	3
1	2	3	1	1	1	1	1	1	3
9	9	9	9	9	9	9	9	9	9
9	9	9	9	9	9	9	9	9	9
9	9	9	9	9	9	9	9	9	9
9	9	9	9	9	9	9	9	9	9
9	9	9	9	9	9	9	9	9	9
9	9	9	9	9	9	9	9	2	2
9	9	9	9	9	9	9	9	9	9
9	9	9	9	9	9	9	9	2	9
9	9	9	9	9	9	9	9	9	9
9	9	9	9	9	9	9	9	9	9
9	9	9	9	9	9	9	9	9	9
9	9	9	9	9	9	9	9	9	9
9	9	9	9	9	9	9	9	9	9
9	9	9	9	9	9	9	9	9	9
9	9	9	9	9	9	9	9	1	3
1	2	2	1	1	2	2	2	2	2
9	9	9	9	9	9	9	9	1	1
9	9	9	9	9	9	9	9	9	9
9	9	9	9	9	9	9	9	9	9
9	9	9	9	9	9	9	9	9	9

9	9	9	9	9	9	9	9	9	9
9	9	9	9	9	9	9	9	1	3
9	9	9	9	9	9	9	9	9	9
9	9	9	9	9	9	9	9	9	9
9	9	9	9	9	9	9	9	9	9
9	9	9	9	9	9	9	9	9	9
9	9	9	9	9	9	9	9	1	3
3	2	2	2	2	2	2	2	2	2
9	9	9	9	9	9	9	9	9	9
9	9	9	9	9	9	9	9	9	9
9	9	9	9	9	9	9	9	9	9
9	9	9	9	9	9	9	9	9	9
9	9	9	9	9	9	9	9	9	9
1	1	1	3	2	1	2	2	1	3
1	2	9	1	2	2	2	2	2	2
1	3	3	1	3	2	3	3	2	3
1	1	1	1	3	3	2	3	3	3
2	2	2	1	1	1	2	2	1	2
1	1	1	1	3	1	3	3	2	3
2	2	1	2	1	3	2	1	2	1
1	2	2	1	1	1	2	2	1	3
9	9	9	9	9	9	9	9	3	3
1	2	2	1	1	1	3	3	3	3
1	2	2	9	2	2	2	2	2	3
9	9	9	1	9	9	9	9	1	9
1	2	2	2	2	2	2	2	2	2
9	9	9	9	9	9	9	9	2	3
1	1	2	1	1	1	2	3	2	1
1	3	1	1	1	3	9	2	1	3
3	1	3	1	1	1	2	2	2	3
2	3	2	3	2	3	2	2	3	3
1	2	1	2	1	3	2	1	1	2
9	9	9	1	9	9	9	9	9	1
1	2	1	3	2	2	3	1	1	2
1	9	9	9	9	9	9	9	1	9
1	9	9	9	9	9	9	9	2	9
1	2	2	1	2	1	1	1	1	2
1	2	1	1	1	3	2	2	1	2
1	9	9	1	1	1	9	9	1	9
3	1	1	3	3	3	3	3	9	9
9	9	9	9	9	9	9	9	9	9
1	1	3	1	1	2	1	1	1	3
1	2	1	1	1	1	1	1	1	2
9	9	9	9	9	9	9	9	3	3
9	9	9	9	9	9	9	9	9	9
9	9	9	9	9	9	9	9	2	2
1	2	2	2	2	2	2	2	2	2

9	9	9	1	9	9	9	9	1	9
9	9	9	9	9	9	9	9	9	9
2	3	2	3	2	1	3	3	3	3
1	1	1	1	1	1	1	1	2	1
1	9	9	9	9	9	9	9	1	9
9	2	9	9	9	9	9	9	2	9
1	1	1	1	2	2	2	2	1	2
2	2	2	2	2	2	2	2	2	2
2	2	2	2	2	2	2	2	2	2
2	2	2	2	1	1	1	1	1	1
1	2	2	1	1	1	1	1	2	2
9	9	9	9	9	9	9	9	9	9
9	9	9	9	1	9	9	1	1	9
2	2	2	2	2	2	2	2	2	2
1	2	2	1	2	2	2	2	3	2
1	2	3	1	2	3	2	3	2	3
9	9	9	9	9	9	9	9	3	3
2	1	2	3	3	1	2	1	1	2
1	9	9	1	3	1	2	3	1	1
1	2	2	3	2	1	2	3	9	9
1	1	1	1	1	1	1	1	2	3
2	2	2	2	2	2	2	2	1	2
2	2	2	2	2	2	2	2	2	2
2	2	2	2	2	2	2	2	2	3
2	2	2	2	2	2	2	2	2	2
1	3	3	1	1	2	2	1	2	2
2	2	2	2	2	2	2	2	3	2
1	1	1	1	1	1	1	1	3	3
1	2	2	1	2	1	2	2	2	2
1	1	2	1	1	1	1	1	1	2
3	3	2	2	2	3	3	3	3	3
1	1	2	1	1	1	1	2	2	3
1	1	1	1	1	1	1	1	2	2
1	2	2	1	2	1	1	1	1	2
1	2	9	1	1	2	3	1	2	1
1	3	2	3	1	1	2	3	9	9
1	1	9	1	3	3	2	2	2	2
9	9	1	1	1	2	9	9	9	2
2	9	9	9	9	9	9	9	9	9
1	1	2	1	1	2	2	2	1	3
1	2	1	2	2	2	2	2	2	3
1	1	2	2	2	2	2	2	2	2
1	2	3	1	2	3	1	2	1	2
1	2	1	2	2	1	2	2	1	3
1	1	1	1	1	1	1	1	1	3
1	1	2	2	2	3	2	2	1	2
1	3	3	1	1	2	2	2	1	2

1	1	2	1	1	2	1	1	3	3
1	1	1	1	2	2	3	1	2	1
2	3	2	1	2	3	2	3	2	2
1	2	2	1	2	2	2	2	9	9
1	2	1	2	2	1	2	2	1	3
1	2	2	1	2	1	2	2	1	2
1	2	3	1	3	1	3	3	1	2
2	3	2	9	1	2	2	2	1	2
2	2	2	2	2	2	2	2	2	2
1	1	2	3	3	2	1	2	2	2
2	2	2	2	2	2	2	2	2	2
2	2	2	2	2	2	2	2	2	2
2	2	2	2	2	2	2	2	1	1
2	2	2	2	2	2	2	2	2	2
2	2	2	2	2	2	2	2	9	9
2	3	2	2	2	2	2	2	2	1
3	3	3	3	3	3	3	3	3	3
2	2	2	2	2	2	2	2	2	2
2	2	2	2	2	2	2	2	1	2
1	2	3	1	2	3	1	2	1	2
1	2	1	2	2	2	2	2	1	1
1	1	1	1	1	1	1	1	1	1
2	2	2	2	2	2	2	2	9	9
1	1	1	1	1	1	1	1	9	9
1	1	3	1	3	1	3	1	1	3
9	9	9	9	9	9	9	9	1	3
1	1	1	1	9	3	3	1	2	1
1	2	2	1	1	2	2	2	3	3
3	2	3	1	3	1	3	2	1	3
1	2	1	1	1	2	3	2	3	3
1	2	3	1	1	1	2	3	1	3
1	2	9	1	2	3	1	2	9	9
2	2	2	1	1	1	1	2	1	1
3	2	2	3	3	3	3	3	2	1
1	9	2	3	9	2	1	2	1	1
9	9	9	9	9	9	9	9	9	9
1	1	2	1	1	1	1	1	1	2
2	2	1	1	1	2	1	1	2	2
1	3	3	1	3	2	2	9	2	3
1	2	2	2	2	2	2	2	2	2
1	2	3	1	2	3	2	3	2	3
1	1	2	1	1	2	1	2	1	3
1	2	3	1	1	1	2	3	1	3
9	9	9	9	9	9	9	9	1	3
9	9	9	9	9	9	9	9	9	9
9	9	9	9	9	9	9	9	9	2
1	1	1	1	1	1	1	1	1	1

1	1	2	1	2	3	2	2	3	3
1	2	2	1	2	1	3	1	1	3
1	2	2	1	2	1	2	3	1	3
1	3	1	3	3	1	3	1	1	3
9	9	9	9	9	9	9	9	1	2
1	2	2	1	1	1	3	2	2	3
3	1	3	3	2	3	3	3	3	3
1	2	2	1	2	2	2	3	1	3
1	1	2	1	1	1	2	1	1	3
1	2	3	1	2	3	1	2	3	2
1	2	3	2	1	2	3	1	1	2
2	2	2	2	2	2	2	2	2	2
2	2	2	2	2	2	2	2	2	2
1	1	3	1	1	3	3	3	3	3
2	2	2	2	2	2	2	2	2	2
1	2	9	1	1	2	2	2	3	3
1	3	3	1	1	1	1	1	2	3
1	3	3	2	3	2	2	3	3	3
2	1	3	2	1	1	1	1	1	3
1	2	3	1	1	2	3	1	1	3
1	3	3	2	3	1	2	3	2	1
1	3	3	3	2	1	1	1	1	3
1	3	3	2	2	2	2	2	1	3
1	3	3	2	3	2	2	3	2	3
3	3	3	3	3	3	3	3	1	3
9	9	9	9	9	9	9	9	9	9
1	2	3	1	2	3	1	2	2	1
1	1	2	2	2	2	2	2	3	3
1	2	3	1	2	1	1	1	2	2
1	2	1	1	3	1	1	2	1	2
2	2	2	2	2	2	2	2	2	2
1	9	9	9	9	9	9	9	9	9
1	2	1	1	2	2	2	2	1	3
1	2	3	2	3	1	3	2	3	1
1	1	1	1	2	1	3	2	3	2
2	1	2	1	3	2	1	3	1	9
3	3	3	3	3	3	3	3	1	3
1	3	3	1	3	1	1	2	1	2
1	1	2	1	2	2	2	2	3	3
1	9	9	9	9	9	9	9	2	9
2	2	1	1	2	1	3	2	1	2
2	2	2	2	2	2	2	2	2	2
3	3	3	1	1	1	3	3	3	3
2	2	2	2	2	2	2	2	2	2
2	2	2	2	2	2	2	2	2	2
1	3	2	2	2	2	2	2	1	2
2	2	2	3	3	3	2	2	2	2

1	1	1	1	1	1	1	1	1	3
2	2	2	2	2	2	2	2	2	2
1	3	3	1	3	2	3	2	2	3
1	2	2	1	2	1	2	2	2	2
2	2	2	2	2	2	2	2	1	3
3	3	2	2	2	2	2	2	2	2
1	1	3	1	1	1	1	3	1	3
1	1	3	1	2	1	1	1	1	3
1	1	3	1	1	1	9	1	1	3
1	3	3	1	1	1	3	3	3	3
2	1	3	1	3	1	3	2	3	3
2	2	2	2	2	2	2	2	2	2
2	1	3	1	3	1	3	2	3	3
1	2	3	1	3	2	1	1	1	3
2	2	2	2	2	2	2	2	2	2
1	3	3	1	1	1	2	2	1	3
2	1	3	2	3	2	2	2	9	9
1	2	2	2	2	1	2	2	2	3
3	3	2	1	2	2	2	2	1	2
1	2	3	2	1	1	1	1	2	3
1	3	3	1	1	1	1	1	1	2
1	2	3	1	3	3	3	1	2	3
9	9	9	9	9	9	9	1	9	9
2	2	2	2	2	2	2	2	2	2
2	2	2	2	2	2	2	2	2	2
1	1	2	1	1	1	1	1	3	1
2	2	3	2	2	2	2	2	3	3
2	2	2	2	2	2	2	2	2	3
3	3	3	1	1	1	3	1	1	3
1	1	2	1	1	1	2	2	3	3
1	2	2	2	3	1	3	1	1	3
1	1	2	1	2	2	2	1	1	2
1	2	1	2	1	1	1	1	3	9
9	9	9	9	9	9	9	9	1	2
2	9	9	9	9	9	9	9	2	9
1	1	2	1	1	1	3	1	3	3
2	2	2	2	2	2	2	2	2	2
1	1	1	3	3	3	2	2	1	2
2	2	2	1	1	1	1	1	1	1
2	2	2	2	2	2	2	2	1	1
1	2	3	2	1	3	1	3	1	2
9	9	9	9	9	9	9	9	9	9
9	9	9	9	9	9	9	9	9	9
1	1	1	1	1	1	1	1	1	1
1	2	2	3	2	3	2	3	1	2
9	9	9	9	9	9	9	9	9	9
2	2	2	2	2	2	9	2	1	2

2	2	2	2	2	2	2	2	2	2
1	3	3	3	3	2	2	2	1	2
1	2	3	1	2	3	1	2	1	3
1	9	1	3	1	9	1	3	1	2
1	2	3	3	3	2	2	1	1	3
1	2	3	1	2	3	1	2	1	2
2	2	2	1	2	2	1	2	2	2
2	9	9	9	9	9	9	9	2	9
1	1	1	1	1	2	3	3	2	1
9	9	9	9	9	9	9	9	9	9
1	1	1	1	1	1	1	1	2	2
1	3	3	2	1	1	3	1	3	3
1	2	1	3	1	1	2	2	1	2
1	2	2	3	2	3	2	2	1	2
1	1	1	1	1	1	1	1	1	1
9	9	9	9	9	9	9	9	9	9
9	9	9	9	9	9	9	9	9	9
1	2	2	1	2	1	2	2	2	2
2	2	2	2	2	2	2	2	2	2
2	2	2	1	2	3	3	3	1	1
2	2	2	2	2	2	2	2	2	2
1	9	1	1	1	1	1	1	2	2
3	3	3	2	2	2	2	2	1	3
2	3	2	1	2	3	1	3	2	1
2	2	2	2	9	2	2	2	1	2
9	9	9	1	9	9	9	9	9	1
1	1	1	1	1	1	2	2	1	9
2	1	2	1	1	1	1	1	2	2
2	2	2	2	2	2	2	2	9	9
2	2	3	2	2	2	2	2	3	3
2	1	2	3	1	1	2	2	1	1
2	2	2	2	2	2	2	2	2	2
2	2	2	2	2	2	2	2	2	2
2	2	2	2	2	2	2	2	2	2
1	2	2	1	2	2	2	2	1	1
2	2	2	2	2	2	2	2	2	2
3	3	3	3	3	3	3	3	2	2
9	9	9	1	9	9	9	9	1	9
9	9	9	9	9	9	2	9	9	9
2	2	2	2	2	2	2	2	2	2
2	2	2	2	2	2	2	2	2	2
9	9	9	1	9	9	9	9	9	9
1	1	1	1	1	2	2	2	1	2
2	9	9	9	9	9	9	9	2	9
2	2	2	2	2	2	2	2	2	2
2	1	2	2	1	2	2	1	1	2
2	2	2	2	2	2	2	2	2	2

2	3	1	2	3	1	3	2	2	1
1	9	9	9	9	9	9	9	1	9
2	2	2	2	2	2	2	2	2	2
1	1	2	1	2	3	2	2	2	3
2	2	2	2	2	2	2	3	2	2
2	2	1	2	1	1	2	2	1	3
1	1	2	1	2	1	1	1	2	1
1	2	2	3	1	2	2	2	1	2
2	2	2	2	2	2	2	2	2	2
9	9	9	9	9	9	9	9	1	1
9	9	9	1	9	9	9	9	3	3
2	2	2	2	2	2	2	2	2	2
1	1	9	1	9	1	2	3	2	2
1	2	2	1	3	2	3	1	2	3
1	2	1	1	2	1	1	1	1	3
1	1	1	1	1	1	1	1	2	1
2	2	2	2	2	2	2	2	2	2
2	2	2	2	2	2	2	2	2	2
3	3	1	1	1	1	2	3	3	3
2	2	2	1	2	2	2	1	1	3
1	1	2	1	1	1	2	1	2	2
1	2	2	1	2	2	2	2	2	3
1	1	3	3	3	2	1	2	1	2
9	9	9	9	9	9	9	9	9	9
3	3	3	3	3	3	3	3	3	2
9	9	9	1	9	9	9	9	1	9
9	9	9	9	9	9	9	9	9	9
9	9	9	9	9	9	9	9	1	9
1	1	1	2	2	2	2	3	3	1
1	1	2	1	1	1	1	1	1	3
2	2	2	2	2	2	2	2	3	3
1	2	9	9	1	9	2	1	1	3
1	2	1	1	2	2	2	2	1	2
2	2	2	2	2	2	2	2	2	2
1	1	2	1	1	1	1	1	2	2
1	1	1	1	1	1	2	2	1	3
1	3	1	2	1	2	1	3	2	3
2	3	2	1	2	9	3	2	2	9
1	2	2	1	1	1	2	1	2	3
1	1	2	1	2	2	2	3	3	3
3	3	2	2	3	3	2	2	1	2
2	1	9	2	3	9	2	1	3	2
9	9	9	9	9	9	9	1	1	9
9	9	2	9	9	9	9	9	3	9
1	2	1	3	2	3	1	2	2	3
2	2	2	2	2	2	2	2	2	2
2	2	2	2	2	2	2	2	2	2

3	3	3	1	1	1	2	2	1	3
9	9	9	9	9	9	9	9	9	9
9	9	9	9	9	9	9	9	9	9
9	9	9	9	9	9	9	9	9	9
9	9	9	9	9	9	9	9	2	2
9	9	1	9	9	9	9	9	1	9
9	9	9	9	9	9	9	9	9	9
9	9	9	9	9	9	9	9	9	9
9	9	9	9	9	9	9	9	9	9
9	9	9	1	9	9	9	9	9	9
2	2	2	2	2	2	2	2	2	2
1	2	2	2	2	3	3	3	1	1
1	2	2	1	1	1	1	2	1	2
3	2	2	3	3	2	3	2	2	2
2	2	2	2	2	2	2	2	1	1
1	2	3	2	3	2	3	2	1	2
2	3	2	3	2	2	2	2	1	1
1	2	2	2	2	2	2	2	2	9
2	1	1	2	1	1	1	1	2	3
2	3	3	2	2	1	2	2	3	2
1	2	2	3	2	1	3	2	3	3
3	3	2	1	2	9	1	1	3	3
1	1	1	1	1	1	1	1	1	1
2	2	2	2	2	2	2	2	2	2
2	2	2	2	2	2	2	2	2	2
3	3	3	3	3	3	3	3	2	2
3	3	3	3	3	3	3	3	2	2
1	1	1	1	1	1	2	2	1	1
2	2	2	2	2	2	2	2	9	9
2	2	2	2	2	2	2	2	3	2
9	9	9	9	9	9	9	9	9	9
2	2	2	2	2	2	2	2	2	2
1	2	1	1	1	1	1	1	1	1
9	9	9	1	9	9	9	9	1	9
3	3	3	3	3	3	3	3	3	3
1	1	1	1	1	1	1	1	1	3
2	2	2	2	2	2	2	2	2	2
2	2	2	2	2	2	2	2	2	2
1	1	3	1	2	3	1	3	1	3
9	9	9	9	9	9	9	9	9	9
1	2	3	1	2	2	3	2	1	3
2	2	2	2	2	2	2	2	2	2
2	2	2	2	2	2	2	2	2	2
1	2	2	1	1	1	2	1	3	3
1	2	2	1	1	1	1	2	1	3
9	9	9	9	9	9	9	9	9	9
2	2	2	1	2	2	2	2	2	2

9	9	9	9	9	9	9	9	9	9
3	3	3	1	1	2	2	2	3	3
2	3	3	1	1	1	1	1	3	3
9	9	9	9	9	9	9	9	9	9
9	9	9	9	9	9	9	9	9	9
9	9	9	9	9	9	9	9	9	9
9	9	9	9	9	9	9	9	9	9
9	9	9	9	9	9	9	9	9	9
9	9	9	9	9	9	9	9	9	9
9	9	9	9	9	9	9	9	9	9
9	9	9	9	9	9	9	9	9	9
9	9	9	9	9	9	9	9	9	9
9	9	9	9	9	9	9	9	9	9
9	9	9	9	9	9	9	9	9	9
9	9	9	9	9	9	9	9	9	9
9	9	9	9	9	9	9	9	9	9
9	9	9	9	9	9	9	9	9	9
9	9	9	9	9	9	9	9	9	9
9	9	9	9	9	9	9	9	9	9
9	9	9	9	9	9	9	9	9	9
9	9	9	9	9	9	9	9	9	9
9	9	9	9	9	9	9	9	9	9
9	9	9	9	9	9	9	9	9	9
9	9	9	9	9	9	9	9	9	9
2	1	1	2	1	3	2	1	1	2
2	2	1	2	2	2	2	2	2	2
1	1	2	1	1	2	2	2	2	2
1	2	2	1	2	2	2	2	1	2
1	3	3	2	2	2	3	2	1	3
1	9	9	9	9	9	9	9	1	9
2	2	2	2	2	2	2	2	2	2
1	2	2	1	1	1	9	2	1	3
1	2	2	2	2	2	2	2	1	1
2	2	1	3	1	2	1	3	1	2
1	1	1	1	1	1	1	1	2	3
1	2	2	1	2	2	2	2	1	9
1	2	1	1	1	1	2	1	1	3
1	2	2	1	1	1	3	1	1	3
1	1	1	1	1	2	2	2	1	3
1	9	2	1	1	1	2	1	1	3
1	9	9	9	9	9	9	9	1	9
1	2	2	1	2	1	2	2	1	3
1	1	3	1	1	1	3	1	1	2
1	3	9	9	3	9	3	9	1	9
1	2	2	1	1	2	2	2	1	3
1	2	2	1	1	3	3	1	1	3
1	2	2	2	2	2	2	2	2	2
1	3	3	2	3	2	3	2	2	2

1	1	2	1	1	1	2	1	1	2
1	1	1	1	1	1	2	1	1	3
3	3	3	1	1	1	3	1	1	3
1	2	2	1	1	2	2	2	3	3
1	2	1	2	2	1	2	2	2	1
1	9	9	9	9	9	9	9	1	9
1	2	1	1	1	1	2	1	1	3
9	9	9	1	9	9	9	9	1	9
1	9	9	9	9	9	9	9	1	9
1	9	9	9	9	9	9	9	1	9
9	9	9	1	9	9	9	9	1	9
9	9	9	1	9	9	9	9	1	9
9	9	9	1	9	9	9	9	9	1
9	9	9	1	9	9	9	9	1	9
1	9	9	9	9	9	9	9	9	1
1	1	3	1	3	1	3	3	3	3
1	3	3	3	3	1	1	3	3	3
1	3	1	2	2	2	2	1	1	3
1	9	9	9	9	9	9	9	9	9
1	3	3	1	3	1	3	1	3	3
9	9	9	1	9	9	9	9	1	9
1	9	9	9	9	9	9	9	3	3
9	9	9	1	9	9	9	9	1	9
9	9	9	1	9	9	9	9	3	9
9	9	9	1	9	9	9	9	1	9
1	2	3	2	1	2	3	2	1	2
1	1	3	3	1	1	3	3	1	9
1	2	3	1	2	1	2	3	2	3
1	1	2	1	1	1	2	1	1	3
2	2	2	2	2	2	2	2	2	2
1	2	3	1	1	1	1	1	1	3
1	1	3	1	9	1	3	3	1	3
3	3	3	3	3	3	3	3	1	3
1	3	1	1	3	1	3	1	9	9
1	2	3	1	2	1	2	1	2	3
1	9	9	9	9	9	9	9	1	9
2	2	2	2	2	2	2	2	2	2
2	2	2	2	2	2	2	2	3	3
2	3	3	2	1	2	3	2	3	2
2	1	2	1	1	1	2	3	2	3
1	2	2	1	1	1	2	2	1	2
1	1	2	9	1	2	2	2	9	3
1	1	1	1	1	3	3	1	1	9
3	3	3	2	2	2	1	1	2	2
1	1	2	1	1	2	2	2	2	1
9	9	9	1	9	9	9	9	2	9
9	9	9	9	1	9	9	9	1	9

2	1	1	1	1	1	2	2	3	3
2	2	2	1	9	3	2	3	1	3
1	1	2	1	1	1	1	1	1	3
9	9	9	1	9	9	9	1	1	9
2	9	9	1	2	9	9	3	2	9
1	1	3	1	1	1	2	1	1	3
1	1	2	1	1	1	3	2	1	3
9	9	9	9	9	9	9	9	1	3
2	9	9	9	9	9	9	9	2	9
2	2	2	2	2	3	3	3	1	3
2	2	2	2	2	2	2	2	2	2
9	9	9	9	9	9	9	9	1	1
1	1	2	1	1	1	2	2	1	3
2	3	9	1	9	9	1	3	3	9
9	9	9	9	9	1	9	9	9	9
3	3	3	3	3	3	3	3	1	3
9	9	9	1	9	9	9	9	9	9
9	9	9	9	9	9	9	9	1	3
2	2	2	2	2	2	2	2	3	3
1	2	1	1	1	1	2	2	1	2
1	3	3	1	1	1	3	2	3	3
1	1	1	1	1	1	1	1	1	3
3	1	3	3	3	3	3	3	3	3
1	1	2	1	1	1	2	1	1	3
1	2	2	1	2	1	2	2	2	2
9	9	9	9	9	9	9	9	9	9
9	9	9	1	9	9	9	9	1	3
1	3	3	1	1	1	3	1	1	3
3	3	3	3	3	3	3	3	1	3
1	3	3	1	1	1	1	1	3	1
9	9	9	9	9	9	9	9	9	9
9	9	9	9	9	9	9	9	9	9
9	9	9	9	9	9	9	9	9	9
9	9	9	9	9	9	9	9	9	9
9	9	9	9	9	9	9	9	9	9
9	9	9	9	9	9	9	9	9	9
9	9	9	9	9	9	9	9	9	9
9	9	9	9	9	9	9	9	9	9
9	9	9	9	9	9	9	9	9	9
9	9	9	9	9	9	9	9	9	9
9	9	9	9	9	9	9	9	9	9
9	9	9	9	9	9	9	9	9	9
9	9	9	9	9	9	9	9	9	9
9	9	9	9	9	9	9	9	9	9
9	9	9	9	9	9	9	9	9	9
9	9	9	9	9	9	9	9	9	9
9	9	9	9	9	9	9	9	9	9

9	9	9	9	9	9	9	9	9	9
9	9	9	9	9	9	9	9	9	9
9	9	9	9	9	9	9	9	9	9
9	9	9	9	9	9	9	9	9	9
9	9	9	9	9	9	9	9	9	9
9	9	9	9	9	9	9	9	9	9
9	9	9	9	9	9	9	9	9	9
9	9	9	9	9	9	9	9	9	9
9	9	9	9	9	9	9	9	9	9
9	9	9	9	9	9	9	9	9	9
9	9	9	9	9	9	9	9	9	9
9	9	9	9	9	9	9	9	9	9
9	9	9	9	9	9	9	9	9	9
9	9	9	9	9	9	9	9	9	9
9	9	9	9	9	9	9	9	9	9
9	9	9	9	9	9	9	9	9	9
9	9	9	9	9	9	9	9	9	9
9	9	9	9	9	9	9	9	9	9
9	9	9	9	9	9	9	9	9	9
1	2	1	1	2	1	2	2	1	2
9	9	9	9	9	9	9	9	9	9
9	9	9	9	9	9	9	9	9	9
9	9	9	9	9	9	9	9	9	9
9	9	9	9	9	9	9	9	9	9
9	9	9	9	9	9	9	9	9	9
9	9	9	9	9	9	9	9	9	9
9	9	9	9	9	9	9	9	9	9
9	9	9	9	9	9	9	9	9	9
9	9	9	9	9	9	9	9	9	9
9	9	9	9	9	9	9	9	9	9
9	9	9	9	9	9	9	9	9	9
9	9	9	9	9	9	9	9	9	9
9	9	9	9	9	9	9	9	9	9
9	9	9	9	9	9	9	9	9	9
9	9	9	9	9	9	9	9	9	9
2	2	2	2	2	2	2	2	2	9
1	3	3	3	3	3	3	3	1	1
1	3	3	1	1	2	3	3	2	3
1	1	1	1	1	3	3	1	3	3
3	3	3	3	3	3	3	3	3	3
1	1	1	1	1	1	1	1	3	3
9	9	9	9	9	9	9	9	1	3
1	9	9	9	9	9	9	9	3	9
9	9	9	9	9	9	9	9	1	3
2	2	2	2	1	3	1	2	2	2
1	1	2	1	1	1	1	1	1	2
1	2	3	1	2	2	2	2	1	3

1	9	2	1	1	1	2	1	1	3
1	1	2	1	1	1	2	2	1	1
1	1	2	1	1	9	2	2	9	9
1	3	3	1	2	1	2	2	1	3
2	2	2	1	1	1	2	1	2	3
1	2	1	1	1	1	1	1	1	2
1	1	2	1	1	1	2	1	1	2
1	1	1	1	1	2	2	2	1	3
2	1	2	9	3	2	2	1	2	3
1	3	3	1	1	2	1	1	3	2
9	9	9	1	1	9	9	9	1	9
1	3	3	1	3	1	1	1	2	3
1	1	2	1	1	1	2	2	3	3
1	1	2	1	2	1	2	2	2	2
2	1	3	1	1	1	2	1	2	2
1	3	1	1	1	1	2	1	3	3
1	1	2	1	1	2	2	1	2	2
1	3	3	1	3	3	3	3	2	2
1	1	1	1	1	1	2	1	2	1
1	1	1	1	1	1	1	1	1	2
1	2	1	1	9	2	3	2	2	9
1	2	2	1	1	1	2	2	1	3
1	1	2	1	1	1	2	2	1	3
9	9	9	9	9	1	9	9	1	9
3	3	3	3	3	3	3	3	2	2
1	1	1	2	1	1	2	1	2	1
1	2	1	1	9	1	9	1	2	2
3	3	1	1	1	2	2	2	3	3
2	2	2	2	2	2	2	2	2	2
1	2	1	1	1	1	2	1	2	2
2	2	3	2	3	3	3	3	1	2
1	2	1	1	1	1	2	2	2	2
1	2	1	1	1	1	2	1	2	2
9	9	9	9	9	9	9	9	9	9
9	9	9	9	9	9	9	9	9	9
9	9	9	9	9	9	9	9	2	2
9	9	9	9	9	9	9	9	2	1
9	9	9	9	9	9	9	9	3	1
9	9	9	9	9	9	9	9	2	1
9	9	9	9	9	9	9	9	9	9
1	1	2	3	1	2	3	2	1	1
1	2	2	1	2	2	2	2	1	3
1	1	1	1	2	9	1	1	1	3
1	3	3	1	3	1	3	1	3	3
1	1	1	1	1	1	1	1	1	3
3	1	3	3	3	3	3	3	1	1
1	3	3	1	3	1	3	1	1	3

2	2	2	2	2	2	2	2	1	2
2	3	3	1	2	3	2	1	2	3
2	2	2	2	2	2	2	2	1	2
1	1	3	1	3	3	9	1	1	3
2	2	2	2	3	1	1	1	1	2
1	2	2	1	2	3	9	2	3	3
1	9	3	1	9	1	9	1	1	1
1	1	2	3	2	1	2	2	3	2
1	1	3	1	2	1	1	1	2	3
9	9	9	1	9	9	9	9	1	1
2	2	2	1	2	2	2	2	1	3
2	1	1	3	2	1	1	3	1	3
1	1	3	1	2	3	2	2	1	1
1	3	3	1	3	1	1	1	1	3
1	1	1	3	1	2	2	2	3	1
1	1	1	1	2	1	1	2	1	3
3	2	2	2	2	2	3	2	1	2
1	9	9	9	9	9	9	9	9	9
1	2	3	1	1	2	3	1	1	3
9	9	9	1	9	9	9	9	9	1
3	2	1	1	1	2	3	2	3	3
2	2	2	1	2	1	2	1	2	2
1	3	3	1	9	1	1	1	1	1
1	3	1	1	3	1	2	2	1	3
1	2	1	1	2	2	1	1	1	2
1	3	3	1	3	1	3	3	1	3
1	2	1	1	3	1	2	3	1	2
3	3	3	3	3	3	3	3	1	3
1	1	2	2	2	2	2	2	1	1
1	1	3	2	3	1	2	3	3	3
1	1	3	1	3	2	2	2	1	3
1	2	3	2	1	2	3	1	3	2
1	1	1	1	2	3	1	1	1	1
1	3	3	1	3	1	3	3	1	3
1	1	1	1	1	1	1	1	1	1
1	1	1	1	3	3	1	3	3	3
1	2	3	1	2	3	2	1	1	2
9	9	9	9	9	9	9	9	9	9
1	9	1	1	1	3	1	1	1	3
1	3	3	1	1	3	1	2	1	3
1	1	1	3	3	1	1	1	1	3
1	2	3	1	2	3	1	2	2	2
1	3	1	1	3	1	1	3	1	3
2	2	2	1	2	3	2	2	3	1
2	2	2	2	2	2	2	2	2	2
1	3	9	2	2	1	1	2	2	3
3	3	3	3	3	3	3	3	2	2

1	3	3	3	3	3	2	2	1	2
1	1	1	2	9	1	1	2	9	9
1	2	2	2	2	2	2	2	1	3
2	1	3	3	2	2	2	1	2	2
1	1	2	2	2	2	2	1	2	2
2	2	2	3	3	3	2	2	1	1
2	2	2	2	2	2	2	2	2	2
9	9	9	9	9	9	9	9	3	3
9	9	9	9	9	9	9	9	9	9
1	2	3	2	1	2	1	3	1	9
2	2	2	2	2	2	2	2	2	2
2	2	2	2	2	2	2	2	2	2
2	2	2	2	2	2	2	2	2	2
2	2	1	1	1	1	1	1	2	2
2	2	2	2	2	2	2	2	2	2
2	2	2	2	2	2	2	2	2	2
2	2	2	2	2	2	2	2	2	2
1	2	2	2	2	2	2	2	2	2
2	2	3	2	2	2	2	3	2	2
3	2	2	2	2	2	2	2	2	2
2	2	2	1	1	2	2	2	2	3
2	2	2	2	2	2	2	2	2	2
2	2	2	2	2	2	2	2	2	2
2	2	1	2	3	3	2	2	2	2
2	2	2	2	2	2	2	2	2	2
2	2	2	2	2	2	2	2	2	2
2	2	2	2	2	2	2	2	2	2
1	2	3	1	1	2	2	3	1	1
1	2	3	2	2	3	1	1	2	2
2	2	2	2	2	2	2	2	2	2
2	2	2	2	2	2	2	2	2	2
2	2	2	2	2	2	2	2	2	2
2	2	2	2	2	2	2	2	2	2
2	2	2	2	2	2	2	2	2	2
2	2	2	2	2	2	2	2	2	2
2	2	2	2	2	2	2	2	2	2
2	2	2	2	2	2	9	2	1	9
2	2	2	2	2	2	2	2	2	2
2	2	2	2	2	2	2	2	2	2
3	3	1	1	1	1	3	3	1	1
2	2	2	2	2	2	2	2	2	2
2	2	2	2	2	2	2	2	2	2
1	2	2	1	3	3	3	3	2	1
2	2	2	2	2	2	2	2	2	2
2	1	1	2	2	3	2	2	1	1
2	2	2	2	2	2	2	2	2	2
2	2	2	2	2	2	2	2	2	2

2	2	2	2	2	2	2	2	1	2
1	2	2	1	1	2	2	2	1	2
1	1	2	1	1	2	2	2	2	2
2	2	2	2	2	2	2	2	2	2
1	2	3	2	1	2	2	3	1	2
2	2	2	2	2	2	2	2	2	2
9	9	9	9	9	9	9	9	1	9
1	1	1	3	1	3	1	1	1	1
1	9	9	9	9	9	9	9	1	9
2	2	2	2	2	2	2	2	2	2
3	3	3	3	3	3	3	3	2	2
2	2	2	2	2	2	1	1	1	2
1	1	1	1	1	1	1	1	3	2
3	9	9	9	9	9	9	9	3	9
2	2	2	2	2	2	2	2	2	2
1	2	2	1	2	1	2	1	1	2
1	1	2	2	2	2	2	2	2	2
1	1	1	1	1	1	1	1	1	9
1	1	9	9	9	9	9	9	1	2
2	2	2	2	2	2	2	2	2	2
2	3	1	1	1	1	1	2	1	9
9	9	9	9	9	9	9	1	1	9
1	9	9	9	9	9	9	9	1	1
2	2	9	3	1	2	3	2	2	9
1	2	3	2	3	2	3	1	1	2
2	1	1	1	1	2	2	2	2	1
1	2	3	1	1	2	2	1	2	3
1	1	3	3	9	1	1	1	3	2
1	2	1	2	1	9	1	3	2	2
1	1	1	1	1	1	1	1	1	9
1	1	1	1	1	1	1	1	9	9
2	2	3	3	3	3	3	3	2	2
2	2	2	2	2	2	2	2	2	2
1	9	9	9	9	9	2	9	2	9
1	2	2	2	2	2	2	2	1	1
1	9	9	9	9	9	9	9	9	9
1	1	1	1	3	1	1	3	1	3
2	2	2	2	2	2	2	2	2	2
2	2	2	2	2	2	2	2	2	2
2	2	2	2	2	2	2	2	2	2
2	3	9	1	1	1	2	9	1	1
1	1	2	1	2	1	2	1	2	2
9	2	9	9	9	9	9	9	9	9
2	2	1	9	2	1	1	1	1	1
1	2	2	2	2	2	2	2	1	1
1	1	1	1	1	1	1	1	2	2
1	9	9	9	9	9	9	9	2	9

1	1	1	1	1	1	1	1	1	1
2	2	2	2	2	2	2	2	1	2
1	1	1	1	1	1	1	1	1	1
2	2	2	2	2	2	2	2	2	2
2	9	9	9	9	9	9	9	2	9
1	2	1	3	2	2	3	1	2	3
3	3	3	3	3	3	3	3	1	3
1	1	1	1	1	1	1	1	1	1
1	1	1	1	1	1	1	1	2	2
1	1	1	3	3	3	3	3	1	1
3	3	3	3	3	3	3	3	2	2
2	2	2	2	2	2	2	2	1	1
2	2	2	2	2	2	2	2	2	2
2	2	2	2	2	2	2	2	2	2
2	2	2	2	2	2	2	2	2	2
1	1	2	1	2	1	2	1	2	2
1	1	1	1	1	1	1	1	1	1
2	1	1	1	1	1	1	1	3	1
9	9	9	9	9	9	9	9	9	9
1	2	1	2	1	2	1	2	1	2
1	1	1	1	1	1	2	2	1	1
1	1	3	1	1	2	2	2	1	3
1	1	1	1	1	1	1	1	1	3
1	2	9	1	2	1	2	2	3	3
9	9	9	9	9	9	9	9	3	3
2	1	1	1	1	1	1	1	2	2
2	1	1	2	3	2	1	2	1	2
3	2	2	2	1	2	1	2	2	2
2	1	1	1	1	2	9	3	1	9
2	2	2	2	2	2	2	2	2	2
3	3	3	3	3	3	3	3	3	3
2	2	2	2	2	2	2	2	1	3
3	3	3	3	3	3	3	3	3	3
1	2	1	3	2	1	2	3	1	2
2	2	2	2	2	2	2	2	2	2
3	3	3	3	3	3	3	3	2	2
2	3	2	3	2	1	2	1	2	3
3	3	3	3	3	3	2	2	2	2
1	3	2	1	2	3	1	2	3	2
3	3	3	3	3	3	3	3	3	3
1	1	1	1	1	1	1	1	1	2
2	1	2	1	3	2	1	2	2	1
9	9	9	9	9	9	9	9	9	9
3	3	3	3	3	3	3	3	3	3
2	2	1	1	1	1	1	1	1	1
2	2	2	2	2	2	2	9	2	2
1	2	2	1	1	1	2	3	1	3

2	2	2	2	2	2	2	2	1	1
1	1	3	3	3	1	3	3	3	3
3	3	3	3	3	3	3	3	3	3
1	1	3	3	3	1	3	3	3	3
1	1	2	1	3	1	1	9	2	1
2	2	2	2	9	9	2	2	2	2
2	2	2	2	2	2	2	2	1	2
2	2	2	2	2	2	2	2	2	2
2	2	2	2	2	2	2	2	9	9
3	3	2	1	3	2	1	2	3	3
2	2	2	2	2	2	2	2	2	2
1	3	2	3	2	3	2	3	3	3
2	2	2	2	2	2	2	2	2	2
1	2	2	1	3	1	1	1	3	3
2	2	2	2	2	2	2	2	2	2
1	2	2	1	1	2	2	2	2	2
2	2	2	2	2	2	2	2	2	2
1	1	3	1	3	2	3	2	2	1
1	1	2	9	2	2	2	2	2	1
1	2	2	3	2	2	2	2	1	9
2	1	2	1	2	1	2	2	2	2
2	2	2	2	2	2	2	2	3	3
1	1	2	1	1	2	2	2	3	3
2	2	2	2	2	2	2	2	1	2
1	1	1	1	1	1	1	1	1	1
1	3	3	1	1	3	2	1	9	9
1	1	3	3	2	1	1	1	1	2
3	3	3	1	3	1	3	1	1	2
1	3	2	2	3	1	2	3	1	1
1	2	2	1	2	1	2	3	2	3
1	3	3	1	1	2	2	3	2	3
2	2	2	2	2	2	2	2	9	9
2	2	2	2	2	2	2	2	1	3
1	9	2	3	1	3	2	3	3	3
1	3	3	1	3	1	3	3	1	1
1	2	2	1	2	1	2	1	1	2
2	2	2	2	2	2	2	2	1	1
9	9	9	9	9	9	9	9	2	2
2	2	2	2	2	2	2	1	2	2
1	3	2	2	1	2	3	2	2	3
2	2	1	2	1	2	3	2	2	3
2	2	2	2	2	2	2	2	2	2
2	2	2	2	2	2	2	2	2	2
2	2	2	2	2	2	2	2	2	2
2	1	1	1	1	2	3	1	3	1
1	1	2	1	3	1	1	9	1	3
2	2	2	2	2	2	2	2	1	3

9	9	9	9	9	9	9	9	9	9
1	2	1	2	1	2	3	1	1	2
1	1	2	9	3	1	1	2	2	1
1	1	1	1	3	1	3	3	2	1
1	2	2	1	1	2	3	1	2	1
1	1	1	1	2	2	2	2	2	2
1	3	2	2	1	2	1	3	2	3
9	9	9	9	9	9	9	9	1	1
1	1	2	2	2	2	2	2	1	3
1	1	2	1	3	3	3	1	1	3
1	1	2	1	1	1	2	2	2	3
1	1	2	2	2	3	2	3	3	2
1	2	2	2	2	2	2	2	2	2
1	1	1	1	1	1	1	1	1	1
2	2	2	3	1	2	2	1	1	2
2	2	2	2	2	2	2	2	2	2
2	1	2	3	1	2	1	2	3	1
1	1	9	1	2	3	3	3	3	1
9	9	9	9	9	9	9	1	1	3
2	2	2	2	2	2	2	2	3	3
9	9	9	9	9	9	9	9	9	9
1	2	1	3	1	2	3	2	1	2
2	2	2	1	1	3	3	3	2	3
3	3	2	2	1	1	2	3	1	2
1	1	2	2	2	2	3	3	1	1
2	2	2	2	3	3	2	3	2	2
1	2	2	2	3	2	3	2	3	2
1	2	1	3	2	1	1	2	2	2
1	3	3	1	3	1	3	3	2	2
1	2	3	1	1	1	2	2	1	1
3	3	2	2	2	1	1	1	1	2
2	3	1	2	3	2	1	3	1	2
1	1	3	3	3	1	3	3	1	3
3	3	3	3	3	3	3	3	2	2
2	2	2	2	2	2	2	2	2	2
2	2	2	2	2	2	2	2	2	2
9	9	9	9	9	9	9	9	3	3
2	2	2	2	2	2	2	2	2	2
2	2	2	1	1	1	1	1	2	2
2	2	2	1	1	1	1	1	2	3
2	2	2	2	2	2	2	2	1	1
1	2	3	2	1	2	3	2	2	3
1	1	1	1	1	1	2	3	2	3
2	3	3	2	3	2	2	2	2	2
9	9	9	9	9	9	9	9	2	9
3	3	3	3	3	3	3	3	1	3
1	1	3	1	2	1	2	2	2	3

3	3	3	3	3	3	3	3	3	3
1	1	1	1	3	3	9	2	3	3
1	2	3	2	3	2	1	1	2	3
9	9	9	9	9	9	9	9	9	9
1	1	1	1	2	2	3	2	2	3
3	3	3	3	3	3	3	3	3	3
1	1	1	1	1	1	1	1	1	1
3	3	3	3	3	3	3	3	2	2
2	2	3	2	1	2	2	3	3	2
1	9	1	1	2	2	1	2	1	3
2	2	2	2	2	2	2	2	2	2
1	1	2	1	2	2	2	2	1	3
1	1	1	2	1	2	1	2	1	1
9	9	9	9	1	9	9	9	1	9
2	2	2	2	2	2	2	2	2	2
3	3	3	2	2	3	2	3	2	2
1	1	2	1	1	1	1	2	3	3
2	2	2	2	2	2	2	2	2	2
2	2	2	2	2	2	2	2	2	2
1	1	1	1	1	1	2	2	2	2
1	2	2	1	2	1	3	2	3	3
3	3	3	3	3	2	3	2	2	2
1	2	2	1	2	2	2	1	2	2
1	1	2	1	3	2	2	2	1	2
1	2	3	1	2	3	1	2	1	2
1	2	3	3	3	3	3	3	1	2
1	2	3	2	2	1	2	1	3	2
1	1	2	3	2	1	2	3	2	3
2	3	2	1	3	2	1	2	3	1
1	1	1	1	1	1	1	1	2	2
3	3	3	3	3	3	3	3	3	1
1	1	2	2	2	2	2	2	3	2
1	1	1	1	1	1	1	1	1	2
1	1	1	1	1	2	2	2	1	1
1	3	3	3	1	1	2	2	3	3
1	3	1	3	1	2	9	2	1	3
1	3	1	3	2	3	2	3	1	3
1	2	3	1	3	1	1	1	3	1
1	1	3	2	2	2	1	2	3	3
1	1	1	3	1	1	3	2	1	3
3	3	3	3	3	3	3	3	2	2
1	3	3	1	1	1	1	1	1	1
3	2	3	1	2	1	2	3	2	1
1	1	2	1	9	1	9	3	3	2
2	3	9	2	3	2	9	2	9	9
2	1	3	2	3	2	1	2	2	1
1	3	3	1	3	1	3	1	1	1

1	2	2	1	2	2	2	2	1	2
1	3	2	1	2	1	2	3	2	3
2	1	1	2	1	2	2	2	1	2
1	1	1	1	1	1	1	1	1	1
1	2	3	1	2	3	1	3	1	3
3	2	2	2	2	2	2	2	1	1
1	2	3	1	2	2	1	3	1	3
1	2	3	1	2	3	3	2	1	3
1	3	3	1	3	1	3	1	1	2
1	3	3	1	3	1	3	2	2	3
1	2	3	2	3	2	3	1	1	2
1	2	3	2	1	2	2	1	3	1
1	1	1	1	1	1	1	1	1	1
1	3	9	3	3	1	1	1	3	1
1	1	3	1	1	3	3	2	3	1
1	1	3	3	1	1	3	3	1	1
1	1	2	2	3	3	2	2	1	3
1	1	1	1	1	1	1	1	3	3
3	2	1	3	2	1	1	1	3	2
1	1	2	1	1	1	1	1	1	3
1	2	1	2	2	1	2	2	2	2
1	1	3	3	3	3	1	3	3	3
1	1	1	1	1	1	3	1	3	3
1	2	2	1	2	1	1	2	1	2
1	1	1	2	2	2	2	2	1	2
2	2	2	2	2	2	2	2	2	2
1	1	3	2	1	2	2	1	1	1
1	2	3	1	2	1	2	3	1	2
2	2	2	2	2	2	2	2	3	2
9	9	9	9	9	9	9	9	9	9
9	9	9	9	9	9	9	9	1	2
9	9	9	9	9	9	9	9	2	1
2	2	2	2	2	1	1	1	2	2
9	9	9	1	9	9	9	9	1	9
2	2	2	2	2	2	2	2	2	2
2	2	2	2	2	2	2	2	1	1
2	2	3	2	2	2	3	2	3	2
1	2	2	1	2	2	2	2	9	1
1	9	9	9	9	9	9	9	9	9
2	1	2	2	2	2	3	2	3	1
2	3	3	1	2	2	2	3	2	3
1	1	3	1	1	2	1	2	2	2
3	3	3	3	3	3	3	3	1	3
3	3	3	3	3	3	3	3	1	3
1	1	3	2	3	2	1	1	1	2
2	2	2	2	2	2	2	2	2	2
1	1	3	2	1	2	2	1	1	1

1	2	2	2	1	1	3	3	1	9
1	2	2	2	2	2	1	1	2	2
2	2	2	2	2	2	2	2	3	2
1	2	3	1	3	2	2	1	1	2
2	2	3	2	2	2	3	2	3	2
2	2	3	2	2	2	3	2	3	2
9	9	9	1	9	2	9	2	3	1
1	1	2	1	3	2	2	2	3	2
1	3	3	1	2	1	2	3	1	3
3	3	3	3	3	3	3	3	2	3
2	3	2	1	3	2	2	1	2	3
1	3	2	1	3	2	3	2	3	2
1	3	1	2	9	1	3	2	1	1
2	2	2	2	2	2	2	2	2	2
2	2	2	2	2	2	2	2	2	2
1	1	2	2	2	2	2	2	1	3
2	2	2	2	2	2	2	2	2	2
2	2	2	2	2	2	2	2	2	2
2	2	2	2	2	2	2	2	2	2
9	2	9	9	9	9	9	9	1	3
1	3	1	1	1	1	1	1	1	9
1	3	1	1	1	1	1	1	1	9
1	9	9	1	1	1	1	1	1	9
1	2	2	1	1	1	2	2	1	2
1	3	3	1	1	1	1	1	1	3
1	9	9	1	9	1	1	1	1	9
1	3	3	1	1	1	1	1	1	2
1	3	3	1	1	1	1	1	1	2
1	2	2	1	1	1	1	1	1	2
9	9	9	1	9	1	1	9	9	9
1	9	9	9	1	1	1	1	1	9
1	9	9	9	9	9	9	9	1	9
1	3	3	1	1	1	1	1	1	2
1	3	3	1	1	1	1	1	1	3
1	9	9	1	1	1	1	1	1	9
1	3	3	1	1	1	1	1	1	3
1	9	9	1	1	1	1	1	1	9
1	3	9	1	1	1	1	1	1	9
1	3	3	1	1	1	1	1	1	9
1	3	3	1	1	1	1	1	1	3
1	3	2	1	1	1	1	1	1	3
1	9	9	1	9	9	9	9	9	9
1	3	3	1	1	1	1	1	1	9
9	9	9	1	9	9	9	9	3	1
1	9	9	1	9	1	1	1	1	9
1	3	3	1	1	1	1	1	1	3
1	3	9	1	1	1	1	1	1	2

1	3	1	1	9	1	1	1	1	9
1	3	1	1	1	1	1	1	1	2
1	3	3	1	1	1	1	1	1	3
1	1	1	1	1	1	1	1	1	1
1	3	3	1	1	1	1	1	1	9
1	3	3	1	1	1	1	1	1	9
1	1	1	1	1	1	1	1	1	1
1	1	2	1	2	1	2	2	1	1
1	3	3	9	1	1	1	1	1	9
1	9	9	1	1	1	1	1	1	9
1	3	2	3	1	1	1	1	1	2
1	3	1	1	1	1	2	2	1	2
1	3	3	1	1	1	1	1	1	9
1	3	1	1	1	1	1	1	1	9
1	9	1	1	1	1	1	1	1	9
1	3	3	1	1	1	1	1	1	9
1	3	1	1	1	1	9	9	1	9
1	3	1	1	1	1	9	9	1	9
1	3	3	1	1	1	1	1	1	9
1	1	1	1	1	9	9	9	1	2
9	9	9	9	9	9	2	9	1	9
9	9	9	9	9	9	9	9	1	9
1	2	3	1	1	1	1	1	1	3
1	3	3	1	1	1	1	1	1	9
1	3	3	1	1	1	1	1	1	2
1	3	3	1	3	1	1	1	1	3
1	3	1	1	1	1	3	3	1	3
1	3	1	3	3	3	3	3	1	9
1	3	3	1	1	1	1	1	1	3
1	9	9	9	1	1	1	1	1	3
1	1	3	1	1	1	1	1	1	2
1	3	3	1	3	1	1	3	1	1
1	9	9	1	1	1	1	1	1	9
1	9	9	1	1	1	1	1	1	9
1	3	3	1	1	1	1	1	1	9
1	3	3	1	3	1	1	1	1	3
9	9	9	1	9	1	1	9	1	9
1	3	3	1	1	1	1	1	1	9
1	3	3	1	3	1	3	1	1	3
1	3	3	1	3	1	1	1	1	3
1	3	3	1	1	1	1	1	1	3
1	3	1	1	1	1	1	1	1	2
1	9	9	1	9	1	1	1	1	9
1	9	1	1	1	1	1	1	1	9
1	3	3	1	1	1	1	1	9	9
1	3	3	1	1	1	1	1	3	3
1	9	9	1	9	1	1	9	1	9

1	3	3	1	1	1	1	1	1	3
1	3	3	1	1	1	1	1	1	3
1	3	3	1	2	1	1	1	1	3
1	3	3	1	1	1	1	1	1	3
1	9	9	1	1	1	9	9	1	9
9	9	9	1	9	1	1	9	1	9
1	3	3	1	2	3	3	3	1	3
1	3	1	1	1	1	9	9	1	3
1	3	1	1	1	1	9	9	1	3
1	3	3	1	1	1	3	2	1	3
2	2	3	1	2	1	2	2	2	3
1	9	9	1	9	9	9	9	1	3
3	3	3	2	1	1	3	9	1	9
1	2	2	1	2	2	2	2	3	3
2	2	2	2	2	2	2	2	2	2
1	1	9	1	1	1	2	2	1	2
1	9	9	9	9	9	9	9	1	3
9	9	9	1	9	9	9	9	1	9
1	1	1	1	1	1	1	1	1	3
1	2	1	1	1	1	1	3	1	3
1	1	3	1	1	1	2	2	1	3
2	2	3	1	1	2	3	2	1	3
1	2	3	1	2	2	3	2	1	3
1	1	2	1	1	2	2	2	1	3
1	9	9	9	9	9	9	9	1	3
1	2	3	1	2	1	3	2	1	3
9	9	9	9	9	9	9	9	1	3
1	1	3	2	1	2	1	3	3	3
1	2	2	1	1	2	2	2	1	3
2	2	3	1	1	1	2	2	1	2
2	2	2	1	2	2	2	2	1	2
1	2	2	1	1	2	3	2	1	3
2	2	3	1	2	2	2	2	2	1
9	9	9	1	9	9	9	9	1	9
1	1	3	1	2	2	3	2	1	3
1	1	1	1	2	2	2	2	1	3
1	1	1	1	9	1	2	2	1	9
1	1	1	1	2	9	1	2	1	9
1	1	1	1	2	1	1	2	1	9
1	3	3	1	3	1	3	2	3	3
1	1	3	1	1	1	3	1	1	3
1	2	3	1	1	1	2	1	1	3
1	1	2	1	2	2	2	2	1	3
3	3	3	3	3	3	3	3	2	2
1	1	1	2	9	2	2	2	1	3
1	2	3	1	1	1	2	1	1	9
9	9	9	1	9	9	9	9	2	3

1	3	3	1	1	1	1	1	1	9
2	2	2	1	1	2	2	2	1	1
1	2	2	1	2	1	2	1	1	3
2	2	2	1	2	2	2	2	1	2
1	9	1	1	1	1	9	1	1	2
2	1	1	1	2	2	2	2	1	2
2	2	2	2	2	2	2	2	9	9
1	2	2	1	2	2	2	2	2	3
1	2	3	1	1	1	3	2	1	9
1	2	3	1	1	2	2	2	3	3
2	2	3	1	3	1	1	1	2	3
3	3	3	1	3	3	3	3	1	3
1	3	2	1	1	2	2	2	3	3
1	1	2	1	1	1	2	2	1	3
1	9	1	2	9	1	2	2	1	9
9	9	9	9	1	9	9	9	1	9
1	1	2	1	1	1	3	1	1	2
1	2	2	1	1	2	2	1	1	2
1	1	1	1	1	1	3	3	1	3
1	1	2	1	1	1	2	1	1	3
3	9	9	9	9	9	9	9	1	9
1	1	2	9	1	1	2	1	1	9
1	2	2	2	2	2	2	2	1	2
1	3	3	3	3	3	3	3	1	3
1	2	1	1	3	2	1	3	1	3
1	2	1	1	3	2	1	3	1	3
9	9	9	1	1	9	9	1	1	9
9	9	9	9	9	9	9	9	9	9
9	9	9	9	9	9	9	9	1	9
1	2	3	1	2	3	1	2	1	3
1	9	9	9	1	1	9	9	1	9
1	1	2	1	1	2	2	1	1	3
1	9	3	1	1	1	1	1	1	9
1	9	9	9	1	1	1	1	1	3
9	3	3	1	1	1	1	1	1	3
1	9	9	1	1	1	1	1	1	9
1	1	3	1	9	1	1	1	1	2
3	3	3	1	1	1	3	1	1	3
1	3	3	1	1	1	1	1	1	3
1	2	3	1	1	1	1	1	1	2
1	3	1	1	1	1	1	2	1	1
1	9	9	1	9	1	1	1	1	9
1	3	3	1	2	1	1	1	1	3
1	9	9	1	9	1	1	9	1	9
1	3	3	1	1	1	1	1	1	3
1	3	3	1	1	1	1	1	3	9
1	9	9	1	9	9	9	9	1	9

1	3	3	1	1	1	3	1	1	3
1	9	9	1	9	1	1	9	1	9
9	9	9	9	9	9	9	9	9	9
9	9	9	9	9	9	9	9	1	9
1	1	1	9	2	9	2	9	1	3
1	3	3	1	1	1	3	3	1	3
9	9	9	1	9	9	9	9	1	2
1	9	9	9	9	9	9	9	1	9
1	9	9	9	9	9	9	9	9	9
9	9	9	1	9	9	9	9	1	9
1	3	1	1	3	3	3	3	1	3
1	2	2	2	2	2	2	2	1	1
1	2	2	1	1	2	2	1	1	3
9	9	9	1	9	9	9	9	1	9
1	1	1	1	2	1	2	2	1	1
1	1	2	1	1	2	2	2	1	3
9	9	9	1	9	9	9	1	1	9
9	9	9	1	9	9	9	9	1	9
9	9	9	1	9	9	9	9	1	9
3	3	3	3	1	3	3	3	1	3
1	1	3	1	2	1	2	1	1	3
9	9	9	1	9	9	9	9	1	9
9	9	9	1	9	9	9	9	1	9
1	1	2	1	1	1	1	1	1	3
1	9	9	1	9	1	9	1	1	9
1	1	2	1	1	1	2	1	1	2
3	2	9	1	9	9	9	1	1	3
1	2	1	1	2	3	3	3	2	3
1	1	2	2	3	1	2	2	1	3
1	1	2	2	3	1	2	2	1	3
1	1	2	1	1	2	3	2	1	3
1	2	1	1	1	1	2	3	1	2
1	1	9	1	1	1	2	2	1	3
1	3	3	1	1	3	3	3	1	3
1	1	2	1	1	2	2	2	2	3
9	9	9	1	9	9	9	9	1	9
9	9	9	1	9	9	9	9	1	9
1	9	9	1	1	9	9	9	1	9
1	1	1	1	1	1	2	3	1	3
1	2	2	1	1	1	1	1	1	3
2	3	2	1	2	3	2	3	2	2
1	2	3	1	2	3	2	2	1	3
9	9	9	1	9	9	9	9	1	9
9	9	9	1	9	9	9	9	1	9
9	9	9	1	1	9	9	9	1	9
9	9	9	1	9	9	9	9	1	3
2	9	9	1	1	1	9	1	1	3

1	3	1	1	1	1	3	1	1	3
1	1	2	1	1	3	3	2	1	3
9	9	9	1	9	9	9	9	1	9
2	2	1	1	2	2	2	2	1	3
1	9	1	1	3	1	3	3	1	3
1	9	9	9	9	9	9	9	1	9
2	3	3	2	2	2	2	2	1	2
1	2	2	1	2	1	2	2	1	3
1	2	2	1	1	1	2	2	1	3
1	1	1	1	2	2	1	2	1	3
2	1	2	1	2	1	2	1	1	3
1	9	9	9	9	9	9	9	1	3
1	9	9	9	9	9	9	9	1	9
9	9	9	1	9	9	9	9	1	9
9	9	9	1	9	9	9	9	1	9
9	9	9	1	9	9	9	9	1	9
3	9	3	1	3	3	3	3	1	3
1	2	2	1	2	2	2	2	1	3
1	9	9	9	9	9	9	9	1	3
1	1	2	1	1	1	2	2	1	9
1	1	1	1	1	2	2	1	1	3
1	2	2	1	1	1	2	2	1	3
2	2	2	1	1	1	2	2	1	3
2	3	3	1	2	1	2	3	1	2
2	3	3	2	2	1	3	3	1	2
9	9	9	1	9	9	9	9	1	3
1	2	2	1	1	1	9	3	1	3
1	2	9	1	2	2	2	2	1	3
1	9	9	1	1	1	9	9	1	3
9	9	9	1	9	9	9	9	1	9
1	2	2	1	1	2	3	2	1	3
1	9	9	9	9	9	9	9	1	9
9	9	9	9	9	1	9	9	1	9
1	2	3	1	1	2	2	2	1	2
1	1	2	1	2	1	2	1	1	2
3	3	3	1	3	3	3	3	1	3
1	2	2	1	2	1	2	2	2	3
1	3	3	1	1	1	1	1	1	3
9	9	9	1	1	1	1	1	9	9
9	3	3	1	9	1	1	9	1	9
1	3	3	3	2	2	3	3	1	3
1	2	2	2	2	9	1	2	1	3
1	2	2	1	1	2	2	2	1	9
1	1	2	1	1	1	2	2	1	2
1	1	2	2	2	2	1	2	2	2
9	9	9	1	9	9	9	9	1	9
1	1	3	1	1	1	9	1	2	3

1	1	3	1	1	1	3	1	1	3
2	2	3	1	1	1	2	2	2	1
1	1	2	1	2	1	2	2	1	3
1	2	2	1	1	1	2	2	1	2
1	1	2	1	2	1	3	2	1	2
1	2	3	1	1	3	3	3	1	2
1	3	2	1	3	1	3	1	1	2
1	2	3	1	1	1	1	2	1	2
2	3	3	1	3	1	2	1	1	2
1	2	2	1	1	1	3	3	3	3
1	2	2	1	1	2	2	2	1	2
1	2	2	1	2	2	2	2	1	3
1	2	2	1	1	1	1	1	1	3
1	1	3	1	1	1	2	2	2	2
1	1	1	1	1	2	2	2	2	2
1	2	3	1	1	1	1	1	1	3
1	2	1	2	2	2	2	2	1	2
2	2	2	2	2	2	2	2	2	2
2	2	2	2	2	2	2	2	1	3
1	1	1	1	1	1	2	2	1	3
1	2	3	1	2	1	2	3	1	2
2	2	3	2	2	3	2	2	2	2
2	2	2	1	1	1	2	2	1	9
1	2	2	1	2	1	2	2	3	3
1	2	9	1	2	1	2	2	2	2
2	2	3	1	1	1	2	2	9	9
1	1	2	1	1	1	1	1	1	3
1	2	9	1	2	1	2	2	2	2
1	2	2	1	2	1	2	2	2	2
1	1	1	1	2	3	3	1	1	2
1	2	2	1	2	1	2	2	1	2
1	1	2	1	2	1	2	1	1	3
1	9	9	9	9	9	9	9	1	2
1	9	9	9	9	9	9	9	1	2
9	9	9	1	9	9	9	9	1	9
9	9	9	1	9	9	9	9	9	9
1	9	9	9	9	9	9	9	2	2
1	9	9	9	9	9	9	9	9	9
9	9	9	1	9	9	9	9	9	9
9	9	9	1	9	3	9	9	2	2
1	3	1	1	2	1	2	2	2	3
1	9	9	9	9	9	9	9	1	9
1	9	9	9	9	9	9	9	1	9
2	2	3	1	2	2	2	2	2	2
9	9	9	9	9	1	9	9	9	9
1	9	9	9	9	9	9	9	1	9
1	9	9	9	9	9	9	9	1	9

1	1	1	1	1	1	2	1	1	3
2	1	1	3	2	3	2	3	3	1
1	9	9	9	9	9	9	9	1	9
9	9	9	1	9	9	9	9	3	2
9	9	9	1	9	9	9	9	1	3
1	9	9	9	9	9	9	9	1	9
9	9	9	1	9	9	9	9	1	9
9	9	9	1	9	9	9	9	1	9
9	9	9	9	9	1	9	9	9	9
1	9	9	9	9	9	9	9	1	9
1	1	1	1	1	3	9	1	1	3
1	3	3	1	1	1	3	2	1	2
1	3	3	1	1	1	3	2	1	3
1	1	1	1	1	1	3	3	1	2
1	1	1	1	1	1	1	1	1	3
1	9	9	1	1	1	1	3	1	3
1	9	9	1	1	1	1	3	1	3
1	1	2	1	1	1	9	3	1	3
1	3	3	1	1	1	3	2	1	2
1	2	2	1	2	2	2	2	2	2
1	2	2	1	1	2	2	2	1	2
1	3	3	1	1	1	1	1	2	2
9	9	9	1	9	9	9	9	1	9
1	1	3	2	3	1	2	9	2	2
1	1	3	9	1	2	1	1	1	3
1	1	2	1	2	2	1	1	1	3
3	3	3	3	3	3	3	3	3	3
1	1	1	1	1	1	1	2	3	3
1	1	3	1	1	1	1	1	1	3
1	1	1	2	2	2	3	2	2	2
1	1	1	1	1	1	1	2	1	3
1	1	3	1	1	2	2	2	1	3
1	2	2	1	1	1	3	3	1	3
1	2	2	1	2	1	2	2	1	2
1	1	1	1	1	1	1	1	3	3
1	1	1	1	1	1	1	1	2	1
1	1	3	1	1	1	3	1	3	3
1	1	3	1	1	1	3	1	3	3
1	1	3	1	3	1	3	3	3	3
1	3	3	1	2	1	2	2	3	3
1	1	1	1	2	1	1	2	1	3
1	1	3	1	1	1	1	1	1	3
1	1	1	1	1	2	2	1	3	3
1	3	3	1	1	1	1	1	3	3
1	3	3	1	1	1	3	1	3	3
1	3	3	1	1	1	3	1	3	3
1	2	2	1	1	1	2	1	3	3

1	1	1	1	1	2	2	2	1	2
1	1	1	1	1	1	1	1	1	2
1	1	1	1	1	1	2	2	3	3
1	3	3	1	1	1	3	1	3	3
1	3	3	1	1	1	3	1	3	3
1	3	3	1	1	1	3	1	3	3
1	2	2	1	1	1	2	1	3	3
1	2	2	1	1	1	2	1	1	3
1	2	2	1	1	1	3	1	2	3
1	1	9	1	1	1	9	1	3	9
1	1	3	1	1	1	2	2	1	3
1	9	9	1	1	1	1	1	1	9
1	2	2	1	1	2	2	1	1	1
1	2	3	1	1	1	2	1	1	2
1	3	3	1	3	1	3	1	1	3
2	2	2	2	2	2	2	2	2	2
1	1	9	1	2	1	9	1	1	1
2	2	2	1	2	2	2	2	1	1
1	3	1	1	3	3	1	3	1	3
1	1	2	1	1	1	2	1	1	2
1	3	3	1	3	2	3	3	1	3
1	1	3	1	3	1	1	1	1	3
1	1	2	1	1	1	1	1	2	9
1	1	2	1	1	1	1	1	2	2
1	1	2	1	1	1	1	1	2	2
1	1	2	1	2	1	2	2	3	3
1	2	2	1	2	1	2	3	1	3
1	1	2	1	2	1	1	2	2	2
1	2	2	1	1	1	2	2	2	2
1	2	3	1	2	2	3	2	1	3
3	3	2	2	2	3	3	3	2	3
2	3	3	2	2	3	2	3	2	3
1	2	1	1	1	1	1	1	1	3
1	1	3	1	1	1	1	1	1	1
1	1	2	1	1	1	1	1	9	9
1	3	3	1	1	1	3	1	3	3
2	3	3	1	3	3	2	2	1	3
2	2	2	2	2	2	2	2	1	9
1	3	3	1	1	1	1	1	1	9
1	1	3	1	1	1	2	2	1	3
1	2	1	2	3	2	2	2	1	3
1	1	1	1	3	1	1	1	9	9
1	1	1	1	3	1	1	1	1	9
1	1	1	1	1	1	3	3	3	3
1	1	1	1	1	3	9	2	1	3
1	1	2	1	2	1	2	2	1	3
1	1	2	1	1	2	2	2	3	3

1	1	2	2	3	1	2	1	1	3
2	2	2	2	2	2	2	2	2	2
1	1	1	1	1	1	3	3	3	3
1	2	2	1	2	1	2	1	1	3
1	2	1	1	1	1	2	2	1	3
1	2	2	1	2	1	2	2	3	3
1	2	3	1	2	3	3	3	1	3
1	2	2	1	2	2	2	2	2	3
1	1	2	1	3	3	2	2	2	1
1	1	1	1	1	1	3	3	1	3
1	1	2	1	2	2	2	2	3	3
2	2	2	2	2	3	2	3	1	3
9	9	9	1	9	9	9	9	1	3
1	2	1	2	1	2	2	2	1	3
1	1	3	1	3	3	3	3	1	3
1	2	2	1	2	1	1	1	2	2
1	2	1	1	3	1	2	3	1	2
1	2	2	1	1	1	2	2	2	3
1	2	9	1	2	1	3	2	2	3
1	1	1	1	1	2	2	2	1	3
1	1	1	1	1	2	2	1	1	2
1	1	2	1	1	2	3	2	1	2
1	3	3	1	1	1	1	1	1	3
1	1	1	1	2	2	2	1	1	9
1	3	3	1	1	1	1	1	2	3
1	1	3	1	1	1	3	1	3	3
1	3	3	1	1	1	1	3	1	3
2	1	2	2	1	3	2	3	2	2
1	9	9	9	9	9	9	9	2	9
9	2	9	1	9	9	9	9	9	2
2	2	3	2	2	3	2	2	2	2
1	1	2	1	1	1	2	2	1	1
2	2	2	1	2	2	3	1	1	3
1	1	2	1	1	1	2	1	3	3
9	9	9	1	9	9	9	1	2	3
1	1	1	1	1	1	2	2	1	2
2	2	2	1	1	2	2	2	2	2
1	9	9	9	9	9	9	9	9	9
1	3	1	1	2	1	3	2	1	2
1	2	1	1	1	3	3	3	2	3
9	9	9	1	9	9	9	9	2	3
1	1	2	1	1	1	1	2	2	3
1	2	1	1	1	1	2	1	1	1
9	9	9	1	9	9	9	9	2	3
1	2	2	1	1	1	2	2	2	3
1	3	3	1	2	3	2	2	2	2
1	2	2	1	1	1	2	1	2	3

1	2	2	1	2	1	2	2	2	3
1	1	1	1	1	2	2	2	1	3
1	1	2	1	1	2	2	3	1	3
2	3	3	1	1	2	2	2	2	2
1	2	1	2	2	3	2	3	1	2
1	1	2	1	2	1	2	2	1	3
1	2	1	1	2	1	2	1	1	3
1	9	9	1	9	9	9	9	9	9
1	2	2	2	2	2	2	2	1	3
1	2	2	1	2	1	1	2	3	3
1	2	2	1	1	2	2	2	1	2
1	9	9	9	9	9	9	9	2	3
1	1	1	2	1	2	2	2	2	2
1	2	2	2	2	2	2	2	1	2
2	3	3	1	3	1	1	1	1	3
2	3	3	1	3	3	2	2	1	3
1	1	1	1	1	1	1	1	1	2
1	1	3	1	1	1	3	1	1	1
1	2	2	1	2	2	1	2	1	9
1	9	9	1	1	2	3	1	3	3
1	3	3	1	1	2	3	1	3	3
1	1	2	1	1	1	1	1	1	3
1	2	2	1	2	2	2	2	1	2
1	2	3	2	1	1	3	2	1	3
1	3	2	1	2	1	3	3	3	3
1	1	1	1	9	1	1	1	1	9
1	2	2	1	1	2	3	2	2	1
1	2	3	1	1	1	1	1	2	1
1	1	1	1	1	1	1	1	2	1
1	9	9	9	9	9	9	9	1	9
1	2	3	1	1	1	1	1	2	1
1	2	1	3	2	2	2	1	1	2
1	2	2	1	1	1	1	1	1	9
1	2	2	1	2	1	2	2	1	3
1	1	2	1	2	1	3	3	1	3
1	2	1	1	1	2	1	2	1	2
1	1	2	1	1	1	2	2	1	2
9	9	9	9	9	9	9	9	1	9
1	2	3	1	1	1	3	1	3	3
1	3	3	1	1	1	3	2	1	1
1	2	2	1	1	1	2	2	1	3
1	1	3	1	1	1	3	1	1	3
1	2	2	1	2	2	2	2	2	2
1	3	3	1	3	1	2	3	1	3
1	3	3	1	3	1	3	1	1	1
1	3	3	1	3	1	3	1	1	1
1	2	3	1	1	1	1	1	1	3

1	3	3	1	3	1	3	1	1	1
1	3	3	1	3	1	3	1	1	1
1	3	3	1	1	1	3	2	1	3
1	2	2	1	1	1	1	1	1	2
1	3	3	1	3	1	3	1	1	3
1	3	3	1	3	1	3	1	1	2
1	1	3	1	1	1	3	1	1	1
1	1	3	1	2	1	2	3	1	3
1	3	3	1	1	1	1	2	1	3
1	3	3	1	1	1	3	2	1	2
1	3	3	1	9	1	3	9	1	3
1	1	3	1	1	1	3	2	1	3
1	1	1	1	1	1	2	1	1	3
1	1	1	1	2	3	2	1	3	3
1	1	1	1	1	2	2	1	3	1
1	1	1	1	1	1	2	3	2	3
1	1	3	1	2	1	1	2	1	1
2	2	3	1	1	1	2	2	1	3
1	1	2	1	2	1	1	1	1	9
1	1	1	9	3	9	9	9	1	3
1	1	1	2	2	1	1	2	2	1
3	3	3	1	1	1	3	3	1	2
9	9	9	9	1	9	9	9	1	3
9	9	9	1	9	9	9	9	3	2
1	9	9	9	9	9	9	9	2	9
9	9	9	9	9	1	9	9	9	9
3	3	3	1	1	2	3	3	1	9
9	9	9	1	9	9	1	9	9	9
2	9	9	9	9	9	9	9	3	9
9	9	9	9	9	1	9	9	9	9
9	9	9	1	9	9	9	9	9	9
9	9	9	1	9	9	9	9	9	9
1	9	9	9	9	9	9	9	2	9
9	9	9	1	9	9	9	9	1	9
2	2	3	1	1	9	9	9	9	9
1	1	3	1	1	1	2	2	1	3
1	1	2	1	1	1	1	1	2	2
1	1	3	1	1	1	3	1	3	1
1	2	2	1	1	2	9	2	3	3
1	2	2	1	1	2	3	2	2	1
1	9	1	1	1	1	1	1	1	2
1	3	3	1	1	1	3	2	2	3
1	2	1	1	2	3	3	2	1	2
1	2	2	1	1	1	1	2	2	3
2	2	3	2	3	2	2	2	1	2
1	3	1	3	1	1	1	3	1	3
1	1	2	1	1	2	2	1	1	2

1	1	1	2	2	1	1	2	3	2
1	1	2	1	2	1	2	2	2	3
2	2	3	1	2	2	2	2	1	2
1	1	3	1	1	1	1	1	2	2
1	1	2	2	2	3	2	2	1	2
1	3	1	1	1	1	1	1	1	9
2	2	2	2	2	2	2	9	9	9
1	3	1	1	3	2	2	2	2	2
1	2	1	1	2	2	2	2	1	2
2	2	2	2	2	2	2	2	1	2
1	2	1	1	2	2	2	2	3	3
1	2	1	1	1	1	2	2	2	3
1	1	1	1	2	3	3	3	2	1
3	3	3	3	3	3	3	3	9	9
1	2	2	2	2	2	2	2	2	2
1	2	2	1	2	1	1	2	3	1
2	1	9	2	1	2	2	2	2	1
1	2	1	2	9	1	2	1	3	3
1	1	1	1	1	1	1	1	2	1
1	1	1	1	1	1	1	1	2	2
1	1	1	1	1	1	1	1	3	3
1	1	1	3	3	2	2	2	1	2
1	2	1	1	2	2	2	2	1	2
1	1	2	1	1	1	1	1	2	2
1	1	2	1	1	1	2	2	3	3
2	2	1	3	1	2	3	1	1	2
1	1	2	1	2	2	2	1	1	3
1	1	1	1	1	1	1	1	1	1
2	2	2	2	2	2	2	2	1	1
2	2	2	2	2	2	2	2	2	2
9	9	9	1	9	9	9	9	9	1
2	9	1	3	2	9	1	3	1	2
2	2	2	2	2	2	2	2	1	9
2	2	3	1	9	2	2	2	1	3
9	9	9	1	9	9	9	9	9	9
9	2	2	2	2	2	2	2	1	3
2	2	2	2	2	2	2	2	2	2
2	2	3	2	2	1	2	2	1	3
1	2	3	1	2	2	2	2	1	1
1	2	2	1	1	1	2	1	1	3
1	2	2	1	2	1	2	2	1	3
1	2	2	1	2	1	2	2	1	3
2	9	9	9	9	9	9	9	3	9
1	9	9	9	9	9	9	9	3	9
1	9	9	9	9	9	9	9	3	9
1	1	1	3	3	3	1	2	2	3
2	2	2	2	3	3	1	2	2	2

1	2	1	3	3	1	3	2	2	3
1	2	1	3	3	1	3	2	2	3
1	2	1	3	3	1	3	2	2	3
3	3	3	1	1	1	3	3	3	1
2	2	2	3	3	3	1	1	3	1
3	9	2	3	2	1	1	2	1	1
2	1	3	3	2	3	2	1	3	1
3	2	1	1	3	2	1	3	2	1
1	2	3	2	3	3	2	1	2	3
2	2	1	1	3	9	2	3	2	3
2	2	2	2	2	2	2	2	3	1
3	2	2	2	2	1	3	3	3	2
1	1	1	2	2	2	2	2	2	2
2	2	2	1	1	1	3	1	2	2
1	1	1	2	2	2	3	3	3	1
1	1	1	2	2	1	2	2	2	2
1	1	2	3	3	3	1	1	2	2
1	1	2	3	3	3	1	1	2	2
1	2	1	3	3	1	3	2	2	3
1	1	1	3	3	1	3	2	2	2
3	3	3	1	1	1	3	3	9	9
2	2	2	2	2	2	2	2	2	2
2	2	2	2	2	2	2	2	2	2
3	3	3	1	1	1	3	3	1	3
3	3	3	1	1	1	3	3	1	3
2	2	2	2	2	2	2	2	2	2
2	2	2	2	2	2	2	2	1	3
3	9	9	9	9	9	9	9	9	9
1	1	1	2	2	2	3	3	3	1
1	1	2	3	3	3	1	1	2	2
1	1	2	3	3	3	1	1	2	2
1	1	2	3	3	3	1	1	2	2
1	1	2	3	3	3	1	1	2	2
9	1	2	2	3	9	2	2	3	9
1	2	3	2	1	3	3	2	3	3
1	3	2	1	3	2	1	3	3	2
2	1	1	2	2	3	1	1	3	1
2	2	2	1	2	3	1	1	3	2
2	3	3	2	3	3	1	1	3	2
2	1	2	3	2	3	1	2	3	2
1	1	2	1	3	3	1	1	3	3
1	2	3	2	3	2	1	1	3	2
2	2	2	2	2	2	2	2	9	9
9	9	9	3	9	9	9	9	3	9
9	9	9	9	9	9	9	9	9	3
3	3	3	1	1	1	3	3	2	3
1	2	2	1	9	1	3	3	1	9

1	2	2	3	9	3	1	1	3	9
1	1	1	3	3	3	1	1	3	1
1	3	3	3	3	3	2	2	3	1
2	2	2	2	2	2	2	2	2	2
2	2	2	2	2	2	2	2	9	2
9	9	9	2	9	9	9	9	2	9
2	9	9	9	9	9	9	9	3	9
1	9	9	9	9	9	9	9	3	9
1	1	2	3	3	3	3	3	3	2
1	2	2	3	3	3	2	1	2	2
2	2	2	2	2	1	3	3	3	1
1	9	9	9	9	9	9	9	3	9
9	9	9	9	2	9	9	9	3	9
9	2	9	9	9	9	9	9	2	9
2	2	2	2	2	2	2	2	1	3
2	9	9	9	9	9	9	9	2	9
1	2	2	3	2	3	2	2	3	2
9	9	9	9	9	9	9	9	2	2
1	3	2	3	3	2	1	3	3	3
1	9	9	9	9	9	9	9	3	9
2	1	1	3	3	3	1	1	3	1
2	2	2	2	2	2	2	2	2	1
2	2	2	2	2	2	2	2	3	1
2	2	2	2	2	2	2	2	9	9
1	1	1	3	3	2	2	2	3	1
1	1	1	3	3	3	1	1	3	1
1	1	1	3	3	3	2	2	3	2
1	9	9	9	9	9	9	9	3	9
1	9	9	9	9	9	9	9	3	9
1	9	9	9	9	9	9	9	3	9
1	9	9	9	9	9	9	9	3	9
1	9	9	9	9	9	9	9	3	9
9	2	9	9	9	9	9	9	2	9
1	2	2	3	2	2	3	3	2	1
1	2	3	3	2	1	2	3	3	3
9	1	9	9	9	9	9	9	9	9
1	2	2	3	2	3	2	2	2	2
9	9	9	9	2	9	9	9	3	9
1	2	2	3	1	3	2	1	3	3
1	1	9	3	3	3	1	1	2	2
1	2	2	3	2	3	2	2	3	3
1	9	9	9	9	9	9	9	2	9
3	3	3	1	1	1	3	3	1	3
1	2	1	2	3	3	2	2	2	2
1	9	9	9	9	9	9	9	3	9
2	1	3	1	1	1	3	3	2	2
1	9	9	3	3	9	9	1	9	9

1	9	9	9	9	9	9	9	3	9
1	9	9	9	9	9	9	9	3	9
2	2	2	2	3	3	1	1	9	9
1	9	9	9	9	9	9	9	3	9
1	1	2	3	3	3	3	3	3	2
1	9	9	9	9	9	9	9	3	9
1	3	3	2	2	2	1	2	1	3
1	1	1	2	2	1	3	3	3	1
1	1	1	3	3	3	1	1	3	3
1	2	3	3	2	1	1	2	3	2
1	1	1	3	3	3	1	1	3	1
3	3	3	1	1	1	3	3	3	3
1	1	2	3	3	3	1	1	3	3
1	1	1	3	2	1	3	3	3	2
1	1	1	3	3	3	1	1	3	2
1	1	1	3	3	3	1	1	3	1
1	2	2	3	3	3	1	1	3	2
1	1	2	3	3	3	3	1	3	3
1	2	3	2	3	2	3	2	2	3
1	2	3	1	9	2	9	1	3	2
9	9	9	9	9	9	3	9	9	9
1	9	9	9	9	9	9	9	3	9
9	9	9	3	9	9	9	9	3	9
9	9	1	9	9	9	9	9	3	9
1	9	9	9	9	9	9	9	2	9
9	9	3	9	9	9	9	9	3	9
9	9	9	9	3	9	9	9	3	9
2	9	9	9	9	9	9	9	2	9
2	9	9	9	9	9	9	9	2	9
2	9	9	9	9	9	9	9	2	9
2	9	9	9	9	9	9	9	2	9
2	9	9	9	9	9	9	9	2	9
2	9	9	9	9	9	9	9	3	9
9	9	9	9	9	9	9	9	3	9
1	1	2	3	3	3	2	2	3	9
1	1	2	3	3	3	3	1	3	2
9	9	2	2	9	1	3	3	3	9
1	9	9	9	9	9	9	9	9	1
3	2	2	2	2	9	1	2	3	2
1	9	9	9	9	9	9	9	3	9
1	9	9	9	9	9	9	9	3	9
1	9	9	9	9	9	9	9	3	9
2	9	9	9	9	9	9	9	3	9
2	9	9	9	9	9	9	9	3	9
2	2	2	2	2	2	2	2	3	2
2	1	2	3	2	3	2	1	2	1
1	2	1	2	3	2	1	2	2	1

2	2	2	2	2	2	2	2	3	2
3	1	1	2	3	3	2	2	3	1
1	1	1	2	2	1	3	2	3	1
1	1	1	3	2	3	1	1	3	1
1	9	9	9	9	9	9	9	9	9
1	9	9	9	9	9	9	9	3	9
1	9	9	9	9	9	9	9	3	9
3	9	9	9	9	9	9	9	9	1
9	9	3	9	9	9	9	9	9	9
3	9	9	9	1	9	9	9	9	9
1	9	9	2	1	1	3	3	2	9
2	9	9	1	9	2	9	9	2	9
2	2	2	2	9	2	2	9	3	9
9	2	9	9	9	9	9	9	3	9
1	9	9	9	9	9	9	9	9	1
2	2	2	2	2	2	2	2	2	2
1	2	2	1	1	3	3	2	2	3
1	9	9	9	9	9	9	9	3	9
1	2	2	2	2	2	2	2	3	2
1	2	2	3	1	3	1	1	3	3
1	1	2	3	2	2	2	2	1	3
9	9	9	3	9	9	9	9	3	9
1	2	2	3	1	3	2	1	3	3
1	1	1	2	2	2	2	2	1	3
1	9	9	9	9	9	9	9	2	9
2	2	1	3	3	3	1	1	9	9
9	9	9	3	9	9	9	9	3	9
1	2	2	3	3	3	1	1	3	3
2	2	2	3	3	3	2	2	3	2
1	2	2	3	2	3	1	2	2	3
2	1	3	2	2	2	2	2	2	2
9	9	9	2	9	9	1	9	2	9
9	9	9	3	9	9	9	9	3	3
2	2	2	2	2	2	2	2	2	9
9	9	9	3	9	9	9	9	3	9
1	1	2	3	1	3	2	2	3	3
1	2	2	3	2	3	2	1	2	2
1	2	2	2	2	3	1	1	3	1
1	2	2	3	1	2	2	2	2	1
1	1	2	3	1	3	1	2	3	2
1	2	2	3	2	3	1	2	3	3
1	3	3	3	3	3	1	1	1	3
1	2	1	3	2	3	1	1	3	1
9	9	9	9	9	9	9	1	9	9
9	9	9	9	9	9	9	9	3	2
2	2	2	2	2	2	2	2	1	3
1	1	3	3	1	3	3	3	3	3

1	1	1	3	3	3	1	1	3	3
1	1	2	3	3	3	1	1	1	3
9	9	9	9	9	9	9	9	2	1
1	2	3	3	3	3	1	2	9	9
2	2	2	2	2	2	2	2	2	2
1	2	2	3	2	2	2	2	3	3
1	2	2	3	2	2	1	2	2	2
1	2	3	3	2	1	1	2	2	2
1	2	2	3	2	3	2	2	2	1
1	1	2	3	2	2	2	2	2	2
1	1	2	3	3	2	2	2	3	3
1	2	2	2	2	2	2	2	3	3
1	1	2	2	2	3	1	1	3	2
1	1	1	3	3	3	2	1	3	3
1	2	1	3	2	3	2	2	3	3
1	1	2	3	1	2	2	2	2	1
2	3	3	3	1	2	2	2	2	3
2	2	2	2	2	2	2	2	2	2
2	2	2	2	2	2	2	2	2	2
2	2	2	2	2	2	2	2	2	2
1	2	2	3	2	2	2	2	2	2
2	2	2	2	2	2	2	2	2	2
2	2	2	2	2	2	2	2	2	2
2	2	2	2	2	2	2	2	9	9
1	1	1	3	3	3	1	1	1	3
2	9	9	9	9	9	9	9	2	9
1	1	1	3	2	3	2	1	2	1
9	9	9	3	9	3	1	1	3	3
1	1	1	2	3	1	3	3	3	3
9	9	9	3	9	9	9	9	9	9
1	1	1	3	3	3	1	1	3	3
2	3	3	3	3	3	3	2	3	3
1	2	2	3	1	2	2	1	2	2
1	1	3	9	3	3	1	2	1	3
1	2	3	3	2	2	2	2	3	3
9	9	9	9	9	9	9	9	2	1
1	2	2	3	3	3	2	2	3	2
1	2	1	1	2	3	2	3	3	1
1	2	1	3	3	3	2	1	2	2
1	1	1	3	3	3	1	1	2	2
9	9	9	9	9	9	9	9	3	9
1	1	1	3	3	3	1	2	3	3
1	1	1	3	2	3	2	2	3	3
2	3	9	3	2	3	1	2	9	9
1	2	3	3	3	2	3	2	3	2
1	1	3	3	2	2	2	2	2	3
1	2	2	3	2	3	2	1	1	2

1	1	2	3	3	3	1	1	3	3
1	2	3	2	1	2	3	2	1	3
1	2	1	3	2	2	1	2	3	3
2	2	2	2	2	2	2	2	2	9
1	2	3	3	1	9	3	3	1	3
9	1	9	9	9	9	9	9	9	9
1	2	1	3	2	3	2	2	3	3
1	1	1	2	2	3	1	2	1	2
2	2	2	2	2	2	2	2	2	2
1	2	2	3	2	2	3	3	2	3
2	2	2	2	2	2	2	2	2	2
1	1	9	3	3	1	3	3	3	3
1	9	9	9	9	9	9	9	3	9
1	2	2	3	2	2	2	2	2	2
2	2	3	2	2	2	2	2	2	2
2	2	2	2	2	2	2	2	2	2
1	2	2	3	3	3	2	1	3	2
1	2	3	2	2	2	2	2	1	3
1	2	1	3	2	3	1	1	9	9
1	9	9	9	9	9	9	9	3	9
1	2	2	3	3	2	2	2	2	3
2	3	9	3	2	2	2	2	2	2
1	3	3	3	2	3	2	2	2	2
1	2	2	3	2	2	2	2	3	3
2	2	2	2	2	2	2	2	3	2
1	1	2	2	3	2	2	1	1	3
1	1	1	3	2	2	2	2	2	2
1	1	1	3	3	2	2	2	3	2
2	2	2	3	2	2	2	2	3	3
1	1	2	3	3	3	2	2	3	3
1	2	2	3	3	3	2	2	3	3
9	1	9	9	9	9	9	9	3	9
2	2	2	3	2	3	2	1	3	3
1	1	3	3	2	1	3	2	3	9
9	9	9	3	9	9	9	9	3	2
2	2	2	2	2	2	2	2	2	2
1	1	2	3	2	3	2	2	3	3
1	2	2	2	2	2	2	2	3	2
2	2	2	2	2	2	2	2	3	3
2	2	2	2	2	3	2	2	2	3
1	1	3	3	1	3	1	2	3	3
2	2	2	2	2	2	2	2	2	2
2	2	2	2	2	2	2	2	2	2
2	2	2	2	2	2	2	2	2	2
1	2	2	2	2	3	2	2	3	2
2	1	2	2	2	2	2	2	2	2
3	3	2	3	1	2	1	2	3	2

9	9	9	9	9	9	9	9	9	9
1	1	3	3	1	3	1	3	3	1
1	9	9	3	9	3	9	9	1	9
1	9	9	9	9	9	9	9	2	9
3	1	9	2	9	3	2	2	3	1
2	3	2	1	2	1	2	3	3	2
9	9	9	3	9	9	9	9	9	9
1	1	2	3	3	3	2	3	3	9
1	2	2	3	2	3	2	1	3	3
1	9	9	9	9	9	9	9	3	9
2	2	2	1	2	3	9	3	2	9
1	1	2	3	2	2	2	2	3	3
3	3	3	3	1	1	3	3	3	3
1	9	9	9	9	9	9	9	9	1
1	3	3	3	1	1	3	3	3	3
3	2	1	2	1	1	3	3	1	1
1	3	3	3	1	3	1	1	3	3
1	9	9	9	9	9	9	9	2	9
1	2	3	3	2	3	2	2	2	3
1	1	1	3	2	1	2	2	2	2
1	1	2	3	3	3	1	1	3	2
9	9	9	2	9	9	9	9	2	9
1	3	3	2	2	2	2	1	2	3
1	1	2	3	3	3	3	2	3	3
1	1	3	9	3	3	1	1	2	2
1	2	2	3	3	3	2	2	3	3
1	3	3	3	3	3	1	1	2	9
9	9	9	3	9	9	9	9	2	9
1	1	2	3	2	3	2	2	3	3
1	2	2	3	3	2	9	1	3	3
2	3	3	1	1	1	3	3	3	2
3	3	3	1	1	1	3	3	3	3
1	1	1	2	1	2	1	2	3	2
1	9	9	9	3	9	9	9	3	9
2	2	1	3	1	1	3	3	2	2
1	2	1	3	3	2	2	2	3	9
9	9	9	9	3	9	9	9	3	3
9	9	9	3	9	9	9	9	9	9
9	9	9	9	3	9	9	9	3	9
1	1	1	2	2	1	1	3	2	2
1	9	9	3	3	3	9	9	3	9
1	1	3	3	1	3	1	1	3	3
3	2	2	2	2	2	2	2	3	3
1	3	1	3	3	2	9	3	3	3
9	9	9	9	3	9	9	9	3	9
2	9	9	9	9	9	9	9	3	3
1	9	9	9	3	9	9	9	3	9

1	2	2	3	3	2	3	2	3	9
1	1	2	3	3	1	2	2	1	3
9	9	9	3	9	9	9	9	3	9
2	9	1	1	2	3	2	3	2	2
2	3	3	2	2	2	2	1	2	3
9	9	1	9	9	9	9	9	9	9
9	9	9	3	9	3	9	9	1	9
1	2	2	2	1	1	2	1	3	2
1	2	2	3	3	3	2	2	3	3
1	1	3	3	3	3	1	3	3	3
1	2	2	3	3	3	2	1	1	3
1	1	1	3	3	3	2	2	3	2
1	1	2	3	3	3	3	1	3	3
1	9	9	3	9	3	9	1	3	3
1	1	1	3	3	3	2	1	3	2
1	1	1	3	3	3	2	1	9	9
1	2	2	3	3	3	2	1	3	2
1	2	2	3	3	3	2	1	3	3
1	9	9	9	9	9	9	9	3	9
2	1	1	2	2	3	2	2	3	2
1	2	3	3	3	3	1	2	3	2
1	9	9	9	9	9	9	9	3	9
1	3	3	3	3	3	3	3	3	3
9	9	9	3	9	9	9	9	9	9
1	1	2	3	2	3	2	1	3	2
9	9	9	3	9	9	9	9	3	9
1	2	1	3	3	3	2	2	3	3
1	9	9	9	9	1	3	3	3	3
1	9	9	3	3	3	9	9	3	9
9	9	9	3	9	9	9	9	3	9
1	3	3	3	3	1	3	1	9	9
9	9	9	3	9	9	9	9	3	9
3	3	3	1	1	1	3	3	9	3
1	2	2	3	3	3	1	2	2	3
1	3	3	3	2	2	2	3	2	3
2	3	3	3	3	2	2	2	3	2
1	9	9	9	9	9	9	9	3	9
1	9	9	3	9	3	9	9	3	9
9	2	9	9	9	9	9	9	2	9
9	9	9	9	3	9	9	9	3	9
1	9	1	9	9	9	9	9	9	1
1	9	9	9	9	9	9	9	3	9
3	3	9	3	3	3	3	3	3	3
1	1	2	3	3	3	2	2	2	2
2	2	3	3	3	2	2	2	2	3
1	2	2	3	3	2	2	2	2	3
1	1	2	3	3	3	2	2	1	3

9	9	9	3	9	9	9	9	3	9
1	2	3	3	3	3	3	1	9	3
1	1	3	3	3	3	3	2	1	3
1	1	3	3	2	2	2	2	3	3
1	2	3	3	3	1	2	1	3	3
1	9	9	9	9	9	9	9	9	9
2	1	2	2	3	2	1	2	2	3
9	9	9	3	9	9	9	9	2	9
1	9	9	9	9	9	9	9	2	9
9	9	9	3	9	9	9	9	3	9
1	1	2	3	2	2	2	2	2	2
1	2	2	3	2	1	2	3	3	3
2	2	2	2	2	2	2	2	2	9
1	2	3	3	3	3	2	2	3	3
1	9	9	9	9	9	9	1	3	9
2	3	1	2	1	3	2	3	3	3
2	2	2	3	2	2	3	3	3	3
1	1	2	2	2	2	2	2	3	3
1	3	3	2	2	3	3	2	3	9
1	1	2	3	3	3	2	3	3	3
1	9	9	9	9	9	9	9	3	9
1	3	3	3	3	2	3	3	3	3
3	3	3	3	1	1	3	3	3	3
1	9	9	9	9	9	9	9	3	9
1	2	2	3	3	3	3	3	3	3
1	2	2	3	3	3	2	2	1	3
1	2	3	3	3	2	3	3	3	3
1	3	3	3	1	3	3	3	3	9
1	2	2	3	2	3	2	1	3	2
2	3	3	3	3	3	2	2	3	3
9	3	9	3	9	1	9	2	9	1
1	9	9	9	9	9	9	9	3	9
3	3	3	2	2	2	3	2	1	3
1	2	3	3	3	2	2	2	3	3
1	2	9	3	3	3	2	1	3	3
2	9	9	3	9	9	9	1	2	9
1	1	1	3	3	3	1	1	3	3
1	1	1	3	3	3	1	1	3	3
1	1	2	3	3	3	2	2	3	3
2	9	2	1	2	9	2	3	2	9
1	1	3	2	2	2	2	2	3	1
1	1	9	3	1	3	3	1	3	9
1	2	2	3	3	3	2	2	1	2
2	2	2	3	1	3	1	1	1	3
1	2	3	3	2	3	2	2	3	3
2	2	3	3	2	3	2	2	3	2
1	9	9	9	9	9	9	9	3	9

1	9	1	9	9	3	1	1	3	1
1	2	3	3	3	2	2	2	1	3
1	9	9	3	3	3	9	9	1	3
1	1	2	3	3	3	2	3	3	1
1	9	9	3	9	3	9	9	3	9
1	2	1	2	1	3	1	2	3	2
1	2	1	3	1	3	3	3	9	9
9	9	9	9	3	9	9	9	9	9
2	2	2	2	2	2	2	2	2	2
1	2	2	3	3	3	1	2	3	3
9	9	1	9	9	9	9	9	9	9
1	1	3	3	3	3	1	9	3	3
3	3	3	1	1	1	3	3	2	2
3	3	3	2	2	3	2	2	1	3
1	2	3	3	3	2	3	3	3	3
1	2	2	3	2	3	2	2	1	3
1	1	3	3	3	3	1	1	3	3
2	1	1	2	3	1	2	2	2	1
9	9	9	3	9	9	9	9	9	9
1	1	2	3	3	1	2	3	9	1
2	2	2	3	3	2	2	2	2	2
2	2	2	3	3	2	2	2	2	2
2	2	3	1	2	2	1	2	1	3
1	9	9	9	9	9	9	9	9	1
2	3	2	2	2	2	2	2	3	3
1	1	1	3	3	3	2	1	1	3
1	2	3	3	2	2	2	2	3	3
1	1	1	3	2	3	2	3	1	3
1	2	2	3	3	2	3	2	3	2
1	2	3	3	2	2	2	1	2	2
1	9	9	3	9	9	9	1	3	9
1	9	9	3	9	3	9	9	3	3
1	1	1	3	2	3	2	1	3	1
1	9	9	9	9	9	9	9	3	9
1	1	1	3	3	3	1	1	3	1
9	9	9	3	9	9	9	9	3	9
1	9	9	9	9	9	9	9	3	9
3	3	3	1	1	1	3	3	2	2
9	9	1	9	9	9	9	9	3	9
1	2	2	3	3	2	2	2	3	2
1	1	2	3	3	3	2	2	2	2
1	1	2	3	3	3	2	1	2	2
1	2	3	3	3	3	1	1	2	1
1	1	1	3	3	3	1	1	3	2
9	9	9	3	9	9	9	9	3	9
1	9	9	3	3	3	9	1	3	9
1	1	1	3	3	3	3	1	3	3

1	9	9	3	9	3	9	9	3	9
1	2	3	3	3	3	2	2	2	3
1	1	3	3	3	3	3	1	3	3
1	1	2	2	3	2	2	2	2	2
1	2	2	3	2	9	2	3	2	3
1	1	3	3	1	1	3	3	3	3
1	1	3	3	3	3	3	3	3	9
9	9	9	3	9	9	9	9	3	9
1	1	2	3	2	3	2	2	2	2
1	1	2	3	3	3	3	3	3	3
1	3	3	3	1	3	2	1	3	3
1	2	2	3	3	2	2	2	3	3
1	1	1	3	3	3	2	2	3	3
1	2	3	3	9	3	3	1	3	3
1	3	3	3	3	3	3	3	3	3
1	1	3	3	3	3	1	2	9	3
1	1	2	3	3	3	1	1	1	3
1	9	9	9	9	9	9	9	3	3
2	2	2	9	9	9	9	9	9	9
3	1	1	3	3	3	1	1	3	3
1	2	3	1	1	1	3	3	1	3
1	2	3	3	3	9	2	3	9	1
1	2	2	3	3	3	2	2	3	3
9	9	9	3	9	9	9	9	9	9
1	2	3	3	2	3	2	2	3	9

WomenInt	WomenPre	WomenMe	CABreastH	Daily	Weekly	Monthly	SixMonthly	Yearly	Anytime
2	2	2	2	3	3	3	3	3	3
2	2	2	2	2	2	2	2	2	2
2	2	2	2	2	2	2	2	2	2
2	2	2	2	2	2	2	2	2	2
2	2	1	2	1	2	2	2	3	1
2	2	2	2	2	2	2	2	2	2
2	2	2	3	3	3	3	1	2	2
3	2	3	2	3	2	1	1	3	2
1	2	2	2	1	1	2	2	2	1
3	2	1	2	2	2	2	2	2	3
3	3	3	1	3	3	1	3	3	3
2	3	2	1	1	1	2	2	3	1
2	2	2	2	1	1	1	1	1	2
2	2	2	1	2	1	2	2	2	3
2	2	2	2	2	2	2	2	2	2
2	2	2	2	3	3	3	2	2	3
2	2	2	2	2	2	2	2	2	1
3	3	3	3	1	3	3	1	3	3
2	2	2	2	2	2	2	2	2	1
3	3	3	2	2	1	1	2	1	1
1	1	3	3	3	3	1	3	3	3
2	3	2	3	2	3	1	3	3	3
3	3	3	3	2	3	1	3	3	3
2	2	2	2	2	2	1	2	3	2
1	2	2	2	2	2	2	2	2	2
3	3	3	1	3	3	3	1	3	3
9	9	9	1	9	9	1	9	9	1
3	3	3	3	3	3	1	1	1	1
3	3	3	3	3	2	1	1	2	2
3	2	1	3	3	3	1	2	1	3
3	3	3	1	3	3	1	2	2	1
3	3	3	1	2	1	2	2	2	2
3	3	3	3	1	3	3	3	3	1
9	1	3	1	3	1	1	1	2	3
1	9	1	9	1	2	2	1	1	2
2	2	3	2	3	3	2	3	3	3
2	3	3	2	1	1	1	2	3	1
1	2	3	3	2	1	2	1	1	1
3	3	3	3	1	1	1	2	3	3
2	2	3	2	1	1	1	2	2	1
2	2	1	3	2	1	1	2	3	2
9	9	9	9	9	1	9	9	9	9
9	9	9	9	9	1	9	9	9	1
3	3	2	1	3	1	9	2	3	1
3	3	3	3	3	3	3	1	3	2
9	9	9	9	9	9	9	9	9	9

2	2	2	2	1	1	2	3	3	1
2	2	3	2	1	1	2	2	3	1
2	2	1	2	2	2	1	2	2	2
1	2	2	2	2	2	1	2	2	2
3	3	3	3	3	3	3	3	1	1
3	1	3	1	3	1	1	3	3	3
2	2	2	1	2	2	2	2	2	3
3	3	3	1	3	3	2	1	1	3
3	3	9	9	3	3	3	1	2	9
2	3	3	1	3	3	3	3	1	2
9	9	9	9	9	9	9	1	9	9
2	2	2	3	1	2	2	2	3	1
2	2	2	2	1	1	2	2	2	2
1	1	2	2	1	1	2	2	2	2
2	2	2	1	1	1	2	2	2	1
2	2	2	2	1	1	1	1	2	1
3	2	2	3	3	1	2	2	2	3
3	3	2	3	3	3	1	1	1	2
2	2	2	1	2	2	2	2	2	2
2	2	2	2	2	2	2	2	2	3
3	3	3	3	3	3	3	3	3	3
1	2	3	2	2	2	1	2	3	2
2	2	2	2	1	2	2	2	2	2
1	1	2	3	1	2	2	3	3	1
2	2	2	1	1	2	2	3	3	1
2	2	2	2	1	2	2	2	2	2
3	3	3	1	2	1	1	2	3	1
3	2	2	1	1	2	2	3	2	2
3	3	3	3	2	1	1	3	3	3
3	2	1	1	3	3	3	3	1	1
9	9	9	9	9	9	9	9	9	9
3	3	3	3	3	3	3	1	3	2
9	3	2	2	3	1	1	2	3	1
2	2	2	2	2	2	2	2	2	2
3	9	1	2	3	1	3	3	3	3
3	2	1	1	3	3	3	2	1	2
3	3	3	2	2	1	2	1	2	2
2	2	2	3	2	2	2	1	3	1
3	3	3	3	3	3	1	1	3	3
2	2	1	1	2	2	2	1	2	1
3	3	3	2	3	2	1	1	2	2
3	3	3	3	2	2	2	2	2	2
1	1	3	1	1	2	1	1	1	1
2	3	2	2	3	2	2	1	1	1
2	3	3	3	3	3	3	2	1	1
1	2	3	2	1	2	2	2	2	3
9	9	9	9	2	2	1	2	2	2

1	3	3	1	2	2	3	1	1	2
3	3	3	1	3	3	1	3	3	3
2	3	3	2	3	3	3	2	1	2
3	1	2	3	1	2	2	1	3	1
2	1	2	2	1	1	2	2	2	2
2	3	2	1	2	2	1	3	2	3
3	3	2	3	1	3	3	2	2	1
3	2	3	3	1	3	9	2	2	3
1	2	3	1	1	3	3	3	3	1
3	3	1	2	2	9	1	9	9	3
2	1	1	1	9	9	9	9	9	9
3	3	3	1	3	3	1	1	2	1
2	2	3	2	1	2	2	3	3	1
2	2	2	2	2	2	2	2	2	1
1	1	3	3	3	3	1	3	3	2
1	1	3	3	3	3	1	3	3	2
3	3	2	1	3	1	2	1	3	1
2	2	2	2	3	2	1	9	1	1
2	2	9	1	9	1	9	9	9	9
2	3	2	2	1	2	2	2	3	2
1	3	3	3	3	3	3	3	3	3
3	3	3	1	3	3	3	1	2	3
2	2	3	2	1	1	2	2	3	2
2	2	2	2	1	1	1	1	1	2
3	3	3	3	2	2	2	2	2	2
3	3	3	3	1	2	2	3	3	1
1	2	2	1	1	1	2	2	2	1
2	1	2	1	1	2	1	1	1	2
1	1	2	1	2	2	2	1	3	2
3	2	3	3	1	1	2	2	3	2
1	2	1	1	1	1	2	2	2	1
1	2	2	1	2	1	3	3	3	1
3	3	3	3	3	2	1	2	1	2
3	1	2	1	1	1	1	1	2	1
2	3	3	3	2	1	3	1	1	1
1	1	1	1	2	2	1	2	2	2
9	9	9	9	9	9	1	9	9	2
3	2	2	1	3	2	2	1	2	3
3	3	3	3	3	3	1	3	3	1
3	3	3	1	3	2	3	9	2	3
2	2	2	2	1	2	1	1	1	1
9	9	9	9	9	9	9	9	9	9
2	2	2	2	1	2	2	1	1	1
1	9	2	2	2	2	2	2	3	1
3	3	3	3	1	2	3	3	3	3
3	3	3	2	2	3	2	1	3	1
9	3	2	1	1	1	2	2	3	1

1	2	2	3	1	9	3	2	3	3
3	3	3	3	3	3	3	3	3	3
3	3	3	3	3	3	3	3	3	3
3	3	3	1	1	3	3	1	3	1
1	2	3	1	3	3	1	2	2	1
1	2	2	1	3	1	2	3	2	3
9	9	9	1	9	2	9	1	2	2
3	2	1	2	2	1	2	1	3	1
2	2	2	2	2	2	2	2	2	2
2	2	2	1	2	2	2	2	2	2
1	1	2	1	2	3	1	3	1	1
2	2	3	1	1	9	9	3	3	9
2	9	2	1	2	2	2	2	2	2
3	3	3	3	3	3	3	3	3	2
2	1	1	2	2	2	1	1	1	1
2	1	1	1	2	2	1	1	1	1
2	1	1	1	1	1	2	2	2	1
2	2	2	1	2	2	1	1	1	1
2	2	2	1	1	2	2	3	3	1
3	3	2	1	2	2	3	2	3	1
9	9	9	9	9	9	2	9	9	9
3	3	2	2	3	1	2	2	2	3
3	3	3	3	2	1	2	2	2	2
1	1	1	1	3	3	2	1	1	3
3	3	3	3	2	2	1	2	2	3
1	1	2	3	3	3	2	3	3	1
3	2	3	3	3	1	1	3	3	1
1	2	2	3	2	1	1	1	2	1
3	3	3	1	2	2	2	1	1	1
1	2	2	1	2	1	1	1	2	1
9	9	9	9	9	9	9	9	9	9
2	2	2	2	2	2	2	2	2	2
2	1	1	2	2	2	1	2	2	1
1	1	1	1	1	1	1	1	1	1
2	2	2	2	3	3	3	3	3	2
1	1	1	1	2	1	1	1	1	2
1	1	1	1	2	2	2	2	2	3
2	3	3	2	3	1	1	2	3	3
3	3	3	1	9	1	1	9	9	1
3	3	9	3	3	3	3	3	3	3
3	9	3	9	1	3	1	1	1	2
2	2	2	2	2	2	2	2	2	2
2	2	3	2	3	1	1	2	3	2
9	9	9	9	9	9	9	9	9	9
9	9	9	9	9	9	9	9	9	9
2	2	1	1	1	2	2	1	1	2
2	2	3	2	2	1	2	2	2	1

9	2	9	9	9	9	9	1	9	9
9	9	9	9	2	9	9	9	9	1
3	3	3	3	9	9	1	9	9	1
9	9	9	9	1	9	9	9	9	1
3	3	3	3	3	3	2	1	3	2
3	3	3	3	2	2	1	2	3	2
3	3	3	3	3	2	2	1	3	2
2	1	3	2	9	9	9	9	9	9
2	1	1	1	3	3	1	3	3	2
1	9	9	9	9	9	2	9	9	9
9	9	2	9	9	9	1	9	9	9
9	9	9	9	9	9	9	9	1	3
1	1	1	2	2	2	2	2	2	2
3	3	1	1	2	2	3	3	1	3
2	9	9	9	9	1	9	9	9	9
3	1	2	2	3	3	3	2	3	2
3	3	3	3	3	3	3	3	3	3
1	1	1	2	2	2	1	1	2	2
2	2	2	3	2	2	1	1	1	1
2	2	2	2	9	9	2	9	9	2
1	2	3	2	1	2	3	3	3	1
2	2	3	2	3	2	2	2	2	1
2	2	2	2	2	2	1	1	2	2
3	3	3	3	2	3	3	3	3	1
2	1	2	2	2	2	2	2	2	2
2	2	2	2	3	2	2	1	2	2
1	1	1	3	1	1	2	2	3	2
1	2	1	1	2	2	2	1	1	2
9	9	9	9	9	9	9	9	9	9
1	2	2	2	1	2	1	2	2	1
2	2	1	2	2	2	2	1	2	2
1	1	1	1	1	1	1	1	1	2
3	2	3	2	3	2	1	3	3	2
3	2	2	3	1	3	3	3	3	3
3	3	3	1	3	3	3	3	1	3
2	1	1	2	2	2	2	2	2	1
3	3	2	2	1	1	2	2	2	1
1	2	2	2	2	2	2	1	2	9
1	2	3	2	1	2	3	2	2	1
1	2	2	2	2	1	2	2	2	2
2	2	2	1	1	2	2	2	2	1
2	1	3	2	3	1	1	2	1	1
1	3	3	3	3	2	1	1	1	1
2	2	2	2	2	2	2	2	2	2
1	3	1	3	2	2	1	2	2	2
1	2	3	3	3	3	2	1	2	3
9	9	9	9	9	9	9	9	9	9

3	3	3	3	1	2	3	9	3	1
3	3	2	1	1	3	2	2	3	1
1	1	2	1	2	2	2	2	2	1
3	3	3	3	3	3	1	3	3	1
3	3	3	3	2	2	2	2	2	2
3	2	2	3	3	9	2	1	3	3
3	1	1	3	1	1	1	3	1	1
1	1	1	1	1	2	2	2	2	2
2	2	2	2	2	2	2	9	9	1
1	1	2	2	1	1	1	1	1	3
1	2	2	1	1	1	1	2	2	2
2	2	1	1	1	1	1	1	1	1
9	9	9	9	2	9	9	9	9	2
2	2	2	2	2	2	2	2	2	2
2	2	2	9	9	2	9	2	2	9
9	9	9	9	9	9	9	9	9	2
9	2	9	2	2	9	9	2	9	2
9	9	2	9	9	2	1	2	9	9
9	2	9	2	2	2	9	2	2	2
9	9	9	9	9	9	9	9	9	9
9	9	9	9	9	9	9	9	9	9
2	2	2	2	2	2	2	2	2	2
2	2	2	2	2	2	2	2	2	2
3	3	3	3	2	2	2	2	3	2
2	2	2	2	2	2	2	2	2	2
2	9	9	2	9	2	9	2	2	2
2	2	2	2	2	2	2	2	2	2
9	9	9	9	9	9	9	9	9	9
9	9	9	9	9	9	9	9	9	9
2	2	2	2	2	2	2	2	2	1
9	9	9	9	9	9	9	9	9	2
9	9	9	9	9	9	9	9	9	9
9	9	9	9	9	9	9	9	9	9
2	9	2	9	2	9	2	9	2	9
9	9	3	9	9	9	2	1	9	9
1	9	3	1	3	1	9	1	2	9
9	9	9	9	9	9	9	9	9	9
9	9	9	9	9	9	2	9	9	9
9	9	2	9	9	2	9	9	2	9
9	9	9	9	3	9	9	9	9	3
1	9	9	9	1	9	9	9	9	9
9	9	9	9	9	9	1	9	9	1
3	3	3	3	3	3	3	3	3	3
2	1	1	2	2	1	1	2	1	1
2	1	2	1	1	2	1	3	2	1
9	2	1	9	9	1	2	1	1	9
1	9	9	9	1	9	9	9	9	9

9	9	9	9	9	9	1	9	9	9
9	9	9	9	9	9	1	9	9	2
1	1	1	2	2	9	9	9	9	3
9	1	2	3	3	2	1	2	3	3
1	1	1	1	1	2	1	2	1	2
1	2	1	1	2	2	2	2	2	2
3	3	3	3	2	1	1	3	3	2
1	1	1	1	3	1	2	2	3	1
9	9	9	9	9	9	9	9	9	9
3	3	3	3	3	3	3	3	3	3
9	9	9	9	9	9	9	9	9	9
9	9	9	9	9	9	9	9	9	9
9	9	9	9	9	9	9	1	9	9
1	1	1	9	1	1	1	1	2	1
1	1	1	1	1	1	1	1	1	1
2	1	1	1	1	2	2	2	2	1
9	9	9	9	9	9	9	9	9	9
1	2	2	1	1	2	2	2	2	1
1	1	1	1	1	1	1	1	1	1
1	3	3	1	3	3	1	2	3	1
1	3	2	2	9	9	1	9	9	3
3	3	3	1	2	3	1	2	3	1
3	3	2	3	3	2	3	1	1	1
2	2	2	2	2	2	2	2	2	2
2	2	2	2	2	2	2	1	2	2
2	2	2	2	2	2	2	2	2	2
2	2	2	2	2	2	2	2	2	2
3	1	2	3	1	1	1	1	1	1
3	2	2	2	2	2	3	3	3	2
2	2	2	2	1	2	2	1	2	1
2	2	2	2	2	2	2	2	2	2
2	2	2	2	2	2	2	2	2	2
2	2	2	3	3	3	2	2	3	2
2	9	3	3	2	2	2	2	2	2
3	3	3	3	2	2	2	2	3	2
3	3	3	2	2	2	3	1	2	1
9	9	9	9	9	9	9	9	9	9
2	1	1	2	3	2	1	2	2	2
3	3	3	3	3	3	3	3	3	3
3	3	3	3	3	3	3	3	3	3
2	2	2	2	2	2	1	1	1	2
2	3	1	2	3	2	1	3	1	2
2	3	3	3	1	3	3	3	1	2
1	2	2	2	1	2	2	2	3	2
1	1	1	1	3	3	3	2	3	2
2	2	2	2	2	2	2	2	2	2
1	1	9	9	2	2	2	9	9	2

1	2	2	2	2	3	1	2	2	2
1	9	9	9	1	9	9	9	9	1
9	9	9	9	9	1	9	9	9	9
3	1	2	3	1	2	3	1	2	1
2	9	9	9	2	9	9	9	9	9
1	1	2	3	3	3	2	9	1	2
1	9	9	9	9	2	9	9	9	3
9	9	9	9	9	9	9	1	9	9
2	2	2	9	2	2	2	2	2	2
9	9	9	9	2	9	9	9	9	9
9	9	9	9	9	2	9	9	9	9
9	9	9	9	9	9	2	9	9	1
2	9	9	9	9	2	9	9	9	9
9	9	9	9	9	9	3	9	9	1
9	9	9	9	9	1	9	9	9	1
2	9	9	9	2	9	9	9	9	9
2	1	2	3	2	3	2	1	3	3
3	2	1	1	3	2	1	3	2	1
2	1	1	2	1	2	1	1	2	1
2	2	3	2	2	2	2	2	2	2
3	3	2	3	3	2	1	2	3	1
2	2	3	3	1	1	1	2	3	1
3	2	1	1	1	1	1	1	1	1
1	2	2	2	3	1	2	2	1	1
3	3	9	3	2	2	1	3	3	2
3	3	3	3	1	3	3	3	3	3
1	3	3	2	2	2	2	2	2	2
3	1	3	2	1	3	3	3	3	1
1	2	3	2	9	2	1	1	3	9
1	3	3	3	3	1	2	2	2	2
2	1	2	1	1	1	3	1	1	1
2	2	2	2	2	2	2	2	2	2
1	2	2	3	1	1	1	1	1	2
9	9	9	9	2	9	9	9	9	1
1	3	3	3	9	9	9	9	9	9
3	3	3	3	2	2	2	2	2	1
9	9	9	9	9	9	9	9	9	9
1	2	2	1	2	1	1	1	1	1
1	1	2	2	2	2	2	2	2	2
2	2	2	2	2	2	2	2	2	2
9	9	9	9	9	2	9	9	9	1
3	3	3	3	1	2	3	3	3	3
9	9	9	9	9	9	9	1	9	1
2	3	2	1	3	3	1	1	2	3
2	2	2	2	2	2	2	2	2	2
3	1	2	3	3	3	3	1	2	1
2	2	2	2	2	2	2	9	2	2

1	2	3	3	3	2	2	1	2	3
9	9	9	1	9	9	1	9	9	9
9	9	9	9	9	9	1	9	9	9
3	1	2	3	1	2	1	3	2	1
3	3	3	3	3	3	3	3	3	1
2	2	2	2	9	9	1	9	9	2
1	2	2	2	3	2	2	1	2	2
1	2	2	2	2	2	2	1	2	1
9	9	9	9	9	9	9	9	9	9
3	3	3	2	3	3	2	1	2	1
3	3	3	1	3	3	3	1	3	3
9	9	9	9	9	9	2	9	9	1
2	2	2	1	2	1	1	2	3	9
9	9	9	9	1	9	9	9	9	2
2	3	3	2	9	9	9	1	9	9
3	3	2	2	3	3	3	1	3	3
9	9	9	9	9	9	9	9	9	9
9	9	9	9	3	9	9	9	9	1
9	9	9	9	9	9	1	9	9	9
2	9	2	3	2	2	2	3	2	3
2	2	2	2	2	2	2	2	2	2
3	3	2	1	3	2	2	2	1	2
2	3	2	1	2	2	2	2	2	2
9	9	9	9	9	9	9	1	9	9
9	9	9	9	9	9	9	1	9	9
1	2	2	1	1	1	1	1	1	2
1	1	1	1	1	1	1	1	1	1
2	3	1	2	1	2	3	1	1	2
2	3	3	3	3	2	2	2	2	2
9	9	9	9	3	9	9	9	9	2
9	9	9	9	9	9	9	9	9	9
2	2	1	1	1	2	1	1	1	1
2	2	2	2	2	2	1	2	2	3
3	3	3	3	1	1	2	2	2	1
3	3	3	3	3	1	3	1	1	1
1	2	1	2	1	2	2	1	2	1
2	3	3	3	1	2	2	1	3	9
2	1	1	2	1	1	2	2	2	3
2	1	2	1	2	3	2	3	1	3
2	2	1	2	1	1	1	1	1	2
3	2	2	3	2	2	2	2	2	1
2	2	3	3	2	2	2	2	2	2
3	3	2	2	3	3	3	2	2	2
3	2	3	1	3	2	1	1	1	1
2	2	2	2	9	3	1	9	9	3
2	2	2	2	2	2	2	2	2	2
2	2	2	2	2	2	1	2	2	3

2	2	2	2	1	2	2	2	2	2
9	9	9	9	1	9	9	9	9	9
9	9	1	9	9	9	9	9	1	9
9	9	9	9	9	1	9	9	9	9
2	2	2	2	3	2	1	1	1	2
3	3	2	2	2	3	3	3	2	3
2	2	2	2	2	2	2	2	2	2
2	2	2	2	2	9	2	2	2	2
9	9	9	9	9	9	9	9	9	9
2	9	9	9	9	9	2	9	9	9
2	2	2	2	2	2	2	2	2	2
2	2	2	2	2	2	2	2	2	2
2	9	9	9	2	2	2	9	9	2
2	2	2	2	2	2	2	2	2	2
2	2	2	2	2	2	2	2	2	2
9	9	9	9	2	9	9	9	9	2
3	3	3	3	3	3	3	3	3	3
9	9	9	9	1	9	9	9	9	1
9	9	9	9	2	9	9	9	9	2
9	9	9	9	2	9	9	9	9	2
9	9	9	9	9	9	9	9	9	9
9	9	9	9	2	9	9	9	9	2
2	2	2	2	3	3	3	3	3	2
2	2	2	2	2	2	2	2	2	2
9	9	9	9	9	9	9	9	9	9
9	9	9	9	9	9	1	9	9	9
1	9	9	9	1	9	1	9	9	1
9	9	9	9	9	1	1	9	9	9
3	3	3	3	3	3	2	3	3	3
2	9	2	2	2	9	2	2	2	2
2	1	2	2	2	9	2	9	2	2
3	3	3	3	3	3	1	3	3	3
1	9	1	9	3	1	3	2	1	1
1	9	9	9	9	9	9	9	1	1
2	2	2	2	2	2	2	2	2	3
9	9	1	9	9	1	9	2	1	2
9	2	2	2	2	9	9	2	2	2
2	2	2	2	2	2	2	2	2	1
3	2	3	3	2	1	3	1	3	1
2	2	2	2	2	2	2	2	2	2
9	9	9	9	9	1	9	9	9	1
3	2	3	3	1	2	3	3	3	1
1	2	3	2	1	1	2	9	3	2
3	2	2	2	2	3	2	3	1	1
3	3	3	1	3	3	1	3	3	3
2	3	2	1	1	2	1	2	1	1
3	3	2	2	1	2	3	3	3	2

9	9	9	9	9	9	1	9	9	9
3	2	1	3	2	1	3	2	1	1
2	2	2	2	2	2	2	2	2	9
9	9	9	9	1	9	9	9	9	1
2	2	1	2	2	2	2	2	2	2
2	3	3	2	3	2	2	1	1	2
3	3	3	3	3	3	3	3	3	3
2	3	3	2	3	2	2	1	1	2
2	3	3	1	3	2	2	3	3	1
2	2	2	1	1	9	9	1	9	2
3	2	3	3	2	2	9	2	2	1
3	3	2	2	2	2	2	2	3	1
9	9	9	9	9	1	9	9	9	9
3	2	2	2	1	3	2	1	3	9
9	9	9	9	2	9	9	9	9	9
2	2	2	2	2	1	1	1	1	1
3	2	3	2	2	2	1	3	3	2
3	3	3	3	2	3	3	3	3	1
3	3	3	3	2	1	2	3	2	3
3	3	3	2	2	2	1	2	3	1
9	9	9	9	9	9	1	9	9	1
3	3	3	3	3	3	9	1	3	3
9	3	2	2	3	2	2	3	2	1
9	9	9	9	9	9	9	1	9	1
9	9	9	9	9	9	1	9	9	1
1	1	1	1	1	2	2	2	2	2
9	9	9	9	9	9	2	9	9	9
2	1	2	9	1	1	1	2	1	2
9	9	9	9	9	9	9	9	9	9
9	9	9	9	9	9	1	9	9	9
3	3	3	3	3	3	3	1	3	3
3	2	3	2	2	3	2	1	3	2
2	3	1	2	9	9	9	9	9	9
1	2	9	9	9	9	1	9	9	9
2	1	1	1	2	1	1	2	2	2
2	3	2	3	9	9	1	9	9	9
1	3	1	2	2	1	9	9	9	1
1	1	1	1	1	1	1	1	1	1
3	3	3	3	3	3	3	1	3	2
2	2	2	2	2	2	2	2	2	2
9	9	9	9	9	9	1	9	9	9
3	3	3	3	9	9	9	9	3	1
2	3	1	3	9	9	9	1	9	1
3	3	3	3	3	3	2	2	2	1
2	3	3	1	2	3	1	2	2	2
3	1	2	3	2	2	3	2	2	2
1	1	1	3	3	3	3	3	3	1

9	9	1	9	9	9	9	2	9	9
2	2	2	2	2	2	2	2	2	1
2	1	1	2	3	1	1	1	1	2
3	3	3	3	2	2	2	2	2	1
1	2	1	1	2	2	2	2	2	2
9	9	9	9	9	9	9	9	9	9
9	9	9	9	9	9	9	9	9	9
2	2	2	2	9	9	1	9	9	1
2	2	2	2	1	2	1	2	2	2
2	2	2	2	9	9	2	9	9	9
2	2	2	2	2	2	2	2	2	3
2	2	2	2	2	2	2	2	2	9
3	3	2	3	2	1	1	3	3	2
2	2	2	2	2	2	2	2	2	2
2	2	2	2	3	3	1	2	3	3
2	9	2	2	2	9	2	9	2	2
9	9	2	9	2	9	9	2	9	2
9	9	9	9	9	9	9	9	9	9
9	9	9	9	1	9	9	9	9	1
2	9	2	2	2	9	2	9	9	2
9	9	9	9	2	9	9	9	9	2
9	9	9	9	9	9	9	9	9	9
2	2	2	2	2	2	2	2	2	2
9	9	9	9	2	9	9	9	9	2
9	9	9	9	9	9	9	9	9	9
9	9	9	9	3	9	9	9	9	3
9	9	9	9	9	9	9	9	9	9
1	1	1	1	1	1	1	1	1	1
9	9	3	3	2	9	9	2	3	2
9	9	9	3	3	9	9	9	3	3
9	9	9	9	3	9	9	9	9	3
2	2	2	2	2	2	2	2	2	2
1	1	1	1	1	1	1	1	1	1
9	9	9	9	3	9	9	9	9	3
2	2	2	2	2	2	2	2	2	2
9	9	9	9	9	9	9	9	9	9
9	9	9	9	2	9	9	9	9	2
9	9	9	9	9	9	9	9	9	9
9	2	9	3	1	9	2	9	3	3
9	9	9	9	1	9	9	9	9	1
2	2	2	2	2	2	2	2	2	2
9	9	9	9	2	9	9	9	9	2
9	9	9	9	2	9	9	9	9	2
9	2	9	9	9	1	9	9	2	2
2	2	2	2	2	2	2	2	2	2
2	2	2	2	2	2	2	2	2	2
9	9	9	9	9	9	9	9	9	9

1	1	1	1	1	1	1	1	1	1
2	2	2	2	2	2	2	2	2	2
9	9	9	1	2	9	9	1	2	1
3	1	2	2	2	2	1	3	3	2
9	9	9	9	2	9	9	9	9	2
9	9	9	9	2	9	9	9	9	2
9	9	9	9	2	9	9	9	9	2
9	9	9	9	1	9	9	9	9	2
9	9	9	9	9	9	9	9	9	9
9	9	9	9	9	9	9	9	9	9
9	9	9	9	9	9	9	9	9	9
9	9	9	9	9	9	9	9	9	9
9	9	9	9	9	9	9	9	9	9
9	9	9	9	9	9	9	9	9	9
2	2	2	2	2	2	2	2	2	2
2	2	2	2	2	2	2	2	2	2
2	2	2	2	2	2	2	2	2	2
2	2	2	2	2	2	2	2	2	2
2	2	2	2	2	2	2	2	2	2
2	2	2	2	2	2	2	2	2	2
2	2	2	2	2	2	2	2	2	2
9	9	9	9	2	9	9	9	9	2
9	9	9	9	1	9	9	9	9	9
2	2	2	2	2	2	2	2	2	2
2	2	2	2	2	2	2	2	2	2
2	2	2	2	2	2	1	2	2	1
9	9	9	9	9	9	9	9	9	9
3	3	3	3	2	1	9	2	2	1
9	9	9	9	9	9	9	9	9	9
2	2	2	2	3	3	3	3	3	2
1	1	1	1	1	1	1	1	1	1
1	1	2	1	2	1	2	3	2	2
1	1	1	1	1	1	1	1	1	1
1	1	1	1	1	1	3	3	3	1
1	1	1	1	1	1	1	1	1	1
9	9	9	9	9	9	1	9	9	1
9	9	9	3	3	9	9	9	3	3
2	2	2	2	2	2	2	2	2	2
1	1	1	1	1	1	1	1	1	1
9	9	9	3	3	9	9	9	3	3
9	9	9	9	2	9	9	9	9	2
9	9	9	9	9	9	9	9	9	9
2	2	2	2	2	2	2	2	2	2
9	9	9	9	9	9	1	9	9	9
9	1	9	1	1	9	1	9	1	1
9	9	9	2	2	9	9	9	2	2
9	2	9	2	2	9	2	9	2	2

9	9	9	9	2	9	1	9	9	2
9	9	9	9	9	9	9	9	9	9
9	3	9	1	2	9	9	1	3	2
9	9	9	9	3	9	9	9	9	3
9	9	9	9	9	9	9	9	9	9
9	9	9	9	2	9	9	9	9	2
9	9	9	9	9	1	1	9	9	9
9	9	9	9	2	9	9	9	9	2
9	2	9	2	1	1	9	9	1	9
1	9	9	9	2	1	1	1	2	1
9	9	9	9	2	9	9	9	9	9
9	9	9	9	9	9	9	9	9	9
1	3	3	3	3	3	3	1	3	3
9	9	9	9	9	9	9	9	9	9
3	3	3	3	2	1	1	9	3	2
1	9	2	2	2	2	3	1	1	3
9	1	9	9	9	9	9	9	1	2
9	9	9	1	1	2	2	2	2	2
2	3	3	2	2	2	2	2	2	2
2	2	2	2	2	2	9	2	9	9
3	3	3	2	1	2	2	2	2	1
9	9	9	9	9	1	9	9	9	9
3	3	3	3	3	3	3	1	2	1
2	3	2	1	2	1	2	1	2	1
3	3	3	3	2	2	1	2	2	3
3	3	2	3	2	2	1	2	2	2
3	3	3	3	3	1	3	3	3	1
3	3	3	3	2	1	3	3	3	1
2	3	2	2	2	1	1	2	3	3
2	2	2	2	2	2	2	2	2	1
9	9	9	9	2	9	9	9	9	1
9	9	9	9	1	9	9	9	9	1
3	3	3	3	2	3	2	2	3	1
9	9	2	9	9	9	3	9	9	9
9	9	9	9	2	9	9	9	9	1
9	9	9	9	9	9	9	9	9	9
9	9	9	9	9	9	9	9	9	9
9	9	2	2	9	2	9	9	2	9
9	9	9	9	9	9	9	9	9	9
2	9	2	2	2	9	2	9	2	2
9	2	9	2	2	9	9	2	2	2
2	2	2	2	2	2	2	2	2	2
2	2	2	2	2	2	2	2	2	2
2	2	2	2	2	2	2	2	2	2
2	2	2	2	2	2	2	2	2	2
2	2	2	2	2	2	2	2	2	2
1	2	1	2	2	2	2	2	1	2

2	3	2	2	3	2	1	1	3	2
2	2	2	1	2	2	2	1	3	2
2	2	2	1	9	9	9	1	9	9
3	3	3	3	3	1	3	1	2	1
3	3	3	3	3	3	3	3	3	3
3	3	9	1	2	2	2	1	1	1
2	2	2	1	2	2	1	1	2	2
3	3	3	3	3	3	3	3	3	3
2	2	2	2	2	2	2	2	2	2
2	2	2	2	2	2	2	2	2	2
2	2	2	2	2	2	2	2	2	2
2	2	2	2	2	2	2	2	2	2
3	3	3	3	3	1	3	3	3	1
2	2	3	3	1	1	1	2	3	2
1	1	2	3	2	2	1	2	2	1
2	2	2	2	2	2	2	2	2	2
2	3	2	2	2	3	2	3	2	2
2	2	3	3	2	2	1	2	3	2
3	3	3	2	1	2	2	3	3	2
2	3	2	1	2	2	1	3	3	1
3	3	2	2	2	1	1	1	2	1
1	2	3	2	3	2	2	1	3	3
1	2	2	2	2	1	3	1	2	1
2	1	3	1	3	3	2	1	2	3
3	3	3	3	3	3	1	3	3	1
2	2	2	2	2	3	2	2	2	9
3	3	3	2	1	2	2	2	3	1
3	3	2	2	1	2	3	3	2	2
3	3	2	1	3	1	1	2	3	3
3	3	3	3	1	1	1	1	1	1
3	3	3	1	3	3	3	1	2	1
2	1	1	2	2	1	2	2	2	2
3	3	3	3	3	3	3	3	3	3
3	3	3	2	2	2	2	2	2	2
1	9	9	9	9	1	9	9	9	9
3	2	3	3	3	2	1	3	2	2
3	2	2	3	1	1	1	1	1	2
1	1	1	1	1	1	1	1	1	1
3	2	2	3	1	1	1	1	1	2
9	9	9	9	9	9	9	9	9	9
2	2	3	1	3	2	2	2	3	1
9	9	9	9	9	1	9	9	9	9
2	2	3	3	3	3	1	2	3	3
2	2	3	3	3	3	1	2	3	3
2	3	2	2	2	3	2	3	2	2
2	3	2	3	3	3	2	2	2	2
2	2	3	2	2	3	2	3	3	2

1	1	2	2	1	2	2	2	2	2
3	3	3	3	3	3	3	3	3	3
3	1	3	1	2	3	9	1	1	1
3	3	3	3	3	2	2	2	3	1
3	1	2	2	1	2	1	2	3	1
3	3	3	3	3	3	3	3	3	1
2	2	1	9	1	2	2	2	2	3
2	3	1	2	1	2	3	2	1	1
3	3	3	1	3	2	1	1	2	3
3	3	3	3	3	3	3	1	3	2
3	2	3	2	3	2	2	2	2	2
3	3	3	1	3	3	2	2	3	1
2	9	2	2	1	9	1	2	3	1
2	1	2	3	2	2	1	1	1	3
3	2	2	1	2	1	1	1	3	1
2	3	2	2	2	2	2	2	2	1
3	3	2	3	3	3	3	3	3	3
3	3	3	3	2	2	2	2	2	2
2	2	2	2	3	3	3	3	3	2
2	2	2	3	2	9	9	9	9	2
2	1	1	2	2	2	2	1	2	2
1	1	2	1	2	1	2	2	9	1
3	2	3	3	3	2	3	2	3	3
2	2	2	1	1	1	1	2	3	3
3	3	3	3	3	3	3	3	3	3
3	2	1	2	2	1	3	2	1	1
2	2	2	2	9	9	9	9	1	1
2	2	2	2	3	3	3	3	3	2
2	2	2	2	2	3	2	2	2	2
2	2	2	2	2	2	2	2	2	2
1	3	2	3	2	1	2	2	1	1
9	9	9	9	3	9	9	9	9	2
3	2	3	2	3	1	2	2	2	1
3	3	3	3	2	2	2	2	2	1
3	3	2	1	1	1	2	2	3	1
3	3	2	1	3	1	2	3	2	1
3	3	3	3	1	1	3	3	3	3
2	3	1	1	2	3	2	2	3	2
3	3	3	3	3	3	1	2	2	3
3	2	3	2	2	1	2	2	2	1
1	3	3	1	3	1	3	3	3	1
2	2	2	2	2	2	2	2	2	2
2	2	2	2	2	2	2	2	2	2
2	2	2	2	2	2	2	2	2	2
3	2	3	2	3	1	2	2	2	1
3	3	3	2	2	1	1	1	2	2
3	3	3	2	2	2	2	2	2	1

3	2	2	2	2	2	2	2	2	3
3	3	2	3	9	1	2	2	2	1
3	3	3	2	2	2	2	2	2	1
3	3	3	3	2	2	1	3	3	2
9	9	9	9	9	9	9	9	9	9
3	3	2	3	3	1	2	2	2	1
3	3	2	3	3	1	2	2	2	1
2	3	2	1	2	2	2	2	2	2
3	2	2	1	2	2	2	2	2	2
9	9	9	9	9	9	9	9	9	9
3	3	2	2	3	2	1	3	2	1
3	3	2	2	3	2	1	3	2	1
1	9	9	9	9	9	9	1	9	9
1	9	9	9	9	9	9	9	9	9
1	2	3	2	3	3	3	2	2	2
9	9	9	1	9	9	1	9	9	9
1	3	1	1	9	9	1	9	9	9
9	9	9	9	9	1	9	9	9	1
9	9	9	9	9	9	9	9	9	9
2	3	2	1	2	2	2	2	1	2
3	2	3	1	1	3	1	3	3	1
9	9	9	9	9	9	9	9	1	9
2	1	1	1	2	1	2	3	1	1
2	2	2	1	1	2	2	2	2	1
2	2	2	2	3	3	1	3	3	3
2	2	2	2	2	2	2	2	2	1
2	2	9	1	3	2	3	1	1	3
2	3	2	3	9	1	9	9	9	9
2	2	2	2	2	2	2	2	2	2
2	9	1	2	2	1	3	2	1	2
9	9	9	9	9	9	9	9	9	9
3	9	2	1	1	2	1	2	2	1
3	3	3	3	3	3	2	2	2	1
2	2	2	9	3	3	1	3	3	2
1	2	2	1	2	2	2	2	2	2
2	2	2	2	2	2	2	2	2	1
9	9	9	9	9	9	9	1	9	9
2	3	2	2	2	2	2	2	2	2
3	3	2	2	2	2	2	2	2	2
1	1	1	1	1	1	1	1	1	2
1	2	2	2	3	3	2	1	1	1
2	3	3	3	2	2	2	2	2	2
1	1	1	1	1	1	1	2	3	1
9	9	9	9	1	9	9	9	9	9
3	3	3	3	3	2	3	3	2	2
9	9	9	9	9	9	9	9	9	9
3	2	3	3	3	2	2	2	3	3

2	2	1	3	2	2	1	2	3	1
2	2	2	2	2	2	2	2	2	2
3	2	1	2	3	3	3	3	3	2
1	2	3	2	2	2	3	2	3	1
3	2	3	2	2	3	1	3	2	1
1	1	9	1	3	3	3	3	3	2
9	9	9	9	9	9	1	9	9	9
3	3	3	3	3	3	3	3	3	3
3	3	3	9	2	3	2	3	9	9
2	2	1	3	2	2	3	1	2	2
2	2	2	2	2	2	2	2	2	2
3	3	3	3	3	3	3	1	3	9
2	1	1	2	2	2	1	1	3	2
3	3	3	3	1	9	9	9	9	1
9	9	9	9	9	9	1	9	9	1
9	9	1	9	1	9	9	9	9	9
2	2	3	3	2	1	1	3	3	1
3	1	1	1	3	3	1	3	3	1
3	3	3	2	3	2	1	2	3	1
3	3	3	3	1	2	3	3	3	1
2	2	2	2	2	2	2	2	2	2
3	3	3	1	1	2	3	2	3	1
2	2	2	2	2	2	2	2	2	2
2	2	2	2	2	2	2	2	2	2
2	2	2	2	1	3	3	3	3	1
2	2	2	2	2	2	2	2	2	2
3	3	3	3	2	2	2	2	2	2
9	9	9	9	2	9	9	9	9	2
3	3	3	3	2	2	2	2	2	2
2	2	2	2	2	2	2	2	2	2
1	1	1	1	1	1	1	1	1	1
9	9	9	9	1	2	1	2	3	3
2	1	2	1	2	2	2	9	1	1
2	2	2	2	2	2	2	2	2	1
3	2	2	2	3	3	2	3	3	2
3	2	2	2	2	3	1	2	2	2
1	1	1	1	2	2	2	2	2	1
3	3	9	3	1	3	3	3	3	1
3	2	2	3	1	2	3	2	3	3
3	3	3	2	1	2	3	3	3	1
9	9	9	1	9	9	1	9	9	1
2	2	2	2	1	2	1	2	2	1
2	2	2	2	3	2	1	2	2	1
3	3	3	3	2	2	3	3	3	1
3	3	3	3	3	3	3	3	1	3
1	2	3	3	1	3	2	2	3	1
9	9	9	9	3	9	9	9	9	3

3	3	9	2	3	3	1	3	2	2
3	2	2	1	2	1	3	2	3	3
3	2	3	2	2	3	2	3	2	3
3	3	3	2	1	2	2	2	3	1
3	2	1	2	1	2	2	2	1	1
2	1	2	3	3	2	1	1	1	1
2	3	3	1	3	2	1	3	2	3
9	9	9	9	1	9	9	9	9	1
9	9	9	9	1	9	9	9	9	1
9	9	9	9	1	9	9	9	9	9
2	2	3	1	2	2	2	1	2	2
3	3	9	9	3	9	9	9	9	3
3	3	3	1	3	2	2	1	2	1
9	9	9	9	1	9	9	9	9	2
2	2	2	2	1	2	2	2	2	2
9	9	9	9	9	9	9	1	9	9
1	2	1	2	3	1	1	3	3	3
1	9	9	9	9	9	1	9	9	9
1	9	9	9	9	1	9	9	9	9
2	9	9	9	9	9	2	9	9	9
1	1	1	1	1	1	1	1	1	1
3	3	3	3	2	2	1	1	3	2
2	2	2	2	2	3	2	2	2	2
3	2	3	3	3	2	2	3	2	1
2	3	3	3	1	3	3	3	3	2
3	3	3	2	2	1	3	3	3	1
3	1	3	3	3	3	3	3	1	3
9	9	9	9	9	9	9	1	9	9
2	1	2	2	2	2	2	2	2	2
3	9	3	3	9	9	9	9	9	1
1	1	3	1	3	3	3	3	1	3
9	9	9	9	1	9	9	9	9	1
3	3	3	3	3	3	3	3	3	3
9	9	9	1	2	9	9	9	9	2
9	9	9	9	1	9	9	9	9	1
2	2	2	2	1	1	2	1	2	1
9	9	9	9	9	9	9	9	9	1
3	1	1	9	1	1	1	1	1	2
3	3	3	3	3	2	2	3	3	1
2	2	2	2	2	2	2	2	2	2
2	2	2	2	2	2	2	2	2	2
2	2	2	2	2	2	2	2	2	2
3	3	3	3	3	3	3	3	3	3
2	2	2	2	9	2	9	9	2	9
1	1	1	1	2	2	2	2	2	1
1	1	1	1	2	2	2	2	2	2
1	3	1	1	1	3	1	3	1	1

3	2	1	1	1	9	3	1	9	3
9	2	2	2	2	2	2	2	2	1
9	2	9	2	9	2	2	2	9	9
9	9	9	9	9	9	9	9	9	9
3	2	1	1	1	9	2	1	9	1
1	2	1	1	1	3	1	3	1	1
9	2	2	9	9	2	9	2	2	3
1	9	2	9	9	1	3	2	1	3
2	9	2	2	9	2	2	2	2	1
1	1	1	1	2	2	2	2	2	1
2	2	2	2	2	2	2	2	2	2
9	9	2	2	2	9	9	2	9	2
2	2	2	2	2	2	2	2	2	2
3	3	3	3	3	3	3	3	3	3
2	2	2	2	2	2	2	2	2	2
9	9	9	9	9	9	9	9	9	9
9	2	9	2	2	9	2	9	2	9
2	2	2	2	2	2	2	2	2	2
2	9	2	1	3	1	9	3	1	9
2	2	2	2	2	2	2	2	2	2
2	2	2	2	2	2	2	2	2	2
2	2	2	2	2	2	2	2	2	2
9	2	1	3	2	2	1	9	1	2
9	1	2	9	2	1	1	3	1	1
2	2	3	2	3	2	2	2	3	3
3	3	3	3	3	3	3	3	3	3
3	2	1	1	1	2	3	2	1	2
3	2	2	1	2	2	1	2	2	1
3	3	1	2	1	3	1	2	1	1
2	3	2	2	2	2	1	2	3	1
3	2	2	2	2	2	3	2	3	2
2	2	3	2	9	9	9	9	9	2
1	2	2	2	3	3	1	1	1	1
9	3	3	3	3	1	9	9	9	2
3	3	3	3	1	1	1	3	3	2
1	1	1	2	1	9	2	1	2	1
3	3	3	3	3	1	3	3	3	1
2	2	2	1	2	2	1	2	2	2
2	2	3	2	1	2	2	2	3	2
3	2	3	3	3	3	2	2	3	3
1	3	1	1	3	3	3	3	1	1
2	3	2	3	3	9	1	2	3	1
3	3	2	2	3	2	2	1	2	3
9	9	9	9	9	9	9	9	9	9
9	9	9	9	1	2	2	1	2	1
2	2	9	2	2	2	2	2	2	1
1	3	1	1	9	9	1	9	9	9

1	1	2	2	3	2	3	1	1	3
2	2	2	2	9	9	9	9	9	9
1	1	1	1	2	2	2	2	2	1
2	2	2	2	2	2	2	2	2	2
1	1	2	2	1	1	1	1	1	1
2	2	2	2	2	2	2	2	2	2
1	2	2	2	1	1	1	9	9	2
3	3	3	3	3	1	3	3	3	1
3	3	3	3	3	3	3	3	3	3
1	1	2	2	1	1	1	1	1	1
1	1	1	2	1	1	1	1	2	1
1	1	1	1	9	1	9	2	2	1
1	1	1	1	9	1	2	2	2	2
2	2	2	1	9	1	2	2	2	1
1	1	2	2	2	2	1	1	9	2
2	1	1	1	9	2	2	2	2	1
1	1	1	1	1	2	1	1	2	1
2	2	1	1	1	1	1	2	2	1
1	1	1	1	9	1	2	2	2	1
2	2	2	2	2	2	2	2	2	2
1	2	1	2	1	1	1	1	1	2
2	2	2	2	2	2	2	2	2	2
9	9	9	9	9	9	9	9	9	9
1	1	1	1	9	1	2	2	2	1
1	1	1	1	1	1	1	1	1	1
2	1	1	1	1	2	1	1	2	2
1	1	1	1	1	2	1	2	1	1
1	1	1	2	1	1	1	2	2	1
2	9	2	2	2	9	2	2	2	1
1	1	1	1	1	1	1	1	1	1
2	2	2	2	2	2	2	2	2	2
2	2	2	2	2	2	2	2	2	2
1	1	1	1	1	1	1	1	1	1
1	1	1	1	1	1	1	1	1	2
1	1	1	1	2	2	2	2	2	2
2	2	2	2	2	2	2	2	2	2
2	2	1	1	1	1	1	1	1	1
1	1	1	1	1	1	2	2	1	1
9	9	9	9	9	9	9	9	9	9
2	2	2	2	2	2	9	2	2	2
2	2	2	2	2	2	2	2	2	2
2	2	2	2	2	2	2	2	2	2
1	1	1	1	9	1	2	2	2	2
2	2	2	2	2	2	2	2	2	2
1	1	2	1	1	1	1	2	1	1
2	2	2	2	2	2	2	2	2	2
1	1	1	1	9	1	9	9	9	2

1	1	1	2	2	2	2	2	2	2
2	2	2	2	2	2	2	2	2	2
1	1	1	1	9	1	2	2	2	1
1	1	1	9	1	1	2	2	1	1
1	1	1	1	1	1	1	1	1	1
2	2	2	2	2	2	2	2	2	2
1	1	1	1	1	1	1	1	1	1
1	2	2	1	9	1	2	2	2	1
2	9	2	2	2	9	2	2	2	9
1	1	1	1	1	1	1	1	1	1
2	9	2	2	2	2	2	9	2	9
1	9	1	1	9	1	1	1	1	1
2	2	2	2	2	9	2	2	2	9
1	1	1	1	1	1	1	1	1	1
1	1	1	1	9	1	9	9	9	1
2	2	2	2	2	2	2	2	2	2
1	1	1	1	2	1	2	2	2	1
9	9	9	9	9	9	9	9	9	9
2	2	2	2	2	2	2	2	2	2
3	3	3	3	3	2	1	3	3	1
3	3	3	3	3	2	1	3	3	1
3	3	3	3	3	2	1	3	3	1
3	3	3	3	3	2	1	3	3	1
3	3	3	3	3	2	1	3	3	1
3	3	3	3	3	2	1	3	3	1
3	3	3	3	2	2	1	3	3	1
3	3	3	3	3	2	1	3	3	1
9	9	9	9	3	2	1	9	3	1
3	3	3	3	3	2	1	3	3	1
3	3	3	3	3	2	1	3	3	1
3	9	3	3	3	2	1	3	3	1
3	9	3	3	3	2	1	3	3	1
3	3	3	3	3	3	2	1	3	1
3	9	3	3	3	2	1	3	3	1
3	3	3	3	3	2	1	3	3	1
3	3	3	3	3	2	1	3	3	1
3	3	3	3	3	2	1	3	3	1
3	3	3	3	3	2	1	3	3	1
3	3	3	3	3	2	1	3	3	1
3	3	3	3	3	2	1	3	3	1
3	3	3	3	3	2	1	3	3	1
3	3	3	3	3	2	1	3	3	1
3	3	3	3	3	2	1	3	3	1
3	3	3	3	3	2	1	3	3	1
9	9	9	9	9	9	9	9	9	9
9	9	9	9	9	9	9	9	9	9
9	9	9	9	9	9	9	9	9	9

9	9	9	9	9	9	9	9	9	9
2	2	3	3	3	2	1	2	2	9
9	9	9	9	9	9	9	9	9	9
9	9	9	9	9	9	9	9	9	9
9	9	9	9	9	9	9	9	9	9
9	9	9	9	9	9	9	9	9	9
9	9	9	9	9	9	9	9	9	9
9	9	9	9	9	9	9	9	9	9
9	9	9	9	9	9	9	9	9	9
9	9	9	9	9	9	9	9	9	9
9	9	9	9	9	9	9	9	9	9
9	9	9	9	9	9	9	9	9	9
2	2	2	1	1	2	2	2	3	1
9	9	9	9	9	9	9	9	9	9
9	9	9	9	9	9	9	9	9	9
9	9	9	9	9	9	9	9	9	9
1	1	1	1	1	2	1	1	3	9
9	9	9	9	9	9	9	9	9	9
9	9	9	9	9	9	9	9	9	9
3	3	3	3	3	2	1	3	3	1
3	3	3	3	3	2	1	3	3	1
1	3	3	3	3	2	1	3	3	1
3	3	3	3	3	2	1	3	3	1
3	3	3	3	3	2	1	3	3	1
3	3	3	3	3	2	1	3	3	1
3	3	3	3	3	2	1	3	3	1
3	3	3	3	3	2	1	3	3	1
3	3	3	3	3	2	1	3	3	1
3	3	3	3	3	2	1	3	3	1
3	3	3	3	3	1	3	3	3	1
3	3	3	3	3	2	1	3	3	1
3	3	3	3	3	3	1	3	3	1
3	3	3	3	3	2	1	3	3	1
3	3	3	9	3	2	1	3	3	1
3	3	3	3	3	3	1	3	3	1
9	9	9	9	3	9	9	9	9	1
3	3	3	3	3	3	1	3	3	1
3	3	3	3	3	3	1	3	3	1
3	3	3	3	3	3	1	3	3	1
3	3	3	3	3	2	1	3	3	1
3	3	3	9	3	9	1	3	3	1
3	3	3	3	3	2	1	3	3	1
3	3	3	3	3	2	1	3	3	1
3	3	3	3	3	2	1	3	3	1
3	3	3	3	3	3	1	3	3	1
3	3	3	3	3	2	1	3	3	1
3	3	3	3	3	2	1	3	3	1

3	3	3	3	3	2	1	3	3	1
3	3	3	3	3	2	1	3	3	1
3	3	3	3	3	2	1	3	3	1
3	3	3	3	3	2	1	3	3	1
3	3	3	3	3	2	1	3	3	3
3	3	3	3	3	2	1	3	3	1
3	3	3	3	3	2	1	3	3	1
3	3	3	3	3	3	2	3	3	1
3	3	3	3	3	3	2	3	3	1
3	3	3	3	3	2	1	3	3	1
3	3	3	3	3	2	1	3	3	1
3	3	3	3	3	2	1	3	3	1
3	3	3	3	3	2	1	3	3	1
3	3	3	3	3	3	1	3	3	1
3	3	3	3	3	3	1	3	3	1
3	3	3	3	3	2	1	3	3	1
3	3	3	3	3	3	1	3	3	1
3	3	3	3	3	2	1	3	3	1
3	3	3	3	9	2	1	3	3	1
3	3	3	3	3	2	1	3	3	1
3	3	3	3	3	2	1	3	3	1
3	3	3	3	3	2	1	3	3	1
3	3	3	3	3	3	1	3	3	1
3	3	3	3	3	3	1	3	3	1
3	3	3	3	3	2	1	3	3	1
3	3	3	3	3	3	1	3	3	1
3	3	3	3	3	2	1	3	3	1
3	3	3	3	1	3	1	3	3	1
3	3	3	3	3	3	1	3	3	1
3	3	3	3	3	3	1	3	3	1
3	3	3	3	3	2	1	3	3	1
3	3	3	3	3	2	1	3	3	1
3	3	3	3	9	9	9	9	9	1
3	3	3	3	3	2	1	3	3	1
3	3	3	3	2	3	1	3	3	9
3	3	3	3	3	2	1	3	3	1
3	3	3	3	3	3	1	3	3	1
3	3	3	3	3	3	1	3	3	1
3	3	3	3	3	3	3	3	3	1
3	3	3	3	3	2	1	3	3	1
3	3	3	3	1	2	1	3	3	1
3	3	3	3	3	2	1	3	3	1
3	3	3	3	3	3	1	3	3	1
3	3	3	3	3	3	1	3	3	1
3	3	3	3	3	2	1	3	3	1
3	3	3	3	3	2	1	3	3	1
3	3	3	3	3	2	1	3	3	1

3	3	3	3	3	2	1	3	3	1
3	3	3	3	3	2	1	3	3	1
3	3	3	3	3	2	1	3	3	1
3	3	3	3	3	2	1	3	3	1
3	3	3	3	3	2	1	3	3	1
3	3	3	3	3	2	1	3	3	1
3	3	3	3	3	2	1	3	3	1
3	3	3	3	3	2	1	3	3	1
3	3	3	3	3	2	1	3	3	1
3	3	3	3	3	2	1	3	3	1
3	3	3	3	3	2	1	3	3	1
3	3	3	3	3	2	1	3	3	1
3	3	3	3	3	2	1	3	3	1
1	3	3	3	3	2	3	3	3	1
3	3	3	3	3	3	1	3	3	1
3	3	3	3	3	2	1	3	3	1
3	3	3	3	3	2	1	3	3	1
3	3	3	3	3	2	1	3	3	1
3	3	3	3	3	2	1	3	3	1
9	9	9	9	9	9	9	9	9	9
3	3	3	3	3	3	1	3	3	1
3	3	3	3	3	2	1	3	3	1
3	3	3	3	3	2	1	3	3	1
3	3	3	3	3	2	1	3	3	1
3	3	1	9	9	9	9	9	9	1
3	3	3	3	3	2	1	3	3	1
3	3	3	3	3	2	1	3	3	1
3	9	3	3	3	2	1	3	3	1
3	3	3	3	3	2	1	3	3	1
3	3	3	3	3	2	1	3	3	1
3	3	3	3	3	2	1	3	3	1
3	3	3	3	3	2	1	3	3	1
3	3	3	3	3	3	1	3	3	1
3	3	3	3	3	3	1	3	3	1
3	3	3	3	3	2	1	3	3	1
3	3	3	3	3	2	1	3	3	1
3	3	3	3	1	2	1	3	3	1
3	3	3	3	3	2	1	3	3	1
3	3	3	3	3	2	1	3	3	1
3	3	3	3	3	2	1	3	3	1
3	3	3	3	3	2	1	3	3	1
9	9	9	9	9	9	9	9	9	9
3	3	3	3	3	2	1	3	3	1
3	3	3	3	3	2	1	3	3	1
3	3	3	3	3	2	1	3	3	1
3	3	3	3	3	2	1	3	3	1
3	3	3	3	3	2	1	3	3	1

3	3	3	3	3	2	1	3	3	1
3	3	3	3	3	2	1	3	3	1
3	3	3	3	3	2	1	3	3	1
3	3	3	3	3	2	1	3	3	1
3	3	3	3	3	2	1	3	3	1
3	3	3	3	3	2	1	3	3	1
3	3	3	3	3	2	1	3	3	1
3	3	3	3	3	9	1	3	3	1
3	3	3	3	3	3	1	3	3	1
3	3	3	3	3	2	1	3	3	1
3	3	3	3	3	2	1	3	3	1
3	3	3	3	3	2	1	3	3	1
3	3	3	3	3	2	1	3	3	1
3	3	3	3	3	2	1	3	3	1
3	3	3	3	3	2	1	3	3	1
3	3	3	3	3	2	1	3	3	1
3	3	3	3	3	2	1	3	3	1
3	3	3	3	3	2	1	3	3	1
3	3	3	3	3	2	1	3	3	1
3	3	3	3	3	2	1	3	3	1
3	3	3	3	3	2	1	3	3	1
3	3	3	3	3	2	1	3	3	1
3	3	3	3	3	2	1	3	3	1
3	3	3	3	3	2	1	3	3	1
3	3	3	3	3	2	1	3	3	1
3	3	3	3	3	2	1	3	3	1
3	3	3	3	3	2	1	3	3	3
2	9	9	9	9	9	9	9	9	9
2	9	9	9	9	9	9	9	9	9
2	9	9	9	9	9	9	9	9	9
2	9	9	9	9	9	9	9	9	9
9	9	9	9	9	9	9	9	9	9
2	2	2	2	2	2	9	9	9	9
9	9	9	9	9	9	9	9	9	9
9	9	9	9	9	9	9	9	9	9
9	9	9	9	9	9	9	9	9	9
9	9	9	9	9	9	9	9	9	9
9	9	9	9	9	9	9	9	9	9
9	9	9	9	9	9	9	9	9	9
9	9	9	9	9	9	9	9	9	9
9	9	9	9	9	9	9	9	9	9
3	3	9	9	9	9	9	9	9	9
1	3	3	1	3	2	1	2	3	1
1	1	1	1	1	1	1	1	1	1
9	9	9	9	9	9	9	9	9	9
9	9	9	9	9	9	9	9	9	9
9	9	9	9	9	9	9	9	9	9

9	9	9	9	9	9	9	9	9	9
3	3	3	9	9	9	9	9	9	9
9	9	9	9	9	9	9	9	9	9
9	1	9	9	9	9	9	1	9	1
9	9	9	9	9	9	9	9	9	9
1	9	9	9	9	2	9	9	9	9
3	3	1	2	1	1	1	1	3	1
2	2	2	1	3	3	1	3	3	2
9	9	9	9	9	9	9	9	9	9
9	9	9	9	9	9	9	9	9	9
9	9	9	9	9	9	9	9	9	9
9	9	9	9	9	9	9	9	9	9
9	9	9	9	9	9	9	9	9	9
3	1	1	3	3	1	1	2	1	9
2	2	2	2	2	2	2	2	2	2
3	3	3	3	2	2	1	2	2	2
3	1	3	1	3	3	2	1	1	3
3	3	3	3	1	3	2	2	3	1
3	3	9	1	2	3	2	3	3	1
3	1	3	1	1	3	1	1	3	1
1	3	3	1	2	1	2	1	3	2
3	3	3	3	1	1	1	1	1	1
2	3	2	2	3	2	3	2	3	1
3	3	3	3	3	3	3	3	3	3
9	9	9	9	9	9	1	9	9	2
2	2	2	2	2	2	2	2	2	2
2	2	2	2	2	2	2	2	2	3
1	1	1	3	3	1	3	3	3	1
3	3	9	1	2	3	1	3	2	1
2	2	2	2	2	2	2	3	3	1
3	3	3	3	3	3	3	3	3	2
3	1	2	1	1	2	3	1	2	1
9	9	9	9	9	9	1	9	9	9
2	3	2	2	3	2	2	2	2	3
9	9	9	9	1	9	9	9	9	9
9	9	9	9	9	9	9	1	9	2
1	2	2	1	2	2	2	1	2	1
9	3	3	1	3	3	2	1	2	3
9	9	9	1	9	1	9	9	9	1
9	9	9	9	1	2	2	2	2	1
9	9	9	9	9	9	9	9	9	9
1	3	3	1	1	9	1	2	3	1
1	2	2	1	3	3	1	3	3	2
3	3	3	3	9	9	9	9	9	1
9	9	9	1	1	1	1	1	9	9
2	2	2	1	9	9	9	1	9	2
2	2	2	2	2	2	2	2	2	2

9	9	9	9	2	9	9	9	9	9
9	9	9	9	9	9	9	9	9	9
3	3	3	3	2	3	1	2	2	1
2	2	2	1	1	1	1	3	3	1
9	9	9	9	1	9	9	9	9	2
9	9	9	9	9	9	2	9	9	9
2	2	2	2	2	2	1	2	2	2
2	2	2	2	2	2	2	2	2	2
2	2	2	2	2	2	2	2	2	2
1	1	1	1	1	2	2	2	2	2
1	1	2	2	2	2	2	2	2	2
9	9	9	9	9	9	9	9	9	9
9	9	9	9	9	9	1	9	9	1
2	2	2	2	2	2	2	2	2	2
3	2	2	2	2	2	2	3	3	1
3	3	3	3	2	2	2	3	2	3
3	3	3	3	1	1	1	1	1	1
2	3	2	1	3	9	1	2	1	1
9	1	2	1	1	2	1	1	1	1
9	9	9	9	2	1	3	2	3	1
3	3	2	2	2	2	2	2	2	2
2	2	2	2	2	2	2	2	2	2
2	2	2	2	2	2	2	2	2	2
2	2	2	2	3	2	1	2	2	2
2	2	2	2	2	2	2	2	2	3
1	1	2	3	3	2	2	2	9	1
2	3	2	2	3	3	2	2	2	3
3	3	2	2	2	2	2	2	2	3
3	2	2	2	2	2	2	2	2	1
1	1	1	2	1	1	1	2	2	2
3	3	3	3	1	3	3	3	3	3
3	3	3	3	3	1	2	3	3	2
2	2	2	1	2	1	1	1	1	1
2	2	2	2	3	2	2	1	1	1
3	2	1	1	2	2	2	2	2	2
9	9	9	9	9	9	9	9	9	3
2	2	2	2	2	3	2	2	2	3
2	2	9	9	3	3	3	3	3	3
9	9	9	9	9	9	9	9	9	2
3	3	3	3	1	3	3	3	3	1
3	2	2	3	3	2	2	2	3	3
2	3	3	2	2	2	2	2	2	1
3	1	2	3	1	2	3	1	2	1
3	3	9	3	2	3	3	3	3	3
1	3	3	1	1	1	3	3	3	1
2	2	2	2	1	1	1	1	1	1
2	2	2	2	1	2	2	2	2	1

2	2	2	2	2	2	2	2	3	2
2	3	2	3	3	2	3	2	3	1
2	2	2	2	1	1	1	1	1	2
9	9	9	9	9	9	9	9	9	2
3	3	3	3	2	3	3	3	3	2
2	2	2	2	1	2	2	1	2	1
2	2	3	2	1	2	2	3	2	2
2	2	2	2	2	1	2	2	2	2
2	2	2	2	2	2	2	2	2	2
1	3	2	3	1	3	2	3	2	1
2	2	2	2	2	2	2	2	2	2
2	2	2	2	2	2	2	2	2	2
1	1	1	1	2	2	2	2	2	1
2	2	2	2	2	2	2	2	2	2
9	9	9	9	9	9	9	9	9	2
2	2	2	2	1	2	2	2	2	2
3	3	3	1	3	3	3	3	3	3
2	2	2	2	2	2	2	2	2	2
2	2	2	2	2	2	2	2	2	2
3	1	2	1	1	2	3	1	2	1
1	3	3	3	2	2	2	2	2	2
1	1	2	1	2	2	2	1	1	1
9	9	9	9	9	9	9	9	9	2
9	9	9	9	9	9	9	9	9	1
3	3	3	3	3	3	3	3	3	3
1	1	1	1	2	1	1	2	1	9
2	3	1	1	1	1	2	3	3	2
3	3	3	3	1	3	3	3	3	3
3	1	1	1	1	3	1	3	2	1
3	3	2	2	1	2	2	2	2	1
3	2	2	2	1	2	2	2	3	1
9	9	1	9	9	9	9	9	2	2
2	3	3	3	3	2	2	2	3	2
3	2	1	2	2	2	2	2	2	1
1	1	1	1	9	9	9	1	9	3
9	9	9	9	9	9	9	9	9	9
1	2	3	1	1	1	3	3	3	1
2	1	2	3	2	2	2	2	2	1
2	3	2	3	3	1	2	1	1	2
2	2	2	2	2	2	2	2	2	2
2	3	3	2	2	1	3	3	2	1
1	3	1	1	3	2	1	2	3	1
3	2	2	2	1	2	2	2	3	1
2	3	9	9	9	9	9	9	9	9
9	9	9	9	9	9	9	9	9	9
9	9	9	9	9	9	9	9	9	9
1	1	1	1	9	9	9	1	9	3

2	3	2	2	1	2	2	3	3	1
2	3	3	2	2	1	1	3	3	3
3	3	1	2	3	2	2	1	2	3
3	3	3	1	1	3	3	3	3	1
2	2	2	2	2	2	2	1	2	2
3	2	3	2	2	1	3	3	3	1
3	3	3	1	3	3	3	3	3	3
3	2	2	3	3	3	3	3	3	1
1	1	1	1	1	2	1	1	2	1
2	2	2	2	2	3	1	2	1	1
1	3	1	2	1	2	3	1	2	1
2	2	2	2	1	1	3	3	3	2
2	2	2	2	2	2	2	2	2	2
3	3	3	1	1	1	2	3	3	1
2	2	2	2	2	2	2	2	2	2
3	3	3	3	1	2	2	2	2	1
1	1	3	1	3	3	3	1	1	1
1	3	3	1	2	1	3	3	3	3
3	3	3	3	1	1	1	3	3	3
3	2	3	1	9	9	9	9	9	1
3	1	2	3	3	3	2	1	1	3
3	1	2	1	3	1	2	3	3	1
3	3	3	3	1	3	3	3	3	2
3	3	3	1	1	3	3	3	3	3
2	2	3	3	1	3	2	1	3	1
9	9	9	9	9	9	9	9	9	9
2	1	2	1	1	2	2	1	2	2
3	3	3	1	2	2	1	3	3	2
2	2	2	2	2	2	3	3	2	3
1	2	2	3	3	3	1	2	2	2
2	2	2	2	3	2	2	2	2	2
9	9	9	1	9	9	1	9	9	1
3	3	1	2	3	2	1	3	3	3
2	2	3	2	3	3	3	1	1	1
3	1	2	2	1	2	3	3	3	3
1	2	3	2	2	1	2	3	2	2
9	3	9	9	1	2	2	2	2	2
2	2	2	2	3	2	1	1	2	2
2	3	3	3	1	3	3	3	3	1
9	9	9	9	2	9	9	9	9	9
3	1	2	1	1	2	3	2	1	3
2	2	2	2	2	2	2	2	2	2
3	3	3	3	2	2	2	2	2	2
2	2	2	2	2	2	2	2	2	2
2	2	2	2	2	2	2	2	2	2
2	3	9	1	2	2	1	1	1	2
2	3	2	3	2	2	2	1	1	2

3	3	2	3	1	1	2	2	3	1
2	2	2	2	2	2	2	2	2	2
3	3	2	3	3	1	2	3	3	1
3	3	3	2	2	2	1	2	3	2
3	1	3	3	2	2	2	2	2	2
2	3	3	3	2	2	2	2	2	3
3	3	2	3	1	1	3	3	3	1
3	3	2	3	2	1	2	2	1	1
3	3	3	1	1	1	3	3	3	1
3	3	3	3	3	3	3	3	3	1
3	3	3	3	1	3	3	3	2	2
2	2	2	2	3	3	3	3	3	1
3	3	3	3	3	3	3	3	3	2
3	3	3	1	3	2	1	1	3	1
2	2	2	2	1	1	1	1	1	2
2	3	2	2	2	2	2	2	2	2
9	9	9	3	9	9	9	1	9	1
2	3	2	2	2	2	2	2	2	1
3	2	2	1	2	2	2	2	2	2
3	2	1	1	2	1	1	1	3	2
2	2	1	1	2	2	1	1	1	1
1	3	2	2	3	3	1	3	3	3
9	9	9	1	9	2	9	9	9	2
2	2	2	2	3	3	1	3	3	3
2	2	2	2	9	9	9	1	9	1
2	2	2	1	2	2	2	2	2	1
2	2	2	3	1	1	3	3	3	1
2	2	2	2	2	2	2	2	2	1
3	3	3	3	3	3	1	3	3	3
1	2	2	1	3	2	1	2	2	2
3	3	3	3	3	3	1	3	3	3
2	2	2	2	1	2	2	2	2	1
2	1	1	1	3	2	2	2	2	1
2	2	2	2	1	2	2	2	2	2
9	9	9	9	2	9	9	9	9	2
3	9	1	3	3	3	1	3	3	3
1	2	2	2	2	2	2	2	2	2
1	1	2	1	1	1	1	1	1	2
1	1	1	1	1	1	1	1	1	1
1	2	2	2	2	2	2	2	2	2
2	3	2	1	1	2	3	2	1	1
9	9	9	9	9	9	9	9	9	9
9	9	9	9	9	9	9	9	9	9
1	1	1	1	1	1	1	1	1	1
3	2	2	3	1	2	3	2	3	1
9	9	9	9	9	9	9	9	9	9
2	2	2	2	1	1	1	1	1	1

2	2	2	2	2	2	2	2	2	2
1	2	2	1	1	1	1	1	1	1
2	3	2	2	1	2	3	1	2	1
3	2	1	2	1	2	3	2	1	1
3	3	3	3	3	3	1	3	3	2
3	1	2	3	1	2	3	1	2	1
1	2	2	2	1	2	2	2	2	1
9	9	9	9	1	9	9	9	9	1
3	3	2	1	1	1	1	2	3	1
9	9	9	9	9	9	9	9	9	9
2	2	2	2	1	1	1	1	1	1
3	2	2	3	2	2	1	3	3	3
2	2	2	2	2	2	1	3	1	1
2	2	2	2	2	2	2	2	2	1
1	1	1	1	1	1	1	1	1	1
9	9	9	9	9	9	9	9	9	9
9	9	9	9	9	9	9	9	9	9
1	1	1	2	2	3	3	3	3	3
2	2	2	2	2	2	2	2	2	2
1	2	2	2	2	9	9	9	9	2
2	2	2	2	2	2	2	2	2	2
2	2	2	2	9	9	9	9	9	2
3	3	3	2	3	3	3	3	1	2
3	2	1	2	2	1	2	3	2	2
9	2	3	2	2	9	1	3	3	1
9	9	9	9	9	9	1	9	9	1
1	9	2	2	2	2	1	2	2	2
2	2	2	2	9	1	9	9	9	2
9	9	9	9	9	9	9	9	9	2
3	3	3	2	3	2	1	2	2	3
2	1	2	1	1	2	1	2	1	1
2	2	2	2	2	2	2	2	2	2
2	2	2	2	2	2	2	2	2	2
2	2	2	2	2	2	2	2	2	2
2	1	2	1	1	2	2	2	2	1
2	2	2	2	2	2	2	2	2	2
2	2	2	2	3	3	3	3	3	3
9	9	9	9	9	9	1	9	9	1
9	9	9	2	9	9	9	9	2	9
2	2	2	2	2	2	2	2	2	2
2	2	2	2	2	2	2	2	2	2
1	9	9	9	9	1	9	9	9	1
1	2	1	2	2	2	3	1	2	1
9	9	9	9	2	9	9	9	9	2
2	2	2	2	2	2	2	2	2	2
1	2	2	1	2	1	2	1	2	1
2	2	2	2	2	2	2	2	2	2

2	1	2	3	3	9	9	9	9	3
9	9	9	9	9	9	1	9	9	1
2	2	2	2	2	2	2	2	2	2
3	2	2	1	3	3	1	2	3	2
2	2	2	2	3	3	3	3	3	2
2	3	3	3	3	3	2	2	1	2
1	2	3	1	2	2	1	1	2	2
3	1	2	3	2	1	2	3	1	1
2	2	2	2	2	2	2	2	2	2
1	1	1	1	9	9	9	9	1	3
3	3	3	1	3	3	3	3	1	9
2	2	2	2	2	2	2	2	2	2
3	2	3	2	3	2	2	2	2	1
2	3	3	3	3	2	2	3	3	2
1	2	2	2	1	2	3	2	2	2
2	3	2	2	1	2	3	2	2	2
2	2	2	2	2	1	2	2	2	2
2	2	2	2	2	2	2	2	2	2
3	3	2	1	3	2	3	1	1	3
3	2	2	2	1	2	3	1	3	1
2	2	2	2	2	1	2	3	3	2
3	3	2	3	2	2	1	2	2	1
3	1	3	2	1	2	1	3	2	2
9	9	9	9	9	9	9	9	9	9
3	3	3	1	2	2	2	2	2	2
9	9	9	9	9	9	9	1	9	1
9	9	9	9	9	9	9	9	1	9
9	9	9	9	9	9	9	1	9	9
2	3	3	3	2	2	2	3	3	1
3	3	3	3	2	2	2	2	2	2
3	3	3	3	2	2	2	2	2	2
1	1	3	9	1	9	9	9	9	1
1	1	3	1	2	2	2	2	2	1
2	2	2	2	2	2	2	2	2	2
2	9	9	9	9	9	1	9	9	9
1	3	2	3	2	2	1	1	2	1
3	3	2	1	1	2	9	3	1	1
1	2	3	3	1	2	1	2	1	1
1	2	2	1	2	1	3	3	3	2
3	3	3	3	3	3	1	3	3	3
3	2	2	2	1	2	3	2	1	1
3	1	2	1	9	9	9	9	1	1
9	9	9	9	9	9	1	9	9	9
9	9	9	9	9	9	1	9	9	9
1	2	1	3	1	3	2	3	1	2
2	2	2	2	2	2	2	2	2	2
2	2	2	2	2	2	2	2	2	2

2	1	3	2	1	2	3	1	2	2
9	9	9	9	9	9	9	9	9	9
9	9	9	9	9	9	9	9	9	9
9	9	9	9	9	9	9	9	9	9
2	2	2	2	9	9	1	9	9	1
9	9	9	1	9	9	1	9	9	9
9	9	9	9	9	9	9	9	9	9
9	9	9	9	9	9	9	9	9	9
9	9	9	9	9	9	9	9	9	9
1	9	9	9	1	9	9	9	9	9
2	1	2	1	1	2	1	2	1	2
2	1	1	2	1	1	1	1	1	1
2	1	2	1	1	1	2	3	3	1
2	3	2	2	2	2	2	2	2	1
2	1	2	1	2	2	2	2	2	1
2	2	2	2	1	2	2	2	2	1
1	1	1	2	1	2	1	9	9	1
2	2	2	2	1	2	2	2	2	1
2	2	3	2	1	1	2	3	3	1
1	2	2	1	3	3	2	9	1	2
2	2	2	3	3	2	1	3	3	2
2	2	2	3	3	2	3	1	3	2
1	1	1	1	3	9	3	3	3	2
2	2	2	2	3	3	3	3	3	2
2	2	2	2	2	2	2	2	2	2
2	2	2	2	2	2	2	2	2	3
2	2	2	2	2	2	2	2	2	2
1	1	2	2	2	1	1	2	2	2
9	9	9	9	9	9	9	9	9	2
2	2	2	2	2	2	2	2	2	2
9	9	9	9	9	9	9	9	9	9
2	2	2	2	2	2	2	2	2	2
1	1	1	1	1	1	1	1	1	1
9	9	9	9	1	9	9	9	9	9
3	3	3	3	3	3	3	3	3	3
1	2	1	1	1	1	1	3	3	1
2	2	2	2	2	2	2	2	2	2
2	2	2	2	2	2	2	2	2	2
2	1	1	3	3	1	3	3	3	1
9	9	9	9	9	9	9	9	9	9
2	3	1	1	2	1	1	2	1	2
2	2	2	2	3	1	3	3	3	1
2	2	2	2	2	2	2	2	2	2
3	3	3	3	2	1	3	3	3	2
3	3	2	2	2	2	1	2	2	2
9	9	9	9	9	9	9	9	9	9
2	2	2	2	2	2	2	2	2	2

9	9	9	9	9	9	9	9	9	9
1	2	1	3	2	2	2	2	2	2
3	3	3	3	1	1	1	9	1	9
9	9	9	9	9	9	9	9	9	9
9	9	9	9	9	9	9	9	9	9
9	9	9	9	9	9	9	9	9	9
9	9	9	9	9	9	9	9	9	9
9	9	9	9	9	9	9	9	9	9
9	9	9	9	9	9	9	9	9	9
9	9	9	9	9	9	9	9	9	9
9	9	9	9	9	9	9	9	9	9
9	9	9	9	9	9	9	9	9	9
9	9	9	9	9	9	9	9	9	9
9	9	9	9	9	9	9	9	9	9
9	9	9	9	9	9	9	9	9	9
9	9	9	9	9	9	9	9	9	9
9	9	9	9	9	9	9	9	9	9
9	9	9	9	9	9	9	9	9	9
9	9	9	9	9	9	9	9	9	9
9	9	9	9	9	9	9	9	9	9
9	9	9	9	9	9	9	9	9	9
9	9	9	9	9	9	9	9	9	9
9	9	9	9	9	9	9	9	9	9
9	9	9	9	9	9	9	9	9	9
3	2	1	3	1	2	3	1	2	1
2	2	2	2	2	2	2	2	2	2
2	2	2	2	1	2	2	2	2	1
3	2	3	3	2	1	1	2	2	2
3	3	3	3	3	3	1	3	3	3
9	9	9	9	9	9	1	9	9	2
2	2	2	2	2	2	2	2	2	2
9	3	3	3	3	3	1	3	3	3
1	1	1	3	9	9	1	9	9	1
2	2	2	2	3	3	1	2	2	1
3	3	3	3	1	3	3	3	3	1
9	9	9	9	9	9	1	9	9	9
2	3	2	3	1	1	3	3	3	3
1	1	1	1	3	3	1	3	3	3
3	2	2	2	1	1	2	2	2	2
2	3	2	3	1	1	3	3	3	3
9	9	9	9	9	9	1	9	9	9
3	3	3	3	1	2	3	2	3	1
3	3	1	3	3	3	1	3	3	1
9	2	9	9	3	9	1	9	9	1
1	3	3	3	3	3	1	3	1	3
3	3	3	3	3	3	1	3	3	3
2	2	2	2	1	2	3	2	3	2
2	2	2	2	2	2	1	2	2	3

1	1	1	1	1	2	2	1	3	1
3	3	3	1	3	1	1	1	1	3
3	3	3	3	9	9	1	9	9	3
3	3	3	1	3	2	2	3	1	3
2	1	2	3	2	1	1	9	2	3
9	9	9	9	1	9	9	9	9	1
2	9	2	3	1	1	2	3	3	3
9	9	9	9	9	9	9	9	1	1
9	9	9	9	2	9	9	9	9	1
9	9	9	9	2	9	9	9	9	1
9	9	9	9	9	9	1	9	9	1
9	9	9	9	9	9	1	9	9	9
9	9	9	9	9	9	9	1	9	9
9	9	9	9	9	9	9	1	9	9
9	9	9	9	9	1	9	9	9	9
3	3	1	3	3	3	3	1	3	3
3	3	3	3	3	3	3	3	3	3
2	2	3	3	2	2	2	2	2	2
1	9	9	9	9	9	9	1	9	9
1	3	3	3	1	3	3	3	3	3
9	9	9	9	9	9	1	9	9	9
3	3	1	2	3	3	1	3	3	1
9	9	9	9	9	9	1	9	9	9
9	9	9	9	9	1	9	9	9	1
9	9	9	9	9	9	1	9	9	9
1	2	3	3	1	2	3	2	1	1
1	1	3	3	3	3	1	1	3	1
3	3	3	2	1	2	3	2	3	1
3	3	3	2	3	1	2	2	3	1
2	2	2	2	2	2	2	2	2	2
3	3	1	1	2	2	9	2	1	3
3	3	3	1	1	3	1	9	3	3
3	3	3	3	3	3	1	3	3	1
9	9	9	9	1	3	1	1	3	1
2	3	3	2	2	3	1	1	3	2
9	9	9	9	9	1	9	9	9	9
2	2	2	2	2	2	2	2	2	2
3	3	3	2	2	2	2	2	2	2
2	2	2	1	2	2	2	2	2	2
2	2	2	1	2	1	1	2	3	2
2	2	2	3	2	2	1	2	3	2
2	2	2	1	2	1	2	2	2	2
9	9	9	9	3	1	3	3	3	3
2	2	2	2	3	3	3	3	1	2
3	2	2	3	3	1	2	1	2	2
9	9	9	9	9	1	9	9	9	1
9	9	9	9	9	9	1	9	9	9

1	1	1	2	2	2	2	1	3	2
3	3	3	3	3	3	1	3	3	3
3	3	3	3	3	3	1	3	3	1
9	9	9	9	1	9	9	9	9	1
1	9	1	3	1	9	9	9	2	2
1	3	2	3	3	3	1	1	1	2
3	3	3	2	3	2	1	1	3	3
3	2	2	3	2	9	9	9	9	9
9	9	9	9	2	9	9	9	9	2
3	2	2	2	1	9	9	9	9	1
2	2	2	2	2	2	2	2	2	2
3	1	1	1	1	9	9	9	9	3
1	3	3	2	3	1	2	3	3	9
1	3	1	9	3	9	1	9	2	3
9	9	9	1	9	9	9	1	9	1
2	2	2	1	3	2	1	3	3	1
9	9	1	9	9	1	9	9	9	9
3	9	1	9	3	1	2	9	3	3
3	3	3	1	3	3	3	3	3	2
1	2	1	2	2	2	2	2	2	2
3	3	3	1	3	3	2	2	1	3
3	3	3	3	1	3	3	2	3	3
1	3	3	2	3	3	3	3	3	3
3	3	3	3	2	2	2	2	2	3
2	2	2	2	1	2	2	2	2	2
9	9	9	9	9	9	9	9	9	9
1	3	1	1	1	9	9	9	9	1
1	2	1	1	3	3	1	3	3	1
3	3	3	3	3	2	2	2	2	3
2	2	2	1	9	9	1	9	9	2
9	9	9	9	9	9	9	9	9	9
9	9	9	9	9	9	9	9	9	9
9	9	9	9	9	9	9	9	9	9
9	9	9	9	9	9	9	9	9	9
9	9	9	9	9	9	9	9	9	9
9	9	9	9	9	9	9	9	9	9
9	9	9	9	9	9	9	9	9	9
9	9	9	9	9	9	9	9	9	9
9	9	9	9	9	9	9	9	9	9
9	9	9	9	9	9	9	9	9	9
9	9	9	9	9	9	9	9	9	9
9	9	9	9	9	9	9	9	9	9
9	9	9	9	9	9	9	9	9	9
9	9	9	9	9	9	9	9	9	9
9	9	9	9	9	9	9	9	9	9
9	9	9	9	9	9	9	9	9	9
9	9	9	9	9	9	9	9	9	9

9	9	9	9	9	9	9	9	9	9
9	9	9	9	9	9	9	9	9	9
9	9	9	9	9	9	9	9	9	9
9	9	9	9	9	9	9	9	9	9
9	9	9	9	9	9	9	9	9	9
9	9	9	9	9	9	9	9	9	9
9	9	9	9	9	9	9	9	9	9
9	9	9	9	9	9	9	9	9	9
9	9	9	9	9	9	9	9	9	9
9	9	9	9	9	9	9	9	9	9
9	9	9	9	9	9	9	9	9	9
9	9	9	9	9	9	9	9	9	9
9	9	9	9	9	9	9	9	9	9
9	9	9	9	9	9	9	9	9	9
9	9	9	9	9	9	9	9	9	9
9	9	9	9	9	9	9	9	9	9
9	9	9	9	9	9	9	9	9	9
9	9	9	9	9	9	9	9	9	9
9	9	9	9	9	9	9	9	9	9
2	2	2	2	2	2	1	2	2	2
9	9	9	9	9	9	9	9	9	9
9	9	9	9	9	9	9	9	9	9
9	9	9	9	9	9	9	9	9	9
9	9	9	9	9	9	9	9	9	9
9	9	9	9	9	9	9	9	9	9
9	9	9	9	9	9	9	9	9	9
9	9	9	9	9	9	9	9	9	9
9	9	9	9	9	9	9	9	9	9
9	9	9	9	9	9	9	9	9	9
9	9	9	9	9	9	9	9	9	9
9	9	9	9	9	9	9	9	9	9
9	9	9	9	9	9	9	9	9	9
9	9	9	9	9	9	9	9	9	9
9	9	9	9	9	9	9	9	9	9
9	9	9	9	9	9	9	9	9	9
9	9	9	9	2	2	9	9	9	2
1	1	1	1	1	3	3	3	3	1
3	3	3	3	1	1	2	1	3	1
1	3	3	2	3	1	2	3	3	1
3	3	3	3	3	3	3	3	3	3
3	3	3	3	3	3	3	3	3	1
3	3	1	1	2	1	3	3	3	1
9	9	9	9	2	9	9	9	9	2
3	3	1	1	2	1	3	3	3	1
2	2	1	3	2	2	1	2	3	2
1	1	3	1	1	2	3	1	2	1
3	3	3	3	3	2	2	2	2	2

2	3	2	3	1	1	3	3	3	3
1	1	1	1	3	3	1	3	3	1
9	9	9	9	1	1	2	3	3	1
3	3	3	3	3	1	3	3	3	2
3	3	3	3	2	2	2	1	2	2
9	1	1	3	2	1	1	3	3	3
2	2	1	3	2	2	1	2	2	3
2	2	2	2	3	2	1	3	3	3
3	2	2	2	3	2	1	2	2	2
1	1	2	3	3	2	1	2	1	2
9	9	9	9	9	9	1	9	9	9
3	3	2	2	2	2	2	2	3	1
3	3	3	3	1	2	3	2	3	1
3	2	2	2	2	2	2	2	1	2
3	2	2	2	2	2	2	1	3	2
3	3	3	3	3	3	1	3	3	2
2	2	2	3	3	3	2	1	2	2
2	2	2	2	1	2	3	1	2	1
1	1	3	1	1	1	1	3	3	9
3	1	1	1	1	1	1	1	1	1
9	9	9	9	9	2	9	9	9	1
2	2	2	1	3	2	1	2	3	2
3	3	2	3	3	3	2	1	3	3
9	9	9	9	9	9	1	9	9	9
2	2	2	2	3	1	3	3	3	3
2	1	2	1	3	3	3	1	2	3
2	2	1	1	2	2	2	2	2	1
1	3	2	1	1	3	1	3	1	3
2	2	2	2	2	2	2	2	2	1
2	2	2	2	2	2	2	2	2	2
1	2	3	1	2	9	9	9	9	9
2	2	2	2	1	2	2	2	2	2
2	2	2	2	2	2	2	2	2	2
9	9	9	9	9	9	1	9	9	9
9	9	9	9	9	9	1	9	9	9
2	2	2	2	9	9	1	9	9	9
2	1	2	2	9	9	9	9	9	9
2	2	2	2	2	2	2	2	2	2
1	2	2	1	2	2	2	2	2	9
9	9	9	9	9	9	9	9	9	9
1	1	3	2	3	3	1	1	2	3
1	2	3	2	2	2	1	1	3	2
3	3	3	3	1	1	1	1	3	1
3	3	3	1	3	3	1	1	3	1
1	1	1	1	1	1	1	1	1	1
1	3	3	1	3	1	3	3	3	1
3	1	1	1	3	1	1	1	3	3

2	2	2	2	3	2	1	1	1	1
2	2	3	2	2	3	1	3	2	1
2	2	2	1	1	2	3	1	2	1
1	3	1	1	1	1	9	9	9	1
1	2	2	2	1	2	3	2	1	1
2	3	3	2	3	2	3	3	3	1
3	3	1	1	1	1	1	3	3	1
1	3	2	1	1	2	3	2	3	3
3	3	3	3	2	1	2	2	3	1
1	1	1	1	1	9	9	9	9	1
3	3	3	3	2	3	1	3	3	1
1	1	3	1	1	1	3	3	2	1
1	1	1	1	2	2	2	2	2	1
1	1	3	1	1	1	3	3	2	1
1	1	1	1	1	1	1	1	1	3
3	3	3	3	3	1	1	1	1	1
2	3	2	3	2	2	2	3	3	1
9	1	9	9	9	1	9	9	9	9
3	2	2	1	1	2	2	3	1	1
9	9	9	9	9	1	9	9	9	9
2	1	3	2	2	3	3	2	2	1
3	2	3	1	9	9	1	9	9	2
1	1	3	1	1	3	2	1	1	9
1	2	3	1	3	3	1	2	2	1
2	3	3	1	1	3	1	1	2	1
3	3	3	3	1	3	3	3	3	3
3	1	1	1	1	1	1	1	3	1
1	3	1	3	1	3	3	1	3	1
1	3	1	1	1	1	1	3	3	3
2	3	2	1	3	2	2	1	2	1
3	3	3	3	2	3	3	1	3	1
1	2	3	1	1	2	3	3	2	1
1	3	3	1	1	3	3	3	3	3
3	3	3	1	1	3	3	3	3	3
3	3	1	1	1	1	1	9	3	1
3	3	3	1	3	3	1	3	3	1
1	3	2	1	1	2	3	3	2	1
9	9	9	9	9	9	9	9	9	9
3	3	3	9	3	3	3	1	3	1
1	2	3	1	3	1	1	2	3	1
1	3	3	1	1	1	1	1	3	1
2	2	2	2	1	1	1	1	1	1
3	3	3	1	3	1	1	1	3	1
3	3	1	1	2	3	3	3	3	3
2	2	2	2	2	2	2	2	2	2
2	3	2	3	1	3	3	2	2	1
3	3	9	9	3	3	2	9	3	3

2	1	2	3	3	2	1	1	2	1
9	9	9	9	9	9	9	9	9	1
3	3	3	3	2	2	1	3	3	2
2	2	2	2	2	2	2	2	2	1
2	2	2	2	1	1	2	2	2	2
1	2	2	3	2	2	2	1	1	2
2	2	2	2	2	2	2	2	2	2
2	2	2	2	3	1	2	2	2	9
9	9	9	9	9	9	9	9	9	9
2	2	3	1	1	2	2	2	3	1
2	2	2	2	2	2	2	2	2	2
2	2	2	2	2	2	2	2	2	2
2	2	2	2	2	2	2	2	2	2
2	2	2	2	1	1	2	2	2	1
2	2	2	2	2	2	2	2	2	2
2	2	2	2	2	2	2	2	2	2
2	2	2	2	2	2	2	2	2	2
2	2	2	2	2	2	2	2	2	2
2	2	2	2	1	2	2	2	2	2
2	2	2	2	2	1	2	2	2	1
2	2	2	3	2	2	2	1	1	2
2	2	2	2	2	2	2	2	2	2
2	2	1	2	2	2	2	2	1	2
2	2	2	2	2	2	2	2	2	2
2	2	2	2	2	2	2	2	2	2
2	2	2	2	2	2	2	2	2	2
2	2	2	2	2	2	2	2	2	2
1	1	1	1	2	2	1	2	2	1
2	2	9	1	1	2	2	3	1	1
2	2	2	2	2	2	2	2	2	2
2	2	2	2	2	2	2	2	2	2
2	2	2	2	2	2	2	2	2	2
2	2	2	2	2	2	2	2	2	2
2	2	2	2	2	2	2	2	2	2
2	2	2	2	2	2	2	2	2	1
2	2	2	2	2	2	2	2	2	2
2	2	2	2	2	2	2	2	2	2
2	2	2	2	2	2	2	2	2	2
2	2	2	2	2	2	2	2	2	2
1	1	1	1	3	3	1	3	1	1
2	2	2	2	2	2	2	2	2	2
2	2	2	2	9	9	9	9	9	2
1	3	2	2	1	2	3	3	3	2
2	2	2	2	2	2	2	2	2	2
2	2	3	2	1	2	9	9	9	1
2	2	2	2	2	2	2	2	2	3
2	2	2	2	2	2	2	2	2	2

2	3	2	2	3	3	2	1	2	2
1	2	2	3	3	3	1	2	1	2
2	2	2	2	2	2	2	2	2	2
2	2	2	2	2	2	2	2	2	2
3	2	1	2	1	2	3	2	1	1
2	2	2	2	2	2	2	2	2	2
9	9	9	9	9	9	9	9	9	3
1	2	3	2	1	2	3	1	2	1
9	9	9	9	9	9	1	9	9	1
2	2	2	2	2	2	2	2	2	2
2	2	2	2	2	2	2	2	2	3
2	2	2	2	2	2	1	2	2	1
3	3	2	3	3	3	1	2	3	3
9	9	9	9	3	9	9	9	9	3
9	9	9	1	2	2	2	2	1	2
1	1	2	2	2	1	2	2	2	2
2	2	2	2	2	2	1	2	2	1
1	1	1	1	1	1	9	2	1	1
1	9	9	9	9	9	1	9	9	1
2	2	2	2	2	2	1	2	2	1
2	3	1	1	2	1	9	2	1	1
9	9	9	9	9	9	1	9	9	9
9	9	9	9	9	9	1	9	9	1
9	9	9	9	3	3	9	2	3	2
3	1	2	1	3	1	3	1	2	1
2	2	1	2	3	2	2	2	3	2
3	1	1	3	3	3	2	1	2	2
2	2	1	1	3	1	3	9	1	1
2	3	2	3	3	3	3	1	1	1
9	9	9	9	1	9	9	1	9	1
9	9	9	9	9	9	9	9	9	3
2	2	2	2	2	2	2	2	2	2
2	2	2	2	2	2	2	2	2	2
2	9	9	9	2	9	2	9	9	1
1	1	1	1	9	9	9	9	9	1
1	9	9	9	9	9	1	9	9	9
1	1	1	1	3	1	3	1	3	1
2	2	2	2	2	2	2	2	2	3
2	2	2	2	2	2	2	2	2	2
2	2	2	2	2	2	2	2	2	2
9	2	3	1	2	3	9	1	2	2
1	2	2	2	2	2	2	2	2	1
9	9	9	2	9	1	9	9	9	9
1	1	1	1	1	1	1	1	1	3
1	1	1	1	3	3	1	3	3	1
3	2	3	2	2	2	2	2	2	3
9	9	9	9	9	9	9	9	1	2

1	1	1	1	1	1	1	1	1	1
2	2	2	2	2	2	1	2	2	2
1	1	1	1	1	1	1	1	1	1
2	2	2	2	2	2	2	2	2	2
9	9	9	9	9	9	2	9	9	2
2	1	2	3	2	1	2	1	3	1
3	3	3	3	3	3	3	3	3	3
1	1	1	1	1	1	1	1	1	1
2	2	2	2	2	2	1	1	1	1
1	1	1	1	3	1	1	1	1	1
2	2	2	2	2	2	2	2	2	2
1	1	1	1	1	1	1	1	1	2
2	2	2	2	2	2	2	2	2	2
2	2	2	2	2	2	2	2	2	2
2	2	2	2	2	2	2	2	2	2
2	2	2	2	2	2	2	2	2	2
1	1	1	1	1	1	1	1	1	1
3	3	2	3	3	1	2	2	2	2
9	9	9	9	9	9	9	9	9	9
9	1	2	1	1	2	1	2	1	1
2	2	1	1	1	1	2	2	3	1
3	3	3	3	3	3	1	3	3	1
3	3	3	3	3	3	3	3	3	3
3	3	3	1	2	2	1	1	3	2
3	3	3	3	3	3	3	3	3	3
1	2	1	2	1	2	2	1	2	2
1	1	1	1	2	1	1	1	1	1
2	2	2	2	2	2	2	2	2	2
9	9	9	9	2	9	9	9	9	3
3	2	1	1	2	2	2	2	2	2
3	3	3	3	2	2	2	2	2	1
3	3	3	3	1	2	3	2	2	2
3	3	3	3	3	3	3	3	3	3
9	1	2	3	2	1	3	2	1	1
2	2	2	2	2	2	2	2	2	2
2	3	3	2	2	2	2	2	2	2
9	1	2	3	1	2	3	2	1	2
2	2	2	2	2	2	2	2	2	3
2	3	3	2	1	2	3	1	2	1
3	3	3	3	3	3	3	3	3	3
1	3	3	1	1	2	3	1	2	1
3	2	1	2	1	2	2	3	1	1
9	9	9	9	9	9	9	9	9	9
3	3	3	3	3	3	3	3	3	3
1	1	1	2	2	2	3	1	1	1
3	2	2	2	2	2	2	2	3	2
3	3	2	2	1	2	3	3	3	1

1	1	1	1	2	2	2	2	2	1
3	3	3	3	3	3	1	3	3	1
3	3	3	3	2	2	2	2	2	3
3	3	3	3	3	3	1	3	3	1
3	2	1	2	3	1	2	2	3	3
2	2	2	2	2	2	2	2	2	2
1	2	3	2	2	2	2	2	2	2
2	2	2	2	2	2	2	2	2	2
9	9	9	9	9	9	9	9	9	2
9	1	3	3	1	3	2	1	3	1
2	2	2	2	2	2	2	2	2	2
1	3	2	1	1	3	1	2	3	3
2	2	2	2	2	2	2	2	2	2
3	1	3	3	3	3	1	1	3	9
2	2	2	2	2	2	2	2	2	2
2	2	2	2	2	2	2	2	2	2
2	2	2	2	2	2	2	2	2	2
3	3	3	3	2	1	1	2	3	2
2	3	1	2	2	2	2	2	2	2
9	9	9	9	3	2	2	2	1	2
2	2	2	1	3	3	1	2	3	1
1	2	1	2	2	1	3	2	1	2
1	2	1	2	2	1	3	2	1	2
3	2	2	1	2	2	2	2	2	2
1	1	1	1	1	1	1	1	1	1
9	9	9	9	9	9	9	9	9	1
3	1	3	3	3	2	1	1	3	1
3	3	3	3	3	2	3	1	1	3
3	9	2	3	9	9	1	9	9	1
3	2	3	3	2	1	2	3	3	1
2	1	3	1	3	1	1	1	1	9
9	9	9	9	9	9	9	9	9	2
1	2	2	2	2	2	2	2	2	1
2	2	3	3	2	3	2	3	2	3
1	2	1	3	2	2	1	3	1	1
1	1	2	9	2	2	2	1	2	2
1	1	1	1	2	2	2	2	2	2
2	2	2	2	9	9	9	9	9	9
2	2	2	1	1	2	2	2	2	2
3	3	3	2	3	2	2	3	3	3
3	3	3	2	3	2	9	3	3	3
2	2	2	2	2	2	2	2	2	2
2	2	2	2	2	2	2	2	2	2
2	2	2	2	2	2	2	2	2	2
9	9	1	3	3	2	9	3	2	2
1	2	1	2	9	9	9	9	9	3
1	2	3	3	2	2	2	2	2	2

9	9	9	9	9	9	9	9	9	9
2	3	1	3	1	2	2	9	2	1
3	2	1	2	3	1	2	2	3	1
3	3	3	3	3	3	1	3	3	1
3	2	2	3	2	3	9	1	3	1
3	3	3	2	2	3	2	2	2	3
2	3	1	2	3	3	3	1	1	1
1	1	1	2	2	2	2	2	2	2
2	2	2	1	2	1	2	2	2	3
2	3	3	3	3	2	1	3	3	3
3	3	3	2	2	2	3	3	3	1
3	3	1	2	3	3	3	1	3	1
2	2	2	3	2	2	2	2	2	2
1	1	1	1	9	9	1	9	9	1
3	9	1	2	3	2	2	1	3	1
2	2	2	2	2	2	2	2	2	2
3	1	2	1	1	2	3	2	3	1
1	3	3	1	3	3	3	3	3	1
9	3	3	3	3	2	9	1	9	9
3	3	3	3	2	2	2	2	2	2
9	9	9	9	9	9	9	9	9	9
9	2	1	3	3	2	1	3	1	1
1	1	1	2	1	2	2	2	2	1
1	2	2	2	1	1	2	2	3	1
2	2	3	3	3	3	1	2	1	2
3	3	3	3	3	2	2	2	2	3
1	3	2	3	9	9	9	9	1	3
1	1	3	2	1	1	2	1	1	1
2	1	3	2	2	2	1	3	3	3
1	1	2	1	1	1	2	2	2	1
3	2	1	2	1	2	3	2	2	2
1	3	2	2	3	2	2	1	1	2
3	3	3	3	3	2	3	3	3	1
2	2	2	2	2	2	2	2	2	2
2	2	2	2	2	2	2	2	2	2
2	2	2	2	2	2	2	2	2	2
3	3	3	3	3	3	3	3	3	3
2	2	2	2	3	3	1	2	2	1
1	2	1	1	2	3	2	1	2	3
1	2	1	1	2	3	2	1	2	3
1	1	1	1	2	2	2	2	2	3
1	3	2	1	2	2	2	2	2	2
2	2	1	3	2	3	1	1	2	1
2	2	2	2	2	2	2	2	2	2
9	9	9	9	3	3	3	1	2	1
3	3	3	3	3	3	3	3	3	3
2	3	3	3	2	2	2	3	1	2

1	2	3	2	2	1	1	3	2	2
1	3	3	3	3	3	3	1	3	1
2	1	2	2	2	2	3	1	2	1
9	9	9	9	9	9	9	9	9	9
2	3	2	3	2	3	2	3	2	2
3	3	3	3	3	3	3	3	3	3
1	3	1	1	1	1	1	1	1	1
2	2	2	2	3	3	3	3	3	3
2	3	1	2	2	3	3	2	1	1
2	3	1	2	2	3	1	9	3	1
2	2	2	2	2	2	2	2	2	2
3	3	3	3	3	3	3	1	2	1
1	1	1	1	1	1	1	1	1	1
9	9	9	9	9	9	1	9	9	1
2	9	1	1	1	1	1	1	1	2
2	2	2	2	3	3	3	3	3	2
3	3	1	1	1	1	1	1	1	3
2	2	2	2	1	9	1	9	1	2
2	9	2	2	1	2	3	2	3	1
1	1	3	1	2	2	3	2	3	1
1	2	3	9	3	1	2	3	2	1
1	1	1	2	2	2	2	3	3	3
3	3	3	3	2	2	2	2	2	2
3	1	2	1	2	2	1	3	3	2
3	1	2	3	1	2	3	1	2	1
1	3	3	3	2	2	1	2	1	3
3	1	2	1	3	3	3	1	3	2
3	3	3	3	3	3	2	1	2	1
2	3	2	1	1	2	3	1	2	1
2	2	2	2	2	2	2	2	2	2
2	1	1	2	3	3	2	2	3	1
2	2	2	2	1	1	1	1	1	2
3	2	3	2	1	3	3	3	3	1
1	3	1	2	1	3	3	1	1	1
1	1	3	2	3	1	1	3	3	2
3	3	3	1	1	3	3	3	3	3
3	3	2	3	1	1	1	3	3	1
1	3	3	1	3	3	3	3	3	3
1	3	1	1	1	3	3	3	3	3
3	3	2	3	1	1	2	2	2	1
2	2	2	2	2	2	2	2	2	2
2	3	1	3	3	1	3	3	3	3
2	2	3	2	3	2	1	3	2	1
3	2	2	1	1	1	2	3	2	3
9	9	9	9	9	9	9	9	9	1
1	2	3	2	3	3	3	3	3	2
3	1	9	3	1	3	1	3	1	1

2	2	3	2	2	1	2	2	2	1
1	2	3	1	1	2	3	2	3	1
2	2	2	2	2	2	2	2	2	2
1	3	1	1	1	1	1	1	1	1
1	2	1	3	1	2	3	1	2	1
1	3	2	2	3	1	2	2	2	1
3	2	3	1	1	1	2	3	3	1
3	3	3	2	3	2	1	2	1	2
3	1	2	1	1	2	1	2	1	1
3	1	3	1	3	2	1	3	3	3
3	1	2	3	1	2	3	2	3	1
1	3	3	1	3	3	9	1	2	1
1	1	1	1	3	1	1	1	1	1
1	3	1	1	3	1	3	1	3	3
2	2	3	2	3	2	2	2	3	1
3	1	1	1	3	3	1	1	1	9
1	2	3	1	2	2	2	1	3	1
3	3	3	3	3	3	3	3	3	1
1	3	3	3	3	1	2	3	3	1
2	9	1	2	3	3	3	1	2	1
3	2	3	2	2	2	2	2	2	1
3	2	3	1	3	1	1	1	3	3
3	9	3	3	3	1	3	1	3	3
1	2	2	1	2	2	2	2	2	1
2	1	1	2	2	1	1	3	3	2
2	2	2	2	2	2	2	2	2	2
3	3	3	3	1	3	1	3	2	1
3	1	9	9	1	3	1	2	2	2
2	2	2	2	3	2	2	2	3	2
9	9	9	9	9	3	9	9	9	9
1	3	3	1	9	9	9	9	9	9
9	9	9	9	9	9	9	9	9	9
2	2	2	2	1	1	2	2	2	1
9	9	9	9	9	9	1	9	9	9
2	2	2	2	3	3	3	3	3	3
1	1	1	1	2	2	2	2	2	2
2	3	3	2	3	3	2	2	2	3
9	9	9	9	1	9	9	9	9	2
9	9	9	9	3	3	3	2	2	3
3	2	3	3	2	2	2	2	2	3
2	3	2	1	2	1	3	2	3	1
3	1	2	1	1	2	1	1	3	2
3	3	3	1	3	2	2	1	1	1
3	3	2	2	1	1	2	1	1	1
3	1	2	2	1	2	1	2	3	1
2	2	2	2	1	2	1	2	1	1
3	3	3	3	1	3	3	3	2	1

9	9	9	9	9	9	1	9	9	2
1	2	1	1	1	1	1	1	1	1
2	3	2	3	3	2	2	2	3	2
3	1	2	1	2	1	1	3	3	2
2	2	2	2	3	3	2	2	3	2
2	3	3	2	3	2	3	3	3	3
3	3	3	3	9	9	9	1	9	1
3	2	3	3	3	2	3	1	1	1
3	3	2	1	3	2	2	1	2	1
1	1	3	3	3	2	3	1	3	1
2	1	2	3	2	3	2	1	3	3
9	2	1	1	3	2	1	1	3	3
1	1	3	2	1	1	1	1	1	1
2	2	2	2	2	2	2	2	2	2
2	2	2	2	2	2	2	2	2	2
3	3	2	2	9	9	9	9	9	2
2	1	2	1	3	2	2	2	2	2
2	2	2	2	2	2	2	2	2	2
2	2	2	2	2	2	2	2	2	2
2	3	3	1	3	2	3	1	2	1
9	9	9	9	1	2	3	9	9	9
9	9	9	9	1	9	9	9	9	9
9	9	9	9	1	9	9	9	9	9
2	2	2	1	1	1	2	2	2	1
3	3	3	3	1	3	3	3	3	3
9	9	9	9	1	9	9	9	9	9
1	2	2	1	1	9	9	9	9	9
1	2	2	2	1	2	2	2	2	1
1	2	2	2	2	2	2	2	2	1
9	9	9	9	9	9	9	9	9	9
9	9	9	9	1	9	9	9	9	9
9	9	9	9	9	1	9	9	9	1
2	2	1	1	1	3	3	3	3	2
3	3	3	3	1	3	3	3	3	1
9	9	9	9	1	9	9	9	9	9
2	2	3	3	1	9	9	9	9	1
9	9	9	9	1	1	9	9	9	9
9	9	9	9	1	9	9	9	9	9
9	9	9	9	1	9	9	9	9	9
1	3	3	9	1	3	3	3	3	9
3	3	3	1	1	3	3	3	3	1
9	9	1	9	1	9	9	9	9	1
9	9	9	9	1	9	9	9	9	9
2	3	9	9	1	9	9	1	9	9
9	9	9	9	1	9	9	9	9	9
1	1	2	1	1	1	1	3	3	1
2	3	2	2	1	2	2	2	2	3

9	9	9	1	1	9	9	9	9	9
2	3	3	2	1	2	2	2	2	2
3	3	3	3	1	2	2	2	2	1
1	1	1	1	1	1	1	1	1	1
9	9	9	9	1	9	9	9	9	9
9	9	9	9	1	9	9	9	9	9
1	1	1	1	1	9	9	9	9	1
2	2	2	2	2	2	1	2	2	2
9	9	9	9	1	9	9	9	9	9
9	9	9	9	1	9	9	9	9	9
2	2	2	2	1	9	9	9	9	2
2	2	2	2	1	9	9	9	9	2
9	9	9	9	1	9	9	9	9	9
9	9	9	9	1	9	9	9	9	9
9	9	9	9	1	9	9	9	9	1
9	9	9	9	1	9	9	9	9	9
9	9	9	9	1	9	9	9	9	9
9	9	9	9	1	9	9	9	9	9
9	9	9	9	1	9	9	9	9	9
2	3	3	3	1	9	9	9	9	1
9	9	9	9	1	9	9	9	9	1
9	9	9	9	1	9	9	9	9	9
3	3	3	3	3	3	3	3	3	1
9	9	9	9	1	9	9	9	9	9
1	2	2	1	1	9	9	9	9	1
3	3	3	9	3	3	9	9	1	3
3	3	3	3	1	3	3	3	3	3
9	9	9	9	1	9	9	9	9	9
3	3	3	3	1	3	3	3	3	9
3	3	3	1	1	9	9	9	9	9
2	2	1	1	1	2	2	2	2	2
3	3	3	1	1	3	3	3	3	3
9	9	9	9	1	9	9	9	9	9
9	9	9	9	9	9	9	9	9	9
9	9	9	9	1	9	9	9	9	9
3	3	3	3	1	3	3	3	3	3
9	9	9	9	1	9	9	9	9	9
9	9	9	9	1	9	9	9	9	9
3	3	3	3	1	3	3	3	3	9
3	3	3	3	1	2	2	1	2	1
3	3	3	3	3	1	1	1	3	1
3	3	3	3	1	1	3	3	3	1
9	9	9	9	1	9	9	9	9	9
9	9	9	9	1	9	9	9	9	9
9	9	9	9	9	9	9	9	9	9
3	3	2	3	1	9	9	9	9	9
9	3	9	9	1	9	9	9	9	9

1	3	1	3	1	9	9	9	9	9
3	3	3	1	3	3	3	3	1	3
3	3	2	3	1	9	9	9	9	9
1	3	1	1	1	9	9	9	9	1
9	9	9	9	1	9	9	9	9	9
9	9	9	9	1	9	9	9	9	9
3	3	1	1	3	1	1	1	3	3
3	3	3	3	3	3	1	3	3	9
3	3	3	3	3	3	1	3	3	9
3	3	3	3	3	3	1	3	3	3
3	3	3	3	2	2	1	1	1	1
3	3	3	3	9	9	9	1	9	9
9	9	9	9	9	9	9	9	1	9
2	2	3	3	3	2	2	1	2	2
2	2	2	2	2	2	2	2	2	1
1	2	2	2	3	3	1	1	2	2
3	9	9	9	9	9	9	1	9	9
9	9	9	9	9	9	9	1	9	9
3	2	9	3	1	1	1	1	2	1
3	2	3	2	1	1	1	1	2	1
1	3	3	2	3	1	1	3	3	1
2	2	2	2	2	1	3	2	3	2
3	3	3	3	3	3	3	2	1	3
3	3	3	3	3	3	3	1	3	1
3	3	3	3	3	3	3	2	1	9
3	3	3	3	9	9	1	9	9	2
2	3	2	3	2	3	1	1	2	2
2	9	1	2	3	2	2	3	3	3
3	3	3	3	3	2	3	3	3	2
2	2	1	1	2	2	2	1	1	1
2	2	2	2	2	2	1	2	2	1
1	2	2	3	2	1	1	1	3	1
3	2	1	3	2	2	1	2	1	3
9	9	9	9	9	9	9	1	9	9
3	3	3	3	3	3	3	2	1	3
3	3	2	1	3	3	2	2	1	1
9	9	9	9	9	1	9	9	9	2
9	9	9	9	9	1	9	9	9	2
9	9	9	9	9	1	9	9	9	2
2	9	1	3	1	1	3	2	3	1
3	3	3	3	3	3	3	3	1	3
3	3	3	3	1	1	3	3	3	3
3	3	3	2	2	2	2	3	3	2
2	2	2	2	2	2	2	2	2	1
3	3	3	3	2	2	2	3	3	2
9	9	9	9	9	9	3	9	9	9
3	3	3	3	9	9	9	1	9	2

9	9	9	9	1	9	9	9	9	9
2	2	3	2	3	2	2	1	2	1
3	3	3	1	2	2	2	2	1	2
2	2	2	2	2	2	2	2	1	1
3	2	9	3	3	3	2	1	1	1
2	3	1	9	3	3	3	3	3	1
9	9	9	9	2	1	2	2	2	1
3	3	3	1	3	3	1	3	3	1
9	9	9	9	9	9	9	9	1	9
3	3	3	3	9	9	1	9	9	1
3	3	3	3	2	2	2	2	3	3
3	3	3	3	3	1	3	3	3	3
3	3	3	3	3	1	2	3	3	3
3	2	3	3	9	1	9	9	9	3
9	9	9	9	9	1	9	9	9	3
9	9	9	9	9	1	9	9	9	9
1	2	3	3	1	1	3	3	3	1
2	2	2	2	2	1	2	2	2	2
3	3	3	3	3	1	3	3	3	3
2	3	1	3	9	1	9	9	9	9
9	9	9	9	9	1	9	9	9	3
9	9	9	9	1	9	9	9	9	1
2	2	2	3	9	1	9	9	9	2
3	3	3	1	1	3	3	3	3	3
3	2	9	3	2	1	3	3	3	9
3	2	9	3	2	1	3	3	3	9
9	9	9	9	9	1	9	9	9	9
9	9	9	9	9	9	9	9	9	9
9	9	9	9	1	9	9	9	9	9
2	2	2	9	1	1	1	3	2	2
9	9	9	9	3	1	3	3	3	9
3	9	2	3	2	1	3	3	3	3
9	9	9	9	1	9	9	9	9	9
3	3	3	3	1	9	3	9	9	3
3	3	3	3	1	2	2	2	2	3
9	9	9	9	1	9	9	9	9	1
3	3	3	3	1	1	1	1	3	2
3	3	3	3	1	3	3	3	3	1
3	3	3	3	1	1	3	3	3	3
3	3	3	3	1	1	1	1	3	2
1	1	3	1	1	9	9	9	9	1
9	9	9	9	1	9	9	1	9	9
3	3	3	3	1	3	3	3	3	3
9	9	9	9	1	9	9	1	9	9
2	3	3	3	1	3	3	3	3	3
9	9	9	9	9	1	9	9	9	9
9	9	9	9	1	9	9	9	9	9

3	3	3	3	1	3	3	3	3	3
9	9	9	9	1	9	9	9	9	9
9	9	9	9	9	9	9	9	9	9
9	9	9	9	9	9	9	9	1	1
9	9	9	9	9	1	9	9	9	1
3	3	3	1	3	3	1	1	3	2
1	2	1	2	2	1	2	9	2	9
9	9	9	9	9	1	9	9	9	9
9	9	9	9	9	9	9	9	9	9
9	9	9	9	9	1	9	9	9	9
3	3	3	3	1	3	3	3	3	1
9	9	9	9	1	9	9	9	9	2
3	3	3	3	1	2	2	2	3	3
9	9	9	9	9	1	9	9	9	9
3	1	1	1	9	1	2	1	1	1
3	3	3	3	3	1	2	3	3	3
9	9	9	9	9	1	9	9	9	9
9	9	9	9	9	1	9	9	9	9
9	9	9	9	9	1	9	9	9	9
3	3	3	3	3	1	3	3	3	3
3	3	3	1	1	3	3	9	3	3
9	9	9	9	9	1	9	9	9	9
9	9	9	9	9	1	9	9	9	9
3	3	3	3	1	9	9	9	9	1
9	9	9	9	9	1	9	9	9	9
2	3	1	1	2	1	1	3	1	2
3	3	3	3	9	1	9	9	9	9
2	3	1	3	2	1	2	3	3	1
3	1	1	2	9	1	9	9	9	1
3	3	3	3	1	1	3	3	3	1
3	3	3	3	2	3	3	3	3	9
2	1	1	2	3	1	3	3	3	2
3	3	2	2	1	1	1	1	1	1
3	3	3	3	3	1	3	3	3	3
2	3	3	3	1	2	1	1	3	2
9	9	9	9	9	1	9	9	9	9
9	9	9	9	9	1	9	9	9	9
1	9	9	9	9	1	9	9	9	9
3	3	3	1	3	1	2	3	3	3
3	3	1	1	3	1	3	3	3	1
2	3	2	2	2	1	2	3	3	2
3	2	3	3	2	1	3	3	3	1
9	9	9	9	9	9	1	9	9	9
9	9	9	9	9	1	1	9	9	9
9	9	9	9	1	9	9	9	9	1
3	3	3	3	1	9	9	9	9	1
2	3	2	1	9	1	9	9	9	9

3	3	3	1	3	1	3	3	3	3
1	1	2	1	9	1	9	9	9	1
9	9	9	9	9	1	9	9	9	9
3	3	3	2	2	1	1	2	2	2
3	3	3	3	9	1	9	9	9	3
9	9	9	9	9	1	9	9	9	1
2	2	2	2	2	1	2	2	3	3
2	2	2	1	2	1	2	2	2	1
3	3	3	3	3	1	3	2	3	2
3	2	3	2	3	1	2	2	3	2
2	2	2	3	3	1	2	2	3	3
3	3	3	3	9	1	9	9	9	9
9	9	9	9	9	1	9	9	9	9
9	9	9	9	9	1	9	9	9	9
9	9	9	9	9	1	9	9	9	9
9	9	9	9	9	1	9	9	9	9
3	3	3	3	3	1	3	3	3	3
2	2	1	3	2	1	2	2	2	3
3	3	3	3	9	9	9	9	9	9
9	9	9	9	9	1	9	9	9	3
3	3	3	3	2	1	3	3	3	2
3	2	2	2	3	1	2	9	9	3
3	3	3	2	2	1	1	2	2	2
3	3	3	3	1	1	2	2	3	2
2	2	2	2	3	1	3	3	3	3
3	9	9	9	9	1	9	9	9	9
2	3	3	3	3	1	1	1	3	3
3	3	3	3	2	1	2	2	2	3
3	3	3	3	1	1	9	9	9	9
9	9	9	9	9	1	9	9	9	9
3	3	3	3	3	1	3	3	3	2
9	9	9	9	9	1	9	9	9	9
9	9	9	9	9	1	9	9	9	9
3	3	2	1	2	1	2	2	3	1
3	3	3	3	2	1	2	2	1	9
3	3	1	1	2	1	2	2	2	3
3	3	3	2	2	1	3	3	3	2
3	3	3	3	3	3	1	2	2	2
9	9	9	3	9	9	9	1	9	3
9	9	9	9	1	9	9	9	9	9
2	3	3	3	3	2	2	3	2	3
3	3	2	3	2	2	2	2	2	2
9	9	9	9	9	9	9	1	9	9
1	1	2	1	2	2	2	1	1	1
3	3	2	2	1	1	2	3	2	2
9	9	9	9	9	1	9	9	9	9
3	3	3	1	3	3	1	1	3	3

1	3	3	3	3	3	1	3	3	3
1	2	2	2	3	2	1	2	2	3
2	2	2	2	3	3	2	1	2	1
2	2	2	2	2	2	1	3	3	2
2	2	2	1	3	2	1	2	3	2
2	1	2	2	3	3	1	3	3	3
1	3	2	1	1	1	3	1	2	1
2	2	2	2	2	2	1	2	3	2
3	2	3	3	2	1	1	2	2	2
3	3	3	3	3	3	2	1	2	1
2	2	2	3	2	2	1	2	2	1
2	2	2	2	9	2	9	9	9	2
3	3	3	3	3	2	1	2	2	3
2	2	2	2	2	2	2	2	2	1
2	2	2	2	2	2	2	2	2	1
1	2	2	1	3	2	2	1	1	2
2	2	2	2	2	2	2	2	2	1
2	2	2	2	2	2	2	2	2	2
3	3	3	3	2	2	2	2	2	2
2	2	3	2	3	1	2	2	3	1
3	2	3	3	2	3	1	2	1	2
3	3	3	2	2	1	2	2	1	1
9	9	9	9	9	9	1	9	9	1
3	2	2	3	1	1	2	3	3	1
1	1	1	2	2	2	1	1	1	2
9	9	9	9	9	9	9	9	9	1
3	1	1	1	2	3	3	3	3	1
1	1	1	2	9	1	9	9	9	2
1	1	1	2	9	9	1	9	9	2
2	2	9	3	1	9	2	9	3	1
3	2	2	3	2	3	1	1	2	2
2	2	2	1	2	1	2	2	2	2
2	2	3	1	2	2	1	2	1	2
2	2	3	1	2	2	1	2	1	2
9	9	9	9	9	9	9	1	9	9
9	9	1	9	9	9	9	9	1	9
1	1	9	2	1	9	9	9	9	1
1	9	9	9	1	9	9	9	9	1
9	9	9	9	9	1	9	9	9	9
1	1	1	9	9	9	1	9	9	1
2	2	3	2	1	2	2	3	3	1
9	9	9	9	9	9	9	9	1	1
9	9	9	9	9	9	9	9	1	1
3	3	3	2	2	1	2	2	2	1
1	9	9	9	1	9	9	9	9	1
9	9	9	9	9	1	9	9	9	1
9	9	9	9	9	1	9	9	9	1

1	3	3	3	1	1	1	3	3	1
2	3	1	2	1	3	2	1	2	1
9	9	9	9	9	1	9	9	9	1
1	3	2	3	3	2	1	2	3	1
2	2	2	1	2	2	1	2	3	1
9	9	9	9	9	9	1	9	9	2
9	9	9	9	9	9	1	9	9	9
9	9	9	9	9	9	1	9	9	9
9	9	9	9	9	9	1	9	9	9
9	9	9	9	1	9	9	9	9	1
3	3	3	3	2	1	2	3	3	3
1	2	3	3	3	1	3	3	3	1
3	3	3	3	2	1	3	3	3	1
3	3	3	3	1	2	2	2	2	3
3	3	3	3	3	3	1	3	3	1
3	3	3	3	3	3	3	1	3	3
3	3	3	3	3	3	3	1	3	3
2	3	3	3	3	3	3	1	3	3
1	2	3	3	3	1	3	3	3	1
1	2	3	1	2	1	1	2	1	2
1	2	2	2	1	1	2	2	2	1
2	2	2	1	2	2	2	2	2	1
9	9	9	9	9	9	1	9	9	9
1	1	2	1	1	2	3	2	1	1
1	3	3	3	3	2	3	3	3	3
1	3	2	3	3	1	1	1	2	3
3	3	3	3	9	9	9	9	9	9
3	3	3	3	2	2	2	2	2	2
3	3	3	3	3	3	1	3	3	2
1	2	2	2	9	9	9	9	9	9
1	3	3	3	2	2	1	1	3	3
2	2	2	2	3	3	1	2	2	9
1	3	3	3	3	1	1	3	3	1
1	2	3	3	2	2	1	2	2	3
3	3	3	3	2	3	1	3	3	1
3	3	2	3	3	1	1	1	2	1
1	3	3	3	1	3	3	3	3	3
1	3	3	3	3	1	3	3	3	3
1	3	3	3	3	3	1	3	3	3
3	3	2	3	2	1	1	2	3	3
1	3	3	3	1	9	9	9	9	3
1	3	3	3	3	1	1	1	3	1
1	2	2	3	3	2	1	2	3	3
1	3	3	3	3	9	1	3	3	1
1	3	3	3	3	9	1	3	3	1
1	3	3	3	3	3	1	3	3	1
3	2	3	3	1	2	1	1	2	1

3	2	3	2	1	2	2	3	2	2
3	1	2	1	1	1	1	2	3	1
1	2	2	1	2	2	2	2	2	1
1	3	3	3	3	3	1	3	3	1
1	3	3	3	3	3	1	3	3	1
1	3	3	3	3	3	1	3	3	1
3	2	3	3	1	3	1	3	3	1
3	3	3	3	3	2	1	3	3	1
3	3	2	2	2	1	1	3	3	3
9	9	9	9	9	9	9	1	9	1
2	2	1	9	1	1	3	3	2	3
9	9	9	9	9	9	9	1	9	1
2	2	1	1	2	2	1	2	2	1
3	2	2	2	3	3	1	2	2	1
3	3	1	1	3	2	1	1	3	3
2	2	2	2	2	2	2	2	2	2
9	1	2	2	2	1	9	1	9	1
2	2	2	2	3	3	1	2	2	1
3	1	1	3	1	3	2	1	3	1
1	1	3	1	1	1	3	2	2	1
3	3	3	1	1	3	3	3	3	1
3	3	3	3	1	3	1	1	3	1
1	2	2	9	2	1	2	1	3	2
1	2	3	1	2	1	2	2	3	2
1	2	3	1	3	1	2	2	3	3
3	2	2	3	2	1	1	3	2	2
3	3	2	1	3	2	3	3	3	1
2	2	2	2	1	2	2	2	2	2
2	3	2	2	1	2	2	3	3	1
2	2	2	2	2	2	1	2	2	2
3	2	2	3	2	2	2	2	2	2
3	2	2	3	3	3	1	1	2	2
3	3	3	3	2	2	9	1	2	9
1	9	3	2	1	3	1	1	3	3
1	2	2	9	2	1	2	1	3	2
1	3	3	3	3	3	1	3	3	1
3	3	3	3	3	3	1	2	3	3
3	3	3	3	2	1	1	2	3	3
9	9	9	9	9	9	9	1	9	1
3	3	2	2	3	3	1	3	2	2
3	3	3	3	1	2	2	2	2	1
9	9	9	9	9	9	9	9	9	1
3	3	1	1	1	3	2	1	3	1
3	3	3	2	3	3	1	3	3	3
3	3	3	3	3	3	2	2	2	2
2	3	3	3	3	3	3	3	2	2
1	2	1	3	3	3	1	3	3	2

2	2	1	3	3	1	1	1	2	2
2	2	2	2	2	2	2	2	2	2
3	3	3	2	3	3	1	3	3	3
2	2	3	3	2	2	2	2	1	2
3	3	2	3	3	3	1	1	2	1
2	3	3	2	2	3	3	3	3	1
3	3	2	9	9	9	9	1	9	1
3	3	3	3	3	3	2	2	2	2
3	2	2	2	1	1	2	2	2	1
3	3	3	2	3	3	1	2	2	2
1	3	3	3	2	3	3	2	1	1
2	2	3	3	3	1	3	3	3	2
2	2	3	3	9	9	1	9	9	2
3	2	2	2	1	2	3	3	3	1
3	3	3	3	3	1	2	3	3	1
2	2	2	2	2	2	2	2	2	2
1	1	3	1	3	1	2	1	2	2
3	3	3	3	1	2	2	3	3	1
3	3	3	3	1	2	1	2	2	1
1	3	3	3	3	3	1	3	3	1
3	2	3	1	2	2	1	1	3	2
2	2	2	3	9	1	9	9	9	1
3	3	3	3	3	3	1	3	3	3
9	9	9	9	3	3	1	1	3	3
3	3	3	3	3	3	2	3	3	3
2	1	2	3	3	1	1	3	3	3
1	3	3	3	3	3	3	1	3	3
1	1	1	2	2	9	1	2	2	1
9	9	9	9	1	9	9	9	9	2
9	2	9	9	3	1	9	9	9	9
3	3	3	2	2	1	2	2	1	1
2	2	3	2	1	2	3	3	3	1
2	2	3	2	3	2	1	1	3	2
3	3	3	3	9	9	1	9	9	2
2	3	3	2	9	9	9	9	1	9
2	2	3	2	2	2	1	2	2	1
2	2	2	2	2	2	2	2	2	2
1	9	9	9	9	3	9	9	9	9
3	2	3	2	2	1	1	1	1	2
3	2	3	2	2	1	2	3	3	2
3	3	3	3	9	9	1	9	9	3
2	3	2	3	3	2	1	1	3	1
2	1	2	1	2	1	1	2	2	2
2	3	2	2	2	1	2	2	3	2
2	2	2	1	3	2	3	3	3	3
3	3	3	2	2	2	2	2	2	2
3	3	3	3	1	1	2	2	3	1

2	2	2	2	1	3	3	3	3	1
3	3	3	2	1	1	1	1	1	1
3	3	3	3	2	1	1	2	3	2
1	2	2	1	3	2	1	2	3	1
3	2	2	3	1	2	3	1	3	1
3	3	2	3	1	3	3	2	3	2
3	3	3	1	2	2	1	3	3	2
9	9	9	1	9	9	9	1	9	3
3	3	3	1	2	2	2	2	2	2
2	2	2	2	3	2	2	2	2	2
2	2	2	2	2	2	1	3	3	2
2	2	3	2	3	2	9	9	9	9
2	2	2	2	2	2	2	2	2	2
1	2	2	2	1	2	2	2	2	1
3	3	3	3	3	3	1	2	3	3
3	3	3	3	3	3	1	2	3	3
2	2	3	1	9	9	9	1	9	1
2	1	2	1	3	1	1	1	1	3
9	9	9	9	9	9	1	9	9	9
3	3	3	3	3	3	1	3	3	1
3	3	3	3	3	3	1	3	3	3
1	2	1	1	3	2	1	1	2	2
2	2	2	2	2	2	1	2	1	2
3	3	3	2	3	3	1	3	3	2
3	3	3	3	3	2	1	3	3	1
1	2	2	1	1	1	1	1	2	2
2	3	3	1	3	1	1	1	2	1
2	3	3	1	2	2	2	2	2	1
2	3	3	1	3	3	1	1	1	1
9	9	9	9	9	9	1	9	9	9
2	1	1	1	3	3	1	1	2	1
3	1	1	2	1	1	2	1	2	2
9	9	9	1	1	1	1	2	2	1
3	3	2	1	2	1	1	2	2	1
3	3	2	1	3	3	3	1	2	3
2	2	2	2	1	1	1	2	2	1
2	2	2	2	1	1	1	2	2	1
9	9	9	9	9	9	9	1	9	1
3	3	3	3	3	3	1	3	3	3
2	2	1	1	2	1	1	2	3	1
2	3	3	3	1	1	1	1	1	1
1	1	1	1	3	1	1	1	1	3
2	2	2	2	2	2	2	2	2	1
3	3	2	3	3	3	1	2	3	1
3	1	1	1	3	1	1	1	3	1
2	3	1	1	2	2	1	1	1	1
3	2	1	1	3	1	3	3	3	1

3	2	1	1	2	1	1	2	2	1
3	2	1	1	2	1	1	2	2	1
2	2	3	3	3	3	1	3	1	3
3	2	2	1	2	2	1	1	1	3
3	3	1	1	2	1	1	2	2	1
1	2	1	3	2	1	1	1	2	1
1	3	3	1	3	2	1	3	1	1
3	2	3	3	1	1	3	2	3	1
1	3	3	3	3	3	1	3	3	1
1	2	3	3	3	1	3	3	3	1
1	3	3	3	3	3	1	3	3	1
1	3	3	3	3	3	1	3	3	1
1	2	3	3	3	3	1	2	2	1
3	3	2	3	3	2	3	2	3	2
3	3	3	3	3	3	3	1	3	3
3	3	9	2	1	2	3	3	3	1
2	2	2	1	1	2	2	2	2	1
2	2	3	2	3	1	1	2	1	1
9	9	9	9	9	9	1	9	9	9
1	3	3	3	9	9	9	9	9	3
2	1	1	3	2	1	3	2	1	2
2	2	3	2	1	1	2	3	3	1
2	2	2	1	2	1	2	2	2	1
1	3	2	3	3	2	1	2	3	1
9	9	9	9	9	1	9	9	9	1
9	9	9	9	9	9	9	1	9	9
9	9	9	9	9	9	1	9	9	1
9	9	9	1	9	9	9	9	1	9
9	9	9	9	3	9	9	9	9	2
9	9	9	1	9	9	1	9	9	9
9	9	9	1	9	9	9	1	9	1
9	9	9	1	9	9	9	9	1	1
9	9	9	9	1	9	9	9	9	2
9	9	9	9	9	1	9	9	9	1
9	9	9	9	2	1	1	2	2	1
1	3	3	3	3	3	1	3	3	3
2	2	1	1	2	1	1	2	2	1
2	1	2	1	3	1	1	1	1	3
3	3	3	2	1	1	1	2	2	1
2	3	3	1	3	1	1	1	2	1
2	1	1	1	9	9	9	1	9	1
3	3	3	3	1	9	9	9	9	1
1	2	2	3	2	2	9	1	1	2
3	3	3	3	2	2	1	2	2	1
2	1	3	2	2	2	2	2	2	3
1	3	3	3	3	3	3	1	3	1
3	2	1	1	3	3	1	3	3	2

1	2	2	3	2	2	1	2	3	1
2	2	3	2	2	3	3	2	2	2
2	2	2	2	1	2	2	2	2	1
3	3	3	2	1	1	2	1	2	3
2	3	2	2	2	2	1	1	1	1
9	9	9	9	9	9	1	9	9	2
9	9	9	9	9	9	9	9	9	2
1	3	3	2	2	2	2	2	2	2
3	3	3	2	3	3	2	2	2	2
2	2	2	2	2	2	3	2	2	2
3	3	3	3	2	2	2	2	2	1
3	3	3	3	3	2	1	1	2	2
2	2	2	2	2	2	2	2	1	1
9	1	9	9	9	9	1	9	9	3
2	2	2	2	2	2	2	2	2	2
1	2	3	1	2	2	2	2	2	2
2	1	9	1	2	2	2	2	2	1
1	3	3	2	3	3	1	1	3	1
1	1	2	3	3	3	1	3	3	2
2	2	2	2	2	2	2	2	2	1
3	3	3	1	3	3	3	3	3	1
2	2	2	2	2	2	2	2	2	2
3	3	3	2	3	3	2	2	2	2
2	2	2	2	3	3	1	3	3	1
3	3	3	1	3	3	1	3	3	1
3	3	3	3	1	9	9	9	9	2
2	3	2	3	2	1	1	3	3	1
1	1	1	1	2	1	2	3	3	1
1	1	1	1	2	2	2	2	2	2
2	2	2	2	2	2	2	2	2	2
9	9	9	9	9	9	9	1	9	9
2	1	1	1	1	1	1	1	1	1
9	9	9	9	3	3	1	3	3	3
3	3	2	2	1	3	3	3	3	1
1	9	9	9	1	9	9	9	9	1
2	2	2	2	2	1	1	2	2	9
2	2	2	2	2	2	2	2	2	1
3	2	2	3	3	2	1	2	1	3
2	3	3	3	2	9	1	2	3	1
3	3	3	3	3	3	3	2	3	1
3	2	2	3	2	1	1	2	2	1
3	2	2	3	2	1	1	2	3	1
9	9	9	9	1	9	9	9	9	1
9	9	9	9	1	9	9	9	9	1
9	9	9	9	1	9	9	9	9	1
2	1	1	2	1	9	9	9	9	1
1	1	1	2	1	2	2	1	2	2

3	3	3	2	3	1	2	1	2	3
3	3	3	2	3	1	2	1	2	3
3	3	3	2	3	1	2	1	2	3
1	2	2	2	2	2	2	3	3	3
1	2	2	2	3	3	1	2	2	2
1	2	3	9	2	3	3	2	1	2
2	3	2	2	2	2	3	3	2	2
2	1	1	2	1	2	1	2	3	1
1	2	1	2	2	1	1	2	1	2
1	2	1	2	1	2	3	2	3	1
1	2	2	2	1	2	2	2	3	2
2	2	2	2	3	3	1	3	2	2
2	2	2	2	2	2	1	3	3	2
2	2	2	3	3	2	2	3	3	2
1	2	2	2	3	3	2	2	2	1
1	3	3	2	2	1	2	3	3	1
3	2	1	1	2	2	3	1	2	2
3	2	1	1	2	2	3	1	2	2
3	3	3	2	3	1	2	1	2	3
3	3	3	2	3	1	2	2	2	3
9	9	9	9	9	9	9	9	9	3
2	2	2	2	3	3	1	3	3	2
2	2	2	2	3	3	1	3	3	3
3	3	3	3	3	3	1	3	3	2
3	3	3	3	2	2	2	2	2	3
2	2	2	2	3	3	1	3	3	2
3	3	3	3	2	2	2	2	2	2
9	9	9	9	3	3	1	3	3	9
1	3	3	3	2	2	1	3	3	1
3	3	2	2	3	2	3	3	3	2
3	3	2	2	3	2	3	3	3	2
3	3	2	2	3	2	3	3	3	2
3	3	2	2	3	2	3	3	3	2
9	9	9	9	9	9	9	9	1	1
2	1	3	2	1	2	1	2	3	1
2	3	2	3	1	2	1	1	2	2
2	2	2	2	2	2	2	2	2	1
2	2	2	2	1	1	3	2	2	2
3	2	1	1	1	2	3	2	2	1
2	1	2	1	1	1	2	3	1	1
1	1	1	1	1	1	2	3	1	1
1	2	3	2	9	2	3	3	2	9
9	9	9	1	9	9	2	9	9	1
9	9	9	9	9	9	3	9	9	9
3	2	2	3	3	3	2	2	2	9
3	3	3	3	3	3	1	3	3	3
9	9	9	9	9	9	3	9	9	1

9	9	9	9	9	9	3	9	9	1
1	1	1	1	1	1	3	1	1	1
1	3	2	3	9	9	3	9	9	3
2	2	2	2	2	2	2	1	2	2
1	2	3	2	2	1	2	9	1	2
9	9	9	9	2	9	9	9	9	2
9	9	9	9	1	9	9	9	9	1
9	9	9	9	9	1	9	9	9	9
2	2	2	1	9	1	3	9	9	2
1	2	2	1	2	1	2	2	3	2
1	1	1	1	2	2	2	2	2	1
9	9	9	9	9	1	9	9	9	2
9	9	9	9	9	1	9	9	9	9
9	9	9	9	2	9	9	9	9	2
3	2	2	1	2	2	2	2	2	2
9	9	9	9	1	9	9	9	9	1
3	3	3	3	1	1	2	2	3	1
9	1	2	1	1	9	9	9	9	1
1	1	1	3	1	3	3	1	1	1
9	9	9	9	1	9	9	9	9	9
1	1	1	1	1	1	3	2	2	1
2	2	2	2	1	1	3	1	1	2
1	3	3	3	2	2	2	2	2	1
9	9	9	9	1	1	3	1	1	2
1	1	1	1	1	2	2	2	2	2
1	1	1	1	1	1	3	1	1	2
1	2	1	2	1	1	3	2	2	1
9	9	9	9	9	1	9	9	9	9
9	9	9	9	1	9	9	9	9	9
9	9	9	9	1	9	9	9	9	9
9	9	9	9	2	9	9	9	9	1
9	9	9	9	1	9	9	9	9	9
9	9	9	9	2	9	9	9	9	2
2	1	2	1	2	3	2	1	2	2
1	2	3	2	1	3	2	1	2	1
1	9	9	9	9	9	9	9	1	1
2	1	2	1	1	2	2	2	3	1
9	9	9	9	9	9	3	9	9	9
2	3	3	3	2	1	1	3	3	1
3	3	3	3	3	3	1	1	2	9
3	2	9	3	1	2	1	2	3	2
9	9	9	9	9	9	3	9	9	1
3	3	3	3	3	3	1	3	3	3
2	2	2	2	2	2	2	2	2	2
9	9	9	9	9	9	3	9	9	9
2	2	2	2	2	2	2	2	2	3
9	9	9	9	9	9	9	1	9	9

9	9	9	9	1	9	9	9	9	9
9	9	9	9	1	9	9	9	9	9
9	9	1	9	1	1	3	1	1	1
9	9	9	9	1	9	9	9	9	9
2	3	3	2	3	3	1	1	2	3
9	9	9	9	1	9	9	9	9	9
3	3	3	3	3	1	9	2	2	2
1	2	2	3	3	1	2	1	3	1
3	3	3	3	2	2	3	2	2	1
1	2	1	1	1	2	3	1	2	1
2	3	3	3	2	2	2	2	2	1
2	2	2	3	2	2	1	2	3	2
3	2	2	3	1	1	2	3	3	1
2	3	2	1	1	2	2	1	2	1
2	2	2	1	2	1	3	1	1	1
2	2	2	2	1	2	2	2	3	1
1	1	1	1	2	2	3	2	2	1
2	1	3	3	2	3	3	3	3	1
2	3	2	3	2	2	2	2	2	1
9	2	1	2	1	2	9	3	2	1
9	2	9	9	9	9	3	9	9	9
9	9	9	9	9	9	3	9	9	1
9	9	9	9	9	9	3	9	9	9
9	9	9	9	9	9	3	9	9	9
9	9	9	9	1	9	9	9	9	9
9	9	9	9	2	9	9	9	9	9
9	9	9	9	1	9	9	9	9	9
9	9	9	9	2	9	9	9	9	2
9	9	9	9	2	9	9	9	9	2
9	9	9	9	2	9	9	9	9	2
9	9	9	9	2	9	9	9	9	2
9	9	9	9	2	9	9	9	9	2
9	9	9	9	1	9	9	9	9	2
9	9	9	9	9	9	3	9	9	9
9	9	9	9	9	9	3	9	9	9
1	1	3	1	1	1	3	2	2	1
1	9	1	1	1	2	1	3	3	1
9	9	9	9	1	9	9	9	9	9
1	2	1	2	2	1	2	1	2	1
9	9	9	9	1	9	9	9	9	1
9	9	9	9	1	9	9	9	9	1
9	9	9	9	1	9	9	9	9	1
9	9	9	9	1	9	9	9	9	1
9	9	9	9	2	9	9	9	9	1
1	2	2	2	2	1	2	2	1	2
2	1	2	2	1	2	3	2	1	1
2	1	2	1	2	1	2	1	2	1

1	2	2	2	2	2	2	2	2	2
2	2	3	3	2	2	3	1	1	1
2	2	2	2	3	3	2	1	1	1
2	2	2	2	3	2	3	2	2	1
1	9	9	9	9	9	9	9	1	1
9	9	9	9	1	9	9	9	9	1
9	9	9	9	1	9	9	9	9	1
9	9	9	9	1	9	9	9	9	2
9	9	9	9	9	9	9	9	9	9
9	9	9	9	9	9	9	9	9	3
9	9	9	9	2	9	3	9	9	9
9	2	9	9	1	9	9	9	3	1
9	9	9	9	1	9	9	9	9	1
9	9	9	9	2	9	9	9	9	9
9	9	9	9	9	9	3	9	9	9
2	1	2	3	1	2	2	2	2	2
2	3	1	2	2	3	1	1	2	2
9	9	9	9	9	9	9	1	9	9
2	2	2	2	2	2	2	1	2	2
1	2	3	2	2	1	3	3	3	1
3	3	3	3	3	3	2	2	2	2
9	9	9	9	9	9	9	1	9	9
3	3	3	3	3	1	2	3	3	2
3	3	3	3	2	2	2	2	2	1
9	9	9	9	9	9	9	9	2	2
1	9	9	9	9	1	1	2	3	1
9	9	9	9	9	9	3	9	9	2
3	2	3	3	3	3	3	2	2	1
1	1	1	1	3	3	1	2	1	1
3	1	2	1	3	3	3	2	3	1
2	2	2	2	3	3	2	2	1	2
9	9	9	9	9	9	9	2	9	9
3	3	3	2	1	9	9	9	9	1
9	9	9	9	2	9	9	9	9	2
9	9	9	9	9	9	3	9	9	2
3	3	2	3	3	1	3	1	2	2
2	2	2	2	2	2	2	2	2	2
1	3	1	1	1	2	2	1	2	1
2	3	2	1	2	3	2	1	2	2
3	3	3	3	2	1	1	3	3	2
2	2	3	1	3	2	2	1	3	3
3	3	3	1	3	3	3	1	3	3
1	3	9	1	3	1	3	3	3	1
9	9	9	1	9	9	9	1	9	9
2	2	2	2	2	2	2	2	2	2
2	2	2	2	2	2	2	2	2	2
3	3	3	3	3	3	3	3	3	1

1	1	1	1	1	1	1	3	3	1
1	2	2	1	3	3	2	2	1	2
1	3	2	2	3	3	9	1	2	1
9	9	9	1	9	9	9	1	9	1
2	2	2	2	2	2	2	2	2	2
3	3	2	3	2	2	2	1	2	2
1	2	2	2	2	2	2	1	3	2
2	2	2	2	2	2	2	2	2	2
2	1	2	2	1	1	1	3	3	1
2	2	3	3	2	2	3	2	2	2
3	3	3	2	2	2	2	2	2	2
3	3	3	3	2	2	3	3	3	2
2	2	2	2	1	2	2	2	2	1
3	3	3	3	1	3	1	3	3	9
1	3	3	1	1	1	1	3	3	1
1	2	2	1	3	3	3	2	2	1
3	3	3	2	3	2	3	2	3	3
2	2	2	2	2	2	2	2	2	2
2	2	2	2	2	2	2	2	2	2
2	2	2	2	2	2	2	2	2	2
2	2	2	2	2	2	3	2	2	2
2	2	2	2	2	2	2	2	2	2
2	2	2	2	2	2	2	2	2	2
9	9	9	9	9	9	9	9	9	2
3	3	3	3	1	3	1	3	3	3
9	9	9	9	9	9	3	9	9	1
1	3	1	2	1	2	3	1	1	1
3	3	3	1	9	9	3	9	9	9
1	2	2	3	1	1	1	1	2	2
1	9	9	9	9	9	3	9	9	9
3	3	3	3	9	9	3	9	9	1
3	3	3	3	3	3	1	1	3	1
3	3	3	2	3	1	3	1	2	2
2	9	1	2	3	2	2	1	3	1
1	3	3	3	2	2	2	1	2	1
2	1	2	1	3	3	1	1	2	9
2	2	2	1	1	1	2	2	2	1
3	3	1	1	1	2	1	2	1	3
2	2	2	2	1	2	2	2	2	1
2	2	2	2	2	2	2	2	2	2
9	9	9	9	9	1	9	9	9	9
2	2	2	2	2	1	2	2	2	1
3	3	3	3	2	1	9	1	3	1
9	9	9	9	2	1	2	3	2	1
3	2	1	2	3	2	1	3	1	1
3	3	3	2	3	3	3	2	3	1
1	2	2	1	2	2	3	2	2	1

2	1	2	2	2	1	1	2	2	2
3	3	3	3	9	9	3	9	9	3
3	2	2	3	2	3	2	1	2	9
2	2	2	2	2	2	2	2	2	2
3	3	3	3	2	2	2	2	3	2
9	9	9	1	9	9	3	9	9	9
2	2	2	2	1	1	2	2	3	1
2	3	3	2	2	2	2	3	3	1
2	2	2	2	2	2	2	2	2	2
3	3	2	3	2	2	1	3	3	2
2	2	2	2	2	2	2	2	2	2
3	1	3	3	3	3	2	1	2	3
9	9	9	9	9	9	2	9	9	9
2	2	2	2	2	2	2	2	2	2
2	2	2	2	2	2	2	2	2	2
2	2	2	2	2	2	2	2	2	2
2	2	2	1	2	1	3	2	3	9
2	3	9	2	2	2	2	2	2	2
1	9	9	9	3	2	3	3	3	2
9	9	9	9	9	9	9	1	9	1
1	2	1	2	2	2	2	2	2	2
2	2	2	1	2	2	2	2	2	2
2	2	3	3	3	1	3	3	3	2
2	3	2	2	2	2	2	2	2	2
1	3	2	2	2	2	2	2	2	1
2	3	3	2	1	1	2	3	3	1
3	3	3	1	3	2	3	1	3	2
1	1	1	1	2	1	2	2	2	1
2	3	2	3	2	1	3	3	3	2
3	3	3	3	9	9	9	9	3	2
3	3	3	3	3	1	1	3	3	2
9	9	9	9	9	9	9	9	1	9
3	1	3	1	3	1	2	2	2	1
9	9	9	9	9	9	3	9	9	3
2	2	2	2	1	3	1	3	3	2
2	2	2	2	2	2	2	2	2	2
1	2	2	1	1	1	2	3	3	2
2	2	2	2	2	1	2	2	2	1
1	3	1	3	2	3	3	2	2	2
9	3	2	2	3	1	2	1	2	3
3	2	2	2	2	2	2	2	2	2
2	2	2	2	2	2	2	2	2	2
2	2	2	2	2	2	2	2	2	2
2	2	2	2	2	2	2	2	2	2
2	3	3	2	3	2	2	2	2	2
2	2	2	2	2	2	2	2	2	1
3	3	3	3	3	2	3	2	1	3

9	9	9	9	9	9	9	9	9	9
3	1	1	3	3	1	3	3	1	1
9	9	9	9	1	9	9	9	2	1
9	9	9	9	1	9	9	9	9	9
9	3	2	1	2	3	3	3	3	3
9	1	2	1	2	2	2	2	2	2
9	9	9	9	9	9	3	9	9	1
9	9	9	9	9	9	3	9	9	2
1	3	9	1	3	9	9	1	9	9
9	9	9	9	9	9	3	9	9	1
2	3	9	1	2	9	9	9	1	9
2	2	3	2	3	1	3	3	3	2
3	3	3	3	3	3	3	3	3	1
9	9	9	9	3	9	9	9	9	9
3	3	3	3	3	1	1	3	3	3
2	3	3	3	3	3	1	3	3	1
3	3	1	1	3	3	1	1	3	1
9	9	9	9	9	1	9	9	9	2
3	2	2	2	1	2	1	2	3	1
2	2	2	2	2	2	2	2	2	2
3	3	2	2	2	2	2	2	2	1
9	9	9	9	9	9	2	9	9	9
3	3	2	2	2	2	3	2	2	1
3	3	3	2	1	3	1	3	3	3
2	2	2	2	2	2	3	1	2	1
3	3	3	2	2	1	2	2	3	3
9	9	9	9	2	9	9	9	9	2
9	9	9	9	2	9	9	9	9	2
3	3	2	3	2	1	2	3	3	1
2	3	2	3	3	1	3	2	3	1
2	3	3	1	2	1	2	3	1	3
3	3	3	3	3	3	1	3	3	1
9	3	2	2	2	1	1	2	2	3
9	9	9	9	9	9	9	1	9	9
3	3	3	2	2	2	2	2	2	2
9	9	9	1	2	1	2	1	2	1
1	3	1	1	3	3	1	1	3	1
9	9	9	9	9	1	9	9	9	9
9	9	9	9	9	1	9	9	9	9
1	2	3	3	2	2	3	2	3	3
9	9	9	9	9	1	9	9	9	9
1	1	3	1	3	2	3	2	3	1
3	3	3	3	3	3	3	3	3	1
3	3	3	3	1	3	1	3	3	2
9	9	9	9	9	1	9	9	9	9
2	3	3	1	9	1	9	9	9	9
9	9	9	9	9	1	9	9	9	1

9	9	9	9	9	9	3	9	9	9
3	3	2	3	3	1	2	1	2	1
9	9	9	9	9	9	9	1	9	9
1	2	3	1	2	2	1	2	3	2
3	3	3	3	2	2	2	2	2	2
9	9	9	1	9	1	9	9	9	1
9	9	9	9	9	9	3	9	9	9
2	2	1	1	1	2	2	2	2	1
2	3	2	3	3	2	3	3	3	1
1	3	3	3	3	1	3	3	3	1
3	3	3	3	1	1	1	3	3	1
2	2	3	3	1	1	1	3	3	1
3	2	2	1	2	2	3	3	3	2
3	3	3	1	9	9	3	9	9	9
1	1	1	1	2	1	3	3	3	9
9	9	9	9	2	1	3	3	3	1
1	3	3	2	1	1	1	3	3	1
3	3	3	3	1	1	1	3	3	1
9	9	9	9	1	9	9	9	9	9
2	1	2	1	1	2	2	1	2	1
2	1	2	1	1	1	1	3	3	2
9	9	9	9	9	9	9	1	9	1
3	3	3	3	3	3	1	3	3	3
9	9	9	1	9	9	9	1	9	9
2	2	2	2	2	2	3	2	2	2
9	9	9	9	9	1	9	9	9	1
3	3	3	3	2	1	1	3	3	9
3	3	3	3	1	3	1	3	3	1
9	9	9	9	9	1	9	9	9	9
9	9	9	9	9	9	3	9	9	1
9	9	1	9	1	9	9	9	9	1
9	9	9	9	2	9	9	9	9	9
9	9	9	9	2	2	2	2	1	3
3	3	3	3	1	2	2	2	2	2
3	3	3	3	1	9	9	9	9	2
3	3	1	3	9	9	3	9	9	1
9	9	9	9	1	9	9	9	9	1
9	9	9	9	9	1	9	9	9	1
2	9	9	9	1	9	3	9	9	2
9	9	9	9	9	9	3	9	9	1
9	9	9	9	9	1	9	9	9	1
9	9	9	9	2	9	9	9	9	9
3	3	3	3	3	3	1	3	3	3
2	2	2	2	2	2	2	2	2	2
3	3	3	2	3	1	3	3	3	1
2	2	2	2	2	2	3	2	2	1
3	3	2	3	2	1	1	3	3	3

9	9	9	9	9	9	3	9	9	9
1	9	3	3	9	1	3	3	3	2
3	3	2	2	2	2	2	2	2	3
9	9	9	9	9	9	9	1	9	2
3	3	3	3	2	2	2	1	3	1
9	9	9	1	9	1	9	9	9	1
3	3	3	2	2	1	2	2	3	3
9	9	9	9	2	9	9	9	9	2
9	9	9	9	1	9	9	9	9	1
9	9	9	9	9	9	3	9	9	9
2	2	2	2	2	2	2	2	2	2
2	2	1	3	2	2	2	1	3	2
9	9	9	9	2	9	9	9	9	2
3	3	3	3	1	2	2	2	2	2
9	9	9	9	1	9	9	9	9	1
3	2	2	1	1	2	1	3	3	3
2	2	3	3	3	2	2	1	2	1
3	2	2	2	2	2	2	2	2	2
9	9	9	9	9	9	3	9	9	3
1	3	3	1	3	1	3	3	1	2
9	9	9	9	1	9	9	9	9	9
2	1	3	1	1	1	2	3	3	1
3	3	3	3	3	1	1	3	3	3
9	9	9	9	9	9	3	9	9	9
3	3	3	3	1	1	1	3	3	3
2	2	3	3	3	3	1	1	3	2
3	3	3	3	3	3	2	1	3	1
9	9	9	9	1	1	1	3	3	1
1	3	1	1	3	1	3	3	2	2
2	2	2	2	1	1	3	3	3	2
1	2	2	1	2	9	9	9	9	2
9	9	9	9	9	1	9	9	9	9
2	2	3	2	3	3	2	3	3	2
2	2	2	2	1	2	2	3	3	2
3	3	2	2	2	2	2	2	2	2
9	9	9	9	9	1	9	9	9	2
3	3	3	3	1	3	1	3	3	3
3	3	3	3	1	3	1	3	3	3
3	3	3	3	2	2	2	2	2	1
2	9	2	2	3	9	1	2	9	3
2	1	1	3	1	3	3	1	1	1
9	9	9	9	9	1	9	9	9	9
3	3	3	3	2	1	3	2	1	1
3	3	3	3	2	2	2	2	2	1
3	2	2	2	3	2	3	2	3	9
2	3	3	1	1	2	2	3	3	2
9	9	9	9	9	1	9	9	9	9

1	1	1	1	1	1	3	1	1	1
1	2	3	1	2	2	2	1	2	3
3	3	3	3	3	3	1	3	3	1
2	1	3	2	3	3	1	1	3	2
9	9	9	9	9	9	3	9	9	9
3	9	2	3	1	3	2	1	2	1
9	9	9	9	3	3	3	3	3	1
9	9	1	9	9	1	9	9	9	1
2	2	2	1	2	2	2	2	2	2
3	3	3	3	2	1	1	3	3	3
9	9	1	9	9	9	3	9	9	1
3	3	3	3	3	3	1	3	3	3
2	3	1	1	2	3	9	3	3	2
3	3	1	3	1	3	1	3	3	1
2	3	2	3	2	1	1	2	3	1
3	2	3	3	9	9	3	9	9	2
3	3	3	3	3	3	3	1	3	3
1	1	2	2	1	2	3	2	1	3
9	9	9	1	9	9	9	1	9	9
3	2	2	3	1	2	2	3	3	1
2	2	2	2	2	2	2	2	2	2
2	2	2	2	2	2	2	2	2	2
2	3	2	1	3	1	3	1	3	3
9	9	9	9	9	1	9	9	9	9
3	3	2	2	3	3	2	1	2	3
3	3	3	3	2	2	3	2	2	2
3	3	2	2	3	3	2	1	2	3
3	3	3	3	1	1	1	3	3	2
2	3	3	1	3	2	3	2	2	1
2	3	3	2	3	1	1	3	3	3
9	9	9	9	9	9	3	9	9	9
9	1	3	9	3	3	1	1	3	9
2	1	2	3	1	1	3	1	2	1
9	9	9	9	9	9	3	9	9	9
1	1	1	1	1	1	3	1	1	1
9	9	9	9	9	9	9	9	1	1
9	9	9	9	9	9	3	9	9	3
2	2	2	2	2	2	2	2	2	2
9	9	9	9	9	9	3	9	9	9
1	2	2	2	1	1	1	3	3	2
2	2	2	2	2	2	2	2	2	2
2	2	2	2	2	2	2	2	2	2
2	3	3	2	3	1	3	3	3	1
1	3	1	2	3	1	1	3	3	2
9	9	9	9	9	9	9	1	9	9
9	1	2	9	1	9	9	9	9	1
1	2	2	3	2	1	3	3	3	2

9	9	9	9	1	9	9	9	9	9
2	3	1	2	1	2	2	3	3	1
3	3	3	2	3	3	3	3	3	1
2	2	2	1	9	9	9	1	9	1
3	3	3	3	3	1	2	3	3	1
3	3	3	3	1	1	1	3	3	1
9	9	9	9	9	1	9	9	9	1
9	9	9	9	1	9	9	9	9	9
1	1	2	3	2	1	2	1	1	2
2	2	3	3	2	1	1	3	3	2
3	3	3	3	3	1	1	3	3	1
3	2	2	3	2	1	1	3	3	2
3	3	3	3	3	9	3	3	3	2
2	1	3	3	3	1	3	1	3	9
3	3	3	3	3	1	1	3	3	9
3	3	3	3	2	1	3	3	3	1
3	3	3	1	1	1	2	3	2	1
3	3	3	3	3	1	1	3	3	1
9	9	1	9	9	9	9	9	2	9
3	3	2	1	2	1	3	3	3	1
2	3	1	2	3	2	2	2	2	3
9	1	9	3	9	1	9	9	9	1
2	2	3	3	1	1	2	1	3	3
9	9	1	9	9	9	3	9	9	9
9	9	9	9	9	9	3	9	9	1

DuringPeri	SeventoTe	MidCycle	SeventoTe	EarlyPerioc	LatePeriod	NoBabies	ManyBabie	HighFat	BirthContr
3	3	3	3	2	2	2	2	2	2
2	2	2	2	2	2	2	2	2	2
2	2	2	2	2	2	2	2	2	2
2	2	2	2	2	2	2	2	2	2
2	2	3	3	1	2	3	2	2	2
2	2	2	2	2	2	2	2	2	2
2	2	2	2	2	2	2	2	2	2
2	2	2	2	2	2	2	2	2	2
1	1	1	2	1	2	2	2	2	2
2	2	3	2	2	1	2	3	1	2
2	1	3	1	3	3	3	3	1	1
2	2	3	3	1	1	2	2	3	3
2	2	2	2	3	2	1	2	2	2
1	1	2	2	1	2	3	2	2	1
2	2	2	2	2	2	2	2	2	2
2	3	3	3	2	2	2	2	2	2
2	2	2	2	2	3	3	3	2	9
3	1	3	3	2	2	2	2	2	2
2	2	2	3	2	3	3	3	1	2
2	2	1	1	2	2	2	2	2	2
2	1	2	2	2	2	2	3	2	3
2	1	3	3	2	2	2	2	2	2
3	1	2	3	2	2	3	3	2	2
2	1	3	1	1	2	2	2	2	2
2	2	2	2	2	2	2	2	3	2
3	1	3	3	3	1	3	1	3	3
9	9	9	9	9	9	9	9	9	9
3	3	3	3	3	3	3	1	1	2
2	2	2	2	2	2	3	2	2	2
2	1	1	3	2	1	2	3	2	1
2	2	2	2	3	3	3	3	3	3
2	2	2	2	2	2	2	2	2	1
3	2	3	3	9	9	9	9	9	9
1	1	2	1	9	1	3	1	2	1
1	2	1	1	2	9	1	2	9	3
3	3	2	3	1	1	1	2	3	3
1	2	3	2	2	2	2	2	1	3
2	3	3	2	3	3	3	3	2	1
2	2	2	2	3	3	2	3	2	2
2	2	3	3	1	2	1	2	2	3
1	2	2	2	2	2	2	3	2	2
2	9	9	9	3	9	2	9	9	9
9	9	9	9	9	9	9	9	9	9
2	3	2	2	3	3	3	3	2	1
2	2	1	3	2	2	1	1	1	2
9	9	9	9	9	9	9	9	9	9

2	2	3	3	1	2	2	3	2	2
2	2	3	3	1	2	2	1	2	2
1	2	2	2	2	1	2	2	2	2
1	1	2	2	2	2	1	2	2	2
1	3	9	3	3	3	3	3	3	1
1	3	2	3	3	3	3	3	1	3
2	3	2	3	3	2	2	2	2	2
2	9	9	3	1	3	3	3	3	3
1	1	1	1	9	9	1	1	1	9
2	1	2	1	2	2	1	2	3	9
9	9	2	9	2	9	2	9	9	9
1	2	2	2	1	1	2	2	2	2
2	1	1	1	1	1	1	1	2	2
1	1	1	1	1	1	3	3	2	2
2	2	2	2	1	1	2	2	2	1
1	1	2	2	1	1	1	2	2	2
2	2	2	1	2	3	3	1	1	2
3	2	1	1	9	9	2	9	1	3
2	2	2	2	2	2	2	2	2	2
3	3	3	3	2	3	3	2	9	2
3	3	3	3	3	3	3	3	3	3
1	1	2	2	1	2	3	3	2	2
2	2	1	1	1	1	1	1	2	2
2	2	3	2	1	2	2	3	2	1
2	2	3	2	1	2	2	1	1	2
1	3	2	2	2	2	3	2	1	2
1	3	2	1	3	1	3	3	1	2
1	1	2	2	1	3	2	3	1	2
1	2	1	2	3	3	3	3	1	2
1	3	3	3	3	3	3	3	9	1
9	9	9	9	9	9	9	9	9	9
2	2	2	2	2	2	2	2	2	2
1	2	3	3	3	1	2	9	9	9
1	2	2	2	2	1	2	2	2	2
3	1	3	2	3	3	1	9	1	2
2	2	2	1	2	2	2	2	2	2
3	1	1	1	1	3	3	3	2	1
2	2	3	2	2	1	1	2	2	1
1	1	1	3	1	3	3	2	2	2
2	2	2	2	1	2	2	1	1	2
3	1	3	2	2	1	3	2	1	2
2	2	2	2	9	9	9	9	9	9
1	3	2	3	1	2	3	3	1	1
2	2	2	2	1	2	2	3	3	2
2	2	3	2	2	2	3	3	2	2
2	3	1	2	2	2	2	2	2	2
2	2	2	2	2	2	3	3	2	2

3	1	1	2	1	2	1	2	3	1
2	2	3	2	1	3	9	1	9	3
2	2	2	3	2	2	2	2	2	2
2	2	3	2	1	1	1	1	2	2
2	1	1	3	1	1	2	3	2	1
2	2	2	1	2	1	1	1	1	1
2	2	2	2	2	2	3	2	2	2
2	3	2	1	3	2	3	1	2	3
2	2	2	2	2	2	2	2	2	1
1	3	2	3	3	3	3	3	2	2
9	9	9	9	9	9	9	9	9	9
1	2	2	1	1	2	2	1	1	2
2	1	1	1	1	1	1	2	3	2
2	2	2	2	2	2	2	2	2	2
2	2	2	2	2	2	3	1	2	1
2	2	2	2	2	2	3	1	9	1
1	2	3	2	1	2	1	3	1	1
3	3	3	3	3	3	2	2	2	2
9	1	2	1	3	3	3	3	3	3
1	2	2	1	1	1	1	2	2	1
3	3	3	3	1	3	3	3	2	3
3	2	1	2	2	2	3	3	3	2
2	2	1	1	1	1	1	1	1	2
2	2	2	2	2	2	2	2	2	2
2	2	2	2	2	2	2	2	2	2
2	3	2	2	3	3	3	3	3	2
2	2	2	2	1	1	2	2	3	1
1	2	1	2	2	1	2	1	2	1
2	1	2	1	1	2	1	3	3	2
1	1	1	2	3	2	3	3	3	2
1	2	3	2	1	2	1	2	2	2
2	2	1	2	2	2	2	2	2	1
2	3	2	1	1	2	3	2	2	1
2	9	1	2	1	2	1	2	1	2
2	3	3	1	3	2	2	1	2	1
9	1	3	1	1	2	3	2	9	2
9	9	9	9	2	9	9	9	9	9
2	1	2	3	2	3	2	2	1	1
3	3	2	2	3	1	9	9	9	9
2	9	2	2	1	3	9	1	3	3
2	2	3	2	1	1	2	1	1	1
9	9	9	9	9	9	9	9	9	9
2	1	1	1	1	2	2	1	2	2
2	2	3	3	1	2	2	3	2	2
3	2	2	1	2	2	2	2	1	2
3	2	2	3	2	2	2	2	2	2
2	2	1	1	2	1	2	3	1	2

3	9	1	2	3	2	3	9	2	1
3	3	3	3	3	3	3	3	3	3
3	3	3	3	3	3	3	2	9	3
1	2	2	3	2	2	2	2	2	2
2	2	3	1	2	1	1	3	3	1
3	3	3	3	3	3	1	2	3	3
9	1	9	9	2	3	1	2	1	9
2	3	1	2	1	2	3	1	2	3
2	2	2	2	1	9	9	9	9	9
2	2	2	2	3	1	2	2	2	2
1	1	1	1	2	1	2	3	2	1
9	9	9	9	2	2	2	2	2	3
2	2	2	2	2	2	2	2	2	2
2	2	2	2	1	1	2	2	2	3
2	2	1	1	1	1	1	1	1	1
1	2	2	2	1	2	1	2	2	2
2	2	1	1	2	2	2	2	2	2
1	2	2	2	1	2	2	3	2	2
2	2	3	2	2	2	3	3	2	2
2	3	1	3	2	1	1	3	1	2
2	9	9	9	9	9	9	9	9	9
3	3	3	3	3	3	2	3	2	2
2	2	2	2	3	3	3	3	3	3
2	1	1	1	2	3	2	2	2	1
1	2	2	2	3	3	2	3	1	2
3	9	2	3	3	3	1	2	3	3
3	3	2	2	2	3	3	3	2	2
2	2	2	3	9	9	9	9	9	9
2	3	2	2	3	3	2	3	2	2
2	2	1	1	1	1	2	2	1	1
9	9	9	9	2	2	2	3	1	2
2	2	2	2	2	2	2	2	2	2
1	2	2	2	1	2	2	1	1	2
1	1	1	1	1	3	3	3	3	3
3	2	2	2	2	2	2	2	2	3
2	2	1	1	2	1	1	1	1	1
3	3	3	3	1	2	2	2	2	2
1	2	3	3	2	2	3	3	3	1
9	9	9	9	9	9	9	9	9	9
3	3	3	3	3	3	3	3	3	3
1	1	2	1	3	2	2	1	2	1
2	2	2	2	2	2	2	2	2	2
1	1	1	3	1	3	2	3	1	2
9	9	9	9	9	9	9	9	9	9
9	9	9	9	9	9	9	9	9	9
1	1	1	1	1	2	2	1	1	2
2	2	3	3	1	1	1	1	1	2

9	9	1	9	9	9	9	9	9	2
9	9	9	9	9	9	9	9	9	2
3	2	2	2	1	1	3	2	1	1
9	9	9	9	1	9	9	9	9	9
2	2	2	2	3	2	2	3	3	2
1	2	2	2	9	9	9	9	9	9
2	2	2	2	3	3	3	2	2	2
9	9	9	9	3	1	2	3	1	2
1	3	2	1	1	2	1	3	2	2
9	9	1	9	9	2	9	9	9	9
2	9	9	9	9	9	9	9	1	9
9	9	9	9	9	2	9	9	9	9
2	2	2	2	2	2	1	2	2	1
2	3	1	2	2	2	3	2	1	3
9	3	9	9	2	9	9	9	9	9
2	3	3	3	3	3	3	3	3	3
3	3	3	3	3	3	3	3	3	3
1	1	1	1	2	1	1	1	2	2
2	1	2	2	1	2	2	2	1	2
2	2	2	3	3	3	3	3	3	2
2	2	3	2	2	2	2	2	1	9
2	3	1	2	3	2	2	2	3	2
2	2	2	2	3	3	2	2	2	2
2	1	9	2	1	3	3	3	2	1
2	2	2	2	2	2	2	2	2	2
1	1	1	1	2	1	2	2	3	3
1	1	1	3	2	2	1	2	2	2
2	1	3	2	2	2	2	1	2	2
9	9	9	9	3	3	3	3	3	3
1	2	2	1	1	1	2	2	2	2
2	2	2	1	2	2	2	2	2	2
2	2	2	2	1	1	2	2	2	3
3	3	2	1	3	9	9	9	9	9
1	2	2	2	2	2	2	3	3	2
1	2	9	1	3	1	3	3	3	2
1	2	2	2	2	2	2	2	2	1
2	2	2	3	2	2	3	1	1	1
2	9	9	9	2	1	1	1	1	2
2	2	3	3	2	2	3	2	2	1
2	1	2	3	1	1	1	1	2	2
1	2	2	1	2	1	2	2	1	1
1	1	1	1	1	1	3	9	2	1
2	3	1	2	1	1	3	3	3	2
2	2	2	2	9	9	9	9	9	9
2	2	2	2	2	2	2	2	1	3
1	2	9	3	1	1	3	3	1	1
9	9	9	9	9	9	9	9	9	9

1	3	3	3	3	3	2	3	1	1
1	3	3	2	1	3	3	3	1	3
1	1	1	2	1	1	1	1	1	2
2	2	1	3	1	3	3	3	1	1
2	2	2	2	3	3	3	3	2	2
3	2	3	2	3	2	3	3	3	3
2	1	2	1	3	3	3	3	3	3
3	2	2	2	1	2	2	2	1	1
2	2	1	2	1	1	1	2	1	2
2	2	1	1	2	2	1	1	1	1
1	9	2	2	2	2	2	2	3	2
2	2	2	2	2	9	9	9	9	9
9	9	9	9	2	9	9	9	9	9
2	2	2	2	2	2	2	2	2	2
1	9	9	2	9	3	1	9	9	3
9	9	9	2	2	2	9	2	9	2
9	9	9	1	9	1	1	9	2	3
3	1	9	9	9	1	9	9	9	1
9	2	9	2	2	9	2	9	2	9
9	9	9	9	9	9	9	9	9	9
9	9	9	9	9	9	9	9	9	9
2	2	2	2	2	2	2	2	2	2
2	2	2	2	2	2	2	2	2	2
2	3	3	3	3	3	3	3	3	3
2	2	2	2	1	1	2	2	2	2
9	2	9	2	2	9	2	9	9	2
2	2	2	2	2	2	2	2	2	2
9	9	9	9	9	9	9	9	9	9
9	9	9	9	9	9	9	9	9	9
2	1	1	1	2	2	2	2	2	2
9	9	9	9	9	2	9	9	9	9
9	9	9	9	9	9	9	9	9	9
9	9	9	9	9	9	9	9	9	9
2	2	9	2	2	9	2	9	9	2
9	3	1	9	9	3	1	9	9	3
1	9	3	1	9	9	1	9	9	1
9	9	9	9	9	9	9	9	9	9
2	9	9	9	9	2	9	9	9	9
9	2	1	9	9	9	1	9	3	2
9	9	9	9	3	9	9	9	9	9
9	1	9	9	2	9	9	9	9	9
9	9	9	9	9	9	9	9	9	1
3	3	3	3	3	3	3	3	3	3
1	2	1	1	2	1	1	1	1	9
2	3	1	2	1	2	1	3	1	2
1	2	9	9	1	1	1	9	1	1
9	1	9	9	3	9	9	9	9	9

9	2	9	9	2	9	9	9	9	9
9	9	9	9	9	9	9	9	2	9
9	9	9	9	9	2	9	3	3	3
1	2	1	3	3	2	1	3	2	2
1	3	3	2	2	3	3	2	2	2
2	1	1	2	2	2	2	2	2	2
3	3	3	2	2	2	3	3	3	2
2	3	1	1	2	2	1	1	2	1
9	9	9	9	9	9	9	9	9	9
3	3	3	3	3	3	3	3	3	3
9	9	9	9	9	9	9	9	9	9
9	9	9	9	9	9	9	9	9	9
1	9	9	9	1	9	9	9	9	9
2	1	3	1	1	1	1	1	2	2
1	1	1	1	1	1	1	1	1	1
1	2	1	1	1	2	1	1	1	2
9	9	9	9	9	9	9	9	9	9
2	2	2	2	1	2	2	2	1	1
1	1	1	1	1	1	1	1	1	1
2	2	2	2	3	3	3	9	2	3
1	2	1	3	3	1	2	1	2	3
1	2	2	3	2	2	3	3	3	2
2	1	3	2	2	3	3	2	3	2
2	2	2	2	2	2	2	2	2	2
2	2	2	2	2	2	2	2	2	2
2	2	2	2	2	2	2	2	2	2
2	2	2	2	2	2	2	2	2	2
2	3	1	2	1	2	3	1	2	3
2	2	2	2	2	2	2	2	2	2
2	3	2	3	1	1	1	2	2	2
2	2	2	2	2	2	2	2	2	2
2	2	2	2	2	2	2	2	2	2
2	1	2	1	2	2	3	2	3	2
3	3	3	2	2	2	2	2	2	2
3	2	3	1	3	2	1	3	3	3
2	9	3	3	1	2	3	2	3	1
9	9	9	9	9	9	9	9	9	9
2	2	2	2	2	2	2	2	2	2
3	3	3	3	3	3	3	3	3	3
3	3	3	3	3	3	3	3	3	3
1	1	2	2	2	9	9	9	9	9
3	1	2	3	2	3	1	3	2	1
2	1	1	1	3	3	1	1	1	2
2	1	1	1	1	1	1	1	1	1
3	3	3	3	1	1	1	2	2	2
2	2	2	2	2	2	2	2	2	2
2	2	2	2	2	2	2	2	2	2

3	1	2	3	2	3	1	2	3	1
9	9	9	9	1	9	9	9	9	9
1	9	9	9	9	9	9	9	9	1
9	9	9	9	1	9	9	9	9	9
9	9	9	1	2	9	9	9	9	9
2	2	1	1	1	1	1	1	1	1
9	9	9	9	2	9	9	9	9	9
9	2	9	9	9	9	9	9	2	9
2	2	2	2	2	2	2	2	2	1
2	9	9	9	9	9	9	9	9	9
9	2	9	9	9	9	9	9	2	9
9	9	9	9	9	9	9	9	9	2
2	9	9	9	2	9	9	9	9	9
9	9	9	9	9	9	2	9	9	9
9	9	9	9	1	9	9	9	9	9
9	9	9	2	9	9	2	9	9	9
2	9	9	9	9	2	9	9	9	9
2	3	1	3	2	1	3	1	2	3
2	2	1	2	1	2	1	3	1	2
2	2	2	2	2	2	2	2	2	3
1	2	2	3	3	2	2	2	1	1
2	1	1	2	3	2	3	3	1	2
1	1	1	1	1	2	1	3	1	2
2	3	2	1	2	3	2	9	2	2
1	3	2	2	3	3	3	2	3	2
3	3	3	3	3	3	3	3	3	3
2	2	2	2	2	2	2	1	2	1
2	2	3	2	3	2	3	3	3	3
1	2	1	2	1	1	2	2	1	2
2	2	1	1	1	1	1	1	2	9
3	2	1	3	1	2	2	2	2	2
2	2	2	2	3	3	1	1	2	1
2	3	3	2	2	2	1	1	2	1
1	1	1	1	1	1	2	2	1	1
9	9	9	9	9	9	9	9	9	9
2	2	2	2	3	3	3	3	2	2
9	9	9	9	9	9	9	9	9	9
1	1	1	2	2	2	1	1	2	1
2	1	2	2	1	1	2	2	2	1
2	2	2	2	2	2	2	2	2	2
9	9	1	9	9	9	9	9	9	9
3	3	3	3	3	3	3	3	3	3
9	9	9	9	3	3	3	3	1	3
2	2	1	1	3	3	2	2	3	2
2	2	2	2	2	2	2	2	2	2
2	3	1	2	1	2	2	3	2	2
2	2	2	2	2	2	2	2	2	2

1	2	2	3	1	2	2	2	2	2
9	2	9	9	9	9	9	9	9	9
9	2	9	9	2	2	2	2	2	2
2	3	1	2	1	2	2	9	1	2
1	1	1	2	2	2	2	2	2	2
9	9	9	9	2	9	9	9	9	9
1	1	2	2	1	1	2	2	2	2
1	2	2	2	1	2	2	9	3	2
9	9	9	9	9	9	9	9	9	9
2	2	2	2	3	3	2	3	2	3
3	1	3	3	3	3	3	3	3	3
9	9	9	9	9	9	9	9	9	9
9	9	9	9	2	9	9	9	9	9
9	9	9	9	2	1	2	2	3	2
9	2	9	9	2	9	9	9	9	9
2	2	1	2	2	3	1	9	3	2
9	9	9	9	9	9	9	9	9	9
9	9	9	9	2	9	9	9	9	9
9	1	9	9	9	9	9	2	9	9
2	2	2	3	3	3	3	3	3	3
2	2	2	2	2	2	2	2	2	2
2	2	2	2	3	3	3	3	2	2
1	2	2	2	2	2	2	1	2	2
9	9	1	9	9	9	9	9	1	9
9	1	9	9	9	9	9	9	9	9
1	1	1	1	1	2	2	2	2	1
1	1	1	1	1	1	1	1	1	1
1	1	2	1	2	3	1	2	3	2
2	2	2	2	2	2	2	3	3	3
9	9	9	9	9	9	9	9	9	9
9	9	9	9	2	1	1	2	2	2
2	1	2	1	2	1	2	3	2	1
1	1	2	2	2	2	3	2	2	2
1	2	3	3	2	2	2	2	2	2
3	3	2	2	3	3	3	1	1	1
2	1	2	3	1	2	1	2	1	1
9	9	9	9	9	9	9	9	9	9
3	2	2	1	1	1	2	2	2	2
1	2	2	2	2	3	3	2	3	9
2	2	2	2	1	1	1	1	1	1
2	2	1	3	2	2	2	2	2	2
2	2	2	2	2	2	2	2	2	2
2	2	2	2	2	2	2	2	2	2
2	3	2	1	2	1	2	1	2	2
2	3	2	3	3	3	3	3	3	3
2	2	2	2	2	2	2	2	2	2
3	3	3	3	3	3	3	3	3	3

2	2	2	2	2	9	9	9	9	9
1	9	9	9	9	9	9	9	9	9
9	1	9	9	9	9	9	1	9	9
1	9	9	9	1	9	9	9	9	9
2	2	1	2	9	9	9	9	9	9
2	2	3	2	3	2	2	9	1	3
2	2	2	2	2	2	2	2	2	2
9	2	2	2	2	2	9	2	2	2
9	9	9	9	9	9	9	9	9	9
9	1	9	9	9	9	9	9	9	9
2	2	2	2	2	2	2	2	2	2
2	2	2	2	2	2	2	2	2	2
2	2	2	9	2	2	2	2	2	2
2	2	2	2	2	2	2	2	2	2
2	2	2	2	2	2	2	2	2	2
9	9	9	9	2	9	9	9	9	9
3	3	3	3	3	3	3	3	3	3
9	9	9	9	1	9	9	9	9	9
9	9	9	9	2	9	9	9	9	9
9	9	9	9	2	9	9	9	9	9
9	9	9	9	9	9	9	9	9	9
9	9	9	9	2	9	9	9	9	9
2	2	2	2	2	2	2	2	2	2
2	2	2	2	2	2	2	2	2	9
9	9	9	9	9	9	9	9	9	9
9	1	9	9	2	9	9	9	9	9
9	2	9	9	9	9	9	9	9	9
9	1	9	9	2	2	2	9	2	9
3	1	3	3	2	2	2	2	2	2
9	2	9	2	9	9	9	9	9	9
9	2	9	2	2	9	2	9	2	9
3	1	9	3	2	2	2	2	2	2
9	3	1	9	1	9	1	9	1	9
9	9	9	2	9	9	9	9	9	9
3	3	3	3	3	3	3	3	3	3
9	1	9	1	9	9	9	9	9	9
2	2	2	2	1	1	9	2	9	2
2	2	2	2	9	9	9	9	9	9
1	3	2	1	2	1	3	3	1	1
2	2	2	2	2	2	2	2	2	2
9	9	9	9	9	9	9	9	2	9
2	2	2	2	2	1	3	3	1	2
3	2	3	2	2	3	2	2	1	3
2	3	2	3	2	3	2	3	2	3
1	3	3	3	3	3	3	3	3	3
2	2	9	1	1	1	1	2	2	2
2	2	2	2	9	9	9	9	9	1

9	9	1	9	9	9	9	9	1	9
3	3	2	1	1	2	2	3	3	2
9	9	9	9	2	2	2	2	2	2
9	9	9	9	1	1	1	1	1	1
2	2	2	2	2	2	2	2	2	2
3	2	1	3	2	3	2	2	3	1
3	3	3	3	3	3	3	3	3	3
3	2	1	3	2	3	2	2	3	1
3	2	3	3	2	3	3	3	1	3
2	2	2	2	2	2	2	2	2	2
2	2	2	2	2	3	3	3	3	3
2	9	2	3	2	1	3	2	2	2
9	1	9	9	3	3	3	3	3	3
9	1	2	2	2	2	2	2	2	2
9	9	9	2	9	9	9	2	9	9
1	1	2	2	2	2	1	1	1	2
2	2	2	2	2	2	3	3	2	3
2	2	2	2	2	2	2	2	1	2
1	2	3	1	3	3	3	3	3	2
2	3	2	3	2	2	2	2	2	2
9	9	9	9	2	2	2	2	2	2
3	1	3	3	1	1	1	1	2	2
3	2	9	1	1	2	1	9	3	2
9	9	9	9	9	9	1	9	9	9
9	9	9	9	9	9	9	9	9	9
2	2	2	2	1	1	1	1	1	1
2	9	9	9	9	2	2	2	2	2
2	1	1	1	3	3	3	3	3	3
9	9	9	9	9	9	9	9	9	9
9	9	1	9	9	9	9	9	1	9
2	3	2	1	2	2	2	2	1	2
3	1	3	2	3	2	3	2	3	1
9	9	9	9	9	9	9	9	9	9
1	9	9	9	9	9	9	9	9	1
2	1	1	2	1	2	2	2	1	1
9	9	9	9	9	9	9	9	9	9
1	2	2	1	1	3	3	2	2	1
1	1	1	1	3	3	3	3	3	3
1	2	2	2	3	1	2	2	3	2
2	2	2	2	2	2	2	2	2	2
9	9	1	9	2	1	2	3	2	1
3	3	3	3	3	3	3	3	3	3
9	9	9	9	2	9	9	9	9	9
3	3	2	3	2	2	2	3	3	3
1	2	9	1	2	1	1	2	3	3
2	2	2	2	2	2	2	2	2	2
3	3	3	2	2	3	3	3	2	2

9	9	9	2	9	9	9	9	9	9
2	2	2	2	2	2	2	2	2	2
1	1	2	2	3	3	2	3	1	2
2	2	2	2	2	2	2	2	2	2
2	2	2	2	2	3	3	3	2	3
9	9	9	9	9	9	9	9	9	9
9	9	9	9	1	1	2	1	1	2
9	9	9	9	2	2	2	2	2	2
1	2	1	1	2	2	2	1	1	2
9	9	9	1	2	2	2	2	2	2
3	3	3	3	2	2	2	2	2	2
2	9	9	9	3	1	3	2	2	3
2	1	1	2	3	3	3	3	2	2
2	2	2	2	2	2	2	2	2	2
1	3	1	1	3	1	3	3	3	3
9	2	9	2	3	3	3	3	3	3
9	2	2	2	9	9	9	9	9	9
9	9	9	9	9	9	9	9	9	9
9	9	9	9	1	9	9	9	9	9
9	2	9	2	9	9	9	2	9	9
9	9	9	9	2	9	9	9	9	9
9	9	9	9	9	9	9	9	9	9
2	2	2	2	2	2	2	2	2	2
9	9	9	9	2	9	9	9	9	9
9	9	9	9	9	9	9	9	9	9
9	9	9	9	3	9	9	9	9	9
9	9	9	9	9	9	9	9	9	9
1	1	1	1	1	9	1	1	1	9
9	3	9	3	3	3	3	9	9	9
9	9	9	3	9	9	9	9	9	9
9	9	9	9	3	9	9	3	9	9
2	2	2	2	2	2	2	2	2	2
1	1	1	1	1	1	1	1	1	1
9	9	9	9	3	9	9	9	9	9
2	9	2	2	2	1	9	2	1	9
9	9	9	9	9	9	9	9	9	9
9	9	9	9	2	9	9	9	9	9
9	9	9	9	9	9	9	9	9	9
9	2	9	1	1	9	9	1	9	2
9	9	9	9	1	9	9	9	9	9
2	2	2	2	2	2	2	2	2	2
9	9	9	9	2	9	9	9	9	9
9	9	9	9	2	9	9	9	9	9
9	9	9	9	2	9	9	9	9	9
2	2	2	2	2	2	2	2	2	2
2	2	2	2	9	9	9	9	9	9
9	9	9	9	9	9	9	9	9	9

1	1	3	2	9	9	9	9	9	9
2	2	2	2	1	2	2	2	2	2
9	2	9	1	9	2	9	9	9	2
2	2	2	2	2	9	2	9	9	2
9	9	9	9	9	9	9	9	9	9
9	9	9	9	9	9	9	9	9	9
9	9	9	9	9	9	9	9	9	9
9	9	9	9	2	9	9	9	9	9
9	9	9	9	9	9	9	9	9	9
9	9	9	9	9	9	9	9	9	9
9	9	9	9	9	9	9	9	9	9
9	9	9	9	9	9	9	9	9	9
9	9	9	9	9	9	9	9	9	9
9	9	9	9	9	9	9	9	9	9
2	2	2	2	2	2	2	2	2	2
2	2	2	2	2	2	2	2	2	2
2	2	2	2	2	2	2	2	2	2
2	2	2	2	2	2	2	2	2	2
2	2	2	2	9	9	9	9	9	9
2	2	2	2	2	2	2	2	2	2
2	2	2	2	9	9	9	9	9	9
9	9	9	9	2	2	9	9	9	9
9	9	9	9	9	9	9	9	9	9
2	2	2	2	9	9	9	9	9	9
2	2	2	2	2	2	2	2	2	2
2	2	2	2	2	2	2	2	2	2
9	9	9	9	9	9	9	9	9	9
2	2	2	2	1	2	1	2	1	2
9	9	9	9	9	9	9	9	9	9
2	2	2	2	2	2	2	2	2	2
1	1	1	1	1	1	1	1	1	1
1	3	1	2	2	2	2	2	2	2
2	2	2	2	9	9	9	9	9	9
2	1	2	1	2	1	2	3	2	2
1	1	1	1	9	9	9	9	9	9
9	9	9	9	2	2	2	2	2	2
9	9	9	3	2	9	9	9	9	9
2	2	2	2	2	2	9	2	9	2
1	1	1	1	1	1	1	1	1	1
9	9	9	3	3	9	9	9	9	9
9	9	9	9	9	9	9	9	9	9
9	9	9	9	9	9	9	9	9	9
2	2	2	2	2	2	2	2	2	2
9	1	9	9	9	2	9	2	9	9
9	1	9	1	1	9	3	1	9	1
9	9	9	2	2	9	9	9	9	9
9	2	9	2	2	9	9	2	9	9

9	9	1	9	2	9	9	9	9	9
9	9	9	9	9	9	9	9	9	9
9	1	9	3	2	9	9	9	1	9
9	9	9	9	3	9	9	9	9	9
9	9	9	9	9	9	9	9	9	9
9	9	9	9	9	9	9	9	9	9
1	1	1	9	2	9	9	9	9	9
9	9	9	9	2	9	9	9	9	9
3	1	9	9	1	2	9	2	9	2
1	2	2	2	1	1	1	9	1	9
9	9	9	9	9	9	9	9	9	9
9	9	9	9	9	9	9	9	9	9
3	3	1	3	9	9	9	9	9	9
9	9	9	9	9	9	9	9	9	9
3	2	9	2	3	3	3	2	2	9
2	3	1	2	3	3	2	1	3	1
9	9	9	9	9	9	9	9	9	9
2	1	9	9	2	2	2	2	2	2
2	2	2	2	2	2	2	2	2	2
1	2	9	9	2	2	9	2	9	9
9	9	9	9	2	9	9	9	9	9
9	2	9	9	1	9	9	9	9	9
2	2	2	2	3	3	3	3	1	2
2	2	3	3	1	2	3	1	2	3
3	1	2	2	2	2	3	2	2	2
2	1	2	2	2	3	3	3	2	2
1	3	3	2	3	3	3	3	2	2
3	2	2	2	2	2	2	2	2	2
1	1	3	2	3	2	3	3	2	2
2	2	2	2	2	2	2	2	2	2
2	2	2	2	2	9	9	9	9	9
9	9	9	9	2	9	9	9	9	9
2	2	9	3	3	2	3	3	1	3
9	9	2	9	9	9	9	9	9	9
9	9	9	9	1	9	9	9	9	9
9	9	9	9	9	9	9	9	9	9
9	9	9	9	9	9	9	9	9	9
9	1	9	1	9	2	9	9	2	9
9	9	9	9	9	9	9	9	9	9
9	2	9	2	2	9	2	9	9	2
9	9	2	9	2	9	9	2	9	9
2	2	2	2	2	2	2	2	2	2
2	2	2	2	2	2	2	2	2	2
2	2	2	2	2	2	2	2	2	2
2	2	2	2	9	9	9	9	9	9
2	2	2	2	2	2	2	2	2	2
1	3	2	2	2	3	2	2	3	2

2	1	2	2	2	1	3	3	2	2
2	2	3	2	2	2	3	3	3	2
9	9	9	1	9	9	9	9	1	9
2	1	2	2	2	2	3	2	3	2
3	3	3	3	3	3	3	3	3	3
2	2	2	2	2	3	3	2	2	2
2	1	2	2	3	3	2	2	2	1
3	3	3	3	3	3	3	3	3	3
2	2	2	2	2	2	2	2	2	2
2	2	2	2	2	2	2	2	2	2
2	2	2	2	2	9	9	2	9	9
2	2	2	2	2	2	2	2	2	2
1	2	2	1	2	2	2	2	2	2
1	2	3	2	3	2	1	2	3	1
2	2	3	3	2	1	2	3	3	2
2	2	2	2	2	2	2	2	2	2
1	2	3	1	2	2	2	1	2	1
2	2	3	2	2	2	3	2	2	2
2	2	2	2	2	2	3	3	3	1
1	1	1	3	3	3	1	2	3	3
1	2	2	2	2	1	2	2	3	3
2	1	3	2	2	2	1	3	1	2
2	3	1	2	2	2	3	2	2	3
2	2	2	1	2	3	3	3	2	2
3	3	3	3	2	2	2	2	1	2
1	2	2	2	3	3	3	3	2	2
2	2	2	2	2	2	2	2	2	2
9	2	2	3	2	3	2	2	2	3
2	2	2	1	3	3	9	2	9	1
2	1	1	1	1	1	2	3	1	2
3	3	3	3	3	3	3	3	3	3
2	1	2	2	3	3	3	3	3	3
3	3	3	3	3	3	3	3	3	3
2	2	2	2	2	2	2	2	2	2
1	9	9	9	9	1	9	9	9	9
3	2	1	2	3	2	3	2	1	3
2	2	2	2	3	3	2	2	2	2
1	1	1	1	2	2	2	2	2	2
2	2	2	2	3	3	2	2	2	2
9	9	9	9	9	9	9	9	9	9
3	2	2	2	2	2	2	3	2	2
9	9	1	9	9	9	9	9	9	1
2	2	2	3	2	1	2	1	2	1
2	1	2	3	2	9	9	1	9	9
3	3	2	2	2	1	2	1	2	3
2	2	3	2	2	2	3	2	3	3
3	2	3	3	2	3	1	2	1	2

1	1	1	2	2	1	2	2	1	2
3	3	3	3	3	3	3	3	3	3
1	1	3	1	3	1	3	1	1	3
3	3	2	3	2	2	1	3	3	3
1	2	1	3	1	1	2	3	3	1
2	1	1	3	2	3	3	3	3	3
3	3	3	3	2	2	2	2	2	2
2	1	3	3	2	1	2	2	1	2
3	2	1	2	3	3	3	3	1	2
2	2	2	2	2	2	2	2	2	2
1	2	1	2	3	2	2	3	3	3
3	2	2	2	2	2	2	3	2	2
9	2	1	2	2	1	2	1	2	2
9	3	2	2	1	1	3	2	1	3
2	2	2	2	3	2	3	3	2	2
2	2	2	2	3	2	2	3	2	2
2	2	3	2	1	2	3	3	3	3
3	2	3	3	2	3	2	3	2	3
2	2	2	2	2	2	2	2	2	2
9	9	9	9	9	2	9	9	9	9
1	2	2	2	2	1	2	1	1	2
2	1	1	2	2	1	2	2	1	2
3	2	2	3	2	3	3	2	3	2
2	1	2	1	2	2	1	3	2	2
3	3	3	3	3	3	3	3	3	3
2	1	3	2	1	1	2	3	1	2
9	9	9	9	9	9	9	9	9	9
2	2	2	2	2	2	2	2	2	2
2	2	2	2	2	2	2	2	2	2
2	2	2	2	2	2	2	2	2	2
3	2	2	1	2	1	3	9	2	3
9	9	9	9	2	3	2	9	1	2
2	2	2	2	3	3	3	3	2	2
3	2	2	2	3	3	3	3	3	2
3	2	3	2	3	2	3	3	2	3
2	2	3	2	2	2	3	2	9	1
1	1	1	1	3	2	2	2	2	2
3	2	3	2	2	3	2	3	2	3
3	3	3	1	3	3	3	3	2	1
1	2	2	2	3	3	3	2	1	2
2	2	2	2	2	2	2	2	2	2
2	2	2	2	2	2	2	2	2	2
2	2	2	2	2	2	2	2	2	2
2	2	2	2	2	2	2	2	2	2
2	2	2	2	3	3	3	3	2	2
2	1	1	2	3	2	3	3	2	3
2	2	2	2	2	2	2	2	2	2

1	2	2	2	3	2	2	1	2	3
2	2	2	2	3	3	3	3	2	2
3	3	3	3	2	2	2	2	2	2
2	2	2	2	2	2	3	3	1	2
9	9	9	9	2	2	2	2	2	2
2	2	2	2	3	3	3	3	2	2
2	2	2	2	3	3	3	3	2	2
2	2	2	2	2	2	2	2	2	2
3	2	2	2	2	2	3	2	2	2
9	9	9	9	9	9	9	9	9	9
1	3	2	2	1	2	3	2	2	1
1	3	2	2	1	2	3	2	1	2
9	9	2	9	9	9	9	9	9	1
9	9	9	9	9	9	9	9	9	2
3	2	2	2	3	1	3	2	2	2
9	9	9	1	9	9	9	9	9	9
1	9	9	9	9	9	9	9	9	9
9	9	9	9	9	9	9	1	9	9
9	9	9	9	9	9	9	9	9	9
2	2	2	2	2	2	2	2	2	2
2	3	2	3	3	3	3	9	1	1
1	9	9	9	9	9	9	9	9	9
2	1	2	1	9	9	9	9	9	9
2	2	2	2	2	2	2	2	2	2
3	3	1	3	2	2	2	1	3	2
2	2	2	2	2	2	2	2	2	2
1	2	9	3	2	3	3	1	9	2
9	9	9	9	9	9	9	9	9	9
2	2	2	2	2	2	2	2	2	2
2	2	1	2	2	2	2	2	3	2
9	9	9	9	9	9	9	9	9	9
2	2	1	3	1	2	3	1	2	1
2	2	2	2	1	1	2	3	2	3
2	2	2	2	2	1	2	2	2	2
2	2	2	1	2	1	2	2	2	1
2	2	2	2	2	2	2	2	2	2
1	9	9	9	9	1	9	9	9	9
2	2	2	2	1	3	3	2	1	1
2	2	2	2	2	2	2	2	2	2
2	2	2	2	3	2	1	1	2	1
1	1	1	1	2	2	1	1	1	2
1	3	2	3	2	3	3	3	1	1
2	2	3	2	2	2	2	3	1	1
9	9	9	9	3	3	3	2	2	1
3	1	2	3	3	2	3	2	3	1
9	9	9	9	2	1	9	3	1	3
3	3	3	3	3	3	3	3	3	3

1	3	3	2	3	3	1	2	2	3
2	2	2	2	2	2	2	2	2	2
3	2	3	1	2	2	9	3	3	3
2	2	2	2	2	1	2	2	2	2
3	2	2	3	3	2	3	2	3	2
2	2	2	2	2	1	2	2	2	2
9	1	9	9	9	9	9	9	9	9
3	3	3	3	3	3	3	3	3	3
3	2	3	9	3	3	3	3	3	3
1	2	2	2	2	1	2	2	3	2
2	2	2	2	2	1	3	9	3	2
9	9	9	9	9	9	9	9	9	9
3	1	1	1	1	1	2	2	2	1
9	9	9	9	9	9	9	9	1	9
9	9	9	9	9	9	9	9	9	9
9	9	9	9	9	9	9	9	9	9
2	1	2	1	2	3	1	1	2	1
3	9	2	3	1	3	3	3	1	1
3	3	2	3	3	3	3	3	3	2
2	2	2	2	2	2	3	3	2	2
9	3	2	1	2	3	9	2	2	2
1	1	1	1	1	1	1	3	1	3
2	2	2	2	2	2	2	2	2	2
2	2	2	2	2	2	2	2	2	2
1	1	1	1	3	3	3	3	1	3
2	2	2	2	2	2	2	2	2	2
2	2	2	2	2	2	2	2	2	3
9	9	9	9	2	9	9	9	9	9
2	2	2	2	2	2	2	2	2	2
2	2	2	2	2	2	2	2	9	2
1	1	1	1	1	1	1	1	1	1
2	1	3	2	2	3	2	1	3	1
2	2	2	1	2	2	1	1	1	1
2	2	2	2	2	2	2	2	2	2
1	2	2	2	2	1	3	9	2	2
1	2	3	1	2	9	1	3	2	1
1	1	1	1	1	1	1	1	1	1
3	3	3	3	2	2	3	3	2	2
9	3	2	1	1	3	2	2	2	1
2	2	3	2	3	2	3	2	2	3
9	9	9	9	9	9	9	9	9	9
2	2	1	1	2	2	2	9	1	1
2	2	2	2	2	2	2	2	2	2
1	2	2	2	2	2	2	2	2	2
3	3	3	3	3	3	3	3	3	3
1	2	1	2	3	3	1	3	3	2
9	9	9	9	3	3	3	3	3	3

3	9	1	2	3	3	3	3	3	3
2	1	2	2	3	2	3	2	3	2
2	3	2	3	3	3	2	2	3	2
2	2	2	2	2	2	2	2	1	2
2	3	1	2	1	2	9	9	1	3
9	9	9	9	9	9	1	9	9	9
2	1	2	3	3	3	3	3	2	2
9	9	9	9	9	9	9	9	9	9
9	9	9	9	9	9	9	9	9	9
9	9	9	9	9	9	9	9	9	9
2	1	9	2	2	1	2	2	1	2
9	9	9	9	9	9	9	2	9	9
2	3	2	2	3	3	3	3	2	2
9	9	9	9	2	9	9	9	9	9
2	2	2	2	2	2	2	2	1	2
9	9	9	9	9	9	2	9	9	9
1	3	2	1	2	3	3	2	2	2
9	1	9	9	9	9	1	9	9	9
9	1	9	9	9	9	1	9	9	9
9	2	9	9	9	9	2	9	9	9
1	1	1	1	1	1	1	1	1	1
2	1	2	3	2	2	1	1	2	2
2	2	2	2	2	2	2	2	2	2
2	1	1	2	3	3	3	2	3	2
3	3	3	3	1	1	1	1	2	9
3	2	2	2	3	3	3	2	3	3
3	3	3	1	3	1	1	1	3	3
9	1	9	9	9	9	9	9	1	9
2	2	2	2	2	2	2	2	2	2
1	3	3	3	9	1	3	3	3	3
1	3	1	3	1	1	3	1	1	3
9	9	9	9	3	9	9	9	9	9
3	3	3	3	3	3	3	3	3	3
9	9	9	9	3	9	9	9	9	9
9	9	9	9	1	9	9	9	9	9
2	9	9	9	1	1	2	1	1	2
9	9	9	9	1	1	1	1	1	1
3	1	1	1	9	9	9	9	9	9
3	2	2	2	9	9	9	9	9	9
2	2	2	2	2	2	2	2	2	2
2	2	2	2	2	2	2	2	2	2
2	2	2	2	2	2	2	2	2	2
3	3	3	3	3	3	3	3	3	3
2	9	2	9	2	9	9	9	2	9
2	2	2	2	2	2	2	2	2	2
2	2	2	2	2	2	2	2	2	2
9	1	9	1	1	9	1	3	1	3

1	3	1	9	9	1	9	9	9	1
1	1	1	1	2	2	9	9	2	9
2	2	2	2	2	2	2	9	2	9
9	9	9	9	1	1	1	1	1	1
9	9	1	9	1	2	9	3	1	9
9	1	3	1	1	9	1	9	3	1
1	9	2	9	9	1	9	9	9	2
1	3	2	1	2	1	3	9	1	3
9	1	1	1	1	9	2	9	2	9
1	1	1	1	2	2	2	2	2	2
2	2	2	2	2	2	2	2	2	2
9	9	2	9	2	9	2	9	9	2
2	2	2	2	2	2	2	2	2	2
3	3	3	3	9	9	9	9	9	9
2	2	2	2	2	2	2	2	2	2
9	9	9	9	9	9	9	9	9	9
2	9	2	2	2	9	9	9	2	9
2	2	2	2	9	9	9	9	9	9
1	9	3	1	3	1	3	2	1	9
2	2	2	2	2	2	2	2	2	2
2	2	2	2	2	2	2	2	2	2
2	2	2	2	2	2	2	2	2	2
1	1	3	2	1	2	2	1	3	2
1	2	9	1	1	2	1	1	1	9
2	3	2	3	2	3	2	3	2	2
3	3	3	3	3	3	3	3	3	3
9	1	1	2	3	1	2	1	2	3
2	2	2	2	2	2	2	2	1	2
1	3	1	3	1	3	2	1	1	1
2	2	2	2	2	2	3	3	1	1
9	2	3	2	2	2	2	2	2	2
2	3	3	3	3	2	1	2	3	3
1	2	2	2	2	2	2	2	2	2
9	1	1	1	1	2	1	2	1	1
1	1	3	3	1	2	1	2	2	2
1	3	2	3	1	1	1	1	1	1
3	3	3	3	3	1	2	3	1	1
1	2	2	2	3	3	9	1	1	1
2	2	1	3	1	2	3	3	2	2
2	2	9	2	2	2	3	3	9	2
2	3	1	1	3	1	1	1	3	3
3	2	1	2	3	2	2	3	2	3
2	2	2	2	2	3	2	2	2	3
9	9	9	9	9	9	9	9	9	9
2	1	1	2	9	9	9	9	9	9
2	2	2	2	2	2	2	2	2	2
1	9	9	9	3	1	1	3	2	9

9	9	9	9	1	3	3	1	2	2
9	9	9	9	1	3	3	2	2	2
2	2	2	2	2	2	2	2	2	2
2	2	2	2	2	2	2	2	2	2
1	1	2	2	2	2	2	2	2	2
2	2	2	2	2	2	2	2	2	2
2	2	2	2	1	1	1	1	1	1
1	1	1	1	1	2	1	1	1	1
3	3	3	3	3	3	3	3	3	3
2	1	2	1	1	1	2	1	2	1
1	1	1	1	1	1	1	1	1	1
9	9	9	9	2	2	2	2	2	2
2	2	2	2	2	2	2	2	2	2
2	2	2	2	2	2	2	2	2	2
2	2	2	2	2	2	2	2	2	2
9	9	9	9	2	2	2	2	2	2
2	1	2	1	1	1	2	1	1	1
1	2	2	2	1	1	1	2	1	1
1	1	1	1	2	2	2	2	2	2
2	2	2	2	2	2	2	2	2	2
1	2	1	2	2	3	2	1	9	1
2	2	2	2	2	2	2	2	2	2
9	9	9	9	9	9	9	9	9	9
9	9	9	9	2	2	2	2	2	2
1	1	1	1	1	1	1	9	1	9
1	1	1	1	1	2	1	2	1	1
1	1	2	1	1	1	2	2	1	2
2	1	1	2	1	2	1	1	2	9
2	2	9	2	2	2	2	9	2	2
1	1	1	1	1	1	1	1	1	1
2	2	2	2	2	2	2	2	2	2
2	2	2	2	2	2	2	2	2	2
1	1	1	1	2	2	2	2	2	2
2	2	2	2	2	2	2	2	2	2
2	2	2	2	2	2	2	2	2	2
2	2	9	2	9	9	9	9	9	9
1	1	1	2	1	1	1	1	1	1
2	2	9	1	1	2	1	1	1	1
9	9	9	9	2	2	2	2	9	2
2	2	9	2	2	2	2	2	2	2
2	2	2	2	2	2	2	2	2	2
2	2	2	2	2	2	2	2	2	2
2	2	9	2	2	2	2	2	2	2
2	2	2	2	2	2	2	2	2	2
1	1	2	1	1	1	2	1	1	2
2	2	2	2	2	2	2	2	2	2
1	1	9	2	9	1	2	2	2	2

2	2	2	9	1	2	1	1	1	1
2	2	2	2	2	2	2	2	9	2
1	2	2	2	2	2	2	2	2	2
1	1	2	9	1	1	1	2	1	1
1	1	1	1	1	1	1	1	1	1
2	2	2	2	2	2	2	2	2	2
2	2	2	2	2	2	2	2	2	2
2	2	2	2	1	1	1	1	2	2
2	2	9	2	9	9	9	9	9	9
1	9	1	1	2	2	2	9	2	9
9	9	9	9	2	2	9	2	2	2
9	1	9	1	1	1	9	1	1	9
9	9	9	9	9	9	9	9	9	9
1	1	1	1	1	1	1	1	1	2
9	9	9	9	1	1	1	1	9	1
2	2	2	2	2	2	2	2	2	2
1	1	1	1	9	9	9	9	9	9
9	9	9	9	9	9	9	9	9	9
2	2	2	2	2	2	2	2	2	2
2	3	2	3	1	3	1	3	1	1
2	3	2	3	1	3	1	3	1	1
2	3	2	3	1	3	1	3	1	1
2	3	2	3	1	3	1	3	1	1
2	2	2	3	1	3	1	9	1	1
2	3	2	3	1	3	1	9	1	1
2	3	2	3	2	3	1	3	1	1
2	3	2	3	1	3	1	3	1	1
2	3	2	3	1	3	1	1	1	1
2	3	2	3	1	3	1	3	1	1
3	3	2	3	1	3	2	3	1	1
2	3	2	3	1	3	1	3	1	1
2	2	2	3	1	3	1	3	1	1
2	3	2	3	1	3	1	3	1	1
2	3	2	3	1	3	1	3	1	1
2	3	2	3	1	3	1	3	1	1
2	3	2	3	1	3	1	3	1	1
2	3	2	3	1	3	1	3	1	1
2	3	2	3	1	3	1	1	1	1
2	3	2	3	1	3	1	1	1	1
2	3	2	3	1	3	1	1	1	1
2	3	2	3	1	3	1	3	1	1
2	3	2	3	1	3	1	3	1	1
2	3	2	3	1	3	1	3	1	1
2	3	2	3	1	3	3	3	1	1
9	9	9	9	9	9	9	9	9	9
9	9	9	9	9	9	9	9	9	9
9	9	9	9	9	9	9	9	9	9

9	9	9	9	9	9	9	9	9	9
9	9	9	9	9	9	9	9	9	9
9	9	9	9	9	9	9	9	9	9
9	9	9	9	9	9	9	9	9	9
9	9	9	9	9	9	9	9	9	9
9	9	9	9	9	9	9	9	9	9
9	9	9	9	9	9	9	9	9	9
9	9	9	9	9	9	9	9	9	9
9	9	9	9	9	9	9	9	9	9
9	9	9	9	9	9	9	9	9	9
9	9	9	9	9	9	9	9	9	9
9	9	9	9	9	9	9	9	9	9
2	9	2	2	9	9	9	9	9	9
9	9	9	9	9	9	9	9	9	9
9	9	9	9	9	9	9	9	9	9
9	9	9	9	9	9	9	9	9	9
9	9	9	9	9	9	9	9	9	9
9	9	9	9	9	9	9	9	9	9
9	9	9	9	9	9	9	9	9	9
2	3	2	3	1	3	1	3	1	1
2	3	2	3	1	3	1	3	1	1
2	3	2	3	1	3	1	3	1	1
2	3	2	3	1	3	1	3	1	1
2	2	2	2	1	3	1	3	1	1
2	3	2	3	1	3	1	3	1	1
2	3	2	3	1	3	1	3	1	1
2	3	2	3	1	3	1	3	1	1
2	3	2	3	1	3	1	3	1	1
3	2	2	3	1	3	1	3	1	1
3	2	2	3	1	3	1	3	1	1
2	3	2	3	1	3	1	3	1	1
2	3	2	3	1	3	1	3	1	1
2	3	2	3	1	3	1	3	1	1
2	2	2	3	1	3	1	3	1	1
2	2	2	2	1	3	1	3	1	1
2	9	2	3	2	9	9	3	3	9
2	3	2	3	1	3	1	3	1	1
2	3	2	3	1	3	1	3	1	9
2	3	2	3	1	3	1	3	1	1
2	3	2	3	3	9	1	3	1	1
2	3	2	3	1	3	1	3	1	9
2	3	2	3	1	3	1	3	1	1
2	3	2	3	1	3	3	9	1	1
2	3	2	3	1	1	3	3	1	1
3	3	2	3	1	3	1	9	3	1
2	3	2	3	1	3	1	3	1	1
2	3	2	3	1	3	1	3	1	1

2	3	2	3	1	3	1	3	1	1
2	3	2	3	1	1	1	9	9	9
2	3	2	3	1	3	1	3	1	1
2	3	2	3	1	3	1	3	1	1
3	2	9	3	1	3	1	3	1	1
2	9	2	3	1	1	3	3	1	1
2	3	2	3	1	3	1	1	1	1
9	3	2	9	1	3	1	3	1	1
2	3	2	3	1	3	1	3	1	1
2	3	2	3	1	3	1	3	1	1
2	3	2	3	1	3	1	3	1	1
3	2	3	2	1	3	1	3	1	1
2	3	2	3	1	3	1	3	1	1
2	3	2	3	1	3	1	3	1	1
2	3	2	3	1	3	1	3	1	1
2	3	2	3	1	3	1	3	1	1
2	3	2	3	1	3	1	1	1	1
2	3	2	3	1	3	1	3	1	1
2	3	2	3	3	1	3	1	1	1
2	3	2	3	1	3	1	3	1	1
2	3	2	3	1	3	1	3	1	1
2	3	2	3	1	1	3	1	1	1
2	3	2	3	1	3	1	3	1	1
2	3	2	3	1	3	1	3	1	1
2	2	2	3	1	3	2	3	1	1
2	3	2	3	1	3	1	3	1	1
2	3	9	2	1	1	3	1	1	1
2	3	2	3	1	3	1	3	1	1
2	3	3	3	1	3	1	3	1	1
9	3	9	3	9	3	1	3	1	1
2	3	2	3	1	3	1	3	1	1
2	9	2	3	1	3	1	3	1	1
2	9	2	3	1	3	1	3	1	1
2	3	2	3	1	3	1	3	1	1
9	9	9	9	9	9	9	9	9	9
2	2	2	3	1	9	1	3	1	1
3	3	2	3	1	3	3	3	1	1
3	3	3	3	1	3	1	3	1	1
3	3	9	3	9	3	1	1	1	1
2	9	2	3	1	9	9	9	9	9
3	2	3	2	1	3	1	3	1	1
2	3	9	3	1	3	1	3	1	1
3	3	3	3	3	3	1	3	1	1
2	3	2	3	1	3	1	3	1	9
2	3	2	3	1	3	1	3	1	1
2	3	2	3	1	3	1	3	1	1
2	3	2	3	1	3	1	3	1	1

2	3	2	3	1	3	1	3	1	1
2	3	2	3	1	3	1	3	1	1
2	3	2	3	1	3	1	3	1	1
2	3	2	3	1	3	1	3	1	1
2	3	2	3	1	3	1	3	1	1
2	3	2	3	1	3	1	3	1	1
2	3	2	3	1	3	1	3	1	1
2	3	2	3	1	3	1	9	1	1
2	3	2	3	1	3	1	3	1	1
2	3	2	3	1	3	1	3	1	1
2	3	2	3	1	3	1	3	1	1
2	3	2	3	1	3	1	3	1	1
2	3	2	3	1	3	1	3	1	1
2	3	2	3	1	3	1	3	1	1
2	3	2	3	1	3	1	9	1	1
2	3	2	3	1	3	1	3	1	1
2	3	2	3	1	3	1	3	1	1
2	3	2	3	1	3	1	3	1	1
2	3	2	3	1	3	1	3	1	1
9	9	9	9	9	9	9	9	9	9
2	3	2	3	1	3	1	3	1	1
2	3	2	3	1	3	1	3	1	1
2	3	2	3	1	3	1	3	1	1
2	3	2	3	1	3	1	3	1	1
2	3	1	3	1	3	2	9	9	9
2	3	2	3	1	3	1	3	1	1
2	3	2	3	1	3	1	1	1	1
2	3	2	3	1	3	1	3	1	1
2	3	3	3	1	3	1	3	1	1
2	3	2	3	1	3	1	3	1	1
2	3	2	3	1	3	1	3	1	1
2	3	2	3	1	3	1	3	1	1
2	3	2	3	1	1	3	3	1	1
2	3	2	3	1	1	3	3	1	1
2	2	2	3	1	3	1	3	1	1
2	2	2	3	1	3	1	3	1	1
2	3	2	3	1	3	3	1	3	1
2	3	2	3	1	3	1	1	1	1
2	3	2	3	1	3	1	3	1	1
2	3	2	3	1	3	1	3	1	1
2	3	2	3	1	3	1	3	1	1
9	9	9	9	9	9	9	9	9	9
2	3	2	3	1	3	1	3	1	9
2	3	2	3	1	3	1	3	1	1
2	3	2	3	1	3	9	3	1	1
2	3	2	3	1	3	1	3	1	1
2	3	2	3	1	3	1	3	1	1

2	3	2	3	1	3	1	3	1	1
2	3	2	3	1	3	1	3	1	1
2	3	2	3	1	3	1	3	1	1
2	3	2	3	1	3	1	3	1	1
2	3	2	3	1	3	1	3	1	1
2	3	2	3	1	3	1	3	1	1
2	3	2	3	1	3	1	3	1	1
3	2	2	3	1	3	9	3	3	9
2	3	2	3	1	3	1	3	1	1
2	3	2	3	1	3	1	3	1	1
2	3	2	3	1	3	1	3	1	1
2	3	2	3	1	3	1	3	1	1
2	3	2	3	1	3	1	3	1	1
2	2	3	3	1	3	1	3	1	1
2	3	2	3	1	3	1	3	3	1
2	3	2	3	1	3	1	3	1	1
2	3	2	3	1	3	1	3	1	1
2	3	2	3	1	3	1	3	1	1
3	2	2	3	1	3	1	3	1	1
2	3	2	3	1	3	1	3	1	1
2	3	2	3	1	3	1	3	1	1
2	3	2	3	1	3	1	3	1	1
2	3	2	3	1	3	1	3	1	1
2	3	2	3	1	3	1	3	1	3
2	3	2	3	1	3	1	3	1	1
2	3	2	3	1	3	1	3	1	1
2	3	2	3	1	3	1	3	1	1
9	9	9	9	9	9	9	9	9	9
9	9	9	9	9	9	9	9	9	9
9	9	9	9	9	9	9	9	9	9
9	9	9	9	9	9	9	9	9	9
9	9	9	9	9	9	9	9	9	9
9	9	9	9	9	9	9	9	9	9
9	9	9	9	9	9	9	9	9	9
9	9	9	9	9	9	9	9	9	9
9	9	9	9	9	9	9	9	9	9
9	9	9	9	9	9	9	9	9	9
9	9	9	9	9	9	9	9	9	9
9	9	9	9	9	9	9	9	9	9
9	9	9	9	9	9	9	9	9	9
9	9	9	9	9	9	9	9	9	9
9	9	9	9	9	9	9	9	9	9
2	2	3	1	1	2	1	2	1	2
1	1	1	1	1	9	1	1	1	9
9	9	9	9	9	9	9	9	9	9
9	9	9	9	9	9	9	9	9	9
9	9	9	9	9	9	9	9	9	9

9	9	9	9	9	9	9	9	9	9
9	9	9	9	9	9	9	9	9	9
9	9	9	9	9	9	9	9	9	9
9	9	9	9	9	9	9	9	9	9
9	9	9	9	9	9	9	9	9	9
9	9	9	9	9	9	9	9	9	9
9	9	9	9	9	9	9	9	9	9
2	2	2	2	2	3	3	3	1	2
9	9	9	9	9	9	9	9	9	9
9	9	9	9	9	9	9	9	9	9
9	9	9	9	9	9	9	9	9	9
9	9	9	9	9	9	9	9	9	9
9	9	9	9	9	9	9	9	9	9
9	9	9	9	1	2	3	1	1	1
2	2	2	2	2	2	2	2	2	2
1	2	2	2	2	2	2	2	2	2
3	9	2	1	3	1	1	3	3	1
2	2	3	2	2	2	2	2	2	2
2	2	2	2	3	1	1	3	3	3
2	1	2	3	2	1	1	2	3	3
2	1	2	1	1	1	2	1	1	2
3	1	3	1	1	3	1	3	1	3
3	3	2	3	1	3	2	2	3	3
2	3	3	3	2	2	3	3	9	3
9	9	9	9	9	9	9	1	9	9
2	2	2	2	2	2	2	2	2	2
2	2	2	2	2	9	9	9	9	9
1	1	2	2	3	2	3	3	1	3
3	2	3	1	3	3	3	3	2	1
3	3	3	3	3	3	3	3	2	2
2	3	3	2	2	2	3	2	2	3
2	1	3	2	1	2	1	2	1	2
9	1	9	9	9	9	9	9	9	9
2	2	2	2	3	3	2	3	1	1
1	9	9	9	9	9	1	9	9	9
9	9	9	9	2	9	9	9	9	9
2	2	1	1	1	2	2	2	1	2
3	2	1	1	3	1	2	3	1	2
9	1	9	9	9	9	9	9	1	9
1	1	9	3	1	1	2	2	3	2
9	9	9	9	9	9	9	9	9	9
9	2	1	3	1	3	3	1	1	2
1	2	2	2	2	2	2	3	9	2
1	1	1	1	1	1	1	1	1	1
9	9	9	9	9	9	9	9	9	9
1	2	2	2	9	9	9	9	9	9
2	2	2	2	2	2	2	3	2	2

9	9	2	9	9	9	9	9	9	9
9	9	9	9	9	9	9	9	9	9
2	2	1	2	2	2	1	2	2	2
3	3	2	2	2	2	2	2	2	1
9	9	9	9	9	9	9	9	9	9
2	9	9	9	9	9	9	9	2	9
2	2	2	2	2	2	2	2	2	2
2	2	2	2	2	2	2	2	2	2
2	2	2	2	2	2	2	2	2	2
2	3	1	1	2	2	2	1	1	1
3	1	2	3	1	2	3	2	3	2
9	9	9	9	9	9	9	9	9	9
9	9	9	9	9	9	9	9	9	9
2	2	2	2	2	2	2	2	2	2
2	2	2	2	2	2	2	2	2	2
1	2	2	2	2	1	3	2	3	3
3	1	3	1	1	3	1	3	1	3
1	1	1	1	2	2	2	2	3	2
2	2	1	1	2	1	1	1	2	1
2	1	2	3	9	9	2	2	9	2
2	2	2	2	2	2	2	2	2	2
2	1	2	2	2	2	2	2	2	2
2	2	2	2	2	2	2	2	2	2
2	2	2	2	2	2	2	2	2	2
3	3	2	2	3	2	2	2	2	2
1	1	2	2	2	2	2	1	3	3
3	2	2	2	2	2	2	2	2	2
2	2	2	2	2	2	2	3	3	3
2	2	2	2	2	2	3	2	2	2
2	1	1	1	2	1	3	2	1	1
3	2	1	2	3	3	3	3	3	3
3	2	2	2	1	1	1	3	2	1
2	1	2	1	1	1	1	1	1	1
2	1	2	1	2	2	1	1	1	1
1	2	1	2	2	2	1	2	1	2
3	3	3	3	2	2	2	3	2	3
2	2	2	2	3	2	2	2	2	2
1	3	3	3	2	2	2	9	1	2
9	9	9	9	2	9	9	9	9	9
3	3	3	3	3	3	3	3	3	3
3	3	2	2	2	2	2	2	2	3
2	2	3	2	2	2	2	2	2	2
2	3	1	2	1	2	3	1	2	3
2	2	2	3	2	1	1	3	3	2
1	1	1	1	1	1	1	1	1	1
3	2	2	2	1	1	3	9	2	2
2	2	2	2	2	2	2	2	2	2

2	2	2	2	2	2	2	2	2	2
2	3	1	3	2	2	2	3	1	2
3	2	2	2	2	2	2	2	9	1
2	3	1	2	2	3	3	3	2	3
1	2	2	2	3	3	2	3	3	2
2	2	2	2	2	2	2	2	2	1
2	3	2	3	2	2	1	3	3	3
1	2	3	1	3	2	1	3	2	1
2	2	2	2	2	2	2	2	2	2
2	3	2	2	3	2	1	3	2	3
2	2	2	2	2	2	2	2	2	2
2	2	2	2	2	2	2	2	2	2
1	1	1	1	2	2	2	2	2	2
2	2	2	2	2	2	2	2	2	2
2	2	2	2	2	2	2	2	2	2
1	1	2	1	2	1	2	1	2	2
3	3	3	3	3	3	3	3	3	3
2	2	2	2	2	2	2	2	2	2
2	2	2	2	2	2	2	2	2	2
2	3	1	2	1	2	3	1	2	3
2	2	1	2	1	2	3	1	1	2
1	1	3	3	1	1	1	1	1	1
2	2	2	2	2	2	2	2	2	2
3	1	1	1	1	1	1	1	1	1
3	3	3	3	3	3	3	3	1	1
9	9	9	9	9	9	9	9	9	9
1	3	2	1	1	2	3	3	2	1
1	3	3	2	2	2	2	2	3	2
2	1	2	1	1	1	1	2	2	1
1	2	2	3	3	2	1	3	2	1
2	2	2	2	2	2	3	3	2	1
9	2	9	1	1	3	2	9	1	1
2	2	3	3	2	2	2	3	3	3
1	1	1	1	1	2	2	1	2	2
2	1	2	2	1	1	2	3	1	2
9	9	9	9	9	9	9	9	9	9
1	3	2	3	1	3	3	1	1	1
2	1	2	3	2	2	2	2	2	2
3	3	3	2	2	2	3	3	3	2
1	1	2	1	2	2	1	2	1	2
2	3	3	2	2	2	3	2	3	3
3	2	1	2	2	1	2	1	2	1
2	2	2	2	2	2	3	3	2	1
9	9	9	9	9	9	9	9	9	9
9	9	9	9	9	9	9	9	9	9
9	9	9	9	9	9	9	9	9	9
2	1	2	2	1	2	3	2	2	3

2	3	2	2	3	2	3	2	2	3
3	2	9	2	3	9	3	9	2	1
2	1	2	2	3	2	3	3	2	3
3	3	2	3	3	3	3	3	1	1
2	2	1	2	9	9	9	9	9	9
3	3	3	2	2	2	1	1	3	3
3	3	3	3	2	3	3	2	1	3
1	2	2	2	2	2	1	2	1	1
1	1	1	1	2	1	2	3	1	1
2	3	1	2	1	2	3	1	2	3
3	1	2	1	2	2	2	1	1	3
2	2	2	2	2	2	2	2	2	2
2	2	2	2	2	2	2	2	2	2
3	1	3	1	1	1	3	3	1	1
2	2	2	2	2	2	2	2	2	2
2	2	2	2	2	2	2	2	1	2
2	1	1	3	3	3	1	1	3	3
2	3	2	2	3	2	3	2	1	2
3	1	2	2	3	1	1	2	2	1
3	3	2	3	3	1	1	2	3	1
2	3	1	2	3	2	3	2	1	2
3	3	2	3	3	1	1	2	3	1
2	2	2	2	2	2	2	2	2	2
2	3	2	2	1	3	3	3	2	3
3	2	1	2	3	3	3	3	3	3
9	9	9	9	9	9	9	9	9	9
2	3	1	2	2	2	1	3	2	2
3	3	2	1	3	3	1	3	3	3
3	3	3	3	2	2	2	2	2	2
3	1	2	3	1	2	1	2	1	2
2	2	2	2	2	2	2	2	2	2
9	9	9	9	9	2	9	9	9	9
2	1	2	2	3	2	2	1	2	2
1	3	2	2	2	2	2	2	1	3
2	3	3	2	2	1	3	3	2	1
3	1	2	1	2	1	2	1	3	2
2	2	2	2	2	2	2	2	2	2
2	1	3	1	2	2	1	1	1	1
2	2	2	2	3	2	1	2	3	2
9	2	9	9	2	9	9	9	9	9
2	1	2	3	1	2	2	1	2	2
2	2	2	2	2	2	2	2	2	2
2	2	2	2	2	2	2	2	2	2
2	2	2	2	2	2	2	2	2	2
2	2	2	2	2	2	2	2	2	2
3	2	2	2	1	2	3	1	1	1
2	2	2	2	2	2	2	2	2	2

3	2	1	2	1	3	1	2	2	1
2	2	2	2	2	2	2	2	2	2
3	2	2	3	3	3	3	3	3	3
2	2	2	2	2	2	1	3	2	2
2	2	2	2	2	2	2	2	2	2
3	3	3	3	2	2	2	2	2	2
3	2	2	3	1	1	3	3	3	3
2	1	1	2	1	1	3	3	3	2
1	1	1	1	3	3	3	3	1	1
3	3	3	3	3	3	3	3	3	2
3	3	2	1	3	3	2	3	1	1
2	2	2	2	2	2	2	2	2	2
3	3	2	1	3	3	2	3	1	1
1	2	2	2	3	3	3	2	1	1
2	2	2	2	2	2	2	2	2	2
2	2	2	2	2	2	2	2	2	2
2	2	2	2	2	2	2	9	2	3
1	2	2	2	2	2	2	2	2	2
2	2	2	2	2	2	2	2	2	2
2	2	1	2	2	2	2	2	2	2
1	1	2	1	1	1	1	3	1	1
3	1	3	3	1	3	1	1	3	3
9	9	9	9	9	9	9	9	2	9
3	1	3	3	1	2	2	2	2	2
2	2	2	2	2	2	2	2	2	2
2	2	2	2	2	2	3	2	2	2
2	2	2	2	3	3	3	2	2	2
2	2	2	2	3	3	3	2	2	2
3	1	3	3	1	3	1	1	1	3
2	1	2	1	1	1	1	1	1	1
3	1	3	3	1	3	1	1	3	3
1	1	2	2	3	3	3	3	1	1
2	2	3	2	1	2	2	2	3	2
2	1	2	2	1	2	2	2	2	2
9	9	9	9	2	9	9	9	9	9
3	1	3	3	1	3	1	1	3	1
2	2	2	2	2	2	2	2	2	2
3	1	1	1	1	1	1	1	3	3
1	2	2	2	1	1	2	2	2	2
2	2	2	2	2	2	2	2	2	2
2	3	2	2	1	2	2	3	2	2
9	9	9	9	9	9	9	9	9	9
9	9	9	9	9	9	9	9	9	9
1	1	1	1	1	1	1	1	1	1
2	2	3	3	1	2	2	3	3	2
9	9	9	9	9	9	9	9	9	9
2	2	2	2	1	1	1	1	1	2

2	2	2	2	2	2	2	2	2	2
3	3	3	2	1	1	1	1	1	1
2	3	1	2	1	2	3	1	2	3
2	3	2	1	1	2	3	2	1	2
2	1	3	3	1	2	3	1	2	3
2	3	1	2	1	2	3	1	2	3
2	3	2	2	2	2	3	2	2	2
9	9	9	9	1	9	9	9	9	9
1	1	1	1	2	1	3	1	1	1
9	9	9	9	9	9	9	9	9	9
1	1	1	1	2	2	2	2	2	2
3	1	2	2	2	3	3	3	2	2
1	1	1	1	2	1	1	2	3	1
2	2	3	2	2	2	2	2	2	2
1	1	1	1	1	1	1	1	1	1
9	9	9	9	9	9	9	9	9	9
9	9	9	9	9	9	9	9	9	9
3	3	3	3	2	2	2	2	2	2
2	2	2	2	2	2	2	2	2	2
2	2	1	3	1	2	2	1	2	1
2	2	2	2	2	2	2	2	2	2
2	2	2	2	2	2	2	9	9	2
2	9	1	2	2	2	1	2	3	2
1	2	3	2	1	2	3	2	1	2
9	2	9	2	2	2	2	2	9	2
9	9	9	9	9	9	9	9	9	1
1	2	2	2	2	2	2	2	1	1
2	2	2	2	2	2	2	9	1	2
2	2	2	2	1	1	2	2	1	1
2	1	2	2	3	2	3	3	2	2
2	1	2	3	1	1	2	2	3	3
2	2	2	2	2	2	2	2	2	2
2	2	2	2	2	2	2	2	2	2
2	2	2	2	2	2	2	2	2	2
2	2	2	2	2	2	2	2	2	2
2	2	2	2	2	2	2	2	2	2
3	3	3	3	3	3	3	3	3	3
9	9	9	9	9	9	9	9	9	1
9	9	9	2	9	9	9	9	9	9
2	2	2	2	2	2	2	2	2	2
2	2	2	2	2	2	2	2	2	2
9	9	9	9	9	9	9	9	1	9
2	2	2	1	1	2	1	2	1	2
9	9	9	9	2	9	9	9	9	9
2	2	2	2	2	2	2	2	2	2
2	1	2	2	2	1	2	1	1	9
2	2	2	2	2	2	2	2	2	2

2	9	2	3	1	2	1	2	1	2
9	9	9	9	9	9	9	9	1	9
2	2	2	2	1	1	1	1	1	1
1	2	1	2	2	1	3	2	2	2
3	2	3	2	2	2	3	3	3	3
2	2	1	2	2	1	1	2	2	2
1	1	2	2	2	2	1	1	1	1
2	3	2	2	2	2	1	1	3	3
2	2	2	2	2	2	2	2	2	2
3	3	3	1	9	9	9	1	9	9
9	9	9	1	9	9	9	9	9	9
2	2	2	2	2	2	2	2	2	2
2	2	3	3	9	3	2	2	2	2
1	2	3	3	2	2	2	2	2	3
2	2	1	2	2	2	1	2	2	1
2	9	1	2	2	2	1	2	2	9
2	2	2	2	2	2	2	2	2	2
2	2	2	2	2	2	2	2	2	2
2	2	2	3	2	3	2	3	2	3
2	1	3	1	1	3	1	2	2	1
1	2	1	2	2	1	1	2	1	2
2	2	2	2	2	2	1	3	3	3
1	3	2	2	1	2	1	3	3	2
9	9	9	9	9	9	9	9	9	9
2	1	2	2	3	3	3	3	3	3
9	9	9	9	9	9	9	9	1	9
9	9	9	9	9	9	9	9	9	9
9	1	9	9	9	9	9	9	9	9
2	2	2	1	1	2	2	2	3	3
2	2	2	2	3	3	3	3	3	3
2	2	2	2	2	2	2	2	2	2
9	9	9	9	1	9	9	9	9	9
2	3	3	2	2	2	2	3	1	1
2	2	2	2	2	2	2	2	2	2
9	1	9	9	9	9	9	9	1	9
1	1	2	2	2	2	2	2	1	1
2	2	3	1	1	3	1	2	3	1
2	9	2	1	1	2	9	9	3	1
3	1	2	3	1	2	3	1	2	3
3	1	3	3	1	3	1	1	1	2
2	3	2	2	2	2	2	2	2	2
2	3	9	1	1	2	2	2	2	3
9	1	9	9	1	9	9	9	9	9
9	9	9	1	9	9	9	1	9	9
3	1	2	3	1	2	1	3	1	2
2	2	2	2	2	2	2	2	2	2
2	2	2	2	2	2	2	2	2	2

1	3	1	2	2	2	9	1	1	1
9	9	9	9	9	9	9	9	9	9
9	9	9	9	9	9	9	9	9	9
9	9	9	9	9	9	9	9	9	9
9	9	9	9	9	9	9	9	9	9
9	1	9	9	9	9	9	9	9	1
9	9	9	9	9	9	9	9	9	9
9	9	9	9	9	9	9	9	9	9
9	9	9	9	9	9	9	9	9	9
9	1	9	9	9	9	9	9	1	9
2	2	1	2	2	2	1	2	2	2
2	1	1	1	1	1	1	2	9	1
3	1	9	3	2	1	2	2	1	1
2	2	2	2	2	2	2	2	2	2
2	2	2	2	2	2	2	2	2	2
2	2	2	2	1	2	2	2	2	2
3	3	3	3	1	1	1	1	2	3
2	2	2	2	2	2	2	2	1	2
9	1	2	2	1	1	2	9	1	3
2	2	3	3	2	3	2	3	2	1
1	2	1	3	1	3	2	3	2	9
1	2	1	3	1	3	2	3	1	2
2	2	2	2	3	3	3	3	3	3
2	2	2	2	2	2	2	2	2	2
2	2	2	2	3	3	3	3	3	3
3	3	3	3	3	3	3	3	3	3
2	2	2	2	2	2	2	2	2	2
3	1	2	3	1	2	3	1	2	1
2	2	2	2	2	2	2	2	2	2
2	2	2	2	2	2	2	2	2	2
9	9	9	9	9	9	9	9	9	9
1	2	2	2	2	2	2	1	2	2
1	1	1	1	2	1	1	1	1	1
9	1	9	9	1	9	9	9	9	9
3	3	3	3	3	3	3	3	3	3
1	1	1	1	2	2	2	2	2	2
2	2	2	2	2	2	2	2	2	2
2	2	2	2	2	2	2	2	2	2
2	1	2	3	1	1	3	1	3	3
9	9	9	9	9	9	9	9	9	9
2	1	1	1	1	1	2	3	2	3
1	2	2	2	2	2	2	2	2	2
2	2	2	2	2	2	2	2	2	2
2	1	2	1	2	2	2	2	2	2
2	1	2	2	3	3	2	1	2	2
9	9	9	9	9	9	9	9	9	9
2	2	2	2	2	2	2	1	2	2

9	9	9	9	9	9	9	9	9	9
2	2	2	2	3	2	2	2	2	2
1	9	9	9	1	9	9	9	9	9
9	9	9	9	9	9	9	9	9	9
9	9	9	9	9	9	9	9	9	9
9	9	9	9	9	9	9	9	9	9
9	9	9	9	9	9	9	9	9	9
9	9	9	9	9	9	9	9	9	9
9	9	9	9	9	9	9	9	9	9
9	9	9	9	9	9	9	9	9	9
9	9	9	9	9	9	9	9	9	9
9	9	9	9	9	9	9	9	9	9
9	9	9	9	9	9	9	9	9	9
9	9	9	9	9	9	9	9	9	9
9	9	9	9	9	9	9	9	9	9
9	9	9	9	9	9	9	9	9	9
9	9	9	9	9	9	9	9	9	9
9	9	9	9	9	9	9	9	9	9
9	9	9	9	9	9	9	9	9	9
9	9	9	9	9	9	9	9	9	9
9	9	9	9	9	9	9	9	9	9
9	9	9	9	9	9	9	9	9	9
9	9	9	9	9	9	9	9	9	9
9	9	9	9	9	9	9	9	9	9
9	9	9	9	9	9	9	9	9	9
2	3	2	2	1	2	1	3	1	2
2	2	2	2	2	2	2	2	2	2
2	3	3	3	2	1	1	2	1	3
2	1	1	2	1	1	1	2	1	2
3	3	1	3	1	1	3	2	2	2
3	1	2	2	1	2	2	1	2	2
2	2	2	2	2	2	2	2	2	2
3	1	9	3	1	3	3	2	3	2
3	2	2	2	1	2	2	2	2	2
2	2	2	2	2	2	2	2	2	2
3	2	2	2	3	3	1	2	1	1
9	1	9	9	1	3	1	3	2	2
3	1	3	2	1	2	1	1	2	3
3	1	3	3	1	3	1	1	1	3
3	2	2	1	2	2	2	1	1	2
3	1	3	2	1	2	1	1	1	1
1	9	9	9	1	9	9	9	9	9
2	1	2	2	1	2	2	1	3	2
3	1	3	3	1	3	1	3	1	3
9	9	9	9	9	9	9	9	1	9
3	1	3	3	1	1	1	1	3	1
3	1	3	1	1	3	1	3	2	1
2	2	2	2	1	2	2	2	2	2
3	2	2	1	2	2	2	2	2	2

1	1	2	2	1	1	1	2	1	2
3	1	2	2	1	2	1	2	2	2
1	1	3	1	1	1	1	1	1	1
3	1	2	2	1	1	1	2	1	2
2	3	2	2	2	1	2	1	2	3
9	9	9	9	1	9	9	9	9	9
1	2	2	3	1	2	1	1	1	2
9	9	9	9	1	9	9	9	9	9
9	9	9	9	2	9	9	9	9	9
9	9	9	9	2	9	9	9	9	9
9	9	9	9	9	9	1	9	9	9
9	1	9	9	1	9	9	9	9	9
9	1	9	9	9	9	1	9	9	9
9	1	9	9	9	9	9	1	9	9
9	1	9	9	9	9	9	9	9	9
3	3	3	1	1	1	3	3	3	3
3	3	3	3	3	3	3	3	3	1
2	2	2	2	2	2	2	2	2	2
9	1	9	9	9	1	9	9	9	9
3	3	3	3	1	3	3	1	1	3
9	1	9	9	1	9	9	9	9	9
9	9	9	9	9	9	9	1	9	9
9	1	9	9	1	9	9	9	9	9
9	9	9	9	9	9	9	9	9	9
9	1	9	9	1	9	9	9	9	9
2	3	2	1	2	2	3	2	1	1
3	3	3	1	1	1	3	1	1	3
2	3	2	2	2	2	3	2	2	2
3	1	3	3	2	2	2	2	3	1
2	2	2	2	2	2	2	2	2	2
3	2	9	9	3	3	2	2	3	1
3	1	3	1	1	1	9	9	9	9
3	3	3	3	1	3	3	3	1	3
3	1	2	1	3	1	2	2	1	1
2	2	2	3	3	3	2	3	3	3
9	1	9	9	9	1	9	9	9	9
2	2	2	2	2	2	2	2	2	2
2	2	2	2	2	2	2	2	2	2
2	2	2	2	3	3	1	2	3	1
2	2	2	2	2	1	3	2	2	2
2	1	2	1	1	2	1	1	1	2
2	1	2	1	1	2	1	1	1	2
3	1	1	3	1	1	3	3	3	1
2	3	2	1	2	2	2	2	2	2
3	1	1	1	1	3	3	1	3	3
9	9	9	9	9	9	9	9	1	9
9	1	9	9	9	9	1	9	9	9

1	2	9	3	1	1	1	1	1	1
2	1	3	3	2	3	1	3	3	3
3	1	3	3	1	3	3	3	1	3
9	9	9	9	9	9	9	1	9	9
9	1	9	1	3	2	1	9	9	2
1	1	2	1	3	1	2	3	1	2
1	1	3	2	1	2	1	1	1	1
9	9	9	9	9	9	9	9	9	9
9	9	9	9	2	9	9	9	9	9
2	1	1	2	3	3	3	3	3	3
2	2	2	2	2	2	2	2	2	2
3	1	3	2	3	3	3	1	1	3
1	1	1	2	1	1	2	1	1	2
9	1	9	2	1	3	1	2	9	1
9	9	9	9	1	9	9	9	9	9
1	2	2	2	2	1	2	2	2	2
9	1	9	9	9	9	9	9	9	9
1	1	9	1	9	9	9	9	9	9
3	2	3	2	3	3	3	3	3	3
1	1	2	2	1	2	1	1	1	2
3	1	3	2	3	3	3	3	2	2
2	3	3	2	3	1	3	2	3	1
3	2	3	3	3	2	3	3	3	3
3	1	2	2	1	1	1	1	1	2
2	1	2	2	2	2	2	2	2	2
9	9	9	9	9	9	9	9	9	9
3	1	2	2	1	2	3	1	3	1
3	3	1	3	3	3	3	3	1	3
3	3	3	3	3	3	3	3	3	3
3	1	2	3	1	2	3	2	2	3
9	9	9	9	9	9	9	9	9	9
9	9	9	9	9	9	9	9	9	9
9	9	9	9	9	9	9	9	9	9
9	9	9	9	9	9	9	9	9	9
9	9	9	9	9	9	9	9	9	9
9	9	9	9	9	9	9	9	9	9
9	9	9	9	9	9	9	9	9	9
9	9	9	9	9	9	9	9	9	9
9	9	9	9	9	9	9	9	9	9
9	9	9	9	9	9	9	9	9	9
9	9	9	9	9	9	9	9	9	9
9	9	9	9	9	9	9	9	9	9
9	9	9	9	9	9	9	9	9	9
9	9	9	9	9	9	9	9	9	9
9	9	9	9	9	9	9	9	9	9
9	9	9	9	9	9	9	9	9	9
9	9	9	9	9	9	9	9	9	9

9	9	9	9	9	9	9	9	9	9
9	9	9	9	9	9	9	9	9	9
9	9	9	9	9	9	9	9	9	9
9	9	9	9	9	9	9	9	9	9
9	9	9	9	9	9	9	9	9	9
9	9	9	9	9	9	9	9	9	9
9	9	9	9	9	9	9	9	9	9
9	9	9	9	9	9	9	9	9	9
9	9	9	9	9	9	9	9	9	9
9	9	9	9	9	9	9	9	9	9
9	9	9	9	9	9	9	9	9	9
9	9	9	9	9	9	9	9	9	9
9	9	9	9	9	9	9	9	9	9
9	9	9	9	9	9	9	9	9	9
9	9	9	9	9	9	9	9	9	9
9	9	9	9	9	9	9	9	9	9
9	9	9	9	9	9	9	9	9	9
9	9	9	9	9	9	9	9	9	9
9	9	9	9	9	9	9	9	9	9
2	1	2	2	1	2	2	2	2	2
9	9	9	9	9	9	9	9	9	9
9	9	9	9	9	9	9	9	9	9
9	9	9	9	9	9	9	9	9	9
9	9	9	9	9	9	9	9	9	9
9	9	9	9	9	9	9	9	9	9
9	9	9	9	9	9	9	9	9	9
9	9	9	9	9	9	9	9	9	9
9	9	9	9	9	9	9	9	9	9
9	9	9	9	9	9	9	9	9	9
9	9	9	9	9	9	9	9	9	9
9	9	9	9	9	9	9	9	9	9
9	9	9	9	9	9	9	9	9	9
9	9	9	9	9	9	9	9	9	9
9	9	9	9	9	9	9	9	9	9
9	9	9	9	9	9	9	9	9	9
2	2	2	2	2	2	2	2	2	2
3	3	3	3	3	3	3	3	3	3
2	9	9	3	3	2	3	3	2	3
3	1	3	1	1	1	1	1	1	1
3	3	3	3	3	3	3	3	3	3
3	3	3	3	1	1	1	1	1	1
2	2	3	2	9	9	1	9	9	9
9	9	9	9	1	9	9	9	9	9
2	2	3	2	9	9	1	9	9	9
2	2	2	3	2	2	2	3	1	2
2	1	1	1	1	2	1	1	1	2
2	2	2	2	3	2	3	2	2	2

3	1	3	2	1	2	1	1	1	1
2	2	2	1	1	2	3	2	1	2
2	1	1	3	1	2	1	1	1	2
2	1	2	2	1	3	1	3	2	2
2	2	2	2	1	2	2	1	2	2
3	1	2	1	1	2	1	9	3	9
2	1	2	2	1	1	1	1	1	2
3	1	3	3	1	2	2	2	2	2
3	2	1	2	2	1	3	2	2	2
1	1	3	2	1	2	1	3	2	1
9	1	9	9	1	9	9	9	9	9
2	3	3	3	2	2	3	2	2	9
2	1	2	2	1	2	1	1	3	2
1	2	2	2	1	3	3	2	2	2
1	2	1	2	2	2	3	3	2	2
2	2	9	1	3	3	2	2	2	2
2	1	2	2	1	2	1	2	1	2
2	3	2	1	2	1	1	2	3	2
1	1	1	1	1	1	3	3	1	2
2	1	2	1	2	3	3	3	2	1
2	3	9	1	2	1	2	2	2	2
2	1	2	1	1	2	1	1	1	2
1	1	9	3	1	1	2	2	2	1
9	1	9	9	9	9	9	9	9	9
3	3	3	3	3	3	3	3	3	3
3	3	1	1	1	2	3	1	2	2
1	1	1	1	1	1	1	2	9	1
2	1	3	1	3	2	1	2	2	1
2	2	2	2	2	2	2	2	2	2
2	2	2	2	2	2	2	2	2	2
9	9	9	9	2	2	3	2	2	2
2	2	2	2	2	2	2	2	2	2
2	2	2	2	2	2	2	2	2	2
9	9	9	9	9	9	9	9	9	9
9	9	9	9	9	9	9	9	9	9
9	9	9	9	9	9	9	9	9	9
9	9	9	9	9	9	9	9	9	9
1	2	2	2	9	9	9	9	9	9
9	9	9	9	9	9	9	9	9	9
9	9	9	9	9	9	9	9	9	9
2	2	2	2	2	2	1	3	2	1
2	2	1	1	2	2	2	2	2	2
1	1	1	1	1	2	2	2	1	1
3	3	1	3	3	3	3	3	1	1
1	1	2	3	1	3	1	3	1	3
3	3	3	3	3	1	3	3	3	3
1	3	1	3	1	3	3	1	3	1

1	2	1	2	2	2	2	2	2	2
2	3	2	1	1	2	3	1	2	1
2	2	2	2	2	2	2	2	2	2
1	1	1	3	1	3	1	9	1	1
1	1	2	2	1	3	3	1	2	2
2	3	1	2	3	2	1	3	2	1
1	1	1	1	1	1	9	1	1	1
1	2	3	1	2	1	3	2	1	9
1	1	3	2	3	1	2	2	2	2
9	9	9	9	1	9	9	9	9	9
3	3	3	3	2	2	2	3	3	1
3	1	1	2	1	2	3	1	1	3
1	1	1	1	3	1	3	1	1	1
3	1	1	2	1	3	3	3	2	3
1	3	2	1	1	2	1	3	1	1
3	1	1	1	3	1	2	3	2	3
2	2	1	1	2	2	2	2	2	1
9	9	9	1	9	9	1	9	9	9
2	3	2	1	1	2	1	2	1	3
9	1	9	9	9	9	9	9	1	9
3	2	3	2	1	1	2	3	2	1
2	1	2	2	2	2	1	2	2	3
3	1	1	1	1	9	9	3	2	3
3	1	2	2	1	1	1	2	2	1
2	3	1	2	1	3	2	3	3	1
3	3	3	1	3	1	3	3	3	3
1	2	3	1	1	2	1	2	2	2
1	3	1	1	1	3	3	1	1	1
1	2	2	1	1	1	2	2	1	2
2	2	3	2	1	2	3	3	2	3
1	3	3	3	2	1	3	1	3	3
3	2	3	1	2	1	2	3	1	2
3	1	1	1	1	3	2	2	2	2
3	3	3	1	3	3	1	3	3	3
1	1	1	3	1	1	3	1	1	1
3	9	1	1	1	3	1	3	3	3
2	3	1	3	1	2	1	3	2	3
9	9	9	9	9	9	9	9	9	9
3	3	3	3	1	3	3	3	1	3
2	1	2	3	3	1	3	1	3	1
3	1	3	1	1	3	3	3	1	3
1	2	2	3	1	2	2	1	3	3
3	1	3	1	3	1	3	3	1	3
2	2	1	1	1	2	2	3	1	2
2	2	2	2	2	2	2	2	2	2
1	2	2	3	9	9	2	9	9	9
3	2	2	2	3	3	3	3	3	3

1	2	3	1	2	1	2	2	2	1
3	1	1	1	3	1	2	3	2	3
3	3	1	1	2	2	9	2	9	1
1	1	2	3	2	2	2	2	1	1
2	2	2	2	1	1	1	1	1	2
2	2	3	3	2	2	3	3	2	1
2	2	2	2	2	2	2	2	2	2
9	9	9	9	9	9	9	9	9	9
9	9	9	9	9	9	9	9	9	9
1	2	1	3	1	2	3	2	1	2
2	2	2	2	2	2	2	2	2	2
2	2	2	2	2	2	2	2	2	2
2	2	2	2	2	2	2	2	2	2
1	1	1	2	2	2	2	2	2	2
2	2	2	2	2	2	2	2	2	2
2	2	2	2	2	2	2	2	2	2
2	2	2	2	2	2	2	2	2	2
2	2	2	2	2	3	2	3	2	1
2	2	2	2	2	2	2	2	2	2
2	2	2	2	2	2	2	2	2	2
2	2	3	1	2	2	2	2	2	2
2	2	2	2	2	2	2	2	2	2
2	2	2	2	2	2	2	2	2	2
2	2	2	2	2	2	2	2	2	2
2	2	2	2	2	2	2	2	2	2
2	2	2	2	2	2	2	2	2	2
2	2	2	2	2	2	2	2	2	2
2	3	1	2	1	2	1	2	9	1
1	2	2	2	2	2	1	1	3	3
2	2	2	9	2	2	2	2	2	2
2	2	2	2	2	2	2	2	2	2
2	2	2	2	2	2	2	2	2	2
2	2	2	2	2	2	2	2	2	2
2	2	2	2	2	2	2	2	2	2
2	2	2	2	2	2	2	2	2	2
2	2	2	2	2	2	2	2	2	2
2	2	2	2	2	2	2	2	2	2
2	2	2	2	2	2	2	2	2	2
2	2	2	2	2	2	2	2	2	2
3	3	1	1	3	3	3	3	1	1
2	2	2	2	2	2	2	2	2	2
2	2	2	2	2	2	2	2	2	2
1	1	2	3	2	2	1	1	3	3
2	2	2	2	2	2	2	2	2	2
9	9	9	9	2	1	2	1	2	1
3	3	3	3	3	3	3	3	3	3
2	2	2	2	2	9	2	2	2	2

2	2	2	2	2	2	2	2	2	2
2	2	2	2	2	2	2	2	2	2
2	2	2	2	2	2	2	2	2	2
2	2	2	2	2	2	2	2	2	2
2	3	2	1	2	3	2	1	2	3
2	2	2	2	2	2	2	2	2	2
3	1	1	3	1	9	9	9	9	9
1	1	1	1	1	2	3	1	3	3
9	9	9	9	9	9	9	9	9	9
2	2	2	2	2	2	2	2	2	2
3	3	3	3	3	3	3	3	3	3
1	1	1	1	1	1	1	1	1	1
3	2	1	2	2	2	3	3	3	3
9	9	9	9	3	9	9	9	9	9
2	2	2	2	2	2	2	2	2	2
1	1	2	2	2	1	2	2	2	1
2	2	2	2	2	2	2	2	2	2
1	1	2	1	1	1	1	1	1	9
9	9	9	9	9	9	9	9	9	9
2	3	3	3	2	2	2	2	2	2
2	1	1	2	1	2	1	3	2	2
1	9	9	9	9	9	9	9	9	9
9	9	9	9	1	9	9	9	9	9
2	9	2	2	2	2	2	2	2	2
2	3	1	2	1	3	2	3	1	3
1	3	2	2	2	2	3	3	3	3
1	2	2	2	1	1	3	1	2	9
1	3	9	1	1	1	1	3	9	1
2	9	1	3	3	1	2	9	1	2
1	1	1	1	1	1	1	1	1	1
3	3	3	3	3	3	3	3	3	3
2	2	2	2	2	2	2	2	2	2
2	2	2	2	2	2	2	2	2	2
9	1	9	9	9	3	2	9	9	9
3	3	2	2	2	2	2	2	2	2
9	1	9	9	9	9	9	9	9	9
3	1	1	3	1	3	1	1	1	1
3	3	3	3	2	2	2	2	2	2
2	2	2	2	2	2	2	2	2	2
2	2	2	2	2	2	2	2	2	2
9	3	1	2	2	2	2	2	2	3
2	2	2	2	2	2	2	2	2	2
9	9	9	2	9	9	3	9	9	9
3	3	1	1	1	1	1	1	1	1
3	3	2	2	2	2	2	2	2	2
2	2	3	3	2	2	2	2	2	2
2	2	2	2	2	2	2	2	2	2

1	1	1	1	1	1	1	1	1	1
2	2	2	2	2	2	2	2	2	2
1	1	1	1	1	1	1	1	1	1
2	2	2	2	2	2	2	2	2	2
9	9	9	9	9	9	9	9	9	9
2	2	1	3	2	3	2	1	2	3
9	9	9	9	3	3	3	3	3	3
1	1	1	1	1	1	1	1	1	1
2	3	9	1	1	1	2	2	1	3
1	1	1	1	3	3	3	3	3	3
2	2	2	2	1	1	1	1	1	1
2	2	2	2	2	2	2	2	2	2
2	2	2	2	2	2	2	2	2	2
2	2	2	2	2	2	2	2	2	2
2	2	2	2	2	2	2	2	2	2
2	2	2	2	2	2	2	2	2	3
1	1	1	1	1	1	1	1	1	1
3	3	3	3	1	1	3	3	2	3
9	9	9	9	9	9	9	9	9	9
2	1	2	1	1	2	1	2	3	1
1	1	2	2	1	1	1	1	1	2
3	3	3	3	3	3	3	2	2	2
3	3	3	3	2	2	3	2	9	2
2	1	2	2	1	1	1	3	3	3
3	3	3	3	3	3	3	3	3	3
2	2	1	2	2	2	1	1	1	1
2	3	2	1	1	1	1	1	2	2
2	2	2	2	2	1	2	2	2	1
2	2	2	3	1	2	1	2	1	2
2	2	2	2	2	2	2	2	2	2
1	1	1	1	2	2	2	2	2	2
2	2	2	2	2	2	2	2	2	2
3	3	3	3	3	3	3	3	3	3
1	1	3	3	2	1	3	1	2	1
2	2	2	2	2	2	2	2	2	2
2	2	2	2	2	2	2	2	2	2
3	1	2	3	2	1	3	2	2	2
3	3	3	3	2	2	2	2	2	2
2	3	1	2	1	2	3	1	2	3
3	3	3	3	3	3	3	3	3	3
1	1	1	1	1	2	1	2	1	2
3	2	3	2	1	1	1	1	1	1
9	9	9	9	9	9	9	9	9	9
3	3	3	3	3	3	9	9	9	3
1	1	1	1	1	1	1	1	1	1
2	2	2	2	2	2	2	2	2	2
2	1	3	3	3	3	3	2	1	2

1	1	1	9	1	1	1	1	1	1
3	3	3	3	3	3	3	3	1	3
3	3	3	3	2	2	2	2	9	1
3	3	3	3	3	3	3	3	1	3
1	2	2	2	1	1	2	1	1	1
2	2	2	2	2	2	2	2	2	2
2	2	2	2	2	2	2	2	2	2
2	2	2	2	2	2	2	2	2	2
2	2	2	2	1	1	1	1	1	1
1	1	1	1	2	3	1	1	2	9
2	2	2	2	1	1	1	1	1	1
2	1	2	1	1	2	3	3	2	1
2	2	2	2	2	2	2	2	2	2
9	9	9	9	1	3	2	1	3	2
2	2	2	2	2	2	2	2	2	2
2	2	2	2	2	2	2	2	2	2
2	2	2	2	2	2	2	2	2	2
2	2	2	2	3	2	3	2	2	1
2	2	2	2	2	2	2	2	2	2
1	3	3	2	3	3	1	2	1	2
2	2	2	1	2	2	2	2	2	2
1	1	2	3	2	2	1	2	1	1
1	1	2	3	1	3	3	2	2	1
2	2	2	2	2	2	2	2	2	2
1	1	1	1	1	1	1	1	1	1
1	1	3	1	3	1	3	1	3	3
1	3	1	1	1	1	2	1	1	2
3	3	3	2	1	2	3	2	1	3
3	3	1	2	1	3	2	1	2	2
2	3	2	3	3	3	3	2	2	1
1	9	2	2	2	2	2	2	2	2
2	2	2	2	2	2	2	2	2	2
2	2	2	1	2	2	2	2	2	1
3	3	3	2	1	2	3	2	1	3
1	2	1	3	1	2	3	1	3	1
2	2	2	2	2	2	2	2	2	2
2	2	2	2	2	2	2	2	2	2
9	9	9	9	9	9	9	9	9	9
2	2	2	1	1	2	2	1	2	1
2	3	1	2	2	3	2	2	2	9
2	3	2	1	2	2	1	2	3	2
2	2	2	2	2	2	2	2	2	2
2	2	2	2	2	2	2	2	2	2
2	2	2	2	2	2	2	2	2	2
9	1	2	3	2	3	1	2	3	3
1	2	2	2	1	1	2	1	1	1
1	2	2	2	2	2	2	2	2	2

9	9	9	9	9	9	9	9	9	9
2	9	3	9	1	2	3	1	2	3
1	2	2	2	1	1	2	1	1	1
3	1	9	1	1	3	3	3	3	3
3	1	1	3	3	1	2	2	3	1
2	1	2	2	3	1	3	2	2	2
2	3	2	1	1	2	2	1	1	3
1	2	2	2	9	9	9	9	9	9
3	3	3	3	3	3	3	3	1	3
3	3	3	3	3	3	3	3	1	1
2	2	2	2	2	2	2	2	1	2
3	2	2	3	1	2	2	2	2	2
2	2	2	2	2	2	2	2	2	2
2	2	1	1	1	1	1	1	1	1
2	3	2	3	1	2	3	2	1	3
2	2	2	2	2	2	2	2	2	2
2	1	1	2	1	2	2	1	1	2
1	3	1	9	3	1	2	2	1	2
9	9	9	1	9	9	9	9	9	9
2	2	2	2	2	2	2	2	2	2
9	9	9	9	9	9	9	9	9	9
2	3	1	2	1	2	3	3	2	1
3	3	2	2	3	2	1	2	1	3
1	3	3	3	1	1	3	3	3	2
2	1	1	3	3	3	2	2	2	1
3	3	2	2	2	2	3	3	3	3
1	2	2	2	1	2	2	3	2	1
1	2	3	2	1	1	2	2	2	1
1	2	2	1	2	2	3	1	3	2
2	2	2	3	2	2	3	3	1	2
2	2	2	2	1	1	1	2	2	2
2	1	2	2	2	1	3	1	3	2
3	3	3	3	1	3	3	3	1	3
2	2	2	2	2	2	2	2	2	2
2	2	2	2	2	2	2	2	2	2
2	2	2	2	2	2	2	2	3	3
3	3	3	3	3	3	3	3	3	3
2	1	3	2	2	2	2	3	1	2
2	1	2	2	2	1	1	1	1	1
2	1	1	2	2	1	1	1	1	1
3	3	3	3	1	1	1	1	1	1
2	3	3	3	1	1	1	2	3	3
2	1	3	2	2	3	2	1	1	2
2	2	3	3	2	2	2	2	2	2
9	9	9	9	9	9	9	9	9	9
3	3	3	3	3	3	3	3	3	3
2	2	3	3	2	2	2	2	2	2

3	3	3	3	1	1	2	3	3	3
1	3	2	3	3	3	3	3	3	3
2	3	1	2	2	3	2	2	2	3
9	9	9	9	9	9	9	9	9	9
3	2	3	2	2	2	3	2	2	2
3	3	3	3	3	3	3	3	3	3
1	9	2	2	2	2	2	2	2	2
3	3	3	3	3	3	3	3	3	3
2	2	2	3	3	3	3	3	2	2
2	3	2	1	1	2	3	3	3	1
2	2	2	2	2	2	2	2	2	2
3	2	3	2	2	2	3	1	3	2
1	1	1	1	1	2	1	2	1	2
9	9	9	9	1	9	9	9	9	9
1	1	1	2	2	2	1	1	1	1
3	3	3	3	2	3	3	2	2	3
3	3	1	3	1	1	1	1	1	2
2	2	2	2	2	2	2	2	2	2
1	2	2	2	3	3	2	2	3	2
1	2	9	1	3	2	3	2	1	3
3	2	3	2	3	1	2	3	2	3
3	9	3	3	3	3	3	3	3	3
2	2	2	2	2	2	2	2	2	2
2	2	2	2	2	1	3	2	2	2
2	3	1	2	1	2	3	1	2	3
2	2	3	1	1	3	3	3	3	3
1	2	2	3	3	1	2	3	3	3
3	3	2	2	2	2	3	9	1	2
3	1	2	3	2	3	9	3	1	2
2	2	2	2	2	2	2	2	2	2
1	1	2	3	1	2	1	2	1	2
2	2	2	2	2	2	2	2	2	2
2	3	2	1	1	2	3	1	2	3
2	3	3	3	1	1	2	3	3	3
3	1	2	3	1	2	3	1	2	3
3	3	1	1	1	3	2	3	2	3
3	3	9	1	1	2	2	2	3	2
3	3	3	3	2	2	2	1	2	1
1	3	1	1	1	1	1	2	2	2
3	3	1	2	1	1	2	2	3	9
1	2	2	2	2	1	2	2	1	2
1	2	3	2	1	3	1	3	1	3
2	2	2	1	1	1	2	3	2	3
2	3	1	3	3	2	1	3	9	2
2	3	2	2	2	2	3	2	2	3
1	2	1	2	1	3	2	2	1	2
3	3	3	3	1	3	9	1	3	1

1	2	2	2	3	3	3	3	2	2
2	3	1	3	1	3	2	1	3	1
2	2	2	2	2	2	2	2	2	2
1	3	1	3	1	1	3	1	1	1
2	3	1	2	1	2	3	1	2	3
1	3	1	2	2	2	2	2	2	2
3	2	3	1	2	1	2	3	2	1
3	2	1	3	2	1	2	3	1	2
2	2	1	3	1	1	2	1	2	1
1	3	3	3	3	3	3	3	1	1
2	3	2	3	1	2	3	2	3	2
3	2	1	3	1	2	3	1	2	3
1	1	1	1	1	1	1	1	1	1
3	1	1	3	1	3	1	1	1	3
2	2	3	2	1	2	3	2	2	1
1	3	1	3	3	1	1	1	3	3
1	2	2	3	1	1	3	3	2	1
3	2	9	9	3	3	3	3	3	3
1	3	2	3	1	2	3	1	2	1
3	1	2	1	3	1	1	1	2	1
2	2	2	2	2	2	2	2	2	2
1	3	3	3	2	1	3	3	3	1
1	3	2	1	1	3	2	1	1	3
2	2	2	3	2	2	3	3	1	2
2	2	2	2	2	2	3	1	3	2
2	2	2	2	2	2	2	2	2	2
1	1	3	3	1	1	3	2	1	1
1	3	2	3	2	1	2	1	2	1
2	2	2	2	2	2	2	2	2	2
9	9	9	9	9	9	9	9	9	9
9	9	9	9	9	9	9	9	9	9
9	9	9	9	9	9	9	9	9	9
2	2	2	2	2	2	2	2	2	2
1	9	9	9	1	9	9	9	9	9
3	3	3	3	2	2	2	2	2	2
2	2	2	2	2	2	2	2	2	2
3	3	1	2	2	2	3	2	2	2
2	2	2	2	2	2	2	2	2	2
2	9	2	3	9	2	9	2	9	9
2	3	2	2	2	2	3	2	3	2
3	2	1	2	3	2	3	2	3	2
1	1	2	2	3	1	1	2	2	2
3	3	2	3	3	3	2	2	2	1
3	3	2	3	3	3	2	2	2	1
2	3	2	1	1	2	3	3	3	2
1	1	1	1	2	2	2	2	2	2
1	1	3	3	1	1	3	2	1	1

2	2	2	2	9	9	9	9	9	9
2	1	2	2	1	2	3	2	2	2
2	2	2	2	2	2	2	2	2	2
1	2	1	1	1	3	3	2	1	1
2	2	2	2	3	2	2	2	2	2
3	3	1	2	2	2	3	2	2	2
2	1	3	2	1	2	1	1	2	3
3	2	2	3	2	3	3	1	2	3
3	1	2	1	1	2	1	3	3	3
3	3	3	2	3	3	3	3	2	2
1	2	3	2	2	3	2	1	2	3
1	2	3	2	3	2	1	2	3	2
1	2	1	1	1	1	3	1	1	9
2	2	2	2	2	2	2	2	2	2
2	2	2	2	2	2	2	2	2	2
3	3	3	3	3	3	2	2	2	3
2	2	2	2	3	1	2	2	2	2
2	2	2	2	2	2	2	2	2	2
2	2	2	2	2	2	2	2	2	2
2	3	1	2	3	2	3	1	1	9
9	2	9	9	9	1	1	9	9	9
9	1	9	9	9	1	1	9	9	9
9	1	9	9	9	1	1	9	9	9
1	2	2	2	1	1	1	1	1	1
3	1	3	3	3	1	1	3	3	3
9	1	9	9	9	1	1	9	9	9
9	1	9	9	2	1	1	2	2	2
2	1	2	1	1	1	1	9	9	9
2	1	9	2	1	2	1	2	1	2
9	9	9	9	9	9	1	9	9	9
9	1	9	9	1	9	1	9	9	9
9	9	9	9	9	9	1	9	9	9
2	1	3	3	3	3	1	3	3	3
3	3	3	3	3	3	1	3	3	3
9	1	9	9	9	1	1	9	9	9
3	1	2	2	2	1	1	3	3	3
9	1	9	9	9	1	1	9	9	9
9	1	9	9	9	1	1	9	9	9
9	1	9	9	9	1	1	9	9	9
9	1	9	9	9	1	1	9	9	9
3	1	3	3	1	3	1	3	3	3
9	9	9	9	1	9	1	1	9	9
9	1	9	9	9	1	1	9	9	9
9	9	9	9	1	9	9	9	9	9
9	1	9	9	9	1	1	9	9	9
3	1	3	2	3	1	1	3	3	2
2	1	2	2	9	1	1	9	9	9

9	1	9	9	9	1	1	9	9	9
2	1	2	9	3	1	1	1	3	2
2	2	2	1	1	1	1	1	1	2
1	1	1	1	1	1	1	1	1	1
9	1	9	9	9	9	1	9	9	9
9	1	9	9	9	9	1	9	9	9
1	1	1	1	1	1	1	1	1	1
2	2	2	2	1	3	1	1	1	1
9	1	9	9	9	1	1	9	9	9
9	1	9	9	3	1	1	3	9	9
2	1	2	2	2	1	1	2	2	2
2	1	2	2	2	1	1	2	2	1
9	1	9	9	1	9	1	9	9	9
9	1	9	9	9	1	1	9	9	9
9	1	9	9	9	1	1	9	9	9
9	1	9	9	9	1	1	9	9	9
9	1	9	9	9	9	1	9	9	9
9	1	9	9	9	9	1	9	9	9
9	1	9	9	9	1	1	9	9	9
9	9	9	9	1	1	1	1	9	9
9	9	9	9	9	1	9	9	9	9
9	1	9	9	9	9	1	9	9	9
1	1	1	1	3	3	1	1	1	1
9	9	9	1	9	9	1	9	9	9
1	1	1	1	3	3	1	3	3	1
3	1	3	3	3	3	1	3	3	3
3	1	3	3	3	3	1	3	3	3
9	1	9	9	3	3	1	9	3	3
9	1	9	9	3	1	1	3	3	3
9	1	9	9	9	9	1	9	9	9
2	1	2	2	9	1	1	1	1	1
3	1	3	3	3	1	1	3	3	3
9	1	9	9	9	1	1	9	9	9
9	1	9	9	9	1	1	9	9	9
9	1	9	9	9	1	1	9	9	9
3	1	3	3	3	3	1	1	1	2
9	1	9	9	9	9	1	9	9	9
9	1	9	9	9	9	1	9	9	9
9	1	9	9	3	1	1	3	3	3
3	2	3	2	3	1	1	1	1	1
1	1	3	2	3	1	1	3	3	1
2	2	2	2	1	3	1	3	1	1
9	1	9	9	9	1	1	9	9	9
9	1	9	9	9	9	1	3	1	1
9	1	9	9	3	1	1	3	3	3
9	1	9	9	1	3	1	3	3	3
9	1	9	9	9	1	1	9	9	9

9	1	9	9	9	1	1	9	9	9
3	1	3	3	1	1	1	9	9	9
9	1	9	9	9	1	1	9	9	9
3	1	3	1	1	1	1	1	3	1
9	1	9	9	9	1	1	1	9	9
1	9	9	9	9	1	9	9	9	9
3	1	3	1	1	3	9	9	9	9
9	1	9	9	9	9	1	9	9	9
9	1	9	9	9	9	1	9	9	9
3	1	3	3	1	3	1	1	1	3
1	2	2	2	1	3	1	1	1	2
1	9	9	9	9	9	9	9	1	9
9	9	1	9	9	9	1	9	1	9
1	2	2	2	3	2	1	2	2	2
1	2	2	2	1	3	1	2	2	2
1	2	1	2	1	2	1	1	1	2
9	9	1	9	9	9	9	9	1	9
9	9	1	9	1	9	9	9	9	9
2	2	1	2	2	2	2	1	1	2
1	2	1	1	1	1	1	1	1	1
3	3	3	3	3	3	3	3	2	2
2	2	2	1	3	3	2	9	1	3
2	2	9	2	1	3	1	1	1	2
3	3	3	3	1	2	1	1	1	1
9	9	9	9	9	3	9	9	9	9
2	1	2	2	1	1	1	1	1	2
3	2	1	3	1	2	1	3	2	1
3	2	2	2	2	3	1	1	1	2
1	1	2	1	2	2	2	1	1	2
2	2	2	2	1	3	1	1	1	3
2	2	2	2	2	2	1	2	2	2
2	2	1	3	1	2	2	1	2	1
2	2	9	2	1	1	1	1	1	1
9	1	9	9	9	9	9	9	9	9
2	2	2	2	1	3	1	1	1	2
2	2	2	1	1	1	1	2	1	1
1	1	2	2	1	9	1	1	2	2
1	1	2	2	1	2	1	1	2	2
1	1	2	2	1	9	1	1	2	2
1	2	3	2	1	3	1	1	1	3
3	1	2	3	1	3	1	1	1	2
3	1	3	1	1	3	1	1	1	2
1	2	2	2	1	2	3	3	2	2
1	2	2	2	1	1	2	9	2	2
9	2	2	2	1	2	3	3	9	2
9	9	1	9	1	1	1	1	1	1
9	9	9	9	9	9	9	9	1	9

9	1	9	9	3	1	1	3	1	2
2	1	2	1	1	2	1	1	1	1
2	2	2	2	1	1	1	9	1	2
2	2	2	2	2	2	2	2	2	2
1	2	3	2	9	9	9	1	9	9
2	2	2	2	1	2	3	1	2	2
2	2	2	2	2	2	2	2	2	2
2	3	3	3	1	2	1	1	2	2
2	9	9	9	3	1	1	3	1	3
9	9	9	9	1	3	3	1	1	2
2	3	9	3	1	3	1	1	1	2
3	3	3	1	3	3	3	3	3	3
9	1	2	2	2	2	3	2	2	9
3	1	3	3	9	9	9	9	9	9
2	1	2	2	2	9	9	1	2	2
9	1	9	9	9	9	9	9	9	9
2	1	2	2	2	2	3	1	1	2
9	1	2	1	9	9	9	9	9	9
3	1	3	3	3	2	3	1	1	1
9	1	9	9	1	2	3	3	2	2
9	9	9	9	2	9	9	9	9	9
9	9	9	9	9	9	9	9	9	9
2	2	2	1	2	2	2	1	2	2
3	3	3	3	1	3	3	3	3	3
3	1	2	3	2	2	3	1	2	1
3	1	2	2	1	2	3	1	2	9
9	1	9	9	9	9	9	1	1	9
9	1	9	9	9	9	9	9	9	9
9	1	9	9	9	9	9	9	9	9
3	1	2	2	3	2	3	2	1	2
9	1	9	9	3	3	3	1	9	9
2	1	3	1	2	3	3	1	1	1
9	1	9	9	9	1	1	9	9	9
9	1	3	3	1	9	1	3	9	3
2	2	2	1	1	3	1	3	3	3
9	9	9	9	9	9	9	9	9	9
2	1	2	2	1	1	1	3	1	3
3	1	3	2	2	2	3	3	3	3
3	1	3	3	3	1	1	3	3	3
2	1	2	2	1	1	1	3	1	3
2	1	2	1	2	1	1	3	2	2
9	1	9	9	9	9	1	9	9	9
1	1	3	1	1	1	1	3	3	3
9	1	9	9	9	9	1	9	9	9
3	1	3	3	3	1	1	3	3	3
9	1	9	9	9	1	1	9	9	9
9	1	9	9	1	9	1	9	9	9

3	1	3	3	3	3	1	3	3	3
9	1	9	1	9	9	1	9	9	9
9	9	9	9	9	9	9	9	9	9
9	9	9	9	9	9	9	9	9	9
2	9	9	9	9	1	9	3	3	3
2	1	2	2	2	2	3	1	1	1
9	1	9	9	9	9	9	1	9	9
9	1	9	9	9	1	9	9	9	9
9	1	9	9	9	9	9	9	1	9
9	1	9	9	9	9	9	9	9	9
3	3	3	1	3	3	3	3	3	3
2	1	2	2	2	2	2	1	2	2
9	1	3	1	3	3	3	1	1	2
9	1	9	9	9	9	9	9	9	9
3	1	1	1	1	1	3	1	1	3
2	1	2	1	3	2	2	1	1	2
9	1	9	9	9	9	9	9	9	9
1	9	9	9	2	2	3	1	3	9
9	1	9	9	9	9	9	1	9	9
3	1	3	3	3	3	3	1	2	3
3	1	3	3	1	2	1	3	3	1
9	1	9	9	1	9	9	9	9	9
9	1	9	9	1	9	9	9	9	9
3	1	1	3	2	2	2	2	2	1
1	9	9	9	9	9	1	1	1	9
9	1	1	2	2	1	2	1	1	2
9	1	3	9	9	9	9	1	9	9
2	1	3	2	3	2	3	1	2	3
1	2	2	1	1	3	3	2	2	2
2	2	2	1	1	2	3	2	2	2
1	2	1	1	2	3	3	3	1	2
9	1	1	2	2	2	2	1	2	1
1	1	9	9	3	3	3	2	1	3
3	1	3	3	3	3	3	1	1	3
2	1	3	1	3	3	3	2	2	3
9	1	9	9	9	1	9	9	9	9
9	1	9	9	3	3	3	3	9	9
9	1	9	9	9	9	9	9	9	1
3	3	2	1	2	2	2	9	1	1
3	1	3	2	3	3	3	3	9	2
3	1	3	2	2	3	3	1	2	3
3	1	3	3	3	3	3	3	3	2
9	1	9	9	9	1	9	9	9	9
9	1	9	9	9	1	9	9	9	9
9	9	9	9	9	9	9	1	9	1
9	9	9	9	9	9	9	9	9	9
9	1	9	9	1	9	9	1	9	9

3	3	3	1	3	3	1	1	3	3
9	1	9	9	2	2	3	1	1	1
9	1	9	9	9	9	9	9	9	9
1	1	2	2	3	2	1	1	3	3
3	1	3	3	3	3	1	1	1	1
9	9	9	9	2	9	9	9	9	9
2	1	2	2	2	2	2	1	3	3
2	2	2	2	2	2	2	2	2	2
2	1	2	2	3	3	3	3	3	3
1	1	2	1	2	2	2	1	1	2
2	1	3	2	3	3	3	1	1	2
1	9	9	9	9	9	9	9	9	1
9	1	9	9	9	9	9	9	9	1
9	1	9	1	9	9	9	1	9	9
9	1	9	9	9	9	9	9	9	9
9	1	9	9	9	9	9	1	9	9
3	1	3	3	9	9	9	9	9	9
3	1	3	2	2	2	2	2	2	2
1	9	9	9	9	9	9	9	9	1
3	1	2	2	2	1	2	2	2	1
1	2	3	1	3	3	3	1	1	1
2	1	2	2	2	2	3	2	3	2
2	1	2	2	2	2	3	2	2	3
3	1	2	3	3	2	3	1	2	2
3	1	3	3	3	3	3	2	3	3
9	1	9	9	9	9	9	1	9	9
3	1	3	3	2	2	3	3	3	3
3	1	3	3	2	2	2	1	2	2
9	1	9	9	9	9	9	1	9	9
9	1	9	9	9	9	9	9	1	9
2	1	2	1	3	2	3	1	2	2
9	1	1	9	9	9	9	9	9	9
9	1	9	9	9	9	9	9	1	9
2	9	2	1	3	1	1	3	1	1
9	1	9	9	3	9	1	9	9	9
3	1	3	3	2	3	3	2	2	2
2	2	2	2	1	3	1	2	1	2
2	1	2	2	3	1	1	1	1	9
9	9	9	9	9	9	3	1	9	9
9	1	9	9	9	1	1	9	9	9
2	1	2	3	3	3	1	2	2	2
3	3	2	2	1	2	3	3	3	3
9	9	1	9	1	2	1	1	1	2
1	1	1	1	1	2	1	2	2	2
1	2	2	1	2	1	2	2	1	2
9	1	9	9	9	9	9	9	9	1
2	1	1	1	3	3	3	2	3	9

3	1	3	3	3	3	3	1	2	3
3	1	3	3	2	3	3	3	2	1
2	2	1	2	3	2	1	2	2	1
2	2	2	1	3	3	3	1	1	1
2	2	1	2	3	3	2	1	2	2
1	2	1	2	2	2	2	1	1	3
3	2	2	3	3	3	3	1	3	2
2	2	2	2	9	2	2	1	2	2
3	2	1	3	3	3	3	1	1	1
2	2	2	2	3	2	1	1	2	1
1	2	2	2	2	2	3	3	1	2
2	2	2	2	2	2	2	2	2	2
1	1	2	2	2	3	3	1	3	3
1	2	2	2	3	1	1	2	2	1
2	2	2	2	2	2	2	1	2	1
2	2	2	2	3	3	2	2	2	3
2	2	2	2	2	2	2	2	2	2
2	2	2	2	2	2	2	2	2	2
2	2	2	2	2	2	2	2	2	2
1	1	1	1	2	2	3	1	1	2
3	1	2	3	3	2	3	1	2	1
2	2	3	2	3	3	3	1	2	3
2	2	2	2	9	1	3	2	2	3
3	2	2	2	2	2	2	1	2	1
2	1	2	1	2	2	2	1	2	2
2	2	3	2	3	3	3	1	2	3
2	2	3	2	2	2	1	1	2	2
2	1	9	1	2	2	2	1	2	2
2	1	2	1	2	2	2	1	2	2
1	3	9	3	3	2	9	1	2	9
1	2	2	2	3	3	3	1	1	2
2	1	2	1	3	3	2	2	3	3
1	2	2	1	9	9	9	9	9	9
1	2	2	1	1	9	9	9	9	9
9	1	9	9	9	9	9	9	9	9
1	9	9	9	1	9	9	9	9	9
9	9	9	9	9	9	9	9	9	9
9	9	9	9	9	9	9	9	9	9
1	9	9	9	9	9	9	1	9	9
2	2	3	1	3	3	3	1	2	2
2	3	2	1	3	2	3	1	2	1
9	9	9	9	9	9	9	9	1	9
9	9	9	9	9	9	9	9	1	9
2	2	3	2	3	3	3	1	2	3
9	9	9	9	9	9	9	9	9	9
9	9	9	9	1	9	9	9	9	9
9	9	9	9	1	9	9	9	9	9

2	2	2	2	3	3	3	2	2	2
3	2	2	3	3	1	3	2	3	3
9	9	9	9	1	9	9	9	9	9
9	9	9	9	9	9	9	9	9	9
9	9	9	9	9	9	9	9	9	9
9	9	9	9	9	9	9	9	9	9
9	1	9	9	9	9	9	1	9	9
9	1	9	9	9	9	9	1	9	9
9	9	9	1	9	9	9	1	9	9
9	9	9	9	9	9	9	9	9	1
3	1	3	1	3	3	3	3	1	3
3	3	3	3	3	3	3	3	3	3
3	1	3	3	3	3	3	3	1	1
3	1	3	3	3	3	3	2	3	1
1	1	2	1	3	3	3	3	3	1
3	1	3	3	3	3	3	1	3	1
3	1	3	3	3	3	3	1	3	1
3	1	3	3	3	3	3	1	3	1
3	3	3	3	3	3	3	3	3	1
2	2	2	1	2	2	2	2	2	1
1	1	2	2	1	2	3	1	2	2
3	1	2	3	1	1	3	3	1	1
9	9	9	1	9	9	9	9	1	9
1	1	2	1	2	3	1	2	2	1
3	3	3	1	3	3	3	3	3	3
3	1	1	3	3	3	2	1	1	1
1	9	9	9	9	1	9	1	3	3
1	1	2	3	3	3	3	1	3	3
3	1	1	3	3	3	1	3	3	3
1	2	2	3	2	1	2	1	3	2
2	1	3	1	3	2	2	3	2	3
9	9	9	9	2	2	1	9	1	1
3	1	1	3	2	2	3	3	3	2
3	1	3	3	2	2	3	3	3	1
1	2	2	3	2	2	2	3	1	1
3	1	1	1	1	3	3	3	3	3
3	1	3	3	3	3	3	1	3	1
3	1	3	1	3	3	3	3	3	1
3	3	3	1	3	3	3	3	9	3
1	1	1	1	3	3	3	1	2	1
2	1	3	3	1	1	2	1	3	3
1	1	9	1	3	3	1	3	3	1
3	1	2	3	2	3	2	3	3	2
3	3	3	3	3	3	3	1	1	3
3	3	3	3	3	3	3	1	1	3
3	3	3	3	3	3	1	3	3	3
1	1	2	2	2	3	3	2	2	1

3	1	2	3	3	2	1	1	9	9
1	1	1	1	3	3	1	1	1	1
1	1	2	1	1	2	3	2	9	1
3	3	3	3	3	3	3	1	3	3
3	3	3	3	3	3	3	1	1	3
3	3	3	3	3	3	3	1	3	3
1	1	2	2	2	3	3	2	2	1
1	1	9	1	3	2	2	2	2	9
2	1	2	2	2	2	2	2	2	2
1	1	1	1	1	1	9	9	9	1
1	2	2	2	1	2	2	2	2	2
9	2	2	2	1	1	1	1	9	1
2	9	9	9	9	1	1	2	2	2
2	2	3	2	1	3	1	3	2	2
1	1	1	1	1	1	1	3	1	3
2	2	2	2	2	2	2	2	2	2
1	1	9	1	2	2	9	1	9	9
2	2	2	2	2	2	3	3	2	2
3	1	3	1	1	3	2	1	1	2
1	1	2	2	2	3	3	2	2	2
2	2	2	2	3	3	1	1	2	3
1	1	1	1	3	3	3	3	1	3
3	1	2	3	2	2	2	2	1	2
3	1	2	3	2	2	2	2	1	2
2	1	2	3	2	2	2	2	1	3
2	1	1	2	1	2	2	1	2	3
2	2	2	2	3	2	3	2	2	2
2	2	2	2	3	3	1	1	2	2
2	2	2	2	2	2	2	1	2	2
2	2	1	2	2	2	2	2	1	2
3	2	2	2	2	3	2	2	3	2
2	1	2	2	2	3	3	3	2	2
9	9	9	9	2	2	1	1	1	3
1	2	3	3	3	3	3	1	1	3
3	1	2	3	2	2	2	2	1	2
3	3	3	3	3	3	1	3	3	3
2	1	2	9	3	3	2	2	3	3
2	2	2	2	3	3	2	2	3	1
9	9	9	9	3	3	3	1	3	3
2	1	1	2	1	3	3	1	3	1
2	2	2	2	1	2	3	1	2	2
1	3	3	3	3	9	1	1	2	3
1	3	3	3	3	1	1	1	2	3
1	2	2	1	1	1	2	1	1	2
2	2	2	2	3	3	3	2	1	3
2	2	2	2	3	3	3	3	2	2
1	2	2	2	3	1	3	1	3	1

1	2	9	1	1	1	2	2	2	3
2	2	2	2	2	2	2	2	2	2
1	2	2	1	1	1	2	1	1	2
2	2	2	1	2	2	2	1	3	1
2	2	1	3	1	2	1	1	1	1
1	2	2	3	2	2	1	1	2	2
9	9	9	9	3	3	3	9	2	2
2	2	2	2	3	3	3	3	2	2
2	2	2	2	1	2	2	2	2	2
2	1	1	1	3	3	3	1	3	3
1	2	3	3	1	3	3	3	1	1
1	2	1	2	3	1	1	1	3	1
9	9	9	9	9	9	9	9	9	9
2	2	2	2	2	2	3	2	2	1
3	3	3	3	1	3	3	1	3	3
2	2	2	2	2	2	2	2	2	2
2	1	2	1	3	3	2	1	3	1
2	2	2	2	2	2	1	1	2	1
1	2	2	2	2	2	2	1	2	1
2	9	1	3	2	2	2	1	1	1
1	1	3	1	3	3	3	1	1	1
1	1	2	2	1	2	3	2	1	2
3	1	3	3	3	3	3	1	2	3
3	1	3	3	3	2	3	2	3	1
3	1	3	3	3	3	3	3	3	3
3	1	3	3	3	3	3	3	3	1
3	1	3	3	3	3	3	1	3	1
2	1	3	2	3	3	3	1	2	3
2	1	1	1	1	1	1	1	1	2
2	1	9	9	9	9	2	9	9	9
2	2	3	2	3	3	3	1	2	3
1	3	2	3	3	3	3	1	2	1
1	2	2	1	2	2	3	1	1	2
2	2	2	2	2	2	3	1	1	3
9	1	9	9	9	9	9	1	9	9
2	2	2	2	3	3	3	1	3	1
2	2	2	2	2	2	2	2	2	2
1	9	9	9	9	9	2	9	9	9
3	1	1	1	2	1	1	2	3	2
1	2	2	1	3	3	3	3	1	2
9	9	9	9	9	9	9	9	9	9
2	2	2	2	1	3	3	1	2	3
1	1	2	1	1	2	2	1	1	2
1	2	3	3	2	3	2	9	2	1
2	3	2	2	3	3	3	3	1	1
2	2	1	3	3	3	3	2	2	3
3	2	2	2	3	2	3	2	2	3

2	3	3	3	1	2	3	1	2	2
2	2	2	2	2	2	2	1	2	2
2	2	2	2	2	2	2	2	2	2
2	1	2	2	2	2	2	2	2	2
2	2	1	2	1	2	2	1	2	2
2	3	2	2	2	3	3	3	2	3
2	2	2	2	3	3	3	1	1	1
9	9	9	9	9	9	9	9	9	9
2	2	2	2	2	2	2	1	2	2
2	3	9	2	3	3	3	2	2	2
1	1	2	1	2	2	2	1	2	2
9	1	9	9	9	9	9	9	9	9
1	1	2	1	1	1	3	3	3	9
2	2	2	2	2	2	2	2	2	2
9	9	3	9	3	3	2	2	3	3
2	1	2	1	3	3	2	2	3	3
2	1	1	1	1	2	2	2	2	2
3	1	1	1	2	1	1	9	1	9
1	9	9	9	9	9	9	9	9	9
1	1	3	1	1	1	1	1	1	1
1	1	3	1	1	1	1	1	1	2
1	2	2	2	1	1	2	2	1	1
1	2	2	2	1	2	2	2	2	2
3	2	2	2	1	2	2	2	2	2
2	3	3	3	1	1	2	3	1	2
1	2	2	2	1	1	1	9	1	9
1	2	2	2	1	2	1	1	1	2
1	1	2	2	1	2	1	1	1	2
1	1	1	2	1	2	2	1	1	2
9	9	9	1	3	9	9	9	9	9
1	1	2	2	1	2	1	1	1	2
3	3	2	1	2	2	1	1	1	2
1	1	1	1	1	2	1	2	1	2
2	2	2	2	3	3	1	1	2	2
1	1	2	1	1	2	3	1	2	2
1	1	2	1	1	2	1	2	2	2
1	1	2	2	1	1	1	2	2	1
2	2	2	2	1	1	1	1	2	2
3	1	3	3	1	1	1	3	1	3
1	2	2	2	1	3	1	2	2	2
2	2	2	2	2	2	1	1	2	2
3	1	1	1	1	1	1	1	1	3
2	2	2	2	2	2	1	2	1	2
1	3	2	3	3	3	3	1	1	3
1	2	1	1	1	1	1	3	3	3
1	1	2	2	1	2	1	1	3	3
3	1	2	1	1	1	1	1	1	2

1	2	3	3	1	3	1	3	3	3
1	2	3	3	1	3	1	3	3	3
1	1	2	2	1	3	1	3	1	3
1	1	2	1	1	3	1	1	1	2
1	2	3	3	1	3	1	3	3	3
2	2	2	2	1	1	1	1	2	2
1	3	1	3	3	1	3	1	3	3
1	1	2	2	3	2	1	1	2	2
3	3	3	3	3	3	3	3	3	3
3	1	3	3	3	3	3	3	1	1
1	3	3	3	3	3	3	3	3	3
1	3	3	3	3	3	3	3	3	3
1	1	1	2	2	2	2	1	1	2
3	1	2	3	3	2	3	2	2	3
2	1	3	3	1	2	3	1	1	2
1	2	2	2	2	2	2	1	1	2
2	2	2	2	2	2	3	1	2	3
1	2	2	2	2	1	1	2	1	1
9	1	9	9	3	3	3	3	3	1
3	3	3	1	3	3	3	3	3	3
1	2	2	1	3	2	1	3	1	2
2	2	2	2	9	1	9	9	9	9
9	9	9	9	9	9	9	9	9	9
9	9	9	9	9	9	9	9	9	9
9	9	9	9	9	9	9	9	1	9
9	9	9	1	9	9	9	1	9	9
2	2	2	2	3	3	3	1	2	2
9	1	9	9	9	9	9	9	9	9
9	9	9	9	2	9	9	9	9	9
9	1	9	9	9	9	9	9	9	9
9	9	9	9	9	9	9	9	9	9
9	9	9	9	9	9	9	9	9	9
9	9	9	9	3	9	9	9	9	9
9	9	9	9	3	2	2	3	2	1
2	2	3	2	3	3	3	1	2	3
3	1	3	3	2	3	3	1	2	2
2	2	2	2	1	1	1	1	2	2
3	1	1	1	1	1	1	1	1	3
1	1	2	2	1	2	1	1	1	2
1	1	2	2	1	2	1	1	1	2
2	2	2	2	1	1	1	1	2	2
3	3	3	3	2	2	3	1	3	3
2	1	1	2	1	2	2	1	2	2
2	2	2	2	2	2	3	1	2	2
3	3	2	3	3	3	2	1	3	2
3	3	3	1	1	3	3	1	3	3
1	2	1	1	3	2	2	1	1	2

2	2	1	3	2	2	2	1	2	2
2	2	2	2	2	2	3	2	2	2
2	2	3	2	3	3	3	3	3	3
3	3	3	1	3	1	2	2	1	2
1	1	1	2	1	2	2	2	3	2
1	1	1	1	2	2	2	1	1	1
2	2	2	2	2	2	3	2	2	2
2	2	2	2	2	1	2	2	2	2
2	2	3	2	2	2	3	1	1	2
3	1	2	3	2	2	2	2	2	2
2	2	2	2	3	3	2	2	1	2
2	2	1	2	3	2	3	2	2	1
2	1	2	2	2	1	2	1	1	1
3	3	1	3	3	3	3	3	3	3
2	2	2	2	2	2	2	2	2	2
2	2	2	2	2	2	2	2	2	2
2	2	2	2	3	3	2	1	2	1
2	1	2	2	3	1	3	9	2	3
2	1	3	1	1	1	1	1	1	1
1	1	1	1	1	1	1	1	1	1
1	2	2	2	1	1	1	1	1	1
2	2	2	2	1	1	1	1	2	3
2	2	3	2	2	2	3	1	1	2
3	3	3	3	2	1	2	1	3	1
1	2	2	2	1	1	2	1	2	2
2	1	2	1	2	3	2	1	2	3
2	2	2	2	2	1	2	1	1	1
2	1	1	1	2	2	3	1	1	1
2	2	2	2	2	2	2	1	2	2
2	2	2	2	9	9	9	1	9	9
9	9	9	1	9	9	9	9	9	9
2	1	2	1	1	2	2	1	1	1
3	1	3	3	1	1	1	1	1	1
1	2	2	3	3	3	3	1	3	2
3	3	3	3	3	3	3	3	3	3
1	2	2	2	9	2	2	2	2	2
1	2	3	2	2	2	2	2	2	2
2	1	2	2	3	3	2	2	2	2
2	3	2	3	3	3	3	2	1	1
2	1	2	1	2	2	2	2	2	1
2	2	2	2	2	2	2	1	2	2
2	2	2	2	2	2	2	1	2	2
9	9	9	9	3	9	9	9	9	9
9	9	9	9	2	9	9	9	9	9
9	9	9	9	3	9	9	9	9	9
1	1	2	2	2	2	3	1	2	2
2	2	1	1	2	1	3	1	2	1

2	3	2	1	1	2	2	3	3	3
2	3	2	1	1	2	2	3	3	3
2	3	2	1	1	2	2	3	3	3
3	1	3	3	2	2	2	2	2	2
2	2	2	3	1	3	1	2	2	2
1	2	1	2	3	3	2	1	1	2
2	3	2	3	2	1	2	3	1	1
2	1	1	3	3	2	3	3	2	3
1	2	1	3	3	2	1	1	2	1
3	3	2	3	2	1	2	1	3	2
2	2	2	2	2	2	2	2	2	2
2	2	2	2	1	3	1	2	2	2
2	2	2	1	3	1	3	2	2	2
2	3	1	3	2	2	2	2	3	3
2	2	3	3	1	3	1	3	2	2
2	2	2	2	2	2	1	1	3	3
2	2	1	2	3	2	2	2	2	2
2	2	1	2	3	2	2	2	2	2
2	3	2	1	1	2	2	3	3	3
2	3	2	1	1	2	2	3	3	3
3	1	3	3	2	2	2	2	2	2
2	2	2	2	1	3	1	3	3	3
3	1	3	3	1	3	1	3	3	3
2	2	2	2	2	2	2	2	2	2
3	1	3	3	2	2	2	2	2	2
2	2	2	2	1	3	1	3	3	3
2	2	3	3	2	2	2	2	3	3
9	9	9	9	1	9	9	9	9	9
1	3	2	2	2	2	2	1	1	1
2	2	1	1	2	2	2	3	3	3
2	2	1	1	2	2	2	3	3	3
2	2	1	1	2	2	2	3	3	3
2	2	1	1	2	2	2	3	3	3
9	9	1	9	3	2	3	3	9	2
2	1	1	3	3	3	3	9	1	2
3	1	2	3	2	3	3	2	3	2
1	2	2	2	3	1	3	2	2	2
1	3	1	2	2	1	1	3	2	2
2	2	1	1	3	2	2	2	1	2
2	3	3	2	3	2	3	3	2	1
1	2	3	1	3	2	2	9	1	2
9	3	9	9	3	2	2	9	1	2
1	3	1	1	1	3	1	3	3	3
9	3	9	9	9	9	9	9	1	9
9	9	9	9	9	9	9	9	9	9
3	1	3	3	1	3	1	3	3	3
9	2	2	2	3	9	9	1	9	1

9	2	2	2	3	2	9	1	9	1
1	3	1	1	3	1	3	1	1	1
1	2	1	1	2	2	2	2	2	2
2	2	2	2	2	9	2	9	2	2
3	9	2	2	3	2	2	2	2	2
9	9	9	9	2	9	9	9	9	9
9	9	9	9	9	9	3	9	9	9
9	3	9	9	9	9	9	9	9	9
1	2	1	2	1	3	2	2	2	2
1	2	2	2	2	2	2	2	2	2
1	3	1	1	3	1	3	1	1	1
2	2	2	2	2	2	2	1	2	3
2	9	9	9	9	9	9	1	9	9
9	9	9	9	2	9	9	9	9	9
2	2	2	2	1	3	1	2	2	2
9	9	9	9	2	9	9	9	9	9
2	2	2	2	2	2	3	2	2	2
2	9	9	9	9	9	9	9	9	9
3	3	2	1	3	3	3	1	3	1
1	9	9	9	3	9	9	9	9	9
1	2	1	2	3	1	3	1	1	1
2	2	2	2	2	1	3	1	1	1
1	3	1	1	3	1	3	1	1	1
2	2	2	2	3	1	3	1	1	1
2	2	2	2	2	2	2	2	2	2
2	2	2	2	2	2	2	2	1	1
1	2	2	2	3	1	3	1	1	1
1	9	9	9	3	9	9	9	9	9
1	9	9	9	3	9	9	9	9	9
1	9	9	9	3	9	9	9	9	9
9	9	9	9	3	9	9	9	9	9
1	9	9	9	3	9	9	9	9	9
9	9	9	9	2	9	9	9	9	9
1	2	2	1	3	2	1	1	1	2
3	3	2	3	3	2	3	2	3	1
9	9	9	9	9	9	9	9	9	9
2	2	9	9	2	2	2	2	2	2
9	2	9	9	9	9	9	9	2	9
2	2	2	2	2	2	2	2	1	1
9	3	9	9	9	9	3	9	1	1
1	1	2	2	3	2	2	1	2	3
9	9	9	9	2	9	9	9	9	9
3	1	3	3	1	3	1	3	3	3
2	2	2	2	2	2	2	2	2	2
9	3	9	9	3	9	9	9	9	9
1	2	9	2	1	1	3	2	1	9
1	9	9	9	3	9	9	9	9	9

1	9	9	9	3	9	9	9	9	9
1	9	9	9	9	9	9	9	9	9
1	3	1	1	2	2	2	1	1	1
1	9	9	9	3	9	9	9	9	9
2	2	1	2	1	2	1	2	2	2
1	9	9	9	3	9	9	9	9	9
2	2	2	2	2	2	2	2	2	2
1	3	1	1	3	1	3	2	2	2
1	3	1	1	3	2	3	3	1	3
2	3	2	1	3	2	1	1	2	1
1	3	1	1	3	1	3	1	1	1
3	1	3	3	1	3	1	1	1	1
1	3	2	2	3	2	2	1	3	2
1	2	2	3	3	2	1	1	1	2
2	2	2	2	3	1	3	2	2	2
2	2	2	3	3	1	2	2	2	2
2	2	2	2	2	2	1	3	2	1
2	2	2	3	3	3	2	2	1	1
2	2	2	3	3	2	1	2	1	2
2	1	2	1	3	2	1	2	1	2
9	2	9	9	9	9	9	9	9	1
9	9	9	9	9	1	9	9	9	9
1	9	9	9	9	9	9	9	9	9
9	3	9	9	9	9	3	9	9	9
9	2	9	9	9	9	9	9	9	1
9	9	9	1	9	9	9	9	9	2
9	3	9	9	9	9	9	2	9	9
9	9	9	9	2	9	9	9	9	9
9	9	9	9	2	9	9	9	9	9
9	9	9	9	2	9	9	9	9	9
9	9	9	9	2	9	9	9	9	9
9	9	9	9	2	9	9	9	9	9
9	9	9	9	2	9	9	9	9	9
9	3	9	9	9	9	9	9	9	9
9	3	9	9	9	9	9	9	9	1
1	2	1	1	3	1	2	2	2	1
3	1	3	3	1	3	2	3	9	9
9	2	9	9	9	9	9	1	9	9
3	2	2	1	2	1	1	2	1	2
9	9	9	9	3	9	9	9	9	9
9	9	9	9	3	9	9	9	9	9
9	9	9	9	3	9	9	9	9	9
9	9	9	9	3	9	9	9	9	9
9	9	9	9	3	9	9	9	9	9
2	2	2	2	2	2	2	2	2	2
2	3	2	1	3	2	3	2	1	2
2	3	2	1	2	1	2	1	2	1

2	2	2	2	2	2	2	2	2	2
1	2	2	3	3	1	2	2	3	3
1	2	2	3	3	1	2	2	3	2
1	2	2	2	1	2	2	2	3	1
9	9	9	9	3	9	9	9	9	9
9	9	9	9	2	9	9	9	9	9
9	9	9	9	3	9	9	9	9	9
9	9	9	9	2	9	9	9	9	9
9	2	9	9	9	1	9	9	9	9
9	9	9	9	2	9	9	9	9	9
1	1	9	1	9	1	1	9	2	2
9	1	3	9	1	3	9	9	9	3
9	9	9	9	9	2	9	9	2	9
9	9	2	9	9	9	9	2	9	9
9	9	9	9	9	9	9	9	9	9
2	3	2	3	2	2	2	2	1	3
2	3	3	3	1	2	3	2	2	3
9	9	9	1	9	9	9	9	9	9
2	2	2	2	2	2	2	2	2	2
3	3	1	1	3	1	2	1	2	1
2	2	2	2	2	2	2	2	2	2
9	9	9	9	9	9	9	9	9	1
2	2	2	2	2	2	2	2	1	2
2	2	2	2	2	2	2	2	2	2
9	9	9	9	9	9	9	2	9	9
9	3	9	1	2	1	2	2	9	1
9	9	9	9	9	9	9	9	9	9
3	1	2	3	1	2	3	2	3	2
3	1	3	3	2	2	2	2	3	2
2	2	2	3	2	2	2	1	1	2
1	1	2	1	3	3	1	2	1	2
2	9	9	9	9	2	9	9	9	9
9	9	9	9	9	9	9	9	9	9
2	2	2	2	2	2	2	2	2	2
9	9	9	9	9	9	9	9	9	9
1	3	2	1	3	2	1	2	1	2
2	2	2	2	2	2	2	1	2	2
2	2	2	2	2	2	2	2	1	2
2	2	2	2	2	2	2	1	1	2
2	2	2	2	2	2	3	2	3	2
1	2	2	3	2	2	3	3	2	3
1	3	1	3	1	3	3	3	1	3
1	3	1	1	3	2	3	2	1	2
1	9	9	9	9	9	9	9	9	9
2	2	2	2	2	2	2	2	9	9
2	2	2	2	2	2	2	2	2	2
3	1	3	3	1	3	1	1	1	1

1	1	3	1	3	1	3	1	3	1
2	2	2	2	1	3	2	2	2	2
1	2	2	1	3	1	2	2	2	1
2	2	3	1	2	2	2	2	1	2
2	2	2	2	2	2	2	2	2	2
2	2	2	2	2	2	2	2	2	2
1	2	3	3	3	2	2	2	2	2
2	2	2	2	3	2	3	2	1	2
2	2	2	2	2	2	2	2	2	2
2	2	2	2	1	3	2	2	2	2
9	9	9	9	2	2	1	3	3	2
2	2	2	2	2	2	1	2	2	2
2	2	2	2	3	3	2	2	1	1
9	9	9	9	2	2	2	2	1	2
1	2	2	2	3	3	1	2	2	1
2	2	2	2	2	2	2	2	1	1
2	2	2	2	2	2	2	3	2	2
2	2	2	2	2	2	2	2	2	2
2	2	2	2	2	2	2	2	2	2
2	2	2	2	2	2	2	2	2	2
2	2	2	2	2	2	2	2	2	2
2	2	2	2	2	2	2	2	2	2
2	2	2	2	2	2	2	2	2	2
2	2	2	2	2	2	2	2	2	2
3	1	3	3	2	2	2	2	2	2
9	9	2	9	9	9	9	9	9	9
1	3	1	1	3	2	3	1	1	2
1	9	2	9	1	2	1	2	3	3
1	2	2	1	2	1	2	2	1	3
1	9	9	9	9	9	9	9	9	9
3	1	3	3	3	1	3	1	1	1
2	2	2	2	2	3	1	3	2	3
2	1	1	1	3	2	2	1	2	3
2	2	1	1	1	2	2	1	1	1
2	2	2	2	1	3	1	3	2	2
9	9	9	9	9	9	9	9	9	9
2	2	2	2	2	2	2	2	2	2
2	3	2	3	3	2	1	2	1	2
2	2	2	2	2	2	3	1	2	2
2	2	2	2	2	2	2	2	2	2
1	9	9	9	9	9	9	9	9	9
2	2	2	2	2	2	2	2	2	2
2	2	2	2	3	2	2	2	1	1
2	2	2	2	1	3	2	2	2	2
2	1	2	2	2	2	1	3	2	3
3	2	2	2	2	3	1	3	2	2
2	2	2	2	2	2	2	2	2	2

1	3	3	1	3	1	2	1	2	1
3	1	3	3	1	3	3	3	3	2
9	9	9	9	3	2	1	2	2	2
2	2	2	2	2	2	2	2	2	2
2	2	2	2	2	2	2	2	2	2
9	9	3	9	9	3	9	9	9	9
2	2	2	2	2	2	2	2	2	2
2	2	2	2	2	2	2	2	3	2
2	2	2	2	2	2	2	2	2	2
3	3	2	3	2	2	1	2	2	1
2	2	2	2	2	2	2	2	2	2
3	3	2	2	1	3	1	3	3	3
9	9	2	9	9	9	9	9	9	9
2	2	2	2	2	2	2	2	2	2
2	2	2	2	2	2	2	2	2	2
2	2	2	2	2	2	2	2	2	2
9	9	9	9	1	2	3	2	1	1
2	2	2	2	1	3	2	2	3	3
2	3	2	1	3	3	3	2	1	1
9	9	9	9	9	9	9	9	9	9
2	2	2	2	2	2	2	2	2	2
2	2	2	2	2	2	2	2	2	2
2	2	2	2	2	2	1	2	3	2
2	2	2	2	2	2	2	2	1	2
3	2	2	1	3	3	2	2	2	2
1	2	2	1	1	3	1	2	2	2
2	2	2	2	1	2	1	2	2	2
1	2	2	2	3	2	2	2	2	1
1	3	1	1	2	2	2	2	2	2
2	2	1	2	2	1	1	1	1	1
1	2	2	2	2	2	2	2	1	1
9	3	9	9	9	9	9	9	9	1
2	2	2	2	3	2	2	2	1	2
2	9	1	3	3	3	1	2	2	1
2	2	2	2	2	9	9	9	9	9
2	2	2	2	2	2	2	2	2	2
1	3	2	1	2	1	1	3	2	3
2	2	2	2	2	2	2	2	2	2
2	2	2	2	2	2	2	2	2	2
2	2	2	2	2	2	2	3	2	1
2	2	2	2	1	3	2	2	2	2
2	2	2	2	2	2	2	2	2	2
2	2	2	2	2	2	2	2	2	2
2	2	2	2	2	2	2	2	2	2
2	2	2	2	2	2	2	2	2	2
2	2	2	2	2	2	2	2	2	2
3	2	3	3	1	1	1	3	3	3

9	9	9	9	9	9	9	9	9	9
3	1	1	1	1	1	1	3	1	1
9	9	9	9	3	9	9	1	9	9
9	3	9	9	9	1	9	9	9	9
2	9	2	1	1	2	1	2	1	2
2	2	2	2	2	1	2	3	1	2
9	9	9	9	9	9	9	1	9	9
2	2	2	1	3	1	3	2	2	1
9	3	9	9	9	9	9	9	9	2
9	9	9	9	3	9	9	9	9	9
9	9	9	9	2	2	2	9	3	1
3	3	2	1	2	2	3	1	1	2
3	1	3	3	1	3	1	3	3	3
9	1	9	9	9	1	9	9	9	9
3	1	3	1	1	3	3	3	3	3
2	2	2	2	2	3	2	2	3	3
3	3	1	1	1	3	3	1	1	1
9	9	9	9	9	1	9	9	9	9
2	2	2	2	2	2	3	1	2	2
2	2	2	1	9	9	9	9	9	9
2	2	2	2	1	3	2	2	1	1
9	2	9	9	9	9	2	2	2	9
9	2	9	9	2	3	3	1	9	9
9	2	2	1	2	2	3	1	3	1
1	3	2	2	3	1	3	1	1	3
2	3	2	2	2	2	3	1	2	2
3	1	3	3	2	2	3	2	2	2
9	9	9	9	9	9	3	9	9	9
2	2	2	2	2	2	3	3	2	2
1	3	2	3	2	1	3	1	1	1
3	2	3	2	1	3	1	3	3	2
2	2	2	2	2	2	2	2	2	2
2	9	3	2	2	2	2	3	1	2
9	3	9	9	9	9	9	1	9	9
2	2	2	2	2	2	1	2	2	3
3	1	2	3	2	1	1	1	3	3
9	9	9	9	9	9	9	2	9	9
9	9	1	9	9	9	3	9	9	9
9	3	9	9	3	9	9	9	1	9
3	3	3	3	2	2	2	1	2	2
9	9	9	1	3	9	9	9	9	9
3	3	1	3	1	1	3	1	1	3
2	2	2	2	2	3	2	2	2	3
2	3	3	1	1	2	1	2	2	2
9	3	9	9	9	9	9	3	9	9
9	9	9	1	2	9	9	9	9	9
9	9	9	9	9	9	9	1	9	9

9	3	9	9	2	2	2	2	2	2
2	3	2	3	1	2	3	1	1	3
9	3	9	9	9	9	9	9	9	9
2	1	2	3	2	1	3	2	2	9
2	2	2	2	2	2	2	2	2	2
9	9	9	9	3	9	9	9	9	9
1	9	9	9	9	9	9	9	9	9
2	2	1	3	2	1	3	2	2	3
1	2	2	2	3	2	2	2	2	2
1	3	3	1	1	3	1	3	3	1
1	2	2	2	2	2	2	2	2	2
1	3	1	1	2	2	2	1	1	1
1	3	2	1	3	2	3	1	1	2
9	9	1	9	9	9	3	1	9	9
1	3	1	2	3	2	3	1	1	2
2	3	2	2	1	2	1	2	1	2
1	9	2	1	1	3	3	1	1	1
2	2	2	2	1	3	1	2	2	1
9	9	9	2	9	9	9	1	9	9
1	2	2	2	3	2	3	1	2	2
2	2	1	3	1	3	2	1	1	1
9	9	9	9	2	9	9	9	9	9
3	9	1	3	1	3	1	1	3	1
9	3	9	9	9	9	9	9	9	9
1	3	2	2	2	2	3	2	2	2
9	9	9	9	9	9	9	9	1	9
1	9	9	9	1	3	1	1	3	3
3	1	3	3	1	3	1	3	3	3
1	9	9	9	9	9	9	1	9	9
9	9	9	9	9	9	9	9	9	9
9	9	9	9	2	2	2	2	2	9
1	9	9	9	9	1	9	9	9	9
3	9	9	2	2	3	9	1	3	2
2	2	2	2	2	2	3	2	1	2
2	1	3	3	1	3	1	3	3	2
1	9	9	9	9	9	9	9	9	9
9	9	9	9	9	9	9	9	9	9
9	9	9	9	9	9	3	1	9	9
9	9	9	9	1	9	9	9	9	9
9	9	9	9	9	9	9	9	9	9
9	9	9	9	9	9	9	1	1	9
1	9	9	9	3	9	9	9	9	9
3	3	3	1	1	3	1	9	9	9
2	2	2	2	2	2	3	2	2	2
2	2	2	2	1	2	2	3	2	2
2	2	2	2	1	3	3	3	3	2
3	3	2	1	3	2	3	1	1	2

9	3	9	9	9	9	9	1	9	9
9	3	3	1	1	3	3	1	2	1
1	1	1	1	9	2	3	1	3	1
2	2	2	2	9	9	3	3	3	3
2	2	2	2	2	3	3	1	2	2
9	9	9	9	9	9	9	9	9	9
2	3	2	2	2	2	3	2	2	3
9	9	9	9	2	9	9	9	9	9
9	9	9	9	2	9	9	1	9	9
9	3	9	9	9	9	9	1	9	9
2	2	2	2	2	2	2	2	2	2
2	2	2	2	3	2	3	2	3	2
2	2	2	2	2	2	2	2	2	2
2	2	2	2	1	3	2	3	2	1
9	9	9	1	3	9	9	9	9	1
2	3	2	3	3	2	1	3	1	2
2	3	1	2	2	2	3	1	1	2
2	2	2	2	2	2	2	2	2	2
3	3	2	2	1	3	1	1	2	3
2	3	1	3	3	1	3	1	1	2
1	9	9	9	9	9	9	9	9	1
2	2	1	3	1	3	1	1	1	2
1	1	3	3	1	3	3	3	3	3
9	3	9	9	9	9	9	9	9	9
3	3	2	1	1	3	1	1	2	1
2	2	2	2	2	2	1	2	3	1
2	2	2	1	1	3	3	3	1	2
2	1	1	1	1	3	1	3	1	1
1	2	1	2	1	1	3	2	1	1
2	3	2	2	2	2	2	2	1	2
3	3	9	2	9	9	1	1	2	9
9	9	9	1	9	9	9	1	9	9
2	2	2	2	2	2	2	2	3	2
2	3	2	2	1	3	1	1	2	1
1	2	2	3	3	1	2	2	1	2
9	9	9	9	9	9	3	1	9	9
1	1	1	1	3	1	3	1	1	1
1	1	1	1	3	1	3	1	1	1
2	2	2	2	3	2	3	1	2	1
9	1	3	3	2	9	1	3	9	2
3	3	3	3	1	2	2	3	2	2
9	9	1	9	1	3	3	1	3	1
2	2	1	2	1	3	3	3	3	2
2	2	2	2	3	2	3	1	2	2
9	2	9	9	2	2	1	3	2	3
2	2	2	2	2	2	3	1	2	2
9	3	9	9	9	9	9	1	9	9

9	3	9	9	3	9	9	1	9	1
3	1	3	3	2	3	1	1	2	3
1	9	9	9	9	9	9	9	9	9
1	3	3	2	2	3	3	2	1	1
9	3	9	9	9	9	9	1	9	9
3	2	3	2	3	3	2	3	1	2
3	2	2	2	2	2	1	3	3	1
9	9	9	9	9	9	9	1	9	9
2	2	2	2	2	2	2	2	2	2
2	2	2	2	3	3	1	1	2	3
9	9	9	9	9	9	9	1	9	9
3	3	3	1	3	1	1	1	1	1
2	9	1	1	2	9	2	9	2	2
3	1	2	2	3	2	2	1	2	2
2	2	3	2	1	2	3	1	2	3
2	2	2	2	2	2	3	1	1	1
3	9	1	9	3	3	3	1	2	3
2	2	1	1	3	1	2	1	2	3
9	9	1	9	9	9	9	1	9	9
2	1	1	2	3	1	2	3	1	2
2	2	2	2	3	2	3	2	2	2
2	2	2	2	3	2	3	2	2	2
1	3	9	2	3	1	3	3	1	1
1	9	9	9	9	9	9	9	9	9
2	2	2	1	1	3	1	3	3	3
3	2	1	1	1	3	3	3	2	2
2	2	2	1	1	3	1	3	1	2
1	1	3	2	2	2	2	2	2	2
2	2	2	2	1	2	3	2	2	2
2	2	2	1	1	3	3	2	2	3
9	9	9	1	1	1	3	3	1	9
9	9	9	9	9	9	3	9	9	9
2	9	1	1	3	2	3	2	2	2
1	9	9	9	9	9	9	9	9	1
1	3	1	1	3	1	3	1	1	1
9	9	9	9	3	9	9	9	9	9
9	3	1	1	3	1	3	1	1	1
3	2	2	2	2	2	2	1	2	1
9	9	9	9	3	1	2	9	9	9
1	3	3	2	2	2	3	1	1	3
2	2	2	2	2	2	2	2	2	2
2	2	2	2	2	2	2	2	2	2
2	1	3	1	3	1	2	1	1	1
1	3	1	1	3	1	3	1	1	2
9	9	9	1	9	9	9	9	9	9
9	9	9	9	2	9	2	9	9	9
2	3	2	1	3	1	3	1	1	1

9	3	9	9	9	9	9	9	9	1
1	2	2	1	2	2	2	1	1	1
3	1	3	3	2	3	3	1	1	1
2	2	2	2	2	2	2	1	2	1
2	1	2	3	1	3	2	3	3	3
3	1	3	3	1	3	1	3	3	3
9	9	9	9	9	9	9	1	9	9
9	9	9	1	9	9	9	1	9	9
2	3	2	2	1	3	2	1	1	1
3	3	2	1	2	2	3	1	3	1
3	1	3	3	1	3	1	3	3	3
1	2	9	3	2	1	2	1	1	1
2	3	2	2	1	3	1	1	1	2
9	3	9	9	1	3	9	1	3	2
1	9	9	9	1	3	1	1	3	3
3	1	3	3	1	3	1	1	1	1
1	2	2	2	3	2	3	1	1	1
9	9	9	9	9	9	9	9	9	9
9	3	9	9	9	9	9	9	9	1
2	3	2	2	2	2	1	1	1	1
3	3	2	2	1	3	1	2	3	1
1	9	9	2	1	3	2	9	1	1
2	3	1	2	2	2	2	3	1	2
9	3	9	9	9	9	9	9	9	1
9	9	9	9	2	9	9	9	9	9

FamilyBrea	EarlyInterc	UTI	BSEnoneec	BSEnecessa	BSEshy	BSEpainful	BSEfearofC	BSEtimewa	BSEdirty
2	2	2	2	2	2	2	2	9	3
2	2	2	2	2	2	2	2	2	2
2	2	2	2	2	2	2	2	2	2
2	2	2	2	2	2	3	3	3	3
1	2	2	1	3	2	2	1	1	2
2	2	2	2	2	2	2	2	2	2
2	2	2	2	2	2	2	2	2	2
2	2	2	1	1	3	3	3	3	3
2	2	2	1	2	3	3	3	2	2
3	2	2	3	2	1	2	2	3	1
1	1	2	3	1	3	3	3	3	1
2	2	2	1	3	2	2	2	2	1
2	2	2	1	1	1	2	2	2	1
2	2	2	3	3	3	3	3	2	2
2	2	2	3	2	1	2	2	2	2
2	2	2	3	2	3	3	3	3	3
3	2	2	1	2	1	2	2	2	1
2	1	2	2	1	3	3	1	2	1
2	2	3	1	3	3	2	2	1	3
2	2	2	2	1	1	2	3	2	1
3	2	2	3	1	3	1	3	3	3
2	2	2	3	1	3	2	1	3	3
3	1	1	3	1	3	3	3	3	3
2	2	2	2	1	2	2	3	3	3
2	2	2	2	2	2	2	2	2	2
3	3	3	3	3	3	3	3	9	1
1	9	9	3	1	3	3	3	3	3
3	2	2	1	1	3	3	9	3	3
3	3	2	2	2	1	2	3	3	2
2	3	1	1	9	1	1	1	1	1
1	2	2	2	2	1	1	1	1	2
1	2	2	2	1	3	3	3	3	3
9	1	9	3	3	3	1	3	3	3
3	1	1	1	2	3	1	1	3	3
9	1	1	3	1	3	1	1	1	9
3	3	3	3	3	3	2	3	2	3
2	2	2	3	1	3	3	3	3	2
2	3	1	1	2	3	2	1	2	3
3	2	3	3	1	3	3	3	3	3
1	2	3	2	3	3	2	1	2	3
2	2	2	2	2	1	2	3	3	2
2	1	9	3	3	2	9	9	9	9
9	9	9	9	9	9	9	9	9	9
2	2	2	1	1	2	2	3	2	2
2	2	2	1	1	3	3	3	1	2
9	9	9	9	9	9	9	9	9	9

3	3	2	1	2	1	2	2	2	2
3	2	2	1	3	2	2	1	1	1
2	2	2	2	1	1	1	1	1	1
2	2	1	3	3	3	2	3	1	3
3	3	3	3	1	3	3	3	3	3
1	3	3	3	1	3	1	3	3	3
2	3	2	3	2	1	3	2	9	1
1	1	1	2	1	3	1	1	3	3
9	9	1	1	3	1	1	1	1	1
1	3	2	3	1	2	2	2	2	3
9	9	9	1	9	9	2	9	9	9
2	1	1	1	3	1	2	2	3	2
2	1	1	2	2	2	2	1	1	1
2	1	1	1	2	1	1	2	2	1
1	1	1	1	1	1	1	1	2	2
1	1	1	1	3	1	2	2	1	2
2	2	2	3	2	2	2	2	3	3
1	1	3	3	1	3	3	1	3	2
2	2	2	2	2	2	2	9	2	2
3	3	3	3	3	3	3	3	3	3
3	3	2	1	3	2	3	2	2	3
2	3	2	2	3	3	1	2	2	2
1	1	1	1	2	1	1	1	2	2
2	2	2	1	3	1	2	2	3	2
2	2	2	1	2	1	2	2	3	2
1	1	1	3	9	9	2	2	3	3
1	1	1	3	3	1	3	2	3	1
1	1	1	3	3	3	1	1	1	1
1	1	1	2	1	1	1	1	3	1
3	3	3	3	1	3	3	3	3	3
9	9	9	9	9	9	9	9	9	9
2	2	2	2	2	3	2	3	2	2
9	9	9	9	9	9	9	1	9	9
2	1	1	2	3	1	3	3	3	2
9	1	1	3	3	1	9	1	1	1
1	1	2	1	2	1	2	2	2	2
2	1	1	1	3	3	1	2	1	1
2	1	3	3	1	3	3	3	2	2
2	3	2	3	1	2	3	1	3	2
2	1	2	3	1	2	1	1	1	2
3	2	1	3	3	1	1	1	1	1
9	9	9	9	9	9	9	9	9	9
1	1	1	1	1	2	1	1	3	3
2	1	2	3	1	3	3	2	1	1
1	2	1	1	2	3	9	2	1	3
2	2	2	1	1	1	1	1	1	1
2	2	2	1	1	1	2	3	3	3

1	2	3	1	2	1	2	3	2	1
3	3	3	1	1	3	1	1	1	1
2	2	2	1	3	3	3	2	3	2
2	1	3	3	1	1	1	2	2	1
1	1	1	1	1	1	2	2	1	1
1	2	1	1	2	2	2	1	1	1
2	2	2	3	1	3	2	3	2	2
3	1	1	1	3	1	1	1	3	3
2	1	2	2	1	1	3	3	3	3
3	1	1	1	1	3	2	2	3	3
9	9	9	1	3	3	1	2	1	1
2	2	2	2	1	1	1	2	2	2
2	1	1	1	2	1	1	1	2	2
2	2	1	2	1	2	3	1	3	3
1	1	3	1	1	3	2	3	3	3
1	1	3	1	1	3	2	3	3	3
3	1	1	1	2	1	1	1	3	2
2	2	2	2	9	2	3	3	3	3
3	3	1	3	1	3	3	3	1	3
1	1	2	1	3	2	2	2	1	1
1	1	1	1	1	3	3	3	3	3
1	2	3	1	1	1	3	1	3	3
2	1	1	2	1	1	1	1	2	2
2	2	2	1	1	2	2	2	2	2
2	2	2	2	2	2	2	2	2	2
3	3	3	1	1	3	3	3	3	3
1	1	1	3	2	2	2	2	2	2
2	2	1	2	1	2	9	1	2	2
2	2	2	1	2	2	1	2	2	2
3	1	2	3	3	1	2	3	1	1
2	1	1	1	2	1	1	1	1	2
2	1	1	3	1	3	2	2	2	1
3	2	2	3	1	2	2	1	1	2
1	2	1	1	9	2	1	2	1	1
3	3	2	3	1	3	2	3	9	3
2	2	3	1	3	1	2	2	2	2
9	9	9	2	2	2	2	2	2	2
1	1	3	1	2	1	1	1	1	2
9	9	9	9	9	9	9	9	9	9
3	3	3	1	1	3	1	1	1	1
1	1	1	1	2	1	1	1	2	1
9	9	9	9	9	9	9	9	9	9
2	1	1	1	2	2	2	2	2	2
2	2	2	1	2	1	2	1	1	2
2	2	2	3	1	3	1	3	3	3
2	2	2	3	1	3	1	3	3	3
1	1	2	1	3	3	3	1	2	1

1	3	2	1	1	2	2	2	9	1
3	3	3	9	3	3	3	3	3	3
3	9	3	1	1	1	1	1	1	1
2	2	2	3	2	3	2	2	2	2
1	1	2	1	1	3	3	1	1	1
9	1	3	1	2	2	2	3	2	1
1	9	9	1	9	9	9	9	9	9
1	2	3	1	2	2	2	3	3	1
9	9	9	1	3	1	1	1	2	1
2	2	2	9	9	9	9	9	9	9
2	3	1	1	2	2	1	3	3	2
1	1	1	1	2	3	3	3	3	3
2	2	2	2	2	2	2	2	2	2
3	3	3	1	1	2	2	2	2	2
2	2	1	1	2	1	2	2	1	2
2	1	2	2	1	1	1	1	1	2
2	1	1	1	2	1	2	2	1	1
2	2	2	1	2	2	2	2	2	2
1	2	1	1	2	3	9	2	1	3
2	3	1	1	3	1	9	1	1	1
9	2	9	9	9	9	9	1	9	9
2	3	1	1	1	3	3	3	3	3
2	3	3	1	3	3	3	3	1	1
1	2	1	3	3	1	2	1	2	2
3	3	2	1	1	3	3	3	3	3
2	1	3	1	2	2	9	1	3	3
2	3	2	3	1	3	3	3	3	3
9	1	9	1	3	3	2	3	3	3
2	1	1	9	9	9	9	9	9	9
1	1	1	2	1	1	1	1	1	1
2	2	2	2	2	2	2	2	2	2
2	2	2	1	1	2	2	2	2	2
2	2	2	1	2	3	3	2	2	2
3	1	3	1	1	1	1	1	1	1
2	3	2	3	1	1	1	2	2	2
1	1	1	1	1	1	1	3	1	1
2	2	2	1	1	1	1	1	1	1
3	1	2	3	1	1	1	1	2	2
1	1	9	3	1	3	3	3	3	3
3	3	3	1	2	2	2	2	2	2
2	1	1	2	1	2	9	9	2	2
2	2	2	2	2	2	2	2	2	2
2	2	2	1	3	3	1	3	2	2
9	9	9	9	9	9	9	9	9	9
9	9	9	9	9	9	9	9	9	9
2	1	1	1	2	1	1	1	1	2
2	2	2	2	2	1	1	1	1	2

9	9	9	9	9	9	9	9	9	1
9	9	9	2	1	3	3	3	3	3
1	1	3	3	1	3	3	3	3	3
9	9	9	2	1	3	3	2	3	3
3	1	3	1	1	3	3	9	9	9
9	9	9	9	9	9	9	9	9	9
1	1	1	3	3	3	2	1	2	1
3	2	1	3	3	3	1	1	2	3
2	2	3	1	2	3	2	2	3	2
9	9	9	9	9	9	9	1	9	9
9	9	9	9	1	9	9	9	9	9
9	9	9	9	9	2	9	9	9	9
1	2	3	1	3	1	2	3	2	1
1	3	2	2	3	3	3	1	1	2
9	9	9	9	1	9	9	9	9	9
3	3	3	2	2	2	2	2	2	2
3	3	3	3	3	3	3	3	3	3
2	2	2	2	1	1	1	2	2	1
2	2	2	1	1	3	2	2	3	3
2	3	1	3	3	2	2	2	2	2
2	1	2	1	2	3	3	3	3	3
2	2	1	2	2	9	2	3	1	2
3	2	3	1	1	2	1	1	1	3
2	3	2	1	1	3	2	3	3	1
2	2	2	1	2	2	2	2	2	2
2	2	2	3	1	3	3	3	2	3
2	2	2	1	2	2	2	1	2	2
2	2	2	2	3	1	2	2	2	2
3	3	3	3	1	1	3	3	2	3
1	1	1	3	1	2	3	1	3	1
2	1	2	3	1	1	1	1	2	3
3	1	3	3	1	3	3	3	3	3
2	9	9	1	2	1	1	2	9	9
1	2	1	1	1	1	3	1	2	1
1	3	1	3	1	2	1	9	1	1
2	2	2	3	3	3	3	2	3	2
1	1	9	1	2	2	1	2	3	2
2	2	2	3	1	3	2	1	1	1
1	1	2	1	2	2	1	3	2	1
2	1	1	1	3	1	2	2	1	1
1	1	1	1	2	9	1	1	1	1
1	1	2	1	1	3	1	2	1	1
2	2	1	2	1	3	2	1	2	1
9	9	9	2	1	1	1	1	3	1
1	2	3	9	2	2	2	2	2	2
2	3	3	3	1	3	1	3	2	1
9	9	9	9	9	9	9	9	9	9

3	3	1	3	1	3	3	3	3	3
1	3	1	1	1	3	1	3	3	3
2	1	2	3	3	3	3	1	3	3
3	2	3	1	1	3	2	3	3	3
2	3	3	2	1	3	2	3	3	3
2	2	2	2	3	1	2	2	9	1
3	3	3	1	1	3	3	3	3	2
1	1	1	1	2	2	1	1	2	1
2	2	2	2	1	2	1	2	2	1
1	2	2	1	1	1	1	1	2	3
2	2	2	2	2	2	2	2	2	2
9	9	9	1	9	9	9	9	9	9
9	9	9	2	9	9	9	9	9	9
2	2	2	2	2	2	2	2	2	2
1	9	9	3	1	9	9	3	1	9
9	2	9	2	2	2	9	2	9	9
1	9	9	9	1	1	9	9	1	9
9	9	9	9	1	9	9	1	2	9
2	9	2	9	2	2	9	9	2	9
9	9	9	9	9	9	9	9	9	9
9	9	9	9	9	9	9	9	9	9
2	2	2	2	2	2	2	2	2	2
2	2	2	2	2	2	2	2	2	2
3	3	3	1	2	1	1	1	1	2
1	2	2	2	2	2	2	2	2	2
9	2	9	9	2	9	9	2	9	2
2	2	2	2	2	2	2	2	2	2
9	9	9	9	9	9	9	9	9	9
9	9	9	9	9	9	9	9	9	9
2	2	2	1	1	2	2	1	2	2
9	9	9	9	2	9	9	9	9	9
9	9	9	9	9	9	9	9	9	9
9	9	9	9	9	9	9	9	9	9
9	9	2	9	9	9	9	9	9	9
1	9	9	3	1	9	9	1	2	9
9	9	9	1	2	9	1	2	9	9
9	9	9	9	9	9	9	9	9	9
9	2	9	9	2	9	9	9	2	9
9	1	2	1	2	9	9	9	1	2
9	9	9	3	9	9	9	9	9	9
9	9	9	9	3	9	9	9	9	9
9	9	9	1	9	9	9	9	9	9
3	3	3	3	3	3	3	3	3	3
1	3	3	3	3	3	3	3	3	1
1	2	1	1	2	1	3	1	2	1
1	1	1	1	9	9	9	1	9	3
9	9	9	1	9	9	9	9	9	9

9	9	9	9	1	9	9	9	9	9
9	9	9	2	9	9	9	9	9	9
9	2	2	1	3	2	2	2	1	1
1	3	2	2	1	2	3	1	2	3
2	3	3	1	2	2	1	1	2	1
1	2	2	1	2	2	2	2	2	2
2	3	2	1	1	3	1	3	3	2
2	1	1	2	1	1	2	2	3	1
9	9	9	9	9	9	9	9	9	9
3	3	3	3	3	3	3	3	3	3
9	9	9	9	9	9	9	9	9	9
9	9	9	9	9	9	9	9	9	9
9	9	9	9	1	9	9	9	9	9
3	1	2	1	9	9	9	1	9	9
1	1	1	1	1	1	1	1	1	1
2	2	2	3	3	1	1	1	2	2
9	9	9	9	9	9	9	9	9	9
2	1	1	1	2	2	2	3	3	2
1	1	1	1	1	1	1	1	1	1
3	1	2	3	3	1	1	1	1	1
1	2	1	3	1	1	3	1	3	1
1	2	1	1	1	3	1	3	3	3
2	2	3	3	1	2	1	2	3	2
2	2	2	2	2	2	2	2	2	2
2	2	2	1	2	2	2	2	2	2
2	2	2	2	2	2	2	2	2	2
2	2	2	2	2	2	2	2	2	2
1	2	3	1	2	1	2	3	1	2
2	2	2	2	3	2	3	9	2	3
3	3	3	1	1	2	2	2	1	1
2	2	2	1	2	2	2	2	2	2
2	2	2	2	2	2	2	2	2	2
3	1	2	2	1	2	3	2	3	3
2	2	2	3	2	2	2	2	2	2
3	3	3	1	3	1	1	1	1	1
2	3	2	2	2	2	2	2	2	2
9	9	9	9	9	9	9	9	9	9
2	1	2	3	3	3	2	2	2	2
3	3	3	3	3	3	3	3	3	3
3	3	3	3	3	3	3	3	3	3
9	9	9	2	9	2	9	9	9	9
2	1	3	1	1	2	1	2	1	2
2	1	1	9	1	9	9	9	9	2
1	1	1	2	2	2	2	2	2	2
2	1	1	3	3	3	2	2	1	1
2	2	2	2	2	2	2	2	2	2
2	2	2	2	3	2	2	2	1	1

2	3	1	2	1	2	3	1	2	3
9	9	9	1	9	1	9	9	9	9
9	9	9	9	9	9	9	9	1	9
9	9	9	9	2	9	9	9	1	9
9	9	9	2	9	9	9	1	9	9
1	1	1	1	2	1	1	1	2	1
9	9	9	1	9	9	9	9	9	9
9	9	9	9	1	9	9	9	9	9
2	2	2	2	1	1	2	2	1	1
9	1	9	1	9	9	9	9	9	9
9	9	9	3	9	9	9	9	3	9
9	9	9	3	9	9	3	9	9	9
9	9	9	2	9	9	9	9	9	9
9	9	9	9	3	9	9	1	9	9
9	9	9	2	9	9	9	9	9	9
9	9	9	9	9	9	9	9	9	9
9	9	9	3	9	9	9	2	9	9
1	2	3	2	3	2	3	1	2	3
2	1	2	1	2	1	1	3	1	2
3	3	3	1	2	2	2	2	2	2
3	1	2	3	1	1	2	1	2	3
3	1	1	2	1	3	1	3	3	3
1	3	2	1	2	1	2	3	1	2
3	2	2	2	1	3	1	2	2	2
2	1	2	1	1	3	2	2	3	3
3	3	3	2	3	2	9	2	3	3
3	1	2	2	3	2	1	1	2	1
1	1	1	1	1	3	1	2	3	3
2	2	2	3	2	1	2	2	2	1
1	1	1	2	1	1	1	1	1	1
1	2	1	2	3	1	2	1	3	1
3	2	2	1	2	2	2	2	2	2
1	1	1	2	2	1	1	2	2	2
1	1	1	2	2	1	2	2	2	2
9	9	9	9	9	9	9	9	9	9
3	3	2	3	9	3	3	2	3	3
9	9	9	9	9	9	9	9	9	9
1	2	1	1	1	1	1	2	2	2
2	2	2	3	2	2	1	2	2	2
2	2	2	2	2	2	2	2	2	2
9	9	9	1	1	3	3	2	3	3
3	3	3	1	3	3	2	3	3	3
3	3	3	1	3	3	3	1	3	3
3	2	1	3	1	3	3	2	3	3
2	2	2	2	2	2	2	2	2	2
3	3	1	3	1	1	1	2	2	2
2	2	2	2	2	2	2	2	2	2

3	3	2	3	1	2	1	2	3	2
9	1	9	1	1	1	9	9	9	9
9	3	3	9	2	2	2	2	2	2
1	3	2	2	3	1	2	3	1	2
2	2	2	3	3	3	3	3	3	3
9	9	9	2	2	9	9	9	9	9
1	1	1	3	1	3	1	1	3	3
2	1	1	3	2	3	1	1	3	3
9	9	9	9	9	9	9	9	9	9
1	3	2	3	1	3	3	1	3	3
3	1	3	3	1	3	3	1	3	2
9	9	2	9	1	9	9	9	9	9
9	9	9	2	9	9	9	9	9	9
2	2	2	1	2	1	2	2	3	3
9	9	9	9	1	9	9	9	9	9
2	3	1	2	2	3	2	2	1	2
9	9	9	9	9	9	9	9	9	9
9	9	9	9	9	9	9	9	9	9
9	9	9	9	1	9	9	2	9	9
3	3	3	1	3	3	2	1	1	1
2	2	2	2	2	2	2	2	2	2
1	1	1	1	3	1	1	1	3	1
2	3	2	3	2	2	2	9	2	2
9	9	9	9	9	9	9	9	9	9
1	9	9	2	1	3	3	3	3	3
1	1	2	3	1	1	2	2	1	1
1	1	1	2	1	1	1	1	1	1
1	1	1	1	2	1	2	3	1	2
3	3	3	2	2	2	2	2	2	2
1	9	9	9	2	9	9	9	9	1
2	1	2	3	2	2	2	2	2	2
2	2	2	1	1	1	2	3	1	2
2	2	2	1	3	1	2	2	2	2
1	2	2	1	1	2	2	2	2	2
1	1	1	1	1	3	3	1	1	1
2	2	2	1	2	2	2	1	2	1
9	9	9	9	9	9	9	9	9	9
2	2	2	9	9	9	9	9	9	9
2	3	2	2	9	2	3	2	3	1
1	1	1	2	1	1	9	2	1	2
2	2	2	2	1	1	9	1	9	1
2	2	2	3	1	3	3	3	3	3
2	2	1	2	2	2	2	2	3	3
2	1	1	1	2	1	1	2	3	2
3	3	3	2	2	2	2	2	2	2
2	2	2	1	1	3	3	2	3	2
3	3	3	1	1	2	2	2	2	2

9	9	9	3	3	3	3	3	3	3
1	9	9	2	1	3	3	3	2	2
9	9	9	1	9	9	9	1	9	9
9	9	9	9	1	9	9	9	9	9
9	9	1	2	3	1	1	2	2	1
3	3	3	1	1	3	2	2	3	3
2	2	2	2	2	2	2	2	2	2
2	9	2	2	2	2	9	2	9	2
9	9	9	9	9	9	9	9	9	9
9	9	9	9	9	9	9	9	9	9
2	2	2	9	3	2	2	2	2	2
2	2	2	9	3	2	2	2	2	2
2	2	9	9	3	2	2	2	2	2
2	2	2	9	9	9	9	9	9	9
2	2	2	9	3	2	2	2	2	2
9	9	9	2	9	9	9	9	9	9
3	3	3	3	3	3	3	3	3	3
9	9	9	1	9	9	9	9	9	9
9	9	9	2	9	9	9	9	9	9
9	9	2	2	9	9	9	9	9	9
9	9	9	9	9	9	9	9	9	9
9	9	9	2	9	9	9	9	9	9
2	2	2	2	2	2	2	2	2	2
2	2	2	9	9	9	9	9	9	9
9	9	9	9	9	9	9	9	9	9
9	9	9	9	9	9	9	9	9	9
9	9	9	9	9	9	9	9	9	9
9	2	9	3	3	3	3	3	3	3
2	2	2	1	1	3	3	3	3	3
9	9	9	9	9	9	9	9	9	9
2	9	2	2	9	2	9	2	9	2
2	2	2	2	2	2	2	2	2	2
1	9	1	1	9	1	9	1	9	9
9	9	9	9	9	9	9	9	9	9
3	3	3	3	3	3	3	3	3	3
9	9	9	9	9	9	9	9	9	9
9	2	2	1	9	9	9	9	9	9
9	9	9	2	2	2	2	2	2	2
3	2	1	2	1	1	1	3	2	3
2	2	2	2	1	1	2	3	2	2
9	9	9	9	1	1	9	9	9	9
3	2	2	3	1	1	3	2	9	1
1	2	1	1	1	3	2	3	1	2
1	2	3	1	2	1	2	3	1	2
3	3	1	1	1	1	3	1	3	2
1	2	1	1	2	1	2	1	2	1
9	9	9	2	1	1	3	2	3	3

9	9	9	9	1	9	9	9	9	9
3	3	2	1	1	2	3	2	1	2
2	2	2	3	3	9	1	1	1	1
1	1	1	1	1	1	1	1	1	1
2	2	2	2	2	2	2	2	2	2
2	3	2	1	2	2	1	3	3	2
3	3	3	3	3	3	3	3	3	3
3	2	2	3	2	2	2	2	2	1
1	1	2	3	1	3	3	3	3	3
2	1	1	3	3	3	3	9	1	1
3	2	2	2	2	2	2	2	2	2
2	1	2	2	2	2	2	2	2	2
3	3	3	3	3	3	3	1	2	3
2	2	2	2	2	2	2	2	2	2
9	9	9	9	9	2	9	9	9	9
1	1	2	2	3	3	2	2	3	2
2	2	2	2	1	1	2	2	2	3
2	2	2	3	1	3	1	3	1	1
3	3	2	2	3	1	3	3	3	1
2	2	2	2	2	2	2	2	2	2
3	3	2	2	1	3	2	2	9	3
1	3	1	1	1	3	3	3	1	1
1	2	1	2	3	3	1	2	9	3
9	9	9	1	9	9	9	9	9	9
1	9	9	9	1	9	9	9	9	9
1	1	1	1	1	1	1	1	1	1
2	2	2	9	9	1	9	9	9	9
3	3	3	2	1	3	3	3	3	3
9	9	9	9	9	9	9	9	9	9
9	9	9	9	1	9	9	9	9	9
2	2	2	3	1	3	1	1	1	3
2	3	2	2	3	3	2	3	2	3
9	9	9	9	9	9	9	9	9	9
9	9	9	1	9	9	9	9	9	9
1	9	1	1	1	3	2	1	3	2
9	9	9	9	9	9	9	9	9	9
3	3	2	2	2	2	3	2	3	2
3	3	3	1	1	1	1	1	1	2
3	2	2	1	1	1	1	2	2	2
2	2	2	2	1	3	2	2	2	2
1	2	1	1	1	3	3	2	3	3
3	3	3	2	2	2	2	2	2	2
9	9	9	2	9	3	2	1	2	3
3	3	3	3	2	2	2	3	3	3
3	3	3	1	1	2	2	3	3	2
2	3	3	3	2	2	3	2	2	2
2	3	1	1	1	1	3	2	2	3

9	2	9	9	1	2	9	9	9	9
2	2	2	3	1	3	1	3	1	1
2	2	2	1	3	3	2	3	3	3
2	2	2	9	9	9	9	9	9	9
2	1	1	2	1	3	2	1	3	3
9	9	9	9	9	9	9	9	9	9
2	2	2	9	9	9	9	9	9	9
2	2	2	2	2	2	2	2	2	2
2	2	1	1	2	3	3	3	3	3
2	2	2	2	1	3	2	2	3	2
2	2	2	1	1	1	1	1	1	1
2	3	2	2	2	3	3	3	3	2
3	2	3	2	1	3	2	2	3	3
2	2	2	2	2	2	2	2	2	2
3	3	2	1	1	3	1	2	3	2
3	3	3	3	3	3	3	3	3	3
9	9	9	9	9	9	9	9	9	9
9	9	9	9	9	9	9	9	9	9
9	9	9	1	9	9	9	9	9	9
9	9	9	2	9	9	9	9	9	9
9	9	9	9	9	9	9	9	9	9
9	9	9	9	9	9	9	9	9	9
2	2	2	2	2	2	9	2	2	2
9	9	9	2	9	9	9	9	9	9
9	9	9	9	9	9	9	9	9	9
9	9	9	3	9	9	9	9	9	9
9	9	9	9	9	9	9	9	9	9
2	2	2	1	1	1	1	1	1	1
2	2	2	2	9	2	2	3	9	9
9	9	9	3	9	9	9	3	9	9
9	9	3	9	9	9	9	9	9	9
2	2	2	2	2	2	2	2	2	2
1	1	1	9	9	9	9	9	9	9
9	9	9	3	9	9	9	3	9	9
1	9	2	1	9	9	1	2	2	1
9	9	9	9	9	9	9	9	9	9
9	9	9	2	9	9	9	9	9	9
9	9	9	9	9	9	9	9	9	9
9	9	3	1	9	2	9	9	1	9
9	9	9	1	9	9	9	9	9	9
2	2	2	2	2	2	2	2	2	2
9	9	9	2	9	9	9	9	9	9
9	9	9	2	9	9	9	9	9	9
9	9	9	2	9	9	9	9	9	9
2	2	2	9	9	9	9	9	9	9
9	9	9	9	3	2	2	2	2	2
9	9	9	9	9	9	9	9	9	9

9	9	9	9	9	9	9	9	9	9
2	2	2	2	2	2	2	2	2	2
9	9	9	2	9	9	2	9	9	9
9	9	9	2	9	9	2	9	9	2
9	9	9	9	9	9	9	9	9	9
9	9	9	9	9	9	9	9	9	9
9	9	9	9	9	9	9	9	9	9
9	9	9	2	9	9	9	9	9	9
9	9	9	9	9	9	9	9	9	9
9	9	9	9	9	9	9	9	9	9
9	9	9	9	9	9	9	9	9	9
9	9	9	9	9	9	9	9	9	9
9	9	9	9	9	9	9	9	9	9
9	9	9	9	9	9	9	9	9	9
2	2	2	9	3	2	2	2	2	2
2	2	2	2	2	2	2	2	2	2
2	2	2	9	3	2	2	2	2	2
2	2	2	2	2	2	2	2	2	2
9	9	9	2	2	2	2	2	2	2
2	2	2	2	2	2	2	2	2	2
9	9	9	9	9	9	9	9	9	9
9	9	9	1	9	9	9	9	9	9
9	9	9	9	1	2	2	2	9	9
9	9	9	9	2	2	2	9	2	2
2	2	2	2	2	2	2	2	2	2
2	2	2	9	9	9	9	9	9	9
9	9	9	9	9	9	9	9	9	9
1	1	1	1	2	3	1	2	2	9
9	9	9	9	9	9	9	9	9	9
2	2	2	9	9	9	9	9	9	9
1	1	1	1	1	1	1	1	1	1
2	2	2	3	2	1	3	2	9	1
9	9	9	9	9	9	9	9	9	9
1	1	1	1	2	2	2	2	2	2
9	9	9	9	9	9	9	9	9	9
2	2	2	1	9	1	9	9	9	1
9	2	2	2	9	9	9	2	9	9
2	9	2	2	2	2	2	2	2	2
1	1	1	1	1	1	1	1	1	1
9	9	3	3	9	9	9	9	9	9
9	9	9	9	9	9	9	9	9	9
9	9	9	9	9	9	9	9	9	9
2	2	2	2	2	2	2	2	2	2
9	9	9	3	1	3	9	9	9	9
9	3	1	1	9	9	1	9	9	1
9	9	2	2	9	9	9	9	9	9
2	9	2	2	9	9	9	2	9	9

9	9	9	2	9	9	9	9	9	9
9	9	9	9	9	9	9	9	9	9
9	9	3	2	9	9	1	9	3	2
9	9	9	3	9	9	9	9	9	9
9	9	9	9	9	9	9	9	9	9
9	9	9	9	9	9	9	9	9	9
9	9	9	2	9	9	9	9	9	9
9	9	9	2	9	9	9	9	9	9
9	2	9	2	9	9	2	9	9	2
9	9	9	2	2	2	2	2	9	9
9	9	9	9	9	9	9	9	9	9
9	9	9	9	9	9	9	9	9	9
9	9	9	9	9	9	9	9	9	9
9	9	9	9	9	9	9	9	9	9
1	2	1	1	1	3	3	3	2	3
2	1	1	1	1	3	1	1	3	2
9	2	9	1	9	9	9	9	9	9
2	2	2	3	1	1	3	1	3	2
2	2	2	1	1	3	3	3	3	2
9	9	9	2	2	2	2	2	2	2
9	9	9	9	1	9	9	9	9	9
9	9	9	3	9	9	9	9	9	3
3	3	3	2	3	3	2	2	2	3
1	2	3	1	2	3	1	2	3	1
3	2	2	2	1	3	3	3	3	3
2	2	2	2	1	3	3	1	3	3
2	3	3	1	9	3	3	3	3	3
2	2	2	3	1	3	3	3	3	3
3	1	1	2	1	3	3	2	3	3
2	2	2	1	1	3	3	3	3	3
9	9	9	1	9	9	9	9	9	9
9	9	9	3	9	9	9	9	9	9
3	1	1	1	9	9	9	9	9	9
2	9	9	9	9	9	9	9	9	9
9	9	9	2	9	9	9	9	9	9
9	9	9	9	9	9	9	9	9	9
9	9	9	9	9	9	9	9	9	9
9	9	2	9	2	9	2	9	9	2
9	9	9	9	9	9	9	9	9	9
9	9	2	9	2	9	9	2	9	2
2	9	2	2	9	2	9	2	9	2
2	2	2	2	2	2	2	2	2	2
2	2	2	2	2	2	2	2	2	2
2	2	2	2	2	2	2	2	2	2
9	9	9	9	9	9	9	9	9	9
2	2	2	1	3	2	2	2	2	2
2	3	2	1	1	2	3	2	2	1

2	1	1	2	2	3	2	3	3	2
2	1	2	2	1	3	3	2	3	2
9	9	9	3	3	3	3	3	1	3
2	3	2	3	2	3	3	1	2	1
3	3	3	3	1	3	3	3	3	3
1	2	2	3	1	3	1	2	3	3
1	2	1	2	1	3	1	2	1	1
3	3	3	3	3	3	3	3	3	3
2	2	2	2	2	2	2	2	2	2
2	2	2	2	2	2	2	2	2	2
9	2	9	9	2	9	2	9	9	2
2	2	2	2	2	2	2	2	2	2
2	1	2	1	3	3	1	1	2	2
2	1	3	3	1	2	3	1	2	2
3	2	2	2	2	2	3	3	3	3
2	2	2	2	2	2	2	2	2	2
2	1	2	2	2	1	2	1	2	3
3	3	3	1	2	3	3	3	2	3
3	2	2	9	1	3	3	3	3	3
2	2	3	2	1	1	3	1	3	2
3	2	2	1	3	3	2	3	2	1
1	3	1	1	2	3	1	2	3	1
1	1	2	1	3	2	1	1	2	2
3	1	2	3	1	1	2	3	3	3
1	2	1	1	2	3	3	3	2	3
2	2	2	3	1	2	2	2	3	3
1	1	1	3	1	2	1	2	3	3
3	2	2	3	1	3	2	9	2	2
2	3	2	1	1	3	2	2	3	2
3	3	3	2	1	3	3	3	3	3
3	3	2	2	3	3	1	3	3	3
3	1	2	2	2	3	1	3	3	3
3	3	3	3	3	3	3	3	3	3
2	2	2	1	1	3	3	3	3	3
9	9	9	1	9	9	9	9	9	9
3	3	3	3	1	2	3	1	2	3
2	2	2	3	3	1	1	1	2	1
2	2	2	2	2	2	2	2	2	2
2	2	2	3	3	1	1	1	1	1
9	9	9	9	9	9	9	9	9	9
2	2	2	1	1	3	3	3	3	3
9	9	9	1	1	3	3	3	3	3
2	1	2	1	1	2	1	3	2	2
9	1	2	1	1	1	2	3	2	1
2	3	1	2	2	2	3	2	3	2
3	3	3	3	3	3	3	3	3	3
3	2	3	2	2	3	3	2	3	2

1	1	2	1	2	1	3	2	2	2
3	3	3	3	3	3	3	3	3	3
1	3	3	3	1	3	3	9	3	1
3	3	2	1	1	2	3	3	3	3
3	2	2	1	3	3	3	2	2	3
2	3	2	1	1	3	1	3	3	3
2	3	3	3	2	3	2	2	3	3
9	1	3	2	2	2	3	1	3	1
1	1	1	1	3	1	1	2	1	1
2	2	2	2	2	1	2	2	2	2
2	2	3	9	2	2	2	2	3	3
2	2	2	1	1	3	3	3	3	3
2	2	1	1	3	2	1	2	2	2
3	2	3	1	1	1	2	3	2	2
2	1	2	3	1	3	2	3	3	3
2	2	2	1	9	3	3	3	9	1
2	2	2	3	3	1	1	1	2	2
3	2	2	1	2	3	2	1	2	1
2	2	2	1	2	2	2	2	2	2
9	9	9	1	9	9	9	9	9	9
2	2	1	1	1	1	2	2	2	2
2	2	2	1	1	1	1	2	1	2
3	2	2	2	2	2	3	2	3	2
3	1	3	2	3	2	3	1	3	2
3	3	3	3	3	3	3	3	3	3
2	3	3	1	1	2	3	2	1	3
1	9	9	2	2	1	2	2	2	1
2	2	2	2	2	2	2	2	2	2
2	2	2	3	3	2	2	2	2	2
2	2	2	2	2	2	2	2	2	2
2	3	1	3	1	1	3	1	3	2
9	9	1	1	3	2	1	2	2	2
2	1	1	1	1	3	2	2	1	1
1	3	2	1	1	2	3	3	1	3
1	2	2	1	1	3	2	3	3	3
2	3	3	2	3	2	3	9	2	9
1	2	2	1	1	3	3	3	3	3
2	1	2	1	1	2	2	3	1	2
3	3	2	2	1	3	3	3	3	3
2	1	1	2	3	2	3	2	2	1
2	1	2	1	1	3	2	2	3	3
2	2	2	2	2	2	2	2	2	2
2	2	2	1	2	2	2	2	2	2
2	2	2	2	2	2	2	2	2	2
2	1	1	1	1	3	2	2	1	1
3	3	3	3	1	1	1	1	3	3
2	2	2	2	1	2	2	1	3	3

2	1	1	1	2	1	3	1	1	2
2	1	1	1	1	3	2	2	1	1
2	2	2	2	1	2	2	1	3	3
2	3	2	1	3	2	2	1	3	1
1	2	1	2	1	1	2	2	9	2
2	1	1	1	1	3	2	2	1	1
2	1	1	1	1	3	2	2	1	1
2	2	2	2	2	2	2	2	2	2
2	2	2	1	1	2	2	2	2	2
9	9	9	9	9	9	9	9	9	9
3	1	2	1	3	3	3	3	3	3
1	3	1	1	3	3	3	3	3	3
9	9	9	2	1	3	3	1	2	3
9	1	9	1	1	1	2	2	3	2
2	2	2	2	3	3	2	2	2	1
9	9	1	9	1	9	9	9	9	9
9	9	9	9	9	9	9	9	9	9
9	9	9	1	9	9	9	2	9	9
9	9	9	9	9	9	9	9	9	9
2	2	2	3	3	3	3	3	3	3
1	3	3	1	1	1	1	3	3	2
9	1	9	9	9	9	9	9	9	9
9	9	9	9	9	9	9	9	9	9
2	2	2	3	3	2	2	2	2	2
2	3	3	1	1	2	1	2	2	2
2	2	2	3	3	2	2	2	2	2
2	3	3	3	3	2	1	2	1	3
9	9	9	1	3	2	1	9	3	1
2	2	2	1	2	3	2	1	1	3
1	3	1	1	3	2	2	3	1	2
9	9	9	9	9	9	9	9	9	9
2	1	3	1	1	2	1	2	3	1
3	3	3	3	1	3	2	2	1	2
2	1	1	2	2	2	2	2	2	2
2	3	3	2	2	2	2	9	2	9
2	2	2	3	3	2	2	2	2	2
9	9	9	1	1	9	9	9	9	9
2	2	2	1	1	2	2	2	2	2
2	2	2	1	1	3	3	3	2	3
2	3	3	2	2	1	2	1	1	1
3	1	1	2	1	1	1	1	1	3
1	1	1	1	1	3	3	1	3	1
2	3	2	9	1	3	1	2	1	1
1	2	2	1	2	2	1	9	1	1
3	3	3	3	1	2	3	1	2	3
1	1	3	1	1	2	1	1	1	9
3	3	3	3	2	3	3	2	3	2

2	1	9	2	2	1	2	3	2	3
2	2	2	2	2	2	2	2	2	2
3	1	3	3	3	3	3	2	2	2
2	2	2	2	2	1	2	2	2	2
3	1	2	2	3	2	2	1	2	3
2	2	2	2	2	2	2	2	2	2
9	9	1	9	1	3	9	9	9	9
3	3	2	1	1	3	3	1	2	3
3	3	3	3	9	9	9	9	9	9
2	2	2	3	1	2	2	2	1	3
2	2	3	1	1	2	2	2	2	2
9	9	9	1	2	1	1	1	1	1
1	1	2	2	3	1	1	1	2	2
9	9	9	1	9	9	1	9	1	9
9	9	9	2	9	9	9	9	9	9
9	9	9	1	1	1	1	1	9	3
2	2	2	1	1	3	3	3	3	3
3	3	2	1	1	1	3	3	3	3
2	2	2	3	3	3	1	3	2	1
2	3	2	3	1	3	3	3	3	3
2	2	2	1	2	2	2	3	2	2
3	3	9	3	3	1	1	3	3	3
3	1	2	2	1	3	3	1	3	3
2	2	2	2	2	2	2	2	2	2
3	9	1	3	1	3	3	3	3	3
2	2	2	2	2	2	2	2	2	2
2	2	2	1	1	2	2	2	2	2
9	9	9	9	1	9	9	9	9	1
2	3	2	2	2	2	3	2	3	2
3	2	2	2	1	3	3	3	3	3
1	1	1	1	1	1	1	1	1	1
2	1	3	2	1	1	2	3	1	2
2	1	3	1	2	2	2	1	2	1
2	2	2	3	1	3	1	2	2	2
2	2	2	1	3	1	3	9	1	1
2	2	1	1	2	2	3	1	3	1
1	1	1	1	1	1	1	1	1	1
2	3	2	3	1	3	3	3	3	3
3	3	2	1	1	1	2	1	3	2
2	3	2	2	2	3	3	1	1	2
9	1	9	1	3	3	1	1	1	1
2	1	2	1	2	3	3	9	3	3
2	2	2	1	1	3	3	2	9	3
2	2	2	1	1	3	3	2	3	3
3	3	3	9	9	9	9	9	9	9
3	3	2	2	1	3	9	3	3	3
3	3	3	3	3	3	3	3	3	3

3	3	1	3	1	3	1	2	3	2
1	2	1	3	1	3	3	2	3	1
3	2	3	2	2	2	2	2	2	2
2	2	2	2	2	2	2	2	2	2
2	1	9	3	9	1	2	9	3	2
9	9	9	9	1	9	9	9	9	9
3	2	2	1	1	3	3	3	3	2
9	1	9	1	9	9	9	9	9	9
9	1	9	1	9	9	9	9	9	9
9	9	1	1	1	3	3	3	3	3
1	9	9	1	9	3	9	9	9	9
9	9	9	9	1	9	9	9	9	9
3	3	2	1	1	3	3	1	9	9
9	9	9	9	3	9	9	9	9	9
1	1	2	1	1	2	2	2	3	3
9	9	9	9	9	9	9	9	9	9
3	2	3	1	2	9	9	9	9	9
9	9	9	1	9	9	9	9	9	9
9	9	9	1	9	9	9	9	9	9
9	9	9	9	2	9	2	9	9	9
1	1	1	3	1	1	1	1	1	1
2	1	2	2	3	1	1	2	2	2
2	2	2	1	1	2	2	2	3	3
2	3	1	1	2	3	3	3	2	3
2	1	1	3	3	3	9	3	1	2
3	2	1	3	1	3	3	3	3	3
1	1	3	1	3	1	3	1	1	1
9	9	9	2	9	9	9	9	9	9
2	2	2	1	2	3	1	2	1	2
1	1	1	3	1	3	3	3	1	1
1	1	3	3	1	3	3	2	3	3
9	9	9	1	9	9	9	9	9	9
3	3	3	1	1	1	1	1	1	1
9	9	9	9	1	9	9	9	9	9
9	9	9	1	9	9	9	9	9	9
2	2	1	1	2	1	1	1	1	2
1	1	1	1	1	1	1	1	1	1
9	9	9	9	9	9	9	9	9	9
9	9	9	9	9	9	9	9	9	9
2	2	2	2	2	2	2	2	2	2
2	2	2	2	2	2	2	2	2	2
2	2	2	2	2	2	2	2	2	2
3	3	3	3	3	3	3	3	3	3
9	2	9	9	2	9	9	2	9	9
2	2	2	2	2	2	2	2	2	2
2	2	2	2	2	2	2	2	2	2
1	9	1	9	1	3	1	3	2	1

3	1	9	9	1	9	3	1	9	9
2	2	9	1	2	9	9	1	2	9
9	2	9	2	9	9	1	9	9	1
1	1	1	2	2	2	2	2	2	2
3	9	1	1	9	1	3	3	1	9
9	1	9	9	1	9	3	1	9	9
1	3	1	1	2	9	3	1	3	9
9	1	2	9	1	9	9	9	2	1
2	9	2	2	2	9	2	9	2	9
2	2	9	2	2	2	2	2	2	2
2	2	2	2	2	2	2	2	2	2
9	9	2	2	9	9	2	9	9	2
2	2	2	2	2	2	2	2	2	2
9	9	9	9	9	9	9	9	9	9
2	2	2	2	2	2	2	2	2	2
9	9	9	9	9	9	9	9	9	9
9	2	9	9	9	9	9	9	9	9
9	9	9	9	9	9	9	9	9	9
3	1	9	9	1	9	3	1	9	9
2	2	2	2	2	2	2	2	2	2
2	2	2	2	1	3	2	2	2	2
2	2	2	2	2	2	2	2	2	2
1	3	2	1	2	2	9	1	3	2
2	1	2	1	2	1	3	1	2	9
2	3	3	3	3	2	2	2	2	3
3	3	3	2	2	1	1	2	3	3
1	2	3	2	3	3	2	1	3	1
2	2	2	1	2	3	3	3	3	3
3	3	1	1	1	3	3	1	2	1
2	2	2	1	1	3	3	2	3	3
2	2	2	2	2	3	3	9	2	2
2	1	2	3	2	2	3	1	2	3
2	2	2	1	2	2	2	2	2	2
1	1	1	2	1	2	2	2	2	2
2	1	3	1	2	1	1	2	3	1
1	1	1	2	2	2	2	2	2	2
3	3	3	3	3	3	3	3	3	3
1	1	3	1	1	1	1	1	1	1
2	2	3	1	3	3	3	3	3	3
3	2	2	3	3	1	2	2	3	2
1	1	3	1	3	1	3	1	1	1
3	2	3	3	3	9	9	9	9	9
3	2	2	1	3	2	3	3	2	3
9	9	9	2	2	2	1	1	3	3
9	9	9	9	9	9	9	9	9	9
2	2	2	3	3	2	2	2	2	2
1	2	3	3	9	2	3	1	1	3

2	3	3	3	2	2	2	1	2	2
3	2	2	1	1	2	1	3	9	1
2	2	2	2	2	2	2	2	2	2
2	2	2	2	2	2	2	2	2	2
2	2	2	2	2	2	2	2	2	2
2	2	2	2	2	2	2	2	2	2
1	1	1	1	1	1	1	1	1	1
1	2	3	1	1	1	2	3	3	3
3	3	3	3	3	3	3	3	3	3
2	1	2	1	2	1	1	1	1	1
1	9	9	1	1	1	1	1	1	9
2	2	2	2	2	2	2	2	2	2
2	2	2	2	2	2	2	2	2	2
2	2	2	2	2	2	2	2	2	2
2	2	2	2	2	2	2	2	2	2
2	2	2	1	1	1	1	9	1	1
1	9	1	1	1	1	1	1	1	1
1	2	2	1	1	1	2	2	1	2
2	2	2	2	2	2	2	2	2	2
2	2	2	2	2	2	2	2	2	2
1	1	1	1	2	1	2	3	2	1
2	2	2	2	2	2	2	2	2	2
9	9	9	9	9	9	9	9	9	9
2	2	2	2	2	2	2	2	2	2
1	1	1	9	9	9	9	9	9	9
1	1	2	1	2	1	2	1	2	1
1	1	1	1	1	1	1	1	1	1
1	1	1	9	1	1	1	1	1	1
2	2	2	2	2	2	9	2	2	2
1	9	1	1	1	1	9	1	1	1
2	2	2	2	2	2	2	2	2	2
2	2	2	2	2	2	2	2	2	2
2	2	9	2	2	2	2	2	2	2
2	2	2	3	1	2	3	3	2	3
2	2	2	2	2	2	2	2	2	2
9	9	9	9	9	9	9	9	9	9
1	1	2	1	1	1	1	1	2	2
1	1	1	1	1	2	1	1	1	1
2	9	2	9	1	1	1	1	1	1
2	2	2	2	2	2	2	2	2	2
2	2	2	2	2	2	2	2	2	2
2	2	2	1	1	1	1	1	1	2
2	2	2	2	2	2	2	2	2	2
2	2	2	2	2	2	2	2	2	2
1	1	2	1	2	1	1	1	1	1
2	2	2	2	2	2	2	2	2	2
2	2	2	3	1	2	2	2	9	9

1	1	1	1	1	2	1	1	2	1
9	2	2	9	9	1	1	9	1	1
2	2	2	3	1	1	1	1	1	1
1	1	1	9	1	1	2	1	1	1
1	1	1	2	1	1	1	1	2	2
2	2	2	2	2	2	2	2	2	2
2	2	2	3	1	2	2	2	2	2
1	2	2	3	1	2	2	2	2	2
9	9	9	9	9	9	9	9	9	9
2	2	2	1	1	1	9	1	9	1
9	2	2	9	9	2	2	9	2	2
1	1	1	1	1	1	1	1	9	1
9	9	9	9	9	9	9	9	9	9
2	2	2	1	2	1	1	1	1	1
9	9	9	1	1	1	1	1	1	1
2	2	2	2	2	2	2	9	9	2
9	9	9	9	9	9	9	9	9	9
9	9	9	9	9	9	9	9	9	9
2	2	2	2	2	2	2	2	2	2
1	1	1	3	1	3	3	3	3	3
1	1	1	3	1	3	3	3	3	3
1	1	1	3	1	3	3	3	3	3
1	1	1	3	1	3	3	3	3	3
1	1	1	3	1	3	3	3	3	3
1	1	1	3	1	3	3	3	3	3
1	1	1	3	1	3	3	3	3	3
1	1	1	9	1	3	3	3	3	3
1	1	1	3	1	3	3	3	3	3
1	1	1	3	1	3	3	3	3	3
1	1	1	3	1	3	3	3	3	3
1	1	1	3	1	3	3	3	3	3
1	1	1	3	1	3	3	3	3	3
1	1	1	3	1	3	3	3	3	3
1	1	1	3	1	3	3	3	3	3
1	1	1	3	1	3	3	3	3	3
1	1	1	1	3	1	3	3	3	3
1	1	1	3	1	3	3	3	3	3
1	1	1	3	1	3	3	3	3	3
1	1	1	9	1	3	3	3	3	3
1	1	1	3	1	3	3	3	3	3
1	1	1	3	1	3	3	3	3	3
1	1	1	3	1	3	3	3	3	3
1	1	1	3	1	3	3	3	3	3
1	1	1	3	9	1	3	3	3	3
9	9	9	9	9	9	9	9	9	9
9	9	9	9	9	9	9	9	9	9
9	9	9	9	9	9	9	9	9	9

9	9	9	9	9	9	9	9	9	9
9	9	9	9	9	9	9	9	9	9
9	9	9	9	9	9	9	9	9	9
9	9	9	9	9	9	9	9	9	9
9	9	9	9	9	9	9	9	9	9
9	9	9	9	9	9	9	9	9	9
9	9	9	9	9	9	9	9	9	9
9	9	9	9	9	9	9	9	9	9
9	9	9	9	9	9	9	9	9	9
9	9	9	9	9	9	9	9	9	9
9	9	9	9	9	9	9	9	9	9
9	9	9	9	9	9	9	9	9	9
9	9	9	9	9	9	9	9	9	9
9	9	9	9	9	9	9	9	9	9
9	9	9	9	9	9	9	9	9	9
9	9	9	9	9	9	9	9	9	9
9	9	9	9	9	9	9	9	9	9
9	9	9	9	9	9	9	9	9	9
9	9	9	9	9	9	9	9	9	9
1	1	1	3	1	3	3	3	3	3
1	1	1	3	1	3	3	3	3	3
1	1	1	3	1	3	3	3	3	3
1	1	1	3	1	3	3	3	3	3
1	1	1	3	1	3	3	3	3	3
1	1	1	3	1	3	3	3	3	3
1	1	1	3	1	3	3	3	3	3
1	1	1	3	1	3	3	3	3	3
1	1	1	3	1	3	3	3	3	3
1	1	1	3	1	3	3	3	3	3
1	1	1	3	1	3	3	3	3	3
1	1	1	3	1	3	3	3	3	3
1	1	1	3	1	3	3	3	3	3
3	1	1	3	1	3	3	3	3	3
1	1	1	3	1	3	3	3	3	3
1	1	1	3	1	3	3	3	3	3
9	9	9	9	1	9	9	9	9	9
1	1	1	3	1	3	3	3	3	3
1	1	1	3	1	3	3	3	3	3
1	1	1	3	1	3	3	3	3	3
1	9	9	3	1	3	3	3	3	3
9	9	1	3	1	3	3	3	3	3
1	1	1	3	1	3	3	3	3	3
3	1	1	3	3	3	3	3	3	3
1	1	1	3	1	3	3	3	3	3
1	1	1	3	1	3	3	3	3	3
1	1	1	3	1	3	3	3	3	3
1	1	1	3	1	3	3	3	3	3

1	1	1	3	1	3	3	3	3	3
9	9	9	3	1	3	3	3	3	3
1	1	1	3	1	3	3	3	3	1
1	1	1	3	1	3	3	3	3	3
1	1	1	3	1	3	3	3	3	3
1	1	1	3	1	3	3	3	3	3
1	1	1	3	1	3	3	3	3	1
1	1	1	3	1	3	3	3	3	3
1	1	1	3	1	3	3	3	3	3
1	1	1	3	1	3	3	3	3	3
1	1	1	3	1	3	3	3	3	3
1	1	1	3	1	3	3	3	3	3
1	1	1	3	1	3	3	3	3	3
1	1	1	3	1	3	3	3	3	3
3	1	1	3	1	3	3	3	3	3
1	1	1	3	1	3	3	3	3	3
1	1	1	3	1	3	3	3	3	3
1	1	1	3	1	3	3	3	3	3
1	1	1	3	1	3	3	3	3	3
1	1	1	3	1	3	3	3	3	3
1	1	1	3	1	3	3	3	3	3
1	1	1	3	1	3	3	3	3	3
1	1	1	1	3	1	3	3	3	3
1	1	1	3	1	3	3	3	3	3
1	1	1	3	1	3	3	3	3	3
9	1	1	3	1	3	3	3	3	3
1	1	1	3	1	3	3	3	3	3
1	1	1	3	1	3	3	3	3	3
1	1	1	3	1	3	3	3	3	3
1	1	1	3	1	3	3	3	3	3
1	1	1	3	1	3	3	3	3	3
1	1	1	3	1	3	3	3	3	3
1	1	1	3	1	3	3	3	3	3
1	1	1	3	1	3	3	3	3	3
9	9	9	9	9	9	9	9	9	9
1	1	1	3	1	3	3	3	3	3
1	3	1	3	1	3	3	3	3	3
1	1	1	3	1	3	3	3	3	3
1	1	1	3	1	3	3	3	3	3
9	9	9	3	1	3	3	3	3	3
1	1	1	3	1	3	3	3	3	3
1	1	1	3	1	3	3	3	3	3
1	1	1	1	1	3	3	3	3	3
1	1	9	3	1	3	3	3	3	3
1	1	1	3	1	3	3	3	1	3
1	1	1	3	1	3	3	3	3	3
1	1	1	3	1	3	3	3	3	3

1	1	1	1	1	3	3	3	3	3
1	1	1	3	1	3	3	3	3	3
1	1	1	3	1	3	3	3	3	3
1	1	1	3	1	3	3	3	3	3
1	1	1	3	1	3	3	3	3	3
1	1	1	3	1	3	3	3	3	3
1	1	1	3	1	3	3	3	3	3
1	1	1	3	1	3	3	3	3	3
1	1	1	3	1	3	3	3	3	3
1	1	1	3	1	3	3	3	3	3
1	1	1	9	1	3	3	3	3	3
1	1	1	3	1	3	3	3	3	3
1	1	1	3	1	3	3	3	3	3
1	1	1	3	1	3	3	3	3	3
1	1	1	9	9	9	9	9	9	9
1	1	1	3	1	3	3	3	3	3
1	1	1	3	1	3	3	3	3	3
1	1	1	3	1	3	3	3	3	3
1	1	1	3	1	3	3	3	3	3
9	9	9	9	9	9	9	9	9	9
1	1	1	3	1	3	3	3	3	3
1	1	1	3	1	3	3	3	3	3
1	1	1	3	1	3	3	3	3	3
1	1	1	3	1	3	3	3	3	3
9	9	9	9	9	9	9	9	9	9
1	1	1	3	1	3	3	3	3	3
1	1	1	3	1	3	3	3	3	3
1	1	1	3	1	3	3	3	3	3
1	1	1	3	1	3	3	3	3	3
1	1	1	3	1	3	3	3	3	3
1	1	1	3	1	3	3	3	3	3
1	1	1	3	1	3	3	3	3	3
1	1	1	3	9	1	3	3	3	3
1	1	1	3	9	1	3	3	3	3
1	1	1	3	1	3	3	3	3	3
1	1	1	3	1	3	3	3	3	3
1	1	1	3	1	3	3	3	3	3
1	1	1	3	1	3	3	3	3	3
1	1	1	3	1	3	3	3	3	3
1	1	1	3	1	3	3	3	3	3
1	1	1	3	1	3	3	3	3	3
9	9	9	9	9	9	9	9	9	9
1	1	1	3	1	3	3	3	3	3
1	1	1	9	1	3	3	3	3	3
1	1	1	3	1	3	3	3	3	3
1	1	1	3	1	3	3	3	3	3
1	1	1	3	1	3	3	3	3	3

1	1	1	3	1	3	3	3	3	3
1	1	1	3	1	3	3	3	3	3
1	1	1	3	1	3	3	3	3	3
1	1	1	3	1	3	3	3	3	3
1	1	1	3	1	3	3	3	3	3
1	1	1	3	1	3	3	3	3	3
1	1	1	3	1	3	3	3	3	3
2	3	3	3	1	3	3	3	3	3
1	1	1	3	1	3	3	3	3	3
1	1	1	3	1	3	3	3	3	3
1	1	1	3	1	3	3	3	3	3
1	1	1	3	1	3	3	3	3	3
1	1	1	3	1	3	3	3	3	3
1	1	1	3	1	3	3	3	3	3
1	1	1	3	1	3	3	3	3	3
1	1	1	3	1	3	3	3	3	3
1	1	1	3	1	3	3	3	3	3
1	1	1	3	1	3	3	3	3	3
1	1	1	3	1	3	3	3	3	3
1	1	1	3	1	3	3	3	3	3
1	1	1	3	1	3	3	3	3	3
1	1	1	3	1	3	3	3	3	3
1	1	1	3	1	3	3	3	3	3
1	1	1	3	1	3	3	3	3	3
1	1	1	3	1	3	3	3	3	3
1	1	1	3	1	3	3	3	3	3
1	1	1	3	1	3	3	1	3	3
9	9	9	9	9	9	9	9	9	9
9	9	9	9	9	9	9	9	9	9
9	9	9	9	9	9	9	9	9	9
9	9	9	9	9	9	9	9	9	9
9	9	9	9	9	9	9	9	9	9
9	9	9	9	9	9	9	9	9	9
9	9	9	9	9	9	9	9	9	9
9	9	9	9	9	9	9	9	9	9
9	9	9	9	9	9	9	9	9	9
9	9	9	9	9	9	9	9	9	9
9	9	9	9	9	9	9	9	9	9
9	9	9	9	9	9	9	9	9	9
9	9	9	9	9	9	9	9	9	9
9	9	9	9	9	9	9	9	9	9
9	9	9	9	9	9	9	9	9	9
1	1	1	1	1	1	3	3	3	2
9	9	9	1	1	1	1	1	1	1
9	9	9	9	9	9	9	9	9	9
9	9	9	9	9	9	9	9	9	9
9	9	9	9	9	9	9	9	9	9

9	9	9	9	9	9	9	9	9	9
9	9	9	9	9	9	9	9	9	9
9	9	9	9	9	9	9	9	9	9
9	9	9	9	9	9	9	9	9	9
9	9	9	9	9	9	9	9	9	9
9	9	9	9	9	9	9	9	9	9
9	9	9	9	9	9	9	9	9	9
3	3	2	1	1	3	9	9	9	9
9	9	9	9	9	9	9	9	9	9
9	9	9	9	9	9	9	9	9	9
9	9	9	9	9	9	9	9	9	9
9	9	9	9	9	9	9	9	9	9
9	9	9	9	9	9	9	9	9	9
1	1	2	3	3	3	3	1	3	3
2	2	2	1	2	2	2	2	3	2
1	2	1	1	3	3	3	3	1	3
3	3	3	1	2	2	3	3	2	1
3	2	2	1	1	1	2	3	3	3
3	3	3	3	3	1	1	1	3	3
3	3	3	1	3	1	2	1	2	3
2	1	1	2	1	3	3	3	3	3
1	3	1	1	3	1	3	1	3	1
3	2	2	2	1	3	1	9	2	2
3	3	3	2	3	2	2	3	2	3
9	9	9	2	1	3	3	2	3	3
2	2	2	2	2	2	2	2	2	2
9	9	9	9	9	9	9	9	9	9
2	1	1	3	1	3	3	3	3	3
1	1	1	1	1	3	3	1	3	1
2	2	2	3	1	3	3	3	3	3
1	3	1	2	3	2	3	9	2	2
1	2	1	1	2	1	2	1	3	1
9	1	9	9	1	9	9	9	9	9
2	2	2	1	1	3	2	3	3	3
9	9	9	3	1	3	1	3	3	3
9	9	9	2	9	2	9	9	9	9
1	2	1	1	2	2	2	2	2	2
1	1	3	1	1	2	1	1	2	2
9	1	1	9	1	3	3	1	2	3
3	2	1	1	1	3	9	1	9	1
9	9	9	9	9	9	9	9	9	9
3	1	1	1	2	2	1	1	1	1
2	2	2	2	1	2	2	2	2	2
1	1	1	1	1	1	1	1	1	1
9	9	9	9	9	9	9	9	9	9
9	9	9	9	9	9	9	9	9	9
2	2	2	1	3	2	2	1	2	2

9	1	9	1	9	9	1	9	9	9
9	9	9	9	9	9	9	9	9	9
3	3	2	1	3	2	2	3	2	1
3	2	1	1	1	3	2	3	3	3
1	9	9	1	1	3	3	1	1	3
9	9	9	1	9	9	9	9	9	9
2	2	2	1	1	9	9	9	9	9
2	2	2	2	2	2	2	2	2	2
2	2	2	2	2	2	2	2	2	2
3	3	2	3	3	1	1	1	1	1
3	2	3	2	3	3	3	1	2	1
9	9	9	9	9	9	9	9	9	9
9	1	9	1	1	9	9	9	9	9
2	2	2	3	2	2	2	1	2	2
2	1	2	2	2	1	2	2	3	1
1	1	2	1	1	2	3	3	3	1
1	3	1	1	3	1	3	1	3	1
3	1	2	1	1	3	1	1	3	3
1	1	1	1	9	1	1	2	1	3
2	1	1	3	1	1	2	3	9	2
2	2	2	2	2	2	2	2	2	2
2	2	2	2	2	2	2	2	2	2
2	2	2	2	2	2	2	2	2	2
2	2	2	2	2	2	2	2	2	2
3	2	2	2	2	2	2	2	2	2
2	2	1	2	1	3	3	3	3	3
2	2	2	2	2	2	2	2	2	2
3	3	3	3	1	1	2	3	3	2
9	3	2	2	3	1	2	3	3	1
2	1	1	3	1	2	3	3	1	3
3	2	2	3	1	3	3	3	3	3
1	2	2	2	1	3	2	3	3	1
1	1	1	2	1	3	2	3	3	3
3	3	3	3	1	2	1	1	2	3
1	2	2	3	1	3	2	2	2	2
1	2	1	3	2	2	3	3	3	3
2	2	2	1	1	2	3	3	3	3
9	9	9	1	1	1	1	1	1	2
9	9	9	2	9	9	9	9	9	9
3	3	3	3	1	3	3	3	3	3
3	3	3	2	2	2	3	3	3	3
2	2	2	2	1	2	1	2	1	2
1	2	3	1	2	1	2	3	1	2
3	3	3	2	1	3	2	2	3	2
1	1	1	1	1	1	1	1	1	1
2	3	3	1	1	1	3	3	3	3
2	2	2	2	1	2	2	2	2	2

3	2	2	2	2	2	2	2	2	2
3	2	3	2	1	2	2	2	3	3
1	1	1	2	2	1	1	1	1	1
2	3	3	1	2	1	2	3	2	2
2	2	1	3	1	3	3	3	3	3
2	2	2	1	1	3	3	3	3	2
3	2	3	1	1	3	3	3	3	3
1	2	3	1	3	2	3	3	2	1
2	2	2	2	2	2	2	2	2	2
2	3	2	1	1	1	2	2	1	2
2	2	2	2	2	2	2	2	2	2
2	2	2	1	2	2	2	2	2	2
2	2	2	2	3	3	3	3	3	3
2	2	2	2	2	2	2	2	2	2
2	2	2	2	2	2	2	2	2	2
2	2	2	2	2	2	2	2	2	2
3	3	3	3	3	3	3	3	3	3
2	2	2	2	2	2	2	2	2	2
2	2	2	2	2	2	2	2	2	2
1	2	3	1	2	1	2	3	1	2
3	2	3	2	2	2	2	2	2	2
1	1	3	1	1	2	2	2	2	1
2	2	2	9	2	2	2	2	2	2
1	1	1	3	3	1	1	1	1	1
3	3	3	9	1	3	3	3	3	3
9	9	9	9	9	9	9	9	9	9
1	2	3	1	1	3	1	1	2	3
2	1	2	2	2	3	2	2	2	2
2	3	2	2	1	1	2	1	2	1
3	3	2	3	1	2	3	3	3	3
3	2	2	2	1	2	2	2	3	3
2	3	2	1	1	1	2	9	2	1
3	2	2	3	3	3	3	2	2	3
2	2	2	1	1	1	1	1	1	1
1	2	1	1	9	1	2	9	1	9
9	9	9	9	9	9	9	9	9	9
1	1	1	1	1	1	3	2	3	3
1	1	1	3	1	3	2	2	1	2
3	3	9	9	9	9	9	9	9	9
1	2	1	1	1	1	1	1	1	1
2	3	2	1	1	3	3	3	3	3
1	1	2	3	1	3	1	1	3	1
3	2	2	2	1	2	2	2	3	3
9	9	9	9	9	9	9	9	9	9
9	9	9	9	9	9	9	9	9	9
9	9	9	9	9	9	9	9	9	9
2	1	1	9	9	9	9	9	9	9

3	2	1	3	1	1	3	1	3	1
2	3	2	9	9	9	9	9	9	9
2	2	2	2	1	3	3	2	3	3
3	1	3	1	3	1	1	3	9	3
9	9	9	9	9	9	9	9	9	9
3	2	2	3	1	3	3	3	3	3
2	2	3	3	1	3	2	3	3	3
2	2	2	3	1	3	3	3	2	2
1	1	1	3	1	3	3	2	1	3
1	2	3	2	2	2	2	2	2	2
2	1	2	9	2	2	2	2	1	3
2	2	2	2	2	2	2	2	2	2
2	2	2	1	2	2	2	2	2	2
1	3	1	1	1	1	1	3	1	1
2	2	2	2	2	2	2	2	2	2
2	1	1	3	1	3	3	3	3	3
1	1	3	1	3	2	3	1	1	3
1	1	2	3	1	3	3	1	3	3
2	3	9	9	1	3	9	1	9	1
3	2	1	1	2	1	3	1	3	1
1	1	2	3	1	3	3	1	3	3
2	3	1	1	1	2	1	3	3	3
2	2	2	3	1	3	3	3	3	3
2	2	3	3	1	9	9	9	9	9
3	3	3	1	1	3	2	3	3	3
9	9	9	9	9	9	9	9	9	9
1	1	1	1	2	1	2	1	3	1
3	3	3	1	1	1	1	1	1	1
2	2	2	1	3	3	3	3	3	3
3	1	1	1	3	2	1	3	2	2
2	2	2	3	2	3	2	3	1	2
1	9	9	9	1	9	9	9	9	9
2	2	2	2	1	3	3	2	3	3
3	3	1	2	1	1	3	2	2	2
1	3	1	3	1	2	9	1	1	1
1	2	3	1	2	3	1	2	1	3
2	2	2	2	2	2	2	2	2	2
3	3	2	3	1	3	1	2	1	3
3	2	2	3	1	3	3	3	3	1
9	9	9	3	9	1	9	9	9	9
3	3	2	9	9	9	9	9	9	9
2	2	2	1	2	1	2	2	2	2
2	2	2	3	2	3	3	3	3	3
2	2	2	1	2	2	2	2	2	2
2	2	2	1	2	1	2	2	2	2
2	2	9	1	1	3	1	1	3	3
2	2	2	2	2	2	2	2	2	2

1	3	1	3	2	1	1	1	2	3
2	2	2	1	2	2	2	2	2	2
3	1	3	1	1	3	2	2	1	3
2	1	1	3	1	3	3	3	3	3
2	2	2	2	2	2	2	2	2	2
2	2	2	2	2	2	2	2	3	3
3	3	1	1	3	3	2	2	2	2
1	1	3	1	1	3	3	3	3	3
1	1	1	1	1	1	1	3	1	3
3	3	3	3	1	3	3	3	3	3
2	1	2	2	2	3	3	3	3	3
2	2	2	3	3	3	3	3	3	3
2	1	2	2	2	3	3	3	3	3
3	3	2	1	2	3	3	3	3	3
2	2	2	1	1	3	3	3	3	3
3	2	2	2	1	2	2	2	2	2
2	3	1	2	1	9	2	3	2	3
2	2	2	2	2	2	3	2	2	2
2	2	2	1	2	2	2	2	3	2
2	2	1	1	1	3	1	3	3	3
1	1	1	3	1	3	3	1	3	3
3	1	3	3	1	1	2	2	3	3
9	9	9	3	1	3	3	2	3	3
2	2	2	2	2	2	2	2	2	2
2	2	2	2	2	2	2	2	2	2
2	1	1	3	1	3	3	3	3	3
3	2	2	1	1	3	3	3	3	2
2	2	2	1	1	3	3	3	3	3
1	1	3	3	1	3	3	3	3	3
1	1	1	2	1	3	2	2	3	3
3	1	2	3	1	3	1	3	3	3
1	1	1	2	2	2	2	2	2	2
2	2	1	1	3	1	2	1	2	1
2	2	2	1	2	2	2	2	2	2
9	9	9	2	2	2	2	2	2	2
1	1	1	2	1	3	3	9	3	3
2	2	2	2	2	2	2	2	2	2
3	3	3	1	2	1	2	2	2	1
2	3	2	1	2	2	2	2	2	2
2	2	2	2	2	2	2	2	2	2
1	2	3	1	3	2	2	1	3	2
9	9	9	9	9	9	9	9	9	9
9	9	9	9	9	9	9	9	9	9
1	1	1	1	1	1	1	1	1	1
2	3	2	1	2	3	2	3	1	2
9	9	9	9	9	9	9	9	9	9
2	2	2	9	9	9	9	9	9	9

2	2	2	2	2	2	2	2	2	2
1	1	1	3	1	1	3	2	3	1
1	2	3	1	2	1	2	3	1	2
3	2	1	1	2	2	3	1	2	2
2	3	3	1	2	2	2	1	1	1
1	2	3	1	2	1	2	3	1	2
2	2	2	1	2	1	2	3	2	2
9	9	9	1	9	1	9	9	9	9
2	2	2	1	1	1	2	2	1	2
9	9	9	9	9	9	9	9	9	9
2	2	2	1	1	1	1	1	1	1
2	3	3	3	3	3	3	3	3	3
3	2	1	1	1	1	2	3	2	3
2	3	2	1	2	2	2	2	2	3
1	1	1	1	1	1	1	1	1	1
9	9	9	9	9	9	9	9	9	9
9	9	9	9	9	9	9	9	9	9
2	2	2	1	1	2	3	3	3	3
2	2	2	2	2	2	2	2	2	2
2	1	3	2	2	1	1	1	2	2
2	2	2	2	2	2	2	2	2	2
2	2	2	2	2	2	2	2	2	2
1	2	2	3	1	1	2	2	1	3
3	1	2	1	2	2	1	3	2	3
9	2	9	1	2	2	2	9	9	1
9	9	9	9	1	9	9	9	9	9
1	1	1	3	1	3	3	3	3	3
2	2	1	1	1	3	3	9	2	9
1	1	1	1	1	1	1	1	1	1
2	2	2	3	1	9	9	9	9	9
2	1	1	1	1	1	2	1	2	2
2	2	2	2	2	2	2	2	2	2
2	2	2	2	2	2	2	2	2	2
2	2	2	2	2	2	2	2	2	2
2	2	2	3	1	1	1	1	1	1
2	2	2	2	2	2	2	2	2	2
3	3	3	3	3	3	3	3	2	2
9	9	9	1	9	9	9	9	9	9
9	2	9	9	9	9	9	9	9	9
2	2	2	2	2	2	2	2	2	2
2	2	2	2	2	2	2	2	2	2
9	9	9	9	1	9	9	9	9	9
2	2	1	1	2	1	2	1	2	1
9	9	9	1	1	2	2	9	9	1
2	2	2	2	2	2	2	2	2	2
1	2	2	2	2	1	2	2	1	2
2	2	2	2	2	2	2	2	2	2

1	3	2	2	1	2	3	1	3	1
9	9	9	1	9	9	9	9	9	9
1	1	1	2	2	2	2	2	2	2
2	2	2	3	3	3	3	3	3	3
3	3	3	2	2	2	2	2	2	2
1	2	9	3	3	2	2	2	1	1
2	1	1	2	1	3	2	2	2	3
2	1	2	2	1	1	2	2	2	2
2	2	2	2	2	2	2	2	2	2
9	9	9	1	1	3	3	3	3	3
1	9	9	1	1	3	3	3	3	3
2	2	2	2	2	2	2	2	2	2
2	3	1	2	2	9	3	1	1	1
3	2	3	3	1	2	2	1	3	3
2	1	2	1	2	1	1	1	2	1
1	1	2	1	2	1	1	1	2	1
2	2	2	1	1	2	2	2	2	2
2	2	2	2	2	2	2	2	2	2
2	3	2	1	1	1	2	1	1	1
2	1	2	2	1	2	2	1	2	1
1	1	1	1	1	1	2	2	2	2
2	2	2	1	1	2	2	2	2	3
3	1	2	1	2	2	1	1	2	1
9	9	9	9	9	9	9	9	9	9
3	3	3	2	9	2	2	2	2	2
9	9	9	9	1	9	9	9	9	9
9	9	9	9	9	9	9	9	9	9
9	9	9	9	9	9	9	9	9	9
3	3	3	1	2	2	2	2	2	1
2	3	3	3	1	1	3	1	3	3
2	2	2	2	2	2	2	2	2	2
9	9	9	1	9	9	9	1	3	1
3	2	1	1	1	2	2	2	2	2
2	2	2	2	2	2	2	2	2	2
9	9	9	1	1	2	3	3	3	3
1	1	1	2	1	3	3	3	3	3
1	2	1	1	2	1	2	1	3	3
2	3	1	1	2	2	1	2	1	2
2	1	1	1	1	2	2	3	2	1
1	1	1	3	1	3	3	3	3	3
2	2	2	1	2	2	2	3	3	2
1	2	1	2	2	2	2	2	2	2
9	9	9	1	9	9	9	9	9	9
9	9	9	2	9	9	9	9	9	1
3	1	2	1	2	1	2	1	3	1
2	2	2	2	2	2	2	2	2	2
2	2	2	2	2	2	2	2	2	2

3	3	3	1	1	1	1	1	3	3
9	9	9	9	9	9	9	9	9	9
9	9	9	9	9	9	9	9	9	9
9	9	9	9	9	9	9	9	9	9
9	9	9	9	9	9	9	9	9	9
9	9	9	1	9	9	9	9	9	9
9	9	9	9	9	9	9	9	9	9
9	9	9	9	9	9	9	9	9	9
9	9	9	9	9	9	9	9	9	9
9	9	9	3	1	3	3	3	3	2
2	2	2	2	2	2	2	2	2	2
2	1	2	1	2	1	2	1	3	1
1	1	1	3	1	3	3	3	3	3
2	2	2	1	2	2	2	2	2	2
2	2	2	2	1	3	3	3	2	2
1	2	1	1	2	2	3	1	3	1
1	3	2	2	2	1	1	2	2	1
2	2	2	3	1	3	3	2	3	3
2	2	3	2	1	1	1	1	1	2
2	1	2	3	2	1	2	1	1	2
1	2	1	1	2	1	2	9	2	3
1	2	3	1	2	1	2	3	2	1
3	3	3	1	1	1	1	1	1	1
2	2	2	2	2	2	2	2	2	2
3	3	3	2	2	2	2	2	2	2
3	3	3	2	2	2	2	2	2	2
2	2	2	3	3	3	3	3	3	3
3	2	1	2	1	1	2	1	2	1
2	2	2	2	2	2	2	2	2	2
2	2	2	2	2	2	2	2	2	2
9	9	9	9	9	9	9	9	9	9
2	2	2	2	2	2	2	2	2	2
1	1	1	1	1	1	1	1	1	1
9	9	9	9	1	1	9	9	9	9
3	3	3	3	3	2	2	2	2	2
2	2	2	3	2	2	2	2	2	2
2	2	2	2	2	2	2	2	2	2
2	2	2	2	2	2	2	2	2	2
2	2	3	3	1	1	1	3	1	2
9	9	9	9	9	9	9	9	9	9
3	1	2	2	1	3	1	1	3	3
2	2	2	2	2	2	2	2	2	2
2	2	2	2	2	2	2	2	2	2
2	2	2	2	1	3	3	3	3	3
3	1	1	1	1	3	3	3	3	3
9	9	9	9	9	9	9	9	9	9
2	2	2	2	2	2	2	2	2	2

9	9	9	9	9	9	9	9	9	9
2	2	2	2	2	3	3	3	3	2
9	9	9	9	1	9	9	9	9	9
9	9	9	9	9	9	9	9	9	9
9	9	9	9	9	9	9	9	9	9
9	9	9	9	9	9	9	9	9	9
9	9	9	9	9	9	9	9	9	9
9	9	9	9	9	9	9	9	9	9
9	9	9	9	9	9	9	9	9	9
9	9	9	9	9	9	9	9	9	9
9	9	9	9	9	9	9	9	9	9
9	9	9	9	9	9	9	9	9	9
9	9	9	9	9	9	9	9	9	9
9	9	9	9	9	9	9	9	9	9
9	9	9	9	9	9	9	9	9	9
9	9	9	9	9	9	9	9	9	9
9	9	9	9	9	9	9	9	9	9
9	9	9	9	9	9	9	9	9	9
9	9	9	9	9	9	9	9	9	9
9	9	9	9	9	9	9	9	9	9
9	9	9	9	9	9	9	9	9	9
9	9	9	9	9	9	9	9	9	9
9	9	9	9	9	9	9	9	9	9
9	9	9	9	9	9	9	9	9	9
1	3	2	1	2	1	2	3	2	1
2	2	2	1	2	2	2	2	2	2
3	3	3	3	1	3	3	3	3	3
2	2	2	3	1	3	3	3	3	3
3	2	1	3	1	3	3	3	3	3
2	1	1	9	1	3	3	3	3	3
2	2	2	1	1	3	2	2	2	2
2	2	2	3	1	3	3	3	3	3
2	2	2	2	1	1	1	1	1	1
2	2	2	1	2	2	3	2	3	1
3	3	3	3	1	3	3	3	3	3
1	1	2	3	1	3	3	3	3	3
1	1	1	3	1	3	2	3	3	3
2	1	3	3	1	3	2	2	3	3
2	2	2	3	1	3	2	2	3	3
2	9	1	1	1	1	3	3	3	1
9	9	9	1	9	9	9	9	9	9
2	1	2	1	1	3	1	3	3	3
3	1	3	1	1	3	3	3	3	3
9	9	9	1	9	1	9	9	2	9
1	1	3	3	1	3	3	3	1	3
2	1	3	3	1	3	3	1	3	3
2	2	2	3	3	3	2	2	2	2
2	2	2	3	1	3	3	3	3	9

2	1	2	2	1	3	3	3	3	3
1	2	1	2	1	3	3	3	3	3
1	1	1	1	1	3	3	3	3	3
2	2	2	3	1	3	3	2	3	3
2	1	3	2	2	3	2	2	2	3
9	9	3	1	9	9	9	9	9	9
2	1	2	3	1	3	3	3	3	3
9	9	9	9	1	9	9	9	9	9
9	9	9	3	1	3	3	2	3	3
9	9	9	3	1	3	3	2	3	3
9	9	9	2	1	9	9	9	9	9
9	9	9	2	1	9	9	9	9	9
9	9	9	1	9	9	1	9	9	9
9	9	9	1	9	9	9	9	9	9
9	1	9	1	1	3	3	3	3	3
3	3	3	1	1	3	3	3	3	3
9	1	1	1	1	3	1	3	3	3
2	2	2	1	2	2	2	2	2	2
9	9	9	9	1	9	9	9	9	9
1	1	3	3	1	3	3	3	3	3
9	9	9	2	1	1	2	3	3	3
9	9	9	1	9	9	9	9	9	9
9	9	9	1	1	1	3	1	3	3
9	1	9	9	1	9	9	9	9	9
9	9	9	9	1	9	9	9	9	9
2	3	3	1	2	2	2	1	2	3
3	1	3	3	1	1	3	3	1	3
3	3	1	3	1	3	3	3	3	3
1	3	3	2	1	3	3	2	3	3
2	2	2	2	2	2	2	2	2	2
1	3	1	3	1	1	3	3	3	3
9	9	9	3	1	3	3	3	3	3
3	3	3	2	1	1	2	3	1	2
1	1	1	3	1	1	1	1	3	3
2	3	1	3	1	3	1	1	3	3
9	9	9	1	9	9	9	9	9	9
2	2	2	2	2	2	2	2	2	2
2	2	2	3	9	3	3	3	3	3
2	2	2	2	3	2	2	3	2	3
1	3	2	2	2	2	2	2	2	2
2	1	2	2	1	2	3	2	2	3
2	1	2	2	1	3	1	2	2	2
3	1	1	3	3	3	3	3	3	3
2	2	3	2	2	2	1	1	3	3
3	1	1	3	1	3	3	3	3	3
9	9	9	3	1	3	3	2	3	3
9	9	9	3	1	3	3	3	3	3

1	1	1	1	1	1	1	3	3	3
3	3	3	2	1	2	2	3	2	2
3	1	3	1	1	3	3	3	3	3
9	9	9	3	1	3	1	1	1	1
9	1	9	2	9	9	2	9	9	9
3	3	2	2	3	1	1	2	3	1
1	1	1	3	1	3	3	3	3	3
9	9	9	9	9	9	9	9	9	9
9	9	9	2	9	2	9	9	9	9
3	3	3	1	1	1	1	1	1	1
2	2	2	2	2	2	2	2	2	2
1	3	3	3	1	3	3	3	3	3
2	1	1	2	1	2	3	2	3	2
9	9	9	9	9	9	9	9	9	9
9	9	9	9	9	9	9	9	9	9
2	1	3	3	3	3	3	3	3	3
1	9	9	9	1	9	9	9	1	9
9	9	9	9	9	9	9	9	9	9
3	3	3	2	3	3	3	3	2	2
1	2	2	2	1	3	3	1	2	3
1	3	1	1	1	3	3	3	3	3
1	1	1	1	1	1	1	1	1	1
1	3	1	3	2	3	3	3	1	1
1	1	1	3	1	3	3	3	3	3
2	2	2	3	1	1	1	2	2	1
9	9	9	9	9	9	9	9	9	9
9	9	9	2	9	9	9	9	9	9
1	1	1	3	1	3	3	3	3	3
3	3	3	3	3	3	3	3	3	3
2	3	2	2	3	3	3	3	1	3
9	9	9	9	9	9	9	9	9	9
9	9	9	9	9	9	9	9	9	9
9	9	9	9	9	9	9	9	9	9
9	9	9	9	9	9	9	9	9	9
9	9	9	9	9	9	9	9	9	9
9	9	9	9	9	9	9	9	9	9
9	9	9	9	9	9	9	9	9	9
9	9	9	9	9	9	9	9	9	9
9	9	9	9	9	9	9	9	9	9
9	9	9	9	9	9	9	9	9	9
9	9	9	9	9	9	9	9	9	9
9	9	9	9	9	9	9	9	9	9
9	9	9	9	9	9	9	9	9	9
9	9	9	9	9	9	9	9	9	9
9	9	9	9	9	9	9	9	9	9
9	9	9	9	9	9	9	9	9	9
9	9	9	9	9	9	9	9	9	9

9	9	9	9	9	9	9	9	9	9
9	9	9	9	9	9	9	9	9	9
9	9	9	9	9	9	9	9	9	9
9	9	9	9	9	9	9	9	9	9
9	9	9	9	9	9	9	9	9	9
9	9	9	9	9	9	9	9	9	9
9	9	9	9	9	9	9	9	9	9
9	9	9	9	9	9	9	9	9	9
9	9	9	9	9	9	9	9	9	9
9	9	9	9	9	9	9	9	9	9
9	9	9	9	9	9	9	9	9	9
9	9	9	9	9	9	9	9	9	9
9	9	9	9	9	9	9	9	9	9
9	9	9	9	9	9	9	9	9	9
9	9	9	9	9	9	9	9	9	9
9	9	9	9	9	9	9	9	9	9
9	9	9	9	9	9	9	9	9	9
9	9	9	9	9	9	9	9	9	9
9	9	9	9	9	9	9	9	9	9
2	1	2	2	1	2	2	2	2	2
9	9	9	9	9	9	9	9	9	9
9	9	9	9	9	9	9	9	9	9
9	9	9	9	9	9	9	9	9	9
9	9	9	9	9	9	9	9	9	9
9	9	9	9	9	9	9	9	9	9
9	9	9	9	9	9	9	9	9	9
9	9	9	9	9	9	9	9	9	9
9	9	9	9	9	9	9	9	9	9
9	9	9	9	9	9	9	9	9	9
9	9	9	9	9	9	9	9	9	9
9	9	9	9	9	9	9	9	9	9
9	9	9	9	9	9	9	9	9	9
9	9	9	9	9	9	9	9	9	9
9	9	9	9	9	9	9	9	9	9
9	9	9	9	9	9	9	9	9	9
2	2	2	2	9	2	9	9	9	9
3	3	3	1	1	3	1	3	3	1
3	2	3	3	1	3	9	1	3	3
3	1	1	3	1	3	3	3	3	3
3	3	3	2	2	2	2	2	2	2
1	1	1	1	1	1	1	1	1	1
9	9	9	1	1	3	2	3	3	2
9	9	9	9	1	9	9	9	9	9
9	9	9	1	1	3	2	3	3	2
3	2	1	2	2	2	2	2	2	2
3	1	1	1	1	1	3	3	3	3
2	2	2	3	1	3	3	3	3	3

2	9	1	1	1	1	3	3	3	1
3	1	1	3	1	3	3	3	3	3
1	1	2	1	1	3	1	2	3	3
1	1	3	2	1	3	2	2	3	3
1	1	2	3	1	3	1	3	3	3
1	1	1	2	2	1	2	3	3	3
3	2	2	3	1	3	3	3	3	3
2	2	2	2	1	3	3	3	3	3
1	3	2	2	2	1	2	3	2	1
1	2	1	1	3	3	3	3	1	3
9	9	9	9	1	9	9	1	9	9
2	3	3	3	1	3	2	1	3	2
2	1	1	1	1	3	3	3	3	3
1	1	2	2	1	3	1	2	3	3
2	2	1	2	1	3	1	2	3	3
2	2	1	3	1	3	3	3	3	3
1	2	2	9	9	9	9	9	9	9
1	3	3	3	1	3	1	1	3	3
1	1	2	1	1	1	2	1	2	2
1	1	1	2	9	1	1	2	1	3
3	1	9	3	1	3	3	1	3	3
2	1	2	2	1	3	3	3	2	3
1	1	1	3	1	1	3	3	3	3
9	1	9	3	1	3	3	3	3	3
3	3	3	3	3	3	3	3	3	3
1	2	1	1	3	1	1	1	1	2
1	1	2	1	1	1	1	1	1	1
3	1	2	1	2	3	1	3	2	1
2	2	2	2	2	2	2	2	2	2
2	2	2	3	1	2	2	2	2	2
3	3	2	2	2	2	3	2	3	2
2	2	2	2	1	3	2	2	2	2
2	2	2	3	1	2	2	2	2	2
9	9	9	9	9	9	9	9	9	9
9	9	1	9	9	9	9	9	9	9
9	9	9	9	9	9	9	9	9	9
9	9	9	1	1	2	3	1	2	1
9	9	9	1	1	2	2	1	2	1
9	9	9	1	1	9	9	9	9	9
9	9	9	9	9	9	9	9	9	9
2	2	2	1	2	3	1	2	2	2
2	2	2	3	2	1	2	1	3	1
2	1	2	3	1	3	3	3	3	3
1	1	3	1	3	3	1	3	3	3
1	1	1	3	1	1	1	3	3	3
3	3	3	3	1	3	1	3	3	3
3	1	3	3	1	3	3	3	9	3

2	2	2	2	2	2	2	2	2	2
2	3	1	1	3	2	1	1	3	2
2	2	2	1	2	2	1	2	2	1
1	1	3	1	3	3	1	1	1	1
2	3	2	1	1	2	3	3	3	2
2	1	2	1	3	1	2	3	1	2
3	3	3	1	1	3	1	9	3	9
2	3	1	3	2	2	3	1	2	1
1	1	1	3	1	3	3	3	3	3
9	9	9	1	9	9	9	9	9	9
3	3	3	3	1	3	1	3	3	3
3	1	1	1	3	1	3	1	2	1
1	3	3	3	1	1	3	1	3	2
3	1	1	1	1	3	3	3	3	3
2	1	3	3	1	1	1	1	1	1
2	3	2	1	9	3	1	1	1	3
2	1	2	2	2	2	1	3	2	2
9	9	9	9	1	9	9	9	9	9
3	2	1	1	1	3	3	1	1	1
9	9	9	1	1	3	3	3	3	3
2	3	1	1	2	1	1	2	2	3
1	1	2	1	3	2	1	1	1	1
3	1	1	1	1	3	3	3	3	3
1	1	3	1	3	3	1	1	3	1
2	1	2	1	2	2	3	1	1	2
3	1	3	1	1	3	3	3	3	3
1	1	1	1	1	3	3	3	3	3
3	3	3	1	1	1	2	1	3	2
2	2	2	1	1	1	3	3	3	1
1	2	1	1	3	1	2	3	1	3
3	3	3	2	3	1	2	1	1	1
1	2	3	1	1	1	2	3	1	2
1	1	1	1	1	2	1	3	3	3
3	1	3	1	1	3	3	3	3	3
3	3	3	1	1	1	1	1	1	1
3	1	1	3	1	3	3	3	3	1
1	2	1	1	2	1	2	3	1	2
9	9	9	9	9	9	9	9	9	9
3	3	3	3	1	3	3	1	3	3
1	3	2	3	1	1	9	3	3	3
1	1	1	1	1	1	1	3	3	3
2	3	2	1	2	1	2	3	1	2
3	1	1	1	1	1	1	1	3	1
2	2	2	2	2	2	1	2	3	2
2	2	2	2	2	2	2	2	2	2
9	9	9	3	3	2	1	2	3	3
3	3	3	3	3	3	3	3	3	3

2	3	1	1	1	2	3	1	2	2
2	3	2	1	3	1	1	1	1	1
2	2	2	3	1	2	2	2	2	2
3	2	3	1	2	1	2	2	3	2
2	2	2	1	1	2	2	2	2	2
1	1	2	1	1	2	2	2	1	1
2	2	2	2	2	2	2	2	2	2
9	9	9	9	9	9	9	9	9	9
9	9	9	9	9	9	9	9	9	9
3	2	1	1	2	1	2	1	2	1
2	2	2	1	1	2	2	2	2	2
2	2	2	2	2	2	2	2	2	2
2	2	2	1	3	2	2	2	2	2
2	2	2	2	2	1	1	1	1	1
2	2	2	2	2	3	2	2	2	2
2	2	2	2	2	2	2	2	2	2
2	2	2	2	2	2	2	2	3	3
2	2	2	2	2	2	2	2	2	2
2	2	2	1	2	1	3	2	2	2
2	2	2	3	2	2	2	2	2	2
2	3	2	1	1	2	2	2	2	2
2	2	2	2	2	2	2	2	2	2
2	2	2	2	2	2	2	2	2	2
2	2	2	1	3	2	2	1	2	2
2	2	2	2	2	2	2	2	2	2
2	2	2	1	2	2	1	2	2	2
2	2	2	1	1	2	2	2	2	2
2	3	2	1	2	1	2	3	1	2
3	3	3	2	2	2	2	2	2	2
2	2	2	2	2	2	2	2	2	2
2	2	2	2	2	2	2	2	2	2
2	2	2	1	1	2	2	2	2	2
2	2	2	2	2	2	2	2	2	2
2	2	2	2	2	2	2	2	2	2
2	2	2	1	2	2	2	2	2	2
2	2	2	1	1	2	2	2	2	2
2	2	2	2	2	2	2	2	2	2
2	2	2	2	2	2	2	2	2	2
2	2	2	2	2	2	2	2	2	2
3	1	1	1	1	3	3	3	1	3
2	2	2	1	1	2	2	2	2	3
2	2	2	2	2	2	2	2	2	2
3	1	2	2	2	1	1	3	1	3
2	2	2	2	2	2	2	2	2	2
1	2	2	1	2	2	2	1	1	1
3	3	3	2	2	2	2	2	2	2
2	2	2	3	3	3	3	3	3	3

2	2	2	2	2	2	2	2	2	2
2	2	2	1	3	2	2	2	2	2
2	2	2	1	2	2	2	2	2	2
2	2	2	2	2	2	2	2	2	2
2	1	2	1	2	3	2	1	2	3
2	2	2	2	2	2	2	2	2	2
9	9	9	3	1	1	1	1	1	1
3	9	1	1	1	1	1	1	1	1
9	1	9	1	9	9	9	9	9	9
2	2	2	2	2	2	2	2	2	2
2	2	2	2	2	2	2	3	3	3
1	1	1	1	1	1	1	1	1	1
3	3	3	2	2	2	3	3	2	3
9	9	9	1	1	1	3	9	9	9
2	2	2	1	3	2	2	2	2	2
2	1	2	1	9	2	2	2	1	2
2	2	2	1	1	3	2	2	2	2
3	2	1	1	1	1	1	1	1	1
9	1	9	1	9	9	9	9	9	9
1	3	3	1	1	2	2	1	1	3
1	1	2	1	1	1	2	1	2	2
9	9	1	1	9	9	9	9	9	9
9	9	9	9	1	9	9	9	9	9
2	2	2	1	3	2	3	3	1	1
2	2	3	1	2	1	3	2	9	1
3	3	3	2	2	1	1	1	1	2
2	2	1	3	1	2	2	1	2	3
9	2	1	1	1	1	3	3	3	1
1	2	3	1	2	1	2	9	1	3
1	1	1	1	1	1	1	1	1	1
3	3	3	3	3	1	2	1	1	2
2	2	2	2	3	3	2	1	3	2
2	2	2	2	2	2	2	2	2	2
9	9	9	1	2	9	2	9	9	9
2	2	1	1	1	3	3	3	3	3
9	9	1	1	9	9	9	9	9	9
3	3	3	1	1	1	1	3	3	3
2	2	2	1	1	1	1	1	1	1
2	2	2	2	2	2	2	2	2	2
2	2	2	2	2	2	2	2	2	2
3	1	1	9	9	9	9	9	9	9
2	2	2	1	1	2	2	2	2	2
9	9	9	9	1	9	9	9	9	9
1	1	1	1	1	1	1	1	1	1
2	2	1	1	1	2	3	2	2	2
2	2	2	2	1	2	2	2	2	2
2	2	2	2	1	2	1	2	2	2

1	1	1	1	1	1	1	1	1	1
2	2	2	1	1	2	2	2	2	2
1	1	1	1	1	1	1	1	1	1
2	2	2	2	2	2	2	2	2	2
9	2	9	9	1	9	9	1	9	9
1	2	3	3	3	1	2	2	3	2
3	3	3	3	3	3	3	3	3	3
1	1	1	1	2	2	2	2	2	2
1	2	3	1	1	2	2	2	2	2
3	3	3	1	1	1	1	1	1	1
1	1	1	1	2	1	2	3	2	1
2	2	2	3	3	3	3	3	3	3
2	2	2	1	3	1	1	1	1	1
2	2	2	1	3	1	1	1	1	1
2	2	2	2	2	2	2	2	2	2
3	2	2	3	2	3	2	2	2	2
1	1	1	1	1	1	1	1	1	1
3	3	3	1	1	3	3	3	3	3
9	9	9	9	9	9	9	9	9	9
2	1	2	1	2	2	1	2	1	3
2	2	1	3	3	1	1	1	1	1
3	1	2	1	3	2	2	2	2	2
3	3	3	1	1	1	1	1	1	1
3	2	2	2	1	1	2	9	1	1
3	3	3	9	3	9	3	9	3	9
2	2	2	1	2	2	1	1	1	2
2	3	3	1	1	1	2	9	1	2
2	1	2	2	2	2	2	1	2	1
2	2	3	1	9	2	9	9	9	9
2	2	2	2	3	1	2	1	2	2
2	2	2	1	2	1	1	9	2	2
2	2	2	2	2	2	2	2	2	2
3	3	3	3	3	3	3	3	3	3
3	1	2	1	1	2	2	3	1	2
2	2	2	2	2	2	2	2	2	2
2	2	2	3	2	3	2	3	2	3
3	1	3	1	3	2	3	9	2	3
2	2	2	2	2	3	3	9	2	2
1	2	3	1	2	3	1	2	1	2
3	3	3	3	3	3	3	3	3	3
3	1	1	1	1	1	2	1	2	3
1	1	1	2	2	3	2	3	2	3
9	9	9	9	9	9	9	9	9	9
3	3	3	3	3	3	3	3	3	3
1	1	1	1	2	1	1	2	2	1
2	2	2	2	2	2	2	2	2	2
2	2	1	2	1	3	3	3	3	3

1	1	1	1	1	1	1	1	1	1
1	3	3	1	1	3	3	3	3	1
1	1	1	1	1	1	1	1	1	1
1	3	3	1	1	3	3	3	3	1
1	1	1	1	3	1	1	2	3	1
2	2	2	2	2	2	2	2	2	2
2	2	2	2	2	2	2	2	2	2
2	2	2	2	2	2	2	2	2	2
1	1	1	1	1	2	2	2	2	1
1	3	2	1	3	1	2	1	1	2
1	1	1	2	2	2	2	2	2	2
2	3	1	1	3	2	3	2	1	2
2	2	2	2	3	2	3	2	2	2
1	3	1	2	1	2	3	2	2	3
2	2	2	2	2	2	2	2	2	2
2	2	2	1	1	3	3	3	3	2
2	2	2	2	2	2	2	2	2	2
3	2	2	2	3	2	1	3	1	1
2	1	2	1	2	2	1	2	2	2
2	3	3	3	1	3	3	2	3	1
2	2	2	1	1	1	1	1	1	1
2	3	2	1	2	3	1	1	1	1
1	1	1	1	2	3	1	1	1	3
2	2	2	2	2	2	2	2	2	2
1	1	1	1	1	1	1	1	1	1
3	3	3	2	3	2	2	2	2	2
3	3	3	1	2	1	1	1	1	1
2	3	1	3	1	1	3	3	3	3
1	2	2	1	9	2	3	2	2	2
2	2	3	2	1	2	3	2	3	3
2	2	2	1	1	3	1	2	3	1
1	2	2	1	3	3	2	1	2	3
2	2	2	1	1	1	1	1	1	1
2	2	3	2	2	2	2	2	2	2
3	3	3	1	2	1	1	1	3	1
1	2	2	1	2	1	2	3	2	1
2	2	2	1	2	2	2	2	2	2
9	9	9	9	9	9	9	9	9	9
2	2	2	1	2	2	2	2	2	1
1	3	3	1	3	2	3	3	1	1
3	3	2	1	3	2	3	3	1	1
2	2	2	2	2	2	2	2	2	2
2	2	2	2	2	2	2	2	2	2
2	2	2	2	2	2	2	2	2	2
9	3	2	9	9	9	9	9	9	9
1	1	1	1	3	1	1	2	3	1
2	2	2	2	2	2	2	2	2	2

9	9	9	9	9	9	9	9	9	9
1	3	1	1	2	2	3	1	3	1
1	1	1	1	1	2	3	1	2	3
3	1	3	2	1	3	3	1	1	1
1	9	1	3	3	3	3	3	3	3
3	3	3	1	1	3	2	1	3	3
2	3	1	1	1	3	3	3	3	3
9	9	9	9	9	9	9	9	9	9
2	1	2	1	2	2	1	1	2	1
3	3	1	1	1	3	1	3	3	3
2	3	2	2	2	2	2	2	1	2
2	2	3	1	3	1	2	3	2	3
2	2	2	2	1	9	9	9	9	9
1	1	1	1	1	3	1	2	3	2
1	2	2	1	2	3	1	3	2	2
2	2	2	2	2	2	2	2	2	2
3	2	3	1	2	2	1	2	3	2
2	3	2	1	9	3	3	3	2	2
9	1	9	1	9	9	9	9	9	9
2	2	2	2	2	2	2	2	2	2
9	9	9	9	9	9	9	9	9	9
2	3	2	1	2	1	2	2	1	2
3	3	2	1	2	2	2	1	1	2
2	2	1	1	2	2	3	3	2	1
1	3	3	1	1	2	2	2	1	1
3	3	3	3	3	3	3	3	2	3
2	2	3	1	2	2	3	2	1	3
2	1	2	1	1	2	2	2	2	2
2	1	2	1	1	3	2	2	3	1
1	1	2	1	1	3	3	3	3	3
3	3	3	1	1	2	2	3	3	3
9	2	1	1	2	2	1	2	3	2
3	3	3	3	3	3	1	3	3	3
2	2	2	2	2	2	2	2	2	2
2	2	2	2	2	2	2	2	2	2
3	3	3	2	2	2	9	1	2	2
3	3	3	1	3	3	3	3	3	3
3	2	1	2	2	2	3	3	3	1
1	1	1	1	2	1	2	3	1	2
1	1	1	1	2	2	1	3	2	2
1	1	1	1	3	1	1	2	1	1
3	2	1	2	2	2	2	2	2	2
3	2	1	3	1	3	3	2	2	1
3	3	3	1	2	3	3	2	2	2
9	9	9	9	9	9	9	9	9	9
3	3	3	3	3	3	3	3	3	3
3	3	2	1	1	3	3	3	3	3

3	3	3	3	3	3	3	3	2	2
1	9	1	1	1	1	1	3	3	1
2	1	2	3	1	2	1	2	3	1
9	9	9	1	2	2	2	2	2	2
3	3	3	1	3	2	2	3	2	3
3	3	3	3	3	3	3	3	3	3
2	2	2	1	1	1	1	1	1	1
3	3	3	3	3	3	3	3	3	3
2	2	1	2	3	1	2	1	2	1
2	3	1	1	1	3	2	3	1	3
2	2	2	2	2	2	2	2	2	2
3	2	3	2	2	2	2	2	2	2
1	2	1	1	2	2	2	2	1	9
9	9	9	9	1	9	9	9	9	9
1	1	1	1	1	9	1	9	2	2
3	3	3	3	3	3	3	3	3	3
1	1	1	1	1	1	1	1	1	1
2	2	2	2	2	2	2	2	2	2
2	2	2	2	2	2	2	2	2	2
1	1	1	1	1	1	2	2	2	3
1	2	1	1	3	1	3	1	1	1
3	3	3	9	9	9	9	9	9	9
2	2	2	3	3	3	1	3	3	1
3	3	1	2	1	2	3	1	3	2
1	2	3	1	2	3	1	2	3	1
3	1	1	1	3	3	1	1	3	1
1	2	2	1	1	1	1	1	1	1
3	2	1	1	3	1	2	3	1	2
1	3	2	2	1	2	1	3	1	2
2	2	2	1	1	3	3	3	3	3
3	3	2	1	1	2	3	2	3	2
2	2	2	3	3	3	3	3	3	3
1	2	3	1	2	2	2	1	2	2
3	3	3	1	3	2	2	3	3	3
1	2	3	1	1	1	3	1	1	3
1	1	2	1	1	1	2	1	3	2
3	2	3	1	1	1	2	2	1	2
1	1	1	1	1	3	3	9	2	3
2	1	1	1	3	1	1	1	1	1
1	1	2	3	1	1	2	1	2	1
2	2	2	2	2	2	2	2	2	2
1	1	3	1	1	1	1	1	1	1
2	1	2	1	2	1	2	1	2	1
2	2	1	1	1	3	9	2	9	3
2	2	3	1	2	2	1	2	1	2
1	3	2	2	2	1	2	1	3	2
2	3	3	3	1	1	3	3	3	1

3	1	3	2	3	3	3	1	3	1
2	1	2	1	3	1	2	3	2	1
2	2	9	1	2	2	2	2	2	2
3	3	1	3	1	1	1	3	1	3
1	2	3	1	2	1	2	3	1	2
2	2	2	1	1	1	2	2	3	3
2	2	1	1	3	2	1	2	3	1
3	1	2	1	2	1	3	9	1	2
2	1	3	3	3	1	1	1	3	1
2	2	2	2	3	1	2	2	2	1
3	2	2	1	2	1	2	1	2	3
2	1	2	1	1	2	3	1	1	2
1	1	1	3	1	1	1	1	3	3
3	1	3	3	1	3	1	1	3	1
1	2	2	3	3	1	1	9	9	2
3	3	3	3	3	3	3	3	1	1
1	1	1	1	3	1	3	1	1	1
3	3	3	1	1	1	3	9	2	3
1	1	1	1	1	3	2	2	2	2
1	1	1	1	1	1	1	1	2	1
1	2	2	1	9	1	1	1	2	1
3	1	1	3	3	3	3	3	3	3
1	1	2	1	1	3	3	3	3	3
1	1	1	1	1	1	1	1	1	3
3	2	2	1	3	2	2	2	2	2
2	2	2	2	2	2	2	2	2	2
3	3	3	1	3	3	1	1	2	1
3	2	1	1	9	2	1	3	2	1
2	2	2	2	2	2	2	2	2	2
9	9	9	9	9	9	9	9	9	9
9	9	9	9	9	9	9	9	9	9
9	9	9	9	9	9	9	9	9	9
2	2	2	1	1	2	2	2	2	2
9	9	9	1	1	9	9	9	9	9
2	2	2	2	2	2	2	2	2	2
2	2	2	1	1	1	3	3	3	3
3	2	3	3	2	2	1	1	2	1
2	2	2	1	3	3	3	3	3	9
2	2	2	3	1	2	9	9	9	9
3	3	2	1	1	2	2	1	1	1
3	2	3	3	1	3	3	3	3	3
3	3	1	1	1	1	1	3	1	1
3	3	3	3	1	3	3	2	3	2
3	3	3	3	1	3	3	2	3	2
1	3	2	1	1	3	1	2	1	3
2	2	2	1	3	1	2	1	2	1
3	3	3	1	3	3	1	1	2	1

9	2	9	3	3	3	2	1	1	1
1	1	2	1	2	1	2	2	2	2
2	2	2	3	3	2	2	3	2	2
1	2	1	3	3	3	1	1	1	3
2	2	2	3	1	3	3	9	3	3
3	2	3	3	2	2	1	2	1	2
9	9	9	1	9	1	9	2	9	9
1	2	1	1	3	2	3	1	1	1
3	1	1	3	1	3	3	2	3	3
3	2	1	3	3	3	3	2	3	3
2	2	1	1	2	3	2	1	2	3
3	2	3	1	3	3	3	3	3	1
3	1	2	1	1	1	2	9	2	3
2	2	2	2	2	2	2	2	2	2
2	2	2	2	2	2	2	2	2	2
9	9	9	1	9	9	2	2	2	2
2	2	2	1	1	3	3	3	3	3
2	2	2	2	2	2	2	2	2	2
2	2	2	2	2	2	2	2	2	2
9	9	9	2	2	2	2	2	2	2
1	3	9	1	1	1	2	1	3	1
1	3	9	1	1	3	9	9	9	9
1	3	9	9	1	9	9	9	9	9
1	1	2	3	1	3	3	3	3	3
1	3	3	3	1	3	3	3	3	3
1	9	9	9	1	9	9	9	9	9
2	2	2	3	1	3	3	3	3	3
1	3	3	2	1	3	9	9	9	9
1	1	2	2	1	3	2	2	2	2
1	3	3	3	1	3	3	3	3	3
1	3	3	3	1	3	9	9	9	9
9	9	9	9	1	3	9	9	9	9
1	3	3	3	1	3	3	3	3	3
1	3	3	1	1	3	3	3	3	3
1	9	9	3	1	3	9	9	9	9
1	3	3	3	1	3	3	3	3	3
1	9	9	3	1	3	9	9	9	9
1	9	9	9	1	3	9	9	9	9
1	3	3	3	1	3	3	3	9	9
1	3	9	1	1	3	9	9	9	9
1	3	3	3	1	3	3	3	3	3
2	1	9	9	1	9	9	9	9	9
1	3	3	2	1	3	3	3	3	3
9	9	9	1	1	3	9	3	3	3
1	3	3	3	1	3	9	9	9	9
1	3	3	3	1	3	3	3	3	3
1	3	3	3	1	3	9	9	9	9

1	9	9	9	1	9	9	9	9	9
1	9	2	3	1	3	2	2	2	2
1	3	3	2	1	3	2	3	2	3
1	1	1	1	1	1	1	1	1	1
1	3	3	3	1	3	9	9	9	9
1	3	3	3	1	3	9	9	9	9
1	1	1	2	1	3	1	1	1	1
1	1	2	2	1	2	2	2	2	2
1	3	3	2	1	3	9	9	9	9
1	9	9	3	1	3	3	3	3	3
1	3	3	3	1	3	3	3	3	3
2	1	2	3	1	3	3	2	3	3
1	3	3	3	1	3	9	9	9	9
1	3	3	3	1	3	9	9	9	9
1	3	3	3	1	3	9	9	9	9
1	3	9	3	1	3	9	9	9	9
1	9	9	3	1	3	3	2	9	9
1	9	9	9	1	3	9	9	9	9
1	3	3	3	1	3	9	9	9	9
9	9	9	3	1	9	9	9	9	9
9	9	9	9	1	3	1	3	2	1
1	1	9	2	1	3	9	9	9	9
1	1	2	3	1	3	2	2	3	3
1	3	3	2	1	3	9	9	9	9
1	2	1	1	1	3	3	3	3	3
3	3	3	1	1	1	1	1	1	1
3	3	3	1	1	3	3	1	3	3
1	3	3	1	1	3	3	2	3	3
1	3	3	2	1	3	2	3	3	3
9	9	9	3	1	3	9	9	9	9
1	3	3	3	1	3	1	1	1	2
1	3	3	3	1	3	2	3	3	3
1	3	3	3	1	3	9	9	9	9
1	9	9	2	1	3	9	9	9	9
1	9	9	3	1	3	9	9	3	9
1	1	1	3	1	3	3	2	3	3
1	9	9	2	1	3	2	2	2	2
1	3	1	2	1	3	3	3	3	3
1	9	9	3	1	3	3	3	3	3
1	1	3	3	1	3	3	3	3	3
1	1	3	3	1	3	3	3	3	3
1	3	3	2	1	3	3	3	3	3
1	1	9	2	1	3	9	9	9	9
1	1	1	3	1	3	3	3	9	9
1	3	3	3	1	3	2	3	3	3
1	3	3	3	1	1	3	3	3	3
1	9	9	3	1	3	3	3	3	3

1	3	3	9	1	3	3	1	3	3
1	1	3	3	1	3	3	3	3	3
1	3	3	2	1	3	9	3	3	3
1	1	1	9	1	3	3	3	3	3
1	1	9	3	1	3	3	3	3	3
1	9	9	3	1	3	3	3	3	3
9	9	9	3	1	1	3	3	3	3
1	9	9	1	9	3	9	9	9	9
1	9	9	1	9	3	9	9	9	9
3	3	3	2	1	3	3	3	3	3
2	1	2	3	1	3	1	2	3	3
9	9	9	9	1	9	9	9	9	9
2	9	9	3	1	3	3	3	3	3
2	2	2	2	1	1	2	3	3	3
2	2	2	3	3	3	3	3	2	3
2	1	1	3	1	3	3	3	3	3
9	9	9	9	9	9	9	9	9	9
9	9	9	1	1	3	9	3	3	3
1	2	3	3	1	3	3	3	3	2
1	1	1	1	1	3	3	3	1	1
3	1	3	2	1	3	3	3	3	3
2	2	3	3	1	3	3	2	1	2
1	2	2	2	1	3	2	2	3	3
1	1	1	3	1	3	3	3	3	3
9	9	9	1	1	3	3	3	3	3
2	3	1	1	1	3	3	3	3	3
2	2	1	3	2	2	3	3	2	2
3	1	2	3	1	1	1	1	3	3
1	2	2	3	1	3	3	3	3	3
1	1	2	2	1	3	3	2	9	3
2	2	2	3	1	3	3	1	2	3
1	3	2	1	1	9	9	9	9	9
1	1	2	3	1	2	3	3	2	2
9	1	9	1	1	3	3	3	3	3
1	2	2	2	1	3	2	2	3	3
1	2	2	1	1	3	3	1	3	3
1	9	9	1	1	3	1	3	1	3
2	1	2	1	1	3	1	3	3	3
1	9	9	1	1	3	1	3	1	3
3	1	2	1	1	3	3	3	3	3
1	1	1	3	1	3	3	3	3	3
1	1	2	3	1	3	3	3	3	3
2	2	2	3	1	3	3	2	2	3
2	2	2	1	1	2	2	2	2	2
2	2	2	3	1	3	3	2	2	1
1	1	2	3	1	3	3	3	3	3
9	9	9	1	1	9	9	1	3	1

1	3	3	3	1	3	3	9	3	3
1	1	2	1	1	3	2	2	2	3
1	1	2	3	1	3	3	3	3	3
2	2	2	3	1	3	2	2	3	3
9	1	1	3	1	3	1	3	3	3
3	3	3	1	3	1	1	1	1	2
2	2	2	2	1	3	3	2	3	3
1	1	2	3	2	2	3	3	2	2
1	1	2	1	1	3	3	3	3	3
1	1	3	3	1	3	3	3	3	3
1	1	2	3	1	2	3	3	3	3
1	3	3	3	1	3	3	3	3	3
1	1	2	3	1	3	3	3	3	3
9	9	9	2	1	3	3	2	3	3
2	1	2	2	1	3	3	3	3	3
9	9	9	9	1	9	9	9	9	9
2	1	2	3	1	3	3	3	3	3
9	9	9	2	1	1	2	2	2	2
1	3	3	3	1	3	3	3	3	3
1	1	2	3	1	3	3	2	3	3
9	9	9	1	9	9	9	9	9	9
9	9	9	9	1	3	3	3	3	3
2	2	2	3	1	3	3	9	9	9
1	1	3	1	1	1	1	3	3	3
3	2	3	3	1	3	3	2	3	3
3	2	3	9	1	3	3	9	3	3
9	1	9	3	1	3	1	1	3	3
9	9	9	9	9	9	9	9	9	9
9	9	9	9	9	9	9	9	9	9
2	3	1	3	1	3	2	2	3	2
9	9	1	3	1	3	3	3	3	3
3	1	3	3	1	3	3	3	3	3
1	9	9	3	1	3	3	3	3	3
1	1	9	9	1	3	3	3	3	3
1	3	3	2	1	3	9	9	9	9
1	9	1	2	1	3	3	3	3	2
1	1	1	3	1	3	3	3	3	3
2	3	3	2	1	3	2	3	3	2
1	3	3	3	1	3	3	3	3	3
1	1	1	3	1	3	3	3	3	3
1	3	2	3	1	3	1	3	3	3
1	9	9	3	1	3	2	3	3	2
1	1	3	3	1	3	3	3	3	3
9	3	9	2	9	3	9	9	9	9
1	3	3	3	1	3	3	3	3	3
1	3	3	3	1	3	3	3	3	9
1	9	9	3	1	3	3	3	3	3

1	3	3	3	1	3	3	3	3	3
1	1	3	2	1	3	9	3	9	3
9	9	9	9	9	9	9	9	9	9
9	1	9	3	1	3	2	3	3	1
3	2	9	1	9	1	1	3	3	3
3	1	1	3	1	3	3	3	3	1
9	9	9	9	1	1	1	2	2	2
9	9	9	3	1	3	3	2	3	3
9	9	9	1	9	9	9	9	9	9
9	9	9	9	1	9	9	9	9	9
1	1	1	1	1	3	3	3	3	3
1	1	1	3	1	3	2	3	2	3
3	1	1	3	1	3	2	3	3	3
9	9	9	9	1	9	9	9	9	9
1	1	1	3	1	1	1	3	1	1
3	1	1	3	1	3	3	3	3	3
9	9	9	9	1	9	9	9	9	9
3	2	9	3	1	3	3	3	3	3
9	9	9	3	1	3	3	3	3	3
3	3	2	3	1	3	3	1	3	3
1	1	1	3	1	3	3	3	3	3
9	9	9	3	1	3	3	3	3	3
9	9	9	3	1	3	3	3	3	3
1	1	1	3	1	3	3	3	1	3
1	9	9	1	1	9	9	3	9	9
2	1	2	1	1	1	1	1	1	1
9	9	9	9	1	3	2	3	3	3
3	1	1	2	1	1	1	2	3	2
9	1	1	1	1	1	1	1	1	1
3	1	1	1	1	1	1	1	1	1
3	1	2	2	1	3	3	2	3	2
2	1	1	3	1	3	3	2	1	2
2	2	3	3	1	3	2	3	3	2
3	1	3	3	1	3	3	3	3	3
3	1	1	3	1	3	3	3	3	3
9	9	9	9	1	9	9	9	9	9
9	1	9	9	1	3	3	3	3	3
9	1	9	9	1	9	9	9	9	9
1	2	2	2	1	3	9	1	3	3
3	3	2	1	1	3	3	3	3	3
3	1	2	3	1	3	3	3	2	3
2	2	2	9	9	3	3	3	3	3
9	9	9	9	1	9	9	9	9	9
9	9	9	9	1	9	9	9	9	9
9	1	9	3	1	3	2	3	3	3
9	1	9	9	1	3	9	1	9	9
9	1	9	3	1	3	9	9	9	9

3	3	3	3	1	3	3	3	3	3
3	1	1	9	1	9	9	9	9	9
9	1	9	1	1	1	1	3	3	3
3	1	1	2	1	3	2	1	3	3
1	1	3	3	1	3	3	3	3	3
9	9	9	2	1	3	2	3	3	3
2	1	2	3	1	3	3	3	3	3
2	2	2	1	1	3	3	1	3	3
3	3	3	3	1	3	9	3	3	3
3	1	1	2	1	3	2	2	2	3
3	3	3	3	1	3	1	3	3	3
9	9	9	3	1	9	9	9	9	9
9	9	9	3	1	3	3	3	3	9
9	9	9	9	1	9	9	9	9	9
9	1	9	9	1	9	9	9	9	9
9	9	9	9	1	9	9	9	9	9
9	3	3	3	1	3	3	3	3	3
2	1	2	1	1	3	2	2	2	3
9	9	9	3	1	3	3	3	3	3
1	2	2	3	1	3	3	3	3	3
3	1	1	3	1	1	2	1	3	3
3	2	2	3	1	3	3	2	3	2
3	2	2	3	1	3	3	2	3	1
2	3	3	2	1	3	2	2	3	3
3	1	2	1	1	3	2	3	3	3
9	1	9	9	1	9	9	9	9	9
3	9	3	3	1	3	3	3	3	3
2	2	2	1	1	3	3	3	3	3
9	1	1	3	1	3	3	3	3	3
9	9	9	3	1	3	3	3	3	3
3	2	3	3	1	3	2	3	3	3
9	1	9	9	1	9	9	9	9	9
9	9	9	3	1	3	3	3	3	3
2	2	3	1	1	1	3	3	2	1
9	9	9	2	1	3	3	3	3	3
1	3	3	2	1	3	3	3	3	3
1	2	2	3	1	3	3	3	3	3
1	3	3	2	1	3	2	2	2	3
1	9	9	3	1	3	3	9	9	9
1	3	3	3	1	3	3	3	3	3
2	3	2	2	1	2	3	2	3	2
3	1	2	2	1	2	3	2	9	2
1	1	1	3	1	3	3	2	3	3
1	1	2	3	1	3	3	3	3	3
1	1	1	3	1	3	3	2	3	3
9	9	9	9	1	9	9	9	9	9
1	1	1	3	1	3	3	1	3	3

3	1	3	3	1	3	3	3	3	3
2	1	2	3	1	2	3	3	3	3
1	1	1	3	1	3	3	3	3	3
3	1	1	3	1	3	3	3	3	3
2	2	2	1	1	1	2	3	3	2
3	1	2	2	2	1	2	2	3	3
3	1	1	1	1	3	3	3	2	3
2	1	2	3	1	3	1	2	3	3
3	1	2	3	1	3	2	3	3	3
2	1	2	1	1	1	1	1	2	2
2	1	2	3	1	3	3	3	3	3
2	1	2	1	2	2	2	2	2	2
3	1	1	3	1	3	1	1	3	3
3	1	2	3	1	3	3	3	3	3
2	1	2	1	1	2	2	2	2	2
3	1	1	1	1	3	3	3	3	3
2	2	2	1	2	2	2	3	3	3
2	2	2	2	2	2	2	2	2	2
2	2	2	2	2	2	2	2	2	2
2	1	1	3	1	3	3	3	1	3
2	1	2	2	1	1	3	3	1	3
2	3	2	3	1	3	3	3	1	2
3	2	1	3	1	3	3	2	3	3
1	1	1	3	1	3	3	3	3	3
2	1	1	2	1	3	2	3	3	3
1	3	2	3	1	1	1	2	2	2
1	2	1	2	2	2	2	2	2	2
2	1	1	9	1	3	2	3	3	1
2	1	1	3	1	3	2	3	3	1
1	1	1	3	1	1	3	3	3	3
2	1	3	2	1	2	2	3	3	2
1	1	1	2	1	3	3	3	3	3
9	1	9	9	1	9	9	9	9	9
9	9	9	9	1	9	9	9	9	9
9	1	9	9	9	9	9	9	9	9
9	9	9	9	1	9	9	9	9	9
9	1	9	9	1	9	9	9	9	9
9	1	9	9	1	9	9	9	9	9
9	9	9	9	1	9	9	9	9	9
3	1	3	3	9	9	9	9	9	9
2	1	1	2	1	3	3	2	3	3
9	9	9	9	1	9	9	9	9	9
9	9	9	9	1	9	9	9	9	9
3	1	2	3	1	1	3	3	3	2
9	1	9	9	1	9	9	9	9	9
9	9	9	9	1	9	9	9	9	9
9	9	9	9	1	9	9	9	9	9

3	1	1	3	1	3	3	3	3	3
1	2	3	1	3	2	1	1	3	2
9	9	9	9	1	9	9	9	9	9
9	1	9	9	1	9	9	9	9	9
9	1	9	9	1	9	9	9	9	9
9	1	9	1	9	9	9	9	9	9
9	9	9	9	1	9	9	9	9	9
9	9	9	9	1	9	9	9	9	9
9	9	9	3	1	3	3	3	3	3
9	9	9	9	1	9	9	9	9	9
3	1	1	3	1	3	3	1	3	3
3	1	1	3	1	3	3	3	3	3
2	1	1	2	1	3	3	3	3	3
3	1	2	3	1	3	3	3	3	3
3	1	1	3	1	3	3	1	3	3
3	1	1	3	1	3	3	3	3	3
3	1	1	3	1	3	3	3	3	1
1	1	1	3	3	1	1	1	2	1
1	1	1	3	1	3	3	3	3	3
1	1	1	3	1	3	3	9	3	3
1	1	1	1	1	3	1	3	3	3
1	1	1	1	1	3	1	3	3	3
9	9	9	9	1	9	9	9	9	9
3	1	2	3	2	2	9	1	2	2
3	1	1	1	2	3	1	2	9	1
2	1	1	3	1	3	3	3	3	3
3	3	3	9	9	9	9	9	9	9
3	1	2	3	1	1	3	3	3	3
3	1	1	2	1	3	3	2	3	3
3	1	2	3	1	3	2	3	3	2
3	1	1	3	1	3	2	3	3	3
3	1	3	1	1	3	3	2	3	2
3	1	1	3	1	3	1	3	3	3
3	1	2	1	1	3	3	2	3	3
2	1	1	1	1	1	3	3	3	3
3	1	1	3	1	3	3	3	3	3
3	1	1	3	1	3	3	3	3	3
3	1	3	3	1	3	3	3	3	3
3	1	1	3	1	3	3	1	3	3
3	1	2	3	1	1	1	2	3	3
2	1	3	1	1	3	3	3	3	3
1	1	1	2	1	3	1	3	3	3
2	1	1	3	1	2	3	1	3	3
3	3	3	3	1	3	3	3	3	3
3	3	3	3	1	3	3	3	3	3
3	1	3	3	1	3	3	3	3	3
2	1	2	2	1	3	3	3	3	3

9	9	9	3	1	3	3	3	3	3
2	1	1	3	1	3	1	1	3	3
3	1	1	3	1	1	1	1	3	3
3	1	3	3	1	3	3	3	3	3
3	3	3	3	1	3	3	3	3	3
3	1	3	3	1	3	3	3	3	3
2	1	2	2	1	3	3	3	3	3
9	1	1	3	1	3	3	3	3	3
3	1	2	3	1	3	1	1	3	3
9	1	9	3	1	3	3	1	3	3
1	1	2	3	1	3	3	3	3	3
1	9	9	3	1	3	9	3	3	3
1	1	2	3	1	1	1	3	3	1
1	1	2	3	1	1	1	3	3	1
1	1	3	3	1	3	3	3	3	3
2	2	2	2	2	3	3	3	3	3
1	1	1	1	2	2	3	3	3	3
2	1	2	1	2	1	1	2	2	2
3	2	1	1	3	1	1	3	2	3
2	1	2	3	1	3	3	3	3	3
1	1	1	3	1	3	1	3	3	3
3	1	1	3	1	3	3	3	3	3
1	1	1	3	1	3	1	3	3	3
1	1	1	3	1	3	1	3	3	3
1	1	1	3	1	3	1	3	3	3
3	1	1	3	1	3	2	3	3	3
3	3	2	3	1	3	3	3	3	3
3	9	2	3	1	2	1	3	2	2
2	2	1	3	1	3	3	3	3	3
2	1	2	2	1	3	2	3	3	3
3	2	2	2	2	3	3	2	3	3
2	2	2	3	1	3	1	3	3	3
3	1	1	3	1	1	3	1	2	9
3	1	2	3	1	1	1	3	3	3
1	1	1	3	1	3	1	3	3	3
3	1	3	3	1	3	3	3	3	3
3	1	2	3	1	3	3	3	3	1
3	1	2	2	2	1	3	1	3	3
3	1	1	1	1	3	1	3	3	1
3	1	1	1	1	3	3	3	3	3
3	1	1	3	2	3	2	2	2	2
3	1	3	3	1	3	3	3	3	3
3	1	3	3	1	3	3	3	3	3
3	1	1	3	2	1	2	2	3	2
3	1	1	3	1	3	3	2	3	3
3	3	3	2	1	3	2	2	3	3
3	1	3	1	1	1	3	3	3	3

3	2	2	1	1	3	3	3	3	3
1	2	2	2	2	2	2	2	2	2
3	1	1	3	2	1	2	3	2	1
3	1	1	1	1	3	3	3	3	3
3	1	1	1	1	3	3	2	3	3
2	2	2	3	1	3	3	3	3	3
2	1	2	3	3	3	3	3	9	2
3	1	2	2	1	3	2	3	2	3
2	2	2	2	1	3	3	3	3	2
3	1	1	2	1	3	3	2	3	3
3	1	2	3	1	3	3	3	3	3
2	1	1	3	1	3	3	3	3	3
9	1	9	1	9	9	9	9	9	9
1	2	2	1	1	3	3	1	3	3
3	1	1	1	1	1	3	3	3	3
2	2	2	3	1	3	1	2	2	2
2	1	2	3	1	3	2	1	3	3
1	1	1	3	1	3	3	3	3	3
1	1	2	3	1	3	3	3	3	3
2	1	1	1	3	3	3	3	3	3
3	1	1	2	1	3	3	3	3	3
3	1	1	3	1	1	1	1	3	3
1	1	1	3	1	3	3	3	3	3
2	1	1	3	1	3	3	2	3	3
3	1	3	2	1	1	9	3	9	3
3	1	1	3	1	3	3	3	3	3
3	1	1	3	1	3	3	3	3	3
1	2	1	9	9	9	9	9	9	9
9	2	9	1	9	2	9	9	9	9
9	2	1	2	1	2	9	2	9	2
2	3	2	3	1	3	3	3	1	2
1	1	1	3	1	3	3	3	3	3
3	1	1	3	1	3	3	3	3	3
2	1	1	1	1	3	2	3	3	3
9	1	9	9	1	9	1	9	9	9
3	1	1	2	1	1	1	1	3	1
2	2	2	2	1	1	2	2	2	2
9	9	9	9	2	9	1	9	9	9
1	2	1	1	1	1	2	2	1	1
1	2	2	3	1	3	3	3	3	3
9	1	9	3	1	3	3	3	3	3
2	1	1	3	1	3	3	3	3	3
2	1	1	3	1	1	3	3	3	2
2	9	9	9	1	9	9	9	9	9
3	1	1	1	2	1	2	1	3	3
3	1	2	3	1	3	2	2	2	2
3	1	1	3	1	1	1	1	3	3

3	1	1	3	1	3	3	3	3	3
2	1	1	2	1	3	3	2	2	2
2	2	2	1	1	3	2	3	3	3
1	1	2	3	1	3	2	1	2	2
3	2	2	1	2	2	2	1	2	3
9	1	2	3	1	3	3	3	3	3
1	1	2	1	1	3	1	1	3	3
1	9	9	9	9	9	9	9	9	9
3	1	1	1	2	1	2	2	2	2
2	1	1	3	1	3	3	2	3	3
2	2	3	2	1	1	2	2	2	3
9	1	1	3	1	3	2	2	3	3
1	1	1	3	3	3	3	3	3	3
2	2	2	2	1	2	2	2	2	2
3	2	3	3	1	3	1	3	2	2
3	1	3	3	1	3	3	3	3	1
2	1	1	2	1	3	3	3	3	3
9	9	9	1	1	1	9	1	1	2
9	1	9	3	1	3	3	3	3	3
1	1	3	2	1	3	1	2	9	1
1	1	3	2	1	3	1	2	3	1
1	1	1	1	1	3	1	1	3	3
2	2	2	2	1	3	3	3	3	3
1	1	2	3	1	3	3	3	3	3
2	1	3	3	1	3	3	3	3	3
1	9	2	1	1	3	1	1	1	1
1	2	2	1	1	3	3	3	3	3
1	2	2	1	1	3	2	3	3	3
1	2	2	1	1	3	2	3	3	3
9	9	9	1	9	2	9	9	1	9
1	2	2	1	1	3	2	3	3	3
3	2	1	1	1	2	1	2	3	3
1	1	1	3	1	3	3	2	3	3
1	2	3	3	1	3	3	3	3	3
1	1	2	1	1	9	3	3	3	1
1	1	1	2	1	2	2	2	2	2
1	1	2	2	1	3	3	3	3	3
1	1	2	9	9	9	9	9	9	9
1	1	1	3	1	3	3	3	3	3
1	1	3	2	1	3	3	1	1	3
1	2	2	3	1	3	3	3	3	3
1	1	1	3	1	1	3	3	3	3
2	2	2	3	1	1	2	2	2	2
1	1	2	3	1	3	3	3	3	3
1	2	3	3	1	3	3	1	3	3
1	1	3	3	1	1	3	3	3	3
1	1	1	3	3	1	1	1	1	1

1	1	3	3	1	1	1	1	3	1
1	1	3	3	1	1	1	1	3	1
3	3	3	1	1	3	3	1	3	3
1	1	1	3	1	3	3	2	3	3
1	1	3	3	1	1	1	1	3	1
1	1	2	3	1	1	1	1	3	1
1	1	1	9	1	3	3	3	3	3
3	2	2	2	1	3	3	3	3	3
3	1	1	3	1	3	3	3	3	3
2	1	1	2	1	3	3	3	3	3
3	1	1	3	1	3	3	3	3	3
9	1	1	3	1	3	3	3	3	3
1	1	1	3	1	3	3	2	3	3
2	1	2	3	1	3	3	2	3	3
3	1	1	3	1	3	3	3	3	3
3	1	1	3	1	3	3	3	3	3
2	1	1	3	1	2	3	2	1	1
2	1	2	2	1	1	1	2	3	1
3	1	3	3	1	3	3	3	3	3
3	3	1	3	1	1	3	3	3	3
1	1	1	2	1	3	1	2	1	2
9	1	9	3	1	3	3	2	3	3
9	1	9	9	1	9	9	9	9	9
9	1	9	9	1	9	9	9	9	9
9	9	9	9	1	9	9	9	9	9
9	9	9	3	1	3	3	3	3	3
3	1	1	9	1	3	3	3	3	3
9	1	9	9	9	9	9	9	9	9
9	9	9	1	9	9	9	9	9	9
9	1	9	9	9	9	9	1	9	9
9	1	9	9	9	9	9	9	9	9
9	1	9	9	9	9	9	9	9	9
9	9	9	9	9	9	9	9	9	9
1	1	1	2	3	3	3	3	3	2
1	3	2	3	1	1	1	2	2	2
2	1	1	2	1	1	3	1	3	3
1	1	2	3	1	3	3	3	3	3
1	1	1	3	1	1	3	3	3	3
1	2	2	1	1	3	3	3	9	3
1	2	2	1	1	3	3	3	3	3
1	1	2	3	1	3	9	3	3	3
3	1	1	3	1	3	3	3	3	3
2	1	1	2	1	3	1	9	3	2
2	1	1	1	1	3	2	3	3	3
2	1	1	1	2	3	2	2	2	3
1	1	1	3	3	1	1	1	1	1
2	1	1	3	1	3	3	3	3	3

1	1	2	1	1	1	2	3	3	1
2	2	3	3	3	1	1	1	3	3
3	3	3	3	1	3	3	3	3	3
1	3	2	2	3	3	3	3	3	3
1	1	1	1	1	3	1	1	3	1
2	2	1	2	1	3	1	2	3	3
2	2	2	9	9	9	9	9	9	9
2	2	2	2	1	1	2	2	3	3
2	1	1	2	1	3	3	1	2	2
2	2	2	3	3	1	2	3	1	2
2	2	2	1	2	1	3	3	3	3
2	1	1	3	1	3	3	3	3	3
3	3	3	1	1	1	1	2	1	1
3	3	2	2	2	3	3	1	2	3
2	2	2	3	1	2	1	3	3	3
2	1	1	2	3	3	3	3	3	1
1	1	1	3	2	2	3	3	3	3
1	2	1	1	1	1	2	1	2	1
1	1	1	2	2	3	2	2	1	2
1	1	1	1	1	1	1	1	1	1
3	3	1	1	1	1	1	1	1	2
3	2	2	2	2	2	2	2	2	2
2	1	1	9	9	9	9	9	9	9
2	1	1	3	1	2	3	9	1	2
1	1	1	3	1	1	1	1	3	1
1	3	2	2	2	3	3	2	2	1
2	1	1	3	1	3	2	3	2	3
1	1	1	1	1	3	1	3	3	3
1	2	2	1	2	2	2	2	2	2
9	1	9	1	1	3	3	1	3	3
9	1	9	3	1	3	9	3	3	3
1	3	2	1	1	1	1	1	1	1
2	3	2	3	2	2	9	1	2	3
2	1	1	3	1	3	3	3	3	3
3	3	3	9	1	1	1	3	2	3
2	2	2	9	9	9	9	9	9	9
2	2	2	2	2	2	2	2	2	2
3	1	2	1	1	3	1	2	1	3
3	3	1	1	2	2	3	2	2	3
2	1	1	3	1	3	3	3	3	3
2	2	2	1	1	3	2	2	3	3
2	2	2	1	1	3	2	2	3	3
9	9	9	1	9	9	9	9	1	9
9	9	9	1	9	1	9	9	9	9
9	9	9	1	9	2	9	9	9	9
3	2	1	1	2	1	1	1	1	1
3	1	2	2	2	2	2	2	1	1

1	3	3	3	3	3	3	1	3	3
1	3	3	3	3	3	3	1	3	3
1	3	3	3	3	3	3	1	3	3
2	2	2	2	2	1	1	1	1	2
2	2	2	1	3	2	2	2	2	2
9	2	2	1	2	2	1	3	1	2
2	3	1	2	3	2	2	1	2	2
3	2	3	1	2	1	2	1	2	2
2	1	2	1	2	1	3	1	2	1
2	2	2	1	3	1	2	1	2	2
2	2	2	1	3	1	1	1	1	1
2	2	2	1	3	2	2	3	3	3
2	2	2	2	3	1	1	1	3	3
1	3	3	3	3	2	1	3	3	3
2	2	2	1	2	2	2	2	1	1
3	2	2	1	3	1	2	3	3	3
2	2	3	1	3	1	2	1	1	1
2	2	3	1	3	1	2	1	1	1
1	3	3	3	3	3	3	1	3	3
1	3	3	3	3	3	3	1	3	3
2	2	2	2	2	2	2	2	2	2
1	3	3	2	2	2	2	2	2	2
1	3	3	2	2	2	2	2	2	2
2	2	2	2	2	2	2	2	2	2
2	2	2	2	2	2	2	2	2	2
1	3	3	3	1	2	2	2	2	2
1	3	3	3	1	2	2	2	2	2
9	9	9	3	1	3	3	3	3	3
2	2	2	1	3	1	1	1	2	2
3	2	1	1	3	3	3	3	3	3
3	2	1	1	3	3	3	3	3	3
3	2	1	1	3	3	3	3	3	3
3	2	1	1	3	3	3	3	3	3
9	3	1	2	3	9	1	1	1	3
9	1	3	1	1	1	3	2	3	1
1	1	3	1	2	1	3	1	3	2
2	2	2	1	3	2	2	3	3	2
1	3	3	1	3	1	2	1	2	3
2	1	1	1	2	1	2	1	1	2
3	2	1	1	3	2	1	3	1	2
3	1	1	1	9	2	2	3	1	3
3	2	3	3	2	1	2	1	2	1
1	3	3	3	1	2	9	2	1	1
9	9	9	3	9	9	9	9	9	9
9	9	9	9	9	9	9	9	9	9
1	3	3	3	1	3	3	3	3	3
9	1	2	1	3	3	2	1	9	2

9	1	2	1	3	3	2	1	9	2
3	1	1	3	1	1	1	1	1	1
2	2	2	1	9	3	9	9	9	9
2	2	2	2	2	2	2	2	2	2
2	2	2	1	2	1	3	3	2	2
9	9	9	1	9	9	9	9	9	9
9	9	9	2	2	2	9	9	9	9
9	1	9	9	3	9	9	9	9	9
2	1	1	1	3	1	1	2	2	2
3	1	2	1	3	3	3	2	3	2
2	2	2	3	3	1	2	1	1	1
2	2	2	9	3	3	3	3	3	3
9	9	9	1	9	2	9	9	9	9
9	9	9	1	3	9	9	9	9	9
2	2	2	1	3	3	3	2	2	2
9	9	9	3	2	1	1	1	3	2
2	2	1	2	3	3	3	3	3	3
9	9	9	9	9	9	9	9	9	9
3	1	1	3	3	1	1	3	1	2
9	9	9	1	9	9	9	9	9	9
3	1	1	2	2	2	9	1	1	1
3	1	1	2	2	2	2	2	2	2
3	1	1	1	3	1	1	1	1	1
3	1	1	1	3	1	1	1	1	1
3	1	1	2	2	2	2	2	2	2
3	1	1	3	1	2	2	2	2	2
2	2	2	1	1	2	2	2	2	1
9	9	9	1	9	9	9	9	9	9
9	9	9	1	9	9	9	9	9	9
9	9	9	1	9	9	9	9	9	9
9	9	9	1	9	9	9	9	9	9
9	9	9	1	9	9	9	9	9	9
9	9	9	3	9	2	9	9	9	9
1	1	2	1	1	2	1	1	1	1
2	3	1	1	1	1	2	3	1	2
3	1	9	9	9	9	9	9	9	9
3	2	2	2	2	3	3	1	2	2
9	9	9	9	3	9	9	9	1	9
3	1	1	1	3	3	3	3	3	3
3	9	9	2	3	3	3	1	3	3
2	2	2	1	3	3	3	3	3	3
9	9	9	9	3	9	9	9	9	9
1	3	3	3	1	3	3	3	3	3
1	1	2	3	3	3	1	1	3	3
9	9	9	1	9	9	9	9	9	9
2	3	2	1	9	2	2	9	2	1
3	9	9	9	3	3	3	3	3	3

9	9	9	1	9	9	9	9	9	9
9	9	9	1	9	9	9	9	9	9
3	1	1	1	2	2	2	1	2	1
9	9	9	1	9	9	9	9	9	9
3	2	2	1	3	3	3	3	3	3
9	9	9	1	9	9	9	9	9	9
2	2	2	1	3	3	2	1	2	3
1	3	3	1	2	1	2	1	3	1
3	2	1	1	1	3	3	3	3	3
1	2	3	1	2	1	2	3	3	2
3	1	1	2	2	2	2	2	1	3
2	2	2	2	2	3	3	3	3	3
1	1	2	3	3	3	2	2	3	3
2	1	2	2	3	1	2	1	1	2
3	1	1	1	3	1	2	2	2	2
2	2	3	2	1	1	1	2	2	2
2	3	2	1	3	1	1	2	2	1
3	2	2	1	3	3	3	3	3	3
1	2	1	1	2	1	2	3	2	1
1	2	1	1	2	1	2	3	2	3
9	9	9	9	9	9	9	9	9	9
9	9	9	1	9	9	9	9	9	9
3	9	9	1	9	9	9	9	9	9
9	9	9	1	9	9	9	9	9	9
9	9	9	9	9	9	9	9	9	9
9	9	9	9	9	9	9	9	9	9
9	9	9	2	9	9	9	9	9	9
9	9	9	2	9	9	9	9	9	9
9	9	9	2	9	9	9	9	9	9
9	9	9	2	9	9	9	9	9	9
9	9	9	2	9	9	9	9	9	9
9	9	9	2	9	9	9	9	9	9
9	9	9	2	9	9	9	9	9	9
3	9	1	3	3	1	1	2	1	2
9	9	9	1	3	1	1	2	2	1
3	1	1	1	3	2	1	1	1	2
2	2	2	3	9	9	9	3	3	3
9	9	9	1	9	9	1	9	9	9
3	2	3	3	3	1	1	1	2	2
9	9	9	1	9	1	9	9	9	9
9	9	9	1	9	1	9	9	9	9
9	9	9	1	9	1	9	9	9	9
9	9	9	1	9	1	9	9	9	9
9	9	9	1	9	1	9	9	9	9
2	2	2	2	2	1	1	2	1	2
3	2	1	2	3	2	2	1	2	1
2	1	2	2	2	2	2	2	2	2

2	2	2	2	2	2	2	2	2	2
1	2	2	3	3	2	2	2	3	3
2	1	1	1	2	3	3	2	2	1
3	2	2	2	1	1	1	1	1	1
9	9	9	1	9	1	9	9	9	9
9	9	9	2	9	9	2	9	9	9
9	9	9	1	9	9	2	9	9	9
9	9	9	9	9	1	3	9	9	9
9	9	9	1	9	9	9	9	2	9
9	2	9	1	9	9	9	2	9	9
9	2	9	1	9	2	9	2	9	3
1	9	9	3	9	2	9	9	9	9
2	9	9	2	2	2	2	2	2	9
9	9	9	9	2	9	9	9	9	9
9	9	9	1	1	9	1	9	9	9
2	2	2	2	2	2	2	2	1	1
1	3	2	3	2	2	3	2	3	1
9	1	9	1	9	9	9	9	9	9
2	2	2	3	9	9	9	9	9	9
1	1	1	2	1	1	3	3	3	2
2	2	2	1	3	3	3	3	3	3
9	9	9	1	2	2	9	1	2	1
3	2	3	3	3	3	3	3	3	3
2	2	2	1	2	2	2	2	2	2
9	9	9	3	2	1	2	1	1	1
2	9	1	9	3	3	2	9	9	2
2	9	9	1	9	9	9	9	9	9
1	2	2	1	1	2	3	1	3	1
2	2	2	2	2	3	2	2	2	2
2	2	1	1	3	2	3	3	3	3
2	2	2	1	3	2	1	3	2	1
9	9	9	3	2	2	2	2	2	1
3	9	9	9	3	3	3	2	3	3
2	2	2	2	2	2	2	2	2	2
9	1	9	9	3	9	9	9	9	9
1	1	2	3	2	3	1	9	2	3
3	1	2	3	1	3	3	3	3	1
2	2	2	3	3	3	3	3	3	3
1	1	1	3	3	3	3	2	3	3
2	2	1	1	3	2	2	3	3	3
2	1	2	1	2	2	1	1	2	3
1	1	1	1	3	3	3	3	3	3
1	3	3	1	3	3	1	2	2	2
3	9	9	1	3	1	1	3	1	3
9	9	9	9	9	9	9	9	9	9
1	3	2	1	2	3	2	2	1	1
3	3	3	3	3	3	3	1	3	3

3	1	1	3	3	3	3	3	3	3
3	1	2	1	2	2	2	2	2	2
3	1	1	1	3	1	1	2	2	2
3	3	2	1	2	3	2	1	1	1
2	2	2	1	2	3	3	2	3	3
2	1	1	1	3	2	2	2	1	2
2	2	2	1	3	1	3	2	3	2
1	1	2	1	2	1	2	3	1	2
2	2	2	3	3	9	9	9	9	9
2	2	1	1	3	3	3	3	3	3
2	2	2	1	3	3	3	2	2	2
1	1	2	3	3	1	3	3	3	3
1	2	1	2	2	3	3	3	3	3
2	1	2	3	3	3	3	1	3	3
3	1	2	2	3	3	2	3	3	3
2	1	1	1	3	3	2	3	3	1
1	3	3	3	3	3	2	3	3	3
2	2	2	2	2	2	2	2	2	2
2	2	2	2	2	2	2	2	2	2
2	2	2	2	2	2	2	2	2	2
2	2	2	2	2	2	2	2	2	2
2	2	2	2	2	2	2	2	2	2
2	2	2	2	2	2	2	2	2	2
2	2	2	2	2	2	2	2	2	2
2	2	2	3	3	2	2	2	2	2
9	9	9	2	9	2	9	9	9	9
3	1	1	1	3	1	2	1	2	1
3	3	1	2	3	3	3	3	3	3
1	2	3	2	1	1	2	1	2	1
9	1	9	9	9	9	9	9	9	9
3	1	1	1	1	1	1	1	1	1
3	2	2	1	3	3	2	3	3	3
2	3	1	2	3	2	1	2	3	3
1	2	3	1	3	3	3	1	1	2
2	1	2	2	3	3	3	3	3	3
9	9	9	2	1	2	2	2	1	1
3	2	2	1	3	3	3	3	3	3
1	2	1	2	2	1	1	2	2	3
2	2	2	2	3	2	1	2	1	1
2	2	2	1	3	2	2	2	2	2
9	9	9	9	9	9	9	9	9	9
1	1	3	1	1	3	1	2	2	1
3	1	1	2	3	3	3	2	3	3
3	3	2	3	3	3	2	3	2	2
3	1	1	1	1	3	2	1	3	1
2	2	2	1	1	1	3	2	1	2
2	2	2	1	2	3	3	3	3	3

3	1	1	3	1	2	1	1	3	2
3	3	3	3	3	3	3	3	3	3
1	3	2	1	2	1	1	1	3	3
2	2	2	2	2	2	2	2	2	2
2	2	2	2	3	3	2	3	3	3
9	9	9	1	9	9	9	9	9	9
2	2	2	3	3	2	2	3	3	3
3	1	3	1	2	1	2	3	3	3
2	2	2	2	2	2	1	2	1	2
2	2	2	1	2	1	2	2	1	2
2	2	2	1	3	2	2	2	2	2
1	1	1	1	1	3	3	3	3	3
9	9	2	1	3	3	2	2	3	3
2	2	2	1	3	2	2	2	2	2
2	2	2	1	1	3	2	2	2	1
2	2	2	2	2	3	2	2	2	3
2	3	2	1	9	9	9	9	9	9
1	2	2	2	3	3	2	2	1	2
3	3	1	2	3	1	1	3	3	3
9	1	9	1	9	9	9	9	9	9
2	2	2	1	1	3	2	3	2	3
2	2	2	1	2	2	2	2	2	1
2	3	3	3	1	3	3	3	3	2
2	2	2	3	3	3	3	3	3	3
2	2	2	1	3	2	1	2	1	2
1	1	1	2	1	3	3	3	3	3
2	1	2	1	2	3	2	2	2	2
3	1	1	3	1	3	3	1	3	3
2	2	2	3	3	3	2	2	3	3
1	3	2	1	3	1	3	1	3	2
2	2	1	1	3	3	3	3	3	3
9	9	9	1	9	9	9	9	9	9
2	2	1	3	3	3	3	1	3	3
3	3	2	3	1	3	3	3	1	1
9	9	9	1	9	9	9	9	9	9
2	2	2	1	2	2	2	2	2	2
3	2	2	1	3	3	3	3	3	2
2	2	2	2	3	3	2	1	3	3
2	2	2	2	3	2	2	2	2	2
3	1	1	2	2	2	2	2	2	2
3	2	2	1	1	2	2	2	2	2
2	2	2	2	2	2	2	2	2	2
2	2	2	3	3	3	3	3	3	3
2	2	2	1	2	2	2	2	2	2
2	2	2	3	2	2	2	2	2	2
2	2	2	1	3	1	2	2	2	2
2	1	1	3	2	2	3	2	3	1

9	9	9	9	9	9	9	9	9	9
3	1	3	1	3	1	3	9	3	3
9	9	1	2	3	2	2	3	3	3
9	9	9	3	9	3	3	3	3	9
2	2	2	2	1	2	3	1	2	3
1	3	3	3	2	2	3	3	3	3
9	9	9	9	3	9	9	9	9	9
1	1	3	3	3	3	3	1	2	3
3	1	3	1	3	3	3	3	3	3
9	9	9	1	1	3	2	2	3	2
2	9	3	2	3	2	2	2	2	2
3	1	2	2	3	3	3	3	3	3
3	3	3	3	1	3	3	3	3	3
9	9	9	9	3	9	1	9	9	9
1	1	3	3	3	3	3	3	3	3
2	1	2	1	3	2	1	2	2	2
3	1	3	1	3	3	3	3	1	1
9	9	9	1	3	3	2	3	3	3
2	2	1	3	3	3	3	3	3	3
9	9	9	1	2	2	2	2	2	2
2	3	1	2	3	3	3	3	3	3
9	9	9	1	3	2	3	3	3	3
9	9	9	3	3	3	9	3	3	3
1	1	2	3	3	3	3	1	3	3
3	1	1	1	3	1	3	3	3	3
3	2	3	1	3	1	3	3	3	3
2	2	2	3	3	3	1	2	3	3
9	9	9	1	9	9	9	9	9	9
1	3	2	2	3	3	3	3	3	3
2	1	1	3	3	3	9	3	3	3
1	3	3	2	1	1	2	3	2	2
2	2	2	1	3	3	3	3	3	3
3	2	1	1	2	2	1	3	2	2
9	1	9	3	3	3	3	3	9	2
1	3	2	3	3	3	3	3	3	3
2	1	2	3	3	3	3	2	2	2
9	9	9	1	9	9	9	9	9	9
9	9	9	3	3	3	3	2	3	3
9	9	9	9	9	9	9	1	9	9
3	2	2	3	1	3	3	3	1	1
9	9	9	3	3	2	9	9	9	9
3	1	1	1	3	1	3	3	1	3
1	2	3	1	3	3	3	3	3	3
1	2	1	3	3	3	3	9	3	3
9	9	9	3	3	3	3	3	3	3
9	9	9	2	9	1	9	9	9	9
9	1	1	9	3	9	9	9	9	9

2	2	2	9	3	9	3	9	1	3
1	1	2	3	3	2	1	2	1	1
9	1	9	9	3	9	9	9	9	9
3	2	2	9	9	9	9	9	9	9
3	2	2	3	3	3	3	3	3	3
9	9	9	1	2	3	1	1	9	3
9	9	1	9	9	9	9	9	9	9
1	2	3	9	3	3	1	3	3	3
2	2	2	3	3	3	3	3	2	2
1	1	1	2	3	3	3	1	3	3
3	2	2	2	3	3	3	3	3	3
2	1	1	9	3	3	1	3	3	3
1	2	2	1	3	3	3	3	3	3
3	1	9	2	3	1	2	1	3	3
3	1	2	3	3	3	3	2	3	2
3	1	2	3	3	3	3	2	3	3
3	1	2	1	3	3	3	3	3	3
1	2	3	3	3	3	1	1	3	3
9	9	9	3	3	3	9	9	9	9
3	1	2	9	1	1	2	2	1	1
1	2	1	1	3	3	3	3	3	3
9	9	9	2	9	9	9	9	9	9
1	1	3	1	3	3	3	3	3	3
3	9	9	1	9	3	2	3	3	3
3	2	1	3	1	3	3	3	3	3
9	9	9	3	2	1	1	1	1	1
1	1	3	3	3	3	3	3	3	3
1	3	3	3	3	3	3	3	3	3
9	9	9	3	3	3	3	3	3	3
9	1	9	9	3	9	9	9	9	9
9	1	9	1	3	3	3	3	3	3
9	9	9	1	3	1	3	1	3	3
2	2	2	1	3	2	2	2	2	2
2	2	2	2	3	3	1	2	2	3
2	2	2	3	3	2	3	3	3	2
9	9	9	3	3	3	9	3	3	3
9	1	9	9	9	1	3	3	1	3
3	9	9	1	3	9	9	9	9	9
9	9	9	9	2	2	9	2	9	9
9	1	9	9	3	9	9	9	9	9
9	9	9	9	3	9	9	9	9	9
9	9	9	2	3	2	3	3	3	3
9	9	9	1	3	3	3	3	3	3
3	2	2	3	2	3	3	3	3	3
2	3	2	3	3	3	3	3	3	3
2	3	2	2	3	3	3	3	3	3
3	2	1	3	3	3	3	2	3	3

9	9	9	1	3	1	1	1	1	1
3	3	3	3	3	3	3	3	3	3
3	1	3	1	1	3	3	3	3	3
1	2	2	3	3	3	1	1	3	3
1	1	1	3	3	3	1	3	3	3
3	9	9	9	3	9	9	9	9	9
2	1	2	1	3	1	3	3	3	3
9	9	9	2	9	9	9	9	9	9
9	9	9	9	3	9	9	9	9	9
9	9	9	9	3	9	9	9	9	9
2	2	2	2	3	3	2	2	2	2
3	1	2	3	3	3	3	3	3	3
2	2	2	2	2	2	2	2	2	2
2	3	2	2	3	3	3	3	3	3
3	9	9	1	9	9	9	9	9	9
3	2	3	2	3	3	1	2	3	2
3	1	2	2	3	3	2	2	2	3
2	2	2	9	9	9	9	9	9	9
1	1	2	2	3	2	2	3	3	2
3	1	3	3	3	3	1	1	3	3
9	9	9	1	3	3	3	1	3	1
3	1	2	3	3	3	2	3	1	3
1	3	3	3	3	3	3	3	3	3
9	9	9	9	3	9	9	9	9	9
1	1	1	3	3	3	3	3	3	3
3	1	2	3	3	3	3	3	3	3
2	1	3	1	3	3	2	3	2	3
1	2	2	1	3	3	3	3	3	3
2	1	2	1	3	3	3	1	3	2
2	1	2	3	3	3	3	3	3	3
9	1	2	1	3	3	1	3	3	3
9	9	9	3	3	3	3	3	3	3
2	2	2	3	3	3	3	3	1	1
2	1	2	1	3	3	3	3	3	3
1	1	1	3	3	3	3	2	3	3
9	9	9	9	3	9	9	9	9	9
3	1	1	3	3	3	3	3	3	3
3	1	1	3	3	3	3	3	3	3
1	1	2	3	3	1	3	1	3	3
9	9	2	3	1	2	9	2	9	2
1	1	3	1	3	1	1	2	1	2
3	3	3	3	3	3	3	3	3	3
2	3	3	3	3	3	2	3	3	3
2	1	2	3	3	3	2	2	2	3
3	1	2	3	3	3	3	3	3	3
1	2	2	1	3	1	1	2	3	3
9	9	9	1	3	3	3	3	3	3

3	9	9	9	3	9	9	1	9	9
1	1	1	1	3	3	3	3	2	3
9	9	1	1	3	3	2	2	3	3
3	1	3	2	3	3	3	2	3	3
9	1	1	9	3	9	9	9	9	9
1	2	3	1	1	2	1	3	2	3
1	3	3	3	3	1	1	2	2	2
9	9	9	3	3	3	3	3	1	3
2	2	2	2	2	2	2	2	2	2
1	1	2	3	3	3	3	3	3	3
9	9	9	9	3	9	9	9	9	9
3	2	1	3	3	3	3	3	3	1
2	2	2	2	2	2	9	3	2	2
2	1	2	1	3	3	3	3	3	3
2	1	2	1	3	3	3	3	3	9
2	1	2	3	3	3	2	3	3	3
3	1	3	3	3	3	3	3	3	3
1	3	3	1	2	1	1	2	2	1
9	9	9	1	3	9	9	9	9	9
1	3	3	1	3	2	3	1	2	2
2	2	2	3	3	3	1	2	3	3
2	2	2	3	3	3	1	2	3	3
3	1	2	3	3	1	3	3	3	3
9	1	9	9	3	9	9	9	9	9
1	3	3	3	3	3	3	3	3	3
2	9	2	3	3	3	3	3	3	3
3	2	1	3	3	3	3	3	3	3
2	2	2	1	1	3	1	2	2	2
3	2	2	3	3	3	3	2	3	3
2	3	2	3	3	3	3	3	3	3
3	9	9	9	3	9	9	9	9	9
9	9	9	3	3	3	3	3	3	3
3	1	1	1	2	3	3	2	3	3
9	9	9	1	3	1	1	3	1	3
3	1	1	3	1	2	1	2	1	3
9	9	9	3	2	2	1	2	2	2
3	1	1	1	9	1	1	1	1	1
1	3	3	2	2	2	2	9	1	9
9	9	9	1	3	3	3	3	1	1
3	1	2	3	3	3	3	3	3	3
2	2	2	3	1	2	2	2	2	2
2	2	2	3	1	3	3	3	3	3
2	1	2	9	9	9	9	9	9	9
3	1	1	3	3	3	1	3	3	3
3	9	9	3	3	3	3	3	3	3
9	9	3	1	3	3	3	3	3	3
1	1	1	2	3	3	3	1	3	3

9	1	9	1	3	3	3	3	3	3
3	2	1	3	3	3	3	1	2	3
1	1	1	2	3	3	3	3	3	3
2	1	2	2	3	3	3	3	3	3
1	2	1	2	3	2	1	2	3	3
1	1	3	1	3	3	3	2	3	3
9	9	9	3	3	3	3	3	3	3
9	9	9	3	3	3	3	3	3	3
2	1	1	3	3	3	3	2	3	3
3	1	2	2	3	3	3	3	3	3
1	3	3	2	3	3	3	3	3	3
1	1	1	2	3	3	3	3	1	1
1	1	2	3	3	3	3	3	3	3
1	3	3	2	3	3	3	3	3	3
1	1	3	3	3	3	3	3	3	3
1	1	1	3	3	3	2	3	3	3
3	1	3	3	3	2	3	1	2	2
9	9	9	3	3	3	3	3	3	3
9	9	9	1	9	1	9	1	3	2
2	1	1	2	2	1	3	1	3	3
2	3	3	1	2	2	1	3	3	2
1	1	2	9	3	3	3	2	1	2
2	3	1	3	3	3	2	2	3	3
9	9	9	9	3	9	9	9	9	9
9	9	9	2	9	1	2	3	2	1

BSEdisliket	BSElumpse	CaBreastCc	CaBreastfr	CaBreastTr	BSEdetects	EverDoneB	StandMirrc	Liedown	RhandtoLb
3	3	1	1	2	2	2	2	2	3
2	2	2	2	2	2	3	2	3	2
2	2	3	3	3	3	2	2	2	2
3	3	2	2	2	2	2	2	2	2
2	3	3	2	2	1	1	2	2	2
2	2	2	2	2	2	2	2	2	2
2	2	1	2	2	2	2	2	2	2
1	3	3	3	3	3	1	2	2	3
1	1	3	2	1	3	1	2	3	2
2	3	1	2	2	3	2	1	2	1
2	1	3	3	1	3	2	2	2	2
2	1	3	2	2	1	1	1	2	2
2	1	1	2	1	3	1	1	1	1
1	1	1	2	2	2	2	2	1	2
2	1	2	2	2	2	2	2	2	2
3	3	2	2	2	2	2	2	2	2
1	1	1	1	1	1	9	9	9	9
1	3	1	3	2	1	2	1	1	1
1	1	1	1	1	1	2	3	2	2
3	1	2	1	3	1	1	2	1	2
3	1	1	1	3	1	1	1	1	1
3	1	1	1	2	1	1	1	1	1
3	1	1	1	3	1	1	1	1	1
1	1	2	1	2	1	1	1	1	1
2	2	3	3	2	2	2	2	2	2
9	1	1	2	3	2	2	1	3	9
3	1	1	1	1	1	9	9	9	9
3	1	1	1	3	1	3	1	1	2
1	2	1	2	3	2	1	2	3	2
1	1	2	1	3	1	1	2	1	1
2	2	1	2	2	1	2	3	2	2
3	1	1	2	2	1	3	1	1	1
3	1	1	2	2	1	9	9	9	9
1	1	1	1	2	1	1	9	1	3
1	2	1	2	1	1	1	2	1	2
3	2	3	3	3	3	3	2	3	1
1	1	1	1	3	1	1	3	1	2
2	1	1	1	3	1	1	2	1	2
3	1	1	1	2	1	3	1	1	1
1	3	1	2	2	3	1	2	2	2
2	1	2	2	2	2	9	9	9	9
9	9	9	9	9	2	9	9	9	9
9	9	9	9	9	9	9	9	9	9
2	2	1	2	2	1	2	2	2	2
2	1	1	1	2	1	2	1	2	2
9	9	9	9	9	9	9	9	9	9

1	1	1	2	2	1	1	1	2	2
1	1	1	1	2	1	1	1	2	2
1	1	2	2	1	1	1	1	2	2
2	1	1	1	2	1	1	1	2	1
3	1	3	1	9	1	3	1	1	3
3	1	3	1	9	1	3	1	1	3
2	1	3	2	1	2	3	2	3	3
3	1	1	1	1	1	2	2	2	2
1	3	1	1	1	1	1	9	1	9
1	2	1	3	1	1	2	1	3	3
9	9	2	9	9	9	9	1	9	9
2	1	1	2	2	2	1	2	2	2
2	2	2	2	1	1	3	3	3	3
1	2	1	1	2	2	1	1	2	2
1	1	1	2	2	2	1	1	2	2
2	2	1	2	2	2	1	2	2	1
3	2	2	2	3	1	1	2	2	3
3	1	3	1	2	1	2	3	2	3
2	1	1	2	2	1	2	2	2	2
3	1	3	3	3	3	9	9	9	9
2	2	2	2	2	2	2	2	2	2
1	1	2	2	2	3	2	2	1	2
1	1	2	2	1	1	1	1	2	2
2	2	1	2	2	2	1	2	2	3
1	2	1	2	3	2	1	2	3	1
3	1	2	2	2	2	1	3	2	2
3	1	1	1	1	3	3	1	1	1
2	1	1	2	3	2	1	2	2	1
1	1	1	1	1	1	2	2	1	1
3	1	3	1	1	1	3	3	1	3
9	9	9	9	9	9	9	9	9	9
3	1	1	3	3	1	3	1	1	1
9	1	9	9	9	1	1	2	1	9
3	1	2	1	3	3	2	2	2	2
1	9	1	3	3	3	3	3	1	3
2	1	1	1	1	2	2	2	2	2
1	1	1	2	2	3	1	3	2	1
2	2	2	2	1	2	1	1	2	2
2	1	1	1	2	1	3	3	3	3
2	1	2	2	2	1	1	1	2	2
1	1	1	1	3	3	2	3	1	2
9	9	9	9	9	9	9	9	9	9
3	1	1	1	1	1	1	2	1	3
2	2	2	2	1	2	2	2	2	2
1	1	2	1	3	1	1	3	1	2
2	3	3	3	2	2	3	3	2	2
3	2	1	1	2	1	2	2	2	2

1	3	1	2	3	1	2	1	3	1
1	1	1	1	1	1	3	1	1	1
2	1	1	1	1	2	1	2	2	2
1	2	2	2	2	1	2	1	1	1
2	3	1	2	2	2	1	2	2	1
1	1	1	1	2	1	1	2	1	2
2	1	2	2	2	1	1	2	2	2
3	3	1	3	3	1	1	3	3	1
2	1	1	1	2	1	2	3	1	2
2	1	1	1	2	1	3	3	1	2
1	1	1	2	2	3	1	3	2	1
1	1	1	2	2	1	1	1	2	2
1	1	1	2	1	1	1	1	1	1
3	1	1	1	2	1	1	1	1	2
3	1	1	2	1	1	1	3	2	1
3	1	1	1	1	1	1	3	2	1
1	1	1	2	1	1	1	2	2	1
3	3	1	1	3	1	1	1	2	2
3	1	1	9	9	9	1	3	1	1
1	1	1	2	2	1	3	1	2	2
1	1	1	1	1	1	3	3	1	1
3	1	2	1	2	1	1	2	1	2
1	1	2	2	1	1	1	1	1	1
2	2	2	2	2	2	1	2	2	1
2	2	2	2	2	2	2	2	2	2
3	1	1	1	3	1	1	3	1	2
1	1	1	1	3	1	1	3	1	1
1	1	1	2	2	1	1	1	1	2
1	2	1	1	2	2	1	2	1	1
3	1	1	1	3	2	1	3	1	2
1	1	1	2	1	1	2	2	1	2
2	1	1	1	2	1	3	3	2	1
2	3	1	2	1	3	3	3	2	3
1	1	2	2	1	2	1	9	1	2
2	1	1	1	3	1	1	2	1	1
3	3	1	2	3	2	1	2	2	2
2	2	3	3	3	3	2	2	2	2
1	1	1	1	2	1	1	1	2	1
9	9	9	9	9	9	3	3	9	9
1	1	1	1	1	1	3	1	1	1
1	1	1	1	2	1	2	2	1	2
9	9	9	9	9	9	9	9	9	9
1	1	1	2	2	3	1	2	3	2
1	1	2	2	1	1	1	2	2	1
3	1	1	1	3	1	2	1	1	2
1	1	1	1	2	2	1	3	3	3
1	1	1	1	2	1	1	3	1	1

3	1	1	1	1	2	1	3	1	2
3	3	3	3	3	3	3	3	3	3
1	1	3	3	3	3	3	3	3	3
2	2	1	2	1	1	3	2	2	1
3	3	1	2	1	2	2	3	1	2
2	3	1	3	1	2	2	3	2	3
9	9	1	2	3	1	2	9	2	1
3	2	1	2	3	1	1	9	2	1
1	1	1	1	3	1	1	9	9	9
9	9	1	3	3	2	2	2	2	2
1	1	2	1	1	3	1	2	1	3
3	1	1	1	2	1	1	3	1	2
2	1	1	2	2	1	2	2	2	2
2	2	3	3	3	3	1	1	1	2
1	1	2	2	1	2	1	1	2	2
2	1	1	2	1	1	1	1	2	2
2	1	1	2	2	1	1	1	2	2
1	1	1	2	1	1	1	2	2	1
1	1	2	1	3	1	1	3	9	9
1	1	1	3	1	1	2	3	3	3
9	9	9	1	9	9	2	2	2	2
3	2	1	2	2	1	1	3	1	1
1	1	2	1	3	3	3	3	3	3
2	1	2	2	1	2	1	3	2	2
3	3	1	1	1	1	3	3	1	2
1	1	2	1	3	2	1	3	1	3
3	1	2	1	3	1	1	3	1	2
2	1	2	1	1	1	3	3	3	3
9	9	9	9	9	9	1	2	1	1
2	1	1	1	2	1	1	1	2	2
2	2	2	2	2	2	1	2	1	2
2	2	2	1	1	1	1	1	1	1
2	2	2	2	2	2	1	1	2	2
1	1	1	1	1	1	1	1	1	1
2	2	1	2	2	2	1	2	2	2
1	1	3	3	3	1	3	3	3	1
1	1	3	3	3	3	2	2	2	2
3	1	1	1	3	1	1	1	1	2
3	3	1	1	1	1	1	3	1	3
2	2	3	3	3	3	2	2	2	2
1	1	2	2	2	2	2	2	1	1
2	2	2	2	2	2	1	3	2	2
2	2	3	1	1	2	1	2	2	2
9	9	9	9	9	9	9	9	9	9
9	9	9	9	9	9	9	9	9	9
1	1	1	1	2	1	1	2	1	1
1	1	2	2	1	1	1	1	2	2

9	9	9	3	9	9	9	9	9	9
3	1	1	1	3	1	3	1	2	2
3	1	1	1	1	1	1	1	1	1
3	1	1	1	2	1	1	9	9	9
9	9	9	9	9	1	9	9	9	9
9	9	9	9	9	9	9	9	9	9
1	1	1	1	1	2	2	2	2	3
1	1	9	9	9	9	9	9	9	9
3	1	2	1	2	2	1	3	2	1
9	9	9	1	9	9	9	9	9	1
9	9	9	2	9	9	1	9	9	9
9	9	9	2	9	9	1	9	9	9
2	2	1	2	2	2	2	2	2	1
2	1	2	2	1	1	2	1	2	3
9	9	9	3	9	9	1	9	9	9
2	2	3	3	3	3	2	2	2	2
3	3	3	3	3	3	3	3	3	3
2	2	1	2	2	2	2	1	1	2
3	1	2	1	2	2	1	2	1	1
2	2	3	3	2	2	2	2	2	2
1	1	2	1	2	3	1	3	3	1
2	9	1	2	2	2	9	9	9	9
1	1	2	1	2	2	1	2	1	1
3	2	1	1	2	1	3	1	1	1
2	2	2	2	2	2	2	2	2	2
3	3	2	3	2	2	2	1	1	2
2	2	2	2	2	1	3	2	1	1
2	2	1	2	2	3	1	1	1	2
3	3	3	3	3	3	3	3	3	3
3	1	1	1	1	1	1	3	1	1
2	3	1	2	2	2	1	2	2	2
3	1	1	1	3	1	2	2	2	2
9	9	1	9	9	9	9	9	9	9
1	1	1	1	1	3	2	2	1	3
1	3	3	1	3	3	3	1	3	3
2	2	1	1	2	3	3	1	1	1
1	3	2	1	2	2	2	3	1	2
1	1	1	1	2	1	1	2	2	1
2	3	2	1	3	2	2	1	2	2
2	2	2	1	2	2	1	1	1	1
2	2	2	3	1	1	1	2	2	1
3	1	2	3	2	1	1	3	2	2
1	1	1	2	3	1	1	2	1	1
1	3	9	9	2	9	2	2	2	2
2	2	3	2	2	2	1	2	3	2
1	2	1	1	3	1	1	3	1	1
9	9	9	9	9	9	9	9	9	9

3	1	1	1	3	1	1	2	1	2
1	1	1	1	3	1	3	3	3	3
3	3	1	1	1	1	1	3	1	3
3	1	1	1	3	1	1	1	2	3
3	1	3	1	2	1	1	1	2	3
2	3	2	3	2	1	2	2	2	1
3	3	1	1	3	1	1	3	3	1
2	2	2	3	2	2	2	2	2	2
2	2	1	2	3	2	2	2	2	2
2	2	2	2	2	2	2	1	2	1
1	2	1	1	1	1	2	2	2	2
9	9	1	9	9	9	2	9	9	9
9	9	2	9	9	9	2	9	9	9
2	2	2	2	2	2	2	2	2	2
3	1	9	1	3	1	2	9	2	9
2	2	2	2	2	2	2	9	9	2
1	1	1	9	1	1	9	9	9	9
1	9	9	2	1	9	9	9	9	9
2	2	2	2	2	2	9	2	9	2
9	9	9	9	9	9	9	9	9	9
9	9	9	9	9	9	9	9	9	9
2	2	2	2	2	2	2	2	2	2
2	2	2	2	2	2	2	2	2	2
1	1	1	1	1	2	1	1	1	2
2	2	2	2	2	2	1	1	2	2
2	2	2	9	2	2	9	9	9	9
2	2	2	2	2	2	2	2	2	2
9	9	9	9	9	9	9	9	9	9
9	9	9	9	9	9	9	9	9	9
2	1	1	1	2	2	1	2	2	1
9	9	9	2	9	9	9	2	9	9
9	9	9	9	9	9	9	9	9	9
9	9	9	9	9	9	9	9	9	9
9	9	9	9	9	9	9	9	9	9
1	2	1	3	2	1	9	9	1	9
1	9	9	9	1	9	9	1	9	9
9	9	9	9	9	9	9	9	9	9
9	2	2	9	9	2	9	2	9	9
9	9	9	1	9	9	9	9	9	9
9	9	3	9	9	9	3	9	9	9
9	1	2	9	9	1	1	9	9	9
9	9	1	2	2	2	9	9	9	9
3	3	3	3	3	3	3	3	3	3
2	3	1	2	2	1	1	2	3	1
2	3	2	1	2	1	2	1	2	3
9	3	1	1	1	2	1	1	2	1
9	9	1	9	9	9	1	9	9	9

9	9	1	9	9	9	2	9	9	9
9	9	9	1	9	9	2	9	9	9
3	3	2	2	3	1	3	2	1	3
1	2	3	1	2	3	2	3	1	2
1	2	2	3	3	2	2	2	2	2
2	2	1	1	1	2	2	2	2	2
3	2	2	1	1	1	2	3	1	2
1	2	1	2	3	1	1	3	1	1
9	9	9	9	9	9	2	3	2	3
3	3	3	3	3	3	3	3	3	3
9	9	9	9	9	9	9	9	9	9
9	9	9	9	9	9	9	9	9	9
9	9	1	9	9	9	9	1	9	9
9	9	9	2	9	9	9	2	9	9
1	1	1	1	1	1	1	1	1	1
3	3	1	1	1	1	1	2	1	1
9	9	9	9	9	9	9	9	9	9
3	2	1	1	1	1	1	2	2	2
1	1	1	1	1	1	1	1	1	1
3	3	1	1	1	2	1	2	2	2
1	3	1	3	1	2	3	3	3	3
2	1	1	1	2	1	2	2	2	2
2	3	1	1	2	3	3	3	2	1
2	2	2	2	2	2	2	2	2	2
2	2	1	2	1	2	2	2	2	2
2	2	1	1	1	1	2	2	2	2
2	2	2	2	2	2	2	2	2	2
3	1	1	2	3	1	1	2	1	3
2	3	3	3	3	3	2	2	2	3
2	1	2	1	2	2	2	3	2	1
2	2	2	2	2	2	2	2	2	2
2	2	2	2	2	2	2	2	2	2
3	2	2	2	3	1	2	3	2	2
2	2	2	3	3	3	2	2	3	3
3	2	2	3	1	3	1	1	1	1
2	2	1	2	3	9	1	2	3	1
9	9	9	9	9	9	9	9	9	9
2	2	9	1	3	2	2	2	2	2
3	3	3	3	3	3	3	3	3	3
3	3	3	3	3	3	3	3	3	3
9	9	2	9	9	9	2	9	9	9
2	1	2	2	1	1	2	1	2	1
9	9	9	2	9	9	9	9	9	9
2	2	1	1	1	1	1	1	1	1
2	2	1	2	2	2	1	2	2	1
2	2	2	2	2	2	2	2	2	2
2	1	2	1	2	2	2	2	2	2

1	2	2	3	1	3	9	9	9	9
9	9	2	9	9	9	1	9	9	9
9	9	9	1	9	9	9	9	1	9
9	9	9	2	9	9	3	9	9	9
9	9	9	9	2	9	9	9	1	9
1	1	1	1	1	1	1	1	1	1
9	9	3	9	9	9	2	9	9	9
9	9	1	9	9	9	9	9	9	9
2	2	1	1	2	2	2	2	2	2
9	9	1	9	9	9	2	9	9	9
9	9	9	2	9	9	9	2	9	9
9	9	9	9	2	9	9	9	9	9
9	9	2	9	9	9	2	9	9	9
9	9	9	2	9	9	9	9	9	2
9	9	1	9	9	9	1	9	9	9
9	9	9	9	9	9	9	9	9	9
9	9	9	2	9	9	9	9	9	9
1	2	3	1	2	1	2	1	2	3
1	1	1	1	2	2	1	1	2	2
2	2	2	2	2	2	2	2	2	2
3	1	1	2	3	1	2	1	1	2
2	1	1	1	3	1	1	2	3	2
3	1	1	1	1	1	1	2	1	2
1	2	2	2	3	1	2	3	1	2
3	1	2	2	3	3	2	2	2	2
3	3	3	3	3	3	2	2	2	9
1	1	1	3	3	2	2	2	2	2
3	1	1	1	1	1	1	3	1	3
3	2	1	1	2	3	1	2	2	2
2	1	1	1	2	1	1	1	1	1
2	2	1	2	3	1	1	2	2	2
2	2	1	1	2	1	1	2	1	1
2	3	2	2	2	2	1	1	1	1
1	1	1	1	1	1	1	1	1	2
9	9	9	9	9	9	9	9	9	9
3	2	1	2	3	2	2	2	2	2
9	9	9	9	9	9	9	9	9	9
2	2	2	2	2	1	1	1	1	1
1	2	3	3	3	3	3	3	3	2
2	2	2	2	2	2	2	2	2	2
3	1	1	1	2	1	2	2	1	2
3	1	1	3	3	1	1	1	1	3
3	1	1	3	3	1	1	1	2	1
3	1	2	1	3	1	1	2	1	1
2	2	2	2	2	2	2	2	2	2
2	1	1	1	2	1	1	2	3	2
2	2	2	2	2	2	2	2	2	2

2	3	2	3	2	3	2	1	3	2
9	9	9	1	9	9	2	2	2	2
2	2	1	9	9	9	3	3	2	2
3	2	1	2	9	3	2	2	1	2
3	3	3	3	3	3	3	3	2	2
9	9	2	9	9	9	3	3	3	2
3	1	1	1	1	1	1	1	1	2
3	1	1	1	2	1	1	1	1	1
9	9	9	9	9	9	9	9	9	9
3	1	1	1	2	2	2	2	1	2
3	1	1	1	3	1	1	1	1	1
9	9	1	9	9	9	9	9	9	9
9	9	1	9	9	9	9	9	9	9
3	3	3	3	3	3	1	9	1	9
9	1	1	1	2	1	9	9	9	9
2	2	2	1	2	2	2	3	2	1
9	9	9	9	9	9	9	9	9	9
9	9	9	9	2	9	9	9	2	9
9	9	9	1	9	9	9	9	9	9
2	2	3	2	3	2	2	2	3	3
2	2	2	2	2	2	2	2	2	2
1	1	1	3	1	2	2	2	2	2
2	1	2	1	3	2	2	1	2	2
9	1	1	9	9	9	9	9	9	9
3	1	1	1	2	2	1	9	9	9
1	1	1	2	2	1	2	2	1	1
1	1	1	1	1	1	1	1	1	1
3	1	1	2	2	3	1	2	3	1
2	2	3	3	3	3	1	2	2	2
9	9	9	2	9	9	2	9	9	9
2	2	2	2	2	2	2	2	2	2
3	1	2	3	1	2	1	2	3	1
1	1	2	3	1	1	3	1	2	2
1	1	1	2	2	2	1	1	1	1
1	1	1	1	3	1	1	3	1	1
2	3	2	1	3	2	9	9	9	9
9	9	9	9	9	9	9	9	9	9
9	9	9	9	9	9	9	9	9	9
2	2	2	3	1	3	3	2	1	2
1	2	1	2	1	2	2	1	2	1
9	1	1	2	1	2	9	1	1	2
3	3	1	2	3	2	1	1	1	1
3	1	1	3	2	2	3	3	3	3
1	2	1	2	3	1	1	3	2	1
2	2	3	3	3	3	2	2	2	2
1	2	1	1	1	2	1	2	2	2
2	2	2	2	2	2	2	2	2	3

3	3	1	2	2	2	1	1	1	1
2	1	2	1	2	1	1	2	1	2
9	9	9	2	9	9	9	2	9	9
9	9	9	9	9	1	9	9	9	9
1	9	1	2	2	1	9	9	9	9
3	3	1	3	2	2	3	2	2	2
2	2	2	2	2	2	2	9	2	2
2	2	2	2	2	2	2	2	9	2
9	9	9	9	9	9	9	9	9	9
9	9	9	9	9	9	9	9	9	9
2	2	2	2	2	2	2	2	2	2
2	2	2	2	2	2	9	9	9	9
2	2	2	2	2	2	2	2	2	2
9	9	9	9	9	9	9	9	9	9
2	2	2	9	9	9	2	2	2	2
9	9	2	9	9	9	2	9	9	9
3	3	3	3	3	3	3	3	3	3
9	9	1	9	9	9	1	9	9	9
9	2	2	9	9	2	2	9	9	9
9	2	2	2	2	2	2	9	9	2
9	9	9	9	9	9	9	9	9	9
9	9	2	9	9	9	2	9	9	9
2	2	2	2	2	2	2	2	2	2
9	9	9	9	9	9	9	9	9	9
9	9	9	9	9	9	9	9	9	9
9	9	9	9	9	9	9	9	9	9
9	9	9	9	9	9	9	9	9	9
2	2	2	2	2	2	3	3	2	9
3	3	1	1	3	1	1	3	1	1
9	9	9	9	9	9	9	9	9	9
9	2	2	2	2	2	2	9	9	9
2	2	2	2	2	2	2	9	2	9
1	9	1	9	1	1	1	9	9	2
9	9	9	9	9	9	9	9	9	9
3	3	3	3	3	3	2	2	2	9
9	9	9	9	9	9	9	9	9	9
9	9	1	9	9	9	1	9	9	9
2	2	2	2	2	2	9	9	9	9
3	1	1	2	2	3	1	1	1	1
3	1	1	3	2	3	2	1	2	3
9	9	1	9	9	9	2	9	9	9
2	9	9	9	9	9	9	9	9	9
3	1	1	3	3	2	2	1	3	2
3	1	1	2	3	2	1	2	1	2
3	1	3	3	3	3	3	3	3	3
3	3	1	2	1	2	1	2	2	1
1	1	1	1	2	2	2	1	1	9

9	9	9	9	9	1	9	1	9	9
1	1	1	2	2	3	2	1	2	3
1	1	1	1	2	2	2	2	2	2
1	1	1	1	3	1	1	1	1	1
2	2	2	1	2	2	2	2	3	2
1	2	2	2	1	3	2	3	1	2
3	3	1	1	1	1	3	3	3	3
1	1	2	2	1	2	2	3	3	2
3	1	3	2	2	1	2	2	2	2
1	1	1	1	2	1	2	2	1	2
2	2	2	2	2	2	2	2	2	3
2	2	1	2	3	9	3	2	9	9
3	1	1	2	3	1	1	3	2	2
2	2	2	2	2	2	2	2	2	2
9	9	9	2	9	9	1	9	9	9
2	1	1	2	1	1	1	2	1	1
2	1	1	1	2	1	3	1	1	2
3	1	1	1	2	3	1	2	2	1
9	9	1	1	3	1	3	1	1	3
2	2	2	2	2	2	2	2	2	2
1	1	1	2	3	3	1	1	2	2
1	1	1	1	1	1	1	9	1	3
3	3	1	1	3	3	1	3	3	2
9	9	1	9	9	9	9	1	9	9
9	1	9	9	9	1	9	9	1	9
1	1	1	1	1	1	1	1	1	1
1	9	9	1	9	9	9	9	2	1
1	1	1	1	3	2	1	1	1	1
9	9	9	9	9	9	9	9	9	9
9	9	9	9	9	1	9	9	9	9
1	2	1	3	2	2	2	3	2	3
2	3	2	3	2	1	3	2	3	2
9	9	9	9	9	9	9	9	9	9
9	9	1	9	9	9	1	9	9	9
2	1	1	2	1	1	1	1	2	2
9	9	9	9	9	9	9	9	9	9
3	2	3	3	2	2	2	3	2	3
1	1	1	1	1	1	1	1	1	1
2	2	2	2	2	2	2	2	2	2
2	1	2	2	2	2	1	1	1	2
3	1	2	2	2	1	2	1	2	2
2	2	2	2	2	2	2	2	2	2
2	3	2	3	2	2	2	2	2	2
3	3	3	3	3	3	2	2	2	2
2	2	1	2	3	3	2	2	2	2
2	2	1	2	2	2	2	2	9	2
2	2	1	9	2	2	2	3	1	2

9	9	9	9	9	1	9	9	9	9
1	1	1	2	2	1	2	2	2	2
1	1	1	1	2	1	1	3	2	2
9	1	9	9	9	1	9	1	9	9
3	1	1	3	2	2	1	9	9	9
9	9	9	9	9	9	9	9	9	9
9	9	9	9	9	9	9	9	9	9
2	2	2	2	2	2	1	9	1	1
3	3	2	2	2	1	2	2	2	2
2	1	1	1	2	1	3	1	1	9
1	1	1	2	2	2	1	2	1	3
2	1	1	1	2	1	9	1	1	2
3	1	1	2	2	1	1	3	1	2
2	2	2	2	2	2	2	2	2	2
1	1	1	3	3	3	2	2	1	3
3	3	3	3	3	3	9	9	9	9
9	9	9	9	9	9	9	9	9	9
9	9	9	9	9	9	9	9	9	9
9	9	1	9	9	9	1	9	9	9
9	9	2	9	9	9	2	9	9	9
9	9	9	9	9	9	9	9	9	9
9	9	9	9	9	9	9	9	9	9
2	2	2	2	2	2	2	2	2	2
9	9	2	9	9	9	2	9	9	9
9	9	9	9	9	9	9	9	9	9
9	9	3	9	9	9	3	9	9	9
9	9	9	9	9	9	9	9	9	9
1	1	1	1	1	1	1	9	1	9
2	2	2	9	2	9	2	9	9	9
9	3	3	9	9	3	3	9	9	9
9	9	9	9	9	9	9	9	9	9
2	2	2	2	2	2	2	2	2	2
9	9	9	9	9	9	9	9	9	9
9	3	3	3	3	3	9	9	9	9
1	1	1	1	9	1	9	9	9	9
9	9	9	9	9	9	9	9	9	9
9	9	2	9	9	9	2	9	9	9
9	9	9	9	9	9	9	9	9	9
2	3	1	2	3	2	9	9	9	9
9	9	1	9	9	9	1	9	9	9
2	2	2	2	2	2	2	2	2	2
9	9	2	9	9	9	2	9	9	9
9	9	2	9	9	9	9	9	9	9
9	9	2	9	9	9	2	9	9	9
9	9	9	9	9	9	9	9	9	9
2	2	2	2	2	2	9	9	9	9
9	9	9	9	9	9	9	9	9	9

9	9	9	9	9	9	9	9	9	9
2	2	9	9	9	9	9	9	9	9
2	9	2	9	9	9	3	9	9	9
9	9	2	9	9	9	9	9	9	9
9	9	9	9	9	9	9	9	9	9
9	9	9	9	9	9	9	9	9	9
9	9	9	9	9	9	9	9	9	9
9	9	1	9	9	9	9	9	9	9
9	9	9	9	9	9	9	9	9	9
9	9	9	9	9	9	9	9	9	9
9	9	9	9	9	9	9	9	9	9
9	9	9	9	9	9	9	9	9	9
9	9	9	9	9	9	9	9	9	9
9	9	9	9	9	9	9	9	9	9
2	2	2	2	2	2	9	9	9	9
2	2	2	2	2	2	9	9	9	9
2	9	2	2	2	2	9	9	9	9
2	2	2	2	2	2	2	2	2	2
2	9	2	9	9	9	9	9	9	9
2	2	2	2	2	2	2	2	2	2
9	9	9	9	9	9	9	9	9	9
9	9	1	9	9	9	2	9	9	9
9	9	2	9	9	9	9	9	9	9
2	9	2	2	2	2	9	9	9	9
2	2	2	2	2	2	9	9	9	9
9	1	9	9	9	1	9	9	9	9
9	9	9	9	9	9	9	9	9	9
3	2	1	2	2	2	2	2	9	2
9	9	9	9	9	9	9	9	9	9
9	9	9	9	9	9	9	9	9	9
1	1	1	1	1	1	1	1	1	1
2	2	1	2	3	2	1	3	3	2
9	9	9	9	9	9	9	9	9	9
2	2	2	1	1	1	2	2	2	2
9	9	9	9	9	9	9	9	9	9
9	9	1	9	9	9	9	9	1	9
9	2	2	9	2	2	9	9	9	9
2	2	2	2	2	2	2	9	9	9
1	1	1	1	1	1	1	1	1	1
9	3	3	9	9	3	3	9	9	9
9	9	9	9	9	9	9	9	9	9
9	9	9	9	9	9	9	9	9	9
2	2	2	2	2	2	2	2	2	2
9	9	1	1	9	9	2	9	9	2
9	1	1	9	1	9	1	9	1	9
9	2	2	9	9	2	2	9	9	9
9	2	2	9	2	2	9	9	9	9

9	9	2	9	9	9	2	9	9	9
9	9	9	9	9	9	9	9	9	9
1	3	1	3	9	2	2	9	9	1
9	9	3	9	9	9	9	9	9	9
9	9	9	9	9	9	9	9	9	9
9	9	9	9	9	9	9	9	9	9
9	9	2	9	9	9	2	9	9	9
9	9	2	9	9	9	2	9	9	9
9	9	2	9	2	9	2	9	9	9
9	9	2	2	2	2	2	2	2	2
9	9	9	9	9	9	9	9	9	9
9	9	9	9	9	9	9	9	9	9
9	9	9	9	9	9	9	9	9	9
9	9	9	9	9	9	9	9	9	9
3	1	3	2	1	1	1	2	2	2
1	1	1	2	2	1	1	1	1	2
9	9	2	9	9	9	2	9	9	9
2	1	2	2	2	2	2	2	2	2
2	2	2	2	2	2	2	2	2	2
2	2	2	2	2	2	2	9	9	3
9	9	1	9	9	9	9	1	9	9
9	9	9	9	1	9	9	9	9	9
2	3	1	1	3	1	1	2	1	3
2	3	1	2	3	1	1	2	3	1
1	1	1	2	3	2	1	1	1	1
3	1	2	2	3	1	1	3	1	2
3	1	1	1	3	1	3	3	3	1
2	3	1	1	3	9	1	2	2	1
2	1	1	1	2	2	9	1	2	3
1	9	1	1	2	1	1	1	1	1
9	9	9	9	9	9	1	1	1	1
9	9	1	9	9	9	1	9	9	9
9	9	1	9	9	9	9	1	9	9
1	9	9	1	9	9	3	9	9	9
9	9	1	9	9	9	2	9	9	9
9	9	9	9	9	9	9	9	9	9
9	9	9	9	9	9	9	9	9	9
9	2	2	9	2	9	9	2	9	9
9	9	9	9	9	9	9	9	9	9
9	2	2	9	2	2	2	9	9	2
9	2	2	9	2	2	9	9	9	9
2	2	2	2	2	2	2	2	2	2
2	2	2	2	2	2	9	9	9	9
2	2	2	2	2	2	9	9	9	9
9	9	9	9	9	9	9	9	9	9
2	2	2	2	2	2	2	2	2	2
2	1	2	2	2	2	3	3	2	2

2	1	1	2	2	1	1	3	1	1
2	1	1	1	3	1	1	2	2	2
3	1	1	2	2	1	1	1	1	1
2	3	2	2	2	2	3	1	1	1
3	3	3	3	3	3	1	1	2	2
3	1	1	1	2	1	1	2	1	2
2	1	1	1	2	1	1	2	2	2
3	3	3	3	3	3	3	3	3	3
2	2	2	2	2	2	2	2	2	2
2	2	2	2	2	2	2	2	2	2
9	2	2	9	9	2	2	9	9	9
2	2	2	2	2	2	2	9	2	9
2	2	2	2	2	2	2	2	2	2
1	1	1	1	1	2	1	3	2	2
3	3	3	3	1	2	1	1	1	1
2	2	2	2	2	2	2	2	2	2
1	2	2	2	1	1	1	1	2	2
3	1	2	1	2	1	2	2	2	2
3	1	1	1	3	1	9	9	9	9
2	1	1	2	2	1	1	3	3	2
2	3	3	2	2	3	2	1	2	3
2	1	1	1	1	1	3	3	1	1
1	1	3	2	2	1	3	1	1	2
2	1	1	1	3	1	1	2	1	2
3	1	1	1	2	2	1	2	2	2
3	1	1	2	3	1	2	2	2	2
3	2	1	1	2	1	1	2	1	1
3	1	2	1	3	1	2	3	1	2
2	1	1	2	2	1	1	3	2	2
3	1	3	1	3	1	3	1	1	1
2	1	1	3	3	1	2	2	3	3
2	3	2	1	3	1	3	3	1	2
3	1	3	3	3	3	3	3	3	3
2	1	2	2	3	3	1	3	2	2
9	9	1	9	9	9	9	9	9	9
3	3	3	3	3	3	3	3	2	2
1	1	9	9	9	9	1	2	1	2
2	2	2	2	2	2	2	2	2	2
1	2	1	1	1	1	3	1	2	2
9	9	9	9	9	9	9	9	9	9
3	1	2	2	2	2	2	2	2	2
3	1	1	1	9	1	1	3	9	9
1	2	1	3	1	2	2	2	1	3
1	2	2	2	3	2	2	2	2	2
2	3	2	3	2	2	2	3	2	3
3	3	2	2	3	2	3	2	3	2
2	3	1	2	3	2	2	3	2	3

2	2	1	1	2	1	9	9	9	9
3	2	3	3	3	3	3	3	3	3
1	1	1	1	1	3	1	1	1	3
3	1	1	1	3	3	3	1	2	2
1	1	2	3	2	1	1	2	1	2
3	1	9	9	9	9	1	3	1	1
3	2	3	3	3	3	2	2	3	2
3	1	2	2	3	3	2	2	2	2
2	1	1	1	1	1	3	1	3	3
2	2	1	1	2	1	2	2	2	2
2	2	3	3	3	3	3	2	2	2
3	1	2	2	2	2	2	2	2	2
2	2	2	3	2	2	9	9	9	9
2	2	2	3	2	2	2	3	2	2
2	1	1	2	2	1	2	1	2	1
3	3	1	1	3	1	2	2	2	2
2	2	2	3	3	3	3	3	2	2
2	2	1	2	2	2	2	2	2	2
2	2	2	2	2	2	2	2	2	2
9	9	1	9	9	9	9	1	9	9
2	1	1	1	1	1	1	9	1	2
1	2	2	1	2	1	1	3	1	2
2	3	2	3	2	3	2	3	3	3
3	2	2	2	2	1	2	2	3	2
3	3	1	1	1	1	3	3	3	3
1	3	1	2	3	2	1	2	3	2
2	2	1	3	2	1	1	9	9	9
2	2	2	2	2	2	2	2	2	2
2	2	2	2	2	2	2	2	2	2
2	2	2	2	2	2	2	2	2	2
2	3	1	3	1	2	2	3	2	2
2	2	2	9	2	2	9	9	9	9
1	2	1	1	2	2	9	9	9	9
3	1	1	2	1	1	1	3	2	2
3	1	1	1	2	1	1	3	3	2
1	2	2	3	2	1	1	9	9	9
3	1	1	1	3	1	1	1	1	2
2	1	1	2	1	2	2	2	2	2
3	1	1	1	3	3	3	2	1	3
1	1	1	1	3	1	1	2	1	2
3	1	1	1	2	1	1	9	9	9
2	2	2	2	2	2	2	2	2	2
2	2	2	2	2	2	2	2	2	2
2	2	2	2	2	2	2	2	2	2
1	2	1	1	2	2	2	2	2	2
3	1	1	1	3	1	1	3	2	2
3	1	1	1	2	1	2	2	2	2

2	2	2	2	3	3	3	2	3	3
1	2	1	1	2	1	2	2	2	2
3	1	1	1	2	1	2	2	2	2
1	3	2	1	3	1	1	2	2	2
2	2	2	1	2	2	2	2	2	2
1	2	1	1	2	1	2	2	2	2
1	2	1	1	2	1	2	2	2	2
2	2	2	2	2	2	2	2	2	2
2	2	2	2	2	2	2	2	2	2
9	9	9	9	9	9	9	9	9	9
3	3	2	1	1	1	2	2	3	3
3	3	1	2	2	1	2	2	1	3
3	1	1	1	3	1	1	2	1	2
2	1	1	3	3	1	1	9	2	1
1	1	3	2	1	3	3	3	3	3
9	9	9	9	9	1	1	9	1	1
9	9	9	9	9	9	9	9	9	9
9	9	9	1	9	9	1	9	9	9
9	9	9	9	9	9	9	9	9	9
3	1	1	2	2	2	2	2	2	2
1	1	1	1	1	1	1	3	1	3
9	9	1	1	3	9	9	9	9	9
9	9	9	9	9	9	9	9	9	9
2	2	1	2	2	1	2	2	2	2
1	1	3	2	1	2	1	1	1	1
2	2	1	2	2	1	2	2	2	2
1	2	1	3	2	1	2	1	3	2
1	1	1	1	1	1	3	1	3	2
1	1	1	3	3	1	1	2	1	2
2	2	2	2	2	2	2	1	3	2
9	9	9	9	9	9	9	9	9	9
1	1	1	2	1	3	1	2	1	3
3	2	1	2	2	3	1	2	3	1
2	3	3	3	1	1	2	2	2	1
2	2	1	2	2	2	2	2	2	2
2	2	1	2	2	1	2	2	2	2
9	9	1	1	2	1	2	2	1	1
2	2	2	2	2	2	2	2	2	2
2	2	2	2	2	2	1	1	2	2
1	1	3	3	3	3	3	3	3	2
2	2	1	2	2	2	1	1	1	1
3	1	1	1	1	1	1	3	1	2
1	1	1	1	2	1	2	2	1	2
1	9	1	1	2	1	2	2	1	2
2	2	3	3	3	3	3	3	2	3
2	1	1	1	1	3	2	1	1	2
3	3	3	2	2	3	3	3	3	2

2	2	2	1	1	3	2	2	1	3
2	2	2	2	2	2	2	2	2	2
2	1	2	2	2	3	2	2	2	2
2	2	2	2	2	2	2	2	2	2
1	3	2	2	1	3	3	3	3	3
2	2	2	2	2	2	2	2	2	2
9	9	9	9	9	1	9	9	9	9
2	1	3	3	3	3	3	3	3	3
9	1	1	2	2	1	9	9	9	9
2	2	1	2	2	2	2	2	1	2
2	2	2	2	2	2	2	2	2	2
1	1	1	1	1	1	1	2	1	1
2	1	2	1	1	2	1	2	2	1
9	9	1	9	9	9	9	9	9	9
9	1	9	9	9	1	9	9	9	9
1	1	1	1	1	1	9	9	1	9
3	1	1	1	3	1	1	2	1	2
2	1	2	3	1	1	3	3	1	1
1	1	1	1	2	1	3	3	3	3
3	1	2	1	2	1	1	2	1	1
2	2	2	1	2	2	1	1	9	2
3	1	1	1	3	1	3	3	9	1
3	1	1	1	2	1	1	3	1	1
2	2	1	2	2	2	2	2	2	2
3	1	1	1	3	1	1	1	1	1
2	2	2	2	2	2	2	2	2	2
2	2	2	2	2	2	2	2	2	2
9	9	2	9	9	9	2	2	2	2
1	2	2	3	2	2	2	2	3	2
3	3	2	2	2	2	3	3	3	3
1	1	1	1	1	1	2	2	2	2
1	3	1	2	2	3	1	3	2	1
1	1	1	1	2	2	2	2	2	2
2	2	2	2	2	2	2	2	2	2
1	1	1	1	2	1	2	2	2	2
2	1	2	3	1	2	2	1	3	2
1	1	1	1	1	1	1	1	1	1
3	1	1	1	2	1	1	3	1	2
3	2	2	3	2	3	1	2	3	2
1	2	1	1	2	1	1	3	2	2
1	1	1	1	1	1	9	1	9	9
3	3	3	3	3	1	1	2	2	2
2	2	2	2	2	2	2	2	2	2
3	1	1	2	2	2	1	2	1	2
9	9	9	9	9	9	2	2	2	2
1	9	2	2	2	1	1	3	2	2
3	3	2	2	2	2	2	2	2	2

2	1	1	2	2	3	1	1	1	1
2	1	1	1	3	1	1	3	1	1
2	2	2	2	3	2	2	3	2	3
2	2	2	2	2	2	2	2	2	2
9	9	2	3	2	3	2	1	2	1
9	9	9	1	9	9	9	1	9	9
3	3	3	2	3	2	2	2	3	2
9	9	9	9	9	1	9	1	9	9
9	9	9	9	9	1	9	1	9	9
3	1	3	3	3	3	9	1	1	1
9	1	9	9	9	2	9	1	9	9
9	9	9	9	9	2	9	9	9	9
9	9	9	9	9	9	9	9	2	9
9	9	9	1	9	9	3	9	9	9
2	1	1	1	2	1	1	3	2	2
9	2	9	9	9	2	2	9	9	9
9	9	9	9	9	9	9	9	9	9
9	9	9	1	9	9	1	9	9	9
9	9	9	1	9	9	1	9	9	9
9	9	9	2	9	9	9	9	9	9
1	1	1	1	1	1	9	9	9	9
1	1	1	1	2	2	2	2	2	2
3	1	1	1	3	1	1	2	2	2
3	2	3	2	2	2	3	3	3	3
3	9	1	1	3	2	1	1	1	3
3	2	1	1	3	1	1	2	1	2
1	1	3	1	1	1	3	1	3	1
9	9	9	9	9	1	2	9	9	9
2	2	2	1	2	2	2	3	2	2
1	1	1	1	1	1	1	3	1	1
1	3	3	1	1	3	3	3	3	3
9	9	1	9	9	9	2	9	9	9
1	1	2	3	2	1	3	3	2	1
9	9	1	9	9	9	9	9	2	9
9	9	1	9	9	9	1	9	9	9
2	2	2	3	2	3	1	1	2	1
1	1	1	1	1	1	1	1	1	1
9	9	9	9	9	9	9	9	9	9
9	9	9	9	9	9	9	9	9	9
2	2	2	2	2	2	2	2	2	2
2	2	2	2	2	2	2	9	2	9
2	2	2	2	2	2	2	2	2	2
3	3	3	3	3	3	3	3	3	3
2	9	2	9	9	2	9	2	9	9
2	2	2	2	2	2	1	1	1	1
2	2	2	2	2	2	2	2	2	2
1	1	1	3	1	1	9	1	3	1

1	9	1	9	1	9	9	9	9	9
1	9	1	2	2	9	9	1	9	9
2	9	9	1	9	1	9	1	9	9
2	2	2	2	2	2	2	2	2	2
1	9	1	9	3	1	9	3	1	3
1	1	2	9	2	2	9	1	9	9
1	2	2	1	3	1	9	9	9	9
2	9	1	9	3	1	9	9	9	9
2	2	2	9	2	2	2	9	9	2
2	2	2	2	2	2	2	2	2	2
2	2	2	2	2	2	9	9	9	9
9	2	9	2	9	2	9	2	9	9
2	2	2	2	2	2	2	2	2	2
9	9	9	9	9	9	9	9	9	9
2	2	2	2	2	2	2	2	2	2
9	9	9	9	9	9	9	9	9	9
9	9	9	9	9	9	9	9	9	9
9	9	9	9	9	9	9	9	9	9
3	1	3	1	3	1	9	9	9	9
2	2	2	2	2	2	9	9	9	9
2	2	2	2	2	2	2	2	2	2
2	2	2	2	2	2	2	2	2	2
2	1	2	2	3	1	1	3	2	1
2	3	1	1	2	3	1	2	2	1
3	3	2	2	2	3	2	2	2	2
2	1	2	2	2	2	1	3	2	2
3	1	2	3	2	1	1	1	1	1
3	1	2	1	2	1	3	3	3	9
1	3	1	1	3	2	1	3	1	1
2	1	2	1	2	1	3	3	2	2
3	3	3	2	2	3	2	2	3	2
2	2	2	1	3	2	2	3	1	2
2	2	1	1	2	2	2	2	2	2
2	2	2	2	2	2	9	9	9	9
1	3	1	1	3	2	1	3	1	2
2	2	3	3	3	3	3	3	3	3
2	3	2	1	2	1	1	3	2	2
1	1	3	3	3	3	1	1	3	1
1	1	1	2	2	2	2	2	2	2
3	2	3	2	3	2	2	2	3	2
1	1	3	1	1	1	3	1	3	1
9	9	9	9	9	9	9	9	9	9
3	2	2	1	3	2	2	2	2	2
1	1	9	9	9	9	9	9	9	9
9	9	9	9	9	9	1	2	2	1
2	2	1	2	2	1	2	2	2	2
1	2	2	3	1	3	3	3	2	3

2	1	1	1	2	2	2	2	1	2
1	2	1	3	2	1	1	1	3	3
2	9	1	1	1	1	2	2	2	2
2	2	2	2	2	2	2	2	2	9
2	2	2	2	2	2	1	1	1	1
2	2	2	2	2	2	1	1	1	1
1	1	1	1	1	1	1	1	1	1
1	2	2	1	1	1	1	2	1	3
3	3	3	3	3	3	3	3	3	3
1	2	2	3	2	1	1	3	1	1
9	9	1	1	1	1	2	2	2	2
2	2	1	1	1	1	2	2	2	2
2	2	2	2	2	2	2	2	2	2
2	2	2	2	2	2	2	2	2	2
2	2	2	2	2	2	9	9	9	9
1	1	1	1	1	1	1	1	1	1
1	2	2	1	1	1	1	1	1	1
1	2	2	1	1	1	1	1	1	2
2	2	2	2	2	2	9	9	9	9
2	2	2	2	2	2	2	2	2	2
2	1	1	2	3	2	1	2	3	1
2	2	2	2	2	2	1	1	1	1
9	9	9	9	9	9	9	9	9	9
2	2	2	2	2	2	2	2	2	2
9	9	9	9	9	9	9	9	9	9
1	1	1	1	2	1	1	1	1	1
2	1	2	1	1	1	1	2	1	1
1	1	1	9	1	1	1	1	1	1
2	2	2	2	2	2	2	2	9	2
1	1	1	1	1	1	2	2	2	2
2	2	2	2	2	2	2	2	2	2
2	2	2	2	2	2	2	2	2	2
2	9	2	2	2	2	2	2	2	2
2	1	1	1	1	1	1	1	1	1
2	2	2	2	2	2	2	2	2	2
9	9	9	9	9	9	9	9	9	9
2	1	1	2	2	2	1	1	1	2
1	2	1	1	1	1	1	2	1	1
1	1	1	1	1	1	9	9	9	9
2	2	2	2	2	2	2	2	2	2
2	2	2	2	2	2	2	2	2	2
2	2	1	1	1	1	9	9	9	9
2	2	2	2	2	2	2	2	2	2
2	2	2	2	2	2	2	2	2	2
1	1	1	2	1	1	1	1	1	1
2	2	2	2	2	2	2	2	2	2
1	1	1	1	3	1	1	1	9	9

1	2	1	2	1	1	1	2	1	1
1	9	1	1	1	9	2	2	9	2
1	1	1	1	1	1	1	1	1	1
2	1	1	2	1	2	1	1	1	1
1	1	1	1	1	1	2	2	2	2
2	2	2	2	2	2	2	2	2	2
2	2	1	1	1	1	1	1	1	1
2	1	2	2	2	2	2	2	2	2
9	9	9	9	9	9	9	9	9	9
1	1	1	1	1	1	9	1	1	1
2	2	2	2	2	2	2	2	9	9
1	1	1	1	1	1	2	2	9	2
9	9	9	9	9	9	9	9	9	9
1	1	1	2	2	2	9	9	9	9
1	9	1	1	1	1	9	9	9	9
2	2	3	3	3	9	9	9	9	9
9	9	9	9	9	9	2	2	2	2
9	9	9	9	9	9	9	9	9	9
2	2	2	2	2	2	2	2	2	2
1	1	1	1	3	1	1	1	1	1
1	1	1	1	3	1	1	1	1	1
1	1	1	1	3	1	1	1	1	1
1	1	1	1	3	1	1	1	1	1
1	1	1	1	3	1	1	1	1	1
1	1	1	1	3	1	1	1	1	1
1	1	1	1	3	1	1	1	1	1
1	1	1	1	3	1	1	1	1	9
3	1	1	1	3	1	1	1	9	9
1	1	1	1	3	1	1	1	1	1
1	1	1	1	3	1	1	1	1	1
1	1	1	1	3	1	1	1	1	1
1	1	1	1	3	1	1	1	1	1
1	1	1	1	3	1	9	1	1	1
1	1	1	1	3	1	1	1	1	1
1	1	1	1	3	1	1	1	1	1
1	1	1	1	3	1	1	1	1	1
1	1	1	1	3	1	1	1	1	1
1	1	1	1	3	1	1	1	1	1
1	1	1	1	3	1	1	1	1	1
1	1	1	1	3	1	1	1	1	9
1	1	1	1	3	1	1	1	1	1
1	1	1	1	3	1	1	1	1	1
1	1	1	1	3	1	1	1	1	1
1	1	1	1	3	1	1	1	1	1
9	9	9	9	9	9	9	9	9	9
9	9	9	9	9	9	9	9	9	9
9	9	9	9	9	9	9	9	9	9

9	9	9	9	9	9	9	9	9	9
9	9	9	9	9	9	9	9	9	9
9	9	9	9	9	9	9	9	9	9
9	9	9	9	9	9	9	9	9	9
9	9	9	9	9	9	9	9	9	9
9	9	9	9	9	9	9	9	9	9
9	9	9	9	9	9	9	9	9	9
9	9	9	9	9	9	9	9	9	9
9	9	9	9	9	9	9	9	9	9
9	9	9	9	9	9	9	9	9	9
9	9	9	9	9	9	9	9	9	9
9	9	9	9	9	9	9	9	9	9
9	9	9	9	9	9	9	9	9	9
9	9	9	9	9	9	9	9	9	9
9	9	9	9	9	9	9	9	9	9
9	9	9	9	9	9	9	9	9	9
9	9	9	9	9	9	9	9	9	9
9	9	9	9	9	9	9	9	9	9
9	9	9	9	9	9	9	9	9	9
1	1	1	1	3	1	1	1	1	3
1	1	1	1	3	1	1	1	1	1
1	1	1	1	3	1	1	1	1	1
1	1	1	1	3	1	1	1	1	1
1	1	1	1	3	1	1	1	1	1
1	1	1	1	3	1	1	1	1	9
1	1	1	1	3	1	1	1	1	1
1	1	1	1	3	1	1	1	1	1
1	1	1	1	3	1	1	1	1	1
1	1	1	1	3	1	1	1	1	1
1	1	1	1	3	1	1	1	1	1
1	1	1	1	3	9	1	1	1	1
1	1	1	1	3	1	1	1	1	1
1	1	1	1	3	1	1	1	1	1
1	1	1	1	3	1	1	1	1	1
1	1	1	1	3	1	1	1	1	1
9	1	9	1	9	1	1	9	1	9
1	1	1	1	3	1	1	1	1	1
1	1	1	1	3	1	1	1	1	1
1	1	1	1	3	1	1	1	1	1
1	1	1	1	3	1	1	1	1	1
3	1	1	1	3	1	1	1	1	1
1	1	1	1	3	1	1	1	1	1
1	1	1	1	3	1	1	1	1	1
1	1	1	1	3	1	1	1	1	9
3	1	1	1	3	1	1	1	1	1
1	1	1	1	3	1	1	1	1	3
1	1	1	1	3	1	1	1	1	1

1	1	1	1	3	1	1	1	1	1
1	1	1	1	3	1	1	1	1	3
1	1	1	3	3	1	1	1	1	1
1	1	1	1	3	1	1	1	1	1
1	1	1	1	3	3	1	1	1	1
1	1	1	1	3	1	1	1	1	1
1	1	1	1	3	1	1	1	1	1
1	1	1	1	3	1	1	1	1	1
1	1	1	1	3	1	1	1	1	1
1	1	1	1	3	1	1	1	1	1
1	1	1	1	3	1	1	1	1	1
1	1	1	1	3	1	1	1	1	1
1	1	1	1	3	1	1	1	1	3
1	1	1	1	3	1	1	1	1	1
1	1	1	1	3	1	1	1	1	1
1	1	1	1	3	1	1	1	1	1
1	1	1	1	3	1	1	1	1	1
1	1	1	1	3	1	1	1	1	1
1	1	1	1	3	1	1	1	1	1
1	1	1	1	3	1	1	1	1	1
1	1	1	1	3	1	1	1	1	1
1	1	1	3	3	1	1	1	1	3
1	1	1	1	3	1	1	1	1	3
1	1	1	1	3	1	1	1	1	1
1	1	1	1	3	1	1	1	1	1
1	1	1	1	3	1	1	1	1	3
1	1	1	1	3	1	1	1	1	1
1	1	1	1	3	1	1	1	1	1
1	1	1	1	3	1	1	1	1	3
1	1	1	1	3	1	1	1	1	1
1	1	1	1	3	1	1	1	1	1
1	1	1	1	3	1	1	1	1	1
1	1	1	1	3	1	1	9	9	9
1	1	1	1	3	1	1	1	1	1
9	9	9	9	9	9	9	9	9	9
1	1	1	1	3	1	1	1	1	1
1	1	1	1	3	1	1	1	1	1
1	1	9	1	3	1	1	1	1	1
1	1	1	1	3	1	1	1	1	1
1	1	1	1	3	1	1	1	1	1
1	1	1	1	3	1	1	1	1	3
1	1	1	1	3	1	1	1	1	3
3	1	1	1	3	1	1	1	1	1
1	1	1	1	3	1	1	1	1	1
1	1	1	1	3	1	1	1	1	1
1	1	1	1	3	1	1	1	1	1
1	1	1	1	3	1	1	1	1	1

1	1	1	1	3	1	1	1	1	1
1	1	1	1	3	1	1	1	1	1
1	1	1	1	3	1	1	1	1	1
1	1	1	1	3	1	1	1	1	1
1	1	1	1	3	1	1	1	1	1
1	1	1	1	3	1	1	1	1	3
1	1	1	1	3	1	1	1	1	1
1	1	1	1	3	1	1	1	1	1
1	1	1	1	3	1	1	1	1	1
1	1	1	1	3	1	1	1	1	1
1	1	1	1	3	1	1	1	1	1
1	1	1	1	3	1	1	1	1	1
1	1	1	1	3	1	1	1	1	3
1	1	1	1	3	1	1	1	1	1
9	9	9	9	9	9	1	1	1	3
1	1	1	1	3	1	1	1	1	1
1	1	1	1	3	1	1	1	1	1
1	1	1	1	3	1	1	1	1	3
1	1	1	1	3	1	1	1	1	3
9	9	9	9	9	9	9	9	9	9
1	1	1	1	3	1	1	1	1	3
1	1	1	1	3	1	1	1	1	1
1	1	1	1	3	1	1	1	1	1
1	1	1	1	3	1	1	1	1	3
9	9	9	9	9	9	1	1	1	2
1	1	1	1	3	1	1	1	1	1
1	1	1	1	3	1	1	1	1	1
1	1	1	1	3	1	1	1	1	1
1	1	1	1	3	1	1	1	1	1
1	1	1	1	3	1	1	1	1	1
1	1	1	1	3	1	1	1	1	1
1	1	1	1	3	1	1	1	1	1
1	1	1	1	3	1	1	1	1	3
1	1	1	1	3	1	1	1	1	1
1	1	1	1	3	1	1	1	1	1
1	1	1	1	3	1	1	1	1	1
1	1	1	1	3	1	1	1	1	1
1	1	1	1	3	1	1	1	1	1
1	1	1	1	3	1	1	1	1	1
1	1	1	1	1	3	1	1	1	1
1	1	1	1	3	1	1	1	1	1
9	9	9	9	9	9	9	9	9	9
1	1	1	1	3	1	1	1	1	1
1	1	1	1	3	1	1	1	1	1
1	1	1	1	3	1	1	1	1	1
1	1	1	1	3	1	1	1	1	1
1	1	1	1	3	1	1	1	1	1

1	1	1	1	3	1	1	1	1	1
1	1	1	1	3	1	1	1	1	1
1	1	1	1	3	1	1	1	1	9
1	1	1	1	3	1	1	1	1	1
1	1	1	1	3	1	1	1	1	1
1	1	1	1	3	1	1	1	1	1
1	1	1	1	3	1	1	1	1	1
1	1	1	1	3	1	1	1	1	1
1	1	1	1	3	1	1	1	1	1
1	1	1	1	9	1	1	1	1	3
1	1	1	1	3	1	1	1	1	1
1	1	1	1	3	1	1	1	1	1
1	1	1	1	3	1	1	1	1	1
1	1	1	1	3	1	1	1	1	1
1	1	1	1	3	1	1	1	1	1
1	1	1	1	3	1	1	1	1	1
1	1	1	1	3	1	1	1	1	1
1	1	1	1	3	1	1	1	1	1
1	1	1	1	3	1	1	1	1	1
1	1	1	1	3	1	1	1	1	1
1	1	1	1	3	1	1	1	1	1
1	1	1	1	3	1	1	1	1	1
1	1	1	1	3	1	1	1	1	1
1	1	1	1	3	1	1	1	1	1
1	1	1	1	3	1	1	1	1	1
1	1	1	1	3	1	1	1	1	1
1	1	1	1	3	1	1	1	1	1
9	9	9	9	9	9	9	9	9	9
9	9	9	9	9	9	9	9	9	9
9	9	9	9	9	9	9	9	9	9
9	9	9	9	9	9	9	9	9	9
9	9	9	9	9	9	9	9	9	9
9	9	9	9	9	9	9	9	9	9
9	9	9	9	9	9	9	9	9	9
9	9	9	9	9	9	9	9	9	9
9	9	9	9	9	9	9	9	9	9
9	9	9	9	9	9	9	9	9	9
9	9	9	9	9	9	9	9	9	9
9	9	9	9	9	9	9	9	9	9
9	9	9	9	9	9	9	9	9	9
9	9	9	9	9	9	9	9	9	9
9	9	9	9	9	9	9	9	9	9
2	1	9	9	9	9	9	9	9	9
1	1	1	1	1	1	9	9	9	9
9	9	9	9	9	9	9	9	9	9
9	9	9	9	9	9	9	9	9	9
9	9	9	9	9	9	9	9	9	9

9	9	9	9	9	9	9	9	9	9
9	9	9	9	9	9	9	9	9	9
9	9	9	9	9	9	9	9	9	9
9	9	9	9	9	9	9	9	9	9
9	9	9	9	9	9	9	9	9	9
9	9	9	9	9	9	9	9	9	9
9	9	9	9	9	9	9	9	9	9
9	9	9	9	9	9	9	9	9	9
9	9	9	9	9	9	9	9	9	9
9	9	9	9	9	9	9	9	9	9
9	9	9	9	9	9	9	9	9	9
9	9	9	9	9	9	9	9	9	9
9	9	9	9	9	9	9	9	9	9
1	1	1	1	3	2	1	2	1	2
2	2	2	2	2	2	2	2	2	2
3	1	1	1	2	1	1	3	1	1
1	3	2	3	1	1	1	1	1	2
3	1	1	1	3	1	1	3	1	2
1	1	1	1	3	1	1	1	3	3
3	2	1	2	3	2	1	2	1	2
2	1	2	1	2	1	1	1	1	1
3	1	1	3	1	3	1	3	1	1
3	1	1	1	3	1	3	3	1	2
3	2	2	3	2	3	2	3	2	2
3	1	1	1	2	1	9	9	9	9
2	2	2	2	2	2	2	2	2	2
9	9	9	9	3	3	9	9	9	9
3	1	1	1	3	3	3	1	3	1
1	1	2	1	3	2	2	2	2	1
3	1	3	2	2	1	2	2	1	1
3	2	2	2	2	3	1	3	3	3
2	1	3	2	1	2	2	1	3	1
9	9	1	9	9	9	1	9	9	9
2	1	1	2	3	3	2	1	2	1
3	1	1	1	3	3	1	3	1	1
9	9	2	9	9	9	1	9	9	9
2	1	1	1	2	1	2	2	1	1
3	3	1	2	2	1	2	1	1	2
3	1	1	3	3	1	3	1	1	3
1	1	1	1	1	1	3	1	1	3
9	9	9	9	9	9	9	9	9	9
1	1	1	1	1	1	9	9	9	9
2	2	1	1	1	1	2	1	1	2
1	1	9	9	9	9	1	1	1	1
9	9	9	9	9	9	9	9	9	9
9	9	9	9	9	9	9	9	9	9
2	1	1	2	2	2	3	3	2	2

9	9	9	9	9	1	9	9	9	9
9	9	9	9	9	9	9	9	9	9
2	3	3	2	2	2	2	1	2	2
3	1	1	1	3	1	3	2	2	1
3	1	1	1	1	1	1	2	2	2
9	9	9	9	9	2	2	9	9	9
9	9	2	2	2	2	2	2	2	2
2	2	2	2	2	2	2	2	2	2
2	2	2	2	2	2	2	2	2	2
2	2	2	2	1	1	1	2	2	2
2	3	1	1	3	3	3	2	1	2
9	9	9	9	9	9	9	9	9	9
9	9	9	9	1	9	1	9	2	9
2	2	2	2	2	2	2	2	2	2
1	1	2	3	2	2	3	1	2	2
3	1	1	2	1	1	2	1	1	3
3	1	1	3	1	3	1	3	1	1
3	1	2	2	2	2	2	2	2	2
1	1	1	2	1	1	2	9	1	1
2	1	1	2	1	3	2	1	9	3
2	2	2	2	2	2	2	2	2	2
2	2	2	2	2	2	2	2	2	2
2	2	2	2	2	2	2	2	2	2
2	2	2	2	2	2	2	2	2	2
2	2	2	2	2	2	2	2	2	2
1	3	2	2	3	1	3	3	1	1
2	2	2	2	2	2	2	2	2	2
3	1	2	2	2	2	2	2	2	2
1	1	2	2	2	1	3	1	2	2
3	1	1	1	2	1	1	1	1	1
3	1	1	1	1	1	3	1	2	3
2	1	1	1	2	1	3	1	1	2
3	1	1	1	1	1	2	1	1	1
2	1	1	1	3	1	1	1	1	1
2	1	1	2	2	1	1	1	2	2
2	2	3	3	3	2	3	2	3	2
2	1	1	2	2	2	2	1	1	1
9	9	2	2	2	2	2	2	2	2
9	9	2	9	9	9	2	9	9	9
3	3	1	1	2	1	3	1	1	2
3	3	2	2	2	2	1	1	1	1
2	2	2	2	2	2	1	1	1	1
3	1	1	2	3	1	1	2	3	1
3	1	3	2	3	1	3	1	2	1
1	1	1	1	1	1	1	1	1	1
2	1	3	1	3	2	1	1	1	1
2	2	1	1	3	1	3	1	1	2

2	2	2	2	2	2	2	1	1	2
1	1	2	3	2	2	2	2	2	3
2	2	2	2	2	1	2	9	1	1
3	2	2	3	2	3	2	3	3	2
3	1	1	1	3	1	2	1	1	3
3	1	1	1	2	1	3	1	1	1
3	1	1	1	2	1	2	1	1	3
3	3	1	1	2	3	2	3	1	2
2	2	2	2	2	2	2	2	2	2
2	1	2	1	2	3	1	2	1	3
2	2	2	2	2	2	2	2	2	2
2	2	2	2	2	2	2	2	2	2
3	3	2	2	2	2	1	1	1	1
2	2	2	2	2	2	2	2	2	2
2	2	2	2	2	2	2	2	2	2
2	2	2	3	2	3	2	3	2	3
3	3	3	3	3	3	3	3	3	3
2	2	2	2	2	2	2	2	2	2
2	2	2	2	2	2	2	2	2	2
3	1	1	2	3	1	1	2	3	1
2	2	1	2	1	2	1	2	1	2
1	1	1	1	1	2	1	1	1	1
2	2	2	2	2	2	2	2	2	2
1	1	1	1	1	1	1	1	1	1
3	1	1	1	3	1	1	1	1	3
9	9	9	9	9	9	9	9	9	9
1	2	2	3	1	3	3	1	2	2
2	1	1	2	2	2	2	2	1	2
1	3	1	2	1	1	1	2	2	1
3	1	1	2	9	1	2	1	1	2
2	1	1	1	3	1	2	1	1	2
3	2	2	2	1	2	1	2	2	3
3	3	2	2	2	2	2	2	2	2
1	1	2	1	2	1	3	1	1	1
9	2	1	2	1	1	1	1	1	1
9	9	9	9	9	9	2	2	2	2
3	1	9	9	9	9	9	9	9	9
2	1	1	2	2	2	1	2	1	2
9	9	9	9	9	9	9	9	9	9
1	1	1	1	2	1	1	1	1	1
2	1	2	1	2	1	1	1	1	2
1	1	3	1	2	1	1	1	1	2
2	1	1	1	3	1	2	1	1	2
9	9	9	9	9	9	9	9	9	9
9	9	9	9	9	9	9	9	9	9
9	9	9	9	9	9	9	9	9	9
9	9	9	2	9	9	1	2	3	2

2	2	1	2	2	3	1	1	1	2
9	9	9	9	9	9	9	9	9	9
3	1	1	2	3	1	2	2	1	3
1	3	1	1	3	3	1	1	1	1
9	9	9	9	9	9	9	9	9	9
3	1	1	1	3	1	3	1	1	2
3	2	2	3	3	3	3	3	2	3
3	1	2	2	2	1	1	2	2	3
3	1	1	1	2	1	1	1	1	1
2	2	1	2	3	2	1	2	3	1
1	2	1	2	1	3	1	2	1	2
2	2	2	2	2	2	2	2	2	2
2	2	2	2	2	2	2	2	2	2
1	3	1	1	3	1	9	9	9	9
2	2	2	2	2	2	2	2	2	2
3	1	1	1	2	1	2	1	1	1
3	3	3	3	3	3	3	3	1	1
2	3	1	3	2	3	3	1	1	2
1	1	1	2	3	2	1	2	1	3
1	2	1	2	1	3	9	9	9	9
2	3	3	1	2	3	3	1	1	2
2	2	2	1	3	2	1	1	1	2
3	1	1	1	3	1	1	1	1	1
9	9	9	9	9	9	3	1	2	3
3	2	2	2	3	2	2	3	3	3
9	9	9	9	9	9	9	9	9	9
3	1	1	1	3	1	1	1	1	1
1	1	2	1	1	1	2	2	2	2
3	1	2	2	3	1	2	2	2	2
1	1	1	2	2	3	1	3	1	2
2	1	2	1	2	2	1	1	2	2
9	9	1	9	9	9	1	9	9	9
3	1	1	2	2	1	3	1	1	2
3	1	1	1	3	2	3	1	1	2
2	1	3	1	2	1	1	3	1	2
2	1	2	1	3	1	1	2	1	2
2	2	2	2	2	2	9	9	9	9
3	1	1	1	3	1	1	2	1	1
3	1	1	1	2	1	3	1	1	2
9	9	9	9	1	9	2	9	9	9
9	9	9	9	9	9	1	3	2	2
2	2	2	2	2	2	2	2	2	2
1	1	1	1	3	1	3	1	2	2
2	2	2	2	2	2	2	2	2	2
2	2	2	2	2	2	2	2	2	2
9	9	1	9	9	9	1	9	2	9
2	2	2	2	2	2	2	2	2	2

3	1	1	1	3	1	1	1	1	3
2	2	2	2	2	2	2	2	2	2
1	1	1	2	3	1	1	3	2	2
3	1	1	1	2	1	3	3	1	2
2	2	2	2	2	2	1	3	1	1
2	2	2	2	3	3	2	2	2	2
2	2	1	1	3	1	1	1	1	1
2	1	1	1	3	1	1	2	1	2
1	1	1	1	3	1	1	1	1	1
3	1	1	1	3	1	2	3	1	3
3	1	3	1	3	3	2	3	1	2
2	2	2	3	2	3	3	3	3	3
3	1	1	1	3	1	2	2	2	2
9	1	1	1	1	1	1	2	1	1
3	1	9	9	9	9	9	9	9	9
2	1	2	2	2	1	1	1	1	1
2	3	2	2	2	3	9	2	2	2
1	1	1	2	2	1	1	2	2	2
2	2	2	2	2	2	2	2	3	3
2	1	1	1	3	1	1	2	2	2
3	1	1	1	2	1	1	3	1	1
2	1	1	1	3	1	1	1	3	1
2	1	1	2	3	2	3	3	3	3
2	2	3	2	3	2	1	1	2	2
2	2	2	2	2	2	2	2	2	2
1	1	1	1	3	1	1	3	2	1
2	1	1	1	3	1	1	3	2	2
3	1	1	1	2	1	1	2	2	2
1	1	1	1	3	1	1	1	1	1
2	1	1	1	3	1	1	1	1	1
3	1	1	1	3	1	1	1	1	1
1	1	1	1	1	1	3	1	1	1
2	1	2	1	1	2	1	2	2	1
2	2	1	2	2	2	9	9	9	9
2	2	2	2	2	2	2	9	9	9
3	1	1	1	3	1	1	1	1	1
2	2	2	2	2	2	2	2	2	2
2	3	1	2	1	2	1	2	1	1
1	1	1	1	1	1	1	1	1	1
2	2	2	2	2	2	2	2	2	2
1	2	1	2	3	2	1	2	3	2
9	9	9	9	9	9	9	9	9	9
9	9	9	9	9	9	9	9	9	9
1	1	1	1	1	1	1	1	1	1
3	2	1	2	3	2	1	1	3	2
9	9	9	9	9	9	9	9	9	9
9	9	9	9	9	9	9	9	9	9

2	2	2	2	2	2	2	2	2	2
1	1	2	3	2	1	2	3	2	3
3	1	1	2	3	1	1	2	3	1
2	3	1	2	3	2	1	2	3	2
3	3	1	2	3	3	1	2	2	2
3	1	1	2	3	1	1	2	3	1
2	2	1	2	3	2	1	2	2	2
9	9	2	9	9	9	1	9	9	9
1	2	2	2	2	2	1	1	2	1
9	9	9	9	9	9	9	9	9	9
1	1	1	1	1	1	1	1	1	1
3	3	1	1	2	3	1	3	2	1
3	1	1	2	2	2	9	9	9	9
2	2	2	3	2	2	9	9	9	9
1	1	1	1	1	1	2	2	2	2
9	9	9	9	9	9	9	9	9	9
9	9	9	9	9	9	9	9	9	9
3	3	1	1	2	1	2	2	2	2
2	2	2	2	2	2	2	2	2	2
1	2	2	2	2	2	3	3	1	2
2	2	2	2	2	2	2	2	2	2
2	2	2	2	2	2	2	9	2	2
1	3	1	1	2	2	1	1	3	3
1	2	2	3	1	2	2	3	1	2
9	2	1	2	2	2	1	1	9	2
9	1	9	9	9	1	9	9	9	9
3	1	1	1	1	1	1	1	1	1
3	3	1	2	2	1	1	1	1	1
1	1	3	3	3	3	1	2	1	2
9	9	1	1	2	2	1	2	1	1
3	1	1	1	1	1	1	1	2	1
2	2	9	9	9	9	2	2	2	2
2	2	2	2	2	2	2	2	2	2
2	2	2	2	2	2	2	2	2	2
1	1	1	1	3	1	1	1	1	1
2	2	2	2	2	2	2	2	2	2
2	2	2	2	2	2	2	2	2	2
9	9	1	9	9	9	1	9	9	9
9	2	9	9	9	2	9	9	9	9
2	2	2	2	2	2	2	2	2	2
2	2	2	2	2	2	2	2	2	2
9	9	1	2	3	1	9	9	9	9
2	2	1	2	3	2	1	2	2	2
1	1	2	9	9	9	1	9	9	9
2	2	2	2	2	2	3	3	3	3
2	9	1	2	2	1	9	9	9	9
2	2	2	2	2	2	2	2	2	2

2	3	3	2	3	1	1	3	2	1
9	9	9	1	9	9	1	9	9	9
2	2	2	2	2	2	2	2	2	2
3	1	1	1	2	2	2	3	3	3
2	2	3	3	3	3	1	1	1	2
1	2	3	3	2	1	2	2	2	2
2	1	1	1	3	1	1	1	1	1
3	2	2	2	1	2	1	2	3	1
2	2	2	2	2	2	2	2	2	2
3	3	9	3	3	3	1	1	1	3
1	1	1	1	2	1	1	3	1	1
2	2	2	2	2	2	2	2	2	2
1	2	2	2	2	2	2	2	2	2
2	3	1	2	3	1	3	3	1	1
1	1	1	1	2	1	1	1	1	2
1	1	1	1	2	1	1	1	1	2
2	2	1	2	2	2	2	2	2	2
2	2	2	2	2	2	2	2	2	2
2	3	2	3	2	2	1	2	2	3
3	2	2	2	1	1	2	2	9	1
2	2	1	2	3	1	1	2	1	1
3	1	1	2	2	1	1	2	1	2
3	3	2	1	2	2	1	1	2	3
9	9	9	9	9	9	9	9	9	9
2	2	2	2	2	2	3	3	3	3
9	9	9	1	9	9	9	1	9	9
9	9	9	9	9	9	9	9	9	9
9	9	9	9	9	9	9	9	9	9
1	1	3	3	3	1	1	2	2	2
3	3	1	1	1	1	1	1	1	1
2	2	2	2	2	2	2	2	2	2
1	3	1	2	9	1	1	3	2	1
2	2	2	2	2	2	2	2	2	2
2	2	2	2	2	2	2	2	2	2
2	1	1	1	1	1	1	9	1	1
3	1	1	1	1	1	1	1	1	1
2	1	1	2	2	1	1	2	1	1
2	3	3	1	2	1	1	9	3	2
3	3	2	3	1	2	1	2	3	2
3	1	1	1	2	1	1	1	1	1
2	1	1	1	1	1	1	1	1	1
2	2	2	2	2	3	2	2	2	2
1	9	9	9	9	1	1	9	9	9
9	9	9	1	9	9	9	9	9	2
2	3	1	3	2	1	1	2	2	3
2	2	2	2	2	2	2	2	2	2
2	2	2	2	2	2	2	2	2	2

2	2	1	1	2	3	1	2	3	2
9	9	9	9	9	9	9	9	9	9
9	9	9	9	9	9	9	9	9	9
9	9	9	9	9	9	9	9	9	9
9	9	9	9	9	9	9	9	9	9
9	1	1	9	9	9	1	9	9	9
9	9	9	9	9	9	9	9	9	9
9	9	9	9	9	9	9	9	9	9
9	9	9	9	9	9	9	9	9	9
2	1	1	2	3	1	9	9	9	9
2	9	2	2	2	2	1	2	2	2
2	1	2	2	2	2	1	2	2	2
3	1	1	1	1	1	1	1	1	1
2	2	2	2	2	2	2	1	2	2
2	2	1	1	2	1	2	2	2	2
2	2	2	1	3	2	1	3	2	3
1	2	1	1	2	1	2	1	1	1
3	1	2	1	3	1	2	2	2	2
1	2	1	1	3	1	1	3	1	1
1	1	1	1	2	1	1	1	1	2
2	1	1	2	1	3	1	2	1	3
2	1	1	2	1	3	1	2	1	3
1	1	2	2	2	2	3	3	3	3
2	2	2	2	2	2	2	2	2	2
2	2	2	2	2	2	2	2	2	2
2	2	2	2	2	2	2	2	2	2
3	3	3	3	3	3	2	2	2	2
2	1	1	2	1	2	1	1	1	2
2	2	2	2	2	2	2	2	2	2
2	2	2	2	2	2	2	2	2	2
9	9	9	9	9	9	9	9	9	9
2	2	2	2	2	2	2	2	2	2
1	1	2	2	2	2	1	1	1	1
9	9	1	9	9	9	1	9	9	9
2	2	3	3	3	3	9	9	9	9
2	2	2	2	2	2	2	2	2	2
2	2	2	2	2	2	2	2	2	2
2	2	2	2	2	2	2	2	2	2
2	1	1	1	1	3	3	3	1	1
9	9	9	9	9	9	9	9	9	9
2	2	1	1	1	1	2	2	1	2
2	2	2	2	2	2	2	2	2	2
2	2	2	2	2	2	2	2	2	2
3	1	1	1	3	1	1	1	1	1
3	1	1	1	3	1	1	1	1	1
9	9	9	9	9	9	9	9	9	9
2	2	1	2	2	1	2	1	2	2

9	9	9	9	9	9	9	9	9	9
2	2	1	2	3	2	3	1	1	1
9	9	9	9	1	9	1	9	9	9
9	9	9	9	9	9	9	9	9	9
9	9	9	9	9	9	9	9	9	9
9	9	9	9	9	9	9	9	9	9
9	9	9	9	9	9	9	9	9	9
9	9	9	9	9	9	9	9	9	9
9	9	9	9	9	9	9	9	9	9
9	9	9	9	9	9	9	9	9	9
9	9	9	9	9	9	9	9	9	9
9	9	9	9	9	9	9	9	9	9
9	9	9	9	9	9	9	9	9	9
9	9	9	9	9	9	9	9	9	9
9	9	9	9	9	9	9	9	9	9
9	9	9	9	9	9	9	9	9	9
9	9	9	9	9	9	9	9	9	9
9	9	9	9	9	9	9	9	9	9
9	9	9	9	9	9	9	9	9	9
9	9	9	9	9	9	9	9	9	9
9	9	9	9	9	9	9	9	9	9
9	9	9	9	9	9	9	9	9	9
9	9	9	9	9	9	9	9	9	9
9	9	9	9	9	9	9	9	9	9
9	9	9	9	9	9	9	9	9	9
2	3	1	2	3	2	1	2	3	2
2	2	1	1	2	1	2	2	2	2
3	1	3	1	1	1	1	1	1	1
3	1	1	2	2	1	1	1	1	1
3	1	1	1	3	1	3	1	1	1
3	1	1	9	9	1	1	1	1	1
2	2	1	2	2	1	1	1	1	1
2	1	1	1	2	1	1	1	1	1
2	1	1	2	2	1	1	1	2	2
1	1	2	1	2	1	1	1	2	2
3	1	1	9	1	1	2	1	1	1
3	1	1	2	2	1	1	1	1	1
3	1	1	1	2	1	1	3	1	1
3	1	1	1	3	1	1	1	1	1
3	1	1	1	2	1	1	1	1	1
3	1	1	1	2	1	1	1	1	1
9	9	1	9	9	9	9	9	9	9
3	1	1	1	2	1	1	1	1	1
3	3	1	1	3	1	1	1	1	2
9	9	1	2	9	9	1	9	9	9
3	1	1	1	2	1	1	1	1	1
3	1	1	1	1	1	1	1	1	1
2	2	1	2	2	2	1	2	2	2
2	1	3	3	2	1	1	1	1	1

3	1	1	1	2	1	1	1	1	1
3	1	1	1	1	1	3	1	1	1
3	1	1	1	3	1	1	1	1	2
3	1	2	1	2	1	1	1	1	1
2	3	2	1	2	3	2	1	2	3
9	9	1	9	9	9	1	9	9	9
3	1	1	1	2	1	1	1	1	1
9	9	1	9	9	9	1	9	9	9
3	1	1	1	2	1	1	2	1	1
3	1	1	1	2	1	1	2	1	1
9	1	1	9	9	9	1	9	9	9
9	1	9	9	9	1	9	9	9	9
9	9	1	9	9	9	1	9	9	9
9	9	9	9	9	1	1	9	9	9
3	1	1	3	1	1	1	9	9	9
3	1	1	3	1	3	9	9	9	9
1	3	1	1	1	1	1	1	1	1
2	2	1	1	2	1	1	2	1	2
9	9	9	9	9	1	1	9	9	9
3	1	1	1	3	1	3	1	3	3
1	1	1	1	3	1	1	1	9	9
9	1	1	9	9	1	3	1	9	9
3	1	1	1	3	1	1	9	9	9
9	9	1	9	9	9	1	9	9	9
9	9	1	9	9	9	1	9	9	9
2	1	1	2	3	2	1	2	2	1
3	1	3	1	3	1	1	1	1	1
3	1	2	2	2	1	1	1	1	2
3	1	1	2	1	1	1	1	1	1
2	2	2	2	2	2	2	2	2	2
3	1	1	1	1	1	1	1	1	1
3	1	3	9	9	1	1	1	1	1
1	3	1	3	3	3	1	2	1	1
1	1	1	3	3	1	1	3	1	3
1	1	1	1	3	1	1	2	1	2
9	9	1	9	9	9	1	9	9	9
2	2	1	2	2	2	1	2	9	1
3	3	2	1	3	2	2	2	3	2
2	2	2	3	2	3	2	3	2	1
2	2	2	2	2	2	2	2	2	2
3	3	1	1	3	3	1	1	1	1
3	1	1	1	3	1	1	1	1	1
9	1	1	1	3	1	1	1	1	1
3	2	2	2	1	3	2	2	2	1
3	1	1	2	3	1	1	1	1	1
3	1	1	1	2	1	9	1	1	9
3	1	1	1	3	1	1	1	1	1

3	1	1	1	2	1	1	1	1	1
2	3	2	1	3	3	1	2	3	2
3	1	1	3	2	1	1	1	1	1
1	3	1	1	3	1	1	9	3	3
9	9	2	3	1	2	1	9	9	9
3	1	1	1	2	1	1	3	2	2
3	1	1	1	3	1	1	1	1	1
9	9	9	9	9	9	9	9	9	9
9	9	2	9	9	9	2	9	9	9
1	1	2	2	2	2	2	2	3	3
2	2	2	2	2	2	2	1	2	2
3	3	1	1	3	1	1	3	3	3
1	1	1	1	1	1	1	1	1	1
9	9	9	9	9	9	9	9	9	9
9	9	9	9	9	9	9	9	9	9
3	3	3	3	3	3	2	2	3	3
9	9	1	9	9	9	1	9	9	9
9	9	9	9	9	9	9	9	9	9
2	1	3	3	3	3	1	1	1	1
3	1	1	1	2	1	1	1	1	1
3	1	2	3	3	2	1	1	1	1
1	1	1	1	1	1	1	1	1	1
1	3	3	3	3	3	3	1	2	2
3	1	1	1	2	1	1	1	1	1
1	1	1	1	1	1	1	1	1	1
9	9	9	9	9	9	9	9	9	9
9	9	9	9	9	9	1	9	9	9
1	1	1	3	3	1	1	1	3	1
3	3	3	3	3	3	3	3	2	2
3	1	1	2	3	2	1	1	1	1
9	9	9	9	9	9	9	9	9	9
9	9	9	9	9	9	9	9	9	9
9	9	9	9	9	9	9	9	9	9
9	9	9	9	9	9	9	9	9	9
9	9	9	9	9	9	9	9	9	9
9	9	9	9	9	9	9	9	9	9
9	9	9	9	9	9	9	9	9	9
9	9	9	9	9	9	9	9	9	9
9	9	9	9	9	9	9	9	9	9
9	9	9	9	9	9	9	9	9	9
9	9	9	9	9	9	9	9	9	9
9	9	9	9	9	9	9	9	9	9
9	9	9	9	9	9	9	9	9	9
9	9	9	9	9	9	9	9	9	9
9	9	9	9	9	9	9	9	9	9
9	9	9	9	9	9	9	9	9	9
9	9	9	9	9	9	9	9	9	9

9	9	9	9	9	9	9	9	9	9
9	9	9	9	9	9	9	9	9	9
9	9	9	9	9	9	9	9	9	9
9	9	9	9	9	9	9	9	9	9
9	9	9	9	9	9	9	9	9	9
9	9	9	9	9	9	9	9	9	9
9	9	9	9	9	9	9	9	9	9
9	9	9	9	9	9	9	9	9	9
9	9	9	9	9	9	9	9	9	9
9	9	9	9	9	9	9	9	9	9
9	9	9	9	9	9	9	9	9	9
9	9	9	9	9	9	9	9	9	9
9	9	9	9	9	9	9	9	9	9
9	9	9	9	9	9	9	9	9	9
9	9	9	9	9	9	9	9	9	9
9	9	9	9	9	9	9	9	9	9
9	9	9	9	9	9	9	9	9	9
9	9	9	9	9	9	9	9	9	9
9	9	9	9	9	9	9	9	9	9
2	2	2	2	2	2	1	1	1	2
9	9	9	9	9	9	9	9	9	9
9	9	9	9	9	9	9	9	9	9
9	9	9	9	9	9	9	9	9	9
9	9	9	9	9	9	9	9	9	9
9	9	9	9	9	9	9	9	9	9
9	9	9	9	9	9	9	9	9	9
9	9	9	9	9	9	9	9	9	9
9	9	9	9	9	9	9	9	9	9
9	9	9	9	9	9	9	9	9	9
9	9	9	9	9	9	9	9	9	9
9	9	9	9	9	9	9	9	9	9
9	9	9	9	9	9	9	9	9	9
9	9	9	9	9	9	9	9	9	9
9	9	9	9	9	9	9	9	9	9
9	9	9	9	9	9	9	9	9	9
9	9	2	9	9	9	2	2	2	2
3	1	1	1	1	1	3	1	1	3
3	1	1	1	3	1	1	1	1	2
3	1	1	1	3	1	1	3	1	1
2	2	3	3	3	3	2	3	3	3
1	1	1	1	1	1	3	1	1	1
3	1	1	2	3	1	1	3	1	1
9	1	1	9	9	9	1	9	9	9
3	1	1	2	3	1	1	3	1	1
1	2	2	2	1	2	2	2	2	1
1	1	1	1	2	2	1	1	1	1
3	1	1	1	2	1	3	2	3	2

3	1	1	1	2	1	1	1	1	1
3	1	1	1	3	1	1	1	1	1
2	1	1	3	3	1	1	1	1	1
3	1	1	1	1	1	1	1	1	1
3	1	1	1	3	1	1	1	1	2
3	1	1	1	2	1	1	1	1	1
3	1	1	1	3	1	1	9	1	1
2	1	1	1	3	1	1	1	1	1
3	2	3	2	1	2	3	2	2	2
3	1	1	1	1	1	1	1	1	1
9	9	9	9	9	9	1	1	1	1
2	1	2	2	3	1	1	2	2	2
3	3	1	1	2	1	1	1	1	1
3	1	2	2	2	1	1	1	1	1
2	1	2	2	2	1	1	1	1	2
1	1	1	1	2	1	1	1	1	1
9	9	1	1	2	1	1	1	1	1
9	1	1	1	3	1	1	1	1	2
1	1	1	1	3	1	1	1	1	1
1	3	1	2	1	1	3	1	2	1
3	1	1	1	3	1	1	1	1	9
3	1	1	1	3	1	1	1	1	1
3	1	1	1	3	1	1	1	1	1
3	1	3	1	1	1	1	1	3	3
3	3	3	3	3	3	3	3	3	3
1	2	1	2	1	2	1	2	1	2
1	1	2	2	2	2	1	1	1	1
2	1	3	2	1	1	1	2	3	1
2	2	2	2	2	2	2	2	2	2
2	2	2	2	2	2	2	2	2	2
2	1	2	2	3	2	2	2	2	1
2	2	1	1	2	1	2	2	2	2
2	2	1	2	2	1	2	2	2	2
9	9	9	9	9	9	9	9	9	9
9	9	9	9	9	9	9	9	9	9
9	9	9	9	9	9	9	9	9	9
2	3	2	1	2	1	9	9	9	9
1	3	9	9	9	9	9	9	9	9
9	9	2	1	3	1	9	9	9	9
9	9	9	9	9	9	9	9	9	9
3	1	1	1	2	1	1	2	3	2
2	1	1	1	2	1	9	9	9	9
3	1	1	1	3	1	1	2	1	1
3	1	1	1	3	1	3	3	3	3
3	3	3	1	1	1	1	1	1	1
3	9	3	3	3	1	3	3	3	3
1	1	1	1	1	3	1	3	1	1

2	2	3	2	3	2	2	3	2	3
2	3	2	3	2	2	1	1	1	1
2	1	1	2	2	2	2	1	3	1
1	3	1	1	1	1	1	3	1	1
2	2	2	1	2	1	2	2	1	2
1	2	1	2	1	3	1	2	3	1
1	1	1	1	3	1	3	3	1	1
2	1	2	3	2	1	3	2	3	1
3	1	1	1	1	1	1	1	1	1
9	9	1	9	9	9	1	9	9	2
3	1	1	1	3	1	3	3	1	3
3	1	1	2	1	2	1	2	1	2
2	1	1	1	2	1	1	2	1	2
3	1	1	1	3	1	3	1	1	1
1	1	1	1	1	1	3	1	1	1
1	9	2	2	2	3	1	1	2	3
2	2	2	2	3	2	2	2	2	3
9	1	9	9	9	1	9	9	9	9
2	2	1	1	1	2	2	3	1	2
3	1	3	1	3	1	3	3	2	2
3	1	3	2	3	2	3	2	1	1
2	1	3	1	1	2	3	3	3	3
3	1	1	1	3	9	3	1	1	1
3	1	1	1	2	1	3	3	1	2
2	1	1	2	3	1	1	2	1	1
1	3	1	1	1	1	1	3	1	1
3	1	1	1	3	1	1	2	1	1
3	3	1	2	3	1	1	1	2	3
3	1	1	3	1	1	1	1	3	3
2	1	1	1	2	1	1	3	2	3
1	1	1	1	2	1	1	2	1	1
2	1	1	2	3	1	1	2	3	1
9	1	1	1	1	3	2	3	2	2
1	3	1	1	1	1	1	3	1	1
1	1	1	3	1	1	1	1	1	1
1	1	1	3	3	1	1	1	1	3
1	3	1	2	1	3	1	2	3	1
9	9	9	9	9	9	9	9	9	9
1	1	1	3	3	1	1	3	3	3
1	1	1	3	3	1	1	3	1	1
3	1	1	1	1	1	1	3	1	1
3	1	1	3	2	3	1	2	3	1
1	1	9	1	1	1	1	3	1	1
2	2	3	2	1	1	2	3	3	2
2	2	2	2	2	2	2	2	2	2
2	1	2	3	2	1	1	1	1	3
3	3	3	3	3	9	3	3	3	3

1	2	1	1	2	1	1	2	1	2
1	1	1	1	1	1	9	9	9	9
3	1	2	2	2	2	2	2	3	1
2	1	2	2	3	3	1	1	1	2
2	2	1	1	1	2	3	3	3	3
1	3	1	2	3	1	1	1	2	2
2	2	2	2	2	2	2	2	2	2
9	9	9	9	9	9	9	9	9	9
9	9	9	9	9	9	9	9	9	9
2	2	1	2	2	1	1	2	2	1
2	2	2	2	2	2	1	2	2	2
2	2	2	2	2	2	2	2	2	2
2	3	2	3	3	3	2	2	2	2
1	2	2	2	2	2	2	1	1	1
2	1	2	2	2	2	1	1	1	1
2	2	2	2	2	2	2	2	1	1
2	3	2	2	2	2	2	2	2	2
2	2	2	2	2	2	1	2	2	2
2	2	2	2	2	2	1	2	1	2
2	2	2	2	2	2	2	2	2	2
2	2	1	1	2	2	2	2	2	2
2	2	2	2	2	2	2	2	2	2
2	2	2	2	2	2	2	2	2	2
2	2	2	2	2	2	1	1	2	2
2	2	2	2	2	2	2	2	2	2
2	2	2	2	2	2	2	2	2	2
2	2	2	2	2	2	1	2	2	2
1	2	1	2	3	2	1	2	2	1
2	2	2	2	2	2	1	1	1	2
2	2	2	2	2	2	2	2	2	2
2	2	2	2	2	2	2	2	2	2
2	2	2	2	2	2	1	2	2	2
2	2	2	2	2	2	2	2	2	2
2	2	2	2	2	2	2	2	2	2
2	1	2	2	2	2	1	2	2	2
2	1	2	2	2	2	1	2	2	2
2	2	2	2	2	2	2	2	2	2
2	2	2	2	2	2	2	2	2	2
2	2	2	2	2	2	2	2	2	2
2	1	3	3	3	1	1	3	1	1
3	1	2	2	2	2	1	3	2	2
2	2	2	2	2	2	2	2	2	2
2	2	2	1	2	2	2	2	1	1
2	2	2	2	2	2	2	2	2	2
1	3	1	2	3	3	2	1	1	2
2	2	2	2	2	2	3	3	3	3
3	3	9	3	3	3	3	3	3	3

2	2	2	2	2	2	2	2	2	2
2	2	1	1	2	2	2	2	2	2
2	2	2	2	2	2	1	2	2	2
2	2	2	2	2	2	2	2	2	2
2	1	2	3	3	2	1	2	3	3
2	2	2	2	2	2	2	2	2	2
1	3	1	1	3	3	9	9	9	9
1	1	1	2	3	1	1	1	1	1
9	9	1	1	3	1	1	9	9	9
2	2	2	2	2	2	2	2	2	2
3	2	2	2	2	2	2	3	2	3
1	1	2	2	1	1	3	3	3	2
3	2	2	2	2	2	2	2	3	2
9	9	1	9	9	9	3	9	9	9
2	2	1	1	1	1	2	2	2	2
2	1	2	1	2	2	1	1	1	1
2	2	1	1	1	1	1	1	2	2
1	1	1	2	1	1	1	1	1	1
9	9	9	9	9	1	9	1	9	9
3	2	3	1	1	1	1	1	1	2
2	2	2	2	2	2	1	2	2	2
9	9	1	9	9	9	1	9	9	9
9	9	1	9	9	9	1	9	9	9
2	2	1	1	2	2	1	3	1	3
1	2	1	2	3	1	2	3	9	1
3	3	1	2	1	1	1	9	3	3
1	1	1	3	1	1	1	2	3	3
1	1	1	1	3	1	1	9	3	9
1	3	1	2	3	1	1	2	3	1
1	1	1	1	1	1	1	1	1	1
2	2	2	2	2	2	3	3	3	3
2	3	2	2	2	2	2	2	2	2
2	2	2	2	2	2	2	2	2	2
2	9	1	2	2	9	1	9	9	9
3	1	1	1	1	1	1	2	2	2
9	9	1	9	9	9	9	9	9	9
3	1	1	1	3	3	1	3	1	1
1	1	3	3	3	3	2	2	2	2
2	2	2	2	2	2	2	2	2	2
2	2	2	2	2	2	2	2	2	2
9	9	9	9	9	9	1	3	3	2
2	2	2	2	2	2	2	2	2	2
9	9	9	1	9	9	9	9	9	9
1	1	2	2	3	1	2	2	2	2
2	2	1	2	2	2	1	1	1	1
2	2	1	1	3	1	3	9	2	3
2	2	1	1	1	1	2	2	2	2

1	1	1	1	1	2	1	1	1	1
2	2	2	2	2	2	2	2	2	2
1	1	1	1	1	1	1	1	1	1
2	2	2	2	2	2	2	2	2	2
9	9	1	9	9	9	9	9	1	9
1	1	3	2	2	1	1	1	2	3
3	3	3	9	9	9	3	3	3	3
2	2	1	1	1	1	1	1	1	1
2	2	2	2	2	2	2	2	2	2
1	1	1	1	3	3	1	1	1	1
2	3	2	2	2	2	3	3	3	3
3	3	3	3	3	3	1	1	1	1
1	2	2	2	2	2	2	2	2	2
1	2	2	2	2	2	2	2	2	2
2	2	2	2	2	2	2	2	2	2
2	2	2	2	2	2	1	1	1	1
1	1	1	1	1	1	1	1	1	1
3	3	1	2	2	3	1	3	3	3
9	9	9	9	9	9	9	9	9	9
1	1	1	2	1	3	9	9	9	9
2	2	1	1	2	2	1	1	2	2
2	2	2	2	2	2	2	2	2	2
1	1	1	1	1	1	1	1	1	1
1	1	1	2	2	2	1	2	2	2
9	3	9	3	9	3	3	3	3	3
2	1	1	2	2	2	1	1	1	1
1	2	1	1	2	3	1	2	2	1
2	1	2	2	2	2	2	1	2	1
9	9	2	9	2	9	1	2	1	9
1	1	2	2	2	2	2	2	3	3
2	1	1	2	1	2	2	9	1	9
2	2	2	2	2	2	2	2	2	2
3	3	3	3	3	3	1	9	1	1
3	1	2	3	1	2	1	2	3	1
2	2	2	2	2	2	2	2	2	2
2	3	2	2	3	2	2	2	2	2
3	3	2	2	2	2	1	2	3	3
2	2	3	2	2	2	2	2	2	2
1	2	1	2	3	1	1	2	3	1
3	3	3	3	3	3	3	3	3	3
1	2	1	2	3	1	1	2	1	2
2	1	2	1	3	1	2	2	2	2
9	9	9	9	9	9	9	9	9	9
3	3	3	3	3	3	3	9	9	9
1	2	1	1	2	2	1	1	1	1
2	2	2	2	2	2	2	2	2	2
3	1	1	1	2	1	1	1	2	2

1	1	2	2	2	9	1	1	1	1
1	1	3	3	3	1	1	3	3	1
1	1	2	1	1	1	2	2	2	2
1	1	3	3	3	1	1	3	3	1
1	1	1	1	1	3	1	1	2	1
2	2	2	2	2	2	2	2	2	2
2	2	2	2	2	2	2	2	2	2
2	2	2	2	2	2	2	2	2	2
1	1	1	1	1	1	2	2	2	2
1	2	2	1	2	2	2	1	2	2
1	2	2	2	2	2	2	2	2	2
3	1	1	2	2	3	1	2	3	1
2	2	1	2	3	1	1	2	2	1
2	1	3	3	3	3	1	1	1	1
2	2	2	2	2	2	2	2	2	2
2	1	1	2	3	2	3	2	1	2
2	2	1	2	2	2	1	2	2	2
2	1	1	1	1	2	2	2	2	2
2	1	1	2	2	2	2	2	2	2
1	1	1	1	3	1	2	2	1	1
2	1	2	2	2	1	1	2	2	2
1	1	2	2	2	2	2	2	2	2
1	1	1	2	1	1	3	1	1	1
2	2	2	2	2	2	2	2	2	2
1	1	1	1	1	1	1	1	1	1
2	2	2	2	2	2	2	2	2	2
1	3	1	1	9	3	1	1	3	3
1	3	1	1	1	1	2	3	3	3
3	2	2	2	3	1	1	2	2	2
3	1	1	2	3	1	1	3	1	2
1	1	1	3	2	2	1	3	3	1
2	2	2	1	3	2	1	2	2	3
2	1	2	2	2	2	2	2	2	2
2	2	2	2	2	2	2	2	2	2
1	1	1	1	3	3	1	2	2	1
2	3	1	2	2	1	3	3	3	3
2	2	2	2	2	2	1	9	2	2
9	9	9	9	9	9	9	9	9	9
1	9	2	2	1	2	2	2	2	2
2	1	1	1	2	1	2	2	1	2
2	1	1	1	2	1	2	2	1	2
2	2	2	2	2	2	2	2	2	2
2	2	2	2	2	2	2	2	2	2
2	2	2	2	2	2	2	2	2	2
9	9	1	1	1	3	9	9	9	9
1	1	1	1	1	3	1	1	2	1
2	2	9	1	3	2	2	2	2	2

9	9	9	9	9	9	9	9	9	9
3	1	1	1	2	3	1	2	2	3
1	2	1	1	1	2	3	3	1	2
1	1	1	1	3	2	1	3	1	3
3	1	1	1	3	1	1	3	3	1
3	1	1	1	3	1	1	3	9	3
3	1	1	1	3	1	1	3	1	3
9	9	9	9	9	9	9	9	9	9
1	2	2	2	3	2	3	3	3	3
3	3	3	2	3	2	3	3	3	2
2	1	1	2	2	2	2	2	2	2
3	2	1	2	2	3	1	3	2	2
9	9	9	9	9	9	1	2	2	2
3	1	1	1	3	1	1	1	1	1
2	3	1	2	3	2	1	2	3	2
2	2	2	2	2	2	2	2	2	2
2	1	2	3	2	2	1	2	2	1
1	2	2	2	2	2	2	2	1	3
9	9	9	1	9	9	1	9	9	9
2	2	2	2	2	2	9	9	9	9
9	9	9	9	9	9	9	9	9	9
3	3	1	2	2	3	3	2	3	1
3	2	1	2	3	2	2	1	1	1
1	2	3	2	1	2	1	1	2	2
2	3	1	2	3	1	1	1	2	2
2	2	3	3	3	3	2	3	3	3
1	2	2	1	2	2	2	2	2	3
2	2	1	1	1	2	2	2	2	2
3	2	1	1	2	3	2	3	2	1
3	1	2	1	1	1	1	3	1	2
2	2	2	2	3	1	3	3	3	2
1	2	2	3	2	1	1	3	2	2
3	1	1	1	3	3	3	3	2	2
2	2	3	3	3	3	2	2	2	2
2	2	2	2	2	2	2	2	2	2
2	2	2	2	2	2	2	2	2	2
3	3	3	3	3	3	3	3	3	3
2	3	9	9	9	9	2	2	2	2
3	1	1	2	3	3	9	9	9	9
3	2	1	1	2	3	9	9	9	9
1	1	3	3	3	3	1	2	3	1
2	2	2	2	2	2	2	2	2	2
2	3	1	2	3	1	3	2	1	2
3	2	1	3	3	2	2	1	2	3
9	9	9	9	9	9	9	9	9	9
3	3	3	3	3	3	3	3	3	3
2	3	1	2	3	2	1	3	1	1

2	2	1	1	1	1	1	1	1	1
2	1	1	1	3	1	2	2	1	1
2	3	2	1	2	1	1	2	3	1
2	2	1	1	2	1	2	2	2	2
2	3	2	3	2	3	1	2	1	3
3	3	3	3	3	3	1	3	3	3
1	1	1	1	1	1	1	1	1	1
3	3	3	3	3	3	3	3	3	3
1	1	2	2	1	2	2	2	3	3
3	1	2	1	3	1	1	1	1	2
2	2	2	2	2	2	2	2	2	2
2	2	2	2	2	2	1	1	1	1
1	2	1	2	1	2	9	9	2	3
9	9	9	1	9	9	1	2	3	3
2	1	2	2	3	3	2	2	2	2
3	3	2	2	2	2	3	3	3	3
1	1	1	1	1	1	3	1	1	1
2	2	1	2	3	1	1	2	3	1
2	2	1	2	2	2	2	2	2	2
1	2	1	1	2	2	2	3	2	1
3	1	1	1	3	3	1	2	3	2
9	9	9	9	9	9	3	3	3	3
2	1	1	3	3	2	3	3	3	3
2	1	1	1	2	2	2	2	3	3
2	3	1	2	3	2	1	2	3	1
1	1	1	1	1	3	2	2	2	3
1	1	1	1	2	3	2	2	3	3
1	2	1	2	3	2	1	2	3	1
1	1	2	1	3	1	2	3	1	2
3	3	1	1	2	2	1	1	1	1
1	3	1	2	3	1	1	2	3	1
3	3	2	2	2	2	1	1	1	1
2	2	2	2	2	2	2	2	2	2
1	1	1	1	3	3	1	1	1	1
1	2	1	2	3	1	1	2	3	1
3	2	1	1	3	1	3	2	3	2
2	1	1	3	2	3	2	1	3	2
1	1	1	3	3	3	3	2	1	2
1	1	1	1	3	1	1	1	1	1
2	3	1	1	1	3	1	2	1	2
3	3	3	3	2	2	1	2	2	2
1	1	1	1	3	1	3	3	1	1
2	3	1	2	3	2	1	2	1	2
1	1	2	1	2	2	1	2	1	1
2	1	1	2	3	1	1	2	3	2
1	2	3	2	1	2	1	2	3	1
1	3	3	3	1	1	1	1	3	3

1	2	1	1	1	1	2	3	3	2
2	3	1	2	3	1	1	3	1	2
2	2	3	3	1	2	2	2	2	2
3	3	1	1	1	1	1	1	1	1
3	1	1	2	3	1	1	2	3	1
2	2	1	1	2	2	1	3	2	1
2	1	1	2	1	3	1	1	2	3
2	1	1	3	2	1	1	2	1	2
1	3	3	1	1	1	1	3	1	3
1	1	3	1	1	1	3	3	1	1
1	3	1	2	3	1	1	2	3	1
1	3	2	2	3	1	1	2	3	3
3	3	1	1	1	1	1	1	1	1
3	1	1	1	3	3	1	1	3	3
1	2	3	3	2	2	1	1	1	2
1	1	1	1	1	1	1	3	1	1
1	1	1	1	1	1	1	3	1	2
1	1	1	1	1	3	3	3	3	2
1	1	1	1	2	3	1	2	1	1
1	1	1	2	2	1	2	1	1	1
1	1	1	2	2	1	1	1	2	2
3	1	1	1	3	3	1	1	1	1
1	1	1	1	1	1	1	3	1	1
1	1	1	1	1	1	1	9	1	1
2	2	1	1	2	2	2	2	2	2
2	2	2	2	2	2	2	2	2	2
1	1	1	3	3	1	3	3	2	3
2	1	3	2	1	1	1	2	3	1
2	2	2	2	2	2	2	2	2	2
9	9	9	9	9	9	9	9	9	9
9	9	9	9	9	9	9	9	9	9
9	9	9	9	9	9	9	9	9	9
2	2	1	1	2	2	2	2	2	1
9	9	9	9	9	1	9	1	9	9
2	2	3	3	3	3	2	2	2	2
3	3	2	2	2	2	9	9	9	9
1	2	1	2	2	1	1	2	1	2
9	9	9	9	9	2	1	9	9	9
1	1	9	1	1	1	1	2	1	1
2	2	1	3	3	2	2	2	3	2
2	1	1	2	3	1	9	9	9	9
1	1	3	1	3	1	1	3	3	1
2	2	3	1	2	1	3	1	2	2
2	2	3	3	2	1	3	1	2	2
1	3	1	2	3	1	1	3	1	2
3	2	1	2	3	2	1	2	3	2
1	1	1	3	3	1	3	3	2	3

9	9	1	1	2	2	1	2	2	2
2	2	1	1	2	2	2	2	2	1
2	3	3	2	3	2	2	2	2	2
3	1	1	3	1	1	1	2	1	1
1	1	1	1	9	1	2	2	2	2
1	2	2	1	3	1	1	2	1	2
9	1	9	9	9	1	9	9	9	9
1	2	2	2	1	3	2	2	1	1
3	1	1	1	3	1	3	1	2	2
3	3	3	3	3	3	3	3	3	3
3	1	1	1	2	3	2	3	1	2
3	1	1	3	2	3	3	2	2	1
1	2	1	2	2	2	1	3	2	1
2	2	2	2	2	2	2	2	2	2
2	2	2	2	2	2	2	2	2	2
9	9	1	2	9	2	9	1	2	2
3	3	2	2	2	2	2	2	2	2
2	2	2	2	2	2	2	2	2	2
2	2	2	2	2	2	2	2	2	2
2	2	2	2	2	2	9	2	2	2
1	1	1	1	1	1	1	1	1	1
9	1	1	1	1	1	1	1	1	1
9	1	1	1	1	1	1	1	1	1
3	1	1	1	1	1	1	1	1	1
3	1	1	1	1	1	1	1	1	1
9	9	1	9	9	9	1	1	1	1
3	9	1	1	2	2	3	3	1	3
9	1	1	1	1	1	1	1	1	1
1	1	1	1	1	1	1	2	1	2
3	1	1	9	1	1	1	1	1	1
9	9	1	1	1	1	1	1	1	1
9	9	9	1	9	2	1	9	9	9
3	1	1	1	1	1	1	1	1	1
3	1	1	1	1	1	1	1	1	1
9	9	1	1	1	1	1	1	1	1
3	1	1	1	1	1	1	1	1	1
9	1	1	1	1	1	1	1	1	1
9	9	1	1	1	1	9	1	1	1
9	9	1	9	1	1	1	1	1	1
9	9	1	1	1	1	1	1	1	1
3	1	1	1	1	1	1	1	1	1
9	1	1	1	1	1	1	9	1	9
3	3	1	1	1	1	1	1	1	1
9	9	9	9	9	9	1	1	1	1
9	9	1	1	1	1	9	1	1	1
3	1	1	3	1	1	1	1	1	1
9	9	1	9	1	1	3	1	1	3

9	1	1	1	1	1	1	1	1	1
2	1	1	1	1	1	3	3	2	2
3	1	1	1	1	1	1	1	1	1
1	1	1	1	1	1	1	1	1	1
9	9	1	1	1	1	1	1	1	1
9	9	1	1	1	1	1	1	1	1
1	1	1	1	1	1	1	1	1	1
2	1	1	1	1	1	1	3	1	1
9	9	1	1	1	1	1	1	1	1
3	1	1	1	1	1	1	1	1	1
3	1	1	1	1	1	1	1	1	1
3	1	1	1	1	1	1	1	1	3
9	9	1	1	1	1	1	1	1	1
9	9	1	1	1	1	1	1	1	1
9	9	1	1	1	1	1	1	1	3
9	9	1	1	1	1	9	1	1	1
9	9	1	1	1	1	1	1	1	1
9	9	1	1	1	1	1	1	1	1
9	9	1	1	1	1	1	1	1	1
9	9	1	9	9	1	1	3	1	1
1	1	1	9	9	9	1	1	9	1
9	9	1	9	9	1	1	1	1	3
3	1	1	1	1	1	1	1	1	1
9	1	1	1	1	1	1	1	1	1
3	1	1	1	1	1	3	1	1	1
1	1	1	1	1	1	1	3	1	1
3	3	1	3	1	1	1	1	1	1
3	2	1	1	1	1	1	3	1	1
3	1	1	1	1	1	1	1	1	1
9	9	1	1	1	1	9	1	9	9
1	1	1	1	1	1	1	1	1	3
3	1	1	1	1	1	1	1	1	1
9	9	1	1	1	1	9	1	1	1
9	9	1	9	1	1	1	1	1	1
9	1	1	1	1	1	1	1	1	1
3	1	1	1	1	1	1	1	1	1
2	1	1	9	1	9	1	1	1	9
3	1	1	1	1	1	1	1	1	1
3	1	1	1	1	1	1	1	1	1
3	1	1	1	1	1	1	1	1	1
3	1	1	1	1	1	1	1	1	1
3	1	1	1	2	1	1	1	1	1
9	9	1	9	9	1	1	1	1	3
9	1	1	1	1	1	1	1	1	1
3	1	1	1	1	1	1	1	1	1
3	1	1	1	1	1	1	1	1	1
3	1	1	1	1	1	1	1	1	1

3	1	1	1	1	1	1	1	1	1
3	1	1	1	1	1	1	1	1	1
3	1	1	1	1	1	1	1	1	1
3	1	1	1	1	1	1	1	1	1
3	1	1	1	1	1	1	1	1	1
9	1	1	1	1	1	1	1	1	1
3	3	1	1	1	2	9	9	1	3
9	9	1	1	1	1	1	1	1	1
9	9	1	1	1	1	1	1	1	1
1	1	1	1	1	1	1	1	1	1
3	1	3	1	2	1	1	3	1	2
9	9	9	1	9	9	1	9	9	9
3	1	1	1	3	1	1	1	1	1
2	1	2	2	3	1	1	2	2	1
2	2	1	1	1	2	2	2	2	2
3	1	1	1	2	1	1	2	1	1
9	1	9	9	9	1	9	9	9	9
3	1	1	1	1	1	9	9	9	9
1	1	1	1	3	1	1	1	1	1
3	1	1	1	1	1	1	1	1	1
3	1	1	1	3	1	1	3	1	1
2	1	1	1	3	2	2	2	1	1
3	2	1	2	3	2	3	3	3	3
3	1	1	1	1	1	1	3	1	3
1	1	1	1	3	1	1	1	1	1
1	1	1	1	2	1	1	3	9	1
2	1	2	1	1	1	1	3	1	2
2	2	1	1	3	2	3	1	1	2
3	1	1	1	2	1	1	1	1	1
2	1	1	1	1	1	1	1	1	1
3	1	1	1	2	1	2	1	1	1
9	1	9	1	9	9	1	9	9	9
3	1	1	1	3	1	1	2	1	1
1	1	1	1	2	1	1	3	1	1
3	2	1	2	3	2	3	3	3	3
3	1	1	1	2	1	1	2	1	1
1	1	1	1	3	1	2	1	9	3
1	1	1	1	3	1	2	1	1	3
1	1	1	1	3	1	2	1	1	3
3	1	1	1	3	1	1	1	1	1
3	1	1	1	1	1	1	1	1	1
3	1	1	1	3	1	1	1	1	1
1	1	1	1	2	1	1	2	1	2
2	2	3	3	3	3	2	2	2	2
1	1	1	9	1	1	1	2	1	2
3	3	1	1	1	1	1	3	1	1
2	1	1	1	3	9	1	9	9	9

3	1	1	1	1	1	1	1	1	1
3	1	1	1	3	1	1	2	1	1
3	1	1	1	2	1	1	3	1	1
3	1	1	2	2	1	9	1	2	2
1	1	1	1	3	1	9	9	9	9
1	3	3	1	1	1	1	1	2	2
3	3	1	2	2	2	1	1	1	1
2	1	1	1	1	1	1	2	1	2
3	1	1	1	3	1	1	3	1	3
3	1	1	1	3	1	1	1	1	1
3	1	1	1	1	1	1	1	1	1
3	3	1	1	3	1	1	3	1	1
3	1	1	2	2	1	1	1	1	2
3	3	1	1	3	2	1	3	1	1
2	1	1	1	2	2	2	1	1	1
9	9	1	9	9	9	9	9	9	9
3	1	1	1	3	1	1	1	2	2
1	1	1	1	2	1	1	2	1	2
3	1	1	1	1	1	1	1	1	1
3	1	1	1	3	1	1	1	1	2
9	9	1	9	9	9	9	9	9	1
1	1	1	1	3	1	1	1	1	1
9	9	9	9	9	9	9	1	2	2
3	3	3	3	3	3	3	3	1	3
3	1	1	1	3	1	1	3	1	1
3	1	1	1	3	1	1	3	1	1
1	1	1	1	9	1	9	9	1	1
9	9	9	9	9	9	9	9	9	9
9	9	9	9	9	9	9	9	9	9
2	1	1	1	2	1	1	3	1	2
3	1	1	1	3	1	1	3	1	3
3	1	1	1	3	1	1	2	1	1
3	1	1	1	1	1	1	1	1	1
3	3	1	9	1	1	1	1	1	9
9	9	1	1	1	1	1	1	1	1
3	1	1	1	1	1	1	1	1	1
3	1	1	1	1	1	1	1	1	1
3	1	1	1	1	2	1	1	1	1
3	1	1	3	1	1	1	1	1	3
3	1	1	1	1	1	1	1	1	1
1	1	1	1	1	1	1	1	1	2
3	1	1	1	1	1	1	1	1	1
3	1	1	1	1	1	1	1	1	1
9	9	1	1	1	1	1	1	1	1
3	1	1	1	1	1	1	1	1	1
9	1	1	1	1	1	1	1	1	1
3	1	1	9	9	9	1	1	1	1

3	1	1	1	1	1	1	1	1	1
3	3	1	1	1	1	1	1	1	3
9	9	9	9	9	9	9	9	9	9
3	1	1	1	3	1	3	3	1	1
3	1	1	1	3	1	1	1	1	1
3	1	1	1	3	1	1	1	1	1
1	1	1	1	2	1	9	9	1	1
2	1	9	9	9	1	1	3	1	1
9	9	1	9	9	9	1	9	9	9
9	1	1	1	3	1	9	9	9	9
1	3	1	1	3	1	1	1	1	1
9	9	1	1	2	1	1	1	1	1
3	1	1	1	2	1	2	3	2	2
9	1	9	9	9	1	9	9	9	9
3	1	1	1	3	1	1	2	1	1
3	1	1	1	3	1	2	1	1	1
9	9	1	9	9	1	1	1	1	1
3	1	1	1	3	1	3	1	1	1
3	1	1	1	2	1	1	1	1	1
3	1	1	1	3	1	2	2	1	1
3	1	1	1	3	1	1	3	1	3
3	1	1	1	3	1	1	3	2	1
3	1	1	1	3	1	1	3	2	1
3	1	1	1	1	1	1	1	1	1
9	1	1	1	2	1	1	9	1	1
1	1	1	2	2	1	1	1	1	1
3	1	1	1	3	1	9	9	1	1
3	1	1	1	3	1	1	2	1	2
1	1	1	2	2	1	1	1	1	1
1	1	1	2	3	1	1	1	1	1
3	1	2	3	3	1	1	1	1	3
3	1	1	1	2	1	1	2	1	1
2	1	1	1	2	1	1	9	1	2
3	1	1	1	3	1	1	3	1	1
3	1	1	1	3	1	1	1	1	1
9	9	1	9	9	1	1	3	1	2
3	1	1	3	3	1	3	1	3	1
9	1	1	9	9	1	1	9	9	1
3	1	1	1	2	1	1	2	1	1
3	1	1	1	3	1	1	1	1	1
3	1	1	2	2	2	1	3	1	2
3	3	1	1	2	1	2	1	1	2
9	9	1	9	9	9	9	9	9	9
9	9	1	9	9	9	9	9	9	9
3	1	1	1	3	1	1	3	1	1
1	1	1	1	3	1	1	1	1	1
9	1	1	1	3	1	9	9	9	9

3	1	1	1	3	1	1	3	1	3
9	1	1	1	3	1	2	1	1	1
9	1	1	1	1	1	2	2	1	2
2	1	1	1	3	1	3	1	1	1
3	1	1	1	3	1	3	3	1	1
3	1	1	1	2	1	1	1	2	2
2	2	1	2	2	1	2	1	2	2
2	1	1	1	3	1	1	1	1	1
3	1	1	1	3	1	1	1	1	1
3	1	1	1	2	1	1	2	2	3
3	1	1	1	2	1	1	1	1	3
9	9	1	9	9	9	1	9	9	9
9	9	1	9	9	9	1	9	9	9
9	9	1	9	9	9	9	9	9	9
9	9	1	9	9	9	1	9	9	9
9	9	1	9	9	9	9	9	9	9
3	3	3	1	3	1	3	1	1	9
2	1	1	1	3	1	3	9	1	3
3	1	1	1	3	1	9	9	9	9
1	1	1	1	3	1	3	1	1	1
3	1	1	1	1	1	1	2	9	1
2	1	1	1	2	1	3	3	1	1
2	1	1	1	2	1	3	2	1	1
3	1	1	2	3	2	1	2	3	1
3	1	1	1	2	1	3	3	1	3
9	1	1	1	9	1	2	9	1	1
1	1	1	1	3	1	1	2	1	1
1	1	1	1	3	1	1	3	1	2
3	1	1	1	2	1	9	9	1	9
3	1	1	1	3	1	3	3	1	1
3	1	1	1	2	1	1	2	1	1
9	1	9	1	9	9	1	1	1	1
3	1	1	9	9	9	1	1	1	1
3	1	1	1	2	1	1	2	1	1
3	1	1	1	3	1	1	1	1	2
3	3	2	2	2	1	1	2	1	1
3	1	1	2	1	1	1	3	1	2
2	1	1	1	1	1	1	1	1	1
9	9	1	1	1	1	1	1	3	1
9	1	1	1	1	1	1	1	1	1
3	2	1	2	1	2	1	3	1	3
2	2	1	2	1	2	1	3	1	2
2	1	1	1	2	1	1	3	1	2
1	1	1	1	1	1	1	2	2	1
1	1	1	1	1	1	1	1	1	1
9	1	1	1	2	1	1	1	1	1
3	3	1	1	3	1	1	1	1	1

3	1	1	1	3	1	1	1	1	1
3	1	1	1	2	1	1	9	1	1
3	1	1	1	2	1	1	2	1	1
3	1	1	1	2	1	1	2	2	2
2	1	2	1	1	1	1	3	1	3
3	1	1	2	3	1	2	2	2	2
2	1	1	1	2	2	1	3	1	1
2	1	1	1	3	1	1	2	1	2
2	1	1	1	2	2	1	2	1	1
3	1	1	1	3	1	1	1	1	2
2	1	1	1	2	1	1	3	1	2
2	2	2	2	2	2	2	2	2	2
1	2	1	1	3	1	2	3	2	2
3	1	1	1	3	1	1	3	1	1
2	2	1	1	2	2	2	2	2	2
3	1	1	1	2	1	1	3	1	1
3	2	1	1	2	1	2	2	2	2
2	2	2	2	2	2	2	2	2	2
2	2	2	2	2	2	1	2	1	1
3	1	1	1	3	1	1	2	1	1
3	1	1	1	3	1	1	2	1	2
3	1	1	1	3	2	1	9	9	9
3	1	1	2	2	1	1	3	1	1
3	1	1	2	1	1	1	3	1	3
3	1	1	2	2	1	1	1	1	1
3	1	1	1	2	1	1	1	1	3
2	2	1	1	1	1	1	1	2	2
9	9	1	2	9	1	1	1	1	2
9	9	1	1	3	1	1	3	3	3
3	1	3	1	3	1	3	3	1	1
2	1	1	1	2	1	1	2	1	1
3	1	2	1	2	1	1	2	1	1
9	9	9	9	9	1	1	9	9	9
9	1	9	9	9	1	9	9	9	9
9	1	9	9	9	1	9	9	9	9
9	1	9	9	9	1	9	9	9	9
9	9	9	9	9	1	1	9	9	9
9	9	9	9	9	1	1	9	9	9
9	9	1	9	9	9	1	9	9	9
9	9	1	1	1	1	9	9	9	9
3	1	1	1	2	1	1	3	1	3
9	9	9	9	9	1	1	9	9	9
9	9	9	9	9	1	1	9	9	9
3	1	1	2	3	1	1	1	1	1
9	9	1	9	9	9	1	9	9	9
9	9	1	9	9	9	1	9	9	9
9	9	1	9	9	9	1	9	9	9

3	1	1	1	3	1	1	2	1	1
3	2	2	1	2	3	2	3	1	3
9	9	1	9	9	9	1	9	9	9
9	9	9	9	9	1	1	9	9	9
9	9	9	9	9	1	1	9	9	9
9	1	9	9	9	1	1	9	9	9
9	9	1	9	9	9	9	9	1	9
9	9	1	9	9	9	1	9	1	9
3	1	1	1	3	1	1	9	9	9
9	9	1	9	9	9	1	9	9	9
3	1	1	1	3	1	1	3	1	1
3	1	1	1	1	1	1	3	1	1
3	1	1	1	1	1	1	3	1	1
3	1	1	1	3	1	1	1	1	1
3	1	1	1	3	1	1	1	1	1
3	1	1	1	3	1	1	1	1	1
3	1	1	1	3	1	1	1	1	1
1	1	1	1	3	2	1	1	1	1
3	1	1	1	1	1	1	3	1	1
3	1	1	1	1	1	1	1	1	1
3	1	1	1	3	1	1	1	1	1
3	2	1	3	1	1	1	3	1	3
9	9	9	9	9	1	9	9	1	9
3	1	1	2	3	1	2	1	2	2
3	1	3	1	2	2	3	2	1	1
1	1	1	1	2	2	1	3	1	1
9	9	9	1	1	9	3	3	3	3
3	1	1	1	3	1	1	1	1	1
3	1	1	1	3	1	1	3	1	1
2	2	1	1	3	1	1	3	1	1
3	3	1	1	3	1	1	3	1	1
3	1	1	2	3	1	1	3	2	2
3	1	1	1	2	1	1	3	1	1
3	1	1	2	3	1	1	3	1	1
1	2	1	1	1	1	1	3	1	3
3	1	1	1	3	1	1	1	1	1
1	1	1	1	3	1	1	3	1	1
3	1	1	1	3	1	1	3	1	1
3	1	1	1	3	1	1	3	1	1
3	1	1	3	3	1	1	2	1	1
3	1	1	1	3	1	1	3	1	1
3	1	3	1	3	1	1	1	1	1
1	1	1	1	2	3	1	2	2	2
3	1	1	3	3	1	1	3	1	1
3	1	1	3	3	1	1	3	1	1
3	1	9	3	3	1	1	3	1	1
3	1	1	2	3	1	1	2	1	1

3	1	2	2	3	1	1	1	1	1
3	1	1	1	3	1	1	1	1	1
1	1	1	1	2	1	1	2	1	1
3	1	1	3	3	1	1	3	1	1
3	1	1	3	3	1	1	3	1	1
3	1	1	3	3	1	1	3	1	1
3	1	1	2	3	1	1	2	1	1
3	1	1	2	3	1	1	1	1	3
3	1	1	1	3	1	1	2	1	1
3	3	1	1	3	1	1	1	1	1
3	1	1	1	2	1	1	1	1	1
1	1	1	1	2	1	1	1	1	3
1	1	1	1	3	1	1	1	1	1
1	1	1	1	3	1	1	1	1	1
3	1	3	1	3	1	1	1	1	1
3	1	1	1	1	1	2	1	1	1
2	1	1	1	3	2	1	1	1	3
2	2	1	2	2	2	1	2	1	2
1	1	3	3	3	1	3	1	3	1
3	1	1	1	3	1	1	2	1	1
3	1	1	1	2	1	1	3	1	1
3	1	1	1	1	1	1	1	1	1
3	1	1	1	1	1	1	3	1	1
3	1	1	1	1	1	2	3	1	1
3	1	1	1	1	1	9	1	1	1
3	1	1	1	2	1	2	2	1	2
1	1	1	2	3	1	2	2	2	2
2	1	2	1	2	2	2	2	2	2
3	1	1	2	2	1	1	1	1	3
2	1	1	1	2	1	2	2	2	2
3	2	1	2	2	2	2	2	2	3
3	1	1	2	3	1	1	2	1	3
1	9	1	1	3	1	1	3	1	2
3	1	1	1	3	1	1	2	1	1
3	1	1	1	1	1	1	3	1	1
3	1	1	3	3	1	1	3	1	1
2	1	1	1	3	1	1	3	3	3
1	1	3	3	3	2	1	3	2	2
3	1	1	1	3	1	3	3	1	1
1	1	1	1	3	1	1	3	1	3
2	1	1	1	3	1	2	2	2	2
3	1	1	1	3	1	1	2	1	1
3	1	1	1	3	1	1	2	1	1
2	1	1	1	2	2	1	3	1	2
3	1	1	1	1	1	1	2	1	1
3	1	1	2	2	1	1	2	1	2
1	2	1	1	3	1	3	3	2	2

1	1	1	1	3	1	2	3	1	3
1	2	1	1	1	1	2	2	2	2
1	1	1	1	3	1	1	2	1	1
1	1	1	1	3	1	1	3	1	3
3	1	1	1	2	1	1	1	1	2
3	1	1	1	1	2	2	1	1	2
3	1	1	1	2	1	3	3	3	3
3	1	1	1	3	2	2	2	2	2
3	3	3	3	3	1	1	2	2	2
3	1	1	1	3	1	2	1	1	1
3	1	1	2	2	1	2	1	1	2
3	1	1	1	3	1	1	2	1	1
9	9	1	9	9	9	9	9	9	9
1	1	1	1	2	1	1	3	2	2
3	1	1	1	3	3	1	1	3	1
2	2	1	1	1	1	3	3	1	2
3	1	1	1	2	1	1	3	1	2
3	1	1	1	1	1	1	1	1	3
3	1	1	1	1	1	1	1	1	1
3	1	1	1	2	2	1	2	1	2
1	1	1	3	3	1	1	3	1	2
3	1	1	2	2	1	2	1	1	1
3	3	1	3	3	1	1	1	1	1
3	1	1	1	3	1	1	3	1	1
2	2	1	1	3	3	1	1	1	2
3	1	1	1	3	1	1	1	1	3
3	1	1	1	3	1	1	1	1	1
9	9	9	9	9	9	1	1	1	3
9	9	1	9	9	9	1	1	1	1
9	2	1	2	2	1	1	9	9	9
3	1	1	1	3	2	2	3	2	2
3	1	1	1	3	1	1	2	1	3
1	1	1	1	2	1	1	2	2	2
3	1	1	1	2	1	1	2	1	1
9	1	1	3	3	1	1	9	9	9
1	1	1	1	3	1	1	2	1	1
2	2	1	2	2	2	2	2	2	2
9	9	3	9	9	9	9	9	9	2
1	1	1	1	2	1	1	1	1	2
3	1	1	1	2	1	1	2	1	1
2	1	1	1	1	1	9	9	9	9
3	1	3	1	3	1	1	2	1	2
2	1	1	1	2	1	1	2	1	1
9	1	9	9	9	1	9	9	9	9
3	2	1	1	1	1	2	2	2	2
2	2	2	2	2	2	2	3	1	2
1	1	1	2	3	1	1	1	1	1

3	1	1	1	3	1	3	2	2	2
2	1	1	1	2	1	1	1	1	2
3	1	1	2	2	1	1	2	1	2
1	1	1	2	2	1	1	2	2	2
2	2	1	2	2	2	1	2	1	1
3	1	1	1	3	1	3	2	2	2
3	1	2	1	3	3	1	1	1	1
9	1	1	9	9	9	9	1	9	9
2	2	2	1	2	2	2	2	2	2
2	1	1	3	1	1	1	2	3	2
3	1	1	1	2	1	1	1	1	2
2	1	2	1	2	2	2	2	1	1
3	1	1	1	1	1	1	3	1	2
2	2	2	2	2	2	2	2	2	2
1	1	1	1	3	1	1	3	3	2
1	1	1	1	3	1	1	3	3	3
3	1	1	1	3	1	1	2	1	1
1	1	1	1	1	1	1	1	1	1
3	1	1	1	2	1	1	1	2	2
1	1	1	1	1	1	1	1	1	9
1	1	1	1	1	1	1	1	1	9
1	1	1	1	2	1	1	1	1	1
3	1	2	1	2	2	1	1	2	1
2	1	3	1	3	1	3	1	1	3
2	1	1	1	2	1	2	1	2	1
1	2	1	1	1	2	1	1	1	1
3	1	1	1	3	1	1	1	1	2
2	1	1	1	2	1	1	1	1	1
2	1	1	1	2	1	1	1	1	1
9	9	1	9	2	9	9	9	9	9
2	1	1	1	2	1	1	1	1	1
3	1	1	1	1	1	1	1	1	1
2	1	1	1	1	1	1	1	1	1
3	1	1	1	1	1	1	1	2	1
2	1	1	2	3	1	1	2	1	3
1	1	1	1	2	1	1	1	1	1
1	1	1	1	2	1	1	1	1	1
9	9	9	9	9	9	1	1	1	3
3	1	1	1	3	1	1	1	1	1
3	1	1	1	3	1	1	1	1	2
3	1	1	1	1	1	2	1	1	1
3	1	1	1	3	1	1	1	1	1
1	1	1	1	3	1	1	1	1	2
3	1	1	1	1	1	1	1	1	1
1	1	1	1	1	1	1	1	1	3
3	1	1	1	3	1	3	1	1	3
1	1	1	1	1	1	2	1	1	1

1	1	1	1	1	1	1	1	1	3
1	1	1	1	1	1	1	1	1	3
3	1	1	1	3	1	1	1	1	1
2	1	1	1	2	1	1	1	1	1
1	1	1	1	1	1	1	1	1	1
1	1	1	1	1	1	1	1	1	1
9	1	1	1	3	1	3	1	1	3
1	1	3	1	3	1	1	1	1	1
3	1	1	1	1	1	1	3	1	1
3	1	1	1	1	1	1	3	1	1
3	1	1	1	3	1	1	3	1	1
3	1	1	1	3	1	1	3	1	1
3	1	1	1	2	1	3	3	1	1
3	1	3	1	1	3	1	3	1	3
3	1	1	1	3	1	1	3	1	1
3	1	1	1	3	1	1	3	1	1
2	1	1	1	2	2	1	2	2	1
1	1	1	1	3	1	1	3	1	1
3	1	1	1	3	1	1	3	1	1
3	1	1	1	1	1	1	3	3	1
1	1	1	3	1	1	1	1	1	2
3	1	1	2	2	1	1	3	3	3
9	9	9	9	9	1	1	9	9	9
9	9	9	9	9	1	1	9	9	9
9	9	9	9	1	9	9	9	1	9
3	1	1	1	3	1	1	9	9	9
1	1	1	1	3	1	1	3	3	3
9	1	9	9	9	1	9	9	9	9
9	9	2	9	9	9	2	9	9	9
9	9	1	9	9	9	9	9	9	9
9	1	9	1	9	9	1	9	9	9
9	1	9	1	9	9	1	9	9	9
9	1	9	9	2	9	1	9	9	9
1	2	1	2	1	1	1	9	9	9
3	1	1	1	3	1	1	1	1	3
3	1	1	1	3	1	1	1	1	1
3	1	1	1	1	1	2	1	1	1
3	1	1	1	3	1	1	3	1	1
3	1	1	1	3	1	1	1	1	2
3	1	1	1	3	1	1	1	1	2
1	1	1	1	2	1	1	1	1	3
3	1	1	1	3	1	1	3	1	1
3	1	1	1	2	2	2	3	1	1
3	1	2	1	2	1	2	3	1	1
3	1	1	1	2	2	1	2	3	3
1	1	1	1	1	3	1	1	3	3
3	1	1	1	1	1	1	3	2	1

1	1	2	2	1	2	1	2	2	2
3	1	1	1	2	2	2	1	1	2
9	1	1	3	2	1	2	3	1	1
3	1	1	1	3	1	3	3	1	3
1	1	1	1	1	1	1	1	1	1
3	1	1	1	3	1	1	1	1	1
9	9	9	9	9	9	9	9	9	9
2	3	1	1	3	2	2	2	1	2
3	1	1	1	1	1	1	3	2	2
3	1	2	2	2	2	3	3	3	3
3	3	1	1	1	1	1	1	2	2
3	1	1	1	2	1	1	1	1	1
1	1	2	2	2	1	1	3	1	2
2	2	3	3	3	3	2	2	3	3
3	1	1	1	3	1	1	3	1	1
1	1	1	9	3	1	2	2	2	2
3	1	1	1	1	1	1	1	1	1
2	1	9	9	9	9	1	2	3	1
1	2	1	2	3	1	1	2	3	2
1	1	1	1	1	1	1	1	1	1
1	2	1	1	1	1	1	2	1	1
2	2	3	3	2	2	2	2	2	2
9	9	9	9	9	9	1	3	2	2
2	1	1	1	1	1	2	9	1	1
1	1	1	1	1	1	1	1	1	1
1	3	2	3	2	1	1	2	3	2
3	1	2	1	2	3	1	2	1	2
3	1	1	1	1	1	1	1	1	1
2	2	2	2	2	2	2	2	2	2
3	1	1	1	3	2	2	2	2	2
3	1	1	1	3	1	1	1	1	1
1	1	1	1	1	1	1	1	1	1
1	2	2	2	1	3	2	2	2	2
3	1	1	2	3	1	2	1	1	2
2	1	1	1	3	2	2	2	1	2
9	9	9	9	9	9	1	1	2	2
2	2	2	2	2	2	2	2	2	2
1	1	1	1	3	1	1	3	1	2
2	1	1	1	3	1	3	3	2	3
3	1	1	1	1	1	2	1	1	1
2	1	1	1	2	1	1	2	1	2
2	1	1	1	2	1	1	2	1	2
9	9	3	9	9	9	3	9	9	9
9	9	3	9	9	9	3	9	9	9
9	9	3	9	9	9	3	9	9	9
1	3	3	3	2	2	2	2	3	3
2	2	2	2	2	2	2	2	2	2

1	3	3	3	3	3	1	3	3	1
1	3	3	3	3	3	1	3	3	1
1	3	3	3	3	3	1	3	3	1
2	2	1	1	3	1	2	2	2	2
3	1	1	1	3	2	2	2	2	1
1	2	2	1	2	1	3	2	1	2
1	2	2	2	3	3	3	2	3	2
1	1	3	2	3	2	2	1	3	3
3	3	3	3	3	2	3	2	2	3
1	2	3	2	2	2	2	3	2	3
2	2	2	2	2	1	2	2	2	2
3	1	2	2	2	2	2	2	2	2
3	2	2	2	1	3	2	2	2	2
1	2	2	3	2	1	2	2	3	3
1	1	2	2	2	2	2	2	2	2
3	3	2	3	1	3	2	2	3	3
1	3	2	3	2	3	1	2	3	3
1	3	2	3	2	3	1	2	3	3
1	3	3	3	3	3	1	3	3	1
1	3	3	3	3	3	1	3	3	1
2	2	1	1	3	1	2	2	2	2
2	2	1	1	3	1	2	2	2	2
2	2	2	2	2	2	2	2	2	2
2	2	1	1	3	1	2	2	2	2
2	2	2	2	3	1	1	1	1	1
2	2	1	1	3	1	2	2	2	2
2	2	2	2	2	2	2	2	2	2
3	1	1	1	3	1	1	1	1	1
2	3	2	2	3	1	3	3	3	3
3	3	3	3	1	3	3	3	3	3
3	3	3	3	1	3	3	3	3	3
3	3	3	3	1	3	3	3	3	3
3	3	3	3	1	3	3	3	3	3
1	1	2	2	3	3	3	2	1	2
2	1	3	2	2	1	3	2	1	1
3	3	2	3	3	2	3	1	2	2
2	3	3	3	2	3	3	3	3	3
3	1	2	2	3	3	2	3	3	2
2	3	3	2	1	2	3	3	3	2
2	2	3	2	2	3	3	2	2	1
1	3	3	3	3	3	3	2	1	3
2	2	3	3	2	1	3	2	3	3
2	3	2	3	2	1	2	9	3	2
9	3	9	9	9	3	9	9	3	9
9	9	9	9	9	9	9	9	9	9
3	1	1	1	3	1	1	1	9	9
2	3	3	3	1	3	3	3	3	3

2	3	3	3	1	3	3	3	3	3
1	3	3	3	1	3	3	3	3	3
9	9	9	9	9	9	2	3	2	2
2	2	2	2	2	2	2	2	2	2
2	2	2	2	2	2	2	2	2	2
9	9	2	9	9	9	2	9	9	9
9	9	3	9	9	9	3	2	9	3
9	9	3	9	9	9	3	9	9	9
2	3	3	2	1	3	3	3	3	3
2	3	3	3	2	2	3	3	3	2
1	3	2	2	2	2	2	2	2	2
3	3	2	3	3	3	2	2	2	2
9	9	3	9	9	9	9	9	9	9
9	3	9	9	9	3	3	9	9	9
2	2	2	1	2	1	3	2	1	3
2	3	3	1	3	2	9	2	9	9
3	3	3	3	2	3	3	2	3	3
9	9	9	9	9	9	9	9	9	9
1	3	3	3	3	1	3	3	1	3
9	9	3	9	9	9	9	3	9	9
1	3	3	3	1	3	2	3	3	3
2	2	3	3	1	3	9	9	9	9
1	3	3	3	1	3	3	3	3	3
1	3	3	3	1	3	3	3	3	2
2	2	2	2	2	2	3	3	3	3
2	2	2	2	2	2	2	2	2	2
1	3	3	2	2	2	2	2	2	2
9	9	3	9	9	9	9	3	9	9
9	9	3	9	9	9	9	3	9	9
9	9	3	9	9	9	9	3	9	9
9	9	3	9	9	9	3	9	9	9
9	9	3	9	9	9	9	3	9	9
9	9	2	9	9	9	2	9	9	9
1	1	3	2	3	2	2	1	1	1
3	3	3	2	3	3	3	2	1	3
9	9	9	9	9	9	9	9	9	9
2	3	3	2	2	3	3	3	3	3
9	9	9	2	9	9	3	2	9	9
3	3	3	3	2	3	3	2	3	3
3	3	3	3	3	2	3	2	3	3
3	3	3	3	3	2	3	3	2	3
9	9	3	9	9	9	2	3	9	9
3	1	1	1	3	1	1	1	1	1
3	3	3	2	3	3	3	1	3	3
9	9	3	9	9	9	2	9	9	9
2	3	3	1	3	3	2	3	1	3
3	3	3	3	1	3	3	3	3	2

9	9	3	9	9	9	3	9	9	9
9	9	3	9	9	9	9	3	9	9
1	3	3	2	2	2	3	3	3	3
9	9	3	9	9	9	9	3	9	9
3	3	2	2	2	2	3	2	3	3
9	9	3	9	9	9	3	9	9	9
1	2	2	3	3	3	3	3	3	2
3	3	3	3	1	2	3	3	3	3
3	1	1	3	3	2	3	2	3	2
1	2	2	1	2	3	3	2	3	2
3	1	2	1	3	1	3	3	3	3
3	1	3	1	2	2	1	1	1	1
3	3	3	3	1	2	3	1	1	1
2	1	3	3	2	2	3	2	3	3
2	2	3	3	1	3	3	3	3	3
2	2	3	3	1	2	3	3	3	3
1	3	3	2	2	2	3	3	3	3
1	3	3	3	1	2	3	3	3	3
2	1	3	2	3	2	3	2	1	2
1	1	2	2	2	2	3	3	3	3
9	1	9	9	1	9	9	9	9	3
9	9	9	3	9	9	9	9	9	9
9	9	3	9	9	9	3	9	9	9
9	9	9	3	9	9	9	9	9	9
1	9	9	2	9	9	9	9	9	9
3	9	9	9	2	9	9	9	9	1
1	9	9	3	9	9	2	9	9	9
9	9	2	9	9	9	2	9	9	9
9	9	2	9	9	9	2	9	9	9
9	9	2	9	9	9	2	9	9	9
9	9	2	9	9	9	2	9	9	9
9	9	2	9	9	9	2	9	9	9
9	9	2	9	9	9	2	9	9	9
1	3	3	3	1	2	3	3	2	2
3	3	3	2	1	2	3	2	3	3
1	2	3	2	1	2	3	3	3	3
3	2	1	1	9	1	9	9	9	9
1	9	3	9	3	1	1	9	9	9
1	2	2	3	1	3	3	2	2	2
9	9	2	9	9	9	3	9	9	9
9	9	3	9	9	9	3	9	9	9
9	9	3	9	9	9	3	9	9	9
9	9	3	9	9	9	3	9	9	9
9	9	3	9	9	9	2	9	9	9
1	2	3	2	1	2	3	2	3	2
2	3	2	3	2	3	3	2	3	2
2	2	2	2	2	2	2	2	2	2

2	2	2	2	2	2	2	2	2	2
1	3	3	3	1	2	3	2	2	2
1	3	3	2	2	1	3	2	2	2
1	2	3	2	1	3	3	3	3	2
9	9	3	9	9	9	3	9	9	9
9	9	3	9	9	9	3	9	9	9
9	9	3	9	9	9	3	9	9	9
9	9	1	9	1	9	3	9	9	9
9	9	3	9	9	9	3	9	9	9
9	9	9	9	9	9	9	9	9	9
3	1	2	2	2	2	9	9	1	9
9	9	9	3	9	9	9	9	1	1
1	9	3	1	3	3	3	9	9	9
9	9	9	2	9	9	3	2	2	2
9	9	2	9	9	9	3	9	9	9
2	3	2	2	2	3	2	2	2	2
2	2	3	2	2	1	2	3	2	3
9	9	9	3	9	9	2	9	9	9
9	9	3	9	9	9	2	2	2	2
3	3	2	3	3	2	3	1	1	2
3	3	2	2	1	3	3	1	3	2
1	3	3	3	2	2	2	2	1	3
3	3	3	3	3	3	3	3	3	3
2	2	2	3	2	2	3	3	3	2
2	2	2	2	3	2	2	1	3	2
2	3	3	2	3	2	2	2	2	2
9	9	9	9	9	3	9	3	9	9
1	3	3	1	3	2	1	3	2	2
2	2	2	2	2	2	2	2	2	2
2	3	3	2	2	3	3	1	3	2
2	3	2	2	1	2	2	2	3	1
1	3	2	3	2	2	2	2	2	2
3	3	3	2	1	3	3	9	9	9
2	2	2	2	2	2	2	2	2	2
9	9	3	2	1	3	3	3	3	3
2	3	3	3	2	3	3	2	1	2
3	3	3	3	2	3	3	3	3	9
3	3	2	3	3	3	3	1	1	2
3	3	3	2	3	3	3	2	3	2
2	1	2	3	1	2	1	2	2	3
3	3	3	3	2	3	2	3	3	2
3	3	3	3	1	3	3	1	2	1
2	3	3	3	3	3	3	2	2	2
1	3	3	1	3	3	1	1	2	2
9	9	9	9	9	9	9	9	9	9
1	3	3	2	2	2	2	2	2	2
1	3	3	3	3	3	3	3	3	1

3	3	3	3	3	3	3	3	3	3
2	2	2	3	1	3	3	1	3	3
1	3	3	2	3	3	2	2	3	2
2	3	3	3	1	3	2	2	3	3
2	3	2	2	2	2	3	2	2	2
2	3	3	3	2	3	3	2	2	2
2	1	2	2	2	3	2	2	3	2
3	3	3	2	3	3	2	2	2	2
9	9	9	9	9	9	3	2	2	2
3	1	3	2	3	3	3	2	2	2
2	3	2	2	2	3	3	2	3	3
3	3	3	3	3	3	3	1	3	3
3	1	3	3	1	3	2	3	3	3
1	3	3	3	3	3	3	1	3	3
3	3	3	3	2	3	3	2	3	3
3	3	3	2	2	1	3	2	2	3
3	3	2	3	3	3	2	2	2	2
2	2	2	2	2	2	2	2	2	2
2	2	2	2	2	2	2	2	2	2
2	2	2	2	2	2	2	2	2	2
2	2	3	2	2	2	2	2	2	2
2	2	2	2	2	2	2	2	2	2
2	2	2	2	2	2	2	2	2	2
2	2	2	2	2	2	2	2	2	2
2	2	2	2	2	2	3	2	3	2
9	9	9	2	9	9	9	9	9	9
1	2	3	3	1	3	9	9	9	9
3	3	3	3	3	3	3	1	3	3
3	1	3	2	3	2	3	2	9	1
1	9	3	9	9	3	9	9	9	9
1	3	3	3	1	3	3	1	3	3
3	3	2	3	3	3	1	3	3	3
2	3	3	1	2	3	3	2	3	3
1	2	3	2	2	1	3	2	2	9
3	1	3	2	3	3	3	2	3	3
1	2	3	2	1	2	3	2	2	2
3	3	3	3	1	3	3	2	3	3
3	3	3	2	1	2	1	1	3	3
1	3	3	3	3	3	3	2	3	3
2	2	3	2	2	3	2	2	2	2
9	9	9	9	9	9	3	3	3	3
3	3	3	3	2	2	2	2	2	2
3	3	3	3	1	3	3	1	3	3
3	3	3	3	2	3	3	3	3	1
2	3	3	2	2	2	3	1	2	3
2	3	1	3	2	3	2	1	3	3
3	1	3	3	2	3	1	1	1	1

1	3	3	2	3	3	2	3	3	3
3	1	3	1	2	3	3	1	3	2
2	3	3	3	2	3	3	1	3	2
2	2	3	2	2	2	2	2	2	2
3	3	3	3	3	1	2	2	2	2
9	9	9	1	9	9	9	9	9	9
2	3	3	3	2	3	3	2	2	2
1	3	3	3	2	3	2	2	2	2
2	2	3	2	2	2	2	2	2	2
3	2	2	2	1	1	3	2	3	2
2	2	2	2	2	2	2	2	2	2
3	3	3	1	3	3	3	1	3	1
3	3	3	3	3	3	2	2	2	2
2	3	2	3	2	3	3	2	2	2
2	3	2	3	2	2	2	1	2	2
2	3	2	2	2	3	3	2	2	2
9	9	2	2	3	3	3	1	3	3
2	1	3	3	2	2	3	2	3	2
1	3	3	1	1	3	3	1	3	3
9	3	9	3	9	9	3	9	9	9
2	2	3	3	2	3	3	2	3	2
1	3	3	2	3	3	2	2	2	2
2	2	3	3	3	3	2	2	2	2
3	3	3	3	2	3	2	2	2	2
2	3	3	2	2	2	2	9	2	3
1	1	1	2	3	2	3	1	3	2
2	3	3	2	2	2	3	2	2	2
1	3	3	1	3	3	2	2	2	2
3	3	3	3	2	3	3	2	3	2
3	3	1	1	1	3	3	3	3	3
3	3	3	3	2	3	1	9	3	9
9	9	9	9	9	3	3	9	9	9
3	3	3	3	1	3	3	2	2	2
3	3	3	3	2	3	3	1	3	3
9	9	9	1	9	9	2	2	2	2
2	2	3	2	2	2	2	2	2	2
2	2	3	2	1	3	2	2	3	3
3	3	2	3	2	2	3	2	3	3
2	2	2	2	2	2	2	2	2	2
2	2	3	3	1	3	3	3	2	2
2	2	3	2	1	1	9	9	9	9
2	2	3	2	2	2	2	2	2	2
3	1	1	3	2	2	3	1	3	2
2	2	2	2	2	2	2	2	2	2
2	2	2	2	2	2	2	2	2	2
2	2	2	2	2	2	2	1	2	2
1	3	2	2	1	2	3	2	2	3

9	9	9	9	9	9	9	9	9	9
1	3	3	3	3	3	1	1	1	1
2	3	3	2	3	3	9	9	3	9
9	9	9	3	9	3	9	9	3	9
1	1	3	2	3	2	2	2	2	2
3	1	2	2	2	2	1	1	1	1
9	9	9	9	9	9	9	3	9	9
2	3	3	3	3	3	3	3	3	3
3	3	3	3	2	3	9	9	9	9
1	3	3	2	2	3	3	2	2	2
2	2	2	2	2	3	9	9	3	9
3	3	3	3	1	3	9	9	9	9
3	3	3	3	3	3	3	1	3	3
9	9	9	9	9	3	3	3	2	2
3	1	3	1	3	1	9	9	9	9
2	1	1	3	3	1	1	3	1	2
3	1	3	3	1	2	3	3	2	3
2	3	3	1	2	3	3	3	3	3
9	3	3	2	2	3	3	2	3	3
2	2	3	3	3	3	9	9	9	9
3	2	3	2	3	3	2	3	3	3
3	3	3	3	3	3	3	9	3	1
3	3	2	2	2	2	2	3	2	2
3	3	3	3	3	3	3	1	3	2
3	3	3	3	2	3	3	1	1	3
3	3	3	2	1	3	3	3	3	3
1	1	3	2	2	3	3	3	3	1
1	9	9	9	9	3	9	9	9	9
3	3	3	3	3	3	3	2	3	3
3	3	3	3	2	3	3	1	3	3
2	1	2	2	3	1	2	2	2	3
3	1	3	1	3	1	3	3	3	3
3	3	3	2	3	3	3	2	1	2
1	3	3	3	2	3	3	3	3	3
1	3	3	2	3	2	2	2	3	3
2	3	2	2	3	3	9	9	9	9
9	9	3	9	9	9	9	9	9	9
3	1	3	3	3	3	3	3	3	3
9	9	3	3	3	9	9	9	3	9
2	1	3	9	9	9	9	9	9	9
9	9	9	3	9	9	9	9	9	9
1	1	3	1	1	3	3	1	3	3
3	1	3	3	3	3	3	2	2	1
3	3	3	3	3	3	3	1	3	3
3	3	3	3	3	3	1	9	3	3
9	9	3	9	9	9	9	9	3	9
9	3	3	3	3	3	3	9	3	3

1	3	3	3	2	3	2	3	3	2
2	3	3	2	1	1	3	1	3	3
9	3	9	9	9	3	3	9	9	9
9	9	9	9	9	9	2	2	3	2
3	3	3	3	3	3	3	3	3	3
3	9	9	3	9	9	3	3	3	2
9	3	3	9	9	9	3	3	3	9
3	1	3	3	2	1	3	3	3	3
3	3	3	2	1	3	3	3	3	3
3	1	3	3	3	2	3	2	3	3
3	3	3	3	1	3	3	1	3	3
3	3	3	3	2	3	3	2	3	3
3	3	3	3	2	3	3	3	3	3
2	3	3	2	1	3	3	2	3	3
2	3	3	3	2	3	3	3	3	2
3	3	3	3	3	3	3	3	3	3
3	3	3	3	2	3	3	3	3	3
1	3	3	3	2	3	2	3	3	3
9	9	3	3	9	2	3	1	3	3
3	1	3	2	2	2	3	2	2	3
1	3	3	2	3	3	3	3	3	3
9	9	2	9	9	9	9	9	9	9
3	3	3	3	3	3	3	1	3	1
3	3	3	3	3	3	1	3	3	3
3	3	3	3	2	3	3	2	3	3
1	3	3	3	1	2	3	1	2	2
3	3	3	3	3	3	9	9	9	9
3	3	1	3	3	3	3	1	3	3
3	3	3	3	3	1	9	9	9	9
9	3	9	9	9	3	9	9	9	9
3	3	3	3	1	3	3	3	3	9
3	3	3	1	3	2	1	1	2	1
2	2	1	2	2	1	3	3	2	2
2	3	3	3	2	3	2	3	3	2
9	3	3	2	2	3	2	3	2	2
9	3	9	9	9	9	9	9	9	9
3	3	9	3	9	9	9	3	9	9
9	9	3	9	9	9	9	3	3	3
9	9	2	2	9	9	3	9	9	9
9	3	9	9	9	3	9	9	9	9
9	9	9	9	9	3	9	3	3	3
3	3	3	3	1	3	1	1	3	2
3	9	9	9	3	3	3	1	3	9
2	2	3	2	1	3	2	2	2	2
3	3	3	3	3	3	1	3	3	3
3	2	3	2	3	3	3	3	3	2
3	3	3	3	1	3	3	3	3	3

1	3	3	3	3	3	1	3	3	3
3	3	3	2	3	3	3	2	3	2
3	3	3	3	3	2	3	1	3	2
3	3	3	3	3	3	1	2	3	2
3	3	3	2	2	3	2	3	2	3
9	3	3	9	9	9	9	9	3	9
3	2	2	2	1	2	3	2	2	3
9	9	3	9	9	9	2	9	9	9
9	9	9	2	9	9	3	3	9	9
9	3	3	9	9	9	3	9	9	9
2	3	3	2	2	2	2	2	2	3
9	3	3	3	1	3	3	3	3	3
2	2	2	2	2	2	2	2	2	2
3	3	3	3	2	3	3	3	3	2
9	3	3	9	9	3	9	3	3	9
1	3	2	2	1	1	2	1	2	3
2	3	3	3	1	3	3	2	3	3
9	9	3	3	3	3	3	3	3	3
2	3	3	3	2	3	3	2	3	3
3	3	3	3	2	3	3	3	3	3
1	3	3	3	3	3	3	3	3	3
2	3	3	3	2	3	3	1	3	2
3	3	3	3	3	3	3	1	3	3
9	9	9	9	9	9	3	9	9	9
3	3	3	3	3	3	3	2	3	3
3	3	3	3	1	3	3	3	3	3
3	3	3	2	2	3	3	3	3	3
3	3	1	2	1	3	3	2	3	3
3	3	3	3	3	3	3	3	3	3
3	3	2	3	3	3	3	2	3	3
3	3	3	3	2	2	3	9	2	9
3	3	3	3	3	3	3	9	3	3
1	3	3	3	3	2	2	2	2	2
3	3	3	3	2	3	2	2	3	2
3	3	3	3	9	1	3	2	2	2
9	3	3	9	9	3	9	9	3	9
3	3	3	3	1	3	3	3	3	3
3	3	3	3	1	3	3	3	3	3
3	3	3	3	3	3	3	2	3	2
3	2	1	2	2	1	1	9	2	9
1	3	3	3	2	3	3	1	3	3
3	3	3	3	1	3	3	3	3	3
2	3	2	3	2	2	2	1	2	2
2	3	2	3	3	3	3	2	3	2
3	3	3	3	1	3	3	1	3	3
3	3	3	3	2	3	3	1	3	3
3	3	3	3	1	3	3	3	3	3

9	9	3	9	9	9	3	9	9	9
3	3	2	1	3	3	1	1	3	3
9	3	3	3	2	3	1	9	9	9
3	3	3	3	2	3	3	1	3	2
9	3	3	3	9	3	9	3	3	9
1	2	1	2	1	2	3	2	2	1
1	3	3	3	3	2	2	3	3	2
3	1	3	1	3	3	3	3	3	3
2	2	2	3	1	2	2	2	2	2
3	3	3	3	3	3	3	2	3	3
9	3	3	2	3	3	3	9	9	9
3	3	3	3	1	3	3	3	3	3
2	2	1	1	2	2	2	2	2	2
3	3	3	1	3	3	3	2	1	3
3	3	3	3	3	3	3	3	3	3
3	3	3	3	2	3	1	2	3	3
3	3	3	3	1	3	9	3	3	3
2	3	3	3	2	2	3	2	2	3
9	3	3	3	1	3	3	3	3	3
1	3	3	3	3	2	3	3	3	2
2	3	3	3	2	3	3	2	2	2
2	3	3	3	2	3	3	2	2	2
1	3	3	2	2	3	3	3	3	3
9	9	3	9	9	9	3	3	3	3
3	3	2	2	1	2	3	3	3	3
3	2	3	2	2	3	3	9	9	9
2	3	3	3	2	3	3	1	3	3
2	2	2	3	3	1	1	2	3	2
2	3	3	3	2	3	3	2	3	3
3	3	3	2	1	3	3	2	3	3
9	3	3	3	2	3	3	9	3	9
3	3	3	3	1	3	3	1	3	9
3	3	3	3	1	3	3	2	3	2
1	3	3	3	1	3	3	3	3	1
3	1	3	3	1	3	2	2	2	2
1	3	2	3	1	3	2	2	9	2
1	3	3	9	9	9	2	3	3	3
3	9	1	1	2	2	2	9	2	2
1	3	3	2	1	1	9	9	9	9
3	3	3	3	2	3	2	2	3	3
2	2	3	2	1	2	2	2	2	2
3	3	3	2	2	3	3	2	2	2
9	9	3	2	1	3	3	3	3	3
3	3	3	3	1	3	9	9	9	9
3	3	3	3	3	3	1	3	3	3
3	3	3	3	1	3	9	3	3	3
3	3	3	3	1	3	3	2	3	3

3	3	3	3	3	3	3	2	3	2
3	3	3	3	2	3	3	3	3	3
3	3	3	3	3	3	3	1	3	1
3	3	3	3	3	1	3	3	3	3
3	3	3	3	2	3	3	2	3	3
3	3	3	3	3	3	3	3	1	1
3	3	3	3	3	3	3	3	3	3
3	3	3	3	3	3	3	3	3	1
3	3	3	2	2	1	3	2	3	2
3	3	3	3	1	3	2	2	3	3
3	3	3	3	2	3	3	1	3	3
3	3	3	3	3	3	3	3	3	3
3	3	3	3	3	3	3	1	3	2
3	3	1	3	3	3	3	1	3	3
3	3	3	3	3	3	3	1	3	3
3	3	3	3	3	3	2	2	2	2
2	3	3	3	1	3	3	2	3	3
3	3	3	3	3	3	3	1	3	3
2	3	3	3	3	2	2	2	3	3
3	1	3	3	3	3	3	3	3	2
3	1	2	3	3	3	3	2	1	1
3	3	3	3	3	3	3	2	3	3
3	3	3	2	2	3	3	2	3	1
1	3	3	9	9	9	9	9	3	9
1	3	3	2	2	2	2	2	3	3

Fingerpads	Fingernails	Palms	Onefinger	Threemidd	FingersinCi	FingersVer	FingersWe	PressNippl	ExamineAr
3	3	1	1	1	2	2	2	2	2
2	3	9	3	9	2	3	2	2	3
2	2	2	2	2	2	2	2	2	2
2	2	2	2	2	2	2	2	2	2
1	1	2	2	2	2	2	2	2	2
2	3	3	3	9	3	3	3	3	3
2	2	2	2	2	2	2	2	2	2
2	2	1	2	2	2	2	2	1	1
2	2	9	1	1	3	3	3	3	3
2	3	2	2	1	3	2	1	3	2
2	2	2	2	2	2	2	2	2	2
1	2	2	2	2	2	2	1	2	2
1	1	1	1	2	2	2	2	2	2
2	2	1	2	2	2	1	2	2	1
2	2	2	2	2	2	2	2	2	2
2	2	2	2	2	2	2	2	2	2
9	9	9	9	9	9	9	9	9	9
3	2	2	2	1	1	1	1	1	1
2	2	2	2	2	2	2	2	3	9
2	1	2	3	3	2	2	2	3	9
2	2	2	1	1	1	1	1	1	1
3	3	2	2	1	2	2	9	1	1
2	3	1	2	1	1	3	1	1	1
2	1	2	2	2	2	1	1	1	1
2	2	2	2	2	2	2	2	2	2
3	2	2	2	2	3	2	2	2	2
9	9	9	9	9	9	1	9	9	9
3	2	2	9	1	1	1	1	1	3
3	2	9	1	2	1	1	1	2	3
3	9	2	3	1	2	1	3	1	2
2	2	2	2	2	2	2	2	2	2
1	2	3	3	2	2	2	1	3	3
9	9	9	9	9	9	1	9	9	9
3	2	3	3	1	1	9	1	1	2
3	1	2	9	1	2	3	1	9	1
3	2	2	3	2	3	2	3	1	3
2	2	2	2	2	2	2	2	2	2
1	2	2	1	1	1	1	1	2	2
3	2	2	1	1	3	1	2	1	3
3	3	2	3	2	9	1	3	1	1
9	9	9	9	9	9	9	9	9	9
9	9	9	9	9	9	9	9	9	9
9	9	9	9	9	9	9	9	9	9
2	1	2	2	2	1	2	2	2	2
2	2	2	1	2	1	2	2	2	1
9	9	9	9	9	9	9	9	9	9

3	2	2	2	2	3	2	2	3	2
1	1	2	2	3	2	2	2	2	1
2	1	1	2	2	2	1	2	1	2
3	2	2	1	1	2	2	1	1	2
3	1	1	3	1	1	1	1	1	1
3	1	1	3	1	1	1	1	1	9
2	3	3	9	9	3	3	3	2	3
2	2	2	2	2	2	2	2	2	2
1	9	9	1	1	1	1	1	1	2
1	3	2	1	2	2	3	1	3	1
9	9	9	9	9	1	9	9	9	9
1	1	2	2	2	1	1	1	2	2
3	3	3	3	3	2	2	1	1	2
1	1	2	2	2	1	1	2	2	1
2	2	3	2	1	2	2	2	1	2
2	2	2	3	2	2	2	3	2	2
3	2	3	3	2	3	3	3	2	2
2	1	3	2	1	1	1	1	2	3
2	2	2	2	2	2	2	2	2	2
9	9	9	9	9	9	9	9	9	9
2	2	2	2	2	2	2	2	2	2
3	2	3	9	2	3	1	2	9	2
1	1	2	2	2	2	2	2	2	2
2	2	2	3	2	1	2	2	3	1
2	2	1	2	1	2	1	2	2	1
1	3	3	9	2	2	3	2	3	2
3	3	2	1	1	2	9	3	1	1
2	3	2	1	2	3	3	3	2	1
2	2	3	2	3	2	1	1	1	1
3	1	3	3	3	1	1	1	3	1
9	9	9	9	9	9	9	9	9	9
3	3	3	1	1	2	2	1	1	1
9	1	3	3	9	2	2	1	1	1
2	2	2	2	2	2	2	2	2	2
3	3	3	3	3	3	3	3	1	1
2	2	2	2	2	2	2	2	2	2
3	2	2	2	3	3	1	1	3	1
1	1	1	1	1	2	3	2	1	1
3	1	2	3	2	3	2	1	1	2
1	2	1	2	2	2	1	1	2	2
3	2	2	3	1	2	1	1	1	1
9	9	9	9	9	9	9	9	9	9
1	1	9	9	9	9	9	9	9	9
2	2	1	2	2	1	1	2	2	3
3	2	1	1	1	1	2	2	2	1
2	2	3	3	3	3	3	2	2	2
2	2	2	2	2	2	1	2	2	2

2	3	1	2	3	1	2	3	2	1
1	1	3	9	2	1	2	3	3	3
2	2	2	2	2	2	2	2	1	2
1	2	1	1	1	2	1	1	2	3
2	2	1	2	2	3	2	1	2	2
2	1	1	2	2	2	1	1	2	2
2	2	2	2	2	2	2	2	2	2
3	1	3	3	3	1	9	1	2	9
2	3	2	2	1	1	2	2	1	2
9	2	9	3	1	2	2	1	2	1
3	1	2	1	3	3	9	9	9	9
3	2	1	2	2	3	2	1	2	2
2	1	1	1	2	1	1	1	1	1
2	2	2	2	2	2	2	1	1	1
1	2	3	3	1	2	1	1	1	1
1	2	3	3	1	2	3	1	2	2
2	2	1	2	2	1	2	2	3	2
3	3	3	3	3	3	3	1	1	3
1	3	9	9	1	1	1	1	1	1
2	2	2	3	2	2	2	2	3	2
1	1	1	1	3	1	1	1	3	3
2	1	3	3	2	2	2	1	2	1
1	1	2	1	1	1	2	1	1	1
3	2	2	2	3	2	1	2	2	2
2	2	2	2	2	2	2	2	2	2
2	3	3	3	9	2	1	1	2	2
3	3	1	9	1	1	1	1	1	1
1	2	1	2	1	2	1	2	1	1
1	2	2	2	2	2	1	2	2	1
3	1	2	3	2	3	2	1	1	1
1	1	2	2	1	1	2	1	1	3
1	1	1	2	3	2	1	2	1	1
1	2	9	1	1	3	1	1	2	1
2	1	2	2	1	1	2	1	2	1
1	1	2	3	2	1	2	1	2	1
1	3	2	1	1	2	2	3	1	2
2	2	2	2	2	2	2	2	2	2
1	1	2	2	1	1	2	2	2	3
9	9	9	9	9	9	9	9	9	9
1	2	3	9	2	1	2	3	3	3
2	2	2	1	1	3	2	2	2	3
9	9	9	9	9	9	9	9	9	9
2	2	1	2	2	1	2	2	1	2
2	3	2	1	2	2	2	2	2	2
3	3	3	9	1	1	1	1	1	1
2	1	2	1	3	2	1	1	1	1
2	1	3	2	1	1	1	1	2	1

9	1	2	9	3	1	2	2	3	1
3	3	3	3	3	3	3	3	3	3
3	3	3	3	3	3	3	3	3	3
1	1	1	2	2	2	2	2	2	2
2	1	1	2	3	3	2	1	3	1
2	2	3	2	2	2	2	1	2	3
2	1	3	1	9	3	1	9	1	9
2	9	1	1	2	2	1	1	9	1
9	9	9	9	9	9	9	9	9	9
2	2	2	2	2	2	2	2	2	2
1	2	2	2	2	2	2	1	1	1
2	2	2	2	2	2	1	1	3	3
2	2	2	9	2	2	2	2	2	2
2	2	3	3	3	3	3	3	2	2
3	3	2	2	2	2	2	2	1	1
1	1	2	2	2	2	1	2	2	3
2	2	1	2	2	3	2	2	1	1
1	1	2	2	1	1	2	2	1	1
9	9	9	9	9	9	9	9	9	9
3	3	3	2	3	3	3	1	1	1
3	2	3	2	1	2	1	2	3	1
2	2	2	2	2	1	2	2	1	1
3	3	3	3	3	3	3	3	3	2
2	2	2	2	1	2	2	1	2	1
3	1	1	1	1	1	2	3	3	3
2	3	3	1	2	1	1	1	3	3
2	3	2	2	1	2	2	1	2	1
3	3	3	3	3	2	2	2	2	1
1	2	3	3	2	2	2	2	2	2
2	2	2	2	2	1	2	3	2	1
1	2	1	1	1	2	2	1	2	1
1	1	2	2	2	2	1	1	1	2
2	2	2	2	2	2	2	2	2	1
1	1	3	3	3	1	1	3	3	3
3	2	2	2	2	1	2	2	1	2
1	2	2	1	1	1	1	2	1	1
2	2	2	2	2	2	2	2	2	2
3	1	3	3	1	2	3	1	1	2
1	1	3	3	3	1	1	1	1	1
2	2	2	2	2	2	2	2	2	2
2	2	1	1	2	2	2	2	2	1
2	2	2	2	2	2	2	2	2	2
2	2	2	2	2	2	2	2	3	3
9	9	9	9	9	9	9	9	9	9
9	9	9	9	9	9	9	9	9	9
1	2	2	1	1	1	2	1	1	1
1	1	1	2	2	2	2	2	2	2

9	9	9	9	9	1	9	9	9	9
1	2	3	3	2	2	2	3	3	3
1	1	2	1	1	1	1	1	1	1
9	9	9	9	9	9	9	9	9	9
9	9	9	9	9	9	9	1	9	9
9	9	9	9	9	9	9	9	9	9
3	3	3	2	2	2	3	1	1	1
9	9	9	9	9	9	9	9	9	9
2	2	3	2	2	3	2	1	2	3
9	9	9	9	9	9	9	9	9	9
9	9	9	9	9	9	9	9	9	9
9	9	9	9	9	9	9	9	9	9
2	2	1	1	2	2	1	2	2	2
2	1	2	3	1	2	2	2	2	1
9	9	9	9	9	9	9	9	9	9
3	3	3	3	3	2	3	3	3	3
3	3	3	3	3	3	3	3	3	3
2	1	1	2	2	2	1	2	2	2
1	2	1	2	1	1	2	1	2	1
2	2	3	2	2	3	2	2	2	2
3	1	3	3	3	2	2	2	2	9
9	9	9	9	9	9	9	9	9	9
1	1	1	1	1	1	1	1	1	1
1	1	2	2	1	1	1	1	1	1
2	2	2	2	2	2	2	2	2	2
2	1	1	1	1	1	1	1	1	1
2	1	1	1	1	1	1	1	1	2
2	2	1	1	1	1	1	1	1	1
3	3	2	2	2	2	2	2	3	3
1	3	3	3	1	3	3	3	3	3
2	2	2	2	2	2	2	2	2	2
1	2	2	2	2	1	2	2	2	1
9	9	9	9	9	9	9	9	9	9
3	2	2	2	3	3	2	3	2	2
3	1	1	1	1	1	2	1	1	1
1	1	1	1	1	1	1	1	2	2
3	9	2	1	2	2	1	2	1	2
1	2	1	1	1	2	1	1	2	2
1	1	1	3	3	3	1	2	3	2
1	1	2	3	2	1	1	2	2	2
1	1	2	2	2	2	2	2	2	2
1	3	2	1	3	1	3	2	1	3
2	3	3	2	2	1	2	1	2	3
2	2	2	2	2	2	2	2	2	2
1	2	2	1	3	3	3	3	3	3
1	2	9	1	3	2	1	2	3	2
9	9	9	9	9	9	9	9	9	9

2	1	3	1	1	1	2	3	3	2
3	3	3	3	3	3	1	2	1	1
2	3	3	3	1	1	1	1	3	1
2	2	2	1	2	3	3	2	2	9
3	2	2	2	2	2	2	2	2	2
2	2	2	2	2	3	2	3	2	3
1	3	1	3	3	3	3	3	1	2
2	2	2	1	1	2	2	2	2	2
1	2	1	1	1	1	1	1	1	1
1	2	1	1	1	1	2	2	2	1
2	2	2	2	2	2	1	1	1	1
9	9	9	9	9	9	9	9	9	9
9	9	9	9	9	9	9	9	9	9
2	2	2	2	2	2	2	2	2	2
9	2	9	2	9	9	2	9	9	2
9	9	9	9	9	2	9	9	9	9
9	9	9	9	9	9	9	9	9	9
9	9	9	9	9	9	9	9	9	9
9	9	9	9	9	2	9	9	9	2
9	9	9	9	9	9	9	9	9	9
9	9	9	9	9	9	9	9	9	9
2	2	2	2	2	2	2	2	2	2
2	2	2	2	2	2	2	2	2	2
2	2	2	2	2	2	2	1	1	1
1	1	1	1	1	2	2	2	2	1
9	9	9	9	9	9	9	9	9	9
2	2	2	2	2	2	2	2	2	2
9	9	9	9	9	9	9	9	9	9
9	9	9	9	9	9	9	9	9	9
2	2	1	2	2	1	2	2	1	1
9	2	9	9	9	9	2	9	9	9
9	9	9	9	9	9	9	9	9	9
9	9	9	9	9	9	9	9	9	9
9	9	9	9	9	9	9	9	9	9
9	3	1	9	9	3	1	9	3	1
2	1	9	2	1	9	2	1	9	1
9	9	9	9	9	9	9	9	9	9
9	9	9	2	9	9	9	9	9	2
9	9	9	9	9	9	9	9	9	9
9	9	9	9	9	9	9	9	9	9
9	9	9	9	2	9	9	9	9	9
9	9	9	9	9	9	9	9	9	1
3	3	3	3	3	3	3	3	3	3
2	1	2	1	1	3	1	2	1	2
1	2	1	3	1	2	2	1	3	2
1	1	1	9	1	9	9	2	9	9
9	9	9	9	9	9	9	9	9	9

9	9	9	9	9	9	9	9	9	9
9	9	9	9	9	9	9	9	9	9
3	1	2	1	1	1	1	1	2	2
3	1	2	3	1	2	3	1	2	3
2	3	3	3	3	3	3	3	3	3
2	2	3	3	3	3	3	3	2	2
3	2	2	3	2	2	2	1	2	1
1	3	2	1	3	3	2	1	1	1
2	3	2	3	2	3	2	3	2	2
3	3	3	3	3	3	3	3	3	3
9	9	9	9	9	9	9	9	9	9
9	9	9	9	9	9	9	9	9	9
9	9	9	9	9	9	9	9	9	9
9	9	9	9	9	9	2	9	9	9
1	1	1	1	1	1	1	1	1	1
3	3	3	3	3	2	2	1	1	2
9	9	9	9	9	9	9	9	9	9
2	2	2	2	2	1	2	1	2	2
1	1	1	1	1	1	1	1	1	1
2	2	2	2	2	2	2	2	2	2
3	3	3	3	3	1	2	2	3	1
2	2	2	2	2	2	2	2	2	2
2	3	1	2	3	2	1	3	1	2
2	2	2	2	2	2	2	2	2	2
2	2	2	2	2	2	2	2	2	2
2	2	2	2	2	2	2	2	2	2
2	2	2	2	2	2	2	2	2	2
3	3	1	2	1	2	3	1	2	3
2	2	2	3	3	3	9	2	2	2
2	1	2	1	1	1	2	1	1	2
2	2	2	2	2	2	2	2	2	2
2	2	2	2	2	2	2	2	2	2
2	2	2	3	2	1	2	2	3	2
9	2	2	2	2	2	3	3	3	3
3	3	9	3	1	3	1	2	9	1
2	3	1	2	3	2	2	2	2	2
9	9	9	9	9	9	9	9	9	9
2	2	2	2	2	2	2	2	2	2
3	3	3	3	3	3	3	3	3	3
3	3	3	3	3	3	3	3	3	3
9	9	9	9	9	2	9	9	9	9
2	3	1	2	3	2	3	9	9	9
9	9	1	9	9	9	9	9	9	9
1	1	1	1	1	1	1	1	1	1
1	2	2	2	2	2	2	2	2	2
2	2	2	2	2	2	2	2	2	2
2	3	3	3	3	3	2	3	3	3

9	9	9	2	9	9	9	9	9	9
9	9	9	9	9	9	9	9	9	9
9	9	9	9	9	9	9	9	9	9
9	9	9	9	1	9	9	9	9	9
9	9	9	9	9	9	9	9	9	9
1	1	1	1	1	1	1	1	1	1
9	9	9	9	9	9	9	9	9	9
1	9	9	9	2	9	9	9	9	9
1	1	2	2	1	2	2	1	1	1
9	9	9	9	9	9	9	9	9	9
9	9	9	9	9	9	9	9	9	9
2	9	9	9	9	9	9	9	9	2
9	9	9	9	9	9	9	9	9	9
9	9	9	9	9	9	9	9	9	9
9	9	9	9	9	9	9	9	9	9
9	9	9	9	9	9	9	9	9	9
9	2	9	9	9	9	9	2	9	3
2	1	2	3	1	2	3	1	2	3
2	3	2	2	2	1	1	1	2	3
2	2	2	2	2	2	2	2	2	2
3	2	3	2	1	2	2	1	1	2
3	3	3	2	1	1	2	1	2	1
3	2	1	2	2	2	2	2	2	2
3	2	2	2	3	2	2	2	1	2
2	2	2	2	2	2	2	2	3	9
2	2	2	9	2	2	2	2	2	2
2	2	2	2	2	2	2	2	2	2
3	3	3	3	2	2	2	2	2	2
2	3	1	2	1	2	3	1	2	1
1	1	1	1	1	1	1	1	1	1
3	2	1	2	1	1	1	1	2	3
1	3	3	2	1	1	3	3	3	3
1	2	2	2	2	2	2	3	3	3
2	2	2	2	2	2	1	1	1	2
9	9	9	9	9	9	9	9	9	9
2	2	2	2	2	2	2	2	2	2
9	9	9	9	9	9	9	9	9	9
2	2	2	1	2	2	2	2	2	2
3	3	2	3	2	2	3	2	2	3
2	2	2	2	2	2	2	2	2	2
2	2	2	9	1	2	2	2	1	1
1	2	2	1	1	3	3	2	3	1
3	3	3	2	2	3	1	1	3	1
2	2	3	2	1	1	2	1	1	1
2	2	2	2	2	2	2	2	2	2
2	1	2	2	3	1	2	2	2	2
2	2	2	2	2	2	2	2	2	2

9	1	2	2	1	2	3	1	2	2
2	2	2	2	2	2	2	2	2	2
2	9	1	1	1	1	2	2	1	1
3	1	2	3	1	2	3	1	2	3
2	2	2	2	2	2	2	2	2	2
2	2	2	2	2	2	2	3	3	2
2	2	1	1	1	2	2	3	3	9
1	2	3	1	1	1	1	1	1	1
9	9	9	9	9	9	9	9	9	9
2	2	2	2	2	2	1	3	3	3
2	1	3	1	2	1	1	1	1	1
9	9	9	9	9	9	9	9	9	9
9	9	9	9	9	9	9	9	9	9
1	9	1	1	1	9	1	9	1	1
9	9	9	2	9	9	9	9	9	9
1	2	2	2	3	3	1	1	1	2
9	9	9	9	9	9	9	9	9	9
9	9	9	9	9	9	9	9	9	9
9	9	9	9	9	1	9	9	9	9
2	3	2	2	3	2	3	2	3	2
2	2	2	2	2	2	2	2	2	2
2	2	2	2	2	2	2	2	2	3
2	2	1	2	2	2	2	2	2	2
9	9	9	9	9	9	9	9	9	9
9	9	9	9	9	9	9	9	9	9
2	2	1	1	1	1	1	1	2	2
2	3	1	1	2	1	1	1	1	1
1	1	2	2	2	3	3	3	2	1
2	3	3	2	3	2	3	3	2	2
9	9	9	9	9	9	9	9	9	9
2	2	2	2	2	2	2	2	2	2
2	3	1	2	3	1	2	3	1	2
1	1	1	2	1	1	1	1	1	1
3	3	3	3	3	3	3	3	3	3
3	1	1	3	1	1	1	1	1	1
9	9	9	9	9	9	9	9	9	9
9	9	9	9	9	9	9	9	9	9
9	9	9	9	9	9	9	9	9	9
3	1	9	3	2	1	2	3	2	2
2	1	2	1	2	1	2	1	2	1
3	2	3	2	1	1	1	1	3	1
3	3	3	3	1	2	3	2	1	3
2	2	2	2	2	2	2	2	2	2
2	1	1	2	3	2	3	1	2	2
2	2	2	2	2	3	3	3	3	3
2	2	2	2	2	2	2	2	2	2
3	2	2	2	2	2	2	3	3	3

1	1	1	2	2	2	2	2	2	2
3	9	2	3	2	1	2	3	1	2
9	9	9	9	9	9	9	9	9	9
9	9	9	9	9	9	1	9	9	9
9	9	9	9	1	9	9	9	9	9
3	2	3	2	2	3	1	2	2	1
9	2	9	9	2	9	2	9	2	9
2	9	9	2	2	9	2	9	2	9
9	9	9	9	9	9	9	9	9	9
9	9	9	9	9	9	9	9	9	9
2	2	2	2	2	2	2	2	2	2
9	9	9	9	9	9	9	9	9	9
2	2	2	9	9	9	9	9	9	9
9	9	9	9	9	9	9	9	9	9
2	2	9	9	9	9	9	9	9	9
9	9	9	9	9	9	9	9	9	9
3	3	3	3	3	3	3	3	3	3
9	9	9	9	9	9	9	9	9	9
9	9	9	9	9	9	9	9	9	9
9	9	2	9	2	9	2	9	2	9
9	9	9	9	9	9	9	9	9	9
9	9	9	9	9	9	9	9	9	9
2	2	2	2	2	2	2	2	2	2
9	9	9	9	9	9	9	9	9	9
9	9	9	9	9	9	9	9	9	9
9	9	9	9	9	9	9	9	9	9
9	9	9	9	9	9	9	9	9	9
2	9	9	9	2	9	9	9	9	9
3	3	3	1	1	9	9	9	9	9
9	9	9	9	9	9	9	9	9	9
9	9	2	9	9	9	9	9	9	9
2	9	2	2	2	9	2	9	2	9
1	9	1	9	9	1	9	1	9	1
9	9	9	9	9	9	9	9	9	9
2	9	2	2	2	2	2	2	2	2
9	9	9	9	9	9	9	9	9	9
9	2	9	9	9	9	9	9	9	9
9	9	9	9	9	9	9	9	9	9
1	2	3	1	1	1	3	1	1	1
1	3	2	2	2	1	2	1	3	2
9	9	9	9	9	9	9	9	9	9
9	9	9	9	9	9	9	9	9	9
2	1	3	2	1	2	3	2	1	3
2	3	2	3	2	1	2	3	1	2
3	3	3	3	3	3	3	3	3	3
2	2	1	2	2	1	2	1	2	1
9	9	9	9	9	9	9	9	9	9

9	9	9	9	9	9	9	9	9	9
3	2	1	1	3	2	1	1	2	2
2	2	2	2	2	2	2	2	2	2
1	1	1	1	1	1	1	1	1	1
2	2	2	2	2	2	2	2	2	2
2	2	3	2	1	2	1	1	3	3
3	3	3	3	3	3	3	3	3	3
2	1	1	2	3	2	3	2	2	2
2	2	2	2	3	3	3	2	2	2
2	2	2	2	2	2	2	1	2	1
3	3	3	3	3	3	2	2	2	2
9	9	9	9	9	9	9	1	9	9
2	1	3	3	9	9	9	9	9	9
2	2	2	2	2	2	2	2	2	2
9	9	9	9	9	9	9	9	9	9
3	2	3	3	1	1	2	2	2	2
3	2	2	2	2	1	2	1	1	1
2	2	3	3	1	1	1	1	1	1
2	1	3	2	1	2	3	1	1	1
2	2	2	2	2	2	2	2	2	2
2	2	2	2	2	2	2	2	2	2
3	3	3	3	3	2	2	1	3	2
3	3	3	3	2	3	2	1	1	1
9	9	9	9	9	9	9	9	9	9
9	9	9	9	9	9	9	9	9	9
1	2	2	2	2	2	2	2	2	2
9	9	3	9	9	9	9	3	9	9
2	2	2	2	2	1	2	2	2	2
9	9	9	9	9	9	9	9	9	9
9	9	9	9	9	9	9	2	9	9
2	9	2	9	2	2	3	9	2	2
3	1	3	1	3	1	2	3	2	3
9	9	9	9	9	9	9	9	9	9
9	9	9	9	9	9	9	9	9	9
3	3	3	2	1	2	2	3	2	1
9	9	9	9	9	9	9	9	9	9
2	1	1	2	3	2	1	2	3	2
1	1	1	1	1	1	1	1	1	1
2	2	2	2	2	2	2	2	2	2
2	2	2	2	2	2	2	2	2	2
2	2	2	2	2	2	2	2	2	2
2	2	2	2	2	2	2	2	2	2
2	2	2	2	2	2	2	2	2	2
2	2	2	2	2	2	2	2	2	2
2	2	2	2	2	2	2	2	2	2
9	2	9	9	9	2	9	2	9	2
3	2	2	2	2	2	2	3	3	2

9	2	9	9	9	9	9	9	9	9
2	2	2	2	2	2	2	2	2	2
3	1	3	3	3	2	1	1	3	1
9	9	9	9	9	9	9	9	9	9
9	9	9	9	9	9	9	9	9	9
9	9	9	9	9	9	9	9	9	9
9	9	9	9	9	9	9	9	9	9
2	2	2	2	1	1	2	2	2	2
2	2	2	2	2	2	2	2	2	2
9	9	9	9	9	9	9	9	9	9
3	3	3	3	2	2	2	2	2	2
2	1	2	1	2	1	2	2	2	3
2	2	2	3	1	2	2	1	1	2
2	2	2	2	2	2	2	2	2	2
2	1	2	3	1	3	3	2	3	1
9	9	9	9	9	9	9	9	9	9
9	9	9	9	9	9	9	9	9	9
9	9	9	9	9	9	9	9	9	9
9	9	9	9	9	9	9	9	9	9
9	9	9	9	9	9	9	9	9	9
9	9	9	9	9	9	9	9	9	9
9	9	9	9	9	9	9	9	9	9
2	2	2	2	2	2	2	2	2	2
9	9	9	9	9	9	9	9	9	9
9	9	9	9	9	9	9	9	9	9
9	9	9	9	9	9	9	9	9	9
9	9	9	9	9	9	9	9	9	9
2	9	9	1	9	9	1	9	9	9
9	2	9	9	9	9	2	9	9	9
9	9	9	9	3	9	9	9	9	9
9	9	9	9	9	9	9	9	9	9
2	2	9	2	9	9	9	9	2	9
9	9	9	9	9	9	9	9	9	9
9	9	9	9	9	9	9	9	9	9
9	9	9	9	9	9	9	9	9	9
9	9	9	9	9	9	9	9	9	9
9	9	9	9	9	9	9	9	9	9
9	9	9	9	9	9	9	9	9	9
9	9	9	9	9	9	9	9	9	9
9	9	9	9	9	9	9	9	9	9
2	2	2	2	2	2	2	2	2	2
9	9	9	9	9	9	9	9	9	9
9	9	9	9	9	9	9	9	9	9
9	9	9	9	9	9	9	9	9	9
9	9	9	9	9	9	9	9	9	9
9	9	9	9	9	9	9	9	9	9
9	9	9	9	9	9	9	9	9	9

9	9	9	9	9	9	9	9	9	9
9	9	9	9	9	9	9	9	9	9
9	9	9	9	9	9	9	9	9	9
9	9	9	9	9	9	9	9	9	9
9	9	9	9	9	9	9	9	9	9
9	9	9	9	9	9	9	9	9	9
9	9	9	9	9	9	9	9	9	9
9	9	9	9	9	9	9	9	9	9
9	9	9	9	9	9	9	9	9	9
9	9	9	9	9	9	9	9	9	9
9	9	9	9	9	9	9	9	9	9
9	9	9	9	9	9	9	9	9	9
9	9	9	9	9	9	9	9	9	9
9	9	9	9	9	9	9	9	9	9
9	9	9	9	9	9	9	9	9	9
9	9	9	9	9	9	9	9	9	9
9	9	9	9	9	9	9	9	9	9
2	2	2	2	2	2	2	2	2	2
9	9	9	9	9	9	9	9	9	9
2	2	2	2	2	2	2	2	2	2
9	9	9	9	9	9	9	9	9	9
9	9	9	9	9	9	9	9	9	9
9	9	9	9	9	9	9	9	9	9
9	9	9	9	9	9	9	9	9	9
9	9	9	9	9	9	9	9	9	9
9	9	9	9	9	9	9	9	9	9
9	9	9	9	9	9	9	9	9	9
9	3	2	9	9	2	9	3	9	9
9	9	9	9	9	9	9	9	9	9
9	9	9	9	9	9	9	9	9	9
1	1	1	1	1	1	1	1	1	1
2	1	1	2	2	3	2	9	1	1
9	9	9	9	9	9	9	9	9	9
2	2	2	2	2	2	1	1	1	1
9	9	9	9	9	9	9	9	9	9
9	9	9	9	9	9	9	9	9	1
9	9	9	9	9	9	9	9	9	9
9	9	9	9	9	9	9	9	9	9
1	1	1	1	1	1	1	1	1	1
9	9	9	9	9	9	9	9	9	9
9	9	9	9	9	9	9	9	9	9
9	9	9	9	9	9	9	9	9	9
2	2	2	2	2	2	2	3	3	3
9	9	9	9	9	9	9	9	9	9
9	9	1	9	1	2	9	1	9	9
9	9	9	9	9	9	9	9	9	9
9	9	9	9	9	9	9	9	9	9

9	9	9	9	2	9	9	9	9	9
9	9	9	9	9	9	9	9	9	9
9	3	9	9	1	9	2	3	9	1
9	9	9	9	9	9	9	9	9	9
9	9	9	9	9	9	9	9	9	9
9	9	9	9	9	9	9	9	9	9
9	9	9	9	9	9	9	9	9	9
9	9	9	9	9	9	9	9	9	9
9	2	9	9	9	2	9	9	9	9
2	2	2	2	9	9	9	9	9	9
9	9	9	9	9	9	9	9	9	9
9	9	9	9	9	9	9	9	9	9
9	9	9	9	9	9	9	9	9	9
9	9	9	9	9	9	9	9	9	9
2	2	2	2	2	2	2	2	2	2
1	3	1	1	1	1	1	2	1	1
9	9	9	9	9	9	9	9	9	9
2	2	2	2	2	2	2	2	2	2
2	2	2	2	2	2	2	2	2	2
9	9	9	9	2	9	9	3	9	9
9	9	9	9	9	9	9	9	9	9
9	1	9	9	9	9	9	9	9	9
3	2	1	2	3	1	2	1	3	3
2	3	1	2	3	1	2	3	1	2
1	2	3	2	1	1	1	1	2	1
3	2	2	2	2	2	2	1	2	2
1	1	1	1	1	1	3	3	1	1
2	1	3	2	2	2	2	2	2	2
2	2	2	2	2	2	2	2	2	2
1	1	1	1	1	1	1	1	1	1
1	1	1	1	9	9	9	9	9	9
9	9	9	9	9	9	9	9	9	9
9	9	9	9	9	9	9	9	9	9
9	9	9	9	9	9	9	9	9	9
9	9	9	9	9	9	9	9	9	9
9	9	9	9	9	9	9	9	9	9
9	9	9	9	9	9	9	9	9	9
2	9	9	9	2	9	9	2	9	9
9	9	9	9	9	9	9	9	9	9
9	9	9	2	9	2	9	2	9	9
9	9	9	9	9	9	9	9	9	9
2	2	2	2	2	2	2	2	2	2
9	9	9	9	9	9	9	9	9	9
9	9	9	9	9	9	9	9	9	9
9	9	9	9	9	9	9	9	9	9
2	2	2	2	2	2	2	2	2	2
3	2	2	2	3	2	3	2	3	3

2	1	9	2	1	1	1	1	2	1
2	2	2	2	2	2	2	2	1	1
1	1	2	2	1	1	1	1	1	1
2	1	3	2	2	3	2	3	2	1
3	3	3	3	3	3	3	3	3	3
2	1	2	1	2	1	1	1	2	2
2	1	3	3	2	1	1	1	1	1
3	3	3	3	3	3	3	3	3	3
2	2	2	2	2	2	2	2	2	2
2	2	2	2	2	2	2	2	2	2
2	9	9	9	2	9	9	2	9	9
9	9	9	1	9	2	9	1	9	1
2	2	2	2	2	2	2	2	2	2
1	3	2	2	2	2	2	3	1	3
1	2	2	1	3	2	3	3	2	2
2	2	2	2	2	2	2	2	2	2
1	3	2	3	2	3	1	2	1	3
2	2	2	2	2	2	2	2	2	2
9	9	9	9	9	9	9	9	9	9
2	2	2	2	2	2	2	1	3	2
2	2	2	3	3	2	3	3	3	3
2	3	2	1	1	2	3	1	1	2
3	1	2	3	2	2	3	1	2	1
3	2	1	2	2	2	2	1	2	1
2	1	2	2	1	1	1	1	1	1
2	2	2	2	2	2	2	2	1	1
2	2	3	1	1	1	2	1	1	1
2	3	2	2	2	2	2	2	2	2
2	2	2	2	1	1	2	1	3	1
3	2	3	1	1	3	2	1	3	1
3	3	3	3	2	2	3	3	2	2
2	3	2	2	2	2	2	2	2	2
3	3	3	3	3	3	3	3	3	3
2	2	2	2	2	2	2	2	2	2
1	9	9	9	9	9	9	9	9	9
3	2	2	3	3	3	3	2	2	2
3	2	1	2	1	1	2	2	2	1
2	2	2	2	2	2	2	2	2	2
2	2	2	1	1	2	1	1	2	3
9	9	9	9	9	9	9	9	9	9
2	2	2	2	2	2	2	2	2	2
9	9	9	9	9	9	9	9	9	1
3	3	3	2	1	3	3	2	2	1
2	2	2	2	2	2	2	2	2	2
2	3	1	2	1	1	1	2	3	1
3	2	3	2	3	2	3	2	3	3
3	1	2	3	2	3	2	2	2	2

9	9	9	9	9	9	9	9	9	9
3	3	3	3	3	3	3	3	3	3
3	3	1	1	1	1	1	3	3	1
2	2	3	2	1	1	9	1	2	2
1	2	3	3	3	2	1	1	2	1
3	3	3	2	1	1	1	1	1	1
2	2	2	2	2	2	2	2	2	2
2	2	2	2	2	2	3	3	9	1
3	3	3	2	2	2	1	1	1	3
2	2	2	2	2	2	2	2	1	1
2	2	2	2	3	3	3	2	2	3
2	9	2	9	2	2	2	2	2	2
9	9	9	9	9	9	9	9	9	9
2	2	2	2	2	3	3	2	3	2
2	2	2	2	1	1	1	2	2	1
2	2	2	2	2	2	2	2	2	2
2	2	2	2	2	2	3	3	3	1
2	2	2	2	2	2	2	2	2	2
2	2	2	2	2	2	2	2	2	2
9	9	9	9	9	9	9	9	9	9
9	1	2	2	2	2	2	1	2	1
1	2	1	2	2	1	1	1	1	2
3	3	3	2	3	2	3	2	2	2
3	1	2	3	2	3	2	2	2	9
3	3	3	3	3	3	3	3	3	3
1	2	3	3	2	2	1	1	2	3
9	9	9	9	9	9	9	9	9	9
2	2	2	2	2	2	2	2	2	2
2	2	2	2	2	2	2	2	2	2
2	2	2	2	2	2	2	2	2	2
1	3	2	3	2	3	1	1	2	2
9	9	9	9	9	9	9	9	9	9
9	9	9	9	9	9	9	9	9	9
2	2	2	2	2	1	1	1	1	1
3	1	3	3	1	2	2	1	2	3
9	9	9	9	9	9	9	9	9	9
2	2	2	2	3	3	3	3	3	3
2	2	2	2	2	2	1	1	1	1
3	3	3	3	1	2	1	2	1	3
1	1	2	2	2	2	1	1	2	2
9	9	9	9	9	9	9	9	9	9
2	2	2	2	2	2	2	2	2	2
2	2	2	2	2	2	2	2	2	2
2	2	2	2	2	2	2	2	2	2
2	2	2	2	2	2	2	2	2	2
2	2	2	2	2	2	1	1	2	3
2	2	2	2	2	2	2	2	2	2

3	3	2	1	3	2	2	1	1	3
2	2	2	2	2	2	2	2	2	2
2	2	2	2	2	2	2	2	2	2
2	2	2	2	2	2	2	2	2	2
2	2	2	2	2	2	1	2	2	2
2	2	2	2	2	2	2	2	2	2
2	2	2	2	2	2	2	2	2	2
2	2	2	2	2	2	2	2	2	2
2	2	2	2	2	2	2	2	2	2
9	9	9	9	9	9	9	9	9	9
3	3	3	3	3	3	2	1	1	1
2	3	1	2	2	1	3	1	1	2
2	2	2	2	1	2	2	2	2	1
2	2	1	2	1	2	2	2	1	2
3	3	3	3	3	3	3	2	2	2
1	2	1	2	1	9	1	2	1	1
9	9	9	9	9	9	9	9	9	9
9	9	2	9	9	9	9	2	9	9
9	9	9	9	9	9	9	9	9	9
2	2	2	2	2	2	2	2	2	2
2	2	2	2	2	2	2	3	9	2
9	9	9	9	9	9	9	9	9	9
9	9	9	9	9	9	9	9	9	9
2	2	2	2	2	2	2	2	2	2
1	1	2	2	1	1	2	2	2	1
2	2	2	2	2	2	2	2	2	2
1	2	2	1	3	3	1	1	2	3
3	3	3	2	3	3	3	3	2	2
1	1	2	1	1	2	1	3	1	2
2	2	2	3	2	1	2	3	1	2
9	9	9	9	9	9	9	9	9	9
1	1	1	3	1	2	1	3	1	2
2	3	2	1	3	2	1	3	1	3
2	2	2	2	2	2	3	3	3	2
2	2	2	2	2	2	2	2	2	2
2	2	2	2	2	2	2	2	2	2
1	2	9	9	9	9	9	9	9	9
2	2	2	2	2	2	2	2	2	2
2	2	2	2	2	2	2	2	2	2
2	2	2	3	3	3	3	3	3	3
1	3	1	1	3	1	1	1	1	1
3	2	2	2	3	1	1	1	1	1
2	2	2	2	2	2	2	2	2	2
2	2	2	2	2	2	2	2	2	2
2	3	1	2	2	2	3	2	3	1
3	1	3	1	3	1	1	2	1	2
3	3	2	3	2	2	2	3	3	3

3	2	3	1	1	2	2	2	1	2
2	2	2	2	2	2	2	2	2	2
3	9	1	9	1	3	2	9	2	2
2	2	2	2	2	2	2	2	2	2
2	3	2	3	1	2	3	3	3	3
2	2	2	2	2	2	2	2	2	2
9	9	9	9	9	1	9	9	9	9
3	3	3	3	3	3	3	3	3	3
9	9	9	9	9	9	9	9	9	9
2	2	3	3	3	3	2	2	2	2
2	2	2	2	2	2	2	2	2	2
3	3	2	9	1	1	2	1	1	1
1	2	2	1	1	2	3	3	2	2
9	9	9	9	9	9	9	9	9	1
9	9	9	1	9	1	9	9	9	9
9	9	9	9	9	9	9	9	9	9
3	1	2	2	1	2	2	1	1	1
1	1	3	1	1	1	1	1	1	1
3	1	3	9	2	2	2	1	2	1
2	2	3	2	1	1	2	1	1	1
9	2	2	2	2	2	2	2	2	2
3	1	3	1	3	1	3	1	3	1
3	2	2	1	9	2	2	1	2	1
2	2	2	2	2	2	2	2	2	2
1	1	3	1	1	1	1	1	1	1
2	2	2	2	2	2	2	2	2	2
2	2	2	2	2	2	2	2	2	2
2	2	2	2	2	2	2	2	2	2
3	2	3	2	2	1	2	3	2	3
3	3	3	3	3	3	3	3	3	3
2	2	1	2	1	2	2	1	2	3
3	2	1	2	3	2	2	3	1	2
2	2	2	2	2	2	2	2	3	3
2	2	2	2	2	2	2	2	2	2
1	1	1	2	2	2	2	2	1	2
3	2	2	2	3	2	2	2	3	2
1	1	1	1	1	1	1	1	1	1
2	2	2	2	2	2	2	2	2	2
3	2	3	1	3	1	3	2	3	2
2	2	2	2	2	2	2	2	2	2
9	9	9	9	1	3	1	3	1	1
1	1	2	1	2	2	2	2	2	2
2	2	2	2	2	2	2	2	2	2
3	1	3	2	1	1	1	1	2	1
2	2	2	2	2	2	2	2	2	2
3	2	3	1	2	2	2	3	2	2
2	2	2	2	2	2	2	2	2	2

1	1	2	3	2	1	3	1	2	2
3	3	3	1	1	1	2	1	2	1
2	3	2	3	2	3	2	3	2	3
2	2	2	2	2	2	2	2	2	2
2	3	1	3	3	1	9	2	1	2
9	9	9	9	9	9	9	9	9	9
2	2	2	3	2	2	2	3	2	2
9	9	9	9	9	9	9	9	9	9
9	9	9	9	9	9	9	9	9	9
1	1	3	3	3	3	3	3	3	3
9	9	9	9	9	9	9	9	9	9
9	2	9	9	9	9	9	9	9	9
9	9	9	9	9	9	9	9	9	9
9	9	9	9	9	9	9	9	9	9
2	1	2	3	2	2	2	2	1	1
9	9	9	9	9	9	9	9	9	9
9	9	9	9	9	9	9	9	9	9
9	9	9	9	9	9	9	9	9	9
9	9	9	9	9	9	9	9	9	9
2	9	9	9	9	9	9	9	9	9
9	9	9	9	9	9	9	9	9	9
2	2	2	2	2	2	2	2	2	2
2	2	2	2	2	2	2	2	2	2
3	3	3	2	2	2	3	1	3	2
2	2	2	1	2	1	1	3	3	3
2	3	2	2	2	3	2	2	1	1
1	3	1	1	1	3	3	3	1	2
9	9	9	9	9	9	9	9	9	9
2	2	2	2	2	2	2	2	3	2
1	3	9	1	1	1	1	3	3	1
3	1	3	1	3	3	2	1	3	3
9	9	9	9	9	9	9	9	9	9
2	2	2	2	2	2	2	2	2	2
9	9	9	9	9	9	9	9	9	9
9	9	9	9	9	9	9	9	9	9
2	1	2	2	1	1	2	3	2	1
1	1	1	1	1	1	1	1	1	1
9	9	9	9	9	9	9	9	9	9
9	9	9	9	9	9	9	9	9	9
2	2	2	2	2	2	2	2	2	2
2	9	9	2	9	2	9	2	2	2
2	2	2	2	2	2	2	2	2	2
3	3	3	3	3	3	3	3	3	3
9	9	2	9	9	9	9	9	2	9
1	1	1	1	1	1	1	1	2	2
2	2	2	2	2	2	2	2	2	2
9	3	1	9	3	1	9	3	1	3

9	9	9	9	9	9	9	9	9	9
3	1	9	9	9	3	1	9	3	1
9	3	1	9	9	9	1	9	9	9
2	2	2	2	2	2	2	2	2	2
9	2	1	2	1	9	3	2	1	9
3	1	3	1	3	2	1	9	9	2
9	9	9	9	9	9	9	9	9	9
9	9	9	9	9	9	9	9	9	9
9	2	9	9	2	9	2	9	2	9
2	2	2	2	2	2	2	2	2	2
9	9	9	9	9	9	9	9	9	9
2	9	9	2	9	9	2	9	9	2
2	2	2	2	2	2	2	2	2	2
9	9	9	9	9	9	9	9	9	9
2	2	2	2	2	2	2	2	2	2
9	9	9	9	9	9	9	9	9	9
9	9	9	9	9	9	9	9	9	9
9	9	9	9	9	9	9	9	9	9
9	9	9	9	9	9	9	9	9	9
9	9	9	9	9	9	9	9	9	9
2	2	2	2	2	2	2	2	2	2
2	2	2	2	2	2	2	2	2	2
3	2	2	3	2	1	2	1	3	9
3	1	1	1	2	3	1	1	2	1
2	2	2	2	2	3	3	3	3	3
2	2	2	2	2	2	2	2	2	2
1	1	1	1	1	1	1	1	1	1
2	3	2	2	3	3	1	1	1	2
3	3	1	1	1	1	2	3	1	2
2	2	2	2	2	2	2	2	2	2
3	2	2	2	2	2	3	3	3	2
1	2	3	3	2	2	3	3	2	2
2	2	2	2	2	2	2	2	2	2
9	9	9	9	9	9	9	9	9	9
2	1	2	1	1	2	1	1	2	1
3	3	3	3	3	3	3	3	3	3
2	2	2	2	2	2	2	2	2	2
3	1	2	1	2	1	2	2	2	1
2	2	2	2	2	2	2	2	2	2
2	3	3	2	3	3	2	2	2	2
1	9	3	2	1	1	1	2	2	1
9	9	9	9	9	9	9	9	9	9
2	2	2	2	2	2	2	2	2	2
9	9	9	9	9	9	9	9	9	9
2	1	2	1	2	2	1	2	1	2
2	2	2	2	2	2	2	2	2	2
3	2	3	2	2	2	2	2	2	2

3	2	2	2	1	3	2	1	1	1
3	3	2	9	1	1	2	1	1	3
2	2	2	2	2	2	2	2	2	2
9	2	2	2	2	2	2	2	2	2
1	1	1	2	2	2	2	2	2	2
1	1	1	9	2	2	2	2	2	2
1	1	1	1	1	1	1	1	1	1
3	3	1	1	2	1	2	1	2	1
3	3	3	3	3	3	3	3	3	3
3	3	3	1	1	1	2	1	1	1
2	2	2	2	2	2	2	2	2	2
2	2	2	2	2	2	2	2	1	1
2	2	2	2	2	2	2	1	1	1
2	2	1	1	1	1	1	1	1	1
9	9	9	9	9	9	9	9	9	9
9	1	9	9	1	1	1	2	2	2
1	1	1	1	1	2	2	2	2	9
2	2	2	1	1	1	1	2	1	1
9	9	9	9	9	9	9	9	9	9
2	2	2	2	2	2	2	2	2	2
1	2	2	1	1	2	1	2	1	1
1	2	2	2	2	2	2	2	2	2
9	9	9	9	9	9	9	9	9	9
2	2	2	2	2	2	2	2	2	2
9	9	9	9	9	9	9	9	9	9
1	1	1	1	1	2	2	1	1	1
1	1	1	2	2	1	2	1	1	1
1	1	9	1	1	1	2	2	2	2
9	2	2	9	2	2	2	9	2	9
2	2	2	2	2	2	9	2	2	9
2	2	2	2	2	2	9	2	2	2
2	2	2	2	2	2	2	2	2	2
2	2	2	2	2	2	2	2	2	2
1	1	1	1	1	1	1	1	1	1
2	2	2	2	2	2	2	2	2	2
9	9	9	9	9	9	9	9	9	9
2	1	1	1	1	2	2	2	2	1
1	1	1	1	2	2	1	1	1	1
9	9	9	9	9	9	9	9	9	9
2	2	2	2	2	2	2	2	2	2
2	2	2	2	2	9	2	9	9	9
9	9	9	9	9	9	9	9	9	9
2	2	2	2	2	2	2	2	2	2
2	2	2	2	2	2	2	2	2	2
1	1	1	1	1	2	1	1	2	3
2	2	2	2	2	9	2	2	9	2
9	1	9	9	9	9	9	9	9	9

2	1	2	1	2	2	1	1	1	1
9	2	9	9	2	2	9	9	2	9
1	1	1	1	1	1	1	1	1	1
1	1	1	1	2	1	2	1	1	1
2	2	2	2	2	2	2	2	2	2
2	2	2	2	2	2	2	2	2	2
1	1	1	1	1	1	1	1	1	1
2	2	2	2	2	2	2	2	2	2
9	9	9	9	9	9	9	9	9	9
1	9	1	9	1	9	9	2	2	9
2	9	2	9	2	9	2	2	9	2
2	9	2	9	2	2	2	2	9	2
9	9	9	9	9	9	9	9	9	9
9	9	9	9	9	9	9	9	9	9
9	9	9	9	9	9	9	9	9	9
9	9	9	9	9	9	9	9	9	9
2	9	2	2	9	9	9	9	9	9
9	9	9	9	9	9	9	9	9	9
2	2	2	2	2	2	2	2	2	2
3	3	3	1	1	1	1	3	1	1
3	3	3	1	1	1	1	3	1	1
3	3	3	1	1	1	1	3	1	1
9	3	3	1	1	1	1	1	1	1
3	3	1	3	1	1	1	3	1	1
3	3	3	1	1	1	1	3	1	1
3	3	3	1	1	1	1	1	1	1
3	3	3	1	1	1	1	3	1	1
3	3	1	3	1	1	1	3	1	1
3	9	3	1	1	1	1	3	1	1
3	3	3	1	1	1	1	3	1	1
3	3	3	1	1	1	1	3	1	1
3	3	3	1	1	1	3	1	1	1
3	3	3	9	1	1	1	3	1	1
3	3	3	1	1	1	1	3	1	1
3	3	3	1	1	1	1	3	1	1
3	3	3	3	1	1	3	3	1	1
3	3	3	3	1	1	1	3	1	1
3	3	3	1	1	1	1	3	1	1
9	3	3	1	1	9	1	3	1	1
3	3	3	1	1	1	1	3	1	1
3	3	3	1	1	1	1	3	1	1
3	3	3	1	1	1	1	3	1	1
3	3	3	1	1	1	1	3	1	1
3	3	3	1	1	1	3	1	1	1
9	9	9	9	9	9	9	9	9	9
9	9	9	9	9	9	9	9	9	9
9	9	9	9	9	9	9	9	9	9

9	9	9	9	9	9	9	9	9	9
9	9	9	9	9	9	9	9	9	9
9	9	9	9	9	9	9	9	9	9
9	9	9	9	9	9	9	9	9	9
9	9	9	9	9	9	9	9	9	9
9	9	9	9	9	9	9	9	9	9
9	9	9	9	9	9	9	9	9	9
9	9	9	9	9	9	9	9	9	9
9	9	9	9	9	9	9	9	9	9
9	9	9	9	9	9	9	9	9	9
9	9	9	9	9	9	9	9	9	9
9	9	9	9	9	9	9	9	9	9
9	9	9	9	9	9	9	9	9	9
9	9	9	9	9	9	9	9	9	9
9	9	9	9	9	9	9	9	9	9
9	9	9	9	9	9	9	9	9	9
9	9	9	9	9	9	9	9	9	9
9	9	9	9	9	9	9	9	9	9
9	9	9	9	9	9	9	9	9	9
3	3	3	1	1	1	1	3	1	1
3	3	3	1	1	1	1	3	1	1
3	3	3	1	1	1	1	3	1	1
3	3	3	1	1	1	1	3	1	1
3	3	3	1	1	3	1	3	1	1
3	3	3	3	1	3	1	3	1	1
2	2	3	1	1	1	1	3	1	1
3	3	3	1	1	1	1	3	1	1
3	3	3	1	1	1	1	1	3	1
3	3	3	1	1	1	1	3	1	1
3	3	1	1	1	1	1	3	1	1
3	3	3	1	1	1	3	1	1	2
3	3	3	1	1	1	1	1	1	1
3	3	3	1	1	1	1	3	1	1
3	3	3	1	1	1	3	3	1	1
3	3	9	1	1	1	1	3	1	1
9	9	9	9	9	9	9	9	9	9
3	3	3	1	1	1	1	3	1	1
3	3	3	1	1	1	1	1	1	1
3	3	3	1	1	1	1	1	1	1
3	3	3	1	1	1	9	3	1	1
3	3	1	3	1	1	1	3	1	1
3	3	3	1	1	1	1	3	1	1
3	3	9	1	1	1	1	3	1	1
3	3	3	1	1	1	1	3	1	1
1	9	3	1	1	1	3	1	1	1
3	9	9	1	1	1	1	1	1	1
3	3	3	1	1	1	1	1	1	1

3	3	1	3	1	1	3	1	1	1
3	3	3	1	1	1	1	1	1	2
3	3	3	1	1	1	1	3	1	1
3	3	3	1	1	1	1	3	1	1
3	3	3	1	1	1	1	3	1	1
3	3	3	1	1	1	1	3	1	1
1	3	3	3	1	1	1	1	1	1
3	3	3	1	1	1	3	3	1	1
3	3	3	1	1	1	1	3	1	1
3	3	3	1	1	1	1	3	1	1
3	3	3	1	1	1	3	3	1	1
3	9	3	1	1	1	1	3	1	1
3	3	3	1	1	1	1	3	1	1
3	3	3	1	1	1	1	3	1	1
3	3	9	1	1	1	1	3	1	1
3	3	3	1	1	1	1	3	1	1
3	3	3	1	1	1	1	3	1	1
3	3	3	1	1	1	1	3	1	1
3	3	3	1	1	1	1	3	2	1
3	3	3	1	1	1	1	3	1	1
3	3	3	1	1	1	1	3	1	1
3	3	1	1	1	1	1	3	1	1
3	3	3	3	1	1	3	1	1	1
1	3	3	1	1	1	1	3	1	1
3	3	3	1	1	1	1	3	1	1
3	3	9	1	1	1	1	3	1	1
3	3	3	1	1	1	1	3	1	1
1	3	3	3	1	1	1	9	1	1
3	3	3	1	1	3	1	3	1	1
3	3	3	1	1	1	1	3	1	1
3	3	3	1	1	1	1	3	1	1
3	3	3	3	1	1	1	1	3	1
9	9	9	9	9	9	9	9	9	9
3	3	9	1	1	1	1	3	1	1
9	9	9	9	9	9	9	9	9	9
3	3	3	1	1	3	1	3	1	1
1	3	3	1	1	1	1	3	9	3
3	3	3	1	1	1	1	3	1	1
1	3	3	1	1	1	1	3	1	1
3	3	3	1	1	1	3	1	3	1
3	3	1	1	1	3	3	1	1	1
3	3	3	1	1	1	1	1	1	1
3	3	3	1	1	1	1	3	1	1
3	3	3	1	1	3	1	3	1	1
3	3	3	1	1	1	1	3	1	1
3	3	3	1	1	1	1	3	1	1
3	3	3	1	1	1	1	3	1	1

3	3	3	1	1	1	1	3	1	1
3	3	3	1	1	1	1	3	1	1
3	3	3	1	1	1	1	3	1	1
3	3	3	1	1	1	1	3	1	1
3	3	3	1	1	1	3	1	1	2
1	3	3	1	1	1	1	3	1	1
3	3	3	1	1	1	1	3	1	1
3	3	3	1	1	3	3	3	1	2
1	3	3	1	1	1	1	3	1	1
3	3	3	1	1	1	1	3	1	1
3	3	3	1	1	1	1	3	1	1
3	3	3	1	1	1	1	3	1	1
3	3	3	1	1	1	1	3	1	1
3	3	3	1	1	1	1	3	1	1
3	3	3	9	9	9	9	9	9	9
3	3	3	1	1	1	1	3	1	1
3	3	3	1	1	1	1	1	1	1
3	3	3	1	1	1	1	1	1	1
3	3	3	1	1	1	1	1	1	1
9	9	9	9	9	9	9	9	9	9
3	3	3	1	1	1	1	3	1	1
3	3	3	1	1	1	3	1	1	1
3	3	3	1	1	1	1	3	1	1
3	9	3	1	1	1	1	3	1	1
1	2	2	1	9	9	9	9	1	1
3	3	9	3	1	1	1	3	1	9
3	3	3	1	1	1	1	3	1	1
1	3	3	1	1	1	1	3	1	1
3	3	3	1	1	1	1	3	1	2
3	1	3	1	1	1	1	1	1	1
3	3	3	1	1	1	1	3	1	1
3	3	3	1	1	1	1	3	1	1
3	3	1	1	1	1	3	1	1	2
3	3	3	1	1	1	1	3	1	1
3	3	3	1	1	1	1	3	1	1
3	3	3	1	1	1	9	3	1	1
3	3	3	1	1	1	1	3	1	1
3	3	3	1	1	1	1	3	1	1
3	3	3	1	1	1	1	3	1	1
3	3	3	1	1	1	1	3	1	1
3	3	3	1	1	1	1	3	1	1
9	9	9	9	9	9	9	9	9	9
3	3	3	1	1	1	1	3	1	1
3	3	3	1	1	1	1	3	1	1
3	3	1	1	1	1	1	3	1	1
3	3	3	1	1	1	1	3	1	1
3	3	3	1	1	1	1	3	1	1

3	3	3	1	1	1	1	3	1	1
3	3	3	1	1	1	1	3	1	1
3	3	3	1	1	1	1	3	1	1
3	3	1	1	1	3	1	3	1	1
3	3	3	1	1	1	1	3	1	1
3	3	3	1	1	3	1	3	1	1
3	3	3	1	1	3	3	3	1	1
3	3	3	1	1	1	1	3	1	1
3	3	3	1	1	3	3	3	1	1
3	1	1	1	1	1	3	1	1	1
3	3	3	1	1	1	1	3	1	1
3	3	3	1	1	1	1	3	1	1
3	3	3	1	1	1	1	3	1	1
3	3	3	1	1	1	1	3	1	1
3	1	3	9	1	1	1	3	1	1
3	3	3	1	1	1	1	3	1	1
3	3	3	1	1	1	1	3	1	1
3	3	3	1	1	1	1	3	1	1
3	3	3	1	1	1	1	3	1	1
3	3	3	1	1	1	1	1	1	1
3	3	3	1	1	1	1	3	1	1
1	3	3	3	1	1	1	3	1	1
3	3	3	1	1	1	1	3	1	1
3	3	3	1	1	1	1	3	1	1
3	3	3	1	1	1	1	1	1	1
1	3	3	1	1	1	1	3	1	1
1	3	3	1	1	1	1	1	1	1
9	9	9	9	9	9	9	9	9	9
9	9	9	9	9	9	9	9	9	9
9	9	9	9	9	9	9	9	9	9
9	9	9	9	9	9	9	9	9	9
9	9	9	9	9	9	9	9	9	9
9	9	9	9	9	9	9	9	9	9
9	9	9	9	9	9	9	9	9	9
9	9	9	9	9	9	9	9	9	9
9	9	9	9	9	9	9	9	9	9
9	9	9	9	9	9	9	9	9	9
9	9	9	9	9	9	9	9	9	9
9	9	9	9	9	9	9	9	9	9
9	9	9	9	9	9	9	9	9	9
9	9	9	9	9	9	9	9	9	9
9	9	9	9	9	9	9	9	9	9
9	9	9	9	9	9	9	9	9	9
9	9	9	9	9	9	9	9	9	9
9	9	9	9	9	9	9	9	9	9
9	9	9	9	9	9	9	9	9	9
9	9	9	9	9	9	9	9	9	9

9	9	9	9	9	9	9	9	9	9
9	9	9	9	9	9	9	9	9	9
9	9	9	9	9	9	9	9	9	9
9	9	9	9	9	9	9	9	9	9
9	9	9	9	9	9	9	9	9	9
9	9	9	9	9	9	9	9	9	9
9	9	9	9	9	9	9	9	9	9
9	9	9	9	9	9	9	9	9	9
9	9	9	9	9	9	9	9	9	9
9	9	9	9	9	9	9	9	9	9
9	9	9	9	9	9	9	9	9	9
9	9	9	9	9	9	9	9	9	9
9	9	9	9	9	9	9	9	9	9
2	1	1	2	1	1	2	3	1	2
2	2	2	2	2	2	2	2	2	2
2	2	2	2	2	2	2	3	2	2
3	1	2	3	2	1	3	1	1	3
3	1	2	1	3	2	1	1	1	1
3	3	3	3	1	1	1	1	1	1
3	1	2	2	1	3	2	1	1	1
1	3	3	1	1	1	1	1	1	1
1	1	1	3	2	1	2	3	1	2
3	9	9	9	9	9	9	9	9	9
3	3	2	2	2	3	3	3	2	2
9	1	9	9	9	9	9	9	9	9
3	3	3	3	3	3	2	2	2	3
9	9	9	9	9	9	9	9	9	9
1	1	3	3	3	3	3	3	1	1
3	2	3	1	2	1	1	1	1	2
3	3	3	1	1	1	2	2	1	2
3	3	3	3	3	3	3	3	3	3
2	3	1	2	3	1	2	3	2	3
9	9	9	9	9	9	9	9	9	9
2	3	1	2	1	2	3	2	3	1
3	1	3	1	1	1	1	1	1	1
9	9	9	9	9	9	9	9	9	9
2	2	2	2	2	2	2	2	2	2
3	2	1	1	1	3	3	2	2	2
3	1	3	3	3	2	1	1	1	1
1	1	1	1	1	1	3	2	1	1
9	9	9	9	9	9	9	9	9	9
9	9	9	9	9	9	9	9	9	9
2	2	1	2	2	2	2	2	2	2
1	9	1	9	1	1	1	9	1	1
9	9	9	9	9	9	9	9	9	9
9	9	9	9	9	9	9	9	9	9
2	2	3	3	2	2	2	2	2	2

9	1	9	9	9	9	9	9	9	9
9	9	9	9	9	9	9	9	9	9
3	2	2	3	2	3	2	3	2	1
2	2	2	2	2	2	1	2	1	2
1	1	2	2	1	1	1	1	1	1
9	9	9	9	9	9	9	9	9	9
2	2	2	2	2	2	2	2	2	2
2	2	2	2	2	2	2	2	2	2
2	2	2	2	2	2	2	2	2	2
3	3	3	1	1	1	1	2	2	3
9	2	9	2	1	2	2	1	3	3
9	9	9	9	9	9	9	9	9	9
9	9	9	9	9	9	9	9	9	9
2	2	2	2	2	2	2	2	2	2
2	2	2	2	2	2	2	2	2	2
2	1	2	3	1	1	2	1	3	1
1	1	1	3	2	1	2	3	1	2
2	2	2	2	2	2	2	2	2	2
1	1	3	2	2	1	1	1	3	3
9	3	9	9	9	9	9	9	9	9
2	3	2	2	2	2	2	3	2	2
2	2	2	2	2	2	2	2	2	2
2	2	2	2	2	2	2	2	2	2
2	2	2	2	2	2	2	2	2	2
2	2	2	2	2	2	2	2	2	2
2	2	1	1	3	3	2	2	1	1
2	2	2	2	2	2	2	2	2	2
2	2	2	2	2	2	2	2	2	2
2	1	2	2	2	2	2	2	2	2
2	1	2	2	1	1	1	1	1	1
3	3	3	3	1	1	2	2	2	1
3	2	3	2	1	2	3	1	1	1
1	1	2	1	1	1	2	1	1	1
2	1	3	3	1	1	1	1	2	1
2	2	2	1	1	2	1	2	1	2
3	2	3	1	3	2	3	2	3	2
1	1	2	2	2	2	2	2	2	2
2	2	2	2	2	2	2	2	2	2
9	9	9	9	9	9	9	9	9	9
2	1	3	3	1	1	1	2	1	3
1	1	2	2	2	2	2	2	2	2
2	1	2	2	2	2	2	2	1	2
2	3	1	2	3	1	2	3	1	2
3	2	3	2	2	2	2	1	2	3
3	1	2	1	1	1	1	1	1	1
1	1	2	2	2	3	3	3	2	9
3	3	3	2	1	2	2	1	1	1

2	2	2	2	2	2	2	2	1	1
3	2	2	2	3	2	2	3	2	3
2	2	2	2	2	2	2	2	2	2
3	2	3	2	3	2	2	3	2	9
1	1	1	1	2	3	2	2	2	2
1	1	1	1	1	1	2	2	2	2
3	1	3	3	1	3	3	1	3	2
2	3	2	2	1	2	2	2	1	3
2	2	2	2	2	2	2	2	2	2
1	2	1	2	3	1	2	3	2	2
2	2	2	2	2	2	2	2	2	2
2	2	2	2	2	2	2	2	2	2
1	1	1	1	1	1	1	1	1	1
2	3	2	2	2	2	2	2	2	3
2	2	2	2	2	2	2	2	2	2
2	3	2	2	3	2	2	3	2	3
3	3	3	3	3	3	3	3	3	3
2	2	2	2	2	2	2	2	2	2
2	2	2	2	2	2	2	2	2	2
2	3	1	2	3	1	2	3	1	2
1	2	1	2	1	1	2	1	1	2
1	2	2	2	2	2	2	1	1	1
2	2	2	2	2	2	2	2	2	2
3	3	3	3	3	3	1	1	1	1
1	3	3	1	1	1	1	1	1	1
9	9	9	9	9	9	9	9	9	9
3	2	3	1	2	1	2	1	2	3
2	2	2	2	2	2	2	2	2	2
3	1	2	3	2	1	1	1	1	1
2	1	2	1	1	2	1	2	1	1
3	2	3	2	1	2	2	1	1	1
1	2	1	2	2	1	2	2	3	2
2	2	3	3	3	3	3	3	3	3
1	1	1	1	2	2	1	2	1	3
1	2	2	9	9	1	1	1	3	3
2	2	2	2	2	9	9	9	9	9
9	9	9	9	9	9	9	9	9	9
2	2	2	2	2	1	1	2	2	1
9	9	9	9	9	9	9	9	9	9
2	1	2	2	2	2	2	1	2	2
2	2	3	3	9	2	2	2	2	2
2	1	1	2	1	2	1	1	2	1
3	2	1	2	2	1	1	1	2	1
9	9	9	9	9	9	9	9	9	9
9	9	9	9	9	9	9	9	9	9
9	9	9	9	9	9	9	9	9	9
2	2	1	1	1	1	1	3	3	3

1	3	3	9	9	9	9	9	9	9
9	9	9	9	9	9	9	9	9	9
2	2	3	3	2	3	2	1	2	2
1	1	1	1	3	3	3	3	3	3
9	9	9	9	9	9	9	9	9	9
3	1	3	3	3	2	2	3	3	3
3	2	2	2	3	2	3	3	3	2
2	2	3	3	3	2	2	2	2	2
2	1	3	2	1	1	1	1	1	1
2	3	1	2	3	1	2	3	1	2
1	2	3	1	2	1	3	2	1	2
2	2	2	2	2	2	2	2	2	2
2	2	2	2	2	2	2	2	2	2
9	9	9	9	9	9	9	9	9	9
2	2	2	2	2	2	2	2	2	2
2	2	2	2	1	9	1	1	1	1
1	1	1	1	1	3	1	1	3	1
3	3	2	3	3	1	1	2	2	2
1	3	1	2	2	2	1	2	3	1
9	9	9	9	9	9	9	9	9	9
3	3	3	3	1	1	2	2	1	3
2	3	3	1	3	2	1	2	3	1
3	1	2	3	3	3	3	3	2	2
3	2	1	3	1	2	3	2	2	3
2	3	2	9	3	2	3	3	2	2
9	9	9	9	9	9	9	9	9	9
1	1	1	2	1	3	2	3	2	2
2	2	2	2	2	2	2	2	2	2
2	2	2	3	3	3	3	3	3	3
2	1	2	2	3	1	2	1	2	3
2	2	2	2	2	2	2	2	2	2
9	9	9	9	9	9	9	9	9	9
3	3	1	3	1	1	3	3	2	2
2	3	3	2	2	2	2	3	2	2
3	1	9	3	1	2	2	2	3	1
3	1	2	1	2	1	3	1	2	1
9	9	9	9	9	9	9	9	9	9
3	1	3	2	1	1	1	1	2	1
3	1	3	3	1	1	1	2	1	1
9	9	9	9	9	9	9	9	9	9
2	1	1	2	3	2	1	2	3	1
2	2	2	2	2	2	2	2	2	2
2	2	2	2	2	2	2	2	2	2
2	2	2	2	2	2	2	2	2	2
2	2	2	2	2	2	2	2	2	2
9	9	9	9	9	9	9	9	9	9
2	2	2	2	2	2	2	2	2	9

3	2	3	3	1	1	1	1	1	1
2	2	2	2	2	2	2	2	2	2
2	2	2	2	2	2	2	2	2	2
2	1	2	3	3	3	2	2	2	2
3	3	3	3	3	3	1	1	3	1
2	2	2	3	3	2	3	3	3	2
1	1	3	1	1	1	1	1	1	1
3	1	3	3	1	2	1	2	2	2
1	1	3	1	1	1	1	1	1	1
3	1	3	3	1	1	2	3	3	2
2	3	3	2	2	2	2	2	2	2
3	3	3	3	3	3	3	2	2	2
2	2	2	2	2	2	2	2	2	2
2	1	3	2	2	1	1	1	2	1
9	9	9	9	9	9	9	9	9	9
2	2	2	2	1	2	1	1	1	1
2	3	1	3	3	2	2	1	2	3
2	2	2	2	2	2	1	2	2	1
2	2	2	2	2	3	2	2	3	2
2	2	2	2	2	1	1	1	1	1
3	1	3	3	1	1	1	1	1	1
3	3	3	1	9	9	9	1	3	1
3	3	3	3	1	2	2	3	3	2
2	2	2	2	2	2	2	2	2	2
2	2	2	2	2	9	3	2	9	2
1	2	2	3	2	2	1	1	1	1
2	1	3	2	2	3	1	1	2	1
2	2	3	3	2	2	2	2	2	2
3	3	3	1	1	3	3	1	1	3
3	3	3	1	1	2	2	1	1	1
2	3	3	1	1	1	1	1	1	1
1	2	2	2	2	2	1	1	1	2
1	2	2	2	2	2	1	1	1	1
9	9	9	9	9	9	9	9	9	9
9	9	9	9	9	9	9	9	9	9
3	3	3	1	1	3	1	1	1	1
2	2	2	2	2	2	2	2	2	2
1	1	1	1	2	2	3	3	3	3
1	1	1	1	2	2	2	2	2	1
2	2	2	2	2	2	2	2	2	2
3	1	2	3	1	1	2	2	3	2
9	9	9	9	9	9	9	9	9	9
9	9	9	9	9	9	9	9	9	9
1	1	1	1	1	1	1	1	1	1
3	1	2	2	3	2	3	2	2	2
9	9	9	9	9	9	9	9	9	9
9	9	9	9	9	9	9	9	9	9

2	2	2	2	2	2	2	2	2	2
1	3	1	1	1	1	1	1	1	1
2	3	1	2	3	1	2	3	1	2
1	2	3	1	2	3	1	3	2	1
3	3	3	1	1	2	2	2	2	2
2	3	1	2	3	1	2	3	1	2
2	3	2	1	2	3	2	1	2	2
9	9	9	9	9	9	9	9	9	9
1	2	3	2	2	1	2	2	2	2
9	9	9	9	9	9	9	9	9	9
1	1	1	1	1	1	1	1	1	1
1	1	3	1	1	1	1	1	1	1
9	9	9	9	9	9	9	9	9	9
9	9	9	9	9	9	9	9	9	9
2	1	2	2	2	2	2	2	2	2
9	9	9	9	9	9	9	9	9	9
9	9	9	9	9	9	9	9	9	9
2	2	2	2	2	2	2	2	2	2
2	2	2	2	2	2	2	2	2	2
2	2	2	2	2	9	1	1	1	2
2	2	2	2	2	2	2	2	2	2
2	2	2	2	2	2	2	2	2	2
3	3	2	3	3	2	2	3	3	2
3	2	1	2	1	2	3	2	1	2
9	2	2	2	9	9	3	3	9	9
1	9	9	9	9	9	9	9	9	9
2	2	2	1	1	1	1	1	1	1
1	1	1	1	1	1	1	1	1	1
1	1	1	1	1	1	1	1	1	1
1	1	3	1	1	1	1	1	1	1
2	2	2	1	1	2	1	2	1	1
2	2	2	2	2	2	2	2	2	2
2	2	2	2	2	2	2	2	2	2
2	2	2	2	2	2	2	2	2	2
1	1	1	1	1	1	1	1	1	1
2	2	2	2	2	2	2	2	2	2
2	2	2	2	2	2	2	2	2	2
9	9	9	9	9	9	9	9	9	9
9	9	9	9	9	9	9	9	9	9
2	2	2	2	2	2	2	2	2	2
2	2	2	2	2	2	2	2	2	2
9	9	9	9	9	9	9	9	9	9
2	2	2	2	2	2	2	2	2	2
9	9	9	9	9	9	9	9	9	9
3	3	3	3	3	3	3	3	3	3
9	9	9	9	9	9	9	9	9	9
2	2	2	2	2	2	2	2	2	2

3	1	2	1	2	1	3	1	2	3
9	9	9	9	9	9	9	9	9	9
2	2	2	2	2	2	2	2	2	2
3	3	3	2	2	2	3	2	2	1
2	2	2	2	2	2	2	2	3	3
2	2	1	1	3	1	2	2	2	2
1	1	2	2	2	2	2	1	1	2
2	2	2	3	2	2	2	2	3	1
2	2	2	2	2	2	2	2	2	2
3	3	3	3	3	2	1	1	1	1
3	3	3	3	1	1	3	1	1	1
2	2	2	2	2	2	2	2	2	2
2	3	3	2	2	2	2	2	2	2
2	3	2	2	3	2	3	2	2	2
2	1	1	1	2	1	1	1	1	2
2	1	1	1	2	1	1	1	1	2
2	2	2	2	2	1	2	2	2	2
2	2	2	2	2	2	2	2	2	2
2	3	2	3	2	2	2	3	3	3
9	2	9	2	3	2	1	2	2	2
1	2	3	2	1	2	1	1	1	2
2	1	2	2	2	2	2	1	1	1
3	2	1	2	3	2	2	1	1	2
9	9	9	9	9	9	9	9	9	9
3	3	3	3	3	3	3	3	3	3
9	9	9	9	9	9	9	9	9	9
9	9	9	9	9	9	9	9	9	9
9	9	9	9	9	9	9	9	9	9
2	2	2	2	2	3	3	3	3	3
1	1	1	1	1	1	1	1	1	1
2	2	2	2	2	2	2	2	2	2
1	1	9	1	3	1	2	1	1	1
2	2	2	2	2	2	2	2	2	2
2	2	2	2	2	2	2	2	2	2
1	1	1	1	1	1	1	1	1	1
3	3	3	1	1	1	1	1	1	1
3	1	1	3	2	2	1	1	2	1
9	2	1	2	1	3	2	1	2	3
3	1	2	1	1	1	3	2	2	3
3	3	3	1	1	2	1	1	1	1
1	1	1	1	1	1	1	1	1	1
2	2	2	2	2	2	2	2	2	2
9	9	9	9	9	9	9	9	9	9
9	9	9	9	9	9	9	9	9	9
1	2	3	2	3	1	2	1	2	3
2	2	2	2	2	2	2	2	2	2
2	2	2	2	2	2	2	2	2	2

1	2	2	2	3	3	1	1	1	2
9	9	9	9	9	9	9	9	9	9
9	9	9	9	9	9	9	9	9	9
9	9	9	9	9	9	9	9	9	9
9	9	9	9	9	9	9	9	9	9
9	9	9	9	9	9	9	9	9	9
9	9	9	9	9	9	9	9	9	9
9	9	9	9	9	9	9	9	9	9
9	9	9	9	9	9	9	9	9	9
9	9	9	9	9	9	9	9	9	9
2	2	2	2	2	2	2	2	2	2
2	2	3	2	2	3	2	2	2	1
1	3	3	3	1	1	2	1	1	1
2	2	2	2	2	3	2	3	2	2
2	2	2	2	2	2	2	2	2	2
2	2	2	3	2	3	2	3	2	2
1	1	2	2	1	9	2	1	2	3
2	2	2	2	2	2	2	2	2	2
1	1	3	2	1	1	2	2	2	1
3	3	3	3	3	2	2	1	1	1
2	2	2	3	2	1	2	3	1	3
2	2	3	2	1	2	3	1	2	3
9	3	3	3	3	3	3	3	3	3
2	2	2	2	2	2	2	2	2	2
2	2	2	2	2	2	2	2	2	2
2	2	2	2	2	2	2	2	2	2
2	2	2	2	2	9	3	3	3	3
2	3	2	1	1	2	1	1	2	2
2	2	2	2	2	2	2	2	2	2
2	2	2	2	2	2	2	2	2	2
9	9	9	9	9	9	9	9	9	9
2	2	2	2	2	2	2	2	2	2
1	1	1	1	1	1	1	1	1	1
9	9	9	9	9	9	9	9	9	9
9	9	9	9	9	9	9	9	9	9
2	2	2	2	2	2	2	2	2	2
2	2	2	2	2	2	2	2	2	2
2	2	2	2	2	2	2	2	2	2
1	1	3	2	1	3	2	1	1	3
9	9	9	9	9	9	9	9	9	9
2	1	2	1	1	1	2	1	2	1
2	2	2	2	2	2	2	2	2	2
2	2	2	3	3	3	3	2	2	2
3	3	3	1	1	1	1	1	1	1
3	3	9	2	2	2	1	1	1	1
9	9	9	9	9	9	9	9	9	9
2	2	1	2	1	2	2	2	1	2

9	9	9	9	9	9	9	9	9	9
1	2	1	1	1	1	1	2	2	2
9	9	9	9	9	9	9	9	9	9
9	9	9	9	9	9	9	9	9	9
9	9	9	9	9	9	9	9	9	9
9	9	9	9	9	9	9	9	9	9
9	9	9	9	9	9	9	9	9	9
9	9	9	9	9	9	9	9	9	9
9	9	9	9	9	9	9	9	9	9
9	9	9	9	9	9	9	9	9	9
9	9	9	9	9	9	9	9	9	9
9	9	9	9	9	9	9	9	9	9
9	9	9	9	9	9	9	9	9	9
9	9	9	9	9	9	9	9	9	9
9	9	9	9	9	9	9	9	9	9
9	9	9	9	9	9	9	9	9	9
9	9	9	9	9	9	9	9	9	9
9	9	9	9	9	9	9	9	9	9
9	9	9	9	9	9	9	9	9	9
9	9	9	9	9	9	9	9	9	9
9	9	9	9	9	9	9	9	9	9
9	9	9	9	9	9	9	9	9	9
9	9	9	9	9	9	9	9	9	9
1	2	3	2	1	2	3	2	1	2
2	2	2	2	2	2	2	2	2	2
3	3	3	3	1	1	1	1	2	2
3	3	3	2	2	1	1	1	1	1
3	9	3	3	2	1	2	1	1	9
3	2	2	1	1	2	3	2	2	1
1	1	2	1	1	1	2	1	2	1
3	1	3	3	3	1	1	2	2	3
1	2	2	2	1	2	2	1	1	1
2	2	2	9	2	1	1	1	1	1
3	3	1	1	1	1	1	1	1	1
2	1	2	3	1	2	3	1	2	1
3	2	3	3	1	1	1	1	1	2
3	3	3	2	1	2	2	1	1	1
3	3	3	1	1	1	1	1	1	2
3	1	2	1	1	1	2	1	1	1
9	9	9	9	9	9	9	9	9	9
2	2	3	3	1	1	1	1	2	1
2	3	3	1	1	1	2	1	1	1
9	9	9	9	9	1	9	9	9	9
3	1	3	3	1	1	2	2	1	1
3	1	3	1	1	3	3	1	1	1
2	2	2	2	2	3	2	3	3	3
2	3	1	9	1	2	2	3	2	3

2	2	2	2	1	1	1	1	1	1
1	1	1	1	1	1	1	1	1	1
1	3	3	2	1	1	2	1	1	2
3	3	2	2	1	2	2	1	1	1
2	1	2	3	2	3	2	1	2	1
9	9	9	9	9	9	9	9	9	9
1	1	1	2	1	1	1	1	1	1
9	9	9	9	9	9	9	9	9	9
1	1	2	3	1	1	1	1	1	1
1	1	2	9	1	1	1	1	1	1
9	9	9	9	9	9	9	9	9	9
1	9	9	9	9	9	9	9	9	9
9	9	9	9	9	9	9	9	9	9
9	9	9	9	9	9	9	9	9	9
9	9	9	9	9	9	9	9	9	9
9	9	9	9	9	9	9	9	9	9
3	3	3	3	3	3	1	1	1	1
2	2	2	2	2	2	2	2	2	2
9	9	9	9	9	9	9	9	9	9
3	1	3	1	1	1	3	3	3	3
9	9	9	9	9	9	9	9	9	9
9	9	9	9	9	9	9	9	9	9
9	9	9	9	9	9	9	9	9	9
9	9	9	9	9	9	9	9	9	9
9	9	9	9	9	9	9	9	9	9
2	3	3	2	1	2	2	3	3	2
3	3	3	1	3	1	3	1	1	1
3	2	2	2	1	9	2	2	3	2
2	1	2	1	1	1	2	1	1	1
2	2	2	2	2	2	2	2	2	2
1	1	1	2	1	2	1	1	1	1
3	3	3	1	1	1	3	1	1	1
3	3	3	2	1	3	3	3	3	1
1	1	3	1	2	3	1	1	1	1
2	2	3	2	2	2	3	2	9	2
9	9	9	9	9	9	9	9	9	9
1	2	1	2	2	2	2	2	1	2
2	2	2	2	2	2	2	2	2	2
2	1	2	3	1	2	3	3	1	2
2	2	2	2	2	2	2	2	2	2
3	3	3	3	3	3	1	1	1	1
1	3	1	2	3	3	1	1	1	1
1	1	3	3	1	1	1	1	1	1
1	1	3	3	3	2	2	1	1	2
3	3	3	1	1	1	1	1	1	2
9	9	9	9	9	1	9	1	1	1
3	2	3	1	1	1	1	3	1	1

3	3	3	1	1	1	2	3	1	1
3	2	3	9	3	3	3	3	3	3
3	3	3	1	1	1	2	2	1	1
3	3	3	1	9	3	3	3	1	1
9	9	9	1	9	9	2	9	9	9
3	1	3	2	2	2	2	1	1	1
3	1	3	1	1	1	1	1	1	1
9	9	9	9	9	9	9	9	9	9
9	9	9	9	9	9	9	9	9	9
2	2	2	2	2	2	2	2	2	2
2	2	2	2	2	2	2	2	2	2
3	3	3	3	1	2	3	3	3	3
2	2	2	1	1	1	1	1	1	1
9	9	9	9	9	9	9	9	9	9
9	9	9	9	9	9	9	9	9	9
3	3	9	9	1	1	1	1	1	1
9	9	9	9	9	9	9	9	9	9
9	9	9	9	9	9	9	9	9	9
1	1	1	1	1	1	1	1	1	1
3	3	3	3	3	3	1	1	1	1
3	1	2	1	1	1	2	1	1	1
1	1	1	1	1	1	1	1	1	1
2	2	3	2	2	2	2	1	3	1
3	2	3	1	2	1	2	3	1	3
1	1	1	1	1	1	1	1	1	1
9	9	9	9	9	9	9	9	9	9
9	9	9	9	9	9	9	9	9	9
3	3	1	1	1	1	1	1	1	1
2	2	2	2	2	1	1	1	1	2
3	2	3	1	1	3	3	1	1	1
9	9	9	9	9	9	9	9	9	9
9	9	9	9	9	9	9	9	9	9
9	9	9	9	9	9	9	9	9	9
9	9	9	9	9	9	9	9	9	9
9	9	9	9	9	9	9	9	9	9
9	9	9	9	9	9	9	9	9	9
9	9	9	9	9	9	9	9	9	9
9	9	9	9	9	9	9	9	9	9
9	9	9	9	9	9	9	9	9	9
9	9	9	9	9	9	9	9	9	9
9	9	9	9	9	9	9	9	9	9
9	9	9	9	9	9	9	9	9	9
9	9	9	9	9	9	9	9	9	9
9	9	9	9	9	9	9	9	9	9
9	9	9	9	9	9	9	9	9	9
9	9	9	9	9	9	9	9	9	9
9	9	9	9	9	9	9	9	9	9

9	9	9	9	9	9	9	9	9	9
9	9	9	9	9	9	9	9	9	9
9	9	9	9	9	9	9	9	9	9
9	9	9	9	9	9	9	9	9	9
9	9	9	9	9	9	9	9	9	9
9	9	9	9	9	9	9	9	9	9
9	9	9	9	9	9	9	9	9	9
9	9	9	9	9	9	9	9	9	9
9	9	9	9	9	9	9	9	9	9
9	9	9	9	9	9	9	9	9	9
9	9	9	9	9	9	9	9	9	9
9	9	9	9	9	9	9	9	9	9
9	9	9	9	9	9	9	9	9	9
9	9	9	9	9	9	9	9	9	9
9	9	9	9	9	9	9	9	9	9
9	9	9	9	9	9	9	9	9	9
9	9	9	9	9	9	9	9	9	9
9	9	9	9	9	9	9	9	9	9
9	9	9	9	9	9	9	9	9	9
2	2	2	2	2	2	2	2	2	2
9	9	9	9	9	9	9	9	9	9
9	9	9	9	9	9	9	9	9	9
9	9	9	9	9	9	9	9	9	9
9	9	9	9	9	9	9	9	9	9
9	9	9	9	9	9	9	9	9	9
9	9	9	9	9	9	9	9	9	9
9	9	9	9	9	9	9	9	9	9
9	9	9	9	9	9	9	9	9	9
9	9	9	9	9	9	9	9	9	9
9	9	9	9	9	9	9	9	9	9
9	9	9	9	9	9	9	9	9	9
9	9	9	9	9	9	9	9	9	9
9	9	9	9	9	9	9	9	9	9
9	9	9	9	9	9	9	9	9	9
9	9	9	9	9	9	9	9	9	9
2	2	2	2	2	2	2	2	2	2
3	3	3	3	3	3	3	3	3	3
2	3	2	2	1	3	2	1	1	1
3	1	3	1	1	1	3	1	1	1
3	3	3	3	3	3	3	3	3	3
1	1	1	1	1	1	1	1	1	1
3	3	2	1	3	1	3	1	1	1
9	9	9	9	9	9	9	9	9	9
3	3	2	1	3	1	3	1	1	1
3	1	2	2	2	2	1	3	2	2
2	2	2	2	2	2	1	1	1	2
2	2	2	2	2	2	2	2	2	2

3	1	2	1	1	1	2	1	1	1
2	3	3	1	1	1	3	1	1	1
1	3	3	1	1	1	2	2	1	1
3	2	3	9	1	2	2	1	1	1
2	2	2	2	1	2	2	1	1	1
2	2	2	1	1	1	1	1	1	1
3	3	3	3	1	1	1	1	1	2
3	1	3	3	2	1	1	1	1	2
1	9	2	1	2	3	1	1	3	2
1	2	3	1	1	2	2	1	2	2
9	9	9	1	1	9	9	9	1	9
3	2	2	2	3	1	2	2	3	3
3	3	3	1	2	1	2	2	1	1
2	1	2	2	1	2	2	1	1	2
2	1	2	3	2	2	2	1	1	1
2	2	2	2	1	1	1	1	1	1
2	2	2	2	2	2	2	2	9	9
2	2	2	2	1	1	3	9	1	1
2	1	2	2	1	1	1	1	1	1
2	1	1	1	2	2	1	1	3	3
2	2	2	2	2	9	2	1	1	1
3	3	3	3	3	3	1	1	1	1
1	1	3	3	1	1	1	2	2	1
3	9	9	1	1	1	1	1	1	1
3	3	3	3	3	3	3	3	3	3
1	2	1	2	1	2	1	2	1	2
1	1	1	1	1	2	2	2	2	2
3	1	2	3	1	1	2	3	3	2
2	2	2	2	2	2	2	2	2	2
2	2	2	2	2	9	9	9	9	9
1	2	2	2	2	1	2	3	2	3
2	2	2	2	2	2	2	2	2	2
2	2	2	2	2	2	2	2	2	2
9	9	9	9	9	9	9	9	9	9
9	9	9	9	9	9	9	9	9	9
9	9	9	9	9	9	9	9	9	9
9	9	9	9	9	9	9	9	9	9
9	9	9	9	9	9	9	9	9	9
9	9	9	9	9	9	9	9	9	9
9	9	9	9	9	9	9	9	9	9
1	2	2	2	2	1	1	1	2	3
9	9	9	9	9	9	9	9	9	9
1	2	1	2	1	1	1	1	1	1
3	3	3	3	1	1	1	3	3	3
3	1	1	1	1	1	1	1	1	1
3	3	3	3	1	3	3	3	3	3
3	1	3	1	1	1	3	1	1	1

2	3	2	3	2	3	2	3	2	3
1	1	1	1	2	1	2	2	3	3
2	3	2	2	2	2	2	3	2	2
1	1	3	3	1	3	1	3	9	1
1	9	3	3	2	2	2	1	2	2
2	3	2	1	2	3	2	1	2	3
1	3	3	1	3	1	3	3	1	1
2	1	2	1	2	3	3	2	1	2
1	3	3	3	1	1	2	2	1	1
9	9	9	9	2	9	1	9	2	9
3	3	3	3	3	3	3	3	3	3
1	1	3	9	1	3	1	1	2	1
1	1	1	2	2	2	1	2	2	2
3	3	3	3	1	1	1	1	3	2
1	1	1	1	1	1	1	1	1	1
1	9	1	9	1	3	2	3	2	3
2	2	2	2	2	9	2	3	2	2
9	9	9	1	9	9	9	9	9	9
2	1	2	3	2	1	2	1	2	3
1	1	2	1	1	1	3	1	2	1
1	2	3	2	2	1	2	3	2	3
3	3	3	3	3	3	3	3	3	3
3	3	3	1	1	1	1	9	9	1
3	3	1	2	1	1	1	1	3	1
1	2	3	1	1	2	2	1	1	2
1	3	1	3	3	3	1	3	1	1
2	3	1	9	1	3	1	1	2	1
1	1	3	1	1	1	1	2	2	1
3	2	2	2	2	2	2	2	1	1
3	3	2	2	1	2	2	1	3	1
2	2	2	2	2	2	2	2	1	1
2	3	1	2	3	1	2	3	1	2
2	2	2	2	2	2	2	2	2	2
3	1	3	3	3	3	3	1	3	1
1	3	1	1	1	1	1	1	1	9
3	1	3	3	3	3	3	3	1	1
2	3	1	1	2	2	1	2	3	3
9	9	9	9	9	9	9	9	9	9
9	1	3	3	3	3	1	1	1	1
3	9	2	1	1	3	1	1	1	1
1	3	1	3	9	1	1	1	3	1
2	3	9	1	2	3	1	2	3	1
1	3	1	1	1	9	1	1	3	1
1	2	3	3	1	1	1	1	1	1
2	2	2	2	2	2	2	2	2	2
3	3	3	3	3	3	3	3	3	3
3	3	3	3	3	3	3	3	3	3

2	3	3	3	2	1	2	3	2	3
9	9	9	9	9	9	9	9	9	9
3	2	2	2	2	2	2	2	2	3
2	2	2	3	3	3	3	2	2	1
3	2	2	2	2	2	1	1	3	2
3	3	2	2	1	2	3	2	1	2
2	2	2	2	2	2	2	2	2	2
9	9	9	9	9	9	9	9	9	9
9	9	9	9	9	9	9	9	9	9
2	3	1	2	3	2	1	2	3	2
2	2	2	2	2	2	2	2	2	2
2	2	2	2	2	2	2	2	2	2
2	2	1	1	1	2	2	1	1	2
1	2	2	2	2	2	2	2	2	2
1	1	1	1	1	1	1	1	1	1
1	1	1	1	1	1	1	1	1	1
2	2	2	2	3	3	3	2	2	2
2	2	2	2	2	2	2	2	2	2
2	2	2	2	2	1	2	3	3	3
2	2	2	2	2	3	2	2	2	3
2	2	1	1	1	3	2	2	2	3
2	2	2	2	2	2	2	2	2	2
2	2	2	2	2	2	2	2	2	2
2	2	2	2	2	2	2	2	1	2
2	2	2	2	2	2	2	2	2	2
2	2	2	2	2	2	2	2	2	2
2	2	2	2	2	2	2	2	2	2
2	2	1	2	1	3	1	2	3	1
2	2	1	2	1	1	1	1	1	2
2	2	2	2	2	2	2	2	2	2
2	2	2	2	2	2	2	2	2	2
2	2	2	2	2	2	2	2	2	2
2	2	2	2	2	2	2	2	2	2
2	2	2	2	2	2	2	2	2	2
2	2	2	2	2	2	2	2	2	2
2	2	2	2	2	2	2	2	2	2
2	2	2	2	2	2	2	2	2	2
2	2	2	2	2	2	2	2	2	2
2	2	2	2	2	2	2	2	2	2
1	1	3	3	1	1	1	1	1	1
2	2	2	2	2	2	2	2	2	2
2	2	2	2	2	2	2	2	2	2
1	2	3	2	2	1	1	3	2	2
2	2	2	2	2	2	2	2	2	2
2	1	1	2	3	3	3	2	2	3
3	3	3	3	3	3	3	3	3	3
3	3	3	3	3	3	3	3	3	3

2	2	2	2	2	2	2	2	2	2
2	2	2	2	2	2	2	2	2	2
2	2	2	2	2	2	2	2	2	2
2	2	2	2	2	2	2	2	2	2
3	3	2	2	2	1	1	1	1	2
2	2	2	2	2	2	2	2	2	2
9	9	9	9	9	9	9	9	9	9
1	1	1	1	1	1	1	3	1	3
9	9	9	9	9	9	1	9	9	9
2	2	2	2	2	2	2	2	2	2
2	3	2	3	2	2	1	2	2	2
2	2	1	1	1	3	3	3	2	2
2	2	3	2	3	3	3	3	2	3
9	9	9	9	9	9	9	9	9	9
2	2	2	2	2	2	2	2	2	2
2	2	9	9	9	9	9	9	9	9
2	2	2	2	2	2	2	2	2	2
1	1	1	1	1	1	1	1	1	1
9	9	9	9	9	9	9	9	9	9
2	2	2	2	2	2	2	2	2	2
1	1	2	2	2	2	2	1	9	2
9	9	9	9	9	9	9	9	9	9
9	9	9	9	9	9	9	9	9	9
3	3	3	3	3	3	2	3	3	2
2	3	1	2	3	1	2	1	2	3
3	3	3	3	2	3	3	3	3	3
3	2	3	3	2	3	3	3	3	3
1	3	1	1	1	1	1	1	1	1
2	3	9	1	3	1	2	9	1	2
1	1	1	1	1	1	1	1	1	1
2	2	2	2	2	2	2	2	2	2
2	3	3	3	3	2	2	2	3	2
2	2	2	2	2	2	2	2	2	2
1	9	2	9	2	9	9	9	9	2
2	2	2	2	2	2	2	2	2	2
9	9	9	9	9	9	9	9	9	9
3	3	3	1	1	1	1	1	1	3
2	2	2	2	2	2	2	2	2	2
2	2	2	2	2	2	2	2	2	2
2	2	2	2	2	2	2	2	2	2
2	1	2	3	1	3	2	1	2	3
2	2	2	2	2	2	2	2	2	2
9	9	9	2	9	9	9	9	9	9
3	3	2	1	1	1	3	2	2	1
1	3	2	2	1	1	2	2	2	2
3	3	3	9	2	3	3	3	2	3
2	2	2	2	1	2	1	2	1	2

1	1	1	1	1	1	1	1	1	1
2	2	2	2	2	2	2	2	2	2
1	1	1	1	1	1	1	1	1	1
2	2	2	2	2	2	2	2	2	2
9	9	9	9	9	9	9	9	9	9
2	1	2	2	1	1	2	3	2	3
3	3	3	3	3	3	3	3	3	3
1	1	1	1	1	1	2	9	2	2
2	2	2	2	2	2	2	2	2	2
1	1	1	1	1	1	1	1	1	3
3	3	3	3	3	3	3	3	3	3
1	1	1	1	1	1	1	1	1	1
2	2	2	2	2	2	2	2	2	2
2	2	2	2	2	2	2	2	2	2
2	2	2	2	2	2	2	2	2	2
1	1	1	1	1	1	1	1	1	1
1	1	1	1	1	1	1	1	1	1
3	3	1	1	1	1	1	3	3	3
9	9	9	9	9	9	9	9	9	9
9	9	9	9	9	9	9	9	9	9
1	1	1	2	1	1	1	9	2	2
2	2	2	2	2	2	2	2	2	2
1	1	1	1	1	1	1	1	1	1
2	2	2	2	2	2	2	2	2	2
3	3	3	3	3	3	3	3	3	9
1	2	1	1	1	1	1	1	1	1
1	3	1	2	2	1	1	1	2	3
2	2	2	2	2	2	1	2	1	2
9	9	9	2	9	9	2	9	9	9
3	3	3	3	3	3	3	3	3	3
2	9	1	2	1	2	9	2	1	2
2	2	2	2	2	3	3	2	2	2
1	1	1	1	1	1	1	1	1	1
3	2	1	2	2	2	2	2	2	2
2	2	2	2	2	2	2	2	2	2
3	9	2	3	3	2	2	2	2	3
3	3	3	3	2	2	2	2	2	2
2	2	2	2	2	2	2	2	2	2
2	3	1	2	3	1	2	3	1	2
3	3	3	3	3	3	3	3	3	3
9	1	2	9	1	1	2	1	1	3
2	2	2	2	2	3	3	3	3	1
9	9	9	9	9	9	9	9	9	9
9	9	9	9	9	9	9	9	9	3
2	2	2	2	2	1	1	1	1	1
2	2	2	2	2	2	2	2	2	2
2	2	2	1	2	2	2	1	1	9

1	1	1	1	1	1	1	1	1	1
1	3	3	3	1	3	1	1	1	3
2	2	1	1	1	1	1	3	3	3
1	3	3	3	1	3	1	1	1	3
2	3	9	9	1	2	1	2	1	1
2	2	2	2	2	2	2	2	2	2
2	2	2	2	2	2	2	2	2	2
2	2	2	2	2	2	2	2	2	2
2	2	2	2	2	1	1	1	1	1
1	9	2	2	1	1	1	2	3	1
2	2	2	2	2	1	2	3	2	2
3	3	2	3	1	3	1	3	1	2
2	2	2	2	2	2	2	2	2	2
1	3	3	2	2	2	3	2	1	1
2	2	2	2	2	2	2	2	2	2
2	2	2	2	2	2	2	2	2	2
2	2	2	2	2	2	2	2	2	2
2	2	2	2	2	2	2	2	2	2
2	2	9	2	2	2	2	2	2	2
2	2	2	2	2	2	2	3	2	2
2	2	2	2	2	2	2	2	2	2
2	2	2	2	2	2	2	2	2	2
9	2	3	3	1	2	1	3	1	1
2	2	2	2	2	2	2	2	2	2
1	1	1	1	1	1	1	1	1	1
2	2	2	2	2	2	2	2	2	2
2	2	2	3	1	1	1	2	3	1
3	3	3	3	3	2	2	2	2	2
3	2	3	2	2	3	1	3	2	1
2	9	3	3	2	1	2	1	2	1
2	2	2	2	2	1	1	2	2	2
1	1	2	3	3	2	2	2	3	3
2	2	2	2	2	2	2	2	2	2
2	2	2	2	2	2	2	2	2	2
2	2	1	2	2	2	1	1	1	1
3	3	3	3	3	3	3	3	3	3
2	2	2	2	2	2	2	2	2	2
9	9	9	9	9	9	9	9	9	9
2	2	2	2	2	2	2	2	2	2
3	3	2	3	3	2	1	1	2	1
3	3	2	3	2	1	1	2	1	2
2	2	2	2	2	2	2	2	2	2
2	2	2	2	2	2	2	2	2	2
2	2	2	2	2	2	2	2	2	2
9	9	9	9	9	9	9	9	9	9
2	3	9	9	1	2	1	2	1	1
2	2	2	2	2	2	2	2	2	2

9	9	9	9	9	9	9	9	9	9
1	2	1	1	2	1	3	1	3	1
3	2	1	2	3	1	1	3	2	1
3	2	2	3	1	1	1	1	3	2
3	1	3	3	1	1	3	1	1	1
3	2	2	2	1	2	2	1	2	1
3	3	3	3	3	3	3	3	3	3
9	9	9	9	9	9	9	9	9	9
2	2	2	2	2	3	3	3	3	3
1	2	3	2	3	2	3	2	3	2
2	2	1	2	2	2	2	1	1	1
3	2	3	2	3	1	1	2	2	1
2	2	2	2	2	2	2	1	2	1
1	1	1	1	1	1	1	1	1	1
2	1	2	3	2	1	2	2	1	2
2	2	2	2	2	2	2	2	2	2
3	2	1	1	2	1	2	3	3	3
2	3	2	2	2	2	2	2	2	2
9	9	9	9	9	9	9	9	9	9
9	9	9	9	9	9	9	9	9	9
9	9	9	9	9	9	9	9	9	9
3	2	3	1	3	2	2	2	1	2
2	3	2	1	2	2	2	1	2	3
1	1	3	2	2	2	1	1	1	1
2	1	1	1	2	3	2	1	1	2
3	3	3	3	3	3	3	3	3	3
1	3	2	2	1	2	3	1	2	2
1	2	2	2	1	2	1	2	3	3
2	3	1	3	2	2	2	2	3	3
1	3	2	2	1	2	1	1	1	1
2	2	2	1	1	1	1	3	3	3
2	2	2	2	1	1	1	1	3	3
3	3	3	3	2	2	2	2	3	3
2	2	2	2	2	2	2	2	2	2
2	2	2	2	2	2	2	2	2	2
2	2	2	2	2	2	2	2	2	2
3	3	3	3	3	3	3	3	3	3
2	2	2	2	2	2	2	3	3	1
9	9	9	9	9	9	9	9	9	9
9	9	9	9	9	9	9	9	9	9
2	3	1	2	3	1	2	3	1	2
2	2	2	2	2	2	2	2	2	2
3	2	1	2	3	2	1	2	1	3
2	2	2	3	3	3	2	1	1	2
9	9	9	9	9	9	9	9	9	9
3	3	3	3	3	3	3	3	3	3
3	3	3	3	3	3	3	3	2	1

1	1	1	1	1	1	1	1	1	1
3	1	9	2	1	2	3	1	1	1
2	3	1	2	3	1	2	3	3	2
2	1	2	2	2	2	2	2	2	2
2	2	2	2	3	2	3	2	3	2
3	3	3	3	3	3	3	3	3	3
1	1	1	1	1	1	1	1	1	1
3	3	3	3	3	3	3	3	3	3
3	2	2	3	2	2	2	2	2	2
3	2	3	2	3	2	1	1	1	2
2	2	2	2	2	2	2	2	2	2
1	1	3	3	3	2	1	2	3	1
2	2	2	2	2	2	2	2	3	2
3	9	1	2	1	1	1	2	1	9
2	2	2	2	2	2	2	2	2	2
3	3	3	3	3	3	3	3	2	2
1	2	3	1	1	1	3	3	1	1
2	1	2	3	1	2	3	1	2	3
2	2	2	2	2	2	2	2	2	2
2	1	1	2	2	3	1	2	2	2
1	3	2	2	1	3	2	1	3	3
3	3	3	3	3	3	3	3	3	3
3	3	3	3	3	3	3	3	3	3
2	1	1	1	1	1	2	1	3	3
2	3	1	2	3	1	2	3	1	2
3	3	3	3	2	3	3	2	3	3
3	3	3	3	3	3	2	3	2	3
2	3	2	1	2	3	2	1	2	2
9	3	1	2	1	2	1	3	1	2
1	1	2	2	2	2	2	2	2	2
2	3	1	2	3	2	2	1	2	3
1	1	1	1	1	2	2	2	2	2
2	2	2	2	2	2	2	2	2	2
3	3	3	2	2	1	1	1	1	1
2	3	1	2	1	2	3	1	2	3
1	1	1	1	2	3	2	2	1	1
2	1	1	2	3	3	3	1	2	1
3	2	3	3	3	3	3	3	3	3
2	2	1	1	2	2	1	1	2	2
1	2	3	3	2	1	2	2	2	3
2	2	2	2	2	2	2	2	2	2
1	1	1	1	1	1	1	1	1	1
3	2	1	2	1	2	1	2	1	2
2	2	2	2	1	2	1	2	2	1
2	2	2	2	1	2	2	2	2	2
2	3	1	2	2	3	2	1	2	2
3	3	1	1	1	2	1	2	1	3

2	2	1	2	1	2	2	1	1	1
3	1	2	3	2	3	2	2	1	2
2	1	1	1	2	2	2	2	2	2
1	3	3	3	1	1	1	1	1	1
2	3	1	2	3	1	2	3	1	2
3	1	3	2	1	1	1	2	3	3
2	1	2	3	2	2	1	3	2	1
1	3	1	2	3	2	3	2	3	3
1	3	3	2	1	1	1	1	1	1
3	3	3	3	3	3	2	1	2	1
3	2	3	2	2	1	2	3	2	3
1	2	2	3	2	1	2	3	1	2
1	1	1	1	1	1	1	1	1	1
1	3	1	1	1	3	3	1	1	1
2	3	2	3	2	3	1	2	3	2
1	1	3	3	1	1	1	1	3	1
3	1	2	2	1	2	1	1	1	1
2	2	3	2	2	2	1	2	9	1
1	1	3	2	2	2	2	1	1	1
1	2	2	9	1	1	1	1	1	1
3	3	3	3	2	2	3	3	9	2
2	1	2	2	3	3	3	1	3	3
3	1	3	3	3	1	1	1	1	3
3	3	1	2	1	1	2	1	2	1
2	2	2	2	2	2	2	2	2	2
2	2	2	2	2	2	2	2	2	2
3	2	2	2	1	2	1	1	3	2
2	1	2	1	3	2	1	3	1	2
2	2	2	2	2	2	2	2	2	2
9	9	9	9	9	9	9	9	9	9
9	9	9	9	9	9	9	9	9	9
9	9	9	9	9	9	9	9	9	9
1	1	1	1	2	2	2	2	1	1
9	9	9	9	9	9	9	9	9	9
2	2	2	2	2	2	2	2	2	2
9	9	9	9	9	9	9	9	9	9
2	2	2	2	1	2	3	2	2	1
9	9	9	9	9	9	9	9	9	9
1	9	9	9	1	2	2	1	2	9
3	2	1	2	3	2	3	3	3	2
9	9	9	9	9	9	9	9	9	9
3	1	2	2	1	1	1	2	3	1
2	2	2	3	2	3	2	1	3	3
2	2	2	2	2	3	2	3	1	3
3	3	2	2	1	2	3	2	3	1
1	2	3	2	2	2	1	2	3	2
3	2	2	2	1	2	1	1	3	2

1	2	2	3	2	1	2	1	9	2
2	2	2	1	2	1	2	2	2	2
2	2	2	2	2	2	2	2	2	2
2	2	1	2	1	1	2	1	2	1
2	2	2	2	2	2	2	2	2	2
3	1	3	1	2	2	1	2	3	1
9	9	9	9	9	9	9	1	9	9
3	1	2	3	2	1	2	3	2	1
2	2	3	2	2	1	1	1	2	3
3	3	3	3	3	3	3	2	2	2
3	1	2	3	1	2	3	1	2	3
2	3	1	2	3	1	2	3	2	3
1	3	9	2	1	2	1	3	1	3
2	2	2	2	2	2	2	2	2	2
2	2	2	2	2	2	2	2	2	2
9	9	9	9	9	3	9	2	9	9
2	2	2	2	2	2	2	2	2	2
2	2	2	2	2	2	2	2	2	2
2	2	2	2	2	2	2	2	2	2
2	2	2	2	2	2	2	2	2	2
1	1	1	9	1	1	1	1	1	1
1	1	9	9	1	1	1	1	1	1
1	1	9	9	1	1	1	1	1	1
2	2	2	2	1	1	1	1	1	1
3	1	3	3	1	1	1	1	1	1
3	1	3	3	1	1	1	1	1	1
3	3	3	1	1	1	1	1	1	1
2	2	2	2	1	1	2	1	1	1
2	2	2	1	1	1	2	1	1	1
3	1	3	3	1	1	1	1	1	1
3	1	3	3	1	1	1	1	1	1
9	9	9	9	9	9	9	9	9	9
3	1	3	3	1	1	1	1	1	1
3	1	3	3	1	1	1	1	1	1
3	1	3	3	1	1	1	1	1	1
3	1	3	3	1	1	1	1	1	1
3	1	3	3	1	1	1	1	1	1
3	1	3	3	1	1	1	1	1	1
3	1	3	3	1	1	1	1	1	1
3	1	3	3	1	1	1	1	1	1
1	3	3	9	1	1	1	1	1	1
9	9	9	9	9	9	9	9	9	1
1	1	3	3	1	1	1	1	1	1
3	3	9	9	9	1	1	1	1	1
3	1	3	3	1	1	1	1	1	1
3	1	3	3	1	1	1	1	1	1
1	1	3	1	1	9	9	1	1	1

3	1	3	3	1	1	1	1	1	1
2	2	9	3	1	1	1	1	1	1
3	1	3	3	1	1	1	1	1	1
1	1	1	1	1	1	1	1	1	1
3	1	3	3	1	1	1	1	1	1
3	1	3	3	1	1	1	1	1	1
1	1	2	2	1	1	1	1	1	1
1	2	2	2	1	1	1	1	1	1
3	1	3	3	1	1	1	1	1	1
3	1	3	3	1	1	1	1	1	1
3	1	3	3	1	1	1	1	1	1
1	3	3	2	1	1	1	1	1	1
3	3	3	3	1	1	1	1	1	1
3	1	3	3	1	1	1	1	1	1
1	3	3	1	1	1	1	1	1	1
3	1	3	3	1	1	1	1	1	1
1	1	1	3	1	1	1	1	1	1
1	3	1	1	1	1	1	1	1	1
3	1	3	3	1	1	1	1	1	1
1	1	1	1	1	1	1	1	1	1
9	3	2	2	2	2	3	3	1	9
9	1	1	2	1	1	3	3	3	3
3	1	1	3	1	1	1	1	1	1
3	1	3	3	1	1	1	1	1	1
3	3	3	1	1	1	1	1	1	1
3	1	3	3	1	1	1	1	1	1
1	1	3	3	1	1	1	1	1	1
3	9	1	3	2	2	1	3	3	3
3	1	3	3	1	1	1	1	1	1
9	9	1	3	1	9	9	1	1	1
3	1	3	3	1	1	1	1	1	1
3	1	3	3	1	1	1	1	1	1
3	1	3	3	1	1	1	1	1	1
3	1	3	3	1	1	1	1	1	1
3	1	3	3	1	1	1	1	1	1
3	1	3	3	1	1	1	1	1	1
9	9	9	9	9	9	9	9	1	9
3	1	3	3	1	1	1	1	1	1
3	1	3	3	1	1	1	1	1	1
1	1	1	1	1	1	1	1	2	1
3	1	3	3	1	1	1	1	1	1
1	1	3	3	1	1	1	1	1	1
9	1	3	3	1	1	1	1	1	1
3	1	3	3	1	1	1	1	1	1
3	1	3	3	1	1	1	1	1	1
3	1	3	3	1	1	1	1	1	1
2	1	2	2	1	1	1	1	1	1

3	1	3	3	1	1	1	1	1	1
3	1	3	3	1	1	1	1	1	1
3	1	1	3	1	1	1	1	1	1
3	1	3	3	1	1	1	1	1	1
3	3	3	1	1	1	1	1	1	1
1	1	1	1	1	1	1	1	1	1
3	1	3	3	2	1	1	1	1	1
1	1	1	1	1	1	1	1	1	1
1	3	1	1	1	1	1	1	1	1
3	3	3	1	1	1	3	1	1	1
3	1	2	2	1	1	3	2	2	1
9	9	9	9	9	9	9	9	9	9
1	1	3	3	1	1	1	1	1	1
1	3	2	2	2	2	2	2	2	2
9	3	1	3	1	2	2	2	2	2
3	2	1	1	1	1	1	1	1	1
9	9	9	9	9	9	9	9	9	9
9	9	9	9	9	9	9	9	9	9
1	1	1	1	1	1	1	1	1	1
1	1	1	1	1	1	1	1	1	1
3	1	3	3	1	1	1	1	1	1
2	2	3	9	2	1	2	2	1	1
3	3	3	3	2	3	2	2	2	1
1	3	1	3	2	1	2	1	3	2
3	1	1	3	3	3	3	3	1	1
2	2	2	2	1	1	2	1	1	2
1	2	3	2	1	3	2	1	2	1
1	1	1	1	2	1	1	1	1	1
2	2	2	1	1	1	2	2	1	1
2	1	3	1	1	1	2	2	1	2
2	2	1	1	2	2	2	2	2	2
9	1	9	9	9	1	9	9	1	9
3	2	1	2	2	1	2	2	2	2
3	1	3	3	2	1	2	1	2	1
3	3	3	3	2	3	1	1	1	2
2	2	3	1	1	1	1	1	2	1
3	3	2	1	1	1	1	2	2	2
3	3	2	2	2	2	2	2	2	2
3	3	2	2	2	1	2	3	1	1
1	2	2	1	1	2	1	2	3	2
3	9	3	1	1	1	1	1	1	1
3	3	3	1	1	1	1	1	1	1
1	1	2	2	2	1	2	1	1	2
1	2	2	2	1	1	1	1	2	2
1	1	2	2	2	1	2	1	1	2
3	3	3	3	3	1	1	1	1	1
9	9	9	9	9	2	1	1	1	1

3	1	3	3	1	1	1	1	1	1
1	1	1	1	1	2	1	1	1	3
2	1	2	2	1	1	1	1	2	2
2	2	2	2	2	2	2	2	2	2
9	9	9	9	9	9	9	9	9	9
2	3	3	3	2	1	1	2	2	2
1	1	1	1	1	1	1	9	1	1
2	2	2	2	2	9	9	9	1	1
3	1	3	3	2	2	1	2	2	2
3	2	3	2	1	2	1	1	1	2
2	1	2	3	1	3	1	1	3	3
3	3	3	1	1	1	1	1	1	1
1	2	2	2	2	1	1	1	1	1
3	3	3	1	1	3	2	1	1	1
2	2	2	1	2	1	1	2	2	1
9	9	9	9	9	1	9	9	9	9
2	2	3	1	1	1	1	1	1	1
1	2	2	2	1	2	2	1	2	1
3	1	1	1	1	1	1	1	1	1
2	2	3	1	1	1	1	1	1	1
9	9	9	9	9	9	9	9	9	9
1	2	3	9	1	1	2	1	1	1
2	2	2	2	2	2	2	2	2	2
3	3	3	3	3	1	1	1	1	1
2	3	3	2	1	1	1	1	2	1
2	3	3	2	2	1	1	1	1	1
2	2	2	2	2	1	1	1	1	1
9	9	9	9	9	9	9	9	9	9
9	9	9	9	9	9	9	9	9	9
3	1	3	2	1	1	3	2	3	1
3	3	3	1	1	3	3	1	1	1
2	1	2	1	1	1	1	2	1	1
1	1	1	9	1	1	1	1	1	1
9	9	9	9	1	1	1	1	1	1
3	1	3	3	1	1	1	1	1	1
1	1	1	3	2	1	1	1	1	1
3	1	3	3	1	1	1	1	1	1
1	1	3	1	1	1	1	1	1	1
3	1	3	3	1	1	1	1	1	1
1	1	3	1	1	1	1	1	1	1
1	3	2	3	1	1	1	1	1	1
3	1	3	3	1	1	1	1	1	1
3	1	3	3	1	1	1	1	1	1
3	9	3	3	1	1	1	1	1	1
3	1	3	3	1	1	1	1	1	1
1	1	1	3	3	1	1	1	1	1
3	3	9	9	1	1	1	1	1	1

1	1	3	9	1	1	1	1	1	1
1	3	3	3	3	1	1	1	1	1
9	9	9	9	9	9	9	9	9	9
3	3	9	1	1	1	1	1	9	1
2	9	9	1	9	1	9	9	9	1
3	3	3	3	1	1	1	1	1	1
1	2	1	2	1	2	3	1	2	1
3	3	3	1	1	1	2	1	1	1
9	9	9	9	9	9	9	9	9	9
9	9	9	9	9	9	9	9	9	9
1	3	1	3	1	3	3	3	3	3
3	1	9	2	1	1	1	1	2	2
3	3	1	1	2	2	2	1	1	1
9	9	9	9	9	9	9	9	9	9
2	1	2	2	2	1	2	1	1	1
3	1	3	3	2	1	2	1	1	1
9	9	9	1	1	9	1	1	1	1
2	2	9	1	2	1	1	1	1	1
1	3	3	1	1	1	1	1	1	1
2	3	2	2	1	1	2	3	1	2
3	1	3	3	1	1	3	3	3	1
1	1	3	1	1	1	1	1	1	1
1	1	1	3	1	1	1	1	1	1
3	3	1	1	1	1	1	1	1	1
9	9	9	9	1	9	1	1	9	1
1	3	1	2	1	1	1	1	1	1
9	2	2	1	1	9	9	1	1	1
1	1	3	3	1	1	1	3	2	2
1	1	3	1	1	1	1	1	1	1
1	3	1	1	1	1	1	1	1	1
3	3	2	2	1	1	1	2	3	1
2	1	2	3	1	1	1	1	1	1
2	2	2	2	1	1	1	3	1	1
1	1	3	3	1	1	1	3	1	1
3	3	3	2	1	3	2	3	1	1
3	1	3	2	1	1	1	1	3	1
3	3	3	1	1	1	3	1	1	1
9	9	9	9	9	9	9	9	1	9
1	2	2	2	2	2	2	1	1	2
1	1	3	2	1	1	1	1	1	1
1	3	3	2	2	1	2	2	1	3
3	1	3	3	1	1	3	2	3	1
9	9	9	9	9	9	9	9	9	9
9	9	9	9	9	9	9	9	9	9
3	2	3	2	1	1	1	1	1	1
3	1	3	9	3	3	1	1	1	1
9	9	9	9	9	9	9	9	9	9

3	1	3	1	1	1	1	1	1	1
1	3	3	1	1	1	1	1	2	1
2	2	2	2	1	2	1	1	1	2
2	2	2	2	9	1	1	2	1	1
1	1	3	1	1	1	3	1	1	1
1	2	2	2	2	2	1	1	1	1
2	1	2	2	1	2	2	3	2	2
1	2	1	1	1	1	2	1	2	1
2	2	2	1	1	1	1	1	1	1
2	2	2	2	1	2	2	2	3	1
1	1	2	2	1	1	2	1	1	1
9	9	9	9	9	9	9	9	9	9
9	9	9	9	9	9	9	9	9	9
9	9	9	9	9	9	9	9	1	9
9	9	9	9	9	9	9	9	9	9
9	9	9	9	9	9	9	9	1	9
1	3	3	1	1	3	3	3	1	3
3	3	3	3	1	1	3	1	1	1
9	9	9	9	1	9	9	9	9	9
2	3	3	1	1	1	2	2	2	1
3	2	3	3	1	1	2	1	1	1
3	3	1	2	3	2	2	1	1	1
3	3	1	1	1	1	1	1	1	1
2	1	3	1	1	2	2	1	2	3
2	3	3	1	2	1	2	1	1	2
3	3	3	2	1	1	3	1	1	1
3	2	2	2	1	3	1	1	1	1
3	2	3	3	1	1	2	3	3	1
9	9	9	1	1	9	9	9	1	1
3	9	9	9	1	1	1	2	1	1
2	2	2	2	1	1	2	1	1	1
1	9	9	9	1	9	9	9	1	9
3	3	3	3	1	1	2	1	3	1
2	3	3	2	1	1	1	1	1	2
2	2	2	2	1	1	2	1	2	1
3	3	3	1	1	2	2	1	3	3
3	1	3	3	1	2	2	1	2	1
3	1	3	3	1	1	1	1	1	1
3	3	9	3	9	9	1	1	1	1
3	1	3	3	1	1	1	1	1	1
3	2	3	2	3	2	3	2	3	2
2	2	2	2	2	1	2	2	2	2
3	3	2	1	1	2	1	1	1	1
2	2	2	2	2	2	2	1	1	1
1	1	2	2	1	1	1	1	1	2
9	9	9	1	1	9	1	1	1	1
1	1	1	1	1	1	1	1	1	1

3	1	3	3	1	1	1	1	1	1
1	1	2	1	1	1	1	1	1	1
1	2	2	2	2	2	2	2	1	2
2	1	2	3	1	2	3	1	1	1
3	1	2	3	2	2	2	2	2	1
2	2	2	3	2	2	1	2	2	2
2	2	2	2	2	2	9	1	2	1
2	2	2	2	1	2	2	1	1	1
2	1	1	1	2	2	2	2	2	2
2	1	2	2	1	1	1	1	1	1
3	1	2	2	2	3	2	2	1	1
2	2	2	2	2	2	2	2	2	2
2	2	2	2	1	2	2	1	1	1
3	1	2	2	1	1	2	1	1	1
2	2	2	2	2	2	2	2	2	2
3	1	2	2	1	1	1	1	1	1
3	2	2	2	2	2	2	2	2	2
2	2	2	2	2	2	2	2	2	2
2	1	2	2	2	2	2	2	2	2
2	2	2	2	2	2	2	2	2	2
2	2	2	2	2	2	2	2	2	2
9	9	9	9	9	9	9	9	9	9
3	3	1	3	1	2	1	1	1	1
3	3	1	3	2	2	2	3	3	1
1	2	1	1	2	1	2	1	1	1
3	3	3	1	2	2	1	1	2	1
1	1	1	2	2	1	1	1	1	2
1	2	1	1	1	9	1	9	9	9
3	3	3	9	9	9	2	2	1	1
2	3	2	3	1	2	2	2	3	3
2	2	2	2	1	1	1	1	1	1
2	2	2	2	1	1	1	1	1	1
9	9	9	9	9	9	9	9	9	9
9	9	9	9	9	9	9	9	9	9
9	9	9	9	9	9	9	9	9	9
9	9	9	9	9	9	9	9	9	9
9	9	9	9	9	9	9	9	9	9
9	9	9	9	9	9	9	9	9	9
9	9	9	9	9	9	9	9	9	9
9	9	9	9	9	9	9	9	9	9
3	3	3	2	2	3	2	1	2	1
9	9	9	9	9	9	9	9	9	9
9	9	9	9	9	9	9	9	9	9
2	2	3	3	2	2	2	2	2	1
9	9	9	9	9	9	9	9	9	9
9	9	9	9	9	9	9	9	9	9
9	9	9	9	9	9	9	9	9	9

1	1	1	1	1	1	1	2	1	1
2	1	3	2	3	3	3	3	3	2
9	9	9	9	9	9	9	9	9	9
9	9	9	9	9	9	9	9	9	9
9	9	9	9	9	9	9	9	9	9
9	9	9	9	9	9	9	9	9	9
9	9	9	9	9	9	9	9	9	9
9	9	9	9	9	9	9	9	9	9
9	9	9	9	9	9	9	9	9	9
9	9	9	9	9	9	9	9	9	9
1	1	3	3	1	1	3	1	1	1
3	2	3	3	1	1	1	1	1	1
3	2	3	3	1	1	1	1	1	1
2	1	2	2	1	1	1	1	1	1
1	1	1	1	1	1	1	1	1	1
1	1	3	1	1	1	1	1	1	1
1	1	3	1	1	1	1	1	1	1
1	1	1	1	1	1	1	1	1	1
3	2	3	3	1	1	1	1	1	1
1	1	2	1	1	1	2	1	1	1
1	1	2	1	1	1	2	1	1	1
3	3	2	2	2	3	2	1	1	2
9	9	9	9	9	9	9	9	9	9
2	1	1	3	1	1	1	1	2	1
2	3	9	1	3	1	2	2	9	9
3	3	3	1	1	1	1	1	2	1
3	3	3	3	3	1	2	1	2	1
3	1	3	3	1	1	2	1	1	1
1	1	1	1	1	1	1	1	1	1
2	1	2	2	1	1	2	2	2	1
1	1	2	2	1	1	9	1	1	1
3	3	2	2	1	2	2	1	1	1
3	3	1	3	1	1	1	1	2	2
1	1	3	2	1	1	1	1	1	1
3	1	2	2	2	2	2	1	1	2
1	1	3	1	1	1	1	1	1	1
1	1	3	9	1	1	1	1	1	1
1	1	3	3	1	1	1	1	1	1
1	1	3	3	1	1	1	1	1	1
3	1	2	2	1	1	1	1	2	1
3	3	9	1	1	1	2	1	1	1
3	1	3	1	1	1	1	1	1	1
1	3	1	1	1	2	3	3	3	2
1	3	2	9	2	3	1	1	2	1
1	1	2	3	1	1	2	1	1	1
1	3	1	3	1	3	1	1	1	1
1	1	1	1	1	1	1	1	1	1

1	1	1	1	1	1	1	1	1	1
1	1	1	1	1	1	1	1	1	1
3	1	1	1	1	1	1	1	1	1
1	9	3	2	1	3	2	1	2	1
1	1	2	3	1	1	2	1	1	1
1	1	3	9	2	1	9	2	1	2
1	1	1	1	1	1	1	1	1	1
1	9	2	3	1	1	2	1	1	9
2	1	3	2	1	1	2	1	1	1
1	1	1	1	1	1	1	1	1	1
1	2	2	2	1	1	1	1	1	1
3	3	1	1	1	1	1	1	1	1
2	2	2	2	1	1	1	1	1	1
1	2	2	2	1	1	1	1	1	1
3	3	3	1	1	1	1	1	1	3
1	1	2	1	1	1	1	1	1	1
3	1	2	2	2	2	2	1	1	1
2	2	2	2	2	2	2	2	2	1
2	1	3	9	1	3	1	1	2	1
1	1	3	3	3	3	1	1	1	1
3	3	3	3	1	1	2	3	3	2
1	1	1	3	1	1	1	1	1	1
1	1	1	2	1	2	2	1	2	1
1	1	2	2	1	1	2	1	1	1
1	1	1	1	1	1	1	1	1	1
3	3	2	2	2	2	2	1	1	1
2	1	1	2	1	1	1	1	1	1
2	2	2	2	2	2	2	2	2	2
2	2	2	2	2	1	1	1	1	1
1	1	2	2	1	1	1	2	2	1
2	2	2	3	3	3	3	2	2	2
3	2	2	1	2	2	2	1	1	3
2	2	2	2	2	2	2	1	1	1
9	3	2	1	1	1	2	1	1	1
1	1	9	9	9	9	9	9	9	9
1	1	1	2	1	2	2	1	2	1
3	1	3	3	2	2	2	1	3	1
2	2	2	2	2	2	2	2	2	2
1	3	3	3	1	1	1	1	1	1
3	1	3	3	2	1	1	1	1	1
2	2	2	2	2	2	2	2	2	2
1	3	3	1	1	1	1	1	3	1
1	3	3	1	1	1	1	1	3	1
2	2	2	2	1	2	2	1	2	1
3	3	1	2	1	1	2	1	1	1
2	2	1	2	2	2	1	1	2	1
2	2	2	2	2	2	2	2	2	2

3	3	3	2	3	2	2	3	3	2
2	2	2	2	2	2	2	2	2	2
2	1	3	3	1	2	2	3	1	1
3	1	3	3	3	3	1	1	3	1
2	1	3	2	2	2	2	1	1	2
2	1	3	3	3	2	2	2	2	2
3	3	3	3	1	2	2	3	3	3
2	2	2	2	2	2	2	2	2	2
2	1	2	2	2	2	3	2	3	1
3	2	2	1	1	1	1	1	1	1
1	1	2	2	2	2	1	2	1	1
1	2	3	2	1	1	1	1	3	1
9	9	9	9	9	9	9	9	9	1
3	3	3	3	2	2	2	1	2	1
3	3	3	3	1	3	3	3	3	3
2	2	2	2	2	2	2	2	2	2
3	3	1	3	2	2	1	1	1	1
2	2	2	2	1	1	1	1	1	1
2	2	3	1	1	1	1	1	2	3
3	1	1	2	1	2	1	1	1	1
1	1	3	3	1	3	1	1	1	1
1	1	3	2	2	1	1	1	1	1
3	1	3	1	1	1	1	1	1	1
1	3	3	1	1	1	2	2	1	3
2	1	2	2	3	2	2	1	1	1
3	1	2	3	1	1	2	1	1	1
1	1	3	1	1	1	1	1	1	1
3	3	3	1	2	2	1	1	2	1
2	2	3	3	2	2	2	2	2	1
2	9	1	9	9	9	9	9	9	9
2	2	2	1	3	1	1	1	2	3
3	2	2	3	3	3	3	3	1	1
3	2	2	2	2	3	3	2	1	1
2	1	2	2	2	2	2	1	1	1
9	9	9	9	1	9	1	1	1	9
2	1	1	2	1	1	2	2	1	1
2	2	2	9	1	3	2	2	2	1
9	9	9	9	9	9	9	9	9	9
3	1	3	3	3	2	1	1	1	3
3	3	3	1	1	2	2	1	1	2
9	1	9	9	9	9	1	9	9	9
2	1	3	1	1	2	1	1	1	1
2	1	3	3	1	1	1	1	1	1
9	9	9	9	1	9	9	9	9	9
2	3	2	2	2	2	1	1	3	2
2	2	3	3	2	2	2	2	2	2
1	1	2	2	1	1	1	1	1	1

3	1	3	2	1	3	1	1	1	2
2	2	2	2	1	1	1	1	1	1
2	2	2	1	1	1	1	1	1	1
2	1	2	2	1	1	2	1	1	2
2	2	2	3	3	3	2	2	2	2
3	1	3	2	2	1	2	1	1	1
2	2	2	1	1	1	1	1	1	2
9	9	9	9	9	9	9	9	9	9
2	2	2	2	2	2	2	2	2	2
3	2	2	2	2	2	2	2	2	2
2	1	2	3	2	2	2	2	1	1
2	2	2	2	2	1	2	1	2	2
3	3	1	1	2	2	1	1	1	2
2	2	2	2	2	2	2	2	2	2
3	1	3	2	2	2	2	2	2	2
3	1	3	3	2	2	2	1	3	1
1	2	2	1	1	1	1	1	1	1
3	3	1	9	1	1	1	1	1	1
2	1	3	3	2	1	1	1	1	1
3	2	2	2	2	9	1	1	1	1
3	2	2	2	1	2	1	1	1	1
2	1	1	1	1	1	1	1	1	1
2	2	2	2	1	1	1	1	2	2
3	3	3	3	1	1	1	1	2	2
3	3	3	3	2	1	1	1	1	2
2	1	1	1	1	1	1	1	1	1
2	2	3	2	1	2	2	1	1	1
3	1	1	1	1	1	1	1	1	1
3	1	1	1	1	1	1	1	1	1
9	9	9	9	9	9	9	9	9	9
3	1	3	1	1	1	1	1	1	1
2	1	2	2	1	1	1	1	1	1
2	1	2	2	1	1	1	1	1	1
3	2	3	3	1	1	2	1	2	2
2	1	2	1	9	9	9	9	9	9
2	1	2	1	1	1	1	1	1	1
2	1	2	1	1	1	1	1	1	1
3	3	1	1	1	1	1	1	1	1
3	3	3	1	1	1	1	1	1	1
3	3	3	2	1	1	1	1	1	2
2	1	2	2	1	2	2	1	1	1
3	3	1	9	1	1	1	1	1	1
2	2	2	2	3	3	3	2	2	2
3	3	3	3	1	3	3	1	3	1
3	1	3	3	1	1	1	1	3	1
3	3	3	3	1	1	1	3	3	3
3	1	3	1	1	1	1	1	1	1

3	1	3	3	1	1	1	1	3	1
3	1	3	3	1	1	1	1	3	1
3	3	3	3	1	1	1	1	1	1
3	1	3	2	1	1	1	1	1	1
3	3	3	1	1	1	1	1	1	1
3	3	3	1	1	1	1	1	1	1
9	3	3	3	1	1	1	1	1	1
2	1	1	1	1	1	2	1	2	1
3	2	3	3	1	1	1	1	1	1
3	2	3	3	1	1	1	1	1	1
1	1	3	3	1	1	1	1	1	1
1	1	3	3	1	1	1	1	1	1
3	1	3	3	1	1	1	1	1	1
3	1	3	1	3	2	1	1	3	1
3	1	3	3	1	1	2	1	9	1
3	1	3	3	1	1	1	1	1	1
2	1	1	2	2	3	2	1	2	1
3	2	2	2	1	1	1	1	1	2
1	1	1	1	1	1	1	1	1	1
1	1	1	1	1	1	1	1	1	1
2	2	2	1	1	1	1	1	2	2
3	3	3	3	1	1	3	3	1	3
9	9	9	9	9	9	9	9	9	9
9	9	9	9	9	9	9	9	9	9
9	9	9	9	9	9	9	9	9	9
9	9	9	9	9	9	9	9	9	9
3	3	3	3	1	1	1	3	1	9
9	9	9	9	9	9	9	9	9	9
9	9	9	9	9	9	9	9	9	9
9	9	9	9	9	9	9	1	9	9
9	9	9	9	9	9	9	9	9	9
9	9	9	9	9	9	9	9	9	9
9	9	9	9	9	9	9	9	9	9
9	9	9	9	9	9	9	9	9	9
3	3	3	1	2	2	1	1	2	1
1	1	3	3	1	1	1	1	1	1
1	1	2	2	1	1	1	1	1	1
1	1	1	3	1	1	1	1	1	1
2	3	2	1	2	2	2	1	1	1
2	2	3	2	1	2	2	1	1	1
3	3	1	1	1	1	1	1	1	1
1	3	2	2	1	2	1	2	2	1
2	1	3	1	1	1	2	2	1	1
3	3	3	1	1	3	2	2	2	2
3	1	3	2	1	2	2	3	3	2
3	3	3	1	1	1	1	1	3	1
3	2	1	2	1	1	1	2	1	1

2	1	1	1	2	2	2	2	1	1
2	2	2	2	2	2	2	2	2	2
3	1	3	3	1	1	1	1	1	1
1	1	1	1	3	2	2	3	2	3
1	1	2	2	1	1	1	1	1	2
1	2	2	2	1	1	1	2	2	2
9	9	9	9	9	9	9	9	9	9
2	2	2	2	2	2	2	2	2	2
2	1	2	3	3	2	1	1	1	2
3	1	2	3	1	1	1	1	1	2
2	2	2	2	2	2	2	2	2	2
2	2	2	2	1	1	1	1	1	1
2	2	3	1	2	2	2	2	1	1
3	3	3	3	3	2	1	2	3	3
1	2	2	2	2	2	2	2	2	2
2	2	2	2	2	2	2	2	2	2
1	1	1	2	2	2	2	2	2	2
2	1	2	2	1	2	1	2	1	3
2	1	2	2	1	1	1	1	1	1
1	1	1	1	1	1	1	1	1	1
1	1	2	2	3	3	1	2	3	1
2	2	2	2	3	3	2	2	2	2
2	1	2	3	3	2	1	1	1	2
1	1	1	1	1	1	2	1	1	1
1	1	1	1	1	1	1	1	1	1
2	3	2	3	1	2	3	2	1	2
3	1	2	1	2	1	2	1	2	2
1	1	2	2	1	1	1	1	1	1
2	2	2	2	2	2	2	2	2	2
2	2	2	2	2	2	2	2	2	2
1	1	1	1	1	1	1	1	1	1
1	1	1	1	1	1	1	1	1	1
2	2	2	2	2	2	2	2	2	2
1	2	3	1	1	1	1	1	1	2
2	2	2	2	1	1	3	3	2	2
3	3	3	3	3	3	2	2	2	2
2	2	2	2	2	2	2	2	2	2
3	1	3	2	2	2	2	2	1	2
2	2	2	2	2	2	2	2	2	2
1	1	3	1	1	1	1	1	1	1
1	1	2	2	1	1	2	1	1	1
1	1	2	2	1	1	2	1	1	1
9	9	9	9	9	9	9	9	9	9
9	9	9	9	9	9	3	9	9	9
9	9	9	9	9	9	9	9	9	9
9	1	2	3	3	2	3	2	3	3
1	1	1	3	3	2	3	3	3	2

3	1	3	3	3	3	3	3	3	3
3	1	3	1	3	3	3	3	3	3
3	1	3	1	3	3	3	3	3	3
3	3	3	1	2	2	2	2	2	1
3	3	3	1	2	2	2	2	2	2
2	3	2	3	2	3	3	2	3	2
3	2	1	2	3	1	2	3	1	2
2	1	2	3	2	2	3	2	1	2
3	1	2	3	1	2	1	3	1	3
2	1	2	3	3	2	3	2	1	2
2	2	2	1	1	1	1	1	1	1
2	3	3	1	1	3	3	3	3	3
2	2	1	3	3	3	3	1	1	1
3	3	3	2	2	2	2	2	3	3
2	2	2	2	2	2	2	1	1	1
2	2	2	3	3	3	2	2	3	3
1	1	1	3	3	3	3	3	3	3
1	1	1	3	3	3	3	3	3	3
3	1	3	1	3	3	3	3	3	3
3	1	3	1	3	3	3	3	3	3
2	2	2	2	2	2	2	2	2	2
2	2	2	2	2	2	2	2	2	2
2	2	2	2	2	2	2	2	2	2
2	2	2	2	2	2	2	2	2	2
3	3	3	1	1	1	1	1	1	1
2	2	2	2	2	2	2	2	2	2
2	2	2	2	1	1	1	1	1	1
3	3	3	1	1	1	1	1	1	1
2	2	2	2	2	3	3	3	3	3
2	1	3	3	3	3	3	3	3	3
2	1	3	3	3	3	3	3	3	3
2	1	3	3	3	3	3	3	3	3
2	1	3	3	3	3	3	3	3	3
3	9	2	9	1	2	3	1	3	9
2	9	3	2	1	9	1	9	2	9
9	3	1	1	3	1	2	1	3	2
1	2	2	2	2	2	1	1	1	1
1	2	2	1	1	2	1	2	1	2
1	2	1	2	3	3	2	3	2	3
3	2	2	3	2	1	2	3	2	2
1	1	1	2	1	3	2	1	3	3
2	3	9	2	3	3	2	3	3	1
9	1	2	3	2	3	2	3	2	3
9	9	9	9	9	9	9	9	9	9
9	9	9	9	9	9	9	9	9	9
9	9	9	9	9	9	9	9	9	9
9	1	1	3	9	9	3	3	3	9

9	1	1	3	3	3	2	3	3	9
1	9	1	2	3	3	3	3	3	3
2	2	2	2	2	2	2	2	2	2
2	2	2	2	2	2	2	2	2	2
2	2	2	2	2	2	2	2	2	2
9	9	9	9	9	9	9	9	9	9
2	9	3	1	1	1	2	2	2	2
9	9	9	9	9	9	9	9	9	9
2	2	2	2	3	3	3	2	3	3
3	2	1	1	3	2	3	3	2	3
2	2	2	2	2	2	2	2	2	2
2	2	2	2	2	2	2	2	2	2
9	9	9	9	9	2	9	9	9	9
9	9	9	9	9	9	9	9	9	9
2	3	1	2	1	3	2	1	3	2
9	9	9	9	9	9	9	9	9	9
2	2	2	2	3	3	3	3	3	3
9	9	9	9	9	9	9	9	9	9
1	3	1	2	3	3	1	3	3	1
9	9	9	9	9	9	9	9	9	9
1	1	1	3	3	3	3	3	3	3
9	9	9	9	9	9	9	9	9	9
1	1	2	2	2	2	2	2	2	2
2	2	2	2	2	2	2	2	2	2
1	1	1	3	3	3	2	2	2	2
2	2	2	2	2	2	2	2	3	3
2	2	2	3	3	3	3	1	1	1
9	9	9	9	9	9	9	9	9	9
9	9	9	9	9	9	9	9	9	9
9	9	9	9	9	9	9	9	9	9
9	9	9	9	9	9	9	9	9	9
9	9	9	9	9	9	9	9	9	9
9	9	9	9	9	9	9	9	9	9
3	2	2	2	3	2	2	2	3	2
2	3	1	2	1	3	2	1	3	2
9	9	9	9	9	9	9	9	9	9
1	1	1	3	3	3	3	3	3	3
9	2	9	9	9	9	2	9	9	9
1	2	2	3	3	3	3	3	2	3
2	2	1	3	3	3	3	3	3	3
2	3	2	3	2	2	1	2	2	1
9	9	9	9	9	9	9	9	9	9
3	3	3	1	9	3	2	3	1	1
1	1	3	1	3	3	3	3	3	3
9	9	9	9	9	9	9	9	9	9
2	3	2	9	3	2	2	3	3	2
1	3	1	1	3	3	1	3	3	3

9	9	9	9	9	9	9	9	9	9
9	9	9	9	9	9	9	9	9	9
1	1	1	3	3	3	3	3	3	3
9	9	9	9	9	9	9	9	9	9
3	1	2	3	3	3	3	3	3	3
9	9	9	9	9	9	9	9	9	9
2	2	2	2	2	2	2	2	2	2
1	2	2	2	2	1	1	1	1	1
9	1	2	9	3	9	3	2	3	3
1	2	3	2	3	3	2	2	3	2
3	3	3	1	1	1	1	2	2	1
3	3	3	1	1	2	2	3	3	3
3	3	3	1	1	2	2	3	3	2
2	2	3	2	3	2	1	2	3	2
1	1	1	3	3	3	3	3	3	3
1	1	1	3	3	3	2	2	2	3
1	1	2	3	3	2	2	2	3	3
2	2	2	2	3	2	3	2	9	3
1	2	3	2	3	2	1	2	3	2
1	1	1	3	3	3	3	3	3	3
9	9	9	9	9	9	9	9	9	9
9	1	9	9	9	9	9	9	9	9
9	9	9	9	9	9	9	9	9	9
9	9	9	3	9	9	9	9	9	9
9	9	9	9	9	9	9	9	9	9
9	9	9	9	9	9	9	9	9	9
9	9	9	9	9	9	9	9	9	9
9	9	9	9	9	9	9	9	9	9
9	9	9	9	9	9	9	9	9	9
9	9	9	9	9	9	9	9	9	9
9	9	9	9	9	9	9	9	9	9
9	9	9	9	9	9	9	9	9	9
9	9	9	9	9	9	9	9	9	9
1	1	2	3	2	1	3	2	3	1
1	2	1	3	2	1	3	2	3	3
1	2	2	2	3	3	3	3	3	2
9	9	9	9	1	9	9	1	9	9
3	9	9	9	1	9	9	9	1	9
2	2	1	2	3	2	2	2	2	3
9	9	9	9	9	9	9	9	9	9
9	9	9	9	9	9	9	9	9	9
9	9	9	9	9	9	9	9	9	9
9	9	9	9	9	9	9	9	9	9
9	9	9	9	9	9	9	9	9	9
1	2	1	2	3	2	3	2	3	2
1	2	1	2	3	2	3	2	3	2
2	2	2	2	2	2	2	2	2	2

2	2	2	2	2	2	2	2	2	2
2	1	2	3	1	1	3	2	2	2
1	1	1	1	1	1	1	2	2	2
2	3	3	2	2	2	3	3	3	2
9	9	9	9	9	9	9	9	9	9
9	9	9	9	9	9	9	9	9	9
9	9	9	9	9	9	3	9	9	9
1	9	9	9	9	2	9	9	9	1
9	9	9	9	9	3	9	9	9	9
9	9	9	9	9	9	9	9	9	9
9	9	9	1	9	2	9	1	9	2
9	9	1	9	1	1	9	1	9	9
9	9	9	9	9	9	9	9	9	9
2	2	2	2	2	9	3	3	3	1
9	9	9	9	9	9	9	9	9	9
2	2	2	2	2	2	2	2	2	2
3	2	3	1	2	2	1	1	2	3
9	9	9	9	9	9	9	9	9	9
2	2	2	2	2	2	2	2	2	2
1	2	1	3	2	1	3	3	1	2
2	2	2	2	2	2	2	2	2	2
2	2	2	2	2	2	2	2	2	2
1	1	1	3	3	3	3	3	2	3
2	2	2	2	2	2	2	3	2	2
2	1	2	2	3	1	2	1	2	1
9	1	1	9	3	3	9	3	2	2
9	9	9	9	9	9	9	9	9	9
2	2	2	2	2	2	2	2	2	2
2	2	2	2	2	2	2	2	2	2
3	3	3	2	2	3	2	3	2	2
2	1	3	2	3	1	3	1	3	2
2	2	2	2	2	2	2	2	2	2
9	9	9	9	9	9	9	9	9	9
2	2	2	2	2	2	2	2	2	2
3	3	1	9	9	9	9	9	9	9
1	2	1	1	1	1	3	3	2	2
2	1	2	2	3	3	3	3	2	3
2	2	2	2	2	2	2	2	2	2
3	2	3	2	3	2	1	2	3	2
2	2	2	2	2	2	2	2	2	2
2	1	2	3	2	3	3	2	2	3
1	3	3	1	3	3	1	3	3	3
2	2	2	2	2	2	2	2	2	1
2	2	2	2	3	3	3	3	3	3
9	9	9	9	9	9	9	9	9	9
2	2	2	2	2	2	2	2	2	2
3	3	3	1	3	3	3	3	1	1

3	3	3	3	3	3	3	3	3	3
2	1	2	2	2	3	2	3	3	3
2	2	2	2	3	3	2	3	3	3
3	2	2	3	2	2	1	1	1	2
2	2	2	2	2	2	2	2	2	2
2	2	2	2	2	2	2	2	2	2
3	3	2	2	1	3	3	2	2	2
2	2	2	2	2	2	2	3	3	2
2	2	2	2	2	2	2	2	2	2
2	2	2	2	2	2	2	2	2	2
2	2	2	2	3	2	3	3	3	2
3	3	3	1	3	3	3	3	2	2
1	1	1	3	3	3	2	2	2	3
3	1	2	2	3	3	3	3	3	3
3	3	2	9	3	1	1	3	3	3
2	1	2	2	3	2	3	3	2	3
2	2	3	1	2	2	2	1	2	2
2	2	2	2	2	2	2	2	2	2
2	2	2	2	2	2	2	2	2	2
2	2	2	2	2	2	2	2	2	2
2	2	2	2	2	2	2	2	2	2
2	2	2	2	2	2	2	2	2	2
2	2	2	2	2	2	2	2	2	2
2	2	2	2	2	2	2	2	2	2
1	2	1	2	2	2	1	2	1	2
9	9	9	9	9	9	9	9	9	9
9	9	9	9	9	9	9	9	9	9
1	3	3	1	3	3	1	3	3	1
9	1	2	1	3	2	3	1	2	3
9	1	9	9	9	9	9	9	9	9
1	1	1	3	3	3	3	3	3	3
2	3	3	3	3	2	3	3	3	3
2	1	3	3	2	3	2	1	3	2
9	9	9	9	9	9	9	9	9	9
1	1	2	1	3	3	3	3	3	3
1	1	2	2	3	2	3	2	3	1
2	2	2	2	3	3	3	3	3	3
3	3	2	3	2	1	3	2	1	3
2	2	1	3	3	3	2	3	3	3
2	2	2	2	2	2	2	2	2	2
1	1	1	3	3	9	9	9	9	9
2	2	2	2	2	2	2	2	2	2
1	3	3	1	3	3	3	3	3	3
3	2	3	1	2	2	2	3	2	3
3	1	3	1	3	3	2	2	3	3
2	1	3	1	1	3	3	3	2	3
2	2	3	3	2	2	2	3	2	3

1	2	2	3	3	3	3	2	3	3
2	2	2	2	2	2	2	3	2	2
3	1	3	1	2	2	2	3	3	3
2	2	2	2	2	2	2	2	2	2
2	2	2	2	2	2	2	2	2	2
9	9	2	9	9	9	9	9	9	9
2	2	2	2	3	3	3	3	3	3
2	2	2	1	1	1	2	2	2	3
2	2	2	2	2	2	2	2	2	2
1	2	1	2	3	1	3	2	2	3
2	2	2	2	2	2	2	2	2	2
3	3	3	1	1	1	1	1	3	1
2	2	2	2	2	2	2	3	2	3
2	2	2	2	2	2	2	2	2	2
2	2	2	2	2	2	2	2	2	2
2	2	2	2	2	2	2	2	2	2
2	2	2	2	3	3	3	3	3	2
2	3	2	9	2	2	2	3	2	3
1	1	1	3	1	1	3	3	3	3
9	9	9	9	9	9	9	9	9	9
2	1	3	1	3	2	3	3	2	3
2	2	2	2	2	2	2	2	2	2
2	2	2	2	2	2	2	2	2	3
2	2	2	2	2	2	2	2	2	2
3	2	9	2	9	2	1	2	2	2
3	3	3	1	3	2	1	3	3	2
2	2	2	3	2	2	2	1	1	2
2	2	2	2	2	2	2	2	2	2
2	1	2	2	2	2	2	2	2	2
1	1	3	1	3	3	3	3	3	3
9	1	3	1	3	3	3	3	3	3
9	9	9	9	9	9	9	9	9	9
2	2	2	2	3	2	3	3	2	2
2	3	3	2	3	2	3	3	3	3
2	2	2	2	2	2	2	2	2	2
2	2	2	2	2	2	2	2	2	2
1	2	2	2	2	2	2	2	2	2
2	2	2	2	2	3	2	3	2	2
2	2	2	2	2	2	2	2	2	2
2	2	2	2	2	2	2	2	2	2
9	9	9	9	9	9	9	9	9	9
2	2	2	2	2	2	2	2	2	2
2	2	2	2	2	2	2	2	2	1
2	2	2	2	2	2	2	2	2	2
2	2	2	2	2	2	2	2	2	3
2	2	2	2	2	2	2	2	2	2
1	1	1	3	3	3	2	2	2	2

9	9	9	9	9	9	9	9	9	9
1	1	1	1	1	1	3	3	3	3
9	9	9	9	9	3	3	3	3	2
9	9	9	9	9	9	9	9	9	9
2	9	1	3	3	3	3	2	2	2
3	3	3	1	1	1	1	1	1	1
9	9	9	9	9	9	9	9	9	9
2	1	2	2	3	2	1	3	3	2
9	9	9	9	9	9	9	9	9	9
2	3	3	2	2	2	2	2	2	3
2	9	2	9	1	2	9	1	9	2
9	9	9	9	9	9	9	9	9	9
3	9	3	3	3	3	3	1	1	1
9	9	9	2	1	1	2	9	3	9
9	9	9	9	9	9	9	9	9	9
3	3	2	3	1	1	2	3	2	1
3	3	3	3	3	3	3	3	3	3
9	1	2	9	9	9	9	9	9	9
2	2	2	2	3	3	3	3	2	3
9	9	9	9	9	9	9	9	9	9
1	1	1	3	2	2	2	3	3	1
3	1	3	1	2	3	2	2	2	2
2	2	2	1	2	2	2	2	2	2
2	1	2	1	2	2	1	3	3	3
1	3	3	1	3	3	3	3	3	3
3	1	2	2	3	3	3	3	3	2
3	1	3	1	1	1	2	3	3	3
9	9	9	9	9	9	9	3	9	9
3	1	3	2	3	2	2	3	3	3
2	3	2	2	2	3	3	3	3	3
1	1	1	3	2	2	2	2	3	3
1	2	2	2	2	2	2	2	2	2
1	1	2	2	2	2	1	3	1	2
3	2	1	3	3	3	3	3	3	3
2	3	2	2	3	3	2	1	3	2
9	9	9	9	9	9	9	9	9	9
9	9	9	9	9	9	9	9	3	9
1	1	1	3	3	3	3	3	3	3
9	9	9	9	9	9	9	9	9	9
9	9	9	9	9	9	9	9	9	9
9	9	9	9	9	9	9	9	9	9
3	1	1	3	1	3	3	3	3	1
3	2	2	2	2	2	2	2	2	1
3	3	3	2	3	3	3	2	3	3
3	3	3	3	1	3	1	3	3	3
9	9	9	3	3	3	9	3	3	9
3	3	1	9	3	3	3	3	3	3

2	2	2	3	3	3	3	3	3	2
1	1	1	3	2	3	3	2	2	3
9	9	9	9	9	9	9	9	9	9
2	2	2	3	2	2	2	3	2	2
3	3	3	3	3	3	3	3	3	3
2	1	1	3	3	3	3	3	3	3
9	9	9	9	9	9	9	9	9	9
1	2	1	1	2	3	2	9	3	9
1	2	2	2	3	3	2	3	3	3
2	3	1	3	3	3	3	3	3	2
3	3	3	3	3	3	3	3	3	3
2	1	3	3	3	3	3	3	3	3
2	1	2	3	3	3	3	3	3	3
3	2	2	2	2	3	3	3	3	3
2	1	2	3	3	3	3	2	3	3
3	1	2	3	3	3	3	3	3	3
1	1	1	3	3	3	3	3	3	3
3	3	2	3	3	2	3	3	3	3
1	3	9	9	9	9	9	9	9	9
1	1	1	3	2	2	3	2	2	2
3	1	3	9	3	3	2	2	2	3
9	9	9	9	3	9	9	9	9	9
3	1	3	1	3	3	1	3	3	3
1	1	3	2	3	3	3	2	3	3
2	2	2	2	2	3	3	3	3	3
2	2	3	2	2	3	2	2	2	3
9	9	9	9	9	9	9	9	9	3
1	3	9	3	3	1	9	9	9	9
9	9	9	9	9	9	9	9	9	3
9	9	9	9	9	9	9	9	9	3
3	3	1	3	3	3	3	3	3	1
2	2	3	2	2	2	2	2	2	1
1	2	2	2	2	2	2	2	2	2
2	2	2	2	3	2	2	3	3	3
2	2	2	2	2	2	2	2	2	2
9	9	9	9	9	9	9	9	9	9
9	9	9	9	9	9	9	9	9	9
9	9	9	9	9	9	9	3	9	9
9	3	9	9	9	2	9	9	9	9
9	9	9	9	9	9	9	9	9	3
9	9	9	9	9	9	9	9	9	9
2	2	2	2	2	2	2	2	2	1
9	9	9	9	9	9	1	3	3	3
2	2	2	2	2	2	2	2	2	2
3	3	3	2	3	3	2	1	3	3
2	3	3	1	2	2	3	2	2	2
3	1	3	2	2	2	3	3	3	3

1	1	3	1	3	3	3	1	1	3
3	1	2	1	3	3	3	3	3	1
2	1	2	9	3	1	2	2	3	2
3	3	2	3	3	2	2	2	2	2
3	3	3	1	1	9	1	2	1	2
9	9	9	9	9	9	9	9	9	9
2	2	1	2	3	2	3	2	1	2
9	9	9	9	9	9	9	9	9	9
9	9	9	9	9	9	9	9	9	9
9	9	9	9	9	9	9	9	9	9
2	2	2	2	2	2	2	3	2	2
2	2	2	1	3	3	3	2	2	3
2	2	2	2	2	2	2	2	2	2
2	3	3	3	1	3	2	2	3	2
9	9	9	9	9	9	3	9	9	9
1	2	1	1	2	2	3	1	2	1
2	3	2	2	2	2	3	3	3	3
2	2	2	2	3	3	3	3	2	2
3	2	3	3	3	3	2	3	2	3
3	2	2	2	3	3	3	2	3	3
3	1	3	3	3	3	3	3	3	3
3	2	3	3	3	2	2	3	3	2
3	3	3	1	3	3	3	3	3	3
9	9	9	9	9	9	9	9	9	9
1	3	1	9	3	3	3	3	3	2
1	1	2	2	3	3	2	3	3	3
1	3	3	1	3	3	2	3	3	2
3	3	3	3	3	3	2	2	2	3
3	2	9	3	2	2	2	3	3	3
3	3	3	1	3	2	1	3	3	2
1	9	2	9	1	2	1	9	3	9
3	1	3	3	3	3	3	1	2	2
2	2	2	2	2	2	2	2	2	2
3	3	3	3	3	3	2	2	3	3
2	2	3	9	1	9	3	3	3	3
9	9	9	9	3	3	9	9	9	3
1	1	1	3	3	3	3	3	3	3
1	1	1	3	3	3	3	3	3	3
2	2	2	2	3	3	3	3	3	2
9	2	9	2	9	2	9	9	1	1
2	2	1	3	2	2	3	3	3	3
3	1	3	1	3	3	3	3	3	3
3	2	3	1	1	2	2	2	2	1
3	2	2	2	2	2	2	2	1	1
1	2	3	2	3	3	2	3	2	3
3	2	2	3	3	2	2	3	3	3
2	2	2	3	3	3	3	3	2	3

1	1	1	3	3	3	3	3	3	3
3	1	3	1	3	3	3	3	3	2
9	9	9	9	9	9	9	9	9	9
3	1	2	2	3	2	2	3	3	3
9	9	9	9	3	3	3	3	3	3
9	2	9	3	1	3	2	1	9	3
2	3	2	2	1	2	2	2	2	2
2	3	1	2	3	3	2	2	3	2
2	2	2	2	2	2	2	2	2	2
2	2	2	2	3	3	3	3	3	3
9	9	9	9	9	9	9	9	9	9
1	3	3	3	3	3	1	3	3	3
2	9	1	3	2	3	2	2	2	2
1	2	2	2	2	2	3	3	3	2
1	9	3	3	2	3	2	2	9	2
3	2	2	3	3	3	2	3	3	3
1	9	9	9	3	3	3	3	3	3
1	2	1	2	1	1	3	2	9	1
1	1	9	9	3	3	9	3	9	3
2	3	3	2	3	3	2	1	9	2
2	2	2	2	2	2	2	2	2	2
2	2	2	2	2	2	2	2	2	2
2	1	1	3	3	3	3	2	2	3
9	1	3	1	9	9	9	9	9	9
1	1	9	2	2	2	1	3	2	3
9	9	9	9	9	9	9	9	9	9
2	3	3	1	3	3	2	3	3	3
2	2	2	1	1	1	1	1	1	1
3	2	2	3	3	2	3	3	3	3
3	2	1	2	3	1	2	3	2	3
9	1	9	9	9	9	9	3	9	9
3	3	3	1	9	9	9	3	3	3
2	1	2	2	3	2	2	3	9	3
9	3	1	1	3	3	1	1	3	3
2	2	2	2	2	2	2	2	2	2
2	2	2	2	2	2	2	3	3	3
1	9	2	3	3	3	3	3	3	3
9	2	9	2	9	3	3	3	3	3
9	9	9	9	9	9	9	9	9	9
3	3	3	2	3	3	2	2	3	2
2	2	2	2	2	2	2	2	2	2
2	2	2	2	2	2	2	2	2	2
1	1	1	3	3	3	3	3	3	3
9	9	9	9	2	3	3	3	3	3
3	3	3	3	3	3	3	1	3	3
3	3	1	3	9	3	1	1	1	1
3	2	3	1	3	3	3	3	3	3

2	1	2	2	3	3	3	3	3	3
3	2	2	2	3	3	2	3	3	2
3	1	1	3	3	3	3	3	3	1
3	1	2	2	3	3	3	3	3	3
1	2	2	1	1	2	3	3	3	3
3	1	3	1	1	1	1	1	1	1
3	3	3	2	3	3	1	1	3	3
3	3	3	1	1	1	3	3	3	3
1	2	2	3	3	3	3	2	3	3
3	3	3	3	3	3	3	3	3	3
3	1	3	3	3	1	3	3	2	3
3	3	3	2	3	3	3	2	3	3
2	1	3	2	2	2	2	2	3	2
3	2	3	1	3	3	3	3	3	3
3	1	3	3	3	3	3	3	3	3
2	2	2	2	2	2	2	2	2	2
1	2	2	3	3	3	3	3	3	3
3	3	3	1	3	3	1	3	1	1
1	3	2	1	3	2	3	9	9	3
2	1	3	1	3	3	3	3	3	3
3	2	2	1	1	2	2	1	1	2
1	2	1	2	2	3	2	3	3	3
2	3	3	2	2	2	3	3	3	3
9	9	9	9	9	9	9	9	9	9
2	2	2	3	1	3	2	3	1	2

BreastSize	Nippledevi	Raisopposi	Undressto	HeardCerv	CervicalCA	PapSmear	HPVandVa	Papdetect	PapforMar
2	2	3	1	2	2	2	2	2	2
2	3	3	1	2	2	2	2	2	3
2	2	2	1	2	2	2	2	2	2
2	2	2	1	2	2	2	2	3	2
2	2	1	2	2	1	1	2	1	2
3	3	2	1	2	2	2	2	2	2
2	2	2	1	2	2	2	2	2	2
2	2	2	1	1	2	2	1	1	1
3	3	3	1	2	2	1	1	1	1
1	3	2	2	3	2	1	3	2	3
2	2	2	1	2	2	2	1	2	2
2	2	3	2	2	1	2	1	1	2
2	2	2	1	2	1	1	1	1	1
2	2	2	2	2	2	2	2	1	2
2	2	2	1	3	3	2	2	2	2
2	2	2	1	3	1	3	3	2	2
9	9	9	1	9	9	9	9	9	9
1	1	1	2	3	1	3	2	2	2
9	9	9	1	9	9	9	9	9	9
2	3	3	2	1	2	2	3	9	9
9	1	1	4	1	1	1	2	1	3
1	1	1	1	1	1	1	1	1	3
1	1	1	5	1	1	1	1	1	3
1	1	1	5	1	1	1	1	1	2
2	2	2	2	3	2	2	2	2	9
2	2	2	1	9	9	9	9	2	2
9	9	9	1	1	1	1	1	1	3
2	1	1	7	1	1	1	1	2	2
2	2	1	1	2	2	2	3	1	2
1	1	3	4	1	2	2	2	9	9
2	2	2	1	3	1	1	1	1	2
2	1	1	2	1	1	2	2	1	3
9	9	9	3	2	2	2	1	1	2
1	1	2	2	1	1	3	2	1	2
2	3	1	9	2	2	1	1	1	2
2	2	2	1	1	1	2	1	3	2
2	2	2	5	2	1	1	2	2	1
1	2	1	1	2	2	2	2	3	2
2	1	1	4	1	1	1	3	1	3
2	9	2	9	9	9	9	9	1	2
9	9	9	9	9	9	9	9	1	2
2	9	9	2	1	2	2	1	9	9
9	9	9	9	2	2	2	2	9	9
2	2	2	1	1	2	2	2	2	2
2	2	2	2	2	2	3	2	2	2
9	9	9	9	9	9	9	9	9	9

2	2	2	2	1	1	1	1	1	1
2	2	2	2	2	1	1	1	1	1
2	2	2	1	1	2	1	2	2	2
2	1	2	3	1	1	2	2	2	2
3	3	3	5	3	2	1	1	2	3
3	3	3	5	3	2	1	1	2	3
2	3	2	1	2	2	2	3	2	3
2	2	2	1	2	2	2	2	2	2
1	1	1	2	2	1	1	1	1	1
2	1	3	1	2	2	1	2	3	2
9	1	9	1	9	9	9	9	1	9
2	1	1	1	1	2	1	1	1	1
2	1	1	1	2	2	2	2	1	2
1	2	2	1	1	2	1	1	1	1
2	2	2	1	2	1	1	2	1	2
2	2	2	1	2	1	1	1	1	1
2	3	3	9	2	2	2	2	2	2
1	3	2	1	2	2	2	2	1	3
2	2	2	1	2	2	2	2	2	2
9	9	9	9	9	9	9	9	2	2
2	2	2	1	1	2	2	2	3	2
2	2	2	1	3	2	2	1	2	2
2	2	2	9	2	1	1	1	1	1
2	2	2	1	1	1	2	1	1	1
2	2	2	9	1	2	2	1	1	1
2	3	2	1	2	2	2	2	1	2
3	1	3	1	3	3	1	1	1	3
1	2	3	9	2	2	1	2	1	2
1	1	2	1	2	2	2	2	1	3
1	1	3	5	3	1	3	3	3	3
9	9	9	9	9	9	9	9	9	9
3	1	3	1	2	2	2	2	2	2
1	1	9	2	2	2	2	2	1	3
2	2	2	1	2	2	2	2	2	2
3	3	3	1	3	2	2	2	2	2
2	2	2	1	2	2	2	2	2	2
1	3	3	1	1	1	3	1	1	2
2	2	1	1	1	2	2	2	1	2
2	3	3	7	2	2	2	2	3	3
2	2	1	1	1	2	2	2	1	2
1	1	2	1	2	3	1	2	1	2
9	9	9	9	9	9	9	9	9	9
9	9	9	1	2	2	3	2	1	2
2	2	1	1	1	2	2	2	1	1
1	2	2	1	3	2	3	3	2	2
3	2	3	9	1	1	1	1	3	2
1	3	3	1	3	2	2	2	1	2

2	3	1	3	1	2	3	1	1	2
3	3	3	1	3	3	3	3	3	3
2	2	2	1	2	3	2	2	2	2
2	1	1	1	1	2	2	2	1	2
2	2	2	1	1	2	1	1	1	1
3	3	2	1	2	2	2	2	3	2
2	2	2	2	1	2	1	2	1	2
1	3	3	9	9	9	9	9	9	9
2	2	2	1	2	2	2	2	2	2
1	1	2	4	1	3	1	2	1	2
9	9	2	9	1	1	9	2	1	2
2	3	3	1	1	2	1	1	1	2
2	1	1	1	1	1	2	1	1	1
1	2	2	2	3	3	2	2	2	2
1	2	2	1	1	2	2	1	2	2
1	2	2	1	1	9	2	2	2	2
2	2	1	1	2	1	1	1	1	1
1	1	3	1	2	2	2	2	1	2
1	3	3	3	2	9	2	2	2	9
2	1	1	1	2	2	2	2	3	1
1	1	3	9	1	2	2	1	1	3
2	1	3	1	2	2	1	3	1	2
2	1	1	1	2	1	1	2	2	2
2	2	2	1	1	1	1	1	2	2
2	2	2	2	2	3	3	2	1	2
2	2	2	1	2	2	2	2	1	2
1	1	3	2	1	1	2	2	1	3
2	1	2	2	1	1	1	2	1	2
2	2	1	1	2	2	2	2	1	2
2	1	3	1	1	1	3	2	2	2
2	1	1	1	1	2	1	2	1	1
1	1	2	1	2	2	2	2	1	2
2	2	1	1	3	2	2	3	3	9
2	1	1	7	1	1	2	2	1	2
1	1	2	1	2	2	2	2	1	2
2	1	3	9	1	1	2	2	1	1
3	3	3	1	2	2	2	9	2	3
2	2	2	1	2	1	1	1	1	2
9	9	9	9	9	9	9	9	9	9
3	3	3	1	2	2	2	2	3	3
2	1	1	1	2	1	1	1	1	2
9	9	9	9	9	9	9	9	9	9
2	2	2	1	2	1	1	1	1	2
2	1	2	2	2	1	1	1	1	2
3	3	3	3	2	2	2	2	9	9
2	1	1	3	1	1	1	3	1	3
1	1	1	1	2	2	2	2	1	2

2	3	1	1	2	2	2	2	2	2
3	3	3	9	2	2	2	2	1	1
3	3	3	1	1	1	1	1	3	3
2	2	2	1	3	2	2	2	1	2
3	2	1	1	2	2	1	2	1	2
2	3	2	1	2	3	2	2	2	3
9	9	2	9	2	1	2	1	2	9
2	9	3	9	1	1	2	2	3	2
9	9	9	1	3	2	2	2	2	9
2	2	2	9	2	2	2	2	2	2
1	3	3	2	1	1	2	3	1	2
3	3	3	1	2	2	2	1	1	2
2	2	2	9	1	2	2	2	2	2
2	2	2	1	2	2	2	1	2	2
2	2	3	1	2	1	1	1	1	2
3	3	3	1	1	1	1	1	1	2
1	1	1	1	2	1	1	1	1	2
1	2	2	1	2	1	1	1	1	2
9	9	9	1	3	2	2	2	2	9
1	3	2	1	2	2	2	2	3	9
2	1	2	1	1	2	3	3	2	3
1	2	2	1	3	2	2	3	2	2
3	3	2	1	3	3	2	2	2	2
2	2	2	1	3	2	2	2	1	2
3	2	2	5	1	1	2	2	2	2
1	3	1	1	2	2	2	2	3	3
1	2	2	1	2	2	2	2	2	3
2	2	3	1	3	1	1	1	2	2
9	2	2	9	2	2	2	2	2	2
2	1	1	1	1	1	2	2	1	2
2	1	2	7	1	1	1	2	1	2
2	3	3	7	2	2	2	2	1	1
2	1	1	2	1	1	1	1	1	1
3	2	3	2	1	3	1	2	3	3
3	3	3	1	1	1	1	1	2	2
1	1	1	7	1	1	1	1	3	3
2	2	2	1	1	1	1	1	1	1
1	1	1	2	1	1	1	1	1	3
1	1	3	2	2	2	2	2	1	3
2	2	2	9	1	2	1	1	1	3
1	1	1	9	9	2	2	1	9	9
2	2	2	4	1	1	1	2	2	2
1	2	2	1	1	2	2	2	2	2
9	9	9	9	9	9	9	9	9	9
9	9	9	9	9	9	9	9	9	9
1	1	2	1	2	1	1	2	1	2
2	1	1	2	1	2	1	1	1	2

9	9	9	2	1	1	3	2	1	9
3	3	3	1	2	2	2	2	2	2
1	1	1	5	1	2	2	2	2	9
9	9	9	3	1	2	2	2	1	9
9	9	9	1	2	2	2	2	9	9
9	9	9	9	9	9	9	9	9	9
1	2	2	1	1	1	2	2	2	2
9	9	9	9	9	9	9	9	9	9
3	2	3	1	1	3	2	2	1	2
9	9	9	4	2	3	1	1	9	9
9	9	9	1	2	2	2	2	9	9
9	9	9	4	2	2	3	3	9	9
2	2	1	2	1	2	3	3	1	1
1	2	3	9	9	9	9	9	1	2
9	9	9	9	2	1	1	1	3	9
2	2	3	1	2	1	2	1	3	2
3	3	3	9	1	1	1	1	3	3
2	2	2	2	1	2	1	1	3	3
1	1	1	1	3	3	3	3	2	2
2	3	2	1	2	2	2	2	2	2
9	9	9	1	2	1	2	2	1	1
9	9	9	9	9	9	9	9	9	9
1	1	1	1	3	2	2	2	3	3
1	1	2	1	2	2	2	2	1	3
2	2	2	1	3	2	2	2	2	2
1	1	1	1	2	2	2	9	1	1
2	1	1	5	1	2	2	2	2	2
1	1	1	1	1	2	2	3	3	3
3	3	3	1	1	1	1	1	1	3
3	3	3	2	1	1	1	1	1	3
2	2	2	1	3	2	2	2	2	2
1	2	2	1	2	2	1	2	1	1
9	9	9	9	9	9	9	9	9	9
2	2	2	1	2	2	2	2	3	2
1	1	3	2	2	2	2	1	2	3
2	2	2	2	1	2	2	1	1	1
1	3	3	9	2	1	2	1	2	9
2	2	2	1	1	2	2	3	2	2
3	1	9	1	3	2	1	2	2	9
1	1	3	1	2	2	2	2	1	1
2	2	2	1	1	2	1	3	1	2
2	1	3	1	1	1	1	1	3	1
3	2	2	3	1	2	1	3	1	1
2	2	2	1	1	1	2	2	2	2
2	1	1	1	3	3	3	3	2	2
1	2	3	2	1	1	1	2	3	2
9	9	9	9	9	9	9	9	9	9

2	2	2	3	2	2	2	1	1	1
1	1	1	1	1	2	2	2	1	3
1	1	1	1	2	2	2	2	2	1
2	3	2	3	2	9	9	9	9	9
2	2	2	2	1	2	2	2	2	2
2	2	2	1	2	2	3	3	2	3
3	3	3	5	1	1	1	2	1	3
2	2	2	1	3	2	1	3	3	2
1	1	1	1	2	1	2	2	1	1
1	1	1	1	1	2	2	2	3	3
1	1	1	9	2	2	2	3	2	2
9	9	9	9	1	1	2	3	3	9
9	9	9	1	2	2	2	2	2	9
2	2	2	1	1	1	1	1	1	9
9	2	2	9	2	2	2	2	2	9
2	9	2	9	2	2	2	2	9	9
9	9	9	9	9	9	9	9	9	9
9	9	9	9	9	9	9	9	9	9
9	2	2	1	2	2	2	2	2	9
9	9	9	9	9	9	9	9	9	9
9	9	9	9	9	9	9	9	9	9
2	2	2	1	1	2	1	1	1	1
2	2	2	1	2	1	2	1	1	2
2	1	1	1	2	1	1	1	1	1
1	2	2	1	2	2	1	1	1	1
9	9	9	9	9	9	9	9	9	9
2	2	2	1	1	2	1	2	1	2
9	9	9	9	9	9	9	9	9	9
9	9	9	9	9	9	9	9	9	9
2	2	2	1	1	1	2	1	1	2
9	9	9	1	2	2	2	2	9	9
9	9	9	9	9	9	9	9	9	9
9	9	9	9	9	9	9	9	9	9
9	9	9	9	9	9	9	9	9	9
9	3	1	1	2	2	2	2	9	3
9	3	1	1	2	2	2	2	9	1
9	9	9	9	9	9	9	9	9	9
9	9	9	1	9	9	9	9	9	9
9	9	9	9	9	9	9	9	9	9
9	9	9	1	9	9	9	9	3	9
9	9	2	9	9	9	9	9	3	9
9	9	9	1	2	2	2	2	9	9
3	3	3	1	1	1	1	1	3	3
3	2	2	9	2	2	2	1	1	3
1	3	1	1	1	1	1	1	3	2
1	2	1	2	1	1	9	9	1	9
9	9	9	1	1	2	2	1	1	9

9	9	9	1	3	2	2	2	1	9
9	9	9	1	1	2	2	2	9	9
2	3	3	4	1	1	2	2	2	2
1	3	2	1	1	2	1	1	3	1
3	3	2	9	9	9	9	9	2	2
2	2	2	1	1	2	3	9	2	2
2	1	2	1	1	1	2	1	2	1
1	1	1	1	2	2	2	2	2	1
2	2	2	9	3	3	3	3	2	2
3	3	3	1	1	1	1	1	3	3
9	9	9	9	9	9	9	9	9	9
9	9	9	9	9	9	9	9	9	9
9	9	9	2	2	2	2	2	9	9
9	9	9	9	1	1	1	1	9	1
1	1	1	1	1	2	1	1	2	9
3	2	2	9	1	1	1	2	1	2
9	9	9	9	9	9	9	9	9	9
2	1	2	1	1	2	2	2	2	2
1	1	1	9	1	1	1	1	1	1
2	2	2	1	2	2	2	2	1	3
2	3	3	4	1	2	2	2	3	1
2	2	2	1	1	2	2	2	2	2
3	3	3	1	2	2	2	1	2	3
2	2	2	1	1	2	1	1	2	2
2	2	2	1	2	1	2	2	2	2
2	2	2	1	1	1	1	1	2	2
2	2	2	1	2	2	2	2	2	2
2	3	1	2	1	1	1	1	1	1
2	2	2	9	2	3	3	3	2	2
1	2	1	1	1	2	2	1	1	2
2	2	2	1	3	3	3	3	2	2
2	2	2	1	3	3	3	3	2	2
1	2	3	4	2	2	2	2	2	1
9	2	2	9	2	2	2	3	3	3
3	3	3	9	1	1	1	2	1	3
2	2	2	9	2	1	3	3	1	2
9	9	9	9	9	9	9	9	9	9
2	2	2	1	2	2	2	2	2	2
3	3	3	1	2	2	2	2	3	3
3	3	3	9	2	2	2	2	3	3
9	9	9	9	3	3	3	3	2	9
9	9	9	9	9	9	9	9	9	9
9	9	9	9	2	2	2	9	9	9
1	1	1	7	1	1	2	3	2	2
3	2	2	2	2	2	1	3	2	2
2	2	2	9	2	2	2	2	2	2
3	3	3	4	1	1	1	1	2	2

9	9	9	9	1	2	3	2	9	9
9	9	9	1	2	2	1	1	2	9
9	9	9	1	2	2	9	2	9	9
9	9	9	3	2	3	1	1	9	9
9	9	9	1	1	3	3	3	9	9
1	1	1	1	1	1	1	1	3	3
9	9	9	1	3	2	3	3	9	9
9	9	9	1	2	2	2	1	2	9
1	1	2	1	2	2	2	2	2	9
9	9	9	1	2	2	2	2	2	9
9	9	9	9	2	3	2	2	9	9
9	9	9	9	1	9	2	2	9	9
9	9	9	9	2	2	2	2	2	9
9	9	9	9	3	2	3	1	9	2
9	9	9	2	2	2	2	3	2	9
9	9	9	9	9	9	9	9	9	9
9	9	2	3	2	2	2	2	9	9
2	1	2	5	2	2	1	1	2	9
1	3	3	1	1	1	1	1	1	2
2	2	2	1	2	2	2	2	2	2
1	1	1	2	1	1	1	2	1	3
2	2	3	5	9	9	9	9	1	3
2	2	2	1	2	2	2	2	2	2
2	3	2	7	3	3	1	3	2	3
1	2	3	7	2	3	3	3	2	3
2	2	2	1	9	9	9	9	9	9
2	2	2	1	3	2	2	2	2	2
2	2	2	1	2	2	2	2	2	2
3	2	2	1	1	3	1	3	1	2
1	1	1	1	1	1	1	1	1	1
2	2	1	1	1	1	1	1	1	2
3	3	3	1	2	2	2	2	2	2
3	3	3	2	2	1	1	2	2	3
2	1	1	1	2	2	2	2	1	2
9	9	9	9	9	9	9	9	9	9
2	2	2	1	1	2	2	2	2	2
9	9	9	9	9	9	9	9	9	9
2	2	2	3	2	2	1	2	1	2
3	2	3	2	2	2	2	2	9	9
2	2	2	9	1	1	1	1	2	2
2	1	2	1	2	2	2	2	1	2
1	2	3	2	2	2	2	2	3	3
1	1	3	1	2	2	2	1	1	9
1	1	2	1	2	3	2	2	2	2
2	2	2	1	2	2	2	2	3	3
2	2	2	1	1	1	2	2	1	1
2	2	2	1	1	1	1	1	2	2

1	1	3	1	1	1	1	1	1	3
2	2	2	1	2	9	2	2	2	9
1	2	2	1	1	1	1	1	1	1
1	2	3	1	2	2	2	3	2	1
2	2	2	1	2	2	2	2	2	2
2	3	2	1	2	2	2	2	2	2
2	2	2	9	1	1	2	2	1	2
2	2	2	1	1	1	3	2	1	3
9	9	9	9	9	9	9	9	9	9
1	2	2	1	2	2	2	2	2	2
1	1	2	2	1	2	2	2	2	2
9	9	2	5	2	2	2	2	1	9
9	9	9	1	9	9	9	9	1	9
9	1	9	9	2	2	2	1	1	1
9	9	2	1	2	1	2	2	1	9
2	2	2	1	2	2	2	2	2	2
9	9	9	9	9	9	9	9	9	9
9	9	9	1	9	3	2	1	2	9
9	9	9	1	2	3	3	3	9	9
3	2	3	1	3	2	2	2	2	3
2	2	2	9	3	3	3	3	2	2
3	2	3	1	2	2	2	2	2	2
2	2	2	1	2	2	2	2	2	2
9	9	1	1	2	2	2	2	9	9
9	9	9	1	2	2	2	2	2	9
2	2	2	3	2	2	2	2	1	1
1	1	1	3	1	1	1	1	1	2
3	1	1	2	1	1	1	1	2	3
2	2	2	1	9	9	9	9	9	9
9	9	9	4	2	3	1	1	3	9
2	2	2	9	2	2	2	2	2	2
3	1	2	1	2	2	2	2	1	2
1	1	1	1	2	2	2	2	2	2
3	3	3	2	1	2	2	2	3	2
1	1	3	1	1	2	2	2	1	1
9	9	9	9	9	9	9	9	1	1
9	9	9	9	9	9	9	9	9	9
9	9	9	9	9	9	9	9	9	9
1	1	3	2	1	1	1	3	3	1
2	1	2	1	1	1	1	2	1	2
1	1	1	1	1	2	1	2	2	2
3	3	3	6	2	2	2	2	2	2
2	2	2	1	2	2	2	2	2	2
1	1	2	1	2	2	2	2	1	2
3	3	3	9	9	9	9	9	3	3
2	2	2	2	1	2	3	3	2	2
3	3	3	6	1	2	3	3	3	3

2	2	2	2	2	2	2	3	3	3
3	1	2	4	1	1	1	1	1	3
9	9	9	2	3	1	2	1	9	9
9	9	9	2	2	2	2	2	1	9
9	9	9	1	2	2	3	3	2	2
2	3	2	1	1	1	1	1	3	3
9	2	9	1	2	2	2	2	2	9
2	2	2	1	2	2	2	2	3	9
9	9	9	9	9	9	9	9	9	9
9	9	9	9	9	9	9	9	9	9
2	2	2	1	2	2	2	2	9	9
9	9	9	1	2	2	2	2	9	9
9	9	9	1	2	2	2	2	9	9
9	9	9	9	9	9	9	9	9	9
9	9	9	1	2	2	2	2	9	9
9	9	9	1	2	2	2	2	2	9
3	3	3	1	3	3	3	3	3	3
9	9	9	1	1	1	1	1	1	9
9	9	9	1	1	1	1	1	2	9
2	9	2	1	1	1	1	1	2	9
9	9	9	9	9	9	9	9	9	9
9	9	9	1	2	2	2	2	2	9
2	2	2	9	2	2	2	2	2	2
9	9	9	9	9	9	9	9	9	9
9	9	9	9	9	9	9	9	9	9
9	9	9	9	1	1	1	1	2	9
9	9	9	9	9	9	9	9	9	9
9	9	9	1	1	1	1	1	1	2
9	9	9	5	1	1	1	1	1	3
9	9	9	9	9	9	9	9	9	9
9	9	2	1	9	9	9	9	9	9
2	2	2	1	1	1	1	1	2	2
9	1	1	1	9	9	9	9	9	9
9	9	9	9	9	9	9	9	9	9
2	2	1	9	9	9	9	9	9	9
9	9	9	9	9	9	9	9	9	9
2	1	9	1	2	2	2	2	9	9
9	9	9	1	9	9	9	9	9	9
1	2	2	1	2	2	2	2	2	3
1	2	1	4	1	2	3	2	3	1
9	9	9	1	2	2	2	2	9	9
9	9	9	9	9	9	9	9	9	9
2	1	2	1	2	2	2	2	2	3
3	1	2	1	2	2	2	2	1	2
3	3	3	1	1	2	2	2	3	3
3	2	1	9	1	2	1	1	1	2
9	9	9	2	9	9	9	9	2	3

9	9	9	9	1	1	2	2	1	9
3	3	1	2	1	1	3	2	2	3
2	2	2	1	2	2	2	2	2	2
1	1	1	4	1	1	1	1	1	1
2	2	2	1	2	2	2	2	2	2
2	2	2	5	3	3	3	1	2	2
3	3	3	2	2	1	1	1	9	9
2	2	2	1	3	3	2	2	3	3
2	2	2	1	3	3	3	3	2	2
1	1	2	3	1	2	2	2	1	2
3	2	2	4	2	2	9	2	2	2
9	9	9	1	3	3	2	2	9	9
9	9	9	1	2	2	2	2	9	9
2	2	2	1	1	2	1	1	9	9
9	9	9	2	1	2	2	2	2	9
2	2	2	5	3	3	1	2	2	2
2	1	3	1	2	2	2	2	3	3
1	1	2	3	2	2	2	2	1	3
1	1	1	1	2	2	2	2	1	1
2	2	2	1	1	1	1	3	2	2
2	2	2	1	2	2	2	2	2	2
3	2	3	1	9	9	9	9	9	9
1	1	1	1	2	2	2	1	3	3
9	9	9	2	1	2	2	2	2	9
9	9	9	2	2	3	2	2	9	9
2	2	2	1	1	1	1	1	1	1
9	9	9	9	1	9	2	2	9	9
2	2	1	3	2	2	2	2	2	3
9	9	9	9	9	9	9	9	9	9
9	9	9	1	1	1	1	1	9	9
2	2	2	1	1	1	1	2	2	3
2	1	3	1	1	2	2	2	2	3
9	9	9	9	9	9	9	9	9	9
9	9	9	4	1	1	1	1	1	9
2	2	1	1	1	2	2	1	1	2
9	9	9	9	9	9	9	9	9	9
3	2	1	1	3	3	2	9	2	3
1	1	1	3	1	1	1	1	1	1
2	2	2	1	3	3	3	3	2	2
2	2	2	2	1	1	2	2	9	9
2	2	2	1	2	2	2	2	2	2
2	2	2	9	2	2	2	2	1	2
2	2	2	1	2	2	2	2	2	2
2	2	2	1	2	2	2	2	2	2
2	2	2	1	2	2	2	2	2	3
2	2	2	1	2	2	2	2	3	3
3	2	2	2	3	3	3	3	2	2

9	9	9	1	2	3	3	2	2	9
2	2	2	5	3	1	3	3	2	2
1	3	3	1	2	2	2	2	2	2
9	9	9	5	1	1	2	1	9	9
9	9	9	1	2	2	2	2	3	9
9	9	9	9	9	9	9	9	9	9
9	9	9	9	9	9	9	9	9	9
2	2	2	9	2	2	2	2	2	2
2	2	2	1	1	1	1	1	2	2
9	9	9	1	2	2	2	2	9	9
2	2	2	1	1	1	1	1	2	2
2	3	3	1	1	1	1	2	2	3
1	2	2	4	1	1	1	1	1	2
2	2	2	1	2	2	2	2	2	2
2	2	3	1	2	2	3	1	9	9
9	9	9	1	9	9	9	9	9	9
9	9	9	9	9	9	9	9	9	9
9	9	9	9	9	9	9	9	9	9
9	9	9	1	2	2	2	2	1	9
9	9	9	1	2	2	2	2	2	9
9	9	9	9	9	9	9	9	9	9
9	9	9	9	9	9	9	9	9	9
2	2	2	1	2	2	2	2	2	2
9	9	9	1	2	2	2	2	2	9
9	9	9	9	9	9	9	9	9	9
9	9	9	9	2	2	2	2	3	9
9	9	9	9	9	9	9	9	9	9
9	9	1	1	2	2	2	2	9	9
2	9	2	1	2	2	2	2	2	9
9	9	3	1	2	9	2	2	3	9
9	9	9	9	9	9	9	9	9	9
2	2	2	1	2	2	2	2	2	2
9	9	9	9	9	9	9	9	9	9
9	9	9	1	9	9	9	9	9	9
9	9	9	1	2	2	2	2	9	9
9	9	9	9	9	9	9	9	9	9
9	9	9	1	2	2	2	2	9	9
9	9	9	9	9	9	9	9	9	9
9	9	9	1	9	9	9	9	9	9
9	9	9	1	9	9	9	9	9	9
2	2	2	1	1	1	1	1	2	2
9	9	9	1	1	1	1	1	2	9
9	9	9	1	9	9	9	9	9	9
9	9	9	1	2	2	2	2	9	9
9	9	9	9	9	9	9	9	9	9
9	9	9	1	2	2	2	2	9	9
9	9	9	9	9	9	9	9	9	9

9	9	9	9	9	9	9	9	9	9
9	9	9	9	9	9	9	9	3	3
9	9	9	1	2	2	2	2	2	9
9	9	9	1	9	9	9	9	9	9
9	9	9	9	9	9	9	9	9	9
9	9	9	9	9	9	9	9	9	9
9	9	9	9	9	9	9	9	9	9
9	9	9	1	9	9	9	9	9	9
9	9	9	9	9	9	9	9	9	9
9	9	9	9	9	9	9	9	9	9
9	9	9	9	9	9	9	9	9	9
9	9	9	9	9	9	9	9	9	9
9	9	9	9	9	9	9	9	9	9
9	9	9	9	9	9	9	9	9	9
9	9	9	1	2	2	2	2	9	9
9	9	9	1	2	2	2	2	9	9
9	9	9	1	9	9	9	9	9	9
2	2	2	1	2	2	2	2	2	2
9	9	9	1	9	9	9	9	9	9
2	2	2	1	2	2	2	2	9	9
9	9	9	9	9	9	9	9	9	9
9	9	9	1	2	2	9	2	9	9
9	9	9	1	9	9	9	9	9	9
9	9	9	1	2	2	2	2	9	9
9	9	9	1	2	2	2	2	2	2
9	9	9	1	2	2	2	2	9	9
9	9	9	9	9	9	9	9	9	9
2	2	1	1	2	2	3	2	1	9
9	9	9	9	9	9	9	9	9	9
9	9	9	9	9	9	9	9	9	9
1	1	1	1	1	1	1	1	1	1
3	2	1	1	2	2	2	2	2	2
9	9	9	9	9	9	9	9	9	9
2	1	2	1	2	2	2	2	3	3
9	9	9	9	9	9	9	9	9	9
9	1	9	1	2	2	2	2	2	2
9	9	9	1	1	1	1	1	2	9
9	9	9	1	9	9	9	9	9	9
1	1	1	1	2	2	2	2	1	1
9	9	3	1	2	2	2	2	3	9
9	9	9	9	9	9	9	9	9	9
9	9	9	9	9	9	9	9	9	9
3	3	3	2	2	2	2	2	2	2
9	9	9	1	1	1	1	1	1	2
9	9	1	1	2	2	2	2	9	1
9	9	2	1	9	9	9	9	9	9
9	9	9	1	9	9	9	9	9	9

9	9	2	1	3	3	3	3	2	9
9	9	9	9	9	9	9	9	9	9
9	2	3	1	2	2	2	2	2	9
9	9	9	1	9	9	9	9	9	9
9	9	9	9	9	9	9	9	9	9
9	9	9	9	9	9	9	9	9	9
9	9	9	1	1	1	1	1	2	9
9	9	9	9	2	2	2	2	2	9
9	9	2	1	1	1	1	1	2	9
9	9	9	1	2	2	2	2	9	9
9	9	9	9	2	2	2	2	9	9
9	9	9	9	9	9	9	9	9	9
9	9	9	9	9	9	9	9	9	9
9	9	9	9	9	9	9	9	9	9
2	2	2	1	2	2	2	2	1	2
2	1	1	1	2	2	2	2	3	2
9	9	9	1	2	2	2	2	9	9
2	2	2	1	2	2	2	2	2	2
2	2	2	1	2	2	2	2	2	2
9	9	9	3	1	1	1	1	1	9
9	9	9	3	1	1	2	2	1	9
9	9	9	2	1	1	9	1	1	9
2	2	1	1	2	2	2	2	3	2
3	1	2	1	2	2	2	2	1	2
2	1	2	2	2	2	2	2	2	2
2	1	2	1	1	1	2	2	2	2
3	1	1	2	2	2	2	2	1	3
2	2	2	2	3	3	3	1	2	2
2	2	2	3	2	2	3	1	2	1
1	1	1	1	2	2	2	2	1	2
9	9	9	1	2	2	2	2	1	2
9	9	9	1	1	1	9	3	2	9
9	9	9	4	1	2	1	1	9	9
9	9	9	5	2	2	3	2	2	9
9	9	9	1	1	9	2	1	2	9
9	9	9	9	9	9	9	9	9	9
9	9	9	9	9	9	9	9	9	9
9	2	9	1	1	1	1	1	9	2
9	9	9	9	9	9	9	9	9	9
2	9	2	9	2	2	2	2	2	9
9	9	9	1	9	9	9	9	9	9
2	2	2	1	1	1	1	1	2	2
9	9	9	1	9	9	9	9	9	9
9	9	9	1	9	9	9	9	9	9
9	9	9	9	9	9	9	9	9	9
2	2	2	1	2	2	2	2	2	2
2	3	3	1	3	3	2	2	1	2

1	2	2	2	1	3	3	3	1	1
1	2	2	1	1	1	2	2	2	2
1	1	1	2	1	1	3	3	2	1
3	2	1	1	9	9	9	9	3	3
3	3	3	4	3	2	2	2	3	2
2	2	1	1	3	1	2	2	1	2
1	9	1	1	2	2	2	2	2	2
3	3	3	1	2	2	2	2	3	3
2	2	2	1	2	2	2	2	9	9
2	2	2	1	1	1	1	1	2	2
9	2	9	1	2	2	2	2	2	9
9	9	1	1	2	2	2	2	9	9
2	2	2	9	1	2	2	1	2	2
9	1	2	1	1	2	2	1	1	2
1	2	3	1	2	2	3	3	3	2
2	2	2	1	2	2	2	2	2	2
2	1	2	9	2	2	2	2	2	1
2	2	2	9	3	3	3	3	2	3
9	9	9	4	9	9	9	9	9	9
2	3	2	2	1	1	2	2	2	2
2	2	2	1	1	1	2	2	3	1
3	2	3	2	2	1	2	2	1	1
2	3	3	5	1	3	2	2	2	1
1	1	2	1	1	3	1	2	1	2
2	2	2	1	2	2	2	2	1	3
1	1	1	2	2	2	2	2	2	2
2	1	1	2	1	3	2	2	2	3
3	2	2	1	2	2	2	2	2	2
2	2	3	1	2	1	2	2	2	2
2	1	1	2	2	2	2	2	1	1
3	3	3	1	3	2	2	2	3	3
2	2	2	3	2	3	3	3	9	9
3	3	3	1	2	2	2	2	3	3
3	3	2	1	3	3	2	2	2	2
9	9	9	1	3	3	3	3	9	3
3	3	2	1	3	3	3	3	3	3
1	1	2	1	2	2	2	2	2	2
2	2	2	3	1	1	1	1	1	1
3	3	3	1	2	2	1	2	2	2
9	9	9	9	9	9	9	9	9	9
2	2	2	3	1	2	2	2	2	2
9	9	9	5	3	1	2	1	9	9
2	3	1	1	2	1	2	3	1	2
2	2	2	1	9	2	2	2	2	2
3	2	3	1	2	3	3	3	2	3
2	3	2	1	3	2	2	1	9	9
3	3	1	1	2	3	3	2	3	3

9	9	9	2	9	9	9	9	9	9
3	3	3	3	2	2	2	2	3	3
3	1	3	1	1	2	2	1	1	3
3	3	3	3	2	2	2	2	2	2
1	2	2	2	3	1	3	1	1	2
9	9	9	1	9	9	9	9	9	9
2	2	2	4	1	2	2	2	2	2
3	1	2	3	3	3	3	3	1	2
1	2	1	1	2	2	2	1	1	3
2	2	2	1	2	3	3	3	1	2
3	2	3	9	2	3	3	3	1	2
2	2	2	3	1	2	2	2	2	2
9	9	9	1	9	9	9	9	9	9
2	2	3	1	2	2	2	2	3	2
2	3	3	2	1	1	3	1	1	2
2	2	2	1	2	2	2	2	2	2
1	1	1	1	2	2	2	2	2	2
2	2	2	1	1	3	2	2	2	2
2	2	2	1	9	9	9	9	2	2
9	9	9	3	1	3	3	1	2	9
2	1	1	2	9	9	9	9	9	9
2	1	2	9	9	9	9	9	1	2
2	3	3	1	2	2	2	2	2	2
1	3	2	1	2	2	2	3	2	3
3	3	3	9	9	9	9	9	9	9
2	1	2	1	2	3	3	2	1	2
9	9	9	1	2	2	2	2	2	9
2	2	2	9	2	2	3	2	2	2
2	2	2	1	3	3	3	3	2	2
2	2	2	7	3	3	3	3	2	2
1	3	1	2	2	3	1	2	3	2
9	9	9	1	9	9	9	9	9	9
9	9	9	9	9	9	9	9	9	9
1	1	2	1	2	1	1	2	1	3
3	3	2	3	1	1	2	2	1	3
9	9	9	9	9	9	9	9	9	9
3	3	3	1	9	9	9	9	9	9
2	2	2	1	2	2	2	2	2	3
2	1	1	5	1	1	1	2	1	3
1	1	2	3	2	2	2	2	1	2
9	9	9	2	2	2	2	2	2	2
2	2	2	1	2	2	2	2	2	2
2	2	2	1	2	2	2	2	2	2
2	2	2	1	3	3	3	3	2	2
2	2	2	1	2	2	2	2	2	2
2	3	3	1	3	2	2	2	2	2
2	2	2	1	2	2	1	2	2	2

3	3	3	5	1	2	1	2	3	3
2	2	2	1	2	2	2	2	2	2
2	2	2	1	2	2	1	2	2	2
2	2	2	1	1	1	1	1	1	1
2	2	2	4	3	2	2	2	2	2
2	2	2	1	2	2	2	2	2	2
2	2	2	1	2	2	2	2	2	2
2	2	2	9	2	2	2	2	2	2
2	2	2	1	2	2	2	2	2	2
9	9	9	9	9	9	9	9	9	9
1	2	2	1	1	1	2	1	1	1
3	3	3	1	2	2	3	2	2	2
1	2	2	1	2	2	2	2	1	2
1	1	2	1	1	3	1	2	3	2
2	2	2	1	2	2	2	2	3	3
2	1	2	1	1	1	2	1	9	9
9	9	9	9	9	9	9	9	9	9
9	9	2	3	2	3	3	3	9	2
9	9	9	9	9	9	9	9	9	9
2	2	2	9	9	9	9	9	2	2
2	1	1	1	2	2	3	2	1	3
9	9	9	9	9	9	9	9	9	9
9	9	9	9	9	9	9	9	9	9
2	2	2	9	3	3	3	3	2	2
1	1	1	2	1	2	2	1	2	1
2	2	2	1	3	3	3	3	2	2
1	2	3	9	1	2	1	2	1	1
2	3	2	1	1	3	3	2	3	2
1	2	3	1	2	2	2	2	2	1
3	2	3	2	2	1	3	1	2	2
9	9	9	9	9	9	9	9	9	9
1	3	1	3	9	9	9	9	1	2
2	1	2	2	1	2	2	1	2	3
2	2	1	2	1	9	1	1	2	2
2	2	2	1	1	2	2	2	2	2
2	2	2	1	3	3	3	3	2	2
9	9	9	1	9	9	9	9	9	9
2	2	2	9	2	2	2	2	2	2
2	2	2	1	3	3	3	3	2	2
3	3	3	1	1	2	3	2	3	1
1	1	1	1	1	2	2	1	1	2
1	1	1	1	1	2	2	1	2	1
2	2	2	1	2	2	2	2	1	2
2	2	2	1	1	3	3	3	1	2
2	2	3	1	2	2	2	2	2	3
2	2	1	1	1	2	2	2	1	2
3	2	3	1	1	2	2	2	3	3

1	3	3	9	9	9	9	9	2	9
2	2	2	1	2	3	3	3	2	2
9	3	3	1	2	2	1	2	2	2
2	2	2	1	2	3	3	3	2	2
2	3	2	1	1	3	3	3	3	2
2	2	2	1	9	9	9	9	9	9
9	9	9	1	9	9	9	2	9	9
3	3	3	2	2	2	2	2	2	2
9	9	9	9	9	9	9	9	9	9
2	3	1	9	9	9	9	9	9	9
2	2	2	1	2	2	2	2	2	2
1	1	2	9	3	1	2	2	1	2
1	1	2	2	1	1	1	1	2	1
9	9	9	1	3	3	1	1	9	9
9	9	9	1	2	2	2	2	9	9
9	9	9	1	2	2	2	2	1	3
1	2	1	3	1	1	2	3	1	2
1	1	1	4	9	9	9	9	9	9
1	2	2	1	1	2	3	3	1	3
1	1	1	3	1	1	1	3	2	2
9	1	2	3	1	2	3	9	2	1
1	3	1	1	2	2	2	2	9	9
1	2	2	1	2	2	2	2	2	2
2	2	2	1	3	2	2	2	2	2
1	1	1	3	2	2	2	2	2	2
2	2	2	1	3	3	3	3	2	2
2	2	2	1	3	2	2	2	2	2
2	2	2	1	2	2	2	2	2	2
9	1	2	1	3	3	3	3	2	3
3	3	3	1	2	2	2	2	3	3
1	2	1	2	2	2	1	3	2	1
1	2	2	3	1	1	1	1	1	2
3	3	3	1	2	2	2	2	2	2
2	2	2	1	2	2	3	3	2	2
2	2	2	1	1	2	9	2	2	2
1	2	1	1	2	3	1	2	2	2
1	1	1	3	1	1	2	1	1	1
2	2	2	1	2	2	2	2	2	2
3	1	1	1	1	1	2	1	2	9
2	2	2	3	2	2	2	2	2	3
1	1	9	1	1	1	1	1	1	1
2	2	2	2	1	2	2	2	2	2
2	2	2	9	2	2	2	2	2	2
2	1	1	3	1	2	2	2	2	2
2	2	2	9	1	2	2	2	2	2
2	9	2	4	2	2	2	1	1	2
2	2	2	9	2	2	2	2	2	2

2	3	2	1	3	1	2	3	2	3
2	1	3	5	1	2	2	1	1	2
2	3	2	1	1	1	2	2	2	3
2	2	2	1	1	1	1	1	2	2
1	2	2	4	2	3	3	1	2	9
9	9	9	2	2	1	2	2	9	9
2	2	2	1	3	3	3	3	3	2
9	9	9	1	1	1	1	1	1	9
9	9	9	1	1	1	1	1	1	9
3	3	3	4	9	9	9	9	9	9
9	9	9	2	2	2	2	2	9	9
9	9	9	1	2	1	9	3	9	9
9	9	9	9	2	1	2	1	2	9
9	9	9	1	2	3	2	1	9	9
1	2	2	1	2	2	2	2	2	2
9	9	9	1	2	2	9	9	2	9
9	9	9	9	9	9	9	9	9	9
9	9	9	1	2	2	2	2	9	9
9	9	9	1	2	2	2	2	9	9
9	9	9	3	9	9	9	9	9	9
9	9	9	1	9	9	9	9	9	9
2	2	2	1	2	3	3	3	2	2
2	3	3	1	2	2	2	2	2	2
1	2	3	1	2	2	2	2	2	2
2	2	1	2	2	2	2	1	1	2
1	2	2	1	1	2	2	9	9	9
2	2	2	1	2	2	2	2	1	3
9	9	9	1	2	2	2	2	1	9
2	2	2	1	2	2	2	2	3	3
1	3	3	2	1	2	1	2	1	1
3	3	3	2	1	2	2	2	3	3
9	9	9	1	2	2	2	2	3	9
2	2	2	7	2	2	1	1	2	2
9	9	9	1	2	2	2	2	2	9
9	9	9	9	1	1	1	1	1	9
1	3	2	9	9	9	9	9	3	2
1	1	1	9	1	1	1	1	1	1
9	9	9	9	9	9	9	9	9	9
9	9	9	9	2	2	2	2	9	9
2	2	2	9	3	3	3	3	2	2
2	2	2	9	3	3	3	3	9	9
2	2	2	9	1	1	1	3	2	2
3	3	3	1	2	2	2	2	3	3
9	2	9	1	2	2	2	2	9	9
2	9	1	3	1	1	1	1	2	2
2	2	2	3	1	1	1	1	2	2
1	9	1	9	2	2	2	2	9	1

9	9	9	1	9	9	9	9	9	9
9	3	1	1	2	2	2	2	2	2
1	9	9	1	9	9	9	9	9	9
2	2	2	3	1	1	1	1	2	2
3	1	9	1	2	2	2	2	9	3
1	9	2	1	2	2	2	2	9	1
9	9	9	1	2	2	2	2	9	9
9	9	9	1	9	9	9	9	9	9
2	9	2	1	2	2	2	2	2	9
2	2	2	3	1	1	1	1	2	2
9	9	9	1	9	9	9	9	9	9
9	2	9	1	2	2	2	2	2	9
2	2	2	1	2	2	2	2	2	2
9	9	9	9	9	9	9	9	9	9
2	2	2	1	1	1	1	1	2	2
9	9	9	9	9	9	9	9	9	9
9	9	9	9	9	9	9	9	9	9
9	9	9	9	9	9	9	9	9	9
9	9	9	1	9	9	9	9	9	9
9	9	9	1	9	9	9	9	9	9
2	2	2	1	2	2	2	1	2	2
2	2	2	1	2	2	2	2	2	2
2	1	2	2	2	2	2	2	1	2
2	1	1	1	1	1	2	1	1	1
3	3	3	1	2	1	2	2	2	3
2	2	2	1	3	2	3	3	2	2
1	1	1	1	1	1	1	1	1	1
2	2	3	1	2	2	2	2	2	2
3	1	3	2	2	3	3	1	1	3
2	2	2	9	2	2	2	2	2	2
2	3	2	9	1	2	2	2	2	2
2	2	3	1	3	3	2	2	2	3
1	2	2	1	3	3	3	3	2	2
9	9	9	2	9	9	9	9	9	9
1	1	1	1	2	2	2	2	3	3
3	3	3	2	1	1	1	1	3	1
2	2	2	1	2	2	2	2	2	2
1	1	3	1	1	2	1	1	2	2
2	2	2	1	2	2	2	2	2	2
2	2	2	1	2	2	2	2	2	2
1	1	1	1	1	1	2	2	2	2
9	9	9	9	9	9	9	9	9	9
2	2	2	1	1	2	2	2	2	2
9	9	9	9	9	9	9	9	9	9
1	2	1	9	2	2	1	1	9	9
2	2	2	1	3	3	3	3	2	2
2	3	3	5	1	3	2	2	9	9

1	2	1	2	2	3	3	3	2	2
1	2	2	9	9	9	9	9	1	1
2	2	2	5	1	1	1	1	1	1
2	2	2	3	1	1	1	1	1	1
2	2	2	3	1	1	1	1	2	2
2	2	2	9	1	1	1	1	1	1
1	1	1	3	1	1	1	1	1	1
2	1	2	3	1	1	1	1	1	2
3	3	3	1	2	2	2	2	3	3
1	1	1	5	1	1	1	1	1	1
2	2	2	4	3	3	3	3	9	9
1	1	1	4	1	1	1	1	2	2
1	9	1	5	1	1	1	1	2	2
1	1	1	4	1	1	1	1	2	2
9	9	9	9	1	1	1	1	2	2
2	2	1	3	1	1	1	1	1	1
1	1	1	4	1	1	1	1	1	2
1	2	2	4	1	1	1	1	1	2
9	9	9	9	1	1	1	1	1	1
2	2	2	9	3	3	3	3	9	9
1	2	1	9	1	1	1	1	1	2
2	2	2	1	2	2	2	3	2	2
9	9	9	9	9	9	9	9	9	9
2	2	2	1	1	1	1	1	1	1
9	9	9	9	9	9	9	9	9	9
1	1	2	9	1	1	1	1	1	1
1	1	2	1	1	1	1	1	1	2
1	1	1	1	1	1	3	1	1	1
2	2	2	9	1	1	1	1	9	9
2	9	2	4	1	1	1	1	2	2
2	2	2	2	3	3	3	3	9	9
2	2	2	1	1	1	2	2	9	9
2	2	2	2	1	1	1	1	2	2
1	1	1	4	1	1	1	1	1	1
2	2	2	1	1	1	2	3	2	2
9	9	9	9	9	9	9	9	9	9
1	1	1	9	1	1	1	1	1	1
1	1	1	9	1	1	1	1	1	1
9	9	9	2	9	9	9	9	9	9
2	2	2	9	3	3	3	3	9	9
9	9	9	9	1	1	1	1	2	2
9	9	9	9	9	9	9	9	9	9
2	2	2	9	1	1	1	1	2	2
2	2	2	9	1	1	1	1	2	2
2	1	1	1	1	1	1	1	1	1
9	2	2	1	2	2	2	2	2	2
9	9	9	2	1	1	1	1	1	2

1	1	1	1	1	1	1	1	1	2
2	2	2	1	2	2	2	2	2	9
1	1	1	2	1	1	1	1	3	3
1	1	2	9	1	1	1	1	1	1
2	2	2	3	1	1	1	1	2	2
2	2	2	1	3	9	9	9	9	9
1	1	1	3	1	1	1	1	2	2
2	2	2	1	1	1	1	1	2	2
9	9	9	9	3	3	3	3	2	2
2	2	9	9	1	1	1	1	1	1
2	2	9	4	3	3	3	3	9	9
9	2	2	2	1	1	1	1	9	9
9	9	9	9	9	9	9	9	9	9
9	9	9	9	1	1	1	1	9	9
9	9	9	9	3	3	3	3	9	9
9	9	9	9	9	9	9	9	9	9
9	9	9	9	1	1	1	1	1	1
9	9	9	9	9	9	9	9	9	9
2	2	2	9	3	3	3	3	2	2
2	1	1	4	1	1	1	1	1	3
2	1	1	2	1	1	1	1	1	3
2	1	1	4	1	1	1	1	1	3
2	1	1	4	1	1	1	1	1	3
2	1	1	4	1	1	1	1	1	3
2	1	1	4	1	1	1	1	1	3
2	1	1	2	1	1	1	1	1	3
2	1	1	4	2	1	1	1	1	3
2	1	1	1	1	1	1	1	1	3
2	1	1	4	1	1	1	1	1	3
2	1	1	1	2	1	1	1	1	3
1	9	1	9	2	1	1	1	1	3
2	1	1	9	2	1	1	1	1	3
2	1	1	4	1	1	1	1	1	3
2	1	1	4	2	1	1	1	1	3
2	1	1	4	1	1	1	1	1	3
2	1	1	4	2	1	1	1	1	3
2	1	1	4	1	1	1	1	1	3
2	1	1	4	9	9	9	9	1	3
2	1	1	4	1	1	1	1	1	3
2	1	1	4	1	1	1	1	1	3
2	1	1	1	1	1	1	1	1	3
2	1	1	4	1	1	1	1	1	3
2	1	1	4	1	1	1	1	1	3
2	1	1	4	1	1	1	1	3	1
9	9	9	9	9	9	9	9	9	9
9	9	9	9	9	9	9	9	9	9
9	9	9	9	9	9	9	9	9	9

9	9	9	9	9	9	9	9	9	9
9	9	9	9	9	9	9	9	9	9
9	9	9	9	9	9	9	9	9	9
9	9	9	9	9	9	9	9	9	9
9	9	9	9	9	9	9	9	9	9
9	9	9	9	9	9	9	9	9	9
9	9	9	9	9	9	9	9	9	9
9	9	9	9	9	9	9	9	9	9
9	9	9	9	9	9	9	9	9	9
9	9	9	9	9	9	9	9	9	9
9	9	9	9	9	9	9	9	9	9
9	9	9	9	9	9	9	9	9	9
9	9	9	9	9	9	9	9	9	9
9	9	9	9	9	9	9	9	9	9
9	9	9	9	9	9	9	9	9	9
9	9	9	9	9	9	9	9	9	9
9	9	9	9	9	9	9	9	9	9
9	9	9	9	9	9	9	9	9	9
9	9	9	9	9	9	9	9	9	9
2	1	1	4	1	9	1	1	1	3
2	1	1	4	1	1	1	1	1	3
2	1	1	1	1	1	1	1	1	3
2	1	1	1	1	1	1	1	1	3
2	1	1	1	1	1	1	1	1	3
2	1	1	1	1	1	2	1	1	3
2	1	1	1	1	2	1	1	1	3
2	1	1	1	1	1	1	1	1	3
2	1	1	4	1	2	1	1	1	3
2	1	1	2	1	1	1	1	1	1
2	1	1	1	1	1	1	1	1	1
1	1	1	1	1	1	1	1	1	3
2	1	1	4	1	1	1	1	1	3
2	1	1	4	1	1	1	1	1	3
2	1	1	5	1	1	1	1	1	3
2	1	1	1	1	1	1	1	1	3
9	1	9	1	2	2	2	2	1	9
2	1	1	1	1	1	1	1	1	3
2	1	1	4	1	1	1	1	1	3
2	1	1	4	1	1	1	1	1	3
2	1	1	1	2	1	1	1	1	3
2	1	1	4	1	1	1	1	1	3
2	1	1	4	1	2	1	1	1	3
2	1	1	4	1	1	1	1	1	3
2	1	1	9	1	1	1	1	1	1
2	1	1	1	1	1	1	3	1	3
2	1	1	1	1	1	1	1	1	3
2	1	1	1	9	1	1	1	1	3

2	1	1	1	1	1	1	1	1	3
2	1	1	1	9	1	1	1	1	3
2	1	1	4	3	1	1	1	1	3
2	1	1	1	1	1	1	1	1	3
2	1	1	1	1	1	1	1	1	3
2	1	1	4	1	1	1	1	1	3
2	1	1	1	1	1	1	1	1	3
2	1	1	1	1	1	1	1	1	3
2	1	1	1	1	1	1	1	1	3
2	1	1	4	1	1	1	1	1	3
2	1	1	1	1	1	1	1	1	3
2	1	1	1	1	1	1	1	1	3
2	1	1	4	1	1	1	1	1	3
2	1	1	3	1	1	1	1	1	3
2	1	1	9	2	1	1	1	1	3
2	1	1	4	1	1	1	1	1	3
1	1	1	9	2	1	1	1	1	3
2	1	1	4	1	1	1	1	1	3
2	1	1	4	1	1	1	1	1	3
2	1	1	4	1	1	1	1	1	3
2	1	1	4	1	1	1	1	1	3
2	1	3	4	2	1	1	1	1	3
2	1	1	1	1	1	1	1	1	3
2	1	1	1	1	1	1	1	1	3
2	1	1	1	1	1	1	1	1	3
2	1	1	4	1	1	1	1	1	3
2	1	1	1	1	1	1	1	1	3
2	1	1	4	1	1	1	1	1	3
2	1	1	1	1	1	1	1	1	3
9	1	1	1	1	1	1	1	1	3
2	1	1	2	1	1	1	1	1	3
2	1	1	1	1	9	9	9	1	1
9	9	9	9	9	9	9	9	1	1
2	1	1	4	1	1	1	1	1	3
9	9	9	9	9	9	9	9	9	9
2	1	1	1	2	1	1	1	1	3
1	2	1	1	1	9	1	1	1	3
2	1	1	4	9	9	1	1	1	3
2	1	1	4	1	9	1	1	1	3
2	1	1	9	2	1	1	1	1	3
2	1	1	4	1	1	1	1	1	3
2	1	1	1	1	1	1	1	1	3
2	1	1	1	2	2	1	1	1	3
2	1	1	4	1	1	1	1	1	3
2	1	1	4	1	1	1	1	1	3
2	1	1	4	1	1	1	1	1	3
2	1	1	4	1	1	1	1	1	3

2	1	1	4	1	1	1	1	1	3
2	1	1	4	1	1	1	1	1	3
2	1	1	4	1	1	1	1	1	3
2	1	1	4	1	1	1	1	1	3
1	1	1	4	1	1	1	1	1	3
2	1	1	4	1	1	1	9	1	3
2	1	1	4	1	1	1	1	1	3
2	1	1	9	1	1	1	1	1	3
2	1	1	4	1	1	1	1	1	3
2	1	1	4	1	1	1	1	1	3
2	1	1	4	1	1	1	1	1	3
2	1	1	4	1	1	1	1	1	3
2	1	1	4	1	1	1	1	1	3
2	1	1	4	1	1	1	1	1	3
9	9	9	4	1	1	1	1	1	3
2	1	1	4	1	1	1	1	1	3
2	1	1	4	1	1	1	1	1	3
2	1	1	4	1	1	1	1	1	3
2	1	1	4	1	1	1	1	1	3
9	9	9	9	9	9	9	9	1	3
2	1	1	4	2	1	1	1	1	3
2	1	1	4	1	1	1	1	1	3
2	1	1	4	1	1	1	1	1	3
2	1	1	4	1	1	1	1	1	3
1	1	1	4	1	1	1	1	1	3
1	2	1	1	1	1	1	1	1	3
2	1	1	9	1	1	1	1	1	3
2	1	1	4	1	1	1	1	1	3
1	1	1	1	1	1	1	1	1	3
3	1	1	1	1	1	1	1	1	3
2	1	1	1	1	1	1	1	1	3
2	1	1	1	1	1	1	1	1	3
1	1	2	1	1	1	1	1	3	1
2	1	1	1	1	1	1	1	3	1
2	1	1	1	1	1	1	1	1	3
2	1	1	2	1	1	1	1	1	3
2	1	1	1	1	1	1	1	1	3
2	1	1	4	1	1	1	1	1	3
2	1	1	4	1	1	1	1	1	3
2	1	1	4	1	1	1	1	1	3
2	1	1	3	1	1	1	1	1	3
9	9	9	9	9	9	9	9	9	9
2	1	1	4	1	1	1	1	1	3
2	1	1	3	1	1	1	1	1	3
2	1	1	4	1	1	1	1	1	3
2	1	1	4	1	1	1	1	1	3
2	1	1	4	1	1	1	1	1	3

2	1	1	4	1	1	1	1	1	3
2	1	1	4	1	1	1	1	1	3
2	1	1	1	1	1	1	1	1	3
2	1	1	4	1	1	1	1	1	3
2	1	1	4	1	1	1	1	1	3
2	1	1	4	1	1	1	1	1	3
2	1	1	1	1	1	1	1	1	3
2	1	1	1	9	1	2	1	1	3
2	1	1	1	1	1	1	1	1	3
1	1	1	9	9	9	9	9	9	9
2	1	1	4	1	1	1	1	1	3
2	1	1	4	1	1	1	1	1	3
2	1	1	4	1	1	1	1	1	3
2	1	1	4	1	1	1	1	1	3
2	1	1	4	1	1	1	1	1	3
2	1	1	4	1	1	1	1	1	3
2	1	1	4	1	1	1	1	1	3
2	1	1	4	1	1	1	1	1	3
2	1	1	4	1	1	1	1	1	3
2	1	1	4	1	1	1	1	1	3
2	1	1	4	1	1	1	1	1	3
2	1	1	4	1	1	1	1	1	3
2	1	1	4	1	1	1	1	1	3
2	1	1	1	1	1	1	1	1	3
2	1	1	4	1	1	1	1	1	3
2	1	1	4	1	1	1	1	1	3
2	1	1	4	9	9	9	9	1	3
9	9	9	9	9	9	9	9	9	9
9	9	9	9	9	9	9	9	9	9
9	9	9	9	9	9	9	9	9	9
9	9	9	9	9	9	9	9	9	9
9	9	9	9	9	9	9	9	9	9
9	9	9	9	9	9	9	9	9	9
9	9	9	9	9	9	9	9	9	9
9	9	9	9	9	9	9	9	9	9
9	9	9	9	9	9	9	9	9	9
9	9	9	9	9	9	9	9	9	9
9	9	9	9	9	9	9	9	9	9
9	9	9	9	9	9	9	9	9	9
9	9	9	9	9	9	9	9	9	9
9	9	9	9	9	9	9	9	9	9
9	9	9	9	9	9	9	9	9	9
9	9	9	9	9	9	9	9	9	9
9	9	9	9	9	9	9	9	9	9
9	9	9	9	9	9	9	9	9	9
9	9	9	9	9	9	9	9	9	9
9	9	9	9	9	9	9	9	9	9

9	9	9	9	9	9	9	9	9	9
9	9	9	9	9	9	9	9	9	9
9	9	9	9	9	9	9	9	9	9
9	9	9	9	9	9	9	9	9	9
9	9	9	9	9	9	9	9	9	9
9	9	9	9	9	9	9	9	9	9
9	9	9	9	9	9	9	9	9	9
9	9	9	9	9	9	9	9	9	9
9	9	9	9	9	9	9	9	9	9
9	9	9	9	9	9	9	9	9	9
9	9	9	9	9	9	9	9	9	9
9	9	9	9	9	9	9	9	9	9
9	9	9	9	9	9	9	9	9	9
1	3	3	1	1	1	1	1	3	3
2	2	2	9	3	3	3	3	2	2
2	2	2	2	2	2	2	2	2	2
3	2	2	9	9	9	9	9	1	1
1	1	1	5	1	1	2	1	1	2
3	3	3	9	9	9	9	9	1	1
3	2	2	9	1	1	1	1	1	2
2	1	1	4	1	1	2	1	1	2
3	1	3	5	2	2	3	3	2	2
9	9	9	9	9	9	9	9	9	9
2	2	3	1	3	3	3	3	2	3
9	9	9	1	2	2	2	2	9	9
3	3	3	9	1	1	1	1	2	2
9	9	9	9	9	9	9	9	9	9
1	3	3	2	2	2	2	2	1	1
3	3	2	9	2	3	1	2	3	2
2	2	3	2	1	1	2	2	2	2
3	3	3	1	2	2	2	2	3	3
1	2	3	1	2	2	2	3	2	1
9	9	9	3	1	1	2	2	1	9
3	3	3	9	1	1	2	2	1	3
1	1	1	2	1	1	1	1	1	1
9	9	9	1	1	1	2	1	2	9
2	2	2	1	1	3	1	2	2	2
1	1	1	2	3	2	1	1	1	2
1	1	1	3	1	1	2	1	2	2
1	1	1	9	1	1	1	1	1	1
9	9	9	9	9	9	9	9	9	9
9	9	9	9	9	9	9	9	9	9
2	1	1	2	1	1	1	1	1	1
1	1	1	9	1	1	3	2	1	1
9	9	9	9	9	9	9	9	9	9
9	9	9	9	9	9	9	9	9	9
2	2	2	1	3	3	9	9	9	9

9	9	9	5	1	2	1	2	9	9
9	9	9	9	9	9	9	9	9	9
2	3	2	1	3	3	2	1	2	2
1	2	2	1	1	1	2	2	2	2
1	2	3	2	1	1	2	2	1	2
9	9	9	3	1	1	2	1	9	2
2	2	2	9	2	2	1	1	2	2
2	2	2	9	2	1	1	1	2	2
2	2	2	9	2	2	2	2	2	2
3	3	2	9	1	2	2	2	2	2
3	3	3	1	1	2	3	3	3	3
9	9	9	9	9	9	9	9	9	9
9	9	9	4	2	2	2	2	1	9
2	2	2	1	3	3	3	3	2	2
2	2	2	1	3	3	2	2	2	2
2	3	3	1	2	2	2	2	2	2
3	1	3	1	2	2	3	3	2	2
2	2	2	9	1	1	1	1	2	2
2	1	9	9	1	1	1	1	1	1
9	9	2	9	9	9	9	9	9	9
2	2	2	3	1	2	2	2	2	2
2	2	2	9	9	9	9	9	2	2
2	2	2	9	2	3	3	3	2	2
2	2	2	9	2	2	1	3	2	2
2	2	2	9	9	9	9	9	2	2
3	1	1	1	2	1	1	2	2	2
2	2	2	9	2	2	2	2	2	2
2	2	2	5	2	2	2	3	2	2
2	2	2	1	2	2	2	2	2	2
2	1	2	1	1	1	1	1	1	3
1	2	3	2	1	1	3	1	1	3
1	2	3	1	1	2	2	2	1	2
1	1	1	1	1	2	3	3	1	2
1	1	1	4	1	1	1	1	1	2
1	2	2	9	1	1	2	2	2	1
2	3	1	1	2	2	1	2	1	2
2	2	2	1	2	2	2	2	2	2
2	2	2	9	1	3	3	1	1	1
9	9	9	1	3	9	3	2	2	9
2	1	1	2	2	2	2	2	2	2
1	1	1	2	1	2	2	2	3	3
2	2	2	1	1	2	2	2	2	2
3	1	2	1	1	1	2	2	1	2
2	1	1	1	1	1	2	2	1	2
1	1	1	1	1	1	1	1	1	1
3	2	2	2	1	1	1	1	1	1
2	1	2	2	2	2	2	9	2	2

1	2	1	1	2	2	2	2	2	2
9	1	1	2	2	2	2	2	1	2
2	2	2	9	1	2	2	2	2	2
3	3	2	9	3	1	2	2	2	2
2	2	2	2	1	1	1	1	2	2
2	2	2	2	1	1	1	1	2	2
2	1	3	1	2	2	2	2	1	2
3	1	3	9	3	3	3	3	2	3
2	2	2	1	3	3	3	3	2	2
3	1	3	1	1	2	2	3	1	2
2	2	2	2	2	2	2	2	2	2
2	2	2	1	3	3	3	3	2	2
1	1	1	9	2	2	2	1	2	2
2	2	2	9	9	9	9	9	2	2
2	2	2	9	1	1	1	1	2	2
2	2	2	1	2	2	2	3	2	2
3	3	3	4	1	1	1	1	2	2
2	2	2	1	1	1	2	2	2	2
2	2	2	9	9	9	9	9	2	2
1	2	1	2	2	1	1	3	1	2
1	2	2	2	2	2	2	2	2	2
1	1	2	1	2	2	2	2	1	1
2	2	2	3	2	2	2	2	2	2
1	1	1	9	1	1	1	1	1	1
1	1	1	2	1	1	1	2	1	3
9	9	9	9	9	9	9	9	9	9
2	1	2	2	2	2	2	9	9	9
2	2	1	1	2	2	2	2	2	2
3	1	3	9	1	1	1	1	9	9
2	1	1	2	3	2	2	1	2	1
2	1	1	2	2	2	2	2	1	2
2	1	2	9	1	2	1	2	1	3
3	3	3	9	2	2	2	1	2	2
2	2	2	9	1	1	1	1	2	2
9	2	1	9	1	1	1	1	9	9
9	9	9	9	9	9	9	9	2	2
9	9	9	9	9	9	9	9	9	9
2	2	2	9	9	9	9	9	1	2
9	9	9	9	9	9	9	9	9	9
2	2	2	1	1	1	2	2	2	2
3	3	2	1	2	2	2	2	2	3
1	1	3	3	1	1	1	2	1	1
2	1	2	2	2	2	2	2	1	2
9	9	9	9	9	9	9	9	9	9
9	9	9	9	9	9	9	9	9	9
9	9	9	9	9	9	9	9	9	9
3	3	3	3	1	1	1	1	1	1

9	9	9	9	9	9	9	9	9	9
9	9	9	9	9	9	9	9	9	9
2	2	2	3	1	2	2	1	1	2
3	3	3	9	9	9	9	9	9	9
9	9	9	9	9	9	9	9	9	9
3	3	1	1	1	1	3	3	2	2
3	2	3	1	2	1	3	2	3	1
2	2	2	9	9	9	9	9	9	9
2	1	2	1	1	1	1	1	1	2
3	1	2	2	3	3	3	3	1	2
3	1	2	2	1	2	3	2	2	3
2	2	2	9	3	3	3	3	2	2
2	2	2	1	3	3	3	3	2	2
9	9	9	9	9	9	9	9	9	9
2	2	2	9	3	3	3	3	2	2
1	9	3	1	2	2	2	3	2	2
1	1	3	5	1	3	1	2	3	3
1	2	3	1	2	2	3	2	2	3
2	3	3	9	1	1	1	1	1	2
9	9	9	9	9	9	9	9	9	9
1	2	3	1	2	2	3	2	2	3
2	3	1	2	1	3	1	1	2	1
2	2	2	1	2	2	2	2	2	2
1	2	3	1	2	2	3	2	9	2
3	2	3	9	9	9	9	9	3	3
9	9	9	9	9	9	9	9	9	9
2	2	2	1	2	2	2	2	3	3
2	2	2	9	2	2	2	2	9	9
2	2	2	4	2	2	2	1	2	2
1	1	2	4	2	2	2	3	1	2
2	2	2	1	2	2	2	2	2	2
9	9	9	2	2	2	2	2	1	9
2	2	2	1	2	2	2	2	1	9
2	2	2	5	9	9	9	9	2	2
2	1	2	2	2	1	2	2	2	1
3	1	2	9	9	9	9	9	2	1
9	9	9	9	9	9	9	9	2	2
1	1	1	3	1	1	2	3	1	2
2	1	1	2	2	2	2	2	2	2
9	9	9	1	3	2	2	2	2	9
3	2	2	9	9	9	9	9	1	2
2	2	2	1	1	3	3	3	2	2
2	2	2	1	2	2	2	2	3	3
2	2	2	1	2	2	3	2	2	2
2	2	2	1	1	3	3	3	2	2
9	9	9	9	2	2	1	2	1	9
3	3	3	1	3	3	3	3	2	2

1	1	3	2	1	1	1	1	1	3
2	2	2	9	3	3	3	3	2	2
3	1	1	1	1	2	2	2	2	3
2	1	2	1	2	2	2	2	1	2
1	1	2	1	2	2	2	2	2	2
2	2	2	9	2	2	3	3	2	2
1	1	1	1	2	2	2	2	1	1
2	1	2	1	1	1	2	2	2	1
1	1	3	1	1	1	2	2	1	3
1	3	3	2	2	2	2	3	3	3
2	2	2	1	1	1	2	2	2	2
2	2	2	2	3	2	2	2	2	2
2	2	2	1	1	1	2	2	2	2
1	1	3	1	1	1	2	2	1	3
9	9	9	9	2	2	2	2	2	1
1	1	2	3	2	2	2	2	2	2
1	2	3	1	3	2	2	2	2	2
1	2	2	9	9	9	9	9	1	2
3	2	2	1	2	2	2	3	3	2
1	1	2	1	2	2	2	2	1	1
1	1	1	1	2	2	2	2	1	3
1	3	3	9	1	1	1	1	9	9
2	3	3	1	2	2	2	2	2	2
2	2	2	9	1	1	1	1	2	2
2	9	2	1	1	1	2	2	2	2
1	1	1	2	2	2	2	2	1	3
1	2	1	2	3	3	3	1	2	2
1	2	1	2	1	3	3	1	2	2
1	1	1	4	1	1	1	1	1	3
1	1	2	1	1	1	1	1	1	3
2	3	1	1	1	1	1	1	1	3
1	1	1	3	2	2	2	1	2	3
2	1	1	2	1	1	1	2	1	2
9	9	9	9	9	9	9	9	9	9
9	9	9	2	3	3	3	3	3	9
3	1	3	1	1	1	1	1	1	3
2	2	2	9	9	9	9	9	2	2
3	1	1	2	1	1	1	1	1	1
1	1	1	9	1	1	1	1	1	1
2	2	2	4	1	1	1	3	2	2
3	2	2	2	1	1	1	1	1	2
9	9	9	9	9	9	9	9	9	9
9	9	9	9	9	9	9	9	9	9
1	1	1	9	1	1	1	1	1	1
2	2	2	2	1	1	1	1	1	2
9	9	9	9	9	9	9	9	9	9
9	9	9	9	9	9	9	9	9	9

2	2	2	9	9	9	9	9	2	2
1	1	1	1	1	1	1	1	1	1
3	1	2	3	1	1	1	1	1	2
2	1	2	2	2	2	2	2	2	3
2	1	1	1	2	2	2	2	1	2
3	1	2	1	1	2	3	1	1	2
1	2	2	9	2	2	1	1	1	2
9	9	9	9	2	9	1	3	1	9
1	2	1	1	1	1	1	1	2	3
9	9	9	9	9	9	9	9	9	9
1	1	1	9	1	1	1	1	2	2
1	1	1	4	3	3	3	3	2	2
9	9	9	9	9	9	9	9	1	1
9	9	9	9	9	9	9	9	9	9
2	2	2	9	1	1	1	1	2	2
9	9	9	9	9	9	9	9	9	9
9	9	9	9	9	9	9	9	9	9
2	2	2	2	2	1	1	1	2	2
2	2	2	9	2	3	3	3	2	2
3	3	2	3	1	1	2	2	2	2
2	2	2	1	2	2	2	2	2	2
2	2	2	9	1	1	1	1	2	2
1	2	3	5	1	3	3	3	2	3
3	2	3	4	1	3	2	1	2	3
9	9	9	9	3	2	2	2	1	1
9	9	9	3	1	2	2	2	1	9
1	1	1	4	2	9	2	2	2	2
1	1	1	2	9	9	9	9	2	3
1	1	1	1	1	1	1	2	1	1
1	1	1	5	3	3	3	3	1	2
1	1	9	3	2	1	1	1	1	1
2	2	2	1	2	3	3	3	2	2
2	2	2	9	9	9	9	9	2	2
2	2	2	9	3	3	3	3	2	2
1	1	1	2	1	1	1	1	2	2
2	2	2	9	3	3	3	3	2	2
2	2	2	9	2	2	2	2	3	3
9	9	9	2	1	2	2	2	9	9
2	9	9	9	2	2	2	2	9	9
2	2	2	2	9	9	9	9	2	2
2	2	3	9	3	2	2	2	3	3
9	1	9	3	1	3	3	3	2	2
2	2	2	9	9	9	9	9	1	2
9	9	9	2	2	2	1	3	2	9
3	3	3	1	1	1	2	2	2	2
9	9	9	9	9	9	9	9	9	9
2	2	2	2	2	2	2	2	2	2

1	2	1	1	3	3	3	3	1	2
9	9	9	1	2	2	2	2	9	9
2	2	2	9	9	9	9	9	2	2
2	2	2	1	2	2	2	2	2	2
3	3	3	9	1	2	1	1	3	3
2	2	2	9	1	2	3	3	2	2
1	2	1	4	2	2	2	2	1	2
1	3	2	1	9	9	9	9	9	9
2	2	2	9	1	2	2	2	2	2
1	1	1	9	2	2	2	2	1	1
1	3	3	2	2	2	2	2	2	2
2	2	2	9	3	3	3	3	2	2
2	2	2	2	1	2	1	2	2	3
3	2	3	1	2	2	2	2	2	3
1	1	1	1	1	2	1	2	2	1
1	1	1	1	1	2	1	2	2	1
2	2	2	9	2	2	2	2	2	2
2	2	2	9	1	2	2	2	2	2
2	2	3	5	9	9	9	9	9	9
9	3	1	1	9	9	9	9	9	9
1	2	2	1	2	2	2	2	3	2
2	2	2	1	1	1	2	2	1	2
2	1	1	1	1	2	1	2	9	9
9	9	9	9	9	9	9	9	9	9
3	3	3	9	3	3	3	3	3	3
9	9	9	1	3	3	3	3	9	9
9	9	9	9	9	9	9	9	9	9
9	9	9	9	9	9	9	9	9	9
1	1	1	1	9	9	9	9	1	2
1	1	1	4	2	2	2	2	1	1
2	2	2	4	2	2	2	2	2	2
1	1	1	4	2	2	2	1	1	2
2	2	2	1	2	2	2	2	1	2
2	2	2	9	3	3	3	3	2	2
1	1	1	3	2	2	2	2	1	2
1	1	1	4	1	2	2	2	2	2
9	3	3	9	1	2	1	3	1	2
2	1	2	2	2	2	1	2	1	2
1	1	1	1	3	3	1	3	2	1
1	1	1	4	1	1	1	1	1	3
1	1	1	9	1	1	2	2	1	1
2	2	2	2	2	2	2	1	2	2
9	9	9	1	1	1	2	1	1	9
9	9	9	2	1	1	1	3	9	9
2	2	1	3	1	2	1	3	1	3
2	2	2	9	3	3	3	3	2	2
2	2	2	1	3	3	2	2	2	2

3	1	1	2	1	2	3	1	1	1
9	9	9	9	9	9	9	9	9	9
9	9	9	9	9	9	9	9	9	9
9	9	9	9	9	9	9	9	9	9
9	9	9	9	9	9	9	9	9	9
9	9	9	3	1	1	2	1	9	9
9	9	9	9	9	9	9	9	9	9
9	9	9	9	9	9	9	9	9	9
9	9	9	9	9	9	9	9	9	9
9	9	1	3	3	3	3	3	9	9
2	2	2	4	1	2	3	3	1	2
2	1	2	4	1	2	1	2	1	2
1	1	1	1	9	9	9	9	9	9
3	2	9	4	1	2	2	2	1	2
2	2	2	1	1	1	2	2	2	2
1	1	3	2	9	9	9	9	9	9
3	2	3	4	1	1	1	1	1	1
2	2	2	3	2	2	2	2	2	2
1	1	2	1	2	1	1	2	2	2
2	2	2	1	3	2	2	2	2	2
2	1	3	3	1	2	3	2	1	3
1	2	3	1	1	2	2	2	3	3
3	3	3	9	1	2	2	1	3	3
2	2	2	9	1	1	1	2	2	2
2	2	2	1	2	2	2	2	2	2
2	2	2	9	1	2	2	1	2	2
3	3	3	1	1	1	2	2	2	2
1	1	1	3	1	1	1	1	1	2
2	2	2	1	2	2	2	2	2	2
2	2	2	9	3	3	3	3	2	2
9	9	9	9	9	9	9	9	9	9
2	2	2	9	3	3	3	3	2	2
1	1	1	9	1	1	1	1	3	2
9	9	9	1	1	1	1	1	1	9
9	9	9	9	9	9	9	9	3	3
2	2	2	9	3	3	3	3	2	2
2	2	2	9	3	9	3	3	2	2
2	2	2	1	1	2	1	2	2	2
2	1	3	4	1	1	1	2	1	3
9	9	9	9	9	9	9	9	9	9
2	1	2	3	3	2	2	1	1	2
2	2	2	3	3	3	3	3	2	2
2	2	2	1	2	2	3	2	2	2
1	1	3	2	1	1	1	1	1	3
1	2	2	4	1	1	1	1	1	2
9	9	9	9	9	9	9	9	9	9
2	2	2	9	1	2	2	9	9	9

9	9	9	9	9	9	9	9	9	9
2	2	2	1	1	2	2	3	2	2
9	9	9	5	1	1	1	2	1	9
9	9	9	9	9	9	9	9	9	9
9	9	9	9	9	9	9	9	9	9
9	9	9	9	9	9	9	9	9	9
9	9	9	9	9	9	9	9	9	9
9	9	9	9	9	9	9	9	9	9
9	9	9	9	9	9	9	9	9	9
9	9	9	9	9	9	9	9	9	9
9	9	9	9	9	9	9	9	9	9
9	9	9	9	9	9	9	9	9	9
9	9	9	9	9	9	9	9	9	9
9	9	9	9	9	9	9	9	9	9
9	9	9	9	9	9	9	9	9	9
9	9	9	9	9	9	9	9	9	9
9	9	9	9	9	9	9	9	9	9
9	9	9	9	9	9	9	9	9	9
9	9	9	9	9	9	9	9	9	9
9	9	9	9	9	9	9	9	9	9
9	9	9	9	9	9	9	9	9	9
9	9	9	9	9	9	9	9	9	9
9	9	9	9	9	9	9	9	9	9
3	2	1	3	3	2	1	2	1	2
2	2	2	1	1	2	2	2	2	2
2	2	2	2	1	1	1	1	1	2
2	1	1	3	1	1	2	1	1	2
2	3	2	4	1	3	2	1	2	2
1	1	1	3	1	1	3	3	1	9
2	1	2	3	1	1	2	2	2	2
1	1	1	4	1	1	1	1	1	1
1	2	3	1	3	2	3	3	2	2
1	2	1	1	1	3	2	2	2	2
1	1	1	4	1	1	2	2	9	9
2	2	1	4	1	1	3	3	1	3
2	1	2	1	1	1	1	1	1	3
2	2	1	4	1	1	2	2	9	9
2	1	2	1	1	1	1	1	2	2
1	1	1	1	1	1	1	1	1	3
9	1	9	1	1	9	2	1	1	9
2	1	2	3	1	1	1	1	1	2
1	1	1	1	1	1	2	1	1	1
9	9	9	1	1	2	1	1	1	9
2	1	1	1	1	1	2	1	2	2
1	1	1	1	1	1	1	1	1	3
3	3	3	1	2	2	2	2	2	2
2	2	1	3	1	1	1	2	2	2

2	1	1	3	1	1	1	1	1	2
1	1	1	3	1	1	1	1	1	2
2	1	1	4	1	1	2	2	1	3
2	1	1	5	1	1	1	1	1	2
2	3	1	2	2	1	2	1	2	1
9	9	9	1	2	1	2	1	1	9
1	1	2	3	1	1	2	1	2	2
9	9	9	2	1	1	1	1	1	9
2	2	2	1	2	2	2	2	2	2
2	2	2	9	2	2	2	2	2	2
9	9	9	1	1	1	2	1	9	9
9	9	9	1	1	1	1	1	1	9
9	9	9	5	1	1	1	2	9	9
9	9	9	1	9	9	9	9	9	9
9	9	9	4	1	1	1	1	9	9
9	9	9	9	9	9	9	9	9	9
1	3	3	2	9	9	9	9	9	9
2	2	2	9	2	1	2	2	2	2
9	9	9	9	1	1	2	1	9	9
3	3	3	3	1	1	1	1	1	1
9	9	9	4	1	1	1	1	1	2
9	9	9	4	1	3	2	1	9	9
9	9	9	3	1	1	1	1	1	9
9	9	9	3	1	2	2	1	9	9
9	9	9	4	1	1	1	1	1	9
3	1	2	9	1	1	1	1	2	3
3	3	1	4	1	1	1	1	1	3
2	1	2	2	2	2	1	1	2	2
1	1	1	1	2	1	2	2	1	2
2	2	2	1	9	9	9	9	9	9
2	1	2	1	1	1	2	2	2	2
2	1	3	3	1	1	1	1	3	3
3	2	1	4	3	2	2	2	3	3
1	1	3	1	2	2	2	1	1	1
3	1	1	3	1	1	2	2	1	3
9	9	9	3	1	2	2	2	9	1
2	1	2	1	1	1	2	2	2	2
2	2	2	1	1	2	1	2	1	3
2	3	2	1	2	3	2	3	2	3
2	2	2	1	2	2	2	2	2	1
1	1	2	1	1	1	1	1	1	3
1	1	1	1	1	1	1	1	1	3
1	1	3	1	1	1	2	1	1	3
3	2	1	1	3	3	3	3	2	3
2	1	1	2	9	1	1	1	1	3
9	1	1	3	1	1	2	2	1	2
1	1	1	4	1	1	1	1	1	3

2	1	1	5	1	1	1	1	1	2
3	3	3	1	1	1	1	1	1	2
2	1	1	3	1	1	2	2	9	9
1	1	1	2	1	1	2	1	1	3
9	9	2	1	9	9	9	9	9	9
1	1	1	4	1	1	1	1	1	3
1	1	1	1	1	1	1	1	2	2
9	9	9	9	9	9	9	9	9	9
9	9	9	2	1	1	2	2	2	9
2	2	2	2	3	3	3	3	2	2
2	2	2	1	1	1	2	2	2	2
3	3	3	3	1	1	2	2	1	3
2	1	3	3	1	1	1	1	1	3
9	9	9	1	9	9	9	9	9	9
9	9	9	9	9	9	9	9	9	9
2	2	2	1	1	2	1	1	3	9
9	9	9	1	1	1	1	1	1	9
9	9	9	9	9	9	9	9	9	9
1	1	1	1	3	3	3	3	3	2
1	1	3	1	1	1	1	1	1	3
1	1	1	1	1	1	1	1	2	2
1	1	1	2	1	1	1	1	1	1
2	2	3	3	3	3	1	3	2	1
1	1	1	1	1	1	1	1	1	3
1	1	1	1	1	3	3	3	2	2
9	9	9	9	9	9	9	9	9	9
9	9	9	9	1	1	1	1	1	9
1	1	1	1	1	1	1	1	1	3
2	2	2	1	2	2	1	1	3	3
1	1	1	1	1	1	2	1	2	1
9	9	9	9	9	9	9	9	9	9
9	9	9	9	9	9	9	9	9	9
9	9	9	9	9	9	9	9	9	9
9	9	9	9	9	9	9	9	9	9
9	9	9	9	9	9	9	9	9	9
9	9	9	9	9	9	9	9	9	9
9	9	9	9	9	9	9	9	9	9
9	9	9	9	9	9	9	9	9	9
9	9	9	9	9	9	9	9	9	9
9	9	9	9	9	9	9	9	9	9
9	9	9	9	9	9	9	9	9	9
9	9	9	9	9	9	9	9	9	9
9	9	9	9	9	9	9	9	9	9
9	9	9	9	9	9	9	9	9	9
9	9	9	9	9	9	9	9	9	9
9	9	9	9	9	9	9	9	9	9
9	9	9	9	9	9	9	9	9	9

9	9	9	9	9	9	9	9	9	9
9	9	9	9	9	9	9	9	9	9
9	9	9	9	9	9	9	9	9	9
9	9	9	9	9	9	9	9	9	9
9	9	9	9	9	9	9	9	9	9
9	9	9	9	9	9	9	9	9	9
9	9	9	9	9	9	9	9	9	9
9	9	9	9	9	9	9	9	9	9
9	9	9	9	9	9	9	9	9	9
9	9	9	9	9	9	9	9	9	9
9	9	9	9	9	9	9	9	9	9
9	9	9	9	9	9	9	9	9	9
9	9	9	9	9	9	9	9	9	9
9	9	9	9	9	9	9	9	9	9
9	9	9	9	9	9	9	9	9	9
9	9	9	9	9	9	9	9	9	9
9	9	9	9	9	9	9	9	9	9
9	9	9	9	9	9	9	9	9	9
9	9	9	9	9	9	9	9	9	9
2	2	2	5	1	1	1	1	2	2
9	9	9	9	9	9	9	9	9	9
9	9	9	9	9	9	9	9	9	9
9	9	9	9	9	9	9	9	9	9
9	9	9	9	9	9	9	9	9	9
9	9	9	9	9	9	9	9	9	9
9	9	9	9	9	9	9	9	9	9
9	9	9	9	9	9	9	9	9	9
9	9	9	9	9	9	9	9	9	9
9	9	9	9	9	9	9	9	9	9
9	9	9	9	9	9	9	9	9	9
9	9	9	9	9	9	9	9	9	9
9	9	9	9	9	9	9	9	9	9
9	9	9	9	9	9	9	9	9	9
9	9	9	9	9	9	9	9	9	9
9	9	9	9	9	9	9	9	9	9
2	2	2	2	3	3	3	3	2	2
3	3	3	2	9	9	9	9	9	9
2	2	3	1	1	1	3	1	1	1
1	1	1	3	1	1	1	1	1	3
2	2	3	9	3	3	3	3	3	3
1	1	1	2	9	9	9	9	9	9
1	1	3	9	1	2	3	1	9	9
9	9	9	3	1	1	1	1	1	9
1	1	3	3	1	2	3	1	9	9
2	3	1	1	2	2	3	3	2	2
3	2	2	1	2	2	2	2	9	9
2	2	2	3	1	1	2	1	2	2

1	1	1	2	1	1	2	1	1	3
1	1	1	9	1	1	2	2	2	2
2	1	1	3	1	1	1	1	1	2
1	1	1	5	1	1	2	2	1	2
2	1	2	1	1	1	1	1	1	2
1	1	1	3	1	1	1	2	1	2
2	1	1	1	1	1	1	2	1	2
2	1	3	4	1	1	2	2	1	2
1	2	1	2	2	2	2	1	2	2
2	1	2	1	1	1	3	3	2	1
9	1	9	4	1	1	2	2	1	9
2	3	2	1	1	2	3	2	2	2
1	1	1	1	9	9	9	9	9	9
2	2	2	3	1	3	2	2	2	2
1	2	2	3	1	3	2	2	2	2
1	1	1	4	1	1	2	2	1	3
9	9	9	9	9	9	9	9	9	9
2	1	2	1	1	1	2	2	1	2
1	2	1	3	3	1	1	2	1	3
2	2	3	9	1	2	3	1	1	1
2	1	2	2	1	1	1	2	1	2
1	1	3	1	1	1	1	1	1	3
1	1	3	1	1	1	1	1	1	3
1	1	1	4	1	1	1	1	1	3
3	3	3	9	1	2	1	1	3	3
1	2	1	2	2	2	2	2	1	2
2	2	2	9	2	2	2	2	1	1
2	3	2	9	9	9	9	9	1	3
2	2	2	9	2	1	1	2	2	2
9	9	9	9	9	9	9	9	9	9
3	2	3	1	1	9	9	9	9	9
2	2	2	2	1	1	3	1	2	2
2	2	2	1	1	1	1	1	2	2
9	9	9	9	2	2	2	2	9	9
9	9	9	9	9	9	9	9	9	9
9	9	9	9	2	2	2	2	9	9
9	9	9	9	1	1	1	1	9	9
9	9	9	9	1	1	1	1	2	1
9	9	9	9	1	1	1	1	9	9
9	9	9	9	9	9	9	9	9	9
2	1	3	1	1	1	1	1	1	2
9	9	9	4	1	1	2	2	9	9
1	1	1	1	1	1	1	1	1	1
3	3	1	1	1	1	2	1	3	3
1	1	3	1	1	2	3	2	1	1
3	3	3	3	1	2	1	2	3	3
3	1	3	1	2	2	2	2	1	3

2	3	2	9	1	1	2	2	2	3
2	2	2	9	9	9	9	9	1	2
3	2	2	2	2	2	2	1	1	2
1	1	1	2	1	3	1	1	1	1
1	2	3	5	1	3	3	3	2	2
1	2	2	1	1	2	2	1	2	3
2	2	3	1	1	9	1	1	1	1
3	1	2	3	2	1	1	3	2	3
1	1	1	4	1	2	2	1	1	1
3	2	9	9	9	9	9	9	1	9
3	3	3	2	1	1	1	1	1	3
1	3	1	9	1	2	2	2	1	1
2	1	2	1	1	2	1	1	2	1
1	3	2	3	2	2	2	2	1	2
1	1	1	9	2	2	2	2	1	1
3	3	3	1	1	1	3	1	1	3
2	3	2	1	1	3	1	1	2	2
9	9	9	2	1	2	2	2	1	9
2	1	2	9	1	3	2	3	1	2
1	1	1	2	3	3	3	2	1	2
2	2	1	9	1	3	3	3	1	1
3	3	3	4	1	1	2	2	1	1
2	1	2	9	1	1	1	1	1	3
1	3	1	1	2	2	2	2	2	2
3	1	2	1	2	1	3	3	1	2
1	3	3	1	2	1	1	2	3	2
1	1	1	1	2	2	2	2	1	2
3	1	1	2	1	1	1	1	1	1
2	2	1	2	2	1	2	2	2	1
1	2	3	2	2	2	2	2	2	3
2	1	2	4	3	2	2	2	1	3
3	1	2	2	1	1	1	1	1	2
2	2	2	1	1	2	2	2	2	2
1	3	3	1	2	2	2	2	2	1
1	3	1	5	1	1	1	1	1	1
3	1	3	4	2	2	2	2	1	3
2	1	2	1	2	3	1	2	1	1
9	9	9	9	9	9	9	9	9	9
1	3	3	5	1	1	2	1	1	3
3	3	3	1	1	2	1	2	1	3
1	1	3	1	1	2	2	2	1	3
2	3	1	1	1	1	1	1	1	2
1	1	3	1	1	2	2	2	1	3
3	1	2	2	1	1	1	1	1	3
2	2	2	9	1	1	1	1	2	2
3	3	3	1	2	2	2	2	2	3
3	3	3	1	2	2	2	2	3	3

3	2	2	2	1	1	1	1	1	1
9	9	9	1	9	9	9	9	1	1
2	3	3	1	1	1	2	1	1	2
1	1	1	2	1	2	1	3	1	2
2	2	2	3	1	1	1	1	1	1
3	2	1	2	2	2	1	1	1	1
2	2	2	9	2	2	2	2	2	2
9	9	9	9	9	9	9	9	9	9
9	9	9	9	9	9	9	9	9	9
1	2	3	1	2	2	1	1	1	3
2	2	1	1	1	1	1	1	2	2
2	2	2	9	2	2	2	2	2	2
2	2	2	1	2	2	2	2	2	2
2	2	2	3	1	1	1	1	1	1
1	1	1	1	2	2	2	2	2	2
1	1	1	1	2	2	2	2	2	2
2	2	2	1	2	2	2	2	2	2
2	2	2	1	2	3	3	3	2	2
3	2	2	2	1	1	1	1	2	2
3	3	3	1	2	2	2	2	2	2
3	2	2	9	2	2	2	2	2	3
2	2	2	1	1	1	2	2	2	2
2	2	2	1	1	1	2	2	2	2
2	2	2	9	9	9	9	9	2	2
2	2	2	9	3	3	3	3	2	2
2	2	2	1	2	2	3	1	2	2
2	2	1	1	1	1	1	1	1	2
2	1	2	2	1	1	1	2	1	2
2	2	2	3	1	1	1	1	1	1
2	2	2	9	2	9	2	2	2	2
2	2	2	9	2	2	2	2	2	2
2	2	1	1	1	1	1	1	1	2
2	2	2	9	2	2	2	2	2	2
2	2	2	1	2	2	2	2	2	2
2	2	1	1	1	1	1	1	2	2
2	2	1	1	1	1	1	1	2	2
2	2	2	9	2	1	2	1	2	2
2	2	2	1	2	2	1	1	2	2
2	2	2	9	2	2	2	2	2	2
1	3	1	2	9	9	9	9	1	3
2	2	1	1	1	2	1	1	2	2
2	2	2	1	1	1	2	2	2	2
3	2	1	1	1	1	1	1	2	3
2	2	2	9	9	9	9	9	2	2
3	3	3	1	1	1	1	2	1	2
3	3	3	9	2	2	1	1	2	2
3	3	3	1	3	3	2	2	2	2

2	2	2	9	1	2	2	2	2	2
2	2	2	1	2	1	2	2	2	2
2	2	2	1	1	2	2	2	1	2
2	2	2	1	2	1	1	1	2	2
2	3	3	1	1	1	2	1	2	2
2	2	2	9	2	2	2	2	2	2
9	9	9	1	1	2	2	2	9	9
1	3	1	2	1	1	1	1	1	1
9	9	9	3	1	1	2	2	9	9
2	2	2	3	2	2	2	2	2	2
2	1	2	9	1	1	1	1	1	2
2	1	1	9	1	1	1	1	1	1
2	1	2	1	3	3	3	3	2	3
9	9	9	1	1	2	2	2	9	9
2	2	2	1	2	2	3	2	2	2
9	9	9	1	2	2	2	2	2	3
2	2	2	1	1	1	1	1	1	2
1	1	1	9	1	1	1	1	1	1
9	9	9	1	1	1	1	1	2	9
2	2	2	9	2	2	1	3	1	1
2	2	1	1	1	1	2	2	1	2
9	9	9	1	1	1	1	1	9	9
9	9	9	4	3	3	1	9	9	9
3	3	3	1	1	2	2	2	3	3
1	2	3	9	1	1	1	1	1	2
3	2	2	9	1	2	2	2	3	3
3	2	2	1	1	2	9	9	2	2
9	3	3	1	1	1	1	2	1	1
9	1	3	2	1	1	2	2	1	1
1	1	1	1	1	1	1	1	1	1
2	2	2	9	2	3	3	3	2	2
2	2	2	1	2	2	2	2	2	2
2	2	2	1	3	3	3	3	2	2
9	9	9	1	1	3	2	2	1	9
2	2	2	9	1	3	2	2	2	2
1	9	9	1	1	1	1	1	1	9
3	3	3	9	1	2	2	2	3	1
2	2	2	9	1	1	2	3	1	1
2	2	2	9	3	3	3	3	2	2
2	2	2	9	3	3	3	3	2	2
1	2	3	1	9	3	2	3	2	3
2	2	2	9	1	2	2	2	2	2
9	9	9	3	3	9	2	3	9	9
1	1	1	1	1	1	1	1	1	1
2	2	2	1	1	1	2	2	1	1
3	3	3	9	2	3	3	3	3	3
2	1	2	1	2	3	3	1	1	1

1	1	1	1	2	1	1	2	9	9
2	2	2	1	3	3	3	3	2	2
1	1	1	9	1	1	1	1	1	1
2	2	2	1	3	3	3	3	2	2
9	9	9	4	3	3	3	3	9	1
2	3	2	4	1	1	1	1	1	2
3	3	3	1	2	2	2	2	3	3
3	9	2	9	1	2	1	1	2	2
2	2	2	9	1	2	2	1	2	2
3	3	3	1	1	1	1	1	1	2
3	3	3	3	2	1	3	3	9	9
1	1	1	3	1	1	1	1	1	1
2	2	2	1	1	2	2	1	2	2
2	2	2	9	1	2	2	2	2	2
2	2	2	9	2	2	2	2	2	2
1	1	1	1	2	2	2	2	3	3
1	1	1	9	1	2	2	2	1	1
3	1	3	3	1	1	1	1	3	3
9	9	9	9	9	9	9	9	9	9
9	9	9	9	9	9	9	9	1	2
2	2	2	3	1	1	3	3	2	2
2	2	2	1	1	2	2	1	2	2
1	1	1	1	1	1	1	1	1	1
2	2	2	9	2	2	2	1	2	2
3	9	3	9	2	2	2	2	3	3
1	1	1	3	1	2	2	1	1	2
2	2	1	9	1	1	1	1	1	9
1	2	3	1	3	1	1	1	2	2
2	9	1	2	1	1	2	2	1	2
3	3	3	1	2	2	2	2	2	2
9	2	1	9	1	2	2	1	1	2
2	2	3	2	2	2	2	2	2	2
1	1	1	9	1	3	3	3	1	1
2	2	2	1	2	2	1	1	1	1
2	2	2	3	3	3	3	3	2	2
3	3	3	9	3	3	3	3	2	2
2	2	2	1	1	1	2	1	1	1
2	2	2	2	2	1	1	1	3	3
3	1	2	9	1	2	3	1	1	2
3	3	3	9	2	2	2	2	3	3
1	1	1	3	1	1	1	1	1	1
1	1	1	9	9	9	9	9	2	2
9	9	9	9	9	9	9	9	9	9
3	3	3	9	2	2	2	9	3	3
1	2	2	1	1	2	1	1	9	9
2	2	2	9	3	3	3	3	2	2
2	2	1	5	1	1	1	1	1	2

1	1	1	1	1	1	1	1	1	1
3	3	3	4	1	1	1	1	1	3
3	3	3	3	9	9	9	9	9	9
3	3	3	4	1	1	1	1	1	3
1	1	1	9	1	2	1	1	1	1
2	2	2	5	3	3	3	3	2	2
2	2	2	1	3	3	3	3	2	2
2	2	2	5	9	9	9	9	2	2
1	1	1	5	2	1	1	3	3	2
2	2	3	1	1	2	1	1	1	3
1	2	2	5	2	2	3	2	2	2
2	3	2	1	1	2	2	2	2	1
2	2	2	3	1	1	1	1	2	2
2	2	2	5	1	1	1	1	1	3
2	2	2	5	3	3	3	3	2	2
2	2	2	1	1	2	2	2	2	2
2	2	2	4	2	3	2	2	2	2
2	2	2	1	3	3	3	3	2	2
2	2	2	1	3	3	3	3	2	2
2	2	1	4	1	1	3	3	2	2
2	2	2	1	1	1	3	3	2	2
2	2	2	1	2	2	2	2	3	3
3	2	3	1	1	2	2	1	2	2
2	2	2	1	2	2	2	2	2	2
1	1	1	1	1	1	1	1	1	1
2	2	2	5	1	3	3	3	2	2
1	2	2	2	1	2	1	2	1	1
2	2	2	1	1	2	3	1	2	2
2	2	2	3	2	1	3	3	2	2
2	3	2	1	2	2	2	2	2	2
3	3	1	3	3	3	2	2	2	2
2	1	1	5	1	1	1	2	1	2
2	2	2	1	2	2	2	3	2	2
2	2	2	5	9	9	9	9	2	2
1	1	2	5	1	1	1	2	1	2
3	3	3	5	2	1	2	1	1	2
2	2	2	1	2	2	2	2	9	9
9	9	9	9	9	9	9	9	9	9
2	2	2	1	1	3	1	2	2	2
2	3	1	2	2	2	2	2	2	2
2	3	1	1	9	9	9	9	2	2
2	2	2	1	2	1	2	2	2	2
2	2	2	1	2	2	2	2	2	2
2	2	2	5	9	9	9	9	2	2
9	9	9	9	9	9	9	9	9	9
1	1	1	1	1	2	1	1	1	1
2	2	2	1	3	3	3	3	2	2

9	9	9	9	9	9	9	9	9	9
2	2	2	1	1	1	2	1	1	2
2	3	2	1	3	2	1	2	2	1
1	1	3	1	9	9	9	9	1	3
1	3	3	1	2	2	2	2	1	3
1	2	1	1	1	1	2	2	1	1
3	3	3	9	9	9	9	9	2	2
9	9	9	9	9	9	9	9	9	9
3	3	3	3	2	2	2	2	2	2
2	2	3	1	2	2	3	2	3	2
1	1	1	1	1	2	3	3	2	2
3	2	1	9	1	1	1	1	1	1
1	1	1	1	1	1	2	2	2	2
1	1	1	4	2	2	2	2	1	1
3	2	1	9	1	3	3	1	1	2
2	2	2	9	3	3	3	3	2	2
2	2	1	9	3	3	2	2	1	2
2	2	2	1	2	2	2	2	2	2
9	9	9	1	9	9	9	9	9	9
9	9	9	9	9	9	9	9	9	9
9	9	9	9	9	9	9	9	9	9
3	2	1	1	1	2	3	3	1	2
2	1	2	1	3	2	2	2	2	2
2	2	1	9	9	9	9	9	1	1
2	3	1	9	9	9	9	9	1	1
3	3	2	9	2	2	2	2	3	3
1	3	2	4	3	3	1	2	1	3
3	2	2	1	1	2	2	3	2	1
1	2	1	4	2	1	3	2	1	2
1	2	3	1	3	2	2	2	1	2
2	2	2	9	2	2	2	3	9	9
3	2	1	1	2	2	2	3	2	2
3	3	3	1	1	1	2	1	1	3
2	2	2	9	2	2	2	3	2	2
2	2	2	9	1	1	1	1	3	3
2	2	2	2	2	2	2	2	2	2
3	3	3	9	2	2	1	2	3	3
2	2	3	9	1	1	1	2	2	2
9	9	9	9	9	9	9	9	9	9
9	9	9	9	9	9	9	9	9	9
3	1	2	9	1	2	1	3	1	2
2	2	2	9	2	2	2	2	2	2
1	2	2	2	1	2	1	1	2	3
3	2	1	1	2	2	2	2	1	2
9	9	9	9	2	2	2	2	9	9
3	3	3	9	1	2	2	1	3	3
2	3	1	9	2	2	2	2	2	2

1	1	1	9	9	9	9	9	1	1
1	2	2	1	2	2	2	2	2	2
1	2	3	1	2	1	3	1	2	1
2	2	2	9	2	2	2	2	1	9
2	2	3	1	2	2	2	2	2	3
3	3	3	9	1	2	1	2	3	3
1	1	1	9	1	3	3	1	1	1
3	3	3	9	1	1	1	1	3	3
3	2	2	1	2	2	2	2	2	3
1	1	1	9	9	9	9	9	2	1
2	2	2	1	3	3	3	3	2	2
2	2	2	5	3	3	2	2	1	2
2	2	2	9	9	9	9	9	1	1
1	2	1	3	1	1	2	2	1	2
2	2	2	9	1	1	2	2	1	2
2	2	2	9	2	3	2	2	3	3
1	1	3	1	1	1	2	2	3	3
1	2	1	1	3	3	3	3	1	2
2	2	2	1	3	3	3	3	9	9
3	2	1	9	1	2	2	2	2	3
2	2	1	9	9	9	9	9	1	2
3	3	3	3	2	2	1	2	3	3
3	3	3	1	2	2	2	1	2	2
3	3	3	1	2	1	2	3	1	2
3	1	2	9	2	2	2	2	1	2
3	3	2	1	2	2	2	2	1	2
3	3	2	1	3	2	2	2	2	2
1	2	3	1	2	2	2	2	1	2
3	1	2	5	1	1	1	3	1	2
2	2	2	1	2	2	2	2	2	2
2	2	2	5	2	3	1	2	1	1
2	2	2	9	2	2	2	2	2	2
2	2	2	2	3	3	3	3	2	2
1	1	1	9	1	1	2	1	1	2
1	2	3	9	1	2	2	2	1	2
2	3	1	1	1	1	1	1	1	2
3	1	2	4	1	1	1	2	1	2
3	3	3	9	2	2	2	2	3	3
1	1	2	1	1	2	2	2	1	1
2	1	2	1	1	2	1	2	2	1
2	2	2	9	2	2	2	2	2	2
1	1	1	1	1	2	1	1	1	2
1	2	1	3	1	2	2	3	1	2
2	1	2	2	1	1	1	1	1	1
2	3	3	9	1	2	3	3	1	2
1	2	2	1	3	3	2	2	2	1
3	1	3	9	1	1	2	2	3	1

1	1	3	1	1	2	2	2	2	2
3	1	2	3	1	1	3	3	1	3
2	2	2	9	1	2	2	2	2	2
1	1	1	2	1	1	1	1	1	1
3	1	2	3	2	2	2	2	1	2
3	3	2	1	1	1	1	1	1	2
2	3	2	4	1	1	2	2	1	2
2	2	1	4	1	1	1	2	2	1
1	3	2	3	1	1	1	2	1	2
1	2	3	1	2	2	2	2	3	3
1	2	3	9	1	3	2	1	1	2
3	2	1	1	2	2	2	2	2	1
1	1	1	5	1	1	1	1	1	1
1	3	3	1	1	2	1	2	1	3
3	2	3	1	2	2	2	2	2	2
1	1	3	1	1	2	2	1	1	3
3	1	3	1	2	2	2	2	1	1
1	3	3	1	1	2	2	2	1	1
2	1	1	4	1	1	2	3	1	2
3	3	2	5	2	1	2	1	2	3
2	3	3	1	1	2	3	3	3	3
3	1	3	1	2	2	2	2	3	3
9	3	1	3	1	2	2	2	3	2
2	1	1	4	1	1	2	1	1	3
2	2	2	2	2	2	2	2	2	2
2	2	2	9	9	9	9	9	2	2
1	3	1	4	1	1	1	1	1	3
1	3	2	1	1	2	3	1	2	1
2	2	2	9	9	9	9	9	2	2
9	9	9	9	9	9	9	9	9	9
9	9	9	9	9	9	9	9	9	9
9	9	9	9	9	9	9	9	9	9
2	2	2	3	1	1	1	1	2	1
9	9	9	1	9	9	9	9	9	9
2	2	2	9	9	9	9	9	3	3
9	9	9	9	9	9	9	9	9	9
3	2	3	1	1	2	2	2	2	2
9	9	9	1	2	2	2	2	9	9
1	9	1	9	9	9	9	9	1	1
1	3	2	3	1	1	1	1	2	3
9	9	9	9	9	9	9	9	9	9
2	2	2	2	1	2	2	2	2	2
2	3	1	1	1	2	1	1	1	9
2	3	1	9	9	9	9	9	1	3
2	1	1	9	1	3	2	1	1	3
3	1	2	1	1	2	1	1	1	1
1	3	1	4	1	1	1	1	1	3

1	2	1	9	1	2	2	1	1	2
2	2	1	3	2	2	2	9	1	2
2	2	2	9	9	9	9	9	9	9
1	1	1	3	2	2	2	1	2	1
2	2	2	1	2	9	2	1	2	1
2	2	1	1	1	2	1	1	1	2
9	9	9	1	2	1	3	1	9	9
1	2	3	1	1	2	3	3	1	2
1	2	3	4	1	2	1	2	1	1
3	2	3	1	1	1	1	1	3	3
1	2	3	1	2	2	1	2	2	3
2	2	2	9	3	2	2	1	2	1
2	3	1	9	1	1	1	3	1	3
2	2	2	1	2	2	2	2	2	2
2	2	2	1	2	2	2	2	2	2
1	1	1	9	9	9	9	9	9	9
2	2	2	3	2	3	3	3	2	2
2	2	2	1	3	3	3	3	2	2
2	2	2	1	2	2	2	2	2	2
2	2	2	9	2	2	2	2	2	2
1	1	9	1	1	1	1	1	1	3
1	1	9	1	1	1	1	1	1	3
1	1	9	1	1	1	1	1	1	3
1	1	1	1	1	1	1	1	1	2
1	1	1	3	1	1	1	1	1	3
1	1	3	4	1	1	1	1	1	3
1	1	3	2	1	1	1	1	1	3
2	1	2	1	1	1	1	1	1	3
2	1	2	1	1	1	1	1	1	2
1	1	9	1	1	1	2	1	1	3
1	1	9	2	1	1	1	1	1	3
9	9	9	3	3	1	2	3	9	2
1	1	1	3	1	1	1	1	1	2
1	1	3	1	1	1	1	1	1	3
1	1	9	1	1	2	2	1	2	2
1	1	9	2	1	1	1	1	1	2
1	1	1	9	1	1	3	1	1	2
1	1	9	1	1	1	1	1	1	3
1	1	1	1	1	1	1	1	9	9
1	1	3	1	1	1	2	1	1	3
1	1	3	3	1	1	1	1	1	1
9	1	1	1	1	1	1	1	1	9
1	1	3	1	1	1	1	1	1	1
1	1	1	1	1	1	1	1	1	3
1	1	1	2	1	1	1	1	1	1
1	1	3	4	1	1	1	1	1	3
1	1	9	9	1	1	1	1	1	2

1	1	9	2	1	1	1	1	1	2
1	1	3	1	1	1	1	1	1	2
1	1	1	1	1	1	1	1	1	3
1	1	1	1	1	1	1	1	1	1
1	1	9	1	1	1	1	1	1	3
1	1	9	1	1	1	1	1	1	3
1	1	1	1	1	1	2	2	2	2
2	1	1	1	1	1	1	1	1	2
1	1	3	1	1	1	1	1	9	9
1	1	1	1	1	1	1	1	1	3
1	1	2	1	1	1	2	2	9	3
1	1	2	2	1	3	3	1	2	2
1	1	9	1	1	1	1	1	1	3
1	1	3	1	1	1	1	1	1	3
1	1	3	1	1	1	1	1	1	3
1	1	9	1	1	1	1	1	1	3
1	1	9	1	1	1	3	1	1	3
1	1	1	1	1	1	2	2	1	9
1	1	9	1	1	1	1	1	1	3
1	1	1	1	1	1	3	1	2	9
2	1	2	2	1	2	2	2	3	1
3	3	3	9	1	2	2	2	1	3
1	1	3	1	1	1	1	1	1	1
1	1	9	1	1	1	1	1	1	3
1	1	1	1	1	2	2	2	3	2
1	1	1	1	1	1	1	1	1	3
1	1	9	1	1	3	2	1	1	1
3	3	2	1	1	1	1	1	1	3
1	1	3	1	1	1	1	2	1	3
1	1	9	3	1	1	2	1	1	3
1	1	2	1	1	1	2	2	1	2
1	1	3	1	1	1	1	1	1	3
1	1	9	2	1	1	1	1	9	9
1	1	9	4	1	1	1	1	1	3
1	1	1	1	1	1	1	1	1	3
1	1	1	1	1	1	1	1	1	3
1	9	9	2	1	1	1	1	1	3
1	1	9	1	1	1	1	1	1	3
1	1	3	1	1	1	1	1	1	3
1	1	1	1	1	1	1	1	1	3
1	1	3	4	1	1	1	1	1	3
1	1	1	1	1	1	1	1	1	3
1	1	9	1	1	1	1	1	1	3
1	1	1	1	1	1	1	1	1	3
1	1	2	1	1	1	1	1	1	3
1	1	1	1	1	1	1	1	1	2
1	1	9	1	1	1	1	1	1	3

1	1	9	1	1	2	2	1	1	3
1	1	9	1	1	1	1	1	1	3
1	1	2	2	1	1	1	1	1	3
1	1	9	1	1	1	1	1	1	1
1	1	1	1	1	1	1	1	1	3
3	1	1	1	1	2	2	1	1	1
1	1	1	5	1	1	2	1	3	1
1	1	1	9	9	9	9	9	1	9
1	1	1	9	1	1	9	9	1	9
1	1	1	4	1	1	1	1	1	3
1	2	1	4	2	2	1	1	1	3
9	9	9	1	1	1	1	1	9	9
1	3	1	5	1	1	2	1	1	3
2	2	1	1	1	1	2	1	1	3
2	3	1	1	2	2	3	3	2	9
1	1	3	4	1	1	2	2	1	2
9	9	9	1	1	1	1	1	9	9
9	9	9	1	1	1	1	1	9	9
1	1	1	4	1	1	1	1	1	2
1	1	1	3	1	1	2	1	2	2
1	1	1	3	1	1	1	1	1	3
2	2	3	3	1	1	1	1	1	3
2	3	3	9	1	1	1	1	1	3
2	3	3	3	1	1	1	1	1	3
3	3	1	4	1	1	2	2	1	3
1	1	1	1	1	1	2	2	1	3
2	2	3	1	1	1	2	3	2	3
1	1	2	1	1	1	1	1	2	2
1	1	1	1	1	1	1	1	1	2
1	1	1	1	1	1	1	1	1	2
1	1	1	1	1	1	1	1	1	2
9	9	9	2	1	1	1	2	2	3
2	1	1	1	1	1	2	1	2	2
2	1	1	5	1	1	1	1	1	3
2	1	1	5	1	1	1	1	1	1
1	2	1	3	1	1	1	1	2	2
2	1	1	9	1	1	3	9	9	9
2	2	2	9	1	1	3	1	1	1
1	1	2	9	1	1	3	1	1	1
2	1	1	2	1	1	1	1	1	3
1	1	1	4	1	1	1	1	1	3
2	1	1	3	1	1	1	1	1	3
2	1	1	3	1	1	2	2	1	2
2	2	2	9	9	9	9	9	2	2
9	1	1	3	1	1	2	2	1	2
1	1	1	1	1	2	2	1	1	3
1	1	2	5	1	1	1	1	1	3

1	1	1	1	1	1	1	1	1	3
2	1	1	1	1	1	2	1	1	2
2	1	1	1	1	1	3	1	1	3
2	2	2	1	1	1	2	1	1	2
9	9	9	4	1	1	1	1	1	1
2	3	3	2	2	1	2	2	2	3
1	1	1	2	1	1	1	1	1	2
2	1	1	4	1	1	2	1	9	9
2	1	1	5	1	1	1	1	9	9
2	1	1	1	1	1	1	1	1	2
3	1	1	1	1	1	1	1	1	3
1	1	1	4	1	1	1	1	1	3
2	1	1	1	1	1	2	2	2	2
2	2	1	3	1	1	1	1	1	2
2	1	2	3	3	1	2	1	2	2
9	9	9	3	1	1	1	1	9	9
1	1	2	3	1	1	1	1	1	2
2	1	1	1	1	1	1	1	1	2
1	1	1	2	1	1	1	1	1	3
1	1	1	3	1	1	1	1	1	3
9	9	9	3	1	1	2	1	9	9
2	1	2	3	1	1	1	1	1	1
2	3	2	3	1	1	1	1	9	9
1	1	1	2	1	1	1	1	1	2
1	1	1	2	1	1	1	1	1	3
1	1	1	9	1	1	1	1	1	3
9	1	9	9	1	1	1	1	2	2
9	9	9	9	9	9	9	9	9	9
9	9	9	9	9	9	9	9	9	9
3	1	1	2	1	1	2	1	1	3
1	1	1	3	1	1	1	1	1	2
2	1	1	4	1	1	1	1	1	2
1	1	9	1	1	1	1	1	9	9
1	1	9	9	1	1	1	1	9	9
1	1	3	1	1	1	1	1	1	3
1	1	9	1	1	1	1	1	1	2
1	1	1	1	1	1	1	1	1	3
1	1	1	2	1	1	1	1	1	3
1	1	1	1	1	1	1	1	1	3
1	1	2	4	1	1	1	1	1	3
1	1	9	1	1	1	2	1	1	1
1	1	9	1	1	1	2	1	1	3
1	1	3	1	1	1	1	1	1	3
1	1	1	1	1	1	3	1	3	1
1	1	1	1	1	1	1	1	1	2
1	1	1	9	1	1	2	2	3	3
1	1	9	1	1	1	1	3	1	3

1	1	1	2	1	1	1	1	1	3
1	1	9	1	1	1	2	2	1	3
9	9	9	9	9	9	9	9	9	9
1	1	9	3	1	9	1	1	1	3
9	1	9	1	1	1	1	1	1	3
1	3	3	3	1	1	1	1	1	2
3	1	1	1	1	1	3	1	1	1
2	1	1	5	1	1	1	1	1	3
9	9	9	3	1	1	1	1	2	9
9	9	9	9	1	1	1	1	1	3
3	3	3	9	1	1	9	9	1	3
1	1	1	2	1	1	3	1	1	1
1	1	2	1	1	1	1	1	1	2
9	1	9	4	1	1	1	1	9	9
1	2	1	3	1	1	1	1	1	2
2	1	1	3	1	1	1	1	1	3
9	1	1	1	1	1	9	1	9	9
2	1	3	3	1	2	1	1	9	9
1	1	2	3	1	1	1	1	1	2
2	1	2	1	1	1	1	1	1	3
1	1	3	1	1	1	1	1	1	3
1	1	3	3	1	1	1	1	1	3
1	1	3	3	1	1	1	1	1	3
1	1	1	2	1	1	1	1	1	3
1	1	1	9	1	1	9	1	1	9
1	1	3	3	1	1	1	1	1	2
2	1	1	1	1	1	1	1	1	3
3	1	1	3	1	2	1	1	1	3
1	1	3	3	1	1	1	1	1	2
1	1	3	3	1	1	1	1	1	2
2	1	1	3	1	2	2	1	1	2
1	1	2	2	1	1	1	1	1	2
2	1	1	3	1	1	1	1	1	3
1	1	1	3	1	1	1	1	1	2
2	1	2	4	1	1	1	1	1	3
2	1	1	3	1	1	1	1	9	9
1	1	1	4	1	1	1	1	1	3
1	1	9	3	1	1	1	1	1	9
2	2	2	3	1	1	2	1	1	1
1	1	1	2	3	1	3	1	1	3
2	2	3	2	1	1	3	3	2	3
3	3	1	3	1	1	1	1	1	3
9	1	9	1	1	1	1	1	1	9
9	1	9	1	1	1	1	1	9	9
1	1	1	3	1	1	1	1	1	2
1	1	1	2	1	1	1	1	1	3
9	9	9	9	1	1	1	1	1	9

3	1	3	3	1	1	1	1	1	3
2	1	1	3	1	1	1	1	1	2
2	1	2	1	1	1	1	1	2	2
2	1	1	1	1	1	1	1	1	3
3	1	1	1	1	1	1	1	1	2
2	1	3	1	1	1	1	1	1	2
3	2	3	3	1	1	9	1	2	2
2	1	1	1	1	1	1	1	1	2
1	2	2	3	1	1	1	3	1	2
1	2	2	1	1	1	1	1	1	2
3	2	2	3	1	1	1	1	1	2
9	9	9	4	1	1	1	1	1	9
9	9	9	3	1	1	1	1	1	9
9	9	9	1	1	1	1	1	1	9
9	9	9	1	1	1	1	1	1	9
9	9	9	1	1	1	1	1	1	9
3	3	3	3	1	1	9	1	1	3
1	1	1	4	2	2	2	2	1	2
9	9	9	3	1	1	1	1	9	9
2	1	1	3	1	1	1	1	1	3
1	1	1	3	1	1	1	1	1	3
2	1	3	3	1	1	2	1	9	9
2	1	2	4	1	1	2	1	1	3
2	2	3	3	1	1	2	1	2	3
2	1	1	3	1	1	1	1	1	2
1	1	1	3	1	1	1	1	1	2
1	1	1	3	1	1	3	1	1	2
1	1	1	2	1	1	1	1	1	3
9	1	1	4	1	1	1	1	1	2
2	1	1	3	1	1	1	1	1	3
1	1	1	3	1	1	1	1	1	2
1	1	9	9	1	1	1	1	1	9
2	2	3	3	1	1	1	1	1	3
1	1	2	3	1	1	1	1	1	3
2	1	1	3	1	1	1	1	1	2
2	2	1	3	1	1	1	1	1	2
2	1	1	3	1	1	2	1	2	2
1	1	1	1	1	1	1	1	1	1
1	1	1	1	1	2	1	1	9	9
1	1	1	1	1	1	1	1	1	3
3	2	3	3	1	1	3	3	1	2
2	2	2	9	1	1	3	3	1	2
2	1	1	5	1	1	1	1	1	3
1	1	1	5	1	1	1	1	1	2
2	2	2	2	1	1	1	1	1	3
9	1	1	2	1	1	1	1	1	9
1	1	1	1	1	1	1	1	1	3

1	1	1	3	1	1	1	1	1	3
1	1	1	4	1	1	1	1	1	3
1	1	1	5	1	1	1	1	1	2
1	2	2	4	1	1	1	1	1	2
2	3	3	4	1	1	1	1	1	1
2	2	2	3	1	1	1	1	1	3
1	2	2	2	1	1	1	1	1	3
1	2	1	3	1	1	1	1	1	2
2	2	2	4	1	1	1	1	2	1
1	1	2	4	1	1	1	1	1	2
2	1	3	3	1	1	1	1	1	3
2	2	2	9	1	1	1	1	2	2
1	2	2	1	1	1	1	1	1	3
1	2	1	1	1	1	1	1	1	3
2	2	2	1	1	1	1	1	1	1
1	1	1	3	1	1	1	1	1	1
2	2	2	2	1	1	2	1	2	2
2	2	2	1	1	1	2	1	2	2
2	2	2	4	3	3	3	3	2	3
2	3	3	3	1	1	1	1	1	3
2	3	3	4	1	1	1	1	1	3
9	9	9	9	9	9	9	9	1	3
1	2	1	4	1	1	1	1	1	3
2	1	3	3	1	1	1	1	1	3
2	1	2	2	1	1	1	1	1	2
2	1	1	1	1	9	9	9	9	9
1	1	1	1	9	9	9	9	9	9
9	9	9	3	1	1	1	1	1	9
2	3	9	1	9	9	9	9	1	3
3	3	3	2	1	1	1	1	3	9
1	1	1	4	1	1	1	1	1	2
1	1	1	4	1	1	1	1	1	2
9	9	9	9	1	1	1	1	9	9
9	1	9	1	9	9	9	9	9	9
9	1	9	1	1	2	1	1	9	9
9	1	9	1	1	1	1	1	9	9
9	9	9	1	1	1	1	1	9	9
9	9	9	1	1	1	1	1	9	9
9	9	9	3	1	1	1	1	1	9
9	9	9	9	1	1	1	3	3	2
1	1	1	3	1	1	1	1	1	3
9	9	9	3	1	1	1	1	1	9
9	9	9	5	1	1	1	1	1	9
1	2	2	2	1	1	1	1	2	2
9	9	9	1	1	1	1	1	9	9
9	9	9	3	1	1	1	1	1	9
9	9	9	3	1	1	1	1	1	9

1	1	1	2	1	1	1	1	1	3
1	2	1	9	1	1	2	1	9	9
9	9	9	3	1	1	1	1	9	1
9	9	9	1	1	1	1	1	1	9
9	9	9	1	1	1	1	1	1	9
9	9	9	1	1	1	1	1	1	9
9	9	9	9	1	1	1	1	1	9
9	9	9	3	1	1	1	1	1	9
9	9	9	4	1	1	1	1	9	9
9	9	9	1	1	1	1	1	1	9
1	3	3	3	1	1	1	1	1	3
1	1	1	3	1	1	1	1	1	3
1	1	1	3	1	1	1	1	1	3
1	1	1	4	1	1	2	1	1	2
1	1	1	4	1	1	1	1	1	1
1	3	1	1	1	1	2	1	9	9
1	3	1	1	1	1	2	1	1	1
1	1	1	1	1	2	1	1	1	1
1	1	1	3	1	1	1	1	1	3
1	1	1	3	1	9	1	1	1	3
1	1	1	3	1	1	1	1	1	3
2	9	2	1	1	1	2	1	1	1
9	9	9	1	1	1	1	1	1	9
1	1	1	9	1	1	1	1	1	9
9	9	9	9	1	1	1	1	3	1
1	1	1	3	1	1	1	1	1	3
2	1	2	1	2	1	2	1	2	3
1	1	1	2	1	1	1	1	1	3
1	1	2	1	1	1	1	1	1	3
2	1	2	1	1	1	2	2	2	2
1	1	2	3	1	1	1	1	1	2
2	1	1	3	1	1	3	1	1	2
2	2	1	1	1	1	1	1	1	1
1	1	1	4	1	1	1	1	1	2
1	2	2	3	1	1	1	1	1	2
1	1	1	2	1	1	1	1	1	3
1	1	3	2	1	1	1	1	1	3
1	1	3	3	1	1	1	1	1	3
1	1	1	1	1	1	1	1	1	3
1	1	3	1	1	1	1	1	2	3
1	1	2	4	1	1	3	1	1	3
1	1	1	3	1	2	2	1	1	2
2	1	3	1	1	3	2	1	1	2
1	1	1	3	1	1	1	1	1	3
1	1	1	9	1	1	1	1	1	9
1	3	3	3	1	1	1	1	1	3
1	1	1	2	1	1	1	1	1	3

1	1	1	2	1	1	1	1	1	3
1	1	1	2	1	1	1	1	1	3
1	1	1	3	1	1	1	1	1	2
1	1	1	4	1	3	3	1	1	3
1	1	1	3	1	1	1	1	1	3
1	1	1	4	1	3	1	1	1	3
1	1	1	2	1	1	1	1	1	3
1	1	1	4	1	1	1	1	1	3
1	1	3	2	1	1	1	1	1	2
1	1	1	9	1	1	1	1	1	3
1	1	1	2	1	1	1	1	1	2
1	1	1	4	1	1	1	1	1	3
1	1	3	3	1	1	1	1	1	3
1	1	3	4	1	1	1	1	1	3
1	1	1	1	1	1	1	1	1	3
1	1	1	2	1	1	1	1	1	3
1	1	3	3	1	2	2	2	1	2
2	2	2	2	1	1	1	1	1	2
3	1	3	1	2	1	1	2	3	1
1	1	2	3	1	1	1	1	1	1
3	1	1	2	1	1	1	1	1	3
1	1	1	2	1	1	1	1	1	3
1	1	1	4	1	3	3	1	1	3
2	1	1	1	1	1	2	1	1	2
2	1	1	1	1	1	2	1	1	2
1	3	2	3	1	1	1	1	1	3
1	2	2	1	1	1	2	2	2	2
2	2	2	1	1	2	2	2	2	2
1	1	1	3	1	1	1	1	1	2
2	2	3	3	1	1	1	1	1	2
3	3	2	1	1	1	2	2	2	2
2	2	2	1	1	1	1	1	1	2
1	1	2	1	1	1	1	1	1	3
1	1	2	1	1	1	1	1	1	1
9	9	9	9	1	3	3	1	1	3
1	1	1	4	1	3	3	1	1	3
1	2	3	5	1	1	1	1	3	3
2	2	2	1	1	1	1	1	3	3
1	1	3	4	1	1	1	1	1	1
1	2	2	2	1	1	1	1	1	3
2	2	2	1	1	1	1	1	1	3
1	1	3	5	1	1	1	1	1	1
1	1	3	9	1	1	1	1	1	1
2	2	2	3	1	1	1	1	1	2
1	1	1	1	1	1	1	1	1	3
1	2	1	2	1	1	1	1	1	2
2	2	2	1	1	1	1	1	1	3

3	3	3	5	1	1	1	1	2	2
2	2	2	1	1	1	1	1	1	3
2	1	2	1	1	1	1	1	1	3
1	1	1	1	1	1	1	1	3	3
1	1	1	4	1	1	1	1	1	3
2	2	2	1	1	1	1	1	1	2
2	3	3	4	1	1	1	1	1	3
2	2	1	1	1	1	1	1	2	2
1	2	2	3	1	1	1	1	3	1
2	1	1	4	1	1	1	1	1	3
2	2	1	5	1	1	2	1	2	2
1	3	2	3	1	1	1	1	1	3
9	9	9	9	1	1	1	9	1	9
1	2	3	1	1	1	1	1	1	3
3	3	1	1	1	1	1	1	1	3
2	2	2	1	2	2	2	2	1	2
2	2	1	4	1	1	1	1	1	2
1	1	1	3	1	1	1	1	1	3
2	1	1	2	1	1	1	1	1	3
1	2	2	1	1	1	2	1	1	2
1	1	1	4	1	1	1	1	1	3
1	1	1	3	1	1	1	1	1	3
3	1	1	4	1	1	1	1	1	1
2	1	1	4	1	1	1	1	1	3
1	1	2	4	1	1	1	1	1	2
1	1	1	4	1	1	1	1	1	3
1	3	1	1	1	1	2	1	1	3
2	1	1	9	1	1	1	3	3	2
1	2	2	9	1	1	1	1	2	2
9	9	9	9	1	1	1	1	2	9
2	3	2	1	1	1	1	1	1	2
2	3	3	2	1	1	1	1	9	9
2	2	2	1	1	9	2	1	1	2
1	1	1	1	1	1	2	1	1	2
9	9	9	1	1	1	1	1	1	3
2	1	2	1	1	3	3	1	1	2
1	1	3	9	3	2	2	3	2	2
9	9	9	3	1	1	3	1	9	9
2	1	2	5	1	1	1	1	1	2
2	1	2	3	1	1	2	2	2	2
9	1	9	4	1	2	2	1	2	2
1	2	3	3	1	1	1	1	3	3
1	1	1	3	1	1	2	1	3	3
1	9	9	4	1	1	2	1	1	9
1	2	3	1	1	1	2	2	2	2
2	2	2	4	1	1	1	1	2	2
1	1	1	5	1	1	2	1	1	3

2	2	2	2	1	1	2	1	1	3
2	2	3	3	1	1	1	1	1	2
1	2	1	1	1	1	2	1	2	2
2	2	2	2	1	1	2	1	2	2
3	1	1	5	1	1	2	2	1	2
2	2	2	2	1	1	2	1	1	3
1	1	1	5	1	1	2	1	1	3
9	9	9	1	1	1	1	1	1	9
2	2	2	1	1	1	2	1	1	3
2	2	2	1	1	1	2	1	1	2
1	1	2	4	1	1	1	1	1	3
3	2	2	1	1	1	1	1	2	2
1	2	2	2	1	1	2	1	1	2
2	2	2	2	1	1	1	1	2	2
2	2	2	1	1	1	1	1	1	3
1	3	3	4	1	1	1	1	1	3
1	1	1	1	1	1	1	1	2	2
1	1	3	1	1	1	1	1	1	3
1	1	2	4	1	1	1	1	1	3
1	1	2	9	1	1	1	1	1	2
1	1	2	4	1	1	1	1	1	2
1	1	2	3	1	1	1	1	1	2
2	2	2	4	3	1	3	1	1	2
2	2	2	4	1	1	1	1	1	3
3	2	3	4	1	1	1	1	1	2
1	1	2	1	1	1	1	1	1	2
1	1	2	4	1	1	1	1	1	2
1	1	3	4	1	1	1	1	1	2
1	1	3	4	1	1	1	1	1	2
9	9	9	9	1	2	1	1	9	9
1	1	3	4	1	1	1	1	1	2
1	2	1	2	1	1	1	1	1	3
1	2	1	2	1	1	1	1	1	1
2	2	9	3	1	1	1	1	1	1
9	9	9	4	1	9	1	1	9	9
1	1	2	4	1	1	1	1	1	2
1	1	1	4	1	1	1	1	1	2
1	1	1	4	9	9	9	9	1	9
1	1	1	4	1	1	1	1	1	3
2	2	3	1	1	1	1	1	1	2
1	2	1	9	1	1	1	1	1	3
1	1	3	1	1	1	1	1	1	3
2	2	3	3	1	1	2	2	2	2
1	3	3	1	1	1	1	1	1	3
1	1	1	4	1	1	1	1	1	3
1	3	1	4	1	1	1	1	1	3
1	1	1	1	1	1	1	1	1	3

1	1	1	3	1	1	1	1	1	3
1	1	1	3	1	1	1	1	1	3
9	2	1	1	1	1	1	1	1	3
1	1	1	3	1	1	1	1	1	3
1	1	1	9	1	1	1	1	1	3
1	1	1	4	1	1	1	1	1	3
1	3	1	4	1	1	1	1	1	3
3	2	1	3	1	1	1	1	2	1
1	1	1	3	1	1	1	1	1	3
1	1	1	3	1	1	1	1	1	3
1	1	3	1	1	1	1	1	1	3
1	1	3	4	1	1	3	1	1	3
1	1	3	4	1	1	1	1	1	3
1	1	1	4	1	1	1	1	1	2
1	1	1	3	1	1	1	1	1	3
1	1	1	2	1	1	1	1	1	3
1	2	3	1	1	1	1	3	1	1
1	1	2	3	1	1	1	1	1	2
1	1	1	4	1	1	1	1	1	3
1	1	1	2	1	1	3	1	1	3
1	1	2	9	1	1	3	1	1	2
3	3	3	1	1	1	1	1	1	3
9	9	9	1	1	1	1	1	1	9
9	9	9	9	1	1	1	1	9	2
9	9	9	2	1	1	1	1	1	9
9	9	9	4	1	1	1	1	9	9
9	9	9	4	1	1	1	1	1	3
9	1	9	1	1	2	1	1	9	9
9	9	9	1	1	1	1	1	1	9
9	9	9	1	2	2	2	2	9	9
9	9	9	1	1	1	1	1	9	9
9	9	9	1	1	1	1	1	9	9
9	9	9	1	1	1	2	1	2	9
9	9	9	3	1	1	1	1	1	9
2	1	1	3	1	1	1	1	2	2
1	1	2	4	1	1	1	1	1	3
1	1	2	2	1	1	1	1	1	2
1	1	3	1	1	1	1	1	1	3
1	1	3	4	1	1	1	1	1	2
1	1	3	4	1	1	1	1	1	2
1	1	1	9	1	1	1	1	1	3
1	1	2	5	1	1	1	1	2	2
2	1	2	1	1	1	2	1	1	2
2	2	2	1	1	3	1	1	1	3
3	2	2	9	1	1	2	1	2	2
1	1	3	5	1	1	1	1	1	3
1	1	3	4	1	1	1	1	1	2

2	2	2	3	1	1	2	1	2	2
1	2	1	1	1	1	1	1	2	2
2	1	3	1	1	1	1	1	1	2
1	1	3	9	1	1	2	1	3	2
1	2	2	3	2	2	2	2	1	1
2	2	2	3	1	1	1	1	1	3
9	9	9	9	9	9	9	9	9	9
2	2	2	1	1	1	2	1	2	2
2	3	2	4	1	1	2	1	1	2
2	2	2	4	1	1	2	1	2	2
2	1	1	9	1	2	2	1	2	2
1	1	1	3	1	1	1	1	1	2
2	1	2	1	1	2	1	1	1	2
2	2	3	1	1	1	2	2	3	3
2	2	2	5	1	1	1	1	1	2
2	2	2	5	1	1	1	1	1	1
2	2	2	9	9	9	9	9	9	9
1	2	2	1	1	2	1	1	1	2
1	1	1	9	1	1	2	1	2	2
1	1	1	1	1	1	1	1	1	1
1	1	2	9	1	1	1	1	1	1
2	2	2	1	1	1	1	1	2	2
2	2	2	9	1	1	2	1	1	2
1	1	1	1	2	1	2	2	2	2
1	1	1	1	1	1	1	1	1	1
2	1	2	2	1	2	2	2	2	3
2	3	1	3	1	1	1	1	1	2
1	1	1	3	1	1	1	1	1	2
2	2	2	1	2	2	2	2	2	2
2	2	2	1	1	1	1	1	2	2
1	1	1	2	1	1	1	1	1	1
1	1	1	1	1	1	2	2	2	2
3	2	1	9	1	1	2	1	1	3
2	2	2	4	1	1	1	1	1	2
2	2	2	1	1	2	2	1	9	9
2	2	2	2	1	1	3	9	2	2
2	2	2	1	1	1	1	1	2	2
2	2	2	3	1	1	2	1	2	2
2	2	2	1	1	1	2	1	2	2
1	9	2	1	1	1	2	1	1	3
1	2	1	1	1	1	2	1	2	2
1	2	1	1	1	1	2	1	2	2
9	9	9	1	1	1	1	1	3	9
9	9	9	3	1	1	1	1	3	9
9	9	9	4	1	1	1	1	2	9
3	3	2	1	1	1	1	1	2	2
2	3	2	1	1	1	1	1	2	2

3	3	2	3	1	1	1	1	2	2
3	2	2	3	1	1	1	1	2	2
3	2	2	3	1	1	1	1	1	1
1	1	1	1	1	1	1	3	2	2
2	2	2	4	1	1	3	3	2	2
3	2	2	3	1	1	1	1	2	1
1	2	3	1	1	1	1	1	1	2
1	2	1	3	1	1	1	1	2	1
2	2	3	9	1	1	1	1	3	2
1	1	1	1	1	1	3	3	2	2
2	2	2	9	1	3	1	3	2	2
3	3	3	9	1	1	1	3	2	2
1	1	1	9	1	1	1	1	2	2
3	1	1	5	1	1	1	1	2	2
1	1	3	9	1	1	3	3	2	2
2	1	1	1	1	1	1	1	2	2
2	2	2	1	1	1	1	1	1	2
2	2	1	1	1	1	1	1	1	2
3	2	2	3	1	1	1	1	2	2
3	3	2	3	1	1	1	1	2	2
2	2	2	1	3	3	3	3	2	2
2	2	2	1	3	3	3	3	2	2
2	2	2	1	3	3	3	3	1	3
2	2	2	9	3	3	3	3	1	3
1	1	1	9	3	3	3	3	1	3
2	2	2	1	3	3	3	3	1	3
1	1	1	1	3	3	3	3	1	3
1	1	1	1	3	3	1	1	1	3
2	2	2	1	2	3	2	3	3	1
3	3	3	4	1	1	1	1	3	2
3	3	3	4	1	1	1	1	3	2
3	3	3	4	1	1	1	1	3	2
3	3	3	4	1	1	1	1	3	2
3	2	2	1	1	3	3	2	3	9
3	1	3	9	1	3	3	3	3	9
3	1	3	6	2	2	2	9	3	3
1	3	2	9	1	1	1	1	3	1
2	1	2	9	1	1	1	1	3	2
2	3	2	9	1	1	1	1	3	2
3	3	2	9	1	1	1	1	3	2
2	1	3	5	2	2	2	2	3	3
3	3	3	4	2	2	2	2	3	3
2	3	2	3	2	3	2	3	3	2
9	9	9	6	1	1	1	1	3	9
9	9	9	9	9	9	9	9	9	9
9	9	9	9	9	9	9	9	2	2
3	2	2	4	1	1	1	1	3	9

3	2	2	5	1	1	1	1	3	9
3	3	3	1	1	2	3	1	3	1
2	3	2	1	3	3	3	3	9	9
2	2	2	9	1	2	2	2	2	2
2	2	2	9	1	2	2	2	2	2
9	9	9	1	2	2	2	1	2	9
3	3	3	3	1	2	2	2	3	2
9	9	9	4	2	1	2	2	2	9
2	2	2	1	1	3	3	1	3	2
2	3	1	1	1	2	1	1	3	2
2	2	2	9	3	3	3	3	3	1
2	2	2	9	1	1	2	1	2	3
9	9	9	1	1	2	1	1	2	9
9	9	9	1	1	1	1	1	3	3
1	3	2	1	1	1	1	1	3	2
9	9	9	1	1	9	2	1	9	9
3	3	3	1	1	1	1	1	3	2
9	9	9	9	9	9	9	9	9	9
3	3	1	5	2	2	2	2	3	3
9	9	9	1	2	2	2	2	2	9
3	3	3	3	1	1	1	1	3	1
9	9	9	9	9	9	9	9	2	2
2	2	2	5	3	3	3	3	2	2
2	2	2	3	3	2	2	2	2	2
2	2	2	5	3	3	2	2	3	1
3	3	3	4	3	3	2	2	2	2
1	1	1	9	1	2	1	1	3	1
9	9	9	1	2	2	2	2	3	9
9	9	9	1	2	2	2	2	2	9
9	9	9	9	2	2	2	2	2	9
9	9	9	9	1	9	9	1	3	9
9	9	9	1	2	2	2	2	2	9
9	9	3	1	2	3	2	3	2	9
1	1	3	1	1	1	2	2	2	1
1	3	2	1	1	1	1	1	3	2
9	9	9	9	9	9	9	9	9	9
3	3	3	1	1	1	3	1	2	2
2	9	9	3	2	2	2	2	2	1
3	3	3	5	1	1	1	1	3	2
3	3	2	6	1	1	1	1	2	2
2	2	2	1	1	1	2	1	2	2
9	9	9	5	1	1	1	1	3	9
1	1	1	1	2	2	2	1	1	3
3	3	3	4	2	2	2	2	2	2
9	9	9	1	2	2	2	2	2	9
2	2	2	9	3	9	3	3	3	2
3	3	3	6	1	1	1	1	9	9

9	9	9	1	2	2	2	2	2	9
9	9	9	1	2	2	2	2	2	9
3	3	3	1	1	1	2	1	3	2
9	9	9	9	2	2	2	2	2	9
3	3	3	4	1	3	3	1	2	2
9	9	9	1	2	2	2	0	2	9
2	2	2	9	3	2	2	1	2	9
1	2	2	2	2	1	1	1	3	1
3	3	3	1	2	3	1	1	3	3
1	2	3	1	2	1	2	1	3	2
1	3	3	3	1	1	1	1	3	2
3	3	3	3	1	1	1	1	3	1
3	1	2	3	1	1	1	1	3	1
3	2	3	1	2	3	1	1	3	1
3	3	3	4	1	2	2	2	3	1
3	3	3	5	1	2	1	2	3	1
3	3	3	4	1	3	1	2	3	2
1	2	3	5	1	2	2	2	3	3
9	1	2	9	1	1	2	3	3	3
3	3	3	9	1	1	1	1	3	1
9	9	9	3	1	1	1	3	9	9
9	9	9	4	1	9	1	9	9	9
9	9	9	5	1	2	1	1	3	9
9	9	9	3	2	9	2	2	9	9
9	9	3	1	2	1	3	3	9	9
9	9	9	4	3	2	3	3	9	9
9	9	9	3	2	3	2	1	9	9
9	9	9	9	1	2	1	1	2	9
9	9	9	1	1	2	2	2	2	9
9	9	9	1	1	2	1	1	2	9
9	9	9	9	1	2	2	2	2	9
9	9	9	4	2	1	2	1	2	9
9	9	9	1	1	1	2	2	2	9
2	3	2	1	1	1	1	1	3	1
3	2	3	1	1	1	1	1	3	2
1	1	1	3	1	1	1	1	3	1
1	9	9	1	2	3	1	1	1	9
9	9	9	1	2	2	3	2	9	9
3	2	2	3	1	1	1	1	3	2
9	9	9	1	1	2	1	1	3	9
9	9	9	1	1	1	2	1	3	9
9	9	9	1	1	1	2	1	3	9
9	9	9	1	1	2	1	1	3	9
9	9	9	1	9	9	9	9	3	9
3	2	2	1	1	1	1	1	2	2
3	2	3	1	1	1	1	1	3	2
2	2	2	1	1	1	1	1	2	1

2	2	2	1	1	1	1	1	2	2
3	1	2	9	1	1	2	2	3	2
2	3	1	1	1	2	2	2	3	1
2	2	3	9	1	2	3	1	3	1
9	9	9	1	1	9	1	1	3	9
9	9	9	5	1	3	3	3	3	9
9	9	9	1	1	3	3	3	3	9
9	9	1	3	1	1	1	1	9	9
9	9	9	1	2	1	1	1	2	9
9	9	9	9	9	9	9	9	9	9
9	9	9	1	2	1	1	1	9	9
1	9	9	1	1	2	3	3	1	9
9	9	9	3	2	2	1	1	9	9
9	9	2	3	2	3	3	3	9	9
9	9	9	1	1	1	1	2	2	9
2	2	2	9	2	2	3	2	2	2
3	2	2	4	2	2	2	1	2	1
9	9	9	6	2	2	2	3	2	9
2	2	2	1	1	1	2	2	3	2
2	3	1	6	1	2	2	2	2	1
2	2	2	1	1	1	2	1	2	2
2	2	2	1	1	2	2	2	3	2
3	3	3	1	1	1	1	1	3	3
2	2	2	1	1	2	3	2	2	2
3	2	1	9	3	3	3	2	2	1
3	2	3	3	1	1	2	2	3	2
9	9	9	1	2	2	2	1	2	9
2	2	2	1	2	2	2	2	2	2
2	2	2	1	2	2	2	2	2	2
1	1	1	1	2	2	2	2	2	1
1	2	2	1	2	2	2	2	2	1
2	2	2	1	1	2	2	2	3	2
9	9	9	1	1	2	3	3	3	9
2	2	2	1	2	2	2	2	2	2
9	9	9	5	1	1	3	1	3	3
3	2	1	3	1	1	2	1	3	2
3	2	3	1	1	2	2	1	3	2
2	2	2	1	1	2	2	2	2	2
2	1	2	6	3	2	3	2	3	2
2	2	2	1	1	1	1	2	3	2
2	3	3	1	2	2	2	2	3	2
3	1	3	1	1	1	2	1	3	3
1	1	1	4	1	1	2	2	2	1
3	3	3	1	1	1	1	1	3	1
9	9	9	9	9	9	9	9	9	9
2	2	2	1	3	3	3	3	2	2
3	2	3	1	1	2	3	1	3	2

3	3	3	4	3	1	3	1	3	1
3	2	2	1	1	3	2	2	3	2
3	3	1	5	2	2	2	2	3	1
2	3	2	1	2	2	2	2	3	1
2	2	2	1	1	1	2	2	2	2
2	2	2	1	1	1	2	3	2	2
2	2	1	6	3	2	2	2	3	2
2	2	2	1	1	1	2	2	3	2
2	2	2	9	1	2	1	1	3	2
2	2	2	5	1	1	2	2	2	2
3	3	2	1	1	1	3	1	3	2
2	2	2	1	1	1	1	1	3	3
3	3	3	1	1	1	3	3	2	2
3	3	3	1	3	3	1	1	2	3
3	3	3	4	1	3	2	2	3	2
3	2	2	1	1	1	2	1	2	2
2	2	1	1	1	2	1	1	2	2
2	2	2	1	3	3	3	3	2	2
2	2	2	5	1	1	3	1	2	2
2	2	2	1	1	2	2	2	2	2
2	2	2	1	1	2	2	2	2	2
2	2	2	1	1	1	1	2	2	2
2	2	2	1	1	2	3	1	2	2
2	2	2	1	3	3	3	3	2	2
2	1	3	1	1	1	2	1	2	3
9	9	9	9	9	9	9	9	9	9
9	9	9	9	9	9	9	9	9	9
3	1	1	1	3	2	3	3	9	9
2	3	1	1	2	3	3	2	1	2
9	9	9	1	1	1	2	1	3	9
3	3	3	9	9	9	9	9	9	9
3	2	1	1	1	1	1	2	3	3
3	1	2	1	1	2	2	3	9	9
9	9	9	9	9	9	9	9	9	9
3	3	3	1	1	2	2	2	2	2
3	1	2	1	2	2	2	2	3	2
2	2	2	3	1	1	1	1	3	1
1	2	3	9	1	1	1	1	3	3
3	3	2	1	1	1	2	2	2	2
2	2	2	1	2	2	2	2	9	9
9	9	9	9	1	2	2	2	9	9
2	2	2	1	1	2	2	1	2	2
3	3	2	1	1	1	1	1	3	3
3	3	2	1	1	1	1	1	3	2
2	1	2	3	2	3	2	1	3	3
3	1	1	1	1	1	2	2	2	2
2	3	2	1	1	1	2	2	2	2

3	2	3	4	1	1	1	2	3	3
2	2	2	1	1	1	2	1	2	2
2	3	1	1	2	2	2	2	3	2
2	2	2	1	1	2	2	1	2	2
2	2	2	9	1	3	3	3	2	2
9	9	9	1	2	2	2	2	9	9
2	2	3	4	1	1	2	2	3	2
3	2	2	1	1	1	2	2	3	3
2	2	2	9	3	1	1	1	2	2
2	1	2	1	1	2	2	2	3	2
2	2	2	1	2	2	2	2	2	2
1	1	3	1	1	1	1	1	3	3
3	2	2	1	1	2	2	2	3	3
2	2	2	1	1	2	2	2	2	2
2	2	2	1	9	9	9	9	9	9
2	2	2	1	2	2	2	2	2	2
2	2	3	9	2	2	2	1	3	2
2	1	3	1	1	1	2	2	1	2
3	3	3	1	3	3	3	3	3	3
9	9	9	1	3	2	2	2	9	9
3	2	2	1	1	1	2	2	2	2
2	2	2	1	2	2	2	2	2	2
2	2	2	4	3	1	1	3	3	1
2	2	2	1	1	2	2	2	2	2
2	3	2	1	2	2	2	1	2	1
2	1	3	4	2	2	2	2	2	2
3	3	2	1	1	1	3	3	2	2
2	2	2	4	2	2	2	2	3	3
2	2	2	1	9	9	9	9	2	2
3	3	1	1	1	1	1	1	3	2
3	3	1	4	1	1	2	2	3	3
9	9	9	1	1	1	3	2	2	2
2	2	2	1	1	1	1	1	3	2
3	3	2	1	1	2	2	2	2	3
2	2	2	4	1	1	1	1	3	3
2	2	2	1	3	3	3	3	2	2
2	2	2	1	2	2	2	3	2	2
2	2	2	1	2	1	2	3	2	2
2	2	2	1	1	2	2	2	2	2
2	2	2	1	1	2	2	2	2	2
9	9	9	9	9	9	9	9	1	2
2	2	2	9	3	3	2	2	2	2
3	2	1	9	1	1	1	2	3	3
2	2	2	1	3	3	3	3	2	2
2	2	2	2	2	3	3	3	2	2
2	2	2	9	9	9	9	9	2	2
3	3	3	1	1	3	1	1	3	2

9	9	9	9	9	9	9	9	9	9
1	3	1	4	1	1	1	2	3	3
3	9	1	1	1	1	1	1	3	9
9	9	9	9	9	9	9	9	9	9
2	3	3	9	1	1	1	1	3	2
1	1	1	9	1	1	1	1	2	3
9	9	9	5	1	1	1	1	9	9
2	3	2	6	1	1	1	1	3	1
9	9	9	9	1	1	2	1	2	2
3	2	2	9	1	2	2	2	2	2
1	2	1	1	2	2	2	2	2	2
9	9	9	9	9	9	9	9	3	1
1	3	1	5	1	1	1	1	3	3
2	1	2	9	1	2	2	2	3	9
9	9	9	9	9	9	9	9	3	3
3	1	1	6	1	1	1	1	3	1
3	3	3	1	1	2	2	1	3	1
9	9	9	4	1	1	2	2	9	9
2	3	3	1	1	1	1	1	3	3
9	9	9	1	1	1	3	1	3	2
1	3	3	1	1	1	2	2	2	2
2	2	3	1	1	1	1	1	3	3
1	1	2	9	1	1	1	1	2	9
3	2	3	4	1	1	1	1	3	3
3	3	2	1	1	1	1	1	3	2
2	3	3	4	9	9	9	9	9	9
1	1	1	5	1	1	1	1	2	2
9	9	9	4	1	1	3	3	2	9
3	3	3	4	1	1	1	1	3	3
3	3	3	5	1	1	1	1	3	3
2	2	2	9	1	1	2	3	2	2
2	2	2	1	1	1	1	1	3	1
3	2	1	3	2	2	2	1	2	1
3	3	3	6	1	1	1	1	3	3
2	2	2	4	9	9	9	9	2	2
9	9	9	9	9	9	9	9	1	3
9	9	9	1	1	1	1	1	3	9
3	3	3	4	1	1	1	1	3	3
9	9	9	1	1	1	1	1	9	9
9	9	9	9	9	9	9	9	3	2
9	9	9	9	9	9	9	9	3	2
2	3	2	1	1	1	1	1	3	1
1	1	1	1	1	1	1	1	3	3
2	1	1	3	1	1	1	1	3	3
3	3	3	6	1	1	1	1	3	3
3	3	3	4	1	1	3	2	9	9
3	3	3	5	1	1	1	1	3	9

2	3	3	1	1	1	1	1	3	3
2	2	3	4	1	1	1	1	3	1
9	9	9	6	9	9	9	9	9	9
2	2	2	1	1	1	1	1	3	2
3	3	3	3	1	1	1	1	3	3
3	3	3	4	1	1	1	1	3	2
9	9	9	4	1	1	1	1	3	9
2	2	3	3	1	2	1	1	3	2
2	3	3	4	1	1	1	1	3	1
2	3	2	4	1	1	1	1	2	2
3	3	3	4	1	1	1	1	3	2
3	3	3	3	1	1	1	1	3	2
2	3	3	5	1	1	1	1	3	2
2	3	3	1	1	1	1	1	3	2
2	3	3	4	1	1	1	1	3	3
3	3	3	4	1	1	1	1	3	3
3	3	2	4	1	1	1	1	3	2
3	3	3	1	1	1	1	1	3	3
9	9	9	1	1	2	2	3	9	9
2	2	1	3	9	9	9	9	9	9
3	3	2	9	1	1	2	2	2	9
9	9	9	1	1	1	2	2	9	9
1	3	1	4	9	9	9	9	9	9
3	3	2	5	1	2	1	1	3	2
2	3	2	1	1	1	1	1	3	2
3	2	2	1	1	1	1	1	3	3
9	9	9	1	1	1	2	1	9	9
9	9	9	1	1	1	9	1	9	9
9	9	9	1	1	1	2	1	9	9
9	9	9	1	1	1	2	1	9	9
2	1	1	4	1	1	1	1	9	9
2	2	2	6	2	2	2	2	2	2
2	2	2	1	1	2	1	1	2	2
2	3	3	5	1	1	1	1	3	2
2	3	2	1	1	1	1	1	3	2
9	9	9	9	9	9	9	9	9	9
9	9	9	3	1	1	1	1	9	9
9	9	9	4	1	1	1	1	3	9
9	9	9	1	1	1	2	1	3	9
9	9	9	1	1	1	2	1	9	9
9	9	9	1	1	1	1	1	3	9
2	2	2	4	3	1	1	1	2	9
9	3	1	9	1	1	1	1	9	9
2	2	2	1	1	1	2	1	2	2
1	3	1	4	1	1	1	1	3	3
2	2	1	4	1	1	1	1	2	2
3	3	3	4	1	1	1	1	3	3

1	3	1	5	1	1	1	1	3	3
2	3	1	4	1	1	1	1	3	3
1	2	2	9	9	9	9	9	2	2
2	2	2	4	2	2	2	2	2	2
9	2	2	5	1	1	1	1	3	3
9	9	9	4	1	1	1	1	3	9
3	2	1	9	1	2	2	1	2	3
9	9	9	1	1	2	2	3	2	9
2	9	9	9	9	2	2	3	9	9
9	9	9	4	1	1	1	1	9	9
2	2	2	1	1	1	1	1	2	2
2	3	3	6	1	1	1	1	3	3
2	2	2	1	2	2	2	2	2	2
2	3	3	4	3	3	2	2	2	2
9	2	9	3	1	1	1	1	2	2
3	2	2	1	2	1	2	2	2	1
3	3	3	1	1	1	1	1	3	2
3	3	2	1	3	1	2	2	2	2
2	3	3	3	1	1	1	1	3	3
2	3	3	5	1	1	1	1	3	3
3	3	3	4	1	1	1	1	3	3
1	3	1	4	1	1	3	1	2	2
3	3	3	4	1	1	1	1	3	3
9	9	9	4	1	1	1	1	9	9
3	3	1	4	1	1	1	1	2	2
2	2	2	3	1	1	1	1	3	2
1	3	3	6	1	1	1	1	3	1
3	3	3	4	1	1	1	1	3	3
2	3	3	4	1	1	1	1	3	2
2	3	3	4	1	1	1	1	3	2
2	1	3	1	1	1	1	1	2	9
2	2	2	9	1	1	1	1	3	3
2	2	2	1	1	1	1	1	3	2
2	3	3	5	1	1	1	1	3	2
3	2	2	9	1	1	1	1	3	2
9	3	3	4	1	3	2	1	3	9
3	3	3	1	1	1	1	1	3	9
3	3	3	1	1	1	1	1	3	3
3	2	2	3	1	1	1	1	3	2
2	2	2	3	1	2	2	2	1	9
3	3	3	4	1	1	1	1	3	1
9	3	3	4	1	1	1	1	3	3
2	2	2	1	1	1	1	1	3	1
1	2	1	1	1	1	1	1	3	3
2	3	3	4	1	1	1	1	3	2
3	3	3	1	1	1	3	1	3	1
3	3	3	1	1	1	1	1	3	3

3	3	3	4	1	9	1	9	3	9
1	1	1	6	2	2	1	1	2	1
9	9	9	9	9	9	9	9	2	2
3	3	2	5	1	1	1	1	3	3
3	3	3	5	9	9	9	9	9	9
2	1	2	9	1	1	1	2	3	2
2	2	2	5	1	1	3	3	2	2
3	3	2	4	1	1	2	2	9	9
2	2	2	1	2	3	2	3	2	2
3	3	3	4	1	1	1	1	3	2
9	9	9	4	1	1	1	1	3	9
3	3	3	5	1	1	1	1	3	3
1	1	2	1	1	3	3	3	2	2
3	2	2	1	2	2	2	1	2	2
2	2	2	4	3	3	1	1	2	2
2	3	2	5	1	1	1	1	3	2
3	3	3	4	1	1	1	1	3	3
3	2	3	6	1	3	1	1	3	1
3	3	3	4	9	9	9	9	2	2
1	1	1	3	3	3	3	3	2	3
2	2	2	9	1	2	1	1	3	2
2	2	2	9	1	2	1	1	3	2
3	3	2	5	1	2	2	1	3	1
9	9	9	3	1	1	2	1	2	1
3	1	3	1	1	2	3	3	2	2
9	9	9	9	9	9	9	9	3	3
2	2	2	6	1	1	1	1	3	3
1	1	1	1	1	1	2	1	2	2
2	3	3	4	1	1	1	1	3	3
2	3	3	1	1	1	1	1	3	2
9	3	9	5	1	1	1	1	3	9
9	3	1	5	1	1	1	1	3	3
2	3	3	1	1	1	1	1	3	2
3	3	1	9	1	1	1	1	3	9
2	2	2	9	3	3	3	2	2	2
3	2	2	1	1	1	1	1	3	2
3	3	3	1	1	2	2	1	3	9
3	3	3	1	2	3	3	3	2	2
9	9	9	9	1	1	1	1	9	9
1	3	3	3	1	1	1	1	3	3
2	2	2	1	1	1	2	1	2	2
2	2	2	1	1	1	1	1	3	3
3	3	3	9	1	1	2	2	9	9
3	3	3	3	1	1	1	1	3	3
3	3	1	5	1	2	1	1	3	3
1	1	1	4	1	1	1	1	3	3
3	3	3	5	1	1	1	1	3	2

3	3	3	5	1	1	2	2	3	3
3	3	3	3	1	1	1	1	3	2
1	3	3	4	1	1	2	1	9	9
3	3	3	4	1	3	1	1	9	9
3	3	3	4	1	2	2	1	9	9
1	1	1	1	1	1	1	1	1	3
1	3	3	4	1	1	1	1	3	3
2	3	2	3	1	1	1	1	1	3
2	3	3	4	1	1	1	1	3	2
3	3	3	4	1	1	1	1	3	3
3	3	3	4	1	1	1	1	3	3
2	3	3	4	1	1	1	1	3	2
2	2	2	4	1	1	1	1	3	2
1	3	3	4	1	1	1	1	3	1
3	3	1	4	1	1	1	1	3	3
2	2	2	5	1	1	1	1	3	2
3	3	3	6	1	1	1	1	3	2
1	3	3	4	1	1	1	1	3	3
1	1	3	3	1	1	1	1	3	3
3	3	3	4	1	1	1	1	3	3
3	1	2	5	1	1	2	1	9	9
3	3	2	5	1	3	2	3	2	3
2	3	3	4	1	1	2	3	3	2
9	9	9	5	1	1	1	1	9	9
3	2	2	1	3	1	2	3	2	2

PapOverEi	PapTwoYe	PapfromM	PapHistory	PapTwoYe	PapOnceor	HPVVaccin	HPVVaccin	EarlyOnset	MultiplePa
2	2	2	2	2	2	2	2	1	2
2	2	2	3	2	3	2	3	2	3
2	2	2	2	2	2	2	2	2	2
2	2	2	2	2	2	2	3	2	2
2	1	2	2	1	2	2	2	1	2
2	2	2	2	2	2	2	2	2	2
2	2	2	2	2	2	2	2	1	2
2	2	3	3	2	2	2	2	1	2
2	3	3	3	3	3	3	3	2	1
1	2	3	1	3	2	1	2	3	2
2	2	2	2	2	2	2	2	2	2
2	2	3	3	2	2	2	2	1	1
2	2	2	1	2	1	2	2	2	2
2	2	1	2	2	2	1	1	3	2
2	2	2	2	2	2	2	2	2	2
2	2	2	2	2	2	2	2	2	2
9	9	9	9	9	9	9	9	9	9
2	2	2	2	2	2	2	2	1	1
9	9	9	9	9	9	9	9	9	9
9	9	9	9	9	9	9	9	9	9
1	1	2	3	2	3	1	1	1	2
2	3	3	3	3	3	2	2	2	1
1	2	3	2	2	3	1	1	1	2
1	2	1	1	1	2	1	2	1	1
9	9	9	9	9	9	9	9	2	9
1	2	2	2	2	1	1	1	1	1
3	3	3	1	9	3	3	1	1	3
2	2	2	2	2	2	2	2	2	2
3	2	1	3	2	1	2	3	1	2
9	9	9	9	9	9	9	9	2	1
3	3	3	3	3	3	1	3	3	2
2	2	2	2	2	2	2	2	1	1
2	3	3	3	3	3	3	3	3	3
9	2	9	1	1	1	2	1	1	9
1	2	3	1	2	3	2	1	1	1
3	3	2	2	3	2	2	2	3	3
2	2	2	2	2	2	2	2	1	2
1	2	3	2	2	2	2	2	2	9
2	2	2	2	1	3	1	1	2	2
1	2	3	2	3	3	2	2	3	1
2	2	2	3	1	3	1	2	2	2
9	9	2	9	9	9	3	9	2	9
9	9	9	9	9	9	9	9	3	9
2	2	2	2	2	2	2	2	2	2
2	2	2	2	2	2	2	2	2	2
9	9	9	9	9	9	9	9	9	9

2	2	1	2	1	2	2	2	2	2
2	2	1	2	1	2	2	2	1	2
2	2	1	1	2	2	2	2	2	2
2	1	1	2	1	1	2	2	2	1
3	3	3	3	1	3	3	3	1	9
3	3	3	3	1	1	3	3	1	9
9	2	9	2	2	2	3	2	2	3
2	2	2	2	2	2	2	2	2	2
1	1	2	2	2	9	3	3	1	1
3	2	1	3	3	3	3	1	1	3
9	9	9	9	9	9	9	9	9	2
2	2	1	1	2	2	2	2	1	1
2	1	2	2	2	3	3	3	2	2
2	2	1	1	1	2	2	2	1	1
2	1	1	2	2	1	1	1	1	1
2	2	3	2	1	2	2	3	1	2
2	2	3	3	3	3	3	3	2	2
1	1	3	2	3	1	1	2	1	1
2	2	2	2	2	2	2	2	1	2
2	2	2	2	2	2	2	2	2	2
2	2	2	2	2	2	2	2	2	2
2	2	2	2	2	2	2	2	1	2
2	2	2	1	1	1	1	1	1	1
2	2	1	2	1	2	2	2	1	1
2	2	3	2	1	2	2	1	1	2
1	1	2	2	1	2	2	2	2	1
3	3	3	3	3	3	1	3	3	3
3	3	3	2	2	1	1	2	1	2
1	2	2	3	1	3	1	1	1	1
3	3	3	3	3	3	3	3	3	3
9	9	9	9	9	9	9	9	9	9
3	3	3	2	2	2	1	2	2	1
3	2	2	9	9	9	9	1	1	3
2	2	2	2	2	2	2	2	2	2
2	2	3	2	1	3	3	1	1	1
2	2	2	2	2	2	2	2	1	2
2	9	9	9	9	9	9	9	1	1
2	2	1	2	2	3	2	2	2	1
2	2	2	2	2	2	2	2	1	3
2	1	2	2	1	2	3	3	2	1
2	2	3	2	1	2	1	2	1	1
9	9	9	9	9	9	9	9	9	9
2	3	2	3	1	2	3	1	1	1
1	1	1	1	2	2	1	1	3	1
1	2	3	2	2	3	3	2	1	1
2	3	1	3	2	3	2	3	3	2
2	2	2	2	2	2	1	2	2	3

1	3	2	1	3	2	1	2	1	2
3	3	3	3	3	3	3	3	3	3
2	2	2	2	2	2	2	2	1	1
2	1	1	2	2	2	2	3	2	1
2	3	2	1	1	1	1	1	1	1
2	2	2	2	1	2	2	3	2	2
2	2	2	2	2	2	2	2	2	2
9	9	9	9	9	9	9	9	9	9
2	2	2	2	2	2	2	2	9	1
1	3	3	2	2	3	1	2	1	1
2	1	1	3	2	1	3	1	1	1
2	1	2	2	1	2	2	2	1	1
2	2	3	3	3	3	2	2	1	2
2	2	2	2	2	2	2	2	1	2
2	2	2	2	2	2	2	2	3	1
2	2	2	2	2	2	2	2	3	9
1	1	2	2	1	2	2	1	1	1
2	2	3	3	3	2	2	2	2	1
2	2	2	1	9	2	9	1	1	1
2	2	1	2	2	1	1	1	1	1
1	3	1	3	1	3	1	3	1	3
1	1	2	1	1	3	1	1	1	2
2	2	1	1	2	2	1	1	1	2
2	2	2	2	2	2	2	2	2	2
3	2	2	1	1	2	2	3	2	2
3	3	2	3	9	3	1	1	1	2
1	1	3	3	3	3	1	1	1	1
1	2	1	2	2	1	1	2	2	1
2	2	2	3	3	3	3	3	1	2
2	2	3	2	3	3	2	1	2	3
2	1	1	2	3	3	3	3	2	2
2	1	2	3	2	1	2	2	1	2
3	2	2	3	2	2	2	2	3	2
2	1	1	1	1	1	1	2	2	1
2	1	3	2	1	3	1	3	1	3
1	1	1	1	1	1	1	1	1	1
2	3	2	3	2	3	2	3	2	2
2	1	2	2	3	2	2	2	1	2
9	9	9	9	9	9	9	9	9	9
3	3	3	3	3	3	3	3	3	3
3	2	2	1	2	2	2	2	1	2
9	9	9	9	9	9	9	9	9	9
2	1	2	2	2	2	1	1	1	1
2	1	2	3	2	2	1	2	2	2
9	9	9	9	9	9	9	9	9	9
2	2	2	3	1	3	1	2	3	2
2	1	2	2	1	1	1	1	1	1

9	1	3	2	9	1	3	1	3	3
1	1	3	2	1	3	1	1	1	1
3	3	3	3	3	3	3	3	3	3
2	2	2	2	2	2	2	2	2	2
2	2	3	2	2	2	2	3	2	2
2	2	1	2	3	2	2	3	2	2
1	2	3	2	9	1	3	3	1	9
9	1	3	2	2	2	1	3	1	2
9	9	9	9	9	9	9	9	2	9
2	2	2	2	2	2	2	2	2	2
3	1	2	3	1	1	2	3	1	3
2	1	2	2	1	3	1	1	1	1
2	2	2	2	2	2	2	2	2	2
2	2	2	2	1	1	1	1	1	1
2	3	2	1	2	2	2	2	1	2
3	2	2	1	2	1	1	1	1	1
3	2	2	1	1	1	1	1	1	2
2	2	1	1	1	2	1	1	1	2
9	9	9	9	9	9	9	9	2	9
2	3	2	1	1	2	2	1	1	1
1	2	2	3	1	2	2	1	1	1
2	3	2	2	2	2	3	2	1	1
2	2	2	3	2	3	2	9	3	3
1	2	1	2	1	1	2	1	1	1
2	2	2	9	9	9	9	9	3	2
3	3	3	3	3	3	3	3	3	9
1	3	2	2	3	2	1	3	1	2
2	2	2	2	2	2	2	2	1	2
2	2	2	2	2	2	2	2	1	2
2	1	2	2	2	2	2	1	1	1
2	2	1	1	2	2	1	2	1	2
2	2	2	1	1	1	3	3	2	2
1	2	2	2	2	1	2	2	1	2
3	1	1	1	3	3	3	3	3	3
2	2	3	2	2	2	2	2	2	2
3	3	1	1	1	1	1	1	3	3
1	1	1	1	1	1	1	1	3	3
1	3	3	3	2	3	1	1	1	1
1	1	3	3	1	1	1	1	1	1
3	3	3	3	3	3	3	3	2	2
9	9	9	9	9	9	9	9	9	9
2	2	2	2	2	2	2	2	2	2
2	2	2	2	2	2	2	2	2	2
9	9	9	9	9	9	9	9	9	9
9	9	9	9	9	9	9	9	9	9
2	2	1	1	2	2	1	2	1	2
2	1	2	2	3	3	3	3	1	2

9	9	9	9	9	9	9	9	9	9
2	2	9	9	9	9	9	9	9	9
9	9	9	9	9	9	9	9	9	1
9	9	9	9	9	9	9	9	9	9
2	9	9	9	9	9	9	9	1	9
9	9	9	9	9	9	9	9	9	9
2	2	2	2	2	2	2	2	1	1
9	9	9	9	9	9	9	9	9	9
1	1	2	2	1	2	1	2	1	1
9	2	9	9	9	9	9	9	9	9
9	9	1	9	9	9	9	9	9	9
9	2	9	9	9	9	9	9	9	9
2	2	1	1	1	1	2	1	1	1
2	2	2	1	1	2	2	1	1	2
9	9	9	9	9	9	9	9	9	1
3	2	3	2	3	3	3	2	2	2
3	3	3	3	3	3	3	3	3	3
2	1	1	1	1	1	1	1	3	1
2	2	2	2	2	2	2	2	1	1
2	2	2	2	2	2	2	2	2	2
1	2	2	2	2	2	2	2	1	2
9	9	9	9	9	9	9	9	9	9
3	3	3	3	3	3	2	2	2	2
1	2	3	1	2	2	1	3	1	1
2	2	2	2	2	2	2	2	2	2
2	2	1	1	1	1	1	1	1	2
2	2	2	2	3	2	1	1	1	1
2	2	3	1	1	1	1	1	1	1
3	3	3	3	3	3	3	3	3	3
3	1	1	1	1	3	1	1	1	1
2	2	2	2	2	2	2	2	2	2
2	2	1	1	2	2	2	2	1	1
9	9	9	9	9	9	9	9	9	9
1	1	1	2	3	3	3	3	2	2
1	3	1	3	1	3	3	1	1	1
2	2	1	1	1	1	1	1	1	1
1	2	3	2	2	3	1	2	2	3
1	1	1	2	1	1	1	1	1	2
9	9	9	9	9	9	9	9	9	1
1	1	1	2	1	1	2	1	2	2
2	1	1	2	1	1	1	1	1	1
3	1	2	1	3	9	1	3	1	2
1	2	2	2	3	3	3	2	2	2
2	2	2	2	2	2	2	2	2	2
2	2	2	2	2	2	2	2	1	1
2	2	2	2	2	2	2	2	1	1
9	9	9	9	9	9	9	9	9	9

1	1	1	1	1	1	1	1	1	1
2	2	1	1	2	1	1	2	1	1
1	1	1	1	1	3	1	2	1	1
9	9	9	9	9	9	9	9	9	9
2	2	2	2	2	2	2	2	2	2
2	9	1	2	2	3	2	1	2	2
1	1	2	2	1	3	1	1	2	3
2	1	1	1	1	1	1	1	2	1
1	1	2	3	2	3	2	2	2	2
1	2	1	1	1	1	1	1	1	2
1	2	2	2	2	2	2	2	1	1
9	9	9	9	9	9	9	9	3	9
9	9	9	9	9	9	9	9	2	9
9	9	9	9	9	9	9	9	1	9
9	9	2	9	9	2	9	2	2	9
9	9	9	9	9	9	9	9	9	9
9	9	9	9	9	9	9	9	9	9
9	9	9	9	9	9	9	9	9	9
2	9	2	9	2	9	2	2	9	2
9	9	9	9	9	9	9	9	9	9
9	9	9	9	9	9	9	9	9	9
1	2	2	2	2	2	2	2	2	2
2	1	1	2	2	2	2	2	2	2
2	2	1	1	1	1	1	1	1	1
2	2	1	1	1	1	1	1	1	1
9	9	9	9	9	9	9	9	9	9
2	1	2	2	2	1	2	2	2	2
9	9	9	9	9	9	9	9	9	9
9	9	9	9	9	9	9	9	9	9
2	3	2	1	2	2	2	1	1	2
9	9	9	9	9	9	9	9	9	9
9	9	9	9	9	9	9	9	9	9
9	9	9	9	9	9	9	9	9	9
9	9	9	9	9	9	9	9	9	9
2	1	3	1	9	1	3	1	9	3
9	3	1	9	1	9	9	1	9	1
9	9	9	9	9	9	9	9	9	9
9	9	9	9	9	9	9	9	9	9
9	9	9	9	9	9	9	9	9	9
9	9	9	9	9	9	9	9	3	9
9	9	9	9	9	9	9	9	1	9
9	9	9	9	9	9	9	9	9	9
3	3	3	3	3	3	3	3	3	3
1	3	2	2	1	2	2	1	1	2
1	2	1	2	1	2	3	1	1	2
9	9	9	9	9	2	9	9	2	9
9	9	9	9	9	9	9	9	1	9

9	9	9	9	9	9	9	9	9	2
9	2	9	9	9	9	9	9	3	9
2	2	3	3	3	3	3	3	3	3
2	3	1	2	3	1	2	3	3	1
1	1	1	1	1	1	1	1	3	2
2	2	3	3	3	3	2	2	2	2
3	2	1	1	2	3	2	2	2	1
3	1	1	2	2	2	3	1	1	3
2	2	2	2	2	2	2	2	2	2
3	3	3	3	3	3	3	3	3	3
9	9	9	9	9	9	9	9	9	9
9	9	9	9	9	9	9	9	9	9
9	9	9	9	9	9	9	9	9	9
9	9	9	9	9	9	9	9	9	1
2	2	1	1	1	3	3	3	1	1
2	3	3	3	2	1	2	2	1	1
9	9	9	9	9	9	9	9	9	9
1	2	1	1	1	2	2	2	1	1
1	1	1	1	1	1	1	1	1	1
3	2	3	2	2	2	2	2	1	1
3	2	1	2	3	1	2	1	1	2
2	2	2	2	2	2	2	2	2	2
2	3	3	2	1	2	2	3	3	9
2	2	2	2	2	2	2	2	2	2
2	2	2	2	2	2	2	2	2	2
2	2	2	2	2	2	2	2	2	2
2	2	2	2	2	2	2	2	2	2
1	2	3	1	1	1	1	3	3	2
2	2	2	3	3	3	3	3	2	2
1	2	1	2	1	1	1	1	1	2
2	2	2	2	2	2	2	2	2	2
2	2	2	2	2	2	2	2	2	2
3	2	2	1	2	2	2	3	3	3
3	3	3	3	3	3	3	3	3	2
2	1	3	1	1	3	3	2	3	3
3	1	2	3	1	2	3	2	2	2
9	9	9	9	9	9	9	9	9	9
2	2	2	2	2	2	2	2	2	2
3	3	3	3	3	3	3	3	3	3
3	3	3	3	3	3	3	3	3	3
9	9	9	2	9	9	9	9	2	9
9	9	9	9	9	9	9	9	9	9
9	3	9	9	9	9	9	9	9	9
2	2	2	2	2	2	2	2	1	1
2	1	2	2	1	2	2	2	1	2
2	2	2	2	2	2	2	2	2	2
1	1	1	1	2	3	3	3	1	1

9	9	2	9	9	9	9	9	9	9
9	9	9	9	9	9	9	9	1	1
1	9	9	9	9	9	9	9	9	9
2	9	9	9	9	9	9	9	9	9
9	9	3	9	9	9	9	9	9	9
9	2	2	2	2	2	1	1	3	3
9	9	9	9	2	9	9	9	9	9
9	9	9	9	9	9	9	9	2	9
9	9	9	9	9	9	9	9	2	9
9	9	9	9	9	9	9	9	2	9
9	2	9	9	9	9	9	9	9	9
9	9	2	9	9	9	9	9	9	9
9	9	9	9	9	9	9	9	2	9
9	9	9	9	9	9	9	9	9	9
9	9	9	9	9	9	9	9	9	9
9	9	9	9	9	9	9	9	9	9
2	9	2	9	9	3	9	9	9	9
2	1	2	3	1	2	2	9	2	1
2	1	2	2	1	2	1	2	1	2
2	2	2	2	2	2	2	2	2	2
1	2	3	3	3	3	1	1	1	1
2	2	2	1	3	2	3	2	2	2
2	2	2	2	2	2	2	2	2	2
2	1	3	2	2	3	2	3	2	3
9	2	3	3	2	3	2	3	2	3
9	9	9	9	9	9	9	9	9	9
2	2	2	2	2	2	2	2	1	1
2	2	2	2	2	2	2	2	3	3
2	2	1	2	3	1	2	2	2	1
1	1	1	1	1	1	1	2	2	1
2	2	1	3	2	1	2	1	1	1
2	2	2	2	2	2	2	2	2	2
3	2	2	2	2	1	1	1	1	1
2	2	1	1	1	1	1	1	2	2
9	9	9	9	9	9	9	9	9	9
2	2	2	2	2	2	2	2	2	2
9	9	9	9	9	9	9	9	9	9
2	2	1	1	1	2	2	2	2	2
9	9	9	9	9	9	9	9	9	9
2	2	2	2	2	2	2	2	2	2
2	2	2	1	2	3	1	2	1	2
3	3	3	3	3	3	3	3	3	3
2	9	2	3	1	3	3	3	1	1
2	2	2	2	2	2	2	2	2	2
3	3	3	2	2	2	2	2	3	3
1	2	2	3	2	2	2	2	1	3
2	2	2	2	2	2	2	2	2	2

1	2	2	2	3	2	3	2	1	1
9	9	9	9	9	9	9	9	9	9
2	2	9	1	1	3	3	3	9	9
2	3	2	1	2	1	3	2	2	3
2	2	2	2	2	2	2	2	3	3
2	2	2	2	2	3	3	3	3	3
1	1	3	1	3	1	1	1	1	1
2	1	2	1	1	3	1	1	1	1
9	9	9	9	9	9	9	9	9	9
2	2	2	2	2	2	2	2	2	2
2	2	2	2	2	2	2	2	1	1
9	9	9	9	9	9	9	9	1	9
9	9	9	9	9	9	9	9	1	9
1	1	9	1	1	1	9	1	2	2
9	9	9	9	9	9	9	9	9	9
3	3	3	2	2	2	2	2	1	1
9	9	9	9	9	9	9	9	9	9
9	9	9	9	9	9	9	9	2	9
2	9	9	9	9	9	9	9	9	9
2	3	2	3	2	3	2	3	3	2
2	2	2	2	2	2	2	2	2	2
2	2	2	2	2	2	2	2	1	1
2	2	2	2	2	2	2	2	2	2
9	9	9	9	9	9	1	9	9	9
9	9	9	9	9	9	9	9	2	9
1	1	1	1	2	2	2	1	2	2
2	1	2	2	2	2	2	2	1	2
1	2	3	2	1	2	2	1	1	2
9	9	9	9	9	9	9	9	9	9
9	9	9	9	9	9	9	9	1	2
2	2	2	2	2	2	2	2	2	2
3	1	2	3	1	2	3	1	2	3
2	3	3	2	1	1	1	1	1	1
1	1	2	2	1	1	1	1	1	2
1	1	1	1	1	1	1	1	1	1
1	2	1	2	2	2	1	2	2	1
9	9	9	9	9	9	9	9	9	9
9	9	9	9	9	9	9	9	9	9
3	2	3	1	3	2	1	3	2	2
1	2	1	2	1	2	2	1	1	2
2	2	2	2	2	2	2	2	2	2
2	2	2	2	2	2	2	2	2	2
2	2	2	2	2	2	2	2	2	2
1	3	1	2	1	3	1	2	1	2
3	3	3	3	3	3	3	3	2	2
2	2	2	2	2	2	2	2	2	2
2	2	2	2	2	2	3	3	3	3

2	3	3	3	3	3	3	3	1	1
2	1	2	3	1	2	3	1	1	2
1	9	9	9	9	9	9	9	9	9
9	9	9	9	9	9	9	9	9	9
3	2	3	2	2	3	1	2	1	9
2	3	2	1	2	3	2	3	2	2
9	9	2	9	9	9	2	9	2	9
9	3	9	3	9	3	3	3	3	9
9	9	9	9	9	9	9	9	9	9
9	9	9	9	9	9	9	9	9	9
9	9	9	9	9	9	9	9	9	9
9	9	9	9	9	9	9	9	9	9
9	9	9	9	9	9	9	9	9	9
9	9	9	9	9	9	9	9	9	9
9	9	9	9	9	9	9	9	9	9
9	9	9	9	9	9	9	9	2	9
3	3	3	3	3	3	3	3	3	3
9	9	9	9	9	9	9	9	1	9
9	9	9	9	9	9	9	9	2	9
9	9	2	9	9	2	9	2	2	9
9	9	9	9	9	9	9	9	9	9
9	9	9	9	9	9	9	9	2	9
2	2	2	2	2	2	2	2	2	2
9	9	9	9	9	9	9	9	9	9
9	9	9	9	9	9	9	9	9	9
2	9	9	9	9	9	9	9	2	9
9	9	9	9	9	9	9	9	9	9
2	2	9	2	9	2	9	2	2	9
1	2	2	2	2	2	2	2	2	2
9	9	9	9	9	9	9	9	9	9
9	9	9	9	9	9	9	9	9	9
2	2	2	2	2	2	2	2	2	2
9	9	9	9	9	9	9	9	9	9
9	9	9	9	9	9	9	9	9	9
9	9	9	9	9	9	9	9	9	9
9	9	9	9	9	9	9	9	9	9
9	9	9	9	9	9	9	9	9	9
9	9	9	9	9	9	9	9	9	9
2	3	1	2	1	3	1	2	2	1
2	3	1	2	2	1	2	1	2	1
9	9	9	9	9	9	2	9	1	3
9	9	9	9	9	9	9	9	9	9
1	2	3	1	2	3	3	2	2	1
3	1	2	3	1	2	3	2	1	2
3	3	3	3	3	1	1	1	3	3
2	1	2	3	2	3	2	2	2	1
1	2	3	2	9	9	9	9	1	1

9	9	9	9	9	9	9	9	9	9
3	2	1	2	1	1	3	2	2	3
2	2	2	2	2	2	2	2	2	2
1	1	1	1	1	1	1	1	1	1
2	2	2	2	2	2	2	2	2	2
2	2	2	3	3	3	3	3	2	2
9	9	9	9	9	9	9	9	9	9
3	3	2	2	2	2	2	2	1	3
2	2	2	2	2	2	2	2	2	2
1	2	2	2	2	2	2	2	1	1
9	3	2	2	2	2	2	2	2	2
9	9	9	2	9	9	9	9	9	9
9	9	9	9	1	9	9	9	9	9
9	9	9	9	9	9	1	9	9	9
9	9	9	9	9	9	9	9	9	9
2	2	2	2	2	2	2	2	1	1
2	2	2	2	2	2	2	2	2	2
3	3	3	3	2	3	1	2	2	2
2	3	3	3	3	3	3	2	3	3
2	2	2	2	2	2	2	2	2	2
2	2	2	2	2	2	2	2	2	2
9	9	9	9	9	9	9	9	9	9
3	3	1	1	1	1	3	1	2	3
9	9	9	9	9	9	9	9	9	9
9	9	9	9	9	9	1	9	9	9
1	1	1	1	1	1	1	1	2	2
9	1	9	9	2	9	9	9	9	9
3	2	2	1	1	1	2	2	2	2
9	9	9	9	9	9	9	9	9	9
9	9	9	9	9	9	1	9	9	9
2	3	3	3	2	2	1	3	1	1
2	3	1	3	1	3	2	3	2	1
9	9	9	9	9	9	9	9	9	9
9	9	9	9	9	9	9	9	1	9
1	2	2	3	3	1	2	2	1	2
9	9	9	9	9	9	9	9	9	9
1	2	3	2	3	2	1	2	1	2
1	1	1	1	1	1	1	1	1	1
2	2	2	2	2	2	2	2	2	2
9	9	9	9	9	9	9	9	9	9
2	2	2	2	2	2	2	2	2	2
1	2	2	2	2	2	2	2	1	1
2	2	2	2	2	2	2	2	2	2
2	2	2	2	2	2	2	2	2	2
3	2	3	2	3	2	2	3	2	3
2	2	2	2	2	2	2	2	2	2
2	2	3	3	3	3	3	2	2	2

9	9	9	9	9	9	9	9	9	1
2	2	2	2	2	2	2	2	2	2
2	2	2	2	2	2	2	2	2	2
9	9	2	9	9	9	9	9	1	9
9	9	9	9	9	9	9	9	2	9
9	9	9	9	9	9	9	9	9	9
9	9	9	9	9	9	9	9	9	9
2	2	2	2	2	2	2	2	1	1
2	2	2	2	2	2	2	2	2	2
9	9	9	9	9	9	9	9	9	9
2	2	2	2	2	2	2	2	2	2
3	3	2	2	2	3	2	3	2	2
2	2	2	2	2	2	2	2	1	1
2	2	2	2	2	2	2	2	2	2
9	9	9	9	9	9	9	9	9	9
9	9	9	9	9	9	9	9	9	9
9	9	9	9	9	9	9	9	9	9
9	9	9	9	9	9	9	9	9	9
9	9	9	9	9	9	9	9	1	9
9	9	9	9	9	9	9	9	2	9
9	9	9	9	9	9	9	9	9	9
9	9	9	9	9	9	9	9	9	9
2	2	2	2	2	2	2	2	2	9
9	9	9	9	9	9	9	9	2	9
9	9	9	9	9	9	9	9	9	9
9	9	9	9	9	9	9	9	3	9
9	9	9	9	9	9	9	9	9	9
9	9	9	9	9	9	9	9	9	9
9	9	9	2	9	9	9	2	2	9
9	9	9	9	9	9	9	9	3	9
9	9	9	9	9	9	9	9	9	9
2	2	2	2	2	2	2	2	2	2
9	9	9	9	9	9	9	9	9	9
9	9	9	9	9	9	9	9	9	9
9	9	9	9	9	9	9	9	9	9
9	9	9	9	9	9	9	9	9	9
9	9	9	9	9	9	9	9	9	9
9	9	9	9	9	9	9	9	9	9
9	9	9	9	9	9	9	9	9	9
9	9	9	9	9	9	9	9	9	9
2	2	2	2	2	2	2	2	2	9
9	9	9	9	9	9	9	9	2	9
9	9	9	9	9	9	9	9	9	9
9	9	9	9	9	9	9	9	9	9
9	9	9	9	9	9	9	9	9	9
9	9	9	9	9	9	9	9	9	9
9	9	9	9	9	9	9	9	9	9

9	9	9	9	9	9	9	9	9	9
3	3	3	3	3	3	3	3	3	3
9	9	9	9	9	9	9	9	9	9
9	9	9	9	9	9	9	9	9	9
9	9	9	9	9	9	9	9	9	9
9	9	9	9	9	9	9	9	9	9
9	9	9	9	9	9	9	9	9	9
9	9	9	9	9	9	9	9	9	9
9	9	9	9	9	9	9	9	9	9
9	9	9	9	9	9	9	9	9	9
9	9	9	9	9	9	9	9	9	9
9	9	9	9	9	9	9	9	9	9
9	9	9	9	9	9	9	9	9	9
9	9	9	9	9	9	9	9	9	9
9	9	9	9	9	9	9	9	9	9
9	9	9	9	9	9	9	9	9	9
9	9	9	9	9	9	9	9	9	9
2	2	2	2	2	2	2	2	2	2
9	9	9	9	9	9	9	9	9	9
9	9	9	9	9	9	9	9	9	9
9	9	9	9	9	9	9	9	9	9
9	9	9	9	9	9	9	9	9	9
9	9	9	9	9	9	9	9	9	9
9	9	9	9	9	9	9	9	9	9
2	2	2	2	2	2	2	2	2	2
9	9	9	9	9	9	9	9	9	9
9	9	9	9	9	9	9	9	9	9
2	2	1	1	2	2	1	2	1	9
9	9	9	9	9	9	9	9	9	9
9	9	9	9	9	9	9	9	9	9
1	1	1	1	1	1	1	1	1	1
2	2	1	1	3	3	1	1	2	2
9	9	9	9	9	9	9	9	9	9
3	3	3	3	3	3	3	3	3	3
9	9	9	9	9	9	9	9	9	9
2	2	2	2	2	2	2	2	2	2
9	9	9	9	9	9	9	9	2	9
9	9	9	9	9	9	9	9	9	9
1	1	1	1	1	1	1	1	1	1
9	9	9	9	9	9	9	3	3	9
9	9	9	9	9	9	9	9	9	9
9	9	9	9	9	9	9	9	9	9
2	2	2	2	2	2	2	2	2	2
2	2	2	2	2	2	2	2	2	2
9	1	9	1	9	1	9	1	9	1
9	9	9	9	9	9	9	9	9	9
9	9	9	9	9	9	9	9	9	9

9	9	9	2	9	9	9	2	2	9
9	9	9	9	9	9	9	9	9	9
9	9	1	3	9	1	2	3	2	9
9	9	9	9	9	9	9	9	9	9
9	9	9	9	9	9	9	9	9	9
9	9	9	9	9	9	9	9	9	9
9	9	9	9	9	9	9	9	2	9
9	9	9	9	9	9	9	9	2	9
9	2	9	9	2	9	9	2	2	9
9	9	9	9	9	9	9	9	9	9
9	9	9	9	9	9	9	9	9	9
9	9	9	9	9	9	9	9	9	9
9	9	9	9	9	9	9	9	9	9
9	9	9	9	9	9	9	9	9	9
2	1	3	3	3	3	2	2	1	1
1	2	3	1	2	3	1	2	1	2
9	9	9	9	2	9	9	9	9	9
2	2	2	2	2	2	2	2	2	2
2	3	2	2	3	2	2	2	2	2
9	9	9	9	9	9	9	9	1	9
9	9	9	9	9	9	9	9	9	9
9	9	9	9	9	9	9	9	1	9
2	2	2	2	2	2	2	2	3	3
3	1	2	3	1	2	3	1	2	3
2	2	2	2	1	3	2	2	2	2
2	2	2	2	2	2	2	2	2	2
3	3	2	9	3	3	3	3	2	2
3	3	3	2	2	2	2	2	1	1
1	3	3	3	2	1	1	3	2	2
2	2	2	2	2	2	2	2	2	2
2	2	2	2	2	2	2	2	2	2
9	9	9	9	9	9	9	9	2	9
9	9	9	9	9	9	9	9	1	9
9	9	9	9	9	9	9	9	2	9
9	9	9	9	9	9	9	9	1	9
9	9	9	9	9	9	9	9	9	9
9	9	9	9	9	9	9	9	9	9
9	9	9	2	9	9	9	2	9	9
9	9	9	9	9	9	9	9	9	9
2	9	2	9	2	9	2	2	2	9
9	9	9	9	9	9	9	9	9	9
2	2	2	2	2	2	2	2	2	2
9	9	9	9	9	9	9	9	9	9
9	9	9	9	9	9	9	9	9	9
9	9	9	9	9	9	9	9	9	9
2	2	2	2	2	2	2	2	2	2
3	2	1	3	2	3	2	2	2	3

1	2	2	2	2	2	2	2	2	2
2	2	2	2	2	2	2	2	2	1
2	2	2	2	2	2	2	2	2	2
3	3	3	1	3	1	2	3	1	1
3	3	3	3	3	3	3	3	3	3
1	2	2	2	1	3	1	1	1	2
2	2	2	2	2	2	2	2	2	2
3	3	3	3	3	3	3	3	3	3
9	9	9	9	9	9	9	9	9	9
2	2	2	2	2	2	2	2	2	2
2	9	2	9	2	9	9	2	9	2
1	9	9	1	9	1	9	1	9	2
2	2	2	2	2	2	2	2	2	2
3	2	2	1	1	1	3	3	2	1
3	3	3	3	3	3	3	3	3	2
2	2	2	2	2	2	2	2	2	2
3	2	3	2	1	2	9	1	2	3
3	3	3	3	2	3	3	3	2	3
9	9	9	9	9	9	9	9	9	9
2	1	3	2	1	2	2	1	1	2
2	1	3	2	3	1	2	1	2	3
2	3	1	3	1	3	1	1	1	1
1	2	2	2	2	1	2	2	2	1
2	1	2	1	2	3	1	1	1	1
1	2	2	3	2	3	1	1	1	1
2	2	2	2	2	2	2	2	2	2
1	2	3	2	1	3	2	1	1	1
2	2	2	2	3	3	3	2	2	3
2	2	2	2	2	2	2	2	2	2
1	2	3	2	2	3	1	1	1	1
3	3	3	3	3	3	3	3	3	3
9	9	9	9	9	9	9	9	9	9
3	3	3	3	3	3	3	3	3	3
2	2	2	2	2	2	2	2	2	2
9	9	9	9	9	9	9	9	9	1
3	3	3	3	2	2	2	2	3	2
2	2	2	2	2	2	2	2	2	2
1	1	1	1	1	1	1	1	1	1
3	2	2	2	2	2	3	2	1	2
9	9	9	9	9	9	9	9	9	9
2	2	2	2	2	2	2	2	2	2
9	9	9	9	9	9	1	9	9	9
1	3	1	2	1	2	1	1	2	2
2	2	2	2	2	2	2	2	2	2
2	3	2	3	2	3	2	3	2	3
9	9	9	9	9	9	9	9	9	9
3	2	2	1	2	2	2	2	3	2

9	9	9	9	9	9	9	9	9	9
3	3	3	3	3	3	3	3	3	3
1	1	1	3	3	1	1	3	1	1
3	2	2	3	2	2	2	3	2	3
1	1	3	2	1	3	1	3	1	1
9	9	9	9	9	9	9	9	9	9
2	2	2	2	2	2	2	2	2	2
3	3	1	2	3	2	1	3	3	3
3	3	1	3	2	3	1	1	1	1
2	2	2	2	2	2	1	1	2	2
1	2	3	2	2	2	1	2	2	1
2	2	2	2	2	2	2	2	2	2
9	9	9	9	9	9	9	9	9	9
2	2	3	3	3	3	3	2	2	2
2	2	2	2	1	3	1	1	1	1
2	2	2	2	2	2	2	2	2	2
2	2	2	2	2	2	2	2	2	2
2	2	2	2	2	3	2	2	3	2
2	2	2	2	2	2	2	2	2	2
9	9	9	9	9	9	9	9	9	2
9	9	9	9	9	9	9	9	9	9
1	3	2	2	1	2	1	2	2	1
2	3	3	3	2	3	2	3	2	3
1	2	2	3	2	3	2	3	2	3
9	9	9	9	9	9	9	9	9	9
3	3	3	2	3	2	1	1	1	2
9	9	9	9	9	9	9	9	9	9
2	2	2	2	2	2	2	2	2	2
2	2	2	2	2	2	2	2	2	2
2	2	2	2	2	2	2	2	2	2
1	3	2	1	2	2	1	1	3	3
9	9	9	9	9	9	9	9	9	9
9	9	9	9	9	9	9	9	9	9
2	2	2	2	2	2	1	2	2	1
2	2	3	1	3	3	1	2	1	1
9	9	9	9	9	9	9	9	9	9
9	9	9	9	9	9	9	9	9	9
3	3	3	2	2	2	2	2	2	3
2	2	3	3	9	2	1	2	2	2
1	2	2	2	1	2	2	2	1	1
2	2	2	2	2	2	2	2	2	2
2	2	2	2	2	2	2	2	2	2
2	2	2	2	2	2	2	2	2	2
2	2	2	2	2	2	2	2	2	2
2	2	2	2	2	2	2	2	2	2
2	2	3	2	1	2	3	2	2	2
2	2	2	2	2	2	2	2	2	2

3	2	1	2	3	3	3	3	3	3
2	2	2	2	2	2	2	2	2	2
2	2	2	2	2	2	2	2	2	2
1	1	1	1	1	2	3	2	2	1
2	2	2	2	2	2	2	2	2	2
2	2	2	2	2	2	2	2	2	2
2	2	2	2	2	2	2	2	2	2
2	2	2	2	2	2	2	2	2	2
2	2	2	2	2	2	2	2	2	2
9	9	9	9	9	9	9	9	9	9
3	1	2	2	2	2	2	2	3	3
2	1	3	2	3	3	1	2	1	3
1	1	2	1	1	3	1	1	1	1
1	2	2	1	1	2	2	1	1	1
3	3	3	3	3	3	3	3	3	3
9	9	9	9	1	9	9	9	9	9
9	9	9	9	9	9	9	9	9	9
9	9	9	9	1	9	9	9	9	9
9	9	9	9	9	9	9	9	9	9
2	2	2	2	2	2	2	2	2	2
2	2	1	2	9	1	3	2	1	2
9	9	9	9	9	9	9	9	9	9
9	9	9	9	9	9	9	9	9	9
2	2	2	2	2	2	2	2	2	2
2	1	3	1	1	1	1	1	1	1
2	2	2	2	2	2	2	2	2	2
2	2	3	2	2	1	1	2	2	2
9	3	3	2	2	2	2	2	2	1
2	1	3	1	2	1	2	2	2	1
1	2	1	2	2	1	3	2	2	1
9	9	9	9	9	9	9	9	9	9
1	2	1	3	1	3	1	2	1	2
3	3	3	1	1	1	2	2	2	1
2	2	1	2	2	2	2	2	3	3
1	1	2	2	2	2	2	2	1	2
2	2	2	2	2	2	2	2	2	2
9	9	9	9	9	9	9	9	9	9
2	2	2	2	2	2	2	2	2	2
2	2	2	2	2	2	2	2	2	2
2	1	2	3	2	2	3	2	1	3
2	3	3	3	3	3	3	3	3	3
2	3	1	2	1	2	2	2	1	1
2	2	2	2	2	1	1	2	2	2
2	2	2	2	2	2	2	2	1	1
2	2	1	2	2	2	3	3	2	1
1	1	1	1	2	2	1	3	1	1
2	2	2	2	3	3	2	2	3	1

1	3	2	2	1	1	3	3	2	2
2	2	2	2	2	2	2	2	2	2
2	2	2	3	3	3	3	3	2	2
2	2	2	2	2	2	2	2	2	2
3	3	3	3	3	2	3	2	3	3
9	9	9	9	9	9	9	9	9	9
9	9	1	9	9	9	9	9	1	9
3	3	3	3	3	3	3	3	3	3
9	9	9	9	9	9	9	9	9	9
9	9	9	9	9	9	9	9	9	9
2	2	2	2	2	2	2	2	2	2
2	2	2	1	2	2	1	2	1	1
1	2	2	1	1	2	1	2	2	1
1	9	9	9	9	9	9	1	9	9
2	9	9	9	9	9	9	1	1	2
2	1	2	3	3	1	9	1	2	2
1	2	2	2	1	3	1	1	1	1
9	9	9	9	9	9	9	9	9	9
2	2	2	2	1	3	2	2	2	1
2	2	2	2	2	2	2	2	2	2
2	2	2	2	2	2	2	2	2	3
9	9	9	9	9	9	9	9	9	9
2	2	2	2	2	2	2	2	2	2
2	2	2	2	2	2	2	2	2	2
2	2	2	2	2	2	2	2	2	2
2	2	2	2	2	2	2	2	2	2
2	2	2	2	2	2	2	2	2	2
2	2	2	2	2	2	2	2	2	2
2	2	3	2	3	2	3	1	2	2
2	3	2	3	2	3	3	2	2	2
2	2	3	1	2	1	2	2	2	3
3	2	2	3	3	2	3	2	1	2
1	1	2	2	2	2	1	1	1	2
2	2	2	2	2	2	2	2	2	2
2	2	1	2	3	2	2	2	2	1
1	3	2	1	2	2	3	1	2	2
1	1	1	1	1	1	1	1	2	2
2	2	3	3	3	3	3	3	2	2
2	1	2	2	3	2	3	2	1	1
2	3	3	2	1	3	1	1	3	3
1	1	1	1	3	3	1	1	1	1
2	2	2	2	3	3	3	3	1	1
2	2	2	2	2	2	2	2	2	2
2	2	2	2	2	2	2	2	1	2
2	2	2	2	2	2	2	2	2	2
2	2	3	2	3	2	3	1	1	2
2	2	2	2	2	2	2	2	2	2

2	2	3	2	1	3	1	1	2	3
1	1	2	2	1	2	1	1	1	1
2	3	2	3	2	3	2	3	2	3
2	2	2	2	2	2	2	2	2	2
1	9	9	9	9	9	9	9	3	9
9	9	1	9	9	9	9	9	9	9
3	2	3	2	3	2	3	2	3	2
9	9	9	9	9	9	9	9	1	9
9	9	9	9	9	9	9	9	1	9
9	9	9	9	9	9	9	9	9	9
9	9	9	3	9	9	9	9	9	9
2	9	9	9	9	9	9	9	9	9
9	9	9	9	9	9	9	9	1	9
9	9	1	9	9	9	9	9	9	9
2	2	2	2	2	2	2	2	1	1
9	9	9	9	9	9	9	9	1	9
9	9	9	9	9	9	9	9	9	9
1	9	9	9	9	9	9	9	9	9
1	9	9	9	9	9	9	9	9	9
9	3	9	9	9	9	9	9	9	9
9	9	9	9	9	9	9	9	9	9
2	2	2	2	2	2	2	2	2	2
2	2	2	2	2	2	2	2	2	2
2	3	1	1	1	3	9	2	2	2
9	1	3	3	3	3	3	3	2	2
9	9	9	9	9	9	9	9	9	9
2	1	2	1	1	1	3	2	2	1
9	9	9	9	9	9	9	9	9	9
3	2	2	2	3	3	3	2	1	1
1	1	1	1	1	3	1	1	1	1
3	3	3	3	3	3	3	3	3	1
9	9	9	9	9	9	9	9	1	9
2	2	2	2	2	2	3	2	2	2
9	9	9	9	9	9	9	9	9	9
9	9	9	9	9	9	9	9	1	9
1	1	2	1	1	1	2	1	1	2
1	1	9	1	2	1	2	1	1	3
9	9	9	9	9	9	9	9	9	9
9	9	9	9	9	9	9	9	9	9
2	2	2	2	2	2	2	2	2	2
9	9	9	9	9	9	9	9	9	9
2	2	2	2	2	2	2	2	2	2
3	3	3	3	3	3	3	3	3	3
2	9	9	9	2	9	9	9	9	9
2	2	2	2	2	2	2	2	2	2
2	2	2	2	2	2	2	2	2	2
9	3	1	9	9	1	9	9	9	9

9	9	9	9	9	9	9	9	9	9
9	9	2	9	2	9	2	2	9	2
9	9	9	9	9	9	9	9	9	9
2	2	2	2	2	2	2	2	2	2
1	9	3	1	3	1	3	1	9	1
9	3	1	3	1	9	1	2	3	1
9	9	9	9	9	9	9	9	9	9
9	9	9	9	9	9	9	9	9	9
9	2	9	2	9	9	2	2	9	2
2	2	2	2	2	2	2	2	2	2
9	9	9	9	9	9	9	9	9	9
9	2	9	2	9	2	9	2	2	9
2	2	2	2	2	2	2	2	2	2
9	9	9	9	9	9	9	9	9	9
2	2	2	2	2	2	2	9	2	2
9	9	9	9	9	9	9	9	9	9
9	9	9	9	9	9	9	9	9	9
9	9	9	9	9	9	9	9	9	9
9	9	9	9	9	9	9	9	9	9
9	9	9	9	9	9	9	9	9	9
2	2	2	2	2	2	2	2	2	2
2	2	2	2	2	2	2	2	2	2
3	1	3	2	1	2	1	3	1	3
1	1	1	1	1	1	1	1	2	1
3	3	3	3	3	3	3	3	2	2
2	2	2	2	2	2	2	2	2	2
1	1	1	1	1	1	1	1	1	1
2	2	2	2	2	2	2	2	2	2
1	3	1	1	1	1	2	3	1	2
2	2	2	2	2	2	2	2	2	2
2	3	3	3	2	2	3	2	2	2
2	3	2	3	2	3	3	2	2	2
2	2	2	2	2	2	2	2	1	1
9	9	9	9	9	9	9	9	9	9
3	2	1	3	1	2	3	2	1	2
1	1	1	1	1	1	1	1	2	2
2	2	2	2	2	2	2	2	2	2
2	2	2	2	2	2	2	2	1	1
2	2	2	3	2	3	2	3	1	2
2	2	2	2	2	2	2	2	1	2
2	3	3	3	3	2	2	2	1	1
9	9	9	9	9	9	9	9	9	9
2	9	9	9	9	9	9	9	2	2
9	9	9	9	9	9	9	9	9	9
9	9	9	9	9	9	9	9	1	1
2	2	2	2	2	2	2	2	2	2
9	9	9	9	9	9	9	9	9	9

2	2	2	1	1	3	2	3	2	2
1	2	3	3	3	3	3	9	2	2
1	1	1	2	2	2	2	2	2	2
1	1	9	9	2	2	2	2	2	2
2	2	2	2	2	2	9	9	1	1
1	1	1	1	2	2	2	2	1	1
1	1	9	1	1	1	1	1	1	1
1	2	1	3	3	3	2	1	1	9
3	3	3	3	3	3	3	3	3	3
1	1	1	1	1	2	1	3	1	1
9	9	9	9	9	9	9	9	9	9
2	2	2	2	2	2	2	2	2	2
2	2	2	2	2	2	2	2	2	2
2	2	2	2	2	2	2	2	2	2
2	2	2	2	2	2	2	2	2	2
1	1	1	1	1	1	2	2	1	1
1	1	2	1	1	2	1	2	2	1
1	1	1	1	2	2	1	2	1	1
1	1	1	1	1	1	1	1	1	1
9	9	9	9	9	9	9	9	9	9
1	2	1	1	1	1	2	1	1	2
2	2	2	2	2	2	2	2	2	2
9	9	9	9	9	9	9	9	9	9
1	1	1	2	2	2	2	2	1	1
9	9	9	9	9	9	9	9	9	9
2	1	2	1	1	1	1	1	1	1
1	1	1	1	2	2	2	1	1	1
2	2	2	2	2	2	2	2	1	1
9	9	9	9	9	9	9	9	9	9
2	2	2	2	9	2	2	2	2	9
9	9	9	9	9	9	9	9	9	9
9	9	9	9	9	9	9	9	9	9
2	2	2	2	2	2	2	2	2	2
1	1	1	1	1	1	1	1	1	1
2	2	2	2	2	2	2	2	2	2
9	9	9	9	9	9	9	9	9	9
1	2	1	1	2	1	2	1	1	1
1	1	1	1	2	1	1	1	1	2
9	9	9	9	9	9	9	9	9	9
9	9	9	9	9	9	9	9	9	9
2	2	2	2	2	2	2	2	2	2
9	9	9	9	9	9	9	9	9	9
2	2	2	2	2	2	2	9	2	2
2	2	2	2	2	2	2	2	9	9
2	1	1	1	2	2	2	1	1	2
2	2	2	2	2	2	2	2	2	2
1	1	1	1	1	1	1	1	1	1

1	1	2	1	1	1	1	2	1	1
2	2	2	9	2	9	9	9	2	9
3	9	3	3	3	3	3	3	2	2
1	1	2	2	2	2	1	1	1	1
2	2	2	2	2	2	2	2	2	2
9	9	9	9	9	9	9	9	9	9
2	2	2	2	2	2	2	2	1	1
2	2	2	2	2	2	2	2	2	2
9	2	9	2	2	9	2	2	9	9
9	1	1	1	1	1	2	2	9	1
9	9	9	9	9	9	9	9	9	9
9	9	9	9	9	9	9	9	9	9
9	9	9	9	9	9	9	9	9	9
9	9	9	9	9	9	9	9	9	9
9	9	9	9	9	9	9	9	9	9
9	9	9	9	9	9	9	9	9	9
1	1	1	1	1	1	1	1	2	2
9	9	9	9	9	9	9	9	9	9
2	2	2	2	2	2	2	2	2	2
3	3	3	3	1	3	3	1	1	1
3	3	3	3	1	2	1	1	1	1
3	3	3	3	1	2	1	1	1	1
3	3	3	3	1	2	1	1	1	1
3	9	3	3	1	2	1	1	1	1
3	1	3	3	1	2	1	1	1	1
3	3	3	3	1	2	1	1	1	1
3	3	3	3	1	2	1	1	1	1
3	3	3	3	1	2	1	1	1	1
3	1	3	3	1	9	1	1	1	1
3	3	3	3	1	2	1	1	1	1
3	3	3	3	1	2	1	1	1	1
3	3	3	3	1	2	1	1	1	1
3	3	3	3	1	2	1	1	1	1
3	3	3	3	1	2	1	1	1	1
3	9	3	3	1	2	1	1	1	1
3	3	3	3	1	2	3	1	1	1
3	3	3	3	1	2	1	1	1	1
3	1	3	3	1	2	1	1	1	1
3	1	3	3	1	2	1	1	1	1
3	3	3	3	1	2	1	1	1	1
3	3	3	3	1	2	1	1	1	1
3	3	3	3	1	2	1	1	1	1
3	3	3	3	1	2	1	1	1	1
3	3	3	3	1	2	1	1	1	1
9	9	9	9	9	9	9	9	9	9
9	9	9	9	9	9	9	9	9	9
9	9	9	9	9	9	9	9	9	9

9	9	9	9	9	9	9	9	9	9
9	9	9	9	9	9	9	9	9	9
9	9	9	9	9	9	9	9	9	9
9	9	9	9	9	9	9	9	9	9
9	9	9	9	9	9	9	9	9	9
9	9	9	9	9	9	9	9	9	9
9	9	9	9	9	9	9	9	9	9
9	9	9	9	9	9	9	9	9	9
9	9	9	9	9	9	9	9	9	9
9	9	9	9	9	9	9	9	9	9
9	9	9	9	9	9	9	9	9	9
9	9	9	9	9	9	9	9	9	9
9	9	9	9	9	9	9	9	9	9
9	9	9	9	9	9	9	9	9	9
9	9	9	9	9	9	9	9	9	9
9	9	9	9	9	9	9	9	9	9
9	9	9	9	9	9	9	9	9	9
9	9	9	9	9	9	9	9	9	9
9	9	9	9	9	9	9	9	9	9
3	3	3	3	1	2	1	1	1	1
3	3	3	3	1	2	1	1	1	1
3	3	3	3	1	2	1	1	1	1
3	3	3	3	1	2	1	1	1	1
3	3	3	3	1	2	1	1	1	1
3	3	3	3	1	2	1	1	1	1
3	1	3	3	1	2	1	1	1	1
3	1	3	3	1	2	1	1	1	1
3	3	3	3	1	2	1	1	1	1
3	3	3	3	1	2	1	1	1	1
3	3	3	3	1	2	1	1	1	1
3	1	3	3	3	2	1	1	1	1
3	3	3	3	1	2	1	1	1	1
3	1	3	3	1	2	1	1	1	1
3	3	3	9	9	2	1	1	1	1
3	3	3	3	1	3	1	1	1	1
9	9	9	9	9	9	1	9	9	1
3	3	3	3	1	2	1	1	1	1
3	3	3	3	1	2	1	1	1	1
3	3	3	3	1	2	1	1	1	1
3	3	3	3	1	2	1	1	1	1
3	3	3	3	1	2	1	1	1	1
3	3	3	3	1	2	1	1	1	1
3	1	3	3	1	2	1	1	1	1
3	3	3	3	9	2	1	1	1	1
3	1	3	3	1	2	1	1	1	1
3	3	3	3	1	2	1	1	1	1
3	3	3	3	1	2	1	1	1	1

3	9	3	3	1	2	1	1	1	1
3	3	3	1	1	2	1	1	1	1
3	3	3	3	1	2	1	1	1	1
3	9	3	1	1	2	1	1	1	1
3	3	3	3	1	2	1	1	1	1
3	3	3	3	1	2	1	1	1	1
3	3	3	1	2	2	1	1	1	1
1	3	3	3	3	2	1	1	1	1
3	3	3	3	1	2	1	1	1	1
3	3	3	3	1	2	1	1	1	1
3	3	3	1	1	2	1	1	1	1
3	3	3	3	1	2	1	1	1	1
3	1	3	3	1	2	1	1	1	1
3	3	3	1	2	2	1	1	1	1
3	3	3	3	1	2	1	1	1	1
3	1	3	3	1	2	1	1	1	1
3	3	3	3	1	2	1	1	1	1
3	3	3	3	1	2	1	1	1	1
3	3	3	3	1	2	1	1	1	1
3	9	3	3	1	2	1	1	1	1
3	3	3	3	1	2	1	1	1	1
3	3	3	3	3	2	3	1	1	1
3	3	3	3	1	2	1	1	1	1
3	3	3	3	1	2	1	1	1	1
3	9	3	3	1	2	1	1	1	1
3	3	3	3	1	2	1	1	1	1
3	3	1	3	1	2	1	1	1	1
3	3	3	3	1	2	1	1	1	1
3	3	3	3	1	2	1	1	1	1
3	3	3	3	1	9	1	1	1	1
3	9	3	3	1	2	1	1	1	1
3	3	3	3	9	9	1	1	1	1
1	1	9	9	9	9	9	9	9	9
3	3	3	3	1	2	1	1	1	1
9	9	9	9	9	9	9	9	9	9
3	3	3	3	1	2	1	1	1	1
3	9	3	3	1	2	1	1	1	1
3	9	3	1	9	2	1	1	1	1
3	3	3	3	1	2	1	1	1	1
3	3	1	3	1	2	1	1	1	1
3	3	3	3	1	2	1	1	1	1
3	1	3	3	1	2	1	1	1	1
3	3	3	3	1	2	1	1	1	1
3	3	3	3	1	2	1	1	1	1
3	3	3	3	1	2	1	1	1	1
3	1	3	3	1	2	1	1	1	1
3	3	3	3	1	2	1	1	1	1

3	3	3	3	1	2	1	1	1	1
3	3	3	3	1	2	1	1	1	1
3	1	3	3	1	2	1	1	1	1
3	3	3	3	1	2	1	1	1	1
3	1	3	3	1	2	1	1	1	1
3	1	3	3	1	2	1	1	1	1
3	1	3	3	1	2	1	1	1	1
3	3	3	3	1	2	1	1	1	1
3	3	3	3	1	2	1	1	1	1
3	1	3	3	1	2	1	1	1	1
3	9	3	3	1	2	1	1	1	1
3	3	3	3	1	2	1	1	1	1
3	3	3	3	1	2	1	1	1	1
3	3	3	3	1	2	1	1	1	1
3	9	3	9	9	9	1	1	1	1
3	3	3	3	1	2	1	1	1	1
3	3	3	3	1	2	1	1	1	1
3	3	3	3	1	2	1	1	1	1
3	3	3	3	1	2	1	1	1	1
3	9	3	3	1	2	1	1	1	1
3	9	3	3	1	2	1	1	1	1
3	3	3	3	1	2	1	1	1	1
3	3	3	3	1	2	1	1	1	1
3	3	3	3	1	2	1	1	1	1
9	3	1	9	9	9	9	1	1	1
3	1	3	3	1	3	1	1	1	1
3	1	3	3	1	2	1	1	1	1
3	3	1	3	1	2	1	1	1	1
3	3	3	3	1	3	1	1	1	1
3	3	3	3	1	2	1	1	1	1
3	3	3	3	1	3	1	1	1	1
3	3	3	3	1	2	1	1	1	1
3	3	3	3	1	2	1	1	1	1
3	3	3	3	1	2	1	1	1	1
3	3	3	3	1	2	1	1	1	1
3	3	3	3	1	2	1	1	1	1
3	3	3	3	1	2	1	1	1	1
3	9	9	3	1	2	9	9	1	1
3	3	3	3	1	2	1	1	1	1
3	3	3	3	1	2	1	1	1	1
3	1	3	3	1	2	1	1	1	1
9	9	9	9	9	9	9	9	9	9
3	1	3	3	1	2	1	1	1	1
3	1	3	3	1	3	1	1	1	1
3	1	3	3	1	2	1	1	1	1
3	1	3	3	3	2	1	1	1	1
3	3	3	3	3	2	1	1	1	1

3	1	1	3	1	3	1	1	1	1
3	1	3	3	1	2	1	1	1	1
3	3	3	3	1	2	1	1	1	1
3	3	3	3	1	2	1	1	1	1
3	3	3	3	1	2	1	1	1	1
3	3	3	3	1	2	1	1	1	1
3	3	3	3	1	2	1	1	1	1
3	1	3	3	1	2	1	1	1	1
3	3	3	3	1	2	1	1	1	1
9	9	9	9	9	9	9	9	9	9
3	3	3	3	1	2	1	1	1	1
3	1	3	3	1	2	1	1	1	1
3	1	3	3	1	2	1	1	1	1
3	3	3	3	1	2	1	1	1	1
3	3	3	3	1	2	1	1	1	1
3	3	3	3	1	2	1	1	1	1
3	3	3	3	1	2	1	1	1	1
3	3	3	3	3	2	2	1	1	1
3	3	3	3	1	2	1	1	1	1
3	1	3	3	1	2	1	1	1	1
3	3	3	3	1	2	1	1	1	1
3	3	3	3	1	2	1	1	1	1
3	3	3	3	1	2	1	1	1	1
3	3	3	3	1	2	1	1	1	1
3	1	3	3	1	2	1	1	1	1
3	1	3	3	1	2	1	1	1	1
3	1	3	3	1	2	1	1	1	1
9	9	9	9	9	9	9	9	9	9
9	9	9	9	9	9	9	9	9	9
9	9	9	9	9	9	9	9	9	9
9	9	9	9	9	9	9	9	9	9
9	9	9	9	9	9	9	9	9	9
9	9	9	9	9	9	9	9	9	9
9	9	9	9	9	9	9	9	9	9
9	9	9	9	9	9	9	9	9	9
9	9	9	9	9	9	9	9	9	9
9	9	9	9	9	9	9	9	9	9
9	9	9	9	9	9	9	9	9	9
9	9	9	9	9	9	9	9	9	9
9	9	9	9	9	9	9	9	9	9
9	9	9	9	9	9	9	9	9	9
9	9	9	9	9	9	9	9	9	9
9	9	9	9	9	9	9	9	9	9
9	9	9	9	9	9	9	9	9	9
9	9	9	9	9	9	9	9	9	9
9	9	9	9	9	9	9	9	9	9
9	9	9	9	9	9	9	9	9	9

9	9	9	9	9	9	9	9	9	9
9	9	9	9	9	9	9	9	9	9
9	9	9	9	9	9	9	9	9	9
9	9	9	9	9	9	9	9	9	9
9	9	9	9	9	9	9	9	9	9
9	9	9	9	9	9	9	9	9	9
9	9	9	9	9	9	9	9	9	9
9	9	9	9	9	9	9	9	9	9
9	9	9	9	9	9	9	9	9	9
9	9	9	9	9	9	9	9	9	9
9	9	9	9	9	9	9	9	9	9
9	9	9	9	9	9	9	9	9	9
9	9	9	9	9	9	9	9	9	9
1	2	1	2	2	3	2	2	1	1
2	2	2	2	2	2	2	2	2	2
2	2	2	2	2	2	2	2	2	2
3	1	3	2	1	2	1	1	1	3
1	3	2	1	2	3	2	1	1	2
1	1	1	1	2	2	2	2	2	2
3	1	1	1	1	2	3	3	1	2
1	1	2	1	2	2	1	1	1	1
2	2	2	2	2	2	2	1	3	2
9	9	9	9	9	9	9	9	9	9
2	2	2	2	3	2	2	3	2	3
9	9	9	9	9	9	9	1	9	9
3	3	3	3	3	2	2	2	2	2
9	9	9	9	9	9	9	9	9	9
1	1	1	1	1	1	1	1	1	1
1	3	2	1	3	1	1	1	1	1
2	2	2	2	2	2	2	2	2	2
3	3	3	3	3	3	3	1	1	1
2	3	2	1	3	2	3	2	2	3
9	9	9	9	9	9	9	9	9	1
2	2	2	1	2	1	2	2	2	2
1	1	1	1	1	1	1	1	1	1
9	9	9	9	9	9	9	9	1	9
2	2	2	2	2	2	2	2	2	1
1	3	2	1	2	3	1	1	2	1
1	2	2	3	1	1	1	2	3	1
1	1	1	1	1	1	1	1	1	3
9	9	9	9	9	9	9	9	9	9
9	9	9	9	9	9	9	9	9	9
2	2	2	2	2	2	2	2	1	2
1	1	1	1	1	1	1	1	1	1
9	9	9	9	9	9	9	9	9	9
9	9	9	9	9	9	9	9	9	9
9	9	9	9	9	9	9	9	9	9

9	9	9	9	9	9	9	1	9	9
9	9	9	9	9	9	9	9	9	9
1	3	2	2	1	2	2	3	2	2
2	2	2	2	2	2	2	2	1	2
2	3	3	3	1	3	1	1	1	2
9	9	9	9	9	9	9	9	9	9
2	2	2	2	2	2	2	2	2	2
2	2	2	2	2	2	2	2	2	2
2	2	2	2	2	2	2	2	2	2
2	2	2	1	1	1	2	2	2	2
2	2	3	3	3	3	3	3	3	9
9	9	9	9	9	9	9	9	9	9
9	9	9	9	9	9	9	9	9	3
2	2	2	2	2	2	2	2	2	2
2	2	2	2	2	2	2	2	1	1
2	2	2	2	2	2	2	2	2	2
2	2	2	2	2	2	2	1	1	2
2	2	2	2	2	2	2	2	2	2
1	1	3	1	3	1	1	1	1	2
9	9	9	9	9	9	9	9	9	9
2	2	2	2	2	2	2	2	2	2
2	2	2	2	2	2	2	2	2	2
2	2	2	2	2	2	2	2	2	2
2	2	2	2	2	2	2	2	2	2
2	2	2	2	2	2	2	2	2	2
1	1	2	2	1	1	2	2	1	1
2	2	2	2	2	2	2	2	2	2
2	2	2	2	2	2	2	2	2	2
2	2	2	2	2	2	2	2	1	9
1	1	2	2	1	2	1	1	1	1
3	3	3	3	2	3	1	1	3	3
2	2	2	2	2	3	1	2	1	1
1	1	2	1	1	1	1	1	1	1
2	2	2	2	1	3	3	1	1	1
2	1	3	2	1	3	2	3	2	2
2	2	1	1	1	2	2	2	1	2
2	2	2	1	2	2	2	2	3	3
1	9	9	9	9	9	9	9	2	2
9	9	9	9	9	9	9	9	2	9
2	2	2	2	2	2	2	2	2	2
3	3	3	1	1	1	1	1	1	2
2	2	2	2	1	2	2	2	2	2
3	1	2	3	1	2	3	1	1	2
2	3	3	3	2	2	2	9	9	9
1	1	1	1	1	1	1	1	1	1
1	1	2	2	2	1	1	1	1	3
2	2	2	2	2	2	2	2	1	1

2	2	2	2	2	2	2	2	2	2
3	1	2	3	1	2	3	1	1	1
2	2	2	2	2	2	2	2	3	3
3	3	2	3	2	2	3	2	2	3
2	2	2	2	2	2	2	2	2	2
2	2	2	2	2	2	2	2	2	2
2	2	2	2	2	2	1	2	2	2
2	2	3	1	2	2	3	3	2	2
2	2	2	2	2	2	2	2	2	2
3	1	2	1	3	1	2	3	3	1
2	2	2	2	2	2	2	2	2	2
2	2	2	2	2	2	2	2	2	2
2	2	2	2	2	2	2	2	2	2
2	3	3	3	3	3	3	3	3	3
2	2	2	2	2	2	2	2	2	2
1	2	1	2	2	1	2	2	2	2
2	2	2	2	2	2	2	2	2	2
2	2	2	2	2	2	2	2	2	2
2	2	2	2	2	2	2	2	2	2
3	1	2	3	1	2	3	1	1	2
2	2	1	2	1	2	1	2	1	2
1	1	1	1	1	3	3	3	1	1
2	2	2	2	2	2	2	2	2	2
1	3	3	3	3	3	3	3	1	1
3	1	3	3	1	3	1	1	1	1
9	9	9	9	9	9	9	9	9	9
9	9	9	9	9	9	9	9	9	9
2	2	2	2	2	2	2	2	2	2
9	9	9	9	9	9	9	9	9	9
1	1	3	3	2	1	1	2	3	3
1	1	2	2	2	2	1	2	2	1
2	1	2	2	2	1	2	2	1	3
2	2	3	3	3	3	3	3	9	9
2	2	2	2	2	2	2	2	1	1
1	9	9	9	9	9	9	9	9	2
2	2	2	2	2	2	2	2	2	2
9	9	9	9	9	9	9	9	9	9
1	2	2	1	2	2	1	1	3	3
9	9	9	9	9	9	9	9	9	9
2	2	2	2	2	2	2	2	2	2
3	2	2	2	3	2	3	2	2	3
1	1	2	1	1	2	2	2	2	2
1	1	2	1	2	2	1	2	2	3
9	9	9	9	9	9	9	9	9	9
9	9	9	9	9	9	9	9	9	9
9	9	9	9	9	9	9	9	9	9
1	1	1	1	1	2	2	2	1	1

9	9	9	9	9	9	9	9	9	9
9	9	9	9	9	9	9	9	9	9
2	2	2	3	2	3	1	1	3	1
9	9	9	9	9	9	9	9	9	9
9	9	9	9	9	9	9	9	9	9
2	2	3	3	2	2	2	2	3	3
2	2	2	3	3	2	2	3	2	2
9	9	9	9	9	9	9	9	9	9
1	1	2	2	1	3	1	1	2	1
3	1	2	3	1	2	3	1	1	2
1	1	2	3	1	2	3	1	1	2
2	2	2	2	2	2	2	2	2	2
2	2	2	2	2	2	2	2	2	2
9	9	9	9	9	9	9	9	9	9
2	2	2	2	2	2	2	2	2	2
2	2	2	2	2	2	2	2	2	2
2	3	3	3	1	3	1	1	1	1
1	3	3	3	2	1	2	2	2	3
3	3	2	1	3	2	1	2	1	2
9	9	9	9	9	9	9	9	9	9
1	3	3	3	2	1	2	2	2	3
1	3	2	1	1	1	1	1	3	3
2	2	2	2	2	2	2	2	2	2
1	3	3	3	2	2	1	2	2	3
3	3	3	3	3	3	3	3	2	3
9	9	9	9	9	9	9	9	9	9
2	3	3	2	2	2	2	1	1	2
9	9	9	9	9	9	9	9	9	9
2	2	2	2	2	2	2	2	3	3
1	2	3	2	1	2	3	2	1	1
2	2	2	2	2	2	2	2	2	2
9	9	9	9	9	9	9	9	9	9
9	9	9	9	9	9	9	9	9	9
2	2	2	2	2	2	2	2	2	2
2	1	2	2	2	1	2	2	1	2
3	1	3	1	2	1	2	3	2	1
2	2	2	2	2	2	2	9	2	2
2	2	2	2	1	3	2	2	1	1
2	2	2	2	2	2	2	2	2	2
9	9	9	9	9	9	9	9	2	9
2	2	2	1	1	2	2	2	1	1
2	2	2	2	2	2	2	2	2	2
1	2	2	2	2	2	2	2	2	2
2	2	2	2	2	2	2	2	2	2
2	2	2	2	2	2	2	2	2	2
9	9	9	9	9	9	9	9	1	9
3	3	2	2	3	2	2	3	2	3

1	2	1	1	1	1	1	1	1	1
2	2	2	2	2	2	2	2	2	2
1	1	3	3	1	3	2	2	1	1
2	2	2	2	2	2	1	2	2	2
2	2	2	2	2	2	2	2	2	2
2	2	2	3	3	3	2	2	2	2
1	3	2	2	1	2	3	1	1	2
3	2	1	2	2	1	2	2	1	2
1	1	3	3	1	3	1	1	1	1
3	2	3	3	3	3	1	1	1	1
2	2	2	2	2	2	2	2	2	2
2	2	2	2	2	2	2	2	2	2
2	2	2	2	2	2	2	2	2	2
1	2	3	3	1	3	1	1	1	1
9	2	1	3	1	9	3	1	3	2
2	2	2	2	2	2	2	2	2	2
3	2	3	2	3	2	3	2	2	9
2	2	1	2	1	2	1	2	2	2
2	2	2	3	2	2	3	2	2	2
1	1	2	2	1	1	1	1	1	1
1	1	2	1	1	3	1	1	1	1
9	9	9	9	9	9	9	9	9	9
2	2	2	2	2	2	2	2	2	2
2	2	2	2	2	2	2	2	2	2
2	2	2	2	2	2	2	2	2	2
1	1	2	2	2	2	2	2	2	1
3	2	2	2	2	2	2	1	1	2
2	2	2	2	2	2	1	1	1	2
1	3	3	3	3	3	1	1	1	1
1	1	2	2	2	2	1	1	2	1
3	3	3	3	3	3	1	1	1	1
2	1	2	2	1	1	1	1	2	1
1	2	1	2	1	2	2	2	3	2
9	9	9	9	9	9	9	9	9	9
9	9	9	9	9	9	9	9	9	9
1	3	3	3	1	3	1	1	1	1
2	2	2	2	2	2	2	2	2	2
1	2	1	2	1	2	1	1	1	1
1	1	1	1	1	1	1	1	1	1
2	2	2	2	2	2	2	2	2	2
3	1	2	1	2	2	2	2	1	2
9	9	9	9	9	9	9	9	9	9
9	9	9	9	9	9	9	9	9	9
1	1	1	1	1	1	1	1	1	1
3	1	2	2	1	3	2	3	1	2
9	9	9	9	9	9	9	9	9	9
9	9	9	9	9	9	9	9	9	9

2	2	2	2	2	2	2	2	2	2
1	1	1	2	2	2	3	3	3	3
3	1	2	3	1	2	3	1	1	2
2	2	3	2	2	3	2	2	2	2
2	2	3	3	3	3	1	1	1	2
3	1	2	3	1	2	3	1	1	2
2	3	2	2	2	2	2	2	2	2
9	9	9	9	9	9	9	9	9	9
1	1	1	2	2	1	2	2	1	2
9	9	9	9	9	9	9	9	9	9
2	2	2	2	2	2	2	2	1	1
3	3	3	3	3	2	2	2	2	1
1	1	2	2	3	3	2	1	2	2
9	9	9	9	9	9	9	9	9	9
2	2	2	2	2	2	2	2	1	1
9	9	9	9	9	9	9	9	9	9
9	9	9	9	9	9	9	9	9	9
2	2	2	2	2	2	2	2	2	2
2	2	2	2	2	2	2	2	2	2
1	2	1	2	2	2	1	2	2	2
2	2	2	2	2	2	2	2	2	2
2	2	2	2	2	2	2	2	2	2
3	3	3	3	3	3	3	3	2	2
1	2	3	2	3	2	1	2	1	2
1	1	9	2	9	9	3	9	3	3
9	9	9	9	9	9	9	9	2	9
2	2	2	2	2	2	2	2	2	2
2	2	2	2	2	2	2	2	1	1
1	1	1	1	1	1	1	1	2	2
3	3	3	3	3	3	1	1	3	1
1	1	2	1	1	2	1	2	1	1
2	2	2	2	2	2	2	2	2	2
2	2	2	2	2	2	2	2	2	2
2	2	2	2	2	2	2	2	2	2
2	2	2	2	2	2	2	2	2	2
2	2	2	2	2	2	2	2	2	2
3	3	3	3	3	3	3	3	2	3
9	9	9	1	9	9	9	9	9	9
9	9	9	9	9	9	9	2	9	9
2	2	2	2	2	2	2	2	2	2
3	3	3	3	3	3	3	3	3	3
2	2	2	2	2	2	2	2	9	1
2	2	2	2	2	2	2	2	1	2
9	9	9	9	9	9	9	9	2	9
2	2	2	2	2	2	2	2	3	3
9	9	9	9	9	9	9	9	9	9
2	2	2	2	2	2	2	2	2	2

1	3	1	3	1	2	9	3	3	1
9	9	9	9	9	9	1	9	9	9
2	2	2	2	2	2	2	2	2	2
2	2	2	2	2	2	2	2	1	2
3	2	2	2	2	2	2	2	2	2
2	2	2	2	2	2	2	2	2	2
1	2	2	2	1	3	1	2	1	2
9	9	9	9	9	9	9	9	9	9
2	2	2	2	2	2	2	2	2	2
1	1	2	2	2	2	2	2	2	2
2	2	2	2	2	2	2	2	2	2
2	2	2	2	2	2	2	2	2	2
9	2	3	9	2	3	3	2	2	2
3	2	2	3	3	3	2	2	1	2
2	1	1	1	2	2	1	3	2	3
2	1	1	2	1	1	2	1	2	3
2	2	2	2	2	2	2	2	2	2
2	2	2	2	2	2	2	2	2	2
9	9	9	9	9	9	9	9	9	9
9	9	9	9	9	9	9	9	9	9
2	3	2	3	1	2	2	3	1	1
2	2	2	2	2	2	1	1	1	1
9	9	9	9	9	9	9	9	9	9
9	9	9	9	9	9	9	9	9	9
3	3	3	3	3	3	3	3	3	3
9	9	9	9	9	9	9	9	9	9
9	9	9	9	9	9	9	9	9	9
9	9	9	9	9	9	9	9	9	9
3	1	2	2	2	3	1	1	1	2
1	1	1	1	1	2	1	1	2	2
2	2	2	2	2	2	2	2	2	2
1	9	1	2	1	9	1	1	1	1
2	1	2	3	1	2	2	3	3	2
2	2	2	2	2	2	2	2	2	2
2	2	2	2	2	2	2	2	2	2
2	2	2	2	2	2	2	2	1	1
2	1	2	2	1	3	3	2	1	2
3	2	3	2	3	1	2	3	1	2
3	2	1	3	1	1	2	2	2	2
1	2	2	2	2	2	2	2	1	1
2	2	3	3	2	2	1	1	2	2
2	2	2	2	2	2	2	1	2	2
9	9	9	9	9	9	9	9	1	9
9	1	9	9	9	9	9	9	9	9
2	1	2	1	3	2	2	3	1	2
2	2	2	2	2	2	2	2	2	2
2	2	2	2	2	2	2	2	2	2

1	2	2	2	2	3	3	3	2	2
9	9	9	9	9	9	9	9	9	9
9	9	9	9	9	9	9	9	9	9
9	9	9	9	9	9	9	9	9	9
9	9	9	9	9	9	9	9	9	9
9	9	9	9	9	9	9	1	9	9
9	9	9	9	9	9	9	9	9	9
9	9	9	9	9	9	9	9	9	9
9	9	9	9	9	9	9	9	9	9
9	9	9	9	9	1	9	9	1	9
2	2	2	2	2	2	2	2	2	2
2	2	2	2	3	2	3	2	2	1
9	9	9	9	9	1	1	9	1	2
2	2	2	2	2	2	2	2	2	3
2	2	2	2	2	2	2	2	2	2
9	9	9	9	9	9	9	9	9	9
1	2	2	2	2	3	3	3	2	2
2	2	2	2	2	2	2	2	2	2
1	2	2	2	2	2	2	9	2	2
2	2	2	2	2	2	2	2	1	1
2	1	2	3	1	2	3	1	1	2
2	3	2	1	2	3	2	3	1	3
3	3	3	3	3	3	3	3	2	2
2	2	2	2	2	2	2	2	2	2
2	2	2	2	2	2	2	2	2	2
2	2	2	2	2	2	2	2	2	2
2	2	2	2	2	2	2	2	3	3
2	1	2	1	2	1	2	1	1	1
2	2	2	2	2	2	2	2	2	2
2	2	2	2	2	2	2	2	2	2
9	9	9	9	9	9	9	9	9	9
2	2	2	2	2	2	2	2	2	2
2	2	1	1	1	1	2	2	2	2
9	9	9	9	9	9	9	9	1	9
3	3	3	3	3	3	3	3	9	9
2	2	2	2	2	2	2	2	2	2
2	2	2	2	2	2	2	2	2	2
2	2	2	2	2	2	2	2	2	2
3	2	3	2	3	1	3	1	1	3
9	9	9	9	9	9	9	9	9	9
1	2	3	2	3	1	3	3	1	1
2	2	2	2	2	2	2	2	2	2
2	2	2	2	2	2	2	2	2	2
2	2	2	2	1	3	1	1	1	1
1	2	2	2	1	1	1	1	2	2
9	9	9	9	9	9	9	9	9	9
9	9	9	9	9	9	9	9	9	9

9	9	9	9	9	9	9	9	9	9
2	2	2	2	2	2	2	2	1	1
9	9	9	9	9	9	9	9	2	9
9	9	9	9	9	9	9	9	9	9
9	9	9	9	9	9	9	9	9	9
9	9	9	9	9	9	9	9	9	9
9	9	9	9	9	9	9	9	9	9
9	9	9	9	9	9	9	9	9	9
9	9	9	9	9	9	9	9	9	9
9	9	9	9	9	9	9	9	9	9
9	9	9	9	9	9	9	9	9	9
9	9	9	9	9	9	9	9	9	9
9	9	9	9	9	9	9	9	9	9
9	9	9	9	9	9	9	9	9	9
9	9	9	9	9	9	9	9	9	9
9	9	9	9	9	9	9	9	9	9
9	9	9	9	9	9	9	9	9	9
9	9	9	9	9	9	9	9	9	9
9	9	9	9	9	9	9	9	9	9
9	9	9	9	9	9	9	9	9	9
9	9	9	9	9	9	9	9	9	9
9	9	9	9	9	9	9	9	9	9
9	9	9	9	9	9	9	9	9	9
9	9	9	9	9	9	9	9	9	9
9	9	9	9	9	9	9	9	9	9
3	1	2	3	2	1	3	2	1	1
2	2	2	2	2	2	2	2	2	2
1	1	2	2	2	2	2	2	1	1
2	2	2	2	2	2	2	2	1	1
1	2	1	3	2	2	1	2	1	2
9	9	9	9	9	9	9	9	1	2
2	2	2	2	2	2	2	2	2	2
1	1	1	1	2	2	3	3	1	1
2	2	2	2	2	2	2	2	3	2
1	1	2	2	3	3	3	3	1	1
9	9	9	9	9	9	9	9	1	1
3	3	2	2	2	2	2	2	2	1
1	2	3	3	1	3	1	1	1	1
9	9	9	9	9	9	9	9	1	1
2	2	2	2	2	2	2	2	1	1
1	1	2	1	1	3	1	2	1	1
9	9	9	9	9	9	9	9	1	9
1	2	2	2	1	2	2	1	1	1
2	1	2	2	2	3	1	1	2	1
9	9	9	9	9	9	9	9	1	9
1	2	2	3	1	2	1	2	1	1
3	2	3	3	1	3	1	1	1	1
2	2	2	2	2	2	2	2	1	2
2	3	2	3	2	3	2	3	3	1

1	2	2	2	2	3	1	1	1	1
2	2	2	2	2	2	1	2	1	1
2	2	3	3	1	3	1	1	1	1
2	2	2	1	1	2	1	2	1	1
2	3	2	3	1	3	2	1	3	2
9	9	9	9	9	9	9	9	3	9
3	2	1	1	2	1	1	2	1	1
9	9	9	9	9	9	9	9	1	9
2	2	2	2	2	2	2	2	2	2
2	2	2	2	2	2	2	2	2	2
9	1	9	9	9	9	9	9	9	9
9	9	9	9	9	9	9	9	1	9
9	9	1	9	9	9	9	9	1	9
9	1	9	9	9	9	9	9	1	9
9	9	9	9	9	9	9	9	9	1
9	9	9	9	9	9	9	9	9	9
9	9	9	9	9	9	9	9	9	9
2	2	2	2	2	2	2	2	9	9
9	9	9	9	9	9	1	9	9	9
1	3	1	3	1	3	3	1	1	1
1	1	2	2	1	2	2	2	9	1
9	9	1	9	9	1	9	9	9	1
9	9	9	9	9	9	9	9	9	1
9	9	9	2	9	9	9	9	9	1
9	9	9	9	9	9	9	9	1	9
1	2	3	1	3	1	2	3	1	2
1	1	3	1	1	1	3	1	3	1
3	2	1	2	2	3	3	2	2	3
2	2	2	2	2	2	1	2	2	2
9	9	9	9	9	9	9	9	9	9
3	3	2	1	2	1	2	1	2	1
1	1	3	1	3	1	1	1	1	1
3	3	3	3	3	3	3	3	3	3
3	1	3	1	1	3	1	1	1	3
1	1	3	3	1	3	1	1	1	2
9	9	9	9	9	9	9	9	3	9
2	2	3	2	2	2	2	2	2	2
1	1	3	3	1	1	1	1	1	1
2	3	2	3	1	2	3	2	2	2
2	1	1	3	1	2	1	1	2	1
1	2	3	3	2	3	1	2	1	1
1	2	2	3	2	3	1	1	1	1
1	1	3	3	1	3	1	1	1	3
3	3	3	2	2	1	1	2	1	1
1	9	2	1	1	3	2	2	2	1
9	9	9	9	9	9	9	9	2	2
1	1	1	2	1	2	1	1	1	1

1	2	1	2	2	1	1	1	1	1
3	2	3	2	3	3	3	3	2	1
9	9	9	9	9	9	9	9	9	9
3	3	3	3	3	3	1	1	9	1
9	9	9	9	9	9	9	9	9	9
1	1	2	1	1	3	1	1	1	1
1	1	2	3	1	3	1	1	1	1
9	9	9	9	9	9	9	9	9	9
9	9	9	9	9	9	9	9	2	9
2	2	2	2	2	2	2	2	2	2
1	3	2	3	2	1	1	2	2	3
3	3	3	1	3	3	3	1	9	1
1	2	3	3	1	1	1	1	1	2
9	9	9	9	9	9	9	9	9	9
9	9	9	9	9	9	9	9	9	9
2	2	9	3	3	3	2	2	2	2
9	9	9	9	9	9	9	9	1	9
9	9	9	9	9	9	9	9	9	9
9	2	3	2	2	2	3	3	3	3
1	2	3	3	3	2	3	2	1	1
2	2	2	2	2	2	2	2	2	2
1	1	1	1	1	1	1	1	1	1
1	3	3	2	3	1	3	3	2	1
1	3	3	3	2	3	2	1	1	1
2	2	2	2	2	2	2	2	2	2
9	9	9	9	9	9	9	9	9	9
9	9	9	9	9	9	9	9	2	9
1	1	3	1	1	3	3	1	1	1
3	3	3	2	1	1	2	2	3	3
2	3	2	1	3	2	1	3	2	3
9	9	9	9	9	9	9	9	9	9
9	9	9	9	9	9	9	9	9	9
9	9	9	9	9	9	9	9	9	9
9	9	9	9	9	9	9	9	9	9
9	9	9	9	9	9	9	9	9	9
9	9	9	9	9	9	9	9	9	9
9	9	9	9	9	9	9	9	9	9
9	9	9	9	9	9	9	9	9	9
9	9	9	9	9	9	9	9	9	9
9	9	9	9	9	9	9	9	9	9
9	9	9	9	9	9	9	9	9	9
9	9	9	9	9	9	9	9	9	9
9	9	9	9	9	9	9	9	9	9
9	9	9	9	9	9	9	9	9	9
9	9	9	9	9	9	9	9	9	9
9	9	9	9	9	9	9	9	9	9
9	9	9	9	9	9	9	9	9	9

9	9	9	9	9	9	9	9	9	9
9	9	9	9	9	9	9	9	9	9
9	9	9	9	9	9	9	9	9	9
9	9	9	9	9	9	9	9	9	9
9	9	9	9	9	9	9	9	9	9
9	9	9	9	9	9	9	9	9	9
9	9	9	9	9	9	9	9	9	9
9	9	9	9	9	9	9	9	9	9
9	9	9	9	9	9	9	9	9	9
9	9	9	9	9	9	9	9	9	9
9	9	9	9	9	9	9	9	9	9
9	9	9	9	9	9	9	9	9	9
9	9	9	9	9	9	9	9	9	9
9	9	9	9	9	9	9	9	9	9
9	9	9	9	9	9	9	9	9	9
9	9	9	9	9	9	9	9	9	9
9	9	9	9	9	9	9	9	9	9
9	9	9	9	9	9	9	9	9	9
9	9	9	9	9	9	9	9	9	9
2	2	2	2	2	2	2	2	9	9
9	9	9	9	9	9	9	9	9	9
9	9	9	9	9	9	9	9	9	9
9	9	9	9	9	9	9	9	9	9
9	9	9	9	9	9	9	9	9	9
9	9	9	9	9	9	9	9	9	9
9	9	9	9	9	9	9	9	9	9
9	9	9	9	9	9	9	9	9	9
9	9	9	9	9	9	9	9	9	9
9	9	9	9	9	9	9	9	9	9
9	9	9	9	9	9	9	9	9	9
9	9	9	9	9	9	9	9	9	9
9	9	9	9	9	9	9	9	9	9
9	9	9	9	9	9	9	9	9	9
9	9	9	9	9	9	9	9	9	9
9	9	9	9	9	9	9	9	9	9
2	2	2	2	2	2	2	2	2	2
9	9	9	9	9	9	9	9	9	9
1	1	2	3	1	3	1	1	3	3
1	3	1	1	1	1	1	1	1	1
3	3	3	3	3	3	3	3	3	3
9	9	9	9	9	9	9	9	9	9
9	9	9	9	9	9	9	9	2	2
9	9	9	9	9	9	9	9	9	1
9	9	9	9	9	9	9	9	2	2
2	2	2	1	3	2	2	2	2	2
9	1	9	9	9	9	9	9	9	1
2	2	2	2	2	2	2	2	2	2

1	1	2	1	1	3	1	2	1	1
2	1	2	2	2	2	2	2	1	1
2	2	2	2	2	2	1	2	1	1
2	2	2	2	2	3	1	1	1	1
2	1	3	3	1	3	1	1	1	1
1	2	2	3	1	3	3	1	1	1
1	1	2	2	2	2	2	2	1	1
2	2	2	2	2	2	2	2	1	2
1	3	1	2	2	2	3	2	2	3
2	1	3	3	1	2	2	1	1	1
9	9	9	9	9	9	9	9	9	1
3	2	2	3	1	2	3	3	2	9
9	9	9	9	9	9	9	9	9	9
3	2	2	2	2	2	2	2	1	1
3	2	2	2	2	2	2	2	2	1
2	2	2	3	2	2	1	2	2	2
9	9	9	9	9	9	9	9	9	9
9	9	9	9	9	9	9	9	1	1
1	1	2	1	1	2	1	1	1	1
1	1	1	1	1	3	1	1	3	1
1	3	3	1	9	3	1	1	1	1
1	2	3	3	2	3	1	2	1	1
1	2	2	2	1	2	2	1	1	1
1	3	3	3	1	3	1	1	1	1
3	3	3	3	3	3	3	3	3	3
1	2	1	2	1	2	1	2	1	3
1	1	1	1	1	1	1	1	1	1
2	3	1	2	3	2	3	1	1	2
2	2	2	2	2	2	2	2	2	2
9	9	9	9	9	9	9	9	9	9
9	9	9	9	9	9	9	9	9	9
2	2	2	2	2	2	2	2	1	1
2	2	2	2	2	2	2	2	2	2
9	9	9	9	9	9	9	9	9	9
9	9	9	9	9	9	9	9	9	9
9	9	9	9	9	9	9	9	9	9
9	9	9	9	9	9	9	9	1	1
3	2	1	2	3	2	1	2	1	1
9	9	9	9	9	9	9	9	9	9
9	9	9	9	9	9	9	9	9	9
2	2	1	1	2	1	2	1	1	1
9	9	9	9	9	9	9	9	9	9
1	3	3	3	2	3	1	1	1	1
3	3	3	3	1	3	3	1	3	3
1	1	2	1	1	3	2	3	1	1
3	3	3	3	3	3	1	3	3	3
1	1	3	1	1	1	3	1	1	1

3	2	3	2	3	2	3	2	2	3
1	2	1	2	1	2	3	2	1	1
1	2	2	1	2	2	1	3	1	2
1	2	2	2	3	3	1	1	1	2
2	3	3	2	2	3	2	1	1	2
2	2	3	2	1	2	3	2	1	2
1	1	1	2	2	3	3	3	1	2
1	2	3	1	2	3	1	2	1	3
1	1	1	1	2	1	1	2	1	1
2	9	1	9	2	3	9	2	1	2
3	3	3	3	3	3	3	3	3	3
2	1	1	1	2	3	1	1	1	1
1	2	2	1	2	1	2	2	3	2
3	1	2	3	1	2	3	1	1	2
1	1	1	1	1	1	1	1	1	1
2	2	3	3	3	3	1	2	1	1
2	3	2	3	2	2	3	2	2	2
9	9	9	9	9	9	9	9	9	1
1	1	2	2	2	1	3	2	1	2
2	3	3	3	3	3	3	1	1	2
2	2	3	3	2	2	2	2	1	2
3	2	1	1	2	2	2	2	1	1
2	1	1	3	1	2	2	1	1	1
2	2	2	2	2	2	2	2	2	2
2	1	1	2	2	2	1	1	2	3
1	1	1	3	1	1	1	1	1	1
1	2	3	2	1	3	1	1	1	1
2	2	2	1	3	1	2	1	1	3
1	2	2	2	2	1	3	2	1	1
2	2	3	1	2	2	1	1	1	1
2	2	2	2	2	2	2	3	3	3
3	1	2	3	1	2	3	1	1	2
2	2	2	2	2	2	2	2	1	1
1	3	3	1	3	3	1	3	1	1
3	1	2	3	1	2	3	1	1	2
3	3	3	3	3	3	1	1	1	1
2	2	1	1	3	2	1	2	1	2
9	9	9	9	9	9	9	9	9	9
1	1	3	1	3	3	1	9	1	1
9	1	2	1	3	1	1	3	1	2
3	3	1	3	1	3	1	1	1	3
3	1	2	3	1	2	3	1	1	2
1	1	1	3	1	1	1	1	1	1
2	2	3	2	1	3	1	2	2	2
2	2	2	2	2	2	2	2	2	2
3	3	3	3	3	3	3	3	3	1
3	3	3	3	3	3	9	9	9	9

2	3	1	2	3	2	1	2	1	2
1	1	1	1	1	1	1	1	1	1
2	2	2	2	3	3	3	3	1	1
2	3	2	2	1	2	3	2	1	2
1	2	2	2	2	2	1	1	1	1
1	2	2	3	3	3	2	2	1	1
2	2	2	2	2	2	2	2	2	2
9	9	9	9	9	9	9	9	9	9
9	9	9	9	9	9	9	9	9	9
2	3	2	1	2	3	2	1	1	2
2	2	2	2	2	2	2	2	1	1
2	2	2	2	2	2	2	2	2	2
2	2	2	2	1	1	1	1	1	1
2	2	2	1	2	1	2	2	2	1
2	2	2	2	2	2	2	2	2	2
2	2	2	2	2	2	2	2	2	2
2	2	2	2	2	2	2	2	2	2
2	2	2	2	2	2	2	2	3	2
2	2	3	1	1	1	2	3	2	2
2	2	2	2	2	2	2	2	2	2
3	2	2	2	2	2	2	2	2	2
2	2	2	2	2	2	2	2	2	2
2	2	2	2	2	2	2	2	2	2
2	1	2	2	2	3	2	2	3	2
2	2	2	2	2	2	2	2	2	2
2	2	2	2	2	2	2	2	2	2
2	2	2	2	2	2	2	2	1	1
9	1	2	1	2	3	1	2	1	2
1	1	1	2	2	2	2	2	2	3
2	2	2	2	2	2	2	2	2	2
2	2	2	2	2	2	2	2	2	2
2	2	2	2	2	2	2	2	1	1
2	2	2	2	2	2	2	2	2	2
2	2	2	2	2	2	2	2	2	2
2	2	2	2	2	2	2	2	2	2
2	2	2	2	2	2	2	2	2	1
2	2	2	2	2	2	2	2	2	2
2	2	2	2	2	2	2	2	2	2
2	2	2	2	2	2	2	2	2	2
3	1	1	1	3	3	1	1	1	1
2	2	2	2	2	2	2	2	2	2
2	2	2	2	2	2	2	2	2	2
2	2	2	1	1	3	3	9	1	1
2	2	2	2	2	2	2	2	2	2
3	3	3	2	2	3	3	2	2	1
2	2	2	2	2	2	2	2	1	1
2	2	2	2	2	2	2	2	2	2

2	2	2	2	2	2	2	2	2	2
2	2	2	2	2	2	2	2	2	2
2	2	2	2	2	2	2	2	1	1
2	2	2	2	2	2	2	2	2	2
2	2	2	2	2	2	2	2	2	2
2	2	2	2	2	2	2	2	2	2
9	9	9	9	9	9	9	9	9	9
1	1	1	1	1	1	1	1	1	3
9	9	9	9	9	9	9	1	9	1
2	2	2	2	2	2	2	2	3	3
1	1	3	2	2	3	2	1	1	2
1	1	1	1	1	1	1	1	2	2
2	2	3	2	3	3	2	2	3	2
9	9	9	9	9	9	9	9	3	3
2	2	2	2	2	2	2	2	9	9
1	2	2	1	2	2	1	2	1	1
2	2	2	2	2	2	2	2	1	2
1	1	1	1	1	1	1	1	1	1
9	9	9	9	9	9	9	9	2	9
9	2	2	2	1	1	2	2	3	3
2	2	2	2	2	2	2	2	2	3
9	9	9	9	9	9	9	1	9	9
1	9	9	9	9	9	9	9	9	1
3	3	3	3	3	3	3	3	9	1
3	1	2	3	1	2	3	2	1	2
3	3	3	2	1	1	3	1	1	1
2	2	2	2	2	2	2	3	2	3
1	3	3	3	2	9	2	1	1	1
1	1	1	1	1	1	9	2	1	1
1	2	2	2	2	2	2	2	1	9
2	2	2	2	2	2	2	2	2	2
2	2	3	3	3	3	3	3	3	2
2	2	2	2	2	2	2	2	2	2
9	9	1	9	9	9	9	9	1	9
2	2	2	2	2	2	2	2	2	2
9	9	9	9	9	9	9	9	1	9
1	3	3	1	1	3	3	3	1	3
1	1	1	1	1	1	1	1	3	3
2	2	2	2	2	2	2	2	2	2
2	2	2	2	2	2	2	2	2	2
9	3	9	2	9	2	1	9	2	9
2	2	2	2	2	2	2	2	2	2
9	9	2	9	9	9	9	9	9	9
1	1	1	1	2	3	3	3	3	3
1	1	2	2	2	2	2	2	1	2
3	3	3	3	3	3	3	3	2	2
1	2	2	1	1	2	2	2	1	1

9	9	9	9	9	1	9	9	9	9
2	2	2	2	2	2	2	2	2	2
1	1	1	1	1	1	1	1	1	1
2	2	2	2	2	2	2	2	2	2
9	9	9	9	9	9	9	9	1	9
2	2	3	3	2	1	1	1	1	1
3	3	3	3	3	3	3	3	3	3
2	2	2	2	2	2	2	2	2	2
2	2	2	2	2	2	2	2	2	2
2	2	2	2	3	3	3	3	1	1
9	9	9	9	9	9	9	9	9	9
1	1	1	1	1	1	1	1	1	1
2	2	2	2	2	2	2	2	2	2
2	2	2	2	2	2	2	2	2	2
2	2	2	2	2	2	2	2	2	2
3	3	3	3	3	3	3	3	2	2
1	1	1	1	1	1	1	1	1	1
3	3	1	1	1	1	2	2	1	1
9	9	9	9	9	9	9	9	9	9
1	3	1	3	1	2	1	2	1	2
2	2	2	2	2	1	1	1	1	1
2	2	2	2	2	2	2	2	1	1
1	1	1	1	1	1	1	1	1	1
2	2	2	2	2	2	2	2	2	2
9	3	3	3	3	3	3	3	3	3
2	2	1	2	1	2	2	1	1	1
1	2	3	2	1	2	3	1	1	1
2	2	2	2	2	2	2	2	2	2
2	9	2	2	1	9	2	2	1	2
2	2	2	2	2	2	2	2	2	2
9	3	1	2	3	1	3	2	1	3
2	2	3	3	3	2	2	2	2	2
1	1	1	1	1	1	1	1	1	1
1	2	2	2	2	2	2	3	2	9
2	2	2	2	2	2	2	2	2	2
2	2	2	2	2	2	2	2	2	2
1	1	1	1	1	1	1	1	1	1
3	3	2	2	2	2	1	1	1	1
3	1	2	3	1	2	3	1	1	2
3	3	3	3	3	3	3	3	3	3
1	1	2	1	2	3	1	2	1	2
9	3	3	3	3	1	1	1	2	2
9	9	9	9	9	9	9	9	9	9
3	3	3	3	3	3	3	3	3	3
9	9	9	9	9	9	9	9	9	9
2	2	2	2	2	2	2	2	2	2
2	2	2	1	2	3	2	1	1	1

1	1	1	1	1	1	1	9	2	2
3	3	3	3	3	3	1	1	3	3
9	9	9	9	9	9	9	9	9	9
3	3	3	3	3	3	1	1	3	3
9	1	2	9	1	1	1	9	1	2
2	2	2	2	2	2	2	2	2	2
2	2	2	2	2	2	2	2	2	2
2	2	2	2	2	2	2	2	2	2
1	2	2	2	2	3	3	3	1	1
2	2	1	2	1	1	2	2	2	1
2	2	2	2	2	2	2	2	1	2
2	3	1	3	2	1	3	2	1	2
2	2	2	2	2	2	2	2	2	2
2	3	2	3	2	3	1	2	1	3
2	2	2	2	2	2	2	2	2	2
2	2	2	2	2	2	2	2	2	2
2	2	2	2	2	2	2	2	2	2
2	2	2	2	2	2	2	2	2	2
2	2	2	2	2	2	2	2	2	2
1	3	3	2	1	9	2	3	3	2
2	2	2	2	2	2	2	2	2	2
3	3	3	3	3	3	3	3	2	2
2	2	2	2	2	2	2	2	2	2
2	2	2	2	2	2	2	2	2	2
1	1	1	1	1	1	1	1	1	1
2	2	2	2	2	2	2	2	2	2
3	1	1	2	2	1	2	2	1	3
2	2	2	2	2	2	2	2	2	2
3	2	1	1	1	2	3	2	1	1
2	2	2	2	2	2	2	2	1	1
2	3	2	1	2	2	2	2	1	1
3	2	2	3	3	2	3	2	1	1
2	2	2	2	2	2	2	2	2	2
2	2	2	2	2	2	2	2	3	2
2	2	2	1	1	3	3	3	3	1
3	3	2	2	3	2	3	2	1	2
9	9	9	9	9	9	9	9	9	9
9	9	9	9	9	9	9	9	9	9
2	2	2	2	2	2	2	2	3	3
2	2	2	2	2	2	2	2	2	2
3	2	2	2	2	2	2	3	9	2
2	2	2	2	2	2	2	2	2	2
2	2	2	2	2	2	2	2	2	2
2	2	2	2	2	2	2	2	2	2
9	9	9	9	9	9	9	9	9	9
9	1	2	1	1	1	1	3	1	2
2	2	2	2	2	2	2	2	2	2

9	9	9	9	9	9	9	9	9	9
2	3	1	2	2	3	2	1	1	2
3	1	3	2	1	3	2	3	3	1
2	1	3	1	1	3	2	2	1	1
1	1	3	3	1	9	1	1	1	1
3	2	1	2	1	1	2	3	1	2
3	1	2	2	1	3	2	3	1	1
9	9	9	9	9	9	9	9	9	9
2	2	2	2	2	2	2	2	2	2
2	2	3	3	3	2	2	3	3	2
2	2	2	2	2	2	2	2	2	3
1	2	2	3	9	3	3	3	3	1
2	1	2	2	3	3	1	1	1	1
1	2	1	1	1	1	1	2	1	1
3	2	1	3	2	2	2	3	2	1
2	2	2	2	2	2	2	2	2	2
2	1	1	1	2	2	1	2	3	2
2	2	2	2	2	2	2	2	2	2
9	9	9	9	9	9	9	9	9	9
9	9	9	9	9	9	9	9	9	9
9	9	9	9	9	9	9	9	9	9
3	2	1	3	2	1	1	2	1	2
2	3	3	1	2	2	3	2	2	2
2	2	2	3	3	3	2	1	3	3
2	2	3	3	3	2	1	1	1	1
3	2	2	2	2	2	2	3	3	3
2	1	1	2	2	3	2	1	2	3
2	3	3	2	2	2	2	1	1	2
3	3	2	1	2	2	1	3	1	2
2	2	3	1	2	3	1	2	1	2
9	9	9	9	9	9	9	9	1	1
2	2	2	2	2	2	2	2	1	1
3	3	3	3	3	3	3	2	3	3
2	2	2	2	2	2	2	2	3	3
3	3	3	3	3	3	3	3	3	3
2	2	2	2	2	2	2	2	2	2
3	3	3	3	3	3	3	3	3	3
2	2	2	2	2	2	2	2	2	2
9	9	9	9	9	9	9	9	9	9
9	9	9	9	9	9	9	9	9	9
3	1	1	1	2	2	3	3	1	1
2	2	2	2	2	2	2	2	2	2
1	2	1	1	2	3	1	2	2	1
3	2	3	2	2	3	2	1	3	1
9	9	9	9	9	9	9	9	9	9
3	3	3	3	3	3	3	3	3	3
2	2	3	2	1	3	2	2	2	2

1	1	1	1	1	1	1	1	1	1
2	2	2	2	2	2	2	2	2	1
3	2	1	2	3	1	2	3	2	2
9	3	9	9	1	9	3	2	1	1
2	3	2	3	2	9	3	1	1	1
3	3	3	3	3	3	3	3	2	3
1	1	1	1	1	1	1	1	2	2
3	3	3	3	3	3	3	3	3	3
3	3	2	2	2	2	3	2	1	2
2	3	1	2	3	2	1	2	1	3
2	2	2	2	2	2	2	2	2	2
1	2	1	3	1	2	3	2	1	2
1	2	2	3	2	3	2	3	1	9
1	3	1	2	3	1	1	3	1	1
2	2	2	2	2	2	2	2	2	2
3	3	2	2	2	3	3	3	3	3
1	1	1	1	1	1	1	1	1	1
3	1	2	3	1	2	3	1	2	2
9	9	9	9	9	9	9	9	9	9
2	1	2	1	2	3	1	2	3	1
3	2	3	2	1	2	1	2	1	3
3	3	3	3	2	3	2	3	2	3
2	2	2	2	2	2	2	2	1	1
1	3	3	1	2	2	2	3	1	1
3	1	2	3	1	2	3	1	1	2
2	2	2	2	3	2	3	3	2	1
2	2	2	2	2	2	2	2	1	1
3	2	1	3	1	2	3	2	1	2
1	3	2	1	2	1	3	1	1	2
2	2	2	2	2	2	2	2	2	2
3	3	1	2	3	1	1	1	3	3
2	2	2	2	2	2	2	2	2	2
2	2	2	2	2	2	2	2	2	2
3	3	3	1	1	2	3	3	1	1
3	1	2	3	1	2	3	1	1	2
2	1	2	2	1	2	2	1	1	2
3	1	3	2	1	2	3	1	1	1
3	3	3	3	3	3	3	3	3	3
1	2	2	1	1	1	1	9	1	2
1	2	3	2	3	1	2	1	2	1
2	2	2	2	2	2	2	2	1	1
3	1	2	3	1	2	3	1	1	2
2	1	1	2	2	3	2	3	1	2
1	1	1	1	1	1	1	1	1	1
2	3	3	2	3	3	3	2	2	3
2	2	1	2	3	1	2	3	1	2
3	3	1	3	1	1	3	1	1	2

1	1	1	2	2	2	2	2	2	2
3	3	1	2	3	2	1	2	1	2
2	2	1	2	1	2	1	2	2	2
1	1	1	1	1	1	1	1	1	1
3	1	2	3	1	2	3	1	1	2
3	2	1	3	2	1	3	2	1	1
3	2	1	2	2	1	2	1	1	2
2	1	2	1	2	1	2	2	1	2
1	2	1	1	1	2	3	3	1	1
3	3	3	3	3	3	3	3	3	3
3	2	3	2	3	1	2	3	1	3
3	2	1	2	1	1	3	1	1	2
1	1	1	1	1	1	1	1	1	1
1	1	1	3	1	1	1	3	1	3
3	2	3	1	1	2	2	3	1	2
1	1	1	1	1	3	1	1	1	1
1	1	2	1	2	3	2	3	1	1
3	3	3	3	3	3	3	3	1	1
3	2	1	3	2	1	3	2	1	2
2	2	1	2	3	1	2	3	2	3
3	3	3	3	3	3	3	3	2	2
3	3	3	3	3	3	3	3	3	3
3	2	1	3	1	2	1	3	3	3
1	1	1	2	1	1	1	1	1	1
2	2	2	2	2	2	2	2	2	2
2	2	2	2	2	2	2	2	2	2
3	3	1	1	2	2	1	3	1	1
3	2	1	3	2	1	3	2	2	1
2	2	2	2	2	2	2	2	2	2
9	9	9	9	9	9	9	9	9	9
9	9	9	9	9	9	9	9	9	9
9	9	9	9	9	9	9	9	9	9
1	1	3	3	3	3	3	3	2	2
9	9	9	9	9	9	9	9	9	9
3	3	3	2	2	2	2	2	2	2
9	9	9	9	9	9	9	9	9	9
2	2	2	2	2	2	2	2	1	1
9	9	9	9	9	9	9	1	9	1
1	9	9	9	1	1	2	1	9	9
2	3	2	3	2	3	1	2	2	2
9	9	9	9	9	9	9	9	9	9
2	2	2	2	1	2	2	1	1	1
1	3	2	3	2	3	2	2	3	3
3	2	3	2	1	2	2	1	3	3
2	1	2	2	1	2	3	1	1	1
2	1	3	1	2	1	2	1	3	1
3	3	1	1	2	2	1	3	1	1

1	1	2	2	1	2	3	2	1	1
2	2	2	2	2	1	1	2	1	2
9	9	9	9	9	9	9	9	9	9
3	2	1	2	3	3	2	1	2	1
3	9	2	1	3	2	1	9	2	1
2	1	3	2	1	2	1	1	1	2
2	9	9	9	9	2	9	9	9	9
3	3	2	1	2	3	2	1	1	2
1	2	3	3	1	1	1	1	1	2
3	3	3	3	3	3	3	3	3	3
1	2	3	1	2	3	1	2	2	3
3	1	2	1	2	1	3	2	3	2
1	2	1	3	1	2	1	3	1	3
2	2	2	2	2	2	2	2	2	2
2	2	2	2	2	2	2	2	2	2
9	9	9	9	9	9	9	1	3	9
2	2	2	2	2	2	2	2	2	2
2	2	2	2	2	2	2	2	2	2
2	2	2	2	2	2	2	2	2	2
2	2	2	2	2	2	2	2	2	2
1	1	1	1	1	9	1	1	1	1
1	1	1	1	1	9	1	1	1	1
1	1	1	1	1	9	1	1	1	1
1	2	2	3	1	3	1	1	1	1
1	1	3	3	1	3	1	1	1	1
1	1	3	3	1	3	1	1	1	1
1	2	2	2	2	1	2	1	2	2
3	2	3	2	2	1	3	1	1	1
1	2	2	1	2	2	1	1	1	1
1	2	2	1	1	3	1	1	1	1
1	1	9	2	1	9	1	1	1	1
9	9	9	9	9	9	9	9	9	9
1	2	2	1	1	2	1	1	1	1
1	3	3	3	1	3	1	1	1	1
2	2	2	2	2	2	2	2	2	2
2	3	3	2	1	1	2	2	3	3
2	3	3	2	1	1	2	2	3	3
2	1	1	3	9	2	1	9	9	1
1	3	1	9	1	9	1	1	1	1
1	2	2	1	1	1	1	3	1	1
1	1	3	3	1	3	1	1	1	1
9	9	1	1	1	9	1	9	1	1
1	2	2	2	2	2	9	1	1	1
1	1	3	3	1	1	1	1	1	1
1	2	2	2	2	1	2	2	2	1
1	1	2	1	1	1	9	1	1	1
2	1	2	1	2	3	1	2	2	1

2	3	3	2	1	1	2	2	3	3
2	1	2	1	2	2	1	2	1	9
1	1	3	3	1	3	1	1	1	1
1	1	1	1	1	1	1	1	1	1
2	2	3	2	3	9	1	1	1	1
2	2	3	2	3	3	1	1	1	1
2	2	1	2	2	2	2	2	2	2
2	2	1	1	1	3	1	1	1	1
9	9	9	9	9	9	9	9	9	9
1	2	2	2	1	2	1	1	2	2
1	1	3	1	1	2	2	2	1	1
2	2	2	2	2	2	2	2	1	1
2	2	3	2	3	3	1	1	1	1
2	2	3	3	2	3	1	1	1	1
2	2	3	3	2	3	1	1	1	1
2	2	3	3	2	3	1	1	1	1
1	2	3	3	1	3	2	1	1	1
1	3	3	1	1	1	9	3	1	3
2	2	3	3	2	3	1	1	1	1
2	2	2	2	3	2	1	1	1	2
3	1	1	1	3	2	1	3	1	2
3	3	3	3	3	2	2	2	1	2
1	1	1	1	1	3	1	1	1	1
1	3	3	1	1	3	1	1	3	3
1	2	1	1	1	1	1	1	3	1
3	3	3	2	1	3	1	1	1	1
1	3	3	3	3	3	3	3	3	3
1	3	3	3	3	3	1	1	1	1
1	2	2	3	1	3	1	2	1	1
1	2	1	3	2	2	1	1	1	1
2	2	1	2	2	1	1	1	1	1
1	3	3	3	3	3	1	3	1	1
9	9	9	9	9	9	9	9	1	1
1	2	2	9	9	3	1	1	1	1
1	3	3	3	2	3	1	1	1	1
1	3	3	3	2	3	1	1	1	1
1	2	2	9	1	3	1	9	9	1
1	3	9	3	1	3	1	1	1	1
1	3	3	3	1	3	9	1	1	1
1	1	3	3	1	3	1	1	1	1
1	2	3	3	1	3	1	1	1	1
1	3	3	3	3	1	1	1	1	3
1	9	1	1	9	9	9	9	3	3
1	3	3	3	1	3	1	1	1	1
2	3	2	2	2	1	1	1	1	1
1	2	2	3	1	3	1	1	1	1
9	9	9	1	1	3	1	1	1	1

1	1	2	3	1	3	1	1	1	1
1	3	3	3	1	9	1	1	1	1
1	1	2	3	1	3	1	1	1	1
1	1	1	1	1	1	3	3	1	1
1	1	3	3	1	3	1	1	1	1
1	1	1	1	1	1	2	1	1	1
1	3	1	1	1	1	1	1	9	9
1	3	3	9	1	1	1	9	1	3
1	9	3	3	1	1	1	3	1	3
1	1	3	3	1	3	1	1	1	1
2	3	3	3	1	3	2	2	1	1
9	9	9	9	9	9	1	9	1	1
1	1	1	3	1	3	1	1	1	1
3	3	2	2	2	3	2	1	1	1
1	3	2	1	2	2	2	2	2	2
2	2	2	3	2	2	1	1	1	1
9	9	9	9	9	9	1	9	9	1
9	9	9	9	9	9	1	9	9	1
2	3	2	2	2	1	1	2	1	1
2	2	2	2	2	2	2	2	1	1
1	2	3	3	1	3	1	1	1	1
2	1	2	2	3	3	1	2	1	1
2	3	3	3	2	3	1	3	3	1
2	9	1	2	2	9	1	1	1	1
3	3	1	3	1	1	1	1	3	1
3	3	1	3	1	1	1	1	3	1
1	3	2	2	1	3	2	1	1	2
2	2	2	2	2	2	2	2	2	2
2	1	2	2	2	2	2	2	1	2
1	2	1	2	1	3	1	1	1	1
2	2	2	2	2	2	1	2	2	1
1	2	3	1	3	1	1	1	1	1
2	2	1	2	1	3	1	2	1	1
1	1	3	3	1	3	1	1	1	1
1	1	1	3	3	1	1	1	3	3
2	1	2	1	2	2	3	2	1	1
9	9	9	9	9	9	9	9	1	1
1	1	2	1	1	1	1	1	1	1
1	1	1	2	1	1	1	1	1	1
2	1	1	3	2	2	1	1	1	1
1	1	3	3	1	3	1	1	1	1
1	1	3	1	3	3	1	1	1	1
1	2	2	2	2	3	2	2	1	2
2	2	2	2	2	2	2	2	9	9
1	2	2	2	2	3	2	2	9	2
1	1	1	3	3	3	1	1	1	1
1	1	2	1	1	2	1	1	1	1

1	1	2	1	1	3	1	1	1	1
1	2	3	2	1	1	1	1	1	1
1	2	2	2	2	2	1	1	1	1
2	2	2	2	1	2	1	1	1	1
1	1	1	1	1	1	1	9	1	1
2	3	2	3	3	2	1	1	2	2
2	2	2	2	2	2	2	2	9	1
9	9	9	9	9	9	9	9	1	1
9	9	9	9	9	9	9	9	9	9
2	2	2	3	2	3	1	1	1	1
2	3	3	1	1	3	1	1	1	1
1	3	3	3	3	3	1	1	1	1
2	2	2	2	2	2	2	2	2	2
1	3	3	1	2	1	2	1	1	1
2	2	2	2	2	2	2	2	9	1
9	9	9	9	9	9	1	9	1	9
1	1	3	2	1	3	1	1	1	1
1	2	2	2	2	2	1	1	1	1
1	2	3	3	1	3	1	1	1	1
2	2	1	2	2	3	1	1	1	1
9	9	9	9	9	9	2	9	1	9
1	1	3	3	1	3	1	1	1	1
9	9	9	9	9	9	9	9	1	2
1	1	2	3	1	2	1	1	1	1
1	2	2	3	2	3	1	1	1	1
1	2	3	2	2	2	1	1	1	1
1	1	2	2	1	9	1	1	1	1
9	9	9	9	9	9	9	9	9	9
9	9	9	9	9	9	9	9	9	9
3	1	2	3	1	3	1	2	1	1
2	1	1	3	1	3	1	1	1	1
1	1	3	3	1	3	1	3	1	1
1	3	1	9	9	9	9	9	1	1
9	9	9	9	9	9	9	9	1	1
1	1	3	3	1	3	1	1	1	1
1	3	3	2	1	3	1	2	2	1
1	1	2	3	1	3	1	1	1	1
1	1	1	1	1	1	1	1	1	1
1	3	3	3	1	3	1	1	1	1
1	1	2	3	1	3	1	1	1	1
2	1	2	3	1	3	2	2	2	2
1	1	2	3	1	3	1	2	2	1
1	1	3	1	1	3	1	1	1	1
1	3	9	9	9	9	9	9	9	9
2	3	3	2	2	2	1	1	1	1
3	3	3	3	3	3	3	3	2	2
1	3	3	3	2	2	2	1	9	3

1	1	1	1	1	1	1	1	1	1
1	3	9	9	9	9	9	9	1	1
9	9	9	9	9	9	9	9	9	9
1	3	3	3	2	3	1	1	1	1
1	1	3	9	1	1	1	2	9	9
1	2	2	2	1	2	1	1	1	1
1	1	2	2	2	3	1	1	9	1
2	2	3	2	1	3	1	1	1	1
9	9	2	9	9	9	9	9	9	2
1	3	3	3	2	2	3	2	1	1
3	3	1	1	1	3	1	1	1	1
1	2	2	2	2	2	2	2	2	2
1	1	2	3	2	2	1	1	1	1
1	9	1	9	9	9	9	1	9	1
1	1	2	1	2	2	1	1	1	1
1	2	3	3	2	3	1	2	1	1
9	9	9	9	9	9	9	9	1	1
9	9	9	9	9	9	1	9	1	1
1	1	3	3	1	3	1	1	1	1
3	2	2	2	2	3	1	2	2	1
1	1	3	3	1	3	1	1	1	1
3	3	3	3	3	3	1	3	1	1
3	3	3	3	3	3	1	3	1	1
1	1	3	3	1	2	1	1	1	1
1	2	2	2	1	3	1	1	1	1
2	3	1	1	2	2	1	1	1	1
1	2	2	3	1	2	1	1	1	1
1	1	1	3	2	1	1	1	2	1
2	3	1	1	2	2	1	1	1	1
2	2	1	1	1	1	1	1	1	1
3	1	2	2	3	3	1	1	2	1
1	1	2	2	1	1	3	1	1	1
1	1	3	3	1	9	1	1	1	1
1	1	2	2	1	1	1	1	1	1
1	1	1	3	2	3	1	1	1	1
9	9	9	9	9	9	1	9	9	1
1	3	3	3	2	9	1	1	1	1
1	9	1	9	9	9	1	9	1	1
2	2	2	2	2	2	2	2	2	1
1	2	2	2	2	2	1	2	2	2
2	2	3	2	2	3	3	2	1	3
1	3	3	3	1	1	3	1	1	1
9	9	9	9	9	9	9	9	9	1
1	9	9	9	9	9	9	9	9	1
1	1	3	3	1	3	1	1	1	1
1	1	3	3	3	3	1	1	1	1
9	9	9	9	9	9	3	1	9	1

1	1	3	3	3	3	1	1	1	1
1	2	1	1	1	3	1	1	1	1
2	2	2	2	1	9	1	1	1	1
1	1	2	3	1	2	1	1	1	1
2	2	2	3	3	3	2	2	1	1
1	2	2	3	1	3	1	1	1	1
2	2	2	2	3	2	2	2	1	1
1	2	2	2	2	2	2	2	2	1
1	2	2	2	2	2	2	2	2	1
1	2	2	2	2	2	1	1	1	1
1	2	2	2	1	3	1	1	1	1
9	9	9	9	9	9	9	9	9	1
9	9	9	9	9	9	9	9	1	9
9	9	9	9	9	9	9	9	9	9
9	9	9	9	9	9	9	9	9	9
9	9	9	9	9	9	9	9	9	9
1	3	2	3	2	3	1	3	9	3
2	3	3	3	2	2	3	2	1	3
1	9	9	9	9	9	9	9	9	9
1	2	2	3	2	3	1	1	1	1
1	2	3	3	1	2	1	1	1	1
9	9	9	9	9	9	9	9	1	1
1	3	2	2	1	1	1	1	1	1
2	3	3	2	2	3	2	3	2	1
2	3	2	2	1	3	1	1	1	1
1	1	3	3	1	3	1	1	1	1
1	1	2	9	3	3	1	1	1	1
3	3	3	3	2	3	2	2	3	1
2	2	2	2	1	3	1	1	1	1
9	1	3	3	1	3	1	1	1	1
1	2	2	2	2	2	1	1	1	1
1	9	1	9	1	9	1	1	9	1
1	3	3	3	1	3	1	2	1	1
1	2	3	1	2	2	1	1	1	1
1	2	2	3	1	3	1	1	1	1
2	1	1	1	3	3	2	1	2	1
2	2	2	2	2	2	1	2	2	1
3	1	3	3	1	3	1	2	1	1
9	9	9	9	9	9	9	9	9	9
1	1	3	3	1	3	1	1	9	1
3	2	3	2	3	2	2	3	2	3
3	2	3	2	2	3	1	1	2	3
1	2	2	3	1	3	1	2	1	1
1	1	2	2	1	2	1	1	1	1
1	2	3	3	2	3	1	2	1	1
1	1	3	3	1	2	1	1	1	1
1	3	3	3	1	1	1	1	1	1

1	3	3	3	3	3	1	1	1	1
1	2	2	1	1	1	1	1	1	1
1	2	2	3	3	3	1	1	1	1
1	1	3	3	1	3	1	1	1	1
1	2	2	3	3	3	1	1	9	9
1	2	2	2	1	3	1	1	1	1
2	3	3	3	2	2	1	1	1	1
1	2	3	3	1	3	1	1	1	1
2	2	2	2	2	2	3	3	2	2
2	2	3	1	2	1	1	1	1	1
1	2	2	3	2	2	1	1	1	1
2	2	2	2	2	2	2	2	2	2
1	1	3	3	1	2	1	1	1	1
1	2	2	2	2	2	1	2	1	1
1	2	1	2	2	2	1	1	1	1
1	1	2	2	1	2	1	1	1	1
2	2	2	2	2	2	2	2	1	1
2	2	2	2	2	2	2	2	2	2
2	2	3	3	2	2	2	2	1	2
1	3	3	3	3	3	1	1	1	1
1	3	3	3	3	3	1	1	1	1
3	1	3	3	1	3	1	1	1	1
2	1	2	3	2	3	1	1	1	1
3	2	2	2	2	1	2	2	3	3
1	2	2	2	1	2	1	1	1	1
9	9	9	9	9	9	9	9	9	9
9	9	9	9	9	9	9	9	9	9
9	9	9	9	9	9	9	9	1	9
2	2	3	3	1	3	1	1	1	1
1	9	2	3	9	2	3	3	1	1
1	1	2	3	1	3	1	1	1	1
1	1	2	3	1	3	1	1	1	1
9	9	9	9	9	9	1	9	9	1
9	9	9	9	9	9	9	1	9	1
9	9	9	9	9	9	9	1	9	1
9	9	9	9	9	9	9	1	9	1
9	9	9	9	9	9	1	9	9	1
9	9	9	9	9	9	1	9	9	1
9	9	9	9	9	9	9	9	1	9
2	3	2	3	1	3	1	1	1	1
2	2	3	3	1	3	1	1	1	1
9	9	9	9	9	9	9	9	9	1
9	9	9	9	9	9	9	9	9	1
2	2	3	2	2	2	1	2	1	1
9	9	9	9	9	9	9	9	9	9
9	9	9	9	9	9	9	9	9	9
9	9	9	9	9	9	9	9	9	9

1	1	2	3	1	3	1	1	1	1
9	9	9	9	9	9	9	9	9	9
9	9	9	9	9	9	9	9	9	9
2	3	3	3	2	2	1	2	9	1
2	3	3	3	2	2	1	2	9	1
9	9	9	9	9	9	9	9	9	9
9	9	9	9	9	9	9	9	9	1
9	9	9	9	9	9	9	9	9	1
1	9	9	9	9	9	9	9	9	1
9	9	9	9	9	9	9	9	9	1
1	1	3	3	3	3	1	1	1	1
3	3	3	1	1	1	1	3	1	1
1	1	3	1	1	3	1	1	1	1
2	2	2	2	2	2	1	2	1	2
9	3	3	3	3	3	1	1	1	1
9	9	9	9	9	9	9	9	1	1
1	2	2	2	3	3	1	1	1	1
1	2	2	2	3	3	1	1	1	1
3	3	3	1	1	1	1	3	1	1
2	3	3	3	2	2	1	1	1	2
2	3	3	3	2	2	1	1	1	2
1	1	1	1	1	1	1	1	2	1
9	9	9	9	9	9	9	9	1	9
1	1	2	2	2	1	1	1	1	1
2	3	2	2	3	3	3	3	3	2
1	1	2	3	1	1	1	1	1	1
3	3	2	3	3	3	3	9	2	2
2	2	3	3	2	3	1	1	1	1
1	2	2	2	3	3	1	1	1	1
1	2	2	1	1	3	2	1	1	1
1	2	2	3	2	3	1	1	1	1
1	1	3	2	1	3	2	2	1	1
1	2	2	2	3	3	1	1	1	1
1	1	2	3	2	3	1	1	1	2
2	2	2	2	1	1	1	1	1	1
2	2	3	3	3	2	1	1	1	1
1	3	3	3	3	3	1	1	1	1
1	1	3	3	1	3	1	1	1	1
1	3	3	3	1	3	1	1	1	1
3	3	3	3	2	3	1	1	1	1
1	1	2	9	9	9	9	9	9	9
1	1	1	1	1	3	1	1	1	1
1	3	3	3	2	2	1	2	1	2
2	2	2	2	1	3	2	2	1	1
2	2	2	2	1	3	2	2	1	1
1	1	1	3	1	1	1	1	1	1
1	2	2	2	2	3	1	1	1	1

2	2	3	3	2	3	1	2	2	1
1	1	3	1	1	3	1	1	1	1
2	2	2	3	3	3	1	1	1	1
2	2	2	2	1	3	2	2	1	1
2	2	2	2	1	3	2	2	1	1
2	2	2	2	1	3	2	2	1	1
1	2	2	2	2	3	1	1	1	1
2	2	3	3	1	3	1	1	1	1
1	1	2	3	1	3	1	1	1	1
1	1	3	3	1	1	1	1	1	1
2	2	2	1	2	2	1	1	1	1
3	2	9	3	3	1	1	1	1	1
1	2	2	3	2	3	1	1	9	1
1	2	2	3	2	3	1	1	1	1
1	1	3	3	3	3	1	3	1	1
3	2	3	3	3	1	1	1	1	1
1	2	2	2	2	2	2	2	2	2
2	2	2	2	2	2	2	2	2	1
3	1	3	3	2	3	1	3	1	3
1	1	2	1	2	3	1	1	1	1
1	2	3	2	1	3	1	1	1	1
1	1	3	3	1	3	1	1	1	1
2	2	2	1	3	3	2	2	1	1
1	1	2	2	2	2	1	1	1	1
1	1	2	2	2	2	1	1	1	1
1	2	3	3	2	3	2	1	1	1
1	2	3	3	3	3	1	1	2	3
2	2	2	2	2	2	2	2	3	1
1	2	2	2	1	3	1	1	1	1
1	1	2	2	1	2	1	2	1	1
3	3	2	3	2	2	3	2	2	2
2	2	3	2	3	3	1	1	1	1
1	1	3	3	1	2	1	1	1	1
1	2	2	2	1	1	1	1	1	1
2	2	2	1	3	2	2	2	1	1
2	2	2	1	3	3	2	2	1	1
3	3	2	2	2	2	2	2	2	2
3	3	2	2	2	2	2	2	2	2
3	1	3	1	1	1	1	1	1	1
1	2	3	1	3	3	1	1	1	1
1	2	3	2	1	1	1	1	1	1
1	1	3	3	1	3	1	1	1	1
1	1	3	3	1	3	1	1	1	1
2	2	2	2	2	2	1	1	1	1
3	3	3	1	1	3	1	1	1	1
2	2	2	2	1	3	2	1	1	1
1	3	3	3	3	3	9	1	1	1

2	2	2	2	2	2	2	2	3	2
2	2	2	1	1	2	2	2	1	1
2	3	3	3	3	3	1	1	1	1
1	3	3	2	1	2	1	2	1	1
1	2	3	2	1	3	1	1	1	1
2	2	3	2	2	3	2	2	1	1
1	3	3	2	1	3	1	2	1	1
2	2	2	2	2	2	2	2	1	1
3	3	2	1	2	1	1	1	2	1
1	2	3	3	1	3	1	1	1	1
2	2	2	2	2	2	2	2	1	1
3	3	3	3	1	3	1	2	1	1
9	9	9	9	9	9	9	9	1	9
3	3	2	2	1	2	1	2	1	1
1	3	3	3	3	3	1	1	1	1
2	2	2	2	2	2	2	2	2	2
1	2	3	3	1	3	1	1	1	1
1	2	3	3	1	3	1	1	1	1
1	2	3	1	1	3	1	1	1	1
2	2	2	2	2	3	1	1	1	1
1	3	1	1	3	3	1	1	1	1
2	3	3	3	2	2	1	1	1	2
1	1	3	1	1	3	1	1	1	1
1	2	3	3	1	3	1	2	1	1
1	2	2	2	2	2	1	1	2	1
1	2	3	3	2	3	1	1	1	1
1	1	3	3	1	3	1	1	1	1
2	3	2	3	1	3	1	1	1	1
2	2	3	2	2	2	1	2	1	1
9	9	9	9	9	9	9	9	9	1
2	1	3	1	2	2	2	1	1	1
9	9	9	9	9	9	9	9	1	1
2	3	1	2	2	2	2	2	1	1
1	1	2	1	2	3	2	1	2	1
3	2	3	3	2	1	1	1	1	1
1	1	2	1	1	1	1	1	1	1
2	2	2	2	2	1	2	1	9	9
9	9	9	9	2	9	9	9	9	9
3	1	2	1	3	2	3	1	2	3
2	2	2	2	2	2	2	1	2	2
2	2	2	2	2	2	2	2	1	1
1	3	3	3	1	3	3	1	1	1
1	1	3	3	1	2	1	1	1	1
2	2	9	3	9	9	1	2	2	1
2	2	2	2	1	2	2	3	2	2
2	2	2	2	2	2	1	1	2	1
2	2	1	3	3	1	1	1	1	1

2	3	3	2	1	2	1	1	1	1
2	2	2	2	2	1	1	1	1	1
2	2	2	2	2	2	2	2	1	1
2	2	2	2	1	2	1	2	2	1
2	1	2	2	2	1	2	2	1	2
2	2	3	3	1	3	2	2	1	1
1	2	3	1	1	2	1	1	1	1
9	9	9	9	9	9	9	9	9	9
1	3	3	2	2	2	1	1	1	1
2	2	2	2	2	2	1	1	1	1
1	1	2	1	1	2	3	2	1	1
2	1	2	3	2	3	2	3	2	3
2	1	2	2	2	2	2	2	1	1
2	2	2	2	2	2	2	2	2	2
3	3	3	3	1	3	3	1	1	1
3	3	3	3	1	3	3	1	1	1
1	1	2	2	2	2	1	1	1	1
1	1	1	1	3	1	1	2	1	1
3	2	3	2	1	3	1	3	9	9
2	2	2	1	1	9	1	1	1	1
1	2	2	3	1	3	3	1	1	1
2	1	1	1	1	1	1	1	1	1
2	2	2	2	2	2	2	2	2	2
1	1	1	1	1	3	1	1	1	1
2	2	3	3	1	3	1	2	1	1
2	1	1	1	1	1	1	1	1	1
1	2	1	1	2	1	1	1	1	1
1	2	1	1	1	2	1	1	1	2
1	2	1	1	1	2	1	1	1	2
9	9	1	9	9	9	9	9	9	9
1	2	1	1	1	2	1	1	2	1
3	1	1	1	1	2	1	1	1	1
1	1	1	1	1	2	1	1	1	1
1	2	2	2	2	3	1	2	1	1
9	9	9	9	9	9	9	9	9	9
1	1	2	1	1	1	1	1	1	1
1	1	2	1	1	1	1	1	1	1
9	2	9	9	9	1	1	1	1	1
3	3	2	3	3	1	1	1	1	1
2	1	2	1	1	3	1	1	1	1
2	2	2	2	1	3	1	2	1	1
1	1	1	1	1	3	1	2	1	1
2	2	2	2	2	2	2	2	2	1
1	1	3	3	3	1	3	1	1	1
1	3	3	3	1	3	1	1	1	1
1	1	1	1	1	1	1	1	1	1
3	2	3	3	1	3	2	2	1	1

1	3	3	1	1	3	1	1	1	1
1	3	3	1	1	3	1	1	1	1
1	2	3	2	1	3	1	1	1	1
1	1	1	1	1	2	1	1	1	1
3	2	3	3	1	1	1	1	1	1
3	2	3	3	3	1	1	1	1	1
1	1	1	1	3	3	1	1	9	1
1	2	2	2	2	2	1	1	1	1
3	3	3	1	9	1	1	9	1	1
1	1	1	1	3	1	1	1	1	1
1	1	3	3	3	3	1	1	1	1
1	1	1	1	1	3	3	1	1	1
1	1	1	1	1	3	3	1	1	1
3	3	3	1	1	1	1	3	1	1
1	1	2	3	1	3	1	2	1	1
1	1	3	3	1	3	1	1	1	1
1	1	2	1	1	3	2	1	2	2
1	1	2	3	1	2	1	1	1	1
1	1	3	3	1	3	1	1	1	1
1	1	3	3	3	3	1	3	9	9
2	1	1	1	2	2	1	1	1	1
3	1	3	3	1	3	1	1	1	1
9	3	3	3	2	2	1	2	9	1
9	3	3	3	2	2	1	2	9	1
9	9	9	9	9	9	9	9	9	9
1	9	9	9	9	9	9	9	9	1
3	1	3	3	1	3	1	1	1	1
9	9	9	9	9	9	9	1	9	1
9	9	9	9	9	9	9	9	1	9
1	9	9	9	9	9	9	9	9	9
1	9	9	9	9	9	9	9	1	9
1	9	9	9	9	9	9	9	1	9
9	9	9	9	9	9	9	9	3	9
9	9	9	9	9	9	9	9	1	9
1	2	1	2	1	3	3	1	1	1
1	1	3	3	1	3	1	2	1	1
1	1	2	2	1	2	1	1	1	1
1	1	1	1	3	1	1	2	1	1
1	2	1	1	2	1	1	1	1	1
1	2	1	1	2	1	1	1	1	1
3	2	9	3	3	1	1	1	9	9
2	3	1	1	1	1	1	1	9	9
1	2	3	2	2	2	1	1	1	1
2	2	2	2	1	2	2	2	1	1
1	1	1	3	2	2	2	2	1	1
1	1	3	1	1	3	1	1	1	1
1	1	9	9	2	2	9	1	1	1

1	2	2	1	1	2	1	1	1	1
2	2	2	2	2	2	2	2	1	1
3	3	3	2	2	2	1	1	1	1
1	2	2	2	3	1	2	1	2	2
2	1	1	2	2	2	1	1	1	1
1	2	3	3	1	3	1	1	1	1
9	9	9	9	9	9	9	9	9	9
2	2	2	2	2	2	2	2	1	1
1	1	2	2	2	2	1	3	1	1
2	2	2	2	2	3	3	1	2	2
2	2	2	2	2	2	2	2	1	1
1	1	3	3	1	3	1	1	1	1
2	2	3	2	3	2	2	2	2	3
3	3	3	3	3	3	3	3	3	2
1	1	2	2	2	2	2	2	1	2
1	1	3	1	1	1	1	1	1	1
9	9	9	9	9	9	9	9	9	9
1	3	2	1	2	3	2	2	1	2
1	2	1	1	2	1	2	1	1	2
1	1	1	1	1	1	1	1	1	1
1	1	1	1	1	1	1	1	1	1
2	2	2	2	2	2	2	2	2	2
1	1	2	2	2	2	1	3	2	1
2	2	2	2	2	2	2	2	2	2
1	1	1	1	1	1	1	1	1	1
1	2	3	2	1	3	2	1	1	2
1	2	3	1	2	3	2	1	1	1
1	1	2	1	2	2	1	1	1	1
2	2	2	2	2	2	2	2	1	1
2	2	2	2	2	2	2	2	1	1
1	3	1	1	1	1	1	1	1	1
2	2	2	2	2	2	2	2	1	1
1	1	2	2	2	2	2	2	1	2
2	1	2	2	1	2	1	2	1	1
9	9	9	9	9	9	1	1	9	9
1	1	1	1	1	1	1	1	1	1
2	2	2	2	2	2	2	2	2	2
2	2	2	2	2	2	1	2	1	1
2	1	3	3	1	2	1	2	2	1
2	2	2	3	2	3	9	1	1	1
2	2	2	2	2	2	1	2	1	1
2	2	2	2	2	2	1	2	1	1
9	9	9	9	9	9	9	9	3	9
9	9	9	9	9	9	9	9	3	9
9	9	9	2	9	9	9	9	3	9
3	2	2	1	3	1	3	2	2	2
3	3	2	1	3	2	2	3	2	2

3	3	1	2	1	3	1	2	3	2
1	3	1	1	2	3	1	1	3	2
3	3	3	1	3	2	1	3	3	3
2	2	3	3	1	3	1	1	2	2
2	2	2	3	1	3	1	1	2	2
2	2	1	3	2	1	1	3	3	2
3	1	1	2	1	2	1	2	3	2
2	1	2	1	1	1	2	3	2	1
2	3	2	1	1	1	1	2	3	2
2	3	3	1	1	2	2	1	2	1
2	2	2	2	2	1	3	3	3	3
2	2	2	1	3	1	3	3	2	2
2	3	1	1	3	3	1	1	2	2
2	2	2	3	1	3	1	1	2	2
2	2	1	1	3	1	3	3	2	2
2	2	2	2	2	2	2	2	9	3
2	3	1	1	2	2	2	2	2	3
2	3	1	1	3	1	3	3	2	3
1	3	1	1	2	3	1	1	3	2
1	3	1	1	2	3	1	1	3	2
2	2	2	2	2	2	2	2	1	1
2	2	2	2	2	2	2	2	1	1
1	1	3	3	1	3	1	1	1	1
1	1	3	3	1	3	1	1	1	1
1	1	3	3	1	3	1	1	2	2
1	1	3	3	1	3	1	1	2	2
1	1	3	3	1	3	1	1	2	2
1	1	3	3	1	3	1	1	1	1
3	3	1	1	2	2	2	2	3	3
2	2	2	1	2	1	2	2	2	3
2	2	2	1	2	1	2	2	2	3
2	2	2	1	2	1	2	2	2	3
2	2	2	1	2	1	2	2	2	3
2	2	1	2	9	2	9	2	3	9
2	2	3	2	9	3	2	2	3	9
9	3	2	3	1	1	3	2	3	2
2	2	3	3	2	2	3	3	3	3
3	2	1	2	3	2	3	2	2	1
3	3	2	1	2	1	2	3	2	2
2	3	2	1	2	3	1	1	1	2
3	1	1	1	1	1	3	3	9	9
3	3	2	1	3	3	3	3	3	3
1	2	1	2	1	2	2	1	2	9
9	9	9	9	9	9	9	9	3	9
9	9	9	9	9	9	9	9	9	9
2	2	2	2	2	2	2	2	2	2
9	3	1	9	3	9	2	2	3	9

3	2	2	2	2	9	3	3	9	3
3	3	1	1	3	1	3	3	3	3
9	9	9	9	9	9	9	9	9	9
2	2	2	2	2	2	2	2	2	2
2	2	2	2	2	2	2	2	2	2
9	9	9	9	9	9	9	9	2	9
3	1	2	1	1	2	3	1	2	2
2	9	9	9	9	9	9	9	3	9
3	3	2	2	2	2	2	2	3	3
1	3	1	2	2	3	1	3	3	2
3	3	1	1	2	2	2	1	2	2
2	2	2	2	2	2	2	2	9	3
9	9	9	9	9	9	9	9	3	9
9	9	9	9	9	9	9	9	9	9
1	3	2	3	3	2	1	3	3	2
9	9	9	9	9	1	9	9	9	9
3	2	2	3	3	2	3	3	3	3
9	9	9	9	9	9	9	9	9	9
3	1	1	1	3	3	3	1	3	3
9	9	9	9	9	9	9	9	2	9
3	3	1	2	2	1	3	2	3	3
2	2	2	2	3	1	3	3	3	3
2	2	2	2	3	1	3	3	3	3
2	2	2	2	2	2	2	2	3	3
3	2	2	2	2	2	2	2	3	3
2	2	2	2	2	2	2	2	2	2
2	2	2	2	2	2	2	2	3	3
9	9	9	9	9	9	9	9	2	9
9	9	9	9	9	9	9	9	2	9
9	9	9	9	9	9	9	9	2	9
9	9	9	9	9	9	9	9	3	9
9	9	9	9	9	9	9	9	2	9
9	9	9	9	9	9	9	9	1	9
2	3	3	2	3	2	3	2	2	1
1	3	2	3	3	2	1	3	3	2
9	9	9	9	9	9	9	9	9	9
2	2	2	2	2	2	2	2	3	3
1	3	2	3	2	1	2	1	2	2
2	2	2	1	2	3	2	2	3	1
3	2	2	9	2	2	1	3	2	2
1	2	3	2	1	2	1	2	3	3
9	9	9	9	9	9	9	9	3	9
1	1	3	3	1	3	1	1	1	1
2	2	2	2	2	2	2	2	2	2
9	9	9	9	9	9	9	9	2	9
3	1	2	3	3	2	9	3	1	3
9	9	9	9	9	9	9	9	9	9

9	9	9	9	9	9	9	9	2	9
9	9	9	9	9	9	9	9	2	9
3	2	1	1	3	1	3	3	3	3
9	9	9	9	9	9	9	9	2	9
2	2	2	1	2	3	3	3	3	3
9	9	9	9	9	9	9	9	2	9
3	1	2	1	2	2	1	3	2	3
3	2	2	2	2	1	3	3	3	3
3	2	1	2	3	3	3	2	3	3
9	3	2	3	3	2	1	2	1	2
2	2	2	3	1	3	1	1	3	3
3	3	2	3	2	2	1	1	3	2
3	1	3	3	2	2	2	2	1	2
2	3	2	3	2	1	2	3	2	2
3	3	1	1	3	1	3	3	3	3
3	2	2	3	2	2	3	3	2	2
2	3	3	3	1	2	3	3	2	2
2	3	1	2	3	2	3	3	2	3
3	1	1	2	3	2	3	2	3	2
3	3	1	1	3	1	3	3	3	3
9	9	9	1	9	9	9	9	9	9
9	9	9	9	3	9	9	9	9	9
9	9	9	9	9	9	9	9	9	9
9	9	9	1	9	9	9	9	9	9
9	9	9	9	9	9	9	3	9	9
9	9	9	9	9	9	9	1	9	9
9	9	1	9	9	9	9	9	9	9
9	9	9	9	9	9	9	9	2	9
9	9	9	9	9	9	9	9	2	9
9	9	9	9	9	9	9	9	2	9
9	9	9	9	9	9	9	9	2	9
9	9	9	9	9	9	9	9	2	9
9	9	9	9	9	9	9	9	2	9
3	2	1	2	1	3	1	3	3	3
3	3	1	2	3	2	3	3	3	2
3	3	1	2	2	1	3	2	3	3
9	9	9	2	9	9	9	9	9	9
9	3	9	9	9	1	9	9	3	9
3	2	2	1	2	1	2	2	2	3
9	9	9	9	9	9	9	9	2	9
9	9	9	9	9	9	9	9	3	9
9	9	9	9	9	9	9	9	3	9
9	9	9	9	9	9	9	9	3	9
9	9	9	9	9	9	9	9	3	9
2	2	2	2	2	2	2	2	2	2
3	2	1	2	3	2	3	2	3	2
2	3	2	1	2	1	2	3	3	2

2	2	2	2	2	2	2	2	2	2
2	2	3	3	1	2	2	3	3	3
2	2	2	1	3	2	2	1	3	2
3	2	2	2	1	3	2	1	3	3
9	9	9	9	9	9	9	9	3	9
9	9	9	9	9	9	9	9	3	9
9	9	9	9	9	9	9	9	3	9
9	9	9	1	9	3	9	1	2	9
9	9	9	9	9	9	9	9	3	9
9	3	9	9	9	9	9	9	2	9
9	2	9	3	9	3	9	9	1	9
9	9	3	9	9	3	9	1	9	9
1	9	1	3	9	2	9	9	3	9
9	3	9	9	9	9	9	9	9	9
9	9	9	9	9	9	9	9	2	9
2	2	2	2	2	2	2	2	2	2
1	2	2	2	2	2	1	1	2	2
9	9	9	9	9	9	9	9	9	9
2	2	2	2	2	2	2	2	2	3
1	2	2	1	1	1	2	2	2	3
2	2	2	2	2	2	2	2	2	2
2	3	2	2	2	2	2	2	2	3
3	2	3	3	3	3	3	3	3	3
2	2	2	2	2	2	2	2	2	3
2	2	2	3	2	9	3	1	3	2
2	2	2	2	2	2	2	2	2	2
9	9	9	9	9	9	9	9	9	9
2	2	2	2	2	2	2	2	2	2
2	2	2	2	2	2	2	2	2	2
1	3	2	1	1	2	3	2	2	3
1	3	3	2	3	2	1	2	2	2
2	2	2	2	2	2	3	3	3	3
9	9	9	9	9	9	9	9	3	9
2	2	2	2	2	2	2	2	2	2
3	2	2	2	3	3	3	3	3	3
1	3	2	3	3	2	3	2	3	2
2	2	2	3	3	3	3	3	3	3
2	2	2	2	2	2	2	2	3	3
1	1	2	1	2	3	2	2	3	2
1	2	1	2	1	2	3	2	3	2
3	3	2	2	3	3	3	3	3	3
1	1	3	3	1	3	3	1	3	3
3	3	1	1	2	2	2	2	2	2
3	3	1	1	3	1	3	3	3	3
9	9	9	9	9	9	9	9	9	9
2	2	2	2	2	2	2	2	2	2
1	1	2	3	2	2	3	3	3	3

3	3	1	1	3	1	3	3	3	3
3	3	2	1	3	2	3	3	3	3
2	3	2	1	3	2	2	3	2	2
3	2	1	3	1	9	3	2	3	3
2	2	2	2	2	2	2	2	2	2
2	2	2	2	2	2	2	2	2	2
2	2	3	2	2	1	2	2	3	2
2	2	2	3	2	1	2	2	2	2
3	2	2	2	3	3	3	3	3	3
2	2	2	2	2	2	2	2	2	2
2	2	2	2	2	2	2	2	2	3
3	3	3	3	1	3	3	3	3	3
2	2	2	2	2	2	2	2	2	1
3	3	3	3	2	3	2	3	3	3
3	2	2	3	2	2	2	3	3	3
2	2	2	2	2	2	2	2	3	3
2	2	2	3	2	2	2	2	2	2
2	2	2	2	2	2	2	2	2	2
2	2	2	2	2	2	2	2	2	2
2	2	2	2	2	2	2	2	2	2
2	2	2	2	2	2	2	2	3	3
2	2	2	2	2	2	2	2	2	2
2	2	2	2	2	2	2	2	2	2
2	2	2	2	2	2	2	2	2	2
3	2	1	1	1	3	3	2	2	2
9	9	9	9	9	9	9	9	9	9
9	9	9	9	9	9	9	9	9	9
9	9	9	9	9	9	9	9	9	9
2	2	3	1	2	1	3	3	3	1
9	9	9	9	9	9	9	9	9	3
9	9	9	9	9	9	9	9	9	9
2	3	3	3	3	3	3	3	9	9
9	9	9	9	9	9	9	9	9	9
9	9	9	9	9	9	9	9	9	9
2	2	2	2	2	2	2	2	3	3
2	3	2	1	3	2	1	3	3	2
3	3	2	1	3	2	3	3	3	3
9	9	9	9	9	9	9	9	9	9
2	2	2	2	2	2	2	2	2	2
9	9	9	9	9	9	9	9	9	9
9	9	9	9	9	9	9	9	9	9
2	2	2	2	2	2	2	2	2	2
1	1	3	3	1	3	3	3	3	3
2	2	1	1	3	2	3	2	3	3
2	2	3	1	2	1	2	1	2	3
2	2	1	3	2	3	2	2	1	1
2	2	2	2	2	2	2	2	3	2

3	3	2	2	3	2	2	2	3	2
2	2	2	2	2	2	2	2	1	1
3	3	3	2	2	3	3	2	3	3
2	2	2	2	2	2	2	2	2	3
2	2	2	2	2	2	2	2	2	3
9	9	9	1	9	9	9	9	9	9
3	2	2	3	3	3	3	3	3	3
1	1	3	3	2	2	1	2	2	2
2	2	2	2	2	2	2	2	2	3
2	1	2	1	2	3	2	2	3	1
2	2	2	2	2	2	2	2	2	2
1	1	3	3	3	3	3	3	3	3
2	2	2	3	3	2	3	2	2	2
2	2	2	2	2	2	2	2	2	2
9	9	9	9	9	9	9	9	9	9
2	2	2	2	2	2	2	3	3	3
3	2	1	1	3	3	3	3	1	3
2	2	2	2	1	2	1	1	1	2
2	3	1	1	2	2	3	3	3	3
3	9	9	9	9	9	9	9	9	9
2	2	2	2	2	2	2	2	2	2
2	2	2	2	2	2	2	2	2	2
3	3	2	2	1	3	3	3	3	3
2	2	2	2	2	2	2	2	2	2
2	2	2	2	3	1	1	1	3	1
2	2	2	2	2	2	2	2	2	2
2	2	3	3	2	3	2	2	3	2
3	1	1	1	3	3	1	3	3	3
2	2	2	2	2	2	2	2	2	2
3	3	1	1	3	1	3	3	3	3
2	2	3	3	3	3	3	3	3	3
2	9	9	9	9	9	9	9	9	9
3	3	2	2	2	2	2	2	3	2
3	2	2	2	1	3	1	2	3	3
1	9	2	2	3	2	3	2	2	2
2	2	2	2	2	2	2	2	2	2
2	2	2	2	2	2	2	2	2	2
2	2	2	2	2	2	2	2	2	2
2	2	2	2	2	2	2	2	2	2
2	2	2	2	2	2	2	2	3	3
2	2	2	2	2	3	2	2	2	2
2	2	2	2	2	2	2	2	2	2
2	2	3	2	2	2	2	2	3	3
2	2	2	2	2	2	2	2	2	2
2	2	2	2	2	2	2	2	2	2
2	2	2	2	2	2	2	2	2	2
3	2	2	3	3	3	3	3	3	3

9	9	9	9	9	9	9	9	9	9
3	3	3	1	3	3	1	1	1	3
9	9	9	9	9	9	3	9	9	9
9	9	9	9	9	9	9	9	9	9
2	3	2	1	2	2	3	2	2	2
2	1	2	3	2	3	2	1	2	2
3	9	9	9	9	9	9	9	9	3
3	2	3	3	3	2	1	3	3	2
2	2	2	2	2	2	2	2	2	2
2	2	2	2	2	2	2	2	3	3
2	2	2	2	2	2	2	2	2	2
3	3	3	3	3	3	3	1	3	3
1	1	3	3	3	3	3	3	1	1
9	9	9	9	9	9	9	9	2	9
1	1	3	1	1	3	3	1	1	3
3	2	1	1	2	2	3	3	1	3
3	3	2	2	1	9	3	1	3	3
9	9	9	9	9	9	9	9	9	9
3	2	3	3	3	3	3	3	3	3
2	2	2	2	2	2	2	2	3	3
2	2	2	2	2	2	2	2	2	2
3	2	2	2	2	1	3	1	3	3
9	2	9	9	9	9	9	9	2	9
1	2	3	3	3	3	3	1	1	2
2	3	1	1	3	1	3	3	3	3
9	9	9	9	9	9	9	9	9	9
2	2	2	2	2	2	2	2	2	2
9	9	9	9	9	9	9	9	9	3
2	1	3	3	2	3	3	2	2	3
3	2	2	2	3	3	3	2	3	3
2	2	2	2	3	1	3	2	2	2
2	2	2	2	2	1	3	3	3	2
2	3	2	2	2	3	2	1	3	2
2	3	2	3	3	3	2	2	2	3
2	2	2	2	2	2	2	2	2	3
1	1	3	3	1	3	3	1	9	3
9	9	9	9	9	9	9	9	2	3
3	2	2	2	3	3	3	3	3	3
9	9	9	9	9	9	3	9	3	3
3	3	2	3	2	1	3	3	2	3
2	2	1	1	2	1	1	1	3	3
2	3	3	1	3	1	2	3	3	1
1	1	3	3	1	3	1	1	3	1
1	1	3	3	1	3	3	1	3	3
3	3	3	3	3	3	3	3	3	3
9	9	9	9	9	9	9	9	9	9
3	3	9	9	3	9	3	2	3	3

3	2	2	3	3	3	3	3	3	3
2	3	1	2	3	3	3	1	3	3
9	9	9	9	9	9	9	9	9	9
2	2	2	2	3	1	2	3	3	3
2	2	2	3	2	3	3	1	3	3
1	9	9	9	9	9	9	9	2	2
9	9	9	9	9	9	9	9	9	2
2	2	1	2	3	2	3	1	3	2
2	2	2	1	3	1	2	2	3	3
2	2	2	2	2	2	2	2	2	2
3	3	2	2	3	1	3	3	3	3
3	2	2	2	3	3	3	3	3	3
3	3	2	2	3	2	3	3	3	3
2	2	3	3	2	3	3	3	3	3
3	2	2	2	3	2	3	3	3	3
3	1	2	2	3	3	3	3	3	3
3	3	3	3	3	3	3	3	3	3
3	3	2	2	3	3	3	3	3	3
9	9	9	2	9	9	9	9	9	3
9	9	9	9	9	9	9	9	9	9
9	9	9	9	9	9	9	9	3	2
9	9	9	9	9	9	9	9	9	9
9	9	9	9	9	9	9	9	9	9
2	3	2	2	3	2	3	3	3	3
3	2	2	2	2	2	2	2	2	2
2	2	2	2	1	1	3	2	3	3
9	9	9	9	9	9	3	9	9	3
9	9	9	9	9	9	9	9	9	9
9	9	9	9	9	9	3	9	9	3
9	9	9	9	9	9	3	9	9	3
9	9	9	9	9	9	9	9	1	1
2	2	2	2	2	2	2	2	2	2
3	2	9	2	3	3	2	3	3	9
2	3	2	2	3	2	3	3	3	3
1	2	2	2	3	2	3	3	9	3
9	9	9	9	9	9	9	9	9	9
9	9	1	9	9	9	9	9	9	3
9	9	9	9	9	9	9	9	3	3
9	9	9	9	9	9	9	9	3	9
9	9	9	9	9	9	3	9	9	3
9	9	9	9	9	9	9	9	9	9
2	1	2	2	2	2	2	2	2	3
9	9	9	9	9	9	9	9	9	9
2	2	2	2	2	2	2	2	2	2
3	2	2	2	2	3	3	2	1	2
2	2	2	2	2	2	2	2	2	3
3	3	2	2	3	2	3	3	3	3

1	1	3	3	3	3	3	1	3	3
2	3	2	2	2	2	3	3	2	3
2	2	2	2	9	1	1	3	3	3
3	2	3	3	2	3	2	1	9	2
2	2	2	1	2	2	3	2	3	3
9	9	9	9	9	9	3	9	3	9
2	3	2	1	2	1	2	2	3	9
9	2	9	9	9	9	9	9	2	9
2	9	9	9	9	9	9	9	9	9
9	9	9	9	3	9	9	9	9	9
2	2	2	2	2	2	2	2	2	3
3	2	1	2	2	2	3	3	3	3
2	2	2	2	2	2	2	2	9	2
2	2	2	2	2	2	2	2	2	2
2	2	2	9	9	9	9	9	3	3
2	1	2	1	1	1	2	1	3	2
2	2	3	3	2	3	3	2	3	3
1	2	2	2	2	2	2	2	3	3
2	2	3	2	2	1	3	3	3	3
3	3	2	2	3	3	3	3	3	3
3	3	1	3	3	1	3	3	3	3
2	2	2	2	2	2	3	3	3	3
3	2	3	3	2	3	3	2	3	3
9	9	9	9	9	9	9	9	3	9
2	3	2	2	3	3	3	3	3	3
2	2	2	2	2	3	3	2	3	3
2	3	2	1	3	2	1	1	3	3
2	2	2	3	3	3	3	2	3	3
2	3	2	1	3	3	3	3	3	3
3	1	2	2	2	9	9	9	3	3
2	9	1	9	2	3	2	2	2	3
3	1	2	2	2	3	1	2	9	9
1	2	3	2	2	2	2	1	1	2
2	2	2	2	2	2	2	2	2	2
2	9	2	1	2	2	2	2	2	2
9	9	9	9	9	9	3	9	9	3
3	3	9	9	3	9	3	3	3	3
3	3	3	3	3	3	3	3	3	3
2	2	2	3	3	2	3	3	3	3
2	9	9	2	9	2	9	2	2	9
3	2	1	3	3	2	2	2	3	3
3	3	3	3	3	3	3	3	3	3
3	3	2	2	3	2	3	3	3	3
2	1	3	3	1	3	2	1	3	3
3	3	2	2	3	2	3	3	3	3
3	3	2	2	3	2	3	3	3	3
3	3	3	3	3	3	3	3	3	3

3	9	9	9	9	1	9	3	3	9
3	1	2	1	1	1	3	3	3	3
2	2	2	2	2	2	2	2	2	2
3	2	3	1	3	2	3	3	3	3
9	9	9	9	9	9	9	9	9	9
1	2	2	2	1	2	3	2	3	2
2	2	2	2	2	2	2	2	2	2
9	9	9	9	9	9	9	9	9	9
2	2	2	2	2	2	2	2	2	2
2	2	2	3	3	2	3	2	3	3
9	9	9	9	9	9	9	9	3	9
1	3	3	3	1	3	1	1	3	1
2	2	2	2	2	2	2	2	2	3
2	2	3	3	2	2	1	2	2	2
2	2	1	2	2	2	1	1	2	1
3	2	2	2	3	2	3	2	3	3
3	2	3	3	1	3	3	1	3	3
2	3	1	2	2	1	1	2	3	2
2	2	2	2	2	2	2	2	3	3
1	2	2	1	2	1	1	2	2	2
2	2	2	2	2	2	2	2	2	2
2	2	2	2	2	2	2	2	2	2
3	2	1	2	2	2	2	2	3	3
3	3	1	2	3	1	3	3	3	3
2	2	1	1	3	3	1	1	2	2
2	2	2	2	2	2	3	2	2	2
2	2	3	2	3	3	3	2	2	3
2	2	2	2	2	2	2	2	2	2
3	2	2	2	3	3	3	2	3	3
1	2	3	2	1	2	3	2	3	3
9	9	9	9	9	9	9	3	9	9
3	3	1	1	3	3	3	3	3	3
3	3	2	2	3	2	3	3	3	3
3	3	9	9	3	9	3	3	3	3
3	3	1	1	3	2	3	2	2	2
2	2	9	1	2	2	2	3	2	2
9	9	9	9	9	9	9	9	3	9
2	2	2	2	2	2	2	2	2	2
9	9	9	9	9	9	9	9	9	9
3	2	2	2	2	2	3	3	2	3
2	2	2	2	2	2	2	2	2	2
3	1	3	3	1	3	2	3	3	3
9	9	9	9	9	9	9	9	3	3
3	3	2	3	2	3	3	3	3	3
3	3	3	1	3	3	3	3	3	3
3	1	3	3	1	3	1	1	9	9
3	3	2	3	3	3	1	3	3	3

3	1	3	3	3	3	3	3	3	2
2	3	3	1	3	2	3	3	2	3
9	9	9	9	9	9	9	9	9	9
9	9	9	9	9	9	9	9	9	9
9	9	9	9	9	9	9	9	9	9
1	1	3	3	1	3	1	1	1	1
3	1	3	2	2	2	2	2	3	3
3	1	3	1	1	2	3	3	2	2
2	2	1	2	1	1	2	2	3	2
2	3	2	3	3	2	3	2	3	3
2	1	3	3	3	3	3	2	3	3
3	2	2	3	3	3	3	3	3	3
2	2	2	2	2	2	3	1	3	3
3	1	2	3	3	3	3	3	3	3
3	1	3	3	1	3	3	3	3	3
2	3	2	1	2	1	3	3	3	3
3	3	2	3	1	2	3	3	3	3
1	1	3	3	1	3	1	3	3	3
9	9	9	9	9	9	9	9	9	9
3	2	2	3	3	3	3	3	3	3
9	9	9	9	9	9	9	9	9	9
3	2	1	3	2	3	3	2	2	3
2	3	3	2	3	3	3	3	3	3
9	9	9	9	3	9	9	9	9	9
3	2	3	2	3	2	2	2	2	2

Smoking	Alcohol	OverFourB	Hightfattyc	BirthPill	Spiritualatt	FamilyHxC	VaginalWa	UrineInfect	STD
2	2	2	2	2	2	2	2	2	2
2	3	2	3	2	3	2	3	2	3
2	2	2	2	2	2	2	2	2	2
2	2	2	2	2	2	2	2	2	2
2	2	1	1	2	2	2	3	2	2
2	2	2	2	2	2	2	2	2	2
3	2	1	2	3	2	1	3	2	1
2	3	3	2	2	2	2	3	2	2
1	9	1	1	2	9	1	2	1	2
1	9	1	3	1	2	3	2	3	2
2	2	2	2	2	2	1	2	2	2
2	2	2	2	3	2	2	2	2	2
2	3	3	3	1	1	1	1	2	1
1	2	3	3	3	3	2	2	2	2
2	2	2	2	2	2	2	2	2	2
2	2	2	2	2	2	2	2	2	2
9	9	9	9	9	9	9	9	9	9
1	1	2	3	2	2	2	2	2	2
9	9	9	9	9	9	9	9	9	9
9	9	9	9	9	9	9	9	9	9
1	1	1	1	1	1	1	1	1	1
2	2	2	2	2	2	2	1	2	1
2	2	1	2	2	2	3	1	1	1
1	1	1	1	1	1	1	1	1	1
9	9	9	9	9	9	9	9	9	9
1	1	2	2	1	1	1	1	2	2
3	1	3	3	1	3	1	3	3	1
2	2	2	2	2	2	2	2	2	2
1	2	1	2	3	1	2	3	1	2
3	2	1	1	2	2	3	1	2	3
2	2	2	3	3	3	1	2	1	3
1	1	3	2	2	2	2	2	2	1
3	3	3	3	3	3	3	3	3	3
1	2	1	1	1	1	1	1	2	1
1	1	2	2	2	1	1	1	2	2
2	2	2	3	3	3	3	2	2	2
2	2	2	2	2	2	2	2	2	2
1	1	2	1	3	3	3	1	1	2
1	1	2	2	1	3	3	1	1	1
2	2	2	2	2	2	3	3	9	1
2	2	2	2	2	2	2	2	2	2
9	9	9	1	9	2	9	9	9	9
9	9	9	9	9	9	9	9	9	9
1	2	2	2	2	2	2	2	1	2
2	1	2	2	2	2	3	2	2	2
9	9	9	9	9	9	9	9	9	9

2	2	2	2	2	2	3	3	2	2
2	2	2	2	2	1	2	3	2	3
2	2	1	1	2	2	2	2	2	2
2	2	2	1	2	2	2	1	1	2
1	3	3	3	3	3	3	1	1	1
9	3	3	3	3	3	3	1	1	1
3	2	2	2	3	2	2	3	2	2
2	2	2	2	2	2	2	2	2	2
1	1	1	1	1	1	1	1	1	1
1	2	3	1	2	3	1	2	3	1
9	9	9	9	9	9	9	1	9	9
2	2	2	2	1	1	2	3	2	2
2	2	2	1	1	1	1	1	2	2
2	2	2	3	3	2	2	2	1	1
2	2	2	1	1	1	2	3	2	3
2	3	2	2	1	1	2	2	3	2
1	1	1	2	3	3	3	3	2	2
3	3	2	3	2	9	1	1	1	3
2	2	2	2	2	2	2	2	2	2
2	2	2	3	9	2	2	3	3	9
2	2	2	2	2	2	2	2	2	2
2	1	2	3	3	3	2	3	2	1
1	1	1	2	2	3	3	2	2	2
2	2	1	1	2	2	1	2	2	1
2	1	2	2	1	2	2	3	3	2
1	1	2	2	2	1	2	1	1	1
3	3	3	3	1	3	3	1	1	1
2	3	3	1	1	1	1	2	2	1
2	2	3	1	2	2	1	1	1	1
1	3	3	3	1	3	3	3	1	1
9	9	9	9	9	9	9	9	9	9
1	2	3	3	2	1	3	2	2	2
9	9	9	9	9	9	2	1	9	1
2	2	2	2	2	2	2	2	2	2
2	3	3	3	9	9	3	1	2	2
1	1	2	2	2	2	2	2	2	2
1	1	2	2	2	9	2	1	1	1
2	2	3	2	2	1	2	2	3	2
3	2	2	1	2	3	2	2	1	3
3	2	2	2	1	1	2	2	2	2
1	1	2	2	2	1	2	1	2	1
9	9	9	9	9	9	9	9	9	9
1	3	9	9	9	1	1	1	1	1
2	2	2	2	3	2	2	1	1	2
1	1	2	2	2	1	1	2	2	1
1	1	2	3	2	3	2	3	2	1
2	3	3	2	2	2	2	2	2	3

3	1	2	3	1	1	2	2	1	3
3	3	3	3	3	3	3	3	3	3
1	1	2	1	2	1	1	2	2	1
2	2	1	1	2	1	2	3	2	1
2	1	1	1	1	1	1	1	1	1
2	2	1	1	2	1	3	2	1	1
1	1	2	2	2	2	2	2	2	2
9	9	9	9	9	9	9	9	9	9
1	1	1	2	2	2	2	1	1	2
1	1	3	2	2	3	2	1	1	1
1	1	2	3	2	2	3	1	1	1
2	2	1	1	2	3	2	2	1	2
3	2	2	2	1	1	2	2	1	2
1	2	2	2	2	1	2	2	2	2
2	2	2	3	3	1	1	2	2	1
2	2	2	1	1	3	2	2	1	9
2	2	1	1	1	1	2	1	1	1
1	1	3	3	2	3	2	2	2	2
2	2	2	9	2	2	9	1	2	1
2	2	1	1	2	2	1	1	2	2
1	3	1	3	1	3	1	3	1	3
1	1	2	2	2	2	1	1	2	2
3	1	1	1	2	1	1	1	2	1
2	2	2	2	2	2	2	2	2	2
2	2	2	2	2	2	2	2	2	2
1	2	2	1	3	3	3	1	2	2
1	1	3	1	3	1	3	1	1	1
1	2	1	2	1	2	1	2	1	2
1	2	2	1	2	2	1	1	1	1
2	1	3	2	2	1	3	2	2	1
1	1	2	3	2	1	2	2	3	3
3	1	2	2	2	2	2	3	3	3
2	2	3	1	1	2	1	9	1	2
1	2	1	2	1	2	1	2	1	1
2	2	2	1	9	3	2	1	9	3
1	1	1	1	3	1	2	1	3	2
1	1	2	1	2	9	1	2	2	9
2	2	1	1	2	3	3	2	2	2
9	9	9	9	9	9	9	9	9	9
3	3	3	3	3	3	3	3	3	3
2	2	3	2	1	2	1	2	2	1
9	9	9	9	9	9	9	9	9	9
2	2	1	1	2	2	2	1	1	2
1	2	2	3	2	2	2	1	2	2
9	9	9	9	9	9	9	9	9	9
1	1	2	3	3	3	3	2	2	2
2	9	1	1	2	1	1	1	2	1

3	3	3	9	1	1	1	2	3	2
1	1	1	1	1	1	3	1	1	1
3	3	3	3	3	3	3	3	3	3
2	2	2	2	2	2	2	2	2	2
2	2	2	2	2	3	3	2	2	2
9	2	3	2	3	2	3	2	3	2
3	1	3	1	3	1	3	3	1	9
3	1	3	2	1	3	1	3	1	9
9	9	9	9	9	9	9	9	9	9
2	2	2	2	2	2	2	2	2	2
1	2	2	2	2	3	3	3	3	2
2	2	2	2	2	2	1	1	1	1
2	2	2	2	2	2	2	2	2	2
1	1	2	1	2	3	2	2	2	2
2	3	2	2	2	2	1	2	2	2
1	2	3	2	1	2	3	2	2	2
2	1	1	1	2	2	1	1	2	1
3	2	1	2	2	1	2	1	2	2
9	9	9	9	9	9	9	9	9	9
1	1	3	1	2	2	1	2	3	2
1	1	2	2	2	1	2	2	1	1
1	1	2	2	2	3	2	2	1	1
3	3	3	3	3	3	3	3	3	3
1	1	9	2	1	1	1	1	1	1
1	1	1	2	3	3	3	2	2	2
1	2	1	3	2	3	3	3	3	1
2	2	3	3	2	9	9	9	9	9
2	2	3	3	3	3	3	3	3	3
2	1	2	2	3	3	2	2	1	2
2	2	3	3	3	3	3	3	3	3
2	1	1	1	2	2	2	1	1	2
2	2	2	1	1	2	2	1	1	2
2	2	1	1	2	2	2	1	1	2
3	3	3	1	1	1	1	3	3	3
1	1	1	1	1	1	3	3	2	2
3	3	3	1	1	1	1	1	1	1
3	3	3	3	3	3	3	3	3	3
3	3	3	3	1	3	3	1	1	1
1	1	3	3	3	1	1	1	3	2
2	2	2	2	2	2	2	2	2	2
9	9	9	9	9	9	9	9	9	9
2	2	2	2	2	2	2	2	2	2
2	2	2	2	2	2	2	2	2	2
9	9	9	9	9	9	9	9	9	9
9	9	9	9	9	9	9	9	9	9
2	3	3	3	3	2	2	1	2	1
2	3	3	3	3	3	2	1	2	2

9	1	9	9	9	9	9	9	9	9
9	9	9	9	2	9	9	9	9	9
9	9	9	9	9	9	9	9	9	9
9	9	9	9	1	9	9	9	9	9
9	9	9	9	9	9	9	9	9	9
9	9	9	9	9	9	9	9	9	9
1	1	2	2	2	2	1	1	2	1
9	9	9	9	9	9	9	9	9	9
1	2	3	3	3	2	2	2	3	1
9	9	1	9	9	9	9	9	9	9
9	9	9	2	9	9	9	9	9	9
9	9	1	9	9	9	9	9	9	9
1	1	1	1	1	1	1	1	1	2
3	1	2	3	1	2	3	1	2	1
9	9	9	9	9	9	9	9	9	9
2	2	2	2	3	3	3	3	3	3
3	3	3	3	3	3	3	3	3	3
1	2	2	2	2	2	2	2	2	2
1	2	1	2	2	9	2	2	2	2
2	2	2	2	2	2	2	2	2	2
2	2	3	1	2	2	2	1	2	2
9	9	9	9	9	9	9	9	9	9
2	2	3	1	1	2	2	1	1	1
1	2	3	2	1	2	2	2	1	1
2	2	2	2	2	2	2	2	2	2
1	1	1	2	1	1	2	2	2	2
2	2	3	2	2	2	2	2	1	1
1	2	2	1	1	1	1	2	1	1
3	3	3	3	3	3	3	3	3	3
1	1	3	3	3	1	3	3	1	1
2	2	2	2	2	2	2	2	2	2
2	2	2	2	2	1	1	1	2	1
9	9	9	9	9	9	9	9	9	9
2	2	2	2	2	1	1	1	1	2
1	1	1	3	3	1	1	1	1	1
1	2	2	3	2	1	2	3	2	1
1	2	3	1	2	3	2	1	2	1
2	1	1	1	1	1	1	1	1	1
9	9	9	9	9	9	9	9	9	9
1	1	2	2	2	1	2	2	2	2
1	2	2	3	2	2	2	2	2	1
1	3	1	3	2	2	1	1	1	1
1	1	2	3	3	3	2	1	1	1
2	2	2	2	2	2	2	2	2	2
1	1	1	1	1	1	1	1	1	1
2	2	3	3	2	2	1	1	3	2
9	9	9	9	9	9	9	9	9	9

1	9	1	1	1	1	1	1	2	9
1	1	3	3	3	2	1	1	1	9
1	1	1	1	1	1	1	1	1	1
9	9	9	9	9	9	9	9	9	9
2	2	2	2	2	2	2	2	2	2
1	1	3	2	2	2	2	3	2	3
3	3	3	3	3	2	1	1	1	2
2	2	3	2	2	2	2	1	1	2
1	2	2	3	2	2	2	2	2	2
2	1	1	1	1	1	2	1	2	3
2	2	2	2	2	2	1	2	2	1
9	9	9	9	9	9	9	9	9	9
9	9	9	9	9	9	9	9	9	9
9	9	9	9	9	9	9	9	9	9
9	9	9	2	9	9	2	9	9	2
9	9	9	9	9	9	9	9	9	9
9	9	9	9	9	9	9	9	9	9
9	9	9	9	9	9	9	9	9	9
9	2	9	9	2	9	2	9	9	2
9	9	9	9	9	9	9	9	9	9
9	9	9	9	9	9	9	9	9	9
2	1	1	1	1	1	1	1	1	1
2	2	1	1	2	2	1	1	2	2
1	2	2	1	1	1	1	1	1	1
2	1	1	1	1	1	1	2	2	1
9	9	9	9	9	9	9	9	9	9
2	1	1	2	3	2	1	2	1	2
9	9	9	9	9	9	9	9	9	9
9	9	9	9	9	9	9	9	9	9
2	1	2	1	2	2	1	2	2	2
9	9	9	9	9	9	9	9	9	9
9	9	9	9	9	9	9	9	9	9
9	9	9	9	9	9	9	9	9	9
9	9	9	9	9	9	9	9	9	9
1	9	1	9	2	9	3	1	2	9
9	9	9	1	9	9	9	9	1	9
9	9	9	9	9	9	9	9	9	9
9	9	9	9	9	9	9	9	9	9
9	9	9	9	9	9	9	9	9	9
9	9	9	9	9	9	9	9	9	9
9	9	9	9	9	9	9	9	9	9
9	9	9	9	9	9	9	9	9	9
3	3	3	3	3	3	3	3	3	3
3	1	2	3	1	2	1	2	3	2
3	1	2	3	1	1	2	1	3	1
9	9	9	9	1	9	9	9	9	2
9	9	9	9	9	9	9	9	9	9

9	9	9	9	9	9	9	9	9	9
9	9	9	9	9	9	9	9	9	9
3	3	3	3	2	2	2	2	2	1
2	3	1	2	3	1	2	3	1	2
2	2	3	2	3	2	3	2	2	3
2	2	2	2	2	3	3	3	3	3
3	3	2	1	2	1	3	2	1	2
1	2	2	2	3	1	2	1	2	2
2	2	2	2	3	3	3	3	2	2
3	3	3	3	3	3	3	3	3	3
9	9	9	9	9	9	9	9	9	9
9	9	9	9	9	9	9	9	9	9
9	9	9	9	9	9	9	9	9	9
9	9	9	9	9	9	9	9	9	9
1	1	1	1	1	1	1	1	1	1
1	2	3	3	3	3	2	3	1	1
9	9	9	9	9	9	9	9	9	9
1	1	2	1	1	1	2	1	1	1
1	1	1	1	1	1	1	1	1	1
1	1	1	1	1	1	1	1	1	1
1	3	1	2	3	1	2	1	2	3
2	2	2	2	2	2	1	2	1	1
9	9	9	9	9	9	9	9	9	9
2	2	2	2	2	2	2	2	2	2
2	2	2	2	2	3	3	3	2	2
2	2	2	2	2	2	2	2	2	2
2	2	2	2	2	2	2	2	2	2
2	2	2	1	3	3	3	2	2	2
2	2	2	2	2	2	2	9	3	3
2	1	1	1	1	2	1	2	1	1
2	2	2	2	2	2	2	2	2	2
2	2	2	2	2	2	2	2	2	2
3	3	3	3	3	3	3	2	3	2
9	2	2	2	2	2	2	2	2	2
2	2	1	1	3	3	3	1	1	3
2	3	9	1	2	3	2	3	2	2
9	9	9	9	9	9	9	9	9	9
2	9	2	1	2	2	2	2	2	2
3	3	3	3	3	3	3	3	3	3
3	3	3	3	3	3	3	3	3	3
9	9	9	9	9	9	9	9	9	9
9	9	9	9	9	9	9	9	9	9
9	3	9	9	9	9	9	9	9	9
1	1	1	2	1	1	1	2	1	1
2	2	2	1	1	2	2	1	1	1
2	2	2	2	2	2	2	2	2	2
1	2	2	2	2	2	3	3	3	3

9	9	9	9	2	9	9	9	9	9
9	9	9	9	9	9	9	9	9	9
9	9	9	1	9	9	9	9	9	9
1	9	9	9	9	9	9	9	9	9
9	9	9	2	9	9	9	9	1	9
2	2	2	2	1	1	2	2	1	2
9	9	9	9	2	9	9	9	9	9
9	9	9	9	9	9	9	9	9	9
9	9	9	9	9	9	9	9	9	9
9	9	9	9	9	9	9	9	9	9
9	9	9	9	9	2	9	9	9	9
9	9	9	9	9	9	3	9	9	9
9	9	9	9	9	9	9	9	9	9
9	9	9	9	3	9	9	9	9	9
1	9	9	9	9	9	9	9	9	9
9	9	9	9	9	9	9	9	9	9
9	2	9	9	9	9	9	1	9	9
3	2	3	2	1	2	1	2	9	2
2	1	2	3	2	2	1	2	1	2
2	2	2	2	2	2	2	2	2	2
2	3	3	2	1	3	3	1	2	2
1	2	2	2	1	2	2	1	3	2
2	2	2	2	2	2	2	2	2	2
2	2	2	3	2	3	2	3	2	1
1	2	1	2	2	3	2	1	2	1
9	9	9	9	9	9	9	9	9	9
1	2	2	3	2	1	3	2	2	2
3	3	3	3	3	3	2	2	2	2
2	2	2	2	2	1	3	2	1	2
1	1	1	1	1	1	2	2	2	1
1	1	1	1	2	9	3	2	2	1
2	2	2	2	2	2	2	2	2	2
2	2	2	2	2	3	3	3	3	3
2	2	2	2	2	1	1	1	1	1
9	9	9	9	9	9	9	9	9	9
2	2	2	2	2	2	2	2	2	2
9	9	9	9	9	9	9	9	9	9
2	2	1	1	1	2	2	1	1	2
9	9	9	9	9	9	9	9	9	9
2	2	2	2	2	2	2	2	2	2
2	2	2	3	3	1	1	2	2	3
3	3	3	3	3	3	3	3	3	3
1	1	9	2	1	3	2	1	1	2
1	1	2	2	2	3	3	2	1	2
3	9	2	2	2	2	2	3	9	2
2	2	2	3	3	1	2	2	3	1
2	2	2	2	2	2	2	2	2	2

1	2	2	3	3	3	2	2	2	2
9	9	2	9	9	9	9	9	9	9
9	1	2	2	1	1	2	2	2	3
2	1	3	2	3	1	3	2	3	2
2	9	1	1	1	1	1	1	1	1
2	2	2	2	2	2	3	3	3	3
1	1	2	2	1	2	1	2	2	2
1	1	2	2	1	2	2	2	2	2
9	9	9	9	9	9	9	9	9	9
2	2	2	2	2	2	2	2	2	2
2	2	3	3	2	3	3	2	2	3
9	9	9	9	9	9	9	9	9	9
9	9	9	9	9	9	9	9	9	9
2	2	2	1	1	1	1	1	3	3
9	9	9	9	9	9	9	9	9	9
2	2	3	3	3	3	3	2	2	2
9	9	9	9	9	9	9	9	9	9
9	9	9	9	9	9	9	9	9	9
9	9	9	1	9	9	9	9	9	9
3	2	3	2	3	2	3	2	3	2
2	2	2	2	2	2	2	2	2	2
2	2	2	2	2	2	1	2	2	2
1	2	2	2	2	2	2	2	2	2
9	9	9	9	9	9	9	1	9	9
9	9	9	9	9	9	9	9	9	9
2	1	1	1	2	1	1	2	2	1
3	2	1	2	3	3	3	3	3	3
3	1	2	3	2	1	1	1	2	3
9	9	9	9	9	9	9	9	9	9
1	2	3	9	9	9	9	9	9	9
2	2	2	2	2	2	2	2	2	2
1	2	3	1	2	3	1	2	3	1
2	3	1	1	1	1	2	2	2	2
2	3	2	2	1	1	2	2	2	2
1	1	3	9	1	3	3	1	1	1
2	1	2	2	2	3	3	3	3	3
9	9	9	9	9	9	9	9	9	9
9	9	9	9	9	9	9	9	9	9
3	1	3	9	1	2	2	2	3	2
1	2	1	2	1	2	1	2	1	2
2	2	2	2	2	2	2	2	2	2
2	2	2	2	2	2	2	2	2	2
2	2	2	2	2	2	2	2	2	2
1	3	1	2	1	2	2	1	2	3
2	2	2	2	2	2	2	2	2	2
2	2	2	2	2	2	2	2	2	2
3	2	2	2	2	2	3	3	3	3

2	2	2	1	1	1	1	1	1	1
3	1	2	1	2	3	1	2	3	1
9	9	9	9	9	9	9	9	1	9
9	9	9	9	9	9	9	9	1	9
9	9	9	9	9	9	9	9	9	9
9	1	3	2	2	3	2	2	3	2
9	9	2	9	9	2	9	9	2	9
3	9	9	3	9	9	3	9	3	9
9	9	9	9	9	9	9	9	9	9
9	9	9	9	9	9	9	9	9	9
9	9	9	9	9	9	9	9	9	9
9	9	9	9	9	9	9	9	9	9
9	9	9	9	9	9	9	9	9	9
9	9	9	9	9	9	9	9	9	9
9	9	9	9	9	9	9	9	9	9
9	9	9	9	9	9	9	9	9	9
3	3	3	3	3	3	3	3	3	3
9	9	9	9	9	9	9	9	9	9
9	9	9	9	9	9	9	9	9	9
9	2	9	9	2	9	2	9	2	9
9	9	9	9	9	9	9	9	9	9
9	9	9	9	9	9	9	9	9	9
2	2	2	2	2	2	2	2	2	2
9	9	9	9	9	9	9	9	9	9
9	9	9	9	9	9	9	9	9	9
9	9	9	9	9	9	9	9	9	9
9	9	9	9	9	9	9	9	9	9
2	9	9	2	9	9	2	9	2	9
2	2	2	9	2	9	2	9	2	9
9	9	9	9	9	9	9	9	9	9
9	9	9	9	9	9	9	9	9	9
2	9	2	9	9	2	9	2	2	2
9	9	9	9	9	9	9	9	9	9
9	9	9	9	9	9	9	9	9	9
9	9	9	9	9	9	9	9	9	9
9	9	9	9	9	9	9	9	9	9
9	9	9	9	9	9	9	9	9	9
9	9	9	9	9	9	9	9	9	9
3	1	2	1	3	2	1	2	1	3
3	2	2	3	2	1	3	2	3	1
3	2	2	9	9	2	9	9	2	9
9	9	9	9	9	9	9	9	9	9
3	2	1	3	2	2	1	2	1	3
3	2	3	1	2	1	2	3	1	2
3	3	3	3	3	1	1	1	1	1
2	2	3	1	2	2	1	2	1	2
1	2	2	2	1	2	3	2	2	3

9	9	9	9	9	9	9	1	9	9
2	1	1	2	1	1	2	3	3	3
2	2	2	2	2	2	2	2	2	2
1	1	1	1	1	1	1	1	1	1
2	2	2	2	3	3	3	3	3	3
3	2	1	2	3	1	2	2	2	2
9	9	9	9	9	9	9	9	9	9
2	2	2	2	3	2	1	1	2	1
2	2	2	2	2	2	2	2	2	2
1	1	2	2	1	2	2	1	1	1
9	2	9	2	2	2	2	2	2	2
9	9	9	9	9	9	9	9	9	2
9	9	9	9	9	9	9	1	9	9
1	9	9	9	9	9	9	9	9	9
1	9	9	9	9	9	9	9	9	9
1	1	1	1	1	1	1	2	1	1
2	2	2	2	2	2	9	9	9	9
2	3	3	2	2	3	3	2	2	2
1	3	3	3	3	3	3	3	3	2
2	2	2	2	2	2	2	2	2	2
2	2	2	2	2	2	2	2	2	2
9	9	9	9	9	9	9	9	9	9
1	3	2	1	3	1	1	1	1	1
9	2	9	9	9	9	9	9	9	9
9	9	9	9	9	9	9	1	9	9
3	3	3	2	1	2	2	2	2	3
9	2	9	9	9	9	9	9	9	9
1	1	3	3	3	2	2	2	1	1
9	9	9	9	9	9	9	9	9	9
9	9	9	9	9	9	9	1	9	9
2	3	2	3	2	3	1	3	2	1
3	2	3	2	3	2	3	2	3	2
9	9	9	9	9	9	9	9	9	9
9	9	9	9	9	9	9	9	9	9
1	3	2	3	1	2	2	3	2	2
9	9	9	9	9	9	9	9	9	9
3	2	2	3	2	3	2	1	3	1
2	9	9	9	9	9	9	9	9	9
2	2	2	2	2	2	2	2	2	2
9	9	9	9	9	9	9	9	9	9
2	2	2	2	2	2	2	1	2	2
1	1	2	2	1	2	2	2	2	2
2	2	2	2	2	2	2	2	2	2
2	2	2	2	2	2	2	2	2	2
3	3	2	3	2	3	3	3	2	3
2	2	2	2	2	2	2	2	2	2
2	2	3	3	2	3	2	3	2	2

9	9	9	9	9	9	9	9	9	9
2	2	2	2	2	2	2	2	2	2
2	2	2	2	2	2	2	2	2	2
9	9	9	9	9	9	9	9	9	9
9	9	9	9	9	9	9	9	9	9
9	9	9	9	9	9	9	9	9	9
9	9	9	9	9	9	9	9	9	9
2	2	2	2	2	2	2	2	2	2
1	1	2	1	2	1	2	2	1	1
1	1	9	9	9	9	9	9	9	9
1	1	1	1	2	2	2	2	2	2
2	2	2	2	2	3	3	1	1	1
2	1	2	2	2	2	2	2	2	2
2	2	2	2	2	2	2	2	2	2
9	9	9	9	9	9	9	9	9	9
9	9	9	9	9	9	9	9	9	9
9	9	9	9	9	9	9	9	9	9
9	9	9	9	9	9	9	9	9	9
9	9	9	9	9	9	9	9	9	9
9	9	9	9	9	9	9	9	9	9
9	9	9	9	9	9	9	9	9	9
9	9	9	9	9	9	9	9	9	9
2	2	2	2	9	2	2	2	2	2
9	9	9	9	9	9	9	9	9	9
9	9	9	9	9	9	9	9	9	9
9	9	9	9	9	9	9	9	9	9
9	9	9	9	9	9	9	9	9	9
9	9	9	9	9	9	9	9	9	9
9	9	9	9	9	9	2	9	9	9
9	9	9	9	9	9	9	9	9	9
9	9	9	9	9	9	9	9	9	9
2	2	2	2	2	2	2	2	2	2
9	9	9	9	9	9	9	9	9	9
9	9	9	9	9	9	9	9	9	9
9	9	9	9	9	9	9	9	9	9
9	9	9	9	9	9	9	9	9	9
9	9	9	9	9	9	9	9	9	9
9	9	9	9	9	9	9	9	9	9
9	9	9	9	9	9	9	9	9	9
9	9	9	9	9	9	9	9	9	9
2	9	2	2	2	2	2	2	2	2
9	9	9	9	9	9	9	9	9	9
9	9	9	9	9	9	9	9	9	9
9	9	9	9	9	9	9	9	9	9
9	9	9	9	9	9	9	9	9	9
9	9	9	9	9	9	9	9	9	9
9	9	9	9	9	9	9	9	9	9

9	9	9	9	9	9	9	9	9	9
3	3	3	3	3	3	3	3	3	3
9	9	9	9	9	9	9	9	9	9
9	9	9	9	9	9	9	9	9	9
9	9	9	9	9	9	9	9	9	9
9	9	9	9	9	9	9	9	9	9
9	9	9	9	9	9	9	9	9	9
9	9	9	9	9	9	9	9	9	9
9	9	9	9	9	9	9	9	9	9
9	9	9	9	9	9	9	9	9	9
9	9	9	9	9	9	9	9	9	9
9	9	9	9	9	9	9	9	9	9
9	9	9	9	9	9	9	9	9	9
9	9	9	9	9	9	9	9	9	9
9	9	9	9	9	9	9	9	9	9
9	9	9	9	9	9	9	9	9	9
9	9	9	9	9	9	9	9	9	9
2	2	2	2	2	2	2	2	2	2
9	9	9	9	9	9	9	9	9	9
9	9	9	9	9	9	9	9	9	9
9	9	9	9	9	9	9	9	9	9
9	9	9	9	9	9	9	9	9	9
9	9	9	9	9	9	9	9	9	9
9	9	9	9	9	9	9	9	9	9
2	2	2	2	2	2	2	2	2	2
9	9	9	9	9	9	9	9	9	9
9	9	9	9	9	9	9	9	9	9
9	9	1	9	9	9	1	9	2	2
9	9	9	9	9	9	9	9	9	9
9	9	9	9	9	9	9	9	9	9
1	1	1	1	1	1	1	1	1	1
1	2	1	1	1	1	1	2	2	2
9	9	9	9	9	9	9	9	9	9
2	2	2	2	2	2	2	2	2	2
9	9	9	9	9	9	9	9	9	9
2	2	2	2	2	2	2	2	2	2
9	9	9	9	9	9	9	9	9	9
9	9	9	9	9	9	9	9	9	9
1	1	1	1	1	1	1	1	1	1
9	9	9	9	9	9	9	9	9	9
9	9	9	9	9	9	9	9	9	9
9	9	9	9	9	9	9	9	9	9
2	2	2	2	2	2	2	2	2	2
2	2	2	2	2	2	2	2	2	2
9	3	1	9	3	1	9	1	9	1
9	9	9	9	9	9	9	9	9	9
9	9	9	9	9	9	9	9	9	9

9	9	9	9	2	9	9	9	9	9
9	9	9	9	9	9	9	9	9	9
9	1	9	2	3	1	9	2	3	1
9	9	9	9	9	9	9	9	9	9
9	9	9	9	9	9	9	9	9	9
9	9	9	9	9	9	9	9	9	9
9	9	9	9	9	9	9	9	9	9
9	9	9	9	9	9	9	9	9	9
9	9	9	2	9	9	9	2	9	9
9	9	9	9	9	9	9	9	9	9
9	9	9	9	9	9	9	9	9	9
9	9	9	9	9	9	9	9	9	9
9	9	9	9	9	9	9	9	9	9
9	9	9	9	9	9	9	9	9	9
1	1	3	2	2	1	2	2	1	3
3	2	2	3	1	3	2	3	1	1
2	9	9	9	9	9	9	9	9	9
2	2	2	2	2	2	2	2	2	2
2	3	2	2	2	2	3	3	3	3
9	9	9	9	9	9	9	9	9	9
9	2	9	9	9	9	9	9	9	9
9	9	9	9	9	9	9	9	9	9
3	3	3	3	3	3	3	3	3	3
1	2	3	1	2	3	1	2	3	1
3	3	3	3	2	3	2	2	2	2
2	2	2	2	2	2	2	2	2	2
2	2	2	2	2	3	3	3	3	3
1	1	2	2	2	3	9	2	2	1
1	3	3	1	2	2	3	3	2	2
2	2	2	2	2	2	2	2	2	2
2	2	2	2	2	2	2	2	2	2
9	9	9	9	9	9	9	9	9	9
9	9	9	9	9	9	9	9	9	9
9	9	9	9	9	9	9	9	9	9
9	9	9	9	9	9	9	9	9	9
9	9	9	9	9	9	9	9	9	9
9	9	9	9	9	9	9	9	9	9
2	9	9	9	9	2	9	9	9	9
9	9	9	9	9	9	9	9	9	9
9	2	9	2	9	9	2	2	9	2
9	9	9	9	9	9	9	9	9	9
2	2	2	2	2	2	2	2	2	2
9	9	9	9	9	9	9	9	9	9
9	9	9	9	9	9	9	9	9	9
9	9	9	9	9	9	9	9	9	9
2	2	2	2	2	2	2	2	2	2
2	3	2	3	2	3	3	2	2	9

2	2	2	2	2	2	2	2	2	2
2	2	2	2	2	2	2	2	2	1
2	2	2	2	2	2	2	2	2	2
3	1	3	2	1	2	1	1	1	1
3	3	3	3	3	3	3	3	3	3
1	1	2	2	2	1	1	1	2	2
2	2	2	2	2	2	2	2	2	2
3	3	3	3	3	3	3	3	3	3
9	9	9	9	9	9	9	9	9	9
2	2	2	2	2	2	2	2	2	2
9	9	9	2	9	9	9	9	2	9
1	9	1	9	3	1	9	3	1	9
2	2	2	2	2	2	2	2	2	2
2	1	2	1	2	3	1	2	3	2
2	2	2	2	2	2	2	2	3	3
2	2	2	2	2	2	2	2	2	2
1	2	2	1	2	3	3	3	2	2
1	1	3	2	2	3	3	3	2	3
9	9	9	9	9	9	9	9	9	9
2	3	1	1	2	1	1	2	3	2
3	3	3	2	2	1	2	2	2	2
1	1	2	2	3	2	1	1	2	2
2	1	2	2	1	1	3	2	3	2
2	2	3	2	2	3	1	1	2	3
1	1	3	1	1	2	2	2	2	1
1	1	2	2	2	2	2	2	2	2
1	1	2	2	2	2	2	1	2	1
2	3	2	2	2	3	2	2	3	2
2	2	2	2	2	2	2	2	2	2
2	2	2	2	2	2	3	2	1	2
3	3	3	3	3	3	3	3	3	3
9	9	9	9	9	9	9	9	9	9
3	3	3	3	3	3	3	3	3	3
2	2	2	2	2	2	2	2	2	2
9	9	9	9	9	9	9	9	9	9
2	3	2	3	2	2	2	2	2	3
2	2	2	1	1	1	1	2	2	2
1	1	1	1	1	1	1	1	1	1
2	2	1	1	1	1	1	1	2	2
9	9	9	9	9	9	9	9	9	9
2	2	2	2	2	2	2	2	2	2
9	9	9	9	9	9	9	9	9	9
3	1	2	1	3	3	2	2	3	2
2	2	2	2	2	2	2	2	2	2
2	3	2	3	2	3	2	3	2	3
9	9	9	9	9	9	9	9	9	9
2	3	3	3	2	3	2	3	3	2

9	9	9	9	9	9	9	9	9	9
3	3	3	3	3	3	3	3	3	3
1	1	1	3	3	1	3	1	1	1
3	3	3	3	2	3	2	3	2	2
1	1	2	2	2	2	3	3	3	1
9	9	9	9	9	9	9	9	9	9
2	2	2	2	2	2	2	2	2	2
3	3	3	3	3	3	2	1	1	2
1	1	3	1	1	2	2	1	1	2
2	2	2	2	2	2	1	2	2	2
1	3	3	2	2	2	2	2	2	2
2	2	2	2	2	2	2	2	2	2
9	9	9	9	9	9	9	9	9	9
3	3	2	2	2	2	3	2	2	3
1	2	3	2	2	2	3	2	1	1
2	2	2	2	2	2	2	2	2	2
2	3	3	3	1	3	3	3	3	3
2	3	9	2	3	2	3	2	3	2
2	2	2	2	2	2	2	2	2	2
9	9	9	9	9	9	9	9	9	9
9	9	9	9	9	9	9	9	9	9
1	1	2	1	2	1	2	1	2	1
2	2	2	2	2	2	3	2	3	3
2	3	2	3	2	3	2	3	2	3
9	9	9	9	9	9	9	9	9	9
2	2	3	2	1	2	2	3	1	3
1	9	9	9	9	9	9	9	9	9
2	2	2	2	2	2	2	2	2	2
2	2	2	2	2	2	2	2	2	2
2	2	2	2	2	2	2	2	2	2
3	2	1	2	3	2	1	2	1	3
9	9	9	9	9	9	9	9	9	9
9	9	9	9	9	9	9	9	9	9
1	2	2	3	2	2	2	1	2	1
2	3	3	2	2	3	1	1	1	2
9	9	9	9	9	9	9	9	9	9
9	9	9	9	9	9	9	9	9	9
2	2	2	2	3	2	3	2	3	2
2	1	2	1	1	3	3	2	1	3
1	1	2	2	2	1	2	1	1	2
2	2	2	2	2	2	2	2	2	2
2	2	2	2	2	2	2	2	2	2
2	2	2	2	2	2	2	2	2	2
2	2	2	2	2	2	2	2	2	2
1	1	2	3	2	3	3	2	1	1
2	2	3	3	2	3	2	2	3	2
2	2	2	2	2	2	2	2	2	2

2	3	2	1	1	1	3	3	3	3
1	1	3	2	2	2	2	2	1	1
2	2	2	2	2	2	2	2	2	2
2	2	1	2	3	2	1	3	1	1
2	2	2	2	2	2	2	2	2	2
1	1	3	3	2	3	3	2	1	1
1	1	3	3	2	3	2	2	1	1
2	2	2	2	2	2	2	2	2	2
2	2	2	2	2	2	2	2	2	2
9	9	9	9	9	9	9	9	9	9
3	3	3	3	3	3	3	3	3	3
1	2	1	1	1	2	3	1	2	1
1	2	2	3	2	1	2	1	1	1
2	1	1	2	2	1	1	2	2	2
3	3	1	3	3	3	1	2	3	9
9	9	9	9	9	9	9	9	9	9
9	9	9	9	9	9	9	9	9	9
9	9	9	9	1	9	9	9	3	9
9	9	9	9	9	9	9	9	9	9
2	9	9	9	9	9	9	9	9	9
3	1	2	9	3	2	2	1	1	1
9	9	9	9	9	9	9	9	9	9
9	9	9	9	9	9	9	9	9	9
2	2	2	2	2	2	2	2	2	2
1	9	1	1	1	1	1	1	1	1
2	2	2	2	2	2	2	2	2	2
3	3	1	1	2	3	3	3	2	2
1	1	3	2	3	2	3	2	9	9
1	2	3	1	1	2	2	1	1	2
2	3	1	2	3	1	2	2	2	3
9	9	9	9	9	9	9	9	9	9
1	3	1	2	1	2	3	2	1	2
2	1	1	2	3	2	3	1	2	3
3	1	1	1	1	1	1	2	2	2
2	2	2	2	2	2	1	1	3	3
2	2	2	2	2	2	2	2	2	2
9	9	9	9	9	9	9	9	9	9
2	2	2	2	2	2	2	2	2	2
2	2	2	2	2	2	2	2	2	2
2	3	3	3	2	3	2	1	1	1
3	3	3	3	3	3	3	3	3	9
2	3	2	1	2	2	3	1	2	1
2	1	1	1	1	2	2	2	2	1
1	1	1	1	2	2	2	2	3	3
9	3	2	3	1	3	2	1	2	3
1	2	3	2	1	1	1	1	1	1
1	1	2	2	2	3	3	3	3	3

3	1	2	2	1	1	1	2	2	2
2	2	2	2	2	2	2	2	2	2
2	2	2	2	2	2	3	3	3	3
2	2	2	2	2	2	2	2	2	2
3	3	2	3	3	2	3	3	2	3
9	9	9	9	9	9	9	9	9	9
9	9	9	9	9	9	9	9	9	9
3	3	3	3	3	3	3	3	3	3
9	9	9	9	9	9	9	9	9	9
9	9	9	9	9	9	9	9	9	9
2	2	2	2	2	2	2	2	2	2
1	1	2	2	2	2	1	2	1	1
2	2	1	1	1	2	2	2	2	2
9	1	9	9	9	9	9	9	9	9
9	9	9	9	9	9	9	9	9	9
3	9	3	3	1	3	3	3	1	1
1	1	1	2	2	2	2	2	2	1
9	9	9	9	9	9	9	9	9	9
1	1	2	2	2	2	2	2	2	1
2	2	2	2	2	2	2	2	2	2
2	2	3	2	2	3	2	3	2	2
9	9	9	9	9	9	9	9	9	9
2	2	2	2	2	2	2	2	2	2
2	2	2	2	2	2	2	2	2	2
2	2	2	2	2	2	2	2	2	2
2	2	2	2	2	2	2	2	2	2
2	2	2	2	2	2	2	2	2	2
2	2	2	2	2	2	2	2	2	2
9	2	9	1	2	3	2	3	2	3
2	2	2	2	2	2	2	2	2	2
2	1	2	2	3	1	2	2	1	2
2	3	2	3	2	2	3	3	2	2
2	2	1	2	1	1	2	2	3	3
2	2	2	2	2	2	2	2	2	2
1	1	2	2	1	1	1	1	1	1
1	2	2	2	3	3	1	1	2	2
2	2	2	2	2	2	2	2	2	2
2	2	2	2	2	2	2	2	2	2
1	1	9	1	9	2	3	2	3	3
9	2	2	2	2	3	2	2	2	2
3	3	3	1	1	3	3	1	1	1
1	1	2	1	1	1	2	3	1	1
2	2	2	2	2	2	2	2	2	2
2	2	2	2	2	2	2	2	2	2
2	2	2	2	2	2	2	2	2	2
2	2	2	3	2	2	2	2	2	1
2	2	2	2	2	2	2	2	2	2

1	1	2	1	3	2	1	3	1	3
2	2	3	2	3	2	2	3	1	1
2	3	2	3	2	3	2	3	2	3
2	2	2	2	2	2	2	2	2	2
9	9	9	9	9	9	9	9	9	9
9	9	1	9	9	9	9	9	9	9
3	2	3	2	3	2	3	2	2	3
9	9	9	9	9	9	9	9	9	9
9	9	9	9	9	9	9	9	9	9
9	9	9	9	9	9	9	9	9	9
9	9	9	9	9	3	9	9	9	9
2	9	9	9	9	9	9	9	9	9
9	9	9	9	9	9	9	9	9	9
9	9	9	9	9	1	9	9	9	9
1	1	3	2	2	2	1	1	2	1
9	9	9	9	9	9	9	9	9	9
9	9	9	9	9	9	9	9	9	9
9	9	9	1	9	9	9	9	9	9
9	9	9	1	9	9	9	9	9	9
9	2	9	9	9	9	9	9	9	9
9	9	9	9	9	9	9	9	9	9
2	2	2	2	2	2	2	2	2	2
2	2	2	2	2	2	2	2	2	2
3	3	3	3	2	2	1	1	1	2
2	2	2	2	3	1	3	1	2	1
9	9	9	9	9	9	9	9	9	9
1	2	2	2	1	3	2	2	2	2
9	9	9	2	9	9	9	9	9	9
1	1	1	1	2	2	2	3	3	3
1	1	3	1	1	1	3	1	1	1
9	1	9	2	3	2	1	3	1	1
9	9	9	9	9	9	9	9	9	9
2	2	2	2	2	2	2	2	2	2
2	9	9	9	9	9	9	9	9	9
9	9	9	9	9	9	9	9	9	9
3	9	1	1	1	1	2	1	1	1
3	2	1	1	1	1	1	1	1	1
9	9	9	9	9	9	9	9	9	9
9	9	9	9	9	9	9	9	9	9
2	2	2	2	2	2	2	2	2	2
9	9	9	9	9	9	9	9	9	9
2	2	2	2	2	2	2	2	2	2
3	3	3	3	3	3	3	3	3	3
9	2	9	9	9	9	9	9	2	9
2	2	2	2	2	2	2	2	2	2
2	2	2	2	2	2	2	2	2	2
9	9	9	9	9	9	9	9	9	9

9	9	9	9	9	9	9	9	9	9
9	9	2	9	9	2	9	2	9	9
9	9	9	9	9	9	9	9	9	9
2	2	2	2	2	2	2	2	2	2
9	9	9	2	1	9	9	9	9	9
3	2	1	3	1	3	1	9	3	1
9	9	9	9	9	9	9	9	9	9
9	9	9	9	9	9	9	9	9	9
9	9	2	9	9	9	2	9	9	2
2	2	2	2	2	2	2	2	2	2
9	9	9	9	9	9	9	9	9	9
9	2	9	9	2	9	9	2	9	9
2	2	2	2	2	2	2	2	2	2
9	9	9	9	9	9	9	9	9	9
2	2	2	2	2	2	2	2	2	2
9	9	9	9	9	9	9	9	9	9
9	9	9	9	9	9	9	9	9	9
9	9	9	9	9	9	9	9	9	9
9	9	9	9	9	9	9	9	9	9
9	9	9	9	9	9	9	9	9	9
2	2	2	2	2	2	2	2	2	2
2	2	2	2	2	2	2	2	2	2
2	3	2	1	1	2	3	1	2	2
2	2	3	3	2	1	2	3	1	3
2	2	2	2	3	3	2	2	3	3
2	2	2	2	2	2	2	2	2	2
1	1	1	1	1	1	1	1	1	1
2	2	2	2	2	2	2	2	2	2
3	1	1	1	1	2	3	2	1	2
2	2	2	2	2	2	2	2	2	2
2	2	2	3	3	3	3	3	2	2
2	3	3	2	1	2	2	1	3	2
1	1	2	2	2	2	2	2	2	2
9	9	9	9	9	9	9	9	9	9
3	2	1	1	2	2	3	2	1	2
2	2	2	2	2	2	2	2	2	2
2	2	2	2	2	2	2	2	2	2
1	1	1	1	1	1	1	1	1	1
3	1	2	3	2	3	2	3	2	3
1	3	1	3	2	1	2	3	1	2
1	2	2	2	2	1	1	1	1	1
9	9	9	9	9	9	9	9	9	9
2	2	3	9	9	9	9	9	9	9
9	9	9	9	9	9	9	9	9	9
1	2	1	2	1	2	1	2	1	2
2	2	2	2	2	2	2	2	2	2
9	9	9	9	9	9	9	9	9	9

2	2	2	2	2	2	2	1	2	1
2	2	2	1	1	1	2	2	2	1
2	2	2	2	9	3	9	1	1	1
2	2	2	2	2	2	2	2	2	2
1	9	1	9	1	9	9	9	9	1
1	1	1	1	1	1	1	2	2	2
1	1	1	1	1	1	2	2	3	3
1	1	1	1	1	3	3	3	1	1
3	3	3	3	3	3	3	3	3	3
1	1	1	2	1	1	3	1	2	1
9	9	9	9	9	9	9	9	9	9
2	2	2	2	2	2	2	2	2	2
2	2	2	2	2	2	2	2	2	2
2	2	2	2	2	2	2	2	2	2
2	2	2	2	2	2	2	2	2	2
1	1	1	1	1	1	1	1	1	1
1	1	1	2	1	2	1	1	1	1
1	1	2	2	2	2	2	1	1	1
1	1	1	1	1	1	1	1	1	1
9	9	9	9	9	9	9	9	9	9
1	1	1	2	2	1	1	2	2	1
2	2	2	9	9	2	2	2	9	9
9	9	9	9	9	9	9	9	9	9
1	1	1	1	1	1	1	1	1	1
9	9	9	9	9	9	9	9	9	9
2	2	2	1	1	1	1	1	2	1
1	2	1	1	1	1	1	1	2	9
1	1	1	1	1	1	1	1	1	1
9	9	9	9	9	9	9	9	9	9
2	2	9	2	9	2	2	2	2	2
9	9	9	9	9	9	9	9	9	9
9	9	9	9	9	9	9	9	9	9
2	2	2	2	2	2	2	2	2	2
2	2	9	2	2	2	2	2	2	2
2	2	2	2	2	2	2	2	2	2
9	9	9	9	9	9	9	9	9	9
1	1	1	1	1	1	1	1	1	1
1	1	2	1	1	1	1	1	1	1
9	9	9	9	9	9	9	9	9	9
9	9	9	9	9	9	9	9	9	9
9	2	9	2	2	9	2	2	2	9
9	9	9	9	9	9	9	9	9	9
2	9	2	2	2	2	2	9	2	2
9	9	9	9	9	9	9	9	9	9
1	1	1	1	1	1	1	2	1	2
2	2	9	9	2	9	2	9	2	9
9	1	1	9	9	1	9	1	1	1

1	1	1	2	1	1	1	1	2	2
2	2	9	2	2	2	9	2	9	2
2	2	2	2	2	2	2	2	2	2
1	1	2	2	2	1	1	1	1	1
2	2	2	2	2	2	2	2	2	2
9	9	9	9	9	9	9	9	9	9
1	1	1	1	1	1	1	1	1	1
2	2	2	2	2	2	2	2	2	2
2	9	2	9	2	9	9	2	9	9
1	1	9	1	1	9	1	1	2	2
9	9	9	9	9	9	9	9	9	9
9	9	9	9	9	9	9	9	9	9
9	9	9	9	9	9	9	9	9	9
9	9	9	9	9	9	9	9	9	9
9	9	9	9	9	9	9	9	9	9
9	9	9	9	9	9	9	9	9	9
2	2	2	2	2	2	2	2	2	2
9	9	9	9	9	9	9	9	9	9
2	2	2	2	2	2	2	2	2	2
1	1	2	2	1	3	3	1	1	1
1	1	2	2	1	3	3	1	1	1
1	1	2	2	1	3	3	1	1	1
1	1	2	2	1	3	3	1	1	1
1	1	2	2	1	3	3	1	1	1
1	1	2	2	1	3	3	1	1	1
1	1	2	2	1	3	3	1	1	1
1	1	2	2	1	3	3	1	1	1
1	1	2	2	3	3	3	1	1	1
1	1	2	2	1	3	3	1	1	1
1	1	2	1	1	3	3	1	1	3
1	1	2	2	1	3	3	1	1	1
1	1	2	2	1	3	3	1	1	1
1	1	2	2	1	3	3	1	1	1
1	1	2	2	1	3	3	1	1	1
1	1	2	2	1	3	3	1	1	1
1	1	2	2	1	3	3	1	1	1
1	1	2	2	1	3	3	1	1	1
1	1	2	2	1	3	3	1	1	1
1	1	2	2	1	3	3	1	1	1
1	1	2	2	1	3	3	1	1	1
1	1	2	2	1	3	3	1	1	1
1	1	2	2	1	3	3	1	1	1
1	1	2	2	1	3	3	1	1	1
1	1	2	2	1	3	3	1	1	1
9	9	9	9	9	9	9	9	9	9
9	9	9	9	9	9	9	9	9	9
9	9	9	9	9	9	9	9	9	9

9	9	9	9	9	9	9	9	9	9
9	9	9	9	9	9	9	9	9	9
9	9	9	9	9	9	9	9	9	9
9	9	9	9	9	9	9	9	9	9
9	9	9	9	9	9	9	9	9	9
9	9	9	9	9	9	9	9	9	9
9	9	9	9	9	9	9	9	9	9
9	9	9	9	9	9	9	9	9	9
9	9	9	9	9	9	9	9	9	9
9	9	9	9	9	9	9	9	9	9
9	9	9	9	9	9	9	9	9	9
9	9	9	9	9	9	9	9	9	9
9	9	9	9	9	9	9	9	9	9
9	9	9	9	9	9	9	9	9	9
9	9	9	9	9	9	9	9	9	9
9	9	9	9	9	9	9	9	9	9
9	9	9	9	9	9	9	9	9	9
9	9	9	9	9	9	9	9	9	9
9	9	9	9	9	9	9	9	9	9
1	1	2	2	1	3	3	1	1	1
1	1	2	2	1	3	3	1	1	1
1	1	2	2	1	3	3	1	1	1
1	1	2	2	1	3	3	1	1	1
1	1	2	2	1	3	3	1	1	1
1	1	2	2	1	3	3	1	1	1
1	1	2	2	1	3	3	1	1	1
1	1	2	2	1	3	3	1	1	1
1	1	2	2	1	3	3	1	1	1
1	1	2	1	1	3	3	1	1	1
1	2	2	3	3	9	1	1	1	1
1	1	2	2	1	3	3	1	1	1
1	2	2	1	1	3	3	1	1	1
1	1	2	2	1	3	3	1	1	1
1	2	2	2	1	3	3	1	1	1
1	1	2	2	1	3	3	1	1	1
9	9	3	9	9	3	1	9	1	1
1	1	2	2	1	3	3	1	1	1
1	2	2	1	1	3	3	1	1	1
1	2	2	1	1	3	3	1	1	1
1	1	2	2	1	3	3	1	1	3
1	1	2	2	1	3	3	1	1	1
1	1	2	2	1	3	3	1	1	1
1	1	2	2	1	3	3	1	1	1
1	2	9	2	1	3	3	1	1	1
1	1	2	2	1	3	3	1	1	1
1	1	2	2	1	3	3	1	1	1
1	1	2	2	1	3	3	1	1	1

1	1	2	2	1	3	3	1	1	1
1	1	2	2	1	3	1	9	9	9
1	1	2	2	1	3	3	1	1	1
1	1	2	2	9	3	3	1	1	1
1	1	2	2	1	3	3	1	1	1
1	1	2	2	1	3	3	1	1	1
1	1	2	2	1	3	3	1	1	1
1	1	2	2	1	3	3	1	1	1
1	1	2	2	1	3	3	1	1	1
1	1	2	2	1	3	3	1	1	1
1	1	2	2	1	3	3	1	1	1
1	1	2	2	1	3	3	1	1	1
1	1	2	2	1	3	3	1	1	1
1	2	2	1	3	3	1	1	1	1
1	1	2	2	1	3	3	1	2	1
1	1	2	2	1	3	3	1	1	1
1	2	2	1	9	1	3	1	3	1
1	1	2	2	1	3	3	1	1	1
1	1	2	2	1	3	3	1	1	1
1	1	2	2	1	3	3	1	1	1
1	1	2	2	1	3	3	1	1	1
1	1	2	2	3	3	1	1	3	1
1	1	2	2	3	3	1	1	1	1
1	1	2	2	1	3	3	1	1	1
1	1	2	2	1	3	3	1	1	1
1	1	2	2	1	3	3	1	1	1
1	1	2	2	1	1	3	1	1	1
1	1	2	2	1	3	3	1	1	1
1	1	2	2	1	3	3	1	1	1
1	1	9	1	1	3	3	1	1	1
1	1	2	2	2	3	3	1	1	1
1	1	2	2	1	3	3	1	1	1
9	9	9	9	9	3	3	1	9	1
1	1	2	2	1	3	3	1	1	1
9	9	9	9	9	9	9	9	9	9
1	1	2	2	1	3	3	1	1	1
1	1	2	2	1	3	3	1	1	1
1	1	2	2	1	3	3	1	1	1
1	1	2	2	1	3	3	1	1	1
1	1	9	9	9	9	9	9	9	9
1	1	2	2	1	3	3	1	1	1
1	1	2	2	1	3	1	1	1	1
1	1	2	2	1	3	3	2	1	1
1	1	2	2	1	3	3	1	1	1
1	1	2	2	1	3	3	1	1	1
1	1	2	2	1	3	3	1	1	1
1	1	2	2	1	3	3	1	1	1

1	1	2	2	1	3	3	1	1	1
1	1	2	2	1	3	3	1	1	1
1	1	2	2	1	3	3	1	1	1
1	1	2	2	1	3	3	1	1	1
1	1	2	2	1	3	3	1	1	1
1	1	2	2	1	3	3	1	1	1
1	1	2	2	1	3	3	1	1	1
1	1	2	2	1	3	3	1	1	1
1	1	2	2	1	3	3	1	1	1
1	1	2	2	1	3	3	1	1	1
1	1	2	2	1	3	3	1	1	1
1	1	2	2	1	3	1	1	1	1
1	1	2	2	1	3	3	1	1	1
1	1	2	2	1	3	3	1	1	1
1	2	1	2	1	3	3	1	1	1
1	1	2	2	1	3	3	1	1	1
1	1	2	2	1	3	3	1	1	1
1	1	2	2	1	3	3	1	1	1
1	1	2	2	1	3	3	1	1	1
1	1	1	2	3	3	9	1	1	1
1	1	2	2	1	3	3	1	1	1
1	1	2	2	1	3	3	1	1	1
1	1	2	2	1	3	3	1	1	1
1	1	2	3	1	3	3	1	1	1
1	1	2	2	1	3	3	1	1	1
1	9	1	2	2	2	9	1	1	1
1	1	2	2	1	3	3	1	1	1
1	1	2	2	1	3	3	1	1	1
1	1	2	2	1	3	3	1	1	1
1	1	2	2	1	3	3	1	1	1
1	1	2	2	2	3	3	1	1	1
1	1	2	2	1	3	3	1	1	1
1	1	1	2	1	3	3	1	1	1
1	1	2	2	1	3	3	1	1	1
1	1	2	2	1	3	3	1	1	1
1	1	2	1	3	3	9	1	1	1
1	1	2	2	1	3	3	1	1	1
1	1	2	2	1	3	3	1	1	1
1	1	2	2	1	3	3	1	1	1
1	1	2	2	1	3	3	1	1	1
1	1	2	2	1	3	3	1	1	1
9	9	9	9	9	9	9	9	9	9
1	1	2	2	1	1	3	3	2	9
1	1	2	2	1	3	3	1	1	1
1	1	2	2	1	3	3	1	1	1
1	2	2	1	3	3	1	1	1	1
1	2	2	1	3	3	1	1	3	3

1	1	2	3	1	3	3	1	1	1
1	1	2	2	1	3	3	1	1	1
1	1	2	2	1	3	3	1	1	1
1	1	2	2	1	3	3	1	1	1
1	1	2	2	1	3	3	1	1	1
1	1	2	2	1	3	3	1	1	1
1	1	2	2	3	3	3	1	1	1
1	1	2	2	1	3	3	1	1	1
1	1	2	1	1	3	3	1	1	1
9	9	9	9	9	9	9	9	9	9
1	1	2	2	1	3	3	1	1	1
1	1	2	1	1	3	3	1	1	1
1	1	2	2	1	3	3	1	1	1
1	1	2	2	1	3	3	1	1	1
1	1	2	2	1	3	3	1	1	1
1	1	2	2	1	3	3	1	1	1
1	1	2	2	1	3	3	1	1	1
1	1	2	2	1	3	3	1	1	1
1	1	2	2	1	3	3	1	1	1
1	1	2	2	1	3	3	1	1	1
1	1	2	2	9	3	1	1	1	1
1	2	1	1	2	3	3	1	1	1
1	1	2	2	1	3	3	1	1	1
1	1	2	2	1	3	3	1	1	1
1	1	2	2	1	3	3	1	1	1
1	1	2	2	1	3	3	1	1	1
1	1	2	2	1	3	3	1	1	1
9	9	9	9	9	9	9	9	9	9
9	9	9	9	9	9	9	9	9	9
9	9	9	9	9	9	9	9	9	9
9	9	9	9	9	9	9	9	9	9
9	9	9	9	9	9	9	9	9	9
9	9	9	9	9	9	9	9	9	9
9	9	9	9	9	9	9	9	9	9
9	9	9	9	9	9	9	9	9	9
9	9	9	9	9	9	9	9	9	9
9	9	9	9	9	9	9	9	9	9
9	9	9	9	9	9	9	9	9	9
9	9	9	9	9	9	9	9	9	9
9	9	9	9	9	9	9	9	9	9
9	9	9	9	9	9	9	9	9	9
9	9	9	9	9	9	9	9	9	9
9	9	9	9	9	9	9	9	9	9
9	9	9	9	9	9	9	9	9	9
9	9	9	9	9	9	9	9	9	9
9	9	9	9	9	9	9	9	9	9
9	9	9	9	9	9	9	9	9	9

9	9	9	9	9	9	9	9	9	9
9	9	9	9	9	9	9	9	9	9
9	9	9	9	9	9	9	9	9	9
9	9	9	9	9	9	9	9	9	9
9	9	9	9	9	9	9	9	9	9
9	9	9	9	9	9	9	9	9	9
9	9	9	9	9	9	9	9	9	9
9	9	9	9	9	9	9	9	9	9
9	9	9	9	9	9	9	9	9	9
9	9	9	9	9	9	9	9	9	9
9	9	9	9	9	9	9	9	9	9
9	9	9	9	9	9	9	9	9	9
9	9	9	9	9	9	9	9	9	9
1	2	2	1	1	1	1	1	2	9
2	2	2	2	2	2	2	2	2	2
2	2	2	2	2	2	2	2	2	2
1	9	1	2	1	1	1	1	3	3
2	1	2	2	2	1	2	2	2	1
2	2	2	2	2	2	2	2	2	2
1	3	2	1	1	1	2	2	2	2
2	2	1	1	2	2	2	2	1	1
3	2	3	3	3	3	3	3	3	3
9	9	9	9	9	9	9	9	9	9
1	2	3	2	3	3	3	3	3	3
9	9	9	1	9	9	9	9	9	9
2	2	2	2	3	3	3	3	3	2
9	9	9	9	9	9	9	9	9	9
1	1	1	1	1	1	1	1	1	1
1	1	2	3	2	2	2	1	3	2
2	2	2	2	2	2	2	2	2	2
1	2	2	1	3	3	3	1	1	1
1	2	3	2	1	2	1	2	3	1
9	9	9	9	9	9	9	9	9	9
1	1	2	1	1	2	1	2	2	2
3	9	1	1	1	1	9	9	1	1
9	9	9	9	9	9	9	9	9	9
2	2	1	1	1	1	1	3	3	3
1	3	3	3	1	1	1	2	2	3
1	1	2	1	2	1	2	2	1	2
2	1	3	1	2	2	2	1	2	3
9	9	9	9	9	9	9	9	9	9
9	9	9	9	9	9	9	9	9	9
1	1	2	2	2	2	2	2	2	2
1	1	1	1	1	1	1	1	1	1
9	9	9	9	9	9	9	9	9	9
9	9	9	9	9	9	9	9	9	9
9	9	9	9	9	9	9	9	9	9

9	9	9	1	9	9	9	9	9	9
9	9	9	9	9	9	9	9	9	9
3	2	2	3	2	3	2	1	2	3
2	2	2	2	2	2	1	2	1	1
3	3	3	3	2	2	1	1	1	1
9	9	9	9	1	9	9	9	9	9
2	2	2	2	2	2	2	2	2	2
2	2	2	2	2	2	2	2	2	2
2	2	2	2	2	2	2	2	2	2
3	3	3	3	1	1	1	1	2	2
2	3	2	3	3	3	3	3	3	2
9	9	9	9	9	9	9	9	9	9
9	9	9	9	9	9	9	9	9	9
2	2	2	2	2	2	2	2	2	2
2	2	2	2	2	2	2	2	2	2
2	2	2	2	2	2	2	2	2	2
3	1	2	3	1	2	3	1	2	3
2	2	2	2	2	2	2	2	2	2
9	1	9	1	1	1	1	1	9	1
9	9	9	9	9	9	9	9	9	9
2	2	2	2	2	2	2	2	2	2
2	2	2	2	2	2	2	2	2	2
2	2	2	2	2	2	2	2	2	2
2	2	2	2	2	2	2	2	2	2
2	2	2	2	2	2	2	2	2	2
2	2	2	1	1	3	3	1	1	3
2	2	2	2	2	2	2	2	2	2
2	2	2	2	2	2	2	2	2	2
9	9	9	9	9	9	9	9	9	9
1	1	2	1	2	1	3	1	1	1
1	1	3	3	2	3	3	3	2	2
1	1	2	2	2	2	2	2	2	2
2	2	2	1	1	2	1	1	1	1
2	2	1	1	1	2	2	2	2	2
2	2	2	2	2	2	2	2	2	2
2	2	2	1	1	3	3	3	3	2
3	2	2	2	2	2	2	2	2	2
2	2	1	1	1	9	9	9	9	9
9	9	9	9	9	9	9	9	9	9
2	2	2	2	2	2	2	2	2	2
2	2	2	2	2	3	3	3	3	3
2	2	2	2	3	3	2	2	3	2
3	1	2	3	1	2	3	1	2	3
9	9	9	9	9	9	9	9	9	9
1	1	1	1	1	1	1	1	1	1
3	3	3	3	3	3	9	2	2	2
3	3	3	2	2	3	3	2	2	3

2	2	2	2	2	2	2	2	2	2
1	1	2	3	2	3	2	2	2	3
3	3	3	3	3	3	3	3	3	3
2	3	2	3	3	3	2	1	2	3
2	2	2	2	2	2	2	2	2	2
2	2	2	2	2	2	2	2	2	2
2	2	2	2	2	2	2	2	2	2
3	3	3	3	2	2	1	1	1	2
2	2	2	2	2	2	2	2	2	2
2	3	2	3	2	1	2	3	1	1
2	2	2	2	2	2	2	2	2	2
2	2	2	2	2	2	2	2	2	2
2	2	2	2	2	2	2	2	2	2
3	3	3	3	3	2	3	3	3	3
2	2	2	2	2	2	2	2	2	2
2	2	3	2	3	2	3	2	3	2
2	2	2	2	2	2	2	2	2	2
2	2	2	2	2	2	2	2	2	2
2	2	2	2	2	2	2	2	2	2
3	1	2	3	1	2	3	1	2	3
1	2	1	2	1	2	1	2	1	2
1	1	1	1	1	1	2	2	2	2
2	2	2	2	2	2	2	2	2	2
1	1	1	3	3	3	3	3	1	1
1	1	1	1	3	3	9	1	1	1
9	9	9	9	9	9	9	9	9	9
9	9	9	9	9	9	9	9	9	9
2	2	2	2	2	2	2	2	2	2
9	9	9	9	9	9	9	9	9	9
2	3	1	1	1	3	3	2	1	2
2	2	2	2	2	3	3	2	2	2
2	1	2	3	1	2	3	2	2	2
9	9	9	9	9	9	9	9	9	9
1	1	1	1	1	1	1	1	1	1
1	1	2	9	1	1	2	2	2	3
2	2	2	2	2	2	2	2	2	2
9	9	9	9	9	9	9	9	9	9
2	2	1	2	1	1	1	2	2	2
9	9	9	9	9	9	9	9	9	9
2	2	2	2	2	2	2	2	2	2
2	3	2	3	2	3	2	2	3	2
1	1	2	1	1	1	2	2	2	1
2	2	3	1	2	2	1	2	2	3
9	9	9	9	9	9	9	9	9	9
9	9	9	9	9	9	9	9	9	9
9	9	9	9	9	9	9	9	9	9
1	1	1	1	1	2	2	2	2	2

9	9	9	9	9	9	9	9	9	9
9	9	9	9	9	9	9	9	9	9
2	3	2	1	2	2	2	2	2	2
9	9	9	9	9	9	9	9	9	9
9	9	9	9	9	9	9	9	9	9
3	3	3	3	3	3	3	2	2	2
2	3	3	2	2	1	1	2	3	2
9	9	9	9	9	9	9	9	9	9
1	1	2	1	1	1	2	1	1	1
3	1	2	3	1	2	3	1	2	3
3	1	2	3	1	2	3	1	2	3
2	2	2	2	2	2	2	2	2	2
2	2	2	2	2	2	2	2	2	2
9	9	9	9	9	9	9	9	9	9
2	2	2	2	2	2	2	2	2	2
2	2	2	2	2	2	2	2	2	2
1	1	1	1	1	1	1	1	1	1
2	2	1	2	3	3	2	2	1	2
2	3	3	2	1	1	1	2	2	2
9	9	9	9	9	9	9	9	9	9
2	2	1	2	3	3	2	2	3	2
3	3	2	9	1	9	1	3	2	1
2	2	2	2	2	2	2	2	2	2
2	2	2	1	2	3	3	2	2	3
3	3	2	3	3	3	2	2	3	3
9	9	9	9	9	9	9	9	9	9
3	3	3	1	3	3	1	2	1	2
9	9	9	9	9	9	9	9	9	9
3	3	3	3	3	3	3	3	3	3
2	1	2	2	2	1	1	2	3	2
2	2	2	2	2	2	2	2	2	2
9	9	9	9	9	9	1	9	9	9
9	9	9	9	9	9	1	9	9	9
2	2	2	2	2	2	2	2	2	2
2	2	2	1	1	3	3	3	9	2
3	2	1	2	3	2	1	3	1	2
2	2	2	9	9	9	9	9	9	9
2	2	3	3	3	2	2	1	1	1
2	2	2	2	2	2	2	2	2	2
9	9	9	9	9	9	9	9	9	9
1	1	2	2	2	1	2	2	1	2
2	2	2	2	2	2	2	2	2	2
2	2	2	1	1	2	2	2	2	2
2	2	2	2	2	2	2	2	2	2
2	2	2	2	2	2	2	2	2	2
9	9	9	9	9	9	9	9	9	9
3	2	2	2	2	2	2	2	2	2

1	1	3	1	1	1	1	1	1	1
2	2	2	2	2	2	2	2	2	2
3	3	3	3	3	3	3	2	2	2
2	2	2	2	2	2	2	2	2	2
2	2	2	2	2	2	2	2	2	2
2	2	2	2	2	2	3	3	3	3
3	3	3	1	1	2	2	2	1	1
2	3	2	1	2	3	2	1	2	1
1	1	2	1	1	2	1	1	1	1
2	2	3	3	3	3	2	2	1	1
2	2	3	2	3	2	2	2	2	2
2	2	2	2	2	2	1	1	1	1
2	2	2	2	2	2	2	2	2	2
1	1	2	1	1	2	3	1	2	3
1	9	9	2	9	1	2	1	9	1
2	2	2	2	2	2	2	2	2	2
2	9	2	9	2	2	2	2	3	2
2	2	2	1	1	9	2	9	1	2
2	3	2	2	2	3	2	2	2	2
1	1	2	2	2	2	1	1	1	1
1	1	2	1	1	1	1	1	1	1
9	9	9	9	9	9	9	9	9	9
2	2	2	2	2	2	2	2	2	2
2	2	2	2	2	2	2	2	2	2
2	2	9	1	2	9	2	2	2	2
2	2	2	2	2	2	2	1	1	1
1	1	2	2	2	1	2	2	2	2
1	1	2	2	2	2	2	2	2	2
1	1	1	9	3	3	3	1	3	1
1	1	1	1	1	3	1	1	1	2
1	1	1	3	1	3	3	1	1	1
1	2	1	2	1	2	1	2	1	2
2	2	2	2	2	1	1	1	3	2
9	9	9	9	9	9	9	9	9	9
9	9	9	2	9	9	9	9	9	9
1	1	1	3	1	3	3	1	1	1
2	2	2	2	2	2	2	2	2	2
1	1	1	2	3	2	2	1	1	1
1	1	1	1	1	9	2	2	2	2
2	2	2	2	2	2	2	2	2	2
2	2	3	2	3	2	3	2	2	3
9	9	9	9	9	9	9	9	9	9
9	9	9	9	9	9	9	9	9	9
1	1	1	1	1	1	1	1	1	1
2	3	3	3	2	2	2	2	2	2
9	9	9	9	9	9	9	9	9	9
9	9	9	9	9	9	9	9	9	9

2	2	2	2	2	2	2	2	2	2
3	3	3	2	2	2	1	1	1	1
3	1	2	3	1	2	3	1	2	3
2	2	2	2	2	2	2	2	2	2
2	2	2	1	1	1	1	1	1	3
3	1	2	3	1	2	3	1	2	3
1	2	2	2	1	2	1	2	2	1
9	9	9	9	1	9	9	9	9	9
2	2	3	3	3	1	1	2	1	3
9	9	9	9	9	9	9	9	9	9
1	1	1	1	1	1	1	1	1	1
1	1	2	2	2	1	2	1	1	1
1	1	1	1	1	2	2	2	2	1
9	9	9	9	9	9	9	9	9	9
1	1	1	1	1	1	1	1	1	1
9	9	9	9	9	9	9	9	9	9
9	9	9	9	9	9	9	9	9	9
2	2	2	2	2	2	2	2	2	2
2	2	2	2	2	2	2	2	2	2
2	1	2	2	1	2	1	2	2	2
2	2	2	2	2	2	2	2	2	2
2	2	2	2	2	2	2	2	2	2
2	2	2	2	2	2	2	2	2	2
1	3	2	1	2	3	2	1	2	3
3	9	3	3	3	3	9	3	3	3
9	9	9	9	9	9	9	9	9	9
2	2	2	2	2	2	2	2	2	2
1	1	1	9	2	2	2	2	2	2
1	1	1	1	1	1	1	1	1	1
1	1	3	2	2	1	1	1	1	1
1	1	1	2	2	2	2	2	2	2
2	2	2	2	2	2	2	2	2	2
2	2	2	2	2	2	2	2	2	2
2	2	2	2	2	2	2	2	2	2
2	2	2	2	2	2	2	2	2	2
2	2	2	2	2	2	2	2	2	2
2	1	3	1	3	1	2	3	2	1
9	9	9	9	1	9	9	9	9	9
9	9	9	9	2	9	9	9	9	9
2	2	2	2	2	2	2	2	2	2
3	3	3	3	3	3	3	3	3	2
9	9	9	9	9	9	9	9	9	9
2	2	2	2	2	2	2	2	2	2
9	9	9	9	9	9	9	9	9	9
3	3	3	3	3	3	3	3	3	3
9	9	9	9	9	9	9	9	9	9
2	2	2	2	2	2	2	2	2	2

2	1	2	1	3	1	3	2	3	2
9	9	9	9	9	9	9	9	9	9
2	2	2	2	2	2	2	2	2	2
1	1	2	1	2	2	2	2	2	2
2	2	2	2	2	2	2	2	2	2
2	3	2	2	2	2	2	2	2	2
2	2	1	1	1	1	1	1	1	1
9	9	9	9	9	9	9	9	9	9
2	2	2	2	2	2	2	2	2	2
2	2	2	2	2	2	2	2	2	2
2	2	2	2	2	2	2	2	2	2
2	2	2	2	2	2	2	9	2	2
2	2	2	2	2	2	2	2	3	3
3	2	2	2	3	3	3	2	2	1
1	3	2	2	1	3	2	2	3	1
1	9	3	1	2	1	1	1	1	1
2	2	2	2	2	2	2	2	2	2
2	2	2	2	2	2	2	2	2	2
9	9	9	9	9	9	9	9	9	9
9	9	9	9	9	9	9	9	9	9
1	1	2	1	2	1	2	1	1	1
2	2	2	2	2	3	2	1	1	1
9	9	9	9	9	9	9	9	9	9
9	9	9	9	9	9	9	9	9	9
3	3	3	3	3	3	3	3	3	3
9	9	9	9	9	9	9	9	9	9
9	9	9	9	9	9	9	9	9	9
9	9	9	9	9	9	9	9	9	9
1	1	1	3	3	1	2	2	2	3
2	2	2	2	2	2	2	2	2	2
2	2	2	2	2	2	2	2	2	2
1	9	2	2	1	1	1	1	1	1
1	1	2	3	1	2	3	3	2	1
2	2	2	2	2	2	2	2	2	2
2	2	2	2	2	2	2	2	2	2
1	2	1	1	1	2	1	1	1	1
1	1	2	2	1	2	3	3	3	1
3	9	2	3	9	2	3	1	2	1
1	1	2	3	1	1	2	1	1	3
1	1	1	1	2	3	3	1	1	2
1	1	2	2	3	3	2	2	1	1
2	2	2	2	2	2	2	2	2	2
9	9	9	9	9	9	9	9	9	9
9	9	1	9	9	9	9	9	9	9
1	2	3	2	1	2	2	3	1	2
2	2	2	2	2	2	2	2	2	2
2	2	2	2	2	2	2	2	2	2

2	2	9	1	1	1	1	3	3	3
9	9	9	9	9	9	9	9	9	9
9	9	9	9	9	9	9	9	9	9
9	9	9	9	9	9	9	9	9	9
9	9	9	9	9	9	9	9	9	9
9	9	9	1	9	9	9	9	9	9
9	9	9	9	9	9	9	9	9	9
9	9	9	9	9	9	9	9	9	9
9	9	9	9	9	9	9	9	9	9
9	9	9	9	9	9	9	9	9	9
2	2	2	2	2	2	2	2	2	2
3	2	1	2	3	3	2	3	2	2
1	1	2	1	1	2	1	1	1	1
2	3	2	2	2	2	2	2	2	2
2	2	2	2	2	2	2	2	2	2
9	9	9	9	9	9	9	9	9	9
2	2	2	2	2	2	2	2	3	3
2	2	2	2	2	2	2	2	2	2
2	2	2	2	2	2	2	2	2	2
2	2	2	2	3	2	3	3	2	2
3	2	2	3	1	3	1	2	2	1
2	3	1	2	3	1	1	3	2	1
2	2	2	2	2	2	2	2	2	2
2	2	2	2	2	2	2	2	2	2
2	2	2	2	2	2	2	2	2	2
2	2	2	2	2	2	2	2	2	2
3	3	3	3	3	3	3	3	3	3
1	2	2	2	2	2	2	2	1	1
2	2	2	2	2	2	2	2	2	2
2	2	2	2	2	2	2	2	2	2
9	9	9	9	9	9	9	9	9	9
2	2	2	2	2	2	2	2	2	2
2	1	1	1	1	2	2	2	3	3
9	9	9	9	9	9	9	9	9	9
9	9	9	9	9	9	9	9	9	9
2	2	2	2	2	2	2	2	2	2
2	2	2	2	2	2	2	2	2	2
2	2	2	2	2	2	2	2	2	2
3	3	3	2	3	3	3	3	2	1
9	9	9	9	9	9	9	9	9	9
2	1	1	1	1	2	2	3	3	1
2	2	2	2	2	2	2	2	2	2
2	2	2	2	2	2	2	2	2	2
1	1	1	2	1	1	2	1	2	1
1	1	1	1	2	2	2	1	1	2
9	9	9	9	9	9	9	9	9	9
9	9	9	9	9	9	9	9	9	9

9	9	9	9	9	9	9	9	9	9
1	1	1	2	2	3	3	2	2	1
9	9	9	9	9	9	9	9	9	9
9	9	9	9	9	9	9	9	9	9
9	9	9	9	9	9	9	9	9	9
9	9	9	9	9	9	9	9	9	9
9	9	9	9	9	9	9	9	9	9
9	9	9	9	9	9	9	9	9	9
9	9	9	9	9	9	9	9	9	9
9	9	9	9	9	9	9	9	9	9
9	9	9	9	9	9	9	9	9	9
9	9	9	9	9	9	9	9	9	9
9	9	9	9	9	9	9	9	9	9
9	9	9	9	9	9	9	9	9	9
9	9	9	9	9	9	9	9	9	9
9	9	9	9	9	9	9	9	9	9
9	9	9	9	9	9	9	9	9	9
9	9	9	9	9	9	9	9	9	9
9	9	9	9	9	9	9	9	9	9
9	9	9	9	9	9	9	9	9	9
9	9	9	9	9	9	9	9	9	9
9	9	9	9	9	9	9	9	9	9
9	9	9	9	9	9	9	9	9	9
9	9	9	9	9	9	9	9	9	9
9	9	9	9	9	9	9	9	9	9
1	1	1	1	1	2	2	2	1	2
2	2	2	2	2	2	2	2	2	2
1	1	2	1	2	2	1	2	2	2
1	1	1	1	2	2	2	2	2	2
3	3	2	2	3	1	2	1	1	2
1	2	2	2	2	3	3	1	2	2
2	2	2	2	2	2	2	2	2	2
1	1	2	2	3	9	1	9	2	2
2	2	2	2	2	2	2	2	2	2
1	2	2	2	3	3	3	2	2	1
1	1	1	1	1	3	2	1	1	1
2	2	2	2	2	3	2	1	1	1
2	1	1	2	2	2	1	1	1	1
1	1	1	1	2	2	2	2	1	1
1	1	1	1	1	2	1	1	1	1
2	1	1	2	2	3	2	1	1	1
9	9	9	9	9	9	9	9	9	9
1	2	1	3	2	2	2	2	1	1
1	1	2	2	2	1	3	3	2	1
9	9	9	1	9	9	9	9	9	9
1	1	1	1	2	2	2	2	2	1
1	1	1	1	1	3	1	1	1	1
2	2	2	2	2	2	2	2	2	2
2	1	2	1	1	9	1	2	3	9

1	1	1	1	1	2	2	1	1	1
1	1	2	1	1	3	1	1	2	1
1	1	1	1	1	2	2	1	1	1
1	1	1	1	2	2	2	2	2	2
1	3	2	1	1	1	1	2	2	3
9	9	9	9	9	9	9	9	9	9
2	1	1	1	3	3	2	2	2	1
9	9	9	9	9	9	9	9	9	9
2	2	2	2	2	2	2	2	2	2
2	2	2	2	2	2	2	2	2	2
9	1	9	9	9	9	9	9	9	9
9	9	9	9	9	9	9	9	9	9
9	9	9	9	9	9	9	9	9	9
9	9	9	9	9	9	9	9	9	9
9	9	9	9	9	9	9	9	9	9
9	9	9	9	9	9	9	9	9	9
9	9	9	9	9	9	9	9	9	9
9	9	9	9	9	9	9	9	9	9
1	9	9	9	9	9	9	9	9	9
1	3	1	1	3	3	3	1	3	1
1	1	1	1	1	2	2	2	2	1
9	9	9	9	9	9	9	9	9	9
9	9	9	9	9	9	9	9	9	9
9	9	9	9	9	9	9	9	9	9
9	9	9	9	9	9	9	9	9	9
3	2	1	2	3	1	2	3	1	3
3	1	1	1	3	3	3	3	1	3
1	1	2	2	2	3	3	2	1	2
2	2	2	2	2	2	2	2	2	2
9	9	9	9	9	9	9	9	9	9
1	1	2	3	2	2	1	1	2	2
3	3	1	3	3	3	3	1	3	3
3	3	3	3	3	3	3	3	3	3
1	3	1	1	2	3	2	3	1	1
3	3	3	3	3	3	1	1	1	1
9	9	9	9	9	9	9	9	9	9
2	2	2	2	2	2	2	2	2	2
1	1	1	2	2	3	2	2	2	2
2	2	2	2	3	3	3	3	3	3
1	1	2	3	1	3	3	3	1	1
1	1	1	1	2	2	2	2	2	1
1	1	1	1	2	2	2	2	2	1
1	1	1	1	1	3	3	1	1	1
2	2	3	3	3	3	2	2	1	2
1	1	1	1	3	3	2	2	2	3
1	1	3	1	1	3	2	1	2	2
1	1	1	2	1	3	2	1	1	1

1	1	1	1	1	3	1	1	1	1
2	9	2	2	3	9	2	3	2	3
9	9	9	9	9	9	9	9	9	9
1	1	1	1	1	3	9	9	1	1
9	9	9	9	9	9	9	9	9	9
1	1	1	1	2	3	2	1	2	2
1	2	1	1	1	2	3	1	1	1
9	9	9	9	9	9	9	9	9	9
9	9	9	9	9	9	9	9	9	9
2	2	2	2	2	2	2	2	2	2
1	1	2	1	2	3	2	2	2	3
1	1	1	1	3	3	1	1	3	3
2	1	1	2	9	1	2	1	3	1
9	9	9	9	9	9	9	9	9	9
9	9	9	9	9	9	9	9	9	9
1	2	1	1	2	1	2	2	2	2
9	9	9	9	9	9	9	9	9	9
9	9	9	9	9	9	9	9	9	9
3	3	3	3	3	3	3	3	3	3
1	1	1	1	1	1	1	1	1	1
2	2	2	2	2	2	2	2	2	2
1	1	1	1	1	1	1	1	1	1
3	3	3	2	1	1	1	3	3	2
1	1	1	1	2	3	1	2	1	1
2	2	2	2	2	2	2	2	2	2
9	9	9	9	9	9	9	9	9	9
9	9	9	9	9	9	9	9	9	9
1	1	3	1	3	3	1	1	1	3
2	2	2	2	2	2	3	3	3	3
2	3	2	3	3	9	1	2	3	3
9	9	9	9	9	9	9	9	9	9
9	9	9	9	9	9	9	9	9	9
9	9	9	9	9	9	9	9	9	9
9	9	9	9	9	9	9	9	9	9
9	9	9	9	9	9	9	9	9	9
9	9	9	9	9	9	9	9	9	9
9	9	9	9	9	9	9	9	9	9
9	9	9	9	9	9	9	9	9	9
9	9	9	9	9	9	9	9	9	9
9	9	9	9	9	9	9	9	9	9
9	9	9	9	9	9	9	9	9	9
9	9	9	9	9	9	9	9	9	9
9	9	9	9	9	9	9	9	9	9
9	9	9	9	9	9	9	9	9	9
9	9	9	9	9	9	9	9	9	9
9	9	9	9	9	9	9	9	9	9
9	9	9	9	9	9	9	9	9	9

9	9	9	9	9	9	9	9	9	9
9	9	9	9	9	9	9	9	9	9
9	9	9	9	9	9	9	9	9	9
9	9	9	9	9	9	9	9	9	9
9	9	9	9	9	9	9	9	9	9
9	9	9	9	9	9	9	9	9	9
9	9	9	9	9	9	9	9	9	9
9	9	9	9	9	9	9	9	9	9
9	9	9	9	9	9	9	9	9	9
9	9	9	9	9	9	9	9	9	9
9	9	9	9	9	9	9	9	9	9
9	9	9	9	9	9	9	9	9	9
9	9	9	9	9	9	9	9	9	9
9	9	9	9	9	9	9	9	9	9
9	9	9	9	9	9	9	9	9	9
9	9	9	9	9	9	9	9	9	9
9	9	9	9	9	9	9	9	9	9
9	9	9	9	9	9	9	9	9	9
9	9	9	9	9	9	9	9	9	9
9	9	9	9	9	9	9	9	9	9
9	9	9	9	9	9	9	9	9	9
9	9	9	9	9	9	9	9	9	9
9	9	9	9	9	9	9	9	9	9
9	9	9	9	9	9	9	9	9	9
9	9	9	9	9	9	9	9	9	9
9	9	9	9	9	9	9	9	9	9
9	9	9	9	9	9	9	9	9	9
9	9	9	9	9	9	9	9	9	9
9	9	9	9	9	9	9	9	9	9
9	9	9	9	9	9	9	9	9	9
9	9	9	9	9	9	9	9	9	9
9	9	9	9	9	9	9	9	9	9
9	9	9	9	9	9	9	9	9	9
9	9	9	9	9	9	9	9	9	9
9	9	9	9	9	9	9	9	9	9
2	2	2	2	2	2	2	2	2	2
9	9	9	9	9	9	9	9	9	9
1	1	3	2	3	3	3	2	2	3
1	1	1	1	1	1	3	1	1	1
3	3	3	3	3	3	3	3	3	3
9	9	9	9	9	9	9	9	9	9
1	1	2	3	2	3	3	1	1	1
9	9	9	9	9	9	9	9	9	9
1	1	2	3	2	3	3	1	1	1
2	2	1	3	2	2	2	2	2	1
9	9	1	1	9	9	9	9	9	9
2	2	2	2	2	2	2	2	2	2

2	1	1	2	2	3	2	1	1	1
1	2	2	1	2	1	3	2	2	2
1	1	2	1	2	1	1	1	2	2
3	3	1	9	2	2	1	1	1	1
1	1	1	2	2	3	2	2	1	1
1	1	1	2	3	3	2	1	1	1
1	1	1	1	2	2	3	2	2	2
1	2	2	2	2	2	3	1	2	1
2	1	3	9	2	3	1	2	3	2
1	2	3	2	3	9	2	1	2	3
9	9	9	9	9	9	9	9	9	9
2	3	2	2	9	2	2	1	1	2
9	9	9	9	9	9	9	9	9	9
1	2	9	9	2	2	2	1	1	1
1	2	3	3	2	2	2	1	1	1
2	2	2	2	2	2	2	2	2	2
9	9	9	9	9	9	9	9	9	9
9	9	9	9	9	9	9	9	9	9
1	1	1	1	1	2	1	1	1	1
2	1	1	1	2	2	2	1	9	3
1	1	1	2	2	3	2	2	1	1
1	1	1	1	2	2	2	2	2	1
1	1	1	3	1	1	1	1	2	9
1	1	1	3	3	3	1	1	1	1
3	3	3	2	3	2	3	1	2	2
2	2	1	3	1	2	1	2	1	2
1	1	1	1	1	1	1	1	1	1
1	3	3	3	1	1	3	2	2	1
2	2	2	2	2	2	2	2	2	2
9	9	9	9	9	9	9	9	9	9
9	9	9	9	9	9	9	9	9	9
2	2	2	2	2	2	2	1	1	1
2	2	2	2	2	2	2	2	2	2
9	9	9	9	9	9	9	9	9	9
9	9	9	9	9	9	9	9	9	9
9	9	9	9	9	9	9	9	9	9
1	1	3	1	1	3	2	3	2	1
1	1	2	2	2	2	2	2	2	9
9	9	9	9	9	9	9	9	9	9
9	9	9	9	9	9	9	9	9	9
1	2	2	2	3	3	3	3	3	2
9	9	9	9	9	9	9	9	9	9
1	1	2	1	2	2	3	1	2	2
1	1	3	1	3	1	1	1	3	3
1	3	3	1	2	1	1	1	1	1
3	3	1	3	3	3	3	3	3	3
3	1	3	3	1	1	3	1	3	1

2	3	2	3	2	3	2	3	2	3
2	2	2	2	3	3	2	2	1	1
3	1	3	2	1	2	1	2	2	2
1	1	1	1	2	1	1	1	1	1
3	2	1	2	2	1	2	2	3	2
3	2	1	3	1	2	3	2	1	2
3	1	1	1	1	1	1	1	1	2
1	2	3	1	2	2	3	2	1	2
1	1	1	1	3	1	1	1	1	1
9	9	1	9	2	9	9	9	2	1
3	3	3	3	1	1	3	1	3	3
2	3	1	1	1	2	1	1	3	3
3	9	1	1	1	3	1	2	2	2
3	3	2	1	2	3	3	2	1	1
3	3	1	3	1	3	1	3	1	1
1	2	3	2	2	2	2	2	2	2
2	2	2	3	2	2	1	2	2	2
9	9	9	9	9	9	9	9	9	9
3	2	1	2	3	2	1	2	2	9
2	2	3	3	1	2	2	2	2	1
3	1	2	3	1	1	1	1	2	3
1	1	1	2	2	2	1	1	1	1
1	1	2	3	3	3	2	2	1	1
2	2	2	2	2	2	2	2	2	2
3	2	3	1	2	3	1	2	3	1
3	3	3	1	3	3	1	1	3	1
1	1	2	2	2	1	2	1	1	1
1	1	1	2	3	3	3	3	2	2
1	1	2	2	2	2	2	2	2	1
2	1	3	2	2	3	1	2	1	2
1	1	1	1	3	1	2	2	2	2
1	3	2	1	2	3	2	1	2	3
2	2	3	3	1	1	1	2	2	1
1	3	9	9	9	1	1	3	1	1
3	1	2	3	1	2	3	1	2	3
1	1	3	3	1	1	3	1	1	1
3	3	2	1	2	3	2	2	3	1
9	9	9	9	9	9	9	9	9	9
1	1	3	1	1	3	3	1	1	1
1	1	3	1	2	2	1	1	1	3
1	1	3	3	3	3	3	3	2	1
3	1	2	3	1	2	3	1	2	3
3	3	3	3	1	1	1	1	3	3
3	2	3	2	3	2	2	2	3	1
2	2	2	2	2	2	2	2	2	2
3	3	3	3	3	3	3	3	2	3
9	9	9	9	9	9	9	9	9	9

9	2	1	1	2	1	1	2	3	2
1	1	1	1	1	1	1	1	1	1
2	2	2	2	1	1	3	3	3	2
2	2	1	1	3	3	2	2	1	2
1	1	2	2	2	2	2	1	2	2
2	2	3	2	1	2	3	2	1	1
2	2	2	2	2	2	2	2	2	2
9	9	9	9	9	9	9	9	9	9
9	9	9	9	9	9	9	9	9	9
3	2	3	1	2	2	1	1	2	3
1	1	2	2	2	3	2	2	2	2
2	2	2	2	2	2	2	2	2	2
2	2	2	2	2	2	1	1	1	1
1	1	1	1	1	1	1	2	2	2
2	2	2	2	2	2	2	2	2	2
2	2	2	2	2	2	2	2	2	2
2	2	2	2	2	2	2	3	2	2
2	3	2	3	2	2	2	2	2	2
2	2	2	2	2	2	2	2	2	2
2	2	2	2	2	2	2	3	3	2
1	2	2	2	1	2	2	1	1	1
2	2	2	2	2	2	2	2	2	2
2	2	2	2	2	2	2	2	2	2
2	1	2	3	2	2	9	2	1	2
2	2	2	2	2	2	2	2	2	2
2	2	2	2	2	2	2	2	2	2
1	1	2	2	2	3	2	2	2	2
2	9	1	9	2	9	2	3	1	2
3	3	3	1	1	1	2	2	2	2
2	2	2	2	2	2	2	2	2	2
2	2	2	2	2	2	2	2	2	2
1	1	2	2	2	3	2	2	2	2
2	2	2	2	2	2	2	2	2	2
2	2	2	2	2	2	2	2	2	2
2	2	2	2	2	2	2	2	2	1
1	1	2	2	2	2	2	2	2	2
2	2	2	2	2	2	2	2	2	2
2	2	2	2	2	2	2	2	2	2
2	2	2	2	2	2	2	2	2	2
1	1	3	1	1	2	3	3	1	1
2	2	2	2	2	2	2	2	2	2
2	2	2	2	2	2	2	2	2	2
1	3	3	3	2	2	2	1	1	3
2	2	2	2	2	2	2	2	2	2
1	1	1	1	2	3	2	3	1	1
1	1	1	1	1	1	1	1	1	1
2	2	2	2	2	2	2	2	2	2

2	2	2	2	2	2	2	2	2	2
2	2	2	2	2	2	2	2	2	2
1	1	3	2	2	3	2	2	2	2
2	2	2	2	2	2	2	2	2	2
2	2	2	2	2	2	2	2	2	2
2	2	2	2	2	2	2	2	2	2
9	9	9	9	9	9	9	9	9	9
1	3	1	1	1	1	1	1	1	1
9	9	9	9	9	9	9	9	9	9
3	3	3	3	3	3	3	3	3	3
2	2	2	1	3	3	3	1	2	1
2	2	2	2	2	2	2	2	2	2
2	2	3	3	2	2	2	2	2	3
3	3	3	3	3	3	3	3	3	3
9	9	9	9	9	9	9	9	9	9
2	2	2	2	1	2	1	1	1	1
2	2	2	2	2	2	2	2	2	2
1	1	1	1	1	1	1	1	1	1
9	9	9	9	9	9	9	9	9	9
3	2	2	2	2	2	2	2	2	2
2	2	1	1	2	2	2	2	1	3
9	9	9	1	9	9	9	9	9	9
9	9	9	9	9	9	9	9	9	9
1	3	3	3	3	3	9	3	3	3
3	3	2	1	2	1	2	1	2	3
2	9	1	1	1	1	1	1	1	1
3	3	3	3	3	3	3	3	3	3
2	9	1	3	9	1	2	1	1	1
1	1	3	2	3	9	1	2	1	2
1	1	1	1	3	3	3	3	3	3
2	2	2	2	2	2	2	2	2	2
2	2	2	2	2	2	2	2	2	2
2	2	2	2	2	2	2	2	2	2
9	9	1	9	9	9	1	9	1	9
2	2	2	2	2	2	2	2	2	2
9	9	9	9	9	9	9	9	9	9
2	2	2	1	2	9	1	1	1	1
3	3	3	3	3	3	3	3	3	3
2	2	2	2	2	2	2	2	2	2
2	9	2	2	2	2	2	2	2	2
2	2	2	2	2	2	2	9	1	1
2	2	2	2	2	2	2	2	2	2
9	1	9	9	9	9	9	9	9	9
3	3	3	2	1	1	1	1	1	2
1	1	2	2	2	2	2	2	2	2
2	2	2	2	2	2	2	2	2	2
1	1	2	2	2	2	2	2	2	2

1	9	9	9	9	9	9	9	9	9
2	2	2	2	2	2	2	2	2	2
1	1	1	1	1	1	1	1	1	1
2	2	2	2	2	2	2	2	2	2
9	9	9	9	9	9	9	9	9	9
1	2	2	3	3	3	2	2	1	1
3	3	3	3	3	3	3	3	3	3
2	2	2	2	2	2	2	2	2	2
2	2	2	2	2	2	2	2	2	2
1	1	1	3	3	3	3	3	3	3
9	9	9	9	9	9	9	9	9	9
1	1	1	1	1	1	1	1	1	1
2	2	2	2	2	2	2	2	2	2
2	2	2	2	2	2	2	2	2	2
2	2	2	2	2	2	2	2	2	2
2	2	2	2	2	2	2	2	2	2
1	1	1	1	1	1	1	1	1	1
1	1	1	1	1	1	1	1	3	3
9	9	9	9	9	9	9	9	9	9
1	2	3	1	2	1	3	1	2	1
1	1	1	1	1	1	1	1	1	1
3	3	3	3	3	1	1	1	1	1
1	1	1	1	1	1	1	1	1	1
2	1	2	2	2	1	2	2	2	2
9	3	9	3	9	3	3	3	3	3
1	1	1	1	2	1	1	2	1	1
2	2	3	1	1	1	2	3	1	2
2	2	2	2	2	2	2	2	2	2
1	2	1	9	1	3	1	1	2	1
2	2	2	2	2	2	2	2	2	2
2	9	1	2	3	1	2	1	2	9
2	2	2	2	3	3	3	3	2	2
1	3	3	3	3	3	3	3	3	3
2	3	2	2	2	9	3	3	9	2
2	2	2	2	2	2	2	2	2	2
2	2	2	2	2	2	2	2	2	2
1	1	1	1	1	1	1	1	1	1
2	2	2	2	2	2	2	2	2	2
3	1	2	3	1	2	1	2	1	2
3	3	3	3	3	3	3	3	3	3
3	1	2	1	3	1	3	1	2	1
2	2	2	2	2	2	1	1	3	3
9	9	9	9	9	9	9	9	9	9
3	3	3	3	3	3	9	9	9	9
9	9	9	9	9	9	9	9	9	9
2	2	2	2	2	2	2	2	2	2
1	2	1	1	2	3	3	3	2	2

2	2	2	2	2	2	2	2	2	2
3	3	3	1	3	1	3	1	3	3
9	9	9	9	9	9	9	9	9	9
3	3	3	1	3	1	3	1	3	3
3	1	2	1	1	3	2	1	3	3
2	2	2	2	2	2	2	2	2	2
2	2	2	2	2	2	2	2	2	2
2	2	2	2	2	2	2	2	2	2
1	1	1	1	1	1	1	1	1	1
2	3	2	3	2	1	2	2	3	1
2	1	2	3	2	1	2	2	2	3
3	1	3	9	1	3	2	2	3	1
2	2	2	2	2	2	2	2	2	2
2	3	2	1	2	3	2	3	3	3
2	2	2	2	2	2	2	2	2	2
1	1	2	2	2	2	2	2	2	2
2	2	2	2	2	2	2	2	2	2
2	2	2	2	2	2	2	2	2	2
2	2	2	2	2	2	2	2	2	2
1	2	1	2	2	2	2	2	2	2
2	2	2	2	2	2	2	2	2	2
2	2	2	2	2	2	2	2	2	2
2	2	2	2	2	2	2	2	1	2
2	2	2	2	2	2	2	2	2	2
1	1	1	1	1	1	1	1	1	1
2	2	2	2	2	2	2	2	2	2
3	1	1	1	2	2	2	2	1	1
2	2	2	2	2	2	2	2	2	2
1	1	3	3	2	3	2	3	1	1
2	3	3	2	2	1	1	2	3	2
1	1	3	9	2	2	1	1	1	1
2	3	3	3	3	3	2	2	1	1
2	2	2	2	2	2	2	2	2	2
3	2	3	3	2	3	2	3	2	3
3	3	3	3	2	1	2	2	2	3
2	1	2	3	3	2	3	2	1	3
9	9	9	9	9	9	9	9	9	9
9	9	9	9	9	9	9	9	9	9
3	3	3	3	3	3	3	3	3	3
2	2	2	2	2	2	2	2	2	2
1	2	2	2	2	2	2	2	3	1
2	2	2	2	2	2	2	2	2	2
2	2	2	2	2	2	2	2	2	2
2	2	2	2	2	2	2	2	2	2
9	9	9	9	9	9	9	9	9	9
3	1	2	1	1	1	2	1	2	2
2	2	2	2	2	2	2	2	2	2

9	9	9	9	9	9	9	9	9	9
2	2	2	1	2	3	1	2	2	9
2	3	2	1	1	3	2	1	3	3
3	3	2	2	2	3	1	9	2	2
3	3	3	3	3	3	3	3	3	1
2	3	1	1	1	2	3	1	1	2
1	2	2	3	3	3	3	2	2	1
9	9	9	9	9	9	9	9	9	9
2	2	2	2	2	2	2	2	2	2
2	3	2	2	3	3	2	2	1	1
1	1	2	1	2	2	2	2	2	1
1	2	3	1	2	2	3	2	1	1
2	2	3	1	2	1	2	1	2	1
1	1	1	1	1	1	1	1	1	1
3	2	2	3	1	2	3	1	2	3
2	2	2	2	2	2	2	2	2	2
1	2	2	1	2	3	2	1	2	2
2	2	2	2	2	2	2	2	2	2
9	9	9	9	9	9	9	9	9	9
9	9	9	9	9	9	9	9	9	9
9	9	9	9	9	9	9	9	9	9
3	1	2	3	2	1	2	3	2	1
2	3	3	3	1	1	2	1	2	3
3	3	2	2	1	1	1	1	1	2
1	2	2	2	2	3	3	3	2	2
3	3	3	3	3	3	3	3	3	3
2	2	1	2	1	3	2	1	2	3
1	2	9	2	3	3	3	3	3	3
3	2	3	2	3	1	2	2	1	2
1	1	2	2	2	2	1	1	2	1
1	3	2	2	2	2	2	3	3	3
1	1	2	2	2	2	3	3	3	3
2	2	3	3	9	2	3	3	9	1
3	3	3	3	3	3	3	3	3	3
3	3	3	3	3	3	3	3	3	3
2	2	2	2	2	2	2	2	2	2
3	3	3	3	3	3	3	3	3	3
2	2	2	2	2	2	2	2	2	2
9	9	9	9	9	9	9	9	9	9
9	9	9	9	9	9	9	9	9	9
1	1	1	1	1	1	1	1	1	1
2	2	2	2	2	2	2	2	2	2
1	2	1	3	3	2	1	2	3	2
3	2	2	3	2	3	2	2	3	3
1	1	9	9	9	9	9	9	9	9
3	3	3	3	3	3	3	3	3	3
2	2	2	1	1	2	2	2	2	3

1	1	1	1	1	1	1	1	1	1
2	3	2	2	3	1	2	1	1	3
3	1	2	1	3	2	1	2	3	1
1	3	9	2	2	2	2	2	1	2
2	3	2	3	2	1	2	2	1	3
9	2	2	2	3	3	3	3	3	3
2	2	2	2	2	2	2	2	2	2
3	3	3	3	3	3	3	3	3	3
2	3	2	2	3	2	2	3	3	2
2	1	2	2	1	3	1	1	3	2
2	2	2	2	2	2	2	2	2	2
3	3	2	3	3	3	3	3	1	3
9	2	2	2	3	2	3	2	2	2
1	1	1	2	3	1	1	1	1	2
2	2	1	1	1	1	1	1	1	1
3	3	3	3	3	3	2	2	3	3
1	1	1	1	1	1	3	3	3	3
2	2	2	2	2	2	2	2	2	2
9	9	9	9	9	9	9	9	9	9
2	3	1	2	2	1	2	1	2	1
2	3	1	2	3	1	2	1	2	1
2	2	3	3	2	3	2	3	2	3
1	1	2	2	2	2	3	2	2	2
1	2	2	3	2	2	1	2	2	1
3	1	2	3	1	2	3	1	2	3
1	2	3	2	2	3	2	3	3	3
2	2	2	2	2	2	2	2	2	2
3	1	2	2	1	2	3	2	2	1
3	1	2	1	3	1	2	3	1	2
2	2	2	2	2	2	2	2	2	2
3	1	2	1	1	1	2	2	3	2
2	2	2	2	2	2	2	2	2	2
2	2	2	2	2	2	2	2	2	2
1	3	3	2	2	3	3	2	2	3
3	1	2	3	1	2	3	1	2	3
3	2	2	1	1	1	1	2	3	1
2	2	3	3	1	2	3	1	2	1
3	3	3	3	3	3	3	3	3	3
2	2	2	3	3	3	3	3	3	3
3	1	3	1	2	1	3	2	3	1
1	1	1	1	1	2	2	2	2	2
3	1	2	3	1	2	1	2	3	1
3	2	3	2	3	2	9	2	2	2
1	1	1	1	1	1	1	1	1	1
3	3	3	3	3	3	2	2	2	2
2	3	2	2	3	2	3	2	2	1
1	1	2	1	1	2	1	2	1	1

2	2	3	3	3	3	2	2	2	3
3	1	2	1	2	3	2	3	1	2
3	2	3	2	2	2	2	2	2	2
1	1	1	1	1	1	1	1	1	1
1	2	3	1	2	3	1	2	3	1
1	2	2	2	2	3	3	3	3	2
1	2	2	1	2	3	2	1	2	2
3	1	2	1	2	3	1	2	3	1
2	1	2	1	3	1	1	3	1	3
2	3	3	2	2	3	2	3	2	3
2	3	2	3	2	3	2	3	2	3
2	2	1	2	1	3	2	1	2	1
1	1	1	1	1	1	1	1	1	1
1	3	3	3	1	1	1	3	1	1
3	1	2	3	1	2	3	2	1	3
3	3	3	3	3	3	1	1	1	1
1	1	1	2	1	1	1	2	2	3
1	1	3	3	3	1	1	1	1	1
3	2	2	3	1	2	1	3	3	2
1	2	1	3	2	2	2	2	2	2
2	3	2	3	2	3	2	3	2	3
3	3	3	3	3	3	3	3	3	3
2	1	3	3	1	2	3	1	3	1
2	2	2	2	2	2	1	1	1	1
2	2	2	2	2	2	2	2	2	2
2	2	2	2	2	2	2	2	2	2
1	1	3	3	3	2	3	2	2	1
1	1	2	3	2	3	1	2	2	3
2	2	2	2	2	2	2	2	2	2
9	9	9	9	9	9	9	9	9	9
9	9	9	9	9	9	9	9	9	9
9	9	9	9	9	9	9	9	9	9
3	3	3	3	3	3	3	3	3	3
9	9	9	9	9	9	9	9	9	9
2	2	2	2	2	2	2	2	2	2
9	9	9	9	9	9	9	9	9	9
1	2	1	1	2	1	2	2	2	2
9	9	9	9	9	9	9	9	9	9
1	2	2	2	1	1	1	9	1	9
2	2	2	3	2	3	2	2	2	2
9	9	9	9	9	9	9	9	9	9
1	1	1	1	1	1	3	1	1	3
3	3	3	3	3	3	3	3	3	3
3	3	3	3	3	3	3	3	3	3
1	3	2	2	2	1	2	1	9	1
2	1	2	3	2	3	1	2	3	1
1	1	3	3	3	2	3	2	2	1

1	1	3	3	3	1	3	2	2	3
2	2	2	2	2	2	2	2	2	2
9	9	9	9	9	9	9	9	9	9
2	2	1	1	2	3	2	1	2	1
1	2	2	1	3	9	9	2	9	9
1	2	1	3	1	2	1	2	1	3
1	9	9	9	9	9	9	9	9	9
3	2	1	2	1	3	2	3	1	2
1	1	2	3	2	2	3	3	3	2
3	3	3	3	3	3	3	3	3	3
1	2	3	1	2	3	1	2	3	1
1	2	3	3	2	1	2	3	2	1
2	1	1	2	9	1	2	2	1	2
2	2	2	2	2	2	2	2	2	2
2	2	2	2	2	2	2	2	2	2
9	9	2	9	9	9	9	9	9	9
2	2	2	2	2	2	2	2	2	2
2	2	2	2	2	2	2	2	2	2
2	2	2	2	2	2	2	2	2	2
2	2	2	2	2	2	2	2	2	2
1	1	9	9	9	1	1	1	1	1
1	1	9	9	9	1	1	1	1	1
1	1	1	9	9	9	1	1	1	1
1	1	1	1	1	2	1	1	2	2
1	1	1	1	2	2	1	1	2	1
1	9	9	9	9	9	9	9	9	9
2	1	2	3	3	2	3	3	2	1
3	1	1	1	3	3	1	2	3	1
1	1	2	1	3	2	1	1	2	2
1	1	1	1	1	3	1	1	1	1
1	1	1	1	3	3	3	1	1	9
1	1	9	9	9	9	1	9	9	9
1	1	1	1	2	2	2	1	1	1
1	1	1	2	3	2	1	1	1	1
2	2	3	2	1	2	1	1	2	2
2	2	3	2	2	3	1	2	3	3
2	2	3	2	2	3	1	2	3	3
1	1	1	9	9	9	1	9	9	1
1	1	1	3	3	3	1	1	1	1
1	1	1	1	1	1	1	1	1	1
1	1	1	3	3	3	1	1	1	1
1	1	1	9	9	9	9	9	9	1
1	2	2	2	2	9	1	1	1	1
1	1	1	1	1	1	1	1	1	1
1	1	1	3	3	3	1	1	1	1
1	2	1	3	2	3	1	1	3	3
1	1	2	3	3	3	2	1	1	2

2	2	3	2	2	3	1	2	3	3
3	3	9	9	9	9	9	1	9	3
1	1	1	2	2	2	2	1	2	1
1	1	1	1	1	1	1	1	1	1
1	9	1	2	2	3	9	1	1	9
1	1	1	2	2	3	9	1	1	9
2	2	2	2	2	2	2	2	2	2
1	1	1	1	1	1	1	1	1	1
9	9	9	9	9	9	9	9	9	9
1	1	1	1	2	3	1	2	2	2
1	1	1	3	2	2	1	2	2	2
2	2	1	2	2	2	2	2	2	2
1	9	1	2	2	3	9	1	1	9
1	9	1	2	2	3	9	1	1	3
1	9	1	2	2	9	3	1	1	3
1	1	1	2	2	9	9	1	1	3
2	2	1	2	2	3	3	1	1	1
2	1	1	1	2	3	3	1	1	3
1	1	1	2	2	3	9	1	1	3
1	2	3	2	3	1	2	1	3	1
3	1	2	3	1	2	1	3	2	1
9	2	9	2	2	1	1	3	3	3
1	1	1	1	1	3	1	1	1	1
1	1	1	1	3	3	1	2	1	1
1	1	1	1	2	2	1	1	1	1
1	1	3	1	1	1	1	1	1	1
3	3	1	3	3	3	3	3	3	3
1	1	1	1	1	3	1	1	1	1
2	2	1	3	2	3	3	2	2	1
1	1	1	1	1	1	1	1	1	1
1	1	3	2	2	2	2	2	2	2
3	3	1	3	3	3	1	1	3	3
9	9	1	1	9	9	1	9	9	9
1	9	1	1	9	9	9	9	9	1
1	1	1	1	9	3	1	1	9	1
1	1	1	1	2	3	2	1	2	1
1	1	1	1	2	9	1	1	1	1
1	1	1	1	3	3	1	9	1	1
1	1	1	1	3	9	1	9	1	1
1	1	1	1	3	3	1	1	1	1
1	1	1	1	1	3	1	1	1	1
1	1	3	1	1	3	3	3	1	1
1	1	9	1	2	3	1	1	2	1
1	1	1	1	1	3	1	1	3	1
1	1	1	1	2	3	1	1	1	1
1	1	1	2	3	3	1	1	2	1
1	9	1	3	9	9	9	9	9	9

3	3	1	3	1	3	2	1	1	1
1	1	1	1	3	3	1	1	1	1
1	2	1	3	2	3	2	1	2	1
1	3	1	3	3	3	1	1	1	1
1	1	1	1	1	3	1	1	1	1
1	1	1	1	3	3	1	1	1	1
9	1	9	9	9	9	9	9	9	9
9	1	1	1	9	1	1	1	1	1
2	1	1	1	3	1	1	1	1	1
1	1	1	1	1	3	1	1	1	1
1	1	1	1	1	2	2	2	2	1
1	1	1	1	9	9	9	9	9	9
1	1	1	1	1	3	3	1	1	1
1	1	1	3	2	3	3	1	1	3
2	2	2	2	1	1	1	1	3	3
1	1	1	1	1	2	2	1	1	1
1	1	1	1	9	9	9	9	9	9
1	1	1	1	9	9	9	9	9	9
1	1	1	1	1	1	3	1	1	1
1	1	1	1	1	2	1	2	2	1
1	1	3	3	3	1	2	1	2	1
1	2	1	2	2	3	3	2	1	2
1	1	1	1	1	3	1	2	2	1
1	1	1	1	1	2	2	1	1	1
1	1	3	1	2	2	2	1	1	1
1	1	3	1	3	3	3	1	1	3
3	3	3	3	2	2	2	2	3	2
1	1	2	2	2	2	2	2	2	2
1	1	1	1	1	1	1	1	1	1
1	1	1	1	3	3	1	1	2	3
2	2	1	2	2	2	2	2	2	2
9	1	2	2	3	1	1	2	2	2
1	1	1	1	2	3	1	2	1	1
2	3	1	2	2	1	3	1	1	1
3	3	1	1	1	1	1	1	1	1
1	1	1	2	1	2	2	1	1	1
1	1	1	1	2	2	3	3	3	3
2	1	2	1	2	3	3	3	3	3
2	1	1	9	2	3	3	3	3	3
1	1	1	1	3	3	3	2	2	2
1	1	1	1	3	3	1	1	1	1
1	1	1	1	3	3	3	1	1	1
2	2	3	2	2	3	2	2	2	1
9	9	9	9	9	9	9	9	9	9
2	2	2	3	2	2	2	2	1	9
1	1	1	1	3	3	3	1	1	1
1	1	1	2	1	2	2	1	2	1

1	1	1	1	2	3	1	1	1	1
1	1	1	1	1	3	2	1	2	1
1	1	1	1	2	2	2	2	2	1
1	1	1	1	2	2	1	1	2	1
1	1	2	2	2	1	2	1	1	1
3	3	2	3	2	2	3	1	2	1
1	1	9	9	1	9	9	9	9	9
1	1	1	1	2	2	2	2	2	2
9	9	9	9	9	9	9	9	9	9
1	1	1	1	2	2	1	2	2	9
1	1	1	1	2	2	1	1	2	2
1	1	1	2	2	3	2	1	1	1
2	2	2	2	2	2	2	2	2	2
1	1	1	2	2	3	3	3	1	1
1	2	2	2	9	1	2	2	2	1
9	9	9	9	9	9	9	9	9	9
2	2	1	2	2	2	2	1	2	2
1	1	1	2	1	2	2	1	2	1
1	1	1	1	1	1	1	1	1	1
1	2	1	2	2	2	2	1	1	1
9	9	9	9	9	9	9	9	9	9
3	3	1	3	1	3	3	1	3	1
2	2	2	2	2	3	3	2	2	2
1	1	1	1	1	3	1	1	1	1
1	1	1	1	1	2	2	1	1	1
1	1	3	2	1	1	2	2	1	1
1	1	1	2	2	2	2	1	1	1
9	9	9	9	9	9	9	9	9	9
9	9	9	9	9	9	9	9	9	9
2	3	2	2	1	3	2	1	1	2
1	1	1	1	1	3	3	1	1	1
1	1	1	1	1	2	2	2	1	1
1	1	1	1	3	1	3	1	1	1
1	1	1	1	3	3	1	9	9	9
1	1	1	3	3	3	1	1	1	1
1	2	1	1	2	3	1	2	2	1
1	1	1	1	3	2	1	1	1	1
1	1	1	3	3	1	1	1	1	1
1	1	1	1	3	3	1	3	1	3
1	1	1	1	3	2	1	1	1	1
2	2	1	3	2	1	1	1	1	2
1	1	1	2	2	3	1	1	2	3
1	1	1	1	3	3	1	1	2	1
9	9	9	9	9	9	9	9	9	9
2	2	1	2	2	3	1	1	1	1
1	2	2	2	2	2	2	1	1	2
1	9	2	1	2	2	2	2	1	1

1	1	1	3	3	3	1	1	1	1
1	2	1	2	2	2	1	1	2	2
9	9	9	9	9	9	9	9	9	9
1	1	1	1	2	3	3	1	1	1
1	1	1	9	9	9	3	9	9	9
2	2	1	1	1	2	2	1	1	1
9	9	1	1	2	3	2	1	2	1
3	3	1	3	1	3	1	1	2	1
9	9	9	9	2	9	9	9	9	9
2	2	1	2	2	2	2	1	2	1
3	1	1	3	3	2	3	3	3	1
2	2	1	2	2	2	2	2	2	2
1	1	1	2	1	3	3	1	2	1
1	1	1	9	9	9	1	9	9	1
2	1	2	1	2	1	1	1	1	1
2	2	1	2	3	3	3	1	1	1
9	9	1	9	9	9	9	1	9	1
1	9	1	9	9	9	9	9	9	1
1	1	1	2	1	2	2	1	2	1
1	1	1	3	3	2	3	3	2	2
1	3	3	3	2	9	1	1	3	1
1	1	1	1	1	3	3	1	1	1
1	1	1	1	1	3	3	1	1	1
1	1	1	1	1	3	1	1	1	1
9	1	1	1	1	2	2	1	1	1
3	3	1	1	2	3	3	1	2	1
1	2	1	2	1	9	3	1	1	1
3	3	1	2	2	3	3	1	1	1
3	3	1	1	2	1	3	2	1	1
3	3	1	1	2	1	3	1	1	1
1	1	1	1	3	2	3	1	2	1
1	1	1	2	1	2	2	1	1	1
3	3	1	3	3	2	2	1	2	1
2	2	2	1	2	2	2	2	3	1
1	1	1	2	2	3	3	1	1	2
9	9	1	9	9	9	1	1	9	9
3	3	1	3	1	3	3	1	3	3
9	9	1	9	9	9	9	9	9	9
1	1	2	2	2	1	9	1	1	1
1	1	2	2	2	2	2	2	2	1
2	3	2	3	2	2	3	2	3	2
3	2	3	3	2	2	3	3	2	2
9	9	9	9	9	9	9	9	9	9
9	9	9	9	9	9	9	9	9	9
1	1	1	1	1	2	3	1	1	1
1	1	1	1	1	3	3	1	1	1
1	9	1	9	9	9	9	1	9	1

1	3	1	3	3	3	3	1	1	1
1	1	1	1	1	2	2	1	1	1
9	1	1	1	1	9	9	1	1	1
1	1	1	1	3	3	3	2	1	1
1	1	1	1	1	2	2	1	1	1
1	2	1	2	2	2	3	2	2	1
2	2	2	2	2	2	2	2	2	2
2	2	2	2	2	2	2	1	1	1
1	2	2	2	2	2	2	2	2	2
2	2	1	1	2	2	3	1	2	1
1	1	2	2	2	2	2	3	2	1
9	9	9	9	9	9	9	9	9	9
9	9	9	9	9	9	9	9	9	9
9	1	9	9	9	9	9	9	9	9
9	9	9	9	9	9	9	9	1	9
9	9	1	9	9	9	9	9	9	9
1	1	3	3	2	2	3	1	3	3
2	3	2	1	2	1	2	3	2	2
1	9	9	9	9	9	9	9	9	9
1	1	1	1	2	2	2	1	1	2
1	1	1	1	1	3	3	1	1	1
2	2	9	2	2	3	2	1	2	1
2	2	1	2	2	3	2	1	2	1
1	1	1	2	2	3	3	2	1	1
2	2	1	2	2	2	3	2	1	1
1	1	1	1	1	3	2	1	1	1
1	9	1	3	3	3	3	1	1	1
2	2	1	2	3	3	3	1	2	2
1	1	1	9	9	9	9	1	1	1
1	1	1	9	9	9	1	1	1	1
1	1	1	2	1	1	2	1	1	1
1	1	9	1	9	9	9	1	1	1
1	1	1	2	2	2	3	1	1	1
1	1	1	3	3	3	1	1	1	1
3	3	1	2	2	3	3	1	1	1
1	1	1	2	1	2	1	1	1	1
1	1	1	1	2	2	2	1	2	1
1	3	1	3	3	3	2	2	2	1
9	9	9	9	9	9	9	9	9	9
1	1	1	1	3	3	1	1	1	2
2	2	2	2	3	3	3	1	2	1
2	2	2	2	2	9	1	3	1	1
1	1	1	1	1	3	1	1	1	1
1	2	1	2	2	2	1	1	1	1
1	1	1	1	2	2	1	1	2	1
1	1	1	2	1	2	2	1	1	1
1	1	1	3	1	1	3	1	1	1

1	3	1	3	3	3	3	1	3	1
1	1	1	2	1	2	1	1	1	1
2	1	3	2	3	1	2	1	1	1
1	1	1	1	2	2	3	3	1	1
9	9	9	9	9	9	9	9	9	9
1	1	1	3	3	3	3	1	1	1
3	3	1	3	3	3	3	1	1	1
1	1	1	1	2	2	3	1	1	1
2	1	1	2	2	2	9	1	1	3
1	1	1	2	1	1	2	2	1	1
1	1	1	1	2	2	3	1	1	1
2	2	2	2	1	2	1	1	2	2
2	1	1	2	3	2	3	1	1	1
1	1	1	3	3	3	3	1	1	1
2	3	1	2	1	3	3	1	3	2
2	1	1	2	2	2	2	1	1	1
1	1	1	2	2	2	2	1	1	2
9	2	1	2	1	2	2	1	1	1
2	1	1	3	3	3	3	1	2	2
1	1	1	1	2	2	2	2	1	1
1	1	1	1	2	2	2	2	1	1
2	2	1	2	1	3	9	9	9	9
1	1	2	2	2	3	2	1	1	2
1	1	1	2	2	3	1	1	1	1
1	1	1	2	2	2	2	1	1	2
9	9	9	9	9	9	9	9	9	9
9	9	9	9	9	9	9	9	9	9
9	9	9	9	9	9	9	9	9	9
2	2	1	2	1	3	2	1	1	1
1	2	1	1	2	3	3	2	9	2
1	1	1	2	2	1	2	2	1	1
1	1	1	2	2	1	9	2	1	1
9	9	9	9	9	9	9	9	9	9
9	9	9	9	9	9	9	9	9	9
9	9	9	9	9	9	9	9	9	9
9	9	9	9	9	9	9	9	9	9
9	9	9	9	9	9	9	9	9	9
9	9	9	9	9	9	9	9	9	9
9	9	9	9	9	9	9	9	9	9
2	2	1	1	3	2	1	1	1	3
2	2	1	2	1	3	2	1	1	1
9	9	9	9	9	9	9	9	9	9
9	9	9	9	9	9	9	9	9	9
1	1	1	1	2	3	3	1	1	1
9	9	9	9	9	9	9	9	9	9
9	9	1	9	9	9	9	9	9	9
9	9	1	9	9	9	9	9	9	9

1	1	1	2	3	3	3	1	1	1
9	9	9	9	9	9	9	9	9	9
9	1	9	9	9	9	9	9	9	9
3	9	1	3	2	2	3	1	1	1
3	9	1	3	2	2	3	1	1	1
9	1	9	9	9	9	9	9	9	9
9	9	9	9	9	9	9	9	9	9
9	9	9	9	9	9	9	9	9	9
9	9	9	9	9	9	9	9	9	9
9	9	9	9	9	9	9	9	9	9
1	1	1	1	2	3	3	3	1	3
1	1	1	1	1	3	1	1	3	1
1	3	1	1	1	3	1	1	1	1
1	2	2	2	2	2	2	1	1	1
1	1	1	3	1	3	3	1	3	3
3	3	1	3	1	1	3	1	1	1
3	3	3	3	3	1	1	1	1	1
3	3	1	2	2	2	1	1	1	1
1	1	1	1	1	3	1	1	3	1
1	1	2	1	2	1	2	1	1	1
1	1	2	1	2	1	2	1	1	1
2	2	2	2	2	2	2	1	1	1
9	9	9	9	9	9	9	9	9	9
2	1	1	1	1	1	1	1	2	3
9	1	1	2	3	3	3	3	3	2
1	1	1	1	2	3	3	2	1	1
2	2	2	3	3	3	3	3	3	9
1	1	1	3	2	2	3	1	1	1
1	1	3	2	2	2	3	1	1	1
3	2	2	3	2	3	2	1	2	3
1	1	2	2	2	3	3	1	1	1
3	3	2	2	1	3	3	2	2	1
3	3	1	2	2	2	3	1	1	1
2	2	2	2	2	2	3	2	1	1
1	2	2	2	2	2	3	1	1	1
1	1	1	2	1	1	3	1	1	1
3	3	1	9	9	9	9	1	9	1
3	3	3	3	1	3	3	3	1	1
3	3	3	3	3	3	3	1	1	1
1	1	3	2	1	1	3	1	2	1
9	9	9	9	9	9	9	9	9	9
1	1	3	3	1	1	1	1	1	1
2	3	3	3	3	2	3	2	2	2
1	1	1	2	2	2	2	1	2	2
1	1	1	2	2	2	2	1	1	2
1	1	1	1	1	1	1	1	1	1
2	2	2	2	1	3	3	1	1	2

1	1	2	3	1	3	3	1	1	1
1	1	1	1	1	1	1	1	1	1
1	1	1	1	1	1	1	1	1	1
1	1	1	2	2	2	2	1	1	1
1	1	1	2	2	2	2	1	1	2
1	1	1	2	2	2	2	1	1	9
2	2	2	2	1	3	3	1	1	2
1	1	2	2	1	1	3	2	1	1
2	2	2	2	1	2	2	1	1	1
3	3	3	3	1	3	3	1	9	1
1	1	1	2	2	2	1	1	1	1
1	1	3	3	3	9	1	9	1	1
1	1	1	2	3	2	1	2	3	3
1	1	2	3	2	2	1	2	3	3
3	3	3	1	1	1	1	1	1	1
2	2	3	3	3	1	2	2	9	1
2	2	2	2	2	2	2	2	2	2
2	2	2	2	2	2	2	2	2	2
3	1	1	1	3	1	3	3	1	3
1	1	2	2	2	3	2	2	2	1
1	1	1	2	3	3	2	1	1	1
1	1	1	3	3	3	1	1	1	1
1	1	2	2	2	2	1	1	1	1
1	1	2	1	1	2	1	1	1	1
1	1	2	1	2	1	1	1	1	1
1	1	1	1	2	3	3	2	1	1
3	2	2	3	3	1	1	2	2	2
3	1	1	1	1	2	3	1	2	1
1	1	1	2	2	2	2	1	1	1
2	2	2	1	3	3	2	1	1	1
3	3	3	3	3	2	2	3	3	2
1	1	1	3	3	3	3	3	2	2
1	1	1	1	3	3	3	1	1	1
3	3	1	2	3	3	3	1	2	2
1	1	2	2	2	2	2	1	1	1
1	1	2	2	2	2	1	1	1	1
1	3	2	3	3	3	3	3	3	2
1	3	1	3	3	3	3	3	3	3
1	1	1	2	3	3	3	1	2	2
1	2	1	3	1	3	3	1	1	2
1	1	1	3	2	3	3	1	1	1
3	3	1	2	3	1	3	1	1	1
3	3	1	2	3	9	3	1	1	1
1	1	2	2	1	2	1	2	2	2
1	1	1	3	3	3	1	1	1	1
1	1	2	2	2	2	2	1	2	2
1	1	1	1	1	3	1	3	1	1

2	2	3	2	2	2	3	2	2	2
9	9	1	9	9	9	9	9	9	1
1	1	1	1	2	2	3	3	1	1
2	2	1	1	2	3	2	2	2	2
1	1	1	1	1	2	2	2	1	1
2	1	1	2	2	2	1	2	2	1
3	3	1	2	1	3	3	2	2	1
1	1	2	2	1	2	2	1	2	2
2	3	3	3	3	3	2	2	1	1
3	1	1	3	2	3	2	1	1	1
2	2	1	2	2	2	2	1	2	1
3	3	1	3	3	3	3	1	3	1
9	9	9	9	9	9	9	9	9	9
3	3	3	3	1	3	2	2	1	1
1	1	1	1	3	3	3	3	1	1
2	2	2	2	2	2	2	2	2	2
3	3	1	3	1	2	1	1	3	2
1	1	1	2	2	2	1	1	1	1
1	1	1	2	1	2	1	1	1	1
2	1	1	1	1	2	1	1	1	1
1	1	1	2	1	1	2	1	2	1
1	1	2	1	2	1	2	1	1	1
1	1	1	1	3	3	3	1	1	1
2	2	2	1	1	2	3	2	2	2
1	9	1	1	1	1	2	1	2	1
1	3	1	3	1	3	3	1	1	1
3	3	1	3	1	1	3	1	1	1
2	2	1	1	3	2	1	1	1	3
1	1	1	2	3	3	9	1	1	1
9	9	9	9	9	9	9	9	9	9
2	2	1	3	1	2	3	2	1	2
1	1	1	3	2	1	2	1	1	1
1	1	1	2	2	2	2	2	2	1
1	1	1	1	2	1	2	1	1	1
1	1	1	2	2	2	2	1	1	1
1	1	1	2	2	1	2	1	1	2
9	9	9	9	9	9	9	9	9	9
9	1	9	9	9	9	9	9	9	9
1	1	2	2	2	2	2	3	2	1
2	2	2	2	1	2	2	1	1	1
1	1	2	3	2	2	1	1	2	1
1	1	1	2	2	3	2	1	1	1
1	1	1	1	2	2	2	1	1	1
1	9	1	2	3	9	2	9	9	1
2	2	2	2	2	2	2	3	2	9
1	2	1	2	2	2	2	1	2	2
2	2	1	1	3	3	2	1	1	1

1	2	1	2	1	3	2	1	1	1
1	1	1	2	1	2	2	1	1	1
1	1	1	2	2	2	2	2	2	2
1	2	2	1	2	2	1	1	2	1
2	2	1	2	2	2	1	1	1	1
1	1	1	2	3	9	3	1	1	1
1	1	1	1	1	3	1	1	1	1
9	9	9	1	9	9	9	9	9	9
1	1	1	2	2	3	1	1	1	1
1	1	2	2	2	2	2	1	1	1
1	1	1	2	2	3	9	1	2	1
2	3	2	2	3	2	2	3	2	3
1	1	2	1	3	2	1	1	1	2
2	2	2	2	2	2	2	2	2	2
2	3	1	3	1	3	3	1	1	1
2	3	1	3	1	3	3	1	1	1
1	2	1	1	1	1	2	1	1	1
1	1	1	1	2	1	1	1	1	1
9	1	9	9	9	9	9	9	9	9
1	1	3	3	3	2	1	1	1	1
2	2	1	2	2	3	1	1	1	1
1	1	2	1	1	2	1	1	1	1
2	1	2	2	2	1	2	2	2	2
1	1	1	1	2	3	1	1	1	1
2	1	1	1	2	3	3	1	3	1
1	1	9	1	1	9	1	1	1	1
1	1	1	2	1	3	1	1	1	1
3	1	1	1	1	3	1	1	1	2
3	1	1	1	1	3	1	1	1	2
1	9	9	9	9	9	9	9	9	9
3	1	1	1	1	3	1	1	1	2
1	1	2	9	1	1	2	1	1	1
1	1	2	1	1	3	1	1	1	1
1	2	1	2	2	2	1	2	1	1
9	9	9	9	9	9	9	9	9	9
1	1	2	2	2	2	1	1	1	1
1	1	2	2	1	2	1	1	1	1
1	1	3	3	3	9	1	9	1	1
1	1	3	3	3	3	1	1	1	1
1	1	2	2	2	3	3	1	2	1
1	1	1	2	2	3	1	1	2	2
1	1	1	1	2	3	1	1	1	1
2	1	2	2	2	2	2	2	2	2
1	1	1	1	3	3	1	1	1	1
3	3	3	3	3	3	3	1	3	3
1	1	1	1	3	3	1	3	3	1
1	1	1	1	1	3	1	1	1	1

1	3	3	3	3	3	1	1	1	1
1	3	3	3	3	3	1	1	1	1
3	3	3	3	3	3	3	1	2	2
1	1	2	1	2	3	1	1	1	1
1	1	3	3	3	3	1	1	1	1
1	1	3	3	3	3	2	1	1	1
1	1	1	3	1	3	1	1	1	1
1	1	1	1	1	1	1	1	2	1
1	1	1	1	9	2	3	1	3	1
1	3	1	1	1	3	1	1	1	1
1	1	1	1	1	3	3	1	1	1
1	1	1	1	2	2	1	1	1	1
1	1	1	1	1	1	1	1	1	1
1	3	3	3	1	1	1	1	3	3
2	1	1	1	1	3	3	1	1	1
1	1	1	1	3	3	3	1	1	1
1	2	1	2	1	2	2	1	2	2
1	2	1	1	2	2	2	1	1	1
1	1	1	3	1	3	3	1	1	1
9	9	9	9	9	9	9	9	9	9
1	1	3	1	1	1	1	2	1	1
2	2	1	2	1	3	2	1	1	1
3	9	1	3	9	9	3	9	1	1
3	9	1	3	2	2	3	9	1	1
9	9	9	1	9	9	9	9	9	9
9	9	9	9	9	9	9	9	9	9
1	1	1	2	2	3	3	1	1	1
9	9	9	9	9	9	9	9	9	9
9	9	9	9	9	9	9	9	9	9
1	9	9	9	9	9	9	9	9	9
9	9	9	9	9	9	9	9	9	9
9	9	9	9	9	9	9	9	9	9
9	9	9	9	9	9	9	9	9	9
9	9	9	9	9	9	9	9	9	9
1	1	1	1	1	3	2	2	2	1
1	2	1	2	1	2	2	2	3	1
1	1	1	1	2	2	1	2	1	1
1	1	1	1	2	1	1	1	1	1
1	1	1	2	1	3	1	1	1	1
1	1	1	2	1	3	1	1	1	1
9	9	9	9	9	9	9	9	9	9
2	2	3	2	2	2	2	2	1	1
1	1	1	2	1	2	2	1	1	1
1	1	1	2	2	3	2	2	2	2
2	2	1	2	2	3	3	2	2	1
1	1	1	3	1	3	1	1	1	1
1	1	1	1	2	2	1	1	1	1

1	1	2	2	2	2	2	2	2	2
1	2	1	2	2	2	2	1	2	1
3	1	2	2	9	3	2	1	1	1
3	2	3	2	9	1	2	1	2	3
2	1	2	2	1	1	1	2	1	2
1	1	1	3	3	2	3	1	1	1
9	9	9	9	9	9	9	9	9	9
2	2	2	2	2	2	3	2	2	2
1	1	1	1	2	2	2	2	1	1
2	2	2	2	2	2	2	2	2	2
1	1	1	2	2	2	2	1	1	1
1	1	1	1	1	1	2	1	1	1
2	3	2	2	3	1	2	3	2	1
2	3	3	3	2	3	3	3	2	3
1	9	1	3	1	3	3	1	2	1
1	1	1	1	3	3	3	1	1	1
9	9	9	9	9	9	9	9	9	9
1	2	1	3	2	1	2	2	3	2
1	2	1	1	1	2	2	1	1	1
1	1	1	1	1	1	1	1	1	1
1	1	1	1	1	1	1	1	1	1
2	2	3	3	3	2	2	2	2	2
1	3	2	2	1	3	2	2	3	3
2	2	2	2	2	2	2	2	2	2
1	1	1	1	1	1	1	1	1	1
3	2	1	2	2	2	1	2	1	3
1	2	2	1	2	3	2	2	1	1
1	1	2	1	1	2	1	1	1	1
1	1	1	2	2	2	2	2	2	2
1	1	1	2	2	2	2	2	2	2
1	1	1	2	2	3	1	1	1	1
1	1	1	1	1	1	1	1	1	1
1	2	1	3	2	3	1	1	2	2
1	1	1	3	1	2	2	1	1	1
9	9	1	9	9	9	9	9	9	9
1	2	2	2	2	2	1	1	1	1
2	2	2	2	2	2	2	2	2	2
1	1	1	2	1	2	2	2	2	2
1	1	1	1	1	1	3	1	3	2
1	1	1	2	2	2	1	1	1	1
1	1	1	1	2	2	2	1	2	1
1	1	1	1	2	2	2	1	2	1
9	9	9	9	9	1	9	9	9	9
9	9	9	9	9	9	9	9	9	9
9	9	9	9	9	9	9	9	9	9
3	1	2	1	3	2	1	3	2	1
3	1	2	2	3	1	1	2	1	2

3	3	3	1	2	3	3	1	1	1
3	3	3	1	1	2	3	3	1	1
2	1	1	1	2	1	1	1	2	3
2	2	2	2	2	3	3	3	3	3
2	2	1	3	1	3	3	3	1	1
3	2	1	2	2	3	2	1	2	9
3	2	3	2	3	1	9	2	3	3
2	1	3	2	3	3	2	1	3	2
1	1	2	3	3	2	3	1	2	1
2	2	1	1	2	1	2	3	2	1
3	1	3	1	3	1	1	1	2	2
2	3	1	3	1	3	1	1	1	1
2	2	2	3	1	3	3	3	2	2
2	2	3	1	3	1	1	1	1	1
2	2	2	2	2	2	3	3	3	3
3	1	2	1	2	3	3	3	3	3
3	1	3	1	3	9	1	1	1	1
3	1	3	1	3	9	1	1	1	1
3	3	3	1	2	3	3	1	1	1
3	1	2	1	9	2	3	2	1	1
1	3	1	3	1	3	3	3	3	3
1	3	1	3	1	3	3	3	3	3
1	3	1	3	1	3	3	3	3	3
1	3	1	3	1	3	3	3	3	3
2	2	2	2	2	2	2	2	2	2
2	2	2	2	2	2	2	2	2	2
2	2	2	2	2	2	2	2	2	2
1	3	1	3	1	3	3	3	3	3
3	1	2	2	2	2	3	3	3	3
2	3	1	3	1	3	2	2	1	1
2	3	2	3	1	3	2	2	1	1
2	3	2	3	1	3	2	2	1	1
2	3	2	3	1	3	2	2	1	1
1	1	3	2	1	9	1	2	2	3
3	2	9	2	2	9	2	9	2	9
1	2	1	2	2	3	2	2	2	3
2	2	1	3	2	2	1	1	2	2
2	1	2	2	1	2	2	1	2	2
3	1	2	1	2	3	3	3	2	2
3	2	1	2	3	2	3	2	2	1
9	9	9	9	9	9	9	9	9	9
1	2	3	2	3	1	2	1	1	1
3	2	3	2	1	2	1	2	1	2
9	9	9	9	9	9	9	9	9	9
9	9	9	9	9	9	9	9	9	9
2	2	2	2	2	2	2	2	2	2
3	2	3	2	2	9	1	9	1	3

3	1	3	2	2	2	9	1	2	2
3	1	3	1	3	1	1	1	1	1
9	9	9	9	9	9	9	9	9	9
2	2	2	2	2	2	2	2	2	2
2	2	2	2	2	2	2	2	2	2
9	9	9	9	9	9	9	9	9	9
9	1	2	2	2	1	1	2	1	2
9	9	9	9	9	1	9	9	9	9
3	1	2	2	2	2	2	1	1	1
1	2	2	3	1	3	1	1	1	1
2	2	2	2	2	2	2	2	2	2
2	3	3	3	1	3	2	2	2	2
9	9	9	9	9	9	9	9	9	9
9	9	9	9	9	9	9	9	9	9
1	1	2	3	3	2	3	1	2	3
3	9	9	9	9	9	9	9	9	9
1	3	2	2	2	3	2	1	1	1
9	9	9	9	9	9	9	9	9	9
3	3	3	3	3	3	1	3	1	1
9	9	9	9	9	9	9	9	9	9
3	1	3	1	3	2	9	1	2	1
3	1	3	1	3	1	1	1	1	1
3	1	3	1	3	1	1	1	1	1
3	2	2	2	2	2	2	2	2	2
3	1	3	1	3	1	2	2	2	2
2	1	3	1	3	1	1	1	1	1
3	1	3	1	3	2	2	2	3	3
9	9	9	9	9	9	9	9	9	9
9	9	9	9	9	9	9	9	9	9
9	9	9	9	9	9	9	9	9	9
9	9	9	9	9	9	9	9	9	9
9	9	9	9	9	9	9	9	9	9
9	9	9	9	9	9	9	9	9	9
3	1	3	1	3	3	3	2	3	1
1	1	2	3	3	2	3	1	2	3
9	9	9	9	9	9	9	9	9	9
2	2	3	2	2	2	2	1	2	2
2	2	2	2	1	3	3	3	3	1
2	1	2	3	3	2	3	1	2	3
2	2	2	2	2	3	3	3	2	1
2	2	3	3	2	3	3	3	3	2
9	9	9	9	9	9	9	9	9	9
1	3	1	3	1	3	3	3	3	3
2	2	2	2	2	2	2	2	2	2
9	9	9	9	9	9	9	9	9	9
2	2	2	2	1	9	2	1	2	1
9	9	9	9	9	9	9	9	9	9

9	9	9	9	9	9	9	9	9	9
9	9	9	9	9	9	9	9	9	9
3	1	3	1	3	1	1	1	1	1
9	9	9	9	9	9	9	9	9	9
2	2	1	3	1	2	2	2	1	1
9	9	9	9	9	9	9	9	9	9
2	2	1	1	2	1	2	3	1	3
3	1	3	1	3	9	2	2	2	2
3	1	3	1	2	2	2	2	3	3
3	2	2	1	2	3	2	1	2	3
3	1	3	1	3	1	1	1	1	1
2	2	2	2	2	2	1	1	1	2
2	1	3	1	3	1	1	1	1	1
3	1	2	3	2	2	1	2	1	2
3	1	3	2	2	2	2	1	2	2
2	2	2	1	3	3	3	3	2	2
2	2	1	2	2	2	3	3	3	3
3	3	2	2	3	1	1	2	2	1
1	2	3	2	1	2	1	2	3	2
3	1	3	1	3	1	1	1	1	1
9	9	9	9	2	9	9	9	9	9
9	9	9	9	9	9	1	9	9	9
3	9	9	9	9	9	9	9	9	9
9	9	9	1	9	9	9	9	9	9
9	9	2	9	9	9	9	9	9	9
9	9	9	2	9	9	9	9	9	9
1	9	9	9	9	9	9	9	9	9
9	9	9	9	9	9	9	9	9	9
9	9	9	9	9	9	9	9	9	9
9	9	9	9	9	9	9	9	9	9
9	9	9	9	9	9	9	9	9	9
9	9	9	9	9	9	9	9	9	9
9	9	9	9	9	9	9	9	9	9
2	3	1	2	3	1	2	2	1	1
3	1	2	2	2	3	1	1	1	1
3	2	3	2	1	3	3	2	1	1
9	3	9	9	2	9	9	9	1	9
9	9	9	9	3	9	9	9	9	9
3	1	3	2	2	1	1	2	2	2
9	9	9	9	9	9	9	9	9	9
9	9	9	9	9	9	9	9	9	9
9	9	9	9	9	9	9	9	9	9
9	9	9	9	9	9	9	9	9	9
9	9	9	9	9	9	9	9	9	9
2	2	2	2	2	2	2	2	2	2
3	2	3	2	3	2	1	2	1	2
3	2	3	2	3	2	1	2	1	2

2	2	2	2	2	2	2	2	2	2
2	2	1	3	2	1	1	1	1	2
2	2	3	3	1	3	2	1	1	1
2	3	1	2	1	3	2	2	2	3
9	9	9	9	9	9	9	9	9	9
9	9	9	9	9	9	9	9	9	9
9	9	9	9	9	9	9	9	9	9
9	2	9	9	9	2	9	9	2	9
9	9	9	9	9	9	9	9	9	9
9	3	9	9	9	3	9	9	3	9
9	3	9	9	1	9	3	9	9	9
9	9	9	9	9	9	9	9	9	9
9	9	9	2	9	9	2	9	3	9
9	9	9	2	9	9	9	9	9	9
9	9	9	9	9	9	9	1	9	9
2	2	2	2	2	2	2	2	2	2
1	3	1	2	2	3	3	1	1	2
3	9	9	9	9	9	9	9	9	9
2	2	2	2	2	1	1	2	2	2
3	1	3	2	3	2	3	2	2	3
2	2	2	2	2	2	2	2	2	2
2	2	2	2	2	2	2	2	2	2
3	1	2	1	2	2	1	1	2	1
3	1	3	2	2	2	2	2	1	1
2	3	2	2	1	2	2	1	2	1
3	2	2	2	2	1	2	2	2	2
2	9	9	9	9	9	9	9	9	9
2	2	2	2	2	2	2	2	2	2
2	2	2	2	2	2	2	2	2	2
2	2	1	1	2	3	2	2	1	2
2	2	2	2	2	3	1	2	3	2
3	1	2	2	2	2	2	2	2	2
9	9	9	9	9	9	9	9	9	9
2	2	2	2	2	2	2	2	2	2
3	1	2	2	2	2	2	2	1	1
3	2	1	2	2	3	2	2	1	2
2	2	2	2	3	3	1	1	2	1
3	1	3	2	2	2	2	2	2	2
3	2	3	2	1	2	2	3	2	1
1	2	3	2	1	2	1	1	2	3
3	2	2	1	2	2	1	2	1	1
3	3	1	1	1	1	1	1	1	3
3	1	2	2	2	2	2	2	2	2
3	1	3	1	3	3	1	1	1	1
9	9	9	9	9	9	9	9	9	9
2	2	2	2	2	2	2	2	2	2
3	1	2	3	2	3	3	1	1	1

3	1	3	1	3	1	1	1	1	1
3	1	1	2	2	3	1	2	1	1
3	1	3	1	3	2	2	1	1	1
3	1	2	2	2	2	2	2	3	1
2	2	2	2	2	2	2	2	2	2
2	2	2	2	2	2	2	2	2	2
2	2	2	2	2	2	2	2	2	2
2	2	2	1	3	2	2	2	2	2
2	2	2	1	3	3	1	1	1	1
2	2	2	1	2	3	2	2	2	2
3	2	2	3	2	2	3	1	2	2
3	1	2	2	2	3	3	1	2	1
3	1	3	1	3	1	1	1	1	1
2	2	2	2	2	3	2	1	1	1
3	1	3	1	2	1	1	1	1	1
3	2	2	2	2	2	2	1	1	1
3	1	2	2	2	2	3	2	2	2
2	2	2	2	2	2	2	2	2	2
2	2	2	2	2	2	2	2	2	2
2	2	2	2	2	2	2	2	2	2
2	2	2	2	2	2	2	2	2	2
2	2	2	2	2	2	2	2	2	2
2	2	2	2	2	2	2	2	2	2
2	2	2	2	2	2	2	2	2	2
2	2	1	3	1	3	1	1	1	1
9	9	9	9	9	9	9	9	9	9
9	9	9	9	9	9	9	9	9	9
9	9	9	9	9	9	9	9	9	9
2	1	3	1	1	2	1	2	2	3
9	9	9	9	9	9	9	9	9	9
9	9	9	9	9	9	9	9	9	9
9	9	9	9	9	9	9	9	9	9
9	9	9	9	9	9	9	9	9	9
9	9	9	9	9	9	9	9	9	9
3	2	2	2	2	2	2	1	1	1
9	1	1	1	3	2	1	2	1	2
2	2	2	2	2	2	1	1	1	1
9	9	9	9	9	9	9	9	9	9
2	3	1	1	2	2	3	3	3	3
9	9	9	9	9	9	9	9	9	9
9	9	9	9	9	9	9	9	9	9
2	2	2	2	2	2	2	2	2	2
3	1	2	1	2	2	1	1	1	1
3	2	2	2	2	2	2	1	2	1
2	3	3	3	2	2	2	1	3	1
2	2	1	2	2	3	3	3	2	2
2	2	2	3	1	3	2	2	2	2

3	1	1	1	1	3	2	1	1	1
1	3	1	3	1	3	3	3	3	3
3	1	1	3	2	2	3	1	2	1
3	2	2	2	2	1	2	2	2	1
3	1	3	2	9	1	2	2	2	1
9	9	9	9	9	1	9	9	9	9
2	2	2	2	2	2	2	1	1	1
2	2	2	2	2	2	1	2	2	2
3	2	2	2	2	1	1	2	2	2
2	1	2	3	2	1	2	3	2	1
2	2	2	2	2	2	2	2	2	2
3	1	1	3	1	3	3	1	3	1
2	1	2	2	2	2	3	1	1	1
2	2	2	2	2	2	2	2	2	2
9	9	9	9	9	9	9	9	9	9
2	2	2	2	2	2	2	2	1	1
3	1	1	1	3	1	3	1	2	1
3	2	2	3	2	2	3	2	2	3
3	2	1	2	3	3	1	1	2	1
3	9	9	9	9	9	9	9	9	9
2	2	2	2	2	2	2	2	2	2
2	2	2	2	2	2	2	2	2	2
3	1	2	2	2	2	2	2	2	2
2	2	2	2	2	2	2	2	2	2
2	2	3	2	1	1	2	2	1	1
2	2	2	2	2	2	2	2	2	2
3	1	1	3	2	3	3	3	2	2
3	1	1	3	1	3	1	1	1	1
2	2	2	2	2	1	2	2	2	2
2	2	2	2	2	2	2	2	2	9
3	1	3	1	3	3	2	1	1	1
9	1	9	9	9	9	9	9	9	9
3	1	2	1	3	2	1	1	2	2
3	1	1	3	3	3	3	2	1	2
2	1	2	1	3	1	2	1	2	1
2	2	2	2	2	2	2	2	2	2
2	2	2	2	2	2	2	2	2	2
2	2	2	2	2	2	2	2	2	2
2	2	2	2	2	2	2	2	2	2
3	1	2	2	2	2	2	1	1	1
1	3	1	2	2	3	1	2	2	2
2	2	2	2	2	2	2	2	2	2
3	2	2	2	1	2	2	2	2	2
2	2	2	2	2	2	2	2	2	2
2	2	2	2	2	2	2	2	2	2
3	2	2	2	2	2	2	2	2	2
3	1	3	2	2	1	2	2	1	2

9	9	9	9	9	9	9	9	9	9
3	3	3	1	1	2	1	3	1	1
3	1	9	9	9	9	9	1	9	9
9	9	9	9	9	9	9	9	9	9
2	2	2	2	2	2	1	1	1	1
2	2	2	2	2	2	2	2	2	2
9	9	9	9	9	9	9	9	9	9
3	2	3	1	3	3	1	1	2	2
2	2	2	2	2	2	2	2	2	2
3	1	2	2	2	2	2	2	2	2
2	2	2	2	2	2	2	2	2	2
2	2	2	1	2	2	1	1	1	1
1	1	1	3	1	3	1	1	1	3
9	9	9	9	9	9	9	9	9	9
3	1	3	3	1	3	3	3	3	3
2	2	2	3	1	3	1	2	2	3
1	2	3	1	1	3	2	1	2	1
9	9	9	9	9	9	9	9	9	9
3	1	3	2	2	2	2	1	2	1
3	2	2	2	2	9	3	1	1	1
2	2	2	2	2	2	2	2	2	2
3	1	3	1	3	3	2	1	2	1
2	9	2	2	9	9	9	9	2	2
2	2	3	2	2	3	3	2	3	2
3	1	3	3	9	1	1	1	1	1
9	9	9	9	9	9	9	9	9	9
2	2	2	2	2	2	2	2	2	2
9	9	9	9	9	9	9	9	9	9
3	1	3	2	2	3	3	1	1	1
3	1	3	2	2	2	2	1	1	1
2	2	2	2	2	1	2	2	2	1
2	2	2	2	3	1	1	1	1	1
1	2	2	3	2	2	1	1	2	2
2	2	3	1	1	3	2	1	2	2
3	1	3	2	2	3	3	1	1	2
1	3	1	3	1	3	3	3	3	3
1	2	3	2	1	1	3	1	3	1
3	1	2	2	3	1	2	1	1	1
9	9	9	9	9	9	9	9	9	1
3	1	3	2	2	2	2	1	1	1
3	1	1	3	1	2	1	1	3	2
3	1	2	2	3	1	2	1	1	1
1	1	2	2	2	2	2	3	3	3
2	1	2	2	2	3	2	1	1	1
1	2	3	2	3	3	3	1	1	1
9	9	9	9	9	9	9	9	9	9
9	9	3	9	3	9	9	1	1	1

2	2	3	2	3	2	2	1	1	1
3	1	3	2	3	1	1	2	2	1
9	9	9	9	9	9	9	9	9	9
3	1	3	1	3	2	1	2	1	2
2	2	3	2	2	2	1	1	1	1
2	2	2	2	2	2	2	2	2	2
9	9	9	9	9	9	9	9	9	9
2	2	2	1	3	2	2	1	1	1
3	1	2	2	1	3	2	1	2	1
2	2	2	2	2	2	2	2	2	2
2	2	3	2	3	2	1	1	1	1
3	1	3	1	3	2	2	1	1	1
3	1	3	1	3	3	2	1	1	1
2	2	2	2	3	3	2	2	2	2
3	1	3	1	2	2	2	2	1	1
3	1	3	1	3	2	3	1	1	1
3	1	3	1	3	3	2	1	1	1
2	2	3	2	3	2	3	1	1	1
2	9	9	2	9	3	9	3	9	3
9	9	9	9	9	9	9	9	9	9
3	1	3	1	3	1	3	1	1	2
9	9	9	9	9	9	9	9	9	9
9	9	9	9	9	9	9	9	9	9
3	1	3	1	2	2	1	1	1	1
2	2	2	2	2	2	2	2	2	2
3	1	2	1	3	1	1	1	1	1
9	9	9	9	9	9	9	9	9	9
9	9	9	9	9	9	9	9	9	9
9	9	9	9	9	9	9	9	9	9
9	9	9	9	9	9	9	9	9	9
9	9	9	9	9	9	9	9	9	9
2	2	2	2	2	2	2	2	2	2
2	9	3	2	3	2	1	2	2	1
3	2	3	2	2	1	2	1	1	2
3	1	3	9	9	9	9	1	9	9
9	9	9	9	9	9	9	9	9	9
9	9	9	9	9	9	9	9	9	9
9	9	9	9	9	9	9	9	9	9
9	9	9	9	9	9	9	9	9	9
9	9	9	9	9	9	9	9	9	9
9	9	3	9	9	9	9	9	9	9
2	2	3	1	1	3	2	1	3	2
9	9	9	9	9	9	9	9	9	9
2	2	2	2	2	2	2	2	2	2
2	2	9	9	9	9	9	9	9	9
2	2	2	3	2	2	2	1	1	1
3	1	3	1	1	1	1	1	1	1

3	1	3	3	1	1	1	1	1	1
3	1	3	2	2	2	3	2	2	1
3	2	1	2	1	2	2	2	2	1
2	9	3	1	3	1	1	1	2	2
2	1	3	2	2	3	3	1	2	2
3	9	9	9	9	9	9	9	9	1
2	1	2	2	3	2	3	2	1	2
2	9	2	2	9	9	9	9	2	2
9	2	9	9	9	9	9	9	9	9
9	9	3	9	9	9	9	9	9	9
3	1	2	2	2	2	2	2	2	2
3	1	3	2	2	3	1	2	2	2
9	9	9	9	9	9	9	9	9	9
2	2	2	2	2	2	2	2	2	2
3	9	9	9	9	9	1	9	9	9
1	1	2	3	2	3	1	1	2	3
3	1	3	1	2	2	1	1	2	2
3	1	3	2	2	3	1	2	2	2
2	3	3	2	2	2	2	1	2	2
3	1	3	2	2	2	2	1	1	1
3	1	3	1	9	3	3	3	1	1
3	1	3	2	3	2	2	3	1	1
2	2	3	2	2	3	2	3	1	1
9	9	9	9	9	9	9	9	9	9
3	1	3	2	3	3	3	1	1	1
2	1	2	2	3	2	2	1	1	1
3	2	2	2	1	2	1	1	2	3
2	1	1	2	2	2	3	2	1	1
2	1	3	2	3	3	3	1	1	1
1	3	3	2	2	2	2	2	9	1
2	2	1	3	2	2	2	3	3	9
9	9	9	9	9	9	9	9	9	9
2	3	1	2	2	2	3	2	3	3
2	2	2	2	2	2	3	2	2	1
9	1	2	2	9	1	3	3	2	1
3	1	9	9	9	9	9	9	9	9
3	1	3	1	3	9	1	1	1	1
3	1	3	1	3	3	1	1	1	1
3	1	3	2	3	2	3	1	1	1
2	9	2	9	1	9	2	2	3	2
3	2	3	2	3	1	3	1	2	2
3	1	3	3	1	3	1	1	1	1
3	2	9	1	2	9	9	1	1	1
3	1	3	1	2	3	1	2	2	2
2	1	2	3	1	2	1	1	1	1
3	2	3	2	3	1	3	1	1	1
2	2	3	2	1	2	3	1	1	1

9	9	9	9	9	9	9	9	9	9
2	2	3	2	1	3	2	1	2	1
2	2	2	2	2	2	2	2	1	1
2	2	1	2	3	2	1	1	1	1
9	9	9	9	9	9	9	9	9	9
1	2	2	9	1	2	9	2	1	9
1	3	2	2	2	3	2	2	2	2
9	9	9	9	9	9	9	9	9	9
2	2	2	2	2	2	2	2	2	2
2	1	3	1	1	2	3	1	1	1
9	9	9	9	9	9	9	9	9	9
1	3	1	3	1	1	1	1	1	3
3	1	2	3	2	2	2	2	2	2
1	3	1	2	2	2	3	3	2	2
1	1	3	1	1	3	3	2	1	3
3	1	3	1	2	2	2	2	2	2
1	1	3	3	1	3	3	1	1	1
3	2	1	2	3	2	3	3	1	2
3	1	3	1	3	3	1	2	2	2
2	3	1	3	3	1	2	1	2	2
2	2	2	2	2	2	2	2	2	2
2	2	2	2	2	2	2	2	2	2
3	1	3	1	2	3	3	1	1	1
3	1	1	2	2	2	1	1	1	1
1	2	2	1	3	1	1	2	2	2
3	2	2	2	2	2	2	2	2	2
2	3	2	3	2	9	2	9	1	2
2	2	2	2	2	2	2	2	2	2
2	2	3	2	2	2	2	1	2	1
3	1	3	1	2	2	2	1	2	2
3	9	9	9	9	9	9	1	9	9
1	1	3	1	3	3	1	1	1	1
3	2	2	2	3	2	1	1	2	1
3	1	3	1	3	3	1	1	1	1
2	1	2	2	2	2	1	2	2	2
2	1	2	2	2	2	2	2	2	2
9	9	9	9	9	9	9	9	9	9
2	2	2	2	2	2	2	2	2	2
9	9	9	9	9	9	9	9	9	9
3	1	3	1	2	2	2	2	2	2
2	2	2	2	2	2	2	2	2	2
3	1	3	2	2	2	2	2	2	1
3	1	3	1	3	1	2	1	1	1
3	1	3	1	2	3	1	1	1	1
3	1	3	3	1	3	1	1	1	1
3	1	9	9	9	9	9	9	9	9
3	1	3	1	2	3	3	1	1	1

2	2	2	2	3	3	3	2	2	2
3	1	3	1	3	3	2	1	2	2
9	9	9	9	9	9	9	9	9	9
3	9	3	9	3	9	2	9	9	9
9	9	9	9	9	9	9	9	9	9
1	3	1	3	1	3	3	3	3	3
1	3	3	3	3	3	3	1	1	1
2	3	1	2	2	2	3	2	2	1
3	3	3	1	3	2	3	1	1	3
3	1	3	2	3	3	2	3	2	1
3	1	1	2	1	1	3	1	1	1
2	2	3	1	2	2	3	1	1	1
3	1	3	1	3	1	1	1	2	2
1	3	3	3	1	3	3	1	1	1
1	3	3	3	1	3	3	1	3	3
3	1	3	1	3	2	3	2	1	1
3	1	3	1	3	3	1	1	1	1
1	9	3	3	1	3	3	3	3	3
9	9	9	9	9	9	9	9	9	9
2	2	3	1	3	3	2	1	1	1
9	9	9	9	9	9	9	9	9	9
3	1	3	2	2	3	3	2	2	2
2	2	3	3	1	3	2	1	1	1
9	9	9	9	3	9	9	9	9	9
2	3	9	3	3	1	2	2	2	2

Poison	InheritedC.	OffensiveP	PostCoitalE	Dysmenorr	Menorrhag	Asymptom	VaginalRas	Vaginalswe	WillLikePa
2	2	2	2	2	2	2	2	2	2
2	3	2	2	2	2	2	2	2	3
2	2	2	2	2	2	2	2	2	2
2	2	2	1	1	9	3	3	3	3
3	3	1	2	2	2	3	2	2	1
2	3	3	3	3	3	3	3	3	2
2	2	1	2	3	1	3	2	1	2
2	2	1	2	2	2	3	2	2	1
3	1	2	2	2	2	2	2	2	3
1	2	1	1	2	2	3	1	2	2
2	2	2	2	2	2	2	2	2	2
2	2	1	1	2	2	2	2	3	1
2	1	1	1	2	2	3	3	1	1
2	2	2	2	2	2	2	2	1	1
2	2	2	2	2	2	2	2	2	2
2	2	2	2	2	2	2	2	2	2
9	9	9	9	9	9	9	9	9	9
2	2	2	2	2	2	2	2	2	1
9	9	9	9	9	9	9	9	9	9
9	9	9	9	9	9	9	9	9	9
1	1	1	1	1	1	1	1	1	1
1	2	1	2	1	1	2	2	1	2
2	2	1	1	1	1	2	1	1	1
1	1	1	1	1	1	1	1	1	1
9	9	2	9	9	9	9	9	9	9
3	3	1	2	2	2	2	2	2	1
3	3	1	3	1	1	1	1	1	1
2	2	2	2	2	2	2	2	2	1
3	1	1	2	3	3	2	3	1	1
1	3	1	3	3	2	1	1	1	2
3	2	1	2	3	3	1	1	1	1
2	2	2	2	2	2	2	2	2	1
1	3	3	3	3	1	3	3	3	3
1	1	1	1	2	1	1	1	1	3
2	9	2	1	1	1	1	2	3	1
2	3	3	3	2	3	2	3	1	2
2	2	2	2	2	2	2	2	2	2
3	2	2	1	2	2	2	1	1	2
3	3	2	2	2	2	2	1	1	1
3	1	1	3	3	1	1	2	3	3
3	2	2	2	3	2	2	2	2	1
9	3	2	2	1	3	2	1	2	9
9	9	9	9	9	9	9	9	9	9
2	2	2	2	2	2	2	2	2	2
2	2	2	2	2	1	2	2	2	2
9	9	9	9	9	9	9	9	9	9

3	3	1	1	2	2	3	2	2	2
1	2	1	2	2	1	1	2	2	2
1	1	2	2	2	1	3	2	2	1
2	2	1	1	1	2	2	1	1	2
3	1	3	3	3	3	3	3	3	3
3	1	3	3	3	3	3	3	3	3
3	3	2	2	3	2	3	3	2	2
2	2	2	1	3	1	2	1	1	1
1	1	1	2	1	2	3	1	2	3
2	1	1	9	9	9	9	9	9	9
9	9	1	9	9	9	9	9	9	1
2	2	2	2	2	2	1	1	1	1
2	2	1	1	1	1	1	1	1	1
1	1	1	1	2	2	2	3	3	1
2	2	3	2	1	2	2	1	1	1
2	2	1	1	2	2	3	3	3	1
2	3	9	9	9	9	9	9	9	3
1	1	1	3	3	3	2	3	3	3
2	2	2	1	2	2	2	2	2	1
2	3	9	9	9	9	9	9	9	2
2	2	2	3	3	3	3	3	3	3
3	3	1	2	2	2	2	1	2	2
2	2	1	1	2	2	3	3	3	1
1	1	1	1	2	2	1	1	1	1
2	2	2	2	2	2	1	1	1	1
2	2	2	2	2	1	2	1	1	1
3	3	3	3	3	3	3	1	1	3
1	2	1	2	3	3	2	2	1	1
2	1	1	1	2	2	3	1	1	1
3	3	3	3	3	3	3	3	3	3
9	9	9	9	9	9	9	9	9	9
3	1	1	2	1	1	2	2	1	3
9	1	3	1	1	9	9	1	1	1
2	2	2	2	2	2	2	2	2	2
9	2	2	2	1	9	1	3	1	1
2	2	2	2	2	2	2	2	2	2
1	1	3	9	9	9	9	9	9	9
2	2	1	1	1	2	2	1	1	2
3	2	1	2	2	2	2	2	2	2
2	2	2	2	2	2	2	1	2	1
1	2	2	2	1	2	3	2	1	1
9	9	9	9	9	9	9	9	9	9
1	3	1	1	2	3	1	1	1	2
2	2	1	1	1	1	1	1	1	1
1	1	2	1	2	2	2	2	2	3
2	2	1	2	1	2	3	3	3	3
3	2	2	2	3	2	2	2	2	1

2	1	1	2	1	2	1	2	3	1
3	3	3	3	3	3	3	3	3	3
1	1	2	2	2	2	2	2	2	2
2	2	2	2	2	2	2	1	2	2
1	1	1	2	1	1	2	3	2	1
2	1	2	2	2	1	1	1	1	1
3	3	2	2	2	2	2	2	2	2
9	9	9	9	9	9	9	9	9	9
2	2	2	1	1	1	2	1	2	2
3	2	1	2	3	3	2	1	1	1
1	1	1	2	1	3	2	2	3	1
2	3	1	1	2	2	2	1	1	2
2	2	1	2	2	3	3	3	3	1
2	2	1	2	2	2	2	2	2	2
1	2	2	3	3	1	2	1	1	2
2	1	3	1	2	1	2	1	1	2
1	1	1	1	2	3	2	1	1	1
3	3	2	2	2	2	2	2	2	3
2	9	1	2	2	2	2	1	1	1
2	2	1	1	1	2	2	2	1	2
1	3	1	1	1	1	1	1	1	1
2	1	2	3	3	2	2	2	2	1
1	1	1	2	3	2	1	1	2	1
2	2	2	2	2	2	2	2	2	1
2	2	2	2	2	2	2	2	2	2
3	3	2	2	2	2	2	2	2	1
1	3	1	1	3	3	3	1	1	1
1	2	1	2	1	2	1	2	1	2
2	2	1	2	2	2	1	1	2	1
3	1	1	3	2	2	1	2	3	1
3	3	2	2	2	3	2	1	1	1
3	1	1	2	3	3	2	1	2	3
2	1	2	2	2	1	9	1	3	3
2	2	2	1	1	2	1	2	1	2
2	3	1	2	3	2	2	1	2	1
3	1	1	1	1	3	1	1	1	1
3	3	2	2	2	2	2	2	2	3
2	2	1	1	1	2	2	1	1	1
9	9	9	9	9	9	9	9	9	9
3	3	3	3	3	3	3	3	3	3
1	1	1	2	1	1	2	1	1	1
9	9	9	9	9	9	9	9	9	9
2	2	1	1	2	2	2	1	1	1
2	2	1	2	2	3	2	2	2	1
9	9	9	9	9	9	9	9	9	9
2	3	1	3	2	2	3	2	1	2
1	1	1	1	1	1	2	1	1	1

3	1	1	3	3	1	2	2	1	2
1	2	1	1	1	1	2	1	1	3
3	3	3	3	3	3	3	3	3	3
2	2	1	1	1	1	1	1	1	2
3	3	1	2	2	2	2	3	3	1
2	2	2	3	2	3	2	3	2	2
1	2	1	2	9	1	9	3	1	2
1	2	1	2	9	1	2	3	1	2
9	9	1	9	9	9	9	9	9	1
2	2	2	2	2	2	2	2	2	2
2	1	1	1	2	3	2	1	3	2
2	1	2	1	2	2	2	2	1	2
2	2	2	2	2	2	2	2	2	2
2	1	2	2	2	2	3	2	2	1
2	2	1	2	2	2	2	1	1	1
2	2	1	2	2	1	1	1	1	1
1	1	1	1	2	2	1	1	2	1
2	2	2	2	2	2	1	1	1	1
9	9	1	9	9	9	9	9	9	1
1	1	2	1	2	3	2	2	1	2
1	2	2	1	2	2	3	3	2	1
2	2	2	2	2	2	2	2	2	2
3	3	3	3	3	3	3	3	3	3
2	2	2	2	2	2	2	2	2	2
1	3	1	2	1	1	3	2	1	1
1	3	1	3	3	1	1	3	3	2
9	9	9	9	9	9	9	9	9	9
3	3	2	2	2	2	2	2	2	2
2	2	2	3	1	1	3	2	2	3
2	2	1	2	2	2	1	1	2	1
2	2	2	1	1	1	2	2	2	2
2	1	1	1	1	2	2	2	2	2
1	1	1	1	1	2	3	3	3	1
3	3	3	3	3	3	3	2	3	3
2	2	2	2	1	2	2	2	2	2
1	1	3	3	3	1	1	1	1	1
3	3	2	2	2	2	2	2	2	3
3	3	1	1	2	2	3	1	1	1
1	1	1	1	1	1	2	2	2	1
2	2	2	2	2	2	2	2	2	2
9	9	9	9	9	9	9	9	9	9
2	2	2	2	2	2	2	2	2	2
2	2	2	2	2	2	2	2	2	2
9	9	9	9	9	9	9	9	9	9
9	9	9	9	9	9	9	9	9	9
2	2	1	2	2	2	2	2	2	1
3	3	1	1	2	2	2	2	2	1

9	9	2	9	9	9	9	9	9	9
9	1	9	9	2	9	9	9	9	9
9	1	2	1	1	3	1	1	1	2
9	9	2	9	9	9	9	9	9	1
9	9	3	3	3	3	3	3	3	9
9	9	9	9	9	9	9	9	9	9
2	1	1	1	1	1	1	2	2	9
9	9	9	9	9	9	9	9	9	9
2	2	3	2	1	2	2	1	1	2
9	9	9	9	9	9	1	9	9	9
9	9	1	9	9	9	9	9	9	9
9	9	9	9	9	9	9	2	9	9
1	1	2	2	1	1	2	2	3	2
3	2	2	2	2	3	3	3	3	3
9	9	9	9	2	9	9	9	9	9
3	3	2	3	2	2	3	3	3	2
3	3	1	1	1	1	1	1	1	1
2	2	1	2	2	2	2	2	2	1
2	2	2	1	1	1	2	2	2	2
2	2	2	2	2	2	2	2	3	2
2	2	1	1	2	3	1	1	1	1
9	9	9	9	9	9	9	9	9	9
1	1	2	2	1	1	1	1	1	2
2	2	1	2	1	1	1	1	1	1
2	2	2	2	2	2	2	2	2	2
2	2	1	1	1	2	2	3	3	2
1	1	1	1	1	1	2	2	1	1
1	1	1	1	2	3	2	2	2	2
3	3	3	3	3	3	3	3	3	3
1	1	1	1	3	1	3	1	1	1
2	2	2	2	2	2	2	2	2	2
1	1	1	2	1	1	2	2	1	1
9	9	9	9	9	9	9	9	9	9
2	1	1	2	2	2	1	1	1	1
1	1	1	3	1	1	1	3	3	1
2	2	1	2	3	2	2	1	1	2
2	3	1	2	3	9	9	9	9	9
1	1	2	1	1	2	2	1	1	2
9	9	2	9	9	9	9	9	9	2
2	2	2	1	1	1	2	2	3	2
1	1	1	1	2	2	1	1	1	2
3	2	1	3	2	2	1	1	1	1
1	1	2	2	2	2	2	2	2	1
2	2	2	2	2	2	2	2	2	2
1	1	3	3	3	3	3	3	3	2
2	1	2	2	2	2	2	2	2	2
9	9	9	9	9	9	9	9	9	9

2	2	1	1	1	1	1	1	1	1
2	2	1	2	1	1	1	1	1	1
1	1	1	1	1	1	1	1	1	1
9	9	9	9	9	9	9	9	9	9
2	2	2	2	2	2	2	2	2	2
2	3	1	1	1	1	1	2	2	2
2	3	1	1	3	2	2	1	1	3
2	2	1	3	2	2	1	1	1	3
2	2	1	2	1	1	1	1	1	2
2	1	2	1	1	1	2	2	2	1
1	2	2	2	2	3	2	2	1	2
9	9	2	9	9	9	9	9	9	3
9	9	2	9	9	9	9	9	9	2
9	9	1	9	9	9	9	9	9	1
9	2	2	9	9	9	2	9	9	9
9	9	9	9	9	9	9	9	9	9
9	9	9	9	9	9	9	9	9	9
9	9	9	9	9	9	9	9	9	9
9	2	9	2	2	9	9	2	2	9
9	9	9	9	9	9	9	9	9	9
9	9	9	9	9	9	9	9	9	9
1	2	1	2	2	1	1	1	1	1
2	2	2	2	2	2	2	2	2	1
1	1	2	1	1	1	1	1	1	1
1	1	2	2	2	1	1	1	1	1
9	9	9	9	9	9	9	9	9	9
2	2	1	1	2	2	2	2	2	1
9	9	9	9	9	9	9	9	9	9
9	9	9	9	9	9	9	9	9	9
2	2	1	1	2	2	2	2	2	1
9	9	9	9	9	9	9	9	9	9
9	9	9	9	9	9	9	9	9	9
9	9	9	9	9	9	9	9	9	9
9	9	9	9	9	9	9	9	9	9
9	9	9	9	9	9	9	9	9	9
9	9	9	2	1	9	3	1	9	9
9	9	9	9	9	9	9	9	9	9
9	9	9	9	9	9	9	9	9	9
9	9	9	9	9	9	9	9	9	9
9	9	3	9	9	9	9	9	9	3
9	9	2	9	9	9	9	9	9	1
9	9	9	9	9	9	9	9	9	9
3	3	3	3	3	3	3	3	3	3
1	9	1	1	1	1	1	1	1	3
3	1	2	1	2	1	2	1	3	1
9	1	1	1	1	1	9	2	3	1
9	9	2	9	9	9	9	9	9	1

9	9	9	2	9	9	9	9	9	1
9	9	9	9	3	9	9	9	9	3
1	1	3	3	1	2	2	2	3	2
3	1	1	2	3	1	2	3	1	2
2	3	2	1	2	2	2	2	2	2
2	2	2	2	2	3	3	3	3	2
3	2	1	1	1	1	1	1	1	1
2	2	1	1	2	2	3	2	1	1
2	2	2	2	2	2	2	2	2	2
3	3	3	3	3	3	3	3	3	3
9	9	9	9	9	9	9	9	9	9
9	9	9	9	9	9	9	9	9	9
9	9	9	9	9	9	9	9	9	9
9	9	1	9	9	9	9	9	9	9
1	1	2	2	2	2	2	2	2	3
1	1	1	1	1	2	3	3	3	1
9	9	9	9	9	9	9	9	9	9
1	1	1	2	1	1	2	1	1	2
1	1	1	1	1	1	1	1	1	1
1	1	1	1	1	1	1	1	1	2
1	2	1	2	2	2	2	2	2	2
2	2	2	2	2	2	2	2	2	1
9	9	1	9	9	9	9	9	9	1
2	2	2	2	2	2	2	2	2	2
3	3	2	2	2	2	2	2	2	2
2	2	1	2	1	2	1	2	1	2
2	2	2	2	2	2	2	2	2	2
2	1	1	1	1	1	1	1	1	3
3	3	2	2	2	2	2	3	3	3
2	1	2	2	2	2	1	1	1	1
2	2	2	2	2	2	2	2	2	2
2	2	2	2	2	2	2	2	2	2
3	1	1	2	3	1	2	3	2	2
2	2	2	2	2	2	3	3	3	2
1	2	1	1	1	1	1	1	1	3
2	3	3	3	2	2	2	2	2	1
9	9	9	9	9	9	9	9	9	9
2	2	2	2	2	2	2	2	2	2
3	3	3	3	3	3	3	3	3	3
3	3	3	3	3	3	3	3	3	3
9	9	2	9	9	9	9	9	9	2
9	9	9	9	9	9	9	9	9	9
9	9	9	9	9	1	9	9	9	9
1	1	1	1	1	1	1	1	1	2
2	1	1	2	2	2	2	2	2	2
2	2	2	2	2	2	2	2	2	2
3	3	3	3	2	1	1	1	1	2

9	9	2	9	9	9	9	9	9	9
9	9	1	9	9	9	9	9	9	2
9	9	9	9	1	9	9	9	9	1
9	9	9	9	9	1	9	9	9	9
9	3	9	1	9	9	9	9	9	9
1	2	3	3	2	1	1	1	2	3
9	9	2	9	9	9	9	9	9	2
9	9	1	9	9	9	9	9	9	3
9	9	2	9	9	9	9	9	9	2
9	9	2	9	9	9	9	9	9	2
9	9	2	9	9	9	9	9	9	9
9	9	9	9	2	9	9	9	9	9
9	9	2	9	9	9	9	9	9	2
9	9	9	9	2	9	9	9	9	9
9	9	1	9	9	9	9	9	9	1
9	9	9	9	9	9	9	9	9	9
9	9	2	9	9	2	9	9	2	9
1	3	3	2	1	3	9	2	1	1
2	2	1	2	3	2	2	2	2	1
2	2	2	2	2	2	2	2	2	2
3	3	1	2	2	2	3	1	2	2
3	2	1	1	2	2	2	2	2	9
2	2	2	2	2	2	2	2	2	2
2	3	2	3	1	9	2	3	2	2
2	2	2	3	1	3	2	2	1	3
9	9	9	9	9	9	9	9	9	9
2	3	2	1	1	2	2	2	2	2
2	2	2	2	2	2	2	2	2	9
3	2	1	2	3	1	2	2	3	1
1	2	1	1	1	1	1	2	2	1
2	3	1	2	1	3	2	2	1	1
2	2	2	2	2	2	2	2	2	2
3	3	1	1	1	1	1	1	1	2
1	1	1	1	1	1	1	1	1	1
9	9	9	9	9	9	9	9	9	9
2	2	2	2	2	2	2	2	2	2
9	9	9	9	9	9	9	9	9	9
2	2	1	2	2	1	1	1	2	1
9	9	9	9	9	9	9	9	9	9
2	2	2	2	2	2	2	2	2	2
2	2	1	2	2	2	1	2	3	1
3	3	3	3	3	3	3	3	3	3
3	3	2	3	9	3	2	2	2	1
2	2	2	2	3	2	3	2	2	2
2	2	2	2	2	2	2	2	2	2
3	2	1	1	2	2	2	2	3	1
2	2	2	2	2	2	2	2	2	2

2	1	1	1	1	2	2	2	3	1
9	9	3	9	9	9	9	9	9	9
3	2	2	2	2	2	2	2	2	2
3	1	2	9	1	3	1	2	3	2
1	1	1	1	3	2	2	2	2	3
3	2	2	2	2	3	3	3	3	2
2	2	1	2	2	1	2	2	2	1
2	2	1	1	2	2	2	2	2	1
9	9	9	9	9	9	9	9	9	9
2	2	2	2	2	2	2	2	2	2
2	3	2	2	2	2	2	2	2	2
9	9	1	9	9	9	9	9	9	1
9	9	1	9	9	9	9	9	9	1
3	3	3	3	3	1	1	1	1	2
9	2	9	9	9	9	9	9	2	1
2	2	1	1	1	1	2	2	2	2
9	9	9	9	9	9	9	9	9	9
9	9	1	9	9	9	9	9	9	1
9	9	9	9	9	1	9	9	9	9
3	2	3	2	3	2	3	2	3	2
2	2	2	2	2	2	2	2	2	2
1	2	2	2	3	3	1	2	2	3
2	2	2	2	2	2	2	2	2	2
9	9	9	9	9	9	9	1	9	9
9	9	2	9	9	9	9	9	9	2
2	2	1	2	2	2	2	2	2	1
3	3	1	1	1	1	1	1	1	1
3	1	2	2	1	1	3	3	1	2
9	9	9	9	9	9	9	9	9	9
9	9	1	9	9	9	9	9	9	3
2	2	2	2	2	2	2	2	2	2
2	3	1	2	3	1	2	3	1	2
2	2	1	2	2	2	2	2	3	2
2	2	2	2	1	2	2	2	2	1
3	1	1	1	3	3	3	1	1	1
2	1	1	2	3	1	2	1	3	1
9	9	9	9	9	9	9	9	9	9
9	9	9	9	9	9	9	9	9	9
1	2	3	2	1	3	1	2	3	3
1	2	1	2	1	2	1	2	1	1
2	2	2	2	2	2	2	2	2	2
2	2	2	2	2	2	2	2	2	2
2	2	2	2	2	2	2	2	2	1
2	1	1	2	3	1	2	3	1	1
2	2	1	1	1	1	1	1	1	2
2	2	2	2	2	2	2	2	2	2
3	3	3	3	3	3	2	2	2	2

1	1	2	2	2	2	2	2	2	1
3	1	1	2	3	9	2	2	2	1
9	9	9	9	9	1	9	9	9	9
9	9	9	9	9	9	9	9	1	9
9	9	2	9	9	9	9	9	9	9
2	3	3	2	9	2	3	1	2	2
9	2	2	9	2	9	9	2	2	2
3	9	2	9	9	2	9	2	2	2
9	9	9	9	9	9	9	9	9	9
9	9	9	9	9	9	9	9	9	9
9	9	9	9	9	9	9	9	9	9
9	9	9	9	9	9	9	9	9	9
9	9	9	9	9	9	9	9	9	9
9	9	9	9	9	9	9	9	9	9
9	9	9	9	9	9	9	9	9	9
9	9	2	9	9	9	9	9	9	2
3	3	3	3	3	3	3	3	3	3
9	9	1	9	9	9	9	9	9	1
9	9	2	9	9	9	9	9	9	2
9	2	2	9	9	2	9	9	2	2
9	9	9	9	9	9	9	9	9	9
9	9	2	9	9	9	9	9	9	2
2	2	2	2	2	2	2	2	2	2
9	9	9	9	9	9	9	9	9	9
9	9	9	9	9	9	9	9	9	9
9	9	2	9	9	9	9	9	9	2
9	9	9	9	9	9	9	9	9	9
9	2	2	9	2	9	2	9	2	2
2	9	2	1	3	2	3	3	2	9
9	9	9	9	9	9	9	9	9	9
9	9	9	9	9	9	9	9	9	9
9	2	2	9	2	9	2	9	2	2
9	9	9	9	9	9	9	9	9	9
9	9	9	9	9	9	9	9	9	9
9	9	9	9	9	9	9	9	9	9
9	9	9	9	9	9	9	9	9	9
9	9	9	9	9	9	9	9	9	9
9	9	9	9	9	9	9	9	9	9
2	2	1	2	3	1	2	3	2	1
2	3	2	3	1	2	3	1	2	2
2	1	2	9	9	9	9	9	9	9
9	9	9	9	9	9	9	9	9	9
2	1	2	1	3	2	1	3	2	2
3	2	1	2	2	2	2	2	2	3
1	1	1	1	1	3	3	3	3	1
3	2	1	2	1	1	3	2	1	2
1	1	2	3	3	3	3	2	1	1

9	9	9	9	9	9	9	9	1	9
1	3	1	2	2	1	3	3	2	1
2	2	2	2	2	2	2	2	2	2
1	1	1	1	1	1	1	1	1	1
3	3	2	3	2	3	2	3	2	2
2	2	2	2	2	1	2	1	2	2
9	9	9	9	9	9	9	9	9	9
2	1	1	2	3	1	2	2	1	3
2	2	2	2	2	2	2	2	2	2
2	2	1	1	1	1	2	1	1	1
2	2	2	3	3	2	3	2	3	2
9	9	9	9	1	9	9	9	9	9
9	9	9	9	9	9	9	1	9	9
9	9	2	9	9	9	9	9	9	1
9	9	9	9	9	9	9	9	2	9
1	1	1	1	1	1	2	1	1	9
9	9	3	2	2	2	2	2	2	2
3	1	2	2	2	2	2	2	1	1
3	3	1	3	3	3	3	2	3	1
2	2	2	2	2	2	2	2	2	2
2	2	2	2	2	2	2	2	2	2
9	9	9	9	9	9	9	9	9	9
1	1	3	1	1	3	3	1	1	3
9	9	9	2	9	9	9	9	9	9
9	9	9	9	9	9	9	1	1	9
3	3	1	1	1	1	1	1	1	2
9	9	9	9	2	9	9	9	9	9
1	1	1	1	2	2	2	2	2	3
9	9	9	9	9	9	9	9	9	9
9	9	9	9	9	9	9	9	1	1
2	3	3	2	3	1	2	9	9	1
3	2	3	2	3	2	3	2	1	3
9	9	9	9	9	9	9	9	9	9
9	9	1	9	9	9	9	9	9	1
3	2	1	2	3	2	2	2	2	1
9	9	9	9	9	9	9	9	9	9
2	3	9	9	9	9	9	9	9	9
9	9	9	1	9	9	9	9	9	1
2	2	2	2	2	2	2	2	2	2
9	9	9	9	9	9	9	9	9	9
2	2	2	2	2	2	2	2	2	2
2	2	1	1	1	1	2	1	1	1
2	2	2	2	3	3	3	3	3	2
2	2	2	2	2	2	2	2	2	2
3	2	3	2	3	9	2	3	3	2
2	2	2	2	2	2	2	2	2	2
2	2	2	2	2	2	2	2	2	3

9	9	2	9	9	9	9	9	9	9
2	2	2	2	2	2	2	2	2	2
2	2	2	2	2	2	2	2	2	2
9	9	9	9	9	9	9	9	9	9
9	9	2	9	9	9	9	9	9	9
9	9	9	9	9	9	9	9	9	9
9	9	9	9	9	9	9	9	9	9
2	2	2	2	2	2	2	2	2	2
1	2	1	1	2	2	2	1	1	1
9	9	9	9	9	9	9	9	9	9
2	2	2	2	2	2	2	2	2	1
1	1	2	2	2	2	2	2	3	1
2	2	1	1	1	2	2	2	2	2
2	2	2	2	2	2	2	2	2	2
9	9	9	9	9	9	9	9	9	9
9	9	9	9	9	9	9	9	9	9
9	9	9	9	9	9	9	9	9	9
9	9	9	9	9	9	9	9	9	9
9	9	1	9	9	9	9	9	9	1
9	9	2	9	9	9	9	9	9	2
9	9	9	9	9	9	9	9	9	9
9	9	9	9	9	9	9	9	9	9
2	2	2	2	2	2	2	2	2	2
9	9	2	9	9	9	9	9	9	2
9	9	9	9	9	9	9	9	9	9
9	9	9	9	9	9	9	9	9	9
9	9	9	9	9	9	9	9	9	9
9	9	9	9	9	9	9	9	9	9
9	2	9	9	9	9	9	9	9	9
9	9	3	9	9	9	9	9	9	3
9	9	9	9	9	9	9	9	9	9
2	2	9	9	9	9	9	9	9	9
9	9	9	9	9	9	9	9	9	9
9	9	9	9	9	9	9	9	9	9
9	9	9	9	9	9	9	9	9	9
9	9	9	9	9	9	9	9	9	9
9	9	9	9	9	9	9	9	9	9
9	9	9	9	9	9	9	9	9	9
9	9	9	9	9	9	9	9	9	9
9	9	9	9	9	9	9	9	9	9
2	2	2	2	2	2	2	2	2	2
9	9	2	9	9	9	9	9	9	2
9	9	9	9	9	9	9	9	9	9
9	9	9	9	9	9	9	9	9	9
9	9	9	9	9	9	9	9	9	9
9	9	9	9	9	9	9	9	9	9
9	9	9	9	9	9	9	9	9	9

9	9	9	9	9	9	9	9	9	9
3	3	2	2	2	2	2	2	2	2
9	9	9	9	9	9	9	9	9	9
9	9	9	9	9	9	9	9	9	9
9	9	9	9	9	9	9	9	9	9
9	9	9	9	9	9	9	9	9	9
9	9	9	9	9	9	9	9	9	9
9	9	9	9	9	9	9	9	9	9
9	9	9	9	9	9	9	9	9	9
9	9	9	9	9	9	9	9	9	9
9	9	9	9	9	9	9	9	9	9
9	9	9	9	9	9	9	9	9	9
9	9	9	9	9	9	9	9	9	9
9	9	9	9	9	9	9	9	9	9
9	9	9	9	9	9	9	9	9	9
9	9	9	9	9	9	9	9	9	9
9	9	9	9	9	9	9	9	9	9
2	2	2	2	2	2	2	2	2	2
9	9	9	9	9	9	9	9	9	9
9	9	9	9	9	9	9	9	9	9
9	9	9	9	9	9	9	9	9	9
9	9	9	9	9	9	9	9	9	9
9	9	9	9	9	9	9	9	9	9
9	9	9	9	9	9	9	9	9	9
2	2	2	2	2	2	2	2	2	2
9	9	9	9	9	9	9	9	9	9
9	9	9	9	9	9	9	9	9	9
2	2	1	1	1	1	1	1	1	1
9	9	9	9	9	9	9	9	9	9
9	9	9	9	9	9	9	9	9	9
1	1	1	1	1	1	1	1	1	1
2	2	1	2	3	2	1	2	2	1
9	9	9	9	9	9	9	9	9	9
2	2	2	2	2	2	2	2	2	2
9	9	9	9	9	9	9	9	9	9
2	2	2	2	2	2	2	2	2	2
9	9	9	9	9	9	9	9	9	9
9	9	9	9	9	9	9	9	9	9
1	1	1	1	1	1	1	1	1	1
9	3	3	9	9	9	9	9	9	3
9	9	9	9	9	9	9	9	9	9
9	9	9	9	9	9	9	9	9	9
2	2	2	2	2	2	2	2	2	2
2	2	2	2	2	2	2	2	2	2
9	1	1	9	1	9	3	1	1	1
9	9	9	9	9	9	9	9	9	9
9	9	9	9	9	9	9	9	9	9

9	2	2	9	9	2	9	9	2	2
9	9	9	9	9	9	9	9	9	9
2	3	9	9	9	9	9	9	9	9
9	9	9	9	9	9	9	9	9	9
9	9	9	9	9	9	9	9	9	9
9	9	9	9	9	9	9	9	9	9
9	9	2	9	9	9	9	9	9	2
9	9	2	9	9	9	9	9	9	2
2	9	2	9	2	9	2	9	2	2
9	9	9	9	9	9	9	9	9	9
9	9	9	9	9	9	9	9	9	9
9	9	9	9	9	9	9	9	9	9
9	9	9	9	9	9	9	9	9	9
9	9	9	9	9	9	9	9	9	9
2	3	2	3	3	2	2	2	2	1
3	2	1	2	3	1	2	1	3	1
9	9	9	9	9	9	2	9	9	9
2	2	2	2	2	2	2	2	2	2
2	2	2	2	2	3	3	2	2	2
9	9	1	9	9	9	9	9	9	2
9	9	2	9	9	9	9	9	9	1
9	9	1	9	9	9	9	9	9	1
3	3	1	1	1	1	1	1	1	2
2	3	1	2	3	1	2	3	1	2
3	3	2	2	2	2	2	2	2	2
2	2	2	2	2	2	2	2	2	2
3	3	9	9	9	9	9	9	9	9
2	3	2	2	2	2	2	2	2	1
9	2	2	2	2	2	2	2	2	1
2	2	2	2	2	2	2	2	9	2
2	2	2	2	2	2	2	2	2	2
9	9	9	9	9	9	9	9	9	9
9	9	1	9	9	9	9	9	9	1
9	9	9	9	9	9	9	9	1	9
9	9	1	9	9	9	9	9	9	9
9	9	9	9	9	9	9	9	9	9
9	9	9	9	9	9	9	9	9	9
2	9	9	2	9	9	2	9	2	9
9	9	9	9	9	9	9	9	9	9
9	2	2	9	9	2	9	9	2	2
9	9	9	9	9	9	9	9	9	9
2	2	2	2	2	2	2	2	2	2
9	9	9	9	9	9	9	9	9	9
9	9	9	9	9	9	9	9	9	9
9	9	9	9	9	9	9	9	9	9
2	2	1	1	2	2	2	2	2	2
1	3	2	1	2	1	2	2	1	2

2	2	2	9	1	1	1	1	1	2
2	2	1	2	2	2	3	2	2	1
2	2	1	2	2	2	2	2	2	1
3	3	1	1	2	1	2	2	3	1
3	3	3	3	3	3	3	3	3	3
1	1	2	2	2	2	2	2	2	1
2	2	2	2	2	2	2	1	1	1
3	3	3	3	3	3	3	3	3	3
9	9	9	9	9	9	9	9	9	9
2	2	2	2	2	2	2	2	2	2
9	2	2	9	9	2	9	9	2	1
3	1	3	1	1	2	2	1	1	3
2	2	2	2	2	2	2	2	2	2
1	2	1	1	2	2	3	3	1	1
3	3	3	3	3	3	3	3	3	3
2	2	2	2	2	2	2	2	2	2
2	1	2	1	2	3	1	2	3	1
3	3	2	3	3	3	3	2	2	2
9	9	9	9	9	9	9	9	9	9
2	2	1	2	2	1	1	2	2	2
2	2	2	2	3	1	2	2	2	2
1	3	1	1	2	2	3	1	1	1
1	2	2	2	2	1	3	1	1	2
2	3	2	1	1	2	2	3	2	2
2	2	2	2	2	2	1	2	2	1
2	2	2	2	2	2	2	2	2	1
1	2	2	2	2	2	2	2	2	2
2	2	2	2	2	2	2	3	2	3
2	2	2	2	2	2	2	2	1	2
3	3	1	2	1	1	3	3	3	3
3	3	3	3	3	3	3	3	3	3
9	9	9	9	1	1	9	9	9	9
3	3	3	3	3	3	3	3	3	3
2	2	2	2	2	2	2	2	2	2
9	9	9	2	9	9	9	9	9	9
3	3	3	2	2	3	3	2	3	3
2	2	3	3	3	3	3	3	3	3
1	1	1	1	1	1	1	1	1	1
2	2	2	2	1	2	1	2	2	2
9	9	9	9	9	9	9	9	9	9
2	2	2	2	2	2	2	2	2	2
9	9	9	9	9	9	9	9	9	9
1	1	2	3	2	2	3	2	3	2
2	2	2	2	2	2	2	2	2	2
2	3	1	2	3	1	2	2	2	2
9	9	9	9	2	9	9	9	9	2
2	2	2	2	3	2	3	3	3	1

9	9	9	9	9	9	9	9	9	9
3	3	3	3	3	3	3	3	3	3
2	1	9	3	3	1	3	3	3	1
3	3	3	3	2	2	2	2	2	2
2	1	1	1	2	2	1	2	3	1
9	9	9	9	9	9	9	9	9	9
2	2	3	3	3	3	3	3	3	2
3	3	2	3	2	3	1	2	3	1
3	1	2	1	2	2	2	1	1	1
2	2	2	2	2	2	2	2	2	2
2	2	2	2	2	2	2	2	2	1
2	2	2	2	2	2	2	2	2	2
9	9	9	9	9	9	9	9	9	9
3	3	3	3	2	3	2	3	2	2
3	2	2	2	2	2	2	2	2	1
2	2	2	2	2	2	2	2	2	2
3	3	2	2	2	2	2	2	2	2
3	2	2	2	2	2	2	2	2	3
2	2	2	2	2	2	2	2	2	2
9	9	9	9	9	9	2	9	9	1
9	9	9	9	9	9	9	9	9	9
2	2	2	2	2	2	2	2	2	1
3	2	2	2	2	2	2	2	2	2
2	3	2	3	2	2	3	2	2	1
9	9	9	9	9	9	9	9	9	9
2	1	1	3	2	3	1	2	1	1
9	9	1	9	9	9	9	9	9	1
2	2	2	2	2	2	2	2	2	2
2	2	2	2	2	2	2	2	2	2
2	2	2	2	2	2	2	2	2	2
1	2	1	2	3	2	3	2	3	1
9	9	9	9	9	9	9	9	9	9
9	9	9	9	9	9	9	9	9	9
2	2	1	2	2	2	2	2	1	1
3	2	2	2	2	3	3	2	1	3
9	9	9	9	9	9	9	9	9	9
9	9	9	9	9	9	9	9	9	9
3	2	2	2	2	2	1	1	1	2
3	3	2	2	2	2	3	2	2	1
2	1	1	1	2	2	1	1	2	2
2	2	2	2	2	2	2	2	2	2
2	2	2	2	2	2	2	2	2	2
2	2	2	2	2	2	2	2	2	2
2	2	2	2	2	2	2	2	2	2
3	2	2	2	2	3	2	2	2	2
3	2	2	1	1	1	2	1	1	2
2	2	2	2	2	2	2	2	2	2

3	3	3	3	2	1	2	3	3	3
3	2	2	2	2	3	2	2	2	2
2	2	2	2	2	2	2	2	2	2
1	1	1	1	1	1	1	1	1	2
2	2	2	2	2	2	2	2	2	2
3	2	2	2	2	3	2	2	2	2
3	2	2	2	2	2	2	2	2	2
2	2	2	2	2	2	2	2	2	2
2	2	2	2	2	2	3	2	2	1
9	9	9	9	9	9	9	9	9	9
3	3	2	2	2	2	2	2	2	2
1	3	1	2	1	1	2	3	2	1
1	2	1	1	1	1	1	2	1	1
1	1	1	1	1	1	1	1	1	1
2	3	3	3	3	3	3	3	3	2
9	1	9	9	1	9	2	9	2	1
9	9	9	9	9	9	9	9	9	9
9	9	9	9	9	9	1	9	9	9
9	9	9	9	9	9	9	9	9	9
9	9	9	9	9	9	9	9	9	9
1	1	2	1	3	3	1	1	2	3
9	9	9	9	9	9	9	9	9	9
9	9	9	9	9	9	9	9	9	9
2	2	2	2	2	2	2	2	2	2
1	1	2	2	2	2	2	2	2	2
2	2	2	2	2	2	2	2	2	2
2	3	2	2	3	3	1	1	2	1
9	9	9	9	9	9	9	9	9	9
1	2	2	1	2	2	2	2	1	2
1	3	2	3	2	2	2	2	1	1
9	9	9	9	9	9	9	9	9	9
3	1	1	2	1	2	1	2	1	1
2	1	1	2	3	2	1	2	2	1
9	1	2	2	2	3	3	1	1	3
2	2	1	1	2	2	2	1	1	2
2	2	2	2	2	2	2	2	2	2
9	9	9	9	9	9	9	9	9	9
2	2	2	2	2	2	2	2	2	2
2	2	2	2	2	2	2	2	2	2
1	1	1	2	3	2	1	1	1	2
2	1	2	2	2	2	2	2	2	2
3	2	2	2	1	1	1	1	1	2
1	2	1	1	1	2	2	2	3	1
3	3	1	1	1	1	2	2	2	1
2	3	1	2	3	1	3	2	2	2
1	2	1	1	1	1	1	1	1	2
3	3	3	3	3	2	2	2	2	3

2	2	2	2	3	3	3	2	2	1
2	2	2	2	2	2	2	2	2	2
3	3	2	2	3	1	9	1	2	3
2	2	2	2	2	2	2	2	2	2
2	3	3	3	3	2	3	2	3	3
9	9	9	9	9	9	9	9	9	9
9	9	1	9	9	9	9	9	9	9
3	3	2	2	2	2	2	2	2	2
9	9	9	9	9	9	9	9	9	9
9	9	9	9	9	9	9	9	9	9
2	2	2	3	1	1	2	2	2	2
1	1	2	2	2	2	2	2	2	1
2	1	1	2	2	2	3	3	3	2
9	9	2	9	1	9	9	9	9	1
9	9	2	2	2	2	2	2	2	9
3	1	3	3	3	3	1	3	3	1
1	2	1	2	2	2	1	2	1	1
9	9	9	9	9	9	9	9	9	9
1	2	2	2	2	2	2	2	2	1
2	2	1	1	3	3	2	2	2	2
2	3	2	2	2	2	2	2	2	3
9	9	9	9	9	9	9	9	9	9
2	2	2	2	2	2	2	2	2	2
2	2	2	2	2	2	2	2	2	2
2	2	2	2	2	2	2	2	2	2
2	2	2	2	2	2	2	2	2	2
2	2	2	2	2	2	2	2	2	2
2	2	2	2	2	2	2	2	2	2
2	1	2	1	2	2	2	3	2	2
2	2	2	2	2	2	2	2	2	2
2	3	1	1	1	1	1	2	2	1
1	2	1	2	2	3	3	2	3	1
1	2	2	3	3	9	1	1	1	1
2	2	2	2	2	2	2	2	2	2
1	1	2	1	1	1	2	1	1	1
2	2	1	2	3	2	1	2	2	1
2	2	2	2	2	2	2	2	2	1
2	2	2	2	2	2	2	2	2	2
2	2	2	2	2	2	2	2	2	1
3	2	2	2	2	2	3	2	2	1
3	1	1	1	1	1	1	1	1	1
2	2	2	3	2	2	1	3	3	2
2	2	2	2	2	2	2	2	2	2
2	2	2	2	2	2	2	2	2	2
2	2	2	2	2	2	2	2	2	2
2	2	1	1	2	2	2	2	2	1
2	2	2	2	2	2	2	2	2	2

2	3	2	1	3	2	3	1	2	3
3	2	2	1	2	2	3	2	2	1
2	3	2	3	2	3	2	3	2	2
2	2	2	2	2	2	2	2	2	2
9	9	2	9	9	9	9	9	9	1
9	9	9	9	1	9	9	9	9	9
2	3	2	3	2	3	2	3	2	3
9	9	1	9	9	9	9	9	9	1
9	9	1	9	9	9	9	9	9	1
9	9	9	9	9	9	9	9	9	9
9	9	9	9	9	9	3	9	9	9
9	9	9	9	9	9	2	9	9	9
9	9	9	9	9	9	9	9	2	1
9	9	9	1	9	9	9	9	9	9
2	3	3	3	3	3	1	2	1	2
9	9	1	9	9	9	9	9	9	9
9	9	9	9	9	9	9	9	9	9
9	9	9	9	9	1	9	9	9	9
9	9	9	9	9	1	9	9	9	9
9	9	9	9	9	9	2	9	9	9
9	9	9	9	9	9	9	9	9	9
2	2	2	2	2	2	2	2	2	2
2	2	2	3	2	2	2	2	2	1
2	2	2	1	1	1	1	1	1	1
3	2	1	1	2	2	2	1	1	1
9	9	9	9	9	9	9	9	9	9
2	2	1	2	2	2	3	3	3	3
9	9	9	9	9	9	9	9	2	2
2	3	2	2	2	2	2	2	2	1
1	1	1	1	1	1	1	1	1	1
3	2	1	3	3	3	3	3	3	3
9	9	2	9	9	9	9	9	9	1
2	2	2	2	2	2	2	2	2	2
9	9	2	9	9	9	9	9	9	2
9	9	3	9	9	9	9	9	9	3
1	1	1	1	2	2	1	1	1	1
1	9	1	1	1	1	1	1	1	1
9	9	9	9	9	9	9	9	9	9
9	9	9	9	9	9	9	9	9	9
2	2	2	2	2	2	2	2	2	2
9	9	9	9	9	9	9	9	9	9
2	2	2	2	2	2	2	2	2	2
3	3	3	3	3	3	3	3	3	3
9	9	9	9	2	9	9	9	9	9
2	2	2	2	2	2	2	2	2	2
2	2	2	2	2	2	2	2	2	2
9	9	9	9	9	9	9	9	9	9

9	9	9	9	9	9	9	9	9	9
2	9	2	9	2	9	9	2	2	9
9	9	9	9	9	9	9	9	9	9
2	2	2	2	2	2	2	2	2	2
9	9	9	9	9	9	9	9	9	9
3	1	3	1	3	2	1	3	1	9
9	9	9	9	9	9	9	9	9	9
9	9	9	9	9	9	9	9	9	9
9	2	9	2	9	9	2	9	2	9
2	2	9	9	9	9	9	9	9	9
9	9	9	9	9	9	9	9	9	9
2	9	9	2	9	9	2	9	2	2
2	2	2	2	2	2	2	2	2	2
9	9	9	9	9	9	9	9	9	9
2	2	2	2	2	2	2	2	2	2
9	9	9	9	9	9	9	9	9	9
9	9	9	9	9	9	9	9	9	9
9	9	9	9	9	9	9	9	9	9
9	9	9	9	9	9	9	9	9	9
9	9	9	9	9	9	9	9	9	9
2	2	2	2	2	2	2	2	2	2
2	2	2	2	2	2	2	2	2	2
3	2	3	3	3	3	3	3	3	3
2	2	2	1	1	1	1	2	3	1
3	3	2	2	2	2	2	2	2	2
2	2	2	2	2	2	2	2	2	2
1	1	1	1	1	1	1	1	1	1
2	2	1	2	2	2	2	2	2	2
1	1	1	1	1	2	3	3	1	1
2	2	1	2	2	2	2	2	2	2
2	3	2	2	2	1	1	1	1	2
1	3	2	2	2	1	3	2	3	2
2	2	2	2	2	2	2	2	2	2
9	9	9	9	9	9	9	9	9	9
3	2	1	1	1	2	2	2	2	1
2	2	2	2	2	2	2	2	2	2
2	2	2	2	2	2	2	2	2	2
1	1	1	1	1	1	1	1	1	3
2	3	1	2	3	1	2	3	1	1
3	1	2	2	2	9	2	2	2	2
1	1	9	9	9	9	9	9	9	9
9	9	9	9	9	9	9	9	9	9
9	9	3	2	2	2	2	2	2	2
9	9	9	9	9	9	9	9	9	9
1	2	9	9	1	9	2	1	9	1
2	2	2	2	2	2	2	2	2	2
9	9	9	9	9	9	9	9	9	9

3	3	2	2	3	3	3	3	3	3
1	3	1	1	1	2	2	3	3	1
1	1	9	9	9	9	9	9	9	9
2	2	2	2	2	2	2	2	2	2
1	1	9	9	9	9	9	9	9	9
2	2	1	1	1	1	1	1	1	2
3	3	1	1	1	1	2	2	2	1
1	1	1	2	9	3	2	2	2	1
3	3	3	3	3	3	3	3	3	3
3	2	1	1	1	1	1	1	2	1
9	9	9	9	9	9	9	9	9	9
2	2	2	2	2	2	2	2	2	2
2	2	2	2	2	2	2	2	2	2
2	2	2	2	2	2	2	2	2	2
2	2	9	9	9	9	9	9	9	2
1	1	2	2	2	9	1	1	1	1
1	1	2	1	2	1	2	1	1	1
1	2	1	1	2	1	2	1	2	1
1	1	1	1	1	1	1	1	1	1
9	9	9	9	9	9	9	9	9	9
1	2	1	1	2	2	1	2	1	1
2	2	2	2	2	2	2	2	2	2
9	9	9	9	9	9	9	9	9	9
1	1	2	2	2	2	2	2	2	2
9	9	9	9	9	9	9	9	9	9
2	1	1	1	1	1	1	1	1	1
1	1	1	1	1	1	1	9	2	1
1	9	1	1	9	1	1	1	1	1
9	9	9	9	9	9	9	9	9	9
9	2	9	9	9	9	9	9	9	9
9	9	9	9	9	9	9	9	9	9
9	9	9	9	9	9	9	9	9	9
2	2	1	9	1	1	1	1	1	1
2	2	1	1	1	1	1	1	1	1
2	2	2	2	2	2	2	2	2	2
9	9	9	9	9	9	9	9	9	9
1	1	1	2	2	2	2	3	1	1
1	1	1	1	1	1	1	1	1	1
9	9	9	9	9	9	9	9	9	9
9	9	9	9	9	9	9	9	9	9
9	9	9	9	9	9	9	9	9	9
9	9	9	9	9	9	9	9	9	9
2	9	1	1	1	1	1	1	1	2
9	9	9	9	9	9	9	9	9	9
1	2	1	1	2	1	2	1	2	1
2	2	2	2	2	2	2	2	2	2
1	1	1	1	1	1	1	1	1	1

2	2	1	1	1	1	1	2	2	1
2	9	2	2	9	2	2	2	2	9
2	2	2	2	2	2	2	2	2	2
1	1	1	1	1	1	2	1	1	1
2	2	2	2	2	2	2	2	2	2
9	9	9	9	9	9	9	9	9	9
3	1	3	2	2	2	2	9	1	2
2	2	2	2	2	2	2	2	2	2
2	9	9	9	9	9	9	9	9	9
2	2	9	9	9	9	9	9	9	9
9	9	9	9	9	9	9	9	9	9
9	9	9	9	9	9	9	9	9	9
9	9	9	9	9	9	9	9	9	9
9	9	9	9	9	9	9	9	9	9
9	9	9	9	9	9	9	9	9	9
9	9	9	9	9	9	9	9	9	9
2	2	9	9	9	9	9	9	9	9
9	9	9	9	9	9	9	9	9	9
2	2	2	2	2	2	2	2	2	2
3	3	1	1	3	3	2	2	1	1
3	3	1	1	3	3	2	1	2	1
3	3	1	1	3	3	2	1	2	1
3	3	1	1	3	3	2	1	2	1
3	3	1	1	3	3	2	2	1	1
3	3	1	1	3	3	2	2	1	1
3	3	1	1	1	3	2	2	1	1
3	3	1	1	3	3	2	1	1	1
3	3	1	1	3	3	2	1	1	1
3	3	1	1	3	3	2	2	1	1
3	3	1	1	3	3	2	1	1	1
3	3	1	1	3	3	2	1	1	1
3	3	1	1	3	3	2	1	1	1
3	3	1	1	3	3	2	2	1	1
3	3	1	1	3	9	2	1	1	1
3	3	1	1	3	3	2	2	9	1
3	3	1	1	3	3	2	1	1	1
3	3	1	1	3	3	2	2	2	1
3	3	1	1	3	3	2	2	1	1
3	3	1	1	3	3	2	1	1	1
3	3	1	1	3	3	2	1	1	1
3	3	1	1	3	3	2	1	1	1
3	3	1	1	3	3	2	2	3	1
3	3	1	1	3	3	2	2	2	1
3	3	1	1	1	3	3	2	1	1
9	9	9	9	9	9	9	9	9	9
9	9	9	9	9	9	9	9	9	9
9	9	9	9	9	9	9	9	9	9

9	9	9	9	9	9	9	9	9	9
9	9	9	9	9	9	9	9	9	9
9	9	9	9	9	9	9	9	9	9
9	9	9	9	9	9	9	9	9	9
9	9	9	9	9	9	9	9	9	9
9	9	9	9	9	9	9	9	9	9
9	9	9	9	9	9	9	9	9	9
9	9	9	9	9	9	9	9	9	9
9	9	9	9	9	9	9	9	9	9
9	9	9	9	9	9	9	9	9	9
9	9	9	9	9	9	9	9	9	9
9	9	9	9	9	9	9	9	9	9
9	9	9	9	9	9	9	9	9	9
9	9	9	9	9	9	9	9	9	9
9	9	9	9	9	9	9	9	9	9
9	9	9	9	9	9	9	9	9	9
9	9	9	9	9	9	9	9	9	9
9	9	9	9	9	9	9	9	9	9
9	9	9	9	9	9	9	9	9	9
3	3	1	1	3	3	2	2	1	1
3	3	1	1	3	3	2	1	1	1
3	3	1	1	3	3	2	2	1	1
3	3	1	1	3	3	2	2	1	1
3	3	1	1	3	3	2	2	1	1
3	3	1	1	3	3	2	2	9	1
3	3	1	1	3	3	2	1	2	2
3	3	1	1	3	3	2	2	2	1
3	3	1	1	3	3	2	2	2	1
3	3	1	1	3	3	2	1	2	1
3	3	1	1	3	3	3	1	2	1
3	3	1	1	3	3	2	2	1	3
3	3	1	1	3	3	2	1	1	1
3	3	1	1	3	3	2	2	1	1
3	3	1	1	3	3	1	2	1	1
3	3	1	1	3	3	2	1	1	1
9	1	1	9	3	3	9	1	1	1
3	3	1	1	3	3	2	1	2	1
3	3	1	1	3	3	2	1	1	1
3	3	1	1	3	3	2	1	1	1
3	3	1	1	3	3	2	1	1	1
3	3	1	1	3	2	9	1	1	1
3	3	1	1	3	3	2	2	1	1
3	3	1	1	3	3	2	1	1	1
3	3	1	1	3	3	2	2	9	1
3	3	1	1	3	3	2	2	9	1
3	3	1	1	3	3	2	1	2	1
3	3	1	1	3	3	2	1	1	1

3	3	1	1	3	3	2	1	1	1
9	9	1	1	3	3	2	1	1	1
3	3	1	1	3	3	3	2	2	1
3	3	1	1	3	3	2	1	3	1
3	3	1	1	3	3	2	2	1	1
3	3	1	1	3	3	2	1	1	1
3	3	1	1	3	3	2	1	2	1
3	3	1	1	3	3	2	1	2	1
3	3	1	1	3	3	9	1	2	1
3	3	1	1	3	3	2	3	1	1
3	3	1	1	3	3	2	1	1	1
3	3	1	1	3	3	2	1	3	1
3	3	1	1	3	3	2	2	1	1
3	3	1	1	1	3	1	1	2	1
3	3	1	1	3	3	2	1	2	1
3	3	1	1	3	3	2	1	2	1
3	3	1	9	3	3	2	1	2	1
3	3	1	1	3	3	2	1	2	1
3	3	1	1	3	3	2	2	1	1
3	3	1	1	3	3	2	2	1	1
3	3	1	1	3	3	2	2	1	1
3	3	1	1	3	3	2	1	1	1
3	3	1	1	1	2	2	1	1	1
3	3	1	1	3	3	2	2	1	1
3	3	1	1	3	3	2	2	1	3
3	3	1	1	3	3	2	1	2	1
3	3	1	1	3	3	1	1	2	1
3	3	1	1	3	3	2	2	1	1
3	3	1	1	1	3	2	1	9	1
3	3	1	1	1	3	9	3	1	1
3	3	1	1	3	3	2	2	2	1
3	3	1	1	3	3	2	1	1	1
9	9	1	3	3	3	2	1	1	1
3	3	1	1	3	3	2	2	1	1
9	9	9	9	9	9	9	9	9	9
3	3	1	1	3	3	2	1	1	1
3	3	1	1	3	3	2	2	1	1
3	3	1	1	3	3	2	1	1	1
3	3	1	1	3	3	2	1	2	1
9	9	1	1	3	3	2	1	2	1
3	3	1	1	3	3	2	1	2	1
1	3	1	1	3	3	2	1	2	1
3	3	1	1	3	3	2	2	1	1
3	3	1	1	3	3	2	1	1	1
3	3	1	1	3	3	2	2	1	1
3	3	1	1	3	3	2	1	2	1
3	3	1	1	3	3	2	2	9	1

3	3	1	1	1	3	3	2	1	1
3	3	1	1	3	3	2	2	1	1
3	3	1	1	3	3	2	2	1	1
3	3	1	1	3	3	2	2	1	3
3	3	1	1	3	3	2	2	1	1
3	3	1	1	3	3	2	1	2	1
3	3	1	1	3	3	2	2	1	1
3	3	1	1	3	3	2	1	1	1
3	3	1	1	3	3	2	2	1	1
3	3	1	1	3	3	2	2	1	1
3	3	1	1	3	3	2	2	1	1
3	1	1	1	3	3	2	2	1	1
3	3	1	1	3	3	2	1	1	1
3	3	1	1	3	3	2	2	1	1
3	3	1	1	3	3	9	2	1	1
3	3	1	1	3	3	2	2	1	1
3	3	1	1	3	3	2	1	2	1
3	3	1	1	3	3	2	1	2	1
3	3	1	1	3	3	2	1	2	1
3	3	1	3	3	9	3	1	1	1
3	3	1	1	3	3	2	1	1	1
3	3	1	1	3	3	2	2	1	1
3	3	1	1	3	3	2	2	1	1
3	3	1	1	3	3	2	2	1	3
3	3	1	1	3	3	2	2	1	3
3	3	1	1	3	3	2	1	1	1
3	3	1	1	3	3	2	1	2	1
3	3	1	1	3	3	2	2	9	1
3	3	1	1	3	3	2	1	2	1
3	3	1	1	3	3	2	2	1	1
3	3	1	1	3	3	2	1	2	1
3	3	1	1	3	3	2	1	1	1
3	3	1	1	1	3	3	2	1	3
3	3	1	1	1	3	3	2	1	3
3	3	1	1	3	3	2	2	3	1
3	3	1	1	3	3	2	2	3	1
3	3	1	1	3	3	2	2	3	1
3	3	1	1	3	3	2	2	1	1
3	3	1	1	3	3	2	2	1	1
3	3	1	1	3	3	2	2	1	1
3	3	1	1	3	3	2	2	1	1
9	9	9	9	9	9	9	9	9	9
1	3	1	1	3	3	2	1	1	1
3	3	1	1	3	3	2	2	1	1
3	3	1	1	3	3	2	2	1	1
3	3	1	1	3	3	2	2	1	3
3	3	1	1	3	3	2	2	1	3

3	3	1	1	3	3	2	2	1	1
3	3	1	1	3	3	2	2	1	1
3	3	1	1	3	1	2	2	1	1
3	3	1	1	3	3	2	2	1	1
3	3	1	1	3	3	2	2	1	1
3	3	1	1	3	3	2	2	1	1
3	3	1	1	3	3	2	1	1	1
3	3	1	1	3	3	2	2	3	1
3	3	1	1	3	3	2	1	1	1
9	9	9	9	9	9	9	9	9	9
3	3	1	1	3	3	2	2	1	1
3	3	1	1	3	3	2	2	1	1
3	3	1	1	3	3	2	1	1	1
3	3	1	1	3	3	2	2	9	1
3	3	1	1	3	3	2	2	9	1
3	3	1	1	3	3	2	2	1	1
3	3	1	1	3	3	2	2	3	1
3	3	1	1	3	3	2	1	3	1
3	3	1	1	3	3	2	2	1	1
3	3	1	1	3	3	2	2	1	1
3	3	1	1	3	3	2	2	1	1
3	3	1	1	3	3	2	1	2	1
3	3	1	1	3	3	2	1	2	1
3	3	1	1	3	3	2	1	1	1
3	3	1	1	3	3	2	2	1	1
3	3	1	1	3	3	2	2	1	1
3	3	1	1	3	3	2	2	1	1
9	9	9	9	9	9	9	9	9	9
9	9	9	9	9	9	9	9	9	9
9	9	9	9	9	9	9	9	9	9
9	9	9	9	9	9	9	9	9	9
9	9	9	9	9	9	9	9	9	9
9	9	9	9	9	9	9	9	9	9
9	9	9	9	9	9	9	9	9	9
9	9	9	9	9	9	9	9	9	9
9	9	9	9	9	9	9	9	9	9
9	9	9	9	9	9	9	9	9	9
9	9	9	9	9	9	9	9	9	9
9	9	9	9	9	9	9	9	9	9
9	9	9	9	9	9	9	9	9	9
9	9	9	9	9	9	9	9	9	9
9	9	9	9	9	9	9	9	9	9
9	9	9	9	9	9	9	9	9	9
9	9	9	9	9	9	9	9	9	9
9	9	9	9	9	9	9	9	9	9
9	9	9	9	9	9	9	9	9	9
9	9	9	9	9	9	9	9	9	9

9	9	9	9	9	9	9	9	9	9
9	9	9	9	9	9	9	9	9	9
9	9	9	9	9	9	9	9	9	9
9	9	9	9	9	9	9	9	9	9
9	9	9	9	9	9	9	9	9	9
9	9	9	9	9	9	9	9	9	9
9	9	9	9	9	9	9	9	9	9
9	9	9	9	9	9	9	9	9	9
9	9	9	9	9	9	9	9	9	9
9	9	9	9	9	9	9	9	9	9
9	9	9	9	9	9	9	9	9	9
9	9	9	9	9	9	9	9	9	9
9	9	9	9	9	9	9	9	9	9
1	1	1	3	3	2	2	1	1	1
2	2	2	2	2	2	2	2	2	2
2	2	2	2	2	2	2	2	2	2
2	2	1	1	1	1	2	2	2	1
1	1	1	2	2	2	2	1	1	2
2	1	2	3	3	3	3	3	3	2
2	2	9	9	9	9	9	9	9	2
2	2	2	2	2	2	3	1	1	1
3	3	2	3	3	3	2	1	1	2
9	9	9	9	9	9	9	9	9	9
3	3	2	2	2	3	2	2	3	2
9	9	9	9	9	1	9	9	9	9
2	2	2	2	2	3	3	3	3	2
9	9	9	9	9	9	9	9	9	9
1	1	1	1	1	1	1	1	1	1
1	2	1	2	1	3	3	3	2	2
2	2	2	2	2	2	2	2	2	1
2	2	1	1	2	2	2	3	3	3
2	2	3	2	2	3	1	2	3	1
9	9	1	9	9	9	9	9	9	1
2	2	2	2	3	2	3	3	2	1
1	1	1	9	3	1	1	1	1	1
9	9	2	9	9	9	9	9	9	2
3	3	1	2	1	2	2	2	2	1
2	1	2	1	1	3	2	1	2	1
1	2	1	2	2	2	2	2	1	1
3	3	1	1	1	1	1	1	1	1
9	9	9	9	9	9	9	9	9	9
9	9	9	9	9	9	9	9	9	9
2	2	1	2	3	3	2	2	2	2
1	1	9	9	9	9	9	9	9	9
9	9	9	9	9	9	9	9	9	9
9	9	9	9	9	9	9	9	9	9
9	9	9	9	9	9	9	9	9	9

9	9	9	9	1	9	9	9	9	9
9	9	9	9	9	9	9	9	9	9
2	1	2	2	2	2	3	3	3	3
1	1	1	1	3	3	2	2	2	1
2	1	1	1	1	1	3	1	1	3
9	9	1	9	9	9	9	9	9	2
2	2	2	2	2	2	2	2	2	2
2	2	2	2	2	2	2	2	2	2
2	2	2	2	2	2	2	2	2	2
1	1	1	1	1	2	2	3	3	1
2	2	3	3	3	3	3	3	3	2
9	9	9	9	9	9	9	9	9	9
9	9	1	9	9	9	9	9	9	9
2	2	2	2	2	2	2	2	2	2
3	2	2	2	2	2	2	2	2	1
2	2	1	1	1	2	2	1	2	2
1	3	1	1	1	1	1	1	1	1
2	2	9	9	9	9	9	9	9	1
1	1	2	1	1	1	1	2	1	1
9	9	9	9	9	9	9	9	9	9
2	2	2	2	2	2	2	2	2	2
2	2	2	2	2	2	2	2	2	2
2	2	2	2	2	2	2	2	2	2
2	2	2	2	2	2	2	2	2	2
2	2	2	2	2	2	2	2	2	2
3	1	1	2	1	2	3	2	1	1
2	2	2	2	2	2	2	2	2	2
2	2	2	3	1	2	3	1	2	1
9	9	9	9	9	9	9	9	9	9
1	1	1	1	1	1	3	1	1	1
3	3	2	3	3	3	2	3	1	1
2	2	1	3	2	2	2	1	1	3
2	1	1	1	1	1	1	1	1	1
2	2	2	2	2	2	2	2	2	1
2	2	2	1	3	2	3	2	1	2
2	2	1	1	1	2	9	2	2	2
2	2	2	2	2	2	2	2	2	1
9	9	1	1	1	9	9	9	9	2
9	9	2	9	9	9	9	9	9	2
2	2	2	2	2	2	2	2	2	2
3	3	3	3	3	3	3	3	3	3
2	2	2	2	2	2	2	1	2	2
1	2	1	2	3	1	2	3	1	1
9	9	9	9	9	9	9	9	9	9
1	1	1	1	1	1	1	1	1	1
3	3	1	1	2	2	2	2	2	1
3	3	2	3	2	2	3	2	2	2

2	2	2	2	2	2	2	2	2	2
2	3	2	2	2	3	3	3	3	2
3	3	1	1	1	1	1	1	1	2
1	9	2	3	3	3	2	2	3	2
2	2	2	2	2	2	2	2	2	2
2	2	2	2	2	2	2	2	2	2
2	2	2	2	2	2	2	1	3	2
1	3	2	2	3	3	3	2	2	3
2	2	2	2	2	2	2	2	2	2
2	3	2	1	3	2	2	3	1	2
2	2	2	2	2	2	2	2	2	2
2	2	2	2	2	2	2	2	2	2
2	2	1	1	1	1	1	1	1	1
3	3	2	2	2	2	2	2	2	2
2	2	2	2	2	2	2	2	2	2
3	2	2	2	2	2	2	2	2	3
2	2	3	3	3	3	3	3	3	3
2	2	2	2	2	2	2	2	2	2
2	2	2	2	2	2	2	2	2	2
1	2	1	2	3	1	2	3	1	1
1	2	1	2	1	2	1	2	1	1
2	2	1	1	1	1	1	1	2	1
2	2	2	9	1	3	1	1	3	1
1	1	1	1	1	1	3	3	3	1
3	1	1	1	3	3	3	1	1	1
9	9	9	9	9	9	9	9	9	9
9	9	9	9	9	9	9	9	9	9
2	2	2	2	2	2	2	2	2	2
9	9	1	1	2	3	2	2	1	1
2	2	1	3	2	1	1	2	1	1
3	3	2	2	2	2	2	2	2	2
2	2	2	9	1	2	1	2	2	1
9	9	2	2	2	2	2	2	2	1
1	1	1	1	1	1	1	1	1	3
1	9	1	1	1	3	2	1	1	9
2	2	9	2	2	2	2	2	9	2
9	9	9	9	9	9	9	9	9	9
3	1	9	9	9	9	9	9	9	9
9	9	9	9	9	9	9	9	9	9
2	2	2	2	2	2	2	2	2	1
3	2	2	3	3	2	3	2	2	3
2	1	3	1	1	3	3	1	1	1
2	1	2	1	1	1	2	2	2	2
9	9	9	9	9	9	9	9	9	9
9	9	9	9	9	9	9	9	9	9
9	9	9	9	9	9	9	9	9	9
2	3	1	1	1	2	2	2	3	1

9	9	9	9	9	9	9	9	9	9
9	9	9	9	9	9	9	9	9	9
3	2	2	2	1	2	2	1	1	3
9	9	9	9	9	9	9	9	9	9
9	9	9	9	9	9	9	9	9	9
2	2	3	3	3	3	3	3	3	2
2	2	1	2	3	2	2	2	2	1
9	9	9	9	9	9	9	9	9	9
1	1	1	1	2	2	3	1	1	1
1	2	1	1	3	1	2	3	1	2
1	2	1	2	3	1	2	3	2	2
2	2	2	2	2	2	2	2	2	2
2	2	2	2	2	2	2	2	2	2
9	9	9	9	9	9	9	9	9	9
2	2	2	2	2	2	2	2	2	2
2	2	2	2	2	2	2	2	2	2
1	1	1	1	1	2	3	3	3	1
3	3	3	3	3	1	2	3	2	2
2	2	2	2	2	9	1	1	3	1
9	9	9	9	9	9	9	9	9	9
3	3	3	3	3	1	2	3	2	2
3	1	1	3	1	3	2	3	2	1
2	2	2	2	2	2	2	2	2	2
2	3	3	3	3	2	1	3	2	2
2	3	2	3	2	3	3	2	2	3
9	9	9	9	9	9	9	9	9	9
1	1	1	1	1	1	1	1	1	1
9	9	9	9	9	9	9	9	9	9
3	3	3	3	3	3	3	3	3	3
1	2	2	1	3	1	2	3	1	1
2	2	2	2	2	2	2	2	2	2
9	9	9	9	9	9	9	9	1	9
9	9	9	9	9	9	9	9	1	9
2	2	2	2	2	2	2	2	2	2
2	2	1	1	9	2	2	2	2	2
2	3	2	2	1	2	3	2	2	2
9	9	9	9	9	9	9	9	9	9
2	1	1	2	2	2	2	2	2	1
2	2	2	2	2	2	2	2	2	2
9	9	2	9	9	9	9	9	9	2
1	2	9	9	9	9	9	9	9	9
2	2	2	2	2	2	2	2	2	2
2	2	2	3	3	3	1	3	3	2
2	2	2	2	2	2	2	2	2	2
2	2	2	2	2	2	2	2	2	2
9	9	1	9	9	9	9	9	9	3
2	2	2	2	2	2	2	2	2	2

1	1	2	2	2	2	2	2	2	2
2	2	2	2	2	2	2	2	2	2
2	3	3	2	3	2	3	2	2	1
2	2	2	2	2	2	2	2	2	1
2	2	2	2	2	2	2	2	2	2
3	3	2	2	2	2	2	2	3	2
3	3	3	2	2	2	3	2	3	1
2	3	1	1	1	2	2	1	2	1
2	1	1	1	2	3	3	1	1	1
3	3	2	3	3	3	3	2	2	1
2	2	3	2	2	3	1	2	3	2
1	1	3	3	3	3	3	3	3	3
2	2	2	2	2	2	2	2	2	1
2	2	1	1	1	2	3	1	2	1
3	1	1	9	1	1	9	1	1	2
2	2	2	2	2	2	2	2	2	2
3	2	2	3	1	2	2	1	2	2
9	2	2	1	2	3	3	2	1	2
3	2	2	2	2	2	3	2	2	2
1	2	1	1	1	1	2	2	2	2
2	1	1	1	1	1	1	1	1	1
9	9	9	9	9	9	9	9	9	9
2	2	2	2	2	2	2	2	2	2
2	2	2	2	2	2	2	2	2	2
2	9	1	9	2	9	2	9	2	2
3	3	1	1	2	1	2	1	1	1
2	2	2	3	3	3	2	2	2	2
2	2	2	3	2	2	2	2	2	2
3	3	1	3	3	3	1	3	1	1
3	2	2	2	2	2	2	2	2	1
3	3	1	1	3	3	3	1	3	1
1	3	1	1	1	2	1	2	1	2
1	2	3	2	1	1	1	1	2	2
9	9	9	9	9	9	9	9	9	9
9	9	2	9	9	9	9	9	9	2
3	3	1	2	3	3	3	1	2	1
2	2	2	2	2	2	2	2	2	2
1	2	1	1	1	2	1	2	1	1
2	2	1	1	1	1	1	1	1	2
2	2	2	2	2	2	2	2	2	2
3	2	1	2	3	2	3	2	3	1
9	9	9	9	9	9	9	9	9	9
9	9	9	9	9	9	9	9	9	9
1	1	1	1	1	1	1	1	1	1
2	2	1	2	2	2	2	2	2	2
9	9	9	9	9	9	9	9	9	9
9	9	9	9	9	9	9	9	9	9

2	2	2	2	2	2	2	2	2	2
1	1	1	1	1	1	1	1	1	1
1	2	9	9	9	9	9	9	9	9
2	2	1	1	1	1	1	1	1	3
3	3	1	2	3	3	3	3	3	2
1	2	1	2	3	1	2	3	1	1
2	3	1	2	3	2	2	1	2	1
9	9	1	9	9	9	9	9	9	9
1	2	1	2	2	2	2	2	3	1
9	9	9	9	9	9	9	9	9	9
1	1	1	1	1	1	1	1	1	1
2	1	1	3	3	3	2	1	1	1
3	3	1	1	1	1	1	1	1	2
9	9	9	9	9	9	9	9	9	9
1	1	1	1	1	1	1	1	1	1
9	9	9	9	9	9	9	9	9	9
9	9	9	9	9	9	9	9	9	9
2	2	2	2	2	2	2	2	2	2
2	2	2	2	2	2	2	2	2	2
2	2	2	2	1	3	2	2	2	2
2	2	2	2	2	2	2	2	2	2
2	2	2	2	2	2	2	2	2	2
2	2	2	2	2	3	3	3	3	3
2	1	2	3	1	2	3	1	2	1
3	9	1	9	2	2	3	9	2	2
9	9	1	9	9	9	9	9	9	2
2	2	1	1	1	2	2	2	2	1
2	2	1	2	2	2	2	2	2	3
1	1	1	1	1	1	1	1	1	1
1	2	1	3	3	3	2	1	1	3
2	2	1	1	2	1	2	2	1	1
2	2	2	2	2	2	2	2	2	2
2	2	2	2	2	2	2	2	2	2
2	2	2	2	2	2	2	2	2	2
2	2	2	2	2	2	2	2	2	2
2	2	2	2	2	2	2	2	2	2
3	2	2	3	1	2	3	2	1	1
9	9	9	9	9	9	1	9	9	1
9	9	9	9	9	9	9	1	9	1
2	2	2	2	2	2	2	2	2	2
3	2	2	2	2	2	2	2	2	3
1	9	2	9	9	9	9	9	9	2
2	2	1	2	1	2	2	2	3	1
9	9	2	9	9	9	9	9	9	2
3	3	2	2	2	2	2	2	2	2
9	9	9	9	9	9	9	9	9	9
2	2	2	2	2	2	2	2	2	2

3	2	3	2	1	2	3	2	1	9
9	9	1	9	9	9	9	9	9	1
2	2	1	1	1	1	1	1	1	2
2	2	2	2	2	2	2	2	2	2
2	2	2	2	2	2	2	2	2	2
2	2	2	2	2	2	2	2	2	2
1	2	2	1	1	2	3	1	1	1
9	9	9	9	9	9	9	9	9	9
2	2	2	2	2	2	2	2	2	2
2	2	2	2	2	2	2	2	2	2
2	2	2	2	2	2	2	2	2	2
2	2	2	2	2	2	2	2	2	2
2	2	2	2	2	2	2	2	2	3
2	2	1	2	3	3	3	2	2	1
3	1	1	2	1	1	2	1	3	2
1	1	1	2	1	1	2	1	1	2
2	2	2	2	2	2	2	2	2	2
2	2	2	2	2	2	2	2	2	2
9	9	9	9	9	9	9	9	9	9
9	9	9	9	9	9	9	9	9	9
1	2	3	2	3	2	3	2	3	3
3	3	1	2	2	2	2	2	2	1
9	9	9	9	9	9	9	9	9	9
9	9	9	9	9	9	9	9	9	9
3	3	3	3	3	3	3	3	3	3
9	9	9	9	9	9	9	9	9	9
9	9	9	9	9	9	9	9	9	9
9	9	9	9	9	9	9	9	9	9
1	1	1	1	1	1	3	1	1	1
2	2	1	1	1	1	1	1	1	1
2	2	2	2	2	2	2	2	2	2
1	1	1	2	2	9	2	1	9	1
1	2	2	2	2	2	2	2	2	2
2	2	2	2	2	2	2	2	2	2
2	2	2	2	2	2	2	2	2	2
2	1	1	2	2	2	2	2	2	1
2	3	1	2	2	1	3	3	2	1
3	2	1	3	2	1	2	2	1	3
2	3	2	1	2	3	2	1	2	1
3	3	1	1	1	1	1	1	1	1
2	2	1	2	3	9	3	9	3	1
2	2	2	2	2	2	2	2	2	2
9	9	1	9	9	9	9	9	9	9
9	9	9	2	9	9	9	9	9	9
3	2	1	3	2	3	2	3	2	1
2	2	2	2	2	2	2	2	2	2
2	2	2	2	2	2	2	2	2	2

3	3	1	1	1	1	2	9	3	2
9	9	9	9	9	9	9	9	9	9
9	9	9	9	9	9	9	9	9	9
9	9	9	9	9	9	9	9	9	9
9	9	9	9	9	9	9	9	9	9
9	9	9	9	9	9	1	9	9	9
9	9	9	9	9	9	9	9	9	9
9	9	9	9	9	9	9	9	9	9
9	9	9	9	9	9	9	9	9	9
9	9	9	1	9	9	9	9	9	3
2	2	2	2	2	2	2	2	2	2
2	2	1	2	2	3	3	2	2	2
2	1	1	1	2	2	2	1	1	1
2	2	2	2	2	2	2	2	2	2
2	2	2	2	2	2	2	2	2	2
9	9	9	9	9	9	9	9	9	9
3	3	2	2	2	2	2	3	2	2
2	2	2	2	2	2	2	2	2	2
2	2	2	2	2	2	2	2	2	2
2	2	2	3	2	3	1	2	2	2
3	2	1	3	1	2	1	3	2	1
3	2	3	2	1	3	3	2	1	2
2	2	2	2	2	2	2	2	2	2
2	2	2	2	2	2	2	2	2	2
2	2	2	2	2	2	2	2	2	2
2	2	1	1	1	1	1	1	1	2
3	3	3	3	3	3	3	3	3	3
2	2	1	2	2	2	1	2	1	1
2	2	2	2	2	2	2	2	2	2
2	2	2	2	2	2	2	2	2	2
9	9	9	9	9	9	9	9	9	9
2	2	2	2	2	2	2	2	2	2
3	3	1	1	1	1	1	1	1	2
9	9	9	9	9	9	1	9	9	9
9	9	3	3	3	3	3	3	3	3
2	2	2	2	2	2	2	2	2	2
2	2	2	2	2	2	2	2	2	2
2	2	2	2	2	2	2	2	2	2
1	1	1	2	1	2	3	1	2	1
9	9	9	9	9	9	9	9	9	9
2	2	1	1	1	1	2	2	2	1
2	2	2	2	2	2	2	2	2	2
2	2	2	2	2	2	2	2	2	2
2	2	1	2	1	2	2	2	1	1
2	2	1	2	1	2	2	1	1	1
9	9	9	9	9	9	9	9	9	9
9	9	9	9	9	9	9	9	9	9

9	9	9	9	9	9	9	9	9	9
3	2	2	2	2	2	2	2	2	2
9	9	2	9	9	9	9	9	9	1
9	9	9	9	9	9	9	9	9	9
9	9	9	9	9	9	9	9	9	9
9	9	9	9	9	9	9	9	9	9
9	9	9	9	9	9	9	9	9	9
9	9	9	9	9	9	9	9	9	9
9	9	9	9	9	9	9	9	9	9
9	9	9	9	9	9	9	9	9	9
9	9	9	9	9	9	9	9	9	9
9	9	9	9	9	9	9	9	9	9
9	9	9	9	9	9	9	9	9	9
9	9	9	9	9	9	9	9	9	9
9	9	9	9	9	9	9	9	9	9
9	9	9	9	9	9	9	9	9	9
9	9	9	9	9	9	9	9	9	9
9	9	9	9	9	9	9	9	9	9
9	9	9	9	9	9	9	9	9	9
9	9	9	9	9	9	9	9	9	9
9	9	9	9	9	9	9	9	9	9
9	9	9	9	9	9	9	9	9	9
9	9	9	9	9	9	9	9	9	9
9	9	9	9	9	9	9	9	9	9
3	2	1	1	1	1	2	1	2	1
2	2	2	1	2	2	2	2	2	2
2	2	1	1	1	2	2	1	2	2
2	1	1	1	1	1	2	2	2	1
3	2	1	1	1	1	2	2	1	1
3	2	1	2	1	2	2	1	1	1
2	2	2	2	2	2	2	2	2	2
2	2	9	3	1	2	3	1	1	1
2	2	1	9	1	2	9	1	2	2
3	2	1	2	1	2	2	3	3	2
3	2	1	1	1	3	3	1	1	1
3	3	1	1	3	3	3	1	1	1
3	1	1	1	1	1	2	1	1	1
2	2	1	1	2	2	1	2	2	1
2	2	2	2	2	2	2	2	2	2
3	1	1	1	2	2	3	1	1	1
9	9	3	1	2	1	3	1	2	1
2	2	1	1	1	1	2	1	1	1
2	3	1	1	1	1	1	1	1	2
9	9	1	9	9	9	9	9	9	1
2	2	2	1	1	2	2	2	1	1
3	1	1	1	1	3	3	1	1	1
2	2	2	2	2	2	2	2	2	2
2	2	1	1	1	1	1	1	1	2

2	2	1	1	2	1	2	1	1	1
3	1	1	2	3	2	3	3	3	1
3	1	1	1	3	3	3	1	1	1
2	2	9	9	9	9	9	9	9	2
2	1	2	3	3	2	2	1	2	3
9	9	1	9	9	9	9	9	9	1
3	2	1	2	2	2	1	1	1	2
9	9	1	9	9	9	9	1	3	1
2	2	2	2	2	2	2	2	2	2
2	2	2	2	2	2	2	2	2	2
9	9	9	9	9	9	1	9	9	9
9	9	9	9	9	9	1	9	9	9
9	9	1	9	9	9	9	9	9	1
9	9	9	9	9	9	1	9	9	9
9	9	1	9	9	9	9	9	9	1
9	9	9	9	9	9	9	9	9	9
9	9	9	9	9	9	9	9	9	9
9	9	9	9	9	9	9	9	9	9
9	9	9	1	9	9	9	9	9	9
3	3	3	1	3	3	3	3	3	1
2	2	1	9	9	9	9	9	9	1
9	9	1	9	9	9	9	9	9	9
9	9	1	9	9	9	9	9	9	1
9	9	9	9	9	9	9	9	1	9
9	9	1	9	9	9	9	9	9	1
2	1	1	2	3	2	1	3	2	1
3	3	3	9	9	9	9	9	9	9
2	3	2	3	2	2	2	1	1	1
2	2	2	2	2	2	2	2	2	1
9	9	9	9	9	9	9	9	9	9
2	1	1	2	2	2	2	2	2	2
9	3	3	3	3	3	3	3	3	1
3	3	2	9	9	9	9	9	9	3
3	1	9	9	9	9	9	9	9	1
3	3	3	3	3	3	3	3	3	1
9	9	2	9	9	9	9	9	9	3
2	2	2	2	2	2	2	2	2	2
3	2	2	2	2	2	2	2	1	1
1	2	2	3	1	1	2	3	2	1
3	3	3	1	1	1	2	1	1	1
2	3	1	1	1	2	3	2	1	1
3	3	1	1	1	1	3	2	1	1
3	3	3	3	1	9	3	3	1	1
2	2	2	2	1	3	3	2	1	3
3	3	2	2	2	2	2	1	9	2
3	2	2	2	3	2	3	2	1	1
3	2	1	2	3	3	2	1	1	1

3	2	9	9	9	9	9	9	9	9
3	3	1	9	9	9	9	9	9	2
9	9	9	9	9	9	9	9	9	9
3	3	1	1	3	3	1	1	1	1
9	9	9	9	9	9	9	9	9	9
3	3	1	1	2	2	2	1	1	1
3	3	1	1	2	2	1	1	1	1
9	9	9	9	9	9	9	9	9	9
9	9	2	9	9	9	9	9	9	2
2	2	2	2	2	2	2	2	2	2
2	2	2	2	1	2	3	1	1	2
3	1	1	1	3	3	1	1	1	1
2	2	1	2	2	2	3	1	2	1
9	9	9	9	9	9	9	9	9	9
9	9	9	9	9	9	9	9	9	9
3	3	2	3	3	2	3	2	1	1
9	9	9	9	9	9	1	9	9	9
9	9	9	9	9	9	9	9	9	9
3	3	3	3	3	3	3	3	3	3
1	1	1	1	1	1	2	2	1	1
2	2	2	2	2	2	2	2	2	2
1	1	1	1	1	1	1	1	1	2
1	3	2	1	3	3	3	3	2	1
2	1	1	1	3	2	1	1	2	1
2	2	2	2	2	2	2	2	2	2
9	9	9	9	9	9	9	9	9	9
9	9	1	9	9	9	9	9	9	1
3	1	1	1	1	1	1	3	1	3
3	3	2	2	3	1	1	1	1	3
2	1	1	2	1	1	3	1	1	2
9	9	9	9	9	9	9	9	9	9
9	9	9	9	9	9	9	9	9	9
9	9	9	9	9	9	9	9	9	9
9	9	9	9	9	9	9	9	9	9
9	9	9	9	9	9	9	9	9	9
9	9	9	9	9	9	9	9	9	9
9	9	9	9	9	9	9	9	9	9
9	9	9	9	9	9	9	9	9	9
9	9	9	9	9	9	9	9	9	9
9	9	9	9	9	9	9	9	9	9
9	9	9	9	9	9	9	9	9	9
9	9	9	9	9	9	9	9	9	9
9	9	9	9	9	9	9	9	9	9
9	9	9	9	9	9	9	9	9	9
9	9	9	9	9	9	9	9	9	9
9	9	9	9	9	9	9	9	9	9
9	9	9	9	9	9	9	9	9	9

9	9	9	9	9	9	9	9	9	9
9	9	9	9	9	9	9	9	9	9
9	9	9	9	9	9	9	9	9	9
9	9	9	9	9	9	9	9	9	9
9	9	9	9	9	9	9	9	9	9
9	9	9	9	9	9	9	9	9	9
9	9	9	9	9	9	9	9	9	9
9	9	9	9	9	9	9	9	9	9
9	9	9	9	9	9	9	9	9	9
9	9	9	9	9	9	9	9	9	9
9	9	9	9	9	9	9	9	9	9
9	9	9	9	9	9	9	9	9	9
9	9	9	9	9	9	9	9	9	9
9	9	9	9	9	9	9	9	9	9
9	9	9	9	9	9	9	9	9	9
9	9	9	9	9	9	9	9	9	9
9	9	9	9	9	9	9	9	9	9
9	9	9	9	9	9	9	9	9	9
9	9	9	9	9	9	9	9	9	9
9	9	9	9	9	9	9	9	9	9
9	9	9	9	9	9	9	9	9	9
9	9	9	9	9	9	9	9	9	9
9	9	9	9	9	9	9	9	9	9
9	9	9	9	9	9	9	9	9	9
9	9	9	9	9	9	9	9	9	9
9	9	9	9	9	9	9	9	9	9
9	9	9	9	9	9	9	9	9	9
9	9	9	9	9	9	9	9	9	9
9	9	9	9	9	9	9	9	9	9
9	9	9	9	9	9	9	9	9	9
9	9	9	9	9	9	9	9	9	9
9	9	9	9	9	9	9	9	9	9
9	9	9	9	9	9	9	9	9	9
9	9	9	9	9	9	9	9	9	9
9	9	9	9	9	9	9	9	9	9
2	2	2	2	2	2	2	2	2	2
9	9	9	9	9	9	9	9	9	9
9	3	2	3	3	3	3	1	1	1
3	3	1	1	3	3	3	1	1	1
3	3	3	3	3	3	3	3	3	3
9	9	9	9	9	9	9	9	9	9
2	3	9	9	9	9	9	9	9	9
9	9	1	9	9	9	9	9	9	2
2	3	9	9	9	9	9	9	9	9
2	3	2	2	2	1	3	1	3	9
1	1	9	9	9	9	9	9	9	9
2	2	2	2	2	2	2	2	2	2

3	1	1	1	2	2	3	1	1	1
2	2	2	2	2	2	2	2	2	2
2	1	2	1	1	2	3	2	2	1
2	1	1	2	3	2	2	2	1	1
3	2	1	1	2	2	2	1	1	1
3	2	1	1	3	3	3	1	1	1
3	2	1	1	1	1	2	1	1	1
3	2	1	2	1	2	3	1	1	2
2	1	2	1	2	2	1	3	2	2
1	1	2	3	1	2	3	2	1	2
9	9	9	9	9	9	9	1	9	1
3	3	2	3	2	3	3	2	3	1
9	9	9	9	9	9	9	9	9	9
2	2	2	1	2	2	2	1	1	2
2	2	2	1	2	2	2	1	1	2
2	2	1	1	1	2	2	1	1	1
9	9	9	9	9	9	9	9	9	9
9	9	1	1	2	2	2	1	1	1
1	1	1	1	1	1	1	1	1	1
3	3	1	2	1	2	1	2	3	1
3	2	1	1	2	2	2	1	1	1
2	3	1	1	1	1	3	2	1	1
2	2	1	1	1	1	2	1	1	1
1	1	1	3	3	3	3	1	1	1
3	3	3	3	3	3	3	3	3	3
1	2	3	2	3	2	3	2	3	2
1	1	2	2	2	2	2	2	2	2
2	1	3	2	1	3	1	1	3	1
2	2	2	2	2	2	2	2	2	2
9	9	9	9	9	9	9	9	9	9
9	9	9	9	9	9	9	9	9	9
2	1	2	2	2	2	2	2	2	2
2	2	2	2	2	2	2	2	2	2
9	9	9	9	9	9	9	9	9	9
9	9	9	9	9	9	9	9	9	9
9	9	9	9	9	9	9	9	9	9
2	3	2	2	3	2	3	1	3	1
2	3	1	1	2	1	9	9	9	1
9	9	1	1	1	9	1	2	2	1
9	9	9	9	9	9	9	9	9	9
2	2	2	2	2	2	2	1	1	1
9	9	9	9	9	9	9	9	9	9
2	3	1	1	1	1	3	1	1	1
1	1	1	1	3	3	3	1	1	1
1	1	1	1	1	3	3	3	3	1
3	3	3	3	1	3	3	3	3	3
1	3	1	1	1	1	3	1	1	1

2	3	2	3	2	3	3	2	2	3
2	2	1	2	3	1	2	3	2	1
2	2	2	2	1	2	3	1	2	2
1	1	1	2	1	1	1	1	1	1
1	2	2	2	3	3	3	3	2	1
2	1	1	2	3	1	2	3	1	1
3	1	1	2	3	1	2	3	1	1
1	3	2	1	2	1	2	3	2	2
1	1	1	1	1	1	3	1	1	1
9	2	1	2	9	1	2	2	2	1
1	1	1	3	1	3	3	3	3	1
1	2	1	3	2	1	1	2	1	1
3	2	2	1	1	2	2	2	2	2
2	3	1	2	2	3	2	1	1	1
1	1	1	3	9	2	1	1	3	1
2	2	2	3	1	1	1	1	1	1
2	2	2	2	2	3	2	3	2	2
9	9	9	1	9	9	9	9	9	1
2	1	1	2	2	1	2	2	3	1
2	2	3	2	3	3	1	1	1	3
3	3	1	1	2	2	2	2	2	3
1	1	1	1	2	2	2	1	2	1
1	2	1	1	1	2	3	1	2	1
2	2	2	2	2	2	2	2	2	1
2	2	1	2	2	2	3	3	3	3
1	3	1	3	3	3	1	1	1	1
2	2	1	1	1	2	3	2	2	1
2	2	1	1	1	1	2	3	3	3
2	3	1	1	1	1	1	1	1	2
3	2	1	2	1	2	3	1	1	1
1	1	3	2	2	2	2	2	2	1
2	1	1	2	1	2	3	2	1	1
1	1	2	3	3	3	2	1	1	1
1	3	2	3	3	3	1	2	2	1
1	2	1	2	3	1	2	3	1	1
1	1	1	1	1	1	3	1	1	1
1	2	1	2	3	2	1	2	3	1
9	9	9	9	9	9	9	9	9	9
3	1	1	1	1	1	3	1	1	1
1	3	1	3	3	1	1	3	1	1
3	1	1	3	1	1	3	1	1	1
1	2	1	2	3	1	2	3	1	1
1	3	1	1	1	1	3	1	1	1
1	3	2	1	2	3	1	2	2	1
2	2	2	2	2	2	2	2	2	2
3	3	2	3	3	2	3	3	1	3
9	9	9	9	9	9	9	9	9	9

2	2	2	1	2	3	1	2	2	1
1	9	1	1	1	1	1	1	1	1
2	1	2	2	2	2	2	2	1	1
2	3	1	2	2	2	1	2	3	1
2	2	2	2	2	2	2	2	2	1
2	3	1	1	1	1	2	2	3	2
2	2	2	2	2	2	2	2	2	2
9	9	9	9	9	9	9	9	9	9
9	9	9	9	9	9	9	9	9	9
2	1	1	2	3	2	1	2	3	1
2	2	1	2	2	2	2	1	1	1
2	2	2	2	2	2	2	2	2	2
1	1	1	1	2	2	2	2	2	1
2	2	2	2	2	2	2	2	2	2
2	2	2	2	2	2	2	2	2	2
2	2	2	2	2	2	2	2	2	2
2	2	2	2	2	2	2	2	2	2
2	2	2	2	2	2	2	2	2	2
2	2	2	3	3	3	3	2	2	2
2	2	2	2	2	2	2	2	2	3
2	2	2	2	2	2	2	3	1	2
2	2	2	2	2	2	2	2	2	2
2	2	2	2	2	2	2	2	2	2
1	2	1	2	2	3	2	2	2	2
2	2	2	2	2	2	2	2	2	2
2	2	2	2	2	2	2	2	2	2
2	3	1	2	2	2	2	1	1	1
3	2	1	2	1	2	1	2	3	1
2	2	1	2	2	2	2	2	1	1
2	2	2	2	2	2	2	2	2	2
2	2	2	2	2	2	2	2	2	2
3	2	1	2	2	2	2	1	1	1
2	2	2	2	2	2	2	2	2	2
2	2	2	2	2	2	2	2	2	2
3	3	2	2	2	2	2	2	2	1
2	2	1	2	2	2	2	1	1	1
2	2	2	2	2	2	2	2	2	2
2	2	2	2	2	2	2	2	2	2
2	2	2	2	2	2	2	2	2	2
1	2	1	1	2	2	3	1	1	1
2	2	2	2	2	2	2	2	2	1
2	2	2	2	2	2	2	2	2	2
2	3	3	2	1	2	3	3	2	1
2	2	2	2	2	2	2	2	2	2
1	1	2	1	1	2	3	3	2	2
1	1	2	2	2	2	2	2	2	2
2	2	3	3	3	3	3	3	3	2

2	2	2	2	2	2	2	2	2	2
2	3	2	2	2	2	2	2	2	3
2	2	2	2	2	2	2	2	2	2
2	2	2	2	2	2	2	2	2	2
2	2	2	2	2	2	2	2	2	2
2	2	2	2	2	2	2	2	2	2
9	9	1	3	1	3	3	1	1	1
1	1	1	1	1	1	1	1	1	1
9	9	1	9	9	9	9	9	9	9
3	3	2	2	2	2	2	2	2	2
3	3	1	1	1	1	1	1	1	2
2	2	1	1	1	1	1	1	1	2
2	3	2	2	2	2	2	2	2	2
3	3	3	3	3	3	3	3	3	9
9	9	2	2	2	2	2	2	2	2
2	1	1	2	2	1	2	2	2	2
2	2	1	1	1	1	1	1	1	2
1	1	1	1	1	1	1	1	1	1
9	9	2	9	9	9	9	9	9	2
2	2	1	3	3	1	2	2	2	2
3	2	1	9	2	2	2	2	2	3
9	9	9	9	9	9	1	9	9	9
9	9	1	9	9	9	9	9	9	1
3	3	2	2	2	2	2	2	2	2
2	1	1	2	3	1	2	3	2	1
1	1	3	1	1	2	2	1	1	1
3	3	1	1	1	1	2	2	2	1
1	1	1	1	1	2	2	9	1	1
1	2	1	2	9	2	9	2	3	9
3	3	1	1	1	1	1	1	1	1
2	2	3	3	3	3	3	3	3	2
2	2	2	2	2	2	2	2	2	2
2	2	2	2	2	2	2	2	2	2
9	9	1	9	9	9	2	9	9	9
1	2	2	2	2	2	2	2	2	2
9	9	9	9	1	9	9	9	9	9
1	1	1	1	1	3	9	3	3	1
3	3	2	2	2	2	2	2	2	3
2	2	2	2	2	2	2	2	2	2
2	2	2	2	2	2	2	2	2	2
1	1	2	9	9	2	9	2	3	1
2	2	2	2	2	2	2	2	2	2
9	9	9	9	9	2	9	9	9	9
2	2	1	1	1	2	2	2	3	2
2	2	2	2	2	2	2	2	2	2
2	2	3	3	3	3	3	3	3	2
2	2	1	1	2	2	2	2	2	1

9	9	9	9	1	9	9	9	9	9
2	2	2	2	2	2	2	2	2	2
1	1	1	1	1	1	1	1	1	1
2	2	2	2	2	2	2	2	2	2
9	9	9	9	9	9	9	9	1	9
2	2	1	1	2	2	3	3	2	1
3	3	3	3	3	3	3	3	3	3
2	2	1	1	1	1	1	1	1	1
2	2	2	2	2	2	2	2	2	2
3	3	1	1	1	3	3	3	3	1
9	9	9	9	9	9	9	9	9	9
1	1	1	1	1	1	1	1	1	1
2	2	2	2	2	2	2	2	2	2
2	2	2	2	2	2	2	2	2	2
2	2	2	2	2	2	2	2	2	2
2	2	2	2	2	2	2	2	2	2
1	1	1	1	1	1	1	1	1	1
3	3	1	1	1	1	3	3	3	1
9	9	9	9	9	9	9	9	9	9
2	3	1	2	3	1	2	1	3	2
1	1	2	2	2	2	2	2	2	1
1	2	1	1	1	1	2	1	1	2
1	1	1	1	1	1	1	1	1	1
2	2	1	1	1	1	1	1	1	2
3	3	3	3	3	3	3	3	3	3
1	1	1	1	1	2	2	2	2	1
1	2	1	1	2	9	1	2	1	1
2	2	2	2	2	2	2	2	2	2
2	2	1	2	2	2	2	3	3	2
2	2	2	2	2	2	2	2	2	2
1	3	1	2	9	9	2	1	3	1
2	2	2	2	2	2	2	2	2	2
3	3	3	3	3	3	3	3	3	3
3	1	1	2	9	3	2	2	1	2
2	2	2	2	2	2	2	2	2	2
2	2	3	3	3	3	3	3	3	3
1	1	1	1	1	1	1	1	1	2
2	2	2	2	2	2	2	2	2	2
1	2	1	2	3	1	3	1	2	1
3	3	3	3	3	3	3	3	3	3
2	1	1	1	1	1	1	1	1	1
3	2	2	2	2	2	2	2	2	2
9	9	9	9	9	9	9	9	9	9
9	9	9	3	3	3	3	3	3	3
9	9	9	9	9	9	9	9	9	9
2	2	2	2	2	2	2	2	2	2
2	2	1	1	1	1	1	2	2	1

2	2	2	2	2	2	2	2	2	3
1	1	1	1	1	3	3	1	1	1
9	9	9	9	9	9	9	9	9	9
1	1	1	1	1	3	3	1	1	1
1	2	1	2	2	3	2	3	2	1
2	2	2	2	2	2	2	2	2	2
2	2	2	2	2	2	2	2	2	2
2	2	2	2	2	2	2	2	2	2
1	1	2	2	2	2	2	2	2	1
2	1	9	9	9	9	9	9	9	2
2	3	2	2	2	2	2	2	2	2
2	1	3	2	1	3	1	1	3	1
2	2	2	2	2	2	2	2	2	2
3	2	1	2	3	3	3	2	1	1
2	2	2	2	2	2	2	2	2	2
2	2	2	2	2	2	2	2	2	2
2	2	2	2	2	2	2	2	2	2
2	2	2	2	2	2	2	2	2	2
2	2	2	2	2	2	2	2	2	2
2	1	1	2	2	2	2	2	2	2
2	1	2	2	2	2	2	2	2	2
2	2	2	2	2	2	2	2	2	2
1	2	2	2	2	2	2	2	2	2
2	2	2	2	2	2	2	2	2	2
1	1	1	1	1	1	1	1	1	1
2	2	2	2	2	2	2	2	2	2
1	2	1	1	1	1	3	3	3	1
2	2	2	2	2	2	2	2	2	2
3	1	3	1	2	2	1	1	1	1
2	3	1	1	2	2	2	1	3	1
1	1	1	1	1	1	1	1	1	1
1	1	1	1	1	2	2	3	3	3
2	2	2	2	2	2	2	2	2	2
2	3	2	3	1	2	3	1	3	2
1	3	1	2	1	2	2	2	3	1
2	1	1	2	3	2	3	3	2	2
9	9	9	9	9	9	9	9	9	9
9	9	9	9	9	9	9	9	9	9
3	3	3	3	3	1	3	2	1	2
2	2	2	2	2	2	2	2	2	2
2	1	2	1	3	2	2	3	2	2
2	2	2	2	2	2	2	2	2	2
2	2	2	2	2	2	2	2	2	2
2	2	2	2	2	2	2	2	2	2
9	9	9	9	9	9	9	9	9	9
1	2	1	1	2	1	2	2	1	1
2	2	2	2	2	2	2	2	2	2

9	9	9	9	9	9	9	9	9	9
1	3	1	2	2	1	2	3	3	9
1	2	1	2	2	3	2	3	2	1
3	3	9	9	9	9	9	9	9	9
3	3	3	9	3	3	3	3	3	1
2	2	1	1	1	3	1	2	2	1
1	2	2	2	1	1	3	3	2	1
9	9	9	9	9	9	9	9	9	9
2	2	2	1	2	2	2	1	1	2
2	3	1	1	1	1	2	1	1	2
1	1	2	2	2	2	2	2	2	2
1	3	1	1	1	2	2	3	3	1
2	2	1	1	2	2	2	2	1	1
1	3	1	1	1	1	3	1	1	1
1	1	1	1	2	3	1	3	2	1
2	2	2	2	2	2	2	2	2	2
3	2	2	2	1	1	2	2	1	1
2	2	3	3	3	2	2	2	2	2
9	9	9	9	9	9	9	9	9	9
9	9	9	9	9	9	9	9	9	9
9	9	9	9	9	9	9	9	9	9
3	2	1	2	3	2	3	1	2	1
2	1	2	3	3	3	3	3	2	2
2	3	1	2	2	2	1	1	3	1
1	3	1	2	2	3	3	3	2	3
3	2	3	2	2	2	3	2	3	3
2	1	1	2	3	1	2	2	3	3
2	2	1	1	1	1	2	9	3	1
3	2	3	3	2	2	1	3	2	1
2	1	1	2	1	2	3	2	1	1
3	3	3	9	2	2	2	2	2	2
2	1	1	1	2	2	3	3	2	1
3	3	1	3	9	2	2	3	3	2
3	3	2	2	2	2	2	2	2	3
3	3	2	2	2	2	2	2	2	2
2	2	2	2	2	2	2	2	2	2
3	3	3	3	3	3	3	3	3	3
2	2	2	2	2	2	2	2	2	2
9	9	9	9	9	9	9	9	9	9
9	9	9	9	9	9	9	9	9	9
1	1	2	2	2	2	2	2	2	3
2	2	2	2	3	3	3	3	3	3
1	2	2	2	2	3	2	2	2	2
2	3	1	1	1	2	3	2	2	3
9	9	2	2	2	2	9	9	9	9
3	3	1	3	3	2	3	9	2	2
3	2	1	1	1	2	2	2	2	2

1	1	1	1	1	1	1	9	1	1
2	2	1	1	3	2	3	2	1	1
2	3	2	3	1	3	2	1	2	9
3	1	1	3	2	2	2	3	1	1
2	3	1	2	2	2	3	1	3	2
3	3	3	3	3	3	3	3	3	3
2	2	9	1	1	1	1	1	1	1
3	3	3	3	3	3	3	3	3	3
2	2	1	2	2	2	3	2	2	2
3	1	1	2	2	1	3	2	1	1
2	2	2	2	2	2	2	2	2	2
3	3	1	2	1	2	3	1	2	1
3	2	2	2	3	2	2	2	2	2
2	2	1	1	1	1	1	1	1	1
1	1	1	1	1	1	1	2	2	2
3	3	3	3	3	3	3	2	2	2
3	1	1	3	1	1	1	3	3	1
2	2	1	1	1	1	1	1	1	3
9	9	9	9	9	9	9	9	9	9
2	3	1	1	2	1	3	3	2	1
3	2	1	1	1	1	1	1	1	1
2	2	2	1	2	3	3	2	3	2
2	2	1	1	1	1	1	1	1	1
3	2	2	2	2	2	2	2	1	1
1	2	1	2	3	1	2	3	1	1
1	3	3	2	2	3	3	2	2	1
2	2	1	1	1	1	2	3	2	3
1	2	1	2	3	2	2	2	3	1
1	3	2	1	3	1	3	2	1	1
2	2	2	2	2	2	2	2	2	1
1	2	1	2	1	2	1	2	1	1
2	2	2	2	2	2	2	2	2	2
2	2	2	2	2	2	2	2	2	2
2	2	1	2	3	3	3	3	3	1
1	2	1	2	3	1	2	3	1	1
2	3	1	1	2	2	3	2	1	1
1	1	1	2	3	1	3	1	2	2
3	3	3	3	3	3	3	3	3	3
1	1	1	1	2	2	3	3	1	1
1	3	2	1	2	3	3	2	1	1
2	2	3	3	2	2	2	2	2	2
2	3	1	2	3	1	2	3	1	1
3	2	1	2	3	3	2	2	1	1
1	1	1	1	1	1	1	1	1	1
2	2	1	2	2	2	3	3	2	1
2	2	1	2	3	1	2	1	2	1
2	2	1	1	2	3	1	2	1	1

3	2	2	1	1	1	2	1	2	1
1	2	1	2	3	1	2	2	1	1
2	2	1	2	2	2	2	2	2	2
1	1	1	1	1	1	1	1	1	1
2	3	1	2	3	1	2	3	1	1
2	2	1	2	3	2	3	1	2	1
1	3	1	2	3	2	3	1	2	1
2	3	1	2	3	1	2	3	2	1
1	3	1	1	2	1	2	1	2	1
3	3	2	2	2	2	2	3	3	3
1	2	1	2	3	1	2	3	1	1
2	1	2	1	2	1	3	2	1	1
1	1	1	1	1	1	1	1	1	3
1	3	1	3	3	3	1	3	1	1
2	3	2	3	2	3	1	2	3	2
3	1	1	1	1	3	3	3	3	3
3	1	1	1	1	1	2	2	3	1
2	1	1	1	1	1	1	1	1	1
2	2	1	1	3	2	3	2	2	1
2	2	2	2	2	2	2	2	2	3
2	3	3	3	3	3	3	3	3	2
3	3	3	3	3	3	3	3	3	3
3	2	3	3	2	3	1	3	2	2
1	1	1	2	1	1	2	2	2	1
2	2	2	2	2	2	2	2	2	2
2	2	2	2	2	2	2	2	2	2
2	2	3	3	2	2	1	1	1	1
1	2	1	3	1	2	1	3	2	3
2	2	2	2	2	2	2	2	2	2
9	9	9	9	9	9	9	9	9	9
9	9	9	9	9	9	9	9	9	9
9	9	9	9	9	9	9	9	9	9
2	2	1	1	1	1	2	2	2	2
9	9	9	9	9	9	9	9	9	9
2	2	1	1	1	1	1	1	1	2
9	9	9	9	9	9	9	9	9	9
1	1	1	2	2	3	2	3	3	2
9	9	9	9	9	9	9	9	9	9
1	2	1	9	9	1	9	1	1	9
2	2	1	2	1	2	1	2	1	1
9	9	9	9	9	9	9	9	9	9
1	1	1	1	1	1	3	1	1	1
3	3	3	3	3	3	3	3	3	1
3	3	3	3	3	3	3	3	3	3
2	1	1	3	2	2	2	1	2	1
2	3	1	2	3	2	1	3	2	1
2	2	3	3	2	2	1	1	1	1

1	2	2	2	2	2	1	2	2	1
2	2	1	1	1	2	2	2	1	1
9	9	9	9	9	9	9	9	9	9
1	1	1	1	1	2	9	1	1	1
9	1	2	3	1	9	2	3	2	1
1	2	1	1	2	1	2	2	1	2
9	9	9	9	9	1	9	9	9	9
1	2	1	2	3	2	1	2	2	2
2	1	1	2	3	3	1	1	2	1
3	3	3	3	3	3	3	3	3	3
2	3	1	2	3	1	2	3	1	1
2	3	3	2	1	2	3	2	1	1
9	1	1	2	3	2	1	2	1	1
2	2	2	2	2	2	2	2	2	2
2	2	2	2	2	2	2	2	2	2
9	9	1	9	9	2	9	9	2	3
2	2	2	2	2	2	2	2	2	2
2	2	2	2	2	2	2	3	2	2
2	2	2	2	2	2	2	2	2	2
2	2	2	2	2	2	2	2	2	2
9	1	1	1	1	3	3	1	1	1
9	1	1	1	1	3	3	1	1	1
9	1	1	1	1	1	3	1	1	1
2	1	1	1	2	2	2	1	1	1
3	3	1	1	3	3	3	1	1	1
9	9	1	9	3	3	3	1	1	1
2	2	2	1	2	2	2	1	1	2
3	1	3	1	3	3	2	1	1	1
2	1	1	1	2	1	3	1	1	1
3	1	1	1	1	1	1	1	1	1
9	3	9	1	9	9	9	9	9	1
9	9	9	9	9	9	2	9	9	9
3	2	1	3	3	1	3	1	1	1
3	1	2	1	1	2	3	1	1	1
2	1	9	2	3	3	1	9	9	9
3	2	2	2	1	1	1	1	2	2
3	2	2	1	1	1	1	2	2	2
9	1	1	9	9	9	9	1	1	1
3	1	1	1	1	1	1	3	1	1
1	1	1	1	1	1	1	1	1	1
3	1	1	1	1	1	3	3	1	1
9	9	9	1	9	9	9	9	9	1
1	1	1	1	1	1	1	1	1	1
1	1	1	1	3	3	1	1	1	1
3	1	1	1	1	1	3	1	1	1
2	1	2	1	2	2	1	2	2	1
3	3	2	2	2	2	2	2	2	2

3	2	2	2	1	1	1	1	2	2
9	1	9	2	9	3	2	3	1	2
2	1	1	1	2	1	2	1	1	1
1	1	1	1	1	1	1	1	1	1
3	1	1	1	9	2	1	2	2	1
3	1	1	1	9	2	1	2	2	1
2	2	1	1	1	1	1	1	1	2
3	1	1	1	2	2	2	1	1	1
9	9	9	9	9	9	9	9	9	9
3	1	3	3	3	3	3	1	1	1
2	1	2	2	2	2	2	2	2	2
2	2	2	2	2	2	2	2	2	2
3	1	1	1	9	2	1	2	2	1
3	1	1	1	9	2	1	2	2	1
3	1	1	1	9	2	1	2	2	1
3	1	1	1	9	2	1	2	2	1
3	2	9	9	9	9	9	1	1	1
1	1	1	2	1	1	1	1	1	1
3	1	1	1	9	2	1	2	2	1
2	1	2	1	3	2	2	1	2	1
2	3	9	9	1	9	9	9	9	1
3	3	1	3	2	2	1	2	1	1
1	1	1	1	2	2	3	2	1	1
3	1	1	1	3	3	3	2	1	1
2	1	1	1	2	2	1	1	1	1
1	1	1	1	1	1	1	1	1	1
3	3	1	1	1	1	1	1	1	3
3	1	1	1	1	1	3	1	1	2
2	2	2	1	2	2	2	2	2	1
1	1	1	1	1	3	3	1	1	1
2	2	1	1	2	1	1	1	2	1
3	1	1	1	3	3	3	3	1	1
9	1	1	1	9	9	9	1	9	1
9	9	1	1	9	1	9	9	9	1
3	1	1	1	2	2	2	1	1	1
3	1	1	1	3	3	3	1	1	1
2	9	9	1	9	1	9	1	1	2
3	1	1	1	1	1	3	3	1	1
9	3	1	1	1	1	3	3	1	1
3	1	1	1	3	3	3	1	1	1
3	1	1	1	3	3	3	1	1	1
3	1	1	1	1	3	3	1	1	1
3	1	1	1	2	2	3	2	1	1
3	1	3	1	1	1	3	1	1	1
3	2	1	1	3	3	3	1	1	1
3	2	1	1	3	3	3	1	1	1
9	9	2	3	1	3	3	2	2	1

2	1	1	1	1	1	3	1	1	1
3	1	1	1	3	1	3	1	1	1
3	2	2	1	2	2	3	2	2	1
1	1	1	1	1	3	3	3	3	1
3	1	1	1	3	3	3	1	1	1
1	1	1	1	3	2	2	1	1	1
9	9	9	9	9	9	9	9	1	1
1	1	1	1	1	1	1	1	1	9
1	1	1	1	1	1	9	1	1	1
3	1	1	1	3	1	3	1	1	1
2	1	2	2	2	2	2	2	2	1
9	1	9	1	9	9	9	9	9	1
3	3	1	1	3	3	1	1	1	3
3	3	1	1	1	2	3	1	1	1
2	2	2	2	2	2	2	2	2	3
2	1	1	1	1	1	2	1	1	1
9	1	9	1	9	9	9	9	9	1
9	1	9	1	9	9	9	9	9	1
3	3	1	1	1	1	1	1	1	1
2	1	1	1	1	1	1	1	1	2
2	2	1	2	3	3	3	2	1	1
2	3	9	9	9	9	9	9	9	9
3	1	3	1	1	2	3	2	1	1
3	1	1	1	2	1	3	1	1	1
2	2	3	1	1	1	3	3	3	3
1	3	3	1	1	1	3	3	3	3
3	1	1	2	3	2	1	3	1	1
2	2	2	2	2	2	2	2	2	2
1	1	1	1	2	2	2	2	2	1
3	1	1	1	2	2	2	2	2	1
2	2	1	1	2	2	2	2	2	1
2	1	1	1	2	2	1	1	2	1
3	1	1	1	3	1	2	1	1	2
2	2	1	2	3	2	3	1	1	1
1	1	3	3	1	1	3	1	1	1
2	1	1	1	1	2	2	2	1	2
3	3	9	9	9	9	9	9	9	1
3	3	2	2	3	3	3	3	3	1
3	3	1	2	1	2	2	1	2	1
2	3	2	3	3	3	2	2	1	1
3	1	1	1	3	1	3	1	1	1
3	1	1	1	3	3	3	1	1	1
2	3	1	2	2	2	3	1	1	1
9	9	2	2	2	2	1	1	1	9
2	3	1	2	2	9	3	1	1	1
3	1	1	1	3	3	3	1	1	1
3	3	9	9	9	9	1	9	9	1

2	1	1	1	3	1	3	2	2	1
3	2	2	1	3	2	2	2	2	2
2	1	2	2	2	2	2	2	2	1
2	1	2	1	2	2	3	2	2	1
3	1	1	1	3	3	1	2	1	9
1	1	1	2	2	2	2	3	3	1
9	9	9	9	9	9	9	9	1	9
2	2	2	1	2	2	2	2	2	9
9	9	9	9	9	9	9	9	9	9
3	1	1	2	2	2	3	1	1	1
2	1	1	1	2	2	3	2	1	1
3	2	1	1	2	9	2	1	1	1
2	2	2	2	3	3	3	2	2	2
3	2	1	1	2	2	2	1	2	1
2	2	2	2	1	2	2	2	2	2
9	9	1	9	9	9	9	9	9	9
2	2	1	1	2	1	1	1	1	1
2	2	1	1	2	2	2	1	1	1
1	1	1	1	2	1	1	1	1	1
3	3	1	1	2	2	2	1	2	1
9	9	1	9	9	1	9	9	9	9
3	3	1	1	1	1	3	1	1	1
2	2	1	2	2	2	2	2	2	1
3	1	1	1	1	1	1	1	1	1
1	3	1	1	1	1	2	1	1	1
1	3	1	1	1	1	1	1	1	1
9	9	1	1	2	2	2	1	1	1
9	9	9	9	9	9	9	9	9	9
9	9	9	9	9	9	9	9	9	9
3	1	1	2	2	2	3	1	1	1
3	3	1	1	1	2	3	1	1	1
2	3	1	1	1	2	3	2	1	1
3	1	1	1	1	9	9	9	9	9
9	9	1	1	9	3	3	3	1	9
3	1	1	3	1	2	3	1	1	1
2	1	1	1	3	3	3	1	1	1
2	1	1	1	3	2	3	1	1	1
1	1	1	1	1	1	3	1	1	1
3	1	9	1	3	3	3	3	1	1
2	1	1	1	3	2	3	1	1	1
1	2	1	2	2	2	2	2	9	2
3	1	2	1	1	2	3	2	2	1
3	1	1	1	3	1	3	1	1	1
9	9	9	9	9	9	9	9	9	9
1	3	1	2	1	1	1	1	1	1
2	1	2	2	2	2	2	2	2	1
1	1	1	2	9	1	2	1	1	1

3	1	1	1	1	1	3	1	1	1
3	2	2	1	2	2	1	2	2	9
9	9	9	9	9	9	9	9	9	9
3	1	1	1	2	2	1	1	1	1
9	9	9	1	1	9	9	9	9	1
2	2	1	2	2	2	1	1	1	1
2	1	2	3	3	2	1	1	2	1
3	2	1	1	2	2	3	2	2	9
9	9	2	9	9	9	9	2	9	9
2	2	1	1	2	2	1	2	2	1
3	3	1	1	1	1	3	1	3	1
3	2	2	2	2	1	2	1	2	2
2	3	1	1	1	2	2	1	1	1
9	9	1	9	9	9	9	1	1	1
2	1	1	1	1	2	2	1	2	1
2	2	1	2	2	2	3	2	1	1
9	9	1	1	9	1	9	1	9	9
9	9	1	1	9	9	9	1	1	1
2	2	1	1	1	2	2	1	1	1
2	2	1	3	2	2	3	1	1	2
3	1	2	1	3	2	1	2	2	3
3	1	1	1	3	1	1	1	1	1
3	1	1	1	3	1	1	1	1	1
3	2	1	1	1	1	2	1	1	1
2	2	1	1	1	1	2	1	1	2
3	2	1	1	1	1	2	1	3	1
2	3	1	2	2	2	1	1	1	1
3	3	2	1	1	2	2	1	1	1
2	3	1	1	1	1	2	1	3	1
2	3	1	1	1	1	2	3	3	1
3	2	1	1	1	2	1	1	1	2
2	3	1	1	2	1	1	1	1	1
2	3	1	1	1	1	2	1	1	1
3	3	1	3	3	3	2	1	1	1
3	1	1	2	3	2	3	1	1	1
9	9	1	1	2	2	9	1	1	1
3	3	1	2	2	2	3	3	1	1
9	9	1	1	1	9	9	9	9	1
1	2	1	1	1	1	2	2	1	1
2	2	2	2	1	1	1	1	1	1
3	2	2	2	3	2	1	2	3	2
2	2	1	1	3	3	2	3	2	2
9	9	1	9	9	9	9	9	9	1
9	9	1	9	9	9	9	9	9	1
2	2	1	1	1	1	2	1	1	2
3	1	1	1	1	1	3	1	1	1
9	9	1	1	9	9	9	1	1	1

3	3	1	1	3	3	3	1	1	1
2	2	1	1	2	1	1	1	1	1
9	9	1	1	2	2	2	9	1	1
2	3	1	1	2	2	2	2	1	1
2	2	1	1	1	1	2	1	1	1
3	3	1	2	2	1	2	1	1	1
2	2	2	2	2	2	2	2	2	2
2	3	1	2	1	2	1	1	1	2
2	2	1	2	3	3	2	3	3	9
3	2	1	2	1	2	2	1	1	1
3	3	1	1	1	1	3	1	1	1
9	9	1	9	9	9	9	9	9	1
9	9	9	9	9	9	9	1	9	1
9	9	9	9	9	9	9	9	1	1
9	9	9	9	9	9	9	9	1	1
9	9	9	9	9	9	9	9	1	1
3	3	3	3	9	2	2	3	1	2
1	2	2	3	3	2	1	3	1	1
9	9	1	9	9	9	9	9	9	1
2	1	1	2	1	1	1	1	1	1
2	2	1	1	1	2	1	1	1	1
2	2	1	2	2	2	3	1	1	1
2	2	1	2	2	2	3	1	1	1
3	3	1	1	2	2	2	2	1	1
3	2	1	1	1	2	2	1	2	1
3	2	1	1	1	1	3	1	1	1
3	3	1	3	3	2	1	1	2	1
3	3	1	1	1	3	3	1	1	1
9	2	1	1	2	2	1	1	1	1
3	3	1	1	1	2	3	1	1	1
2	3	1	1	2	2	2	1	1	1
9	9	1	1	9	9	9	1	1	1
2	3	1	2	3	2	3	1	1	1
3	2	1	1	1	1	3	2	1	1
3	3	1	3	2	2	1	1	1	1
2	2	1	1	2	2	3	1	1	1
2	2	1	2	2	1	3	2	2	1
3	2	1	1	9	1	3	2	2	2
9	9	9	9	9	9	9	9	9	9
3	1	2	2	2	2	1	1	2	1
1	2	1	2	2	1	1	2	3	1
2	1	1	2	2	1	1	2	3	1
3	1	2	1	3	3	3	1	1	1
2	1	1	1	1	1	2	1	1	1
2	2	1	1	2	2	2	2	2	1
2	2	1	1	2	2	3	1	2	1
1	1	1	1	3	1	3	1	1	1

3	3	1	1	1	1	1	1	1	1
3	1	1	1	3	1	3	1	1	1
3	2	1	1	3	1	2	2	1	1
2	3	1	1	3	2	3	1	1	1
9	9	1	2	3	1	3	1	1	3
3	3	1	1	3	1	3	2	1	1
3	2	1	1	2	1	2	1	1	1
3	2	1	1	3	1	3	1	1	1
3	3	1	1	3	1	3	1	1	1
3	3	1	1	2	1	3	1	1	1
3	2	1	1	1	1	2	1	1	1
1	2	2	1	2	2	2	2	2	2
2	3	1	1	1	1	3	2	1	2
3	3	1	1	3	1	3	1	1	1
2	2	2	1	1	1	2	2	1	1
2	2	1	1	2	2	2	1	1	1
2	2	2	2	2	2	2	2	2	2
2	2	2	2	2	2	2	2	2	2
3	3	1	3	2	2	2	2	1	2
2	2	1	1	1	2	2	2	2	1
2	2	1	1	1	2	2	2	2	1
9	9	1	1	3	3	3	1	1	1
3	2	1	2	2	2	2	1	1	1
2	2	1	2	2	2	3	1	1	1
2	2	1	1	1	1	2	1	1	1
9	9	9	9	9	9	9	9	9	9
9	9	9	9	9	9	9	9	9	9
9	9	1	9	9	9	9	9	9	9
3	2	1	1	3	3	3	1	1	1
9	2	3	3	9	2	9	1	1	3
2	2	1	1	1	1	2	1	1	1
2	2	1	1	1	1	2	1	1	1
9	9	1	9	9	9	9	9	9	1
9	9	9	1	9	9	9	9	9	1
9	9	9	1	9	9	9	9	9	1
9	9	9	1	9	9	9	9	9	1
9	9	1	9	9	9	9	9	9	1
9	9	1	9	9	9	9	9	9	1
9	9	1	9	9	9	9	9	9	1
3	2	1	2	3	3	1	1	1	1
3	2	1	1	3	3	3	1	1	1
9	9	1	9	9	9	9	9	9	9
9	9	1	9	9	9	9	9	9	1
2	3	1	1	3	2	3	2	2	1
9	9	1	9	9	9	9	9	9	2
9	9	1	9	9	9	9	9	9	9
9	9	1	9	9	9	9	9	9	9

3	3	1	1	3	1	3	1	1	1
9	9	9	9	9	9	9	9	9	9
9	9	1	9	9	9	9	9	9	9
1	3	1	2	2	2	3	1	1	1
1	3	1	2	2	2	3	1	1	1
9	9	1	9	9	9	9	9	9	9
9	9	9	9	9	9	9	1	9	1
9	9	9	9	9	9	9	1	9	1
9	9	1	9	9	9	9	9	9	9
9	9	1	9	9	9	9	9	9	1
3	3	1	1	2	2	1	1	1	1
3	3	1	1	9	1	3	1	1	1
3	2	1	1	1	3	1	1	1	1
2	2	1	2	2	1	2	1	1	1
3	3	1	1	1	1	1	1	1	1
1	3	1	1	3	3	1	1	1	1
3	1	1	1	3	3	3	3	3	1
3	1	1	1	3	1	1	1	1	1
3	3	1	1	9	1	3	1	1	1
1	3	1	1	2	1	1	1	1	1
1	3	1	1	2	1	1	1	1	1
1	1	1	1	1	1	1	1	1	2
9	9	1	9	9	9	9	9	9	9
2	3	1	1	1	1	3	1	1	1
2	1	1	2	3	3	3	3	3	2
3	2	1	1	3	3	1	1	1	1
3	9	3	3	3	3	3	3	3	3
1	3	1	1	3	1	1	1	1	1
2	2	1	1	3	2	3	1	1	1
2	2	2	1	1	1	2	1	1	2
3	2	1	1	2	1	2	1	1	1
3	2	1	1	2	2	3	1	1	1
3	1	1	1	3	1	1	1	1	1
2	2	1	2	2	2	3	2	1	1
2	3	1	1	1	1	1	1	1	1
2	2	1	1	1	3	2	1	1	1
9	3	1	1	3	1	3	1	1	1
3	3	1	1	3	1	3	1	1	1
3	3	1	1	3	1	3	1	1	1
2	3	1	1	2	3	1	1	1	1
9	9	3	3	3	1	1	1	1	1
1	1	1	1	3	1	2	1	1	1
2	3	3	2	3	1	1	2	2	1
2	2	1	1	1	1	3	1	1	1
2	2	1	1	1	1	3	1	1	1
1	1	3	3	3	1	1	1	1	1
3	3	1	1	1	1	2	1	1	1

1	1	1	1	3	3	2	1	1	1
1	1	1	1	3	1	1	1	1	1
1	1	1	1	1	1	1	1	1	1
2	2	1	1	1	1	2	1	1	1
2	2	1	1	1	1	3	1	1	1
2	2	1	1	1	1	3	1	1	1
3	3	1	1	1	1	2	1	1	1
1	3	1	1	2	1	1	1	1	1
2	2	1	1	2	1	2	1	1	1
3	2	1	1	3	1	1	1	1	1
3	1	1	2	3	2	2	2	2	1
3	1	1	1	3	1	3	1	1	1
2	1	1	1	2	9	2	1	2	1
2	1	2	1	2	2	2	2	2	1
3	1	1	1	3	3	3	1	1	1
2	1	1	1	3	1	3	1	1	1
2	2	2	2	2	2	2	2	2	2
2	2	2	2	2	1	2	2	2	1
3	3	1	3	3	1	1	3	1	3
3	3	1	1	3	1	3	2	1	1
3	2	1	1	2	1	2	1	1	1
3	3	1	1	1	1	1	1	1	1
2	2	1	1	1	1	1	2	1	1
2	1	1	1	1	1	2	1	1	1
2	1	1	1	1	1	2	1	1	1
3	3	1	1	3	1	2	1	1	1
1	2	2	3	2	2	3	3	3	1
3	1	1	1	1	1	2	3	2	2
2	1	1	1	3	1	3	2	2	1
3	2	1	1	2	1	2	1	1	1
2	3	2	2	2	3	3	3	3	2
2	3	1	2	2	2	3	3	2	1
3	3	1	1	1	1	3	1	1	1
3	2	1	1	3	1	2	1	1	3
2	2	1	1	1	1	2	1	1	1
2	2	1	1	1	1	2	1	1	1
2	2	2	1	1	2	2	2	2	2
3	3	2	1	1	2	2	2	2	2
3	3	1	1	1	1	3	1	1	3
3	3	1	1	3	1	2	2	1	1
3	2	2	2	2	1	3	1	1	1
3	3	1	1	1	1	3	3	1	1
3	3	1	1	1	1	3	3	1	1
2	1	2	2	2	2	2	2	2	1
3	3	2	2	3	2	2	2	2	1
2	1	1	2	2	1	3	2	1	9
1	1	1	1	2	1	1	1	1	1

2	2	2	2	2	2	2	2	2	2
9	9	1	2	3	2	2	1	1	1
2	3	1	1	1	1	2	1	1	1
3	2	1	1	1	1	2	2	2	2
2	1	2	2	2	2	3	1	2	1
9	1	2	1	2	1	2	1	1	2
3	3	2	1	3	2	3	1	1	1
2	2	2	2	2	1	3	2	2	1
2	2	2	1	1	2	2	2	1	2
3	3	1	1	3	1	3	1	1	1
2	2	1	1	1	1	2	1	1	1
3	3	1	1	3	1	3	1	1	1
9	9	9	9	9	1	9	9	9	1
3	2	2	1	1	3	2	2	2	1
3	3	1	1	1	1	3	1	1	1
2	2	2	2	2	2	2	2	2	2
3	2	1	1	1	1	3	2	2	1
3	1	1	1	3	3	1	2	2	1
3	1	1	1	3	1	2	2	2	1
9	1	1	2	1	2	2	1	1	1
2	1	1	1	1	1	1	1	1	1
1	3	1	1	2	1	1	1	1	1
3	1	1	1	3	1	3	1	1	1
2	3	1	1	2	1	3	1	1	1
2	2	1	1	1	1	2	1	1	2
1	1	1	1	1	1	3	1	1	1
1	3	1	1	3	3	3	1	1	1
3	2	1	2	3	3	1	1	1	1
2	3	1	1	3	2	3	2	2	1
9	9	1	9	9	9	9	9	9	1
3	2	2	2	3	9	2	2	3	1
1	1	1	2	3	3	2	1	1	9
2	2	1	2	2	2	2	2	2	1
2	3	1	1	2	1	2	1	1	1
2	2	1	2	2	2	2	1	1	1
1	2	1	2	2	2	3	1	1	1
9	9	2	2	2	2	2	2	2	2
9	9	9	9	9	1	9	9	9	9
1	1	1	1	2	3	1	3	3	1
2	1	1	2	2	2	2	1	1	1
2	2	1	2	1	2	2	1	1	1
2	2	1	1	2	2	2	1	1	1
2	2	1	2	2	2	2	1	1	1
9	9	1	9	9	9	1	1	9	9
2	3	2	2	2	2	2	2	2	2
1	2	1	2	2	2	2	2	2	2
3	2	1	1	2	2	3	1	1	1

3	3	1	1	1	2	3	1	1	1
2	2	1	1	1	2	2	1	1	1
2	3	1	2	2	2	2	2	1	2
2	2	1	2	2	2	2	1	1	1
1	2	1	2	2	1	2	2	2	1
3	2	1	2	1	2	9	2	2	1
3	1	1	3	3	1	2	1	1	1
9	9	9	1	9	9	9	9	9	9
3	1	1	3	3	1	2	1	1	2
2	2	1	2	2	2	2	1	1	1
3	2	1	2	3	3	3	1	1	1
2	3	2	3	2	3	2	3	2	2
9	1	1	1	2	2	2	2	2	1
2	2	2	2	2	2	2	2	1	2
3	2	3	1	3	1	3	1	1	1
3	2	3	1	3	1	3	1	1	1
1	2	1	1	1	1	1	1	1	1
3	1	1	1	3	3	2	2	1	1
9	9	9	9	9	9	9	1	9	1
9	1	1	1	3	1	3	1	1	1
3	1	1	3	3	2	3	1	1	1
2	1	1	1	2	2	2	1	1	1
2	2	2	2	2	2	2	2	2	3
3	2	1	2	2	2	2	1	1	2
3	3	1	1	1	2	3	1	1	1
1	1	1	1	1	1	1	1	1	1
3	1	1	1	3	2	2	1	1	1
2	1	1	1	3	3	2	1	1	2
2	1	1	1	3	3	2	1	1	2
9	9	9	9	1	9	9	9	9	9
2	1	1	1	3	3	2	1	1	2
2	1	1	1	3	3	3	1	1	1
2	1	1	1	3	3	3	1	1	1
2	1	1	1	3	2	3	1	1	1
9	9	9	9	9	9	9	9	9	9
2	1	1	1	1	1	1	1	1	1
2	1	1	1	1	1	1	1	1	1
1	1	3	1	3	9	3	3	1	9
3	1	1	1	3	1	3	1	1	1
3	1	1	1	2	2	3	1	1	1
2	2	1	1	2	2	2	3	2	1
3	1	1	1	3	3	2	2	1	1
2	2	2	1	2	2	2	2	2	2
3	3	1	3	3	3	1	1	1	1
3	1	1	3	3	3	3	3	3	1
3	1	1	1	3	3	3	1	1	1
2	1	1	1	3	2	2	3	3	1

3	1	3	1	3	3	3	3	1	1
3	1	3	1	3	3	3	3	1	1
3	3	1	2	3	2	2	1	1	1
1	1	1	2	1	1	3	1	1	1
3	1	1	1	3	1	3	1	1	1
1	1	1	1	1	1	3	1	1	1
3	1	1	1	3	1	3	1	1	3
2	1	1	1	1	1	2	1	2	9
3	3	1	2	3	1	1	1	1	1
3	2	1	1	1	1	1	1	1	1
3	3	1	1	1	1	3	1	1	1
1	1	1	2	3	1	1	1	1	1
1	1	1	2	3	1	1	1	1	1
3	3	1	1	1	3	3	1	3	9
1	2	1	1	2	1	1	1	1	1
3	3	1	1	1	1	2	1	1	1
1	2	1	1	1	2	3	1	1	2
2	2	1	1	2	1	3	2	1	1
3	3	1	1	3	3	1	1	1	1
9	9	9	9	9	9	9	9	9	9
1	1	1	1	1	2	2	1	1	1
3	2	1	2	2	2	3	1	1	1
1	1	1	2	2	2	3	1	1	1
1	3	1	2	2	2	3	1	1	1
9	9	9	1	9	9	9	9	9	9
9	9	1	9	9	9	9	9	9	9
3	3	1	1	3	3	3	1	1	1
9	9	9	1	9	9	9	9	9	1
9	9	1	9	9	9	9	9	9	9
9	9	1	9	9	9	9	9	9	9
9	9	9	9	9	1	9	9	9	9
9	9	9	9	9	1	9	9	9	9
9	9	1	9	9	9	9	9	9	1
9	9	1	9	9	9	9	9	9	9
2	3	9	9	9	9	9	9	9	9
3	2	1	1	2	1	1	1	1	1
2	2	1	1	1	1	1	1	1	2
3	1	1	1	3	3	2	2	1	1
3	1	1	1	3	3	2	1	1	1
3	1	1	1	3	3	2	1	1	1
9	9	1	1	3	1	3	1	1	1
9	3	1	1	3	3	3	1	1	1
2	1	1	2	2	1	3	1	1	1
2	2	1	1	2	2	2	2	2	2
2	3	1	1	2	2	2	2	2	2
3	3	1	1	3	1	3	1	1	1
2	1	1	1	1	1	2	1	1	1

2	2	2	1	2	2	1	2	1	2
2	2	2	2	9	1	2	2	2	1
1	3	2	2	2	2	2	2	2	1
2	1	3	2	2	2	2	1	1	2
1	1	1	1	2	1	1	2	1	1
2	2	1	2	2	1	2	1	1	1
9	9	9	9	9	9	9	9	9	9
2	2	2	2	2	2	2	2	2	2
2	2	1	2	2	2	2	1	1	1
2	2	2	2	2	2	1	3	1	1
2	3	1	2	1	2	2	1	1	1
1	2	1	1	1	1	2	1	1	1
2	3	2	2	2	3	2	2	1	1
2	1	3	3	3	3	3	3	3	3
3	1	2	2	2	2	2	2	2	2
3	3	2	1	1	1	2	1	1	1
9	9	9	9	9	9	9	9	9	9
2	3	1	2	1	2	1	2	1	2
3	3	1	2	2	2	3	1	1	1
1	1	1	1	1	1	1	1	1	1
1	1	3	1	1	1	1	1	1	1
2	2	2	2	2	2	2	2	2	3
1	1	2	2	1	1	3	3	2	1
2	2	2	2	2	2	2	2	2	1
1	1	1	1	1	1	1	1	1	1
3	2	1	2	2	2	2	2	2	2
2	2	3	3	2	2	1	1	1	2
2	1	1	1	1	1	1	1	1	1
2	2	2	2	2	2	2	2	2	2
2	2	2	2	2	2	2	2	2	2
2	3	1	1	3	3	3	1	1	1
1	1	3	3	3	3	3	3	3	1
2	2	1	2	3	2	1	2	2	1
2	2	1	1	2	1	2	1	1	1
9	9	1	9	9	9	9	9	1	9
1	2	1	1	1	1	1	1	1	1
2	2	2	2	2	2	2	2	2	2
2	2	2	2	2	2	2	2	2	9
2	1	2	2	2	2	3	1	1	2
2	1	1	1	1	2	2	1	1	1
2	2	1	1	2	2	2	2	1	1
3	3	1	1	2	2	2	2	1	1
9	9	1	9	9	9	9	9	9	3
9	9	9	2	9	9	9	9	9	9
9	9	1	9	9	9	9	9	9	3
2	3	2	2	2	2	9	2	2	2
1	2	2	2	2	2	3	2	2	2

3	2	1	1	3	3	3	2	1	3
1	2	1	1	3	3	3	2	1	3
3	1	1	1	1	1	3	1	2	3
3	3	3	1	3	3	1	3	3	2
1	1	1	3	1	1	2	2	2	2
1	2	2	1	2	1	2	3	2	2
3	1	9	9	9	9	9	9	9	2
3	3	1	2	3	2	3	3	2	2
3	2	1	9	1	3	2	1	2	3
3	1	2	3	2	3	2	1	3	3
2	2	2	2	2	2	2	2	2	2
1	1	2	2	2	2	2	2	2	3
2	2	2	2	2	2	3	1	1	2
1	1	2	2	2	2	2	2	2	3
3	3	2	2	2	2	1	3	3	1
3	1	2	2	2	3	1	3	3	1
3	1	1	3	2	2	2	1	2	2
9	1	1	3	2	2	2	1	2	2
2	2	1	1	3	3	3	2	1	3
2	2	1	1	3	3	3	2	1	3
3	3	2	2	2	2	2	2	2	1
3	3	2	2	2	2	2	2	2	1
3	3	2	2	2	2	2	2	2	2
3	3	3	1	3	3	1	3	3	2
2	2	2	2	2	2	2	2	2	1
2	2	3	1	3	3	1	3	3	2
2	2	3	1	3	3	1	3	3	2
3	3	3	1	3	3	1	3	3	1
3	3	1	3	1	2	2	2	2	3
3	2	2	1	3	3	1	1	1	3
3	2	2	1	3	3	1	1	1	3
3	1	2	1	3	3	1	1	1	3
3	2	2	1	3	3	1	1	1	3
1	3	1	2	9	1	1	3	1	3
2	1	1	9	2	3	2	1	3	3
9	2	1	2	3	9	1	1	2	2
2	3	1	2	2	2	3	1	1	3
1	2	1	3	2	2	3	1	1	2
3	3	2	2	2	1	3	1	3	3
1	1	1	3	1	1	2	2	2	3
9	9	1	3	3	1	2	1	2	3
1	1	1	3	2	1	2	1	1	3
1	2	1	2	1	2	2	1	2	2
9	9	1	9	9	9	9	9	9	9
9	9	9	9	9	9	9	9	9	9
2	2	2	2	2	2	2	2	2	2
2	3	1	9	2	3	3	2	1	3

2	2	1	3	1	2	9	2	2	2
1	1	1	3	2	2	3	1	1	2
9	9	9	9	9	9	9	9	9	9
2	2	2	2	2	2	2	2	2	2
2	2	2	2	2	2	2	2	2	2
9	9	2	9	9	9	9	9	9	2
2	2	1	2	2	2	2	3	1	3
9	9	9	3	9	9	9	9	9	2
1	1	2	3	3	2	1	2	2	3
1	1	1	3	1	2	3	3	3	3
2	2	1	3	1	1	3	1	1	3
2	3	2	2	2	2	2	2	2	2
9	9	1	9	9	9	9	9	9	2
9	9	9	9	9	9	9	9	9	9
1	2	1	2	3	1	2	3	1	3
9	9	9	9	9	2	9	9	9	9
3	2	1	3	2	2	1	2	1	3
9	9	9	9	9	9	9	9	9	9
3	1	1	1	1	1	1	1	3	3
9	9	2	9	9	9	9	9	9	2
2	1	1	3	1	1	3	1	1	2
1	1	1	3	1	1	3	1	1	3
1	1	2	2	2	2	2	2	2	2
2	2	1	3	1	1	3	1	1	3
2	2	2	2	2	2	2	2	2	3
1	1	3	1	2	2	2	2	2	2
3	3	1	1	2	3	9	2	1	1
9	9	2	9	9	9	9	9	9	2
9	9	2	9	9	9	9	9	9	2
9	9	2	9	9	9	9	9	9	2
9	9	2	9	9	9	9	9	9	2
9	9	2	9	9	9	9	9	9	2
9	9	2	9	9	9	9	9	9	2
3	3	2	2	1	3	1	2	2	2
1	2	1	2	3	1	2	3	1	3
9	9	9	9	9	9	9	9	9	9
2	2	1	2	3	3	1	1	2	2
1	2	1	2	2	2	2	3	3	3
1	3	1	3	1	1	2	2	1	3
1	1	1	3	1	1	3	1	1	2
2	2	2	2	2	2	2	2	2	2
9	9	1	9	9	9	9	9	9	2
3	3	3	1	3	3	1	3	3	1
2	2	1	3	1	1	3	1	1	2
9	9	2	9	9	9	9	9	9	2
2	2	2	9	3	9	2	3	1	3
9	9	9	9	9	9	9	9	9	9

9	9	2	9	9	9	9	9	9	2
9	9	2	9	9	9	9	9	9	2
1	1	1	3	1	1	3	1	1	3
9	9	2	9	9	9	9	9	9	2
3	1	2	1	2	2	1	1	1	3
9	9	2	9	9	9	9	9	9	2
2	3	1	2	1	3	3	2	2	3
3	3	1	3	1	1	3	1	2	3
3	3	1	3	1	2	2	3	3	3
2	1	1	2	1	2	2	2	2	2
1	1	1	2	3	3	1	3	3	3
3	2	1	3	1	1	3	1	1	3
1	1	1	3	2	3	2	2	2	2
3	2	2	3	2	2	3	2	1	2
2	2	1	3	1	1	3	1	1	2
1	1	1	3	1	1	2	1	1	2
3	2	1	2	1	1	3	1	1	2
3	1	2	2	2	2	2	2	2	3
1	3	1	2	3	2	2	3	3	3
1	1	1	3	1	1	3	1	1	3
9	9	9	9	9	9	9	9	3	9
9	9	9	9	9	1	9	9	9	9
9	9	9	3	9	9	9	9	9	3
9	9	9	9	9	9	3	9	9	9
9	9	9	9	2	9	9	9	9	9
9	9	9	9	9	9	9	9	3	9
9	9	9	9	9	9	9	2	9	9
9	9	2	9	9	9	9	9	9	2
9	9	2	9	9	9	9	9	9	2
9	9	2	9	9	9	9	9	9	2
9	9	2	9	9	9	9	9	9	2
9	9	2	9	9	9	9	9	9	2
9	9	2	9	9	9	9	9	9	2
1	2	1	3	1	1	2	2	3	3
3	1	1	3	2	1	3	1	1	2
1	1	1	3	1	2	3	2	1	3
9	1	2	9	3	9	1	9	2	2
9	9	1	9	9	9	9	3	3	2
1	1	1	3	1	2	1	2	1	3
9	9	1	9	9	9	9	9	9	3
9	9	1	9	9	9	9	9	9	3
9	9	1	9	9	9	9	9	9	3
9	9	1	9	9	9	9	9	9	3
9	9	1	9	9	9	9	9	9	2
2	2	2	2	2	2	2	2	2	2
1	2	1	2	1	2	3	2	1	3
1	2	1	2	1	2	3	2	1	3

2	2	2	2	2	2	2	2	2	2
2	2	1	2	2	2	2	1	1	3
2	3	1	3	2	2	1	3	2	3
3	2	1	2	2	2	1	3	1	3
9	9	1	9	9	9	9	9	9	9
9	9	1	9	9	9	9	9	9	3
9	9	1	9	9	9	9	9	9	3
9	9	1	9	9	1	9	1	9	3
9	9	1	9	9	9	9	9	9	3
9	9	2	9	9	9	9	9	9	2
9	9	3	9	9	9	1	9	9	1
9	9	9	1	9	9	1	9	9	1
9	9	2	2	9	3	1	3	2	2
9	9	9	9	1	9	9	9	9	2
9	9	1	9	9	9	9	9	9	2
2	2	2	2	2	2	2	2	2	2
2	3	2	1	3	3	2	2	2	2
9	9	9	9	9	9	3	9	9	3
2	2	1	2	2	2	2	2	1	3
2	3	2	3	1	3	2	1	1	3
2	2	2	2	2	2	2	2	2	2
2	2	2	2	2	2	2	2	2	2
9	2	1	3	1	1	3	1	1	3
2	2	1	2	1	1	3	1	2	2
2	1	2	3	2	3	3	2	2	2
2	2	1	9	1	2	9	1	2	3
9	9	9	9	9	2	9	9	9	3
2	2	2	2	2	2	2	2	2	2
2	2	2	2	2	2	2	2	2	2
3	3	2	2	2	2	2	2	2	2
1	2	2	1	1	2	1	2	1	9
2	2	2	2	2	2	2	2	2	3
9	9	1	9	9	9	9	9	9	3
2	2	2	2	2	2	2	2	2	2
2	3	1	3	3	3	1	1	1	2
1	2	1	2	1	2	1	1	2	3
3	1	1	2	1	2	2	2	2	3
2	2	1	2	3	2	2	2	2	2
2	3	2	2	3	2	3	2	2	2
2	1	1	2	3	2	3	2	3	3
2	2	1	3	2	2	3	2	2	3
3	1	1	3	1	1	1	3	3	3
2	2	1	3	1	2	2	1	2	3
1	1	1	3	1	1	1	1	1	3
9	9	9	9	9	9	9	9	9	9
2	2	2	2	2	2	2	2	2	2
3	1	1	2	3	2	3	1	1	3

1	1	1	3	1	1	3	1	1	3
2	2	1	2	2	2	1	2	1	3
1	2	2	3	1	2	3	1	1	3
2	1	1	3	1	2	3	2	1	3
2	2	2	2	2	2	2	2	2	2
2	2	2	2	2	2	2	2	2	2
2	2	1	3	1	2	1	2	3	2
1	2	1	3	1	1	2	2	2	3
3	3	1	3	1	2	2	2	2	3
2	1	2	2	2	2	2	2	2	2
2	3	1	2	2	2	2	2	1	3
3	3	1	3	2	2	1	1	1	3
2	2	1	3	1	1	3	1	1	3
3	3	1	1	2	2	2	2	2	3
1	2	1	3	1	1	2	1	1	3
1	2	1	2	1	1	2	2	1	3
3	3	2	2	2	2	1	3	3	3
2	2	2	2	2	2	2	2	2	2
2	2	2	2	2	2	2	2	2	2
2	2	2	2	2	2	2	2	2	2
2	2	1	2	2	2	2	2	2	2
2	2	2	2	2	2	2	2	2	2
2	2	1	2	3	1	2	3	1	3
2	2	2	2	2	2	2	2	2	2
1	2	1	2	1	3	2	1	3	3
9	9	9	9	9	9	9	9	9	9
9	9	9	9	9	9	9	9	9	9
9	9	9	9	9	9	9	9	9	9
3	3	3	2	1	3	3	2	1	3
9	9	9	9	1	9	9	9	9	3
9	9	9	9	9	9	9	9	9	9
9	9	9	9	9	9	9	9	9	9
9	9	9	9	9	9	9	9	9	9
9	9	9	9	9	9	9	9	9	9
1	1	1	3	1	2	1	1	1	2
1	2	1	2	3	1	2	2	3	3
2	1	1	3	1	1	1	2	1	3
9	9	9	9	9	9	9	9	9	9
3	2	2	2	2	2	2	2	2	3
9	9	9	9	9	9	9	9	9	9
9	9	9	9	9	9	9	9	9	9
2	2	1	3	1	2	2	2	2	2
1	1	1	2	1	1	3	1	1	3
2	2	2	3	1	2	2	1	1	3
3	2	1	9	2	1	2	3	2	3
3	2	3	1	3	9	1	2	2	1
2	2	2	2	2	2	2	2	2	2

3	3	1	2	1	3	3	2	1	3
3	3	2	2	2	2	2	2	2	3
3	3	2	2	2	1	1	1	1	3
1	2	2	2	2	2	2	2	2	2
2	2	2	2	2	2	2	2	2	3
1	9	9	9	9	9	9	1	9	9
3	3	1	2	2	2	3	2	1	3
2	3	1	2	2	2	2	2	2	3
2	2	2	2	2	2	2	2	2	2
2	3	2	3	3	2	1	3	3	3
2	2	2	2	2	2	2	2	2	2
3	3	1	3	3	3	1	1	1	3
2	1	2	2	2	2	2	2	1	3
2	2	1	2	2	2	2	2	2	2
9	9	9	9	9	9	9	9	9	9
3	2	2	2	2	2	2	1	1	3
1	3	1	3	1	1	1	1	1	3
2	2	3	2	9	2	2	2	2	2
1	1	1	3	1	1	1	1	1	3
9	9	9	9	9	9	9	9	1	9
2	2	2	2	2	2	2	2	2	2
2	2	2	2	2	2	2	2	2	2
2	2	2	2	2	2	2	1	2	2
2	2	2	2	2	2	2	2	2	3
2	3	2	2	2	2	2	2	2	3
2	2	2	2	2	2	2	2	2	2
3	2	2	2	1	2	1	2	3	2
3	1	1	3	1	3	1	1	1	3
1	2	2	2	2	2	2	2	1	2
1	1	1	3	1	1	3	1	1	2
1	2	1	3	1	2	1	2	2	3
9	9	9	9	1	9	9	9	9	2
2	2	1	2	2	2	2	2	1	3
3	1	1	3	2	2	1	3	2	3
1	2	1	3	1	1	2	1	1	3
2	2	2	2	2	2	2	2	2	2
2	2	2	2	2	2	2	2	2	2
2	2	2	2	2	2	2	2	2	2
2	2	2	2	2	2	2	2	2	2
1	1	1	3	1	1	3	1	1	2
2	2	2	2	2	2	2	2	2	2
2	2	2	2	2	2	2	2	2	2
2	2	1	3	1	1	2	2	2	2
2	2	2	2	2	2	2	2	2	2
2	2	2	2	2	2	2	2	2	2
2	2	2	1	3	2	2	2	2	2
2	1	1	3	1	2	3	2	1	3

9	9	9	9	9	9	9	9	9	9
3	3	3	3	3	3	3	1	3	3
9	9	9	9	9	9	9	1	1	3
9	9	9	9	9	9	9	9	9	9
1	1	2	3	1	1	3	2	1	2
2	2	1	3	1	1	3	1	1	3
9	9	9	9	9	9	9	9	1	9
2	1	1	3	2	2	3	1	2	3
2	2	1	3	1	3	3	1	2	3
2	2	2	2	2	2	2	2	2	2
2	2	9	9	1	2	2	2	2	2
2	1	1	3	1	2	3	1	1	3
3	1	1	1	3	3	1	1	1	3
9	9	3	9	9	9	9	9	9	3
3	1	1	1	3	3	1	1	1	3
2	2	1	1	3	3	1	1	1	3
1	1	1	3	1	1	3	3	2	2
9	9	9	9	9	9	9	9	9	9
2	2	1	2	1	2	1	1	1	3
3	2	1	2	2	1	1	1	1	3
2	2	2	2	2	2	2	2	2	2
3	1	1	3	1	2	9	2	2	1
2	2	2	9	2	9	9	9	9	2
3	3	2	2	3	2	2	2	2	3
9	1	1	3	1	1	3	1	1	3
9	9	9	9	9	9	9	9	9	9
2	2	1	2	9	9	9	9	9	9
9	9	9	3	9	9	9	9	9	2
3	3	1	2	1	3	1	1	2	3
3	2	1	3	1	1	3	1	1	3
1	1	9	9	9	9	9	9	9	1
2	2	1	3	1	1	2	2	2	3
3	1	1	2	3	1	2	3	2	3
3	2	1	3	3	2	1	2	2	2
3	1	1	2	1	2	2	1	1	2
3	3	1	1	3	3	1	3	3	3
2	3	9	9	9	9	9	9	1	3
1	2	1	3	2	2	3	2	1	3
9	3	1	9	9	9	9	9	1	9
3	3	1	2	3	3	1	1	1	3
1	2	1	3	1	1	2	2	2	2
1	1	1	2	1	3	3	1	1	3
3	3	1	1	3	3	1	3	3	1
3	1	2	2	3	3	2	2	1	3
3	3	1	3	1	2	1	1	1	3
9	9	9	9	9	9	9	9	9	9
9	2	1	3	1	9	2	1	1	3

2	2	1	3	2	2	1	1	1	3
1	3	1	2	1	1	2	1	2	3
9	9	9	9	9	9	9	9	9	9
2	2	2	2	2	2	2	2	2	2
2	1	1	3	1	2	3	1	2	3
2	2	1	3	1	1	3	1	1	3
9	9	1	9	9	9	9	9	9	9
2	2	2	2	2	2	3	2	2	9
2	1	1	3	2	2	2	2	1	3
2	2	2	2	2	2	2	2	2	2
2	2	1	3	2	1	3	1	1	3
2	2	1	3	2	2	3	1	1	3
3	2	1	3	1	1	2	1	1	3
3	2	1	2	2	3	2	1	1	3
2	3	1	3	1	1	3	1	1	3
2	3	1	3	1	1	2	1	1	3
3	1	1	3	1	1	3	1	1	3
3	2	1	3	2	2	2	1	1	3
9	2	3	9	2	9	2	9	2	9
9	9	9	9	9	9	9	9	9	9
2	2	1	3	1	1	2	9	2	9
9	9	9	9	9	9	9	9	9	9
9	9	9	9	9	9	9	9	9	9
2	1	1	3	2	2	2	2	1	3
2	2	2	2	2	2	2	2	2	2
1	1	1	3	1	1	3	1	1	3
9	9	1	9	9	9	9	9	9	9
9	9	9	9	9	9	9	9	9	9
9	9	1	9	9	9	9	9	9	9
9	9	1	9	9	9	9	9	9	9
9	9	1	3	1	1	3	1	1	3
2	2	2	2	2	2	2	2	2	2
2	2	2	2	2	2	2	2	2	3
2	1	1	3	1	2	2	1	1	3
9	9	1	3	1	9	9	9	1	9
9	9	9	9	9	9	9	9	9	9
9	9	1	9	9	9	9	9	9	9
9	9	1	9	9	9	9	9	9	3
9	9	3	9	9	9	9	9	9	9
9	9	1	9	9	9	9	9	9	9
9	9	1	9	9	9	9	9	9	9
2	2	2	2	3	2	2	2	2	1
9	9	9	9	9	9	9	9	9	9
2	2	2	2	2	2	2	2	2	2
9	9	9	9	9	9	9	9	9	9
3	2	1	2	2	2	1	1	1	3
3	1	1	3	1	1	2	1	1	3

3	1	1	3	3	3	1	3	1	3
2	2	1	3	3	2	2	1	1	3
2	9	1	2	2	2	1	3	2	3
2	2	1	2	2	1	2	1	2	1
3	3	1	2	3	3	1	3	3	2
9	9	9	9	1	9	3	9	1	9
1	2	1	2	2	1	2	1	2	3
9	2	2	9	2	9	9	9	9	2
9	9	9	9	9	9	2	9	9	9
9	9	1	9	9	9	9	9	9	3
2	2	2	2	2	2	2	2	2	3
3	1	1	2	2	3	1	2	1	3
9	9	2	2	2	2	2	2	2	2
2	2	2	2	2	2	2	2	2	3
9	9	1	3	1	1	9	9	9	3
1	2	2	3	3	1	3	2	3	3
2	1	1	3	2	2	2	2	1	3
3	3	2	2	2	2	2	2	2	2
2	2	1	2	2	2	2	1	1	3
2	2	1	3	3	2	1	1	1	3
9	1	1	3	1	1	1	1	1	3
2	1	1	2	3	2	3	1	1	3
3	2	1	3	1	2	1	1	1	1
9	9	1	9	9	9	9	9	9	9
3	3	1	3	1	1	1	1	1	3
2	2	1	3	1	1	1	1	2	3
2	3	1	2	3	3	2	1	1	2
3	1	1	2	1	1	2	1	1	3
3	3	1	2	1	2	1	1	1	3
2	2	1	3	2	2	2	2	2	9
3	2	1	2	3	9	2	9	3	2
9	9	9	9	9	9	9	9	9	9
2	2	2	2	2	2	2	2	2	2
3	2	1	3	1	1	2	1	1	2
1	2	1	2	2	3	3	1	1	3
9	9	1	9	9	9	9	1	9	3
9	1	1	3	1	1	1	1	1	3
3	1	1	3	3	1	1	1	1	3
3	3	1	2	2	2	1	2	2	3
3	2	3	2	3	2	9	2	3	2
2	2	1	3	2	2	2	2	2	3
3	3	1	3	1	3	9	1	1	3
2	2	1	3	2	1	2	2	1	3
2	1	1	3	3	3	1	3	3	2
2	2	1	1	2	2	1	1	1	3
2	2	1	3	2	1	2	2	1	3
3	1	1	3	1	1	2	1	1	3

9	9	1	9	9	9	9	9	9	3
1	3	1	3	3	1	1	2	2	3
2	2	1	2	2	2	2	2	2	3
2	3	1	3	3	2	2	1	1	3
9	9	9	9	9	9	9	9	9	9
2	3	1	2	1	3	2	3	2	2
2	2	9	9	9	9	9	9	9	9
9	9	9	9	9	9	9	9	9	9
2	2	2	2	2	2	2	2	2	3
1	1	1	3	2	2	1	1	1	3
9	9	9	9	9	9	9	9	1	3
3	3	1	1	1	3	3	1	1	3
2	2	2	2	1	1	3	2	2	2
2	1	3	2	3	2	2	2	3	2
3	2	2	1	2	2	3	2	1	2
2	2	2	2	2	2	3	2	2	3
3	1	1	3	1	3	9	1	1	3
2	2	1	2	1	2	1	3	3	3
2	1	1	3	1	1	3	1	1	3
3	3	1	3	1	2	2	3	3	2
2	2	2	1	3	2	2	2	2	3
2	2	2	1	2	2	2	2	2	3
3	3	1	3	1	2	1	1	1	3
2	3	1	3	1	1	3	1	1	2
2	2	2	2	2	2	2	2	2	3
2	2	1	2	3	2	2	2	2	3
3	2	1	2	2	2	2	2	2	3
2	2	1	2	2	2	2	2	2	2
2	2	1	2	2	2	1	1	1	3
2	2	1	3	2	2	1	1	1	3
9	9	1	9	9	9	9	9	9	3
3	1	1	3	1	1	1	1	1	3
1	2	1	3	2	2	2	2	2	3
9	1	1	3	3	1	3	1	1	3
2	2	1	3	1	1	3	1	1	3
1	1	1	3	2	2	2	2	2	2
9	9	2	9	9	9	9	9	9	3
2	2	2	2	2	2	3	9	1	2
9	9	9	9	9	9	9	9	9	9
2	2	1	2	1	2	2	1	1	3
2	2	2	2	2	2	2	2	2	2
2	2	1	3	2	2	2	1	1	3
1	1	1	3	1	1	3	1	1	9
3	1	1	3	1	1	2	1	1	3
3	1	1	3	3	3	3	3	1	3
9	9	1	2	1	1	3	1	1	3
2	2	1	3	2	2	2	1	1	3

3	2	9	9	9	9	9	9	9	3
3	3	1	2	1	1	1	2	2	3
9	9	9	9	9	9	9	9	9	9
9	9	2	2	2	2	2	2	2	9
9	9	9	9	9	9	9	9	9	9
3	3	3	1	3	3	1	3	3	1
3	3	1	3	3	3	1	1	1	3
2	3	9	9	1	9	9	9	9	2
3	3	2	3	2	1	2	3	2	2
2	3	1	3	3	3	2	3	2	2
3	3	1	3	3	3	1	1	1	3
3	3	1	2	2	2	2	1	1	3
1	2	1	2	2	2	3	1	1	3
3	3	1	3	3	3	1	1	1	3
3	3	1	3	3	3	3	1	1	3
1	3	1	3	1	1	3	1	1	3
3	2	1	2	2	2	1	2	1	3
3	3	1	1	3	3	1	3	1	3
9	9	9	9	9	9	9	9	9	9
3	1	1	3	2	2	9	1	1	3
9	9	9	9	9	9	9	9	9	9
3	3	2	2	2	2	9	3	3	3
3	2	1	3	2	2	1	1	1	3
9	9	1	3	9	9	9	9	9	9
2	1	2	2	2	2	2	2	2	1

AfraidPapS	LiketoKnov	WilldoPapi	WillLikePa	Embarrase	WorriedAb	NoTimefor	PapSmearN	PapAgainst	LikeHPVVa
2	2	2	2	2	2	2	2	2	2
3	3	3	3	3	3	3	3	3	2
2	2	2	2	2	2	2	2	2	2
3	3	3	2	2	2	2	2	2	2
2	2	3	2	2	2	2	3	3	1
2	2	2	2	3	3	3	3	3	3
1	3	1	2	3	1	3	2	2	2
2	2	3	3	2	2	1	2	2	1
3	9	3	3	3	3	3	3	3	3
3	2	3	2	3	3	2	2	2	3
2	1	2	3	2	3	1	2	2	2
2	2	2	2	3	1	2	2	2	1
2	3	1	2	3	1	2	3	2	1
1	2	2	2	1	2	2	2	2	1
2	2	2	2	2	2	2	2	2	9
2	2	2	2	2	2	2	2	2	2
9	9	9	9	9	9	9	9	9	9
1	1	1	3	1	1	1	1	1	1
9	9	9	9	9	9	9	9	9	9
9	9	9	9	9	9	9	9	9	9
1	1	1	1	1	1	1	3	3	1
2	2	2	2	2	2	2	2	2	2
2	1	1	1	3	3	3	1	3	1
1	1	1	1	2	1	1	1	1	1
9	9	2	9	9	9	9	9	9	9
1	1	2	2	2	1	1	3	1	1
1	1	3	3	3	1	1	1	3	1
1	1	1	2	1	1	2	3	3	1
3	2	1	2	3	3	2	1	1	1
1	2	9	1	1	3	1	3	2	2
1	1	1	3	1	1	2	2	3	1
2	1	2	1	2	3	1	2	3	1
1	1	1	1	1	1	1	1	3	1
1	1	1	1	2	1	1	1	1	1
2	2	1	1	2	1	1	1	1	1
2	1	3	1	2	1	3	1	2	3
1	1	3	2	2	2	2	3	3	2
1	1	1	2	1	1	3	2	3	1
3	1	2	1	3	3	3	3	3	1
3	9	2	3	2	2	3	3	3	3
2	1	2	1	2	2	3	3	3	1
2	9	9	9	1	3	9	9	1	1
9	9	9	9	9	9	9	9	9	9
2	2	2	2	2	2	2	2	2	2
2	1	2	2	2	2	1	2	2	1
9	9	9	9	9	9	9	9	9	3

2	2	3	3	2	2	2	1	2	1
2	2	1	1	2	2	2	3	3	1
2	2	2	2	1	1	2	2	2	1
2	2	2	1	1	2	1	2	2	2
3	1	3	3	3	1	3	3	3	1
3	1	3	3	1	3	3	3	3	1
2	2	2	3	3	3	3	2	2	2
3	1	1	1	3	3	3	3	3	1
2	1	3	2	1	3	2	1	3	1
9	9	9	9	3	9	9	9	1	3
9	9	9	9	9	9	9	9	1	2
1	2	2	3	3	3	2	2	2	2
1	2	2	1	2	1	1	2	1	1
1	1	2	2	3	3	3	3	3	1
2	2	3	2	1	2	2	1	2	1
1	2	2	1	2	2	2	2	3	1
1	2	3	2	1	2	3	2	2	1
2	1	2	1	3	3	1	2	1	1
1	1	2	2	3	2	2	3	3	1
2	2	2	3	3	3	3	3	2	3
2	1	2	2	1	2	3	3	3	1
3	1	2	1	1	2	2	2	2	2
2	2	3	3	2	2	2	2	2	1
1	1	2	2	1	2	2	2	2	1
2	2	3	2	2	2	2	1	1	1
2	2	2	1	1	3	1	2	1	2
1	1	3	3	1	1	3	1	3	1
1	2	2	3	3	1	2	3	2	1
2	9	1	2	3	2	3	3	3	1
1	9	3	3	3	3	3	3	3	3
9	9	9	9	9	9	9	9	9	9
2	1	3	3	3	3	3	3	3	3
9	1	2	2	3	3	3	2	3	1
2	2	2	2	2	2	2	2	2	1
2	1	1	3	2	3	2	3	2	1
2	2	2	2	2	2	2	2	2	2
9	9	9	9	9	9	9	9	9	1
2	1	2	2	1	2	2	3	2	1
2	2	2	2	2	2	2	2	2	2
2	2	1	1	2	1	2	2	1	2
2	1	1	3	2	3	2	9	2	1
9	9	9	9	9	9	9	9	9	9
1	1	3	2	3	2	1	1	1	2
1	1	1	2	3	2	2	2	2	1
2	2	2	3	2	1	2	3	2	2
2	2	3	2	2	3	1	2	2	2
2	1	3	2	2	1	2	3	3	1

2	3	1	1	2	3	1	2	1	1
3	3	3	3	3	3	3	3	3	1
2	2	2	2	2	2	2	2	2	1
1	1	2	2	2	1	2	2	2	2
1	1	1	1	2	2	1	1	2	1
1	1	1	1	2	1	1	1	1	2
2	1	1	2	2	2	2	2	3	1
9	9	9	9	9	9	9	9	9	9
2	2	2	2	2	2	2	2	2	2
1	1	2	1	3	3	2	2	3	1
1	1	9	2	2	3	1	2	1	9
2	2	2	3	2	2	2	1	1	2
2	2	3	3	3	3	3	2	2	1
2	2	2	2	2	2	2	2	2	1
3	1	3	2	3	3	3	2	3	2
3	1	3	2	2	3	2	3	3	2
1	2	2	1	1	2	2	2	1	1
2	1	1	2	1	2	1	1	1	1
3	3	3	1	3	1	3	1	3	1
2	1	2	1	2	2	2	2	1	2
3	1	3	1	3	1	3	1	3	1
1	1	2	3	2	1	2	2	3	1
2	2	2	1	1	2	2	2	1	1
2	3	1	2	2	2	2	2	2	2
2	2	2	2	2	2	2	2	2	2
2	1	1	2	2	1	2	1	3	1
3	1	3	1	3	3	3	3	3	1
1	1	1	2	2	2	2	2	2	1
2	2	2	2	1	1	2	1	1	2
1	3	1	2	1	3	3	1	2	1
1	2	2	3	2	1	2	2	2	2
2	2	1	3	2	2	1	1	1	1
2	9	2	2	1	1	2	3	2	3
1	2	2	1	1	2	1	2	2	1
2	3	3	3	2	2	1	2	3	1
1	1	1	1	9	2	1	1	1	1
3	3	3	3	3	3	3	2	2	2
1	2	2	1	2	1	1	1	1	1
9	9	9	9	9	9	9	9	9	9
3	3	3	3	3	3	3	3	3	1
1	2	2	3	2	1	2	1	1	1
9	9	9	9	9	9	9	9	9	9
1	2	1	1	1	1	2	1	1	1
2	2	3	2	1	1	1	1	1	1
9	9	9	9	9	9	9	9	9	1
1	1	3	1	3	2	3	3	3	1
1	1	3	2	1	3	3	1	2	1

3	2	3	2	3	1	2	3	1	2
1	1	1	1	3	3	3	3	3	3
3	3	3	3	3	3	3	3	3	3
2	2	2	2	2	2	2	2	2	2
2	2	2	3	3	2	2	2	2	2
3	1	2	3	2	3	2	3	2	2
9	1	9	1	2	3	9	2	1	9
9	1	9	1	2	1	9	2	1	9
9	9	9	9	9	9	9	9	9	2
2	2	2	2	2	2	2	2	2	2
2	2	1	1	1	3	3	2	3	1
2	1	2	2	2	3	3	3	3	1
2	2	2	2	2	2	2	2	9	2
2	1	2	1	1	2	2	3	3	1
2	3	2	1	2	2	2	2	2	1
2	2	1	1	1	1	2	2	3	1
1	2	2	1	2	2	1	1	2	1
1	2	2	3	2	1	2	2	2	1
9	9	9	9	9	9	9	9	9	2
3	1	2	2	1	2	3	1	3	2
1	1	1	1	2	1	3	2	2	2
2	2	2	2	1	1	2	2	2	1
3	3	3	3	3	3	3	3	3	9
2	2	2	2	2	2	2	2	1	2
3	1	1	2	3	3	3	3	3	1
2	2	1	3	3	3	1	3	2	2
9	9	9	9	9	9	9	9	9	9
2	2	2	2	2	2	2	2	2	3
1	1	2	2	2	2	1	2	3	3
2	2	1	2	2	2	2	2	2	1
2	2	1	1	1	2	1	2	1	9
2	1	1	1	1	2	2	2	2	2
1	1	1	2	2	2	1	1	1	1
3	3	1	3	3	1	3	2	3	3
1	1	1	1	3	3	3	3	2	1
1	1	1	1	1	1	1	1	1	1
3	3	3	3	3	3	3	3	3	3
1	1	2	2	1	1	2	3	3	1
1	1	3	1	3	3	3	1	2	1
2	2	2	2	2	2	2	2	2	2
9	9	9	9	9	9	9	9	9	9
2	2	2	2	2	2	2	2	2	2
2	2	2	2	2	2	2	2	3	2
9	9	9	9	9	9	9	9	9	9
9	9	9	9	9	9	9	9	9	9
2	2	3	2	1	2	2	2	2	1
2	2	3	2	2	2	1	1	1	1

9	9	3	9	9	9	9	9	9	9
9	9	9	9	9	9	9	9	9	9
1	2	3	3	3	3	3	3	3	1
9	9	9	9	9	9	9	9	9	1
9	9	9	9	9	9	9	9	9	1
9	9	9	9	9	9	9	9	9	9
9	9	9	9	9	9	9	9	9	9
9	9	9	9	9	9	9	9	9	9
3	2	2	2	1	2	1	3	2	2
9	9	9	9	9	1	9	9	9	9
9	2	9	9	9	9	9	9	9	9
9	9	9	9	9	9	9	2	9	9
1	1	2	2	1	1	2	1	2	1
3	9	3	2	2	1	3	1	3	1
9	9	9	9	1	9	9	9	9	9
2	2	2	2	3	3	3	3	3	3
1	1	1	1	1	1	1	1	1	1
2	2	2	2	2	2	2	2	2	2
2	2	2	2	2	2	2	2	2	2
2	2	2	2	2	2	2	2	2	2
1	2	1	1	1	1	1	1	1	1
9	9	9	9	9	9	9	9	9	9
1	1	1	1	2	1	1	1	2	2
3	1	2	1	2	1	3	3	3	1
2	2	2	2	2	2	2	2	2	2
1	1	2	2	2	2	2	2	2	1
1	1	1	1	1	2	3	3	2	2
1	2	2	1	1	2	2	2	2	1
3	3	3	3	3	3	3	3	3	9
3	1	1	1	3	3	3	1	1	1
2	2	2	2	2	2	2	2	2	2
1	2	2	1	1	1	1	1	1	1
9	9	9	9	9	9	9	9	9	9
1	2	2	2	1	1	1	1	1	2
1	1	3	1	1	1	3	1	1	3
1	1	2	2	1	2	2	1	2	1
2	9	9	9	9	9	9	9	9	2
1	1	1	1	1	2	2	1	1	3
9	9	9	9	9	9	9	9	9	3
2	1	1	1	1	2	2	2	2	2
2	1	1	3	2	2	2	2	2	1
1	1	1	2	2	3	3	3	1	1
1	1	1	2	2	2	2	1	1	1
2	2	2	2	2	2	2	2	2	2
2	2	2	2	2	2	2	2	2	1
1	2	3	3	2	1	2	3	1	1
9	9	9	9	9	9	9	9	9	9

1	9	2	1	1	1	1	3	2	1
3	2	2	1	1	2	1	1	1	1
1	1	1	1	1	1	1	1	1	1
9	9	9	9	9	9	9	9	9	9
2	2	2	2	2	2	2	2	2	2
3	3	2	2	2	2	2	1	1	2
3	1	1	3	1	3	2	1	3	3
2	2	1	2	2	2	2	2	2	2
1	1	1	2	1	1	1	1	1	1
1	1	3	1	1	1	1	1	3	1
2	2	2	1	2	2	1	1	2	1
9	9	9	9	9	9	9	9	9	3
9	9	9	9	9	9	9	9	9	1
9	9	9	9	9	9	9	9	9	1
2	9	2	9	9	2	9	9	2	2
9	9	9	9	9	9	9	9	9	9
9	9	9	9	9	9	9	9	9	9
9	9	9	9	9	9	9	9	9	9
2	9	2	9	2	2	9	2	2	9
9	9	9	9	9	9	9	9	9	9
9	9	9	9	9	9	9	9	9	9
2	2	2	2	2	2	2	2	2	1
1	2	2	1	2	2	2	1	2	2
1	2	2	1	1	1	1	1	1	2
2	1	1	2	2	1	1	1	1	2
9	9	9	9	9	9	9	9	9	9
2	2	1	1	2	2	2	2	2	2
9	9	9	9	9	9	9	9	9	9
9	9	9	9	9	9	9	9	9	9
2	2	3	2	2	2	2	2	2	2
9	9	9	9	9	9	9	9	9	9
9	9	9	9	9	9	9	9	9	9
9	9	9	9	9	9	9	9	9	9
9	9	9	9	9	9	9	9	9	9
9	9	9	9	9	9	9	9	9	9
9	1	9	9	3	1	9	9	1	9
9	9	9	9	9	9	9	9	9	9
9	9	9	9	9	9	9	9	9	9
9	9	9	9	9	9	9	9	9	9
9	9	9	9	9	9	9	9	9	3
9	9	9	9	9	9	9	9	2	3
9	9	9	9	9	9	9	9	9	9
3	3	3	3	3	3	3	3	3	3
3	3	3	3	3	3	3	3	3	3
2	1	2	1	3	1	3	1	2	1
1	1	1	1	9	9	9	3	2	2
9	9	9	9	9	9	9	9	9	1

9	9	9	9	9	9	9	9	9	1
9	9	9	9	9	9	9	9	9	3
2	2	2	1	1	3	3	3	3	3
3	1	2	3	1	3	1	2	3	3
2	2	2	2	2	2	2	2	2	9
2	2	2	2	3	3	3	3	3	2
1	1	1	1	1	1	1	1	1	9
2	2	2	1	2	3	1	3	2	2
2	3	2	3	1	2	2	2	2	2
3	3	3	3	3	3	3	3	3	3
9	9	9	9	9	9	9	9	9	9
9	9	9	9	9	9	9	9	9	9
9	9	9	9	9	9	9	9	9	9
1	9	9	9	9	9	9	9	9	9
3	3	3	3	3	3	3	3	3	2
1	1	1	1	1	2	2	2	3	1
9	9	9	9	9	9	9	9	9	9
2	1	1	2	2	2	2	3	3	1
1	1	1	1	1	1	1	1	1	1
2	2	2	2	2	2	2	2	2	2
2	2	2	2	2	2	2	2	3	2
3	9	1	2	1	3	3	3	1	1
9	9	9	9	9	9	9	9	9	1
2	2	2	2	2	2	2	2	2	2
2	2	2	2	3	3	3	3	3	2
2	2	2	2	2	2	2	2	2	1
2	2	2	2	2	2	2	2	2	2
2	2	2	2	1	1	1	1	1	1
2	2	2	2	2	2	2	2	2	3
2	1	1	1	1	2	1	1	2	1
2	2	2	2	2	2	2	2	2	2
2	2	2	2	2	2	2	2	2	2
2	2	2	3	3	3	3	3	3	3
2	2	2	2	2	2	2	2	2	3
3	1	1	1	3	3	3	3	3	1
2	3	1	2	3	1	2	3	2	1
9	9	9	9	9	9	9	9	9	9
2	2	1	2	2	2	2	2	2	3
3	3	3	3	3	3	3	3	3	3
3	3	3	3	3	3	3	3	3	3
9	9	9	9	9	9	9	9	9	2
9	9	9	9	9	9	9	9	9	9
9	9	9	9	9	1	9	9	9	2
2	2	2	2	2	2	2	3	3	1
2	2	2	2	2	1	1	1	1	1
2	2	2	2	2	2	2	2	2	2
2	3	2	2	1	1	2	2	2	1

9	9	9	1	9	9	9	9	9	9
9	9	9	9	9	9	9	9	9	1
9	9	9	9	9	9	9	9	9	9
9	2	9	9	9	9	9	9	1	9
1	9	9	9	9	9	9	9	1	9
2	2	2	2	1	1	1	2	2	1
9	9	9	9	9	9	9	9	9	2
9	9	9	9	9	9	9	9	9	2
9	9	9	9	9	9	9	9	9	2
9	9	9	9	9	9	9	9	9	2
9	1	9	9	9	9	9	9	9	9
9	9	9	2	9	9	9	9	2	9
9	9	9	9	9	9	9	9	9	2
9	9	9	3	9	9	9	9	9	9
9	9	9	9	9	9	9	9	9	1
9	9	9	9	9	9	9	9	9	9
2	9	9	9	9	9	9	9	2	9
2	3	2	1	2	3	9	2	1	3
1	2	2	1	1	2	2	1	2	1
2	2	2	3	3	3	2	3	3	3
1	1	2	2	1	1	2	1	3	1
1	2	1	2	1	2	9	2	2	1
2	2	1	2	2	2	2	3	2	2
3	2	3	2	1	2	3	3	3	9
1	2	2	3	2	2	9	1	2	2
9	9	9	9	9	9	9	9	9	9
1	1	2	2	2	2	2	2	2	1
9	9	9	9	9	9	9	9	1	9
2	2	2	3	2	2	3	1	1	1
1	2	2	1	1	1	1	1	2	9
2	2	1	2	2	1	2	2	1	1
2	2	2	2	2	2	2	2	2	1
2	2	2	3	3	3	3	3	3	1
1	1	1	1	1	1	1	1	1	1
9	9	9	9	9	9	9	9	9	9
2	2	2	2	2	2	2	2	2	2
9	9	9	9	9	9	9	9	9	9
2	2	1	1	3	2	2	2	2	1
9	9	9	9	9	9	9	9	9	9
2	2	2	2	2	2	2	2	2	2
2	1	1	2	1	2	2	1	1	1
1	3	3	3	3	3	3	3	3	3
1	1	1	1	3	3	3	3	3	1
2	2	2	2	3	3	3	3	3	2
2	2	2	2	2	2	2	2	2	3
2	2	1	2	2	2	2	2	2	2
3	3	3	3	9	2	2	2	3	2

2	2	1	1	1	2	2	3	3	1
9	3	9	9	9	9	9	9	9	9
2	2	2	2	2	2	2	2	2	1
1	2	1	2	1	2	1	2	1	1
2	2	2	2	2	1	1	1	1	1
2	2	2	2	2	2	3	3	3	2
1	1	1	2	2	1	3	3	3	1
2	1	2	1	2	1	3	3	3	9
9	9	9	9	9	9	9	9	9	9
3	1	1	1	2	2	3	3	3	2
2	2	2	2	2	2	2	2	2	2
9	9	9	9	9	9	9	9	9	1
9	9	9	9	9	9	9	9	9	1
2	1	1	9	1	1	9	9	9	1
9	9	9	9	9	9	9	9	9	1
2	2	2	2	2	2	2	2	2	2
9	9	9	9	9	9	9	9	9	9
9	9	9	9	9	9	9	9	9	2
9	9	9	9	9	9	2	9	9	9
3	2	3	2	3	2	3	2	3	2
2	2	2	2	2	2	2	2	2	2
1	3	3	3	2	2	2	1	1	2
2	2	3	3	2	2	2	2	2	2
9	9	1	9	9	9	9	9	9	9
9	9	9	9	9	9	9	9	9	9
2	2	1	1	2	2	2	2	2	1
1	1	2	2	2	1	1	1	1	1
1	2	1	2	1	1	2	3	2	2
9	9	9	9	9	9	9	9	9	9
9	9	9	9	9	9	9	9	9	1
2	2	2	2	2	2	2	2	2	9
3	1	2	3	1	2	3	1	2	3
2	2	2	1	2	2	3	3	3	2
1	2	2	1	1	2	2	2	2	1
1	1	1	2	2	2	2	2	2	1
2	2	1	2	2	2	1	2	2	1
9	9	9	9	9	9	9	9	9	9
9	9	9	9	9	9	9	9	9	9
2	3	9	1	3	2	3	1	2	3
2	1	2	1	2	1	2	1	2	1
2	2	2	2	2	2	2	2	2	2
2	2	2	2	2	2	2	2	3	1
2	1	1	3	2	3	3	3	3	1
2	3	2	1	3	2	3	2	2	1
2	9	3	3	3	2	2	3	2	2
2	2	2	2	2	2	2	2	2	2
2	2	2	2	3	3	3	3	3	2

2	1	1	1	1	1	1	1	2	1
2	9	2	2	3	2	3	2	2	2
9	1	9	9	9	9	9	9	9	1
9	9	9	1	9	9	9	9	9	1
9	1	9	9	9	9	9	9	9	9
9	2	9	1	2	1	2	1	2	1
9	2	9	9	2	9	9	9	2	2
9	2	9	2	2	2	2	2	2	2
9	9	9	9	9	9	9	9	9	9
9	9	9	9	9	9	9	9	9	9
9	9	9	9	9	9	9	9	9	9
9	9	9	9	9	9	9	9	9	9
9	9	9	9	9	9	9	9	9	9
9	9	9	9	9	9	9	9	9	9
9	9	9	9	9	9	9	9	9	9
9	9	9	9	9	9	9	9	9	2
3	3	3	3	3	3	3	3	3	3
9	9	9	9	9	9	9	9	9	1
9	9	9	9	9	9	9	9	9	2
9	9	2	9	2	9	9	2	2	9
9	9	9	9	9	9	9	9	9	9
9	9	9	9	9	9	9	9	9	2
2	2	2	2	2	2	2	2	2	2
9	9	9	9	9	9	9	9	9	9
9	9	9	9	9	9	9	9	9	9
9	9	9	9	9	9	9	9	9	2
9	9	9	9	9	9	9	9	9	9
9	9	2	9	9	2	9	2	9	2
9	9	9	9	9	9	9	9	9	9
9	9	9	9	9	9	9	9	9	9
9	9	9	9	9	9	9	9	9	9
9	2	9	2	9	2	9	2	9	9
9	9	9	9	9	9	9	9	9	9
9	9	9	9	9	9	9	9	9	9
9	9	9	9	9	9	9	9	9	9
9	9	9	9	9	9	9	9	9	9
9	9	9	9	9	9	9	9	9	9
9	9	9	9	9	9	9	9	9	9
2	3	2	1	2	3	2	2	2	2
2	2	3	2	3	1	2	3	2	2
9	1	9	9	9	9	9	9	9	9
9	9	9	9	9	9	9	9	9	9
1	3	1	2	1	3	1	3	2	1
3	3	3	3	2	2	2	2	2	1
1	1	1	3	3	3	3	3	3	1
2	1	1	1	3	2	1	2	2	2
2	9	2	3	3	2	1	3	9	9

9	1	9	9	9	9	9	9	9	1
2	2	1	3	3	3	2	3	3	1
2	2	2	2	2	2	2	2	2	9
1	1	1	1	1	1	1	1	1	1
2	2	2	2	2	2	2	2	2	2
1	2	1	2	2	2	2	2	2	2
9	9	9	9	9	9	9	9	9	9
3	2	2	3	2	1	2	3	2	1
2	2	2	2	2	2	2	2	2	2
1	1	1	1	3	1	3	3	3	1
2	1	2	1	1	2	2	3	3	1
9	2	9	9	9	9	9	9	9	9
9	1	9	9	9	9	9	9	9	9
9	9	9	9	9	9	9	9	9	1
9	9	2	9	9	9	9	9	9	2
9	1	9	9	9	9	9	9	9	9
2	2	2	2	2	2	2	2	2	2
1	1	2	9	3	1	3	3	3	1
1	1	2	3	3	9	2	3	3	3
2	2	2	2	2	2	2	2	2	2
2	2	2	2	2	2	2	2	2	2
9	9	9	9	9	9	9	9	9	9
1	2	1	2	1	2	2	1	1	3
2	9	9	9	9	9	9	9	9	9
9	1	9	9	9	9	9	9	9	1
2	3	3	2	2	3	2	3	2	1
9	9	1	9	9	9	9	9	9	9
3	2	2	2	2	2	2	2	2	2
9	9	9	9	9	9	9	9	9	9
9	9	9	9	9	9	9	9	9	1
3	2	3	3	3	2	3	2	9	3
3	2	3	2	3	2	1	3	3	3
9	9	9	9	9	9	9	9	9	9
9	9	9	9	9	9	9	9	9	9
2	3	2	2	1	2	2	3	1	1
9	9	9	9	9	9	9	9	9	9
9	9	9	9	9	9	9	9	9	9
9	1	9	1	1	1	1	1	1	1
2	2	2	2	2	2	2	2	2	2
9	9	9	9	9	9	9	9	9	9
2	2	2	2	2	2	2	2	2	9
1	1	1	1	2	1	2	2	2	1
2	2	2	2	2	2	2	2	2	3
2	2	2	2	2	2	2	2	2	2
2	1	3	2	3	2	3	2	3	1
2	2	2	2	2	2	2	2	9	2
3	3	3	3	2	2	1	2	2	2

9	1	9	9	9	9	9	9	9	1
2	2	2	2	2	2	2	2	2	2
2	2	2	2	2	2	2	2	2	1
9	1	9	9	9	9	9	9	9	9
9	1	9	9	9	9	9	9	9	9
9	9	9	9	9	9	9	9	9	9
9	9	9	9	9	9	9	9	9	9
2	2	2	2	2	2	2	2	2	2
2	1	2	1	2	2	2	2	3	1
9	9	9	9	9	9	9	9	9	9
1	1	1	2	1	1	2	1	2	1
1	1	1	2	2	2	3	3	3	2
2	2	2	2	2	2	2	2	3	2
2	2	2	2	2	2	2	2	2	2
9	9	9	9	9	9	9	9	9	9
9	9	9	9	9	9	9	9	9	9
9	9	9	9	9	9	9	9	9	9
9	9	9	9	9	9	9	9	9	9
9	9	9	9	9	9	9	9	9	1
9	9	9	9	9	9	9	9	9	2
9	9	9	9	9	9	9	9	9	9
9	9	9	9	9	9	9	9	9	9
2	9	2	2	2	9	2	2	2	2
9	9	9	9	9	9	9	9	2	2
9	9	9	9	9	9	9	9	9	9
9	9	9	9	9	9	9	9	9	9
9	9	9	9	9	9	9	9	9	9
9	9	9	9	9	9	9	9	9	9
9	9	9	9	9	9	9	9	9	9
9	9	9	9	9	9	9	9	9	3
9	9	9	9	9	9	9	9	9	9
9	9	9	9	9	9	9	9	9	9
9	9	9	9	9	9	9	9	9	9
9	9	9	9	9	9	9	9	9	9
9	9	9	9	9	9	9	9	9	9
9	9	9	9	9	9	9	9	9	9
9	9	9	9	9	9	9	9	9	9
9	9	9	9	9	9	9	9	9	9
9	9	9	9	9	9	9	9	9	9
9	9	9	9	9	9	9	9	9	9
2	2	2	2	2	2	2	2	2	2
9	9	9	9	9	9	9	9	9	2
9	9	9	9	9	9	9	9	9	9
9	9	9	9	9	9	9	9	9	9
9	9	9	9	9	9	9	9	9	9
9	9	9	9	9	9	9	9	9	9
9	9	9	9	9	9	9	9	9	9

9	9	9	9	9	9	9	9	9	9
2	2	2	2	2	2	2	2	2	2
9	9	9	9	9	9	9	9	9	9
9	9	9	9	9	9	9	9	9	9
9	9	9	9	9	9	9	9	9	9
9	9	9	9	9	9	9	9	9	9
9	9	9	9	9	9	9	9	9	9
9	9	9	9	9	9	9	9	9	9
9	9	9	9	9	9	9	9	9	9
9	9	9	9	9	9	9	9	9	9
9	9	9	9	9	9	9	9	9	9
9	9	9	9	9	9	9	9	9	9
9	9	9	9	9	9	9	9	9	9
9	9	9	9	9	9	9	9	9	9
9	9	9	9	9	9	9	9	9	9
9	9	9	9	9	9	9	9	9	9
9	9	9	9	9	9	9	9	9	9
2	2	2	2	2	2	2	2	2	9
9	9	9	9	9	9	9	9	9	9
9	9	9	9	9	9	9	9	9	9
9	9	9	9	9	9	9	9	9	9
9	9	9	9	9	9	9	9	9	9
9	9	9	9	9	9	9	9	9	9
9	9	9	9	9	9	9	9	9	9
2	2	2	2	2	2	2	2	2	2
9	9	9	9	9	9	9	9	9	9
9	9	9	9	9	9	9	9	9	9
1	1	1	1	1	1	1	1	1	1
9	9	9	9	9	9	9	9	9	9
9	9	9	9	9	9	9	9	9	9
1	1	1	1	1	1	1	1	1	1
1	9	1	1	1	1	1	1	1	3
9	9	9	9	9	9	9	9	9	9
2	2	2	2	2	2	2	2	2	9
9	9	9	9	9	9	9	9	9	9
2	2	2	2	2	2	2	2	2	2
9	9	9	9	9	9	9	9	9	9
9	9	9	9	9	9	9	9	9	9
1	1	1	1	1	1	1	1	1	1
9	9	9	9	9	9	9	9	3	3
9	9	9	9	9	9	9	9	9	9
9	9	9	9	9	9	9	9	9	9
2	2	2	2	2	2	2	2	2	2
2	2	2	2	2	2	2	2	2	2
9	9	1	9	1	9	1	9	1	1
9	9	9	9	9	9	9	9	9	9
9	9	9	9	9	9	9	9	9	9

9	9	9	2	9	9	9	2	2	9
9	9	9	9	9	9	9	9	9	9
9	9	9	9	9	9	9	9	9	9
9	9	9	9	9	9	9	9	9	9
9	9	9	9	9	9	9	9	9	9
9	9	9	9	9	9	9	9	9	9
9	9	9	9	9	9	9	9	9	9
9	9	9	9	9	9	9	9	9	2
9	2	9	2	9	2	9	2	2	2
9	9	9	9	9	9	9	9	9	9
9	9	9	9	9	9	9	9	9	9
9	9	9	9	9	9	9	9	9	9
9	9	9	9	9	9	9	9	9	9
9	9	9	9	9	9	9	9	9	9
1	2	2	2	1	1	2	2	1	2
3	2	1	2	1	1	2	2	3	2
9	1	9	9	9	9	9	9	9	9
2	2	2	2	2	2	2	2	2	2
2	2	2	2	3	3	3	2	2	2
9	9	9	9	9	9	9	9	9	1
9	9	9	9	9	9	9	9	9	1
9	9	9	9	9	9	9	9	9	1
2	2	2	2	2	2	2	2	2	3
3	1	2	3	1	2	3	1	2	1
2	2	2	2	2	2	2	2	2	1
2	2	2	2	2	2	2	2	2	1
9	1	3	2	3	3	3	3	3	1
3	1	3	1	1	1	3	3	3	1
2	1	2	1	2	3	3	3	3	1
2	2	2	2	2	2	2	2	2	2
2	2	2	2	2	2	2	2	9	2
9	9	9	9	9	9	9	9	2	9
9	9	9	9	9	9	9	9	9	1
9	9	9	9	9	9	9	9	1	9
2	9	9	9	9	9	9	9	9	9
9	9	9	9	9	9	9	9	9	9
9	9	9	9	9	9	9	9	9	9
2	9	9	9	2	9	9	2	9	9
9	9	9	9	9	9	9	9	9	9
9	9	9	2	9	2	9	2	2	2
9	9	9	9	9	9	9	9	9	9
2	2	2	2	2	2	2	2	2	2
9	9	9	9	9	9	9	9	9	9
9	9	9	9	9	9	9	9	9	9
9	9	9	9	9	9	9	9	9	9
2	2	2	2	2	2	2	2	2	2
2	3	9	1	1	3	2	2	2	1

1	1	1	1	1	1	1	1	2	1
2	1	2	2	1	2	3	2	2	2
2	1	3	2	1	2	2	2	2	2
1	1	3	3	3	2	2	1	3	1
3	3	3	3	3	3	3	3	3	3
1	1	2	2	2	2	3	3	3	1
2	2	2	2	2	2	2	2	2	1
3	3	3	3	3	3	3	3	3	3
9	9	9	9	9	9	9	9	9	9
2	2	2	2	2	2	2	2	2	2
9	2	9	9	2	9	2	9	2	2
1	9	1	2	3	1	3	1	1	1
2	2	2	2	2	2	2	2	2	1
1	2	2	1	2	3	3	2	2	1
3	3	2	2	3	3	3	3	3	2
2	2	2	2	2	2	2	2	2	2
2	2	3	1	2	3	1	3	2	2
2	2	2	2	2	2	2	2	2	9
9	9	9	9	9	9	9	9	9	9
2	1	2	3	1	2	2	1	2	1
2	2	2	2	2	2	2	2	2	2
1	1	2	2	3	1	2	3	2	1
2	2	2	2	2	2	2	2	2	1
1	2	3	2	2	2	2	2	1	2
3	9	2	2	3	2	2	3	3	2
2	1	2	2	2	2	2	2	2	1
2	2	2	2	2	2	2	2	2	2
2	1	3	2	3	3	2	2	2	2
2	2	2	2	2	2	2	2	2	1
3	9	2	2	9	2	9	2	2	3
3	2	3	3	3	3	3	3	3	1
9	9	9	9	9	9	9	9	9	9
3	3	3	3	3	3	3	3	3	3
2	2	2	2	2	2	2	2	2	2
1	9	9	9	9	9	9	9	9	9
2	2	3	2	2	2	3	3	2	2
3	3	3	2	1	1	2	2	3	2
1	1	1	1	1	1	1	1	1	1
2	2	2	2	2	2	2	2	2	1
9	9	9	9	9	9	9	9	9	9
2	2	2	2	2	2	2	2	2	2
9	9	9	9	9	9	9	9	9	9
1	2	2	1	3	2	1	3	2	2
2	2	2	2	2	2	2	2	2	2
2	2	2	3	1	2	3	2	3	2
1	2	2	2	2	2	3	2	3	2
2	3	2	2	3	2	3	3	2	2

9	9	9	9	9	9	9	9	9	9
3	3	3	3	3	3	3	3	3	3
3	1	1	3	3	1	3	3	1	1
2	1	2	2	3	3	3	3	3	2
2	2	2	1	1	3	3	3	3	1
9	9	9	9	9	9	9	9	9	9
2	2	2	2	2	2	2	2	2	2
2	2	1	2	3	2	3	1	2	2
2	1	1	1	1	1	2	2	1	1
2	2	2	2	2	2	2	2	2	1
1	1	1	1	1	1	1	2	3	1
2	2	2	2	2	2	2	2	2	2
9	9	9	9	9	9	9	9	9	9
3	3	9	2	3	2	3	2	3	3
3	1	1	2	1	2	3	2	3	1
2	2	2	2	2	2	2	2	2	2
2	2	2	2	2	2	2	2	2	2
3	3	3	3	3	2	2	9	2	2
2	2	2	2	2	2	2	2	2	2
9	9	9	9	9	9	9	9	9	1
9	9	9	9	9	9	9	9	9	9
1	1	2	1	2	1	2	1	2	2
3	3	3	3	3	2	3	2	2	2
2	3	1	2	2	3	3	2	2	2
9	9	9	9	9	9	9	9	9	9
2	1	3	2	2	1	3	2	1	1
9	9	9	9	9	9	9	9	9	9
2	2	2	2	2	2	2	2	2	2
2	2	2	2	2	2	2	2	2	2
2	2	2	2	2	2	2	2	2	2
2	2	3	1	2	3	2	3	2	9
9	9	9	9	9	9	9	9	9	9
9	9	9	9	9	9	9	9	9	9
1	1	1	1	2	1	3	3	3	2
9	9	9	9	9	9	9	9	9	9
9	9	9	9	9	9	9	9	9	9
9	9	9	9	9	9	9	9	9	9
2	1	2	2	2	1	2	2	2	2
3	1	3	1	3	3	2	2	3	9
1	1	1	2	2	1	1	2	2	1
2	2	2	2	2	2	2	2	2	1
2	2	2	2	2	2	2	2	2	2
2	2	2	2	2	2	2	2	2	2
2	2	2	2	2	2	2	2	2	2
1	2	2	2	2	2	2	2	2	2
3	2	1	2	3	2	3	1	2	2
2	2	2	2	2	2	2	2	2	2

2	3	3	3	3	3	2	1	2	2
1	2	2	2	2	2	2	2	9	2
2	2	2	2	2	2	2	2	2	2
1	1	1	2	1	1	1	2	1	2
2	2	2	2	2	2	2	2	2	2
1	2	2	2	2	2	2	2	2	2
1	2	2	2	2	2	2	2	2	2
2	2	2	2	2	2	2	2	2	2
2	2	2	2	2	2	2	2	2	2
9	9	9	9	9	9	9	9	9	9
2	2	2	2	2	2	2	2	2	2
2	9	2	1	2	3	1	1	3	1
3	1	3	1	3	3	3	3	3	9
2	9	1	2	1	2	1	2	1	1
2	2	2	2	2	2	2	2	2	2
3	2	9	1	1	2	1	3	1	9
9	9	9	9	9	9	9	9	9	9
9	9	9	2	9	9	9	1	9	9
9	9	9	9	9	9	9	9	9	9
9	9	9	9	9	9	9	9	9	9
3	2	2	2	2	2	1	1	9	1
9	9	9	9	9	9	9	9	9	9
9	9	9	9	9	9	9	9	9	9
1	1	1	2	1	1	2	2	2	1
2	2	2	2	2	2	2	2	2	3
1	1	1	2	2	1	1	2	2	1
1	2	3	9	2	1	1	2	1	3
9	9	9	9	9	9	9	9	9	9
3	2	3	1	2	1	3	1	2	2
2	1	1	2	2	1	2	2	2	2
9	9	9	9	9	9	9	9	9	9
1	2	3	2	1	2	3	1	2	1
2	3	2	1	3	2	3	1	9	2
3	3	3	2	2	2	2	2	2	1
1	2	2	1	2	2	1	2	2	1
1	1	1	2	2	1	1	2	2	1
9	9	9	9	9	9	9	9	9	9
2	2	2	2	2	2	2	2	2	1
2	2	2	2	2	2	2	2	2	2
2	2	2	2	2	2	2	2	2	1
2	2	2	2	2	2	2	2	2	2
2	2	2	3	3	3	1	1	1	1
1	1	1	2	2	2	3	3	3	1
1	1	1	2	2	2	3	3	2	2
3	3	3	3	2	2	3	3	2	3
1	1	1	1	1	2	2	2	3	1
3	2	2	2	3	2	3	2	3	3

1	2	2	1	1	2	3	2	9	2
2	2	2	2	2	2	2	2	9	2
3	2	3	3	9	2	2	3	2	3
2	2	2	2	2	2	2	2	2	2
3	3	3	2	3	2	3	3	2	2
9	9	9	9	9	9	9	9	9	9
9	9	9	9	1	9	9	9	9	1
3	3	2	3	3	3	3	3	2	3
9	9	9	9	9	9	9	9	9	9
9	9	9	9	9	9	9	9	9	9
2	2	2	2	2	2	2	2	2	2
1	2	1	1	2	2	1	2	2	9
3	2	2	2	2	1	1	1	2	2
1	1	9	9	9	9	9	9	9	2
9	1	9	9	9	9	9	9	9	1
3	1	1	1	1	1	3	1	1	1
1	1	3	3	3	1	3	3	3	1
9	9	9	9	9	9	9	9	9	9
2	1	3	1	1	2	3	3	3	1
2	2	2	2	2	2	2	2	2	1
3	3	3	3	3	3	3	3	2	2
9	9	9	9	9	9	9	9	9	9
2	2	2	2	2	2	2	2	2	2
1	1	2	2	2	2	2	2	2	2
2	2	2	2	2	2	2	2	2	2
2	2	2	2	2	2	2	2	2	2
1	1	1	2	2	2	2	2	2	2
2	2	2	2	2	2	2	2	2	2
2	2	9	3	3	2	3	2	2	2
2	2	2	2	2	2	2	2	2	2
3	2	1	2	3	2	1	2	2	1
2	3	2	1	2	2	2	3	2	1
2	1	2	2	1	1	2	1	3	1
2	2	2	2	2	2	2	2	2	1
1	2	1	1	1	1	2	1	1	1
2	3	1	2	3	1	2	3	2	3
1	1	1	1	1	1	1	1	1	3
2	2	2	2	2	2	2	2	2	2
2	3	3	2	3	2	3	2	3	1
1	1	1	3	1	1	1	1	1	1
1	1	1	1	3	3	1	3	3	3
2	2	3	3	1	1	3	3	2	2
2	2	2	2	2	2	2	2	2	2
2	2	2	2	2	2	2	2	2	2
2	2	2	2	2	2	2	2	2	2
2	1	2	2	3	3	3	3	3	1
2	2	2	2	2	2	2	2	2	2

1	2	1	3	2	1	3	2	1	2
1	1	1	1	3	1	3	2	3	1
3	2	3	2	3	2	3	2	3	2
2	2	2	2	2	2	2	2	2	2
9	9	9	9	9	9	9	9	2	2
9	9	9	2	9	9	9	9	9	9
2	3	2	3	2	3	2	3	2	1
9	9	9	9	9	9	9	9	9	1
9	9	9	9	9	9	9	9	9	1
9	9	9	9	9	9	9	9	9	9
9	9	9	9	3	9	9	9	9	9
9	9	9	2	9	9	9	9	9	9
9	9	9	9	9	9	9	9	9	9
3	9	9	9	9	9	9	9	9	3
2	2	2	2	2	2	2	2	2	1
9	9	9	9	9	9	9	9	1	1
9	9	9	9	9	9	9	9	9	9
9	9	9	9	1	9	9	9	9	9
9	9	9	9	1	9	9	9	9	9
9	9	9	9	2	9	9	9	9	9
9	9	9	9	9	9	9	9	9	9
2	2	2	2	2	2	2	2	2	2
2	1	1	1	1	1	1	2	3	1
1	2	2	3	2	1	2	3	2	3
2	3	1	2	3	2	1	2	1	3
9	9	9	9	9	9	9	9	9	9
2	2	2	2	2	2	2	2	2	2
9	9	9	9	9	9	9	9	9	1
2	1	2	3	2	2	2	3	3	2
1	1	1	1	1	1	1	1	1	1
3	3	3	3	3	3	3	3	9	3
9	9	9	9	9	9	9	9	9	1
2	2	3	3	2	3	1	3	3	1
9	9	9	9	9	9	9	9	9	9
9	9	9	9	9	9	9	9	9	1
1	2	2	2	2	2	2	2	1	1
1	1	1	1	1	1	1	1	1	1
9	9	9	9	9	9	9	9	9	9
9	9	9	9	9	9	9	9	9	9
2	2	2	2	2	2	2	2	2	9
9	9	9	9	9	9	9	9	9	9
2	2	2	2	2	2	2	2	2	2
3	3	3	3	3	3	3	3	3	3
2	9	9	9	9	2	9	9	9	9
2	2	2	2	2	2	2	2	2	9
2	2	2	2	2	2	2	2	2	9
9	9	9	9	9	9	9	9	9	9

9	9	9	9	9	9	9	9	9	9
2	9	2	9	2	2	9	9	2	9
9	9	9	9	9	9	9	9	9	9
2	2	2	2	2	2	2	2	2	9
9	9	9	9	9	9	9	9	9	9
1	3	9	1	9	3	1	1	1	9
9	9	9	9	9	9	9	9	9	9
9	9	9	9	9	9	9	9	9	9
2	9	2	9	2	9	2	2	2	2
9	9	9	9	9	9	9	9	9	9
9	9	9	9	9	9	9	9	9	9
9	9	2	9	2	9	9	2	2	9
2	2	2	2	2	2	2	2	2	2
9	9	9	9	9	9	9	9	9	9
2	2	2	2	2	2	2	2	2	2
9	9	9	9	9	9	9	9	9	9
9	9	9	9	9	9	9	9	9	9
9	9	9	9	9	9	9	9	9	9
9	9	9	9	9	9	9	9	9	9
9	9	9	9	9	9	9	9	9	9
2	2	2	2	2	2	2	2	2	2
2	2	2	2	2	2	2	2	2	2
3	9	2	3	3	3	1	3	3	3
3	2	9	1	2	3	1	3	1	1
2	2	2	2	2	2	2	2	2	2
2	2	2	2	2	2	2	2	2	1
1	1	1	1	1	1	1	1	1	1
2	2	2	2	2	2	2	2	2	2
2	3	1	1	2	3	1	1	1	1
2	2	2	2	2	2	2	2	2	2
2	2	9	1	1	1	3	3	2	3
3	1	3	2	3	2	2	3	2	2
2	2	2	2	2	2	2	2	2	2
9	9	9	9	9	9	9	9	9	9
2	3	9	1	3	1	3	1	2	1
2	2	2	2	2	2	2	2	2	3
2	2	2	2	2	2	2	2	2	2
3	3	3	3	1	1	1	1	1	2
2	3	1	2	3	1	3	2	2	1
2	2	2	2	2	1	1	1	1	2
9	9	9	9	9	9	9	9	9	9
9	9	9	9	9	9	9	9	9	9
2	2	2	2	2	2	2	2	2	2
9	9	9	9	9	9	9	9	9	9
2	1	2	1	2	1	2	1	1	9
1	1	1	2	2	1	1	2	2	1
9	9	9	9	9	9	9	9	9	9

2	3	2	1	1	1	2	2	2	1
1	1	1	1	3	3	3	3	2	1
9	9	9	9	9	9	9	9	9	9
2	2	2	2	2	2	2	2	9	9
9	9	9	9	9	9	9	9	9	9
2	2	2	2	2	2	2	2	2	1
1	1	1	1	2	2	2	2	2	2
2	1	1	1	1	1	1	2	1	1
3	3	3	3	3	3	3	3	3	3
1	1	2	1	2	3	2	1	2	1
9	9	9	9	9	9	9	9	9	9
2	2	2	2	2	2	2	2	2	2
2	2	2	2	2	2	2	2	2	9
2	2	2	2	2	2	2	2	2	2
2	2	2	2	2	2	2	2	2	9
1	1	1	1	1	1	9	9	9	1
1	9	2	1	1	2	9	1	1	1
1	1	2	1	2	1	1	2	1	1
1	1	1	1	1	1	1	1	1	2
9	9	9	9	9	9	9	9	9	9
1	1	1	2	1	1	2	1	1	1
2	2	2	2	2	2	2	2	9	2
9	9	9	9	9	9	9	9	9	9
2	2	2	2	2	2	2	2	2	2
9	9	9	9	9	9	9	9	9	9
2	1	2	2	1	1	2	2	1	1
1	1	1	1	1	2	1	2	1	1
1	1	1	1	1	1	1	1	9	1
9	9	9	9	9	9	9	9	9	9
9	9	9	9	9	9	9	9	9	9
9	9	9	9	9	9	9	9	9	9
9	9	9	9	9	9	9	9	9	9
1	1	1	1	1	1	1	1	9	9
2	2	2	2	2	2	2	2	2	1
2	2	2	2	2	2	2	2	2	2
9	9	9	9	9	9	9	9	9	9
1	1	1	1	1	1	1	1	2	1
1	1	2	1	1	1	1	1	1	1
9	9	9	9	9	9	9	9	9	9
9	9	9	9	9	9	9	9	9	9
9	9	9	9	9	9	9	9	9	9
9	9	9	9	9	9	9	9	9	9
2	2	9	1	1	1	1	1	9	1
9	9	9	9	9	9	9	9	9	9
2	1	9	1	2	1	1	2	2	1
2	2	2	2	2	2	2	2	2	2
9	9	9	9	9	9	9	9	9	1

1	1	1	1	2	2	2	2	2	1
2	9	2	9	2	2	9	2	2	9
2	2	2	2	2	2	2	2	2	3
1	1	2	2	1	2	1	2	1	1
2	2	2	2	2	2	2	2	2	9
9	9	9	9	9	9	9	9	9	9
2	2	2	2	2	2	2	2	2	9
2	2	2	2	2	2	2	2	2	2
9	9	9	9	9	9	9	9	9	9
9	9	9	9	9	9	9	9	9	9
9	9	9	9	9	9	9	9	9	9
9	9	9	9	9	9	9	9	9	9
9	9	9	9	9	9	9	9	9	9
9	9	9	9	9	9	9	9	9	9
9	9	9	9	9	9	9	9	9	9
9	9	9	9	9	9	9	9	9	9
9	9	9	9	9	9	9	9	9	9
9	9	9	9	9	9	9	9	9	9
2	2	2	2	2	2	2	2	2	2
3	1	1	2	2	2	3	3	3	1
3	1	2	2	2	2	3	3	3	1
3	1	1	2	2	2	3	3	3	1
3	1	2	2	2	2	3	3	3	1
3	1	2	2	2	2	3	3	3	1
3	1	1	2	2	2	3	3	3	1
3	1	2	2	2	2	3	3	3	1
3	1	1	2	2	2	3	3	3	1
3	1	1	1	2	2	3	3	3	1
3	1	1	2	2	2	3	3	3	1
3	1	3	2	2	2	3	3	3	1
3	1	2	2	2	2	3	3	3	2
3	1	1	2	2	2	3	3	3	1
3	1	1	2	2	2	3	3	3	1
3	1	3	1	2	2	3	3	3	1
3	9	1	2	3	2	3	3	3	1
3	1	2	2	2	3	3	3	3	1
3	1	1	2	2	2	3	3	3	1
3	1	2	2	2	2	3	3	3	1
3	1	1	2	2	2	3	3	3	3
3	1	2	2	2	2	3	3	3	1
3	1	1	2	2	2	3	3	3	3
3	1	1	2	2	2	3	3	3	1
3	1	2	2	2	2	3	3	3	1
3	1	1	2	2	2	3	3	3	1
9	9	9	9	9	9	9	9	9	9
9	9	9	9	9	9	9	9	9	9
9	9	9	9	9	9	9	9	9	9

9	9	9	9	9	9	9	9	9	9
9	9	9	9	9	9	9	9	9	9
9	9	9	9	9	9	9	9	9	9
9	9	9	9	9	9	9	9	9	9
9	9	9	9	9	9	9	9	9	9
9	9	9	9	9	9	9	9	9	9
9	9	9	9	9	9	9	9	9	9
9	9	9	9	9	9	9	9	9	9
9	9	9	9	9	9	9	9	9	9
9	9	9	9	9	9	9	9	9	9
9	9	9	9	9	9	9	9	9	9
9	9	9	9	9	9	9	9	9	9
9	9	9	9	9	9	9	9	9	9
9	9	9	9	9	9	9	9	9	9
9	9	9	9	9	9	9	9	9	9
9	9	9	9	9	9	9	9	9	9
9	9	9	9	9	9	9	9	9	9
9	9	9	9	9	9	9	9	9	9
9	9	9	9	9	9	9	9	9	9
3	1	1	2	2	2	3	3	3	1
3	1	2	2	2	2	3	3	3	1
3	1	1	2	2	2	3	3	3	1
3	1	1	2	2	2	3	3	3	1
3	1	1	2	2	2	3	3	3	1
3	1	1	2	2	2	3	3	3	1
3	1	2	2	2	2	3	3	3	1
3	1	2	2	2	2	3	3	3	1
3	1	1	2	2	2	1	3	3	1
3	1	1	2	2	2	3	3	3	1
3	1	1	2	2	2	3	3	3	1
1	1	2	2	2	2	3	3	3	1
3	1	1	2	2	2	1	3	3	1
3	1	1	2	2	2	3	3	3	1
3	1	1	2	2	2	9	3	3	1
3	1	1	2	2	2	3	3	3	1
9	9	9	9	9	9	9	9	9	1
3	1	1	2	2	2	3	3	3	1
3	1	1	2	2	2	1	1	3	1
3	1	1	2	2	2	1	1	3	1
3	1	1	2	2	3	3	3	3	1
3	1	1	2	2	2	3	3	3	1
3	1	1	2	2	2	3	3	3	1
3	1	1	2	2	2	3	3	3	1
3	1	1	3	2	2	3	3	3	1
3	1	1	2	2	2	3	3	3	1
3	1	1	2	2	2	3	3	3	1
3	1	3	1	2	2	2	3	3	1

2	1	1	2	2	2	3	3	3	1
3	1	3	1	2	2	2	3	3	1
3	1	3	1	1	2	3	3	3	1
3	1	1	2	2	3	3	3	3	1
3	1	1	2	2	2	3	3	3	1
3	1	2	2	2	2	3	3	3	1
3	1	3	1	2	2	2	3	3	1
3	1	1	2	2	2	3	3	3	1
3	1	1	2	2	2	3	3	3	1
3	1	3	2	2	2	3	3	3	1
3	1	1	2	2	2	3	3	3	1
1	2	2	2	3	3	3	3	3	1
3	1	2	2	2	2	3	3	3	1
3	1	1	2	2	2	3	3	3	1
3	1	1	2	2	2	3	3	3	1
3	1	2	2	2	2	3	3	3	1
3	1	1	2	2	2	3	3	3	1
3	1	1	2	2	2	3	3	3	1
3	1	1	2	2	2	3	3	3	1
3	1	2	2	2	2	3	3	3	1
3	1	2	2	2	2	3	3	3	1
3	1	1	2	2	2	3	3	3	1
3	1	2	2	2	3	3	3	3	1
3	1	1	2	2	2	3	3	3	1
1	9	1	2	2	2	3	3	3	1
3	1	2	2	2	2	2	3	3	1
3	1	1	2	2	2	3	3	3	1
1	1	2	2	2	3	9	3	3	1
3	1	1	2	2	2	2	3	3	1
3	1	1	9	9	9	3	3	3	1
3	1	1	2	2	2	3	3	3	3
3	1	1	2	2	2	3	3	3	1
3	1	1	2	2	2	3	3	3	1
3	1	1	2	2	2	3	3	3	1
9	9	9	9	9	9	9	9	9	9
3	1	1	2	2	2	3	3	3	1
3	1	2	2	3	3	3	3	3	3
3	1	1	2	2	2	3	3	3	9
3	1	2	2	3	3	3	3	3	1
3	1	1	2	2	2	3	3	3	1
3	1	1	2	2	3	3	3	3	1
3	1	1	2	2	2	3	3	3	1
3	1	1	2	2	2	3	3	9	1
3	1	1	2	2	2	9	9	3	1
3	1	2	2	2	2	3	3	3	1
3	1	2	2	2	2	3	3	3	1
3	1	1	2	2	2	3	3	3	1

3	1	1	2	2	2	3	3	3	1
3	1	1	2	2	2	3	3	3	1
3	1	1	2	2	2	3	3	3	1
3	1	2	2	2	2	3	3	3	1
1	1	2	2	2	2	2	3	3	1
3	1	2	2	2	2	3	3	3	1
3	1	1	2	2	2	3	3	3	1
3	1	2	2	2	2	3	3	3	1
3	1	1	2	2	2	3	3	3	1
3	1	1	2	2	2	3	3	3	1
3	1	1	1	2	2	3	3	3	1
3	1	1	2	2	2	3	3	3	1
3	1	1	1	2	2	3	3	3	1
3	1	1	2	2	2	2	3	3	1
3	1	1	2	2	9	9	9	3	1
3	9	2	2	2	2	3	3	3	1
3	1	1	2	2	2	3	3	3	1
1	2	2	2	2	3	3	2	3	1
3	1	1	1	2	2	3	3	3	1
3	1	1	2	2	2	3	3	3	1
3	1	1	2	2	2	3	3	3	1
3	1	1	2	2	2	3	3	3	1
3	1	2	2	2	2	3	3	3	1
3	1	1	1	2	2	3	3	3	1
2	1	2	1	1	1	1	1	9	1
3	1	2	2	2	2	3	3	3	3
3	1	1	2	2	2	3	3	3	3
3	1	2	2	2	2	3	3	3	1
3	1	1	2	2	2	3	3	3	2
3	1	1	2	2	2	3	3	3	3
3	1	1	2	2	2	3	3	3	2
2	1	1	2	2	2	3	3	3	1
1	3	1	2	2	2	3	3	3	1
1	3	1	2	2	2	3	3	3	1
3	1	1	2	2	2	3	3	3	1
3	1	1	2	2	9	3	3	3	1
3	1	1	2	2	2	3	3	3	1
3	1	1	2	2	2	3	3	3	1
3	1	1	2	2	2	3	3	3	1
3	1	1	2	2	2	3	3	3	1
3	1	2	2	2	2	3	3	3	1
9	9	9	9	9	9	9	9	9	9
3	1	1	2	2	2	3	3	3	1
3	1	1	2	2	2	3	3	3	1
3	1	1	2	2	2	3	3	3	1
1	2	2	2	2	3	3	3	3	1
1	2	2	2	2	3	3	3	3	1

3	1	1	2	2	2	3	3	3	1
3	1	1	2	2	2	3	3	3	1
3	1	1	2	2	2	3	3	3	1
3	1	1	2	2	2	3	3	3	1
3	1	1	2	2	2	3	3	3	1
3	1	1	2	2	2	3	3	3	1
3	1	1	2	2	2	3	3	3	1
3	1	1	2	2	2	3	3	3	1
3	1	1	2	2	2	3	3	3	1
9	9	9	9	9	9	9	9	9	9
3	1	2	2	2	2	3	3	3	1
3	1	1	2	2	2	3	3	3	1
3	1	2	2	2	2	3	3	3	1
3	1	2	2	2	2	3	3	3	1
3	1	2	2	2	2	3	3	3	1
3	1	2	2	2	2	3	3	3	1
3	1	2	2	2	2	3	3	3	1
3	1	1	2	2	2	3	3	3	1
3	1	2	2	2	2	3	3	3	1
3	1	1	2	2	2	3	3	3	1
3	1	1	2	2	2	3	3	3	1
3	1	1	2	2	2	3	3	3	1
3	1	2	2	2	2	3	3	3	1
3	1	2	2	2	2	3	3	3	1
3	1	2	2	2	2	3	3	3	1
3	1	1	2	2	2	3	3	3	1
3	1	1	2	2	2	3	3	3	3
9	9	9	9	9	9	9	9	9	9
9	9	9	9	9	9	9	9	9	9
9	9	9	9	9	9	9	9	9	9
9	9	9	9	9	9	9	9	9	9
9	9	9	9	9	9	9	9	9	9
9	9	9	9	9	9	9	9	9	9
9	9	9	9	9	9	9	9	9	9
9	9	9	9	9	9	9	9	9	9
9	9	9	9	9	9	9	9	9	9
9	9	9	9	9	9	9	9	9	9
9	9	9	9	9	9	9	9	9	9
9	9	9	9	9	9	9	9	9	9
9	9	9	9	9	9	9	9	9	9
9	9	9	9	9	9	9	9	9	9
9	9	9	9	9	9	9	9	9	9
9	9	9	9	9	9	9	9	9	9
9	9	9	9	9	9	9	9	9	9
9	9	9	9	9	9	9	9	9	9
9	9	9	9	9	9	9	9	9	9
9	9	9	9	9	9	9	9	9	9

9	9	9	9	9	9	9	9	9	9
9	9	9	9	9	9	9	9	9	9
9	9	9	9	9	9	9	9	9	9
9	9	9	9	9	9	9	9	9	9
9	9	9	9	9	9	9	9	9	9
9	9	9	9	9	9	9	9	9	9
9	9	9	9	9	9	9	9	9	9
9	9	9	9	9	9	9	9	9	9
9	9	9	9	9	9	9	9	9	9
9	9	9	9	9	9	9	9	9	9
9	9	9	9	9	9	9	9	9	9
9	9	9	9	9	9	9	9	9	9
9	9	9	9	9	9	9	9	9	9
1	9	1	2	1	2	3	2	3	3
2	2	2	2	2	2	2	2	2	2
2	2	2	2	2	2	2	2	2	2
2	9	2	1	1	1	3	3	1	3
2	2	2	2	2	1	1	1	1	1
2	2	2	2	2	2	2	2	2	1
1	2	1	1	2	2	1	2	1	3
2	1	3	1	2	2	2	2	3	9
3	2	3	2	1	2	2	2	3	2
9	9	9	9	9	9	9	9	9	9
3	2	3	2	2	2	2	2	2	3
9	1	9	9	9	9	9	9	9	9
2	2	2	3	3	3	3	3	2	2
9	9	9	9	9	9	9	9	9	9
1	1	1	1	1	1	1	1	1	1
2	2	1	1	1	3	3	3	1	2
1	1	1	3	1	1	3	3	3	1
3	2	9	1	2	2	3	2	1	3
2	1	2	3	2	3	2	3	1	1
9	9	9	9	9	9	9	9	9	1
1	1	2	1	2	1	3	3	3	1
1	1	1	9	1	1	9	1	1	9
9	9	9	9	9	9	9	9	9	2
1	1	2	2	2	2	2	2	3	1
2	2	1	1	3	3	1	1	2	1
2	1	1	2	2	1	2	2	2	1
1	1	1	1	1	1	3	1	2	1
9	9	9	9	9	9	9	9	9	9
9	9	9	9	9	9	9	9	9	9
2	2	2	2	2	2	2	2	2	9
9	9	9	9	9	9	9	9	9	1
9	9	9	9	9	9	9	9	9	9
9	9	9	9	9	9	9	9	9	9
9	9	9	9	9	9	9	9	9	9

9	9	9	9	9	9	9	1	9	1
9	9	9	9	9	9	9	9	9	9
1	2	3	2	3	2	1	3	9	2
2	2	2	2	1	1	2	2	2	1
2	1	1	2	1	1	1	1	2	1
9	9	9	9	9	9	9	9	9	3
2	2	2	2	2	2	2	2	2	2
2	2	2	2	2	2	2	2	2	2
2	2	2	2	2	2	2	2	2	2
1	1	1	2	2	3	3	3	1	1
2	2	2	2	2	2	2	2	2	2
9	9	9	9	9	9	9	9	9	9
2	9	9	9	9	9	9	9	9	1
2	2	2	2	2	2	2	2	2	2
2	1	1	2	1	1	3	3	3	1
2	1	1	1	2	3	1	2	3	1
1	1	1	1	1	1	1	1	1	1
1	1	3	1	3	3	2	2	2	2
1	1	1	2	1	1	1	1	2	1
9	9	9	9	9	9	9	9	9	9
2	2	2	2	2	2	2	2	2	2
2	2	2	2	2	2	2	2	2	2
2	2	2	2	2	2	2	2	2	2
2	2	2	2	2	2	2	2	2	2
2	2	2	2	2	2	2	2	2	2
1	1	2	1	1	2	2	3	1	1
2	2	2	2	2	2	2	2	2	2
2	3	9	2	1	2	3	2	1	1
9	9	9	9	9	9	9	9	9	9
3	1	1	3	3	1	3	3	3	1
3	1	3	1	3	3	3	3	3	1
3	9	2	3	1	1	1	2	2	1
2	1	2	1	2	2	2	2	2	1
1	1	1	1	1	1	2	3	3	1
1	3	3	2	9	1	2	2	2	1
2	2	2	2	2	1	1	1	2	9
1	1	1	2	3	3	3	3	3	1
2	2	2	9	9	9	9	9	9	1
9	9	9	9	9	9	9	9	3	9
2	2	2	2	2	2	2	2	2	2
3	3	3	2	2	2	2	2	2	3
2	2	2	2	2	1	2	3	3	1
2	3	1	2	3	1	2	3	1	1
9	9	9	9	9	9	9	9	9	9
1	1	1	1	1	1	1	1	1	1
1	1	1	2	2	2	2	2	1	1
2	1	2	3	2	2	2	3	3	1

2	2	2	2	2	2	2	2	2	2
2	2	2	3	2	1	2	1	2	1
2	2	2	2	2	2	2	2	2	2
2	2	2	2	2	2	2	2	2	9
2	2	2	2	2	2	2	2	2	9
2	2	2	2	2	2	2	2	2	2
3	1	2	2	3	3	3	3	3	1
2	1	2	3	2	1	3	2	2	2
2	2	2	2	2	2	2	2	2	9
1	3	1	2	3	2	3	1	2	1
2	2	2	2	2	2	2	2	2	2
2	2	2	2	2	2	2	2	2	2
1	1	1	1	1	1	1	1	1	1
2	2	3	3	3	3	3	3	3	2
2	2	2	2	2	2	2	2	2	9
2	2	2	2	2	2	2	2	2	2
3	3	3	3	3	3	3	3	3	3
2	2	2	2	2	2	2	2	2	2
2	2	2	2	2	2	2	2	2	2
2	3	1	2	3	1	2	3	1	1
2	1	2	1	2	1	2	1	2	1
1	1	2	2	2	2	1	1	3	1
2	1	3	1	1	2	1	3	1	9
1	1	1	1	1	1	1	1	3	9
1	1	3	3	3	3	3	3	3	1
9	9	9	9	9	9	9	9	9	9
9	9	9	9	9	9	9	9	9	9
2	1	1	1	1	1	2	2	2	2
2	3	1	1	2	2	2	2	1	1
1	1	2	1	3	1	1	1	3	1
2	2	2	2	2	2	2	2	3	1
1	2	1	2	2	2	2	2	1	1
1	1	1	1	1	1	1	1	1	2
3	3	3	3	3	3	3	3	3	1
9	9	9	9	9	9	9	9	9	9
2	2	2	2	2	2	2	2	2	2
9	9	9	9	9	9	9	9	9	9
9	9	9	9	9	9	9	9	9	9
9	9	9	9	9	9	9	9	9	9
1	1	1	1	1	1	1	1	1	1
2	3	3	9	2	3	2	2	2	2
2	2	9	1	1	3	1	3	3	3
1	2	1	2	2	2	2	1	2	2
9	9	9	9	9	9	9	9	9	9
9	9	9	9	9	9	9	9	9	9
9	9	9	9	9	9	9	9	9	9
1	1	1	1	2	2	2	2	3	1

9	9	9	9	9	9	9	9	9	9
9	9	9	9	9	9	9	9	9	9
2	9	2	2	2	2	2	2	2	9
9	9	9	9	9	9	9	9	9	9
9	9	9	9	9	9	9	9	9	9
3	3	3	2	2	2	3	3	3	9
1	1	2	3	2	2	2	2	3	1
9	9	9	9	9	9	9	9	9	9
1	1	1	2	2	1	3	3	3	1
1	3	2	3	1	2	3	1	2	1
3	1	2	1	3	2	1	2	1	1
2	2	2	2	2	2	2	2	2	2
2	2	2	2	2	2	2	2	2	2
9	9	9	9	9	9	9	9	9	9
2	2	2	2	2	2	2	2	9	2
2	2	2	2	2	2	2	2	2	2
3	3	3	3	1	1	2	2	1	1
3	1	3	3	2	1	2	3	2	2
3	2	1	3	2	2	3	2	2	1
9	9	9	9	9	9	9	9	9	9
3	1	3	3	2	1	2	3	2	2
3	3	2	2	1	2	1	2	3	1
2	2	2	2	2	2	2	2	2	2
3	1	3	3	1	2	3	2	2	2
3	3	2	2	3	2	3	2	3	3
9	9	9	9	9	9	9	9	9	9
2	3	2	1	1	2	1	2	1	1
9	9	9	9	9	9	9	9	9	9
3	3	3	3	3	3	3	3	3	3
2	3	2	2	2	2	2	2	2	1
2	2	2	2	2	2	2	2	2	2
9	1	9	9	9	9	9	9	9	9
9	1	9	9	9	9	9	9	9	9
2	2	2	2	2	2	2	2	2	2
2	2	3	3	3	3	3	3	2	2
3	1	2	2	1	2	1	2	1	2
9	9	9	9	9	9	9	9	9	2
2	1	1	1	3	2	2	3	3	1
2	2	2	2	2	2	2	2	2	2
9	9	9	9	9	9	9	9	1	2
9	9	9	9	9	9	9	9	9	1
2	2	2	2	2	2	2	2	2	2
2	2	2	2	2	2	2	2	2	2
2	2	2	2	2	2	2	2	2	2
2	2	2	2	2	2	2	2	2	2
1	9	9	9	1	9	9	9	9	1
2	2	2	2	2	2	2	2	2	2

2	2	2	2	2	2	2	2	2	2
2	2	2	2	2	2	2	2	2	2
3	1	3	1	3	3	3	3	3	1
3	1	2	2	3	3	3	3	3	1
2	2	2	2	2	2	2	2	2	2
2	2	2	3	3	3	3	3	2	2
1	2	3	1	1	1	3	2	2	1
2	3	2	1	2	1	2	1	3	1
2	1	2	2	3	3	3	2	3	1
3	1	3	1	1	3	3	3	3	1
2	2	2	2	2	2	2	2	2	3
3	3	3	2	2	2	2	2	2	2
1	1	1	2	2	2	2	2	2	1
2	1	1	3	3	3	3	3	3	1
9	1	2	3	1	2	3	1	3	3
2	2	2	2	2	2	2	2	2	1
2	2	2	2	3	3	3	3	3	2
1	2	2	2	2	1	3	2	1	2
2	2	2	2	3	2	2	2	3	2
1	1	1	3	3	3	3	3	3	1
3	1	2	1	1	2	3	2	2	1
9	9	9	9	9	9	9	9	9	9
2	2	2	2	2	2	2	2	3	2
2	2	2	2	2	2	2	2	2	2
2	2	2	2	2	2	2	2	2	2
2	1	1	1	3	1	3	2	3	1
1	1	1	2	1	1	2	3	3	2
1	1	1	2	1	1	2	1	3	2
3	1	3	1	3	3	3	3	3	1
3	1	3	2	3	3	3	3	3	1
1	1	3	3	3	3	3	3	3	1
2	2	2	2	2	2	2	2	1	1
1	2	2	2	2	2	2	2	2	2
9	9	9	9	9	9	9	9	9	9
9	9	9	9	9	9	9	9	9	1
3	9	3	1	3	3	3	1	3	1
2	2	2	2	2	2	2	2	2	2
2	2	1	2	1	2	1	1	1	1
2	1	2	2	1	2	2	9	2	1
2	2	2	2	2	2	2	2	2	2
2	3	2	1	2	3	1	3	2	2
9	9	9	9	9	9	9	9	9	9
9	9	9	9	9	9	9	9	9	9
1	1	1	1	1	1	1	1	1	1
2	2	9	9	9	9	9	9	9	9
9	9	9	9	9	9	9	9	9	9
9	9	9	9	9	9	9	9	9	9

2	2	2	2	2	2	2	2	2	2
1	3	1	3	3	3	2	1	2	1
9	9	9	9	9	9	9	9	9	9
3	3	3	3	3	3	3	3	2	1
2	1	1	1	2	1	2	3	1	2
2	3	1	2	3	1	2	3	1	1
2	2	3	2	1	2	2	2	1	1
9	9	9	1	9	9	9	9	9	1
1	2	1	3	3	2	1	2	2	1
9	9	9	9	9	9	9	9	9	9
1	1	1	1	1	1	1	1	1	1
1	1	1	3	1	1	3	3	3	1
2	1	1	2	9	1	9	9	9	9
9	9	9	9	9	9	9	9	9	9
1	1	1	1	1	1	1	1	1	1
9	9	9	9	9	9	9	9	9	9
9	9	9	9	9	9	9	9	9	9
2	2	2	2	2	2	2	2	2	2
2	2	2	2	2	2	2	2	2	2
2	3	2	3	2	2	2	2	2	1
2	2	2	2	2	2	2	2	2	2
2	2	2	2	2	2	2	2	2	2
2	2	2	2	2	2	2	2	2	1
2	3	2	1	3	2	1	3	2	1
9	2	2	9	2	2	2	2	1	1
9	9	9	9	9	9	9	9	9	1
3	1	3	1	1	3	3	3	3	1
3	3	9	2	2	2	2	2	2	3
3	3	3	3	2	1	1	1	1	9
1	1	1	1	1	1	3	2	3	2
1	1	1	2	1	2	1	1	2	1
2	2	2	2	2	2	2	2	2	2
2	2	2	2	2	2	2	2	9	2
2	2	2	2	2	2	2	2	2	2
2	2	2	2	2	2	2	2	2	2
2	2	2	2	2	2	2	2	2	2
2	1	3	2	1	3	2	3	2	2
9	9	9	9	9	9	9	9	9	1
9	9	9	9	9	9	9	9	9	9
2	2	2	2	2	2	2	2	2	1
3	3	3	3	3	3	3	3	2	2
1	9	9	9	9	9	3	9	9	9
2	1	2	2	2	2	2	2	2	1
9	9	9	9	9	9	9	9	9	1
2	2	2	2	3	3	3	3	3	3
9	9	9	9	9	9	9	9	9	9
2	2	2	2	2	2	2	2	2	2

9	9	9	9	9	9	9	9	9	2
9	9	9	9	9	9	9	9	9	1
2	2	2	2	2	2	2	2	2	1
2	2	2	2	3	3	2	1	3	3
2	2	2	2	2	2	2	2	2	1
2	2	2	2	2	2	2	2	2	2
2	2	2	1	1	2	2	3	3	1
9	9	9	9	9	9	9	9	9	9
2	2	2	2	2	2	2	2	2	2
2	2	2	2	2	2	2	2	2	3
2	2	2	2	2	2	2	2	2	2
2	2	2	2	2	2	2	2	2	2
3	3	3	3	3	3	3	3	3	1
2	2	1	3	3	3	2	2	3	2
1	1	2	1	2	2	3	1	3	3
1	1	2	1	2	2	3	1	3	3
2	2	2	2	2	2	2	2	2	2
2	2	2	2	2	2	2	2	2	2
9	9	9	9	9	9	9	9	9	9
9	9	9	9	9	9	9	9	9	9
2	3	3	3	3	3	3	3	3	2
2	1	2	1	2	2	3	3	3	1
9	9	9	9	9	9	9	9	9	9
9	9	9	9	9	9	9	9	9	9
3	3	3	3	3	3	3	3	3	3
9	9	9	9	9	9	9	9	9	9
9	9	9	9	9	9	9	9	9	9
9	9	9	9	9	9	9	9	9	9
2	3	1	2	3	1	2	3	1	3
1	1	1	1	1	1	1	1	1	1
2	2	2	2	2	2	2	2	2	2
1	2	9	1	2	1	1	1	9	1
2	2	2	2	2	2	2	2	2	2
2	2	2	2	2	2	2	2	2	2
2	2	2	2	2	2	2	2	2	2
3	1	3	1	3	3	3	3	3	1
3	1	2	2	1	2	3	3	1	3
2	1	3	2	1	3	1	2	2	2
1	2	3	3	1	2	2	2	2	1
3	1	2	2	3	1	2	3	2	1
2	9	2	2	3	3	2	2	2	1
2	2	2	2	2	2	2	2	2	3
9	1	9	9	9	9	9	9	9	1
2	9	9	9	9	9	9	9	9	9
2	1	3	2	2	3	2	1	2	2
2	2	2	2	2	2	2	2	2	2
2	2	2	2	2	2	2	2	2	2

2	2	3	3	3	1	1	1	2	1
9	9	9	9	9	9	9	9	9	9
9	9	9	9	9	9	9	9	9	9
9	9	9	9	9	9	9	9	9	9
9	9	9	9	9	9	9	9	9	9
1	9	9	9	9	9	9	9	9	1
9	9	9	9	9	9	9	9	9	9
9	9	9	9	9	9	9	9	9	9
9	9	9	9	9	9	9	9	9	9
9	9	9	9	9	3	9	9	9	3
2	2	2	2	2	2	2	2	2	2
2	2	3	2	3	2	3	2	2	1
3	1	3	1	3	3	3	3	3	1
2	2	2	2	2	2	2	2	2	2
2	2	2	2	2	2	2	2	2	2
9	9	9	9	9	9	9	9	9	9
2	2	2	2	2	2	2	2	2	1
2	2	2	2	2	2	2	2	2	2
2	2	2	2	2	2	2	2	2	2
2	1	1	2	2	1	1	2	2	3
3	1	2	1	2	3	1	3	2	1
3	1	2	3	2	1	3	2	1	3
2	2	2	2	2	2	2	2	2	1
2	2	2	2	2	2	2	2	2	2
2	2	2	2	2	2	2	2	2	2
2	2	2	2	2	2	2	2	2	2
3	3	3	3	3	3	3	3	3	2
2	2	2	1	1	2	2	1	1	1
2	2	2	2	2	2	2	2	2	9
2	2	2	2	2	2	2	2	2	2
9	9	9	9	9	9	9	9	9	9
2	2	2	2	2	2	2	2	2	2
2	9	1	1	1	1	1	1	1	1
9	9	9	9	1	9	9	9	9	9
3	3	3	3	3	3	3	9	3	3
2	2	2	2	2	2	2	2	2	2
2	2	2	2	2	2	2	2	2	2
2	2	2	2	2	2	2	2	2	2
3	1	1	1	1	2	1	1	2	3
9	9	9	9	9	9	9	9	9	9
1	1	1	2	1	1	1	1	2	2
2	2	2	2	2	2	2	2	2	2
2	2	2	2	2	2	2	2	2	2
3	1	2	1	2	2	2	2	3	1
2	1	1	2	3	2	2	2	3	1
9	9	9	9	9	9	9	9	9	9
9	9	9	9	9	9	9	9	9	9

9	9	9	9	9	9	9	9	9	9
2	2	2	2	2	2	9	2	3	2
9	9	9	9	9	9	9	9	9	1
9	9	9	9	9	9	9	9	9	9
9	9	9	9	9	9	9	9	9	9
9	9	9	9	9	9	9	9	9	9
9	9	9	9	9	9	9	9	9	9
9	9	9	9	9	9	9	9	9	9
9	9	9	9	9	9	9	9	9	9
9	9	9	9	9	9	9	9	9	9
9	9	9	9	9	9	9	9	9	9
9	9	9	9	9	9	9	9	9	9
9	9	9	9	9	9	9	9	9	9
9	9	9	9	9	9	9	9	9	9
9	9	9	9	9	9	9	9	9	9
9	9	9	9	9	9	9	9	9	9
9	9	9	9	9	9	9	9	9	9
9	9	9	9	9	9	9	9	9	9
9	9	9	9	9	9	9	9	9	9
9	9	9	9	9	9	9	9	9	9
9	9	9	9	9	9	9	9	9	9
9	9	9	9	9	9	9	9	9	9
9	9	9	9	9	9	9	9	9	9
2	3	2	1	2	3	2	1	2	1
2	2	2	2	2	2	2	2	2	2
2	2	2	2	2	2	2	2	2	2
3	1	2	1	3	2	1	3	3	1
2	3	2	2	1	2	2	1	1	9
2	1	1	1	2	2	3	2	3	1
2	2	2	2	2	2	2	2	2	2
1	1	1	2	1	1	1	2	2	1
2	2	2	2	2	2	2	2	3	1
2	1	2	1	3	2	3	1	1	1
1	1	3	1	3	1	3	3	3	1
2	1	3	1	3	3	3	3	3	1
3	1	1	2	2	1	1	3	3	1
2	1	2	2	2	2	2	2	3	1
2	2	2	2	2	2	2	2	2	1
2	1	1	2	3	3	3	3	3	2
9	9	9	9	9	9	9	9	1	1
2	1	3	1	3	3	2	3	3	1
1	2	1	3	2	2	2	2	2	1
9	9	9	9	9	9	9	9	9	1
2	1	3	1	1	3	3	3	3	1
1	1	3	1	1	3	3	3	3	1
2	2	2	2	2	2	2	2	2	2
1	2	1	3	2	3	2	1	3	1

2	1	3	1	3	3	3	3	3	1
2	1	1	3	2	1	2	3	3	1
3	1	1	2	1	3	3	3	3	1
2	1	1	2	3	1	3	3	3	1
2	1	2	3	2	1	2	1	2	2
9	9	9	9	9	9	9	9	9	1
2	1	3	2	3	3	3	3	3	1
9	9	9	9	9	9	9	9	9	1
2	2	2	2	2	2	2	2	2	2
2	2	2	2	2	2	2	2	2	2
9	1	9	9	9	9	9	9	9	9
9	1	9	9	9	9	9	9	9	9
9	9	9	9	9	9	9	9	9	1
9	9	9	9	1	9	9	9	9	1
1	1	1	3	1	1	1	1	3	9
9	9	9	9	9	9	9	9	9	9
9	9	9	9	9	9	9	9	9	9
9	9	9	9	9	9	9	9	9	9
1	9	9	9	9	9	9	9	9	9
3	3	3	1	3	3	3	9	3	3
9	9	9	9	9	9	9	9	9	1
9	1	9	9	9	9	9	9	9	9
9	9	9	9	9	9	9	9	9	1
9	9	9	1	9	9	9	9	9	1
9	9	9	9	9	9	9	9	9	1
2	3	1	2	3	1	3	2	1	2
9	9	9	9	9	9	9	9	9	9
3	1	2	1	3	3	3	3	3	1
2	1	3	2	2	3	3	2	3	1
9	9	9	9	9	9	9	9	9	9
2	2	1	2	2	2	2	2	3	1
3	3	1	3	3	3	3	1	3	1
2	1	3	3	3	3	3	3	3	3
1	1	3	1	3	1	3	3	3	1
1	1	1	1	1	1	1	1	3	1
2	1	2	3	1	2	3	2	3	3
2	2	2	2	3	2	2	2	3	2
2	1	1	1	2	1	2	2	3	1
2	3	3	3	3	2	1	2	2	2
1	1	1	1	3	3	3	3	1	1
2	1	2	1	2	2	2	3	3	1
1	1	1	1	2	1	2	3	3	1
3	1	3	1	3	3	3	3	3	9
1	2	1	1	3	3	2	2	2	2
3	1	3	1	3	1	3	3	3	2
2	1	3	3	3	3	3	3	3	3
2	1	3	1	3	3	3	3	3	1

9	9	9	9	9	9	9	9	9	9
9	1	9	2	2	1	1	2	9	1
9	9	9	9	9	9	9	9	9	9
9	1	3	1	2	3	3	1	3	1
9	9	9	9	9	9	9	9	9	9
2	1	3	1	3	3	3	3	3	1
3	1	3	3	1	1	1	3	3	1
9	9	9	9	9	9	9	9	9	9
9	9	9	9	9	9	9	9	2	2
2	2	2	2	2	2	2	2	2	2
1	1	1	1	3	1	1	1	2	2
3	1	3	1	3	3	3	3	3	9
2	1	2	2	1	2	1	1	1	1
9	9	9	9	9	9	9	9	9	3
9	9	9	9	9	9	9	9	9	9
1	2	3	1	2	1	2	3	3	3
9	1	9	9	9	9	9	9	9	1
9	9	9	9	9	9	9	9	9	9
3	2	2	2	2	2	2	2	3	2
1	1	1	1	1	2	1	1	2	1
2	2	2	2	2	2	2	2	2	9
1	3	1	1	1	1	1	1	2	1
2	3	2	2	3	2	1	3	3	2
2	1	1	9	9	9	9	9	9	9
2	2	2	2	2	2	2	2	2	1
9	9	9	9	9	9	9	9	9	9
9	9	9	9	9	9	9	9	9	1
3	9	3	1	1	1	1	1	1	1
3	3	3	3	2	1	1	1	2	1
3	9	1	3	3	1	3	3	3	1
9	9	9	9	9	9	9	9	9	9
9	9	9	9	9	9	9	9	9	9
9	9	9	9	9	9	9	9	9	9
9	9	9	9	9	9	9	9	9	9
9	9	9	9	9	9	9	9	9	9
9	9	9	9	9	9	9	9	9	9
9	9	9	9	9	9	9	9	9	9
9	9	9	9	9	9	9	9	9	9
9	9	9	9	9	9	9	9	9	9
9	9	9	9	9	9	9	9	9	9
9	9	9	9	9	9	9	9	9	9
9	9	9	9	9	9	9	9	9	9
9	9	9	9	9	9	9	9	9	9
9	9	9	9	9	9	9	9	9	9
9	9	9	9	9	9	9	9	9	9
9	9	9	9	9	9	9	9	9	9
9	9	9	9	9	9	9	9	9	9

9	9	9	9	9	9	9	9	9	9
9	9	9	9	9	9	9	9	9	9
9	9	9	9	9	9	9	9	9	9
9	9	9	9	9	9	9	9	9	9
9	9	9	9	9	9	9	9	9	9
9	9	9	9	9	9	9	9	9	9
9	9	9	9	9	9	9	9	9	9
9	9	9	9	9	9	9	9	9	9
9	9	9	9	9	9	9	9	9	9
9	9	9	9	9	9	9	9	9	9
9	9	9	9	9	9	9	9	9	9
9	9	9	9	9	9	9	9	9	9
9	9	9	9	9	9	9	9	9	9
9	9	9	9	9	9	9	9	9	9
9	9	9	9	9	9	9	9	9	9
9	9	9	9	9	9	9	9	9	9
9	9	9	9	9	9	9	9	9	9
9	9	9	9	9	9	9	9	9	9
9	9	9	9	9	9	9	9	9	9
9	9	9	9	9	9	9	9	9	9
9	9	9	9	9	9	9	9	9	9
9	9	9	9	9	9	9	9	9	9
9	9	9	9	9	9	9	9	9	9
9	9	9	9	9	9	9	9	9	9
9	9	9	9	9	9	9	9	9	9
9	9	9	9	9	9	9	9	9	9
9	9	9	9	9	9	9	9	9	9
9	9	9	9	9	9	9	9	9	9
9	9	9	9	9	9	9	9	9	9
9	9	9	9	9	9	9	9	9	9
9	9	9	9	9	9	9	9	9	9
9	9	9	9	9	9	9	9	9	9
9	9	9	9	9	9	9	9	9	9
9	9	9	9	9	9	9	9	9	9
9	9	9	9	9	9	9	9	9	9
2	2	2	2	2	2	2	2	2	2
9	9	9	9	9	9	9	9	9	9
2	9	2	2	3	3	3	3	3	9
3	1	3	1	3	3	3	1	3	1
3	3	3	3	3	3	3	3	3	3
9	9	9	9	9	9	9	9	9	9
9	9	9	9	9	9	9	9	9	9
9	9	9	9	9	9	9	9	9	1
9	9	9	9	9	9	9	9	9	9
9	9	9	9	9	9	9	9	9	2
9	9	9	9	9	9	9	9	9	1
2	2	2	2	2	2	2	2	2	1

2	1	1	2	3	3	3	3	3	2
2	2	2	2	2	2	2	2	2	1
2	1	1	3	1	1	3	3	3	1
2	1	2	2	3	2	3	3	3	1
2	1	2	3	3	2	3	3	3	1
1	1	1	1	1	1	3	2	3	1
2	1	2	1	3	3	3	3	3	2
2	1	2	1	3	2	3	2	3	1
2	9	3	2	2	1	2	3	2	2
2	2	1	1	3	1	2	3	3	1
9	9	1	9	9	9	9	9	9	1
2	2	2	1	2	3	3	1	2	2
9	9	9	9	9	9	9	9	9	9
1	1	1	1	3	1	3	3	3	1
1	1	1	1	3	1	3	3	3	1
3	1	3	1	3	3	3	3	3	1
9	9	9	9	9	9	9	9	9	9
2	1	2	3	3	2	3	3	3	1
1	1	2	1	2	1	2	3	3	1
3	2	2	2	3	3	1	2	2	2
9	1	9	2	3	3	3	3	3	1
2	1	3	1	2	2	2	3	3	1
1	1	1	2	1	1	2	2	1	1
3	1	3	1	3	3	3	3	3	1
3	3	3	3	3	3	3	3	3	3
2	2	1	2	1	2	1	2	1	1
2	1	1	1	1	1	1	1	2	1
2	1	3	1	3	2	3	1	3	1
2	2	2	2	2	2	2	2	2	2
9	9	9	9	9	9	9	9	9	9
9	9	9	9	9	9	9	9	9	9
2	2	2	2	2	2	2	2	2	9
2	2	2	2	2	2	2	2	2	9
9	9	9	9	9	9	9	9	9	9
9	9	9	9	9	9	9	9	9	9
9	9	9	9	9	9	9	9	9	9
3	1	1	1	2	3	2	1	2	1
2	3	1	2	3	2	3	2	1	9
2	1	2	1	2	1	2	1	2	9
9	9	9	9	9	9	9	9	9	9
1	1	1	1	1	1	3	2	3	1
9	9	9	9	9	9	9	9	9	9
3	1	3	1	3	3	3	3	3	1
1	1	3	1	3	1	3	3	3	3
3	3	3	1	1	1	1	2	1	1
1	3	3	3	3	3	3	3	3	3
3	1	1	3	1	1	3	1	1	1

2	3	2	3	3	3	2	1	1	2
2	1	9	2	2	2	1	2	2	1
2	1	2	1	2	1	2	2	1	2
1	1	2	3	3	3	3	3	3	1
2	2	9	1	2	2	1	2	2	1
2	3	1	2	3	1	2	3	2	1
2	1	1	1	2	1	2	2	1	3
3	2	1	2	1	3	2	1	2	2
3	1	1	1	3	1	1	2	3	1
2	9	1	2	3	3	3	2	1	1
1	1	1	1	1	1	1	2	3	1
2	3	1	2	1	2	3	1	2	1
2	2	2	1	1	2	1	2	2	2
1	1	2	3	3	2	1	2	2	1
1	1	1	1	1	1	1	1	2	3
1	1	1	1	1	1	1	1	3	1
2	3	2	2	2	2	2	2	2	2
9	9	9	9	9	9	9	9	9	9
2	3	1	2	2	1	2	2	3	1
3	1	3	3	3	3	3	1	3	2
3	3	3	2	2	1	2	2	2	1
1	2	2	3	2	2	2	1	2	3
2	3	1	2	3	1	2	3	1	1
1	1	3	1	1	1	1	2	3	1
2	2	2	3	3	3	3	2	2	2
9	3	3	3	3	2	2	2	1	1
3	1	3	1	3	3	3	1	3	1
1	3	3	1	1	2	1	1	1	1
2	2	2	2	2	2	2	2	2	2
2	2	3	1	1	2	2	3	3	1
1	1	1	2	2	2	2	2	3	1
2	3	1	2	2	2	3	2	1	1
1	2	2	3	3	3	2	1	2	2
1	3	3	3	3	2	2	2	1	2
2	3	9	1	2	3	1	2	3	1
1	1	1	1	3	3	3	1	3	3
3	2	1	3	2	1	2	3	2	2
9	9	9	9	9	9	9	9	9	9
1	1	1	1	3	1	1	1	3	1
1	3	1	1	3	1	3	3	1	1
3	1	3	1	1	1	1	1	1	1
2	3	9	1	2	3	1	2	3	1
1	1	1	1	3	3	3	2	2	1
1	1	3	1	3	1	1	1	2	2
2	2	2	2	2	2	2	2	2	2
3	3	3	3	3	3	3	3	1	3
9	9	9	9	9	9	9	9	9	9

2	3	3	2	2	2	1	2	2	1
1	9	3	3	3	3	3	3	3	9
3	3	3	3	3	3	3	3	2	3
1	1	1	3	2	2	2	3	3	2
1	2	2	2	2	2	1	1	2	1
2	2	1	2	2	1	1	1	2	1
2	2	2	2	2	2	2	2	2	2
9	9	9	9	9	9	9	9	9	9
9	9	9	9	9	9	9	9	9	9
2	2	2	3	3	2	1	1	1	1
2	2	2	2	2	2	2	2	3	1
2	2	2	2	2	2	2	2	2	2
1	2	2	2	2	2	1	2	2	2
2	2	2	2	2	2	2	2	2	1
2	2	2	2	2	2	2	2	2	2
2	2	2	2	2	2	2	2	2	2
2	2	2	2	2	2	2	2	2	2
2	2	1	2	2	2	2	3	2	3
2	1	1	2	2	2	2	2	1	2
2	2	2	2	2	2	2	2	2	1
2	2	2	2	2	2	2	2	2	2
2	2	2	2	2	2	2	2	2	2
2	2	2	2	2	2	2	2	2	2
1	1	2	2	1	2	2	2	2	1
2	2	2	2	2	2	2	2	2	2
2	2	2	2	2	2	2	2	2	2
2	2	2	2	2	2	2	2	3	1
2	1	2	3	2	1	2	1	2	9
1	1	1	1	1	1	1	1	1	2
2	2	2	2	2	2	2	2	2	2
2	2	2	2	2	2	2	2	2	2
2	2	2	2	2	2	2	2	3	1
2	2	2	2	2	2	2	2	2	9
2	2	2	2	2	2	2	2	2	2
2	2	2	2	2	2	2	2	3	2
2	2	2	2	2	2	2	2	3	2
2	2	2	2	2	2	2	2	2	2
2	2	2	2	2	2	2	2	2	2
2	2	2	2	2	2	2	2	2	2
3	1	3	1	3	1	1	3	3	9
2	2	2	2	2	2	2	2	3	2
2	2	2	2	2	2	2	2	2	2
2	3	2	1	2	3	2	2	2	1
2	2	2	2	2	2	2	2	2	2
1	1	2	1	1	3	2	2	3	1
2	2	2	2	2	2	2	2	2	9
2	2	2	2	2	2	2	2	2	3

2	2	2	2	2	2	2	2	2	2
2	2	3	2	3	2	2	3	2	3
2	2	2	2	2	2	2	2	2	2
2	2	2	2	2	2	2	2	2	2
2	2	2	2	2	2	2	2	2	2
2	2	2	2	2	2	2	2	2	2
3	1	1	3	3	3	3	3	3	1
1	1	1	1	1	1	1	1	1	1
1	9	9	9	9	9	9	9	9	1
2	2	2	2	2	2	2	2	2	2
3	2	3	2	1	1	1	1	1	1
2	2	2	2	9	1	1	1	1	1
3	2	3	2	3	2	2	2	3	1
9	9	9	9	9	9	9	9	9	9
2	2	2	2	2	2	2	2	2	2
2	1	2	2	3	2	2	1	2	1
2	2	2	2	2	2	2	2	3	1
1	1	2	2	2	3	2	1	1	1
9	9	9	9	9	9	9	9	9	1
2	2	2	2	2	2	2	2	1	1
1	2	2	1	3	2	2	2	2	1
9	9	9	9	9	1	9	9	9	9
9	9	9	9	9	9	9	9	9	1
2	2	2	2	2	2	2	2	2	2
2	1	2	3	2	2	1	3	2	1
2	2	1	1	1	2	2	1	1	1
2	2	2	1	2	2	2	2	1	1
1	9	1	1	1	2	2	2	1	1
9	9	9	9	9	9	9	9	9	9
1	1	1	1	1	1	2	2	2	9
2	2	2	2	2	2	2	2	2	9
2	2	2	2	2	2	2	2	2	3
2	2	2	2	2	2	2	2	2	2
1	9	9	9	2	9	9	9	9	1
2	9	2	2	3	3	3	2	3	1
9	9	9	9	1	9	9	9	9	1
3	3	1	2	2	1	1	3	3	1
3	3	3	3	3	3	3	3	9	1
2	2	2	2	2	2	2	2	2	2
2	2	2	2	2	2	2	2	2	2
2	9	2	1	3	1	2	1	3	2
2	2	2	2	2	2	2	2	2	2
9	9	9	9	9	3	9	9	9	9
2	2	2	2	2	2	2	2	2	2
2	2	2	2	2	2	2	2	2	1
2	2	2	1	3	3	3	2	3	3
1	1	2	1	2	1	2	1	2	3

9	9	1	9	9	9	9	9	9	9
2	2	2	2	2	2	2	2	2	2
1	1	1	1	1	1	1	1	1	1
2	2	2	2	2	2	2	2	2	2
9	9	9	9	9	9	1	9	9	1
1	1	1	2	2	2	2	2	3	1
3	3	3	3	3	3	3	3	3	3
1	1	1	1	1	1	1	1	1	1
2	2	2	2	2	2	2	2	2	1
1	1	1	1	3	3	3	3	3	1
9	9	9	9	9	9	9	9	9	2
1	1	1	1	1	1	1	1	1	1
2	2	2	2	2	2	2	2	2	2
2	2	2	2	2	2	2	2	2	2
2	2	2	2	2	2	2	2	2	2
2	2	2	2	2	2	2	2	2	2
1	1	1	1	1	1	1	1	1	1
1	1	3	3	3	3	3	3	3	1
9	9	9	9	9	9	9	9	9	9
1	2	3	1	2	3	2	1	2	1
1	1	1	1	1	1	1	1	1	2
2	2	2	2	2	2	2	2	2	1
1	1	1	1	1	1	1	1	9	9
2	2	2	2	2	2	2	2	2	1
3	3	3	3	3	3	3	3	3	3
1	1	1	1	1	1	1	1	1	1
1	1	1	1	2	3	2	1	2	1
2	2	2	2	2	2	2	2	2	2
3	2	2	2	1	1	1	1	2	1
2	2	2	2	2	2	2	2	2	2
1	1	2	2	3	1	1	2	2	1
2	2	3	3	3	3	2	2	2	2
3	3	3	3	3	3	3	3	3	3
2	3	3	3	1	1	1	9	1	1
2	2	2	2	2	2	2	2	2	2
3	3	3	3	3	3	3	2	2	2
2	2	2	2	3	3	3	3	3	1
2	2	2	2	2	2	2	2	2	2
2	3	1	2	3	1	2	3	1	1
3	3	3	3	3	3	3	3	3	3
1	1	1	1	1	3	1	2	1	1
2	2	2	2	2	2	2	2	2	2
9	9	9	9	9	9	9	9	9	9
3	3	3	3	3	3	3	3	3	9
9	9	9	9	9	9	9	9	9	9
2	2	2	2	2	2	2	2	2	2
3	9	3	3	2	1	1	2	3	1

3	3	3	3	3	3	3	3	3	1
1	1	1	1	3	3	3	3	3	1
9	9	9	9	9	9	9	9	9	9
1	1	1	3	3	3	3	3	3	1
2	3	1	2	3	1	2	3	2	1
2	2	2	2	2	2	2	2	2	2
2	2	2	2	2	2	2	2	2	2
2	2	2	2	2	2	2	2	2	2
1	1	1	1	1	1	1	1	1	9
2	1	1	2	1	2	1	2	1	1
3	3	2	3	2	3	2	3	2	2
2	1	3	1	3	2	3	1	3	1
2	2	2	2	2	2	2	2	2	2
2	3	3	2	2	3	2	3	2	1
2	2	2	2	2	2	2	2	2	2
2	2	2	2	2	2	2	2	2	2
2	2	2	2	2	2	2	2	9	2
2	2	2	2	2	2	2	2	2	2
2	2	2	2	2	2	2	2	2	2
2	2	3	1	2	2	1	3	2	2
2	2	2	2	2	2	2	2	1	1
2	2	2	2	2	2	2	2	2	2
2	2	2	2	2	2	2	2	2	9
2	2	2	2	2	2	2	2	2	2
1	1	1	1	1	1	1	1	1	1
2	2	2	2	2	2	2	2	2	9
3	2	3	1	1	1	1	1	1	1
2	2	2	2	2	2	2	2	2	2
2	2	1	2	2	3	2	1	1	2
2	2	2	2	2	1	2	2	2	1
1	1	9	2	1	1	2	2	2	2
3	2	2	3	3	2	2	1	2	9
2	2	2	2	2	2	2	2	2	2
2	2	2	2	2	2	2	2	2	3
2	1	3	2	1	1	2	3	3	1
3	2	2	3	2	2	1	2	1	1
9	9	9	9	9	9	9	9	9	9
9	9	9	9	9	9	9	9	9	9
1	9	2	3	2	2	1	3	1	2
2	2	2	2	2	2	2	2	2	2
9	2	3	2	1	2	1	2	2	2
2	2	2	2	2	2	2	2	2	9
2	2	2	2	2	2	2	2	2	2
2	2	2	2	2	2	2	2	2	2
9	9	9	9	9	9	9	9	9	9
2	1	1	1	1	2	2	1	2	9
2	2	2	2	2	2	2	2	3	2

9	9	9	9	9	9	9	9	9	9
9	9	9	9	9	9	9	9	9	1
2	3	1	2	3	1	2	3	2	1
9	9	9	9	9	9	9	9	9	9
3	1	1	1	1	3	1	3	1	1
1	2	2	3	1	2	1	2	3	1
2	3	2	1	2	3	2	1	3	1
9	9	9	9	9	9	9	9	9	9
2	2	2	2	2	2	2	2	2	2
2	2	2	3	3	2	2	2	3	2
2	2	2	2	2	2	2	2	2	1
1	1	2	2	1	2	3	3	1	1
2	2	2	2	2	2	2	2	2	2
2	2	1	1	1	3	3	3	3	1
2	3	1	2	3	1	2	3	1	1
2	2	2	2	2	2	2	2	2	2
2	1	2	2	3	2	2	3	2	1
2	2	2	2	2	2	2	3	2	2
9	9	9	9	9	9	9	9	9	9
9	9	9	9	9	9	9	9	9	9
9	9	9	9	9	9	9	9	9	9
2	3	2	3	1	2	1	2	1	1
1	2	3	2	1	3	1	3	1	1
2	3	3	2	2	3	1	2	3	1
3	3	1	1	2	3	3	3	1	3
3	3	3	2	2	2	3	3	3	3
1	2	2	1	2	2	2	2	2	1
2	3	3	3	3	3	9	2	2	2
2	3	3	2	2	1	2	1	1	1
3	1	1	1	3	2	3	2	3	1
2	2	1	1	1	1	1	1	1	1
1	1	1	2	2	2	2	3	1	2
1	2	3	3	3	2	3	2	2	2
3	3	3	3	3	3	3	3	3	2
2	2	2	2	2	2	2	2	2	2
2	2	2	2	2	2	2	2	2	2
3	3	3	3	3	3	3	3	3	3
2	2	2	2	2	2	2	2	2	3
9	9	9	9	9	9	9	9	9	9
9	9	9	9	9	9	9	9	9	9
3	3	3	3	3	3	3	3	3	1
3	3	3	2	2	2	2	1	1	2
3	2	3	1	2	3	1	2	3	1
3	2	3	3	2	3	2	2	1	3
9	9	9	9	9	9	9	9	9	9
9	3	9	3	3	3	3	3	3	3
2	2	2	2	1	3	3	2	2	1

1	1	1	1	1	1	1	1	1	1
2	3	2	2	3	3	1	1	2	1
9	9	9	9	9	9	9	9	9	2
9	9	2	9	9	2	9	1	3	2
3	2	3	2	3	1	2	3	2	3
3	3	3	3	3	3	3	3	3	3
1	1	1	1	1	1	1	1	1	1
3	3	3	3	3	3	3	3	3	3
2	2	3	3	3	2	2	2	2	2
2	2	2	1	1	2	2	3	3	1
2	2	2	2	2	2	2	2	2	2
2	2	1	2	3	1	2	3	2	3
2	3	3	3	3	2	2	3	2	2
1	1	1	1	1	1	1	1	1	1
2	1	1	1	1	1	2	2	2	1
2	2	2	3	3	3	3	3	3	2
1	1	1	3	3	3	3	3	1	1
3	3	3	3	3	3	3	3	3	2
9	9	9	9	9	9	9	9	9	9
2	3	1	2	3	1	2	2	1	2
1	1	1	1	1	1	1	1	1	1
3	2	3	2	3	2	3	2	3	2
1	1	1	1	1	1	1	1	1	1
2	2	2	1	3	3	2	1	9	1
2	3	1	2	3	1	2	3	1	1
2	2	3	3	3	3	2	2	3	2
3	2	3	2	2	3	3	3	1	1
2	3	2	1	2	2	3	2	2	1
2	1	2	3	1	1	2	1	2	1
3	1	1	1	3	3	3	3	3	1
2	3	2	3	2	2	3	3	2	1
2	2	2	2	2	2	2	2	2	2
2	2	2	2	2	2	2	2	9	2
1	1	1	1	2	1	2	2	3	1
2	3	1	2	3	1	2	3	1	1
2	3	1	2	1	2	1	2	3	3
2	2	2	1	1	3	3	9	1	1
3	3	3	3	3	3	3	3	3	3
1	1	2	2	3	3	1	9	1	1
2	1	3	2	1	2	3	1	2	2
2	2	2	2	2	2	2	2	2	2
2	3	1	2	1	2	1	2	1	1
2	1	2	1	2	1	3	2	2	1
1	1	1	1	1	1	1	1	1	1
2	2	2	2	9	3	2	2	2	9
2	3	1	2	2	2	1	2	2	1
3	1	1	2	1	1	3	2	1	3

1	2	2	1	2	2	3	3	2	1
3	2	3	2	3	2	1	2	1	1
2	2	2	2	1	1	1	1	2	2
1	1	1	1	1	1	1	1	1	1
2	3	1	2	3	1	2	3	1	1
2	3	1	2	3	2	1	3	2	1
2	3	1	3	2	1	2	3	3	1
2	2	2	1	2	1	1	2	2	1
2	1	2	1	2	1	2	2	1	1
3	3	3	3	3	3	3	3	1	3
2	2	3	2	3	2	2	3	1	1
2	3	2	2	1	3	2	3	1	1
3	2	1	1	1	2	1	3	3	1
3	3	3	1	1	3	3	1	1	1
3	2	1	2	3	2	1	2	2	2
1	3	3	3	3	3	3	1	3	1
1	1	1	3	3	3	2	3	2	1
3	2	1	1	2	1	1	1	3	1
1	1	3	2	2	2	1	1	2	2
2	1	2	2	2	3	3	2	1	1
2	1	1	3	2	2	3	1	1	1
3	3	3	3	1	3	3	1	3	1
1	3	1	3	2	3	1	9	1	1
1	1	1	2	1	2	1	1	1	1
2	2	2	2	2	2	2	2	2	2
2	2	2	2	2	2	2	2	2	2
1	2	2	1	1	3	3	2	3	1
2	1	2	3	2	1	3	2	1	2
2	2	2	2	2	2	2	2	2	2
9	9	9	9	9	9	9	9	9	9
9	9	9	9	9	9	9	9	9	9
9	9	9	9	9	9	9	9	9	9
2	2	2	3	3	3	3	3	2	1
9	9	9	9	9	9	9	9	9	9
2	2	2	2	2	2	2	2	2	9
9	9	9	9	9	9	9	9	9	9
2	2	2	2	1	2	2	2	3	2
9	1	9	9	9	9	9	9	9	3
1	2	3	3	3	1	1	2	1	2
2	2	2	3	3	3	3	3	2	2
9	9	9	9	9	9	9	9	9	9
1	1	1	1	1	1	1	1	3	2
1	2	3	2	1	2	3	2	1	3
2	1	3	2	1	1	2	3	3	1
3	2	1	3	2	2	3	1	1	1
2	3	2	1	2	3	1	3	2	1
1	2	2	1	1	3	3	2	3	1

3	1	2	2	2	2	2	2	1	2
1	1	2	2	2	2	2	2	1	1
9	9	9	9	9	9	9	9	9	9
2	1	1	1	2	1	1	1	3	1
9	9	9	9	3	9	2	2	2	1
1	2	1	2	1	2	3	1	1	1
9	9	9	1	9	9	9	9	9	9
2	1	2	3	1	2	3	2	2	2
1	2	1	2	3	3	3	2	3	1
3	3	3	3	3	3	3	3	9	3
2	3	1	2	3	1	2	3	2	2
3	2	3	1	2	1	2	1	3	1
2	1	1	3	3	1	1	3	1	1
2	2	2	2	2	2	2	2	2	2
2	2	2	2	2	2	2	2	2	2
9	9	9	1	9	9	9	9	1	9
2	2	2	2	2	2	2	2	2	2
3	9	2	1	2	1	3	2	2	2
2	2	2	2	2	2	2	2	2	2
2	2	2	2	2	2	2	2	2	2
3	1	9	1	9	9	9	9	9	1
3	1	9	1	9	9	9	9	9	1
9	1	9	1	9	9	9	9	9	1
3	1	3	1	3	3	3	3	3	1
3	1	3	1	3	3	3	3	3	1
9	9	9	1	3	3	3	3	3	1
2	1	2	1	3	3	3	2	3	1
2	1	2	1	3	2	3	2	3	1
2	1	2	1	3	2	3	2	3	1
1	1	1	1	1	1	1	1	9	9
9	9	9	9	9	9	9	9	9	9
9	1	9	9	9	9	9	9	3	9
3	1	3	1	3	3	3	3	3	1
3	1	3	1	3	3	2	3	3	1
9	1	1	3	9	9	9	9	9	1
2	1	1	3	2	2	2	2	3	1
2	1	1	3	3	2	2	2	3	1
3	1	9	1	9	9	9	9	9	9
3	1	1	1	1	1	3	3	9	1
1	1	1	3	3	3	3	3	3	1
3	1	3	1	3	3	3	3	3	1
9	9	9	1	9	9	9	9	9	1
1	1	1	1	1	1	1	1	1	1
3	1	3	1	3	3	3	3	3	1
2	2	2	2	3	3	3	2	2	1
3	1	3	1	3	3	3	3	3	1
3	1	3	2	3	3	3	3	2	3

2	1	1	3	2	2	2	2	3	1
2	1	1	1	3	1	2	2	2	1
3	1	3	1	3	3	3	3	3	1
1	1	1	1	1	1	1	1	1	1
3	1	3	1	3	1	3	3	3	1
3	1	3	1	3	1	3	3	3	1
2	1	2	2	1	2	2	2	2	1
2	1	1	1	1	1	2	2	2	1
9	9	9	9	9	9	9	9	9	1
3	1	3	1	3	3	3	3	3	1
2	1	1	2	2	1	2	3	2	1
2	1	2	2	2	2	2	2	2	2
3	1	3	1	3	1	3	3	3	1
3	1	3	1	3	1	3	3	3	1
3	1	3	1	3	1	3	3	3	1
3	1	3	1	3	1	3	3	1	1
2	1	3	1	3	1	3	3	3	1
3	9	1	2	1	1	1	3	1	1
3	1	3	1	3	1	3	3	3	1
3	2	3	2	3	1	3	2	2	3
9	2	9	3	3	3	3	3	3	1
9	2	3	2	2	3	3	3	2	3
2	1	2	1	3	2	3	3	3	1
3	1	3	1	3	3	3	3	3	2
3	1	1	1	1	1	2	1	3	1
1	1	1	1	1	1	1	1	3	1
3	3	3	3	3	3	3	3	3	1
2	1	2	2	2	2	2	2	3	1
3	1	3	1	3	1	3	2	3	1
3	1	3	1	3	1	3	3	3	1
1	1	1	2	2	3	2	2	1	2
3	1	1	1	3	3	3	3	3	1
9	9	9	9	9	9	9	9	9	9
3	1	3	1	3	3	3	3	3	1
1	1	2	1	3	1	3	3	3	1
3	1	3	1	3	3	3	3	3	1
9	1	1	1	1	1	3	2	9	1
3	1	3	1	3	1	3	3	3	1
3	1	3	1	3	3	3	3	3	1
3	1	1	1	3	3	3	3	3	1
3	1	3	1	3	3	3	3	3	1
3	1	3	2	3	3	3	3	3	1
2	1	1	1	1	1	1	1	3	1
3	1	3	1	1	3	3	1	3	1
3	1	2	1	3	3	3	3	3	9
3	3	1	1	3	3	3	3	3	1
3	3	1	3	3	3	3	3	3	1

1	1	3	1	3	1	3	3	3	1
3	1	1	1	3	3	3	3	3	1
3	1	3	1	1	2	3	3	3	1
3	1	1	1	1	3	1	2	2	3
3	1	3	1	3	3	3	3	3	1
1	1	1	1	1	1	1	1	1	3
9	9	9	9	9	9	9	9	9	1
1	1	1	9	3	3	3	3	3	1
3	1	1	1	3	3	3	3	3	1
3	1	1	3	1	3	3	3	3	1
2	1	3	1	3	3	3	3	3	1
9	9	9	9	9	9	9	9	9	1
1	1	1	1	3	3	3	3	3	1
2	1	3	1	3	3	2	3	3	1
3	3	3	3	3	3	3	3	3	2
3	1	3	1	3	3	3	3	3	1
9	9	9	9	9	9	9	9	9	1
9	9	9	9	9	9	9	9	9	1
1	1	1	1	2	2	2	2	3	2
2	2	2	2	2	2	2	2	9	3
3	1	2	1	3	1	3	1	3	1
9	9	9	9	9	9	9	9	9	9
1	1	2	1	3	2	3	2	1	1
3	1	2	1	3	3	3	2	3	1
3	3	3	1	1	1	1	1	1	1
3	3	3	1	1	1	1	1	1	3
3	1	1	1	3	1	2	1	2	2
2	2	2	2	2	2	2	2	2	2
3	1	1	1	3	3	3	3	3	1
2	1	2	1	2	2	2	2	3	1
2	1	2	1	2	2	2	2	3	1
1	1	9	3	1	2	1	2	2	2
3	9	1	9	3	3	2	2	3	1
3	1	1	2	3	3	3	3	3	1
1	3	3	3	2	2	1	1	1	1
1	1	1	1	3	3	2	2	3	1
2	1	3	3	3	3	3	3	3	2
1	1	1	1	1	3	3	3	3	1
2	1	2	3	3	3	3	3	3	1
3	2	3	1	1	1	3	3	3	1
3	1	1	1	3	3	3	3	3	1
3	1	1	3	3	3	3	3	3	1
1	1	3	1	3	1	3	3	3	1
1	1	1	1	1	1	1	1	9	1
1	1	3	9	1	9	3	3	3	2
3	1	1	3	3	3	3	3	3	1
1	9	3	1	3	1	3	3	3	1

3	1	3	1	3	3	3	3	3	1
2	1	1	1	2	2	3	2	3	1
2	1	1	2	3	1	2	3	3	1
2	2	2	2	2	2	2	2	3	1
1	1	3	3	1	1	2	3	1	1
2	1	3	3	3	2	2	2	2	1
9	9	1	9	9	9	9	9	9	1
9	9	9	9	9	9	9	9	9	2
9	9	9	9	9	9	9	9	9	9
2	1	1	3	3	3	3	3	3	1
3	1	2	1	2	2	2	3	3	1
3	1	3	1	3	3	3	3	3	1
2	2	2	2	2	2	2	2	2	2
2	1	2	3	2	2	3	1	2	1
9	9	1	2	1	2	2	2	2	2
9	9	9	1	9	9	9	9	9	1
2	1	3	1	3	3	3	3	3	1
1	1	1	1	1	1	2	2	2	1
3	1	3	1	3	3	3	3	3	1
3	1	3	1	3	3	3	3	3	1
9	9	1	9	9	9	9	9	9	1
3	1	2	1	2	2	2	1	2	1
2	2	2	2	2	3	2	2	9	9
2	9	3	1	3	3	3	3	3	1
3	1	1	2	3	1	1	3	3	1
2	1	1	2	3	1	1	3	3	1
1	1	3	1	3	1	3	2	3	1
9	9	9	9	9	9	9	9	9	9
9	9	9	9	9	9	9	9	9	9
3	1	3	1	2	3	3	1	3	1
3	1	3	1	3	3	3	3	3	1
3	1	3	1	3	3	3	3	3	1
9	9	9	9	9	9	9	9	9	9
9	9	9	9	9	9	9	9	9	1
3	1	3	1	3	3	3	3	3	1
1	1	1	1	2	1	3	3	3	2
3	1	3	1	3	3	3	3	3	1
9	1	9	1	2	2	1	1	2	1
3	3	1	1	3	3	3	3	3	1
3	1	3	1	3	3	3	3	3	1
1	1	2	1	3	3	3	2	3	1
3	1	2	1	3	3	3	3	3	1
3	1	3	1	3	3	3	3	3	1
9	9	9	9	9	9	9	9	9	9
3	1	1	1	3	1	3	3	3	3
2	1	3	1	3	3	3	3	3	1
3	1	1	3	3	3	3	3	3	1

9	1	9	1	9	9	9	9	9	1
2	1	2	9	1	1	1	3	2	3
9	9	9	9	9	9	9	9	9	9
1	1	1	1	1	1	2	1	1	1
3	1	9	1	1	1	9	9	9	9
1	1	2	2	1	2	3	3	3	1
1	1	1	1	2	1	3	2	3	1
1	1	2	2	3	2	3	3	3	1
9	2	9	9	9	9	9	9	9	9
2	1	2	1	2	3	3	3	3	1
1	1	1	1	1	1	1	1	3	1
2	2	2	2	2	2	2	2	3	1
2	1	3	2	3	3	3	3	3	3
9	1	1	1	9	1	9	9	9	1
1	2	2	3	1	1	2	1	2	1
3	1	3	1	3	1	3	3	3	1
9	9	9	9	9	9	9	9	9	9
9	1	9	1	9	3	9	9	9	1
1	1	1	1	1	3	3	3	3	1
2	2	2	2	2	2	2	2	2	2
1	2	3	1	2	3	1	2	3	1
3	1	3	1	1	3	3	1	3	1
3	1	3	1	1	3	3	1	3	1
1	1	3	1	3	3	3	3	3	1
3	1	2	3	3	3	3	3	3	1
1	1	1	1	1	1	1	1	2	1
3	1	1	2	3	3	3	3	3	1
2	1	1	1	2	2	3	2	3	1
1	1	1	1	1	1	1	1	3	1
1	1	1	1	1	1	1	1	1	1
3	1	3	1	3	3	3	3	3	3
2	1	2	1	2	3	2	3	3	3
2	1	1	2	2	2	3	3	2	1
3	1	1	1	3	3	3	3	3	1
3	1	2	1	3	3	3	3	3	1
3	1	1	2	2	1	3	3	3	1
3	1	3	1	3	3	3	3	3	1
9	1	1	1	9	9	9	9	9	9
1	1	1	2	1	2	1	1	2	1
2	1	1	1	1	1	1	1	1	1
2	3	2	1	2	3	2	2	2	1
2	3	1	1	1	1	3	2	3	9
9	9	9	9	9	9	9	9	9	1
9	9	9	9	9	9	9	9	9	1
2	1	1	2	2	3	2	3	3	1
1	1	1	9	2	1	1	2	3	1
9	1	9	9	9	9	9	9	9	1

1	1	3	3	3	3	3	3	3	1
3	1	1	1	3	3	3	3	3	1
9	1	9	1	9	1	9	9	9	1
2	1	3	1	3	1	3	3	3	1
3	1	1	1	3	3	3	3	3	1
2	1	2	1	3	3	3	3	3	1
2	2	2	2	2	2	2	2	2	2
2	1	2	2	2	2	2	2	2	1
9	9	9	9	9	9	9	9	9	9
2	1	1	1	2	1	3	3	3	1
3	1	3	1	3	3	3	3	3	1
9	9	9	9	9	9	9	9	9	9
9	9	9	9	9	9	9	9	9	9
9	9	9	9	9	9	9	9	9	1
9	9	9	9	9	9	9	9	9	1
9	9	9	9	9	9	9	9	9	1
3	1	2	2	2	3	3	2	3	3
2	3	3	2	1	2	3	2	1	1
3	1	3	1	3	3	3	3	3	1
2	1	2	2	1	2	1	2	2	1
2	1	9	1	3	1	2	3	3	1
2	1	1	1	3	3	3	1	3	1
2	1	2	2	1	1	3	1	3	1
2	1	1	2	9	2	2	3	2	1
2	1	2	1	3	2	3	3	3	1
3	1	1	1	3	1	3	3	3	1
3	1	3	1	3	3	3	2	3	1
3	1	3	1	3	3	3	3	3	1
2	1	2	3	3	3	3	9	3	1
3	1	3	1	3	3	3	3	3	1
2	1	2	1	2	3	3	2	2	1
9	1	9	1	9	1	9	9	9	1
3	1	3	1	3	1	3	3	3	1
1	1	3	1	1	1	3	3	3	1
1	1	1	2	3	1	3	2	3	1
3	1	1	1	3	1	3	3	3	1
3	1	3	2	3	2	3	3	3	1
2	1	3	2	2	2	3	3	3	2
9	9	9	9	9	9	9	9	9	9
2	1	1	1	3	3	3	3	3	9
2	3	2	2	2	1	2	1	2	2
2	3	2	2	2	1	2	1	2	2
3	1	2	2	3	3	3	3	3	1
2	1	2	1	2	1	2	3	3	1
2	2	2	1	1	2	3	3	3	1
3	1	1	2	3	3	3	3	3	1
1	1	3	1	1	1	3	3	3	1

3	1	3	1	3	3	3	3	3	1
2	1	2	1	3	2	3	3	3	1
1	1	3	1	1	1	3	3	3	1
3	1	3	1	3	3	3	3	3	1
2	2	3	3	2	2	3	2	3	1
2	1	1	2	2	2	2	3	3	1
2	1	2	1	2	2	1	2	2	1
2	1	2	3	2	1	3	3	3	1
3	2	2	2	2	2	3	2	9	9
2	1	1	1	1	1	3	3	2	1
2	1	2	2	2	2	2	2	2	1
2	2	2	2	2	2	2	2	2	2
1	1	3	1	1	1	3	3	3	1
1	1	1	3	1	1	3	2	3	1
1	1	2	2	2	2	2	2	2	1
1	1	1	1	3	1	3	2	3	1
2	1	1	2	2	2	2	2	2	2
2	2	2	2	2	2	2	2	2	1
2	2	2	2	2	2	2	2	2	2
2	1	3	1	3	3	3	3	3	1
2	1	3	1	3	3	3	3	3	1
2	1	2	1	3	2	3	3	3	1
2	1	3	1	3	1	2	3	3	1
3	1	3	1	3	3	3	1	3	2
3	1	1	2	3	3	3	3	3	1
9	9	9	9	9	9	9	9	9	9
9	9	9	9	9	9	9	9	9	9
9	1	9	9	9	9	9	9	9	9
9	1	9	9	9	9	9	9	9	1
9	2	9	2	3	3	3	9	3	3
3	1	3	1	3	3	3	3	3	1
3	1	3	3	3	3	3	3	3	1
9	9	9	9	9	9	9	9	9	1
9	9	9	9	9	9	9	9	9	1
9	9	9	9	9	9	9	9	9	1
9	9	9	9	9	9	9	9	9	1
9	9	9	9	9	9	9	9	9	1
9	9	9	9	9	9	9	9	9	1
9	9	9	9	9	9	9	9	9	9
2	1	2	3	3	2	3	3	3	1
2	1	2	1	3	2	3	3	3	1
1	9	9	9	9	9	9	9	9	9
9	9	9	9	9	9	9	9	9	9
2	1	1	1	3	3	3	2	3	1
9	9	9	9	9	9	9	9	9	1
9	9	9	9	9	9	9	9	1	1
9	9	9	9	9	9	9	9	1	1

2	1	1	1	2	3	3	3	3	1
9	9	9	9	9	9	9	9	9	1
9	1	9	9	9	9	9	9	9	9
3	3	1	1	3	1	3	3	3	1
3	3	1	1	3	1	3	3	3	1
9	9	9	9	9	9	9	9	1	1
9	9	9	9	9	9	9	9	9	1
9	9	9	9	9	9	9	9	9	1
9	9	9	1	9	9	9	9	9	1
9	9	9	9	9	9	9	9	9	1
1	1	3	1	3	1	3	3	3	1
3	1	3	1	3	1	3	3	3	1
3	1	3	1	1	3	3	3	3	1
3	1	9	1	3	3	3	9	3	1
3	1	3	1	3	1	3	3	3	1
1	1	1	3	1	3	3	1	3	1
9	1	1	1	3	1	3	1	3	1
9	1	1	1	3	1	3	1	3	1
3	1	3	1	3	1	3	3	3	1
1	1	1	1	1	1	2	3	3	1
1	1	1	1	1	1	2	3	3	1
1	2	2	2	2	2	2	2	1	1
9	9	1	9	9	9	9	9	9	9
1	1	1	1	3	1	1	3	3	1
1	3	3	3	2	2	2	2	1	2
1	1	1	2	1	1	1	1	3	1
3	3	9	2	3	3	3	2	3	2
3	1	3	1	1	3	3	3	3	1
1	1	1	1	1	1	3	3	3	1
1	1	1	1	3	3	3	3	2	2
1	1	3	3	3	3	3	3	3	2
1	1	3	1	1	3	3	3	3	3
3	1	1	1	3	1	3	1	3	1
2	1	2	1	1	1	3	3	3	1
3	1	1	1	3	3	3	3	3	1
3	1	3	1	3	1	3	3	3	1
3	1	3	1	3	3	3	3	3	1
3	1	3	1	3	3	3	3	3	1
3	1	3	1	3	1	3	1	3	1
3	2	3	1	3	1	3	1	3	1
3	1	3	1	3	1	3	3	3	3
2	1	3	1	3	1	3	3	3	1
1	1	3	3	3	2	2	2	2	1
3	1	3	1	3	1	3	3	3	1
3	1	3	1	9	1	3	3	3	1
3	1	3	1	1	1	3	1	3	1
3	1	3	1	3	3	3	3	3	1

3	1	3	3	1	1	3	3	3	1
1	1	3	1	1	1	1	3	3	1
1	2	3	3	3	2	2	2	2	9
3	1	3	1	3	3	3	3	3	1
3	1	3	1	3	1	3	3	3	1
3	1	3	1	3	3	3	3	3	1
2	1	3	1	3	3	3	2	3	1
3	1	3	1	3	2	3	3	3	1
3	1	3	1	3	1	3	3	3	1
3	1	3	3	1	1	1	1	1	1
2	1	2	1	3	1	3	3	3	1
3	1	3	1	3	3	3	3	3	9
2	1	1	1	3	3	3	3	3	1
1	1	1	1	3	3	3	3	3	1
3	3	3	1	3	3	3	3	3	1
2	2	2	2	2	2	2	2	2	2
2	2	2	2	2	2	2	2	2	2
2	2	2	2	2	2	2	2	9	1
1	2	1	1	1	3	1	3	1	3
2	1	3	1	3	1	3	3	3	1
3	1	3	1	3	3	3	3	3	1
3	1	1	1	3	3	3	3	3	1
3	3	3	1	3	3	3	3	3	1
2	2	2	1	2	2	2	3	2	1
2	2	2	1	2	2	2	3	2	1
3	3	3	2	3	3	2	1	3	1
1	1	3	1	2	1	3	3	3	1
2	2	2	2	2	2	2	2	3	2
3	1	3	1	1	3	3	3	3	1
3	1	3	1	2	2	2	3	3	1
2	1	2	2	2	2	2	2	2	2
2	1	3	1	1	3	2	2	3	1
2	1	2	1	1	1	3	3	3	1
2	1	2	9	9	9	9	9	9	9
3	1	3	1	3	3	3	3	3	1
3	1	3	1	3	3	3	3	3	1
2	2	2	2	2	2	2	2	2	2
2	2	2	2	2	2	2	2	2	2
3	1	3	1	3	3	3	1	3	1
3	9	2	2	1	3	3	2	3	1
2	1	2	1	3	3	3	3	3	1
3	3	1	1	3	3	3	3	3	1
3	3	1	1	3	3	3	3	3	1
2	2	2	2	2	2	2	2	2	1
3	1	3	1	3	3	3	3	3	1
3	1	3	1	2	2	3	3	3	1
3	1	3	1	3	3	3	1	3	1

2	2	2	2	2	2	2	2	2	1
1	1	3	1	1	2	3	1	2	1
1	1	1	3	2	1	3	3	3	1
2	1	1	1	2	1	2	2	3	1
1	1	2	1	3	3	3	2	3	1
2	1	2	1	3	3	3	3	3	3
3	1	3	1	3	3	3	1	3	1
2	1	3	1	2	2	3	3	3	1
1	2	1	2	2	2	2	2	2	1
3	1	2	2	1	2	3	3	3	1
2	1	2	1	2	1	2	2	2	1
3	2	3	1	3	3	3	3	3	1
9	9	9	9	9	9	9	9	9	1
3	1	1	1	3	3	3	3	3	1
1	1	3	1	1	1	3	3	3	1
2	2	2	2	2	2	2	2	2	2
3	1	2	1	3	2	3	2	3	1
3	1	3	1	3	3	3	3	3	1
3	1	3	1	3	3	3	3	3	1
2	1	2	1	3	3	3	3	3	1
3	1	9	1	3	1	3	3	3	1
1	1	1	1	1	1	2	3	3	1
3	1	3	1	3	3	3	3	3	1
2	1	3	1	3	2	3	3	3	9
1	1	2	2	1	2	2	3	3	1
3	1	2	1	3	3	3	3	3	1
1	1	3	1	3	3	3	3	3	1
2	1	2	3	3	2	3	3	3	1
2	1	1	1	3	3	3	2	3	1
9	9	9	9	9	9	9	9	9	1
2	2	2	3	1	2	2	3	3	9
9	9	9	9	9	9	9	9	9	9
2	1	1	2	2	2	2	2	3	1
3	1	2	1	3	3	3	3	3	1
1	1	2	1	1	1	2	3	3	1
1	1	1	1	2	2	2	3	3	1
2	2	2	2	2	1	1	2	2	2
9	1	9	9	9	9	9	9	2	9
1	2	2	1	3	2	3	1	2	2
3	1	2	2	3	3	3	3	3	1
1	1	1	1	3	3	3	1	3	1
2	1	3	1	1	3	3	3	3	1
2	1	2	1	2	2	3	3	3	1
9	9	1	9	9	1	9	2	9	1
2	2	2	2	2	2	2	2	2	2
2	2	2	2	2	2	2	2	2	9
1	1	3	9	1	1	3	3	3	1

2	1	1	2	2	2	3	3	3	1
2	1	1	2	2	1	2	2	2	1
2	2	2	2	2	2	2	2	2	1
2	1	2	2	2	2	2	3	2	1
1	2	2	3	1	3	2	3	2	2
3	1	1	1	3	3	3	3	3	1
1	1	1	2	1	1	3	1	3	1
9	9	9	1	9	9	9	9	9	9
1	1	2	2	1	1	3	1	3	1
1	1	1	2	2	2	3	3	3	1
2	1	1	1	1	1	1	1	3	1
3	2	3	2	2	1	3	2	2	3
2	2	1	2	1	2	3	1	2	1
2	2	2	2	2	2	2	2	2	2
3	1	1	2	1	3	3	3	3	1
3	1	1	2	1	3	3	3	3	1
2	1	3	1	3	3	3	1	3	1
3	1	3	1	3	3	3	3	3	1
9	9	9	9	9	9	9	9	9	9
3	1	9	1	2	2	3	3	3	1
2	1	1	2	2	2	3	3	3	1
1	1	1	1	2	1	1	1	1	1
2	3	2	2	2	3	3	3	3	3
3	1	1	1	3	3	3	3	3	1
3	1	2	1	3	3	3	3	3	1
2	1	1	1	1	1	1	1	2	1
3	1	1	1	3	3	3	3	3	1
3	1	3	1	3	1	3	3	3	1
3	1	3	1	3	1	3	3	3	1
9	2	9	9	9	9	9	9	1	1
3	1	3	1	3	1	3	3	3	1
1	1	1	2	2	2	1	3	3	1
1	1	1	2	2	2	1	3	3	1
2	1	3	1	2	3	3	3	3	1
9	9	9	9	9	9	9	9	9	2
2	1	2	1	1	1	2	1	9	1
2	1	2	1	2	1	2	2	2	1
9	9	9	9	9	9	9	9	9	9
3	1	3	1	3	3	3	3	3	1
3	1	1	1	1	1	3	3	3	1
3	1	3	1	3	3	3	3	3	1
3	1	3	1	3	3	3	3	3	1
2	2	2	2	2	2	2	2	2	2
3	3	3	1	3	3	3	3	3	1
1	1	3	3	3	1	3	1	3	1
3	3	1	1	3	1	3	3	3	1
1	1	1	1	3	1	3	3	3	1

1	1	1	1	1	1	3	3	3	1
1	1	1	1	1	1	3	3	3	1
3	1	3	1	3	3	3	3	3	1
1	1	2	1	2	2	2	2	2	1
1	1	1	1	1	1	3	3	3	1
3	1	9	1	1	1	3	3	3	1
1	1	3	1	3	1	3	3	3	1
9	9	9	9	9	9	9	9	9	9
3	1	1	1	3	3	3	3	3	1
3	1	9	9	9	9	9	9	9	1
3	1	3	1	3	3	3	3	3	1
3	1	3	1	3	3	3	3	3	1
3	1	3	1	3	3	3	3	3	1
9	9	9	9	9	9	9	9	9	9
2	3	3	1	3	1	3	3	3	1
2	2	3	1	3	3	3	3	3	1
2	1	2	2	1	1	2	1	1	1
1	1	2	1	1	1	1	2	3	1
3	1	3	1	3	1	3	3	3	1
9	9	9	9	9	9	9	9	9	9
1	2	1	2	2	1	1	1	1	1
3	1	3	1	3	3	3	3	3	1
3	3	1	1	1	1	3	3	3	1
3	3	1	1	1	1	3	3	3	1
9	1	9	9	9	9	9	9	9	1
9	9	9	1	9	9	9	9	9	1
3	1	1	1	3	2	3	3	3	1
9	9	9	9	9	9	9	9	9	1
9	1	9	9	9	9	9	9	9	9
9	1	9	9	9	9	9	9	9	9
9	1	9	9	9	9	9	9	9	1
9	1	9	9	9	9	9	9	9	1
9	9	9	9	9	9	9	9	9	1
9	1	9	9	9	9	9	9	9	9
9	9	9	9	9	9	9	9	9	9
1	1	1	2	1	1	3	3	3	1
2	1	2	2	2	2	2	2	2	1
3	1	3	1	3	3	3	3	3	1
3	1	1	1	3	3	3	3	3	1
3	1	1	1	3	3	3	3	3	1
3	1	3	1	3	3	3	3	3	9
1	1	1	1	2	2	2	3	3	1
1	1	1	2	1	1	2	2	3	1
2	2	2	2	2	2	2	2	2	1
2	1	2	2	3	2	2	3	1	2
3	1	3	1	3	3	3	3	3	1
3	1	2	1	2	2	3	3	3	1

2	2	2	2	2	2	2	2	2	1
1	2	2	2	2	2	2	2	2	2
2	1	2	1	2	2	1	1	1	1
2	1	2	3	1	3	2	3	2	1
1	1	1	1	2	1	2	1	1	1
3	1	1	1	1	1	1	1	3	1
9	9	9	9	9	9	9	9	9	9
2	2	2	2	2	2	2	2	2	2
2	3	2	2	2	1	3	3	3	1
2	2	3	2	3	2	3	3	2	2
2	1	1	1	1	1	2	3	1	1
3	1	3	1	2	3	3	3	3	1
1	1	2	3	2	2	2	3	2	1
3	3	3	3	3	3	2	2	1	3
2	2	2	2	2	2	2	2	2	2
2	1	1	2	3	1	3	1	3	1
9	9	9	9	9	9	9	9	9	9
1	2	1	2	1	2	1	2	3	1
1	1	2	2	2	2	2	2	3	1
1	1	1	1	1	1	1	1	1	1
2	1	2	1	2	1	2	1	2	1
3	9	1	2	2	2	1	1	1	2
2	2	3	3	3	2	2	1	1	2
2	1	1	1	1	2	2	3	3	1
1	1	1	1	1	1	1	1	1	1
3	2	2	2	2	2	2	3	3	1
2	1	2	3	1	2	2	2	1	3
3	9	1	2	1	1	2	1	2	1
2	2	2	2	2	2	2	2	2	2
2	2	2	2	2	2	2	2	2	1
3	9	3	1	3	2	3	1	3	1
1	1	1	1	1	1	1	1	3	1
2	2	2	1	2	2	2	1	1	2
2	1	1	1	2	2	2	2	3	1
9	1	9	9	9	9	9	9	9	1
1	1	1	1	1	1	1	1	1	1
2	2	2	2	2	2	2	2	2	2
9	9	9	9	9	9	9	9	9	9
2	3	9	2	1	9	2	3	1	1
1	1	1	1	1	1	1	1	1	1
2	1	2	1	2	2	3	3	3	1
2	1	2	1	2	2	3	3	3	1
9	9	9	9	9	9	9	9	9	3
9	3	9	9	9	9	9	9	9	3
9	9	9	9	9	9	9	9	9	3
2	2	1	3	2	3	3	2	2	2
2	3	1	2	1	1	1	2	2	2

2	3	1	2	3	3	1	3	1	3
2	3	1	3	2	3	3	1	1	3
1	3	2	1	3	1	1	1	1	3
2	2	2	2	2	2	2	2	1	1
2	2	2	1	3	3	3	3	3	2
1	2	3	2	2	1	3	2	2	3
1	1	9	2	2	3	9	2	1	1
1	2	1	2	3	2	1	3	1	2
2	2	3	3	2	2	3	1	1	3
2	3	2	3	3	2	3	1	1	3
2	1	3	1	3	1	1	1	1	3
1	3	1	2	2	2	2	2	2	2
3	1	3	1	3	1	1	1	1	2
1	3	2	1	3	2	2	2	2	2
3	1	2	2	1	1	1	2	2	3
3	1	3	3	1	1	1	2	2	3
2	3	2	2	2	1	2	2	2	1
2	3	2	2	2	1	2	2	2	1
2	3	1	3	2	3	3	1	1	3
2	3	1	3	2	3	3	1	9	3
3	1	3	1	3	3	3	3	3	9
3	1	3	1	3	3	3	3	3	1
2	2	2	2	2	2	2	2	2	1
2	2	2	2	2	2	2	2	2	9
3	1	3	1	3	3	3	3	3	9
2	2	2	2	2	2	2	2	2	2
2	2	2	2	2	2	2	2	2	2
3	1	3	1	3	3	3	3	3	1
1	3	2	2	2	2	1	1	1	3
3	3	1	3	1	3	3	3	3	3
3	3	1	3	1	3	3	3	3	3
3	3	1	3	1	3	3	3	3	3
3	3	1	3	1	3	3	3	3	3
1	3	2	2	9	1	9	3	3	3
2	9	3	9	2	9	2	3	2	3
1	2	3	9	1	9	3	2	2	3
2	2	1	3	2	1	3	3	2	3
2	1	3	1	1	1	1	1	1	9
1	3	2	2	2	3	3	3	1	9
2	3	2	1	2	1	2	2	1	3
3	2	1	3	1	3	1	2	1	3
1	2	2	1	1	1	1	1	1	3
1	2	3	2	1	2	1	2	1	9
9	3	9	9	9	9	9	9	9	3
9	9	9	9	9	9	9	9	9	9
2	2	2	2	2	2	2	2	2	2
9	2	1	9	1	2	2	1	2	3

2	2	2	2	2	2	2	2	2	3
2	3	2	3	3	1	1	1	1	3
9	9	9	9	9	9	9	9	9	9
2	2	2	2	2	2	2	2	2	2
2	2	2	2	2	2	2	2	2	2
9	9	9	9	9	9	9	9	9	2
2	2	2	2	2	2	2	2	2	2
9	9	9	9	2	9	9	9	9	3
1	2	3	3	2	2	2	3	2	3
2	1	3	1	3	2	2	2	1	3
2	2	2	2	2	2	2	2	2	3
2	9	3	2	2	2	3	9	9	2
9	9	9	9	9	9	9	9	9	2
9	9	9	9	9	9	9	9	9	9
2	1	1	2	3	1	2	3	1	3
9	3	9	9	9	9	9	9	9	9
3	1	3	3	3	3	3	3	3	3
9	9	9	9	9	9	9	9	9	9
1	1	1	3	1	3	1	3	1	3
9	9	9	9	9	9	9	9	9	2
2	1	1	3	1	1	1	1	1	3
1	3	1	3	1	1	1	1	1	1
2	2	2	2	2	2	2	2	1	3
1	3	1	3	1	1	1	1	1	3
1	3	1	3	2	2	3	3	3	3
1	3	1	3	1	1	2	2	2	3
3	1	3	1	3	2	2	2	1	3
9	9	9	9	9	9	9	9	9	2
9	9	9	9	9	9	9	9	9	2
9	9	9	9	9	9	9	9	9	2
9	9	9	9	9	9	9	9	9	2
9	9	9	9	9	9	9	9	9	2
9	9	9	9	9	9	9	9	2	9
3	3	1	1	3	2	1	2	1	1
2	1	1	2	3	1	2	3	1	3
9	9	9	9	9	9	9	9	9	9
2	2	2	2	2	2	2	2	2	2
1	3	2	1	1	2	3	1	2	3
1	2	2	3	2	1	1	2	3	3
2	2	2	2	2	2	2	2	2	2
2	2	2	2	2	2	2	2	2	2
9	9	9	9	9	9	9	9	9	2
3	1	3	1	3	3	3	3	3	1
2	2	2	2	2	2	2	2	2	3
9	9	9	9	9	9	9	9	9	2
2	9	1	2	9	1	9	2	1	3
9	9	9	9	9	9	9	9	9	9

9	9	9	9	9	9	9	9	9	2
9	9	9	9	9	9	9	9	9	2
1	3	1	3	1	1	1	1	1	3
9	9	9	9	9	9	9	9	9	2
3	3	1	3	3	3	3	3	3	3
9	9	9	9	9	9	9	9	9	2
2	1	2	3	2	1	3	2	1	2
1	3	1	2	2	2	3	3	3	3
1	3	3	1	2	2	2	1	2	3
3	2	1	2	3	2	1	2	2	2
3	3	2	2	1	1	3	3	2	2
1	3	1	3	1	1	1	1	1	3
3	2	2	2	2	2	2	2	3	3
1	2	2	3	2	1	2	1	2	3
2	2	2	2	2	2	9	1	2	2
2	2	2	2	2	1	1	1	2	3
1	3	1	3	2	2	2	3	3	2
2	3	1	3	1	1	1	2	2	3
2	3	2	2	1	2	2	9	1	3
1	3	1	3	1	1	1	1	1	3
9	9	9	9	9	9	9	9	1	9
9	9	9	9	1	9	9	9	9	9
9	9	9	9	9	9	9	9	9	9
9	9	9	9	1	9	9	9	9	9
1	9	9	9	9	9	9	9	9	9
9	9	9	9	9	9	2	9	9	9
9	9	9	9	3	9	9	9	9	9
9	9	9	9	9	9	9	9	9	2
9	9	9	9	9	9	9	9	9	2
9	9	9	9	9	9	9	9	9	2
9	9	9	9	9	9	9	9	9	2
9	9	9	9	9	9	9	9	9	2
9	9	9	9	9	9	9	9	9	2
1	2	2	3	3	1	2	3	2	3
1	3	2	3	2	1	1	1	2	3
1	3	1	2	3	3	3	2	1	3
9	2	9	9	9	2	9	9	9	2
9	9	9	2	9	2	9	9	9	9
1	3	1	3	2	2	2	2	1	3
9	9	9	9	9	9	9	9	9	2
9	9	9	9	9	9	9	9	9	3
9	9	9	9	9	9	9	9	9	3
9	9	9	9	9	9	9	9	9	9
9	9	9	9	9	9	9	9	9	3
2	2	2	2	2	2	2	2	2	3
2	3	2	3	2	1	2	1	2	3
2	3	2	3	2	1	2	1	2	3

2	2	2	2	2	2	2	2	2	2
1	2	2	2	2	1	3	3	3	9
1	2	2	1	3	3	2	2	2	3
1	2	1	3	2	3	3	2	1	3
1	9	9	9	9	9	9	9	1	3
9	9	9	9	9	9	9	9	3	3
9	9	9	9	9	9	9	9	9	3
9	3	1	3	1	1	9	9	1	3
9	9	9	9	9	9	9	9	9	9
9	9	9	1	9	9	3	9	9	9
9	9	9	9	3	9	9	3	9	1
2	9	9	9	9	2	3	9	9	1
3	1	3	2	9	9	2	9	9	3
9	9	9	9	9	9	9	9	9	9
9	9	9	9	9	9	9	9	1	9
2	2	2	2	2	2	2	2	2	1
3	2	2	3	2	3	2	1	2	1
9	9	9	9	9	9	9	9	9	3
2	2	2	2	2	2	2	2	2	9
1	2	1	1	2	1	1	1	1	1
2	2	2	2	2	2	2	2	2	2
2	2	2	2	2	2	2	2	1	3
3	3	2	2	3	2	3	3	3	9
2	2	2	2	2	2	2	2	2	2
2	1	1	1	2	2	3	2	1	2
2	2	9	2	2	2	2	9	2	2
9	9	9	9	9	9	9	9	9	2
2	2	2	2	2	2	2	2	2	2
2	2	2	2	2	2	2	2	2	2
2	2	2	2	2	2	2	2	2	2
9	9	9	9	9	9	9	9	9	2
2	3	2	2	2	2	2	2	2	3
9	9	9	9	9	9	9	9	9	9
2	2	2	2	2	2	2	2	2	2
2	2	2	2	1	2	2	3	2	2
2	2	2	3	2	1	2	3	1	3
3	3	3	3	3	3	3	3	3	3
2	2	2	2	2	2	2	2	2	3
1	3	1	2	3	1	2	1	2	3
2	1	2	3	2	3	2	1	2	3
1	3	2	2	1	1	1	2	3	3
1	3	1	1	1	1	1	3	3	3
1	3	1	3	2	2	2	1	2	2
1	9	1	3	3	1	3	3	3	3
9	9	9	9	9	9	9	9	9	9
2	2	2	2	2	2	2	2	2	2
2	3	1	3	1	1	3	3	3	3

1	3	1	3	1	1	1	1	1	3
2	2	2	2	2	2	2	2	2	2
2	3	2	2	1	1	1	2	1	3
2	3	2	2	1	2	1	2	3	3
2	2	2	2	2	2	2	2	2	2
2	2	2	2	2	2	2	2	2	2
3	2	2	3	2	3	2	2	2	2
2	3	2	3	2	3	1	2	2	3
1	3	1	2	2	2	2	3	3	3
2	2	2	2	3	3	3	2	3	3
2	3	1	2	2	2	2	2	2	3
3	3	3	2	2	2	3	3	3	3
2	2	2	2	2	2	2	2	2	2
2	3	3	2	1	1	3	3	3	1
3	3	2	3	3	3	3	3	3	3
2	3	2	3	2	2	3	2	3	3
3	2	2	2	2	2	3	2	3	2
2	2	2	2	2	2	2	2	2	2
2	2	2	2	2	2	2	2	2	2
2	2	2	2	2	2	2	2	2	2
2	2	2	2	2	2	2	2	2	2
2	2	2	2	2	2	2	2	2	2
2	1	1	2	3	2	3	1	2	2
2	2	2	2	2	2	2	2	2	2
2	3	3	3	2	3	2	1	2	2
9	9	9	9	9	9	9	9	9	9
9	9	9	9	9	9	9	9	9	9
9	9	9	9	9	9	9	9	9	9
3	2	3	3	2	1	2	1	2	3
9	9	9	9	9	9	9	9	9	9
9	9	9	9	9	9	9	9	9	9
9	9	9	9	9	9	9	9	9	9
9	9	9	9	9	9	9	9	9	9
9	9	9	9	9	9	9	9	9	9
2	2	2	2	2	2	2	3	3	3
1	2	9	3	1	2	3	1	1	3
2	3	1	2	2	2	2	2	2	3
9	9	9	9	9	9	9	9	9	9
2	2	2	2	2	2	2	2	2	1
9	9	9	9	9	9	9	9	9	9
9	9	9	9	9	9	9	9	9	9
2	2	2	2	2	2	2	2	2	3
2	3	2	3	1	3	2	3	3	3
3	3	1	9	2	2	2	3	2	3
3	2	1	2	1	3	1	3	1	3
3	2	2	2	2	2	3	2	2	2
2	2	2	2	2	2	2	2	2	2

2	3	1	2	1	1	1	1	3	3
2	1	1	2	3	1	2	2	2	3
2	3	3	3	3	1	3	3	2	3
2	2	2	2	2	2	2	2	2	2
3	3	1	1	1	3	3	3	3	3
9	9	9	9	9	9	9	1	9	9
3	3	3	3	3	2	3	3	3	3
2	3	3	3	3	2	3	3	3	3
3	2	2	2	1	2	3	2	2	2
2	1	3	2	2	2	2	1	2	2
2	2	2	2	2	2	2	2	2	2
3	3	3	3	3	3	3	3	3	3
1	3	3	1	3	3	3	2	3	3
2	3	2	2	2	2	2	2	2	2
9	9	9	9	9	9	9	9	9	9
2	3	2	2	1	3	2	3	3	3
1	3	2	3	3	3	3	3	3	3
2	1	3	2	2	3	2	2	2	2
3	3	1	1	3	3	1	3	3	3
9	3	9	9	9	9	9	9	9	3
2	2	2	2	2	2	2	2	2	3
2	2	2	2	2	2	2	2	2	2
2	3	2	2	3	3	3	3	3	3
2	3	2	2	2	2	2	9	2	3
1	2	2	1	2	3	2	1	9	9
2	2	2	2	2	2	2	2	2	2
1	3	3	2	2	2	1	2	3	2
3	3	1	3	3	3	3	1	3	3
2	2	2	2	2	2	2	2	3	3
1	3	1	3	1	1	2	1	2	1
3	3	1	1	3	3	3	3	3	3
9	9	9	9	9	9	9	9	9	3
1	3	1	3	1	1	3	1	9	3
1	3	1	3	3	1	3	3	3	3
3	3	1	3	3	2	2	2	2	9
2	2	2	2	2	2	2	2	2	2
2	2	2	2	2	2	2	2	2	2
2	2	2	2	2	2	2	2	2	2
2	2	2	2	2	2	2	2	2	2
2	2	2	2	2	2	2	2	2	2
3	3	2	2	2	2	2	3	3	3
2	2	2	2	2	2	2	2	2	9
2	2	2	2	2	2	2	2	9	2
2	2	2	2	2	2	2	2	2	2
2	2	2	2	2	2	2	2	2	2
1	2	2	2	2	2	2	2	2	9
1	3	2	2	3	3	2	2	3	3

9	9	9	9	9	9	9	9	9	9
3	3	1	1	1	1	3	1	1	3
9	9	9	2	3	9	9	9	9	2
9	9	9	9	9	9	9	9	9	9
2	2	1	3	1	1	2	2	2	2
1	3	1	3	1	1	1	1	1	2
9	3	9	9	9	9	9	9	9	9
1	3	2	2	1	1	1	1	1	3
3	3	1	1	1	3	3	3	3	9
2	3	2	2	2	2	2	2	2	1
2	2	2	2	2	2	2	2	2	1
2	3	3	3	3	2	3	1	3	3
3	3	3	3	3	3	3	3	3	3
9	9	9	9	9	9	9	9	9	9
3	1	3	3	3	3	3	3	3	3
3	1	3	3	3	3	3	3	3	3
1	3	3	3	2	1	3	3	1	3
9	9	9	9	9	9	9	9	9	9
3	3	1	2	3	1	2	2	3	9
1	3	9	9	2	2	2	3	3	9
2	2	2	2	2	2	2	2	2	2
2	9	1	2	2	1	1	1	3	2
9	2	9	9	2	9	2	9	2	2
3	3	3	3	3	3	3	3	3	3
3	3	1	3	3	1	1	1	1	9
9	9	9	9	9	9	9	9	9	9
9	9	9	9	9	9	9	9	9	9
9	9	9	9	9	9	9	9	9	2
3	3	3	3	3	3	3	3	3	3
3	9	2	1	3	3	3	3	3	3
2	1	1	3	1	2	2	2	1	2
1	3	1	2	2	2	1	1	1	9
2	1	1	3	1	1	1	2	3	3
3	3	3	2	1	3	1	1	3	3
2	3	2	3	3	1	2	1	3	2
3	1	3	1	3	3	3	3	3	3
3	2	3	3	2	3	1	2	3	3
2	3	3	3	2	2	3	3	3	3
1	9	9	9	9	9	9	9	9	3
3	3	1	3	3	1	1	2	3	3
2	2	2	3	1	1	2	2	2	9
2	3	3	3	1	2	1	2	1	3
2	2	2	2	2	2	3	3	3	2
3	3	1	2	3	3	3	3	3	3
3	3	3	3	3	3	3	3	3	3
9	9	9	9	9	9	9	9	9	9
9	3	9	9	9	9	9	9	9	3

1	3	1	3	1	1	2	3	3	3
2	3	1	2	1	1	2	2	1	3
9	9	9	9	9	9	9	9	9	3
2	2	2	2	2	2	2	2	2	2
3	3	3	3	3	3	3	3	3	3
1	3	1	3	1	1	1	1	1	2
9	3	9	9	9	9	9	9	9	3
2	3	1	3	2	1	3	1	1	3
2	2	2	2	2	2	2	2	2	3
2	2	2	2	2	2	2	2	2	2
2	3	3	3	3	3	3	3	3	3
2	3	2	2	3	2	3	3	3	3
3	3	1	2	3	3	3	3	3	9
2	3	3	2	2	2	2	2	3	3
2	3	1	2	1	2	1	2	3	3
3	3	3	3	3	3	3	3	3	3
3	3	1	3	1	1	2	2	3	9
3	3	1	1	3	1	3	3	3	3
2	1	9	1	9	3	3	9	9	3
9	9	9	9	9	9	9	9	9	9
9	9	9	9	9	9	9	9	9	9
9	9	9	9	9	9	9	9	9	9
9	9	9	9	9	9	9	9	9	9
2	9	2	3	2	2	2	2	2	3
2	2	2	2	2	2	2	2	2	2
1	3	1	3	1	9	9	9	9	9
9	3	9	9	9	9	9	9	9	9
9	9	9	9	9	9	9	9	9	9
9	3	9	9	9	9	9	9	9	3
9	3	9	9	9	9	9	9	9	3
3	3	3	3	3	3	3	3	3	9
1	3	1	2	2	2	2	2	1	9
1	3	2	2	2	2	2	2	1	1
2	3	1	2	2	2	2	2	2	3
9	3	9	9	9	9	9	9	9	9
9	9	9	9	9	9	9	9	9	9
9	3	9	9	9	9	9	9	9	3
9	9	9	9	9	9	9	9	9	3
9	3	9	9	9	9	9	9	9	3
9	3	9	9	9	9	9	9	9	3
9	9	9	3	9	9	9	9	9	9
1	2	2	2	2	2	1	1	1	9
9	9	9	9	9	9	9	9	9	9
2	2	2	2	2	2	2	2	2	2
9	9	9	9	9	9	9	9	9	3
3	3	2	3	3	3	3	3	3	2
3	3	3	3	2	1	3	3	3	9

1	3	3	1	3	1	1	1	3	3
3	3	2	2	2	2	2	2	2	3
2	3	3	2	2	2	1	2	1	2
3	2	3	1	2	2	2	1	1	3
9	3	1	2	1	2	3	2	2	9
9	9	9	3	9	9	9	9	9	3
2	9	2	3	1	2	3	2	1	9
9	2	9	9	9	9	2	9	2	2
9	2	9	9	9	9	9	9	9	9
9	9	9	9	9	9	9	9	9	3
2	3	1	3	2	2	2	2	2	2
3	3	3	3	2	3	3	3	3	9
2	2	2	2	2	2	2	2	2	2
3	3	1	2	3	3	3	3	3	3
9	3	1	3	1	1	1	9	9	9
1	2	3	3	2	1	1	2	3	9
2	3	2	2	3	2	3	2	3	3
2	2	2	2	2	2	2	2	2	2
2	3	2	3	2	2	2	2	3	3
3	3	3	3	3	1	3	3	3	3
1	3	1	3	3	1	1	1	3	3
2	3	3	3	2	2	3	2	3	3
1	1	1	1	3	3	3	3	3	3
9	9	9	9	9	9	9	9	9	3
3	3	3	3	3	3	3	3	3	3
3	3	3	3	1	3	3	3	3	3
3	2	1	3	2	3	2	3	3	2
3	3	2	2	3	3	3	3	3	9
1	3	3	2	2	1	3	3	2	3
2	2	9	3	9	9	9	9	3	3
1	3	1	3	1	3	3	3	2	3
9	9	9	9	9	9	9	9	9	9
3	2	3	2	2	2	3	3	2	2
2	2	2	2	2	2	2	2	2	3
2	2	1	1	2	2	2	2	2	3
9	3	9	9	9	9	9	9	9	9
3	3	1	3	1	1	1	1	1	9
3	3	1	3	1	1	1	1	1	3
3	3	3	2	3	2	3	3	3	9
3	2	3	2	3	2	3	2	2	1
2	2	1	2	2	2	2	2	2	2
3	3	2	3	1	2	3	3	3	3
2	3	1	2	2	1	2	1	3	3
2	3	2	2	2	2	2	2	3	3
3	3	1	3	3	3	3	3	3	3
2	3	1	2	2	1	2	1	3	3
3	3	1	1	1	3	1	1	1	3

3	3	9	9	9	1	9	1	9	3
2	2	1	1	3	3	3	3	3	3
2	3	2	2	2	2	2	2	3	2
1	3	1	3	2	1	3	2	1	3
9	9	9	9	9	9	9	9	9	9
2	3	9	2	3	2	1	3	2	3
9	9	9	9	9	9	9	9	9	9
9	9	9	9	9	9	9	9	9	9
9	3	1	3	2	1	2	2	2	3
1	3	3	3	3	3	3	3	3	3
9	9	9	9	9	9	9	9	9	3
3	3	3	3	3	3	3	1	3	3
1	3	1	3	1	1	1	1	1	2
2	2	2	2	2	3	3	2	3	2
2	2	1	2	2	1	2	2	1	1
1	3	3	3	1	2	3	2	3	9
3	3	1	3	3	3	3	3	3	3
2	1	1	2	2	2	2	2	9	9
3	3	3	3	3	3	3	3	3	3
2	2	1	1	2	3	2	3	2	3
1	3	3	3	3	3	3	2	3	3
1	3	3	3	3	3	3	2	3	3
2	3	1	1	3	3	3	3	3	9
1	3	3	3	1	1	1	1	1	1
1	3	2	2	2	2	2	2	9	2
2	3	3	3	3	3	3	3	3	3
2	1	2	2	2	3	3	1	2	3
2	2	2	2	2	2	2	2	2	2
2	3	2	2	3	2	2	3	3	9
2	3	2	2	3	2	2	1	3	3
9	9	9	9	9	9	9	9	9	3
3	3	9	1	3	3	3	3	3	9
2	3	1	3	2	2	3	2	2	3
9	3	1	3	1	1	1	1	1	3
1	3	1	3	1	1	2	2	2	3
1	2	2	3	1	2	2	3	2	2
9	9	9	9	9	9	9	9	9	9
9	9	3	9	2	3	3	3	3	2
9	9	9	9	9	9	9	9	9	9
3	3	3	3	3	3	3	3	3	3
2	2	2	2	2	2	2	2	2	2
2	2	2	2	2	2	2	2	2	9
9	9	9	9	9	9	9	9	9	2
3	3	3	3	3	3	3	3	3	3
3	3	3	3	3	3	3	3	3	3
9	9	9	9	9	1	9	9	9	3
2	3	2	2	2	1	3	3	3	3

2	3	1	3	1	1	1	1	1	3
1	3	1	3	2	1	2	3	3	3
9	9	9	9	9	9	9	9	9	9
9	9	9	9	9	9	9	9	9	2
9	9	9	9	9	9	9	9	9	9
3	1	3	1	3	3	3	3	3	1
3	3	3	3	3	3	3	3	3	3
3	3	1	3	3	3	1	2	3	3
2	3	1	2	1	1	1	1	3	9
2	3	3	2	3	3	3	3	3	3
3	3	3	3	3	3	3	3	3	3
2	3	3	3	3	3	3	2	3	3
2	2	2	2	2	2	3	3	2	2
3	1	3	2	3	3	3	3	3	3
3	3	3	3	3	3	3	3	3	3
2	2	2	2	2	2	2	2	3	3
1	3	2	2	2	2	3	3	3	3
1	3	3	3	3	3	3	1	3	3
9	9	9	9	9	9	9	9	9	9
1	3	1	1	1	3	1	3	3	3
9	9	9	9	9	9	9	9	9	9
2	3	3	2	3	2	3	2	3	3
2	3	1	1	3	2	3	3	3	3
9	9	9	9	9	9	9	9	9	9
1	2	2	2	2	2	2	2	1	2

AfraidofHP	KnowMore	HPVVaccin	HPVVaccin	Embarrass	TooBusyfo	HPVVaccin	HPVAgains	CervCA2	CervScreen
2	2	2	2	2	2	2	2	2	2
2	2	2	2	2	2	2	2	2	2
2	2	2	2	2	2	3	2	2	2
2	2	2	2	2	2	2	2	2	2
2	2	2	3	2	2	2	3	2	1
3	3	3	3	3	3	3	2	2	2
1	3	2	3	2	1	3	2	2	2
2	2	1	1	2	2	2	2	1	2
3	3	3	3	3	1	1	1	2	2
2	1	2	3	2	1	2	2	2	2
2	2	2	2	2	2	2	2	2	2
2	2	1	2	2	3	2	3	2	1
2	9	2	2	2	3	3	3	2	1
2	2	2	2	3	2	2	2	2	2
9	9	9	9	9	9	9	9	2	2
2	2	2	2	2	2	2	2	2	1
9	9	9	9	9	9	9	9	#NULL!	#NULL!
1	2	2	2	1	1	1	1	2	1
9	9	9	9	9	9	9	9	#NULL!	#NULL!
9	9	9	9	9	9	9	9	1	2
1	1	3	1	3	3	3	3	1	1
2	2	2	2	2	2	2	3	1	1
2	1	1	1	2	1	2	2	1	1
2	9	1	2	1	2	1	1	1	1
9	9	9	2	9	9	9	9	2	2
1	1	3	3	2	2	1	1	#NULL!	#NULL!
3	9	1	1	1	3	3	3	1	1
2	1	2	2	1	3	3	3	1	1
2	3	3	2	1	1	2	3	2	2
3	1	2	3	1	1	2	1	1	2
1	1	1	2	1	2	3	3	2	1
2	1	2	1	2	1	3	3	1	1
1	1	1	1	1	1	1	1	2	2
3	2	1	1	1	2	1	9	1	1
1	2	1	2	1	2	2	1	2	2
2	3	9	2	2	3	3	3	1	1
2	2	2	2	2	9	2	2	2	1
1	1	1	1	1	1	2	3	2	2
3	1	1	2	3	3	3	3	1	1
2	3	2	3	2	1	1	2	#NULL!	#NULL!
2	1	2	1	2	3	3	3	#NULL!	#NULL!
2	3	9	2	1	9	1	2	1	2
9	9	9	9	9	9	9	9	2	2
2	2	2	2	2	2	2	2	1	2
2	2	3	2	2	2	2	2	2	2
2	1	2	3	2	1	3	1	#NULL!	#NULL!

1	2	2	3	2	2	1	1	1	1
2	2	1	2	2	2	3	3	2	1
2	2	2	1	2	2	2	2	1	2
2	1	2	2	2	1	1	2	1	1
1	1	3	3	1	3	3	3	2	2
1	1	3	3	1	3	3	3	2	2
3	2	3	2	3	2	3	2	2	2
3	1	1	1	3	3	3	3	2	2
9	1	9	1	1	9	1	2	2	1
9	9	9	1	3	9	9	1	2	2
9	9	9	9	9	9	9	3	#NULL!	#NULL!
2	2	2	3	3	2	2	1	1	2
1	1	1	1	1	1	1	1	2	2
1	2	2	2	3	3	2	2	1	2
1	2	2	2	2	2	3	3	2	1
1	2	2	2	3	3	3	2	2	1
2	2	3	2	3	3	2	2	2	2
2	1	1	2	1	1	3	3	2	2
2	1	2	1	2	3	3	3	2	2
3	3	3	3	3	3	3	3	#NULL!	#NULL!
2	1	1	2	2	2	2	2	1	2
2	1	3	2	2	2	2	3	2	2
1	2	2	1	1	2	2	1	2	1
2	2	2	3	1	2	2	2	1	1
2	2	1	2	2	2	2	2	1	2
2	3	2	1	2	2	3	1	2	2
1	1	9	3	3	1	9	3	2	2
2	3	1	2	3	3	2	1	2	2
2	9	1	2	1	3	3	2	2	2
1	1	3	3	3	3	3	3	2	1
9	9	9	9	9	9	9	9	#NULL!	#NULL!
3	1	3	3	2	3	3	3	2	2
2	1	3	2	2	3	9	3	2	2
3	1	1	2	2	2	2	2	2	2
1	1	2	3	2	9	2	3	2	2
2	2	2	2	2	2	2	2	2	2
3	1	3	1	2	3	2	3	1	1
2	2	1	2	2	3	2	2	1	2
2	2	2	2	2	2	2	9	2	2
1	2	2	3	2	1	1	2	1	2
2	1	2	1	3	1	2	3	2	2
9	9	9	9	9	9	9	9	#NULL!	#NULL!
2	1	3	2	1	3	2	1	2	2
1	1	2	2	1	1	1	2	1	2
1	1	1	1	1	2	2	3	2	2
2	1	2	3	1	2	1	2	1	1
3	1	1	1	2	2	3	3	2	2

2	3	1	2	3	1	2	1	1	2
3	2	3	3	2	3	3	1	2	2
1	1	2	3	1	1	2	3	2	2
2	1	2	2	1	1	1	1	1	2
2	1	2	1	2	2	2	2	1	2
2	2	1	2	2	2	2	2	2	2
2	1	1	2	2	2	2	3	1	2
9	9	9	9	9	9	9	9	#NULL!	#NULL!
2	1	2	1	3	3	2	2	2	2
2	1	2	2	1	2	2	3	1	2
9	9	9	9	9	9	9	9	1	1
2	1	1	2	2	2	2	1	1	2
2	2	3	2	2	2	2	2	1	1
1	1	2	1	3	3	3	3	2	2
3	3	2	2	3	3	2	3	1	2
3	3	2	2	3	3	2	3	1	#NULL!
1	1	2	2	1	2	2	1	2	1
2	1	1	3	1	2	1	3	2	2
3	1	9	1	3	3	1	3	2	#NULL!
2	1	2	2	1	1	1	1	2	2
3	1	3	1	3	1	3	1	1	2
1	1	1	2	3	2	3	3	2	2
1	2	2	2	1	2	1	1	2	1
1	2	1	2	2	2	2	2	1	1
2	2	2	2	2	3	2	2	2	2
2	1	1	1	2	2	2	3	2	2
3	1	3	1	3	3	3	3	1	1
1	2	1	2	1	2	2	9	1	1
2	2	1	1	2	1	2	1	2	2
2	1	1	3	3	1	3	3	1	1
3	3	2	2	2	1	2	2	1	2
2	3	1	2	3	1	2	2	2	2
2	2	1	2	2	3	2	2	2	2
2	1	2	1	2	1	1	2	1	1
2	1	2	2	1	2	3	9	2	2
1	1	1	1	9	1	1	1	1	1
2	2	2	2	3	3	3	3	2	2
2	2	1	2	2	1	2	2	2	1
9	9	9	9	9	9	9	9	#NULL!	#NULL!
3	2	3	3	2	3	3	1	2	2
2	2	1	2	2	2	1	2	2	1
9	9	9	9	9	9	9	9	#NULL!	#NULL!
2	1	1	2	3	2	2	1	2	1
1	2	3	2	1	2	2	1	2	1
3	1	9	3	3	3	3	3	2	2
3	1	3	1	1	3	3	3	1	1
2	1	1	2	2	3	3	3	2	2

3	2	3	2	3	1	2	2	2	2
3	1	3	3	3	3	3	3	2	2
3	3	3	3	3	3	3	3	1	1
2	2	2	2	2	2	2	2	2	2
2	1	2	2	2	2	2	3	2	2
3	2	3	2	3	2	3	2	2	2
9	9	9	9	9	9	9	9	2	1
9	9	9	9	9	9	9	9	1	1
9	9	9	9	9	9	9	9	2	2
2	2	2	2	2	2	2	2	2	2
2	1	2	3	2	1	1	2	1	1
9	1	3	3	3	3	3	3	2	2
2	2	2	2	2	2	2	9	1	2
2	3	2	2	1	2	1	3	2	2
2	2	1	2	1	1	1	1	2	1
2	3	2	1	2	2	2	2	1	1
2	3	2	2	1	1	1	1	2	1
2	3	2	2	1	1	1	1	2	1
9	9	9	9	9	9	9	9	2	2
3	2	2	3	2	3	1	3	2	2
1	1	1	2	2	3	1	2	1	2
1	1	1	1	2	2	2	2	2	2
9	9	9	9	9	9	9	9	2	2
2	2	2	2	2	2	2	2	2	2
3	1	1	2	3	3	3	3	1	1
1	2	1	2	3	2	1	3	2	2
9	9	9	9	9	9	9	9	2	2
3	3	3	3	3	3	3	3	2	1
3	1	1	3	3	1	3	3	2	2
2	3	2	2	2	2	2	2	1	1
9	9	9	9	9	9	9	9	1	1
1	1	1	3	3	3	3	2	2	2
1	1	2	2	1	2	1	1	1	1
3	1	3	3	1	3	2	2	1	2
1	2	1	2	2	1	3	2	1	1
1	1	1	1	1	1	1	1	1	1
3	3	3	3	3	3	3	3	1	1
3	1	3	2	1	1	3	1	1	1
2	1	1	1	3	3	3	3	2	2
2	2	2	2	2	2	2	2	1	2
9	9	9	9	9	9	9	9	#NULL!	2
2	2	2	2	2	2	2	2	1	1
2	2	2	2	2	2	2	9	1	2
9	9	9	9	9	9	9	9	#NULL!	#NULL!
9	9	9	9	9	9	9	9	#NULL!	#NULL!
2	2	3	2	1	2	2	2	2	1
2	2	3	3	2	2	1	2	1	2

9	9	1	9	9	9	9	9	1	1
9	9	9	9	9	9	9	9	2	2
3	1	3	1	3	3	3	3	1	2
9	9	9	9	9	9	9	9	1	2
9	9	9	9	9	9	9	9	2	2
9	9	9	9	9	9	9	9	#NULL!	#NULL!
9	9	9	9	9	9	9	9	1	1
9	9	9	9	9	9	9	9	#NULL!	#NULL!
2	1	3	1	2	2	1	2	1	2
9	9	9	9	9	9	9	9	2	2
9	9	9	2	9	9	9	9	2	2
9	9	9	9	9	9	1	9	2	2
1	2	3	2	1	1	2	1	1	2
2	3	3	2	1	3	2	1	#NULL!	#NULL!
1	9	9	9	9	9	9	2	2	1
3	3	2	3	3	2	2	3	2	1
1	2	1	1	1	1	1	1	1	1
1	1	2	2	1	1	1	1	1	2
2	2	2	2	2	2	2	2	2	2
2	1	2	2	2	2	2	2	2	2
1	2	1	1	1	1	1	1	2	1
9	9	9	9	9	9	9	9	#NULL!	#NULL!
2	3	1	1	1	1	1	2	2	2
3	1	3	1	3	3	3	3	2	2
2	2	2	2	2	2	2	2	2	2
1	1	1	2	1	1	1	2	2	2
2	2	1	1	2	2	2	2	1	2
1	2	2	3	2	2	2	2	1	2
9	9	9	9	9	9	9	9	1	1
3	1	1	1	3	3	3	3	1	1
2	2	2	2	2	2	2	2	2	2
1	2	2	1	1	1	1	1	2	2
9	9	9	9	9	9	9	9	#NULL!	#NULL!
2	2	2	1	1	1	2	2	2	2
3	1	3	3	3	3	2	3	2	2
2	9	3	2	1	2	2	2	1	2
9	9	9	9	9	9	9	9	2	1
2	1	1	1	2	2	2	2	1	2
9	9	9	9	9	9	9	9	2	2
1	2	1	2	1	2	2	3	2	2
1	1	1	1	2	3	3	2	1	2
1	1	3	2	2	1	3	1	1	1
1	1	2	2	1	1	2	2	1	2
2	2	2	2	2	2	2	2	1	1
2	3	1	2	2	2	2	2	2	2
2	3	2	3	2	1	2	9	1	1
9	9	9	9	9	9	9	9	#NULL!	#NULL!

1	1	3	2	2	2	2	2	2	2
2	1	1	1	2	3	1	3	1	2
1	1	1	1	1	1	1	1	2	2
9	9	9	9	9	9	9	9	2	#NULL!
2	2	2	2	2	2	2	2	1	2
3	2	2	3	2	2	2	3	2	2
3	3	1	2	3	1	2	3	1	1
1	2	1	1	2	1	1	2	2	2
1	1	1	1	1	2	1	1	2	1
3	3	2	1	1	2	1	2	1	2
2	2	2	2	2	2	2	2	2	2
9	9	9	9	9	9	9	9	1	1
9	9	9	9	9	9	9	9	2	2
9	9	9	9	9	9	9	9	1	1
9	9	2	9	9	2	2	2	2	2
9	9	9	9	9	9	9	9	2	2
9	9	9	9	9	9	9	9	#NULL!	#NULL!
9	9	9	9	9	9	9	9	#NULL!	#NULL!
9	9	9	9	9	9	9	9	2	2
9	9	9	9	9	9	9	9	#NULL!	#NULL!
9	9	9	9	9	9	9	9	#NULL!	#NULL!
1	2	2	1	1	1	1	1	1	2
2	2	2	2	2	2	2	2	2	1
2	2	2	2	1	1	2	1	2	1
1	2	1	1	2	1	1	1	2	2
9	9	9	9	9	9	9	9	#NULL!	#NULL!
2	2	2	2	1	2	2	2	1	2
9	9	9	9	9	9	9	9	#NULL!	#NULL!
9	9	9	9	9	9	9	9	#NULL!	#NULL!
2	1	2	2	2	2	2	2	1	1
9	9	9	9	9	9	9	9	2	2
9	9	9	9	9	9	9	9	#NULL!	#NULL!
9	9	9	9	9	9	9	9	#NULL!	#NULL!
9	9	9	9	9	9	9	9	#NULL!	#NULL!
9	9	9	9	9	9	9	9	2	2
1	9	9	1	9	9	1	1	2	2
9	9	9	9	9	9	9	9	#NULL!	#NULL!
9	9	9	9	9	9	9	9	#NULL!	#NULL!
9	9	9	9	9	9	9	9	#NULL!	#NULL!
9	9	9	9	9	9	9	9	#NULL!	#NULL!
9	9	9	9	9	9	9	2	#NULL!	#NULL!
9	9	9	9	9	9	9	9	2	2
3	3	3	3	3	3	3	3	1	1
3	3	3	3	3	3	3	3	2	2
2	1	3	2	1	2	1	2	1	1
2	2	1	1	1	1	1	1	1	1
9	9	9	9	9	9	9	9	1	2

9	9	9	9	9	9	9	9	2	2
9	9	9	9	9	9	9	9	1	2
2	2	1	2	3	2	2	3	1	1
2	3	2	3	1	3	2	3	1	2
9	9	9	9	9	9	9	9	#NULL!	#NULL!
2	3	3	2	2	3	2	3	1	2
9	9	9	9	9	9	9	9	1	1
2	1	3	2	1	1	2	1	2	2
2	2	2	2	1	3	2	2	2	2
3	3	3	3	3	3	3	3	1	1
9	9	9	9	9	9	9	9	#NULL!	#NULL!
9	9	9	9	9	9	9	9	#NULL!	#NULL!
9	9	9	9	9	9	9	9	2	2
9	2	9	9	9	9	9	2	1	1
2	2	2	2	2	2	2	2	1	2
2	2	2	2	2	1	1	2	1	1
9	9	9	9	9	9	9	9	#NULL!	#NULL!
1	1	2	3	2	2	2	3	1	2
1	1	1	1	1	1	1	1	1	1
2	2	2	2	2	2	2	2	2	2
2	2	2	2	2	2	2	2	1	2
3	1	1	3	3	2	2	3	1	2
9	9	9	9	9	9	9	9	2	2
2	2	2	2	2	2	2	2	1	2
2	2	2	2	2	2	2	2	2	1
2	2	2	2	2	2	2	2	1	1
2	2	2	2	2	2	2	2	2	2
2	1	1	1	1	1	1	1	1	1
2	3	9	2	3	9	2	2	2	2
1	2	1	1	2	1	2	2	1	2
2	2	2	2	2	2	2	2	2	2
2	2	2	2	2	2	2	2	2	2
2	2	3	2	3	2	3	2	2	2
3	3	3	9	9	2	2	2	2	2
1	1	1	3	3	3	3	3	1	1
2	3	1	2	3	1	2	2	2	1
9	9	9	9	9	9	9	9	#NULL!	#NULL!
2	1	3	2	3	3	3	2	2	2
3	3	3	3	3	3	3	3	2	2
3	3	3	3	3	3	3	3	2	2
9	9	9	9	9	9	9	9	2	2
9	9	9	9	9	9	9	9	#NULL!	#NULL!
1	1	2	1	1	1	1	3	2	2
1	2	2	3	2	2	2	2	1	1
2	1	1	1	1	1	1	1	2	2
2	2	2	2	2	2	2	2	2	2
2	2	2	2	3	2	2	2	1	1

9	9	9	2	9	9	9	2	1	2
9	9	9	9	9	9	9	9	2	2
9	9	1	9	9	9	9	9	2	2
9	9	9	1	9	9	9	3	2	2
2	9	9	9	9	9	1	9	1	2
1	1	1	2	2	2	1	9	1	1
9	9	9	9	9	9	9	9	2	2
9	9	9	9	9	9	9	9	2	2
9	9	9	9	9	9	9	9	2	2
9	9	9	9	9	9	9	9	2	2
9	9	1	9	9	9	9	2	2	2
9	9	2	9	9	9	3	2	1	#NULL!
9	9	9	9	9	9	9	2	2	2
9	2	9	9	9	9	9	9	2	2
9	9	9	9	9	9	9	9	2	2
9	9	9	9	9	9	9	9	#NULL!	#NULL!
9	9	9	2	9	9	9	2	2	2
2	3	2	1	2	9	2	1	2	2
1	2	1	1	2	3	1	1	1	1
3	2	2	2	2	2	2	2	2	2
2	1	3	2	1	2	1	3	1	1
2	1	3	2	3	2	2	3	#NULL!	#NULL!
2	2	2	2	2	2	2	2	2	2
9	9	9	9	9	9	9	9	2	2
2	3	1	2	3	2	2	2	2	2
9	9	9	9	9	9	9	9	#NULL!	#NULL!
1	1	1	3	2	3	1	2	2	2
9	9	9	9	9	9	9	1	2	2
3	2	1	2	1	3	1	2	1	2
9	9	9	9	9	9	9	9	1	1
1	2	2	3	2	2	1	2	1	1
1	1	1	3	1	1	3	2	2	2
2	2	2	1	1	1	1	2	2	1
1	1	1	1	1	1	1	1	2	2
9	9	9	9	9	9	9	9	#NULL!	#NULL!
2	2	2	2	2	2	2	2	1	2
9	9	9	9	9	9	9	9	#NULL!	#NULL!
1	2	2	1	2	2	2	2	2	2
9	9	9	9	9	9	9	9	2	2
2	2	2	2	2	2	2	2	1	1
2	2	1	2	2	1	2	9	2	2
3	3	3	3	3	3	3	3	2	2
1	1	1	3	3	3	3	3	2	2
2	2	2	2	2	2	3	3	2	2
3	3	3	3	3	3	3	2	2	2
2	2	2	2	2	2	2	2	1	1
2	2	2	2	2	2	2	2	1	1

2	2	1	2	2	1	3	3	1	1
9	9	9	9	9	9	9	3	2	#NULL!
2	9	2	2	2	3	2	2	1	1
3	2	9	1	2	3	1	3	2	2
1	1	1	1	1	2	2	2	2	2
2	2	2	2	2	2	2	2	2	2
1	1	2	2	2	3	3	3	1	1
9	9	9	9	9	9	9	9	1	1
9	9	9	9	9	9	9	9	#NULL!	#NULL!
2	1	1	1	2	3	3	3	2	2
2	2	2	2	2	2	2	1	1	2
9	9	9	9	9	9	9	9	2	2
9	9	9	9	9	9	9	9	#NULL!	#NULL!
1	1	1	1	9	1	1	1	2	2
9	9	9	9	9	9	9	9	2	1
2	3	3	2	1	3	2	2	2	2
9	9	9	9	9	9	9	9	#NULL!	#NULL!
9	9	9	9	9	9	9	9	#NULL!	2
9	9	9	9	1	9	9	9	2	2
3	2	3	2	3	2	3	2	2	2
2	2	2	2	2	2	2	2	2	2
1	3	3	3	2	2	1	1	2	2
2	2	2	2	1	2	2	2	2	2
9	1	9	9	9	9	9	9	2	2
3	9	9	9	9	9	9	9	2	2
2	2	2	2	2	2	2	2	2	2
1	1	1	1	1	1	2	3	1	1
2	1	2	3	2	3	1	2	1	1
9	9	9	9	9	9	9	9	#NULL!	#NULL!
9	9	9	9	9	9	9	9	2	2
9	9	9	9	9	9	9	9	2	2
1	2	3	1	2	3	1	2	2	2
2	2	2	1	3	3	3	3	2	2
2	2	1	2	2	2	2	2	1	2
1	1	1	2	2	1	1	1	1	2
1	2	1	2	2	3	2	2	#NULL!	#NULL!
9	9	9	9	9	9	9	9	#NULL!	#NULL!
9	9	9	9	9	9	9	9	#NULL!	#NULL!
2	3	1	3	1	1	2	2	1	1
2	1	2	1	2	1	2	2	1	1
2	2	2	2	2	2	2	2	1	2
2	1	1	2	2	2	3	3	2	2
2	1	1	3	2	3	3	3	2	2
2	2	3	2	1	2	3	3	2	2
2	3	2	2	1	3	1	3	#NULL!	#NULL!
2	2	2	2	2	2	2	2	1	2
2	2	2	2	3	3	3	3	1	2

2	2	2	2	1	1	1	1	2	2
2	1	2	2	3	3	2	2	1	1
9	9	9	9	9	9	9	9	2	1
9	9	9	9	9	9	9	9	2	2
9	1	9	9	9	9	9	9	2	2
1	3	3	1	3	9	1	3	1	1
9	2	2	9	2	9	2	2	2	2
2	2	2	2	2	2	2	2	2	2
9	9	9	9	9	9	9	9	#NULL!	#NULL!
9	9	9	9	9	9	9	9	#NULL!	#NULL!
9	9	9	9	9	9	9	9	2	2
9	9	9	9	9	9	9	9	2	2
9	9	9	9	9	9	9	9	2	2
9	9	9	9	9	9	9	9	#NULL!	#NULL!
9	9	9	9	9	9	9	9	2	2
9	9	9	9	9	9	9	9	2	2
3	3	3	3	3	3	3	3	2	2
9	9	9	9	9	9	9	9	1	1
9	9	9	9	9	9	9	9	1	1
9	9	9	9	9	9	9	9	1	1
9	9	9	9	9	9	9	9	#NULL!	#NULL!
9	9	9	9	9	9	9	9	2	2
2	2	2	2	2	2	2	2	2	2
9	9	9	9	9	9	9	9	#NULL!	#NULL!
9	9	9	9	9	9	9	9	#NULL!	#NULL!
9	9	9	9	9	9	9	9	1	1
9	9	9	9	9	9	9	9	#NULL!	#NULL!
9	9	2	9	2	9	9	9	1	1
9	9	9	9	9	9	9	9	1	1
9	9	9	9	9	9	9	9	#NULL!	#NULL!
9	9	9	9	9	9	9	9	#NULL!	#NULL!
9	9	9	9	9	9	9	9	1	1
9	9	9	9	9	9	9	9	#NULL!	#NULL!
9	9	9	9	9	9	9	9	#NULL!	#NULL!
9	9	9	9	9	9	9	9	#NULL!	#NULL!
9	9	9	9	9	9	9	9	#NULL!	#NULL!
9	9	9	9	9	9	9	9	2	2
9	9	9	9	9	9	9	9	#NULL!	#NULL!
3	1	2	2	2	2	2	2	2	2
2	1	2	3	2	1	2	2	1	2
9	2	9	9	9	9	9	9	2	2
9	9	9	9	9	9	9	9	#NULL!	#NULL!
3	1	3	1	3	3	2	2	2	2
2	3	2	3	2	2	2	2	2	2
3	1	1	1	1	1	1	9	1	2
1	2	2	1	2	1	2	2	1	2
9	1	9	9	9	9	9	9	#NULL!	#NULL!

9	9	9	9	9	9	9	9	1	1
3	2	3	3	1	2	2	1	1	1
9	9	9	9	9	9	9	9	2	2
1	1	1	1	1	1	1	9	1	1
2	2	2	2	2	2	2	2	2	2
1	2	2	1	2	2	2	2	2	2
9	9	9	9	9	9	9	9	2	1
2	2	3	2	1	1	1	2	2	2
2	2	2	2	2	2	2	2	2	2
1	1	1	1	1	3	9	3	1	2
3	2	1	1	3	2	2	1	2	2
9	9	1	9	9	9	9	9	2	2
9	9	9	9	9	9	9	9	2	2
9	9	9	9	9	9	9	9	1	2
9	9	9	9	9	9	9	9	1	2
1	9	9	9	9	9	9	9	2	2
2	2	2	2	2	2	2	2	2	2
1	1	1	1	3	2	3	3	2	2
3	2	9	3	1	3	3	9	2	2
2	2	2	2	2	2	2	2	1	1
2	2	2	2	2	2	2	2	2	2
9	9	9	9	9	9	9	9	#NULL!	#NULL!
1	1	1	3	3	1	1	1	2	2
9	9	9	2	9	9	9	9	1	2
9	9	9	9	9	9	9	9	2	2
1	1	1	1	1	1	1	1	1	1
9	2	9	9	9	9	2	9	1	#NULL!
2	3	3	3	1	1	1	1	2	2
9	9	9	9	9	9	9	9	#NULL!	#NULL!
9	9	9	9	9	9	9	9	1	1
2	1	1	3	1	2	1	2	1	1
2	3	2	2	3	2	3	2	1	2
9	9	9	9	9	9	9	9	#NULL!	#NULL!
9	9	9	9	9	9	9	1	1	1
2	3	2	2	3	3	2	2	1	2
9	9	9	9	9	9	9	9	#NULL!	#NULL!
9	9	9	9	9	9	9	9	2	2
1	1	1	1	1	1	1	1	1	1
2	2	2	2	2	2	2	2	2	2
9	9	9	9	9	9	9	9	1	1
9	9	9	9	9	9	9	9	2	2
1	1	1	1	1	9	3	3	2	2
3	2	2	2	2	2	2	2	2	2
2	2	2	2	2	2	2	2	2	2
2	9	3	2	3	3	2	2	2	2
2	2	2	2	2	2	2	2	2	2
3	2	3	2	3	3	2	2	2	2

9	9	9	9	9	9	9	9	2	2
2	2	2	2	2	2	2	2	2	1
2	2	1	2	2	3	2	3	2	2
9	9	9	1	9	9	9	9	1	1
9	9	1	9	9	9	9	9	2	2
9	9	9	9	9	9	9	9	#NULL!	#NULL!
9	9	9	9	9	9	9	9	#NULL!	#NULL!
2	1	1	2	3	3	3	3	2	2
2	1	2	1	2	2	2	3	1	1
9	9	9	9	9	9	9	9	2	2
1	1	1	2	1	2	1	2	1	1
3	2	3	2	2	1	1	1	1	1
2	2	2	2	2	2	2	2	1	1
2	2	9	9	9	9	9	9	2	2
9	9	9	9	9	9	9	9	2	2
9	9	9	9	9	9	9	9	#NULL!	#NULL!
9	9	9	9	9	9	9	9	#NULL!	#NULL!
9	9	9	9	9	9	9	9	#NULL!	#NULL!
9	9	9	9	9	9	9	9	2	2
9	9	9	9	9	9	9	9	2	2
9	9	9	9	9	9	9	9	#NULL!	#NULL!
9	9	9	9	9	9	9	9	#NULL!	#NULL!
2	2	2	2	2	2	2	2	2	2
9	9	9	9	9	9	9	2	2	2
9	9	9	9	9	9	9	9	#NULL!	#NULL!
9	9	9	9	9	9	9	9	2	2
9	9	9	9	9	9	9	9	#NULL!	#NULL!
9	9	9	9	9	9	9	9	2	2
9	9	9	9	9	9	9	9	2	2
9	9	9	9	9	3	9	9	2	#NULL!
9	9	9	9	9	9	9	9	#NULL!	#NULL!
9	9	9	9	9	9	9	9	2	2
9	9	9	9	9	9	9	9	#NULL!	#NULL!
9	9	9	9	9	9	9	9	#NULL!	#NULL!
9	9	9	9	9	9	9	9	2	2
9	9	9	9	9	9	9	9	#NULL!	#NULL!
9	9	9	9	9	9	9	9	2	2
9	9	9	9	9	9	9	9	#NULL!	#NULL!
9	9	9	9	9	9	9	9	#NULL!	#NULL!
9	9	9	9	9	9	9	9	#NULL!	#NULL!
2	2	2	2	2	2	2	2	1	1
9	9	9	9	9	9	9	2	1	1
9	9	9	9	9	9	9	9	#NULL!	#NULL!
9	9	9	9	9	9	9	9	2	2
9	9	9	9	9	9	9	9	#NULL!	#NULL!
9	9	9	9	9	9	9	9	2	2
9	9	9	9	9	9	9	9	#NULL!	#NULL!

9	9	9	9	9	9	9	9	#NULL!	#NULL!
2	2	2	2	2	2	2	2	#NULL!	#NULL!
9	9	9	9	9	9	9	9	2	2
9	9	9	9	9	9	9	9	#NULL!	#NULL!
9	9	9	9	9	9	9	9	#NULL!	#NULL!
9	9	9	9	9	9	9	9	#NULL!	#NULL!
9	9	9	9	9	9	9	9	#NULL!	#NULL!
9	9	9	9	9	9	9	9	#NULL!	#NULL!
9	9	9	9	9	9	9	9	#NULL!	#NULL!
9	9	9	9	9	9	9	9	#NULL!	#NULL!
9	9	9	9	9	9	9	9	#NULL!	#NULL!
9	9	9	9	9	9	9	9	#NULL!	#NULL!
9	9	9	9	9	9	9	9	#NULL!	#NULL!
9	9	9	9	9	9	9	9	#NULL!	#NULL!
9	9	9	9	9	9	9	9	2	2
9	9	9	9	9	9	9	9	2	2
9	9	9	9	9	9	9	9	#NULL!	#NULL!
9	9	9	9	9	9	9	9	2	2
9	9	9	9	9	9	9	9	#NULL!	#NULL!
9	9	9	9	9	9	9	9	2	2
9	9	9	9	9	9	9	9	#NULL!	#NULL!
9	9	9	9	9	9	9	9	2	2
9	9	9	9	9	9	9	9	#NULL!	#NULL!
9	9	9	9	9	9	9	9	2	2
2	2	2	2	2	2	2	2	2	2
9	9	9	9	9	9	9	9	2	2
9	9	9	9	9	9	9	9	#NULL!	#NULL!
1	1	1	1	1	1	1	1	2	2
9	9	9	9	9	9	9	9	#NULL!	#NULL!
9	9	9	9	9	9	9	9	#NULL!	#NULL!
1	1	1	1	1	1	1	1	1	1
2	3	1	1	3	2	1	1	2	2
9	9	9	9	9	9	9	9	#NULL!	#NULL!
9	9	9	9	9	9	9	9	2	2
9	9	9	9	9	9	9	9	#NULL!	#NULL!
9	9	2	9	2	9	9	2	2	2
9	9	9	9	9	9	9	9	1	1
9	9	9	9	9	9	9	9	#NULL!	#NULL!
1	1	1	1	1	1	1	1	2	2
9	9	9	9	9	9	9	2	2	2
9	9	9	9	9	9	9	9	#NULL!	#NULL!
9	9	9	9	9	9	9	9	#NULL!	#NULL!
2	2	2	2	2	2	2	2	2	2
2	2	2	2	2	2	2	2	1	1
9	9	1	9	1	9	1	1	2	2
9	9	9	9	9	9	9	9	#NULL!	#NULL!
9	9	9	9	9	9	9	9	#NULL!	#NULL!

9	9	9	9	9	9	9	9	2	2
9	9	9	9	9	9	9	9	#NULL!	#NULL!
9	9	9	9	9	9	9	9	2	2
9	9	9	9	9	9	9	9	#NULL!	#NULL!
9	9	9	9	9	9	9	9	#NULL!	#NULL!
9	9	9	9	9	9	9	9	#NULL!	#NULL!
9	9	9	9	9	9	9	9	1	1
9	9	9	9	9	9	9	9	2	2
9	9	2	9	2	9	2	2	1	1
9	9	9	9	9	9	9	9	2	2
9	9	9	9	9	9	9	9	2	2
9	9	9	9	9	9	9	9	#NULL!	#NULL!
9	9	9	9	9	9	9	9	#NULL!	#NULL!
9	9	9	9	9	9	9	9	#NULL!	#NULL!
2	2	2	2	2	1	9	2	2	2
1	2	1	3	3	1	2	3	2	2
9	1	9	9	9	9	9	9	2	2
2	2	1	1	1	2	2	2	2	2
3	3	3	2	2	2	2	3	2	2
9	9	9	9	9	9	9	1	1	1
9	9	9	9	9	9	9	9	1	1
9	9	9	9	9	9	9	9	1	1
3	2	3	1	2	3	1	2	2	2
2	3	1	2	3	1	2	3	2	2
2	2	2	2	2	2	2	2	2	2
3	1	1	3	3	2	2	3	1	1
3	1	9	1	3	3	3	3	2	2
1	1	1	1	2	3	3	3	2	2
1	1	2	1	1	2	2	3	2	2
2	2	2	2	2	2	2	2	2	2
9	9	9	9	9	9	9	9	2	2
9	9	9	2	9	9	9	9	1	1
9	9	9	9	9	9	9	9	1	2
9	9	9	9	9	2	9	9	2	2
2	9	9	9	9	9	9	9	1	#NULL!
9	9	9	9	9	9	9	9	#NULL!	#NULL!
9	9	9	9	9	9	9	9	#NULL!	#NULL!
2	9	9	9	2	9	2	2	1	1
9	9	9	9	9	9	9	9	#NULL!	#NULL!
9	2	9	2	9	9	2	2	2	2
9	9	9	9	9	9	9	9	#NULL!	#NULL!
2	2	2	2	2	2	2	2	1	1
9	9	9	9	9	9	9	9	#NULL!	#NULL!
9	9	9	9	9	9	9	9	#NULL!	#NULL!
9	9	9	9	9	9	9	9	#NULL!	#NULL!
2	2	2	2	2	2	2	2	2	2
2	1	2	2	3	2	2	2	2	2

2	1	1	2	2	2	1	3	1	2
2	1	1	1	2	2	2	2	1	1
2	2	2	2	2	2	2	2	1	1
2	1	2	2	3	1	2	3	#NULL!	#NULL!
3	3	3	3	3	3	3	3	2	2
1	1	2	2	3	3	3	3	2	1
2	1	1	1	1	2	1	1	2	2
3	3	3	3	3	3	3	3	2	2
9	9	9	9	9	9	9	9	2	2
2	2	2	2	2	2	2	2	1	1
9	9	2	9	2	9	9	2	2	2
9	3	1	9	1	9	1	1	2	2
2	1	1	2	3	2	3	3	1	2
2	1	2	3	3	2	3	3	1	2
3	3	2	3	2	3	3	3	2	2
2	2	2	2	2	2	2	2	2	2
1	2	1	2	1	2	1	2	2	2
9	9	9	9	9	9	9	9	2	2
9	9	9	9	9	9	9	9	#NULL!	#NULL!
1	2	2	2	3	1	3	2	1	1
1	1	3	3	3	3	2	3	1	1
1	1	1	1	1	2	2	2	2	1
2	2	3	3	3	2	2	2	1	2
3	2	2	2	1	2	3	2	1	2
2	1	2	2	3	3	3	3	2	2
2	2	2	3	2	2	2	2	2	2
2	2	2	2	2	2	2	3	1	2
2	2	2	2	3	2	2	3	2	2
2	1	2	1	2	1	2	3	2	1
9	3	3	2	9	9	1	1	2	2
3	1	1	1	2	3	2	3	2	2
9	9	9	9	9	9	9	9	2	2
3	3	3	3	3	3	3	3	2	2
2	2	2	2	2	2	2	2	2	2
2	9	9	9	9	9	9	9	2	2
2	3	2	3	2	2	2	2	2	2
1	2	2	3	3	3	3	3	2	2
1	1	1	1	1	1	1	1	1	1
2	1	2	2	1	1	1	1	2	2
9	9	9	9	9	9	9	9	#NULL!	#NULL!
2	2	2	2	2	2	2	2	1	2
9	9	9	9	9	9	9	9	2	1
2	3	2	1	2	2	2	3	2	1
2	2	2	2	2	2	2	2	#NULL!	2
3	2	3	2	3	2	3	2	2	2
3	1	1	1	2	3	3	3	2	2
3	3	3	2	2	1	3	2	2	2

9	9	9	9	9	9	9	9	#NULL!	#NULL!
3	3	3	3	3	3	3	3	2	2
3	1	1	3	3	3	3	1	1	2
3	2	3	2	3	2	3	2	2	2
9	1	2	1	1	1	2	2	2	1
9	9	9	9	9	9	9	9	#NULL!	#NULL!
3	3	2	3	2	2	2	2	1	2
2	3	1	3	2	2	3	2	2	2
3	1	3	1	3	3	3	3	2	2
1	1	1	3	2	1	3	3	2	2
2	1	1	1	1	1	1	3	2	2
2	2	2	2	2	2	2	2	1	2
9	9	9	9	9	9	9	9	#NULL!	#NULL!
3	2	2	2	3	2	3	3	2	2
3	1	2	1	3	3	2	3	1	1
2	2	2	2	2	2	2	2	2	2
2	3	3	3	2	1	1	2	2	2
2	2	2	2	2	2	2	2	1	2
2	2	2	2	2	2	2	2	#NULL!	#NULL!
9	9	9	9	9	9	9	9	1	2
9	9	9	9	9	9	9	9	#NULL!	#NULL!
1	2	1	1	2	1	2	1	#NULL!	#NULL!
2	3	3	3	2	3	2	2	2	2
3	2	3	2	3	2	3	2	2	2
9	9	9	9	9	9	9	9	#NULL!	#NULL!
2	3	2	1	2	3	2	2	2	2
9	9	9	9	9	9	1	9	2	2
2	2	2	2	2	2	2	2	2	2
2	2	2	2	2	2	2	2	2	2
2	2	2	2	2	2	2	2	2	2
9	9	9	9	9	9	9	9	2	2
9	9	9	9	9	9	9	9	#NULL!	#NULL!
9	9	9	9	9	9	9	9	#NULL!	#NULL!
3	2	2	2	3	3	3	3	2	1
9	9	9	9	9	9	9	9	1	1
9	9	9	9	9	9	9	9	#NULL!	#NULL!
9	9	9	9	9	9	9	9	#NULL!	#NULL!
2	3	2	2	2	2	2	2	2	2
9	9	9	9	9	9	9	9	1	1
2	1	1	2	2	2	2	2	2	2
1	1	1	3	3	1	2	3	2	2
2	2	2	2	2	2	2	2	2	2
2	2	2	2	2	2	2	2	2	2
2	2	2	2	2	2	2	2	2	2
1	2	2	2	2	2	2	2	2	2
2	3	9	2	1	2	3	3	2	2
2	2	2	2	2	2	2	2	2	2

3	3	3	2	2	1	1	3	1	2
1	2	1	1	2	2	2	2	2	2
2	2	2	2	2	2	2	2	2	2
2	2	9	1	1	1	1	1	1	1
2	2	2	2	2	2	2	2	2	2
1	2	2	2	2	2	2	2	2	2
1	2	2	2	2	2	2	2	2	2
2	2	2	2	2	2	2	2	2	2
2	2	2	2	2	1	1	3	2	2
9	9	9	9	9	9	9	9	#NULL!	#NULL!
2	2	2	2	2	2	2	3	1	1
2	2	1	3	1	1	2	3	2	2
9	9	9	9	9	9	9	9	2	2
3	2	1	2	1	2	1	1	1	2
2	2	2	2	2	2	3	3	2	2
9	9	9	9	9	9	9	9	1	1
9	9	9	9	9	9	9	9	#NULL!	#NULL!
1	9	9	9	9	9	2	2	2	2
9	9	9	9	9	9	9	9	#NULL!	#NULL!
9	9	9	9	9	9	9	9	#NULL!	#NULL!
1	2	2	2	2	2	2	1	2	2
9	9	9	9	9	9	9	9	#NULL!	#NULL!
9	9	9	9	9	9	9	9	#NULL!	#NULL!
2	1	2	2	2	1	2	2	2	2
3	3	3	3	3	3	3	3	1	2
2	1	2	2	2	1	2	2	2	2
3	2	2	1	1	2	1	2	1	2
9	9	9	9	9	9	9	9	1	2
2	2	2	2	2	2	2	2	2	2
1	3	1	2	2	3	1	2	2	1
9	9	9	9	9	9	9	9	#NULL!	#NULL!
2	1	3	1	2	1	2	2	#NULL!	#NULL!
2	3	2	3	2	2	3	2	1	2
1	1	1	1	1	2	2	2	1	#NULL!
1	1	1	2	2	2	3	3	1	2
2	1	2	2	2	1	2	2	2	2
9	9	9	9	9	9	9	9	#NULL!	#NULL!
1	2	2	2	1	2	1	2	2	2
2	2	2	2	2	2	2	2	2	2
1	1	1	2	2	2	3	3	1	2
2	2	2	2	2	2	2	2	1	2
3	1	2	1	3	1	1	3	1	2
1	1	1	2	2	2	2	3	2	2
2	2	1	1	1	1	2	2	1	2
3	2	3	2	3	3	2	2	2	2
1	1	1	2	2	2	2	2	1	2
2	3	3	3	2	2	3	3	1	2

2	2	2	2	2	2	2	2	#NULL!	#NULL!
2	2	2	2	2	2	2	2	2	2
3	2	2	2	2	2	2	2	2	2
2	2	2	2	2	2	2	2	2	2
3	2	2	3	3	3	2	2	1	2
9	9	9	9	9	9	9	9	#NULL!	#NULL!
9	9	9	9	9	9	9	9	#NULL!	#NULL!
3	3	3	3	3	3	3	3	2	2
9	9	9	9	9	9	9	9	#NULL!	#NULL!
9	9	9	9	9	9	9	9	#NULL!	#NULL!
2	2	2	2	2	2	2	2	2	2
9	9	9	9	9	9	9	9	2	1
1	2	2	1	1	2	2	2	1	1
1	9	9	9	9	9	9	3	2	2
9	1	9	9	9	9	9	9	2	2
1	1	1	1	1	3	3	3	2	2
2	1	1	2	3	1	3	3	1	1
9	9	9	9	9	9	9	9	#NULL!	#NULL!
2	1	3	1	1	3	3	3	1	2
1	1	1	2	2	3	3	3	1	1
2	2	2	2	2	2	2	2	1	2
9	9	9	9	9	9	9	9	2	2
2	2	2	2	2	2	2	2	2	2
1	1	2	2	2	2	2	2	2	2
2	2	2	2	2	2	2	9	2	2
2	2	2	2	2	2	2	2	2	2
2	1	1	2	2	2	9	3	2	2
2	2	2	2	2	2	2	2	2	2
3	2	2	3	2	2	2	2	2	2
2	2	2	2	2	2	2	2	2	2
1	2	1	1	2	1	1	1	2	2
2	3	1	2	2	3	1	2	1	1
1	1	1	2	3	2	2	3	2	2
1	1	1	2	2	2	3	3	2	2
2	1	1	3	1	1	1	2	1	2
2	1	2	2	3	2	1	2	2	2
1	1	1	1	1	1	1	3	1	1
2	2	2	2	2	2	2	2	2	2
1	1	2	2	2	9	2	3	1	1
1	1	1	3	1	1	2	2	2	2
3	1	3	1	3	3	3	3	1	1
1	1	3	1	1	3	3	3	1	2
2	2	2	2	2	2	2	2	2	2
2	2	2	2	2	2	2	2	1	2
2	2	2	2	2	2	2	2	1	2
3	1	3	3	3	3	3	3	2	2
2	2	2	2	2	2	2	2	2	2

1	3	2	1	3	2	1	2	2	1
1	1	1	1	3	3	2	3	1	2
2	9	3	3	2	3	2	2	1	1
2	2	2	2	2	2	2	9	1	1
9	9	9	9	9	9	9	1	2	2
9	9	9	9	9	9	2	9	2	1
2	3	2	3	1	2	2	3	2	2
9	9	9	9	9	9	9	9	1	1
9	9	9	9	9	9	9	9	1	1
9	9	9	9	9	9	9	9	#NULL!	#NULL!
9	9	9	9	9	2	9	9	2	2
9	9	9	9	9	9	2	9	2	1
9	9	9	9	2	9	9	9	2	1
9	9	9	9	9	9	9	9	2	2
2	1	1	1	3	3	3	3	2	2
9	9	9	9	9	9	9	9	2	2
9	9	9	9	9	9	9	9	#NULL!	#NULL!
9	9	9	9	1	9	9	9	2	2
9	9	9	9	1	9	9	9	2	2
9	9	9	9	1	9	9	9	#NULL!	#NULL!
9	9	9	9	9	9	9	9	#NULL!	#NULL!
2	2	2	2	2	2	2	2	2	2
2	2	2	2	2	3	3	3	2	2
3	2	2	2	2	3	1	1	2	2
3	1	3	3	3	2	3	3	2	2
9	9	9	9	9	9	9	9	1	2
2	2	2	2	2	2	2	2	2	2
9	9	9	9	9	9	9	9	2	2
2	3	9	2	2	2	1	1	2	2
1	1	1	1	1	1	1	1	1	2
3	3	3	3	3	2	2	9	1	2
9	9	9	9	9	9	9	9	2	2
1	1	1	1	1	1	1	1	2	2
1	9	9	9	9	9	9	9	2	2
9	9	9	9	9	9	9	9	1	1
2	1	2	1	2	1	1	2	#NULL!	#NULL!
1	1	1	1	1	1	1	1	1	1
9	9	9	9	9	9	9	9	#NULL!	#NULL!
9	9	9	9	9	9	9	9	2	2
9	9	9	9	9	9	9	9	2	2
9	9	9	9	9	9	9	9	2	2
2	2	2	2	2	2	2	2	1	1
3	3	3	3	3	3	3	3	2	2
9	9	9	9	9	9	9	9	2	2
9	9	9	9	9	9	9	9	1	1
9	9	9	9	9	9	9	9	1	1
9	9	9	9	9	9	9	9	2	2

9	9	9	9	9	9	9	9	#NULL!	#NULL!
2	9	2	9	2	2	2	2	2	2
9	9	9	9	9	9	9	9	#NULL!	#NULL!
9	9	9	9	9	9	9	9	1	1
9	9	9	9	9	9	9	9	2	2
9	1	2	9	9	1	9	1	2	2
9	9	9	9	9	9	9	9	2	2
9	9	9	9	9	9	9	9	#NULL!	#NULL!
9	9	2	9	2	9	2	2	2	2
9	9	9	9	9	9	9	9	1	1
9	9	9	9	9	9	9	9	#NULL!	#NULL!
2	9	2	9	9	2	9	2	2	2
2	2	2	2	2	2	2	2	2	2
9	9	9	9	9	9	9	9	#NULL!	#NULL!
2	2	2	2	2	2	2	2	1	1
9	9	9	9	9	9	9	9	#NULL!	#NULL!
9	9	9	9	9	9	9	9	#NULL!	#NULL!
9	9	9	9	9	9	9	9	#NULL!	#NULL!
9	9	9	9	9	9	9	9	#NULL!	#NULL!
9	9	9	9	9	9	9	9	#NULL!	#NULL!
2	2	2	2	2	2	2	2	2	2
2	2	2	2	2	2	2	2	2	2
3	3	3	3	3	3	3	3	2	2
2	1	2	1	2	1	2	1	1	1
2	3	3	3	3	2	2	2	2	1
1	1	1	3	3	3	3	3	2	2
1	1	1	1	1	1	1	1	1	1
2	2	2	2	2	2	2	2	2	2
3	1	1	1	2	3	1	1	2	2
2	2	2	2	2	2	2	2	2	2
9	2	2	3	3	9	2	2	1	2
1	2	3	1	3	2	2	3	2	2
1	2	2	2	2	2	2	2	2	2
9	9	9	9	9	9	9	9	#NULL!	#NULL!
2	3	3	3	2	1	2	1	2	2
3	3	3	3	3	3	3	3	1	1
2	2	2	2	2	2	2	2	2	2
2	2	2	2	2	2	2	2	1	2
2	9	2	2	3	2	2	2	2	2
2	2	2	2	2	2	2	2	2	2
9	9	9	9	9	9	9	9	1	1
9	9	9	9	9	9	9	9	#NULL!	#NULL!
2	2	2	2	2	2	2	2	1	2
9	9	9	9	9	9	9	9	#NULL!	#NULL!
9	9	9	9	9	9	9	9	2	2
2	1	2	2	2	1	2	2	2	2
9	9	9	9	9	9	9	9	1	2

2	2	2	1	1	2	2	2	2	2
1	2	2	2	3	3	3	3	#NULL!	#NULL!
9	9	9	9	9	9	9	9	1	1
9	9	9	9	9	9	9	9	1	1
9	9	9	9	9	9	9	9	1	1
1	1	1	9	9	9	9	9	1	1
2	2	2	2	2	2	2	2	1	1
2	2	2	2	2	2	2	2	1	1
3	3	3	3	3	3	3	3	2	2
1	1	1	1	1	1	1	2	1	1
9	9	9	9	9	9	9	9	2	2
2	2	2	2	2	2	2	2	1	1
9	9	9	9	9	9	9	9	1	1
2	2	2	2	2	2	2	2	1	1
9	9	9	9	9	9	9	9	1	1
1	1	1	1	1	1	1	1	1	1
1	1	2	1	2	1	2	1	1	1
1	2	2	1	1	1	1	2	1	1
2	2	2	2	2	2	2	9	1	1
9	9	9	9	9	9	9	9	2	2
2	2	2	1	1	1	2	1	1	1
2	2	9	2	2	2	2	9	2	2
9	9	9	9	9	9	9	9	#NULL!	#NULL!
2	2	2	2	2	2	2	9	1	1
9	9	9	9	9	9	9	9	#NULL!	#NULL!
1	1	1	2	2	2	1	1	1	1
1	2	2	2	1	1	1	2	1	1
1	1	1	1	1	1	1	9	1	1
9	9	9	9	9	9	9	9	1	1
9	9	9	9	9	9	9	9	1	1
9	9	9	9	9	9	9	9	2	2
9	9	9	9	9	9	9	9	1	1
9	9	9	9	9	9	9	9	1	1
1	1	1	1	3	3	1	3	1	1
2	2	2	2	2	2	2	2	1	1
9	9	9	9	9	9	9	9	#NULL!	#NULL!
1	2	1	1	2	1	2	1	1	1
1	2	1	1	1	1	1	2	1	1
9	9	9	9	9	9	9	9	#NULL!	#NULL!
9	9	9	9	9	9	9	9	2	2
9	9	9	9	9	9	9	9	1	1
9	9	9	9	9	9	9	9	#NULL!	#NULL!
1	1	1	2	2	2	2	9	1	1
9	9	9	9	9	9	9	9	1	1
1	1	1	1	1	2	2	1	1	1
2	2	2	2	2	2	2	2	2	2
9	9	9	9	9	9	9	9	1	1

1	1	1	1	1	1	2	1	1	1
9	9	9	9	9	9	9	9	2	2
3	3	3	3	2	2	2	2	1	1
1	1	1	1	1	1	1	1	1	1
9	9	9	9	9	9	9	9	1	1
9	9	9	9	9	9	9	9	2	#NULL!
9	9	9	9	9	9	9	9	1	1
2	2	2	2	2	2	2	2	1	1
9	9	9	9	9	9	9	9	2	2
9	9	9	9	9	9	9	9	1	1
9	9	9	9	9	9	9	9	2	2
9	9	9	9	9	9	9	9	1	1
9	9	9	9	9	9	9	9	#NULL!	#NULL!
9	9	9	9	9	9	9	9	1	1
9	9	9	9	9	9	9	9	2	2
9	9	9	9	9	9	9	9	#NULL!	#NULL!
9	9	9	9	9	9	9	9	1	1
9	9	9	9	9	9	9	9	#NULL!	#NULL!
2	2	2	2	2	2	2	2	2	2
3	1	1	2	2	3	3	3	1	1
3	1	2	2	2	3	3	3	1	1
3	1	1	2	2	3	3	3	1	1
3	1	1	2	2	2	9	3	1	1
3	1	2	2	2	2	3	3	1	1
3	1	2	2	2	3	3	3	1	1
3	1	1	2	2	2	3	3	1	1
3	1	1	2	2	3	3	3	2	1
3	1	3	2	3	1	2	3	1	1
3	1	1	2	2	3	3	3	1	1
3	1	1	2	2	3	3	3	2	1
3	1	1	2	3	3	3	3	2	1
3	1	1	2	2	3	3	3	2	1
3	1	1	2	2	3	3	3	1	1
3	1	3	2	2	3	3	3	2	1
3	1	1	2	2	3	3	3	1	1
3	1	2	2	2	3	3	3	2	1
3	1	2	2	3	3	3	3	1	1
3	1	2	2	2	3	3	3	#NULL!	#NULL!
3	1	1	2	2	3	3	3	1	1
3	1	2	2	9	3	3	3	1	1
1	1	2	2	2	3	3	3	1	1
3	1	2	2	2	3	3	3	1	1
3	1	2	2	2	3	3	3	1	1
3	1	1	2	2	3	3	3	1	1
9	9	9	9	9	9	9	9	#NULL!	#NULL!
9	9	9	9	9	9	9	9	#NULL!	#NULL!
9	9	9	9	9	9	9	9	#NULL!	#NULL!

9	9	9	9	9	9	9	9	#NULL!	#NULL!
9	9	9	9	9	9	9	9	#NULL!	#NULL!
9	9	9	9	9	9	9	9	#NULL!	#NULL!
9	9	9	9	9	9	9	9	#NULL!	#NULL!
9	9	9	9	9	9	9	9	#NULL!	#NULL!
9	9	9	9	9	9	9	9	#NULL!	#NULL!
9	9	9	9	9	9	9	9	#NULL!	#NULL!
9	9	9	9	9	9	9	9	#NULL!	#NULL!
9	9	9	9	9	9	9	9	#NULL!	#NULL!
9	9	9	9	9	9	9	9	#NULL!	#NULL!
9	9	9	9	9	9	9	9	#NULL!	#NULL!
9	9	9	9	9	9	9	9	#NULL!	#NULL!
9	9	9	9	9	9	9	9	#NULL!	#NULL!
9	9	9	9	9	9	9	9	#NULL!	#NULL!
9	9	9	9	9	9	9	9	#NULL!	#NULL!
9	9	9	9	9	9	9	9	#NULL!	#NULL!
9	9	9	9	9	9	9	9	#NULL!	#NULL!
9	9	9	9	9	9	9	9	#NULL!	#NULL!
9	9	9	9	9	9	9	9	#NULL!	#NULL!
3	1	1	2	2	3	2	2	1	#NULL!
3	1	1	2	2	3	3	3	1	1
3	1	1	2	2	3	3	3	1	1
3	1	1	2	2	3	3	3	1	1
3	1	1	2	2	3	3	3	1	1
3	1	1	2	2	1	2	3	1	1
3	1	2	2	2	3	3	3	1	2
3	1	2	2	2	3	3	3	1	1
3	1	2	2	2	3	3	3	1	2
3	1	2	2	3	3	3	3	1	1
3	1	2	2	3	3	3	3	1	1
3	1	1	2	2	3	3	3	1	1
3	1	1	2	2	3	3	3	1	1
3	1	1	2	2	3	3	3	1	1
3	1	1	2	2	3	3	3	1	1
3	1	1	2	2	3	3	3	1	1
3	1	1	2	9	3	3	3	2	2
3	1	1	2	2	3	3	3	1	1
3	1	1	2	2	3	3	3	1	1
3	1	1	2	2	3	3	3	1	1
3	1	1	9	2	2	3	3	2	1
3	1	1	2	2	3	3	3	1	1
3	1	1	2	2	3	3	3	1	2
3	1	1	2	2	3	3	3	1	1
3	1	1	2	2	3	3	3	1	1
3	1	1	2	2	3	3	3	1	1
3	1	1	2	2	3	3	3	1	1
1	1	2	2	3	3	3	3	#NULL!	1

3	1	1	2	2	3	3	3	1	1
2	3	3	1	2	1	2	3	#NULL!	1
3	1	1	1	3	3	3	3	2	1
3	1	2	2	3	3	3	3	1	1
3	1	1	2	3	3	3	3	1	1
3	1	1	2	2	3	3	3	1	1
3	1	1	2	2	3	3	3	1	1
3	1	1	2	2	3	3	3	1	1
3	1	1	2	2	3	3	3	1	1
3	1	1	2	2	3	3	3	1	1
3	1	1	2	2	3	3	3	1	1
3	1	3	2	2	3	3	3	1	1
3	1	2	2	2	3	3	3	1	1
3	1	2	2	1	3	3	3	1	1
3	1	1	2	2	3	3	3	2	1
3	1	1	2	2	3	3	3	1	1
3	1	1	2	2	3	3	3	2	1
3	1	1	2	2	3	3	3	1	1
3	1	2	2	2	3	3	3	1	1
3	1	1	2	2	3	3	3	1	1
3	1	1	2	2	2	3	3	1	1
3	1	1	9	2	2	3	3	2	1
3	1	1	2	2	3	3	3	1	1
3	1	1	2	2	3	3	3	1	1
3	1	1	2	2	3	3	3	1	1
3	1	1	1	2	3	3	3	1	1
3	1	1	2	2	3	3	3	1	1
3	1	1	2	2	3	3	9	1	1
3	1	1	2	2	3	3	3	1	1
3	1	1	9	9	3	3	3	1	1
1	9	2	2	3	3	3	3	1	1
3	1	1	2	2	3	3	3	1	#NULL!
3	1	1	2	2	3	3	3	#NULL!	#NULL!
3	1	1	2	2	3	3	3	1	1
9	9	9	9	9	9	9	9	#NULL!	#NULL!
3	1	1	2	2	3	3	3	2	1
3	1	1	2	2	3	3	3	1	#NULL!
3	1	1	2	2	3	3	3	#NULL!	#NULL!
3	1	1	2	2	3	3	3	1	#NULL!
3	1	1	2	2	3	3	3	2	1
3	1	1	2	2	3	3	3	1	1
3	1	1	2	2	3	3	3	1	1
3	1	1	2	2	3	3	3	2	2
3	1	1	2	2	3	3	3	1	1
3	1	1	2	2	3	3	3	1	1
3	1	2	2	2	3	3	3	1	1
3	1	2	2	2	3	3	3	1	1

3	1	1	2	2	3	3	3	1	1
3	1	2	2	2	3	3	3	1	1
3	1	1	2	2	3	3	3	1	1
3	1	1	2	2	3	3	3	1	1
3	1	2	2	2	3	3	3	1	1
3	1	2	2	2	3	3	3	1	1
2	1	2	2	2	2	3	3	1	1
3	1	2	2	2	3	3	9	1	1
3	1	9	2	2	3	3	3	1	1
3	1	1	2	2	3	3	3	1	1
3	1	1	2	2	3	3	3	1	1
3	1	1	2	2	3	3	3	1	1
3	1	1	2	2	3	3	3	1	1
3	1	2	2	2	3	3	3	1	1
3	1	1	2	2	3	3	3	1	1
3	1	1	2	2	3	3	3	1	1
3	1	1	2	2	3	3	3	1	1
3	1	1	2	2	3	3	3	1	1
3	1	1	2	2	3	3	3	1	1
3	1	2	2	2	3	3	3	#NULL!	#NULL!
3	1	2	2	2	3	3	3	2	1
3	1	1	2	2	3	3	3	1	1
3	1	1	2	2	3	3	3	1	1
3	1	2	1	3	3	3	3	1	1
9	1	1	2	2	3	3	3	1	1
3	1	1	2	2	3	3	3	1	1
3	1	1	2	2	3	3	3	1	1
3	1	3	2	2	3	3	3	1	1
3	1	2	2	2	3	3	3	1	1
1	1	2	2	3	3	3	3	1	1
3	1	2	2	2	3	3	3	1	1
3	1	1	2	2	3	3	3	1	1
3	1	1	2	2	3	3	3	1	1
3	1	1	2	2	3	3	3	1	1
3	1	1	2	2	3	3	3	1	1
3	1	1	2	3	3	3	3	1	1
3	1	1	2	3	3	3	3	1	1
3	1	1	2	2	3	3	3	1	1
3	1	1	2	2	3	3	3	1	1
1	1	9	2	2	3	3	3	1	1
1	1	2	2	2	3	3	3	1	1
9	9	9	9	9	9	9	9	#NULL!	#NULL!
3	1	2	2	2	3	3	3	1	1
3	1	1	2	2	3	3	3	1	1
3	1	1	2	2	3	3	3	1	1
3	1	2	2	2	3	3	3	1	1
3	1	1	2	2	3	3	3	1	1

3	1	1	2	2	3	3	3	1	1
3	1	1	2	2	3	3	3	1	1
3	1	1	2	2	3	3	3	1	1
3	1	1	3	2	3	3	3	1	1
3	1	1	2	2	3	3	3	1	1
3	1	1	2	2	3	3	3	1	1
3	1	2	2	2	3	3	3	1	1
3	1	1	2	2	3	3	3	#NULL!	1
3	1	2	2	2	3	3	3	1	1
9	9	9	9	9	9	9	9	#NULL!	#NULL!
3	1	1	2	2	3	3	3	1	1
3	1	1	2	2	3	3	3	1	1
3	1	1	2	2	3	3	3	1	1
3	1	2	2	3	3	3	3	1	1
3	1	2	2	2	3	3	3	1	1
3	1	2	2	2	3	3	3	1	1
3	1	2	2	2	3	3	3	1	1
3	1	2	2	2	3	3	3	1	1
3	1	2	1	2	2	3	3	1	1
3	1	1	2	2	3	3	3	1	1
3	1	1	2	2	3	3	3	1	1
3	1	1	2	2	3	3	3	1	1
3	1	1	2	2	3	3	3	1	1
3	1	1	2	2	3	3	3	1	1
3	1	2	2	2	3	3	3	1	1
3	1	1	2	2	3	3	3	1	1
3	1	2	2	2	3	3	3	#NULL!	#NULL!
9	9	9	9	9	9	9	9	#NULL!	#NULL!
9	9	9	9	9	9	9	9	#NULL!	#NULL!
9	9	9	9	9	9	9	9	#NULL!	#NULL!
9	9	9	9	9	9	9	9	#NULL!	#NULL!
9	9	9	9	9	9	9	9	#NULL!	#NULL!
9	9	9	9	9	9	9	9	#NULL!	#NULL!
9	9	9	9	9	9	9	9	#NULL!	#NULL!
9	9	9	9	9	9	9	9	#NULL!	#NULL!
9	9	9	9	9	9	9	9	#NULL!	#NULL!
9	9	9	9	9	9	9	9	#NULL!	#NULL!
9	9	9	9	9	9	9	9	#NULL!	#NULL!
9	9	9	9	9	9	9	9	#NULL!	#NULL!
9	9	9	9	9	9	9	9	#NULL!	#NULL!
9	9	9	9	9	9	9	9	#NULL!	#NULL!
9	9	9	9	9	9	9	9	#NULL!	#NULL!
9	9	9	9	9	9	9	9	#NULL!	#NULL!
9	9	9	9	9	9	9	9	#NULL!	#NULL!
9	9	9	9	9	9	9	9	#NULL!	#NULL!
9	9	9	9	9	9	9	9	#NULL!	#NULL!
9	9	9	9	9	9	9	9	#NULL!	#NULL!

9	9	9	9	9	9	9	9	#NULL!	#NULL!
9	9	9	9	9	9	9	9	#NULL!	#NULL!
9	9	9	9	9	9	9	9	#NULL!	#NULL!
9	9	9	9	9	9	9	9	#NULL!	#NULL!
9	9	9	9	9	9	9	9	#NULL!	#NULL!
9	9	9	9	9	9	9	9	#NULL!	#NULL!
9	9	9	9	9	9	9	9	#NULL!	#NULL!
9	9	9	9	9	9	9	9	#NULL!	#NULL!
9	9	9	9	9	9	9	9	#NULL!	#NULL!
9	9	9	9	9	9	9	9	#NULL!	#NULL!
9	9	9	9	9	9	9	9	#NULL!	#NULL!
9	9	9	9	9	9	9	9	#NULL!	#NULL!
9	9	9	9	9	9	9	9	#NULL!	#NULL!
2	3	2	2	1	2	2	3	1	1
2	2	2	2	2	2	2	2	2	2
2	2	2	2	2	2	2	2	2	2
1	2	1	1	1	3	3	2	#NULL!	#NULL!
1	2	2	2	2	1	2	1	1	1
1	1	1	1	1	1	1	1	#NULL!	#NULL!
3	2	1	1	2	1	1	1	1	1
9	9	9	9	9	9	9	9	1	1
2	3	2	2	2	2	2	2	2	2
9	9	9	9	9	9	9	9	#NULL!	#NULL!
2	2	2	2	2	2	3	2	2	2
9	1	9	9	9	9	9	9	2	2
2	2	3	3	3	2	2	2	1	1
9	9	9	9	9	9	9	9	#NULL!	#NULL!
1	1	1	1	1	1	1	1	2	2
3	2	3	2	3	2	2	2	2	2
1	1	1	3	1	2	1	3	1	1
3	3	3	3	3	3	3	3	2	2
2	3	2	2	2	3	2	2	2	2
9	9	9	9	9	9	9	9	1	1
1	1	1	1	1	3	1	3	1	1
9	9	9	9	9	9	9	9	1	1
9	9	9	9	9	9	9	9	1	1
1	2	2	2	1	3	3	1	1	2
2	1	2	1	2	1	2	1	2	2
2	1	2	1	2	1	1	2	1	1
3	1	2	2	2	2	1	1	1	1
9	9	9	9	9	9	9	9	#NULL!	#NULL!
9	9	9	9	9	9	9	9	#NULL!	#NULL!
9	9	9	9	9	9	9	9	1	1
1	1	1	1	1	1	1	9	1	1
9	9	9	9	9	9	9	9	#NULL!	#NULL!
9	9	9	9	9	9	9	9	#NULL!	#NULL!
9	9	9	9	9	9	9	9	2	2

1	1	1	1	1	1	1	1	1	2
9	9	9	9	9	9	9	9	#NULL!	#NULL!
2	2	2	3	3	1	3	2	2	2
2	1	1	2	3	2	2	2	1	1
3	1	1	1	3	1	2	2	1	1
9	9	9	9	9	9	9	9	1	1
2	2	2	2	2	2	2	2	2	2
2	2	2	2	2	2	2	2	2	1
2	2	2	2	2	2	2	2	2	2
1	1	1	1	1	2	2	2	1	2
2	2	2	2	2	2	2	2	1	2
9	9	9	9	9	9	9	9	#NULL!	#NULL!
9	9	9	9	9	9	9	9	2	2
2	2	2	2	2	2	2	2	2	2
2	1	1	2	3	3	1	3	2	2
1	1	2	1	3	2	3	3	2	2
1	1	1	1	1	1	1	3	2	2
2	2	2	2	2	2	2	2	1	1
2	1	9	1	1	2	1	1	1	1
9	9	9	9	9	9	9	9	#NULL!	#NULL!
2	2	2	2	2	2	2	2	1	2
2	2	2	2	2	2	2	2	#NULL!	#NULL!
2	2	2	2	2	2	2	2	2	2
2	2	2	2	2	2	2	2	2	2
2	2	2	2	2	2	2	2	#NULL!	#NULL!
1	1	2	2	2	2	1	1	2	1
2	2	2	2	2	2	2	2	2	2
2	3	2	1	2	3	2	1	2	2
9	9	9	9	9	9	9	9	2	2
3	1	1	2	2	3	3	3	1	1
3	1	1	1	3	3	3	3	1	1
1	1	1	1	2	2	3	3	1	2
2	1	3	2	3	3	2	2	1	2
1	1	1	1	3	3	3	3	1	1
2	9	3	2	3	2	1	3	1	1
9	9	9	9	9	9	9	9	2	2
3	1	1	2	3	2	2	3	2	2
1	1	1	9	9	9	9	9	1	2
9	9	9	9	9	9	9	9	2	#NULL!
2	2	2	2	2	2	2	2	2	2
3	3	3	3	3	3	3	3	1	2
2	2	2	2	2	2	2	2	1	2
2	3	1	2	3	1	2	3	1	1
9	9	9	9	9	9	9	9	1	1
1	1	1	1	1	1	1	1	1	1
1	2	2	2	2	2	2	1	1	1
2	1	3	3	2	3	3	3	2	2

2	2	2	2	2	2	2	2	2	2
2	3	2	2	2	3	2	9	2	2
2	2	2	2	2	2	2	2	1	2
9	9	9	9	9	9	9	9	2	1
9	9	9	9	9	9	9	9	1	1
2	2	2	2	2	2	2	2	1	1
3	1	3	2	3	3	3	3	2	2
2	2	3	3	1	1	1	1	2	2
9	9	9	9	9	9	9	9	2	2
2	2	3	1	2	2	3	2	1	2
2	2	2	2	2	2	2	2	2	2
2	2	2	2	2	2	2	2	2	2
1	1	1	1	1	1	1	1	2	2
2	2	2	2	2	2	2	2	#NULL!	#NULL!
9	9	9	9	9	9	9	9	1	1
2	2	2	2	2	3	2	2	2	2
3	3	3	3	3	3	3	3	1	1
2	2	2	2	2	2	2	2	1	1
2	2	2	2	2	2	2	2	#NULL!	#NULL!
2	3	1	2	3	1	2	3	2	1
2	1	2	1	2	1	1	1	2	2
1	1	1	1	1	1	2	2	2	2
9	9	9	9	9	9	9	9	2	2
9	9	9	9	9	9	9	9	1	1
3	1	3	3	3	3	3	3	1	1
9	9	9	9	9	9	9	9	#NULL!	#NULL!
9	9	9	9	9	9	9	9	2	2
2	2	2	2	2	2	2	2	2	2
9	2	1	2	1	1	1	1	1	1
1	3	2	2	3	1	2	3	2	2
2	1	2	3	2	2	3	3	2	2
1	1	1	1	1	1	1	1	1	2
2	2	2	2	2	2	2	3	2	2
9	1	2	2	1	9	2	1	1	1
9	9	9	9	9	9	9	9	1	1
2	2	2	2	2	2	2	9	#NULL!	#NULL!
9	9	9	9	9	9	9	9	#NULL!	#NULL!
9	9	9	9	9	9	9	9	#NULL!	#NULL!
9	9	9	9	9	9	9	9	#NULL!	#NULL!
1	1	1	1	1	1	1	1	1	1
3	3	3	2	3	2	2	2	2	2
3	1	3	3	3	3	2	3	1	1
2	2	2	1	2	3	3	2	2	2
9	9	9	9	9	9	9	9	#NULL!	#NULL!
9	9	9	9	9	9	9	9	#NULL!	#NULL!
9	9	9	9	9	9	9	9	#NULL!	#NULL!
1	1	1	1	1	1	1	1	1	1

9	9	9	9	9	9	9	9	#NULL!	#NULL!
9	9	9	9	9	9	9	9	#NULL!	#NULL!
9	9	9	9	9	9	9	9	1	2
9	9	9	9	9	9	9	9	#NULL!	#NULL!
9	9	9	9	9	9	9	9	#NULL!	#NULL!
9	9	9	9	9	9	9	9	1	1
3	2	2	3	1	2	1	3	2	1
9	9	9	9	9	9	9	9	#NULL!	#NULL!
1	1	1	3	3	3	3	3	1	1
2	3	1	2	3	1	2	3	2	2
2	3	2	1	2	3	1	2	1	2
2	2	2	2	2	2	2	2	2	2
2	2	2	2	2	2	2	2	2	2
9	9	9	9	9	9	9	9	#NULL!	#NULL!
2	2	2	2	2	2	2	2	2	2
2	2	2	2	2	2	2	9	2	2
1	1	1	1	1	1	1	1	1	2
3	2	1	3	3	2	1	3	2	2
2	3	2	1	2	2	2	2	1	1
9	9	9	9	9	9	9	9	#NULL!	#NULL!
3	1	2	3	2	1	2	3	2	2
3	1	2	1	3	1	3	3	1	2
2	2	2	2	2	2	2	2	2	2
2	2	2	3	3	3	2	2	2	2
2	3	3	2	3	3	3	2	#NULL!	#NULL!
9	9	9	9	9	9	9	9	#NULL!	#NULL!
1	1	1	1	1	1	1	1	2	2
9	9	9	9	9	9	9	9	2	2
3	3	3	3	3	3	3	3	2	2
2	2	9	2	3	2	2	3	2	2
2	2	2	2	2	2	2	2	2	2
9	1	9	9	9	9	9	9	2	2
9	1	9	9	9	9	9	3	2	2
2	2	2	2	2	2	2	2	#NULL!	#NULL!
2	2	2	2	2	3	3	3	2	1
1	2	1	3	2	1	2	1	#NULL!	#NULL!
2	2	2	2	2	2	2	2	#NULL!	#NULL!
2	1	1	1	2	2	2	3	1	1
2	2	2	2	2	2	2	2	2	2
9	9	9	9	9	9	9	9	2	2
2	2	2	1	2	2	1	2	#NULL!	#NULL!
2	2	2	2	2	2	2	2	1	2
2	2	2	2	2	2	2	3	2	2
2	2	2	2	2	2	2	2	2	2
2	2	2	2	2	2	2	2	1	2
9	1	9	1	3	9	9	9	2	2
2	2	2	2	2	2	2	2	2	2

2	2	2	2	2	2	2	2	1	1
2	2	2	2	2	2	2	2	2	2
3	1	3	1	3	3	3	3	1	2
1	1	2	2	3	3	3	3	2	2
2	2	2	2	2	2	2	2	2	2
2	2	2	3	3	3	2	2	2	2
2	3	1	2	2	1	1	2	2	2
2	2	1	2	2	2	2	9	1	1
3	1	3	3	3	3	3	3	1	1
3	3	3	3	3	3	3	3	2	2
2	2	2	2	2	2	2	2	1	1
2	2	2	2	2	3	3	3	2	2
1	1	1	3	3	3	3	3	1	1
2	1	1	3	3	3	3	3	1	1
1	1	2	1	2	1	2	3	2	2
2	1	2	1	2	2	2	2	2	2
2	2	2	2	3	3	3	3	2	2
1	3	2	1	2	1	3	2	#NULL!	#NULL!
1	2	1	2	1	2	1	2	2	2
3	1	3	1	3	3	3	3	2	2
3	1	3	1	3	3	3	3	2	2
9	9	9	9	9	9	9	9	1	1
2	2	2	2	2	2	2	2	2	2
2	2	2	2	2	2	2	2	1	1
2	2	2	2	2	2	2	2	1	1
3	1	1	1	3	3	3	3	2	2
1	1	1	2	1	2	2	3	2	2
1	1	1	2	1	2	2	3	1	2
3	1	3	1	3	3	3	1	1	1
2	1	3	1	3	3	3	3	1	1
1	2	9	3	3	3	3	3	1	1
1	1	2	2	3	1	2	2	2	2
1	1	1	1	2	1	2	1	1	1
9	9	9	9	9	9	9	9	#NULL!	#NULL!
9	9	9	9	9	9	9	9	2	2
3	3	9	1	3	9	1	3	1	1
2	2	2	1	2	2	2	2	#NULL!	#NULL!
2	1	2	1	2	1	2	1	1	1
1	2	2	2	2	2	2	2	1	1
2	2	2	3	3	2	3	2	1	1
1	2	3	2	1	2	3	2	1	1
9	9	9	9	9	9	9	9	#NULL!	#NULL!
9	9	9	9	9	9	9	9	#NULL!	#NULL!
1	1	1	1	1	1	1	1	1	1
9	9	9	9	9	9	9	9	1	1
9	9	9	9	9	9	9	9	#NULL!	#NULL!
9	9	9	9	9	9	9	9	#NULL!	#NULL!

2	9	9	9	9	9	9	9	#NULL!	#NULL!
1	2	3	1	1	2	1	1	1	1
9	9	9	9	9	9	9	9	1	1
1	2	2	3	3	2	2	2	2	2
2	3	3	3	3	2	2	2	2	2
2	3	1	2	3	1	2	3	1	2
1	1	2	2	2	2	2	2	2	2
9	9	9	9	9	9	9	9	2	#NULL!
1	2	2	1	2	2	2	2	1	1
9	9	9	9	9	9	9	9	#NULL!	#NULL!
1	1	1	1	1	1	1	1	1	1
1	1	1	3	1	3	3	3	2	2
9	9	9	9	9	9	9	9	#NULL!	#NULL!
9	9	9	9	9	9	9	9	#NULL!	#NULL!
1	1	1	1	1	1	1	1	1	1
9	9	9	9	9	9	9	9	#NULL!	#NULL!
9	9	9	9	9	9	9	9	#NULL!	#NULL!
2	2	2	2	2	2	2	2	2	1
2	2	2	2	2	2	2	2	2	2
2	1	2	2	1	2	1	1	1	1
2	2	2	2	2	2	2	2	2	2
2	2	2	2	2	2	2	2	1	1
2	1	1	2	2	2	3	1	1	2
3	2	1	2	1	2	3	2	1	2
1	1	1	1	1	1	2	2	2	2
9	9	9	9	9	9	9	9	1	2
3	1	3	3	3	3	3	3	2	#NULL!
1	2	3	1	1	2	2	3	#NULL!	#NULL!
9	9	9	9	9	9	9	9	1	1
2	2	2	3	2	2	2	3	2	2
1	1	2	1	2	1	2	2	2	1
2	2	2	2	2	2	2	9	2	2
2	2	2	2	2	2	2	2	#NULL!	#NULL!
2	2	2	2	2	2	2	2	2	2
2	2	2	2	2	2	2	2	1	1
2	2	2	2	2	2	2	2	2	2
3	1	3	2	3	2	1	2	2	2
9	9	9	9	9	9	9	9	1	2
9	9	9	9	9	2	9	9	2	2
3	1	1	3	3	3	3	3	#NULL!	#NULL!
3	2	3	2	3	2	3	2	2	2
9	1	9	1	9	9	9	3	1	2
2	2	2	1	2	2	2	1	#NULL!	#NULL!
9	9	9	9	9	9	9	9	2	2
3	3	3	2	2	2	2	2	1	1
9	9	9	9	9	9	9	9	#NULL!	#NULL!
2	2	2	2	2	2	2	2	2	2

1	3	1	3	1	2	2	1	2	2
9	9	9	9	9	9	9	9	2	2
1	1	1	1	1	1	1	1	#NULL!	#NULL!
3	9	2	2	3	3	3	3	2	2
2	3	9	1	3	2	1	9	1	2
2	1	2	2	2	2	2	2	1	2
1	1	1	3	1	2	3	3	2	2
9	9	9	9	9	9	9	9	#NULL!	#NULL!
2	2	2	2	2	2	2	2	1	2
3	3	3	3	3	3	3	3	2	2
2	2	2	2	2	2	2	2	2	2
2	2	2	2	2	2	2	2	2	2
2	9	3	2	3	1	2	3	1	2
2	3	3	3	2	2	3	9	2	2
3	2	2	2	1	2	1	1	1	2
3	2	2	2	1	2	1	1	1	2
2	2	2	2	2	2	2	2	2	2
2	2	2	2	2	2	2	2	1	2
9	9	9	9	9	9	9	9	#NULL!	#NULL!
9	9	9	9	9	9	9	9	#NULL!	#NULL!
2	2	3	3	3	3	3	3	2	2
2	1	2	3	3	3	1	3	1	1
9	9	9	9	9	9	9	9	1	2
9	9	9	9	9	9	9	9	#NULL!	#NULL!
3	3	3	3	3	3	3	3	2	2
9	9	9	9	9	9	9	9	2	2
9	9	9	9	9	9	9	9	#NULL!	#NULL!
9	9	9	9	9	9	9	9	#NULL!	#NULL!
2	1	3	2	1	3	1	2	#NULL!	#NULL!
1	1	1	1	1	1	1	3	2	2
2	2	2	2	2	2	2	2	2	2
1	1	1	2	3	1	2	2	2	2
2	2	2	2	2	2	2	2	2	2
2	2	2	2	2	2	2	2	2	2
9	9	3	3	3	3	3	3	2	2
3	1	3	1	3	3	3	3	1	2
3	1	2	1	3	3	1	2	1	2
1	2	9	1	2	2	2	2	2	2
2	3	1	2	1	1	1	2	2	2
3	1	2	2	3	3	3	2	1	1
2	3	3	3	2	2	2	2	1	1
3	3	3	3	3	9	3	3	2	2
9	9	9	9	9	9	9	9	1	1
9	1	9	9	9	9	9	9	1	1
3	1	2	3	2	1	3	2	1	2
2	2	2	2	2	2	2	2	2	2
2	2	2	2	2	2	2	2	2	2

2	3	1	1	2	9	2	2	1	2
9	9	9	9	9	9	9	9	#NULL!	#NULL!
9	9	9	9	9	9	9	9	#NULL!	#NULL!
9	9	9	9	9	9	9	9	#NULL!	#NULL!
9	9	9	9	9	9	9	9	#NULL!	#NULL!
9	9	9	9	9	9	9	9	1	1
9	9	9	9	9	9	9	9	#NULL!	#NULL!
9	9	9	9	9	9	9	9	#NULL!	#NULL!
9	9	9	9	9	9	9	9	#NULL!	#NULL!
9	9	9	3	9	9	3	3	2	2
2	2	2	2	2	2	2	2	1	2
2	2	1	1	2	1	1	2	1	2
3	1	3	1	3	3	3	3	#NULL!	#NULL!
2	2	2	2	2	2	2	2	1	2
2	2	2	2	2	2	2	2	1	1
9	9	9	9	9	9	9	9	#NULL!	#NULL!
1	1	1	1	1	1	2	2	1	1
2	2	2	2	2	2	2	2	2	2
2	2	2	2	2	2	2	2	2	1
3	3	1	3	1	3	1	2	2	2
2	1	3	2	1	3	1	2	1	2
1	3	3	3	3	2	3	3	1	2
1	1	1	1	1	1	1	1	1	2
2	2	2	2	2	2	2	9	1	1
2	2	2	2	2	2	2	9	2	2
2	2	2	2	2	2	2	2	1	2
2	2	2	2	2	2	2	2	1	1
2	1	2	1	2	1	2	1	1	1
9	9	9	9	9	9	9	9	2	2
2	2	2	2	2	2	2	2	2	2
9	9	9	9	9	9	9	9	#NULL!	#NULL!
2	2	2	2	2	2	2	2	2	2
2	2	3	3	2	1	1	1	1	1
9	1	9	9	9	9	9	9	1	1
3	3	3	3	3	3	3	3	#NULL!	#NULL!
2	2	2	2	2	2	2	2	2	2
2	2	2	2	2	2	2	2	2	#NULL!
2	2	2	2	2	2	2	2	1	2
2	1	3	3	3	3	1	1	1	1
9	9	9	9	9	9	9	9	#NULL!	#NULL!
1	1	9	1	1	2	2	2	2	2
2	2	2	2	2	2	2	2	2	2
2	2	2	2	2	2	2	2	2	2
2	1	2	1	2	2	1	3	1	1
2	1	1	2	2	2	2	3	1	1
9	9	9	9	9	9	9	9	#NULL!	#NULL!
9	9	9	9	9	9	9	9	1	2

9	9	9	9	9	9	9	9	#NULL!	#NULL!
2	2	2	2	2	2	2	2	1	2
9	9	9	9	9	9	9	9	1	1
9	9	9	9	9	9	9	9	#NULL!	#NULL!
9	9	9	9	9	9	9	9	#NULL!	#NULL!
9	9	9	9	9	9	9	9	#NULL!	#NULL!
9	9	9	9	9	9	9	9	#NULL!	#NULL!
9	9	9	9	9	9	9	9	#NULL!	#NULL!
9	9	9	9	9	9	9	9	#NULL!	#NULL!
9	9	9	9	9	9	9	9	#NULL!	#NULL!
9	9	9	9	9	9	9	9	#NULL!	#NULL!
9	9	9	9	9	9	9	9	#NULL!	#NULL!
9	9	9	9	9	9	9	9	#NULL!	#NULL!
9	9	9	9	9	9	9	9	#NULL!	#NULL!
9	9	9	9	9	9	9	9	#NULL!	#NULL!
9	9	9	9	9	9	9	9	#NULL!	#NULL!
9	9	9	9	9	9	9	9	#NULL!	#NULL!
9	9	9	9	9	9	9	9	#NULL!	#NULL!
9	9	9	9	9	9	9	9	#NULL!	#NULL!
9	9	9	9	9	9	9	9	#NULL!	#NULL!
9	9	9	9	9	9	9	9	#NULL!	#NULL!
9	9	9	9	9	9	9	9	#NULL!	#NULL!
9	9	9	9	9	9	9	9	#NULL!	#NULL!
2	3	2	1	2	3	2	1	2	2
2	1	2	2	2	2	2	2	1	2
2	2	2	2	2	2	2	9	1	1
2	1	2	1	2	1	2	3	1	1
9	9	9	9	9	9	9	9	1	2
2	9	2	1	2	3	3	3	1	1
2	2	2	2	2	2	2	2	1	1
3	1	2	1	1	2	1	3	1	1
1	1	2	1	1	1	2	3	2	2
2	1	1	3	3	2	1	3	1	2
1	1	3	1	1	3	3	3	1	1
3	1	3	1	3	3	3	3	1	1
2	1	1	2	2	1	3	3	1	1
2	1	2	2	3	3	3	3	1	1
2	1	1	2	2	2	2	2	1	1
2	1	2	2	3	3	2	3	1	1
9	9	9	9	9	9	9	2	1	#NULL!
1	1	3	1	3	2	3	3	1	1
2	2	2	2	2	2	2	2	1	1
9	9	2	9	9	9	9	9	1	2
2	1	3	1	1	3	3	3	1	1
1	1	3	1	3	3	3	3	1	1
2	2	2	2	2	2	2	2	2	2
2	3	9	1	3	1	3	1	1	1

2	1	3	1	3	3	3	3	1	1
3	1	1	3	3	3	3	3	1	1
3	1	1	2	1	3	3	3	1	1
2	1	3	1	2	2	3	3	1	1
2	3	1	2	3	1	2	3	2	1
9	9	9	9	9	9	9	9	2	1
3	1	3	1	3	3	2	3	1	1
9	9	9	9	9	9	9	9	1	1
2	2	2	2	2	2	2	2	2	2
2	2	2	2	2	2	2	2	2	2
9	9	1	9	9	9	9	9	1	1
9	9	1	9	9	9	9	9	1	1
2	3	1	1	2	3	1	1	1	1
3	1	3	9	2	9	9	9	#NULL!	#NULL!
9	9	9	9	9	9	9	9	1	1
9	9	9	9	9	9	9	9	#NULL!	#NULL!
9	9	9	9	9	9	9	9	#NULL!	#NULL!
9	9	9	9	9	9	9	9	2	1
1	9	9	9	9	9	9	9	1	1
3	9	1	1	3	3	3	3	1	1
9	9	9	9	9	9	9	9	1	1
2	1	9	9	3	9	9	2	1	2
9	9	9	9	9	9	9	9	1	1
9	9	9	9	9	9	9	9	1	2
9	9	9	9	9	9	9	9	1	1
1	2	3	9	1	2	3	2	1	1
9	9	9	9	9	9	9	9	1	1
3	1	9	1	3	3	3	3	2	2
3	1	2	2	3	3	3	3	2	1
9	9	9	9	9	9	9	9	#NULL!	#NULL!
2	1	1	2	3	2	2	3	1	1
3	1	1	3	3	3	3	3	1	1
3	3	3	3	3	3	3	3	2	2
9	1	3	1	3	3	3	1	2	2
1	1	1	1	3	3	1	3	1	1
9	9	9	9	9	9	9	3	1	2
2	2	1	1	2	2	2	3	1	1
1	1	1	1	2	2	2	3	1	2
3	3	2	3	2	3	2	3	2	2
1	1	1	1	3	3	3	1	2	2
2	1	2	2	2	2	3	3	1	1
1	1	1	1	1	1	1	1	1	1
9	9	9	9	9	9	9	9	1	1
3	2	1	1	2	2	3	3	2	2
1	1	2	1	3	3	3	3	#NULL!	1
2	1	3	2	3	3	3	3	1	1
3	1	3	1	3	3	3	3	1	1

9	9	9	9	9	9	9	9	1	1
2	3	2	2	2	2	2	2	1	1
9	9	9	9	9	9	9	9	1	1
3	1	1	3	2	3	3	3	1	1
9	9	9	9	9	9	9	9	#NULL!	#NULL!
1	1	3	1	3	3	3	3	1	1
3	1	1	1	3	3	3	3	1	1
9	9	9	9	9	9	9	9	#NULL!	#NULL!
9	9	9	9	9	9	9	2	1	1
2	2	2	2	2	2	2	2	2	2
1	1	1	1	3	1	2	1	1	1
9	9	9	9	9	9	9	9	1	1
3	2	3	1	2	2	2	1	1	1
2	9	1	9	1	9	9	2	#NULL!	#NULL!
9	9	9	9	9	9	9	9	#NULL!	#NULL!
3	3	9	2	2	2	1	2	1	2
9	9	9	9	9	9	9	9	1	1
9	9	9	9	9	9	9	9	#NULL!	#NULL!
2	3	3	3	3	3	3	9	2	2
2	1	2	2	2	2	3	3	1	1
9	9	9	9	9	9	9	9	1	1
1	1	1	1	2	1	1	3	1	1
2	2	1	2	3	1	3	2	2	2
9	9	9	9	9	9	9	9	1	1
1	1	2	2	2	2	2	2	1	2
9	9	9	9	9	9	9	9	#NULL!	#NULL!
9	9	9	9	9	9	9	9	1	1
3	1	3	1	1	1	1	1	1	1
2	1	1	2	2	2	3	3	2	2
3	1	1	2	3	3	3	3	1	1
9	9	9	9	9	9	9	9	#NULL!	#NULL!
9	9	9	9	9	9	9	9	#NULL!	#NULL!
9	9	9	9	9	9	9	9	#NULL!	#NULL!
9	9	9	9	9	9	9	9	#NULL!	#NULL!
9	9	9	9	9	9	9	9	#NULL!	#NULL!
9	9	9	9	9	9	9	9	#NULL!	#NULL!
9	9	9	9	9	9	9	9	#NULL!	#NULL!
9	9	9	9	9	9	9	9	#NULL!	#NULL!
9	9	9	9	9	9	9	9	#NULL!	#NULL!
9	9	9	9	9	9	9	9	#NULL!	#NULL!
9	9	9	9	9	9	9	9	#NULL!	#NULL!
9	9	9	9	9	9	9	9	#NULL!	#NULL!
9	9	9	9	9	9	9	9	#NULL!	#NULL!
9	9	9	9	9	9	9	9	#NULL!	#NULL!
9	9	9	9	9	9	9	9	#NULL!	#NULL!
9	9	9	9	9	9	9	9	#NULL!	#NULL!
9	9	9	9	9	9	9	9	#NULL!	#NULL!

9	9	9	9	9	9	9	9	#NULL!	#NULL!
9	9	9	9	9	9	9	9	#NULL!	#NULL!
9	9	9	9	9	9	9	9	#NULL!	#NULL!
9	9	9	9	9	9	9	9	#NULL!	#NULL!
9	9	9	9	9	9	9	9	#NULL!	#NULL!
9	9	9	9	9	9	9	9	#NULL!	#NULL!
9	9	9	9	9	9	9	9	#NULL!	#NULL!
9	9	9	9	9	9	9	9	#NULL!	#NULL!
9	9	9	9	9	9	9	9	#NULL!	#NULL!
9	9	9	9	9	9	9	9	#NULL!	#NULL!
9	9	9	9	9	9	9	9	#NULL!	#NULL!
9	9	9	9	9	9	9	9	#NULL!	#NULL!
9	9	9	9	9	9	9	9	#NULL!	#NULL!
9	9	9	9	9	9	9	9	#NULL!	#NULL!
9	9	9	9	9	9	9	9	#NULL!	#NULL!
9	9	9	9	9	9	9	9	#NULL!	#NULL!
9	9	9	9	9	9	9	9	#NULL!	#NULL!
9	9	9	9	9	9	9	9	#NULL!	#NULL!
9	9	9	9	9	9	9	9	#NULL!	#NULL!
9	9	9	9	9	9	9	9	1	1
9	9	9	9	9	9	9	9	#NULL!	#NULL!
9	9	9	9	9	9	9	9	#NULL!	#NULL!
9	9	9	9	9	9	9	9	#NULL!	#NULL!
9	9	9	9	9	9	9	9	#NULL!	#NULL!
9	9	9	9	9	9	9	9	#NULL!	#NULL!
9	9	9	9	9	9	9	9	#NULL!	#NULL!
9	9	9	9	9	9	9	9	#NULL!	#NULL!
9	9	9	9	9	9	9	9	#NULL!	#NULL!
9	9	9	9	9	9	9	9	#NULL!	#NULL!
9	9	9	9	9	9	9	9	#NULL!	#NULL!
9	9	9	9	9	9	9	9	#NULL!	#NULL!
9	9	9	9	9	9	9	9	#NULL!	#NULL!
9	9	9	9	9	9	9	9	#NULL!	#NULL!
9	9	9	9	9	9	9	9	#NULL!	#NULL!
9	9	9	9	9	9	9	9	#NULL!	#NULL!
2	2	2	2	2	2	2	2	2	2
9	9	9	9	9	9	9	9	#NULL!	#NULL!
9	9	9	9	9	9	9	9	1	1
3	3	3	3	3	3	3	3	1	1
3	3	3	3	3	3	3	3	2	2
9	9	9	9	9	9	9	9	#NULL!	#NULL!
9	9	9	9	9	9	9	9	1	2
9	9	9	9	9	9	9	9	1	1
9	9	9	9	9	9	9	9	1	2
2	2	2	2	2	2	2	2	2	2
2	9	1	1	1	1	1	2	2	2
3	9	2	2	3	2	2	3	1	1

2	1	2	2	3	3	2	3	1	1
1	1	2	2	2	2	2	1	1	1
3	1	1	3	1	3	3	3	1	1
2	1	1	2	3	3	3	3	1	1
2	1	3	3	3	3	3	3	1	1
1	1	1	3	1	3	2	3	1	1
3	1	2	2	3	3	3	3	1	1
2	1	2	1	3	3	3	3	1	1
2	2	3	2	1	2	3	2	2	2
2	1	3	1	3	2	3	3	1	1
9	9	9	9	9	9	9	9	1	1
2	2	2	2	2	2	2	2	1	2
9	9	9	9	9	9	9	9	#NULL!	#NULL!
2	1	1	3	3	3	2	3	1	2
2	1	1	3	3	3	2	3	1	2
3	1	3	1	3	3	3	3	1	1
9	9	9	9	9	9	9	9	#NULL!	#NULL!
2	1	3	3	3	3	3	3	1	1
2	1	2	1	1	1	1	3	2	1
2	3	3	3	1	1	1	1	1	2
2	9	3	3	3	3	3	3	1	1
2	1	2	2	2	2	3	3	1	1
3	1	1	1	3	3	3	1	1	1
3	1	3	1	3	3	3	3	1	1
3	3	3	3	3	3	3	3	1	2
2	1	2	1	2	1	2	1	2	2
2	9	1	1	1	1	1	1	2	2
2	1	1	3	3	1	2	1	#NULL!	#NULL!
2	2	2	2	2	2	2	2	2	1
9	9	9	9	9	9	9	9	#NULL!	#NULL!
9	9	9	9	9	9	9	9	1	#NULL!
9	9	9	9	9	9	9	9	1	1
9	9	9	9	9	9	9	9	1	1
9	9	9	9	9	9	9	9	2	2
9	9	9	9	9	9	9	9	#NULL!	#NULL!
9	9	9	9	9	9	9	9	2	2
3	1	1	1	1	2	3	2	1	1
9	9	9	9	9	9	9	9	1	1
9	9	9	9	9	9	9	9	1	1
9	9	9	9	9	9	9	9	#NULL!	#NULL!
2	3	2	3	2	1	1	1	1	1
9	9	9	9	9	9	9	9	1	1
3	1	3	1	3	3	3	3	1	1
3	1	3	1	3	3	3	1	1	1
2	3	3	3	3	3	3	1	1	2
3	3	1	3	3	3	3	3	1	2
3	1	1	1	3	3	3	1	2	2

3	2	2	3	3	2	3	9	1	1
2	2	1	3	2	3	1	2	#NULL!	#NULL!
2	2	2	2	2	2	2	2	2	2
1	1	1	1	1	1	1	3	1	2
2	2	3	2	2	2	2	2	1	2
2	3	1	2	3	1	2	3	1	2
3	3	3	1	1	2	2	1	1	#NULL!
1	2	3	2	3	2	1	2	2	1
3	1	3	1	3	3	3	3	1	2
2	9	1	9	2	3	2	2	#NULL!	#NULL!
1	1	1	1	3	3	2	3	1	1
2	3	1	2	3	1	2	3	1	2
2	2	1	2	1	2	1	2	1	2
1	2	3	2	2	3	2	9	2	2
3	3	2	1	2	2	1	1	2	2
3	2	2	2	2	2	3	1	1	1
3	2	3	2	2	2	2	2	1	2
9	1	9	9	9	9	9	9	1	2
1	2	3	2	2	1	2	2	1	2
1	2	2	2	1	9	1	2	2	2
2	3	3	2	3	2	1	2	1	2
1	1	1	1	3	3	3	3	1	1
2	3	1	2	3	1	2	3	1	1
1	1	1	1	3	3	3	3	2	2
2	1	1	2	1	2	2	1	2	1
3	3	1	1	3	2	2	3	2	1
3	1	3	1	3	3	1	3	2	2
1	2	3	3	3	3	3	2	1	1
2	2	2	2	2	2	2	2	2	1
3	1	2	3	3	2	3	2	2	2
1	3	2	3	3	2	3	3	2	2
2	3	2	1	2	3	1	2	1	1
2	2	2	2	2	3	3	1	1	2
2	2	3	3	3	2	2	1	2	2
2	3	1	2	3	1	2	3	1	1
3	1	1	3	3	3	3	3	2	2
3	2	1	1	2	3	2	2	2	2
9	9	9	9	9	9	9	9	#NULL!	#NULL!
1	1	1	1	1	1	1	1	1	1
3	1	1	3	1	3	1	3	1	2
1	1	1	1	3	1	1	1	1	2
2	3	1	2	3	1	2	3	1	1
3	1	1	1	3	1	2	3	1	2
1	1	3	1	3	2	2	2	1	1
2	2	2	2	2	2	2	2	1	1
3	2	2	1	1	3	3	1	2	2
9	9	9	9	9	9	9	9	2	2

2	1	3	2	3	2	2	2	1	1
9	9	9	9	9	9	9	9	#NULL!	#NULL!
3	3	3	2	2	2	3	3	1	1
2	2	3	3	3	3	2	2	1	2
1	1	1	1	1	1	1	2	1	1
1	2	2	2	3	3	2	2	2	2
2	2	2	2	2	2	2	2	2	2
9	9	9	9	9	9	9	9	#NULL!	#NULL!
9	9	9	9	9	9	9	9	#NULL!	#NULL!
2	2	2	2	1	1	1	3	2	2
2	2	2	2	2	2	2	3	1	1
2	2	2	2	2	2	2	2	2	2
2	2	2	2	2	2	2	1	2	2
1	2	2	2	2	2	2	2	1	1
2	2	2	2	2	2	2	2	2	2
2	2	2	2	2	2	2	2	2	2
2	2	2	2	2	2	2	9	2	2
1	2	2	1	2	2	1	3	2	2
2	2	2	2	2	1	1	3	1	1
2	2	2	2	2	2	2	2	2	2
2	2	1	1	2	2	2	2	2	2
2	2	2	2	2	2	2	2	1	1
2	2	2	2	2	2	2	2	1	1
1	2	2	1	1	2	2	2	#NULL!	#NULL!
2	2	2	2	2	2	2	9	2	2
2	2	2	2	2	2	2	2	2	2
2	2	2	2	2	2	2	3	1	1
9	9	9	9	9	9	9	9	1	1
2	2	2	2	2	2	2	2	1	1
2	2	2	2	2	2	2	2	2	#NULL!
2	2	2	2	2	2	2	9	2	2
2	2	2	2	2	2	2	3	1	1
9	9	9	9	9	9	9	9	2	2
2	2	2	2	2	2	2	2	2	2
2	2	2	2	2	2	2	3	1	1
2	2	2	2	2	2	2	3	1	1
2	2	2	2	2	2	2	2	2	1
2	2	2	2	2	2	2	2	2	2
2	2	2	2	2	2	2	2	2	2
9	9	9	9	9	9	9	9	#NULL!	#NULL!
2	2	2	2	2	2	2	2	1	2
2	2	2	2	2	2	2	2	1	1
1	2	3	2	1	2	3	2	1	1
2	2	2	2	2	2	2	2	#NULL!	#NULL!
2	3	9	2	2	3	2	1	1	1
9	9	9	9	9	9	9	9	2	2
3	3	3	3	3	3	3	3	2	2

2	2	2	2	2	2	2	2	1	2
2	3	2	3	3	2	2	3	2	1
2	2	2	2	2	2	2	2	1	2
2	2	2	2	2	2	2	2	2	1
2	2	2	2	2	2	2	2	1	1
2	2	2	2	2	2	2	2	2	2
3	1	1	3	3	3	3	3	1	2
1	3	1	3	1	3	1	3	1	1
9	9	9	9	9	9	9	9	1	1
2	2	2	2	2	2	2	2	2	2
2	3	3	2	2	2	2	1	1	1
1	1	1	2	2	2	2	2	1	1
1	2	2	3	2	1	1	2	2	2
9	9	9	9	9	9	9	9	1	2
2	2	2	2	2	2	2	2	2	2
2	9	1	1	2	2	2	2	2	2
1	1	1	3	2	2	2	3	1	1
1	1	1	1	1	1	1	1	1	1
9	9	9	9	9	9	9	9	1	1
1	2	2	2	2	2	2	2	2	2
2	2	1	2	2	2	2	2	1	1
9	9	9	9	1	9	9	9	1	1
9	9	9	9	9	9	9	9	2	2
1	1	1	2	1	3	3	9	1	2
3	2	1	2	1	2	3	2	1	1
1	1	1	2	3	2	1	3	1	2
1	1	2	3	2	3	1	9	1	2
1	1	9	2	2	3	3	1	1	1
2	2	1	1	1	1	1	1	1	1
9	1	9	9	9	9	9	2	1	1
9	9	9	9	9	9	9	9	2	2
3	3	3	3	3	3	3	3	2	2
2	2	2	2	2	2	2	2	2	2
9	2	9	2	9	9	2	9	1	2
3	1	2	3	3	3	3	3	1	2
9	9	9	9	9	9	9	9	1	1
1	3	2	2	2	2	2	2	1	2
1	1	1	1	1	1	1	1	1	1
2	2	2	2	2	2	2	2	2	2
2	2	2	2	2	2	2	2	2	2
2	3	9	3	3	9	2	3	#NULL!	2
2	2	2	2	2	2	2	2	1	2
9	9	9	9	9	9	1	9	2	#NULL!
2	2	2	3	3	3	2	2	1	1
1	1	1	1	1	1	1	3	1	1
3	3	3	3	2	3	2	2	2	2
2	3	2	3	2	3	2	2	2	2

1	9	9	9	9	9	9	1	2	1
2	2	2	2	2	2	2	2	2	2
1	1	1	1	1	1	1	1	1	1
2	2	2	2	2	2	2	2	2	2
9	9	9	9	9	9	9	9	2	2
1	1	2	2	2	3	3	3	1	1
3	3	3	3	3	3	3	3	2	2
1	1	1	1	1	1	1	1	1	2
1	1	1	1	1	1	1	1	1	2
1	1	1	1	1	1	1	1	1	1
2	2	2	2	2	2	2	9	2	1
1	1	1	1	1	1	1	1	1	1
2	2	2	2	2	2	2	2	1	2
2	2	2	2	2	2	2	2	1	2
2	2	2	2	2	2	2	2	2	2
2	2	2	2	2	2	2	2	2	2
1	1	1	1	1	1	1	1	1	2
1	1	1	1	1	1	1	3	1	1
9	9	9	9	9	9	9	9	#NULL!	#NULL!
3	1	3	1	3	1	3	1	#NULL!	#NULL!
2	1	1	1	1	1	1	3	1	1
9	2	1	2	2	2	2	2	1	2
9	9	9	9	9	9	9	9	1	1
1	1	1	1	1	1	1	9	2	2
3	3	3	3	3	3	3	3	2	2
2	2	1	1	1	1	2	2	1	2
1	1	1	1	2	2	9	2	1	1
2	2	1	2	1	2	2	1	2	1
2	3	3	2	2	2	2	2	1	1
2	2	2	2	2	2	2	2	2	2
2	9	1	2	9	1	2	2	1	2
2	2	2	2	2	2	2	3	2	2
3	3	3	3	3	3	3	9	1	2
2	1	2	3	2	3	1	2	2	2
2	2	2	2	2	2	2	2	2	2
3	3	3	3	3	3	3	2	2	2
9	3	9	2	2	1	2	2	1	1
2	2	2	2	2	2	2	2	2	1
2	3	1	2	3	1	2	3	1	2
3	3	3	3	3	3	3	3	2	2
2	9	1	2	3	1	2	3	1	1
2	2	2	2	2	2	2	2	#NULL!	#NULL!
9	9	9	9	9	9	9	9	#NULL!	#NULL!
9	9	9	9	9	9	9	9	2	2
9	9	9	9	9	9	9	9	1	2
2	2	2	2	2	2	2	2	2	2
2	2	2	1	3	3	3	3	1	1

1	1	1	1	1	1	1	9	1	1
1	1	3	1	3	3	1	3	1	1
9	9	9	9	9	9	9	9	#NULL!	#NULL!
1	1	3	1	3	3	1	3	1	1
2	3	2	1	2	1	3	9	1	2
2	2	2	2	2	2	2	2	2	2
2	2	2	2	2	2	2	2	2	2
2	2	2	2	2	2	2	9	#NULL!	#NULL!
9	9	9	9	9	9	9	9	2	1
2	2	2	1	2	2	3	9	1	2
2	1	2	2	2	2	2	2	2	2
2	1	1	3	3	1	2	1	1	2
2	2	2	2	2	2	2	2	1	1
3	2	1	2	3	3	2	1	1	1
2	2	2	2	2	2	2	9	2	2
2	2	2	2	2	2	2	2	1	2
2	2	2	2	2	2	2	9	2	2
2	2	2	2	2	2	2	2	2	2
2	2	2	2	2	2	2	2	2	2
1	3	1	3	2	2	2	1	1	1
1	1	1	2	2	1	2	1	1	1
2	2	2	2	2	2	2	2	2	2
9	9	9	9	9	9	9	9	1	2
2	2	2	2	2	2	2	2	2	2
1	1	1	1	1	1	1	1	1	1
9	9	9	9	9	9	9	9	1	2
1	3	2	2	1	1	2	3	1	2
2	2	3	3	3	3	3	3	1	2
2	2	1	1	2	3	1	2	2	1
2	1	1	3	2	2	2	2	2	2
2	1	1	2	3	2	2	2	2	2
9	9	9	9	9	9	9	9	1	1
2	2	2	2	2	2	2	2	2	2
2	3	2	2	2	2	2	2	#NULL!	#NULL!
3	2	1	2	1	2	2	1	1	1
2	2	2	3	2	2	2	2	2	1
9	9	9	9	9	9	9	9	2	2
9	9	9	9	9	9	9	9	#NULL!	#NULL!
1	3	1	3	3	2	2	1	1	2
2	2	2	2	2	2	2	2	2	2
3	2	1	2	3	1	2	2	#NULL!	#NULL!
9	9	9	9	9	9	9	9	2	1
2	2	2	2	2	2	2	2	2	2
2	2	2	2	2	2	2	2	#NULL!	#NULL!
9	9	9	9	9	9	9	9	#NULL!	#NULL!
9	9	9	9	9	9	9	9	1	2
2	2	2	2	2	2	2	3	2	2

9	9	9	9	9	9	9	9	#NULL!	#NULL!
2	3	1	2	2	1	2	2	1	1
2	3	2	1	2	1	3	9	2	2
9	9	9	9	9	9	9	9	#NULL!	#NULL!
3	1	1	1	1	1	3	3	2	2
2	2	3	1	1	2	2	1	1	1
2	3	2	1	2	3	2	1	#NULL!	#NULL!
9	9	9	9	9	9	9	9	#NULL!	#NULL!
2	1	2	2	2	2	2	2	2	2
2	3	2	3	2	3	2	1	2	2
2	1	1	2	2	2	2	2	1	2
2	3	2	2	2	3	3	1	1	1
1	2	2	2	1	1	2	9	1	1
1	1	1	1	2	2	3	3	2	2
2	3	1	2	3	1	2	1	1	2
2	2	2	2	2	2	2	2	2	2
2	2	3	2	2	1	2	3	2	2
2	2	2	2	2	2	2	2	2	2
9	9	9	9	9	9	9	9	#NULL!	#NULL!
9	9	9	9	9	9	9	9	#NULL!	#NULL!
9	9	9	9	9	9	9	9	#NULL!	#NULL!
2	3	2	2	3	2	1	2	1	2
1	2	2	3	3	3	3	1	2	2
2	2	2	1	1	1	2	3	#NULL!	#NULL!
2	2	2	1	1	3	3	1	#NULL!	#NULL!
3	3	3	2	2	3	3	3	2	2
3	2	2	2	3	2	2	2	2	2
2	2	2	3	2	1	1	3	1	2
1	1	1	1	1	1	3	3	2	1
2	1	1	1	3	3	3	3	2	2
1	1	1	2	2	2	2	2	2	2
1	2	3	2	1	2	3	2	2	2
2	2	9	1	3	3	3	1	1	1
3	2	2	3	3	3	3	2	2	2
2	2	2	2	2	2	2	2	1	1
2	2	2	2	2	2	2	2	2	2
3	3	3	3	3	3	3	3	2	2
3	3	3	3	3	2	2	2	1	1
9	9	9	9	9	9	9	9	#NULL!	#NULL!
9	9	9	9	9	9	9	9	#NULL!	#NULL!
2	3	1	2	3	1	2	3	1	2
1	2	3	3	3	2	1	1	2	2
2	3	2	1	3	2	2	3	1	2
1	1	3	2	3	2	1	1	2	2
9	9	9	9	9	9	9	9	2	2
3	3	3	3	3	3	3	3	1	2
2	3	2	1	2	1	2	3	2	2

1	1	1	1	1	1	1	1	#NULL!	#NULL!
1	1	2	3	2	2	2	3	2	2
1	3	2	2	1	3	2	2	2	1
3	1	3	1	3	2	2	3	2	2
2	3	2	3	2	3	2	1	2	2
3	3	3	3	3	3	3	3	1	2
1	1	1	1	1	1	1	1	1	2
3	3	3	3	3	3	3	3	1	1
2	3	3	2	2	3	2	2	2	2
3	1	3	1	3	2	3	2	#NULL!	#NULL!
2	2	2	2	2	2	2	2	2	2
1	3	2	1	3	2	3	1	2	2
2	2	2	1	3	2	1	1	#NULL!	#NULL!
1	2	2	2	2	1	1	1	1	1
1	1	1	2	2	2	2	2	1	1
2	2	2	3	3	3	3	3	2	2
1	1	1	1	1	1	1	3	1	1
3	9	2	1	1	1	2	3	2	2
9	9	9	9	9	9	9	9	2	2
1	2	3	1	2	2	3	2	1	2
2	3	1	3	1	2	3	2	#NULL!	#NULL!
3	2	3	3	2	3	2	3	2	2
1	1	1	1	1	1	1	1	2	2
2	3	2	2	2	2	2	9	2	1
2	3	1	2	3	1	2	3	2	2
1	2	3	2	2	3	3	3	2	2
1	1	1	1	1	1	1	1	2	2
2	2	2	3	2	1	2	2	2	2
2	2	1	3	2	2	1	3	1	1
3	1	1	1	3	3	3	3	2	2
3	1	2	2	2	2	2	1	2	2
2	2	2	2	2	2	2	2	2	2
2	2	2	2	2	2	2	2	2	2
3	2	1	2	2	1	2	1	1	1
2	3	1	2	3	1	2	1	1	2
3	2	3	3	3	2	3	3	1	1
3	9	1	2	2	1	2	2	1	1
3	3	3	3	3	3	3	3	2	2
1	2	2	2	2	1	1	2	1	2
1	1	1	2	3	2	3	2	1	2
2	2	2	2	2	2	2	9	2	2
2	3	1	2	3	1	2	3	1	2
2	3	2	1	2	3	2	2	1	2
1	1	1	1	1	1	1	1	1	1
9	9	9	9	9	9	9	9	1	2
2	3	2	2	3	2	2	2	2	2
1	1	1	3	2	1	1	1	1	1

1	2	2	2	1	3	3	3	1	2
2	3	1	2	3	1	2	3	1	1
2	1	2	2	2	2	2	2	1	2
1	1	1	1	1	1	1	1	1	1
2	3	1	2	3	1	2	3	2	2
2	1	2	2	2	1	3	3	1	1
2	3	1	2	1	2	3	3	1	1
2	3	2	1	2	1	2	2	1	1
2	1	2	2	1	1	1	1	1	1
2	1	1	3	2	1	3	3	2	2
2	3	2	1	2	3	1	1	1	2
2	2	1	1	2	3	2	2	2	2
1	1	1	1	1	1	1	1	1	1
1	1	3	3	1	3	3	1	1	2
2	3	3	2	2	3	1	9	2	2
1	1	3	1	1	3	1	1	1	2
1	1	1	3	3	2	3	2	2	2
1	1	1	3	3	2	3	2	1	2
1	1	2	2	2	2	2	3	1	1
1	1	1	2	2	1	3	1	2	1
2	1	1	2	3	3	3	3	1	2
1	3	3	3	1	3	3	3	2	2
2	2	1	1	2	1	2	1	1	2
2	3	1	1	2	2	1	2	1	1
2	2	2	2	2	2	2	2	2	2
2	2	2	2	2	2	2	2	#NULL!	#NULL!
2	3	1	3	1	3	2	3	1	1
1	3	1	2	3	3	1	2	1	2
2	2	2	2	2	2	2	2	#NULL!	#NULL!
9	9	9	9	9	9	9	9	#NULL!	#NULL!
9	9	9	9	9	9	9	9	#NULL!	#NULL!
9	9	9	9	9	9	9	9	#NULL!	#NULL!
2	3	3	2	1	2	3	2	1	1
9	9	9	9	9	9	9	9	#NULL!	#NULL!
9	9	9	9	9	9	9	9	#NULL!	#NULL!
9	9	9	9	9	9	9	9	#NULL!	#NULL!
2	2	2	2	2	2	2	2	1	2
9	9	9	9	9	9	9	9	2	2
2	9	2	9	9	2	2	1	#NULL!	#NULL!
2	3	3	3	3	3	3	3	1	1
9	9	9	9	9	9	9	9	#NULL!	#NULL!
1	1	1	1	1	1	1	3	1	2
3	3	3	3	3	3	3	3	1	2
2	3	3	2	1	1	3	1	#NULL!	#NULL!
3	2	1	3	2	1	3	1	1	2
2	1	3	2	1	2	1	2	1	2
2	3	1	3	1	3	2	3	1	1

2	2	2	2	1	2	3	2	1	2
2	2	2	2	1	2	2	1	2	2
9	9	9	9	9	9	9	9	#NULL!	#NULL!
2	1	2	1	1	1	2	1	2	2
9	3	2	9	2	2	3	1	2	#NULL!
1	1	2	1	2	3	9	2	1	2
9	9	9	1	9	9	9	9	2	1
2	3	2	3	2	2	2	9	1	2
2	3	3	3	1	2	2	2	1	2
3	3	3	3	3	3	3	3	1	1
3	1	2	3	1	2	3	2	2	2
3	2	3	2	3	2	3	3	2	2
3	1	2	2	1	2	1	3	1	1
2	2	2	2	2	2	2	2	2	2
2	2	2	2	2	2	2	2	2	2
9	2	9	9	9	9	2	2	#NULL!	#NULL!
2	2	2	2	2	2	2	2	2	2
2	2	2	2	2	2	2	2	2	2
2	2	2	2	2	2	2	2	2	2
2	2	2	2	2	2	2	2	2	2
9	1	9	1	9	9	9	9	1	1
9	1	9	1	9	9	9	9	1	1
9	1	9	1	9	9	9	9	1	1
3	1	3	1	3	3	3	3	1	1
3	1	3	1	3	3	3	3	1	1
9	1	9	1	3	3	3	3	1	1
2	1	2	1	3	2	1	1	1	1
2	1	2	1	3	3	3	3	1	1
2	1	2	1	3	3	3	3	1	1
9	9	9	9	9	9	9	9	1	1
9	9	9	9	9	9	9	9	1	1
3	9	9	9	9	9	9	9	2	1
3	1	3	1	3	3	3	3	1	1
3	1	3	1	3	2	3	3	1	1
2	1	1	3	3	3	3	1	1	2
3	1	1	1	3	3	3	3	1	1
3	1	1	1	3	3	3	3	1	1
9	9	9	9	9	9	9	9	1	1
3	3	3	1	3	3	3	3	1	1
1	1	1	1	3	3	3	3	1	1
3	1	3	3	3	3	3	3	1	1
9	1	9	1	9	9	9	9	1	1
3	1	1	1	3	3	3	3	1	1
3	1	1	1	3	3	3	3	1	1
1	2	2	2	2	2	2	2	1	1
3	1	3	1	3	3	3	3	1	1
2	1	3	3	3	3	2	2	1	1

3	1	1	1	3	3	3	3	1	1
2	1	1	2	9	9	2	2	1	1
3	1	3	1	3	3	3	3	1	1
1	1	1	1	1	1	1	1	1	1
1	1	3	1	3	3	3	3	1	1
1	1	3	1	3	3	3	3	1	1
1	1	1	1	1	1	1	1	1	1
2	1	1	1	2	3	3	3	1	1
1	1	1	1	3	3	3	3	1	1
3	1	3	1	3	3	3	3	1	1
1	1	2	1	1	3	2	2	1	1
2	1	2	2	2	2	2	2	1	2
1	1	3	1	3	3	3	3	1	1
1	1	3	1	3	3	3	3	1	1
1	1	3	1	3	3	3	3	1	1
1	1	3	1	3	3	3	3	1	1
1	1	3	1	2	3	3	3	1	1
1	1	1	2	1	9	1	3	1	1
1	1	3	1	3	3	3	3	1	1
2	3	2	3	1	2	3	2	1	1
9	2	9	9	9	9	9	9	1	2
3	3	3	3	3	3	3	3	1	2
1	1	1	1	1	1	1	1	1	1
3	1	3	1	9	3	1	3	1	1
3	1	1	2	1	2	1	3	1	2
1	1	1	1	1	1	3	3	1	1
1	3	3	3	3	3	3	3	1	2
2	1	2	1	2	3	3	3	1	1
2	1	2	1	3	3	3	3	1	1
1	1	3	1	3	3	3	3	1	1
2	2	2	2	2	2	2	2	1	1
3	1	3	1	3	3	3	3	1	1
9	1	9	9	9	9	9	9	1	1
3	1	3	1	3	3	3	3	1	1
1	1	2	1	2	3	3	3	1	1
3	1	3	1	3	3	3	3	1	1
1	1	1	1	1	2	2	2	1	1
3	1	3	1	3	3	3	3	1	1
3	1	3	1	3	3	3	3	1	1
3	1	1	1	3	3	3	3	1	1
3	1	3	1	3	3	3	3	1	1
3	1	1	1	1	1	1	3	1	1
3	1	9	2	3	3	1	3	1	1
3	1	3	1	3	3	3	3	1	1
9	9	9	9	9	9	9	9	1	1
3	3	1	1	3	3	3	3	1	1
3	3	1	3	3	3	3	3	1	1

1	1	3	1	3	3	3	3	1	2
3	1	1	1	3	3	3	3	1	1
1	1	3	1	3	3	3	3	1	1
1	1	1	1	1	1	1	1	1	1
3	1	1	1	3	3	3	3	1	1
1	1	3	3	1	1	1	1	1	2
9	9	9	9	9	9	9	9	1	1
3	1	1	1	3	3	3	3	#NULL!	#NULL!
3	1	1	1	3	3	3	3	1	1
3	1	1	3	1	3	1	3	1	1
3	1	2	1	3	3	3	3	2	2
9	9	9	9	9	9	9	9	1	1
3	1	1	1	3	3	3	3	1	1
2	2	3	1	2	1	3	3	1	1
2	2	1	1	1	3	3	3	2	2
3	1	3	1	3	3	3	3	1	1
9	9	9	9	9	9	9	9	1	1
9	9	9	9	9	9	9	9	1	1
2	2	2	2	2	2	2	2	1	1
3	3	3	3	3	3	3	3	1	1
3	1	2	1	3	3	1	3	1	1
9	9	9	9	9	9	9	9	1	1
1	1	1	1	1	1	1	3	1	1
3	1	3	1	3	3	3	3	1	1
2	1	1	1	2	3	1	3	1	1
3	3	1	3	1	3	1	3	1	1
1	1	1	2	1	1	1	2	1	1
2	2	2	2	2	2	2	2	1	1
3	1	1	1	3	3	3	3	1	1
3	1	3	1	3	2	3	3	1	1
3	1	3	1	3	2	3	3	1	1
1	3	2	1	2	3	1	2	1	1
3	1	3	1	3	2	2	3	1	1
2	1	1	2	3	3	1	3	1	1
1	1	1	1	1	1	1	1	1	1
1	1	1	2	3	2	2	3	1	1
2	9	3	3	3	3	3	3	1	1
1	1	1	1	3	3	3	3	1	1
1	3	3	3	3	3	3	3	1	1
3	1	1	1	3	3	1	3	1	1
3	1	1	1	3	3	3	3	1	1
3	1	3	1	3	3	3	3	1	1
1	1	3	1	3	3	3	3	1	1
2	9	2	2	9	2	2	2	#NULL!	#NULL!
1	9	2	3	3	3	3	3	1	1
3	1	3	1	3	3	3	3	1	2
3	1	3	1	3	3	3	3	1	1

3	1	3	1	3	3	3	3	1	1
2	1	2	1	3	2	3	3	1	1
3	1	1	2	3	2	9	3	1	1
2	2	2	2	2	2	2	3	1	1
2	1	2	3	2	3	2	3	1	1
2	1	2	3	3	2	3	2	2	1
9	9	9	9	9	9	9	9	1	1
2	1	2	2	2	2	2	2	1	1
9	9	9	9	9	9	9	9	1	1
1	1	1	3	3	3	3	3	1	1
2	1	2	1	2	2	2	3	1	1
3	1	3	1	3	3	3	3	1	1
2	2	2	2	2	2	2	2	1	1
2	1	1	1	1	3	2	1	1	1
1	1	2	2	2	2	2	2	2	1
9	9	9	9	9	9	9	9	1	1
3	1	3	1	3	3	3	3	1	1
1	1	1	1	1	2	2	2	1	1
3	1	3	1	3	3	3	3	1	1
3	1	3	1	3	3	3	3	1	1
9	9	9	9	9	9	9	9	1	1
3	1	3	1	3	3	3	3	1	1
9	9	9	9	9	9	9	9	1	1
3	3	3	3	3	3	3	3	1	1
2	1	1	2	2	3	3	3	1	1
2	1	1	2	2	3	3	3	1	1
1	1	2	1	3	3	3	3	1	1
9	9	9	9	9	9	9	9	#NULL!	#NULL!
9	9	9	9	9	9	9	9	#NULL!	#NULL!
2	1	3	1	3	3	2	3	1	1
3	1	3	1	3	3	3	3	1	1
3	1	3	1	3	3	3	3	1	1
9	9	9	9	9	9	9	9	1	1
3	1	1	3	3	9	9	9	1	1
3	1	3	2	3	3	3	3	1	1
2	1	2	1	3	2	1	3	1	1
3	1	3	1	3	3	3	3	1	1
2	1	1	1	1	1	1	1	1	1
3	1	1	3	3	3	3	3	1	1
3	1	3	1	3	3	3	3	1	1
2	1	2	1	3	2	2	3	1	1
3	1	3	1	3	3	3	3	1	1
3	1	3	1	3	3	3	3	1	1
9	9	9	9	9	9	9	9	1	1
3	1	1	1	3	1	2	2	1	1
3	1	3	1	3	3	3	1	1	1
3	1	2	2	3	3	3	3	1	1

9	1	1	9	9	9	9	9	1	1
1	1	2	3	1	1	9	9	1	1
9	9	9	9	9	9	9	9	#NULL!	#NULL!
3	1	1	1	1	2	1	1	1	#NULL!
9	9	9	9	9	9	9	9	1	1
2	1	1	3	3	3	3	3	1	1
2	1	1	1	1	3	1	3	1	1
1	1	2	2	2	3	3	3	1	1
9	9	9	9	9	9	9	9	1	1
2	1	2	1	2	3	3	3	1	1
1	1	1	1	1	1	1	1	1	1
2	1	1	2	2	2	2	3	1	1
1	1	1	2	2	2	2	3	1	1
3	1	1	1	3	1	3	9	1	1
1	2	1	1	2	2	1	1	1	1
3	1	3	1	3	3	3	3	1	1
9	9	9	9	9	9	9	9	1	1
9	9	9	9	9	9	9	9	1	2
1	1	1	1	1	3	3	3	1	1
2	2	2	2	2	2	2	2	1	1
3	1	1	2	2	3	2	1	1	1
3	1	3	3	3	3	3	3	1	1
3	1	3	3	3	3	3	3	1	1
3	1	3	1	3	3	3	3	1	1
1	1	1	2	3	3	3	3	1	1
1	1	1	1	1	1	1	2	1	1
3	1	1	2	3	3	3	3	1	1
2	1	1	1	1	3	1	2	1	2
1	1	1	1	1	1	1	3	1	1
1	1	1	1	1	1	1	9	1	1
3	1	3	3	3	3	2	3	1	2
3	1	1	1	3	1	3	3	1	1
2	1	1	2	2	3	3	2	1	1
3	1	1	1	3	3	3	3	1	1
3	1	2	1	3	3	3	3	1	1
2	1	3	1	3	3	3	3	1	1
3	1	3	1	3	3	3	3	1	1
9	1	1	9	9	9	9	9	1	1
3	1	1	2	1	1	2	1	1	1
1	1	1	1	1	1	1	1	2	1
2	1	2	2	2	3	2	3	1	1
9	9	9	9	9	9	9	9	1	1
9	9	9	9	9	9	9	9	1	1
9	9	9	9	9	9	9	9	1	1
3	1	2	2	3	2	3	3	1	1
3	1	1	2	1	2	1	3	1	1
9	1	9	9	9	9	9	9	1	1

3	1	3	1	3	3	3	3	1	1
3	1	1	1	3	3	3	3	1	1
2	1	9	1	9	2	9	3	1	1
3	1	3	1	3	3	3	3	1	1
3	1	3	1	3	3	3	3	1	1
9	9	9	9	9	9	9	9	1	1
2	2	2	2	2	2	2	9	1	1
1	1	2	2	2	2	2	2	1	1
9	9	9	9	9	9	9	9	1	1
2	1	2	1	3	3	2	3	1	1
3	1	3	1	3	3	3	3	1	1
1	9	9	9	9	9	9	9	1	1
9	9	9	9	9	9	9	9	1	1
9	9	9	9	9	9	9	9	1	1
9	9	9	9	9	9	9	9	1	1
9	9	9	9	9	9	9	9	1	1
3	1	1	2	3	1	1	3	1	1
2	2	3	3	1	2	3	1	2	2
2	1	3	1	3	3	3	3	1	1
2	1	2	2	2	1	3	3	1	1
3	1	1	1	3	3	3	3	1	1
3	1	1	1	3	3	3	3	1	1
2	1	2	1	3	2	3	3	1	1
2	1	1	2	2	1	2	3	1	1
3	1	2	1	3	3	3	3	1	1
3	1	1	3	3	3	3	3	1	1
3	1	1	1	3	1	3	3	1	1
3	1	3	1	3	3	3	3	1	1
3	1	2	3	3	3	3	3	1	1
3	1	3	1	3	3	3	3	1	1
2	1	2	1	2	2	1	3	1	1
9	1	9	1	9	9	9	9	1	1
3	1	3	1	3	3	3	3	1	1
1	1	3	1	1	3	3	3	1	1
2	1	1	2	3	3	1	3	1	1
3	1	1	1	3	3	1	3	1	1
3	1	3	2	3	3	3	3	1	1
2	2	3	2	2	3	3	3	1	1
9	9	9	9	9	9	9	9	1	2
9	9	9	9	9	9	9	9	1	1
1	2	1	2	2	1	3	3	1	1
1	2	1	2	2	1	3	3	1	1
2	1	2	2	3	3	3	3	1	1
2	1	2	1	3	3	3	3	1	1
2	1	1	1	1	3	2	3	1	1
3	1	1	2	3	3	3	3	1	1
1	1	3	1	1	3	3	3	1	1

3	1	3	1	3	3	3	3	1	1
3	1	2	1	3	3	3	3	1	1
1	1	9	1	3	3	3	3	1	1
3	1	3	1	3	3	3	3	1	1
3	1	2	1	3	2	1	3	1	1
2	1	1	2	2	2	3	3	1	1
2	1	2	1	2	2	2	2	1	1
2	1	2	1	2	3	3	3	1	1
9	9	9	9	9	9	9	9	1	1
3	1	2	1	3	3	3	3	1	1
2	2	1	1	2	2	2	2	1	1
2	2	2	2	2	2	2	2	1	1
3	1	3	1	1	3	3	3	1	1
1	1	1	3	1	3	3	3	1	1
1	1	1	2	2	2	2	2	1	1
1	1	1	1	3	3	3	3	1	1
2	2	2	2	2	2	2	3	1	1
2	1	1	1	2	2	2	3	1	1
2	2	2	2	2	2	2	2	2	2
3	1	3	1	3	3	3	3	1	1
3	1	3	1	3	3	3	3	1	1
9	9	9	9	9	9	9	9	#NULL!	#NULL!
2	1	3	1	3	2	3	3	1	1
3	1	3	1	3	3	1	3	1	1
3	1	1	3	3	3	3	3	1	1
9	9	9	9	9	9	9	9	1	#NULL!
9	9	9	9	9	9	9	9	#NULL!	#NULL!
9	1	9	9	9	9	9	9	1	1
3	1	2	1	3	3	3	3	#NULL!	#NULL!
9	3	1	1	1	3	3	9	1	1
3	1	3	1	3	3	3	3	1	1
3	1	3	1	3	3	3	3	1	1
9	9	9	9	9	9	9	9	1	1
9	9	9	9	9	9	9	9	#NULL!	#NULL!
9	9	9	9	9	9	9	9	1	2
9	9	9	9	9	9	9	9	1	1
9	9	9	9	9	9	9	9	1	1
9	9	9	9	9	9	9	9	1	1
9	9	9	1	9	9	9	9	1	1
2	2	2	3	1	2	2	1	1	1
3	1	2	1	3	3	3	3	1	1
9	1	9	9	9	9	9	9	1	1
9	1	9	9	9	9	9	9	1	1
3	1	1	1	3	3	1	3	1	1
9	9	9	9	9	9	9	9	1	1
9	9	9	9	9	9	9	9	1	1
9	9	9	9	9	9	9	9	1	1

3	1	3	1	3	3	3	3	1	1
3	1	3	1	1	3	3	1	1	1
9	9	1	9	9	9	9	9	1	1
3	1	3	1	3	3	3	3	1	1
3	1	3	1	3	3	3	3	1	1
9	9	9	9	9	9	9	9	1	1
9	9	9	9	9	9	9	9	1	1
9	9	9	9	9	9	9	9	1	1
9	9	9	9	9	9	9	9	1	1
9	9	9	9	9	9	9	9	1	1
1	1	3	1	1	3	3	3	1	1
3	1	3	1	3	3	3	3	1	1
3	9	3	1	1	3	3	3	1	1
3	9	9	9	9	9	9	9	1	1
3	1	3	1	3	3	3	3	1	1
3	1	3	1	3	3	3	3	1	1
3	1	1	1	9	1	1	1	1	1
3	1	1	1	9	3	1	1	1	2
3	1	3	1	3	3	3	3	1	1
1	2	1	1	1	3	3	3	1	#NULL!
1	2	1	1	1	3	3	3	1	1
1	1	1	1	1	1	1	3	1	1
9	9	1	9	9	9	9	9	1	1
1	1	1	1	3	1	3	1	1	1
2	3	2	3	3	2	1	2	1	1
1	1	1	1	1	1	1	3	1	1
3	2	3	3	3	3	3	3	2	1
1	1	3	1	1	3	3	3	1	1
1	1	1	1	2	3	3	3	1	1
2	2	3	3	3	3	3	3	1	1
3	1	3	3	3	3	3	3	1	1
3	1	1	2	2	3	1	3	1	1
3	1	1	1	3	3	1	1	1	1
2	1	3	1	3	3	3	3	1	1
3	1	1	2	3	3	3	3	1	1
3	1	3	1	3	3	3	3	1	1
3	1	3	1	3	3	3	3	1	1
3	1	3	1	3	3	3	3	1	1
3	1	3	1	3	3	1	3	1	1
2	1	1	1	2	3	1	1	1	1
9	9	9	9	9	9	9	9	1	1
2	3	3	1	3	3	3	3	1	2
1	2	2	2	2	3	1	1	1	2
3	1	3	1	3	3	3	3	1	1
3	1	3	1	3	3	1	3	1	1
1	1	3	1	3	3	1	3	1	1
3	1	3	1	3	3	3	3	1	1

3	1	3	1	1	3	3	1	1	1
1	1	3	1	1	1	3	3	1	1
9	9	9	9	9	9	9	9	1	1
2	1	3	2	3	3	1	3	1	2
2	1	3	1	3	3	1	3	1	1
2	1	3	2	3	3	1	3	1	2
3	1	3	1	3	3	3	3	1	1
3	3	3	1	3	3	3	3	1	1
3	1	3	1	3	3	3	3	1	1
2	1	3	1	1	1	1	1	1	1
3	1	2	1	3	3	2	3	1	1
9	9	9	9	9	9	9	9	1	1
1	1	1	1	1	3	3	3	1	1
1	1	1	1	3	3	3	3	1	1
3	1	1	1	3	3	3	3	1	1
2	2	2	2	2	2	2	2	1	1
2	2	2	2	2	2	2	2	1	2
9	2	9	2	2	2	2	2	1	1
2	3	1	1	2	3	1	2	2	1
3	1	3	1	3	3	3	3	1	1
3	1	3	1	3	3	3	3	1	1
3	1	1	1	3	3	3	3	1	1
2	1	3	2	3	3	1	3	1	2
2	1	1	2	2	2	3	3	1	1
2	1	1	2	2	2	3	3	1	1
3	2	1	3	3	2	3	2	1	1
2	1	2	1	3	3	3	3	1	1
2	2	2	2	2	2	2	3	1	2
3	1	3	1	3	3	3	3	1	1
2	1	2	1	2	2	3	3	1	1
3	1	2	2	2	2	2	2	1	1
2	1	3	1	1	3	3	3	1	1
2	1	2	1	1	2	3	3	1	1
9	9	9	9	9	9	9	9	1	1
2	1	3	2	3	3	1	3	1	2
2	1	3	2	3	3	1	3	1	2
2	2	2	2	2	2	2	2	1	1
2	2	2	2	2	2	2	2	1	1
1	1	3	1	3	3	3	3	1	1
3	1	3	1	3	2	1	2	1	1
3	1	3	1	3	3	3	3	1	1
3	1	1	3	3	3	3	3	1	1
3	1	1	3	3	3	3	3	1	1
3	1	1	3	3	2	2	2	1	1
3	1	3	1	3	3	3	3	1	1
2	1	2	1	3	3	3	3	1	1
3	1	3	1	1	3	3	3	1	1

2	2	2	2	3	2	3	3	1	1
2	1	2	1	1	3	3	2	1	1
1	1	1	2	1	2	3	3	1	1
2	1	1	2	2	1	1	1	1	1
2	1	3	1	3	3	2	3	1	1
3	1	2	3	3	3	3	3	1	1
3	1	2	3	3	2	3	3	1	1
2	1	2	1	2	3	3	3	1	1
2	1	1	1	1	3	1	2	1	1
3	1	1	2	3	3	3	3	1	1
2	1	2	2	2	2	2	2	1	1
3	1	3	1	3	3	3	3	1	1
9	9	9	9	9	9	9	9	1	1
3	1	1	3	3	3	1	3	1	1
3	1	1	1	3	3	1	3	1	1
2	2	2	2	2	2	2	2	2	2
3	1	2	1	3	3	3	3	1	1
3	1	3	1	3	3	3	3	1	1
3	1	3	1	3	3	3	3	1	1
3	1	2	1	3	3	3	3	1	1
3	1	3	3	3	3	1	3	1	1
1	2	1	1	1	3	3	3	1	1
3	1	3	1	3	3	3	3	1	1
9	9	9	9	9	9	9	9	1	1
2	1	3	1	1	2	1	3	1	1
3	1	3	1	3	3	3	3	1	1
3	1	3	1	3	3	3	3	1	1
2	2	2	3	1	2	2	1	1	1
3	1	1	1	3	3	1	3	1	1
9	9	9	9	9	9	9	9	1	1
9	9	9	9	9	9	9	9	1	1
9	9	9	9	9	9	9	9	1	1
2	1	3	1	3	3	3	3	1	#NULL!
3	1	2	1	3	3	3	3	1	1
1	1	2	1	1	2	1	3	1	1
3	1	1	1	3	3	3	3	1	2
2	2	2	2	3	3	2	2	2	2
9	9	1	9	9	9	9	2	1	1
3	2	1	3	2	1	2	1	1	1
3	1	1	2	3	3	3	3	1	1
9	1	9	2	9	2	9	2	1	2
3	1	3	1	1	3	3	3	1	1
2	1	3	1	3	3	2	2	1	1
3	9	9	2	9	2	1	9	1	1
2	2	2	2	2	2	2	2	1	1
9	9	9	9	9	9	9	9	1	1
1	1	3	1	1	3	9	3	1	1

2	1	1	1	2	3	3	3	1	1
2	1	1	1	1	2	2	2	1	1
2	1	1	1	3	3	3	3	1	1
2	1	1	2	2	2	3	2	1	1
2	3	3	3	2	2	3	2	1	1
3	9	3	1	3	3	3	3	1	1
1	1	1	1	1	3	3	3	1	1
9	9	9	1	9	9	9	9	1	1
1	1	2	2	1	3	2	3	1	1
1	1	2	1	1	3	3	3	1	1
2	1	1	1	2	1	3	1	1	1
3	2	3	2	3	2	1	3	1	1
3	9	3	3	3	3	1	3	1	1
2	2	2	2	2	2	2	2	1	1
3	1	1	3	1	3	3	3	1	1
3	1	1	3	1	3	3	3	1	1
3	1	3	1	3	3	3	3	1	1
3	1	9	1	3	3	3	3	1	1
9	1	9	9	9	9	9	9	1	1
3	1	1	3	9	9	3	3	1	1
3	1	1	2	3	3	3	3	1	1
1	1	2	1	1	1	1	1	1	1
3	2	3	3	2	3	2	3	2	1
3	1	2	2	3	3	3	3	1	1
3	1	3	3	3	3	3	3	1	1
2	1	1	2	1	1	1	1	1	1
3	1	1	1	1	3	3	3	1	1
3	1	1	1	1	3	3	3	1	1
3	1	1	1	1	3	3	3	1	1
9	9	9	2	9	9	9	2	1	2
3	1	3	3	3	3	3	3	1	1
1	1	1	2	2	1	3	3	1	1
1	1	1	2	2	1	3	3	1	1
2	1	3	1	3	3	3	3	1	1
1	3	1	2	2	1	2	1	1	#NULL!
2	1	1	2	1	1	1	1	1	1
2	1	2	1	2	2	2	2	1	1
9	9	9	9	9	9	9	9	#NULL!	#NULL!
1	1	3	1	3	3	3	3	1	1
3	1	1	1	3	3	3	3	1	1
3	1	3	1	3	3	3	3	1	1
3	1	3	1	3	3	3	3	1	1
2	2	2	2	2	2	2	9	1	1
3	1	3	1	3	3	3	3	1	1
1	1	3	3	3	3	3	3	1	1
3	1	1	1	3	3	3	3	1	1
1	1	1	1	3	3	2	3	1	1

1	1	3	3	1	3	3	3	1	1
1	1	3	3	1	3	3	3	1	1
3	1	3	1	3	3	3	3	1	1
1	1	2	1	2	2	2	2	1	1
1	1	3	3	1	3	3	3	1	1
1	1	3	3	1	3	3	3	1	1
1	1	3	1	3	3	3	3	1	1
9	9	9	9	9	9	9	9	1	1
1	3	3	1	3	3	3	1	1	1
3	3	1	1	3	3	9	9	1	1
3	1	3	1	3	3	3	3	1	1
3	1	3	1	3	3	3	1	1	1
3	1	3	1	3	3	3	1	1	1
9	9	9	9	9	9	9	9	1	1
3	3	3	1	3	3	2	2	1	1
3	3	3	1	3	3	3	3	1	1
2	2	1	2	1	1	2	3	1	1
2	1	2	1	3	3	3	3	1	1
3	1	3	1	1	3	3	3	1	1
9	9	9	9	9	9	9	9	1	1
2	3	1	1	1	1	1	1	1	1
3	1	2	1	3	3	3	3	1	1
3	1	3	1	3	3	3	3	1	1
3	1	3	1	3	3	3	3	1	1
9	9	9	9	9	9	9	9	1	1
9	9	9	9	9	9	9	9	1	1
3	1	1	1	3	3	3	3	1	1
9	9	9	9	9	9	9	9	1	2
9	1	9	9	9	9	9	9	1	1
9	1	9	9	9	9	9	9	2	2
9	9	9	9	9	9	9	9	1	1
9	9	9	9	9	9	9	9	1	1
9	9	9	9	9	9	9	9	1	1
9	1	9	9	9	9	9	9	1	1
9	9	9	9	9	9	9	9	1	1
1	1	1	2	1	3	3	3	1	1
3	1	1	1	3	2	2	2	1	1
3	1	3	1	3	3	3	3	1	1
3	1	1	2	1	3	1	2	1	1
3	1	1	1	1	3	3	3	1	1
9	9	9	9	9	9	9	9	1	1
3	1	3	1	3	3	3	3	1	1
2	1	1	1	3	3	3	3	1	1
3	1	3	2	2	2	2	2	1	2
2	1	2	2	3	2	3	2	1	1
3	1	3	1	3	3	3	3	1	1
3	1	1	2	3	3	3	3	1	1

1	2	2	2	2	2	2	2	1	1
2	2	2	2	2	2	2	2	1	1
3	3	1	2	2	2	2	3	1	1
3	1	1	3	3	2	2	2	1	1
1	1	2	1	2	1	2	1	2	2
3	1	1	1	2	3	3	3	1	1
9	9	9	9	9	9	9	9	#NULL!	#NULL!
2	2	2	2	2	2	2	2	1	1
1	2	2	2	1	2	2	3	1	1
2	3	2	1	2	3	1	2	1	1
2	1	1	1	2	3	2	1	1	2
3	1	3	1	2	3	3	3	1	1
1	2	3	2	3	2	2	2	1	2
3	3	3	3	3	3	3	3	1	1
2	2	2	2	2	2	2	2	1	1
1	1	1	1	3	3	3	3	1	1
9	9	9	9	9	9	9	9	#NULL!	#NULL!
1	2	1	2	2	3	3	3	1	2
3	1	1	3	1	3	1	3	1	1
1	1	1	1	1	1	1	1	1	1
1	1	1	1	1	1	1	1	1	1
2	2	2	2	2	2	2	2	1	1
1	3	2	2	1	2	2	3	1	1
2	2	2	2	2	2	2	2	2	1
1	1	1	1	1	1	1	1	1	1
2	3	2	1	2	2	2	2	1	2
2	1	3	2	2	1	2	2	1	1
1	2	2	2	1	2	2	1	1	1
2	2	2	2	2	2	2	2	2	2
2	2	2	2	2	2	2	2	1	1
3	1	3	1	3	3	3	3	1	1
2	9	1	2	1	2	3	2	1	1
2	2	1	2	2	2	2	3	1	1
2	1	1	1	2	1	2	2	1	1
9	1	1	1	9	9	9	3	1	2
1	1	1	1	1	1	1	9	1	1
2	2	2	2	2	2	2	2	1	1
9	9	9	9	9	9	9	9	1	1
1	1	2	2	1	2	3	2	1	1
1	1	1	1	1	1	1	1	1	1
2	1	2	1	2	3	3	3	1	1
2	1	2	1	2	3	3	3	1	1
9	9	9	9	9	9	9	1	1	1
9	9	9	9	9	9	9	1	1	1
9	9	9	9	9	9	9	1	1	1
1	3	1	2	2	2	1	2	1	1
1	3	1	2	1	2	1	2	1	1

1	3	1	2	1	2	1	1	1	1
1	3	1	2	1	2	1	1	1	1
1	3	1	3	2	1	2	1	1	1
3	1	3	1	3	3	2	1	1	1
2	2	2	1	3	3	3	3	1	1
3	3	9	2	9	1	2	2	1	1
1	3	2	3	2	3	2	2	1	1
3	2	1	2	2	1	2	2	1	1
2	1	2	3	2	1	3	2	1	1
2	1	1	2	1	3	1	2	1	1
1	2	2	2	2	2	2	2	1	2
2	2	1	3	1	1	1	9	1	1
2	2	1	3	1	3	3	3	1	1
1	3	1	3	3	3	3	2	1	1
2	2	2	1	3	3	3	9	1	1
1	2	2	2	2	3	3	3	1	1
1	3	1	3	1	2	2	2	1	1
1	3	1	2	3	2	2	2	1	1
1	3	1	2	1	2	1	1	1	1
1	3	1	2	1	2	1	1	1	1
9	9	9	9	9	9	9	9	2	2
3	1	3	1	3	3	3	2	2	2
3	1	3	1	3	3	3	3	2	2
9	9	9	9	9	9	9	9	2	2
9	9	9	9	9	9	9	9	2	2
2	2	2	2	2	2	2	2	2	2
2	2	2	2	2	2	3	3	2	2
3	1	3	1	3	3	3	3	2	2
1	3	1	2	2	2	2	2	2	2
3	3	1	3	3	3	3	3	1	1
3	3	1	3	3	3	3	3	1	1
3	3	1	3	3	3	3	3	1	1
3	3	1	3	3	3	3	3	1	1
2	1	2	9	2	9	2	1	1	2
9	2	9	3	2	2	3	1	1	2
9	9	2	1	1	1	2	3	2	2
1	2	2	1	3	2	2	1	1	1
9	9	9	9	9	9	9	9	1	1
9	9	9	9	9	9	9	9	1	1
2	2	1	3	2	1	1	2	1	1
1	1	1	3	3	1	1	2	2	2
3	3	2	3	1	2	1	1	2	2
1	2	3	3	1	2	1	2	2	2
9	9	9	9	9	9	9	9	1	1
9	9	9	9	9	9	9	9	#NULL!	#NULL!
2	2	2	2	2	2	2	2	#NULL!	#NULL!
3	9	9	3	9	1	9	2	1	1

2	2	2	2	2	2	2	1	1	1
1	3	1	3	1	1	1	1	1	2
9	9	9	9	9	9	9	9	2	2
2	2	2	2	2	2	2	2	1	2
2	2	2	2	2	2	2	2	1	2
9	9	9	9	9	9	9	9	2	2
2	2	2	2	2	2	2	2	1	2
9	9	9	9	9	9	9	9	2	1
2	3	3	3	3	3	3	2	1	2
2	2	1	2	3	3	1	1	1	2
1	3	1	3	1	1	1	1	2	2
2	2	9	3	3	3	3	3	1	1
9	9	9	9	9	9	9	3	1	2
9	9	9	9	9	9	9	9	1	1
2	1	1	2	3	1	2	3	1	1
9	3	9	9	9	9	9	9	1	#NULL!
3	1	3	3	3	3	3	3	1	1
9	9	9	9	9	9	9	9	#NULL!	#NULL!
3	2	3	1	1	2	2	1	2	2
9	9	9	9	9	9	9	9	2	2
2	3	2	2	1	2	1	1	1	1
3	1	1	3	1	1	2	2	#NULL!	#NULL!
1	3	1	3	1	1	1	1	2	2
1	3	1	3	1	1	1	1	2	2
1	3	1	3	1	1	1	1	2	2
1	3	1	3	1	1	2	1	2	2
1	3	1	3	1	1	1	1	1	2
9	9	9	9	9	9	9	9	2	2
9	9	9	9	9	9	9	9	2	2
9	9	9	9	9	9	9	9	2	2
9	9	9	9	9	9	9	9	1	#NULL!
9	9	9	9	9	9	9	9	2	2
9	9	9	9	9	9	9	9	2	2
1	3	2	1	2	1	2	1	1	1
2	1	1	2	3	1	2	3	1	1
9	9	9	9	9	9	9	9	#NULL!	#NULL!
2	3	2	2	2	1	3	2	1	1
2	3	1	1	3	3	3	3	2	2
1	3	1	2	2	2	2	2	1	1
2	2	2	2	2	2	2	2	1	1
2	2	2	2	2	2	2	2	1	1
9	9	9	9	9	9	9	9	1	1
3	1	3	1	3	3	3	3	2	2
1	3	1	3	1	3	3	3	2	2
9	9	9	9	9	9	9	9	2	2
9	9	9	9	9	9	9	9	2	#NULL!
9	9	9	9	9	9	9	9	1	1

9	9	9	9	9	9	9	9	2	2
9	9	9	9	9	9	9	9	2	2
1	3	1	3	1	1	1	1	1	1
9	9	9	9	9	9	9	9	2	2
1	3	1	3	3	3	3	3	1	2
9	9	9	9	9	9	9	9	2	2
3	3	3	3	2	1	2	1	2	2
1	3	2	2	3	3	3	2	2	1
1	3	2	2	2	3	3	2	2	2
3	9	2	1	3	1	2	2	2	1
2	2	3	1	3	3	3	1	1	1
1	3	1	3	1	1	1	1	1	1
1	3	1	3	1	1	1	1	1	1
2	1	1	2	2	3	2	1	2	2
2	9	3	1	1	2	2	2	1	2
1	2	2	2	1	1	1	2	1	2
2	2	2	2	2	2	2	3	1	2
9	1	1	2	1	1	1	3	1	2
2	1	2	3	2	3	2	1	1	1
1	3	1	3	1	1	1	2	1	1
9	9	9	9	1	9	9	9	1	1
9	9	9	3	9	9	9	9	1	#NULL!
1	9	9	9	9	9	9	9	1	2
9	3	9	9	9	9	9	9	2	#NULL!
9	9	9	9	9	9	2	9	2	1
9	9	9	9	9	9	2	9	2	2
9	9	9	2	9	9	9	9	2	2
9	9	9	9	9	9	9	9	1	2
9	9	9	9	9	9	9	9	1	2
9	9	9	9	9	9	9	9	1	2
9	9	9	9	9	9	9	9	1	2
9	9	9	9	9	9	9	9	2	1
9	9	9	9	9	9	9	9	1	1
1	3	2	3	1	2	1	2	1	1
2	3	1	3	2	1	2	1	1	1
1	2	1	2	3	1	1	1	1	1
9	1	9	9	3	9	3	9	2	2
9	9	9	9	9	9	9	9	2	2
2	3	9	2	1	1	1	2	1	1
9	9	9	9	9	9	9	9	1	2
9	9	9	9	9	9	9	9	1	1
9	9	9	9	9	9	9	9	1	1
9	9	2	9	9	9	9	9	1	2
9	9	9	9	9	9	9	9	#NULL!	#NULL!
2	3	2	3	2	1	2	1	1	1
2	3	2	3	2	1	2	2	1	1
2	3	2	3	2	1	2	1	1	1

2	2	2	2	2	2	2	2	1	1
9	9	9	9	9	9	9	9	1	1
1	2	2	2	1	1	1	2	1	2
2	2	2	3	3	3	3	1	1	2
9	9	9	9	9	9	9	2	1	#NULL!
9	9	9	9	9	9	9	2	1	2
9	9	9	9	9	9	9	9	1	2
9	9	1	3	1	9	9	1	1	1
9	9	9	9	9	9	9	9	2	1
9	9	9	9	9	9	9	9	#NULL!	#NULL!
9	9	3	9	9	9	9	9	2	1
9	9	3	9	3	9	9	9	1	2
9	2	2	2	3	3	9	9	2	2
9	9	9	3	9	9	9	9	2	2
9	9	9	9	9	9	9	9	1	1
2	2	2	2	2	2	2	2	2	2
3	2	3	2	3	2	1	2	2	2
9	9	9	9	9	9	9	9	2	2
9	9	9	9	9	9	9	9	1	1
2	3	1	2	3	1	2	2	1	2
2	2	2	2	2	2	2	3	1	1
1	3	2	1	3	2	2	2	1	2
3	3	3	3	2	3	3	3	1	1
1	2	2	2	2	2	2	2	1	2
1	1	2	2	3	2	1	2	2	2
9	3	2	2	2	2	1	1	1	1
9	9	9	9	9	9	9	9	2	2
2	2	2	2	2	2	2	2	2	2
2	2	2	2	2	2	2	2	2	2
2	2	2	2	2	2	2	2	2	2
3	3	3	3	2	3	1	2	2	2
1	3	1	2	2	1	2	2	1	2
9	3	9	9	9	9	9	9	1	2
2	2	2	2	2	2	2	2	2	2
2	3	2	2	2	2	3	3	1	1
2	3	2	3	2	3	3	3	1	1
3	3	3	3	3	3	3	3	1	2
3	3	1	2	2	2	2	2	1	2
2	2	3	2	1	2	2	1	2	2
2	1	2	3	2	3	2	1	1	1
2	2	1	3	2	1	1	1	2	2
3	3	3	1	1	1	3	3	1	1
2	3	1	3	2	2	2	2	1	1
1	3	1	1	3	3	1	3	1	1
9	9	9	9	9	9	9	9	#NULL!	#NULL!
2	2	2	2	2	2	2	2	2	2
2	3	1	3	1	2	3	3	1	2

1	3	1	3	3	1	1	9	2	1
2	2	2	2	2	2	2	2	1	2
2	3	1	2	1	1	1	1	2	2
1	3	3	2	3	3	3	3	2	2
2	2	2	2	2	2	2	2	1	1
2	2	2	2	2	2	2	9	1	1
1	2	1	2	1	2	2	2	2	2
1	3	1	3	1	1	1	3	1	1
2	3	2	2	2	2	2	3	1	2
3	1	3	2	3	2	2	3	1	1
2	3	1	2	2	2	3	2	1	1
3	3	3	3	3	3	3	3	1	1
2	2	1	1	2	2	3	3	1	1
2	1	2	1	3	1	3	3	2	2
3	3	2	3	3	3	3	3	1	2
3	3	2	3	2	2	1	3	1	1
2	3	3	3	2	3	3	3	1	2
2	2	2	2	2	2	2	2	2	2
2	2	2	2	2	2	2	2	1	1
2	2	2	2	2	2	2	3	1	2
2	2	2	2	2	2	2	2	1	2
2	2	2	2	2	2	2	2	1	1
2	2	2	3	2	3	1	2	1	2
2	2	2	2	2	2	2	2	2	2
2	2	2	3	2	1	3	2	1	1
9	9	9	9	9	9	9	9	#NULL!	#NULL!
9	9	9	9	9	9	9	9	#NULL!	#NULL!
9	9	9	9	9	9	9	9	2	2
2	2	1	1	1	3	1	2	2	2
9	9	9	9	9	9	9	9	1	1
9	9	9	9	9	9	9	9	#NULL!	#NULL!
9	9	9	9	9	9	9	9	1	1
9	9	9	9	9	9	9	9	1	2
9	9	9	9	9	9	9	9	#NULL!	#NULL!
3	3	1	1	3	3	1	3	1	2
2	2	2	2	1	1	2	3	2	2
2	3	1	2	2	2	2	2	1	1
9	9	9	9	9	9	9	9	1	1
3	1	3	2	2	2	2	1	1	1
9	9	9	9	9	9	9	9	2	2
9	9	9	9	9	9	9	9	1	2
1	3	2	2	2	2	2	9	1	2
2	3	3	3	3	2	3	3	1	1
2	9	1	3	2	9	2	2	1	1
2	9	1	1	2	2	1	1	2	2
2	2	2	9	1	1	1	1	1	1
2	2	2	2	2	2	2	2	1	1

1	2	1	3	1	2	3	3	1	1
2	1	9	3	2	2	9	2	1	1
1	3	2	3	2	3	3	3	2	2
1	3	2	2	2	2	2	2	1	2
3	3	1	3	3	3	1	3	1	2
9	9	9	9	9	1	9	9	2	2
3	3	3	3	3	3	3	3	1	1
2	2	2	3	3	3	3	3	1	1
2	2	2	2	2	2	2	1	2	1
2	2	2	2	2	2	2	2	1	2
2	2	2	2	2	2	2	2	2	2
3	3	3	3	3	3	3	3	1	1
3	2	2	2	2	3	3	3	1	2
2	2	2	2	2	2	2	2	1	2
9	9	9	9	9	9	9	9	#NULL!	#NULL!
2	3	2	2	3	2	3	3	2	2
3	3	1	3	2	2	2	2	2	2
2	2	3	2	3	2	2	3	1	1
3	3	1	1	3	3	1	3	2	2
9	9	9	9	9	9	9	9	2	2
2	3	2	2	2	2	2	2	1	1
2	2	2	2	2	2	2	2	2	2
1	3	2	2	2	3	3	3	2	1
3	3	3	3	3	3	3	3	1	2
9	9	9	9	9	9	9	9	2	2
2	2	2	2	2	2	2	3	2	2
2	3	2	2	3	2	2	3	1	1
3	3	1	3	3	3	1	3	2	2
1	3	2	2	3	2	2	3	#NULL!	#NULL!
3	3	1	2	3	3	2	1	1	1
3	3	1	1	3	3	3	3	1	1
9	9	9	9	9	9	9	9	1	1
2	3	1	3	3	3	1	3	1	1
1	3	1	3	3	3	3	3	1	2
9	9	9	9	9	9	9	9	1	1
2	2	2	2	2	2	2	2	2	2
2	2	2	2	2	2	2	2	2	2
2	2	2	2	2	2	2	2	2	1
2	2	2	2	2	2	2	2	1	2
2	2	2	2	2	2	2	2	1	2
3	2	2	2	3	3	2	2	#NULL!	#NULL!
9	9	9	9	9	9	9	9	2	2
2	2	2	2	2	2	2	2	1	1
2	2	2	2	2	2	2	2	2	2
2	2	2	2	2	2	2	2	2	2
9	9	9	9	9	9	9	9	#NULL!	#NULL!
2	1	2	2	3	3	3	3	1	2

9	9	9	9	9	9	9	9	#NULL!	#NULL!
1	3	1	1	1	1	1	1	1	1
3	3	9	3	2	3	2	3	1	1
9	9	9	9	9	9	9	9	#NULL!	#NULL!
2	2	2	2	2	2	2	2	1	1
3	2	3	2	2	3	2	3	1	1
1	9	9	9	9	9	9	9	1	1
1	3	2	2	1	1	1	2	1	1
9	9	9	9	9	9	9	9	1	1
9	2	2	2	2	2	2	3	1	2
2	2	2	2	2	2	2	2	2	2
3	3	2	3	3	3	3	3	#NULL!	#NULL!
3	3	1	1	1	3	3	3	1	1
9	9	9	9	9	9	9	9	1	2
3	1	3	3	3	3	3	3	#NULL!	#NULL!
3	1	9	3	3	3	3	3	1	1
1	3	1	3	1	1	3	2	1	2
9	9	9	9	9	9	9	9	1	1
9	9	9	9	9	9	9	9	1	1
2	3	9	2	1	2	3	3	1	1
2	2	2	2	2	2	2	2	1	1
3	3	2	3	2	2	2	1	1	1
9	3	9	9	9	9	2	2	1	1
3	3	3	3	3	3	3	3	1	1
9	9	9	9	9	9	9	9	1	1
9	9	9	9	9	9	9	9	#NULL!	#NULL!
9	9	9	9	9	9	9	9	1	1
9	9	9	9	9	9	9	9	1	1
3	3	3	3	3	3	3	3	1	1
3	3	3	1	3	3	3	3	1	1
2	3	1	2	2	1	2	1	1	1
9	9	9	9	9	9	9	9	1	1
2	1	3	2	2	2	1	1	2	2
3	1	9	3	3	3	3	3	1	1
3	3	2	3	2	2	3	3	#NULL!	#NULL!
3	1	3	1	3	3	3	3	#NULL!	#NULL!
2	3	1	3	3	3	3	1	1	1
2	3	2	3	2	3	3	3	1	1
9	9	9	9	9	9	9	9	1	1
3	3	1	3	3	1	2	3	#NULL!	#NULL!
9	9	9	9	9	9	9	9	#NULL!	#NULL!
1	1	1	3	1	2	1	1	1	1
2	1	2	1	2	3	2	3	1	1
1	3	1	2	3	3	3	3	1	1
3	3	3	3	3	3	3	3	1	1
9	9	9	9	9	9	9	9	1	1
9	3	1	9	9	9	9	9	1	1

1	3	1	3	1	3	3	3	1	1
1	2	1	2	1	2	1	9	1	1
9	9	9	9	9	9	9	9	#NULL!	#NULL!
2	2	2	2	2	2	2	2	1	1
3	3	3	3	3	3	3	3	1	1
2	2	2	2	2	2	2	2	1	1
9	9	9	9	9	9	9	9	1	1
1	3	9	2	2	2	2	9	1	2
2	3	3	3	3	3	3	3	1	1
2	2	2	2	2	2	2	2	1	1
3	3	3	3	3	3	3	3	1	1
2	3	2	2	1	3	3	3	1	1
9	9	9	9	9	9	9	9	1	1
2	3	2	3	2	2	2	3	1	1
3	3	1	2	2	2	1	3	1	1
3	3	3	3	3	3	3	3	1	1
9	9	9	9	9	9	9	9	1	1
2	3	1	1	3	3	3	3	1	1
2	1	9	2	2	3	2	2	1	2
9	9	9	9	9	9	9	9	#NULL!	#NULL!
9	9	9	9	9	9	9	9	1	1
9	9	9	9	9	9	9	9	1	1
9	9	9	9	9	9	9	9	#NULL!	#NULL!
2	3	2	2	2	2	2	2	1	2
2	2	2	2	2	2	2	2	1	1
9	9	9	9	9	9	9	9	1	1
9	9	9	9	9	9	9	9	1	1
9	9	9	9	9	9	9	9	1	1
9	9	9	9	9	9	9	9	1	1
9	9	9	9	9	9	9	9	1	1
9	9	9	9	9	9	9	9	1	1
9	3	2	3	1	2	9	3	2	2
2	3	2	2	2	2	2	2	1	2
2	3	2	3	2	2	2	2	1	1
9	3	9	9	9	9	9	9	1	1
9	9	9	9	9	9	9	9	#NULL!	#NULL!
9	9	9	9	9	9	9	9	1	1
9	9	9	3	9	9	9	9	1	1
9	9	9	9	9	9	9	9	1	1
9	9	9	9	9	9	9	9	1	1
9	3	9	9	9	9	9	9	1	1
9	9	9	9	9	9	9	9	2	1
9	9	9	9	9	9	9	9	1	1
2	2	2	2	2	2	2	2	1	1
3	3	3	3	3	3	3	3	1	1
2	3	1	2	2	2	3	3	1	1
9	9	9	9	9	9	9	9	1	1

1	3	3	1	1	3	1	1	1	1
3	1	2	2	2	2	2	2	1	1
2	3	2	3	2	2	2	2	#NULL!	#NULL!
2	1	2	3	1	2	2	1	2	2
9	9	9	9	9	9	9	9	1	1
1	3	9	3	9	9	2	3	1	1
9	9	9	9	9	9	9	9	1	2
9	3	9	9	9	9	2	2	1	2
9	9	9	9	9	9	9	9	#NULL!	2
9	9	9	9	9	9	9	9	1	1
2	3	2	2	2	2	2	2	1	1
9	9	9	9	9	9	9	9	1	1
2	2	2	2	2	2	2	2	2	2
3	9	1	2	3	3	3	3	2	2
9	9	9	9	9	9	9	9	1	1
9	9	9	9	9	9	9	9	2	1
2	3	2	2	2	3	2	3	1	1
2	2	2	2	2	2	2	2	2	1
2	3	2	3	2	2	2	2	1	1
3	3	3	3	3	3	3	3	1	1
1	3	1	3	3	1	1	3	1	1
2	3	3	3	3	2	3	3	1	1
3	3	3	3	3	3	3	3	1	1
9	3	1	9	9	9	9	9	1	1
1	3	3	3	3	3	3	3	1	1
3	3	3	3	3	3	3	3	1	1
2	3	1	2	2	3	2	3	1	1
9	9	9	9	9	9	9	9	1	1
1	9	3	2	3	3	3	2	1	1
3	9	9	9	9	9	9	9	1	1
2	9	3	9	2	9	2	3	1	1
9	9	9	9	9	9	9	9	1	1
2	1	2	1	2	2	2	2	1	1
3	3	3	3	3	3	3	3	1	1
2	1	1	2	2	2	2	2	1	1
9	9	9	9	9	9	9	9	1	2
9	9	9	9	9	9	9	9	1	1
3	3	1	3	3	3	3	3	1	1
9	9	9	9	9	9	9	9	1	1
9	9	2	1	2	3	3	2	1	2
2	2	2	2	2	2	2	2	1	1
3	3	3	3	3	3	3	3	1	1
2	3	1	2	2	2	2	3	1	1
2	3	2	2	2	2	2	3	1	1
3	3	1	3	3	3	3	3	1	1
2	3	1	2	2	2	2	3	1	1
3	3	1	3	1	1	1	3	1	1

9	9	9	9	3	9	1	2	1	#NULL!
3	3	3	1	3	3	1	1	2	2
2	2	2	2	2	2	2	3	#NULL!	#NULL!
1	3	1	3	1	1	1	3	1	1
9	9	9	9	9	9	9	9	#NULL!	#NULL!
2	2	3	3	2	3	1	3	1	1
9	9	9	9	9	9	9	9	1	1
9	9	9	9	9	9	9	9	1	1
2	3	1	2	2	1	1	2	2	2
1	3	3	3	3	3	3	3	1	1
9	9	9	9	9	9	9	9	1	1
3	3	1	3	3	1	3	3	1	1
2	2	1	2	2	2	2	2	1	2
2	9	1	2	2	2	2	3	2	2
1	3	1	1	2	1	1	3	2	2
9	9	9	9	9	9	9	9	1	1
3	3	3	3	3	3	3	3	1	1
9	9	9	9	9	9	9	9	1	2
3	3	3	3	3	3	3	3	#NULL!	#NULL!
2	2	3	1	2	3	3	2	2	2
3	2	3	3	3	3	3	3	1	2
3	3	3	3	3	3	3	3	1	2
9	9	9	9	9	9	9	9	1	2
1	3	2	3	3	1	2	1	1	1
2	2	2	3	2	1	2	2	1	2
2	2	9	3	2	1	2	1	#NULL!	#NULL!
2	9	1	2	2	2	2	3	1	1
2	2	2	2	2	2	2	2	1	1
9	9	9	9	9	9	9	9	1	1
3	3	3	2	1	3	1	3	1	1
9	3	1	9	9	9	9	9	1	1
9	9	9	9	9	9	9	9	1	1
2	3	1	3	2	2	1	3	1	1
9	3	1	3	9	9	9	9	1	1
1	3	1	3	1	1	1	1	2	2
2	2	2	2	2	2	2	2	1	1
9	9	9	9	9	9	9	9	1	2
2	3	1	3	1	1	2	2	2	2
9	9	9	9	9	9	9	9	1	1
3	3	3	3	3	3	3	3	1	1
2	2	2	2	2	2	2	2	1	1
9	9	9	9	9	9	9	9	1	1
1	3	1	3	1	1	1	1	1	1
2	3	1	3	2	9	3	3	1	1
3	3	3	3	3	3	3	3	1	2
3	9	9	9	9	9	9	9	1	1
2	3	2	2	2	3	3	3	1	1

1	3	3	3	1	1	1	1	1	1
3	3	1	3	2	2	3	3	1	1
9	9	9	9	9	9	9	9	1	1
2	3	2	2	3	3	2	3	1	2
9	9	9	9	9	9	9	9	1	2
3	1	3	1	3	3	3	3	1	1
3	3	3	3	3	3	3	3	1	1
2	3	3	3	3	3	2	3	1	1
9	9	9	9	9	9	9	9	1	1
2	3	3	2	3	3	1	3	1	1
3	3	3	3	3	3	3	3	1	1
3	3	3	3	3	3	3	3	1	1
3	2	3	2	3	2	2	2	1	1
2	3	3	1	3	3	3	3	1	1
3	3	3	1	3	3	3	3	1	1
3	3	1	3	2	3	3	3	1	1
2	3	2	2	2	2	2	2	1	1
1	3	3	3	3	1	3	3	1	1
9	9	9	9	9	9	9	9	1	1
3	3	1	3	2	3	3	3	1	1
9	9	9	9	9	9	9	9	1	1
2	3	2	2	3	3	3	3	1	2
2	3	1	3	3	3	3	3	1	1
9	9	9	9	9	9	9	9	1	1
1	3	1	2	2	1	1	2	2	1

PapSmear2	HPVVacc2	Pap2Detec	Married2	PapOver18	pap2yearsa	menopaus	historyCAC	pap2yearly	onceonly2
2	2	2	2	2	2	2	2	2	2
2	2	2	1	2	2	2	1	2	1
2	2	2	2	2	2	2	2	2	2
2	2	1	2	2	2	2	2	2	2
1	2	2	2	2	2	2	2	2	2
2	2	2	2	2	2	2	2	2	2
2	2	2	2	2	2	2	2	2	2
2	1	2	2	2	2	1	1	2	2
1	1	2	2	2	1	1	1	1	1
1	2	2	1	2	2	1	2	1	2
2	1	2	2	2	2	2	2	2	2
2	1	2	2	2	2	1	1	2	2
1	1	2	2	2	2	2	2	2	2
2	2	2	2	2	2	2	2	2	2
2	2	2	2	2	2	2	2	2	2
2	2	2	2	2	2	2	2	2	2
#NULL!	#NULL!	#NULL!	#NULL!	#NULL!	#NULL!	#NULL!	#NULL!	#NULL!	#NULL!
2	2	2	2	2	2	2	2	2	2
#NULL!	#NULL!	#NULL!	#NULL!	#NULL!	#NULL!	#NULL!	#NULL!	#NULL!	#NULL!
2	2	#NULL!	#NULL!	#NULL!	#NULL!	#NULL!	#NULL!	#NULL!	#NULL!
1	2	2	1	2	2	2	1	2	1
1	1	2	1	2	1	1	1	1	1
1	1	2	1	2	2	1	2	2	1
1	1	2	2	2	2	2	2	2	2
2	2	2	#NULL!	#NULL!	#NULL!	#NULL!	#NULL!	#NULL!	#NULL!
#NULL!	#NULL!	2	2	2	2	2	2	2	2
1	1	2	1	1	1	1	2	#NULL!	1
1	1	2	2	2	2	2	2	2	2
2	2	2	2	1	2	2	1	2	2
2	2	#NULL!	#NULL!	#NULL!	#NULL!	#NULL!	#NULL!	#NULL!	#NULL!
1	1	2	2	1	1	1	1	1	1
2	2	2	1	2	2	2	2	2	2
2	1	2	2	2	1	1	1	1	1
2	2	2	2	#NULL!	2	#NULL!	2	2	2
1	1	2	2	2	2	1	2	2	1
2	1	1	2	1	1	2	2	1	2
1	2	2	2	2	2	2	2	2	2
2	2	1	2	2	2	1	2	2	2
1	2	2	1	2	2	2	2	2	1
#NULL!	#NULL!	2	2	2	2	1	2	1	1
#NULL!	#NULL!	2	2	2	2	2	1	2	1
2	1	#NULL!	#NULL!	#NULL!	#NULL!	2	#NULL!	#NULL!	#NULL!
2	2	#NULL!	#NULL!	#NULL!	#NULL!	#NULL!	#NULL!	#NULL!	#NULL!
2	2	2	2	2	2	2	2	2	2
2	2	2	2	2	2	2	2	2	2
#NULL!	#NULL!	#NULL!	#NULL!	#NULL!	#NULL!	#NULL!	#NULL!	#NULL!	#NULL!

1	1	2	2	2	2	2	2	2	2
1	1	2	2	2	2	2	2	2	2
1	2	2	2	2	2	2	2	2	2
2	2	2	2	2	2	2	2	2	2
1	1	2	1	1	1	1	1	2	1
1	1	2	1	1	1	1	1	2	2
2	2	2	1	#NULL!	2	#NULL!	2	2	2
2	2	2	2	2	2	2	2	2	2
1	1	2	2	2	2	2	2	2	#NULL!
1	2	1	2	1	2	2	1	1	1
#NULL!	#NULL!	2	#NULL!	#NULL!	#NULL!	#NULL!	#NULL!	#NULL!	#NULL!
1	1	2	2	2	2	2	2	2	2
2	2	2	2	2	2	2	2	2	1
1	1	2	2	2	2	2	2	2	2
1	2	2	2	2	2	2	2	2	2
1	1	2	2	2	2	1	2	2	2
2	2	2	2	2	2	1	1	1	1
2	2	2	1	2	2	1	2	1	2
2	2	2	2	2	2	2	2	2	2
#NULL!	#NULL!	2	2	2	2	2	2	2	2
2	2	1	2	2	2	2	2	2	2
2	1	2	2	2	2	2	2	2	2
1	1	2	2	2	2	2	2	2	2
2	1	2	2	2	2	2	2	2	2
2	1	2	2	2	2	1	2	2	2
2	2	2	2	2	2	2	2	2	2
1	1	2	1	1	1	1	1	1	1
1	2	2	2	1	1	1	2	2	2
2	2	2	1	2	2	2	1	2	1
2	2	1	1	1	1	1	1	1	1
#NULL!	#NULL!	#NULL!	#NULL!	#NULL!	#NULL!	#NULL!	#NULL!	#NULL!	#NULL!
2	2	2	2	1	1	1	2	2	2
2	2	2	1	1	2	2	#NULL!	#NULL!	#NULL!
2	2	2	2	2	2	2	2	2	2
2	2	2	2	2	2	1	2	2	1
2	2	2	2	2	2	2	2	2	2
2	1	2	2	2	#NULL!	#NULL!	#NULL!	#NULL!	#NULL!
2	2	2	2	2	2	2	2	2	1
2	2	1	1	2	2	2	2	2	2
2	2	2	2	2	2	2	2	2	2
1	2	2	2	2	2	1	2	2	2
#NULL!	#NULL!	#NULL!	#NULL!	#NULL!	#NULL!	#NULL!	#NULL!	#NULL!	#NULL!
2	2	2	2	2	1	2	1	2	2
2	2	2	2	2	2	2	2	2	2
2	2	2	2	2	2	1	2	2	1
1	1	1	2	2	1	2	1	2	1
2	2	2	2	2	2	2	2	2	2

2	1	2	2	2	1	2	2	1	2
2	2	1	1	1	1	1	1	1	1
2	2	2	2	2	2	2	2	2	2
2	2	2	2	2	2	2	2	2	2
1	1	2	2	2	1	2	2	2	2
2	2	1	2	2	2	2	2	2	2
1	2	2	2	2	2	2	2	2	2
#NULL!	#NULL!	#NULL!	#NULL!	#NULL!	#NULL!	#NULL!	#NULL!	#NULL!	#NULL!
2	2	2	2	2	2	2	2	2	2
1	2	2	2	2	1	1	2	2	1
#NULL!	2	2	2	2	2	2	1	2	2
1	1	2	2	2	2	2	2	2	2
2	1	2	2	2	2	1	1	1	1
2	2	2	2	2	2	2	2	2	2
2	1	2	2	2	2	2	2	2	2
2	2	2	2	2	2	2	2	2	2
1	1	2	2	2	2	2	2	2	2
2	2	2	2	2	2	1	1	1	2
2	2	2	#NULL!	2	2	2	2	#NULL!	2
2	2	1	2	2	2	2	2	2	2
2	1	2	1	2	1	2	1	2	1
1	2	2	2	2	2	2	2	2	1
1	2	2	2	2	2	2	2	2	2
1	1	2	2	2	2	2	2	2	2
2	2	2	2	1	2	2	2	2	2
2	2	2	2	1	1	2	1	#NULL!	1
2	2	2	1	2	2	1	1	1	1
1	2	2	2	2	2	2	2	2	2
2	2	2	2	2	2	2	1	1	1
2	2	2	2	2	2	1	2	1	1
1	2	2	2	2	2	2	2	1	1
2	2	2	2	2	2	2	1	2	2
2	2	1	#NULL!	1	2	2	1	2	2
2	2	2	2	2	2	2	2	2	2
2	2	2	2	2	2	1	2	2	1
2	2	2	2	2	2	2	2	2	2
2	#NULL!	2	1	2	1	2	1	2	1
1	1	2	2	2	2	2	2	1	2
#NULL!	#NULL!	#NULL!	#NULL!	#NULL!	#NULL!	#NULL!	#NULL!	#NULL!	#NULL!
2	2	1	1	1	1	1	1	1	1
1	1	2	2	1	2	2	2	2	2
#NULL!	#NULL!	#NULL!	#NULL!	#NULL!	#NULL!	#NULL!	#NULL!	#NULL!	#NULL!
1	1	2	2	2	2	2	2	2	2
1	1	2	2	2	2	2	1	2	2
2	2	#NULL!	#NULL!	#NULL!	#NULL!	#NULL!	#NULL!	#NULL!	#NULL!
1	2	2	1	2	2	2	1	2	1
2	2	2	2	2	2	2	2	2	2

2	2	2	2	#NULL!	2	1	2	#NULL!	2
2	2	2	2	2	2	1	2	2	1
1	1	1	1	1	1	1	1	1	1
2	2	2	2	2	2	2	2	2	2
1	2	2	2	2	2	1	2	2	2
2	2	2	1	2	2	2	2	1	2
2	1	2	#NULL!	2	2	1	2	#NULL!	2
2	2	1	2	#NULL!	2	1	2	2	2
2	2	2	#NULL!	#NULL!	#NULL!	#NULL!	#NULL!	#NULL!	#NULL!
2	2	2	2	2	2	2	2	2	2
2	2	2	2	1	2	2	1	2	2
2	1	2	2	2	2	2	2	2	1
2	2	2	2	2	2	2	2	2	2
2	1	2	2	2	2	2	2	2	2
1	1	2	2	2	1	2	2	2	2
1	1	2	2	1	2	2	2	2	2
1	1	2	2	1	2	2	2	2	2
1	1	2	2	2	2	2	2	2	2
2	2	2	#NULL!	#NULL!	#NULL!	#NULL!	#NULL!	#NULL!	#NULL!
2	2	1	#NULL!	2	1	2	2	2	2
2	2	2	1	2	2	2	1	2	2
2	2	2	2	2	1	2	2	2	2
2	2	2	2	2	2	2	1	2	1
2	2	2	2	2	2	2	2	2	2
2	2	2	2	2	2	2	#NULL!	#NULL!	#NULL!
2	2	1	1	1	1	1	1	1	1
2	2	2	1	2	1	2	2	1	2
1	1	2	2	2	2	2	2	2	2
2	2	2	2	2	2	2	2	2	2
2	2	2	2	2	2	2	2	2	2
1	2	2	2	2	2	2	2	2	2
2	2	2	2	2	2	2	2	2	2
1	1	2	2	2	2	2	2	2	2
1	2	1	1	1	2	2	2	1	1
1	1	2	2	2	2	1	2	2	2
1	1	1	1	1	1	2	2	2	2
1	1	2	2	2	2	2	2	2	2
1	1	2	1	2	1	1	1	2	1
2	2	2	1	2	2	1	1	2	2
1	1	2	1	1	1	1	1	1	1
2	1	#NULL!	#NULL!	#NULL!	#NULL!	#NULL!	#NULL!	#NULL!	#NULL!
1	2	2	2	2	2	2	2	2	2
2	2	2	2	2	2	2	2	2	2
#NULL!	#NULL!	#NULL!	#NULL!	#NULL!	#NULL!	#NULL!	#NULL!	#NULL!	#NULL!
#NULL!	#NULL!	#NULL!	#NULL!	#NULL!	#NULL!	#NULL!	#NULL!	#NULL!	#NULL!
1	2	2	2	2	2	2	2	2	2
1	1	2	2	2	2	2	2	1	1

2	2	2	#NULL!	#NULL!	#NULL!	#NULL!	#NULL!	#NULL!	#NULL!
2	2	2	2	2	2	#NULL!	#NULL!	#NULL!	#NULL!
2	2	2	#NULL!	#NULL!	#NULL!	#NULL!	#NULL!	#NULL!	#NULL!
2	2	2	#NULL!	#NULL!	#NULL!	#NULL!	#NULL!	#NULL!	#NULL!
2	2	#NULL!	#NULL!	2	#NULL!	#NULL!	#NULL!	#NULL!	#NULL!
#NULL!	#NULL!	#NULL!	#NULL!	#NULL!	#NULL!	#NULL!	#NULL!	#NULL!	#NULL!
2	2	2	2	2	2	2	2	2	2
#NULL!	#NULL!	#NULL!	#NULL!	#NULL!	#NULL!	#NULL!	#NULL!	#NULL!	#NULL!
2	2	2	2	2	2	2	2	2	2
1	1	#NULL!	#NULL!	#NULL!	2	#NULL!	#NULL!	#NULL!	#NULL!
2	2	#NULL!	#NULL!	#NULL!	#NULL!	2	#NULL!	#NULL!	#NULL!
2	2	#NULL!	#NULL!	#NULL!	2	#NULL!	#NULL!	#NULL!	#NULL!
2	2	2	2	2	2	2	2	2	2
#NULL!	#NULL!	2	2	2	2	2	2	2	2
1	1	1	#NULL!	#NULL!	#NULL!	#NULL!	#NULL!	#NULL!	#NULL!
2	1	1	2	1	2	1	2	1	1
1	1	1	1	1	1	1	1	1	1
1	1	1	1	2	2	2	2	2	2
2	2	2	2	2	2	2	2	2	2
2	2	2	2	2	2	2	2	2	2
2	2	2	2	2	2	2	2	2	2
#NULL!	#NULL!	#NULL!	#NULL!	#NULL!	#NULL!	#NULL!	#NULL!	#NULL!	#NULL!
2	2	1	1	1	1	1	1	1	1
2	2	2	1	2	2	1	2	2	2
2	2	2	2	2	2	2	2	2	2
2	#NULL!	2	2	2	2	2	2	2	2
2	2	2	2	2	2	2	2	1	2
2	2	1	1	2	2	1	2	2	2
1	1	2	1	1	1	1	1	1	1
1	1	2	1	1	2	2	2	2	1
2	2	2	2	2	2	2	2	2	2
1	2	2	2	2	2	2	2	2	2
#NULL!	#NULL!	#NULL!	#NULL!	#NULL!	#NULL!	#NULL!	#NULL!	#NULL!	#NULL!
2	2	1	2	2	2	2	2	1	1
2	1	2	1	2	1	2	1	2	1
2	1	2	2	2	2	2	2	2	2
2	1	2	#NULL!	2	2	1	2	2	1
2	2	2	2	2	2	2	2	2	2
1	2	2	#NULL!	#NULL!	#NULL!	#NULL!	#NULL!	#NULL!	#NULL!
2	2	2	2	2	2	2	2	2	2
1	2	2	2	2	2	2	2	2	2
1	1	1	2	1	2	2	2	1	#NULL!
1	2	2	2	2	2	2	2	1	1
2	2	2	2	2	2	2	2	2	2
2	2	2	2	2	2	2	2	2	2
1	2	1	2	2	2	2	2	2	2
#NULL!	#NULL!	#NULL!	#NULL!	#NULL!	#NULL!	#NULL!	#NULL!	#NULL!	#NULL!

2	1	2	2	2	2	2	2	2	2
2	2	2	1	2	2	2	2	2	2
2	2	2	2	2	2	2	2	2	1
#NULL!	#NULL!	#NULL!	#NULL!	#NULL!	#NULL!	#NULL!	#NULL!	#NULL!	#NULL!
2	2	2	2	2	2	2	2	2	2
2	2	2	1	2	#NULL!	2	2	2	1
1	2	2	1	2	2	2	2	2	1
1	2	1	2	2	2	2	2	2	2
2	2	2	2	2	2	2	1	2	1
2	2	1	1	2	2	2	2	2	2
2	2	2	2	2	2	2	2	2	2
2	2	1	#NULL!	#NULL!	#NULL!	#NULL!	#NULL!	#NULL!	#NULL!
2	2	2	#NULL!	#NULL!	#NULL!	#NULL!	#NULL!	#NULL!	#NULL!
1	1	2	#NULL!	#NULL!	#NULL!	#NULL!	#NULL!	#NULL!	#NULL!
2	2	2	#NULL!	#NULL!	#NULL!	2	#NULL!	#NULL!	2
2	2	#NULL!	#NULL!	#NULL!	#NULL!	#NULL!	#NULL!	#NULL!	#NULL!
#NULL!	#NULL!	#NULL!	#NULL!	#NULL!	#NULL!	#NULL!	#NULL!	#NULL!	#NULL!
#NULL!	#NULL!	#NULL!	#NULL!	#NULL!	#NULL!	#NULL!	#NULL!	#NULL!	#NULL!
2	2	2	#NULL!	2	#NULL!	2	#NULL!	2	#NULL!
#NULL!	#NULL!	#NULL!	#NULL!	#NULL!	#NULL!	#NULL!	#NULL!	#NULL!	#NULL!
#NULL!	#NULL!	#NULL!	#NULL!	#NULL!	#NULL!	#NULL!	#NULL!	#NULL!	#NULL!
1	1	2	2	2	2	2	2	2	2
2	1	2	2	2	2	2	2	2	2
1	1	2	2	2	2	2	2	2	2
1	1	2	2	2	2	2	2	2	2
#NULL!	#NULL!	#NULL!	#NULL!	#NULL!	#NULL!	#NULL!	#NULL!	#NULL!	#NULL!
1	2	2	2	2	2	2	2	2	2
#NULL!	#NULL!	#NULL!	#NULL!	#NULL!	#NULL!	#NULL!	#NULL!	#NULL!	#NULL!
#NULL!	#NULL!	#NULL!	#NULL!	#NULL!	#NULL!	#NULL!	#NULL!	#NULL!	#NULL!
2	1	2	2	2	1	2	2	2	2
2	2	#NULL!	#NULL!	#NULL!	#NULL!	#NULL!	#NULL!	#NULL!	#NULL!
#NULL!	#NULL!	#NULL!	#NULL!	#NULL!	#NULL!	#NULL!	#NULL!	#NULL!	#NULL!
#NULL!	#NULL!	#NULL!	#NULL!	#NULL!	#NULL!	#NULL!	#NULL!	#NULL!	#NULL!
#NULL!	#NULL!	#NULL!	#NULL!	#NULL!	#NULL!	#NULL!	#NULL!	#NULL!	#NULL!
2	2	#NULL!	1	2	2	1	2	#NULL!	2
2	2	#NULL!	2	#NULL!	1	2	#NULL!	2	#NULL!
#NULL!	#NULL!	#NULL!	#NULL!	#NULL!	#NULL!	#NULL!	#NULL!	#NULL!	#NULL!
#NULL!	#NULL!	#NULL!	#NULL!	#NULL!	#NULL!	#NULL!	#NULL!	#NULL!	#NULL!
#NULL!	#NULL!	#NULL!	#NULL!	#NULL!	#NULL!	#NULL!	#NULL!	#NULL!	#NULL!
#NULL!	#NULL!	1	#NULL!	#NULL!	#NULL!	#NULL!	#NULL!	#NULL!	#NULL!
#NULL!	#NULL!	1	#NULL!	#NULL!	#NULL!	#NULL!	#NULL!	#NULL!	#NULL!
2	2	#NULL!	#NULL!	#NULL!	#NULL!	#NULL!	#NULL!	#NULL!	#NULL!
1	1	1	1	1	1	1	1	1	1
2	1	2	1	2	1	2	2	2	2
1	1	1	2	2	2	2	2	2	2
#NULL!	#NULL!	2	#NULL!	#NULL!	#NULL!	#NULL!	#NULL!	#NULL!	2
2	1	2	#NULL!	#NULL!	#NULL!	#NULL!	#NULL!	#NULL!	#NULL!

2	2	2	#NULL!	#NULL!	#NULL!	#NULL!	#NULL!	#NULL!	#NULL!
2	2	#NULL!	#NULL!	#NULL!	2	#NULL!	#NULL!	#NULL!	#NULL!
2	2	2	2	2	2	1	1	1	1
1	1	1	2	2	1	2	2	1	2
#NULL!	#NULL!	2	2	2	2	2	2	2	2
2	#NULL!	2	2	2	2	1	1	1	1
2	1	2	2	1	2	2	2	2	1
2	2	2	2	1	2	2	2	2	2
2	2	2	2	2	2	2	2	2	2
1	1	1	1	1	1	1	1	1	1
#NULL!	#NULL!	#NULL!	#NULL!	#NULL!	#NULL!	#NULL!	#NULL!	#NULL!	#NULL!
#NULL!	#NULL!	#NULL!	#NULL!	#NULL!	#NULL!	#NULL!	#NULL!	#NULL!	#NULL!
2	2	#NULL!	#NULL!	#NULL!	#NULL!	#NULL!	#NULL!	#NULL!	#NULL!
1	1	#NULL!	2	#NULL!	#NULL!	#NULL!	#NULL!	#NULL!	#NULL!
1	1	2	#NULL!	2	2	2	2	2	1
1	2	2	2	2	1	1	1	2	2
#NULL!	#NULL!	#NULL!	#NULL!	#NULL!	#NULL!	#NULL!	#NULL!	#NULL!	#NULL!
2	2	2	2	2	2	2	2	2	2
1	1	2	2	2	2	2	2	2	2
2	2	2	1	1	2	1	2	2	2
2	2	1	2	1	2	2	2	1	2
2	2	2	2	2	2	2	2	2	2
2	1	2	1	2	1	1	2	2	2
1	1	2	2	2	2	2	2	2	2
2	2	2	2	2	2	2	2	2	2
1	1	2	2	2	2	2	2	2	2
2	2	2	2	2	2	2	2	2	2
1	1	2	2	2	2	1	2	2	2
2	2	2	2	2	2	2	1	1	1
2	1	2	2	2	2	2	2	2	2
2	2	2	2	2	2	2	2	2	2
2	2	2	2	2	2	2	2	2	2
2	2	2	2	1	2	2	2	2	2
2	2	1	1	1	1	1	1	1	1
1	2	2	1	2	2	1	2	2	1
2	2	2	2	1	2	2	1	2	2
#NULL!	#NULL!	#NULL!	#NULL!	#NULL!	#NULL!	#NULL!	#NULL!	#NULL!	#NULL!
2	2	2	2	2	2	2	2	2	2
2	2	1	1	1	1	1	1	1	1
2	2	1	1	1	1	1	1	1	1
2	2	2	#NULL!	#NULL!	#NULL!	#NULL!	2	#NULL!	#NULL!
#NULL!	#NULL!	#NULL!	#NULL!	#NULL!	#NULL!	#NULL!	#NULL!	#NULL!	#NULL!
2	#NULL!	#NULL!	#NULL!	#NULL!	1	#NULL!	#NULL!	#NULL!	#NULL!
2	2	2	2	2	2	2	2	2	2
1	2	2	2	2	2	2	2	2	2
2	2	2	2	2	2	2	2	2	2
1	1	2	2	2	2	2	2	2	1

2	2	#NULL!	#NULL!	#NULL!	#NULL!	2	#NULL!	#NULL!	#NULL!
1	1	2	#NULL!	#NULL!	#NULL!	#NULL!	#NULL!	#NULL!	#NULL!
#NULL!	2	#NULL!	#NULL!	2	#NULL!	#NULL!	#NULL!	#NULL!	#NULL!
1	1	#NULL!	#NULL!	2	#NULL!	#NULL!	#NULL!	#NULL!	#NULL!
2	2	#NULL!	#NULL!	#NULL!	#NULL!	1	#NULL!	#NULL!	#NULL!
1	1	1	1	#NULL!	2	2	2	2	2
2	2	#NULL!	#NULL!	#NULL!	#NULL!	#NULL!	#NULL!	2	#NULL!
2	1	2	#NULL!	#NULL!	#NULL!	#NULL!	#NULL!	#NULL!	#NULL!
2	2	2	#NULL!	#NULL!	#NULL!	#NULL!	#NULL!	#NULL!	#NULL!
2	2	2	#NULL!	#NULL!	#NULL!	#NULL!	#NULL!	#NULL!	#NULL!
2	2	#NULL!	#NULL!	#NULL!	2	#NULL!	#NULL!	#NULL!	#NULL!
2	2	#NULL!	#NULL!	#NULL!	#NULL!	2	#NULL!	#NULL!	#NULL!
2	2	2	#NULL!	#NULL!	#NULL!	#NULL!	#NULL!	#NULL!	#NULL!
2	1	#NULL!	2	#NULL!	#NULL!	#NULL!	#NULL!	#NULL!	#NULL!
2	2	2	#NULL!	#NULL!	#NULL!	#NULL!	#NULL!	#NULL!	#NULL!
#NULL!	#NULL!	#NULL!	#NULL!	#NULL!	#NULL!	#NULL!	#NULL!	#NULL!	#NULL!
2	2	#NULL!	#NULL!	2	#NULL!	2	#NULL!	#NULL!	1
1	1	2	#NULL!	2	2	2	1	2	2
1	1	2	2	2	2	2	2	2	2
2	2	2	2	2	2	2	2	2	2
1	2	2	1	2	2	1	1	1	1
#NULL!	#NULL!	2	1	2	2	2	2	1	2
2	2	2	2	2	2	2	2	2	2
1	2	2	1	2	2	1	2	2	1
2	2	2	1	#NULL!	2	1	1	2	1
#NULL!	#NULL!	#NULL!	#NULL!	#NULL!	#NULL!	#NULL!	#NULL!	#NULL!	#NULL!
2	2	2	2	2	2	2	2	2	2
2	2	2	2	2	2	2	2	2	2
1	2	2	2	2	2	2	2	1	2
1	1	2	2	2	2	2	2	2	2
1	1	2	2	2	2	2	1	2	2
2	2	2	2	2	2	2	2	2	2
1	2	2	1	1	2	2	2	2	2
2	2	2	2	2	2	2	2	2	2
#NULL!	#NULL!	#NULL!	#NULL!	#NULL!	#NULL!	#NULL!	#NULL!	#NULL!	#NULL!
2	2	2	2	2	2	2	2	2	2
#NULL!	#NULL!	#NULL!	#NULL!	#NULL!	#NULL!	#NULL!	#NULL!	#NULL!	#NULL!
1	2	2	2	2	2	2	2	2	2
2	2	#NULL!	#NULL!	#NULL!	#NULL!	#NULL!	#NULL!	#NULL!	#NULL!
1	1	2	2	2	2	2	2	2	2
2	2	2	2	2	2	2	2	2	1
2	2	1	1	1	1	1	1	1	1
2	1	2	#NULL!	2	#NULL!	2	1	2	1
2	2	2	2	2	2	2	2	2	2
2	2	1	1	1	1	1	2	2	2
2	2	2	2	2	2	2	1	2	2
1	1	2	2	2	2	2	2	2	2

1	1	2	1	2	2	2	2	1	2
2	2	2	#NULL!	#NULL!	#NULL!	#NULL!	#NULL!	#NULL!	#NULL!
1	1	2	2	2	2	#NULL!	2	2	1
2	2	2	2	2	1	2	2	2	2
2	2	2	2	2	2	2	2	2	2
2	2	2	2	2	2	2	2	2	1
2	2	2	2	2	2	1	2	1	2
2	2	2	1	2	2	2	2	2	1
#NULL!	#NULL!	#NULL!	#NULL!	#NULL!	#NULL!	#NULL!	#NULL!	#NULL!	#NULL!
2	2	2	2	2	2	2	2	2	2
2	2	2	2	2	2	2	2	2	2
2	2	2	#NULL!	#NULL!	#NULL!	#NULL!	#NULL!	#NULL!	#NULL!
#NULL!	#NULL!	2	#NULL!	#NULL!	#NULL!	#NULL!	#NULL!	#NULL!	#NULL!
2	1	2	2	2	2	#NULL!	2	2	2
2	2	2	#NULL!	#NULL!	#NULL!	#NULL!	#NULL!	#NULL!	#NULL!
2	2	2	2	1	1	1	2	2	2
#NULL!	#NULL!	#NULL!	#NULL!	#NULL!	#NULL!	#NULL!	#NULL!	#NULL!	#NULL!
2	1	2	#NULL!	#NULL!	#NULL!	#NULL!	#NULL!	#NULL!	#NULL!
2	2	#NULL!	#NULL!	2	#NULL!	#NULL!	#NULL!	#NULL!	#NULL!
2	2	2	1	2	1	2	1	2	1
2	2	2	2	2	2	2	2	2	2
2	2	2	2	2	2	2	2	2	2
2	2	2	2	2	2	2	2	2	2
2	2	#NULL!	#NULL!	#NULL!	#NULL!	#NULL!	#NULL!	#NULL!	#NULL!
2	2	2	#NULL!	#NULL!	#NULL!	#NULL!	#NULL!	#NULL!	#NULL!
2	2	2	2	2	2	2	2	2	2
1	1	2	2	2	2	2	2	2	2
1	1	2	1	2	2	1	2	2	2
#NULL!	#NULL!	#NULL!	#NULL!	#NULL!	#NULL!	#NULL!	#NULL!	#NULL!	#NULL!
1	1	1	#NULL!	#NULL!	#NULL!	#NULL!	#NULL!	#NULL!	#NULL!
2	2	2	2	2	2	2	2	2	2
2	2	2	2	1	2	2	1	2	2
2	2	2	2	2	1	1	2	2	2
2	2	1	2	2	2	2	2	2	2
2	2	2	2	2	2	2	2	2	2
#NULL!	#NULL!	2	2	2	2	2	2	2	2
#NULL!	#NULL!	#NULL!	#NULL!	#NULL!	#NULL!	#NULL!	#NULL!	#NULL!	#NULL!
#NULL!	#NULL!	#NULL!	#NULL!	#NULL!	#NULL!	#NULL!	#NULL!	#NULL!	#NULL!
1	2	1	2	1	2	1	2	1	2
1	2	2	2	2	2	2	2	2	2
1	2	2	2	2	2	2	2	2	2
2	2	2	2	2	2	2	2	2	2
2	2	2	2	2	2	2	2	2	2
2	2	2	2	2	1	2	2	2	1
#NULL!	#NULL!	1	1	1	1	1	1	1	1
2	2	2	2	2	2	2	2	2	2
2	2	1	1	2	2	2	2	2	2

2	2	1	1	2	1	1	1	1	1
1	1	2	1	2	2	2	1	2	2
2	1	#NULL!	#NULL!	2	#NULL!	#NULL!	#NULL!	#NULL!	#NULL!
2	2	2	#NULL!	#NULL!	#NULL!	#NULL!	#NULL!	#NULL!	#NULL!
2	2	2	2	1	2	1	2	2	1
1	1	1	1	2	1	2	2	2	1
2	2	2	#NULL!	#NULL!	#NULL!	2	#NULL!	#NULL!	#NULL!
2	2	1	#NULL!	#NULL!	1	#NULL!	1	#NULL!	1
#NULL!	#NULL!	#NULL!	#NULL!	#NULL!	#NULL!	#NULL!	#NULL!	#NULL!	#NULL!
#NULL!	#NULL!	#NULL!	#NULL!	#NULL!	#NULL!	#NULL!	#NULL!	#NULL!	#NULL!
2	2	#NULL!	#NULL!	#NULL!	#NULL!	#NULL!	#NULL!	#NULL!	#NULL!
2	2	#NULL!	#NULL!	#NULL!	#NULL!	#NULL!	#NULL!	#NULL!	#NULL!
2	2	#NULL!	#NULL!	#NULL!	#NULL!	#NULL!	#NULL!	#NULL!	#NULL!
#NULL!	#NULL!	#NULL!	#NULL!	#NULL!	#NULL!	#NULL!	#NULL!	#NULL!	#NULL!
2	2	#NULL!	#NULL!	#NULL!	#NULL!	#NULL!	#NULL!	#NULL!	#NULL!
2	2	2	#NULL!	#NULL!	#NULL!	#NULL!	#NULL!	#NULL!	#NULL!
2	2	1	1	1	1	1	1	1	1
1	1	2	#NULL!	#NULL!	#NULL!	#NULL!	#NULL!	#NULL!	#NULL!
1	1	2	#NULL!	#NULL!	#NULL!	#NULL!	#NULL!	#NULL!	#NULL!
1	1	2	#NULL!	#NULL!	#NULL!	2	#NULL!	#NULL!	2
#NULL!	#NULL!	#NULL!	#NULL!	#NULL!	#NULL!	#NULL!	#NULL!	#NULL!	#NULL!
2	2	2	#NULL!	#NULL!	#NULL!	#NULL!	#NULL!	#NULL!	#NULL!
2	2	2	2	2	2	2	2	2	2
#NULL!	#NULL!	#NULL!	#NULL!	#NULL!	#NULL!	#NULL!	#NULL!	#NULL!	#NULL!
#NULL!	#NULL!	#NULL!	#NULL!	#NULL!	#NULL!	#NULL!	#NULL!	#NULL!	#NULL!
1	1	2	#NULL!	2	#NULL!	#NULL!	#NULL!	#NULL!	#NULL!
#NULL!	#NULL!	#NULL!	#NULL!	#NULL!	#NULL!	#NULL!	#NULL!	#NULL!	#NULL!
1	1	2	2	2	2	#NULL!	2	#NULL!	2
1	1	2	1	2	2	2	2	2	2
#NULL!	#NULL!	#NULL!	#NULL!	#NULL!	#NULL!	#NULL!	#NULL!	#NULL!	#NULL!
#NULL!	#NULL!	#NULL!	#NULL!	#NULL!	#NULL!	#NULL!	#NULL!	#NULL!	#NULL!
1	1	2	2	2	2	2	2	2	2
#NULL!	#NULL!	#NULL!	#NULL!	#NULL!	#NULL!	#NULL!	#NULL!	#NULL!	#NULL!
#NULL!	#NULL!	#NULL!	#NULL!	#NULL!	#NULL!	#NULL!	#NULL!	#NULL!	#NULL!
#NULL!	#NULL!	#NULL!	#NULL!	#NULL!	#NULL!	#NULL!	#NULL!	#NULL!	#NULL!
#NULL!	#NULL!	#NULL!	#NULL!	#NULL!	#NULL!	#NULL!	#NULL!	#NULL!	#NULL!
2	2	#NULL!	#NULL!	#NULL!	#NULL!	#NULL!	#NULL!	#NULL!	#NULL!
#NULL!	#NULL!	#NULL!	#NULL!	#NULL!	#NULL!	#NULL!	#NULL!	#NULL!	#NULL!
2	2	2	1	2	1	2	2	2	1
2	2	1	2	2	1	2	2	2	2
2	2	#NULL!	#NULL!	#NULL!	#NULL!	#NULL!	#NULL!	#NULL!	#NULL!
#NULL!	#NULL!	#NULL!	#NULL!	#NULL!	#NULL!	#NULL!	#NULL!	#NULL!	#NULL!
2	2	2	1	2	2	1	2	2	1
2	2	2	2	1	2	2	1	2	2
2	2	1	1	1	1	1	1	1	2
1	1	2	2	2	2	2	1	2	1
#NULL!	#NULL!	2	1	2	2	1	2	#NULL!	#NULL!

2	2	2	#NULL!	#NULL!	#NULL!	#NULL!	#NULL!	#NULL!	#NULL!
2	2	2	1	1	2	2	2	2	2
2	2	2	2	2	2	2	2	2	2
1	1	2	2	2	2	2	2	2	2
2	2	2	2	2	2	2	2	2	2
2	1	2	2	2	2	2	1	1	1
1	1	#NULL!	#NULL!	#NULL!	#NULL!	#NULL!	#NULL!	#NULL!	#NULL!
2	2	1	1	1	1	2	2	2	2
2	2	2	2	2	2	2	2	2	2
2	2	2	2	2	2	2	2	2	2
#NULL!	2	2	2	#NULL!	1	2	2	2	2
2	2	#NULL!	#NULL!	#NULL!	#NULL!	#NULL!	2	#NULL!	#NULL!
2	2	#NULL!	#NULL!	#NULL!	#NULL!	#NULL!	#NULL!	2	#NULL!
1	1	#NULL!	#NULL!	#NULL!	#NULL!	#NULL!	#NULL!	#NULL!	#NULL!
2	2	2	#NULL!	#NULL!	#NULL!	#NULL!	#NULL!	#NULL!	#NULL!
1	2	2	2	2	2	2	2	2	2
2	2	1	1	2	2	2	2	2	2
2	2	2	1	1	1	1	1	2	1
2	2	2	2	2	1	1	1	1	1
1	2	2	2	2	2	2	2	2	2
2	2	2	2	2	2	2	2	2	2
#NULL!	#NULL!	#NULL!	#NULL!	#NULL!	#NULL!	#NULL!	#NULL!	#NULL!	#NULL!
2	1	1	1	1	1	2	2	2	2
2	2	2	#NULL!	#NULL!	#NULL!	#NULL!	#NULL!	#NULL!	#NULL!
2	2	#NULL!	#NULL!	#NULL!	#NULL!	#NULL!	#NULL!	#NULL!	#NULL!
1	1	2	2	2	2	2	2	2	2
2	2	#NULL!	#NULL!	#NULL!	2	#NULL!	#NULL!	2	#NULL!
2	2	2	1	1	2	2	2	2	2
#NULL!	#NULL!	#NULL!	#NULL!	#NULL!	#NULL!	#NULL!	#NULL!	#NULL!	#NULL!
1	1	#NULL!	#NULL!	#NULL!	#NULL!	#NULL!	#NULL!	#NULL!	#NULL!
1	2	2	1	2	1	1	1	2	2
2	2	2	1	2	1	2	1	2	1
#NULL!	#NULL!	#NULL!	#NULL!	#NULL!	#NULL!	#NULL!	#NULL!	#NULL!	#NULL!
1	1	2	#NULL!	#NULL!	#NULL!	#NULL!	#NULL!	#NULL!	#NULL!
2	1	2	2	2	2	2	1	1	2
#NULL!	#NULL!	#NULL!	#NULL!	#NULL!	#NULL!	#NULL!	#NULL!	#NULL!	#NULL!
2	#NULL!	2	1	2	2	1	2	1	2
1	1	2	2	2	2	2	2	2	2
2	2	2	2	2	2	2	2	2	2
2	2	#NULL!	#NULL!	#NULL!	#NULL!	#NULL!	#NULL!	#NULL!	#NULL!
2	2	2	2	2	2	2	2	2	2
2	2	2	2	2	2	2	2	2	2
2	2	2	2	2	2	2	2	2	2
2	2	2	2	2	2	2	2	2	2
2	2	2	1	1	2	1	2	1	2
2	2	1	1	2	2	2	2	2	2
2	2	2	2	2	2	1	1	1	1

2	2	2	#NULL!	#NULL!	#NULL!	#NULL!	#NULL!	#NULL!	#NULL!
2	2	2	2	2	2	2	2	2	2
2	2	2	2	2	2	2	2	2	2
2	1	#NULL!	#NULL!	#NULL!	#NULL!	2	#NULL!	#NULL!	#NULL!
2	2	1	#NULL!	#NULL!	#NULL!	#NULL!	#NULL!	#NULL!	#NULL!
#NULL!	#NULL!	#NULL!	#NULL!	#NULL!	#NULL!	#NULL!	#NULL!	#NULL!	#NULL!
#NULL!	#NULL!	#NULL!	#NULL!	#NULL!	#NULL!	#NULL!	#NULL!	#NULL!	#NULL!
2	2	2	2	2	2	2	2	2	2
1	1	2	2	2	2	2	2	2	2
2	2	#NULL!	#NULL!	#NULL!	#NULL!	#NULL!	#NULL!	#NULL!	#NULL!
1	1	2	2	2	2	2	2	2	2
1	2	2	1	1	1	2	2	2	1
1	1	2	2	2	2	2	2	2	2
2	2	2	2	2	2	2	2	2	2
2	1	#NULL!	#NULL!	#NULL!	#NULL!	#NULL!	#NULL!	#NULL!	#NULL!
#NULL!	#NULL!	#NULL!	#NULL!	#NULL!	#NULL!	#NULL!	#NULL!	#NULL!	#NULL!
#NULL!	#NULL!	#NULL!	#NULL!	#NULL!	#NULL!	#NULL!	#NULL!	#NULL!	#NULL!
#NULL!	#NULL!	#NULL!	#NULL!	#NULL!	#NULL!	#NULL!	#NULL!	#NULL!	#NULL!
2	2	2	#NULL!	#NULL!	#NULL!	#NULL!	#NULL!	#NULL!	#NULL!
2	2	2	#NULL!	#NULL!	#NULL!	#NULL!	#NULL!	#NULL!	#NULL!
#NULL!	#NULL!	#NULL!	#NULL!	#NULL!	#NULL!	#NULL!	#NULL!	#NULL!	#NULL!
#NULL!	#NULL!	#NULL!	#NULL!	#NULL!	#NULL!	#NULL!	#NULL!	#NULL!	#NULL!
2	2	2	2	2	2	2	2	2	2
2	2	2	#NULL!	#NULL!	#NULL!	#NULL!	#NULL!	#NULL!	#NULL!
#NULL!	#NULL!	#NULL!	#NULL!	#NULL!	#NULL!	#NULL!	#NULL!	#NULL!	#NULL!
2	2	1	#NULL!	#NULL!	#NULL!	#NULL!	#NULL!	#NULL!	#NULL!
#NULL!	#NULL!	#NULL!	#NULL!	#NULL!	#NULL!	#NULL!	#NULL!	#NULL!	#NULL!
2	2	#NULL!	#NULL!	#NULL!	#NULL!	#NULL!	#NULL!	#NULL!	#NULL!
2	2	2	#NULL!	#NULL!	#NULL!	#NULL!	2	#NULL!	#NULL!
2	2	1	#NULL!	#NULL!	#NULL!	#NULL!	#NULL!	#NULL!	#NULL!
#NULL!	#NULL!	#NULL!	#NULL!	#NULL!	#NULL!	#NULL!	#NULL!	#NULL!	#NULL!
2	2	2	2	2	2	2	2	2	2
#NULL!	#NULL!	#NULL!	#NULL!	#NULL!	#NULL!	#NULL!	#NULL!	#NULL!	#NULL!
#NULL!	#NULL!	#NULL!	#NULL!	#NULL!	#NULL!	#NULL!	#NULL!	#NULL!	#NULL!
2	2	#NULL!	#NULL!	#NULL!	#NULL!	#NULL!	#NULL!	#NULL!	#NULL!
#NULL!	#NULL!	#NULL!	#NULL!	#NULL!	#NULL!	#NULL!	#NULL!	#NULL!	#NULL!
2	2	#NULL!	#NULL!	#NULL!	#NULL!	#NULL!	#NULL!	#NULL!	#NULL!
#NULL!	#NULL!	#NULL!	#NULL!	#NULL!	#NULL!	#NULL!	#NULL!	#NULL!	#NULL!
#NULL!	#NULL!	#NULL!	#NULL!	#NULL!	#NULL!	#NULL!	#NULL!	#NULL!	#NULL!
#NULL!	#NULL!	#NULL!	#NULL!	#NULL!	#NULL!	#NULL!	#NULL!	#NULL!	#NULL!
1	1	2	2	2	2	2	2	2	2
1	1	2	#NULL!	#NULL!	#NULL!	#NULL!	#NULL!	#NULL!	#NULL!
#NULL!	#NULL!	#NULL!	#NULL!	#NULL!	#NULL!	#NULL!	#NULL!	#NULL!	#NULL!
2	2	#NULL!	#NULL!	#NULL!	#NULL!	#NULL!	#NULL!	#NULL!	#NULL!
#NULL!	#NULL!	#NULL!	#NULL!	#NULL!	#NULL!	#NULL!	#NULL!	#NULL!	#NULL!
2	2	#NULL!	#NULL!	#NULL!	#NULL!	#NULL!	#NULL!	#NULL!	#NULL!
#NULL!	#NULL!	#NULL!	#NULL!	#NULL!	#NULL!	#NULL!	#NULL!	#NULL!	#NULL!

#NULL!	#NULL!	#NULL!	#NULL!	#NULL!	#NULL!	#NULL!	#NULL!	#NULL!	#NULL!
#NULL!	#NULL!	1	1	1	1	1	1	1	1
2	2	2	#NULL!	#NULL!	#NULL!	#NULL!	#NULL!	#NULL!	#NULL!
#NULL!	#NULL!	#NULL!	#NULL!	#NULL!	#NULL!	#NULL!	#NULL!	#NULL!	#NULL!
#NULL!	#NULL!	#NULL!	#NULL!	#NULL!	#NULL!	#NULL!	#NULL!	#NULL!	#NULL!
#NULL!	#NULL!	#NULL!	#NULL!	#NULL!	#NULL!	#NULL!	#NULL!	#NULL!	#NULL!
#NULL!	#NULL!	#NULL!	#NULL!	#NULL!	#NULL!	#NULL!	#NULL!	#NULL!	#NULL!
#NULL!	#NULL!	#NULL!	#NULL!	#NULL!	#NULL!	#NULL!	#NULL!	#NULL!	#NULL!
#NULL!	#NULL!	#NULL!	#NULL!	#NULL!	#NULL!	#NULL!	#NULL!	#NULL!	#NULL!
#NULL!	#NULL!	#NULL!	#NULL!	#NULL!	#NULL!	#NULL!	#NULL!	#NULL!	#NULL!
#NULL!	#NULL!	#NULL!	#NULL!	#NULL!	#NULL!	#NULL!	#NULL!	#NULL!	#NULL!
#NULL!	#NULL!	#NULL!	#NULL!	#NULL!	#NULL!	#NULL!	#NULL!	#NULL!	#NULL!
#NULL!	#NULL!	#NULL!	#NULL!	#NULL!	#NULL!	#NULL!	#NULL!	#NULL!	#NULL!
#NULL!	#NULL!	#NULL!	#NULL!	#NULL!	#NULL!	#NULL!	#NULL!	#NULL!	#NULL!
2	2	#NULL!	#NULL!	#NULL!	#NULL!	#NULL!	#NULL!	#NULL!	#NULL!
2	2	#NULL!	#NULL!	#NULL!	#NULL!	#NULL!	#NULL!	#NULL!	#NULL!
#NULL!	#NULL!	#NULL!	#NULL!	#NULL!	#NULL!	#NULL!	#NULL!	#NULL!	#NULL!
2	2	2	2	2	2	2	2	2	2
#NULL!	#NULL!	#NULL!	#NULL!	#NULL!	#NULL!	#NULL!	#NULL!	#NULL!	#NULL!
2	2	#NULL!	#NULL!	#NULL!	#NULL!	#NULL!	#NULL!	#NULL!	#NULL!
#NULL!	#NULL!	#NULL!	#NULL!	#NULL!	#NULL!	#NULL!	#NULL!	#NULL!	#NULL!
#NULL!	2	#NULL!	#NULL!	#NULL!	#NULL!	#NULL!	#NULL!	#NULL!	#NULL!
#NULL!	#NULL!	#NULL!	#NULL!	#NULL!	#NULL!	#NULL!	#NULL!	#NULL!	#NULL!
2	2	#NULL!	#NULL!	#NULL!	#NULL!	#NULL!	#NULL!	#NULL!	#NULL!
2	2	2	2	2	2	2	2	2	2
2	2	#NULL!	#NULL!	#NULL!	#NULL!	#NULL!	#NULL!	#NULL!	#NULL!
#NULL!	#NULL!	#NULL!	#NULL!	#NULL!	#NULL!	#NULL!	#NULL!	#NULL!	#NULL!
2	2	2	#NULL!	2	2	2	2	2	2
#NULL!	#NULL!	#NULL!	#NULL!	#NULL!	#NULL!	#NULL!	#NULL!	#NULL!	#NULL!
#NULL!	#NULL!	#NULL!	#NULL!	#NULL!	#NULL!	#NULL!	#NULL!	#NULL!	#NULL!
1	1	2	2	2	2	2	2	2	2
2	2	2	2	2	2	2	2	1	1
#NULL!	#NULL!	#NULL!	#NULL!	#NULL!	#NULL!	#NULL!	#NULL!	#NULL!	#NULL!
2	2	1	1	1	1	1	1	1	1
#NULL!	#NULL!	#NULL!	#NULL!	#NULL!	#NULL!	#NULL!	#NULL!	#NULL!	#NULL!
2	2	2	2	2	2	2	2	2	2
1	1	2	#NULL!	#NULL!	#NULL!	#NULL!	#NULL!	#NULL!	#NULL!
#NULL!	#NULL!	#NULL!	#NULL!	#NULL!	#NULL!	#NULL!	#NULL!	#NULL!	#NULL!
2	2	2	2	2	2	2	2	2	2
2	2	1	#NULL!	#NULL!	#NULL!	#NULL!	#NULL!	#NULL!	#NULL!
#NULL!	#NULL!	#NULL!	#NULL!	#NULL!	#NULL!	#NULL!	#NULL!	#NULL!	#NULL!
#NULL!	#NULL!	#NULL!	#NULL!	#NULL!	#NULL!	#NULL!	#NULL!	#NULL!	#NULL!
2	2	2	2	2	2	2	2	2	2
1	1	2	2	2	2	2	2	2	2
2	2	#NULL!	2	#NULL!	2	#NULL!	2	#NULL!	2
#NULL!	#NULL!	#NULL!	#NULL!	#NULL!	#NULL!	#NULL!	#NULL!	#NULL!	#NULL!
#NULL!	#NULL!	#NULL!	#NULL!	#NULL!	#NULL!	#NULL!	#NULL!	#NULL!	#NULL!

2	2	2	#NULL!	#NULL!	#NULL!	#NULL!	2	#NULL!	#NULL!
#NULL!	#NULL!	#NULL!	#NULL!	#NULL!	#NULL!	#NULL!	#NULL!	#NULL!	#NULL!
2	2	2	#NULL!	#NULL!	#NULL!	2	1	#NULL!	2
#NULL!	#NULL!	#NULL!	#NULL!	#NULL!	#NULL!	#NULL!	#NULL!	#NULL!	#NULL!
#NULL!	#NULL!	#NULL!	#NULL!	#NULL!	#NULL!	#NULL!	#NULL!	#NULL!	#NULL!
#NULL!	#NULL!	#NULL!	#NULL!	#NULL!	#NULL!	#NULL!	#NULL!	#NULL!	#NULL!
1	1	2	#NULL!	#NULL!	#NULL!	#NULL!	#NULL!	#NULL!	#NULL!
2	2	2	#NULL!	#NULL!	#NULL!	#NULL!	#NULL!	#NULL!	#NULL!
1	1	2	#NULL!	#NULL!	2	#NULL!	#NULL!	2	#NULL!
2	2	#NULL!	#NULL!	#NULL!	#NULL!	#NULL!	#NULL!	#NULL!	#NULL!
2	2	#NULL!	#NULL!	#NULL!	#NULL!	#NULL!	#NULL!	#NULL!	#NULL!
#NULL!	#NULL!	#NULL!	#NULL!	#NULL!	#NULL!	#NULL!	#NULL!	#NULL!	#NULL!
#NULL!	#NULL!	#NULL!	#NULL!	#NULL!	#NULL!	#NULL!	#NULL!	#NULL!	#NULL!
#NULL!	#NULL!	#NULL!	#NULL!	#NULL!	#NULL!	#NULL!	#NULL!	#NULL!	#NULL!
2	2	2	2	2	2	1	1	1	1
2	2	1	2	2	2	1	2	2	1
2	2	#NULL!	#NULL!	#NULL!	#NULL!	#NULL!	#NULL!	2	#NULL!
2	2	2	2	2	2	2	2	2	2
2	2	2	2	2	1	2	2	1	2
1	1	2	#NULL!	#NULL!	#NULL!	#NULL!	#NULL!	#NULL!	#NULL!
2	2	2	#NULL!	#NULL!	#NULL!	#NULL!	#NULL!	#NULL!	#NULL!
#NULL!	1	2	#NULL!	#NULL!	#NULL!	#NULL!	#NULL!	#NULL!	#NULL!
2	2	1	2	2	2	2	2	2	2
2	2	2	2	1	2	2	1	2	2
2	2	2	2	2	2	2	2	2	1
2	2	2	2	2	2	2	2	2	2
2	2	2	1	1	1	2	#NULL!	1	1
2	1	2	2	1	1	1	2	2	2
2	1	2	2	2	1	1	1	2	2
2	2	2	2	2	2	2	2	2	2
2	2	2	2	2	2	2	2	2	2
#NULL!	2	2	#NULL!	#NULL!	#NULL!	#NULL!	#NULL!	#NULL!	#NULL!
1	1	#NULL!	#NULL!	#NULL!	#NULL!	#NULL!	#NULL!	#NULL!	#NULL!
2	2	2	#NULL!	#NULL!	#NULL!	#NULL!	#NULL!	#NULL!	#NULL!
2	1	2	#NULL!	#NULL!	#NULL!	#NULL!	#NULL!	#NULL!	#NULL!
#NULL!	#NULL!	#NULL!	#NULL!	#NULL!	#NULL!	#NULL!	#NULL!	#NULL!	#NULL!
#NULL!	#NULL!	#NULL!	#NULL!	#NULL!	#NULL!	#NULL!	#NULL!	#NULL!	#NULL!
1	1	#NULL!	2	#NULL!	#NULL!	#NULL!	2	#NULL!	#NULL!
#NULL!	#NULL!	#NULL!	#NULL!	#NULL!	#NULL!	#NULL!	#NULL!	#NULL!	#NULL!
2	2	2	#NULL!	2	#NULL!	2	#NULL!	2	#NULL!
#NULL!	#NULL!	#NULL!	#NULL!	#NULL!	#NULL!	#NULL!	#NULL!	#NULL!	#NULL!
1	1	2	2	2	2	2	2	2	2
#NULL!	#NULL!	#NULL!	#NULL!	#NULL!	#NULL!	#NULL!	#NULL!	#NULL!	#NULL!
#NULL!	#NULL!	#NULL!	#NULL!	#NULL!	#NULL!	#NULL!	#NULL!	#NULL!	#NULL!
#NULL!	#NULL!	#NULL!	#NULL!	#NULL!	#NULL!	#NULL!	#NULL!	#NULL!	#NULL!
2	2	2	2	2	2	2	2	2	2
2	2	2	2	1	2	2	1	2	1

2	2	2	2	2	2	2	2	2	2
2	2	2	2	2	2	2	2	2	2
2	2	2	2	2	2	2	2	2	2
#NULL!	#NULL!	1	1	1	1	1	2	1	2
2	2	1	2	1	1	1	1	1	1
2	2	2	2	2	2	2	2	2	1
2	2	2	2	2	2	2	2	2	2
2	2	1	1	1	1	1	1	1	1
2	2	#NULL!	#NULL!	#NULL!	#NULL!	#NULL!	#NULL!	#NULL!	#NULL!
1	1	2	2	2	2	2	2	2	2
2	2	2	#NULL!	2	#NULL!	2	#NULL!	2	#NULL!
2	2	#NULL!	#NULL!	2	#NULL!	#NULL!	2	#NULL!	2
2	1	2	2	2	2	2	2	2	2
2	1	2	2	1	2	2	2	2	2
2	2	1	2	1	1	1	1	1	1
2	2	2	2	2	2	2	2	2	2
2	2	2	2	1	2	1	2	2	2
2	2	2	1	1	1	1	1	2	1
#NULL!	#NULL!	#NULL!	#NULL!	#NULL!	#NULL!	#NULL!	#NULL!	#NULL!	#NULL!
2	2	2	2	2	2	1	2	2	2
2	2	1	2	2	2	1	2	1	2
2	2	2	2	2	1	2	1	2	1
2	2	2	2	2	2	2	2	2	2
1	2	2	2	2	2	2	2	2	1
2	2	2	1	2	2	2	1	2	1
2	2	2	2	2	2	2	2	2	2
2	2	2	1	2	2	1	2	2	1
2	2	2	2	2	2	2	2	1	1
2	2	2	2	2	2	2	2	2	2
2	2	2	2	2	2	1	2	2	1
2	2	1	1	1	1	1	1	1	1
2	2	#NULL!	#NULL!	#NULL!	#NULL!	#NULL!	#NULL!	#NULL!	#NULL!
2	2	1	1	1	1	1	1	1	1
2	2	2	2	2	2	2	2	2	2
2	2	#NULL!	1	#NULL!	#NULL!	#NULL!	#NULL!	#NULL!	#NULL!
2	2	1	1	1	1	1	1	2	2
2	2	2	2	2	2	2	2	2	2
1	1	2	2	2	2	2	2	2	2
1	2	2	2	1	2	2	2	2	2
#NULL!	#NULL!	#NULL!	#NULL!	#NULL!	#NULL!	#NULL!	#NULL!	#NULL!	#NULL!
2	2	2	2	2	2	2	2	2	2
2	1	#NULL!	#NULL!	#NULL!	#NULL!	#NULL!	#NULL!	#NULL!	#NULL!
2	2	2	2	2	1	2	2	2	2
2	2	2	2	2	2	2	2	2	2
2	2	2	1	2	1	2	1	2	1
2	1	#NULL!	#NULL!	#NULL!	#NULL!	#NULL!	#NULL!	#NULL!	#NULL!
2	2	1	1	1	2	2	2	2	2

#NULL!	#NULL!	#NULL!	#NULL!	#NULL!	#NULL!	#NULL!	#NULL!	#NULL!	#NULL!
2	2	1	1	1	1	1	1	1	1
2	1	2	1	2	2	2	1	1	2
2	2	2	2	1	2	2	1	2	2
2	1	2	2	2	2	1	2	2	1
#NULL!	#NULL!	#NULL!	#NULL!	#NULL!	#NULL!	#NULL!	#NULL!	#NULL!	#NULL!
2	2	2	2	2	2	2	2	2	2
2	2	2	2	1	1	2	2	1	2
2	1	2	1	1	1	2	1	2	1
2	2	2	2	2	2	2	2	2	2
2	2	2	2	2	2	1	2	2	2
2	2	2	2	2	2	2	2	2	2
#NULL!	#NULL!	#NULL!	#NULL!	#NULL!	#NULL!	#NULL!	#NULL!	#NULL!	#NULL!
2	2	1	2	2	2	1	1	1	1
2	1	2	2	2	2	2	2	2	1
2	2	2	2	2	2	2	2	2	2
2	2	2	2	2	2	2	2	2	2
2	2	2	2	2	2	2	2	2	1
#NULL!	#NULL!	2	2	2	2	2	2	2	2
2	1	2	#NULL!	#NULL!	#NULL!	#NULL!	#NULL!	#NULL!	#NULL!
#NULL!	#NULL!	#NULL!	#NULL!	#NULL!	#NULL!	#NULL!	#NULL!	#NULL!	#NULL!
#NULL!	#NULL!	2	2	2	1	2	2	2	2
2	2	2	2	2	1	1	1	2	1
2	2	2	1	2	2	2	1	2	1
#NULL!	#NULL!	#NULL!	#NULL!	#NULL!	#NULL!	#NULL!	#NULL!	#NULL!	#NULL!
2	2	2	2	1	1	1	2	1	2
2	2	2	#NULL!	#NULL!	#NULL!	#NULL!	#NULL!	#NULL!	#NULL!
2	2	2	2	2	2	2	2	2	2
2	2	2	2	2	2	2	2	2	2
2	2	2	2	2	2	2	2	2	2
1	2	1	2	2	1	2	2	2	2
#NULL!	#NULL!	#NULL!	#NULL!	#NULL!	#NULL!	#NULL!	#NULL!	#NULL!	#NULL!
#NULL!	#NULL!	#NULL!	#NULL!	#NULL!	#NULL!	#NULL!	#NULL!	#NULL!	#NULL!
1	2	2	1	2	2	2	2	2	2
2	2	2	1	2	2	1	2	1	1
#NULL!	#NULL!	#NULL!	#NULL!	#NULL!	#NULL!	#NULL!	#NULL!	#NULL!	#NULL!
#NULL!	#NULL!	#NULL!	#NULL!	#NULL!	#NULL!	#NULL!	#NULL!	#NULL!	#NULL!
2	2	2	1	1	1	1	2	2	2
1	2	2	1	2	2	1	1	#NULL!	2
2	2	2	2	2	2	2	2	2	2
2	2	2	2	2	2	2	2	2	2
2	2	2	2	2	2	2	2	2	2
2	2	2	2	2	2	2	2	2	2
2	2	2	2	2	2	2	2	2	2
2	2	2	2	2	2	2	2	2	2
2	2	2	2	2	2	1	2	2	2
1	2	2	2	2	2	2	2	2	2

1	2	1	1	1	2	2	2	1	1
2	2	2	2	2	2	2	2	2	2
1	2	2	2	2	2	2	2	2	2
1	1	2	2	2	2	2	2	2	2
2	2	2	2	2	2	2	2	2	2
2	2	2	2	2	2	2	2	2	2
2	2	2	2	2	2	2	2	2	2
2	2	2	2	2	2	2	2	2	2
2	2	2	2	2	2	2	2	2	2
#NULL!	#NULL!	#NULL!	#NULL!	#NULL!	#NULL!	#NULL!	#NULL!	#NULL!	#NULL!
2	1	2	2	1	2	2	2	2	2
2	2	2	2	2	2	1	2	1	1
2	2	2	2	2	2	2	2	2	1
1	2	1	2	2	2	2	2	2	2
2	2	1	1	1	1	1	1	1	1
2	1	#NULL!	#NULL!	#NULL!	#NULL!	#NULL!	#NULL!	2	#NULL!
#NULL!	#NULL!	#NULL!	#NULL!	#NULL!	#NULL!	#NULL!	#NULL!	#NULL!	#NULL!
2	2	#NULL!	2	#NULL!	#NULL!	#NULL!	#NULL!	2	#NULL!
#NULL!	#NULL!	#NULL!	#NULL!	#NULL!	#NULL!	#NULL!	#NULL!	#NULL!	#NULL!
#NULL!	#NULL!	2	2	2	2	2	2	2	2
2	2	2	1	2	2	2	2	#NULL!	2
#NULL!	#NULL!	#NULL!	#NULL!	#NULL!	#NULL!	#NULL!	#NULL!	#NULL!	#NULL!
#NULL!	#NULL!	#NULL!	#NULL!	#NULL!	#NULL!	#NULL!	#NULL!	#NULL!	#NULL!
2	2	2	2	2	2	2	2	2	2
2	1	2	2	2	2	1	2	2	2
2	2	2	2	2	2	2	2	2	2
1	2	2	2	2	2	1	2	2	2
2	2	1	2	#NULL!	1	1	2	2	2
2	2	2	2	2	2	1	2	2	2
2	1	2	2	2	2	2	2	2	2
#NULL!	#NULL!	#NULL!	#NULL!	#NULL!	#NULL!	#NULL!	#NULL!	#NULL!	#NULL!
#NULL!	#NULL!	2	2	2	2	2	1	2	1
2	1	2	1	1	1	1	2	2	2
1	1	2	2	2	2	2	2	2	2
2	2	2	2	2	2	2	2	2	2
2	2	2	2	2	2	2	2	2	2
#NULL!	#NULL!	#NULL!	#NULL!	#NULL!	#NULL!	#NULL!	#NULL!	#NULL!	#NULL!
2	2	2	2	2	2	2	2	2	2
2	2	2	2	2	2	2	2	2	2
2	2	1	2	2	2	2	1	2	2
2	1	2	2	2	1	1	1	1	1
2	1	2	2	2	1	2	2	2	2
2	2	2	2	2	2	2	2	2	2
2	2	2	2	2	2	2	2	2	2
2	2	2	1	2	2	2	2	2	2
2	2	2	2	2	2	2	2	2	2
2	2	1	1	2	2	2	2	1	1

#NULL!	#NULL!	2	#NULL!	2	1	2	2	2	2
2	2	2	2	2	2	2	2	2	2
1	2	2	2	2	2	2	1	1	1
2	2	2	2	2	2	2	2	2	2
2	2	1	2	1	1	1	1	1	2
#NULL!	#NULL!	#NULL!	#NULL!	#NULL!	#NULL!	#NULL!	#NULL!	#NULL!	#NULL!
#NULL!	2	#NULL!	#NULL!	#NULL!	#NULL!	2	#NULL!	#NULL!	#NULL!
2	2	2	2	1	1	1	1	1	1
#NULL!	#NULL!	#NULL!	#NULL!	#NULL!	#NULL!	#NULL!	#NULL!	#NULL!	#NULL!
#NULL!	#NULL!	#NULL!	#NULL!	#NULL!	#NULL!	#NULL!	#NULL!	#NULL!	#NULL!
2	2	2	2	2	2	2	2	2	2
2	2	2	2	2	2	2	2	2	2
1	1	2	2	2	2	2	2	2	2
1	1	#NULL!	#NULL!	2	#NULL!	#NULL!	#NULL!	#NULL!	#NULL!
2	2	#NULL!	#NULL!	2	#NULL!	#NULL!	#NULL!	#NULL!	#NULL!
2	2	2	1	2	2	2	1	1	2
2	2	2	2	2	2	2	2	2	1
#NULL!	#NULL!	#NULL!	#NULL!	#NULL!	#NULL!	#NULL!	#NULL!	#NULL!	#NULL!
2	2	2	1	2	2	2	2	2	1
1	2	2	2	2	2	2	2	2	2
2	#NULL!	2	2	2	2	2	2	2	2
2	2	#NULL!	#NULL!	#NULL!	#NULL!	#NULL!	#NULL!	#NULL!	#NULL!
2	2	2	2	2	2	2	2	2	2
2	2	2	2	2	2	2	2	2	2
2	2	2	2	2	2	2	2	2	2
2	2	2	2	2	2	2	2	2	2
2	2	2	2	2	2	2	2	2	2
2	2	2	2	2	2	2	2	2	2
2	2	2	1	2	2	1	2	1	2
2	2	1	1	2	1	2	1	2	1
1	2	2	2	2	2	1	2	2	2
1	1	2	2	1	2	2	1	1	2
2	2	2	2	2	2	2	2	2	2
2	2	2	2	2	2	2	2	2	2
#NULL!	2	2	2	2	2	2	2	1	2
1	2	2	2	2	1	2	2	2	2
2	1	2	2	2	2	2	2	2	2
2	2	2	2	2	2	1	1	1	1
2	1	2	#NULL!	2	2	2	2	1	2
2	2	2	1	2	1	1	2	2	1
1	1	2	2	2	2	2	2	1	1
2	2	2	2	2	2	2	2	1	1
2	2	2	2	2	2	2	2	2	2
2	2	2	2	2	2	2	2	2	2
2	2	2	2	2	2	2	2	2	2
2	1	2	2	2	2	1	2	1	2
2	2	2	2	2	2	2	2	2	2

2	2	2	1	2	2	1	2	2	1
2	1	2	2	2	2	2	2	2	2
2	2	2	1	2	1	2	1	2	1
1	1	2	2	2	2	2	2	2	2
2	1	2	#NULL!	2	#NULL!	#NULL!	#NULL!	#NULL!	#NULL!
2	2	#NULL!	#NULL!	#NULL!	#NULL!	2	#NULL!	#NULL!	#NULL!
2	2	1	2	1	2	1	2	1	2
1	1	2	#NULL!	#NULL!	#NULL!	#NULL!	#NULL!	#NULL!	#NULL!
1	1	2	#NULL!	#NULL!	#NULL!	#NULL!	#NULL!	#NULL!	#NULL!
#NULL!	#NULL!	#NULL!	#NULL!	#NULL!	#NULL!	#NULL!	#NULL!	#NULL!	#NULL!
2	2	#NULL!	#NULL!	#NULL!	#NULL!	#NULL!	1	#NULL!	#NULL!
#NULL!	2	#NULL!	#NULL!	2	#NULL!	#NULL!	#NULL!	#NULL!	#NULL!
2	1	2	#NULL!	#NULL!	#NULL!	#NULL!	#NULL!	#NULL!	#NULL!
2	1	#NULL!	#NULL!	#NULL!	#NULL!	2	#NULL!	#NULL!	#NULL!
2	2	2	2	2	2	2	2	2	2
#NULL!	#NULL!	2	#NULL!	#NULL!	#NULL!	#NULL!	#NULL!	#NULL!	#NULL!
#NULL!	#NULL!	#NULL!	#NULL!	#NULL!	#NULL!	#NULL!	#NULL!	#NULL!	#NULL!
2	2	#NULL!	#NULL!	2	#NULL!	#NULL!	#NULL!	#NULL!	#NULL!
2	2	#NULL!	#NULL!	2	#NULL!	#NULL!	#NULL!	#NULL!	#NULL!
#NULL!	#NULL!	#NULL!	#NULL!	#NULL!	1	#NULL!	#NULL!	#NULL!	#NULL!
#NULL!	#NULL!	#NULL!	#NULL!	#NULL!	#NULL!	#NULL!	#NULL!	#NULL!	#NULL!
2	2	2	2	2	2	2	2	2	2
2	2	2	2	2	2	2	2	2	2
2	2	2	2	2	1	2	2	2	1
2	1	2	2	#NULL!	2	1	1	1	1
2	#NULL!	#NULL!	#NULL!	#NULL!	#NULL!	#NULL!	#NULL!	#NULL!	#NULL!
2	2	2	1	2	2	2	2	2	2
2	2	2	#NULL!	#NULL!	#NULL!	#NULL!	#NULL!	#NULL!	#NULL!
2	2	1	1	1	2	2	2	1	1
1	2	2	2	2	2	2	2	2	1
2	2	1	1	1	1	1	1	1	1
2	2	1	#NULL!	#NULL!	#NULL!	#NULL!	#NULL!	#NULL!	#NULL!
1	1	2	2	2	2	2	2	2	2
2	2	2	#NULL!	#NULL!	#NULL!	#NULL!	#NULL!	#NULL!	#NULL!
1	1	2	#NULL!	#NULL!	#NULL!	#NULL!	#NULL!	#NULL!	#NULL!
#NULL!	#NULL!	1	2	2	2	2	2	2	2
1	1	2	2	2	2	#NULL!	2	2	2
#NULL!	#NULL!	#NULL!	#NULL!	#NULL!	#NULL!	#NULL!	#NULL!	#NULL!	#NULL!
2	2	#NULL!	#NULL!	#NULL!	#NULL!	#NULL!	#NULL!	#NULL!	#NULL!
2	2	2	2	2	2	2	2	2	2
2	2	#NULL!	#NULL!	#NULL!	#NULL!	#NULL!	#NULL!	#NULL!	#NULL!
1	2	2	2	2	2	2	2	2	2
2	2	1	1	1	1	1	1	1	1
2	2	#NULL!	#NULL!	2	#NULL!	#NULL!	#NULL!	2	#NULL!
1	1	2	2	2	2	2	2	2	2
1	1	2	2	2	2	2	2	2	2
2	2	#NULL!	2	#NULL!	1	2	#NULL!	#NULL!	2

#NULL!	#NULL!	#NULL!	#NULL!	#NULL!	#NULL!	#NULL!	#NULL!	#NULL!	#NULL!
2	2	2	2	#NULL!	#NULL!	2	#NULL!	2	#NULL!
#NULL!	#NULL!	#NULL!	#NULL!	#NULL!	#NULL!	#NULL!	#NULL!	#NULL!	#NULL!
1	1	2	2	2	2	2	2	2	2
2	2	#NULL!	1	2	#NULL!	1	2	1	2
2	2	#NULL!	2	#NULL!	1	2	1	2	#NULL!
2	2	#NULL!	#NULL!	#NULL!	#NULL!	#NULL!	#NULL!	#NULL!	#NULL!
#NULL!	#NULL!	#NULL!	#NULL!	#NULL!	#NULL!	#NULL!	#NULL!	#NULL!	#NULL!
2	2	2	#NULL!	#NULL!	2	#NULL!	2	#NULL!	#NULL!
1	1	2	2	2	2	2	2	2	2
#NULL!	#NULL!	#NULL!	#NULL!	#NULL!	#NULL!	#NULL!	#NULL!	#NULL!	#NULL!
2	2	2	#NULL!	#NULL!	2	#NULL!	2	#NULL!	2
2	2	2	2	2	2	2	2	2	2
#NULL!	#NULL!	#NULL!	#NULL!	#NULL!	#NULL!	#NULL!	#NULL!	#NULL!	#NULL!
1	1	2	2	2	2	2	2	2	2
#NULL!	#NULL!	#NULL!	#NULL!	#NULL!	#NULL!	#NULL!	#NULL!	#NULL!	#NULL!
#NULL!	#NULL!	#NULL!	#NULL!	#NULL!	#NULL!	#NULL!	#NULL!	#NULL!	#NULL!
#NULL!	#NULL!	#NULL!	#NULL!	#NULL!	#NULL!	#NULL!	#NULL!	#NULL!	#NULL!
#NULL!	#NULL!	#NULL!	#NULL!	#NULL!	#NULL!	#NULL!	#NULL!	#NULL!	#NULL!
#NULL!	#NULL!	#NULL!	#NULL!	#NULL!	#NULL!	#NULL!	#NULL!	#NULL!	#NULL!
2	1	2	2	2	2	2	2	2	2
2	2	2	2	2	2	2	2	2	2
2	2	2	2	1	2	1	2	2	2
2	1	2	2	2	2	2	2	2	2
2	2	2	1	1	1	1	1	1	1
2	2	2	2	2	2	2	2	2	2
1	1	2	2	2	2	2	2	2	2
2	2	2	2	2	2	2	2	2	2
2	1	2	1	2	1	2	2	2	2
2	2	2	2	2	2	2	2	2	2
2	2	2	2	2	1	1	1	2	2
2	2	2	1	2	1	2	1	2	1
2	2	2	2	2	2	2	2	2	2
#NULL!	#NULL!	#NULL!	#NULL!	#NULL!	#NULL!	#NULL!	#NULL!	#NULL!	#NULL!
2	2	1	1	1	2	2	1	2	2
1	1	1	2	2	2	2	2	2	2
2	2	2	2	2	2	2	2	2	2
1	1	2	2	2	2	2	2	2	2
2	2	2	2	2	2	2	1	2	1
2	2	2	2	2	2	2	2	2	2
2	2	2	2	2	1	1	1	1	2
#NULL!	#NULL!	#NULL!	#NULL!	#NULL!	#NULL!	#NULL!	#NULL!	#NULL!	#NULL!
2	2	2	2	2	#NULL!	#NULL!	#NULL!	#NULL!	#NULL!
#NULL!	#NULL!	#NULL!	#NULL!	#NULL!	#NULL!	#NULL!	#NULL!	#NULL!	#NULL!
1	1	#NULL!	#NULL!	#NULL!	#NULL!	#NULL!	#NULL!	#NULL!	#NULL!
2	2	2	2	2	2	2	2	2	2
2	2	#NULL!	#NULL!	#NULL!	#NULL!	#NULL!	#NULL!	#NULL!	#NULL!

2	2	2	2	2	2	2	2	2	1
#NULL!	#NULL!	2	2	2	2	1	1	1	1
1	1	2	2	2	2	2	2	2	2
1	1	2	2	2	2	#NULL!	#NULL!	2	2
1	1	2	2	2	2	2	2	2	2
1	1	2	2	2	2	2	2	2	2
1	1	2	2	2	2	#NULL!	2	2	2
1	1	2	2	2	2	2	1	1	1
2	2	1	1	1	1	1	1	1	1
1	1	2	2	2	2	2	2	2	2
2	2	#NULL!	#NULL!	#NULL!	#NULL!	#NULL!	#NULL!	#NULL!	#NULL!
1	1	2	2	2	2	2	2	2	2
1	1	2	2	2	2	2	2	2	2
1	1	2	2	2	2	2	2	2	2
1	1	2	2	2	2	2	2	2	2
1	1	2	2	2	2	2	2	2	2
1	1	2	2	2	2	2	2	2	2
1	1	2	2	2	2	2	2	2	2
1	1	2	2	2	2	2	2	2	2
2	2	#NULL!	#NULL!	#NULL!	#NULL!	#NULL!	#NULL!	#NULL!	#NULL!
1	1	2	2	2	2	2	2	2	2
2	2	2	2	2	2	2	2	2	2
#NULL!	#NULL!	#NULL!	#NULL!	#NULL!	#NULL!	#NULL!	#NULL!	#NULL!	#NULL!
1	1	2	2	2	2	2	2	2	2
#NULL!	#NULL!	#NULL!	#NULL!	#NULL!	#NULL!	#NULL!	#NULL!	#NULL!	#NULL!
1	1	2	2	2	2	2	2	2	2
1	1	2	2	2	2	2	2	2	2
2	1	2	2	2	2	2	2	2	2
1	1	#NULL!	#NULL!	#NULL!	#NULL!	#NULL!	#NULL!	#NULL!	#NULL!
1	1	2	2	2	2	2	2	#NULL!	2
2	2	#NULL!	#NULL!	#NULL!	#NULL!	#NULL!	#NULL!	#NULL!	#NULL!
2	2	#NULL!	#NULL!	#NULL!	#NULL!	#NULL!	#NULL!	#NULL!	#NULL!
1	1	2	2	2	2	2	2	2	2
1	1	2	2	2	2	2	2	2	2
2	2	2	2	2	2	2	2	2	2
#NULL!	#NULL!	#NULL!	#NULL!	#NULL!	#NULL!	#NULL!	#NULL!	#NULL!	#NULL!
1	1	2	2	2	2	2	2	2	2
1	1	2	2	2	2	2	2	2	2
#NULL!	#NULL!	#NULL!	#NULL!	#NULL!	#NULL!	#NULL!	#NULL!	#NULL!	#NULL!
2	2	#NULL!	#NULL!	#NULL!	#NULL!	#NULL!	#NULL!	#NULL!	#NULL!
1	1	2	2	2	2	2	2	2	2
#NULL!	#NULL!	#NULL!	#NULL!	#NULL!	#NULL!	#NULL!	#NULL!	#NULL!	#NULL!
1	1	2	2	2	2	2	2	2	2
1	1	2	2	2	2	2	2	2	2
1	1	2	2	2	2	2	2	2	2
2	2	2	2	2	2	2	2	2	2
1	1	2	2	2	2	2	2	2	2

1	1	2	2	2	2	2	2	2	2
2	2	2	#NULL!	2	2	2	#NULL!	2	#NULL!
1	1	1	1	1	#NULL!	1	1	1	1
1	1	2	2	2	2	2	2	2	2
1	1	2	2	2	2	2	2	2	2
#NULL!	#NULL!	#NULL!	#NULL!	#NULL!	#NULL!	#NULL!	#NULL!	#NULL!	#NULL!
1	1	2	2	2	2	2	2	2	2
1	1	2	2	2	2	2	2	2	2
2	2	2	2	#NULL!	2	#NULL!	2	2	#NULL!
1	1	2	2	#NULL!	2	2	2	2	2
2	2	#NULL!	#NULL!	#NULL!	#NULL!	#NULL!	#NULL!	#NULL!	#NULL!
1	1	#NULL!	#NULL!	#NULL!	#NULL!	#NULL!	#NULL!	#NULL!	#NULL!
#NULL!	#NULL!	#NULL!	#NULL!	#NULL!	#NULL!	#NULL!	#NULL!	#NULL!	#NULL!
1	1	#NULL!	#NULL!	#NULL!	#NULL!	#NULL!	#NULL!	#NULL!	#NULL!
2	2	#NULL!	#NULL!	#NULL!	#NULL!	#NULL!	#NULL!	#NULL!	#NULL!
#NULL!	#NULL!	#NULL!	#NULL!	#NULL!	#NULL!	#NULL!	#NULL!	#NULL!	#NULL!
1	1	2	2	2	2	2	2	2	2
#NULL!	#NULL!	#NULL!	#NULL!	#NULL!	#NULL!	#NULL!	#NULL!	#NULL!	#NULL!
2	2	2	2	2	2	2	2	2	2
1	1	2	1	1	1	1	1	2	1
1	1	2	1	1	1	1	1	2	2
1	1	2	1	1	1	1	1	2	2
1	1	2	1	1	1	1	1	2	2
1	1	2	1	1	#NULL!	1	1	2	2
1	1	2	1	1	2	1	1	2	2
1	1	2	1	1	1	1	1	2	2
1	1	2	1	1	1	1	1	2	2
1	1	2	1	1	1	1	1	2	2
1	1	2	1	1	2	1	1	2	#NULL!
1	1	2	1	1	1	1	1	2	2
1	1	2	1	1	1	1	1	2	2
1	1	2	1	1	1	1	1	2	2
1	1	2	1	1	1	1	1	2	2
1	1	2	1	1	1	1	1	2	2
1	1	2	1	1	#NULL!	1	1	2	2
1	1	2	1	1	1	1	1	2	2
1	1	2	1	1	1	1	1	2	2
#NULL!	#NULL!	2	1	1	2	1	1	2	2
1	1	2	1	1	2	1	1	2	2
1	1	2	1	1	1	1	1	2	2
1	1	2	1	1	1	1	1	2	2
1	1	2	1	1	1	1	1	2	2
1	1	2	1	1	1	1	1	2	2
1	1	1	2	1	1	1	1	2	2
#NULL!	#NULL!	#NULL!	#NULL!	#NULL!	#NULL!	#NULL!	#NULL!	#NULL!	#NULL!
#NULL!	#NULL!	#NULL!	#NULL!	#NULL!	#NULL!	#NULL!	#NULL!	#NULL!	#NULL!
#NULL!	#NULL!	#NULL!	#NULL!	#NULL!	#NULL!	#NULL!	#NULL!	#NULL!	#NULL!

#NULL!	#NULL!	#NULL!	#NULL!	#NULL!	#NULL!	#NULL!	#NULL!	#NULL!	#NULL!
#NULL!	#NULL!	#NULL!	#NULL!	#NULL!	#NULL!	#NULL!	#NULL!	#NULL!	#NULL!
#NULL!	#NULL!	#NULL!	#NULL!	#NULL!	#NULL!	#NULL!	#NULL!	#NULL!	#NULL!
#NULL!	#NULL!	#NULL!	#NULL!	#NULL!	#NULL!	#NULL!	#NULL!	#NULL!	#NULL!
#NULL!	#NULL!	#NULL!	#NULL!	#NULL!	#NULL!	#NULL!	#NULL!	#NULL!	#NULL!
#NULL!	#NULL!	#NULL!	#NULL!	#NULL!	#NULL!	#NULL!	#NULL!	#NULL!	#NULL!
#NULL!	#NULL!	#NULL!	#NULL!	#NULL!	#NULL!	#NULL!	#NULL!	#NULL!	#NULL!
#NULL!	#NULL!	#NULL!	#NULL!	#NULL!	#NULL!	#NULL!	#NULL!	#NULL!	#NULL!
#NULL!	#NULL!	#NULL!	#NULL!	#NULL!	#NULL!	#NULL!	#NULL!	#NULL!	#NULL!
#NULL!	#NULL!	#NULL!	#NULL!	#NULL!	#NULL!	#NULL!	#NULL!	#NULL!	#NULL!
#NULL!	#NULL!	#NULL!	#NULL!	#NULL!	#NULL!	#NULL!	#NULL!	#NULL!	#NULL!
#NULL!	#NULL!	#NULL!	#NULL!	#NULL!	#NULL!	#NULL!	#NULL!	#NULL!	#NULL!
#NULL!	#NULL!	#NULL!	#NULL!	#NULL!	#NULL!	#NULL!	#NULL!	#NULL!	#NULL!
#NULL!	#NULL!	#NULL!	#NULL!	#NULL!	#NULL!	#NULL!	#NULL!	#NULL!	#NULL!
#NULL!	#NULL!	#NULL!	#NULL!	#NULL!	#NULL!	#NULL!	#NULL!	#NULL!	#NULL!
#NULL!	#NULL!	#NULL!	#NULL!	#NULL!	#NULL!	#NULL!	#NULL!	#NULL!	#NULL!
#NULL!	#NULL!	#NULL!	#NULL!	#NULL!	#NULL!	#NULL!	#NULL!	#NULL!	#NULL!
#NULL!	#NULL!	#NULL!	#NULL!	#NULL!	#NULL!	#NULL!	#NULL!	#NULL!	#NULL!
#NULL!	#NULL!	#NULL!	#NULL!	#NULL!	#NULL!	#NULL!	#NULL!	#NULL!	#NULL!
1	1	2	1	1	1	1	1	2	2
1	1	2	1	1	1	1	1	2	2
1	1	2	1	1	1	1	1	2	2
1	1	2	1	1	1	1	1	2	2
1	1	2	1	1	1	1	1	2	2
2	1	2	1	1	1	1	1	2	2
1	1	2	1	1	2	1	1	2	2
1	1	2	1	1	2	1	1	2	2
1	1	2	1	1	1	1	1	2	2
1	1	2	2	1	1	1	1	2	2
1	1	2	2	1	1	1	1	2	2
1	1	2	1	1	2	1	1	1	2
1	1	2	1	1	1	1	1	2	2
1	1	2	1	1	2	1	1	2	2
1	1	2	1	1	1	1	#NULL!	#NULL!	2
1	1	2	1	1	1	1	1	2	1
2	2	2	#NULL!	#NULL!	#NULL!	#NULL!	#NULL!	#NULL!	#NULL!
1	1	2	1	1	1	1	1	2	2
1	1	2	1	1	1	1	1	2	2
1	1	2	1	1	1	1	1	2	2
1	1	2	1	1	1	1	1	2	2
1	1	2	1	1	1	1	1	2	2
1	1	2	1	1	1	1	1	2	2
1	1	2	1	1	2	1	1	2	2
1	1	2	2	1	1	1	1	#NULL!	2
1	2	2	1	1	2	1	1	2	2
1	1	2	1	1	1	1	1	2	2
1	1	2	1	1	1	1	1	2	2

1	1	2	1	1	#NULL!	1	1	2	2
1	1	2	1	1	1	1	2	2	2
1	1	2	1	1	1	1	1	2	2
1	1	2	1	1	#NULL!	1	2	2	2
1	1	2	1	1	1	1	1	2	2
1	1	2	1	1	1	1	1	2	2
1	1	2	1	1	1	1	2	2	2
1	1	2	1	2	1	1	1	1	2
1	1	2	1	1	1	1	1	2	2
1	1	2	1	1	1	1	1	2	2
1	1	2	1	1	1	1	2	2	2
1	1	2	1	1	1	1	1	2	2
1	1	2	1	1	2	1	1	2	2
1	1	2	1	1	1	1	2	2	2
1	1	2	1	1	1	1	1	2	2
1	1	2	1	1	2	1	1	2	2
1	1	2	1	1	1	1	1	2	2
1	1	2	1	1	1	1	1	2	2
1	1	2	1	1	1	1	1	2	2
1	1	2	1	1	#NULL!	1	1	2	2
1	1	2	1	1	1	1	1	2	2
1	1	2	1	1	1	1	1	1	2
1	1	2	1	1	1	1	1	2	2
1	1	2	1	1	1	1	1	2	2
1	1	2	1	1	#NULL!	1	1	2	2
1	1	2	1	1	1	1	1	2	2
1	1	2	1	1	1	2	1	2	2
1	1	2	1	1	1	1	1	2	2
1	1	2	1	1	1	1	1	2	2
1	1	2	1	1	1	1	1	2	#NULL!
1	1	2	1	1	#NULL!	1	1	2	2
#NULL!	#NULL!	2	2	1	1	1	1	#NULL!	#NULL!
#NULL!	#NULL!	2	2	2	2	#NULL!	#NULL!	#NULL!	#NULL!
1	1	2	1	1	1	1	1	2	2
#NULL!	#NULL!	#NULL!	#NULL!	#NULL!	#NULL!	#NULL!	#NULL!	#NULL!	#NULL!
1	1	2	1	1	1	1	1	2	2
1	1	2	1	1	#NULL!	1	1	2	2
1	1	2	1	1	#NULL!	1	2	#NULL!	2
1	1	2	1	1	1	1	1	2	2
1	1	2	1	1	1	2	1	2	2
1	1	2	1	1	1	1	1	2	2
1	1	2	1	1	2	1	1	2	2
1	1	2	1	1	1	1	1	2	2
1	1	2	1	1	1	1	1	2	2
1	1	2	1	1	1	1	1	2	2
1	1	2	1	1	2	1	1	2	2
1	1	2	1	1	1	1	1	2	2

1	1	2	1	1	1	1	1	2	2
1	1	2	1	1	1	1	1	2	2
1	1	2	1	1	2	1	1	2	2
1	1	2	1	1	1	1	1	2	2
1	1	2	1	1	2	1	1	2	2
1	#NULL!	2	1	1	2	1	1	2	2
1	1	2	1	1	2	1	1	2	2
1	1	2	1	1	1	1	1	2	2
1	1	2	1	1	1	1	1	2	2
1	1	2	1	1	2	1	1	2	2
1	1	2	1	1	#NULL!	1	1	2	2
1	1	2	1	1	1	1	1	2	2
1	1	2	1	1	1	1	1	2	2
1	1	2	1	1	1	1	1	2	2
1	1	2	1	1	#NULL!	1	#NULL!	#NULL!	#NULL!
1	1	2	1	1	1	1	1	2	2
1	1	2	1	1	1	1	1	2	2
1	1	2	1	1	1	1	1	2	2
1	1	2	1	1	1	1	1	2	2
#NULL!	#NULL!	2	1	1	#NULL!	1	1	2	2
1	1	2	1	1	#NULL!	1	1	2	2
1	1	2	1	1	1	1	1	2	2
1	1	2	1	1	1	1	1	2	2
1	1	2	1	1	1	1	1	2	2
1	1	2	1	#NULL!	1	2	#NULL!	#NULL!	#NULL!
1	1	2	1	1	2	1	1	2	1
1	1	2	1	1	2	1	1	2	2
1	1	2	1	1	1	2	1	2	2
1	1	2	1	1	1	1	1	2	1
1	1	2	1	1	1	1	1	2	2
1	1	2	1	1	1	1	1	2	1
1	1	2	1	1	1	1	1	2	2
1	1	1	2	1	1	1	1	2	2
1	1	1	2	1	1	1	1	2	2
1	1	2	1	1	1	1	1	2	2
1	1	2	1	1	1	1	1	2	2
1	1	2	1	1	1	1	1	2	2
1	1	2	1	1	#NULL!	#NULL!	1	2	2
1	1	2	1	1	1	1	1	2	2
1	1	2	1	1	1	1	1	2	2
1	1	2	1	1	2	1	1	2	2
#NULL!	#NULL!	#NULL!	#NULL!	#NULL!	#NULL!	#NULL!	#NULL!	#NULL!	#NULL!
1	1	2	1	1	2	1	1	2	2
1	1	2	1	1	2	1	1	2	1
1	1	2	1	1	2	1	1	2	2
1	1	2	1	1	2	1	1	1	2
1	1	2	1	1	1	1	1	1	2

1	1	2	1	1	2	2	1	2	1
1	1	2	1	1	2	1	1	2	2
1	1	2	1	1	1	1	1	2	2
1	1	2	1	1	1	1	1	2	2
1	1	2	1	1	1	1	1	2	2
1	1	2	1	1	1	1	1	2	2
1	1	2	1	1	1	1	1	2	2
2	1	2	1	1	2	1	1	2	2
1	1	2	1	1	1	1	1	2	2
#NULL!	#NULL!	#NULL!	#NULL!	#NULL!	#NULL!	#NULL!	#NULL!	#NULL!	#NULL!
1	1	2	1	1	1	1	1	2	2
1	1	2	1	1	2	1	1	2	2
1	1	2	1	1	2	1	1	2	2
1	1	2	1	1	1	1	1	2	2
1	1	2	1	1	1	1	1	2	2
1	1	2	1	1	1	1	1	2	2
1	1	2	1	1	1	1	1	2	2
1	1	2	1	1	1	1	1	1	2
1	1	2	1	1	1	1	1	2	2
1	1	2	1	1	2	1	1	2	2
1	1	2	1	1	1	1	1	2	2
1	1	2	1	1	1	1	1	2	2
1	1	2	1	1	1	1	1	2	2
1	1	2	1	1	1	1	1	2	2
1	1	2	1	1	2	1	1	2	2
1	1	2	1	1	2	1	1	2	2
#NULL!	#NULL!	2	1	1	2	1	1	2	2
#NULL!	#NULL!	#NULL!	#NULL!	#NULL!	#NULL!	#NULL!	#NULL!	#NULL!	#NULL!
#NULL!	#NULL!	#NULL!	#NULL!	#NULL!	#NULL!	#NULL!	#NULL!	#NULL!	#NULL!
#NULL!	#NULL!	#NULL!	#NULL!	#NULL!	#NULL!	#NULL!	#NULL!	#NULL!	#NULL!
#NULL!	#NULL!	#NULL!	#NULL!	#NULL!	#NULL!	#NULL!	#NULL!	#NULL!	#NULL!
#NULL!	#NULL!	#NULL!	#NULL!	#NULL!	#NULL!	#NULL!	#NULL!	#NULL!	#NULL!
#NULL!	#NULL!	#NULL!	#NULL!	#NULL!	#NULL!	#NULL!	#NULL!	#NULL!	#NULL!
#NULL!	#NULL!	#NULL!	#NULL!	#NULL!	#NULL!	#NULL!	#NULL!	#NULL!	#NULL!
#NULL!	#NULL!	#NULL!	#NULL!	#NULL!	#NULL!	#NULL!	#NULL!	#NULL!	#NULL!
#NULL!	#NULL!	#NULL!	#NULL!	#NULL!	#NULL!	#NULL!	#NULL!	#NULL!	#NULL!
#NULL!	#NULL!	#NULL!	#NULL!	#NULL!	#NULL!	#NULL!	#NULL!	#NULL!	#NULL!
#NULL!	#NULL!	#NULL!	#NULL!	#NULL!	#NULL!	#NULL!	#NULL!	#NULL!	#NULL!
#NULL!	#NULL!	#NULL!	#NULL!	#NULL!	#NULL!	#NULL!	#NULL!	#NULL!	#NULL!
#NULL!	#NULL!	#NULL!	#NULL!	#NULL!	#NULL!	#NULL!	#NULL!	#NULL!	#NULL!
#NULL!	#NULL!	#NULL!	#NULL!	#NULL!	#NULL!	#NULL!	#NULL!	#NULL!	#NULL!
#NULL!	#NULL!	#NULL!	#NULL!	#NULL!	#NULL!	#NULL!	#NULL!	#NULL!	#NULL!
#NULL!	#NULL!	#NULL!	#NULL!	#NULL!	#NULL!	#NULL!	#NULL!	#NULL!	#NULL!
#NULL!	#NULL!	#NULL!	#NULL!	#NULL!	#NULL!	#NULL!	#NULL!	#NULL!	#NULL!
#NULL!	#NULL!	#NULL!	#NULL!	#NULL!	#NULL!	#NULL!	#NULL!	#NULL!	#NULL!
#NULL!	#NULL!	#NULL!	#NULL!	#NULL!	#NULL!	#NULL!	#NULL!	#NULL!	#NULL!
#NULL!	#NULL!	#NULL!	#NULL!	#NULL!	#NULL!	#NULL!	#NULL!	#NULL!	#NULL!
#NULL!	#NULL!	#NULL!	#NULL!	#NULL!	#NULL!	#NULL!	#NULL!	#NULL!	#NULL!
#NULL!	#NULL!	#NULL!	#NULL!	#NULL!	#NULL!	#NULL!	#NULL!	#NULL!	#NULL!
#NULL!	#NULL!	#NULL!	#NULL!	#NULL!	#NULL!	#NULL!	#NULL!	#NULL!	#NULL!

#NULL!	#NULL!	#NULL!	#NULL!	#NULL!	#NULL!	#NULL!	#NULL!	#NULL!	#NULL!
#NULL!	#NULL!	#NULL!	#NULL!	#NULL!	#NULL!	#NULL!	#NULL!	#NULL!	#NULL!
#NULL!	#NULL!	#NULL!	#NULL!	#NULL!	#NULL!	#NULL!	#NULL!	#NULL!	#NULL!
#NULL!	#NULL!	#NULL!	#NULL!	#NULL!	#NULL!	#NULL!	#NULL!	#NULL!	#NULL!
#NULL!	#NULL!	#NULL!	#NULL!	#NULL!	#NULL!	#NULL!	#NULL!	#NULL!	#NULL!
#NULL!	#NULL!	#NULL!	#NULL!	#NULL!	#NULL!	#NULL!	#NULL!	#NULL!	#NULL!
#NULL!	#NULL!	#NULL!	#NULL!	#NULL!	#NULL!	#NULL!	#NULL!	#NULL!	#NULL!
#NULL!	#NULL!	#NULL!	#NULL!	#NULL!	#NULL!	#NULL!	#NULL!	#NULL!	#NULL!
#NULL!	#NULL!	#NULL!	#NULL!	#NULL!	#NULL!	#NULL!	#NULL!	#NULL!	#NULL!
#NULL!	#NULL!	#NULL!	#NULL!	#NULL!	#NULL!	#NULL!	#NULL!	#NULL!	#NULL!
#NULL!	#NULL!	#NULL!	#NULL!	#NULL!	#NULL!	#NULL!	#NULL!	#NULL!	#NULL!
#NULL!	#NULL!	#NULL!	#NULL!	#NULL!	#NULL!	#NULL!	#NULL!	#NULL!	#NULL!
#NULL!	#NULL!	#NULL!	#NULL!	#NULL!	#NULL!	#NULL!	#NULL!	#NULL!	#NULL!
1	1	1	1	2	2	2	2	2	1
2	2	2	2	2	2	2	2	2	2
2	2	2	2	2	2	2	2	2	2
#NULL!	#NULL!	2	2	1	2	1	2	2	2
2	1	2	2	2	1	2	2	2	1
#NULL!	#NULL!	2	2	2	2	2	2	2	2
1	1	2	2	1	2	2	2	2	2
2	1	2	2	2	2	2	2	2	2
2	2	2	2	2	2	2	2	2	2
#NULL!	#NULL!	#NULL!	#NULL!	#NULL!	#NULL!	#NULL!	#NULL!	#NULL!	#NULL!
2	2	2	1	2	2	2	2	1	2
2	2	#NULL!	#NULL!	#NULL!	#NULL!	#NULL!	#NULL!	#NULL!	#NULL!
1	1	2	2	1	1	1	1	1	2
#NULL!	#NULL!	#NULL!	#NULL!	#NULL!	#NULL!	#NULL!	#NULL!	#NULL!	#NULL!
2	2	2	2	2	2	2	2	2	2
1	2	1	2	2	1	2	2	1	2
2	2	2	2	2	2	2	2	2	2
2	2	1	1	1	1	1	1	1	1
2	2	2	2	2	1	2	2	1	2
2	2	2	#NULL!	#NULL!	#NULL!	#NULL!	#NULL!	#NULL!	#NULL!
2	2	2	1	2	2	2	2	2	2
1	1	2	2	2	2	2	2	2	2
2	1	2	#NULL!	#NULL!	#NULL!	#NULL!	#NULL!	#NULL!	#NULL!
1	2	2	2	2	2	2	2	2	2
1	1	2	2	2	1	2	2	2	1
2	1	2	2	2	2	2	1	2	2
1	1	2	2	2	2	2	2	2	2
#NULL!	#NULL!	#NULL!	#NULL!	#NULL!	#NULL!	#NULL!	#NULL!	#NULL!	#NULL!
#NULL!	#NULL!	#NULL!	#NULL!	#NULL!	#NULL!	#NULL!	#NULL!	#NULL!	#NULL!
1	1	2	2	2	2	2	2	2	2
2	2	2	2	2	2	2	2	2	2
#NULL!	#NULL!	#NULL!	#NULL!	#NULL!	#NULL!	#NULL!	#NULL!	#NULL!	#NULL!
#NULL!	#NULL!	#NULL!	#NULL!	#NULL!	#NULL!	#NULL!	#NULL!	#NULL!	#NULL!
#NULL!	#NULL!	#NULL!	#NULL!	#NULL!	#NULL!	#NULL!	#NULL!	#NULL!	#NULL!

1	2	#NULL!	#NULL!	#NULL!	#NULL!	#NULL!	#NULL!	#NULL!	#NULL!
#NULL!	#NULL!	#NULL!	#NULL!	#NULL!	#NULL!	#NULL!	#NULL!	#NULL!	#NULL!
2	1	2	2	2	1	2	2	2	2
2	2	2	2	2	2	2	2	2	2
2	2	2	2	2	1	1	1	2	1
2	1	#NULL!	2	#NULL!	#NULL!	#NULL!	#NULL!	#NULL!	#NULL!
1	1	2	2	2	2	2	2	2	2
1	1	2	2	2	2	2	2	2	2
2	2	2	2	2	2	2	2	2	2
2	2	2	2	2	2	2	2	2	2
2	2	1	1	2	2	1	1	1	1
#NULL!	#NULL!	#NULL!	#NULL!	#NULL!	#NULL!	#NULL!	#NULL!	#NULL!	#NULL!
2	2	2	#NULL!	#NULL!	#NULL!	#NULL!	#NULL!	#NULL!	#NULL!
2	2	2	2	2	2	2	2	2	2
2	2	2	2	2	2	2	2	2	2
2	2	2	2	2	2	2	2	2	2
2	2	2	2	2	2	2	2	2	2
1	1	2	2	2	2	2	2	2	2
1	1	2	2	2	2	1	2	1	2
#NULL!	#NULL!	#NULL!	#NULL!	#NULL!	#NULL!	#NULL!	#NULL!	#NULL!	#NULL!
2	2	2	2	2	2	2	2	2	2
#NULL!	#NULL!	2	2	2	2	2	2	2	2
2	2	2	2	2	2	2	2	2	2
1	2	2	2	2	2	2	2	2	2
#NULL!	#NULL!	2	2	2	2	2	2	2	2
1	2	2	2	2	2	2	2	2	2
2	2	2	2	2	2	2	2	2	2
2	2	2	2	2	2	2	2	2	2
2	2	2	2	2	2	2	2	2	2
1	1	2	1	2	2	2	2	2	2
2	1	2	1	1	1	1	1	2	1
2	2	2	2	2	2	2	2	2	1
2	2	2	2	2	2	2	2	2	2
1	1	2	2	2	2	2	2	2	1
2	2	2	2	2	2	1	2	2	1
1	2	2	2	2	2	2	2	2	2
2	2	2	2	2	2	2	2	2	2
2	1	2	2	2	#NULL!	#NULL!	#NULL!	#NULL!	#NULL!
2	2	2	#NULL!	#NULL!	#NULL!	#NULL!	#NULL!	#NULL!	#NULL!
2	2	2	2	2	2	2	2	2	2
2	2	1	1	1	1	1	2	2	2
2	2	2	2	2	2	2	2	2	2
2	2	2	2	1	2	2	1	2	2
2	2	2	2	2	1	1	1	2	2
1	1	2	2	2	2	2	2	2	2
1	1	2	2	2	2	2	2	2	2
2	#NULL!	2	2	2	2	2	2	2	2

2	2	2	2	2	2	2	2	2	2
2	2	2	2	1	2	2	1	2	2
2	2	2	2	2	2	2	2	2	2
2	2	2	2	1	1	2	1	2	2
1	1	2	2	2	2	2	2	2	2
1	1	2	2	2	2	2	2	2	2
2	2	2	2	2	2	2	2	2	2
2	2	2	1	2	2	1	2	2	2
2	2	2	2	2	2	2	2	2	2
2	2	2	2	1	2	2	2	1	2
2	2	2	2	2	2	2	2	2	2
2	2	2	2	2	2	2	2	2	2
2	1	2	2	2	2	2	2	2	2
#NULL!	#NULL!	2	2	2	1	1	1	1	1
1	1	2	2	2	2	2	2	2	2
2	2	2	2	2	2	2	2	2	2
1	1	2	2	2	2	2	2	2	2
2	2	2	2	2	2	2	2	2	2
#NULL!	#NULL!	2	2	2	2	2	2	2	2
1	2	2	2	1	2	2	1	2	2
2	2	2	2	2	2	2	2	2	2
2	2	2	2	2	2	2	2	2	1
2	2	2	2	2	2	2	2	2	2
1	1	2	2	2	1	1	1	1	1
1	2	2	1	1	2	1	1	2	1
#NULL!	#NULL!	#NULL!	#NULL!	#NULL!	#NULL!	#NULL!	#NULL!	#NULL!	#NULL!
2	#NULL!	#NULL!	#NULL!	#NULL!	#NULL!	#NULL!	#NULL!	#NULL!	#NULL!
2	2	2	2	2	2	2	2	2	2
1	1	#NULL!	#NULL!	#NULL!	#NULL!	#NULL!	#NULL!	#NULL!	#NULL!
2	1	2	2	2	2	1	1	2	2
2	2	2	2	2	2	2	2	2	2
1	2	2	1	2	2	2	2	2	2
2	1	2	2	2	2	1	1	1	1
1	1	2	2	2	2	2	2	2	2
1	1	#NULL!	#NULL!	2	#NULL!	#NULL!	#NULL!	#NULL!	#NULL!
#NULL!	#NULL!	2	2	2	2	2	2	2	2
#NULL!	#NULL!	#NULL!	#NULL!	#NULL!	#NULL!	#NULL!	#NULL!	#NULL!	#NULL!
#NULL!	#NULL!	2	2	2	2	2	2	2	2
#NULL!	#NULL!	#NULL!	#NULL!	#NULL!	#NULL!	#NULL!	#NULL!	#NULL!	#NULL!
2	2	2	2	2	2	2	2	2	2
2	2	2	1	1	2	2	2	1	2
1	2	2	2	2	2	2	2	2	2
2	2	2	2	2	2	2	2	2	2
#NULL!	#NULL!	#NULL!	#NULL!	#NULL!	#NULL!	#NULL!	#NULL!	#NULL!	#NULL!
#NULL!	#NULL!	#NULL!	#NULL!	#NULL!	#NULL!	#NULL!	#NULL!	#NULL!	#NULL!
#NULL!	#NULL!	#NULL!	#NULL!	#NULL!	#NULL!	#NULL!	#NULL!	#NULL!	#NULL!
1	1	2	2	2	2	2	2	2	2

#NULL!	#NULL!	#NULL!	#NULL!	#NULL!	#NULL!	#NULL!	#NULL!	#NULL!	#NULL!
#NULL!	#NULL!	#NULL!	#NULL!	#NULL!	#NULL!	#NULL!	#NULL!	#NULL!	#NULL!
2	1	2	2	2	2	2	1	2	1
#NULL!	#NULL!	#NULL!	#NULL!	#NULL!	#NULL!	#NULL!	#NULL!	#NULL!	#NULL!
#NULL!	#NULL!	#NULL!	#NULL!	#NULL!	#NULL!	#NULL!	#NULL!	#NULL!	#NULL!
2	2	2	2	2	2	1	1	2	2
2	2	1	2	2	2	2	1	1	2
#NULL!	#NULL!	#NULL!	#NULL!	#NULL!	#NULL!	#NULL!	#NULL!	#NULL!	#NULL!
1	1	2	2	2	2	2	2	2	1
2	2	2	2	1	2	2	1	2	2
2	2	2	1	2	2	2	1	2	2
2	2	2	2	2	2	2	2	2	2
2	2	2	2	2	2	2	2	2	2
#NULL!	#NULL!	#NULL!	#NULL!	#NULL!	#NULL!	#NULL!	#NULL!	#NULL!	#NULL!
2	2	2	2	2	2	2	2	2	2
2	2	2	2	2	2	2	2	2	2
1	2	1	1	2	1	1	1	2	1
2	2	2	1	2	1	1	1	2	2
1	1	2	2	1	1	2	2	1	2
#NULL!	#NULL!	#NULL!	#NULL!	#NULL!	#NULL!	#NULL!	#NULL!	#NULL!	#NULL!
2	2	2	1	2	1	1	1	2	2
1	1	2	2	2	1	2	2	2	2
2	2	2	2	2	2	2	2	2	2
2	2	#NULL!	2	2	1	1	1	2	2
#NULL!	#NULL!	1	1	1	1	1	1	1	1
#NULL!	#NULL!	#NULL!	#NULL!	#NULL!	#NULL!	#NULL!	#NULL!	#NULL!	#NULL!
2	2	1	1	2	1	1	2	2	2
2	2	#NULL!	#NULL!	#NULL!	#NULL!	#NULL!	#NULL!	#NULL!	#NULL!
2	1	2	2	2	2	2	2	2	2
2	2	2	2	2	2	1	2	2	2
2	2	2	2	2	2	2	2	2	2
2	2	2	#NULL!	#NULL!	#NULL!	#NULL!	#NULL!	#NULL!	#NULL!
2	2	2	#NULL!	#NULL!	#NULL!	#NULL!	#NULL!	#NULL!	#NULL!
#NULL!	#NULL!	2	2	2	2	2	2	2	2
2	2	2	2	2	2	2	2	2	2
#NULL!	#NULL!	2	2	1	2	1	2	2	2
#NULL!	#NULL!	2	2	2	2	2	2	2	2
2	2	2	2	2	2	2	2	2	1
2	2	2	2	2	2	2	2	2	2
2	2	2	#NULL!	#NULL!	#NULL!	#NULL!	#NULL!	#NULL!	#NULL!
#NULL!	#NULL!	2	2	2	2	2	2	2	2
2	2	2	2	2	2	2	2	2	2
2	2	1	1	2	2	2	2	2	2
2	2	2	2	2	2	2	2	2	2
2	2	2	2	2	2	2	2	2	2
1	2	2	#NULL!	#NULL!	#NULL!	#NULL!	#NULL!	#NULL!	#NULL!
2	2	2	2	1	1	2	2	1	2

1	1	2	1	2	2	2	2	2	2
2	2	2	2	2	2	2	2	2	2
2	2	2	1	2	2	1	1	2	1
2	2	2	2	2	2	2	2	2	2
2	2	2	2	2	2	2	2	2	2
2	2	2	2	2	2	2	1	1	1
2	2	2	2	2	1	2	2	2	2
2	2	2	2	1	2	2	2	2	2
2	2	2	1	2	2	1	1	2	1
2	2	1	1	1	2	1	1	1	1
2	2	2	2	2	2	2	2	2	2
2	2	2	2	2	2	2	2	2	2
2	2	2	2	2	2	2	2	2	2
2	2	2	1	2	2	1	1	2	1
2	2	2	2	#NULL!	2	2	1	2	#NULL!
2	2	2	2	2	2	2	2	2	2
2	2	2	2	1	2	1	2	1	2
#NULL!	#NULL!	2	2	2	2	2	2	2	2
2	2	1	2	2	2	2	1	2	2
2	2	2	2	2	2	2	2	2	2
2	2	2	1	2	2	2	2	2	1
1	1	#NULL!	#NULL!	#NULL!	#NULL!	#NULL!	#NULL!	#NULL!	#NULL!
2	2	2	2	2	2	2	2	2	2
1	1	2	2	2	2	2	2	2	2
2	2	2	2	2	2	2	2	2	2
2	2	2	1	2	2	2	2	2	2
2	1	2	2	1	2	2	2	2	2
2	1	2	2	2	2	2	2	2	2
1	1	2	1	2	1	1	1	1	1
1	1	2	1	2	2	2	2	2	2
1	1	2	1	1	1	1	1	1	1
2	1	2	1	2	2	2	2	2	2
1	2	2	2	2	2	2	2	2	2
#NULL!	#NULL!	#NULL!	#NULL!	#NULL!	#NULL!	#NULL!	#NULL!	#NULL!	#NULL!
2	2	1	#NULL!	#NULL!	#NULL!	#NULL!	#NULL!	#NULL!	#NULL!
1	1	2	1	2	1	1	1	2	1
#NULL!	#NULL!	2	2	2	2	2	2	2	2
1	1	2	2	2	2	2	2	2	2
1	1	2	2	2	2	2	2	2	2
1	2	2	2	2	2	2	2	2	2
1	1	2	2	1	2	2	2	2	2
#NULL!	#NULL!	#NULL!	#NULL!	#NULL!	#NULL!	#NULL!	#NULL!	#NULL!	#NULL!
#NULL!	#NULL!	#NULL!	#NULL!	#NULL!	#NULL!	#NULL!	#NULL!	#NULL!	#NULL!
1	1	2	2	2	2	2	2	2	2
1	1	2	2	1	2	2	2	2	1
#NULL!	#NULL!	#NULL!	#NULL!	#NULL!	#NULL!	#NULL!	#NULL!	#NULL!	#NULL!
#NULL!	#NULL!	#NULL!	#NULL!	#NULL!	#NULL!	#NULL!	#NULL!	#NULL!	#NULL!

#NULL!	#NULL!	2	2	2	2	2	2	2	2
1	1	2	2	2	2	2	2	2	2
1	1	2	2	1	2	2	1	2	2
2	2	2	1	2	2	1	2	2	1
2	2	2	2	2	2	1	1	1	1
2	1	2	2	1	2	2	1	2	2
1	1	2	2	2	1	2	2	2	2
1	2	2	#NULL!	#NULL!	#NULL!	#NULL!	#NULL!	#NULL!	#NULL!
1	1	2	1	2	2	2	2	2	2
#NULL!	#NULL!	#NULL!	#NULL!	#NULL!	#NULL!	#NULL!	#NULL!	#NULL!	#NULL!
1	1	2	2	2	2	2	2	2	2
2	2	2	2	1	1	1	1	1	2
#NULL!	#NULL!	2	2	2	2	2	2	1	1
#NULL!	#NULL!	#NULL!	#NULL!	#NULL!	#NULL!	#NULL!	#NULL!	#NULL!	#NULL!
1	1	2	2	2	2	2	2	2	2
#NULL!	#NULL!	#NULL!	#NULL!	#NULL!	#NULL!	#NULL!	#NULL!	#NULL!	#NULL!
#NULL!	#NULL!	#NULL!	#NULL!	#NULL!	#NULL!	#NULL!	#NULL!	#NULL!	#NULL!
1	1	2	2	2	2	2	2	2	2
2	2	2	2	2	2	2	2	2	2
2	2	2	2	2	2	2	2	2	2
2	2	2	2	2	2	2	2	2	2
1	1	2	2	2	2	2	2	2	2
2	2	2	1	1	1	1	1	1	1
2	1	2	1	2	2	1	2	1	2
2	2	2	2	2	2	#NULL!	2	#NULL!	#NULL!
2	2	2	#NULL!	#NULL!	#NULL!	#NULL!	#NULL!	#NULL!	#NULL!
2	2	2	2	2	2	2	2	2	2
#NULL!	#NULL!	2	1	2	2	2	2	2	2
1	2	2	2	2	2	2	2	2	2
2	2	2	2	1	1	1	1	1	1
1	1	2	2	2	2	2	2	2	2
2	2	2	2	2	2	2	2	2	2
#NULL!	#NULL!	2	2	2	2	2	2	2	2
2	2	2	2	2	2	2	2	2	2
1	1	2	2	2	2	2	2	2	2
2	2	2	2	2	2	2	2	2	2
2	2	1	1	1	1	1	1	1	1
2	2	#NULL!	#NULL!	#NULL!	#NULL!	#NULL!	2	#NULL!	#NULL!
2	2	#NULL!	#NULL!	#NULL!	#NULL!	#NULL!	#NULL!	#NULL!	#NULL!
#NULL!	#NULL!	2	2	2	2	2	2	2	2
2	2	1	1	1	1	1	1	1	1
2	2	2	2	2	2	2	2	2	2
#NULL!	#NULL!	2	2	2	2	2	2	2	2
1	2	2	#NULL!	#NULL!	#NULL!	#NULL!	#NULL!	#NULL!	#NULL!
2	2	2	2	2	2	2	2	2	2
#NULL!	#NULL!	#NULL!	#NULL!	#NULL!	#NULL!	#NULL!	#NULL!	#NULL!	#NULL!
2	2	2	2	2	2	2	2	2	2

2	2	2	2	2	1	2	1	2	2
2	2	#NULL!	#NULL!	#NULL!	#NULL!	#NULL!	#NULL!	#NULL!	#NULL!
#NULL!	#NULL!	2	2	2	2	2	2	2	2
2	2	2	2	2	2	2	2	2	2
1	1	1	1	1	2	2	2	2	2
2	2	2	2	2	2	2	2	2	2
2	2	2	2	2	2	2	2	2	1
#NULL!	#NULL!	#NULL!	#NULL!	#NULL!	#NULL!	#NULL!	#NULL!	#NULL!	#NULL!
2	2	2	2	2	2	2	2	2	2
2	2	2	2	2	2	2	2	2	2
2	2	2	2	2	2	2	2	2	2
2	2	2	2	2	2	2	2	2	2
1	2	2	1	#NULL!	2	1	#NULL!	2	1
2	2	2	1	1	2	2	1	1	1
1	2	2	2	2	2	2	2	2	2
1	2	2	2	2	2	2	2	2	2
2	2	2	2	2	2	2	2	2	2
2	2	2	2	2	2	2	2	2	2
#NULL!	#NULL!	#NULL!	#NULL!	#NULL!	#NULL!	#NULL!	#NULL!	#NULL!	#NULL!
#NULL!	#NULL!	#NULL!	#NULL!	#NULL!	#NULL!	#NULL!	#NULL!	#NULL!	#NULL!
2	2	1	2	2	1	2	1	2	2
2	2	2	2	2	2	2	2	2	2
1	2	#NULL!	#NULL!	#NULL!	#NULL!	#NULL!	#NULL!	#NULL!	#NULL!
#NULL!	#NULL!	#NULL!	#NULL!	#NULL!	#NULL!	#NULL!	#NULL!	#NULL!	#NULL!
2	2	1	1	1	1	1	1	1	1
2	2	#NULL!	#NULL!	#NULL!	#NULL!	#NULL!	#NULL!	#NULL!	#NULL!
#NULL!	#NULL!	#NULL!	#NULL!	#NULL!	#NULL!	#NULL!	#NULL!	#NULL!	#NULL!
#NULL!	#NULL!	#NULL!	#NULL!	#NULL!	#NULL!	#NULL!	#NULL!	#NULL!	#NULL!
#NULL!	#NULL!	2	2	1	2	2	2	2	1
2	2	2	2	2	2	2	2	2	2
2	2	2	2	2	2	2	2	2	2
2	1	2	2	2	#NULL!	2	2	2	#NULL!
2	2	2	2	2	2	2	1	2	2
2	2	2	2	2	2	2	2	2	2
2	2	2	2	2	2	2	2	2	2
2	2	2	2	2	2	2	2	2	2
1	2	2	2	2	2	2	2	2	1
1	2	2	2	1	2	1	2	1	2
1	2	2	2	1	2	2	1	2	2
1	1	2	1	2	2	2	2	2	2
2	2	2	2	2	2	1	1	2	2
2	1	2	2	2	2	2	2	2	2
2	1	2	#NULL!	#NULL!	#NULL!	#NULL!	#NULL!	#NULL!	#NULL!
1	2	#NULL!	#NULL!	#NULL!	2	#NULL!	#NULL!	#NULL!	#NULL!
1	2	2	1	2	2	2	2	1	2
2	2	2	2	2	2	2	2	2	2
2	2	2	2	2	2	2	2	2	2

2	1	2	2	2	2	2	2	2	1
#NULL!	#NULL!	#NULL!	#NULL!	#NULL!	#NULL!	#NULL!	#NULL!	#NULL!	#NULL!
#NULL!	#NULL!	#NULL!	#NULL!	#NULL!	#NULL!	#NULL!	#NULL!	#NULL!	#NULL!
#NULL!	#NULL!	#NULL!	#NULL!	#NULL!	#NULL!	#NULL!	#NULL!	#NULL!	#NULL!
#NULL!	#NULL!	#NULL!	#NULL!	#NULL!	#NULL!	#NULL!	#NULL!	#NULL!	#NULL!
2	1	#NULL!	#NULL!	#NULL!	#NULL!	#NULL!	#NULL!	#NULL!	#NULL!
#NULL!	#NULL!	#NULL!	#NULL!	#NULL!	#NULL!	#NULL!	#NULL!	#NULL!	#NULL!
#NULL!	#NULL!	#NULL!	#NULL!	#NULL!	#NULL!	#NULL!	#NULL!	#NULL!	#NULL!
#NULL!	#NULL!	#NULL!	#NULL!	#NULL!	#NULL!	#NULL!	#NULL!	#NULL!	#NULL!
2	2	#NULL!	#NULL!	#NULL!	#NULL!	#NULL!	#NULL!	#NULL!	2
2	2	2	2	2	2	2	2	2	2
1	2	2	2	2	2	2	2	1	2
#NULL!	#NULL!	#NULL!	#NULL!	#NULL!	#NULL!	#NULL!	#NULL!	#NULL!	2
2	2	2	2	2	2	2	2	2	2
2	2	2	2	2	2	2	2	2	2
#NULL!	#NULL!	#NULL!	#NULL!	#NULL!	#NULL!	#NULL!	#NULL!	#NULL!	#NULL!
1	1	2	2	2	2	2	2	2	1
2	2	2	2	2	2	2	2	2	2
1	2	2	2	2	2	2	2	2	2
2	2	2	2	2	2	2	2	2	2
2	2	2	1	2	2	2	1	2	2
2	2	1	1	2	1	2	2	2	1
2	1	1	1	1	1	1	1	1	1
1	2	2	2	2	2	2	2	2	2
2	2	2	2	2	2	2	2	2	2
2	1	2	2	2	2	2	2	2	2
2	2	2	2	2	2	2	2	2	2
1	1	2	2	2	2	2	2	2	2
2	2	2	2	2	2	2	2	2	2
2	2	2	2	2	2	2	2	2	2
#NULL!	#NULL!	#NULL!	#NULL!	#NULL!	#NULL!	#NULL!	#NULL!	#NULL!	#NULL!
2	2	2	2	2	2	2	2	2	2
1	1	1	2	2	2	2	2	2	2
1	1	2	#NULL!	#NULL!	#NULL!	#NULL!	#NULL!	#NULL!	#NULL!
#NULL!	#NULL!	1	1	1	1	1	1	1	1
2	2	2	2	2	2	2	2	2	2
2	2	2	2	2	2	2	2	2	2
1	2	2	2	2	2	2	2	2	2
1	2	2	1	1	2	1	2	1	2
#NULL!	#NULL!	#NULL!	#NULL!	#NULL!	#NULL!	#NULL!	#NULL!	#NULL!	#NULL!
2	1	2	2	2	2	1	2	1	2
2	2	2	2	2	2	2	2	2	2
2	2	2	2	2	2	2	2	2	2
1	1	2	1	2	2	2	2	2	1
1	1	2	2	2	2	2	2	2	2
#NULL!	#NULL!	#NULL!	#NULL!	#NULL!	#NULL!	#NULL!	#NULL!	#NULL!	#NULL!
2	#NULL!	#NULL!	#NULL!	#NULL!	#NULL!	#NULL!	#NULL!	#NULL!	#NULL!

#NULL!	#NULL!	#NULL!	#NULL!	#NULL!	#NULL!	#NULL!	#NULL!	#NULL!	#NULL!
2	2	2	2	2	2	2	2	2	2
1	2	2	#NULL!	#NULL!	#NULL!	#NULL!	#NULL!	#NULL!	#NULL!
#NULL!	#NULL!	#NULL!	#NULL!	#NULL!	#NULL!	#NULL!	#NULL!	#NULL!	#NULL!
#NULL!	#NULL!	#NULL!	#NULL!	#NULL!	#NULL!	#NULL!	#NULL!	#NULL!	#NULL!
#NULL!	#NULL!	#NULL!	#NULL!	#NULL!	#NULL!	#NULL!	#NULL!	#NULL!	#NULL!
#NULL!	#NULL!	#NULL!	#NULL!	#NULL!	#NULL!	#NULL!	#NULL!	#NULL!	#NULL!
#NULL!	#NULL!	#NULL!	#NULL!	#NULL!	#NULL!	#NULL!	#NULL!	#NULL!	#NULL!
#NULL!	#NULL!	#NULL!	#NULL!	#NULL!	#NULL!	#NULL!	#NULL!	#NULL!	#NULL!
#NULL!	#NULL!	#NULL!	#NULL!	#NULL!	#NULL!	#NULL!	#NULL!	#NULL!	#NULL!
#NULL!	#NULL!	#NULL!	#NULL!	#NULL!	#NULL!	#NULL!	#NULL!	#NULL!	#NULL!
#NULL!	#NULL!	#NULL!	#NULL!	#NULL!	#NULL!	#NULL!	#NULL!	#NULL!	#NULL!
#NULL!	#NULL!	#NULL!	#NULL!	#NULL!	#NULL!	#NULL!	#NULL!	#NULL!	#NULL!
#NULL!	#NULL!	#NULL!	#NULL!	#NULL!	#NULL!	#NULL!	#NULL!	#NULL!	#NULL!
#NULL!	#NULL!	#NULL!	#NULL!	#NULL!	#NULL!	#NULL!	#NULL!	#NULL!	#NULL!
#NULL!	#NULL!	#NULL!	#NULL!	#NULL!	#NULL!	#NULL!	#NULL!	#NULL!	#NULL!
#NULL!	#NULL!	#NULL!	#NULL!	#NULL!	#NULL!	#NULL!	#NULL!	#NULL!	#NULL!
#NULL!	#NULL!	#NULL!	#NULL!	#NULL!	#NULL!	#NULL!	#NULL!	#NULL!	#NULL!
#NULL!	#NULL!	#NULL!	#NULL!	#NULL!	#NULL!	#NULL!	#NULL!	#NULL!	#NULL!
#NULL!	#NULL!	#NULL!	#NULL!	#NULL!	#NULL!	#NULL!	#NULL!	#NULL!	#NULL!
#NULL!	#NULL!	#NULL!	#NULL!	#NULL!	#NULL!	#NULL!	#NULL!	#NULL!	#NULL!
#NULL!	#NULL!	#NULL!	#NULL!	#NULL!	#NULL!	#NULL!	#NULL!	#NULL!	#NULL!
#NULL!	#NULL!	#NULL!	#NULL!	#NULL!	#NULL!	#NULL!	#NULL!	#NULL!	#NULL!
1	2	2	2	1	2	2	1	2	2
2	2	2	2	2	2	2	2	2	2
1	1	2	2	2	2	2	2	2	2
2	1	2	2	2	2	2	2	2	2
2	1	2	2	2	2	2	1	2	2
2	2	2	#NULL!	#NULL!	#NULL!	#NULL!	#NULL!	#NULL!	#NULL!
2	2	2	2	2	2	2	2	2	2
1	1	2	2	2	2	2	2	2	2
2	2	2	2	2	2	2	2	2	2
2	2	2	2	2	2	2	2	1	1
2	2	#NULL!	#NULL!	#NULL!	#NULL!	#NULL!	#NULL!	#NULL!	#NULL!
2	2	2	1	1	1	2	2	2	2
1	1	2	1	2	2	1	1	2	1
2	2	#NULL!	#NULL!	#NULL!	#NULL!	#NULL!	#NULL!	#NULL!	#NULL!
1	1	2	2	2	2	2	2	2	2
1	1	2	1	2	2	2	2	2	1
2	1	2	#NULL!	#NULL!	#NULL!	#NULL!	#NULL!	#NULL!	#NULL!
1	1	2	2	2	2	2	2	2	2
2	1	2	2	2	2	2	2	2	1
1	1	2	#NULL!	#NULL!	#NULL!	#NULL!	#NULL!	#NULL!	#NULL!
2	1	2	2	2	2	2	1	2	2
1	1	2	1	1	2	1	1	2	1
2	2	2	2	2	2	2	2	2	2
1	2	2	2	2	1	2	1	2	1

1	1	2	2	2	2	2	2	2	1
1	1	2	2	2	2	2	2	2	2
2	2	2	1	2	2	1	1	2	1
1	1	2	2	2	2	2	2	2	2
2	1	2	2	2	1	2	1	2	1
2	1	2	#NULL!	#NULL!	#NULL!	#NULL!	#NULL!	#NULL!	#NULL!
2	1	2	2	1	2	2	2	2	2
1	1	2	#NULL!	#NULL!	#NULL!	#NULL!	#NULL!	#NULL!	#NULL!
2	2	2	2	2	2	2	2	2	2
2	2	2	2	2	2	2	2	2	2
2	1	#NULL!	#NULL!	#NULL!	2	#NULL!	#NULL!	#NULL!	#NULL!
1	1	2	#NULL!	#NULL!	#NULL!	#NULL!	#NULL!	#NULL!	#NULL!
1	2	#NULL!	#NULL!	#NULL!	#NULL!	2	#NULL!	#NULL!	#NULL!
#NULL!	#NULL!	#NULL!	#NULL!	#NULL!	2	#NULL!	#NULL!	#NULL!	#NULL!
1	1	#NULL!	#NULL!	#NULL!	#NULL!	#NULL!	#NULL!	#NULL!	#NULL!
#NULL!	#NULL!	#NULL!	#NULL!	#NULL!	#NULL!	#NULL!	#NULL!	#NULL!	#NULL!
#NULL!	#NULL!	#NULL!	#NULL!	#NULL!	#NULL!	#NULL!	#NULL!	#NULL!	#NULL!
2	2	2	2	2	2	2	2	2	2
2	1	#NULL!	#NULL!	#NULL!	#NULL!	#NULL!	#NULL!	#NULL!	#NULL!
1	1	2	2	2	1	2	1	2	1
1	1	2	2	2	2	2	2	2	2
2	1	#NULL!	#NULL!	#NULL!	#NULL!	2	#NULL!	#NULL!	2
1	1	2	#NULL!	#NULL!	#NULL!	#NULL!	#NULL!	#NULL!	#NULL!
2	1	#NULL!	#NULL!	#NULL!	#NULL!	#NULL!	2	#NULL!	#NULL!
1	1	2	#NULL!	#NULL!	#NULL!	#NULL!	#NULL!	#NULL!	#NULL!
1	1	2	1	2	2	1	2	1	2
1	1	2	1	2	2	1	2	2	2
1	1	2	2	1	2	2	2	2	1
2	2	2	2	2	2	2	2	2	2
#NULL!	#NULL!	#NULL!	#NULL!	#NULL!	#NULL!	#NULL!	#NULL!	#NULL!	#NULL!
2	2	2	2	1	1	2	2	2	2
1	1	1	1	2	2	1	2	1	2
2	2	1	1	1	1	1	1	1	1
2	1	2	2	1	2	1	2	2	1
2	2	2	1	2	2	1	1	2	1
2	2	#NULL!	2	#NULL!	#NULL!	#NULL!	#NULL!	#NULL!	#NULL!
2	2	2	2	2	2	1	2	2	2
1	2	2	1	2	2	1	1	2	2
2	2	2	1	2	1	2	1	2	2
2	2	2	2	2	2	2	1	2	2
1	1	2	1	2	2	1	1	2	1
1	1	2	1	2	2	2	1	2	1
2	1	2	1	2	2	1	1	2	1
2	2	2	1	1	1	1	2	2	2
1	1	2	1	2	#NULL!	2	2	2	1
2	2	2	2	#NULL!	#NULL!	#NULL!	#NULL!	#NULL!	#NULL!
1	1	2	1	2	2	2	2	2	2

1	1	2	2	2	2	2	2	2	2
1	1	2	2	1	2	1	2	1	1
2	2	#NULL!	#NULL!	#NULL!	#NULL!	#NULL!	#NULL!	#NULL!	#NULL!
2	1	2	1	1	1	1	1	1	1
#NULL!	#NULL!	#NULL!	#NULL!	#NULL!	#NULL!	#NULL!	#NULL!	#NULL!	#NULL!
1	1	2	1	2	2	2	2	2	1
1	1	2	2	2	2	2	1	2	1
#NULL!	#NULL!	#NULL!	#NULL!	#NULL!	#NULL!	#NULL!	#NULL!	#NULL!	#NULL!
2	2	2	#NULL!	#NULL!	#NULL!	#NULL!	#NULL!	#NULL!	#NULL!
2	2	2	2	2	2	2	2	2	2
2	2	2	2	2	1	2	1	2	2
2	2	2	1	1	1	1	2	1	1
1	1	2	1	2	2	1	1	2	2
#NULL!	#NULL!	#NULL!	#NULL!	#NULL!	#NULL!	#NULL!	#NULL!	#NULL!	#NULL!
#NULL!	#NULL!	#NULL!	#NULL!	#NULL!	#NULL!	#NULL!	#NULL!	#NULL!	#NULL!
1	1	1	#NULL!	2	2	#NULL!	1	1	1
1	1	2	#NULL!	#NULL!	#NULL!	#NULL!	#NULL!	#NULL!	#NULL!
#NULL!	#NULL!	#NULL!	#NULL!	#NULL!	#NULL!	#NULL!	#NULL!	#NULL!	#NULL!
2	2	1	2	#NULL!	2	1	2	2	2
1	1	2	1	2	2	1	1	1	2
1	1	2	2	2	2	2	2	2	2
1	1	2	2	2	2	2	2	2	2
1	2	2	2	2	1	1	2	1	2
1	1	2	1	2	1	1	1	2	1
2	2	2	2	2	2	2	2	2	2
#NULL!	#NULL!	#NULL!	#NULL!	#NULL!	#NULL!	#NULL!	#NULL!	#NULL!	#NULL!
1	1	2	#NULL!	#NULL!	#NULL!	#NULL!	#NULL!	#NULL!	#NULL!
1	1	2	1	2	2	1	2	2	1
1	1	1	1	1	1	1	2	2	2
2	1	2	2	2	1	2	2	1	2
#NULL!	#NULL!	#NULL!	#NULL!	#NULL!	#NULL!	#NULL!	#NULL!	#NULL!	#NULL!
#NULL!	#NULL!	#NULL!	#NULL!	#NULL!	#NULL!	#NULL!	#NULL!	#NULL!	#NULL!
#NULL!	#NULL!	#NULL!	#NULL!	#NULL!	#NULL!	#NULL!	#NULL!	#NULL!	#NULL!
#NULL!	#NULL!	#NULL!	#NULL!	#NULL!	#NULL!	#NULL!	#NULL!	#NULL!	#NULL!
#NULL!	#NULL!	#NULL!	#NULL!	#NULL!	#NULL!	#NULL!	#NULL!	#NULL!	#NULL!
#NULL!	#NULL!	#NULL!	#NULL!	#NULL!	#NULL!	#NULL!	#NULL!	#NULL!	#NULL!
#NULL!	#NULL!	#NULL!	#NULL!	#NULL!	#NULL!	#NULL!	#NULL!	#NULL!	#NULL!
#NULL!	#NULL!	#NULL!	#NULL!	#NULL!	#NULL!	#NULL!	#NULL!	#NULL!	#NULL!
#NULL!	#NULL!	#NULL!	#NULL!	#NULL!	#NULL!	#NULL!	#NULL!	#NULL!	#NULL!
#NULL!	#NULL!	#NULL!	#NULL!	#NULL!	#NULL!	#NULL!	#NULL!	#NULL!	#NULL!
#NULL!	#NULL!	#NULL!	#NULL!	#NULL!	#NULL!	#NULL!	#NULL!	#NULL!	#NULL!
#NULL!	#NULL!	#NULL!	#NULL!	#NULL!	#NULL!	#NULL!	#NULL!	#NULL!	#NULL!
#NULL!	#NULL!	#NULL!	#NULL!	#NULL!	#NULL!	#NULL!	#NULL!	#NULL!	#NULL!
#NULL!	#NULL!	#NULL!	#NULL!	#NULL!	#NULL!	#NULL!	#NULL!	#NULL!	#NULL!
#NULL!	#NULL!	#NULL!	#NULL!	#NULL!	#NULL!	#NULL!	#NULL!	#NULL!	#NULL!
#NULL!	#NULL!	#NULL!	#NULL!	#NULL!	#NULL!	#NULL!	#NULL!	#NULL!	#NULL!
#NULL!	#NULL!	#NULL!	#NULL!	#NULL!	#NULL!	#NULL!	#NULL!	#NULL!	#NULL!

#NULL!	#NULL!	#NULL!	#NULL!	#NULL!	#NULL!	#NULL!	#NULL!	#NULL!	#NULL!
#NULL!	#NULL!	#NULL!	#NULL!	#NULL!	#NULL!	#NULL!	#NULL!	#NULL!	#NULL!
#NULL!	#NULL!	#NULL!	#NULL!	#NULL!	#NULL!	#NULL!	#NULL!	#NULL!	#NULL!
#NULL!	#NULL!	#NULL!	#NULL!	#NULL!	#NULL!	#NULL!	#NULL!	#NULL!	#NULL!
#NULL!	#NULL!	#NULL!	#NULL!	#NULL!	#NULL!	#NULL!	#NULL!	#NULL!	#NULL!
#NULL!	#NULL!	#NULL!	#NULL!	#NULL!	#NULL!	#NULL!	#NULL!	#NULL!	#NULL!
#NULL!	#NULL!	#NULL!	#NULL!	#NULL!	#NULL!	#NULL!	#NULL!	#NULL!	#NULL!
#NULL!	#NULL!	#NULL!	#NULL!	#NULL!	#NULL!	#NULL!	#NULL!	#NULL!	#NULL!
#NULL!	#NULL!	#NULL!	#NULL!	#NULL!	#NULL!	#NULL!	#NULL!	#NULL!	#NULL!
#NULL!	#NULL!	#NULL!	#NULL!	#NULL!	#NULL!	#NULL!	#NULL!	#NULL!	#NULL!
#NULL!	#NULL!	#NULL!	#NULL!	#NULL!	#NULL!	#NULL!	#NULL!	#NULL!	#NULL!
#NULL!	#NULL!	#NULL!	#NULL!	#NULL!	#NULL!	#NULL!	#NULL!	#NULL!	#NULL!
#NULL!	#NULL!	#NULL!	#NULL!	#NULL!	#NULL!	#NULL!	#NULL!	#NULL!	#NULL!
#NULL!	#NULL!	#NULL!	#NULL!	#NULL!	#NULL!	#NULL!	#NULL!	#NULL!	#NULL!
#NULL!	#NULL!	#NULL!	#NULL!	#NULL!	#NULL!	#NULL!	#NULL!	#NULL!	#NULL!
#NULL!	#NULL!	#NULL!	#NULL!	#NULL!	#NULL!	#NULL!	#NULL!	#NULL!	#NULL!
#NULL!	#NULL!	#NULL!	#NULL!	#NULL!	#NULL!	#NULL!	#NULL!	#NULL!	#NULL!
#NULL!	#NULL!	#NULL!	#NULL!	#NULL!	#NULL!	#NULL!	#NULL!	#NULL!	#NULL!
#NULL!	#NULL!	#NULL!	#NULL!	#NULL!	#NULL!	#NULL!	#NULL!	#NULL!	#NULL!
1	1	2	2	2	2	2	2	2	2
#NULL!	#NULL!	#NULL!	#NULL!	#NULL!	#NULL!	#NULL!	#NULL!	#NULL!	#NULL!
#NULL!	#NULL!	#NULL!	#NULL!	#NULL!	#NULL!	#NULL!	#NULL!	#NULL!	#NULL!
#NULL!	#NULL!	#NULL!	#NULL!	#NULL!	#NULL!	#NULL!	#NULL!	#NULL!	#NULL!
#NULL!	#NULL!	#NULL!	#NULL!	#NULL!	#NULL!	#NULL!	#NULL!	#NULL!	#NULL!
#NULL!	#NULL!	#NULL!	#NULL!	#NULL!	#NULL!	#NULL!	#NULL!	#NULL!	#NULL!
#NULL!	#NULL!	#NULL!	#NULL!	#NULL!	#NULL!	#NULL!	#NULL!	#NULL!	#NULL!
#NULL!	#NULL!	#NULL!	#NULL!	#NULL!	#NULL!	#NULL!	#NULL!	#NULL!	#NULL!
#NULL!	#NULL!	#NULL!	#NULL!	#NULL!	#NULL!	#NULL!	#NULL!	#NULL!	#NULL!
#NULL!	#NULL!	#NULL!	#NULL!	#NULL!	#NULL!	#NULL!	#NULL!	#NULL!	#NULL!
#NULL!	#NULL!	#NULL!	#NULL!	#NULL!	#NULL!	#NULL!	#NULL!	#NULL!	#NULL!
#NULL!	#NULL!	#NULL!	#NULL!	#NULL!	#NULL!	#NULL!	#NULL!	#NULL!	#NULL!
#NULL!	#NULL!	#NULL!	#NULL!	#NULL!	#NULL!	#NULL!	#NULL!	#NULL!	#NULL!
#NULL!	#NULL!	#NULL!	#NULL!	#NULL!	#NULL!	#NULL!	#NULL!	#NULL!	#NULL!
#NULL!	#NULL!	#NULL!	#NULL!	#NULL!	#NULL!	#NULL!	#NULL!	#NULL!	#NULL!
#NULL!	#NULL!	#NULL!	#NULL!	#NULL!	#NULL!	#NULL!	#NULL!	#NULL!	#NULL!
2	2	2	2	2	2	2	2	2	2
#NULL!	#NULL!	#NULL!	#NULL!	#NULL!	#NULL!	#NULL!	#NULL!	#NULL!	#NULL!
2	1	2	2	2	2	2	1	2	1
1	1	2	1	2	1	2	2	2	2
2	2	1	1	1	1	1	1	1	1
#NULL!	#NULL!	#NULL!	#NULL!	#NULL!	#NULL!	#NULL!	#NULL!	#NULL!	#NULL!
2	1	#NULL!	#NULL!	#NULL!	#NULL!	#NULL!	#NULL!	#NULL!	#NULL!
1	1	2	#NULL!	#NULL!	#NULL!	#NULL!	#NULL!	#NULL!	#NULL!
2	1	#NULL!	#NULL!	#NULL!	#NULL!	#NULL!	#NULL!	#NULL!	#NULL!
2	2	2	2	2	2	2	2	1	2
2	2	#NULL!	#NULL!	#NULL!	2	#NULL!	#NULL!	#NULL!	#NULL!
2	1	2	2	2	2	2	2	2	2

2	1	2	1	2	2	2	2	2	1
2	2	2	2	2	2	2	2	2	2
1	1	2	2	2	2	2	2	2	2
2	2	2	2	2	2	2	2	2	1
1	1	2	2	2	2	1	1	2	1
1	2	2	2	2	2	2	1	2	1
1	2	2	2	2	2	2	2	2	2
2	2	2	2	2	2	2	2	2	2
2	1	2	2	2	1	2	2	2	2
2	2	2	2	2	2	1	1	2	2
2	2	2	#NULL!	#NULL!	#NULL!	#NULL!	#NULL!	#NULL!	#NULL!
2	2	2	2	1	2	2	1	2	2
#NULL!	#NULL!	#NULL!	#NULL!	#NULL!	#NULL!	#NULL!	#NULL!	#NULL!	#NULL!
2	2	2	2	1	2	2	2	2	2
2	2	2	2	1	2	2	2	2	2
2	2	2	1	2	2	2	1	2	2
#NULL!	#NULL!	#NULL!	#NULL!	#NULL!	#NULL!	#NULL!	#NULL!	#NULL!	#NULL!
2	2	2	2	#NULL!	#NULL!	#NULL!	#NULL!	#NULL!	#NULL!
1	2	2	1	2	2	2	2	2	2
2	1	2	2	2	2	2	2	2	1
1	2	2	2	2	1	1	2	#NULL!	1
1	1	2	1	2	2	1	1	2	1
1	1	2	1	2	2	2	2	2	2
1	1	2	1	2	1	1	1	2	1
1	1	1	1	1	1	1	1	1	1
2	2	2	2	2	2	2	2	2	2
2	2	2	2	2	2	2	2	2	2
#NULL!	#NULL!	2	1	2	1	2	2	1	2
1	2	2	2	2	2	2	2	2	2
#NULL!	#NULL!	#NULL!	#NULL!	#NULL!	#NULL!	#NULL!	#NULL!	#NULL!	#NULL!
#NULL!	#NULL!	#NULL!	#NULL!	#NULL!	#NULL!	#NULL!	#NULL!	#NULL!	#NULL!
2	1	2	2	2	2	2	2	2	2
1	1	2	2	2	2	2	2	2	2
2	2	#NULL!	#NULL!	#NULL!	#NULL!	#NULL!	#NULL!	#NULL!	#NULL!
#NULL!	#NULL!	#NULL!	#NULL!	#NULL!	#NULL!	#NULL!	#NULL!	#NULL!	#NULL!
2	2	#NULL!	#NULL!	#NULL!	#NULL!	#NULL!	#NULL!	#NULL!	#NULL!
1	1	#NULL!	#NULL!	#NULL!	#NULL!	#NULL!	#NULL!	#NULL!	#NULL!
1	1	2	2	1	2	2	2	1	2
1	1	#NULL!	#NULL!	#NULL!	#NULL!	#NULL!	#NULL!	#NULL!	#NULL!
#NULL!	#NULL!	#NULL!	#NULL!	#NULL!	#NULL!	#NULL!	#NULL!	#NULL!	#NULL!
1	1	2	2	2	2	2	2	2	2
2	2	#NULL!	#NULL!	#NULL!	#NULL!	#NULL!	#NULL!	#NULL!	#NULL!
1	1	2	2	2	1	1	1	2	1
2	1	1	1	1	1	1	1	2	1
2	2	2	2	2	2	2	2	2	1
1	2	1	1	1	1	1	1	1	1
2	2	2	1	2	2	1	2	2	2

2	2	2	1	1	2	1	2	1	2
#NULL!	#NULL!	2	2	2	2	2	2	2	2
2	1	2	2	2	2	2	2	2	2
1	1	2	2	2	2	2	2	1	1
2	2	2	2	2	1	1	2	2	1
2	1	2	1	2	2	1	2	2	2
1	1	2	2	2	2	2	2	2	1
1	2	2	1	2	2	1	2	2	1
2	1	2	2	2	2	2	2	2	2
#NULL!	#NULL!	2	#NULL!	2	#NULL!	2	#NULL!	2	1
1	1	2	1	1	1	1	1	1	1
2	2	2	2	2	2	2	2	2	1
1	1	2	2	2	2	2	2	2	2
2	2	2	2	1	2	2	1	2	2
2	2	2	2	2	2	2	2	2	2
2	1	2	1	2	2	1	1	1	1
1	1	2	2	2	1	2	1	2	2
2	2	2	#NULL!	#NULL!	#NULL!	#NULL!	#NULL!	#NULL!	#NULL!
2	2	2	2	2	2	2	2	2	2
2	2	2	2	2	1	1	1	1	1
2	2	2	2	2	2	1	1	2	2
2	2	2	2	1	2	2	2	2	2
1	1	2	1	2	2	2	1	2	2
2	2	2	2	2	2	2	2	2	2
2	2	2	2	2	2	2	2	2	2
1	2	1	2	2	2	2	1	2	2
2	2	2	2	2	2	1	2	2	1
1	1	2	2	2	2	2	2	1	2
2	2	2	2	2	2	2	2	2	2
2	2	2	1	2	2	1	2	2	2
2	2	2	1	2	2	2	2	2	2
1	1	2	2	1	2	2	1	2	2
2	2	2	2	2	2	2	2	2	2
2	2	2	2	2	1	1	2	1	1
1	1	2	2	1	2	2	1	2	2
2	2	2	1	1	1	1	1	1	1
1	2	2	2	2	2	2	2	1	2
#NULL!	#NULL!	#NULL!	#NULL!	#NULL!	#NULL!	#NULL!	#NULL!	#NULL!	#NULL!
2	1	2	1	2	2	1	2	1	1
1	2	2	1	#NULL!	2	2	2	1	2
2	2	2	1	1	1	2	1	2	1
1	1	2	2	1	2	2	1	2	2
2	2	2	1	2	2	2	1	2	2
1	1	2	1	2	2	1	2	2	1
1	1	2	2	2	2	2	2	2	2
2	2	2	1	1	1	1	1	1	1
2	2	1	1	1	1	1	1	1	1
[truncated: 510,229 more chars]
